# Genome and Transcriptome sequence of Finger millet (*Eleusine coracana* (L.) Gaertn.) provides insights into drought tolerance and nutraceutical properties

**DOI:** 10.1186/s12864-017-3850-z

**Published:** 2017-06-15

**Authors:** Shailaja Hittalmani, H. B. Mahesh, Meghana Deepak Shirke, Hanamareddy Biradar, Govindareddy Uday, Y. R. Aruna, H. C. Lohithaswa, A. Mohanrao

**Affiliations:** 1 Marker Assisted Selection Laboratory, Department of Genetics and Plant Breeding, University of Agricultural Sciences, Bengaluru, 560065 India; 2 Manipal University, Manipal, 576104 India; 3 Department of Genetics and Plant Breeding, College of Agriculture, V. C. Farm, University of Agricultural Sciences, Mandya, 571405 India

**Keywords:** Next-generation sequencing, Whole genome assembly, Functional annotation, RNA-sequencing, microsatellites, C4 pathway

## Abstract

**Background:**

Finger millet (*Eleusine coracana* (L.) Gaertn.) is an important staple food crop widely grown in Africa and South Asia. Among the millets, finger millet has high amount of calcium, methionine, tryptophan, fiber, and sulphur containing amino acids. In addition, it has C4 photosynthetic carbon assimilation mechanism, which helps to utilize water and nitrogen efficiently under hot and arid conditions without severely affecting yield. Therefore, development and utilization of genomic resources for genetic improvement of this crop is immensely useful.

**Results:**

Experimental results from whole genome sequencing and assembling process of ML-365 finger millet cultivar yielded 1196 Mb covering approximately 82% of total estimated genome size. Genome analysis showed the presence of 85,243 genes and one half of the genome is repetitive in nature. The finger millet genome was found to have higher colinearity with foxtail millet and rice as compared to other *Poaceae* species. Mining of simple sequence repeats (SSRs) yielded abundance of SSRs within the finger millet genome. Functional annotation and mining of transcription factors revealed finger millet genome harbors large number of drought tolerance related genes. Transcriptome analysis of low moisture stress and non-stress samples revealed the identification of several drought-induced candidate genes, which could be used in drought tolerance breeding.

**Conclusions:**

This genome sequencing effort will strengthen plant breeders for allele discovery, genetic mapping, and identification of candidate genes for agronomically important traits. Availability of genomic resources of finger millet will enhance the novel breeding possibilities to address potential challenges of finger millet improvement.

**Electronic supplementary material:**

The online version of this article (doi:10.1186/s12864-017-3850-z) contains supplementary material, which is available to authorized users.

## Background

Finger millet (*Eleusine coracana* (L.) Gaertn.) is an allotetraploid (2n = 4× = 36, AABB) annual cereal millet crop that includes two distinct subspecies: subsp. *coracana* (cultivated finger millet or ragi) and subsp. *africana* (wild finger millet) [1, 2]. Based on morphological, cytogenetic, and molecular evidences, it is believed that modern finger millet (*E. coracana* subsp. *coracana*) is domesticated from wild finger millet (*E. coracaca* subsp. *africana*) populations [2–5]. The *E. coracana* subsp. *coracana* was domesticated around 5000 years ago in western Uganda and the Ethiopian highlands. Subsequently, finger millet was introduced into Western Ghats of India around 3000 BC. Thus India became the secondary centre of diversity for finger millet. It is the fourth most important millet after sorghum, pearl millet and foxtail millet [6]. It occupies 12% of global millet’s area and is cultivated in more than 25 countries in African and Asian continents [7]. Finger millet is potentially a climate-resilient and nutritious crop with high nutraceutical and antioxidant properties [8]. Very importantly, finger millet grain is gluten-free, rich in calcium, fiber and iron, has excellent malting qualities, with low glycemic index (GI) and because of these properties, finger millet is a choice food for diabetics [9–11]. In sub-Saharan Africa and South Asia, millets are the survival food crops for resource-poor people. It produces reasonable grain and fodder yields under low input crop production systems and even survives on soils of low fertility. Finger millet grain is used to prepare diverse food cuisines and is highly nutritious. Apart from having rich nutritional value, it has an efficient carbon concentrating mechanism through C4 pathway. Genes involved in C4 mechanism could be one of the reasons for its hardy nature under low moisture and hot environmental conditions. It is worth comparing genes involved in C4 carbon assimilation network with already characterized genes from other cereal crops [12–14] and understand the variation at genomic level. In spite of its immense importance in human diet, development of genomic resources and high throughput breeding efforts have been limited. Considering these, we developed the whole genome sequence data for the short duration (110–115 days), high yielding (5.0–5.5 tonnes per hectare), drought tolerant finger millet variety ML-365, which was bred and released in 2008 [15]. The present study describes the first *de novo* genome assembly of orphan nutri-cereal finger millet using combination of Illumina and SOLiD sequencing technologies. The whole genome assembly, SSRs, C4 pathway genes, resistance genes, drought responsive genes and calcium transport and regulation genes will be invaluable genomic resources for the future finger millet studies.

## Methods

### Genome size estimation of finger millet and wild species

The ML-365 finger millet variety was developed through recombination breeding by crossing IE1012 and Indaf-5 in our laboratory earlier. Seeds of ML-365 (*Eleusine coracana* (L.) Gaertn. subsp. *coracana*), EC516253 (*Eleusine jaegeri* Pilg.), EC516251 (*Eleusine multiflora* Hochst. Ex A. Rich.), EC516248 (*Eleusine tristachya* Lam.), EC516245, EC516243 (*Eleusine indica* (L.) Gaertn.), EC541533, and EC541538 (*Eleusine coracana subsp. africana*) were grown in pots containing red soil and fertilizer mix. Young leaves were collected and chopped into pieces using sterilized blade and stained using nuclear isolation buffer (NIB). The NIB composed of hypotonic Propidium Iodide (PI), 50 μg/ml in 3 g/L trisodium citrate dihydride containing 0.05% (*v*/v) of Nonidet P-40 containing 2 mg/mL RNaseA stored in a dark amber bottle in a refrigerator. The liquid was filtered through 30 μm nylon mesh and samples were processed for ploidy estimation as per the protocol suggested by Krishan [16]. Stained nuclei were analyzed using BD FACS cell sorter at Central Imaging and Flow Cytometry Facility (CIFF), Centre for Cellular and Molecular Platforms (C-CAMP), NCBS, Bengaluru, India. Genome sizes of all *Eleusine* species were estimated by comparing with *Pisum sativum* as an internal standard since, its nuclear genome is more stable [17, 18] and ease in preparation of high quality nuclei suspension, which appear to be free of compounds interfere with PI staining [19]. The genome size was derived by multiplying the 1C value (pg) with 965 Mb (1 pg equivalent value) [20].

### Nucleic acid isolation

Genomic DNA (gDNA) was isolated from all cultivated and wild species as per the manufacturer’s instruction (Cat # 69104, DNAeasy Plant Mini Kit) and DNA quality was checked by Nanodrop. The ML-365 finger millet variety was grown in well-watered (WW) and low moisture stress (LMS) conditions in pots under green house condition. Moisture stress was induced on the 80^th^ day after sowing for 10 days [21]. Leaf tissue was sampled on 91^st^ day from WW (soil moisture level was 18.43%) and LMS (soil moisture level was 9.65%) plants. Total RNA was isolated using TRIzol reagent (Catalog #15596026, Invitrogen) and genomic DNA contamination was removed by DNase (Catalogue # AM1906, Ambion) treatment. The RNA Integrity and quantity was checked on Bioanalyzer using Agilent RNA 6000 nano chip.

### Paired-end, mate pair library preparation and sequencing

Genomic DNA was used to prepare two independent Illumina paired-end (PE) libraries (2 × 151, and 2 × 150 nts) by following manufacturer’s instruction using NEXTFlex DNA sequencing kit (catalog # 5140–02, Bioo Scientific). Pired-end libraries were sequenced using Illumina HiSeq4000 and NextSeq500. Also, three Illumina mate-pair (MP) libraries of insert size 2.5 Kb were prepared using Illumina Nextera Mate Pair sample preparation gel plus protocol (catalog # FC-132-1001, Illumina). To prepare SOLiD mate pair library, 3 μg of gDNA was fragmented on Covaris S220 and fragmented sample was size selected on 1% Agarose gel at the range of 3–4 Kb. The size distribution was checked on Agilent Bioanalyzer high sensitivity chip. Then SOLiD MP library was prepared as per the SOLiD mate pair library prep protocol and library was sequenced (2 × 61 nts) on SOLiD 5500 platform. All the sequencing work was carried out at Genotypic Technology Private Limited, Bengaluru, India.

### DNA-seq data pre-processing and *de novo* genome assembly

The raw reads of PE and MP libraries were trimmed for adapter sequence contamination and low-quality bases (PHRED score of <Q30) using FASTX-ToolKit (http://hannonlab.cshl.edu/fastx_toolkit/). The SOAPdenovo2 [22] assembler was used to assemble all the high-quality PE and MP reads. The contigs were further scaffolded using SSPACE [23] and gaps in the scaffolds were closed by GapCloser module in the SOAPdenovo2 [22]. The genome completeness of assembly was checked by CEGMA [24].

### Gene prediction and functional annotation

The assembled scaffolds were used to predict the genes using AUGUSTUS [25] by *Zea mays* as a reference gene model. In addition, RNA-seq data was incorporated to AUGUSTUS to assist the gene prediction. The gene ontology annotation was carried out by performing a BLAST with the protein sequences of Viridiplantae retrieved from UniProt database. The pathway analysis of genes was carried out by KAAS server [26] using *Arabidopsis thaliana* (ath), *Glycine max* (gmx), *Oryza sativa japonica* (osa), and *Vitis vinifera* (vvi) as reference organisms. The protein domain structures of all protein coding genes were identified using InterProScan5 software [27].

### Gene families, phylogenetic analysis and mining of transcription factors

The protein sequences of maize (ftp://ftp.ensemblgenomes.org/pub/plants/release-22/fasta/zea_mays/pep/), sorghum (ftp://ftp.ensemblgenomes.org/pub/plants/release-31/fasta/sorghum_bicolor/pep/), rice (ftp://ftp.plantbiology.msu.edu/pub/data/Eukaryotic_Projects/o_sativa/annotation_dbs/pseudomolecules/version_7.0/all.dir/), foxtail millet (http://foxtailmillet.genomics.org.cn/page/species/download.jsp), and *Brachypodium (* ftp://ftp.ensemblgenomes.org/pub/plants/release-31/fasta/brachypodium_distachyon/pep/) were downloaded. The paralogs and orthologs among finger millet, rice, sorghum, maize, foxtail millet, and *Brachypodium* were identified using OrthoMCL [28]. The groups having at least one copy of gene from each genome were considered as core orthologous groups (COGs). Based on orthoMCL clustering, protein sequences of single copy ortholog gene groups from six species were aligned and inferred phylogenetic relationship as described previously [29]. The consensus phylogenetic tree was drawn using FigTree V1.4.2 (http://tree.bio.ed.ac.uk/software/figtree/). Scaffolds of finger millet genome was concatenated and syntenic relationship among *Poaceae* species was inferred by Symap tool [30]. Protein sequences of plant transcription factors (TFs) were retrieved from plant transcription factor database (http://planttfdb.cbi.pku.edu.cn/index.php) and used as a reference to identify TFs in ML-365 genes by BLASTP (default e-value cutoff = 10^−10^, minimum identity = 40% and minimum query coverage = 50%) alignments.

### Analysis of calcium transporter, disease resistant and C4 pathway genes

We retrieved gene sequences from NCBI database involved in calcium transporter process based on functional annotation like Ca(2+)/H(+) antiporter (CAX1), two pore channel (TPC1), CaM-stimulated type IIB Ca(2+) ATPase, Calmodulin dependent protein kinase 1 and 2 (CaMK) based on previous studies [31, 32]. The predicted Finger millet protein sequences were queried against these protein sequences using BLASTP analysis. Also, the genes containing leucine rich repeats (LRR), coiled-coil (CC), protein kinases (PKs) and NB-ARC domains were identified in finger millet predicted gene set based on protein family (Pfam) annotation using InterProScan5 software [27]. In order to understand the genes involved in C4 mechanism, previously characterized C4 pathway genes from different cereals were used as a reference [12, 13] to identify C4 genes in finger millet genome. Homologs with highest identity were selected from finger millet sequenced data for each of the seven C4 pathway genes viz., phosphoenolpyruvate carboxylase (PEPC), PEPC kinase (PPCK), NADP-malate dehydrogenase (NADP-MDH), NADP-malic enzyme (NADP-ME), pyruvate orthophosphate dikinase (PPDK), PPDK-regulatory protein (PPDK-RP), and carbonic anhydrase (CA). Ortholog genes from finger millet genome were used for multiple sequence alignment (MSA) and phylogenetic analyses with other C4 cereal genes [33].

### RNA-seq library preparation and data pre-processing

Around 200 ng of total RNA from well-watered (WW) and low moisture stress (LMS) samples was taken and independent paired-end (PE) libraries were prepared using SureSelect Strand Specific RNA library Prep ILM kit (Cat# 5500–0135, Agilent Technologies). The final libraries were quantified using Qubit and quality was validated on Agilent D1000 ScreenTape system. Finally, the adapter containing fragments were quantified using KAPA library quantification kit (Catalog # KK4824, KAPA Biosystems) and RNA-seq libraries were sequenced (2 × 150 nts) using Illumina NextSeq500. The raw reads were pre-processed for adapter contamination and low-quality bases (PHRED score < 30) using FASTX-ToolKit (http://hannonlab.cshl.edu/fastx_toolkit/).

### Transcriptome assembly, differential gene analysis and functional annotation

High quality pre-processed reads from WW and LMS samples were assembled independently using Trinity [34]. Unigenes were identified by clustering (90% homology and 95% query coverage) the putative transcripts (PUTs) of WW and LMS samples using CD-Hit [35–37]. RNA-seq reads were mapped to unigenes set using Bowtie2 [38] and number of reads mapped per unigenes was counted by in-house script. Differential gene expression between WW and LMS samples was analyzed using DESeq [39]. Functional annotation of putative transcripts was performed by comparing Viridiplantae protein sequences in UniProt database (http://www.uniprot.org/taxonomy/33090). Transcripts with more than 30% protein homology were considered for downstream analysis.

### Validation of drought responsive genes through qRT-PCR

Equal amount of total RNA of WW and LMS samples was used to synthesize complementary DNA (cDNA) using RevertAid First Strand cDNA synthesis kit (K1622, ThermoFisher Scientific). Twenty micro litres of reaction mixture containing SYBR Premix Ex Taq (RR420A, TaKaRa), ROX reference dye, 10 μM of each of forward and reverse primers, and template cDNA was used to perform qRT-PCR in Corbett RotorGene 6000. Two biological replicates and three technical replicates were amplified for all samples. All the ‘Ct’ values were normalized based on ‘Ct’ value of elongation factor housekeeping gene and differential gene expression (fold change) was calculated as per 2^-ΔΔCt^ method.

### Simple and complex repeat prediction

Transposable elements (TEs) were identified in finger millet genome using a combination of *de novo* and reference based approaches. Finger millet repeat database was built using RepeatModeler and repeats were predicted using RepeatMasker 4.0.5 (http://www.repeatmasker.org). Similar approach was followed to identify TEs in Trinity assembled transcripts from RNA-seq data. The simple sequence repeats (SSRs) were predicted genome-wide using Microsatellite Identification tool [40] (http://pgrc.ipk-gatersleben.de/misa/misa.html) and categorized as Class I and II based on the previous report [41].

### Primer designing for SSRs and electronic PCR

Primers were designed for flanking sequences of SSRs using Primer3 tool (the criteria set were like GC = 50–55%, Tm = 55–65 °C and product length = 100–350 bp). Primer sequences overlapping with repetitive elements were eliminated. The efficacy of designed primers was checked by e-PCR [42] (five bases mismatches were allowed at a primer binding site) and primer sequences hitting multiple sites in the genome were eliminated. Randomly, 35 SSRs comprising di-, tri-, tetra-, penta-, and hexa-nucleotide types were selected for wet lab validation.

### Oligo synthesis and genotyping of finger millet accessions

Oligos were synthesized at Eurofins Genomics Private Limited, Bengaluru, India. DNA from young leaf samples was isolated using CTAB method [43]. We set up 10 μl volume PCR reaction containing 20 ng of genomic DNA, 5 μl of emeraldAMP GT PCR master mix (Cat # RR310A, Takara), 0.5 μl of 10 mM of each forward and reverse primers. PCR amplification was carried out in Eppendorf vapo protect with initial denaturation temperature at 95^o^ C for 3 min followed by 30 cycles with 30 s at 95^o^ C, 30 s of annealing temperature (55^o^ C), 1 min of 72^o^ C, final extension for 5 min at 72^o^ C. PCR products were resolved on 3.5% agarose and 8% PAGE (only for monomorphic primers) stained with Ethidium bromide (final concentration at 0.5 μg/mL) and visualized using gel documentation unit.

## Results and Discussion

### Estimation of genome size of ML-365 finger millet variety and related eleusine species

ML-365 variety was developed in our laboratory as a part of collaborative research funded by the McKnight Foundation, USA (http://www.ccrp.org/sites/default/files/finger_millet___year_4___development_of_high-yielding_disease-resistant_and_drought-tolerant_finger_millet_genotypes.pdf) through farmer’s participatory varietal selection approach and released for commercial cultivation in 2008 in India. It is a drought tolerant, blast disease resistant variety possessing good cooking qualities preferred by farmers and consumers. Over the years, it has spread across several thousands of hectares and survives very well under long spells of drought.

**Fig. 1:**
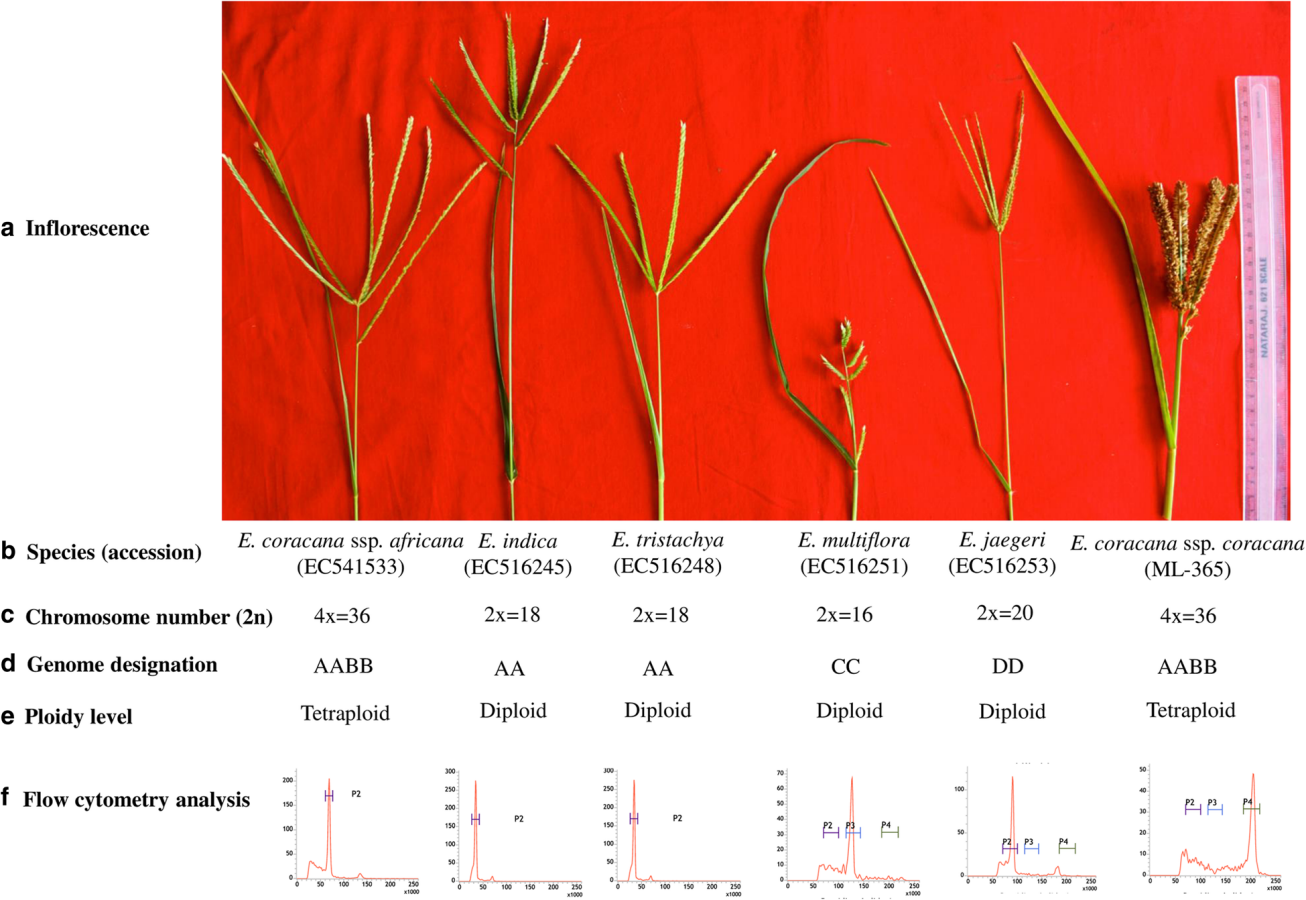
The species of *Eleusine* and their chromosome number, genome designation, ploidy level and flow cytometry analysis. In case of flow cytometry results ‘x’ and ‘y’ axes represents nuclei counts and relative fluorescence intensity, respectively

In order to estimate the genome size of cultivated and wild species of *Eleusine,* the samples were subjected to flow cytometry analysis. One accession each of *E. jaegeri*, *E. multiflora,* and ML-365, and two accessions of *E. triastachya*, *E. indica*, *E. coracana* subsp. *africana* species were chosen. The average DNA content (2C value) of *E. jaegeri*, *E. multiflora*, *E. tristachya*, *E. indica*, *E. coracana* subsp. *africana* was 1.20 pg, 1.84 pg, 1.14 pg, 1.21 pg, 2.52 pg, respectively. The genome size of wild species ranged from 580 Mb (*E. jaegeri*) to 1217 Mb (*E. coracana* subsp. *africana*). The total DNA content (2C value) and genome size of ML-365 (*E. coracana*) were 3.01 pg and 1453 Mb, respectively (Fig. 1). The genome sizes of *E. coracana* subsp. *coracana* and *E. coracana* subsp*. africana* are relatively similar. This could be because, finger millet was domesticated from *E. coracana* subsp*. africana* [2–5]. Several studies indicated that *E. indica* could be the maternal donor of ‘A’ genome of *E. coracana* subsp. *coracana* and ‘B’ genome donor is yet to be deciphered [44, 45]. Few studies showed that *E. floccifolia* is the ‘B’ genome donor [46–48]. However, waxy sequence and multicolor genomic in situ hybridization (McGISH) studies revealed that ‘B’ genome donor must have become extinct or yet to be uncovered [49]. Genome sequencing of wild species of ‘A’ and ‘B’ genomes, comparison with tetraploid cultivated species (*E. coracana* subsp. *coracana*) resolve the ambiguity of the progenitors.

### Genome sequencing and genome assembly

Around 45 Gb of paired-end and 21 Gb of mate-pair data was generated using Illumina and SOLiD sequencing technologies (Additional file 1). Combination of paired-end and mate pair reads were used to assemble the ML-365 genome followed by gap closing yielded 1196 Mb of consensus genome size, representing 82.31% of the estimated finger millet genome (Additional file 2). The assembly consisted of 525,759 scaffolds (>200 bp) with N50 length of 23.73 Kb (Table 1) and the average length of scaffolds was 2275 bp. Genome assembly was filtered for mitochondrion, chloroplast, fungal, and bacterial sequence contaminations. We further evaluated the ML-365 scaffolds for genome completeness by Core Eukaryotic Gene Mapping Approach (CEGMA) tool. Results showed that around 94.35% of core eukaryotic genes (CEG) were present in the ML-365 genome (Additional file 3). Highly self-pollinated behavior, small flower size, narrow genetic base of germplasm, lack of precise information on trait values of wild species for their inclusion in finger millet improvement, highly neglected in terms of research as compared to crops like rice, wheat and maize at national and international level, are the major factors behind limited breeding improvements of this crop [50–52]. However, hybridization between Indian and African germplasm resulted into development of several ‘Indaf’ series of finger millet varieties, which have gained popularity in the farmer’s field. Later on, the first major milestone in finger millet genomics is the development of first detailed genetic linkage map in F_2_ population by crossing *E. coracana* subsp. *coracana* cv. Okhale-1 and its wild progenitor *E. coracana* subsp. *africana* acc. MD-20 [53]. Since then, few researchers have concentrated on assessing the genetic variability among finger millet population and QTL mapping for important traits [4, 54–56]. However, gene cloning, Marker Assisted Selection (MAS), genome-wide association studies in finger millet are inadequate. The availability of whole genome assembly of ML-365 will expedite the improvement of finger millet.

**Table 1:** Genome assembly statistics of ML-365

Details	Value
Total length of sequence (Mb)	1196.06
No. contigs/scaffolds	525,759
Minimum length of contigs/scaffolds (bp)	200
Maximum length of contigs/scaffolds (bp)	454,778
Average length of contigs/scaffolds (bp)	2274.92
N50 (bp)	23,732
GC content (%)	44.76
No. of genes predicted	85,243
a. Non-TE related genes	78,647
b. TE related genes	6596

Mb Million bases

bp base pairs

### Gene prediction and functional annotation of genes

**Fig. 2:**
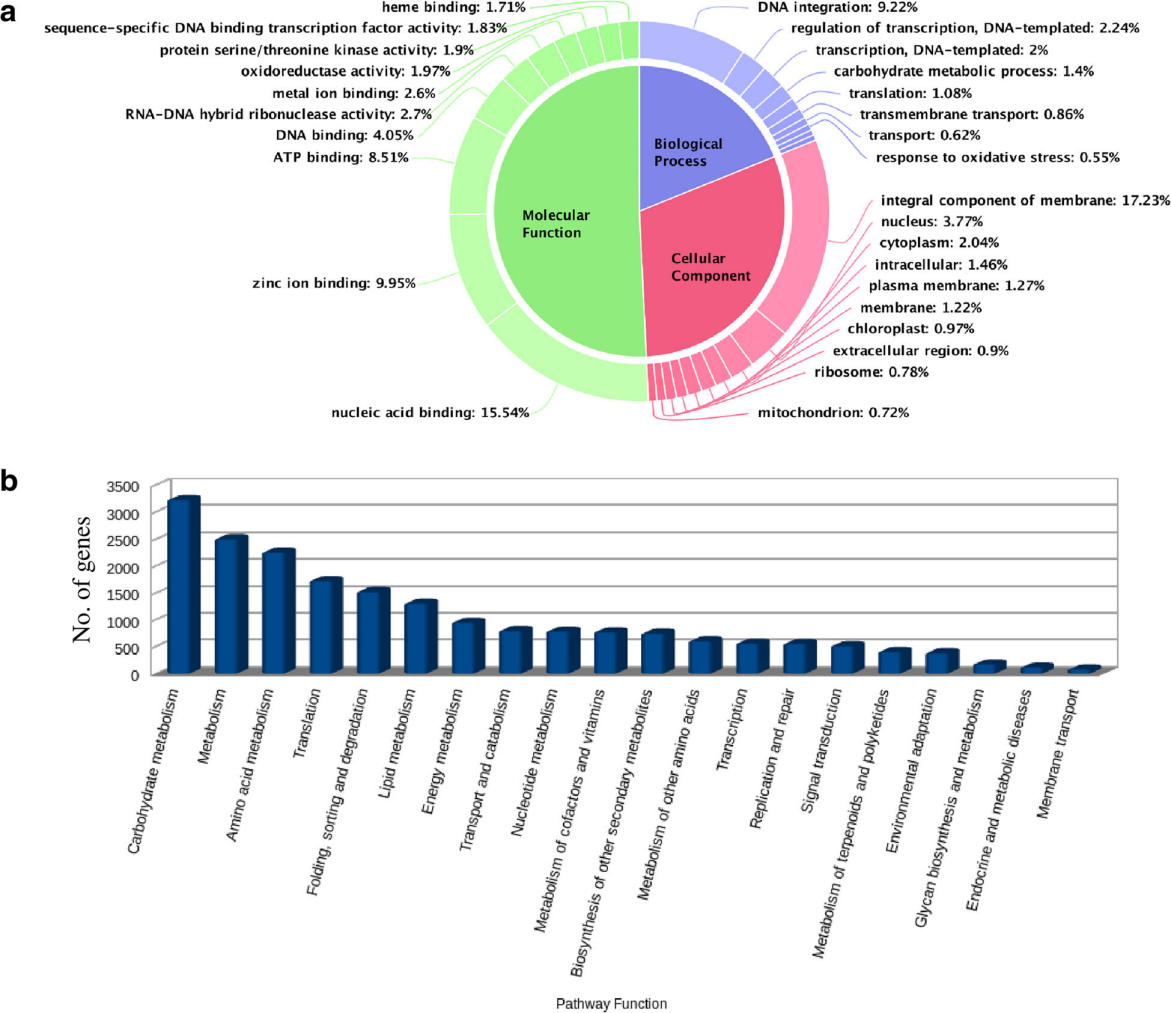
The gene ontology (**a**) and pathway prediction (**b**) of finger millet

We predicted, 78,647 non-TE related and 6596 TE related genes summing up to a total of 85,243 genes in ML-365 genome based on *de novo* method of gene prediction using Augustus. Functional annotation confirmed that majority of the genes had homologs with known functions in UniProt protein database. The gene ontology (GO) annotation of genes revealed that more number of genes were involved in molecular function followed by cellular and biological processes. Majority of the genes were involved in nucleic acid binding, zinc ion binding, and ATP binding activities under molecular function. Similarly, DNA integration and integral components of membrane were major functions related to biological and cellular processes, respectively (Fig. 2a). The KAAS server based pathway prediction showed that majority of genes were involved in carbohydrate metabolism, amino acid metabolism, translation, lipid metabolism and folding, sorting, and degradation pathways (Fig. 2b). Out of 85,243, 52,541 genes contained Pfam domain as per InterProScan results and these genes were distributed across 3254 gene families. Among 3254, retrotransposon gag protein, gag-polypeptide of LTR copia-type, reverse transcriptase, zinc knuckle, putative gypsy type transposon, protein kinase, cytochrome P450, and integrase core domain were the major gene families.

### Gene family orthologs and synteny with other sequenced *Poaceae* species

**Fig. 3:**
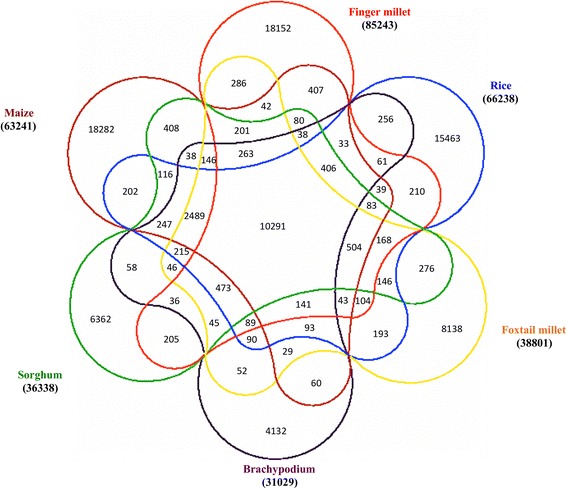
Distribution of orthologous gene families among major *Poaceae* species. Numbers in parentheses indicate the number of genes used for clustering

**Fig. 4:**
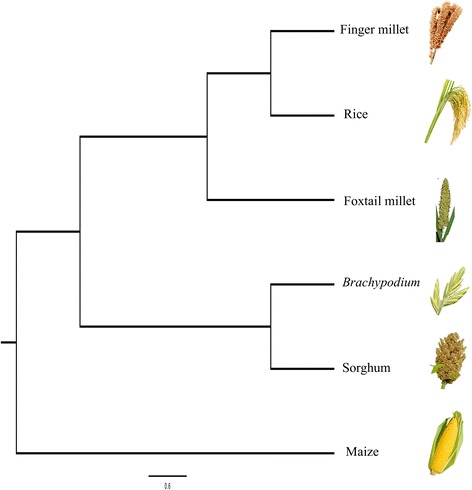
Phylogenetic relationship of six *Poaceae* species revealed based on single copy ortholog genes

The ortholog gene families across species provide insight into co-evolution of plant species. Clustering of genes of six *Poaceae* species (Finger millet, Rice, Foxtail millet, Sorghum, Maize, and *Brachypodium*) using OrthoMCL tool resulted in 10,291 core orthologous groups (COGs) gene families shared by all six species (Fig. 3). These families could have arisen due to whole genome duplication event. Among COGs gene families, 23,332 (35.22%) of rice, 24,427 (28.66%) of finger millet, 17,212 (47.37%) of sorghum, 27,401 (43.33%) of maize, 15,927 (41.05%) of foxtail millet and 17,888 (57.65%) of *Brachypodium* genes formed core gene families common to all species. However, within those COGs, 6107 (9.22%) of rice, 2976 (3.49%) of finger millet, 7337 (20.19%) of sorghum, 4403 (6.96%) of maize, 7879 (20.31%) of foxtail millet and 6999 (22.56%) of *Brachypodium* genes have retained single copy. Remaining genes were in multiple copies and these expansions or contractions of gene families have a significant role in diversification of flowering plants [57, 58]. The expanded gene families belonged to zinc knuckle, reverse transcriptase, aspartyl protease, leucine rich repeat, gag-polypeptide, gypsy type transposon, integrase, and ATHILA ORF-1 family protein domain containing genes. Single copy orthologous gene (766 genes across six species) based phylogeny among six species revealed that finger millet is closer to rice followed by foxtail millet (Fig. 4).

**Fig. 5:**
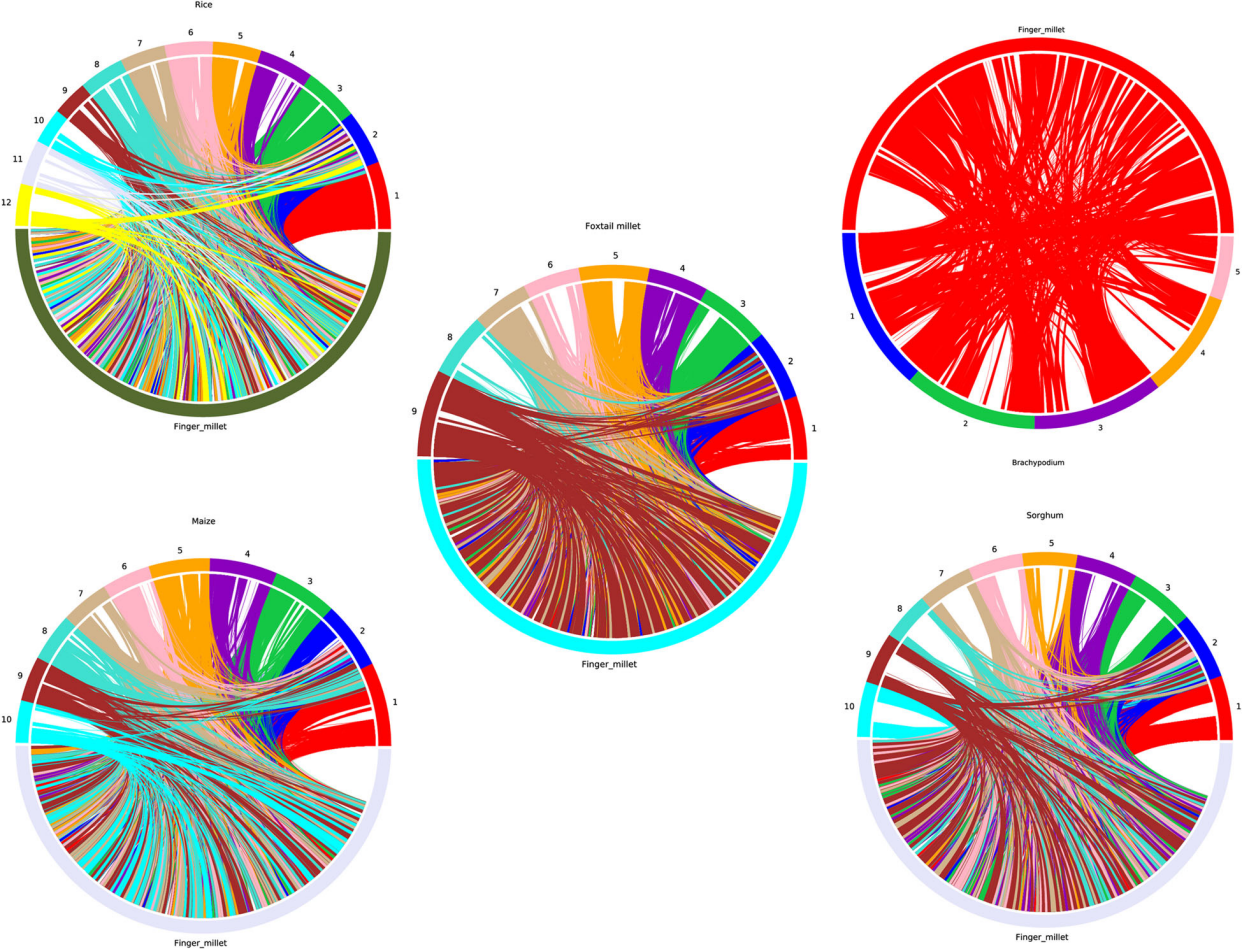
Syntenic genomic blocks of Finger millet with other *Poaceae* species (Rice, Sorghum, Maize, Foxtail millet and *Brachypodium*)

Intergenome collinear analyses between finger millet, *Brachypodium*, foxtail millet, sorghum, maize, and rice showed highly conserved genomic regions, signifying close evolutionary relationship among these grass species. In total, we identified 1592 large collinear blocks between finger millet and rice; 1709 between finger millet and foxtail millet; 1693 between finger millet and sorghum; 1613 between finger millet and maize; 436 between finger millet and *Brachypodium*, indicating 97%, 98%, 95%, 96%, and 82% of finger millet genome is colinear with these grass species, respectively (Fig. 5). Previous comparative analysis also revealed more number of conserved genomic regions between finger millet and rice genomes [59]. This syntenic relationship of finger millet with other cereal crops will enable us to map orthologous candidate Quantitative Trait Loci (QTLs) or genes of interest [60–63].

### Repeat content in finger millet genome

Transposable elements are the major components of eukaryotic genomes and their integration in the genome play a vital role in genome evolution and duplication. The assembled scaffolds were analysed for repeat content using RepeatModeller and RepeatMasker tools. We annotated finger millet genome using *de novo* constructed repeat library indicating around 49.92% of finger millet genome is repetitive. The retroelements constitutes around 35.56%, LTR elements were the major components (33.26%) among retroelements. Subsequently, unclassified repeats and DNA transposons were found in minor fractions of 9.73% and 4.48%, respectively (Table 2). Previous report also indicated the richness of finger millet genome with repeats based on DNA reassociation kinetics [64]. This repetitive nature of the finger millet genome is attributed to larger lengths of interspersed DNA repeats as reported in pearl millet [64, 65]. We predicted the repeat content in a similar manner like genome in Trinity assembled putative transcripts, the overall repeat content was found to be 4.87%. Most of this fraction (4.28%) was constituted unclassified repeats and remaining was simple and low complexity repeats.

**Table 2:** Repeat content in ML-365 finger millet genome

Repeat type	No. of copies	Percent (%)
Total interspersed repeats		49.77
A. Retroelements	754,889	35.563
SINEs:	8647	0.127
LINEs:	69,277	2.175
LTR elements:	676,965	33.261
ERV_classI	1430	0.025
ERV_classII	1318	0.044
B. DNA transposons	196,245	4.477
C. Unclassified:	432,219	9.734
Small RNA	8450	0.130
Satellites	182	0.002
Simple repeats	1404	0.012
Total		49.92

*SINEs* Short Interspersed Elements

*LINEs* Long Interspersed Elements

*LTR* Long Terminal Repeat

**Fig. 6:**
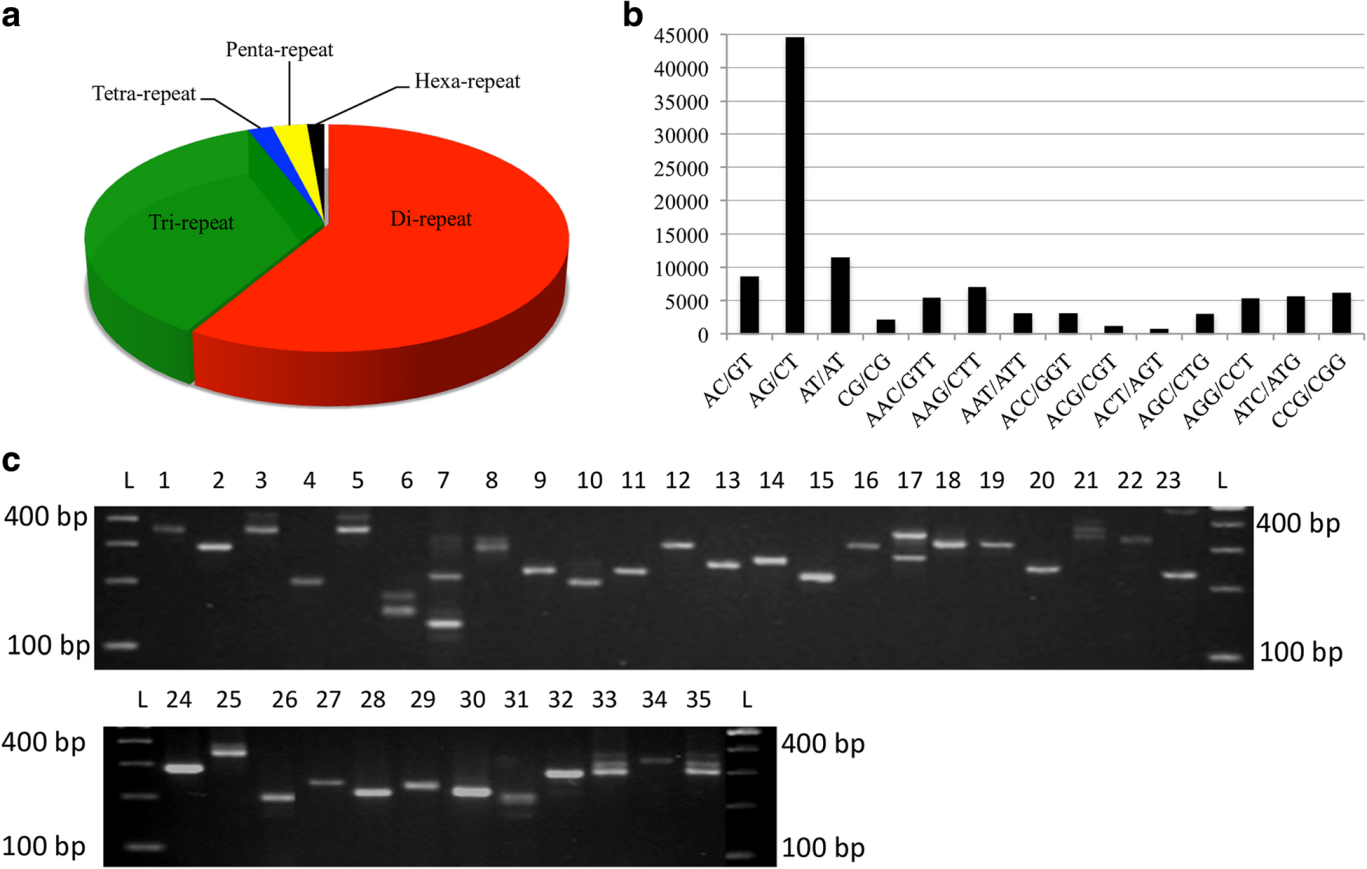
Distribution of SSRs (**a**), (**b**) and allelic variation (**c**) of selected SSRs in ML-365 finger millet variety

Simple sequence repeats (SSRs) play an evolutionary relationship in genome evolution. The scaffolds of ML-365 genome were subjected for identification of SSRs using MISA. A total of 114,083 SSRs were distributed across ML-365 genome signifying the abundance of SSRs [66], of which 66,805 (58.56%), 40,578 (35.57%), 2179 (1.91%), 3010 (2.64%) and 1511 (1.32%) were di-, tri-, tetra-, penta-, and hexa- types, respectively. Among di-repeats, AG/CT (66.64%) were highest followed by AT/AT (17.21%), AC/GT (12.96%) and CG/CG (3.20%) types. Similarly, AAG/CTT were the highest among tri-repeats types followed by CCG/CGG (15.13%), ATC/ATG (14%), AGG/CCT (13.19%), AAC/GTT (13.30%), ACC/GGT (7.55%), AAT/ATT (7.54%), AGC/CTG (7.47%), ACG/CGT (2.78%) and ACT/AGT (1.82%) (Fig. 6). In case of tetra type repeats, AAAT/ATTT (21.48%) and AAAG/CTTT (19.55%) were higher as compared to other types of tetra type repeats. The penta repeats, AAAAG/CTTTT (10.76%) and AAAAT/ATTTT (10.20%), and among the hexa repeats, AACACC/GGTGTT (13.70%) were found to be in higher proportion. Primers were designed for di-, tri-, tetra-, penta-, and hexa- types of SSRs using Primer3 software. Primer sequence flanking the repetitive regions and primers generating multiple amplimers were eliminated using ePCR and finally 18,514 SSRs were shortlisted for future applications (Additional file 4). The wet lab PCR validation of randomly chosen 35 SSRs in ML-365 confirmed the *in silico* e-PCR results (Fig. 6). Also, fingerprinting of 26 finger millet accessions, 14 wild species revealed minor allelic variation on 3.5% agarose gel for 35 SSRs (Additional file 5). However, when PCR amplified products were resolved on PAGE, the extent of polymorphism observed was much higher. In addition, we observed higher polymorphism in case of wild species of finger millet as compared to *Eleusine coracana* germplasm on PAGE gels. The inherent drawback of low polymorphism in finger millet was observed in previous studies [67, 68]. However, SSRs identified in this study can be further effectively used in diversity studies, linkage map construction, association mapping, QTL mapping of agronomically important traits, and marker assisted breeding programmes.

### Transcriptome assembly and gene expression study in contrasting moisture regimes

**Fig. 7:**
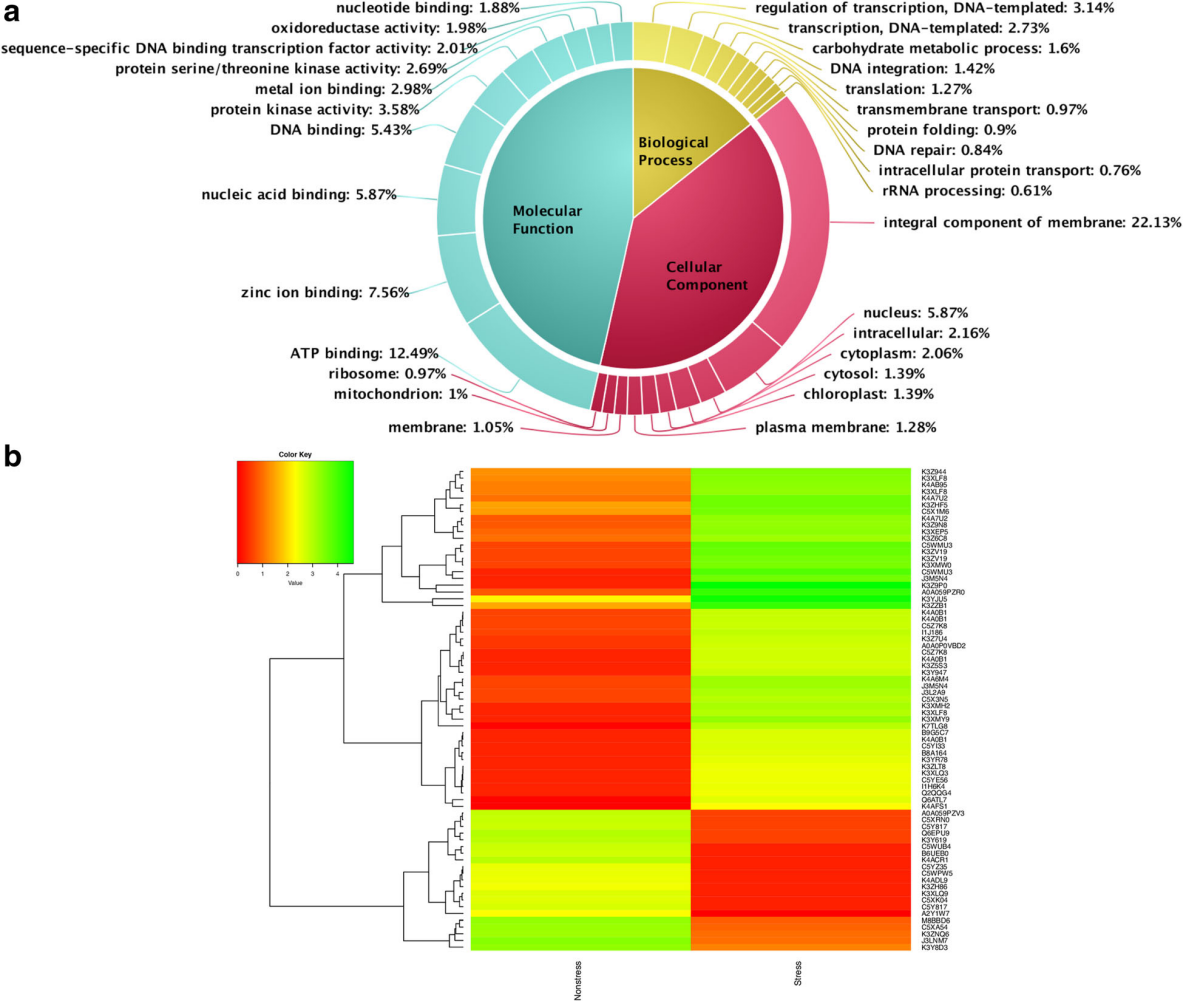
Gene ontology annotation of putative transcripts (**a**) and differential gene expression (**b**) in low moisture stress and control samples

Independent transcriptome assembly was performed for well-watered (WW) and low moisture stress (LMS) samples using Trinity. The number of transcripts assembled in WW and LMS samples were 53,300 and 100,046, respectively. These transcripts were clustered (based on 90% homology and 95% query coverage using CD-Hit) to make unique putative transcripts (PUTs)/unigenes and finally 126,312 PUTs were used for differential gene expression. Around 64% of PUTs were annotated against viridiplantae protein sequences from UniProt database. Large number of transcripts were known to be of ATP binding and zinc ion binding types under molecular function. Similarly, transcripts involved in membrane integral component and transcription regulation activities were more for cellular and biological processes, respectively (Fig. 7).

Differential gene expression between WW and LMS samples showed that 111,096 unigenes were expressed in both the samples. Few unigenes were expressed only in specific conditions like WW (2287 unigenes) and LMS (12,893 unigenes) conditions. Out of 111,096 common genes, 25,796, 23,210 and 62,090 genes were up, down and neutral in regulation, respectively. Around 4859 genes were significantly (‘*p*’ value less than 0.05) expressed between WW and LMS conditions. Out of which, 2333 were up-regulated and 2526 were down-regulated in LMS as compared to WW condition. The protein domain (Pfam) annotation of these genes showed that only 1099 genes in up-regulated and 1883 genes in down-regulated had Pfam designation. Among up-regulated genes, majority belonged to protein kinase domain (PF00069), Myb-like DNA-binding (PF00249), pectinacetylesterase (PF03283), protein tyrosine kinase (PF07714), zinc-binding (PF13966), Hsp20/alpha crystalline family (PF00011), protein phosphatase 2C (PF00481), and late embryogenesis abundant protein (PF02987). Also, protein kinase, protein tyrosine kinase, cytochrome P450, NB-ARC, UDP-glucoronosyl and UDP-glucosyl transferase Pfam domains were in majority among down-regulated genes. Three up (g161426.t1, g77173.t1 and g86441.t1) and two down (g134601.t1 and g40229.t1) regulated drought responsive genes were validated by qRT-PCR in WW and LMS samples for the proof of concept. The pattern of their regulation was in concordance with the RNA-seq results (Additional file 6). Several drought responsive genes have been characterized in finger millet using various methodologies. However, their deployment in breeding drought tolerant finger millet genotypes have not been attempted [21, 69–71]. Hence, genes identified in this study could make a remarkable impact in drought tolerance breeding. Characterization of these genes further would provide insights on the importance of these genes to utilize them in finger millet or other food crops to impart abiotic stress-tolerance.

### Mining of plant transcription factors (TFs) in finger millet

**Fig. 8:**
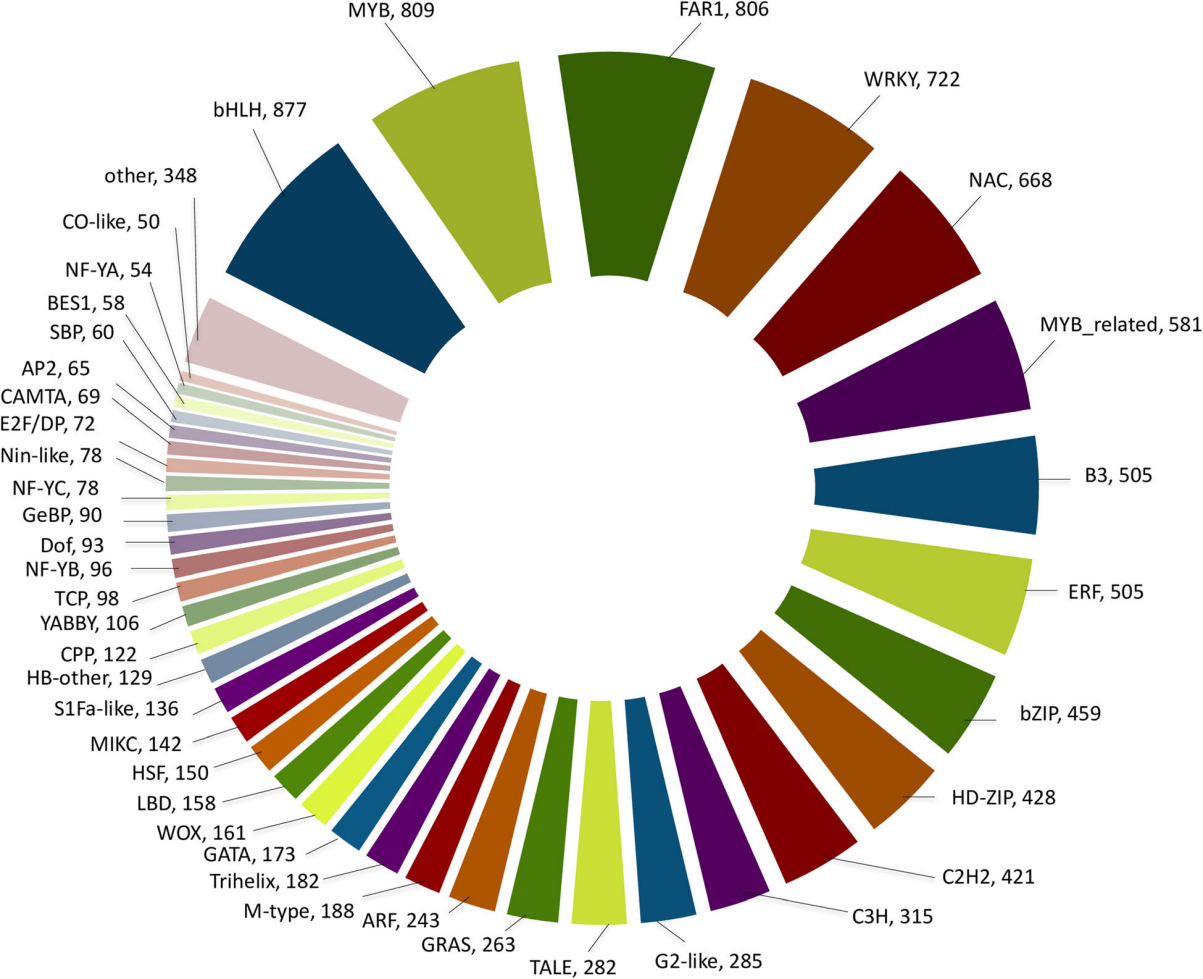
Distribution of transcription factors in finger millet genome

The protein-protein homology analysis of genes of ML-365 with plant TFs protein database revealed 56 various families of TFs distributed across 11,125 genes in finger millet. Among them, bHLH, MYB, FAR1, WRKY, NAC, MYB related, B3, ERF, bZIP, HD-ZIP, C2H2, C3H, G2-like, TALE, GRAS, ARF, M-type, Trihelix, GATA, WOX, LBD, HSF, MIKC, S1Fa-like, HB other, CPP, and YABBY were majorly distributed TFs in the finger millet genome (Fig. 8). These 11,125 genes were found to have homology with 75 plant species and foxtail millet, rice, wild species of rice, apple, *Brachypodium*, maize, sorghum, wheat and wild species of wheat (Additional file 7) were major among other plant species.

### Mining of drought responsive and disease resistance genes

Finger millet is a drought tolerant cereal crop and mining for drought responsive genes will hasten future breeding activities to develop varieties for drought prone areas. The Pfam based identification of drought responsive genes revealed that 2866 genes were distributed across 19 Pfam domains. The protein kinases (PF00069), protein tyrosine kinases (PF07714), BTB/POZ (PF00651), NAD dependent epimerase/dehydratase family (PF01370), U-box (PF04564), universal stress protein family (PF00582), and DCPS (PF00571) domains containing genes were majorly distributed in ML-365 genome (Table 3). Most of these genes were associated with WRKY, MYB, MYC, ZFHD, NAC, ABF, AREB, GRF, and NF-Y transcription factors, which are responsible for drought tolerance [72–75]. Utilization of these TFs to study the binding sites of TFs and analyzing *cis*-acting elements will enhance further understanding of drought tolerance in finger millet. Hence, identification and prediction of *cis*- regulatory elements through promoter analysis is a crucial step in functional analysis and signaling networks. Several novel technologies like overexpression, RNAi, zinc finger nucleases (ZFNs), transcription activator-like effector nucleases (TALENs), and clustered regulatory interspaced short palindromic repeats (CRISPRs) technologies to understand the role of these TFs in finger millet will have major impact in breeding for abiotic stress tolerant varieties [76, 77].

**Table 3:** Distribution of protein family associated with drought tolerance genes in ML-365 genome

Pfam ID	Pfam domain name	Gene count
PF00069	Protein kinase domain	1386
PF07714	Protein tyrosine kinase	546
PF00651	BTB/POZ domain	351
PF01370	NAD dependent epimerase/dehydratase family	175
PF04564	U-box domain	86
PF00582	Universal stress protein family	82
PF00571	CBS domain containing proteins (DCPS)	77
PF05699	hAT family dimerisation domain	36
PF08879	WRC	32
PF00999	Sodium/hydrogen exchanger family	20
PF03061	Thioesterase superfamily	19
PF08880	QLQ	16
PF04185	Phosphoesterase family	12
PF07649	C1-like domain	10
PF00257	Dehydrin	10
PF03107	C1 domain	3
PF02637	GatB domain	3
PF04147	Nop14-like family	2

Finger millet blast caused by an *Ascomycetes* fungus, *Magnaporthe grisea* is a devastating disease. Blast pathogen attacks the plant at three different plant growth stages viz., seedling, finger and neck [78]. The estimated yield loss due to blast is around 28%, but under favorable condition it may be up to 80–90% [79–81]. So far, no resistance genes (R-genes) have been mapped in finger millet except few recent studies [82, 83]. We looked for R-genes in the predicted gene set of ML-365. Totally, 1766 genes were identified with domains of NB-ARC (250), LRR (471), CC (6) and PKs (1044) (Additional file 8). Seventy-six NB-ARC genes identified in this study have homologs with previously identified genes (ABW04964.1, ABW04969.1, ABW04972.1, ABW04973.1, ABW04975.1, ABW04976.1, ABW04983.1, ABW04991.1 and ADB12239.1) [84, 85]. Hence, these R-gene sequences could be used for allele mining and mapping of resistance genes in the finger millet accessions.

### Calcium transport and accumulation genes

Finger millet grain contains rich sources of nutrients, specifically it possess 5–10 times higher calcium in grains as compared to other cereals [8]. Homology based analysis identified 330 calcium transport and accumulation related genes. Among 330, 28 CaM ATPase, 145 CaMK1, 125 CaMK2, 29 CAX1 and 3 TPC1 genes were identified (Additional file 9). Out of 330, six genes (g5694.t1, g73960.t1, g89161.t1, g107035.t1, g135510.t1, and g146823.t1) were found to be homologs to Calcium transport and regulation genes identified previously [31]. Large number of finger millet germplasm remain uncharacterized for several important traits, however small scale analysis of nutritional value of cultivated and wild species of *Eleusine* showed wider variations for protein, iron and calcium [86]. Genes identified in this study will help in exploring finger millet germplasm for calcium uptake, translocation and accumulation in various tissues in near future.

### C4 photosynthetic pathway genes and phylogenetic relationship

Water scarcity has led to frequent droughts coupled with higher air temperature in many parts of the world. As a consequence, crops productivity has been negatively affected. In nature, some plants have evolved an efficient carbon concentration mechanism through C4 pathway to perform well under arid and hot climate. Therefore, characterization of genes associated with C4 pathways and their deployment could improve the efficiency of both water and nitrogen [87]. In this study, 146 C4 pathway genes were identified that belong to seven key enzymes by functional annotation of genes of ML-365. Among 146 genes, 34 CA, 31 NAD-MDH, 21 NADP-ME, 27 PEPC, 17 PPDK, 7 PPDK-RP and 9 PPCK genes were identified in the finger millet genome (Additional file 10). Protein-protein homology based analysis with C4 genes of five cereals [12, 13] (Rice, Sorghum, Maize, Foxtail millet and *Brachypodium*) showed that finger millet genome has conserved C4 gene sequences by forming separate groups in majority cases. The phylogenetic tree results of phosphoenolpyruvate carboxylase (PEPC) genes showed four out of five finger millet genes formed a unique group with rice and one PEPC gene had higher homology with *Brachypodium* PEPC gene (Additional file 11). One of the PEPC kinase (PPCK) genes of finger millet showed homology with maize and sorghum, while remaining three genes shared ancestry with rice and sorghum (Additional file 11). Similarly, phylogenetic results of pyruvate orthophosphate dikinase (PPDK) enzyme showed that maximum number of PPDK genes were homologous to maize and sorghum (Additional file 11). Likewise, genomic comparison for PPDK-regulatory protein (PPDK-RP) showed three finger millet genes shared maximum homology with foxtail millet and two genes with rice and *Brachypodium*. Here, sorghum and maize formed separate clade indicating their distinct origin from common ancestor (Additional file 11). Phylogenetic analysis from NADP-malate dehydrogenase (NADP-MDH), NADP-malic enzyme (NADP-ME), and carbonic anhydrase (CA) sequences revealed that finger millet genes formed a distinct group as compared to the homologous genes from other cereals under the study and shared common ancestor with sorghum, maize, foxtail millet, *Brachypodium*, and rice (Additional file 11). This clearly indicates that finger millet has unique copies of genes for these proteins. The presence of multiple copies of these genes indicate the possible gene duplication during the evolution of C4 pathway [88]. However, confirmation of gene numbers needs further experiments with tissue-specific transcriptome analysis. Understanding of core genes involved in C4 pathway, associated transporters [13, 89] and their functional characterization could help to decipher the C4 carbon fixation in improving drought tolerance.

## Conclusion

The present study of whole genome sequencing and annotation of finger millet crop is the first report. The results on combination of paired-end and mate pair reads with variable read lengths generated from Illumina and SOLiD platforms enabled to assemble around 82% of total finger millet genome. Interestingly, almost half of the genome is interspersed with transposable elements based on *de novo* repeat prediction strategy. Mining of transcription factors (TFs), core C4 pathway genes, and mRNA sequencing identified large number of drought related TFs and drought responsive gene repertoire. These findings enable plant breeders towards advancement in deploying new breeding technologies to develop drought tolerant finger millet varieties, which could survive better in moisture stress regimes without compromising for its net productivity. Highly repetitive nature of finger millet genome and probable progenitors needs to be studied in future with the third generation sequencing technologies and sequencing of wild species, respectively. The availability of genomic resources from this study is likely to enable NGS-based allele discovery, genetic mapping, and identification of candidate genes for agronomically important traits. The genomic resources developed in this sequencing effort have been made available to the research community that will have significant impact in the near future.

## Additional files

Additional file 1: Sequence data generated for ML-365 finger millet variety. (PDF 51 kb)

Additional file 2: NGS data analysis workflow followed for ML-365 genome and transcriptome. (PDF 118 kb)

Additional file 3: The CEGMA results for whole genome assembly of ML-365. (PDF 44 kb)

Additional file 4: Primer sequences for SSRs identified in ML-365 genome. (PDF 4301 kb)

Additional file 5: List of SSRs used for fingerprinting of finger millet germplasm accessions. (PDF 525 kb)

Additional file 6: Validation of differentially expressed drought responsive genes through qRT-PCR. (PDF 116 kb)

Additional file 7: Gene counts of ML-365 genome shared across other species of plants. (PDF 66 kb)

Additional file 8: Nucleotide sequences of resistance genes identified in ML-365 genome. (PDF 3136 kb)

Additional file 9: Nucleotide sequences of calcium accumulation and transportation genes identified in ML-365 genome. (PDF 485 kb)

Additional file 10: Protein Sequences of C4 photosynthetic genes identified in ML-365 genome. (PDF 119 kb)

Additional file 11: Phylogenetic tree of C4 pathway depicting the sharing of common ancestry among different cereals. (PDF 1716 kb)

## Abbreviations

ATP: Adenosine triphosphate
BAC: Bacterial artificial chromosome
CC: Coiled-coil
CDPK: Calmodulin dependent protein kinase
CEG: Core eukaryotic genes
Gb: Giga bases
GI: Glycemic index
GO: Gene ontology
Kb: Kilo bases
LMS: Low moisture stress
LRR: Leucine rich repeats
LTR: Long terminal repeats
Mb: Mega bases
pg: Pico gram
PKs: Protein kinases
PUTs: Putative transcripts
SSRs: Simple sequence repeats
TFs: Transcription factors
WW: Well-watered
