# Supplementary material for: Genome and Transcriptome sequence of Finger millet (Eleusine coracana (L.) Gaertn.) provides insights into drought tolerance and nutraceutical properties
Source: BMC Genomics. 2017 Jun 15;18:465. doi: 10.1186/s12864-017-3850-z (PMC5472924; doi:10.1186/s12864-017-3850-z)
Supplement: Supplementary file 1 — Sequence data generated for ML-365 finger millet variety. (PDF 51 kb) [file 12864_2017_3850_MOESM1_ESM.pdf]

Supplement File 1: Sequence data generated for ML-365 finger millet variety

Molecule type	Platform	Instrument model	Library type	Read length	Total reads
DNA	Illumina	HiSeq 4000	Paired-end	2x151	196296544
DNA	Illumina	NextSeq 500	Paired-end	2x150	98584208
DNA	Illumina	NextSeq 500	Mate pair	2x76	25779780
DNA	Illumina	NextSeq 500	Mate pair	2x75	63714252
DNA	Illumina	NextSeq 500	Mate pair	2x75	104927118
DNA	ABI SoLiD	ABI 5500xl Genetic Analyzer	Mate pair	2x61	106081606
RNA	Illumina	NextSeq 500	Paired-end	2x150	115678148
