# Supplementary figures and images for: Genome and Transcriptome sequence of Finger millet (Eleusine coracana (L.) Gaertn.) provides insights into drought tolerance and nutraceutical properties
Source: BMC Genomics. 2017 Jun 15;18:465. doi: 10.1186/s12864-017-3850-z (PMC5472924; doi:10.1186/s12864-017-3850-z)

**Supplement File 2:** NGS data analysis workflow followed for ML-365 genome and transcriptome.

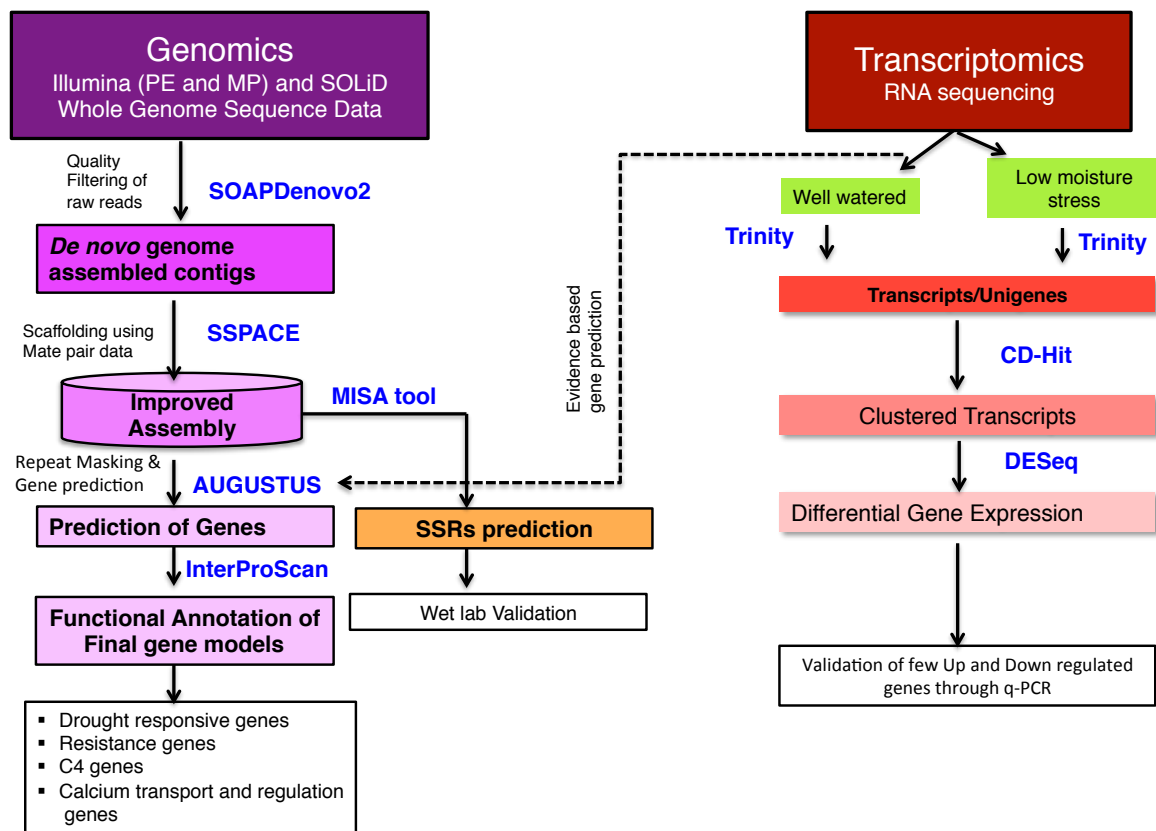

Supplement: Supplementary file 2 — NGS data analysis workflow followed for ML-365 genome and transcriptome. (PDF 118 kb) [file 12864_2017_3850_MOESM2_ESM.pdf]
