# Supplementary material for: Genome and Transcriptome sequence of Finger millet (Eleusine coracana (L.) Gaertn.) provides insights into drought tolerance and nutraceutical properties
Source: BMC Genomics. 2017 Jun 15;18:465. doi: 10.1186/s12864-017-3850-z (PMC5472924; doi:10.1186/s12864-017-3850-z)
Supplement: Supplementary file 3 — The CEGMA results for whole genome assembly of ML-365. (PDF 44 kb) [file 12864_2017_3850_MOESM3_ESM.pdf]

Supplement File 3: CEGMA results for whole genome assembly of ML-365

	No. of proteins	% Completeness	#Total	Average	% Ortho
Complete	214	86.29	652	3.05	84.58
Group 1	60	90.91	157	2.62	80
Group 2	48	85.71	144	3	85.42
Group 3	51	83.61	156	3.06	82.35
Group 4	55	84.62	195	3.55	90.91
Partial	234	94.35	809	3.46	91.88
Group 1	63	95.45	191	3.03	87.3
Group 2	55	98.21	186	3.38	89.09
Group 3	56	91.8	200	3.57	96.43
Group 4	60	92.31	232	3.87	95
