# Supplementary material for: Genome and Transcriptome sequence of Finger millet (Eleusine coracana (L.) Gaertn.) provides insights into drought tolerance and nutraceutical properties
Source: BMC Genomics. 2017 Jun 15;18:465. doi: 10.1186/s12864-017-3850-z (PMC5472924; doi:10.1186/s12864-017-3850-z)
Supplement: Supplementary file 4 — Primer sequences for SSRs identified in ML-365 genome. (PDF 4301 kb) [file 12864_2017_3850_MOESM4_ESM.pdf]

Supplement File 4: Primer sequences for SSRs identified in ML-365 genome

SSR_ID	Scaffold	SSR_Type	SSR_Motif	SSR Class	SSR_Start	SSR_End	Forward sequence	Reverse sequence	Product_size
FMgSSR-7062	scaffold471	p2	(AC)10	Class I	38299	38318	AAAGCGGAACCGTCAGGGGAAA	AACTGCAGCAGCAAGTGCGT	342
FMgSSR-7063	scaffold7799	p2	(AC)10	Class I	12634	12653	ACACACGCGCACACACACAA	ACCCGGCAGGGCATTGTTTT	289
FMgSSR-7064	scaffold93877	p2	(AC)10	Class I	1222	1241	ACACAGAACATAAGGTCGTGCGCT	CCACGTTCAGTTTTGTGTCCTAGCC	319
FMgSSR-7070	scaffold814	p2	(AC)10	Class I	35030	35049	AGAGCTGTGCTTCAGAGCAGGA	TGCTAACACGTCAGCCCCTT	310
FMgSSR-7071	scaffold3245	p2	(AC)10	Class I	51745	51764	AGAGGCATCACAACCCTGCAA	TGCTAAGGCGCTCTCACAATCA	272
FMgSSR-7073	scaffold20847	p2	(AC)10	Class I	752	771	AGCCCGTGTTCTTCTACGGCAT	TTGAAGGCGAGAGGCTTGGA	344
FMgSSR-7075	scaffold5513	p2	(AC)10	Class I	13357	13376	AGGACCAGCAAAAGACAGCTCCT	TCTGAAAGCTGATGCGGCGA	272
FMgSSR-7083	scaffold1175	p2	(AC)10	Class I	66870	66889	GCTGATCGATTCCCCAAGAAAGAC	ATTGGGATCTACACGACAGCCCCT	268
FMgSSR-7085	scaffold15800	p2	(AC)10	Class I	7833	7852	GTGGCAGTGTGGTCACTTTCAT	AGCACGCTGCCGCTTGTTTA	348
FMgSSR-7091	scaffold10151	p2	(AC)10	Class I	11875	11894	TCGGCCTCCGAGTGAACTTT	AGTGCACCTCTGTTGGTTGGTGA	347
FMgSSR-7092	scaffold3029	p2	(AC)10	Class I	20890	20909	TCTGCTCGGTCAAGGGTGTTCA	ACTGGCATGAAGTGGCGGAGAA	298
FMgSSR-7093	scaffold8377	p2	(AC)10	Class I	20618	20637	TGAGCGAAACATGGACGAAGCG	TAGACCAAGACCACGGATGCCA	347
FMgSSR-7094	scaffold4783	p2	(AC)10	Class I	14843	14862	TGAGCTCGGCAAGGAAGATGT	GCTCCATGCTGTGGGAACATGA	343
FMgSSR-7095	scaffold9693	p2	(AC)10	Class I	23110	23129	TGAGGCCCGTGAATGATGCGAA	AAGGTGGCCTGTCAATCAGCGA	277
FMgSSR-7096	scaffold3524	p2	(AC)10	Class I	5036	5055	TGCCACAGCAAGCACGTTCA	CTGTTGTTGAGCTGGTGCTCTTGA	344
FMgSSR-7097	scaffold2938	p2	(AC)10	Class I	31796	31815	TGCCACTGTTACTTGGACCCTGT	AGCCTGGCATGGACACAACCTT	326
FMgSSR-7099	scaffold79135	p2	(AC)10	Class I	873	892	TGCGAGCCGACGGTTGTTCTAT	TACCGACCTTCGTTGGCGTCTT	333
FMgSSR-7100	scaffold2505	p2	(AC)10	Class I	35712	35731	TGCTGCAAGCTGTCGCATTT	AAAAGCTGCTCACTCGGTCGCT	248
FMgSSR-7101	scaffold1155	p2	(AC)10	Class I	44497	44516	TGGCCAACCACACGGATGTCAA	ACCTGTAGCTGCAAGCCAATGGA	214
FMgSSR-7102	scaffold8880	p2	(AC)10	Class I	20760	20779	TGGCTATCCCTAGATGTGGTCTGC	AGTTAGACAAATTGCCCCCGC	228
FMgSSR-7103	scaffold106978	p2	(AC)10	Class I	1259	1278	TGGGCTGACAAAAACCAGCAGA	ACAAGGACGGTGCCATTGACGA	343
FMgSSR-7106	scaffold5024	p2	(AC)10	Class I	28258	28277	TGTCACGACTTCACCACCCGAA	TTGGTGGTGGATGGTGGGTTCA	338
FMgSSR-7107	scaffold24529	p2	(AC)10	Class I	3266	3285	TGTTGGTAGCACCAGACCTCCT	TTGACGTTCTGCTGACGGCT	299
FMgSSR-7109	scaffold898	p2	(AC)10	Class I	75026	75045	TTCTGCCCCTTGTTGAGGCCAT	AGGCGCGGTTCAGCAAACAA	240
FMgSSR-7111	scaffold611	p2	(AC)10	Class I	112324	112343	TTGCTGACAACAACGGGGCT	TGTTTTCACCACTACGGCGGCT	274
FMgSSR-7114	scaffold3787	p2	(AC)10	Class I	890	909	TTGCTTTTCTGGCCCACCCT	AATCCGTTGGCTGCGAATGGTC	258
FMgSSR-7116	scaffold42402	p2	(AC)11	Class I	3753	3774	AAAGCACAAGCCCTTATGCCCC	CACTGACTGCACATTGCGCTGA	239

SSR_ID	Scaffold	SSR_Type	SSR_Motif	SSR Class	SSR_Start	SSR_End	Forward sequence	Reverse sequence	Product_size
FMgSSR-7119	scaffold3867	p2	(AC)11	Class I	33850	33871	AACCACCCCACAACACACCCAA	AAACACGCACTTCGTGCCTCCT	339
FMgSSR-7120	scaffold2623	p2	(AC)11	Class I	27170	27191	AACCTCGTCAGCGACAACAGCA	ACGAGGTGTCCCGTGGTGTTTA	345
FMgSSR-7121	scaffold346267	p2	(AC)11	Class I	229	250	AACGTCGGGAAATCCAGGACCA	CCCCTGAATCCATGCTTCTTCGGT	287
FMgSSR-7129	scaffold579	p2	(AC)11	Class I	17060	17081	ACTTTGGCCATTCCAGCCAGA	TCTGGACAGGACCACCATTGCAT	200
FMgSSR-7131	scaffold545	p2	(AC)11	Class I	3958	3979	AGGACGAGTAACAAGCATGGCAGA	TTTCGGAGCCTGCAGCTACTGT	295
FMgSSR-7132	scaffold10223	p2	(AC)11	Class I	14537	14558	AGGGGTTCTCCTACCAGACGTTCA	ACACCGCAGCACTTTGCACT	267
FMgSSR-7135	scaffold586	p2	(AC)11	Class I	42209	42230	ATGGCAAGGAGTACGTTGGCGT	TGGTCACCAAAAGGCCGGGAAA	329
FMgSSR-7136	scaffold13963	p2	(AC)11	Class I	20258	20279	ATTGGAGGAGCGGAGGTAGCTT	CTCTCTCATTTCCTTCCATCGAGC	299
FMgSSR-7142	scaffold33565	p2	(AC)11	Class I	918	939	GAGATGCCTGTATCTGCTATCTGC	TGTCTGAATCAGCCCGCTACGA	231
FMgSSR-7143	scaffold18569	p2	(AC)11	Class I	4961	4982	GCCGTTCTTTTCACAAGGGGCA	AGGGACTGAAGCGGTAGCAA	332
FMgSSR-7144	scaffold4918	p2	(AC)11	Class I	34527	34548	GCGCGACCAACAAGCTGTCAAT	AGAAAACCTCGGCGTTGCGT	284
FMgSSR-7146	scaffold106846	p2	(AC)11	Class I	601	622	TCCGGGATGCATGCTGTGTTGA	ACTCAGCTCAAAGCCTTGCCTC	256
FMgSSR-7149	scaffold18180	p2	(AC)11	Class I	8199	8220	TGCCTGTTGCAGGACGCAAA	ATTTGGGAGACCGTCAGCAGCA	282
FMgSSR-7151	scaffold14097	p2	(AC)11	Class I	9924	9945	TGGCTGCAAGCAGAACAGCCAT	TGGCTCCCAAAGCTGTAAGGCA	254
FMgSSR-7152	scaffold3946	p2	(AC)11	Class I	25030	25051	TGGTGTCACGGCAGATGGAA	TGGTGGTGACTGGATTCAAGCGT	336
FMgSSR-7153	scaffold8212	p2	(AC)11	Class I	8443	8464	TGTCGGATGGGAACTGTTGGT	AGGGGAAGTTTTGTCGAGGCCTAT	342
FMgSSR-7156	scaffold6229	p2	(AC)11	Class I	37675	37696	TTCATGAGCTGCAACGGGCA	TGATGGACATCTCTGAATCCTGGC	323
FMgSSR-7159	scaffold17159	p2	(AC)12	Class I	8799	8822	AAGGGGCGCAGGAACATAGACA	TGCAGGAGTGTTGTGCGGTT	348
FMgSSR-7161	scaffold25226	p2	(AC)12	Class I	1358	1381	ACGCTTCCTTCCAAACGAGCGA	TGAAGTACCACCCGCAGACTT	269
FMgSSR-7162	scaffold1543	p2	(AC)12	Class I	33166	33189	ACTGGCTTAACCTGCTCACACGA	ACCCCGGTGCAAGCGTTTTT	270
FMgSSR-7163	scaffold2884	p2	(AC)12	Class I	51508	51531	AGCTTAGCTTGCTTGCTCCCCT	AGCCGGCACTCTTTGCTCAACA	282
FMgSSR-7167	scaffold803	p2	(AC)12	Class I	61575	61598	CCCTGTGCCAAGCTGGATGAAA	TTTTGTCTCGGTCATGGTGGGACG	205
FMgSSR-7168	scaffold161	p2	(AC)12	Class I	74105	74128	CCGCTTCCGCAATCAACAGACA	AGGTTCATCAGGCACGCACA	200
FMgSSR-7169	scaffold1718	p2	(AC)12	Class I	8481	8504	CGTGAGTTCATGATCAAAGGGCCA	TGGTGCCTTCAATCCTACCGCT	268
FMgSSR-7171	scaffold4887	p2	(AC)12	Class I	8820	8843	TCGCTAGAAGCCGCATCCTT	TGCATTGATGACACGGCGTTG	200
FMgSSR-7174	scaffold5961	p2	(AC)12	Class I	4676	4699	TGAACGCCTAGATGCTGCCA	TCAAGGGGTTGGAGCCAATGCT	238
FMgSSR-7176	scaffold15	p2	(AC)12	Class I	165475	165498	TGCTCCAGCGAACGCAGTACAA	ATTCGCGAGCACCACCATGA	221
FMgSSR-7181	scaffold1770	p2	(AC)13	Class I	47363	47388	ACCTTTTCCGCTGGTTGCCA	AGCTAGCCAGCCATTTGTCCA	320

SSR_ID	Scaffold	SSR_Type	SSR_Motif	SSR Class	SSR_Start	SSR_End	Forward sequence	Reverse sequence	Product_size
FMgSSR-7184	scaffold8629	p2	(AC)13	Class I	17102	17127	AGGGCGCCAAAGCACAAGAT	CGGCGCTGAAATTGACTTGCGT	349
FMgSSR-7185	scaffold14371	p2	(AC)13	Class I	16627	16652	ATGGTTGTCCTTGCGGTGTCGT	AGCAAACGCCGACTCTGGCTTA	207
FMgSSR-7187	scaffold836	p2	(AC)13	Class I	93997	94022	ATTGCTTGTTCGACGTCAGGCG	AAGCAGCGCTTCCAGAACCA	321
FMgSSR-7188	scaffold2217	p2	(AC)13	Class I	32270	32295	GCAACGCGCACGGATCATTT	AGCTTGCCGCTGGAGTAAGTGT	345
FMgSSR-7189	scaffold2612	p2	(AC)13	Class I	40465	40490	GGGGGTTAGGGAGATTGGACAAGA	GGTCCACCTAGTTTGGCTGCAA	338
FMgSSR-7191	scaffold10920	p2	(AC)13	Class I	3724	3749	TGAAACCTGCATGCACCCCT	AGCAGCAACAACGTCCTGCCTA	254
FMgSSR-7192	scaffold3130	p2	(AC)13	Class I	48845	48870	TGAAGTCTCCGCGGTGAGTGAT	TCCCTTGCTGCCTTGCATGA	245
FMgSSR-7197	scaffold1180	p2	(AC)14	Class I	53688	53715	AAACACGCGCATAATCGCTGGG	TCATCGCAACCAGGAGCAAGGT	323
FMgSSR-7203	scaffold7122	p2	(AC)14	Class I	18443	18470	AGCGCATTGCGTGCAACGAT	TGTAAGGCAAATGCCCAAGGGA	219
FMgSSR-7205	scaffold930	p2	(AC)14	Class I	69621	69648	ATCGCTTCTACGCCGCATTGGA	AGCAAGAATGGCCACAGCGT	236
FMgSSR-7209	scaffold4	p2	(AC)14	Class I	116787	116814	TCACCCCTTGTTGGCTTCCAGT	ATACGGCGGTGCCGCAAATA	253
FMgSSR-7214	scaffold15855	p2	(AC)14	Class I	5524	5551	TGTGGGCGACCTGTTTTCGAT	TGACTGCAGGTGGAGTGCAT	336
FMgSSR-7218	scaffold17912	p2	(AC)15	Class I	5971	6000	ACCAGCCAGTCCTTTTAGGCTCT	GCAGCTCCAAGAAACAATGAGCAC	337
FMgSSR-7228	scaffold3029	p2	(AC)15	Class I	55591	55620	TGCGGCACAAGTGTGTGCAT	ACCGAATAAGAGAGACGCACGA	301
FMgSSR-7229	scaffold6125	p2	(AC)15	Class I	22727	22756	TGGCTGGTTACAAGGTTGCCGA	TCAGGTTGGTGTGTGGCGAT	220
FMgSSR-7234	scaffold1866	p2	(AC)16	Class I	50460	50491	AGCCGCCCGCATAAAATCCA	ACTGTGGCTTGGAAAGGGCA	337
FMgSSR-7237	scaffold1898	p2	(AC)16	Class I	64123	64154	ATCCACACCACCCACAATGGCA	AGGGGAATCGCCATCAGCATCA	234
FMgSSR-7243	scaffold4626	p2	(AC)17	Class I	12754	12787	AACTTCAGGCGAGCACGAGACA	TGCCAAGAGGTACAACCCGT	202
FMgSSR-7246	scaffold439	p2	(AC)17	Class I	57517	57550	TCGATGAACTGCCAGCGAGACA	TCTTCAGCGGCATTGGTGAGGA	322
FMgSSR-7248	scaffold4395	p2	(AC)18	Class I	32190	32225	AGCCTGCATGTCCCTGTCCTTT	ACGGAGCAAGCTAAAGAGCCCA	263
FMgSSR-7250	scaffold2943	p2	(AC)18	Class I	30920	30955	AGGATCGGCAGTTTCCCAATGGA	ACAGCAGACAGTTCGCGTTGGA	234
FMgSSR-7251	scaffold678	p2	(AC)18	Class I	101693	101728	GGAGGTGCTTTTTGTGACTTTGGC	CGCGTCATTTAGCGCTGTCGTA	345
FMgSSR-7253	scaffold126	p2	(AC)18	Class I	59949	59984	TGCTCTCTGAAGCACGTCACAC	GTCAGTTGCTTACAACGACGCTGA	296
FMgSSR-7255	scaffold15228	p2	(AC)18	Class I	308	343	TTCCCTAACCGTTCACTGGGCA	TGCACTTCACTGCTTCTGCCA	238
FMgSSR-7256	scaffold443	p2	(AC)18	Class I	21670	21705	TTCCTTCATTGGCGCGTGGA	TGAACGGAACCTTTCGTGACGC	301
FMgSSR-7259	scaffold114906	p2	(AC)19	Class I	155	192	GCCCATAGGAAATGGTTCCTGAGA	AAAGGAGGTTTTGCGTGCGTGC	217
FMgSSR-7263	scaffold20861	p2	(AC)19	Class I	7435	7472	TGCTGCCAGTTTTGCCACCAT	TGGCCTTTCTGACTGCCTGCAA	293
FMgSSR-7264	scaffold3559	p2	(AC)19	Class I	46458	46495	TGGCTAATCTTCCACGAGTATCGG	TTCGATGCCCTTGTGGGCCTAA	328

SSR_ID	Scaffold	SSR_Type	SSR_Motif	SSR Class	SSR_Start	SSR_End	Forward sequence	Reverse sequence	Product_size
FMgSSR-7266	scaffold11992	p2	(AC)20	Class I	8926	8965	AGTGCTGGGCGCTTCGTAAA	TGCAGATGCTTGAGGGCATGGT	328
FMgSSR-7267	scaffold4149	p2	(AC)20	Class I	30390	30429	ATGGCCTTGACACTTGCTGC	TCGCACATCGGACAACAGAACTCA	200
FMgSSR-7269	scaffold6949	p2	(AC)20	Class I	24231	24270	TGTGTGCGTGAACGCATGTGA	AGCCAGGAATAAATCCCGCCCA	200
FMgSSR-7270	scaffold9057	p2	(AC)21	Class I	29680	29721	TGACTTATGCGCAGCGTCTTGA	TTGGCTGACGCCAAACTTGT	235
FMgSSR-7276	scaffold822	p2	(AC)22	Class I	43704	43747	ATGCTGGAAGACGGGCTTGT	GGAGCTGCCAATGGAAAGTCGT	254
FMgSSR-7277	scaffold8880	p2	(AC)22	Class I	13628	13671	TAGTGGCATCGGCAGCACAACA	TGCCACCAAACCCCAATGCT	296
FMgSSR-7279	scaffold8250	p2	(AC)23	Class I	18816	18861	TGTCCGCGGGTGTGGTATATGT	AGTGTGTCCAGGCAAATCCAGC	235
FMgSSR-7283	scaffold1155	p2	(AC)25	Class I	46977	47026	TCAGCATTACCCATGCAGTTCC	TTTGGTCACGTGCCCCTAGTGT	200
FMgSSR-7288	scaffold19239	p2	(AC)27	Class I	2528	2581	ACGTGAGCAAGGCTGGGATGAT	TGCAGGGTCAACGGGACAATGA	338
FMgSSR-7290	scaffold4982	p2	(AC)27	Class I	4571	4624	CCATTGATGTGGCGCATAACAC	TGGCATCGTTGGGATCTTGGA	254
FMgSSR-7295	scaffold966	p2	(AC)30	Class I	95437	95496	GTCAACCAACTAGCTATAGCACCC	AGGACCCATATAGTTCCCTGTCCCT	284
FMgSSR-7301	scaffold251	p2	(AC)6	Class II	111153	111164	AAAAAGACCACGCACGCACG	TCAGCGGTCAAAACACGCCT	302
FMgSSR-7302	scaffold840	p2	(AC)6	Class II	2349	2360	AAAAGAGAGGCCGGCAAACG	GCTGTGTGTAGTACTCCTCGACTT	348
FMgSSR-7305	scaffold7010	p2	(AC)6	Class II	25066	25077	AAACGCACCACGTATTGCACCC	TCGTGCATGCGCTTTGATGC	209
FMgSSR-7308	scaffold4901	p2	(AC)6	Class II	18883	18894	AAAGACAACCCCCATTCCTCGC	TGGCGGAGCCAGATGCATAA	350
FMgSSR-7310	scaffold4081	p2	(AC)6	Class II	12862	12873	AAAGCTTGCAGACGGCGAGT	TTTGCGTGGTGTGGTGTGGT	336
FMgSSR-7312	scaffold983	p2	(AC)6	Class II	10762	10773	AAAGGCTGCTCCGCGAACAA	TTTAGAGCTAAGGCCCTTGCCAGC	325
FMgSSR-7314	scaffold377	p2	(AC)6	Class II	91948	91959	AAAGTCGCGTCGTGCTGAGT	TCCCGCTATCAAGTTGTCGGCT	244
FMgSSR-7316	scaffold2128	p2	(AC)6	Class II	34829	34840	AAATCCGATCTGAGGAAGCGGTCG	ATCCGTGGTGGATTTAGGATGCCG	261
FMgSSR-7322	scaffold31254	p2	(AC)6	Class II	3673	3684	AACAATGCGTGGCATCAGGC	AAGTTTGGCGACAAGGCCGA	274
FMgSSR-7326	scaffold1406	p2	(AC)6	Class II	12053	12064	AACCCTGCAGCAGCTTGTGT	AAGTGCAAGCACACTGCCCT	338
FMgSSR-7327	scaffold7243	p2	(AC)6	Class II	36472	36483	AACCTCTGAGTGCAGGCCGTTT	TGTTAGTGATGCCGTGCGCT	295
FMgSSR-7328	scaffold6345	p2	(AC)6	Class II	14205	14216	AACGAGCCGGGTGTTCATCT	TCGTTGTTGCCTAGTGAGGCCAT	230
FMgSSR-7329	scaffold12582	p2	(AC)6	Class II	18936	18947	AACGCACGGGCAAAGTGCTA	ACCAAACGGGGCCCAATGAA	267
FMgSSR-7333	scaffold15470	p2	(AC)6	Class II	14168	14179	AAGCATGCCCACACCACACA	ATCAGGCTGGCTTGTCGTTGCT	287
FMgSSR-7349	scaffold36450	p2	(AC)6	Class II	619	630	AATTGTCGCGTATCGGCGCT	ATGGCCTTTTGGACCTGCGTGA	255
FMgSSR-7351	scaffold16198	p2	(AC)6	Class II	3464	3475	ACAACCTCGCCGAATCGAACCA	TCTGTGCAACTGCAACGCCA	350
FMgSSR-7352	scaffold5948	p2	(AC)6	Class II	1839	1850	ACAACGCCCGACATCAAGCA	AGGGACCGTCACAACAACGAGT	226

SSR_ID	Scaffold	SSR_Type	SSR_Motif	SSR Class	SSR_Start	SSR_End	Forward sequence	Reverse sequence	Product_size
FMgSSR-7357	scaffold80733	p2	(AC)6	Class II	1426	1437	ACACACACACACTCCCAGCGTT	TGCCTGATCTGCGGCTGTAA	226
FMgSSR-7363	scaffold16198	p2	(AC)6	Class II	3350	3361	ACACATCCGCACACATGCGA	AGGTTGTCATCGCCGTTCGTGT	221
FMgSSR-7370	scaffold1389	p2	(AC)6	Class II	71407	71418	ACACCTGCAGTAGCAAACAGC	AAGGTGCATGGCAACAACGACG	256
FMgSSR-7371	scaffold767	p2	(AC)6	Class II	60704	60715	ACACCTGCCCATCACCGATTCA	AAGGCCAAGCGCATCCTTGA	232
FMgSSR-7380	scaffold407	p2	(AC)6	Class II	14489	14500	ACAGGTTTGCCAGTTTTTGGGGC	AGGAGAATGCGAGCGGTTGTGT	314
FMgSSR-7385	scaffold9586	p2	(AC)6	Class II	11005	11016	ACATCCACACACACTCGTCCA	ACGGCGTCTCGCTTCAATCA	266
FMgSSR-7391	scaffold426286	p2	(AC)6	Class II	148	159	ACCACCACCACCTGGAGATTCA	AGCTGGGTTCAGGTCAGGTAGTGT	205
FMgSSR-7395	scaffold14435	p2	(AC)6	Class II	10275	10286	ACCAGTTGCCCCTTGTGCAT	TACTGCTGTTCCTGTGGCCCTT	314
FMgSSR-7396	scaffold4324	p2	(AC)6	Class II	25438	25449	ACCCAACCACGGGTTAGAAGA	CCGTGAAAGCCTTGCAGCAAAT	319
FMgSSR-7420	scaffold3672	p2	(AC)6	Class II	49935	49946	ACCTTGTTCTGAGTCCTTGTCTCC	AGTCTCCAGGCCTACCGAGTCTAT	318
FMgSSR-7429	scaffold588	p2	(AC)6	Class II	45874	45885	ACGAGAGCTTGGTGTCTCACCT	TTGGTCGCTTGCTTCGTGCT	264
FMgSSR-7430	scaffold126877	p2	(AC)6	Class II	719	730	ACGAGATGAACGTCGCTGGCAA	TGGAAAACGCTGCCCCCATT	247
FMgSSR-7432	scaffold3280	p2	(AC)6	Class II	27600	27611	ACGAGCCATCGATACAGGCCAT	TTGATGGGGACCTGACTTGCCT	336
FMgSSR-7435	scaffold5511	p2	(AC)6	Class II	30758	30769	ACGATGCCGCAAAGTGGTGT	TCGAGAACTTCATGCGCCAGGT	291
FMgSSR-7436	scaffold88	p2	(AC)6	Class II	155974	155985	ACGCAAAACCGCTTGCAACA	TTAACCGCTCCGCTCCGAATCT	254
FMgSSR-7438	scaffold1108	p2	(AC)6	Class II	80942	80953	ACGCACACATGCTAAGAGCG	CCGGCGCGACTGTAATCACAAA	339
FMgSSR-7447	scaffold350	p2	(AC)6	Class II	110530	110541	ACGCTTAACCGCGAACTCCCTT	ACGCGTGAAAGATCGCCACT	273
FMgSSR-7450	scaffold2732	p2	(AC)6	Class II	22150	22161	ACGGCCCATACAACACACACGA	TGCACGCATGATGCCTCAACAG	299
FMgSSR-7452	scaffold12767	p2	(AC)6	Class II	17953	17964	ACGGCCTAGGGGCATTCACAAT	TTCAAGCCCACTCAGCTCACCA	340
FMgSSR-7455	scaffold2861	p2	(AC)6	Class II	22527	22538	ACGGTGCAAGCAAACAGCACA	AGCCGAGGTTCGTCAGGGAAAT	315
FMgSSR-7456	scaffold2889	p2	(AC)6	Class II	7200	7211	ACGTCGTGTCCTTATGGGCT	GTAGCAGCATTGCAACAAGCAC	263
FMgSSR-7458	scaffold121	p2	(AC)6	Class II	73769	73780	ACGTGCAGACGTGCTTACTAGC	AGCCGGTTGCACGGGTTTTT	219
FMgSSR-7461	scaffold4369	p2	(AC)6	Class II	12594	12605	ACTAAGCAAGCTTTCCGGCCA	TGCAGCAGCTCTATGAGCCA	323
FMgSSR-7463	scaffold19277	p2	(AC)6	Class II	12977	12988	ACTAGGCTGTCAGCACGAAGCA	TGCGTTGACCCCTTCACGGTAA	340
FMgSSR-7465	scaffold1230	p2	(AC)6	Class II	56537	56548	ACTCCTAGCTTACCCACAGCCA	TGCGATCAGTAGTCATGCACGC	324
FMgSSR-7466	scaffold132	p2	(AC)6	Class II	41043	41054	ACTCGCCTTCATCACGCTCACA	ATGGGCATCTGCCTGTGCAA	254
FMgSSR-7469	scaffold40519	p2	(AC)6	Class II	333	344	ACTGCTTGGCAGCTGTGACT	AGTGTCCCATGCCTCCTTGAGA	273
FMgSSR-7471	scaffold12481	p2	(AC)6	Class II	6993	7004	ACTGTACTGTGGACCATGGCTT	AGCACATTGCGTGCCATTCT	320

SSR_ID	Scaffold	SSR_Type	SSR_Motif	SSR Class	SSR_Start	SSR_End	Forward sequence	Reverse sequence	Product_size
FMgSSR-7474	scaffold1316	p2	(AC)6	Class II	48518	48529	AGAAATGCGGCTGCAGGCAA	ACGAAAGACACTCTGTTGCTCGGT	250
FMgSSR-7475	scaffold2470	p2	(AC)6	Class II	41501	41512	AGAACTTCGTCCGGTGGGCATA	AGGCATGGAACACATGCGGA	336
FMgSSR-7477	scaffold2489	p2	(AC)6	Class II	3163	3174	AGACCCACCCGCACAGTTTTCT	TTGCGGTACTGAGCTTGGCT	312
FMgSSR-7482	scaffold192	p2	(AC)6	Class II	27144	27155	AGCAAGGGATGACACGTGCAGA	GGCCTCCTTGCTTTGCCAACAT	334
FMgSSR-7493	scaffold398	p2	(AC)6	Class II	131174	131185	AGCAGCTGTTTCCAGCACAAGGA	TGCGGCATCGGTGCATGAAA	300
FMgSSR-7496	scaffold17927	p2	(AC)6	Class II	5659	5670	AGCCAAGAGCAAGAAGCACCCA	AAACAACCGCAGCTACCGCA	282
FMgSSR-7502	scaffold31955	p2	(AC)6	Class II	4568	4579	AGCGACGAGTGACATCTTCATCCA	ACCACAGCCACAACCAAGTCCA	332
FMgSSR-7505	scaffold7437	p2	(AC)6	Class II	33747	33758	AGCGTGGCGCCTCGTATTACTT	TGTGCCGGGTGAAAGACCCAAA	348
FMgSSR-7506	scaffold4052	p2	(AC)6	Class II	13756	13767	AGCTCCTTCTCGTTGCCACCTT	ACTGGTGCGACATTGCCACT	337
FMgSSR-7515	scaffold5469	p2	(AC)6	Class II	15914	15925	AGGCACAGCTCGCAACATCA	TGGCACGCGTGTTCTCTGACTT	239
FMgSSR-7516	scaffold3837	p2	(AC)6	Class II	20236	20247	AGGCCTTCAAGAAACCCCTCTCCT	ACGCTCCACATTGCTCTCCCTT	340
FMgSSR-7517	scaffold8747	p2	(AC)6	Class II	18775	18786	AGGCGCCAAGTACAACCACACA	TATTTGCCTCCTATGAAGGCCGGG	346
FMgSSR-7522	scaffold81196	p2	(AC)6	Class II	1319	1330	AGGCTGACTTTGGCTTGGGTGA	GCTGCAACAAACGACGGCAT	282
FMgSSR-7530	scaffold4675	p2	(AC)6	Class II	40722	40733	AGTACCACCGCTGCAAAGCA	TTCTGGTTGTTGGGCCGTGT	210
FMgSSR-7536	scaffold9046	p2	(AC)6	Class II	17085	17096	AGTGAAGCACAGGGCTGGACAT	GGCTGCAAATTTACGCGTGCCT	348
FMgSSR-7541	scaffold1781	p2	(AC)6	Class II	55603	55614	AGTTGCTCCCTGGGCAAGTA	GGATGCTTGGCCAGTACGGTTT	334
FMgSSR-7545	scaffold3528	p2	(AC)6	Class II	16305	16316	ATATGCACGGTCAGGCTCCGAA	ACCAGGTTGTGCCTCGACAT	245
FMgSSR-7547	scaffold1203	p2	(AC)6	Class II	54374	54385	ATCAGCGCAAGTGCTACATCGG	TGGCTTGGTGTCGCAATCCAAC	324
FMgSSR-7549	scaffold22681	p2	(AC)6	Class II	6253	6264	ATCCACCGTTTAAGCAGCCCGT	AGCTGAGACAGGTGGTGTCGTT	215
FMgSSR-7554	scaffold8221	p2	(AC)6	Class II	10270	10281	ATCGACGAAAGCACCTTGCCCA	AGTTGTCGGCCACCTGCAAA	347
FMgSSR-7555	scaffold561	p2	(AC)6	Class II	67349	67360	ATCGAGGCGTTAGCTAGGCA	AATTTGCTGCCACGACAAGCCC	320
FMgSSR-7557	scaffold15059	p2	(AC)6	Class II	205	216	ATCGCCGAGGAATCAAGCTGGT	ACTGGGGCCTTCAGAACAGT	263
FMgSSR-7558	scaffold1228	p2	(AC)6	Class II	29125	29136	ATCGCGAGCGGAGCAATCAT	TGTTGCCGTTCGAAGTGCCT	221
FMgSSR-7561	scaffold5942	p2	(AC)6	Class II	35031	35042	ATGACCGAGACGGTGGCGAAAT	TCCGCGCCCCATAAAACTCGAA	265
FMgSSR-7562	scaffold21794	p2	(AC)6	Class II	8947	8958	ATGAGCCGAAGCTTGCAGGAGT	AGTGCAGGAAGCAACGCCAA	242
FMgSSR-7566	scaffold2430	p2	(AC)6	Class II	24354	24365	ATGCCCGTTTGGCCATGTCA	TGCGCGGTTGCACTGTGTTT	306
FMgSSR-7575	scaffold327	p2	(AC)6	Class II	29353	29364	ATTCCAGTACGGTCGTCTACGCCT	AACCAAAAGGCCACGCACCA	343
FMgSSR-7576	scaffold8956	p2	(AC)6	Class II	30050	30061	ATTGATCGTTTCGGGCCGGT	TGCAGCTCAACGCCTGCTTTT	293

SSR_ID	Scaffold	SSR_Type	SSR_Motif	SSR Class	SSR_Start	SSR_End	Forward sequence	Reverse sequence	Product_size
FMgSSR-7578	scaffold84	p2	(AC)6	Class II	156248	156259	ATTTCGGCGGAGTGCAGCAA	ACCGCGCTGTGAAGAGAGTGAT	284
FMgSSR-7581	scaffold19292	p2	(AC)6	Class II	11129	11140	CACACACACAGCTGCTGCAA	AGCAGAAGAAGCAGGGAAGGCT	285
FMgSSR-7585	scaffold14197	p2	(AC)6	Class II	16862	16873	CCACCATGTGTTGTTGGCAGGT	TGCTGGGGGCACTTACTTCT	230
FMgSSR-7587	scaffold892	p2	(AC)6	Class II	5582	5593	CCAGAGTCCTGCTTCATGCCAA	TAGCACGAAGGGTTCCTGTGGA	282
FMgSSR-7591	scaffold2479	p2	(AC)6	Class II	32893	32904	CCTGCAGACAATCTGGAAAGATCC	AGGCAAGCACAGGCTACTACA	325
FMgSSR-7592	scaffold173	p2	(AC)6	Class II	46785	46796	CGCAAGCCGACAACCAACCATA	CCAAGGGGCGGCGCAAAATAAA	203
FMgSSR-7595	scaffold11756	p2	(AC)6	Class II	13405	13416	CGCACTGTCCGTTCCAAAGCAA	TGGGAAATTTCTGCCGACCGCT	335
FMgSSR-7596	scaffold1342	p2	(AC)6	Class II	18047	18058	CGCCACGCCAACTCATCACAAA	ATGACGTGGCGTTCAACGTG	240
FMgSSR-7597	scaffold654	p2	(AC)6	Class II	51517	51528	CGGCGTCCGCTGGTTTACAAAA	CGGCGGCAACTATGGTGCAATTT	268
FMgSSR-7598	scaffold96095	p2	(AC)6	Class II	159	170	CGGGTTGCGTGCATCGACAAAA	GGGGGTGTATGAAACGTCATGTGT	334
FMgSSR-7599	scaffold7594	p2	(AC)6	Class II	29510	29521	CGTCAGCTCGCAAAAACGAGCA	TCGCCACTATGGCTTGGCAACT	200
FMgSSR-7603	scaffold11817	p2	(AC)6	Class II	9118	9129	CTTGCCGCCCTGCATTTCAT	TGGTGAAGGATGATGCCCGAGA	320
FMgSSR-7605	scaffold2753	p2	(AC)6	Class II	32462	32473	GAAGTGGATCGGAGTTGCTACAAG	AGGAGTCGTTTGTTGGTCGTCTCG	315
FMgSSR-7607	scaffold1781	p2	(AC)6	Class II	63579	63590	GCAACCTGCTCCCTTCTATTCGACA	ACAGGGTCGTGGTTGCACTTCT	345
FMgSSR-7608	scaffold7271	p2	(AC)6	Class II	9077	9088	GCAACTTTGGATCACCGTCGCT	TCAGTGTACCTGAAGGAGTTGGGGA	220
FMgSSR-7609	scaffold18162	p2	(AC)6	Class II	11291	11302	GCAATCATGACTTCCACGCGCA	AACCACGCGTGCATGAAAGAGC	337
FMgSSR-7612	scaffold132758	p2	(AC)6	Class II	511	522	GCAGCAAGCCATGCACTGTAGA	TTGCTGTTCACTGACGTGGGGA	299
FMgSSR-7614	scaffold18	p2	(AC)6	Class II	196482	196493	GCAGGTGCGTTTTCCACTGTGT	ATTTGCCTGGTCCCTGTCGT	288
FMgSSR-7616	scaffold2612	p2	(AC)6	Class II	25534	25545	GCCAATGGTCGCCTGTACCAAA	ACTGTGCTGAAGTAGGAACCGCT	267
FMgSSR-7617	scaffold66	p2	(AC)6	Class II	129408	129419	GCCATAGAAAGTCTCGTTGTGGAGC	AATGCGGCATGTGGCAAGGT	204
FMgSSR-7619	scaffold22967	p2	(AC)6	Class II	2797	2808	GCCCACAAAGCACGGCTCATTT	AAGGAGTCGGTGTCGCTTGCAT	252
FMgSSR-7622	scaffold125	p2	(AC)6	Class II	175774	175785	GCGAAAGCGATGGCGTTGTT	TCGTCCATGTCTGTTGTGCGCT	236
FMgSSR-7623	scaffold14464	p2	(AC)6	Class II	7263	7274	GCGAGCTTGTGTTCGCCTTCTT	TGGCGCCATTGTACACCACT	280
FMgSSR-7625	scaffold226	p2	(AC)6	Class II	106076	106087	GCGCTTCGATCTGGTATGCACT	GGCTGAACTGCTTTCAGCTTTCGG	313
FMgSSR-7626	scaffold9233	p2	(AC)6	Class II	17063	17074	GCTTTTCAACTAGACACCAGCCAG	GAACAAACGCAGGTGCTTTCAG	344
FMgSSR-7627	scaffold1620	p2	(AC)6	Class II	48301	48312	GGCCGCGCCCAGAAAAGAAAAA	AAAAGCGAAGGCGAGGAAGCGA	281
FMgSSR-7628	scaffold15849	p2	(AC)6	Class II	18464	18475	GGTATTATGTATCTGGGACTGGGGG	ACGTACCCAGCACTGGCATT	337
FMgSSR-7629	scaffold439	p2	(AC)6	Class II	9508	9519	GGTCATTCATGCAGTACCAGTCCCA	TTTCGAGCTTTGGTGCGGGA	309

SSR_ID	Scaffold	SSR_Type	SSR_Motif	SSR Class	SSR_Start	SSR_End	Forward sequence	Reverse sequence	Product_size
FMgSSR-7630	scaffold27156	p2	(AC)6	Class II	4570	4581	GTGCAAACCAACTCAGCACCCA	CGGTGATGAGCTAGCTAGAAGCCT	307
FMgSSR-7638	scaffold9449	p2	(AC)6	Class II	21147	21158	TACTTGCGGTGTGGCACGAT	TTGCGAGGAGTTCAGGCTGT	299
FMgSSR-7639	scaffold8783	p2	(AC)6	Class II	31398	31409	TAGGCATTCATGGCACTGCACC	TCCCAGCTCTTGATTTTGCAGGC	206
FMgSSR-7640	scaffold46	p2	(AC)6	Class II	149364	149375	TAGGCGCTCAACGTTGCCAT	TGGTGCAGAGACCCGTTGGAAA	277
FMgSSR-7643	scaffold229834	p2	(AC)6	Class II	59	70	TAGGTTCCCCACCTCGGCACATTA	GACCGATCTCACTGGTACACCGTT	200
FMgSSR-7644	scaffold421	p2	(AC)6	Class II	120290	120301	TATGGTCTGTTCCATCTCTGGCCG	GCTCTGTCACTGTGCACGTGTTT	300
FMgSSR-7645	scaffold7457	p2	(AC)6	Class II	10456	10467	TCAAAACACGCGCGCACGAA	ACGAGCGCAAGCCACATGAA	330
FMgSSR-7648	scaffold2703	p2	(AC)6	Class II	9186	9197	TCACAACTGATGCAGCACCGA	CAGCTGCTTATTATGGTCCTGTGC	330
FMgSSR-7656	scaffold5423	p2	(AC)6	Class II	16325	16336	TCACGGTGAAACGCAACGCT	TTTTCCTGCCTGCCTGGCTT	316
FMgSSR-7657	scaffold549	p2	(AC)6	Class II	26244	26255	TCACGTGACGAGCCAGTGTT	TCCTTCTCATCGCGACGACGTT	287
FMgSSR-7658	scaffold220	p2	(AC)6	Class II	14362	14373	TCACTTACCTTCCGTGCCTGGT	TTCAATCTTGAGGACGAGCAGC	335
FMgSSR-7659	scaffold26649	p2	(AC)6	Class II	5056	5067	TCACTTACGTTGACCAAGCCCT	GTTTCCAGAGTACTGCCCTCAATC	294
FMgSSR-7661	scaffold4706	p2	(AC)6	Class II	28500	28511	TCAGAAACAGGTGGGAGAGCTGA	GCCTGCGACTCGAGGTGTCTATTA	220
FMgSSR-7664	scaffold263	p2	(AC)6	Class II	14244	14255	TCAGCCGGGCTAGCTATGAGAA	GGGCGCATAATGCTGGAGTT	341
FMgSSR-7666	scaffold5129	p2	(AC)6	Class II	21761	21772	TCAGCGTCACCACTCTCCTGTT	ATGATGTGCCACCGTGCGAT	318
FMgSSR-7670	scaffold4197	p2	(AC)6	Class II	18791	18802	TCATCGGACTCACATGTCACCG	AGGTAGTAGTACAACGGGTGGGT	211
FMgSSR-7672	scaffold12999	p2	(AC)6	Class II	14090	14101	TCCACCCAAACACATGCACCCT	GCCCGCAACCAAGTCATCAACA	284
FMgSSR-7676	scaffold169	p2	(AC)6	Class II	138912	138923	TCCCGCCTCGCACACAAAAGAT	AATATCGCCTGCGTGCGGTT	241
FMgSSR-7682	scaffold2995	p2	(AC)6	Class II	41250	41261	TCCGCGGAGCAAATGTCCATCA	AGATGTGATGTGACGGGGTGGA	243
FMgSSR-7683	scaffold5692	p2	(AC)6	Class II	15172	15183	TCCGCTCGCTGAACAAACCT	TAGCAACACGATTGCGCCGT	324
FMgSSR-7687	scaffold3955	p2	(AC)6	Class II	12772	12783	TCCTTCACTGCTTGCGCCAT	TGCGGATTGTCCGGCAAGTT	286
FMgSSR-7690	scaffold1254	p2	(AC)6	Class II	9107	9118	TCGACGCCTCGTCGTACAGATA	TTGGTCGCTTCGATTGCGTTCC	340
FMgSSR-7691	scaffold2094	p2	(AC)6	Class II	11497	11508	TCGACGTCATTGAACACCACGC	AACGAAAATTGCGGCCACGC	288
FMgSSR-7694	scaffold1411	p2	(AC)6	Class II	7393	7404	TCGAGCTTGTGCACGTCCTT	GAGCGCATATGACTTAGAACCGAC	267
FMgSSR-7698	scaffold23869	p2	(AC)6	Class II	6583	6594	TCGCGCATGCCAACTCACAA	CCATAGACCCATACTCCCATCCCA	264
FMgSSR-7701	scaffold316	p2	(AC)6	Class II	79437	79448	TCGGCTCTCTCGTCAACCAA	ATGCGTATGCCACCCAACCAGT	342
FMgSSR-7703	scaffold5029	p2	(AC)6	Class II	34954	34965	TCGGTCAATGTACGCTGCTGCT	AGCATGCGACCAAATCGGAGGA	211
FMgSSR-7711	scaffold3016	p2	(AC)6	Class II	17492	17503	TCTGCCCTTGGCGCCTTTTT	ACGCTCTGGAAGTGGCTGAACA	306

SSR_ID	Scaffold	SSR_Type	SSR_Motif	SSR Class	SSR_Start	SSR_End	Forward sequence	Reverse sequence	Product_size
FMgSSR-7712	scaffold88571	p2	(AC)6	Class II	970	981	TCTGTGCCTGGCATTGAGAGCA	TTAACACAGGCCGGTGAGAGT	324
FMgSSR-7714	scaffold2600	p2	(AC)6	Class II	12817	12828	TCTTGCGCGAAAACAGCGGA	TATGGCCGCTGCGGTTCAAT	339
FMgSSR-7716	scaffold5794	p2	(AC)6	Class II	36990	37001	TGAACCTCTGCGCGTTCACA	AGCTTGCGACCCGTTCGTTCTT	330
FMgSSR-7717	scaffold40519	p2	(AC)6	Class II	1552	1563	TGAAGGGCACCTGTTCGTTCCA	TGTCCCATGCCTCCTTGAGAGT	337
FMgSSR-7721	scaffold24258	p2	(AC)6	Class II	816	827	TGACGACACGAGAGCAACAGCA	ATCGAAAGGGCCGTGTTGTCCT	284
FMgSSR-7724	scaffold17627	p2	(AC)6	Class II	15115	15126	TGAGATGCGGCCCTTTCACT	AAACGGCGCCATCCGTTGTA	335
FMgSSR-7728	scaffold65239	p2	(AC)6	Class II	1425	1436	TGATGCCCAGCGTCATGCATCT	TGAAATCCCTCGGGAGTACCGGAT	255
FMgSSR-7729	scaffold3069	p2	(AC)6	Class II	5413	5424	TGATGTTCATGGCCTGCACGGT	AGCAAGGGCAGTTGGGAGCTTT	327
FMgSSR-7732	scaffold5540	p2	(AC)6	Class II	33402	33413	TGATTGGAGTTGCACGCGGT	AGCCGACAAGAACGCAACGA	221
FMgSSR-7733	scaffold660	p2	(AC)6	Class II	961	972	TGCAACCTGCTTGCTTCCCCTT	ATCGCCGCCATGCATGAACT	216
FMgSSR-7743	scaffold5164	p2	(AC)6	Class II	45756	45767	TGCACTGCACGTGGAACACT	ACCTGCAACACCCAACCCAACA	259
FMgSSR-7744	scaffold10300	p2	(AC)6	Class II	6645	6656	TGCACTGCACTGGGCTCCTTTT	ACATGGCTGGCTTGGCTCCATT	340
FMgSSR-7748	scaffold15443	p2	(AC)6	Class II	18111	18122	TGCATTTCTGACGCACCCGT	TCGCTGCTTAGTGTTGCTGC	272
FMgSSR-7752	scaffold5392	p2	(AC)6	Class II	23998	24009	TGCCATTTTGGAGTTGGACGCA	GCGCCACTGCGTCAATTTCGTT	308
FMgSSR-7764	scaffold2708	p2	(AC)6	Class II	63031	63042	TGCCGACATGCAGAAGTTTGCG	AGCTGCCTTCTGCCCCTTGTTT	200
FMgSSR-7767	scaffold5169	p2	(AC)6	Class II	20259	20270	TGCCGCAAAGAGCAGCTACA	ACCTGCGCTGGAGTGTGATT	335
FMgSSR-7770	scaffold1295	p2	(AC)6	Class II	12364	12375	TGCCTTGCCGAAAACGTTGC	GGTTGGCTTGAAGGGCAGCAAA	345
FMgSSR-7771	scaffold2286	p2	(AC)6	Class II	7929	7940	TGCGAACTGGGAACATGCCA	TCCTCTCAAACCATGGGTGGCT	272
FMgSSR-7773	scaffold20695	p2	(AC)6	Class II	14317	14328	TGCGCGAGAAAGAACACGGA	ATGGCGATGCTCGGTTAAGGCA	284
FMgSSR-7774	scaffold4333	p2	(AC)6	Class II	35189	35200	TGCGTCCGTCTTGTTCAGTCGT	TGGGCGCATCAGCAGATTGT	316
FMgSSR-7777	scaffold3232	p2	(AC)6	Class II	18053	18064	TGCGTTTTGCTCCTCAGGCA	TCGATTTGTGTGCGGGCGTT	306
FMgSSR-7787	scaffold3455	p2	(AC)6	Class II	42117	42128	TGGAATTGTCGCACGCTGCAT	CGGCACAAGGCATTGTTCTAGC	298
FMgSSR-7788	scaffold1880	p2	(AC)6	Class II	4444	4455	TGGACCACCAACCACCATGT	AAATTGAAGCCCACCTGGCCGA	309
FMgSSR-7790	scaffold713	p2	(AC)6	Class II	51016	51027	TGGACTGGGGAACCTTTGCAGT	TTAAGGTCGGTTCGGGCTTTGC	296
FMgSSR-7795	scaffold360	p2	(AC)6	Class II	62002	62013	TGGCATGCACCGTGACAAGT	TCCTAGCGCGCGTTACTCTTGA	214
FMgSSR-7799	scaffold22	p2	(AC)6	Class II	253234	253245	TGGCCGTCGCTTCTTGTTCCAA	AGGTTGTTCTGACGTGCGCT	317
FMgSSR-7801	scaffold3109	p2	(AC)6	Class II	49502	49513	TGGCTGGAGAGAACAACGGT	TTGGTGCGCACACTCCTCTGTT	346
FMgSSR-7804	scaffold23006	p2	(AC)6	Class II	7641	7652	TGGGCAACCTGCAATACCGAGT	AACCCAACAAGCCCGCATCT	333

SSR_ID	Scaffold	SSR_Type	SSR_Motif	SSR Class	SSR_Start	SSR_End	Forward sequence	Reverse sequence	Product_size
FMgSSR-7806	scaffold210	p2	(AC)6	Class II	142905	142916	TGGGCTCATACGCCCGTTGTTT	ACGAGAGAAGCGCATGGAACGA	278
FMgSSR-7808	scaffold804	p2	(AC)6	Class II	10292	10303	TGGTAGCGAGTTTCAGCATTCCCA	ACCCCTTGTCGACTTCGTTGCT	340
FMgSSR-7809	scaffold273	p2	(AC)6	Class II	32627	32638	TGGTAGTCAGTGGGGACGGTATGT	CCGTCATCAATTTCGTGCACCC	297
FMgSSR-7817	scaffold2106	p2	(AC)6	Class II	21816	21827	TGTCCCACTTTGGCAGGGCTAT	TTGAGCACGAGCACTCCCAACT	293
FMgSSR-7819	scaffold1993	p2	(AC)6	Class II	20194	20205	TGTCGTTGGAGCAATGAACTGACG	TTTTCATCGGGAGGGGCAAGGA	329
FMgSSR-7820	scaffold6977	p2	(AC)6	Class II	2324	2335	TGTGACACTCGCTCGTCATCGT	CGCCGCTGCAAGCTTGTTTT	261
FMgSSR-7827	scaffold5599	p2	(AC)6	Class II	43008	43019	TGTTGATCAGCAATGCAGCCGA	TGCATGTTTGTGTGCTGCCTGA	338
FMgSSR-7829	scaffold21	p2	(AC)6	Class II	226807	226818	TGTTGCGTCACAAGGCCACT	ACCATCAAACCTGTGTGCCCGT	266
FMgSSR-7835	scaffold1223	p2	(AC)6	Class II	60424	60435	TTCCACCACGCGAGAGGACAAA	ACTGGTTTGGTGTGCGGTGGAT	285
FMgSSR-7840	scaffold17	p2	(AC)6	Class II	202684	202695	TTCCGGCTCAACCGCACAAT	GCTTGATTCGGCTCTGATCGTTGG	313
FMgSSR-7842	scaffold25063	p2	(AC)6	Class II	9758	9769	TTCCGTCACGTTCTCCACCGAT	TCCGGGTTGTGTCTTCTCTCGT	256
FMgSSR-7846	scaffold1503	p2	(AC)6	Class II	72407	72418	TTCCTCTACGGCTCTACGCCAA	AGCCACCAAATGGGAGCACA	317
FMgSSR-7848	scaffold2739	p2	(AC)6	Class II	34601	34612	TTCGAGGCCTCCACGCGAAAAT	ATTGCACGACGCGGAAGGAA	303
FMgSSR-7849	scaffold345	p2	(AC)6	Class II	38608	38619	TTCGCACAGTTGCCAGCCTT	TTCCACGTCAACACCATCCCCA	312
FMgSSR-7852	scaffold2282	p2	(AC)6	Class II	33726	33737	TTCGGCGGTGACCTCAAACACA	TGCAGGGGTGGAGCCAAAAACT	221
FMgSSR-7855	scaffold9500	p2	(AC)6	Class II	12288	12299	TTCTTCTGTGGGCCGCGAAA	ACACCCAGGCGTTCGGAATGAA	326
FMgSSR-7858	scaffold577	p2	(AC)6	Class II	51339	51350	TTGACACGCCTGCTTTCGTTGC	TATTCGCCGGCGCGTGTTTT	264
FMgSSR-7866	scaffold120	p2	(AC)6	Class II	89996	90007	TTGCCATCCTTGAAGCGGCA	TGCAACCGTGGTCCTCCAATGT	260
FMgSSR-7867	scaffold80733	p2	(AC)6	Class II	1308	1319	TTGCCGTGGTTGACCTACTGGT	AACGCTGGGAGTGTGTGTGTGT	296
FMgSSR-7871	scaffold436	p2	(AC)6	Class II	65999	66010	TTGCGCAGATTTCAGGCGCT	TTTTATCCGCCGGGAAGCAGCA	270
FMgSSR-7875	scaffold14	p2	(AC)6	Class II	116148	116159	TTGCTTGCATGCGTGTGTGT	ATGGCCAGTTGGGCCTCTCAAT	212
FMgSSR-7878	scaffold12925	p2	(AC)6	Class II	14976	14987	TTGGACGCTTGCCTGGCATT	ACCGCGGCAGATAAAGCTCGTT	267
FMgSSR-7880	scaffold13152	p2	(AC)6	Class II	14919	14930	TTGGCGGGCACAAGAACCAA	TGGCCTGGTTCACTTGTTTGCG	313
FMgSSR-7883	scaffold4292	p2	(AC)6	Class II	23651	23662	TTGGTGTTTGGTCTCTCGCGCA	TGTCCGTATGACGCTGCCCTTT	345
FMgSSR-7884	scaffold10062	p2	(AC)6	Class II	3947	3958	TTGGTTTGGGCAGATCCAGCGT	TTCCCAACCGCACTTCACGA	300
FMgSSR-7896	scaffold1059	p2	(AC)6	Class II	86549	86560	TTTGCTTGCGGCTTGCCACT	CCACGACCACGAGCAAGCTTTT	273
FMgSSR-7898	scaffold21195	p2	(AC)6	Class II	9170	9181	TTTGGCAACTCCGTTACCCCCA	ATCAAGTGGTGTGGTGGCCT	289
FMgSSR-7899	scaffold8975	p2	(AC)6	Class II	31947	31958	TTTGGTGCTGCCACGTTTGC	AATAGTTGCGCACAGGTCCAGG	295

SSR_ID	Scaffold	SSR_Type	SSR_Motif	SSR Class	SSR_Start	SSR_End	Forward sequence	Reverse sequence	Product_size
FMgSSR-7900	scaffold20534	p2	(AC)6	Class II	13248	13259	TTTGGTTGCGGTTGTGCGGT	TGCGTGCAAAAGCTTCGCTGA	345
FMgSSR-7903	scaffold5376	p2	(AC)6	Class II	34722	34733	TTTTGCTTTCGCGTGTGGCG	TCGCAAATGAGCGTGTCGGT	248
FMgSSR-7908	scaffold6345	p2	(AC)7	Class II	15047	15060	AAAGACATCCAAGGGAGGGGGT	TGTGTGGGTGTCCTATGGGCTT	283
FMgSSR-7910	scaffold1157	p2	(AC)7	Class II	85095	85108	AAATAAACGGCGGTCTTCCCCG	TTTGCTCGGTACTCGCGGTGTT	238
FMgSSR-7912	scaffold13152	p2	(AC)7	Class II	13882	13895	AACAACACAGCCCCACCAACCA	TAAGTGAAACCGCAACGGCGGA	322
FMgSSR-7918	scaffold12671	p2	(AC)7	Class II	12232	12245	AACTGACCACGATGTGCGGTGT	TCGGTCACTGGGGTGCAAATGT	203
FMgSSR-7920	scaffold1156	p2	(AC)7	Class II	52239	52252	AAGAGGAGACCCGACCCGAAAA	TGGCGGCAATGCACAGAGTT	274
FMgSSR-7923	scaffold4543	p2	(AC)7	Class II	16012	16025	AAGCCCAAGAAGGCGACGTT	TGCTCAAGGAGCTTGGTGGA	320
FMgSSR-7925	scaffold4722	p2	(AC)7	Class II	37564	37577	AAGCTGCAAGCACAACACACGG	AGTTTTCACACCTGCGGCAACG	229
FMgSSR-7927	scaffold2067	p2	(AC)7	Class II	25506	25519	AAGGCGATGGAACGAGGTGT	AGGGCGCAACCCCTCAAAGAAA	346
FMgSSR-7932	scaffold8629	p2	(AC)7	Class II	17263	17276	AATTTCAGCGCCGCACACCA	AGGTGCTTGCATGCAGGAGTGT	309
FMgSSR-7934	scaffold3837	p2	(AC)7	Class II	50408	50421	ACAAGGTTAGCATGGCTGCGT	ACCAGCCGAAACCAATACCGCA	277
FMgSSR-7936	scaffold10654	p2	(AC)7	Class II	7635	7648	ACACACCCCCACCCACCAAAAA	TTCATATTCGAGAGCAGGCGGC	323
FMgSSR-7945	scaffold165	p2	(AC)7	Class II	23140	23153	ACACGGCAGTTCAAACCCCA	TAGCGGGTGCAAAAATGTGGGC	243
FMgSSR-7948	scaffold15172	p2	(AC)7	Class II	8164	8177	ACAGTTCTCCTGCGCATTCAAG	AAGCATTCCTGGCAGTAGCAGC	293
FMgSSR-7963	scaffold6922	p2	(AC)7	Class II	17218	17231	ACCGCAACCCACGACACACTAA	GGCGACAGGAAGTCGTTGTT	269
FMgSSR-7964	scaffold4343	p2	(AC)7	Class II	10882	10895	ACCGGAGGGTGGCATGGAAAAA	TGCCACGTCTAGTTCATCACTTCCC	346
FMgSSR-7965	scaffold12966	p2	(AC)7	Class II	1174	1187	ACGAATCGATAGCCGGTTCGCA	ACGCCTCTTGTGAGGAATTGGC	307
FMgSSR-7970	scaffold13820	p2	(AC)7	Class II	14302	14315	ACGCACGCCTGAAATGCAGA	GCACACGCTGCTGGTGGTTTTT	338
FMgSSR-7973	scaffold1520	p2	(AC)7	Class II	33996	34009	ACGGCGATCAACAACGGAGA	TGGCAGTTGGGGTTGCGTTT	281
FMgSSR-7974	scaffold265	p2	(AC)7	Class II	93021	93034	ACGGTGCGTGAGGTGAAACA	TAAAGAACTCGCGGCGCTCA	325
FMgSSR-7975	scaffold10406	p2	(AC)7	Class II	18521	18534	ACGTAAGTGTGTAAGGCCCGA	TGCGCCAGACGTTATTCAGGA	247
FMgSSR-7976	scaffold394	p2	(AC)7	Class II	44904	44917	ACGTCAAACGGCAACAGTGC	TGCATCGATCGGCATGTGAGTG	305
FMgSSR-7980	scaffold324	p2	(AC)7	Class II	102526	102539	ACTAGCTTGGGGGAGCAACA	AACTGGGAAAGCGCACTGCT	338
FMgSSR-7983	scaffold20082	p2	(AC)7	Class II	6572	6585	ACTGCAACACACGCCAGTAAC	AGTACAGGCCACACCATGCTT	321
FMgSSR-7984	scaffold13726	p2	(AC)7	Class II	4093	4106	ACTGCACAGTACTTGCTCGGTA	CGCCACAACAATTGCAATGACACC	302
FMgSSR-7985	scaffold1463	p2	(AC)7	Class II	63252	63265	ACTGTTTGATCGCCACGGCT	TCACAGGCCGCAGGAAATTGGT	318
FMgSSR-7986	scaffold1022	p2	(AC)7	Class II	65621	65634	ACTTCCAATCGTGGGCACCGAA	ACGGAAATGCTCCACGCCTA	267

SSR_ID	Scaffold	SSR_Type	SSR_Motif	SSR Class	SSR_Start	SSR_End	Forward sequence	Reverse sequence	Product_size
FMgSSR-7987	scaffold4589	p2	(AC)7	Class II	28096	28109	ACTTTGTTAGAGGGGGTGCAGG	TGGTTCTCGCGCGTGCTTTT	276
FMgSSR-7988	scaffold13482	p2	(AC)7	Class II	3996	4009	AGAACTCACCCACGCACTCTCA	TGCATGCCATGAGGCACAACA	237
FMgSSR-7989	scaffold309093	p2	(AC)7	Class II	307	320	AGAACTTGCCCGCGCGTTTA	AGCGTCCCGTTGGCCATTCTAT	346
FMgSSR-7997	scaffold28753	p2	(AC)7	Class II	4724	4737	AGCACTGTAGATGGCCGGTT	ACTGCTGGTGGCAGAGACTCAA	338
FMgSSR-7998	scaffold46	p2	(AC)7	Class II	91665	91678	AGCAGTTGAACCAACCGACCT	TGCTCCGCTGCATTTTCATCGC	215
FMgSSR-8002	scaffold2513	p2	(AC)7	Class II	28883	28896	AGCCGGAATCAGCTACCAGT	ACCCCACCGAGTAGGCAAATCT	339
FMgSSR-8008	scaffold42985	p2	(AC)7	Class II	4595	4608	AGCGCAGAGACTGACCTTTGGT	TCGTCGCACGCATGACAGAGAA	265
FMgSSR-8009	scaffold4227	p2	(AC)7	Class II	30849	30862	AGCGGAAGCAAGGGCAGGAAAT	AGCACTGCAAAGTGAGAGTGGG	296
FMgSSR-8015	scaffold596	p2	(AC)7	Class II	42709	42722	AGGCGGCGCAAGAACTTCAA	GCACCTAGTATTTTCACGCGCACA	258
FMgSSR-8020	scaffold12926	p2	(AC)7	Class II	22758	22771	AGTCACAGTGCAGCGGAATCCT	ATGTCCAGCCGGTTCAACGTGT	261
FMgSSR-8024	scaffold15050	p2	(AC)7	Class II	10650	10663	AGTTGCACTTCACAGGAGGCGT	GGAAGGACAGATCTCTCACAGCCA	335
FMgSSR-8028	scaffold2436	p2	(AC)7	Class II	27410	27423	ATCATCACTTCCCATTGCCGCC	AACCGTTTCATCCACGCGCA	280
FMgSSR-8038	scaffold886	p2	(AC)7	Class II	19725	19738	CATGCATGTGCCTGGTTGGT	TGGATGGATGCGTCGGGGAAAA	294
FMgSSR-8040	scaffold9704	p2	(AC)7	Class II	4107	4120	CCACCTGGCGTAACGCAACAAA	CGCGCTTGATCGTGGTACAT	260
FMgSSR-8041	scaffold20170	p2	(AC)7	Class II	4015	4028	CCCACTACCGCTCCAACTGTAGAA	AGAAAGCGCATGGAGTCGCA	347
FMgSSR-8042	scaffold6045	p2	(AC)7	Class II	3080	3093	CCCATGAGCATACTGTGGTGGTGT	AACTTGATTGGCCCCCACGA	226
FMgSSR-8045	scaffold19083	p2	(AC)7	Class II	362	375	CCTTGCGCGCCGCATTATACTA	TCTGCCCCGTGATCTGTTTGGA	312
FMgSSR-8048	scaffold6231	p2	(AC)7	Class II	2067	2080	CGCCATGTCCAACGCTTGAT	TGCCGATCACCAATCACCACCA	328
FMgSSR-8050	scaffold626	p2	(AC)7	Class II	40555	40568	CGGCCAGCCGGCCATTTTATATT	ATGATCCGTGCACGAGTAGGGT	330
FMgSSR-8051	scaffold3189	p2	(AC)7	Class II	17109	17122	GAAGCCCAACCAGCAGTGATT	TGCCAAATTGCTAGCGCACA	350
FMgSSR-8052	scaffold2315	p2	(AC)7	Class II	64192	64205	GACGTGACAATCGTTCCGCA	AGACAGCGATGCCACCTACT	350
FMgSSR-8053	scaffold1363	p2	(AC)7	Class II	16215	16228	GCAAGCCGATGGCGATGTTT	TTCTCGCTCCCAATCGTCCAGA	335
FMgSSR-8055	scaffold2001	p2	(AC)7	Class II	49534	49547	GCACAAGTTCGCTCGTTTTGGGT	AGCCCCAGACTCCCAGAGCTTAAA	292
FMgSSR-8056	scaffold17761	p2	(AC)7	Class II	4385	4398	GCAGAGGGTAAGGTAACAGTTTCGG	ACAGTCAGTGCCTATCGCTGCT	209
FMgSSR-8058	scaffold131	p2	(AC)7	Class II	141651	141664	GCCATTGAACTATAGGAACCCCAC	TCGTGCTTACTCGGGTTCAGCTT	250
FMgSSR-8059	scaffold5112	p2	(AC)7	Class II	43082	43095	GCCGCAATTGGATCAAGCCGTT	TTGTTGGTTAGCATCCGCCAGC	224
FMgSSR-8060	scaffold62142	p2	(AC)7	Class II	2610	2623	GCGTGATGAGACCAGACGCTTA	AGGGTGCCCACGGATATACCTAGT	346
FMgSSR-8061	scaffold5173	p2	(AC)7	Class II	8561	8574	GCGTTGAAAGAAAACTCCGGCGA	AGGAAGGTTCTGGGGAGGTAGT	347

SSR_ID	Scaffold	SSR_Type	SSR_Motif	SSR Class	SSR_Start	SSR_End	Forward sequence	Reverse sequence	Product_size
FMgSSR-8063	scaffold3104	p2	(AC)7	Class II	36119	36132	GGGCGTTGTCCACGTTACTGAT	ACGAGAGGTGTCCCCCACAAAA	327
FMgSSR-8064	scaffold14181	p2	(AC)7	Class II	18127	18140	GGTGCATCAATTGAAACTGGCACG	TGTTGGCGCAGAAGTTGGGT	286
FMgSSR-8065	scaffold589	p2	(AC)7	Class II	54298	54311	GTCACGGCCACCCATTTTAGCA	TGCCCATGACGTATCTGGGT	350
FMgSSR-8068	scaffold261	p2	(AC)7	Class II	127171	127184	TACAGCATCATCCATCGTCGGGGA	TCGCCGTGTTGTTAATGGAAGCG	333
FMgSSR-8070	scaffold169	p2	(AC)7	Class II	18344	18357	TATTGCAACGGCCGAGGGGATT	TGGCTTTGGGGTGCAAACAAGG	301
FMgSSR-8071	scaffold1523	p2	(AC)7	Class II	45410	45423	TCAAATATCTCGCCGCCGGAGT	TCGACGAACTTCCCTTCCTCTCGT	303
FMgSSR-8074	scaffold5117	p2	(AC)7	Class II	33950	33963	TCACCATGACGTTTTCTTCTCGGG	TGCAGAGAAGGATTCTCGAGCTGT	230
FMgSSR-8076	scaffold660	p2	(AC)7	Class II	97123	97136	TCACCGCTGACCCTGTTCCTTA	AGCATCCACCGCGTGACAAT	317
FMgSSR-8086	scaffold30	p2	(AC)7	Class II	232980	232993	TCCGTCGTCTCTACCTCCGATTCA	TTGTTGCTGCGACGGAGATCGT	334
FMgSSR-8088	scaffold9015	p2	(AC)7	Class II	10587	10600	TCCTCTTCTGTTCCAAGTCCCTCCT	TGTGTGTTTTGGGGTGGGGCTA	336
FMgSSR-8091	scaffold10502	p2	(AC)7	Class II	942	955	TCGAAACCAAGGGCCCGACAAA	TGTGTGCGGTTGGCACCCTATT	335
FMgSSR-8092	scaffold1382	p2	(AC)7	Class II	34704	34717	TCGAAGGTTGGCGTTGGCAT	TCGTCGGCTGATGACCTCAA	318
FMgSSR-8094	scaffold75284	p2	(AC)7	Class II	1438	1451	TCGACTGCCTGATGTTGCCA	TCAGTTCGGTTGCTGGGTCCAA	287
FMgSSR-8097	scaffold524	p2	(AC)7	Class II	82988	83001	TCGGCCCCACTGAAACGTCAAA	ATCGATTGCTACGCGCCCAT	202
FMgSSR-8100	scaffold390	p2	(AC)7	Class II	113642	113655	TCGTCTTTCTCTGTCTCGCCGT	ACTTGGCAGTGTCGTTGCGA	290
FMgSSR-8101	scaffold1353	p2	(AC)7	Class II	10204	10217	TCGTGCCGTGCAAACAAGGA	AGCGTGCAGCCACGTGTAGATT	336
FMgSSR-8102	scaffold1693	p2	(AC)7	Class II	75486	75499	TCTAGTGCCGCCACTCAACT	TGCAGGCAACACACACGCAT	347
FMgSSR-8106	scaffold74	p2	(AC)7	Class II	60162	60175	TCTTCGGTGCTCATGTCTGCCA	TCCACCCTAAGTACCAGGCCAA	344
FMgSSR-8115	scaffold356	p2	(AC)7	Class II	59708	59721	TGCACAACAAACCCCGACCGTA	TTGTCTTGATCTGCCGGCCACT	287
FMgSSR-8123	scaffold44981	p2	(AC)7	Class II	905	918	TGCCAAACTTTCGGTGCTTGC	TGCACCGATGGATCGCACAA	314
FMgSSR-8124	scaffold76948	p2	(AC)7	Class II	1042	1055	TGCCACCCACAATCAAGGCA	GCTGGTTTTGGTCGTTGGTCGT	305
FMgSSR-8128	scaffold1644	p2	(AC)7	Class II	20053	20066	TGCCCCTTTGGGTTCATCGTGT	TTGGGCACAAATGAGACCGGGA	322
FMgSSR-8129	scaffold13623	p2	(AC)7	Class II	12705	12718	TGCCCGCATCCATCTGTTTCCT	ACTGCAGCACAAGAGCAGTCGT	349
FMgSSR-8131	scaffold476	p2	(AC)7	Class II	73341	73354	TGCGGATTGCGGTGATTGTCCA	CGTAGCAGCGTACGGGCTTTTA	299
FMgSSR-8132	scaffold714	p2	(AC)7	Class II	59471	59484	TGCGGCAAATCACTCAGTCATGC	TACGCCCTGGTTCTGGTGCAAA	325
FMgSSR-8134	scaffold10813	p2	(AC)7	Class II	7881	7894	TGCGTCGAAACCAAAGGCCA	AAAGGGATTGCGACGCTCCTCA	350
FMgSSR-8135	scaffold11102	p2	(AC)7	Class II	26371	26384	TGCGTCTCGTGTGTCTCGAACT	TGGCTCCAAACGGCTTGTGT	347
FMgSSR-8138	scaffold5124	p2	(AC)7	Class II	35214	35227	TGCTGGTTCGACTATGGTGAAAGGG	ATCGGTGGAATGCAGGTCGGTT	222

SSR_ID	Scaffold	SSR_Type	SSR_Motif	SSR Class	SSR_Start	SSR_End	Forward sequence	Reverse sequence	Product_size
FMgSSR-8141	scaffold535	p2	(AC)7	Class II	94494	94507	TGGAAACCTAACCAGCGCCT	TCGGCACGCAGCAAGATGAA	298
FMgSSR-8144	scaffold2106	p2	(AC)7	Class II	18708	18721	TGGAGCGTGGTGACTTGCTTGT	TTCCCGCCAAAAGAACGAGGGA	339
FMgSSR-8147	scaffold3390	p2	(AC)7	Class II	9018	9031	TGGCATCGGTTCGTTTGGCT	TGTTGCAGCGAGTTGGTGCT	318
FMgSSR-8151	scaffold3761	p2	(AC)7	Class II	29022	29035	TGGGAAAGACGACATGCAAGCCA	TCAAGCCCAAACCTGCCCTTCA	256
FMgSSR-8155	scaffold13260	p2	(AC)7	Class II	10369	10382	TGGGCTTTGCGAACGCATACA	AGGCGCCAGGACGAGTTTTT	202
FMgSSR-8156	scaffold10564	p2	(AC)7	Class II	5661	5674	TGGGGTGCACCGTACCAATTCA	TTGCATCGGATCGGGTGACGTT	262
FMgSSR-8159	scaffold12926	p2	(AC)7	Class II	24072	24085	TGTCAGCGCCAATCTCAGCA	TCCTGTTCCAAGACGAGAACCT	349
FMgSSR-8160	scaffold1332	p2	(AC)7	Class II	59687	59700	TGTGCAACAAGGACACGAGGGA	ACGACACGCGACACGTCAAA	334
FMgSSR-8163	scaffold311	p2	(AC)7	Class II	38739	38752	TTCATCGAGCTTGTGGCTGGTG	ATGGCCGACCAGGACACATCTT	347
FMgSSR-8167	scaffold6000	p2	(AC)7	Class II	6841	6854	TTCTGCCGCGGGATCTTTGA	TGGCATTGAACAAGCGACATCCG	348
FMgSSR-8168	scaffold11519	p2	(AC)7	Class II	22224	22237	TTGCAACCACCGTCTCCCAA	TGGTGGTTTCAGGCTTTCGGCT	282
FMgSSR-8169	scaffold8161	p2	(AC)7	Class II	22136	22149	TTGCAGCATGATGGGTGTGGGT	GCTCTCTGATTTGGCTGTCATTCGC	230
FMgSSR-8170	scaffold114	p2	(AC)7	Class II	166146	166159	TTGCAGGCCCCACTTCCCATTT	TGGCCAGACTGACGCATGTTGT	299
FMgSSR-8172	scaffold10199	p2	(AC)7	Class II	21512	21525	TTGCCACATCCTGCGTGCAT	TTTGGTGTGGTCTCGTGCTCGT	309
FMgSSR-8174	scaffold3046	p2	(AC)7	Class II	35126	35139	TTGGACGTAACCGCATCGCA	TTGCGTGGCCATACAGACCACA	255
FMgSSR-8175	scaffold1286	p2	(AC)7	Class II	49017	49030	TTGTCACCGCCGCATTGGTT	ACACTCCGCGGCACGTATTACA	293
FMgSSR-8184	scaffold5961	p2	(AC)8	Class II	31079	31094	AACCGTGTGGCTGTTACGGGTT	ACGACTGGAGACTGAGGTACCAA	320
FMgSSR-8185	scaffold7758	p2	(AC)8	Class II	16196	16211	AACGTGAGACAAGGGGGCTCAA	ACAAGCTGCTCGTCGACCTCAT	249
FMgSSR-8191	scaffold2816	p2	(AC)8	Class II	11186	11201	AAGCGCGCATTGGACCACAA	AGCCATTTAGCAACGCGTCACC	286
FMgSSR-8192	scaffold21052	p2	(AC)8	Class II	8577	8592	AAGGAAACTGGAGGGCATGCGA	TTGTTGCGCGCTCGAGTAGGTA	259
FMgSSR-8194	scaffold29566	p2	(AC)8	Class II	6562	6577	AAGTGCACTGGTCTGCTGGT	TGGCAGTGCTGCTCATGATGT	272
FMgSSR-8195	scaffold4111	p2	(AC)8	Class II	38077	38092	AATAGCAACTGCACCGGAGCCT	GTTGTTGCTTGTCTGTGAGCCA	345
FMgSSR-8196	scaffold10058	p2	(AC)8	Class II	11587	11602	ACAAAGTGCGGAAGTGGCGA	AGGCGCGTAGGCATCTTCTT	226
FMgSSR-8197	scaffold9884	p2	(AC)8	Class II	17899	17914	ACAACGCGACAAAAGCGCAGT	CCTGATCATGCATCACCACACA	348
FMgSSR-8201	scaffold12474	p2	(AC)8	Class II	17960	17975	ACAGGCCACGGTTACAAAACGC	TGCGCGCTGCCAAGCAAAAA	348
FMgSSR-8202	scaffold11010	p2	(AC)8	Class II	24193	24208	ACATCGCGTTGGTATGACCGCT	TCTCGGTGCGGAAACCTGATGT	207
FMgSSR-8205	scaffold239986	p2	(AC)8	Class II	337	352	ACCAAAACAGGGCGCTTGCT	TCGCCTCACATTTGCCTCTCCT	241
FMgSSR-8208	scaffold2284	p2	(AC)8	Class II	42171	42186	ACCATGCATTGTTGGGTAGCGT	TGTCTTCCCTAACAGCACCCCT	335

SSR_ID	Scaffold	SSR_Type	SSR_Motif	SSR Class	SSR_Start	SSR_End	Forward sequence	Reverse sequence	Product_size
FMgSSR-8212	scaffold599	p2	(AC)8	Class II	24967	24982	ACCTCGTAGAACCCACGCTT	TTCCTTGCGATGCTCGTGCT	312
FMgSSR-8214	scaffold150	p2	(AC)8	Class II	61442	61457	ACCTGTGCTGCGAGTCGTAT	TGCTGCTGCTGCTGATGCTT	255
FMgSSR-8215	scaffold168	p2	(AC)8	Class II	63422	63437	ACGAACGCAACGGACATCGT	TGGGCAAGAAGGGCAAAGCA	302
FMgSSR-8221	scaffold491	p2	(AC)8	Class II	60213	60228	ACGCGAAGTCAAAGCGAACGA	TGGTACGTCCACAACAGGAGGA	224
FMgSSR-8222	scaffold12	p2	(AC)8	Class II	57474	57489	ACGCGCATGGACAAGGACAA	TGCATGCAACACAACTGGAGCG	310
FMgSSR-8225	scaffold294	p2	(AC)8	Class II	24146	24161	ACGCTTGTTCAGCGACGCAA	ATCGATCCACGGTAGGAGGTTGGA	317
FMgSSR-8226	scaffold2571	p2	(AC)8	Class II	40674	40689	ACGGCAACAAGCTCAAAGTCACG	AGCGCATGCATGTGGCTGAT	220
FMgSSR-8231	scaffold6812	p2	(AC)8	Class II	30059	30074	AGAGAGGCAGAGAGGTAAGCCA	TGTTGTCCTCCCCTGCTGAGTT	344
FMgSSR-8235	scaffold2523	p2	(AC)8	Class II	60117	60132	AGCCATGACTCTCGAGGCAACA	GCGTGCCTTACACTGCCACTTT	318
FMgSSR-8237	scaffold1879	p2	(AC)8	Class II	34690	34705	AGCCGGAATCAGCTACCAATGC	ACCCCACCGAGTAGGCAAAT	332
FMgSSR-8240	scaffold2439	p2	(AC)8	Class II	22758	22773	AGCTGTAGGAGGCACGCAAGAA	TCCACGCCTGCATGCATCACAT	344
FMgSSR-8242	scaffold1471	p2	(AC)8	Class II	70776	70791	AGCTTGTCGGACTGGTGGATGT	ACGGTTGTAGAAGCGTGTGACCT	348
FMgSSR-8243	scaffold61855	p2	(AC)8	Class II	317	332	AGGACGTCGTCTTGCTGTGAGT	TGTCAAGGAACGGGTCGGACAA	246
FMgSSR-8246	scaffold730	p2	(AC)8	Class II	95769	95784	AGGCATCACCATGCAGGCATCA	TTTGTTCCATCCGCGTCGCT	306
FMgSSR-8247	scaffold211716	p2	(AC)8	Class II	128	143	AGGGACGACGGTCTCCTTTTTGT	TTTGAGCCTCGGCTCCTGTGAA	218
FMgSSR-8248	scaffold9704	p2	(AC)8	Class II	2083	2098	AGGTTGCAACTGGCCGGAAGAA	ACCAACAGGTGTGCAACGGA	339
FMgSSR-8254	scaffold48202	p2	(AC)8	Class II	3768	3783	ATCGTCGGCCGAGAATTGGTCT	ACGCAGACAGCACAACGCAT	349
FMgSSR-8255	scaffold2493	p2	(AC)8	Class II	36741	36756	ATGCGCTCGCGCTGAACTTA	TGGTGTACTGCCTCCATTCAAGTG	347
FMgSSR-8256	scaffold599	p2	(AC)8	Class II	108694	108709	ATGCGGCCTTTCCAATCTTGCG	AGATGGCAGCGGTCAGTCATCA	292
FMgSSR-8257	scaffold1802	p2	(AC)8	Class II	26519	26534	ATGCTGCATGGTTGGCGTGT	ATCAGCAAAGGCATGGCGTCCT	330
FMgSSR-8258	scaffold1769	p2	(AC)8	Class II	75490	75505	ATGGCGCGATGGCTGGTAACTA	ACCCGCCTATACGTGCTGCATT	239
FMgSSR-8259	scaffold71837	p2	(AC)8	Class II	1476	1491	ATGGGCGACCACATCCATATCTGC	TCTGGTGCTCCAAGCATCCATT	291
FMgSSR-8260	scaffold3999	p2	(AC)8	Class II	33713	33728	ATTCCGACAACACTGCGTCGCT	TATCCGCCTTCGTATGGGCA	341
FMgSSR-8261	scaffold3837	p2	(AC)8	Class II	8262	8277	ATTGAGCCCCATCGCAACAGGT	ATATGCGATTGCTGCTGCTGGC	305
FMgSSR-8263	scaffold530	p2	(AC)8	Class II	51358	51373	CAACACCACAAACCCCAACACA	TGGACACCAAGGAAGCTAGTTGAGG	333
FMgSSR-8265	scaffold1621	p2	(AC)8	Class II	29573	29588	CCGCGGTCAAATCAACTGGCAA	AATGGAACACACATGTCCCCGC	311
FMgSSR-8266	scaffold14260	p2	(AC)8	Class II	21902	21917	CGACCATCATGCATATGCGGTTAC	ACTCTGGGTTGATCGCTTGAGGT	310
FMgSSR-8272	scaffold14680	p2	(AC)8	Class II	4482	4497	CTGGTAGCCTGTAAAAAGCCTGTG	ACCGCTTCTCTGATTCCTTGCG	238

SSR_ID	Scaffold	SSR_Type	SSR_Motif	SSR Class	SSR_Start	SSR_End	Forward sequence	Reverse sequence	Product_size
FMgSSR-8274	scaffold2690	p2	(AC)8	Class II	26252	26267	GCGCACACACTTTTCGTTTCCG	AATCACACGCGCACAGCGAT	265
FMgSSR-8275	scaffold8247	p2	(AC)8	Class II	11792	11807	GCGCGCCGCTATAAATTGAC	TGCGCAATGGGATGGTGCTT	264
FMgSSR-8278	scaffold2622	p2	(AC)8	Class II	23260	23275	TAACGCCATTCACAGCTCGCCT	GGTGATGGATGACAAAGCACCCCT	314
FMgSSR-8282	scaffold460	p2	(AC)8	Class II	90720	90735	TCAACCCACCCGGCTCAAAT	GCTGGTGAATCCAACATTCCACCT	307
FMgSSR-8285	scaffold1	p2	(AC)8	Class II	118272	118287	TCACGCGTGCAAGTTGTGGA	AGCTTCGGCCGGCAGTGTTATT	314
FMgSSR-8289	scaffold137809	p2	(AC)8	Class II	806	821	TCAGGTCAAGCACATCCACGCT	TGAGGTCGGTGCTTGCCAAA	261
FMgSSR-8292	scaffold934	p2	(AC)8	Class II	98919	98934	TCATGCGCCTAAGAGCGGAACA	TGCAACACGCTGAGACGGAA	268
FMgSSR-8297	scaffold760	p2	(AC)8	Class II	107188	107203	TCCTGCCTGTGATCGTGTGA	TTTCCTGGCCTGCTGGACACTT	222
FMgSSR-8300	scaffold9525	p2	(AC)8	Class II	18789	18804	TCGACACCAAGTCGACGTAGCA	TCCAGCTTCCTTGCTGCTGA	282
FMgSSR-8301	scaffold650	p2	(AC)8	Class II	105838	105853	TCGCCGCTATCGTCATGTCCTT	TACGATGGGAAAGAAGCGGCCA	303
FMgSSR-8305	scaffold1928	p2	(AC)8	Class II	8378	8393	TCGGCCATCGTGGCTAAGAA	AATGCCGGGCTGGGTCAAGTAA	338
FMgSSR-8314	scaffold330049	p2	(AC)8	Class II	272	287	TGCAGCAACGCACACGAGAA	TGGGTGGTAACATAGGCAGCACA	342
FMgSSR-8315	scaffold9114	p2	(AC)8	Class II	1052	1067	TGCAGGCAATGGCGAAGTCA	TTGCTGCTGCATCCCACAGGTT	202
FMgSSR-8316	scaffold1376	p2	(AC)8	Class II	83801	83816	TGCATCACTGACAGGGTGGGAT	TGTGGCTTGTGCATGGAACTGC	346
FMgSSR-8317	scaffold2467	p2	(AC)8	Class II	43136	43151	TGCCACAACAACCCTGCACA	TCCGGGCTGTCAAACCTGAA	328
FMgSSR-8318	scaffold51169	p2	(AC)8	Class II	727	742	TGCCACACATCTGTTGACCTGT	AGGCAAGCCTGTTGCGATGACT	345
FMgSSR-8325	scaffold5901	p2	(AC)8	Class II	21907	21922	TGCGGTGCGGGCACAGTAATAA	TCCCAACGTGTCAACGGTCTCT	305
FMgSSR-8334	scaffold4986	p2	(AC)8	Class II	8940	8955	TGGGCAATGTAATGTCTGACCCGT	TTCACATTTCGGCCAGGACGCT	246
FMgSSR-8336	scaffold20820	p2	(AC)8	Class II	9469	9484	TGGTATCATGTGCCCTGAGCGA	AACGCAGCGAGTCCGGTGTTAT	216
FMgSSR-8342	scaffold586	p2	(AC)8	Class II	32790	32805	TGTCATGGTACGCAAGTGTAGCCC	GTGCGACAAAGCTAGCAACAACC	287
FMgSSR-8345	scaffold6953	p2	(AC)8	Class II	11807	11822	TGTGCTGGCTTGGGTTGTGT	AACATGGCTGGGCATTGTGCCT	256
FMgSSR-8346	scaffold30960	p2	(AC)8	Class II	3012	3027	TGTGGTGCCAATTTTCACGCGG	TTGTTCTTGGCAGACGCGTGGT	319
FMgSSR-8349	scaffold8401	p2	(AC)8	Class II	20993	21008	TTAGAAGCGCTCGTCTTCCTCTGC	ATGGCGCGCGTGAAGAAAGA	292
FMgSSR-8353	scaffold536	p2	(AC)8	Class II	49365	49380	TTCGCGGTACCCCATGATGA	TGGAAACCAGAATGCTGCGGTG	337
FMgSSR-8362	scaffold5735	p2	(AC)8	Class II	19158	19173	TTGCGTTGCGTGCGAGTAGT	TGACCTTGGGTCAGCTAGCAA	220
FMgSSR-8363	scaffold500	p2	(AC)8	Class II	87059	87074	TTGGCTGGATGACGGGACGAAA	ACCCTTCCCATCTGCACGGAAA	345
FMgSSR-8365	scaffold164	p2	(AC)8	Class II	36953	36968	TTTCAGCAGCCACACACCCACA	AGTGGCAGTGGCAAACAAGCA	285
FMgSSR-8368	scaffold64	p2	(AC)8	Class II	169047	169062	TTTCCCGAGAACGTGACTGCCT	AGCACTCTGTCACGCTAGTGTTC	222

SSR_ID	Scaffold	SSR_Type	SSR_Motif	SSR Class	SSR_Start	SSR_End	Forward sequence	Reverse sequence	Product_size
FMgSSR-8370	scaffold13869	p2	(AC)8	Class II	12436	12451	TTTCTCGCTACGCCGCTGTT	TTGCACTTGCACGCAGGAGA	301
FMgSSR-8371	scaffold358	p2	(AC)8	Class II	120483	120498	TTTGAGCAATCGCCCCCTTAGC	GCAAGCACTTGTCGACGACGTTA	217
FMgSSR-8372	scaffold104	p2	(AC)8	Class II	96821	96836	TTTGGCAGTAACTCGGCCTGCT	TGCTTGCAGGGATGCAGGTGTT	312
FMgSSR-8373	scaffold11728	p2	(AC)8	Class II	15043	15058	TTTTCGACGGCATGCTTGTGGC	CGTGTGTGCCGTCGTCAATGTT	346
FMgSSR-8377	scaffold24	p2	(AC)9	Class II	142542	142559	AACGCCCACACCTGTTACGGTT	TGCAAAAAGAGGAGCGAGAGC	308
FMgSSR-8381	scaffold588	p2	(AC)9	Class II	75413	75430	ACACGAGCTTGGTGTGTCACCT	TCACCTTTGGCCGCATGCTT	276
FMgSSR-8383	scaffold129	p2	(AC)9	Class II	15935	15952	ACAGGTGATCCTCACGCACGAA	ACCCGAAAGCAACGGCCAAT	263
FMgSSR-8390	scaffold507	p2	(AC)9	Class II	9723	9740	ACGCAATGAACCACCTCAGCCT	TGGTGCTTCGAACTCGCATGA	268
FMgSSR-8393	scaffold102	p2	(AC)9	Class II	3945	3962	ACTGCAGGCGCCAAGTGATT	TCCGGTTCTTTCGTCGGTCTGA	338
FMgSSR-8394	scaffold385	p2	(AC)9	Class II	31841	31858	ACTGCGTCGTGTGTTGTGCT	TGGTTCAGTGTCTGGCGACGTT	287
FMgSSR-8395	scaffold1406	p2	(AC)9	Class II	44857	44874	ACTTGCACGCCTCATTGGCT	TGGGCTGGGTTGTTGTTGAGCA	232
FMgSSR-8396	scaffold3242	p2	(AC)9	Class II	55268	55285	AGAAAGCCGACGGTTGCACT	ATCGCCAACATCCTCGCTGT	295
FMgSSR-8398	scaffold7413	p2	(AC)9	Class II	19215	19232	AGACGTGCAATGAGGCAGCAA	TGTCCTCGTCACACATCGGA	334
FMgSSR-8400	scaffold1090	p2	(AC)9	Class II	30240	30257	AGCACTTCTCCACCAACGGCAT	TTTAGCGCACACACCGCACA	285
FMgSSR-8402	scaffold2192	p2	(AC)9	Class II	40555	40572	AGCGTGAGCAACGGTTACATGC	ACCATGCGTGGGTGATCCAAGA	314
FMgSSR-8403	scaffold53318	p2	(AC)9	Class II	2601	2618	AGCTGACACAGTTCTTCGCGCT	GGTTACCTTTCTCATGGCTTGCTC	305
FMgSSR-8404	scaffold7768	p2	(AC)9	Class II	24308	24325	AGCTTCGGCATGCTGACCTGTT	TGCTTTGCAGCGCTTTCTGCT	271
FMgSSR-8407	scaffold1808	p2	(AC)9	Class II	37704	37721	AGGGGTGAGGGAGCAGATTTCA	ACGGGTCAAAACAGCAGCCA	318
FMgSSR-8408	scaffold1258	p2	(AC)9	Class II	47414	47431	ATACACACACCGTCGACAGCCA	TTGACGTGGTGTAAGCCGCA	321
FMgSSR-8409	scaffold1124	p2	(AC)9	Class II	11396	11413	ATCGGTCCTGCAAGGGCTTCTT	TTGGGTTGGGTTCGGTGCAA	280
FMgSSR-8410	scaffold7184	p2	(AC)9	Class II	34026	34043	ATCTAGCGCAAATGGACCGTGG	ATGCATGCGTGCGTAACTGC	268
FMgSSR-8412	scaffold2493	p2	(AC)9	Class II	36868	36885	CACTTGAATGGAGGCAGTACACCA	TAAGCCAACGGATCGAGCAGCA	217
FMgSSR-8413	scaffold2903	p2	(AC)9	Class II	30685	30702	CCACGCGCGCACTTGATACAAA	TGGTCGTGCTGATCCTGCCAAA	333
FMgSSR-8415	scaffold2084	p2	(AC)9	Class II	51898	51915	CCCTTGGTATGGCATCTTCCAGCA	ACTTCTGCTCTGTGGGGTAAGGGT	347
FMgSSR-8416	scaffold87284	p2	(AC)9	Class II	162	179	CGCTCACTGCTGCAAAGCCTTA	AGAACGCTCTTCTGGGCGTA	321
FMgSSR-8417	scaffold14	p2	(AC)9	Class II	132796	132813	CGTACGCGTGGAACGGAACAAA	TCGTCCATCTCTGCGCTGCATT	230
FMgSSR-8418	scaffold13817	p2	(AC)9	Class II	15137	15154	CGTAGTACGTGAAGTCCTCTGGT	ACGCAGTCCACATACTGTGCT	311
FMgSSR-8419	scaffold3489	p2	(AC)9	Class II	53279	53296	CGTCGGGTCAAAATTACTCGCAACG	ACACCATGGCACCATCCTCCAA	236

SSR_ID	Scaffold	SSR_Type	SSR_Motif	SSR Class	SSR_Start	SSR_End	Forward sequence	Reverse sequence	Product_size
FMgSSR-8421	scaffold1233	p2	(AC)9	Class II	17527	17544	GCGCTGTGCTGCTGGATCAAAA	TGGCCTGTCCACATGCATCA	234
FMgSSR-8422	scaffold713	p2	(AC)9	Class II	11037	11054	GCTTGTTTGTTCTGCATCGTGCCA	TCACGGACATAAAGGATGGGCACC	319
FMgSSR-8423	scaffold4259	p2	(AC)9	Class II	3637	3654	GGAGTTGCTGGTTACCAATCTCCA	ACCATGGCCTTCATCATTTCAGGC	313
FMgSSR-8428	scaffold131	p2	(AC)9	Class II	41334	41351	TCACTAACGTGTGGGAGCGCAT	AACTTTAGGCTGGCTGGACCGT	244
FMgSSR-8432	scaffold129351	p2	(AC)9	Class II	505	522	TCCACCTGCTTGACCTTCTGCT	ATCCCGCGTCCGAATACTGCAT	217
FMgSSR-8433	scaffold2172	p2	(AC)9	Class II	49575	49592	TCCAGCTCTCGTGATTTGCCCT	TGTGGCGTTGTGAGCAACAT	310
FMgSSR-8440	scaffold3810	p2	(AC)9	Class II	22990	23007	TCGCTTGACCAACACACGGA	ACGTGCCCCATCGATCAAAACCA	300
FMgSSR-8441	scaffold4879	p2	(AC)9	Class II	41963	41980	TCGCTTTCGTGCGTCGAGAT	TGTGTACGATTAATGCCGCCAGC	221
FMgSSR-8447	scaffold4396	p2	(AC)9	Class II	43851	43868	TGATTAACGCGCCACGCCAA	ACGCTCACTAACTGGGCTGCTA	348
FMgSSR-8449	scaffold10971	p2	(AC)9	Class II	933	950	TGCACTGCTGTGGCCAATGA	ACACGTCAACCAAGCCAGCA	296
FMgSSR-8450	scaffold777	p2	(AC)9	Class II	92564	92581	TGCCAGAGTGCACCGACATGTAT	ATCTCGTGCCTCGGGCTTGATT	348
FMgSSR-8453	scaffold242	p2	(AC)9	Class II	6348	6365	TGCCGTTTAATCAACCGCGAGC	TTGTCTATCGGCAGTCGGCAGT	280
FMgSSR-8454	scaffold5599	p2	(AC)9	Class II	39053	39070	TGCGCCATCGTGCACGTAAA	TGTTCTGTGCCTCGCCTGCTAT	341
FMgSSR-8458	scaffold24207	p2	(AC)9	Class II	5151	5168	TGCTGGCACACCACAGATGTTCA	GTCTGCTTGTTGCCAAAGTTGCAG	217
FMgSSR-8463	scaffold10188	p2	(AC)9	Class II	3422	3439	TGGTCGGTGTTGATCGACGCTT	ATTGGAAAGGCGGGGTGCAA	331
FMgSSR-8464	scaffold6237	p2	(AC)9	Class II	31745	31762	TGGTTAGCCTGTGACTGCGAGA	AGCCGAGGGAGCAAGGTTTT	349
FMgSSR-8466	scaffold6750	p2	(AC)9	Class II	24666	24683	TGTCAGGTCTTTGGCATCAGCGA	AGCATGCCAAACCGTCCGAA	262
FMgSSR-8469	scaffold2576	p2	(AC)9	Class II	5222	5239	TGTGCGACCCAAATGTGAGCA	TGTGGCATAGAACCAAGGCGA	314
FMgSSR-8471	scaffold122	p2	(AC)9	Class II	46978	46995	TTGCCTCGGTCCCTTTGTGA	AGGGCCACCATACAAGCCACTA	349
FMgSSR-8475	scaffold55031	p2	(AC)9	Class II	708	725	TTGTGTGCCCCTTTGCGCTT	TGCTGCAGAAGAAGGGCATGTG	320
FMgSSR-8477	scaffold2380	p2	(AC)9	Class II	59933	59950	TTTCCTCTCCGTTGGTCATTGGGC	TGCTGCAGCTTGGTTGGCACTA	311
FMgSSR-8478	scaffold7930	p2	(AC)9	Class II	30864	30881	TTTTGGCGCCGTTCGTGGTA	ATAACCCCCACGATCCACGCAT	342
FMgSSR-8479	scaffold16535	p2	(AG)10	Class I	11918	11937	AAAACGGGTAGGCGCTGCTCAT	TACCGCACCACACCACACTA	266
FMgSSR-8480	scaffold19967	p2	(AG)10	Class I	10705	10724	AAAATGGGGCCACGAGGACT	GCATGGAATTGGTTCGCTTGGCT	285
FMgSSR-8482	scaffold11602	p2	(AG)10	Class I	4415	4434	AAATCGAGCGTCCTGTGGCTCA	TGCAGCGAATTCCAGCCCAA	323
FMgSSR-8485	scaffold1999	p2	(AG)10	Class I	39429	39448	AACAAAAGGAGGGGAAGCGCGA	TTCCGCTTCCATCCCAACGA	214
FMgSSR-8486	scaffold5888	p2	(AG)10	Class I	35273	35292	AACAGGCAAGATGCACACCCCT	TGACAGGCGCAGAACGTCAGAA	311
FMgSSR-8490	scaffold15708	p2	(AG)10	Class I	823	842	AACCAAGTCTGTGCGCCTCA	ACACCTTACGCACGCATTGGT	332

SSR_ID	Scaffold	SSR_Type	SSR_Motif	SSR Class	SSR_Start	SSR_End	Forward sequence	Reverse sequence	Product_size
FMgSSR-8491	scaffold509	p2	(AG)10	Class I	18164	18183	AACGACAGCCCTTTTCGTCCGT	TTGGGCACTGTTTGGTCGCA	317
FMgSSR-8492	scaffold1163	p2	(AG)10	Class I	34532	34551	AACGACGTCATCGAAGCAGGGT	TGTGGAGGTCACTCCATCCCCTTT	323
FMgSSR-8498	scaffold25200	p2	(AG)10	Class I	3814	3833	AAGATGGCGGCGGACGAAGAAT	ATGCACGAAGCTACGTCAGAGC	291
FMgSSR-8499	scaffold1265	p2	(AG)10	Class I	32867	32886	AAGCCAAATGACGCGGAAGGGA	ACATTTGGTCGCCACAACGGAC	250
FMgSSR-8503	scaffold52289	p2	(AG)10	Class I	553	572	AAGGGCCGACCACCGTGAAAAA	AAAACGCAGATTTGCGGGCG	346
FMgSSR-8504	scaffold13215	p2	(AG)10	Class I	1015	1034	AAGGGCGAAAGCGAAAGGCT	ACGAACGGGAAAGGCACGATGT	258
FMgSSR-8508	scaffold2001	p2	(AG)10	Class I	55018	55037	AAGTTTTAGGCGGAAGGCGTGG	TTCAGACGCCGCGCATAGAGAA	243
FMgSSR-8512	scaffold282509	p2	(AG)10	Class I	222	241	AATTCCCTGCTCCGCAGCTACT	AGCGGGATGAAGATCCAGTGCAA	323
FMgSSR-8515	scaffold2229	p2	(AG)10	Class I	11503	11522	ACACCTAGCGAAGCAAGCCT	TCGCTAGGAGGAGGAGGACCTATT	347
FMgSSR-8518	scaffold101771	p2	(AG)10	Class I	826	845	ACAGGTGATCGTTGTGCACGGA	TAGCGGCCAGCTGTCTTTTGCT	293
FMgSSR-8520	scaffold10977	p2	(AG)10	Class I	8875	8894	ACCAACCAGGGCCAGTGCTTTT	ACGCCACGAGCACAACAAGT	255
FMgSSR-8522	scaffold9867	p2	(AG)10	Class I	13256	13275	ACCACCTCGCATTGGCCAAGAT	AAGACACCTCGTCGTCATGCCA	246
FMgSSR-8525	scaffold20695	p2	(AG)10	Class I	10425	10444	ACCAGCACAGCAGCATGTAGTGA	ACCCCGCACTGCTCACATTGAA	297
FMgSSR-8526	scaffold2456	p2	(AG)10	Class I	11676	11695	ACCAGCAGCACACACGTTCA	TTTCGAGCGACTGGTACAGGGT	312
FMgSSR-8532	scaffold894	p2	(AG)10	Class I	16441	16460	ACCCGTTGCCGTTGGTTCTT	ACGGATGAGTGAATGAGGCGCA	287
FMgSSR-8538	scaffold2468	p2	(AG)10	Class I	42749	42768	ACGAACTGGCGATGAGAGCA	TCGAATCGAGTCATCTCGCCCT	342
FMgSSR-8540	scaffold2053	p2	(AG)10	Class I	65035	65054	ACGAGTGCGTGGCCCCTTAAAT	AGGAAAGGGAGCTCAGCGTTGT	325
FMgSSR-8541	scaffold129	p2	(AG)10	Class I	160635	160654	ACGCAGCAGGCTGCAAACAA	AACAACGCGCACCGATTCGT	331
FMgSSR-8546	scaffold857	p2	(AG)10	Class I	11631	11650	ACGTGAGGATCCATGAGCGAGA	CGTCGTGTGCGTGCAAAGTT	325
FMgSSR-8547	scaffold714	p2	(AG)10	Class I	56634	56653	ACGTGCTACACGTTGGTTGGA	TACCGGCAGTGTACGCAGCATT	206
FMgSSR-8550	scaffold920	p2	(AG)10	Class I	48016	48035	ACTCACGGTGCCACTTCCAA	ATGGTGTGCTCTCCCTCTCTCA	305
FMgSSR-8564	scaffold92383	p2	(AG)10	Class I	936	955	AGAGGAAGACAAGTCGTCGCCA	TGAGATCCAAACGCCATCACCG	292
FMgSSR-8566	scaffold5913	p2	(AG)10	Class I	11193	11212	AGCAAGCGAAGCGAAGCGAA	ACAGTCCCAGCACAAGCAAGCA	298
FMgSSR-8568	scaffold10151	p2	(AG)10	Class I	23323	23342	AGCAGTGGTTGTGACCGTCCAT	ATTTTGCCCCCAAGTGCTGC	260
FMgSSR-8569	scaffold3972	p2	(AG)10	Class I	11282	11301	AGCATCAGGATGGTCAGGAGGA	TTTGCACCGGCAGCGTTTTT	239
FMgSSR-8571	scaffold13521	p2	(AG)10	Class I	5231	5250	AGCATGGCTGAGGATATCTGGACC	ACCAACGAACCAGGGAGCATGA	271
FMgSSR-8584	scaffold1575	p2	(AG)10	Class I	45094	45113	AGGCTCGGGATTTTCCCCTT	GCTGCGTGCGTGACAACAAA	251
FMgSSR-8586	scaffold1931	p2	(AG)10	Class I	67232	67251	AGGGCAGTAGCGGGCATTCAAA	ACCGTGTGCATCAGACAACCCA	231

SSR_ID	Scaffold	SSR_Type	SSR_Motif	SSR Class	SSR_Start	SSR_End	Forward sequence	Reverse sequence	Product_size
FMgSSR-8591	scaffold1266	p2	(AG)10	Class I	30631	30650	AGTCAACGACAAGCCGACGA	ACCAGCAAACCAGCCGTCTCTT	342
FMgSSR-8592	scaffold156	p2	(AG)10	Class I	3227	3246	AGTCCCGGCAATCTTGCCATTG	AGGCGTGTTGTCCTGTGGTCAT	211
FMgSSR-8596	scaffold535	p2	(AG)10	Class I	93528	93547	AGTTGCCGTTGGTGCCATCT	TTAGCGACGCCCATGCGTTT	228
FMgSSR-8607	scaffold4917	p2	(AG)10	Class I	40310	40329	ATGCGTACAGGGAACGGGAGAA	TGAAAGAAGCAGGTCAGCAGC	340
FMgSSR-8609	scaffold989	p2	(AG)10	Class I	60861	60880	ATGCGTGGGCGAGTCTGTGAAA	TGAAACCAGCGCCCAGCAAT	314
FMgSSR-8610	scaffold12021	p2	(AG)10	Class I	8516	8535	ATGGACGCGCCATTGGGAAA	AACGGATCACACACACAGCCCA	262
FMgSSR-8612	scaffold2319	p2	(AG)10	Class I	32493	32512	ATTACCCAAGCCATCGGCCA	GCATGCGAGGAGGCATTGTCAT	304
FMgSSR-8613	scaffold6476	p2	(AG)10	Class I	33104	33123	ATTCGCGCACGAGGCCATCATT	TTTCTCTTGCTCTCTGGCGACG	200
FMgSSR-8615	scaffold3550	p2	(AG)10	Class I	1835	1854	ATTGCGTTCCCTCGACGGTGTT	TCCCGCAAGTTGATGCTGGT	221
FMgSSR-8617	scaffold357	p2	(AG)10	Class I	45492	45511	ATTGGTGTGTGCGTGGATGGGA	TGCTTGCCTGCTCTGCCAAA	217
FMgSSR-8618	scaffold12691	p2	(AG)10	Class I	5880	5899	ATTTGTGCTCGTGCGCTGCT	AACCCTAACCGGTGTCCCTCAA	343
FMgSSR-8619	scaffold3121	p2	(AG)10	Class I	8232	8251	CACGCATCTAATTGCCGCACCA	AGTGGCAAGGTTGGCATTAACCG	269
FMgSSR-8623	scaffold5797	p2	(AG)10	Class I	21461	21480	CCAGATTCTGTTTGCCGTTGGACC	TCCACTTTTCCGTGGGGGTT	260
FMgSSR-8626	scaffold10332	p2	(AG)10	Class I	5945	5964	CCTACAAGGTGCCAAGATCAAACG	AGAGATGTGCACTCGCCTGT	256
FMgSSR-8628	scaffold5348	p2	(AG)10	Class I	21990	22009	CGAAGTTGCACCACGTAACCGA	AGCATCGCGCACGAAAACCA	290
FMgSSR-8632	scaffold2950	p2	(AG)10	Class I	26469	26488	CGGCTGCATGGTTTCTGTTGGT	GCTCGTTTGTCGTCTGGCTGTT	322
FMgSSR-8633	scaffold3958	p2	(AG)10	Class I	10221	10240	CGGGCTCCATTAATCGCAAATCGG	GCAAGTGATGCAAACGTCACGA	221
FMgSSR-8636	scaffold19678	p2	(AG)10	Class I	1588	1607	CGTGAATGAACAGGTTAGGCGT	AAACAACGCGCAACGCAAGC	254
FMgSSR-8640	scaffold20	p2	(AG)10	Class I	134016	134035	GCAGCAGCCACGCTGCATTATT	GCGGCCATGGTTGTGTTTGA	298
FMgSSR-8642	scaffold1842	p2	(AG)10	Class I	27754	27773	GCCCGCCCAAATTCGTTTCGTT	TGTGGCCATGCCGATGATGA	323
FMgSSR-8643	scaffold226	p2	(AG)10	Class I	137864	137883	GCCCGTTGCAGTGCCGTAAAAA	ATTTGCCATCCCGGCTGAACCT	335
FMgSSR-8644	scaffold439	p2	(AG)10	Class I	84015	84034	GCCGAAGAGGAAGGCAAAAGCA	ACACGACGCGCGGGTAGAAATA	335
FMgSSR-8645	scaffold6860	p2	(AG)10	Class I	882	901	GCCGGATACGAAGGAAAGGAGT	AATAGACACGCGTGGCCTGCAA	336
FMgSSR-8647	scaffold4206	p2	(AG)10	Class I	25487	25506	GCGAAAGGATTCTGTCATCCCAAGG	GGCAACCAAAGCCCTTTGCACAT	318
FMgSSR-8650	scaffold387	p2	(AG)10	Class I	6967	6986	GGTTTGGCCGTTTGGGCTTTGT	TCATTCGACTGCCTCGCCTGTT	324
FMgSSR-8653	scaffold5966	p2	(AG)10	Class I	6554	6573	TAACCTTGTGCCAAACGGGCCT	TTCATGCCGCCGTGAACTACGA	242
FMgSSR-8654	scaffold12804	p2	(AG)10	Class I	3961	3980	TACCACTCACAGGATCCACCAC	GCCTGGCTTTAAAGGTGACGCA	346
FMgSSR-8657	scaffold17970	p2	(AG)10	Class I	2446	2465	TCAAAAGGCAACGGCACGCA	AGCTGCTTAGTTGCGAGAGCTGA	324

SSR_ID	Scaffold	SSR_Type	SSR_Motif	SSR Class	SSR_Start	SSR_End	Forward sequence	Reverse sequence	Product_size
FMgSSR-8659	scaffold29194	p2	(AG)10	Class I	5993	6012	TCAAGCAAGCCAGACATGGAGACC	ACACCAAGTGAATGGAGGCACC	272
FMgSSR-8660	scaffold1006	p2	(AG)10	Class I	33449	33468	TCACACAAACGAAGCCGCCA	ACCAGCTCCGTCGTGTCGTATT	208
FMgSSR-8662	scaffold1179	p2	(AG)10	Class I	47861	47880	TCACGGCTGACACAAGCAAAC	GGGCGTGCGTCACATTTCAGTT	347
FMgSSR-8670	scaffold7446	p2	(AG)10	Class I	24321	24340	TCCCAGGTGCACTGACCCAAAA	TGGAGATCGCACAGCGTCACAA	248
FMgSSR-8677	scaffold1028	p2	(AG)10	Class I	10836	10855	TCGAAGAGCCGGTCTTGTTTGGT	TGGCGTCGGAGGTTCACTAT	327
FMgSSR-8679	scaffold1582	p2	(AG)10	Class I	37542	37561	TCGATGACGAGGCGGCAAAA	AGCAGCCAGCAGCAACTTCA	295
FMgSSR-8680	scaffold5244	p2	(AG)10	Class I	16961	16980	TCGCCAGGTTTGACGAGCAA	GGTGGGTCTGCTGACTGCTGTAAT	278
FMgSSR-8681	scaffold3509	p2	(AG)10	Class I	35256	35275	TCGCCATCACCGTCATCTCA	AATGGACGGACGTGGCGAGAAA	200
FMgSSR-8691	scaffold1394	p2	(AG)10	Class I	74643	74662	TCGGCGTTGAGGGAAGCATT	AAGACGAGCACGTCATGGCACT	304
FMgSSR-8692	scaffold2326	p2	(AG)10	Class I	61272	61291	TCGGGAACGCGGGATTTTATCG	TGCATGCAAAGCGAAGCCGT	350
FMgSSR-8693	scaffold342081	p2	(AG)10	Class I	175	194	TCGGGCATCATGGGAGACGAAA	TTGCAGATGACCCACCGCATGT	243
FMgSSR-8694	scaffold212169	p2	(AG)10	Class I	324	343	TCGGTGACAATCATGCCTGCTG	GCAACCACTGCGCGCACTTAAA	284
FMgSSR-8696	scaffold3648	p2	(AG)10	Class I	10703	10722	TCGTCAGTGAAACTCCTAACCGGA	TACCCCATGCCTTGCTTTGGGA	275
FMgSSR-8700	scaffold2852	p2	(AG)10	Class I	46724	46743	TGACCCAACCGAAACCGCAA	TCCCCAATGCAAGGCTCCAA	278
FMgSSR-8701	scaffold736	p2	(AG)10	Class I	35080	35099	TGACGCGCGCAACACAACAA	TGCTCGTCGGCATTTCCCTCTT	326
FMgSSR-8704	scaffold2947	p2	(AG)10	Class I	12465	12484	TGATTTTCTGGCTCCGCCCT	AATTCAGCACGTTAGGCGCCAG	308
FMgSSR-8708	scaffold1352	p2	(AG)10	Class I	8995	9014	TGCACCGGAGAACCAAGCATGT	ACAGTCGCCCAGATTGTGCTCT	212
FMgSSR-8709	scaffold6077	p2	(AG)10	Class I	29035	29054	TGCACGCGTCAAAAACGTCG	AGGGCTTAGCCGAGCAACAACA	263
FMgSSR-8713	scaffold465	p2	(AG)10	Class I	102235	102254	TGCATGATGGCATTTGACGGCG	TCATTGCGGTGCAGGTATCCGA	319
FMgSSR-8716	scaffold1030	p2	(AG)10	Class I	75691	75710	TGCCAGGATCACGCAGTAGCAT	TAAGCTCGGGCGCAACACAA	314
FMgSSR-8720	scaffold3133	p2	(AG)10	Class I	41484	41503	TGCGCAGTGTGAATGGCTGA	AAAAGTGCTCAACCCACCCCCA	335
FMgSSR-8725	scaffold8410	p2	(AG)10	Class I	23921	23940	TGCGTCTGCTACAACCAGCA	GCTGGTTCAGCGCGGCAATAAT	294
FMgSSR-8727	scaffold12366	p2	(AG)10	Class I	20368	20387	TGCTCGTCGGTTTCTGGCTT	AGTTCGAGATTGGGTGTGGCGT	315
FMgSSR-8729	scaffold6040	p2	(AG)10	Class I	32999	33018	TGCTGCCTCGACTTCTGTCA	TCGTACAGCCAAGCTCGCAA	335
FMgSSR-8731	scaffold5991	p2	(AG)10	Class I	16153	16172	TGCTTCTGCTGTCAACTCTCTGGC	GCTATGTCCCTAACACAGGCGT	350
FMgSSR-8732	scaffold12681	p2	(AG)10	Class I	20548	20567	TGGAGGACACCAAACACGGTCA	TGCTGTCCTACGGTCATCGTCA	347
FMgSSR-8737	scaffold2486	p2	(AG)10	Class I	26568	26587	TGGCGAAATCCACCACTGCGTA	TTTCCGCAAAAGGACGCCGCTA	326
FMgSSR-8742	scaffold2573	p2	(AG)10	Class I	60748	60767	TGGCTTTCGTTGGTTTGCAGGG	TTGCTTCCCTCCCCATCTTCCA	293

SSR_ID	Scaffold	SSR_Type	SSR_Motif	SSR Class	SSR_Start	SSR_End	Forward sequence	Reverse sequence	Product_size
FMgSSR-8744	scaffold173	p2	(AG)10	Class I	157247	157266	TGGGTGGAAAGCTGGACACA	ATATGCCCAGACCGTCCTCGAT	317
FMgSSR-8748	scaffold1573	p2	(AG)10	Class I	41946	41965	TGGTCATGGAGACCTAGACAACAC	TGAGCGCCACCTAGTTTTCTGC	341
FMgSSR-8749	scaffold2006	p2	(AG)10	Class I	48978	48997	TGGTCCGCCGATCAGATGTTGA	TCCGTCTTGCATTGGTGCCCTT	334
FMgSSR-8753	scaffold2635	p2	(AG)10	Class I	7119	7138	TGTCCACGCTCGTTTGGTGA	TTGGGGCTTTCACGAGGCATGT	326
FMgSSR-8755	scaffold532	p2	(AG)10	Class I	17848	17867	TGTCTGCGAGAAACTCGTGGCA	ACACGGATTCGTTCAGGCTGGT	304
FMgSSR-8757	scaffold338	p2	(AG)10	Class I	2457	2476	TGTGCTCCTAGCGATGCAATGT	TGGCTCATGTTTAGCAGCTTCC	341
FMgSSR-8763	scaffold17958	p2	(AG)10	Class I	9223	9242	TGTGTGCTAGCTTGCATTGCCC	TTTTCCTGATCGCTGCCGACCT	341
FMgSSR-8764	scaffold14408	p2	(AG)10	Class I	11186	11205	TGTTAATCGGGTGCCTGCCT	CATTTTTGCCGGCGTGTGCT	260
FMgSSR-8766	scaffold4031	p2	(AG)10	Class I	55358	55377	TGTTGCCATGCAAGGTGCAGT	TTGCGACATGGGAGGGGAAA	250
FMgSSR-8769	scaffold417	p2	(AG)10	Class I	97843	97862	TGTTTGCACGAGCCAACCGT	GCTGATAAGTGTGGAAGACGGCCT	313
FMgSSR-8775	scaffold1816	p2	(AG)10	Class I	33693	33712	TTCCCCCAATCTTCGTGCTGCT	AACACCGCCGCCATTGTTGT	223
FMgSSR-8781	scaffold15049	p2	(AG)10	Class I	14311	14330	TTGCAAGAACCGAGGAGCCGAA	TGGGCTCTTTCGTGGGGACAAA	338
FMgSSR-8782	scaffold5325	p2	(AG)10	Class I	26320	26339	TTGCACCTGCCACCTCATCGTA	TCTGTCCCATGAAGCCCACGAA	230
FMgSSR-8783	scaffold6396	p2	(AG)10	Class I	35980	35999	TTGCAGCGGGAGGAAAGGTTCA	TCGCGTAATGGGCCTAATGGCA	251
FMgSSR-8784	scaffold15074	p2	(AG)10	Class I	14091	14110	TTGCAGTCACCCTCGTCCCAAA	AAAAGAAAACGTGCCCACCGGC	219
FMgSSR-8785	scaffold2623	p2	(AG)10	Class I	6325	6344	TTGCCACACGTGCACACAGA	GCTGCAGCTGAAGTTTGCTGGT	349
FMgSSR-8786	scaffold3398	p2	(AG)10	Class I	10561	10580	TTGCCGGTGCGCCAAAAGTT	ACAACAAACCCGCCAATGCCA	301
FMgSSR-8787	scaffold11907	p2	(AG)10	Class I	15125	15144	TTGCCGGTTAGGCTATGCCA	TGCTGCGCAAGACAGATGTGA	333
FMgSSR-8796	scaffold34707	p2	(AG)10	Class I	3768	3787	TTGTGCCACCACACTTCGCT	AGCTGCACACTCGCCAACAT	270
FMgSSR-8804	scaffold7682	p2	(AG)10	Class I	4126	4145	TTTGGTAGAGCGCAGCAGCA	GCCGCGCATGAAGAAGAAGCAA	310
FMgSSR-8805	scaffold21260	p2	(AG)10	Class I	6228	6247	TTTGTTGTCGGTGCTCCCTGCT	TGTGTCACCCGTGGGTTGCATT	267
FMgSSR-8806	scaffold9	p2	(AG)10	Class I	255407	255426	TTTTCACGGCAAAGGCGGAAGC	TAAACCAAACGTGCGTCGGGGA	287
FMgSSR-8811	scaffold8969	p2	(AG)11	Class I	2251	2272	AAATCGTCGTTCGTTGCGGC	TCGCATGCCGTGTGCGTTTT	292
FMgSSR-8812	scaffold31783	p2	(AG)11	Class I	4220	4241	AACATCGCACCGACGTGTCA	TTTGGCTTTGTTGCGACCACCG	284
FMgSSR-8813	scaffold13364	p2	(AG)11	Class I	7508	7529	AACCAGGGAGGGCGTCTTCTTT	TGCCATCACGTACACACGCA	221
FMgSSR-8815	scaffold2205	p2	(AG)11	Class I	38279	38300	AACCCTATCATGCCGCCGCTTT	AGACGTACAGGCTGCTGCAA	318
FMgSSR-8818	scaffold112	p2	(AG)11	Class I	37054	37075	AACGGCACGTTTTGCGTTCG	TTCGCCGGACATGCACACAA	213
FMgSSR-8819	scaffold10391	p2	(AG)11	Class I	15128	15149	AACGGCCGGATTGCAAGGTT	TTTGCACTGTGTTCGCCGTTCG	295

SSR_ID	Scaffold	SSR_Type	SSR_Motif	SSR Class	SSR_Start	SSR_End	Forward sequence	Reverse sequence	Product_size
FMgSSR-8824	scaffold894	p2	(AG)11	Class I	80530	80551	AAGCCCATTTATGGTGCCCCGA	ATTGCGTGCAAGCAGCAGGA	300
FMgSSR-8829	scaffold3023	p2	(AG)11	Class I	34798	34819	AATCCCACGCGCTCACATCACT	AAGTGAACTCCGTTCCGCCA	334
FMgSSR-8836	scaffold22659	p2	(AG)11	Class I	3918	3939	ACAATGTCAATGCGCAGGCGT	GCAGCGGATCAAGCAACACCAA	203
FMgSSR-8838	scaffold5079	p2	(AG)11	Class I	16315	16336	ACACCGTATCCCTGGTTGGCAT	TGCGTGCGCGTGTTTATTGCT	326
FMgSSR-8839	scaffold3108	p2	(AG)11	Class I	23873	23894	ACACTCGATCGCGCTGGAAA	TGGAAGCTGCATGGCATTTGGT	271
FMgSSR-8843	scaffold6559	p2	(AG)11	Class I	6421	6442	ACAGGCCTGAGCCTTTCCCATT	TGCATTTTGCGCTTCACGCAG	289
FMgSSR-8845	scaffold21483	p2	(AG)11	Class I	3106	3127	ACCAACGCTGCAACGCATCA	ACTGGCAGTCTCGTGTGCAA	208
FMgSSR-8848	scaffold58870	p2	(AG)11	Class I	2402	2423	ACCCTTACACATCGACCACGCT	ACGACGAGCCCCACTTGAACTT	340
FMgSSR-8851	scaffold3097	p2	(AG)11	Class I	54068	54089	ACCGCAGACGCCGTTTTCAT	CAGTGCAGTGTTTCCTGTGCTGA	203
FMgSSR-8855	scaffold2992	p2	(AG)11	Class I	44709	44730	ACGACTGCCTGCCTACTACTGT	TGTGCTGCCAGTCAGCTGTT	327
FMgSSR-8858	scaffold1	p2	(AG)11	Class I	103480	103501	ACGCCAACACGCAGGGTGATTA	AACCGAAAGTAAGCCCCGCA	348
FMgSSR-8859	scaffold8692	p2	(AG)11	Class I	4457	4478	ACGCGTTGCGGACGCTTTAT	AGCGTTGTAGTCATAGCCCTGC	349
FMgSSR-8860	scaffold2351	p2	(AG)11	Class I	16789	16810	ACGCTGCGGAACATGCACAA	ACACATGCGTTGCCCGTACA	314
FMgSSR-8861	scaffold35689	p2	(AG)11	Class I	2162	2183	ACGTCGAAGCTGCCGTTGTT	CGCCATCGCCCGAAACATTGAA	319
FMgSSR-8880	scaffold3050	p2	(AG)11	Class I	17678	17699	AGCCGGCAAACACGAAAGCA	ACGACAGACGAGCGCATACACA	241
FMgSSR-8881	scaffold8649	p2	(AG)11	Class I	1150	1171	AGCCTCATGCTGCCCGTTTT	TGCCCATCTCCACGATTCCACT	303
FMgSSR-8891	scaffold4225	p2	(AG)11	Class I	16177	16198	AGGATGGCAACTTCTGCAGGCAT	TGAACCCCCTCTGCACATTGCT	316
FMgSSR-8893	scaffold3	p2	(AG)11	Class I	90846	90867	AGGCAGGACAGTTCACAGAACA	TCTTGCATGCTCCTGAAGCCCT	314
FMgSSR-8894	scaffold17377	p2	(AG)11	Class I	1898	1919	AGGCATGATGCATTATTGCGGCG	AACGGGCGGGAAAAACGGAA	241
FMgSSR-8897	scaffold530	p2	(AG)11	Class I	92705	92726	AGGCGCTTTACGAAGCCACA	TGGCTGTGGCTGCACGAACTAA	209
FMgSSR-8899	scaffold9522	p2	(AG)11	Class I	24648	24669	AGGGCAAGCTTGGGCATCCTTT	TCTTGTAGTGCGCAAGGAGGT	343
FMgSSR-8900	scaffold74046	p2	(AG)11	Class I	865	886	AGGGTATTGCTTCGCACTGACA	TGGCAGTGTCCCAATTCTTGCT	232
FMgSSR-8902	scaffold7630	p2	(AG)11	Class I	20306	20327	AGTAGCGCCCCCTCCAAAAA	TGTCGGTCCAACAGCAGTCTCA	321
FMgSSR-8905	scaffold12769	p2	(AG)11	Class I	5022	5043	AGTGACTAACACTCGGTGCCT	ACGCAATTGCATGGGCGTGA	344
FMgSSR-8907	scaffold340	p2	(AG)11	Class I	74032	74053	AGTGTGTTGCAGCAGATGGCA	AGCCTGGTGGCAACGTCTTT	278
FMgSSR-8910	scaffold670	p2	(AG)11	Class I	90525	90546	ATACACGCACGTGAAGGGCA	TCCGCCACTGTCTGACCATACT	265
FMgSSR-8912	scaffold26865	p2	(AG)11	Class I	2620	2641	ATCGGAGAAAAGGCGCCGAA	TTCTATAGCGGTGCAGCCCA	267
FMgSSR-8918	scaffold15764	p2	(AG)11	Class I	4150	4171	ATGCCGGACGGATTTGCTCCTT	ACAAGAGGCACAAGGTGTGCGA	219

SSR_ID	Scaffold	SSR_Type	SSR_Motif	SSR Class	SSR_Start	SSR_End	Forward sequence	Reverse sequence	Product_size
FMgSSR-8920	scaffold16274	p2	(AG)11	Class I	8193	8214	ATGGTCCGAGAAATGGCACCGT	TGGATTCGTGGTACACCTGTCG	347
FMgSSR-8924	scaffold104491	p2	(AG)11	Class I	1246	1267	ATTCGGAGCGTGAGACGTGA	TCATGTTTGCCGCCCTTGTGT	238
FMgSSR-8929	scaffold9158	p2	(AG)11	Class I	30923	30944	CAGCACAGGAAGCACAACAAGA	ATCAAAGGCACACTCGCCCA	264
FMgSSR-8939	scaffold299	p2	(AG)11	Class I	43086	43107	CTGTGTGGTTTCACTTGGCACA	AAGTGCAAAGCTGAGGCCCTGT	209
FMgSSR-8941	scaffold361	p2	(AG)11	Class I	76658	76679	GCAGCAGCGTCGATGGAAAT	ATGCACTACCCGTTCCCTTCGT	253
FMgSSR-8943	scaffold8798	p2	(AG)11	Class I	22305	22326	GCAGTGAACCGTGGCAGCAAAA	TGCCTGATGAACTTGGGCAGCA	343
FMgSSR-8944	scaffold10666	p2	(AG)11	Class I	5789	5810	GCCCGGCCGGTTCAGTTATTTT	TACTTACCACGGCTGCGTTGCT	343
FMgSSR-8953	scaffold15125	p2	(AG)11	Class I	6415	6436	GCTGTAAGCGATCACTGGTTCA	CTTACAGCTCTGGTGCGTTTCA	338
FMgSSR-8957	scaffold4970	p2	(AG)11	Class I	6508	6529	GTGCAACCGCACAAGAGCAT	GCAGTCTGCACAGAAGGACGAGAA	309
FMgSSR-8969	scaffold4868	p2	(AG)11	Class I	25640	25661	TCCACAGTTGGACCCTTCCCAA	TGCACAGGTCTGAACTTCAAGGGC	235
FMgSSR-8970	scaffold629	p2	(AG)11	Class I	17454	17475	TCCACCTCGGGTCGCAGAAAAA	TGGCTGCCGCATGCATTAGA	325
FMgSSR-8974	scaffold30719	p2	(AG)11	Class I	6743	6764	TCGAGGACCTTTGCATCCTGTGA	GGGCACTGATGACTGTTGACAAACG	341
FMgSSR-8976	scaffold3890	p2	(AG)11	Class I	50939	50960	TCGCAGGTGAACACGAAGGT	ACACAAGCGTTGGCAGAGCA	254
FMgSSR-8977	scaffold4936	p2	(AG)11	Class I	5165	5186	TCGCCAAGCAGCCGGATAATCA	AGTGCCGCCCATTTCAGTTCCA	218
FMgSSR-8979	scaffold5941	p2	(AG)11	Class I	16359	16380	TCGTGAGGTGGTTCAGTGGCAA	ACACCCCTGACTAAAGAGGGACT	344
FMgSSR-8981	scaffold2140	p2	(AG)11	Class I	21331	21352	TCTACGCCATCACGCAACTTGT	TCCAAACACAAGAGCCCGCTGA	262
FMgSSR-8983	scaffold3396	p2	(AG)11	Class I	9187	9208	TGAAGTAGAGAAGCAGAGCGAGGA	AACCCCATCCCTCAAGCCTCAA	313
FMgSSR-8984	scaffold889	p2	(AG)11	Class I	16975	16996	TGACGAAAGGGACGCGTTGA	ACTCGACGACGGCATTGCAT	344
FMgSSR-8987	scaffold3676	p2	(AG)11	Class I	11589	11610	TGCAACACGCAATGCCTCGAA	TGCACCAGACTGCATTGCCA	216
FMgSSR-8988	scaffold2344	p2	(AG)11	Class I	15744	15765	TGCAACTGCAGCACAGCACA	TGCTGGTGACCGGTGAAGAT	203
FMgSSR-8992	scaffold61297	p2	(AG)11	Class I	1914	1935	TGCACGGTTTGAGTTGTACGCA	TTGAAGCCCAGCTCACTCAGCA	253
FMgSSR-8993	scaffold659	p2	(AG)11	Class I	22474	22495	TGCAGGGCTGAAATTCCGCTT	AGCAACCGCCGCTTCAGAAA	254
FMgSSR-8994	scaffold14235	p2	(AG)11	Class I	18528	18549	TGCAGGGTGGCTGCTTACAACA	TGAGAGACATGAAACGGACGCTGT	307
FMgSSR-8995	scaffold12657	p2	(AG)11	Class I	7552	7573	TGCATCAGTGCGCGGTATGT	CGGTGGCCGGTTCAGCTATAAT	326
FMgSSR-9000	scaffold3689	p2	(AG)11	Class I	31195	31216	TGCCGTTTCCGGCAAAGCAT	AACGCGATGCGAGTGGCAAT	330
FMgSSR-9010	scaffold758	p2	(AG)11	Class I	7588	7609	TGCTGCTCGTCCAATCCTTGT	GCCTGTTTGAGGCCCATGTT	345
FMgSSR-9011	scaffold3992	p2	(AG)11	Class I	12194	12215	TGCTGCTGCTTTTCCACCACAC	ACCACCGGCGTGTCCTCTTTTT	223
FMgSSR-9012	scaffold4826	p2	(AG)11	Class I	21509	21530	TGCTGGTGGCGAACGAGTTT	TGCGCGCATAGCAAACCTGA	267

SSR_ID	Scaffold	SSR_Type	SSR_Motif	SSR Class	SSR_Start	SSR_End	Forward sequence	Reverse sequence	Product_size
FMgSSR-9013	scaffold8211	p2	(AG)11	Class I	22721	22742	TGCTGTGTCACGAGGGAGGAAA	AAGGCAGCCGACAATCAACAGG	266
FMgSSR-9014	scaffold19136	p2	(AG)11	Class I	1168	1189	TGCTTCTGCTGCAAGTCGTGGA	ATGCGTGGGCAAAGCGTGTT	293
FMgSSR-9015	scaffold5475	p2	(AG)11	Class I	27632	27653	TGCTTGCATGGCCCCTCTTCTT	AACAACGGCAATGGCGCATC	278
FMgSSR-9016	scaffold14138	p2	(AG)11	Class I	10692	10713	TGGAGAGCGACGTGTTCCTGTT	TGCGTGTTGCCAGGGAAAGT	321
FMgSSR-9021	scaffold17481	p2	(AG)11	Class I	3110	3131	TGGCGCAACTTTGTGCACCT	ACAGCATCGTCGTCAGGTCAGT	328
FMgSSR-9025	scaffold22319	p2	(AG)11	Class I	4927	4948	TGGTCGGGGTTGGCAATGTT	ATTCGGGCGTAGCACACCACAT	241
FMgSSR-9033	scaffold894	p2	(AG)11	Class I	50895	50916	TGTGCAAGCCCACACCATCT	TGCACAGGCAAAAACCTTCCCG	290
FMgSSR-9035	scaffold2759	p2	(AG)11	Class I	25435	25456	TGTGCGATCCACGTAGTGGTGT	TGCATCTGAGCTTACATGGCAGAG	216
FMgSSR-9041	scaffold4081	p2	(AG)11	Class I	43915	43936	TTAATTGCCTCCGGCGCTGT	TGCCCGGAGAATGGGGAATTATCG	303
FMgSSR-9042	scaffold3197	p2	(AG)11	Class I	14029	14050	TTACGCAGGCAAGGCAGCTT	TGTGGCGACGAGACTTTCACGTT	347
FMgSSR-9043	scaffold53085	p2	(AG)11	Class I	1863	1884	TTAGCGATCTTGCTGGCGCT	TTACACGGCCATGCGACGAA	336
FMgSSR-9044	scaffold8910	p2	(AG)11	Class I	27309	27330	TTAGCGGGGCATCAAGTCCA	AACTCCAATCGCTATCGCCGCT	280
FMgSSR-9048	scaffold12	p2	(AG)11	Class I	59389	59410	TTCACTGTGCTGTGCTCCAACG	AAGGCGAGCCGGATCATTTCGT	282
FMgSSR-9050	scaffold2136	p2	(AG)11	Class I	4842	4863	TTCGAGAGGAACGCGAAACCGT	TTCACACGCAGCGCATTGGT	211
FMgSSR-9054	scaffold6412	p2	(AG)11	Class I	5389	5410	TTGATGTCGGCGTGGCTTGT	ATTCTTGGCATGCCCGTGCT	237
FMgSSR-9057	scaffold1237	p2	(AG)11	Class I	50117	50138	TTGCATTGCGCAGGCCGATA	AGATCATGCACTCTCGCGGT	216
FMgSSR-9062	scaffold832	p2	(AG)11	Class I	51471	51492	TTGTGCCGGGCTAGTGCTAGTT	ACATCTCGGCTCCACCCAACAA	240
FMgSSR-9063	scaffold19447	p2	(AG)11	Class I	9257	9278	TTGTGTGGCCCTCCAATCCGTT	TGTTGGACACTTCAACACTGGG	273
FMgSSR-9064	scaffold7179	p2	(AG)11	Class I	39333	39354	TTTCCGCGTTTCGGGTTCGT	TCCAACGCAACGACGGTACTGA	310
FMgSSR-9069	scaffold4050	p2	(AG)12	Class I	27267	27290	AAAAGAACCACCACCTGCGGCT	ATCTTTCTCCGCTGCCTGCCAA	219
FMgSSR-9070	scaffold2474	p2	(AG)12	Class I	57488	57511	AAAAGGCGCCCCCAAGTACACA	ATTTGTGGGGAGGAAGAGGGGA	350
FMgSSR-9072	scaffold4327	p2	(AG)12	Class I	34955	34978	AACGTGCACATCAGCAGCGA	TGCGTCTGCCGGCACAAAAT	299
FMgSSR-9074	scaffold15586	p2	(AG)12	Class I	15502	15525	AAGCAGCAGCAGCTAGACCT	TGTTGACAATGCACAACCGGC	205
FMgSSR-9076	scaffold1096	p2	(AG)12	Class I	28486	28509	AAGGCGGCCAACGATGATGT	TGCAGGCCGATGTTCAGGTCAA	215
FMgSSR-9081	scaffold25191	p2	(AG)12	Class I	8612	8635	AATCAATGCGTCGTCCCCGTCA	ACCGAAAGTGCAGCCCACAA	227
FMgSSR-9082	scaffold9006	p2	(AG)12	Class I	18393	18416	AATCTGAAAGGAGCCGCCGTCA	TGCCCGATCTTCATGCACCA	259
FMgSSR-9085	scaffold2047	p2	(AG)12	Class I	7261	7284	ACACGCACTGCACACACTGA	TGTGATGCTGTTGTGGCATCGC	316
FMgSSR-9089	scaffold1950	p2	(AG)12	Class I	69666	69689	ACCGCTTCGGTGAACGCATTT	ATCGGCCGCATGCTCTGTTT	317

SSR_ID	Scaffold	SSR_Type	SSR_Motif	SSR Class	SSR_Start	SSR_End	Forward sequence	Reverse sequence	Product_size
FMgSSR-9100	scaffold6612	p2	(AG)12	Class I	17093	17116	ACGGCCGTACGTGTAAGCTTGT	GCCGATTTCCGGAATACACGCA	205
FMgSSR-9101	scaffold36	p2	(AG)12	Class I	219940	219963	ACGTCGGCGACTCAGCAAAT	CGTCACGCAATGCAAATCGCAC	227
FMgSSR-9111	scaffold1301	p2	(AG)12	Class I	26907	26930	AGCATACCTCAAGGCCACACGA	ACCAGCACACATGACTCACCT	309
FMgSSR-9113	scaffold471	p2	(AG)12	Class I	41427	41450	AGCCGAAGCGAAAAGCGTGT	TCCACGAACCAACCACTGCGAA	279
FMgSSR-9116	scaffold234451	p2	(AG)12	Class I	123	146	AGCGCCGGTAATGGTGATGT	AGGACTGCCGCCGCAAAATA	216
FMgSSR-9119	scaffold1263	p2	(AG)12	Class I	42031	42054	AGCTTGTGTGGCTTTGGCTGGT	TATTGATAGCTTCCCGCGCGTC	230
FMgSSR-9123	scaffold343	p2	(AG)12	Class I	76701	76724	AGGCTAAAACCAACGCAGGGGA	ACGCCTGCAACGACCACTTT	212
FMgSSR-9130	scaffold631	p2	(AG)12	Class I	84454	84477	AGTGAAGGAAGCCACCAAAGTG	TTTGTGACGTGGAGCTCGGA	344
FMgSSR-9132	scaffold14952	p2	(AG)12	Class I	14848	14871	AGTTCGTGTGCTGCTGGCAA	ACATCGCCACTTGCCCACTT	340
FMgSSR-9133	scaffold3737	p2	(AG)12	Class I	43223	43246	ATAGCTGAGGGAGTCGTGGCTA	TGATGTCAGCATGTCCGTCGCT	285
FMgSSR-9146	scaffold1735	p2	(AG)12	Class I	49979	50002	CCCCCTGCGTTTTCTCTTGGTT	ACTGCCCTCCTGCATCTGTCTA	309
FMgSSR-9149	scaffold7237	p2	(AG)12	Class I	13363	13386	CGGTGTTGATGCACACCCAAGA	TGACCGCTTGCTGGTGGTTGTA	350
FMgSSR-9150	scaffold55	p2	(AG)12	Class I	155348	155371	CGTGGCAACACACAGACACACA	AGCAGCAGCACTCGGGTTTT	346
FMgSSR-9151	scaffold10736	p2	(AG)12	Class I	21789	21812	CGTGTTGTTTCCGCTGATGCGT	GCTTTTCATCAGCCGTTGCCGT	334
FMgSSR-9152	scaffold4091	p2	(AG)12	Class I	19698	19721	CGTTGCGCCAGTTCACGTTCTT	AGCATCTGCAGCAGCCCAAT	249
FMgSSR-9153	scaffold14636	p2	(AG)12	Class I	13712	13735	GCAAAAAGGCGCCGTTGGTTT	AATAGCTTGCCGTGCGAGTGCT	314
FMgSSR-9154	scaffold396	p2	(AG)12	Class I	127368	127391	GCCAAAGTTGGGCTGAGCGAAT	ATGCACAAAGTACGCGTGAGGC	300
FMgSSR-9158	scaffold5704	p2	(AG)12	Class I	43435	43458	GCCGTGCCGTGCATTCAGAAAT	GGCAAAAGGCCGAACAGCGAAT	226
FMgSSR-9159	scaffold12999	p2	(AG)12	Class I	4417	4440	GCCTCAGGAACAGAAAAGGAAGCC	AAGCGTGCACTCTCCCTTCT	292
FMgSSR-9161	scaffold144	p2	(AG)12	Class I	137000	137023	GCTCTCTTCTGCAGGCACTACT	TTTCCAGTGCAACTGCGCGA	234
FMgSSR-9162	scaffold38453	p2	(AG)12	Class I	2861	2884	GGCATGCTTTGGCAAGGAAGGA	AGACCACGCCTTGACTGCAA	214
FMgSSR-9167	scaffold629	p2	(AG)12	Class I	3236	3259	TAGCCGGGGCAACCCATATT	AGCATCGGAGTGCGCTAGCTTT	331
FMgSSR-9171	scaffold12357	p2	(AG)12	Class I	11482	11505	TCACCTGCCAGACAAACCAGT	ATTTTTGGACGTGGGGCTCTGG	314
FMgSSR-9173	scaffold935	p2	(AG)12	Class I	32936	32959	TCACGCCTCAACCACAACACT	TTGTTGGTTGGTCAGCGCACCT	329
FMgSSR-9178	scaffold653	p2	(AG)12	Class I	64624	64647	TCCCAAGGAGTGGGCGCAAATA	AGCGCTTCAGGAAGCACACA	252
FMgSSR-9179	scaffold97	p2	(AG)12	Class I	77687	77710	TCCCATCGACGCTTGACAACGA	CCGCTTGTCTTCTCTCTCTTCGCA	323
FMgSSR-9182	scaffold102	p2	(AG)12	Class I	58003	58026	TCCTGCGACTCTCCCAATCGAA	CCGAACCGAGCAAAGCAAAGCA	338
FMgSSR-9183	scaffold857	p2	(AG)12	Class I	52609	52632	TCCTGCTCGCCTCAATCCTT	AGCGCCTTTGAGTTGCCTCCTT	321

SSR_ID	Scaffold	SSR_Type	SSR_Motif	SSR Class	SSR_Start	SSR_End	Forward sequence	Reverse sequence	Product_size
FMgSSR-9184	scaffold5410	p2	(AG)12	Class I	18430	18453	TCCTGCTTTGGCACGTCCTCTT	AAACTCTGCGTTCGCCAGCA	243
FMgSSR-9189	scaffold538	p2	(AG)12	Class I	14502	14525	TCTCTACGTTCACCGTGCGT	TGCGCTCCTTGCCGCTTTTT	341
FMgSSR-9190	scaffold1968	p2	(AG)12	Class I	40543	40566	TCTGAGTCGTGTTCCGATCGCT	TGGGGCTATTCCCTTTACGCGA	245
FMgSSR-9192	scaffold2441	p2	(AG)12	Class I	31851	31874	TGAACCCCGGAAGAGCTAAGCA	TTGTGGCATTCGCTTGGTCC	338
FMgSSR-9193	scaffold14809	p2	(AG)12	Class I	12272	12295	TGAACCTCATCCATGGTCCAGCA	TGCGTTTCGTGTCGTGCCAT	214
FMgSSR-9194	scaffold1401	p2	(AG)12	Class I	58924	58947	TGAATCCAACCGCCCCAACAGT	ACCAGAACAATAACACCGGCCT	328
FMgSSR-9198	scaffold948	p2	(AG)12	Class I	73578	73601	TGAGCGAGCTAAGCAAGCGACT	ATCGCAGACACGAGAATGGCGT	317
FMgSSR-9204	scaffold1010	p2	(AG)12	Class I	18805	18828	TGCCCAACCGCTTCAATGCAA	TGAAGATTCGCCGTTCGCGT	336
FMgSSR-9210	scaffold10058	p2	(AG)12	Class I	2734	2757	TGCGCCATAACTGTGACCTAGA	TTCTCCCCGGATGCCGTTTGAT	337
FMgSSR-9213	scaffold17771	p2	(AG)12	Class I	9231	9254	TGCGTGCCTAGATTGCACGTTG	AGGAGACGTGAGTTGGGCAT	241
FMgSSR-9216	scaffold2208	p2	(AG)12	Class I	52792	52815	TGGCATGGACAGGACGCTTT	TCCATTCCATCACGGCACGACT	334
FMgSSR-9217	scaffold20461	p2	(AG)12	Class I	15739	15762	TGGCCCCTGCTACACACAATCA	TGCCGGTGAGCAAATGGCTT	341
FMgSSR-9219	scaffold20540	p2	(AG)12	Class I	13709	13732	TGGGCTTGCACTGTAGCGTT	TAGCTCCTCCTTCCTCCTCTGA	321
FMgSSR-9222	scaffold25341	p2	(AG)12	Class I	10093	10116	TGGTGCAGAAGCAAAGCAAGCA	TGGCATCAGCTGCTCAAAGCTC	229
FMgSSR-9224	scaffold3395	p2	(AG)12	Class I	43659	43682	TGGTGGTGAGAGCCAAAAGGGT	AGCTTTCTCTGTTCGCGTCCGA	350
FMgSSR-9231	scaffold3242	p2	(AG)12	Class I	53470	53493	TGTGGTTGCAGTAAGATGCGCC	TGCTCCAAACACCCCCATTGCT	280
FMgSSR-9239	scaffold2071	p2	(AG)12	Class I	48087	48110	TGTTTGGGCGCGTATGCTGA	TTTGCGGGCATGCATAGTTGGC	228
FMgSSR-9240	scaffold1066	p2	(AG)12	Class I	32066	32089	TTAGTGGCGGAGGGCAAACT	AGGATGGTGTCTGTCCAGGCTT	256
FMgSSR-9242	scaffold1926	p2	(AG)12	Class I	449	472	TTCATGTCGGCGCGAAAGCA	TGTCGCTATCAGGGCTCCGTAA	280
FMgSSR-9247	scaffold196	p2	(AG)12	Class I	138818	138841	TTGCGTTGCAGCTGCTGTGT	AACCCTGCAACGAAGCTCGTCA	339
FMgSSR-9253	scaffold1005	p2	(AG)13	Class I	48407	48432	AAAGCATGGGTATGCCCCCT	CGGAGAAACATGCATTCGTGCCA	346
FMgSSR-9256	scaffold860	p2	(AG)13	Class I	48236	48261	AACCGACCACACAAACAGCAGG	AATCCTCACAACCGCCTCCGAA	348
FMgSSR-9262	scaffold5636	p2	(AG)13	Class I	14130	14155	AAGCAGCATTGGCAGCAGGT	AATTGTTCAGCGCTCACCTGGG	324
FMgSSR-9264	scaffold4903	p2	(AG)13	Class I	42671	42696	AAGGTTGACAGCCCGATTCCCT	ATCGTCCGGTCGTTTTCAGGCA	301
FMgSSR-9267	scaffold17	p2	(AG)13	Class I	13978	14003	AATTGTGAGCGGCGACAAAGCC	GGGCGCGAGCTTTGTGTTGATT	215
FMgSSR-9268	scaffold6890	p2	(AG)13	Class I	9581	9606	ACAAAATTTGGGCGCGGGTGT	TGGTGAGGCATGACAAGCAC	324
FMgSSR-9269	scaffold14992	p2	(AG)13	Class I	3596	3621	ACACCGTGTGCCTCGTGAAACT	ATAGCCGGCAGCGTGTGTTT	302
FMgSSR-9270	scaffold2324	p2	(AG)13	Class I	56753	56778	ACACGCACACGCATACACCT	ACATCGATCGGCTTGTTGCTGC	252

SSR_ID	Scaffold	SSR_Type	SSR_Motif	SSR Class	SSR_Start	SSR_End	Forward sequence	Reverse sequence	Product_size
FMgSSR-9273	scaffold567	p2	(AG)13	Class I	15378	15403	ACATTCCTGGCCTCTAGACTGG	TGTGCGGGTATGTGCAGGTT	318
FMgSSR-9274	scaffold11408	p2	(AG)13	Class I	5274	5299	ACCACCACCGGAAACACACGAA	TGCAACAGCATCAGCACCGA	315
FMgSSR-9280	scaffold2164	p2	(AG)13	Class I	18935	18960	ACGACGAAGACGACGAGGACAT	AGCAGCATCAATGGTGCCAGGT	294
FMgSSR-9281	scaffold22707	p2	(AG)13	Class I	7323	7348	ACGCGAGCAGCTCTTTGTCGTA	TGCATGCAGGAGCTTCGTCCTT	209
FMgSSR-9282	scaffold12546	p2	(AG)13	Class I	3899	3924	ACGGCCCGAGAAACAAGATGGT	AGGGGCCATCGAAGTAATGAATGC	311
FMgSSR-9285	scaffold1773	p2	(AG)13	Class I	66713	66738	ACGTAGCTCGTAGCTGTCGTCT	AATGCGCCACACGCACAACA	312
FMgSSR-9288	scaffold3260	p2	(AG)13	Class I	7422	7447	ACTTGGCACAGTCCAACCGT	AGGCAACGTGCCGTGTTCAT	327
FMgSSR-9290	scaffold8458	p2	(AG)13	Class I	8422	8447	AGAGAGAGAGAGAAACAGGGAAGC	AAGAATCACCTCACGCCGCT	319
FMgSSR-9292	scaffold336	p2	(AG)13	Class I	84194	84219	AGAGGAGAGCGGCAGCATTT	GCTGCTGGTTCCTAGTTATCTCCGT	262
FMgSSR-9293	scaffold578	p2	(AG)13	Class I	89906	89931	AGATGCCAGGTCAAGCAGGA	ATCCGATGGTAGGTGGAACAGGGT	259
FMgSSR-9295	scaffold31869	p2	(AG)13	Class I	5267	5292	AGCAATGGGCGGCAATGTTCA	ACGATGGGCAAACCGCTTGT	287
FMgSSR-9296	scaffold100759	p2	(AG)13	Class I	286	311	AGCACGCATTAAGGTGTCCAGG	GCAAGCACACTGTTAGTGACCTGT	313
FMgSSR-9297	scaffold2586	p2	(AG)13	Class I	39449	39474	AGCAGCAAAGCAAGGCAAAGC	TGCACACCGGGAACATATGCGA	323
FMgSSR-9298	scaffold52284	p2	(AG)13	Class I	2938	2963	AGCAGCATGCAGCAAAGGAGGA	ACGGCATGTGAACACCCTGGAT	326
FMgSSR-9300	scaffold8333	p2	(AG)13	Class I	16610	16635	AGCCACCAATTCACGGCCTACA	TGGGTTCATCACGACACCGTCA	317
FMgSSR-9303	scaffold40	p2	(AG)13	Class I	161121	161146	AGCGCCAACGAGGAACAGGAAA	ACGCCACTGTGTCTGTCTGTCT	247
FMgSSR-9306	scaffold194	p2	(AG)13	Class I	52150	52175	AGCTAAGCCGGTGGGATCTTGT	TGGAAGCTGCTGCTCAAGTGGA	300
FMgSSR-9316	scaffold21936	p2	(AG)13	Class I	4423	4448	AGTGAGTGCCCCAATTGCACGA	ACAACAACACGCGAGGCTGA	344
FMgSSR-9319	scaffold193	p2	(AG)13	Class I	115964	115989	AGTTCGTGCGGGGGAGAAAGAA	AGGAGCGTCGTGCAGCTACAAA	340
FMgSSR-9322	scaffold15492	p2	(AG)13	Class I	14964	14989	ATCGCCATTTGGCGGAAGAGGA	TGCACGCCTCCATGTTCTTGT	222
FMgSSR-9324	scaffold2191	p2	(AG)13	Class I	27116	27141	ATGCAAGCGTGGCTTCATTCGG	ACGGCGGGTACATGTCCAAT	317
FMgSSR-9325	scaffold29686	p2	(AG)13	Class I	5692	5717	ATGCACAGTTGGGCAGGCAA	TGCTTGCATCGGTGTGCTAACG	207
FMgSSR-9326	scaffold4458	p2	(AG)13	Class I	11303	11328	ATGCAGCACACGTTGCCACT	ACCAGGCGCGATGACAGAACAT	290
FMgSSR-9333	scaffold874	p2	(AG)13	Class I	91050	91075	CCTCCAGTGTTGCATTGAGAAGGC	TTTCCCGTCGCAGCTCAACCTT	321
FMgSSR-9337	scaffold2086	p2	(AG)13	Class I	60397	60422	GAGCTGCAGATCGATGAACACA	ACACACGTGATCTCCTCCAGCA	325
FMgSSR-9343	scaffold179	p2	(AG)13	Class I	66797	66822	GCGGGTTCTGTGTGTTTTCGCT	ATTACTACACCCGCGCCGTTCA	224
FMgSSR-9345	scaffold128646	p2	(AG)13	Class I	141	166	GCTTTGCTTGGGGAAACGACCA	TTTTGCGTCGCGTAGCTCGT	240
FMgSSR-9348	scaffold15	p2	(AG)13	Class I	216377	216402	TACGCGACGCGGATGAGTTT	AGTAGCAGCGACCACCTTCCTT	324

SSR_ID	Scaffold	SSR_Type	SSR_Motif	SSR Class	SSR_Start	SSR_End	Forward sequence	Reverse sequence	Product_size
FMgSSR-9350	scaffold914	p2	(AG)13	Class I	19021	19046	TCCAAATCCGACCGTGGCTACT	AAGCGACACAGTTGCCTGCT	310
FMgSSR-9351	scaffold2554	p2	(AG)13	Class I	8982	9007	TCCAACCACCAATACCGTCGCA	GCAGATGACGTGTTTTGGGAGTCT	288
FMgSSR-9352	scaffold7346	p2	(AG)13	Class I	14339	14364	TCCAACTTTGCAGGCCCATGCT	GCGCTGGATCGTTCGTTTCGTT	234
FMgSSR-9356	scaffold2427	p2	(AG)13	Class I	47283	47308	TCGACCGGCCTTCGTTCTCTTT	TGCTACCACGAGCAGTCGCAAT	293
FMgSSR-9358	scaffold1759	p2	(AG)13	Class I	63059	63084	TCGCGAAATTTTGGAGCGCGA	CATGTTTAAACCAGCGCGGC	214
FMgSSR-9359	scaffold2622	p2	(AG)13	Class I	69695	69720	TCGCGCGGGGAAGAAGAAAGAA	TGTCGTGAACCAGTGGCCAACA	333
FMgSSR-9361	scaffold5892	p2	(AG)13	Class I	5833	5858	TCTCGATGTGCCGGCGAAAA	TTGGCCAGTTTTGGCCCTTGGA	315
FMgSSR-9363	scaffold1590	p2	(AG)13	Class I	57246	57271	TGACGGCAACCTGCTGCAAA	ATCGTGAGGCCCACAAGCCATA	334
FMgSSR-9367	scaffold777	p2	(AG)13	Class I	47757	47782	TGATGGCATCTGGAGTCTGCCT	ACGTTGTTTGCTTTGGGCTGGT	347
FMgSSR-9374	scaffold4613	p2	(AG)13	Class I	17893	17918	TGCGTGCAGCTGGACAAGAA	ATAGCTGCCCCGATCTGCCTAA	342
FMgSSR-9375	scaffold2633	p2	(AG)13	Class I	12333	12358	TGCTCATGTGCTCTCCTCTGCT	TGGTGCTGACACTTGTGCACCT	262
FMgSSR-9376	scaffold71	p2	(AG)13	Class I	52395	52420	TGCTGCAACTCCCAATCCGTCA	AAATGTTCGGCGGCGGGTTA	315
FMgSSR-9377	scaffold1015	p2	(AG)13	Class I	3331	3356	TGGAAAAGCAAGCCACGGACA	ACAACAGCAACAGCCAGCCT	332
FMgSSR-9380	scaffold18	p2	(AG)13	Class I	194372	194397	TGGTGGGTTCCACAGGCTTTCT	ACTCCCGTACGTTTCCTCCCTT	315
FMgSSR-9382	scaffold345268	p2	(AG)13	Class I	164	189	TGTGCGGCAGATCGTTTTGGTG	ATCGAGGTAGTTCGTGCTCATCGC	223
FMgSSR-9387	scaffold6818	p2	(AG)13	Class I	11512	11537	TTCCCGCCAAACACCCTCAAGT	ATTCTGCCTGCTCCCCCAAACT	310
FMgSSR-9388	scaffold509	p2	(AG)13	Class I	77656	77681	TTCCGCAGTGCGTTGACTGT	TTACGCGAGCAGTTCGGCACAT	302
FMgSSR-9390	scaffold22695	p2	(AG)13	Class I	10242	10267	TTGCACGCATGAGAGCCTCGAA	AGTTGGCCATGGCGTCCAAGAA	327
FMgSSR-9391	scaffold24598	p2	(AG)13	Class I	7269	7294	TTGCACGCGTTTTGCTGGTG	AGCTGCTCATTCTGCCCTCCTT	303
FMgSSR-9394	scaffold10504	p2	(AG)13	Class I	16145	16170	TTGTTGCAGTCCCCACAGTCCA	TTCGGCTGGCAGCAGGAAGAAA	286
FMgSSR-9395	scaffold22457	p2	(AG)13	Class I	3871	3896	TTTCCTTTGCGTGCCATGCG	TGCCGTAGATGAGAAGGGCGAA	277
FMgSSR-9398	scaffold8921	p2	(AG)13	Class I	12079	12104	TTTGTTGGTGGTGCTGCCGA	CACCAGAAAAGGTGCTGCACGA	278
FMgSSR-9401	scaffold29543	p2	(AG)14	Class I	3903	3930	AAAGCACACGCGCACGCATA	ACGGCGAATGCCAAGGATTT	201
FMgSSR-9403	scaffold2666	p2	(AG)14	Class I	50862	50889	AACCCTTGACTGGTCGGACCTT	AGGAGCGTGTTGCCTTGGTT	207
FMgSSR-9404	scaffold5690	p2	(AG)14	Class I	17629	17656	AAGACAAGCGATGCGAGCGA	TCGATGACAGAAGGGTGGCA	237
FMgSSR-9409	scaffold398	p2	(AG)14	Class I	108093	108120	AAGTGGCTAGATCTGTCCGCGA	AGCAGCCTGCCGCTCCAATAAA	307
FMgSSR-9411	scaffold460	p2	(AG)14	Class I	50941	50968	AATCCGACCGTGGCTGCTGTAA	ACAACCGACACAGTTGCCTGCT	287
FMgSSR-9412	scaffold91	p2	(AG)14	Class I	88271	88298	AATGACAAACGGCCGTGCAGTG	ATGGAGCCGATCTGCGTGTGTT	310

SSR_ID	Scaffold	SSR_Type	SSR_Motif	SSR Class	SSR_Start	SSR_End	Forward sequence	Reverse sequence	Product_size
FMgSSR-9413	scaffold5057	p2	(AG)14	Class I	33560	33587	AATGATCGCGGCGAGGAACA	ACCAGGCCGCTAGTTGTTGT	254
FMgSSR-9414	scaffold2631	p2	(AG)14	Class I	19195	19222	AATGGGTGCATCATGACCGGGA	TGTTGGGGCAAGCAGCAGTT	340
FMgSSR-9418	scaffold8528	p2	(AG)14	Class I	11942	11969	ACAGCAGGCCGGCAGAACTTTA	TGAGCATGGGCATGGTGGTGTT	253
FMgSSR-9419	scaffold7043	p2	(AG)14	Class I	14852	14879	ACCAAGGAACGGAGAAACGGCT	AGGTTGCCAATGATGCCGCT	200
FMgSSR-9429	scaffold106	p2	(AG)14	Class I	18840	18867	ACGCGTTCTCCGACGATGATGA	AGGGACGAGTCATTTGCCACCA	330
FMgSSR-9430	scaffold26	p2	(AG)14	Class I	136963	136990	ACGCGTTGGGTGGAGAAAGT	TCAGCGATCTCATGCAAGTTGTGC	313
FMgSSR-9432	scaffold4618	p2	(AG)14	Class I	5213	5240	ACGGGAGCGAAAAAGGCATCCA	AGGCTCCATATGCCTTCGTCTCCT	236
FMgSSR-9436	scaffold2571	p2	(AG)14	Class I	28159	28186	ACTCGCTCCTGTGAGCGCATAA	ACGGGCCAATCGTTCCCTTT	291
FMgSSR-9445	scaffold2010	p2	(AG)14	Class I	11452	11479	AGGAAGGCCACCATTACCTGGA	TCTCCTTCCGTTGCGTGGATGT	301
FMgSSR-9449	scaffold2249	p2	(AG)14	Class I	19071	19098	AGGTCATCCCAGCCATGTTGGA	ATAGTTGTCACGGGATCCCTCC	311
FMgSSR-9455	scaffold4309	p2	(AG)14	Class I	36536	36563	ATAGCCACCGGAGCTTGCCAAT	ATTCCCCACAAATCCGGCGT	304
FMgSSR-9456	scaffold30	p2	(AG)14	Class I	22065	22092	ATCCAAAATCCGATCGGCCGT	ATCGGCGGATGCGAAAACCA	350
FMgSSR-9457	scaffold2951	p2	(AG)14	Class I	54690	54717	ATCCAGTGCTGCTGAGCCTTCT	AACGCAGCAGGGCTGAAACA	231
FMgSSR-9462	scaffold5540	p2	(AG)14	Class I	8789	8816	CCCGATCTCAGCTGCCACATTT	AAAGAGGTAGCGGCCAATGCCA	253
FMgSSR-9463	scaffold6003	p2	(AG)14	Class I	6045	6072	GACCACGAAATAGGCACAGAAGCA	TGAGCAAAGCAAGCACACCA	309
FMgSSR-9465	scaffold1308	p2	(AG)14	Class I	20454	20481	GCAAAAGGTACTAACCGGATGAGG	TCTCAGGTTAGTGAGTGGTCCGT	317
FMgSSR-9469	scaffold9712	p2	(AG)14	Class I	8713	8740	GCAGCAGCAAAGCAAGCGAA	TTTTCTGGCGCACGAAACGC	321
FMgSSR-9471	scaffold242	p2	(AG)14	Class I	16893	16920	GGGACACCAAATTGCATGCTGGA	ACGCATGCAGGATAATGGCGTG	252
FMgSSR-9473	scaffold35688	p2	(AG)14	Class I	2936	2963	GGTGGCCTTGCCCTCTTTGTTT	ACCGGGTTAACGCGTGATGGAT	325
FMgSSR-9474	scaffold2025	p2	(AG)14	Class I	28421	28448	TACGCCCGTCTTTCATTGCGGT	ACCTGCTATGTTTGGGCTTGGCT	240
FMgSSR-9475	scaffold1258	p2	(AG)14	Class I	55416	55443	TAGCATGGCAACACGAGCCA	ATTGGAGAAGGAAGCGAGCGGT	334
FMgSSR-9481	scaffold568	p2	(AG)14	Class I	52911	52938	TCCAACCTACCTCGCCCTCAAA	TCCAAGACTCTTCTGGCATGTGGC	335
FMgSSR-9487	scaffold22469	p2	(AG)14	Class I	5689	5716	TCGCAGCAGAATAGGATGGCGA	AAGGAGGAAGGAAGTGGTGCGT	213
FMgSSR-9490	scaffold13557	p2	(AG)14	Class I	10233	10260	TCGTGCATGTGGTGTTGGCT	AGGCAGAATTCGTGGAGCTCTGT	341
FMgSSR-9491	scaffold1578	p2	(AG)14	Class I	46790	46817	TCGTGCTTTCACCGTGGCTT	AAGCGGCGAGGTTTGGCTTT	206
FMgSSR-9493	scaffold782	p2	(AG)14	Class I	3300	3327	TCTGCTGCTGCGATTTCCGA	TGCGAAAGACTTGCGGCGAA	301
FMgSSR-9495	scaffold23619	p2	(AG)14	Class I	5172	5199	TGAGAGGCATAGGATAGCAGGGCA	TCGGAAACGGGACAGAATGGCA	268
FMgSSR-9496	scaffold9407	p2	(AG)14	Class I	15593	15620	TGCAAGCTGCAGGGTCAAGACT	ACTCCTTGGGTCTCAGTTGGCT	348

SSR_ID	Scaffold	SSR_Type	SSR_Motif	SSR Class	SSR_Start	SSR_End	Forward sequence	Reverse sequence	Product_size
FMgSSR-9497	scaffold1970	p2	(AG)14	Class I	52923	52950	TGCACACACACACACACGCA	GCATGGCCTTGGCACGTCAAAA	346
FMgSSR-9499	scaffold16958	p2	(AG)14	Class I	7778	7805	TGCAGGCCATTGGTTGCACA	TCGCATAGGCACAACACGGT	349
FMgSSR-9500	scaffold10681	p2	(AG)14	Class I	5664	5691	TGCAGGCGTGAAGAAGTCAAGG	TGCATGCATCCAGCATTCTAGC	259
FMgSSR-9501	scaffold694	p2	(AG)14	Class I	13801	13828	TGCAGGTCTGCCCATTATTCCACC	ATGCTTTGAGGAAGGACAGCTCCC	326
FMgSSR-9502	scaffold10783	p2	(AG)14	Class I	2070	2097	TGCAGTGTTGCCGCCCATTT	ACTGTTCTCGCAAACCGGGAGT	339
FMgSSR-9504	scaffold3029	p2	(AG)14	Class I	18175	18202	TGCCAGATGAGGTCCATGGATTGG	ATGCGTGCTTATGAGTGGCAGG	348
FMgSSR-9508	scaffold32692	p2	(AG)14	Class I	1432	1459	TGCCTGCAATGAGCTCAGCA	AGAGCAGTGCCCTGATCGTT	250
FMgSSR-9510	scaffold800	p2	(AG)14	Class I	47610	47637	TGCGATCCGTTCCTGCCTTT	ATGCAGTCTGTTTCAGCGCGAG	339
FMgSSR-9511	scaffold169	p2	(AG)14	Class I	3671	3698	TGCGGCCTCGCGTCAAATAA	TTCTGCAACGCCACTAGCCA	325
FMgSSR-9520	scaffold343	p2	(AG)14	Class I	7370	7397	TGCTGTCACCACCCCACAAA	GCTCTGCTTCTCTTAAAGCACGCC	330
FMgSSR-9521	scaffold3121	p2	(AG)14	Class I	6062	6089	TGGAACAGGAATGGCGGCTTGA	TTGCACAGTCGCCATCAGCA	307
FMgSSR-9522	scaffold9229	p2	(AG)14	Class I	13351	13378	TGGACGAAGCGATGACGGAT	TGCAGTGGCGGAGAAGTGAT	349
FMgSSR-9524	scaffold4657	p2	(AG)14	Class I	27284	27311	TGGCAAAGTCCATAGCGTGGCA	AGGTGCGCTCGACAACACCAAT	315
FMgSSR-9525	scaffold1015	p2	(AG)14	Class I	73536	73563	TGGCACCAACCAGCCATCAT	ACTGGGTTGTGGTTGAGCAGCA	293
FMgSSR-9526	scaffold799	p2	(AG)14	Class I	66095	66122	TGGCAGGATTGTCCTCGGAGTT	GGTAACGCCCATGTACCTCTATGT	302
FMgSSR-9527	scaffold4543	p2	(AG)14	Class I	43295	43322	TGGCTCGCCGACGTATAAGCAA	ACCAGGCCATGACTGCTCCATT	242
FMgSSR-9528	scaffold1122	p2	(AG)14	Class I	69797	69824	TGGCTTTGTGCCATAGGCGA	TCGAAGTGTCACCGTGCGAA	278
FMgSSR-9533	scaffold412	p2	(AG)14	Class I	130175	130202	TGTGGTGCAGTGTCCGACTT	TTTGGCATAGAGAGGGCCACCTGA	330
FMgSSR-9540	scaffold751	p2	(AG)14	Class I	45203	45230	TTGAATCGAAGAGGAGGGAAGGGG	TCTATTGCGGCGTGAAGGCA	345
FMgSSR-9549	scaffold4375	p2	(AG)15	Class I	22458	22487	AAATTCAGGCGGGGATGCTCCT	AAAAGCAGTGGTGGAGCCGA	245
FMgSSR-9550	scaffold6223	p2	(AG)15	Class I	2061	2090	AACAGAGATGCAAGGTGGGGGA	TCATCGACGAACATGGCGCA	303
FMgSSR-9554	scaffold3840	p2	(AG)15	Class I	11202	11231	AACGGGAGAACACCCAGCAAGT	TTTGAGGCGGCCTTTCGTCT	277
FMgSSR-9556	scaffold16283	p2	(AG)15	Class I	3774	3803	AAGCCCAGACCCAAGGCAACAA	TATGCCGCTGCGCTTGCTTT	303
FMgSSR-9560	scaffold1677	p2	(AG)15	Class I	24238	24267	AAGGTCTCCAAAGGGGAAGGCT	CCCTCCCCCTCCCCTATAATTCTT	232
FMgSSR-9561	scaffold74	p2	(AG)15	Class I	73968	73997	ACACCACCCCTTTGTGGGGTTA	ATGCTCTATCCTGTCGCCGCTA	334
FMgSSR-9562	scaffold5896	p2	(AG)15	Class I	26958	26987	ACACGAATGCACGACGACCCAT	AGCGCCCTCTTTGGCATTTCCT	348
FMgSSR-9564	scaffold7873	p2	(AG)15	Class I	12480	12509	ACCCCTTATGCGCCTCGAGTTT	ACGGTGTTTGCGAGGTAGCTAGA	276
FMgSSR-9565	scaffold28165	p2	(AG)15	Class I	7567	7596	ACCGGCAAGCACCAAAGCAA	CGCAGCTGAGCGTCTGCAAAAA	348

SSR_ID	Scaffold	SSR_Type	SSR_Motif	SSR Class	SSR_Start	SSR_End	Forward sequence	Reverse sequence	Product_size
FMgSSR-9572	scaffold2404	p2	(AG)15	Class I	48875	48904	ACTCACTGCAGTCTTGTTCGCA	AGCCGTCAATTCCAACCGAGAGT	350
FMgSSR-9573	scaffold282	p2	(AG)15	Class I	140475	140504	ACTCCGATTCAGGAACGACGCT	CCATGAGCTTGCTGCACGCATT	314
FMgSSR-9574	scaffold15850	p2	(AG)15	Class I	14212	14241	AGCACAACCCAGATCCGCTTGA	TTGGCCTTGAGCGCTTGATGGA	348
FMgSSR-9584	scaffold3377	p2	(AG)15	Class I	23231	23260	AGCGTGGTCGTGTTTCCGACTT	TGTGCACACGGCTCCAAGTCAT	336
FMgSSR-9591	scaffold20777	p2	(AG)15	Class I	974	1003	AGGGTGATGTAGAATGGAGCGCA	AGAAACAGCCGCCGGTACAA	349
FMgSSR-9593	scaffold12819	p2	(AG)15	Class I	1031	1060	AGTCTTCCATGAGTCGCAGCCA	AGATCACCGCTGCTCACCATCA	256
FMgSSR-9595	scaffold44	p2	(AG)15	Class I	77642	77671	AGTTGCGTCCAGCCTGCAAA	ACAGCCGCACGACAGAGAGATT	274
FMgSSR-9598	scaffold8015	p2	(AG)15	Class I	25933	25962	ATCTTCGAGTGGAACGCGGCTT	TCGCCGCACCCAGGAATCATAA	235
FMgSSR-9601	scaffold4360	p2	(AG)15	Class I	11727	11756	ATGGGTGCCTCGTCGTGCTAAT	ACGCATACGGTTGACCAGCTT	240
FMgSSR-9602	scaffold164210	p2	(AG)15	Class I	593	622	ATGGGTTCATGCTGGCAGGCTT	ACCAGTTGACCGCACCACTT	293
FMgSSR-9605	scaffold46572	p2	(AG)15	Class I	2351	2380	ATTCCCCGCACGGAGCATCTAA	AGCCACATGATCCAGCAAGC	345
FMgSSR-9608	scaffold5475	p2	(AG)15	Class I	14648	14677	CCCGGCCCACATTTCTTCGTTT	GTGGATAAGTGCACACACGTCA	310
FMgSSR-9611	scaffold156	p2	(AG)15	Class I	175455	175484	GAGAGAGAGATTAGGGGAGGGAGA	ACTGTGCATTGCCGGCTTGT	330
FMgSSR-9612	scaffold6608	p2	(AG)15	Class I	26865	26894	GCAGCTACCGACAGGTGCATTA	TCAAGCTATCCTTCGCCCGCAA	288
FMgSSR-9614	scaffold9361	p2	(AG)15	Class I	31820	31849	GCTCCGACTGTGGCTTCCAAAA	TGTGCGCAGGTGTATTCCACA	295
FMgSSR-9623	scaffold790	p2	(AG)15	Class I	34166	34195	TCAGCAAGAAGGCCAGAAGGGA	TAATACGCGGGCCTGCTGTCAA	204
FMgSSR-9625	scaffold4651	p2	(AG)15	Class I	11372	11401	TCATCGCCTCCACACTGTCA	TGCAGTGCACCCAACGATACCA	340
FMgSSR-9632	scaffold2468	p2	(AG)15	Class I	42523	42552	TCCGCACAAACAATGCACCA	AAGCGTCATCGCCTCGTCTA	340
FMgSSR-9633	scaffold10933	p2	(AG)15	Class I	18091	18120	TCCTCAAGAGCCTTCTGGATGTCCT	ACGCAACTTCAACTGCACCA	285
FMgSSR-9635	scaffold2519	p2	(AG)15	Class I	35154	35183	TCGAAGCAACTCGGTGTACGACT	AATGAACAGACGCGGCCAGT	271
FMgSSR-9647	scaffold24036	p2	(AG)15	Class I	528	557	TGCCTTGTCGTTGCAGCTCTGA	TCCTCAACAGATCCAGCCACCT	244
FMgSSR-9650	scaffold15210	p2	(AG)15	Class I	13237	13266	TGCGCCACGTCAGCTTTGTT	TGCCTGCAGTGACCATGTGTGT	298
FMgSSR-9655	scaffold533	p2	(AG)15	Class I	29526	29555	TGCTTGTAAGGCCCTGCCAT	TGCTTGTACCTTGCGTTGGGGA	307
FMgSSR-9656	scaffold329	p2	(AG)15	Class I	94463	94492	TGGCACGATGGCAAGATGCT	GCGGCAGCTTCAAGGTCCTTAT	307
FMgSSR-9657	scaffold2240	p2	(AG)15	Class I	33016	33045	TGGCACTGCGTGACCAAGTGAA	ACCACTCGCTGCTAAAAGGCCA	232
FMgSSR-9658	scaffold10837	p2	(AG)15	Class I	8471	8500	TGGCACTGGCAAACTGGCAT	ACGTGATGCGTGAGCAAGTGGA	210
FMgSSR-9661	scaffold19677	p2	(AG)15	Class I	7085	7114	TGGGGCAGCTCTTGTTGGAA	ACTGGTGACCTTCTGACATTGCC	232
FMgSSR-9662	scaffold455	p2	(AG)15	Class I	26312	26341	TGGTGGCATGAGAGGAGGGAAT	AGCTCACGCTGTTGCAAGGA	348

SSR_ID	Scaffold	SSR_Type	SSR_Motif	SSR Class	SSR_Start	SSR_End	Forward sequence	Reverse sequence	Product_size
FMgSSR-9672	scaffold58237	p2	(AG)15	Class I	1490	1519	TTAGCATTGGCACCTTGCACCG	ATTCTTCGGTTGCTCCACCCGT	203
FMgSSR-9673	scaffold4370	p2	(AG)15	Class I	19902	19931	TTCGGGCGGTTGCTGTTGTT	TGGCGTGCGAAAGAAGCCAA	268
FMgSSR-9674	scaffold13033	p2	(AG)15	Class I	12326	12355	TTGCCGCGAGCTTCTATGCCTT	TCGCGTCCACGACGTGTTTT	277
FMgSSR-9682	scaffold2970	p2	(AG)16	Class I	58309	58340	AAACAGATGGCAGTGTGCCGGA	AGGATGCGCTCAGCTGGTGATT	310
FMgSSR-9684	scaffold5709	p2	(AG)16	Class I	17693	17724	AAAGAGAGGAGGCGTGAGGCAT	ATTTGCTGCCCTCGTCGTGA	326
FMgSSR-9688	scaffold2182	p2	(AG)16	Class I	61046	61077	AACGCACTTGGATGGCCGAA	ACGAAATGGCACGTCACCCT	315
FMgSSR-9695	scaffold250	p2	(AG)16	Class I	121346	121377	ACAGCCTAAAGCGCCAACGA	TAACGCGAACGACAAACGGGGA	220
FMgSSR-9696	scaffold107204	p2	(AG)16	Class I	1341	1372	ACAGTGCCAGGATTCACGAGCA	TGCCGTTGTCAAATCGCTGCTG	268
FMgSSR-9697	scaffold4845	p2	(AG)16	Class I	20415	20446	ACCCATCCAAATCCGTCCCTGA	ATGAACGGCTCGCCAGCAAA	317
FMgSSR-9699	scaffold351	p2	(AG)16	Class I	56923	56954	ACCGAAGCCCACGACGAAAA	TGACGGGCGATGACAACACCAA	322
FMgSSR-9701	scaffold27928	p2	(AG)16	Class I	9217	9248	ACGAAGCCTCTGATCCATGCCA	ATTGCTGCGCCTCGAGATGT	347
FMgSSR-9703	scaffold2841	p2	(AG)16	Class I	34202	34233	ACGCACGACGAGGTCAACAA	AACGGTTCGGCGAGAGTTCGTT	334
FMgSSR-9711	scaffold359572	p2	(AG)16	Class I	71	102	AGCACAGCTGACTGCACAAGGA	AGTTGTGGCCTCGTGGACAGTT	285
FMgSSR-9712	scaffold42577	p2	(AG)16	Class I	3245	3276	AGCACGCAGCGAAGGATGAT	TGGGCCGTTGGGCCATAGATTA	228
FMgSSR-9713	scaffold9218	p2	(AG)16	Class I	4045	4076	AGCATCGCCTGCTACAGCTACA	ATGTGGCACGACGCGATGAT	259
FMgSSR-9716	scaffold1418	p2	(AG)16	Class I	26795	26826	AGCGGGAGGAGTTTTGGAGTGT	TTTGTCACTTGTGCTGTGCCGC	230
FMgSSR-9728	scaffold176	p2	(AG)16	Class I	158840	158871	ATCGGATCTAGAGGAAAGGAGGCG	TTCACACTAACCGTCGTGCCCT	282
FMgSSR-9731	scaffold1619	p2	(AG)16	Class I	11571	11602	ATGCAGGAGTTCGACAAGCGGT	AGCCTTGCCCTCCAACATTCT	210
FMgSSR-9732	scaffold50419	p2	(AG)16	Class I	3508	3539	ATGCGCTACCAAATGCCGGA	TCCTCAGATCCAGAAGACCCGT	265
FMgSSR-9734	scaffold3992	p2	(AG)16	Class I	13558	13589	ATGGCACTTGCGAGCTCTTGGT	AACGTCGCCAAAGGTTGCGT	261
FMgSSR-9735	scaffold8864	p2	(AG)16	Class I	5393	5424	ATTGGCCGAGCAACGAAGGA	TTAGGGCGCCACGAGAAACCAA	230
FMgSSR-9737	scaffold7016	p2	(AG)16	Class I	25487	25518	CAGCCTATCACCACGTGCACAA	TGCCATGCATGTGCTGCTGT	333
FMgSSR-9743	scaffold737	p2	(AG)16	Class I	85718	85749	GCAGCACGGCCATTATCGACAT	TCTACGTGTGTAGGGCGGTT	279
FMgSSR-9749	scaffold2730	p2	(AG)16	Class I	39597	39628	GGACAAAGTGAATACAGTGCAGCC	TCCACCATTCCATCCAGCCA	330
FMgSSR-9751	scaffold4109	p2	(AG)16	Class I	43352	43383	TACGCCGTGCGTGTCTACAT	TGCACGCGCTCGTACATTGA	321
FMgSSR-9757	scaffold18444	p2	(AG)16	Class I	6853	6884	TCCCGCCAGAAATCCAACCACA	TCATTGTCCCAATCGCCCCCAA	315
FMgSSR-9762	scaffold2	p2	(AG)16	Class I	211046	211077	TCGATGATGCCCGTGTTGCTGA	TTCGCTCATCTCGCTTGCTCCT	296
FMgSSR-9765	scaffold349	p2	(AG)16	Class I	125555	125586	TCGTCACATCGTCTCAACGCTCA	CGGCAAGCAAGGCACTTCTCAT	308

SSR_ID	Scaffold	SSR_Type	SSR_Motif	SSR Class	SSR_Start	SSR_End	Forward sequence	Reverse sequence	Product_size
FMgSSR-9768	scaffold6344	p2	(AG)16	Class I	3588	3619	TGACACCTTTGCTCGCCACGTA	TGAAGAACACCGCGCGAAGGTA	211
FMgSSR-9774	scaffold1945	p2	(AG)16	Class I	65283	65314	TGCATGCTCGCCTTGCATTGT	TGCACGAGGAACGCCTTGTT	228
FMgSSR-9789	scaffold24397	p2	(AG)16	Class I	5629	5660	TGGCTCGTCATCGTGTGTCCAA	AAGGCTGGTGGATGCAGAGTGT	250
FMgSSR-9796	scaffold315	p2	(AG)16	Class I	119912	119943	TGTCAACTGGAGAAAGGATGGCTG	ACGTTGAGCCTTGAGCCTACACT	241
FMgSSR-9803	scaffold24417	p2	(AG)16	Class I	4447	4478	TGTTCTTCGTAGCGGCGCAT	AGGCACACACCTCTTGTAGCCA	216
FMgSSR-9804	scaffold26639	p2	(AG)16	Class I	6969	7000	TGTTGTAGCTTAGAGGGGTGCT	ATCCTTGGTGGCTTGGTGCTGA	249
FMgSSR-9808	scaffold1099	p2	(AG)16	Class I	19114	19145	TTGATCGGTCGGCTATCGCA	ATCAACCAAGCCAACGCCCA	340
FMgSSR-9812	scaffold113	p2	(AG)16	Class I	87041	87072	TTGTGTGGGTCTCGCTCTGCAT	TGCTGCGGGAGCTGTAGCTTTT	343
FMgSSR-9815	scaffold6644	p2	(AG)17	Class I	24398	24431	AAACCGTCCCCTTGCTTGTCCT	CGATTGGCGCCATCCATGTGAA	327
FMgSSR-9818	scaffold273	p2	(AG)17	Class I	79069	79102	AACAAAGGTAGTGGCGGCGA	AAGGGACACGCAACGAACTGGA	304
FMgSSR-9819	scaffold1184	p2	(AG)17	Class I	61249	61282	AACACTCTGCCCATTTCACCCC	TGGTGCCTCACACTGATTAGCCT	348
FMgSSR-9820	scaffold594	p2	(AG)17	Class I	16485	16518	AACCAGCCACGGGTTTCCACAT	TCCAGAGCTTCTGTCGTCCCTT	229
FMgSSR-9822	scaffold6514	p2	(AG)17	Class I	7719	7752	AAGTACCTTGAGGAGGGGGTTGGA	AGCCACCTACGATCCGAAGTCA	308
FMgSSR-9825	scaffold2784	p2	(AG)17	Class I	51964	51997	AATGCCCTTTGGTCCAGGTGGATG	TGAAGTGAGGGTGCATGGCT	265
FMgSSR-9828	scaffold10235	p2	(AG)17	Class I	16190	16223	ACACGCATGAGCGCGATTGT	ACGCAGCTGGAACGCTACAT	281
FMgSSR-9829	scaffold5465	p2	(AG)17	Class I	32160	32193	ACATGCACAACCCACCGTCTCA	TGGTGGTCACCGGGCATTTT	334
FMgSSR-9843	scaffold20876	p2	(AG)17	Class I	3837	3870	AGGCTTTCAGCACTGCGGTAACT	AATCTGTGGGCGTGGCGATT	291
FMgSSR-9847	scaffold6847	p2	(AG)17	Class I	27318	27351	AGGTCGCGCGGCTTATGTTT	ATCCGCGCGCGAGAGAAAAA	232
FMgSSR-9850	scaffold84	p2	(AG)17	Class I	174517	174550	AGTCACCGCGTCGAACACTT	GCGCCGAAAACTTTCCTGCCAA	330
FMgSSR-9851	scaffold6994	p2	(AG)17	Class I	13505	13538	AGTCCCTCCTGCTCTCCACTTT	ATGAGCCCATGTCGGTTGCCAT	322
FMgSSR-9859	scaffold68009	p2	(AG)17	Class I	274	307	ATGTGTGTGTGCGCGTCCAA	ACGTCGAAGATGGCTCGTGTGA	332
FMgSSR-9862	scaffold13872	p2	(AG)17	Class I	4685	4718	CCACATCAGTCAACGGATCTAGCA	ATGGCGGAGCTGATCGAGCATT	266
FMgSSR-9863	scaffold3004	p2	(AG)17	Class I	30628	30661	CCCCTGCAGCACAGCAAGAAAA	CGCGCCTGCATTGCATGTGTAT	264
FMgSSR-9866	scaffold10320	p2	(AG)17	Class I	1793	1826	CGATGCGAACGTGCATCTGTGA	ATCCACCCTTCTCGGCTTCTCT	212
FMgSSR-9869	scaffold6130	p2	(AG)17	Class I	10537	10570	GCATGTGCCATGTGCTCTTGTCT	ATGGCATGCATCGCCGTGAA	348
FMgSSR-9870	scaffold369	p2	(AG)17	Class I	76641	76674	GCGACTGGCAGCATCCATAAGA	GCAACCAGATGAGTCAGCATGAGA	341
FMgSSR-9873	scaffold10911	p2	(AG)17	Class I	13827	13860	GTTGTTGCCTACTCAGCTGCAC	CCCCAAAACCCTAACCCTAATCCT	350
FMgSSR-9874	scaffold268	p2	(AG)17	Class I	43513	43546	TAACGCGGACTGATGGCACACA	GCCATGGCCATTGGTGACAA	272

SSR_ID	Scaffold	SSR_Type	SSR_Motif	SSR Class	SSR_Start	SSR_End	Forward sequence	Reverse sequence	Product_size
FMgSSR-9875	scaffold3354	p2	(AG)17	Class I	38031	38064	TAGCCTGAGGCACGCTTTGACA	AAGCCAGACGGACCGGATGAAA	244
FMgSSR-9878	scaffold5375	p2	(AG)17	Class I	21861	21894	TCAGGCCGAGCATGATGGGAAA	TCGTGTAGACTTCGCGGCTTGT	212
FMgSSR-9883	scaffold9657	p2	(AG)17	Class I	28523	28556	TCGACGACTTGCCGAAACCA	TGAAGCCATTCACAGCCCACT	296
FMgSSR-9884	scaffold1051	p2	(AG)17	Class I	14158	14191	TCGATGGAGTGAGGCGGCAAAT	TGCAGCATGCATGTGGCTCA	266
FMgSSR-9888	scaffold1928	p2	(AG)17	Class I	56031	56064	TCGTGGCCACCCATGATTGA	AAGGGTTGGTTTCTGCCGCT	248
FMgSSR-9894	scaffold3036	p2	(AG)17	Class I	23427	23460	TGATCGCCGACCAGGACAAGAA	AGCGTTGAAAGCCCCTTTTGCC	346
FMgSSR-9895	scaffold8460	p2	(AG)17	Class I	30983	31016	TGCAAGTCGGACGATTCCCTCA	ACCTGCTGTCATCGCAAGCTCA	247
FMgSSR-9897	scaffold16213	p2	(AG)17	Class I	9548	9581	TGCAGGCATCCAATACATGCAACG	ACCCTGCAGCCAACCTTCTTCT	271
FMgSSR-9899	scaffold501	p2	(AG)17	Class I	2096	2129	TGCATGCAGTCGTGGTCAAGGT	TCGTTTTCGCGTGACCTCGT	343
FMgSSR-9902	scaffold9671	p2	(AG)17	Class I	23021	23054	TGCCTCAAAATTTGCCGGGCT	TGTCTTCGCAGCTCAAGTCCCA	220
FMgSSR-9906	scaffold1508	p2	(AG)17	Class I	37251	37284	TGCTCTGCTTGCCCCTGATTGT	TCGCCACTTTGCTAGCCATTCTGA	282
FMgSSR-9907	scaffold4714	p2	(AG)17	Class I	27083	27116	TGCTCTTGGCAAGTCTGCGA	TCCTTGGGCTTGAGCAGCTTGA	298
FMgSSR-9919	scaffold8118	p2	(AG)17	Class I	3014	3047	TTCTTCCCAAGCCGAGAGGCAA	TGGTGGGCTACTGTCATGCAA	313
FMgSSR-9925	scaffold1906	p2	(AG)17	Class I	34211	34244	TTTGCGGTTCACCGAAGCGA	TTCAGCAGCGGCAGCAGGTTTT	283
FMgSSR-9930	scaffold65763	p2	(AG)18	Class I	1011	1046	AAATTGTTCGGCACCCGCCT	ATCCTCAGTGTCAGCTCCCCTGTT	218
FMgSSR-9932	scaffold250	p2	(AG)18	Class I	88413	88448	AACGTCTCGGACTTCTCCAGCA	TTGCTACTGCCCTCGCTCTTCA	349
FMgSSR-9941	scaffold2990	p2	(AG)18	Class I	9710	9745	ACACCAATGGAGCCTAGATCACC	GGACAAAACAGCAGCTCCTGAACA	347
FMgSSR-9942	scaffold9556	p2	(AG)18	Class I	25454	25489	ACAGAATTCGACCAGCGCGT	AATGCCACTGGCCTACGAGTCT	311
FMgSSR-9943	scaffold944	p2	(AG)18	Class I	9262	9297	ACAGCGGGACACTCTCAAAACCA	ACTGAACGGCAGGAGCAGGAAT	350
FMgSSR-9947	scaffold30507	p2	(AG)18	Class I	5950	5985	ACCAGCGAATCAACAGGCGA	ATTGCAGTTGGTGGTGTCCG	276
FMgSSR-9953	scaffold7021	p2	(AG)18	Class I	29923	29958	ACGTGCCACCCAACAAACGA	ACGTTGTCGAAGATGGTCCGCT	247
FMgSSR-9954	scaffold10911	p2	(AG)18	Class I	14028	14063	ACTGTAGCTCGGGGTGAAGAGA	TCACGAAATTGCCAGCGCCT	301
FMgSSR-9956	scaffold18868	p2	(AG)18	Class I	12481	12516	AGACAATGCCGCACAAGCCT	TTGCGCCTCACGTAACCCTGAA	221
FMgSSR-9957	scaffold431	p2	(AG)18	Class I	63821	63856	AGCCACGCCTTATCGCTGAGTT	CCATTGGCAACCATGCCACAGA	333
FMgSSR-9959	scaffold111	p2	(AG)18	Class I	132614	132649	AGCTGGCGTTGCTTGGTTGA	GCGCAGGATTCGTGGATGCAAA	251
FMgSSR-9962	scaffold5129	p2	(AG)18	Class I	11508	11543	AGGCCTTGTGCATGGTACGTCA	CGCCTGCAAAATCACCATCCGT	258
FMgSSR-9963	scaffold2522	p2	(AG)18	Class I	9129	9164	AGGGAACCCCACGATGCTTT	ACCGATTAGGCGAGGCGATCAA	340
FMgSSR-9970	scaffold12166	p2	(AG)18	Class I	4307	4342	ATGGACGAGAGCGTGCATGAGT	ACGCCATATCTGGGAGCAGCAA	275

SSR_ID	Scaffold	SSR_Type	SSR_Motif	SSR Class	SSR_Start	SSR_End	Forward sequence	Reverse sequence	Product_size
FMgSSR-9971	scaffold18988	p2	(AG)18	Class I	5182	5217	ATGGGAAGAAAACCCGCTGCGA	CGGCTCAAAGTTGGTGGCCATT	308
FMgSSR-9973	scaffold9296	p2	(AG)18	Class I	7922	7957	CACATCCTCATAGCGTGCAATCCA	GGTACTTGGATGTATAGCTGCCTGC	342
FMgSSR-9975	scaffold282	p2	(AG)18	Class I	121704	121739	CGCCGCCATCCTTCTTCACTTT	AATCAATCGGGCAACCCTGCCT	248
FMgSSR-9979	scaffold2742	p2	(AG)18	Class I	16824	16859	GCCATACGGCATGAATCAGCTT	ATCTGCTCCGCCGCAATGAGAA	301
FMgSSR-9983	scaffold10058	p2	(AG)18	Class I	8566	8601	GGGGCGCATTTCTCGCACAAAA	CGCCGCAAAGCAAACAGCAA	236
FMgSSR-9986	scaffold540	p2	(AG)18	Class I	11600	11635	TCACGTGCAGTTTGGCTTGTGA	ATTGCTGGCCCTTGCATGAGGT	318
FMgSSR-9988	scaffold19678	p2	(AG)18	Class I	5475	5510	TCCATGCTACAACGGGCACA	TACAAAGGAAGCGTGCGGGGAA	350
FMgSSR-9995	scaffold21	p2	(AG)18	Class I	63650	63685	TCGTCCCTGCAGAATCAGAGCA	TGCGCGCATCTCAAAGCACA	342
FMgSSR-9996	scaffold8733	p2	(AG)18	Class I	25043	25078	TCTGTGGTAACTTCCTTGTGCTGG	TCATCCGCGATGAAGGGATGGT	343
FMgSSR-9998	scaffold68923	p2	(AG)18	Class I	341	376	TGCAAAGCGCTAGGTGAGGACA	AGCTTGGTCCTTGCACACCGAT	325
FMgSSR-9999	scaffold3656	p2	(AG)18	Class I	10431	10466	TGCATGCGTTTCGCTCGTCA	AAGCCATCCACCTTGACCGCAA	284
FMgSSR-10005	scaffold266	p2	(AG)18	Class I	26976	27011	TGCCGTGCAGCGACAAGTTT	CGCCCATGCATGTTCATGGCTA	318
FMgSSR-10006	scaffold6074	p2	(AG)18	Class I	38529	38564	TGCCTGCCGTTTTTCATCAGCG	TGCGCGTGTGCCGCTAAAAT	350
FMgSSR-10013	scaffold32178	p2	(AG)18	Class I	1770	1805	TGGAACAAACTCATCTCGCCAC	TGCGCCGATCTCTGCAGTTCAT	320
FMgSSR-10014	scaffold2573	p2	(AG)18	Class I	60891	60926	TGGAAGATGGGGAGGGAAGCAA	TGCCAGAATAAGGCGCTGGT	322
FMgSSR-10017	scaffold12813	p2	(AG)18	Class I	13010	13045	TGGCATGGAATGCGCACTGA	ATTCTTGCGTGGCAGCCGTA	335
FMgSSR-10018	scaffold363	p2	(AG)18	Class I	105003	105038	TGGCTGCACACTTCACTCAGCA	TGCAAGGCTAAAAACGCGCGAA	309
FMgSSR-10024	scaffold17035	p2	(AG)18	Class I	15850	15885	TGTTTGATCAGGCGGCAGTGGA	TCACGCTGAAACATGGGAGGACA	202
FMgSSR-10025	scaffold721	p2	(AG)18	Class I	45739	45774	TGTTTTGCTGCGCCGATCGAA	ACAACACCCAAAGAGCCGACGA	333
FMgSSR-10030	scaffold11575	p2	(AG)18	Class I	18117	18152	TTGATTGGTCGGGCACACTCCT	ACGGGCTCACAAGTCACAACCA	224
FMgSSR-10031	scaffold3455	p2	(AG)18	Class I	42657	42692	TTGGAAGCGACGCGAAGGAA	CGGCTGAAACGCAATTGTGCCA	207
FMgSSR-10033	scaffold743	p2	(AG)18	Class I	51748	51783	TTTCCCTTGCTTGCCGGGTT	TTGCGCGCCATGAAGAACAC	347
FMgSSR-10037	scaffold2324	p2	(AG)19	Class I	41492	41529	AACGCTTGCAGGAGCACGAA	AGTGGGCTGCATTCGCTTACCA	319
FMgSSR-10038	scaffold209551	p2	(AG)19	Class I	389	426	AAGAAAGGTCCACGGCGTCTGT	TCGCCGGATCGAATCAGACA	241
FMgSSR-10039	scaffold2305	p2	(AG)19	Class I	6487	6524	AAGCAAGCGGCTTTTGCTGC	TTTGGTGCGAGTTGGACCCAGT	308
FMgSSR-10045	scaffold387	p2	(AG)19	Class I	37256	37293	ACAGGCAAGCACCAACATGTGC	TGTTGTCCACTTGTCCTGGGT	249
FMgSSR-10050	scaffold30180	p2	(AG)19	Class I	3769	3806	ACGCACGAACACCTGCGAAA	TTGCTCAGAGCGCACACACA	306
FMgSSR-10058	scaffold48036	p2	(AG)19	Class I	374	411	AGGTGCACTCATGACTGGGAGT	TCCCCAACTCCTTGCTTCAGGT	305

SSR_ID	Scaffold	SSR_Type	SSR_Motif	SSR Class	SSR_Start	SSR_End	Forward sequence	Reverse sequence	Product_size
FMgSSR-10070	scaffold9655	p2	(AG)19	Class I	17459	17496	CTAGCACGACGGCACATGAA	TTTTCCACCGGACGCCTTCTCT	323
FMgSSR-10072	scaffold3267	p2	(AG)19	Class I	24150	24187	GCCCTGCCAGACAAGGAAGATT	ACCCTTCCCCGGACAAAGTGAA	323
FMgSSR-10080	scaffold765	p2	(AG)19	Class I	65863	65900	TAGAAACGCAGGGGCTCACT	AACCACCACCTTGTCGTCGTCT	211
FMgSSR-10081	scaffold1794	p2	(AG)19	Class I	49561	49598	TCAAGCTTGTGTGCTGTGTGGT	TAACGCAGTGTGGTTGGGGTGT	277
FMgSSR-10082	scaffold62533	p2	(AG)19	Class I	926	963	TCAGGTAACTTGCCCAGCAGGA	TGGTAGAGGTTGGCCTCTCTCTCT	314
FMgSSR-10089	scaffold18620	p2	(AG)19	Class I	11206	11243	TCCTTGCCGTCACGCAGTTT	AGGGACGAACAAGAGAATGGCCT	201
FMgSSR-10090	scaffold25076	p2	(AG)19	Class I	9571	9608	TCGCCTCTCCATGGTCGAATCT	TCAACTCGATTCCGGTGTCGCT	328
FMgSSR-10092	scaffold2014	p2	(AG)19	Class I	34864	34901	TCGGAAAAGAAAGGGCGTCGCT	TTGGCCAACTTCTCCTCCACCA	265
FMgSSR-10097	scaffold4885	p2	(AG)19	Class I	35013	35050	TGCAATAGACGCGCGTACACA	TCGCGGCTCTCTGCAAAACT	328
FMgSSR-10102	scaffold8708	p2	(AG)19	Class I	22864	22901	TGCTTGCACACGCGTTCACT	ATTGCCCACAGCCGACATTCCT	325
FMgSSR-10104	scaffold15730	p2	(AG)19	Class I	11142	11179	TGGCAGCACACTGTCCTCTTCT	TCACGCCGATTCAACCTCCA	345
FMgSSR-10106	scaffold341	p2	(AG)19	Class I	121807	121844	TGGCCGCCGTGATTTACTCCAA	ACTGTGTGCCCGCCCTCATAAA	251
FMgSSR-10110	scaffold13264	p2	(AG)19	Class I	8471	8508	TGTGCCGCCCGTAACATCAT	TTGGATTGCCAAGCTCCCCCAA	343
FMgSSR-10113	scaffold1996	p2	(AG)19	Class I	33412	33449	TGTTTGTTGGGTTGGGTCGGGT	TTGTTGGTGCGCTCGCGTTT	275
FMgSSR-10115	scaffold73	p2	(AG)19	Class I	165296	165333	TTCGCTCGAAAGGAGGCAGTGA	GCAAACTGGCATCTCACAACCCA	334
FMgSSR-10116	scaffold20658	p2	(AG)19	Class I	10470	10507	TTCGGGGCAAGGGCTAGGATTT	TTGAGCCATGGCCATCGTGGAT	314
FMgSSR-10118	scaffold5735	p2	(AG)19	Class I	21763	21800	TTGTCAGCAGGGCGTGTATGGT	TCGCTGTCGCCGTTCCAAAT	242
FMgSSR-10119	scaffold983	p2	(AG)19	Class I	77749	77786	TTTCACCGAAATGGCACCACCC	AAGCGCACTTCATCGCGCTCAT	318
FMgSSR-10126	scaffold129	p2	(AG)20	Class I	143464	143503	ACAAGGAGTCCGGAGTCATCCA	ATGGGGACGGCAAGTGCAAGAT	201
FMgSSR-10128	scaffold9051	p2	(AG)20	Class I	28807	28846	ACACTGTGATGATGCATGGGGG	AAGCTCAGGGAGATGATGCGGT	288
FMgSSR-10129	scaffold5263	p2	(AG)20	Class I	30856	30895	ACGTGGTACCGCCTTGACTT	TCGCCATGCACACAACAAAGCC	267
FMgSSR-10131	scaffold11692	p2	(AG)20	Class I	22614	22653	ACTGGTGTGGGGATGCAACT	AACCAGCGCGTTCACACAGT	247
FMgSSR-10132	scaffold15527	p2	(AG)20	Class I	10276	10315	AGACGTTGGGTAGCTCGCAT	TGGCGGGTGCTAAAATCAGCGA	241
FMgSSR-10133	scaffold3268	p2	(AG)20	Class I	29452	29491	AGAGAGGCAAGGAGAGGAGTGT	GCAAATCCAGGCAAGCTTGTCAAC	265
FMgSSR-10134	scaffold607	p2	(AG)20	Class I	20060	20099	AGCACGAACAAAGTCTCCAACAGC	TGCTGCGTCGGTCAGTCAAT	342
FMgSSR-10144	scaffold310	p2	(AG)20	Class I	7398	7437	AGTGTGCACCAAGGGTCGATGT	TCGGACCAGCAATCCTACGTCA	350
FMgSSR-10146	scaffold3986	p2	(AG)20	Class I	43375	43414	ATCTTGGGCGGTGCCATCATGT	AGCAAGAAGCAGCAACGCCT	276
FMgSSR-10150	scaffold2488	p2	(AG)20	Class I	29377	29416	ATTGGTCGAGAGGAGCAGCA	TGGCGGGGCAGCTAGATTTTGA	309

SSR_ID	Scaffold	SSR_Type	SSR_Motif	SSR Class	SSR_Start	SSR_End	Forward sequence	Reverse sequence	Product_size
FMgSSR-10151	scaffold3448	p2	(AG)20	Class I	48447	48486	CAACGTCCTCGCTAGCAAAAGT	ACGCGTTCCACTTCATCGGACA	212
FMgSSR-10153	scaffold2300	p2	(AG)20	Class I	20416	20455	GCCACCATCACTTTGCACAGCA	TGCATGCACACACGGACAGT	343
FMgSSR-10155	scaffold370	p2	(AG)20	Class I	125834	125873	GCCCGGCCAGTAATTGTATGCT	TGCATGCATTGCCGTTGCAG	299
FMgSSR-10157	scaffold30707	p2	(AG)20	Class I	685	724	GCGAATCTCGCGTGCATGATGA	AGCGCCGTTGCAACCTTGTCTA	228
FMgSSR-10158	scaffold34894	p2	(AG)20	Class I	2836	2875	GCTTATACCTCCCGTGACCTCGAA	ATACTTAATCTCCGGTCGTGCCCC	346
FMgSSR-10160	scaffold2634	p2	(AG)20	Class I	25579	25618	GGCACGTTGCACGCTATTGTCA	TCGCAGTTGGTGTCTACGCA	348
FMgSSR-10161	scaffold17309	p2	(AG)20	Class I	7874	7913	GGTTGAAACGGGGCAACAGCAA	ACGTATGCCTGGGCTTTGCAT	299
FMgSSR-10163	scaffold751	p2	(AG)20	Class I	19399	19438	TAATGGCGGCTCGGTCGTTGAT	ACAGCTTCACCGTCCCGTTT	292
FMgSSR-10164	scaffold1591	p2	(AG)20	Class I	63815	63854	TAGGTGGCGGCAAAATGGCT	AAAACCGTCATCGGGGGCTTCA	230
FMgSSR-10168	scaffold24452	p2	(AG)20	Class I	7153	7192	TCAGCACTTCTGTCGCCCAT	AGGTGTTTCCACGCACCACA	339
FMgSSR-10169	scaffold1436	p2	(AG)20	Class I	16581	16620	TCATCCATGGCTGCACGAGT	TGCCACTCTGAACCTCTTCTGGT	250
FMgSSR-10170	scaffold23810	p2	(AG)20	Class I	4805	4844	TCCAGCACCACCTAGTTTTCGT	TCACCCACACCCACAACGTTCA	262
FMgSSR-10174	scaffold16590	p2	(AG)20	Class I	10589	10628	TCTGCATCACCATAACCCCTTGC	TCAGCCAAATTCGGCCCAGAGA	248
FMgSSR-10180	scaffold3711	p2	(AG)20	Class I	26425	26464	TGCATGCATCCGCGTATGGTACT	TGGGGCTTGTCCGCATTGTT	327
FMgSSR-10181	scaffold6965	p2	(AG)20	Class I	10347	10386	TGCCAAGAGGCAAAACATGGCG	TCCTATGTGGCACAGTGGGTCT	332
FMgSSR-10182	scaffold7640	p2	(AG)20	Class I	27322	27361	TGCCGAACAAACCGTCACTCCT	GCCCTTGACAGCATCGTGAAGT	320
FMgSSR-10186	scaffold1430	p2	(AG)20	Class I	71710	71749	TTCACACCACCGGATTGCATCG	TCTGGCGGCGCAAAACAAAC	331
FMgSSR-10190	scaffold1802	p2	(AG)20	Class I	32464	32503	TTGGGTCTTTCTGCAGCGCCAT	AAGAGCAAAAGGGCCGCCAA	332
FMgSSR-10192	scaffold756	p2	(AG)20	Class I	96146	96185	TTTGCCAACGCCAAACGCTG	AGGCGGGCTCAACATCGAAGAA	338
FMgSSR-10197	scaffold20638	p2	(AG)21	Class I	3309	3350	AAACGGCAGCTGTGCTTCCA	TTGCCGTTTGCCGTCGATACCT	283
FMgSSR-10206	scaffold26278	p2	(AG)21	Class I	3148	3189	ACCAGCACAAGAAAGGCGTCA	AGCATGCCAAGCTCCACACCAT	269
FMgSSR-10215	scaffold7922	p2	(AG)21	Class I	21649	21690	AGGGAAACACATGGATGCTCGCT	TGTTCGACGCCGCTGAAAGA	314
FMgSSR-10216	scaffold340	p2	(AG)21	Class I	95608	95649	AGGGAGGGTGTACATTGGCTGT	GTGCAACTCATTTGGTGCAACCTG	220
FMgSSR-10219	scaffold6634	p2	(AG)21	Class I	2075	2116	AGTGTGCCTTGGATCCTCTGCT	TCACAATGGTGAGCACGACACA	275
FMgSSR-10220	scaffold2296	p2	(AG)21	Class I	29710	29751	AGTTGCCACTGCTGCGTCAGAT	ACTCCCCTCACTTCACTCCTCCTT	302
FMgSSR-10226	scaffold2069	p2	(AG)21	Class I	67797	67838	ATGGTGGTGCCCACACAAGT	TCAATAGCGGCGGCCCAAAA	296
FMgSSR-10227	scaffold11652	p2	(AG)21	Class I	17350	17391	ATTTTGCGCAGGGTGCTGCT	AGGCTTGGCGTCTCCTCATCAT	298
FMgSSR-10229	scaffold784	p2	(AG)21	Class I	10436	10477	CCTAAGAGATGCTAATCCCAAGCG	TGCCCGGCCTCTTGCTTATGTT	296

SSR_ID	Scaffold	SSR_Type	SSR_Motif	SSR Class	SSR_Start	SSR_End	Forward sequence	Reverse sequence	Product_size
FMgSSR-10231	scaffold2138	p2	(AG)21	Class I	34154	34195	GCAACAAGTTGCAACGGGGCATA	TTGGCGGCATGCATGGTTCA	344
FMgSSR-10233	scaffold874	p2	(AG)21	Class I	8568	8609	GCATGCAGATTCACGTGGGCAA	TTTATACCGGTTGGCGGGTGCT	319
FMgSSR-10235	scaffold8344	p2	(AG)21	Class I	30915	30956	GGCAACCACTGTGCGTGACAAA	ACCTGAAGTGCCCAACAAGCA	207
FMgSSR-10243	scaffold4075	p2	(AG)21	Class I	27778	27819	TCCTCCCTAGTGCACCTTGCAT	AAAACCCACACGACCGAGCA	317
FMgSSR-10244	scaffold182672	p2	(AG)21	Class I	555	596	TCGACACATGGCCACACCTCAA	ACCGCCCTCTCCCTTTCTCTTCAT	256
FMgSSR-10247	scaffold1510	p2	(AG)21	Class I	56944	56985	TGAAGCGCACCTTCTTGCACT	ACATGCCACACAGCTCATGCCT	270
FMgSSR-10248	scaffold391	p2	(AG)21	Class I	70171	70212	TGCAGAGAGCAGAATGCGCTGA	ATTCCGCTTTGCGCGACTGA	284
FMgSSR-10252	scaffold2148	p2	(AG)21	Class I	42417	42458	TGCTGCATCCTGCACACAGT	CGCAGCTTGGACGATGGCATTT	305
FMgSSR-10255	scaffold7682	p2	(AG)21	Class I	24340	24381	TGTGCAGCGCCGCAGATATT	AGCGTCTCTGCCACCAAGAAGT	314
FMgSSR-10257	scaffold2488	p2	(AG)21	Class I	28364	28405	TGTTGCTTGGTCGTGCACCT	AAAGCGGGTGTTTGGACGGA	305
FMgSSR-10258	scaffold224	p2	(AG)21	Class I	88558	88599	TTAACTTCGGCGAGGAAGGCGT	AGTGGCTAAAGATCGACGCGAGA	274
FMgSSR-10259	scaffold17184	p2	(AG)21	Class I	9339	9380	TTAGCCTCTGTTGTGGCGCA	TTTCTCACGCGCTGGAGCTT	270
FMgSSR-10260	scaffold175	p2	(AG)21	Class I	56980	57021	TTGCAACGGCCCTGTCTACACT	TGTGCCTGACTCCTTTCTTCCCT	308
FMgSSR-10265	scaffold13594	p2	(AG)22	Class I	9681	9724	AACCGCGCACATGCCTTTGT	ATAGCAGAAGCTCGATGGCGCA	223
FMgSSR-10266	scaffold5325	p2	(AG)22	Class I	27273	27316	AAGATAGTCGGGGAAAGAGGCGCT	TATGTTACACGGGCACTGGGCA	349
FMgSSR-10277	scaffold2134	p2	(AG)22	Class I	23245	23288	ATACACAAGGTTGTCGCCGC	TGTGTTTCTCCCCCTTCACTCTCT	314
FMgSSR-10279	scaffold7847	p2	(AG)22	Class I	14218	14261	ATGCCAGCTGCACCAACACT	AACTAGGGTTCCTCACACCGCCTA	313
FMgSSR-10280	scaffold5304	p2	(AG)22	Class I	36177	36220	ATGCGAGCAGCCACCACTACTT	AGGATCGTGCAAGTGAAGGCGA	219
FMgSSR-10285	scaffold6485	p2	(AG)22	Class I	26640	26683	CACGTCACGCATGCTGAATCCT	AGCGGCGGCTTCTATTGCAT	315
FMgSSR-10288	scaffold1629	p2	(AG)22	Class I	20592	20635	CGGCAACTCAGAGTAAAATAGGCG	TGCACGCCGCATGTACCATT	305
FMgSSR-10292	scaffold59260	p2	(AG)22	Class I	470	513	TAGCACCACGAGTAGGCAAGGA	ATGTCGTCGCCCTATTGGCTCT	278
FMgSSR-10295	scaffold38945	p2	(AG)22	Class I	237	280	TCGAACTGCACGGCACAGAA	TGCTCTTCTACGTGCGCCTCAA	303
FMgSSR-10296	scaffold4120	p2	(AG)22	Class I	29527	29570	TCGGCAACAAACATGCGGCT	ACTGGGACGGCAATCACGTTCA	275
FMgSSR-10297	scaffold20830	p2	(AG)22	Class I	7023	7066	TCGTCCGAGAAGGAAGGAGCAA	TCCTTTCCGTCGACGTTGGT	234
FMgSSR-10302	scaffold2056	p2	(AG)22	Class I	39043	39086	TGCAGGCCCATGCCTATGTTCA	CGATGCGCTGGATCGTTCGTTT	294
FMgSSR-10308	scaffold2515	p2	(AG)22	Class I	64505	64548	TGTCACCGGCGGGATGCTTAAA	TGGCCGTGTGTGGCAGCATTAT	314
FMgSSR-10310	scaffold42	p2	(AG)22	Class I	153285	153328	TTAATAACCCGTGCCGCCGTGA	TGCCTTGCTTTGCTTGCGGT	219
FMgSSR-10313	scaffold22644	p2	(AG)23	Class I	938	983	AAAGAAGCGAGCGCCCTCACAA	TGCTCGTTCGATTTCGCCCA	306

SSR_ID	Scaffold	SSR_Type	SSR_Motif	SSR Class	SSR_Start	SSR_End	Forward sequence	Reverse sequence	Product_size
FMgSSR-10319	scaffold18180	p2	(AG)23	Class I	10491	10536	ACAAGCCACTGCCGCAACTA	ACTCGCGCTTCAGTGTGTAGCA	289
FMgSSR-10320	scaffold4646	p2	(AG)23	Class I	24663	24708	ACATGCATGCACAGCAAGCG	ATTGGCAGCCACGAGTGCAA	286
FMgSSR-10322	scaffold6575	p2	(AG)23	Class I	22043	22088	ACCACTTTAGAGCTGCCCCACA	TGCGCGAATGAGGAATGCCA	298
FMgSSR-10323	scaffold1578	p2	(AG)23	Class I	14902	14947	ACCAGTCCAAAAGCAACACGC	TCATATCCTCCCAGGTGCTGGT	242
FMgSSR-10324	scaffold25794	p2	(AG)23	Class I	4127	4172	ACCATTCTTGCGAGAGCGTCA	TGAGAGTGTGGGGAGTCATTGAGGT	261
FMgSSR-10327	scaffold159	p2	(AG)23	Class I	64820	64865	ACTGCACTCGGGAGGCTTTTGA	AGCACAGGTCTTCCTGCCCTTT	333
FMgSSR-10329	scaffold7739	p2	(AG)23	Class I	14309	14354	AGCCTATCGCACTGCACGTT	ACGTGTGAGGTGCCGATGGTAA	311
FMgSSR-10330	scaffold15132	p2	(AG)23	Class I	12046	12091	AGCTCGCGTTGTAGCAGAGGAA	TGCATGGGCGGGAGATCTAA	301
FMgSSR-10331	scaffold204	p2	(AG)23	Class I	99471	99516	AGGGAACTACATTCCAGACGACGA	TGGGCGGCAAGTGTGTTCTT	284
FMgSSR-10333	scaffold9756	p2	(AG)23	Class I	7107	7152	CGACATGAAGCCGTTTGAGGCA	ATGGTGGCCTCCCCACAATGAT	349
FMgSSR-10334	scaffold10106	p2	(AG)23	Class I	3485	3530	CGGCGATGTCAAATCCTTGGGT	TCCGTTCATGCCCCTGATGT	350
FMgSSR-10335	scaffold3922	p2	(AG)23	Class I	37400	37445	CGGTAAAACCACCGCACCGAAA	AACCCGCTGCTGCAGTGAAA	318
FMgSSR-10337	scaffold15597	p2	(AG)23	Class I	10134	10179	TATGCACCGGCATGGCCTTCTT	TCATGGCCTGCAATGTGTGC	283
FMgSSR-10339	scaffold313	p2	(AG)23	Class I	59069	59114	TCGATCGATGGGACAGGTTGAGGA	ATGCGTTCGTGTTGCGCTCT	325
FMgSSR-10344	scaffold7573	p2	(AG)23	Class I	10921	10966	TGCTATGCAACTGATGGAGGAGC	CGGTGTTGATGCTCCACTGCAA	247
FMgSSR-10347	scaffold12911	p2	(AG)23	Class I	6478	6523	TTAAGCGACGTCCAGTGCTGCT	TTCGACCGTTTCGAGGCCATCA	348
FMgSSR-10349	scaffold2418	p2	(AG)23	Class I	39897	39942	TTTTTCCCCAGGCTGCAGGT	AGCAGACTGAGGCCGTCTAGTAA	278
FMgSSR-10350	scaffold12370	p2	(AG)24	Class I	8479	8526	AAAGCTGCTGCCGTTTTGGTGG	TCGTATCTATTGGCCGTGCCGT	275
FMgSSR-10355	scaffold21627	p2	(AG)24	Class I	4697	4744	ACCACACAACACACACGCACAC	TTGAAGCGCAAGCCATCGCA	301
FMgSSR-10357	scaffold7947	p2	(AG)24	Class I	20803	20850	AGATTCATGCTCCGTTGACGCC	GATTTTGTCCCCACAACGGTCA	330
FMgSSR-10360	scaffold287	p2	(AG)24	Class I	91976	92023	AGCGACTGCATCTACGTCTGGA	AGGCTTCCCAGAGTGACAACGA	288
FMgSSR-10363	scaffold10592	p2	(AG)24	Class I	3375	3422	ATGGTAGATCTTTGGCGGGCGA	TGCGACCTTCTCGTCGACTAAC	259
FMgSSR-10364	scaffold3659	p2	(AG)24	Class I	43220	43267	CGCCGCCTGCGAATTTCATT	TCCCAGGTAAACAACCCACCCA	205
FMgSSR-10365	scaffold4886	p2	(AG)24	Class I	29369	29416	GCACGTGCATCGCAAAAACG	CGATTTGGTTAGGCGCCGCAAT	210
FMgSSR-10366	scaffold27832	p2	(AG)24	Class I	3835	3882	TACCAGCCCATACCACCAAGCA	TGTGCTCCAGCTGCCTTGTGAA	300
FMgSSR-10368	scaffold15871	p2	(AG)24	Class I	4504	4551	TCATTGCCGCACAATGCCCT	AGGGCAAGCTCGACTTGGACATA	237
FMgSSR-10369	scaffold34847	p2	(AG)24	Class I	1460	1507	TCTTTGGCTGCGGGGGATGATT	TTTTAAGGCGGTAAGGCGAGGC	311
FMgSSR-10371	scaffold764	p2	(AG)24	Class I	88168	88215	TGCACAGCCGCACACATGAA	TCGATAACCCGCCGTGCAAA	240

SSR_ID	Scaffold	SSR_Type	SSR_Motif	SSR Class	SSR_Start	SSR_End	Forward sequence	Reverse sequence	Product_size
FMgSSR-10372	scaffold724	p2	(AG)24	Class I	15010	15057	TGCCCATCCAAACACGTCTCCA	TTCCAACTCTTCGCCGGGCTAT	343
FMgSSR-10374	scaffold8535	p2	(AG)24	Class I	21731	21778	TGGATCCATCATCACGCGCT	TCTCTCACACACCACCAAACCG	327
FMgSSR-10377	scaffold394	p2	(AG)24	Class I	59450	59497	TGTTTTGCCACGGACCGATGTG	GCATGTGGACCCAATAGTGCAAGG	231
FMgSSR-10381	scaffold49	p2	(AG)25	Class I	99963	100012	AAAATGGCATCGTAGGGGCGCA	TTGTTCGCTGTTCGGACGCA	326
FMgSSR-10382	scaffold454	p2	(AG)25	Class I	72150	72199	AACACGGTAAACCAGGCGGT	TGCAGCAACTGGCGCTTTCA	345
FMgSSR-10387	scaffold6211	p2	(AG)25	Class I	35027	35076	AGATCCTTCTTGCGTGCCGA	ACGGCCAAGTGGTCCTACTCAT	305
FMgSSR-10389	scaffold5393	p2	(AG)25	Class I	19179	19228	AGCGCACAAGACGAAGCGAA	TGATTTCATCGCACACGCCG	329
FMgSSR-10391	scaffold23224	p2	(AG)25	Class I	6648	6697	AGGGGAAAGGGGAAGCTCACAT	TTGCTGGCGTCCATGAGGTTGA	284
FMgSSR-10392	scaffold253	p2	(AG)25	Class I	69181	69230	ATGCACGCAAGAGGAGCCAAGT	TAATTGCCGCCTCGCCTTGT	229
FMgSSR-10393	scaffold30	p2	(AG)25	Class I	171400	171449	CCAGTCGGATAGAGCTTTGCTGCT	GGATCTTCTCCATCTTCTCTCCCTC	273
FMgSSR-10395	scaffold906	p2	(AG)25	Class I	35703	35752	CGCAAGATGCATGTACTGATCCCGT	TCCATGGTGCCACTATCCCAGT	268
FMgSSR-10396	scaffold16856	p2	(AG)25	Class I	4717	4766	TAAATCGCGCCCAGCCACTCAT	TGACGGGTGCTCACATGTCACT	303
FMgSSR-10397	scaffold4096	p2	(AG)25	Class I	46762	46811	TCAAAGTCGTACAGGTCAGCGAGG	TCAGGATTGCGATGAGGTGCGT	323
FMgSSR-10398	scaffold5461	p2	(AG)25	Class I	20889	20938	TCCTCTTCGGCGGTGAAGTT	ACCCAAACAAAGTGCCCCGGTT	324
FMgSSR-10400	scaffold179084	p2	(AG)25	Class I	202	251	TGACACAGAGAGTCGCGGAAGT	TTGACATGGGCCACTTGTGCCT	267
FMgSSR-10401	scaffold1995	p2	(AG)25	Class I	57681	57730	TGCGACGAAGAATGCCGAGT	TCAATGCCGGCACATACACG	338
FMgSSR-10403	scaffold3089	p2	(AG)25	Class I	43590	43639	TGCTTCATCTTCCTCCTCGGCT	TGTTGGCCGAAACATGGCGT	321
FMgSSR-10404	scaffold49830	p2	(AG)25	Class I	2925	2974	TTGCGTGGGAGCGTTTGTCT	ACAACAGCCTCACCCAAAGTGG	325
FMgSSR-10406	scaffold17048	p2	(AG)26	Class I	7780	7831	AAAGCACACGCCAACCACCA	TCCCTCCAGCATGTGTGCAA	277
FMgSSR-10413	scaffold253	p2	(AG)26	Class I	85532	85583	CCAGTTGCAATCGTCTTCTTCCCA	GTGAACATCCGTGCATGTCCCT	341
FMgSSR-10414	scaffold2431	p2	(AG)26	Class I	65021	65072	CCGGAGTTCTTGCGCCGTTAAT	TGGCTGCAAGCAGACATGTTTTGG	272
FMgSSR-10418	scaffold25923	p2	(AG)26	Class I	1683	1734	TGCAAGCCAGCAACAGAGCA	ACACTTTGGGCGCGTGTTCTT	278
FMgSSR-10421	scaffold32805	p2	(AG)27	Class I	5854	5907	AATCGGAGAAGGAGGAGGTGGTGT	TTTTGTAGGGGCGGCCCATGTT	267
FMgSSR-10422	scaffold3098	p2	(AG)27	Class I	51415	51468	ACAAGCACACCCCTCGCTTCTT	TGGCGTCAGTGTACTCTCCTTCCT	349
FMgSSR-10424	scaffold10930	p2	(AG)27	Class I	21327	21380	CAGTCGAGGATGATAAGCCCTAGT	TCACCACGGTAAGTGGCCATGAT	337
FMgSSR-10431	scaffold8256	p2	(AG)27	Class I	16462	16515	TGCCGTTGCGTGACATCGAA	AGGCTGCCATTTAGCCCAACCA	331
FMgSSR-10436	scaffold617	p2	(AG)28	Class I	23814	23869	CGAATCAAATTGGGGTGCGCCAT	TTGCGCACTGTCCCACAAGT	204
FMgSSR-10438	scaffold4438	p2	(AG)28	Class I	41613	41668	TACTACGCTCGCCACGAACTGT	TGCCATCCACCCTTTTGTCCCT	226

SSR_ID	Scaffold	SSR_Type	SSR_Motif	SSR Class	SSR_Start	SSR_End	Forward sequence	Reverse sequence	Product_size
FMgSSR-10441	scaffold501	p2	(AG)28	Class I	38018	38073	TGGAGTCTGATCCGCCCATGTT	TGCACCACTTCAACCAGACCCA	275
FMgSSR-10447	scaffold50617	p2	(AG)29	Class I	2654	2711	AGCCCAGCACTAGCTCGTTGTA	GGCCAGCCATTGGCAACCAAAT	279
FMgSSR-10450	scaffold3693	p2	(AG)29	Class I	50745	50802	TACTGCTCATAGCCGTTGCGCT	TAATCCCTTGCACACCCCCTCT	302
FMgSSR-10451	scaffold334	p2	(AG)29	Class I	102287	102344	TCACAGCCATTCGCGCACAA	ACACCTGGACGAATACAGAAGGACC	299
FMgSSR-10453	scaffold4335	p2	(AG)29	Class I	32441	32498	TCCACCGAAGGGCGAACCTAAA	TTCGACGAGTGGCCGTGATGTT	251
FMgSSR-10454	scaffold7430	p2	(AG)29	Class I	17845	17902	TCCTCCCAGCACGCACAACTTA	ACGCAAGGTTGAGCAGGACT	339
FMgSSR-10458	scaffold7055	p2	(AG)30	Class I	22142	22201	TCGATGGACAAACCTGCTGCCT	TTGTGCTGGCGAAGATCACAGC	345
FMgSSR-10459	scaffold3572	p2	(AG)30	Class I	17693	17752	TGCAAGCTCTGCAAGAACACG	ACAGAAAATCCTGCGTCCGCCA	323
FMgSSR-10464	scaffold7385	p2	(AG)31	Class I	29928	29989	AGCGAATTGCCGCAGCAGAA	AAAGCCGTGTTGTGCCAACTGC	330
FMgSSR-10466	scaffold14537	p2	(AG)31	Class I	16157	16218	GCACCACCAAATCGAGTCCGAA	TGCGCTGAAAAATTGGCTCCCG	347
FMgSSR-10467	scaffold387	p2	(AG)31	Class I	13323	13384	GTGTTGAACCATGTCGCTGCGT	AACCTCAAGAGTGCGTGCGT	221
FMgSSR-10470	scaffold27757	p2	(AG)32	Class I	8500	8563	AAGCTTCTCCAACCGTGCCCAT	TCACGGGGAAAAGTGTGGGCTT	224
FMgSSR-10471	scaffold243	p2	(AG)32	Class I	108634	108697	TAAACTTGCACGGCTCGGACCA	AAAACGATCGCCCTCTCCTTGG	347
FMgSSR-10472	scaffold20925	p2	(AG)32	Class I	3155	3218	TCAACCAAGCAAAAGGCGGCA	ACAAACAAGCCCACACCCCACA	246
FMgSSR-10476	scaffold7192	p2	(AG)33	Class I	24049	24114	TGGGGGAAAAGGCTTCCGTT	TGCAGAGTGCAGACAACAGGTG	222
FMgSSR-10477	scaffold513	p2	(AG)34	Class I	119418	119485	ACCAACGTGGTCGTAGAGCTTCA	TCGTGAGTCCTCTCTCTCTCTCT	279
FMgSSR-10478	scaffold9516	p2	(AG)35	Class I	11357	11426	TCGCGCCCGTATTCTTGAAGGT	TCTTGGTGCCGTTGTCGGAA	281
FMgSSR-10479	scaffold21702	p2	(AG)35	Class I	8038	8107	TGCCGACCCATCGTTCAAGCAT	CGGATAAGACGTGCGTGTGCAT	325
FMgSSR-10480	scaffold394	p2	(AG)35	Class I	47300	47369	TGCTGCACAGGTCCAAGTGT	ACGCCATTCATGCGTGCAGAT	256
FMgSSR-10483	scaffold22209	p2	(AG)37	Class I	6271	6344	TGACGGATTGTAGAGAGAGAGCGA	TTGGTCAGCAGCTCGTCGAT	326
FMgSSR-10484	scaffold54965	p2	(AG)39	Class I	1885	1962	TTTGCTCATCGCCTGCCGTT	AAAACATCGAGTGCGGCTCCCT	313
FMgSSR-10490	scaffold4504	p2	(AG)54	Class I	5306	5413	ATTTGCACCATCTCCGGCCGTT	ATAACCGACGCCTGGGTGTCTT	248
FMgSSR-10492	scaffold25341	p2	(AG)6	Class II	9652	9663	AAAACACGCGCGCACACACA	AGCTGCAACTCCACCGCTTCAT	210
FMgSSR-10493	scaffold3524	p2	(AG)6	Class II	1921	1932	AAAACCCACAGGAGAGGGTGGA	ATTGGTTGCTCTCGATGCGTGC	311
FMgSSR-10494	scaffold3653	p2	(AG)6	Class II	27942	27953	AAAACCTGGCCGGTGTCCAA	AGTTACTCAATTGGGCCGGCGT	227
FMgSSR-10503	scaffold22224	p2	(AG)6	Class II	5597	5608	AAACAAGCGACGACGCACGA	TGCAGCGACGTTGCGAGTGATA	309
FMgSSR-10505	scaffold186	p2	(AG)6	Class II	32540	32551	AAACACCGGAGACTAGAGGCCA	TTTGGTTGGTTGGGAAGCACGC	322
FMgSSR-10509	scaffold1205	p2	(AG)6	Class II	7703	7714	AAACCAAGCCAGCACCAGGA	TGATTGGTTCGTTCTCCCCGCA	342

SSR_ID	Scaffold	SSR_Type	SSR_Motif	SSR Class	SSR_Start	SSR_End	Forward sequence	Reverse sequence	Product_size
FMgSSR-10523	scaffold5212	p2	(AG)6	Class II	25152	25163	AAAGCAACGGCAGCGTTGTG	TCGAGACTGGGATGGTGATCGTGA	237
FMgSSR-10524	scaffold6242	p2	(AG)6	Class II	18848	18859	AAAGCAGGCAGGCAGAGACA	ATCGTATTGTGGGTGCGCAGGT	284
FMgSSR-10539	scaffold128	p2	(AG)6	Class II	121257	121268	AAATGGAGCGCTACGGACGA	AGGGCGCAGATCTAGCTCAA	322
FMgSSR-10542	scaffold247	p2	(AG)6	Class II	19062	19073	AACACGTGTCTGCGTTGTCCGA	TTGCAACACCCAAAAACCGGGC	344
FMgSSR-10543	scaffold5840	p2	(AG)6	Class II	15364	15375	AACAGAGCATGCCACTGCGA	ATGTGGGCGGCAGATCAACTCA	246
FMgSSR-10546	scaffold733	p2	(AG)6	Class II	102868	102879	AACCCTGCTGCAAAAGGTGGCT	TCGTTCGTTCGTTGCCTCGT	270
FMgSSR-10551	scaffold4951	p2	(AG)6	Class II	10529	10540	AACCTGCAAGCGCCGTAGAA	AGTGCTGCTGTGTTCCCCTT	328
FMgSSR-10554	scaffold738	p2	(AG)6	Class II	28036	28047	AACGAACCTCGGCCTGCATT	AGCTTGCCGCATTGGTGAGT	259
FMgSSR-10557	scaffold1458	p2	(AG)6	Class II	25558	25569	AACGCACTGCACGCAAGCAA	TTGACATGCAAGAGCACCCCCT	231
FMgSSR-10558	scaffold8682	p2	(AG)6	Class II	10844	10855	AACGCCAACAACTTCCGCTTGC	AGCCACCAACACGCAAAGCA	350
FMgSSR-10559	scaffold5016	p2	(AG)6	Class II	35047	35058	AACGCCCAGGAAAAGTCGCA	ACCCTAACACTCCCCTCAGCTT	296
FMgSSR-10560	scaffold223	p2	(AG)6	Class II	20640	20651	AACGCGGCCTCAAACACCAT	AGTGTTGTTGCTGCTGGCGA	314
FMgSSR-10562	scaffold2437	p2	(AG)6	Class II	49884	49895	AACGGGTTCTCCGAATTGGC	ACAGGACGAAAGGTAGGAGCGT	349
FMgSSR-10563	scaffold233	p2	(AG)6	Class II	8297	8308	AACGGTCGCGTTCGTCGTTT	TCGATTGAGTGCCGATCCACGA	211
FMgSSR-10564	scaffold1680	p2	(AG)6	Class II	35040	35051	AACGTGCCATGCGTGTCGAA	ATAGCTGCATCAACGAACCGGG	260
FMgSSR-10565	scaffold3433	p2	(AG)6	Class II	57816	57827	AACGTGCTTGCGCTGGTCAT	TGCTGCTTGTTGTTGTTGGGGC	293
FMgSSR-10572	scaffold1615	p2	(AG)6	Class II	29928	29939	AACTTAGGGCATCTCCAGCAGT	AAGCTCACCACGCACACACA	306
FMgSSR-10576	scaffold2979	p2	(AG)6	Class II	26507	26518	AAGACGGTGATGGCGTGGCATT	TGTCGACGTCGCCTTTCGAAGT	345
FMgSSR-10590	scaffold1455	p2	(AG)6	Class II	36618	36629	AAGCTCGGCATTGCATGGGGAA	ACAAACAACAGAAGGCCCCGGA	230
FMgSSR-10596	scaffold25905	p2	(AG)6	Class II	1363	1374	AAGGATAGGAGCGTTGGGGCTGTA	ATGATCTTGCCAGCACCGTCCA	307
FMgSSR-10598	scaffold550	p2	(AG)6	Class II	49014	49025	AAGGCACCCACCAACAGAGAGA	TCGCGTGCTAGCTGCACAAA	228
FMgSSR-10602	scaffold19297	p2	(AG)6	Class II	3145	3156	AAGGTGCTCAGCAGCGGGATAA	ACGTGCACACAAGGGTGGTT	213
FMgSSR-10603	scaffold223592	p2	(AG)6	Class II	432	443	AAGGTTTTCCGGCCCGATTCCA	AAATTCAGGCACCGCACACAGC	345
FMgSSR-10610	scaffold9541	p2	(AG)6	Class II	12085	12096	AATAGCCCGCTCCCAACGAAGA	TGGCATCTGCGGAACAACCT	322
FMgSSR-10612	scaffold1662	p2	(AG)6	Class II	72590	72601	AATCGCAGGGGAACGTGCAT	AACGACGTCACCATCACCGGAA	204
FMgSSR-10615	scaffold46	p2	(AG)6	Class II	59175	59186	AATGAAGGAACCGAGCGGCA	AATTGCCGTTACGGTGGTGGGT	348
FMgSSR-10622	scaffold62046	p2	(AG)6	Class II	1243	1254	AATTGCGCCACCCACTGTGA	AGGCTACGCACTGTTGTTACGGT	334
FMgSSR-10627	scaffold2700	p2	(AG)6	Class II	40813	40824	ACAAGCTCGTCAAGGCGCAGAT	GCACCACCTTGAACAGGTAGTCGT	242

SSR_ID	Scaffold	SSR_Type	SSR_Motif	SSR Class	SSR_Start	SSR_End	Forward sequence	Reverse sequence	Product_size
FMgSSR-10630	scaffold2106	p2	(AG)6	Class II	18886	18897	ACACCCGCGCACATGAGAAT	ACCATCAACGGAACGGAACGGA	298
FMgSSR-10636	scaffold5042	p2	(AG)6	Class II	13352	13363	ACACGGCATGCTACACCTCA	ACGCTTGCCTATCTCCAGCACA	314
FMgSSR-10640	scaffold20004	p2	(AG)6	Class II	11793	11804	ACAGCAATCGGCCCAGAGAACA	GCTGCGGCTATTGTTTGCTGCT	295
FMgSSR-10644	scaffold3937	p2	(AG)6	Class II	37038	37049	ACAGCAGCGTGTAAAGCCCA	ACGCGCAACCACAAAAACGC	288
FMgSSR-10647	scaffold324	p2	(AG)6	Class II	117745	117756	ACAGCGCGAAGCACAAGACA	ATACCTGCTGCGTGCAGTGA	259
FMgSSR-10651	scaffold10840	p2	(AG)6	Class II	6350	6361	ACATCGCGCACGCAACTTGT	AACATCCAGCGACCTACCTGCT	236
FMgSSR-10656	scaffold8265	p2	(AG)6	Class II	25001	25012	ACATGTCCATACGCACGCTCCA	TTCGTTGCCCAACTCCCACGTT	202
FMgSSR-10657	scaffold10994	p2	(AG)6	Class II	22845	22856	ACCAAGCGATTGGCATTCGAGA	TCCGGACAGCCTCAATGTTGCAT	239
FMgSSR-10663	scaffold22643	p2	(AG)6	Class II	6715	6726	ACCAGCCCACTGAACAAGCTCT	ACACACCATCGAAAGCCTCGCT	300
FMgSSR-10668	scaffold11670	p2	(AG)6	Class II	14132	14143	ACCCACAGGTTGCTTAGCCTTGT	ACTCATGCAACCCCCATGGACA	334
FMgSSR-10669	scaffold9157	p2	(AG)6	Class II	20390	20401	ACCCATCTTCTCCGGCGAGGTTAT	CGCACGTACATTATGCGCCGTT	276
FMgSSR-10677	scaffold3783	p2	(AG)6	Class II	26908	26919	ACCCTGCGGTTGAAAGCACTGA	TGGTGGCATTAGGGGAACCTTCT	288
FMgSSR-10682	scaffold5223	p2	(AG)6	Class II	33487	33498	ACCGCACAAACCCACAAATGCC	AGCATGGCCTGGTTGGTGACAT	333
FMgSSR-10690	scaffold13702	p2	(AG)6	Class II	18042	18053	ACCGGCGGTGTTCATGTGAT	TGTTCTCGCTCAGGCTGCCATT	222
FMgSSR-10692	scaffold13	p2	(AG)6	Class II	56907	56918	ACCTCCGTGCCTCTTTTGCCTT	AGAGTTGGCGAGTGCGTGAA	212
FMgSSR-10695	scaffold7346	p2	(AG)6	Class II	9577	9588	ACCTGCAACAGCAGCCTCTT	TCGCGAGTCGATTGGCAGTGTT	343
FMgSSR-10696	scaffold112850	p2	(AG)6	Class II	677	688	ACCTGGATTCCTGATGGTGCAGA	AGCACTGTCACGCCATCCTCTT	318
FMgSSR-10700	scaffold3858	p2	(AG)6	Class II	41252	41263	ACCTTTGGGCGGTTTGTGTGGA	TGCTTATTGCAGGCGCGTTG	203
FMgSSR-10701	scaffold8444	p2	(AG)6	Class II	17375	17386	ACGAAACCCCAACCCAAGAGCA	ACATTGTGCAGATCGCCGGT	309
FMgSSR-10705	scaffold5564	p2	(AG)6	Class II	18455	18466	ACGACAAACTAGGGAGACGGAGGA	TTTGTGTGACCCACCTGTCAGC	283
FMgSSR-10714	scaffold4628	p2	(AG)6	Class II	15482	15493	ACGATGTGCTCACCTTCGTGGA	TCCTCTCATTCCCCTTCCACCT	336
FMgSSR-10715	scaffold2678	p2	(AG)6	Class II	8645	8656	ACGCACAGAGGAAGCAACCA	AGTGGCCCATAGCGCTTTTCCT	340
FMgSSR-10719	scaffold6340	p2	(AG)6	Class II	10708	10719	ACGCCGCAGATTGATGGTGT	GCAGAGTGTGGAGCCAGCAAAT	210
FMgSSR-10720	scaffold84	p2	(AG)6	Class II	74271	74282	ACGCCGCATGATGTAGCCTT	TCAATCTCCCCGTCGGCATT	349
FMgSSR-10723	scaffold981	p2	(AG)6	Class II	4463	4474	ACGCCTGACGCTCAAGCAGAAT	AAACACGTGCGACAGAGCCT	232
FMgSSR-10733	scaffold361	p2	(AG)6	Class II	15596	15607	ACGGAACAATGAAACCGGGCT	AGGCGCGCCAATTTGCATGT	295
FMgSSR-10737	scaffold246	p2	(AG)6	Class II	39930	39941	ACGGAGAGGAACACGCAAGCAA	TACCAGGCGGCCATCAACAACA	217
FMgSSR-10738	scaffold65082	p2	(AG)6	Class II	1603	1614	ACGGCCGAACATGTGGTGTT	AAGCGTTTTGCTCGTCGCCA	328

SSR_ID	Scaffold	SSR_Type	SSR_Motif	SSR Class	SSR_Start	SSR_End	Forward sequence	Reverse sequence	Product_size
FMgSSR-10740	scaffold6472	p2	(AG)6	Class II	17504	17515	ACGGCGAGAAGCGGCAATTA	AACTGCCTGCCTGCTTTGCT	255
FMgSSR-10742	scaffold3462	p2	(AG)6	Class II	11326	11337	ACGGGATGGGTTTGGCAGAAGA	ACCCTCACAACACTACCACCACT	221
FMgSSR-10744	scaffold9521	p2	(AG)6	Class II	6767	6778	ACGGGCGGTGGTGAGAAAGATT	ACGGTCATGGTCACAAAACGCT	238
FMgSSR-10748	scaffold307526	p2	(AG)6	Class II	251	262	ACGTACGTATGCGCGTGTTC	ACGGCCATACGGGTGTTGAA	289
FMgSSR-10749	scaffold6460	p2	(AG)6	Class II	38822	38833	ACGTCACACGCGCTACACTT	AGCGCAGAGAAACTTTGCCCCT	266
FMgSSR-10754	scaffold8466	p2	(AG)6	Class II	4722	4733	ACGTGCCGATGCCATGTGTA	TTTGGCCGCGGAAGGCTTTT	321
FMgSSR-10755	scaffold4898	p2	(AG)6	Class II	26208	26219	ACGTGCTGCTGTTGGCCTAGAA	TTTCATGCACTATCACGCCCGC	338
FMgSSR-10759	scaffold713	p2	(AG)6	Class II	52409	52420	ACGTTCATCCATTGGGAGGCGA	AAGGACAGCAGCAGTAGCCGTA	326
FMgSSR-10760	scaffold5290	p2	(AG)6	Class II	21199	21210	ACGTTGCAGCTAGCGGATGAGT	TTTGTCCGCACGCTCTGCTT	204
FMgSSR-10764	scaffold6911	p2	(AG)6	Class II	2509	2520	ACTGCTCGCTGGCAGTTTCA	GCACTGTTCTTGTTCCACCGCT	314
FMgSSR-10767	scaffold1604	p2	(AG)6	Class II	27692	27703	ACTGTAGCAACCACCTTGCGGT	AGCAATGTCTTGGGCTTGGTCA	261
FMgSSR-10772	scaffold12078	p2	(AG)6	Class II	2267	2278	ACTTGCGTGGTTTGAGGCCA	TCCAACGACGTGGAATGGCT	207
FMgSSR-10777	scaffold8759	p2	(AG)6	Class II	27165	27176	ACTTTGCACCGAAAACGGCT	TCGTGCAAGCACCACCAACA	263
FMgSSR-10779	scaffold791	p2	(AG)6	Class II	48860	48871	AGAACACGGGCACGCTCAAA	TAAGCGGCGGCTGGCTTATGAA	236
FMgSSR-10780	scaffold7615	p2	(AG)6	Class II	14649	14660	AGAAGAGGAGGAAGGATGGATGGA	TACCAGATCTAGGCCCGCTCAT	329
FMgSSR-10792	scaffold5436	p2	(AG)6	Class II	25991	26002	AGCAAATGCGGGTGCACTGT	TGTGTTGCAATCCGCCCTGA	201
FMgSSR-10794	scaffold77050	p2	(AG)6	Class II	379	390	AGCAACTTGCATCAGGGTCGCA	TGATGAGCGAAAACGGCAGCAC	317
FMgSSR-10797	scaffold118	p2	(AG)6	Class II	127705	127716	AGCAAGCAGAGGCTAAGGCCAA	TGCCAACCTGGCATCCACAGTA	207
FMgSSR-10801	scaffold1181	p2	(AG)6	Class II	40274	40285	AGCACAACCCAACAGAGCACA	AACCCCTGTTTAGCGGCGGAAT	201
FMgSSR-10818	scaffold367	p2	(AG)6	Class II	29994	30005	AGCAGTGTGGACTCTGGAGCAA	AGAGCCTTCTCCGACTGATGCT	336
FMgSSR-10819	scaffold8499	p2	(AG)6	Class II	15035	15046	AGCATAAATAGCAGCGGGGCGT	AACCCGTGCACGAACACGAT	249
FMgSSR-10820	scaffold6158	p2	(AG)6	Class II	26791	26802	AGCATCAAGGAACCAACGGGCA	ACACCGTCACGATTGGCTCTGT	310
FMgSSR-10825	scaffold256	p2	(AG)6	Class II	10029	10040	AGCATTGCGCGCCATCATTGT	AGCATCATGCGTGTGTCGGT	339
FMgSSR-10826	scaffold23724	p2	(AG)6	Class II	13487	13498	AGCCACAGCCATGGGTTTGATG	ACTGGCTGCTGTCCAAGTGCAT	342
FMgSSR-10835	scaffold42110	p2	(AG)6	Class II	2420	2431	AGCCGCCTTGAATACCGTTGA	AGTCCATTTTAGAGGACGCCGCA	290
FMgSSR-10839	scaffold6812	p2	(AG)6	Class II	4075	4086	AGCGAGATCCAAGCAAGTGCCA	GCGTGCGCTACGATGTAAACCA	310
FMgSSR-10847	scaffold320886	p2	(AG)6	Class II	178	189	AGCGCCATGCTCTCTGTGTCAA	ACGCGAGTGAGGATGAAAGCGA	275
FMgSSR-10851	scaffold4701	p2	(AG)6	Class II	30693	30704	AGCGCTGCAGGGCGATTTTT	TTGAGTGTGCCCTTGCAGTGGT	215

SSR_ID	Scaffold	SSR_Type	SSR_Motif	SSR Class	SSR_Start	SSR_End	Forward sequence	Reverse sequence	Product_size
FMgSSR-10856	scaffold16128	p2	(AG)6	Class II	2402	2413	AGCGTGATGATCCTCGTACGCAA	AGGGCGTGCGTTTGGAACAA	335
FMgSSR-10857	scaffold17462	p2	(AG)6	Class II	3566	3577	AGCGTTGTATGGGCCGCAAT	GCGTCCACACTTGGAACGTGAA	228
FMgSSR-10871	scaffold11406	p2	(AG)6	Class II	22464	22475	AGCTGTCTGGGAGAAGAAGCCA	AGAAGAACCGTGAAGCCAGGGA	323
FMgSSR-10873	scaffold7131	p2	(AG)6	Class II	19417	19428	AGCTGTTCTGCCATGGAGCCAA	TTGAAGAGGGGAGAGAGAGGGAGA	350
FMgSSR-10874	scaffold4051	p2	(AG)6	Class II	16686	16697	AGCTGTTTGAGTGGGTATACGGCT	ACCGTGCATAAGAAGACACCGCT	269
FMgSSR-10876	scaffold1361	p2	(AG)6	Class II	65258	65269	AGCTTCCGTGGCGCTGAAAA	ATCGCCACGTGCAACACCTT	337
FMgSSR-10879	scaffold318843	p2	(AG)6	Class II	249	260	AGCTTGTGGGCTATCCAGGTCA	TGGCTTTCGCAGAGTCTTGCT	239
FMgSSR-10885	scaffold10196	p2	(AG)6	Class II	22506	22517	AGGAGGCAACAAGGCACGTGAA	AAAGCCGGGGTGAAAATCCCCT	304
FMgSSR-10887	scaffold1766	p2	(AG)6	Class II	41308	41319	AGGCAAGACATTCGCGGTTGGA	AAGTAGTCGTCCTTGCGCACCT	219
FMgSSR-10888	scaffold31084	p2	(AG)6	Class II	2668	2679	AGGCAGTCAGGAAATTGGCCGT	TCGCAGGCTTTAACCTCGATGC	324
FMgSSR-10891	scaffold3444	p2	(AG)6	Class II	1062	1073	AGGCCTACACGTTGGCATCGAA	ATGGGCTCGTCAGGGCACTAAA	336
FMgSSR-10892	scaffold316580	p2	(AG)6	Class II	62	73	AGGCCTTCCAAGCCTCTTCACT	ACACGGATGCTTCGGTCACT	218
FMgSSR-10900	scaffold753	p2	(AG)6	Class II	41820	41831	AGGCTAGGGCAGCAAGAAAGGT	AAATCGCTTGGCTCCACCCACA	270
FMgSSR-10902	scaffold2297	p2	(AG)6	Class II	41873	41884	AGGCTGCTCATGGCAAGATCTGA	ATTCTCTCTCTATCCCCGCACGCA	327
FMgSSR-10903	scaffold17503	p2	(AG)6	Class II	10330	10341	AGGCTTAGGCAGCACCCCATTT	TTTGGACCGTTCCCCCATGA	287
FMgSSR-10905	scaffold10903	p2	(AG)6	Class II	1067	1078	AGGGAAAGAGGTGATTCGGGTGA	AGCACCGTAGGAGCAAATCCA	346
FMgSSR-10906	scaffold4608	p2	(AG)6	Class II	33963	33974	AGGGAAGAGATGGGAAAAGGGGA	CACACACCACTTTCTTGTGACGTG	279
FMgSSR-10910	scaffold10942	p2	(AG)6	Class II	20145	20156	AGGGCCATTCAAACGTAGCGGT	TGCACCCCAACGTGGCAAAA	280
FMgSSR-10919	scaffold615	p2	(AG)6	Class II	97602	97613	AGGGTTGATCGAGAACGCCCAT	ATTTGCAGTCCATTGCGGCGTC	303
FMgSSR-10923	scaffold769	p2	(AG)6	Class II	53599	53610	AGGTGCTTGTGGCTCTGGTTCT	TCACGCATTAATCGCGCACCT	293
FMgSSR-10926	scaffold9429	p2	(AG)6	Class II	20755	20766	AGGTTAGAGGCAGGAAACGCA	TGTCTTGCTCCTGGTCGTGT	334
FMgSSR-10930	scaffold738	p2	(AG)6	Class II	42106	42117	AGTACCTCGCATTGCCTAGCCT	AGCGTAGCGGGCATGTTTGGAT	348
FMgSSR-10940	scaffold396	p2	(AG)6	Class II	5344	5355	AGTGAAGTGCAATGCGGCCA	ACTGCGGCTAGGCTGATTGTGT	294
FMgSSR-10950	scaffold25042	p2	(AG)6	Class II	1870	1881	AGTGTGCAAGCGCTGCTTGTTT	TCGCCCGCATCATCATGAGAGAA	305
FMgSSR-10956	scaffold11658	p2	(AG)6	Class II	16294	16305	AGTTGCCCTTTGGCGAGCAGTA	TTGTTGCGCTTGGGAACTGGGT	350
FMgSSR-10958	scaffold6734	p2	(AG)6	Class II	39882	39893	AGTTTGGGCTCCTTACCGCT	CGGTCGCGAATTCTTGTGCTGT	200
FMgSSR-10961	scaffold2708	p2	(AG)6	Class II	2911	2922	ATACCTTCATCGCAGCCACGCA	ATCCGCCGCAACCATTTCCT	284
FMgSSR-10963	scaffold504	p2	(AG)6	Class II	23867	23878	ATAGCCGCGTTGCAAGTTCACG	TCGACGCTGCGACCTAGACATT	224

SSR_ID	Scaffold	SSR_Type	SSR_Motif	SSR Class	SSR_Start	SSR_End	Forward sequence	Reverse sequence	Product_size
FMgSSR-10965	scaffold1309	p2	(AG)6	Class II	14708	14719	ATATTGGTGGAGCGATGGCAGC	TGAACCACCAGCTAGGCCATGT	330
FMgSSR-10978	scaffold798	p2	(AG)6	Class II	78431	78442	ATGAAGTGGCGGCGGATCTT	AGGGTTGTGGAGGCATGACGAA	300
FMgSSR-10980	scaffold6061	p2	(AG)6	Class II	19879	19890	ATGAGGCATTGAGGGCTGCACA	TGCTTGCTTCCATGAGGTTCCCT	347
FMgSSR-10981	scaffold10564	p2	(AG)6	Class II	20594	20605	ATGCATGCAGAGACAACCGCCT	TGCATGCACTGGACCGGTAGTT	338
FMgSSR-10982	scaffold738	p2	(AG)6	Class II	92018	92029	ATGCATGCCTCCACCGGAAT	TGTCCACGGTTCGAACAAGCCA	318
FMgSSR-10983	scaffold4533	p2	(AG)6	Class II	12670	12681	ATGCCAGCAAGGCTCCAACA	TCGTCGGTTCCGTCCAATCGAA	327
FMgSSR-10985	scaffold358	p2	(AG)6	Class II	88476	88487	ATGCGCACGCTTCGGTTTGA	AAGGAAGTTTCCGACACGCC	301
FMgSSR-10994	scaffold1303	p2	(AG)6	Class II	60395	60406	ATGGGCGCATTTAGGCGAGA	ACGCGATCGAGCAACGTCTT	324
FMgSSR-10998	scaffold20368	p2	(AG)6	Class II	10888	10899	ATGGTGACCACTTCACTGTCGAGG	GTGAACGCAGAAGCTAATGTGC	287
FMgSSR-10999	scaffold480	p2	(AG)6	Class II	120918	120929	ATGTCTGCAGGCAAGGCAAGGT	TTCCGAGCATTTGGGTTCCGCT	244
FMgSSR-11003	scaffold18510	p2	(AG)6	Class II	16356	16367	ATGTGGCGCTCAATGCGGTA	ACGAGTTTTCCCCACAAGTCCCT	228
FMgSSR-11005	scaffold4370	p2	(AG)6	Class II	17091	17102	ATTATCATGACCGCCGCCGA	GGCAACAAGCAACGGCTCCAAA	215
FMgSSR-11008	scaffold109562	p2	(AG)6	Class II	1270	1281	ATTGATGGGCGTGAGTGCTCGT	TCTTTTCCCCGCCCCTTCTTCT	202
FMgSSR-11011	scaffold2083	p2	(AG)6	Class II	8852	8863	ATTGCTGTTGCCACTGTGTGCC	TGCCCTCCACAATGGATAGCGA	303
FMgSSR-11013	scaffold52980	p2	(AG)6	Class II	2451	2462	ATTGTTGCTTCGTCCAGCTGCC	TGCGGGCTCGCTTCATTCACTT	337
FMgSSR-11018	scaffold4752	p2	(AG)6	Class II	35769	35780	ATTTTCGTGGCGGGCTCCAA	TCGATCGTGCATATCCCCTGCT	342
FMgSSR-11019	scaffold5348	p2	(AG)6	Class II	20773	20784	CAAAATATGTGTGCCGGCTGGA	TGAGACGCTAGTCGCTGAGTTGA	203
FMgSSR-11022	scaffold1097	p2	(AG)6	Class II	25652	25663	CACGGATCATGCAGTTTCGTATGG	ATGCAGGTTCCCCTATCTGCTACC	253
FMgSSR-11028	scaffold10306	p2	(AG)6	Class II	24490	24501	CAGCTACAAGTCTGCACTCGCA	ATTGCTGGCGCCCATTGAGA	322
FMgSSR-11031	scaffold1192	p2	(AG)6	Class II	32372	32383	CCAACGCATGCTATGGCACGTT	AATTTCCGTGCAGGATGGGGCA	344
FMgSSR-11038	scaffold659	p2	(AG)6	Class II	62463	62474	CCAGTCGCAATTTGCTGTGCCT	TGATTGTGGTGGGCAGCAGT	350
FMgSSR-11043	scaffold22869	p2	(AG)6	Class II	8020	8031	CCCCTGCCGATCAACGTTATGT	GCACTGTTTGGCAAATCTGGGT	320
FMgSSR-11058	scaffold73989	p2	(AG)6	Class II	1908	1919	CCTGGGTTCCTTGCGCTTGTTT	AGCGGCGTTAAAGCTCAGCA	252
FMgSSR-11062	scaffold17466	p2	(AG)6	Class II	4021	4032	CGATTCCCATCCATTTGAACCACG	TGTCTTCCAAGTCCCATACGTGCG	328
FMgSSR-11074	scaffold3659	p2	(AG)6	Class II	3954	3965	CGCTTTTCAGAAGGATGCGCCA	ACGGTCTGCAGTATGGGATGCT	261
FMgSSR-11078	scaffold2170	p2	(AG)6	Class II	34317	34328	CGGCCTCTTGCATGCCAAATCT	TGGCATGGATTTTGGGCAACGC	335
FMgSSR-11084	scaffold18550	p2	(AG)6	Class II	10182	10193	CTTTTGACCAAAAAGGGGCACG	TCCTCACACGCCCTCTCTTTCT	214
FMgSSR-11085	scaffold8125	p2	(AG)6	Class II	22701	22712	GAAAAGCATTGCGGCGAGCA	ACGGTGCATGTCAAACGAAGCA	342

SSR_ID	Scaffold	SSR_Type	SSR_Motif	SSR Class	SSR_Start	SSR_End	Forward sequence	Reverse sequence	Product_size
FMgSSR-11086	scaffold176	p2	(AG)6	Class II	142039	142050	GACAATTTAACCGTCCACGCATCG	AGCTGCACTGTCGTAGCACT	259
FMgSSR-11094	scaffold15857	p2	(AG)6	Class II	14427	14438	GCAATCAGCAAGCGGCGAAT	TGGTCGGATCTTGAGCTGGGTT	298
FMgSSR-11095	scaffold5068	p2	(AG)6	Class II	2960	2971	GCACACGTATAGCTAGGGTGGA	TGAGCCCAGCTTCTAGAGCGAA	350
FMgSSR-11096	scaffold9896	p2	(AG)6	Class II	1910	1921	GCACGAAGTTTCAGTGGCCCTT	TGCCTGTTTTTCCCGCTGCT	349
FMgSSR-11098	scaffold441	p2	(AG)6	Class II	113822	113833	GCACGCACGCTTCTCCTCAAAA	ACAACAACCATCGGTCGCCACT	329
FMgSSR-11099	scaffold5068	p2	(AG)6	Class II	2262	2273	GCACGTGCAAGATAAAGAGGCTCG	ACGGAGGCATCGTTTCTGCCTT	334
FMgSSR-11100	scaffold2256	p2	(AG)6	Class II	17212	17223	GCACTGGCAACGCTCGTCTTTT	TGCGCGCGGAACATATGCAA	282
FMgSSR-11101	scaffold3365	p2	(AG)6	Class II	40539	40550	GCAGAGAAGATGAGACAGAAACGG	CGCTGACATGGGGGCAAAACAA	328
FMgSSR-11102	scaffold3667	p2	(AG)6	Class II	31999	32010	GCAGAGACTGAACGAACCCTTGGT	AGCTGGAGCTGTTGACGAGAGT	272
FMgSSR-11103	scaffold2498	p2	(AG)6	Class II	57519	57530	GCAGCAAACCAACAAAGCGGGA	GCTGCTGCCGATGCGTTTACTT	326
FMgSSR-11106	scaffold10655	p2	(AG)6	Class II	8645	8656	GCAGGCACGCTAAGCATGTGAA	TATTATCCCGTGCCGGTGGACA	328
FMgSSR-11107	scaffold14213	p2	(AG)6	Class II	6462	6473	GCAGTAATGGAGGACAAACCAGTG	AGCGCAAGGTTAGTGTGATTCGC	325
FMgSSR-11112	scaffold47	p2	(AG)6	Class II	201007	201018	GCATCTTTTCTGCCGCCCATCA	AATCGGCCCATCAAGCTCCT	350
FMgSSR-11117	scaffold122231	p2	(AG)6	Class II	588	599	GCCACGACCAAACAGAAAAGCA	ACAGAGGTTCTTTCCGCCACCA	308
FMgSSR-11120	scaffold751	p2	(AG)6	Class II	50867	50878	GCCGCAAGCCTTGTTGTGTT	TGATCTGTCAGGTGCCTGCT	350
FMgSSR-11123	scaffold2091	p2	(AG)6	Class II	32889	32900	GCCTTCTTCACGGGAACATCCA	AGCGTATGTTTCGTGCGCCT	298
FMgSSR-11132	scaffold10511	p2	(AG)6	Class II	17944	17955	GCGGCTTCATGCACGTAACT	AGCGCCGCTATACCTCGTTT	202
FMgSSR-11137	scaffold9395	p2	(AG)6	Class II	24379	24390	GCGTTCGTTTGCCGTTTTCCCT	ACCGAATACTCAGTTGCCCTCGT	293
FMgSSR-11139	scaffold1124	p2	(AG)6	Class II	53289	53300	GCTCCCTCTCCTTCCTCCACTAAT	TCCTTCAAGGCCTCTTTGCGGA	306
FMgSSR-11141	scaffold151418	p2	(AG)6	Class II	287	298	GCTGCAGGGTGTGCAGCTTTTT	TTGTGCCCGTTGCACGTACT	201
FMgSSR-11144	scaffold18104	p2	(AG)6	Class II	7273	7284	GCTTGCCAACGAGTGACGCAAA	TTGAACTGTGTGCTGCTGGC	216
FMgSSR-11145	scaffold23702	p2	(AG)6	Class II	2877	2888	GCTTGGGGCACTCCACAAGAAT	ACCTGCACGGCACATCAAGA	344
FMgSSR-11151	scaffold202	p2	(AG)6	Class II	18769	18780	GGACTTTCTCTCGTCAACGACGCA	GGGGGTGAGGGGTGTTGTTTTT	245
FMgSSR-11152	scaffold2202	p2	(AG)6	Class II	18091	18102	GGAGAGAGGCAGAGGAAGAAGAGA	TGCAGGTACCGCAAACGGAGAT	308
FMgSSR-11155	scaffold2757	p2	(AG)6	Class II	44561	44572	GGCATGAGCCCCCTTAACTCCTTT	TGACCCAAATGCCCACCTCTCT	306
FMgSSR-11156	scaffold10957	p2	(AG)6	Class II	21099	21110	GGCATTGCAGCATTAGAGCATCCA	AGCAACGGTGATTCGACTGTACCA	343
FMgSSR-11158	scaffold22357	p2	(AG)6	Class II	12357	12368	GGCCGATGCCTGCTGTTTTT	ACCGACACCTGGGTGTTTTTCTGT	290
FMgSSR-11165	scaffold4203	p2	(AG)6	Class II	26835	26846	GGCTGCTCGATAAGTGCTCGAT	TGAGACAAAACGAGGGCAGACT	303

SSR_ID	Scaffold	SSR_Type	SSR_Motif	SSR Class	SSR_Start	SSR_End	Forward sequence	Reverse sequence	Product_size
FMgSSR-11166	scaffold1515	p2	(AG)6	Class II	5939	5950	GGCTGTTAAGGCGTTGATTGATGC	TCCTATGCAACACAATGGAGTCCC	341
FMgSSR-11175	scaffold12564	p2	(AG)6	Class II	22143	22154	GGGCTGATGTGGCATTGGACTT	TGGCATTTGCCGCTTGGCAT	220
FMgSSR-11178	scaffold5436	p2	(AG)6	Class II	14556	14567	GGTGCGCGCGTTAAACAAGT	ATTGGTCGCCATCCCATCGGTA	325
FMgSSR-11189	scaffold1300	p2	(AG)6	Class II	26903	26914	GTGGTGGATCCAGACTACAGCTCA	TTGGCGCAGTGGCGGATTTA	348
FMgSSR-11194	scaffold5088	p2	(AG)6	Class II	26546	26557	TAATTCTGTGCCGCTGGCTGCT	TACACGGGCCAAAAGCATGGGT	226
FMgSSR-11198	scaffold8013	p2	(AG)6	Class II	17843	17854	TACGTCCTTGTGAGCAGCGCAA	GCCGCAACGTTTGTGGGTTT	335
FMgSSR-11199	scaffold2297	p2	(AG)6	Class II	41480	41491	TAGAGTTGATGTCGCCGGGT	TTGCCATGAGCAGCCTCTACCT	273
FMgSSR-11200	scaffold7405	p2	(AG)6	Class II	17699	17710	TAGATGGCGCGCCGTTGTTA	AGCGGGCGAAATGCCTTTGT	323
FMgSSR-11201	scaffold140	p2	(AG)6	Class II	122252	122263	TAGCACAGAGCCAAAGAGGGGA	TGGTGCCCGACCAGTCAACATA	224
FMgSSR-11203	scaffold13979	p2	(AG)6	Class II	10194	10205	TAGCCGCGTTCCAGTTGTTGGA	AGCAATCGACCTGCTGCACT	235
FMgSSR-11207	scaffold265	p2	(AG)6	Class II	81989	82000	TAGCTGCGGCTTCCTTGTGAGA	GCGCTTCACCGTGCATGTTA	344
FMgSSR-11208	scaffold20637	p2	(AG)6	Class II	10575	10586	TAGCTTGTCGCTATCCCCGGAA	TGTTGCCAACCTTTGCGCCT	292
FMgSSR-11217	scaffold1630	p2	(AG)6	Class II	55964	55975	TATGCTCAGATCCTGGCTCCACCA	TCGACACCTTTCCGTCGTGCTT	242
FMgSSR-11218	scaffold1510	p2	(AG)6	Class II	38960	38971	TATGGCAGCAATGAGTGCGGGT	AGGCCTTTGTGACAGACAGGGA	262
FMgSSR-11219	scaffold1187	p2	(AG)6	Class II	17930	17941	TATGGCCGATTCTTGGCCGCAT	AAAACGCTCTGCGTCCCTCCAA	342
FMgSSR-11223	scaffold12911	p2	(AG)6	Class II	12464	12475	TCAACAACTGCTGCCCCGTT	ATGCCGGCCGTGCAAATCTT	331
FMgSSR-11231	scaffold365	p2	(AG)6	Class II	118507	118518	TCAAGGTGGCGTGTTCGGTT	AAAAGCAGGCACGCACGACA	265
FMgSSR-11235	scaffold14001	p2	(AG)6	Class II	13958	13969	TCACCCTCGAAAACGTGAGCGAA	GTGGGCGCCGTTGCTTTATTCA	217
FMgSSR-11236	scaffold28073	p2	(AG)6	Class II	1807	1818	TCACCGTCGCCGATTTCGTT	GGGACCAAGAAATGCCGCCAAA	279
FMgSSR-11239	scaffold4202	p2	(AG)6	Class II	42631	42642	TCACTTAGGGAGGTTGGTGCAT	ATATCTGGCGGCTGGCACAACA	211
FMgSSR-11240	scaffold3038	p2	(AG)6	Class II	40834	40845	TCAGCCAGCGCCACAAAGAA	AGCTGCAGGATGACGACGTT	258
FMgSSR-11243	scaffold317	p2	(AG)6	Class II	80767	80778	TCAGTTCATTGCGGCAGCGT	TTCCGACAGCAAGGCTGCTA	307
FMgSSR-11256	scaffold9982	p2	(AG)6	Class II	26640	26651	TCATTGTTGCCTTGCGCCGAA	ACTTGACTCCCACTCCTAGTCGCA	272
FMgSSR-11259	scaffold8081	p2	(AG)6	Class II	6009	6020	TCCAAATCCGCGGTGCACAA	TACGAGCTGCAAAACCGAGTGC	215
FMgSSR-11276	scaffold10607	p2	(AG)6	Class II	282	293	TCCGACGATCCAAATTGCTCGC	TGGCACAGTGCTCGGCATTT	205
FMgSSR-11280	scaffold3222	p2	(AG)6	Class II	10775	10786	TCCGCATGGCAGAAGTGTGA	ATATTTGCATGGCGGAGGCCCA	347
FMgSSR-11285	scaffold1120	p2	(AG)6	Class II	44102	44113	TCCGTGATGTCGCAGTAGCA	TGGATAAAGGCACCAGCTCCGA	288
FMgSSR-11288	scaffold18380	p2	(AG)6	Class II	3279	3290	TCCTCCCATTTGCCCACCATCA	TCTTGGCGTTGCACTGCTCT	293

SSR_ID	Scaffold	SSR_Type	SSR_Motif	SSR Class	SSR_Start	SSR_End	Forward sequence	Reverse sequence	Product_size
FMgSSR-11289	scaffold1602	p2	(AG)6	Class II	51746	51757	TCCTCCCCCGTGACCGTAAAAA	AGTGCGTGCCACCAGAATCA	225
FMgSSR-11297	scaffold8874	p2	(AG)6	Class II	18740	18751	TCGAACAATGCCGGGTCTGA	CCCCATTTTGCCCAACCCCAAT	203
FMgSSR-11302	scaffold1352	p2	(AG)6	Class II	47870	47881	TCGAGGCCTAGATTCCTGCGAA	AGGATTTGGATTGGTGGAGCGACA	311
FMgSSR-11307	scaffold9181	p2	(AG)6	Class II	22237	22248	TCGCAAGCTTTGGCAAGACCCT	ACCAACGGTGACATCACAGGCA	343
FMgSSR-11309	scaffold3827	p2	(AG)6	Class II	3459	3470	TCGCATGAACGGGGAAAGGT	CGGCCCAAGACAGCTTGGTTTT	306
FMgSSR-11310	scaffold579	p2	(AG)6	Class II	30729	30740	TCGCCAAACGAGGCAAAGCA	TCAGCATTTGTCGCGGGACCTT	295
FMgSSR-11311	scaffold3272	p2	(AG)6	Class II	18873	18884	TCGCCACAACAACGTGCTGA	ATTGTAGGCAAACCCACCGGCA	305
FMgSSR-11312	scaffold543	p2	(AG)6	Class II	58974	58985	TCGCCATGCCGGAGTCTTCAAA	GGTTTTCCGCAGCGCCTTTCTT	347
FMgSSR-11316	scaffold25	p2	(AG)6	Class II	66051	66062	TCGCCGAACTGGTAATTTGTGCC	AGGCTGCGCTTGGTACAGTT	349
FMgSSR-11319	scaffold19177	p2	(AG)6	Class II	7905	7916	TCGCCGGTGTTTCCTTCAGCTT	ACTCATTTCCGCTTCCGCACCT	309
FMgSSR-11322	scaffold2981	p2	(AG)6	Class II	5073	5084	TCGCGCAGTCTCACGTCATGTT	TATGCATTTGCATGCCCGCC	327
FMgSSR-11323	scaffold2591	p2	(AG)6	Class II	29216	29227	TCGCGCGCAGTGAAAGTTTG	TTGCGGAATGGCAGCAGTGT	257
FMgSSR-11326	scaffold2444	p2	(AG)6	Class II	34471	34482	TCGCGCTGCTTATCATGCAC	TTCGATCCCGTATCCGCTGCAA	345
FMgSSR-11328	scaffold7952	p2	(AG)6	Class II	10071	10082	TCGCGTCCACGCTGTTGATT	GTCAACGCGCGGCATTACTT	289
FMgSSR-11334	scaffold36836	p2	(AG)6	Class II	1211	1222	TCGGAGCCATCCGTTTGCATGA	ACGATGGCCAATTGGAGCAAGGA	292
FMgSSR-11335	scaffold3741	p2	(AG)6	Class II	48799	48810	TCGGCAAACAGTGGACTGTGGA	CGACATCGCGCGCTTTCAAT	331
FMgSSR-11342	scaffold2161	p2	(AG)6	Class II	42384	42395	TCGGTGAAGCGCGAGAGGAATA	TTGCGCGACACGTGGTGATT	346
FMgSSR-11346	scaffold11663	p2	(AG)6	Class II	6577	6588	TCGTTGCCATCAGCGATGCCTT	AGTGTTGCCTCGTGTCCTTAGC	349
FMgSSR-11348	scaffold37229	p2	(AG)6	Class II	3520	3531	TCTCACCACCGAGCTTGACGAA	ATGGCCGCCTGCTGTTATGT	298
FMgSSR-11359	scaffold563	p2	(AG)6	Class II	87677	87688	TCTGCTGCGCCACATTCTGT	TGAACCTTCGCAGTTCGCACCT	291
FMgSSR-11362	scaffold3536	p2	(AG)6	Class II	9432	9443	TCTTGCATCGAGCTTGCCGT	TTTTGTGGAGCCCGACGCTT	244
FMgSSR-11363	scaffold40892	p2	(AG)6	Class II	4892	4903	TCTTTGCGTGGTACGCGACA	TCTAATGCAAGCGCGCAACG	312
FMgSSR-11372	scaffold1867	p2	(AG)6	Class II	72401	72412	TGACACGAGAGCGAGAAGCA	TGTGTGTGTTTGGGCCTCCCTT	305
FMgSSR-11374	scaffold6644	p2	(AG)6	Class II	39977	39988	TGACCAAGCTCGTTGGTGGGTT	TTAGTTCGGTGCCGTGGCGATT	347
FMgSSR-11376	scaffold2110	p2	(AG)6	Class II	22199	22210	TGACCACAGCTCGCAGCATCTT	TTTCACGACACGTCGCTCATGC	262
FMgSSR-11377	scaffold2438	p2	(AG)6	Class II	29521	29532	TGACCCGACCTTTGTGTTTGCG	TTCTGCCAGTGCCAATTGCTGC	215
FMgSSR-11378	scaffold1776	p2	(AG)6	Class II	27005	27016	TGACCGCCATTTGACATGACCT	TGTGCGGTTCCTATAGTTGCGT	209
FMgSSR-11390	scaffold29386	p2	(AG)6	Class II	4723	4734	TGAGATACACGGCCTAGCCCAA	AACCCAACCTGCGGCCATTT	200

SSR_ID	Scaffold	SSR_Type	SSR_Motif	SSR Class	SSR_Start	SSR_End	Forward sequence	Reverse sequence	Product_size
FMgSSR-11395	scaffold3396	p2	(AG)6	Class II	9609	9620	TGAGGCTTGAGGGATGGGGTTT	AAATCACCGCGGCACTTGCT	281
FMgSSR-11402	scaffold5607	p2	(AG)6	Class II	26099	26110	TGATGAGAGTTTGGCCGCACGA	TCCTGCACGCTTTTGTCCGA	348
FMgSSR-11406	scaffold356	p2	(AG)6	Class II	53610	53621	TGATGGAACACGAGAACCCCGT	TTTGCTGTGCCAACTGCTTCCG	201
FMgSSR-11410	scaffold11561	p2	(AG)6	Class II	6815	6826	TGCAAAAACGAATGGCCCGCA	ATAACTGCCGTGGTCGTGCTGT	263
FMgSSR-11414	scaffold800	p2	(AG)6	Class II	80577	80588	TGCAACAGGAATGGTGGCGT	CCGGTGTGCACCGTTCAGAAAT	272
FMgSSR-11416	scaffold11428	p2	(AG)6	Class II	19022	19033	TGCAAGAGCAACAAACGGGC	TCCACTTTGAGCCGCAGCAA	222
FMgSSR-11417	scaffold804	p2	(AG)6	Class II	96470	96481	TGCAATCTTGTCCCCAGAGCCA	CGCTCGCTGCACGATTCTTCAA	345
FMgSSR-11418	scaffold4754	p2	(AG)6	Class II	18686	18697	TGCAATGATGCGTCCTATGCCC	TGGCACGATTGAACGCCGTGTA	267
FMgSSR-11419	scaffold742	p2	(AG)6	Class II	100758	100769	TGCAATGCATGCAGCAGGTGT	TCGGCAAGCCAGTAACGAACCA	238
FMgSSR-11420	scaffold889	p2	(AG)6	Class II	17320	17331	TGCAATGCCGTCGTCGAGTT	TTCGTGCTCCATCGTGTGTGCT	224
FMgSSR-11421	scaffold1132	p2	(AG)6	Class II	84900	84911	TGCACACATTTGCTCCTGGGGT	AATAGACCGGACGTCGCTTGCT	333
FMgSSR-11426	scaffold30099	p2	(AG)6	Class II	4313	4324	TGCACTCGGACCATTGCTCGTT	TGCTGCTGCTTGGAGACTTG	222
FMgSSR-11436	scaffold9311	p2	(AG)6	Class II	22854	22865	TGCAGGCAAAGGTTGAAGGCA	AAGGCCATCTGTCCCAAGGCAT	219
FMgSSR-11439	scaffold5339	p2	(AG)6	Class II	27423	27434	TGCAGTAGCGACCATCTGTGGA	TCAACGGCCATTTGCAGGACA	286
FMgSSR-11440	scaffold1379	p2	(AG)6	Class II	36742	36753	TGCAGTGGATTTGAGCGGCA	TGCACTCATAGAGGCGTGCGTT	295
FMgSSR-11444	scaffold2380	p2	(AG)6	Class II	58061	58072	TGCATCATCACACACAGGCCA	TGGGTGATCTGACGGTGGTT	316
FMgSSR-11450	scaffold1988	p2	(AG)6	Class II	75975	75986	TGCCAACTGTCCTCAGGCGTAA	AGCGTTGGAGCATGGAGATGGT	338
FMgSSR-11451	scaffold43018	p2	(AG)6	Class II	1955	1966	TGCCAAGTAGCAAATTGGGAAGCC	TGCCTTGTCCTTCAACCCTCCA	200
FMgSSR-11456	scaffold5262	p2	(AG)6	Class II	19612	19623	TGCCAGGCTAACTATCGCTTGTC	ACGCACAGACACGCTCAATCCT	346
FMgSSR-11458	scaffold1712	p2	(AG)6	Class II	65996	66007	TGCCAGTAGCAACAAACTGCAC	TCGTATGGCGGGAGTTTTGGCA	295
FMgSSR-11459	scaffold1252	p2	(AG)6	Class II	27986	27997	TGCCATCGGTGCCACAAGAA	GGCGAGTTGATTTTTGGTCTCGTGC	350
FMgSSR-11461	scaffold2489	p2	(AG)6	Class II	57960	57971	TGCCATGCTGCTTGATGTTGGAC	TGCAGGCTGGAATGGCACATGA	345
FMgSSR-11468	scaffold2663	p2	(AG)6	Class II	30581	30592	TGCCCCTGCCAAAGTAGCAA	ACACACGACGACTGGCCAATAA	224
FMgSSR-11471	scaffold63456	p2	(AG)6	Class II	621	632	TGCCGAAGACGAAGTGGGAGAT	AGATGCTTCCCGTTTCCCAGCA	251
FMgSSR-11474	scaffold2831	p2	(AG)6	Class II	7766	7777	TGCCGAGAGGTCAAACGGGAAA	TGTCGCGTGGTTTCGCACAT	247
FMgSSR-11480	scaffold3317	p2	(AG)6	Class II	56008	56019	TGCCGGTCCTGTGTTTCCGTTT	TGTGTGAACACGGTGGCCTTCA	245
FMgSSR-11485	scaffold4853	p2	(AG)6	Class II	21966	21977	TGCCTCTTGTGGGACCGCATTT	ATGTGTTTGGTTGCCGCCCA	222
FMgSSR-11486	scaffold1139	p2	(AG)6	Class II	28745	28756	TGCCTGCTCAAGCATGTCCAA	AGTTGGTCCCAGTGGTCAAGCA	277

SSR_ID	Scaffold	SSR_Type	SSR_Motif	SSR Class	SSR_Start	SSR_End	Forward sequence	Reverse sequence	Product_size
FMgSSR-11487	scaffold6814	p2	(AG)6	Class II	29891	29902	TGCCTTCTTCTTTTGCGGGGA	TACCTGTTCCGCCATGGTTGGT	322
FMgSSR-11504	scaffold12970	p2	(AG)6	Class II	15605	15616	TGCGGCTAAGCATCCGGTCATT	AACCAACGAAGTGGCCGACA	276
FMgSSR-11505	scaffold305098	p2	(AG)6	Class II	223	234	TGCGGTACGAAAGGTGCTACGA	ACGCGACCCCAAGGCCAATAAT	241
FMgSSR-11518	scaffold21702	p2	(AG)6	Class II	7542	7553	TGCTCAGTTCATCGTGCTGCCT	TCGTGCGCCATGCACTATCT	341
FMgSSR-11519	scaffold1518	p2	(AG)6	Class II	39911	39922	TGCTCCAAAAGCTCTCGCCA	AGCGTGGCAATACTTTGCGCT	347
FMgSSR-11523	scaffold1288	p2	(AG)6	Class II	27452	27463	TGCTGAAGCTGGACACGATGGA	TGCTGTTGAGTACGTGCCCACT	307
FMgSSR-11525	scaffold2748	p2	(AG)6	Class II	50776	50787	TGCTGCCGTAAGCAGCGTTT	GCGCAGCAATGGATTGGAGCTT	254
FMgSSR-11527	scaffold5470	p2	(AG)6	Class II	23194	23205	TGCTGCTGATCAAAGAGCCCA	GGCCCAGACCTTAATTGATTGCAG	343
FMgSSR-11529	scaffold18245	p2	(AG)6	Class II	17717	17728	TGCTGCTGTTGCTGCTTGCT	CGCTCGCATCTTTGAACGAACG	350
FMgSSR-11530	scaffold13259	p2	(AG)6	Class II	8453	8464	TGCTGGCTTCGCTGGAGTAT	CGGCACACGCAAAAGGAAGGAA	244
FMgSSR-11532	scaffold20903	p2	(AG)6	Class II	1359	1370	TGCTGGGTGAATGTAGGCAGGT	TCATCAGTTGCCAGCGCTCCTT	291
FMgSSR-11534	scaffold4214	p2	(AG)6	Class II	5891	5902	TGCTGTTCAGCATCGCCTTGGA	ACAAGCTGTCGCGCCGATTA	252
FMgSSR-11537	scaffold4181	p2	(AG)6	Class II	9505	9516	TGCTTCGAAAGCTTGGCATGACG	ACCTTCGTTGCGTTAGCACCA	210
FMgSSR-11553	scaffold17970	p2	(AG)6	Class II	13921	13932	TGGATTTGGCCGGAGCTGTTGA	AGTGTCGCATTTGCACGGCT	304
FMgSSR-11554	scaffold21597	p2	(AG)6	Class II	15504	15515	TGGCACCATCGCAAAACGGA	TCGCCACATCACGGTTCAGT	297
FMgSSR-11562	scaffold3276	p2	(AG)6	Class II	22503	22514	TGGCATGGTGGATGGAAGTGT	AGCTCATGCGCGTGCGAAAA	340
FMgSSR-11567	scaffold9561	p2	(AG)6	Class II	10442	10453	TGGCCTCTTTCCACTTTGCGA	AACAACAACGCTGTTCGCCG	333
FMgSSR-11571	scaffold6145	p2	(AG)6	Class II	13629	13640	TGGCGAGGAACATTGTGGCA	ACCAGCCTTGGTCAGCACAA	312
FMgSSR-11573	scaffold4886	p2	(AG)6	Class II	38238	38249	TGGCGCGCAATACCATTCCA	ATCCGGCAGGCACTGCATTT	323
FMgSSR-11576	scaffold2086	p2	(AG)6	Class II	45092	45103	TGGCGGCAAACAACACAGCA	AACGCCTCTCATGTCGCTCTCA	221
FMgSSR-11578	scaffold18914	p2	(AG)6	Class II	6186	6197	TGGCGGGCCTTCATTAACACAAC	AGGCTTGCTGTCCTGTGGTCAT	222
FMgSSR-11579	scaffold889	p2	(AG)6	Class II	15502	15513	TGGCGTTTGTTTAGGCGCGA	AATTCCGGGCTTACGTGCTGCT	276
FMgSSR-11582	scaffold961	p2	(AG)6	Class II	68082	68093	TGGCTGCATCGAACACAGCA	TGGAAACACTGTGCCGCCAT	266
FMgSSR-11583	scaffold6644	p2	(AG)6	Class II	24091	24102	TGGCTGGCACACATGGGACAAA	TGCACTGCACAGTGGGGTTAGT	311
FMgSSR-11587	scaffold9698	p2	(AG)6	Class II	15581	15592	TGGCTTGGCGCAGATCTTGGTA	AAACCGGCTCCAAAACCGGA	204
FMgSSR-11590	scaffold1090	p2	(AG)6	Class II	26625	26636	TGGGCCTAGCCAAACGCCAAAA	TCCCTCACTTTCTCTCTCCCGT	267
FMgSSR-11591	scaffold787	p2	(AG)6	Class II	69994	70005	TGGGCGCCATGTGGTTTCAT	TGCTTGTGGTGGCGAGCACTTA	251
FMgSSR-11592	scaffold37176	p2	(AG)6	Class II	3024	3035	TGGGCGCCTTTGTGCTTCAA	AAAGCCCAAACAACCCGGTGCT	262

SSR_ID	Scaffold	SSR_Type	SSR_Motif	SSR Class	SSR_Start	SSR_End	Forward sequence	Reverse sequence	Product_size
FMgSSR-11597	scaffold9077	p2	(AG)6	Class II	4285	4296	TGGGGATAAACACCGGCACA	AGCATTTCAGGTTGGCGAGGCA	308
FMgSSR-11600	scaffold793	p2	(AG)6	Class II	25496	25507	TGGGGCCAATTATGCCTCGCAA	ACGCCCTTTGCTGGAGAACT	328
FMgSSR-11604	scaffold6413	p2	(AG)6	Class II	26449	26460	TGGTACGGTACAGCCACCAAGT	ATTCCGGTCAGTGGTCCAAGCA	297
FMgSSR-11606	scaffold2147	p2	(AG)6	Class II	41537	41548	TGGTGATCCCTTTTCTCGCGTCA	AATGCACCAGGGCTGCAACA	345
FMgSSR-11607	scaffold2076	p2	(AG)6	Class II	66815	66826	TGGTGCAGGGGCTTGAACAGTA	ACAACATGGGCCCCACACAT	259
FMgSSR-11609	scaffold526	p2	(AG)6	Class II	88711	88722	TGGTGCTTGGTGCAAGTGATGC	ACCAAGGGTCCGTGCCAAAA	344
FMgSSR-11618	scaffold2969	p2	(AG)6	Class II	23979	23990	TGGTTGCACCGGGGAACTGTAA	TTGGTGCAACGACAAGCGGA	272
FMgSSR-11621	scaffold686	p2	(AG)6	Class II	1661	1672	TGGTTTGTTGCGGTGTCGGT	GCGCAACGGAGCCGTATTTCTT	223
FMgSSR-11622	scaffold2745	p2	(AG)6	Class II	12594	12605	TGTACATAGCCCGCGAGTCAGT	ATTGACGGTATTCGGTGCTGCC	333
FMgSSR-11624	scaffold11092	p2	(AG)6	Class II	14266	14277	TGTCAAGGGCGTCAAACCCACA	TGACGTACGCTGCAAGCTCA	294
FMgSSR-11625	scaffold1272	p2	(AG)6	Class II	65500	65511	TGTCACCGAGTCCATCTGTCCA	TGTTTGCTGCTTGGCTGGCT	248
FMgSSR-11627	scaffold1845	p2	(AG)6	Class II	4319	4330	TGTCATGCACGGTGAGGGAGAA	ACCTGTTGCCTCATGTTGCCCA	206
FMgSSR-11631	scaffold629	p2	(AG)6	Class II	91942	91953	TGTCCCAACCTTCAGAGCGAGA	AGCGTCACGCACATCTTGTCCT	247
FMgSSR-11639	scaffold179	p2	(AG)6	Class II	68106	68117	TGTCTACTGCTGATTGGCCGCT	GCGCGCACGACAAAACAAGT	220
FMgSSR-11640	scaffold2859	p2	(AG)6	Class II	14453	14464	TGTCTCGTGCCTTTGCGCATT	TGGCAGCAGTCCTTGGCATT	215
FMgSSR-11646	scaffold922	p2	(AG)6	Class II	8819	8830	TGTGCACCGCGGTCTTTGTT	TTCAATCATCTCACCGCCACCG	316
FMgSSR-11649	scaffold9722	p2	(AG)6	Class II	27470	27481	TGTGCCACAGTCAGCACCAACT	ACCCTTTTCTCGCCGAATCCGA	303
FMgSSR-11652	scaffold1	p2	(AG)6	Class II	338944	338955	TGTGGGCGTGCATGGTAGTGAT	TGTCCTCTTCGTGGCGTTGT	257
FMgSSR-11660	scaffold4441	p2	(AG)6	Class II	1140	1151	TGTGTGGACAACAAGACACGGT	AGATGAGTCCACAGACCATAGCGA	311
FMgSSR-11665	scaffold808	p2	(AG)6	Class II	101940	101951	TGTTGACGTCCATGCAGCCTT	GGCTTGCAGGGAATCACACTCT	275
FMgSSR-11666	scaffold10428	p2	(AG)6	Class II	13547	13558	TTAAAGCCGAGCCTGGGAAGCA	TCACGGCCATGGACTGCATCTT	305
FMgSSR-11669	scaffold8266	p2	(AG)6	Class II	31061	31072	TTACAATTTGGGGGTTCCAGGGGG	TATGTGCCATTTTGCCCCTGCC	262
FMgSSR-11672	scaffold20703	p2	(AG)6	Class II	8674	8685	TTAGCTGCGGTGCTAGTCCA	AAAGTTACCAGCCCACGCCAGT	245
FMgSSR-11673	scaffold297	p2	(AG)6	Class II	81241	81252	TTAGGCCGCCGATTTTGCCA	ACAGGACAACAAACGCCCGGAT	292
FMgSSR-11678	scaffold4042	p2	(AG)6	Class II	31031	31042	TTCAAGTCACATGCGGGCGT	ACATGACGCGGTGGAACCACAT	207
FMgSSR-11679	scaffold78	p2	(AG)6	Class II	151740	151751	TTCAAGTGTCCAGGGTGGCAGT	AGTGCAGTGTGCAGCGCTAT	222
FMgSSR-11681	scaffold290	p2	(AG)6	Class II	140523	140534	TTCACCCTAACACACGCACGCT	GTCCGATCCGAATCTAGCGGTT	335
FMgSSR-11684	scaffold2071	p2	(AG)6	Class II	45940	45951	TTCATGTGGGACCCAACCAGCA	TTGAAGAGGGGAGAGAGAGAGG	348

SSR_ID	Scaffold	SSR_Type	SSR_Motif	SSR Class	SSR_Start	SSR_End	Forward sequence	Reverse sequence	Product_size
FMgSSR-11685	scaffold1867	p2	(AG)6	Class II	65002	65013	TTCCAGTTTCGAGGCACGCA	AGGCCTGCTGGGCTGTTTTT	242
FMgSSR-11697	scaffold4811	p2	(AG)6	Class II	44658	44669	TTCGGAGGAGTTGGGACGGATT	ATGCCGAGAGGCAAGATGCCAA	239
FMgSSR-11700	scaffold279	p2	(AG)6	Class II	31942	31953	TTCGGGCGCTTTTACCACGA	TGGGTGATGTTGCCGCTCAA	266
FMgSSR-11708	scaffold39777	p2	(AG)6	Class II	4542	4553	TTCTGAGAACTGCGCCGTGA	AACGGAAGCGATGCGTGTCT	293
FMgSSR-11709	scaffold12176	p2	(AG)6	Class II	12540	12551	TTCTGTCCGTTCATCTGCGGCT	ACAAAAGCACGCGGGAAGAGGA	349
FMgSSR-11710	scaffold388	p2	(AG)6	Class II	52840	52851	TTCTGTTCTTTAGCGGCAGCGG	CGGGCGCGTTATAAAGCCACAA	350
FMgSSR-11723	scaffold3433	p2	(AG)6	Class II	59495	59506	TTGCAGCGGCCCACTGTTTT	TGGCAGAGAAGACAGCGCGAAA	307
FMgSSR-11726	scaffold17571	p2	(AG)6	Class II	8260	8271	TTGCCGTTCTGCGCTTGAGA	TTTGGGCTGTTGAAGGGCCA	342
FMgSSR-11729	scaffold1249	p2	(AG)6	Class II	11244	11255	TTGCGCCGCTCGCGTTTTTA	AGGTGATGTGTGGTCTGCGCTT	200
FMgSSR-11733	scaffold183	p2	(AG)6	Class II	43689	43700	TTGCTCACCGCAGCTACCACAA	TATTCGAATCGCAGCAGGCGGT	225
FMgSSR-11737	scaffold12723	p2	(AG)6	Class II	17084	17095	TTGGAGGCAGGGGATGGGTTTA	ACGATTCGTGGATCCTTAGCCCCT	339
FMgSSR-11741	scaffold2024	p2	(AG)6	Class II	9806	9817	TTGGGCCCTGTTGGGGATTTCA	GCGAGAAAACTCGCTTCGCT	305
FMgSSR-11742	scaffold92514	p2	(AG)6	Class II	1639	1650	TTGGGCCTTTCGTTCGTTGTGG	AGCAAGCAGCAGCAGGGAAA	301
FMgSSR-11743	scaffold8791	p2	(AG)6	Class II	1595	1606	TTGGGGATTAGGGACGCGGAAA	ACGCAACGGCGGACAATCAA	297
FMgSSR-11744	scaffold17889	p2	(AG)6	Class II	8859	8870	TTGGGGCCAAACTGACTAAGGTGG	TCCTGACGGAGTGAGGCTTTCT	222
FMgSSR-11750	scaffold4392	p2	(AG)6	Class II	40050	40061	TTGGTTGCGTCAACGTGGCT	TATGTGTGCAACACCTGCTGGG	240
FMgSSR-11753	scaffold16991	p2	(AG)6	Class II	6703	6714	TTGTCATTGCGGCGAGAAGTGC	TGGCACTCGAATCTGACCGCAT	246
FMgSSR-11758	scaffold787	p2	(AG)6	Class II	68187	68198	TTGTGCCCGAGGACTCGTAA	GCACGTCAATTTGCACCGCA	311
FMgSSR-11760	scaffold127	p2	(AG)6	Class II	126235	126246	TTGTGCTTTGTGGTGCAGTCGC	TCGCGTGGTTCCATTGGACCTT	341
FMgSSR-11765	scaffold27637	p2	(AG)6	Class II	1845	1856	TTTAAGCGGCAGCCGTTGGT	AGTTGCCCAACAAGCTCAACG	281
FMgSSR-11771	scaffold5041	p2	(AG)6	Class II	9272	9283	TTTGAAAGCTCCCGCGCTCTGT	ACAAGCCACACCATCATTGGGGA	339
FMgSSR-11772	scaffold3601	p2	(AG)6	Class II	36608	36619	TTTGAATGCCTTGTCGCCCCCA	ACGCCTACACCCAGATGTTCCA	274
FMgSSR-11773	scaffold10020	p2	(AG)6	Class II	9968	9979	TTTGCAGGAACAGGCGCCAA	ACCGCGTAAAAGACGTCACCTGT	216
FMgSSR-11780	scaffold4396	p2	(AG)6	Class II	10040	10051	TTTGGCTTGTGCGCGAGAGT	TTTTAACGCCGAGAGGCGGACT	270
FMgSSR-11784	scaffold5083	p2	(AG)6	Class II	35901	35912	TTTGTTCCTCCTACTGCGGCCA	TTGCTTATCCATGGCACTGCGG	303
FMgSSR-11788	scaffold7645	p2	(AG)6	Class II	4989	5000	TTTTGAGCCGCGCCTTGGAT	AAATTTGGCCCGGCACGAATGG	269
FMgSSR-11789	scaffold7639	p2	(AG)6	Class II	4972	4983	TTTTGAGCGATGAGCGGCGT	TTTCTGCCCCGTTCTGCTGCAT	267
FMgSSR-11791	scaffold1802	p2	(AG)6	Class II	25784	25795	TTTTGCTTTCGTCGCTCGCTCG	AAACCAAGCCCCATCATGCCCT	247

SSR_ID	Scaffold	SSR_Type	SSR_Motif	SSR Class	SSR_Start	SSR_End	Forward sequence	Reverse sequence	Product_size
FMgSSR-11798	scaffold5723	p2	(AG)7	Class II	36932	36945	AAACCGTGCCTCACGAGCAA	TCGTGCGCAGACTTCACCAA	296
FMgSSR-11800	scaffold6390	p2	(AG)7	Class II	8339	8352	AAACGCAACACGGAGTTCCTGC	TTCAGCAGCTGCGTCGTCAT	215
FMgSSR-11801	scaffold7094	p2	(AG)7	Class II	19808	19821	AAACGGCTGCATGAGGGAGAGA	ATGGCGAGCGAGCGAACAAA	330
FMgSSR-11802	scaffold3228	p2	(AG)7	Class II	16527	16540	AAACGTGTGTGCAGAGAGCGGA	TCAACCGCGCTGCTTAGCTT	265
FMgSSR-11805	scaffold1517	p2	(AG)7	Class II	76180	76193	AAACTTGCACGGCTCGGACGAT	ACCTCCGGACATCACACGGAAA	327
FMgSSR-11809	scaffold2816	p2	(AG)7	Class II	15728	15741	AAAGCGCGCAAGTGCGACAA	TATCGCATCGCACGACCAGCTT	229
FMgSSR-11813	scaffold10471	p2	(AG)7	Class II	17893	17906	AAATCATGGATGGTGGGCGCGT	TGGTCGCGTTGTCATGAGTGGT	320
FMgSSR-11816	scaffold5644	p2	(AG)7	Class II	31058	31071	AAATGCGTGCTCCCAAGTCCCA	ACATTCGCTCCGAAAGCTTGGC	294
FMgSSR-11817	scaffold15798	p2	(AG)7	Class II	13582	13595	AAATGTGCATGCAGCCATGCGG	ACGGCGTAGAGGCAACCAAACA	287
FMgSSR-11818	scaffold14181	p2	(AG)7	Class II	10309	10322	AAATGTGCCAGGCGTGCTGA	TTGGCGGGAGAGCTTCGGTTTT	249
FMgSSR-11820	scaffold494	p2	(AG)7	Class II	42286	42299	AACAACGGAGCGAGAAGGGGAA	TGAAACATGGCCCTGTCGGT	213
FMgSSR-11822	scaffold28575	p2	(AG)7	Class II	3372	3385	AACACCGCAGGGCCAAATCA	TTCGTTCCCGGTTGCTTGCT	267
FMgSSR-11826	scaffold42880	p2	(AG)7	Class II	1056	1069	AACAGCTCGCAGCTCGTGAA	ACTCTCCTCACCCCCACTGTACTA	300
FMgSSR-11827	scaffold4118	p2	(AG)7	Class II	56408	56421	AACCACACCAACCGCAGCAT	ACTGGGAGAGACGATCGAGCAA	201
FMgSSR-11829	scaffold5805	p2	(AG)7	Class II	20735	20748	AACCCTAAGTTGTGTTGGGCAC	ACAAAATGCCCGTGCTACGACT	318
FMgSSR-11836	scaffold186	p2	(AG)7	Class II	148534	148547	AACGCGTGTTTTGATGCGGC	TCACCCCACAGACACACACA	229
FMgSSR-11838	scaffold1069	p2	(AG)7	Class II	76324	76337	AACGGCTGCTTGTTTGGGCT	AGCAGCCCATTCTCCTCTCACT	247
FMgSSR-11839	scaffold11710	p2	(AG)7	Class II	18427	18440	AACGTCAGCTCGTCTGTTGCCA	ACGCATGACACACGTGCACAA	256
FMgSSR-11842	scaffold10320	p2	(AG)7	Class II	1593	1606	AACTTGTTGTCGCCACCCGA	AGTTCACAGATGCACGTTCGCA	339
FMgSSR-11850	scaffold19192	p2	(AG)7	Class II	8078	8091	AAGCATGCTGCGTTGACGGT	CCGCAAGGTCGTAACACAACCA	235
FMgSSR-11851	scaffold7329	p2	(AG)7	Class II	23399	23412	AAGCCAGAGCCACGAGTGAAGA	TGATGCAGGAAGCCACTGCTGA	318
FMgSSR-11853	scaffold13459	p2	(AG)7	Class II	4804	4817	AAGCCTGCTCAGTCATCGTCGT	AAAACCCATCTTGCGCGGTG	229
FMgSSR-11859	scaffold3384	p2	(AG)7	Class II	37796	37809	AAGCGTCGCCTGCCTAAATGGT	AAGGTGGTCCCGAAGACACGAA	279
FMgSSR-11870	scaffold12505	p2	(AG)7	Class II	2372	2385	AAGTGTGCGGGATGGAGAGCTT	TAGTATGGGCGCGAGACACGAA	293
FMgSSR-11873	scaffold133	p2	(AG)7	Class II	58013	58026	AATACTGGCTGGCTGCGTCGTT	ATGCAGGGGAGGCCGTGTATTT	272
FMgSSR-11876	scaffold1600	p2	(AG)7	Class II	74884	74897	AATCGCAGGGGAACGTGCATCA	AACGACGTCACCGTGCATCA	213
FMgSSR-11878	scaffold362	p2	(AG)7	Class II	51232	51245	AATGCAGTGCGGCGCAACAA	TGCAAACTCCGGTCATCGTGT	242
FMgSSR-11886	scaffold1586	p2	(AG)7	Class II	13876	13889	ACAACGAACTGTTGTGGCAGCG	ACCAAATGGCCTCCTGCGCATA	319

SSR_ID	Scaffold	SSR_Type	SSR_Motif	SSR Class	SSR_Start	SSR_End	Forward sequence	Reverse sequence	Product_size
FMgSSR-11888	scaffold3455	p2	(AG)7	Class II	13479	13492	ACAAGGCCGCGGCATCTTTCTA	AGGTTGGACGAAAGGGCGCAAT	238
FMgSSR-11889	scaffold2924	p2	(AG)7	Class II	57763	57776	ACAAGTCCGGTTTGTGGCGA	ATTCAAGGCCCGTCACGCAT	228
FMgSSR-11890	scaffold136	p2	(AG)7	Class II	125923	125936	ACAAGTTGAGAAGGCACTGCGG	TGGATGACATTCTCCCACGCCA	322
FMgSSR-11891	scaffold29083	p2	(AG)7	Class II	1983	1996	ACACAACGATGGGGCAGCAA	TTCAGCATGCAGCAGCGAGA	294
FMgSSR-11893	scaffold5599	p2	(AG)7	Class II	26083	26096	ACACATGCATGCTTGCTGCCA	AGCAGCCGCTCATCATCTTGCT	231
FMgSSR-11895	scaffold2043	p2	(AG)7	Class II	52174	52187	ACACGTACGGGAATGGCGAA	TGGCTTTTCCCGCGGTCTTT	333
FMgSSR-11898	scaffold697	p2	(AG)7	Class II	91670	91683	ACAGAACCAGGTCATGCCCATTCC	TAGAGCAGCAGCAACGGTGGTT	349
FMgSSR-11901	scaffold8353	p2	(AG)7	Class II	4490	4503	ACAGCAGCGTGTGTCATCGT	AGCAGTGACTTGGCTCCGAA	209
FMgSSR-11902	scaffold10889	p2	(AG)7	Class II	6894	6907	ACAGGCCCACAAAGCACCAA	TTGTCACACGCAACGCGAGA	344
FMgSSR-11905	scaffold1269	p2	(AG)7	Class II	86907	86920	ACATGCAAGCCGGGTACGTT	GCGACGCAACACGTGGTTGAAA	230
FMgSSR-11907	scaffold1873	p2	(AG)7	Class II	28141	28154	ACATTGTGGGCCGACGGAAA	TGCGGCCTGGTTCATCCCATAA	236
FMgSSR-11910	scaffold3002	p2	(AG)7	Class II	15415	15428	ACCAACGCTCCATGAGCCCTAA	ACCAACTGCACAGGTAGGCAGA	316
FMgSSR-11911	scaffold275	p2	(AG)7	Class II	111224	111237	ACCACCGAGCTTGACGAACCAA	TGCCAAGAAAAGCACACGGGA	298
FMgSSR-11913	scaffold421	p2	(AG)7	Class II	6445	6458	ACCACGGACTGCAGCATTTCA	TATGCATGCGCTCTGCTTCACG	333
FMgSSR-11916	scaffold14316	p2	(AG)7	Class II	4895	4908	ACCAGCGCACACAACACACA	CGTCGCTGCAAGGACGGAATTT	339
FMgSSR-11917	scaffold19108	p2	(AG)7	Class II	11468	11481	ACCCAAACATTCATCCGGCTCCT	TCCACCATGGCATGCGTCTT	256
FMgSSR-11918	scaffold1148	p2	(AG)7	Class II	34127	34140	ACCCCATTGTGCGGTTTGGGTT	TTGCTTGCTGCGATGGGACA	276
FMgSSR-11924	scaffold8820	p2	(AG)7	Class II	15480	15493	ACCGAGAGCAGCTCCAAGCTTTT	ACAGCGGTGTGCTGAAGATGCT	212
FMgSSR-11930	scaffold3360	p2	(AG)7	Class II	29284	29297	ACCGCGATGCTGGTGCTTTT	TGCACGTGGGTCCCGAAAACAA	334
FMgSSR-11939	scaffold941	p2	(AG)7	Class II	15398	15411	ACCTGACGTGCTGTAAGCCT	TGTGCCCCAGCTATGACGGTTT	254
FMgSSR-11940	scaffold369	p2	(AG)7	Class II	80772	80785	ACCTGCATTGCATGCCGAGA	TTGTTCGTGCCCAAGTCCGAGT	220
FMgSSR-11945	scaffold7877	p2	(AG)7	Class II	4999	5012	ACGACGGAAGATGTCCCAACCA	ATTCCGACAGGCGACAGCACAT	334
FMgSSR-11948	scaffold13134	p2	(AG)7	Class II	18913	18926	ACGCAAGTCTGTGCGACCAA	AAATGAAACGCGCCAGGAAGGG	270
FMgSSR-11951	scaffold2033	p2	(AG)7	Class II	18903	18916	ACGCCAAAGGCAAACATGGCT	TCCGCACGAAGAACGTCGAT	312
FMgSSR-11953	scaffold2856	p2	(AG)7	Class II	1191	1204	ACGCCCCAAGTGAAACCGTTCT	TTGCTGGCTTCGACTTGCGT	330
FMgSSR-11963	scaffold602	p2	(AG)7	Class II	73677	73690	ACGGATAGGCATCGCGACAA	TTGTTACGCGATGGACGGGA	337
FMgSSR-11968	scaffold16150	p2	(AG)7	Class II	7207	7220	ACGGTGTGGATGACCGAGAAGA	ATTGACAAGTGCCGCCAGGACA	349
FMgSSR-11977	scaffold5948	p2	(AG)7	Class II	22907	22920	ACGTGTGCGCTTTGTGGTGT	TGCCCGGGCCATATATATCCGT	309

SSR_ID	Scaffold	SSR_Type	SSR_Motif	SSR Class	SSR_Start	SSR_End	Forward sequence	Reverse sequence	Product_size
FMgSSR-11978	scaffold560	p2	(AG)7	Class II	125414	125427	ACGTTAGGGCGGCCAACACTTA	TCACGCATTCGGCACCAAGA	270
FMgSSR-11987	scaffold7337	p2	(AG)7	Class II	19215	19228	ACTTGGCACATCGCGTCTCA	TGTTCAAACCAGCAAGCTCGGG	290
FMgSSR-11988	scaffold119	p2	(AG)7	Class II	112912	112925	AGAAAAGTCCGAGCGACACCCA	CCGCGCCAAACAGCTGTCATTT	342
FMgSSR-11993	scaffold408	p2	(AG)7	Class II	36721	36734	AGACGCTGCTTGGCTTGGTT	ACACCACTGTCCGTGCGAAA	288
FMgSSR-11997	scaffold3116	p2	(AG)7	Class II	22362	22375	AGAGCACGCGAAGAAACCGA	TGGCACCATTTTAGAAGCCCCA	259
FMgSSR-12003	scaffold250	p2	(AG)7	Class II	54573	54586	AGATATGCCGCTCTTCCCCAT	CATCTCTGATCCTGCTGTTGGGT	350
FMgSSR-12004	scaffold821	p2	(AG)7	Class II	92732	92745	AGATCAGGTTCTTGGATGGACCC	TGGTGCGTGTGTGGTTCACTGT	332
FMgSSR-12007	scaffold4316	p2	(AG)7	Class II	3563	3576	AGATGGCGGAATGGGGCAAT	ATCGCGACGGGAATTTGTTGCG	297
FMgSSR-12008	scaffold1774	p2	(AG)7	Class II	64316	64329	AGCAAAAGCGTCAGGATCAGCG	TCGAATCCGTCGTCTGTTGCCTT	332
FMgSSR-12010	scaffold5870	p2	(AG)7	Class II	14933	14946	AGCAACAACCACCATGCCGT	GAACAAGAACCGCAACAAACCC	300
FMgSSR-12011	scaffold6117	p2	(AG)7	Class II	2888	2901	AGCAACGCGAACGCCATGTTT	TTTGAAACACCGACCACCCCCA	293
FMgSSR-12012	scaffold819	p2	(AG)7	Class II	88195	88208	AGCAAGCAACAAGTGGGACGGA	AAGGCTCTGCTGCTGCTCTTCT	337
FMgSSR-12014	scaffold15214	p2	(AG)7	Class II	12459	12472	AGCAATGCAGTGCGGCTCAA	AAAGTTGCAAACTCCGGTCGCC	250
FMgSSR-12020	scaffold3034	p2	(AG)7	Class II	33196	33209	AGCACGTCGGTGTCATAACG	CGGCTTCGAGATGGCACAGGTAAT	348
FMgSSR-12023	scaffold4140	p2	(AG)7	Class II	29815	29828	AGCAGTGTGCACGCAACGTA	TCCGTGCACCCAAGAGCTTCAA	306
FMgSSR-12027	scaffold3823	p2	(AG)7	Class II	4603	4616	AGCCACTGGATTAGCACTGGCA	TGGCAACAAGGAAGCTGTTGGA	330
FMgSSR-12032	scaffold8174	p2	(AG)7	Class II	10308	10321	AGCCGAGACTTTGCGGCTCTTT	TCGCAAATGGCAAGTGTGGCA	310
FMgSSR-12036	scaffold4712	p2	(AG)7	Class II	41210	41223	AGCCTGCAAAACTGCGCTTGT	ACGCGACCCATGATAGGGAACA	330
FMgSSR-12038	scaffold49864	p2	(AG)7	Class II	465	478	AGCGATCGCGTGCTGCAAAA	ATGAATGCTGCTCCTCTGCAC	283
FMgSSR-12047	scaffold7411	p2	(AG)7	Class II	44	57	AGCGGCTGAAAGAGAGAGAGGGAA	TCCTTCACATGCCCACGCCTTT	251
FMgSSR-12051	scaffold7164	p2	(AG)7	Class II	19876	19889	AGCGTACCTTGCAGCCATACCA	GGCAGCTCAAAGGTGATGCGTT	310
FMgSSR-12052	scaffold3030	p2	(AG)7	Class II	28591	28604	AGCTCCAACTTGCTGATGGCA	ACCACCTCTCCGGATCTCAGAA	331
FMgSSR-12057	scaffold8986	p2	(AG)7	Class II	7602	7615	AGCTTGCACAGTTGGCAGCA	CCTCTTGTTTATTTGCCGAGAGGCG	327
FMgSSR-12058	scaffold26977	p2	(AG)7	Class II	3899	3912	AGCTTGCAGTTGCAGGTCGT	TCGCAGCAGAGACAGAACGCAT	305
FMgSSR-12059	scaffold152	p2	(AG)7	Class II	89412	89425	AGCTTGCATCTCTCGCTCACTCA	TCCCCATTTCAAGAGCAGTAGCCG	313
FMgSSR-12072	scaffold7069	p2	(AG)7	Class II	15223	15236	AGGCTAGCAGCGACTAGTGTGT	AGGGCAAGCATTGCGGCTTT	311
FMgSSR-12081	scaffold14051	p2	(AG)7	Class II	17269	17282	AGGGTGTAACCGTGCAGCAA	GCCGTCACTTATATCCAGCTTCTG	297
FMgSSR-12086	scaffold429	p2	(AG)7	Class II	90073	90086	AGGTTGAACAACACGCACCAGC	TAGCGAGGCGTGGCGTATTACA	305

SSR_ID	Scaffold	SSR_Type	SSR_Motif	SSR Class	SSR_Start	SSR_End	Forward sequence	Reverse sequence	Product_size
FMgSSR-12087	scaffold9119	p2	(AG)7	Class II	5575	5588	AGGTTGACCTTCGATGTCCAGCA	ACGGCACAGCAGATCCGTAAGT	327
FMgSSR-12089	scaffold8859	p2	(AG)7	Class II	28878	28891	AGTACGTGTACGGGGAGAGGTT	TAGCAACGTGGCACCAACGACT	244
FMgSSR-12091	scaffold512034	p2	(AG)7	Class II	138	151	AGTCGGGTGGCTTATCCCGTTT	TGGCCAATGTTAGCTGTCTCGC	200
FMgSSR-12094	scaffold189	p2	(AG)7	Class II	137264	137277	AGTGATGGAGCGGTTCCGTTGA	TCAAGGCCTCAAGCTCAAGCCA	325
FMgSSR-12095	scaffold7293	p2	(AG)7	Class II	16996	17009	AGTGCGGCGAGCTCTTTTGT	AAGTTCAGCCACACCGACCA	291
FMgSSR-12097	scaffold1335	p2	(AG)7	Class II	8805	8818	AGTGGAAACGGCCATGTGCT	AGCCGCGCCTCCATACATTT	239
FMgSSR-12102	scaffold2010	p2	(AG)7	Class II	57163	57176	AGTGTTGCACGCAGCTTCCTCT	ATGTGGGCCACCATGCATCA	328
FMgSSR-12110	scaffold1614	p2	(AG)7	Class II	66522	66535	ATCACCCCCTTTGTGTGTGCGT	TGGCTCTCGTGAGCGTTCCTTT	341
FMgSSR-12117	scaffold8125	p2	(AG)7	Class II	20291	20304	ATCGTGAGTTCACACCGCAGCA	TGGCAATCGCGCTGGGACTAAT	247
FMgSSR-12127	scaffold1067	p2	(AG)7	Class II	5077	5090	ATGCACCGGGTGTTGTTAGC	TTGCCATGGGTTGTTGCGGA	299
FMgSSR-12130	scaffold7466	p2	(AG)7	Class II	31455	31468	ATGCCCCCTTTCTTCTGCGT	TTCACGTCTTCCGCTTCCGT	343
FMgSSR-12134	scaffold832	p2	(AG)7	Class II	47413	47426	ATGCTGTGGCACAAGCCCAT	TTTTTGCGAGCGCCCTTCTG	313
FMgSSR-12147	scaffold1111	p2	(AG)7	Class II	26809	26822	ATTCGCTGCTGCCGAGAACCTT	AGCGACGATGGCTGTTGTGA	300
FMgSSR-12149	scaffold2619	p2	(AG)7	Class II	29656	29669	ATTGCCTACCCTGTCATCGTGC	ACTGCGTGCAGTCCTGTTGT	338
FMgSSR-12150	scaffold19140	p2	(AG)7	Class II	14093	14106	ATTGCCTCCTTCCTCTCAGCCT	TCGTACCAGCGTCATGCTCTCT	228
FMgSSR-12151	scaffold1371	p2	(AG)7	Class II	59139	59152	ATTGCGCAGGAAATCGCGTACC	ACACCCCTCGCATTGCAGTT	301
FMgSSR-12152	scaffold542	p2	(AG)7	Class II	56500	56513	ATTGCGCGAGACGCAAACCA	AACAAAAGCCCACCAAGGCCCA	221
FMgSSR-12154	scaffold214	p2	(AG)7	Class II	32036	32049	ATTGGTTGCTGCTGCAAGGGGA	ACAGCGCATTCACACACGCA	338
FMgSSR-12160	scaffold44650	p2	(AG)7	Class II	3680	3693	ATTTGTGCCGCAAAGGTCGC	ACCGCCAAGGATTTGGGCTT	347
FMgSSR-12161	scaffold3064	p2	(AG)7	Class II	14440	14453	CAAAGCTATGTCTGCCACTGTAGG	CACCGGTTGACCATTCCTGTGA	231
FMgSSR-12164	scaffold3282	p2	(AG)7	Class II	58040	58053	CACGCGAGATTCCACAACGACA	TCCCCGCGCTTAACCGATTCTT	275
FMgSSR-12166	scaffold2276	p2	(AG)7	Class II	39201	39214	CCACTGACTACATATACGTCCTCCC	AAGGCTTGGCCCAGCAGTTGAT	229
FMgSSR-12168	scaffold781	p2	(AG)7	Class II	33736	33749	CCCAATTCGGCAAACCGCAA	AGGAAAATACGGCAGCAGCGGA	307
FMgSSR-12169	scaffold153	p2	(AG)7	Class II	75480	75493	CCCACGGGTTGCTTATTCTGCT	TGCCATCTTGTTGGCTGGGGTT	309
FMgSSR-12170	scaffold8450	p2	(AG)7	Class II	27914	27927	CCCCAACGAGGTACTGCTGTTTGT	ACACGTGGACATAAGGGGACCA	249
FMgSSR-12182	scaffold7615	p2	(AG)7	Class II	15263	15276	CCGCTTCGTTTTCACCCACACA	ACGAACGGAGACGTCAGTCCAT	201
FMgSSR-12197	scaffold172	p2	(AG)7	Class II	16553	16566	CGCCATCGTGCATTTCAGCCAA	AGCCGACAGGGAGTGCCATTAT	260
FMgSSR-12202	scaffold189	p2	(AG)7	Class II	101397	101410	CGGAGCGCAGACGAACGAAAAA	ACGCATGCACTTTTGGCCCT	335

SSR_ID	Scaffold	SSR_Type	SSR_Motif	SSR Class	SSR_Start	SSR_End	Forward sequence	Reverse sequence	Product_size
FMgSSR-12205	scaffold6389	p2	(AG)7	Class II	1153	1166	CGTATTGCCATTGCCTTCCGGT	ACGCTTTGGAATTGCCGCAGA	273
FMgSSR-12209	scaffold955	p2	(AG)7	Class II	17817	17830	CGTGTGGTGCAAAAAGAGGCGT	TAGGGACCATAGGCCACTTAGCTG	225
FMgSSR-12210	scaffold31855	p2	(AG)7	Class II	3243	3256	CTCTGCAGATCGCAAAGCGT	TTGGTTGCACACAAGAACGGCG	282
FMgSSR-12221	scaffold11733	p2	(AG)7	Class II	14989	15002	GCCAGCGGGCCTTGTTCAATTT	TAGCGGCAGCTATAGCCAGAGA	287
FMgSSR-12223	scaffold14664	p2	(AG)7	Class II	15246	15259	GCCCGACCAAGACAGCTTTTGT	GCACAGCTCTACACATTTGGCTCA	350
FMgSSR-12229	scaffold4579	p2	(AG)7	Class II	20971	20984	GCGAGAGAGAGATTGGTGGGTGAT	ACGGTAGCATGGACGGATGCAA	233
FMgSSR-12231	scaffold12974	p2	(AG)7	Class II	4924	4937	GCGCGTCCAACGAAGCAAAA	AACGGTCGTTGCCGTCTTCA	298
FMgSSR-12232	scaffold10075	p2	(AG)7	Class II	3583	3596	GCGGCACAATGCTGCTTTACGA	TGAAGGATGGCGAAGCCGAA	313
FMgSSR-12239	scaffold1250	p2	(AG)7	Class II	76994	77007	GCTAGCACACACACATTCTGGTCC	TGGTGTTGCCGATGAGCGTT	251
FMgSSR-12242	scaffold4788	p2	(AG)7	Class II	24281	24294	GCTGATTCCAAGCTGGTGCCAA	ACTGTGCCACACGCACAATCA	221
FMgSSR-12243	scaffold29492	p2	(AG)7	Class II	2123	2136	GCTGGTGAACAGTAGCCGACAA	ACCTCCACCGATTGCACCAACA	349
FMgSSR-12244	scaffold3405	p2	(AG)7	Class II	4745	4758	GCTTCGCGTTGTGGCAGAGAAA	AGAACGGCTCACGGAGACAA	341
FMgSSR-12245	scaffold1466	p2	(AG)7	Class II	40697	40710	GGAGCAAAGCAAATGCTCGGGA	ACTTACGAACTTGCGGCCGACT	304
FMgSSR-12252	scaffold52968	p2	(AG)7	Class II	1596	1609	GGGAGAGAGGCAGAGGAAGAGAAA	AAAGCGGACATGTCGTTGCTGC	284
FMgSSR-12254	scaffold25721	p2	(AG)7	Class II	3864	3877	GGGTTCTGTGATGAACCCGTGTA	TTGTGTCTGGCTGTTCAGGCGT	347
FMgSSR-12257	scaffold11422	p2	(AG)7	Class II	18917	18930	GGTGGGCCCTTGCCATACCTTAAT	TCCCGTCTTCTTCGTTGGAGAGT	322
FMgSSR-12258	scaffold11854	p2	(AG)7	Class II	2164	2177	GTCAGGAACAATCGCACTGGCA	TGCTTCCCCAAGCAGCATCT	243
FMgSSR-12266	scaffold3174	p2	(AG)7	Class II	38917	38930	TAAAAGAGCAACCACCCTGGCG	TCGCTGCGCTGCTTTTTCGT	305
FMgSSR-12267	scaffold5198	p2	(AG)7	Class II	7848	7861	TAAATTGGTGCGTGCCCTGCCT	TGCCCTGCTTCTTCTCCTGCTT	244
FMgSSR-12270	scaffold4196	p2	(AG)7	Class II	17804	17817	TACATGCATGGGCGCTCTCGAA	ACATCTCGAACGAGGCATCCA	247
FMgSSR-12276	scaffold13978	p2	(AG)7	Class II	19364	19377	TAGAGGCGACGAATCACACCCA	AATCGGTCCCCATTCCAGTACCGA	342
FMgSSR-12278	scaffold3137	p2	(AG)7	Class II	6120	6133	TAGCAATGAAGAGCTGCGGCCA	TCCATCCACAAGTCCAGCTGTGT	329
FMgSSR-12288	scaffold31924	p2	(AG)7	Class II	1630	1643	TATACTACGCGTCAGCCCATCCCA	TGTTGGGTCCGTCGTTCTTGGA	201
FMgSSR-12295	scaffold306	p2	(AG)7	Class II	121156	121169	TCAAAAAGTCACCCGCCGCT	AGGGGCAATGACAGCTCAAGCA	253
FMgSSR-12300	scaffold207927	p2	(AG)7	Class II	418	431	TCAAGGGCCAACGGTACGTTCA	AGGAACCGGCTGGTTGTCAGAA	207
FMgSSR-12302	scaffold91538	p2	(AG)7	Class II	858	871	TCACACGTGGCTTTGTTGCGT	TGCGCACAGCTACAAGCAGA	301
FMgSSR-12303	scaffold6	p2	(AG)7	Class II	281317	281330	TCACGTGACTCTAACTGCTGCCA	TTGCCAGTTTTCGCGCAGGA	330
FMgSSR-12304	scaffold3514	p2	(AG)7	Class II	48499	48512	TCACTGCCGATGCACCCAACAT	AACAACTTGGCGTCGCGTTC	296

SSR_ID	Scaffold	SSR_Type	SSR_Motif	SSR Class	SSR_Start	SSR_End	Forward sequence	Reverse sequence	Product_size
FMgSSR-12311	scaffold8353	p2	(AG)7	Class II	24537	24550	TCATACCGTGCGTTGTGCCA	ACACCAACCTCAGCTCTCTCCT	298
FMgSSR-12325	scaffold26238	p2	(AG)7	Class II	5214	5227	TCCACAAGCACCACTACACCAAGG	TCTCGTCGTCTACCTCAAGCGA	329
FMgSSR-12328	scaffold743	p2	(AG)7	Class II	63958	63971	TCCACGGTTCTGCGAATGCAA	ACATCCTCGTGCGAGCTGCTTT	238
FMgSSR-12331	scaffold1235	p2	(AG)7	Class II	69068	69081	TCCATAGCCAGTTTTTGCGGC	TGCACACATGCGAATTCGGTAAGG	313
FMgSSR-12339	scaffold6212	p2	(AG)7	Class II	39441	39454	TCCGTGGTTCCAGCATCCGATT	ACCTTGGTCTTCCTGCAGTGCT	333
FMgSSR-12341	scaffold1191	p2	(AG)7	Class II	16178	16191	TCCTCGTCCCCATGCCATTTGA	TGCATGGCACTACCGTCAATGT	283
FMgSSR-12343	scaffold15612	p2	(AG)7	Class II	18409	18422	TCCTGTTGTTCCCCACCTTGTGT	TGGATGGCTGACAAGGAAGGCT	266
FMgSSR-12347	scaffold4558	p2	(AG)7	Class II	1012	1025	TCGAGGGTAGCTGTTGCCTT	TGACGCGCGTGCCGTAATAA	345
FMgSSR-12348	scaffold329893	p2	(AG)7	Class II	111	124	TCGATTCCCTGTCTGATGCGGT	TTGAGTTGGCGCAGTTGCATCG	246
FMgSSR-12350	scaffold6945	p2	(AG)7	Class II	17899	17912	TCGCACTGCTATCTCGGTGCAT	AGGCGGTGCTTTGTTCGCTT	347
FMgSSR-12352	scaffold1097	p2	(AG)7	Class II	82202	82215	TCGCCAAGCAATGGAAGGAGGT	AGATGTCACCAACGCCGTGT	210
FMgSSR-12366	scaffold7430	p2	(AG)7	Class II	2936	2949	TCGCTGCTTGCACTGACTGACT	TGCTCCGACGTTGACGTGCTAA	258
FMgSSR-12367	scaffold38098	p2	(AG)7	Class II	2243	2256	TCGGAGTGCGAGGCAATGCTAT	AACGGCTCCCCTGCAAGTCAAA	290
FMgSSR-12374	scaffold3447	p2	(AG)7	Class II	32395	32408	TCGTGTCGTGTGCTGAATCGT	AGTCGTAGCGGCTCCAAATCA	237
FMgSSR-12375	scaffold189	p2	(AG)7	Class II	74343	74356	TCGTTCGGCTCCTTTGTGCAT	AATACGACGATGGCTTGGGGCA	347
FMgSSR-12382	scaffold1085	p2	(AG)7	Class II	15743	15756	TCTCTCTTGGTGGTGGAACGGA	TTAGAGGTGGCGCAGGGTTGTT	245
FMgSSR-12385	scaffold1919	p2	(AG)7	Class II	55546	55559	TCTGCCATGGTCGTGGTCAT	ACCCTGCCGCTGTCTGTAAA	326
FMgSSR-12386	scaffold1361	p2	(AG)7	Class II	41912	41925	TCTGCTTGCTTGGGTGCGTT	AGCGCAGATTTGGACGCGAGTA	317
FMgSSR-12387	scaffold61	p2	(AG)7	Class II	133140	133153	TCTGGCTTTCGCGGGTTTCA	TTCACTTGCCACTACCAGGCGT	246
FMgSSR-12389	scaffold4605	p2	(AG)7	Class II	24111	24124	TCTTGCTGCCCGTTCTATGCCT	GCTGGATCGATTCAACGAAGCACA	280
FMgSSR-12391	scaffold15540	p2	(AG)7	Class II	11932	11945	TGAAGCTACCCATGCAACAGGT	TCACCTCAAAGGCCGCACCAAT	298
FMgSSR-12396	scaffold534	p2	(AG)7	Class II	46922	46935	TGACGGCGACTGCTGTGTTT	ACGACACGAACGTTGCCCAT	278
FMgSSR-12402	scaffold17042	p2	(AG)7	Class II	2911	2924	TGAGCGGCAGTGACTAGGAGAA	GCACGTGCCGTGAAACGAAA	264
FMgSSR-12410	scaffold1953	p2	(AG)7	Class II	23334	23347	TGATTAGCGGGAGCAGAAGCGT	AAAACACGCCGCCGCCAAAA	235
FMgSSR-12411	scaffold11802	p2	(AG)7	Class II	14849	14862	TGATTGTCGTGCGGCAACTTGG	AATGCACGTCCAAATGGGGAGC	261
FMgSSR-12412	scaffold3491	p2	(AG)7	Class II	43174	43187	TGCAAACGGTCGCAACAACCA	AAACACTGGGCGAGAGAGCGAA	237
FMgSSR-12414	scaffold2090	p2	(AG)7	Class II	20407	20420	TGCAACGCGCTGTACGAAGT	ATCATCATGTTGCGGTTGCGCC	307
FMgSSR-12416	scaffold2300	p2	(AG)7	Class II	18343	18356	TGCAAGGAACAGTGGCAGCA	AGCGCAGCGCGAATCCTAAA	330

SSR_ID	Scaffold	SSR_Type	SSR_Motif	SSR Class	SSR_Start	SSR_End	Forward sequence	Reverse sequence	Product_size
FMgSSR-12418	scaffold5878	p2	(AG)7	Class II	11233	11246	TGCACTGCCGTCGTTTTCGT	CCGCGACCAATTTCAGTAGCGT	263
FMgSSR-12421	scaffold8268	p2	(AG)7	Class II	29434	29447	TGCAGGTGCGTACGAAATACCCA	TCGAGACATGGAACCTTGCCACT	294
FMgSSR-12425	scaffold8389	p2	(AG)7	Class II	24538	24551	TGCATTGGCGCGACCTGATT	CGTGATTGTTACGCAGGGCA	350
FMgSSR-12429	scaffold9261	p2	(AG)7	Class II	17080	17093	TGCCACTCGTGTCAGCCTTGAT	ATGGCCGCCGTGATTTTGGT	264
FMgSSR-12432	scaffold131434	p2	(AG)7	Class II	759	772	TGCCCAAGAAGGGTGCATAGAC	TTGAACCACACGCAACGCCA	207
FMgSSR-12434	scaffold3346	p2	(AG)7	Class II	44694	44707	TGCCGCAGGCTTAGGTTAGGAA	AGCTGGGCCTCATGCTGAACAA	324
FMgSSR-12437	scaffold2785	p2	(AG)7	Class II	48028	48041	TGCGACCTGCCTTCCCTTTCTT	TGCTCCTGATTGACGGACAAGAC	202
FMgSSR-12439	scaffold11	p2	(AG)7	Class II	248391	248404	TGCGCACTTTTGCTCTGGATGC	TGCATGTGTCACGCGTCGTT	245
FMgSSR-12440	scaffold7450	p2	(AG)7	Class II	5154	5167	TGCGCATGTTTGTGACCCGA	TGCATGGCGTTTGGACGGTT	322
FMgSSR-12442	scaffold2601	p2	(AG)7	Class II	2664	2677	TGCGCTGTGCTGTTTTGGCT	GGGCTGCATTGCTGTGCATCAT	275
FMgSSR-12444	scaffold5591	p2	(AG)7	Class II	28595	28608	TGCGGCAACGACGTGAAATCT	TGCATTGGCTGTCCGACCACTT	293
FMgSSR-12447	scaffold5800	p2	(AG)7	Class II	4909	4922	TGCGGGCTGCTGCATCTTTA	TACGTGCTATCACACGCGCA	246
FMgSSR-12449	scaffold32569	p2	(AG)7	Class II	4208	4221	TGCGGTTGCTAACACGACACA	TGCCGGCATTGTGGGAGAGAAT	241
FMgSSR-12454	scaffold286263	p2	(AG)7	Class II	230	243	TGCTCCATTCGATCCCCTTTGC	GCCATCACGAAATCTTGTGGTACG	347
FMgSSR-12458	scaffold4497	p2	(AG)7	Class II	27867	27880	TGCTCTGGTTCTTTCCTCGGCA	TGGTGCTGACTGCTGCTCTGTT	246
FMgSSR-12460	scaffold11353	p2	(AG)7	Class II	5165	5178	TGCTGACACCTGTGCAACTCAC	CATGGAACGACCAGTACCTATGTG	253
FMgSSR-12465	scaffold164	p2	(AG)7	Class II	356	369	TGCTGTAGATCAACGTTTCCCG	TGGTGCTTCCCATGCCCAAACT	343
FMgSSR-12467	scaffold5154	p2	(AG)7	Class II	30061	30074	TGCTTCATGCCACGCACTTGT	TGATGTTGATGTCCGGCAGGCT	251
FMgSSR-12468	scaffold16365	p2	(AG)7	Class II	2969	2982	TGCTTGCCAGTGTACTGCCTCT	TCACCGGTAAGGCTGCTGCAAT	259
FMgSSR-12477	scaffold2932	p2	(AG)7	Class II	23461	23474	TGGATGCCTGAGTGCACAACACT	TGACGGTTACCACTGCCCTT	278
FMgSSR-12478	scaffold44186	p2	(AG)7	Class II	1238	1251	TGGCAAATGGGCACTGCGAA	TCATCCGCTCGTGCAAAGCA	303
FMgSSR-12479	scaffold17244	p2	(AG)7	Class II	15293	15306	TGGCAAGGCACATAGTTTCGCC	TCGGTGTTGCTGCGGGATTT	215
FMgSSR-12483	scaffold446	p2	(AG)7	Class II	37009	37022	TGGCACCTTTTCAACGCGCA	TCGGCACCTGTACAGCATAGCA	229
FMgSSR-12485	scaffold12	p2	(AG)7	Class II	130114	130127	TGGCATGAAGGTCATCGACGGT	TCAGAGGCGGTCGTTGGATGAT	284
FMgSSR-12489	scaffold172	p2	(AG)7	Class II	135604	135617	TGGCCTGATTCCATGCCAGT	AGCTTTGCACCAGGGCACTT	323
FMgSSR-12490	scaffold753	p2	(AG)7	Class II	28986	28999	TGGCGATTTGCTCTGCGTCT	AGTCCAGGGGTGGTCGGTATTT	247
FMgSSR-12493	scaffold12570	p2	(AG)7	Class II	18601	18614	TGGCGGGTTTGCTACCGCTAAA	TGCGATTGCTGCCTGTCTCT	295
FMgSSR-12494	scaffold758	p2	(AG)7	Class II	58458	58471	TGGCGTGTCTATGTTGGCGA	TGTGCGCGTTTTGGGTGGAT	258

SSR_ID	Scaffold	SSR_Type	SSR_Motif	SSR Class	SSR_Start	SSR_End	Forward sequence	Reverse sequence	Product_size
FMgSSR-12495	scaffold2377	p2	(AG)7	Class II	7001	7014	TGGCTGCGTGCACGAAAAAGT	AAGCCGCAATCTCCGACTGT	298
FMgSSR-12503	scaffold7593	p2	(AG)7	Class II	19624	19637	TGGGGATGCCTGTTGCATGTGT	GCCGCAGTCGTCGTCTTGTTTT	346
FMgSSR-12505	scaffold8712	p2	(AG)7	Class II	17435	17448	TGGGGCTCAAGCCATAACACA	ACGGCGAGCTCGTAATTTTGCT	349
FMgSSR-12514	scaffold19678	p2	(AG)7	Class II	10174	10187	TGGTGGTGTGAGTTGTTGCGGT	AGCACTCACTCGGCTTCACT	269
FMgSSR-12520	scaffold12714	p2	(AG)7	Class II	12943	12956	TGGTTGTGTTGATGTGTGGTTGGG	TCCTCTTCGCTAGTTTCCACTCGT	286
FMgSSR-12521	scaffold880	p2	(AG)7	Class II	36363	36376	TGGTTTCGTGGCCTTACGCT	TGCGGTTGTGGTCCTGCATT	247
FMgSSR-12523	scaffold2666	p2	(AG)7	Class II	46117	46130	TGTCAAGGACGTCGAACCCT	AAAGGAGCGTGTTGCCCTGGTT	209
FMgSSR-12537	scaffold22307	p2	(AG)7	Class II	11756	11769	TGTGGCCGACAAAATGTGCGT	TCAGTCATTGCCGCTTGCCT	314
FMgSSR-12538	scaffold5769	p2	(AG)7	Class II	5135	5148	TGTGGCGGCGGAAGAACAAA	ACTAGCCCACCAAAGCACCGTT	268
FMgSSR-12539	scaffold33988	p2	(AG)7	Class II	2506	2519	TGTGGTGTTAGGCTGTTGGGT	TTGATCAGCGACCAAGGGAAGC	313
FMgSSR-12546	scaffold5767	p2	(AG)7	Class II	10493	10506	TGTTCTCGCCATGGGAGCTGAT	GCTGCTCTGAATGTCTATGATGCCG	333
FMgSSR-12547	scaffold8995	p2	(AG)7	Class II	2095	2108	TGTTGTTGCTGCGTAGACCCCA	ATGAACGCCGTATGCAGCCA	289
FMgSSR-12548	scaffold977	p2	(AG)7	Class II	52054	52067	TTACACGGACGACGGGCGAATA	TCCGGTGGAGATGTTGGACTGA	225
FMgSSR-12551	scaffold34671	p2	(AG)7	Class II	198	211	TTATGCGCATGGCTGTCCGT	ACAGCAGGCAGTCTAGTGGTGT	307
FMgSSR-12555	scaffold2436	p2	(AG)7	Class II	17711	17724	TTCACTCACCAGGCGCCATTCA	ATCGCAACACCGTCCTCCATGT	340
FMgSSR-12557	scaffold987	p2	(AG)7	Class II	38725	38738	TTCCAAGCCGAAGATTTGGCCG	TGACCGGGAATGCCTTGCTTCA	328
FMgSSR-12562	scaffold3001	p2	(AG)7	Class II	7756	7769	TTCGCATCAACCGCCGCAAA	ACCTTGACCTTGTTGCCCTCCT	231
FMgSSR-12564	scaffold1119	p2	(AG)7	Class II	64561	64574	TTCGTCGTTGACGCTCATCC	AAATTTCGCATCCGCCGCGT	337
FMgSSR-12565	scaffold2021	p2	(AG)7	Class II	16411	16424	TTCTGCCACTTTGTCACCCACG	AGGCGACAAGTGAAGAGGGAGT	294
FMgSSR-12568	scaffold9184	p2	(AG)7	Class II	6563	6576	TTGATCTGTGAGCTGGTGGCGA	TCCGATGCCACGCCATTTCA	319
FMgSSR-12571	scaffold2038	p2	(AG)7	Class II	53067	53080	TTGCCCTACTTGTTGGATGGGC	ACCACGCACGATACGGCAAT	344
FMgSSR-12572	scaffold92	p2	(AG)7	Class II	184376	184389	TTGCCGGTGCAGCCAAAAGT	ACGCAGGTCAGGTGTCAAGTCA	201
FMgSSR-12575	scaffold6930	p2	(AG)7	Class II	7062	7075	TTGCGCGAAGGGAGTGTGAA	AGCATCATTGCACGGTTTGCCG	316
FMgSSR-12579	scaffold140	p2	(AG)7	Class II	75851	75864	TTGCTTCCTTGTGCCACCGA	ACACAAATCTCCGCCGCTTCCT	349
FMgSSR-12584	scaffold230	p2	(AG)7	Class II	107257	107270	TTGGAGGTTGGCACTTTGGCCT	TCATGGCACTGTCGTCGTCGTT	300
FMgSSR-12588	scaffold819	p2	(AG)7	Class II	87771	87784	TTGGGTGGCAGGCGATGATTGA	TCGCCTCTTCCTTGTTTGGCA	342
FMgSSR-12591	scaffold233	p2	(AG)7	Class II	36058	36071	TTGGTGGCACGCCTTTGGAA	TCTGTGACGACGTTCTGCAGTT	249
FMgSSR-12598	scaffold3779	p2	(AG)7	Class II	35689	35702	TTTGCACCTGGCGCACACACTA	TTCGCGTGAGGACGTTCGTA	330

SSR_ID	Scaffold	SSR_Type	SSR_Motif	SSR Class	SSR_Start	SSR_End	Forward sequence	Reverse sequence	Product_size
FMgSSR-12600	scaffold6039	p2	(AG)7	Class II	21757	21770	TTTGCCTGTGGGCTGCTCAT	AACCAATCCACTCCCAGGCTGA	333
FMgSSR-12603	scaffold2297	p2	(AG)7	Class II	35326	35339	TTTGTGGGCTGCAATGCGTA	TGCAGCAGGCTTCACTTGGA	350
FMgSSR-12608	scaffold391	p2	(AG)7	Class II	128153	128166	TTTTCTCGTCGCCGTTCCGT	CGCACACGTCCTGCTGTTTT	217
FMgSSR-12609	scaffold1095	p2	(AG)7	Class II	18998	19011	TTTTGCCAGCCCGCTTCCAT	ACGGTAATTCAACGGCGCTCA	339
FMgSSR-12610	scaffold8784	p2	(AG)7	Class II	2399	2412	TTTTGGCATGTTGGCCTGACCG	ACCACAACCCCCATGACCAAGT	337
FMgSSR-12612	scaffold3949	p2	(AG)7	Class II	14688	14701	TTTTGTGCGCGTGAGAGAGGGA	AACACCCCCTGCCAGCATTT	286
FMgSSR-12614	scaffold102	p2	(AG)8	Class II	144836	144851	AAAAATCGAGCGCCCTGTGGCT	ACGGCGCGGAATTTTGGAGA	337
FMgSSR-12617	scaffold4274	p2	(AG)8	Class II	9859	9874	AAAACGGAACCCCAGGCCACAA	ACGCTTCAGCCCATCAAGCA	297
FMgSSR-12619	scaffold8690	p2	(AG)8	Class II	8278	8293	AAAAGGCGTCAGGCTCGTGTCA	TCAGCAAGAGAGTTGCTGTCCT	254
FMgSSR-12620	scaffold255	p2	(AG)8	Class II	65069	65084	AAAATCGCCAAGGCAGGCGT	AGGCAAAGAACCTCGTGCCA	319
FMgSSR-12621	scaffold2768	p2	(AG)8	Class II	4397	4412	AAAATGAGGAAGGTGGCGGGGA	CACCGTGACATTGATGGCGTGA	258
FMgSSR-12627	scaffold563	p2	(AG)8	Class II	61923	61938	AAACGCTTCTCGTGGTCGTGGA	TTATGTAGGCGCGGCAAGGT	321
FMgSSR-12628	scaffold8130	p2	(AG)8	Class II	6976	6991	AAACTTTTGCCCGGAGGCGA	ACGACGGAAGAGAATGGTCGCT	272
FMgSSR-12630	scaffold807	p2	(AG)8	Class II	73929	73944	AAAGCGGAGAGAGAACGAGCGA	CCCAGTGGCGGACGTAAGATTT	326
FMgSSR-12644	scaffold1573	p2	(AG)8	Class II	2130	2145	AACGCCTTCCTGCCTGTTTCGT	TGCCTGCCAGGGCAAATGTT	220
FMgSSR-12646	scaffold5480	p2	(AG)8	Class II	34652	34667	AACTGCTACCACGACCCCAACT	TGCCGGAATTCGAAGGACACGA	239
FMgSSR-12650	scaffold3553	p2	(AG)8	Class II	19405	19420	AAGCACCGGCAACAAACGGT	ATGCTGAAGCTACGGGATGGCA	258
FMgSSR-12654	scaffold11633	p2	(AG)8	Class II	13492	13507	AAGCTGCTTTGGCTGCGCTT	ATGTCCAGTTCGCCATGGGTGT	342
FMgSSR-12661	scaffold11878	p2	(AG)8	Class II	12004	12019	AAGGCGTGTGTGGCATGATGGT	GGGCATCTCACAGTAGAGGCACTT	232
FMgSSR-12666	scaffold2460	p2	(AG)8	Class II	57025	57040	AATGCATTGCGGACGGACAC	AATCACATGCGACGCAGCAACG	333
FMgSSR-12668	scaffold16693	p2	(AG)8	Class II	2994	3009	AATTGGGCCGTAGGATCCCTGT	AGGCAGTAGCAACGCACAAGA	266
FMgSSR-12669	scaffold426	p2	(AG)8	Class II	57060	57075	ACAACGGAAAGGTGTGCCGA	AACGTTTTGTGGGTCCGGGT	256
FMgSSR-12670	scaffold59033	p2	(AG)8	Class II	884	899	ACAAGCGTGGGTTTCTGGCT	ACCCGATCGCAATGCTGCAA	288
FMgSSR-12673	scaffold6212	p2	(AG)8	Class II	8465	8480	ACACAGTACGGGCGCAACAA	ATGCCTGCGCCTCACTTCACTT	341
FMgSSR-12676	scaffold10366	p2	(AG)8	Class II	14967	14982	ACACCTACAGCGCTAAGCAGCA	ACCCCACAACTCTCTCGCATCA	330
FMgSSR-12679	scaffold3645	p2	(AG)8	Class II	38652	38667	ACAGACTGTCGGCAAGAACCCA	TGCGCACAACGATGCTTGCT	275
FMgSSR-12682	scaffold168816	p2	(AG)8	Class II	633	648	ACAGCCGACAAACACGTCCT	ATGCGTGGTCAGCAGTGTGGTT	325
FMgSSR-12683	scaffold275	p2	(AG)8	Class II	143402	143417	ACAGCGGCACCTTTGGCTTT	AAAGCATCGCCGAAGAGCGA	346

SSR_ID	Scaffold	SSR_Type	SSR_Motif	SSR Class	SSR_Start	SSR_End	Forward sequence	Reverse sequence	Product_size
FMgSSR-12684	scaffold6067	p2	(AG)8	Class II	37660	37675	ACAGCGGCTAACTTGCCAGA	GCCAACAGATTGTGTTCCAACG	255
FMgSSR-12685	scaffold2522	p2	(AG)8	Class II	1160	1175	ACAGCTGCCTTCCTGACTTCCT	TGCTTGCGCTAATGCAGTGT	288
FMgSSR-12688	scaffold7356	p2	(AG)8	Class II	36215	36230	ACAGGGTGGACATACAGACTGG	AGCACTATCAGTAGAGGCTCGCCA	316
FMgSSR-12690	scaffold13864	p2	(AG)8	Class II	14847	14862	ACAGTTCGCGCCCGAGAAAA	TCGACACCGGTCAGTGCCAAAA	264
FMgSSR-12691	scaffold160765	p2	(AG)8	Class II	226	241	ACAGTTGTGGCTGGGATTGGCT	TGGCCCTTTTCACCACCTGT	237
FMgSSR-12692	scaffold306	p2	(AG)8	Class II	76251	76266	ACATGCCCACGTTATAGGCAGC	TGCTTTCTTCCACGGAGCTTCCA	306
FMgSSR-12696	scaffold291	p2	(AG)8	Class II	18164	18179	ACATGTTGGCCCTCCTGGAA	TCACCCACCGGTAACACGTACT	330
FMgSSR-12697	scaffold16209	p2	(AG)8	Class II	3031	3046	ACCAACCAGCCTGCAAAGCA	TCCAACAGCGTTCGCATCGT	323
FMgSSR-12701	scaffold20275	p2	(AG)8	Class II	6429	6444	ACCAAGTCTGGCCACATGCGTT	ACTTGCGCATCCATCCGCTT	228
FMgSSR-12706	scaffold21	p2	(AG)8	Class II	172395	172410	ACCATGCGATGCACTGGTTGA	ACTGCGTTGCTTGGGTTGAGGA	319
FMgSSR-12708	scaffold1272	p2	(AG)8	Class II	85569	85584	ACCGAATGGTGTTTCCGGTGGA	TCATCAATCACGCGCACCCA	249
FMgSSR-12710	scaffold2028	p2	(AG)8	Class II	37090	37105	ACCGGTGAATTCCAGCAGTGTCA	TGGGACACTCATCGGTTCTGCT	347
FMgSSR-12715	scaffold223	p2	(AG)8	Class II	19530	19545	ACGAACAAGAACGTCGGGACGAA	TGGTCGCCGTGGCATTTGAA	339
FMgSSR-12717	scaffold873	p2	(AG)8	Class II	73174	73189	ACGACGTGCTGCAACAGCAAT	TCACGCCCCGCCTTGATAAGAA	248
FMgSSR-12719	scaffold36256	p2	(AG)8	Class II	670	685	ACGATCAAGCACACCCCACA	TTGCAGCACTATGACAGAAGGGGC	344
FMgSSR-12721	scaffold1186	p2	(AG)8	Class II	53303	53318	ACGCACGGTCGCATGAACAA	ACAACTGCAACTTGGGACGGGA	277
FMgSSR-12722	scaffold9	p2	(AG)8	Class II	278621	278636	ACGCCATCGGACCTGTCACAAA	ACGCTCTGCCTCATGTTCTGCT	320
FMgSSR-12724	scaffold47	p2	(AG)8	Class II	14158	14173	ACGCCTTCTCGCACCAAGTA	AATCGAAACCCGACAGCGCA	323
FMgSSR-12729	scaffold17707	p2	(AG)8	Class II	7768	7783	ACGGTACGGTTTGCTTCTGCCA	AGGGCAGGCAGCATGTTTGT	289
FMgSSR-12730	scaffold2888	p2	(AG)8	Class II	3772	3787	ACGGTTGGGTACATGCGTGACA	AAGAGCAGTTCACGTGGGAGCA	256
FMgSSR-12732	scaffold1111	p2	(AG)8	Class II	38700	38715	ACGTAGCAGCAGCAAGCCTCAT	TGCTTGCCTGACTTGTTGGATTGC	305
FMgSSR-12733	scaffold13710	p2	(AG)8	Class II	19627	19642	ACGTCCAAGAAAGGACATACGGGC	GCCATGTGTTGTCATGGGGCAA	271
FMgSSR-12738	scaffold299	p2	(AG)8	Class II	101454	101469	ACTGGCATGGAAGAAGCAGCAGT	ACGCCATGATCGAGAGGGCTTT	322
FMgSSR-12741	scaffold23	p2	(AG)8	Class II	121082	121097	ACTGGTGTGAGTGGCAAGCA	AGCTCTTTTGGGCCGGGCTTTT	285
FMgSSR-12742	scaffold8977	p2	(AG)8	Class II	27436	27451	ACTGTGCAAGTGAACGTTCGGG	TGGGTGTGTTTCATACGTACTGGG	323
FMgSSR-12743	scaffold22653	p2	(AG)8	Class II	9148	9163	ACTTGCCGTCAGCTCACACACA	AAGCGATTTCGCACCCACGA	253
FMgSSR-12744	scaffold4287	p2	(AG)8	Class II	4946	4961	ACTTGGACAGAGCAAGTCGATGG	TCAGGAGATATGCGTGCTCCGT	306
FMgSSR-12749	scaffold617	p2	(AG)8	Class II	113313	113328	AGAAGGCGGAGCTTGTGTGA	GCCGTATGGGCCGAAATACGTT	276

SSR_ID	Scaffold	SSR_Type	SSR_Motif	SSR Class	SSR_Start	SSR_End	Forward sequence	Reverse sequence	Product_size
FMgSSR-12750	scaffold7050	p2	(AG)8	Class II	24458	24473	AGACATGGTCGGGGTTTTTGCT	TTTGCTTCTCGCCTGGTTGGCA	350
FMgSSR-12753	scaffold156622	p2	(AG)8	Class II	104	119	AGAGGCGACATCCCAGTTGGTT	TCCGGAGTCCGAAGAGAAGACA	318
FMgSSR-12760	scaffold841	p2	(AG)8	Class II	98352	98367	AGATCGCGTGCCGCATGATT	ACGCACATGGTAGTTGCTGCTG	345
FMgSSR-12763	scaffold1981	p2	(AG)8	Class II	45856	45871	AGCAAGCCGTGGCACCATTT	AGCACCATGATCGGCAACGACA	219
FMgSSR-12767	scaffold30719	p2	(AG)8	Class II	5579	5594	AGCACATCATGGTGGCAGCAGT	TGGCGTACCGCTTCGAGAACAA	211
FMgSSR-12775	scaffold8592	p2	(AG)8	Class II	7166	7181	AGCAGCACCCTGCGCAAAAT	TGCCTGCCATATGGGTCCTCTT	330
FMgSSR-12778	scaffold16421	p2	(AG)8	Class II	4436	4451	AGCAGCGCGGCAAATGTAGT	CCCAGCTTTCGCACGTGTTGTT	290
FMgSSR-12779	scaffold31864	p2	(AG)8	Class II	5380	5395	AGCAGCGGCGCAAGAGTATT	AGCTCGTGCGGGCAAGAAAA	231
FMgSSR-12783	scaffold20929	p2	(AG)8	Class II	6913	6928	AGCCACGCTTGAATCCTGCAT	CGTGCGCAAACTCCACCAACAA	348
FMgSSR-12784	scaffold2918	p2	(AG)8	Class II	41110	41125	AGCCAGCATGGGATGAACTGCT	ACGATCTACTCCATCTTCTCGACG	299
FMgSSR-12785	scaffold3998	p2	(AG)8	Class II	25339	25354	AGCCGACAACACGCTAAGGA	ACTGTACGAAGGTAGTCCTCGGT	222
FMgSSR-12786	scaffold609	p2	(AG)8	Class II	40817	40832	AGCCGTCGTAGCCCGTTTCATT	GTGGAACTGGAACGTGGTGCAT	308
FMgSSR-12787	scaffold9032	p2	(AG)8	Class II	26169	26184	AGCCTTGATGAACCACTCCCCT	AGGTGGAGTTTGGCACGCTT	201
FMgSSR-12789	scaffold7293	p2	(AG)8	Class II	20545	20560	AGCGAGAACGAAGCAGCACA	CAATGGCGGTGCAGTGAGAT	343
FMgSSR-12792	scaffold5982	p2	(AG)8	Class II	16571	16586	AGCGGGGACCCCTTGACTTTTT	AAGTGGGCCCTTTCGGATGCAA	327
FMgSSR-12796	scaffold1867	p2	(AG)8	Class II	75147	75162	AGCTAGTGTTCCTCGTGGCGTT	TCATACCGAAGCCCGCAACA	286
FMgSSR-12801	scaffold120	p2	(AG)8	Class II	185018	185033	AGCTTGCTGCTTTGCTTGCTGC	AGCTTGGAACGAAGGTCCCTCA	202
FMgSSR-12802	scaffold6847	p2	(AG)8	Class II	24273	24288	AGCTTGCTTTGGTTGGGGCA	ATTAGTGCGCGAGGCGAGATGA	292
FMgSSR-12807	scaffold8064	p2	(AG)8	Class II	19126	19141	AGGAGCTTACCGTTGTTGGTCA	TGGCGGAAGGCCGGAAAACATA	262
FMgSSR-12812	scaffold1734	p2	(AG)8	Class II	25372	25387	AGGCCAGTCGCCCTTAAGAGAA	AGCCCATCTGCCCACAACTCAT	260
FMgSSR-12816	scaffold16115	p2	(AG)8	Class II	15706	15721	AGGCGGCCAGCAATCGAAAA	TGGCTCGCAAGAGGAAGAAGGA	274
FMgSSR-12817	scaffold249	p2	(AG)8	Class II	14965	14980	AGGCTGGGTTCACGATGGTGTT	TCGACACTTCCTGTCTCTCTCGCT	304
FMgSSR-12818	scaffold7025	p2	(AG)8	Class II	17764	17779	AGGCTGGTTCACTGACAGTTCC	TGTACCCATGACTCCACAACCA	217
FMgSSR-12821	scaffold16115	p2	(AG)8	Class II	1061	1076	AGGGAGCACTTCCACTCAAGGA	AACCCCAACCCGAGGTGATGAT	340
FMgSSR-12823	scaffold1424	p2	(AG)8	Class II	68243	68258	AGGGATTTGGTTTGTGGGTCACCA	TGTCTCCACGAGCTAGGAACCTCT	284
FMgSSR-12833	scaffold15011	p2	(AG)8	Class II	20725	20740	AGTATTCAGCAGCGCAGAGCGT	TCCTGTGCATAGGGCGACACAA	310
FMgSSR-12835	scaffold13664	p2	(AG)8	Class II	17974	17989	AGTCGCTAGTCGCGCATGTT	AAGGCAAAGAGCAGCAGGGAGA	318
FMgSSR-12840	scaffold7895	p2	(AG)8	Class II	30144	30159	AGTGCCCCATCCGGCAAAAA	TTTCAAGCCGCACAGCCACT	307

SSR_ID	Scaffold	SSR_Type	SSR_Motif	SSR Class	SSR_Start	SSR_End	Forward sequence	Reverse sequence	Product_size
FMgSSR-12841	scaffold5572	p2	(AG)8	Class II	20081	20096	AGTGCCGACGCAACACGAAA	ACTTCTGCTGCTGCTTGCGTT	322
FMgSSR-12842	scaffold287	p2	(AG)8	Class II	74274	74289	AGTGCGCGTGCAAAGCAAGT	ACGTGCTACCAGGACTGATGT	299
FMgSSR-12844	scaffold4978	p2	(AG)8	Class II	41309	41324	AGTGGCCAGAAGTCAGAGCA	TATCGCACGCCGCATGAAGA	217
FMgSSR-12846	scaffold24141	p2	(AG)8	Class II	8251	8266	AGTGGTGTTGGCTTCGCCTGAA	TTGCTCTGCCGTTGGAGACA	345
FMgSSR-12848	scaffold669	p2	(AG)8	Class II	4941	4956	AGTGTCCCCCTGTGTTGCACTT	CAAAAGCAAAGTGCTGACCCCT	273
FMgSSR-12850	scaffold246296	p2	(AG)8	Class II	452	467	AGTTACCCCTCCCTATCACCGCAT	AACTGCAAGCCGTTCCTTCGGT	219
FMgSSR-12851	scaffold38535	p2	(AG)8	Class II	2674	2689	AGTTCGTGCGAGGGAGGAAGAA	ACCGGTTGTGTGAATGTGTGTGAC	329
FMgSSR-12855	scaffold1389	p2	(AG)8	Class II	8833	8848	ATCAACCTCCGCGAACCACA	TACACAACATGTACGGGCGGCA	325
FMgSSR-12856	scaffold10680	p2	(AG)8	Class II	1708	1723	ATCAGGACTGACGCAAGACCGT	TTGCGTGCGTCGTGTTACTG	308
FMgSSR-12858	scaffold1362	p2	(AG)8	Class II	64146	64161	ATCCGCTTCAATGGTGTGCG	ACCCCGGTCCAATACCTCTCAACT	343
FMgSSR-12859	scaffold669	p2	(AG)8	Class II	90949	90964	ATCGAGCCAAGAACCGGCGAAA	ACGGCAAAAACGAGACGGCA	286
FMgSSR-12860	scaffold303	p2	(AG)8	Class II	83612	83627	ATCGCCCTCCCTCAGTCCAATCTT	TCAGTTCGCCACGGGAAACACA	255
FMgSSR-12865	scaffold522	p2	(AG)8	Class II	54991	55006	ATGACGCGCCTGGTACCTGTTT	AGCAACGCCGATGATGAGCA	305
FMgSSR-12869	scaffold25084	p2	(AG)8	Class II	2901	2916	ATGCCTAAAGCTGCGGTGGGTT	TCCCGTGTCTGCCTCTTCCTTT	325
FMgSSR-12874	scaffold3744	p2	(AG)8	Class II	18253	18268	ATGCGTCAGTTTCTGCCGACGA	TTTGATGGACGAGCAGGCCCAA	332
FMgSSR-12878	scaffold639	p2	(AG)8	Class II	1126	1141	ATGTAACAGCGGCAACCCCTCA	CCGTAACTGACAAGCGCTACGA	256
FMgSSR-12880	scaffold497	p2	(AG)8	Class II	6642	6657	ATGTGTACAGTTGGGAGGGCCA	TGGGAACGGGTCCCTAACATCA	286
FMgSSR-12881	scaffold2044	p2	(AG)8	Class II	39377	39392	ATTAGCCACCGTAGGGCCGATT	AACCGGGCCATGTGCAACAA	236
FMgSSR-12882	scaffold1894	p2	(AG)8	Class II	28035	28050	ATTATTCACGGACGCGCTGCAC	ACGGTCGACAAGCAACAGCTA	341
FMgSSR-12891	scaffold6184	p2	(AG)8	Class II	23861	23876	CAGGCAACAAACTTCCATGGTGCT	AGGTGCTCACTCAACTGTCCCT	312
FMgSSR-12892	scaffold50777	p2	(AG)8	Class II	3071	3086	CCAGCGCATGCAAATGTGCGTAT	GCGGCAGCCAACGGTTTCTTTT	283
FMgSSR-12895	scaffold252	p2	(AG)8	Class II	34764	34779	CCGTTGCAGCATTAGGCGTGTT	AGTGGACTCCGCTTGAAGAAGGT	220
FMgSSR-12898	scaffold714	p2	(AG)8	Class II	41042	41057	CGAGCAAGCCTCGTGTTTAGCA	TCCACACCAAAATCCCCTCCCT	214
FMgSSR-12899	scaffold5169	p2	(AG)8	Class II	23924	23939	CGAGCAAGTTGCCCTGTTTGGT	TACCGTTCCGGCGTGATCTGTT	272
FMgSSR-12906	scaffold5231	p2	(AG)8	Class II	29717	29732	CGCCCACATGGTCGGTAACTTT	ACGGCGGCGAAGGTTTATTGT	216
FMgSSR-12908	scaffold108746	p2	(AG)8	Class II	816	831	CGCTTGTCAGTGCCCAAGACAT	ACGAGGGCCGCATTCATTCTCA	348
FMgSSR-12909	scaffold2435	p2	(AG)8	Class II	44375	44390	CGGCAAGCTGAAGCACTCTTGA	ACCAGTGCTTAACCAGTCTCCCA	318
FMgSSR-12910	scaffold12370	p2	(AG)8	Class II	15532	15547	CGGCGGTCGGCTTTGTTTTT	GGTTTGTCCAAATTCCGCGCCA	236

SSR_ID	Scaffold	SSR_Type	SSR_Motif	SSR Class	SSR_Start	SSR_End	Forward sequence	Reverse sequence	Product_size
FMgSSR-12911	scaffold36	p2	(AG)8	Class II	57788	57803	CGGGGCATTGACGAGCGATTTT	ACGTCGGTGCGAACATGACA	288
FMgSSR-12912	scaffold3720	p2	(AG)8	Class II	11303	11318	CGGGGCTGCCTTTCCAATTTCA	CGTCCGTCCCATAACCGCAAAT	261
FMgSSR-12914	scaffold28094	p2	(AG)8	Class II	5575	5590	CGTGCACAAAAACACAGGGGGT	AATAAGCCAGGTGGTCGGTCA	209
FMgSSR-12915	scaffold8282	p2	(AG)8	Class II	6934	6949	CGTGTTCATACAGAAACGGCAGGC	ACAAAAGCCCGCCGCAAGTT	300
FMgSSR-12916	scaffold2380	p2	(AG)8	Class II	35661	35676	CGTTCACTTTTTGCTCCCCACGA	ACGTTGCCTCGAACTGCTCGTT	221
FMgSSR-12918	scaffold18214	p2	(AG)8	Class II	3520	3535	GAAGCATTGCTGCTGCCGAT	AGCTGCCGCGTCACATTAGA	308
FMgSSR-12919	scaffold12490	p2	(AG)8	Class II	18439	18454	GAAGCTTGAGAGCAACGACAGA	GCCGTAACCATGATCTAAGGCGA	350
FMgSSR-12922	scaffold4138	p2	(AG)8	Class II	7300	7315	GCAACTCGCACAATTAGACAGC	TCCACCCCTTCTGACGTACACT	339
FMgSSR-12923	scaffold1146	p2	(AG)8	Class II	74235	74250	GCAAGCACAACACACGGTGACA	GCAATGCAACGACCCGCGATAA	348
FMgSSR-12924	scaffold31983	p2	(AG)8	Class II	4891	4906	GCAAGCTGCTTCATTCGGGCAT	GCGGCAGCGCCCAGAATAAAAT	246
FMgSSR-12925	scaffold1388	p2	(AG)8	Class II	58121	58136	GCACGTACGCTGCAGCAAAAA	ACGCAGCTGCAATGAGCAGA	332
FMgSSR-12927	scaffold687	p2	(AG)8	Class II	70205	70220	GCACGTGCAAGATAAAGAAGCACG	TTGTCTCGCCCGATCTCATCGT	253
FMgSSR-12935	scaffold8138	p2	(AG)8	Class II	26445	26460	GCCGTTGTGTACTAGTAGGGAAGA	AAAGGAGTGAGGCACCGACGTT	326
FMgSSR-12936	scaffold688	p2	(AG)8	Class II	68667	68682	GCCTTGTGATGCACGTCCGTAT	AAAAATCTCCCCGCGTGACC	275
FMgSSR-12937	scaffold4057	p2	(AG)8	Class II	37098	37113	GCGATTGCAACGAGCCGGATAA	TGCATGAATGGGCCCAACCCAA	251
FMgSSR-12938	scaffold4803	p2	(AG)8	Class II	38361	38376	GCGCAATTGATTCCACGGCA	CGCGCATTTGGGCTGTTCAA	213
FMgSSR-12939	scaffold11652	p2	(AG)8	Class II	17899	17914	GCGCGCGATCAATCGATGCTAT	TGGTCAAGCACCCAGCATGA	240
FMgSSR-12942	scaffold62	p2	(AG)8	Class II	31897	31912	GCGTACGCTAGTCATTAGGTGACA	TGGTGGTACCATCTCCCTCTCT	330
FMgSSR-12946	scaffold4513	p2	(AG)8	Class II	25374	25389	GCTCGTTTTAGCATCCACGTCTGC	AACTGCCCACACCACATGTCCA	245
FMgSSR-12948	scaffold1490	p2	(AG)8	Class II	19142	19157	GCTGCCTCGCCGTATGTTGTAT	TCCGACTTTGTACCATGCATCCG	345
FMgSSR-12955	scaffold14174	p2	(AG)8	Class II	9776	9791	GGAAGGCGAAGGTGCGATTGTT	TTCCATGACGTCCGTGGCAGAA	347
FMgSSR-12956	scaffold152	p2	(AG)8	Class II	146660	146675	GGATGGTATCCATACGCTCGCT	AGGGAAGAAACGCTATGCACGGA	303
FMgSSR-12957	scaffold9712	p2	(AG)8	Class II	11770	11785	GGCAACGCTCCAGCCAAGTTTT	TCCTTTGGCCGCCTCCATTTGT	350
FMgSSR-12959	scaffold260379	p2	(AG)8	Class II	418	433	GGCCAAAGCCAAAGCAGCTACA	AGCTCCACGGCCGCTACAAATA	260
FMgSSR-12964	scaffold13	p2	(AG)8	Class II	162601	162616	GGGCCATGTTATGCACGCATGT	TCTCGTTTTCTTCTCCCCCGCA	334
FMgSSR-12969	scaffold7956	p2	(AG)8	Class II	29161	29176	GGTTCTTGCTTTCACCTGCTCA	CAATAGGAGGGCACATACAGCCT	350
FMgSSR-12974	scaffold285	p2	(AG)8	Class II	15946	15961	GTTGCGCGCGCGAATCAAAT	TGGCGGTGATTGGTACTCTGCT	239
FMgSSR-12976	scaffold4040	p2	(AG)8	Class II	34033	34048	GTTTCACTGAAGCACGGCGA	ACGACTCGTTCGCATCCGCTAT	322

SSR_ID	Scaffold	SSR_Type	SSR_Motif	SSR Class	SSR_Start	SSR_End	Forward sequence	Reverse sequence	Product_size
FMgSSR-12980	scaffold222762	p2	(AG)8	Class II	287	302	TAAACTGTGCCTTCCGCGGTCT	AAACCAGCTGGCCGCTTGAA	328
FMgSSR-12981	scaffold2087	p2	(AG)8	Class II	62923	62938	TAAGAACACCCTTGCCCCTCCT	TTTTTATCGCCGTCACGCCC	306
FMgSSR-12987	scaffold702	p2	(AG)8	Class II	6168	6183	TACTCGAAGAAGCGAGCAAGGC	ACGTTTTGCCGCTCAAGCTCA	259
FMgSSR-12989	scaffold2992	p2	(AG)8	Class II	32331	32346	TAGGGTCACTTTCAGACGCCGA	TCCACCGACAGCTCATGCTCAA	335
FMgSSR-12991	scaffold173	p2	(AG)8	Class II	46455	46470	TATAGGCGCCGCAGACTCAACT	AGGGCAGCACTGTCGAACCAAA	331
FMgSSR-13001	scaffold2830	p2	(AG)8	Class II	30915	30930	TCAGCTTTTTGCCTGCCGCT	AAAAAGGCCCGCCCGTGTTA	309
FMgSSR-13004	scaffold5491	p2	(AG)8	Class II	12275	12290	TCATGACATGTGACGTCCAACC	TTGCGATGGTGCATTGAGCC	334
FMgSSR-13005	scaffold174	p2	(AG)8	Class II	8643	8658	TCATGCGGATGGCCACGAAA	AGCAAACCACCCACCGCTAT	327
FMgSSR-13009	scaffold264960	p2	(AG)8	Class II	372	387	TCCAACCTCAAACCGACGCGAA	ACCACACGCGCCAAGAAAAA	344
FMgSSR-13012	scaffold7050	p2	(AG)8	Class II	22636	22651	TCCACCACATGGGCCACTCAAT	TTTTGCTTCTCGCCTGGTTGGC	321
FMgSSR-13018	scaffold36	p2	(AG)8	Class II	91608	91623	TCCCCAACAGCGAGTTGCAT	AGTCGGGATCGCGAAAGAGGAA	312
FMgSSR-13021	scaffold9371	p2	(AG)8	Class II	14032	14047	TCCCTGCAGATTCCAAGCTGGT	TGCTTATCATTGGCTCTGCGGC	299
FMgSSR-13035	scaffold3945	p2	(AG)8	Class II	50446	50461	TCCTTCATGGGGCTCAAGCCAT	GGTGGATAGAACCGGTAAGAAACCC	299
FMgSSR-13036	scaffold2320	p2	(AG)8	Class II	29237	29252	TCGAACGTGAAGGCGGGTTT	AATCACCTTGCTTCCCGAGCGT	309
FMgSSR-13037	scaffold1103	p2	(AG)8	Class II	63286	63301	TCGAAGTTGTGTGTCGCGGT	TTCGTTCGTGTCGCCCATGT	279
FMgSSR-13041	scaffold1440	p2	(AG)8	Class II	63196	63211	TCGAGTTAGCTACCGTCATCGG	ATCGACACCCCAATCACCAGCA	294
FMgSSR-13043	scaffold1659	p2	(AG)8	Class II	61765	61780	TCGCACTGCACAAACGCACA	TTTTGTGCCGCGAGGGGTAA	259
FMgSSR-13044	scaffold1718	p2	(AG)8	Class II	36265	36280	TCGCCAACGCGATGAAAAGCA	TCCTGCCATTCGCAGCCCTTTT	275
FMgSSR-13053	scaffold1701	p2	(AG)8	Class II	48386	48401	TCGCGCTATCAAGAGCAGGCAA	ATTGGTGTGCCGTGACGGTT	309
FMgSSR-13055	scaffold4091	p2	(AG)8	Class II	5423	5438	TCGCGTCTGCTGATGTTCTCTGA	ACGCTGCGAATTGGAGCTTGTG	242
FMgSSR-13056	scaffold8859	p2	(AG)8	Class II	3567	3582	TCGCTCCCACTCTCTACGTTCTCA	TAGGGGAGAAACAAGTGCAGCG	296
FMgSSR-13057	scaffold5943	p2	(AG)8	Class II	33414	33429	TCGCTGCAGTCATGGGTCAT	AGTGCCATGCACCCTAGTCACA	237
FMgSSR-13067	scaffold29050	p2	(AG)8	Class II	7798	7813	TCGTGTGCGCTGTCGAACAA	AGCTGCCGTCAATCCCAACA	210
FMgSSR-13075	scaffold164	p2	(AG)8	Class II	134205	134220	TCTTTCCAGGCACCAAGATCATCC	TGTGCACAGCACACGTGCAA	304
FMgSSR-13079	scaffold7185	p2	(AG)8	Class II	10991	11006	TGACCGGAGGATGCCACAAT	AGGAAACATCGTTGGTGGCGA	344
FMgSSR-13081	scaffold6767	p2	(AG)8	Class II	15010	15025	TGACTAGCAATCGGCTTCTTCCCA	ATCGACCTCCGCCATTGCAT	335
FMgSSR-13082	scaffold7846	p2	(AG)8	Class II	25833	25848	TGACTGCGGCTCCAAAAGCA	ACTCATTCGCAGAGAAGGCAGGT	223
FMgSSR-13084	scaffold520	p2	(AG)8	Class II	86966	86981	TGAGGAGGGGCATGGAAGTGAA	CGGCCGGTCACATCTCTTCTTT	282

SSR_ID	Scaffold	SSR_Type	SSR_Motif	SSR Class	SSR_Start	SSR_End	Forward sequence	Reverse sequence	Product_size
FMgSSR-13086	scaffold17499	p2	(AG)8	Class II	6265	6280	TGATATCGTGTGTGTGCGTGC	ACGATGACCTGCTCTGGATTTGC	322
FMgSSR-13089	scaffold1263	p2	(AG)8	Class II	43941	43956	TGATGGCGGACTGCAATTTGGC	AAAGGTGCGATGAATGCGACGG	339
FMgSSR-13092	scaffold6961	p2	(AG)8	Class II	34229	34244	TGCAACCAGGCAACTTGTAAGC	TGAGCCACGTGGGTGTGTTTCA	326
FMgSSR-13097	scaffold3864	p2	(AG)8	Class II	46200	46215	TGCAGCGCGGATGCTTCAAA	ATCATCGCGAAACGACAGGGGA	296
FMgSSR-13098	scaffold3193	p2	(AG)8	Class II	30748	30763	TGCAGGACTGATGACACGACCA	TGCCAGCAGACATCACAACCA	302
FMgSSR-13102	scaffold615	p2	(AG)8	Class II	52649	52664	TGCCAAGCTGGTGACCTGATGA	TCCACGCACGACACGGCATATT	322
FMgSSR-13104	scaffold7053	p2	(AG)8	Class II	34786	34801	TGCCACCGAAAAGCCAAGAAGC	TTACCCATGCCCACATCCCACA	289
FMgSSR-13111	scaffold1369	p2	(AG)8	Class II	75243	75258	TGCCGTGTTCTTCGCTTTGT	AGAGCACGTACGAGCGCAAT	273
FMgSSR-13114	scaffold1530	p2	(AG)8	Class II	21925	21940	TGCCTGCCATCAACAGTGCT	TGCGATTCCGAGCTGCCTTT	287
FMgSSR-13115	scaffold1772	p2	(AG)8	Class II	50006	50021	TGCGACGGCGAAAGCTTCTT	TCCCAACCAACCGCATACCCAA	284
FMgSSR-13117	scaffold820	p2	(AG)8	Class II	6050	6065	TGCGCATAGGCACATCCAGCAA	TTGCCGTCAACCCGTGCATT	287
FMgSSR-13120	scaffold117	p2	(AG)8	Class II	124592	124607	TGCGCCACGAGACAGTCAGTTT	ACAATCAGACCGCCGTGCTT	325
FMgSSR-13127	scaffold1537	p2	(AG)8	Class II	10983	10998	TGCGTCATGTTTGTCACTGCCG	GCCATTGCTCCTCGCAACTTGT	261
FMgSSR-13128	scaffold15202	p2	(AG)8	Class II	8392	8407	TGCGTGCGACACCGAAACAA	ATGCGTCGATCTCTGTTGAGGAGC	350
FMgSSR-13129	scaffold1690	p2	(AG)8	Class II	64121	64136	TGCGTGTCTGGTGCCTTGGATT	CTGCTGCGTGCGTGCTTTAT	349
FMgSSR-13135	scaffold11243	p2	(AG)8	Class II	6690	6705	TGCTGCTCGACCACACACACTT	TCGCGAAGCCGATCACCACAAT	202
FMgSSR-13137	scaffold12937	p2	(AG)8	Class II	6980	6995	TGCTGGACACCTTCTCGTCAGA	AGCAATGCAAGCTCGCCACA	316
FMgSSR-13145	scaffold3480	p2	(AG)8	Class II	49242	49257	TGGAAGCACTATCCCTCTGCGA	AACGGGATGAACAGCAGCCGAT	342
FMgSSR-13147	scaffold1833	p2	(AG)8	Class II	8801	8816	TGGACGCATTTTCCGTACTGC	GCCAAGCCACGAATGACAAGCA	309
FMgSSR-13151	scaffold319935	p2	(AG)8	Class II	100	115	TGGAGAGCAAAGCGGTTGGA	TGAATCCATGCACCCAACCCAC	211
FMgSSR-13159	scaffold7393	p2	(AG)8	Class II	28939	28954	TGGCAGCAAGAGAGAGGCAACA	TCACATTGTTCCCGATCCCCGT	292
FMgSSR-13160	scaffold3848	p2	(AG)8	Class II	35836	35851	TGGCAGCCGACAACTTTGTATTGC	TGGCGTACATCTTTGCACTGGA	311
FMgSSR-13165	scaffold140	p2	(AG)8	Class II	119298	119313	TGGCGGAAATCACGCGCAAA	CCTTACAGTAAAGACGCAGCCCT	310
FMgSSR-13167	scaffold431	p2	(AG)8	Class II	110891	110906	TGGCTCAGCGCTCGTAGCTTAT	GCCCAATGACGCCGTTCGTTTA	341
FMgSSR-13170	scaffold27200	p2	(AG)8	Class II	4944	4959	TGGCTGCTGTCCATCCTTGCAT	ATTGACTGGCTCGTGATGGCGT	325
FMgSSR-13171	scaffold11266	p2	(AG)8	Class II	12616	12631	TGGGAGCAAACCCGCGTTCTAA	TGCCGCGCAAAGCAACTCAT	200
FMgSSR-13173	scaffold42450	p2	(AG)8	Class II	2284	2299	TGGGCCAGGAGGTTGGTTTTGT	AATGCCGTTGCCACATGCAC	287
FMgSSR-13177	scaffold3026	p2	(AG)8	Class II	8643	8658	TGGGCTTGTGAACGTGCCAT	TGCCAACAAGCTGGTGCAGA	313

SSR_ID	Scaffold	SSR_Type	SSR_Motif	SSR Class	SSR_Start	SSR_End	Forward sequence	Reverse sequence	Product_size
FMgSSR-13178	scaffold18159	p2	(AG)8	Class II	8860	8875	TGGGGACCAGCAAGTTGCAAAA	ATCCCAGCACCTTTCCTGATCCCT	308
FMgSSR-13179	scaffold1049	p2	(AG)8	Class II	62587	62602	TGGTCGTGTCCCATGTGCCATT	TGCTCGTCGATGCTTGTAGCTGT	291
FMgSSR-13182	scaffold1330	p2	(AG)8	Class II	2147	2162	TGGTGCCCAGGTGTTCAAAGT	TCCCTGGTCAAACCAAAGGTTACG	346
FMgSSR-13189	scaffold16336	p2	(AG)8	Class II	6672	6687	TGTCCATCAGTTGCTCGCCCAT	AACACGCTCCGAGACAACACCA	251
FMgSSR-13190	scaffold2898	p2	(AG)8	Class II	53332	53347	TGTCCCACCAAAGTGTGAAGACAG	AAGGCTTGGGGAACAGCAGTGT	213
FMgSSR-13191	scaffold263232	p2	(AG)8	Class II	188	203	TGTCCCTGGCCAATGAAAGACCA	ATCGCAGCGTGCAAACAGAGGA	203
FMgSSR-13198	scaffold1943	p2	(AG)8	Class II	36551	36566	TGTGCATGTGCATGCCCCTTT	TGAACAAGCACCACCAGCAGGA	268
FMgSSR-13201	scaffold3617	p2	(AG)8	Class II	48591	48606	TGTGCTCTGGCAACCACTGTGT	ATGCCGCCGTTCAAATGAGTCG	238
FMgSSR-13203	scaffold3724	p2	(AG)8	Class II	20798	20813	TGTGGACTTTGCTGGTGGCA	AAGTGCGTGGACCAGGTTGA	302
FMgSSR-13205	scaffold9711	p2	(AG)8	Class II	450	465	TGTGGGGCTTCTTTGGTCCCTA	ACGGCGAGCACCACCATTTT	350
FMgSSR-13206	scaffold2073	p2	(AG)8	Class II	49329	49344	TGTGGTGGAGCCGGACAAAA	ATTGGCCACCAGCATGCGTA	263
FMgSSR-13210	scaffold1356	p2	(AG)8	Class II	64669	64684	TGTGTGTGTGGCGTGTGTTGT	TCCATTGACGGAGCTTGACGA	324
FMgSSR-13212	scaffold1905	p2	(AG)8	Class II	20143	20158	TGTGTTTGTGTCGCGCAGTG	TTGGACGCTTCAGCAGCCTT	339
FMgSSR-13216	scaffold91815	p2	(AG)8	Class II	1563	1578	TGTTGACCAAGCGTTCCTCCCT	TCGCGTTCATTCACGCACCA	203
FMgSSR-13218	scaffold235962	p2	(AG)8	Class II	385	400	TTAACAAGGCAGCAGCGGCA	TACGCTTCGCGAGGTCAAGT	307
FMgSSR-13220	scaffold8138	p2	(AG)8	Class II	11995	12010	TTAAGGGGTGGGCGTTGCCATT	TGACGTGACTGCACCACACA	223
FMgSSR-13221	scaffold9621	p2	(AG)8	Class II	19081	19096	TTAATGCATGGCCGCGACTTGG	ACCACACCAGCACAGCACAA	319
FMgSSR-13222	scaffold83	p2	(AG)8	Class II	167164	167179	TTACACTGACGTGGGGGCTT	TTTTGCTGCCGCCACGGTAT	319
FMgSSR-13229	scaffold5840	p2	(AG)8	Class II	13556	13571	TTCGACGAATCGTGGGCTCTGT	ATGGGTTGCGGGAAGCAATGGA	293
FMgSSR-13232	scaffold4976	p2	(AG)8	Class II	17819	17834	TTCGGCATTGCATGGGGAAGGA	TCCACGAAGCAAACAAGGAGGCA	237
FMgSSR-13233	scaffold5772	p2	(AG)8	Class II	27521	27536	TTCGTCAACGCAGCATCCCGTA	TTTGTCCTCCACACCCCTCGTT	290
FMgSSR-13235	scaffold887	p2	(AG)8	Class II	68451	68466	TTCTGCAATGACTGGTTGGGGC	AGGTTCCATTGCGGGCAACA	237
FMgSSR-13239	scaffold1526	p2	(AG)8	Class II	32846	32861	TTGAGCAGCTGCACGCACAT	ACCGGCTTATTTCCTCAGCACCA	343
FMgSSR-13242	scaffold176	p2	(AG)8	Class II	114695	114710	TTGCAAGACGTTTGGCGCTC	AAGAGAACGCAGCATCCGCA	237
FMgSSR-13243	scaffold36743	p2	(AG)8	Class II	4214	4229	TTGCAGATCGAGTGGCAGGTGA	TCACTAAGCCTCGGAAAGGCGA	331
FMgSSR-13247	scaffold986	p2	(AG)8	Class II	21437	21452	TTGCGGAAGTGATGCGTACCGA	AGCAGAGGCAAGCAAAGCGA	262
FMgSSR-13252	scaffold297	p2	(AG)8	Class II	83267	83282	TTGCTGCAACAAGTACGCACGC	TGCCCAGTTCACCGTGTTCA	254
FMgSSR-13254	scaffold23932	p2	(AG)8	Class II	7064	7079	TTGGCCTCACACATGACGTGCT	TGGAACGAAGCGGACATTCTGGT	286

SSR_ID	Scaffold	SSR_Type	SSR_Motif	SSR Class	SSR_Start	SSR_End	Forward sequence	Reverse sequence	Product_size
FMgSSR-13259	scaffold2309	p2	(AG)8	Class II	47589	47604	TTGTGCAGCTGGCAGACGATGA	GCATGGGCACATGAAGAAAGGCA	291
FMgSSR-13263	scaffold123	p2	(AG)8	Class II	62548	62563	TTTCGGAGGAGTTCAGATCCGC	AGGTGGGAGTTGTATCGGCAACA	290
FMgSSR-13269	scaffold15805	p2	(AG)8	Class II	11167	11182	TTTGGCAATGGCACGCTTCAGG	TCGAGAGGCATCGATCCAGTT	296
FMgSSR-13270	scaffold259	p2	(AG)8	Class II	55973	55988	TTTGTTGCTCCACACAGCGTCG	TGGCCGGTAACTTTCGGTTGGA	266
FMgSSR-13271	scaffold7361	p2	(AG)8	Class II	34601	34616	TTTTATCCGTCCCGTGTGGCGT	TGCGCGGTGGCTCAAAAACA	298
FMgSSR-13272	scaffold2517	p2	(AG)8	Class II	43281	43296	TTTTCCCGCTAGCCATGCGT	ACGGGGCGAACTGCTGAAAT	275
FMgSSR-13273	scaffold161	p2	(AG)8	Class II	100466	100481	TTTTCCGGTGGGTTCCTCGT	TGACATTACCACCAGTCAGGGG	206
FMgSSR-13274	scaffold4507	p2	(AG)8	Class II	23985	24000	TTTTTGTGGCCATGGCGCGT	ACCGTAAAGGCGGATGATGGCA	274
FMgSSR-13276	scaffold6073	p2	(AG)9	Class II	41427	41444	AAACAGCGCTGGTGTGTGGT	AGTGGAGTGTGGAGTGCTGCTT	301
FMgSSR-13277	scaffold24915	p2	(AG)9	Class II	8501	8518	AAACCGTGCATGGGACCGACAT	TTTGAGGCACACCCCTCTCA	231
FMgSSR-13282	scaffold93	p2	(AG)9	Class II	116028	116045	AAATGGCACGCTTTGCGGCT	CCGGCCGGTGCCCAATTATTTT	344
FMgSSR-13285	scaffold756	p2	(AG)9	Class II	73747	73764	AACACATCTGAAAACCCGCCAC	GCATGATAGGTGCGGACATGCT	200
FMgSSR-13286	scaffold4698	p2	(AG)9	Class II	19426	19443	AACAGCTTCCGTTCGGTCCCTT	GCAACACACGGACACGTTGCTA	286
FMgSSR-13288	scaffold197148	p2	(AG)9	Class II	254	271	AACGAAGGAGTGTTGTCCGCCT	CCGCGTCCGCATATCACTTGTT	273
FMgSSR-13295	scaffold273	p2	(AG)9	Class II	24192	24209	AACGGAACAAGTAGCGGCGT	TGCTGCAAGAGCGTGGATGACA	321
FMgSSR-13296	scaffold5149	p2	(AG)9	Class II	7504	7521	AACTCGTGGCAGGTTCAGACGA	AAATGGCAGCCACCTACACTGC	241
FMgSSR-13298	scaffold72	p2	(AG)9	Class II	18479	18496	AACTTCCATGGCCCGTGATGGT	TTGGTGCAGCGCGACTTTTG	346
FMgSSR-13301	scaffold1615	p2	(AG)9	Class II	22064	22081	AAGCAGCACACACAAAGCCACG	AGGTAAGCTGTCAAGGGCGT	247
FMgSSR-13307	scaffold1602	p2	(AG)9	Class II	50306	50323	AAGGTGGTAAGGGCGGTTGACA	ATCGTCATCGCCATTGGGCA	260
FMgSSR-13319	scaffold1694	p2	(AG)9	Class II	28422	28439	ACAACACAATTCGTCCCGGCCT	ACACAAACCGACCTTCTCGGCA	224
FMgSSR-13323	scaffold1410	p2	(AG)9	Class II	89367	89384	ACACCTAAGCAAGGCAGCAGCA	TGAGCCTGCCACAGTATCCCTT	309
FMgSSR-13325	scaffold10586	p2	(AG)9	Class II	13201	13218	ACAGAGACAAAGCCCAAGGCACA	TGTTGACGACACGCAGCTCACT	243
FMgSSR-13328	scaffold34364	p2	(AG)9	Class II	6212	6229	ACAGCGAGATGGTGGCAATGA	GTTGCTTCTCTCTCCCCTCTTTCT	344
FMgSSR-13329	scaffold7846	p2	(AG)9	Class II	23422	23439	ACAGCGCCACCTTTGGCTTT	AGCATAGCAGCAGACACGGCAT	312
FMgSSR-13331	scaffold1828	p2	(AG)9	Class II	29123	29140	ACATCGACGGTGGCCCATTT	AGGCTTCGATGCTGAGCGTGAT	273
FMgSSR-13336	scaffold9	p2	(AG)9	Class II	114891	114908	ACCACACGCGAGAAGCGTTA	TTCTTGCCGAACTTGAGCCCGT	326
FMgSSR-13337	scaffold11536	p2	(AG)9	Class II	10682	10699	ACCACCCCCAAAACCATCTCCA	AATTGTTCAGCGCCAACCTGGG	328
FMgSSR-13338	scaffold8959	p2	(AG)9	Class II	18573	18590	ACCAGAGCCACTGTGAACCA	TTCAAGCCACTCAAGTCCCTGC	209

SSR_ID	Scaffold	SSR_Type	SSR_Motif	SSR Class	SSR_Start	SSR_End	Forward sequence	Reverse sequence	Product_size
FMgSSR-13339	scaffold1354	p2	(AG)9	Class II	49282	49299	ACCATGACAAATGCCCGTCCGA	TTGCCGCTGAGAAAAGCGAACG	275
FMgSSR-13342	scaffold11440	p2	(AG)9	Class II	5330	5347	ACCCTGCAGTCAATGGTGCAGTT	GGACCACGTCCACGTGAGTTTT	296
FMgSSR-13343	scaffold27874	p2	(AG)9	Class II	893	910	ACCCTGGGTGTGAATTAACGCTGT	AGCTTAGCACGAGCACAATGG	321
FMgSSR-13347	scaffold1906	p2	(AG)9	Class II	37716	37733	ACCGGTAGTATGAGGGCGCATT	AAGGCCTGCGTGATGACGTT	298
FMgSSR-13355	scaffold604	p2	(AG)9	Class II	106505	106522	ACGCACACGCATGGCTTCAT	ACGCACACTTCGTCGTCTTTGT	350
FMgSSR-13356	scaffold44727	p2	(AG)9	Class II	2256	2273	ACGCACGTGCAGAAGTGGAA	AGGCCACTTGCCCTGCTCAAAA	348
FMgSSR-13357	scaffold2075	p2	(AG)9	Class II	8245	8262	ACGCCACCACCACACACTTCAT	TGCGTGATCGCGTACGGAAA	320
FMgSSR-13358	scaffold237	p2	(AG)9	Class II	66788	66805	ACGCCACCCTTTTTCCAGCA	TTTGGCGTGAAGACAGGCGT	318
FMgSSR-13359	scaffold18669	p2	(AG)9	Class II	15064	15081	ACGCTGACGCCTGCACAAGATA	TGCACAGAGACAAGGCGGGATT	344
FMgSSR-13360	scaffold1626	p2	(AG)9	Class II	29599	29616	ACGGCAACACAGGGCATGAA	AGCCATCGGGATCAGCCTGAAA	299
FMgSSR-13361	scaffold7775	p2	(AG)9	Class II	20975	20992	ACGGCAGCCTGGCTTGCATATT	TAGGCGAAAATTGTGCGCCACC	336
FMgSSR-13362	scaffold36481	p2	(AG)9	Class II	3579	3596	ACGGCATCATCCAAACGGGT	TTTGTGCGAGCCAGACGGAA	305
FMgSSR-13364	scaffold68	p2	(AG)9	Class II	20921	20938	ACGGGCGCTTTTCATCATGTGC	TTTGACTGTGCGCCGTTCCA	245
FMgSSR-13365	scaffold6378	p2	(AG)9	Class II	19011	19028	ACGTGCAGGAGTGCGAAGAA	ACACAGAAGCCATCACCAGCGA	219
FMgSSR-13368	scaffold114595	p2	(AG)9	Class II	852	869	ACGTGCTCAGGCACAAAGGA	AACCACGTCTGCATCCAAGCGA	210
FMgSSR-13369	scaffold3111	p2	(AG)9	Class II	16114	16131	ACTCAGTTTCGGGCCTTCCT	TGCGTTTGGGGGACAGAAGT	263
FMgSSR-13371	scaffold25939	p2	(AG)9	Class II	2266	2283	ACTCTCAACCGCATGATGTCTCCA	TAGGCACCGCAGAGCTGGTTTT	256
FMgSSR-13373	scaffold3711	p2	(AG)9	Class II	43745	43762	ACTTGACCTCGCGAAGCGTA	AGTTCTTCCCGCCACAAACGGT	313
FMgSSR-13381	scaffold26367	p2	(AG)9	Class II	1194	1211	AGATCTTGAATGGCCGTCGAGC	ATCACCCTTCCGTTGCTGGCAT	223
FMgSSR-13382	scaffold3447	p2	(AG)9	Class II	22963	22980	AGATGCACGCACAAGTCGGT	ATGGACTGCTCTGCTCTGGCTT	203
FMgSSR-13385	scaffold17382	p2	(AG)9	Class II	5786	5803	AGCAAGACCTGCTTTGGCGT	TAGCTTTGGCCGCTTTGCGT	218
FMgSSR-13386	scaffold15002	p2	(AG)9	Class II	10476	10493	AGCAAGTGGGAAGAGACGAAGGT	TCTGTTGAGTCGTTCATCCGCC	232
FMgSSR-13387	scaffold2032	p2	(AG)9	Class II	5700	5717	AGCAATTTCTCACCTCGCCGCT	TGGGCGTTGCATGTGGTGTT	306
FMgSSR-13389	scaffold37759	p2	(AG)9	Class II	1672	1689	AGCACATCAGCAGTCGACCACA	TCCTCATTGCGTCCAGGTGT	245
FMgSSR-13395	scaffold81	p2	(AG)9	Class II	31441	31458	AGCCAAGCCAACAAGGAAGGGA	AGGTGGAAGAGGCTGTTATGGT	239
FMgSSR-13399	scaffold1861	p2	(AG)9	Class II	43237	43254	AGCGACACGTCCGACTTCTT	TCGCTCACACAGCACTTCAGCA	245
FMgSSR-13402	scaffold1157	p2	(AG)9	Class II	59442	59459	AGCGGCAAGCAAAGCAAAGCA	AATTCCATCACCGCATGCTGCC	230
FMgSSR-13404	scaffold5171	p2	(AG)9	Class II	830	847	AGCTAGCCAGGCAGGCATGAAA	ACTAGAGCGGGGGCACAACAAT	350

SSR_ID	Scaffold	SSR_Type	SSR_Motif	SSR Class	SSR_Start	SSR_End	Forward sequence	Reverse sequence	Product_size
FMgSSR-13406	scaffold5952	p2	(AG)9	Class II	11318	11335	AGCTCGTGCACTGCTTAGCCTT	AGGCAAGCCGCAAAGTAGCAA	234
FMgSSR-13407	scaffold3478	p2	(AG)9	Class II	55268	55285	AGCTGATGCGTGGTGGAGAA	ACCGGGCACTTTCGGACACTAA	315
FMgSSR-13411	scaffold30437	p2	(AG)9	Class II	1162	1179	AGCTGTCCGATTGGAGCGAA	AGCAGCATGATGGTACGGCA	295
FMgSSR-13414	scaffold323	p2	(AG)9	Class II	76766	76783	AGGACATGCGGAGGAAGCAA	TTTGGGCTTGAGCCGAACCAGT	272
FMgSSR-13417	scaffold2049	p2	(AG)9	Class II	53695	53712	AGGATTGCAAAGCGCTCGGT	TGGTGTTTGGTCAGACGCTGGA	296
FMgSSR-13421	scaffold3575	p2	(AG)9	Class II	52489	52506	AGGCCGGCCCAAATGACATGAA	TTCCCTTTCCGCGACCCAAACT	350
FMgSSR-13423	scaffold20288	p2	(AG)9	Class II	4237	4254	AGGCGCCCCATTTTTGGGAA	ACGTCGGTGGGTAACGTGCATT	279
FMgSSR-13426	scaffold282614	p2	(AG)9	Class II	402	419	AGGGCGACATCATGGTGCTT	AATGAGGAAGTCCGGGCCGATT	276
FMgSSR-13428	scaffold586	p2	(AG)9	Class II	5746	5763	AGGTGCGAAACAAGGGAGCA	AGCGCTAAAGACCCTGGCATGT	321
FMgSSR-13433	scaffold1168	p2	(AG)9	Class II	30236	30253	AGTCAACAAGCACGGCCACA	TGAGAGTTTGCACCGTGTCGTCT	234
FMgSSR-13439	scaffold1097	p2	(AG)9	Class II	27526	27543	AGTGTGGCGCGCAGCTTAAT	TTTGGTTTGGTGGCGTCTCCGT	345
FMgSSR-13445	scaffold56	p2	(AG)9	Class II	11050	11067	ATCCACCAATCGCCGTGAGTGT	ACCAAAATGACCCAGCCCAACCA	293
FMgSSR-13447	scaffold4659	p2	(AG)9	Class II	31637	31654	ATCGCAGCATTGATCAGGTGGC	AACGGCGATTCGTCTCTGCT	288
FMgSSR-13450	scaffold7749	p2	(AG)9	Class II	31886	31903	ATCGGGCTCTACCTAGACCGAACT	AGGCAACGGATGCTTGTCGAGT	282
FMgSSR-13451	scaffold16436	p2	(AG)9	Class II	18029	18046	ATGACGCCGGTCGAGATTGT	ACGTGCATCGCAGCCTTTCT	254
FMgSSR-13452	scaffold172	p2	(AG)9	Class II	156243	156260	ATGACGTTGCACCATGGCCAGA	ATTGCCACTCAGCCAATGCCCA	282
FMgSSR-13454	scaffold344	p2	(AG)9	Class II	59178	59195	ATGCACTGGAGAGCCGGAAACA	TCGGAGCAGATCAGCAATGCGT	289
FMgSSR-13457	scaffold131	p2	(AG)9	Class II	24316	24333	ATGGACATCCAAGGGCACAGCA	AGGTCAATGCAAGCCGACGTGT	335
FMgSSR-13458	scaffold10766	p2	(AG)9	Class II	9004	9021	ATGGACTTGGGTCTGATGGGGT	TGGCGCTTTCTGTTTCACACCT	262
FMgSSR-13461	scaffold11753	p2	(AG)9	Class II	22904	22921	ATGGCCACAACAAGGAGGGT	TGGCACGCTAAAAGGGTCGTCA	243
FMgSSR-13465	scaffold626	p2	(AG)9	Class II	21181	21198	ATGTGCAGCACCATAGCCGA	GCAGCGGTCAAATTTTTCTGGCCT	287
FMgSSR-13466	scaffold12381	p2	(AG)9	Class II	6382	6399	ATGTGCCGATCCTCCCTTGACA	AAGCCAAGGCCCAAGGACGAAA	267
FMgSSR-13467	scaffold30829	p2	(AG)9	Class II	3363	3380	ATGTGCCTGCATCTTCCGCT	TCAGCAGTTGTGGTCCGTCACA	331
FMgSSR-13470	scaffold1340	p2	(AG)9	Class II	74988	75005	ATTCGGTGCACCTACCAGGCAA	TGTGTCTTCTTGCTCCGCTCCA	350
FMgSSR-13472	scaffold4007	p2	(AG)9	Class II	22106	22123	ATTTGCAAGCGGTGAGGACCGA	TGCGTACGCGTTGATGCATTGG	311
FMgSSR-13473	scaffold4999	p2	(AG)9	Class II	28834	28851	ATTTGGGCGCAACCAACGGA	CCCACGCTTCGCAATTACACCA	305
FMgSSR-13480	scaffold239	p2	(AG)9	Class II	34253	34270	CAGAGACAAAGCGCAAGACACA	TAACGCGCATCTCACTGCCT	236
FMgSSR-13481	scaffold1855	p2	(AG)9	Class II	17964	17981	CATGGCACTCAAAGGATCGTGA	TCGTGCTCGTGCATGGCAAA	327

SSR_ID	Scaffold	SSR_Type	SSR_Motif	SSR Class	SSR_Start	SSR_End	Forward sequence	Reverse sequence	Product_size
FMgSSR-13490	scaffold1204	p2	(AG)9	Class II	42628	42645	CCAGCAAACACATAGACCGTTGCC	CGCCATTTTGCTTGCTTCCTCG	262
FMgSSR-13493	scaffold2582	p2	(AG)9	Class II	56578	56595	CCATCGCAGCGAAGCCAACAAA	AATGAGCTCGCTCTGTCTCTCTCG	247
FMgSSR-13496	scaffold216	p2	(AG)9	Class II	41445	41462	CCTGGAACATAAGCATGCGATGAG	ACACACAAGCGGCGAGATTGGA	320
FMgSSR-13505	scaffold263091	p2	(AG)9	Class II	143	160	CGGTGAAGCAGCACTGCCAAAT	TGGTCGTCGAAATCTGCGCT	240
FMgSSR-13510	scaffold9766	p2	(AG)9	Class II	17174	17191	GAAGGCTGGGTTTGAAGACCCTAT	ACGTTGTGCAGACACGAAGC	296
FMgSSR-13512	scaffold202598	p2	(AG)9	Class II	226	243	GCAATGCACATGCACGCTACCT	AAATTATTCAGGCCCGCAGCCG	289
FMgSSR-13515	scaffold2049	p2	(AG)9	Class II	53336	53353	GCAGCTGCATGCACAAGTCA	ACCGAGCGCTTTGCAATCCT	306
FMgSSR-13520	scaffold20888	p2	(AG)9	Class II	421	438	GCCGGGCCAGGTATATATTCTGTGA	TCGCTCTGATCCAGCTGCACTAT	345
FMgSSR-13521	scaffold1748	p2	(AG)9	Class II	53698	53715	GCCTCCAAATGCTCAAAAAGGCCG	GCATCGTACGTGTGTGGTTTTC	349
FMgSSR-13526	scaffold140	p2	(AG)9	Class II	171445	171462	GCTGCAGCTGGCAGTGAACAAT	TTTTCATGCTCCCCTCTCGCCT	284
FMgSSR-13529	scaffold227	p2	(AG)9	Class II	134036	134053	GGCCTGTTCATGTGGATATGGGCT	AAGCGACGCCAATGGAAGGA	342
FMgSSR-13531	scaffold323	p2	(AG)9	Class II	122201	122218	GGTGCTAAGGAGAGGAAGAAGGCA	ATGGCCGGATTGGCAACTGGAA	321
FMgSSR-13535	scaffold19823	p2	(AG)9	Class II	12018	12035	GTTCAGGAGCAAACAATGTGCG	TCCGTGTGCTGCAACCATGT	231
FMgSSR-13536	scaffold285	p2	(AG)9	Class II	45645	45662	TAAACCAGAACCAGCCGCGT	TTTGTTTGCGCCGTGCCACA	222
FMgSSR-13541	scaffold25561	p2	(AG)9	Class II	9154	9171	TAAGCGCTGGACGGTTGCTT	TGCAGATGGCTACAGGGATGGA	309
FMgSSR-13542	scaffold224	p2	(AG)9	Class II	127006	127023	TAAGCGGGGTGGATTATGCGGA	TGCGTTTCATCATGTGCCGGT	233
FMgSSR-13546	scaffold49221	p2	(AG)9	Class II	674	691	TAGATCTGCCTCCGCTTGCT	AAAGCGCAGTTGCGTGGGTA	213
FMgSSR-13548	scaffold911	p2	(AG)9	Class II	3380	3397	TAGCCCAAAAGCACAAGGGGCA	ATCCTTGTGAAGCGCGACTCGT	343
FMgSSR-13549	scaffold19844	p2	(AG)9	Class II	3650	3667	TATGATTGTGCACGCGGTCCGA	TTTCACTGTTGAGGCACCCGCA	290
FMgSSR-13554	scaffold5883	p2	(AG)9	Class II	31797	31814	TCAAGTGGATGCGTCAGCCT	ACGTCCATCTAGGGTCCAAACACT	299
FMgSSR-13555	scaffold1529	p2	(AG)9	Class II	61092	61109	TCACACAACGACGCAACGGT	AAAATAGCGGGAGCGGCAGAGT	264
FMgSSR-13561	scaffold384	p2	(AG)9	Class II	17195	17212	TCAGACACAGTGCGAATGCAGA	TTCGGCCAGCCGTTCAAGATGA	293
FMgSSR-13563	scaffold4976	p2	(AG)9	Class II	26877	26894	TCAGCGTGCAACGATGGTGA	GCGATCTTCTGCTCCAGGATCT	323
FMgSSR-13566	scaffold1397	p2	(AG)9	Class II	8742	8759	TCATCCGTCTGCGACCTGAACT	CCTGATGCTTGCATCTCCTTTACC	311
FMgSSR-13575	scaffold13152	p2	(AG)9	Class II	10504	10521	TCCATCATCATGGTCATCCTGGGT	TCGTCCCATCCCCATGCAATGA	298
FMgSSR-13576	scaffold5249	p2	(AG)9	Class II	19341	19358	TCCCACCTTGTCACGAGAGGAT	ATGGCACGTTCGTGTGCCAGAT	324
FMgSSR-13577	scaffold9191	p2	(AG)9	Class II	14581	14598	TCCCAGTACTCACTTCTCTGTCCCT	ACGCCTGCACCACAACAGAA	247
FMgSSR-13581	scaffold9488	p2	(AG)9	Class II	11037	11054	TCGAAATGGGAAACCACGGTGC	TTGAGTTGGGGGCCATTGGTGA	266

SSR_ID	Scaffold	SSR_Type	SSR_Motif	SSR Class	SSR_Start	SSR_End	Forward sequence	Reverse sequence	Product_size
FMgSSR-13585	scaffold109893	p2	(AG)9	Class II	968	985	TCGCAAAGTAGCCCAGCTGCAA	TGTGCGTTTGTTGCGTCAGGT	319
FMgSSR-13586	scaffold116	p2	(AG)9	Class II	50951	50968	TCGCAAGTCACGCGTCTTCT	TAAAAGCGAGCCCTGGTGCAGT	314
FMgSSR-13587	scaffold1418	p2	(AG)9	Class II	80654	80671	TCGCACTTGCTCTTGCCTCTGT	AGGTCGTGTGCGCGTGTTTT	253
FMgSSR-13588	scaffold5792	p2	(AG)9	Class II	3643	3660	TCGCAGAATGTTGAAGGCCACA	CACGCCATTGATGACATCGCCA	336
FMgSSR-13589	scaffold4176	p2	(AG)9	Class II	18705	18722	TCGCAGCATAGGTGCCAACA	TCAACTTGCCAACCCTGGCT	279
FMgSSR-13595	scaffold557	p2	(AG)9	Class II	118109	118126	TCGCGCTATGGCAGAGGAACAA	ATTCCGGGAACAGCGAGCAA	313
FMgSSR-13596	scaffold1087	p2	(AG)9	Class II	69052	69069	TCGCGTGGTGGGGTTAATTGGT	ACTGCAAGTCGTTGCGGCAT	279
FMgSSR-13598	scaffold1476	p2	(AG)9	Class II	83732	83749	TCGGCAGCCTTCTTCTTGGCTT	GATAAAGTCGGTTGCCTTGCCA	316
FMgSSR-13599	scaffold22806	p2	(AG)9	Class II	4270	4287	TCGGCCTCGCCTTTTTCCTGTT	ACGAAGGCGGCATTCTGCAA	318
FMgSSR-13600	scaffold4327	p2	(AG)9	Class II	13903	13920	TCGGCTGCGTGTATGGATAGGT	TGCTTTCTCTCACGCCATAGGGA	289
FMgSSR-13605	scaffold8074	p2	(AG)9	Class II	5840	5857	TCGTCGTTGATGGCTGCTTGGT	TTCTTTCCTCAGAAGCCCCGCA	295
FMgSSR-13609	scaffold1101	p2	(AG)9	Class II	36690	36707	TCTAGCGCGTTGGTAGCACA	ATTCACCGGAGAACGATAGGCACG	332
FMgSSR-13611	scaffold546	p2	(AG)9	Class II	110404	110421	TCTGATGTAGGCGCACCCTT	AGTCGGATGCAAGTCGGACCAT	214
FMgSSR-13612	scaffold12315	p2	(AG)9	Class II	14607	14624	TCTGCGCGTGAAGCCAGTTT	ATCGGCGCATCTGTTTGGCT	301
FMgSSR-13624	scaffold6830	p2	(AG)9	Class II	33459	33476	TGCACCACTTTTGTTCGTTGCCC	AGGGCCCAAAAGGGCCAAAA	296
FMgSSR-13628	scaffold9581	p2	(AG)9	Class II	5093	5110	TGCACGGCACCGTATCGAATGA	AAGCATGTGGCGGAAAGGAGCA	323
FMgSSR-13629	scaffold5546	p2	(AG)9	Class II	25810	25827	TGCAGATGGGAGAGAGGTGCAGAA	AGCTTGCTCTCTGCCGCAAT	309
FMgSSR-13630	scaffold167	p2	(AG)9	Class II	150149	150166	TGCAGCAACTGGACACGACA	TGGCCGGGAATCTGGAATGGTT	311
FMgSSR-13631	scaffold14	p2	(AG)9	Class II	106110	106127	TGCATCCACCGTCGTCCTAACA	TTGGGCCGTATCAGCCACATCT	299
FMgSSR-13634	scaffold2278	p2	(AG)9	Class II	61129	61146	TGCCAAAGCGGCGAATCTCT	ACCCGTGCCATTTGTTCCGA	324
FMgSSR-13636	scaffold669	p2	(AG)9	Class II	63142	63159	TGCCCGTTCGAATACACCATGC	AGCAGTTGCAGGCCATCGTCAT	284
FMgSSR-13640	scaffold948	p2	(AG)9	Class II	71754	71771	TGCCTGCACTTCCACGTGTT	AGCAGCCATGCAAGAACGCA	344
FMgSSR-13651	scaffold3233	p2	(AG)9	Class II	25092	25109	TGCGCGTGGACAGCAAAATGA	TCACCAGCTCACGGACCACAAA	339
FMgSSR-13653	scaffold1401	p2	(AG)9	Class II	78247	78264	TGCGGCTGATGTCGGTTTGA	TCGACACCACGGTCCAATGCAA	326
FMgSSR-13654	scaffold10750	p2	(AG)9	Class II	4682	4699	TGCGTCGGTGAAACGTGAGT	TCCCGCAAACAACACTCGCCTA	345
FMgSSR-13662	scaffold1454	p2	(AG)9	Class II	44817	44834	TGCTTGTTTCGCGGCTGGTT	TTGACGACCACCGTAAGCCGTA	311
FMgSSR-13668	scaffold1519	p2	(AG)9	Class II	46357	46374	TGGCCCATGCAAGTGGAGAA	AGCGCTGGATATCCTTGTCTGA	350
FMgSSR-13671	scaffold7	p2	(AG)9	Class II	70020	70037	TGGCGGAAACGGCAAGTACCAT	TTCGATGTCAGCCGCGCTTT	236

SSR_ID	Scaffold	SSR_Type	SSR_Motif	SSR Class	SSR_Start	SSR_End	Forward sequence	Reverse sequence	Product_size
FMgSSR-13675	scaffold13857	p2	(AG)9	Class II	12399	12416	TGGCTTCGTCTGAAACGTCGGT	TGTTCCAATGGACAGCGTGAC	238
FMgSSR-13680	scaffold2850	p2	(AG)9	Class II	12142	12159	TGGGCCTTTTGTGTCTGTCGT	TGACGTGTTCAGTTTGGGCGT	305
FMgSSR-13684	scaffold3917	p2	(AG)9	Class II	7710	7727	TGGTGGCGTGGGTTTGACTGAA	TTCTCTCCCTCCCTCCCTCTTT	347
FMgSSR-13687	scaffold2049	p2	(AG)9	Class II	55646	55663	TGGTTTCGGCGGCGACAGAATA	TGTTGACAAGGCACGCAGGT	232
FMgSSR-13692	scaffold1658	p2	(AG)9	Class II	55617	55634	TGTCATCATGTGTCGACTGGGACA	TTTTGCCTGCGCTTGTGGTC	245
FMgSSR-13695	scaffold1130	p2	(AG)9	Class II	311	328	TGTCCTCGTCATCCGAGCTCAA	TGGCTTTGCCCAGGAACACT	304
FMgSSR-13698	scaffold28406	p2	(AG)9	Class II	617	634	TGTCTGGACCACCTGATGACCA	TTTCCAGCGTCGGCTGATTG	350
FMgSSR-13699	scaffold1407	p2	(AG)9	Class II	59389	59406	TGTGCACGCTTGGATCCGTT	AAACCATGCATTGTCGGCAGCG	299
FMgSSR-13700	scaffold15938	p2	(AG)9	Class II	11795	11812	TGTGCCATCATGCAAGCCGT	TAGGCCCAATGCAACACTGCCT	267
FMgSSR-13703	scaffold1694	p2	(AG)9	Class II	16086	16103	TGTGCGTGCGCGTTTTTGGA	ATGAGCAACGAGACGGGCAT	291
FMgSSR-13704	scaffold1970	p2	(AG)9	Class II	50358	50375	TGTGCTGTTGGGCGCATGTA	TCGCTCGCTTCATGGTGGCTTT	248
FMgSSR-13706	scaffold12682	p2	(AG)9	Class II	13623	13640	TGTGGTCGAAGAAGCCCTGA	AGGAGCGCGAGGTACACAATGA	328
FMgSSR-13711	scaffold114595	p2	(AG)9	Class II	678	695	TGTTCCAACCGGCAGCTGACTT	ACTTCCTTTGTGCCTGAGCACG	266
FMgSSR-13717	scaffold38743	p2	(AG)9	Class II	530	547	TTAATTTGATCTCCCCGCCCGC	AACCCTCACACCCACACACACA	232
FMgSSR-13718	scaffold15176	p2	(AG)9	Class II	4301	4318	TTACCACTTTCGCTGGCCCT	AAGTGGGGCTTTTCCTCGGACT	322
FMgSSR-13719	scaffold347	p2	(AG)9	Class II	83601	83618	TTACGGCACACCAGGTTCAGCA	ACAACAACCGTGCGCATGTGA	348
FMgSSR-13724	scaffold6813	p2	(AG)9	Class II	25123	25140	TTCACTCCCTTGCAACGCCCTT	ACGCACTATGCCGCGCTTAT	322
FMgSSR-13725	scaffold29897	p2	(AG)9	Class II	9739	9756	TTCAGTGGTCCCAGTCGAGCAA	AGAAGTTCGATGCCACGCGA	258
FMgSSR-13730	scaffold1053	p2	(AG)9	Class II	5750	5767	TTCTCTTGCAGCCTCGTGGT	AGCCACATGCGTGTATGTCTCGT	282
FMgSSR-13734	scaffold8222	p2	(AG)9	Class II	23170	23187	TTGAGCGTCGCATGTTGGGGAA	ATCACTCGGAATCGGCTTGCGT	200
FMgSSR-13737	scaffold2009	p2	(AG)9	Class II	6367	6384	TTGCCAACCGCTCCCGAGATTA	ACACAAAGCCCCCGAAACGGTA	252
FMgSSR-13738	scaffold2596	p2	(AG)9	Class II	40716	40733	TTGCCTTGCCGGTTGCCATT	AAGCAACCGAACTCACGCCA	339
FMgSSR-13740	scaffold55	p2	(AG)9	Class II	104207	104224	TTGCGCCTGCGAGAAGTCAA	AGGGTTCGCGAGGCAATGCTTA	223
FMgSSR-13741	scaffold22659	p2	(AG)9	Class II	3646	3663	TTGCGCTGGCTGATACAGATGC	ACGTTACGCCTGCGCATTGA	228
FMgSSR-13745	scaffold11431	p2	(AG)9	Class II	23679	23696	TTGCTGTGCATGTCGCTCCA	GCTTGTTGGCAGCTGCTTTGGT	262
FMgSSR-13746	scaffold16193	p2	(AG)9	Class II	10692	10709	TTGGACATGGGTGTTGCCGA	CACTTTACTCTTCCCTCCTAGCCA	250
FMgSSR-13750	scaffold297	p2	(AG)9	Class II	21970	21987	TTGGCGGGAAATCTATGGCGCT	TGCTGCCGTGGTGAACAAGT	231
FMgSSR-13752	scaffold7474	p2	(AG)9	Class II	35100	35117	TTGGTTGTTGCCACATGCCAGG	ACCAGACAACTGATGTGGGAGT	338

SSR_ID	Scaffold	SSR_Type	SSR_Motif	SSR Class	SSR_Start	SSR_End	Forward sequence	Reverse sequence	Product_size
FMgSSR-13754	scaffold9652	p2	(AG)9	Class II	13657	13674	TTGTGGGGAAGCAGCCAATGGA	AGGAAAGTGGCTCCAGGATGGT	294
FMgSSR-13756	scaffold14504	p2	(AG)9	Class II	8527	8544	TTTACTTTCTCCCGGCACCTCTGC	AAGGGAACAAAGCCCTACGCCA	271
FMgSSR-13757	scaffold12446	p2	(AG)9	Class II	24326	24343	TTTCCATGGCCAGGTAAGCGCA	TTGCGGACGCAGCTGTTGTT	276
FMgSSR-13759	scaffold1407	p2	(AG)9	Class II	46185	46202	TTTCTGCTTCGCATCGGCCA	TGGCGCCAAATGTTCCATCGT	207
FMgSSR-13761	scaffold4244	p2	(AG)9	Class II	20393	20410	TTTGGCCAGCGTCGCAAAGA	TGCCAAACTGCTATCTCCACTCCA	331
FMgSSR-13764	scaffold570	p2	(AG)9	Class II	60447	60464	TTTTCCCAGCAGCAGCACGA	GCATGTTTCCACAGCAACCCCA	211
FMgSSR-13768	scaffold4096	p2	(AT)10	Class I	4205	4224	AAAAGATCCGTCGTCGTCGCGT	TGGTTGGATGGGGTGTGCAT	331
FMgSSR-13769	scaffold12507	p2	(AT)10	Class I	156	175	AAACGGTGTCGGGCAGCCTAAT	AGGGCTTTAGCGCAGTTTCGT	326
FMgSSR-13773	scaffold6191	p2	(AT)10	Class I	28813	28832	AAATTTTCGTGGCCCAGGGGGA	TCGCCTCGTGTGGGTCTTTT	267
FMgSSR-13776	scaffold42454	p2	(AT)10	Class I	4927	4946	AACCTCAGGAAAACCCAACCCAGC	TGGTTGAGGAGCAAGAACGCA	262
FMgSSR-13777	scaffold3964	p2	(AT)10	Class I	28972	28991	AAGCCGGGGCAATAAACTCCCT	TCCATGCTCTGCTCACCACA	202
FMgSSR-13778	scaffold15232	p2	(AT)10	Class I	11654	11673	AAGGAATATGCGCCAGCCAGGA	GCAAAACGTGAGCGGAGCAACT	213
FMgSSR-13786	scaffold3381	p2	(AT)10	Class I	45296	45315	ACACGTCCGTGGTGTCTTTCCT	TCGCAACACACTCACGAGCA	285
FMgSSR-13787	scaffold14076	p2	(AT)10	Class I	7468	7487	ACAGCTGCATATTCTGCCCTTCAG	TTCCCAACGACGTCTCTGCCTT	322
FMgSSR-13788	scaffold23845	p2	(AT)10	Class I	8430	8449	ACAGTCGTCGCCATGCATCT	AAACGAACCCGTCGTGTGTTGG	230
FMgSSR-13789	scaffold389	p2	(AT)10	Class I	38062	38081	ACATCGCCATTGGCACCTCCTT	GGTTGACGCGAATTAGTCCATGCT	301
FMgSSR-13793	scaffold333	p2	(AT)10	Class I	23131	23150	ACCATACGGCGGGACGAAAT	AACCAAGTTGGGACTGCGCT	244
FMgSSR-13795	scaffold2791	p2	(AT)10	Class I	8733	8752	ACCCTGAGACCAGCAACACA	CCAGTCCAGCAGCACTGCTATCAT	306
FMgSSR-13796	scaffold3923	p2	(AT)10	Class I	5532	5551	ACCGAATCTGCCATGATGCCGAA	ACGCGCGTGCAGATACGGAAAT	298
FMgSSR-13798	scaffold2952	p2	(AT)10	Class I	23327	23346	ACCGCCACTCTGCTCGAATCAT	TGCCGACAACGGCAACATCT	288
FMgSSR-13800	scaffold32631	p2	(AT)10	Class I	3423	3442	ACCGGAGAATGGTTGGACCAGA	ACTTGGCGATCAGCGTCTCA	323
FMgSSR-13801	scaffold20819	p2	(AT)10	Class I	5256	5275	ACCTGATCAAGATCCGATGCACCA	TCAAAGTGATGACCGATGAGCCAC	331
FMgSSR-13803	scaffold7025	p2	(AT)10	Class I	478	497	ACGAAGACACCCTGGTTGCGAT	GGAAATGATTCTCAGTTCGCTGCC	319
FMgSSR-13804	scaffold2070	p2	(AT)10	Class I	23403	23422	ACGAGGAGCGAATTGGGTTGGT	GTGTCACACATTTCCTCGGCA	319
FMgSSR-13805	scaffold26314	p2	(AT)10	Class I	5109	5128	ACGCGTCCTTTATGTGCCCACT	AGGCATTCCTTGCATGCCACCT	338
FMgSSR-13808	scaffold9408	p2	(AT)10	Class I	29160	29179	ACTTGCACACACTGACCTTCG	ACGTTTCCACGACGGCATCA	256
FMgSSR-13810	scaffold13701	p2	(AT)10	Class I	11513	11532	AGCAAGCACTGAAAGGACGACT	TCTTGGCCATGTGCCTACCT	342
FMgSSR-13813	scaffold21339	p2	(AT)10	Class I	8031	8050	AGCACGGGCGCAAAGGAAAA	ATGCAGATGCAGATGCAGCCCT	342

SSR_ID	Scaffold	SSR_Type	SSR_Motif	SSR Class	SSR_Start	SSR_End	Forward sequence	Reverse sequence	Product_size
FMgSSR-13815	scaffold10564	p2	(AT)10	Class I	1844	1863	AGCATGGCACTGGAATCTTGGA	TCCATTTGCGGCTAGTCAACCA	339
FMgSSR-13816	scaffold94760	p2	(AT)10	Class I	412	431	AGCCATCTTTGTCGCTGCCA	ACAGCATGACTAGAGGCGTGCAA	320
FMgSSR-13817	scaffold7211	p2	(AT)10	Class I	32009	32028	AGCCGAGTACATAGCGGTCAT	GCTGACCTTGAGGTCCCAAGATGT	305
FMgSSR-13823	scaffold366	p2	(AT)10	Class I	100963	100982	AGCTAGCAGGCCGGTGCAATTT	TTTGTCCTCAGCTTGGCGCCTT	348
FMgSSR-13826	scaffold4297	p2	(AT)10	Class I	28939	28958	AGCTGCAGGTTGAAGGCATGGA	AACGCCAGAGGACACTCGTCAA	262
FMgSSR-13827	scaffold1392	p2	(AT)10	Class I	58843	58862	AGCTTCCAATTCACGCCAGCA	TCCATTCTCAAACGCTGCTCCACT	310
FMgSSR-13828	scaffold2603	p2	(AT)10	Class I	32207	32226	AGCTTCCTTGCTACCTCGGCAA	ACAACCTGGAAGTCCGCCGTTT	313
FMgSSR-13833	scaffold56	p2	(AT)10	Class I	161179	161198	AGGCGGCTGAACAATGGACTCT	TTTGGCAAGTGGGGCCAGAA	319
FMgSSR-13836	scaffold4383	p2	(AT)10	Class I	6712	6731	AGGTGCGGTCAGCAGGTTTTGT	GGTTCAATGGCAGCGTCGACTA	350
FMgSSR-13837	scaffold48330	p2	(AT)10	Class I	2315	2334	AGTCATCACGATGGGCGGGTAA	TTCAGCGCGCAACACGCTAT	349
FMgSSR-13838	scaffold7590	p2	(AT)10	Class I	36735	36754	AGTGCCCTTCCAAAGTGTTGCAC	TCCCATGTGACCCAGCAGATGA	323
FMgSSR-13842	scaffold47342	p2	(AT)10	Class I	152	171	ATGGCTTGGCCTCGTTTTCCCA	TTGGCGCAAAACATGTGCGG	297
FMgSSR-13848	scaffold4207	p2	(AT)10	Class I	29391	29410	CGCTGCACCTAAGATTTGGAGT	TGTCTACAAGCTGACGCGGT	214
FMgSSR-13851	scaffold4984	p2	(AT)10	Class I	34232	34251	GCATGAGGTGAAAGCCAGAACA	TGCAGGCTACCAAACACACCCT	298
FMgSSR-13852	scaffold65	p2	(AT)10	Class I	79062	79081	GCCACCCCAGCCAAAGAAAAGA	GGGTGGCCGATGTACAAAAGGA	279
FMgSSR-13853	scaffold6137	p2	(AT)10	Class I	13544	13563	GCCCACACACCACATTCAGAGA	ATTTGCGGCTGCTTCGTCGT	250
FMgSSR-13856	scaffold7725	p2	(AT)10	Class I	13940	13959	GCCGCCCGCACGAGTAAATAAA	TTTGTTCCGCAACACCGCCA	269
FMgSSR-13857	scaffold1937	p2	(AT)10	Class I	2297	2316	GCGCGACGCGAAAGAACAAA	TGAGTCACGAAGAGACGATGGA	222
FMgSSR-13858	scaffold19530	p2	(AT)10	Class I	12509	12528	GCGTGTGGCATAGCTGGTTT	AGTTGGCACCACGTTCCACA	310
FMgSSR-13859	scaffold12851	p2	(AT)10	Class I	16014	16033	GCGTGTGGCATGATAGCTGGTT	CGCCTTCCATTGAGGGCATT	345
FMgSSR-13860	scaffold1537	p2	(AT)10	Class I	27769	27788	GCGTTGATCACGAACAGACGACA	TTGGCTCCATTATCGGCGGCAT	342
FMgSSR-13865	scaffold8402	p2	(AT)10	Class I	22892	22911	GGGAGGAAATTGGTGGATCGGGTT	TTGCGCAGCACGGACCATAA	347
FMgSSR-13868	scaffold621	p2	(AT)10	Class I	63326	63345	GGTTCAACGACCTAATCCTAGCTG	AGCGCGCAGCGTGTCATTTA	330
FMgSSR-13870	scaffold110	p2	(AT)10	Class I	168780	168799	GTTCCGTTTGCAGGTTCATGCT	TCAGGTTGACAAGCCAGTCCCA	346
FMgSSR-13877	scaffold4691	p2	(AT)10	Class I	7081	7100	TATGCGCTTGCCGTTGCGAT	ACACGGCAACACCTTGTGGT	307
FMgSSR-13878	scaffold3377	p2	(AT)10	Class I	14810	14829	TCAGGCGCGTTCGATGAAGT	TGTCAGGTGAGTGCGGCAAA	331
FMgSSR-13890	scaffold36376	p2	(AT)10	Class I	3055	3074	TCGAAGCGCGCTCTAGTAATGTG	TCCGATCGTGCTCTTTGGCAGT	350
FMgSSR-13892	scaffold3901	p2	(AT)10	Class I	12262	12281	TCGAGAGGAGAAGGCTGCACGTTA	ATGGTTGCCGTGAGGTGGGTAT	217

SSR_ID	Scaffold	SSR_Type	SSR_Motif	SSR Class	SSR_Start	SSR_End	Forward sequence	Reverse sequence	Product_size
FMgSSR-13893	scaffold4324	p2	(AT)10	Class I	30192	30211	TCGCCGCCTTTTCTCGTTCA	CGATGCATCCAAATGCTGGCCT	341
FMgSSR-13895	scaffold7029	p2	(AT)10	Class I	33494	33513	TCGTCGACGCACAACAACAACA	ACCCAACCGCAGACTTTCACCA	286
FMgSSR-13899	scaffold434	p2	(AT)10	Class I	69107	69126	TCTGCCCTTTGCCGATCACA	AATGGAGCGCCATCGGTTCA	254
FMgSSR-13903	scaffold172	p2	(AT)10	Class I	48983	49002	TGCAGCCTATTTTGCTCGCGT	TTTGCAAAGCCTGTGCGTCG	223
FMgSSR-13909	scaffold1443	p2	(AT)10	Class I	66222	66241	TGCCTTGGTGGAACGAACGACT	TTGAGGGATGGGGTTGGGGAAT	321
FMgSSR-13911	scaffold1525	p2	(AT)10	Class I	23696	23715	TGCGCATGTACGCAGAGTGT	ACCAGCTACCGGCAAACACA	329
FMgSSR-13913	scaffold63041	p2	(AT)10	Class I	1957	1976	TGCGTGTCATGTGGTAAGCCCT	GCGCGGTCAACCCAAGCTTTTT	317
FMgSSR-13914	scaffold32	p2	(AT)10	Class I	88247	88266	TGCTGAAACCGACCTGCTGCAT	CCTGTGCTGTGTGCTCGATTCT	348
FMgSSR-13915	scaffold13017	p2	(AT)10	Class I	8131	8150	TGCTGCACAAACGCATGCCA	AGCACCTTCATCACGTCGTCCA	263
FMgSSR-13919	scaffold22406	p2	(AT)10	Class I	4165	4184	TGGCTATCTGCCTTCGTGTGTG	ATGGTCTTGTGGACGCGGTT	344
FMgSSR-13922	scaffold1963	p2	(AT)10	Class I	46793	46812	TGGGACGAACAACCTACCAAGC	TCAGAGCAAAGACACGGTGGA	347
FMgSSR-13929	scaffold32067	p2	(AT)10	Class I	1142	1161	TGGTTGCCCCATGGAACGAT	TTCTCGCGCGAAGTGTGTGT	229
FMgSSR-13932	scaffold1406	p2	(AT)10	Class I	29883	29902	TGTCTTTGTGGGCTTGCTTGCT	AGCTGTTTGCTCATGTCCGCCA	329
FMgSSR-13936	scaffold657	p2	(AT)10	Class I	99324	99343	TTACTTGCTACCTGCTGCGTCG	CAGCGTGCAGCATATATTCTCGGT	326
FMgSSR-13937	scaffold431	p2	(AT)10	Class I	121964	121983	TTCAGCATTGCACAGCACGC	AGCCGCGTTGATGATCTTGTTG	200
FMgSSR-13939	scaffold975	p2	(AT)10	Class I	54085	54104	TTCGTTCGTGGACGTTGGCA	TCGTCGTCCTCAGCAGACCAAA	290
FMgSSR-13949	scaffold29969	p2	(AT)11	Class I	4469	4490	AACACGCGATTCCCAAGCTCCA	TGCTGGTGGAGACACATCCA	309
FMgSSR-13951	scaffold7884	p2	(AT)11	Class I	14797	14818	AACCGACCGACGCGTTGAAT	CATGAGCAAACAACAGATCGGC	296
FMgSSR-13953	scaffold2605	p2	(AT)11	Class I	34764	34785	AACGCTTCGTCGTCGGACTTCA	TGGAAGATGATGGGCAGCGTGT	350
FMgSSR-13956	scaffold2414	p2	(AT)11	Class I	754	775	ACAGCGTGGTGCTTCATTCTGA	AAGGACAGGAACCTCGGCAA	317
FMgSSR-13963	scaffold23727	p2	(AT)11	Class I	1484	1505	ACCGGCGCGCTGAATAACAT	TTATGGTTCCGGCACAGCGT	282
FMgSSR-13964	scaffold30786	p2	(AT)11	Class I	1853	1874	ACCGTGGCTAGGATCGAAGCAT	AGCAAACGCACGCGTTCTTGA	325
FMgSSR-13966	scaffold671	p2	(AT)11	Class I	66273	66294	ACGAACCGGACGACCAAACATGA	TGTTGTTGTGGCCGTGGCAT	343
FMgSSR-13968	scaffold13227	p2	(AT)11	Class I	14273	14294	ACGAGGCGCTACCAATTCGATGT	GTCACGCTCGTCATTTGCACCT	326
FMgSSR-13973	scaffold2306	p2	(AT)11	Class I	53171	53192	ACGTGCTACCGCTGCATCAA	AACAGGGTATCGGTTGCGCT	325
FMgSSR-13976	scaffold150	p2	(AT)11	Class I	34609	34630	AGATAGATGCCGCCTGTGCTGT	TTCCACCCAAGGCATCTCCTGA	246
FMgSSR-13982	scaffold975	p2	(AT)11	Class I	89809	89830	AGGATGATGGAGGTGGATCGGACA	TGACGTGGCTTAGCATGGTTGC	286
FMgSSR-13983	scaffold5309	p2	(AT)11	Class I	10652	10673	AGGCAACTGAGGTCGGCACTAT	GCACAATCGGTCGCTAAAGGCA	350

SSR_ID	Scaffold	SSR_Type	SSR_Motif	SSR Class	SSR_Start	SSR_End	Forward sequence	Reverse sequence	Product_size
FMgSSR-13986	scaffold1503	p2	(AT)11	Class I	22225	22246	AGGGCTAGCTCTTTTGGAACGCA	GGCATTCATCAAGTTCCCAGTGGC	313
FMgSSR-13987	scaffold1866	p2	(AT)11	Class I	52220	52241	AGGGGGTAAGTCAACTTGCTTGAG	TGCTGTTAGCTCTCAGGTGCAA	342
FMgSSR-13989	scaffold30786	p2	(AT)11	Class I	888	909	AGTATCGTAGCCACGGGAGACA	AAGTTCGGCGTGTCGTCGTT	299
FMgSSR-13990	scaffold11220	p2	(AT)11	Class I	15238	15259	AGTGGATTGCACGAAAGTCTCGGT	GGCGTACAACAAATTGCGTCCA	348
FMgSSR-13995	scaffold21912	p2	(AT)11	Class I	994	1015	ATCAAGAGCAGCCAGGGCGAAA	ACCGAGGAGCACCGAATGTGAA	208
FMgSSR-13996	scaffold2060	p2	(AT)11	Class I	11471	11492	ATCACGTGCAGTGGCACACA	ACTCGCCGAGGTCGATTTGT	328
FMgSSR-13998	scaffold873	p2	(AT)11	Class I	99422	99443	ATCCTGTGAATTGGCCCGGCTT	TGCGAAAACAGGTGCGTCCA	279
FMgSSR-14000	scaffold198	p2	(AT)11	Class I	20138	20159	ATGCTCTAGCTGGCATGGTCCT	GCTGGCTCAAATACTGCTGCGT	350
FMgSSR-14009	scaffold23175	p2	(AT)11	Class I	7896	7917	CGGTTCCGTGTTCAACGTTGGTAG	TTGTGGCAGACGAGATGACCGT	342
FMgSSR-14012	scaffold31430	p2	(AT)11	Class I	5470	5491	GAAATAGGGGTACTTTGGACGGGT	TGTGGTTTGCAGTGGATGCTTG	316
FMgSSR-14017	scaffold27392	p2	(AT)11	Class I	1126	1147	GCTGATGTCAGTAGGTTGAAACGC	TCCAGAGGCTTTTTGGAGTTGC	294
FMgSSR-14018	scaffold383	p2	(AT)11	Class I	112049	112070	GCTGATTTACCATCGCCGTGCT	ACTGCTGCACTGACCTTTCCGT	227
FMgSSR-14019	scaffold1721	p2	(AT)11	Class I	7846	7867	GGACAGAGCCTGCAGTCAAGAACA	TCCGGCGCCAACAATTTCCA	272
FMgSSR-14021	scaffold14331	p2	(AT)11	Class I	6150	6171	GGCACCAACTGAATGTGTGACTGC	CGCCAAATCGGCTTATCCATGT	232
FMgSSR-14022	scaffold1871	p2	(AT)11	Class I	60066	60087	GTGGCATCGTGAGTGACACAA	TGGAAAGCCCAGACGTGACA	325
FMgSSR-14028	scaffold7119	p2	(AT)11	Class I	21192	21213	TCATGTACGTGGTGCGTGCTGA	TGCGGAACTGGAAAATGGGAGC	300
FMgSSR-14029	scaffold8572	p2	(AT)11	Class I	30503	30524	TCATGTCTCTTGTGTCCAGCAGC	AGATTAGCCCCTGTCGCTGT	340
FMgSSR-14030	scaffold3993	p2	(AT)11	Class I	21295	21316	TCATGTGGTAGGGTCGTTCGGT	ACATGCTCCAGTCGGTGGAT	212
FMgSSR-14033	scaffold938	p2	(AT)11	Class I	78953	78974	TCCATTTGGTCGTCTCAGGCA	GTGTACAACGCATGTCTATGGTGC	350
FMgSSR-14036	scaffold5560	p2	(AT)11	Class I	22608	22629	TCGGAGCAAACCTGGAGAACA	ACCACGAACCTGACATGGGA	350
FMgSSR-14040	scaffold53955	p2	(AT)11	Class I	2920	2941	TGAGGAGCGCCAGCACAAAA	TCGAACACCTCCATTTGGCCCT	301
FMgSSR-14043	scaffold2152	p2	(AT)11	Class I	18498	18519	TGCAGGCCTAGCTTTATGGGCT	ACCCTGCACCCTTGTCACAT	318
FMgSSR-14046	scaffold856	p2	(AT)11	Class I	31525	31546	TGCCACCTTGCTGATCATCCT	ACAACCCACGGCGAACTTGA	230
FMgSSR-14051	scaffold9367	p2	(AT)11	Class I	26586	26607	TGCCTCCTTCGCATTACCACA	ACACACCACTAATGAGCTGCCGA	263
FMgSSR-14052	scaffold1898	p2	(AT)11	Class I	16765	16786	TGCGCGCGACTTGTTGATACA	AGCTGCATTTCAGGAGACCGCA	293
FMgSSR-14055	scaffold107	p2	(AT)11	Class I	88962	88983	TGCTCCACGTGTTATGGGGA	TGTCCTCCAGTGGTATGAGCATC	312
FMgSSR-14056	scaffold2625	p2	(AT)11	Class I	2292	2313	TGCTCTTCGTCACCTTCCCA	TGTTGCTCGATTGCAGCGGT	345
FMgSSR-14061	scaffold6973	p2	(AT)11	Class I	12892	12913	TGGAGTCATTGCTTGCAACAGC	TCGAAGCGCCATATCGCCAA	326

SSR_ID	Scaffold	SSR_Type	SSR_Motif	SSR Class	SSR_Start	SSR_End	Forward sequence	Reverse sequence	Product_size
FMgSSR-14065	scaffold221	p2	(AT)11	Class I	2220	2241	TGGTGGCTGCCAAATCGCAA	AGCGCTAGGGTTTACGGATCT	267
FMgSSR-14066	scaffold1819	p2	(AT)11	Class I	47410	47431	TGGTTGCAGATAGGGCTTCCGT	TTTGCATCACCAGCGGAGCA	349
FMgSSR-14067	scaffold10467	p2	(AT)11	Class I	16790	16811	TGTCGTGTGAAGCACGGACTCA	TCCGTTCACTGATTTGGCCGCA	324
FMgSSR-14068	scaffold506	p2	(AT)11	Class I	32564	32585	TGTGTAAAATCCGGCTCGCCCT	AGTCACCGAGGTGAACTGTCA	328
FMgSSR-14069	scaffold2097	p2	(AT)11	Class I	12450	12471	TGTTAACGACGTGATCCGCCTG	ATCACGTCCGGCTCCAGTTT	315
FMgSSR-14073	scaffold15643	p2	(AT)11	Class I	4172	4193	TTGGCTTTGTGCCTCCTAACTCCC	TGATCCGGTGAAGCGGTGACAA	350
FMgSSR-14074	scaffold2835	p2	(AT)11	Class I	48383	48404	TTGTCACTCAGTTGCCCACAGC	AGCATTTCCACTACCGCATCCA	325
FMgSSR-14076	scaffold425	p2	(AT)11	Class I	28601	28622	TTTGGCACCTAGGGCGCATTCA	AAATCGCGGGAGCATCTTCGGA	324
FMgSSR-14077	scaffold2028	p2	(AT)12	Class I	9714	9737	AAAGGCCCAGAATGCGCCAA	TGGGCTGGGGAGTGTTGTAA	335
FMgSSR-14078	scaffold1450	p2	(AT)12	Class I	55554	55577	AAATTTCAACACCGCCGCACCC	GCTTCCACACATGGCGCTTTCA	303
FMgSSR-14079	scaffold3638	p2	(AT)12	Class I	10956	10979	AACAAGCGCGACACGTCGTA	AGGTGGCGGAAGCTCTGTTGTT	321
FMgSSR-14080	scaffold25441	p2	(AT)12	Class I	8394	8417	AACACGGAAAGAGTGGCGGCTT	TGGGGAGAAATTTGCGGCTCGT	291
FMgSSR-14083	scaffold868	p2	(AT)12	Class I	72784	72807	AAGAAGGGCGGCTTCATCGTCT	ACACTCCGTCCACCACTTGCAT	309
FMgSSR-14094	scaffold30130	p2	(AT)12	Class I	5360	5383	ACCAACCGACCGACACGCTAAA	GCAATCAAGAGATGGCGTGTGGA	204
FMgSSR-14095	scaffold6507	p2	(AT)12	Class I	34976	34999	ACCACCACCGCAAGTGCTTT	GGCTAGGAAGCTGACTTTTGGTGC	303
FMgSSR-14100	scaffold1029	p2	(AT)12	Class I	65900	65923	ACCTTAGCATGCACGGCTTCA	GCTGAGCAACGGTCAACACGAT	333
FMgSSR-14101	scaffold251	p2	(AT)12	Class I	105214	105237	ACGAACCACGTCAGCAACGAGT	TCGGTTTCTACGCCAAGCACGA	332
FMgSSR-14102	scaffold6324	p2	(AT)12	Class I	6847	6870	ACGCATCCAACGGATTTCTGCT	TGACATCGTGGGATCATGCGCT	266
FMgSSR-14110	scaffold378158	p2	(AT)12	Class I	158	181	AGCACCACCAACTCTACGGT	ACGATCGTGGGCTGAGTTGGTT	223
FMgSSR-14115	scaffold24629	p2	(AT)12	Class I	4194	4217	AGGCAACTTGTCATAGCAGGGA	AGGGCCGTCGGTAAGTGAAA	300
FMgSSR-14118	scaffold160	p2	(AT)12	Class I	52673	52696	AGGGCGTTGACCATCGCCTAAA	TGGCCCATGCACCACACAAA	260
FMgSSR-14121	scaffold18668	p2	(AT)12	Class I	8937	8960	AGTCGAATACTACATCCGCGCCA	AACCCCTGGTCAGGAGCCAAAA	350
FMgSSR-14124	scaffold453	p2	(AT)12	Class I	74899	74922	ATCCAACCACTAGTGACGGACG	TCAAGGCAACAACTCCGCACA	318
FMgSSR-14129	scaffold14527	p2	(AT)12	Class I	2455	2478	CGCATTCACCGACTGCTTCA	AGCGGTTTTCAGAGAGCAGCA	319
FMgSSR-14136	scaffold10553	p2	(AT)12	Class I	16499	16522	GCGCCTGGATGGGCTAGATTTT	TTCCCCTCCAACCAAGGCAA	321
FMgSSR-14137	scaffold28596	p2	(AT)12	Class I	4450	4473	GCGTGGACATTAAGCTATGTTGCGG	TTGCACCCCTATCAAGGCCGTA	261
FMgSSR-14138	scaffold8534	p2	(AT)12	Class I	11140	11163	GCTCTGTTCATCAGGCGGCAAT	AAATCGCCCGGGAATCCTGCAT	350
FMgSSR-14139	scaffold614	p2	(AT)12	Class I	40665	40688	GCTGGGCTCTAGTCCCCTTCATTT	TGCGTAATCAGCCATACCGAGTCC	344

SSR_ID	Scaffold	SSR_Type	SSR_Motif	SSR Class	SSR_Start	SSR_End	Forward sequence	Reverse sequence	Product_size
FMgSSR-14140	scaffold7028	p2	(AT)12	Class I	28979	29002	GGCGTATAAATCAGGTTCGGTCGGA	ACCGTGCGTTTACTTGCCGA	225
FMgSSR-14141	scaffold2001	p2	(AT)12	Class I	22021	22044	GGCTTCCTTTCGAGTGACAAGTGC	TCATCGATTCGTTGCGCCCT	284
FMgSSR-14143	scaffold892	p2	(AT)12	Class I	80441	80464	TATACTAGATGGCCGACAGGC	GTTGGACCGGCACATTGACA	350
FMgSSR-14145	scaffold2790	p2	(AT)12	Class I	32388	32411	TCAGTTGGAAGAAGGCTTCGGCA	AGCTGGATTGGCGCAAGAATCG	275
FMgSSR-14146	scaffold6338	p2	(AT)12	Class I	33997	34020	TCCTGACTTTGCTCGTGGAGAGA	GCACGGCTGATTACATTGACAGGC	341
FMgSSR-14148	scaffold1617	p2	(AT)12	Class I	31591	31614	TCGAGGTGAGCCATTTGCTGGA	AGTTGGTCAGCTCCAACCCT	311
FMgSSR-14149	scaffold4112	p2	(AT)12	Class I	29992	30015	TCGTCACCTAATCTCAGGCCCA	ATGCCACGGAAAGGACACGA	284
FMgSSR-14151	scaffold12522	p2	(AT)12	Class I	10079	10102	TGACCTCGCATGGGTGGTATTGT	CCTCGTGCATATTCGCCAGGTT	232
FMgSSR-14152	scaffold25470	p2	(AT)12	Class I	2470	2493	TGAGAGGCCCCAGAAAGCAAGA	ACCAAAGGCTGGATCTCACCA	263
FMgSSR-14154	scaffold6381	p2	(AT)12	Class I	24436	24459	TGATCACAGGTGGGGGTGCTAA	AGCTGGGTGGCCGGTAAAACAT	225
FMgSSR-14161	scaffold6796	p2	(AT)12	Class I	33487	33510	TGGCAATGCACGGACACTTTGC	ACGAATCGAACAAGCGGCTC	268
FMgSSR-14162	scaffold2056	p2	(AT)12	Class I	11499	11522	TGGCCAGAACAATCAGCTGGAC	GTGGTTTGTGTATGCTCCACTGTC	300
FMgSSR-14164	scaffold72065	p2	(AT)12	Class I	937	960	TGGGCCAGGAACTAGTTGGTCA	TTGGCCTGAGGTGAGTTGGTGA	298
FMgSSR-14166	scaffold3242	p2	(AT)12	Class I	30132	30155	TGTCCATTGGGTCCACCTACCA	ATCCAGGGCAAGCTGTTGCT	298
FMgSSR-14168	scaffold1345	p2	(AT)12	Class I	73862	73885	TTGCGCCACAGGGTTCTGTT	TGACCAGAGGAGGGAAACAAGGGA	335
FMgSSR-14170	scaffold5393	p2	(AT)12	Class I	23838	23861	TTGCTGATTGGTTCGTCGCCCA	TACACGTGCTGCGAGTCTGT	344
FMgSSR-14171	scaffold5072	p2	(AT)12	Class I	33332	33355	TTGCTGGCACATGCACTGCT	TGTGCTTTGCCCGGTGGTTT	255
FMgSSR-14173	scaffold9854	p2	(AT)12	Class I	30774	30797	TTGTTGCTGGCGCAACTGCT	CAGGACAAGTTGGCGCTTCA	265
FMgSSR-14175	scaffold4566	p2	(AT)12	Class I	39459	39482	TTTTCCACACGACGCCTGCT	AATGCGAAGGAGAAGAGAGGAGCG	336
FMgSSR-14181	scaffold2192	p2	(AT)13	Class I	55185	55210	ACCATTGACACCTGCACCGCAT	TGTCCTTCCCCGCACGTACTTT	324
FMgSSR-14182	scaffold13692	p2	(AT)13	Class I	19620	19645	ACCGAAACTCAACGAGACCGTGA	TCGCCCTGTACATGGTTGGT	283
FMgSSR-14183	scaffold855	p2	(AT)13	Class I	44049	44074	ACGCGTTACACGTCATCGGT	AGCTTGTTGCTCAAGGAAGGCCA	226
FMgSSR-14185	scaffold251	p2	(AT)13	Class I	105049	105074	ACGGGTACTGCAACATGCTCGT	ACTCGTTGCTGACGTGGTTCGT	213
FMgSSR-14189	scaffold1774	p2	(AT)13	Class I	30202	30227	ACTGGTGTGTGCTGTGTAATTGGC	ACCGCACCAGCGTTTGCATT	341
FMgSSR-14190	scaffold332	p2	(AT)13	Class I	57253	57278	AGCCAAAACAGCCTGTGCCT	AGGTGTTTGGGTCTGGAATTGTGC	295
FMgSSR-14191	scaffold1756	p2	(AT)13	Class I	66246	66271	AGCGAGCATACCTGAAGCTGGA	TCAGCCAGACTTTGGCGCATCA	346
FMgSSR-14198	scaffold1248	p2	(AT)13	Class I	6752	6777	ATGAAGGATCATCCAGCGGTGC	ACGAGATAGTCCTACTGCCTTGTGG	327
FMgSSR-14199	scaffold9777	p2	(AT)13	Class I	5391	5416	ATGATGCTCATCCCCGCGTT	CCATCATCCAGGGATCCAAAAGGAC	316

SSR_ID	Scaffold	SSR_Type	SSR_Motif	SSR Class	SSR_Start	SSR_End	Forward sequence	Reverse sequence	Product_size
FMgSSR-14200	scaffold1563	p2	(AT)13	Class I	55399	55424	ATGCCTTCGTAGGGAGGTAGCA	TCGGCATGTGCAGCATCTCACT	350
FMgSSR-14201	scaffold1083	p2	(AT)13	Class I	59182	59207	ATGGAACACGTGCACGCCAA	GCAGGGTGGCAAATGAAGCTGT	349
FMgSSR-14204	scaffold2046	p2	(AT)13	Class I	55454	55479	ATGGCGCGTGCATGGGTAAA	ATGCGTCGGTCCTGCATGTT	230
FMgSSR-14206	scaffold2436	p2	(AT)13	Class I	14379	14404	ATTGCCGCGCTTGCAGTACA	ATGGCGGATACTGGACGCGTAA	278
FMgSSR-14211	scaffold6892	p2	(AT)13	Class I	3917	3942	CCAGCGTCGTATTGGTGCATGA	TGCGTTTGGAACTTGATGGGCA	315
FMgSSR-14212	scaffold2752	p2	(AT)13	Class I	8066	8091	CCGACGTGGCAATATTGTTGTG	TCCACGGACATTTCTTGGCGCA	349
FMgSSR-14213	scaffold23942	p2	(AT)13	Class I	6779	6804	CCTTGGAGTAACAGTCCCTTGCACA	TCTGCTAAGCAAGGATGGAACGGT	200
FMgSSR-14215	scaffold8	p2	(AT)13	Class I	203070	203095	CGCCAACCATTCATGCTGAACCA	TGGACGCAACAGGCTTGATGACA	333
FMgSSR-14216	scaffold22305	p2	(AT)13	Class I	4715	4740	CGGCATTTCATGCATGTGCCTTG	AGACCTGTCTCACTCATGCCCT	309
FMgSSR-14217	scaffold14631	p2	(AT)13	Class I	12187	12212	GCAGTTCAAGCGGTTCAATCCGT	ACACCTGACACACACGCACA	323
FMgSSR-14220	scaffold2013	p2	(AT)13	Class I	35863	35888	GGGATATGAACGTGTTGTGCCA	TCACCATTCTTCGGGGTTTGCTCA	216
FMgSSR-14222	scaffold9842	p2	(AT)13	Class I	11519	11544	TAGCGCTGTCGTGTGTGTGTGT	TGCTGATTTCTGCCCCCGTT	344
FMgSSR-14224	scaffold1039	p2	(AT)13	Class I	70310	70335	TCCAACAACGGAGAAGGTGCT	TTGCTAGCCGCACGATGGTT	283
FMgSSR-14228	scaffold11640	p2	(AT)13	Class I	13934	13959	TCGACATCGTGCTCGCCAAA	GCGCGTCATTCGATTGGTGCAT	332
FMgSSR-14233	scaffold2129	p2	(AT)13	Class I	17418	17443	TGACGTGAGGGAATTCGGCGAT	ACGACGGAACTACGTGGACGAT	231
FMgSSR-14234	scaffold12649	p2	(AT)13	Class I	11262	11287	TGATCCTGACGCACACACGTT	TGATGATGTTGCGTGCACCCTG	312
FMgSSR-14238	scaffold329	p2	(AT)13	Class I	52025	52050	TGCCATGCTGGTAGCTTTGCAG	TGGTGACTGACACTGGGCTTGT	296
FMgSSR-14239	scaffold10257	p2	(AT)13	Class I	28801	28826	TGCCCTTGCAAGTAAGCCACCA	ACACAAGGGTGAAGGGCAGT	324
FMgSSR-14240	scaffold131	p2	(AT)13	Class I	69193	69218	TGCTGCAACCAAGTGCCTCA	ATCCTCAGCAGGAGCAGACGTT	224
FMgSSR-14242	scaffold3339	p2	(AT)13	Class I	40058	40083	TGCTTACAGCATGGATCCCCA	ATGAAAGGGAGTGGAGCTGCGGTA	330
FMgSSR-14243	scaffold286	p2	(AT)13	Class I	117082	117107	TGGACATGCGACAGGTCGAGAA	TGAAACCCTGGTACGGGTTCGT	309
FMgSSR-14244	scaffold788	p2	(AT)13	Class I	81184	81209	TGGAGGGTGCATGTGTCAGCAA	TGGGCTGGAACAAACGACCA	320
FMgSSR-14246	scaffold17184	p2	(AT)13	Class I	6668	6693	TGGTAGGTGTTCAACTGAGGGTGT	TGGACAACAGATGGGTAGTTCCAC	323
FMgSSR-14249	scaffold14665	p2	(AT)13	Class I	3279	3304	TGTGTGTGTTGCCTCAGAGTGGA	AAGTTCTTCAGGCCGTGTGCCA	338
FMgSSR-14251	scaffold1715	p2	(AT)13	Class I	54319	54344	TTCAGGTTCATCCGTCTGCGGT	TGCACTGCTGGACGATGGAGAA	334
FMgSSR-14257	scaffold1795	p2	(AT)14	Class I	14563	14590	AAGGCCAAGGTGGACGTCAAGA	AATTCTGCACGGGGGAAACCGT	340
FMgSSR-14258	scaffold623	p2	(AT)14	Class I	68528	68555	AATCCAGCTTGTCGCGTTCCGA	ACCACCAGCACCCACAAAACTCA	341
FMgSSR-14260	scaffold13701	p2	(AT)14	Class I	4847	4874	ACAAAGGCAAGGCGTCGAGA	TGGGAGCAGCATGGAGGAAGAT	339

SSR_ID	Scaffold	SSR_Type	SSR_Motif	SSR Class	SSR_Start	SSR_End	Forward sequence	Reverse sequence	Product_size
FMgSSR-14261	scaffold7238	p2	(AT)14	Class I	987	1014	ACACGCACAAATGCGAGAAGCC	TGGATTCGCGTGCTTTTGGGGA	330
FMgSSR-14262	scaffold1117	p2	(AT)14	Class I	12314	12341	ACACGGAAAATGATCGACGGAC	CCTGTTCATGGCTCGTCTTGGA	350
FMgSSR-14264	scaffold36340	p2	(AT)14	Class I	1737	1764	ACCCGGATGCAATGCAGCAA	ATCAAGCCAGCCCACCACACAA	253
FMgSSR-14268	scaffold114	p2	(AT)14	Class I	193970	193997	ACGGACCATGCCAACACCAA	TGGTGGCACATGGTCAAAGGCA	335
FMgSSR-14270	scaffold834	p2	(AT)14	Class I	77491	77518	ACTCGTTCGTCTTCGTGGTGGA	TTGCAGCTGTTGGACTGCCTGT	225
FMgSSR-14271	scaffold2151	p2	(AT)14	Class I	63239	63266	ACTGCGCTGCCCTATAGGTTGA	ACACCTAGGACTGTTGTTGCCT	333
FMgSSR-14275	scaffold1898	p2	(AT)14	Class I	61665	61692	AGCAAAATGAGTGGCGGAGTGC	TGGCTTGCTGATGCTGAGGAGA	307
FMgSSR-14276	scaffold15568	p2	(AT)14	Class I	10860	10887	AGCAGTGTAGGTTCGGTGTGCAA	AGCCGGCTCATGGGCTACAAAT	323
FMgSSR-14277	scaffold7539	p2	(AT)14	Class I	19492	19519	AGCATGTGATGCTGCTGGCAA	TGCATCTGCCCGCGTTACCATA	350
FMgSSR-14278	scaffold1201	p2	(AT)14	Class I	18757	18784	AGCCATCGCAACGAGCTAAGCA	TTGATGGCATGGAGTTTCGGGC	279
FMgSSR-14280	scaffold6307	p2	(AT)14	Class I	30287	30314	AGGCACCGTCGCCTGCATAAAT	TCGCGACGGTTCACCATCCTTT	248
FMgSSR-14281	scaffold3050	p2	(AT)14	Class I	19317	19344	AGTCAACAGCTACACGTGGCCT	ACAGAGAGCTTGCCCGGATCTT	310
FMgSSR-14282	scaffold1677	p2	(AT)14	Class I	34885	34912	AGTGGGCAGTGGCAACGATT	AAGCGCCCCTCCATCATCCAAA	311
FMgSSR-14284	scaffold2277	p2	(AT)14	Class I	39813	39840	ATCCGCTGCTTGTGCGATTGGA	GCTTGCTCGTACTCCCGTGAAA	350
FMgSSR-14286	scaffold2952	p2	(AT)14	Class I	45358	45385	ATGCAATCGCTGGCTGTCCT	TTGGACGGGTACTTCTTCACGC	348
FMgSSR-14288	scaffold9299	p2	(AT)14	Class I	16545	16572	ATGGGTTCGTAGGGCAAGATGTGC	AAAAGCCGGGTTATCCTCGTTC	327
FMgSSR-14290	scaffold892	p2	(AT)14	Class I	30342	30369	CCATCTTCGCCTTGCCTAGTTCGT	TTGTTCTTGCCTCCCGGGTTCA	344
FMgSSR-14291	scaffold2560	p2	(AT)14	Class I	27830	27857	CCTCATGATGGGTCATCAGCTAAC	AGTCAACGTTGTGGAGGAGGA	329
FMgSSR-14298	scaffold6362	p2	(AT)14	Class I	12007	12034	TAGCGCACACGTCCTTTGCT	TTCGCTTATTCACCCCCTTCGTCC	344
FMgSSR-14301	scaffold10062	p2	(AT)14	Class I	1011	1038	TCCACCACACGTTGATGTGGCT	ACGCGAATATGCGTCCACTTGC	216
FMgSSR-14307	scaffold23331	p2	(AT)14	Class I	246	273	TCCCCAAAGTGCAGGTCGTGTT	TGTTGCGTGTTGGCCGGTAT	217
FMgSSR-14308	scaffold548	p2	(AT)14	Class I	34431	34458	TGAGAGATGTGTGGAGAAGGGCA	AGCCTGATCGATCGTACGTCCT	339
FMgSSR-14309	scaffold12316	p2	(AT)14	Class I	7479	7506	TGAGCTAGTCGGGCTACACCAA	AGCTGGCGAATCTGGAATGGGA	321
FMgSSR-14316	scaffold3132	p2	(AT)14	Class I	14776	14803	TGCCCATGCACGTGAACCTT	AAATTGGCCGGCGCGCTAAA	336
FMgSSR-14318	scaffold153	p2	(AT)14	Class I	68233	68260	TGCGACTCCAGCAGCTCAGATT	TTCAGACGAGCGATGCTTGC	320
FMgSSR-14322	scaffold2653	p2	(AT)14	Class I	36117	36144	TGGGAGCAGCATGGAGGAAGAT	TGTGGGAGGGAAGCAGTGTTGT	303
FMgSSR-14323	scaffold12507	p2	(AT)14	Class I	17397	17424	TGGGCGTATGGTGCTAACCGAA	GCATAGTGGACATGCGACGACT	306
FMgSSR-14327	scaffold3679	p2	(AT)14	Class I	1832	1859	TGTTCCTGTGGGGAGTTGTGTCA	GGCTCAACTCACCAGGTGCTTCAT	348

SSR_ID	Scaffold	SSR_Type	SSR_Motif	SSR Class	SSR_Start	SSR_End	Forward sequence	Reverse sequence	Product_size
FMgSSR-14328	scaffold6612	p2	(AT)14	Class I	15283	15310	TGTTTTGGTGGTGCGGCGAA	ACAGCGCGTCCCTTGCATTT	295
FMgSSR-14331	scaffold10548	p2	(AT)14	Class I	14934	14961	TTGCAGGTGCCACATCTTGGGT	TCTGTTGCGTGACGTGCTACT	350
FMgSSR-14332	scaffold1157	p2	(AT)14	Class I	67831	67858	TTGCGTAGCATGTTGTCCCCGT	TCAAGCACATCGAGTCCGGCAA	296
FMgSSR-14333	scaffold102807	p2	(AT)14	Class I	1361	1388	TTGTTGAGACGGCCACCACAGT	ACGTCTGCCTGCCCTAGTTTGA	300
FMgSSR-14334	scaffold162	p2	(AT)15	Class I	72261	72290	AATCGAAAGCTGCCCCCATCCA	ACGCGATCCAACAGGAGAGA	332
FMgSSR-14335	scaffold1735	p2	(AT)15	Class I	76483	76512	AATGGCGTCGTTGTTCTGGGGA	ATGCGCCGAATGTGCCAACT	271
FMgSSR-14337	scaffold475	p2	(AT)15	Class I	63737	63766	ACAGGATGTGCTCACAGACGCA	AGCCCATTTGATGGTGCCGA	334
FMgSSR-14338	scaffold49979	p2	(AT)15	Class I	3783	3812	ACCATGCACGGTCACCCTTTGA	TGCACAGCTTCCAACCGAACCT	342
FMgSSR-14339	scaffold3231	p2	(AT)15	Class I	53514	53543	ACCCGTTCTTGAAGGGAAGCCT	TCCGGATCGACGACCTTGACCTTA	275
FMgSSR-14340	scaffold2307	p2	(AT)15	Class I	57721	57750	ACCTGCAGCTGTCCTCTGTGTA	AACGGGGCCGAACCCGATATAA	337
FMgSSR-14341	scaffold2010	p2	(AT)15	Class I	20238	20267	ACCTGCTGCAATAGCCACGA	AGAGCTGTGTGGCACGCTAA	264
FMgSSR-14342	scaffold7280	p2	(AT)15	Class I	12598	12627	ACGCCATTGCCATCTCATGCT	TGCCAGCGCATCAACTCTGA	282
FMgSSR-14343	scaffold37194	p2	(AT)15	Class I	3026	3055	ACGCCTTGGACGAAGGTATGCT	GCACTGCACCATGCCGGAAAAA	293
FMgSSR-14344	scaffold19128	p2	(AT)15	Class I	12860	12889	ACGGAAGCGTGGTAATGGGT	TCCTCCACCAGATGACCTTCAACC	328
FMgSSR-14346	scaffold1496	p2	(AT)15	Class I	84825	84854	AGAAGCTGGCCTGTTGCTGA	ACGACCGGCGCGCTGAATAATA	219
FMgSSR-14347	scaffold25600	p2	(AT)15	Class I	3544	3573	AGCAGCAGCGTGGGATCAGTTT	AAGCATTGCAGTGTGCGGGT	275
FMgSSR-14348	scaffold14492	p2	(AT)15	Class I	13983	14012	AGTGCCATGAAGATCGCACCCA	ACCATTTTGCCACAACTGCGCT	347
FMgSSR-14352	scaffold13097	p2	(AT)15	Class I	15387	15416	ATTCAGTCTCGCCCACTGCCAA	AAGCGATTTTTGGTGGACCCGC	301
FMgSSR-14354	scaffold14952	p2	(AT)15	Class I	5773	5802	CCAAACTGGATAGAGGTCCCAAAGC	TGGCCCGATCAGCTACATGCAA	347
FMgSSR-14355	scaffold20706	p2	(AT)15	Class I	12304	12333	CCATGATGGCTCAGCTTGATGTGT	TGAGCCTGTCATCTCGTGTGT	339
FMgSSR-14356	scaffold5134	p2	(AT)15	Class I	1240	1269	CCTGTGACCAATATAGGTGTCGTGC	AGCAAGAATCAATCTAGGCCCTGC	327
FMgSSR-14359	scaffold9747	p2	(AT)15	Class I	14618	14647	GGCAAGGCCACATGAGTTCTCT	AAGCTGGCGCCGGACAAAAA	217
FMgSSR-14363	scaffold2008	p2	(AT)15	Class I	54271	54300	TAATCCTTTCGGCGCCGTCCAT	TCAGAACATGACCATGCCCACA	346
FMgSSR-14364	scaffold129	p2	(AT)15	Class I	37671	37700	TAGTCGTATGCCCTCTTGGCCACT	AAGCAGACGGTGATGTTCGACG	344
FMgSSR-14365	scaffold1720	p2	(AT)15	Class I	57880	57909	TATGCACCAGACTTGCCCCA	GTAGCAAGATGATTCCATAGGCCG	335
FMgSSR-14366	scaffold1034	p2	(AT)15	Class I	92679	92708	TCACGATGGTCTGCTGTACCGA	TGCGTTCATCGGTGGTGCTT	348
FMgSSR-14367	scaffold682	p2	(AT)15	Class I	92643	92672	TCACGCACAGCAGCCAACAA	TGCGGGAACGACATGAGCGAAA	289
FMgSSR-14371	scaffold107368	p2	(AT)15	Class I	190	219	TCGTAGTGGAGAGGTAGTGCAACA	ACTCGTCACGGCAAACAGCA	334

SSR_ID	Scaffold	SSR_Type	SSR_Motif	SSR Class	SSR_Start	SSR_End	Forward sequence	Reverse sequence	Product_size
FMgSSR-14380	scaffold9021	p2	(AT)15	Class I	20872	20901	TGTGTCCTTTCCAGCAAAACGC	TCCAATGGAAGCTCCCCGGAAT	348
FMgSSR-14381	scaffold4887	p2	(AT)15	Class I	3776	3805	TTCCAGCGGTCAACCCAAGA	TGCTGCAAGAATAGCACACCCCT	328
FMgSSR-14386	scaffold534	p2	(AT)16	Class I	57802	57833	ACACGAACAAAGCGTGGCGT	ACCGTCCTCAGGTACACACACA	333
FMgSSR-14387	scaffold9045	p2	(AT)16	Class I	18058	18089	ACAGACACACTGGTACCTTGGAGTG	TGTCGCTATAGCCCTTTCACCT	350
FMgSSR-14390	scaffold10638	p2	(AT)16	Class I	25786	25817	ACGCGCCAGCTGCTTCATTT	AGCCCTCTGATGCGTAACCA	230
FMgSSR-14391	scaffold2992	p2	(AT)16	Class I	39145	39176	ACGCTAATGTGTGGTGGACGCA	ACGTATCATGCGCCGTCGTT	296
FMgSSR-14392	scaffold3969	p2	(AT)16	Class I	14550	14581	ACGCTCGGCCCGTTTTTGGATA	ACCCTAATGTGCCCCAGGCTTT	298
FMgSSR-14393	scaffold8588	p2	(AT)16	Class I	4621	4652	ACGCTTGCCGAGAGGTCAAA	GCAAGTTTGGTCCATCGCGTGT	310
FMgSSR-14397	scaffold1720	p2	(AT)16	Class I	57715	57746	AGCGCATTATGCACCAGACTTGC	AGCAAGATGATTCCATAGGCCGGA	340
FMgSSR-14398	scaffold646	p2	(AT)16	Class I	66910	66941	AGCGCCTCCGGTGTAGCAAATA	TGGCTGTGGAAAGACGCAGACT	276
FMgSSR-14399	scaffold1128	p2	(AT)16	Class I	56290	56321	AGCGGTGTCAGCCTTTCAACCT	ACCGACCGGCACGCTGAATAAT	237
FMgSSR-14403	scaffold1110	p2	(AT)16	Class I	27060	27091	ATAACGCGCTCGACGGATGA	CGGCACAAAGTGGCTTTCCT	349
FMgSSR-14404	scaffold11752	p2	(AT)16	Class I	859	890	ATCAGGGAAACCAGCAGCTCCA	TTGGTGTTGTCGGCCAGGACAT	336
FMgSSR-14407	scaffold5620	p2	(AT)16	Class I	30716	30747	ATTACAGGCAACTGGGCCGGAT	AGCTGGTTTGGTTTGGCTGG	345
FMgSSR-14409	scaffold14165	p2	(AT)16	Class I	10851	10882	CCCAGCAACACCCTGATGATGA	ACCAAGGCGAATGTGGTCAGT	336
FMgSSR-14410	scaffold8450	p2	(AT)16	Class I	27610	27641	CCCTACCCTTCGAAAGATCCAAGT	ACAAACAGCAGTACCTCGTTGGGG	343
FMgSSR-14413	scaffold80	p2	(AT)16	Class I	57398	57429	TCATCACCGAGTCCGCCAAT	AAGCCCACGCATTCCAGGTTCA	315
FMgSSR-14415	scaffold1626	p2	(AT)16	Class I	65371	65402	TCGCGCACATGAACAGCCAGTA	ACCCGGCTGCCAAATTCAACA	203
FMgSSR-14421	scaffold2990	p2	(AT)16	Class I	35102	35133	TGCATCTTTGCGGCAACCCT	AGGCAAGGGGAAGGTTTTGATCCC	294
FMgSSR-14425	scaffold10804	p2	(AT)16	Class I	21159	21190	TGGAATGCAGGCTGGTTCGGTT	TCAACACCGGTAGGCGGTAACT	335
FMgSSR-14428	scaffold319	p2	(AT)16	Class I	10395	10426	TTAACACGTCCGCTGCTGCT	TCATCACGTGGCCCATCTGCTT	272
FMgSSR-14432	scaffold3202	p2	(AT)17	Class I	17323	17356	AGCAAATCCGATCGCACGCA	GGCACGTGGCCACAGAACAAAA	349
FMgSSR-14433	scaffold237	p2	(AT)17	Class I	118748	118781	AGCAGAGCCAGACCCAGAAGTT	CCACTGGAAGTACCACCAAAGGA	333
FMgSSR-14434	scaffold1667	p2	(AT)17	Class I	46680	46713	AGGAGTGCGTTGCATAGCCCAA	CGGTTGCATGCCCTTTGCCAAT	241
FMgSSR-14435	scaffold6761	p2	(AT)17	Class I	7173	7206	AGGCATCTTGGAACAGCTGCGA	AAGAATGTGGTTCGGCCGGT	298
FMgSSR-14442	scaffold638	p2	(AT)17	Class I	33469	33502	CCAAGCTGTGATTTGCTGGCGT	TTCAAGTGGCGGTTGTGGCT	294
FMgSSR-14443	scaffold18	p2	(AT)17	Class I	220786	220819	GACTTGCAGATTCTACTCCCACCA	TTTCCCAGCGGTATCCTGTTGC	245
FMgSSR-14448	scaffold4473	p2	(AT)17	Class I	6693	6726	TCAACTGCCTGCATCTCCCACA	ATGCACTTACTTCAGCCCGCCA	343

SSR_ID	Scaffold	SSR_Type	SSR_Motif	SSR Class	SSR_Start	SSR_End	Forward sequence	Reverse sequence	Product_size
FMgSSR-14453	scaffold18409	p2	(AT)17	Class I	517	550	TGCATAGATGTTTGGCCCAGCAAG	AGGGCCTGCAACTTTTCGCT	264
FMgSSR-14455	scaffold2604	p2	(AT)17	Class I	52396	52429	TGCCAGTGCTGTACACTGCATA	AGGCACAGGTTCCACGTTGCTT	338
FMgSSR-14456	scaffold1282	p2	(AT)17	Class I	33879	33912	TGGGAGCCACATCATGCTCAGT	AGAGCCAGCAAGGTCTTCCTCA	270
FMgSSR-14457	scaffold3991	p2	(AT)17	Class I	29553	29586	TGGGCCCACTAATTCCAAACGTGG	ACGCGCTTTATCACTGGCTCT	331
FMgSSR-14459	scaffold1260	p2	(AT)17	Class I	87594	87627	TGGTGCCTTCCCTCCCTGTATT	ACGACCGATGCAAGGAGATCTTGG	347
FMgSSR-14460	scaffold9027	p2	(AT)17	Class I	1427	1460	TGTCTGGTGAAGATGCACGGA	TGCCTCGTTGGCCTCCAAACAA	223
FMgSSR-14464	scaffold5255	p2	(AT)17	Class I	37382	37415	TGTTCTTCCGGGCATTCCATCT	TTTTGGCTCCACCTTGCGTT	330
FMgSSR-14467	scaffold11320	p2	(AT)17	Class I	9673	9706	TTTCGGTTGGACTTGGACCCCT	TTCGGGTAATGATGGGAGGCA	321
FMgSSR-14468	scaffold7104	p2	(AT)17	Class I	4218	4251	TTTTGCGGACGTGTCTGGCT	TTAGGGCCCGCACACAAGTGAA	348
FMgSSR-14469	scaffold400	p2	(AT)18	Class I	47022	47057	AAAACATGTTGAGGGCGGGAGG	CTGATGCTGGATAACAACGAGAGC	340
FMgSSR-14471	scaffold7802	p2	(AT)18	Class I	26755	26790	AAGGGCGTGTTGGCTGGTTT	AGCTTTCGGTCACGTTGGGACA	319
FMgSSR-14473	scaffold1178	p2	(AT)18	Class I	50166	50201	ACAACACTGCACCAGGAGGT	AAAAGAGGTGACGTGGCTGC	349
FMgSSR-14474	scaffold2303	p2	(AT)18	Class I	10889	10924	ACCCGAGCTGGAATGCTGGTAT	TGCGCAAGAGGAGTGCTGGAAT	313
FMgSSR-14476	scaffold1121	p2	(AT)18	Class I	59673	59708	ACTCGGTAGAAAAGACCCTGCCA	AGCCTATCGGTTGGACATCGAGA	335
FMgSSR-14477	scaffold8359	p2	(AT)18	Class I	26971	27006	ACTCTGCATGCTCAACCCTACG	ACAAGAACGAGTTGACCCCCT	320
FMgSSR-14479	scaffold626	p2	(AT)18	Class I	24063	24098	AGGGCCAAGAGCGAATGGAA	TGCGGGCTGTGAAGAAGAAGCA	325
FMgSSR-14480	scaffold6259	p2	(AT)18	Class I	8969	9004	AGTTTTGCAGGGGTTTGCGCT	AGCTGAGCTGCCGCATGTATGT	331
FMgSSR-14481	scaffold445	p2	(AT)18	Class I	78327	78362	ATCCAACACACAGCCTCGTGCT	TTGCCTGCTTCGAGCCGTTT	257
FMgSSR-14482	scaffold6936	p2	(AT)18	Class I	27969	28004	ATTATGGCCGCGGGGGAGTTTT	TGGCGGCCTTCATTCCTTTGT	317
FMgSSR-14486	scaffold17622	p2	(AT)18	Class I	3382	3417	GTGCAGCAGAGCAGGAGAAAT	CGAGTTCAGATTGAGTGGCTTGTC	350
FMgSSR-14488	scaffold92	p2	(AT)18	Class I	168850	168885	TCGGCCAACAGTTGCCAAAACT	TGCGTTCGGTCGACTCATTG	254
FMgSSR-14489	scaffold558	p2	(AT)18	Class I	64757	64792	TCTCCCCGGCTAGTTGGTTGAT	TCGTTCACGACAAAGTAGGGCA	340
FMgSSR-14490	scaffold2855	p2	(AT)18	Class I	49342	49377	TGACCACCAACAGCAGTGACCA	TTCCTGGCACGAGTACGTGA	338
FMgSSR-14492	scaffold10068	p2	(AT)18	Class I	11617	11652	TGTCACAACGAGTCTTAATGCGGC	TCACGCATCGACTGAACGTC	279
FMgSSR-14494	scaffold2834	p2	(AT)19	Class I	34047	34084	AACGACACGATGGCCGACAA	AGGGGAAACACACACACACACACA	257
FMgSSR-14497	scaffold4638	p2	(AT)19	Class I	2915	2952	ACTCACGTTAACAGCGGACG	TGCTCGCCATACCAAAGTCGCA	342
FMgSSR-14498	scaffold883	p2	(AT)19	Class I	42731	42768	ACTGCAACCCTCCAAGCACA	CGCCGGTCTTTTTCTAGGGCTT	333
FMgSSR-14499	scaffold10248	p2	(AT)19	Class I	10038	10075	AGCAAAAGGTCCACCTCCCTGT	AGGCTGTTGCTGCTGCAAGGAT	311

SSR_ID	Scaffold	SSR_Type	SSR_Motif	SSR Class	SSR_Start	SSR_End	Forward sequence	Reverse sequence	Product_size
FMgSSR-14501	scaffold1522	p2	(AT)19	Class I	11059	11096	AGCTCCATCGATCACCCAACGA	AGACGTCGCGTAGGCATGAGAT	278
FMgSSR-14503	scaffold4936	p2	(AT)19	Class I	35496	35533	AGGCATCGTTGAGCCTGTCTCT	ACCACGAGAGCCCACCATGTTT	346
FMgSSR-14505	scaffold10406	p2	(AT)19	Class I	11331	11368	AGGGCTTAGCCGCTTAAGGA	TCGCCCCACAGCCACTGTAAAT	245
FMgSSR-14506	scaffold1909	p2	(AT)19	Class I	40686	40723	AGGTGGCCATAATAAGCCGCCA	TAGCTTGTTGCCGCCCCTAT	245
FMgSSR-14507	scaffold5465	p2	(AT)19	Class I	20546	20583	CCGTTGACGGCTTAGGTTGTAA	CGATGGCTATGGGACCGTAAATTC	350
FMgSSR-14508	scaffold3087	p2	(AT)19	Class I	4529	4566	CGCATTCGTTGCTGGCTAGA	TGTGCTGAATGGGTGTGGGTGT	320
FMgSSR-14512	scaffold33788	p2	(AT)19	Class I	1767	1804	GCCTGAAAGACAAGGATGACGCGA	TCACAACGCGATCCCAACTCCA	344
FMgSSR-14513	scaffold4712	p2	(AT)19	Class I	30360	30397	GCTGTGAGAAATGAGAAGCGCCA	ACGAGAGGATGGGGCAGAAAA	350
FMgSSR-14515	scaffold19297	p2	(AT)19	Class I	9122	9159	GTCGTCTTGTCATCCTCCAGATTG	TTGTGTGTGTTCCCCCTTGCCA	315
FMgSSR-14517	scaffold15064	p2	(AT)19	Class I	4989	5026	TCAAGCCGTGCGGTGGAAAA	TGATCGGGACGGTTGGTACA	318
FMgSSR-14518	scaffold759	p2	(AT)19	Class I	69219	69256	TCAGGAACTTTGTTGCTGCAGGGT	ACGCTTCCTTCTGAAGTACTCCCAC	339
FMgSSR-14522	scaffold579	p2	(AT)19	Class I	30065	30102	TGCTTGGGGCGAGCAAGTACAA	AAATCGGCCGCGAGAACACA	327
FMgSSR-14525	scaffold1211	p2	(AT)19	Class I	61242	61279	TGTTTCGCACTTTGCAGGCG	CGAGACAATTGCATGATCACTCGG	319
FMgSSR-14527	scaffold589	p2	(AT)19	Class I	55619	55656	TTGTGCTTACCCGCCGTTGT	ACGCGCGTTTGTTTCTCCCA	344
FMgSSR-14532	scaffold265	p2	(AT)20	Class I	91874	91913	ACGCCGTGAAGAAGGCCATTGA	TGCTTGTATCATTCCAGGCGGAGC	350
FMgSSR-14535	scaffold29065	p2	(AT)20	Class I	1716	1755	ACTCCAGTGGCAGAGGATGGAA	ACCTTCTGATCTGGTGGATTGCAG	335
FMgSSR-14536	scaffold3130	p2	(AT)20	Class I	14915	14954	ACTCGCGTGTTTTGTTCGACCC	TGGCACGGCAAGGTTCAGAGTT	318
FMgSSR-14537	scaffold2607	p2	(AT)20	Class I	34128	34167	AGCAAGCACGTCACCATCCA	ATGAGAGCTGCGGCACAGTTGA	217
FMgSSR-14541	scaffold152	p2	(AT)20	Class I	43072	43111	CAGTGGCGTCGAATCTTGTTGA	CTCCGCGGTAATTTGTTGGATCAC	350
FMgSSR-14546	scaffold10936	p2	(AT)20	Class I	25750	25789	CCCTGCAAGTGAACAACACAATGC	TCTGGCCACCTAAGAGCATCCA	343
FMgSSR-14547	scaffold4917	p2	(AT)20	Class I	20332	20371	CGCGCGCGACGAAATTAGGAAA	ATGGCGCCGGGATGATGATTGT	257
FMgSSR-14551	scaffold9587	p2	(AT)20	Class I	22759	22798	GCGTGATGGTGCTGATTTGGCA	TAGCATGGGCCACTGGCTGAAT	238
FMgSSR-14552	scaffold913	p2	(AT)20	Class I	84827	84866	GCTCTATCAGTGGCGTCCAACCAA	TTCTTGGACTCTGCCCTGGTCT	290
FMgSSR-14556	scaffold661	p2	(AT)20	Class I	39968	40007	TCCCGATTCCACATAGAGCCACA	AGCCAGTCATGCCATGTTCCA	330
FMgSSR-14557	scaffold8493	p2	(AT)20	Class I	1864	1903	TCGGCGTGAGTCACACACAT	AAGTGGACGTCTTGTTCCCGCA	335
FMgSSR-14560	scaffold17358	p2	(AT)20	Class I	10200	10239	TGGCGTAGTCTGGCCGTTTT	GGACACCGTTAACTGGCGTTGA	240
FMgSSR-14562	scaffold314	p2	(AT)20	Class I	109983	110022	TGGTGCTCCTCCAATCCAATCTGT	TCCACATACCTGGCGTTCTCA	335
FMgSSR-14564	scaffold8788	p2	(AT)20	Class I	18527	18566	TGTGCCGTCCTTTGCTGAGAGA	AAAGCAGCAGCGGCAACGAT	337

SSR_ID	Scaffold	SSR_Type	SSR_Motif	SSR Class	SSR_Start	SSR_End	Forward sequence	Reverse sequence	Product_size
FMgSSR-14565	scaffold383	p2	(AT)20	Class I	18176	18215	TGTGGGGTACAGAGGCTGGATCAT	ACACAGCAAAGACTCGGCAAGA	342
FMgSSR-14566	scaffold13353	p2	(AT)20	Class I	24368	24407	TTCTTGGCCCGGCACTTACTGA	TCCGTTGTTCATTCGGCCTCCT	332
FMgSSR-14568	scaffold119165	p2	(AT)20	Class I	533	572	TTGTGCCTCTCATGCCTCGTCT	TTGAGACTCGTGCCGTCTCTGT	310
FMgSSR-14569	scaffold8780	p2	(AT)20	Class I	13569	13608	TTTGCTGGCCTTGACACGGT	TGGGCATAGGGCTCGCTTTT	275
FMgSSR-14570	scaffold23868	p2	(AT)21	Class I	6642	6683	AAACTGTGGCCGCAAGCAGA	TTTGGACCCCTTGAGTGAACGC	342
FMgSSR-14571	scaffold32260	p2	(AT)21	Class I	2522	2563	AACAGCTGCCATACAGGCACCA	TGCTGGCACTCTGAAATTGGGC	260
FMgSSR-14573	scaffold1869	p2	(AT)21	Class I	36766	36807	ACACGCTCAGGCACGATTCA	TGTCTCTCCGCCAAACATGC	225
FMgSSR-14574	scaffold2887	p2	(AT)21	Class I	7412	7453	ACATGGAACCAGGCGCTGAACT	ACATGAGTAGCTGAGGATGGACCC	340
FMgSSR-14576	scaffold12413	p2	(AT)21	Class I	19105	19146	ACGCTGGGTAACGTCGTAACAA	CGTGCCGAATTATGCTCATGCCA	344
FMgSSR-14578	scaffold4244	p2	(AT)21	Class I	22110	22151	AGCCGACAGTATTGGACCACGA	CCTGTTTGAGTTGGACTTCTGGCG	329
FMgSSR-14579	scaffold3189	p2	(AT)21	Class I	12417	12458	AGCTTCCACGCCAGATACGACT	TAATTCCGCCGTGTGCGACAGA	331
FMgSSR-14582	scaffold1024	p2	(AT)21	Class I	19204	19245	AGTCGGCAAATCGGCGAACA	AGCATTCGGGTGCCATTCGGTA	344
FMgSSR-14583	scaffold1594	p2	(AT)21	Class I	11147	11188	CCTGTTTTGATACCACACGACACC	GGTGCACTTGCTATCAGTTTGCTGC	323
FMgSSR-14585	scaffold4840	p2	(AT)21	Class I	33661	33702	CTCAAGAGGCACCGGATTATACCA	GACAGGAGAACACCTCAAGGTTCA	347
FMgSSR-14589	scaffold1015	p2	(AT)21	Class I	12337	12378	TAAAGCACCAGCCACCAGCAGA	CGGTTAATTTCTTCTCTGTCCGCCC	345
FMgSSR-14591	scaffold1230	p2	(AT)21	Class I	82159	82200	TGAGCTGCGGCATCATGGGAAT	TCCCTCTCCCTGCAACTGCAAA	348
FMgSSR-14592	scaffold1855	p2	(AT)21	Class I	41894	41935	TGCACATAGCATAGCGCGGA	TTGTACGTGCGTGAGCGTGT	343
FMgSSR-14593	scaffold475	p2	(AT)21	Class I	60374	60415	TGCTCTGCCGTACGGAGCAATAA	TGCTACGCGCTCCCCATGAAAT	294
FMgSSR-14595	scaffold9011	p2	(AT)21	Class I	18745	18786	TTACCATTTTGCTGCGGCACCC	TTCAGCACACTGCGCTCCAT	292
FMgSSR-14596	scaffold1412	p2	(AT)21	Class I	32796	32837	TTGCAGTAGAGCCACGCTGCTT	CGAGGCGCTCATCGATTTTGGT	277
FMgSSR-14597	scaffold21799	p2	(AT)22	Class I	8790	8833	ACGAGCGCGCAATTTCAGACT	TTCGTCTCCCTTTCGGCCTT	277
FMgSSR-14601	scaffold22853	p2	(AT)22	Class I	6336	6379	CACCATCCAAATTCACTCTCCCTC	TGGATGTCAGCCAAGGTCTCTCA	306
FMgSSR-14603	scaffold9016	p2	(AT)22	Class I	1977	2020	GCTTCTCTCTCGTTTTGTCTTCCC	GCCACCAAGGAGTTACAACGAA	348
FMgSSR-14604	scaffold672	p2	(AT)22	Class I	87403	87446	GTCGACGCGGTTAAGCAACTGA	CGCGTGCTCTGGATCCTTCTTT	348
FMgSSR-14605	scaffold2811	p2	(AT)22	Class I	44885	44928	TCGTGTATGCGTGTGGGAACT	TGGTGGGACAGCAGTAGAACAGA	314
FMgSSR-14606	scaffold12015	p2	(AT)22	Class I	11731	11774	TGACAGGTCCATGCTCCACA	TCCTGGTGGTTCAAACGAGAGCA	278
FMgSSR-14607	scaffold630	p2	(AT)22	Class I	22359	22402	TGCACACGTTGGCGAAGCTA	TCAGTCGCGCTTTCCGCTGTAT	342
FMgSSR-14609	scaffold3991	p2	(AT)22	Class I	45045	45088	TGGAATCGAGGAGTGTGTGACT	ACGCTAGCAGCGTCCATCACTA	340

SSR_ID	Scaffold	SSR_Type	SSR_Motif	SSR Class	SSR_Start	SSR_End	Forward sequence	Reverse sequence	Product_size
FMgSSR-14611	scaffold6743	p2	(AT)22	Class I	14802	14845	TTCGGCGTCATCAACATCGTCG	GCCTTTTTCTTTCTCTCCGCTCCA	312
FMgSSR-14615	scaffold8092	p2	(AT)23	Class I	21571	21616	ACGCCCTGAAACACCATGAGACA	CAACGGCGCGCTGATTTTCT	350
FMgSSR-14616	scaffold15169	p2	(AT)23	Class I	1381	1426	ACTTCGCTTACCTGCACTCTGA	CAATATCCGGCATGATGCTGCT	323
FMgSSR-14618	scaffold3808	p2	(AT)23	Class I	8833	8878	AGCCCGCGCACTCTAGATTTAC	TGTAGGGGTTGGACTCCACGTT	271
FMgSSR-14619	scaffold12308	p2	(AT)23	Class I	13754	13799	AGCCCTATGAATGTATGAGCGGCA	GACATGGTGCAAAGTGCAAACC	346
FMgSSR-14625	scaffold7691	p2	(AT)23	Class I	6011	6056	TTCTGGTGCAACGTACGGGT	TCACAGCGTGTGGTGTAGGAGT	315
FMgSSR-14626	scaffold11571	p2	(AT)23	Class I	17249	17294	TTTGGGGCTGGTTGTTGGCA	TATGGGAGTGCCCTAGGCCCTTTT	348
FMgSSR-14628	scaffold45	p2	(AT)24	Class I	86619	86666	AAACGCCCCAGCTATGCCAACT	TTGTCGACATCAAGGAGCCCGA	342
FMgSSR-14631	scaffold532	p2	(AT)24	Class I	104011	104058	AGCTTGGCATCTGTTTGCATGGC	GCTGTGATAGTCAGCTCCTCAGTGT	239
FMgSSR-14632	scaffold55	p2	(AT)24	Class I	186613	186660	CCAGCACCTATACGTACATTCCCCA	TTCGCGTCCGCATTCATGGACT	349
FMgSSR-14633	scaffold37474	p2	(AT)24	Class I	1069	1116	CTACTCTCAAACTACTTGCGCCTC	TTCAGGCTTCCCCTCACCAA	273
FMgSSR-14634	scaffold3805	p2	(AT)24	Class I	37677	37724	GCTGCATGTTGCCTGAAAACCA	TGCCCGTAGCAACACACAGACA	350
FMgSSR-14635	scaffold15802	p2	(AT)24	Class I	7987	8034	TGCAGCATCCACGCCATTGT	TTAGCGCGGGCCATCTTACT	349
FMgSSR-14636	scaffold6469	p2	(AT)24	Class I	19356	19403	TGCTACCTCGGCCGTAAGCTAAA	GCGTGGCTCCACCAGTGGATAATA	346
FMgSSR-14639	scaffold17256	p2	(AT)25	Class I	4966	5015	ACAACCGTGCCGCCCTTTAT	TCGTTAGGAAGACTCCACCAGCA	297
FMgSSR-14641	scaffold5258	p2	(AT)25	Class I	33609	33658	ACGGCTGCCGAAGGCCATATTT	AGCTTGGCTACCACTTGCACA	341
FMgSSR-14643	scaffold2195	p2	(AT)25	Class I	32089	32138	CACGAGGCAAAAGTAGGTCCGT	GCTGTTTCCAATGGCCCAATCGT	348
FMgSSR-14647	scaffold1287	p2	(AT)25	Class I	30923	30972	GCTTAGCTCATACGCTATGTGACC	ACAGTCGATGGTTCGATGGTCC	329
FMgSSR-14649	scaffold15812	p2	(AT)25	Class I	6557	6606	TGGCATGCAGATGTATGAAACGCC	GCTTGCTCCGATCACATTTTGCAC	287
FMgSSR-14651	scaffold27538	p2	(AT)25	Class I	6211	6260	TGTGCGATCCAAGGCAAGAGGA	TGGATCCGGCCGTGCATTTT	349
FMgSSR-14652	scaffold1643	p2	(AT)25	Class I	10227	10276	TTGAGGGTTGACGTCCGAGGTT	ATGCACGCACGCCTTGAGAT	338
FMgSSR-14657	scaffold4233	p2	(AT)26	Class I	31622	31673	AAGGTAGCATGCTGTTTGCGGC	TTGTGGCGTATGGCACTGGA	284
FMgSSR-14664	scaffold6926	p2	(AT)26	Class I	4045	4096	CGTGATGCCGTGTGTTGAGGAA	TCAGCTGTGGGTGCACCTGATT	339
FMgSSR-14665	scaffold12706	p2	(AT)26	Class I	14308	14359	GCCGTCGTCTGATACCAAAAGT	TGCCACACTGAATTTTCACCCC	340
FMgSSR-14668	scaffold14673	p2	(AT)26	Class I	1480	1531	TCTCCTAGACGACTACACCTCTCT	TCCGTATGTGCCACCAAACACA	325
FMgSSR-14670	scaffold7211	p2	(AT)27	Class I	7745	7798	AAAACAACCCCACAGGGGCACA	ACATGCATGCGTGGAAGCCT	278
FMgSSR-14672	scaffold156	p2	(AT)27	Class I	99944	99997	ACAAGGCTTCTCCTTGACACCT	TCTATTGTGCGTCTGGTCAGCA	300
FMgSSR-14673	scaffold1737	p2	(AT)27	Class I	59257	59310	ACCACATTCCCGTTGTGGCA	ATAGCCCAATGTCAACGCCGCT	335

SSR_ID	Scaffold	SSR_Type	SSR_Motif	SSR Class	SSR_Start	SSR_End	Forward sequence	Reverse sequence	Product_size
FMgSSR-14677	scaffold5717	p2	(AT)27	Class I	12571	12624	GCGTCTACGAGCGCCAACAAAT	TGTGGCCTTCCTGCTTGCTT	253
FMgSSR-14680	scaffold8733	p2	(AT)27	Class I	17662	17715	TCAACGCACGTGACACAGCA	GCTCTCACGCGCTTTTCGTT	292
FMgSSR-14681	scaffold670	p2	(AT)27	Class I	76826	76879	TCCAAGCAAACGGCTAAGGCA	ACGTGATCATGGCAGCACGGTT	346
FMgSSR-14687	scaffold3294	p2	(AT)28	Class I	18448	18503	ACCTGCCGAAAGGTCAGTTGCT	TGCCTTGTTGGCTCTCAAGCAGT	324
FMgSSR-14688	scaffold3101	p2	(AT)28	Class I	8159	8214	ACGCCAACGGCAACGTTATG	TCGCCGCTGAGTAGTGTAGCTT	350
FMgSSR-14691	scaffold2460	p2	(AT)28	Class I	46443	46498	GTGCACATTTTACAACCGGCCT	TGCTCTACTCAACCCAGGTTCCA	251
FMgSSR-14693	scaffold1421	p2	(AT)28	Class I	81274	81329	TGCGTCACAAGGCAGTGACA	TGAGCTTCAAGGTTGACCAGCA	350
FMgSSR-14697	scaffold4634	p2	(AT)29	Class I	43495	43552	AAGGCCGATGACTGTGAACACG	CGCCTGTCATACTCCTGATTCTCGT	305
FMgSSR-14698	scaffold5620	p2	(AT)29	Class I	35624	35681	ACCAGCCAACCAGACACGTT	TTGATCCGTCGATCTGCGCT	333
FMgSSR-14699	scaffold310	p2	(AT)29	Class I	4481	4538	ACTGTCGGAGAAGAGGCTCGTTA	GGGTCAAACTGGCCTGTCATCA	339
FMgSSR-14703	scaffold837	p2	(AT)29	Class I	73919	73976	GCGTTGTCCGATTGATGCTACA	GCACCGGTTCCACGAAAAGT	333
FMgSSR-14705	scaffold3930	p2	(AT)29	Class I	8531	8588	TGCAAGCTGGCTGGTGTGTGAA	ATTGTGTGGTGCGGCTGGAA	336
FMgSSR-14707	scaffold6595	p2	(AT)30	Class I	10261	10320	ACCCTTCCCATCTGCACGGAAA	TCTGCTGTTGACGGCCTTGT	220
FMgSSR-14709	scaffold16539	p2	(AT)30	Class I	5068	5127	ATCCGTGGCAGAGCGATCACAA	TACACAACGGCACAACGCCA	302
FMgSSR-14714	scaffold3132	p2	(AT)31	Class I	29269	29330	ACCAAACAACCGGTGCGCTA	TGGTTGTCGGAAAGTCGTCGGA	325
FMgSSR-14716	scaffold17809	p2	(AT)31	Class I	9455	9516	TCAGATCATGGGCCACCGACAT	TGACCATCTCGTCGTCAGTCACA	335
FMgSSR-14717	scaffold1154	p2	(AT)31	Class I	18928	18989	TGCTCCATGCCCAAGCTGAACT	TTGCACGGGGTTACTGACCAGA	341
FMgSSR-14721	scaffold9303	p2	(AT)33	Class I	5869	5934	AGCTTATTCAGTACCCACCAGGG	ATGAGATCGAGACGGAGACAGGCA	338
FMgSSR-14722	scaffold7637	p2	(AT)33	Class I	24564	24629	GGTGAGCAGGAGCTTGAGGATGAA	GCCACATCTTCACCAAAACACGGG	337
FMgSSR-14724	scaffold5352	p2	(AT)33	Class I	9330	9395	TGGGCATCAAACTCGCCGTT	GGCGGTGATGGTGCTACTTGAGAA	346
FMgSSR-14725	scaffold201	p2	(AT)33	Class I	38809	38874	TTATGATTGCGGCCGGTGACCA	AGCGCGCAACGTGCCATTTA	284
FMgSSR-14726	scaffold11255	p2	(AT)34	Class I	18495	18562	ACACTGCTCGAATCTCACGCT	AAGTTGCATGTGTCGGGGCT	308
FMgSSR-14727	scaffold13487	p2	(AT)34	Class I	15924	15991	ACCGGTTTGTCTGCCGAACTCA	GCGGAGCTGCTAGCGTATACAA	349
FMgSSR-14728	scaffold4360	p2	(AT)34	Class I	11267	11334	ACCTGCATATTGTCACAGCGAGGA	ATAGGTTAGTTGTGGCGACGGGGA	343
FMgSSR-14729	scaffold11329	p2	(AT)34	Class I	13735	13802	GTGCGAATGGCGTGCATGAT	GGTCCATCCTTGACGTTTTCTGCC	237
FMgSSR-14730	scaffold6647	p2	(AT)35	Class I	23003	23072	ACAAACGCCCTCGTAACGGT	ATCTCCAGCATGGAGGGCCAAA	227
FMgSSR-14731	scaffold4259	p2	(AT)35	Class I	48273	48342	ACCAGCTCGTTTCGGTCTGCAA	TCGTTGCGCTAGCCTCTTGGAA	348
FMgSSR-14733	scaffold7159	p2	(AT)36	Class I	14699	14770	ACCACACAACCAAACAGCCGA	TGCTTGTGTGATGCCATAGTCC	321

SSR_ID	Scaffold	SSR_Type	SSR_Motif	SSR Class	SSR_Start	SSR_End	Forward sequence	Reverse sequence	Product_size
FMgSSR-14734	scaffold3833	p2	(AT)37	Class I	18449	18522	TCTGGCTCCGGTTGTCCACATT	GGTGCGAGCATAGCTGCGTTTT	338
FMgSSR-14736	scaffold1522	p2	(AT)38	Class I	40011	40086	TTGCTGGCACCTAGACAGACCT	CGTGGACGATTCATCTAGAACGCGA	343
FMgSSR-14737	scaffold22685	p2	(AT)39	Class I	4900	4977	TGGGCTTTCGCATGTGTTGT	ACCGCAAGCATGGGAGACAGAA	241
FMgSSR-14742	scaffold2183	p2	(AT)6	Class II	62906	62917	AAAATCGTGCGTCCGCCTCT	ACGAACTGTTGTGGACGTGCCT	276
FMgSSR-14749	scaffold39	p2	(AT)6	Class II	159368	159379	AAACGAGGGTGATCGGCAGT	ACGTTCTCTTCCGGCTCCGATT	304
FMgSSR-14754	scaffold1048	p2	(AT)6	Class II	45674	45685	AAATCTTCTCCCCAGGAACAGCGG	GTCGACGCTTTTGGCTTGCTGA	243
FMgSSR-14757	scaffold1405	p2	(AT)6	Class II	41372	41383	AACACCGACCACAAGGGCTA	TGTTGGGGGTGAGGGTACTTCT	335
FMgSSR-14761	scaffold1180	p2	(AT)6	Class II	89362	89373	AACATAGTGAGCGGTGCCGA	ATTGCTGCAACTAGCTGGCCGA	299
FMgSSR-14767	scaffold237223	p2	(AT)6	Class II	240	251	AACCGCTACTCCGGGGATGTTT	TGCAGAGCAGTGCACCACCTAT	319
FMgSSR-14769	scaffold709	p2	(AT)6	Class II	57922	57933	AACGAAGGAAGCGCACCGAA	TGCTCCTCGCGCGATCTTCTTA	222
FMgSSR-14771	scaffold331553	p2	(AT)6	Class II	209	220	AACGCACACACGCTTTGCGA	CGTACGAAAGGAGTTGGGTATGGT	278
FMgSSR-14779	scaffold28920	p2	(AT)6	Class II	3375	3386	AACTGGGGGAGACATTCGTTGC	AGCGTGTTGGCTGCTTTGGA	346
FMgSSR-14780	scaffold3356	p2	(AT)6	Class II	45550	45561	AAGAAACGTGCGGCATGGCT	CGGTCTATACATTTGGCCCAACCCA	265
FMgSSR-14781	scaffold18945	p2	(AT)6	Class II	6708	6719	AAGAAGTCGTTCGCCGTCACCA	AGCTCCAAGACTTCTGCGTTGCT	322
FMgSSR-14791	scaffold4579	p2	(AT)6	Class II	24659	24670	AAGCATGGCCCTCTCTCTCTCT	AAGCACGTACCTGTGTTGGGCT	341
FMgSSR-14794	scaffold5672	p2	(AT)6	Class II	41000	41011	AAGCCGGCCTGTTTTGCTGA	TTGGCGCTGTCTTATGCCGGTT	310
FMgSSR-14797	scaffold3446	p2	(AT)6	Class II	24999	25010	AAGCTGCTGGTAGTCGGTGGAT	GGTTCAAGTGCAACAGCTGCGT	341
FMgSSR-14799	scaffold20842	p2	(AT)6	Class II	9166	9177	AAGGCGACCATTTCCGTCGTGT	AGGGCAGCAAATATGTGGCTCGT	256
FMgSSR-14806	scaffold1749	p2	(AT)6	Class II	8107	8118	AAGTTCGGGCTCTCGCCATTGT	TGGAACACACATGTATTCGCCG	299
FMgSSR-14811	scaffold2128	p2	(AT)6	Class II	35756	35767	AATGGGCACACATCGGCACA	TGATGCGCGTCCGTGTTTCT	328
FMgSSR-14816	scaffold1116	p2	(AT)6	Class II	86220	86231	ACAAAGGGGAGGTTGGGGGTAT	CACAACGCAAATGCCATGCTCA	331
FMgSSR-14818	scaffold2062	p2	(AT)6	Class II	39577	39588	ACAACTTTGTTGGGCATGGGC	TGGCTAGTTGCTTTTGTCGCCCT	287
FMgSSR-14821	scaffold38359	p2	(AT)6	Class II	3699	3710	ACAAGGTGCTGTTGGGTTCCGA	AGGTGTGGTTAGAGGACGGAGCAT	302
FMgSSR-14823	scaffold874	p2	(AT)6	Class II	71029	71040	ACAATTCGGAGTGTGGCGTGGT	AGTTGTTTGGCATCGAGGGGCT	346
FMgSSR-14829	scaffold15385	p2	(AT)6	Class II	580	591	ACACCTCTCTCCGACCTTTGCT	TGATGCAACCGGGAACGCAA	332
FMgSSR-14832	scaffold13911	p2	(AT)6	Class II	1200	1211	ACACGGAGAAATTGCAGGCAC	ACAACCTTGTGGCGTGCTGT	322
FMgSSR-14833	scaffold2853	p2	(AT)6	Class II	39836	39847	ACACGGGACAGAAGGAAGTGCT	TGGTGCAACGGCCTGTCAAA	223
FMgSSR-14835	scaffold4801	p2	(AT)6	Class II	18958	18969	ACACGGTGCTGGCCCCTTATTT	ACCACCACTGAATTGTGTCCTCCA	264

SSR_ID	Scaffold	SSR_Type	SSR_Motif	SSR Class	SSR_Start	SSR_End	Forward sequence	Reverse sequence	Product_size
FMgSSR-14836	scaffold12553	p2	(AT)6	Class II	5237	5248	ACACGTGATCGATTCTGGCCT	AAAGCATGGGGAGGAACCCTCT	335
FMgSSR-14840	scaffold165	p2	(AT)6	Class II	76017	76028	ACAGCAACAGTGCGCTTCAGT	ACGGGAAACGTGCCCAGAAA	304
FMgSSR-14842	scaffold418	p2	(AT)6	Class II	115510	115521	ACAGCAGCAAGCTGAGTCAATC	TGCGTGCGTTGTTGGATGCT	305
FMgSSR-14843	scaffold2796	p2	(AT)6	Class II	48982	48993	ACAGCATTTTTGTCTGCGGCAC	AAGTACGAGACGCCGAGCTCAA	344
FMgSSR-14844	scaffold5585	p2	(AT)6	Class II	13043	13054	ACAGGGCATCCTACCAAAAAGGGT	TGGAAGTTGCTGGCTCGCTTCA	338
FMgSSR-14847	scaffold4479	p2	(AT)6	Class II	34772	34783	ACATGAACCTCTTGGCCATGCT	TGTTCAGGCTGGAGGGACCATT	328
FMgSSR-14850	scaffold153	p2	(AT)6	Class II	145078	145089	ACATGCCTGACCAGAAGCCA	AACCATTGGCTATGCCCACCGA	323
FMgSSR-14851	scaffold31596	p2	(AT)6	Class II	2051	2062	ACATGCTCGCGGACGTACACAT	AGCCGCCACTAGATCGAGCAAA	330
FMgSSR-14853	scaffold398	p2	(AT)6	Class II	80934	80945	ACATGGCTGGCTTTTCGGTTGC	TCCTGGAAGCACCCTCACCAAA	306
FMgSSR-14858	scaffold12553	p2	(AT)6	Class II	7145	7156	ACCAACAGCGATGACAGGCA	TCAGTGGTGTGTGCCAACGTGA	319
FMgSSR-14868	scaffold2760	p2	(AT)6	Class II	24851	24862	ACCAGGGTGTTCGGTGGCATTA	TGGCAAGATTACTAGCCGCGCA	319
FMgSSR-14873	scaffold282	p2	(AT)6	Class II	104572	104583	ACCATGCACGTGACCAGCTT	AGCCACAACCCCTTTCCATCCT	316
FMgSSR-14874	scaffold766	p2	(AT)6	Class II	28676	28687	ACCCAAGGCGCATACCACCATT	CCACAAGTGACACATTCCAGAGCA	337
FMgSSR-14875	scaffold244	p2	(AT)6	Class II	118179	118190	ACCCACACTTTGCACATCACCA	ACTTGCAGACATGTTCCTCCGT	212
FMgSSR-14878	scaffold5773	p2	(AT)6	Class II	22534	22545	ACCCAGGCTTTGCTGCTCAA	AACAGCACATGGATGGGTTGGG	324
FMgSSR-14881	scaffold11432	p2	(AT)6	Class II	13859	13870	ACCCGTAGCGTATGGGCAAACA	TTTCTCACCTTCACCACAGCGG	310
FMgSSR-14882	scaffold251	p2	(AT)6	Class II	18740	18751	ACCCGTTAAGAAAGGGCCCAA	ATGCGGTGCTAGCAACAGTGGT	313
FMgSSR-14883	scaffold3030	p2	(AT)6	Class II	17032	17043	ACCCTGTCTTTCTTCCTGCTGC	GCGCGCGAATGATCACTTCTTCA	212
FMgSSR-14885	scaffold29593	p2	(AT)6	Class II	4079	4090	ACCGCAGCAGAACAACGGAT	ACTTTCGACCTAGCTTGCGGGT	324
FMgSSR-14886	scaffold375	p2	(AT)6	Class II	101658	101669	ACCGCCAGTCAGCTCGAATCAT	TGCCGTCTCCAGTCGCAAAA	246
FMgSSR-14890	scaffold122	p2	(AT)6	Class II	126165	126176	ACCGTACCCTTGTCTGTTGCCA	TGGCAGGATCTGTTGCACCT	293
FMgSSR-14892	scaffold1723	p2	(AT)6	Class II	55852	55863	ACCGTTGGGCGTTGTCTAGT	TGGTTCCATTTGTTGTCCCTCCGT	348
FMgSSR-14895	scaffold152	p2	(AT)6	Class II	116250	116261	ACCTCGTCGCCAAAACACGA	ACCAGCTCTGGTCGCATTGA	300
FMgSSR-14896	scaffold6489	p2	(AT)6	Class II	11943	11954	ACCTCGTTGATGAGGGTCGCTA	TGTGACCCTGAAGCCAGTGCAT	312
FMgSSR-14901	scaffold7895	p2	(AT)6	Class II	15703	15714	ACCTGCTCGATCGAAACTGCACA	CGACTCTGCCTCGTCCTTGAAT	277
FMgSSR-14904	scaffold73	p2	(AT)6	Class II	140723	140734	ACCTTCTCCAAGCTGAGGCACA	TGTTCTTAACCCACCGCCAGT	225
FMgSSR-14914	scaffold19676	p2	(AT)6	Class II	3005	3016	ACGACGATGCCGTCTCTTCA	TGACGGTTTGTGACCGCCAA	347
FMgSSR-14917	scaffold12613	p2	(AT)6	Class II	14672	14683	ACGATGCGTGATCAGTCGCT	TGCGCTGCTGATGCGCAAAT	229

SSR_ID	Scaffold	SSR_Type	SSR_Motif	SSR Class	SSR_Start	SSR_End	Forward sequence	Reverse sequence	Product_size
FMgSSR-14920	scaffold1345	p2	(AT)6	Class II	85699	85710	ACGCACACCCTGGTTTGAGGTT	AAGCACTGACGATCCATGGCGT	217
FMgSSR-14927	scaffold13447	p2	(AT)6	Class II	21840	21851	ACGCGTGCTCATGCCTTTTGA	TGTACGAGGTAGGGAAGCCAGT	347
FMgSSR-14932	scaffold9560	p2	(AT)6	Class II	22179	22190	ACGCTGGGGCACTAGCAAGTTT	TTCGCATCGGTTTGTTGTCGCC	326
FMgSSR-14935	scaffold13834	p2	(AT)6	Class II	15499	15510	ACGGAAAGGCGAGTGAAAGCGT	GGCTGGCGTATGTGTCAGCAAT	317
FMgSSR-14941	scaffold176313	p2	(AT)6	Class II	319	330	ACGGCAGCATGCTCTGTCAT	AGCCGAGGCTAACCTGCTAA	320
FMgSSR-14942	scaffold1795	p2	(AT)6	Class II	16352	16363	ACGGCGTTCTTGCATTGCTCA	TGTGCACACAGTGGCATTACGA	212
FMgSSR-14952	scaffold655	p2	(AT)6	Class II	8660	8671	ACGTCTTGTCGCAGAAGAGGCA	ATACGCACGGTGTGGTTCTCCA	326
FMgSSR-14953	scaffold6236	p2	(AT)6	Class II	19204	19215	ACGTGAGGGTCATGGATAGAGGCA	AGCCATGCACCAGATTGTTTCCC	337
FMgSSR-14954	scaffold20058	p2	(AT)6	Class II	4478	4489	ACGTGCAAATTGGGCTCGTGA	TGCAACATGGTTGAGGAGTGGA	342
FMgSSR-14958	scaffold18587	p2	(AT)6	Class II	12585	12596	ACGTGTGAGGCAAGCGCTAA	TCATGTGCTGCTCTTCTGCCA	224
FMgSSR-14960	scaffold1168	p2	(AT)6	Class II	90590	90601	ACGTGTTTGAGGACTGCTCCGA	AGGCCACCGCCTGAACATTA	316
FMgSSR-14967	scaffold936	p2	(AT)6	Class II	52381	52392	ACTCGCACTGGACGTAAGCA	ATCCCGAACATCGGGGCTTT	333
FMgSSR-14968	scaffold8402	p2	(AT)6	Class II	13228	13239	ACTCGCCAATTGCGTTCCCA	AGGCCGTCGCTAATGTGGGTTT	242
FMgSSR-14970	scaffold4817	p2	(AT)6	Class II	5525	5536	ACTCGTGTGTGCATGCGTGA	AACCAGCGCGCCGTACTATT	349
FMgSSR-14973	scaffold600	p2	(AT)6	Class II	71841	71852	ACTGCCGGAATTTGGGGGTA	TGGCAACAAGTACGCAGGGTCA	228
FMgSSR-14976	scaffold6229	p2	(AT)6	Class II	8926	8937	ACTGTGTGTTGTGTCTGGCTTCCT	ACCAGTTCACATGCGTCTGCCT	219
FMgSSR-14977	scaffold5764	p2	(AT)6	Class II	33545	33556	ACTTCCTGTACGCAGATCACTGG	CAAAAGACGGACTATGTGTCCAGG	267
FMgSSR-14978	scaffold15504	p2	(AT)6	Class II	9883	9894	ACTTCTGATAGCTCGCGCCTGA	TCCTCACGCCTCTGATTTGGCT	325
FMgSSR-14981	scaffold48113	p2	(AT)6	Class II	2724	2735	ACTTGGGCAGTTGGCAGACAGT	AGACAGGCCCGTACCTGTTGTT	225
FMgSSR-14984	scaffold9	p2	(AT)6	Class II	112260	112271	AGAAAGCGGTGGCTCGTGAA	AGTTTTTCAGGACGTTGGCCC	347
FMgSSR-14993	scaffold1295	p2	(AT)6	Class II	11195	11206	AGAGATGCACGCACGAATGCT	GTGGTTCGATCGAGAGTAACAAGC	350
FMgSSR-14996	scaffold2264	p2	(AT)6	Class II	7659	7670	AGAGGGATGCAAGTGTGTCGGA	TGGCTCACCGGAAACTTTGGCA	229
FMgSSR-14999	scaffold4379	p2	(AT)6	Class II	40510	40521	AGCAAACAACGAACGGCCGAA	ATTGCGTATCGCCGTACTCCGT	332
FMgSSR-15001	scaffold819	p2	(AT)6	Class II	36956	36967	AGCAAAGCTCCCATAGTCCCA	GCTCGCGACCAACAACAACA	350
FMgSSR-15002	scaffold7476	p2	(AT)6	Class II	5608	5619	AGCAACCACGGCGACAACAA	AGAGATCAGGGCAAGAGACTGGA	268
FMgSSR-15004	scaffold813	p2	(AT)6	Class II	75112	75123	AGCAACCTCACCGTTGCGAA	ATTACTGTAGGCCCTTCTCGCCCA	312
FMgSSR-15008	scaffold5253	p2	(AT)6	Class II	3195	3206	AGCAATTCAGCCCCAAACACTGC	TGGAGGTGTGTCAGTTGGTGTGA	258
FMgSSR-15011	scaffold1801	p2	(AT)6	Class II	39903	39914	AGCACCAACGCCAAGCACTA	AAGTAGTGGAATGCGGCCCTGT	244

SSR_ID	Scaffold	SSR_Type	SSR_Motif	SSR Class	SSR_Start	SSR_End	Forward sequence	Reverse sequence	Product_size
FMgSSR-15013	scaffold3303	p2	(AT)6	Class II	52157	52168	AGCACCGAACATGCAGCACT	TGCAAGGTGACTGCTTTGCTGG	306
FMgSSR-15014	scaffold6528	p2	(AT)6	Class II	6954	6965	AGCACGAGCTTCCTTGTGGGTT	TGTCGACAGAGGTCGCATAGT	317
FMgSSR-15018	scaffold22889	p2	(AT)6	Class II	6232	6243	AGCACTTCATCTCGCTTAGGCCAT	AGGGCGTCCTCAAACACCGAAT	308
FMgSSR-15019	scaffold911	p2	(AT)6	Class II	89467	89478	AGCAGCCGCAATTGCTCTCA	TCCAAATCAAGGGGCGGTTTGC	319
FMgSSR-15034	scaffold16955	p2	(AT)6	Class II	8164	8175	AGCATGCATGTGTCGCTTGTGT	TCCAGCCTCGCAAGCACCATAA	283
FMgSSR-15035	scaffold31855	p2	(AT)6	Class II	7995	8006	AGCATTGCTACACAGGAACACG	GGTTGTAGCTTCATGAGAACCAGC	309
FMgSSR-15037	scaffold20454	p2	(AT)6	Class II	2605	2616	AGCCAACAGTAGGTGGCTTGT	TGCTACAAGATTTCTCCGCCCCT	311
FMgSSR-15042	scaffold535	p2	(AT)6	Class II	1219	1230	AGCCCACGCGCACAAATTGA	GCGTCTTGCGACATGAGCGTTA	348
FMgSSR-15053	scaffold5656	p2	(AT)6	Class II	35102	35113	AGCCTAACCTTCGCAAATCGTGC	ATGGTTCCAGTTGTGCCACG	236
FMgSSR-15055	scaffold3436	p2	(AT)6	Class II	51643	51654	AGCGCACGAGTGCATAGAGACA	TGTGGCCATTTTGATGGCAAGGC	266
FMgSSR-15056	scaffold7949	p2	(AT)6	Class II	16217	16228	AGCGCACGTGCTTACTTGGA	AGACATGTTCACCCGAGCACCA	281
FMgSSR-15062	scaffold2269	p2	(AT)6	Class II	28180	28191	AGCGCTGCAATGTGAGGAGT	TGAGTGTGGAGTCCCTTACGGT	260
FMgSSR-15066	scaffold547	p2	(AT)6	Class II	100638	100649	AGCTAGACTTGACTCCCTGTGAC	TGGAAGGGCAAAACGTCTGCT	233
FMgSSR-15067	scaffold445	p2	(AT)6	Class II	70592	70603	AGCTCACACCACACACAACG	TAAAGCACGGAAAGGAGGGGCA	289
FMgSSR-15073	scaffold4917	p2	(AT)6	Class II	26116	26127	AGCTTCTTGCATGGAGGTGCGT	CGTGTGCTACTCGCAACAAAGT	203
FMgSSR-15074	scaffold12337	p2	(AT)6	Class II	3192	3203	AGCTTGCGCTTGATGTGCGT	ACTTCACTGACGAGGACGACGA	349
FMgSSR-15075	scaffold4546	p2	(AT)6	Class II	36040	36051	AGGAAGAAGCAAGGCGCGTGTA	ACTTGAACCACCAGCGCTCACT	332
FMgSSR-15080	scaffold824	p2	(AT)6	Class II	74887	74898	AGGAGGAGACAAACTGGGGAGT	TTCGCCACCGATGTCAGCTT	330
FMgSSR-15084	scaffold21807	p2	(AT)6	Class II	2465	2476	AGGCAAAGCTCATCTCCGTGT	TGCCATCCCAAGAAACACCAGG	338
FMgSSR-15087	scaffold7810	p2	(AT)6	Class II	12226	12237	AGGCACAAAGGACAGCCTTCT	ACGTGAAACAGACGGACGGACA	344
FMgSSR-15091	scaffold7316	p2	(AT)6	Class II	26281	26292	AGGCCTAGGCGCAATTTCTGTGT	ATTCAACTTGGAAGGAGCGGGC	275
FMgSSR-15095	scaffold4332	p2	(AT)6	Class II	29304	29315	AGGCGGCTGAATGATGGACTCT	ACCGGTTGGAGCATCAAAGGTTG	328
FMgSSR-15096	scaffold189	p2	(AT)6	Class II	3052	3063	AGGCTATCAAGCTGGCCCCTTT	GCACAAGTTTTCGGTGACCGGTTG	333
FMgSSR-15103	scaffold171437	p2	(AT)6	Class II	520	531	AGGGCAAACAAGCGGAAGTCCT	ATTGGAACCGGTGGGAGGAGAGAT	308
FMgSSR-15105	scaffold54062	p2	(AT)6	Class II	1259	1270	AGGGCAATAGTACGCACCTGTCA	TGGATGGGGTCTACACACAGCA	272
FMgSSR-15106	scaffold4595	p2	(AT)6	Class II	30286	30297	AGGGCCAGCTTTAGTGCGAA	AGGGGACTGGCATGGTCTCAAA	322
FMgSSR-15111	scaffold14592	p2	(AT)6	Class II	2070	2081	AGGGGAGCATTCCTAGTAGACCCA	TGCCCATGAGCGAGGATGACAA	252
FMgSSR-15116	scaffold2409	p2	(AT)6	Class II	56391	56402	AGGGTTAAGTGCCGGTGCAGAT	TGGTGTGTCGCTGCTGCATT	235

SSR_ID	Scaffold	SSR_Type	SSR_Motif	SSR Class	SSR_Start	SSR_End	Forward sequence	Reverse sequence	Product_size
FMgSSR-15118	scaffold244	p2	(AT)6	Class II	39340	39351	AGGTCTTGCGCCATGCAAAGT	TTGATGCAGCCACACGGGTCTT	227
FMgSSR-15119	scaffold15467	p2	(AT)6	Class II	4417	4428	AGGTGCTTGCGTCTATGTCCGT	ACATGGTGCGACTCACCCTCTA	275
FMgSSR-15123	scaffold1115	p2	(AT)6	Class II	64415	64426	AGTAGTGGCAATTAGCTGGGTAGC	AGCGACCTACGTGGAACTGA	332
FMgSSR-15127	scaffold4161	p2	(AT)6	Class II	44157	44168	AGTCGCGCCCATAAATGTTGCC	ACGCGAACAACAACTACAGTCTGC	200
FMgSSR-15133	scaffold3776	p2	(AT)6	Class II	16032	16043	AGTGATCGCAGAGGTGCCGAAT	GACAAAAGCATCCATGCCGTGA	326
FMgSSR-15135	scaffold9953	p2	(AT)6	Class II	27561	27572	AGTGCATACACGGTACAACCCT	AGCGACTGACACTGGTCAGAGA	292
FMgSSR-15138	scaffold3199	p2	(AT)6	Class II	18181	18192	AGTGGAACGCAACAGAGCGTGA	TGCCCTGGCCTCGAAAGTCAAA	221
FMgSSR-15140	scaffold346363	p2	(AT)6	Class II	404	415	AGTGGGCGCCATAACCCAAACT	TGGCTGTCATGCATCTGCCGTT	222
FMgSSR-15156	scaffold10	p2	(AT)6	Class II	57089	57100	AGTGTTGCTGCTGACCAACGTCT	TGGTCAGTTGCTAAAACGCCCG	257
FMgSSR-15157	scaffold1530	p2	(AT)6	Class II	24569	24580	AGTTCTCCATGCGCACCATCAT	AGCCAGAAAATTTGGTTGAGGGGC	229
FMgSSR-15161	scaffold11065	p2	(AT)6	Class II	8216	8227	AGTTGTACGTGCGGCTAGGTCA	ACATGCGACGGTAAGCCACA	295
FMgSSR-15168	scaffold241	p2	(AT)6	Class II	22770	22781	ATATTGGTGCCACTGCGCTCCT	AAAGGTGAGCTGCAAGCCCTCT	323
FMgSSR-15170	scaffold3045	p2	(AT)6	Class II	38445	38456	ATCACAGGCGTGGGATGCTT	TTGGTGGCATGGTGAAACCGGA	320
FMgSSR-15171	scaffold9033	p2	(AT)6	Class II	27239	27250	ATCACGGGAGAGGGCAGGTATT	TTGCACGAGCATCCAACGCT	286
FMgSSR-15172	scaffold2439	p2	(AT)6	Class II	31913	31924	ATCCCAACACAGGGGCTACACT	GGCCCACCTGTCATAATTAGACCCA	308
FMgSSR-15176	scaffold19054	p2	(AT)6	Class II	5251	5262	ATCGCAGACGACGTAGCGAAGA	CGTGAGTGTGGAGGGGAGGATTAT	349
FMgSSR-15184	scaffold1978	p2	(AT)6	Class II	61958	61969	ATCTTACGGTGCACTGCCGAGT	ACACCCAAGTGCAAAAAGGCGG	309
FMgSSR-15189	scaffold29469	p2	(AT)6	Class II	956	967	ATGCAACGGCCAAGGCCAAA	GAGTTGACAGGGGAGAGGCATA	346
FMgSSR-15191	scaffold6621	p2	(AT)6	Class II	17474	17485	ATGCAGTAGACAAGGAGCGGCA	TTTGACTGCGTGGTGTCCTGCT	331
FMgSSR-15193	scaffold4228	p2	(AT)6	Class II	44015	44026	ATGCCCTGCTTGAGAGAGGAGA	ACGCGGTGCAGTAGAAACCTGA	289
FMgSSR-15194	scaffold3436	p2	(AT)6	Class II	45791	45802	ATGCCGCAAAGCATGCCAGA	TGCTATTGGGGGTGGATGGCAA	249
FMgSSR-15196	scaffold2619	p2	(AT)6	Class II	28502	28513	ATGCCTTGCCCTTCTTCCCCTT	TATGATGCAGGCTGCGGTTGCT	338
FMgSSR-15200	scaffold196	p2	(AT)6	Class II	105190	105201	ATGCGTGCTACGTTGGAACC	GCAAGCAAATGGTGTCTGTGGTGA	215
FMgSSR-15201	scaffold29627	p2	(AT)6	Class II	8749	8760	ATGCTGCTGCTATGGTGGGTGA	ACCAGCCTTCCAGACCAAATCT	285
FMgSSR-15204	scaffold4503	p2	(AT)6	Class II	23752	23763	ATGGCGACCAGCATCGATCTTC	ACGATCCGCCTAACGCAAAACG	240
FMgSSR-15205	scaffold125930	p2	(AT)6	Class II	854	865	ATGGCGATGTGCGTCAGGAA	TCATGTAAGCCTACCACGGCCA	227
FMgSSR-15207	scaffold15610	p2	(AT)6	Class II	3448	3459	ATGGGAAGGCACATGGGTTGCT	ACTTCCGACTGAACTGGTGCCT	345
FMgSSR-15208	scaffold2755	p2	(AT)6	Class II	7363	7374	ATGGGACGCGTGATATGCCGAT	TGGAGTACACACACACACACACAG	333

SSR_ID	Scaffold	SSR_Type	SSR_Motif	SSR Class	SSR_Start	SSR_End	Forward sequence	Reverse sequence	Product_size
FMgSSR-15209	scaffold25910	p2	(AT)6	Class II	7824	7835	ATGGGTGGGAACGAGCTAGCAA	TGAGCAACATGGCAGTCGGT	269
FMgSSR-15215	scaffold3648	p2	(AT)6	Class II	15681	15692	ATTCACTCACTGGGCCTGGAT	ATGTGTGGTAGGGAGGCACCAT	328
FMgSSR-15218	scaffold128	p2	(AT)6	Class II	50430	50441	ATTCTCCGGCCAAAGGGGAA	TCGTTTGACCTCCTTGTGTGACGG	342
FMgSSR-15220	scaffold30630	p2	(AT)6	Class II	2538	2549	ATTGCAACGGCTCTGGGTGT	GCCTTGCGCACGCAACTCAATA	229
FMgSSR-15222	scaffold6085	p2	(AT)6	Class II	33250	33261	ATTGGCTGCTTCACTCCAAGCG	TCAAACGAGACGCACGTGATGC	335
FMgSSR-15224	scaffold4312	p2	(AT)6	Class II	30246	30257	ATTTACGGGCCGCAAGACTGCT	ACATCTGGTGATGGCAAAGGCG	248
FMgSSR-15225	scaffold14040	p2	(AT)6	Class II	11266	11277	ATTTAGTGCGTTGCGCTCCA	ATGTGTTTGGGCATCGGCGT	339
FMgSSR-15230	scaffold8546	p2	(AT)6	Class II	13133	13144	CAACATTTGCAAACCTGCCAGC	TGCATTCCATTCGTGAAGGAGGGT	286
FMgSSR-15237	scaffold2664	p2	(AT)6	Class II	29905	29916	CAGAGTCCTGAACAACTGCTGC	AGGGTTGCAGATGGTTACTCCT	350
FMgSSR-15243	scaffold9057	p2	(AT)6	Class II	28592	28603	CCACATGCTCCAGTCCATATACGA	TGGATACCCACCACCTGGGTTTT	284
FMgSSR-15244	scaffold328	p2	(AT)6	Class II	84791	84802	CCACCACTATAGGCTGCTTTTCTGG	TCGCCATATCTAGCTTGCACCT	347
FMgSSR-15246	scaffold75343	p2	(AT)6	Class II	927	938	CCAGAGTTCAAGAGCAGGAGCCTA	GCATGCCTTCCTCCTCTAATGTGA	350
FMgSSR-15248	scaffold8737	p2	(AT)6	Class II	20253	20264	CCAGCAGTTCATCCGCATCCAT	ACTGTAACGACCGACACGCA	331
FMgSSR-15257	scaffold3307	p2	(AT)6	Class II	19455	19466	CCGCCCAAATGTTGAACGAGCA	AGACTTCTTCAACCCGGGCA	323
FMgSSR-15266	scaffold168569	p2	(AT)6	Class II	656	667	CCTGCTGACAGGTGGGTCAAAGAA	ACTGCTTGTGGGTGGCAGTA	292
FMgSSR-15269	scaffold353847	p2	(AT)6	Class II	183	194	CCTTGGCGCCATTGCTGCTATT	GCGCTTTTCCTTCCTAATCTGAGC	218
FMgSSR-15273	scaffold1822	p2	(AT)6	Class II	15143	15154	CGCACAAAGCAACAGCTTGAGCA	AACCTTCCGCTGCGACCAAA	339
FMgSSR-15275	scaffold8897	p2	(AT)6	Class II	24680	24691	CGCATCCATGTGTTATCACGTCTC	GCCCAATGCAAGAGACACGCAA	269
FMgSSR-15280	scaffold271	p2	(AT)6	Class II	139046	139057	CGCGCACATCAAAACAGCAGGA	CGACGTTGTTGTTCTGCAGGTAAGC	327
FMgSSR-15283	scaffold41756	p2	(AT)6	Class II	5751	5762	CGGCGACAACAAAGAACAGCGA	TTGCTTCGTACTCCGCTTGCCT	335
FMgSSR-15287	scaffold14779	p2	(AT)6	Class II	13410	13421	CGGGTTGTGGAAGATTTCCTTGCC	TGTGTGTCGGGTGCCATCAT	307
FMgSSR-15291	scaffold520	p2	(AT)6	Class II	83720	83731	CGTGCAGCCGAGGGAATTTGTA	ACCAGTCCGTCAGCGAATGA	342
FMgSSR-15293	scaffold595	p2	(AT)6	Class II	34883	34894	CGTTGCGCACAGTCCTTCACTT	TCACTTGACACCAGCATCACAC	345
FMgSSR-15296	scaffold3104	p2	(AT)6	Class II	34445	34456	CTGTGCAAAGCTATCGATTGACCG	TGCTTGGCGAGGTGAAGCAT	350
FMgSSR-15298	scaffold6294	p2	(AT)6	Class II	20698	20709	GAAGGAGGGGAGGGGGATTTTT	CGCTGCGTGCATGAATCATCGT	283
FMgSSR-15301	scaffold1996	p2	(AT)6	Class II	15751	15762	GACAACTGACGCAGATGCACA	ACCATTTTTGCCCACGTCTCCCT	301
FMgSSR-15307	scaffold16202	p2	(AT)6	Class II	17533	17544	GAGGGTGTGGCATAACCTATAGCA	ATTTGAGGGAACCGTGCTGTGC	336
FMgSSR-15310	scaffold45	p2	(AT)6	Class II	4101	4112	GCAATGGGGTGCCAATGGCTTT	TCGCCACCAACAATCGCAGA	202

SSR_ID	Scaffold	SSR_Type	SSR_Motif	SSR Class	SSR_Start	SSR_End	Forward sequence	Reverse sequence	Product_size
FMgSSR-15313	scaffold19848	p2	(AT)6	Class II	14376	14387	GCACCTGCACGCAGAACAAT	TTGCTCAGGCCACGTTCACACT	319
FMgSSR-15315	scaffold13782	p2	(AT)6	Class II	3743	3754	GCACGATGGTGCATCTCTCAT	ATGACAGCTCCGTCTTGGCA	214
FMgSSR-15316	scaffold16162	p2	(AT)6	Class II	1117	1128	GCACGGTTGTGCATGACCTTCT	AAAAGAGCCCCTTCCTCGTCCT	281
FMgSSR-15321	scaffold8434	p2	(AT)6	Class II	2681	2692	GCCAACCGCACACCACACATTT	TGCGCCGCATCAGTTAGCAA	273
FMgSSR-15322	scaffold2222	p2	(AT)6	Class II	12587	12598	GCCAAGACACACGCCCAAATCA	TCACATGCACATGGTTGCGTCC	321
FMgSSR-15325	scaffold1898	p2	(AT)6	Class II	66968	66979	GCCAGGAAGAAGTCAGGCTTTGGT	ACGCAACCCCCAACAGACACAT	332
FMgSSR-15326	scaffold14088	p2	(AT)6	Class II	12653	12664	GCCAGTTGAAGGAAATGGAAGTCCC	ACTCCAGTGAGGCACAACTCT	345
FMgSSR-15330	scaffold679	p2	(AT)6	Class II	24911	24922	GCCGCCTGCTATAGTAGATCTAAGG	TGTGGCCACCTACTTGTTGGGA	336
FMgSSR-15332	scaffold1145	p2	(AT)6	Class II	17423	17434	GCGAGTTGCACAACAAAGAACC	TTTATACGGCGACGGCCAACGA	316
FMgSSR-15342	scaffold2812	p2	(AT)6	Class II	50991	51002	GCGGACACGTGCGTTGCATATT	AAAGTACGCGAACATCGGGCCA	295
FMgSSR-15347	scaffold241102	p2	(AT)6	Class II	385	396	GCTCTGAGCTGGTTCGGACTTT	ATCAAAGGGCGGTCCAGGTGTT	201
FMgSSR-15349	scaffold22647	p2	(AT)6	Class II	6448	6459	GCTCTTCAAGCATACAGCTTTGGC	AGCAGCATGGCCTGTTACAGAGA	234
FMgSSR-15350	scaffold13936	p2	(AT)6	Class II	4828	4839	GCTGCAGCAACAGAGTAGTGGA	GACAGCATATTGAGGACTTCAGCG	288
FMgSSR-15356	scaffold785	p2	(AT)6	Class II	19189	19200	GGACCCCAGGTGATAAAATGGCGA	TGCAACACGACTTTTGGGGTCT	306
FMgSSR-15358	scaffold783	p2	(AT)6	Class II	75765	75776	GGACTCTGTTCAGCCAAAAGTCTG	TCAGTGGCACCTGAATACCTTCCT	345
FMgSSR-15359	scaffold6645	p2	(AT)6	Class II	37581	37592	GGAGCAATTTTGCAACTGCCACC	TTGTGCGCTGGTGCTGGTTT	344
FMgSSR-15360	scaffold6743	p2	(AT)6	Class II	14998	15009	GGAGCGGAGAGAAAGAAAAAGGCA	TGGCGCCTTCTTCTGATGTGA	205
FMgSSR-15365	scaffold116064	p2	(AT)6	Class II	630	641	GGCCAATTGCCAGATAAATCGC	TGGAGGCATACAGGCCGTTTCA	305
FMgSSR-15370	scaffold5370	p2	(AT)6	Class II	27479	27490	GGCGAACCTGATGTGTTCAGTTGG	ACACTCGCCATACCATGCCTGA	301
FMgSSR-15384	scaffold1191	p2	(AT)6	Class II	35777	35788	GGGGGACTACACACACAAGCAT	TTGCCGTGCCAGTGGAAGTT	237
FMgSSR-15385	scaffold4041	p2	(AT)6	Class II	22188	22199	GGGGGAGAAGGGGAACTGAATCAT	AGGGGTACACCAGTCAGAGTCA	320
FMgSSR-15392	scaffold307	p2	(AT)6	Class II	104644	104655	GGTGCAAACATCATACTCATGCCC	TGAATGTTAAGCGGGCTAGGCA	305
FMgSSR-15393	scaffold12720	p2	(AT)6	Class II	11546	11557	GGTGGTACATGTGGCGGCAAAA	AGGGCGCAACGGAAGAATTCCA	220
FMgSSR-15394	scaffold16955	p2	(AT)6	Class II	9982	9993	GGTTACCTTGACTCCTTGTTGAGG	AGCAGCTGATGATATGCACCCA	223
FMgSSR-15397	scaffold2870	p2	(AT)6	Class II	9148	9159	GTCCGTGAAACCAATGATTGTGGG	AGGACATGGCCCCTCCTTTACA	340
FMgSSR-15398	scaffold7719	p2	(AT)6	Class II	27981	27992	GTCCTTGCAGCCGAAGCTTTCA	AGGAACGCATCACGTCCCAACT	225
FMgSSR-15404	scaffold10860	p2	(AT)6	Class II	3182	3193	GTGGACATGTGTCACAAAAGGGTC	ACTGCCAAGCGCGTCCAGAATA	247
FMgSSR-15406	scaffold13811	p2	(AT)6	Class II	16425	16436	GTGGCTGGTTATTTGAGGTCTCCT	ACGTGAGAGCGTTTGTGCATGT	350

SSR_ID	Scaffold	SSR_Type	SSR_Motif	SSR Class	SSR_Start	SSR_End	Forward sequence	Reverse sequence	Product_size
FMgSSR-15409	scaffold1644	p2	(AT)6	Class II	16060	16071	GTGTGGTTTCCATGTTGTTTCCGC	AGGTGGTTCTCCCTGGTGAGATGA	213
FMgSSR-15416	scaffold17109	p2	(AT)6	Class II	15313	15324	TACAAATATCCTCGGCCGGCGT	TCGGTGTTCACGGCGTCATT	212
FMgSSR-15417	scaffold524	p2	(AT)6	Class II	43285	43296	TACAGACGCGTGCCTGGGTTTT	AAAAGGAGCACGAAGAGATGGCCC	228
FMgSSR-15420	scaffold5005	p2	(AT)6	Class II	5199	5210	TACGCAGGCGCCTATGTTGT	ACCAGCGGTGGGCTCAAGATTT	295
FMgSSR-15427	scaffold3608	p2	(AT)6	Class II	2536	2547	TAGCGCTGTCGTGTGTGTGT	ACGTGCTTTCGCATATTGGCCC	276
FMgSSR-15428	scaffold13813	p2	(AT)6	Class II	20245	20256	TAGCTTGGTGTCGGCAGTCGAT	TGGAGTCATGGAGTCGTGATGTGC	222
FMgSSR-15435	scaffold4111	p2	(AT)6	Class II	28565	28576	TATTCACCGTGAGGTGGCGTGA	ACCCCAGCTGAAAGCCATGACA	241
FMgSSR-15436	scaffold1169	p2	(AT)6	Class II	21338	21349	TATTTCTGCAGGCGCGGGAT	AATTTCACGCGGCGTCCGTT	334
FMgSSR-15437	scaffold242626	p2	(AT)6	Class II	79	90	TCAACCATCCATACAGTGCCAGGA	TCAGGGATCTTTCTGGTGTGCT	331
FMgSSR-15438	scaffold866	p2	(AT)6	Class II	43864	43875	TCAAGCGAGTGGGGGCAAAA	TCGAGCGCAGGAGAGAGAGAAA	237
FMgSSR-15439	scaffold2459	p2	(AT)6	Class II	27810	27821	TCAAGGTGGCGTGACAACAACG	ACGGCGGGTCAACAACTAGA	324
FMgSSR-15442	scaffold2851	p2	(AT)6	Class II	46061	46072	TCACATGGAGATACCACGTCCGA	GGGGCTTTGCTGCATGGTAGTT	259
FMgSSR-15446	scaffold911	p2	(AT)6	Class II	72848	72859	TCACCTTTTCCGCAAGTGGTTC	TCCACAGGCTGCTTATGCGT	279
FMgSSR-15447	scaffold448	p2	(AT)6	Class II	118204	118215	TCACGAAGCCCCTCGTCTATCAA	AGTGATGATGCGGAGAGCCCTT	270
FMgSSR-15450	scaffold487	p2	(AT)6	Class II	14582	14593	TCACGTGAGCCATGTCTGTGCT	GCACATGCAAATCTGCCGGTAT	307
FMgSSR-15454	scaffold3686	p2	(AT)6	Class II	13085	13096	TCAGCTTCACACGCTAAATCCGGT	CAGTGGTGTGTGTCGACAGAATTG	346
FMgSSR-15455	scaffold34	p2	(AT)6	Class II	147347	147358	TCAGCTTTCGCATACTCCCTCCA	TCCAGGCTGTGTGCATGTGTCA	350
FMgSSR-15456	scaffold34488	p2	(AT)6	Class II	3394	3405	TCAGGCTAGCGGGTACATTTGC	ACACATCGTCATTGCCACACTG	224
FMgSSR-15459	scaffold1123	p2	(AT)6	Class II	68278	68289	TCATCGCCAGCAGCAGTGCATA	ATTGTCGCAGCGTCCACTGT	300
FMgSSR-15461	scaffold3616	p2	(AT)6	Class II	4892	4903	TCATCTGAGCCCTGAGTGAGCA	TACGCCCTATGTTGTGGCGACT	263
FMgSSR-15468	scaffold24385	p2	(AT)6	Class II	5922	5933	TCCACATTCAACTCTTGGCGCA	AGGGCGTGACTGCATGGAATGT	222
FMgSSR-15469	scaffold5724	p2	(AT)6	Class II	19563	19574	TCCACCACAAAGCGCAGAGA	TTCAGGGCACCGATCAGCACAA	339
FMgSSR-15471	scaffold20448	p2	(AT)6	Class II	14840	14851	TCCACTCCAAACTCAGGCGT	ATGGTGGCCTGTTAAGCTGCGA	225
FMgSSR-15473	scaffold26442	p2	(AT)6	Class II	8935	8946	TCCAGCGTCAGCACCCAGAAAA	TGTTGGACCTCAGCTCGGTGAT	274
FMgSSR-15475	scaffold4957	p2	(AT)6	Class II	42068	42079	TCCCACACGATCGGTTTCCCAA	TGCCAAAGCGACATCGGAGTGA	268
FMgSSR-15476	scaffold9017	p2	(AT)6	Class II	26167	26178	TCCCCACAACCACAAAGGATACGA	ACCTGCCATGCCATTGACAGCA	251
FMgSSR-15479	scaffold17503	p2	(AT)6	Class II	9021	9032	TCCCCGAAACAAGCCCAAACCA	AATTGACCGAAAGCGGCGGA	296
FMgSSR-15486	scaffold2463	p2	(AT)6	Class II	30178	30189	TCCGCAACGCAAACTCCTGCTA	AACGCGACCGTGAACGTGAA	343

SSR_ID	Scaffold	SSR_Type	SSR_Motif	SSR Class	SSR_Start	SSR_End	Forward sequence	Reverse sequence	Product_size
FMgSSR-15487	scaffold10559	p2	(AT)6	Class II	23019	23030	TCCGCACCACCTGACAAGGAAT	TCCATGCGCGGTAAAACGGT	305
FMgSSR-15489	scaffold4306	p2	(AT)6	Class II	3264	3275	TCCGCGTTGCACGAAAAGGA	CCCATATCAAGGCCAACTTTCAGC	266
FMgSSR-15515	scaffold855	p2	(AT)6	Class II	63788	63799	TCGAGCAGGAAGCTGCAATCCA	TCGCGCCTTGCGTTCTGAAA	285
FMgSSR-15517	scaffold26883	p2	(AT)6	Class II	4603	4614	TCGAGGCAGGGCATTGCCATATT	ACGGAAAGGTGCTGGTTTTCGC	342
FMgSSR-15523	scaffold221	p2	(AT)6	Class II	28175	28186	TCGCCCACCAACACGGTTTACA	TCGCGGGGCTTTTGAAAGACAT	344
FMgSSR-15524	scaffold17619	p2	(AT)6	Class II	10464	10475	TCGCCGCAAAAACTGGTGCT	GCCATGGCGTGCTTTTGCTGAT	346
FMgSSR-15535	scaffold2583	p2	(AT)6	Class II	20521	20532	TCGCTTTGAAGCTGTCGGGA	TGGATGACGGTGGTGCCGAAAT	319
FMgSSR-15536	scaffold11035	p2	(AT)6	Class II	22932	22943	TCGGAATAGTCCCTGTCTGCTTGG	ACCGCAAACCACAGGTTCCA	274
FMgSSR-15537	scaffold2687	p2	(AT)6	Class II	26730	26741	TCGGACCACAAGGTTCGCACAA	ACGCGCATTCATACGGCAGA	268
FMgSSR-15538	scaffold13225	p2	(AT)6	Class II	17382	17393	TCGGATTTGGAGGGAGGCAACT	TGTCGCGGCAAAGCAGATCA	264
FMgSSR-15542	scaffold19171	p2	(AT)6	Class II	7137	7148	TCGGCCGAAGCGTATCCATTGT	TGCTCACGCAACATGCTGGA	285
FMgSSR-15546	scaffold1669	p2	(AT)6	Class II	17510	17521	TCGGTTCCTATCAAACTCGGTGC	TGTCCATCTCCTGTAACCTGGCA	340
FMgSSR-15547	scaffold1523	p2	(AT)6	Class II	53488	53499	TCGGTTGAACGGAGCTGGGATT	AGCTGTCGCTGACTTGTCGTGT	299
FMgSSR-15548	scaffold2444	p2	(AT)6	Class II	8316	8327	TCGTACCGTGACCATGGCTGTT	GCGTATTGGGCCTGATTGTGATGC	294
FMgSSR-15549	scaffold12626	p2	(AT)6	Class II	8726	8737	TCGTACGATGGTGGCGTATTCTGA	TGCACTCTTCTTGCCGGTGGTT	275
FMgSSR-15551	scaffold2971	p2	(AT)6	Class II	38241	38252	TCGTATCAGCGGAAGGTGATGCT	AGGAGCCGTTGCAATCTTCCGA	252
FMgSSR-15553	scaffold7034	p2	(AT)6	Class II	18813	18824	TCGTCGCACTTGGTGTTCCTCA	TGCTTTCCGAGTACCATCATGCCC	343
FMgSSR-15558	scaffold184890	p2	(AT)6	Class II	625	636	TCGTTCGAAGCGCCCAACTT	ACAGTCCATGGCGTGGTCATCA	213
FMgSSR-15563	scaffold3743	p2	(AT)6	Class II	21081	21092	TCTCGCACACACCATGTGCT	TGCTGAGAGCAAAACGCAAGC	298
FMgSSR-15564	scaffold1433	p2	(AT)6	Class II	70639	70650	TCTCGCGCCAATCACTTCGT	TTGTGACATGTGGGGCTCATGC	340
FMgSSR-15566	scaffold589	p2	(AT)6	Class II	73448	73459	TCTGACTGATGCGTCGGTGTCA	AGGCTTGAAATGCATGCGAACG	343
FMgSSR-15568	scaffold550	p2	(AT)6	Class II	108958	108969	TCTGCCTCTGGAAAAGCACCGA	AAAAACACCACACGGGACCCCA	259
FMgSSR-15570	scaffold259	p2	(AT)6	Class II	33909	33920	TCTGCTCCATGAATGCGTTGTG	AGCCTTGCTTGGCTCGTGGATT	274
FMgSSR-15571	scaffold4819	p2	(AT)6	Class II	48653	48664	TCTGCTCCGGTTGCTTTGTGA	TGCCCCTAATCCAACCTGCATGA	275
FMgSSR-15572	scaffold2325	p2	(AT)6	Class II	11842	11853	TCTGGAAACCAGTTCGACCAGC	CCCGCCTCGCCCAAAAATTCAA	270
FMgSSR-15577	scaffold398	p2	(AT)6	Class II	103335	103346	TCTTCATGCGCGTGGAACGAT	TGTGGCGATCAGGTCAGTGCTT	200
FMgSSR-15582	scaffold287	p2	(AT)6	Class II	38443	38454	TCTTTCGAGAAGACGCCGCT	TGCACGTGCTGAGGTATCGTGT	349
FMgSSR-15583	scaffold501	p2	(AT)6	Class II	66323	66334	TGAACGGATTTGCACCGCATGA	AATTGGAGTCCGTGATGCCCGA	301

SSR_ID	Scaffold	SSR_Type	SSR_Motif	SSR Class	SSR_Start	SSR_End	Forward sequence	Reverse sequence	Product_size
FMgSSR-15587	scaffold675	p2	(AT)6	Class II	13254	13265	TGACAAACGCCACAAGCGGA	AAGGCATCGCCATCGGAGTA	312
FMgSSR-15589	scaffold52730	p2	(AT)6	Class II	1966	1977	TGACAAGACAGCGTGTGCGT	TGCTACAAGTGGCGTTGTTGGGA	214
FMgSSR-15595	scaffold2091	p2	(AT)6	Class II	67524	67535	TGACGACAGGGTCTCAGGGTTT	AAAGCCACCGTGCCTGTTTG	331
FMgSSR-15599	scaffold55406	p2	(AT)6	Class II	1454	1465	TGACTCGGGCCCAGCTTTGTTT	TCGTTGTAGCGGCGTTACCAGT	349
FMgSSR-15603	scaffold272	p2	(AT)6	Class II	103794	103805	TGACTTGCAAAGGGAAGGAGAGCA	GCTGCATGTGGTCGAGCAACAAA	248
FMgSSR-15606	scaffold2744	p2	(AT)6	Class II	43450	43461	TGAGATGGCTGTTGGCTGCACT	TGGCTGTGGCTTGCAATGGGAT	285
FMgSSR-15607	scaffold10988	p2	(AT)6	Class II	9325	9336	TGAGCACCTTTGGTGGAGTGCAA	ACGTCAGTGTGTGCATTCGGT	293
FMgSSR-15610	scaffold7595	p2	(AT)6	Class II	14675	14686	TGAGCCAACAAAACGCACGCT	TGCTGCAAGGAGGAGCGTTAGA	342
FMgSSR-15611	scaffold12426	p2	(AT)6	Class II	10071	10082	TGAGGCCTGTGGCCCAATTGTA	TCTGGTGAGCCATGGTGTGGTT	286
FMgSSR-15612	scaffold5900	p2	(AT)6	Class II	26518	26529	TGAGGCGGCTTGGTGGTTGATT	TGGGCCAACTTTCCTATTGGGC	320
FMgSSR-15615	scaffold3034	p2	(AT)6	Class II	32850	32861	TGAGTGGAGCACCAAACCGT	TGTTTCCTGAGATACCCGCCA	246
FMgSSR-15618	scaffold12586	p2	(AT)6	Class II	3021	3032	TGATGGCGAGGGTTTTCCCAGA	ACCTTGTTCTGAGGCCAGCAGT	333
FMgSSR-15620	scaffold16504	p2	(AT)6	Class II	16074	16085	TGCAAAGAAACCGGTGGTGGGA	TGCCCGGTCAGCATTGTTTTGC	340
FMgSSR-15622	scaffold10248	p2	(AT)6	Class II	10752	10763	TGCAAAGGTGGCTGAAGAATCC	TCACATGAAGACCGCAAGGCGA	320
FMgSSR-15629	scaffold18696	p2	(AT)6	Class II	5197	5208	TGCAAGCCCAAGACAGGCAT	ATGCTGCGTCCTCTCTACGACT	323
FMgSSR-15630	scaffold10996	p2	(AT)6	Class II	27277	27288	TGCAATGCCATCAAGGGAGCCT	AATCCGACCTGGCTGCAGAA	319
FMgSSR-15631	scaffold5456	p2	(AT)6	Class II	24148	24159	TGCACAACCCGCATCTTCGT	TGGCCACCAAAAATGGCGGA	305
FMgSSR-15636	scaffold9671	p2	(AT)6	Class II	15944	15955	TGCACGCCATGACCGATGTGAT	TGCTCATCCGTTGCATCCACGA	305
FMgSSR-15640	scaffold6530	p2	(AT)6	Class II	24205	24216	TGCAGCATCTTTTGTCGGCACT	AGAACAACGTGTGGCCTACTGT	286
FMgSSR-15643	scaffold27992	p2	(AT)6	Class II	6991	7002	TGCAGGAGAACCCACAATCACCA	CTTGCATTCCCAGTTCTTGGTCAG	301
FMgSSR-15645	scaffold1548	p2	(AT)6	Class II	14922	14933	TGCAGGCTTCCATAGTTGCG	GGATCTCCTGCAAGTCTGTTTTCC	331
FMgSSR-15647	scaffold4454	p2	(AT)6	Class II	26880	26891	TGCAGTGATGTGTGTGCGAGGT	TTGGTAAAGGCCACCACCCGAT	338
FMgSSR-15652	scaffold11797	p2	(AT)6	Class II	2405	2416	TGCATCACGGGAACCTTCTTGC	AAATGCGCAGGTTGTGCAGC	312
FMgSSR-15653	scaffold128	p2	(AT)6	Class II	14632	14643	TGCATCGCTGATCGACCTCCTT	ATTGGCGAACGCAGCGTCAT	329
FMgSSR-15655	scaffold2935	p2	(AT)6	Class II	40491	40502	TGCATGACGTGACGTTTGAAGC	TCGCGGAAACGGTGAATGTTCG	342
FMgSSR-15657	scaffold4349	p2	(AT)6	Class II	10771	10782	TGCATGGGCCAATCAATCCCCT	TGATGGTGTGCGTGTGTGCT	330
FMgSSR-15659	scaffold8916	p2	(AT)6	Class II	9742	9753	TGCATGTCGGTCGTCACTTGCT	TTAGCCCTTTGGGAGCGCATGA	258
FMgSSR-15660	scaffold24516	p2	(AT)6	Class II	7019	7030	TGCATGTGTGGGTCGGTTGGAA	TTGGAGCTGAATTCCTCACTGCCG	314

SSR_ID	Scaffold	SSR_Type	SSR_Motif	SSR Class	SSR_Start	SSR_End	Forward sequence	Reverse sequence	Product_size
FMgSSR-15661	scaffold29	p2	(AT)6	Class II	96144	96155	TGCATGTTTTGAAGTCCCTGCCTG	ACATTGGCGTAGTCGTGCCCAA	300
FMgSSR-15662	scaffold67462	p2	(AT)6	Class II	1974	1985	TGCATTCTCAGTGGTGGCCTCA	TGTTGGGTTGGGTTGACTGGCA	333
FMgSSR-15666	scaffold3293	p2	(AT)6	Class II	16510	16521	TGCCAACGATCTTTAGCGGACTCA	GCATGGTCACCCGACACCAGTAAT	317
FMgSSR-15668	scaffold5557	p2	(AT)6	Class II	33615	33626	TGCCAACGTGGCATGAGGAAGT	AGGCTGCGATTCAGTGCAAGA	336
FMgSSR-15670	scaffold5667	p2	(AT)6	Class II	9585	9596	TGCCATGTAGCAGGGGTGTTCT	TGGGCCGGACCACAAACATT	348
FMgSSR-15677	scaffold6220	p2	(AT)6	Class II	27515	27526	TGCCGCCCATACCAGCAAAA	AAAACGCACGGATAGCTGGGCT	246
FMgSSR-15680	scaffold255590	p2	(AT)6	Class II	222	233	TGCCGTTCCCAATCACTGTTCA	AAAGTGTTGGCAGTGCAAGGGC	340
FMgSSR-15681	scaffold38496	p2	(AT)6	Class II	1833	1844	TGCCGTTGGGCGTGCTTTTA	TGGGTGGCGTGATTGTTCTCCA	307
FMgSSR-15682	scaffold6983	p2	(AT)6	Class II	26638	26649	TGCCTACGTTTCGAGCGCAT	AATTATGCACAGCCGCACAGGG	258
FMgSSR-15683	scaffold6930	p2	(AT)6	Class II	15660	15671	TGCCTGCAAGCAAGCCACAT	ATTTTCGGCGCACCTCGACT	238
FMgSSR-15685	scaffold793	p2	(AT)6	Class II	71669	71680	TGCCTTCTTGTTGTCCATGCCT	ACTAATGGCTGATACACCTAGGGC	326
FMgSSR-15686	scaffold1231	p2	(AT)6	Class II	64347	64358	TGCCTTTGCCAGCCTACTTGGT	ATTCTCGGGGTTGTTCGGGCTT	271
FMgSSR-15687	scaffold1549	p2	(AT)6	Class II	71639	71650	TGCGAGGTGGATGGCCTTTGTT	AACACCGTCGGGAAACGCAA	298
FMgSSR-15688	scaffold20183	p2	(AT)6	Class II	1550	1561	TGCGCCATTTAGCGCTGTCA	AGCATCCACACCTGTGACCA	324
FMgSSR-15690	scaffold68108	p2	(AT)6	Class II	515	526	TGCGCTTGGCGCATTAAGGA	ATTTGAACTCCGTGGACCGCCT	274
FMgSSR-15694	scaffold1989	p2	(AT)6	Class II	11028	11039	TGCGTCGTTGGTGTTGCTCT	ACCACGATAACACTGTGCCGAC	290
FMgSSR-15695	scaffold29627	p2	(AT)6	Class II	3102	3113	TGCGTGCCATTCCAAACATGC	AACGCGGACTCTCATTCGTGCT	350
FMgSSR-15696	scaffold2974	p2	(AT)6	Class II	19659	19670	TGCGTGTAGCGTGTGAGTAGGT	ACCGTGTGACAGCTGTGTGCTA	293
FMgSSR-15699	scaffold8054	p2	(AT)6	Class II	6272	6283	TGCTATGCTTAGGGATGAAGGGCT	AGTGTGGAATCCATTATCGGGCCT	346
FMgSSR-15703	scaffold140388	p2	(AT)6	Class II	938	949	TGCTCGGCACGGCTACAGATTT	ACGGGCCATGACATCCGAGTTT	218
FMgSSR-15704	scaffold14470	p2	(AT)6	Class II	248	259	TGCTGAAGCCAGTGAGTGCT	GGTTTATGACGTCCGTGCAACT	237
FMgSSR-15705	scaffold21684	p2	(AT)6	Class II	1029	1040	TGCTGACTTACATGCCCAATCG	AGTCGTGGGTTCATGTTGCTGT	307
FMgSSR-15706	scaffold8539	p2	(AT)6	Class II	12193	12204	TGCTGAGATGATGGCTACCTGT	TCAGATCGCTCTGGCAGGAA	329
FMgSSR-15715	scaffold4228	p2	(AT)6	Class II	43866	43877	TGCTGTTTCGTGCGAATGCGA	CCTCTCTCAAGCAGGGCATTGT	350
FMgSSR-15717	scaffold327	p2	(AT)6	Class II	136978	136989	TGCTTGTGCAACAGACAGCCA	TGCTCGCGATTCGTGATCGT	302
FMgSSR-15721	scaffold1191	p2	(AT)6	Class II	61493	61504	TGGAAGAACAACGGGCATGCAG	AGTTTCCGACCTGCCGGATT	323
FMgSSR-15732	scaffold5569	p2	(AT)6	Class II	15730	15741	TGGAGCCTCAAGGAGCATGTGT	ACTCTCACAAGGATCCCAGTTTGC	229
FMgSSR-15733	scaffold8945	p2	(AT)6	Class II	18772	18783	TGGAGGAAAAGGACGATGGCGA	ACAAAGCAAGCATGCCATGGGG	265

SSR_ID	Scaffold	SSR_Type	SSR_Motif	SSR Class	SSR_Start	SSR_End	Forward sequence	Reverse sequence	Product_size
FMgSSR-15736	scaffold4357	p2	(AT)6	Class II	14580	14591	TGGATCAGGCAGTTGTGCTGGA	GGTGATCCATAACCGTAGCAAACCC	333
FMgSSR-15750	scaffold493	p2	(AT)6	Class II	101912	101923	TGGCCACTCTCCAGGTGCATTT	TGTGTCTCGTGGGAGAAGAAAGAG	347
FMgSSR-15754	scaffold794	p2	(AT)6	Class II	17449	17460	TGGCGCATGGCAAACTTGGT	TGTGCATGCTGGAACACAAGGGA	276
FMgSSR-15763	scaffold6849	p2	(AT)6	Class II	2036	2047	TGGCTTATACCCACACCGTGA	GCTCTCTGTCGGACTGCTGATGAA	350
FMgSSR-15767	scaffold5109	p2	(AT)6	Class II	43267	43278	TGGGCACGTTCTTCCCACGATT	ACAAACTTCGACCCCGAGACCT	332
FMgSSR-15768	scaffold7346	p2	(AT)6	Class II	5090	5101	TGGGCAGCGCCTTCAAGGTAAT	ACCAACGGCTGGATTATGGGCT	305
FMgSSR-15769	scaffold52420	p2	(AT)6	Class II	1410	1421	TGGGCGACGAGGAGCACATATT	AGCGAGTGGTCGAAGTGCAA	348
FMgSSR-15774	scaffold161	p2	(AT)6	Class II	18580	18591	TGGGGATGACACTGCAGAATGGA	ATGTGGCGCCGTAGGTAGTACA	326
FMgSSR-15779	scaffold1030	p2	(AT)6	Class II	68350	68361	TGGGGTTCGATGTTTGTGCACTTC	AGGCTGCCACATAGGTGAAACCA	250
FMgSSR-15780	scaffold178	p2	(AT)6	Class II	53844	53855	TGGGGTTGTAACCTGCTTCGGA	ACATGGACACCTCACGCACACA	327
FMgSSR-15785	scaffold790	p2	(AT)6	Class II	94335	94346	TGGTCGACCTTACCAATCAGCCT	CGGATGACCGGCCATTTGTTCT	208
FMgSSR-15786	scaffold788	p2	(AT)6	Class II	81329	81340	TGGTCGTTTGTTCCAGCCCA	ACAGTGCTTCCATGTGGCGA	267
FMgSSR-15798	scaffold43420	p2	(AT)6	Class II	289	300	TGTACATTGGCACCCTAGTCCTGC	TCAGGCTAGAGTTGGTGCTGCAT	264
FMgSSR-15799	scaffold1849	p2	(AT)6	Class II	16906	16917	TGTACGCATGGGCCTGTTCAGT	TTTTGCTTTCCTCTTCGCCCGC	331
FMgSSR-15810	scaffold2893	p2	(AT)6	Class II	26065	26076	TGTCCAGGTTCTGCCACTCCTT	TGGCCTCTGAGAAAATCTGCCACA	265
FMgSSR-15812	scaffold449	p2	(AT)6	Class II	89097	89108	TGTCGCCCTTTCTTCGTGGCTT	AAAGGCATGCACGTCTGCGA	302
FMgSSR-15814	scaffold7353	p2	(AT)6	Class II	18366	18377	TGTCGTTGGGTTGCCATGGAGT	TATGGCTTGCCCGCGTCATT	215
FMgSSR-15816	scaffold7	p2	(AT)6	Class II	42649	42660	TGTGATCTGGAACCCAAACTACCC	GCCGGTGCTGATTGTCTAAGAACT	348
FMgSSR-15819	scaffold929	p2	(AT)6	Class II	99054	99065	TGTGCGGGCATCCAATGCAA	TGTTCGTACTGTGGCTTAGGCG	282
FMgSSR-15823	scaffold1461	p2	(AT)6	Class II	18323	18334	TGTGCTTGAAACGAGAGCACCG	CGTGCGTTGCTACGGACAAACT	316
FMgSSR-15825	scaffold8199	p2	(AT)6	Class II	9269	9280	TGTGGAGGGGGCGGTATTTGTT	ACCTTATGAGAAACGGGGCTTCCA	316
FMgSSR-15833	scaffold161	p2	(AT)6	Class II	153792	153803	TGTTCCTGTCATGCTCGGACAGT	GCCCTGGTGCAAAATTGCCAAA	264
FMgSSR-15834	scaffold224	p2	(AT)6	Class II	124162	124173	TGTTCTGTAACCTGGAGCTGGCA	AGCAGGCTGGTTTGTCCCTT	311
FMgSSR-15837	scaffold11369	p2	(AT)6	Class II	7103	7114	TGTTGCTCTGCTGGTCGGACTT	TCCACCAAGAGAAGCAAGGGGA	219
FMgSSR-15842	scaffold7357	p2	(AT)6	Class II	1928	1939	TGTTGTGTGGGTGCGTGATGGA	TGGTGCATGCAAGGTGTGCT	350
FMgSSR-15857	scaffold1191	p2	(AT)6	Class II	69747	69758	TTCAGCAAAGGGGGTGTCGCTT	AAGGTGCAGGGTGCATCTGGAA	269
FMgSSR-15860	scaffold1179	p2	(AT)6	Class II	49302	49313	TTCAGGTTCCTGGATGTTGGCTCG	ACGAGGTGTTCGTCACCTCCAA	208
FMgSSR-15862	scaffold2287	p2	(AT)6	Class II	49248	49259	TTCCATGCCATCCAACGCCA	TGCTTGTCTCCCGTTGTACGTCT	259

SSR_ID	Scaffold	SSR_Type	SSR_Motif	SSR Class	SSR_Start	SSR_End	Forward sequence	Reverse sequence	Product_size
FMgSSR-15867	scaffold3486	p2	(AT)6	Class II	25244	25255	TTCCTCTGTTGCGTCGCCATGT	TCGGCGCAGTTGTTCACCAAA	217
FMgSSR-15868	scaffold1591	p2	(AT)6	Class II	47817	47828	TTCCTGTTTGGATGCGGGCCTT	TTGGTCAGGAACGCGAAGACCA	309
FMgSSR-15872	scaffold4317	p2	(AT)6	Class II	38484	38495	TTCGACTCAATGGCCATACGCC	ACTGGGATGCACCTGTGTGA	202
FMgSSR-15873	scaffold1010	p2	(AT)6	Class II	14510	14521	TTCGCGGGGGACGATTGGAAAA	GGTGGCCCGTTGCACATTTCTT	299
FMgSSR-15875	scaffold5715	p2	(AT)6	Class II	7149	7160	TTCGGCGTGACCTGACTCAACA	ACTTGCACTGATGGGGTTGCT	265
FMgSSR-15878	scaffold11962	p2	(AT)6	Class II	3909	3920	TTCTTCCCAACCGCGCCCTTTA	ATCGCAGAGATCCATGGCCACA	316
FMgSSR-15879	scaffold9435	p2	(AT)6	Class II	26450	26461	TTGAACCCTCATGGGGCCGAAA	AATCGGTTGCAAGTTGCTCGGC	340
FMgSSR-15880	scaffold15722	p2	(AT)6	Class II	7754	7765	TTGAATCGCTGCCGAACTGTGG	ACGTGCTTTCTATCGACCCCATGC	297
FMgSSR-15884	scaffold1090	p2	(AT)6	Class II	16309	16320	TTGCACACTGGCAACCAGGCTA	TACCCTGCGCGTCTTCTTCT	297
FMgSSR-15887	scaffold4075	p2	(AT)6	Class II	28366	28377	TTGCCCTGCTTGCCCAAGGTAA	AGCGATGCGGAAGGATACCACA	346
FMgSSR-15888	scaffold30722	p2	(AT)6	Class II	5871	5882	TTGCGGGGATTGGCAGCATT	TGGACCGTCCGCGTGTTTTT	272
FMgSSR-15890	scaffold93896	p2	(AT)6	Class II	741	752	TTGCGGGTTCCAGCACACTT	ACCGTTGCCCCCAAACCATT	289
FMgSSR-15891	scaffold1908	p2	(AT)6	Class II	28247	28258	TTGCGTGGGAGACGCACAAA	TTGAGGCCTTGGAGGGTGCAAT	266
FMgSSR-15893	scaffold1067	p2	(AT)6	Class II	56952	56963	TTGCTCGGTGGCAGACAGAA	TACCCAATGATCTGGAGCCTCCGT	258
FMgSSR-15895	scaffold272	p2	(AT)6	Class II	127491	127502	TTGCTTCAGGTCAGGCACTGGT	AACTAGCGCCTGGGTTGTGTGT	294
FMgSSR-15896	scaffold278	p2	(AT)6	Class II	70102	70113	TTGCTTTGACGAAGGCGACCCA	AAGCAGCGCATAAGCTCCCTGA	237
FMgSSR-15897	scaffold17729	p2	(AT)6	Class II	4374	4385	TTGGACCCCCGAAGTACCTTGT	TTGCTGCCAATAGCCTATCCTCCG	300
FMgSSR-15898	scaffold7972	p2	(AT)6	Class II	11149	11160	TTGGATGCCTTCGCACCTGT	AGAAATCCTACGCTGGGCGTGA	298
FMgSSR-15900	scaffold2382	p2	(AT)6	Class II	14614	14625	TTGGCATGTTCTGCAGGGGT	TGCTTGACCGTTTCTGGGTTGGT	293
FMgSSR-15902	scaffold1492	p2	(AT)6	Class II	24078	24089	TTGGCCCTGTTCGGAAAGATTC	CACAGCCATTGCTCGTGCATCA	268
FMgSSR-15903	scaffold2433	p2	(AT)6	Class II	14940	14951	TTGGCGATTGGTGACCTCCTCT	TGTGCCGTTCTCCATTTGCCT	350
FMgSSR-15904	scaffold396	p2	(AT)6	Class II	12789	12800	TTGGCGCATCGCAAATTCGT	ACCGTTGGCGAAAGCTTCTT	289
FMgSSR-15908	scaffold832	p2	(AT)6	Class II	37589	37600	TTGGTGGAGACAACGCCGGATT	GGAGTACAACGATGGGAACACTGA	267
FMgSSR-15909	scaffold6616	p2	(AT)6	Class II	9657	9668	TTGTCAAGCCCGTGCACCAT	TCAGTTGGAGCTGTGTCTCCGT	288
FMgSSR-15910	scaffold8796	p2	(AT)6	Class II	5681	5692	TTGTCCGCGACCTGCTTGTT	AGGTTCCGGCCCAACATTTCCT	295
FMgSSR-15914	scaffold5001	p2	(AT)6	Class II	15065	15076	TTGTGTGCCAAACTGAGCCTCG	TGGCTCCTCGTTTCACGAGAT	306
FMgSSR-15919	scaffold2648	p2	(AT)6	Class II	22720	22731	TTGTTGCTGGCTGCGGAGGATA	AAATCGAGCACGGATGCGCT	286
FMgSSR-15922	scaffold959	p2	(AT)6	Class II	77762	77773	TTTCCCCTCGCTGAAGCACGAA	TGGCCGTTGCCTTGTCAGTT	208

SSR_ID	Scaffold	SSR_Type	SSR_Motif	SSR Class	SSR_Start	SSR_End	Forward sequence	Reverse sequence	Product_size
FMgSSR-15926	scaffold1296	p2	(AT)6	Class II	50145	50156	TTTGCAGGCAATCAGCAGACGC	GTGCGTAGACGAGGTAGATCAGGT	297
FMgSSR-15930	scaffold2352	p2	(AT)6	Class II	24168	24179	TTTGGGTGGCGCACTGAACT	GGTACCAAGTACCATCCAGGTGT	237
FMgSSR-15936	scaffold840	p2	(AT)6	Class II	50956	50967	TTTTCCCCGCGCAGCGTTTT	TTCTGTTCGCTGTGCCGCAT	283
FMgSSR-15937	scaffold29899	p2	(AT)6	Class II	2189	2200	TTTTGATAGGGGGCAAGGGGCA	AATCCATTGGGCCACGGAGTCA	200
FMgSSR-15940	scaffold6009	p2	(AT)7	Class II	8159	8172	AAAACGGAGCCCTTTTCCCTGC	AACTTGGCAACCAGTCTGGCCT	333
FMgSSR-15944	scaffold1255	p2	(AT)7	Class II	52241	52254	AAAGCAACGGGACACGCAGA	ACCAGTACAATGCACCGAGCGA	338
FMgSSR-15947	scaffold1971	p2	(AT)7	Class II	5597	5610	AAATGGTCAGGTCAGCCCGTCT	TGGTCCGAACACTACTTTCTGCCG	312
FMgSSR-15948	scaffold42293	p2	(AT)7	Class II	2724	2737	AAATTGCTGCTGTGCGAACCCG	TGCATGTGCAGTTGGTGTGGA	263
FMgSSR-15950	scaffold2502	p2	(AT)7	Class II	12307	12320	AACAACGGCCATGAGCACGA	AGCATGTAACGACTCGGCGGTT	276
FMgSSR-15951	scaffold4314	p2	(AT)7	Class II	20004	20017	AACCCTGGGTGTGGTTTAGCGT	AATCAAAGGGATGCCTGCGGGT	311
FMgSSR-15954	scaffold4036	p2	(AT)7	Class II	4755	4768	AACCTCAAGCCTGCCTTTGCGT	TGTTGGGGCACGACTGCTTT	347
FMgSSR-15959	scaffold2034	p2	(AT)7	Class II	53884	53897	AAGCCTGACCAACACTGTATGC	CCACATGTAATGAGACCCGAAAGG	322
FMgSSR-15963	scaffold49875	p2	(AT)7	Class II	1745	1758	AAGCTCGGCATCGGCATGAA	ATGCCACAGTTGAGCGGCTA	254
FMgSSR-15964	scaffold2396	p2	(AT)7	Class II	41697	41710	AATAATGCACCGCGGACGCT	GCCAGGCATACCTGAGGGCTATTT	320
FMgSSR-15968	scaffold567	p2	(AT)7	Class II	3690	3703	ACAACCACAAGCAAGGGCCA	TTTTAGTGCGTCCGGCCGGTTA	339
FMgSSR-15973	scaffold647	p2	(AT)7	Class II	78623	78636	ACACAAGCATGCCGCCTTCA	AGCATTGCCAGCCGGTGTAA	281
FMgSSR-15974	scaffold4283	p2	(AT)7	Class II	20285	20298	ACACACGTAGGCAGGCTCAACA	TGTTCCGGCGCATCTAGACTCA	341
FMgSSR-15980	scaffold767	p2	(AT)7	Class II	61768	61781	ACACGGTGCTGCAACCAAGA	ACCCCAGGCGAAGAAGCAAA	288
FMgSSR-15981	scaffold2261	p2	(AT)7	Class II	52716	52729	ACAGACACTCACTCACACGCA	ACATCACGTTGTTCTCGGCCA	259
FMgSSR-15983	scaffold14493	p2	(AT)7	Class II	21886	21899	ACAGATGCGGTGCAATGGTTCA	ACTGAGCACGCACGCAACAA	295
FMgSSR-15986	scaffold9275	p2	(AT)7	Class II	19707	19720	ACATCGTCTTGGGGCGCAGATT	CGCCTTTGGCCCGACAACAAAA	331
FMgSSR-15991	scaffold820	p2	(AT)7	Class II	80366	80379	ACCAAACGTTACTTGGGGGTGT	ACGCCGCACTTGTTGTGTTGT	336
FMgSSR-15992	scaffold173161	p2	(AT)7	Class II	296	309	ACCAACCCCGGCTAAAATTCCA	TGTAACCCCACCTGGCCTTTCT	214
FMgSSR-15994	scaffold5254	p2	(AT)7	Class II	4264	4277	ACCAAGGATCGTGCTATGGCCT	ATGCCAAACCAGACCACCGGAT	301
FMgSSR-15995	scaffold278	p2	(AT)7	Class II	121374	121387	ACCAATAAGCCACAGGGCTGCAA	TGCACAGTTAGCTCTCTGTTCCACG	291
FMgSSR-16003	scaffold7485	p2	(AT)7	Class II	3741	3754	ACCCAAAGTTGTTGCGCGAT	GCGGTGGCAATCAAACGTAATC	300
FMgSSR-16009	scaffold1201	p2	(AT)7	Class II	80966	80979	ACCCTGCACCGAAGAAGTGCAT	TGAGCCATGCTTCAGCACCTGT	239
FMgSSR-16011	scaffold7348	p2	(AT)7	Class II	36387	36400	ACCGATTCCAGCTCCTGCTACA	GTCGTCAAGCAGGGGAAAAAGA	250

SSR_ID	Scaffold	SSR_Type	SSR_Motif	SSR Class	SSR_Start	SSR_End	Forward sequence	Reverse sequence	Product_size
FMgSSR-16013	scaffold11250	p2	(AT)7	Class II	7492	7505	ACCGTCTGGATGCTGGATGTCA	TCGAATTCACCAACTGCGAGACCT	343
FMgSSR-16020	scaffold1812	p2	(AT)7	Class II	43353	43366	ACCTTGTCTTGCAAAGGACCTTCC	TAGCCGTTGCATAAGGTGACCG	287
FMgSSR-16021	scaffold16591	p2	(AT)7	Class II	3123	3136	ACGACATAAACCCGGTAGCGCA	ACACACGTCAACTGTCATGCGG	338
FMgSSR-16023	scaffold621	p2	(AT)7	Class II	67304	67317	ACGAGCTCAGTTGGTTGGGCTA	TGCGCCAATTAATGGCTGAGCA	252
FMgSSR-16024	scaffold224	p2	(AT)7	Class II	105813	105826	ACGATGCTGCTTCCGACGACAA	TGTGGCCACCAAAGTCGTGACA	319
FMgSSR-16026	scaffold21746	p2	(AT)7	Class II	6529	6542	ACGCAGCAAACTGCTCATCCA	ACAACGACGCTCCTTGCTCACA	238
FMgSSR-16027	scaffold4344	p2	(AT)7	Class II	8461	8474	ACGCATGCATGTTCTCACGC	GCTTTTGCATGCCTTTGCTGCC	326
FMgSSR-16031	scaffold2156	p2	(AT)7	Class II	19467	19480	ACGGAGCTTGACTTTTGCTCGC	ATGTTGAGCCCGGTGAAGTCGT	285
FMgSSR-16034	scaffold107057	p2	(AT)7	Class II	275	288	ACGGCAAACACTCGTGCTGA	ATATGACCCCAGGGACTGCGTT	346
FMgSSR-16039	scaffold662	p2	(AT)7	Class II	72559	72572	ACGTTGGCTCAGTCATGGCA	CCCATGTTGTGCGAGCGGTTTT	319
FMgSSR-16041	scaffold9777	p2	(AT)7	Class II	9681	9694	ACTCCTTCCGTTCGTACCCACT	TGGATCTCAGCAGCAAGGGACA	327
FMgSSR-16042	scaffold5004	p2	(AT)7	Class II	47692	47705	ACTCGGCAGGTCAAATTGGCT	ACTGGTGAGCTCGCGAGAAGTA	270
FMgSSR-16043	scaffold23905	p2	(AT)7	Class II	6880	6893	ACTCGGCGGCCAGAAGTAAACA	ATCCTCCCTTCCGTGGTGCATA	284
FMgSSR-16046	scaffold5761	p2	(AT)7	Class II	29151	29164	ACTGCAAGCCCCGGCATTTT	ACCAACCGACCACGCTCTGAAT	287
FMgSSR-16051	scaffold2380	p2	(AT)7	Class II	27241	27254	AGAACATAGCTCCGCCAGTGGT	TCCTCGAGCTTTATGCAGCCT	305
FMgSSR-16053	scaffold13779	p2	(AT)7	Class II	5843	5856	AGACACACCGTGCATGCAAAC	ATAACTGCTGTGCTGCCCGA	347
FMgSSR-16054	scaffold1466	p2	(AT)7	Class II	87125	87138	AGACAGCACACCAAGCTCCCAA	TGTCATGTCACGCGCCTAGT	302
FMgSSR-16055	scaffold1612	p2	(AT)7	Class II	53505	53518	AGAGAGGCTGCAGAATGACCGT	TTTGCCACCTTGGTTCCTGTCG	209
FMgSSR-16062	scaffold14030	p2	(AT)7	Class II	2722	2735	AGCAGATTTTGCTGCGCGTT	TGGACTCGCATGCCCAACTA	300
FMgSSR-16063	scaffold2388	p2	(AT)7	Class II	28491	28504	AGCAGCGCGCATGGAAGAAT	ACGGCCCTCTTCCAGCCTTTTT	315
FMgSSR-16065	scaffold49849	p2	(AT)7	Class II	3211	3224	AGCATGCGTTTGGTTGAGTGGA	ACTGCTTCAGCTATGTCTTCTGCC	322
FMgSSR-16066	scaffold11304	p2	(AT)7	Class II	17059	17072	AGCATGTCGGCAGATTCGCA	TCCATCCAGTGGTTTATGGAGACC	309
FMgSSR-16068	scaffold323	p2	(AT)7	Class II	100385	100398	AGCCAGTTGAAGTCACGCAGGA	AAGAGAACGGGCCATCGAAGCA	305
FMgSSR-16073	scaffold2012	p2	(AT)7	Class II	34633	34646	AGCCTGGCAAGCATTTCACGGA	TCTTCACGGACCAGCACGTT	346
FMgSSR-16075	scaffold9132	p2	(AT)7	Class II	8208	8221	AGCGAGCAGGTTGTGGATTGTCA	CGTACGTTCGTAGGGACGCATT	331
FMgSSR-16077	scaffold10446	p2	(AT)7	Class II	6326	6339	AGCGGAGAAGGGCAGGAAACAA	TGCCGCTTGCGCTGATCATT	252
FMgSSR-16078	scaffold8895	p2	(AT)7	Class II	23176	23189	AGCTAAAGATCCACCGGGTGCT	AATGCAATGGCCCCCATGCT	350
FMgSSR-16080	scaffold23957	p2	(AT)7	Class II	4748	4761	AGCTCTGTCGGGAACGGATGAT	GCGTTCTCGATCGATGTGCCAA	269

SSR_ID	Scaffold	SSR_Type	SSR_Motif	SSR Class	SSR_Start	SSR_End	Forward sequence	Reverse sequence	Product_size
FMgSSR-16081	scaffold19776	p2	(AT)7	Class II	2500	2513	AGCTGCACGCCTTCCATGTT	TTGGCGCCCATTAGAACGCA	324
FMgSSR-16085	scaffold6125	p2	(AT)7	Class II	31954	31967	AGGAAATGATGCGCGGTAGCCT	TCGCCACAGAACGTTCATCCCT	269
FMgSSR-16089	scaffold94690	p2	(AT)7	Class II	1041	1054	AGGCAACGCGTCACGTTTGT	AGTTCCGATCCCCATGCAGT	299
FMgSSR-16094	scaffold31843	p2	(AT)7	Class II	691	704	AGGCTTCCGGCAACAACAAGGA	ACAGGGACCGATCGGACTGAAA	340
FMgSSR-16096	scaffold1968	p2	(AT)7	Class II	53825	53838	AGGGACAGTAGCCAATACCACCA	CCGTACTTTGCTGCCAGCCATT	328
FMgSSR-16099	scaffold1346	p2	(AT)7	Class II	56856	56869	AGGGCATGGGTCAGGTGAAA	AGGGTTGGAGGGCAAGAACT	331
FMgSSR-16101	scaffold437	p2	(AT)7	Class II	46842	46855	AGGGCGTAAGTGGCACTGTCAT	CGGGCAAGCAATTCAAGGCACA	334
FMgSSR-16102	scaffold4446	p2	(AT)7	Class II	27082	27095	AGGGTGCGTGTCGCACGATTAT	ACTACCGGTCTGCCTGGAACAT	290
FMgSSR-16103	scaffold18348	p2	(AT)7	Class II	1634	1647	AGGTGAGCGCGTAGGTTGTGTT	ACCGCCCAAGGGACATGATT	313
FMgSSR-16107	scaffold1249	p2	(AT)7	Class II	17437	17450	AGTCCTACTGAAGCTGAGAGCCGA	ACTTGATGCGCAAACTCGCT	266
FMgSSR-16115	scaffold178	p2	(AT)7	Class II	61005	61018	ATCGGCAGGAGGGCAGAAACTT	AAAGAGACACGGCGACAAGCCT	328
FMgSSR-16118	scaffold327	p2	(AT)7	Class II	139729	139742	ATCTCTTCCGCGTGCTTCCGAT	TGTCATGCTAGTAACGTGGCGG	321
FMgSSR-16120	scaffold607	p2	(AT)7	Class II	104184	104197	ATGATGCGGTGGTTACGTGGGT	AGGCCAGGCCAGAGTTCACAAA	239
FMgSSR-16122	scaffold7029	p2	(AT)7	Class II	26418	26431	ATGCCACATGCCCCGTGAAA	TGGGGACGGCCATCATGTTA	265
FMgSSR-16124	scaffold231173	p2	(AT)7	Class II	50	63	ATGCGGCCAAGGCTTGAACT	TGCCAACGTAGCATGCGTGA	313
FMgSSR-16127	scaffold1071	p2	(AT)7	Class II	65169	65182	ATGTCAATGCTAGTGGCAAGGCTG	TTCAATGAACAGAGGACCCAAGGC	200
FMgSSR-16128	scaffold216958	p2	(AT)7	Class II	145	158	ATGTTTGGGTTGGGTCCGGT	CGCTCGAGTGCTTTTATGAGGT	342
FMgSSR-16129	scaffold645	p2	(AT)7	Class II	16268	16281	ATTCAAAGCGCATGACGCCG	TGTGCGATGCCACGAAATGC	235
FMgSSR-16130	scaffold831	p2	(AT)7	Class II	755	768	ATTCATCACGCTGCGCTCCA	CCGTCTCAATATTTATCGGCCCAC	276
FMgSSR-16134	scaffold3787	p2	(AT)7	Class II	34628	34641	ATTGTTGCTGCCTTGCGACG	TGCCCATTGATATCCCCTCCGT	329
FMgSSR-16138	scaffold1195	p2	(AT)7	Class II	27923	27936	CAGCTGTGAACAAACGGGCGTA	AACGACCAGGCATCACCACT	348
FMgSSR-16140	scaffold8326	p2	(AT)7	Class II	15857	15870	CAGTGTCACAAACTGGACACGCT	TGATGCCTTCGACCCCCAAAGT	286
FMgSSR-16144	scaffold1287	p2	(AT)7	Class II	14266	14279	CCGCATATGCCACTATCTTCAGTG	TTGCTTGACCACTGTCCCCA	342
FMgSSR-16145	scaffold51011	p2	(AT)7	Class II	3402	3415	CCGGCGCCTTGCGGAATAATAA	AACCGACACGATGGCAGTGGAA	246
FMgSSR-16159	scaffold1727	p2	(AT)7	Class II	15005	15018	CTCATGTCCACAAGTGAGCACTAC	ACATCCTCTGCAACGTCCTCT	321
FMgSSR-16160	scaffold6192	p2	(AT)7	Class II	26675	26688	CTGCAGGTACCCTTTCCTCTTTTC	AGCAAGGAATCACCAAACACCACC	318
FMgSSR-16161	scaffold673	p2	(AT)7	Class II	65751	65764	CTTGTCCAGGGGCTCAAAAACA	TCCACGACAAAGTCTACACCACG	259
FMgSSR-16162	scaffold3083	p2	(AT)7	Class II	27365	27378	GAGTTGCATACGTCACCACCTT	AGCCTCTAGTGTGTCTTCCTCGCT	283

SSR_ID	Scaffold	SSR_Type	SSR_Motif	SSR Class	SSR_Start	SSR_End	Forward sequence	Reverse sequence	Product_size
FMgSSR-16165	scaffold1463	p2	(AT)7	Class II	81321	81334	GCAACAAAAACGCCAGAGGCGA	TTGTCGAGGCCCACACATTCCA	283
FMgSSR-16173	scaffold6072	p2	(AT)7	Class II	20905	20918	GCATTGGCTCTGCCCTCAACAA	TCTGATGGTCGGCTTGTCTTACG	338
FMgSSR-16177	scaffold125	p2	(AT)7	Class II	65138	65151	GCCGGCGTCTGACAAGATGATA	AATTGCGCTTCCAAGCATGCCC	345
FMgSSR-16178	scaffold164	p2	(AT)7	Class II	166048	166061	GCCGTCGTGAAGCCTACATCAA	AGCTGACAGATCCAGGTTGCCA	332
FMgSSR-16182	scaffold11713	p2	(AT)7	Class II	3757	3770	GCGCGCACACACATACACAA	TCGCGTGCAAGGAGATGATGA	273
FMgSSR-16185	scaffold3295	p2	(AT)7	Class II	17330	17343	GGACTATGACTCGTATAGCCCAACG	AACGTTGATGCTCACGTCCAGG	347
FMgSSR-16186	scaffold339	p2	(AT)7	Class II	41039	41052	GGCACGTGAGTAATTTTACGGCTG	AGGAAGAGCAGCGGTTGACAGT	328
FMgSSR-16187	scaffold1562	p2	(AT)7	Class II	60070	60083	GGCAGCCAACTTCATTTCGGGT	ACGCGCTTGTCAAGGCTGGTAA	349
FMgSSR-16188	scaffold208	p2	(AT)7	Class II	72796	72809	GGCCCGTCAAACAAACCCGTTA	GCGGCGCAAAACCCTAACTGAA	327
FMgSSR-16191	scaffold383522	p2	(AT)7	Class II	206	219	GGCTTGGCGCTGCTTTCTAT	TTGAATTCACTAGGGTCCTCCCCC	206
FMgSSR-16196	scaffold1982	p2	(AT)7	Class II	27392	27405	GGTCAAGAACATGGTTGAACCGGA	TGCCACACTTACGGTGCCAT	272
FMgSSR-16197	scaffold1082	p2	(AT)7	Class II	69954	69967	GGTGAATAGAATGCCCAGTGGTAGC	TGGCTAGCAACCAAAAGCACCCT	299
FMgSSR-16198	scaffold11932	p2	(AT)7	Class II	7575	7588	GGTGTCACCACCGTAGTCATTG	GGCCATGTGCCAAGTCAAACCA	301
FMgSSR-16216	scaffold21307	p2	(AT)7	Class II	6217	6230	TAGCGTTCGCTATCTGCTATCCGC	GGAGGCTCCAAATAGCGTGTCT	229
FMgSSR-16220	scaffold20058	p2	(AT)7	Class II	3167	3180	TCAAACGACCGATGCGCTGA	TGGTGCTACGCGTGGCTAGAAA	308
FMgSSR-16221	scaffold5296	p2	(AT)7	Class II	20402	20415	TCAAGCAGGGCTCACGATTGGT	ACAGTGTGGCAGAAGCAGCGTA	311
FMgSSR-16222	scaffold190	p2	(AT)7	Class II	70176	70189	TCAAGCCTTGCCGAACTGTCGT	AGTGTGCCTCGGGTTCTCAGTT	350
FMgSSR-16223	scaffold67872	p2	(AT)7	Class II	117	130	TCACAGTGACATAGTATCCCCGCA	AGCCCCGCGTCACAGTGTTAAT	350
FMgSSR-16225	scaffold15066	p2	(AT)7	Class II	12673	12686	TCACGCAAACCTTCCATGTGTGG	AGCAGCAGCAACAACAGCTCA	235
FMgSSR-16229	scaffold2690	p2	(AT)7	Class II	23925	23938	TCAGGCAAAGCAACAGGCACA	AGGCCAAGTAGATGCGGTCGAT	283
FMgSSR-16232	scaffold1359	p2	(AT)7	Class II	47628	47641	TCATGCGCGATCATCAACCCCA	GGGGCATTCAGCAACAAGCCAA	333
FMgSSR-16234	scaffold370	p2	(AT)7	Class II	117285	117298	TCATGTGCATGAGCACGGGT	TGCTTGGTTGGTACTGTGTGCC	227
FMgSSR-16235	scaffold17410	p2	(AT)7	Class II	6175	6188	TCATGTTGTTGCTGCCCCGT	TCCACCCGAAGGTAAAAACACG	336
FMgSSR-16237	scaffold831	p2	(AT)7	Class II	57577	57590	TCCAGCCGGAGCCACTATCAAT	TGTGAACGCCCTTAGTGCTGGA	333
FMgSSR-16246	scaffold5647	p2	(AT)7	Class II	13817	13830	TCCGTTGCGCCAGCTACTTCAA	TCGTTGTGGGTGCTTTCCTGTGA	346
FMgSSR-16254	scaffold18405	p2	(AT)7	Class II	7623	7636	TCGCAGCGACCAAGCATTTCT	ATGGTGCGAAGATGGCCGAA	285
FMgSSR-16261	scaffold23175	p2	(AT)7	Class II	9570	9583	TCGGGTAGCAGTGGAGCTGTAT	ACGCTCCAAAGGATGGACACCA	297
FMgSSR-16266	scaffold1005	p2	(AT)7	Class II	86947	86960	TCGTTGCTGCAGACGGTTACCT	AGCCAGCCGCGGTCAATAAA	236

SSR_ID	Scaffold	SSR_Type	SSR_Motif	SSR Class	SSR_Start	SSR_End	Forward sequence	Reverse sequence	Product_size
FMgSSR-16271	scaffold7893	p2	(AT)7	Class II	13084	13097	TCTCGCGTCTCTAGCGGACAAA	AGATGCATGGGAAATGGGAGGC	313
FMgSSR-16279	scaffold7846	p2	(AT)7	Class II	22414	22427	TGAATTGTGCGAGGGCTGCT	ACGCAAGTGATAAAGCCGGGGT	257
FMgSSR-16280	scaffold332	p2	(AT)7	Class II	134741	134754	TGACGCGTTTCTTTTGCGGGA	TGAAGGGGCGCGTCAGTTTT	261
FMgSSR-16283	scaffold241228	p2	(AT)7	Class II	258	271	TGAGCACCAGCAGCTTCGTT	TTGGCAGCTCGAGGCTGTTT	299
FMgSSR-16287	scaffold2745	p2	(AT)7	Class II	27853	27866	TGCAAGTTCGTCCTGGCACTCA	AATGGAGACGACGCTGAACCGA	342
FMgSSR-16293	scaffold385	p2	(AT)7	Class II	108154	108167	TGCACGCACTCAACCACAAACA	AGCTGCGGTGTCAGCATCAT	261
FMgSSR-16294	scaffold246	p2	(AT)7	Class II	92692	92705	TGCAGCCAGTTCTGTCTCTGGA	TCAGGCCTGGACTTCTGTCGTT	224
FMgSSR-16301	scaffold8493	p2	(AT)7	Class II	1747	1760	TGCGAGATGTCAAGATGTACCGGG	TCAGCTGGTACTTCCGGGTCAA	323
FMgSSR-16302	scaffold288	p2	(AT)7	Class II	97163	97176	TGCGCGGCAATTGTAGGTTT	TTTGCAAGTTGCGCGAGACG	309
FMgSSR-16310	scaffold11470	p2	(AT)7	Class II	13338	13351	TGCTAGTCACGGGAGTCCTCGATT	AAGGTGCTCCTCTGCCCTCCAATA	346
FMgSSR-16311	scaffold1719	p2	(AT)7	Class II	130901	130914	TGCTCATGAGGGCAAAAGCCA	TGGATGATCCTCGAAGCCTCTCA	329
FMgSSR-16313	scaffold8145	p2	(AT)7	Class II	31881	31894	TGCTCGAGGCCATGTTCTTCGT	TGCACGCCCACCTCCAAAAA	271
FMgSSR-16314	scaffold2010	p2	(AT)7	Class II	39645	39658	TGCTGACCTTGTTTCCCAGGGT	GGACAGGATGACCTGTTTGTGTCCA	347
FMgSSR-16315	scaffold2315	p2	(AT)7	Class II	43415	43428	TGCTTCCATGCTGCTTGTTACGC	ATTGGGAAGCACGAGGTACGGT	280
FMgSSR-16316	scaffold3192	p2	(AT)7	Class II	1696	1709	TGCTTGCGTGAGTTTAGGCTGGT	TGTGCATCACGGTGCATACGGT	271
FMgSSR-16317	scaffold2374	p2	(AT)7	Class II	36719	36732	TGCTTTGACGACGCCACTGA	TTGCTTTGCCTGCCCCCACATA	314
FMgSSR-16318	scaffold19848	p2	(AT)7	Class II	16419	16432	TGGAACACGGAAGCGTGCACTA	TCGCATTCCATGGCTCGTCT	209
FMgSSR-16321	scaffold181	p2	(AT)7	Class II	59117	59130	TGGAAGGAAAGCCAGGCCAT	TGCGCCTGCAAACAAGCAGT	289
FMgSSR-16322	scaffold3610	p2	(AT)7	Class II	46078	46091	TGGACGAGCAATCGGCAGGTTT	TGACTTTGTTGCCGACGGGT	207
FMgSSR-16325	scaffold7733	p2	(AT)7	Class II	7184	7197	TGGATCAACCGAACGAGTCCAG	AGGAGTACAAACTGGTGGGAGCA	349
FMgSSR-16331	scaffold2290	p2	(AT)7	Class II	21909	21922	TGGCTAACCACTTGACGTGCCA	ACGGGCGATGCTGCTTTCTT	306
FMgSSR-16334	scaffold1640	p2	(AT)7	Class II	20190	20203	TGGCTGGATGGCTTGTTTGTGC	TGGTGGTTGTTTCGTTGCCA	254
FMgSSR-16340	scaffold10544	p2	(AT)7	Class II	4016	4029	TGGGCCGCGAATCCATTTTGA	TGCCCCCTCTGCCGTTAGGAATTA	319
FMgSSR-16341	scaffold11575	p2	(AT)7	Class II	22148	22161	TGGGGCAAGGCCCTACAGATTA	CGTGACATGCGACTTGCTGCTT	330
FMgSSR-16345	scaffold500	p2	(AT)7	Class II	23326	23339	TGGTCAATGCATGCCGTACAATGC	TGCATGGGCCTTCTACCTGTGA	302
FMgSSR-16352	scaffold21945	p2	(AT)7	Class II	5870	5883	TGGTTTGGTTTGGCTGGGGTGT	AAGAGGACGCAAGGCGAGATGT	327
FMgSSR-16359	scaffold12161	p2	(AT)7	Class II	20216	20229	TGTGCCTGGTGCTGGACATCTT	TTCCAGAACCCAAGAGCCCACT	301
FMgSSR-16362	scaffold9883	p2	(AT)7	Class II	10011	10024	TGTGGACTAAAGACCAACGCCTGG	ACGTGCCGCCAAAAAGCTTCA	283

SSR_ID	Scaffold	SSR_Type	SSR_Motif	SSR Class	SSR_Start	SSR_End	Forward sequence	Reverse sequence	Product_size
FMgSSR-16367	scaffold10418	p2	(AT)7	Class II	19875	19888	TGTTGCCTCACTTGCTTCGGA	TTCGGTGACGCTTGAAGACCCA	237
FMgSSR-16368	scaffold355	p2	(AT)7	Class II	73460	73473	TGTTGCTGGCTACATATCCGGT	TGTGCCTTGTGAATGCTGTCCCT	329
FMgSSR-16370	scaffold75617	p2	(AT)7	Class II	1903	1916	TGTTTCGCTTGGAAGCGTGGT	ATCACATTCGTGGTGCATGCGG	347
FMgSSR-16371	scaffold83707	p2	(AT)7	Class II	1255	1268	TTAAGTGCGGCAACCTGGCA	TCACTCCCTAGCCACTTTGCAT	311
FMgSSR-16375	scaffold1220	p2	(AT)7	Class II	43956	43969	TTAGCCGTTTCGTGCGGCAT	AGAGAGGGCGCAACACAGTT	208
FMgSSR-16377	scaffold30584	p2	(AT)7	Class II	3385	3398	TTCACGGGGTTTGATGTGCCGA	TGTTTGCAGTTCGTGCGCCA	268
FMgSSR-16378	scaffold1501	p2	(AT)7	Class II	31105	31118	TTCCACATGTGCCTCCATGGCT	TTGGCTCTCATGGCCGTTGTCA	295
FMgSSR-16379	scaffold910	p2	(AT)7	Class II	65406	65419	TTCCAGGCGGACGTGAATCTCT	AACGAGGCTGAAGTCAAGCGCA	235
FMgSSR-16380	scaffold8401	p2	(AT)7	Class II	18206	18219	TTCCGTTTCCGGCTACGGTGTT	AGGGCGCTCTAATCCCACGTTT	346
FMgSSR-16384	scaffold18696	p2	(AT)7	Class II	6917	6930	TTGCAAGCCCAAGACAGGCA	AATTCTATGCGCGCGACTCGGT	327
FMgSSR-16387	scaffold1101	p2	(AT)7	Class II	35999	36012	TTGCCTCGACCGCTCGGAAATA	AGCGGCGAAGAATCGCAAGA	342
FMgSSR-16394	scaffold11149	p2	(AT)7	Class II	9802	9815	TTGGTGGGCAAACGAACGGT	TCACGCCTCTCTGCACGTTT	286
FMgSSR-16398	scaffold2625	p2	(AT)7	Class II	22432	22445	TTGTGTGCAGCAGGGAATGC	TACACGCTTCAACCTGGCGT	336
FMgSSR-16401	scaffold13627	p2	(AT)7	Class II	16728	16741	TTTAGGAGGTGGGTGCAGCCTT	CGCGAATTGAGGTGAGACCCAT	276
FMgSSR-16402	scaffold6985	p2	(AT)7	Class II	27304	27317	TTTAGGCTACGAGCCCTGCCAA	ATAATCGGCGCACCCTTGTG	262
FMgSSR-16405	scaffold3234	p2	(AT)7	Class II	45793	45806	TTTGGATCCGACACCGCCAA	TTGCAGCAACTAGCGTGCCT	336
FMgSSR-16407	scaffold22162	p2	(AT)7	Class II	11269	11282	TTTTGTTTGGGGTGGAGTGGGG	TGAGCAAAAGCTGGAGCCCT	298
FMgSSR-16409	scaffold5884	p2	(AT)8	Class II	10493	10508	AAAAGTGCGAGTTCGCGGTGA	TCGTGCAAGCTCTCTAGCAAGTCG	200
FMgSSR-16410	scaffold162851	p2	(AT)8	Class II	756	771	AAACAAAGTAGAGCCCCGGCGA	TGCGAGGAGCAGCGATCATCAA	210
FMgSSR-16412	scaffold5434	p2	(AT)8	Class II	14466	14481	AAACGGAGGCTCCAGATCGT	GGGAGGTTGGTTTGCAAGTGATGC	247
FMgSSR-16415	scaffold3093	p2	(AT)8	Class II	52605	52620	AACATCAACCATGCCTCCCGCT	TGTGCATTGCCGGTCACGTT	283
FMgSSR-16416	scaffold5275	p2	(AT)8	Class II	6857	6872	AACCATCGCAAACGCGACATCC	TTGCCGATGGCCTTGCTCTCAT	272
FMgSSR-16417	scaffold14838	p2	(AT)8	Class II	3969	3984	AACGACTATTTCCAGCCCGCGT	TGCGCACGGCATTGTAACGA	302
FMgSSR-16420	scaffold8358	p2	(AT)8	Class II	31005	31020	AACTCGCGCAGGTGTTCTCCAA	AAGCTGCACACGTGCGATTGT	344
FMgSSR-16421	scaffold594	p2	(AT)8	Class II	1001	1016	AACTTGGCAACCAGTCTGGCCT	TCCCTTGCGATCCAGGTTCA	317
FMgSSR-16423	scaffold2426	p2	(AT)8	Class II	52898	52913	AAGAGCAACATGGAGCTGGCCT	TGTCAGGCCCAACCAAGCAA	281
FMgSSR-16427	scaffold7074	p2	(AT)8	Class II	26386	26401	AAGCGCCCGCGTTATCAAGCTA	CACCCCAATTCCCCAAATGTCA	324
FMgSSR-16430	scaffold67	p2	(AT)8	Class II	178886	178901	AAGCTGCATCCCACATGCCA	TGGCCACACGTCTATACCGACT	327

SSR_ID	Scaffold	SSR_Type	SSR_Motif	SSR Class	SSR_Start	SSR_End	Forward sequence	Reverse sequence	Product_size
FMgSSR-16431	scaffold1590	p2	(AT)8	Class II	39193	39208	AAGGCCTCCAGGACACAAGTGA	TGTGTTGAGTCCATTGCCAGTCG	275
FMgSSR-16433	scaffold53676	p2	(AT)8	Class II	2057	2072	AAGTGCGGCGCCAACAACAA	ATACCCAGTAGCCGTGCACCTA	316
FMgSSR-16438	scaffold2742	p2	(AT)8	Class II	21012	21027	ACAAATGTGGCTTCCCACTGCT	AGGTGAAAGGACCGCATGGA	323
FMgSSR-16439	scaffold4603	p2	(AT)8	Class II	579	594	ACAACGACGTCTTCGATCGTCT	CCATGGTTGCCCTTCTGAAATGCT	328
FMgSSR-16448	scaffold2653	p2	(AT)8	Class II	29501	29516	ACAGTACTACCCGGCCATCTGA	GTGTCTCCCACTATAGCTAGCCTGA	349
FMgSSR-16449	scaffold10043	p2	(AT)8	Class II	9151	9166	ACAGTGGCTGCAGTAGTGCTAA	AATGACCCACGCATCGCTGT	244
FMgSSR-16453	scaffold148892	p2	(AT)8	Class II	348	363	ACCAGGAATGTTCTCTGCCCA	TATGCAACCCTTCCGTTGGC	346
FMgSSR-16456	scaffold11700	p2	(AT)8	Class II	6383	6398	ACCGCCCGAAGTATTCAAGGCT	TGCCAGTTTGGGGTCAGCAGTT	311
FMgSSR-16460	scaffold7141	p2	(AT)8	Class II	2367	2382	ACCTCCAAGGAAATTGGCCTCACC	CGGGACCTGATTTTGGGTGCTT	335
FMgSSR-16461	scaffold5800	p2	(AT)8	Class II	24669	24684	ACCTGGACGGCGTGGTCATTTT	AATTCCCGTGAGCGGCAACA	200
FMgSSR-16466	scaffold2031	p2	(AT)8	Class II	56098	56113	ACGCCCATAGGTTTGCTCTGT	GGGAAGAGGGTGGGAAAAGGATGT	285
FMgSSR-16468	scaffold6554	p2	(AT)8	Class II	16948	16963	ACGCGTGTTTTGAAGCTGGC	ACAATCTCTTGGCGAGACCGCT	256
FMgSSR-16469	scaffold38636	p2	(AT)8	Class II	1310	1325	ACGCTCCGTGTTGCCAAGAA	TGCCAAGTTGGGTGTGATGCCT	300
FMgSSR-16470	scaffold877	p2	(AT)8	Class II	44931	44946	ACGGCATGTCAATAGCGCGT	ACTTTTCTCGTCGCCGGTGTCA	346
FMgSSR-16472	scaffold6362	p2	(AT)8	Class II	6029	6044	ACGGTTGCCTCTATCATGACCCT	TCAAACGACAGGCGCGCTAA	255
FMgSSR-16473	scaffold15263	p2	(AT)8	Class II	1864	1879	ACGTCGCGATCAACAGGGTCAT	ATCAGCTTCACCCACTGCCA	327
FMgSSR-16476	scaffold1438	p2	(AT)8	Class II	26242	26257	ACTCTTCCAGCAGGCTTACCAGT	CGATGCAAATTCGCAAACACACCG	268
FMgSSR-16478	scaffold544	p2	(AT)8	Class II	54448	54463	ACTGCAGCTGAGGCGCAAAA	TCAGCTGGGAAGGGAACAAACG	329
FMgSSR-16482	scaffold1371	p2	(AT)8	Class II	79381	79396	ACTGTCCTTGTGAACTGTGCCA	TGTGGAGTGTTCCATAGTCGTCGG	334
FMgSSR-16484	scaffold800	p2	(AT)8	Class II	74134	74149	AGACCACAAGAAAGGGCTCGT	TGCAGCAATGAACTGAAGCGGC	301
FMgSSR-16494	scaffold20114	p2	(AT)8	Class II	2220	2235	AGCAAGCGCCTGTGGACTTT	TTTCGCACCGGGGGAACAAGAT	305
FMgSSR-16495	scaffold560	p2	(AT)8	Class II	19773	19788	AGCAAGCTTAGTGAGCAACGGA	TGGTCTAATTCGCGGCGTGT	329
FMgSSR-16496	scaffold10122	p2	(AT)8	Class II	10491	10506	AGCAAGTCCGCCGGTGAAGAAA	CCGGCATCATCAGCACCTTGAA	326
FMgSSR-16497	scaffold24823	p2	(AT)8	Class II	3579	3594	AGCACCCAATGCAACCGTCT	TGCAACCCACATGAGAACGCA	237
FMgSSR-16504	scaffold10837	p2	(AT)8	Class II	3469	3484	AGCCAGCCGACGACTAAACA	TGTCGCGAGTCTTCTGCAGCTT	223
FMgSSR-16505	scaffold28	p2	(AT)8	Class II	77064	77079	AGCCCCATGGCAAACCATTCCA	AGCGGTAGTGCTGCTGCTTCAA	325
FMgSSR-16510	scaffold2068	p2	(AT)8	Class II	39084	39099	AGCGCAAACAACAAGCCCGT	TTCCCATTTTTGGACGCTGCCC	317
FMgSSR-16514	scaffold387	p2	(AT)8	Class II	60614	60629	AGCGTCCAACTTTTGGGGTGAC	GTCGGTCGGATGTCTGCTTTGA	246

SSR_ID	Scaffold	SSR_Type	SSR_Motif	SSR Class	SSR_Start	SSR_End	Forward sequence	Reverse sequence	Product_size
FMgSSR-16515	scaffold212258	p2	(AT)8	Class II	440	455	AGCTCAAAGCTCGATGCCGA	AGGCAGCATCAAAGAAGATGGCGA	298
FMgSSR-16516	scaffold1014	p2	(AT)8	Class II	58611	58626	AGCTCACGACCTCCAATAGCCA	AGCTGCACTCGAAGCTCAAACA	285
FMgSSR-16518	scaffold2227	p2	(AT)8	Class II	17889	17904	AGCTGCAATGCCGTTCATGTGT	AAGCTGAGCATCGCCAGCAA	315
FMgSSR-16519	scaffold4714	p2	(AT)8	Class II	4575	4590	AGCTGTGCGCATTGCATGGT	TTCATCTTGCTCGCCACGAGGT	282
FMgSSR-16523	scaffold14066	p2	(AT)8	Class II	23641	23656	AGGATCACATTTCCCTACAGGCCA	TCCTGCCTCCTCCATCATTCTCCT	200
FMgSSR-16524	scaffold16276	p2	(AT)8	Class II	15758	15773	AGGCCGACAAGTCACTCGCAAA	TGGCTAGGCTAAACAGACACCGT	321
FMgSSR-16525	scaffold847	p2	(AT)8	Class II	15475	15490	AGGCTTTGGTGTCCAACGCAGA	TATTCTACACGCGCCGCTGGAT	317
FMgSSR-16526	scaffold18748	p2	(AT)8	Class II	4918	4933	AGGGAAGGAGAGTAGGGTAGGGAA	ACCGACGAGCAGGCGATTTT	331
FMgSSR-16529	scaffold734	p2	(AT)8	Class II	9438	9453	AGTAGGGACCAAGTAGAATGCCCA	AACCACGAGTTCCAGTGTCGGT	247
FMgSSR-16532	scaffold201	p2	(AT)8	Class II	137213	137228	AGTGCATTAGGTGCGACCACA	TGCTGCTTTGGGCCTTGGTT	250
FMgSSR-16535	scaffold78938	p2	(AT)8	Class II	500	515	AGTTCATTGGTGGCCAGGGGTT	TGGCCATAAGGTGCACTGCT	244
FMgSSR-16541	scaffold4276	p2	(AT)8	Class II	40197	40212	ATCTGGCCCCAACATGCTCCTT	AGCCACGGGTGGTGTTGGAAAA	223
FMgSSR-16542	scaffold714	p2	(AT)8	Class II	64936	64951	ATGATAACCGCAGATGGCGTCC	TGTTGCGACGACGATCAGGA	326
FMgSSR-16544	scaffold9990	p2	(AT)8	Class II	17539	17554	ATGCAGTAGAGCCACGCTGCTT	ATGCATGCGCGTGAGGACAT	204
FMgSSR-16551	scaffold22654	p2	(AT)8	Class II	6516	6531	ATTGCGCGTGACACTGACCT	TGTGGTGGATGCTACGATTGGA	327
FMgSSR-16552	scaffold115368	p2	(AT)8	Class II	2284	2299	ATTTTGACGACAGCGCGGCA	CGCGCTGTTATAGTTGTGCGGA	340
FMgSSR-16555	scaffold23471	p2	(AT)8	Class II	8388	8403	CAGGTGGACCATTTGATGTACGCA	GTCAGCAGAAACACAACGGCCA	302
FMgSSR-16557	scaffold3563	p2	(AT)8	Class II	3207	3222	CCGGTAATCGGTACAAGGGTCACA	AGCACAGCAACGATGCCACT	282
FMgSSR-16559	scaffold1804	p2	(AT)8	Class II	16616	16631	CCTGAACGCGCACACACCAAAA	TGACCGTGAAAAACAGCGCGA	290
FMgSSR-16563	scaffold765	p2	(AT)8	Class II	71799	71814	CGAACACCGCTAACATCATCGTGG	CGCAACCCAGTGAAAGATGACACG	327
FMgSSR-16566	scaffold7865	p2	(AT)8	Class II	29736	29751	CGACCAAGGCTAGTGCCTATCCAA	GCGTCCGTTTTGAGTTCATGGT	347
FMgSSR-16567	scaffold8434	p2	(AT)8	Class II	1482	1497	CGCCTCTCGCAAGCACCATAAA	CGCATGTGTCGCTTGCGTTT	295
FMgSSR-16568	scaffold140	p2	(AT)8	Class II	47047	47062	CGCGCCGTGTCATTCTGCTAAT	ACCGAGTGTTGGTCGCACAAT	239
FMgSSR-16569	scaffold2290	p2	(AT)8	Class II	23730	23745	CGCGTGGCTTGTGAGTGTCAAA	TTTGGCGGATACGCCGATCTCA	348
FMgSSR-16572	scaffold149	p2	(AT)8	Class II	20638	20653	GAGGTCCCAAAACCCTAACCCT	TGCTTAGGCCTGCACACGTCAA	216
FMgSSR-16573	scaffold169390	p2	(AT)8	Class II	358	373	GCACGCAACGCAACATGCAA	CGTAGCATGTATAACCCCATGAGCG	342
FMgSSR-16577	scaffold3217	p2	(AT)8	Class II	58681	58696	GCCATGACCAGCCCAAATCTGA	ACAATTGGCATGCTTGGGGCA	323
FMgSSR-16579	scaffold1499	p2	(AT)8	Class II	70912	70927	GCCTATGGTGACACACTAGCAAAC	AATCTGCAATTGGGGGCTCGCT	329

SSR_ID	Scaffold	SSR_Type	SSR_Motif	SSR Class	SSR_Start	SSR_End	Forward sequence	Reverse sequence	Product_size
FMgSSR-16582	scaffold4990	p2	(AT)8	Class II	20067	20082	GCTCACCCTCCATCGCTTGGTAAA	TGATTAGACCCACAGGTGGAACC	310
FMgSSR-16584	scaffold18191	p2	(AT)8	Class II	8070	8085	GCTGCTAGAACTCACTACATGGACC	TCCACCAAGGTGAATGGGCA	345
FMgSSR-16586	scaffold6355	p2	(AT)8	Class II	14526	14541	GGAGGCAGAGAATTTGTCCTCAGCA	TGTGACTCGGCACCTGATCATT	249
FMgSSR-16589	scaffold71	p2	(AT)8	Class II	81940	81955	GGCATCAAGATGTTCAGTGGGCCT	TGGATGATGATCGCCCATGTGT	338
FMgSSR-16592	scaffold1718	p2	(AT)8	Class II	35116	35131	GGGGGATAACGAAGCATATCAGGC	TCGTGTTAGCAGTGTTGGACGG	348
FMgSSR-16595	scaffold1533	p2	(AT)8	Class II	59176	59191	GGTGGGCCGTCAACGGAAATTA	GCACAGCAGACTGAGCACAACA	306
FMgSSR-16598	scaffold2954	p2	(AT)8	Class II	52633	52648	GTGCAAGAGAGATGGTCTGGGACT	ACAAGCCTCGAAGCAGAGGAGA	337
FMgSSR-16601	scaffold2567	p2	(AT)8	Class II	43414	43429	TAAGACTGGCATTGGGAGCCGA	ATAGCCATCGTGTTCGGCGTGT	339
FMgSSR-16606	scaffold38705	p2	(AT)8	Class II	2116	2131	TATAGCGGCGGCGGCAAAAA	TGAGCGCGTTGAATTGCCGA	285
FMgSSR-16612	scaffold5769	p2	(AT)8	Class II	1338	1353	TCACGGGTCGCACCAATTCA	TGTGTGGCTCAACGACACTGGT	340
FMgSSR-16613	scaffold8374	p2	(AT)8	Class II	10018	10033	TCAGAACCCACTCAAGCCTACCA	TCCATTCCGTCAGCCCACTTGT	238
FMgSSR-16621	scaffold846	p2	(AT)8	Class II	18601	18616	TCATGTGTGCACGTGTGACG	ACGGTTCACACACTGCCGGTTA	274
FMgSSR-16622	scaffold10896	p2	(AT)8	Class II	13009	13024	TCCACACGTGGACTAAAACGACGA	AAGCCGGATGCACATACCCACA	272
FMgSSR-16624	scaffold217	p2	(AT)8	Class II	87816	87831	TCCCCTGTAACGGCAAAGGA	GCCATGCACGTTATGTTGCGCT	241
FMgSSR-16626	scaffold686	p2	(AT)8	Class II	7963	7978	TCCCTGCACAAGGGCCAAAT	TTGAAAGGTTCCGGTTCGCGCT	293
FMgSSR-16627	scaffold283	p2	(AT)8	Class II	112349	112364	TCCGCTGCACCTGAACATTGGT	TTGCGCGCTAGCTTCCGTTA	298
FMgSSR-16629	scaffold32201	p2	(AT)8	Class II	303	318	TCCTATCAGCTAGAAGGGGTGC	ACGACGTACGCCTTGGTATGGA	313
FMgSSR-16638	scaffold15492	p2	(AT)8	Class II	14232	14247	TCGGCTCCGTCATTCCCTTCAA	TCGCCACCTGCATCATCACTCA	322
FMgSSR-16639	scaffold196	p2	(AT)8	Class II	74409	74424	TCGGGATTACTGCTGGCTGCAT	TTCCCATCCATCCGCTTGCT	328
FMgSSR-16640	scaffold6775	p2	(AT)8	Class II	25131	25146	TCGTGCTGCTTTGAGCCAGT	ATCGTAGCCGCCGTCACAAA	260
FMgSSR-16641	scaffold1548	p2	(AT)8	Class II	39740	39755	TCGTGGTGTCAACACCGCAACT	TCCTACTACGCTTGTATGCTGGAG	263
FMgSSR-16642	scaffold3135	p2	(AT)8	Class II	63107	63122	TCGTTCAATCTGCTTTCGGGCA	TGCCGAAATGTGGCGGTTTCTG	234
FMgSSR-16644	scaffold23724	p2	(AT)8	Class II	8967	8982	TCTACCGGCCATTTGCTGCT	TATTCGTTTCGACGTCGCGTGC	280
FMgSSR-16647	scaffold1326	p2	(AT)8	Class II	80129	80144	TCTCGCTGTCGGTCCTTGTT	TGTCAGCTCACCAGTCACCA	315
FMgSSR-16649	scaffold2987	p2	(AT)8	Class II	54049	54064	TCTTCGGGGCAACGGTCATCAT	TCGGGTTGGGTCTCCCACAATA	345
FMgSSR-16653	scaffold1774	p2	(AT)8	Class II	13471	13486	TGACACGCTGTGATTTGGCGT	ATACCATGCAACTGCGCCACCA	338
FMgSSR-16655	scaffold68	p2	(AT)8	Class II	78377	78392	TGACGACGGCCAGCAAATCA	TCCGGTCGCCATTTCGATTGT	298
FMgSSR-16658	scaffold7091	p2	(AT)8	Class II	27736	27751	TGATCACGGGTGGGGATGCTAA	TTCGGAAGGGGTATGCGGAA	289

SSR_ID	Scaffold	SSR_Type	SSR_Motif	SSR Class	SSR_Start	SSR_End	Forward sequence	Reverse sequence	Product_size
FMgSSR-16659	scaffold434	p2	(AT)8	Class II	2019	2034	TGATCAGTGTTGCGGCGGTCTA	AACTGGGGAAAGCTGCACCACA	228
FMgSSR-16663	scaffold799	p2	(AT)8	Class II	80570	80585	TGCAACGCTGCTGCCTACTT	GCAAAGGCCAAGACGTTCAAGGA	214
FMgSSR-16665	scaffold5651	p2	(AT)8	Class II	27235	27250	TGCACGCAGCCATCGCTTTT	GGCATCGAACTCTCTTTTGCGTGC	332
FMgSSR-16668	scaffold16270	p2	(AT)8	Class II	10857	10872	TGCATGTGCGATTGTCCAGACG	TCACCGCCACCACGTCAAAA	221
FMgSSR-16670	scaffold1651	p2	(AT)8	Class II	66861	66876	TGCCCTGCATCATCAGCTTG	CGCTCGCGGTTCCTTAAACA	350
FMgSSR-16674	scaffold1714	p2	(AT)8	Class II	55108	55123	TGCGAGGAAACACCGCCTTT	ACACAGCGTCATGGGAACTGA	248
FMgSSR-16675	scaffold6250	p2	(AT)8	Class II	14968	14983	TGCGTGCACTGCATTTTCCCT	ACCTTGGCGCTGTTTTTCGGT	336
FMgSSR-16677	scaffold3057	p2	(AT)8	Class II	2541	2556	TGCTGCAAGTAGGGACGATACA	TTATTGCCCCGACAAGGCCTGA	318
FMgSSR-16678	scaffold325	p2	(AT)8	Class II	83324	83339	TGCTGCCAGAACGTGGCAAT	CGTTTGCAGCTGGCCTGACAAT	265
FMgSSR-16679	scaffold5395	p2	(AT)8	Class II	38158	38173	TGGCATACGGTACGACAAGCGT	AGGCTTGCTATCGTGTCGTGTCA	220
FMgSSR-16682	scaffold3368	p2	(AT)8	Class II	296	311	TGGCTCGGCAAGGAGACAAA	ACAGGTGCATCTTGGCCGTT	278
FMgSSR-16683	scaffold1412	p2	(AT)8	Class II	28488	28503	TGGCTGAGCACATGAGCCCTTT	TTCCGGTTTTCCAACGGGTGGT	281
FMgSSR-16687	scaffold2040	p2	(AT)8	Class II	48569	48584	TGGGCCTTGTCAGTCTGTGTCA	TCGTCATGTGCCTTGAACCTTG	311
FMgSSR-16701	scaffold417	p2	(AT)8	Class II	59720	59735	TGGTTCAGCTAGGTTGTCCTTCCT	AGTCGATCGTCTCGCTTCGT	344
FMgSSR-16702	scaffold2079	p2	(AT)8	Class II	56925	56940	TGTAGCTTGCAGCACTGTAGCG	TACGGGCTTGGTGCTACCTGTT	332
FMgSSR-16707	scaffold3537	p2	(AT)8	Class II	31684	31699	TGTCTCGCTGTTCTTGGCGT	AGCGCAGGCTTCGATCCAACAA	242
FMgSSR-16708	scaffold694	p2	(AT)8	Class II	93880	93895	TGTGATCGGGCGAACACTTG	TGGGCTTGTTCGTCGTCGTT	207
FMgSSR-16711	scaffold28406	p2	(AT)8	Class II	7138	7153	TGTGGCTGGATTGCTCCTCCTT	CGTGCCTGCTTCCTCAACGAAT	334
FMgSSR-16717	scaffold9108	p2	(AT)8	Class II	9288	9303	TGTTGACAGTGGCAGCAAGGA	ACACAAGCACAAGCACCGCT	348
FMgSSR-16722	scaffold4894	p2	(AT)8	Class II	34101	34116	TTCGCCCACGCGGTAATGAA	GCGCGTGCCATGAAGGTGTTAT	311
FMgSSR-16724	scaffold30	p2	(AT)8	Class II	203560	203575	TTCTTGCCCACGCTGGTGTT	TCACAAGCCATCAACGTCCCCA	277
FMgSSR-16725	scaffold324	p2	(AT)8	Class II	44012	44027	TTCTTGTTGCTCGCCACCGT	TACTGTTTGCCATGCGCTGTCG	261
FMgSSR-16729	scaffold5176	p2	(AT)8	Class II	29409	29424	TTGGCCAACTTTCAGTCGCC	AGGAGTGCATGCAACCGTGT	328
FMgSSR-16731	scaffold3106	p2	(AT)8	Class II	36341	36356	TTTAGCATGCTGCGCTCGGA	TTTGCAGCGCTCAGGACCAA	296
FMgSSR-16732	scaffold79	p2	(AT)8	Class II	105410	105425	TTTCAGAGGCAGCCGCAAGT	ACCACCAATCAAGCACAGCCCA	315
FMgSSR-16742	scaffold4357	p2	(AT)9	Class II	18225	18242	AAATGTGCTTGTGTGCCACCCG	TCGCACCACCAGATGCAATGGA	205
FMgSSR-16746	scaffold4195	p2	(AT)9	Class II	32318	32335	AATAGGCGCCCACACACACACA	GGCACATGTACCTGGAAGGTCT	347
FMgSSR-16748	scaffold20804	p2	(AT)9	Class II	13456	13473	AATGCCCCAGCAAATCCCCAGA	TGATGCGGCATTGCGACGTA	345

SSR_ID	Scaffold	SSR_Type	SSR_Motif	SSR Class	SSR_Start	SSR_End	Forward sequence	Reverse sequence	Product_size
FMgSSR-16753	scaffold7913	p2	(AT)9	Class II	8850	8867	ACAGCAACGCGGGTTGTGAAA	TGTGCCTTGGTGATGTGCGT	209
FMgSSR-16754	scaffold127	p2	(AT)9	Class II	51263	51280	ACAGCCGTGGACGACAACATGA	ATCGTGACCGTGGCCGTTTACA	256
FMgSSR-16756	scaffold15609	p2	(AT)9	Class II	12708	12725	ACCAACCTGGCATAGGGGAGTGAT	AGTGAGGAAGCACAAGGCAGCA	291
FMgSSR-16757	scaffold1066	p2	(AT)9	Class II	42863	42880	ACCACTCGTGCGTATAGCCGTT	TTATGCCACGGATACCACCCCT	325
FMgSSR-16763	scaffold10215	p2	(AT)9	Class II	10067	10084	ACCTGTGCATTCCAACGGACT	AGCGCAATCGGCAAGGTTCA	263
FMgSSR-16764	scaffold1	p2	(AT)9	Class II	391874	391891	ACGACAATGGGCATCCGAAGCA	TCCCACGACCTATTTTCGGTCTCC	301
FMgSSR-16766	scaffold5667	p2	(AT)9	Class II	4947	4964	ACGCCAGTTACCTCTGCCATGT	TTCCGTTGCCCCAGATACACGA	269
FMgSSR-16767	scaffold14743	p2	(AT)9	Class II	19823	19840	ACGCGCATGGCCAGGTAATA	AGGTGCCGCATGAACATCCCTT	302
FMgSSR-16768	scaffold249	p2	(AT)9	Class II	16928	16945	ACGCTCCAAAGAATGGACACCA	TGGCAACGCGCGACGATAAA	271
FMgSSR-16769	scaffold98116	p2	(AT)9	Class II	1328	1345	ACGGCTGGCTGGCATGAAAT	AACGTTGGCGGGAAGGAGAA	346
FMgSSR-16770	scaffold1126	p2	(AT)9	Class II	54164	54181	ACGTCAGGTTGCTCCAGTTCGT	AGCATGTGACAGCGACGTTTGG	203
FMgSSR-16771	scaffold77	p2	(AT)9	Class II	21002	21019	ACGTCGTCACTAGGTCCGTT	ACCATCCAGTGAGCAATGTACAGG	346
FMgSSR-16781	scaffold302	p2	(AT)9	Class II	90301	90318	ACTGAACCTGGTTGCCAAACCCT	GGCTTGATGCGCGAAAGCTGAT	350
FMgSSR-16782	scaffold1974	p2	(AT)9	Class II	6762	6779	ACTGACCTTGCTGCCCAAGACA	CCGATGCAAAAGACACCTGGCA	315
FMgSSR-16784	scaffold3752	p2	(AT)9	Class II	27380	27397	AGACACACACATGAAGTCCCCCA	TGTCGCAGAGATTGGGCCTTGT	350
FMgSSR-16787	scaffold21626	p2	(AT)9	Class II	10029	10046	AGATCCAAACAACCGGAGGGT	ACAACACGACAAACGAGCGCA	207
FMgSSR-16789	scaffold4676	p2	(AT)9	Class II	17755	17772	AGCACAGGCGAGTAATGGTCA	TGTGGTGCCTATCGTGTAGCA	238
FMgSSR-16791	scaffold15495	p2	(AT)9	Class II	13369	13386	AGCATCTGGTACACGGGTTGT	ACGCCGTTGAAGTTATACCACCG	347
FMgSSR-16794	scaffold30322	p2	(AT)9	Class II	6043	6060	AGCCATGCATCGTCGTCCACAT	AGCCGCCGCCCTTTGTTTTT	305
FMgSSR-16799	scaffold1994	p2	(AT)9	Class II	61170	61187	AGCTCCTTCGGGAGACGACAAA	TGGACACTTTGCAGTGGGAGGA	265
FMgSSR-16802	scaffold9856	p2	(AT)9	Class II	16726	16743	AGCTGTAATATGCTGGGTGGCCT	ACAGCAGCTGATGAGTGAGTGC	285
FMgSSR-16803	scaffold373	p2	(AT)9	Class II	13859	13876	AGCTTCCGGGCTTCCGAGTTTT	AGATCCCACCACCATGCAGACT	260
FMgSSR-16805	scaffold5244	p2	(AT)9	Class II	20681	20698	AGCTTGGACTCGTCTTCTTCCCT	CCATGGCATGTCACGTGATGCT	349
FMgSSR-16806	scaffold4170	p2	(AT)9	Class II	7310	7327	AGGACCAGCCGACTACAGTCAA	GCGCTGCAAGTGTTGAGCGAAT	346
FMgSSR-16807	scaffold52	p2	(AT)9	Class II	29703	29720	AGGATCCGCAGCTGTGACTGAT	TGAGCCATGGTAAGGTCAGGCT	339
FMgSSR-16810	scaffold16057	p2	(AT)9	Class II	8033	8050	AGGCTCACCTCTTCCATCTGCT	CTCCAAATCCACCTCATTCGCA	349
FMgSSR-16812	scaffold20093	p2	(AT)9	Class II	10888	10905	AGGGACAGTGCGGTATGTGT	TGCATGAGCCCTGTTTGGCCTT	323
FMgSSR-16813	scaffold792	p2	(AT)9	Class II	93102	93119	AGGGCACGCGAGAAAACACA	ACCGTGGTGGAAACCGAGTTGA	235

SSR_ID	Scaffold	SSR_Type	SSR_Motif	SSR Class	SSR_Start	SSR_End	Forward sequence	Reverse sequence	Product_size
FMgSSR-16816	scaffold3596	p2	(AT)9	Class II	26913	26930	AGTATGCGTCTTGCGGCGAA	TGCTCCTGCACAAGATTGGCGA	214
FMgSSR-16817	scaffold11020	p2	(AT)9	Class II	22499	22516	AGTTCCTGATGCCGGAGTGAGA	GGTCACGGTGAGTTTGTTTCCTGC	303
FMgSSR-16818	scaffold11116	p2	(AT)9	Class II	22595	22612	AGTTGGACTGGACTAACCGACC	ATTGGACCATGGGCTGGTTGGA	336
FMgSSR-16819	scaffold55	p2	(AT)9	Class II	127078	127095	ATAATGCGCGTGGAGGCTGAGT	TGGTCATTGGCGTCGGGTTT	295
FMgSSR-16820	scaffold22591	p2	(AT)9	Class II	7846	7863	ATCAGAGCGCCATTCACCCAGT	TGTCCAAACCGGGGGCTAATGA	248
FMgSSR-16821	scaffold2034	p2	(AT)9	Class II	7146	7163	ATCAGCGTACGTGTGGACGA	TGCATGCTCTAACTCGCTTGGGT	314
FMgSSR-16823	scaffold9791	p2	(AT)9	Class II	20289	20306	ATCCCCGGCAACGGCAAAAT	TTATCGGGCATGCGATGCGA	302
FMgSSR-16827	scaffold301	p2	(AT)9	Class II	20531	20548	ATGGAGAAGCAAGGGCGACA	GCATTGTGTGGCTCGACCATGT	322
FMgSSR-16831	scaffold7957	p2	(AT)9	Class II	21269	21286	ATTTTGAGAGGAAGGGCCAGTGGG	ATTTTGCACGCTGGGCAAGTGG	322
FMgSSR-16835	scaffold11161	p2	(AT)9	Class II	4277	4294	CCCAATGGTACCAAAGCCCGAA	TCCTGTCGTTGCCCCACATTCA	207
FMgSSR-16842	scaffold18749	p2	(AT)9	Class II	3673	3690	CGAGATTTCCGGCAACGTGCAT	ACGTGCGCATCGTACATCGT	206
FMgSSR-16844	scaffold9429	p2	(AT)9	Class II	24925	24942	CGGCGGGGAAATGGTTTGGAAAA	AAACCGTTGCAAGGCTCGGT	302
FMgSSR-16845	scaffold2975	p2	(AT)9	Class II	41389	41406	CGGCGTGTGATCACCACAATCA	ATCGCGGACGGCTAGATGAA	297
FMgSSR-16852	scaffold30354	p2	(AT)9	Class II	5211	5228	GCATTCAGCATCTCTTCACCACAC	ACGCCTTTGGGCCATGGAAA	245
FMgSSR-16853	scaffold43376	p2	(AT)9	Class II	356	373	GCCCGACAACTCACTTGCAACA	TGCCGAGCTGATAACGACCTGA	248
FMgSSR-16860	scaffold6565	p2	(AT)9	Class II	1765	1782	GGGGTTATGTTTTGAACCGGGCA	TCCAGGTGCATGCCTGAAGA	339
FMgSSR-16866	scaffold3194	p2	(AT)9	Class II	48121	48138	GTGGCAAGTGACCGGCATCTTAAT	ATGGAAACTTATGCGGGCAGGG	321
FMgSSR-16869	scaffold2374	p2	(AT)9	Class II	1679	1696	TACGTGGCGCAATGCAACCT	TGCGCGTGACCGTAAGTTTT	350
FMgSSR-16870	scaffold4070	p2	(AT)9	Class II	4045	4062	TAGTACGCTTTTCGGCCCTGCT	TTCATGTCATCGGAGCCACCGT	297
FMgSSR-16871	scaffold14527	p2	(AT)9	Class II	1311	1328	TATATGCGGAAAGCGGGGCT	ACGACGGACCACCACTCACAAA	283
FMgSSR-16874	scaffold13903	p2	(AT)9	Class II	18507	18524	TCACGTCGATCAGTCCAGCACA	GTTGGACAAACCCTCACTCTTTGG	342
FMgSSR-16875	scaffold6603	p2	(AT)9	Class II	21797	21814	TCAGCATCCAACACAGGGCT	TCTGCAGAATCTTCTGAGGGGGA	257
FMgSSR-16876	scaffold7768	p2	(AT)9	Class II	22313	22330	TCAGCCGAGTTGACAAGCACA	TCGCAAACGACACAGCCCAA	305
FMgSSR-16877	scaffold12428	p2	(AT)9	Class II	16151	16168	TCAGTCACGATTTGCCGCGT	TACGCCTACCAGAGACGCGAAT	252
FMgSSR-16882	scaffold5534	p2	(AT)9	Class II	34156	34173	TCCATGCACGACACGCAGAA	GCGGCGTCTGTTTCGGTGAAAA	341
FMgSSR-16884	scaffold1496	p2	(AT)9	Class II	262	279	TCCCAGCGACTAAACATAACCCCA	TGGCAAGTCGGGTTGCAAGGAT	304
FMgSSR-16885	scaffold4731	p2	(AT)9	Class II	35627	35644	TCCCATGATTTTCACCCAGGTCCA	AGAGTTGCCTCAACCTCGACCA	319
FMgSSR-16890	scaffold6766	p2	(AT)9	Class II	3917	3934	TCCTTGCTCCATTGCCGTGA	TGCATGAGTGCTCTTGCGGT	346

SSR_ID	Scaffold	SSR_Type	SSR_Motif	SSR Class	SSR_Start	SSR_End	Forward sequence	Reverse sequence	Product_size
FMgSSR-16892	scaffold5819	p2	(AT)9	Class II	11469	11486	TCGCTAGGGGAGGAGAGAAAAACC	TGCGATGCCCACAAAAGCACA	347
FMgSSR-16895	scaffold3361	p2	(AT)9	Class II	10619	10636	TCGGGATTGTTGGTTCGCTGGT	ACTGCTTTGGGCTCGGCTACAT	249
FMgSSR-16897	scaffold48435	p2	(AT)9	Class II	3096	3113	TGAAGCACACATCGCAGTTGTC	TCCTCCTTGGCATTTTGTGCGT	335
FMgSSR-16899	scaffold174	p2	(AT)9	Class II	96969	96986	TGAATTGCGGATCGCCACGA	CGTGGACAAGCATGCAACGA	335
FMgSSR-16900	scaffold16900	p2	(AT)9	Class II	4663	4680	TGACCCGCCTGGTAGTCAAA	ATGGTCGGTGAATGGAGCCT	347
FMgSSR-16905	scaffold4424	p2	(AT)9	Class II	28476	28493	TGCAGCTGTACAACCAGCAACA	TCCCGCGGTATTTGACAGCGTT	286
FMgSSR-16907	scaffold15249	p2	(AT)9	Class II	4278	4295	TGCATTCACCGAACAGTACGCC	GCGTGTGTTTCACGCATGCTCT	241
FMgSSR-16911	scaffold1227	p2	(AT)9	Class II	81517	81534	TGCCGTTTTCACTGCCACGA	TGGCGGAGGATTCAGGTTCCAT	294
FMgSSR-16914	scaffold3207	p2	(AT)9	Class II	50342	50359	TGCGTCAGTTGCCCCATACAGT	AGCCACGAACTTGAGCTGTCCT	236
FMgSSR-16917	scaffold23237	p2	(AT)9	Class II	5196	5213	TGCTGTGGCAGTTGCACGAA	ACTTGCTCTTGGCCTCTTGGGA	309
FMgSSR-16919	scaffold17830	p2	(AT)9	Class II	17234	17251	TGCTTGCGATTAACCAGCCCGA	ATGCGTGCACTAACGATGGGGA	294
FMgSSR-16920	scaffold5045	p2	(AT)9	Class II	25790	25807	TGCTTGCTCCTCCTTCACCCTT	TGCACAAGCACCTCTTGATCGT	347
FMgSSR-16922	scaffold132	p2	(AT)9	Class II	41301	41318	TGGAACGATGCCGAACGTCAGT	CGTTGTTGTCCAGTGCGTCACA	254
FMgSSR-16932	scaffold4270	p2	(AT)9	Class II	20757	20774	TGGCTGCTCCTTAATTCCTGGCA	TCAGCGTCATGTACGCCCTA	259
FMgSSR-16937	scaffold5353	p2	(AT)9	Class II	36662	36679	TGGGGGAACATGACATGGTGCAA	AAGCTGTCCCAAACAGGGCCTA	219
FMgSSR-16938	scaffold2065	p2	(AT)9	Class II	67438	67455	TGGGTTGTGTTGCCAAGGTCA	GCGAGGCATGTGGGTTTTGCAT	346
FMgSSR-16946	scaffold11687	p2	(AT)9	Class II	25055	25072	TGTGCATAACCGCCGCTACGAT	TGGTGGCGTGGCATCAGACAAT	346
FMgSSR-16948	scaffold75650	p2	(AT)9	Class II	1552	1569	TGTGCTTCACCCGAACTTCCCT	TGGTTGTGCATGTAGCGGGAGA	277
FMgSSR-16949	scaffold3044	p2	(AT)9	Class II	3646	3663	TGTGCTTGGTGGGAGGAGAA	AACCCCTGCCCCCTTTTGTTGT	296
FMgSSR-16951	scaffold3186	p2	(AT)9	Class II	20679	20696	TGTGTGCGGGTGTTTGTGAGGA	TCATGATGTAGCGCGGTGCTGT	267
FMgSSR-16954	scaffold4360	p2	(AT)9	Class II	43594	43611	TGTTGCCGAAGGCATGGGAT	AGCTTCGCCGCTGCACTAAA	305
FMgSSR-16959	scaffold1734	p2	(AT)9	Class II	46762	46779	TTGCTCTTGGAGTTGCTAGAGGGC	AGCGGCTAGTGGCAGATGTCAA	218
FMgSSR-16960	scaffold5537	p2	(AT)9	Class II	17621	17638	TTGCTTCGCGGTCTCATCAGGT	AAAGTGGCTGATCGCCTGGCTT	228
FMgSSR-16964	scaffold3334	p2	(AT)9	Class II	25765	25782	TTTTGCTTGCGCCCGTCTCA	TCATTGGCCTCGCTATGCTCCA	327
FMgSSR-16968	scaffold6256	p2	(CA)10	Class I	16746	16765	AAACGGTCACTGCTTGCCCA	TTCGTGTGTGCCGCTTCGAT	224
FMgSSR-16970	scaffold1904	p2	(CA)10	Class I	38127	38146	AAGCTGGCTGTCAGGCAACACA	AAAACACCACCCCTCCTGCT	277
FMgSSR-16975	scaffold1331	p2	(CA)10	Class I	34302	34321	ACCTGGCAGGAATGCTAGTCCA	TGCAAATGTCCGTTACCCCAGA	275
FMgSSR-16978	scaffold8252	p2	(CA)10	Class I	11902	11921	ACGTGGGTCCCAACTCAAGCAA	GCAAGCATGTTTTGTCCGTGCC	306

SSR_ID	Scaffold	SSR_Type	SSR_Motif	SSR Class	SSR_Start	SSR_End	Forward sequence	Reverse sequence	Product_size
FMgSSR-16979	scaffold2954	p2	(CA)10	Class I	29729	29748	ACTGGAGCGGCGAACCTTTT	ACAGGGCTCCGGCATTCAACAA	325
FMgSSR-16981	scaffold1570	p2	(CA)10	Class I	67542	67561	ACTTTTGCGCCGGTGCGTTT	ATCAGATGTGTCGCGAGTGCGT	345
FMgSSR-16992	scaffold14795	p2	(CA)10	Class I	19661	19680	ATGCGGCCAGGATGCTTGAGTT	GGCAGAAGCTTGGCAAGTGCTTT	299
FMgSSR-16993	scaffold669	p2	(CA)10	Class I	47551	47570	ATTGCCTGCTGCCACCATGT	TCGGCCTCCGATGATGAGCATA	329
FMgSSR-16996	scaffold2404	p2	(CA)10	Class I	54188	54207	GTCGCTCTTGCCGCTACTTTCT	TGACCTTGCTCGAAGTTGTCCA	344
FMgSSR-16997	scaffold1295	p2	(CA)10	Class I	3678	3697	TACACGCTGCATCGTCTGACCT	GCTAGCAAGCTTCGAAAGCGA	203
FMgSSR-16998	scaffold2300	p2	(CA)10	Class I	8913	8932	TCCCTTAATCGCGCACACGGAT	ACAACAGGTGTGGCTTGCTGGA	341
FMgSSR-16999	scaffold158	p2	(CA)10	Class I	49256	49275	TGCACGCGGCATTCGTAAAC	ACTCCCGTCTTCTTCGTGTGCT	230
FMgSSR-17001	scaffold463	p2	(CA)10	Class I	33848	33867	TGCCATCGACCCTTCTACTACCCA	TGGGTGCACCACTTGCTTGT	298
FMgSSR-17002	scaffold1013	p2	(CA)10	Class I	82620	82639	TGGACTGCAATGGTCAACACACCA	GCTTTGCTAAGCTCCTCACTACTGC	299
FMgSSR-17005	scaffold6350	p2	(CA)10	Class I	5637	5656	TGGGACAGAAAATGAAGGGTGC	TCGCTGAGGTGATGCAGGCTAA	337
FMgSSR-17007	scaffold14477	p2	(CA)10	Class I	23546	23565	TGTGCAATGACTCAAGCACGGG	TGTGCACCTGGTTTCTTGCTGC	314
FMgSSR-17009	scaffold2068	p2	(CA)10	Class I	4220	4239	TGTGCGATGTGGATTGGACTCCT	AGACCATGCAAACTCACCAGCA	253
FMgSSR-17010	scaffold834	p2	(CA)10	Class I	10601	10620	TTCGCCCACACACCGAGACAAA	TGCCCACGGTGTCCGTTTCTAT	338
FMgSSR-17013	scaffold10268	p2	(CA)10	Class I	3972	3991	TTTGCATCTCGACGGCGGAA	TGCCCCTGCAGGTTGCTGATTT	203
FMgSSR-17025	scaffold9911	p2	(CA)11	Class I	13791	13812	AGTGCAACAGCTCACACCTCCA	ACCACAGCCATGTTGGCTCA	214
FMgSSR-17026	scaffold14665	p2	(CA)11	Class I	3585	3606	ATGGCACACGGCCTGAAGAACT	TCCGAGCACTCGTACATAGCTCCT	296
FMgSSR-17027	scaffold2472	p2	(CA)11	Class I	47367	47388	ATGTGCAGCCAAAGCTGGTGGA	TGCGTGCCATTGCCTTTGTC	256
FMgSSR-17029	scaffold65890	p2	(CA)11	Class I	947	968	GGGCAAAAAGGTGATGGCACCAA	TGGTGGCCTTCCTCTAAGTCGT	212
FMgSSR-17030	scaffold1143	p2	(CA)11	Class I	60312	60333	TCGAGAATGAGACGAGACAGATCC	GGTCATTTGATCTCCGTTGGTGGGT	294
FMgSSR-17033	scaffold190951	p2	(CA)11	Class I	278	299	TGCTTGCCATAGCAGTTTGTGC	TTTTTGGTCGCCCACGGGATGA	224
FMgSSR-17038	scaffold4036	p2	(CA)12	Class I	28372	28395	AAGCAAAGGCACACCGGCAA	TCCGGCCTGGGTTTGTTGAA	295
FMgSSR-17039	scaffold42402	p2	(CA)12	Class I	39	62	AAGCACAAGCCCTTATGTCCCC	ATGTCGGAGTATCCGGCAACCT	278
FMgSSR-17042	scaffold11182	p2	(CA)12	Class I	1138	1161	ACCTCAGCCTCGAAACGACCTT	AGGTTGGTGCCGTGTGTGCTTA	210
FMgSSR-17044	scaffold9023	p2	(CA)12	Class I	13440	13463	ACGGTCAACACCTTCCCCTGAA	TCAACGACGACCCGACCAACAA	307
FMgSSR-17057	scaffold332	p2	(CA)12	Class I	131961	131984	TGTTCCCGTACAAGCAGCCCAA	AAGCAGCAGCAGAGACGCAT	329
FMgSSR-17058	scaffold4857	p2	(CA)12	Class I	33842	33865	TTACTTAAACGCCTGCGCCC	CAGCAAGTTGAGGTGAGTGCGT	213
FMgSSR-17061	scaffold8166	p2	(CA)13	Class I	9442	9467	ACCTCGAGCTGCTTGGACCATT	ACCTGGTGAAGGTCATGCGGAA	346

SSR_ID	Scaffold	SSR_Type	SSR_Motif	SSR Class	SSR_Start	SSR_End	Forward sequence	Reverse sequence	Product_size
FMgSSR-17066	scaffold1362	p2	(CA)13	Class I	34405	34430	ACTGGAGTAGGGGGTGGAAAACA	TCCAGGACGCGACGATTAGAGGAT	331
FMgSSR-17067	scaffold1904	p2	(CA)13	Class I	26656	26681	AGCCAACACCCGTTCCTTGTGT	TGGACCGCTTTAACACCCCT	328
FMgSSR-17069	scaffold1372	p2	(CA)13	Class I	66971	66996	AGCTCGAGTGATGTATTGACAGGC	TGGGAGATGCAGATGCAGCCTA	298
FMgSSR-17072	scaffold111491	p2	(CA)13	Class I	720	745	ATGGGATGAGTTGCGAGCACGA	TGGCGTGCGAGTGTAGCAAT	261
FMgSSR-17074	scaffold2784	p2	(CA)13	Class I	53947	53972	TATAGTGACGATGGCCGCGA	TGGTTCCTGGCGCAAGTGATGA	222
FMgSSR-17075	scaffold789	p2	(CA)13	Class I	16921	16946	TCCAAATGTGGGCGACCTGT	AGCCTCTCTAGGAACACTTCACCT	303
FMgSSR-17078	scaffold513	p2	(CA)14	Class I	104143	104170	ACATCATGCGCAACGCCAGA	ATACAGCTCACACGGCTGCT	276
FMgSSR-17081	scaffold1558	p2	(CA)14	Class I	22242	22269	ACGTATGGGCACGGCCAAAT	AGCTGTTGCTGGGCTGGATT	271
FMgSSR-17083	scaffold129	p2	(CA)14	Class I	60719	60746	AGGTTGCAGTTGCCCATACGA	GCTGCGGCAACTTCCAGGTTTT	265
FMgSSR-17084	scaffold77545	p2	(CA)14	Class I	104	131	CCCTTCTTTCTTTGCTCTCGTGTC	GCCCTCTTTTCCGCAAAGCACT	347
FMgSSR-17085	scaffold25827	p2	(CA)14	Class I	4370	4397	CCGGTCATGTTCGACTAGCGTT	ACCTCGTCTTGCATGCCTTGCT	256
FMgSSR-17090	scaffold28307	p2	(CA)14	Class I	1172	1199	TTTGCGACGAAGGTGATGGTGC	ACGCATGACCCGAACGGAAA	319
FMgSSR-17092	scaffold153	p2	(CA)15	Class I	24162	24191	ACACGAGTGCAACAGCACCA	TCCAGCAAGGTGTCTTGATTGGCT	314
FMgSSR-17099	scaffold2002	p2	(CA)15	Class I	52924	52953	TCCACCAATCACGGGAACACACA	CGTGAGCAGGTTGAATTTTGCTGC	345
FMgSSR-17100	scaffold324	p2	(CA)15	Class I	14212	14241	TCCTTGCTTCCAAAACACGCCA	TGGGCAATCAAAGCTCCTGCCA	293
FMgSSR-17105	scaffold9301	p2	(CA)16	Class I	14826	14857	ACACGGCAGTGAGGACAACAGT	TGTTCTCGAGCACCTCCACA	239
FMgSSR-17108	scaffold8791	p2	(CA)16	Class I	29825	29856	GCCGCGCCATTGAATTGTCCTT	ACCGTTTTCGCTGCGTTTCGT	295
FMgSSR-17110	scaffold5656	p2	(CA)16	Class I	41096	41127	TTTTCGCGGCTGGAGCTTCT	AAACGTTGTGTCGTCGGTCGGT	348
FMgSSR-17111	scaffold8236	p2	(CA)17	Class I	9692	9725	AACACCGGCGCAAAGCGTTA	ACCAAGCCTTTGGTGCAGCTCT	264
FMgSSR-17112	scaffold26652	p2	(CA)17	Class I	6442	6475	AGTTGTTTATCCGCCCACCCCA	TAAACCACCAGCTGCACAGTCG	334
FMgSSR-17115	scaffold148066	p2	(CA)17	Class I	125	158	TTTAGCGGCCTACTCCATGCGT	TGTTATCGACGGCGATTGGCGA	205
FMgSSR-17118	scaffold2649	p2	(CA)18	Class I	18896	18931	ACCGAATGCAAGCCGCTCAT	ACACGGGCACCCCCTGAATAAA	261
FMgSSR-17120	scaffold586	p2	(CA)18	Class I	38219	38254	AGCACGCAAGCCAGAATGCCTA	TAGCAACAACCGGAGTGTGCGT	309
FMgSSR-17123	scaffold577	p2	(CA)18	Class I	57780	57815	ATATCCCGCAGCAACGCTCA	TTCGGTTTACACCCCAGCTCCA	281
FMgSSR-17128	scaffold10313	p2	(CA)19	Class I	4559	4596	TGACCACCCTGGTACTTGACAGA	TCCATGTGCCCAGGGACTTT	323
FMgSSR-17129	scaffold121064	p2	(CA)19	Class I	657	694	TTGCCCTGGTTTGAGGTTGCCA	GCCAGTGGCTCATATGACTGAGGT	231
FMgSSR-17132	scaffold21967	p2	(CA)20	Class I	1274	1313	AGGAATACCAGGCTTTGCTGC	TGTTTCATCCCTCGCTGCGTCA	229
FMgSSR-17134	scaffold13116	p2	(CA)20	Class I	6721	6760	TCCATTTCCCCCTTTGGTGGCA	TGGCTGGGCTACACAAAAAGGA	201

SSR_ID	Scaffold	SSR_Type	SSR_Motif	SSR Class	SSR_Start	SSR_End	Forward sequence	Reverse sequence	Product_size
FMgSSR-17135	scaffold198	p2	(CA)20	Class I	85851	85890	TGCATCTTTCTGGACCCAAGGCT	TGACGGGCAATGACCTGTTCGT	252
FMgSSR-17138	scaffold390122	p2	(CA)21	Class I	125	166	ATTCCAGCGGGATCCTTGTGCT	TGGACGAACGATGGAACAAGCCT	214
FMgSSR-17145	scaffold6050	p2	(CA)24	Class I	24103	24150	TTGGCCTCTTTCTGGTGCAAGC	TCCTTGGCATACTCCTTGTTCACG	331
FMgSSR-17147	scaffold14795	p2	(CA)27	Class I	16913	16966	TGCAGAAAACAACCTTCCCCAGC	TGTCAGCCCAATTGAAGAGGCTCA	246
FMgSSR-17149	scaffold4640	p2	(CA)30	Class I	3614	3673	GCCCATGAGTGAATTGTGCGCT	TGTGCTCGCGGAAGGAAAGGAA	250
FMgSSR-17151	scaffold3376	p2	(CA)39	Class I	26060	26137	TGGCTGCTGACTGTAGGTTCACA	AATGGGACGCCTTGGTGGTCAA	229
FMgSSR-17156	scaffold2103	p2	(CA)6	Class II	46929	46940	AAAATGTGTGGCAGCGACGAGC	TGGCAACACAAATCGCAGGCA	211
FMgSSR-17162	scaffold22380	p2	(CA)6	Class II	6120	6131	AAACCGGCAACTCCCAACCACA	AGATCGAACAAGGCGAGCGA	263
FMgSSR-17164	scaffold8831	p2	(CA)6	Class II	26087	26098	AAACGTGTCGTCTCGAACCGCT	ACCGCAACTTGATGCACGGA	318
FMgSSR-17165	scaffold342073	p2	(CA)6	Class II	84	95	AAAGCAGTCCAGCTCCAGCACT	AACACGCTGGGATGGAAATCGC	323
FMgSSR-17167	scaffold3518	p2	(CA)6	Class II	44583	44594	AAAGCTCGCTCACACACGCA	AGTCACGTCCTGTTTGCGCT	303
FMgSSR-17169	scaffold268	p2	(CA)6	Class II	107359	107370	AAATCAATGCGCGCCGACCT	ACGTGCTGGATGACGACGAT	272
FMgSSR-17171	scaffold291	p2	(CA)6	Class II	1609	1620	AACAACGACGTCGCCAACCT	TGTTGCTGCTTGACCTCAGCGA	301
FMgSSR-17172	scaffold8011	p2	(CA)6	Class II	29096	29107	AACAAGCCTAGCCACACGCA	GCAATGATGGTGTTGCCTCGCA	322
FMgSSR-17173	scaffold4583	p2	(CA)6	Class II	22712	22723	AACACACTCCCAAGCGTCCACA	ACCATTGTGTGCCGGCGTAT	310
FMgSSR-17177	scaffold11314	p2	(CA)6	Class II	24610	24621	AACAGCCTAAGGCATCCTCGCT	GTGGTGCTCTTGTGTGCACGAT	327
FMgSSR-17181	scaffold3031	p2	(CA)6	Class II	31157	31168	AACCAGCAGCCCGAGACAGTAT	AGTCAGGTGAGTTAAGGGGCTGA	276
FMgSSR-17182	scaffold455	p2	(CA)6	Class II	114713	114724	AACCATAGCCAAAGCGAACGGG	AAGCAGTAGCACCGACCAGT	334
FMgSSR-17183	scaffold12966	p2	(CA)6	Class II	514	525	AACCATCAAACCATGCGGGCAC	ATCCTAGACTCGCCACTGGTGT	245
FMgSSR-17184	scaffold3671	p2	(CA)6	Class II	48770	48781	AACCGAGAACACAGCAGGCA	TGCGATTTCGATCCAGCGGTGA	318
FMgSSR-17186	scaffold9314	p2	(CA)6	Class II	18098	18109	AACGCCAAACAAAAGCGGGC	TGAAGGCGCGCAATTTCCCA	306
FMgSSR-17187	scaffold2227	p2	(CA)6	Class II	33970	33981	AACGCGTGGGGAGCGTTTAAGT	TGACTGTGCGCTTGTTCCGT	230
FMgSSR-17191	scaffold11744	p2	(CA)6	Class II	6291	6302	AACTAATCATCGCCCAGCGGCA	TTTGCTTTGGTGAGGGCGGT	279
FMgSSR-17197	scaffold370	p2	(CA)6	Class II	126327	126338	AAGAGAACCGCGACAGCAGCAT	TGCAGTCAGGCGGCATTCAA	257
FMgSSR-17199	scaffold3672	p2	(CA)6	Class II	19312	19323	AAGCAAGAGCGCCTGCATGGAT	AAGCCAAGTGCGTGTGACGA	227
FMgSSR-17200	scaffold13701	p2	(CA)6	Class II	19418	19429	AAGCACTACAGGGCCGACATGA	AACGAAACTTGCTCTGCGCTGC	277
FMgSSR-17203	scaffold793	p2	(CA)6	Class II	20178	20189	AAGCCGCCTGCAATGTCTCTCA	GCATTGCTGTTGTTGGAAAGCG	350
FMgSSR-17206	scaffold510382	p2	(CA)6	Class II	174	185	AAGCGTGGCTCTCCCACGGTAAAA	TAGCGATCGAGTTTCGCAGCGT	203

SSR_ID	Scaffold	SSR_Type	SSR_Motif	SSR Class	SSR_Start	SSR_End	Forward sequence	Reverse sequence	Product_size
FMgSSR-17209	scaffold17985	p2	(CA)6	Class II	337	348	AAGGAGAACGAGGCAAGCGGTA	ACTGCATCATGGCGAGGCTT	237
FMgSSR-17212	scaffold2709	p2	(CA)6	Class II	13316	13327	AAGGCGCTGCAGCATCATTC	ACAGCGAGCTGTCGTGGTTCAA	332
FMgSSR-17215	scaffold1753	p2	(CA)6	Class II	27176	27187	AATAAGGTCGGCCGAGTTGGCT	TGTCGTCACCGAGCACACTGTT	226
FMgSSR-17216	scaffold4618	p2	(CA)6	Class II	9383	9394	AATCCGTCTCGCGCCAAGAT	GGCTGCTGCTGCTTGCAGATTT	303
FMgSSR-17224	scaffold8477	p2	(CA)6	Class II	20526	20537	ACAAGAACAGGCACCAACCACA	TGGTGTTCGGTCGGTTGCCATA	208
FMgSSR-17225	scaffold179494	p2	(CA)6	Class II	359	370	ACAAGCACAGAGAGCGCATGT	AACCGTTTTCAGGCCGAGCA	253
FMgSSR-17227	scaffold163114	p2	(CA)6	Class II	463	474	ACACAAAGCGGGCAGACGAT	TAAAGTCCGCCATGGGCTGGTT	312
FMgSSR-17229	scaffold7426	p2	(CA)6	Class II	931	942	ACACACGCGGGACTTTTGCT	TGTGTGTGTGCACCATGAACCG	349
FMgSSR-17232	scaffold32	p2	(CA)6	Class II	199910	199921	ACACGTCCCAGGCATAAGGTGT	TCACCCATGTGTTCACTGCTGCT	216
FMgSSR-17234	scaffold35460	p2	(CA)6	Class II	3747	3758	ACACTCAGGCAAGGGTTCATCT	TCACTAAGGAGAGCAGCTAGAGCA	350
FMgSSR-17235	scaffold10312	p2	(CA)6	Class II	26313	26324	ACACTCAGGTCTCAGACGCATGT	ACCCCTGCTGCATCTTCCAT	333
FMgSSR-17237	scaffold3804	p2	(CA)6	Class II	5989	6000	ACACTTGTCGGTGATGGCGAGT	AACAGTGGCGGACCAAGCAA	238
FMgSSR-17240	scaffold11869	p2	(CA)6	Class II	11397	11408	ACAGCGCTAGCACGGCTAAACT	TCACTTGCACCGCTCGTGTT	236
FMgSSR-17241	scaffold2529	p2	(CA)6	Class II	44119	44130	ACAGCTGGATGGTGCAAGGCAT	TGACGCCATGGGTTGATGGA	297
FMgSSR-17242	scaffold16880	p2	(CA)6	Class II	6558	6569	ACAGGCTGGACGGAATTAGCACA	AGCGAAGCACGCATTTTGGA	208
FMgSSR-17243	scaffold2767	p2	(CA)6	Class II	30006	30017	ACATACATTGCGCGGCACCA	ACGTGACGACGTGCAGATGAGA	307
FMgSSR-17245	scaffold6823	p2	(CA)6	Class II	18169	18180	ACATCAACTCCCCACTGTCCCT	AGTCGGCATTTATGGGCCTGCT	345
FMgSSR-17247	scaffold155	p2	(CA)6	Class II	114335	114346	ACATGCGTTGTGCACCGTGA	ATGAAACCGCCGCGAAAGCA	297
FMgSSR-17250	scaffold16136	p2	(CA)6	Class II	5290	5301	ACATTTGCTCGACCCAGAGCCA	GTCCACCACCTCAAAACAGAGACA	349
FMgSSR-17254	scaffold6	p2	(CA)6	Class II	118498	118509	ACCACCGAAGCCATGGATGAGA	AAATGGCCTGTGCCGCACTCTA	348
FMgSSR-17255	scaffold109320	p2	(CA)6	Class II	921	932	ACCACTGATGCAGAGGCCAAGT	TCGCGTGCATCATTAGCGTGT	330
FMgSSR-17256	scaffold19962	p2	(CA)6	Class II	12459	12470	ACCATGTTGGCCCAGCTCTCTT	TCAGTCATGCGGTGCACGTT	343
FMgSSR-17257	scaffold2690	p2	(CA)6	Class II	28010	28021	ACCCGAGTTACAGACCACACACA	TGTCGGCGTAAACTCCAGCCAT	322
FMgSSR-17259	scaffold136504	p2	(CA)6	Class II	595	606	ACCCGCCCATGCATGATGAAA	GCGCTGACTTGTTGCTGATGCT	231
FMgSSR-17268	scaffold887	p2	(CA)6	Class II	81344	81355	ACCTCGATTTATTGGCCGGGCT	ATTCATGGCAAAGGCCGCGT	323
FMgSSR-17271	scaffold18416	p2	(CA)6	Class II	5450	5461	ACGAAAGGGACGCCTCAACCAA	TTGTGCCTGCATTGCACCTCGT	349
FMgSSR-17272	scaffold4149	p2	(CA)6	Class II	31290	31301	ACGACTCGGCCAACTACACACA	TGCAGACTTGCTGTCCGGGTTT	260
FMgSSR-17281	scaffold3466	p2	(CA)6	Class II	15216	15227	ACGCCGAGATCTTCCCATCTCA	AGGCGGCACGCTACGTATTCAA	324

SSR_ID	Scaffold	SSR_Type	SSR_Motif	SSR Class	SSR_Start	SSR_End	Forward sequence	Reverse sequence	Product_size
FMgSSR-17289	scaffold24279	p2	(CA)6	Class II	8834	8845	ACGGCATATCGGTGTTGGTGCT	AGTGCATGCGTGAGTTGCCT	319
FMgSSR-17290	scaffold12682	p2	(CA)6	Class II	10267	10278	ACGGCCGTCATTACAGCCAGAA	AAAGATCCTCCTTGCGGCGT	342
FMgSSR-17291	scaffold8223	p2	(CA)6	Class II	21954	21965	ACGGGAGTAACCCCCACCATTT	TGTCTGAGCAACATGACAGTAGGC	308
FMgSSR-17294	scaffold132580	p2	(CA)6	Class II	909	920	ACGTGGCCATTTATGCATTCGGC	TTGTTCATCGGCGCGCTTGT	338
FMgSSR-17295	scaffold5674	p2	(CA)6	Class II	17168	17179	ACGTGTGCAGTGAAGTCGGA	TTTGGAGGATGGGCCAGTGT	330
FMgSSR-17298	scaffold107	p2	(CA)6	Class II	115366	115377	ACTACAGATCAGCCTCCCGCTT	ACGGTTCAAATGGGGTTGCGT	339
FMgSSR-17299	scaffold3128	p2	(CA)6	Class II	64322	64333	ACTGAGCAAGCCGTGTGTGTGA	ACTGCCGATTGCCCATTGCT	213
FMgSSR-17301	scaffold4216	p2	(CA)6	Class II	27984	27995	ACTGCTCGTCTCACTGGGACAT	GGGCTCTGCACAAAACGGATCT	325
FMgSSR-17304	scaffold230	p2	(CA)6	Class II	128385	128396	ACTTGAGGGCCAGCCAAGATCA	AAGCTTGCGCAGATAGGCAGGA	270
FMgSSR-17305	scaffold8775	p2	(CA)6	Class II	4055	4066	ACTTGGGACGATAGCAGGGAGA	CGATTGGCCTCTGACCAACCAA	350
FMgSSR-17306	scaffold4623	p2	(CA)6	Class II	3270	3281	AGAACCAAGGATCCCATGCCCT	TTTCGTCAACGCGTGTCGCT	276
FMgSSR-17307	scaffold224	p2	(CA)6	Class II	17272	17283	AGAAGGGAACAGGAACGCCACA	TCCCCTCAAGTGGATCAATGGCT	350
FMgSSR-17313	scaffold9789	p2	(CA)6	Class II	5356	5367	AGAGGAAGATGAAGCCGGGACA	CGTCAGCCATTGTCTTTTCGGGT	289
FMgSSR-17316	scaffold1243	p2	(CA)6	Class II	51385	51396	AGCAACCAACATCCAGGCGA	AGGCGTGTTGTGTAGGTGAGCA	287
FMgSSR-17317	scaffold1419	p2	(CA)6	Class II	54389	54400	AGCAACCACCACTCACATCGCA	GCTGCTGTGCAGTGACAAGT	307
FMgSSR-17320	scaffold25664	p2	(CA)6	Class II	6704	6715	AGCACCCAACATCCCTTGGT	ATTCGGGCAAAATCACCCGCCT	328
FMgSSR-17321	scaffold2883	p2	(CA)6	Class II	40587	40598	AGCACTTGCATTAGCTCGGCCT	ATGCTGTCCAGCACCAATGCCT	341
FMgSSR-17323	scaffold14592	p2	(CA)6	Class II	13068	13079	AGCAGCGTGCAATGGTGGAT	TCACGTGGTGGTTTGTCGGA	310
FMgSSR-17324	scaffold2792	p2	(CA)6	Class II	25858	25869	AGCAGTCGTGGTGCCTCACAAT	TCGCCAGGAGGTTACATGAGTGT	313
FMgSSR-17331	scaffold36	p2	(CA)6	Class II	43762	43773	AGCCGATGTCCGTTGCTTCT	TTGCACATATCCACAGGCCGCT	208
FMgSSR-17337	scaffold229	p2	(CA)6	Class II	67507	67518	AGCGACACATTCCAGGAACGGA	CGTTGTCGGCGGCAAAAAGT	312
FMgSSR-17339	scaffold709	p2	(CA)6	Class II	25229	25240	AGCGCGAGAGAAGGCTACTTGT	AATTGGCCCAAGCCTACCGTGT	261
FMgSSR-17344	scaffold3260	p2	(CA)6	Class II	7931	7942	AGCGTTGGGTGACCACATCAGT	TGAGCGAGTGCGTGATAACGTGT	349
FMgSSR-17345	scaffold26657	p2	(CA)6	Class II	4594	4605	AGCTATCAGTGGCGAAGCTAGGGA	AGGATGAGCCGAGGTGTTAGGGAA	307
FMgSSR-17346	scaffold9361	p2	(CA)6	Class II	27209	27220	AGCTCCGCCTTGAGTGAGTCTT	TCGACGACGCGACGATTCTT	250
FMgSSR-17348	scaffold50104	p2	(CA)6	Class II	3896	3907	AGCTCGCTTTCCTCTTGCCA	TGACCATGCTGAACGCAGGAGA	293
FMgSSR-17351	scaffold17563	p2	(CA)6	Class II	12186	12197	AGCTGTTCATGCTGCCATGGGT	GGGAAGGCTTGTTTGAGGCGAA	296
FMgSSR-17352	scaffold65197	p2	(CA)6	Class II	601	612	AGCTTGAAGCAGGCAGCCAA	TCCAAACTCCAAAGCACCCGCA	286

SSR_ID	Scaffold	SSR_Type	SSR_Motif	SSR Class	SSR_Start	SSR_End	Forward sequence	Reverse sequence	Product_size
FMgSSR-17357	scaffold4746	p2	(CA)6	Class II	8396	8407	AGGATGCTGTGATGGAGAAGCCT	TACTCGAGTCGCTCTCGTGGAT	309
FMgSSR-17358	scaffold19496	p2	(CA)6	Class II	5618	5629	AGGCACACAACAGGAATGCCCT	TTCTTTGCTGGCCAACCTGC	308
FMgSSR-17360	scaffold54435	p2	(CA)6	Class II	3789	3800	AGGCACCGTTGTTTGTGCCA	GTGGTATAAGTCGGTTGTGCTGAC	315
FMgSSR-17361	scaffold50104	p2	(CA)6	Class II	3745	3756	AGGCATTTGATTCCCCCACCCA	AGGTGGCAAGAGGAAAGCGA	265
FMgSSR-17362	scaffold4838	p2	(CA)6	Class II	13511	13522	AGGCCGGGTGTCGTTTAGGAAA	TTTGGCGCCGCATGCAATGA	322
FMgSSR-17363	scaffold4801	p2	(CA)6	Class II	44726	44737	AGGCGCAAAGGCCCAATGAA	AACGTTCCTGCCTGCTGCTT	281
FMgSSR-17366	scaffold894	p2	(CA)6	Class II	78482	78493	AGGCTGCGAATTGCGTGGTT	TCGCAAGGACCGCGAGGTTTTA	206
FMgSSR-17369	scaffold324	p2	(CA)6	Class II	139487	139498	AGGGCCACCTTTTGGAACAACTGA	TGCCATCCATTTCCATCGGCCA	303
FMgSSR-17371	scaffold25461	p2	(CA)6	Class II	2720	2731	AGGGGCTATATGGCACCAGAGT	TGTGACAGTCGCTAGCGTGGTT	343
FMgSSR-17373	scaffold5450	p2	(CA)6	Class II	24628	24639	AGGGTTTGGGATGGAGTGGCAA	GTGGAAACGACTTCACAGGGCA	349
FMgSSR-17377	scaffold204	p2	(CA)6	Class II	95813	95824	AGGTTTGGCATGTGCATGGCTT	TGCTGCGGCAAAACATGATAGC	257
FMgSSR-17378	scaffold28886	p2	(CA)6	Class II	3897	3908	AGTAAAGAGTGCGTGACCGGCT	ACCTTTGCGGTGTTCATGTCGC	222
FMgSSR-17379	scaffold24239	p2	(CA)6	Class II	3966	3977	AGTCCAGGCCAGCCCATTTTGA	AGTTACGTCGTTGCCGCACA	208
FMgSSR-17383	scaffold2771	p2	(CA)6	Class II	29153	29164	ATAAAACGACGCGCTCCCCT	TGCTTTGCGGCAACGGAAGA	266
FMgSSR-17384	scaffold2459	p2	(CA)6	Class II	44051	44062	ATAACCAAGACGCCCGCCCATA	GGGTGGTCATTTAGTGCTTACAGG	281
FMgSSR-17386	scaffold1242	p2	(CA)6	Class II	85695	85706	ATAGCGTCGCCAGCTGCAAA	AGTGACGTTGCCGGACGAAA	323
FMgSSR-17387	scaffold31176	p2	(CA)6	Class II	4424	4435	ATAGGGCGCCGACCAAAGCAAT	TCGGGAGCGTTGGAACAAGT	343
FMgSSR-17395	scaffold925	p2	(CA)6	Class II	78095	78106	ATGACCGGTGAGGAGTTGGGTT	ACCACGGCAACAAGGATGTCA	313
FMgSSR-17397	scaffold5409	p2	(CA)6	Class II	33898	33909	ATGATGCGTTCGTTGGCTCCGT	AGCAGCAGCGCAAGCGTTTA	237
FMgSSR-17398	scaffold283883	p2	(CA)6	Class II	215	226	ATGCAAACATGGCGGAAGGC	GGGACCAGCTTGTTGCGTTCAA	214
FMgSSR-17399	scaffold144	p2	(CA)6	Class II	51386	51397	ATGCATCCTGCAGCGGACCAAA	TCGGATTCAGCTCTTTGTGGCG	320
FMgSSR-17400	scaffold7250	p2	(CA)6	Class II	21488	21499	ATGCTTGCGCAGTGGCAGTA	ATCGGCGGTGCGTTTTCCTT	313
FMgSSR-17401	scaffold32194	p2	(CA)6	Class II	4511	4522	ATGGCACGCATAAACCGGCA	GTGTCTTGGACACTTTAGCCCTTG	334
FMgSSR-17403	scaffold24084	p2	(CA)6	Class II	6159	6170	ATGGCGGCACTAGCAGTTGT	TGGCGATCACAATCACCATCGGA	324
FMgSSR-17404	scaffold4713	p2	(CA)6	Class II	43147	43158	ATGGGTTGTGCCACACATGCAC	TGGAAGCTGAAGAGACCTGCCA	296
FMgSSR-17410	scaffold3841	p2	(CA)6	Class II	24367	24378	ATTATTTGCGCCGCCCGGTT	CGTGCAGCGACACAATGCAA	301
FMgSSR-17412	scaffold3032	p2	(CA)6	Class II	14392	14403	ATTCCGCAGCTGGCACTGTT	AACAGCCCGCTATGCTAGTCGT	284
FMgSSR-17413	scaffold1970	p2	(CA)6	Class II	22489	22500	ATTGAGGCCATGCTCTCGTGGA	TTACCGCCACAAGCCCACAACT	207

SSR_ID	Scaffold	SSR_Type	SSR_Motif	SSR Class	SSR_Start	SSR_End	Forward sequence	Reverse sequence	Product_size
FMgSSR-17415	scaffold2269	p2	(CA)6	Class II	29391	29402	ATTGCCAAGTAAGCTGTGCCCC	ACGGTGTTGCCTTGCGTTGT	293
FMgSSR-17422	scaffold5645	p2	(CA)6	Class II	16671	16682	CACACCACTTGCAGCAGCATCA	TGTTGATACGACTGCTGGCCCT	345
FMgSSR-17426	scaffold9281	p2	(CA)6	Class II	6394	6405	CCAGCAAGATGCTTCTTCAATGCG	TCCACTGAGTTTGCATGTTGGG	271
FMgSSR-17430	scaffold394753	p2	(CA)6	Class II	42	53	CCTCAACAAAAAGGGACTGGACTG	GCCGGAGTGCAAATTTCTTGCC	234
FMgSSR-17432	scaffold55293	p2	(CA)6	Class II	262	273	CGACTCAATTTGCAGGAGCAGCA	ACCATGGCGTTTCGTGCCTT	271
FMgSSR-17436	scaffold10980	p2	(CA)6	Class II	9409	9420	CGCACAAGCTGCTTCGAGAAAA	TTCGCGCTTGCTCTGAAAAGGC	312
FMgSSR-17439	scaffold5044	p2	(CA)6	Class II	29010	29021	CGCCATTGGATTTGTGGCAGCA	CGCGGACAAAGTTGTGTGTGCAT	295
FMgSSR-17440	scaffold155	p2	(CA)6	Class II	114147	114158	CGCCGCACGTTTTGGTGTTT	TCACGGTGCACAACGCATGT	253
FMgSSR-17442	scaffold426	p2	(CA)6	Class II	48114	48125	CGCGCCACAACACCACAAAGAT	AATCACATGAGCCCGACGGTGA	287
FMgSSR-17444	scaffold1255	p2	(CA)6	Class II	58724	58735	CGGCCAAACTTAGACCGGTATGT	GGGAGGATGGAGAGCTCAACAACAA	348
FMgSSR-17445	scaffold873	p2	(CA)6	Class II	77469	77480	CGGGATCCCCGGATTTTGTGAA	TTGGTCAGCATTGCGTGCGA	209
FMgSSR-17448	scaffold11877	p2	(CA)6	Class II	1158	1169	GACTTTTGCCCTTTTTCCCACCCA	ACTGGGGGCAGATTTGATGGT	300
FMgSSR-17452	scaffold273	p2	(CA)6	Class II	93663	93674	GCACGAAACGATTGGTGGCA	TCACAGGGTTGCTTCAGAAGTCGT	228
FMgSSR-17453	scaffold490	p2	(CA)6	Class II	74328	74339	GCACGCGCGCATGGATTTAT	GGACATGGTCGTTGCCGTAGTT	315
FMgSSR-17461	scaffold1267	p2	(CA)6	Class II	50373	50384	GCCCAAGAACCCAACCAACCAA	TGCGCTGTTCATTGCCCTGT	233
FMgSSR-17463	scaffold7282	p2	(CA)6	Class II	13546	13557	GCCCATGCGGTGAGCCATTTTT	AGTGAAGCTAGGGACGAAGACCA	284
FMgSSR-17466	scaffold9069	p2	(CA)6	Class II	3431	3442	GCCTGTTCAATTGCCGGCGTTA	GGTGTGTGCAGCTGTATGTGCT	341
FMgSSR-17467	scaffold633	p2	(CA)6	Class II	19124	19135	GCCTTGTTATTAGCTTCCCCACCC	TGCGTCCTGCTACGTGCTGATT	311
FMgSSR-17469	scaffold3038	p2	(CA)6	Class II	10115	10126	GCGCTGCTGCGATCTTGCTAAA	TTGCGTGGGTGTGTGTGTGT	283
FMgSSR-17470	scaffold10124	p2	(CA)6	Class II	19968	19979	GCGTGCATACGGGTTGCTTTGT	TTTCGGCATGGGGGCGAAAT	343
FMgSSR-17471	scaffold5967	p2	(CA)6	Class II	33026	33037	GCTAGCCTTGGTATTCCATCAGTGC	ACTGGTTTGCCCCTACGTCA	292
FMgSSR-17472	scaffold9812	p2	(CA)6	Class II	12958	12969	GCTGCAGCACAGCGCACATAAT	AACTGCTGCGTCTGCATCGT	221
FMgSSR-17475	scaffold2187	p2	(CA)6	Class II	11621	11632	GGAAGCCACTTAGTTCATCGGCCA	TGCGTCATGTGATGTTAAGCACGG	213
FMgSSR-17477	scaffold2763	p2	(CA)6	Class II	26407	26418	GGCGACAAAAATGGCTCCAGCA	TGCGTCGTCGTCGTGTCATT	339
FMgSSR-17479	scaffold333	p2	(CA)6	Class II	102336	102347	GGGTCCATCAAAGCTTCTGGGA	CTCGAAGCCTTGAGCACACAT	240
FMgSSR-17481	scaffold919	p2	(CA)6	Class II	69621	69632	GTGCCTGTGAAGAAACGAAAAGCG	AAGCTTCAGCTGCCCCCAAT	233
FMgSSR-17483	scaffold42	p2	(CA)6	Class II	69455	69466	GTGTTTGGATGCACGTGAGCGT	AGTTGTGGGCTTGTGGCGGTAA	313
FMgSSR-17484	scaffold5029	p2	(CA)6	Class II	16775	16786	GTTGCTCGTTTGCGTGCACAT	ACGGAGAGCTGAAGCTGTGGAA	283

SSR_ID	Scaffold	SSR_Type	SSR_Motif	SSR Class	SSR_Start	SSR_End	Forward sequence	Reverse sequence	Product_size
FMgSSR-17486	scaffold5178	p2	(CA)6	Class II	32051	32062	TAACAGCATCACCACCAGGCGA	ACCTGCAGTCCATCGCCCATTT	331
FMgSSR-17491	scaffold3226	p2	(CA)6	Class II	21183	21194	TACCCACGCGCATGCTACCAAA	AACTACGCTCGATCACACCGCT	238
FMgSSR-17492	scaffold17349	p2	(CA)6	Class II	6488	6499	TACCGATGATATGCCCCCGCTA	GTGGCGGGGAGAGAAGTTAGTT	325
FMgSSR-17493	scaffold3883	p2	(CA)6	Class II	19219	19230	TACGACGGACGCGCATAGTACA	ATCGTGGTTGCTGCGGTTGT	228
FMgSSR-17494	scaffold449	p2	(CA)6	Class II	72605	72616	TAGCGCACCGCATCTGTGAA	TGACTGGGACTCTGGGAGCTAT	234
FMgSSR-17495	scaffold977	p2	(CA)6	Class II	86430	86441	TAGGGGCCGCAAAGCAATAGCA	TGGAGAAGAGGTCTCATGCAGC	240
FMgSSR-17496	scaffold3587	p2	(CA)6	Class II	37362	37373	TCAAAGGACGACGGTGTGGGTA	TCTCGACGTTTCGGCAGCTT	278
FMgSSR-17509	scaffold2427	p2	(CA)6	Class II	11097	11108	TCACGCACCGTGTGTACCACTT	AAGCAACGTCCTCATCGTCCCA	276
FMgSSR-17513	scaffold34009	p2	(CA)6	Class II	1214	1225	TCCAAACAATGCTCCCGGCT	ACCCAATTGGTCTTGTCATCGTCC	329
FMgSSR-17516	scaffold9413	p2	(CA)6	Class II	27088	27099	TCCACTTTTGCTGGCGCGAA	AGCTTCGCCCTCACTCACAACA	350
FMgSSR-17517	scaffold5318	p2	(CA)6	Class II	16405	16416	TCCAGCTCTAGGTGATGGATGC	TGGGTAGATGGTGAGAGCTCAAGG	260
FMgSSR-17520	scaffold28941	p2	(CA)6	Class II	2711	2722	TCCCAGAAGACATCAGAGGGAGGA	TCAATGAGCGCGGGCGTTTT	226
FMgSSR-17523	scaffold3113	p2	(CA)6	Class II	22045	22056	TCCGAAGCAAGGACCCACTTGA	TGATTTTGCACACGGGGCGA	221
FMgSSR-17528	scaffold13	p2	(CA)6	Class II	11708	11719	TCCGGCAAGATGACGAGGTTGA	TGTGACCCCCGCCTATAACTCA	350
FMgSSR-17529	scaffold12570	p2	(CA)6	Class II	2571	2582	TCCTCGCCTTGGACGCTTGTTT	TCGCTGCCAAGAAACTGCCA	257
FMgSSR-17532	scaffold1116	p2	(CA)6	Class II	67453	67464	TCCTGTTGGGTGTTGGCGCATA	GCCACTAGGAAAGAGACAGTAGCA	350
FMgSSR-17533	scaffold866	p2	(CA)6	Class II	81179	81190	TCCTTGGTGCGCACTTGTTGT	TGCGGGAATGGTTCATCAGC	348
FMgSSR-17534	scaffold13906	p2	(CA)6	Class II	21524	21535	TCGAAGCACCATGACGGTGAA	TTGTGAAGAGTGGGGTAGCGA	245
FMgSSR-17537	scaffold1007	p2	(CA)6	Class II	91158	91169	TCGCCCCTTGCAAATGGGATCT	TAACACGCCGCCTGCGTTATCT	345
FMgSSR-17538	scaffold2659	p2	(CA)6	Class II	31737	31748	TCGCTCACGCGTTCTCCCTTTT	ACGTTCCTTTCACACGGCGA	339
FMgSSR-17540	scaffold1097	p2	(CA)6	Class II	48171	48182	TCGCTGTTCTTCCGTTGGTGCT	TGGTGTCTCTCCAGACAAGCGA	325
FMgSSR-17541	scaffold11929	p2	(CA)6	Class II	9419	9430	TCGGCACAAGGACGTGTTCACT	TTCGCATCACACGTCGGCAA	321
FMgSSR-17542	scaffold8528	p2	(CA)6	Class II	19310	19321	TCGGCTCGTACGCAACTTCCTT	TTCACGAGCCGTTCGTTCATGC	234
FMgSSR-17545	scaffold832	p2	(CA)6	Class II	59883	59894	TCGTGTTCCACAGGAGCGTT	TTTTGTCGGCAGTGCCTCGT	218
FMgSSR-17549	scaffold6477	p2	(CA)6	Class II	18420	18431	TCGTTTTGCCTCCCTGGACAGT	CGCAACAGAGCTTTGCAACACA	343
FMgSSR-17550	scaffold15064	p2	(CA)6	Class II	15548	15559	TCTAGACGGCAATCCGAGGACA	GCGCTCGATTACGCGGTAGTTT	250
FMgSSR-17554	scaffold247	p2	(CA)6	Class II	73357	73368	TGAAATTCAGGTGGGGGCCTCT	TCGGTCTGCATGTGAGCCAA	313
FMgSSR-17561	scaffold24915	p2	(CA)6	Class II	9601	9612	TGAGATGGAAGAAGGGCGGCAA	AGAAGAAGACAGGCTGCGGT	246

SSR_ID	Scaffold	SSR_Type	SSR_Motif	SSR Class	SSR_Start	SSR_End	Forward sequence	Reverse sequence	Product_size
FMgSSR-17565	scaffold7981	p2	(CA)6	Class II	23032	23043	TGAGTGTGCCATGCTGCCAT	TGCTGACATGCCACACAGGT	342
FMgSSR-17566	scaffold7790	p2	(CA)6	Class II	8897	8908	TGATAACAACCCCGATTGGGTCGG	GCTGGCCTTAAAGCCTTTCGCA	320
FMgSSR-17567	scaffold1549	p2	(CA)6	Class II	62397	62408	TGATCGAAAGACGGGGAATGGC	TTCGCCGACAACAAGGACCGAT	310
FMgSSR-17569	scaffold4875	p2	(CA)6	Class II	21205	21216	TGATGCCTGTTCGGTTGGTGGT	ACAACACGCAAGCAGACGCA	234
FMgSSR-17580	scaffold2861	p2	(CA)6	Class II	50587	50598	TGCAGGCACATCATCGACACT	AACACCACACCCCCAAACCA	348
FMgSSR-17584	scaffold5757	p2	(CA)6	Class II	39010	39021	TGCATGTCGCATGTGCGTGT	TGCACGTACGGTGTCCTTGCTT	327
FMgSSR-17587	scaffold126	p2	(CA)6	Class II	171617	171628	TGCCGCTATCGCTCAAGTCGAA	TTGATGGCTGACCGCTTCCTGA	244
FMgSSR-17591	scaffold16683	p2	(CA)6	Class II	15133	15144	TGCCTTCTCCGCGCTACAAA	ACCAACCACCACGGCATGTA	328
FMgSSR-17592	scaffold8779	p2	(CA)6	Class II	23061	23072	TGCGATGTTCAACCAGCAGACT	AATGTGGCGTTTCTCGTGCC	290
FMgSSR-17593	scaffold2919	p2	(CA)6	Class II	49740	49751	TGCGCGCATGGTCTTTCTCA	TGGATGTGTAGCAGCCCTGTCA	327
FMgSSR-17594	scaffold810	p2	(CA)6	Class II	34316	34327	TGCGGAGCCGACACAATTGACA	ATGATCACGCGACCAACGTGGA	279
FMgSSR-17601	scaffold840	p2	(CA)6	Class II	8476	8487	TGCTCCATCGAACTCAGCCTCA	AATGTGGTCACGGTTGCCGGAT	282
FMgSSR-17607	scaffold1202	p2	(CA)6	Class II	5532	5543	TGCTTGCAAAGCAGGCTCACA	TGTGTAACACTGTCGTCTCGGGT	230
FMgSSR-17608	scaffold79071	p2	(CA)6	Class II	1031	1042	TGCTTGTGAGACAACCCGTGCT	AGCTCGGCTTTCCTGCGTTT	255
FMgSSR-17610	scaffold5407	p2	(CA)6	Class II	32184	32195	TGGAGTCGACGCGACAAACA	AAAGTGCATACGGGCTCGACCA	225
FMgSSR-17611	scaffold5492	p2	(CA)6	Class II	3044	3055	TGGATCTGGATGAAGCAACGGCT	ACAATGACTACGGCAGCAGCGA	281
FMgSSR-17613	scaffold7459	p2	(CA)6	Class II	9589	9600	TGGCATGGGGAGTTGCACTT	TCCTCGCTCATGCTCATCCTGT	326
FMgSSR-17614	scaffold2573	p2	(CA)6	Class II	24396	24407	TGGCCACACCGTGCACAATCTA	CGTGCCAGTCCATCCCTTTTCT	254
FMgSSR-17616	scaffold24042	p2	(CA)6	Class II	6388	6399	TGGCCGTGTTTGACGCAGAA	TACACCTGCGGCCAGCAAAA	307
FMgSSR-17617	scaffold13142	p2	(CA)6	Class II	4624	4635	TGGCGACAAGCCGACAATCA	AGTCGGTCGAAGGAACTGGGAA	205
FMgSSR-17619	scaffold8187	p2	(CA)6	Class II	25595	25606	TGGCGTCGCGTTTGATCTCT	ATTGATGGGCATAGCTCCGGCT	300
FMgSSR-17625	scaffold161	p2	(CA)6	Class II	15953	15964	TGGTACCCGCGGAACTTGAACT	ATCGAGCGGCCTGTTTCAGT	221
FMgSSR-17626	scaffold24942	p2	(CA)6	Class II	10292	10303	TGGTACGGATGGATCACACACT	GGGTAAGCGACTACTGTCGTATCA	350
FMgSSR-17628	scaffold5022	p2	(CA)6	Class II	33449	33460	TGGTGAACGAGGAATCTCGCA	AGTCTTCCGTGCGTGCACTT	227
FMgSSR-17630	scaffold168859	p2	(CA)6	Class II	255	266	TGGTGCTTGCAGGCTACAAAGG	TTTACTGATGCAGCGGCCAGGT	230
FMgSSR-17632	scaffold4459	p2	(CA)6	Class II	25410	25421	TGGTGGTGTCAACAGCAGGT	GCTCCAGCTTGCAGATCCGTTT	280
FMgSSR-17633	scaffold172	p2	(CA)6	Class II	84576	84587	TGTAGCCATGTGAAGCGAGGCT	ACGACAGTGGCCATTGCTGCTA	331
FMgSSR-17637	scaffold40	p2	(CA)6	Class II	68606	68617	TGTCATCAAACTGATCCCCTCCCC	TGGGTGCTGGATCATGCAGTGT	286

SSR_ID	Scaffold	SSR_Type	SSR_Motif	SSR Class	SSR_Start	SSR_End	Forward sequence	Reverse sequence	Product_size
FMgSSR-17649	scaffold3345	p2	(CA)6	Class II	29382	29393	TGTGGAGTTTGGACGTTGGGCT	AAAAGGCGTGCTCCTTCGTCGT	341
FMgSSR-17650	scaffold23724	p2	(CA)6	Class II	2564	2575	TGTGGTTGGCATCCCCAAGTCA	TTGCGCAACCCGGTCCGAATTA	290
FMgSSR-17651	scaffold5555	p2	(CA)6	Class II	2548	2559	TGTTGACGGAGACGCAGACA	TGGCGGAGCAAAAGCTCCAA	223
FMgSSR-17655	scaffold965	p2	(CA)6	Class II	82456	82467	TTATTGTGACGGCGTGAGGAGC	TCCCAAACCAATGACGCGGACA	317
FMgSSR-17668	scaffold4467	p2	(CA)6	Class II	6667	6678	TTGATGCCGTGGCCTCATGTCA	GCGATGTTCCTCCGCCTTCATT	294
FMgSSR-17669	scaffold891	p2	(CA)6	Class II	75166	75177	TTGATGGCGGCTTTCTGCAAGG	AGCATGGGGCTGATCGCTCATT	214
FMgSSR-17670	scaffold429	p2	(CA)6	Class II	61325	61336	TTGATGGGCCGGTTCGGTTT	TGTCACATGTCCGCGTCTAGTCA	337
FMgSSR-17676	scaffold11286	p2	(CA)6	Class II	13329	13340	TTGCATCAGACGCCGACCAA	TGCCCTGCCCGAATCTTGAA	211
FMgSSR-17682	scaffold12565	p2	(CA)6	Class II	17363	17374	TTGCTAACGTGCATCGGCCA	TGGTATGGCCTTGGTGTGCT	251
FMgSSR-17686	scaffold98805	p2	(CA)6	Class II	1010	1021	TTGCTGGCGGAGCACAACAT	TACCACCGAGCGCAGCAATCTT	294
FMgSSR-17694	scaffold3093	p2	(CA)6	Class II	18403	18414	TTTAGCTGCAGTCGCACAGGCT	TGGCCAACTGCCGTTCAAGACA	238
FMgSSR-17695	scaffold1433	p2	(CA)6	Class II	68653	68664	TTTAGCTGCTGACACGCGCA	ACGGGTCTCCTTCTTCTCGTCA	333
FMgSSR-17699	scaffold866	p2	(CA)6	Class II	19671	19682	TTTGTTCTGGCAGGCTCGCA	TTCCGCTGTTTCAGTGGCGT	267
FMgSSR-17700	scaffold385	p2	(CA)6	Class II	116236	116247	TTTTCCGCCTTCCTTGGCCGTT	TGCTGTGCTTTGCATGATTCTGGC	325
FMgSSR-17702	scaffold1970	p2	(CA)6	Class II	57135	57146	TTTGTCGGGCGTCCGCTTT	GCGTTCGAGAGCCCGTCAAATA	340
FMgSSR-17703	scaffold2451	p2	(CA)6	Class II	14874	14885	TTTTTGCGGCGCCGTATCGT	ACACCACCACGTAGGCCAAGAA	291
FMgSSR-17710	scaffold3046	p2	(CA)7	Class II	35892	35905	AAAGGTGCCAACAAGCACCGGA	ACCCATGCCTGGTTGCTCCAAA	249
FMgSSR-17711	scaffold5423	p2	(CA)7	Class II	11263	11276	AAAGTCGATATTGCCCCTCGGC	AGGCTTAGGAATGCCCTCTCCA	306
FMgSSR-17714	scaffold4249	p2	(CA)7	Class II	37911	37924	AACAAAGACGGTAGGGAGCCCA	GCATCGAGCTCTGGATTGAGCA	218
FMgSSR-17716	scaffold3259	p2	(CA)7	Class II	42840	42853	AACACACGCCCACACTACCCAT	CGTGACGTGCTCGATGAACT	243
FMgSSR-17720	scaffold1937	p2	(CA)7	Class II	4660	4673	AACCAATGGCTGGGACCTACAC	TGCTCTGCTGCAGCTATACCCT	296
FMgSSR-17722	scaffold11924	p2	(CA)7	Class II	5096	5109	AACCGCGCTGCAATGATCCA	TCGAGGTGTGGCCTTTGTCA	214
FMgSSR-17723	scaffold6125	p2	(CA)7	Class II	8593	8606	AAGAACGTGCAGCTGTGGCT	ACGCTTTCTCGCCTAACGGA	349
FMgSSR-17725	scaffold11987	p2	(CA)7	Class II	3071	3084	AAGCTCCTGGGGTTTTGCCACA	TCCGAACCGCCCCAACCTTTTT	260
FMgSSR-17728	scaffold6280	p2	(CA)7	Class II	33561	33574	AAGGGCAGAACTGCGTTGGT	TGCGTGTCTGCACACGTCAT	309
FMgSSR-17734	scaffold41779	p2	(CA)7	Class II	1552	1565	ACAAAGCCTTCCCAACCGCA	TTCAGGTCGCGGTTTGGCTT	330
FMgSSR-17742	scaffold9297	p2	(CA)7	Class II	17434	17447	ACCAGCCGGTGTGCGAATTT	TGCTTCTGGCTTCTTGCGTAGC	281
FMgSSR-17743	scaffold25705	p2	(CA)7	Class II	2882	2895	ACCCACCCCAACACAACACA	AGATGCTAGGCCCTCAGTTTGA	204

SSR_ID	Scaffold	SSR_Type	SSR_Motif	SSR Class	SSR_Start	SSR_End	Forward sequence	Reverse sequence	Product_size
FMgSSR-17744	scaffold10996	p2	(CA)7	Class II	18716	18729	ACCCCAATGTGCCTCGCTATGA	TGCGGAACTAAGGCCTGAAGAC	350
FMgSSR-17745	scaffold3	p2	(CA)7	Class II	65735	65748	ACCCTGCAAAATGCATGCCCA	AACAGTGCGAGACAGCACGA	247
FMgSSR-17746	scaffold5166	p2	(CA)7	Class II	11591	11604	ACCGACCATAAGCCAAAACTGGGG	TGCTATTGTTTGGGAGGCCAGG	275
FMgSSR-17747	scaffold7886	p2	(CA)7	Class II	1913	1926	ACCGCATCAAGGCAACGGAA	AGGTGCTGCCGAGCTCTACAAT	306
FMgSSR-17748	scaffold5358	p2	(CA)7	Class II	21481	21494	ACCGGGATCAAAACCATAGGTCTC	GCGAGACCCAATTCTGCAGTAGCA	323
FMgSSR-17749	scaffold5603	p2	(CA)7	Class II	8248	8261	ACCTACCTGCAAGGCTGCAACA	AATGGATGGTGCGGTAAGGGGT	333
FMgSSR-17753	scaffold20087	p2	(CA)7	Class II	7161	7174	ACGCACCGCGTCCTCTCTTTTT	TGGCCATGATGCTCATCCTTCCT	314
FMgSSR-17756	scaffold8193	p2	(CA)7	Class II	24728	24741	ACGCCCAGTCACACGACAAGAA	TGGCTGTTTTAGGTCCGGTGGT	298
FMgSSR-17758	scaffold1780	p2	(CA)7	Class II	9935	9948	ACGTGTTGGCGTTTTGCCAC	GTCAAAATCGAGACTATGGGCCTC	320
FMgSSR-17765	scaffold5608	p2	(CA)7	Class II	34723	34736	AGAAAATCCAGGCGCGGAAGA	AGCAGTCCCCTTAGCCATTCCT	333
FMgSSR-17766	scaffold359	p2	(CA)7	Class II	102730	102743	AGAAGACAGCTGCGCGTACA	TTGCTGCCGTTAGTGCTGCT	208
FMgSSR-17767	scaffold18543	p2	(CA)7	Class II	3726	3739	AGACAGCAACACACACAGGGGA	ACAAGGTTCCGGCTCACAGT	311
FMgSSR-17772	scaffold2748	p2	(CA)7	Class II	21440	21453	AGCAGCTCCGCTCCACTTACAA	TGGCACAAGGCACAAGCTCA	320
FMgSSR-17773	scaffold9569	p2	(CA)7	Class II	7073	7086	AGCATCATGGACAGACGAGCGA	ACGCCATGCATGCTGCTGTA	247
FMgSSR-17774	scaffold6463	p2	(CA)7	Class II	19551	19564	AGCATTGCCGCCACCGTTAT	AGCTGTTGTGACGACGCGAT	291
FMgSSR-17776	scaffold4345	p2	(CA)7	Class II	9417	9430	AGCGACGGGAAATGAGGCGTAA	TGCCAAGCAAAGCAATGCGG	310
FMgSSR-17777	scaffold1687	p2	(CA)7	Class II	62709	62722	AGCGCTGCAGGCTACACATCAT	GCGGGTCGACGATGTTACAGAA	308
FMgSSR-17778	scaffold12942	p2	(CA)7	Class II	9474	9487	AGCTCAGCGTTCACCACGTAGA	TTTGCCCCAGGCATTTCCAGGT	335
FMgSSR-17779	scaffold572	p2	(CA)7	Class II	22288	22301	AGCTCTGATGCACGACGCAA	TTTTATGTTCGCGCGCTGGTGG	310
FMgSSR-17781	scaffold7474	p2	(CA)7	Class II	35753	35766	AGCTTTGTCGTAGTGCCAGGGT	ATGGCCAGCAACTAGCCAGTGA	249
FMgSSR-17784	scaffold2499	p2	(CA)7	Class II	36384	36397	AGGCACAACAACTCGTACCAACCT	AAACGCACACACGCACGCAT	349
FMgSSR-17789	scaffold14477	p2	(CA)7	Class II	11201	11214	AGGGTGGCCACGTTAGCCAAAA	TTCGCTCGTTCGCTCCATCCAA	271
FMgSSR-17792	scaffold632	p2	(CA)7	Class II	42932	42945	ATATGTGGAGGAGCCAGCGGTT	TGCCCAGAGCTGGTGAGATCCATA	302
FMgSSR-17793	scaffold10840	p2	(CA)7	Class II	11570	11583	ATCCCCATCAATGCCTGACCCT	ACGAGCCTGTGACAACCACACA	266
FMgSSR-17797	scaffold1024	p2	(CA)7	Class II	44860	44873	ATGAAGACGTCGCTGCCGAT	ATGCTTCGTTCGTCGTGGGTGT	348
FMgSSR-17798	scaffold9444	p2	(CA)7	Class II	2086	2099	ATGAATGCATGCGGTTGGCAGG	TATGCGCAGTGCCGCGTTAT	309
FMgSSR-17799	scaffold155	p2	(CA)7	Class II	69315	69328	ATGATAACACTGATCCCCCACCCC	AGGAGCTTGGTGTGTGTGTGTGT	327
FMgSSR-17801	scaffold3723	p2	(CA)7	Class II	37150	37163	ATGCAGGAGGGCTGTGTGTT	TGTTTGAGGGGCCCTTGGTCTT	347

SSR_ID	Scaffold	SSR_Type	SSR_Motif	SSR Class	SSR_Start	SSR_End	Forward sequence	Reverse sequence	Product_size
FMgSSR-17803	scaffold13330	p2	(CA)7	Class II	16715	16728	ATGGAACGTATGGACAGGGGAGGA	TGCTTCGCGGCGTTGAATGT	343
FMgSSR-17805	scaffold25018	p2	(CA)7	Class II	8224	8237	ATGGTAGACGGATGGCAATGGCAC	ACTTGCTCGGGCAACCTAAACA	258
FMgSSR-17807	scaffold1549	p2	(CA)7	Class II	6908	6921	ATGTCGCGCTGCGTCTGTTT	TCTGTTGACTTCCAGCTCGCCT	295
FMgSSR-17808	scaffold3274	p2	(CA)7	Class II	41156	41169	ATGTGGCAACAACACCTCGTGC	AAACAGCAGCAGTGGCAGCA	326
FMgSSR-17810	scaffold625	p2	(CA)7	Class II	63312	63325	ATTGACACACACACGCACACGC	AATCACGCCATTAGGCAGCG	310
FMgSSR-17811	scaffold790	p2	(CA)7	Class II	83517	83530	ATTGCTGAGCGGCTGCCATT	TTTCGGCTACAACCACCTCCGT	256
FMgSSR-17815	scaffold6685	p2	(CA)7	Class II	21614	21627	ATTTTAACGGGACGAGGAACGGGG	ACCATTGGTGTGGAGGCAGA	214
FMgSSR-17819	scaffold1714	p2	(CA)7	Class II	1836	1849	CCAACACAGCAAACCACATGCCA	TGCTACGAGCCGAGTGTCAA	202
FMgSSR-17821	scaffold1949	p2	(CA)7	Class II	15078	15091	CCCCCTCTTTGCTCGTGGTTTT	TGGCAATGGCAAGCTGCAAG	287
FMgSSR-17823	scaffold5907	p2	(CA)7	Class II	12278	12291	CCTGCAGAAGCAGCAAACAGCA	TGCCTGCGTCTTGTGGATGGAT	299
FMgSSR-17824	scaffold5883	p2	(CA)7	Class II	14371	14384	CGATCTCATTCTTCCTGAAGCCCT	GGAATGAACATTGGGGCTGCCA	312
FMgSSR-17825	scaffold13979	p2	(CA)7	Class II	12532	12545	CGCATGCTCACCTCTCCAAACA	ACGTAGCCGCACTCGATGATGT	315
FMgSSR-17828	scaffold2118	p2	(CA)7	Class II	63923	63936	CGGTGCCGCGTTGCTTTCATAA	TGCACCGACGAACCCTGTGTTT	240
FMgSSR-17829	scaffold4481	p2	(CA)7	Class II	40971	40984	CGGTGGCGGGTGTTTTCTTTGT	ATGCTGGTGGCCGTCGTTTT	293
FMgSSR-17830	scaffold3279	p2	(CA)7	Class II	15168	15181	CGTCACGGTCATGCTCATGCAA	TGAACGGCGAACTGGTGATCCA	206
FMgSSR-17831	scaffold1572	p2	(CA)7	Class II	4706	4719	CGTGCTTGAACCGGGTTTGCAT	TCGCCATCAGGGGTACGTCTTT	315
FMgSSR-17833	scaffold258	p2	(CA)7	Class II	17579	17592	CTCACATTCTCACTGCACCTCTTC	TCCCCTGTCTCGCTGTGAATGT	305
FMgSSR-17834	scaffold21413	p2	(CA)7	Class II	3629	3642	GAGCGCTTCGACATCTGAATCT	TGTGAGCTGCTGTGAAAGGCA	281
FMgSSR-17835	scaffold8977	p2	(CA)7	Class II	27709	27722	GAGTCGGAAAATGTCATGCGGT	TGGGCATCAGCGTGTTGTCT	323
FMgSSR-17840	scaffold9644	p2	(CA)7	Class II	3549	3562	GCAGTGAGTGCTGGGGCTTTAT	AAGATGAAGTGCCCTGAGGGGT	205
FMgSSR-17842	scaffold6692	p2	(CA)7	Class II	18536	18549	GCCATGCACGCTCACAGCTTTA	TGCTGATCACCGTTGGTCCACT	281
FMgSSR-17843	scaffold800	p2	(CA)7	Class II	62959	62972	GCCGCCAACCATTGTTGCTGTT	ATGCTCGAGTCTTCGTTCGCCT	258
FMgSSR-17844	scaffold392	p2	(CA)7	Class II	92343	92356	GCGGCTGCAGGAAATGCTCAAA	ACACAGCGCCAACCAAGTGT	285
FMgSSR-17846	scaffold2619	p2	(CA)7	Class II	31046	31059	GGCATACAAAGAGGTTGTGTGGGT	TGGTCGGCCAAGATCTCCAACA	326
FMgSSR-17848	scaffold1832	p2	(CA)7	Class II	63991	64004	GGGCTCTAGCCTCTAATGAAGCA	TCGATTATGGGCAACTGGCGCT	345
FMgSSR-17849	scaffold8167	p2	(CA)7	Class II	20202	20215	GGTCCGTTATAATGCACAGACACCC	ACATCCGTGCATAGCGCACCTA	309
FMgSSR-17859	scaffold1212	p2	(CA)7	Class II	776	789	TACATCCTGCCTGGCGTTCCAT	CCACGTCGGACGCCATTTTTGT	262
FMgSSR-17862	scaffold384	p2	(CA)7	Class II	14423	14436	TACGTTGTTGGCGTGGCTTCCT	TCACCTGAACAACGGCAGAGCA	332

SSR_ID	Scaffold	SSR_Type	SSR_Motif	SSR Class	SSR_Start	SSR_End	Forward sequence	Reverse sequence	Product_size
FMgSSR-17864	scaffold9290	p2	(CA)7	Class II	1073	1086	TAGTTTGCTCAAGGCCAGCCCA	CAAGGACACCAGGGATCCAAGT	216
FMgSSR-17867	scaffold1502	p2	(CA)7	Class II	26263	26276	TCACCAGTTCGCAGCCATCCAT	CTGTGACTTGCTCTCTGTTGCAGT	252
FMgSSR-17874	scaffold103807	p2	(CA)7	Class II	785	798	TCAGTGTCCCTGGCTCCCTGTATT	ACAGCTGACAATACCCGCCT	284
FMgSSR-17877	scaffold6503	p2	(CA)7	Class II	25136	25149	TCCCATCTCCAACTCCAACGGA	TGCCGCCAAATCAAACCGCA	213
FMgSSR-17878	scaffold2978	p2	(CA)7	Class II	7562	7575	TCCCTTCCCGCATTGGCAACTT	TACGACCCGAGTGTCCGTAGTCAA	311
FMgSSR-17879	scaffold793	p2	(CA)7	Class II	91104	91117	TCCGAGTGGCGTTTTGTCACCT	ACGCGATCAAAGGAACAGCC	340
FMgSSR-17886	scaffold11520	p2	(CA)7	Class II	9455	9468	TCGATGTCATGGCACCACAAGG	AGCACTCTGGTTGCTAGACCTGT	216
FMgSSR-17887	scaffold835	p2	(CA)7	Class II	89502	89515	TCGCCACGCGTCGGTAATACTT	ACGGATGATTAGCGGTTGGGCA	299
FMgSSR-17893	scaffold4903	p2	(CA)7	Class II	46122	46135	TCGCTCGTCTCTGTCCCTCTTT	TGTGTCCATTTGGCCTTCCTTC	344
FMgSSR-17896	scaffold179	p2	(CA)7	Class II	136829	136842	TCGGAAGTTTTTGCTCCTCCGCT	TGGGCACATGCGCCTAAGAA	200
FMgSSR-17897	scaffold281	p2	(CA)7	Class II	86643	86656	TCGGGTGCAAGAACAGCGAACT	TCGTGGCGTAACGAACGGTAAC	314
FMgSSR-17899	scaffold15787	p2	(CA)7	Class II	12421	12434	TCGTAAAGTGGGGACCAGGAGT	CCTGTGGTGGAACAAAGTCTCCGA	335
FMgSSR-17900	scaffold67121	p2	(CA)7	Class II	1915	1928	TCGTGAGCGATAACGAGTTCACC	AAGGAAGGAGCAACGGTGTGGA	203
FMgSSR-17901	scaffold3233	p2	(CA)7	Class II	12526	12539	TCGTGCGTTACAACCGGAGA	TCGAAACAAAGCTGGCCCGT	335
FMgSSR-17903	scaffold560	p2	(CA)7	Class II	62766	62779	TGAAGCACGTCGCCCGAAAT	GCTAAACGTGTTGCCCTAGCGT	300
FMgSSR-17905	scaffold738	p2	(CA)7	Class II	14253	14266	TGACAGCACAGCACAGCACA	AGGTGCGGATGGAGCTTTCT	290
FMgSSR-17910	scaffold55	p2	(CA)7	Class II	144445	144458	TGAGCCCTTGTGGTAGCCTCTT	ACAGCGCCAGCAAACACACT	350
FMgSSR-17911	scaffold2406	p2	(CA)7	Class II	11065	11078	TGAGGGCGTCTCTTCCAGCATT	AGGCACAAGTTGGGTGTGTG	329
FMgSSR-17914	scaffold40049	p2	(CA)7	Class II	1148	1161	TGCAACCTACCCACCTAACCCA	ATTTGGCGCTGCCGCTCATT	291
FMgSSR-17916	scaffold73155	p2	(CA)7	Class II	2155	2168	TGCACACCGTGCCTCTTGAA	CGAACGTCGCATTGGCTGTTGA	244
FMgSSR-17917	scaffold68	p2	(CA)7	Class II	175194	175207	TGCACATGCACACACTCAACCC	TCGCCATTGGCACTAACTGAGACC	295
FMgSSR-17918	scaffold21721	p2	(CA)7	Class II	4296	4309	TGCACCAAGGATCCTATCGAACCA	GGGGTACGGTGGGAAGGAAAGAAT	349
FMgSSR-17920	scaffold1487	p2	(CA)7	Class II	74925	74938	TGCACGCGAGCTCTACTTGT	ACGATGAACGCTCGTTCGGA	248
FMgSSR-17921	scaffold1045	p2	(CA)7	Class II	1080	1093	TGCAGCCAAGCAAAGCAAAGCA	AGCGAAGAAGGCGAGGCGAAAT	325
FMgSSR-17922	scaffold686	p2	(CA)7	Class II	91701	91714	TGCAGGTGTCGACCAGATGACT	TTATCATCTCGCTGGTCCGCGT	330
FMgSSR-17925	scaffold1286	p2	(CA)7	Class II	72631	72644	TGCATCCTGCAGCGAACCAAA	CGCGCCATTCTACCTTTCCCAA	293
FMgSSR-17928	scaffold6673	p2	(CA)7	Class II	17545	17558	TGCATTTGTGCTGCGCTCGT	CGTGGTCAGTAGTGTGTCGTGTGT	344
FMgSSR-17932	scaffold4242	p2	(CA)7	Class II	40544	40557	TGCCCTCCTTGAACAGAGCCAA	TGTCTCATCACAACCTCCAACGCT	299

SSR_ID	Scaffold	SSR_Type	SSR_Motif	SSR Class	SSR_Start	SSR_End	Forward sequence	Reverse sequence	Product_size
FMgSSR-17934	scaffold729	p2	(CA)7	Class II	99102	99115	TGCCTTCACGATGGCAGGGATT	AGCAACAAGGGACATCAGCAGC	202
FMgSSR-17937	scaffold3751	p2	(CA)7	Class II	41406	41419	TGCGCTCATCACCTGCGAAA	AATTTGCACGAGCCAGGCGA	263
FMgSSR-17942	scaffold23724	p2	(CA)7	Class II	6489	6502	TGGAGCGGTACACGCTGTTGTT	TCGTCGACCATGCAAGCTCTCT	221
FMgSSR-17943	scaffold273	p2	(CA)7	Class II	30876	30889	TGGATACAAACCCACGCGCA	ACGATTCGATGAGTTGCCCCCA	334
FMgSSR-17949	scaffold134	p2	(CA)7	Class II	162639	162652	TGGTGTAATGACGTGGGCAAAGGA	TCCCGGCAAGGTTGCCATTACA	341
FMgSSR-17951	scaffold5149	p2	(CA)7	Class II	31817	31830	TGTAGGCGCTTTCCTTGCTGGT	AACAAGTTGGAACCCCCTCGCT	262
FMgSSR-17955	scaffold3097	p2	(CA)7	Class II	1566	1579	TGTCCACGAGGCGTCGTTTT	AGTCCCGGCAACCGTCTTCTTT	348
FMgSSR-17956	scaffold30059	p2	(CA)7	Class II	2027	2040	TGTCTTGCCCCAAAACCGACGA	TCCGTGACGACGGCAAAATCCT	314
FMgSSR-17958	scaffold148	p2	(CA)7	Class II	123530	123543	TGTGAGTTCACATGCCCATCG	CTGCCAGAACTAAAACTTTGCCCC	339
FMgSSR-17959	scaffold7035	p2	(CA)7	Class II	13533	13546	TGTGCACGCGCATGTTTGCT	GGGCTTTTCACAACCCGGTCAT	248
FMgSSR-17960	scaffold22140	p2	(CA)7	Class II	2078	2091	TGTGCAGTTGCACGTGGTGA	TGGGAACACCAGTTGAATGGCGA	330
FMgSSR-17964	scaffold16340	p2	(CA)7	Class II	5019	5032	TGTGCGGAGCAGAAAATCCCCA	TCTTGCTAGTGCCACCATCGCT	283
FMgSSR-17967	scaffold10111	p2	(CA)7	Class II	14283	14296	TGTGGTGGAGCGTCTGGTAA	AGCTGACGCTGTCCACATCCTA	229
FMgSSR-17968	scaffold2550	p2	(CA)7	Class II	57157	57170	TGTTCTGCATCAGGCAGCCTCT	TACAGGCCTCAAGCCCTCAACT	295
FMgSSR-17969	scaffold270143	p2	(CA)7	Class II	270	283	TGTTGAACGGACCGAAGGGCAA	ACGTCTCGCACTTCACCTGGAA	203
FMgSSR-17970	scaffold211	p2	(CA)7	Class II	70713	70726	TGTTTTTGCGCCTGTGGCCT	GCTTGTACATGCCGTGGGCAAT	311
FMgSSR-17974	scaffold558	p2	(CA)7	Class II	16185	16198	TTCCGAGATCAGGCGCGAAT	TCACTGCACAGCACAGCACA	200
FMgSSR-17977	scaffold16291	p2	(CA)7	Class II	4941	4954	TTCGTCCTTGATCCCGGCCATT	TGCCGACATGTGATGTGCAACT	350
FMgSSR-17982	scaffold120	p2	(CA)7	Class II	171106	171119	TTGACTTCCCGGCGCACAAT	TGCTCCAGCTGCATCACCATGT	244
FMgSSR-17984	scaffold3109	p2	(CA)7	Class II	16328	16341	TTGCGGAGAAGGCGTTGCAT	TGTTATTTCGGCTTGCCGCCAC	307
FMgSSR-17987	scaffold1087	p2	(CA)7	Class II	70979	70992	TTGGCACTGCTGGTGAGGAA	TTTGCGAATGCGGAGTGGCT	349
FMgSSR-17990	scaffold8791	p2	(CA)7	Class II	31245	31258	TTGTCCGTGCTGGTGTCGTT	CGCACACACTGCGAAAAAGGCT	299
FMgSSR-17999	scaffold10942	p2	(CA)7	Class II	20533	20546	TTTGCCACGTTGGGGTGCAA	ACGACCCGGCTTTTATGCGACT	221
FMgSSR-18014	scaffold699	p2	(CA)8	Class II	48149	48164	AAGCGCACGTCACCATGTTTGG	ACCCTTTGATGGCGTGGTGTCA	288
FMgSSR-18017	scaffold20030	p2	(CA)8	Class II	12031	12046	AATCGGTTACTGAACCACCCCC	TTTCGGGCATGTGTTGCCCA	258
FMgSSR-18018	scaffold5516	p2	(CA)8	Class II	4048	4063	AATGGTTGGGGCCTTTGCGT	TAAATTGTCTGGCCCCACCCGA	343
FMgSSR-18025	scaffold2910	p2	(CA)8	Class II	52046	52061	ACACGGCAGTGTCCAAGTAGCA	TCATCGACAAGCTCCATCCCCT	342
FMgSSR-18027	scaffold319	p2	(CA)8	Class II	34557	34572	ACAGAAGGGTGTGGCACTGT	AGGTTTGAACGCGCTACGGCTA	300

SSR_ID	Scaffold	SSR_Type	SSR_Motif	SSR Class	SSR_Start	SSR_End	Forward sequence	Reverse sequence	Product_size
FMgSSR-18030	scaffold141406	p2	(CA)8	Class II	246	261	ACATGGGAGTTGGTGTGTGCGT	GTGGATGGTGCAAAACACCGCA	340
FMgSSR-18032	scaffold23106	p2	(CA)8	Class II	6541	6556	ACCACCACAAGCCTGCACTT	TGGATGTGCGGCTCAACCCTTT	326
FMgSSR-18033	scaffold7756	p2	(CA)8	Class II	20926	20941	ACCACCGCTGACTCATTTGCAC	ACAAGCCAATGCCGGCGAAA	261
FMgSSR-18034	scaffold3687	p2	(CA)8	Class II	46857	46872	ACCCCACCCCTGTTTGCTTTGA	TGTCTACTCAGGCGGTTGCT	325
FMgSSR-18035	scaffold14436	p2	(CA)8	Class II	20175	20190	ACCCTGCAACGGCAAAACACT	ACCTTGGTGATTACGGCGGCAT	350
FMgSSR-18037	scaffold2945	p2	(CA)8	Class II	51134	51149	ACCGGATGCAGGCACATCAT	TGCCTTACCCTCTGGATGGCATA	280
FMgSSR-18039	scaffold1825	p2	(CA)8	Class II	68828	68843	ACGAACGGAGGAAGTTAGCGGA	GCCGCGCACCAAAACTTCAT	349
FMgSSR-18040	scaffold200	p2	(CA)8	Class II	91751	91766	ACGAAGCAAAGGACCGGCTGAA	ACGAATGTGTGCAAACTGGCGA	217
FMgSSR-18044	scaffold2886	p2	(CA)8	Class II	42036	42051	ACGCATAGCTTGACCACGCA	TGCCACCAGCAGGTGCAAAA	289
FMgSSR-18045	scaffold1030	p2	(CA)8	Class II	76291	76306	ACGCATGTCACGGTCAAGGT	TGTGCGTGTGTTGTTGCCGA	316
FMgSSR-18046	scaffold2974	p2	(CA)8	Class II	32227	32242	ACGCATTGCTCACACGTCTCT	ACAGAGTAGCACGGCAGCAT	220
FMgSSR-18048	scaffold5412	p2	(CA)8	Class II	5471	5486	ACGGATTCATTGCGCCAACCA	GGGATGAAATGGAACAAGCCCA	258
FMgSSR-18050	scaffold243072	p2	(CA)8	Class II	405	420	ACGTCCAACATCGACCACTCCT	ATTAGCTGAGTGGCTGGCGA	335
FMgSSR-18051	scaffold4053	p2	(CA)8	Class II	43359	43374	ACTCCAAGTTCCCACACGCT	TGGCTGAGCTAAGGCAGCAT	294
FMgSSR-18052	scaffold5958	p2	(CA)8	Class II	2930	2945	ACTCCACTAGCCTGTGTTGAGC	TTGTGCTACCTTCGCCACCAGT	315
FMgSSR-18054	scaffold12735	p2	(CA)8	Class II	15676	15691	ACTTAGCAGTCTAGTGCAACCCC	AAGCTTGGCTGTCTCTGTGGGT	348
FMgSSR-18056	scaffold1768	p2	(CA)8	Class II	58257	58272	ACTTCAACCGCAACCCAGCA	GGTTGAACCCAGAACCTCTTGACA	269
FMgSSR-18057	scaffold2785	p2	(CA)8	Class II	12154	12169	ACTTTTCACATGGCTGGCCGGA	TCGGCGTCGGGTCGTTATGAAT	216
FMgSSR-18062	scaffold7418	p2	(CA)8	Class II	16409	16424	AGAGAAGTTGGCCGGAAGGAA	GCTGGGGGAGTTGTTGTCAAGT	317
FMgSSR-18064	scaffold134428	p2	(CA)8	Class II	298	313	AGAGGAGCACACAACCCAGAGT	TCAGTGACGGAGCCAACCACAA	277
FMgSSR-18065	scaffold180010	p2	(CA)8	Class II	560	575	AGAGGCCACCAAGGAAAGAGCA	TGTGTGCTCTGTGCTGAACCA	206
FMgSSR-18066	scaffold1466	p2	(CA)8	Class II	70148	70163	AGATTGTCTGTCCGCACCTCA	TGTGTTGCAGGGACCCGCTATT	326
FMgSSR-18067	scaffold41	p2	(CA)8	Class II	13062	13077	AGCATGTGCGTGCGATACAGGA	TGCACCCAACAAAACGCCCT	255
FMgSSR-18068	scaffold1241	p2	(CA)8	Class II	70012	70027	AGCCCACGCTGCTCAAAATCA	CGCCTATGGTTTCGCTACGGAGAA	241
FMgSSR-18069	scaffold925	p2	(CA)8	Class II	46718	46733	AGCCGGTCAGCAAATCGTCGTT	AGCAGGTAGCACGCATCCTCAT	334
FMgSSR-18072	scaffold835	p2	(CA)8	Class II	80533	80548	AGCTCGACTCGAATGTGCAAGA	AGGTGCCGCACATCACTGTT	273
FMgSSR-18074	scaffold5516	p2	(CA)8	Class II	14555	14570	AGGCGCTCGACCAGTATTCGTT	ACCGTCTCAGAACGCCTAGGTT	304
FMgSSR-18076	scaffold550	p2	(CA)8	Class II	82554	82569	AGGTGCAGCTACACGCACAACA	AGTGCTCACCGCATCTGCGTAT	212

SSR_ID	Scaffold	SSR_Type	SSR_Motif	SSR Class	SSR_Start	SSR_End	Forward sequence	Reverse sequence	Product_size
FMgSSR-18077	scaffold77667	p2	(CA)8	Class II	1220	1235	AGGTGCCAGCAGTGCAAGTGAA	TGTTGTGTGCCACGGCTTGA	324
FMgSSR-18079	scaffold4754	p2	(CA)8	Class II	3250	3265	AGGTTGCGTAGCTTGCCGTT	AGGTCGAGAAACTGCACACCGT	337
FMgSSR-18082	scaffold4	p2	(CA)8	Class II	112677	112692	ATATGCTACCCACCACGCCTCA	TGCAACCCGTAAGTGGGATGGT	292
FMgSSR-18084	scaffold7349	p2	(CA)8	Class II	22438	22453	ATCCACGTCCACGCTCGAAGAT	TGTGCTTGTACACGGTGCCTGA	299
FMgSSR-18085	scaffold3052	p2	(CA)8	Class II	35057	35072	ATGAAGGACGCGGCTGCATCAA	TCGGCAAGGACCCAAATCGTGT	327
FMgSSR-18086	scaffold6125	p2	(CA)8	Class II	8944	8959	ATGCAACCTCCCCTTGCTCCAA	GCCACCGTTGCCTTCGCAAATA	320
FMgSSR-18087	scaffold588	p2	(CA)8	Class II	50928	50943	ATGCACTCCAGTCATGCGCT	AAAGTAGGCGCACCGCTTGGAT	291
FMgSSR-18088	scaffold15384	p2	(CA)8	Class II	11902	11917	ATGCGGAAGGCATCACCTCTCT	TGGTTTCACCAAGGCTTTACCGC	287
FMgSSR-18089	scaffold4000	p2	(CA)8	Class II	31811	31826	ATGGTTGAAGCTGGTGGTGCCT	ACCCTTCGTTTGGTCGCTCT	308
FMgSSR-18091	scaffold5884	p2	(CA)8	Class II	13667	13682	ATTAGCCCCTGCCACAAACGCA	GCTGCTGCAAGTGCACAACA	332
FMgSSR-18092	scaffold12553	p2	(CA)8	Class II	21039	21054	ATTCCCTCGCTTCGGCATCA	TCGAAGCCGCATTGGGTTGA	323
FMgSSR-18094	scaffold9761	p2	(CA)8	Class II	5855	5870	ATTGGACACCGGGAGCAGAT	ACCTTGCAAACAAACCTGCTGGG	253
FMgSSR-18096	scaffold4025	p2	(CA)8	Class II	53378	53393	CCTGACAGGTCCATCAGACCAA	AGCAACGGGAAGCAGCAACA	219
FMgSSR-18098	scaffold4623	p2	(CA)8	Class II	30230	30245	CGCCTTTTGCGTTGTTGCGT	AGTCGTGCTCTGTCGCGTTCAT	209
FMgSSR-18102	scaffold4850	p2	(CA)8	Class II	16916	16931	CGTGAAAACGGATGAATCGGCCA	TTTCATGGCTGCGGACGGTAGT	346
FMgSSR-18103	scaffold14011	p2	(CA)8	Class II	13246	13261	CGTTGAGCACAGCTTGCGTT	TTTATGGGTTGACGGCGGAGGA	250
FMgSSR-18105	scaffold2799	p2	(CA)8	Class II	12109	12124	GCATGGTGTTGCAAATGTGGTGC	ACGCTGCTTGCTTGGAAGGT	349
FMgSSR-18110	scaffold287	p2	(CA)8	Class II	47528	47543	GGCATGTGCTGTGTGGATAACAGG	TGTGTGCTCTCCGTTGGACCAT	234
FMgSSR-18115	scaffold120601	p2	(CA)8	Class II	68	83	TAGGCTTATAGCCCCCACCACACA	ACGAGCATGACCAGATCCCTCA	303
FMgSSR-18118	scaffold2095	p2	(CA)8	Class II	31062	31077	TCACACAAGACGCACGCACA	TCGTCAAGGAACCTGGGTGT	249
FMgSSR-18121	scaffold22668	p2	(CA)8	Class II	3334	3349	TCATGTTGTTGCCATTGCCCCC	TTGCTTGAGCCCCTCTGTGACT	337
FMgSSR-18123	scaffold9472	p2	(CA)8	Class II	14111	14126	TCCATCTTCAGGAAGGAGAGCGGA	AGGCACAACTTTGGTCGCCAT	308
FMgSSR-18130	scaffold1120	p2	(CA)8	Class II	28702	28717	TCGTCGACACGGCGAAGAAA	ACCGCGACGTCACCTTGTGTAT	294
FMgSSR-18132	scaffold1155	p2	(CA)8	Class II	59517	59532	TCTCCGTGACTTGCTCCCTTCT	AGTTCCAGAAACCATGGTGGGC	300
FMgSSR-18133	scaffold3441	p2	(CA)8	Class II	4069	4084	TCTTCGTTCCCGCCCTTTCT	CCGCACCCATCTGAGTTGTCTT	339
FMgSSR-18135	scaffold5664	p2	(CA)8	Class II	26674	26689	TGAAGTCTGTAGCACAACCCCC	TGGATGCATGCACCGAACCAAC	309
FMgSSR-18136	scaffold588	p2	(CA)8	Class II	49101	49116	TGCACTCTAGTCATGCGCTTAGCC	AGGTGTGGTTGTGAACGTGGGT	308
FMgSSR-18137	scaffold497	p2	(CA)8	Class II	19012	19027	TGCATGTGCAATGACTCGAGCG	TGTGCACCTGGTTTCTGCTTGC	308

SSR_ID	Scaffold	SSR_Type	SSR_Motif	SSR Class	SSR_Start	SSR_End	Forward sequence	Reverse sequence	Product_size
FMgSSR-18139	scaffold14158	p2	(CA)8	Class II	18657	18672	TGCGCATGTACGTGCTCTCT	TGCAAGGCGTATGTGCGTGT	331
FMgSSR-18140	scaffold287	p2	(CA)8	Class II	69119	69134	TGCGCGTTTTGCAATGTGCC	ACTGCTGCTTGATGTGTGCTAC	341
FMgSSR-18142	scaffold175	p2	(CA)8	Class II	76019	76034	TGCTCCAACAACAGCCGTTCCA	TCACTAATCGCGCGTGGTCAA	203
FMgSSR-18143	scaffold5683	p2	(CA)8	Class II	8884	8899	TGGAGCACCACCACGACAAAGA	AGTTGGCGAAGTTGTTACGGCG	262
FMgSSR-18148	scaffold399	p2	(CA)8	Class II	11072	11087	TGTCAGCAACGTCCTACCACT	AGGCGCTAATGCCATCAGAAAGC	348
FMgSSR-18149	scaffold20	p2	(CA)8	Class II	233948	233963	TGTCGTCTGCACCGGCCTATTT	GCCAGCAACTTTTGAGGGCT	321
FMgSSR-18151	scaffold181	p2	(CA)8	Class II	150961	150976	TGTGCGCCACTGCTTTTGCT	TGCACTGCACTAGTGTGTTCCGT	309
FMgSSR-18152	scaffold1415	p2	(CA)8	Class II	85606	85621	TGTGCTTACCGGCAGTGACTGAA	TCCTCGTCAGGTCGTGTTTGCT	240
FMgSSR-18160	scaffold4052	p2	(CA)8	Class II	11456	11471	TTTCCGGGGAAGTGCGTTCA	ATGGTCCACGCATGTGCATC	338
FMgSSR-18164	scaffold102	p2	(CA)9	Class II	8290	8307	AAAAACGCCAAGTGGCCTGC	GCACACACAAGCCAAGCCTCTA	297
FMgSSR-18167	scaffold446	p2	(CA)9	Class II	26821	26838	AACCTGCAACCGGTGTGTCT	AATAAAGCGCCGCGTTCGTG	247
FMgSSR-18169	scaffold12600	p2	(CA)9	Class II	1832	1849	AACGCACCGCGCCAATTTCT	AACACGTTACGCACGACGCA	202
FMgSSR-18170	scaffold22392	p2	(CA)9	Class II	4361	4378	AACGTGGGAAGCAGGCAACT	TGCTGTTTGCAAGCGCCACA	320
FMgSSR-18176	scaffold191	p2	(CA)9	Class II	111451	111468	AATGAAGGCCGGTAGGCCATGT	TGCAGCGAGAGAGTGGAAACCA	293
FMgSSR-18178	scaffold19048	p2	(CA)9	Class II	3445	3462	AATTATTCGGCCAGCGCCCA	TCTCGGCTAAGTCAGGGTGCAA	278
FMgSSR-18179	scaffold26574	p2	(CA)9	Class II	6884	6901	ACAAAGCGGCACATGACTCG	ATGATCACGCACGCACTCTGGT	328
FMgSSR-18181	scaffold2423	p2	(CA)9	Class II	24285	24302	ACAGAGCACCGCACCAATCGAA	ACGCCAAGATCCACGACAGCAA	238
FMgSSR-18185	scaffold7912	p2	(CA)9	Class II	26353	26370	ACCACCATCGCCATCGTTTCTGT	TCACCTGACTACGAACGCCAGT	325
FMgSSR-18188	scaffold1188	p2	(CA)9	Class II	68687	68704	ACGCGCTTCGCTTCCACTTT	TGGCGGTGTTATTGCTTCGGGT	321
FMgSSR-18189	scaffold5536	p2	(CA)9	Class II	6560	6577	ACGCGTTTCTACTCCGCCGTAA	TTGGCGCCGTGAGGGGAATAAT	345
FMgSSR-18190	scaffold36161	p2	(CA)9	Class II	4831	4848	ACGGTCGCAGTTTGGAGGTCTT	AGGCCATGTTGCGCTGCTAT	268
FMgSSR-18191	scaffold10779	p2	(CA)9	Class II	17438	17455	ACGTGCTTTCGCCGGATCAT	AGGACATCGGGATCGGAAGGAA	200
FMgSSR-18197	scaffold1202	p2	(CA)9	Class II	27931	27948	AGCCCCTCAACGACCAAACAGA	ACTTGAACGGCCTGCACGAT	347
FMgSSR-18198	scaffold10176	p2	(CA)9	Class II	20320	20337	AGCGAGCAACTCGGTGGTAA	AGGGTCCGCTGCTGCAAGAAAA	310
FMgSSR-18204	scaffold847	p2	(CA)9	Class II	73020	73037	ATCAAGGTCCATGTCTCCACGA	ACGCACATCTCACTGATGCCGA	208
FMgSSR-18206	scaffold4667	p2	(CA)9	Class II	23595	23612	ATGCCGCAGTTTGGCTTGCT	AATGGAACTCTCGTGTCGGGCA	321
FMgSSR-18213	scaffold3076	p2	(CA)9	Class II	49720	49737	CGCATGCGCACACGAACAAT	GCGCTGCAGTTAGGAAAGAAAAGC	246
FMgSSR-18214	scaffold2453	p2	(CA)9	Class II	47708	47725	CGGCTGGTCATGCTTTGCACAT	TTGCTGCCTGCCCAGAGTTTCA	249

SSR_ID	Scaffold	SSR_Type	SSR_Motif	SSR Class	SSR_Start	SSR_End	Forward sequence	Reverse sequence	Product_size
FMgSSR-18215	scaffold8166	p2	(CA)9	Class II	7680	7697	GCAGTGCCTCCACAATGTTGAACT	TGAAACTTCCTGGCCAACGGGA	321
FMgSSR-18220	scaffold1570	p2	(CA)9	Class II	53818	53835	TCATGATGTTGCTGCCTGCGTG	ACGGCTCTCGATATGCTCGTCA	268
FMgSSR-18222	scaffold220	p2	(CA)9	Class II	59643	59660	TCCCTTGCATCCACACTGACT	TCGGGTGCGACTGCATTTCA	298
FMgSSR-18226	scaffold7771	p2	(CA)9	Class II	20761	20778	TGACACTATATACCACTGCCGCC	TGCCCCTCAGAACAAATTGGTAGG	295
FMgSSR-18228	scaffold12028	p2	(CA)9	Class II	3731	3748	TGCAAACCTTGGTGTGGTGCT	ATTGCGTTCTCTTAGGTGGGTGGG	283
FMgSSR-18229	scaffold15990	p2	(CA)9	Class II	12948	12965	TGCAATCCAGTAAAGCAGGTACCCC	ACAGACAGGAGACTGTGTCCCA	288
FMgSSR-18233	scaffold2763	p2	(CA)9	Class II	26534	26551	TGCGCGCGCCATATATTACCCA	ACCGTGCTCGGGCTAAGAATGA	203
FMgSSR-18235	scaffold1420	p2	(CA)9	Class II	59090	59107	TGCTCTGGCCCAAAGGTTTGCT	CCATGTCCACTTCATGGCTGGTGA	290
FMgSSR-18236	scaffold9569	p2	(CA)9	Class II	8810	8827	TGGACACACGAGTGAGACAAGAAGG	ACGAAGTGCACCTGCACGCTAT	293
FMgSSR-18237	scaffold1970	p2	(CA)9	Class II	20472	20489	TGGCCATGATTTACGCTCCCCA	ATGAAGCATGGAGCAGCAGCCA	315
FMgSSR-18240	scaffold36836	p2	(CA)9	Class II	2180	2197	TGGCCTTGAGACACCCCATCAT	AGGTGGAGGAAGAAGACAGGCT	272
FMgSSR-18241	scaffold401297	p2	(CA)9	Class II	188	205	TGGCTGCCAGGAAAAGCCAA	AAGAAGCAGAGCGCTGAGCAGT	221
FMgSSR-18242	scaffold5761	p2	(CA)9	Class II	38594	38611	TGGCTTGGCGTTGCTCTTCA	AAGAGGCCGGCACAAAGTGGAA	255
FMgSSR-18245	scaffold937	p2	(CA)9	Class II	21933	21950	TGTCGTCTGCTGCTTGCTCA	TCACTACGGATGTGCGCGAAGT	278
FMgSSR-18248	scaffold174	p2	(CA)9	Class II	65046	65063	TTACACAAGGCCCCAGGTGACA	ACAAAAACCCCGCCAGACGA	317
FMgSSR-18251	scaffold914	p2	(CA)9	Class II	73288	73305	TTGCAGTCGACGACGTGATGGT	TCATGTGCCACCGTTGCAGT	271
FMgSSR-18258	scaffold9248	p2	(CG)6	Class II	9961	9972	AAAAATCCGCGGTCAAGAGGCG	ACGCGCCGAAACGAAAAACG	293
FMgSSR-18260	scaffold15754	p2	(CG)6	Class II	15337	15348	AAAAGGCGATTTCCGGGCGT	GCGAACCAAGTCGCAAGCTCAA	229
FMgSSR-18261	scaffold332	p2	(CG)6	Class II	36652	36663	AAACACGGTGCCGTTTCTGC	GCAAAATGCGTGGCGTTGGA	210
FMgSSR-18262	scaffold1225	p2	(CG)6	Class II	10149	10160	AAACGCTGATGTCGCCGCTA	AGCGCTCACGTGTGTTGGTCTT	231
FMgSSR-18266	scaffold9615	p2	(CG)6	Class II	11504	11515	AAAGGCAAGGCGTTGCGACA	TGTGCGTACGTGCTTCAGCTTG	244
FMgSSR-18268	scaffold62822	p2	(CG)6	Class II	1662	1673	AAAGTGGGTGCGGCGAAGAT	TTGGCCAGCACTTCGGCAAT	320
FMgSSR-18274	scaffold7739	p2	(CG)6	Class II	11859	11870	AACACGTACACGCCGCCTTT	TCTTGCAGGCCCAAGTCGTAGA	350
FMgSSR-18277	scaffold3366	p2	(CG)6	Class II	30917	30928	AACATCATGCCGTGCCCCTT	AGCCGCAAGAGCCTTCCATT	313
FMgSSR-18278	scaffold61873	p2	(CG)6	Class II	1866	1877	AACATCCGCAAGATCCGCAAGC	AACGTGCGTCGGACTTCGACTT	306
FMgSSR-18282	scaffold6364	p2	(CG)6	Class II	27380	27391	AACGAGCTGGACGACGGTTGTT	TTCCCGTGCTTGTTGCTGCT	303
FMgSSR-18283	scaffold177795	p2	(CG)6	Class II	196	207	AACGCAAAGCTCCGTCGTGT	GCGAGCATTCAGAAGCCACCAT	253
FMgSSR-18285	scaffold50141	p2	(CG)6	Class II	1326	1337	AACGTCAAGACCTGCCGCAA	AACGCATCGTTGTACTCTGGCG	291

SSR_ID	Scaffold	SSR_Type	SSR_Motif	SSR Class	SSR_Start	SSR_End	Forward sequence	Reverse sequence	Product_size
FMgSSR-18289	scaffold2240	p2	(CG)6	Class II	7151	7162	AAGACTCGGGCGCTCCAACAAA	ATTCGGCCCAGCAAACACGA	267
FMgSSR-18292	scaffold2363	p2	(CG)6	Class II	67546	67557	AAGCCGTGTGTGTGTGTGGT	AGCAGTCGCAGAGCTTTGGT	260
FMgSSR-18295	scaffold865	p2	(CG)6	Class II	55413	55424	AAGCGTGACCGCACAAACAC	TGCATGTGCAATCTCGGGGT	318
FMgSSR-18299	scaffold53494	p2	(CG)6	Class II	2551	2562	AAGCTGCGCAAGTACGGCAT	TCGATCGCATTCGTGTTGCCGA	203
FMgSSR-18301	scaffold20648	p2	(CG)6	Class II	6877	6888	AAGGCAAGCTGAGGTGTGGCTA	TGAAACCGACTACTCACCCCCT	325
FMgSSR-18307	scaffold1594	p2	(CG)6	Class II	53463	53474	AATTTGCCCGTTGCCTCGCT	TGCCAACGCGAGGGGATTTT	267
FMgSSR-18310	scaffold301	p2	(CG)6	Class II	52446	52457	ACAAGAACCGCTTGCTGCGT	TAAGATTTTGAGGCCCAGGGTGGC	344
FMgSSR-18314	scaffold3689	p2	(CG)6	Class II	47068	47079	ACACGGCAGTACACGAACACGA	ACGCGCACTTTGCCGCTAAT	339
FMgSSR-18318	scaffold7645	p2	(CG)6	Class II	17664	17675	ACAGGCAGCAATCGTTGCGA	TGGTTCCCGGCGCTTTTCTT	228
FMgSSR-18319	scaffold223273	p2	(CG)6	Class II	146	157	ACATTCCGGTGGTGCTGGAT	AGATGACCACCCTTGAGCAGCA	241
FMgSSR-18320	scaffold11017	p2	(CG)6	Class II	20704	20715	ACCAAATGAGCCCATTCCACGC	ATACGCGGGGCTTCCAATTGCT	295
FMgSSR-18329	scaffold29653	p2	(CG)6	Class II	3778	3789	ACCATGTTGGCAACGAGGCA	AGGACGATGAATGGCGGAGCTA	247
FMgSSR-18335	scaffold1713	p2	(CG)6	Class II	25000	25011	ACCGACCGAATCGAACCCAACA	TGCGACAGACGCCGAGAAGATT	218
FMgSSR-18336	scaffold52	p2	(CG)6	Class II	199721	199732	ACCGAGAACGGCTCAATGGGAA	AAATCGGACGGAGTCGGACGTA	306
FMgSSR-18337	scaffold3479	p2	(CG)6	Class II	34102	34113	ACCGATCCAACCGTCTCCGAAT	TGGTGGTCGCTCGCCTTTTT	226
FMgSSR-18339	scaffold17176	p2	(CG)6	Class II	8749	8760	ACCGCAATCGCACGCAAACA	AAAAACCGGCGCACAGAGGT	249
FMgSSR-18341	scaffold5599	p2	(CG)6	Class II	17333	17344	ACCGTCACCTGCTCGTTGAT	TTCACGGCGGGCATGATGAA	329
FMgSSR-18342	scaffold18869	p2	(CG)6	Class II	12266	12277	ACCTCATTCCGTTGCCACGCTT	TTTTTAAATGGCGCCGGGGTGG	307
FMgSSR-18354	scaffold1239	p2	(CG)6	Class II	20743	20754	ACGCATTACTGCAGCGGTGT	TGTGACGACGATTGTGTTCGGC	339
FMgSSR-18356	scaffold805	p2	(CG)6	Class II	59389	59400	ACGCGCAGGTTGTGAGTTGT	ACCAACATGCAGGTTTCACGCA	265
FMgSSR-18358	scaffold24242	p2	(CG)6	Class II	6560	6571	ACGGAGCCGGGCATGGAATTTT	TGCTTAACTGGTGGCGGAGTGA	312
FMgSSR-18360	scaffold37312	p2	(CG)6	Class II	5514	5525	ACGGGTGCAACCAATACGCA	TGGTCGAAATGGAACTCGCGCT	227
FMgSSR-18362	scaffold55251	p2	(CG)6	Class II	2112	2123	ACGTGCTGCAGGACAAGTACGA	TACTTCGCCACATACTCGCGCT	280
FMgSSR-18363	scaffold1228	p2	(CG)6	Class II	28648	28659	ACGTGGCGGGTTGTTTATGCCT	TCGAACAAGGGCTGCACCTTCA	264
FMgSSR-18365	scaffold1191	p2	(CG)6	Class II	11172	11183	ACGTTAGGATGCAAGGAGGCCA	GCGCGATCCAGGGATAAGCATT	295
FMgSSR-18366	scaffold174618	p2	(CG)6	Class II	167	178	ACGTTCGGCGAAGGCAGTTT	ACCGCAACGGAGATCCACGAAT	232
FMgSSR-18375	scaffold288	p2	(CG)6	Class II	73193	73204	ACTGGTACCGCAAACGCCAA	TTCGCGCACGGCTGTATGAA	302
FMgSSR-18376	scaffold3	p2	(CG)6	Class II	284117	284128	ACTGTGCGCTGCGATGTCTT	AAGGCCGGCAAATCTCGCAA	329

SSR_ID	Scaffold	SSR_Type	SSR_Motif	SSR Class	SSR_Start	SSR_End	Forward sequence	Reverse sequence	Product_size
FMgSSR-18381	scaffold698	p2	(CG)6	Class II	94455	94466	AGAGCTCTGCGCCTGCAATAGA	ACAAACAGCAGCAGCAGGCA	338
FMgSSR-18384	scaffold4758	p2	(CG)6	Class II	17699	17710	AGCAGCAAGGGAAAAAGGTGGC	AACGTTGCGTTCGGTGTTGC	225
FMgSSR-18386	scaffold862	p2	(CG)6	Class II	35245	35256	AGCATGCAAAAGGTGGTGGGGT	TGCATGCTGAGAGGAACACCT	237
FMgSSR-18389	scaffold3749	p2	(CG)6	Class II	29956	29967	AGCATGTGTGTGGAGATGCCAAGA	CGCGTGCATTGCAACCAACA	238
FMgSSR-18390	scaffold12044	p2	(CG)6	Class II	23242	23253	AGCCAACAACTGGGCCTGCTAT	TGGCTTGCCACTGGTGGATT	334
FMgSSR-18392	scaffold20688	p2	(CG)6	Class II	8550	8561	AGCCAGCGCCCAACGATAAA	CGTCGCGTGTAAAAAGGCGGAA	332
FMgSSR-18398	scaffold170677	p2	(CG)6	Class II	386	397	AGCGCGCTGGCGTTAAATGA	TGGCTTCACGCAGGGCTTTT	332
FMgSSR-18402	scaffold3856	p2	(CG)6	Class II	145	156	AGCGGCAGCATCCCAATCACAT	GCAGCGTCATTAACCGGTAAACCC	344
FMgSSR-18403	scaffold36825	p2	(CG)6	Class II	2856	2867	AGCGGCGAAGTCAACAGGAT	TGCCCAGAACAGTTTGCCGT	298
FMgSSR-18409	scaffold341	p2	(CG)6	Class II	106981	106992	AGCTTTGCGTTCCGTTGCCA	TGGCCGCTAGCTCGAGACAAAA	282
FMgSSR-18411	scaffold2011	p2	(CG)6	Class II	64961	64972	AGGCAGCAAAACAGGTGCGT	TCGCTCTTTCAAGCGACGGT	308
FMgSSR-18417	scaffold3230	p2	(CG)6	Class II	30183	30194	AGGTTCTAGAGCTTTGGGCGCT	TTTTCACCTGGCGTCTCGGA	291
FMgSSR-18418	scaffold28853	p2	(CG)6	Class II	4789	4800	AGGTTGCGCCGAACTGCAAA	AGCTGCTGCGATTCACTGTCCA	223
FMgSSR-18420	scaffold10942	p2	(CG)6	Class II	24178	24189	AGTGCCGCCAAGGAAACCGTTA	TGCCTCTGCCTGTTCTGCTTCT	349
FMgSSR-18421	scaffold5600	p2	(CG)6	Class II	31464	31475	AGTGCGATTGCGTGCTGCTT	TGTGCGCGTGACACCAGAAA	322
FMgSSR-18424	scaffold7337	p2	(CG)6	Class II	20004	20015	AGTGGCGGTTGAACGAGCTGAA	TTTCCGGCCGTGTTTGCTGT	342
FMgSSR-18433	scaffold5156	p2	(CG)6	Class II	38919	38930	ATAACGCCAAAACGCGCGAC	CGCCAAAAAGCACACAGCACCT	327
FMgSSR-18437	scaffold12132	p2	(CG)6	Class II	13575	13586	ATCGATTCGGTTGGCACGTGGA	TGCGCGTTGCGTTGACGATT	335
FMgSSR-18439	scaffold526	p2	(CG)6	Class II	15308	15319	ATCGGCGCGCGTTACTTCAA	AAGCACTCGCTGCCGTTGAT	245
FMgSSR-18446	scaffold79533	p2	(CG)6	Class II	524	535	ATGCGCGGGCAACGTTTCAT	TCGACGACGACGGGACAAAGAA	272
FMgSSR-18449	scaffold7071	p2	(CG)6	Class II	3233	3244	ATGGATGCGCGCGATTAGCA	TTTTCGTTCTGCTCCGCAGGCT	295
FMgSSR-18454	scaffold1205	p2	(CG)6	Class II	18817	18828	ATGGTGTTTAGCGCCGTCGT	ACAGCGTTTCTGGCCTTCTAGGT	322
FMgSSR-18455	scaffold8586	p2	(CG)6	Class II	2035	2046	ATGTAACCGAGCTCGCAGGCAA	TCGTGCGCTTTTCTTGCCCA	329
FMgSSR-18458	scaffold13305	p2	(CG)6	Class II	11329	11340	ATTGTTGCGACGCTTGAGCA	CGGCGAATCGGATTGGATTGGAT	295
FMgSSR-18459	scaffold1018	p2	(CG)6	Class II	70535	70546	ATTTCCCTCGCAACTGCTCGGT	ATTCGAAAGCGTTTGCGGCG	295
FMgSSR-18464	scaffold4343	p2	(CG)6	Class II	14714	14725	CACGTCGTGGCGTACCATGTTT	AGGCGCAACGCAGCATCAAT	331
FMgSSR-18475	scaffold469	p2	(CG)6	Class II	9306	9317	CCGAGAGGCCAAGTCGAATCACAT	TGGCGACCGACCATTTATCAAC	349
FMgSSR-18477	scaffold7355	p2	(CG)6	Class II	6815	6826	CGACCGCCTTCTCCAGCTAATTGT	TTGTCGGCATCGGCGGATTCTA	344

SSR_ID	Scaffold	SSR_Type	SSR_Motif	SSR Class	SSR_Start	SSR_End	Forward sequence	Reverse sequence	Product_size
FMgSSR-18482	scaffold1684	p2	(CG)6	Class II	67742	67753	CGCGAGCGAAAGAGCAACGAAA	ATTCCAACGTCGTCCGCACT	272
FMgSSR-18494	scaffold22866	p2	(CG)6	Class II	9774	9785	GCATGAGTCACGCGTTCTACCT	CTTGCACAAGCACAACCGCA	200
FMgSSR-18502	scaffold80055	p2	(CG)6	Class II	566	577	GCCGCTGTGACGATGCATACAA	AGCAGCGCCGCGAAATACAT	350
FMgSSR-18506	scaffold540	p2	(CG)6	Class II	82471	82482	GCGTGCACTGCAACCAACAA	AGATGCATGCCAAGAAGGCTGC	225
FMgSSR-18510	scaffold1991	p2	(CG)6	Class II	21458	21469	GCTTTAGTCCAGTCAGCGAGCA	GGTGAAGCGATTTGGGCAAGGT	235
FMgSSR-18511	scaffold13800	p2	(CG)6	Class II	18026	18037	GGAAGGGAAGGAGAAAGCTCGTGT	ACCGTTTGGTGCGGAGCATT	333
FMgSSR-18518	scaffold2890	p2	(CG)6	Class II	47893	47904	GGCGCCCGGGGCAATTAAAAAT	ACTGTGGGCTTGTCGCTTCA	267
FMgSSR-18519	scaffold1197	p2	(CG)6	Class II	51069	51080	GTCCAAACAACCCGGCTCTAGT	TGGCGGCGCATCCAAAAGAA	307
FMgSSR-18520	scaffold614	p2	(CG)6	Class II	38119	38130	GTGCGTGTTTGTTGGACGACGA	TGCGTGGTGCGGCTTTAGTT	337
FMgSSR-18523	scaffold7670	p2	(CG)6	Class II	18250	18261	TAAATTAATGCGCAGGCGGCGG	TGCAAAATGCATGCACCGGCT	243
FMgSSR-18527	scaffold2914	p2	(CG)6	Class II	43104	43115	TAATCTGCGCGCGCTTGTGT	TTTGCGTCGGATTCGGGTAGCA	332
FMgSSR-18528	scaffold246	p2	(CG)6	Class II	31926	31937	TACACGCTGCTCCGTTGACACT	ATGCATGATCGAGCTGTGCCCT	244
FMgSSR-18544	scaffold590	p2	(CG)6	Class II	51948	51959	TCACCGTGAAACGCAGGCAA	GGGGAATCGACACGGCATCTTT	258
FMgSSR-18554	scaffold8694	p2	(CG)6	Class II	22876	22887	TCCAATTGCTCGGAAGGCGA	AGCAACAATTCACGGGGGCA	342
FMgSSR-18555	scaffold3762	p2	(CG)6	Class II	46935	46946	TCCACGCACGGACGAGTTCAAA	AACCGCATCACATCACGGCA	305
FMgSSR-18561	scaffold141598	p2	(CG)6	Class II	139	150	TCCGAAGATTTCCTCGCGCA	TGGTCACCGGCAAGCTGTTCAA	332
FMgSSR-18566	scaffold74	p2	(CG)6	Class II	109016	109027	TCCGGGCTGAGGCTCAAAAA	TGCGATGAACGAACCGTGCT	332
FMgSSR-18568	scaffold49	p2	(CG)6	Class II	72769	72780	TCCGTTTGCGGGTGCGATAA	TCCGGCCAGCTGCAAAATGT	202
FMgSSR-18569	scaffold3101	p2	(CG)6	Class II	29511	29522	TCGAACGAGTTTCCCGGTTGGT	TGGTCATCACGGTTGTGCGT	303
FMgSSR-18573	scaffold32711	p2	(CG)6	Class II	6789	6800	TCGACCGGAATGCAGGAGTCTT	TGTGGGCTGATCAAAACGTGCG	329
FMgSSR-18574	scaffold10323	p2	(CG)6	Class II	16646	16657	TCGACGATTTTCGTGTGCGCT	TGCCGGAGCATCAACGAACTGT	306
FMgSSR-18575	scaffold7973	p2	(CG)6	Class II	8102	8113	TCGACGCTCGCGCAACAATA	GGGCATCGATCGCCTGTTGAAT	215
FMgSSR-18577	scaffold7861	p2	(CG)6	Class II	27716	27727	TCGAGCGCGTGGTATGGTTTGT	AAACCGCGTGACAACCACCA	305
FMgSSR-18579	scaffold33193	p2	(CG)6	Class II	2629	2640	TCGCCGTCGCGTTCTCTCTATT	ACAAACCACTGTGGAGGCCCTT	317
FMgSSR-18580	scaffold7337	p2	(CG)6	Class II	13175	13186	TCGCTCCAAGTCGCGGAATCAA	ACGTGTTTTCGGTGCGCGTT	294
FMgSSR-18587	scaffold2217	p2	(CG)6	Class II	57946	57957	TCGGTCGGTAGATGTGTGCT	ACTATACAGCAACGCGGCCA	210
FMgSSR-18588	scaffold9248	p2	(CG)6	Class II	11885	11896	TCGTCAAAGCCGCGTGTGTT	TAGCACGTCGATTGGCTGGT	216
FMgSSR-18590	scaffold538	p2	(CG)6	Class II	10708	10719	TCGTTTAGTGCGGTCGCGTT	ACGTCGTACGGTCGGTGAAA	211

SSR_ID	Scaffold	SSR_Type	SSR_Motif	SSR Class	SSR_Start	SSR_End	Forward sequence	Reverse sequence	Product_size
FMgSSR-18595	scaffold23237	p2	(CG)6	Class II	10581	10592	TGAAAGGCACGCAGGTGACA	ATCCGCACTGAATGGTTGGCCT	296
FMgSSR-18600	scaffold310718	p2	(CG)6	Class II	230	241	TGACGAAGTGCAGAAGCGCA	ACGGTTGCGAGTTCAGCCATGA	287
FMgSSR-18601	scaffold129633	p2	(CG)6	Class II	314	325	TGACTGCATCTTATCCCGCGCT	ATATCGGGCACCGCATCCTTGA	349
FMgSSR-18602	scaffold337	p2	(CG)6	Class II	7966	7977	TGAGACAAGAGCACGAGGCACT	TGGCTTCTCTGGCGCTTTTCA	229
FMgSSR-18603	scaffold1420	p2	(CG)6	Class II	56503	56514	TGAGCGTGCATGTCTCTCACCA	AGCTGTTCCGAGACCAGTGGAA	250
FMgSSR-18607	scaffold14581	p2	(CG)6	Class II	2536	2547	TGATGCGTCCATGCGGCTTA	AACGTACAGCGCGACGGAAA	283
FMgSSR-18608	scaffold6692	p2	(CG)6	Class II	17064	17075	TGATGGGCGGACTTTGCTCCAA	ACACTCTGCTGGTTGCCCAGAT	296
FMgSSR-18609	scaffold3586	p2	(CG)6	Class II	39526	39537	TGATTGGCAACCGAGCGTGGTA	AACCTGCCCAATTTCCCGTGGT	281
FMgSSR-18611	scaffold1166	p2	(CG)6	Class II	30152	30163	TGCACTCGCAGATGTGTGGCAT	ACAACACGGCGACACGGATT	314
FMgSSR-18612	scaffold8759	p2	(CG)6	Class II	7871	7882	TGCACTGGCTCGTGGCTAAT	TGGGTTGGTTCCTCGACCGAAA	296
FMgSSR-18613	scaffold425	p2	(CG)6	Class II	34672	34683	TGCAGCCATGCACGATCAGAGT	CGCGCACGAGCTCCAAACTTTA	213
FMgSSR-18614	scaffold33352	p2	(CG)6	Class II	889	900	TGCAGTCAGAAGCAGCTCGGAT	AAAATCGGCGGCATCCTCCACT	319
FMgSSR-18619	scaffold4419	p2	(CG)6	Class II	43857	43868	TGCCACAGCGCAAAAGGTGT	AGTGGTGGCGAGCAACTTGA	212
FMgSSR-18628	scaffold851	p2	(CG)6	Class II	98363	98374	TGCCCGACACGAGAGTTCCATT	TGGTCTTGCGGTGCGAGTTT	277
FMgSSR-18629	scaffold17865	p2	(CG)6	Class II	1457	1468	TGCCCGGATTTGGATCGGAACT	AGCGGGTCGTAGAGTGGTATGT	340
FMgSSR-18632	scaffold48203	p2	(CG)6	Class II	5090	5101	TGCCGCCGTCGCTATTGTTT	TATGCTTCCTTCACACGCGGCT	344
FMgSSR-18637	scaffold622	p2	(CG)6	Class II	35304	35315	TGCGTAAACACGCAGCGGAT	ACGGCTATCAACGGGCTTGCTT	273
FMgSSR-18642	scaffold5558	p2	(CG)6	Class II	11510	11521	TGCTTATGCACGGCCACCTT	ATGACACATGCCTGCGCACT	244
FMgSSR-18643	scaffold68	p2	(CG)6	Class II	173017	173028	TGCTTGCTTGGTCCTCAGCAGT	TAAGCTAGAGCTGCTACGCCCT	324
FMgSSR-18644	scaffold115318	p2	(CG)6	Class II	151	162	TGCTTTCGCTCGTTTCATCCGC	TGAGTCGGCGTCGGGTACATAA	330
FMgSSR-18654	scaffold1281	p2	(CG)6	Class II	7962	7973	TGGCGCACGTACCCGAAAAT	CGCGCAATCGCTGTTCGTTACT	263
FMgSSR-18658	scaffold315791	p2	(CG)6	Class II	330	341	TGGTCAAAGGCGGGGTCTTGTT	GCTGCCGCGTCAAATTCGTGAT	233
FMgSSR-18660	scaffold3200	p2	(CG)6	Class II	4023	4034	TGGTCGGCGCGTTGGTGATAAA	AGCGGGAACGGATGACACGAAA	323
FMgSSR-18661	scaffold21245	p2	(CG)6	Class II	5370	5381	TGGTTAGTCGTTGCGCGCTT	TTGCCCCGTCCAGCGAAAAA	215
FMgSSR-18671	scaffold2017	p2	(CG)6	Class II	49960	49971	TGTGCACTTGACGCCTTGACCT	ACCAGTGCACAGCGAGGTTT	230
FMgSSR-18672	scaffold2260	p2	(CG)6	Class II	50555	50566	TGTGTGCCGTGCAAAAACGGA	GGCTCACATGCCTGCTGGAAAT	215
FMgSSR-18673	scaffold780	p2	(CG)6	Class II	64064	64075	TGTGTGCGTGTGTGTTTGCC	TCCATGTCCAATGGCTGGTGCT	330
FMgSSR-18675	scaffold6594	p2	(CG)6	Class II	22184	22195	TGTTGCAAACCGTCACCGATGG	GCCGCTAAGTGGATGCACAACA	203

SSR_ID	Scaffold	SSR_Type	SSR_Motif	SSR Class	SSR_Start	SSR_End	Forward sequence	Reverse sequence	Product_size
FMgSSR-18681	scaffold3597	p2	(CG)6	Class II	24326	24337	TTAGCACCATTGGCAGCAGTCC	ACTCCGTGACAAGCGACTCCAT	340
FMgSSR-18686	scaffold1971	p2	(CG)6	Class II	52802	52813	TTCCCTCCAACCAAAGAGCCGA	ACGCGGCTTCATGGTCACAA	248
FMgSSR-18695	scaffold2757	p2	(CG)6	Class II	28549	28560	TTGCAGGTGCAGGTGAAGGTGA	ACATCTGCATGGGCCACGAGTA	350
FMgSSR-18697	scaffold3310	p2	(CG)6	Class II	33938	33949	TTGCCGGCACACCAATGTCA	TCGCCGTCACCTTCTCTCTGTT	330
FMgSSR-18699	scaffold5758	p2	(CG)6	Class II	38242	38253	TTGGCCACTGCCGCCTTTTT	TGCGGCACGGGAGGAAAAGAAA	340
FMgSSR-18707	scaffold94	p2	(CG)6	Class II	184295	184306	TTGGTAGCATCCCAAGGCCACT	AAACACTGACCGCACCGACTGT	234
FMgSSR-18708	scaffold876	p2	(CG)6	Class II	97161	97172	TTGGTCTACGGGTTTGCGCGAT	TCTCAGCTCGCGCACGTTTT	321
FMgSSR-18712	scaffold932	p2	(CG)6	Class II	46727	46738	TTGTTGCCGGCGAGTTGGTT	ATGCAGTGCACAGGGGTACACA	283
FMgSSR-18721	scaffold6246	p2	(CG)6	Class II	33504	33515	TTTCGCCGTGCGGTTTCGTA	AAAGCCAAGTCGCTCTGTCACG	349
FMgSSR-18725	scaffold9004	p2	(CG)6	Class II	22770	22781	TTTGATGCCGTGCAACCCGT	ACCAACCGTACGCCACTCTT	306
FMgSSR-18736	scaffold1267	p2	(CG)6	Class II	45334	45345	TTTTGCATAAGGCTGGCGCTGC	TGCCTGCATTACCGCCACAT	308
FMgSSR-18737	scaffold1174	p2	(CG)6	Class II	34487	34498	TTTTGCCATGCCACCCTACG	TCGAAGCCGGAGAGGTTGAACT	345
FMgSSR-18742	scaffold257	p2	(CG)7	Class II	62866	62879	AACAAATCCTCGTGGAGCCGGT	TAATCGCTATGCGTGCTGCCGT	313
FMgSSR-18744	scaffold950	p2	(CG)7	Class II	24090	24103	AACCGACAGCAGTACAGCACGA	TCGCGGGGGCTGAACAAAAA	217
FMgSSR-18750	scaffold1361	p2	(CG)7	Class II	69298	69311	ACACGATGCATGGCTTGGCA	ACTGTATGTCGCAGCGCAAGA	275
FMgSSR-18754	scaffold1873	p2	(CG)7	Class II	15969	15982	ACATTGTTTAGCGGGGAGCG	TTTCGTTTCGTGCGTGCTGC	271
FMgSSR-18755	scaffold8729	p2	(CG)7	Class II	11404	11417	ACCAAGTGCAAACGCTCGCT	ACCCGTGGCAGCCAAAACTCTA	290
FMgSSR-18763	scaffold1167	p2	(CG)7	Class II	50694	50707	ACGCTGCAACCTCACACCAGAT	AAGCCCAACACCAACACCCA	345
FMgSSR-18765	scaffold5366	p2	(CG)7	Class II	20869	20882	ACGGCTGGTTGTTTTTGGTCGG	TCACTGTGAGATGGGGCCTTGT	200
FMgSSR-18769	scaffold8061	p2	(CG)7	Class II	14318	14331	ACTGCGGATTGCCGAGGAACAT	TCGCCGCACATGCAAAGAGA	306
FMgSSR-18774	scaffold2983	p2	(CG)7	Class II	34862	34875	AGCACGGCAGAATTAGCACCGA	GGCCGGTCACATCCTGTTCAAT	325
FMgSSR-18780	scaffold119	p2	(CG)7	Class II	133378	133391	AGTGCGCATCGTGTCTTCCGAT	GCGTGAGACGTGCAAGTGCAAA	291
FMgSSR-18781	scaffold601	p2	(CG)7	Class II	59690	59703	AGTTCACCGTCCCGTCAGTT	TTCAAGCTGCGGGACCACTT	343
FMgSSR-18783	scaffold10942	p2	(CG)7	Class II	30528	30541	ATATGAAAGGCCGTCGCGCA	ACAGTGGCGCTGTCAACACA	261
FMgSSR-18786	scaffold191298	p2	(CG)7	Class II	394	407	ATCGTTTCGGTTCGCGTTTCCG	TGAAGGCGACCGTAGAGGAAGT	328
FMgSSR-18791	scaffold39569	p2	(CG)7	Class II	5004	5017	ATTCGTGAAACCCTGCGCCT	ATCACCGGTTTGCCATCGACCA	320
FMgSSR-18793	scaffold161373	p2	(CG)7	Class II	166	179	ATTTTGCGCTATCTGGCCACGG	AAACCAGCGGTTCACGGCAA	291
FMgSSR-18803	scaffold44421	p2	(CG)7	Class II	1610	1623	GCGGCGCGAAAAATTGAACTGC	GCGCGTTAGCGGCTTCAAAA	217

SSR_ID	Scaffold	SSR_Type	SSR_Motif	SSR Class	SSR_Start	SSR_End	Forward sequence	Reverse sequence	Product_size
FMgSSR-18806	scaffold6226	p2	(CG)7	Class II	18759	18772	GGCACGAAGCGCAAATGGAA	TCACCGTGCAATCTGCAGTCCT	315
FMgSSR-18812	scaffold2709	p2	(CG)7	Class II	27457	27470	TCCAAATCAGGGGGCCGTTT	ACCATGAAGGGCAACCGAGCAA	247
FMgSSR-18814	scaffold13141	p2	(CG)7	Class II	4799	4812	TCCTCGTCGCGATCCTTGACTT	AGCAAACTGACACCGCGTGA	341
FMgSSR-18817	scaffold1749	p2	(CG)7	Class II	48095	48108	TCGCATGAGCCATTGGAGTGGT	TGCCACCGATGGCCGAGATTTT	266
FMgSSR-18818	scaffold160627	p2	(CG)7	Class II	592	605	TCGCGTGGCTGCAAAACCAA	TCAGGTCGTTGCCATTCTGC	350
FMgSSR-18821	scaffold4371	p2	(CG)7	Class II	45315	45328	TCGGCGTCGAACTCGAATGA	TGGCAAAGTCAAGCTCGCGT	287
FMgSSR-18827	scaffold1767	p2	(CG)7	Class II	36961	36974	TGCCGCCGTCGTTGTTGTTT	AGTTTCGTACAGGGCCCCGATT	205
FMgSSR-18828	scaffold79533	p2	(CG)7	Class II	759	772	TGCGCGGGCAACGTTTCTTT	TGTCGCCGTGTTTCCTGAGA	343
FMgSSR-18832	scaffold3007	p2	(CG)7	Class II	42353	42366	TTCAACTCGCCTTGCGTCGT	TGAAGACATGCGTCGCCTCCAT	259
FMgSSR-18854	scaffold555	p2	(CG)8	Class II	21021	21036	ACACAGCAAACGGTGCGACT	ATGATCAGAATCTCGCCGCGCA	325
FMgSSR-18855	scaffold7959	p2	(CG)8	Class II	12232	12247	ACCCCAGACTTCTCCGCTTCAA	ACATGCCGTCGACCCAACTT	316
FMgSSR-18856	scaffold349	p2	(CG)8	Class II	61300	61315	ACCGGTGTGGTTACGTGAGCTT	TCTTGACGCGCCAACCGTAA	323
FMgSSR-18865	scaffold5358	p2	(CG)8	Class II	1169	1184	ATTGCCCTCCCCATCATCTCCT	TGCCTGAGCCTGTCCTCAAAGT	311
FMgSSR-18878	scaffold1024	p2	(CG)8	Class II	12068	12083	TTCCGCATCAACCTCCGTTCGT	TCGCGGATGCTCTGCATTGT	275
FMgSSR-18885	scaffold17829	p2	(CT)10	Class I	10831	10850	AAAAGGACAGGCCTGCACACGA	AGGGCTGGAGCGGAGTTTTT	226
FMgSSR-18886	scaffold223	p2	(CT)10	Class I	112642	112661	AAACAAGCCCAACCGTGAACCG	TGGAGCAAAGCAGATCCGGACA	320
FMgSSR-18888	scaffold27244	p2	(CT)10	Class I	5825	5844	AAACGTACTCTTCCGCACGC	TGTCCGATGGGTGCGCTTTT	265
FMgSSR-18889	scaffold1165	p2	(CT)10	Class I	42027	42046	AAACGTCATGGCAGGAGGCACA	AGGGATGCGGCTGCTATTTGTCA	301
FMgSSR-18891	scaffold7443	p2	(CT)10	Class I	21272	21291	AAAGCAGCTTGCCGTCCTCTCA	ATGCAGAATGGCAGAGGGTTGC	322
FMgSSR-18893	scaffold605	p2	(CT)10	Class I	32822	32841	AACACGAATCTCCAGCGAGCCA	ATGTTGAAGTGGAGTGGAGGGGGA	297
FMgSSR-18894	scaffold21168	p2	(CT)10	Class I	9427	9446	AACAGAAGCCTAAGCAGCGAGG	ACCATGCAGTGCGTAGCAGACT	341
FMgSSR-18896	scaffold549	p2	(CT)10	Class I	77320	77339	AACCAGCACACCATCTCAGCCA	ACCGACCGATTCCTCCATCGTA	270
FMgSSR-18899	scaffold188	p2	(CT)10	Class I	113513	113532	AACGGCTTTGTGGGGACCATGA	AGCGGCTGTCTAGTGCAGTTGT	344
FMgSSR-18900	scaffold4297	p2	(CT)10	Class I	25978	25997	AACGTTGGCACGTTGCCGTT	TTGCACGTCGGCGACACATT	222
FMgSSR-18907	scaffold532	p2	(CT)10	Class I	42282	42301	AAGCAGCAGCGACAGCTTGGAA	AGCTGGCCGCCTTCTTCGATTT	328
FMgSSR-18908	scaffold3605	p2	(CT)10	Class I	37565	37584	AAGCCGCCAAAACACCACCA	ACAAGGCGACGAACCTTCCACA	295
FMgSSR-18915	scaffold128	p2	(CT)10	Class I	5340	5359	AAGGGAACAGCAGCGACTCGAA	CACGGCGTTCGCGGATAAAT	296
FMgSSR-18917	scaffold4609	p2	(CT)10	Class I	37047	37066	AATCGGGCGCGTTGCTAAGA	TGAGACCAGCCGAGCAATGCAA	295

SSR_ID	Scaffold	SSR_Type	SSR_Motif	SSR Class	SSR_Start	SSR_End	Forward sequence	Reverse sequence	Product_size
FMgSSR-18921	scaffold41	p2	(CT)10	Class I	207524	207543	AATTGCAACGGGCGAGAGGA	AGTGCCGCAGCGGGATTTTA	207
FMgSSR-18922	scaffold107	p2	(CT)10	Class I	139661	139680	ACAAAGGGCAGGCCCGAATGTA	ACACGATGAACAGCGCACCA	231
FMgSSR-18931	scaffold314	p2	(CT)10	Class I	113696	113715	ACAGAAAGCGGAAAGGCACC	GCATAAACGTGCGGTGGGTTGT	266
FMgSSR-18932	scaffold1829	p2	(CT)10	Class I	53155	53174	ACAGATTCTATGCCCCCGCT	TGCAAGGCGGCTGCATCTTT	303
FMgSSR-18947	scaffold18975	p2	(CT)10	Class I	6739	6758	ACCTCAGCACAATACGCCAGACT	TCGCTGCATGTGGGTTGGGTTA	321
FMgSSR-18950	scaffold3601	p2	(CT)10	Class I	35822	35841	ACGAAGAACGCGTCTTTTGGGC	AAACGGCATGCCTCGGTGAA	205
FMgSSR-18953	scaffold16785	p2	(CT)10	Class I	14460	14479	ACGCCGCAGTTATGGGCTTA	ACTGTGGCCTGCGCAAAAGA	322
FMgSSR-18956	scaffold47	p2	(CT)10	Class I	11761	11780	ACGGCGACGCGTTTGTTGTT	TCACTTGCTACCTGCCTGCCTT	252
FMgSSR-18959	scaffold2301	p2	(CT)10	Class I	33416	33435	ACGTGTGTACGTTCGGCTCA	TGCAACATGCAGTGCCTCGAA	349
FMgSSR-18962	scaffold121	p2	(CT)10	Class I	30995	31014	ACTAGCTTACTTCCAGGCCCCACT	TGCATCGCCAGAGGGAGAAGAGAT	274
FMgSSR-18969	scaffold31230	p2	(CT)10	Class I	3324	3343	ACTTCCATCCGCATTCGCGT	TGACGTCGCTGTCGTGTCCATT	295
FMgSSR-18972	scaffold26751	p2	(CT)10	Class I	1760	1779	AGAAGCACATGGCGGGGTTT	TTGCGGGCTTCCGCATGAAT	334
FMgSSR-18975	scaffold686	p2	(CT)10	Class I	60184	60203	AGATCAGGAACATCGTGGCCGT	GCATCCAGCTGCTGCGAATCTT	341
FMgSSR-18980	scaffold1827	p2	(CT)10	Class I	23363	23382	AGCAGCTAAGCGCACGGTATGT	AGCGTTGCACGGAACAGCAGAT	271
FMgSSR-19001	scaffold4928	p2	(CT)10	Class I	9367	9386	AGGAACACATGCGTGATGGCGT	ACGTACGAGCTGTTTCCACCACT	318
FMgSSR-19002	scaffold15754	p2	(CT)10	Class I	3589	3608	AGGACCCAACCCAATTGACCCA	CCAAAGGTAAGGTGGCCTCATCGT	319
FMgSSR-19005	scaffold16330	p2	(CT)10	Class I	14540	14559	AGGCGAAAGCCGGCAAGAAA	TCCCCAGCCCCTTGCAAAAA	332
FMgSSR-19012	scaffold1487	p2	(CT)10	Class I	58524	58543	AGTACCGGAACGGCAGCAAT	TCGACATCATCGGGACAACGCT	350
FMgSSR-19013	scaffold7009	p2	(CT)10	Class I	26495	26514	AGTCACGTCATTTTCGGTGGCG	GCGGTGGGAAACGGCATTACTA	239
FMgSSR-19014	scaffold512	p2	(CT)10	Class I	17847	17866	AGTGAGGTTCAAGGGCCTACTCCA	ACTGTCCCATGCGGTTCCAGAT	349
FMgSSR-19018	scaffold6199	p2	(CT)10	Class I	28568	28587	AGTTGGGCAAAGCTCTAACCCT	TGCGGTGTCTAACCACGGATGT	317
FMgSSR-19020	scaffold6414	p2	(CT)10	Class I	34699	34718	ATATGCACGCATACCGCCACCA	TGCAGCATCGAGCATCCCAA	272
FMgSSR-19021	scaffold5929	p2	(CT)10	Class I	29980	29999	ATATGCGTGAGTCGTGCACCCA	TGGCAAGCGTTGTACCAGGGAT	294
FMgSSR-19023	scaffold1407	p2	(CT)10	Class I	80148	80167	ATCGAACGACTCGTGCACCGTA	AAGTCTGCAATGGGGGCTCAGA	234
FMgSSR-19025	scaffold7437	p2	(CT)10	Class I	20604	20623	ATGAAAGCTGTGGGCTGTGGCT	TTCGTGCACTCTGCCGTTACCT	277
FMgSSR-19026	scaffold7691	p2	(CT)10	Class I	12929	12948	ATGACTTATGGGAGCTCCACCCCT	CACAAGGCCGAATCTTGCGT	212
FMgSSR-19028	scaffold3623	p2	(CT)10	Class I	22434	22453	ATGCCCAGATGCGCCCAAAA	TCCAAACCTGCATGGGTGACGA	314
FMgSSR-19037	scaffold2075	p2	(CT)10	Class I	31625	31644	ATGTTCGCGGCTTGCTTGCT	ACCTCACGTTGGCGTGCTTT	306

SSR_ID	Scaffold	SSR_Type	SSR_Motif	SSR Class	SSR_Start	SSR_End	Forward sequence	Reverse sequence	Product_size
FMgSSR-19040	scaffold55	p2	(CT)10	Class I	82187	82206	CACAAGCAGGTGTGCGCTTT	TTGTCCATGAAGGTCGCCAACG	301
FMgSSR-19041	scaffold17196	p2	(CT)10	Class I	11433	11452	CACGATTGTTGTTTTCCGTCTGCC	ACAGCGGGACGATCAGTCTT	248
FMgSSR-19043	scaffold805	p2	(CT)10	Class I	92802	92821	CCACTGGTTTCTTGAGTTCCTTCCC	TTAGCGGGAAAAGAGCTTGCTAGGC	200
FMgSSR-19046	scaffold744	p2	(CT)10	Class I	77461	77480	CCGATACCATTGTTGTCGCTAGCCT	ACCTCATGGTATTGAAGTGAGCGG	265
FMgSSR-19047	scaffold71391	p2	(CT)10	Class I	104	123	CCGCCGCCGCTTGCTTTATTTA	TGCTGGTGCTGCTTCTGCACAT	200
FMgSSR-19051	scaffold419	p2	(CT)10	Class I	6622	6641	CGAGCGATGCGGTGTTTGTGTT	CGTCGGATCAACGCCCTTTCAA	281
FMgSSR-19053	scaffold327	p2	(CT)10	Class I	79502	79521	CGCAGCCGCATTCAGGTCAAAT	TGCGTGCATTTTCAGCGAGC	316
FMgSSR-19054	scaffold3076	p2	(CT)10	Class I	34387	34406	CGCCGCATAAAGGAACTCTCGT	ATGTGGCCGTTCTTGCGGAT	347
FMgSSR-19057	scaffold4497	p2	(CT)10	Class I	5453	5472	CGCGTTTGGGATGCACACACAT	TGCCGGTGCATCTCATTGGACA	335
FMgSSR-19058	scaffold378	p2	(CT)10	Class I	9622	9641	CGCTGGAACCCTGTACACGAAA	TCGAGCCTCTTGATCATGGCGA	331
FMgSSR-19067	scaffold2305	p2	(CT)10	Class I	20297	20316	GCAATCGCTCATGATTCGGCCA	ATGGAGCTCAGGCTAGGGTTGT	324
FMgSSR-19070	scaffold76599	p2	(CT)10	Class I	1668	1687	GCACAGCGGCTTCACATGTTGT	TGCTGCCCTTTTGCAGCTTGT	337
FMgSSR-19072	scaffold8634	p2	(CT)10	Class I	18995	19014	GCAGAGCTGTTGCGAAAATGGCA	TGCGTTCGTGCGCGATTTGT	274
FMgSSR-19073	scaffold2000	p2	(CT)10	Class I	30589	30608	GCCATGAATGTTGGCAAGGTCTGG	AGCACAAGTCCAATCCCACAAC	248
FMgSSR-19075	scaffold8606	p2	(CT)10	Class I	17187	17206	GCGAAGCTCAATTCCGCCTTCTT	AGCGCTGACATGGGGTGTACAAT	349
FMgSSR-19079	scaffold10395	p2	(CT)10	Class I	22886	22905	GCGGTTGTGTGCACCTTTGT	AATGAAGCTCACGCACGCCA	330
FMgSSR-19080	scaffold4312	p2	(CT)10	Class I	17083	17102	GCGTGCATGCATGTAGCCAT	AGCCGTGAAAGGCCCAACAA	348
FMgSSR-19084	scaffold613	p2	(CT)10	Class I	65750	65769	GGCCTCACATTTGGCCCTTTCA	TGCTGTGGGATCGCTTGTGTGT	283
FMgSSR-19092	scaffold7474	p2	(CT)10	Class I	671	690	TAGCCGGTGCTTGTGCATGAGT	TGCACCCAAACAAAGGGGCA	286
FMgSSR-19094	scaffold1605	p2	(CT)10	Class I	31313	31332	TCAACAACCTTCGCCCAGACCA	AGGTTTCTCCTCCAGCCGTCTT	322
FMgSSR-19100	scaffold105317	p2	(CT)10	Class I	769	788	TCAGCCACCTGGAATGACCGAA	TGGGCCTTTGGCAACTCACA	268
FMgSSR-19108	scaffold3989	p2	(CT)10	Class I	2588	2607	TCCAAGGCGATCTTCAACGCGA	AGTGCACACGGATGGACAACCA	296
FMgSSR-19109	scaffold1519	p2	(CT)10	Class I	46586	46605	TCCAGCGCTACTTGCATCCAACA	TTTGTCGACCACGCTCTCGT	338
FMgSSR-19110	scaffold176	p2	(CT)10	Class I	46327	46346	TCCAGTGAGGTCGAAGAAGGCA	TGCGCGCACAAAACAACCCA	231
FMgSSR-19111	scaffold24384	p2	(CT)10	Class I	7927	7946	TCCCTTCCCACAGAACAGCTCA	ATTTGCAGCCGCCACGGTAT	289
FMgSSR-19112	scaffold5030	p2	(CT)10	Class I	18011	18030	TCCGCAGAGCCGCGAATGATAA	ACTTGAAGCAGGACCGGTGGAA	304
FMgSSR-19114	scaffold3776	p2	(CT)10	Class I	15473	15492	TCCGCTGTCATTGTTGCAGGT	TCCTGTCGATCCGCGTTACA	346
FMgSSR-19116	scaffold3599	p2	(CT)10	Class I	29767	29786	TCCGTCATTGCACCATCGCT	TCACATTGGCTCTGGGGCAGAA	336

SSR_ID	Scaffold	SSR_Type	SSR_Motif	SSR Class	SSR_Start	SSR_End	Forward sequence	Reverse sequence	Product_size
FMgSSR-19119	scaffold9741	p2	(CT)10	Class I	11846	11865	TCCTGGTTCTGAAGCACCTCCA	CAGTCTTCTATAGTGGTCCCTTCCC	309
FMgSSR-19122	scaffold1803	p2	(CT)10	Class I	46708	46727	TCCTTTCAGATGCACACGGGCT	CGCATCCTTGGCCCTCAGTTTT	258
FMgSSR-19123	scaffold32333	p2	(CT)10	Class I	6415	6434	TCGAAATCACGCGTCACCCA	TTGCGGAAGTAGGGGCCAATGT	313
FMgSSR-19124	scaffold308	p2	(CT)10	Class I	147626	147645	TCGAGTTGCGCACGTTTCGT	TCCTGTGTGCGCCGGTTTTT	262
FMgSSR-19126	scaffold11860	p2	(CT)10	Class I	11418	11437	TCGCCACTGCAACTGCAACA	CGCCTTTTCGGTGCATGTGGAT	266
FMgSSR-19128	scaffold4609	p2	(CT)10	Class I	37475	37494	TCGCGGGTAAATCGGTTGGT	ATGGATTAACAGCGCCCGCGTA	324
FMgSSR-19129	scaffold9139	p2	(CT)10	Class I	12122	12141	TCGCTCTGTACTTGTCACCCGT	AGAGGTGGCGAGCAGAAGAA	307
FMgSSR-19130	scaffold5136	p2	(CT)10	Class I	42078	42097	TCGGAGCCACAACACAATGC	TGGCGCGACGTGTTTCTGTT	272
FMgSSR-19132	scaffold16168	p2	(CT)10	Class I	12826	12845	TCGTGCGCATCGCATCGTAT	AAAATGCGGGCTCTGGGGCTTT	277
FMgSSR-19133	scaffold16966	p2	(CT)10	Class I	13953	13972	TCGTGGACAAACTTGCACGC	TTCCGGGTGATGTGGGAACAGA	346
FMgSSR-19137	scaffold3171	p2	(CT)10	Class I	26803	26822	TCTCGCCCGTTACACTGAACCA	CAACCCTGTGTTTGCGGCAT	318
FMgSSR-19142	scaffold14217	p2	(CT)10	Class I	4104	4123	TCTTCCTTTCCCTCGGCTCCAA	TGTAGTCATTGGGCGCGTCT	310
FMgSSR-19145	scaffold27020	p2	(CT)10	Class I	1252	1271	TCTTTTCTCTGCTCGCTCGC	GATCTCGATAGGGGCCTGACAT	272
FMgSSR-19146	scaffold12968	p2	(CT)10	Class I	19967	19986	TGAATGTGTGTGTGGCTGTGGG	ATCCCCATGCTCTGCGCTTCAA	349
FMgSSR-19147	scaffold979	p2	(CT)10	Class I	80754	80773	TGACGAAGCGCTGACCAACT	TTATCCCAAACTGCTGCGTGCG	317
FMgSSR-19149	scaffold8606	p2	(CT)10	Class I	3963	3982	TGACGTACGTTACGTTGACCCG	TCGTCGTAGCAACGAAGCATGG	327
FMgSSR-19150	scaffold1655	p2	(CT)10	Class I	55608	55627	TGACGTGTACGGCCAATCGGAA	TCCCAGCTGTGCGTACTTGCTA	257
FMgSSR-19151	scaffold1460	p2	(CT)10	Class I	82697	82716	TGAGCGCAAGCGAGATACCCAT	TGATGCATCCATGTCGCTCCT	335
FMgSSR-19152	scaffold2731	p2	(CT)10	Class I	28526	28545	TGAGCTCCACCAGCTTTCGT	TCCTGTTGTCGTGCCTGTCAGT	350
FMgSSR-19153	scaffold17358	p2	(CT)10	Class I	8804	8823	TGAGGGAGCCAACCTGCCATTT	TATGCATGCTGCCTGAGGTCGT	238
FMgSSR-19155	scaffold13078	p2	(CT)10	Class I	7734	7753	TGCAAACGGCAGCCCGATAA	AAAGCAGCAGCGCACAGTCA	276
FMgSSR-19161	scaffold3203	p2	(CT)10	Class I	47929	47948	TGCCATGTAGATAGCGGCGA	GCCAACAGCCGAACAAGCCAAA	315
FMgSSR-19168	scaffold3038	p2	(CT)10	Class I	46555	46574	TGCCGCGGTTCGTGATGAAA	TGGCAAATGGAAGCGTGAACGG	246
FMgSSR-19169	scaffold17871	p2	(CT)10	Class I	13399	13418	TGCGCCGTAGCACATCCAAA	TGCCTCCCGATCGATCCTTTCT	316
FMgSSR-19170	scaffold13855	p2	(CT)10	Class I	10547	10566	TGCGCTACGCTGCACCCATTTA	ACGCCGCTTATTGCTGTGTGGA	349
FMgSSR-19172	scaffold31230	p2	(CT)10	Class I	1618	1637	TGCGGCAGCAGTATCTCTCGTT	TGAAATGCGGGATGGCGAGGAA	245
FMgSSR-19182	scaffold5499	p2	(CT)10	Class I	26792	26811	TGCTGAGCCTTCAGTTTCCACT	TCCAGCCTCTTTCACCTAGTTCAC	317
FMgSSR-19186	scaffold8061	p2	(CT)10	Class I	13155	13174	TGCTGGCACACAATCGCACA	TCAGCCAGTTCCTGAGGCTGTT	248

SSR_ID	Scaffold	SSR_Type	SSR_Motif	SSR Class	SSR_Start	SSR_End	Forward sequence	Reverse sequence	Product_size
FMgSSR-19191	scaffold1195	p2	(CT)10	Class I	13929	13948	TGGCACCAGGCTTTGCCTTT	TTGCAAAAGAGACGGCGGGGTA	332
FMgSSR-19193	scaffold16197	p2	(CT)10	Class I	13369	13388	TGGCCAATCACAACCTGCACAC	CGCTTGCAAAATCCATGGCGCT	344
FMgSSR-19197	scaffold19384	p2	(CT)10	Class I	8004	8023	TGGCGCGTGCGTTATGTGTT	AGGCCATCCATCGGTGTTCGAT	222
FMgSSR-19207	scaffold19303	p2	(CT)10	Class I	16954	16973	TGTCAATCACGACCGTGCCACT	TGGCTGACGGCGACAAATCA	262
FMgSSR-19208	scaffold5151	p2	(CT)10	Class I	21390	21409	TGTCCCCCAACACTTTCGTGCT	TTGCAGCTCCAAAAGGCCGA	215
FMgSSR-19216	scaffold7708	p2	(CT)10	Class I	19848	19867	TTACAGATTCGGCCCAGTGCCA	TTTGGCTCATGGGCACAGCAGA	329
FMgSSR-19218	scaffold1812	p2	(CT)10	Class I	36460	36479	TTAGCGGGCGGTGCAAGAAA	AAAGCGCGCAAACAGCAACG	342
FMgSSR-19220	scaffold8722	p2	(CT)10	Class I	15721	15740	TTCAACGGTAGTGCTGCCCA	ACGCAGTGCCGACGTTTTGA	304
FMgSSR-19222	scaffold5469	p2	(CT)10	Class I	4836	4855	TTCATTGCCCCCAAGACTCCCT	AGAGCAAAGCGGCAGGACAA	331
FMgSSR-19223	scaffold363	p2	(CT)10	Class I	22797	22816	TTCCCAAACAACCAAACCCGCC	TCCACCGCCACTGGCATGAAAT	273
FMgSSR-19225	scaffold4400	p2	(CT)10	Class I	22487	22506	TTCCTGCATAGGATTGCGGC	TGTCGTCCCCACCCAAATCACA	335
FMgSSR-19227	scaffold293	p2	(CT)10	Class I	35798	35817	TTCGCGAGGACAAAAAGCTGGG	TGAATGCCGCTACTGTGGGCTT	205
FMgSSR-19230	scaffold1785	p2	(CT)10	Class I	27566	27585	TTGAACGTGCCGTCACATCGGA	AGCCATTCTCTCGGTTCATAGGGT	208
FMgSSR-19236	scaffold8654	p2	(CT)10	Class I	1416	1435	TTGCTGAGTAGCGGTCAGGCTT	AACAGTTCGCGAATCGTCGTGC	342
FMgSSR-19238	scaffold75402	p2	(CT)10	Class I	567	586	TTGGCTATGGCAGTAGCACCGT	ATGGCCTACCGCTCTGTTGCAT	302
FMgSSR-19239	scaffold936	p2	(CT)10	Class I	19645	19664	TTGGCTTTGGGGGCTAATGGCT	ACGGCGTCAGTCTGAGGTGTAA	206
FMgSSR-19241	scaffold6366	p2	(CT)10	Class I	30272	30291	TTTAGATTCGCCCGCCACAACC	ACTCGCCGTGCTGACAACTT	271
FMgSSR-19247	scaffold16857	p2	(CT)10	Class I	10702	10721	TTTGATGCGCACACACGGGA	ACACTGCGAACCCAGTGCTACA	345
FMgSSR-19255	scaffold64715	p2	(CT)11	Class I	1074	1095	AACATCACTGATCGGCGTGGGA	TTCATGGCGTTGGCCTTGCT	266
FMgSSR-19256	scaffold548	p2	(CT)11	Class I	62292	62313	AACATCCTCGCGCGTTTCGT	TGCGGAACTGTCCGAAAGACA	323
FMgSSR-19260	scaffold4074	p2	(CT)11	Class I	12972	12993	AAGCTTCGCGTTGGTTGTGC	ACAAAAGCCTCAGCCGACGA	336
FMgSSR-19263	scaffold5092	p2	(CT)11	Class I	16779	16800	AATCCACCAGCCACCACGAA	GCTGTTGATTCGTTGCAGGGGA	222
FMgSSR-19269	scaffold6096	p2	(CT)11	Class I	29282	29303	ACACACGACGTCAGATGTGCCA	TGTCCGCAGCTTGCTGAGAT	296
FMgSSR-19271	scaffold1245	p2	(CT)11	Class I	13923	13944	ACACGCACAAGTACTGCCTTGC	TGGCCAATGATGGGGGTGCTTT	304
FMgSSR-19272	scaffold5063	p2	(CT)11	Class I	31941	31962	ACAGAACAGGATCGGCCACGTT	AAGCAGCTGGCGTGGAGTAGTA	304
FMgSSR-19273	scaffold65994	p2	(CT)11	Class I	2071	2092	ACAGCGACATTCAGAGCAGCGA	TTCGTTTCCCCATCTGCTGCGA	348
FMgSSR-19277	scaffold9307	p2	(CT)11	Class I	27516	27537	ACATGGTGAGGGCAGCAACCAA	TCGGCCATTCCAACAACCGT	295
FMgSSR-19278	scaffold805	p2	(CT)11	Class I	92565	92586	ACCAGTTCACCACCGTGTTCCA	AGAGGCATGTTCCATTCGCAC	289

SSR_ID	Scaffold	SSR_Type	SSR_Motif	SSR Class	SSR_Start	SSR_End	Forward sequence	Reverse sequence	Product_size
FMgSSR-19280	scaffold3916	p2	(CT)11	Class I	40064	40085	ACCATGTGCTGTGGTGGCTGAT	TTTTGGTTTGGCAAGGGCCG	350
FMgSSR-19286	scaffold5920	p2	(CT)11	Class I	29023	29044	ACCTACCAAACGCACCGCAA	TGAATTCACCAGCGGAGGCT	346
FMgSSR-19292	scaffold27618	p2	(CT)11	Class I	2688	2709	ACGCCTGGATCTTTGTGCGACT	TCCATTGGTTCCCAGCGTCA	327
FMgSSR-19294	scaffold367	p2	(CT)11	Class I	69096	69117	ACGGCACCAAAAGCGACCAT	ACCCACCGGAACCAAATCCTCT	304
FMgSSR-19299	scaffold1957	p2	(CT)11	Class I	54937	54958	ACGTGTGTTTCCCTGCACCA	ATGTCCGTCAGGCCTGCAAT	314
FMgSSR-19302	scaffold26578	p2	(CT)11	Class I	3463	3484	ACTCAGTCCGATTGCAGCCA	TCGACTAACCAAGAGGGCACA	247
FMgSSR-19303	scaffold2555	p2	(CT)11	Class I	24091	24112	ACTCATTTCGTTACCCCGCAGCA	AGGTCGCTGAATCCACTGCCAT	232
FMgSSR-19306	scaffold11300	p2	(CT)11	Class I	1345	1366	ACTTCCGTGGAGCTTGTTGCCA	TGCAATGCATACTTGCGCGT	289
FMgSSR-19309	scaffold3184	p2	(CT)11	Class I	21704	21725	AGAGCAGCAATGAGCGGCAA	ATCCTGCGAGGAACAAGCACCT	340
FMgSSR-19310	scaffold3198	p2	(CT)11	Class I	28464	28485	AGATGTAACGGCGGCAGCAA	AACAACCCGGATTCTGCTCCCA	308
FMgSSR-19313	scaffold30162	p2	(CT)11	Class I	4773	4794	AGCAGCAGCGATGGTAAGAACG	ATGACCACAGCTTCGACAGCGA	344
FMgSSR-19317	scaffold52761	p2	(CT)11	Class I	2615	2636	AGCCAAGTTGCATGGGAATGCT	ACCGCACAGTGACGTTCTGCAA	256
FMgSSR-19318	scaffold5071	p2	(CT)11	Class I	33407	33428	AGCCCAGCATGTTGGTCCTTGT	AGAACTGCGTCACAGAACAGC	318
FMgSSR-19319	scaffold230717	p2	(CT)11	Class I	261	282	AGCCCCACCCATCCAAAGCAAA	AGTTGTACACAGCGTGCCGA	313
FMgSSR-19324	scaffold2751	p2	(CT)11	Class I	48744	48765	AGCTGCGTTTGTGTGTCCGT	TGCGCACTTTCGGAAGGAAACG	247
FMgSSR-19325	scaffold4282	p2	(CT)11	Class I	28934	28955	AGCTGTTGCCGCATGCTACT	AGCCGACCACCCATGGTTTT	252
FMgSSR-19326	scaffold291	p2	(CT)11	Class I	17211	17232	AGCTGTTGTTGACAGTGGGTCC	ACCAGCTACATTTCTGGGCTGC	200
FMgSSR-19333	scaffold19441	p2	(CT)11	Class I	7349	7370	AGGGGCTTTATCGCTCTGCAT	TCGGCAAACGTCAACCACAA	270
FMgSSR-19334	scaffold2598	p2	(CT)11	Class I	16148	16169	AGGTCTCACTCTCACAGCTTGC	AGATGGGAGAGATGCGCAGAGA	328
FMgSSR-19338	scaffold16598	p2	(CT)11	Class I	2095	2116	ATCGGCCACTCCGATCCTTACA	TCGACCTGTGAATGGGATGGGT	301
FMgSSR-19342	scaffold454	p2	(CT)11	Class I	46038	46059	ATGCCACTTGGGCAGGATGA	ACAGGCCCCATAGACAAGTGGA	348
FMgSSR-19347	scaffold1151	p2	(CT)11	Class I	80700	80721	ATTGCCACTCAGCCAATGCCCA	TGTTGTTGCACGCTGAGGGA	289
FMgSSR-19349	scaffold31700	p2	(CT)11	Class I	3724	3745	CACCAGGCCTGCTTTGAAAACACA	ACGTTCCCTGATGCCACCCATT	217
FMgSSR-19351	scaffold4210	p2	(CT)11	Class I	24457	24478	CAGTGTGAACATGTTGGCGGGA	ACGGCGGGGTTTTTGGTGGATA	251
FMgSSR-19353	scaffold4901	p2	(CT)11	Class I	23415	23436	CCCCTGGATATGACAATGGACTTG	GGTGGTATGTTACAGGGGATCACA	336
FMgSSR-19358	scaffold4546	p2	(CT)11	Class I	5821	5842	CGCGTCAACCAACCAAACCCTA	ATGGCCGTCCGGCTGTAAAA	316
FMgSSR-19361	scaffold1682	p2	(CT)11	Class I	30304	30325	CGTGCTCGCCTCAACGATGAAA	ATCCGTCACAATGCGGTTCACG	241
FMgSSR-19362	scaffold1114	p2	(CT)11	Class I	30672	30693	GACATGTCTTCTTCCTTGCGCT	TTGGGCCATGGAAGACTGGT	222

SSR_ID	Scaffold	SSR_Type	SSR_Motif	SSR Class	SSR_Start	SSR_End	Forward sequence	Reverse sequence	Product_size
FMgSSR-19363	scaffold21031	p2	(CT)11	Class I	10960	10981	GACTCATTTGGCTCCGTAGGAAGA	ACACTCTCTGCAGGGTTCTGACA	350
FMgSSR-19364	scaffold15528	p2	(CT)11	Class I	3720	3741	GCAAACAAACACGCGCAGCA	AGGAGGGAGCACAAGGCAACAA	288
FMgSSR-19368	scaffold1225	p2	(CT)11	Class I	80485	80506	GCACGCTTCGCAGCGCATAATA	CGGCAGTTTGGGTTGCGCTAAA	339
FMgSSR-19371	scaffold24153	p2	(CT)11	Class I	11016	11037	GCCCATGAAGCCCAATTTTTCTGC	AGGTTGTCGCTGTGGAGCTCTT	306
FMgSSR-19372	scaffold9063	p2	(CT)11	Class I	29011	29032	GCCGATCAGCACCACCTTGAAT	TGGCCAAGCAAGCAGCCAAA	251
FMgSSR-19374	scaffold39835	p2	(CT)11	Class I	4274	4295	GGAACAAAGCATTGGGCACGCA	CGTGCGGCTGCAATTACTGA	295
FMgSSR-19375	scaffold3146	p2	(CT)11	Class I	37741	37762	GGAATCCATGCTCCGGAAAACA	TTATGTGCCAACGTGTCAGCGG	303
FMgSSR-19380	scaffold7567	p2	(CT)11	Class I	6433	6454	GTGGTTATGCTTCCGCGATGCT	GCGCCCACAAAAGAGTTGCTGT	244
FMgSSR-19383	scaffold4575	p2	(CT)11	Class I	36022	36043	TAAACCAGCAGCTTGGCGCA	ACGAGTGCGCGCCTGATTTT	238
FMgSSR-19390	scaffold44287	p2	(CT)11	Class I	241	262	TAGTTCGCCGGAGTTGTGCTGA	ACTGCCATGCTTGCCTTGCT	341
FMgSSR-19394	scaffold124184	p2	(CT)11	Class I	862	883	TCAAGGCTCCAAGTACACGGCT	ACAGTGTGACCATGGATCTCTGCG	350
FMgSSR-19403	scaffold4828	p2	(CT)11	Class I	37245	37266	TCCTTCTCTCAATGCAGAGGCGA	ACGACTCAATTGGGTGGGTGCT	212
FMgSSR-19405	scaffold18644	p2	(CT)11	Class I	9253	9274	TCCTTTGAGCTTGGCGATGGCA	ACCAAGCCCGTCGTCATTGT	242
FMgSSR-19410	scaffold4293	p2	(CT)11	Class I	33404	33425	TCGTGCATTGGGGAGAAGGTGA	AGTACCCCCACTTGCTCCAGAT	314
FMgSSR-19412	scaffold3076	p2	(CT)11	Class I	18248	18269	TCTGCCTGTCCTTGCACGTAGT	ATGGGAAGGAATTGCGGCGGAT	306
FMgSSR-19414	scaffold4817	p2	(CT)11	Class I	18483	18504	TGAATCCCGGCTGTACCGATCA	ATGGCAATTAGCAAGGCTGGCG	257
FMgSSR-19415	scaffold6708	p2	(CT)11	Class I	5818	5839	TGACAGAACCGTACGCAAGGCA	TGAGGAGGAGCGGTGTCTGTTT	234
FMgSSR-19418	scaffold1703	p2	(CT)11	Class I	58152	58173	TGAGCCCGCTGGTCAGTTTGTT	GCCAAGATGCTGGATCTGGAGAGA	306
FMgSSR-19423	scaffold975	p2	(CT)11	Class I	99093	99114	TGCAAGTTTGCAACACGGCG	ACACGACGGAGCATGCCAGAAT	333
FMgSSR-19424	scaffold54953	p2	(CT)11	Class I	1602	1623	TGCACCTTGGCCATCTCTCTGA	TCACCTATGCGCTAAGCAAGCC	269
FMgSSR-19428	scaffold8656	p2	(CT)11	Class I	19535	19556	TGCAGCTCAGTTGTGGAGGCAA	TGGCGTTCGACGTCAGGAGAAA	338
FMgSSR-19429	scaffold879	p2	(CT)11	Class I	49976	49997	TGCAGTAATAAAGCGCTCCACC	ACGGTTGGATCAACAGAAGGGA	328
FMgSSR-19431	scaffold8910	p2	(CT)11	Class I	28737	28758	TGCATGGATGCATGGACGACGA	ACGCGTGTGCCTGAAGAAAACT	307
FMgSSR-19432	scaffold488	p2	(CT)11	Class I	101368	101389	TGCCGCGTCTGAGTTGGATCTT	ACATTATGGAGGAGGCCTCGGTGA	223
FMgSSR-19436	scaffold6590	p2	(CT)11	Class I	4545	4566	TGCGCCAATTATCCAAACGGGT	TGTGCCACCATCACACAGGGAT	341
FMgSSR-19437	scaffold839	p2	(CT)11	Class I	56080	56101	TGCGCTCGTTTGACTTGTCCCA	TGTGGCGAAGGTTCCGTTTGGA	315
FMgSSR-19445	scaffold316	p2	(CT)11	Class I	22083	22104	TGGTGCTGGGCTCAACATGGTA	TTTCGCTGGTTGGGAGAGGA	349
FMgSSR-19446	scaffold3672	p2	(CT)11	Class I	51889	51910	TGGTTGGGACGAAGGGGATT	AAATGCTGCGAGGGCGTGAA	302

SSR_ID	Scaffold	SSR_Type	SSR_Motif	SSR Class	SSR_Start	SSR_End	Forward sequence	Reverse sequence	Product_size
FMgSSR-19448	scaffold8039	p2	(CT)11	Class I	9952	9973	TGTCTCGCTGCTTCCTCTTGGA	TGGCGATTCACACCTGGCACAA	280
FMgSSR-19449	scaffold1537	p2	(CT)11	Class I	70167	70188	TGTGAAGTGCTCCGACCGGAAA	CGCCTTGCCCATTCGTCATA	305
FMgSSR-19453	scaffold6542	p2	(CT)11	Class I	24205	24226	TGTGTTTCTTGGCGTCTCGGGT	TCAGCATCTGCTTTCCCGCT	273
FMgSSR-19462	scaffold11523	p2	(CT)11	Class I	12333	12354	TTCGCATCGATCGTGCCCTT	TGGGACTCACATGCCAGCCTAT	241
FMgSSR-19464	scaffold14129	p2	(CT)11	Class I	10734	10755	TTCGCGTGCTTTTGCCGTGT	AAACAGGGGAACTGGTGCCACA	250
FMgSSR-19465	scaffold61519	p2	(CT)11	Class I	1773	1794	TTCTTCAAACCCTGCCCTCTCTGG	TGTGCTGCGCTAGCGATTTGTG	318
FMgSSR-19468	scaffold16160	p2	(CT)11	Class I	3603	3624	TTGAGCTTCGAGCGGGTGAT	TTGCACTTTGTCGCGGTCATGC	285
FMgSSR-19470	scaffold8127	p2	(CT)11	Class I	22439	22460	TTGCATGCATGTGGGATGTGGC	TAACACGGTGGGCAAGGGCATT	302
FMgSSR-19472	scaffold11131	p2	(CT)11	Class I	15611	15632	TTGCGGGGAAAACGGTTGCT	TGCAGCGTATTGTGACGCGA	320
FMgSSR-19473	scaffold2806	p2	(CT)11	Class I	49460	49481	TTGGACGCAAGCGGCTGAAA	TTTTGAACGCGCCGAGGAGA	291
FMgSSR-19474	scaffold11614	p2	(CT)11	Class I	538	559	TTGGAGCGAACCAACAGGGGAA	AGGGGAGGAATCGCCTGTTACAA	350
FMgSSR-19485	scaffold6007	p2	(CT)12	Class I	21563	21586	AACAAGCAGCCAGCCACCTT	ACGCAGAGCGGCAGCATGATTA	223
FMgSSR-19489	scaffold8459	p2	(CT)12	Class I	8942	8965	AACCGCGTGCCTCTACCAGTTT	AACTCGGCGCCAAAACCGAA	278
FMgSSR-19490	scaffold26825	p2	(CT)12	Class I	4275	4298	AACGCAGCGAGCATGGCATA	ACATGTGCGACAAACGATGGCA	233
FMgSSR-19495	scaffold10471	p2	(CT)12	Class I	14880	14903	AAGTGGCAAGGCAACTGGCA	ACTGGAAGACGCGCCTTGTT	301
FMgSSR-19498	scaffold4218	p2	(CT)12	Class I	31780	31803	ACAATCACTTACGCGCGACGA	AGTTGAGAGCACTGTGGCGA	332
FMgSSR-19500	scaffold27185	p2	(CT)12	Class I	9888	9911	ACATGGACGCTGAAACGGCA	ATGTACCTTGGAGAGCGCGT	273
FMgSSR-19504	scaffold11170	p2	(CT)12	Class I	2475	2498	ACCCAAACGTGTCCTGGACGAA	TTTCGCATGCACGCAGTCGT	325
FMgSSR-19505	scaffold2344	p2	(CT)12	Class I	43203	43226	ACCCATTCTTCGAACGGCAACG	ACGGCAGGAGAGGAAAAGCACT	209
FMgSSR-19508	scaffold2810	p2	(CT)12	Class I	1743	1766	ACGAAACGCGTTGCACTGCT	TGCCATTTGCACCGGCTTGT	266
FMgSSR-19513	scaffold2924	p2	(CT)12	Class I	27761	27784	ACGGGCTCGATCCGTGAAAA	AGTCTCAGAGCCAGCTCCTGTCTT	346
FMgSSR-19514	scaffold1134	p2	(CT)12	Class I	90381	90404	ACGTCCTGTTTTGATGCGCGA	AGCTTGCGCGGTTAATTTGGCG	337
FMgSSR-19515	scaffold1042	p2	(CT)12	Class I	58526	58549	ACGTCTGGACACAGCATCGGAA	TTTGCCTGTCCGCCGATCAA	337
FMgSSR-19516	scaffold975	p2	(CT)12	Class I	83245	83268	ACGTGCGTGACGTAAACCGT	GCATGCATGGTTCGCAAGGA	286
FMgSSR-19517	scaffold16598	p2	(CT)12	Class I	3552	3575	ACGTTTTCAAGAAGGTCGCTGC	TCAGCATCCCATAGACGCGA	346
FMgSSR-19518	scaffold390	p2	(CT)12	Class I	42465	42488	ACTAACCAAACGTGCTGCCGA	TCGGTTGGGTAGTTGCCGTT	313
FMgSSR-19519	scaffold11304	p2	(CT)12	Class I	15688	15711	ACTCCCGTGAACGCGTCAGAAA	TCCCGTCCGTGTCGTTTGGTTT	229
FMgSSR-19520	scaffold187089	p2	(CT)12	Class I	237	260	ACTCCGCCATCCACACGTTTCT	TGTGGCTGCACACGACGATT	282

SSR_ID	Scaffold	SSR_Type	SSR_Motif	SSR Class	SSR_Start	SSR_End	Forward sequence	Reverse sequence	Product_size
FMgSSR-19521	scaffold33	p2	(CT)12	Class I	200396	200419	ACTCTTAGTCCTCATCGTCGGCCT	CGCATGATGTGGTGCTGCTGTT	293
FMgSSR-19522	scaffold4437	p2	(CT)12	Class I	4348	4371	ACTGCCGGTGCCGAGAAAAA	TCCTTCCCGGTGAGAGAGAGTTCA	348
FMgSSR-19523	scaffold4054	p2	(CT)12	Class I	41681	41704	ACTTCACTGAGCGCGTCCACAT	TTCTCCATGGGGCTCAAGCCAT	225
FMgSSR-19525	scaffold13804	p2	(CT)12	Class I	5879	5902	AGAGGGCCACCTGCATCAAGTT	TGGGTGCCATCACTCCCATT	253
FMgSSR-19527	scaffold204	p2	(CT)12	Class I	56855	56878	AGCAGGATCCAAGTGAGAGCGA	TTTCACTGCCATCCTTGGCGCA	313
FMgSSR-19529	scaffold11331	p2	(CT)12	Class I	11558	11581	AGCCAGGTTCCACCTATTCACA	ACGTCGTCTCCATCGATCGTCT	245
FMgSSR-19545	scaffold9704	p2	(CT)12	Class I	3662	3685	ATCGGACAGTTCACGGGAGCAA	CCGCCGGATCTTTTTGTTGCGT	306
FMgSSR-19547	scaffold3803	p2	(CT)12	Class I	20819	20842	ATGCCGTACTGGCTTACTGCGA	TTGTCCTCTTTTGCCCCGCGAT	338
FMgSSR-19548	scaffold8154	p2	(CT)12	Class I	9325	9348	ATGCGCACTGGTTGCCACAT	TTGGCAGGGGCTATTGATGGGA	349
FMgSSR-19558	scaffold16232	p2	(CT)12	Class I	7459	7482	GAGCGCGTGCTCTCAAAAGT	TGACTGCCAACACTTGCATGTCC	264
FMgSSR-19559	scaffold27038	p2	(CT)12	Class I	5163	5186	GCACCCAACAATCACCGTGCAA	TCCCCAGGCTCAACAATCATCA	235
FMgSSR-19560	scaffold12188	p2	(CT)12	Class I	14614	14637	GCCAGAAAACAGTTTGTTTCCCCC	GCCTGGCATGCTTTGGCAAGTA	249
FMgSSR-19561	scaffold4598	p2	(CT)12	Class I	29523	29546	GCCGTCGCCAAAAGCTTGAT	AGATCTACGAAGAGGACGGGCT	314
FMgSSR-19564	scaffold2582	p2	(CT)12	Class I	58033	58056	GCGATGGCATGCGGAGCTTATT	AAAACAGTCGCACCGGAGTCCT	305
FMgSSR-19566	scaffold3906	p2	(CT)12	Class I	34381	34404	GCTTGTGAGGTTGTTGGGAACTGC	GGATGTTTGGGTTTGACCGTGTGT	321
FMgSSR-19567	scaffold134	p2	(CT)12	Class I	180510	180533	GGGCCATCCCAAAACAAGAGTCGT	TGCATGACCATGTCACACGCAC	316
FMgSSR-19570	scaffold20943	p2	(CT)12	Class I	3386	3409	GTGCTCCAAAACGAGGCTGCAA	TTTCATCAGTGGGCAAGCAGCG	294
FMgSSR-19575	scaffold2054	p2	(CT)12	Class I	43073	43096	TAATGAATCCCGGGAAAGCGCC	GCGCATAGCAATTGCAGCGTCT	339
FMgSSR-19590	scaffold2536	p2	(CT)12	Class I	22053	22076	TCCGACTGGTTTGTCCTACCAACG	AGGATTAACCCGCTCTCGATACCA	326
FMgSSR-19592	scaffold1145	p2	(CT)12	Class I	71671	71694	TCCGTTTTGCTTGGGCCTGA	ACAGATTCGCCGCCATGGGAAA	280
FMgSSR-19593	scaffold3218	p2	(CT)12	Class I	13965	13988	TCCTAACCCAACCCCTCTCTCTGA	ACCATTGCCATTGATGCCACGC	249
FMgSSR-19596	scaffold7669	p2	(CT)12	Class I	13196	13219	TCGTTGGGTCGTCTTGGGCTTT	TAGCGGAAGAGAACGCACAGCA	298
FMgSSR-19601	scaffold46	p2	(CT)12	Class I	120171	120194	TGAACGCCTGAACAACGCCA	ACAAGTAGTCGCGGGCAAGT	266
FMgSSR-19602	scaffold101	p2	(CT)12	Class I	165363	165386	TGAAGTGGCGAACGGGACACAT	TGCCTGTCATACTCGCCGGATCTA	230
FMgSSR-19603	scaffold3026	p2	(CT)12	Class I	24208	24231	TGCAAAGGCTGGGTGAGCAA	ACGGCGGAGCATGGCAAATA	337
FMgSSR-19606	scaffold2301	p2	(CT)12	Class I	37336	37359	TGCAATGTCACCCGACACCT	TGGCATGAGGCTGTGGAGAA	347
FMgSSR-19613	scaffold44984	p2	(CT)12	Class I	780	803	TGCGTGAATTTAGCCCGCCT	GGAAGGGAATGGCGTCCGTTTT	273
FMgSSR-19618	scaffold8395	p2	(CT)12	Class I	7462	7485	TGGCGGCATGATCTTCGTCCTT	ACAGCGCCTGCTGGAATCCTTT	316

SSR_ID	Scaffold	SSR_Type	SSR_Motif	SSR Class	SSR_Start	SSR_End	Forward sequence	Reverse sequence	Product_size
FMgSSR-19619	scaffold1020	p2	(CT)12	Class I	30757	30780	TGGCGGGTAGGATATCCCTTGA	TGAAAGTCCGTAAGCACGCTCG	350
FMgSSR-19621	scaffold3396	p2	(CT)12	Class I	52345	52368	TGGTGAGGCGACCAAAGTGCAA	GCCCAATGCGTCTGCGTAGAAT	215
FMgSSR-19626	scaffold3490	p2	(CT)12	Class I	33608	33631	TGTGCAAATTGGGGGCAGAGGT	AGCATGTAGAGATGGTGCAGGGA	237
FMgSSR-19631	scaffold156	p2	(CT)12	Class I	96556	96579	TGTGGGATTCGTTGGGTGGACA	ACAGCATTGGCTTCCTGGCT	228
FMgSSR-19634	scaffold1274	p2	(CT)12	Class I	52731	52754	TGTTTGCTGCTACCCACGCA	AGGCAGGTCGGTCAAGCCTTTT	308
FMgSSR-19636	scaffold463	p2	(CT)12	Class I	110597	110620	TTACGCGGTAGGCAGCTCGAAA	ACACCCACTTCGGCCCACTTTA	334
FMgSSR-19638	scaffold13114	p2	(CT)12	Class I	5633	5656	TTCCCACTTTACCGTGGCTGCT	AATGGCACAACGGCATCAGGGT	303
FMgSSR-19640	scaffold3131	p2	(CT)12	Class I	17393	17416	TTCCCTACCTGCAGCACCAACA	AACGCACCACCTGATGCTGA	258
FMgSSR-19645	scaffold14604	p2	(CT)12	Class I	9035	9058	TTCTCACTGAGCAGACAGGCTCAC	AATGGGACTCTCATGCGGTGCT	343
FMgSSR-19649	scaffold11981	p2	(CT)12	Class I	22117	22140	TTGAACGAGGTCGCCATGGACT	AGAGAGGAACGTCCACGTGAT	343
FMgSSR-19651	scaffold8264	p2	(CT)12	Class I	27180	27203	TTGGCGGGCATGAGTTGTGT	ACGGCGCTCACCATTTGCTT	326
FMgSSR-19652	scaffold5434	p2	(CT)12	Class I	30805	30828	TTGGCGTTGCTTTCCCCTCACA	ACGAGCAAGCTTGGCAATGGA	318
FMgSSR-19656	scaffold42	p2	(CT)13	Class I	56148	56173	AAAGCAAGTGTTGGTGTGCGGG	ACTCCTTTGACCTGAAACGGCGA	325
FMgSSR-19658	scaffold6539	p2	(CT)13	Class I	18359	18384	AACAAGCGCACTTTTGGCCG	AAGCCGAGAGGATGGACGCTTT	219
FMgSSR-19659	scaffold7557	p2	(CT)13	Class I	28476	28501	AACCACACGGGAAACAGCCA	ATGTCAACACGGCATGCAGCTC	247
FMgSSR-19660	scaffold321753	p2	(CT)13	Class I	257	282	AACGCTTTCAAAACCCGTCGCC	TGTCGTGTTGGAGTTGGGGA	252
FMgSSR-19665	scaffold10367	p2	(CT)13	Class I	18669	18694	AATGGAAGCGCAGACGAGGCTT	GCTTTGCTTGGGGAAACGACCA	278
FMgSSR-19667	scaffold252	p2	(CT)13	Class I	134727	134752	AATTTTGGGCTTGGGTCGGGCT	AATTCGGTCACAAGGGCCTGGA	327
FMgSSR-19668	scaffold3766	p2	(CT)13	Class I	29788	29813	ACACACACCACGCTGGAGTCAT	TATTCGACGGGTGACCTGTGCT	282
FMgSSR-19669	scaffold15086	p2	(CT)13	Class I	6922	6947	ACACACAGAGGAGATCCACGCA	TCGGTTACAGCCAGGATCGCAA	207
FMgSSR-19671	scaffold29502	p2	(CT)13	Class I	4212	4237	ACACGCTGCTGCTGCTTTCA	AAACAGGTGCCGGTCGTTCT	270
FMgSSR-19673	scaffold1015	p2	(CT)13	Class I	8664	8689	ACAGCTGTGCAAACCCCACT	TGCCTTGGTGTACAGGTCAGAGA	339
FMgSSR-19675	scaffold91006	p2	(CT)13	Class I	683	708	ACATTCGGATCCACCACTCCCT	TTGTTCCGGCTCCGAGCATA	318
FMgSSR-19678	scaffold4375	p2	(CT)13	Class I	18168	18193	ACGAGCGACAGCTTTTCCGT	GTGCACGTTTCCATATGGGGCA	254
FMgSSR-19681	scaffold43	p2	(CT)13	Class I	193839	193864	ACGCGCCACATTACCGTTGT	TCGTGACATGTGCTGTTGCCT	310
FMgSSR-19682	scaffold823	p2	(CT)13	Class I	59085	59110	ACGGAAATTCCTCGATGCCGT	AGTGCCGCTGGCGTTGTAGTAA	315
FMgSSR-19687	scaffold719	p2	(CT)13	Class I	67667	67692	ACTTCGTCGGTACGTACGCTCT	TCACATGGTTCCACTCCGGACA	325
FMgSSR-19688	scaffold20090	p2	(CT)13	Class I	10626	10651	ACTTGGTGCTTGAGAAGAGCTGG	TGGTGTTCGTTTCCCCTCTCCA	318

SSR_ID	Scaffold	SSR_Type	SSR_Motif	SSR Class	SSR_Start	SSR_End	Forward sequence	Reverse sequence	Product_size
FMgSSR-19691	scaffold138	p2	(CT)13	Class I	64532	64557	AGAGCGTCGTTGCGTTGTGT	TGGATGGCCAATGACGTCACCA	203
FMgSSR-19693	scaffold21234	p2	(CT)13	Class I	734	759	AGAGGTGTCTGGTGTTCGTGA	TGCATGCATCAGTTTCCGCTGC	276
FMgSSR-19701	scaffold660	p2	(CT)13	Class I	3187	3212	AGCGACATGCGTGAAAAGGCT	TTGAATGGGGGCCATCCCGAAA	347
FMgSSR-19709	scaffold10603	p2	(CT)13	Class I	5964	5989	AGGGAGCGAACAATAGTGGGTCCT	ATCTGAAGCCCATGGAAGGGGA	225
FMgSSR-19712	scaffold17847	p2	(CT)13	Class I	11274	11299	AGGTTTCGCTTGAGACCCCA	ACACGCTGGTAGCACCAGAAGA	337
FMgSSR-19713	scaffold485	p2	(CT)13	Class I	25515	25540	AGTGCGCAGACAAGGTCGAT	ATTCAAGTGGTCCACCCCGCAA	265
FMgSSR-19715	scaffold25905	p2	(CT)13	Class I	8825	8850	ATCCTCTTCACCCGATGGCACA	TAGGCTTCGGGTTCGGATCTAGCA	311
FMgSSR-19718	scaffold848	p2	(CT)13	Class I	83533	83558	ATGGCACTTGCGTCATGCCT	TGCAGAAGTGGCACCGGGTTTT	294
FMgSSR-19720	scaffold1811	p2	(CT)13	Class I	9367	9392	ATGGGCGTATTTGGTGCGGT	ATGTTCGGCTTGCGTTGCGT	231
FMgSSR-19721	scaffold311	p2	(CT)13	Class I	98678	98703	CACGCTTGCGCGTGTTGAAA	TTGAGCGAACGCACGGTACT	201
FMgSSR-19725	scaffold1466	p2	(CT)13	Class I	24036	24061	CCTCTCTCTCTCTCTCTCTCTCA	ATGGCTGGTTGCGTGTAGCTGT	244
FMgSSR-19727	scaffold463	p2	(CT)13	Class I	105879	105904	CTGTGCTGTTCGCGGTTGAA	TTGCCCTGGGCTTGATGTGGAT	343
FMgSSR-19729	scaffold11013	p2	(CT)13	Class I	11310	11335	GCAAGGAGCAAAACCTCCTCCA	AGTGCTCGACGGCTATGACA	343
FMgSSR-19731	scaffold16826	p2	(CT)13	Class I	338	363	GCGCTGCAGCTTAAGTGGTGTT	AAACCGGGGCCCACATGACATT	330
FMgSSR-19737	scaffold7956	p2	(CT)13	Class I	24034	24059	GTCAACGGGTTCAAGACGAGCA	ACTGCAACTCGCAGCTCTCA	327
FMgSSR-19743	scaffold706	p2	(CT)13	Class I	14871	14896	TCAATCATGCGCGTTCGCCT	CCGCCGCTTTTATCCAGTCTCA	237
FMgSSR-19746	scaffold16421	p2	(CT)13	Class I	12328	12353	TCATGGGTCGCACCGACTGTTA	ACTCGTGGAGCTTGGCAATGGA	217
FMgSSR-19750	scaffold27770	p2	(CT)13	Class I	4113	4138	TCCGTAGACCTCACATGGCAGT	TCGTCGAACAGCAGCAGCAT	281
FMgSSR-19752	scaffold5152	p2	(CT)13	Class I	34493	34518	TCCTTCACATGCATCACGCAGC	TTGCCCGGTCCTTTGCTTGGAT	254
FMgSSR-19755	scaffold10819	p2	(CT)13	Class I	12885	12910	TCGCAGCTTCTTTCACGGGACT	AATTCAGCCCAGCAAGCCCA	299
FMgSSR-19756	scaffold21	p2	(CT)13	Class I	51432	51457	TCGCCCACATTGAACCTGCT	ACAGAGCCGAACCGATGAGT	239
FMgSSR-19757	scaffold17866	p2	(CT)13	Class I	5002	5027	TCGCTACCTTGACGAGCAGAGA	ACCGTCTGGTTGCTTAGTGTGCT	320
FMgSSR-19758	scaffold7052	p2	(CT)13	Class I	15175	15200	TCGCTAGCTGCCTTGTGTCCTT	ACATTCAGGCACGGCGAGGAAT	319
FMgSSR-19762	scaffold19519	p2	(CT)13	Class I	3286	3311	TCTCTCACCCTCCTTCGGATCT	GCCACCGCTGTCGAGCTTTATT	322
FMgSSR-19768	scaffold4699	p2	(CT)13	Class I	6527	6552	TGATGTGTGCGCACGTTTCGT	GCATCCTGTGTGCGCCGTTTTT	317
FMgSSR-19771	scaffold679	p2	(CT)13	Class I	103720	103745	TGCAACGTGTTCCCTTCGCT	TTTTATTTGGCGGCAGGCAGGC	308
FMgSSR-19773	scaffold65	p2	(CT)13	Class I	63767	63792	TGCAATTAGCTGCAGCCGAGGT	CGTGGCGAGTGCAAACACACAA	291
FMgSSR-19777	scaffold3551	p2	(CT)13	Class I	50709	50734	TGCAGCGATGCATACTCTTCAGCA	CCCACGACTGGCCCATCAAAAA	320

SSR_ID	Scaffold	SSR_Type	SSR_Motif	SSR Class	SSR_Start	SSR_End	Forward sequence	Reverse sequence	Product_size
FMgSSR-19781	scaffold4605	p2	(CT)13	Class I	38747	38772	TGCCACAAAACACCACACGCT	TGGTAGGTGTGCATCGGCATCA	311
FMgSSR-19782	scaffold7590	p2	(CT)13	Class I	3351	3376	TGCCCCTCCAAATGCTCTACA	TAATTTGGGCGCAGGAGGTGGT	317
FMgSSR-19787	scaffold14631	p2	(CT)13	Class I	13075	13100	TGCGACGCTCTGTTGTGATGT	ACGCCTAGTCATGCGCCAATCA	308
FMgSSR-19793	scaffold12021	p2	(CT)13	Class I	11987	12012	TGCTGTCAGCGACACAACTGGA	TCCCTTCATCTAGAGAGGCGGCAA	265
FMgSSR-19794	scaffold3269	p2	(CT)13	Class I	31938	31963	TGGAAAACAGGTGCGCGTGA	AAGCAGGTGCGAGTACCATCCA	216
FMgSSR-19795	scaffold37040	p2	(CT)13	Class I	3760	3785	TGGAGCGAGGGCTCACAAAA	AACCCATGCAATGAGCTGGGCA	259
FMgSSR-19799	scaffold7952	p2	(CT)13	Class I	19544	19569	TGGCTTGGTTTTGGCTGTTGTCG	TGGTGTAAACACCCACCATCTGC	329
FMgSSR-19807	scaffold783	p2	(CT)13	Class I	68170	68195	TGTGCACACGGCTGAGTCAT	TGTCGTCGTTGCTGGAATGGGT	298
FMgSSR-19808	scaffold746	p2	(CT)13	Class I	100165	100190	TGTGTGACCATCGAGCTCTTGACA	AATTCAGGGCTTGCAGACCACG	205
FMgSSR-19815	scaffold9433	p2	(CT)13	Class I	27512	27537	TTCTGTGCTTGGGTTGGCGT	TGAATCCTGGCTGGCATCGTCT	205
FMgSSR-19820	scaffold1357	p2	(CT)13	Class I	28728	28753	TTGGGGAAGGTGTATTTGGGCCAG	TCATGGCACTCAAGGATCGTCA	311
FMgSSR-19822	scaffold40386	p2	(CT)13	Class I	5981	6006	TTGTGCTCCAACGTGTGGACTG	AATGCAAGTGCAACGCCGTC	253
FMgSSR-19823	scaffold1171	p2	(CT)13	Class I	31146	31171	TTGTTGCACCCCCTCTTGCCAT	AAGTTGGGTCGATGACCAGCCA	299
FMgSSR-19824	scaffold17847	p2	(CT)13	Class I	12067	12092	TTTCGACAACAGCGGAGCTCGT	GGGCTACAGAAGATCAACAGCCCT	350
FMgSSR-19825	scaffold2094	p2	(CT)13	Class I	64006	64031	TTTGCCGAAACGTGGCCCAGAA	AGCTGGTTACGCCAACGTGGTT	295
FMgSSR-19829	scaffold248114	p2	(CT)14	Class I	208	235	AAAAGCCCATCCGCATCCCACA	AATCCGACCGCTCGCTCATA	287
FMgSSR-19832	scaffold2027	p2	(CT)14	Class I	40441	40468	AAAGAGGACTAGGCACTTGGGC	TGGGAATGGGATGGTGGAACCT	227
FMgSSR-19833	scaffold9214	p2	(CT)14	Class I	20755	20782	AAAGCAAAACACGCCCCGGT	GCGGTGGTGCATTGGCGAATAA	305
FMgSSR-19836	scaffold10364	p2	(CT)14	Class I	14352	14379	AACTGTGGGCCGAGAAAGGT	ACGTGAGCAGAACCAGTGTGGA	314
FMgSSR-19843	scaffold12314	p2	(CT)14	Class I	9480	9507	AAGGCTGTGCGTGCATGGAA	AGCACCAGAACGGAAGGAAGGA	346
FMgSSR-19844	scaffold2829	p2	(CT)14	Class I	22619	22646	AATCGCTCGCTGCTCACCTACA	TGGCACGCGAAAGAACAGGA	295
FMgSSR-19849	scaffold7924	p2	(CT)14	Class I	3796	3823	ACCCGTTAATGCATGGCGGT	CGGCAGTCATAGACCCACAAGT	299
FMgSSR-19854	scaffold4497	p2	(CT)14	Class I	25376	25403	ACCTGCATTGGGGAGCACATGA	TCAATCGCCCGTGTCACCCTAT	338
FMgSSR-19859	scaffold241	p2	(CT)14	Class I	18743	18770	ACGCCAAGTTAGTGCGTTGTGC	TTTCGTAGCTGGTCGGCAGTCA	243
FMgSSR-19862	scaffold3322	p2	(CT)14	Class I	3257	3284	ACGCGCTTTTCCCTTTTCTCGC	ATGGCGACAACGATGCCGAT	272
FMgSSR-19863	scaffold4666	p2	(CT)14	Class I	37350	37377	ACGCTATTCTACGCCACGCT	TGCTTGTTGACACGGTCTGCCA	335
FMgSSR-19866	scaffold13	p2	(CT)14	Class I	225978	226005	ACGGAAATGCACCGAAAGAGGT	TGGACGCATCTTGCACGGAA	303
FMgSSR-19867	scaffold12420	p2	(CT)14	Class I	15163	15190	ACGTCCAAGGGGTGAGCAGTTT	TCCCATGCACTCGAATCGTCCA	294

SSR_ID	Scaffold	SSR_Type	SSR_Motif	SSR Class	SSR_Start	SSR_End	Forward sequence	Reverse sequence	Product_size
FMgSSR-19869	scaffold669	p2	(CT)14	Class I	55190	55217	ACTCGTAGGATGCAGAGCAGT	TCGAGCTGGTGTGTTCTTCATGTG	262
FMgSSR-19871	scaffold58605	p2	(CT)14	Class I	1649	1676	ACTGCTTAGTGCCGCTCTCCAA	ATCCGCGGTTTGACGGTTAGCA	255
FMgSSR-19872	scaffold9771	p2	(CT)14	Class I	25493	25520	ACTTTGCCCCCTCCTTTGACCA	ACCCTAGTCTTTCCGGCATCCT	268
FMgSSR-19873	scaffold425	p2	(CT)14	Class I	76251	76278	AGAGGCACAAATTGAGGTGGGT	TTGCTGCCACCCTCAACCTA	221
FMgSSR-19875	scaffold6937	p2	(CT)14	Class I	29632	29659	AGCCCATCACCCGCTCATTT	ATTGATCGAGCTGCCCGAGGTT	259
FMgSSR-19876	scaffold2751	p2	(CT)14	Class I	21717	21744	AGCCGTTCCCAGCTCTCTCATT	AACTCCACGGCAGGTACGACAT	330
FMgSSR-19879	scaffold1304	p2	(CT)14	Class I	38158	38185	AGCGCGGCAGATTCCACATT	AGGGTTACGCACACGCCAAGAT	348
FMgSSR-19888	scaffold4844	p2	(CT)14	Class I	6657	6684	AGTGATGGCAACGCTTGGTCCT	ACAATGCACGCACGTTCCAACC	345
FMgSSR-19894	scaffold224	p2	(CT)14	Class I	6161	6188	ATGACCACCCATCCATGGTTCTGC	ACTACGTGCCAATGGGTCAGCA	324
FMgSSR-19895	scaffold7065	p2	(CT)14	Class I	1456	1483	ATGACGGCGCACAAGTCTCCTT	AAAGCCTTGCTGTTTGACGGGC	312
FMgSSR-19902	scaffold10643	p2	(CT)14	Class I	22577	22604	CAATGCCGGAGGAGAAGGAGTA	GCTAGCGCACGCATTGCAGATT	309
FMgSSR-19904	scaffold203027	p2	(CT)14	Class I	38	65	CCCCCAACCCCAAAAACAGAAACT	TGGGTGTCACCGGCATAACCAA	315
FMgSSR-19905	scaffold10475	p2	(CT)14	Class I	5895	5922	CCTGGTAGCATTCTTCAGTTGGGT	TCGCATGCAACGAACTTGCCA	340
FMgSSR-19908	scaffold327	p2	(CT)14	Class I	5562	5589	CGGCGCATGCTATGAGGAAACA	AGTCAGGAGCAACGGACTCACA	326
FMgSSR-19915	scaffold894	p2	(CT)14	Class I	74793	74820	GCTCTCTGGTCTGCTCTGGTTT	ATCGGCTTTGGGCTTTGGCT	339
FMgSSR-19918	scaffold2306	p2	(CT)14	Class I	37938	37965	GGGCATGCGCTCTTTTCCGTAT	TTCCTCGACGACAACACGAGCA	245
FMgSSR-19920	scaffold1001	p2	(CT)14	Class I	33765	33792	GGTGTGACCCTGTGCTCTCTTTGT	TTGCGGTGGCATCTGGACAAGT	341
FMgSSR-19926	scaffold3346	p2	(CT)14	Class I	41752	41779	TCATGCTGTGTCCCCTTTCCCA	AACCTGCAAGCGCCGTAGAA	339
FMgSSR-19928	scaffold2703	p2	(CT)14	Class I	19963	19990	TCCACACACACTAGCCACCACA	TTTGCCGGCACTTCTGCTCT	208
FMgSSR-19932	scaffold834	p2	(CT)14	Class I	21655	21682	TCCTTGGCGCGTCTGTTTGT	AGCCTCCATGGCACGAACCAAA	226
FMgSSR-19934	scaffold142359	p2	(CT)14	Class I	463	490	TCGATCTGTCAGGCGAACCCAA	TCAGCGGCTTCTTTCCCTCTGA	275
FMgSSR-19936	scaffold2987	p2	(CT)14	Class I	45381	45408	TCGCGCGTGCATGACTTTGT	CGATCATGATTTGTGGCGGGCT	324
FMgSSR-19941	scaffold51575	p2	(CT)14	Class I	2830	2857	TCTCGTTTCGCCGTTGCATGA	TGCCACAGCCTGCACTCAAA	232
FMgSSR-19943	scaffold5282	p2	(CT)14	Class I	34682	34709	TGAGCACTGGTGCCTCGACATT	AAGTCATCTGCACGGCTGCT	346
FMgSSR-19944	scaffold1526	p2	(CT)14	Class I	75937	75964	TGATGAGGAACGGGCGGAATGT	ATTCAGCCGCAAAACGCTCGAC	226
FMgSSR-19948	scaffold2638	p2	(CT)14	Class I	39600	39627	TGCCCCGCCAACTTACTGTTGA	AGCTTTGGCCACCAGCAGCTTA	267
FMgSSR-19951	scaffold7956	p2	(CT)14	Class I	26067	26094	TGCTCAGATGTTACTGGGCGGA	ATTGGCACGACCGGATGCAGAA	265
FMgSSR-19952	scaffold20	p2	(CT)14	Class I	150242	150269	TGCTGCTGCTTTTTGCGGCT	AATTCTCGCGTGCGACGAAACC	344

SSR_ID	Scaffold	SSR_Type	SSR_Motif	SSR Class	SSR_Start	SSR_End	Forward sequence	Reverse sequence	Product_size
FMgSSR-19956	scaffold10077	p2	(CT)14	Class I	23419	23446	TGGACTGGGCGCAGCTTTTT	AGCACGCACGGTCCCTTTATGT	289
FMgSSR-19966	scaffold4613	p2	(CT)14	Class I	15976	16003	TGTGGCTACCATTGGTCCGCAT	AAGTGTTTGCTGCGTTCCGC	215
FMgSSR-19967	scaffold3321	p2	(CT)14	Class I	9078	9105	TGTGTTTGCACGTGGGCTGT	AATGCTCGTTCTCATGGGGCGT	225
FMgSSR-19969	scaffold1230	p2	(CT)14	Class I	68672	68699	TTCACCGCGTTATTGGCCTGCT	TGGGAAATGCGAAACGTGCTGC	299
FMgSSR-19977	scaffold3799	p2	(CT)14	Class I	16532	16559	TTTACTGGTCTCTGCGCTGGCT	AACTTTGGCCGGATGCGTCT	281
FMgSSR-19981	scaffold4038	p2	(CT)15	Class I	6512	6541	AAAGCACGCTTCGGACGACA	TGGCATGTGGCTTTCGTGGGAT	249
FMgSSR-19983	scaffold17957	p2	(CT)15	Class I	11041	11070	AAATGCACAGACAGCAGCACGG	TGTCCGTGCACCACATTTCGGA	258
FMgSSR-19984	scaffold1496	p2	(CT)15	Class I	56317	56346	AACAGACAGCAAGCCGGACCAT	ACCACGTCAAAGTGCTGCAA	299
FMgSSR-19985	scaffold6886	p2	(CT)15	Class I	33195	33224	AACGGCCGGATGCTACACAA	ATGCATACGCCCGATTGCCA	217
FMgSSR-19988	scaffold14401	p2	(CT)15	Class I	4312	4341	AAGCAGCACCCTGTGTCCAAGT	TCCTGTGGCTGCTTCGAGTTGA	266
FMgSSR-19995	scaffold22047	p2	(CT)15	Class I	8305	8334	AATAGGCCACCGCTACCTCCAA	AGGCCGAAATGCCAACACGGTA	320
FMgSSR-20002	scaffold585	p2	(CT)15	Class I	33336	33365	ACCAGCATTCACGGCACGAA	AGTGTTTGGCGCGTGCACTT	318
FMgSSR-20007	scaffold65	p2	(CT)15	Class I	205345	205374	ACGAAACGAAACGTGGGAGGT	ATCATGCAACCCCCTGCTGTGA	293
FMgSSR-20008	scaffold2212	p2	(CT)15	Class I	25648	25677	ACGCCACATGCCTCGCAGATTA	TTCCCCACACGCAAGCCAAA	320
FMgSSR-20009	scaffold673	p2	(CT)15	Class I	66657	66686	ACGCGCGGAGCTGTTCTCTATT	TTTTAGCCTCCTAGGCCCAGTCCA	242
FMgSSR-20010	scaffold3745	p2	(CT)15	Class I	21790	21819	ACGGATGGAAGCTTTTCCGTGAC	CCACTAGCACCTGACAAAATCAGCG	229
FMgSSR-20011	scaffold13074	p2	(CT)15	Class I	4837	4866	ACGGATGTGACGCCACCTTT	CAGTCGCAAGCATGCACATCGT	225
FMgSSR-20016	scaffold568	p2	(CT)15	Class I	95280	95309	AGCAAGGCCGGCCAAGAGAAAA	TCAGGGGTGGGTGGCAAAAA	228
FMgSSR-20018	scaffold9707	p2	(CT)15	Class I	16944	16973	AGCAGAGGTTGTGGTGACGCTT	GCCCAGGACACGGCCAATTTTT	323
FMgSSR-20019	scaffold3269	p2	(CT)15	Class I	23290	23319	AGCAGCACAAGGCCAAAGCA	TAGCACCCGCCGTGAAAATGGT	242
FMgSSR-20020	scaffold1475	p2	(CT)15	Class I	81898	81927	AGCATAGCACGCCTGCGAAA	AAAGGTGGCTCTCTGCCGGTTA	265
FMgSSR-20024	scaffold3007	p2	(CT)15	Class I	3343	3372	AGCGGCCACTGTTTCCACAA	TGCTCCGGCCTATCTAAGGCTCAT	202
FMgSSR-20025	scaffold9218	p2	(CT)15	Class I	19560	19589	AGGAAGCGCCTTGCAGATCACA	TCGACCATTCCCCATCAGTGGA	265
FMgSSR-20028	scaffold1270	p2	(CT)15	Class I	86596	86625	AGTTTGTGATGGCGGCAGGA	AACCCGTCCACCAATTCGCCAT	261
FMgSSR-20034	scaffold17	p2	(CT)15	Class I	242635	242664	ATCCGCATCAGTGGCTCGTGTT	AACCACTTGCAGTGGCAGGA	337
FMgSSR-20036	scaffold10399	p2	(CT)15	Class I	13399	13428	ATCTGCGCGACATTGGACTGGT	TGTGTCCTGTGCGTCCGCTAAT	330
FMgSSR-20040	scaffold3537	p2	(CT)15	Class I	17420	17449	ATGGCTGGGAAACGACGGAAGA	TGCCGTGAAGGAGCGAAAGT	264
FMgSSR-20042	scaffold42536	p2	(CT)15	Class I	1405	1434	ATGTCTGTGCAACGCTCCCA	TGGCGTCAATGGCGTCATCTCT	277

SSR_ID	Scaffold	SSR_Type	SSR_Motif	SSR Class	SSR_Start	SSR_End	Forward sequence	Reverse sequence	Product_size
FMgSSR-20045	scaffold8	p2	(CT)15	Class I	143091	143120	ATTTGGGTCCCTCTTCTCCGCT	AGTTGCGTCCAGCCTGCAAA	322
FMgSSR-20048	scaffold38015	p2	(CT)15	Class I	664	693	CGCACAAGCCGTAAAGGGCAAA	ATGATTCTCAGCACCACCGCCT	350
FMgSSR-20051	scaffold10406	p2	(CT)15	Class I	10048	10077	GCAGTACGTGCTTGTTTGCCGT	ACTGTGAATGGACGAGCAGCAGT	350
FMgSSR-20054	scaffold2479	p2	(CT)15	Class I	2108	2137	GCCGGCAATAGCGGTAATCTCT	TGCTTGCCCACTGCATCGAA	319
FMgSSR-20057	scaffold9155	p2	(CT)15	Class I	20561	20590	GGCGCCATAAGCCCGTTTTT	AACTGCTGCTGTCGCTAGGTGA	296
FMgSSR-20058	scaffold9116	p2	(CT)15	Class I	13554	13583	GGGGGCATACTTCCATTCCATTGC	TTGTGGTGCGTAGGTGCTCA	301
FMgSSR-20060	scaffold1387	p2	(CT)15	Class I	66306	66335	TAGCCAAAAGCACCGGGTCCAA	CGCATACGTGCGTTGAGCCTTT	301
FMgSSR-20061	scaffold1347	p2	(CT)15	Class I	8596	8625	TCAAGAGCCAGCCCCTTTCCTT	TTCGCCAGCCGGAAACGAAATC	213
FMgSSR-20063	scaffold1612	p2	(CT)15	Class I	2492	2521	TCAGCCACACGGAATTACCGAA	TGAGAGATTGAGAGGAGCAGCGA	328
FMgSSR-20064	scaffold1480	p2	(CT)15	Class I	50864	50893	TCCAAGCCGACGCGACCTAAAA	GCGAGCTGCTGCTGTTGTTGTT	275
FMgSSR-20068	scaffold5037	p2	(CT)15	Class I	14913	14942	TCGCCGCTTGCCTTTTTCGT	TCAAGGTGCCCACCAACCTGAT	264
FMgSSR-20070	scaffold3398	p2	(CT)15	Class I	35343	35372	TCTCTCATGCCATAGCGATGTACG	CTGCGTGCATGCCTAGATGT	291
FMgSSR-20077	scaffold1499	p2	(CT)15	Class I	50381	50410	TGAGATGTGAGATGCCGCGA	TTTGATCGATCGGCGGCCTTGT	289
FMgSSR-20079	scaffold326	p2	(CT)15	Class I	15417	15446	TGCAGGTGCAGCTTGTCTACCA	TGCTTCTCCAACGTGGTCCT	280
FMgSSR-20082	scaffold83788	p2	(CT)15	Class I	622	651	TGCCCATGCCTCCTTGTCATCA	GGCGGAAGAGGAAGAAAGCCAA	229
FMgSSR-20087	scaffold25912	p2	(CT)15	Class I	8595	8624	TGCTCTCAAACACACGCACAGG	TAACCCCTTGCCAGCTGCAGAA	347
FMgSSR-20088	scaffold1380	p2	(CT)15	Class I	79679	79708	TGCTGGGCACACAAAAACGCT	ATTACCTCCGCTGTCAGCCA	223
FMgSSR-20092	scaffold18221	p2	(CT)15	Class I	8598	8627	TGGCATTGCGATGGGCCTTT	TCGAAGCAGTGGACGGAGAAGA	202
FMgSSR-20094	scaffold2503	p2	(CT)15	Class I	61175	61204	TGGTTCAGGGCCAACGAAGTCA	AAGTCGTGCCTCGCCATGAT	207
FMgSSR-20099	scaffold293	p2	(CT)15	Class I	7513	7542	TTAAAGCGGGCGGCATCTCA	TTTCGCCTGCTCCCAAACCT	310
FMgSSR-20103	scaffold48041	p2	(CT)15	Class I	4193	4222	TTCTTGTTGCGCTTGCGGGA	ACGGTCACAACGCAAGGCAT	227
FMgSSR-20113	scaffold21609	p2	(CT)15	Class I	1946	1975	TTTTTCTCCACCCCACCCCACA	TCACCAGGGCCTTCGCATTGAT	336
FMgSSR-20118	scaffold622	p2	(CT)16	Class I	80899	80930	AACCGTGTTGGTTGTGCGCT	TTGCCGGTGCCCATCTGAACTT	273
FMgSSR-20120	scaffold458	p2	(CT)16	Class I	74223	74254	AAGCCAGTGCAAGTGCTGCT	AACTCGGCAACTCCATCGCA	212
FMgSSR-20125	scaffold1323	p2	(CT)16	Class I	62578	62609	AAGTCAGTGCCCACCACATCCA	TCCCAGAGCCATCAACGCACAT	292
FMgSSR-20126	scaffold72	p2	(CT)16	Class I	65470	65501	AAGTCTGTAACGCCCCTGCT	TGCTGCTTATGAAGCTGCGGT	208
FMgSSR-20127	scaffold969	p2	(CT)16	Class I	89511	89542	AATATGCGCAGCCTGCAAGACG	ACACGGCCGGTTGACAACAA	290
FMgSSR-20133	scaffold6199	p2	(CT)16	Class I	37098	37129	ACCCGTAAAGCCACTTCCGT	TCCAGTTGTTGTCGATGCACACG	348

SSR_ID	Scaffold	SSR_Type	SSR_Motif	SSR Class	SSR_Start	SSR_End	Forward sequence	Reverse sequence	Product_size
FMgSSR-20134	scaffold1675	p2	(CT)16	Class I	5339	5370	ACCTCACGCCAAGAACAGACA	ACAGAGAAGTCATGGCCGCA	208
FMgSSR-20135	scaffold22031	p2	(CT)16	Class I	13383	13414	ACCTTCTTTGCTAATCGGCGGC	TGAACACACACCGACTTGCGAC	345
FMgSSR-20136	scaffold883	p2	(CT)16	Class I	72007	72038	ACGACTCAGGTGTTCTGACTCC	AGCACGAACAAAGTCTCCAACAGC	274
FMgSSR-20143	scaffold150	p2	(CT)16	Class I	176953	176984	ACTTCCACTTCTCCCGAACCCT	TCTCGAACCATCGTTGCCGT	225
FMgSSR-20145	scaffold8679	p2	(CT)16	Class I	12105	12136	AGACGGATTGGCCGCCTTTA	AGCTGCTGTTGTCCCGTTGGTT	287
FMgSSR-20146	scaffold6497	p2	(CT)16	Class I	37864	37895	AGATTGCATCGCTTTGGGACGA	TGCAGCATTGCAAAGCCCTGA	301
FMgSSR-20155	scaffold1735	p2	(CT)16	Class I	25684	25715	AGCTCAGCATGCCGAAGAAACCT	TGGCAGGCAGTTGTTGCCTT	205
FMgSSR-20156	scaffold1924	p2	(CT)16	Class I	62155	62186	AGCTCTTCCCTGCAACCCTGTT	TCCGGTGACCAGCTGAGAACAA	214
FMgSSR-20165	scaffold2069	p2	(CT)16	Class I	66281	66312	ATGACCGCTACATCGTCAGCCT	ACGCCATCGGGATGATGCCATA	269
FMgSSR-20169	scaffold29503	p2	(CT)16	Class I	5641	5672	ATTCAATCAAGCTCGGCAGCGG	TGTGGATGCGAGCGAGATGT	277
FMgSSR-20171	scaffold1432	p2	(CT)16	Class I	11103	11134	CAGGTCTCTTTAGTGGCCCGTT	AGGAGTGAAAAGCTGCCTTGCG	231
FMgSSR-20174	scaffold74	p2	(CT)16	Class I	47769	47800	CGTTCCGATCCTGCTGATGGTACA	GTTTTACCGGCGGTCACCAA	350
FMgSSR-20175	scaffold20591	p2	(CT)16	Class I	9860	9891	CTTGCTTGCGCCGGTGTTTT	AGCACGAAACTTCCGCCTGT	346
FMgSSR-20176	scaffold2771	p2	(CT)16	Class I	54359	54390	GCACAACAGGGCGTTTGCTTCA	ACGGAGCATTTCACCGTTGC	311
FMgSSR-20177	scaffold56	p2	(CT)16	Class I	144335	144366	GCCGTGCATGTGGATTGTGTGT	TGGTCAGATCTAGTCGCGTCCT	327
FMgSSR-20187	scaffold538	p2	(CT)16	Class I	26275	26306	TCAGCAGCGATACTGCTCCA	TTTGCCTCGTTTCAGCGCCA	238
FMgSSR-20190	scaffold5583	p2	(CT)16	Class I	16834	16865	TCGAAATCGAGGAACGGGCA	TCGATCTTTCCCCGTGTGGACT	254
FMgSSR-20194	scaffold5226	p2	(CT)16	Class I	11935	11966	TCGGGCCACTGCTTTTTGTGGA	GCACACCGTGAGCTACCAACTGTA	331
FMgSSR-20195	scaffold2681	p2	(CT)16	Class I	10647	10678	TCTCTCTCCACACACCAGTCGT	ACCTGGCAATGGGGACGAAA	309
FMgSSR-20196	scaffold1655	p2	(CT)16	Class I	54348	54379	TCTGTCAGTGGTATGCGCCGAT	ACGAAGCATGAACTGCTGGTGC	338
FMgSSR-20197	scaffold824	p2	(CT)16	Class I	46156	46187	TGACAGTGTGAATGCCTCCGGT	ACAGAAACCCGAAGGAGCCCAA	259
FMgSSR-20198	scaffold9300	p2	(CT)16	Class I	31158	31189	TGACGATGTGCAACAACGACCC	TACGGCGCATGCTTCTTGGA	259
FMgSSR-20199	scaffold6914	p2	(CT)16	Class I	23029	23060	TGAGCGTGTACGTTGGCTCT	AGGCAACAACAACCTTGCCA	296
FMgSSR-20204	scaffold1232	p2	(CT)16	Class I	55099	55130	TGCCAACAAAACAGTGGGCACC	TCTCTCTCGTCGGTCGTCACTT	296
FMgSSR-20206	scaffold8139	p2	(CT)16	Class I	18189	18220	TGCCATGGCCGAAGCATTACCT	ACGAGTTGGACGGGTTTCACGA	313
FMgSSR-20210	scaffold10019	p2	(CT)16	Class I	527	558	TGCTTGTGAGGTTGTTGGGAAC	TGGTAGACTTCCCTTGCTAGAGGC	350
FMgSSR-20212	scaffold599	p2	(CT)16	Class I	27388	27419	TGGCACATTTTCGTACAGGCCC	ACCACCGCCGCTACAAATCCAA	225
FMgSSR-20214	scaffold443	p2	(CT)16	Class I	94578	94609	TGGTCGATTGGACACGTTGCCT	ACACAACTTGCTGGTGGCCAGA	284

SSR_ID	Scaffold	SSR_Type	SSR_Motif	SSR Class	SSR_Start	SSR_End	Forward sequence	Reverse sequence	Product_size
FMgSSR-20215	scaffold267137	p2	(CT)16	Class I	32	63	TGGTGCTGATTCAGTATGTCCTCC	ATAGTGAGCTGCGCGTCGTCAA	312
FMgSSR-20219	scaffold8354	p2	(CT)16	Class I	13757	13788	TGTTTTGGCGGGATTGTTGCCG	GCAAATTTGAGGCTGAGGCGCT	345
FMgSSR-20220	scaffold24	p2	(CT)16	Class I	201635	201666	TTACCGCCGCCAAGAACCTTGT	CGCAGTTCAGCGTGCTTTACCA	305
FMgSSR-20227	scaffold6961	p2	(CT)16	Class I	21479	21510	TTGCAAAGGAGGCAGCAGCA	TTAATTCTGCAGGGCGCACGGT	335
FMgSSR-20229	scaffold27594	p2	(CT)16	Class I	3354	3385	TTGGCGAAGAGCACAGCCAGAA	ACCGTGCGGAGTGCAAGAAGAA	268
FMgSSR-20233	scaffold4640	p2	(CT)17	Class I	22462	22495	AAACCATCGAAAACACCCGCCG	CCACTGCCAGGGAACCAGAAAA	309
FMgSSR-20238	scaffold14360	p2	(CT)17	Class I	12506	12539	AACCGTGTGGCGGCGACTATTA	ATCGTGTCATCCGCCAGTTGCT	328
FMgSSR-20242	scaffold747	p2	(CT)17	Class I	94443	94476	AAGCAACCCCTTCTTGGCGT	ACTGGCGGCGATCAGCAAAA	240
FMgSSR-20246	scaffold791	p2	(CT)17	Class I	73377	73410	AAGGCCCAGGAGCTTGCAGAAT	ATGTCCGGCTTCATCATGGCGT	267
FMgSSR-20248	scaffold309	p2	(CT)17	Class I	59040	59073	AATCTGTGGGCGTGGCGATT	ACTGACCCCAGTTTGCCTGACA	310
FMgSSR-20249	scaffold161	p2	(CT)17	Class I	155685	155718	AATTTGCAACGCCTCGCCTTCC	AAGCGTCACGAATCGAGCCGAA	273
FMgSSR-20252	scaffold1037	p2	(CT)17	Class I	5952	5985	ACAGGCGTACGCAGCAACAA	TGTGAAGTGACTCGTAGTGTGGC	347
FMgSSR-20254	scaffold11614	p2	(CT)17	Class I	3457	3490	ACCACCACGAGAGCATCGACAA	ACCATCCATCCCAATTCCCCGA	278
FMgSSR-20257	scaffold393	p2	(CT)17	Class I	22340	22373	ACCTCAGAGAAACACGGGAGCTT	TCTGACTTTGGCCGACGTGT	337
FMgSSR-20258	scaffold516	p2	(CT)17	Class I	91993	92026	ACCTGCCCGCCGTTTGAAAAA	ATGGTGGCGCATGCTCTTCTGT	288
FMgSSR-20259	scaffold7397	p2	(CT)17	Class I	28090	28123	ACCTGCCTATTCGGCTGTTTGCT	AAGTTCGTTCGCGCTTGCCT	205
FMgSSR-20261	scaffold7573	p2	(CT)17	Class I	16155	16188	ACGGATTGGCAGTAGTCTCTCCCT	TGCAGCTTCTGCCAACGCTA	289
FMgSSR-20265	scaffold4655	p2	(CT)17	Class I	1486	1519	AGCACAAAAGGCGCACAGGA	AGGGCTGGGAACTGTAAGCGTT	315
FMgSSR-20268	scaffold18536	p2	(CT)17	Class I	6942	6975	AGCCCCACCAGCCAAATTCACA	ACTCTCAAAGCCACCGTCCT	266
FMgSSR-20270	scaffold2936	p2	(CT)17	Class I	46857	46890	AGGCTTCCGTGAACAGCAGGTA	GCGGCAATGCAACTCTGCTCAA	339
FMgSSR-20272	scaffold9	p2	(CT)17	Class I	102179	102212	AGGGCTCCCTTTGGAGCATCTT	ACTGTGGACACGGCACATCACA	326
FMgSSR-20274	scaffold475	p2	(CT)17	Class I	93462	93495	AGGGTTTCTCCTCACCCACCAT	TGTTGTCGAGGCAGGCAAGTGT	312
FMgSSR-20281	scaffold221	p2	(CT)17	Class I	115213	115246	AGTTTTGCAGCTTGCTTGGGGG	TGGCAATGGCAGGGAACCAA	341
FMgSSR-20282	scaffold8742	p2	(CT)17	Class I	10791	10824	ATCAATGGGCAATGGCGGTCCT	ACGTACGAGAGGTGTGCGGAAT	320
FMgSSR-20288	scaffold4784	p2	(CT)17	Class I	44638	44671	ATGTGGTCGTCCGCAACTCACT	AAACTGGACGGACGGCAATCA	325
FMgSSR-20290	scaffold18537	p2	(CT)17	Class I	7792	7825	ATTGCGGGCAACTGGAAGGTGA	AGCCTGTTTGGTCCCAGCAT	335
FMgSSR-20293	scaffold3265	p2	(CT)17	Class I	43482	43515	CGATGGCACTGTTGAAAACCGCA	ACGTGCGAAGCTGCTGCATA	336
FMgSSR-20305	scaffold29409	p2	(CT)17	Class I	5097	5130	TCCACCCACCCCCTTGTCTTTT	TCCGTGTCGCCTTGCATAGA	273

SSR_ID	Scaffold	SSR_Type	SSR_Motif	SSR Class	SSR_Start	SSR_End	Forward sequence	Reverse sequence	Product_size
FMgSSR-20310	scaffold88167	p2	(CT)17	Class I	1009	1042	TCCTGTGTCGGCACCATGTCTT	TCACGGGGCGCATGAGAATCAA	344
FMgSSR-20311	scaffold1486	p2	(CT)17	Class I	17130	17163	TCGACCGACGTGTGTTCATCA	TCTTTTGCCCGGCAGTGGTT	271
FMgSSR-20313	scaffold12469	p2	(CT)17	Class I	16375	16408	TCGCTCCATTGCTGCTGCTAGT	ATTCCTCTGCCCCGTTGGCATT	335
FMgSSR-20315	scaffold39487	p2	(CT)17	Class I	4455	4488	TCGTTGCTGCCCATCAGCAT	ACGCTAGTGACAGGTGACGCAT	261
FMgSSR-20316	scaffold548	p2	(CT)17	Class I	69736	69769	TCTGCCGCGATAAGGACATCA	TGTCAAGGCAACCGTCGGTACA	332
FMgSSR-20317	scaffold465	p2	(CT)17	Class I	25520	25553	TCTTCGGCAGTCAACTTCACCA	ACAGTCGAGGATGATAAGCCGT	323
FMgSSR-20318	scaffold93060	p2	(CT)17	Class I	555	588	TGAAGCGCACAGACTGGTCTCA	ATCGCTTGTGGCCTTTCTGCCT	242
FMgSSR-20320	scaffold18	p2	(CT)17	Class I	6254	6287	TGACGCAACTCCCCATCTCA	TGCTGCGGCACCAAACAACA	348
FMgSSR-20325	scaffold508	p2	(CT)17	Class I	24315	24348	TGCTAGTCCATAGCCCAACCGA	AGGTCTGAAGCAGCACCGACAT	241
FMgSSR-20330	scaffold64	p2	(CT)17	Class I	48649	48682	TGTGTCCTTGCCGCCAACTCAT	TATTTTTGAGCCCAGCAGGCCC	232
FMgSSR-20332	scaffold5782	p2	(CT)17	Class I	13504	13537	TGTTGTGCACGGTACGGTCGAT	AACAGCGTACGTCGTTGCGT	335
FMgSSR-20340	scaffold318	p2	(CT)17	Class I	121694	121727	TTGCGCCGTGCAGATTGGTA	AGCACAAAAGCAGGGGTGGT	258
FMgSSR-20343	scaffold2094	p2	(CT)17	Class I	53104	53137	TTTAGCAGCGGCAGCAGGTCTT	TTTGCGATTCACCGACGCGA	303
FMgSSR-20347	scaffold2032	p2	(CT)18	Class I	6459	6494	AAAGCCCGCCGGAAGTTGTA	GCTCCTGAATCGGTTAGGGTTCGT	220
FMgSSR-20349	scaffold13687	p2	(CT)18	Class I	16832	16867	AACCAATCCGCACGCGCTAA	ATGTGTTGCTCCTCCCCAGATG	275
FMgSSR-20350	scaffold8783	p2	(CT)18	Class I	1460	1495	AACCATTGGCCGTTGGAGACCA	TGTGTTGGCGGTGCTAAGGT	232
FMgSSR-20355	scaffold1854	p2	(CT)18	Class I	63859	63894	AAGGCACCATCGATTGGCACGA	TCCTCCACACACACACGAGAGA	214
FMgSSR-20377	scaffold2332	p2	(CT)18	Class I	24894	24929	AGCGCTTTCATTCGCGCAGT	TGCTTGCCAGTGAGGTATGCGA	321
FMgSSR-20384	scaffold8199	p2	(CT)18	Class I	20129	20164	ATAATGCAGCACCCACGGCA	TCAGGATGGTTGGTGTAGTTCGGT	308
FMgSSR-20385	scaffold17308	p2	(CT)18	Class I	15722	15757	ATCAATGCGTGTGCAGTCGAGC	TGTCCAGCACCTGGCTCAGTTT	219
FMgSSR-20390	scaffold260	p2	(CT)18	Class I	123321	123356	ATGTCGTCCACCGCCTTGTCAT	TTTCACCACCCTCAGCAGTGGA	258
FMgSSR-20395	scaffold15764	p2	(CT)18	Class I	8080	8115	GCGCGTCAAGACAACGCTTCAA	TTCAAAGCGCGACCGGACAA	230
FMgSSR-20399	scaffold28463	p2	(CT)18	Class I	133	168	GGCTCACTAGAACTGCATAGGACT	TGTCTCTTGCTCTCCTCTGCCA	270
FMgSSR-20401	scaffold3472	p2	(CT)18	Class I	36435	36470	TAGCAGCATTTGCGGACACGGA	TTTGAACGAACGCATCGACCGC	203
FMgSSR-20402	scaffold4109	p2	(CT)18	Class I	5552	5587	TCAAAAGAGGCAAGGCGCAAGC	TGGTGGCTTTTCTCATGGCAGC	338
FMgSSR-20419	scaffold181	p2	(CT)18	Class I	106275	106310	TGCCGCTTTCACGTCGCAAT	GCGCGCTTGACTCGTTGGTTAT	259
FMgSSR-20420	scaffold2515	p2	(CT)18	Class I	38882	38917	TGCTTTCCACTTCCGCAGCA	TCACGGGAACATCCATGGCTTGA	215
FMgSSR-20423	scaffold1597	p2	(CT)18	Class I	66496	66531	TGGCTTCTGTCTGTGCAGTGGT	ACGAGCAGCATCAATAGCGCGT	337

SSR_ID	Scaffold	SSR_Type	SSR_Motif	SSR Class	SSR_Start	SSR_End	Forward sequence	Reverse sequence	Product_size
FMgSSR-20428	scaffold6651	p2	(CT)18	Class I	22499	22534	TGTGAGATGCGAAGGCGTGT	AACCACTGTCCGAGCTTCTCCT	339
FMgSSR-20429	scaffold4437	p2	(CT)18	Class I	28031	28066	TGTGATCCACTTGCAGCGACCA	TCTTGCACCGCACGGAGAAT	302
FMgSSR-20433	scaffold1173	p2	(CT)18	Class I	72092	72127	TTCACCCAGACTACTTCCCCACCA	AGTGGAGTGCGGTGTTGGAT	310
FMgSSR-20434	scaffold100965	p2	(CT)18	Class I	960	995	TTCATGTGGGCTCCACTGCT	ATGGCACTGACCTCACACGCTT	223
FMgSSR-20436	scaffold48760	p2	(CT)18	Class I	3962	3997	TTCTAGCGGTTGACCCTCGGTT	TGGACGACCGCAAACGAAGA	215
FMgSSR-20438	scaffold13135	p2	(CT)18	Class I	6128	6163	TTCTCGTTTCGGCCGCGTTT	TTCGTGCCGCATTTCTTGCC	307
FMgSSR-20448	scaffold4173	p2	(CT)19	Class I	48419	48456	AAAGTGCCAAAAGGACCAGCGG	AGGTGGCCTGTCACAATGCACA	291
FMgSSR-20462	scaffold13026	p2	(CT)19	Class I	6013	6050	ACAGCCACCCCAATTTCCCT	TGTCGTTGCCCGATGTTGGA	313
FMgSSR-20466	scaffold5445	p2	(CT)19	Class I	14190	14227	ACCCGGTAGAGGCAGTCCAAAA	AGCCTTCATCGACAAGTGGGGA	270
FMgSSR-20467	scaffold2306	p2	(CT)19	Class I	9406	9443	ACCTCGGCTGCAGATCAAGCAA	ACCACCGGCCGCAACTTAAA	218
FMgSSR-20468	scaffold10421	p2	(CT)19	Class I	17879	17916	ACCTCTCTAACATCCCACCAGACCT	AACAGATCCGTGGAGGATGGGGAA	295
FMgSSR-20472	scaffold14249	p2	(CT)19	Class I	18400	18437	ACGCGGCCATGTCTCTGAAA	TAGCTCGAGGAACGATTGGGCA	266
FMgSSR-20474	scaffold1572	p2	(CT)19	Class I	62950	62987	ACTGTCACCAGTGGGCAAAGTGT	TGCTCACGGAAAGCGATGAGACA	277
FMgSSR-20476	scaffold6924	p2	(CT)19	Class I	1592	1629	AGCACCCATCCAACCCCAAAGA	GCATGTTCGCAGAGGCGTTT	320
FMgSSR-20477	scaffold12880	p2	(CT)19	Class I	27011	27048	AGCAGCACACCAAGCTCACT	ACACCAACCTTCACCAGCAAGC	247
FMgSSR-20479	scaffold16	p2	(CT)19	Class I	19411	19448	AGCCACCTCAAGCAAGTGCTCA	AGCCATTGGTGGCTTGGACGAT	321
FMgSSR-20484	scaffold178	p2	(CT)19	Class I	120677	120714	ATGCATGGGCAATGGAGCGA	ATTTACGCGCACACTGGCCT	261
FMgSSR-20485	scaffold12123	p2	(CT)19	Class I	5689	5726	ATGGCGTTATGCCCGTTGCT	ACCAAACGCGAAGCCACACA	251
FMgSSR-20488	scaffold804	p2	(CT)19	Class I	96952	96989	ATTCACTGCCACTGCCAGGACAA	ACCATGTTTCACAGCGAACGCC	316
FMgSSR-20495	scaffold3987	p2	(CT)19	Class I	11147	11184	CGCTGTTTGAGATTCTTGGCAAGGG	TGGGGAAACACTCGTGTGTGCAT	290
FMgSSR-20499	scaffold1214	p2	(CT)19	Class I	6065	6102	GGCGTGTGCCAAATGCTGAGTT	CCGTCGTCCGTCGTTCACTATCAA	290
FMgSSR-20500	scaffold34594	p2	(CT)19	Class I	540	577	TAGCGCACGAGTTCGCATCT	TGGCAAGATGGGGGTCAAGTCT	285
FMgSSR-20504	scaffold97	p2	(CT)19	Class I	156298	156335	TCCAATCGCTCTCATCCCCACA	TGGGTTGTGACGGCCAAGTT	331
FMgSSR-20505	scaffold19048	p2	(CT)19	Class I	6088	6125	TCCAGTTACGCCACTGCTGCTT	TGCTCTATTTGCCCCTGGCT	226
FMgSSR-20506	scaffold505	p2	(CT)19	Class I	8211	8248	TCCATTGACGATTTGGCGTCCG	ACTCAAACGCGGCACTACGCTA	318
FMgSSR-20511	scaffold5256	p2	(CT)19	Class I	30586	30623	TCTAAAGCGGCGGTCAATGC	TTAGGCAACTCACCAGCGTCGT	349
FMgSSR-20514	scaffold2951	p2	(CT)19	Class I	29671	29708	TGACAGGTCACCCAACTAGCGA	TCCATGGCAGACTGCGCTTT	261
FMgSSR-20524	scaffold16056	p2	(CT)19	Class I	14372	14409	TGGGTATGCACTTTGTGTGGCA	ACGGCAACTAGTAGCACATGGACA	341

SSR_ID	Scaffold	SSR_Type	SSR_Motif	SSR Class	SSR_Start	SSR_End	Forward sequence	Reverse sequence	Product_size
FMgSSR-20530	scaffold464	p2	(CT)19	Class I	17505	17542	TTCTCCGCACGCTCCATGAACA	AATTCCTGGTTGGATACGGCGG	200
FMgSSR-20537	scaffold23724	p2	(CT)19	Class I	9419	9456	TTTGCGCGCCGAGTGTATGT	AGTTGGCGCAAACGTGCTGT	306
FMgSSR-20538	scaffold15618	p2	(CT)20	Class I	14391	14430	AAACATCTCCATTCCCTGCCGC	ACAACGCAGGTGAGGTTACGGA	250
FMgSSR-20540	scaffold457	p2	(CT)20	Class I	97907	97946	AACCGTGACTGCGCGAGAAA	TGAACTGCCGCGGATTTGACT	319
FMgSSR-20547	scaffold5016	p2	(CT)20	Class I	20483	20522	ACAGCTCCTGACACACGACA	TGCAACAAAGCACCTTGCAGGC	247
FMgSSR-20549	scaffold8523	p2	(CT)20	Class I	5117	5156	ACCCCGGAATACAAGTGGACCT	TTTTGCTGCTGCGTGCCGAT	211
FMgSSR-20552	scaffold425	p2	(CT)20	Class I	108090	108129	ACCGTGCGCGGGTTCATATCAT	TGCTTGACGCCATGCCTGTT	274
FMgSSR-20554	scaffold3982	p2	(CT)20	Class I	38763	38802	ACGAATGCTCAGGGACGCAA	ATGACTCAATTCCTGGCTCCGC	271
FMgSSR-20560	scaffold1551	p2	(CT)20	Class I	8808	8847	AGATGCGCGAAGATGGTTGCCT	ATCAGCTGGCCCTGAGCTTT	200
FMgSSR-20568	scaffold299029	p2	(CT)20	Class I	117	156	ATCGCTCTGTCGGAAGCATGGA	TGACAGGGCAATGCGTGCAA	270
FMgSSR-20569	scaffold389	p2	(CT)20	Class I	130150	130189	ATGGCATCCCACAAGTTGACGC	AAGCACGCAGGCAAGGGTAA	318
FMgSSR-20570	scaffold149	p2	(CT)20	Class I	132521	132560	ATTCTTCGGTTGCTCCACCCGT	AAGGCAAGGTGTGTTGCCCTCA	242
FMgSSR-20571	scaffold16442	p2	(CT)20	Class I	11615	11654	ATTTCCTAGATCCCCACTGGACGC	GCCAACGATCACAGGAGGCAAA	335
FMgSSR-20576	scaffold6346	p2	(CT)20	Class I	26325	26364	CGGTGGAACATGGGAACCACTT	TGGAGAGGATAGCGGATAGGCA	350
FMgSSR-20579	scaffold161	p2	(CT)20	Class I	117940	117979	GCGAAGCAAATGCTGCACAAGG	TCTCGTTTGGTGACACACGCA	329
FMgSSR-20582	scaffold38015	p2	(CT)20	Class I	2380	2419	GGCTGTTCGTTCCAGGGTCAAA	AGATGCAAGACTCCACAGGCT	347
FMgSSR-20583	scaffold69	p2	(CT)20	Class I	49614	49653	TATCTGCAGTATCGGCGCGCTT	TTTTCCCCTCATTTCGGCGAGC	304
FMgSSR-20589	scaffold387966	p2	(CT)20	Class I	213	252	TCGGTGTACCGGAAACCAGGAT	TTGTGCTCCCTTGCGTGACA	262
FMgSSR-20592	scaffold5004	p2	(CT)20	Class I	9077	9116	TGAAGCGACACTTGCTCGGA	AGTGTGAGGGCACATCAGGT	261
FMgSSR-20594	scaffold1545	p2	(CT)20	Class I	61353	61392	TGAGCTCTCTCTGTCACACACG	TTGCTTGACGTCATCGGAGGCA	224
FMgSSR-20595	scaffold593	p2	(CT)20	Class I	51587	51626	TGCACTGCAGCTCCCAAGATGA	ACAAAACCGAGCCTCAGTGCGA	318
FMgSSR-20596	scaffold1678	p2	(CT)20	Class I	51299	51338	TGCATGCACACACGGACAGT	TCTGCACACGAGGGACAAGGAT	293
FMgSSR-20603	scaffold21657	p2	(CT)20	Class I	5782	5821	TTCTTGAGCCTCACACGCCAGT	TGGGCCCATGATCTAGACACAACA	312
FMgSSR-20610	scaffold4527	p2	(CT)21	Class I	33299	33340	AAGAGTTGCAAGGTCCTGCCGT	CCGTCGCATTTCACGTGCTT	335
FMgSSR-20611	scaffold296	p2	(CT)21	Class I	31612	31653	AAGTCTTTGAGGCCAGCCGA	TGTGCGGTTCAACGTAGGCA	267
FMgSSR-20612	scaffold24700	p2	(CT)21	Class I	3433	3474	AATCAGGCTCAAGCAGCCCACA	CCGCCCAAGATGTGCAACCAAA	213
FMgSSR-20613	scaffold647	p2	(CT)21	Class I	74805	74846	ACACTAGGGTTTACACACCCGTCA	TCGGCAATGGACATGAGGCA	213
FMgSSR-20615	scaffold283	p2	(CT)21	Class I	31441	31482	ACCAACCGGCAACAAGTCGT	ACAAAACGATGCGCCGTGCT	236

SSR_ID	Scaffold	SSR_Type	SSR_Motif	SSR Class	SSR_Start	SSR_End	Forward sequence	Reverse sequence	Product_size
FMgSSR-20616	scaffold4869	p2	(CT)21	Class I	34503	34544	ACCGAGGACGTGGGAGTCAAAT	GCGATTGCACATATGGAAGCTAGGG	340
FMgSSR-20619	scaffold1237	p2	(CT)21	Class I	28591	28632	ACTCAGCTGACGAACGGGTTGA	TGGCTGCTGGTCACTTTGCT	290
FMgSSR-20625	scaffold8511	p2	(CT)21	Class I	11922	11963	CCACGTGTCAGGTCCATGTCATCA	TTGCGACATCTGCACGGTCA	330
FMgSSR-20626	scaffold475	p2	(CT)21	Class I	6450	6491	CCTCAGCACAAGCTGAAGCA	TGCTAGGTGTTAGGCGGATGGT	252
FMgSSR-20627	scaffold6005	p2	(CT)21	Class I	14132	14173	CGCTGCTACCTGTTGAAACCCA	TTCTACTAGTCGGGAGAGAAGGGC	252
FMgSSR-20631	scaffold394	p2	(CT)21	Class I	134355	134396	TATCAAGGGCGTGTCTGCCTGT	AGCATCGGCGAAAACCAGCA	349
FMgSSR-20632	scaffold31629	p2	(CT)21	Class I	1308	1349	TATCTGGTGGGAGCTGACTCGTGA	TTCACCGGCATTGGCAAGGACT	208
FMgSSR-20633	scaffold1245	p2	(CT)21	Class I	36352	36393	TCAACAGGCGCCCACAATCT	ACCACGGTTCATGACCCTCA	350
FMgSSR-20639	scaffold1471	p2	(CT)21	Class I	49372	49413	TCTCACAACCCATGCATGACGC	ACCCCACAACCACACACAGA	315
FMgSSR-20640	scaffold3445	p2	(CT)21	Class I	41877	41918	TCTGCGCCGGTAGTGATTGA	AGCGCTGACACGGTGAATGT	306
FMgSSR-20643	scaffold37194	p2	(CT)21	Class I	2727	2768	TGCCAGCGGAGGCAAACAAA	TGCAACGCCCACGCTCTAAA	226
FMgSSR-20644	scaffold11126	p2	(CT)21	Class I	8817	8858	TGCCATCACGGACGTGGTAA	TTGCTGTTTCAGTGCGCCGT	344
FMgSSR-20650	scaffold7969	p2	(CT)21	Class I	8707	8748	TGGTCCATGCCTACCTTGTGCT	TGCGGCCTGCTCAACTTTGT	278
FMgSSR-20651	scaffold119150	p2	(CT)21	Class I	696	737	TGGTTGGTGGCTGGTGAGAA	TGAGCAAGCTCGCTGTGAGGAA	240
FMgSSR-20653	scaffold2004	p2	(CT)21	Class I	27970	28011	TGTGCCGAGTCCAAATGCCT	AGTGACGTGGTGAGAAGGTGGT	337
FMgSSR-20654	scaffold2015	p2	(CT)21	Class I	26068	26109	TGTGTGGTGGTGCGAATCGT	CCGCACGCAGACGTGATTGAAA	314
FMgSSR-20660	scaffold10032	p2	(CT)21	Class I	16116	16157	TTTGCTCACGAGCTTGGCGT	AGCGCAATCGTGTCCAGCAGAT	285
FMgSSR-20661	scaffold10390	p2	(CT)21	Class I	7669	7710	TTTGGCTTTGTTGCGACCACCC	AGCAGTCAGCAGCAACAGTCGT	235
FMgSSR-20665	scaffold2139	p2	(CT)22	Class I	35578	35621	AAGCCACTCCACCATCGTTCCA	TGCCAATCATCACTGTGGGGCT	291
FMgSSR-20666	scaffold368	p2	(CT)22	Class I	106804	106847	ACACCGAAGACGCTCAATGCCA	TGCATTGGAGCGGAACCAGA	295
FMgSSR-20670	scaffold1471	p2	(CT)22	Class I	47788	47831	ACCACATGCGGAACTGCCCTAA	ACAGCAGCAATCCCCTCATGT	334
FMgSSR-20671	scaffold947	p2	(CT)22	Class I	39040	39083	ACCGCATCCATGTGCGTTCA	CTCTCTTTCTACTCGCTGCTTTCG	300
FMgSSR-20678	scaffold158	p2	(CT)22	Class I	109604	109647	ACTCGCGAACCCGCTTTGTT	ACCTTCTTGCCGCCCATCATCT	305
FMgSSR-20682	scaffold30507	p2	(CT)22	Class I	5287	5330	AGCCCTCAGAGTGTAGCACCAA	ACGTCGCTTGCTGAAATGCTGA	347
FMgSSR-20684	scaffold615	p2	(CT)22	Class I	77404	77447	ATAGCGGGAGCACATCGCTCAA	TTGCCACGCCACCATTTCCA	282
FMgSSR-20691	scaffold19	p2	(CT)22	Class I	190959	191002	GGCAATCTCGAATGCAACCGCT	TCTTGCAGCTTGAGCAGCCT	335
FMgSSR-20692	scaffold480	p2	(CT)22	Class I	66487	66530	GGCAGTTTCCGGCAATCAGT	ACGCGCATGCGTTGATCGTT	305
FMgSSR-20694	scaffold4967	p2	(CT)22	Class I	3649	3692	GGTACAAGCTAACTGACAGAGGGA	GGAGAGGAAGAGAGAAGAGGGAGA	350

SSR_ID	Scaffold	SSR_Type	SSR_Motif	SSR Class	SSR_Start	SSR_End	Forward sequence	Reverse sequence	Product_size
FMgSSR-20700	scaffold3949	p2	(CT)22	Class I	29390	29433	TCCTCCCTATCACCGTTCACCTGT	TGTGGGCCTCTTGTCACTGCAT	319
FMgSSR-20707	scaffold4437	p2	(CT)22	Class I	10192	10235	TGTTAGCGGTGTTCGTCACAGT	TCACTTGGTCCTTTCAACCCGC	288
FMgSSR-20708	scaffold660	p2	(CT)22	Class I	95287	95330	TTGCCGCAGCAACTTCCAGT	AAGCAGCCCGGCCCATTAAA	278
FMgSSR-20709	scaffold956	p2	(CT)22	Class I	21598	21641	TTTCATGCTCCCTCTCGCCT	GCTGCAGCTGGCAGTGAACAAT	307
FMgSSR-20713	scaffold1401	p2	(CT)23	Class I	12503	12548	ACAAGCAGCCCAGGAAGCAA	TGCTTCCATGTTCACGGTCACGA	259
FMgSSR-20721	scaffold146	p2	(CT)23	Class I	90952	90997	AGCACAAGTGGACAATGGGCCT	TGCCCCTTTTGCAAATCACTGGC	207
FMgSSR-20722	scaffold15101	p2	(CT)23	Class I	7020	7065	AGCAGTGCAGCAAGCAAGCA	GGCATCAGGAAACAGCCAAGCA	286
FMgSSR-20726	scaffold2859	p2	(CT)23	Class I	22677	22722	AGCTAGTTGCTGAGGGGCTGTT	TGTTTTGGGGGCAGACGCTT	290
FMgSSR-20727	scaffold14363	p2	(CT)23	Class I	2464	2509	AGCTCACACACACAAACAGAGAGC	AACCTATGCGCGTGCCTCATCA	349
FMgSSR-20728	scaffold23573	p2	(CT)23	Class I	4457	4502	AGCTTCGAGGTCCTGTTGCTCA	ACAGTAGCGAGAAGCCAAGCCA	345
FMgSSR-20731	scaffold9208	p2	(CT)23	Class I	10476	10521	ATCCTACACTCGCGCGCAATCA	ATCAACCTCCGCGAACCAGAGA	271
FMgSSR-20733	scaffold1611	p2	(CT)23	Class I	29032	29077	CACACCACCTATGTTGTGCAGC	ACGCACAAAGGAAAGCCTCGCT	325
FMgSSR-20745	scaffold16433	p2	(CT)23	Class I	5530	5575	TGGGCATGCTTTATCCTAGCCGT	ACAACGAGGGATCCGAAGCA	346
FMgSSR-20749	scaffold568	p2	(CT)23	Class I	86522	86567	TTCCGTGCAATGTGTGTGCG	TCGCCAGACCGGTTTACACA	315
FMgSSR-20753	scaffold11030	p2	(CT)24	Class I	22246	22293	ACCAAACTGAAGCCGCCTCTCT	TTTGGCGCTCAATCCGTCGT	303
FMgSSR-20755	scaffold1513	p2	(CT)24	Class I	34264	34311	ACGGCAATATGGCCCGAGCAAA	AGTCAACGACAAGCCGACGA	340
FMgSSR-20756	scaffold10447	p2	(CT)24	Class I	10224	10271	ATCGGCCAATCGCGTCATCT	TGGCTGGGCAATAAGGAAACGGA	329
FMgSSR-20757	scaffold15254	p2	(CT)24	Class I	9175	9222	CCCCAAAAATCTTGCGGTGGCT	AGGCCGCAACATTAACGTGTCA	302
FMgSSR-20762	scaffold1208	p2	(CT)24	Class I	4828	4875	TCCAGAGTCATGGGCGACAACA	AAGCACCGGTCGATGACGAA	231
FMgSSR-20765	scaffold1615	p2	(CT)24	Class I	23971	24018	TGACAGGCAGGACGGCAGAAAT	AACAGGCAAGATGCACACCCCT	320
FMgSSR-20766	scaffold8060	p2	(CT)24	Class I	9832	9879	TGCCATTTAGCCCAACACCCCA	TATCACCACACCGCCATCGT	340
FMgSSR-20767	scaffold17457	p2	(CT)24	Class I	6056	6103	TTTGGTTCAGCGGGGAAGTGCT	AGGACATACCTTCTGGGCATTCCA	322
FMgSSR-20769	scaffold5000	p2	(CT)25	Class I	23193	23242	AAAGGAACGTCCTATGCGGCCA	TGCAAGGGTGCAAGCGCAAA	291
FMgSSR-20776	scaffold44719	p2	(CT)25	Class I	2323	2372	AGGCTCTGTCAATGCACCATCCA	TGCACCGACAATGAGGGAGA	272
FMgSSR-20783	scaffold11	p2	(CT)25	Class I	236439	236488	TCGTTTTCTCGCGTCTGTCG	AAGCTACCTCAACGCCTCTCCA	220
FMgSSR-20785	scaffold4849	p2	(CT)25	Class I	9262	9311	TGACGCAGGAAAGGCTGTGCTA	CCACTTGCCGCTTTGAGCAACA	335
FMgSSR-20787	scaffold2420	p2	(CT)25	Class I	49712	49761	TGCACGAAAAGGACGGCAACT	GAGAGAACGACTGCTTGTGTGT	337
FMgSSR-20796	scaffold2306	p2	(CT)26	Class I	26757	26808	TCGTTCTGACACGATGGGCA	ACGAGGCAGTCAAATGTGCGT	310

SSR_ID	Scaffold	SSR_Type	SSR_Motif	SSR Class	SSR_Start	SSR_End	Forward sequence	Reverse sequence	Product_size
FMgSSR-20797	scaffold2802	p2	(CT)26	Class I	14877	14928	TCTCTCTCTCTCGTTGGGTCCT	TTGAGCACAAGAGGCGGGAT	335
FMgSSR-20798	scaffold3480	p2	(CT)26	Class I	15907	15958	TGATGAGGGAGCGAACAACAGT	TGCCTCTGCCATTACTTGAGCCT	203
FMgSSR-20800	scaffold10941	p2	(CT)26	Class I	18883	18934	TGGCCCGCCGTTAACATTGA	GGGGGAAGAGGCAAGATAGACA	246
FMgSSR-20801	scaffold1393	p2	(CT)26	Class I	81046	81097	TGTCCCCTTCAGTCACCGTT	GAGAGTACTAGTGGTGTGTCACGA	350
FMgSSR-20802	scaffold2943	p2	(CT)26	Class I	7191	7242	TTCGGCAGGCAATGGCACTT	ACGACCATTGTGCCAGAGCCAA	223
FMgSSR-20803	scaffold3338	p2	(CT)26	Class I	9567	9618	TTTACGTTGCAGCAGAGACCG	ACCGTGCCAGGCTTTCCTTT	303
FMgSSR-20806	scaffold26578	p2	(CT)27	Class I	3294	3347	ACTGCAGCCAGAACCCAAGA	AGCACGCTCCACCCAGTTATGA	225
FMgSSR-20809	scaffold4285	p2	(CT)27	Class I	1584	1637	AGCACGGCACCATCGTCATCAT	AGCCAAAGCCTGCACCAGAT	338
FMgSSR-20810	scaffold8879	p2	(CT)27	Class I	11277	11330	AGCCTTGTGATACTACAGGGACC	CAGAGTACATGGGCAAATGGATCG	300
FMgSSR-20814	scaffold16255	p2	(CT)27	Class I	16134	16187	GCTTCAGCGTGTGTGAGATGTGT	TCGGCCACCACTACAGGAAACA	204
FMgSSR-20817	scaffold670	p2	(CT)27	Class I	75838	75891	TGGTCCATGCCAGCCAACTGAA	AACAGGTTGCACGAAGTCCCGT	332
FMgSSR-20819	scaffold830	p2	(CT)27	Class I	99306	99359	TGTTGCTACTCGCCGCTACCAT	ATGCACACAATGCGCGTCCT	243
FMgSSR-20821	scaffold4568	p2	(CT)27	Class I	20968	21021	TTGGCTCTCGCCAAAACCGAGA	TGATTTGGGCGGCTGGACTTGA	252
FMgSSR-20824	scaffold18	p2	(CT)28	Class I	114307	114362	AGCCCAGCGCGACATAGTCATT	ACCGGATGGCAAGGTGCATT	309
FMgSSR-20825	scaffold2974	p2	(CT)28	Class I	24968	25023	AGGTCCTCACGCGACAAGAA	AGGTTCGATGCGAGTGCCATGT	302
FMgSSR-20829	scaffold11717	p2	(CT)28	Class I	19601	19656	TGACGTCCGTGGTGCTCATCTT	TGCAGCGTGTGTGACTCTGTGT	305
FMgSSR-20830	scaffold12078	p2	(CT)28	Class I	6491	6546	TGCGACCCGTCGGTCATATT	ACAGAAGTCCAAGGACGCACGTT	349
FMgSSR-20833	scaffold4995	p2	(CT)29	Class I	9443	9500	ACCGTCACACGTGGTACATAAGGC	ATTTGTGCGCGGGATCAACG	328
FMgSSR-20835	scaffold8086	p2	(CT)29	Class I	9829	9886	AGCCGGGTAAATGGAGTGGT	GCACACAAGCTCGCTCCTTCTT	291
FMgSSR-20836	scaffold8086	p2	(CT)29	Class I	9025	9082	AGCCGGGTAAATGGAGTGGTTGT	GCCAACTTCACCTCGCTCAACA	338
FMgSSR-20838	scaffold37148	p2	(CT)29	Class I	3647	3704	CGTTGCAAATGCCAAGCGCA	AAGCGAGTGAAGCCGAGTGAGT	294
FMgSSR-20841	scaffold11561	p2	(CT)29	Class I	7273	7330	TCTTCTTTCGCCGCCAGCAT	TTTGCTGAACAGCGGACTGGGT	308
FMgSSR-20850	scaffold11879	p2	(CT)30	Class I	7975	8034	TGGGCGAGGCAACAGCATAA	AGCCGAAAAGAACACAAGCGCA	212
FMgSSR-20851	scaffold15286	p2	(CT)30	Class I	6461	6520	TTGCACATGCGCAGTCATCCAC	TGACAAGGGATGGCCATGAAGGA	348
FMgSSR-20862	scaffold2261	p2	(CT)33	Class I	52530	52595	ACCGTTGTCAGCATCCCCAA	GCGTGTGAGTGAGTGTCTGTATCT	350
FMgSSR-20863	scaffold9910	p2	(CT)33	Class I	4308	4373	TGACCTCTGCGGCTGTTGAA	TAGGGATTGGATGGCGAGAGCA	205
FMgSSR-20864	scaffold468	p2	(CT)35	Class I	11417	11486	AGCAGGGCGTCAGAGTTCACTT	AGCCGCGGAATAGTGCTGAT	272
FMgSSR-20865	scaffold732	p2	(CT)36	Class I	98586	98657	AAAGCACCGGTTTGGCGGAT	AGCCGGCACGAGAACGAAGTTT	274

SSR_ID	Scaffold	SSR_Type	SSR_Motif	SSR Class	SSR_Start	SSR_End	Forward sequence	Reverse sequence	Product_size
FMgSSR-20867	scaffold1491	p2	(CT)37	Class I	44873	44946	AGCCGGCGATAGCAGTTTCGTT	TGCGTGGAATCCTTTTGGGCTG	285
FMgSSR-20868	scaffold595	p2	(CT)39	Class I	110128	110205	ATGCCTTTGTCCGTGAGCCA	TCCATGGCCTCGCCCTCAATTT	303
FMgSSR-20870	scaffold2859	p2	(CT)43	Class I	54904	54989	TATCCTTCTCAAATCGCTCCCGCC	AACCACGCGCAAGCATACGA	305
FMgSSR-20871	scaffold20300	p2	(CT)43	Class I	905	990	TTTCGCGTGCTGCTACTGACGA	ACCGTCTTGGTGGAAGAGGAAGGA	345
FMgSSR-20873	scaffold5580	p2	(CT)6	Class II	30972	30983	AAAAAGCTGACTCGCTCCGCCT	ACGCAAAAGGACGGCTACCACA	287
FMgSSR-20880	scaffold326385	p2	(CT)6	Class II	94	105	AAAACGACTGGCTGTCCGAACG	ACGATGTGCTCACCTTCGTGGA	306
FMgSSR-20884	scaffold6165	p2	(CT)6	Class II	1918	1929	AAAAGCACGCACCGACACGA	AATCGGTGCCAAAGCCACCA	250
FMgSSR-20903	scaffold4422	p2	(CT)6	Class II	25447	25458	AAACGTGCCGCTGTTTGCCA	TCGGCTGTTCCCTCCAGAAA	298
FMgSSR-20915	scaffold323	p2	(CT)6	Class II	74282	74293	AAAGGACCGACCGCATGGTGTT	TTGCGCCATTCATCAGACGGGA	243
FMgSSR-20923	scaffold150478	p2	(CT)6	Class II	645	656	AAAGTTGAGATGGCGGCGTTGG	AGCATCGAATTCGGCAGCCA	340
FMgSSR-20927	scaffold96	p2	(CT)6	Class II	118267	118278	AAATGCGTCGATGCGGCACT	TAAAGCTCACGCACACGGCA	347
FMgSSR-20929	scaffold46	p2	(CT)6	Class II	133678	133689	AAATGGCGACCGCAAAAGGTGG	TTGCCATTGCGGTGAGGAGGAA	341
FMgSSR-20932	scaffold16818	p2	(CT)6	Class II	12303	12314	AACAACAACTCTGCCTCCGGCT	TGTGCTATGGTTGCGCTGGT	300
FMgSSR-20935	scaffold227	p2	(CT)6	Class II	58196	58207	AACACCGACGACACAGTACGCT	TCGTCGTGTTTGCTGCCGAT	343
FMgSSR-20939	scaffold4892	p2	(CT)6	Class II	13124	13135	AACAGGTCGACAAAGCCGCA	TGCACACAAGGAGAGTGGCGTT	334
FMgSSR-20961	scaffold446	p2	(CT)6	Class II	65033	65044	AACGGGGCAAGGGGCAGAAAAA	TTGTCGAAGTGCTTGCCGGAGT	342
FMgSSR-20973	scaffold7830	p2	(CT)6	Class II	10461	10472	AAGAGCAACTCACCGTCGCACA	AGGGAGATGGAGGCTGGTGTTT	299
FMgSSR-20985	scaffold251054	p2	(CT)6	Class II	448	459	AAGCGCAACTGCACAAGCAC	ACGTACTCCATGTCCATCCAGAGA	269
FMgSSR-20994	scaffold4007	p2	(CT)6	Class II	23717	23728	AAGCTGCCGTTTGCTGGCTT	TTCGTCTTTGGTCGCCGCAT	318
FMgSSR-21001	scaffold23522	p2	(CT)6	Class II	7488	7499	AAGTGGATCGAATGCTGCCGCT	AGCGCTCGGGAGAGAAGAGAGATT	277
FMgSSR-21004	scaffold8820	p2	(CT)6	Class II	16152	16163	AAGTTCCACGCTGTTGCCGA	GGTGCTTAGCAGGGAGGAAGAT	288
FMgSSR-21008	scaffold895	p2	(CT)6	Class II	61208	61219	AAGTTGCTTGCTCGGGTGCT	ATTGCGAACAGCAGGGGGACAA	334
FMgSSR-21010	scaffold480	p2	(CT)6	Class II	51895	51906	AATAGGCGCGCAATCGCTGT	TGCGGGTCCCACCGGAAAAATA	222
FMgSSR-21030	scaffold21481	p2	(CT)6	Class II	5264	5275	AATGGTTGCTGCTGAGGCCGAT	TCCGTGGAAATCCCATGTCCGA	347
FMgSSR-21034	scaffold4821	p2	(CT)6	Class II	22931	22942	AATTACCCGCCATGCCACCA	AGGTGCGCTGCATCTTCGTT	324
FMgSSR-21037	scaffold3166	p2	(CT)6	Class II	56361	56372	AATTCGAGTCAGGGGCCCAA	GCCCAACCAAAAGTGGCCAAGA	317
FMgSSR-21046	scaffold1220	p2	(CT)6	Class II	2291	2302	AATTTCTACACCCGGACGCAGC	ACCATGCCGCCGTCGTTTTA	312
FMgSSR-21050	scaffold210	p2	(CT)6	Class II	125222	125233	ACAAGAAACAGACCCGCCGT	GCGGTTTCAGCATCACTCGCAA	340

SSR_ID	Scaffold	SSR_Type	SSR_Motif	SSR Class	SSR_Start	SSR_End	Forward sequence	Reverse sequence	Product_size
FMgSSR-21055	scaffold49437	p2	(CT)6	Class II	3951	3962	ACAAGGTTCCCCGCTCAGTT	AGCAGGGGCGCAATCATCATCA	200
FMgSSR-21069	scaffold240	p2	(CT)6	Class II	10587	10598	ACACCTCGCCGAACGTCTTACT	TGTTGCGCGAGGTTTACATGCC	304
FMgSSR-21076	scaffold1356	p2	(CT)6	Class II	47783	47794	ACACTAATGCGTGTGCCTCAAC	AGGCAGAGCCATCCTTTGACCA	332
FMgSSR-21081	scaffold7873	p2	(CT)6	Class II	26311	26322	ACAGACAGCAGGCAGTGCTGAA	TGCTGCATTCTGCGGGGTTT	231
FMgSSR-21083	scaffold3051	p2	(CT)6	Class II	47381	47392	ACAGATGGCAGCGAAGACAGGT	GCAGGATTGCCAGCCAACCAAA	296
FMgSSR-21086	scaffold800	p2	(CT)6	Class II	65789	65800	ACAGCAGAGATTACAGCTGGTGGC	ACTGTGGCAGCTGTGTGAGCAT	337
FMgSSR-21110	scaffold3252	p2	(CT)6	Class II	43081	43092	ACATTCTCACTCGCGTGCTCCT	TTGAGTGCGCCAGATCCACGTT	236
FMgSSR-21115	scaffold1408	p2	(CT)6	Class II	2221	2232	ACCAAACCAACAAGGGCCGA	ATGTCACTGACCCTGCCTGTCT	323
FMgSSR-21117	scaffold3782	p2	(CT)6	Class II	50600	50611	ACCACACCATCGCTTGGTCA	TCAGCGGTCCAACCCAGATGAT	337
FMgSSR-21118	scaffold544	p2	(CT)6	Class II	55836	55847	ACCACAGCCCACAACTATGCAC	AGAGGCGACGACAAAAGGGT	344
FMgSSR-21125	scaffold13407	p2	(CT)6	Class II	7215	7226	ACCAGAGGCCCGTTTGCTTT	TCCGCAACCACATCAAGCGGAT	349
FMgSSR-21126	scaffold692	p2	(CT)6	Class II	61159	61170	ACCAGTGACGTCAGGATCAGCA	TCGTGCTTGGTGCTTGCAAAGG	235
FMgSSR-21138	scaffold331024	p2	(CT)6	Class II	200	211	ACCCAGTTGGTTGACTTGGGCA	TTTCGTTCCCGGTTGCCTGTGA	212
FMgSSR-21147	scaffold8665	p2	(CT)6	Class II	27565	27576	ACCCTCCTCTCTCTCCCTCTTT	GACAGGAGGAGTCAGTGCAGAT	349
FMgSSR-21150	scaffold10818	p2	(CT)6	Class II	21088	21099	ACCGACGCCTGGGTGTTTTT	ATCCGGCCGATGCCTGCTTTTT	311
FMgSSR-21154	scaffold1774	p2	(CT)6	Class II	75292	75303	ACCGCAGCTTTGGTGAATTGGT	ATTCGCAACGGCAGTAGCCT	341
FMgSSR-21158	scaffold9682	p2	(CT)6	Class II	3562	3573	ACCGGGGTTGCCATCTTGTT	TCAGGAGCTACAAGAACCCTCCA	344
FMgSSR-21162	scaffold96292	p2	(CT)6	Class II	254	265	ACCGTGTGATGTGCTCGCCTAA	ACGGCAAAGACAATGCCGCAA	344
FMgSSR-21168	scaffold6206	p2	(CT)6	Class II	6520	6531	ACCTCTTTCTTCTTGTTGCCCGC	TGGCGGCATTACACATGGGA	311
FMgSSR-21177	scaffold319433	p2	(CT)6	Class II	72	83	ACCTTTGGTGCTTGTTGCGACG	TGATGCCAGGCTCCAGACAGAA	327
FMgSSR-21187	scaffold369	p2	(CT)6	Class II	94585	94596	ACGAGCGGCGTGTGTGTTTT	TCGCACGTGACGGGTTGATT	343
FMgSSR-21191	scaffold211613	p2	(CT)6	Class II	35	46	ACGATGGTGAGCTCTCCCTATTTC	ATCTGCCAAAGGCCGCCGTAAT	200
FMgSSR-21195	scaffold1841	p2	(CT)6	Class II	43509	43520	ACGCAATCGAGAGCCGACAT	ACGCGCCGTTATTCCAACCA	262
FMgSSR-21198	scaffold1956	p2	(CT)6	Class II	39410	39421	ACGCACAGCTATACATGCGG	AATTCCATGGAGACTGCCAGGTCG	334
FMgSSR-21200	scaffold5460	p2	(CT)6	Class II	10646	10657	ACGCAGCGGACCGTTTTCAT	TTGTAGGTGGTGGTACTCGGCA	347
FMgSSR-21211	scaffold20	p2	(CT)6	Class II	33525	33536	ACGCGTCAGCCATCATCTCT	TCACGTTGGTACGGATGCTGAA	251
FMgSSR-21213	scaffold16367	p2	(CT)6	Class II	10510	10521	ACGCGTGTCGCCAAGTGATT	AACACGATAACACGTGCGTGGC	216
FMgSSR-21215	scaffold6261	p2	(CT)6	Class II	33270	33281	ACGCTGGCCACAAACAGGATCA	AAACCCCAACAGTGAGCCCT	238

SSR_ID	Scaffold	SSR_Type	SSR_Motif	SSR Class	SSR_Start	SSR_End	Forward sequence	Reverse sequence	Product_size
FMgSSR-21216	scaffold1698	p2	(CT)6	Class II	23675	23686	ACGGATACGTGCCCATGACA	ACGCGGCCAACAAGCGAAAT	245
FMgSSR-21218	scaffold279	p2	(CT)6	Class II	45058	45069	ACGGCAGATTTGCAGGAGCA	CGTGTACGCGAGGAAACGACAT	304
FMgSSR-21224	scaffold5376	p2	(CT)6	Class II	35099	35110	ACGGCGTAACGCACTCGTTT	AGTGCGGAGGGAGAAATGCAGA	220
FMgSSR-21229	scaffold8385	p2	(CT)6	Class II	13116	13127	ACGGGGCTGGCCTGTAGTTTTT	AGGAGGAAATGTGGCATGGATCGC	350
FMgSSR-21231	scaffold19297	p2	(CT)6	Class II	15344	15355	ACGGTAATTGTTTGGCACCCAGG	TGCTGGCTGCTGTGATTCCA	273
FMgSSR-21233	scaffold3689	p2	(CT)6	Class II	42611	42622	ACGGTGCGTTGCGTTTCCAT	ATTTACGGCCTTGTCCGCGT	217
FMgSSR-21237	scaffold28741	p2	(CT)6	Class II	6782	6793	ACGTCGGTCAATTACCCCGTCA	ACGTAAGGGGCAACGGAAGGAA	337
FMgSSR-21244	scaffold7706	p2	(CT)6	Class II	18221	18232	ACGTTTCACGCAAGCAGGCA	TGCAGCTGACAGGCGATGAA	285
FMgSSR-21252	scaffold60	p2	(CT)6	Class II	142271	142282	ACTCCGAGTCAACTTGCCACT	AGCATGCCGTTTGTGTGCTT	315
FMgSSR-21262	scaffold11687	p2	(CT)6	Class II	4394	4405	ACTGTTCATGCGGCTTGCCA	TGTTCTTTACTGTGCCCGCTGC	337
FMgSSR-21264	scaffold37	p2	(CT)6	Class II	131853	131864	ACTTACTCCTTCCCAGACGCCA	TTCCATCGAGGTTCAGCGGT	347
FMgSSR-21265	scaffold10268	p2	(CT)6	Class II	5964	5975	ACTTGAGTCACGGGAAGCTCCT	AACGTGCAGCAACCAGACCTGA	268
FMgSSR-21266	scaffold510	p2	(CT)6	Class II	41563	41574	ACTTGCTGCTACGCACCGAT	TTGTACCGCGGGGAATGAAGCA	278
FMgSSR-21273	scaffold9765	p2	(CT)6	Class II	16426	16437	AGACGATCCACCGTCTGTTGGT	TGCGCTTGAGACCGAGTGAGAT	217
FMgSSR-21277	scaffold3747	p2	(CT)6	Class II	49446	49457	AGAGATCAGACATGAGCGGTGGT	TCGGGAGATGCTTCCTGCCAAT	317
FMgSSR-21284	scaffold1934	p2	(CT)6	Class II	29547	29558	AGATCCGAATCGCTGACGCA	TGTGATGCTGGATCTGGTTGCCA	343
FMgSSR-21289	scaffold6922	p2	(CT)6	Class II	25532	25543	AGCAACATCAGCGGCACCTT	GAGGAGAATTCCATGTCAGCTGTG	278
FMgSSR-21299	scaffold1378	p2	(CT)6	Class II	15638	15649	AGCACACACGTGCCAGTACAGT	TCCGCATCAATGAGCGCCAA	294
FMgSSR-21312	scaffold12727	p2	(CT)6	Class II	10316	10327	AGCAGACCAGCACCATCCCTTT	TGGCGTACGTGTTGCCAAGT	345
FMgSSR-21326	scaffold285222	p2	(CT)6	Class II	54	65	AGCCCAACATGCCACAACCGAT	GGGACTCAAGTGACCACTCAGTTT	281
FMgSSR-21328	scaffold1930	p2	(CT)6	Class II	15375	15386	AGCCCCAAGGAGCGAGTTTA	GGCCAAAAGAACATGGGCCGTT	329
FMgSSR-21329	scaffold17220	p2	(CT)6	Class II	13022	13033	AGCCCCCTGAACATGTTTGTGCT	TTGCCGACGAGGCATCATGT	256
FMgSSR-21331	scaffold2002	p2	(CT)6	Class II	27392	27403	AGCCGAAGGAAGCAGCACAA	TGCCGGTTTGCACTTGGTCA	224
FMgSSR-21335	scaffold1740	p2	(CT)6	Class II	75615	75626	AGCCGCTCGCAATTTCCACA	AGCCGAGAAGCGCGTGATGATA	294
FMgSSR-21348	scaffold10089	p2	(CT)6	Class II	3618	3629	AGCTCACGGTGTTTGCTTGGA	GCGGTGGCAAAAGGGGCAATTT	313
FMgSSR-21350	scaffold868	p2	(CT)6	Class II	25990	26001	AGCTCGCGGTTAGTTCAATGCG	AGAACCAAGCCTGTGTGCCTCA	290
FMgSSR-21357	scaffold9191	p2	(CT)6	Class II	7119	7130	AGCTTGCAAAAGCTGTTGGCCC	TGGAACCGAGACAAGCTTCCAT	296
FMgSSR-21359	scaffold12178	p2	(CT)6	Class II	5601	5612	AGGAACTGCACGCGTCTTCCAA	TGCTCGATGCAACCATGCCGTA	317

SSR_ID	Scaffold	SSR_Type	SSR_Motif	SSR Class	SSR_Start	SSR_End	Forward sequence	Reverse sequence	Product_size
FMgSSR-21374	scaffold7029	p2	(CT)6	Class II	27608	27619	AGGCAGCTAGTTGTTGTTGCCA	AAACAAGCACTGCGTGGGGA	275
FMgSSR-21378	scaffold486	p2	(CT)6	Class II	104572	104583	AGGCCCTGCACCAAATGCTT	AGGAGGAAGCCCCTTTTTGCCT	220
FMgSSR-21385	scaffold23084	p2	(CT)6	Class II	5489	5500	AGGCGCTGCTCCTTCTGTCAAT	ACCAGGAAACAAGCCGTGGT	349
FMgSSR-21398	scaffold6447	p2	(CT)6	Class II	18613	18624	AGGGCCAGCGGAACATCGAAAA	TGCAAGGACGGTACAGACACCA	223
FMgSSR-21399	scaffold13928	p2	(CT)6	Class II	21615	21626	AGGGCGTCGTCTTGGAAGGATT	AGTTGAACTCGGAGCGCACACA	237
FMgSSR-21407	scaffold14322	p2	(CT)6	Class II	292	303	AGGTGTCAGGTCTTGTGCGA	AGGCGTCGGGGAGCAATTTT	296
FMgSSR-21409	scaffold1092	p2	(CT)6	Class II	40945	40956	AGGTTGCATGCATAGTGGGGCA	TGCCTGTCTTGGGTGTTCCCTT	209
FMgSSR-21412	scaffold12107	p2	(CT)6	Class II	17335	17346	AGTACTGGCGCTCCGCTTCTTA	GGAATTTCCGATATCCGGTGCAGT	348
FMgSSR-21413	scaffold3349	p2	(CT)6	Class II	14131	14142	AGTAGACGCGTGTGCTCTCT	TGTGGGTTGTCCTCTGAAAGCGA	287
FMgSSR-21420	scaffold1496	p2	(CT)6	Class II	59158	59169	AGTGCATGTGCATGGTGGCT	GCCGGTGTTCCCAAAAGCACAA	350
FMgSSR-21422	scaffold898	p2	(CT)6	Class II	74554	74565	AGTGGCTCATTCCGGCACTT	TTGGGTCCTGTCACACTGCCAA	275
FMgSSR-21423	scaffold189	p2	(CT)6	Class II	115858	115869	AGTGGGACGGTGACATGCCTTA	AAAACCTGCACCAGCAAGCACC	348
FMgSSR-21436	scaffold942	p2	(CT)6	Class II	64475	64486	AGTTTGCCCCGTGTGACAAC	TTGGCAAGACGGCAACACATGC	268
FMgSSR-21440	scaffold1313	p2	(CT)6	Class II	1796	1807	ATAATAAGCACCCGGCGCGA	GCACGAAACGAGACGAAAGCCA	350
FMgSSR-21444	scaffold4212	p2	(CT)6	Class II	24392	24403	ATAGCGGCCGGTTCATGACT	ACAGGAGTGCACAAGGAGCTAGA	223
FMgSSR-21447	scaffold200318	p2	(CT)6	Class II	547	558	ATCAAAGGGCGGTCCAGGTGTT	TTTGGACTGTGCTGCCTGCTGT	288
FMgSSR-21451	scaffold3732	p2	(CT)6	Class II	9484	9495	ATCACGTGGGCGTGCTTTCT	TCTAAGGGCATCCACAGGCGTT	242
FMgSSR-21453	scaffold5811	p2	(CT)6	Class II	24950	24961	ATCAGAATCGGGTCGCACAGCA	TCGGCATGCGATTGCAAGTACC	348
FMgSSR-21458	scaffold29183	p2	(CT)6	Class II	1598	1609	ATCGATGTGGGGCTGTGTGAGA	ACAGCAGCAGGGAAACAGAGCA	320
FMgSSR-21460	scaffold214014	p2	(CT)6	Class II	42	53	ATCGCCAGCTGCAACAATGA	CACGCGCATTCCATTTAGAGCA	338
FMgSSR-21464	scaffold4900	p2	(CT)6	Class II	20418	20429	ATCGTTGCAGCGCCGAAGAA	AAGAGATGCCGGTGCCGGTTTT	316
FMgSSR-21465	scaffold109	p2	(CT)6	Class II	92828	92839	ATCTATTAGGTACGGCACTCCCCC	TCGCCACCAATCATCGTGCT	347
FMgSSR-21466	scaffold1669	p2	(CT)6	Class II	9145	9156	ATCTCCTGCCCTCCATTTGAGC	AAGAGCGGCTGCATGTGGATCA	248
FMgSSR-21467	scaffold805	p2	(CT)6	Class II	92330	92341	ATCTGACAAGCTGACGGCCAGA	TGGAACACGGTGGTGAACTGGT	322
FMgSSR-21469	scaffold30984	p2	(CT)6	Class II	2583	2594	ATGAAAATCGCACCCAGCCCCA	ATTTTTGCCCCCATTCCCTGCC	258
FMgSSR-21476	scaffold3603	p2	(CT)6	Class II	50873	50884	ATGCATGGGTGAGCGATCCACT	TGTTTCTCCTTCCCGCATGGCA	296
FMgSSR-21491	scaffold21429	p2	(CT)6	Class II	12023	12034	ATGGAATGTGCTGTAGGAGAGGCG	AGAGCCGTGGAAGTGGAAGACA	340
FMgSSR-21496	scaffold7579	p2	(CT)6	Class II	1789	1800	ATGGGCGATACGTGGTGACGTT	ATACAACGACACTGCGCCACCT	200

SSR_ID	Scaffold	SSR_Type	SSR_Motif	SSR Class	SSR_Start	SSR_End	Forward sequence	Reverse sequence	Product_size
FMgSSR-21497	scaffold19246	p2	(CT)6	Class II	9798	9809	ATGGGCTGTTTGCCCCTGGAAT	TGCGACAAAATCGGGGAGTTGG	277
FMgSSR-21500	scaffold4984	p2	(CT)6	Class II	2017	2028	ATGTGCAGCGCCATCCTCAA	TGAGCAGCATGCACCACACA	305
FMgSSR-21501	scaffold159	p2	(CT)6	Class II	23332	23343	ATGTGCCGATGTGCGTGTGT	AAGCAACCCGGGAAGCAACCTT	348
FMgSSR-21503	scaffold2080	p2	(CT)6	Class II	44769	44780	ATGTGCGTGGCGTGGAAGAT	TCAAGTGGTGGGCGCCAAAT	245
FMgSSR-21504	scaffold1155	p2	(CT)6	Class II	9563	9574	ATTAATAAACCCTGCCGGCCTCCC	ATCCGCGAGCTTGAGCACTAGA	231
FMgSSR-21508	scaffold938	p2	(CT)6	Class II	45857	45868	ATTCCGCAGGGACCTGCAAT	AGCTGTCCAAAGCCACCCTT	327
FMgSSR-21522	scaffold9189	p2	(CT)6	Class II	20754	20765	ATTTACCGGATGTGCCCAATCCCC	AGGAAGAACAGGGAGAGAGGGA	318
FMgSSR-21525	scaffold5410	p2	(CT)6	Class II	25083	25094	ATTTGGTGCGCGGTTTAGGTGC	TGCTGCGGCAACATGGGTTT	235
FMgSSR-21530	scaffold2399	p2	(CT)6	Class II	4463	4474	CAATTCGCAAAGCGCCCCAA	ATGCACATGACAGAGGGCCA	256
FMgSSR-21534	scaffold32289	p2	(CT)6	Class II	5091	5102	CACCACAGCTAGCAACAGTTCA	AGGTGTGTGTGCGTCGGTGTAT	297
FMgSSR-21540	scaffold1060	p2	(CT)6	Class II	3313	3324	CAGTTCCACGACCACATTGGATTG	TCGACTGCTGCCTCCAACTGTTT	329
FMgSSR-21543	scaffold4675	p2	(CT)6	Class II	47328	47339	CATTGTGTCGTGGCTGCTCA	AGGCGCGTTGGAAGAAGAGGAA	350
FMgSSR-21545	scaffold9235	p2	(CT)6	Class II	23157	23168	CCAAGTTGCCCATGAAACACACAC	TGTGCACGATTCCCGAATCCCA	273
FMgSSR-21554	scaffold4208	p2	(CT)6	Class II	16471	16482	CCCAGCTACGTCACTGTTGACAAG	AAGTGCCTGTTTGCCAGACTC	294
FMgSSR-21558	scaffold1829	p2	(CT)6	Class II	75520	75531	CCCTGCGCCGCACCAAAAATTA	TGCTTTGCCTGCCTGCTGAT	342
FMgSSR-21565	scaffold13478	p2	(CT)6	Class II	7395	7406	CCGGGCCTATGTCTCCTTCAACAA	TGCCAACAAAACAGCAGACGCC	350
FMgSSR-21573	scaffold1292	p2	(CT)6	Class II	17123	17134	CGACCGCAACTCTGCCAAACAA	ACGCGCGAGTGTGTGTTGTA	321
FMgSSR-21574	scaffold1561	p2	(CT)6	Class II	2077	2088	CGAGGCGCGCTCTTTTGCTTAT	ACGTTTGTCTCTCTGCGCGT	345
FMgSSR-21576	scaffold354	p2	(CT)6	Class II	87177	87188	CGCAACTGCCAACCAACACT	ACGGTGATGCCTCCTTTCAAGC	347
FMgSSR-21578	scaffold4169	p2	(CT)6	Class II	7259	7270	CGCATGCTGATGTTCAGCCACA	TGCGTCGACAGAGGGAGAAGAGAT	262
FMgSSR-21579	scaffold3693	p2	(CT)6	Class II	41288	41299	CGCCAAGTCACTTCCCACCATT	AGCGGTGGAGGCATAGGATGTT	259
FMgSSR-21593	scaffold140379	p2	(CT)6	Class II	537	548	CGGCCGCCAGAAATTGCATTGA	TTGCCCCACAGCACGTTGTA	246
FMgSSR-21595	scaffold14254	p2	(CT)6	Class II	8516	8527	CGGCGAGGCGCCTTTTTCTTTT	AAACCATGACCAGATCGCCGGA	279
FMgSSR-21597	scaffold3083	p2	(CT)6	Class II	53988	53999	CGGGTCGGGTCTGATTGGTTTT	TTCCGCTGCATTGCACGTTG	241
FMgSSR-21598	scaffold42202	p2	(CT)6	Class II	3028	3039	CGTGAGCACCAATCAGCATCGT	TGGTGGCGGGATGCATTGTT	335
FMgSSR-21599	scaffold1179	p2	(CT)6	Class II	84416	84427	CGTGCAGTGTGCGCGTATTCAT	ACTTTCGTTCCATGGGCCAGCA	218
FMgSSR-21607	scaffold35377	p2	(CT)6	Class II	1091	1102	CTGGAAACGAATCCTAACACCGTC	ACCGAGGGCTGCATCTTGTGTT	328
FMgSSR-21608	scaffold4685	p2	(CT)6	Class II	13098	13109	GACTGGACAAGCGAAACTGTGT	TGCCTTCACTCCCCTTGATCTT	349

SSR_ID	Scaffold	SSR_Type	SSR_Motif	SSR Class	SSR_Start	SSR_End	Forward sequence	Reverse sequence	Product_size
FMgSSR-21621	scaffold125249	p2	(CT)6	Class II	182	193	GCAGAACACGCCATTTGGTGGA	TGAGAGCAAGAAGCCAAGGGGA	307
FMgSSR-21622	scaffold10643	p2	(CT)6	Class II	3990	4001	GCAGCTGCTGGAGATGCACTTA	CAGCTCAGCGCTGTTCTTACA	264
FMgSSR-21625	scaffold1437	p2	(CT)6	Class II	27631	27642	GCAGTCGCATCGCAGCCAAAAT	TGCACGTTGCTGTTGGCCTT	215
FMgSSR-21628	scaffold176721	p2	(CT)6	Class II	534	545	GCATTATTGACCCACCTGGCCACT	AGCCGACCCAACAAGCCAATGA	297
FMgSSR-21633	scaffold1505	p2	(CT)6	Class II	10829	10840	GCCCTACAAAATTGGCGGCACA	TGCCCAGGCATGGAGTTGTT	271
FMgSSR-21639	scaffold58554	p2	(CT)6	Class II	2595	2606	GCCGTTTTCAGTAGGCGTGTGT	TGGGGCGGGGGAACAATAATGA	211
FMgSSR-21646	scaffold1342	p2	(CT)6	Class II	95590	95601	GCGATGAAATGCAGGAACCTTGCC	TCACGGCACAAACGGTACGA	238
FMgSSR-21652	scaffold7156	p2	(CT)6	Class II	19787	19798	GCGGAGCTTGGAAACTGGCAAA	TACGCAAAACGCGAGCGTGA	347
FMgSSR-21655	scaffold787	p2	(CT)6	Class II	14920	14931	GCGGCGCGAAGCCATTTTAT	TTTGGCCACCGCTCCAAACA	296
FMgSSR-21656	scaffold418	p2	(CT)6	Class II	36046	36057	GCGGCGGCAATCAATCATCCAT	ACACACACAGCAGGCACACA	337
FMgSSR-21657	scaffold22313	p2	(CT)6	Class II	9650	9661	GCGGGTTTCACCCAACTTCGAT	TCACTAGCATGTGGTTGCTGGCT	342
FMgSSR-21665	scaffold2749	p2	(CT)6	Class II	1146	1157	GCTCTGCTGTGCACGTTGCTTT	TGGCGTGCTTGCTTTGGCTT	226
FMgSSR-21667	scaffold5516	p2	(CT)6	Class II	27316	27327	GCTGCACGGGCTAATAATACGACA	TCCCTCTCTCCTCTCCAGGTCAAT	350
FMgSSR-21668	scaffold1463	p2	(CT)6	Class II	24153	24164	GCTGTGGGCTGAAACCGCAAAA	AAACGCCTGCTTCCTCAGCA	302
FMgSSR-21672	scaffold3551	p2	(CT)6	Class II	50535	50546	GGATGCCTTCCGCATCCTTGTT	ATGCATCGCTGCAGGAACTGGT	229
FMgSSR-21684	scaffold2916	p2	(CT)6	Class II	57159	57170	GGGCTGCACGCTTGTGTTCATT	TTCTTCCTGCGTTCCTCCCTCT	209
FMgSSR-21685	scaffold24291	p2	(CT)6	Class II	6756	6767	GGGGCAAAGCTGATGCGTAGTT	TGTTGAACCCACTGCGGCAA	328
FMgSSR-21687	scaffold14500	p2	(CT)6	Class II	14215	14226	GGTCCCGCTTGTCAGTCTCTTT	TCAAGCAAGCACGGACGCAT	334
FMgSSR-21692	scaffold332	p2	(CT)6	Class II	33446	33457	GTCCCTGGATGCGTGCAACATA	TGTCATGCTAGTGCACCGCGTA	324
FMgSSR-21694	scaffold1944	p2	(CT)6	Class II	61190	61201	GTGTGTGCTTCGTCATTGGAGT	GCTGCGGCATGTGTCTTAGGAT	256
FMgSSR-21704	scaffold12683	p2	(CT)6	Class II	20550	20561	TAACGGCAGCTCATGGCGTTCT	TAAGAGACGGTTGTTGGCCGCA	267
FMgSSR-21709	scaffold469	p2	(CT)6	Class II	105409	105420	TAAGCCCAGTACGCGGACCAAT	TCGGCCGAAGGAAAGAGAGAGA	272
FMgSSR-21720	scaffold1655	p2	(CT)6	Class II	82523	82534	TACGCCTCGCCTTCTCCTCATT	GTTTGGGGCCCAGGAGAAAGAA	330
FMgSSR-21731	scaffold1046	p2	(CT)6	Class II	22504	22515	TAGGAAATGTGCGTGGCGCT	TGTGTTCGTGTGCGTGTGGA	213
FMgSSR-21733	scaffold4850	p2	(CT)6	Class II	41290	41301	TAGGCAGCTATAGGTGGCATCG	GTGCTAGCTATGACGATCTGAAGG	335
FMgSSR-21734	scaffold30297	p2	(CT)6	Class II	6498	6509	TAGGTGCGGCGTTCACACAACT	ACCTTTCCGATGCACCAAGCA	250
FMgSSR-21738	scaffold10143	p2	(CT)6	Class II	15874	15885	TATGTGTTGTGTCGCCGCCT	AATTTGGCGGGCGAGAGTGA	265
FMgSSR-21739	scaffold1390	p2	(CT)6	Class II	36346	36357	TCAACCCGAGATCCACCACCAA	TCCTGATCGTTGCCGCTTCA	213

SSR_ID	Scaffold	SSR_Type	SSR_Motif	SSR Class	SSR_Start	SSR_End	Forward sequence	Reverse sequence	Product_size
FMgSSR-21746	scaffold25470	p2	(CT)6	Class II	6697	6708	TCAATGCGGTGCTGCAAACG	ATGCACGAGTCCATCCAGCCAA	309
FMgSSR-21747	scaffold3281	p2	(CT)6	Class II	4803	4814	TCAATTTTTGGGGAGCCCACGG	TTCCTTTCCTTGCGACACGACG	292
FMgSSR-21751	scaffold215831	p2	(CT)6	Class II	408	419	TCACCCGGAGTGGGTGAAAA	TGACCGCATCTCCGTCTCCTTT	263
FMgSSR-21752	scaffold19911	p2	(CT)6	Class II	8144	8155	TCACGAAATTGCCGCTCCCT	AGGAGAGCGCACACGAATCA	295
FMgSSR-21758	scaffold3916	p2	(CT)6	Class II	39914	39925	TCAGCAACAGCCAAGGCAGGAA	AAATCCCACGGCAACGCTACCA	347
FMgSSR-21761	scaffold33011	p2	(CT)6	Class II	3880	3891	TCATACCCTCCACCTTCCCCAACA	GCTCTTTTCTTGGCTGCACCAGT	335
FMgSSR-21776	scaffold12	p2	(CT)6	Class II	117588	117599	TCCAACAGATCTGCCGTACTCC	ACGGGGCCATGTATGCAAGT	230
FMgSSR-21777	scaffold15803	p2	(CT)6	Class II	6737	6748	TCCAAGATCGCGTCACGTGGAA	CGTCGTGCTGCTTGCAAACCAA	284
FMgSSR-21778	scaffold225	p2	(CT)6	Class II	105235	105246	TCCAATTGACGCCCACCAACCA	TCCGCACTTTGCACAACCCA	302
FMgSSR-21785	scaffold1797	p2	(CT)6	Class II	68886	68897	TCCACGCTCCCGAATGGTTT	ACGTCTCTCGAGCGCCTTCTTT	350
FMgSSR-21791	scaffold1185	p2	(CT)6	Class II	18615	18626	TCCATCTCCAACAAGGCGCAGA	TTGGGCTGACACTCCTGCAAGT	312
FMgSSR-21795	scaffold17930	p2	(CT)6	Class II	14773	14784	TCCCAGAGCCATTGTAGAGGCA	TAAATGCCTCCGTTCGCCACCA	335
FMgSSR-21798	scaffold15190	p2	(CT)6	Class II	7484	7495	TCCCCCACATGGCTCTTTCTTGT	TCGGCCGAGCAGAATGGTGAAA	247
FMgSSR-21806	scaffold2526	p2	(CT)6	Class II	65451	65462	TCCCGTGTTCGTTTGTGGCA	TGCTGACGAGCCAAGCACAA	298
FMgSSR-21824	scaffold1335	p2	(CT)6	Class II	35261	35272	TCCGCTGCGCATGCAAGAAA	ACACCTTAGTCGTTCCCACGCA	340
FMgSSR-21829	scaffold8656	p2	(CT)6	Class II	11796	11807	TCCGTTCGCTTCATCACGTTGGA	TCGGCAGGCGGAGAGAAAGATT	341
FMgSSR-21831	scaffold34606	p2	(CT)6	Class II	573	584	TCCTACCACGTGAAGCCTTGAT	CATCCCATGATCTCAGTTCCCACA	320
FMgSSR-21834	scaffold5871	p2	(CT)6	Class II	26152	26163	TCCTGCATTCGCCGATGTGACT	AGCTGAGGAGGCAACGCTAAGA	318
FMgSSR-21837	scaffold20037	p2	(CT)6	Class II	13702	13713	TCCTTCACTCCACTGACAGCCCTT	TCCGATCGCCCAAATCACCA	343
FMgSSR-21839	scaffold7644	p2	(CT)6	Class II	23771	23782	TCCTTTCGTCCTTTCCCCTGTGA	TGCCACGTTGTTGTGTAGGGGA	309
FMgSSR-21847	scaffold11899	p2	(CT)6	Class II	11745	11756	TCGAGTTTCCTTGCACGGGT	ACCGAATCTCCTCTACTGCTCG	341
FMgSSR-21855	scaffold3771	p2	(CT)6	Class II	32895	32906	TCGCGAAGTGACAAACCGCA	TGATGTTGGCTCAGGGCTTGCT	279
FMgSSR-21859	scaffold250	p2	(CT)6	Class II	87265	87276	TCGCTACCGAGCAATTTCCCCA	TGTGCGCCTTTCATGCACACAC	347
FMgSSR-21860	scaffold36611	p2	(CT)6	Class II	2452	2463	TCGCTCTTCGTCAAGCACGTCA	TTTGCAGGCGACGACATCCA	339
FMgSSR-21872	scaffold32589	p2	(CT)6	Class II	2927	2938	TCGGCCAACACATCAGGATCA	AGGCGGTGAGCAACATGGAT	276
FMgSSR-21880	scaffold3774	p2	(CT)6	Class II	35637	35648	TCGGCTGGAATTCGTGAGCGTA	ACAATGCTCATGTGGCGGGT	278
FMgSSR-21887	scaffold2157	p2	(CT)6	Class II	54512	54523	TCGGTCACCGCCTGCATTTT	TGGCAACACCGTCTCGCTGATT	303
FMgSSR-21894	scaffold2603	p2	(CT)6	Class II	41625	41636	TCGTAGCGGGTTGCCGAGAAAA	TTACACAATGGCGAGCGCACAG	279

SSR_ID	Scaffold	SSR_Type	SSR_Motif	SSR Class	SSR_Start	SSR_End	Forward sequence	Reverse sequence	Product_size
FMgSSR-21898	scaffold1592	p2	(CT)6	Class II	68204	68215	TCGTTCGCGGGAATGGTGAA	AATCGCGCACGGACAGAGGAAT	321
FMgSSR-21899	scaffold11644	p2	(CT)6	Class II	15036	15047	TCGTTGTGCACGCTGATGGA	TGGTGGGTGAGTGAACCTTGGA	348
FMgSSR-21900	scaffold709	p2	(CT)6	Class II	13612	13623	TCGTTTCGACGGCTTTCGGAGT	ACGGACGAACAGCTGCACAA	348
FMgSSR-21903	scaffold6338	p2	(CT)6	Class II	22113	22124	TCTATTTGCTGACGCCACCGT	AGGAGCGAAGAAAGGTCGAACCA	332
FMgSSR-21905	scaffold9196	p2	(CT)6	Class II	13921	13932	TCTCACACGCGTGCAGCATA	AGAGAAAGAGCAAGAGCGGGCA	288
FMgSSR-21907	scaffold22883	p2	(CT)6	Class II	4011	4022	TCTCCATGATCTTACCCCCGACA	TGAGGCCCACTGGAGAAGAAGA	322
FMgSSR-21911	scaffold12370	p2	(CT)6	Class II	3866	3877	TCTCCTGCAAGCGGCAACAA	CGGCTTTGGCTCGCTCAACAAA	280
FMgSSR-21920	scaffold29733	p2	(CT)6	Class II	2065	2076	TCTGGCTGCAGATTGAAGCACC	TTGCGGGCCTTTTGGGCTTT	208
FMgSSR-21922	scaffold10499	p2	(CT)6	Class II	18542	18553	TCTTCATGCCGTGGACTACGA	AACGCGCGTGTTGACCTGTT	293
FMgSSR-21926	scaffold18348	p2	(CT)6	Class II	17299	17310	TCTTTTTATCCCCGTCCGCCCA	GCCGCACTTAGCTTTTCCTTCGT	284
FMgSSR-21928	scaffold4333	p2	(CT)6	Class II	1535	1546	TGAAAGGAGCATTAGCGCCGGT	TGCGACTGCAACGAGCCGAATA	324
FMgSSR-21929	scaffold2010	p2	(CT)6	Class II	15142	15153	TGAACAGGAGGCGTCATGGA	TGGTGTTGTGCTTGGCCACT	350
FMgSSR-21931	scaffold66514	p2	(CT)6	Class II	581	592	TGAACCGGTGCTGATGGCAA	AGCAACGCTCATTGGGGAGA	276
FMgSSR-21934	scaffold15929	p2	(CT)6	Class II	11593	11604	TGAAGCCGGGAGTTTGTGTGT	ACAAAGGATGGTAGAGACGCGA	249
FMgSSR-21936	scaffold84	p2	(CT)6	Class II	6211	6222	TGAAGTCAGACTCCGGACTGACCA	AAGGCAAAGGCCACAGAGGCAA	323
FMgSSR-21952	scaffold213	p2	(CT)6	Class II	109846	109857	TGAGGTTGGGCCCGGGATTTTT	TACACTTGCTCTCAAGGCGGGT	336
FMgSSR-21953	scaffold17330	p2	(CT)6	Class II	14110	14121	TGAGGTTGTGGATCAGCGGCTT	AAAGCCTTGTCGGCAATGCACC	225
FMgSSR-21954	scaffold1031	p2	(CT)6	Class II	17394	17405	TGAGTGCCCCCGCAATCATCTT	TGGGGAGTGCTAACGCAATCGT	279
FMgSSR-21955	scaffold2271	p2	(CT)6	Class II	39926	39937	TGAGTGCGACGACGAACCAACT	GCTGCCGCGAGTTCTTTTCT	303
FMgSSR-21959	scaffold3821	p2	(CT)6	Class II	39762	39773	TGAGTTTCTACGCCGCCTCACA	TGCATGCTCGTCAAGGGGGAAA	220
FMgSSR-21970	scaffold56	p2	(CT)6	Class II	176697	176708	TGCAAGAGCCTGATGTTTGTGC	AGACCAGCATCCATGGCCAACA	329
FMgSSR-21971	scaffold3428	p2	(CT)6	Class II	29420	29431	TGCAAGCGGATTTCGAGCAAGC	GGGCACGGATATTTGCCTGGAA	346
FMgSSR-21975	scaffold10815	p2	(CT)6	Class II	14731	14742	TGCACATGAAGGCATGAAGCGG	ACCGCCTCCTTGACGCAAAT	244
FMgSSR-21984	scaffold1976	p2	(CT)6	Class II	65501	65512	TGCAGGTCATGCAGGAGGAAA	TGATGCTACTCCGTGCGTGTCA	225
FMgSSR-21985	scaffold363	p2	(CT)6	Class II	79835	79846	TGCAGGTCCCAATTGTTGCGT	TCAGCTACTTTCATGGCGCACG	290
FMgSSR-22006	scaffold8462	p2	(CT)6	Class II	6151	6162	TGCCACGAACATCCGAGTCAA	TGGAGTGGGGACAAAAGCCA	338
FMgSSR-22009	scaffold11573	p2	(CT)6	Class II	14836	14847	TGCCATCCCTATGGATACTGCTGC	TGTGAAGCTGCCGAGAGGAAGT	348
FMgSSR-22015	scaffold130039	p2	(CT)6	Class II	353	364	TGCCCCAACCACCAAAATCCGA	ATGCCAGCACCCTGCTTCTCAA	276

SSR_ID	Scaffold	SSR_Type	SSR_Motif	SSR Class	SSR_Start	SSR_End	Forward sequence	Reverse sequence	Product_size
FMgSSR-22017	scaffold866	p2	(CT)6	Class II	39874	39885	TGCCGACGTGGCAAAGAACA	TGTTGGCCTTCATGTCTCGCGT	277
FMgSSR-22018	scaffold1077	p2	(CT)6	Class II	97849	97860	TGCCGCAATTTCGTTGCCGA	AGCATGACCTGCATCGAGGA	350
FMgSSR-22019	scaffold430	p2	(CT)6	Class II	120078	120089	TGCCGCCATCTCTCACATCTCT	ATTTGTGCCTCCCCGATACGCA	315
FMgSSR-22020	scaffold58475	p2	(CT)6	Class II	1080	1091	TGCCGTCACCGCCACAAAAA	CCATGGTTGCAATGCTGCGGTT	200
FMgSSR-22021	scaffold1005	p2	(CT)6	Class II	89207	89218	TGCCGTGGGCATGCACATTA	ACAGACAGGGGCGTTTAGTGCT	335
FMgSSR-22023	scaffold327	p2	(CT)6	Class II	16434	16445	TGCCTCATTGGACTGAAGCGT	ACGGGGCCAAAACGATTGGT	321
FMgSSR-22025	scaffold2219	p2	(CT)6	Class II	17418	17429	TGCCTTCTCCGCCATCCATGTT	TGCCCAACCAATTTCAAGCGCA	263
FMgSSR-22033	scaffold1752	p2	(CT)6	Class II	28339	28350	TGCGGCCATGTATCCAGGGTTT	ATCTCTCCACCCATGTGCCTGT	240
FMgSSR-22035	scaffold6616	p2	(CT)6	Class II	16219	16230	TGCGGTCTTTGCCTTTGCGT	CCGTCTCACCACGCGTCAAAAA	271
FMgSSR-22039	scaffold10939	p2	(CT)6	Class II	19975	19986	TGCGTTGTGACAGCCAATCTCC	TTGTCGTGCCGACTCGAAGCAT	303
FMgSSR-22045	scaffold14670	p2	(CT)6	Class II	5511	5522	TGCTCGTTGCTGGATGGCAA	AAGTGCCAAGCCCACGACAGTT	289
FMgSSR-22046	scaffold2132	p2	(CT)6	Class II	46863	46874	TGCTGAACCACGGATCCGAAA	ATCGTGGCCATCGAAAGAGGGA	252
FMgSSR-22048	scaffold6330	p2	(CT)6	Class II	17135	17146	TGCTGCTTTGTGGGGATCTGT	TCAACGGCTCACTCTGCACT	275
FMgSSR-22053	scaffold12674	p2	(CT)6	Class II	1051	1062	TGCTTTCCCGTCGTTCACGGAT	AGAGGGAGCAGCTTTGTCTCA	328
FMgSSR-22055	scaffold4571	p2	(CT)6	Class II	20783	20794	TGCTTTGCGATGCCGCTACT	AGGCGTCTCGCCCTTTATGA	296
FMgSSR-22056	scaffold302	p2	(CT)6	Class II	47337	47348	TGCTTTTTCAGCGCGGCTCCTA	AAAACGTGGCACACACCCCA	326
FMgSSR-22059	scaffold19585	p2	(CT)6	Class II	8517	8528	TGGAAGCACGTCCATGTTACGCT	AGGGCGAAGCGGTTGCTTACTT	283
FMgSSR-22065	scaffold31235	p2	(CT)6	Class II	888	899	TGGAGAGGACGATGAATCGGGGAA	ATTGGAGGGTGCAGACCGGAAA	200
FMgSSR-22077	scaffold4690	p2	(CT)6	Class II	26519	26530	TGGCACAACCACACGAAGGCAT	AACAAAGACGCGTACCACGAGC	316
FMgSSR-22091	scaffold6692	p2	(CT)6	Class II	24113	24124	TGGCCGGGTTAGCAGTTGAGAA	TGCCCAAGTCTCGGTTATGAGACC	295
FMgSSR-22108	scaffold2924	p2	(CT)6	Class II	43872	43883	TGGGGATGTCAACAAGGTTTCG	TCGCCAGGGATGAAAGAGAGAGT	308
FMgSSR-22109	scaffold787	p2	(CT)6	Class II	12603	12614	TGGGGTGCTCTGCTCTGTTGTT	TGGCAGAAGCAAAGCGAGACGA	272
FMgSSR-22111	scaffold87854	p2	(CT)6	Class II	52	63	TGGGTGATGTTGCCGCTCAA	TTCCGCTTTCCGTGACGTCCAA	286
FMgSSR-22123	scaffold2540	p2	(CT)6	Class II	60527	60538	TGGTGTATGCGGTGTGCCATGA	ACGCCCTCCAAAGGTTGCGAAT	309
FMgSSR-22133	scaffold9596	p2	(CT)6	Class II	23747	23758	TGGTTTGACAAGTGACGGGGT	ATGGCGCCTCAGACTTAGCCTT	347
FMgSSR-22149	scaffold2136	p2	(CT)6	Class II	16148	16159	TGTCGCTCACAACGTTGAGCCT	GGCAAATGTTTTCCGCACCACCT	239
FMgSSR-22150	scaffold226	p2	(CT)6	Class II	142690	142701	TGTCGGTCGCGAATCCTCTCTT	TTCTGAACTTTCGCGTTGCCGC	236
FMgSSR-22161	scaffold9444	p2	(CT)6	Class II	20946	20957	TGTGGTGCACGCACGGAAAT	TTTACGGAAGAGACCCACACACGC	262

SSR_ID	Scaffold	SSR_Type	SSR_Motif	SSR Class	SSR_Start	SSR_End	Forward sequence	Reverse sequence	Product_size
FMgSSR-22165	scaffold49	p2	(CT)6	Class II	106613	106624	TGTGTGTGTGTGTGGAAGGGA	GTGAGTTTCTAGGAGGTTGGGACA	327
FMgSSR-22169	scaffold64	p2	(CT)6	Class II	191068	191079	TGTTGCTCTGGGCAAGTGGAGA	ACCGTGTAGCACCATCACTGGA	336
FMgSSR-22170	scaffold113706	p2	(CT)6	Class II	405	416	TGTTGGGCTGGGCATCATCCAT	AAGCTCATCAGCGCATGCCA	317
FMgSSR-22179	scaffold388	p2	(CT)6	Class II	53018	53029	TTATAACGCGCCCGCTTGGACT	TTGCCGGACAACAGCGAAGACT	271
FMgSSR-22186	scaffold21905	p2	(CT)6	Class II	11869	11880	TTCACGTGCCGTCGGTTTCA	ACGGCATCGAAGCTTTGGCA	294
FMgSSR-22194	scaffold15767	p2	(CT)6	Class II	11861	11872	TTCCAGTTTCGGCGACCACTCA	AGGCGCACCATGATTGGATGA	331
FMgSSR-22196	scaffold2191	p2	(CT)6	Class II	20751	20762	TTCCCCACATGTTGCCCATCCT	AAAGAGGACAAGGCACCGCCAA	334
FMgSSR-22200	scaffold159	p2	(CT)6	Class II	24690	24701	TTCCGGCAATGTGTCGTTTGCC	AAGAGGCCTTTCCCAGGCATCA	338
FMgSSR-22206	scaffold2252	p2	(CT)6	Class II	58511	58522	TTCGCCCAACGCATCTCTCA	AGCTAGAACGAGCCGGATGCAA	208
FMgSSR-22209	scaffold13613	p2	(CT)6	Class II	15787	15798	TTCGTCGTCTCGTCTGCCAA	AAGTACCAGCACCACCAAGCCA	341
FMgSSR-22220	scaffold12078	p2	(CT)6	Class II	14903	14914	TTCTTCAACCGCTCTGCTCTGC	AAGGCTTTAGACGCCGATGGCA	224
FMgSSR-22226	scaffold315	p2	(CT)6	Class II	102354	102365	TTGACCTTGTTGCCGTCCTCGT	TGTGTTCGCATCAACCGCCA	232
FMgSSR-22227	scaffold834	p2	(CT)6	Class II	94094	94105	TTGACGGCCAAAATGAGTGGGC	AATGCTTGCGCTCGGGAGTT	304
FMgSSR-22228	scaffold4054	p2	(CT)6	Class II	42734	42745	TTGACGGGAGAAACCACACCT	ATGCTGCGGATCTCGAGAAGGA	200
FMgSSR-22231	scaffold21377	p2	(CT)6	Class II	5206	5217	TTGAGCGAGCAGAGCATCCA	AGCCTTTTGCCGCTGCATGT	314
FMgSSR-22232	scaffold34896	p2	(CT)6	Class II	6481	6492	TTGATACATGGCTGGACCGCCT	GCTGGAAGCGAAACAACAGGCA	272
FMgSSR-22237	scaffold13701	p2	(CT)6	Class II	383	394	TTGCCATCAGCACCCACACAGT	TGTGGTGTTGTATGAGCCCCCA	320
FMgSSR-22243	scaffold10504	p2	(CT)6	Class II	26716	26727	TTGCTGCGCGCAAAGTACGA	AGCTCCTGCGCACTTCAGAGTA	328
FMgSSR-22245	scaffold24080	p2	(CT)6	Class II	6846	6857	TTGCTGTTCGGTGGCTACTCCT	TGCTAGGCACGGCAATAACAACG	340
FMgSSR-22247	scaffold5249	p2	(CT)6	Class II	30366	30377	TTGGAAGCCCAGCGGAATAGCA	TATCACAGCTGCTCGGAGGCTT	347
FMgSSR-22249	scaffold3463	p2	(CT)6	Class II	45161	45172	TTGGAGGCCGTCCTTGCATT	TGCGGTCAAAACGACTGCGA	313
FMgSSR-22257	scaffold59	p2	(CT)6	Class II	183098	183109	TTGGCGAGTATGCGCTCGGATT	TGACATGCCACCCGAGAGAACAA	290
FMgSSR-22260	scaffold329	p2	(CT)6	Class II	45874	45885	TTGGCGTTGAAACACGGGCA	TTCAGTCTTTCGGAGCAGCGCA	270
FMgSSR-22263	scaffold1450	p2	(CT)6	Class II	54760	54771	TTGGGCCACGTTACGAACCA	TTTCCCACGAAGCGAAGCGA	347
FMgSSR-22264	scaffold6276	p2	(CT)6	Class II	9660	9671	TTGGGCCAGATGCGATGTTGCT	TTCGTGCCAGCAAGAAGCGA	321
FMgSSR-22269	scaffold27578	p2	(CT)6	Class II	6304	6315	TTGGTGAAACCGCGTGAGAAGC	TGTGGGTCAAGCCGAACAACCA	326
FMgSSR-22270	scaffold31880	p2	(CT)6	Class II	3629	3640	TTGGTGTATGACAGGCGTCCGA	CACTCCAGCGCGTTGCAAAA	304
FMgSSR-22272	scaffold1436	p2	(CT)6	Class II	32534	32545	TTGTCCCCCTGCCACCATTCAA	CGACGGCCTCAAAAGCCCAAAA	350

SSR_ID	Scaffold	SSR_Type	SSR_Motif	SSR Class	SSR_Start	SSR_End	Forward sequence	Reverse sequence	Product_size
FMgSSR-22277	scaffold6271	p2	(CT)6	Class II	19026	19037	TTGTTAGAACAGGAGCCGAGCG	AGTGTCGTGTTTGCCCCGAA	278
FMgSSR-22281	scaffold558	p2	(CT)6	Class II	115645	115656	TTGTTTTTGCGAGCCGTGCG	ACGGCACCGCGCAGATATGATT	281
FMgSSR-22285	scaffold36695	p2	(CT)6	Class II	1987	1998	TTTCAACCTCCCAACGCCCA	AGCTGGAGCTTTAGGGGATAGGGT	206
FMgSSR-22288	scaffold660	p2	(CT)6	Class II	89980	89991	TTTCCAAGTCCACGGCACAGCA	TTGTGATCACGCGCCGACAT	236
FMgSSR-22298	scaffold12725	p2	(CT)6	Class II	1385	1396	TTTCGGTTTGCACGGCTCCT	ACGGTGCAAGCTTGTCGCTA	303
FMgSSR-22306	scaffold1514	p2	(CT)6	Class II	12495	12506	TTTGAGGCCCGTTTAGCGCA	ACCACAACCGTGGTAGCTCT	213
FMgSSR-22319	scaffold20521	p2	(CT)6	Class II	3411	3422	TTTTACCAGCCGGTCCACCT	AGCTGACCTGTCAGTTTGCGGT	342
FMgSSR-22321	scaffold42	p2	(CT)6	Class II	191782	191793	TTTTCCCATCACCCACCAGCCA	AGCATCGAGCTTGAAACGGGCA	314
FMgSSR-22327	scaffold599	p2	(CT)6	Class II	49649	49660	TTTTGGGCGCTGGTCCCTTT	TTGACGCGCGCCCATCCTAAAA	310
FMgSSR-22331	scaffold1626	p2	(CT)6	Class II	19699	19710	TTTTTGGGCCGGCATTGGTTCG	TACACGTGCGCTGAAGAGGT	231
FMgSSR-22343	scaffold4648	p2	(CT)7	Class II	12758	12771	AAACACACTCCACTCCTCCCCT	AGAAGGTTTGGTGCCCGTTG	321
FMgSSR-22346	scaffold1406	p2	(CT)7	Class II	45190	45203	AAACCCTCGCTTCGCTCGTT	TCGTAGTTACGCTGCGGGTGTT	201
FMgSSR-22348	scaffold7382	p2	(CT)7	Class II	9913	9926	AAACTACTGGCCCAGCTGCGAA	TGCGAGGATTGGGGGAAAGCAA	271
FMgSSR-22349	scaffold5460	p2	(CT)7	Class II	28738	28751	AAACTGACCACCGGAAAGCGGT	TTGCTGAGCTCGGCGAAGAACA	278
FMgSSR-22350	scaffold1168	p2	(CT)7	Class II	71844	71857	AAAGAACGGAACAGGCGGCA	AGCCACTTGCCTGTGGAAGA	286
FMgSSR-22355	scaffold4399	p2	(CT)7	Class II	39165	39178	AAAGGAGGAGAAAGCGGTGGGA	AGTGACGTCAGATGCGTGCT	330
FMgSSR-22356	scaffold908	p2	(CT)7	Class II	103263	103276	AAAGGCGGGGCGGGTTTAAT	ATAGCGGGGGCGCTTTTGTT	292
FMgSSR-22361	scaffold1067	p2	(CT)7	Class II	62014	62027	AAATCCGGTGCCCCTCTGTCAT	AGCCACTAGCTCGTTCGCAACA	292
FMgSSR-22362	scaffold6124	p2	(CT)7	Class II	14107	14120	AAATGCACAGACAGCAGCTCGG	TGCAACTGCCACACATGACAGC	344
FMgSSR-22363	scaffold15987	p2	(CT)7	Class II	9925	9938	AACAACACTGACCGCCGCAT	TCATTGGAAAGCGAGAGCGACA	207
FMgSSR-22364	scaffold1748	p2	(CT)7	Class II	25073	25086	AACAACGCTCGCGAACCACA	ATGATTGCTGACCTGGCGGT	278
FMgSSR-22365	scaffold213	p2	(CT)7	Class II	36214	36227	AACACACCCGGTTTCGACCA	AAGGCGAGGAAGACAAGCGTGA	260
FMgSSR-22370	scaffold6708	p2	(CT)7	Class II	25959	25972	AACAGTTCAGGGCACGGTGGAA	GGATCGAAATAGCATCAGCATCGC	325
FMgSSR-22372	scaffold1307	p2	(CT)7	Class II	24591	24604	AACCACGCGACCCACCTTTT	TGTTTGCGGAGGATGGAGGTGA	341
FMgSSR-22379	scaffold850	p2	(CT)7	Class II	88345	88358	AACCGCACATCACGCTACCT	TGTGCACTGCATTCGGGAGAGA	307
FMgSSR-22397	scaffold858	p2	(CT)7	Class II	5875	5888	AACTGACCAAGTCGAGCGACGA	TCCAAGAGCACGTCGTGTGGAA	311
FMgSSR-22398	scaffold1066	p2	(CT)7	Class II	77061	77074	AACTGATGACTGGAGGGCATGG	TGGGGAAGAGGCAACGTGCATT	340
FMgSSR-22399	scaffold1583	p2	(CT)7	Class II	62476	62489	AACTGCAGGCATGGCCTAGT	AACGGGCAGCAGTAGCAAGT	344

SSR_ID	Scaffold	SSR_Type	SSR_Motif	SSR Class	SSR_Start	SSR_End	Forward sequence	Reverse sequence	Product_size
FMgSSR-22402	scaffold867	p2	(CT)7	Class II	60752	60765	AAGAAACTATGGGGCTGCGCGA	GCACGCCAGCTTAGCTAGTACCAT	317
FMgSSR-22404	scaffold24115	p2	(CT)7	Class II	924	937	AAGAAGGCGTCAAGGATCACCG	TTGACTTTGGCGACGCGGTT	325
FMgSSR-22407	scaffold1767	p2	(CT)7	Class II	4581	4594	AAGAGCGAGCCGTGGTTCACAT	ACGGCGTGCATTGTCGCTTT	312
FMgSSR-22409	scaffold1797	p2	(CT)7	Class II	5167	5180	AAGATGCGCGACCAAACAGC	TTGCCAGACGCTGCAAACGA	332
FMgSSR-22413	scaffold287	p2	(CT)7	Class II	63933	63946	AAGCAAGTCCTTGTGTGCGGGA	TGTGCAGTGTTAACGGCCAGACA	270
FMgSSR-22414	scaffold2320	p2	(CT)7	Class II	16298	16311	AAGCACAGGCCATCTGCCACTA	TAGTGGTGGTGGTGTGGTCGAA	329
FMgSSR-22418	scaffold3162	p2	(CT)7	Class II	31997	32010	AAGCCTGCTGCGTTTTGGCT	AAGATCGAGAGGGCGTGTCTGT	314
FMgSSR-22427	scaffold231	p2	(CT)7	Class II	10013	10026	AAGGCCATTCACGCCCCTTCTT	GTGCGTCTCCCAATCGCTTTCT	272
FMgSSR-22428	scaffold10559	p2	(CT)7	Class II	14891	14904	AAGGCCCAGCGCTTATCTCGAA	TTTTCGGATCCGTCGTCGCTGT	251
FMgSSR-22431	scaffold1106	p2	(CT)7	Class II	89378	89391	AAGGGGCGTAACCAGGATTGCT	TTGATGGGAGGCTTGCGTGCAT	319
FMgSSR-22434	scaffold18785	p2	(CT)7	Class II	14142	14155	AAGTCCGTTTGTACGGCCTCGT	TGATCACCACGTCTCCTGCACA	215
FMgSSR-22440	scaffold2660	p2	(CT)7	Class II	25687	25700	AATCGAGCGCGGTACAATCCGT	AAGCGTCTGGAGCAGTCTGCAA	293
FMgSSR-22442	scaffold9311	p2	(CT)7	Class II	12891	12904	AATCGGACTTGCTGCAGCGTCA	TTGTGCAAACGACAACTGGGCG	200
FMgSSR-22445	scaffold191	p2	(CT)7	Class II	128622	128635	AATGCGGCCACTGTTGCTCT	TGCACACCGCAACAGCACAA	281
FMgSSR-22452	scaffold9436	p2	(CT)7	Class II	187	200	ACAAAGCCACGCACGCAGAT	ACGCCATTGCACACTGCAGAAC	297
FMgSSR-22455	scaffold10335	p2	(CT)7	Class II	10670	10683	ACAACGACGCATGTTCCCCT	ATTCATCAACGCGATGGGGGAG	233
FMgSSR-22456	scaffold2025	p2	(CT)7	Class II	47354	47367	ACAACGCCGTGCTTAGGAAGGT	TGCCCGTGATGAAGAGCAGT	240
FMgSSR-22459	scaffold2888	p2	(CT)7	Class II	7275	7288	ACACCAAAGCGCGAATCCCA	AACTGTACGCTGCATCGGTCCT	327
FMgSSR-22462	scaffold45559	p2	(CT)7	Class II	4750	4763	ACACCGAAACCGGGGTCAACTT	AGCCGCTGGTTGCCTTTGAA	279
FMgSSR-22463	scaffold451	p2	(CT)7	Class II	119758	119771	ACACCGCAAGCCTATGCGAA	ACTTGCACTCCTCCGCTCAA	248
FMgSSR-22471	scaffold3779	p2	(CT)7	Class II	44502	44515	ACACGTCGCGGAATACGACA	TGGTTGTTGGTCTGGTCGCCAT	336
FMgSSR-22472	scaffold391	p2	(CT)7	Class II	98091	98104	ACACTGCTGCCGCATTCACT	TTTCTCGCTGCGTCCACTGTGA	244
FMgSSR-22476	scaffold4316	p2	(CT)7	Class II	45152	45165	ACAGCGAGTGCTGTCATTTCGC	ACTCGATGCTCATGGCTTCCCT	294
FMgSSR-22483	scaffold3747	p2	(CT)7	Class II	55317	55330	ACATGCCAACCAAGGAGGGCTA	TCACGCCATAGTTTGGAGCCGT	256
FMgSSR-22487	scaffold3436	p2	(CT)7	Class II	42838	42851	ACCAACCAACGTCATCTTCCATGC	ACGCGCATCGGCAATTGGAT	263
FMgSSR-22488	scaffold6755	p2	(CT)7	Class II	20082	20095	ACCACGCGCTACAACAGTGA	ACAGCCGTGGACTTGGATTGGA	237
FMgSSR-22495	scaffold1147	p2	(CT)7	Class II	15256	15269	ACCCACATAACCACCACGGCAT	ATTGTTCTCCCCAACGCACGCT	261
FMgSSR-22500	scaffold51151	p2	(CT)7	Class II	3265	3278	ACCCGGGCAGCTCAGTTTCATT	TGAGGAGCCAGCAGGTTTAGCA	325

SSR_ID	Scaffold	SSR_Type	SSR_Motif	SSR Class	SSR_Start	SSR_End	Forward sequence	Reverse sequence	Product_size
FMgSSR-22502	scaffold7444	p2	(CT)7	Class II	11438	11451	ACCCGTGGCACTTCTTGACA	TTGGCCGTGGTGTGAGTCCATT	309
FMgSSR-22508	scaffold40444	p2	(CT)7	Class II	3415	3428	ACCCTTTGCCAAGTGCCACTAACA	TGATGGTAGCAATGTGGGTGCG	329
FMgSSR-22512	scaffold753	p2	(CT)7	Class II	60800	60813	ACCGACGTGGTGAGCTGTTCTT	GCGCAGAAATCAAGCGACCACT	205
FMgSSR-22521	scaffold3121	p2	(CT)7	Class II	28058	28071	ACCTGATCAAATCGGTGGGCGT	TTGCCAAATGGACAGCGCCA	225
FMgSSR-22523	scaffold1406	p2	(CT)7	Class II	68994	69007	ACCTGCTCGCTTCTCCTCCATT	TGGACGGGATGTGATTAGGCAAGG	347
FMgSSR-22528	scaffold1920	p2	(CT)7	Class II	50941	50954	ACGAATGCCACAATGGACCTGACA	TGGTGACGCTCCTTCGATGA	336
FMgSSR-22533	scaffold8830	p2	(CT)7	Class II	5694	5707	ACGAGGCGCCGTTACCAGAAAA	ACAAACCTGGATCGGCGGAA	281
FMgSSR-22536	scaffold15	p2	(CT)7	Class II	19157	19170	ACGCAGATCCTCTCGCACTACA	AAAGCCAGCAGCATAGGGGT	295
FMgSSR-22539	scaffold3692	p2	(CT)7	Class II	39586	39599	ACGCCGCTGCAGCTAGTTTT	AGCTCCGAATGGACGCACAA	328
FMgSSR-22541	scaffold3626	p2	(CT)7	Class II	21571	21584	ACGCGTGAGCTTTCTGGTAAAGAG	AAAGAACCTCCCTGCCGCTTCA	320
FMgSSR-22545	scaffold12282	p2	(CT)7	Class II	12961	12974	ACGGAGCGACGGAGTAGGTTTA	TCCGTGGGCAAGAAACGACA	316
FMgSSR-22546	scaffold4338	p2	(CT)7	Class II	42169	42182	ACGGATATGCGTGCAGGAGCTA	TGAGCAACTTGGAGATGCGGTG	207
FMgSSR-22554	scaffold13265	p2	(CT)7	Class II	14500	14513	ACGTCGCGGTAAAGCAGCAA	TTTTTCACGCACGCACGCAC	286
FMgSSR-22556	scaffold1265	p2	(CT)7	Class II	44800	44813	ACGTGTACCGAGGGAAACCA	TCGGACGCTCGGTGAAGTGAAA	258
FMgSSR-22559	scaffold5398	p2	(CT)7	Class II	31985	31998	ACTCAGCACGCACACTTGCAT	ACCATTTCAAAACCCGGCCCA	222
FMgSSR-22561	scaffold713	p2	(CT)7	Class II	52621	52634	ACTCGGAAGCGAAGCGAGTT	TAAGCAAAGCAGCACGAGGCGA	307
FMgSSR-22565	scaffold15352	p2	(CT)7	Class II	14609	14622	ACTGCAAGGACACAAGGCGA	TTCACGCCTGGCTCAAAGCA	228
FMgSSR-22569	scaffold1532	p2	(CT)7	Class II	4475	4488	ACTGCTTGGAAACGCAGGCT	AACATGGATGAGGCAAGCGGGT	250
FMgSSR-22571	scaffold2935	p2	(CT)7	Class II	48830	48843	ACTGGTCCTGTTCACCAGCGTT	TCGTTAGCACTCGCCAGCAGTT	205
FMgSSR-22574	scaffold11566	p2	(CT)7	Class II	865	878	ACTTCCTCTCGGCAGCTTCACA	TGGAGGCAAGGACAACTGGA	336
FMgSSR-22576	scaffold1540	p2	(CT)7	Class II	11996	12009	ACTTGACGTCACGCCCCTTTCA	AGAGGGGGAGACGAAGTGACAA	324
FMgSSR-22589	scaffold3212	p2	(CT)7	Class II	33697	33710	AGCAAAGAGGCGCACACAGT	CCGCGCGTTCGTTTCTTGTGAT	305
FMgSSR-22591	scaffold1869	p2	(CT)7	Class II	10110	10123	AGCAAAGTAGACAGCCCATGCGA	TGATGAGCTCCCGCTTGCACTT	328
FMgSSR-22594	scaffold2181	p2	(CT)7	Class II	23649	23662	AGCAAGGCCATGCCATGTCTA	TCCTCAGCACCACATGCACA	264
FMgSSR-22597	scaffold5469	p2	(CT)7	Class II	17849	17862	AGCACACCGGTTTTTCGGCA	AACGCACCAACGGACCTGACAA	303
FMgSSR-22598	scaffold1684	p2	(CT)7	Class II	18768	18781	AGCAGCACGGGAAAAGTATGGCT	ACGTACGGGAGGGAACCAATGT	243
FMgSSR-22599	scaffold356	p2	(CT)7	Class II	124302	124315	AGCAGGACGCATAGCTTGAGCA	TAGGCGACGCGAAACGGAAA	299
FMgSSR-22600	scaffold98	p2	(CT)7	Class II	150155	150168	AGCATGCAGTCGGGCATCAT	ACATCGCCATGGACGCCATCTT	262

SSR_ID	Scaffold	SSR_Type	SSR_Motif	SSR Class	SSR_Start	SSR_End	Forward sequence	Reverse sequence	Product_size
FMgSSR-22604	scaffold50471	p2	(CT)7	Class II	1744	1757	AGCCAATCCACACCCACAACT	AAGGGCGTCAATGCCCAAGACA	339
FMgSSR-22623	scaffold4722	p2	(CT)7	Class II	26560	26573	AGCCTTCGGCCTAGCCAAACAA	AGAGTTGCTCGGGAGAGAGAGT	244
FMgSSR-22624	scaffold2367	p2	(CT)7	Class II	61189	61202	AGCGAAGTTCAGCAGCGCAA	TTCAGCCCACGGCGTCAAAA	322
FMgSSR-22626	scaffold9648	p2	(CT)7	Class II	13475	13488	AGCGACCATCCAACCAGGCATA	AAGCCCACCGTCAACTTTGCAG	350
FMgSSR-22629	scaffold8916	p2	(CT)7	Class II	5125	5138	AGCGCACCTTCTATCCACGTCA	ACGCTGCTTTCGATCCCAGGTA	331
FMgSSR-22631	scaffold6271	p2	(CT)7	Class II	26082	26095	AGCGCCACCAAGGTGATGATGA	ACGGCGTGTGTTTTTCCCGT	346
FMgSSR-22633	scaffold3828	p2	(CT)7	Class II	46903	46916	AGCGGAGGCCAAATTCCTGA	TCGCTCGCAAAACCAACAGC	203
FMgSSR-22638	scaffold4575	p2	(CT)7	Class II	16878	16891	AGCGTATGCATTGCACGGGA	AGAAGCCGCCGCATACACAT	326
FMgSSR-22640	scaffold2565	p2	(CT)7	Class II	35264	35277	AGCGTTGCATTCGCTGACAT	ATCGGCAACGGTGTTTGGCT	280
FMgSSR-22641	scaffold3575	p2	(CT)7	Class II	20301	20314	AGCTATGAGCTCTGGCCTACTGGA	ACGGCTGCCGACATCTTCAA	275
FMgSSR-22642	scaffold5709	p2	(CT)7	Class II	19177	19190	AGCTCACCCCTTTCCTCTGGAT	TTGTCATGGATCCAGTTCGCCG	303
FMgSSR-22643	scaffold2058	p2	(CT)7	Class II	28425	28438	AGCTCCATTGACCGTACCCGAA	ACGGGTTGGCAATGAGGCAA	218
FMgSSR-22646	scaffold117	p2	(CT)7	Class II	58338	58351	AGCTGCATCTTAGGGTTCCTTGCT	AGGGCCTTATCGACCGCTAACA	229
FMgSSR-22648	scaffold3026	p2	(CT)7	Class II	6607	6620	AGCTGCGTGCAAAGAAGCAGT	GCGGCTGGCTTGTGCTTTTCTT	265
FMgSSR-22649	scaffold1579	p2	(CT)7	Class II	17299	17312	AGCTGGTCCCACCATCATCACT	ACGGTCGTTTGCCCCCATTT	232
FMgSSR-22652	scaffold363	p2	(CT)7	Class II	71593	71606	AGCTTAGCGTGCGTGGGAAA	ACTGGCTCGATGGGCTTCATCA	235
FMgSSR-22655	scaffold11845	p2	(CT)7	Class II	7011	7024	AGGAACATGGGGATTGAGGCGT	ACCCGCTGTCCTCGAAGTTTGT	251
FMgSSR-22658	scaffold11236	p2	(CT)7	Class II	5357	5370	AGGAAGAAGCAGAAGGCGCAGT	ACACGTTCATTGGTCCCTTCTGGG	249
FMgSSR-22659	scaffold2183	p2	(CT)7	Class II	10509	10522	AGGACCCCAATTTGCCATCTGGA	CTCTACCATGTTCTCCCATGCACA	348
FMgSSR-22669	scaffold1230	p2	(CT)7	Class II	586	599	AGGCGCACGATCAATGCGAT	TGCGTGCAGAGAATTCCGCCAT	329
FMgSSR-22672	scaffold1087	p2	(CT)7	Class II	60842	60855	AGGCTAACATTCCCTGGCCACA	CGCATTCTGTTCAGGACGCCAA	288
FMgSSR-22675	scaffold872	p2	(CT)7	Class II	72291	72304	AGGCTTCTTCCATAGGGTGATGCT	AGCTCGCAGCAGATTACAGCAC	308
FMgSSR-22676	scaffold1106	p2	(CT)7	Class II	85134	85147	AGGGCAAAACACGCTGTACTCCT	ACCATGTGTGCTGTGGTCCAA	350
FMgSSR-22688	scaffold14143	p2	(CT)7	Class II	8762	8775	AGTCCCATACTGCAATCGCTCC	TTCAGCGGCACAGTGATTCTCG	276
FMgSSR-22690	scaffold4764	p2	(CT)7	Class II	50256	50269	AGTCGCATGTACCCAGTGCT	GCATCCAGGATTCCGCAGTAGTGA	303
FMgSSR-22691	scaffold23724	p2	(CT)7	Class II	3235	3248	AGTCGCTGACGTGGAAGCAA	TTTGCAGCCGTCTGGTTGGCTA	225
FMgSSR-22694	scaffold1007	p2	(CT)7	Class II	65471	65484	AGTGATGCACTCGGCGAACT	AAGCGAAAGCCTCGTTGCCT	248
FMgSSR-22698	scaffold192	p2	(CT)7	Class II	151901	151914	AGTTCTGGTTGGCCTCGTGCTT	ACTCGATGTTCTAGCCGCTGT	267

SSR_ID	Scaffold	SSR_Type	SSR_Motif	SSR Class	SSR_Start	SSR_End	Forward sequence	Reverse sequence	Product_size
FMgSSR-22700	scaffold11644	p2	(CT)7	Class II	20211	20224	AGTTGGAGGCAGCTTATGCCCT	ATTTCAAGCCACCCAAGCCGGT	301
FMgSSR-22704	scaffold1933	p2	(CT)7	Class II	17967	17980	ATAAATGTGGCGACGCAGCACG	TCGACGGGCATGTGTCTTGTGT	346
FMgSSR-22708	scaffold8540	p2	(CT)7	Class II	3966	3979	ATAGCTTGCGCTTGCTGCCT	CCCAGAAATCATTGCTCGCGGT	285
FMgSSR-22714	scaffold2	p2	(CT)7	Class II	55861	55874	ATCATCATCCGGGGCAAAGGCA	TGCACAACACCGCCTCTCATGT	254
FMgSSR-22716	scaffold9272	p2	(CT)7	Class II	13130	13143	ATCCGATCAGGATCACCAGCCT	CGCACAAGCATGTGTTTACCTGGG	296
FMgSSR-22725	scaffold395	p2	(CT)7	Class II	117029	117042	ATCTTCTCCCGCGTCTTCTCCA	ACGGCATCAGCATCACCACCTT	253
FMgSSR-22726	scaffold1315	p2	(CT)7	Class II	37172	37185	ATGAAACCCTGCAGGAGACCGT	ATGCAGCCATGGCACGTGAA	278
FMgSSR-22728	scaffold1601	p2	(CT)7	Class II	46780	46793	ATGAAGCAAGTGGGCGCCAT	ACCGCCGCATTCGAGATGAGTA	241
FMgSSR-22729	scaffold9241	p2	(CT)7	Class II	20252	20265	ATGACGACCTTGGTGCATCCCT	AATTAGCAGCGGCAACAGCG	290
FMgSSR-22730	scaffold3838	p2	(CT)7	Class II	18153	18166	ATGAGCAGGCCTTCACTAGCCA	ATGTCGCTGACGCTGTGGAT	302
FMgSSR-22735	scaffold7422	p2	(CT)7	Class II	35494	35507	ATGCATGCGCCCCGTCAAAA	TCGAATAGCGACGCGTGTGT	340
FMgSSR-22736	scaffold305	p2	(CT)7	Class II	4142	4155	ATGCCACCCAGCAATTGAGCCT	TGACACCATGCATCACCACCGT	222
FMgSSR-22740	scaffold7133	p2	(CT)7	Class II	28560	28573	ATGCCGCGTATGTGACTGTGGA	AGGTTGAGGGCGCTTACTCCAA	320
FMgSSR-22745	scaffold819	p2	(CT)7	Class II	21065	21078	ATGGCAATCCCCGTGTAGAGCA	GCAACTTGCATTGTCCCTGCCT	267
FMgSSR-22758	scaffold1608	p2	(CT)7	Class II	40058	40071	ATTAGCTCCTGCAGCGCGAAAC	AACGCACCCACATCCGTTCT	330
FMgSSR-22759	scaffold4453	p2	(CT)7	Class II	44878	44891	ATTCATCGCTTCCTCGCGTCCA	TGATTTCGTCGCCTAGATCCGC	232
FMgSSR-22769	scaffold541	p2	(CT)7	Class II	42284	42297	ATTTCTTCCACTCCGCGCGCTA	TGCCCAGTGTCTCCATGCCATA	279
FMgSSR-22771	scaffold2365	p2	(CT)7	Class II	51336	51349	ATTTGGCTCATGCCGCTGCT	CCATGTGGGGTTTTTGTGGGCA	251
FMgSSR-22776	scaffold36	p2	(CT)7	Class II	212569	212582	CACAAGGCCACTAGTATCACCACA	ATGCTGTTGGCCGAGTGCCATA	254
FMgSSR-22785	scaffold1935	p2	(CT)7	Class II	63796	63809	CCCGGTTCTCAGTACACCCAACAA	TGTGGCCATGAGAAACAGTGGA	349
FMgSSR-22786	scaffold124891	p2	(CT)7	Class II	28	41	CCCTCCTTCACTCCAAACTTTTCCC	TCAAAGACGACGGCCCAGAA	338
FMgSSR-22793	scaffold4163	p2	(CT)7	Class II	30414	30427	CCTGCAACTGCAAGACTCTTTCCT	ACGGGACGTGGCAAATGCATGA	297
FMgSSR-22794	scaffold2579	p2	(CT)7	Class II	1064	1077	CCTGCATGGCAATTGGAAGCGT	TTGCGCACCCATCGGTTTCT	326
FMgSSR-22795	scaffold10302	p2	(CT)7	Class II	8559	8572	CCTTATAAATTGGGGCCCGCCT	ATCGTCGTGGCGAAGTTGCAGA	301
FMgSSR-22796	scaffold15	p2	(CT)7	Class II	172631	172644	CGAAGCAATGGCAAGTCGCACA	TGAACCAAGCAAGGGCGCAT	338
FMgSSR-22797	scaffold4759	p2	(CT)7	Class II	15386	15399	CGAGGCCCATTAGTCCGACCATTA	AGGTGATGCGGCGAAATACGGA	332
FMgSSR-22800	scaffold12607	p2	(CT)7	Class II	4953	4966	CGCATGGTGTTCAATGCACAAG	AACTGCCTTTTGTGACCGGCCT	328
FMgSSR-22801	scaffold7242	p2	(CT)7	Class II	5717	5730	CGCGCATGCATTCTGACGTT	AGCACGAATCATGTGGGGCTGT	253

SSR_ID	Scaffold	SSR_Type	SSR_Motif	SSR Class	SSR_Start	SSR_End	Forward sequence	Reverse sequence	Product_size
FMgSSR-22803	scaffold806	p2	(CT)7	Class II	16416	16429	CGCGCGAAATTCGTCGTGAA	ATGGCTATGGCATGGCAGCA	267
FMgSSR-22806	scaffold1270	p2	(CT)7	Class II	63812	63825	CGCGTGTCACACCTGCGATTTT	AGCTCGAACAGGTGTGGTGTGT	292
FMgSSR-22809	scaffold3420	p2	(CT)7	Class II	42307	42320	CGCTGCCTTGACTTTGCGGTTT	TGGTGCTTTGCTGCTTGTGTGC	301
FMgSSR-22820	scaffold94140	p2	(CT)7	Class II	754	767	GCAAGCTGCTGCTACTGCAA	TAAGCGCAGGGGCAAAACCTCA	251
FMgSSR-22822	scaffold90	p2	(CT)7	Class II	128262	128275	GCATGCCGCACGTGCATAGTAA	AAAGCCCGTGAATCCGTCGT	335
FMgSSR-22830	scaffold2025	p2	(CT)7	Class II	17164	17177	GCCGTTGCCAAATCCAAGCCAA	TGCAGAATGGCAAAGGGTCCCA	241
FMgSSR-22833	scaffold5769	p2	(CT)7	Class II	3488	3501	GCCTTCGTTGGATCCCAAACACA	ATTGCCAGAACATCCGCCGT	202
FMgSSR-22836	scaffold8543	p2	(CT)7	Class II	14329	14342	GCGCACACGCGCCAATTTTT	AGAGCACGGATCCAAGCCTACA	298
FMgSSR-22841	scaffold59003	p2	(CT)7	Class II	2827	2840	GCGGATTTGCCGGCAGGTTTTT	TGGCTTCATGCGGGTTGAGAGA	243
FMgSSR-22846	scaffold14618	p2	(CT)7	Class II	14018	14031	GCTGAACCACGGGCCCAAAAAT	AAGCGTTGTACGTCTCCGCCAT	327
FMgSSR-22848	scaffold1621	p2	(CT)7	Class II	27334	27347	GCTTGCATTGCTTGCAGCGT	AGCGCATCTCGTGTACGCAT	350
FMgSSR-22851	scaffold187	p2	(CT)7	Class II	58422	58435	GGCCTTTTTCCCCTGCGCTTAT	ACGGGTCACGTCAGGCCTTTTT	319
FMgSSR-22853	scaffold2387	p2	(CT)7	Class II	30871	30884	GGCGCCAATTCTGTCACTGCTT	TCATTGCCGCCTGCCTGATT	303
FMgSSR-22855	scaffold26442	p2	(CT)7	Class II	9407	9420	GGCTACCTCTGCCCTAACGTCAAA	AAGTGGCATCGATAGCGGTGGT	341
FMgSSR-22861	scaffold782	p2	(CT)7	Class II	85251	85264	GTGCAAGTGCGGCCACTAGTAA	TGCGGTGAACAGTGGAAACGGT	213
FMgSSR-22862	scaffold79	p2	(CT)7	Class II	177801	177814	GTGCTTCGCGGCACCTGTTAAT	AGGCTGCAGCTTCCTTGTGAGA	287
FMgSSR-22872	scaffold330187	p2	(CT)7	Class II	84	97	TAAGCCAGCGCGGTTACTGT	TCGGTCCATGCTTGCTCACT	282
FMgSSR-22878	scaffold75114	p2	(CT)7	Class II	377	390	TAGAATTGGCTGCGCGGGGATT	TCAGAAAGCTCATGCGGCGT	215
FMgSSR-22900	scaffold2756	p2	(CT)7	Class II	32317	32330	TCACCAACCGCCACCTCTCTTT	ATGCACTGCACGACAATGTGCC	254
FMgSSR-22906	scaffold935	p2	(CT)7	Class II	21380	21393	TCACTCTTGCCTCATCGCCA	ACACAGCGTGGCTGATTTTGCC	257
FMgSSR-22915	scaffold7142	p2	(CT)7	Class II	26755	26768	TCCACACTAGCCGTTGTGACCT	ACCACCACCGCCCACATGATTT	272
FMgSSR-22917	scaffold1709	p2	(CT)7	Class II	7797	7810	TCCACCGCCTGCACATCATTGT	TGCCGCCACGACCATCAAAA	308
FMgSSR-22918	scaffold2777	p2	(CT)7	Class II	27014	27027	TCCACCTGCATTTCGTCTCTCT	TGATTTCGGCCACCGCATCT	337
FMgSSR-22921	scaffold183020	p2	(CT)7	Class II	367	380	TCCAGCGTGGACCCAACAAT	CGCTGGAACGCCTGGATTTTGT	263
FMgSSR-22922	scaffold5896	p2	(CT)7	Class II	19650	19663	TCCAGGTTTATGCTGCCCTCCT	AGGCATGGTTAGAGCTGCAAGAGT	330
FMgSSR-22926	scaffold22544	p2	(CT)7	Class II	5984	5997	TCCATGTGATGGCGCGGTATGT	TGCCACCTTGTAACGGAGCACA	292
FMgSSR-22928	scaffold37	p2	(CT)7	Class II	44629	44642	TCCCCCATTTTACCAACCGCGT	TGCTTCAAAGCCATGACGCACG	296
FMgSSR-22933	scaffold13606	p2	(CT)7	Class II	18096	18109	TCCCTTCCCATGATGGCACGAT	ACCGCCCATAGCCAACAACA	323

SSR_ID	Scaffold	SSR_Type	SSR_Motif	SSR Class	SSR_Start	SSR_End	Forward sequence	Reverse sequence	Product_size
FMgSSR-22935	scaffold623	p2	(CT)7	Class II	25599	25612	TCCGAAGTTGCACTGGAATCGC	CCATTGTCATGGCCAAACCTCCA	350
FMgSSR-22939	scaffold24297	p2	(CT)7	Class II	7802	7815	TCCGGCTCGTACTAACCCCAAA	TGTGCACCATCGCGTTGACA	276
FMgSSR-22943	scaffold1820	p2	(CT)7	Class II	47859	47872	TCCTCGGGAAGCCAAATCCACA	AATCAAGGCGTGCGTGCAGA	250
FMgSSR-22949	scaffold19273	p2	(CT)7	Class II	9910	9923	TCGACGACAGCGACCAAAGTGA	TGCGCGGTTCATCTGCCAAA	299
FMgSSR-22951	scaffold151408	p2	(CT)7	Class II	254	267	TCGAGGCAGCATGACAACTGGA	TGCCGAATCCGACATCACCAGA	281
FMgSSR-22954	scaffold1654	p2	(CT)7	Class II	23940	23953	TCGCCATGCAGCTGGTCAACAA	ACAGAAAGCTCTCGTGCCGT	301
FMgSSR-22958	scaffold1122	p2	(CT)7	Class II	56074	56087	TCGCCGACAAGCAATACGCT	AGTGTGCACGGTCAAGGCAA	210
FMgSSR-22961	scaffold42	p2	(CT)7	Class II	35769	35782	TCGCTCGCTTCATGGTGGCTTT	TGCTTCAGCTTGATACGGCCCA	344
FMgSSR-22966	scaffold7627	p2	(CT)7	Class II	19853	19866	TCGGGAATCAGTAGAGGGCTCACA	TCGCGCTATGGCAGAGGAAGAA	324
FMgSSR-22968	scaffold2477	p2	(CT)7	Class II	8675	8688	TCGGGTCTCTGCTTTTTGCGGA	TCAGCACACCTTGAGCTACCAC	304
FMgSSR-22972	scaffold2547	p2	(CT)7	Class II	43548	43561	TCGGTGCGCTGATTGCATGT	TGGTACGCAGACGCAGTTGT	317
FMgSSR-22973	scaffold648	p2	(CT)7	Class II	22539	22552	TCGGTGGCCACGTCAGTCAAAA	TTAAGCCCAACGCGCCACTT	309
FMgSSR-22974	scaffold806	p2	(CT)7	Class II	44319	44332	TCGGTGTACTGGGAATGGTGGT	TTCCCGTGCAAGCCAAGTGAGT	224
FMgSSR-22977	scaffold30	p2	(CT)7	Class II	132215	132228	TCGTCCCTGCACGCAAACACAT	ATGATCTGCGGGAGCAATGGCA	299
FMgSSR-22979	scaffold6144	p2	(CT)7	Class II	21429	21442	TCGTGAAGACTGTGGCCTTTGC	AGCCGTGCTCCTCATCTTTGCT	212
FMgSSR-22980	scaffold25711	p2	(CT)7	Class II	4695	4708	TCGTGTGCCCTTCCCTTCTTCA	AATTGGCGTGCACAGGACGA	203
FMgSSR-22981	scaffold7010	p2	(CT)7	Class II	26149	26162	TCGTTGCCGTCATGGGTGTT	TGGTCAGCCTGGGCCTAACAAT	327
FMgSSR-22984	scaffold66005	p2	(CT)7	Class II	691	704	TCTCATTCACTCCACCGACACC	ATGGTTGCTGCCGAGCACAT	274
FMgSSR-22991	scaffold2131	p2	(CT)7	Class II	15118	15131	TCTGGATCCATGACACGTCCAA	ATGACCTTCCCTTGCACCCGTT	327
FMgSSR-22993	scaffold3827	p2	(CT)7	Class II	26047	26060	TCTGTTTCCACTTGCTGGCGT	AATCCGCTTGCGCTTTCGCT	243
FMgSSR-22998	scaffold22093	p2	(CT)7	Class II	4440	4453	TGAACAAACTCTTGCTCCCCCG	TTGAGCGGAGCACAGGGTAA	338
FMgSSR-22999	scaffold2731	p2	(CT)7	Class II	65642	65655	TGAAGCCACACCCATCACCACA	TCAGTAAAGCATGCCGTCGTGT	337
FMgSSR-23007	scaffold291	p2	(CT)7	Class II	21927	21940	TGACCTTTCCTGACCCAACAGCA	TGGCGATGCAAGGCCTTTTGAC	227
FMgSSR-23008	scaffold491	p2	(CT)7	Class II	89433	89446	TGACGCGTGGCATCGTAACA	TGCGAATCTCATTTGGTTGGCCG	235
FMgSSR-23010	scaffold8131	p2	(CT)7	Class II	16388	16401	TGACTCGCTTCTTCGTCGCT	AAACAAGGGCCGAGTGAGCA	324
FMgSSR-23012	scaffold281499	p2	(CT)7	Class II	266	279	TGAGACGCCGTCAGCTTGGATA	TCACCACCACTCAGAACCCCAA	212
FMgSSR-23014	scaffold42352	p2	(CT)7	Class II	3431	3444	TGAGATTTCCGGGCAGCACTGT	CGTGCGTTCACGTGTGCATT	350
FMgSSR-23019	scaffold498	p2	(CT)7	Class II	27504	27517	TGAGCGTCTGGTTCTATGCCGT	ACGACTGGACGGGGTTTCGTTT	270

SSR_ID	Scaffold	SSR_Type	SSR_Motif	SSR Class	SSR_Start	SSR_End	Forward sequence	Reverse sequence	Product_size
FMgSSR-23023	scaffold12440	p2	(CT)7	Class II	2167	2180	TGCAACATCGCTGGTCCACT	TCGGCATGCGGCAAAACTCT	212
FMgSSR-23028	scaffold13702	p2	(CT)7	Class II	9972	9985	TGCACAGCATGCAACTCTTGGC	AGCACGTGTGCTCTACCGAA	330
FMgSSR-23038	scaffold165830	p2	(CT)7	Class II	165	178	TGCAGCAGACCCATTTTGCG	TGAGGGCTCGTTTGCCTTGA	206
FMgSSR-23043	scaffold3435	p2	(CT)7	Class II	22720	22733	TGCATGCCCTTGGGCACACATA	ACACTCTGTCACGGATGGCACA	295
FMgSSR-23044	scaffold3787	p2	(CT)7	Class II	1798	1811	TGCATGCGCGTGTTGGTTTGA	AGGCAGAGGAAGAAGAGAAGGGA	271
FMgSSR-23045	scaffold841	p2	(CT)7	Class II	31883	31896	TGCATGCGCTTTGCTGTTGC	AGCTATCGATGCTGGCCACAAG	263
FMgSSR-23057	scaffold5027	p2	(CT)7	Class II	45351	45364	TGCCATCACAGTCGCAGCACAA	AACGCGGTTCAGACTTGGTCCT	290
FMgSSR-23058	scaffold1889	p2	(CT)7	Class II	46839	46852	TGCCATGGAGATGCAGGAACACA	TGGCGATGGAAGCGGCTTTT	328
FMgSSR-23060	scaffold10094	p2	(CT)7	Class II	17708	17721	TGCCTCAGTGACGCTTTGGCAT	TCGCGGTGCTCTTCTTTGCT	244
FMgSSR-23062	scaffold3499	p2	(CT)7	Class II	22225	22238	TGCCTGCCCAGTGTGTTCTGTA	AGTCGTGCCAGCAAAGGAGCAT	282
FMgSSR-23069	scaffold379	p2	(CT)7	Class II	86228	86241	TGCGCGTGGTCTTGTGTAAGGT	TGTTGTTGCTCTGTGCCTGGCT	308
FMgSSR-23070	scaffold3465	p2	(CT)7	Class II	5955	5968	TGCGCTGCGGAAGTTGCATA	TGCTGCGGAACGCAGAGATT	233
FMgSSR-23071	scaffold247	p2	(CT)7	Class II	85267	85280	TGCGGAAACCTCAGTGGCGATT	AAGAGCGCGATCATGGCAACCA	263
FMgSSR-23074	scaffold16365	p2	(CT)7	Class II	263	276	TGCGTCCATTCTCCTGCTCTGA	AGGGAAGCAGTTGCAGCAGT	316
FMgSSR-23077	scaffold656	p2	(CT)7	Class II	25331	25344	TGCTCCGTCAGTGGTTCATGCT	ACGACGATGCAGTCGCCATTGA	320
FMgSSR-23079	scaffold642	p2	(CT)7	Class II	29280	29293	TGCTCTGTTTGCTTCCGCCA	ACGAGAAAGGCGAGCACAAAGC	279
FMgSSR-23085	scaffold2477	p2	(CT)7	Class II	31532	31545	TGCTTCCACGACCAGCTTCCAA	TGGTTTTGCAACCGCTGCCT	300
FMgSSR-23088	scaffold10329	p2	(CT)7	Class II	18882	18895	TGCTTGGCTTGTTGGGTGCT	TCGCCATGGAGCACGATTCTCT	230
FMgSSR-23092	scaffold2686	p2	(CT)7	Class II	55728	55741	TGGACTGTTGGTCTCTCGGACA	AACGGAGGACATGCTGCCACAA	220
FMgSSR-23093	scaffold232	p2	(CT)7	Class II	54660	54673	TGGAGACGCAGCAGTGGCTTTT	AATCGCACCCAGCAAAGGGGAA	293
FMgSSR-23096	scaffold11530	p2	(CT)7	Class II	2080	2093	TGGATGGCTGCACGCGTTTT	TATTTGCCGCATGAGCTTCCGC	293
FMgSSR-23099	scaffold465	p2	(CT)7	Class II	83021	83034	TGGCAACGTGCACTGTCGTA	TGGTTGTAAAGCAGCTAGGGGGA	287
FMgSSR-23102	scaffold10084	p2	(CT)7	Class II	13605	13618	TGGCATGAATGGCGAAGCGA	TCCCAATGCCAGGCACCAAA	232
FMgSSR-23103	scaffold2104	p2	(CT)7	Class II	31030	31043	TGGCATTCGTTGCATGCTGCT	CCAAAGAACAATTTGGGCCACCTG	240
FMgSSR-23106	scaffold11269	p2	(CT)7	Class II	13947	13960	TGGCCACAAGCTTGGTGAGAAGT	TCCGCCATCTTGCATCCAACT	317
FMgSSR-23109	scaffold787	p2	(CT)7	Class II	110999	111012	TGGCCGAATGACGAACACCACT	GCAGCTGCTTGCTTGCTTGCTA	287
FMgSSR-23120	scaffold4211	p2	(CT)7	Class II	17307	17320	TGGGCCACTTGTCAATCACACGA	AGGCTGGATGCACTGCCTATGT	271
FMgSSR-23121	scaffold671	p2	(CT)7	Class II	93554	93567	TGGGCCTTTGGGAGGGAATTGT	TGTCTGAGCGCAGCGATGAT	305

SSR_ID	Scaffold	SSR_Type	SSR_Motif	SSR Class	SSR_Start	SSR_End	Forward sequence	Reverse sequence	Product_size
FMgSSR-23127	scaffold2150	p2	(CT)7	Class II	21598	21611	TGGTCCTTGCCTTCCACCGTAA	TTTCAGTGCGATGCGGCAGT	331
FMgSSR-23128	scaffold1498	p2	(CT)7	Class II	35092	35105	TGGTCGAAGTGCCAGAACGGAA	TGCAGCGCTGGATTCAGAATGT	311
FMgSSR-23131	scaffold1763	p2	(CT)7	Class II	31808	31821	TGGTGAAGCAGTGAAGGACGCT	TGCACATGACCGTGAGGCTT	295
FMgSSR-23142	scaffold2290	p2	(CT)7	Class II	45865	45878	TGGTTTGGGGCGAAGTGAGA	AGGCGAAGCCAGCTTTTGAGGT	205
FMgSSR-23159	scaffold2934	p2	(CT)7	Class II	46034	46047	TGTGCGACTCATTTGACTCCGT	TCGAATGGACAACGACCTACAGGA	247
FMgSSR-23160	scaffold177397	p2	(CT)7	Class II	401	414	TGTGCTAGTCTAGGAGTCTTCTCCC	TCGCGTGACGCAGCTGTTTT	273
FMgSSR-23162	scaffold15860	p2	(CT)7	Class II	10703	10716	TGTGGCCAGAAGTGACTCAGGT	ACCCCTGAACACTTGCCTGCAT	202
FMgSSR-23170	scaffold1049	p2	(CT)7	Class II	48938	48951	TGTTGCATGTTTGAGGGCGTGC	ACGACAGCACTCGCCATCAGTT	265
FMgSSR-23180	scaffold3244	p2	(CT)7	Class II	49846	49859	TTCACAACTCGTTCGTGTCCGC	TTGATGTGGTCGTCGTCGGA	306
FMgSSR-23182	scaffold7729	p2	(CT)7	Class II	31967	31980	TTCACGATGTGCGAACCGCT	ATTGCAACCGGACAAGGGGA	281
FMgSSR-23184	scaffold11383	p2	(CT)7	Class II	11236	11249	TTCATCACTGCGGTCTGGCTGT	TGTTGCGCAGCAGTTCGATG	340
FMgSSR-23193	scaffold6390	p2	(CT)7	Class II	35436	35449	TTCCGGATCCACTGCAGCCATA	TCCGCGTGTTGATCTTCTCCCA	218
FMgSSR-23199	scaffold1316	p2	(CT)7	Class II	10900	10913	TTCGAGGTTGCTGCGGTACCTT	TGGCACGAGCCAAACGTCAA	233
FMgSSR-23200	scaffold15130	p2	(CT)7	Class II	5229	5242	TTCGGTGGCGAAAGGAGGGAAA	TAAATTCCGTGGCGTCGCAAGC	337
FMgSSR-23205	scaffold61593	p2	(CT)7	Class II	1194	1207	TTCTCTCGTTCACACAGCCAGC	AATCTCGAACTCCTGGACGGCA	303
FMgSSR-23206	scaffold6755	p2	(CT)7	Class II	21800	21813	TTCTGCAGGACAACGACAGCGT	TGGGCGTGCAATCCTTGTGT	325
FMgSSR-23213	scaffold546	p2	(CT)7	Class II	47485	47498	TTGACGTGCGTGATTGACGGGT	ACCAAAACGCCATCACCACCCT	348
FMgSSR-23214	scaffold268	p2	(CT)7	Class II	111274	111287	TTGATTCGCATGACGCGTGCTC	GCGTTGCTTTCCTTTGCCGT	242
FMgSSR-23215	scaffold19582	p2	(CT)7	Class II	4025	4038	TTGCAGCTGAATAGCCGCGAGA	ATGCCACCTTCGTTGTGCGA	291
FMgSSR-23222	scaffold3355	p2	(CT)7	Class II	24037	24050	TTGCGGTCGAGCAAGATCCA	TTGTTGCCACCCCTGCCAAA	323
FMgSSR-23223	scaffold8844	p2	(CT)7	Class II	29105	29118	TTGCTGCAACCAACAGGCAGGA	ATTCTGGGGCAGGACGAAGGAA	343
FMgSSR-23234	scaffold92514	p2	(CT)7	Class II	1378	1391	TTGGGCTTGAGGTAGTGGCGTA	TGTTGGGCCACAACGAACGA	260
FMgSSR-23236	scaffold15	p2	(CT)7	Class II	232245	232258	TTGGGTGATGTGCGTCAATGGC	TGCAGCGGCGTACATTCCAT	258
FMgSSR-23252	scaffold4075	p2	(CT)7	Class II	10850	10863	TTTCTTCGTCTCCTCCGGTCCT	GCGTTACCTGAGCCTGACTTGGTT	337
FMgSSR-23260	scaffold17619	p2	(CT)7	Class II	13822	13835	TTTGCGCGCGTTGAAGTGCT	TGCATCGCAAGAATCAAGGGGC	348
FMgSSR-23261	scaffold2382	p2	(CT)7	Class II	36959	36972	TTTGGAACCTGGCTCCGTCACT	AAATGCAAGGACCCCTGCAAGC	223
FMgSSR-23263	scaffold495	p2	(CT)7	Class II	16954	16967	TTTGGTGTCATCACCGGCCT	TTGCCTTGCGTAACGTGCGA	348
FMgSSR-23265	scaffold44368	p2	(CT)7	Class II	5676	5689	TTTGTCACTTGTGCTGTGCCGC	TTTGGAGTGTAGTGGACGCGGT	328

SSR_ID	Scaffold	SSR_Type	SSR_Motif	SSR Class	SSR_Start	SSR_End	Forward sequence	Reverse sequence	Product_size
FMgSSR-23269	scaffold2904	p2	(CT)7	Class II	19746	19759	TTTTCACCTCGACCGTGCGT	AAGGGAACAGAACACGCGGA	200
FMgSSR-23273	scaffold221	p2	(CT)7	Class II	29091	29104	TTTTTCCTCCACCTCCGCCCAA	AGCCCTGCTTCTGCTTGTTGCT	334
FMgSSR-23275	scaffold18764	p2	(CT)7	Class II	10643	10656	TTTTTCGGAGGTGAGCGTCG	TGTTGCAGGCACTGGCGAAT	203
FMgSSR-23281	scaffold1063	p2	(CT)8	Class II	43519	43534	AAAACCCGCCAGTAGGTCAGCA	ATGCTCGTGGGCAAGAAGAGGA	336
FMgSSR-23291	scaffold158	p2	(CT)8	Class II	99200	99215	AAACCGGACATCGACCACCT	AGTTCTCCGTCGCGAGAGTGTT	336
FMgSSR-23298	scaffold20956	p2	(CT)8	Class II	9937	9952	AAAGCTGTTGCGGAAGGGAACC	TCAGGCACCATCAGCGTTGT	350
FMgSSR-23300	scaffold13159	p2	(CT)8	Class II	13077	13092	AAAGGCTTCAAGCAGCCGCA	TGGTGAGAGAAGACAGGGAAGCCT	316
FMgSSR-23301	scaffold7202	p2	(CT)8	Class II	22128	22143	AAATAGGCTCGCGTCCGACA	AAGGGATGGGGATTGCGGATGA	246
FMgSSR-23302	scaffold5891	p2	(CT)8	Class II	16808	16823	AAATATCGCTCGGTACTCGGGGCT	GGTGGCTGCAAGAAGAGGTAGA	269
FMgSSR-23308	scaffold99	p2	(CT)8	Class II	46673	46688	AACAGCCAAACCCGTTGCCA	AGCCACCACTAGGGGTAGTATTGT	343
FMgSSR-23312	scaffold2305	p2	(CT)8	Class II	63034	63049	AACCACAAGACGTGCCCTCACA	GCAAGAATTAGCATCACGAGGCCG	332
FMgSSR-23319	scaffold210081	p2	(CT)8	Class II	385	400	AACCGGAATTGCGTGTGGCA	ACGACTTGGCCCTTTGTTGGGT	333
FMgSSR-23329	scaffold486	p2	(CT)8	Class II	64980	64995	AAGCAGGACGCATTACAGGCCA	GGGGCACGGTAACTTGGCAAAA	275
FMgSSR-23335	scaffold21719	p2	(CT)8	Class II	14551	14566	AAGCTCCGGGCCACCTAAGAAA	ACCAATCGAGCGCAGGAGCAAA	275
FMgSSR-23340	scaffold100	p2	(CT)8	Class II	95967	95982	AAGGCCCAGTTGGCACCTTGTA	TTCCAGCGCATCGACTCTGTCA	223
FMgSSR-23341	scaffold1071	p2	(CT)8	Class II	65579	65594	AAGGCGAAACAAACGGTCCAGC	AGCAGCAACTAATGCCCCTCCT	231
FMgSSR-23352	scaffold777	p2	(CT)8	Class II	51569	51584	AATCGCTTTCGCTTCCTCCGCT	AGCAACCTTGTCAAGCTCCCCA	312
FMgSSR-23355	scaffold3971	p2	(CT)8	Class II	23843	23858	AATGGAGACGCTGCCTTGCCTT	ACTTGCACTTGCACGTCGGAGA	287
FMgSSR-23356	scaffold7787	p2	(CT)8	Class II	25700	25715	AATGGCAGCCTGACACGACGAT	GCCGCAGCGGTTCTCTTTCTTT	318
FMgSSR-23361	scaffold319	p2	(CT)8	Class II	21910	21925	ACAAGAGCGGTAGCTTTGCCCT	TACAAAAACCCCGGCAGCGT	307
FMgSSR-23367	scaffold213	p2	(CT)8	Class II	142978	142993	ACACGCGTCAACGGGTGATT	GGCACCCGAGACATGCAAAT	297
FMgSSR-23368	scaffold22859	p2	(CT)8	Class II	4106	4121	ACACGGAACGAAACGCCCTT	AGCTTGGGCGCATCAACTACAC	349
FMgSSR-23374	scaffold27867	p2	(CT)8	Class II	1903	1918	ACAGCGCACGTAGGGATCAT	ATCAAGCAGAACCGCGTCACCT	320
FMgSSR-23383	scaffold26218	p2	(CT)8	Class II	3620	3635	ACATGGCGTTGCTTCTCGGAGT	TGCACCGTGGTTTGTTATGGCA	272
FMgSSR-23384	scaffold3007	p2	(CT)8	Class II	32199	32214	ACATGGTTGTCATGTGCCCTCT	ATTAGTCGGCTAGGAGGGAGGT	234
FMgSSR-23390	scaffold11565	p2	(CT)8	Class II	24856	24871	ACCAAGACAGGGGAGGAGACAA	ACATGCCTGGAAGCTAGAGATCC	201
FMgSSR-23391	scaffold1757	p2	(CT)8	Class II	60929	60944	ACCAAGTGAAGCATGGGGATCA	TTGGAGGGTTCGAGAAGGGAAG	316
FMgSSR-23392	scaffold100	p2	(CT)8	Class II	168345	168360	ACCAAGTGCGCTAGCGGTTTCT	TCGGGCGAGCTAACTATCGCTT	348

SSR_ID	Scaffold	SSR_Type	SSR_Motif	SSR Class	SSR_Start	SSR_End	Forward sequence	Reverse sequence	Product_size
FMgSSR-23393	scaffold398	p2	(CT)8	Class II	121714	121729	ACCAATCGAGCTGCAGCAACCT	CGAAGCTCAAGCGCACACAT	319
FMgSSR-23394	scaffold7860	p2	(CT)8	Class II	14479	14494	ACCACAAGCCTGCGATGGTGAA	ACACTTGGAGGCATTGCGGAGA	342
FMgSSR-23402	scaffold5005	p2	(CT)8	Class II	4434	4449	ACCCAGCAGTGTGTGTGTGT	TACCCTGTGTGGCTCTGCAAGT	345
FMgSSR-23405	scaffold10259	p2	(CT)8	Class II	8218	8233	ACCCGATAGCCGCTAGGAAAGT	AAGCATGCGGCGGAAATGGT	316
FMgSSR-23408	scaffold7397	p2	(CT)8	Class II	1383	1398	ACCGACGGTAACCCGAATCA	CACACATCGCTCTGAGCTCCAT	315
FMgSSR-23415	scaffold12376	p2	(CT)8	Class II	21186	21201	ACCTGATCCGCGCGATCTTA	TCGCTACACTACTCCCATGCCT	334
FMgSSR-23421	scaffold3288	p2	(CT)8	Class II	39236	39251	ACGACAAGTGGTGTGGCGATGT	TGCCGCAATAGTTTGGATGGTC	263
FMgSSR-23422	scaffold5318	p2	(CT)8	Class II	37614	37629	ACGACACTGACGTTGGCGAAT	TCAAAAGCGCAGACGACACGA	322
FMgSSR-23426	scaffold26357	p2	(CT)8	Class II	3477	3492	ACGAGGGCCGCATTCATTCTCA	ACAATGTCGCCGTACCAGTCCT	349
FMgSSR-23433	scaffold26023	p2	(CT)8	Class II	5749	5764	ACGCGAGCGAGACGATTCTT	TCGTTGATTCGTAGCGCCGT	252
FMgSSR-23435	scaffold61	p2	(CT)8	Class II	39812	39827	ACGCTGTTTAGCGCACGCAT	AGCGAGAAGTGCTGTTCGGACT	201
FMgSSR-23438	scaffold2010	p2	(CT)8	Class II	29250	29265	ACGGCAGGAAAACGCTCCTACT	ACAGTGTGAGCTTCGGTCTCT	336
FMgSSR-23442	scaffold1954	p2	(CT)8	Class II	72949	72964	ACGGGGAGGCAGCACCAAAAAT	CGGCAACGGGATCCAACAAT	324
FMgSSR-23445	scaffold106	p2	(CT)8	Class II	7750	7765	ACGTCCGCCCATTATTGTGCCT	AACGGCTGATGGGTTGTGGTGA	319
FMgSSR-23448	scaffold25669	p2	(CT)8	Class II	3944	3959	ACGTGCGTAAACCGGCACAA	AGGAAAGCGAGAGAGGAGGCAA	339
FMgSSR-23461	scaffold24940	p2	(CT)8	Class II	6900	6915	ACTGCCGCACATCCAGTGTT	TGCAACTGATGCGGCTTGCT	329
FMgSSR-23462	scaffold14470	p2	(CT)8	Class II	3341	3356	ACTGCCTGCTCCATTTCGCA	TTGTAGCTTCAGCTCGCTCGGT	216
FMgSSR-23463	scaffold2082	p2	(CT)8	Class II	22504	22519	ACTGGTCATGTCGGCGAAGA	AAAGGTTGAGGTGCTGCGAGGT	347
FMgSSR-23466	scaffold6246	p2	(CT)8	Class II	8565	8580	ACTTGGTGTCCCTTGACTCGCA	CGGTGCCCATAAACTAACTCGC	350
FMgSSR-23470	scaffold21912	p2	(CT)8	Class II	815	830	AGAGCGCAAAGCAAGCGACA	TTCTTTCGCCCTGGCTGCTCTT	322
FMgSSR-23473	scaffold1953	p2	(CT)8	Class II	4043	4058	AGCAAGCAAGCAATGGGGAGA	AAAAACACAGCAGCGGACCAGC	322
FMgSSR-23476	scaffold12440	p2	(CT)8	Class II	6108	6123	AGCACATGACTTAGCCCGTCGT	AATTCGGACGGCAAGCTCGACA	208
FMgSSR-23477	scaffold5434	p2	(CT)8	Class II	34075	34090	AGCACCGACGCAAAGCAAGT	ACCGACGCCTTTCTCCCCAAAA	211
FMgSSR-23478	scaffold2499	p2	(CT)8	Class II	37792	37807	AGCACGTCCGATTTGGCTGT	TAGGCAGAAGACAGGGGCGTTT	328
FMgSSR-23481	scaffold7297	p2	(CT)8	Class II	3106	3121	AGCATAGGTGGTGGTGGCTT	ACTAGGAGGACCTCTTGCTGGA	340
FMgSSR-23482	scaffold9072	p2	(CT)8	Class II	20881	20896	AGCATCCGCGTCTCTTTGGT	AGCATTTGATTTCGCCGCCCAC	222
FMgSSR-23483	scaffold21	p2	(CT)8	Class II	88881	88896	AGCCAAAAGCCACGTCCGAA	CCGCCATGGCCGTTAATAACCT	319
FMgSSR-23488	scaffold7959	p2	(CT)8	Class II	6314	6329	AGCGGACATGTTTGGTCCGT	AAGGAGACAGGGCTTTCGGAT	330

SSR_ID	Scaffold	SSR_Type	SSR_Motif	SSR Class	SSR_Start	SSR_End	Forward sequence	Reverse sequence	Product_size
FMgSSR-23491	scaffold186	p2	(CT)8	Class II	39800	39815	AGCGTCCCCAATCCTTCCCAAA	ATCCTGCCGCCACTACGTGTTA	291
FMgSSR-23494	scaffold9704	p2	(CT)8	Class II	4987	5002	AGCTCACAGCTTGCTGAACGGA	AGCACACAAGACACTAGCGCGT	256
FMgSSR-23495	scaffold27244	p2	(CT)8	Class II	7784	7799	AGCTCTGTTGACGGCTGACCAA	TGCGCCAACAGATAAGGCCA	223
FMgSSR-23497	scaffold15049	p2	(CT)8	Class II	1424	1439	AGCTGCAACTCCACCGCTTCAT	ACTGTCTATCTATGCGCCAGGT	277
FMgSSR-23498	scaffold9512	p2	(CT)8	Class II	21994	22009	AGCTGCAGCCTTCAGTCACTTG	TTGGCTTGGTGGTTGGCATGGA	244
FMgSSR-23505	scaffold5861	p2	(CT)8	Class II	12880	12895	AGCTTTGTTTGAGCGAGGCAGAC	TCCGAGCAAAGGCAAACCATGC	343
FMgSSR-23509	scaffold10681	p2	(CT)8	Class II	10617	10632	AGGACAATGCTCGCCATGCT	ACACCGACGACCGACGTTCAAT	286
FMgSSR-23510	scaffold4269	p2	(CT)8	Class II	23071	23086	AGGACTGAAACAGTGCAGTGGCA	ATACACAGGATGATGGGTGTGGCG	231
FMgSSR-23514	scaffold3601	p2	(CT)8	Class II	31808	31823	AGGCAAAGCTTAGCGTCATGC	CTGATTTGCAGCTTGTCGTAGCCA	318
FMgSSR-23515	scaffold5554	p2	(CT)8	Class II	29310	29325	AGGCACGGTGACATGGACAA	AGCTCAGATTCGGCCAAGCCAA	230
FMgSSR-23517	scaffold2959	p2	(CT)8	Class II	57229	57244	AGGCAGTGAGCGTTCCAGAGTT	AGCTCCGGGATTGCGATCCTTT	336
FMgSSR-23522	scaffold5480	p2	(CT)8	Class II	22170	22185	AGGGTGTGAACCGGTGACTGAT	AGTGTTGCCGGTTACTGACGCA	219
FMgSSR-23523	scaffold287	p2	(CT)8	Class II	116017	116032	AGGTCGTACAGGGGAGTAGTCT	ACCAATGGTCAGTAGCAGGTGGT	282
FMgSSR-23524	scaffold18959	p2	(CT)8	Class II	6279	6294	AGGTGAGGGGAAAAGGTGACCA	TCCACCTAAGCCTTTGCTGCT	330
FMgSSR-23526	scaffold2827	p2	(CT)8	Class II	22442	22457	AGTATGCCCAACACCTGCTCCA	TGAGGCTGCGGAGGCAATAGAA	295
FMgSSR-23529	scaffold813	p2	(CT)8	Class II	28852	28867	AGTCTTGCTCCATCTACCAGTCG	CTACAACGGCTTACTAAAGGGGTC	200
FMgSSR-23531	scaffold4644	p2	(CT)8	Class II	40866	40881	AGTGGCACCGCAGTTTTGGT	TGTGCAGTACACGCGGAAGGAT	294
FMgSSR-23533	scaffold2327	p2	(CT)8	Class II	50972	50987	AGTGGTGGCCTGTACTTGGT	TGGCTCAAACGAGCTACCTGGA	289
FMgSSR-23535	scaffold151	p2	(CT)8	Class II	109427	109442	AGTGTGTGTCCGCGCCATTT	AGTCACCACTCACCACAGCA	254
FMgSSR-23538	scaffold19010	p2	(CT)8	Class II	2878	2893	ATAGTACTGGGCAATGGCGGCT	TGAGGCAGGCAAACAAGGGAGA	331
FMgSSR-23542	scaffold8400	p2	(CT)8	Class II	2698	2713	ATCAGTTCTGAACCTGCCCCGT	AGCTGGACCTTTCCATGCTCTGA	238
FMgSSR-23544	scaffold1676	p2	(CT)8	Class II	39293	39308	ATCCACGCTTGCCGCTCTGATT	AAGCGTGCAGCTTTTCCACTCG	263
FMgSSR-23549	scaffold6335	p2	(CT)8	Class II	23730	23745	ATCGCGGCGGTACCAGAAAT	TGCGAGAAAATGGACGGCCT	305
FMgSSR-23560	scaffold20166	p2	(CT)8	Class II	1186	1201	ATGATGGCTGCTGCTTCGCA	TTCAGTGCGACGAGGCGGAAAA	312
FMgSSR-23564	scaffold28973	p2	(CT)8	Class II	1909	1924	ATGCCCAGACCGTCCTTGAT	TGGAAAGCTGGACACATGTTGCG	271
FMgSSR-23568	scaffold13159	p2	(CT)8	Class II	11227	11242	ATGCTGGCTGCCGCTTACTT	ACCGTGAACTTTTCCCCACCGT	301
FMgSSR-23572	scaffold167475	p2	(CT)8	Class II	306	321	ATGGGTGCGTGTAGCAAGCCAA	TTCTGTTGCTCGCTTCGCCT	332
FMgSSR-23575	scaffold1399	p2	(CT)8	Class II	43152	43167	ATGTATGTGCGCTTGCAGGTGG	AATGCTCTTTCTCACGGGGCGT	236

SSR_ID	Scaffold	SSR_Type	SSR_Motif	SSR Class	SSR_Start	SSR_End	Forward sequence	Reverse sequence	Product_size
FMgSSR-23578	scaffold1920	p2	(CT)8	Class II	17832	17847	ATGTTTCTGGGCCCGAATGCCA	ACCTTGCCGGAAGCTCAGTT	306
FMgSSR-23588	scaffold10672	p2	(CT)8	Class II	98	113	CAGATAACGTGTAACACAGCCAGG	AGAGGTCTGTCGGTGTGTGTGT	239
FMgSSR-23590	scaffold825	p2	(CT)8	Class II	97298	97313	CCAAAAACTCGTCGCTGCGT	CAAATCCGCCATGCTCGCAA	311
FMgSSR-23595	scaffold6061	p2	(CT)8	Class II	27376	27391	CCATGACCGACATCTCATCACTGC	TTTCGTAAAGCATGCAGCGCCC	297
FMgSSR-23596	scaffold30738	p2	(CT)8	Class II	3061	3076	CCCAAATTGCCCTTGTGTGGCA	ACAGTAGCTCGGGTGTGTGTGT	203
FMgSSR-23602	scaffold42	p2	(CT)8	Class II	27503	27518	CCGCTGACACGGCGTTACATTT	TGCTCTGCTCGCCGTTTACT	260
FMgSSR-23605	scaffold25582	p2	(CT)8	Class II	6293	6308	CCGTTTCTGCCCCTTTCATTCACT	ATGAACAGTGGAGAAGCCGTGG	320
FMgSSR-23606	scaffold25042	p2	(CT)8	Class II	7778	7793	CCTCCGCCACTCTGCCATTATT	TGCAGGAACGCGACAGGAATGT	343
FMgSSR-23618	scaffold1325	p2	(CT)8	Class II	19704	19719	CGCGGAACAAAGTACGTTGGCA	AAGACGTGTGGCGCAATGCT	284
FMgSSR-23621	scaffold2449	p2	(CT)8	Class II	47904	47919	CGGTGCAGGCGTTTTTATGCCA	TGTCGCGCTCCTTACTTTCGCT	312
FMgSSR-23622	scaffold1529	p2	(CT)8	Class II	24656	24671	CGTCCCGCTGATGATAAAGCGT	AAGCGACAGAGCCTTTGCCA	335
FMgSSR-23623	scaffold6163	p2	(CT)8	Class II	5377	5392	CTCTGCGCCTTTTGTGTTTCCA	ACACCTCAAGGGGTAGAGAAGTGC	342
FMgSSR-23628	scaffold3168	p2	(CT)8	Class II	27782	27797	GCACATCCATGCTGCCGTGTTT	GCTCGCGCAATTCGACATCA	349
FMgSSR-23631	scaffold2894	p2	(CT)8	Class II	19172	19187	GCACTTTTGGTTCGGTAGATCGCA	AGCCCTAAGACACTTTCACCGCA	336
FMgSSR-23638	scaffold6658	p2	(CT)8	Class II	16854	16869	GCCATGCAAATGCACGGGTA	TCCACACGGGTGTGCTAGTT	227
FMgSSR-23641	scaffold4552	p2	(CT)8	Class II	34511	34526	GCCGTGACCGTGAATGAAGGAA	TCGCAGTCGCAGAAAACGCA	299
FMgSSR-23642	scaffold692	p2	(CT)8	Class II	79757	79772	GCCTTGCAAAGCTGGGCTTCTT	TTTGCACGCGCACAGAATCG	200
FMgSSR-23644	scaffold2095	p2	(CT)8	Class II	27113	27128	GCGAGTCTTGTGGGCTCGAAAT	TCGTGTGACCGGTTCTTGCT	296
FMgSSR-23650	scaffold7048	p2	(CT)8	Class II	2290	2305	GCGCTTGTGGCAGCTTATGT	CGACAGTCTGAGTTTGGGCATCA	350
FMgSSR-23653	scaffold2192	p2	(CT)8	Class II	12034	12049	GCTCTTCACTGATCCGCCGTTT	TTCATCGCTTGCCTGCCGAA	332
FMgSSR-23661	scaffold22418	p2	(CT)8	Class II	2870	2885	GGCGAAGCTTGCCAATTGCT	GCGTAAAGGCATTTGCTCGCCA	335
FMgSSR-23662	scaffold9155	p2	(CT)8	Class II	20795	20810	GGCGCCATAAGCCCCTTTTT	TTCCGAGCGGTCGTGAAAAGCA	229
FMgSSR-23663	scaffold744	p2	(CT)8	Class II	7623	7638	GGCGGCCACTTGCCAAAGAAAA	AAAACCTGCGTGTGCCTGCT	263
FMgSSR-23666	scaffold1659	p2	(CT)8	Class II	53669	53684	GGGTCATGCATTGTACCGCCAA	TGGGCGGCAGATGTTGCTTT	299
FMgSSR-23668	scaffold35894	p2	(CT)8	Class II	4096	4111	GGTGTGCATCATTTCACCGGCA	TTGCGTGCACGTGTCTTCGT	302
FMgSSR-23669	scaffold11816	p2	(CT)8	Class II	19871	19886	GGTTGCGCCGCATTTGTAGT	TAGCTGGCGTTGGTTGGGCTTT	307
FMgSSR-23670	scaffold498	p2	(CT)8	Class II	26944	26959	GTCAGCCAAAACAATGTTGCGCTC	TGAGCAGCGACACGAGCTACTT	233
FMgSSR-23681	scaffold19151	p2	(CT)8	Class II	12546	12561	TACAGCTCCACTTGTTGCCGCT	ACTTGCGTGGTTTGAGGCGA	221

SSR_ID	Scaffold	SSR_Type	SSR_Motif	SSR Class	SSR_Start	SSR_End	Forward sequence	Reverse sequence	Product_size
FMgSSR-23682	scaffold19193	p2	(CT)8	Class II	7896	7911	TACCAAAAGCGACCGACCGGAA	AACGCGCGTATTCGTATTGCCG	337
FMgSSR-23687	scaffold2134	p2	(CT)8	Class II	3289	3304	TAGCGCTGCTAATGGCCAGA	TGTGTGGGATTGGATTTGGGGCT	333
FMgSSR-23692	scaffold7189	p2	(CT)8	Class II	5781	5796	TATCATCGCTTGCGAGTGCGGT	TGTTTCGAGGTCAGCCCTGT	264
FMgSSR-23693	scaffold3656	p2	(CT)8	Class II	43201	43216	TATGGTTTGGGGCGAAGCGAGA	AAACCAGAGGCGAAGGCAGCTT	218
FMgSSR-23695	scaffold4475	p2	(CT)8	Class II	14023	14038	TCAAAAGCCCACCTGCCGCTAA	TGGTACCGCCATGGCCAAATCT	324
FMgSSR-23701	scaffold1913	p2	(CT)8	Class II	37137	37152	TCAATGCCACCAAGCAGCCA	TGCCCACACCCCAATGGACTTT	306
FMgSSR-23702	scaffold6876	p2	(CT)8	Class II	17306	17321	TCACAGCAAAGGCCATCAGCGA	TCCATCATGCTGCCGAACCCAA	277
FMgSSR-23706	scaffold475	p2	(CT)8	Class II	81886	81901	TCACTGCCGGTAGTTCCGTGTT	AGGTCAAAAATGCACAGCCGCC	327
FMgSSR-23709	scaffold9017	p2	(CT)8	Class II	24926	24941	TCATCATCGCTCGTGTGACCCA	TGCTGGATTGGAACGGCTTGCT	287
FMgSSR-23712	scaffold1552	p2	(CT)8	Class II	7687	7702	TCATTGCAGATGTCGCTCGCCT	AGCAAAGCAACACGTCCGCT	219
FMgSSR-23727	scaffold341	p2	(CT)8	Class II	11627	11642	TCCGATCCATGCCCAGCTAA	AACGCAACGCAACGGAACCA	243
FMgSSR-23728	scaffold9910	p2	(CT)8	Class II	13234	13249	TCCGATGTCTCCATCTATCCAACC	ACAAGACAAATTGGCCAGGGCG	252
FMgSSR-23731	scaffold23523	p2	(CT)8	Class II	7465	7480	TCCGGTAAATGTCCGTGTGGCA	TCCGGTTTCCATCTCACGGGTT	333
FMgSSR-23732	scaffold238	p2	(CT)8	Class II	15149	15164	TCCTAACACCTTTCCCTACGTGC	TGATGGATCCGTGCCCGAAA	336
FMgSSR-23734	scaffold40	p2	(CT)8	Class II	32812	32827	TCCTGAACCGGTGGTTAGCAGT	TGCAACAAGCGGACGAACCA	291
FMgSSR-23737	scaffold56	p2	(CT)8	Class II	209522	209537	TCGAAGCAAGCAGTGTGGCA	TGCTTCTGTGGCTGTAACGCGA	279
FMgSSR-23741	scaffold24924	p2	(CT)8	Class II	7330	7345	TCGATCCACCACTTCATCACTCCA	AGTCCGCCGCTACAAACCAA	281
FMgSSR-23742	scaffold4566	p2	(CT)8	Class II	45317	45332	TCGATTTTTCCCGTGAGCGACCT	TAAGGCGACGCTGCTCCTGTTT	335
FMgSSR-23748	scaffold163	p2	(CT)8	Class II	132479	132494	TCGGAGGAACCGATCCAGTTT	AGATTCTCGTGGCATGCGTGA	240
FMgSSR-23749	scaffold302	p2	(CT)8	Class II	78232	78247	TCGGCTCGGTGCTTTGCTTT	TGTCCTGGCTGGCTGCAATAGA	311
FMgSSR-23753	scaffold6250	p2	(CT)8	Class II	19785	19800	TCGGGTGTGTTTGTTGGCGA	ACGGCGTTTGTTGGAGAGGCTT	336
FMgSSR-23760	scaffold6063	p2	(CT)8	Class II	17930	17945	TCGTTGCACTTTCAGCATGTCC	CCTGGACTGCTGTCTGCTTGTT	306
FMgSSR-23762	scaffold813	p2	(CT)8	Class II	1054	1069	TCGTTTCCTCCGTGCGGAATGT	AGCACTGTGTTCCCCGAAGCAA	343
FMgSSR-23763	scaffold137	p2	(CT)8	Class II	51066	51081	TCGTTTGTGCCCTGTCTCGTGT	CCGCTCTTGCCTCTGCGTTTTT	323
FMgSSR-23766	scaffold100942	p2	(CT)8	Class II	1076	1091	TCTCGTCTCGCGCACGATTT	CCGCCGCTGCCAATTTTGAT	341
FMgSSR-23774	scaffold15797	p2	(CT)8	Class II	14545	14560	TGAACTGGACCGGCAACCAAGA	TCATTGCCATGGTGCGTGGA	282
FMgSSR-23779	scaffold1763	p2	(CT)8	Class II	71384	71399	TGAGCAAGGCGGAGCTGAAA	TTCTTGTTGTACGCCTCCCGCA	302
FMgSSR-23783	scaffold5333	p2	(CT)8	Class II	8186	8201	TGAGTCTGGTGACAAGGACACCCT	TGAGCCACGCCATGGTGGATTT	348

SSR_ID	Scaffold	SSR_Type	SSR_Motif	SSR Class	SSR_Start	SSR_End	Forward sequence	Reverse sequence	Product_size
FMgSSR-23785	scaffold783	p2	(CT)8	Class II	9695	9710	TGAGTGCGCAATCCAACAGACTCA	ATTGCTTGCGATTGCGGTTGGG	271
FMgSSR-23787	scaffold463	p2	(CT)8	Class II	104846	104861	TGATGCTTGATGGCGGCGAA	AGCGCTCCATCGAGTCCCTTTT	305
FMgSSR-23788	scaffold2089	p2	(CT)8	Class II	41279	41294	TGATTCGCGCGCTTCGTTGT	AGGGCCCGAGTCAATTTGAAGGT	327
FMgSSR-23789	scaffold54636	p2	(CT)8	Class II	2241	2256	TGCAAAGCGATTCCCACCTGCT	AAAAATCACCAGCCTCGCGACC	330
FMgSSR-23792	scaffold1267	p2	(CT)8	Class II	40115	40130	TGCAACCACATTGCAGCCAA	AATGATGCAGGCAACAAGGGGC	271
FMgSSR-23793	scaffold3189	p2	(CT)8	Class II	15936	15951	TGCAACTATCCCTGTCACGCA	TGTTGTACCAGCCAGATCGGA	224
FMgSSR-23797	scaffold7044	p2	(CT)8	Class II	18401	18416	TGCACATCACCTGCGTCCAGTT	AGTGAGTAACGTAGCGGCCT	305
FMgSSR-23805	scaffold1962	p2	(CT)8	Class II	22528	22543	TGCAGGCGAGTTGGTGTGTT	AGCGGCTTCCACAGCGATAGAT	333
FMgSSR-23806	scaffold4595	p2	(CT)8	Class II	10331	10346	TGCAGTGCTGGACTTGCTGA	GCCGGAGATGGTGGCAAATTGA	304
FMgSSR-23807	scaffold2904	p2	(CT)8	Class II	45047	45062	TGCATCTCAGGTGGCACGTT	ACGAAGACGCGCCTCCATATCT	332
FMgSSR-23812	scaffold10401	p2	(CT)8	Class II	17076	17091	TGCCAGTCGACCATACTGCTCA	ACCCACGAACACGTGGCTTT	305
FMgSSR-23818	scaffold44	p2	(CT)8	Class II	113480	113495	TGCCCGCAGCGCTTAATCAT	TGCTGACGTGCAGCCCCATTTA	309
FMgSSR-23819	scaffold8864	p2	(CT)8	Class II	8019	8034	TGCCCTGGTTCTCTTCTGCCCATA	AGCGACACGTCCGACTTCTT	270
FMgSSR-23822	scaffold7415	p2	(CT)8	Class II	3622	3637	TGCCGTGCATGTCGCAGTTT	ACCAGGATGGTCGCTGTCAACT	269
FMgSSR-23825	scaffold313	p2	(CT)8	Class II	86963	86978	TGCGAGCACGCGAATACACA	ATCGCTCTCCACCACTGCTATG	344
FMgSSR-23827	scaffold18441	p2	(CT)8	Class II	3006	3021	TGCGCACGCAGAAGCGAATA	AACAACCGCTCATCCTTCCGGT	326
FMgSSR-23840	scaffold5029	p2	(CT)8	Class II	33743	33758	TGCTGCCGTCTTGCCACATCAT	TCACGGGCCCAATGTCACAACA	265
FMgSSR-23844	scaffold973	p2	(CT)8	Class II	39581	39596	TGCTTCTTTGATCCTTCCCGGT	GCTGCAAGAGCGTCTAAAACCGT	284
FMgSSR-23863	scaffold3810	p2	(CT)8	Class II	15554	15569	TGGGCCACCAAACAGACAGA	AAAAGAAGCCGGAGCAGGTCGT	320
FMgSSR-23864	scaffold5965	p2	(CT)8	Class II	14728	14743	TGGGCCGCTGCTTTGTTTCT	AAGACGCCAGTACGCGTCTCAA	340
FMgSSR-23865	scaffold12021	p2	(CT)8	Class II	8686	8701	TGGGCTGTGTGTGTGATCCGTT	AGCGACGATGCCAATTGTTGCC	295
FMgSSR-23866	scaffold2799	p2	(CT)8	Class II	58964	58979	TGGGCTTAGATGTAGGTCTGCGA	TGGAGAAACAGTGGCGCACGAT	284
FMgSSR-23868	scaffold960	p2	(CT)8	Class II	50249	50264	TGGGTTCCGCAAACTTGGCT	AGGGGGCAATTGGACAAGCAGT	209
FMgSSR-23872	scaffold2622	p2	(CT)8	Class II	46198	46213	TGGTCGCATTGCATCAGCCT	TTGCACGCATGTCCCAATCCCT	295
FMgSSR-23873	scaffold2054	p2	(CT)8	Class II	48829	48844	TGGTGACGCTGCCACTTCAT	AGGACACGACGACCGTTGCTTA	237
FMgSSR-23876	scaffold4832	p2	(CT)8	Class II	10617	10632	TGGTGCGAAAAGGCCTCACT	TCGAGCTGCCCAAAGTGGGAAA	234
FMgSSR-23884	scaffold887	p2	(CT)8	Class II	45419	45434	TGTACTGCACGCTGACACGA	AACGGCTGCGCCCAAACTAA	295
FMgSSR-23885	scaffold2513	p2	(CT)8	Class II	14321	14336	TGTCAAGCTGCTTCTCGTGGCA	GCCTGCGCACTGCTTCAGTTTT	339

SSR_ID	Scaffold	SSR_Type	SSR_Motif	SSR Class	SSR_Start	SSR_End	Forward sequence	Reverse sequence	Product_size
FMgSSR-23895	scaffold3277	p2	(CT)8	Class II	30247	30262	TGTGGCCGTTGGATCAGCATCA	TCCTTCGCGAACTGAAGCACCA	252
FMgSSR-23897	scaffold2844	p2	(CT)8	Class II	27525	27540	TGTGGTCAACGGGTGGTTCA	GCAGCCCCAAGGCAAAGCAAAT	349
FMgSSR-23901	scaffold97600	p2	(CT)8	Class II	602	617	TGTTCCTTGCCTGGTCCCAA	CGGTTGCCAAGTGAAGCCTACAA	349
FMgSSR-23902	scaffold271	p2	(CT)8	Class II	133621	133636	TGTTGACCGTTGCCTGCGAA	ATCATGCACCGCAGCTTGCTCA	299
FMgSSR-23903	scaffold3533	p2	(CT)8	Class II	29780	29795	TGTTGCCCTAGACACCACCGAA	AAGCGCTTCTTAGGCGCTGTGA	327
FMgSSR-23909	scaffold3588	p2	(CT)8	Class II	15530	15545	TTCAGGCCTTGCGAATACGAGG	ATTGCATCTGGCGGGTCTGGTT	216
FMgSSR-23910	scaffold9682	p2	(CT)8	Class II	7582	7597	TTCATCCCCCAACGAAGCCA	GCCGGCAGCACAAAAGATGTGT	263
FMgSSR-23916	scaffold1374	p2	(CT)8	Class II	35867	35882	TTCCGTTCCGTGGTGCCAGAAT	ACAGTAGGGGAGTGGCAGCAAT	252
FMgSSR-23918	scaffold1632	p2	(CT)8	Class II	41287	41302	TTCCTGCCTGGTGCCACACAAA	AACGCTCAACATCCACCTCCCT	328
FMgSSR-23922	scaffold10473	p2	(CT)8	Class II	17295	17310	TTCGTGCGCAGCATGAGAGT	CCCTCGTCGAGCCAATGGTAAT	304
FMgSSR-23925	scaffold1449	p2	(CT)8	Class II	38899	38914	TTCTGATGCTGGCTGCTGTG	TCAAGGAGCCAATAACACGCGG	253
FMgSSR-23929	scaffold12911	p2	(CT)8	Class II	8477	8492	TTCTTGATGCGGCGGTGGAAGT	GCTGCTCGTGCTCTGTAGTCTGTA	345
FMgSSR-23930	scaffold32488	p2	(CT)8	Class II	2208	2223	TTCTTTTGGGTGCTGGCTGGCT	ATTGGCTTCGCAGGCTATGTCC	324
FMgSSR-23940	scaffold4817	p2	(CT)8	Class II	30043	30058	TTGCTGCTTCATCCGCGCTT	TGCGACGACGAGTGGCATGTAA	221
FMgSSR-23944	scaffold284	p2	(CT)8	Class II	154812	154827	TTGGAAGTCGCAGGAGCGTT	ACATTGCGCTGTGCTCCAGT	236
FMgSSR-23945	scaffold10933	p2	(CT)8	Class II	22704	22719	TTGGAAGTGAGGTCCAGCCCAT	AGGAGTGGCCTTGGGGATTT	339
FMgSSR-23946	scaffold16	p2	(CT)8	Class II	146884	146899	TTGGACCCGGACCCGAAAAT	CCGATTGGATAGGTGGGAGTCA	332
FMgSSR-23948	scaffold209	p2	(CT)8	Class II	27025	27040	TTGGTGCATGGGCAAGGTTGGA	TCCGACGTCAGGCAACAACA	296
FMgSSR-23951	scaffold378	p2	(CT)8	Class II	90255	90270	TTGTCCGCATCTGAGCCACACA	AACATTGGACGCTGTGGACGGT	276
FMgSSR-23955	scaffold8697	p2	(CT)8	Class II	27271	27286	TTTACCTCCTCTTGCAGCCACC	AGCAAGAGGGAAGAGACGAAGGT	243
FMgSSR-23956	scaffold3202	p2	(CT)8	Class II	21293	21308	TTTAGCTGTGCCTGTGCGCT	TCGTCCGTGTGTACGCTCTTGT	298
FMgSSR-23957	scaffold10141	p2	(CT)8	Class II	9299	9314	TTTCACTCCAGCAAGCCGCA	TCCTGCACGCCAACCCAAGAAA	244
FMgSSR-23958	scaffold1316	p2	(CT)8	Class II	11132	11147	TTTCACTGCAAGGGCACGCT	ACGCGGCAAGAACCAAAGTCCT	271
FMgSSR-23961	scaffold9710	p2	(CT)8	Class II	1890	1905	TTTCCCACTAGTCGCCGTGCAA	AACTCCCCGTCCAGAATCACCA	327
FMgSSR-23965	scaffold213	p2	(CT)8	Class II	49833	49848	TTTGCAGAACGTCCGTGGTTCC	ATATGCAGCCGCCAGAACCCAA	306
FMgSSR-23966	scaffold157	p2	(CT)8	Class II	184561	184576	TTTGCCGCTGCGAGCCAAAT	CGCACATGTAAGAGGTGGCGAT	344
FMgSSR-23968	scaffold4952	p2	(CT)8	Class II	31836	31851	TTTGTGTTGGGCCTAGCCCGTT	AAGCGGGGGATGATACGGCAAA	217
FMgSSR-23974	scaffold1855	p2	(CT)9	Class II	45614	45631	AAAAAGGCTGGAGCCAGCGACA	TTTTGCTCGCGTTTCGGGGA	202

SSR_ID	Scaffold	SSR_Type	SSR_Motif	SSR Class	SSR_Start	SSR_End	Forward sequence	Reverse sequence	Product_size
FMgSSR-23977	scaffold4444	p2	(CT)9	Class II	10606	10623	AAAAGCAAGGGAGGAGCGGA	ACAGTTGCTGGTAGCCAGTGCT	240
FMgSSR-23982	scaffold11835	p2	(CT)9	Class II	1507	1524	AAACTGTGCCGCCCGCAAAA	TGCAAAGCGAAAAGGCCAGC	308
FMgSSR-23986	scaffold14271	p2	(CT)9	Class II	267	284	AACAAGCAGAGCAGAGGAGGCA	ACAGAGGTGCGCTTATGACGTG	306
FMgSSR-23987	scaffold38	p2	(CT)9	Class II	3639	3656	AACAGCAACAGCGGAAGCCA	TCGGAAAGCCATCCAACCGA	329
FMgSSR-23991	scaffold12718	p2	(CT)9	Class II	1685	1702	AACCCTCAATCCTAATCAGCCCCC	AAGCCACTCACCAAGCTAGGCA	344
FMgSSR-23996	scaffold417	p2	(CT)9	Class II	15567	15584	AACGGTATCTTGCGCGCGTT	GCAACACGCGACCGCTTTATCA	342
FMgSSR-23998	scaffold3413	p2	(CT)9	Class II	22143	22160	AACGTTGCGAAACACGCGGA	CGCGAGCCAACGGTCATTTCTT	336
FMgSSR-23999	scaffold13016	p2	(CT)9	Class II	7080	7097	AACTCCGCAGCGACACGAAA	TGCTTTCTCTCCCCTTCCTCCCTT	332
FMgSSR-24001	scaffold7518	p2	(CT)9	Class II	16934	16951	AACTGGTGCACGCAAGTGCT	ACACGGCGGATATGCACCAACA	208
FMgSSR-24002	scaffold16687	p2	(CT)9	Class II	14232	14249	AACTTTAGCCCAGCTGAGCCCT	CACACCAAAAGGAGAGGAGGGA	235
FMgSSR-24011	scaffold222	p2	(CT)9	Class II	65459	65476	AAGGCAAGCGAACTGGTGTACG	ACGCCCATAGGAGCAGCAAACA	284
FMgSSR-24018	scaffold2003	p2	(CT)9	Class II	12175	12192	AATCAAAGCGGGTGTGGCTGGT	GCCGCGTTCACACAATCGATCA	257
FMgSSR-24023	scaffold391	p2	(CT)9	Class II	6489	6506	AATGAATCCCGGGCAAGCGA	TGTGCGCATAGCGATTGTAGCC	335
FMgSSR-24031	scaffold724	p2	(CT)9	Class II	50577	50594	ACACGAAGCAGGAAGGTGTGTT	TCTGCCTACATCTGTGCTGCCT	241
FMgSSR-24037	scaffold6809	p2	(CT)9	Class II	10581	10598	ACAGGTGAACAACAACCCGGCA	TGTTGAGCATAACGGCTGCCCA	281
FMgSSR-24041	scaffold597	p2	(CT)9	Class II	61917	61934	ACATGCGTCAGTGTCACAGGCT	TGATGCTCAACGACACAACGCT	348
FMgSSR-24043	scaffold4241	p2	(CT)9	Class II	21206	21223	ACCACACCGCTACAGTGGTTCA	TTGGAAGCAGAGGTCGACGAGT	318
FMgSSR-24062	scaffold5105	p2	(CT)9	Class II	20334	20351	ACCTGTCGTTCACTGCTCGCAA	AACTATGGCGGCACCATTGCCT	319
FMgSSR-24063	scaffold7866	p2	(CT)9	Class II	26862	26879	ACCTTGCGCGCTCTGAATTCTCT	TCCATGCCGATCGACGATGCTT	320
FMgSSR-24067	scaffold4	p2	(CT)9	Class II	283683	283700	ACGATACGGAGCACCCAACAGT	ACCGTCGTCGTCGCTGTTTT	319
FMgSSR-24068	scaffold3975	p2	(CT)9	Class II	34885	34902	ACGATCAGCATGACCCCACCAA	TCTTTCGGCATTGCGGCGAA	293
FMgSSR-24071	scaffold13778	p2	(CT)9	Class II	4294	4311	ACGCCAATCACGCCACACAT	TGACGAGCAAACACATCTCGGA	220
FMgSSR-24076	scaffold627	p2	(CT)9	Class II	42286	42303	ACGCCTTCCGTAATGCTGCT	TGCTCCTTGTCACCGGGATTGA	237
FMgSSR-24081	scaffold744	p2	(CT)9	Class II	48443	48460	ACGGGCTAAGGATCTCCATGATGC	ACGAATTCCTGATGTGCCGCCT	249
FMgSSR-24083	scaffold32787	p2	(CT)9	Class II	4250	4267	ACGTCGAGAAGCAGCCAGAA	GCAGAAGCATTTTGCGGTTCGC	289
FMgSSR-24084	scaffold19647	p2	(CT)9	Class II	15255	15272	ACGTGACGATTGTTGCGGAGAGT	TCATTGGATTGGGCGTCGCA	346
FMgSSR-24090	scaffold3136	p2	(CT)9	Class II	32705	32722	ACTTCTGCTGCTGCTTGCGT	AAAAATACACCGCCCCCGCT	203
FMgSSR-24099	scaffold1681	p2	(CT)9	Class II	70698	70715	AGCAACAGGAGCACCACTCTGA	TGCCATTTCAAGCGAGCGGT	270

SSR_ID	Scaffold	SSR_Type	SSR_Motif	SSR Class	SSR_Start	SSR_End	Forward sequence	Reverse sequence	Product_size
FMgSSR-24100	scaffold787	p2	(CT)9	Class II	112128	112145	AGCAAGCAAGGCAGCTACGGAA	TGCATGCGCACTGGATATCTGGA	264
FMgSSR-24101	scaffold98	p2	(CT)9	Class II	180100	180117	AGCAAGGTCGTAATCCAGCGGAA	ATGCAGTATTACGCCGCCCCAA	283
FMgSSR-24102	scaffold12179	p2	(CT)9	Class II	9127	9144	AGCAATGGCTTGCTCGATCGTC	TGCATAACACCTCCTGACGCCT	267
FMgSSR-24107	scaffold1194	p2	(CT)9	Class II	7204	7221	AGCATGGGACCATCTTGGGCTT	TGGCCAGGTGGGTCAATAATGC	291
FMgSSR-24108	scaffold3146	p2	(CT)9	Class II	31324	31341	AGCCAAAACGCTAGCACCACCA	TCCCATCTTTTGCCAAGCTGCC	257
FMgSSR-24110	scaffold4663	p2	(CT)9	Class II	19465	19482	AGCCACTTGGACAGCAGCAT	ACATGCTGCCACAACCCCTT	307
FMgSSR-24112	scaffold4850	p2	(CT)9	Class II	34070	34087	AGCCCGTCCTTGTTGTGACTGA	AGCTGCAAAGTCTTGTGGCCT	345
FMgSSR-24114	scaffold14350	p2	(CT)9	Class II	5791	5808	AGCCGCTTGCCTCGTGTACATA	AACCCTGGTGAAGTGGTAGCGA	317
FMgSSR-24120	scaffold17258	p2	(CT)9	Class II	11320	11337	AGCGGTGTGATTGCAGGTGA	CCTGCGCACAACGTGTTCGATT	349
FMgSSR-24122	scaffold3132	p2	(CT)9	Class II	29792	29809	AGCTCCGACGACTTTCCGACAA	AGGGATTTGGCCTGCCTGCAAT	322
FMgSSR-24125	scaffold3702	p2	(CT)9	Class II	33049	33066	AGCTTCTGCACAACCACGCA	TGTTCTTGCCGATGAAGGCCGA	280
FMgSSR-24129	scaffold20830	p2	(CT)9	Class II	6169	6186	AGGAAGTCGGGTGATTTGGCGA	AACGTACCCTTGCCTTTGCTGC	271
FMgSSR-24130	scaffold1301	p2	(CT)9	Class II	80128	80145	AGGAATAGCCGCAAGCCTGCAA	TCTGGCACCAACTTCCTTCGCT	332
FMgSSR-24131	scaffold910	p2	(CT)9	Class II	87248	87265	AGGCATTATCTCTCTCTCCCCCT	AGCATGCTGTGCTAGTTACCTCGT	313
FMgSSR-24133	scaffold4917	p2	(CT)9	Class II	37689	37706	AGGCGCAAGCGCAACTTCAT	ATCCGACGATGCTCCATTGGGT	328
FMgSSR-24135	scaffold98	p2	(CT)9	Class II	94714	94731	AGGCTCAAGGAAGCGTCTGA	TGTTGAGCATACCACTGGCAGGA	311
FMgSSR-24143	scaffold28923	p2	(CT)9	Class II	6970	6987	AGTCCACCCAGTCCAGCTTCTT	TGCAGCAGCAGGCAAACAACT	218
FMgSSR-24144	scaffold8606	p2	(CT)9	Class II	15671	15688	AGTGAAGAAGGCCCTAATCCCCCA	ATCAGAATCCTGCTGCGTGTCTGG	331
FMgSSR-24145	scaffold309	p2	(CT)9	Class II	61012	61029	AGTGATTGGTTGCGCGTCGT	TCATGGCAACAATCGCAGGCAC	203
FMgSSR-24147	scaffold1844	p2	(CT)9	Class II	1211	1228	AGTTGGTGCTGCGGGTGAAA	ACCCACCAACGCGGGGTTTAAT	286
FMgSSR-24151	scaffold4021	p2	(CT)9	Class II	45092	45109	ATCACCCACGTGTGCTTTTGCC	TTGGTGGCATCATTGGCTGGCT	347
FMgSSR-24152	scaffold1098	p2	(CT)9	Class II	37569	37586	ATCACCGCTCACCACACGTT	AGCCAGACAACGCACGAACA	209
FMgSSR-24157	scaffold62387	p2	(CT)9	Class II	1729	1746	ATCGAAAAGTGGTAGGCCCCCT	TGAGCGGGCGGGCATTAAAA	337
FMgSSR-24159	scaffold588	p2	(CT)9	Class II	28948	28965	ATCGGTGTTTGTGTGGCCCTGT	TGCTGCCTTGTGAGACCCAT	322
FMgSSR-24164	scaffold3420	p2	(CT)9	Class II	4023	4040	ATGCAAGGCATGGGTGTGCT	TCCATCACGCATGGACACTGCT	298
FMgSSR-24165	scaffold11905	p2	(CT)9	Class II	12292	12309	ATGCAGCAATACAGCCAAGCCG	TCGGCCAAAAATGGCCGGAT	255
FMgSSR-24168	scaffold1056	p2	(CT)9	Class II	30432	30449	ATGCCTAAATCCACCCGGCAGA	AGCCGTAACGGGATCCATCA	226
FMgSSR-24172	scaffold2853	p2	(CT)9	Class II	11578	11595	ATGGGCCGCTTTAGACTAGTTCCG	GCACGTGGCTCCGTTGAAACTT	267

SSR_ID	Scaffold	SSR_Type	SSR_Motif	SSR Class	SSR_Start	SSR_End	Forward sequence	Reverse sequence	Product_size
FMgSSR-24176	scaffold74	p2	(CT)9	Class II	105455	105472	ATTACGCGCCAAGCAAGCCA	TTACCGCGAGACGGCGAAAA	341
FMgSSR-24177	scaffold43403	p2	(CT)9	Class II	1699	1716	ATTAGCCGCACCCTACCCTTCT	AATTGCCTCGCGCTGGAACA	285
FMgSSR-24178	scaffold18800	p2	(CT)9	Class II	7338	7355	ATTCCTGGTGTGGGAAGGCA	TGGCGCTCAATCCATCACGA	300
FMgSSR-24179	scaffold176	p2	(CT)9	Class II	15333	15350	ATTCTCTGCATGCGAGCCGT	ACCTGGAGGTGTGAAGCACGTT	238
FMgSSR-24180	scaffold7242	p2	(CT)9	Class II	2093	2110	ATTGCCCAAATTCGTGACGGCG	ACCCACGGAAGCCCCGTATAAA	343
FMgSSR-24181	scaffold18956	p2	(CT)9	Class II	6097	6114	ATTGCGATGTTGAGGGGTGCGA	ATCGGCCTACCAGATGCAGT	206
FMgSSR-24182	scaffold4225	p2	(CT)9	Class II	32184	32201	ATTGTGCCAGCATCAGCGCA	CGGCGCCCACAAATTGGTTCAA	209
FMgSSR-24185	scaffold34	p2	(CT)9	Class II	173616	173633	ATTTGGGTTCAGGTTGCGGGGA	ACCACACGACACACGCAGATCA	247
FMgSSR-24194	scaffold13287	p2	(CT)9	Class II	5256	5273	CCCAAGAGCGCCGACATCAAAT	TGGGTTTGGACCCATTTGCCA	242
FMgSSR-24196	scaffold26391	p2	(CT)9	Class II	4540	4557	CCGCAATTTGCCACGTCGTT	AGGCAATTTGCACTTGGCACGG	218
FMgSSR-24197	scaffold4159	p2	(CT)9	Class II	14623	14640	CCGTGACAGTCATGCAAAGACACA	CCAACAACTATCCAGATCCAGCAC	217
FMgSSR-24202	scaffold2146	p2	(CT)9	Class II	59709	59726	CGACAGTGTTAGCGTTGCCGTT	AATGCGACAAGAAGGGGGTGCT	344
FMgSSR-24212	scaffold491	p2	(CT)9	Class II	115945	115962	CTGAGAGTGTCTCATGCAAACCCA	AGGGAGAATGTACCAGGAGGCA	233
FMgSSR-24225	scaffold4581	p2	(CT)9	Class II	9598	9615	GGCAGCCAACGAACACAGCAAA	ACGAAACCCTGCACAGGTTCACA	299
FMgSSR-24227	scaffold358	p2	(CT)9	Class II	130950	130967	GGCCTTAACGAATGCGCTGTGT	TGCTTCTGTGCCGCCACTAT	270
FMgSSR-24231	scaffold1369	p2	(CT)9	Class II	88325	88342	TAAGCAAACGTGACGTGTCCGC	AATCCACGCCCGAAGGCAAA	210
FMgSSR-24235	scaffold2139	p2	(CT)9	Class II	16271	16288	TACAGCCATCCTGAAGCCAGCA	GTTTGCGCGCCAAATGGGAA	221
FMgSSR-24236	scaffold152845	p2	(CT)9	Class II	454	471	TACCCAGGCACGTGGAGCATTT	ATGGTGCATCCACACCACCT	239
FMgSSR-24241	scaffold56	p2	(CT)9	Class II	7386	7403	TATGGCCGCTGCTCGCGATAAA	TTGGAAGCACAGAAGCCGCA	213
FMgSSR-24244	scaffold4214	p2	(CT)9	Class II	38853	38870	TATTTGCGGGGCGGGTTTGT	TGGGCTCGACAGAGGAAGAGAA	301
FMgSSR-24246	scaffold9976	p2	(CT)9	Class II	510	527	TCAATCTCTAAAGCGCAGCCCG	TAGGAAGAGAGGGAGCCGATGT	216
FMgSSR-24252	scaffold1265	p2	(CT)9	Class II	31951	31968	TCAGCATGCCGAAGAAGCCT	ACGAGTCCACCACTCTCATGCT	321
FMgSSR-24257	scaffold67267	p2	(CT)9	Class II	1978	1995	TCATCTTCAACGGCCCGCAGAT	ATCACGTTGTGAAGGGGAGCGA	264
FMgSSR-24260	scaffold88	p2	(CT)9	Class II	136995	137012	TCCAAGTGACACGGTGCAAAGC	ATGCGAGCACATGTATGCCACG	301
FMgSSR-24262	scaffold8982	p2	(CT)9	Class II	6456	6473	TCCATGCAGGGGTTATATGGCAC	GCAATGAAACTGCGTGTGGCGT	342
FMgSSR-24265	scaffold23724	p2	(CT)9	Class II	1670	1687	TCCCGCACCCGTAGTGATTT	ACTCGGGTGGGAAAAGCCGAAA	310
FMgSSR-24270	scaffold16359	p2	(CT)9	Class II	11719	11736	TCCGCGCCATCGTTTTGCTT	TGGTCTTTCTGTAGCTGTCACGCA	239
FMgSSR-24273	scaffold20587	p2	(CT)9	Class II	6350	6367	TCCTGCCCGTCAATGATCAGGT	AGCACAAAGAGGGCGCATGT	251

SSR_ID	Scaffold	SSR_Type	SSR_Motif	SSR Class	SSR_Start	SSR_End	Forward sequence	Reverse sequence	Product_size
FMgSSR-24275	scaffold3828	p2	(CT)9	Class II	41578	41595	TCCTGCTGCGAGACTGAACGAA	AACAACAAGAGCAGCGGCGA	270
FMgSSR-24277	scaffold25158	p2	(CT)9	Class II	3144	3161	TCCTTCAGCACATGGCCATCAC	TGCCCCAACCCATGCTATTGCT	257
FMgSSR-24279	scaffold4999	p2	(CT)9	Class II	24909	24926	TCGCAATTACACCCCTGGACA	ATGGACGGCATGGACACCTGAT	292
FMgSSR-24280	scaffold3721	p2	(CT)9	Class II	18638	18655	TCGCACGCTGTAACAGTGAGGT	ATCCTGCTGTCCACTTGCCAGA	229
FMgSSR-24290	scaffold11048	p2	(CT)9	Class II	8030	8047	TCGGCTGCACCAAAGAAGCA	ATCAATCTCCACGCACCGCA	336
FMgSSR-24293	scaffold726	p2	(CT)9	Class II	11312	11329	TCTACTAGTGTGGTTGTGCGCC	TCCGGTGGCAGCAGTGTGAATA	328
FMgSSR-24297	scaffold9293	p2	(CT)9	Class II	8929	8946	TCTCTCCGGGGACTCAAAGGAA	TGCTCGTCAAAAGAGAGCCCGT	316
FMgSSR-24301	scaffold4450	p2	(CT)9	Class II	34509	34526	TCTTTGCGGAGCGCGGTTTT	TCAAAGGTGGACGGCTCTGT	231
FMgSSR-24313	scaffold1827	p2	(CT)9	Class II	32172	32189	TGAGGCCCCAGTGGTTTTTCT	TCTTAGCCCCTCACTGCTGCTT	307
FMgSSR-24314	scaffold5728	p2	(CT)9	Class II	29916	29933	TGAGGGGCAAGAGATGAGGCTT	AGATTTCGGCTGTGCGGCAA	349
FMgSSR-24316	scaffold4242	p2	(CT)9	Class II	48506	48523	TGATTTGGAGAGGCGGCACGTA	TGCAAGCCGTTTTCACTCGTGG	331
FMgSSR-24320	scaffold7210	p2	(CT)9	Class II	24076	24093	TGCACCTACGCAGCCTCCATTT	TGCCATCTCGTCAGATGCCTGT	339
FMgSSR-24324	scaffold4150	p2	(CT)9	Class II	46969	46986	TGCAGGAAACGAAGTCCAGCGA	AGTGAGCGGTCGAGGTCAGATT	262
FMgSSR-24326	scaffold5526	p2	(CT)9	Class II	21098	21115	TGCATCCCTGCTGCACCAAT	AGCCGCGTCCTACTGTTTTGCT	245
FMgSSR-24333	scaffold4840	p2	(CT)9	Class II	35763	35780	TGCCCGAAAATTGCGCCGTA	TCCGTGTCCTTGTGGATGCGAT	295
FMgSSR-24334	scaffold11879	p2	(CT)9	Class II	7523	7540	TGCCCTCTCCACGACTTCATCT	ATGCTATCGGGAGCCGTCAT	244
FMgSSR-24335	scaffold6052	p2	(CT)9	Class II	17747	17764	TGCCCTCTTTGTGTGCCTCTCT	AGCAACGCATAGGTCATCGC	219
FMgSSR-24338	scaffold20540	p2	(CT)9	Class II	5310	5327	TGCCGCCACTGTAGTCAAGCAT	ATGAGGACGGCAGCAGTGAT	322
FMgSSR-24344	scaffold110	p2	(CT)9	Class II	100717	100734	TGCTAACATAGCGACAGCCGGT	AGGGATGCCTCTTTTCGGCACA	204
FMgSSR-24350	scaffold5613	p2	(CT)9	Class II	29750	29767	TGCTCTCCCTCTATCCCCTTCA	ACCAACGTTCATGCCCTGCTCA	309
FMgSSR-24354	scaffold5375	p2	(CT)9	Class II	2299	2316	TGCTGCCGATGCGCTTACTT	ACAAAGCGCAGGGAGTGTTTGC	316
FMgSSR-24357	scaffold341	p2	(CT)9	Class II	125058	125075	TGCTTCGCCCCCGCTAGTTTTT	AGCGCCAACCAGAGGAATGA	342
FMgSSR-24359	scaffold5306	p2	(CT)9	Class II	34501	34518	TGCTTGGTCAAGGTGTGAGTCCT	AGGTTCACCACGCCTATTCCTCCA	336
FMgSSR-24360	scaffold6291	p2	(CT)9	Class II	9110	9127	TGCTTGTGTGAGGTTGCAGTGA	ACCCAAGCAGAATGTCAGGGATGG	317
FMgSSR-24361	scaffold2191	p2	(CT)9	Class II	29062	29079	TGCTTTGGTGCATTGCGACG	AGTCCTGGACCACACTGATGGA	255
FMgSSR-24362	scaffold22659	p2	(CT)9	Class II	10303	10320	TGCTTTGGTGCTTTGCACGGT	TGGCTCTGGCAGGTGCAAAAT	303
FMgSSR-24363	scaffold15307	p2	(CT)9	Class II	9978	9995	TGGACGAACCGCTGGTTTCA	AAGCGCCATCTTCGGGTTCA	342
FMgSSR-24364	scaffold37544	p2	(CT)9	Class II	1768	1785	TGGCAAGTCAGCTCGTAGTCCT	AGCACGCACGTGCACACATA	322

SSR_ID	Scaffold	SSR_Type	SSR_Motif	SSR Class	SSR_Start	SSR_End	Forward sequence	Reverse sequence	Product_size
FMgSSR-24365	scaffold9770	p2	(CT)9	Class II	17590	17607	TGGCAGTGACATGCCCACAA	AGAAAAGCCAAGGGTCCAGCA	318
FMgSSR-24367	scaffold2898	p2	(CT)9	Class II	38488	38505	TGGCGTCACGTCATCTTCCT	ACCATGGAGCGATGCGATGT	332
FMgSSR-24368	scaffold15314	p2	(CT)9	Class II	11744	11761	TGGGCTTGGGCATTGCTGTT	ACGCCGTTTGCCGCACATAA	281
FMgSSR-24370	scaffold15137	p2	(CT)9	Class II	10912	10929	TGGGTGCACGGAACAATCGTCA	ACGACAGGGTGGATTGGGTGTT	277
FMgSSR-24373	scaffold24943	p2	(CT)9	Class II	10313	10330	TGGTTGGCCATCGGGATGAA	TCCGAAGCAACTGACGAAATCGG	342
FMgSSR-24376	scaffold160	p2	(CT)9	Class II	87513	87530	TGTCATCCACAGCGCTGACT	CGCAAGACACGCGAACAGAA	232
FMgSSR-24380	scaffold4502	p2	(CT)9	Class II	30437	30454	TGTCCGTTCAAGCCTCATGCCA	AACTGACGCGGTGCAGACAT	272
FMgSSR-24386	scaffold6660	p2	(CT)9	Class II	2051	2068	TGTGAGCGAGCAAGAATGGGGT	TCTTGCTGCCGCCATGGTAA	266
FMgSSR-24395	scaffold605	p2	(CT)9	Class II	35405	35422	TGTGGGCAACCAACCAGATGT	CCATTGGTGGCCATTGGCTTCA	312
FMgSSR-24398	scaffold7296	p2	(CT)9	Class II	7517	7534	TGTTCGCCCTCTTTGTCGCA	TGCTCACGTCGAACAGGTCA	339
FMgSSR-24402	scaffold59824	p2	(CT)9	Class II	1464	1481	TTACAAACCCGACCATCGCACC	TGGTTGAGGCCGATGTTGTGCT	306
FMgSSR-24403	scaffold20509	p2	(CT)9	Class II	8342	8359	TTAGTTGGGCCTCAGTGTTAGGGC	TGAAGTTATGCGCAGTGACAGGG	226
FMgSSR-24406	scaffold7839	p2	(CT)9	Class II	10621	10638	TTCACGGTCACGCCTTCCACAA	ATGATATGTGGTGGGGCGTCGT	217
FMgSSR-24414	scaffold5064	p2	(CT)9	Class II	14477	14494	TTCGTCGAGGGCACAAGGAT	TCGCTAGTTCCAGAACCACCCT	339
FMgSSR-24415	scaffold4163	p2	(CT)9	Class II	49438	49455	TTCGTCTGCTTCACCGGCAA	AGCAACCACACTCACTCCTCCT	350
FMgSSR-24420	scaffold186	p2	(CT)9	Class II	63020	63037	TTCTTGCCGAGGATGTTGCGGA	TGTGGCTGAACCGCTGAAGAGT	331
FMgSSR-24422	scaffold2492	p2	(CT)9	Class II	44734	44751	TTGACCCCGCTTACGAGCTT	TATGAGGGCAAAGGCGTTGCGA	294
FMgSSR-24426	scaffold12527	p2	(CT)9	Class II	17619	17636	TTGCCACTACCCATGCTGCCAA	ACCAAATGCACCATAGCACGCA	252
FMgSSR-24428	scaffold40	p2	(CT)9	Class II	95116	95133	TTGCGAGTCAAACCGCAGCA	TTTTGTCTGGGGTGTCGGAGCA	229
FMgSSR-24435	scaffold27027	p2	(CT)9	Class II	11281	11298	TTGGAGGGCCACGATTGCTT	AGCGAAGTGCTCTAACGCGA	311
FMgSSR-24444	scaffold8101	p2	(CT)9	Class II	16751	16768	TTGTTTTGGCTGCGGAGCGT	ATGCCCGTGCATGTTCCAGT	298
FMgSSR-24446	scaffold3157	p2	(CT)9	Class II	20152	20169	TTTACCTCACCCTCACCGCA	TTCTTTCTGGTGCGTGTGCGAC	298
FMgSSR-24448	scaffold14307	p2	(CT)9	Class II	17632	17649	TTTAGCCAGCCGCCGCTATTGT	TGTGCGATGGCTCCTTGCCTAA	334
FMgSSR-24452	scaffold1318	p2	(CT)9	Class II	64859	64876	TTTCTTCGCTGCCGCCAACT	GTGCCGTGCATTCTCTCTCACA	296
FMgSSR-24453	scaffold40	p2	(CT)9	Class II	37657	37674	TTTCTTCGGGCTCGCCATCA	TGCCATCCATGCCCTTCACAGA	211
FMgSSR-24458	scaffold1986	p2	(CT)9	Class II	21967	21984	TTTGGGGCATGCTATGCGGT	TGCATCGCTTGTGTCTCCGTCA	253
FMgSSR-24470	scaffold188	p2	(GA)10	Class I	88421	88440	AACGGGAGTGCAGGTCAAGCAA	TCCTTGAGGAAGAACCAGCCGT	317
FMgSSR-24472	scaffold18025	p2	(GA)10	Class I	7686	7705	AAGCCGACCGGTGTGAGAGAAA	AGGATGCGGGGGTGGAAAACAA	349

SSR_ID	Scaffold	SSR_Type	SSR_Motif	SSR Class	SSR_Start	SSR_End	Forward sequence	Reverse sequence	Product_size
FMgSSR-24491	scaffold8161	p2	(GA)10	Class I	3015	3034	ACACATGGTTAGGAGTGTAGCACC	TCCCATTGTGTTGCTGCTGCTG	201
FMgSSR-24494	scaffold3370	p2	(GA)10	Class I	10354	10373	ACACGCTGGTGTGCTCAGAGAT	ACGTCAGCATGCACCAACCCTT	330
FMgSSR-24498	scaffold3550	p2	(GA)10	Class I	6689	6708	ACATCGTCGTTTCTCGGCGT	TAACGGCGAAGTTGCGCTTCTG	210
FMgSSR-24499	scaffold7795	p2	(GA)10	Class I	19191	19210	ACATGAGGCATCCTCGTGCCAT	ATGAAACGTGGGCCGTGGAA	291
FMgSSR-24500	scaffold13499	p2	(GA)10	Class I	15679	15698	ACATGGCGGCTTTGCTTGGT	TAGCATTTGGGCTGCACTGCCT	304
FMgSSR-24501	scaffold2951	p2	(GA)10	Class I	21077	21096	ACATGGTTGTTGGCGAGGTCGT	TCCATGCATGCCACCGATGA	274
FMgSSR-24502	scaffold2488	p2	(GA)10	Class I	850	869	ACATGTAACTGCTCGCCGCTGT	TAGTGGTAAGCTTCCTCCCTCCCT	311
FMgSSR-24504	scaffold3253	p2	(GA)10	Class I	51442	51461	ACCAGCAATGAATCGCGCCAA	TTCTGTCTTTGGGGCCGTGT	256
FMgSSR-24506	scaffold8790	p2	(GA)10	Class I	5441	5460	ACCAGCATGTGAGTTTGAGGGA	GGCAAGATCATGGACTGTGGCA	325
FMgSSR-24512	scaffold206	p2	(GA)10	Class I	110290	110309	ACCGGCCATTGTTGCCTGTT	ACTCTTCGCGCTGAGCATTCA	264
FMgSSR-24517	scaffold688	p2	(GA)10	Class I	77783	77802	ACGCGATAATCACGAGGCGA	TGCGTGTTGGTTCGTCCGAT	291
FMgSSR-24519	scaffold8700	p2	(GA)10	Class I	14744	14763	ACGGGCATGCTGCTCAACAA	TGTTCGGTGTTCGATCGCCTGT	310
FMgSSR-24520	scaffold1663	p2	(GA)10	Class I	11472	11491	ACGTCAGCAGCGGAGAACAA	ACAAACAAAAGGCACACGGGC	264
FMgSSR-24524	scaffold12831	p2	(GA)10	Class I	5868	5887	ACTGCATGCACGCACTTGCT	AGATGGCGCGACACTGGTTT	200
FMgSSR-24526	scaffold874	p2	(GA)10	Class I	24646	24665	ACTGGCTGTTGTTTGGAGACCTGA	ACAGCCTCACACAGGCTTGA	326
FMgSSR-24528	scaffold3522	p2	(GA)10	Class I	43988	44007	AGAAAGCACAGCCGCACATGA	TGGTGGCCTTTGATTCCCCTCA	298
FMgSSR-24533	scaffold660	p2	(GA)10	Class I	30456	30475	AGCAAAGCTTCGCGACGACA	AGGTGAAGTGGGCGTCAAGGAA	304
FMgSSR-24535	scaffold1900	p2	(GA)10	Class I	13761	13780	AGCAGAGCGGCAGAATTGGT	AATGGCCGGCCTTCTGTCTTCT	329
FMgSSR-24537	scaffold14685	p2	(GA)10	Class I	6839	6858	AGCATCCACCGTCCGACGTAAT	TGAGCCAACGAACCTGCCACTT	348
FMgSSR-24542	scaffold1914	p2	(GA)10	Class I	66044	66063	AGCCGTGGACATGAGTGTTGCT	ACACTTTACTCTCCCTCCCAGCCA	251
FMgSSR-24555	scaffold761	p2	(GA)10	Class I	108383	108402	AGGCGAGCAGGTTTGGTGGATT	AGCGCGAGGTCTAGCAAAAGCA	228
FMgSSR-24559	scaffold2810	p2	(GA)10	Class I	48843	48862	AGGTCACATGAGGGTGCGTT	CCATGCCACCCTGACAACAAGT	333
FMgSSR-24562	scaffold4848	p2	(GA)10	Class I	18878	18897	AGTCCCAAACCTGCTGTCCT	ATGGTGCAACTACGCAAAGCGG	210
FMgSSR-24568	scaffold18849	p2	(GA)10	Class I	12069	12088	AGTTACTGACGCACACGGGCAT	TGCGTGGTGGGCAATGAAGT	349
FMgSSR-24570	scaffold18270	p2	(GA)10	Class I	9502	9521	ATAGGAGCAAAAGCCGCCCCAA	TTCACCCAGCCGGGTATGAAGA	232
FMgSSR-24579	scaffold767	p2	(GA)10	Class I	22605	22624	ATGCATCGTCCGGCCAAACA	AGCACGCAGGTTTACGTTCCGT	276
FMgSSR-24581	scaffold2777	p2	(GA)10	Class I	26446	26465	ATGCGTTGGCGAGATGGATGGT	ACGAGCACCGTACAGGCAGAAA	291
FMgSSR-24584	scaffold2729	p2	(GA)10	Class I	33170	33189	ATGGCCCTCCCTGACAAGAGGAAT	ACATGCCGACCCTTTTGCCT	211

SSR_ID	Scaffold	SSR_Type	SSR_Motif	SSR Class	SSR_Start	SSR_End	Forward sequence	Reverse sequence	Product_size
FMgSSR-24586	scaffold1145	p2	(GA)10	Class I	45482	45501	ATGTCGCAGCAGCAGAGGTTGT	TGCATCCAAGTTGTGCGAGTGC	287
FMgSSR-24592	scaffold5848	p2	(GA)10	Class I	14386	14405	ATTCGGCGTTGGAGTGGGTT	TGCACCCCAAAAGCCCACAA	268
FMgSSR-24596	scaffold4488	p2	(GA)10	Class I	26760	26779	CCAGGAGGGAAATGATGCCGAT	ACGGCTCTCCAGTGCCATTCAT	348
FMgSSR-24599	scaffold2376	p2	(GA)10	Class I	43797	43816	CCCTGCTCCTTCTATTAGCTTCCA	ACATTCTTGTTGCACCCCCTCG	341
FMgSSR-24610	scaffold505	p2	(GA)10	Class I	93798	93817	CGTCTGCGCATGCCTTGACTTT	TCACGGCGCTAGCCAATGTT	220
FMgSSR-24611	scaffold253	p2	(GA)10	Class I	36972	36991	CGTGTGTGCCGCAACAATCT	ACGATCGGAGAGAGAGATGCACGA	340
FMgSSR-24613	scaffold463	p2	(GA)10	Class I	94264	94283	GAGATGGTGCATGTTGTCTCTAGG	TGTTGGGGCCGTTTGAGGTT	216
FMgSSR-24614	scaffold3923	p2	(GA)10	Class I	24728	24747	GCAACCCAGGCGAGTGCTTTTT	AAGCGCCCATGGCCTAATGGAT	267
FMgSSR-24615	scaffold8956	p2	(GA)10	Class I	26875	26894	GCAAGCACCAACACGTGCAA	TTGTTGTCCACGTGTCCTGGGT	221
FMgSSR-24618	scaffold11198	p2	(GA)10	Class I	26333	26352	GCCGGCGCTTAGATTTTGCCAT	TGGGCACCATTGTGTTTCCGGT	222
FMgSSR-24621	scaffold6486	p2	(GA)10	Class I	35262	35281	GCGAGCAACAAGTGACAGTGCT	TCGCTTCAAGGAACTGCCCA	311
FMgSSR-24623	scaffold23724	p2	(GA)10	Class I	10145	10164	GCGCGGATGCATGCAGATAGAA	TGCTTGCTTGCTTGCCGGTT	303
FMgSSR-24624	scaffold1981	p2	(GA)10	Class I	30275	30294	GCGGAGAAGGCCCCCGATTTATTT	GTTGTGGCTGTTGGTGGTGT	340
FMgSSR-24625	scaffold5990	p2	(GA)10	Class I	22106	22125	GCTCGTGCAGCAGGACATGTTT	GCACAACTGTGCAGGCAACT	260
FMgSSR-24633	scaffold1134	p2	(GA)10	Class I	19427	19446	TAATCAACGCGCGAGTGCCA	TATTGCTCGATGGTCGCGCT	293
FMgSSR-24649	scaffold2825	p2	(GA)10	Class I	49162	49181	TCCAACCGTACATGCGCAACA	TGGCATCATTGGCAACCTCCCT	339
FMgSSR-24650	scaffold11573	p2	(GA)10	Class I	16593	16612	TCCAAGCGCTGCATGGATGA	AGTGCCAGCAACAACCGCAT	283
FMgSSR-24659	scaffold3262	p2	(GA)10	Class I	15474	15493	TCCGGGAACGGCAATGGAGAAT	TGCAGCTTTGCGCTATGCGA	279
FMgSSR-24665	scaffold145	p2	(GA)10	Class I	46489	46508	TCGACATGCCCTTCTGTCGAT	ACCATTGGCAAGAGGCACTGCT	289
FMgSSR-24669	scaffold1709	p2	(GA)10	Class I	48851	48870	TCGATGATCTGCATGCTGCGGT	ACGCGTGCATTCTATCCAAGG	203
FMgSSR-24674	scaffold2502	p2	(GA)10	Class I	36884	36903	TCGTGTGTCGTTGTTGGGCT	AGTCCTTTGACTCCAGAGGGCA	250
FMgSSR-24678	scaffold804	p2	(GA)10	Class I	21451	21470	TCTCGCGGCAGTAATTGGCA	AGTTTTCGATGGACACGGCG	306
FMgSSR-24681	scaffold14350	p2	(GA)10	Class I	14061	14080	TCTTCACGCCACAGTACAGGGA	TCTTGGGGTGAACCCTTGTTGA	317
FMgSSR-24684	scaffold9117	p2	(GA)10	Class I	14116	14135	TGAACCAGAGTCGGTGATGCCT	GCAAACGCAATTCGGTCCGTGA	317
FMgSSR-24687	scaffold9367	p2	(GA)10	Class I	25446	25465	TGAAGGCGGCGGCATATGAA	TTGCAATGCACGTTGTCGGG	307
FMgSSR-24692	scaffold11950	p2	(GA)10	Class I	12364	12383	TGAGCCACATCAGCGAAGCA	AACAAAAGACGGTCGTGCCCCT	206
FMgSSR-24694	scaffold497	p2	(GA)10	Class I	48663	48682	TGAGGCGGCACCAAAAACCA	TTCTCAGGCGGTTCAGACGACA	268
FMgSSR-24706	scaffold8213	p2	(GA)10	Class I	2252	2271	TGCATGACACAGCACGAGCA	TGTGGAAGCGACGGGCAGATTT	339

SSR_ID	Scaffold	SSR_Type	SSR_Motif	SSR Class	SSR_Start	SSR_End	Forward sequence	Reverse sequence	Product_size
FMgSSR-24708	scaffold6849	p2	(GA)10	Class I	34513	34532	TGCCCAAGTCTCGCTTATGAGACC	ACGGCCGGGTTAGCAGAAACAT	244
FMgSSR-24709	scaffold31	p2	(GA)10	Class I	159732	159751	TGCCCCCTCCCCCTCTATCTAAAA	GCCATTTCTGGATGTAACGCCAGC	345
FMgSSR-24712	scaffold5743	p2	(GA)10	Class I	3601	3620	TGCCTCGTTGCACCAAGAGAGA	TGGACGGCGTCACACTGAAACA	349
FMgSSR-24717	scaffold1539	p2	(GA)10	Class I	15385	15404	TGCGGCGTCACGCTTGTAGAAT	GCATGTGAACCTGCCCACATCA	222
FMgSSR-24723	scaffold23837	p2	(GA)10	Class I	1535	1554	TGCTCAGGAAGGGAACACAGGA	AGCCTGCCGCATTCAATCAAGC	310
FMgSSR-24727	scaffold2454	p2	(GA)10	Class I	56269	56288	TGCTGGCGACTGAAAATGCGT	TGCTTCGTCACACCAACACGGA	201
FMgSSR-24728	scaffold1081	p2	(GA)10	Class I	29595	29614	TGCTGTCTGCGAATGGCATCAGA	TGCATTGCAGCCTGTCACCA	296
FMgSSR-24730	scaffold2210	p2	(GA)10	Class I	35383	35402	TGCTTGTTCGATAGCCCCTGCT	AACGTCACGCGTGCTTGGAA	329
FMgSSR-24732	scaffold13764	p2	(GA)10	Class I	3897	3916	TGGCCATCGTTGCAGAAGCA	ACCATTTTCCTCGGCCCACA	263
FMgSSR-24734	scaffold4302	p2	(GA)10	Class I	44780	44799	TGGCGCCACGTGGTAGAATGAT	CCTTGGCCAATTCAAGTGGACCCT	274
FMgSSR-24735	scaffold136	p2	(GA)10	Class I	69597	69616	TGGCTCAATCGTTCAGAGGGCT	AGGTTCACAGTTGCGTTCTACCCA	331
FMgSSR-24739	scaffold25903	p2	(GA)10	Class I	6921	6940	TGGGTTTTGGTAGCACTGCAGGA	TAACTTTGCCCCAGTGCATCGC	251
FMgSSR-24740	scaffold4496	p2	(GA)10	Class I	25906	25925	TGGTCCTCGGCATCCTGAGATA	TGCCACACTGCAAACGAGAGTG	264
FMgSSR-24746	scaffold17061	p2	(GA)10	Class I	7387	7406	TGTGCGGCTGAAGTGCAACA	AGTCAGTTGTAAGCCCGTGCAGT	297
FMgSSR-24752	scaffold173	p2	(GA)10	Class I	100947	100966	TGTTGGCCAGTGTCTTTGCCA	TGGGCCTGCATATGGTCTCGAT	205
FMgSSR-24753	scaffold144406	p2	(GA)10	Class I	234	253	TTACAACCATGGCGCACGCA	TCCAAAACACACGTCGTCCCCT	326
FMgSSR-24760	scaffold5772	p2	(GA)10	Class I	19192	19211	TTCCGTCGCGCCAATCATGT	ACACGTCATTGATCCACCCCCT	310
FMgSSR-24763	scaffold3751	p2	(GA)10	Class I	49746	49765	TTCGCGCTGCTGCTGTTGAA	TTTCACGTGCCGCTTTCCCT	321
FMgSSR-24771	scaffold8471	p2	(GA)10	Class I	22637	22656	TTGCAGTCGTTCCTGCCACCTT	CCGCTCAAACCCCGTTATGCTT	257
FMgSSR-24775	scaffold16291	p2	(GA)10	Class I	6941	6960	TTGCTGGTAGCCAGTGCTCCAT	GGTCCGACCGACAAAGCACAAA	348
FMgSSR-24780	scaffold3004	p2	(GA)10	Class I	63274	63293	TTTCGCGTCACCAGCCAGATCA	TGCAAAACGGGCAAGGCTGA	270
FMgSSR-24782	scaffold403	p2	(GA)10	Class I	106707	106726	TTTGCTCGTGAGCCATGGGGAT	TGCTGCTGCGAAGGTAAAGCAC	207
FMgSSR-24784	scaffold8709	p2	(GA)10	Class I	8201	8220	TTTGGGGGTGCTACGATTCGCT	ATCAGCCCAAACGAGGAACGCA	285
FMgSSR-24785	scaffold4822	p2	(GA)10	Class I	30338	30357	TTTTGCCCCCGAATGCCGTT	ATCTGCCATGGTGGTGGTGT	274
FMgSSR-24790	scaffold13430	p2	(GA)11	Class I	12562	12583	AAACGTTGCGCTATGCACGG	ACATCAAACGGTTCAGGCCGGT	344
FMgSSR-24791	scaffold994	p2	(GA)11	Class I	33335	33356	AACACCACGCCCGTCAAACA	TCCAAGAAACCGGTGGCCAAGT	299
FMgSSR-24794	scaffold4492	p2	(GA)11	Class I	24344	24365	AACCTCGCGAGTGCTTGCTT	TGCATGCTGCTGGTACTAGTGGT	264
FMgSSR-24795	scaffold924	p2	(GA)11	Class I	27215	27236	AACGCGCGAACCCAAACCAA	AGAGAGGCCGCAGTGTCAAGAA	338

SSR_ID	Scaffold	SSR_Type	SSR_Motif	SSR Class	SSR_Start	SSR_End	Forward sequence	Reverse sequence	Product_size
FMgSSR-24796	scaffold958	p2	(GA)11	Class I	57996	58017	AACTGCGGCGCTCGATCCTAAA	ATCCTCACAGTGGCCGGTTTCT	338
FMgSSR-24799	scaffold32588	p2	(GA)11	Class I	1032	1053	AAGGAGGCGTTTTCGCTGCT	AATTGCCTTTAGGCACTGCCGC	313
FMgSSR-24803	scaffold1450	p2	(GA)11	Class I	49253	49274	ACAACACGAGTGTGCTCCAAAG	TGGACTGGCACCACCGTGATTT	320
FMgSSR-24806	scaffold5813	p2	(GA)11	Class I	24492	24513	ACAATGGTGATTGCGGTGGGGA	TTTAGCGGCCACACAGTTGC	239
FMgSSR-24807	scaffold6005	p2	(GA)11	Class I	27739	27760	ACACGCGCCACACACCTAAT	TGGGCCATTTGGCAGCTTGGTA	329
FMgSSR-24809	scaffold1137	p2	(GA)11	Class I	18495	18516	ACCAGCGCTCTCCTCTTCGATT	AGGCCATTAGAGGCCACTTGGT	321
FMgSSR-24810	scaffold15641	p2	(GA)11	Class I	14104	14125	ACCCTCCTCATTCCCCACAACA	TGGACATGGTGATCAAGGAGGCA	344
FMgSSR-24815	scaffold954	p2	(GA)11	Class I	26914	26935	ACGAGAACAGCGGCTTGACGAA	AGAGACGCTGCCAGTTGACAT	206
FMgSSR-24817	scaffold2105	p2	(GA)11	Class I	16961	16982	ACGATTTGGGGTTGCATTGGCG	TCCAGTTGCAATTCGCCCGT	239
FMgSSR-24818	scaffold131	p2	(GA)11	Class I	128626	128647	ACGCGCACGGCAATCAAACA	AGAAGGAGGCCATGAAGACGCA	316
FMgSSR-24821	scaffold3276	p2	(GA)11	Class I	14772	14793	ACGGCAGCCATTTCACCGTT	TCCGACTGCGACGACACAGAAA	294
FMgSSR-24822	scaffold31869	p2	(GA)11	Class I	3226	3247	ACGGGCGGCGATGTTTGTTT	ATGCGAGCTGGTTGGTGGAT	283
FMgSSR-24825	scaffold22436	p2	(GA)11	Class I	12842	12863	ACGTGGGATCTTACCAGTCAGGCA	AGGAGCGCCGCCTTCTCAATTT	318
FMgSSR-24826	scaffold83	p2	(GA)11	Class I	19908	19929	ACTCCAGTGCCAACGGGAAA	ACTGCCCACCCCATTCGTACTT	320
FMgSSR-24831	scaffold14439	p2	(GA)11	Class I	3584	3605	AGAAGCACTCAAGTCCTGCCA	ACGTTACCCAATTTCCTTCCCCC	253
FMgSSR-24832	scaffold8505	p2	(GA)11	Class I	10685	10706	AGACACGTGCGCATCAGCAT	TGTGGCTGCCGGCTTGTTTA	312
FMgSSR-24834	scaffold689	p2	(GA)11	Class I	5282	5303	AGATGAAGCGCGAGCAGGTT	TGGCCAACTCCTTCTGCACCAT	322
FMgSSR-24836	scaffold1147	p2	(GA)11	Class I	66641	66662	AGCAATGTCCCTTCGTCTGGCA	TCGTGCGTTTTGCCCAGAGT	338
FMgSSR-24839	scaffold1731	p2	(GA)11	Class I	51830	51851	AGCACGTTGTCTGTCGCCAT	AACCAGCTCAGGCCCAAACCAA	332
FMgSSR-24850	scaffold10068	p2	(GA)11	Class I	10805	10826	AGCGTGCTGGTGGGATTTGTGA	ACAGCGGTGAATCACGAGCACT	285
FMgSSR-24851	scaffold4692	p2	(GA)11	Class I	1034	1055	AGCTCAAACGCACGCCCAAA	TCACACGCCGTTAACCACCTCA	344
FMgSSR-24856	scaffold820	p2	(GA)11	Class I	87972	87993	AGGCTTTGCCGCTTTGGCTT	CACAGCGAATGAAGTGGCCCAA	211
FMgSSR-24857	scaffold2670	p2	(GA)11	Class I	48328	48349	AGGGAACAGTGCGGCGTCAAAT	AGTGCGGAAATCAGAGCCGT	289
FMgSSR-24860	scaffold4379	p2	(GA)11	Class I	35563	35584	AGGTAGGCTGTCGTGTGCTTGT	ACAGCGCTCGAGTCCGATGAAT	229
FMgSSR-24861	scaffold11765	p2	(GA)11	Class I	39695	39716	AGTCAGCGGCAAGCAGACAACT	AGCTGAAGGCTAGGCAACCA	290
FMgSSR-24864	scaffold977	p2	(GA)11	Class I	55136	55157	AGTCGACTTCGTCGTGAACAGC	AGGGGCAAACAAGACACACCGT	328
FMgSSR-24871	scaffold18294	p2	(GA)11	Class I	1526	1547	AGTTCTTCTACACGGCCAGCCT	AGCGAGACCGTCTTTGCTTGCT	309
FMgSSR-24872	scaffold12933	p2	(GA)11	Class I	2328	2349	ATCAGATGCCGCGGTTAAAGCC	AACTGTGCTTCTTGCGCCGT	246

SSR_ID	Scaffold	SSR_Type	SSR_Motif	SSR Class	SSR_Start	SSR_End	Forward sequence	Reverse sequence	Product_size
FMgSSR-24873	scaffold114	p2	(GA)11	Class I	36559	36580	ATCATCAAAAGCCCTAGGGGGAGG	TCCATGTTTGACAACCCGGC	259
FMgSSR-24874	scaffold960	p2	(GA)11	Class I	44406	44427	ATCCGACATGAGAGCGGCAT	ACGCACATTGTGGCTTGCTTCA	270
FMgSSR-24876	scaffold25064	p2	(GA)11	Class I	3290	3311	ATGACCACACTGCCACTGCCAT	TACTGGCCGTCGCTTGCTTT	345
FMgSSR-24879	scaffold1612	p2	(GA)11	Class I	65280	65301	ATGCCAAGGATATGCCCGTGCT	TCAGCTCGTGAGGCCTTCTT	300
FMgSSR-24882	scaffold2163	p2	(GA)11	Class I	37475	37496	ATGGCAGGGACACTTGCTGCAT	TCGCAAATACACTCCTGGACGA	299
FMgSSR-24886	scaffold1235	p2	(GA)11	Class I	82346	82367	ATTCGCAAGCAAGACGCACG	ATGCATGCGTGAGCCACAGT	311
FMgSSR-24887	scaffold14453	p2	(GA)11	Class I	6267	6288	ATTGCCGGAATGGCTCTGGTCT	TGGCATGGTGGGTTCGCCATAA	333
FMgSSR-24888	scaffold428	p2	(GA)11	Class I	113665	113686	ATTTGCGCCTGACTCACCACCT	TTCAAAAACGGCGTGGCGCT	286
FMgSSR-24896	scaffold23846	p2	(GA)11	Class I	2495	2516	GAGTTGAAGAAGGGGTCGGTCT	TCCACCGCATTGCAACACGAGA	325
FMgSSR-24898	scaffold62	p2	(GA)11	Class I	29551	29572	GCACTCTTCTTCCGGTGACATCCT	GTGACCTCTCCTTCTCTTTCTCCCA	345
FMgSSR-24902	scaffold830	p2	(GA)11	Class I	34625	34646	GCGTCGCATTTCAGTCCCCAAA	AGAACCGAAGAGCGAAGCGA	297
FMgSSR-24903	scaffold78	p2	(GA)11	Class I	20004	20025	GCGTTAATGTCGCACGCGGAAA	ATCAAAGCACCCCACCGCTT	299
FMgSSR-24905	scaffold3741	p2	(GA)11	Class I	24373	24394	GGCCAAATCCACACGTCGACAA	ATTCGCCGCCGAGATCATCA	294
FMgSSR-24906	scaffold193917	p2	(GA)11	Class I	315	336	GGCGCGGCATGGATTCTAAA	TGCTTAAGCGACGGAGACCA	332
FMgSSR-24910	scaffold13006	p2	(GA)11	Class I	14573	14594	GGTTGGTGGGTTTCTGCGTGTT	TTCGAACCACAAAGCCCCGT	310
FMgSSR-24916	scaffold1148	p2	(GA)11	Class I	54569	54590	TAGCGGCGGCAGATTTCTCT	ATCCTTCCTTCCTCGATCTGGC	312
FMgSSR-24926	scaffold7326	p2	(GA)11	Class I	17571	17592	TCATGACGTCCATGCTCACGCT	AGCACCAGGCCCTTCTTGATCT	207
FMgSSR-24930	scaffold4068	p2	(GA)11	Class I	27950	27971	TCCACAACACCAGCACACAGGA	TCCAGCACAAGCTGCAGTCA	308
FMgSSR-24934	scaffold248	p2	(GA)11	Class I	135065	135086	TCCATCGCCATGATCGTCCT	ACAACACCCACACCCGTAACCA	336
FMgSSR-24935	scaffold20791	p2	(GA)11	Class I	5500	5521	TCCCCCACTCTACTATGCCACGAA	GGCCCAACAAGGAAAAAGCCCA	222
FMgSSR-24936	scaffold178174	p2	(GA)11	Class I	275	296	TCCCCTGTGTCATTTTCCGCCA	GGGCGAAACTTTAGTCCTCGCAGT	297
FMgSSR-24939	scaffold39777	p2	(GA)11	Class I	4387	4408	TCCGAGACGCACTCGTTTGGAT	AGTTCTGTTGTGGGCACTGGT	223
FMgSSR-24941	scaffold15641	p2	(GA)11	Class I	15207	15228	TCCTGCCCACCCTTGTTCAACT	TCAGGTCCGGCGTTGTCAAT	249
FMgSSR-24942	scaffold2377	p2	(GA)11	Class I	39861	39882	TCGACCGATCACAACCACTGCT	TGCATGCCGATGGAGCCTATCA	332
FMgSSR-24943	scaffold1264	p2	(GA)11	Class I	59172	59193	TCGATCTCGAAGGACATCCGCT	CACGTTCATCGCTTCCTTGCGT	311
FMgSSR-24949	scaffold1295	p2	(GA)11	Class I	30916	30937	TCTAGCGCAACTCCCTGCTT	CCCCTTCCCCATTTTAGGAAGTCGT	347
FMgSSR-24951	scaffold13906	p2	(GA)11	Class I	4320	4341	TGAACAGTGCACAGCCAGCA	ACCCAAGCAACTGCTCTCGGAA	297
FMgSSR-24953	scaffold1744	p2	(GA)11	Class I	49592	49613	TGAAGCTGGTTCTCGTCGTCGT	ATTCGGGCGAGGGGTTGTTGTT	338

SSR_ID	Scaffold	SSR_Type	SSR_Motif	SSR Class	SSR_Start	SSR_End	Forward sequence	Reverse sequence	Product_size
FMgSSR-24958	scaffold3253	p2	(GA)11	Class I	38938	38959	TGAGCCGCCAGAGCAATAGAGA	AGTGCTCGACGCGGGTAAAACT	253
FMgSSR-24960	scaffold3765	p2	(GA)11	Class I	39424	39445	TGATAGGCGGCAGAGCAAGCAA	TGCTGTCTTGCCCGTTCGATGT	321
FMgSSR-24962	scaffold5049	p2	(GA)11	Class I	34041	34062	TGCAGGCTGCTCTGAGTTGACA	TCGCCTTGCATGCAGGAACAT	251
FMgSSR-24971	scaffold17558	p2	(GA)11	Class I	4205	4226	TGCGCTCGCACGTGAAATCT	ATATGCACGCCCTTTCCTCGCA	318
FMgSSR-24972	scaffold26816	p2	(GA)11	Class I	6832	6853	TGCGGATGGACAAAAGGGCT	GCAAACAGAGCACGGCAGTT	346
FMgSSR-24973	scaffold168574	p2	(GA)11	Class I	475	496	TGCGGTTGCCAAGTGAAGCCTA	TGGAAGCTGCTACGACGACCTT	305
FMgSSR-24975	scaffold20670	p2	(GA)11	Class I	1998	2019	TGCTCTTTCTCACGGAGCGT	ACACTGGATTAGGCGGTGGTGT	255
FMgSSR-24976	scaffold25105	p2	(GA)11	Class I	7663	7684	TGCTGCTTCTGCATGCTGGT	TGACAGGCTTGAGGGCCTTTCT	202
FMgSSR-24979	scaffold424	p2	(GA)11	Class I	69543	69564	TGCTTGGTGAATCAAGCCTTGTGG	TCGCTTCGCTGTATTGTTGCCT	228
FMgSSR-24982	scaffold15059	p2	(GA)11	Class I	682	703	TGGCAGTGCTCCTACCGAGTTT	TTATGGAACACGTGCGCCGACT	337
FMgSSR-24984	scaffold40327	p2	(GA)11	Class I	2812	2833	TGGGAGATCGATGATGGATGCCT	ACCTCAGCTCTGACCGACCTTT	296
FMgSSR-24987	scaffold1772	p2	(GA)11	Class I	8492	8513	TGGTGTCGTTGTTGCTGCCA	TCAAACCCTAAGCCCCTCAATCCC	312
FMgSSR-24992	scaffold5637	p2	(GA)11	Class I	33092	33113	TGTGTGACTGACTCCCGTATTCTC	TCCATACATCTGAGCCAATCAGCC	207
FMgSSR-25000	scaffold5096	p2	(GA)11	Class I	5263	5284	TTCCGAGGCCACGCAAACTT	TGCCCACCCACTGCCTTTTACT	205
FMgSSR-25005	scaffold2362	p2	(GA)11	Class I	22792	22813	TTGCCGATGGTGGTTGATTGCG	AAAGGCAAAAGCGGTGGCGT	332
FMgSSR-25007	scaffold1115	p2	(GA)11	Class I	72689	72710	TTGCGGTTCGCGAGCAAAGT	AGAAGCTTTTGCGGCGAGTG	253
FMgSSR-25011	scaffold7397	p2	(GA)11	Class I	20078	20099	TTTAGATCCGTGCGACGCGACA	TGAAGGCTCGTACCGACGAACA	276
FMgSSR-25012	scaffold20	p2	(GA)11	Class I	245643	245664	TTTAGATCCTAGCCTCACCCCACC	ACGGTTCAGAGAGGTGCACACA	341
FMgSSR-25016	scaffold12784	p2	(GA)12	Class I	17303	17326	AAACGGAAGAAAGTGCGCCG	AACATGACACCACGCACGGGAA	323
FMgSSR-25018	scaffold1035	p2	(GA)12	Class I	65100	65123	AAAGAAACGGACGCCGCACT	AACCAAAGGGTGCGCCCAAA	234
FMgSSR-25019	scaffold432	p2	(GA)12	Class I	92226	92249	AAAGTACGCGCCCGAACACA	ATTTGGACGACCACGCCGTT	306
FMgSSR-25022	scaffold3135	p2	(GA)12	Class I	12991	13014	AACTCCAGCCATTGCCATCCGA	AGTGCAACAGTGCAAACGGACG	256
FMgSSR-25024	scaffold1093	p2	(GA)12	Class I	68717	68740	AAGGCTGTAAACGCGACGGA	TCGTTATTAGTCGCTGGGCGCT	263
FMgSSR-25028	scaffold6546	p2	(GA)12	Class I	33165	33188	ACACAAAAGCGAGCCGCGTA	TGGTATGCATGGCTGGATGCCT	313
FMgSSR-25030	scaffold13200	p2	(GA)12	Class I	20012	20035	ACACGAAATGGCCCAGAACGGA	AAACGAAGTCGAGGCCGCTGAT	330
FMgSSR-25033	scaffold36946	p2	(GA)12	Class I	775	798	ACCACTTGCAGGGCAGAATCGT	CGTGATCATGACTATCGGTGACTC	318
FMgSSR-25035	scaffold100	p2	(GA)12	Class I	3003	3026	ACCATGCACGATACAGGAGGGA	CAGCGCATGTTTGGGTGCAA	248
FMgSSR-25040	scaffold3086	p2	(GA)12	Class I	16476	16499	ACGCGGTCCGGAGTGAATAGAA	ATGTCCCTTGACCAGCCGGTTT	303

SSR_ID	Scaffold	SSR_Type	SSR_Motif	SSR Class	SSR_Start	SSR_End	Forward sequence	Reverse sequence	Product_size
FMgSSR-25043	scaffold558	p2	(GA)12	Class I	20213	20236	ACGTGGTTGCCGGTTCCTTCTT	ATTCCTGCCCAAACCACCGT	289
FMgSSR-25047	scaffold377	p2	(GA)12	Class I	126518	126541	AGAAAGGGGGACGAATGTGCGA	CCGGCAGCTGGCACCAATTTTT	303
FMgSSR-25051	scaffold1259	p2	(GA)12	Class I	90628	90651	AGCACGAAACTTCCGCCTGT	ACGCCATTCCATGGCCACACAT	212
FMgSSR-25052	scaffold3055	p2	(GA)12	Class I	57999	58022	AGCAGTTGTGCCAGGATGGA	TGTCCTAAACTGCCGTCACAGG	343
FMgSSR-25053	scaffold3156	p2	(GA)12	Class I	11127	11150	AGCATGCCTTCCTCCAGTGA	GCCACCTCCTCCCATCTTTGGATT	296
FMgSSR-25056	scaffold12848	p2	(GA)12	Class I	16531	16554	AGCGCGGCAGATTTGCTTTGA	TCGGGCGCTTTGGCTTTCTT	305
FMgSSR-25059	scaffold3150	p2	(GA)12	Class I	46356	46379	AGCGTCCGCCTCCAAAAGAA	ATTGACTCCCACCTTCCTCGGT	340
FMgSSR-25064	scaffold2549	p2	(GA)12	Class I	47815	47838	AGCTTTGCGAGTGCGGCTAT	TGTGTAACAGTGCTCTCCCTCC	345
FMgSSR-25066	scaffold1240	p2	(GA)12	Class I	13139	13162	AGGCAAGGAAAAGGCTGCATTC	TTGCCTAGCCAGGGTGACGTTT	316
FMgSSR-25069	scaffold9238	p2	(GA)12	Class I	29344	29367	AGGCTTTAGAAGCCGGTCGCAA	AAGAGGGCTCCGACTTCAGCTA	350
FMgSSR-25070	scaffold1	p2	(GA)12	Class I	371353	371376	AGGGATGGGGAATTGGGGAATGGA	ACAGATGCTTCAACGGGCCACA	312
FMgSSR-25076	scaffold3201	p2	(GA)12	Class I	19171	19194	AGTGGGTCACATGACACGAGAGT	TTTGGTCTTGTAGGGCCTGGGT	212
FMgSSR-25077	scaffold22788	p2	(GA)12	Class I	12045	12068	AGTGTAGAGCCAAACGGGCCAA	TTCCCCATCAAGCCGCCATTCT	235
FMgSSR-25078	scaffold4558	p2	(GA)12	Class I	868	891	AGTGTGGCTGTGTGGGATCT	TTCAAGACAGCCATCCTCGGCA	247
FMgSSR-25088	scaffold1809	p2	(GA)12	Class I	20515	20538	ATCAGTGTTGGCCGGCTATGCT	ATGCGGCGTCATTCGCTTCT	321
FMgSSR-25092	scaffold20929	p2	(GA)12	Class I	9337	9360	ATGCACGACTGTGGCTTGGT	ACAAACCAGGGAGCAAGAGCA	347
FMgSSR-25095	scaffold2444	p2	(GA)12	Class I	57407	57430	ATTGCGCGAGATGCAGGGTT	TCGGGGTTTGGTTGTCGGTT	339
FMgSSR-25096	scaffold23123	p2	(GA)12	Class I	960	983	ATTTGAGCCGTCGCCGTTGT	ACTTCACGAGCCCTACGATGAT	344
FMgSSR-25099	scaffold28830	p2	(GA)12	Class I	3979	4002	CGCCCGTTGTGCCGCTTTTTAT	ACAATTCACCGCTACAGGCCCA	272
FMgSSR-25102	scaffold1995	p2	(GA)12	Class I	29306	29329	GCAACGCGACGCACAAACAA	AAAGCACGGGCACGCAACAA	282
FMgSSR-25103	scaffold6746	p2	(GA)12	Class I	11173	11196	GCATGCTGCAAGGACTATCATGGA	AGGTCGTCAATTGCTCCGTCGT	299
FMgSSR-25110	scaffold770	p2	(GA)12	Class I	11069	11092	GTCTGTTGCTTTGCAGTGGAGT	TGGTCGTTGGCAATCGGAGCAT	342
FMgSSR-25112	scaffold281	p2	(GA)12	Class I	69066	69089	TACATCTCAGTGCGCACGTCCA	AAGGCTCCACCGCTAACACT	272
FMgSSR-25117	scaffold1481	p2	(GA)12	Class I	61252	61275	TATAAGTGCGGAAGTGCCGCGT	TGCGGCCGATGATGCTGGATAA	347
FMgSSR-25118	scaffold16072	p2	(GA)12	Class I	1633	1656	TCAAGTCCCTGCACGGGAAACA	ACCCAAAAGCCAGCAAACCCT	345
FMgSSR-25119	scaffold7592	p2	(GA)12	Class I	34229	34252	TCAGCGCACGTACTTCATGGGA	TGTCAACACATGGAGGATGGCACA	347
FMgSSR-25120	scaffold977	p2	(GA)12	Class I	46665	46688	TCCAAGCTTTCATGGGCCAACCA	AGGCTGGAAACGTGAACCAGGA	304
FMgSSR-25124	scaffold38149	p2	(GA)12	Class I	2182	2205	TCCGGCTGTGCTGACTTCAA	TCCTGAGATCCAAAAGCCGTCACC	304

SSR_ID	Scaffold	SSR_Type	SSR_Motif	SSR Class	SSR_Start	SSR_End	Forward sequence	Reverse sequence	Product_size
FMgSSR-25127	scaffold4125	p2	(GA)12	Class I	53961	53984	TCGCCGACAATGGCGAACAA	AGGCAGTCATCTTCTTCCTCCCGA	228
FMgSSR-25130	scaffold522	p2	(GA)12	Class I	35159	35182	TCGGCAGCTGAAAGACACGA	TCAAATTGCAGCCGCGAGCA	308
FMgSSR-25132	scaffold1285	p2	(GA)12	Class I	59013	59036	TCGTCGTCGGCTTCTTCGGATT	TGAATGCTGATCCACCACCCCT	321
FMgSSR-25134	scaffold4541	p2	(GA)12	Class I	42990	43013	TCTGCCAAAAGAGGCGCCAA	TCCTTACAACCGCAGCCACAGT	282
FMgSSR-25135	scaffold136	p2	(GA)12	Class I	133219	133242	TGAGCACCACACATCCACACCA	TTTTCTCCTCCCACGCACCACA	323
FMgSSR-25140	scaffold683	p2	(GA)12	Class I	19813	19836	TGCAACGTGCATGTCTTGCG	AAGGGCAATGCGTGTAGGCT	211
FMgSSR-25141	scaffold7335	p2	(GA)12	Class I	19480	19503	TGCAAGCAATGTATGGGGCCT	TGCTTCTGGGCTGTGTTCGT	331
FMgSSR-25146	scaffold4288	p2	(GA)12	Class I	25584	25607	TGCCCAGACCACGACGTTTGTT	TACGGGCGACCTTAACACCACA	307
FMgSSR-25149	scaffold2734	p2	(GA)12	Class I	53160	53183	TGCCTGCGAATGTGGAATCCCT	TGCTTACGTGGTGCAGGCTT	330
FMgSSR-25155	scaffold15183	p2	(GA)12	Class I	5311	5334	TGCTGATGTTCCAGCAGTAGCCA	AAGCGCCAGCGCAAGATTCA	341
FMgSSR-25156	scaffold10804	p2	(GA)12	Class I	11787	11810	TGCTTGCTTATACGGCGGCT	AGCGGAGCCATCAAAGCTGA	221
FMgSSR-25159	scaffold10823	p2	(GA)12	Class I	1213	1236	TGGCGGCATGCGTCATTCTT	ATTGCTGCTTAGCCCCTCTGGT	272
FMgSSR-25161	scaffold22204	p2	(GA)12	Class I	5560	5583	TGGGATGAGCTCGTCAAAGCCT	AGCCTCGCGTGGTGTTATCCAA	261
FMgSSR-25163	scaffold3257	p2	(GA)12	Class I	2014	2037	TGGGGTTCAACAATGCCAAGCG	AGCAGGCCACCATCGCTTTCTT	231
FMgSSR-25164	scaffold1637	p2	(GA)12	Class I	55613	55636	TGGTCACGATTGGCGTAGCA	TTTGTGTCATGCGACAGGCACC	304
FMgSSR-25165	scaffold775	p2	(GA)12	Class I	82627	82650	TGGTTCTTCGACCGTGCCAA	ACGCATCGACCGGAAAAGAGGT	316
FMgSSR-25169	scaffold9305	p2	(GA)12	Class I	19173	19196	TTACACGGTGCAACTGGATCAGGG	TCCTTGGCTCCTTGCTGTTGCT	247
FMgSSR-25170	scaffold714	p2	(GA)12	Class I	57114	57137	TTACGCAGGTGAAATCGGGCCA	GCGCGTTCATATGCATGGTGCT	234
FMgSSR-25171	scaffold10752	p2	(GA)12	Class I	471	494	TTACGGGCCCAATTAGAGCGCA	AGCGTGTGGTTGGTCGCTTT	244
FMgSSR-25172	scaffold244	p2	(GA)12	Class I	35492	35515	TTCATCTCGCGCTGCTTTCCCT	TTGCGACCCATCGTCAAGAGCA	285
FMgSSR-25177	scaffold5168	p2	(GA)12	Class I	16137	16160	TTGCACGTCGCACCACCATT	AGCTGCCACTCATCCCTTTCCT	272
FMgSSR-25179	scaffold7607	p2	(GA)12	Class I	14767	14790	TTTGCATGGCGAAGCAGCGA	CGTTGCTTATGCTGATGTTGGTGC	295
FMgSSR-25181	scaffold17412	p2	(GA)12	Class I	7829	7852	TTTGCCGTGGAAGCTGGCAT	ACTTGCCTCGTCGCAATGCT	325
FMgSSR-25184	scaffold23056	p2	(GA)13	Class I	3771	3796	AACGCGGTCAGGAACAGTCA	AGGCCAACCATGAGCTACACCT	300
FMgSSR-25185	scaffold1578	p2	(GA)13	Class I	63020	63045	AACTAGGCTGCCGCATGGATCA	TGTGCTCTGCAATGGCATCACC	263
FMgSSR-25190	scaffold31586	p2	(GA)13	Class I	4040	4065	AATGCGTTCTCGTGTAGCGGGT	TGCATGCATGAGGCTTTCGAGG	223
FMgSSR-25191	scaffold4654	p2	(GA)13	Class I	10510	10535	AATTCGTGCGTGCTCGGTGA	ACGTTCAAAGCCTTCGGGCA	288
FMgSSR-25194	scaffold1843	p2	(GA)13	Class I	61684	61709	ACAACTGCGTGACAAAGGCGT	AGGTGCTTTGCTGTGTGCTGGT	291

SSR_ID	Scaffold	SSR_Type	SSR_Motif	SSR Class	SSR_Start	SSR_End	Forward sequence	Reverse sequence	Product_size
FMgSSR-25199	scaffold3162	p2	(GA)13	Class I	20803	20828	ACCACCCTTGCATTGTCATCCCA	AGGCGGTGAAGAGTTTGTGCCA	317
FMgSSR-25203	scaffold606	p2	(GA)13	Class I	85781	85806	ACCGATAATGATTTGTGGCGGGC	ACGCGCATGATTTGTCGGCT	328
FMgSSR-25207	scaffold4330	p2	(GA)13	Class I	11691	11716	ACGCCCAAATCGAAGTTCAGGT	TGTGGAGCGAGCGACACAAACT	219
FMgSSR-25208	scaffold1185	p2	(GA)13	Class I	24743	24768	ACGGACCAAGCAAGCACAGACA	TCGGCGCCGTAACTTCTTGT	325
FMgSSR-25209	scaffold4429	p2	(GA)13	Class I	36127	36152	ACGGCCGGATTCGATCAGCTTT	AAGCAATCTGCACACGCCCT	338
FMgSSR-25210	scaffold602	p2	(GA)13	Class I	15312	15337	ACTGCCTTTGCTTCAGCCCA	AAGCAGCGGGGAATTACGCA	346
FMgSSR-25224	scaffold6816	p2	(GA)13	Class I	2936	2961	AGCTTCATGTTGCGGCCGTT	TGATGAGAGGTGGCAGGCTTGA	321
FMgSSR-25227	scaffold851	p2	(GA)13	Class I	70019	70044	AGGAGCAAAGTCTGCCTCTCAGT	TCAGCTCTAAAGCCCACATGCCT	329
FMgSSR-25232	scaffold9257	p2	(GA)13	Class I	18246	18271	AGGTGTAATAGTCTTGGCGGCACA	ACATGTGGCAGTGGAGCACACA	231
FMgSSR-25233	scaffold12926	p2	(GA)13	Class I	12557	12582	AGGTGTTGTCGGCAACTCACAGA	AACTCGCCTTGCAGCAGCTT	217
FMgSSR-25238	scaffold1993	p2	(GA)13	Class I	14748	14773	ATCAAGGGAGGTGGAGCCACTTGT	AGCCCACCCACCGTTAAAGTCA	287
FMgSSR-25239	scaffold349	p2	(GA)13	Class I	125709	125734	ATGAGAAGTGCCTTGCTTGCCG	AAGGAGCAGCAGCAGCAGAA	335
FMgSSR-25240	scaffold2969	p2	(GA)13	Class I	19195	19220	ATGAGTGCCACCACACTTGGCA	AGTTTAAAGCGCGGGCACATGG	316
FMgSSR-25241	scaffold525	p2	(GA)13	Class I	102946	102971	ATGTTGCAGAGCGCGGAAGA	ACACCACGCAAGCAAAACCCA	211
FMgSSR-25242	scaffold5584	p2	(GA)13	Class I	38043	38068	ATTCGTGGCCTTGCGAGTGA	AGCCCACCAACAAACTGCCA	307
FMgSSR-25248	scaffold175	p2	(GA)13	Class I	57358	57383	CGCAAGGTAAGAAGGGAAACACGC	ACACACTGCCTCTCTCAGTGCT	227
FMgSSR-25251	scaffold1261	p2	(GA)13	Class I	40640	40665	GCAGCCAAGCGACACAGTTGAT	AGGATCGCGTCACGTGGAAA	212
FMgSSR-25252	scaffold357	p2	(GA)13	Class I	15249	15274	GCCAGCGAGATGCTCCTTGAAA	ACCTCTCTTTCTCCATGTCGCTGC	297
FMgSSR-25254	scaffold24002	p2	(GA)13	Class I	5799	5824	GGCGGTCAAATCTGTGCATGGT	TTGGCCATTTGGGCTCACCT	273
FMgSSR-25256	scaffold1537	p2	(GA)13	Class I	29589	29614	GGGCTTTGCTTTGACAGCACCA	ATGAATTGCTCCAGCGCCCA	327
FMgSSR-25259	scaffold3101	p2	(GA)13	Class I	7240	7265	GTGCGCGGCGACGAATTAAA	TCCCTCCTGTTGTGTGTGTGCT	290
FMgSSR-25261	scaffold2410	p2	(GA)13	Class I	30615	30640	TCAAGAGGAGCAGCCAAAGCGT	ATCGGGTCTTCAGAGTGCTCCA	300
FMgSSR-25262	scaffold249	p2	(GA)13	Class I	115851	115876	TCAAGCTGGAGCTGTTGTACGC	AAGCTTCCGGTGCAAGCGAT	293
FMgSSR-25265	scaffold110607	p2	(GA)13	Class I	140	165	TCACGGGTTTGTCCACGTCT	TTCGTCTCCGAGCATGTCACCA	289
FMgSSR-25266	scaffold734	p2	(GA)13	Class I	62455	62480	TCACTCACCACGGTCGTCGTTT	TTTGGTTGCGCGTGACTCGT	247
FMgSSR-25270	scaffold880	p2	(GA)13	Class I	9369	9394	TCCGCTGTGGCCATTTCAGGAT	TGAGTGGAAGAAGTAGGCCCCA	307
FMgSSR-25275	scaffold3665	p2	(GA)13	Class I	23298	23323	TCGAACCATCGTCCTTCGTCGT	ATCGGTTCTTGGCGCTTGCT	210
FMgSSR-25280	scaffold111	p2	(GA)13	Class I	144753	144778	TCTTGGCATGACCGGTTGAGGT	TCGTGGATGGCAAAGTCGCA	271

SSR_ID	Scaffold	SSR_Type	SSR_Motif	SSR Class	SSR_Start	SSR_End	Forward sequence	Reverse sequence	Product_size
FMgSSR-25288	scaffold384	p2	(GA)13	Class I	109692	109717	TGCACAACGGAGGCGTGAAGAA	AGCCGCTCGAATCTGCAAGT	231
FMgSSR-25290	scaffold7069	p2	(GA)13	Class I	12071	12096	TGCATTCACCACCCAAGCGT	AAACAACGGTGCAACCGAGC	243
FMgSSR-25292	scaffold1110	p2	(GA)13	Class I	57922	57947	TGCCGCGAAGCTAAATGCCT	ACAGTCGATGCGATGCCAACCT	343
FMgSSR-25293	scaffold2874	p2	(GA)13	Class I	314	339	TGCGCTGATCTGTGGCGTTT	TGCAGACTCCACTGCCGTTT	323
FMgSSR-25306	scaffold3306	p2	(GA)13	Class I	51432	51457	TGGGTTGGTGGTGTGAGTGCAT	TATGGGCACATGGCACGACT	349
FMgSSR-25308	scaffold468	p2	(GA)13	Class I	105038	105063	TGTGCAAACGGCGAGTGCTT	AAGCGTTCGAAAGCGTGCTG	346
FMgSSR-25309	scaffold12842	p2	(GA)13	Class I	6081	6106	TGTGCAGACAACCACCATCAGA	AGGGGTGAGCCAAAATGCTGGT	238
FMgSSR-25312	scaffold67997	p2	(GA)13	Class I	100	125	TGTGGCTTGGTCCCTATGGAAA	ACACAAACCACACATGCACTGG	296
FMgSSR-25318	scaffold6602	p2	(GA)13	Class I	8847	8872	TTAACCGCCACACCACCCATGT	TCCCGTGACTGATCGAGCGAAA	258
FMgSSR-25319	scaffold4408	p2	(GA)13	Class I	27148	27173	TTCACATGCCTCCTGCACGACA	TGTCACGACGCGAGTAGAAAAGCA	286
FMgSSR-25320	scaffold4481	p2	(GA)13	Class I	41524	41549	TTCACCGGAGCGCATGCAAA	TGCGCTGTCAATGACCACTGT	282
FMgSSR-25322	scaffold1120	p2	(GA)13	Class I	80232	80257	TTGCACGCGCATGGTAGCTT	GCGCCCATGTTTTTAACCCGCT	303
FMgSSR-25325	scaffold29364	p2	(GA)13	Class I	7472	7497	TTGCCTTCCCCTTCCTTGGTCT	AGTTCGATTTGGTGGTGGCGGT	332
FMgSSR-25327	scaffold4171	p2	(GA)13	Class I	7152	7177	TTGCTCAAGCCGAAGACTGCAC	AGTAGAGCACCTCTCCTCCATCCA	337
FMgSSR-25329	scaffold115368	p2	(GA)13	Class I	984	1009	TTGCTTTGCATGCCCCCTAGCA	TTGGCTGGCACACGCATCAT	238
FMgSSR-25332	scaffold86	p2	(GA)13	Class I	68791	68816	TTGGTAGGCACACAAGCGGA	ACGCGAGTGCATGCTTTCCA	233
FMgSSR-25334	scaffold251	p2	(GA)13	Class I	56540	56565	TTTGCGCATATGGGCCCTCGAT	TGCCACTGCCATGCAAGGAT	283
FMgSSR-25335	scaffold2906	p2	(GA)13	Class I	22516	22541	TTTGCGTTCCCTTTTGTCGGCG	AAACGAAGCAGAGTGCGCGA	325
FMgSSR-25340	scaffold1260	p2	(GA)14	Class I	12116	12143	AAGATCGACCGGGAGCTGTTCA	TCCGATCGAACAGGGCCATT	329
FMgSSR-25342	scaffold1953	p2	(GA)14	Class I	61539	61566	AAGCTATGCTGCTCGGTGGAGT	AGGTTTCAAACGACGAGGCGCT	289
FMgSSR-25346	scaffold13604	p2	(GA)14	Class I	18681	18708	AATACATCCGCCCTGCGCCATT	AGGCGGCTCAAAATACACTGCC	315
FMgSSR-25347	scaffold894	p2	(GA)14	Class I	2563	2590	AATCATGTGTCGCGTCCGCA	TTGCGGCTCAGCAATGGCACTA	236
FMgSSR-25350	scaffold50002	p2	(GA)14	Class I	2743	2770	ACAAGCACGTCCCTGGTTCA	AGCTGCTCGAGGAAACAGCA	309
FMgSSR-25356	scaffold7305	p2	(GA)14	Class I	31669	31696	ACCGCTTCTGGGGACAACAA	ACCCGAGCAGACACGGGTTAAT	333
FMgSSR-25357	scaffold21028	p2	(GA)14	Class I	6538	6565	ACGAAGCAACCCGAAACGCA	TCGTCGAGATCCGCATCGACTT	271
FMgSSR-25358	scaffold820	p2	(GA)14	Class I	90154	90181	ACGAGAAGCAGCAGCATGACCA	CGCGCCACAACCAACCAACATA	346
FMgSSR-25363	scaffold835	p2	(GA)14	Class I	86372	86399	ACGGCCATGAAAACCCAACCCA	TGCCTTTTAAGCAACGCGCC	318
FMgSSR-25368	scaffold770	p2	(GA)14	Class I	17473	17500	AGCAGCATACGCCGTTCTCT	ATGGGCATGGACATGAGCCCTT	232

SSR_ID	Scaffold	SSR_Type	SSR_Motif	SSR Class	SSR_Start	SSR_End	Forward sequence	Reverse sequence	Product_size
FMgSSR-25376	scaffold10	p2	(GA)14	Class I	268581	268608	AGGAATGCCATTGAGCAAACCCAC	GCATGCGGCAAACCCCATCAAA	292
FMgSSR-25378	scaffold13143	p2	(GA)14	Class I	16252	16279	AGGCCTGTTGATTGGGCAGCTA	AAATAATCTGCCCCTGCCCACC	350
FMgSSR-25385	scaffold604	p2	(GA)14	Class I	110610	110637	ATCACAACCTTCGCCAGCACCA	ACTCATCGCACTGCTCACACCA	231
FMgSSR-25387	scaffold10831	p2	(GA)14	Class I	15809	15836	ATCGTCGTAGGACGCTATCGCT	TCAACTCAGCCCACAACTGGCA	336
FMgSSR-25388	scaffold5410	p2	(GA)14	Class I	17383	17410	ATGCACCCGCCACGGTTTTT	TCTTGGCGTCGTCACGTTCA	270
FMgSSR-25389	scaffold551	p2	(GA)14	Class I	29286	29313	ATGCCCTGGCTTGGCTTCACTT	AAACTCCCACCCCTTTGCGA	282
FMgSSR-25392	scaffold5368	p2	(GA)14	Class I	41638	41665	ATTGCCTGCTGGTTGCTGGACT	GCCTGTATCCCTCTCTTTCTCACAC	233
FMgSSR-25393	scaffold253053	p2	(GA)14	Class I	199	226	CCACCGATAGCTAGGGTTAGGTTGA	TGTTTTACCCAAGCCGGCCCAA	258
FMgSSR-25395	scaffold118	p2	(GA)14	Class I	79063	79090	CCGTGCTTGTTGGTGGCAAACT	AGGGTACGTTTAGGCTTTTCCAGC	220
FMgSSR-25396	scaffold18236	p2	(GA)14	Class I	14721	14748	GCATACCGGCAATGATGTGGCA	CCCCTAAGCATGCGCCACTTTT	258
FMgSSR-25397	scaffold4973	p2	(GA)14	Class I	18915	18942	GCATCGCGCATTTCGGCATT	TGGTGCTAGGTGCTTGCGTGTA	286
FMgSSR-25398	scaffold10933	p2	(GA)14	Class I	18228	18255	GCGCGTGTGTAAGAGAGAGAGA	AGGTGCGTGTAAATGGCCCAGA	347
FMgSSR-25403	scaffold907	p2	(GA)14	Class I	1239	1266	TCAACCCTAACGCTCGTCGCAA	TGCTATTGCGCAGCCCAGAA	324
FMgSSR-25404	scaffold52156	p2	(GA)14	Class I	3083	3110	TCACACAACGACGGAACGGTGT	TGAAACCATGTGGCGGCCTT	348
FMgSSR-25405	scaffold4171	p2	(GA)14	Class I	12601	12628	TCACGCGGCTGCTTCAATCT	ACGAGCGATGATGGCAGAGGAT	232
FMgSSR-25410	scaffold7442	p2	(GA)14	Class I	14501	14528	TCCCTTTCCTCCTGCCTCTTTCCA	AAGCTAGCGCGCACACACAT	255
FMgSSR-25415	scaffold146108	p2	(GA)14	Class I	294	321	TCGTATCTTGTTGCCGCAGGCT	AGCGTAAGCCGGTGCAGGTAAT	344
FMgSSR-25418	scaffold1514	p2	(GA)14	Class I	48017	48044	TGACGAAGCCTCCTCCATAGCA	TATGGGGCGACAAACAGGCTGA	318
FMgSSR-25420	scaffold5503	p2	(GA)14	Class I	9306	9333	TGATTGATGCCGCTGCGTGT	AGCTTGAGCAGCATTTGGGTGC	269
FMgSSR-25424	scaffold150	p2	(GA)14	Class I	172404	172431	TGCATGGCAACGGCAAGACA	TGCCAATTGAAGGGCCAGCA	202
FMgSSR-25425	scaffold734	p2	(GA)14	Class I	53169	53196	TGCCTAGCTAGAATGGCGCCTAGA	AGAGCCGCATGCATCACTGT	272
FMgSSR-25429	scaffold1585	p2	(GA)14	Class I	51853	51880	TGGATGCACCTTTCATGTCCAGCA	ATCTCACGGCTGCCACGTAT	240
FMgSSR-25430	scaffold7121	p2	(GA)14	Class I	28591	28618	TGGCCGTGGCAGAACAAATGGT	AGGAACGCGCAACCTGACAT	235
FMgSSR-25432	scaffold10543	p2	(GA)14	Class I	13758	13785	TGTACTCCGCTAGTTCCCGAAGGA	ACAACCGTCCGATCCGCATCTT	202
FMgSSR-25438	scaffold630	p2	(GA)14	Class I	76160	76187	TTCTGGCCAGGTTGTTGGCGAT	TGCCACACACGCATGACTCT	278
FMgSSR-25439	scaffold5671	p2	(GA)14	Class I	5913	5940	TTGCACTGCACGCACACGTA	ACTGCCGCTTCAGATATGGCGT	210
FMgSSR-25443	scaffold3552	p2	(GA)14	Class I	29514	29541	TTGGGTGGTGCGATTGGCAT	TTGTGCTGTGCTGGCCACATGA	333
FMgSSR-25446	scaffold179884	p2	(GA)14	Class I	94	121	TTTTCCAACGAGCTTGGCTGGG	AACATGATCTTCACGCCGCCGA	261

SSR_ID	Scaffold	SSR_Type	SSR_Motif	SSR Class	SSR_Start	SSR_End	Forward sequence	Reverse sequence	Product_size
FMgSSR-25447	scaffold25274	p2	(GA)14	Class I	6033	6060	TTTTGTTGGTTCGTGCGTCCCC	AAGCAATCCTTCCGCTCCCT	300
FMgSSR-25452	scaffold975	p2	(GA)15	Class I	35705	35734	AAATCACACGCGCACCCCTT	TGCATGTGGCGACTGAATGC	349
FMgSSR-25462	scaffold6832	p2	(GA)15	Class I	6925	6954	ACGACGTGTTGGTTGGAAGCTCA	TTCACCGCGTTATTGGCCTGCT	342
FMgSSR-25463	scaffold1697	p2	(GA)15	Class I	42149	42178	ACGCGCATCAGCAAAGGCAA	TTTCCGCGGTCTCCTTGTTGCT	292
FMgSSR-25466	scaffold324	p2	(GA)15	Class I	128593	128622	ACTGCAATAATGCGTGTGGGG	TGGTTGCCACCTTCCAGGTTCA	298
FMgSSR-25471	scaffold6603	p2	(GA)15	Class I	6106	6135	AGCAAGCAACCAGCCAGGCTAA	TGGTGAAGGGGAAGTCGTGCAA	303
FMgSSR-25472	scaffold16276	p2	(GA)15	Class I	4227	4256	AGCAGCCAAGGCTGTTTCCCAA	TTATGGTGCTGCGTTCCTGC	321
FMgSSR-25474	scaffold8268	p2	(GA)15	Class I	697	726	AGCCGCAGAATAGTGCTGATTGG	AGGGGTTCAGCGGTTTCAGCTA	350
FMgSSR-25484	scaffold42615	p2	(GA)15	Class I	1479	1508	AGGGTATTTCTTCGCACCGACA	TGGACGCTCTAAATCTGCTCCCA	233
FMgSSR-25486	scaffold768	p2	(GA)15	Class I	96437	96466	AGTGAATGGCCTTGCGCTGA	TGTGGCCCACCACTTGTGTA	258
FMgSSR-25492	scaffold13610	p2	(GA)15	Class I	18432	18461	ATCGCTGCACGGACAAAGGA	ACGGCCTAAAACACCATCCGGT	309
FMgSSR-25494	scaffold31288	p2	(GA)15	Class I	5288	5317	ATGCTAGGGGATAGTTGGGCGA	CCCATCTAACGCCCTGCCTTAAACA	309
FMgSSR-25504	scaffold5973	p2	(GA)15	Class I	29038	29067	CGGGATGCAACGACATTCAGCA	TTGTTGCGTTGGGACCCGAT	326
FMgSSR-25508	scaffold899	p2	(GA)15	Class I	58969	58998	TAGAGAACCCAGCTTGCCGCTT	TTCAACCTGTCTCGCGTCGTCA	295
FMgSSR-25510	scaffold184260	p2	(GA)15	Class I	189	218	TCACGAGGGCTGCAAAAGGTGT	AGCGACAGCAAGTGTTGCGA	207
FMgSSR-25514	scaffold4383	p2	(GA)15	Class I	29767	29796	TCCATTGCCTCGTTGTGCCA	AACCAGGAACTCACAAGTCCAGCC	314
FMgSSR-25519	scaffold380	p2	(GA)15	Class I	60071	60100	TCGTCCTTGATGGTGGTTGCCA	TTAGCTCTCGTCATGCACCCGT	218
FMgSSR-25520	scaffold1974	p2	(GA)15	Class I	22718	22747	TCGTGGTTACGAACGCATCGCT	ACACGCGCGTTAGTTCGGTT	333
FMgSSR-25522	scaffold22621	p2	(GA)15	Class I	2756	2785	TCTGCTACATGAGTGCATCCCT	TCCGTGCAAGGTGATCTGGGAA	281
FMgSSR-25529	scaffold828	p2	(GA)15	Class I	27751	27780	TGATGTCTCACTGTTGCGCCGA	ACCATTCATGCCGCAGGGAACT	342
FMgSSR-25530	scaffold18988	p2	(GA)15	Class I	5026	5055	TGCACAAGCATGCGCAAACG	TGATCGTCCAGTCCAACCCGAA	346
FMgSSR-25531	scaffold1962	p2	(GA)15	Class I	69901	69930	TGCCGCTGATGCTTTTTCCGT	AATCAGCGGATCGCAACGCA	301
FMgSSR-25535	scaffold47	p2	(GA)15	Class I	158344	158373	TGGCCATGAATGTTACGCCCGA	GCACAGACGAAGCATTGCCCAT	278
FMgSSR-25536	scaffold29	p2	(GA)15	Class I	146529	146558	TGGCGAAGCTGCACAGATGA	TGTCGAAAATCGGGTGGGTGTCA	330
FMgSSR-25538	scaffold894	p2	(GA)15	Class I	6734	6763	TGGGTGCTACACGTGCTTCT	AGCAGCGCACAAAAGCCAACT	346
FMgSSR-25543	scaffold537	p2	(GA)15	Class I	108051	108080	TTCCTTCCCCGCCCAACAAAGA	AACGCCCCTTCCACAACGGTTT	333
FMgSSR-25548	scaffold12618	p2	(GA)15	Class I	6219	6248	TTGTGACGGCGTTCGGACAA	AACTCTCCGTTACTGGCCTGCT	321
FMgSSR-25553	scaffold224	p2	(GA)16	Class I	21385	21416	AAAAGCACCGTCCACACTGCCA	TCATAGCGGCATCGCCAAGT	297

SSR_ID	Scaffold	SSR_Type	SSR_Motif	SSR Class	SSR_Start	SSR_End	Forward sequence	Reverse sequence	Product_size
FMgSSR-25559	scaffold16283	p2	(GA)16	Class I	1386	1417	AAGATCCACCGATGACCGTCAGGA	TTGCCCTCCACACACACACT	258
FMgSSR-25560	scaffold5475	p2	(GA)16	Class I	4179	4210	AAGCTGCACGCAAGCATGTC	TGCACGCAAAGGAACACGATGC	237
FMgSSR-25566	scaffold1921	p2	(GA)16	Class I	10019	10050	ACAAATTGGGCCCACCGCCTAT	AATCCAGCGCGGCCACTTCTAT	346
FMgSSR-25568	scaffold343	p2	(GA)16	Class I	34357	34388	ACATCTCAAGGCAAGGCAAAGG	GCGTTGCTTGGAACCGCCATTT	339
FMgSSR-25574	scaffold1189	p2	(GA)16	Class I	48545	48576	ACGGTGGCACAAGGAGAGAGAA	ATGGTGACGCGTGCTAGCTGAA	349
FMgSSR-25577	scaffold240020	p2	(GA)16	Class I	490	521	AGAGCCGCTTCCTCATTGGA	TCCGACAATGGCACACTCTCCT	250
FMgSSR-25581	scaffold186737	p2	(GA)16	Class I	501	532	AGCATGGTCATGGGTCAGCA	TGGTCTGTTAGTCTTGACTGCGCT	312
FMgSSR-25582	scaffold1281	p2	(GA)16	Class I	23649	23680	AGCCAGAGACACAAGCACGCTA	AGCCAGCGTTGTGCGAAACA	282
FMgSSR-25583	scaffold1980	p2	(GA)16	Class I	48922	48953	AGCCTGTTTGGTCCCAGCAT	TCAGCAGAAATCACATTGCGGGC	202
FMgSSR-25588	scaffold12752	p2	(GA)16	Class I	7409	7440	AGGAGGAGGCGTTCATTCCTCT	TGGGCTAGCAGCCACACAACAA	304
FMgSSR-25590	scaffold716	p2	(GA)16	Class I	92905	92936	AGGGACACAGATCGAAACAGAGCA	AGTGCGGTGCAGACAGAACAGT	267
FMgSSR-25594	scaffold1032	p2	(GA)16	Class I	70013	70044	AGTGCTTGATGCCTGTCCGAGA	TCGCATGCGCTTTTCGCAGT	304
FMgSSR-25596	scaffold3306	p2	(GA)16	Class I	51631	51662	ATCCGTGCAACTCGCCAACA	TTTTCACACATGGCACCACCGC	217
FMgSSR-25598	scaffold713	p2	(GA)16	Class I	5200	5231	ATGGTCGGCAACGGCAAGTT	TGCGATACTGGTTTTGCGCCAC	284
FMgSSR-25606	scaffold36575	p2	(GA)16	Class I	4589	4620	CGATGCTTTGTGGTAGGTAGGAGT	ATCGACGACCCGTCCAATGT	248
FMgSSR-25607	scaffold17850	p2	(GA)16	Class I	14798	14829	CGGCATTGCCCCGTTTATGGAT	AGTTATCGCAGCCCACAACTCG	295
FMgSSR-25610	scaffold204	p2	(GA)16	Class I	126007	126038	GCCGAAGTTGTGTTTGTGGGCT	TGGCCTTCCATTGACCCAAAGC	313
FMgSSR-25612	scaffold10770	p2	(GA)16	Class I	8462	8493	GCGGTGGTGCATTGGCGAATAA	AGCCAACACTACCCGGTCTCAA	324
FMgSSR-25620	scaffold12904	p2	(GA)16	Class I	14277	14308	TCATGCATGTGGTGTCGGCT	ATTAGGGCCAAGGTTGGTGGCA	272
FMgSSR-25623	scaffold328	p2	(GA)16	Class I	19341	19372	TCCTGGTGAACTTGAAGGCTCC	AAATGGTGCCATCACTGAGCCC	236
FMgSSR-25625	scaffold53894	p2	(GA)16	Class I	2304	2335	TCGACTCCTGCAAACGTGCTGT	TTGTGTTTGGAGAAGCCGCAGG	250
FMgSSR-25628	scaffold25341	p2	(GA)16	Class I	11470	11501	TGACACACACACCACTTTGCTGC	TCATGTCCACCATCAGCGTCGT	285
FMgSSR-25629	scaffold346	p2	(GA)16	Class I	116641	116672	TGACGCAGGGCCAGCATTTT	TTTTTGTGGGTGGGGGCTTTGG	320
FMgSSR-25644	scaffold17458	p2	(GA)16	Class I	12546	12577	TGGGCAGTTTCTGTCGGCCTTT	TGCGTTTGGGAACCAGCCAA	220
FMgSSR-25645	scaffold6963	p2	(GA)16	Class I	41800	41831	TGGGTATGCAGCATGCGGAGTT	TGCTTTGAAAATTCGACGCCCC	318
FMgSSR-25650	scaffold9978	p2	(GA)16	Class I	22714	22745	TGTGCAACACCAACGATTCTGC	GTTGGACGAGTCAGTGCTGGTA	324
FMgSSR-25654	scaffold2938	p2	(GA)16	Class I	37782	37813	TGTGGCCGAATCTAGCCCAT	TCCGTTGGTCGCTGAATGTCCA	276
FMgSSR-25657	scaffold988	p2	(GA)16	Class I	52365	52396	TTAAAGCACGGTCGACGCTCCA	ACGCCATTGCTTTCCTGCCT	346

SSR_ID	Scaffold	SSR_Type	SSR_Motif	SSR Class	SSR_Start	SSR_End	Forward sequence	Reverse sequence	Product_size
FMgSSR-25660	scaffold379	p2	(GA)16	Class I	114980	115011	TTCTGCATGGGGTAAAGCGGCA	TACTAGCGGCGATCCGTGTTCA	251
FMgSSR-25661	scaffold5173	p2	(GA)16	Class I	8965	8996	TTGACCTCGTTGAAGCGCTCGT	TTTACTGGCTGCCGACGTGTGT	282
FMgSSR-25662	scaffold1712	p2	(GA)16	Class I	54159	54190	TTGATCAACACGTGGCCCCT	ACATGACGTCCAGCAGATCGCA	263
FMgSSR-25665	scaffold751	p2	(GA)16	Class I	12464	12495	TTGCTGGCAAGTTGCGGTGT	TCGGCTGCTGCTTCTCTATTGCT	309
FMgSSR-25666	scaffold5010	p2	(GA)16	Class I	22067	22098	TTGCTGGCGAGCATGTCGAT	ATTGAGCCAAGAGTCCCTCCCA	226
FMgSSR-25668	scaffold254	p2	(GA)16	Class I	109464	109495	TTGGTGCGCGATCGTCAAAG	TCAGGAGCGAGGCTCCAAAA	342
FMgSSR-25669	scaffold1326	p2	(GA)16	Class I	3233	3264	TTTAGTGCAACGGCGCAGGA	TACGCCACAGCGGCTCAATGAA	234
FMgSSR-25677	scaffold161	p2	(GA)17	Class I	13953	13986	AAGTGGCAGCAAGGAGGAAGGT	TCGGCGGCCCATTTATTCACAC	333
FMgSSR-25679	scaffold20160	p2	(GA)17	Class I	9643	9676	ACAAGCATGAAGCACCGGCA	AAGGAAAGGGGCAGAGGAAGGA	224
FMgSSR-25681	scaffold11174	p2	(GA)17	Class I	6614	6647	ACCAACCAACAACATGCAGCGG	AGCGAGCAAGGCAAGTAGGCAA	341
FMgSSR-25684	scaffold424	p2	(GA)17	Class I	80838	80871	ACCGTTTGGGTTGGAGGAGCAT	TAAAAAGTTCGCCCCGCCCT	319
FMgSSR-25688	scaffold6836	p2	(GA)17	Class I	5173	5206	ACGTCAGGGAAAAGAGCTACCG	TCTCTCTCTCAAGCACGCACA	265
FMgSSR-25689	scaffold109	p2	(GA)17	Class I	116448	116481	ACTCGCGCGCACACCAAAAT	TCGAAACATGTCTGCACGGGGT	304
FMgSSR-25694	scaffold10462	p2	(GA)17	Class I	17457	17490	AGCCTGGTCTATGCAACGCA	ACTCAACGCACAGCTTCCTCTGT	253
FMgSSR-25699	scaffold391	p2	(GA)17	Class I	128603	128636	AGGCGGGCAGTAAAAACCGTCA	AAGGATGCTCCACTACGCGGAA	338
FMgSSR-25701	scaffold1720	p2	(GA)17	Class I	5142	5175	AGTCCGCAAGCCATTCACCA	CGCACTCCGTCAAAACATGCCA	326
FMgSSR-25708	scaffold2614	p2	(GA)17	Class I	35758	35791	ATGACTGCGCGCTGCTAACT	ATGGTTGCACGCTCTCGCTT	283
FMgSSR-25709	scaffold13001	p2	(GA)17	Class I	2982	3015	ATGATGCGTTGGTGGTGGTGGT	TCCATCGACGAGCTGCTTGCTT	316
FMgSSR-25710	scaffold2145	p2	(GA)17	Class I	6240	6273	ATGCACACCAGTTGCATGGC	AGCCATGGAGCCTTGCTCTT	228
FMgSSR-25713	scaffold7330	p2	(GA)17	Class I	9962	9995	ATGGTTGTTGTTGCCGGGACGA	ACGAGAGAAGCAAACGGGGTGT	336
FMgSSR-25716	scaffold682	p2	(GA)17	Class I	63046	63079	CCAAAGGTAAGGTGAAAGCACACC	AGGACCCAACCCAAATTGACCCA	327
FMgSSR-25720	scaffold225882	p2	(GA)17	Class I	492	525	CGCGTGCATTGAGGTCGCATAA	AGACCGGAGACGATGTGCTCAA	325
FMgSSR-25722	scaffold1294	p2	(GA)17	Class I	82286	82319	CTTCGATGAAGAACTCACGGAACC	ATGAGCTTGAGCAGCTCCGACA	208
FMgSSR-25724	scaffold3455	p2	(GA)17	Class I	16418	16451	GCAACACAACAGTGCGGCAGAA	ACCCGCGATTGTTTGTCGCT	258
FMgSSR-25731	scaffold13	p2	(GA)17	Class I	47210	47243	TCACTCTGGATGTGTTGCCCCT	ACCCAATCGGCAGCCTGATCTA	248
FMgSSR-25732	scaffold14146	p2	(GA)17	Class I	1459	1492	TCAGCAGGCTTGCGGAAACA	ACAAGTTCGGTGCGCGGTAA	264
FMgSSR-25733	scaffold2404	p2	(GA)17	Class I	33609	33642	TCATCGTCGACGCCGTTCAA	TGTGGTATGCCTGCTCCCTCAT	293
FMgSSR-25735	scaffold7976	p2	(GA)17	Class I	15869	15902	TCGACGAGGACCATTCCTGCAT	TACACCCTCTCTCTCACAGCCT	308

SSR_ID	Scaffold	SSR_Type	SSR_Motif	SSR Class	SSR_Start	SSR_End	Forward sequence	Reverse sequence	Product_size
FMgSSR-25736	scaffold7280	p2	(GA)17	Class I	5679	5712	TCGCATTTCTAAGCGGCGTCCT	GCAGGGGGTGGTCGGTATTTCATT	223
FMgSSR-25740	scaffold1067	p2	(GA)17	Class I	77847	77880	TCGTCGTCAATCTGGCTGCACA	ATGCAAAAGGCGGCTTCCCT	207
FMgSSR-25749	scaffold2896	p2	(GA)17	Class I	10958	10991	TGCCAACCGTGAGCTTTCACTCA	ATGGGCCTTGCTTACATCGGCA	267
FMgSSR-25750	scaffold2430	p2	(GA)17	Class I	35032	35065	TGCCTGCGCGATTGATCGGATA	AGCAAGGCGGGAACTGCTTCAA	276
FMgSSR-25752	scaffold975	p2	(GA)17	Class I	49582	49615	TGCGGAGGAGTTTGATGCTGCT	TCTACGCGGCGATCTCAACA	234
FMgSSR-25753	scaffold2980	p2	(GA)17	Class I	32839	32872	TGCTGGCCTGGCTCAGTACAAA	AAAAGTGGCGGCCAAGATGGGA	311
FMgSSR-25761	scaffold6889	p2	(GA)17	Class I	275	308	TTCGCCGATGTGGATGGTGCAT	TCCAGCAAGCGCAATCGTGT	279
FMgSSR-25762	scaffold21530	p2	(GA)17	Class I	761	794	TTGCAGCTCGCAAACATCGC	CGCTAAGCGTTGTAAGGCGTCA	268
FMgSSR-25766	scaffold17547	p2	(GA)17	Class I	17998	18031	TTGTGCGAGCAGCATGGGTT	ACTGTCAGAGTCAAAACGGCG	318
FMgSSR-25774	scaffold35643	p2	(GA)18	Class I	1800	1835	ACATCGCCCGAGAAGGAGTCTA	TTGGGCCCTCGCCGTTTATTGT	232
FMgSSR-25776	scaffold31618	p2	(GA)18	Class I	4636	4671	ACCCTGCCAAAGGAGAAAACAACG	GCCAACATGATTGATCTATGCGCC	339
FMgSSR-25777	scaffold968	p2	(GA)18	Class I	35122	35157	ACGCACGACAGGCAAAAGCA	TCGCTGCACACTGACGAAGA	285
FMgSSR-25779	scaffold1034	p2	(GA)18	Class I	29958	29993	ACGTAGTCTGGGTTGCGCCAAA	AGCATGCAAGTGCTGCGTGT	231
FMgSSR-25783	scaffold16152	p2	(GA)18	Class I	18755	18790	AGCGTGAGTGTGTTGCTTGCAG	ACCTTTTGCCAGTTTGCTGCCT	257
FMgSSR-25791	scaffold2105	p2	(GA)18	Class I	75073	75108	ATCGGTGCCTGCTTGCCTCTTT	TGCTTGAAGTCCGGCAGTAACG	202
FMgSSR-25800	scaffold11531	p2	(GA)18	Class I	20923	20958	GCGCCCATTGAAAGCCGAAACA	TTTTGGACCAGGTTTGGCCCGT	208
FMgSSR-25805	scaffold1820	p2	(GA)18	Class I	68687	68722	GGTGGCGACTTTGCACGTATGA	AGCTCCTAGAACACTCGTGCTGA	266
FMgSSR-25812	scaffold2850	p2	(GA)18	Class I	20949	20984	TCCAATCACGTGGTGATGCCGA	TGTGGCTACGATTTGGCGCA	263
FMgSSR-25817	scaffold11279	p2	(GA)18	Class I	25065	25100	TCTCTCGGTATGCCTGCCTGAT	TTCCTGGTTTGACCCTGCCT	243
FMgSSR-25818	scaffold2452	p2	(GA)18	Class I	48418	48453	TCTGCTTTGCATGCGGCCAA	TCGACCTTGCGTTTGACGGTGA	214
FMgSSR-25819	scaffold714	p2	(GA)18	Class I	88375	88410	TGATCCGCCTCCATCTCCGTTT	GCGGCTGCCAAAGCAAAAGT	337
FMgSSR-25820	scaffold10428	p2	(GA)18	Class I	14532	14567	TGATGCCGCGCTTGAAGCTA	ACATACGAATGGCAGGGGCA	324
FMgSSR-25829	scaffold4632	p2	(GA)18	Class I	19669	19704	TGTGGCGACCTTGGGAATCA	ACCATTGACGGCATCCACGA	220
FMgSSR-25835	scaffold602	p2	(GA)18	Class I	83001	83036	TTCTCGCACCCGAGCCAAGAAA	AATCTCTTGCGGCCATGTGTGC	206
FMgSSR-25836	scaffold3054	p2	(GA)18	Class I	26979	27014	TTGAACTCGGACGGGAATCGGT	TCGCTCAATGGAAGCCCAACCT	273
FMgSSR-25839	scaffold2401	p2	(GA)19	Class I	45505	45542	AAATAACAGCAGGCACGCACGC	ACAAACAACGGTTGCCAGCCA	332
FMgSSR-25841	scaffold4171	p2	(GA)19	Class I	24911	24948	AACCTCGCCGGTGCTTCGATTT	ATAACGCCGTGCCCATCTGCTT	200
FMgSSR-25848	scaffold1512	p2	(GA)19	Class I	32607	32644	ACCGGCCGGCTATTTAAGAACCA	TGGCGATGTTGCTGTTGCCA	214

SSR_ID	Scaffold	SSR_Type	SSR_Motif	SSR Class	SSR_Start	SSR_End	Forward sequence	Reverse sequence	Product_size
FMgSSR-25850	scaffold4031	p2	(GA)19	Class I	27930	27967	ACGGTTGATGTTATCGCCCCACA	AGACGCTGCTTCAAGCGCAA	333
FMgSSR-25857	scaffold3079	p2	(GA)19	Class I	41695	41732	AGCACGGCCATTTAGGCACA	TGGCCATCGCCCTCTCTATTCT	347
FMgSSR-25859	scaffold7707	p2	(GA)19	Class I	29450	29487	AGCGCTGGAACACAGAAACCCA	TGTGTTGTGCCAACTGCCGA	238
FMgSSR-25864	scaffold894	p2	(GA)19	Class I	30771	30808	AGGCTGCAGCGCAGAAATTAGC	CTGCGGCGTTGTTGTTGCTT	350
FMgSSR-25865	scaffold910	p2	(GA)19	Class I	83714	83751	AGGTGCTTGCTGCAATGCGT	GCAAACCACTCCACCCAATCGT	350
FMgSSR-25871	scaffold2618	p2	(GA)19	Class I	5730	5767	ATGCTGCGGCTTGATCCCTT	TTCAGTCTACTCTCCTCCACTGCG	221
FMgSSR-25872	scaffold31696	p2	(GA)19	Class I	5239	5276	ATGTCATGGGATCGGCCACA	ATTTCTTGGCGCACGCGTTT	294
FMgSSR-25876	scaffold3295	p2	(GA)19	Class I	52507	52544	CCACGTCATTGCTCAACTGCCA	ACAACGTCACCAAGCACCACCA	257
FMgSSR-25879	scaffold2709	p2	(GA)19	Class I	54882	54919	CGGATTTTCTACTCTGCTCACACCC	AGCACGTCCACCGTAGAACTGT	280
FMgSSR-25883	scaffold14647	p2	(GA)19	Class I	2730	2767	GTTTGCGCGCCAAATGGCAA	ATCAGTCCACTTAGCAGAGGGCCA	279
FMgSSR-25885	scaffold18391	p2	(GA)19	Class I	2381	2418	TCATGCCACCTTTGCTCGGTCA	AGCTCACGGTTGGCCTTCTT	347
FMgSSR-25888	scaffold6616	p2	(GA)19	Class I	14363	14400	TCGCATCGCATGACGAACTCAC	TGCATCACATTATCTGCGCCCT	320
FMgSSR-25890	scaffold4752	p2	(GA)19	Class I	12349	12386	TGAGCAAGCTGCTGCCCATAGA	GACAGTTTACTAGCTTGGCAGAGG	337
FMgSSR-25905	scaffold4734	p2	(GA)19	Class I	25286	25323	TGTCTGCCTCCTAGCCATGTCAGT	ACGGATGGGTGGCAAGACATGA	299
FMgSSR-25909	scaffold991	p2	(GA)19	Class I	74830	74867	TTCTTCCAACGCCGCCAACT	TAAAGTGAGCGTGCCGCATCCA	274
FMgSSR-25915	scaffold2432	p2	(GA)20	Class I	33268	33307	AAACAGGACGAAGCTGCAAGCG	TCCTGCAACGGCACAAGAACCA	327
FMgSSR-25916	scaffold3245	p2	(GA)20	Class I	45916	45955	AACCTGCCAGGAGAACATGGGT	ATGGAAGAGGTGGCCCGGAAAT	347
FMgSSR-25919	scaffold22436	p2	(GA)20	Class I	2066	2105	AAGCACATCATGGTGGGCGACT	TGCTCGTTACGGCCACCAATCA	310
FMgSSR-25920	scaffold7034	p2	(GA)20	Class I	15236	15275	AATGCCCCACCGCATAGGGTTT	ACACCTTCTTGCCGTGCTTTGC	295
FMgSSR-25921	scaffold2079	p2	(GA)20	Class I	43986	44025	ACACTGCCAACAGTCCATACTTGC	TGTGCGACTCCTTTGACTCCGT	299
FMgSSR-25923	scaffold137	p2	(GA)20	Class I	106010	106049	ACCACCTGCGTTCTGTAGGCAA	GCGAATACGCAGAAGGCACTGT	349
FMgSSR-25924	scaffold1039	p2	(GA)20	Class I	14533	14572	ACCCGTGGCCTGGTTCAGAATTA	GCGAACGACGCCAACTGAAT	340
FMgSSR-25925	scaffold5268	p2	(GA)20	Class I	31196	31235	ACCGACAGGCCATCCTTTTGCT	TCGCGTGCACTCCATTGACA	310
FMgSSR-25926	scaffold6895	p2	(GA)20	Class I	4146	4185	ACCGCACGTCCAAAACACCA	TAGCTGCTGGCTGGTGCAGATT	309
FMgSSR-25929	scaffold12057	p2	(GA)20	Class I	16287	16326	ACGGAGCTGATTAGTTGCGACG	ACAGACGCAATGGATCGGAGCA	262
FMgSSR-25930	scaffold20644	p2	(GA)20	Class I	7230	7269	ACGTACAGGCAGATGGCACT	TGGCTGCTGAAAGTGTGCGT	317
FMgSSR-25931	scaffold486	p2	(GA)20	Class I	584	623	ACTGGAACAACCACGGTCACCT	TGCTGCTGCTACTGCTGCTGTT	312
FMgSSR-25932	scaffold2357	p2	(GA)20	Class I	33715	33754	ACTGGAAGGTGCCCGCATTTCT	TTCCGTGCCAAGCCAAAAGC	205

SSR_ID	Scaffold	SSR_Type	SSR_Motif	SSR Class	SSR_Start	SSR_End	Forward sequence	Reverse sequence	Product_size
FMgSSR-25934	scaffold35057	p2	(GA)20	Class I	4024	4063	AGATGCAGTGGCGGTTTGGT	ATGATGGGGGCACGTGTCAGAT	337
FMgSSR-25935	scaffold1326	p2	(GA)20	Class I	7157	7196	AGCGAAGACCAGGACAGAATCCA	TACCAGCTCGCCCTGCAAATGT	253
FMgSSR-25937	scaffold2724	p2	(GA)20	Class I	20547	20586	AGCGCACGAACTGGACAATGGA	GCCTGAAACGTTTGCTAGCCT	272
FMgSSR-25938	scaffold56	p2	(GA)20	Class I	91115	91154	AGCGGCCCTCAGCACTTACAAT	TGCGAAAACACACACACTCGC	338
FMgSSR-25945	scaffold16599	p2	(GA)20	Class I	13000	13039	AGTCGGCGACATGCGTCTTT	AAATCACCTTAACCCCACAGGGGC	201
FMgSSR-25950	scaffold48	p2	(GA)20	Class I	109214	109253	ATGCCGACGCAGGCAAAACA	TCGAAGACGTTCGATGGTCGCT	298
FMgSSR-25953	scaffold7707	p2	(GA)20	Class I	22348	22387	ATGTCGGGGCATTATGCGGA	TGCTCGCTGCTGTCTCGCTAAA	299
FMgSSR-25956	scaffold5079	p2	(GA)20	Class I	2458	2497	CGCTAAGGACAGTCTGAGACCACA	ACGAGTTCACCTGCGAGCAA	350
FMgSSR-25957	scaffold16788	p2	(GA)20	Class I	14104	14143	CGTTGGCCATCGTTGCAGAAGT	ACCCGAGCTCCTCCTCCCTAATTT	233
FMgSSR-25958	scaffold3631	p2	(GA)20	Class I	9944	9983	GCGGTTTTCAAGACGCGACA	TCGTTTGTTACCGTGGGTGCCA	223
FMgSSR-25959	scaffold32820	p2	(GA)20	Class I	4301	4340	GCTCGCATTGATTGGACGAGCA	ACAGCAGACACGGAACTCCACT	252
FMgSSR-25962	scaffold1316	p2	(GA)20	Class I	61639	61678	TCATCATGCTGCAGGAGCGTCT	TCCAAACTGTGCATCGCGCAAA	343
FMgSSR-25963	scaffold106372	p2	(GA)20	Class I	1233	1272	TCGGTCAGCAATTGTGCGAGGT	ACCACAGCTCTTCCATGCGACT	303
FMgSSR-25965	scaffold92	p2	(GA)20	Class I	193577	193616	TGAAGTTCGTCGCTGCTGCAT	AACCTTTCTCCCTGTCGCCCTT	350
FMgSSR-25967	scaffold2984	p2	(GA)20	Class I	30509	30548	TGCCGCAAGAAGAGAGGAGACA	AGTCCAAGCGACGCAATGGT	246
FMgSSR-25969	scaffold301	p2	(GA)20	Class I	123446	123485	TGCCGGCAAATGGAGACAAGGA	TGCACACGCTGTTTCGAGGAGT	348
FMgSSR-25970	scaffold804	p2	(GA)20	Class I	61627	61666	TGCGGTGTTCCTTGGACTCACT	TGTGCTTGTTCTTGGTGGGCGA	236
FMgSSR-25973	scaffold22144	p2	(GA)20	Class I	5336	5375	TGGGACTCGTTGCAGCTGTTGT	TGGCGGATAGCACTGTTCAACACA	330
FMgSSR-25974	scaffold9556	p2	(GA)20	Class I	27389	27428	TGGGGGAGCGAAAGATTTGCGT	ACATAACACACAGCGGGCGT	276
FMgSSR-25975	scaffold7284	p2	(GA)20	Class I	4947	4986	TGGGGTCTTGCGTTTGGGTGTA	TTCGCCACCACACATCAGCA	206
FMgSSR-25976	scaffold25989	p2	(GA)20	Class I	3442	3481	TGGTCAGCTTTGATGGGTGGCA	TCTCTCTTCTCCTTCTCATCCCCC	238
FMgSSR-25982	scaffold6703	p2	(GA)20	Class I	25900	25939	TTGAGGGACGCCATCATGCCAA	TTGTTGATGTCAGGGACCGCCT	283
FMgSSR-25987	scaffold18341	p2	(GA)21	Class I	2196	2237	AAATCGAAGTCGTCGGGTCGGT	ACGCGCATGTCTTTCCTGGT	270
FMgSSR-25989	scaffold4225	p2	(GA)21	Class I	30794	30835	AACGGTGTGTTAAACCAACGCGG	TCCTTGAAATGGCGCTTGCTGC	277
FMgSSR-25992	scaffold551	p2	(GA)21	Class I	42493	42534	ACATTGCAGTGCTGTCCCGT	TGTGGTGAAACTTTCCGGTGGCT	294
FMgSSR-25994	scaffold1610	p2	(GA)21	Class I	13026	13067	ACGCCACCGAATCTATGCACCT	ATCACAGCGTACACCACACGCA	253
FMgSSR-25995	scaffold329	p2	(GA)21	Class I	39081	39122	ACGCCACGCCTACGATTGAT	TACCGTGTATGTGCTCGTGCGT	345
FMgSSR-25999	scaffold6836	p2	(GA)21	Class I	5332	5373	AGCAGAGGTTGAGCATCGCA	ACCAAGATCCGGCCCCAAAA	224

SSR_ID	Scaffold	SSR_Type	SSR_Motif	SSR Class	SSR_Start	SSR_End	Forward sequence	Reverse sequence	Product_size
FMgSSR-26005	scaffold11021	p2	(GA)21	Class I	12614	12655	ATGCGAGAGAGAGAGGGAGTTCGT	TCTCATACTCCTTGCCGTCGGT	328
FMgSSR-26007	scaffold16885	p2	(GA)21	Class I	7883	7924	CCAAAAAGCACACGCTTGCACA	TGGCACAGCATTTTGGCGCT	322
FMgSSR-26009	scaffold3017	p2	(GA)21	Class I	51201	51242	CGGGCAAACCCAACCCAAAGTT	ACACATCCCCCTCCCCAAGAAA	347
FMgSSR-26011	scaffold14318	p2	(GA)21	Class I	13869	13910	GGCAGTTACCGAGGCCCTTTTT	AAATTACCGGCCACATCCCGCA	337
FMgSSR-26016	scaffold145	p2	(GA)21	Class I	51471	51512	TCGACATGCCCTTCTGCCAGTT	TGGCATGCACATTCAGTAGAGC	256
FMgSSR-26022	scaffold1568	p2	(GA)21	Class I	12211	12252	TGAGGTTAAAGTTGGTCCCGTCCT	AGGCCCCAAATAGATGCCCA	310
FMgSSR-26023	scaffold12424	p2	(GA)21	Class I	16438	16479	TGATGGCAACGCCGCACTAA	AACACCACGCGTTTCGGCAA	324
FMgSSR-26026	scaffold12911	p2	(GA)21	Class I	7811	7852	TGCGGAGCACACCACACAAGTT	TCGGTACGTACGCTCTTGTGCT	212
FMgSSR-26027	scaffold2984	p2	(GA)21	Class I	39122	39163	TGCTCGACTTCTCTTGTGGCGA	ACGAAGCCCAGACAATGCACA	219
FMgSSR-26030	scaffold377	p2	(GA)21	Class I	82041	82082	TTCGAAGAGGCGGAGAAGGGAA	TTGCCGGTTGATCGAGGCATGT	342
FMgSSR-26032	scaffold1268	p2	(GA)21	Class I	91754	91795	TTCTGGCCAAAGGCAGCAGCTA	GGCGTCAAGGCGTTGAATGA	226
FMgSSR-26033	scaffold390	p2	(GA)21	Class I	70854	70895	TTGGGGTCGTTGTGATGCCGAT	ACCCCGACTGCTTGGTTTCT	249
FMgSSR-26035	scaffold8603	p2	(GA)21	Class I	8384	8425	TTTCTGTGTCGTCCCTGCCGAT	AAAGCCTTCACGTTTCACGGCG	273
FMgSSR-26038	scaffold7313	p2	(GA)22	Class I	7165	7208	AAAATGAGCGGCGGCTGGAA	AAATGCATCACGGCGACACG	350
FMgSSR-26041	scaffold267	p2	(GA)22	Class I	5316	5359	ACCCCCACAAACGACGAGCAAA	TGAACTACGTGCCCAAGTGCT	243
FMgSSR-26042	scaffold450	p2	(GA)22	Class I	126758	126801	ACCGAGCAAAGCAGAACGGT	AGCCCACGACCGCTTTTTACCA	299
FMgSSR-26044	scaffold786	p2	(GA)22	Class I	86806	86849	ACTGAAATCGCCAACGCCATGC	ACTCAGACCCAGCCCAAAAGCA	218
FMgSSR-26046	scaffold403	p2	(GA)22	Class I	22720	22763	AGCACACGCCAGCCACCATTAT	TTTGGGGCTTTGGCCAGCAT	269
FMgSSR-26048	scaffold11027	p2	(GA)22	Class I	24212	24255	AGTGCAAGCGCCTGGATCTCAA	ATAGGCGGCTCGCTTCGTCTTT	290
FMgSSR-26052	scaffold2910	p2	(GA)22	Class I	53949	53992	CCCCACCTTTGTGGAAGGCATA	CAGGGGATCTCGTCACTGCAAA	348
FMgSSR-26054	scaffold16692	p2	(GA)22	Class I	7666	7709	CTGCCAGGAGTTTGTTCCTTACGA	TTCTTGCCGGCCTCTTGCTT	350
FMgSSR-26057	scaffold6928	p2	(GA)22	Class I	2357	2400	GGCAGGACGCGGAAACAAATCA	TCGGTCGTCCTGCGCATAACTT	339
FMgSSR-26061	scaffold1575	p2	(GA)22	Class I	24357	24400	TAACTGCGCATTCAACGCAGCC	TCAACAGTTGTTGGCGCAGC	331
FMgSSR-26063	scaffold5546	p2	(GA)22	Class I	24326	24369	TCCCGCGAACGGATGGTTAAGT	CCGGGCACAAATTGAACGGCAT	267
FMgSSR-26067	scaffold67	p2	(GA)22	Class I	9722	9765	TCGCCGTCGATCATTGCGAGTT	ACAGTTTGGTCCTGCTCGCT	345
FMgSSR-26079	scaffold9135	p2	(GA)22	Class I	10509	10552	TGTGCAAGGTCAACGTCCAGCA	TGCCAGCCATGGCAAACACT	319
FMgSSR-26080	scaffold6004	p2	(GA)22	Class I	14731	14774	TGTGCTCCATGCGCTGAGAT	TGCACAAGTTCTGCCTTCGACC	242
FMgSSR-26082	scaffold1006	p2	(GA)23	Class I	61382	61427	AAAGGCCTTGCGTCGTGTGA	ACCGTTGACACACGCACCACTA	252

SSR_ID	Scaffold	SSR_Type	SSR_Motif	SSR Class	SSR_Start	SSR_End	Forward sequence	Reverse sequence	Product_size
FMgSSR-26083	scaffold2625	p2	(GA)23	Class I	3832	3877	AAGCCATGCTCGCACCTGAA	ACAATGGCACCCACCGCTAA	211
FMgSSR-26085	scaffold4536	p2	(GA)23	Class I	22146	22191	AATGAGCCCCATTCCAACGCCA	AGGATCGCACTTTCCGGATCA	329
FMgSSR-26093	scaffold3083	p2	(GA)23	Class I	53775	53820	AGTTCCGGCCAAACACCGCTAA	CCGGTCCAAAAGAACAGCCCAA	339
FMgSSR-26096	scaffold227	p2	(GA)23	Class I	127566	127611	ATGTGCTTTGCTTGCTGGCTCG	TCGGGTTCTCGATCCGCTTT	342
FMgSSR-26098	scaffold7297	p2	(GA)23	Class I	21886	21931	GTGCAGCGGTTTTGCAGGAACT	GTTGTTGCTCGTCCCCGATT	309
FMgSSR-26100	scaffold3085	p2	(GA)23	Class I	19190	19235	TCACGGCAAGGGACTACGTT	GCTTTTGGCTATGTGCGGGA	350
FMgSSR-26102	scaffold663	p2	(GA)23	Class I	63425	63470	TCCTTGACGCACTCGCATGA	ACCAGTCGAGTCCTCCTCACTT	242
FMgSSR-26108	scaffold1881	p2	(GA)23	Class I	52237	52282	TGCCACATCCATGGAATGCTCCT	TCCCCTACAACGACCTCACACCTT	267
FMgSSR-26119	scaffold351	p2	(GA)24	Class I	40072	40119	AAAACGGCATGTGGTGCGTG	TTCATCAGCCTTGCGTGCAG	275
FMgSSR-26124	scaffold2601	p2	(GA)24	Class I	28120	28167	AGCGCAAAGGTGACCCTTCCAA	AGGAGCAGAGGCAGCAGAAGAA	223
FMgSSR-26129	scaffold701	p2	(GA)24	Class I	87006	87053	ATTCGGCTCTGCGGGAGCTTAT	AGTTGTGCACCAGGCTGTCACT	298
FMgSSR-26132	scaffold9646	p2	(GA)24	Class I	24733	24780	GCCGCAAGTTCTAGCAAGGGTT	TTGGCGAGCACCAGGTTGTT	210
FMgSSR-26135	scaffold12885	p2	(GA)24	Class I	6207	6254	TCCCGTATCCATCGCACAGAGT	TGGCATGGCGACGACAGATT	281
FMgSSR-26136	scaffold6058	p2	(GA)24	Class I	5570	5617	TGCATGTTGCACTCTGGGCT	TGTCGCCAGATCCATGAAGGCT	312
FMgSSR-26139	scaffold1343	p2	(GA)24	Class I	73613	73660	TGTGCTTCAGCCACTTCAGCCT	TCATCTTCCTTCTGCCGCCACT	344
FMgSSR-26144	scaffold4407	p2	(GA)25	Class I	2273	2322	ACACACCACACACACCCCAA	ACCATAAGCCACCCACCGTT	334
FMgSSR-26152	scaffold7524	p2	(GA)25	Class I	13351	13400	ATCGTCACCAGCACCACAACCA	ACCAACAGTGAGGAGCAGCGAT	303
FMgSSR-26161	scaffold4360	p2	(GA)25	Class I	45542	45591	TGCGAGGATGGATGGTGCGTTA	TCGAAACCCTAGCAACTCACGCT	212
FMgSSR-26162	scaffold10727	p2	(GA)25	Class I	22196	22245	TGCGCAGCAGAAAGGCCACATA	AAACGCGAAACAGTGGGCGA	339
FMgSSR-26163	scaffold662	p2	(GA)25	Class I	18537	18586	TGCTGCTGTGAACTCCGCAA	TTGTCTCGGTGGCAAGCGTT	306
FMgSSR-26164	scaffold416	p2	(GA)25	Class I	60193	60242	TGTTGCCTGCGCTCTTGGTT	TGTCCCTACGCATGCTCGTTGT	228
FMgSSR-26166	scaffold424	p2	(GA)25	Class I	77616	77665	TTGGACGGAGGGCAATGTCGAT	AGGCCAATCACCACCCACAA	345
FMgSSR-26167	scaffold6949	p2	(GA)26	Class I	20827	20878	AATTGGTCCGCCTGCTGCTT	ACGCCTTTTCCTCAGCCCAA	346
FMgSSR-26168	scaffold3064	p2	(GA)26	Class I	10674	10725	ACAACACTCATGTGGCGGGT	AGTCCGTTAGGATCCACGGCTA	350
FMgSSR-26180	scaffold13775	p2	(GA)27	Class I	19165	19218	ACATCACCGGCGTATCCGTTGT	ACAGGGCGAACGAAAGGCTGAA	340
FMgSSR-26189	scaffold37396	p2	(GA)27	Class I	3640	3693	TGCACAGGTGCAACGGACAT	TAGTCGGCCCACCCATTACCAT	274
FMgSSR-26193	scaffold5900	p2	(GA)27	Class I	23666	23719	TGTCGACGTGGAGAGGTGCAAA	AATTGCCACTCCACCCCAAACC	230
FMgSSR-26197	scaffold18168	p2	(GA)28	Class I	5628	5683	TCATGGGATGTCACAACGACGA	TGCGCACTGTTCACAAGTCCA	205

SSR_ID	Scaffold	SSR_Type	SSR_Motif	SSR Class	SSR_Start	SSR_End	Forward sequence	Reverse sequence	Product_size
FMgSSR-26200	scaffold355	p2	(GA)28	Class I	94797	94852	TTTGGCTTGCCGCTCACACA	TCTCTTCTCTTGCCGACGCCTT	280
FMgSSR-26201	scaffold22014	p2	(GA)29	Class I	1509	1566	AAGGGACCAACGAGCTTGAGGA	CTCAACCCCTCTCTTATCTCACCA	317
FMgSSR-26203	scaffold5544	p2	(GA)29	Class I	22082	22139	AGAGGATCAGCCATTGCACCGT	AGACTCTCTCCACTCTAAGCCCT	227
FMgSSR-26207	scaffold3577	p2	(GA)30	Class I	54037	54096	AAGCAAGGTGCTGCAGGAGT	ATCCAGCACAATCCCAGGCA	345
FMgSSR-26209	scaffold790	p2	(GA)30	Class I	26314	26373	TAAAATGGGAGAGGGAGGGAGGGT	AGGCCTGACAAGCTTCATCCCA	338
FMgSSR-26211	scaffold272	p2	(GA)30	Class I	85235	85294	TGTCACTAACGACACGAAGGCA	TGGCCGTCATCGAACTTGTCGT	323
FMgSSR-26213	scaffold151563	p2	(GA)31	Class I	444	505	ACTACGAGCGCAAGCAGTGA	TTCGTGGAGATGCAAGCCCAGT	223
FMgSSR-26220	scaffold16000	p2	(GA)33	Class I	16845	16910	AACGGCAAGCAACAGAGAGGGA	CCCGCAAAGTTACGGCCGTTTT	272
FMgSSR-26222	scaffold376	p2	(GA)33	Class I	16462	16527	TAGGCCAGGCTCAGTACAAGCA	TTGCCCACTCTAGTGTCGCCTT	200
FMgSSR-26227	scaffold162	p2	(GA)35	Class I	84759	84828	TGTGGCACACCCTTCTTAGCA	GGCCAGTCACCACTCCAATGACTT	348
FMgSSR-26229	scaffold18408	p2	(GA)37	Class I	6787	6860	ACTCTTGCAGGATGAGTCACCG	AGGTGTTGGTTGGCTTGCCTCT	299
FMgSSR-26231	scaffold10111	p2	(GA)39	Class I	7654	7731	TTGCCACTGCACCACAGAGACA	AACCTCTGCTTTGTGCCCACCT	312
FMgSSR-26232	scaffold4594	p2	(GA)40	Class I	7901	7980	ACAACGCTGTGGTTTCGCGT	AAAAACCAAGTCCCGCCCCCTA	298
FMgSSR-26233	scaffold5739	p2	(GA)41	Class I	29199	29280	ACGACACCCATATGCTCGGT	AAAACGCCTCCACTGCCAAACC	277
FMgSSR-26243	scaffold1800	p2	(GA)6	Class II	19342	19353	AAACACAACCAGCAGCGAACCC	TGCAAATGGCTTCACCTTGCCG	339
FMgSSR-26250	scaffold142869	p2	(GA)6	Class II	282	293	AAACCATCCCCAGCCACCAA	GTCCCTGCTTGCACTTTGCACT	313
FMgSSR-26260	scaffold325	p2	(GA)6	Class II	86930	86941	AAAGCAGTACGGAACCCAGGCA	GGCCAAAATGGGCCGCAAGTAA	205
FMgSSR-26266	scaffold79	p2	(GA)6	Class II	146126	146137	AAAGGAGAGTAGCGTGGAGGAGCA	ATAAACGCCACTCGGCGCAA	348
FMgSSR-26267	scaffold7110	p2	(GA)6	Class II	15902	15913	AAAGGCAAGGCACCAGAGCGAT	TTGCCCCGTTGAAACAGCGA	337
FMgSSR-26268	scaffold5695	p2	(GA)6	Class II	17532	17543	AAATAGACGGAGCGTAGCGCGT	AAATCGGTGCCTCATGGCTTGG	327
FMgSSR-26269	scaffold17662	p2	(GA)6	Class II	2813	2824	AAATATGTCGGCCGCCCAGT	AAATAGTTGCCACCGGATCCCTCC	236
FMgSSR-26273	scaffold2580	p2	(GA)6	Class II	52444	52455	AAATGGGACGTCAGTAGCCCGA	AGCGTTGTCCGGCTCACAAT	345
FMgSSR-26281	scaffold7065	p2	(GA)6	Class II	27758	27769	AACACCGTGGCCCTTTTGGCTA	TGCGAATTGCAGCTTTCTGCC	270
FMgSSR-26291	scaffold2753	p2	(GA)6	Class II	31778	31789	AACATGTGTCCGCCAATGCTGG	ACATGGCCGTGATGGAAGGT	206
FMgSSR-26292	scaffold1356	p2	(GA)6	Class II	50548	50559	AACATGTGTCCTGCCGCCCTTT	TCAGAGACCCTTTGCTTGTGCGT	264
FMgSSR-26295	scaffold33356	p2	(GA)6	Class II	7244	7255	AACCGCAACGTGCCTAACCA	TTGGTCGTCTGGTCTCCAGTCT	318
FMgSSR-26299	scaffold15246	p2	(GA)6	Class II	7456	7467	AACGCACACCTACGCGGTTT	AAGCACCACCGCACTATGCT	236
FMgSSR-26302	scaffold20699	p2	(GA)6	Class II	1475	1486	AACGCTGTGTCGCCATGAGA	TCATGCACATGGCTTCCGACCA	350

SSR_ID	Scaffold	SSR_Type	SSR_Motif	SSR Class	SSR_Start	SSR_End	Forward sequence	Reverse sequence	Product_size
FMgSSR-26307	scaffold79	p2	(GA)6	Class II	114737	114748	AACGGCCTCGCTCTCACAAAGT	TTTAACCATGGACCCGCCA	213
FMgSSR-26314	scaffold288	p2	(GA)6	Class II	75211	75222	AACGTGACTGAGCCACTGCACA	ACGCAACGATGTTTCCGTGGGA	214
FMgSSR-26318	scaffold14329	p2	(GA)6	Class II	21559	21570	AACTGACATGGCGTGAGGTCCA	ACGTATATGCCGTTGCGCTCT	233
FMgSSR-26321	scaffold194	p2	(GA)6	Class II	5409	5420	AACTGCGCAAGAACACGCCT	AACCCCGTCCTTCTTTTCTTGGGC	331
FMgSSR-26333	scaffold610	p2	(GA)6	Class II	106160	106171	AAGCAACAGCAAGCCACGAACC	TTGCACCATTGCGCTCGTGA	275
FMgSSR-26340	scaffold4902	p2	(GA)6	Class II	34714	34725	AAGCATGTTGTGGGCCCGAT	TTCTGAGGCCGAGAGATTTAGGCG	208
FMgSSR-26342	scaffold515	p2	(GA)6	Class II	115489	115500	AAGCCGAACGCGTGAGGAAA	AGCGGCAACATTCCTTGGTGA	304
FMgSSR-26343	scaffold805	p2	(GA)6	Class II	95373	95384	AAGCCTTCTGCATGTCTGCCGT	TGTCGTATGTGCATGCTGAGCAGA	309
FMgSSR-26358	scaffold4296	p2	(GA)6	Class II	16982	16993	AAGGCGGCAACAGTCCGATGAA	TGGGCACCGTTTGCGTTGTT	269
FMgSSR-26374	scaffold2094	p2	(GA)6	Class II	4351	4362	AATACCCGCTCTCCGCAATCGT	GGGTTGGTTTCCGCTGCAACTT	244
FMgSSR-26375	scaffold2562	p2	(GA)6	Class II	33783	33794	AATAGAACGCACTGGTGGCTGG	TTGTATTCAACCGGGGCCGT	273
FMgSSR-26380	scaffold401	p2	(GA)6	Class II	58123	58134	AATCCGCAGCACCGGAAAGAGT	TGCTGACGCGTCTGTGATAGAA	242
FMgSSR-26381	scaffold789	p2	(GA)6	Class II	56012	56023	AATCCGCTCATTGCCGCTCA	ATGGGGGCTGCCCTACTTTCAA	319
FMgSSR-26390	scaffold53	p2	(GA)6	Class II	205426	205437	AATGCAAGAGCTCGTCAAGGGC	ATGTGACAGCTTCGTCTCGCCT	228
FMgSSR-26393	scaffold5757	p2	(GA)6	Class II	18731	18742	AATGCCGTAACCGTCGGTCTGT	AGCTCGCCTCTTGCAAGGTT	237
FMgSSR-26394	scaffold11564	p2	(GA)6	Class II	9725	9736	AATGCGAGCGGGAATCGGTCAA	TGCCCGCTCAAACAGCCAAA	344
FMgSSR-26398	scaffold16252	p2	(GA)6	Class II	7465	7476	AATGGGCGATTCCTCCCCTGAT	ACGCGCGTGCTAAGTCCAAA	232
FMgSSR-26407	scaffold1232	p2	(GA)6	Class II	58329	58340	ACAAACAAGCTGGCCTGCGA	AAAGGACCGGATTGCCCCTTCT	249
FMgSSR-26409	scaffold1097	p2	(GA)6	Class II	86643	86654	ACAAGACCCGGCCTTTTCCACT	TCATTTCCTGGTCGCTTGCCGT	331
FMgSSR-26413	scaffold5782	p2	(GA)6	Class II	45679	45690	ACAAGGCCATCACCAGTTGCCA	TTCCTGCGCCACGTTCAACT	257
FMgSSR-26422	scaffold4512	p2	(GA)6	Class II	23926	23937	ACACATGGACTTCGTTATGGGCCT	TTCCAAAGGCACCCTGAGCA	332
FMgSSR-26424	scaffold8598	p2	(GA)6	Class II	5372	5383	ACACCAATGCGGTCTTGCCT	ACGCTAGGTCCTTTGGCGCATT	304
FMgSSR-26428	scaffold8840	p2	(GA)6	Class II	23442	23453	ACACCTCCCTGCACCAGATTGT	TGCCGCACCTGGCCTTTAAT	301
FMgSSR-26432	scaffold128	p2	(GA)6	Class II	144321	144332	ACAGAGAGCAAAGCAGGGCA	AAAGCGGTGACGGCGAAGAT	348
FMgSSR-26434	scaffold4599	p2	(GA)6	Class II	27537	27548	ACAGATCGACCTTGTGCTCCTG	AGAGTGGCGGATGCGTTTGT	250
FMgSSR-26435	scaffold237701	p2	(GA)6	Class II	432	443	ACAGCACTGACACGAAGGACCA	AATTTCGGGCGATTTGGACGGC	200
FMgSSR-26436	scaffold3226	p2	(GA)6	Class II	20874	20885	ACAGCGGACGATGTTGGTGGAA	TGATGTGCGCGATAACAACCGC	274
FMgSSR-26442	scaffold4408	p2	(GA)6	Class II	29632	29643	ACATACAACGCACAACGCGCA	GCTTCTGTAGCCAGGACCACAA	202

SSR_ID	Scaffold	SSR_Type	SSR_Motif	SSR Class	SSR_Start	SSR_End	Forward sequence	Reverse sequence	Product_size
FMgSSR-26443	scaffold6661	p2	(GA)6	Class II	31320	31331	ACATACGTGCCGTGGCGAAA	TTCGCACCCAACATGAGCGA	298
FMgSSR-26445	scaffold15178	p2	(GA)6	Class II	6814	6825	ACATCATGACCGTTGTTGCGGC	AATTTCTCTTGCGCGCCACTGC	239
FMgSSR-26446	scaffold7756	p2	(GA)6	Class II	8593	8604	ACATCCACCATTCTCGCCGGTT	ACTCGAACATTCTGGTCCGCGA	223
FMgSSR-26447	scaffold1809	p2	(GA)6	Class II	18613	18624	ACATCTGAAACGCTTCGGGCCT	ATGGGTGGTGACAACTGGGA	225
FMgSSR-26451	scaffold12653	p2	(GA)6	Class II	10162	10173	ACCAAAGCTACCACCGTTGC	TCCTCCAAATCCTTGCCTCCCA	340
FMgSSR-26453	scaffold27454	p2	(GA)6	Class II	2145	2156	ACCAATAATTCGGCCCGGTCGT	AGACCCTCTCTTGTGCTGATCG	240
FMgSSR-26458	scaffold68923	p2	(GA)6	Class II	2242	2253	ACCAGAGCATTGCGGCAACA	TGCCGGTGCGCTGTATCTGAAT	342
FMgSSR-26459	scaffold5738	p2	(GA)6	Class II	1965	1976	ACCAGATTTGCTAGAGGGTTGGGC	TATTCCACTGTCCGGATGGCGT	318
FMgSSR-26465	scaffold29972	p2	(GA)6	Class II	6275	6286	ACCCACCGCAGGTGAGATTT	TGCGTGGAATCCGGCTGCTTAT	254
FMgSSR-26467	scaffold15674	p2	(GA)6	Class II	12823	12834	ACCCATGCAGCCATGCCTAAGT	ATAAACGGCTGCCTGTCCCA	334
FMgSSR-26468	scaffold1864	p2	(GA)6	Class II	13999	14010	ACCCGTGCTACACTCAAGCTCA	AGCCGCAGCAAACTACGATGA	325
FMgSSR-26469	scaffold8975	p2	(GA)6	Class II	28365	28376	ACCCTTACACAGTTTGCGGCCT	GCGCTCCTGCTCTTCTGAACAA	246
FMgSSR-26471	scaffold30292	p2	(GA)6	Class II	6015	6026	ACCGACAGCTGCTACGCCTTTA	CGGGCACTCGGCTAGTCAGAATTT	321
FMgSSR-26481	scaffold1557	p2	(GA)6	Class II	35764	35775	ACCGTTCCGTTTGGGACAGCTA	AGCCAAATCGGCTCTCCTTCA	347
FMgSSR-26482	scaffold9455	p2	(GA)6	Class II	1041	1052	ACCGTTGCGCACATACTGCAT	TCGTTCTAGGAATCTGGCCGGT	285
FMgSSR-26483	scaffold233782	p2	(GA)6	Class II	437	448	ACCTAAGTCAACCCTGCTCCCT	TTCTCTCTTCCACGCCGTCT	337
FMgSSR-26484	scaffold2981	p2	(GA)6	Class II	40206	40217	ACCTCGCAATCGACTAGGCA	TCAAGGACTGCTGCCAGCTT	341
FMgSSR-26487	scaffold3413	p2	(GA)6	Class II	13070	13081	ACCTGCACGATCGAAACCAGCA	TTGGCACGATGCAAAAGCGG	252
FMgSSR-26489	scaffold305	p2	(GA)6	Class II	54142	54153	ACCTTTGTCGTGTCGTGCCAGT	TATCCTCCGTCATGCGCTTGGT	348
FMgSSR-26491	scaffold975	p2	(GA)6	Class II	7708	7719	ACGAATGGGGAGCGAAGAGGTA	AAAGCGCAGCGGCCTTTTCA	335
FMgSSR-26493	scaffold18023	p2	(GA)6	Class II	7793	7804	ACGACAGGCAAGCAGAAGCAGT	ACGCGGCTGCAACTGCAAAA	211
FMgSSR-26494	scaffold3247	p2	(GA)6	Class II	29899	29910	ACGACGCGGTCCATGAGTTTCA	TCGTGCCTTGAACACGGTCT	242
FMgSSR-26496	scaffold3338	p2	(GA)6	Class II	35255	35266	ACGACTCATGAACGCACAGGCA	TGGCGTGACGACGCAAAAGA	263
FMgSSR-26497	scaffold410	p2	(GA)6	Class II	4946	4957	ACGAGGCACGTGACGAGAAA	TGGAAGCAGCTGGTCCATTCGT	285
FMgSSR-26498	scaffold1957	p2	(GA)6	Class II	24048	24059	ACGAGTAGGCAAGGAGAACGGT	TTCTTCGGTGCCGAGCCAAGAT	320
FMgSSR-26505	scaffold393	p2	(GA)6	Class II	39389	39400	ACGCCATCTGTTGCGATTCTTG	AGGAGCGACGGGAAGAAAACT	333
FMgSSR-26507	scaffold99547	p2	(GA)6	Class II	533	544	ACGCCGCGATTTGTGTCTGT	AGCGGAGGAGGCAACAATCT	239
FMgSSR-26510	scaffold9062	p2	(GA)6	Class II	26741	26752	ACGCCTTCGTCTTGCTCTGT	AAATACCTACTCGTCGGCCAGC	314

SSR_ID	Scaffold	SSR_Type	SSR_Motif	SSR Class	SSR_Start	SSR_End	Forward sequence	Reverse sequence	Product_size
FMgSSR-26516	scaffold4940	p2	(GA)6	Class II	2104	2115	ACGCTTGATTTGCTTGCGTCCC	AGCCACTGCCGTCGAATTGATGA	258
FMgSSR-26518	scaffold12504	p2	(GA)6	Class II	1775	1786	ACGGAACCGCTTTTGTTGCCT	ACGGTGCGTTGCGTTTCCAT	262
FMgSSR-26522	scaffold11715	p2	(GA)6	Class II	9065	9076	ACGGCACCAACACCGAGATGAT	ACTTAAGCATGCCGCGCACA	327
FMgSSR-26524	scaffold266	p2	(GA)6	Class II	126398	126409	ACGGCAGCGTTGCTTGATGA	ACAATCAGTAGCACGCCCGAGT	282
FMgSSR-26526	scaffold9433	p2	(GA)6	Class II	22428	22439	ACGGCGCCGAGGGAAAATAA	TGCAGACTCTGATTGTGGGAGGT	302
FMgSSR-26529	scaffold2003	p2	(GA)6	Class II	33797	33808	ACGGGATGGTGCATTGGTGA	TGCCCCACACAACCTCCATTGT	337
FMgSSR-26531	scaffold41214	p2	(GA)6	Class II	5426	5437	ACGGGCAATGAGCCCCAATTT	TGGTGTTGGTTGACGATGCGGA	219
FMgSSR-26535	scaffold8890	p2	(GA)6	Class II	27547	27558	ACGGTGAGGAAGCGATGCTGTA	AGCTTGACTTTGGCAGTCGGCA	294
FMgSSR-26540	scaffold2689	p2	(GA)6	Class II	28456	28467	ACGGTTCGAACTATGCCCAGGA	AGCGGCATCTGCTTCCAACT	282
FMgSSR-26544	scaffold18454	p2	(GA)6	Class II	1617	1628	ACGTCACTAGCATGTGGTTGCC	TTGTTTGTACGGCACCCTACCC	314
FMgSSR-26550	scaffold28307	p2	(GA)6	Class II	856	867	ACGTGAGCCCACATGCCACTTA	ACCGCTACAACCGCGTCTTCTT	297
FMgSSR-26557	scaffold3100	p2	(GA)6	Class II	30118	30129	ACTAGATGATGGGGCGGCAGAA	TTAACGGCCATCCACAGTGCCA	301
FMgSSR-26559	scaffold13077	p2	(GA)6	Class II	7864	7875	ACTCCAGCCGTTCAAGGCAA	AGCAGTTGAGTGAAGGGGCAGT	200
FMgSSR-26565	scaffold4366	p2	(GA)6	Class II	14862	14873	ACTCGCCCCAATTCAACGCT	ATTTGTGGTGGACTAGAGGGCT	305
FMgSSR-26566	scaffold22530	p2	(GA)6	Class II	2927	2938	ACTCGGCCATTCGACAACGAGT	AGGCATGCGAGCCAAACCCTAA	350
FMgSSR-26570	scaffold6847	p2	(GA)6	Class II	23731	23742	ACTGAGGCGGTGAAACCGTGAA	AGCTAGCCTGCCCATGCTTTGT	277
FMgSSR-26571	scaffold2642	p2	(GA)6	Class II	27110	27121	ACTGCAGGTGTTGGAAGCGA	TGGTTGACGACGCACAACTCGT	232
FMgSSR-26590	scaffold366	p2	(GA)6	Class II	89539	89550	ACTTTGCGCCATGCACACGA	AGTGGGAGGCAGCGACAAAA	247
FMgSSR-26600	scaffold3366	p2	(GA)6	Class II	39311	39322	AGACTCCTGGCATGTTGAGGTCT	GGTTTCATTGTCGGACGCGTGA	326
FMgSSR-26605	scaffold195	p2	(GA)6	Class II	47416	47427	AGAGCGCCATCGGGATGGAATA	TTAACTTTCCCCCGCGCCCTAT	347
FMgSSR-26606	scaffold11447	p2	(GA)6	Class II	13820	13831	AGAGGAAGCTGCCAACCCTTCT	TCCCCTTCTATTCCTCTGCCACCA	216
FMgSSR-26611	scaffold6625	p2	(GA)6	Class II	6645	6656	AGATCACGCACACACGAGCA	ACAATCCATCCACGCACGCT	307
FMgSSR-26618	scaffold7149	p2	(GA)6	Class II	32485	32496	AGCAAAAAGCCACAACCCGACT	TCACGCAACGTGTTTGCCCT	348
FMgSSR-26623	scaffold926	p2	(GA)6	Class II	76881	76892	AGCAACGCTCGATGATTGGGTG	AACACAGCGGCTTTCTGCGA	257
FMgSSR-26627	scaffold44	p2	(GA)6	Class II	197948	197959	AGCACCTAAGCCTGTCGTGCAT	AATAGCAACGGTGGTTCGGCCA	350
FMgSSR-26630	scaffold5028	p2	(GA)6	Class II	11593	11604	AGCACGTAAGAGGCTGCAACT	ATCAACCCCAGCACCAGCACAA	234
FMgSSR-26643	scaffold13	p2	(GA)6	Class II	96253	96264	AGCAGTGGTGCCTTTGTGTCTG	TGCATTCAGAAGGTGGAGAGAGGG	243
FMgSSR-26650	scaffold1719	p2	(GA)6	Class II	27583	27594	AGCCAACAGAGAGTGGTCAAGCA	AACGGTGGTTGTGATGGCGA	245

SSR_ID	Scaffold	SSR_Type	SSR_Motif	SSR Class	SSR_Start	SSR_End	Forward sequence	Reverse sequence	Product_size
FMgSSR-26654	scaffold1953	p2	(GA)6	Class II	49834	49845	AGCCAGCTCCAAACACTGCCTT	ATCCATCGCGTCGCATCGCATA	302
FMgSSR-26655	scaffold17261	p2	(GA)6	Class II	8334	8345	AGCCATGACGGTGCACACAA	TTTGAAATGGGACGTGGCCCGA	228
FMgSSR-26657	scaffold17757	p2	(GA)6	Class II	6302	6313	AGCCCCAGCATGTTGCCAGAAT	TGACACCCGGTTTTTGCCCT	348
FMgSSR-26686	scaffold475	p2	(GA)6	Class II	18321	18332	AGCGCTCCAACGGAATCACTGT	TGCGAAAGTCCAAATCGTGGGC	330
FMgSSR-26687	scaffold607	p2	(GA)6	Class II	79135	79146	AGCGGAACCATCGGTAGCAA	ATGTTCACTCCAGCAGCGGCAA	339
FMgSSR-26692	scaffold12208	p2	(GA)6	Class II	14619	14630	AGCGTGCAAGTGAGTGTGCT	GCAAGAACAGAATGTACACGACGG	250
FMgSSR-26698	scaffold207936	p2	(GA)6	Class II	35	46	AGCTCGCCGTGATGTGAAGA	AGCGACCTGGCGAATCATCT	233
FMgSSR-26713	scaffold77	p2	(GA)6	Class II	170248	170259	AGCTTAGCTGCTGTTGCTCCGT	ACCAGTGCAGAAGTGCTGGCAT	276
FMgSSR-26717	scaffold931	p2	(GA)6	Class II	60817	60828	AGCTTCGCTGCAACTTCCCT	AGCCCAAGTTGGCCCAGTCTTA	292
FMgSSR-26726	scaffold1008	p2	(GA)6	Class II	52450	52461	AGGAACAGCCGAACTCACCGTT	TCTAAACCCAGACGTGCACCA	319
FMgSSR-26727	scaffold3024	p2	(GA)6	Class II	30924	30935	AGGAACGAAAAGCCGCGGAA	CGATCCTGCGTCACTGCTTT	310
FMgSSR-26733	scaffold3258	p2	(GA)6	Class II	5889	5900	AGGACGAGTCGGCGTCGAATTA	ATTTGCAGGGTGCAACGGGA	258
FMgSSR-26737	scaffold425213	p2	(GA)6	Class II	212	223	AGGAGCTCGTTGGCCTAGACTT	TGCTCTCTCCGTTAGACCTGCT	201
FMgSSR-26738	scaffold1793	p2	(GA)6	Class II	48495	48506	AGGAGGAAATGGCTCGATGCGT	ACGACGCTTGCAATCATACGGC	345
FMgSSR-26741	scaffold8127	p2	(GA)6	Class II	21529	21540	AGGAGGTGCCGTTAGGTTGTGT	TCGAGCTCTCCAACTATGCTGCT	233
FMgSSR-26747	scaffold11311	p2	(GA)6	Class II	16904	16915	AGGCAGCCATTAGCGAAGCGAT	GGCATCGTTGATTGGTGTCGCA	349
FMgSSR-26748	scaffold3653	p2	(GA)6	Class II	17429	17440	AGGCAGCTCGCACTTTGGAA	AAGGCTGGCGCGGAATTGAA	243
FMgSSR-26753	scaffold3212	p2	(GA)6	Class II	54985	54996	AGGCCGTATCGGCGAACATT	ATGCCGTCACGCCGAAAACA	325
FMgSSR-26755	scaffold7387	p2	(GA)6	Class II	7011	7022	AGGCCTTTATTTGCCCCCGTGA	AACTTACCGAAGCCGCTGCTGA	323
FMgSSR-26756	scaffold210	p2	(GA)6	Class II	118429	118440	AGGCGATGGCTTCCACCTTT	TGCAGCTTATCAGGCAGTGGT	327
FMgSSR-26757	scaffold347	p2	(GA)6	Class II	93799	93810	AGGCGCGGCGTCTCTGTTTAAT	TGTGAGCGACCACAAATCGCT	341
FMgSSR-26763	scaffold21198	p2	(GA)6	Class II	9891	9902	AGGCGGCAGCACATTCAACA	TTCGCTCCGGCAAACCCAAT	300
FMgSSR-26768	scaffold29880	p2	(GA)6	Class II	3191	3202	AGGCTGAATGTGCGCGTGAA	TGCCTCCGTGTGAAATGCTCGT	272
FMgSSR-26772	scaffold1067	p2	(GA)6	Class II	22146	22157	AGGGAGAGGCGAAGAACAACGA	ATGCGCTGCTTCTGGGCTTT	287
FMgSSR-26774	scaffold4603	p2	(GA)6	Class II	24722	24733	AGGGAGGTTTTTCGGAATGGAGCC	AAAGCGGAAACCCGAAGTGCGT	346
FMgSSR-26779	scaffold4312	p2	(GA)6	Class II	33335	33346	AGGGCCCAAAGCCACGAACATT	TCGAAACGCGTCGTTGGAAGT	231
FMgSSR-26782	scaffold2071	p2	(GA)6	Class II	47923	47934	AGGGTGCAGGAAAAGCGAGACA	TCAGCATACGCGCCCAAACA	256
FMgSSR-26787	scaffold54965	p2	(GA)6	Class II	1393	1404	AGGTAGCGCACACCCGCTATTA	TGCACGCTGTTGTTGTTGGTGG	319

SSR_ID	Scaffold	SSR_Type	SSR_Motif	SSR Class	SSR_Start	SSR_End	Forward sequence	Reverse sequence	Product_size
FMgSSR-26791	scaffold6664	p2	(GA)6	Class II	33029	33040	AGGTGGGACCTTGATGGAGACA	TCTCACCCGACGGCCAAATCTT	256
FMgSSR-26794	scaffold1412	p2	(GA)6	Class II	20417	20428	AGGTGTGTGTGCATCGGTGT	AAAAACTTCTGGCGCCGGGT	257
FMgSSR-26798	scaffold3210	p2	(GA)6	Class II	2806	2817	AGGTTGACTTGGTGCCTGACCT	AAATTGCCATGTCACTGGCGGC	264
FMgSSR-26802	scaffold3102	p2	(GA)6	Class II	57951	57962	AGTAGGAAGGCAGCAATCCGCT	CACGCACTTGCGCCAATGAT	335
FMgSSR-26809	scaffold1651	p2	(GA)6	Class II	67525	67536	AGTCGCGTTGGAACGCCTAA	TTCCAGGTCGGATCCTTCGCAA	332
FMgSSR-26810	scaffold10	p2	(GA)6	Class II	147136	147147	AGTCGGTTCAGCCTTCAGCCTT	TCGACGCATATTGCTGTCCCTCA	332
FMgSSR-26813	scaffold7160	p2	(GA)6	Class II	21314	21325	AGTCTAGGTTGGCCTGGACCTT	ACGTTTTTCTCCTGTCAGACGC	341
FMgSSR-26818	scaffold31272	p2	(GA)6	Class II	5156	5167	AGTGAGGAGCTTGACAACCCGT	ACAGCTGCGCCAACTGCAAA	243
FMgSSR-26821	scaffold3342	p2	(GA)6	Class II	30303	30314	AGTGCGACAATGCGTGTGGT	CCATTGCATGCAGCCAACTGCT	304
FMgSSR-26828	scaffold12759	p2	(GA)6	Class II	2048	2059	AGTTCGTAGCCGTTGTCCGT	AGCGTGCAGGTGATGCTGTCAT	329
FMgSSR-26835	scaffold34269	p2	(GA)6	Class II	4740	4751	ATAATGGACGCAGCGTGCTGGA	AGCTGTGTGGGCAAAACCGT	335
FMgSSR-26839	scaffold18273	p2	(GA)6	Class II	7093	7104	ATATGCGTGAAGGCGGAGCACT	CGCCTTCGTTGGCGTAAACCAT	242
FMgSSR-26847	scaffold9980	p2	(GA)6	Class II	22179	22190	ATCATAGCACGCTACCTCGCCT	TACGGCCCAACTAAACGGCT	344
FMgSSR-26849	scaffold2010	p2	(GA)6	Class II	40399	40410	ATCCACTCGCCAGCGTCACAAT	AAGCACGGCCCAAAACCTGA	332
FMgSSR-26852	scaffold4465	p2	(GA)6	Class II	44659	44670	ATCCGCTGCCATGGAGGTTGAA	AGCCCAGCTGCAGTCCATTCTT	329
FMgSSR-26855	scaffold15369	p2	(GA)6	Class II	6738	6749	ATCCTCTGCTGGGGTTCTCACA	TGCATGCGCTTTGCTGTTGC	346
FMgSSR-26857	scaffold920	p2	(GA)6	Class II	48432	48443	ATCGACTTTGGTTGGTTGCCGC	ACGATGATGTGAGCTCTCCCTCT	350
FMgSSR-26864	scaffold576	p2	(GA)6	Class II	69481	69492	ATCGGGATTGAGAGAGCAGAGCGA	GCGCAATCGCATGAACGGAT	345
FMgSSR-26865	scaffold7947	p2	(GA)6	Class II	23626	23637	ATCGGTTGGGCAGTTACCGACA	ATGTCGCCTGTTGGCGTGAA	258
FMgSSR-26871	scaffold36114	p2	(GA)6	Class II	4703	4714	ATGACGGCGTTCTGGGGTTTGT	AAGCCGACCAATGCGCAACA	331
FMgSSR-26876	scaffold3473	p2	(GA)6	Class II	29634	29645	ATGCACCCTTTCGGCTTTCCCA	TGGCACTTGCTACGGTGAGA	341
FMgSSR-26879	scaffold12490	p2	(GA)6	Class II	1376	1387	ATGCCACGCTTGCTGCTTCT	ATAAGCCCACCAGGCCGATACA	342
FMgSSR-26881	scaffold29394	p2	(GA)6	Class II	7964	7975	ATGCCGCAGTCCAAAACCAGGA	AGACCACGAGTTTGCTGGGTGA	350
FMgSSR-26884	scaffold106961	p2	(GA)6	Class II	781	792	ATGCCTTGCCCTTGCCTTGT	TGCATACTCATGTGGGCGGTGA	200
FMgSSR-26889	scaffold4736	p2	(GA)6	Class II	29910	29921	ATGCGTGGATTCGAAGGAGGGA	ATGCTTTGCCGCCGTAGGTT	254
FMgSSR-26892	scaffold22114	p2	(GA)6	Class II	958	969	ATGCTCGTCCGCTCTACTGCTT	TTTTCCCTCTCTCCACCCACCCAT	282
FMgSSR-26894	scaffold4138	p2	(GA)6	Class II	51011	51022	ATGGACCATGGCGTGCTCTT	ACGCCAACTGCGCCAACTAA	340
FMgSSR-26895	scaffold2575	p2	(GA)6	Class II	22904	22915	ATGGAGAAAAGCCGAGGCGA	TTCAAGGGGAGCACGCTTGGTT	237

SSR_ID	Scaffold	SSR_Type	SSR_Motif	SSR Class	SSR_Start	SSR_End	Forward sequence	Reverse sequence	Product_size
FMgSSR-26898	scaffold688	p2	(GA)6	Class II	70594	70605	ATGGATTCATGCAGGGCAGGAC	TGGTCCAACTCATCTTATGGCCACG	255
FMgSSR-26901	scaffold1928	p2	(GA)6	Class II	55864	55875	ATGGCCCTCATGAGCCAAAAGC	TGTGACAGAAGCCAAGGGCA	350
FMgSSR-26908	scaffold2861	p2	(GA)6	Class II	27221	27232	ATGGGTGATTCGGCCAGCTTGA	AGGAGGATTCAGGAGCACGGAT	347
FMgSSR-26911	scaffold437	p2	(GA)6	Class II	9585	9596	ATGGTTTTGTGCCCGACTGACG	TGCTTGGCCTTTTGGCGCTT	262
FMgSSR-26916	scaffold2445	p2	(GA)6	Class II	64513	64524	ATTCAAGCCGCCACCACTCACA	AGCGCACATGTTCCTCCCATGA	293
FMgSSR-26928	scaffold718	p2	(GA)6	Class II	25456	25467	ATTTCGCGCCGCCGTCTTTT	ATGTTGTGGTGACCGCGTGA	248
FMgSSR-26931	scaffold8303	p2	(GA)6	Class II	28679	28690	ATTTTCGCCGCTCTTGTGGC	TGGCCGTCTATTGGCCGTTGAT	296
FMgSSR-26939	scaffold5847	p2	(GA)6	Class II	36009	36020	CATGGGCAGATCACACACAACA	TCGTTCCCTGAATCATACATGCCC	308
FMgSSR-26946	scaffold7479	p2	(GA)6	Class II	10045	10056	CCGCCAGTGCAACTGATCAACA	AAGGAACCTTGGCGGCCATT	306
FMgSSR-26957	scaffold8185	p2	(GA)6	Class II	5280	5291	CCTTTGTTATCAGCCACGCGCA	TCTCTCATATCGCTCGCCTCCA	347
FMgSSR-26961	scaffold493184	p2	(GA)6	Class II	143	154	CGACTAAGTTACTTCGAGGCAGAGC	TGACACTACTGCTGTCTGGTGGT	201
FMgSSR-26966	scaffold27815	p2	(GA)6	Class II	5825	5836	CGCACCTGCGCAATCAATCGAA	GTGCACGGATCAACCGATCGAA	309
FMgSSR-26968	scaffold10561	p2	(GA)6	Class II	3058	3069	CGCAGATGTTCATGCCGTGT	GCCAAATGATTCTGCAGCCCGT	213
FMgSSR-26971	scaffold2829	p2	(GA)6	Class II	14843	14854	CGCGAGCAACAAGAAACCCCAA	ACCTCAGTACGCACCACCGAAT	230
FMgSSR-26972	scaffold6272	p2	(GA)6	Class II	4337	4348	CGCGCAATGTTAGTGCAACACG	TGAGCGAGCTAGTGACGACCTT	231
FMgSSR-26977	scaffold221	p2	(GA)6	Class II	56359	56370	CGCTCGACGTTTTTGCGCTT	AGTGGCACCCGCACATTTCA	343
FMgSSR-26982	scaffold5918	p2	(GA)6	Class II	27295	27306	CGGCGCCGATGGCTAATCTAAA	AAAATCACGCCGCCCTCGTT	237
FMgSSR-26987	scaffold2	p2	(GA)6	Class II	175337	175348	CGGTGGCGTTTCCTTCGCTTTT	AATGCCTCTCAAACAAGCCCCC	318
FMgSSR-26988	scaffold161	p2	(GA)6	Class II	44163	44174	CGGTTGAGTTGAGCGCTTGCTT	GCATTGCAAGTGCAAGCCACCT	306
FMgSSR-26989	scaffold2959	p2	(GA)6	Class II	49767	49778	CGTCCTAATCCATATGCAGTGTCC	AGTGAGATGGTGTGTCTGCCA	211
FMgSSR-26991	scaffold3271	p2	(GA)6	Class II	7108	7119	CGTGCCTTGGTCCTTCCTTTGA	TTGGTACGGCAACGGCAACT	201
FMgSSR-27005	scaffold1172	p2	(GA)6	Class II	48629	48640	GACGACGCGTCTCACAAAGA	AAGGGTTTGCTTGGGTACGGCA	335
FMgSSR-27013	scaffold32971	p2	(GA)6	Class II	4202	4213	GCAGAAATGGCGCAAGCGTA	AGGCTGAATCTCCCCCTTCTCCTT	255
FMgSSR-27019	scaffold13737	p2	(GA)6	Class II	20416	20427	GCATCCTATTCGTGCCCTCTCACT	TGCTTGAGCGGTTTGTTCGCA	350
FMgSSR-27020	scaffold1149	p2	(GA)6	Class II	79075	79086	GCATGCGCACTAGCAGATCGAA	ACGTGTTGACGGCGCACAAA	319
FMgSSR-27023	scaffold4496	p2	(GA)6	Class II	21062	21073	GCCAGCCAAACAGTTGCCAT	ACTTGTGGCATGCCTTGTCGGA	306
FMgSSR-27029	scaffold24899	p2	(GA)6	Class II	6274	6285	GCCGCGGCGGGAAATAACAAAA	ATGGCCGACTGGAGCGCTTATT	287
FMgSSR-27033	scaffold778	p2	(GA)6	Class II	69167	69178	GCCTAGGTTCCCACCGCTGATTTT	AGGGCTGGACAAAAGCTACGTGA	340

SSR_ID	Scaffold	SSR_Type	SSR_Motif	SSR Class	SSR_Start	SSR_End	Forward sequence	Reverse sequence	Product_size
FMgSSR-27045	scaffold2909	p2	(GA)6	Class II	5509	5520	GCGGATGCTGCAATGGCTTT	TGCAACTGATTGGTGGGCCT	258
FMgSSR-27046	scaffold13352	p2	(GA)6	Class II	2695	2706	GCGGCAGCAAACTTGCAAAC	TAGTGCACAACTGCACATGGCG	216
FMgSSR-27047	scaffold226	p2	(GA)6	Class II	133152	133163	GCGGTTGTGTTGTGGGCTTTCA	TTTCCTGCATCCCGCATTGCCT	256
FMgSSR-27066	scaffold23103	p2	(GA)6	Class II	7559	7570	GGCGGAAGAACGTCATCAGCAA	CCGATGCTTGACGAGGTGCAAA	232
FMgSSR-27068	scaffold1535	p2	(GA)6	Class II	3437	3448	GGCGGTTCAAGACTAGACAGTGGA	ACTGATGCGGCACCACCCTTTA	330
FMgSSR-27069	scaffold5371	p2	(GA)6	Class II	15572	15583	GGCTGTTGAGGAAGCTGCAGGATA	AATGGCGTAATGCGCGCCAA	308
FMgSSR-27073	scaffold3234	p2	(GA)6	Class II	10046	10057	GGGCAAACAGCTGTGACGGAAA	TCACAGCAAGCCACTGCACA	318
FMgSSR-27074	scaffold20699	p2	(GA)6	Class II	1214	1225	GGGCGCGTACAGCGTAAACAAT	TGAGCTCGGTGACGCATGTGAA	304
FMgSSR-27077	scaffold3180	p2	(GA)6	Class II	17910	17921	GGGTTACTCGCCACATTGCACA	TGGTACGAATTGGCTTCCCCGT	350
FMgSSR-27080	scaffold554	p2	(GA)6	Class II	92705	92716	GGTGAAATTGGCTTCCCGAATGGC	AATGACGCGTTGGATGACGGGT	269
FMgSSR-27082	scaffold11950	p2	(GA)6	Class II	13387	13398	GGTGTGACGCTTGCAGGAAAGT	AGCTTCCCACACCACATCCACA	224
FMgSSR-27084	scaffold900	p2	(GA)6	Class II	19347	19358	GGTGTTGCATCATCACACACATGCC	TCCGACGGTGGTTTGCCTTTCA	332
FMgSSR-27085	scaffold1009	p2	(GA)6	Class II	24853	24864	GTCACCCTTGCGCATGCAACAT	GGCCACCCCAAGCACACAAAAA	271
FMgSSR-27086	scaffold2438	p2	(GA)6	Class II	17102	17113	GTCGGCTGATGCTTCTTGCT	TGTGTGTGCCCCCTTCACTCTT	325
FMgSSR-27092	scaffold7373	p2	(GA)6	Class II	15530	15541	GTGTCCTCATCCCTGTTACCATGC	AGCTGTGTTTGGTGACGCCAGT	215
FMgSSR-27096	scaffold2344	p2	(GA)6	Class II	48199	48210	TAAAGTTTGCTGCCGCCGCT	GCGACGGCGACCATTGTTTCAT	258
FMgSSR-27099	scaffold3197	p2	(GA)6	Class II	36313	36324	TAAGCAAGGAAGCATGGGCG	TGCAGGTTTGCAGCCAAGCAA	235
FMgSSR-27100	scaffold8934	p2	(GA)6	Class II	22313	22324	TACAAGAAAGCCTCGGCAGGCA	TCACACTGGAGCTCAAGCCCAAA	200
FMgSSR-27102	scaffold1442	p2	(GA)6	Class II	70485	70496	TACCTGCATCTCGGTGGCAA	AGTTGCAACGCACACGCTCT	218
FMgSSR-27103	scaffold935	p2	(GA)6	Class II	33995	34006	TACGCTGTACCGGCATGATGGA	TCTCATGCTCCGCAAGGCAGAA	221
FMgSSR-27107	scaffold6211	p2	(GA)6	Class II	5478	5489	TAGCAACCCATGAAGCGCCCAA	ACTCCATCACCGCAAGCCAA	303
FMgSSR-27108	scaffold841	p2	(GA)6	Class II	90920	90931	TAGCACGGTCGGCATGCATCTT	TCCGTTGCTTTGGTGCTCCTGT	296
FMgSSR-27110	scaffold377	p2	(GA)6	Class II	27562	27573	TAGCTGGGCCTGCCATTTGT	ACGCTTGGCCCGAATCTTGA	263
FMgSSR-27114	scaffold1719	p2	(GA)6	Class II	31771	31782	TATCGTCCCGCCTTGCATTGCT	AGGCCGCTCGAACATTCTCTGA	344
FMgSSR-27127	scaffold5795	p2	(GA)6	Class II	29935	29946	TCAACGGGCGTCGGTGTTTT	TCACGGCGGTTAAGTTCGGT	326
FMgSSR-27128	scaffold7591	p2	(GA)6	Class II	16043	16054	TCAACGTGGTAGCCCTCGGTTT	TGAGGCATGTTCCGTATCGCCA	248
FMgSSR-27129	scaffold152484	p2	(GA)6	Class II	101	112	TCAAGCCGCTGGGAAACTATGC	TGCGCTACTTCACATCGATCAC	200
FMgSSR-27131	scaffold8208	p2	(GA)6	Class II	22028	22039	TCAATGCCTTCGGCATCGGTCA	AAACCCATAGCTGCCACGCTCA	218

SSR_ID	Scaffold	SSR_Type	SSR_Motif	SSR Class	SSR_Start	SSR_End	Forward sequence	Reverse sequence	Product_size
FMgSSR-27134	scaffold981	p2	(GA)6	Class II	23891	23902	TCAATTGACCTCGCTTGGGTGC	ACTGTGGCGGGCGATAAACT	285
FMgSSR-27138	scaffold1978	p2	(GA)6	Class II	34276	34287	TCACAGGCCGGCTAAGCAACAA	AAAACACGCGGTCCACCACT	329
FMgSSR-27139	scaffold235	p2	(GA)6	Class II	5856	5867	TCACCCAAGCAGAATGCCTGA	ACGCACAGAGAGCTACCCCAAT	238
FMgSSR-27140	scaffold9221	p2	(GA)6	Class II	5507	5518	TCACGCCGAAGAGAGTGAGTGA	TCAATCCATACGCCGACCCT	269
FMgSSR-27141	scaffold618	p2	(GA)6	Class II	78947	78958	TCACGGCATGGGTGGGATTT	ATGGGCACTATAGCCATGACCC	246
FMgSSR-27143	scaffold2534	p2	(GA)6	Class II	27069	27080	TCAGAGCACGAGCACGGATT	AAGGCAGCTCCAGTTTGGCA	279
FMgSSR-27149	scaffold9232	p2	(GA)6	Class II	7011	7022	TCAGTTGAGCCCATGTACCAGT	ACCCATCTGCTCTGCTCTCTCA	248
FMgSSR-27151	scaffold9321	p2	(GA)6	Class II	12704	12715	TCATCGATGCCCTGCCAAAAGC	ACTTGTTGCTTGTGCCGCGT	263
FMgSSR-27155	scaffold1604	p2	(GA)6	Class II	58875	58886	TCATGGCCGCACCAAATCCA	ACAGCCCACGAAATCGTCGAA	245
FMgSSR-27161	scaffold4737	p2	(GA)6	Class II	5322	5333	TCCCAAGTTTCCAAGCCCACGA	ACCGTCTCAAGCATGGTCCGAA	324
FMgSSR-27168	scaffold4419	p2	(GA)6	Class II	46930	46941	TCCCCCGTCGTCATCTGTTGAT	TGCCAACTTGCAAAAGCGCGA	250
FMgSSR-27175	scaffold4552	p2	(GA)6	Class II	2847	2858	TCCGAGGAAGCTAGGGTTTGCT	TGCAGCCCACTTGTTCGTCTCA	291
FMgSSR-27178	scaffold7449	p2	(GA)6	Class II	16661	16672	TCCGCCAAGGCGTTTCTCACTT	AGTCTGGATATCCCATACCGCTCC	281
FMgSSR-27186	scaffold5469	p2	(GA)6	Class II	5318	5329	TCCGTCAGTGCCCTCTTGCATA	TTGCAGGCCTTTGGAGTCTCGT	308
FMgSSR-27188	scaffold407	p2	(GA)6	Class II	17130	17141	TCCGTGGCCATCATTGTTGCCT	AAGCCCCGTAGCCCATCAAAGT	289
FMgSSR-27192	scaffold2333	p2	(GA)6	Class II	15445	15456	TCCTCGTCACACGCGCAGATTT	ACCCACCTGCAGGCATACAT	318
FMgSSR-27193	scaffold14917	p2	(GA)6	Class II	17019	17030	TCCTCTGCCGATTGCGAGAT	GCACGATGACCGACAACGGTTT	339
FMgSSR-27197	scaffold2329	p2	(GA)6	Class II	29281	29292	TCCTGTTTCCTCTCGCCGATCT	AGCTGGTGGGACCGAGTTCATA	294
FMgSSR-27201	scaffold18534	p2	(GA)6	Class II	17125	17136	TCGAATGCAACCTGCATGCCAC	ATGACACACCGGCCAGCAAA	309
FMgSSR-27202	scaffold3433	p2	(GA)6	Class II	44440	44451	TCGAGCTCCGAACACTCGGATT	TGCAACGGGCCCAACCAACTAA	249
FMgSSR-27204	scaffold13737	p2	(GA)6	Class II	10156	10167	TCGAGGCCTTCAAGCCGCATAA	TGAAGCGCATCCAACCGTCA	212
FMgSSR-27206	scaffold196817	p2	(GA)6	Class II	534	545	TCGAGGTTGTAGGTGGCGAA	AGTCACAGTCTGCGTGATCCGT	265
FMgSSR-27216	scaffold21	p2	(GA)6	Class II	71706	71717	TCGCAATTCTCAGCGCCACCTT	TTCACCTGCAGCCACTCCACTT	283
FMgSSR-27217	scaffold11934	p2	(GA)6	Class II	1689	1700	TCGCATGCCCCCAATACGTT	TCCAACCGAAACGGCTTGCT	286
FMgSSR-27218	scaffold340	p2	(GA)6	Class II	16786	16797	TCGCCACCGGCGTCTTGTTAAT	ATCGCGGTTAATGGTCGGGA	277
FMgSSR-27221	scaffold28	p2	(GA)6	Class II	215596	215607	TCGCCGTCGATTATGACCTGCT	ACATTCACCACGCTTCGCCT	299
FMgSSR-27222	scaffold492	p2	(GA)6	Class II	107015	107026	TCGCCTCTGCAGTGCCAAAA	AATGGAAGGTGGGAGCTGGCAT	337
FMgSSR-27223	scaffold176	p2	(GA)6	Class II	143639	143650	TCGCCTCTGCTTTGTCGATG	AACTCTCTAAAGCGCATCCCGC	303

SSR_ID	Scaffold	SSR_Type	SSR_Motif	SSR Class	SSR_Start	SSR_End	Forward sequence	Reverse sequence	Product_size
FMgSSR-27225	scaffold4673	p2	(GA)6	Class II	14071	14082	TCGCCTTCGTGAGTTGTCATCG	TCTCATCTCATCCAGCGCCA	328
FMgSSR-27231	scaffold20655	p2	(GA)6	Class II	4816	4827	TCGCTACGGGGAGAGAGAGATT	ACTGGAGTGCAATAAGGAAGGACG	344
FMgSSR-27233	scaffold500	p2	(GA)6	Class II	70540	70551	TCGCTGCAAGCTGTTCACCA	TTTGATCCAGCGGTTGCCCA	350
FMgSSR-27234	scaffold5310	p2	(GA)6	Class II	37093	37104	TCGGATCCAAGGCAGAACAACC	AAAGCTGGCCCACGTAGAAGCA	315
FMgSSR-27250	scaffold3786	p2	(GA)6	Class II	53893	53904	TCGGGTTTTCTGCCCAGTGCAA	AGCCGTTCTGTTCTCAACCAGG	307
FMgSSR-27251	scaffold9600	p2	(GA)6	Class II	23471	23482	TCGGTTTCATGGGAGGAGAGGT	TCGTGGACCCACACATCAGCTT	215
FMgSSR-27259	scaffold10853	p2	(GA)6	Class II	5385	5396	TCGTCGCTCGTAAGTCGCAA	AGCATGCCCAGACACAGCAGAA	239
FMgSSR-27260	scaffold10212	p2	(GA)6	Class II	15729	15740	TCGTCGCTGCCAAGGGTAAA	TGCGTGGACCGAGTTTGCTGAT	231
FMgSSR-27264	scaffold12625	p2	(GA)6	Class II	7441	7452	TCGTGCTTTCGTGCATGGAGGT	AGCCCTGGACATGCGCTTCTAA	254
FMgSSR-27267	scaffold970	p2	(GA)6	Class II	51987	51998	TCGTGGTATGCTCGTATGTCCG	GGCTTCAGGTATCAACCCTTTGGC	302
FMgSSR-27268	scaffold2105	p2	(GA)6	Class II	14458	14469	TCGTTCGTCGCTGTTGGGTT	ACCCAAGGCAACATTTCCCGGT	296
FMgSSR-27269	scaffold648	p2	(GA)6	Class II	46983	46994	TCGTTGCCGTTCTCTTCGCA	TGCCTTTTGGGTTCCTTTCCGT	270
FMgSSR-27270	scaffold2226	p2	(GA)6	Class II	62508	62519	TCGTTGTGCACGAGCGAGAT	TTGCGGATTTTCTCGGACGGGT	275
FMgSSR-27273	scaffold8895	p2	(GA)6	Class II	2717	2728	TCTATTGCGCTGCTACGCCT	GCATTCAAGTCGGTTGGCAGCA	319
FMgSSR-27280	scaffold4818	p2	(GA)6	Class II	40084	40095	TCTCGTGGACTTGCACACCA	TGCCATCCCACCGACCAATGTT	214
FMgSSR-27282	scaffold587	p2	(GA)6	Class II	62765	62776	TCTGCAACGTGGTGAGGGAT	ATCATCAGCAGCAGCCACAGCA	247
FMgSSR-27285	scaffold39	p2	(GA)6	Class II	57645	57656	TCTTCACCGAAACGCACACCA	ACGGCAGTGCACGAGCATCTAA	210
FMgSSR-27287	scaffold4393	p2	(GA)6	Class II	28895	28906	TCTTTGTTGCGCTGTCGTGCT	ATGGGTGTGCGTAGCAATGGGT	344
FMgSSR-27288	scaffold30821	p2	(GA)6	Class II	315	326	TGAAACCCTCGCCCACAACA	GGCGCCATGTAGTTTAGGCCAT	258
FMgSSR-27291	scaffold1219	p2	(GA)6	Class II	64503	64514	TGAACACATGCCCACACACACA	TGTGGGGGCACACAAAAAGGT	240
FMgSSR-27292	scaffold2639	p2	(GA)6	Class II	28001	28012	TGAAGGGATTCCACTGACGCGA	ACACGTCACGTCTTGGCCCTTT	304
FMgSSR-27296	scaffold2723	p2	(GA)6	Class II	3414	3425	TGACATCGTGTCACGCGCAA	TGGAGCGCAGTAAAGGCGAA	338
FMgSSR-27299	scaffold6129	p2	(GA)6	Class II	15623	15634	TGACCATTGCCCGCAACTTCCT	TGCATCCACGTCCCCGAATTGT	330
FMgSSR-27304	scaffold18290	p2	(GA)6	Class II	13113	13124	TGACTCCGATGAGACAGCTCCT	TGCAACGGCAAGAACACGACA	329
FMgSSR-27307	scaffold5293	p2	(GA)6	Class II	42191	42202	TGACTTTCGCTGCTGAGCTCGT	GCAATGGCCCCGAGTTTTGTGT	305
FMgSSR-27308	scaffold4337	p2	(GA)6	Class II	16172	16183	TGAGAGAAGTTGGCTTCGATGGGG	ACGTGCATCTTGGTCCTAGCCA	282
FMgSSR-27309	scaffold25470	p2	(GA)6	Class II	1977	1988	TGAGCAAAACACGACCGACCCA	AAGCGCACACCGGATTGAGT	271
FMgSSR-27311	scaffold3817	p2	(GA)6	Class II	12748	12759	TGAGCCAGCAGCAATAGCGGAA	TGCCAAAGCTCTGAACCAAAGCG	333

SSR_ID	Scaffold	SSR_Type	SSR_Motif	SSR Class	SSR_Start	SSR_End	Forward sequence	Reverse sequence	Product_size
FMgSSR-27320	scaffold1039	p2	(GA)6	Class II	73578	73589	TGAGCTTCTTCGCGACGTGT	TGCCGACGCTGTGTTTTCCA	254
FMgSSR-27325	scaffold1870	p2	(GA)6	Class II	54019	54030	TGATCCGCTGTTTCCTGCGT	GCAGCACAAACCGTGCTCTT	350
FMgSSR-27326	scaffold169	p2	(GA)6	Class II	111176	111187	TGATCGTGGAGAACATGGCGGA	AGCTTTACCTGTGTGCGGTGGT	278
FMgSSR-27327	scaffold1111	p2	(GA)6	Class II	55675	55686	TGATCTCAGTGCAATGCGGCCT	AACGACAAATGAGCTGGCGCAC	343
FMgSSR-27331	scaffold987	p2	(GA)6	Class II	33241	33252	TGATGCCCAGATGACACCGTGA	AAGCCCCTCAACGCGACAAA	250
FMgSSR-27336	scaffold14642	p2	(GA)6	Class II	22408	22419	TGATTAGAGCAGCAGCGCGT	ACCTTCGTCCAAGCGCAGGATT	306
FMgSSR-27346	scaffold231130	p2	(GA)6	Class II	324	335	TGCAATACAGACGCTGGCACA	ACCAAAGCTGATGGATGTGTCCTC	350
FMgSSR-27349	scaffold855	p2	(GA)6	Class II	5085	5096	TGCACACTTGCAGTTGCAGTCC	TCAGTGCCGTAACTCTGCCGTT	317
FMgSSR-27350	scaffold119	p2	(GA)6	Class II	174655	174666	TGCACAGTTTCTGCGATGCGT	ACTTGCGAAGCGCAGGTTGA	304
FMgSSR-27351	scaffold833	p2	(GA)6	Class II	72545	72556	TGCACCACTGCACCTTTCGACT	ACGCGATGAGAAGCCGTTACGA	272
FMgSSR-27352	scaffold3138	p2	(GA)6	Class II	9276	9287	TGCACCTTGGTCTTGCCGAACT	CGAGCTCGTCTCGCAGTTGTTT	337
FMgSSR-27356	scaffold452	p2	(GA)6	Class II	1097	1108	TGCAGATCGAGTGGCAAGTGA	TCGCCAAATCATTGCCCGGT	211
FMgSSR-27358	scaffold7292	p2	(GA)6	Class II	32793	32804	TGCAGCATCAACTGCTTCAGGC	ATTGCGTTCACCATCCCGGT	284
FMgSSR-27359	scaffold18446	p2	(GA)6	Class II	5418	5429	TGCAGCCGGACATCTTTGCCTT	ACGTAGGTGCGTGCAAACGA	285
FMgSSR-27360	scaffold5593	p2	(GA)6	Class II	15451	15462	TGCAGCGAGGAAACGGAGAACA	ACCACCGAGCTGCTGTTGTCTT	343
FMgSSR-27363	scaffold754	p2	(GA)6	Class II	76516	76527	TGCAGGCAGCTCCAACTCTTCA	TGAACTGAAGGCGGGCAACA	347
FMgSSR-27364	scaffold11077	p2	(GA)6	Class II	11835	11846	TGCAGGGGTCATGGTGTTTCCA	CCGCTGAACCTCGATGGCTAAA	294
FMgSSR-27365	scaffold5463	p2	(GA)6	Class II	27920	27931	TGCAGGTTGCTGGAATGGCA	TGTGTTTGCACAGCACCCGA	234
FMgSSR-27366	scaffold590	p2	(GA)6	Class II	111324	111335	TGCAGTGGCTTCGCATGTCT	CCAACATCTACCGCCAACAAGAGC	268
FMgSSR-27368	scaffold9514	p2	(GA)6	Class II	9090	9101	TGCATCTTGCACCGCCATAGGT	TTTGGGCTGTAGCCAACCAACG	266
FMgSSR-27369	scaffold1552	p2	(GA)6	Class II	76148	76159	TGCATGCAGTGTGAGAGGGCAA	AGGCGGCACAATTAGCTGGGTT	337
FMgSSR-27370	scaffold45140	p2	(GA)6	Class II	1013	1024	TGCATGCCACGTCGTCGTTT	AGACCGCCGTGTTCTTTGGA	226
FMgSSR-27372	scaffold7069	p2	(GA)6	Class II	15585	15596	TGCATGGCATTGGCAGTGGT	ATGCGGATGCGCAGGTTGTT	324
FMgSSR-27374	scaffold1469	p2	(GA)6	Class II	4246	4257	TGCATTCATGCTTCCGGCGA	TGATCTCGCTCGCCCAACAACT	291
FMgSSR-27376	scaffold44	p2	(GA)6	Class II	74500	74511	TGCATTTTGTTGCGCACGCC	TGCGACGGTGGGGAAATGTT	268
FMgSSR-27384	scaffold2560	p2	(GA)6	Class II	25770	25781	TGCCATCATTTCGGCAGGCA	TGGTTGCCACGCCCAATCTT	341
FMgSSR-27385	scaffold13811	p2	(GA)6	Class II	2025	2036	TGCCCACATAAGCTACTTAGCCGC	TGCCATAGCTGGACCACCGTTA	330
FMgSSR-27389	scaffold204	p2	(GA)6	Class II	88013	88024	TGCCGCCCTTGTTCCATTTGA	AGCAACAATGAGCGGGTGGTCA	318

SSR_ID	Scaffold	SSR_Type	SSR_Motif	SSR Class	SSR_Start	SSR_End	Forward sequence	Reverse sequence	Product_size
FMgSSR-27391	scaffold3566	p2	(GA)6	Class II	23226	23237	TGCCGCTTTGAACTCACGCT	TCGCCGCTGCGTTGTTTTGT	306
FMgSSR-27394	scaffold8268	p2	(GA)6	Class II	1058	1069	TGCCGGATGGGGCATCATAA	TGATGTCAGCGCCTCCGTTT	311
FMgSSR-27396	scaffold1503	p2	(GA)6	Class II	75364	75375	TGCCGTTGGTGACCAGGGAAAT	ACAACCCAGCAACCCGCAAA	242
FMgSSR-27397	scaffold5940	p2	(GA)6	Class II	15898	15909	TGCCTACCAGAGTAGTCATGGGTT	AACTCTCTCTTTCCCTCCCTCTCC	207
FMgSSR-27399	scaffold1543	p2	(GA)6	Class II	34887	34898	TGCCTATGGGGCATGCCTTGAA	TCTTTCACCGGTTGTGCAGGA	321
FMgSSR-27400	scaffold3463	p2	(GA)6	Class II	38313	38324	TGCCTCATCCATGGTCCAGCAA	ATGACAAACAGTGGCACCACGA	200
FMgSSR-27401	scaffold1153	p2	(GA)6	Class II	95079	95090	TGCCTCGCCTTCCCCTCAAAAT	TAGCTAACGTTTGGCCGCTGCT	289
FMgSSR-27416	scaffold22444	p2	(GA)6	Class II	1407	1418	TGCGATGAACCATAGCAGGGTA	TCTTCCACTAGGAGACCTTGACCA	333
FMgSSR-27418	scaffold565	p2	(GA)6	Class II	99054	99065	TGCGCACACAAACTGGCGTA	ACATTCGACGCAACGGTGACCT	239
FMgSSR-27419	scaffold4903	p2	(GA)6	Class II	42466	42477	TGCGCACACACACTCACACA	TGAAACCCGCCCGTTCAATCT	297
FMgSSR-27423	scaffold2706	p2	(GA)6	Class II	18357	18368	TGCGCATCCCCCTCAAAACA	TGCGCGCGGCCGTTTAATAAT	319
FMgSSR-27431	scaffold875	p2	(GA)6	Class II	27793	27804	TGCGCTATCCTTCACCGAGA	AGTTCTCTCGCCTTGCACCCTT	251
FMgSSR-27433	scaffold6922	p2	(GA)6	Class II	6373	6384	TGCGGACTTCGCATCGACAA	GCGCACGGTGGAAAAAGGGAAA	312
FMgSSR-27434	scaffold2785	p2	(GA)6	Class II	56564	56575	TGCGGCCAGGTGCTCAAAAA	TTGGCTGTTGTTACGCCCTCCT	237
FMgSSR-27435	scaffold1060	p2	(GA)6	Class II	22669	22680	TGCGGCCTGTTTTCGCATGT	ACATGATCCTGCACCCGTCCAT	303
FMgSSR-27446	scaffold79	p2	(GA)6	Class II	6098	6109	TGCGTTTGTAGGGTAGGGAACGA	ATGGAGCTCCGGGCACATCAAT	202
FMgSSR-27452	scaffold20149	p2	(GA)6	Class II	9083	9094	TGCTCAGACGAAGGAGCTTTCAGG	TCAGGTCTGCACAGTGGGTTCA	348
FMgSSR-27453	scaffold4853	p2	(GA)6	Class II	22356	22367	TGCTCAGCATGTTCATTCGCCC	TGTCACCAATCTCCCAACCTCGT	347
FMgSSR-27460	scaffold13922	p2	(GA)6	Class II	18554	18565	TGCTGCATCGCTGCTGTGTA	TCCAATGGCGACCACGCAAA	348
FMgSSR-27462	scaffold2497	p2	(GA)6	Class II	50690	50701	TGCTGCCTGCTGCTTGGTTT	TCGGCGTTTTTCTCTCCACCGT	303
FMgSSR-27469	scaffold20695	p2	(GA)6	Class II	5380	5391	TGCTGTCGAAAGAGGTGGCA	TCGCTCTGCCTTGGCCACAATA	272
FMgSSR-27470	scaffold4214	p2	(GA)6	Class II	53147	53158	TGCTGTGGATTCATGGCGGTGT	CGGTGAAAAGCGGTGGCCATTT	229
FMgSSR-27484	scaffold22612	p2	(GA)6	Class II	5249	5260	TGGAGCAGCATGCGGAGTTGAA	CGAGCACGGCAGTGTTACAA	350
FMgSSR-27487	scaffold15766	p2	(GA)6	Class II	8249	8260	TGGAGCTTAGCTTGGGCAGCAA	ACGACGCAGGCTCCCAATTT	239
FMgSSR-27492	scaffold9487	p2	(GA)6	Class II	6668	6679	TGGATCAAGGACATGCGCAATAGG	TCTCCTTCTCTTCCCTTCCTCCAC	347
FMgSSR-27496	scaffold8086	p2	(GA)6	Class II	10278	10289	TGGATTCTTGGTGCTGCTGGGA	ACCACGTTACGTTCTGCCACAGT	247
FMgSSR-27498	scaffold3447	p2	(GA)6	Class II	35726	35737	TGGATTTGCGTCCGGTTCGT	AGGCGAGGTGGATGATGGAAGA	346
FMgSSR-27500	scaffold18280	p2	(GA)6	Class II	16228	16239	TGGCAGCAAGAGGGATGCAA	TCCACTCCATGACCCCATGGTT	326

SSR_ID	Scaffold	SSR_Type	SSR_Motif	SSR Class	SSR_Start	SSR_End	Forward sequence	Reverse sequence	Product_size
FMgSSR-27504	scaffold391	p2	(GA)6	Class II	1704	1715	TGGCCAGTGGCAACGCTTAGTT	TCAATCCGTCCACGCCAACA	282
FMgSSR-27513	scaffold10059	p2	(GA)6	Class II	19611	19622	TGGCGTGATTAGGCACGGTGAT	AGCCGGCCAAGCTGCTATTT	307
FMgSSR-27521	scaffold1096	p2	(GA)6	Class II	84463	84474	TGGCTGCCGACGCTTTCTTT	TGCATGCATGTTACTGCGGC	289
FMgSSR-27527	scaffold62276	p2	(GA)6	Class II	519	530	TGGCTTGGTCGTCCATGCTGAT	CCGTCGGAGAAATTGGTACGGT	239
FMgSSR-27529	scaffold23801	p2	(GA)6	Class II	3311	3322	TGGGACATCCGGGTCTTTAGTCT	TTCCTCTACCACTCTCTCCCCAGT	350
FMgSSR-27532	scaffold821	p2	(GA)6	Class II	5179	5190	TGGGCACTACGCAGCAAAGGAT	ACCGGCGATGTCTTTGCGTA	310
FMgSSR-27539	scaffold7372	p2	(GA)6	Class II	17343	17354	TGGGGTGAAAAGCTGGATGCGT	AGGGACCAACGTCATGCAACGA	341
FMgSSR-27541	scaffold7305	p2	(GA)6	Class II	30730	30741	TGGGTCACGACCAAGCCACAAA	GGCAAGCAGGGACGCAACAAAA	244
FMgSSR-27547	scaffold612	p2	(GA)6	Class II	12021	12032	TGGTCAATCTTGGGATTGCCAGGT	TCGGCCATAGCTTGATGCTGCT	290
FMgSSR-27548	scaffold874	p2	(GA)6	Class II	26976	26987	TGGTCCACGGCTCAGAAAAGT	TGGCGCTGGTTAGACGGCTTAT	344
FMgSSR-27550	scaffold755	p2	(GA)6	Class II	56789	56800	TGGTCGAAACCAAAACGGCA	AGGCCTGGAGCACAAAACCTCA	275
FMgSSR-27551	scaffold74	p2	(GA)6	Class II	51289	51300	TGGTCGCCACATGGGCATGATT	ACCACCAAAACGAAGCGACGAC	252
FMgSSR-27553	scaffold5798	p2	(GA)6	Class II	18975	18986	TGGTCTGCTGATGGCCCGTTTT	GGATTATAGTGTGGTCCCGCAAAC	299
FMgSSR-27554	scaffold8173	p2	(GA)6	Class II	28953	28964	TGGTCTTTCCACTCCCACTTCGT	TGGGGCTGGAACCTTCAGCATT	253
FMgSSR-27556	scaffold12306	p2	(GA)6	Class II	3155	3166	TGGTGGACCACTAGCTACGTAAGA	ACCACCAGTTGCATGCACGA	216
FMgSSR-27557	scaffold7175	p2	(GA)6	Class II	12394	12405	TGGTGGCAACTGCCCACTTT	ACTCTGACTCGCCACCGACAAA	211
FMgSSR-27558	scaffold3187	p2	(GA)6	Class II	38670	38681	TGGTGGCGCCATTGATGATTGC	ACGCAAAACGCCAGCACCAT	324
FMgSSR-27566	scaffold10568	p2	(GA)6	Class II	563	574	TGGTTCGCTTCGCTTCGCTT	TAGCGCATTCACTTGGGCTGGT	244
FMgSSR-27567	scaffold1117	p2	(GA)6	Class II	48777	48788	TGGTTGTGAATGGTGGCTCG	AGGAGCGCGCCTGAATTTCT	270
FMgSSR-27579	scaffold15230	p2	(GA)6	Class II	7837	7848	TGTGAGCATGCAGCAGGACCAT	TGCCCATGGCTGCACTCCATTT	342
FMgSSR-27580	scaffold3461	p2	(GA)6	Class II	32823	32834	TGTGAGGCCCGCTTTTGCTT	TGCACCAATAGGAACACGCCA	327
FMgSSR-27584	scaffold2210	p2	(GA)6	Class II	58228	58239	TGTGCGGATTCGGAAAAGCG	AGCCCAAGACTCAACCCACCAA	247
FMgSSR-27590	scaffold25209	p2	(GA)6	Class II	3447	3458	TGTGGTGTTTTAGAGAGGAGGGGG	GTGGACTGCAGGAACCTTCCTAAT	314
FMgSSR-27592	scaffold10641	p2	(GA)6	Class II	21349	21360	TGTGTCCGTCGCTGTTGAGGAA	CGGCCGCGATTCACGAAAACAA	342
FMgSSR-27595	scaffold7818	p2	(GA)6	Class II	17162	17173	TGTGTGGGAAGAAGGCGACA	TCCTAAGGCCTATCACAACTCGCA	250
FMgSSR-27596	scaffold631	p2	(GA)6	Class II	49192	49203	TGTGTGGGTCAAGATTGATGCCGT	TGCCGGTTGAAGAAGAAGGCGA	245
FMgSSR-27602	scaffold48	p2	(GA)6	Class II	181221	181232	TGTTCTTCGCGTGCGTGCTT	AGAAAGCAGGCGTTGCTGGA	347
FMgSSR-27606	scaffold159	p2	(GA)6	Class II	39921	39932	TGTTGGCCAGGTTTCGTATGAC	ACGGCATTCACGTTGCGCTT	299

SSR_ID	Scaffold	SSR_Type	SSR_Motif	SSR Class	SSR_Start	SSR_End	Forward sequence	Reverse sequence	Product_size
FMgSSR-27607	scaffold2912	p2	(GA)6	Class II	57766	57777	TGTTGTGGTTTGAACGCCCAGT	TGCACCGATGCGATTCGGGTAT	260
FMgSSR-27612	scaffold10942	p2	(GA)6	Class II	30890	30901	TTACTGTGCTCCGTTCGCCGAT	TCGAAACCGCACCACCGAAT	316
FMgSSR-27619	scaffold4838	p2	(GA)6	Class II	13215	13226	TTCACCGGTGGAGCCCAAAA	TTTCCTAAACGACACCCGGCCT	319
FMgSSR-27621	scaffold3549	p2	(GA)6	Class II	3428	3439	TTCACGCAGTTCGGCAGGCTAT	TCGGTCGGTCCAAGTGCAGAAA	328
FMgSSR-27628	scaffold3755	p2	(GA)6	Class II	43385	43396	TTCCAAGTCGCTGTGCCTGGAT	TGCGATCAAAATCTGCGGCGA	323
FMgSSR-27631	scaffold71	p2	(GA)6	Class II	177275	177286	TTCCGCGTACGTTTCGCTCT	TGCTAGTGACACCAGAACCCGT	345
FMgSSR-27632	scaffold6291	p2	(GA)6	Class II	14399	14410	TTCCGTCACCAGCATTCCAGCA	TGGCCTTCGCCTGTTCAACACA	337
FMgSSR-27633	scaffold4808	p2	(GA)6	Class II	37993	38004	TTCCTACAGCGCTTTGCCCCTT	GCCTGTTCGCGCTGTTTCTTCA	258
FMgSSR-27635	scaffold190548	p2	(GA)6	Class II	327	338	TTCGATGCGGGTGAGGTTCTCT	TGCTCCTCTCCTTCTCTGGCTT	272
FMgSSR-27636	scaffold14903	p2	(GA)6	Class II	8442	8453	TTCGCAGGGAGGAAACCGCAAT	TCGTCTGATGCCCCGTTGATGT	281
FMgSSR-27647	scaffold266	p2	(GA)6	Class II	118602	118613	TTCTTCACCGGGCTTCGCCATT	ATTCCAAGCTTGCAGCGCGA	290
FMgSSR-27657	scaffold32755	p2	(GA)6	Class II	2974	2985	TTGCAAACCCCGTGAAACTGGC	AAGGACATCGCCTTCGACCAGA	277
FMgSSR-27659	scaffold5035	p2	(GA)6	Class II	5780	5791	TTGCAAACTGCAAACCGCCG	AAACACCAAGCAGAGGCACGCA	350
FMgSSR-27660	scaffold5883	p2	(GA)6	Class II	20939	20950	TTGCAGGGATGAAGAGCAGCGT	CGGAGGAAAGCAGGGGAAAGTTCA	257
FMgSSR-27663	scaffold6236	p2	(GA)6	Class II	18192	18203	TTGCCCCTTTTGCTCCTGTGCT	TTGCCGATCGTCGAAGTGCT	309
FMgSSR-27676	scaffold22865	p2	(GA)6	Class II	1890	1901	TTGCGGTGCCAGGATTGGAA	AGAAGCCACGTCATCCCACTGT	295
FMgSSR-27684	scaffold15078	p2	(GA)6	Class II	593	604	TTGGCGATTGAGTGGGGTGTGA	ACTGAAGGGTGGAACCCACGTT	346
FMgSSR-27692	scaffold4387	p2	(GA)6	Class II	9752	9763	TTGGGTCGGAAAGAAGGCAGCA	AGCCCATTAATCCGTGGGCCAT	244
FMgSSR-27694	scaffold1144	p2	(GA)6	Class II	60427	60438	TTGGTAGTTGGGAGCGAGACGA	ACCCAAGTGGCCAAGTCTCT	297
FMgSSR-27697	scaffold3624	p2	(GA)6	Class II	9057	9068	TTGTGGATTAATGCCGGCGGTG	TTGTGCGTGACTGGCCTCAA	267
FMgSSR-27699	scaffold11771	p2	(GA)6	Class II	21394	21405	TTTACCTCCTCCCTGCCCAAGT	GCCCAGAAATTGCAGCGCCAAA	312
FMgSSR-27706	scaffold5993	p2	(GA)6	Class II	27415	27426	TTTCCTGACCGCCACCATGT	GATCTCTTCTGTGTTCAACTGGCG	238
FMgSSR-27718	scaffold12183	p2	(GA)6	Class II	4500	4511	TTTGCGTGTCGAGTGGCTGCTA	AAATCACCCGCGTGTCTCCT	286
FMgSSR-27721	scaffold43425	p2	(GA)6	Class II	2460	2471	TTTGGAACCCGGTTTCGGACCA	AGCCGCTCTTCTTCTTCGTGGA	246
FMgSSR-27726	scaffold1789	p2	(GA)6	Class II	70796	70807	TTTGGTTGGACCCGACCGGTTT	TGCCTCTTCGTTGTCGCCTT	262
FMgSSR-27727	scaffold9030	p2	(GA)6	Class II	31343	31354	TTTGTTTGCTCCTGCCTCGGGT	ACGGGCATGCAACCCAATCA	269
FMgSSR-27728	scaffold15133	p2	(GA)6	Class II	13493	13504	TTTTACAAGCGAGGCCGCAG	AAACCGTCAGCGCCTCTTCGTT	294
FMgSSR-27734	scaffold967	p2	(GA)7	Class II	23106	23119	AAAAAGAGCGCGGCATGGCA	TGTTGGTTCGTGCCCGTTGA	224

SSR_ID	Scaffold	SSR_Type	SSR_Motif	SSR Class	SSR_Start	SSR_End	Forward sequence	Reverse sequence	Product_size
FMgSSR-27735	scaffold492	p2	(GA)7	Class II	40459	40472	AAAACGCGGAGCCAGTGGAA	AGGCCCGTTCGTCGAAAACTCA	307
FMgSSR-27742	scaffold437	p2	(GA)7	Class II	10152	10165	AAACCCAGCGCAGGAACGAA	AAGCGCGTTGGAGACAAAAGCC	303
FMgSSR-27745	scaffold115	p2	(GA)7	Class II	100633	100646	AAACGGAGCAGCGGACCAAA	AGGAGGAGCAGAAGTTTTGCGGT	274
FMgSSR-27749	scaffold5637	p2	(GA)7	Class II	15681	15694	AAAGAGCCTTGCGCTGCTGT	CTGCTCCTACCATCTAGCTAAGCA	240
FMgSSR-27757	scaffold1296	p2	(GA)7	Class II	27096	27109	AAATGCACGCGTTCCAAGCA	AGATCGAAGCTGGGAGCGACAA	286
FMgSSR-27758	scaffold25165	p2	(GA)7	Class II	10717	10730	AAATGCGCACTCGCCAAGGT	AACCCAAGCAGCAGCAGCAA	202
FMgSSR-27763	scaffold8798	p2	(GA)7	Class II	6393	6406	AACAGCGACCGCTGATGGTT	AAACCACCTGATTCGCCCCA	319
FMgSSR-27765	scaffold17828	p2	(GA)7	Class II	5284	5297	AACATGTGCAGTGCGTGGAC	AAAATGGTTGGTCTCCCTCTGCCG	294
FMgSSR-27767	scaffold592	p2	(GA)7	Class II	5939	5952	AACCGAAACCGACTCGCTCGAT	AGGAAGTCCAGCTGGTTGCAGT	330
FMgSSR-27771	scaffold995	p2	(GA)7	Class II	85754	85767	AACCGGGATGCAACGGCATT	TTGCATGTGCAAGGACCGCA	273
FMgSSR-27773	scaffold12500	p2	(GA)7	Class II	7130	7143	AACGCCCAGTTGGTCTCTTCGT	TGGGCGCAATGAGTATGCGA	303
FMgSSR-27775	scaffold15249	p2	(GA)7	Class II	3835	3848	AACGGACGGAGAACGTGGTGAA	TCGACCGCTGTTGTTTCGCA	318
FMgSSR-27776	scaffold41976	p2	(GA)7	Class II	1119	1132	AACGGCACGCCCGAACATTT	TTCGCACTTTTCGCTCCCACCT	219
FMgSSR-27782	scaffold6490	p2	(GA)7	Class II	14067	14080	AACTCGGCCACCAACCTTGCTA	TGTGTGCCGTAAGGGGCAAGAA	289
FMgSSR-27783	scaffold40	p2	(GA)7	Class II	46975	46988	AACTGCCAAGTGGCCAAGCA	AGTTGCAGGCTTGCAGGACA	284
FMgSSR-27787	scaffold2950	p2	(GA)7	Class II	5908	5921	AAGAGCTGATCGCACAGTGCCT	ATCGGCGAATACGCAACGGT	249
FMgSSR-27790	scaffold18802	p2	(GA)7	Class II	11136	11149	AAGCACGTGTGACTACCGTCCA	TTGCTGGAGGGCAAGTGAAGCA	242
FMgSSR-27792	scaffold63075	p2	(GA)7	Class II	810	823	AAGCCACACATGTCCCCCTGTT	TGCGGCCAGCCACAAGTAGAAA	306
FMgSSR-27794	scaffold10021	p2	(GA)7	Class II	4364	4377	AAGCGAAAGCGGGCACAATG	AGTGGCGCATCTTCTCAAGCCA	324
FMgSSR-27795	scaffold5917	p2	(GA)7	Class II	20240	20253	AAGCGGGGTTTTGGGGTCCAAT	AGGGTCTGCCAAGTGAATGCGT	260
FMgSSR-27796	scaffold15836	p2	(GA)7	Class II	16563	16576	AAGCGTTGCCGCTTGGATCA	AAAAGGCCCCAACGGCATGT	301
FMgSSR-27801	scaffold43784	p2	(GA)7	Class II	3216	3229	AAGGGAGGAGGAGAGGTAACGA	TGCTAATTGCTACTGGCCGCGT	347
FMgSSR-27806	scaffold2278	p2	(GA)7	Class II	62304	62317	AAGTCGACGCAGGTTTGGCA	TGTCGTCTCTAGCCACAGCAA	305
FMgSSR-27812	scaffold25364	p2	(GA)7	Class II	9057	9070	AATCACGCCTGAGGAAACCACG	GCCGGCGATGGCAGTAAGTTTT	255
FMgSSR-27813	scaffold18225	p2	(GA)7	Class II	5204	5217	AATCAGTTACCCGAGCTGTGCG	ATGGTCCGCTGCTCCAAAGCAT	298
FMgSSR-27815	scaffold11737	p2	(GA)7	Class II	4109	4122	AATCCGTGACTCCGGCTGTT	GCAACGCGGAGATTTGGCTAGT	349
FMgSSR-27816	scaffold660	p2	(GA)7	Class II	14958	14971	AATCGCAGCAGCGACGGAAA	AAGACGCCACGCCATTTTGC	272
FMgSSR-27825	scaffold1450	p2	(GA)7	Class II	24674	24687	ACAAACAGCGGTGGATGGCA	TTGGTTGTGTCGTGCTCCGT	273

SSR_ID	Scaffold	SSR_Type	SSR_Motif	SSR Class	SSR_Start	SSR_End	Forward sequence	Reverse sequence	Product_size
FMgSSR-27838	scaffold11059	p2	(GA)7	Class II	21992	22005	ACAGGAGAGACGGCAAGACGAA	TTGAGCTAGCTATACCACCCCC	227
FMgSSR-27841	scaffold968	p2	(GA)7	Class II	73733	73746	ACAGGCTTAGAGCACTCCAGCA	TGGACAGAGGCTGCCATGGATT	290
FMgSSR-27842	scaffold5727	p2	(GA)7	Class II	31750	31763	ACAGTGCTTCAGGCGGCAAA	ACCTGTACCCGCCCGAAAATCA	212
FMgSSR-27847	scaffold1851	p2	(GA)7	Class II	4104	4117	ACATGCTTTTGCCACCGCCA	ACCCCGTGAGCCAACATCATCA	208
FMgSSR-27862	scaffold5306	p2	(GA)7	Class II	41132	41145	ACCATTCCCAGTTTCTGCTTGC	TGTGTGGCCTCCTGTTCCAA	339
FMgSSR-27865	scaffold3562	p2	(GA)7	Class II	14566	14579	ACCCACTTTTGTTCAGCCCTTGC	TTGAACCAGCGGATCAGAACGG	332
FMgSSR-27874	scaffold5794	p2	(GA)7	Class II	37821	37834	ACCGTCGCTGAGGTTGGTGAAA	TGGCGTAGCTTGCTCTCGCAAT	349
FMgSSR-27875	scaffold5135	p2	(GA)7	Class II	21428	21441	ACCGTGACCTTAAGCCCTGT	GTGCTCATCTTCAACCTTGAGCCA	350
FMgSSR-27877	scaffold60	p2	(GA)7	Class II	15750	15763	ACCTGGAGTACCCATGCCTACCAT	AACTGGCGTATGACCGCATCCT	326
FMgSSR-27880	scaffold5028	p2	(GA)7	Class II	23985	23998	ACGAACGGCAGGCGAGAAAT	ACCATTACGAGCGTTGGGCA	310
FMgSSR-27881	scaffold1334	p2	(GA)7	Class II	60342	60355	ACGAACTGCTCGACAGTCCTGTTA	ACGACGTGTTGTTCGTCTCATTGC	350
FMgSSR-27884	scaffold835	p2	(GA)7	Class II	995	1008	ACGACGATCCATGCGCGCTTTA	TGCCTCGTATTAGCAACAGGCG	329
FMgSSR-27885	scaffold11623	p2	(GA)7	Class II	11614	11627	ACGAGCTTATTGGACATGCCGCT	CGTCGCGTGGAAACTGGAAA	270
FMgSSR-27891	scaffold10704	p2	(GA)7	Class II	6188	6201	ACGCCAACCCGTGAACCAAT	AAACAGAGCAGGCGCCAGAA	341
FMgSSR-27893	scaffold286	p2	(GA)7	Class II	36164	36177	ACGCGACAACAACACCCACA	AAGGCCCACCGGTCATTGTGTT	206
FMgSSR-27894	scaffold2053	p2	(GA)7	Class II	44025	44038	ACGCGGCTGCTTACGCTAAT	AACAGCAGTCACCACTGGCCTT	281
FMgSSR-27895	scaffold6314	p2	(GA)7	Class II	11420	11433	ACGCTCACAGCCGTTGTTGT	TCCTCGTCTTCAACCTTGAGCCA	267
FMgSSR-27898	scaffold351	p2	(GA)7	Class II	92820	92833	ACGCTTTGTCTTTCGCCGCT	AGATGCACGGCAATGGTGGA	273
FMgSSR-27900	scaffold4549	p2	(GA)7	Class II	8005	8018	ACGGGCTTGCTTTTGCGTTG	AAAAAGGGCGTGCTGGTGGT	274
FMgSSR-27902	scaffold12503	p2	(GA)7	Class II	7449	7462	ACGGTGTGCTGCTGATGCAA	TCTGCTGCAACGACGCATGT	291
FMgSSR-27911	scaffold7189	p2	(GA)7	Class II	3054	3067	ACTCGAAAGAGACACCGGGGAA	TCAGCAACGCGTAGGGAATCA	302
FMgSSR-27915	scaffold4225	p2	(GA)7	Class II	2334	2347	ACTGGCAAGGACGCAATCGGAA	ACTGCGCTCCACCACCTACATA	346
FMgSSR-27917	scaffold2344	p2	(GA)7	Class II	19284	19297	ACTTGCAGTTGCAGGGGCAT	TGTCAAATCAGCCACACGCCAC	284
FMgSSR-27920	scaffold449308	p2	(GA)7	Class II	50	63	AGAAACGGCGCCATGGAAGA	CAGCAACGAAATCTCTCATCAGCC	221
FMgSSR-27922	scaffold57628	p2	(GA)7	Class II	845	858	AGACCGCGCTGCACTACAAA	TCGTGGCCTCTCCAATTCACGA	336
FMgSSR-27923	scaffold29879	p2	(GA)7	Class II	5107	5120	AGACGAAACTCGCATTCACCGA	TTGGGCAGCTCAAGGAATGGCA	350
FMgSSR-27925	scaffold28044	p2	(GA)7	Class II	2017	2030	AGAGAGCACGCCATGCTTGA	TTGTTGTTGGGCAGCGGGAA	215
FMgSSR-27930	scaffold90241	p2	(GA)7	Class II	523	536	AGAGGGGTTCGCCACCATTTCT	AAAACGCAGGGGGTGTGCCATA	326

SSR_ID	Scaffold	SSR_Type	SSR_Motif	SSR Class	SSR_Start	SSR_End	Forward sequence	Reverse sequence	Product_size
FMgSSR-27931	scaffold5301	p2	(GA)7	Class II	16049	16062	AGATCAAAGCCCACCACCACCA	TGTAACGTGCCAACGCTGCT	333
FMgSSR-27934	scaffold78601	p2	(GA)7	Class II	1745	1758	AGCAAATGCACCCGCCTGAA	TCGAGCCGATGCTCAGTTGT	306
FMgSSR-27935	scaffold643	p2	(GA)7	Class II	53839	53852	AGCAAGCACCGATCTCACGAGT	TCCCCAATCAAGGCGGATCACT	342
FMgSSR-27938	scaffold17503	p2	(GA)7	Class II	2511	2524	AGCACCCCCAGCTTCTTTTCCT	TGAACAAGCCCCAGGTGGAA	321
FMgSSR-27944	scaffold10428	p2	(GA)7	Class II	23690	23703	AGCAGAACAGGCTTGTGGGCTT	TCCAAAGCGTAAGCGGCATGGA	230
FMgSSR-27949	scaffold10803	p2	(GA)7	Class II	19054	19067	AGCAGCGGCGTCATCATTCT	GTGCGCACACGAAAGGCAAA	336
FMgSSR-27952	scaffold1306	p2	(GA)7	Class II	61500	61513	AGCAGGAGCAGCTAAAACGGCA	ATGCTGCGCTGTGTACGCTA	336
FMgSSR-27954	scaffold540	p2	(GA)7	Class II	74683	74696	AGCAGTATGGGCGACAACCA	ACCGATCGGGTTCGTGTTGA	290
FMgSSR-27959	scaffold26450	p2	(GA)7	Class II	504	517	AGCATGCAGCAAAGAGCATGGC	ACGATGCAAGGATCGGAGGT	300
FMgSSR-27963	scaffold1530	p2	(GA)7	Class II	65188	65201	AGCCAATGAGCGTGAAGGCAGT	AAAAGTGGGCGCGTCCTTGA	312
FMgSSR-27964	scaffold1393	p2	(GA)7	Class II	30572	30585	AGCCCCACGCTGAAAGTTTG	TCGATGGCCCAAAACGACGA	255
FMgSSR-27965	scaffold1401	p2	(GA)7	Class II	21350	21363	AGCCCCCTCCTAACCGCTATAA	AGGGGGCTATGGTCCAAATGACCT	297
FMgSSR-27982	scaffold7508	p2	(GA)7	Class II	13744	13757	AGCGTCATGCAACGGGGAAGAA	CACTTGGAGCTTGATCGCGT	319
FMgSSR-27999	scaffold4387	p2	(GA)7	Class II	32429	32442	AGGAAAGCAAGGGCAGCATTGG	TGAGGCCGACACGCACTAAA	329
FMgSSR-28004	scaffold101801	p2	(GA)7	Class II	275	288	AGGAGAGGGAGAGTGAGAGAAGGA	GCAGCGCGTACTGATGACTTGT	342
FMgSSR-28006	scaffold2602	p2	(GA)7	Class II	32434	32447	AGGCAAGTGTGTGCGTGTGT	ACTTTGAATCCTAGCTCCGCCCT	282
FMgSSR-28009	scaffold396	p2	(GA)7	Class II	59771	59784	AGGCCCATTGTTTCCCTCCCAA	AGCAGTCGTCGCCATGTTGT	282
FMgSSR-28010	scaffold7387	p2	(GA)7	Class II	15238	15251	AGGCCTATCGGTTGTGCAGT	AAATTACATGCCCACGCTGCCC	348
FMgSSR-28013	scaffold3392	p2	(GA)7	Class II	48124	48137	AGGCGGGAGCATTCAGCATT	TGGGTTACAACAACCACCCCCT	245
FMgSSR-28015	scaffold544	p2	(GA)7	Class II	43911	43924	AGGCTCAATGTCGGCTAACACG	TTGCGGCACCTGAAGCTGAA	203
FMgSSR-28017	scaffold853	p2	(GA)7	Class II	2660	2673	AGGGAAGCATTTGCAGGAGGGA	TTAGCCAGCACCTCCTTGACGA	344
FMgSSR-28020	scaffold17433	p2	(GA)7	Class II	2938	2951	AGGGAGGACCGTTGCTTTGCAT	TTGCTTTGTCGGCGCGTTTG	347
FMgSSR-28029	scaffold186	p2	(GA)7	Class II	63177	63190	AGGTGGCGTCATATGCGTTGGA	ACGGTCATCAGTCGCCGTTCAA	274
FMgSSR-28031	scaffold10648	p2	(GA)7	Class II	24361	24374	AGTAAGGCTAGGCTTGGTCCCT	AGCAAACTGCTTCACGATCCCCA	287
FMgSSR-28035	scaffold7803	p2	(GA)7	Class II	14365	14378	AGTGAAACTGTTGCTGGTCGCCT	AGTAGTAACTGCTGTGCCCCCT	285
FMgSSR-28053	scaffold62	p2	(GA)7	Class II	145865	145878	ATACGGCTCCTGCTACGCAACT	AGCCGCTTTGCGCCTTCAAT	245
FMgSSR-28054	scaffold11263	p2	(GA)7	Class II	22138	22151	ATCAACCCAACACGCGCTACGA	TCCATTTTGGCGGTCAACCGGA	302
FMgSSR-28060	scaffold16539	p2	(GA)7	Class II	6025	6038	ATCCCGCAGGTAAAGCCGATGT	TGGCTGCTCTTGTGACTGGCAA	290

SSR_ID	Scaffold	SSR_Type	SSR_Motif	SSR Class	SSR_Start	SSR_End	Forward sequence	Reverse sequence	Product_size
FMgSSR-28061	scaffold25000	p2	(GA)7	Class II	7394	7407	ATCCTTCACCGCTGACCCAACA	ACGTCAACGACCCACTTCACCT	295
FMgSSR-28066	scaffold1655	p2	(GA)7	Class II	52918	52931	ATCGTGCGCAACAAGGCTTCA	TTGACCAAGGTGCGCATGGCTA	220
FMgSSR-28069	scaffold117841	p2	(GA)7	Class II	1060	1073	ATCTCGACCTGCATGTGTGCGT	TGTGCACAGGACGGCCAAAA	295
FMgSSR-28072	scaffold692	p2	(GA)7	Class II	18650	18663	ATCTTGTTGGCCGTTGCGCT	ACGCCGGATTCGAACTCCACAA	345
FMgSSR-28078	scaffold40528	p2	(GA)7	Class II	1765	1778	ATGCAAGTCGCCGCACGTAA	ATGATCTGCAGAGCGAGCCA	329
FMgSSR-28080	scaffold497	p2	(GA)7	Class II	20240	20253	ATGCCGTGCACAGAATCGCA	AATAGCGCCCGACGGAAACACA	304
FMgSSR-28086	scaffold12612	p2	(GA)7	Class II	6957	6970	ATGGACGACAGTACGCCACA	TGTTTCCCCAGCACCCAGGTTT	212
FMgSSR-28087	scaffold1812	p2	(GA)7	Class II	20620	20633	ATGGCACGAAAGCCCAGCAA	AACGCGAGCGTTGCCTTCTT	221
FMgSSR-28093	scaffold943	p2	(GA)7	Class II	86523	86536	ATGTATCAAGCGCCGCAGCA	TGATTGGTCGCCGTGGCATT	297
FMgSSR-28098	scaffold4197	p2	(GA)7	Class II	16238	16251	ATGTCTACTGCGGTGCTGTGCT	AGCGTGAGAAAGGAGTGGCT	322
FMgSSR-28099	scaffold1839	p2	(GA)7	Class II	25745	25758	ATGTGGGTGGAGGCAGGGTTAT	ACAATGAGTCGTTCGCATCCGC	320
FMgSSR-28105	scaffold1150	p2	(GA)7	Class II	72936	72949	ATTCGCCGCACATTCGCTCA	AGCCCAACAGTGGCTTTCACA	292
FMgSSR-28106	scaffold3498	p2	(GA)7	Class II	7826	7839	ATTCTAGACGAGAGAGAGAGGGGG	AGGCAGAGAGGCACGTGTACTT	272
FMgSSR-28109	scaffold56769	p2	(GA)7	Class II	1757	1770	ATTTGCGGCCTCAGAAGGCA	ACAGCATGTTGTTGCTGCGA	319
FMgSSR-28111	scaffold1007	p2	(GA)7	Class II	22039	22052	ATTTGTGAACGCGGTGCGGT	ATGCCATGCCTCTCACCACT	315
FMgSSR-28117	scaffold13462	p2	(GA)7	Class II	22044	22057	CCAACAACAAACACAAACGCGGC	TGCTGCCAGTTAGCACACCTGA	255
FMgSSR-28121	scaffold94552	p2	(GA)7	Class II	931	944	CCCACACCCCCACACAAAAACA	ATGTGTTGTGTTGCCGCCTCCT	323
FMgSSR-28123	scaffold28538	p2	(GA)7	Class II	1198	1211	CCGTGCCTTTCGCTTTGCTT	AAGCACAGGCCATCTGCCACTA	331
FMgSSR-28124	scaffold1144	p2	(GA)7	Class II	7902	7915	CCTCCCATGGAGAGAAGCACAT	AATTGCCACTCCCTGCCTCCAA	316
FMgSSR-28134	scaffold22926	p2	(GA)7	Class II	8762	8775	CGCAACGAGACTGCACAACCAA	ACTCGTAGCAGCTTTTCCTGGCA	325
FMgSSR-28140	scaffold2191	p2	(GA)7	Class II	51212	51225	CGCCGTCGATGGAGCTACAAAT	ATTCTCGTTGGGGCCTGCCAAT	266
FMgSSR-28141	scaffold4686	p2	(GA)7	Class II	41393	41406	CGCGATCTATCGAGAGAGATGACCT	GTGCGAATAAGGGTTGGCAGCA	339
FMgSSR-28143	scaffold12044	p2	(GA)7	Class II	23432	23445	CGCGCGCGCATGTGATGAATTA	TTGAACGCCCATCATGGCTGGA	346
FMgSSR-28147	scaffold1727	p2	(GA)7	Class II	64231	64244	CGGAGCTTGCGTGAGAGAAGAA	TCCTGTCCTCGAAACCCAAAAGC	270
FMgSSR-28150	scaffold5726	p2	(GA)7	Class II	17548	17561	CGGCGGAAGCTCTTGCCAAAAA	TGTGCACATGGCTCACCAACCT	343
FMgSSR-28152	scaffold10967	p2	(GA)7	Class II	13878	13891	CGGGTGTAGCGCACAAGTTCAT	TTCGGCGTTGCCGTCAAACT	247
FMgSSR-28155	scaffold2239	p2	(GA)7	Class II	47766	47779	CGTGCAGTTGCAAAAGCAGCCA	CCACCGGTCAAAAATGCGGTGT	202
FMgSSR-28157	scaffold3854	p2	(GA)7	Class II	53649	53662	CGTTGCACGCTTTGACGTGT	AACAGCTAGGCACTGCCGACAA	256

SSR_ID	Scaffold	SSR_Type	SSR_Motif	SSR Class	SSR_Start	SSR_End	Forward sequence	Reverse sequence	Product_size
FMgSSR-28162	scaffold3010	p2	(GA)7	Class II	25640	25653	GAACGTTGCCGAGCATGCACTT	CCATGCCTGTCAATATCGGCCA	200
FMgSSR-28175	scaffold2402	p2	(GA)7	Class II	16418	16431	GCAGGATGCACATGATCTTGGTGTG	AGAAACCAAGGCAAGCACCCAC	324
FMgSSR-28179	scaffold1794	p2	(GA)7	Class II	17067	17080	GCCATCGCCAGAGCACTTACTT	TGATCCAGCTGGCGTTGCTTCA	278
FMgSSR-28180	scaffold22378	p2	(GA)7	Class II	4691	4704	GCCCACCAACCACTATATGCATCC	TCCGACGGCTTGGGTGTTTT	228
FMgSSR-28181	scaffold19461	p2	(GA)7	Class II	4820	4833	GCCCATGCCGAAATGCATGTGT	TGCTGGTTGTAGCAGACGCTGA	288
FMgSSR-28182	scaffold1010	p2	(GA)7	Class II	57195	57208	GCCCGCCAGTAGAGTTAGGTTTGA	TCGCATGGATGATGTGGCGT	310
FMgSSR-28183	scaffold5660	p2	(GA)7	Class II	1414	1427	GCCGCATCCAACTCCTATCTTCGT	GCAACAGAATGACGCTAATGCACCC	336
FMgSSR-28187	scaffold9242	p2	(GA)7	Class II	13397	13410	GCGCAAACATCCGTCAAGCA	TCCACCGTATTTCTGGCTCCCA	347
FMgSSR-28197	scaffold14767	p2	(GA)7	Class II	8198	8211	GCGTTCGACGAAGGGAGGAACTAA	AATGGGCACACCCGGCTAAACT	333
FMgSSR-28198	scaffold52283	p2	(GA)7	Class II	2014	2027	GCGTTGGAGCCACATTTTCCCA	TCCCCACCCACATGCTCGAAAA	311
FMgSSR-28199	scaffold2067	p2	(GA)7	Class II	35158	35171	GCGTTGTCTTTTAGAGACCAGGGTG	TTACGCACCTCACTGCCACTGT	350
FMgSSR-28206	scaffold1123	p2	(GA)7	Class II	6950	6963	GGCCCCACCTATTAGCTAGCACTT	AGCGAGTGTGTGAGGGCTTTGT	321
FMgSSR-28211	scaffold14549	p2	(GA)7	Class II	19283	19296	GGCTTTTTGCCACCATACACGA	TGCAGCTGTGATTCCGTGCAGT	309
FMgSSR-28213	scaffold11692	p2	(GA)7	Class II	19172	19185	GGGAGAGATAGAGAGATGGGAGACA	CCAGCAGAGTTGTGGGAACTGACA	305
FMgSSR-28220	scaffold10890	p2	(GA)7	Class II	17722	17735	GTCGGAAGATGGCTGTCGTCAA	ACTCACAGACTCCTCCTCTCTCCA	303
FMgSSR-28225	scaffold4339	p2	(GA)7	Class II	22135	22148	GTTGTCCGCCGCCAAATCAT	ATGGGAGTTTGCGACAGGGT	337
FMgSSR-28229	scaffold814	p2	(GA)7	Class II	37110	37123	TAAGGTGGGAAGTGGCGAACCA	AGCGGTGCACACTGCAAACA	301
FMgSSR-28231	scaffold3292	p2	(GA)7	Class II	40984	40997	TACAAAAGGGCTAGGGCTGAGGGA	AAGCTGAGATGTGACCCCACT	270
FMgSSR-28236	scaffold108	p2	(GA)7	Class II	92828	92841	TACTCGGCTCGTTGAATGGGGT	TCTGCCACGCATCAACGCAT	251
FMgSSR-28237	scaffold869	p2	(GA)7	Class II	23872	23885	TACTCTTCGGCAAGGCGTGCAA	AACGTTCTCTCTCTCACCCACCCA	278
FMgSSR-28242	scaffold1306	p2	(GA)7	Class II	72668	72681	TAGCGGTCGCTGTTGTGCAT	TCGACCCGAGACGGTTCACAAA	312
FMgSSR-28250	scaffold8261	p2	(GA)7	Class II	7083	7096	TATGCCGCGCTCATGGATGT	TGCATCTGCACGGGCTAGTGAT	338
FMgSSR-28255	scaffold1041	p2	(GA)7	Class II	10130	10143	TATTCGGGGGTTAGGCCACACT	ACGTGTGCTGGCCTACTTTTGC	271
FMgSSR-28262	scaffold401	p2	(GA)7	Class II	119462	119475	TCACAGTTGGCCTTGCTGTCGT	AAAATGCCCCAGCCTCGCTT	333
FMgSSR-28264	scaffold5999	p2	(GA)7	Class II	9723	9736	TCACCACAGGCTGCAGTTGTCA	TTTCCTTCCCTCGCAAGATCGC	347
FMgSSR-28266	scaffold327	p2	(GA)7	Class II	94834	94847	TCACCGAACCAAACTTGTGCCC	ATTGGGATCTCTCATGGCCGCA	318
FMgSSR-28268	scaffold441	p2	(GA)7	Class II	37130	37143	TCACGTGCCGCCGTTGATAA	TCGACACGACTAGGGCCGATAA	238
FMgSSR-28280	scaffold1189	p2	(GA)7	Class II	44988	45001	TCATGCTGTTGGTGTCGGTGGT	CGCACGCAGAATCAAAGCCACA	330

SSR_ID	Scaffold	SSR_Type	SSR_Motif	SSR Class	SSR_Start	SSR_End	Forward sequence	Reverse sequence	Product_size
FMgSSR-28289	scaffold3291	p2	(GA)7	Class II	10406	10419	TCCACAGCCCTTGGTTGCTT	TGTCTTCCAGCTTCTCGTGGCT	289
FMgSSR-28291	scaffold55	p2	(GA)7	Class II	57208	57221	TCCACTTTCTTGTGAGCACGCC	CCACCGCCGCTTGAGCAATTTT	282
FMgSSR-28293	scaffold4097	p2	(GA)7	Class II	12012	12025	TCCATCACCCGCGTTCTTCACA	AGCCCAATGAGCTTTCACCCGT	212
FMgSSR-28299	scaffold8161	p2	(GA)7	Class II	27283	27296	TCCCTGGTATCGTTTCACCCTAGC	TCAGCGATGAGAGGCGAGTGTT	338
FMgSSR-28302	scaffold11551	p2	(GA)7	Class II	8118	8131	TCCGCTTCCCACCTTCCTTCAA	ATTCAGTACCCAGGCCAGCCAA	332
FMgSSR-28303	scaffold2835	p2	(GA)7	Class II	11545	11558	TCCGGCCCTGAGCATTTCTCTT	ACACAAGACCTCGACCTCAACT	209
FMgSSR-28305	scaffold4523	p2	(GA)7	Class II	25082	25095	TCCGGTTACGCGTTCACATGCT	AGCACGCGGTGCCGATTTCTAT	298
FMgSSR-28307	scaffold7831	p2	(GA)7	Class II	31847	31860	TCCGTTCCACTGCTGCTCACAA	AAGAGAGCAACTGCGTGCGT	244
FMgSSR-28312	scaffold640	p2	(GA)7	Class II	109285	109298	TCGAACTTGCCGTGGTTCTCGT	TGCTTTGCTCCCCGCCATGATT	249
FMgSSR-28317	scaffold26126	p2	(GA)7	Class II	2555	2568	TCGATGGGCGCTTTTGGTCTGA	TTGCAGCAACAGCGATTCCG	337
FMgSSR-28318	scaffold706	p2	(GA)7	Class II	68954	68967	TCGCAGCAACAGCTTCCGTT	AGCCGAAATGATCCCCAGTCGT	282
FMgSSR-28321	scaffold8776	p2	(GA)7	Class II	22350	22363	TCGCCCACCGCATCATCAAA	CCCAAGGTGGACTTGCTTGCAT	339
FMgSSR-28329	scaffold408917	p2	(GA)7	Class II	167	180	TCGGGTCATGTCCCTCTGCTTT	CGGTTGTTCAGGATCCTTGATGGGT	214
FMgSSR-28334	scaffold28996	p2	(GA)7	Class II	2956	2969	TCGTCGAGCTTCAAGGAGGT	TGACCAATGAGATCCGCTGCT	318
FMgSSR-28336	scaffold4708	p2	(GA)7	Class II	12740	12753	TCGTGGTCGAGAAGGTCTCACT	CCCAACTCACAAACTCTTCCACCC	318
FMgSSR-28338	scaffold11435	p2	(GA)7	Class II	13358	13371	TCTATGCCGCACTGCCATCT	ATAGCAGGTCAGCTCGATGGCTAC	216
FMgSSR-28339	scaffold14228	p2	(GA)7	Class II	12673	12686	TCTCAGTGATGCTCAACGGCT	ACGGTCCAGATGAAACCACCGT	253
FMgSSR-28342	scaffold6584	p2	(GA)7	Class II	6897	6910	TCTCGCTTCAAACAGTCCAGCA	TTACATGGGCTGAGTCTCATGGGC	233
FMgSSR-28359	scaffold4654	p2	(GA)7	Class II	34192	34205	TGAGAGCGGCAGCGTGTAGAAA	ATGGCGGCAAAAGCCTCGTT	309
FMgSSR-28368	scaffold1140	p2	(GA)7	Class II	35578	35591	TGATGCCGTTGTGCCATGCT	TCCATGACGTTGCACTGGACCT	210
FMgSSR-28372	scaffold20751	p2	(GA)7	Class II	6457	6470	TGCAACAACCCGATTTCCCGT	AACCGCAGTCTGTCCATGCAGT	334
FMgSSR-28373	scaffold9867	p2	(GA)7	Class II	13501	13514	TGCAACTCTGCGTCCTCATGGA	TGTGCCGTGATGGCCTCTTT	280
FMgSSR-28378	scaffold9084	p2	(GA)7	Class II	6673	6686	TGCACATGTCCCCTACCTGAA	CCGCGACTCAACTACATCATTGGC	350
FMgSSR-28380	scaffold374	p2	(GA)7	Class II	46727	46740	TGCAGATGCACCCACCAGAAGT	TTGTTGCCTCCTGTCCTCGTCA	338
FMgSSR-28382	scaffold789	p2	(GA)7	Class II	45687	45700	TGCAGCGCGAGTACGCTAAA	ATTCTTTCGTCGCCGTCGCT	339
FMgSSR-28388	scaffold18811	p2	(GA)7	Class II	12680	12693	TGCATGTGATCCCACCGCAATG	CGCGGGGCTCTTGTTTTTCCAA	325
FMgSSR-28389	scaffold477	p2	(GA)7	Class II	110279	110292	TGCATTGACTGGCTAGTCTCGCT	TCGCCGAGCTGCTATGCTTT	344
FMgSSR-28390	scaffold1489	p2	(GA)7	Class II	19679	19692	TGCATTTCGCCCCACTGAGAGA	ATGAGGAAGGATGCGTCGCTGT	315

SSR_ID	Scaffold	SSR_Type	SSR_Motif	SSR Class	SSR_Start	SSR_End	Forward sequence	Reverse sequence	Product_size
FMgSSR-28391	scaffold6439	p2	(GA)7	Class II	11899	11912	TGCCACGCACCAGTTGTTGA	AAATCACTGCTACGCGCCACCA	327
FMgSSR-28392	scaffold3513	p2	(GA)7	Class II	31187	31200	TGCCAGAGTTCGCAAGCACA	AGCGCCACTGCCGTGAAAAT	271
FMgSSR-28400	scaffold5355	p2	(GA)7	Class II	43791	43804	TGCCTCAGCGAGTGCGTATT	ATGTGTTCTGGGTTGCGTGGGT	284
FMgSSR-28421	scaffold1808	p2	(GA)7	Class II	70820	70833	TGCTAACTGCTTCCAAACCGGA	TGCGTATTTTCCGACCGACGA	274
FMgSSR-28427	scaffold21241	p2	(GA)7	Class II	6512	6525	TGCTGCGCACATATGGATGTGGA	TGCATCTGCACGGGCTACTGAT	318
FMgSSR-28430	scaffold347	p2	(GA)7	Class II	2875	2888	TGCTGGCTGCTGCTTTTCCT	AGCCCGAAACAAAACGGGCA	208
FMgSSR-28436	scaffold428	p2	(GA)7	Class II	43357	43370	TGGACTTCAAGGTACAGGCGGT	AGGCCACGATGACTTTAGGCGA	337
FMgSSR-28438	scaffold12937	p2	(GA)7	Class II	9228	9241	TGGAGCAATACAAGCAGGCGCT	AGCTCATGAACAGCCAGCGA	328
FMgSSR-28441	scaffold44	p2	(GA)7	Class II	37071	37084	TGGAGTAAGGCTTCCGTGGGAT	GGGTGGAGCCGAAAGCCAATTT	284
FMgSSR-28443	scaffold56	p2	(GA)7	Class II	65023	65036	TGGATGTATGGGAATGGCACTGGC	ATGCTTGCCGTGGACAGTCA	207
FMgSSR-28444	scaffold9820	p2	(GA)7	Class II	9568	9581	TGGCAAAAAGCTTCCGCCGT	TGTGGAACTTTTCCTCCGGTGC	270
FMgSSR-28448	scaffold93377	p2	(GA)7	Class II	178	191	TGGCAGTGACGGGCTTTGTT	ACCGGCTGACCGAACAATCCAA	256
FMgSSR-28450	scaffold1967	p2	(GA)7	Class II	21514	21527	TGGCCGCTTCTTTAACGCCA	ACCCTAACCCTACACGCTTGCT	296
FMgSSR-28462	scaffold8663	p2	(GA)7	Class II	17145	17158	TGGTACTGAACTGACGCGAAGCA	TCGTGGCAGGAGGCCCTTTTAT	205
FMgSSR-28467	scaffold3478	p2	(GA)7	Class II	52461	52474	TGGTGCCTCCATTTGAAGCGA	ATGCTGGCAGCCGATGAAGGAT	260
FMgSSR-28469	scaffold18784	p2	(GA)7	Class II	9805	9818	TGGTGCTGTGGATTTGCACTGG	TGAATGTACTGGCTGAGCAGTCG	330
FMgSSR-28474	scaffold1576	p2	(GA)7	Class II	11812	11825	TGTCCACAGGTAGGGCCAAGTT	TACGCTCTTCCATGTGGGACCCTT	292
FMgSSR-28475	scaffold6950	p2	(GA)7	Class II	6417	6430	TGTCCATGTCACAAGTGGCGGT	TCTTCGAGCATCAACACGCCT	339
FMgSSR-28478	scaffold11853	p2	(GA)7	Class II	7821	7834	TGTCGGCAAACAGTGGTGGAGA	TGGTGGGGCATGACATCACT	327
FMgSSR-28480	scaffold205	p2	(GA)7	Class II	106579	106592	TGTCTCTCGCTCGTCGTTGTCA	AATCCACGAGCACGCAAGGA	205
FMgSSR-28481	scaffold35392	p2	(GA)7	Class II	4873	4886	TGTCTTAACGGCTGGGAGGGAA	TCAATTTCGGCCGCTCTGCT	294
FMgSSR-28483	scaffold1983	p2	(GA)7	Class II	49979	49992	TGTGATTGGCAGAGGGGGAA	CGAACGGTCCACGAAAAAGGCA	315
FMgSSR-28491	scaffold48831	p2	(GA)7	Class II	1593	1606	TGTGGCAATGTCAGCGTGGA	GCAGGAAATTGGTTTCGAGTAGCC	218
FMgSSR-28493	scaffold38	p2	(GA)7	Class II	4284	4297	TGTGGTTTGGTGTGTGTGTGCG	TTGTGATGACGCGCGAGCAA	266
FMgSSR-28505	scaffold6226	p2	(GA)7	Class II	13622	13635	TGTTCGTGCAACACTTGAGCG	AGCAGCAAAGCGGTGCGTTT	349
FMgSSR-28509	scaffold188	p2	(GA)7	Class II	64597	64610	TTATCGAATGGCGTGGGCGA	ATGCGCGGAGCTTGCTTATCCT	322
FMgSSR-28511	scaffold11869	p2	(GA)7	Class II	25379	25392	TTCACACCGACAGAGAGAGAAGCG	AAAACCTTACCCTACTGCCGAGCG	295
FMgSSR-28524	scaffold12553	p2	(GA)7	Class II	3501	3514	TTCCTGATGTGTTCCTCCCCATGC	ACCCATCATGGCCGACAAGGTT	204

SSR_ID	Scaffold	SSR_Type	SSR_Motif	SSR Class	SSR_Start	SSR_End	Forward sequence	Reverse sequence	Product_size
FMgSSR-28529	scaffold5244	p2	(GA)7	Class II	37312	37325	TTCGTCACAGGTCCCGTCTCTT	AGCAGCCTGGGGCGAAATGAAA	322
FMgSSR-28530	scaffold2635	p2	(GA)7	Class II	44562	44575	TTCGTGGGTGATCATGGGACAC	CGCCAGATGCTTGTTACTCTCGGT	294
FMgSSR-28543	scaffold27907	p2	(GA)7	Class II	5454	5467	TTGCAATGGTGGAACCCGGA	TGTCCCTGCTGATCGTGCATCT	223
FMgSSR-28544	scaffold21862	p2	(GA)7	Class II	3465	3478	TTGCAGGCTCGCAGCTCAAT	TACATCGTCCAAGGTGCGTGCT	296
FMgSSR-28547	scaffold3945	p2	(GA)7	Class II	34345	34358	TTGCGGCAAGGAGCTCAATGGT	TTCCTCCATTCGGTTGGGCCTT	335
FMgSSR-28550	scaffold26311	p2	(GA)7	Class II	4013	4026	TTGGAACGTGCGAAGGGGAGAA	AGGCAGTGGCATCGGTGAAAGT	238
FMgSSR-28552	scaffold20366	p2	(GA)7	Class II	10142	10155	TTGGCGCGCAAGCTAGAGTT	TGCTCACATCATCCGATCCCCT	298
FMgSSR-28560	scaffold2199	p2	(GA)7	Class II	22049	22062	TTGTTGCTTTGCGTGCGCCT	AGCGGGCCGGAAAATATCGT	235
FMgSSR-28564	scaffold354	p2	(GA)7	Class II	72006	72019	TTTAACCTGCGGCTGCTGTCCT	ACGAACGGGTATTTGCAGGGGT	322
FMgSSR-28565	scaffold1169	p2	(GA)7	Class II	82403	82416	TTTCGCGCTGAAAAGCGTGC	TGGACCAGGCCGCCTTTTCTTT	324
FMgSSR-28571	scaffold1292	p2	(GA)7	Class II	44615	44628	TTTGCACGAATTTCACCGGGCG	TCGAAACGGCTTGGTCGTCT	278
FMgSSR-28573	scaffold3779	p2	(GA)7	Class II	19781	19794	TTTGCCGTGCCCTTGCTCTT	CATGTTGAGACTTGAGTGGTCGTG	258
FMgSSR-28575	scaffold3564	p2	(GA)7	Class II	3695	3708	TTTGCTGAGGCCTCTGTCGT	ACTGCAGAATCGCAGCCTCA	272
FMgSSR-28576	scaffold4748	p2	(GA)7	Class II	37689	37702	TTTGCTTTCCGCCGGCGTTT	TGCCGCCACTTATGTACTGCT	337
FMgSSR-28581	scaffold14588	p2	(GA)7	Class II	11539	11552	TTTGTGGCCGCAATTGCCCT	AGCGCGTGTCAAGCCTAACCAA	244
FMgSSR-28585	scaffold4675	p2	(GA)8	Class II	39671	39686	AAAAGCAGAGCACGCGCAGA	TCGCCATGGCACTCTCTTCGAT	233
FMgSSR-28588	scaffold2983	p2	(GA)8	Class II	52613	52628	AAAGAGGGGAATGGTTGGTCGC	AGCTTGAGGCAATAGGCTCGCT	345
FMgSSR-28595	scaffold147	p2	(GA)8	Class II	74553	74568	AACACACAACGCTGCTTCAGCC	AAGCGAAACGTCAGCTCTTGCG	338
FMgSSR-28596	scaffold15120	p2	(GA)8	Class II	12219	12234	AACAGGCCACCGTCATGATCCA	ACCGTTTCCGCCCCTCTCATTT	205
FMgSSR-28600	scaffold31	p2	(GA)8	Class II	166572	166587	AACGACAGCACGAGTTGGCA	AATCTTCTTGTTGCACCCTCCCCC	217
FMgSSR-28610	scaffold6746	p2	(GA)8	Class II	10417	10432	AAGAGAGGTGCGGGAAGTATGGGA	ACTCCAGCAATCCAAACCGCCA	323
FMgSSR-28613	scaffold3482	p2	(GA)8	Class II	51108	51123	AAGCACAAAACCATCCTCGCCG	TTGTGCTCTCGACATCACGGCT	290
FMgSSR-28617	scaffold13484	p2	(GA)8	Class II	11715	11730	AAGCGATGAGGAAGCGGTAGCA	GCCGTCCCATAACCGCAAATGA	231
FMgSSR-28630	scaffold2717	p2	(GA)8	Class II	9137	9152	AATCAACGCCAAGGCCGGTACT	TGGCAACTGGGCGGCTTGATTA	260
FMgSSR-28633	scaffold267	p2	(GA)8	Class II	30750	30765	AATGGCACGCACACGAACCT	AACGGAACATCGCCCTTGCTGA	284
FMgSSR-28642	scaffold3112	p2	(GA)8	Class II	23365	23380	ACACATTTTCGGGCTGCGCT	CGGTGCACTGGCTGATTGGAAA	344
FMgSSR-28644	scaffold11312	p2	(GA)8	Class II	17903	17918	ACACGCGACCTTTTCGCCAT	AATACAATTCAACGCGGCGGGC	279
FMgSSR-28652	scaffold89	p2	(GA)8	Class II	27578	27593	ACAGGAAGACAGCAAACGGGGT	AAAAGCAAGGGCCTAGCCTGGT	346

SSR_ID	Scaffold	SSR_Type	SSR_Motif	SSR Class	SSR_Start	SSR_End	Forward sequence	Reverse sequence	Product_size
FMgSSR-28654	scaffold21607	p2	(GA)8	Class II	1991	2006	ACATGCAAGCCCGACCACAA	ACTCAAATCGTTTCTGCCTCCTGC	348
FMgSSR-28657	scaffold1162	p2	(GA)8	Class II	56240	56255	ACCAATGAACCAGGCGGAACGA	AACCTCAGCGGCGATTTCCA	242
FMgSSR-28658	scaffold8712	p2	(GA)8	Class II	25759	25774	ACCACAACCGCTGGGAAAGAACT	TTTGTCCACCATCCCAGCGT	303
FMgSSR-28665	scaffold33193	p2	(GA)8	Class II	3029	3044	ACCCTTGCACAAGACGACAGCA	ATGTTCTGACCATCGCCGCT	294
FMgSSR-28669	scaffold23522	p2	(GA)8	Class II	4796	4811	ACCGCGATGAGAGCAACCATGT	AAGCAGCGTGATGGCATGATGG	259
FMgSSR-28670	scaffold2502	p2	(GA)8	Class II	49038	49053	ACCGCGCGCTTGAGACTTTT	GCGTGGAATAAACAGCGCGCAA	304
FMgSSR-28671	scaffold55378	p2	(GA)8	Class II	395	410	ACCGGCCAAAAACGGACCAA	TTAAATAGCCGGTGCGGCTGGA	331
FMgSSR-28675	scaffold19108	p2	(GA)8	Class II	11072	11087	ACCGTCTTGCAGTTCCAGAGGA	TGAGAACGTGTCCCTCCATACG	350
FMgSSR-28679	scaffold2170	p2	(GA)8	Class II	57485	57500	ACCTTTGCAGCACCTTCGCA	TCATGTGATGTGGCCAGGGTGT	350
FMgSSR-28688	scaffold10474	p2	(GA)8	Class II	16482	16497	ACGATTGGCGTGAGGGTTCA	TGTCGACGCGCATCTTCCTTCA	286
FMgSSR-28689	scaffold4422	p2	(GA)8	Class II	36014	36029	ACGCAAAGCTCAACACGCAT	GGCTCAAGCTTCACCCATGTT	343
FMgSSR-28694	scaffold2893	p2	(GA)8	Class II	2035	2050	ACGGAAGCGGCCAATTCACCAT	TTTGGATGTGAAGCGTGGCGGA	313
FMgSSR-28696	scaffold3607	p2	(GA)8	Class II	4825	4840	ACGGCAAACATCCCCCTCTTGT	TCTCCACCAGTTGCGTTGCT	234
FMgSSR-28698	scaffold3978	p2	(GA)8	Class II	4192	4207	ACGGCCAGGAAACAATCGGA	ATGTGCGTGCGCCTCTAAGACA	326
FMgSSR-28707	scaffold30638	p2	(GA)8	Class II	997	1012	ACTAAACTTGCACGCCGCGA	ACGCAAGCAAGCACAATCGGA	237
FMgSSR-28708	scaffold3447	p2	(GA)8	Class II	54423	54438	ACTAGTGCCGTGTGCTGGTT	ACGTGGACAATCGCCTGGTCAA	310
FMgSSR-28709	scaffold2186	p2	(GA)8	Class II	64981	64996	ACTCCAGCTGCTGATTACAGTGC	TGCAGTTCACCAGTGATTGTGCC	333
FMgSSR-28710	scaffold5255	p2	(GA)8	Class II	8291	8306	ACTCGGTCCTTTCATCCTGCCA	ACCCTCAACCTAACTCCTCACCT	210
FMgSSR-28711	scaffold331	p2	(GA)8	Class II	102083	102098	ACTGACTGCTTTGCGTGCGT	TATGTTGGGTCGGGTGGCCAAA	200
FMgSSR-28717	scaffold61	p2	(GA)8	Class II	16420	16435	ACTGCTGCCGGCTTAGTTCGTT	TCATGCGCGCGCGACTTAAT	200
FMgSSR-28718	scaffold3509	p2	(GA)8	Class II	28322	28337	ACTGCTGCTTGCGCGCTTAT	TCGTCGTCGTGGCCATCTATCT	201
FMgSSR-28719	scaffold11205	p2	(GA)8	Class II	10980	10995	ACTGCTGGCGAGATGTGAGGAA	TGCAAGAGGCAGAAAGCCAACA	350
FMgSSR-28726	scaffold2176	p2	(GA)8	Class II	38418	38433	ACTTGTGCAAGGTGAGTGAGTG	TCTTCTTGTCGACGGTGCGT	350
FMgSSR-28731	scaffold7895	p2	(GA)8	Class II	16078	16093	AGAAGGGCATTCACGGCTTACA	TGCAGACGAGCCAAACATTGGTG	317
FMgSSR-28732	scaffold1582	p2	(GA)8	Class II	76267	76282	AGAAGGTCAGCCCGTTGGTGAA	GAAGCCCTTAGACACCTTTGTGTG	267
FMgSSR-28736	scaffold234635	p2	(GA)8	Class II	449	464	AGAGAGAGAGACTCACAACGAGC	ACCGTGCATGGATTGGAGTGTG	249
FMgSSR-28737	scaffold33945	p2	(GA)8	Class II	1165	1180	AGAGAGAGGGTGTGGGAGAGAA	TTTCGGTGTCACAGTACCCCTCCT	212
FMgSSR-28738	scaffold30173	p2	(GA)8	Class II	826	841	AGAGATGAACGGCAGGCACA	AGCCAGGACTGGATTCACGGTT	311

SSR_ID	Scaffold	SSR_Type	SSR_Motif	SSR Class	SSR_Start	SSR_End	Forward sequence	Reverse sequence	Product_size
FMgSSR-28749	scaffold132	p2	(GA)8	Class II	70420	70435	AGCAATGAGGTGCAGCGTGT	CACAAAACCACGTGCCATGCCT	203
FMgSSR-28750	scaffold14596	p2	(GA)8	Class II	19272	19287	AGCACAAAACCATCCTTGCCGC	AACAGCGGTAGCAACGGTCA	221
FMgSSR-28759	scaffold960	p2	(GA)8	Class II	54489	54504	AGCCACTTCGTCGAAACCGTCA	TTGATGTCAGGCAGCCGTCACT	283
FMgSSR-28764	scaffold241	p2	(GA)8	Class II	23761	23776	AGCCCTTAGTGGAGTTGACCGT	ATCCGCAGACAAGCGCACAT	247
FMgSSR-28766	scaffold1848	p2	(GA)8	Class II	45043	45058	AGCCGTGTGGACTCTTCGCAAT	TGCTCAACAAGCAACTGCGTCG	275
FMgSSR-28770	scaffold411	p2	(GA)8	Class II	106663	106678	AGCGATCGTGACGAGAACGA	TGCTGCAAGGTACTCCCCCTTT	259
FMgSSR-28773	scaffold2216	p2	(GA)8	Class II	60985	61000	AGCGCTTCTTCTTGCCGTGGAT	AGGCAGCGATCAGCGTATGGAA	300
FMgSSR-28774	scaffold3273	p2	(GA)8	Class II	39550	39565	AGCGGCTACGGCATTCAAGT	TCCCTCTTGCCTATGACCCACT	314
FMgSSR-28776	scaffold123378	p2	(GA)8	Class II	843	858	AGCGTTGCTTCCTTGTCCCGAA	ATTAGCATGCACACGGACGC	294
FMgSSR-28779	scaffold3006	p2	(GA)8	Class II	7706	7721	AGCTCTGCAAGCGTCCACCTTT	TGCAGCCCTGCTGATACCTAGA	252
FMgSSR-28783	scaffold3820	p2	(GA)8	Class II	41493	41508	AGCTTTGGCCAGCAATAGGCTC	TTAGGTCCCGGGCTCATGGTACTT	347
FMgSSR-28786	scaffold118921	p2	(GA)8	Class II	264	279	AGGAACCCAGGTGCAAACAGT	TTGGGTTTCCTGCGTTGCCA	278
FMgSSR-28789	scaffold2044	p2	(GA)8	Class II	40245	40260	AGGACGGACTGACGCAACAT	TGACGATGGCATTTGCCGCT	203
FMgSSR-28802	scaffold1390	p2	(GA)8	Class II	52984	52999	AGGGCAAAGCCAACATCGGTGA	TTCAACACATCGGTCGGCTGGT	251
FMgSSR-28803	scaffold1126	p2	(GA)8	Class II	88038	88053	AGGGCCTTTTTCACCACCGT	TGCTAACTGCTTCCGCTGCT	229
FMgSSR-28815	scaffold2995	p2	(GA)8	Class II	28081	28096	AGTAGCTTGTTGCGGGCACTGT	TCGTCGTCGAATTGAAGCGGGT	244
FMgSSR-28817	scaffold172920	p2	(GA)8	Class II	667	682	AGTCGTAGCAAGGAAAGGGCAC	GCTTGCAGGCTTTGGCAAGGTT	279
FMgSSR-28820	scaffold230202	p2	(GA)8	Class II	239	254	AGTGCTGCAACTGCAACGGA	GCTCGCAACTGAAGACAACGCA	281
FMgSSR-28822	scaffold3435	p2	(GA)8	Class II	51257	51272	AGTGGGTTGTGATTTATGCCCGTG	TACGCCGCGCCTATGTGGTTAT	323
FMgSSR-28825	scaffold1677	p2	(GA)8	Class II	56311	56326	AGTGTTGTGCGCTCAGAGCTT	TGGTTCGGCTCGGTTTGATGCT	231
FMgSSR-28826	scaffold202244	p2	(GA)8	Class II	172	187	AGTGTTGTGCTGCCTTCGCA	TCCCATGGAGAACCGGCATTGA	254
FMgSSR-28835	scaffold6891	p2	(GA)8	Class II	4861	4876	ATCCTTTGCTCCAGCTTGCCCT	TGTGTGTGAGCGTGTGGCTGAA	348
FMgSSR-28836	scaffold2978	p2	(GA)8	Class II	10455	10470	ATCGCGCCACTTGAACTAGCCA	TGGCACACGTAGTATGGGGCTT	265
FMgSSR-28839	scaffold200	p2	(GA)8	Class II	50207	50222	ATCGGCGTCACAGGCATGAA	TCGGCGATGCAGCAACGTAA	280
FMgSSR-28846	scaffold5534	p2	(GA)8	Class II	2075	2090	ATGCACTCACCGGCACCAAT	TGGCGCACACCAAACCCTTT	271
FMgSSR-28852	scaffold6894	p2	(GA)8	Class II	9206	9221	ATGGAGCTACACCAAGCAGAGGGA	CGGGCATTTCAAGGCTCGTT	250
FMgSSR-28855	scaffold11487	p2	(GA)8	Class II	20574	20589	ATGGGGAAAGGCGCACAACTGA	AGGTGGGGTTCAACCACGGATT	284
FMgSSR-28856	scaffold284	p2	(GA)8	Class II	28599	28614	ATGGGGTTGCTGGTTGAGGGTT	TTCGTCTACGCGCACTGGTT	309

SSR_ID	Scaffold	SSR_Type	SSR_Motif	SSR Class	SSR_Start	SSR_End	Forward sequence	Reverse sequence	Product_size
FMgSSR-28863	scaffold12205	p2	(GA)8	Class II	7790	7805	ATTCCTGAGCCCGTTGCAGAGT	AAGCTTCGCGTCCGTTTCCT	249
FMgSSR-28864	scaffold6123	p2	(GA)8	Class II	13553	13568	ATTCTCTTGGCCGGATTGGCGT	CGTTGCATTGGCCAGCTGTGAA	342
FMgSSR-28866	scaffold6755	p2	(GA)8	Class II	19616	19631	ATTGATCCCCGAGCGCACGTTA	ATCGTCGAGTTGACAGCCGCAT	283
FMgSSR-28871	scaffold14917	p2	(GA)8	Class II	19384	19399	ATTTGTGCTCCGCATTCTGGCG	TGGCCTCCACCATCCATTGCTA	277
FMgSSR-28873	scaffold909	p2	(GA)8	Class II	26782	26797	CACACACGCCCGTTCCTTTTCA	TTTTCCGCTACCACGCCATCGT	334
FMgSSR-28879	scaffold9206	p2	(GA)8	Class II	10652	10667	CCCTCGTCGAGCCAATGGTAAT	TGCGCTCTCTCTCTCTCTCCAT	350
FMgSSR-28881	scaffold7804	p2	(GA)8	Class II	25048	25063	CCGCGATCCGTGATGTGATGAT	TACGTGGCCATGCACTCCTT	239
FMgSSR-28882	scaffold26053	p2	(GA)8	Class II	5076	5091	CCGTACGCATCGAGCGCATTTT	TGCGCTTGCGACGACTTTCA	290
FMgSSR-28883	scaffold7576	p2	(GA)8	Class II	35349	35364	CCTCTTGCGCTTTGGATCTCGT	ACAGCCCGCAAAGAGTCCCATT	250
FMgSSR-28887	scaffold7020	p2	(GA)8	Class II	7762	7777	CGACAGCATCGGGGGAAAGAAT	TACACGGCTAGAAACTCCGGCA	250
FMgSSR-28888	scaffold13797	p2	(GA)8	Class II	23019	23034	CGAGCGTTTTGCGGCTGAGAAT	ACACACATTCTGCGCTCCTGCT	257
FMgSSR-28893	scaffold764	p2	(GA)8	Class II	74025	74040	CGCTGAAGATCAATCATGTGTGCG	TTTGGACGGCATGTCCGCAT	304
FMgSSR-28896	scaffold212838	p2	(GA)8	Class II	41	56	CGCTGGTCATTATGTTGAGAGTCGC	AGCTCCAGCGTTCTGGTTGA	270
FMgSSR-28903	scaffold1359	p2	(GA)8	Class II	26671	26686	CGTCGAGTTGCTTGGTGCAT	ACGTTCCATTGATCGGTGCC	220
FMgSSR-28904	scaffold1713	p2	(GA)8	Class II	72989	73004	CGTGCCATGTTCAGCGGTTTGA	AGCTGGGATTTCGGGTGCTT	329
FMgSSR-28914	scaffold4000	p2	(GA)8	Class II	22708	22723	GCATCGGCGGGGTGATCAATTT	TTGCAGTGTTGCCTCCTCTGGT	303
FMgSSR-28915	scaffold5138	p2	(GA)8	Class II	2526	2541	GCATGATGCTGCACGCCAAA	TTGGCGTTGTGGAGAGAAGGGT	239
FMgSSR-28918	scaffold820	p2	(GA)8	Class II	38725	38740	GCCAAACGCGACGCAGAAAGAA	AAGCCAGGCGAGCGAGAGAAAT	283
FMgSSR-28919	scaffold17382	p2	(GA)8	Class II	10438	10453	GCCAGAAGAAGAGTACGTAAGGGT	ACGCCATAGCAACAGGCAACT	238
FMgSSR-28921	scaffold196	p2	(GA)8	Class II	80873	80888	GCCTCTTGCTTGCTTGCCTT	TGCAGAAACTGACCTCGTGGGT	335
FMgSSR-28922	scaffold55	p2	(GA)8	Class II	57774	57789	GCCTTTGTGTGTGCCTGCGAAA	GGTCTGCGTGCCGTTGTGTTTT	209
FMgSSR-28924	scaffold25862	p2	(GA)8	Class II	3956	3971	GCGAGCCCTGCCGGATATTTTT	AAATTATGAGCCGCGCCCCCAA	342
FMgSSR-28926	scaffold4568	p2	(GA)8	Class II	14851	14866	GCGCAGAGGCAACATCGAAA	ACTTGACCCAATGGCCACTCCT	309
FMgSSR-28928	scaffold228	p2	(GA)8	Class II	89233	89248	GCGGTTCATCGGTTTCCTGCAA	TTGCTGCTCTCAAGCCCTCA	250
FMgSSR-28929	scaffold21	p2	(GA)8	Class II	42059	42074	GCGTTGGTTCCATGTTGGCAGT	ACAAGCGACGGGCATTGGAT	350
FMgSSR-28932	scaffold22140	p2	(GA)8	Class II	5433	5448	GCTCTCTCTTGCCATCATGTGCCT	GGCTGGCTTTATCACTGGTGACACT	329
FMgSSR-28935	scaffold1450	p2	(GA)8	Class II	7151	7166	GCTGGAGACCGCGATCCTGTATTT	TCCTTGCCCCTGGAGAAAAACGA	338
FMgSSR-28937	scaffold4686	p2	(GA)8	Class II	24889	24904	GGAAACAACGGCTTGCTGCCAT	TAGGTTTGCACGACGAAGGCGT	327

SSR_ID	Scaffold	SSR_Type	SSR_Motif	SSR Class	SSR_Start	SSR_End	Forward sequence	Reverse sequence	Product_size
FMgSSR-28939	scaffold2861	p2	(GA)8	Class II	26287	26302	GGACCATGTGGAGTGTTCCACTTCT	TGCACTCGGAGATCTTGACCCT	347
FMgSSR-28942	scaffold2895	p2	(GA)8	Class II	18642	18657	GGCGCCCACCAAACAGAAAAGT	TGGAAAATCTCAATCGGGCCGC	345
FMgSSR-28943	scaffold14788	p2	(GA)8	Class II	17672	17687	GGCTAGCCATTTGAGGACTGCCAA	ACCTCCCACTTGTTGATGGCGT	210
FMgSSR-28949	scaffold107	p2	(GA)8	Class II	37672	37687	GTCGTTCATGCCATGCCAACCA	GCAACTGCAAGCGACACGGTTA	299
FMgSSR-28952	scaffold14037	p2	(GA)8	Class II	16708	16723	GTGTGGCAGTGCTGGAATATGT	AGACTTGCGGCATGCCTTGT	288
FMgSSR-28959	scaffold3547	p2	(GA)8	Class II	6223	6238	TAAGCGTACTGCACCGCCATGA	CCAAATCATCGTTGCCTCTCGCA	290
FMgSSR-28962	scaffold7207	p2	(GA)8	Class II	4487	4502	TACGTGTGCGGCACCATTGCTA	TGGCACCCAGAGCCATCAAA	320
FMgSSR-28963	scaffold123378	p2	(GA)8	Class II	726	741	TAGCCGTAGCGTAGCGTTGCTT	AAGCCTTACATGCCCACCCA	206
FMgSSR-28964	scaffold9870	p2	(GA)8	Class II	24947	24962	TAGCGCAACAGCTGATCTCACG	CGAACTTACCCAAGGTCAAGCAAG	247
FMgSSR-28966	scaffold6899	p2	(GA)8	Class II	15765	15780	TAGGGGGAAAAGAGCTTGCTAGGC	ACACGCGCGTCTCAAATTGTGT	296
FMgSSR-28970	scaffold12205	p2	(GA)8	Class II	8425	8440	TATGCATGGTTGTTGCCGGGAC	ACGCGTGTTAGCGTGTACGA	249
FMgSSR-28972	scaffold8459	p2	(GA)8	Class II	29081	29096	TATTAGGTCACCCTTGCCGCCT	ACGGCCTTTGCTGATGCACT	326
FMgSSR-28978	scaffold808	p2	(GA)8	Class II	96912	96927	TCAAGGTTGACGAAACGGGCGA	TTTCTGGCGGCCAAGCCAAA	248
FMgSSR-28982	scaffold7627	p2	(GA)8	Class II	17211	17226	TCACCATCGGTGGAGCGAAT	TGGTGCATGTGTTAGTAGGACGGA	346
FMgSSR-28983	scaffold1375	p2	(GA)8	Class II	25090	25105	TCACCCAGACGCGGACGAAAAA	TTGTCCATAACTCCTCTCCCACCC	342
FMgSSR-28984	scaffold7189	p2	(GA)8	Class II	30110	30125	TCAGAGCGTCCAACGACGTA	GCAGCCAATGCTGCTCATGT	318
FMgSSR-28989	scaffold6618	p2	(GA)8	Class II	6730	6745	TCATGCACACCTGCTGAAGGCA	AGCGCCCCTTGAAGCACATT	239
FMgSSR-28994	scaffold20170	p2	(GA)8	Class II	13646	13661	TCCAAGACCACGGATTGACGTG	TAAACGTGTCGGGCCATGTCGT	231
FMgSSR-28995	scaffold451	p2	(GA)8	Class II	25984	25999	TCCAAGCCAAACAGCGGAAGGT	ATGGAGGAGGGTTTGTGGATGC	314
FMgSSR-29000	scaffold7394	p2	(GA)8	Class II	22977	22992	TCCACGGCAACTTCGGTAGCAA	ACCAAAACGCCCTCGTGTGT	234
FMgSSR-29002	scaffold842	p2	(GA)8	Class II	83750	83765	TCCCACGATGCAAATGCGGA	ATGCACCATGCACCAGCACA	304
FMgSSR-29009	scaffold3649	p2	(GA)8	Class II	34167	34182	TCCTGCCTCCTGTTATGTGCCA	TGTCCGCATTGGACCCCCAATTT	328
FMgSSR-29012	scaffold7315	p2	(GA)8	Class II	5607	5622	TCCTTTGAAATGGCGCCAAGCTG	TGCCATTTCGGTTCCTGCTGGT	332
FMgSSR-29020	scaffold10724	p2	(GA)8	Class II	11260	11275	TCGCACCACCCCACTCTAACAT	TAGCTGCGCAAAGCGGAGTGTA	350
FMgSSR-29022	scaffold4228	p2	(GA)8	Class II	13301	13316	TCGCCGATGCGGTTGAAGATGA	AGACAATTCAGGGGGCTCGT	305
FMgSSR-29023	scaffold3152	p2	(GA)8	Class II	48162	48177	TCGCTGCGTGTGGAATTGTGGA	AACCCGGACACAATACGCCAGA	328
FMgSSR-29025	scaffold17707	p2	(GA)8	Class II	2852	2867	TCGGCATCCCGAGATAGAAACAGG	TGCTTGCCACACTGCAAACGA	257
FMgSSR-29027	scaffold1292	p2	(GA)8	Class II	37791	37806	TCGGGGTAGAACTTCCTCGCAA	TGCGTCTGACTCTCGATCCGTT	238

SSR_ID	Scaffold	SSR_Type	SSR_Motif	SSR Class	SSR_Start	SSR_End	Forward sequence	Reverse sequence	Product_size
FMgSSR-29029	scaffold6933	p2	(GA)8	Class II	12968	12983	TCGTAGTAGCCCTGCGTCACAA	TTGCGGCACCACTCGTGATGAT	204
FMgSSR-29035	scaffold3154	p2	(GA)8	Class II	35497	35512	TCGTTGCCAGTGGTTTGGCT	ATGCCGTGAGCCCGTGATTCTT	214
FMgSSR-29043	scaffold330583	p2	(GA)8	Class II	243	258	TGAACGCGGCAACCAGGAGAAT	TTTTCTCAGCGGCGTTCCGT	232
FMgSSR-29046	scaffold7078	p2	(GA)8	Class II	7911	7926	TGAAGGATGGCTGCTGCTGGTT	AGCAAATGAGCAACGGCACACG	325
FMgSSR-29048	scaffold6000	p2	(GA)8	Class II	15610	15625	TGACCAGCATTCATCTGTCCCC	TGTCTTCAACCTCCAGCCACCA	344
FMgSSR-29052	scaffold7385	p2	(GA)8	Class II	20962	20977	TGAGCCCACCATCACGCAAA	TTGTGCAGCTTCTGACGGGA	291
FMgSSR-29055	scaffold631	p2	(GA)8	Class II	88130	88145	TGATGACCTTCTCTTGCACCCA	GCGCGCAATGGATCTTTTCTGGA	350
FMgSSR-29060	scaffold49	p2	(GA)8	Class II	178190	178205	TGCACCTGTGGTGTGAATGCTG	TGGAGTTGGGATGGGAGGTACACA	332
FMgSSR-29061	scaffold1352	p2	(GA)8	Class II	47692	47707	TGCACGACATCGATTCATCCCCT	AGTCGCTCGCGCAACCAATTT	207
FMgSSR-29064	scaffold431	p2	(GA)8	Class II	90482	90497	TGCAGAGGGAGAGAGAGTGAGT	GCAAGCCAAAAGTGGACGATCA	278
FMgSSR-29067	scaffold3322	p2	(GA)8	Class II	21345	21360	TGCAGGATGGTGATGGGGTTGA	AGAGCGTTATCGTTGTGCCC	347
FMgSSR-29070	scaffold7201	p2	(GA)8	Class II	11163	11178	TGCATGCATGGCATGGCAGA	ATGACGGGGCCACGGTCTTTTT	248
FMgSSR-29073	scaffold1602	p2	(GA)8	Class II	43167	43182	TGCATGCGTATGGCACGTTGAC	TGGGCTGCTGGATGCACAAA	281
FMgSSR-29078	scaffold8496	p2	(GA)8	Class II	21376	21391	TGCCACAGCTGCCACATTCA	TCGTGGTGCCGAGCAATCTCAA	348
FMgSSR-29080	scaffold24209	p2	(GA)8	Class II	2585	2600	TGCCACTGGAAAGACACCACCT	ACGATCGTGCTCCCAAACGACT	237
FMgSSR-29081	scaffold977	p2	(GA)8	Class II	17773	17788	TGCCACTTGTGTCACCGAGGAT	AATGGCACCATGGCGGCTAA	318
FMgSSR-29083	scaffold10621	p2	(GA)8	Class II	21886	21901	TGCCATTGCAGCATCACCTCA	TGAGCGCACTACTATAAGGATGGC	234
FMgSSR-29085	scaffold6875	p2	(GA)8	Class II	35376	35391	TGCCGAGTGTTTGTGCTGGT	GTGCACTCTCCAATGGCGCAAA	247
FMgSSR-29086	scaffold17589	p2	(GA)8	Class II	9798	9813	TGCCTCCTTTGACACATGGGGT	AGGCCAATGTCAACGCGACT	330
FMgSSR-29088	scaffold9782	p2	(GA)8	Class II	17844	17859	TGCGCAACAACCAGCAGAGA	TGGGATGCAGGGAGCCATGAAA	205
FMgSSR-29089	scaffold17	p2	(GA)8	Class II	35489	35504	TGCGCACACATGAGCGAAGT	ATACTCCGGCCGGCCAATTT	321
FMgSSR-29097	scaffold11480	p2	(GA)8	Class II	20686	20701	TGCGGTGACTGTTATACACGCC	TGTGGCTGTCCATAACCCCTATGC	292
FMgSSR-29099	scaffold2276	p2	(GA)8	Class II	45835	45850	TGCGTGTGATTCTCACTCTGTCCC	AAGAAACCGAGAACCGTCGCGT	308
FMgSSR-29100	scaffold4060	p2	(GA)8	Class II	7523	7538	TGCGTGTGCATCAGCCCTTACA	ATTGTCATCGTACCGGCGTGCT	244
FMgSSR-29105	scaffold3156	p2	(GA)8	Class II	11327	11342	TGCTCCATGGACGCCAAATCCA	TGTGGGGCCCGCTGATCAATTT	214
FMgSSR-29106	scaffold241	p2	(GA)8	Class II	150450	150465	TGCTCCTGCTCCTGCTTTATCCT	TTGCGCAGATTTGTTACCCGGC	230
FMgSSR-29108	scaffold5607	p2	(GA)8	Class II	21388	21403	TGCTGAACACGTTGAGCGAGGT	TCCGTCTTCTCGCATAGCAGCA	210
FMgSSR-29109	scaffold19912	p2	(GA)8	Class II	2198	2213	TGCTGCATCCGCCAATCGTT	AGGCTCGGAGCTAGATGCACTT	346

SSR_ID	Scaffold	SSR_Type	SSR_Motif	SSR Class	SSR_Start	SSR_End	Forward sequence	Reverse sequence	Product_size
FMgSSR-29110	scaffold1258	p2	(GA)8	Class II	5506	5521	TGCTGCATTGTCGGGGTATGGA	ACGAATGCTTGTCGTTGGATGC	341
FMgSSR-29111	scaffold2621	p2	(GA)8	Class II	49215	49230	TGCTGCCTACACTCGTCGTT	TGGCAGGTGTGTGGCTCAAA	248
FMgSSR-29114	scaffold5440	p2	(GA)8	Class II	1041	1056	TGCTGTGGCGTTGTCACTGT	TGGCCGTTTCTTGGTTCCCCTT	290
FMgSSR-29118	scaffold2150	p2	(GA)8	Class II	36828	36843	TGGAACGCTGTGTCTGGCCATT	TTGGTGTTCACGCCCTCTGCTT	315
FMgSSR-29120	scaffold7591	p2	(GA)8	Class II	27787	27802	TGGACAAGTGTGTAGGTGGGCA	AAACAGAAGCTCTCGCCGAGGT	297
FMgSSR-29121	scaffold12714	p2	(GA)8	Class II	13508	13523	TGGACCTCATGTGAGAGGTCGT	AGCTGGGCATGGGCTTGCTATT	341
FMgSSR-29122	scaffold9206	p2	(GA)8	Class II	10855	10870	TGGAGAGAGAGAGAGAGAGCGCAA	TTCGTGCGCATGAGAGTCGT	289
FMgSSR-29125	scaffold7631	p2	(GA)8	Class II	16533	16548	TGGATCACGCGACGCAAGCTAT	GTAAACGCACGCGCCATTCT	325
FMgSSR-29129	scaffold7716	p2	(GA)8	Class II	15685	15700	TGGCATGATCTTGCGAGGCTGT	GGTGAGCCAGGGGCAAAATGAA	317
FMgSSR-29149	scaffold5499	p2	(GA)8	Class II	606	621	TGGTTCTCGCTTGGGTTTGGGT	AAGAGAAGGCGAGCGAGCAAGT	349
FMgSSR-29151	scaffold1087	p2	(GA)8	Class II	68498	68513	TGGTTGGTGCGCGTACTGAT	AGCGAAATTGACGTGCGGTGT	248
FMgSSR-29152	scaffold20	p2	(GA)8	Class II	179941	179956	TGGTTTCGTCACAAGCGTGGGT	ACGAGCGTACCCGTTACGTACT	200
FMgSSR-29155	scaffold6232	p2	(GA)8	Class II	17640	17655	TGTCGGCAGCATGCATTAGGTG	GGCGATGATGTTGCTGTTGCCA	236
FMgSSR-29156	scaffold3388	p2	(GA)8	Class II	23495	23510	TGTCGGCCTGTCCGTCAACAAT	AAGAGGCCGAAAACAGGCCCAA	290
FMgSSR-29162	scaffold1578	p2	(GA)8	Class II	43071	43086	TGTGCCCCTCATCAGTGCAT	AGCAGTCCTGGCCAACCAAACA	291
FMgSSR-29165	scaffold2852	p2	(GA)8	Class II	50022	50037	TGTGGTGAAGGCGGTTGAGA	TAGTGCGCGCCACCAGAATCAT	293
FMgSSR-29168	scaffold709	p2	(GA)8	Class II	103275	103290	TGTTGTGCAGATGCTGTTGGGC	GCCACGGTTGCGCGTATTGAAA	307
FMgSSR-29178	scaffold2515	p2	(GA)8	Class II	33367	33382	TTCATGCTGCTGCGTCCTCGAT	AGCGGAGCAAACACCGCTTT	242
FMgSSR-29179	scaffold4997	p2	(GA)8	Class II	22640	22655	TTCCACGGTTCATCGCAACCCA	AAGGTGAAGGCGTTCCGTGTCA	291
FMgSSR-29185	scaffold1728	p2	(GA)8	Class II	17891	17906	TTCTTTCACCGCCTCTAGCCGT	TCTTCTAATGCGGCCTCTCCCT	238
FMgSSR-29186	scaffold86	p2	(GA)8	Class II	58810	58825	TTGACAAACTGAGAGGAGGGGG	AGAGGGCAATCCAATAGCCTGC	332
FMgSSR-29188	scaffold2286	p2	(GA)8	Class II	12580	12595	TTGCAAACGGGAACATGCCA	ATGGGACCCCTCAACGTTGGGATA	253
FMgSSR-29190	scaffold11144	p2	(GA)8	Class II	5028	5043	TTGCATCGAAGGCCGGAAGGAA	AGGCCCAACACGTTCGGTCAAA	240
FMgSSR-29195	scaffold826	p2	(GA)8	Class II	2355	2370	TTGCCCAGGTGTTGGTGGTCTT	AGCGACTGTGCCCATCTTCCAT	205
FMgSSR-29198	scaffold6644	p2	(GA)8	Class II	26048	26063	TTGCGTGGACGGAGTGAAGT	TGTTGGTCGCCATGGTGATGCT	303
FMgSSR-29202	scaffold5214	p2	(GA)8	Class II	1724	1739	TTGCTGTGTTGGTGGGGAGA	ATTCGTTCGGCATTGGCCCAGA	344
FMgSSR-29203	scaffold3496	p2	(GA)8	Class II	36386	36401	TTGGAAGGGCGGAGGGCAAAAT	ACCTCGGTTCAGTTCAGCATCGT	297
FMgSSR-29204	scaffold5536	p2	(GA)8	Class II	11009	11024	TTGGCCGTCCGTTGTGTTGT	TTGTCGTCGAGGTGCATCGGAA	235

SSR_ID	Scaffold	SSR_Type	SSR_Motif	SSR Class	SSR_Start	SSR_End	Forward sequence	Reverse sequence	Product_size
FMgSSR-29207	scaffold229	p2	(GA)8	Class II	113696	113711	TTGGGAAAGCAGTCGCCCAT	TTGTGTGCGCACCGGTATGGTT	216
FMgSSR-29208	scaffold2605	p2	(GA)8	Class II	30371	30386	TTGGGCAGGTCAGCCGCTAAAT	TGCTCTCGCTCCGTTGCTACTT	279
FMgSSR-29212	scaffold162	p2	(GA)8	Class II	99343	99358	TTGGTGTCGTCCTGTCGTACATCG	ATGCAGGCCCAAGGCCAAACTT	345
FMgSSR-29227	scaffold7834	p2	(GA)8	Class II	9507	9522	TTTCGGCGCAGCGAAAGCAA	ATAAAGTGTGCCAGTGACCGGG	282
FMgSSR-29228	scaffold6383	p2	(GA)8	Class II	30957	30972	TTTGACGCACATCAGTCGGC	AGCAAGCAAGGAAACAGCTGCG	255
FMgSSR-29230	scaffold8749	p2	(GA)8	Class II	10118	10133	TTTGACGTGAAACGACCGCACG	CCGCAATGCATGCAGCACAT	200
FMgSSR-29237	scaffold4190	p2	(GA)8	Class II	40389	40404	TTTTCCAGTGTGACCTCGCTCG	ACCCGTAGGTAGATGCATCCATGA	348
FMgSSR-29238	scaffold5278	p2	(GA)8	Class II	26126	26141	TTTTCTCACGCGTACCCGCA	TTGAAGTCCCGCATCAACCGCT	307
FMgSSR-29242	scaffold14809	p2	(GA)8	Class II	13128	13143	TTTTTCCGGACCACTACCAGGC	TGAGGGAAGCCAGTGTTTGCAC	332
FMgSSR-29246	scaffold375	p2	(GA)9	Class II	86869	86886	AAAATTGGGGCGCGGATGGT	ACTTGCGAGACGGGGAAAGTGA	316
FMgSSR-29248	scaffold713	p2	(GA)9	Class II	100390	100407	AAAGCATCCGGTGCGTGACT	TCATCCTTGTCCATGTCCGCGT	331
FMgSSR-29250	scaffold23084	p2	(GA)9	Class II	5238	5255	AAATCAGGCGGCAGCGACAT	GCACCTTTCCAACGTCACCT	341
FMgSSR-29257	scaffold62420	p2	(GA)9	Class II	2131	2148	AACGGCAGAGAAAGCAGCCACT	CCAACCGCACAAGCAACTGCAA	242
FMgSSR-29258	scaffold388	p2	(GA)9	Class II	72815	72832	AACGGCCCTAGAACTTCGCT	TCGGATAAGCGCCAGCGATCAA	228
FMgSSR-29263	scaffold5874	p2	(GA)9	Class II	21962	21979	AAGAGGGATAGGGATAGAGGGGGT	AGGGTCATGCTCTCAGGTTGCT	330
FMgSSR-29266	scaffold10766	p2	(GA)9	Class II	9546	9563	AAGCTAGGGGCGAGGAAAGAAACG	ATGCAGTCTTGCTTCCCCACCA	201
FMgSSR-29268	scaffold1204	p2	(GA)9	Class II	42472	42489	AAGGCACGGAGAAACACGCA	GGCAACGGTCTATGTGTTTGCTGG	288
FMgSSR-29269	scaffold95	p2	(GA)9	Class II	173562	173579	AAGGCCCAACCCACCACAACAA	TATCGCCATTGCGGGCTTTCCT	220
FMgSSR-29277	scaffold5792	p2	(GA)9	Class II	25804	25821	AAGTGCAGCCAAGGGCAGATGA	GCAATGCGCAATGAAGGAACGTG	293
FMgSSR-29278	scaffold804	p2	(GA)9	Class II	61314	61331	AAGTGCAGTGCGTGTCGTCT	TCCTCAAAACCAAACCGCGTCG	229
FMgSSR-29279	scaffold10579	p2	(GA)9	Class II	1687	1704	AATCACATGCGGTGGCACGA	ACCGTTGCGTCTCGACTCTTGA	248
FMgSSR-29283	scaffold94875	p2	(GA)9	Class II	193	210	AATGCCACCATCCGGTGGAGAA	AGCAAAGGTAGTAGCAGCGGCA	303
FMgSSR-29286	scaffold3064	p2	(GA)9	Class II	2172	2189	ACAAAAGGGAGGAGCTTGAGGGTG	ACAACCCTACCAGCCACTTATCCC	324
FMgSSR-29287	scaffold1455	p2	(GA)9	Class II	36472	36489	ACAAACCACGCCCGTTGTGA	TTCCCCATGCAATGCCGAGCTT	338
FMgSSR-29290	scaffold1795	p2	(GA)9	Class II	31167	31184	ACAAGAATCAAGCGGCGGCA	TCTCGATCGGTCTCCATGATGC	340
FMgSSR-29291	scaffold73	p2	(GA)9	Class II	26127	26144	ACAAGGGAGAAATGACGGCGCT	TGTCGGGTGACAAGAGTGCTCA	245
FMgSSR-29292	scaffold6889	p2	(GA)9	Class II	516	533	ACACGATTGCGCTTGCTGGA	ACGCTGCGCCGAGAAGATTT	315
FMgSSR-29293	scaffold20442	p2	(GA)9	Class II	4355	4372	ACACGCAGCCCGAGAAAGAA	CGTTCGCGCAGCTTGACATGAT	272

SSR_ID	Scaffold	SSR_Type	SSR_Motif	SSR Class	SSR_Start	SSR_End	Forward sequence	Reverse sequence	Product_size
FMgSSR-29294	scaffold373	p2	(GA)9	Class II	22484	22501	ACAGAAATCGCAGCCACTCGGT	AGCAAGTGGACGTGCAGTGACA	338
FMgSSR-29295	scaffold1904	p2	(GA)9	Class II	60163	60180	ACAGCAAGCGGCATAGTTGGGA	AGCGTACCGCCAATGTTGAACT	200
FMgSSR-29300	scaffold3010	p2	(GA)9	Class II	22264	22281	ACATCAGCAGCAGCATGAGAGC	TGGCACCAGGTCGAACAAGGAA	323
FMgSSR-29304	scaffold5551	p2	(GA)9	Class II	16985	17002	ACCACCAGCACCAGTCTCATCA	TGTTTCTGTGCGCGTTGCGT	323
FMgSSR-29306	scaffold1925	p2	(GA)9	Class II	48357	48374	ACCATGTTGCGGTTCCGAAGGA	TTGCCTGCGCATCTTGTCTGGA	235
FMgSSR-29308	scaffold4445	p2	(GA)9	Class II	4825	4842	ACCCCAGATTCAGGACTTGCAACA	AGTTCGAGGCTGTTGTGCCT	319
FMgSSR-29313	scaffold1496	p2	(GA)9	Class II	83503	83520	ACCGGCCAGAGAAGAACAACACT	ACATCAACATGCACGGGCCA	211
FMgSSR-29315	scaffold105	p2	(GA)9	Class II	145985	146002	ACCGTCCAAGTACTGCGGTT	AGTGTCATGCCGTCACAGCAAA	334
FMgSSR-29320	scaffold5	p2	(GA)9	Class II	32664	32681	ACGCACACATTGTCCAAATCCG	ATGTAGCTCCGTCCCTGGGTACAA	290
FMgSSR-29321	scaffold4142	p2	(GA)9	Class II	4612	4629	ACGGAAGCAGTTGCACCCAGTA	TGCTGCTGGCCATGCTGATT	207
FMgSSR-29324	scaffold26526	p2	(GA)9	Class II	3714	3731	ACGGCTTTTGTGGGACTGGA	AGTTGCTACCTCTGTGCGCT	225
FMgSSR-29325	scaffold13246	p2	(GA)9	Class II	17663	17680	ACGGGTCGGTGCAAAACTGA	AAAAACACGCACGTGACGCC	274
FMgSSR-29328	scaffold8385	p2	(GA)9	Class II	7933	7950	ACGTCAGCATGTCACACCGT	TCCAAGTCACACAGCTGCTACTGC	251
FMgSSR-29330	scaffold811	p2	(GA)9	Class II	907	924	ACGTCCGGTGAAATCGGACCAA	TGAATTTGGTGGCTTGGCAGC	316
FMgSSR-29335	scaffold908	p2	(GA)9	Class II	106097	106114	ACTCAAATGATACCGCCCCGCA	CCCTGCATGAGCAACCGACTTT	215
FMgSSR-29336	scaffold2829	p2	(GA)9	Class II	15859	15876	ACTCAATCCCGCCGTACTGCAT	ACCTTGCTCCAAGCCTCACGAA	220
FMgSSR-29337	scaffold1246	p2	(GA)9	Class II	70970	70987	ACTGCCATAGGCTGGCCATT	ACCGGGTGTCTTTTGTTCGTCCA	249
FMgSSR-29339	scaffold4552	p2	(GA)9	Class II	6647	6664	ACTGCTTTGCCGAAGTCGCT	TCAAGATGGCGAGTTGGCGT	214
FMgSSR-29341	scaffold43618	p2	(GA)9	Class II	831	848	ACTGTGGATTGGTCAACGATGGTG	AAATGGTGCCTGCGCCTATG	324
FMgSSR-29343	scaffold11689	p2	(GA)9	Class II	22764	22781	AGAAAACACGCATGCAAGGGG	AGCTGGTGACACGGGCAGAAAA	237
FMgSSR-29344	scaffold10793	p2	(GA)9	Class II	3164	3181	AGAAAAGAGCTTGGGGGTGGCA	ATTGAGACCGGACGACTCCTGA	290
FMgSSR-29353	scaffold10036	p2	(GA)9	Class II	8675	8692	AGAGTAGCAGAGGCAGAGTCGT	TCGTCGAGCTTCGCTAGCTT	234
FMgSSR-29355	scaffold18211	p2	(GA)9	Class II	6506	6523	AGCAAGGTGATATGACAGGGGAGG	ACGAGCACACCTAACAGGGCTT	281
FMgSSR-29359	scaffold8076	p2	(GA)9	Class II	7196	7213	AGCACTGAGCGGTGCAAGAT	TGACGCAAATCTGTCGGCGT	300
FMgSSR-29363	scaffold14316	p2	(GA)9	Class II	17646	17663	AGCAGCTGGTTCCCCAACAAGT	TGCACGCAAACAGCATCGGT	301
FMgSSR-29372	scaffold4120	p2	(GA)9	Class II	6543	6560	AGCCCCCAAAAACCCAAACTCCT	ACGCGATGCCGTTACATCCA	231
FMgSSR-29378	scaffold8978	p2	(GA)9	Class II	16407	16424	AGCTCGTAAACAAGGTACGCGA	TCTGGGTGCTCCTCCACATCAA	251
FMgSSR-29381	scaffold819	p2	(GA)9	Class II	71894	71911	AGGAGAATAGCAGCGACGTGGT	TCCACCAGCCAGCCACATTTGA	324

SSR_ID	Scaffold	SSR_Type	SSR_Motif	SSR Class	SSR_Start	SSR_End	Forward sequence	Reverse sequence	Product_size
FMgSSR-29388	scaffold4234	p2	(GA)9	Class II	26527	26544	AGGCCGCGACTTTCCTGTTT	TGTGTGCGTGCAGTTGCTGT	278
FMgSSR-29396	scaffold75	p2	(GA)9	Class II	181491	181508	AGGGGCTGTGACAAACTAGGCT	TGGTGCGCTCAGGATTCACA	234
FMgSSR-29397	scaffold20429	p2	(GA)9	Class II	1665	1682	AGGGTGATGGTGTCGTGTCA	AGAGTCAATCACCGCCAAGGT	348
FMgSSR-29398	scaffold8113	p2	(GA)9	Class II	23313	23330	AGGGTGATTAGGTACAAGGTCGGA	AGGAGGAAGAGATGCCGGTGTT	272
FMgSSR-29399	scaffold8051	p2	(GA)9	Class II	17397	17414	AGGTCCAATAGCACCCGCACAA	TGCCCATGCCACAAGAGCTGAT	241
FMgSSR-29400	scaffold17433	p2	(GA)9	Class II	2722	2739	AGGTCGAGGGAAAAGGCCGAAA	TTGTCGGCGCGTTTGGTACA	267
FMgSSR-29401	scaffold16888	p2	(GA)9	Class II	15046	15063	AGGTCTGGAAAGCGCCAAAGT	ACTCCGCATCTGGAAAGCTCA	253
FMgSSR-29402	scaffold1110	p2	(GA)9	Class II	10400	10417	AGGTGAACAGTGAGTGCAGCGT	GCGAAACGAAAATGCAGCAGCG	243
FMgSSR-29403	scaffold326497	p2	(GA)9	Class II	47	64	AGTACTGTGAAGTCAGCTCCCACG	TCGTGTGTGGTGCTAGTGCGAT	263
FMgSSR-29404	scaffold3524	p2	(GA)9	Class II	22832	22849	AGTAGCAGCATGCTCCATTCAC	TAGCTGATGCCACTGCCGCTAT	201
FMgSSR-29412	scaffold5803	p2	(GA)9	Class II	144	161	AGTGGGGCACAAGACTGCCTAA	TCCGGTTCGAGGGGAATTCCAAA	338
FMgSSR-29420	scaffold4558	p2	(GA)9	Class II	25154	25171	ATAACCAGCGAACCCACGGACA	TGCCTGCGTGGATTCTAGCGTA	348
FMgSSR-29421	scaffold7678	p2	(GA)9	Class II	7549	7566	ATAATGCGGCATGGAGGCGT	GTGCGCGCAAGCAAAGCAAA	238
FMgSSR-29428	scaffold158	p2	(GA)9	Class II	143359	143376	ATCGCTGGCGCACTGAGAGAAA	AGGGCGCAGTAGCTGTCAACAT	316
FMgSSR-29431	scaffold1085	p2	(GA)9	Class II	13872	13889	ATCGTGACGGGGAGGTTGGTTT	TGCATAACCTTACCACGCTCCACT	287
FMgSSR-29432	scaffold3999	p2	(GA)9	Class II	12686	12703	ATCTACGCTTGTGTGTCGCCGT	TCCTTTGATCACGTTGCCATCTCC	284
FMgSSR-29437	scaffold540	p2	(GA)9	Class II	5232	5249	ATGGAAGGAGTCGAGTGAGCCA	TGACCCGCGCTTTCCTCAGTTT	274
FMgSSR-29438	scaffold2405	p2	(GA)9	Class II	41609	41626	ATGGACTCCAACGCGTGCTT	ATGCAGCACGAGGTCAAGCGAA	236
FMgSSR-29439	scaffold8154	p2	(GA)9	Class II	18613	18630	ATGGAGCGATGTAGGGAGGCAT	AGGCGTGGTCATCGGTACAACA	333
FMgSSR-29441	scaffold2325	p2	(GA)9	Class II	35850	35867	ATGGCCAACGGACCTTGCAT	TCTGTCGCTCTTGCTCAGTCCT	234
FMgSSR-29450	scaffold3991	p2	(GA)9	Class II	46354	46371	ATGTGGGTGCGTTGCGTGAT	AGCGAGCAATGCTGATGGGACA	290
FMgSSR-29452	scaffold5130	p2	(GA)9	Class II	19125	19142	ATTAGGGCGCGTCGTCTTTGGT	TGGCTCATGTCATCCGTGGAGT	342
FMgSSR-29458	scaffold3821	p2	(GA)9	Class II	18670	18687	ATTGGGAGACGGTAGCGCCATT	TTGTCAGGCACCATTGCCGT	332
FMgSSR-29459	scaffold2560	p2	(GA)9	Class II	20075	20092	ATTGGGTGGAGTTTGCTCCGCT	TCCACGCAGTCAGTGGTTCAGA	293
FMgSSR-29460	scaffold829	p2	(GA)9	Class II	86460	86477	ATTGGTGATGTGCGTGCGTG	AGGTGGGGCAAAGGCTTTCTCA	257
FMgSSR-29467	scaffold5907	p2	(GA)9	Class II	36874	36891	CCGCCCAAGTCGCACTTTTT	AGCTTAACTACCAGCGCTGCCTT	317
FMgSSR-29468	scaffold28755	p2	(GA)9	Class II	6172	6189	CCGTGCTGGGTACTACCACAAAGT	ACATACCACACGGGCAGCTA	318
FMgSSR-29469	scaffold152053	p2	(GA)9	Class II	614	631	CGATGGCGCGCTGCATTATT	ATTCTTGAAAACCCCGGCGCGA	234

SSR_ID	Scaffold	SSR_Type	SSR_Motif	SSR Class	SSR_Start	SSR_End	Forward sequence	Reverse sequence	Product_size
FMgSSR-29470	scaffold929	p2	(GA)9	Class II	76476	76493	CGCAGTCCACGCAGGTTTTGTT	AGAAGCGTCCGAGTCACCGTAT	260
FMgSSR-29472	scaffold10526	p2	(GA)9	Class II	12224	12241	CGCCCGTGATCTACTCAAACCA	AGGACGCGCCTACCACTTTGTT	346
FMgSSR-29476	scaffold4893	p2	(GA)9	Class II	32732	32749	CGCTTGTAGCGCTCAATGTGCT	TGGCCTATGGCAACCAGCTA	227
FMgSSR-29479	scaffold280	p2	(GA)9	Class II	113625	113642	GCACAACGTGTCGTGCCTTGAA	ACCGACGGTAACCCGAATCA	313
FMgSSR-29481	scaffold3083	p2	(GA)9	Class II	46252	46269	GCACACGCGGAGCAAAGGTTAAA	TGCTGCTGCCTGCATTCCAT	331
FMgSSR-29487	scaffold4106	p2	(GA)9	Class II	35512	35529	GCAGCGCTAGCTCAACGATCAA	ACCATCCCCACACCGCTTTCTT	314
FMgSSR-29489	scaffold6939	p2	(GA)9	Class II	9805	9822	GCCAAGCAAATAAAGGCTGGCACA	AACTGAGGAGCGCTGGCATCTT	337
FMgSSR-29490	scaffold5505	p2	(GA)9	Class II	18743	18760	GCCCACAAATCTTGCCTTTGTCTCG	ACACGCAACGTGAAACCGGA	204
FMgSSR-29491	scaffold446	p2	(GA)9	Class II	13068	13085	GCCGCAGAGCTCGAAACCAAAT	TCGCCTTTGCTCGTAGTCCA	256
FMgSSR-29492	scaffold6258	p2	(GA)9	Class II	39602	39619	GCCGGAGCTGTCACCAACTAAA	AGCCAACTTGGAGCATCGGA	350
FMgSSR-29501	scaffold307	p2	(GA)9	Class II	28213	28230	GGGAAACGTCCTTCTGGGCTTT	TGCCCAACTCCTTTCGTCTGCAT	295
FMgSSR-29503	scaffold326	p2	(GA)9	Class II	142123	142140	GGGCATTGCCGGTTGCATAAGA	AGTTCCGAGCCCGACGAAAAGT	350
FMgSSR-29505	scaffold28726	p2	(GA)9	Class II	6735	6752	GGTTGATGGGAGTCTTGCCACA	ACGTACCTCAACCAAAACGCCT	239
FMgSSR-29507	scaffold961	p2	(GA)9	Class II	78863	78880	GTGACGTCCATCATCCATTAGTCC	TATTGCCAGATCAAGGCGGC	261
FMgSSR-29509	scaffold3000	p2	(GA)9	Class II	50204	50221	GTTCTGGCAACACAAACGCCCA	ACCGAACGGCCGCGTATGTTAT	200
FMgSSR-29515	scaffold3837	p2	(GA)9	Class II	2840	2857	TAACGCTTGCAGTTGGCCGT	TGCTCTGGTGCTGCGAGTTT	285
FMgSSR-29517	scaffold16553	p2	(GA)9	Class II	694	711	TACCATCGCTTGAAGCCCCA	ACATACTTGCTCGCGTCTGC	350
FMgSSR-29518	scaffold207	p2	(GA)9	Class II	93024	93041	TACCCGCCGTAGCACATTCGTA	ACTCAAGTCCACACGCCTTGCT	246
FMgSSR-29526	scaffold17688	p2	(GA)9	Class II	3172	3189	TCACACATTGCGGACAGTCGGA	ACGGGCAAGCTTAGGTTTCGGA	233
FMgSSR-29528	scaffold28575	p2	(GA)9	Class II	4270	4287	TCACAGTGCGTGCGTCAGGTTA	AAACTCTCGGGCGGCATGACAA	307
FMgSSR-29529	scaffold10190	p2	(GA)9	Class II	9365	9382	TCACGACGATGACTGAAGCTTTGC	ACAATACGGCGAGCATGGCA	302
FMgSSR-29531	scaffold2523	p2	(GA)9	Class II	8624	8641	TCAGAGGAGCGAAGCTGCAGAA	TGACGGAATCAGGCGAACAGGT	327
FMgSSR-29535	scaffold267	p2	(GA)9	Class II	90510	90527	TCATTGCCGATGGCACCACA	ACGACTTTCGAGGTCGCGTT	285
FMgSSR-29536	scaffold1689	p2	(GA)9	Class II	45919	45936	TCCACCGATGCCCAAAAGCA	TGCATGGCGGGAACAGCAAA	256
FMgSSR-29541	scaffold9564	p2	(GA)9	Class II	10266	10283	TCCATCGTTTACGCCTGCCCAA	TCCTCTGCAGCACAACGCAA	320
FMgSSR-29544	scaffold5540	p2	(GA)9	Class II	8632	8649	TCCCAAGCCACTCGTTGTCCTT	TCGATGGGCTTCTTGGTGGTGA	297
FMgSSR-29546	scaffold3104	p2	(GA)9	Class II	22728	22745	TCCGGCACAACGAGAACGAAGT	ACCACCATCTCACAGCCGTCAA	333
FMgSSR-29552	scaffold7865	p2	(GA)9	Class II	24834	24851	TCGAAACGGTCCTCCTTCGCTT	CACCCACCAACGCAAACCAACA	281

SSR_ID	Scaffold	SSR_Type	SSR_Motif	SSR Class	SSR_Start	SSR_End	Forward sequence	Reverse sequence	Product_size
FMgSSR-29553	scaffold22559	p2	(GA)9	Class II	3427	3444	TCGAACTGTCGCCTGTGAGAGA	CGCTACCGCTACGCCCATTTTA	313
FMgSSR-29555	scaffold7779	p2	(GA)9	Class II	17531	17548	TCGCCAATGCGCAAAGCACT	TTTCGGATCACGGGTGACGGTT	251
FMgSSR-29560	scaffold213347	p2	(GA)9	Class II	128	145	TCGGCCGGCATCATTGCTTT	TGAGCACCACCACCACTGTA	222
FMgSSR-29566	scaffold7126	p2	(GA)9	Class II	36019	36036	TCTGCATCATCCGAGAGCGT	AGAGAGAGAGCAGGCACGTT	324
FMgSSR-29569	scaffold2393	p2	(GA)9	Class II	8916	8933	TGAAGAGGCGTGCGAAGTCGAT	TGTCGAGTGATGCTTCGCGT	317
FMgSSR-29570	scaffold346	p2	(GA)9	Class II	95259	95276	TGACCGAAGCTGCACAGGAA	AAAAGCCCACACGTGCCGTT	313
FMgSSR-29572	scaffold890	p2	(GA)9	Class II	45827	45844	TGACGATATCGACGCTAGGCCA	AAGGCCCGCTGCAGTATTGGTT	233
FMgSSR-29573	scaffold2335	p2	(GA)9	Class II	18657	18674	TGACGCAGGAAACGCATCAACG	TGCCGGTACGCTGGGCTTTTAT	235
FMgSSR-29574	scaffold2848	p2	(GA)9	Class II	13844	13861	TGACGTCCATCATCCATCAGTCCA	TGGACACGGTCGTATCAGAGACA	313
FMgSSR-29578	scaffold6128	p2	(GA)9	Class II	30352	30369	TGATACGCGTCGCATCGCAA	TTGCCCTGGCTTGACTTTCCCT	233
FMgSSR-29579	scaffold4846	p2	(GA)9	Class II	7554	7571	TGATCCGATGAACCTCCGCCTT	AAACTGAGCGTGTGCCGCAT	234
FMgSSR-29586	scaffold256718	p2	(GA)9	Class II	112	129	TGCAACTAAACTTACGCCGCGA	GTCAGGCGCACCGTTGATGAAT	350
FMgSSR-29587	scaffold615	p2	(GA)9	Class II	75930	75947	TGCAAGAGCCTACGTGTGCCTA	ATGTCGCGGTCTGGTGTCCAAA	348
FMgSSR-29594	scaffold117841	p2	(GA)9	Class II	1182	1199	TGCACTTTTGGCCGTCCTGT	ATGTGATGTGCCAGACCTTCGC	253
FMgSSR-29597	scaffold343795	p2	(GA)9	Class II	90	107	TGCAGTTGGCATATCGCTACCA	CGCATGTAAGGCAGCGACGATT	203
FMgSSR-29600	scaffold5440	p2	(GA)9	Class II	11730	11747	TGCATGCAGCTGTGAAGGAGA	ACCATAGCACACCAACCGCT	350
FMgSSR-29601	scaffold3918	p2	(GA)9	Class II	1900	1917	TGCATGGGCTGATGCATGGT	AGCAGCCACGTCTTCTTCCCAA	274
FMgSSR-29605	scaffold1558	p2	(GA)9	Class II	29756	29773	TGCCGGATGCGTCATCCATT	TGTCGGCAATGACGCGATGT	307
FMgSSR-29607	scaffold1653	p2	(GA)9	Class II	74073	74090	TGCCTAGTGTGAAGTCCAGCCT	TCGGTGCGGTGGATCAATGT	325
FMgSSR-29611	scaffold1916	p2	(GA)9	Class II	30130	30147	TGCCTTGCGGCTCTATCACAGT	GGCAGGTCTCTAAAGAAGTCCGCT	330
FMgSSR-29612	scaffold1993	p2	(GA)9	Class II	38490	38507	TGCCTTTGCTATGGTGCAGAGA	TCACGCGCAGGAGCTTAACA	284
FMgSSR-29615	scaffold1898	p2	(GA)9	Class II	67238	67255	TGCGCCACAATTGGTCTGGT	AGGTGGTGATGCCAAAGCCA	309
FMgSSR-29616	scaffold2523	p2	(GA)9	Class II	58185	58202	TGCGGGAACACGCAGCAATA	GCGCGCGCAACTGTGTATTA	321
FMgSSR-29617	scaffold25	p2	(GA)9	Class II	198913	198930	TGCGGGGAAGAGGAAGAAGAGA	ACAAAGGCGCATGTGGCTGA	241
FMgSSR-29624	scaffold2306	p2	(GA)9	Class II	43164	43181	TGCTGCTCTTGGAGCTAGCTGTT	TCCATGGCAGCTGTCCGCTATT	342
FMgSSR-29628	scaffold3317	p2	(GA)9	Class II	45260	45277	TGGACTTTGCTTGCTTGTTGGGC	AAAGCGAACACGAGCAGATGCC	292
FMgSSR-29644	scaffold57497	p2	(GA)9	Class II	673	690	TGGTAATGGCGTCGGGGTTTCT	TTGCCGCGCAAGGAAAGCTA	316
FMgSSR-29646	scaffold8073	p2	(GA)9	Class II	17581	17598	TGGTGCTCATGGCACGAAGT	GCGACAGACGACAATTGAGCGA	335

SSR_ID	Scaffold	SSR_Type	SSR_Motif	SSR Class	SSR_Start	SSR_End	Forward sequence	Reverse sequence	Product_size
FMgSSR-29648	scaffold3252	p2	(GA)9	Class II	22445	22462	TGTAGGCGTGCCGATCTCTT	TGCTCAGGATTGCCGCCTCTTT	263
FMgSSR-29654	scaffold4757	p2	(GA)9	Class II	31968	31985	TGTGCAGTGGGAGAGGAGAAAGGA	ATGGCCCAAAATTCACCGGC	217
FMgSSR-29658	scaffold2	p2	(GA)9	Class II	902	919	TGTGGACGACTTGACCGTGA	TTTCCCCACTTACTTGGACGCCAC	317
FMgSSR-29666	scaffold7616	p2	(GA)9	Class II	10371	10388	TGTTCTGCGTGCATGCGTGT	ACGGAGCGAAGTTGGAAGAGCA	275
FMgSSR-29667	scaffold210251	p2	(GA)9	Class II	231	248	TGTTGTTGGGGGTGCAGAGACA	GGCCCATAAAATGCGTCGCCTA	329
FMgSSR-29669	scaffold71526	p2	(GA)9	Class II	980	997	TTACCGCGGGCAAAAAGGCA	TGCAACACGCAAGGGACGTT	302
FMgSSR-29677	scaffold2711	p2	(GA)9	Class II	41038	41055	TTCGCATCGTTGGTGCTTGTGG	TGTGCGCGTAGCATTTTCACCG	229
FMgSSR-29678	scaffold14181	p2	(GA)9	Class II	11116	11133	TTCGGACCGTTGGATGGATGGA	TCGCAAACGCAACGTCGTCT	332
FMgSSR-29683	scaffold164	p2	(GA)9	Class II	59322	59339	TTGAGGGTTGCCAAGGTCCA	TTGGACTCTCGGTCTCGTTGCT	295
FMgSSR-29686	scaffold615	p2	(GA)9	Class II	41614	41631	TTGCATCGTTCTTCCCGGCTGA	TCACGTGCATGCCATCGACT	320
FMgSSR-29690	scaffold203	p2	(GA)9	Class II	44832	44849	TTGCGTTCTCACGAATCTGCCG	TTGCGCCGGTCAAAGTGGAA	314
FMgSSR-29694	scaffold2705	p2	(GA)9	Class II	32676	32693	TTGGATGGTCCGATTGGGCTGA	ATTGATGACGGCGCACAGGT	344
FMgSSR-29698	scaffold10305	p2	(GA)9	Class II	25607	25624	TTGGGTCGCTCATTCCATGGCT	GGGCGCTTGACCATTTTGCTGT	340
FMgSSR-29701	scaffold26262	p2	(GA)9	Class II	2231	2248	TTGTCTCAGCTTGAGGGTGGTACG	TTTGGTTGGGGTTGGGGGTGAA	306
FMgSSR-29704	scaffold2831	p2	(GA)9	Class II	8944	8961	TTGTGGGGGTGTTCGCCAAA	CGGCAACGACAAAAAGGACGCT	348
FMgSSR-29710	scaffold1056	p2	(GA)9	Class II	76110	76127	TTTCGAGCACCACCGCTGAA	TCTGAAAATCACGCCGCCCT	281
FMgSSR-29711	scaffold6199	p2	(GA)9	Class II	25572	25589	TTTCTGCGAGCGTTGAGAGGCA	CGGCCATGAAAGCTGCCAATGT	220
FMgSSR-29712	scaffold9708	p2	(GA)9	Class II	12774	12791	TTTCTTTGCGGCGACAGGTGGA	ACGAAAGGCAAGAGGAGTGGGT	248
FMgSSR-29720	scaffold30821	p2	(GC)10	Class I	4368	4387	TTTGTACCCCGCGATTCGCCTT	AATCGCGCGCACGCTAACAA	229
FMgSSR-29725	scaffold1169	p2	(GC)6	Class II	100651	100662	AAAACCACGAGACCACGCGA	TGCCGACGCAACTCGATCTTCA	274
FMgSSR-29728	scaffold397	p2	(GC)6	Class II	85997	86008	AAACCAGAGGAAATCGGGGTTCGG	AAAAACCAGCAACGCCTGAGCC	318
FMgSSR-29730	scaffold12448	p2	(GC)6	Class II	22209	22220	AAAGTTTCGGCCCGTGGACT	TCGGCTCTCCATTCCGAACT	303
FMgSSR-29731	scaffold79352	p2	(GC)6	Class II	970	981	AAATGACCGACACGCACACG	AGGCCGTGATAGGTCATCCACT	343
FMgSSR-29741	scaffold6375	p2	(GC)6	Class II	27566	27577	AACGAAAGGAGCAACAAGCGGG	ACTGATCACGACCGGCAAACGA	284
FMgSSR-29757	scaffold571	p2	(GC)6	Class II	6354	6365	AAGGGATCATCCGCGACTGGTT	TAACCAGCAGGCCAACACGTCA	216
FMgSSR-29758	scaffold10510	p2	(GC)6	Class II	24415	24426	AAGTGAAAAGCCATGCGCCG	AGCAGGTCTGCTTCTTGCTGCT	258
FMgSSR-29760	scaffold8265	p2	(GC)6	Class II	23933	23944	AATCACCGCGCGCCCATAAT	AGCTGCACGCACCCCATTTA	271
FMgSSR-29762	scaffold1059	p2	(GC)6	Class II	88637	88648	AATCTTGAGGCTCACGCAGCCA	CACGCACGCACGAATGTTGT	242

SSR_ID	Scaffold	SSR_Type	SSR_Motif	SSR Class	SSR_Start	SSR_End	Forward sequence	Reverse sequence	Product_size
FMgSSR-29763	scaffold1351	p2	(GC)6	Class II	21669	21680	AATGCCGCCGCTTGACATCGTT	ATCCTCGCGCTTGTTAGCGA	200
FMgSSR-29771	scaffold18777	p2	(GC)6	Class II	12337	12348	ACAACACCAGCGCGCAACTA	AGGGCGCCTCACAAATTCCT	248
FMgSSR-29777	scaffold9536	p2	(GC)6	Class II	5596	5607	ACACACGTGCGTCGTGTTTCA	TCGTCGCCACTTGTGCAGTT	216
FMgSSR-29779	scaffold369034	p2	(GC)6	Class II	76	87	ACACGACCAAGCCGCACTACAT	AAAGACGCCGAACGGCAGAT	292
FMgSSR-29788	scaffold1742	p2	(GC)6	Class II	35996	36007	ACATAGTGAGCACACACGACGG	GCTGGTTGCGTGTGAGGATAGT	309
FMgSSR-29791	scaffold2067	p2	(GC)6	Class II	3157	3168	ACCACCATTCCCCACATTCGCA	ACCAAAACCGAACACCGCGA	325
FMgSSR-29794	scaffold765	p2	(GC)6	Class II	72874	72885	ACCACGAGCTGACGAACCCTAT	TGTGCCCCACAGCAACCATCAT	338
FMgSSR-29800	scaffold28	p2	(GC)6	Class II	168155	168166	ACCGTCGATAGGACATCCCTCTCA	AGTTAGCAGCAATCCGGGCA	313
FMgSSR-29802	scaffold122344	p2	(GC)6	Class II	127	138	ACCTATACCGGTCTGTTCACGC	AGCGCATCCTCGACCGTCATTT	284
FMgSSR-29806	scaffold12429	p2	(GC)6	Class II	1710	1721	ACGAACTCAACCTCCGCGAT	ACATTGCTCAACCGGTGCGT	275
FMgSSR-29807	scaffold140885	p2	(GC)6	Class II	149	160	ACGACGATTGTCTTCACGGCGA	ACGCGATTGGTTGCCTGACA	342
FMgSSR-29810	scaffold7560	p2	(GC)6	Class II	13326	13337	ACGATGATTGATGCGCGGGT	ATCGCAACAACAGCTCCGCA	274
FMgSSR-29811	scaffold76	p2	(GC)6	Class II	25624	25635	ACGCATTTATTCGGCGACCCGT	TCAAAACGATGCGCAACCGC	342
FMgSSR-29813	scaffold2441	p2	(GC)6	Class II	52626	52637	ACGCCGCGCAAACATTCTCA	TGGTGACACTATGTCAGATCAGGGC	236
FMgSSR-29816	scaffold136685	p2	(GC)6	Class II	926	937	ACGCTCAATTGCTTCAACCCAC	CCGCGGGTTCGCCGAAAATATAA	249
FMgSSR-29820	scaffold59642	p2	(GC)6	Class II	938	949	ACGGATGAGAACCGCGACAACA	AACACATCTCTCCGCCAGCTCA	306
FMgSSR-29822	scaffold57464	p2	(GC)6	Class II	1782	1793	ACGTCGGCGATGAAGCAGACAT	AACGAATGAACGCCGTCCCA	271
FMgSSR-29823	scaffold202	p2	(GC)6	Class II	72991	73002	ACGTGCCCGCGTTATGTTCT	TGTTCGCATCTCCGAACGCT	347
FMgSSR-29824	scaffold5233	p2	(GC)6	Class II	21072	21083	ACGTGCCGTTGTTGATCGCA	AGCCGCCGTAACTTTGTCCTGT	274
FMgSSR-29825	scaffold395	p2	(GC)6	Class II	12407	12418	ACGTTTCTGCTGCAACGCCA	ACCGAGTGCTGGGTAAGTCCAA	294
FMgSSR-29827	scaffold23619	p2	(GC)6	Class II	9732	9743	ACTACACGTGCGTCATCTGC	ACCCACTCTGCAAACTCTCGAT	220
FMgSSR-29830	scaffold31084	p2	(GC)6	Class II	3979	3990	ACTCATCACACATGCTCCGCGA	AGCAGCCCGTCCAAACCAAA	340
FMgSSR-29837	scaffold891	p2	(GC)6	Class II	73632	73643	ACTGCATATCACATTGGCCCGGT	TCATTCAGCAACGGCAGGCT	342
FMgSSR-29840	scaffold1747	p2	(GC)6	Class II	72353	72364	ACTTGGGCCCGCTCTAGAACAA	TTTCTTTCTCCACCAGCCGCCA	304
FMgSSR-29843	scaffold2858	p2	(GC)6	Class II	27323	27334	AGACAAAAAGCAGGCGTGGC	CGAGTTCGTTGCGTCGTTGGAT	281
FMgSSR-29845	scaffold6925	p2	(GC)6	Class II	6857	6868	AGATCGATCGCTTCAGGGCA	AATTGACGAACGGCCCGGAT	261
FMgSSR-29851	scaffold596	p2	(GC)6	Class II	38656	38667	AGCACCGGGTTGAAGGTGATGT	GGGTTGCAACTTCATTGCCTTCCT	225
FMgSSR-29854	scaffold924	p2	(GC)6	Class II	64129	64140	AGCCACGTATGAAGACGATAGGGA	TCTGGCCGCTCTTTCCAAATG	246

SSR_ID	Scaffold	SSR_Type	SSR_Motif	SSR Class	SSR_Start	SSR_End	Forward sequence	Reverse sequence	Product_size
FMgSSR-29855	scaffold2166	p2	(GC)6	Class II	903	914	AGCCCTCTGTTGTTGGACCAGT	CGGCTTAGTGCAGTCCGGTAAA	265
FMgSSR-29856	scaffold519	p2	(GC)6	Class II	49554	49565	AGCCTACCCGAACGTGTGGAAA	ATGGCGGGTGCGTCTTTGTT	330
FMgSSR-29859	scaffold50258	p2	(GC)6	Class II	876	887	AGCGCCATCATGTCGTCGTA	TGGAAAAACACGCCGCGCAT	320
FMgSSR-29863	scaffold1345	p2	(GC)6	Class II	11178	11189	AGCGTTTGAATGGTGGCGCT	AGCTTGACGTACCCGTTCAGCA	338
FMgSSR-29866	scaffold1725	p2	(GC)6	Class II	43990	44001	AGCTCGTCCTTCTTTCCGCA	GCGGCAAAAGCGCCGATATTTT	282
FMgSSR-29867	scaffold2027	p2	(GC)6	Class II	37629	37640	AGCTGCAGCATCAGCGTTCT	ACAGCAAGCAAGCGCACACA	260
FMgSSR-29873	scaffold2571	p2	(GC)6	Class II	18920	18931	AGGAGCGGGGGAATGAGATGGAAT	TCGCTTCACGGGTTCTCGTT	345
FMgSSR-29875	scaffold6983	p2	(GC)6	Class II	17818	17829	AGGATACGTTTTGCCCGCGA	TACCCAACCCAACCCAAACCCA	228
FMgSSR-29879	scaffold623	p2	(GC)6	Class II	33119	33130	AGGCAGCACTGGATCTTAGGGT	AGCAGCCAAACGGAAGAGGCAA	326
FMgSSR-29880	scaffold3709	p2	(GC)6	Class II	48083	48094	AGGCATAACGGCTACACTGCGT	AGCAGGGTCCATTGCGAACCAT	240
FMgSSR-29886	scaffold28070	p2	(GC)6	Class II	1992	2003	AGGTCAGTGCAGAAGACGCAGA	AGCGCGCTGATGAACAAGGT	206
FMgSSR-29887	scaffold90956	p2	(GC)6	Class II	545	556	AGGTGAGAAGGCGCCAGTCTTT	AGCTGGTATGGCGAAGCAGT	338
FMgSSR-29888	scaffold1799	p2	(GC)6	Class II	4549	4560	AGTCAAGCAGAGAGGCAACCGT	AGAACTGAAGCGGCGAACCA	259
FMgSSR-29889	scaffold12344	p2	(GC)6	Class II	6546	6557	AGTCCAAGGAAGAAGACGCGGT	TGATGACGCGGGCTAGCATTGA	349
FMgSSR-29899	scaffold192746	p2	(GC)6	Class II	107	118	ATCAATGCCGAGGGCCTCAA	ACTTGCGATCGGGAATCAGCGA	288
FMgSSR-29901	scaffold5391	p2	(GC)6	Class II	13605	13616	ATCCGCACACAAGCCAAGGA	ACATGCACGAACGGTGTGGT	275
FMgSSR-29902	scaffold50700	p2	(GC)6	Class II	1075	1086	ATCGACAGCTGCACGATCTCCT	ATCGTCGTGCTCGGCATGTT	236
FMgSSR-29903	scaffold553	p2	(GC)6	Class II	113617	113628	ATCGATCGCGCGTTTGTGGA	TCGCCGACAAGCTCATCAGTCA	347
FMgSSR-29906	scaffold36753	p2	(GC)6	Class II	2829	2840	ATCGTTACATCGGTGGCGCT	ACGCGCTTGTGTTGCTGACT	299
FMgSSR-29913	scaffold6029	p2	(GC)6	Class II	16482	16493	ATGATGCCGCCGTGACTGTGTA	AGCAGCTGGTCAGCAGAGAACA	296
FMgSSR-29916	scaffold9888	p2	(GC)6	Class II	15504	15515	ATGCTCAACATGCTCGGCGTCT	TACCATGGCACGGGGAGGAAAA	233
FMgSSR-29917	scaffold8638	p2	(GC)6	Class II	2503	2514	ATGGACCGCTGCTGCTACCTTT	GCGCGCGCGACTTTATTGAA	343
FMgSSR-29920	scaffold79907	p2	(GC)6	Class II	1444	1455	ATGTGTGGGCATGTGTGCGT	ACAGGCTGGATGGGGATGGTTT	209
FMgSSR-29923	scaffold4570	p2	(GC)6	Class II	13388	13399	ATTCGAAAGCGTTTGCGGCG	CGTCACGCGCAGATCCCAAAAA	334
FMgSSR-29924	scaffold782	p2	(GC)6	Class II	29868	29879	ATTCGACGGTGCTCTCGCTT	ACCTGCTTGAAAACCTCCCGCT	294
FMgSSR-29925	scaffold192639	p2	(GC)6	Class II	93	104	ATTCGCCCGACAGATCGCTT	TGCAGAGAACGAGACGGTTGT	334
FMgSSR-29927	scaffold900	p2	(GC)6	Class II	9687	9698	ATTGCTCTACTGCGTGTGGCGT	CGTGAAGCAATGCCGCTCCAAT	232
FMgSSR-29929	scaffold135	p2	(GC)6	Class II	67687	67698	ATTGGTCGCGTCCGAGTTGA	AGACTGCGGCAGCATCATCA	240

SSR_ID	Scaffold	SSR_Type	SSR_Motif	SSR Class	SSR_Start	SSR_End	Forward sequence	Reverse sequence	Product_size
FMgSSR-29933	scaffold2280	p2	(GC)6	Class II	38877	38888	ATTTTCGTCAACGCCCGCGA	TTCTGGGCGAAGGAGGAGATGA	300
FMgSSR-29938	scaffold96864	p2	(GC)6	Class II	819	830	CACCACGACGGAAATTGAGCGT	TGTCACGCACCGGGATTTCA	346
FMgSSR-29941	scaffold392	p2	(GC)6	Class II	46788	46799	CCGAGCCGATCCCAAACATTCA	TGCATGTCGAGTGCGTGACT	262
FMgSSR-29942	scaffold10215	p2	(GC)6	Class II	19643	19654	CCGGGCCACGTTTTTGGTTGAA	TCGCCGGAAACGCAAAAGCA	328
FMgSSR-29943	scaffold7719	p2	(GC)6	Class II	26277	26288	CCGGTGATTCCGGACATGATGA	AGTTGCACTGGTGGAGTGCCTT	280
FMgSSR-29945	scaffold11535	p2	(GC)6	Class II	356	367	CGACGCGCTGATGGTTGCAAAT	TCGCGACTGTTTTGTGCCTCCA	309
FMgSSR-29948	scaffold4932	p2	(GC)6	Class II	5351	5362	CGCAAACCCAAACCCAAACCCA	AGCAGCACTGCAGCACAACA	212
FMgSSR-29949	scaffold496516	p2	(GC)6	Class II	173	184	CGCCACCGGTTAGGCTATAACA	ACCGTTTGACGCAGGTTTAGGG	209
FMgSSR-29967	scaffold58097	p2	(GC)6	Class II	215	226	GCATCGGTCGAAAAGATGCCGT	ACGTTGGACGTTCTGCGAGT	218
FMgSSR-29983	scaffold5214	p2	(GC)6	Class II	15976	15987	GCGGCATTGTTCCTGCTTCGTT	ACCAGTCGATCGCGCAACTA	222
FMgSSR-29984	scaffold12009	p2	(GC)6	Class II	4993	5004	GCGGCGGACTAAATTCGTGT	AACAGCCTGCCAAGTTCGCT	205
FMgSSR-29987	scaffold180	p2	(GC)6	Class II	84617	84628	GCTGCTTGCATGCCATTGCT	ACGGGTTTCGACGTTTCGCT	296
FMgSSR-29997	scaffold2874	p2	(GC)6	Class II	42387	42398	GTCGACAACGGCTGGATGTT	TGCGGCGGCAAATAGCCAAA	339
FMgSSR-29999	scaffold12941	p2	(GC)6	Class II	10575	10586	GTGTTCCGCGCGCAGTTTTT	GGCGCTCGCAGCTTTAACTCAA	343
FMgSSR-30000	scaffold19447	p2	(GC)6	Class II	3612	3623	TAAACCCATGCGCCACACGACT	ACTTGGGCCGGGCGTAAAGAAT	279
FMgSSR-30006	scaffold1785	p2	(GC)6	Class II	61822	61833	TAGTTCGCACGCACAGGCAT	TTCGCGCCATCTGCTGCATT	275
FMgSSR-30010	scaffold179	p2	(GC)6	Class II	67564	67575	TATTACCGACGGCGACAGCA	TATCAAGCGGGGAATGCGTGGT	249
FMgSSR-30012	scaffold14575	p2	(GC)6	Class II	11540	11551	TCAAATAGCCCCTGGTTTGGGAGC	TCAGCTTGTGGCCGCTGAAT	244
FMgSSR-30013	scaffold1417	p2	(GC)6	Class II	56689	56700	TCAACACGCAGCAGTCAGCA	GTGCCGTGCACGATTGCTTT	222
FMgSSR-30016	scaffold5084	p2	(GC)6	Class II	13187	13198	TCAGCACGTCCCTATTGCTTCCA	ATGCGCTTTCGGTTTGCGGA	250
FMgSSR-30017	scaffold12884	p2	(GC)6	Class II	21165	21176	TCATCATCACTAGAGCGGGCGT	AACACGCAGGCTGTTCCAATGC	256
FMgSSR-30030	scaffold71746	p2	(GC)6	Class II	673	684	TCCGAGACAAGAACGTGAGCACT	TCGCGCGCCTGTAAGCATTT	219
FMgSSR-30034	scaffold26222	p2	(GC)6	Class II	7749	7760	TCCTCCACCCGTTCTTGTCGAA	ACTGCCGCCAGACTCCAAAT	205
FMgSSR-30036	scaffold1701	p2	(GC)6	Class II	11046	11057	TCCTTGCTGGATTTCCCCCTCT	ATGTAACGTGCGGCATCAGGGT	311
FMgSSR-30039	scaffold6925	p2	(GC)6	Class II	6016	6027	TCGACGAGCCGTACGTACCTTA	AGTACGTCGACCCCCTCAAGAACT	321
FMgSSR-30042	scaffold18466	p2	(GC)6	Class II	9231	9242	TCGCCGCTGCTCCTGTTTTT	AGGCCCTGGCCCAATCAAACAA	303
FMgSSR-30043	scaffold7798	p2	(GC)6	Class II	30802	30813	TCGCCTCGCGAAATTGCACA	AACCGGACAACAGGGAGCATGT	344
FMgSSR-30044	scaffold13366	p2	(GC)6	Class II	9335	9346	TCGGATCTCGCGGGGCTTAAAA	TGACTCGCGCCAGCAAAACT	343

SSR_ID	Scaffold	SSR_Type	SSR_Motif	SSR Class	SSR_Start	SSR_End	Forward sequence	Reverse sequence	Product_size
FMgSSR-30049	scaffold199849	p2	(GC)6	Class II	505	516	TCGGGCACGAAGTTGGAGAAGA	ATGGAGCCCCTCGACACATTCA	257
FMgSSR-30052	scaffold321	p2	(GC)6	Class II	105781	105792	TCGTGCAGCTCTACGCATCCAA	TTAACACCGGCGCAAAGCGT	312
FMgSSR-30059	scaffold30474	p2	(GC)6	Class II	2959	2970	TCGTTTCGCTTTCCGACGCT	TCCAGAAAACGTGCCTGCGCTA	217
FMgSSR-30063	scaffold34635	p2	(GC)6	Class II	2928	2939	TCTGGATCGAAGGACGGCATGT	AACTTCCCCGGCGTCATGGATT	317
FMgSSR-30065	scaffold6003	p2	(GC)6	Class II	34522	34533	TGAATGCGTGGCCTGCGAAA	TTCTCAGTGGACGCGTGTTGGT	327
FMgSSR-30066	scaffold373	p2	(GC)6	Class II	58264	58275	TGAGATGCACCGGCTTAGCTGT	TTCGGGCTTCCGGCAGAAAA	304
FMgSSR-30067	scaffold15115	p2	(GC)6	Class II	8701	8712	TGATCGATGAGCGCGGTGAA	AAGAAGCTTCCCAGCAAGGCGA	260
FMgSSR-30068	scaffold3881	p2	(GC)6	Class II	13895	13906	TGATGATGGCTGCACTGCGA	AACGGGCGCTGCACAAAACT	328
FMgSSR-30070	scaffold4605	p2	(GC)6	Class II	965	976	TGATGGCATGGCAGCACACT	TGGGCGTTTTGCGCTTGGAT	319
FMgSSR-30074	scaffold1990	p2	(GC)6	Class II	40896	40907	TGCACACTGCTGTCGTTGGT	TCTGCAGCTCACTCCCGTTT	324
FMgSSR-30075	scaffold36076	p2	(GC)6	Class II	814	825	TGCACAGATGCCAGGTGCATGA	AGCAATGCAGCACTCCTCGTCA	255
FMgSSR-30079	scaffold24242	p2	(GC)6	Class II	6336	6347	TGCATCAAAGGCATCGGTGTCG	AAAATTCCATGCCCGGCTCCGT	261
FMgSSR-30080	scaffold2407	p2	(GC)6	Class II	18159	18170	TGCATTGCCTCAATTCGCTCGC	AGCACGCGCCAAATCAACCA	229
FMgSSR-30081	scaffold1073	p2	(GC)6	Class II	22050	22061	TGCATTTTTCGCCAACCGCCA	TCGGCTCCGGAATCACAAAGGA	321
FMgSSR-30091	scaffold105795	p2	(GC)6	Class II	417	428	TGCCATTCCGAGGCCAAGAAGT	ACAAAAATCCCAGCGTCCCC	235
FMgSSR-30092	scaffold52149	p2	(GC)6	Class II	1316	1327	TGCCGGAGCTGTCGCTTAACAT	ACCGGCAAAGATACTGACGCGA	245
FMgSSR-30094	scaffold2202	p2	(GC)6	Class II	21173	21184	TGCCGTTGCCCATTGCCATT	GATGCTTGCGCTCCGCTTTT	331
FMgSSR-30095	scaffold88	p2	(GC)6	Class II	134014	134025	TGCCTTCTCGGCACCAAAGGAAA	AGAAAGCGATGCGCGACACA	245
FMgSSR-30097	scaffold67773	p2	(GC)6	Class II	1811	1822	TGCCTTTGGAGCTCCTGGAT	GCGCTTTCGCCTTCATCTCGAA	220
FMgSSR-30100	scaffold236540	p2	(GC)6	Class II	365	376	TGCGCGGAGATATGTCGCTTGT	AAAATCCTTGGCCGCGCGTT	291
FMgSSR-30103	scaffold8839	p2	(GC)6	Class II	7939	7950	TGCGTAGCGTTTGAGGCAAC	TGCATTGGTGGGCGAATAGCCT	321
FMgSSR-30111	scaffold5120	p2	(GC)6	Class II	47946	47957	TGCTGCGACCTTGAAACGCT	CGATTGCTGTTGTCTGTGTGCG	261
FMgSSR-30112	scaffold68080	p2	(GC)6	Class II	638	649	TGCTGCTGCACGTAGTGCTT	TCACGTTTGCCGCGCACATT	276
FMgSSR-30113	scaffold2124	p2	(GC)6	Class II	22905	22916	TGCTGCTTCTTCCCCACGAA	AACCAAGCACCGGTTGCACT	295
FMgSSR-30114	scaffold1273	p2	(GC)6	Class II	54269	54280	TGCTTGCTTGCTTGCTGCAC	TGGACATTGGCAGGTCAACGGT	348
FMgSSR-30116	scaffold5748	p2	(GC)6	Class II	16362	16373	TGGAACCTCCCTGCCTTTTCGT	TGGTTGTGCATCATCGGCGT	339
FMgSSR-30117	scaffold3073	p2	(GC)6	Class II	31993	32004	TGGAGATGAAGAAGGCGGCGAA	GGCGCAAACTCGCAAGACAACA	275
FMgSSR-30121	scaffold2489	p2	(GC)6	Class II	21634	21645	TGGCGGAAAGGCAATGTCTCGT	ATTGGCAATCTAGGAGAGGCCG	336

SSR_ID	Scaffold	SSR_Type	SSR_Motif	SSR Class	SSR_Start	SSR_End	Forward sequence	Reverse sequence	Product_size
FMgSSR-30124	scaffold9191	p2	(GC)6	Class II	22904	22915	TGGGCGGCATCTGCGATTTT	TGCCCGTTGCAGGCTTTTTGT	231
FMgSSR-30128	scaffold773	p2	(GC)6	Class II	44908	44919	TGGTAGTAGCTTGCGCGCTT	TGGCTCAGGTTGCCACAAGT	280
FMgSSR-30130	scaffold840	p2	(GC)6	Class II	97462	97473	TGGTTTCGAGGCGTTGTGGGTT	ATGCATGCGCTCTGCGTTGT	307
FMgSSR-30131	scaffold5177	p2	(GC)6	Class II	9051	9062	TGTATCGCAGCCACCTCGTGTT	ACGGTGCTGAAAATTCGCCG	289
FMgSSR-30132	scaffold142739	p2	(GC)6	Class II	577	588	TGTCGATCAACCGCCGCAAT	ACCACATCAATCAGGTCCCGCA	238
FMgSSR-30136	scaffold526	p2	(GC)6	Class II	76065	76076	TGTGCCTGTGCACCCGAAAGAT	TGCGTCCGCGTGCAAATAGT	216
FMgSSR-30140	scaffold475	p2	(GC)6	Class II	91059	91070	TGTGGCGTGCGTACCTGTGTTA	ACGTTGGTGCTCAAACGGCA	279
FMgSSR-30142	scaffold19254	p2	(GC)6	Class II	13290	13301	TGTGTCGCCGCAAAAGCGAA	AAATCAAACGCGGCGCGGAA	315
FMgSSR-30143	scaffold5	p2	(GC)6	Class II	256826	256837	TGTGTTGTTGCCGTCCCTGT	TTGTGCTCACTGTCCACGCA	210
FMgSSR-30147	scaffold55	p2	(GC)6	Class II	99493	99504	TGTTGGCGCAAGAGCACGTT	TGCAGCTAGCTTCCCTGCTAGT	310
FMgSSR-30156	scaffold2720	p2	(GC)6	Class II	39887	39898	TTCAAAGCTGACCCGAGCGT	ATCTGCTCGACATGACAGGCGT	212
FMgSSR-30157	scaffold1158	p2	(GC)6	Class II	8144	8155	TTCAGCAGCAGTTACACCGCGT	ATAATCGTCGCGTCGTCCTCGT	250
FMgSSR-30158	scaffold14536	p2	(GC)6	Class II	8770	8781	TTCATGCCTGGGCCACATTCGT	ACCAACATTGGATGAGCTGGTG	203
FMgSSR-30167	scaffold744	p2	(GC)6	Class II	8273	8284	TTCGGAGAGCGCTGCAAGTT	TTGAATTGGTGTGCGCGGGA	297
FMgSSR-30171	scaffold524	p2	(GC)6	Class II	93899	93910	TTCTCGGTGTTGCAGCAGGT	AAGAAGTCGCGCATCTGGCT	233
FMgSSR-30172	scaffold7361	p2	(GC)6	Class II	17122	17133	TTCTCGTTCCTCTCCAAGGGATGG	GCGTTGCGGATGGCATTGAT	350
FMgSSR-30174	scaffold2131	p2	(GC)6	Class II	48635	48646	TTCTTGAGACTCCGCTTCCGCT	ATGATAGTGGTCGTTGTCGCCG	342
FMgSSR-30175	scaffold16329	p2	(GC)6	Class II	5148	5159	TTGAAGCAAAGCGCGCAAGC	AGCTTGCGTAACGCGGCAAA	286
FMgSSR-30179	scaffold110241	p2	(GC)6	Class II	99	110	TTGCTGGCTGGATCTCGGCAAT	TAATGCATGGTGGAGCCGCTGT	201
FMgSSR-30188	scaffold165	p2	(GC)6	Class II	62794	62805	TTTACACGAGCGCTTGGCCT	TTGGACCATGGCCTGTGCAAGT	344
FMgSSR-30196	scaffold5101	p2	(GC)6	Class II	39664	39675	TTTGCGGCTTCGATCGGTGGAT	ATGAATGCTGTCACGCTCACGC	316
FMgSSR-30212	scaffold4092	p2	(GC)6	Class II	47718	47729	TTTTGAGTGACGCACGCGCT	TGCACCGCTGAATTCTCCCCTT	269
FMgSSR-30227	scaffold167290	p2	(GC)7	Class II	243	256	AAGCCGCCTCGCCAAAAAGA	AGCGTTCCTTCAATGGGCGT	221
FMgSSR-30231	scaffold1200	p2	(GC)7	Class II	22099	22112	AATTCCCCTCGTTTACGCGTCC	ATCGGCAGGCACGACAGAAGAA	334
FMgSSR-30232	scaffold2332	p2	(GC)7	Class II	2646	2659	ACACTCCATTGCCGTCTCCA	AGCATCGCGACACAGGAACT	216
FMgSSR-30233	scaffold3693	p2	(GC)7	Class II	6619	6632	ACAGGCGTAAAAGGAGCGGACT	TTCAAAGCTGACCCGTGCGT	202
FMgSSR-30235	scaffold14224	p2	(GC)7	Class II	6223	6236	ACCGACCGCGTTATTTGTCCCA	ACGTGCTTTGTTGGCTCGTG	290
FMgSSR-30236	scaffold2968	p2	(GC)7	Class II	55329	55342	ACGAAGGTTGAAGCAGGTCAGGA	TCGAGCCGAGGCTGCAATTT	291

SSR_ID	Scaffold	SSR_Type	SSR_Motif	SSR Class	SSR_Start	SSR_End	Forward sequence	Reverse sequence	Product_size
FMgSSR-30237	scaffold117	p2	(GC)7	Class II	17008	17021	ACGGACGCATCACAAGCTCCAA	ATACTCGCGAAGCGGCCAAT	326
FMgSSR-30240	scaffold8488	p2	(GC)7	Class II	24488	24501	ACGTCGTCTTCGTCGCATCA	TGGATCAGCGCCACTTGCAT	242
FMgSSR-30242	scaffold126591	p2	(GC)7	Class II	418	431	ACGTTTTGCCACTGCTCGGT	AACCGCGTCAAAGCGGTCTT	230
FMgSSR-30243	scaffold5726	p2	(GC)7	Class II	34413	34426	ACTGAGGCCTTCATCTCCACCT	TTCTGGATCTTGGACTGCGTGTCG	267
FMgSSR-30250	scaffold126340	p2	(GC)7	Class II	355	368	AGCACACGGGCACCAGAAAA	CCGTGTTGTTGTTCTGCAGCGT	336
FMgSSR-30252	scaffold5455	p2	(GC)7	Class II	6704	6717	AGCGCCACAACAAACCCATAGC	CGCAGAATAATGCGTTGGCGCT	262
FMgSSR-30258	scaffold7574	p2	(GC)7	Class II	3679	3692	AGTGGTTCCGTTTTGTGGTGCC	TGCCAGCGGCTGCACAATTT	343
FMgSSR-30267	scaffold609	p2	(GC)7	Class II	42558	42571	ATGCGCAGGTGTCGTGTCAT	TGATCAAACGTGCATGCGGC	272
FMgSSR-30277	scaffold587	p2	(GC)7	Class II	72691	72704	CTGCAATGTTCACAGCGGCACA	TGCATGGCGCTCGAAAGTGA	200
FMgSSR-30283	scaffold9242	p2	(GC)7	Class II	442	455	GCGAGACGCAGCAAGGAAAACT	AAACCGTGCTGTCTCCGCAA	339
FMgSSR-30291	scaffold156221	p2	(GC)7	Class II	764	777	TCACGCGCGAAGATGGTCCTTA	ACAGCTACGCCCAGGTTGAT	281
FMgSSR-30294	scaffold162086	p2	(GC)7	Class II	206	219	TCGAGCGACACCATCTCGACTT	TGGCGTCTTGTAGCCGCTTT	330
FMgSSR-30298	scaffold4204	p2	(GC)7	Class II	19153	19166	TCTTCTTGACGGGGAAGACGGA	TTCCTGCATACCAACCGGCCTT	260
FMgSSR-30301	scaffold3058	p2	(GC)7	Class II	19625	19638	TGCAGGCCACAAATCGTCAGGT	AACGAGGACGGTTTGCGTGA	216
FMgSSR-30302	scaffold5120	p2	(GC)7	Class II	13278	13291	TGCAGGCGAAGAATGGAAGCCA	TCGCGACAAAACTCGCCGTT	293
FMgSSR-30306	scaffold51961	p2	(GC)7	Class II	1610	1623	TGGGAGAATGTGTCGTGGCAGA	AAAAGCGGCGTGTCGTCGTT	347
FMgSSR-30308	scaffold300	p2	(GC)7	Class II	35533	35546	TGGTTGACCGCACCAGACTCAA	TGGCGGCAATTGCGTGGAAA	336
FMgSSR-30314	scaffold105192	p2	(GC)7	Class II	628	641	TTAGGTGTGCGTGTGTCGTCGT	AAGAAGGCGGCCGCTAAGAAGT	338
FMgSSR-30315	scaffold31420	p2	(GC)7	Class II	1909	1922	TTCGCACCGCCAAAGGAACA	CGAATCATTGGCCGACATGGCT	326
FMgSSR-30319	scaffold924	p2	(GC)7	Class II	39948	39961	TTGAGAACGTGCCGGAGCTT	TGCACTGCAAGCCAGTGTGT	345
FMgSSR-30320	scaffold4364	p2	(GC)7	Class II	5203	5216	TTGCAGCTTTCGGGTGTGGT	AAGAGAACGTTCGCGGCTGT	272
FMgSSR-30322	scaffold594	p2	(GC)7	Class II	71723	71736	TTGCTGTTGCCAAGCCCGGATA	AAAGGCGCTGCGTTAGGTCT	327
FMgSSR-30323	scaffold7251	p2	(GC)7	Class II	31762	31775	TTGTGGATCGGAAAGGCTGGCT	AAAGGGGCCTTCGCGTAGTGAT	350
FMgSSR-30325	scaffold13232	p2	(GC)7	Class II	19939	19952	TTTGGCCCATTGGAAGCGGA	ACAGGCATTTCGCCGAGCAT	298
FMgSSR-30332	scaffold852	p2	(GC)8	Class II	16454	16469	ACGAAACACTGACGGGCACA	AGCTGCTGTACGTGGTGATGCT	318
FMgSSR-30333	scaffold9020	p2	(GC)8	Class II	3771	3786	ACGAGCGAGCAAAGCAGCAA	ATCACGACGTTGACGTGCCA	282
FMgSSR-30334	scaffold9340	p2	(GC)8	Class II	23965	23980	ACGCACAAACGAGGGCACTACT	ACACACCAGTCACGGCGAAA	259
FMgSSR-30336	scaffold3478	p2	(GC)8	Class II	45902	45917	ACTTGCAACGCTTCGCCTGA	AGCGCGCGGTGAATATAGGA	284

SSR_ID	Scaffold	SSR_Type	SSR_Motif	SSR Class	SSR_Start	SSR_End	Forward sequence	Reverse sequence	Product_size
FMgSSR-30338	scaffold320	p2	(GC)8	Class II	74212	74227	AGAGCGCTGCCCACTGTATTGA	ATGGCCAACACACGCTGCAT	331
FMgSSR-30347	scaffold692	p2	(GC)8	Class II	68452	68467	ATTCACACTGGGATCTCGCGCT	ACTTGCAACGCTTCGCCTGA	219
FMgSSR-30354	scaffold16	p2	(GC)8	Class II	169579	169594	GCGCAAAATTTCCGGCACCCTT	ACGAAGTAAAGCAGGAGCCACA	301
FMgSSR-30355	scaffold2005	p2	(GC)8	Class II	46330	46345	TATTCAAAGGGGGTGCCACGCA	TTGGCAATAAAGGCCGGGGT	224
FMgSSR-30356	scaffold174760	p2	(GC)8	Class II	427	442	TCGACGAACTCTGCCTTGCTCA	TCCAGGCAATGTCCAAGCGT	249
FMgSSR-30361	scaffold1919	p2	(GC)8	Class II	11047	11062	TGCGGAAAGTCTCATCGCACT	ACGCCAATTGACCACAGCGT	312
FMgSSR-30366	scaffold3146	p2	(GC)8	Class II	44140	44155	TTGGCGGGTTCTCTTGTTCCCA	TGTTGACCGTGTGGGCCATGAA	318
FMgSSR-30368	scaffold369992	p2	(GC)9	Class II	214	231	ATCGTGGGCCTTGTGTGCAT	AGGGGGTAGAGTAGCCAAACGACA	232
FMgSSR-30374	scaffold7952	p2	(GT)10	Class I	17230	17249	AAGCTTGAAATCCCCGCTGGCT	TTGTAGCGCCGAGCTCCAGAAA	307
FMgSSR-30378	scaffold1221	p2	(GT)10	Class I	91863	91882	ACAGAGGAAAGGATGCAACGGCA	GCCGGAGACGAAAATGGTGCAA	305
FMgSSR-30380	scaffold28327	p2	(GT)10	Class I	5725	5744	ACCATGACCTTGCTGGTCACCT	TGTGTTCCTGCCTGTGATCGT	284
FMgSSR-30384	scaffold32076	p2	(GT)10	Class I	3359	3378	ACCGTCCACAATTGATGATCCAGGG	TGAATTACCCTGTCGCCTCCCA	287
FMgSSR-30386	scaffold9386	p2	(GT)10	Class I	10660	10679	ACTTGAGCCCATCTGTCACCA	TGCCTCATCTTGCCTCAGCACA	280
FMgSSR-30393	scaffold9097	p2	(GT)10	Class I	23765	23784	AGCGATCATCTTGAACATCGGCG	GGCATGCATTTCTTGCTATGTGCC	253
FMgSSR-30398	scaffold576	p2	(GT)10	Class I	98273	98292	AGGTTGAGGTGCCTTGGTGACT	AAGGGCAACCGCAACAGCAA	320
FMgSSR-30405	scaffold12880	p2	(GT)10	Class I	19747	19766	ATGGAGCGGCATCAAGCGAA	AAACGGGGCTCGTGTGTGAA	342
FMgSSR-30407	scaffold230	p2	(GT)10	Class I	106366	106385	ATTGTCGCCGAAAAGCTCGGGA	AGCCAAGCCAGCACCAAGTT	350
FMgSSR-30408	scaffold4883	p2	(GT)10	Class I	23825	23844	ATTTGTTGGGCCAGATAGCCTCCG	ATCGAGGGAGCAGCCGATTGTT	285
FMgSSR-30409	scaffold7026	p2	(GT)10	Class I	29048	29067	ATTTTCTCCGCCTGCGTTGTGC	TCGAACGCGTCATCGAACACCT	258
FMgSSR-30411	scaffold5624	p2	(GT)10	Class I	42769	42788	CAGGGCAAAGAAGTGTGTGCGT	AGCCTGCATCTAATCGTAGCGTGC	332
FMgSSR-30414	scaffold219	p2	(GT)10	Class I	62889	62908	GCGCGATTCTATTCTGCTGCGT	AGCCCATGCCTGGGATTCTCAA	341
FMgSSR-30415	scaffold36579	p2	(GT)10	Class I	6503	6522	TCAGCTTTGCCATTGTCCGGGT	TTGTGCCGTCGCGAACGAAT	337
FMgSSR-30428	scaffold933	p2	(GT)10	Class I	17032	17051	TGCTGGGCCAAAAGGAATGAGC	AGTGCCTAACACACACGGCA	252
FMgSSR-30432	scaffold13689	p2	(GT)10	Class I	12987	13006	TGTGCCAAGATCCCACACTGCT	GCGTGGCGTTTGACGTTGTT	337
FMgSSR-30435	scaffold26312	p2	(GT)10	Class I	5008	5027	TTCCTGCAGCCGATTCTCAGCA	AAAGGATGAAGGCTCCTCCCCA	304
FMgSSR-30436	scaffold907	p2	(GT)10	Class I	67470	67489	TTCGAAACGACCCGGCGAAA	ACGGATCCAACCGCAAACCA	222
FMgSSR-30439	scaffold1230	p2	(GT)10	Class I	41433	41452	TTTGTGATGCCGCACACGCT	TTGCTTAAGCCCCGGGGAACAT	214
FMgSSR-30440	scaffold18294	p2	(GT)11	Class I	9086	9107	AAGCGCTTTGCGAAAAGGACCG	TCGCCACGGAATGCTTTGTTGC	276

SSR_ID	Scaffold	SSR_Type	SSR_Motif	SSR Class	SSR_Start	SSR_End	Forward sequence	Reverse sequence	Product_size
FMgSSR-30442	scaffold16	p2	(GT)11	Class I	237947	237968	ACGACTTCTTCGGCGCCATT	TGCCCGTATGTGGCATGTAGCA	225
FMgSSR-30446	scaffold1908	p2	(GT)11	Class I	5841	5862	AGCTTGAGGAGCTCGGAGTCAA	ATACAAGTTTACGACCCCCTCCCC	280
FMgSSR-30450	scaffold37	p2	(GT)11	Class I	62635	62656	ATCGTTGATCTCGCGCTGGCTT	TGTTGCCATGATCGGCCCAACT	241
FMgSSR-30461	scaffold405	p2	(GT)11	Class I	76527	76548	TGTCCTCACCACGGCCATACAA	AAGTCTGTTGGGACCAAGCCCT	293
FMgSSR-30462	scaffold8027	p2	(GT)11	Class I	20101	20122	TGTGGTGCCTAACTGGAGCA	AAGGTGCTTGTACGGGTGGCTT	326
FMgSSR-30465	scaffold5452	p2	(GT)11	Class I	4159	4180	TTGGCGTTGTGGTCTAACGGGT	CCGCTGCAACAAACCAACCGAA	253
FMgSSR-30466	scaffold876	p2	(GT)11	Class I	9137	9158	TTGTGCTCGCCTTGGTAGGTGT	ACAAAACTCGAGCGAGCGTGGA	260
FMgSSR-30470	scaffold2420	p2	(GT)12	Class I	3097	3120	ACATCTGCTTCGCCCGTTCT	TTTCATGCACGCGTTCCCCA	250
FMgSSR-30473	scaffold2768	p2	(GT)12	Class I	30016	30039	GGGCTTCTTTTTCTTCCCCTGGCT	ACTGCCTGTTTCCCATCCAACCT	332
FMgSSR-30475	scaffold14	p2	(GT)12	Class I	133634	133657	TCGAGGCTTGCGTTGGCTACAA	ATGCGCGCACAGACCTCAAA	232
FMgSSR-30476	scaffold154	p2	(GT)12	Class I	123671	123694	TCGGGATCGCGCGTACGTTAAA	TGCTGGATGTTCACGAGCCACT	341
FMgSSR-30477	scaffold58	p2	(GT)12	Class I	69605	69628	TCGTATCGGGCGCCACACATTT	TCATGGTGGTCGTTGTCGTCGT	333
FMgSSR-30480	scaffold5604	p2	(GT)12	Class I	10711	10734	TGCGTTGTCGTGGGCATTGT	TCTGCATGTGCACTCGCTGTT	286
FMgSSR-30487	scaffold2141	p2	(GT)13	Class I	4889	4914	ACACCATCGTCACCACCACTGA	TTTGTTGATCGGTCGTCGCCGT	348
FMgSSR-30488	scaffold3008	p2	(GT)13	Class I	36443	36468	ACGGATGTGCGCAAAGTGCAA	TCGTCGTGGTTTGCTGGTGGTA	319
FMgSSR-30489	scaffold1152	p2	(GT)13	Class I	61528	61553	AGAGGGTGGAGGCACAAGAAGT	AAGCTCGCTAGCAAGGCCTCAT	346
FMgSSR-30490	scaffold4	p2	(GT)13	Class I	158114	158139	AGCCTCTGTGTTAGGCAAGAGT	TCGAATGACCGTTGGCCTTGCT	244
FMgSSR-30491	scaffold5015	p2	(GT)13	Class I	34440	34465	CGCGCACAGCTTGCTCAAAA	TCGTTGCCCCTGCCATTGGTTA	282
FMgSSR-30495	scaffold2142	p2	(GT)13	Class I	57908	57933	GCGGGTTGAAAGTGTTGAACAGAC	TGGAGTAATGTGCTGCTTGGCCT	300
FMgSSR-30497	scaffold170881	p2	(GT)13	Class I	588	613	TGCGTATTTCCGGGTTGCACA	GCAAGCACCACCACACACACAT	325
FMgSSR-30498	scaffold22018	p2	(GT)13	Class I	10528	10553	TGGCCCTAATTCTGCTGGAGATCG	ACAAAGCGGCCCATGCATCA	327
FMgSSR-30500	scaffold1	p2	(GT)13	Class I	21494	21519	TTGTGAAGCGTGCAGGTCGAGA	ACGGTTCCTGACGTCTGCGTTT	214
FMgSSR-30501	scaffold8132	p2	(GT)13	Class I	26750	26775	TTTGGTAACATGCGCGGCAC	TTTATCCACCGCAGGCAGCA	338
FMgSSR-30502	scaffold2019	p2	(GT)14	Class I	25827	25854	AAAACGTTGTGCCTGGCAACGG	TGCGTGGCATTTTTCTCCGCA	266
FMgSSR-30503	scaffold26776	p2	(GT)14	Class I	2163	2190	ACAGCGCCTCTTCGACAACACA	AACACACGCACACAGAGCAC	335
FMgSSR-30510	scaffold846	p2	(GT)14	Class I	21293	21320	ATTTCTGCAAAGGGGCGGATCG	TGGGATGGTGTGCAGCATGTGT	248
FMgSSR-30515	scaffold505	p2	(GT)14	Class I	70632	70659	TCCTGATTCCTGCTGCGTTCGT	TGCGCTAGCTTGGTTGCTGA	330
FMgSSR-30516	scaffold2736	p2	(GT)14	Class I	24427	24454	TTGCACGCTCAGACGAACTCCA	ATTTGAGTCGCGCTGGTCCT	311

SSR_ID	Scaffold	SSR_Type	SSR_Motif	SSR Class	SSR_Start	SSR_End	Forward sequence	Reverse sequence	Product_size
FMgSSR-30517	scaffold21	p2	(GT)14	Class I	233800	233827	TTGGCTGCTTTGCTTTGCGG	TTGTTTGGCCAGAACGGGCA	328
FMgSSR-30519	scaffold113	p2	(GT)15	Class I	52874	52903	GCCCTCTTGTGGGCTTGCAAAA	AGGCCACACATCATCGGCCATT	331
FMgSSR-30521	scaffold7064	p2	(GT)15	Class I	16246	16275	TCGTCCTTTTGTGGCCTCCTGT	TTGTCAAAGCCGAGGCCCAGAT	340
FMgSSR-30524	scaffold1570	p2	(GT)16	Class I	71899	71930	AACTTGATTGGCCCGCACGA	GCATGATTATGCATGAGCCCCT	334
FMgSSR-30525	scaffold1072	p2	(GT)16	Class I	64748	64779	ACAGACAGGGCAGAGCATGA	CACGCACGCACACGTTGAAA	291
FMgSSR-30526	scaffold1043	p2	(GT)16	Class I	45874	45905	ACGCATCAGTGGAGGTGAGCTT	TTGGTCTCGCTCTATCGCTGCT	295
FMgSSR-30528	scaffold318	p2	(GT)16	Class I	113693	113724	AGTGAGCACGCGAGCTGTATGT	AATGGCCAAGCAACGCACCT	310
FMgSSR-30531	scaffold611	p2	(GT)16	Class I	108130	108161	TAGCTCCCAAACCTCCCACCAA	ATGCGCGCAGCCATGTTGAA	332
FMgSSR-30535	scaffold27397	p2	(GT)16	Class I	1470	1501	TGCATGTGTGTGTCTGTGCG	TTGGCAGCCGTCATTTCCGA	245
FMgSSR-30536	scaffold10223	p2	(GT)16	Class I	7332	7363	TGCCGACGTCGATCAATGGCTT	TGCATGCACGCCTGTTGCTT	336
FMgSSR-30540	scaffold1676	p2	(GT)16	Class I	27815	27846	TTCACAAGCCGTGCCCGTTT	AGCACTTTCCCACCGCGATT	311
FMgSSR-30541	scaffold318	p2	(GT)16	Class I	115046	115077	TTTTTGCCGACGTGGCTGCT	ACCCCCTGAAATGGCCAAGCAA	304
FMgSSR-30544	scaffold634	p2	(GT)18	Class I	94374	94409	AAAAGAAGGCAAGGCAGCGTCG	TCGGACTTGATTCACGCCTGCT	285
FMgSSR-30545	scaffold357	p2	(GT)18	Class I	26748	26783	AAGCCTGTGAAGCATCGCCA	ACGCCAAACAGCACACTCCT	327
FMgSSR-30551	scaffold3918	p2	(GT)18	Class I	32262	32297	TGGCCCTTGATTCTGAGCCT	ACGTGTGTTCACTTGCGCTTGG	255
FMgSSR-30553	scaffold94	p2	(GT)19	Class I	87410	87447	ATTGCCCGCGTGCTGATGAT	TTGGAGTGAAGCACGGAGACCA	325
FMgSSR-30555	scaffold792	p2	(GT)20	Class I	60777	60816	AACGCTTGTGTGTGCGTCGT	AGGAGGAGCGACCACCTGAAAA	322
FMgSSR-30557	scaffold914	p2	(GT)20	Class I	83423	83462	AGCAGGTAAAAGGGAGGAGGGTGT	AACGGTGGGCGTAAAACCTAGC	243
FMgSSR-30560	scaffold47	p2	(GT)20	Class I	216312	216351	AGTGATGTGCATGGGCCACAA	TGCAGCTCCCCCAGTTTCAGTT	307
FMgSSR-30562	scaffold31527	p2	(GT)20	Class I	1152	1191	TGCCCTCTCCTTTAGTCACAGCA	CCGTCTCTAGTGATTTTTCCCGTG	206
FMgSSR-30566	scaffold3890	p2	(GT)21	Class I	18546	18587	AGCCGAAGCTAGCTCTAACTGGA	GCAACAGACTGATCCTGTCCTCGT	289
FMgSSR-30567	scaffold50930	p2	(GT)21	Class I	1797	1838	TGATGGCCAGTGTGTTGTAGCC	ATAAGGCCAAGGCAGAGGATACGG	346
FMgSSR-30574	scaffold460	p2	(GT)25	Class I	96603	96652	GGTTAGTGGAAAGGACCAAGCAGA	ATGTGATGGTTGGGGTAGGGGA	320
FMgSSR-30581	scaffold6000	p2	(GT)28	Class I	8753	8808	TTGTGCCTCGTGTAGCTATGCC	AGCCCCAAGTTCTCACACACCT	347
FMgSSR-30582	scaffold3269	p2	(GT)29	Class I	33536	33593	AATGACGTCACGAGGAGGCAGA	ACACATGCATTGCGCCACGA	308
FMgSSR-30588	scaffold213	p2	(GT)36	Class I	54423	54494	GGCCCTCCTCCATCATTACACGTT	AGCGGAAGCAAGGGCAGGAAAT	337
FMgSSR-30589	scaffold7904	p2	(GT)37	Class I	16200	16273	TGTGCCCAATGTTCAAGCCAAG	ACAACGGCCATTGCATCGGT	241
FMgSSR-30590	scaffold5390	p2	(GT)6	Class II	9633	9644	AAAACGTGGTACTGGCGCTTGC	ACCGTCACATTGCCATCGCT	320

SSR_ID	Scaffold	SSR_Type	SSR_Motif	SSR Class	SSR_Start	SSR_End	Forward sequence	Reverse sequence	Product_size
FMgSSR-30592	scaffold1306	p2	(GT)6	Class II	12212	12223	AAACGCGGGGATCTTTGGCT	TGACACGCCTCGGTTGTATGTGT	266
FMgSSR-30596	scaffold655	p2	(GT)6	Class II	88031	88042	AAAGCATCAGCGAGGGTAGCGT	TTCCGCCCGGCTTCAAAACT	340
FMgSSR-30607	scaffold806	p2	(GT)6	Class II	61523	61534	AACCGTAACCTGACAGCGCCTT	ATACACAAAGCCACGCCATCGG	320
FMgSSR-30611	scaffold39740	p2	(GT)6	Class II	3623	3634	AACGTCGAGATCGTTGAGCCGT	GCTTCATCTCGCGCAAAGCCAA	317
FMgSSR-30612	scaffold11899	p2	(GT)6	Class II	1387	1398	AACTGCGAAGGATTTGGCTGGC	AATAACGCCGGTTGCTGGCT	339
FMgSSR-30614	scaffold279	p2	(GT)6	Class II	2391	2402	AACTTGGAGCTGGCAACCACGA	ATTTCGTGGACGGGAACATGCG	330
FMgSSR-30622	scaffold10915	p2	(GT)6	Class II	1602	1613	AAGCATCCAGGGCTGTGAGGAA	CGCCCATCATCCATCACAAACA	322
FMgSSR-30627	scaffold9328	p2	(GT)6	Class II	26827	26838	AAGGGTGTGCGTGTCTTGCAGT	AATAGAGGGCATCAGGAGCACGGA	281
FMgSSR-30640	scaffold11382	p2	(GT)6	Class II	5564	5575	AAGTGGGGTGCAATGGCGTT	TCCCATCGTTGGTTGAGGGCAT	222
FMgSSR-30652	scaffold625	p2	(GT)6	Class II	36125	36136	ACACGGTGCATTTGCTGTGGT	TCGTGCAGTGCAGAGCTGGAAA	286
FMgSSR-30658	scaffold10944	p2	(GT)6	Class II	7632	7643	ACATGGTGACGGTACATGGATCTG	TCATTAGCTTCCACACACACGC	340
FMgSSR-30664	scaffold278380	p2	(GT)6	Class II	410	421	ACCACCACCACGTCGTTCACAT	TGCCATTGCTCACTCACAGCA	335
FMgSSR-30670	scaffold5226	p2	(GT)6	Class II	15481	15492	ACCATCACCTGACGGTTCTCCT	AGACAGAAGCACCCAAGCCTCA	250
FMgSSR-30675	scaffold873	p2	(GT)6	Class II	80154	80165	ACCCTGATTAACTGTGCGTGGCA	TTTTTGCGTGACAGCCGCGA	322
FMgSSR-30676	scaffold1731	p2	(GT)6	Class II	49863	49874	ACCGAGCCACGGTAAAAGGT	ACGTCATCGTCACCTTGCAGGA	224
FMgSSR-30683	scaffold22390	p2	(GT)6	Class II	6282	6293	ACGACGCCTTTTGGAGATGGCA	TCCAGCTTGTGCACGTCCTT	290
FMgSSR-30685	scaffold12154	p2	(GT)6	Class II	18725	18736	ACGATGACGACAGCTGCAACCA	ACGACCCAGACGTCACATGCTT	343
FMgSSR-30686	scaffold1457	p2	(GT)6	Class II	51882	51893	ACGCACCATTTGTAGGCGTCA	ACGTCAAACGGCAACAGTGC	317
FMgSSR-30687	scaffold41292	p2	(GT)6	Class II	317	328	ACGCCCTGAGATCTGCGACTAA	AGAAGCAGGCCTCCATGTTGGT	275
FMgSSR-30690	scaffold2870	p2	(GT)6	Class II	12822	12833	ACGCGGTACCCATGCGTTTT	AACGGCCCTTGGTGAATGGGAA	307
FMgSSR-30700	scaffold192	p2	(GT)6	Class II	132023	132034	ACGGAAGCTTTGCAGCCGAA	TACGTTAACGCACCCGCACTCA	336
FMgSSR-30702	scaffold1731	p2	(GT)6	Class II	49733	49744	ACGGACACCTTGGTCAAAGCCA	GCTCGGTGCCAGAGCTTTCTTT	212
FMgSSR-30704	scaffold23809	p2	(GT)6	Class II	7577	7588	ACGGAGGAGGATCCTGTGACAA	TCCTCGCCAGGGAAGTTCGTAA	236
FMgSSR-30706	scaffold15306	p2	(GT)6	Class II	19281	19292	ACGGGGCAAATACGAACGCA	GCGAGCTTCTGTTTGGCTGCTT	213
FMgSSR-30710	scaffold77	p2	(GT)6	Class II	19960	19971	ACGTACCACTGCACGTACGCAT	ATGAAGGCCTGAACTGCGGCTT	308
FMgSSR-30711	scaffold6627	p2	(GT)6	Class II	47	58	ACGTGCTCCTCTCTTCTAGCGT	TGTCACAGTTCCGCGAGAGCAA	262
FMgSSR-30713	scaffold3150	p2	(GT)6	Class II	14250	14261	ACTCCAAACCGGCAGCATGA	TTACACGCGGCAGTGTCCAAGT	305
FMgSSR-30715	scaffold5093	p2	(GT)6	Class II	17072	17083	ACTGCTGTTTCGGGTTTCGCT	TTGTTGGGTGGTGGGAGGTCTT	313

SSR_ID	Scaffold	SSR_Type	SSR_Motif	SSR Class	SSR_Start	SSR_End	Forward sequence	Reverse sequence	Product_size
FMgSSR-30718	scaffold174	p2	(GT)6	Class II	11888	11899	ACTGTACGCGTGCAACAACAGC	AATCCTGTCAGCGGGCCAAA	312
FMgSSR-30740	scaffold2527	p2	(GT)6	Class II	37535	37546	AGCACAGCGGCACACGATAA	TGCTTGTGCGTTGCTCTACAGT	295
FMgSSR-30748	scaffold6	p2	(GT)6	Class II	51824	51835	AGCAGCAGCAACACGAGGAA	AACTGCGCGGACCACACAATCT	285
FMgSSR-30753	scaffold999	p2	(GT)6	Class II	39928	39939	AGCATGAGGGGGAAAGGTGT	TGTACGTAATATGGGCGGCGCT	202
FMgSSR-30754	scaffold9077	p2	(GT)6	Class II	3143	3154	AGCCAAGCTACCATCAACACCCA	AATGTGTGCGCCCGAGGTTT	295
FMgSSR-30756	scaffold10548	p2	(GT)6	Class II	22300	22311	AGCCCAAGAGGCTTGTAACCGT	AGTTCACGAAGATGCCGCGT	239
FMgSSR-30762	scaffold8259	p2	(GT)6	Class II	19747	19758	AGCGACGTCGTCATTGCTGT	TTTCGGCGTCGTGGCTTCAA	303
FMgSSR-30763	scaffold408	p2	(GT)6	Class II	1500	1511	AGCGCTGAATAAACCGGTCCGA	TTTCTTCCCCCGCGTTCGTT	293
FMgSSR-30765	scaffold1318	p2	(GT)6	Class II	75336	75347	AGCTACACTACACGGAGCAGCA	ACATGGAGCAAGAAGGCACGGA	335
FMgSSR-30768	scaffold4974	p2	(GT)6	Class II	30480	30491	AGCTATCGCGCACGAGAACCAA	TGCGACTTGTGACAATACCCCGA	340
FMgSSR-30778	scaffold251602	p2	(GT)6	Class II	308	319	AGGACTCTGGCGTGTCATTTGCT	GCCACATACATTTATCGATCCCCC	338
FMgSSR-30788	scaffold808	p2	(GT)6	Class II	100714	100725	AGGGGCGATCGTACCAAACA	ACAACATGCAACGATGGACGC	318
FMgSSR-30789	scaffold10342	p2	(GT)6	Class II	22392	22403	AGGGTGCATTTCGGAAGGGAGAGA	CCAACGCACCTGCGAAAGTGAA	349
FMgSSR-30795	scaffold630	p2	(GT)6	Class II	47000	47011	AGGTTGCTTTGGACTTGCCCGA	AGCATGCCGTTCTGCCATGA	338
FMgSSR-30796	scaffold10761	p2	(GT)6	Class II	18844	18855	AGTATTAAGGTTCATCGCCCCCGC	TGCAACTTGCTGGTTTGCCT	269
FMgSSR-30798	scaffold20552	p2	(GT)6	Class II	5646	5657	AGTGCCACCACAGGTTCCGATT	ACGATCATCTTCTTGCGTGCCC	300
FMgSSR-30800	scaffold9637	p2	(GT)6	Class II	6496	6507	AGTGCCTTGAGGATTGCGCT	AGGTAGCATGCCTCGTGCACTT	241
FMgSSR-30810	scaffold21	p2	(GT)6	Class II	231207	231218	ATAGGGCTGTGATTTTGCCGGG	TGTCCTCAGGGCACGAAATGGT	301
FMgSSR-30811	scaffold4313	p2	(GT)6	Class II	37142	37153	ATAGGTAGCACAGCCACACGCA	CCGTAGTGGCCTTAATCTTACGC	350
FMgSSR-30817	scaffold265	p2	(GT)6	Class II	62946	62957	ATCGATGGGAGAGCGATGCGTT	AGCCCATCCAGGTTTCATCCCT	292
FMgSSR-30819	scaffold140	p2	(GT)6	Class II	173885	173896	ATCGCGCACAAGATAGAGCCGT	ACAGCACGGTTTCCACGCAA	227
FMgSSR-30823	scaffold12858	p2	(GT)6	Class II	14253	14264	ATCGGCAAGCTACAGCCGGTTA	AGCCTGCATGAGGCAACAGCTT	282
FMgSSR-30830	scaffold4615	p2	(GT)6	Class II	30876	30887	ATGCGAAACCACCGTGTGTGTG	AGGTGCAGGTTTGGGCATGTGT	343
FMgSSR-30838	scaffold9605	p2	(GT)6	Class II	17855	17866	ATGGCGGGAAGCGAGAAGGAAT	TGCCATCACGTGTAGTGGTCGT	345
FMgSSR-30839	scaffold326	p2	(GT)6	Class II	133953	133964	ATGGCTTGTTGGCGCGTTCA	TCTTCCTCGCCATCGGTGACTT	322
FMgSSR-30844	scaffold416	p2	(GT)6	Class II	60417	60428	ATGTGCAGTGCGCGGCTTTA	ATTGCCCGCACGGCATTGAA	338
FMgSSR-30845	scaffold1197	p2	(GT)6	Class II	69557	69568	ATGTTGTTGGGGCATCAGGAGC	TCGTGTGCCGCTCTTCTCTCTT	297
FMgSSR-30846	scaffold760	p2	(GT)6	Class II	75180	75191	ATTACGGGAGTGTCTCGTGGGA	TTTATGCCTGACTGGGCGAGCA	200

SSR_ID	Scaffold	SSR_Type	SSR_Motif	SSR Class	SSR_Start	SSR_End	Forward sequence	Reverse sequence	Product_size
FMgSSR-30848	scaffold6648	p2	(GT)6	Class II	23205	23216	ATTGACCAAGGAACCACCGCCA	TTTGAGTGAAGGGCAAGGTCAC	304
FMgSSR-30849	scaffold20364	p2	(GT)6	Class II	8281	8292	ATTGCGTCGCCATGGGTGTT	AAAACCACCCCGGTGCTCTACA	294
FMgSSR-30853	scaffold58481	p2	(GT)6	Class II	330	341	CACAGAGTCACATCCTTGAGGCA	TGATGCTGCACTTGTGACGGGT	350
FMgSSR-30855	scaffold284	p2	(GT)6	Class II	29571	29582	CAGACACCACAAAAAGGTGGTAGG	TCAGACTGCAACACCGACTGT	295
FMgSSR-30862	scaffold32534	p2	(GT)6	Class II	3267	3278	CCGCGTGAAGTTGATGGTCGTT	ACACACGGCTCAGCGAGGAAAA	313
FMgSSR-30865	scaffold728	p2	(GT)6	Class II	65057	65068	CCGTTGCCGTTGAGCAACAT	TGTTACGCCCTTCCTTGCCT	218
FMgSSR-30866	scaffold1655	p2	(GT)6	Class II	26738	26749	CCTGTGAGCAATGCAGCTGAGA	TCAGCGCAGCGCCTCTTTTT	257
FMgSSR-30884	scaffold16512	p2	(GT)6	Class II	5085	5096	GCACGCATGTGCTGCTTCAA	ACACACGTTGGCCTGGTGACTT	315
FMgSSR-30889	scaffold15827	p2	(GT)6	Class II	18868	18879	GCCAAATTCCCTTGACTCGACCCT	ACAGAGGTTCACAGCCCTTGCT	279
FMgSSR-30891	scaffold14669	p2	(GT)6	Class II	17059	17070	GCCTTTCTTTTCGCAAGAGGCA	AGCGCAGGCCACATGCTTAT	273
FMgSSR-30892	scaffold3455	p2	(GT)6	Class II	60556	60567	GCGCACGTTTTCGCTCTGTGAT	ACAAAGTGGTTCGCGAAGTGGC	200
FMgSSR-30894	scaffold50372	p2	(GT)6	Class II	2072	2083	GCGGGAGTTGGAGAAGAGGAGTTT	GCGTGGAAACCTCGGCAAACAA	334
FMgSSR-30896	scaffold4682	p2	(GT)6	Class II	12032	12043	GCTAGCAATCCGACAGTTTTCCAC	TCGCAGTGATCCCCATTCAGCA	255
FMgSSR-30910	scaffold1542	p2	(GT)6	Class II	51013	51024	GCTGAAGCTTTTGACTCGGCGT	GCGTGCGGCAGGCAAGTATTTT	289
FMgSSR-30915	scaffold2256	p2	(GT)6	Class II	21068	21079	GGATCAGATTGCTCCATGCTTTCC	ACCCATTACAGTGAGCACCCCT	338
FMgSSR-30916	scaffold7642	p2	(GT)6	Class II	22582	22593	GGCCGTACATAACTCCAGCACCAT	TGCAAACGCATGACCCTTGG	344
FMgSSR-30920	scaffold14806	p2	(GT)6	Class II	12919	12930	GGGACCAGCTTGTTGCGTTCAA	TGCAAACATGGCGGAAGGTCAC	342
FMgSSR-30924	scaffold2427	p2	(GT)6	Class II	62466	62477	GGTCTTGTTTTGTGCGCGGTGT	AATGGCGTGCCTTCCTCACACA	208
FMgSSR-30935	scaffold71	p2	(GT)6	Class II	116294	116305	TACACTGACGCGCTCTGTTCGT	TGCTGATCTTGGAGCTGGGCAT	340
FMgSSR-30940	scaffold3179	p2	(GT)6	Class II	22381	22392	TAGCCCAAAACGAACGAGCCCT	TGCCACAGCTTTCCGGTGTT	296
FMgSSR-30941	scaffold7955	p2	(GT)6	Class II	16058	16069	TAGCTGAAGGCGTACGCAACCT	ACCACACACGCGTACACACGAA	296
FMgSSR-30945	scaffold2968	p2	(GT)6	Class II	26453	26464	TATTCGCGCTTGGCTGGGTT	AGACGATGCATTTGCCGTGCT	336
FMgSSR-30949	scaffold13305	p2	(GT)6	Class II	18169	18180	TCACCTTGTGCATGCAGCCT	TGGACGAATTTTCAAGGCGCGA	224
FMgSSR-30950	scaffold1230	p2	(GT)6	Class II	63542	63553	TCACTCACTGTCACTGGCGT	AAGCAACTCGAAGCGTGCGT	338
FMgSSR-30952	scaffold311	p2	(GT)6	Class II	107811	107822	TCAGAGGCAGCTTTTGGTGTCGT	TCATTCGTCGTTCTCGCGTTCG	250
FMgSSR-30955	scaffold32596	p2	(GT)6	Class II	3656	3667	TCATCGGCTTTTAGGGTGGTGG	TTCGCGTGACGAGCCAATGT	307
FMgSSR-30956	scaffold131018	p2	(GT)6	Class II	541	552	TCATGCACGCCGGTTTTCCT	TGAACCCCTGGAACACTCGT	350
FMgSSR-30960	scaffold6961	p2	(GT)6	Class II	3131	3142	TCCACTCACAGCAACCAACCCA	CGAAATGCCGCTGTTCGTCA	323

SSR_ID	Scaffold	SSR_Type	SSR_Motif	SSR Class	SSR_Start	SSR_End	Forward sequence	Reverse sequence	Product_size
FMgSSR-30965	scaffold352	p2	(GT)6	Class II	23401	23412	TCCCGCTTTCGCCGTTTTCA	TCGTGGAATCCCGCTCTTGGTT	254
FMgSSR-30970	scaffold14045	p2	(GT)6	Class II	7263	7274	TCCGGCACGTTTGTCACTCGAT	AACTCCCCTGCTCCCTGCAAAA	310
FMgSSR-30972	scaffold253	p2	(GT)6	Class II	26713	26724	TCCGGTTCATGTCAGCGTCACT	AGACCACACGCAGCAAAGCA	260
FMgSSR-30977	scaffold6661	p2	(GT)6	Class II	4610	4621	TCCTCGTCATGCGCTGAAGTCT	TCCCCAAGGCCTGGAGTTTT	214
FMgSSR-30980	scaffold25553	p2	(GT)6	Class II	2486	2497	TCGAGCACGATGCAAGCACA	TGCAGCTCATGGACGTTGGGAA	349
FMgSSR-30993	scaffold856	p2	(GT)6	Class II	60136	60147	TCGCGTGAGTCGTTTCCTGCTT	TTTCTTCGTCCTCCATGCCTCAGC	325
FMgSSR-30998	scaffold1098	p2	(GT)6	Class II	82455	82466	TCGGAGTTTCATCTCAGCTGGC	TCACACAGCAGTTCGCCGTT	290
FMgSSR-31009	scaffold3353	p2	(GT)6	Class II	31929	31940	TCGTTTGGCTGTCCTCGCAA	AACACGAGCCAAGCACCTTCCA	265
FMgSSR-31015	scaffold15037	p2	(GT)6	Class II	16654	16665	TGACAAATGCCAACGCCGGA	ACTGGCTGGCAGCAGCTTTA	290
FMgSSR-31024	scaffold17886	p2	(GT)6	Class II	9799	9810	TGCAAACCGGCACGAACGAA	TCGGTGGCTCAACTCATCACCT	214
FMgSSR-31025	scaffold9353	p2	(GT)6	Class II	18683	18694	TGCAAACGGGCGGACATGAA	TCCCGCAGGGTACCATCACACTTA	299
FMgSSR-31028	scaffold8482	p2	(GT)6	Class II	27226	27237	TGCAATGGGCACCCGTCTTT	TGAATCTCGGTTGTGCCGCCTT	298
FMgSSR-31032	scaffold179	p2	(GT)6	Class II	98688	98699	TGCACGCGTCTCGGTTGATA	TACGTCGCCAGCACCGAAAA	324
FMgSSR-31038	scaffold2778	p2	(GT)6	Class II	11334	11345	TGCAGCAGTCAGTCCCTCCTATGT	AGCACTGGGATGTGCAACAGAC	208
FMgSSR-31040	scaffold8572	p2	(GT)6	Class II	11493	11504	TGCATGCATGGTGTCTGTACTGC	AACGCGAGGGCTTTTTCGCA	308
FMgSSR-31041	scaffold4936	p2	(GT)6	Class II	4344	4355	TGCATGCCAGCTGGAAGGATCA	TACGTGCTCTGTTCGCAGTCCT	308
FMgSSR-31042	scaffold5157	p2	(GT)6	Class II	12136	12147	TGCATGGTAGTTAGGAACGGCGTG	TAGCTGCGCTGTCCTGAAATCC	200
FMgSSR-31048	scaffold2098	p2	(GT)6	Class II	26369	26380	TGCCAAAGAGCTCGGGTGTT	TCCATGGCCTCAGGTAATAGTGC	347
FMgSSR-31051	scaffold2811	p2	(GT)6	Class II	15310	15321	TGCCCATGGTGGTGTTGTCAGT	AAGCACTTGCAGATCGCCGT	299
FMgSSR-31053	scaffold9029	p2	(GT)6	Class II	12872	12883	TGCCGTTGCCGACTTGTCTT	GCAGGGCTTCTAGCGTTGCATT	258
FMgSSR-31061	scaffold6401	p2	(GT)6	Class II	17132	17143	TGCGGAGTGTTTGCCCTTCA	TGTGGCCTCTGCGTTTACGA	242
FMgSSR-31067	scaffold387	p2	(GT)6	Class II	37795	37806	TGCTCCAACCATATGAGCCGCTT	TTCCACTTCCACCCACTGCT	214
FMgSSR-31071	scaffold7803	p2	(GT)6	Class II	8705	8716	TGCTCCTTTGGAATCTGACCAGCA	ACGAGGGGAAGCTTTGCCCTTTA	213
FMgSSR-31072	scaffold1885	p2	(GT)6	Class II	49931	49942	TGCTCGGGCAAGCAATGCAA	AGGTGCACCACGTCACGATCAA	265
FMgSSR-31075	scaffold2039	p2	(GT)6	Class II	75571	75582	TGCTTGCAGGTGGGTTGTGT	AATCGTGCCCGTGTCTGCAT	210
FMgSSR-31077	scaffold8652	p2	(GT)6	Class II	28013	28024	TGCTTGCTGCCTTGCTGTCA	GGGCGGCGGCATGAATAGATTT	312
FMgSSR-31083	scaffold1182	p2	(GT)6	Class II	13258	13269	TGGACACAGTATCAGTTTGGCGAC	ATCGTCAGTCAGCTCCACCTGT	288
FMgSSR-31089	scaffold14952	p2	(GT)6	Class II	6401	6412	TGGCCCTTTCTCAGCAGCCTTT	TGTCCATCGTCAACCAACTCCAGG	317

SSR_ID	Scaffold	SSR_Type	SSR_Motif	SSR Class	SSR_Start	SSR_End	Forward sequence	Reverse sequence	Product_size
FMgSSR-31092	scaffold2419	p2	(GT)6	Class II	22473	22484	TGGCTCAAAAATGCCGCGTCT	TGCAGCTTCTCCCGCGTAAA	228
FMgSSR-31093	scaffold2363	p2	(GT)6	Class II	67419	67430	TGGCTCGTGCTTGGCTGAAA	TTTGATCTCGCATCGCACACGC	212
FMgSSR-31094	scaffold1200	p2	(GT)6	Class II	53139	53150	TGGCTCTAAGTCGAGGAGTTTCC	TGAAGCCCGATCGTCATCCGAA	237
FMgSSR-31096	scaffold145	p2	(GT)6	Class II	90739	90750	TGGCTTCCTCGACATCGCAACA	ATGCTCTGGCTTCCACCTCACA	214
FMgSSR-31098	scaffold507	p2	(GT)6	Class II	13750	13761	TGGGCGAGAGAGAGAAAGAGAGA	AGGACGACGATGACGCTGGTAT	238
FMgSSR-31100	scaffold18669	p2	(GT)6	Class II	14414	14425	TGGGGTGGATGTCTCTAGGTTGA	TTCGTGGAGCGGCTGAAGAT	313
FMgSSR-31101	scaffold1735	p2	(GT)6	Class II	76963	76974	TGGGGTTTACCGTACGTGTTAGGG	CGCGCCAAAGCCAAATGCAA	262
FMgSSR-31104	scaffold294	p2	(GT)6	Class II	75178	75189	TGGTAAACGCCATTGATGCGGC	AACCACTCGGCTCCCGTTTT	247
FMgSSR-31110	scaffold1852	p2	(GT)6	Class II	36506	36517	TGGTGCAGGCTTGCAGTCAT	AAGGTGAAAGTCAGGCCCTGGT	343
FMgSSR-31115	scaffold158454	p2	(GT)6	Class II	603	614	TGTCAGAGAGGTTTGCCCGT	TGACCTGTTTGAGGGCTCCAGT	300
FMgSSR-31117	scaffold4118	p2	(GT)6	Class II	19811	19822	TGTCCCGTGAAACATGCGGT	TCGAACCTAGTCTTTGGCATGCAG	250
FMgSSR-31118	scaffold4163	p2	(GT)6	Class II	32816	32827	TGTCGTCTCTGCTTCGTGTGCT	TCGGTGAACTTCAGTCTCTGCT	347
FMgSSR-31119	scaffold549	p2	(GT)6	Class II	86	97	TGTCTGTCCTGAGGGTCGTGTT	TGGGGTGTTTGCTCTGGTTGGA	208
FMgSSR-31121	scaffold4155	p2	(GT)6	Class II	25973	25984	TGTGCAAAGCCGATTGGCGA	AAAATGGCAGCGACGGTGGA	297
FMgSSR-31123	scaffold5	p2	(GT)6	Class II	163989	164000	TGTGCCTTGAAGCTGCCAAAGA	AGTGGAATGAACCACACACCCGT	344
FMgSSR-31130	scaffold767	p2	(GT)6	Class II	56492	56503	TTACCGGCTGGATTGGTGGA	ACCGAGAACAGATTAGAGCGGACA	335
FMgSSR-31132	scaffold266717	p2	(GT)6	Class II	89	100	TTCATTGCGCTTCCAACGCC	AGCCGCCCAACACCGAAAAA	349
FMgSSR-31134	scaffold70	p2	(GT)6	Class II	25781	25792	TTCCTGATCGCCTGCTCAGT	AACGATGGTAGCCGCGTTGT	226
FMgSSR-31139	scaffold7330	p2	(GT)6	Class II	10768	10779	TTCGCGTTATTGCCCTCTCGCT	AGCGTGTGTAGCACGCAGAGTT	282
FMgSSR-31142	scaffold783	p2	(GT)6	Class II	47235	47246	TTCTTTCGTTGACGGTCGGCCA	AACGGCCGAAGTTGGTGGGAAA	300
FMgSSR-31148	scaffold11079	p2	(GT)6	Class II	2323	2334	TTGAGGCTCAGATACAGAGGGGGA	ATCAGAGAAGCACGCGGTGGAT	348
FMgSSR-31149	scaffold4494	p2	(GT)6	Class II	11416	11427	TTGCACGTGTGTGTCACGTTCG	AAATTTCCGGCGATGGCTGC	309
FMgSSR-31150	scaffold3705	p2	(GT)6	Class II	36109	36120	TTGCCGATGAGGCGTTCGAT	AAAGTCGTCGGGCAGTGATGGT	263
FMgSSR-31164	scaffold4699	p2	(GT)6	Class II	19507	19518	TTTCAGATGCTGCTGCTGCGGT	TCCAAATTTCGGTTCCAGCCGC	293
FMgSSR-31167	scaffold3544	p2	(GT)6	Class II	48360	48371	TTTCGCGCGGTTCACGAGTT	AGGAACGGAGATCAGGCGAGTT	342
FMgSSR-31171	scaffold170360	p2	(GT)6	Class II	186	197	TTTGCGGTTTGCCGGATGGT	TTTTCTCCTGCACCCCGAACGA	293
FMgSSR-31174	scaffold15	p2	(GT)6	Class II	156989	157000	TTTTAGGCACGCCGAGCACA	TGGCCTTGGTGCACGGTTAGAT	315
FMgSSR-31180	scaffold282	p2	(GT)7	Class II	122791	122804	AAACCGGAACGGATTGCGCT	TTAGCAGCAGCACACACGCA	295

SSR_ID	Scaffold	SSR_Type	SSR_Motif	SSR Class	SSR_Start	SSR_End	Forward sequence	Reverse sequence	Product_size
FMgSSR-31181	scaffold2238	p2	(GT)7	Class II	40679	40692	AAACTGCACGTACGATGGAGACCC	ACGTGTGTTGTGCAGCAGTGTG	317
FMgSSR-31184	scaffold416	p2	(GT)7	Class II	110351	110364	AACATCAGGCGTGTGCCGAA	AGCTGAAAGCCAACTGTCTGCC	324
FMgSSR-31187	scaffold12248	p2	(GT)7	Class II	20039	20052	AACCTTCTGGGTGCGTGCTT	TATTCGCGCCCTGGGACAGTTT	316
FMgSSR-31189	scaffold187	p2	(GT)7	Class II	103109	103122	AACGGCGTGATGTCTCGCAT	ATGCCGCCAAACCGCCAAAA	349
FMgSSR-31190	scaffold2760	p2	(GT)7	Class II	6005	6018	AACGGGCATCAAATCGCTCCCT	TCGGCCCATGAGCCTATGAACA	346
FMgSSR-31192	scaffold7995	p2	(GT)7	Class II	17669	17682	AACTGCATCTCTCCTTGCTAGCCG	TGGGATTGCCGCTAAACGTACCA	346
FMgSSR-31193	scaffold8310	p2	(GT)7	Class II	4192	4205	AAGAACTTCCCGTCCCTGCTCT	AACCAAGTGCAGCCTCCAACCT	304
FMgSSR-31194	scaffold27200	p2	(GT)7	Class II	5867	5880	AAGCACACAGCATGCGCCTT	TGTGGCACACTGACGGGAAACA	240
FMgSSR-31196	scaffold139	p2	(GT)7	Class II	112824	112837	AAGCTCATGTGGCCGAAGCACT	TTTACCCAAACAGCGGCGAGGT	308
FMgSSR-31202	scaffold50404	p2	(GT)7	Class II	1982	1995	ACAGGCGCGAATGTGCTTCT	AGCTGCCGTAACTTCACCCT	207
FMgSSR-31204	scaffold17801	p2	(GT)7	Class II	12675	12688	ACATCCTGCAGTTGTGCCTCT	GGGGGTGGTTTTACACCCACTGTT	349
FMgSSR-31210	scaffold480	p2	(GT)7	Class II	54542	54555	ACCCGAGGTATGAAATGGGTTGCT	TGCCAGGGGCAAAAGGCAAA	325
FMgSSR-31211	scaffold1542	p2	(GT)7	Class II	8925	8938	ACCCGTTCGATGCTTCCTTCCT	ATGGCGGAATCCGTTGCACT	236
FMgSSR-31219	scaffold13034	p2	(GT)7	Class II	9173	9186	ACGGCATGGCCGCAAATGAA	GCCATGCCATGACACCCATT	215
FMgSSR-31221	scaffold299	p2	(GT)7	Class II	140880	140893	ACGGGGAATGATGAGGTCTCTTGC	GCCATGCATTTTTACCTCCCCAGC	346
FMgSSR-31223	scaffold12142	p2	(GT)7	Class II	5409	5422	ACGGGTTCAGTCCGTGCCTTTT	CCAGCTGCTGCTTTGCCGATTT	243
FMgSSR-31224	scaffold5031	p2	(GT)7	Class II	38079	38092	ACGGTGCGTCACGGTTTGTT	AGCTTGTCCATGTTGAACGCCG	316
FMgSSR-31240	scaffold27326	p2	(GT)7	Class II	8469	8482	AGAGAGTGCGCACACCAGAA	TTCTTTCAGCACGCAGCATCGC	210
FMgSSR-31241	scaffold3098	p2	(GT)7	Class II	40647	40660	AGATCTCGGAAGCCATGAAGCA	TTTCCACCGCACAAGCGCAA	264
FMgSSR-31243	scaffold4568	p2	(GT)7	Class II	13920	13933	AGCAGGTCGACGAAAGCTCGAT	ATGCACCGACGGCATTGTCT	233
FMgSSR-31246	scaffold7337	p2	(GT)7	Class II	11321	11334	AGCATGCGCACGTCTGTCTA	TTCACTTTGGCAGGCCGTCTGT	329
FMgSSR-31247	scaffold377	p2	(GT)7	Class II	18101	18114	AGCCGTGGCAGATGCAACAGAA	ATGCGAGGTCGTGGAACCGTAT	303
FMgSSR-31249	scaffold261	p2	(GT)7	Class II	18380	18393	AGCGCCAAGTTGCAGCTGAT	AACGAATGCGCATCTGGCGT	321
FMgSSR-31250	scaffold686	p2	(GT)7	Class II	7642	7655	AGCGCCATGCAAGGAGTCACAT	ACCACGAAAACTGATGCCACCC	348
FMgSSR-31262	scaffold73	p2	(GT)7	Class II	15984	15997	AGGAACTCCGCGCCCATCTTTT	AAAGCAACGCAAAAGCGCCG	264
FMgSSR-31269	scaffold13272	p2	(GT)7	Class II	14612	14625	AGGTGGCACCACTGGGTTTCAT	ACAGGAGGAGCTGGCAAAGGAA	286
FMgSSR-31272	scaffold108	p2	(GT)7	Class II	52244	52257	AGTGAGCGAGTTTCGGTCGT	TTTCTATGGCTCGCAGGGGCTA	283
FMgSSR-31275	scaffold2106	p2	(GT)7	Class II	19819	19832	AGTTTGTTCGATGTGGCGCACG	TGCAAACAGATCGACGGCATGT	303

SSR_ID	Scaffold	SSR_Type	SSR_Motif	SSR Class	SSR_Start	SSR_End	Forward sequence	Reverse sequence	Product_size
FMgSSR-31276	scaffold144	p2	(GT)7	Class II	70987	71000	ATAAGAGCGCGCGTAAAGCAGG	ATCTGTGCCGTACAGGTTGTCG	308
FMgSSR-31279	scaffold9437	p2	(GT)7	Class II	6204	6217	ATAGGCCGATATCGTCACGAGG	CACTCGCACGCCAGGAAAAA	337
FMgSSR-31289	scaffold40066	p2	(GT)7	Class II	442	455	ATGGTTGGTGGCGCGCAATA	AGCAGCACGCATTTCTTTCCGC	240
FMgSSR-31290	scaffold10	p2	(GT)7	Class II	238271	238284	ATGTCGTGTGTGTATGTGGGGTGG	GCACGGTAGGACTATGATTGAAGC	272
FMgSSR-31294	scaffold14129	p2	(GT)7	Class II	13899	13912	CAATGCATTTGGTGACCGCTTG	AGTGCCTGGGTTGCTCACTGAT	309
FMgSSR-31303	scaffold2255	p2	(GT)7	Class II	1227	1240	CCGTGTGAAACGACCAAATTGTCC	AGCTATGCTCGCTTGCACTGGA	293
FMgSSR-31307	scaffold5872	p2	(GT)7	Class II	16211	16224	CGCCATGAAAGTCACAAGAGCGT	TGCTCGGTCCTACTCCCTTTGGTA	349
FMgSSR-31308	scaffold3806	p2	(GT)7	Class II	40408	40421	CGCCGACAAGAAAACGCTGTCA	AGAGGCGCAGAACACACCAT	260
FMgSSR-31309	scaffold7504	p2	(GT)7	Class II	17314	17327	CGCGCGCGTTCTTGTTTCAT	AAAGCTTTGCTGGGTACGGCCA	312
FMgSSR-31310	scaffold1499	p2	(GT)7	Class II	79973	79986	CGCGTTCGTCAGCAACTCCATT	TACGCCGGCCTTGTTGTCTTGT	274
FMgSSR-31311	scaffold2910	p2	(GT)7	Class II	39569	39582	CGTTGCGTTGCTGCTGCTAGTT	TGGTCGGTGTTGATCGACGCTT	340
FMgSSR-31312	scaffold605	p2	(GT)7	Class II	70583	70596	CTCGCTCGTTTCGGTGCAAT	GCCAAATCGAACCACCCAAGCA	228
FMgSSR-31313	scaffold6922	p2	(GT)7	Class II	22695	22708	GAGTTGGGGTGGATGTCTATAGGT	GATGCAAGAGAAAGGATTCGGGAC	313
FMgSSR-31316	scaffold37325	p2	(GT)7	Class II	2869	2882	GCATACCCTCAACTGGTGCCAT	AGATCCTCACGCACAAACATGC	303
FMgSSR-31318	scaffold805	p2	(GT)7	Class II	51712	51725	GCATGGCACGTTATGTGGCT	CCAAACATGCCCTAAGAATGCAGC	211
FMgSSR-31320	scaffold629	p2	(GT)7	Class II	108768	108781	GCCAGGACGCCATATCATGTCT	GCCCGATCGTGTGCTTCTCTCTTT	342
FMgSSR-31321	scaffold9119	p2	(GT)7	Class II	6655	6668	GCGATCAGCAGCAGCAGCAATA	ACAGCCCATCCACATCAGCAGT	271
FMgSSR-31330	scaffold6080	p2	(GT)7	Class II	30718	30731	GGGTGCCCAAATTTCACACACA	CGTGGCCTTCAAGATATTCAGGAG	202
FMgSSR-31332	scaffold2831	p2	(GT)7	Class II	62867	62880	TACCCTCGCTGTGAACTCGTGA	GGTGGGTGCCTTCCAGTTTACTCA	218
FMgSSR-31335	scaffold4496	p2	(GT)7	Class II	36141	36154	TCACGTCGAACACGTCTGCTGT	ATTAGAGCCGAATCCCGTGGCA	290
FMgSSR-31349	scaffold20940	p2	(GT)7	Class II	6128	6141	TCCGTGCAACTGCAACGCTA	ACATGCGCACACATGCGACT	337
FMgSSR-31357	scaffold116087	p2	(GT)7	Class II	779	792	TCGGCCATGATCTCCTCGTTGT	ACACGTGCAGTGCAGGAACA	276
FMgSSR-31360	scaffold1664	p2	(GT)7	Class II	30222	30235	TCGTCATGCATTGCCGAGTCA	TTCGTGCGAACAACATCGAGCG	298
FMgSSR-31362	scaffold2344	p2	(GT)7	Class II	54024	54037	TCTCGTGTGTGTGTGATGTCCA	GGCACACATGCTCGTGCCTTTT	345
FMgSSR-31368	scaffold57660	p2	(GT)7	Class II	1330	1343	TGAATTCTTTTCGCCGCCCGTG	TGGCTACCGCAACGTGTTCTGA	346
FMgSSR-31370	scaffold325	p2	(GT)7	Class II	18136	18149	TGACCTCCCCGGTTCAAGCAAT	TGCGGTTTGAACGACAGGCT	240
FMgSSR-31371	scaffold21068	p2	(GT)7	Class II	8316	8329	TGACGTCAAGGCCACGACACAT	TGCATCCTTGACATCCGCTGCT	217
FMgSSR-31373	scaffold304790	p2	(GT)7	Class II	152	165	TGAGGCACTGCTTTCCTTCATCCC	ATTGCCAGGGGTCACAAGCTCA	200

SSR_ID	Scaffold	SSR_Type	SSR_Motif	SSR Class	SSR_Start	SSR_End	Forward sequence	Reverse sequence	Product_size
FMgSSR-31376	scaffold299150	p2	(GT)7	Class II	144	157	TGATGTGGTGCAAGCAGGGGAA	TCGCTGACTGCGCCGAGTTTTA	243
FMgSSR-31379	scaffold1403	p2	(GT)7	Class II	34196	34209	TGCAAGCGAGAACATTTCGGCG	TACGTGGTTGGCCGGTGTTTGA	262
FMgSSR-31383	scaffold3	p2	(GT)7	Class II	62373	62386	TGCAGCCGCGATCGAATACA	TACTTGCAGGCAGCACAGTCGT	289
FMgSSR-31384	scaffold1599	p2	(GT)7	Class II	79488	79501	TGCAGGGCATGGTGTCTCGTTT	CGACAAAATGCCGCTGTCGCTT	339
FMgSSR-31387	scaffold44	p2	(GT)7	Class II	73480	73493	TGCATGTGGTCTCGCGATTCA	TGTCACCCTCTCTCATGTGGAACA	322
FMgSSR-31389	scaffold9974	p2	(GT)7	Class II	17698	17711	TGCCATCTGCTGCTGGCTTT	TGGTGCGCACATTTGCTGAG	209
FMgSSR-31394	scaffold50104	p2	(GT)7	Class II	3420	3433	TGCTCATGCATCCGGTGACACA	TTTGCCCCTCCTGTCCCCAATA	262
FMgSSR-31398	scaffold4663	p2	(GT)7	Class II	18863	18876	TGCTCTCAATGGGCGGGTTT	TGACACCATGACCGACTCCAGA	285
FMgSSR-31403	scaffold48252	p2	(GT)7	Class II	2791	2804	TGGCCACATTGAGAAAGTTGGTGC	TGCTTGCAAACTCCATGGCACA	295
FMgSSR-31404	scaffold987	p2	(GT)7	Class II	55242	55255	TGGCCAGAATTAACTGTTGCCTCG	ATGCACTTGAATGGCAAGCCCG	349
FMgSSR-31405	scaffold104447	p2	(GT)7	Class II	546	559	TGGCGATCAATGGAGGGGTT	AAAGAGACAGCCGCTTGCTGGT	284
FMgSSR-31413	scaffold341	p2	(GT)7	Class II	63074	63087	TGGTTCCTGCAGACGCAACTGA	TCCATTCGTTGGTGCGAGCAGA	329
FMgSSR-31418	scaffold3974	p2	(GT)7	Class II	15092	15105	TGTCGTTTAAGGCGGTTGGCTCT	TGGCATTTGCAGTTGCAGCCA	224
FMgSSR-31420	scaffold6656	p2	(GT)7	Class II	15083	15096	TGTGTGGGTCGCCGCAATTT	AGCAAACACCGTTTGGGCTGT	252
FMgSSR-31424	scaffold5543	p2	(GT)7	Class II	29174	29187	TTCAGCTGCAGTGCACCCTGTT	ATGCCAAGTGCAAACCGCCA	230
FMgSSR-31425	scaffold676	p2	(GT)7	Class II	25097	25110	TTCATTGGCACGTTGCGTGAGC	TTGCAGATGTGGCCCATGACGA	261
FMgSSR-31429	scaffold651	p2	(GT)7	Class II	35264	35277	TTGATGCGTCCGTCCATCACCT	TCGGAGCGCCATAACCACACAA	271
FMgSSR-31433	scaffold5930	p2	(GT)7	Class II	14477	14490	TTGTCATGCCCCAATGGGCT	TGTTGCTGCACACAACTCCTCG	324
FMgSSR-31436	scaffold55496	p2	(GT)7	Class II	1797	1810	TTTCGCACTGTGCTCGGCAA	AACACGCCCGCCGTATCTGATT	337
FMgSSR-31439	scaffold1358	p2	(GT)7	Class II	58609	58622	TTTGCTCTGTGTTCACCCCACG	AAGGTTACGAAGACCGGCGA	309
FMgSSR-31440	scaffold6699	p2	(GT)7	Class II	23435	23448	TTTGGCCTTTTCCGGTTTGGCG	ATCACGTTGCAAAGAGCGCACC	238
FMgSSR-31441	scaffold2451	p2	(GT)7	Class II	8835	8848	TTTGTATGGGGATGGTGCGGCT	TGCACGAACCATGTCACGCA	242
FMgSSR-31445	scaffold11710	p2	(GT)8	Class II	17689	17704	AAAGGCAGGTCGGGAAATGGCA	ACCTCGCACAGTTTGATCGGGA	244
FMgSSR-31447	scaffold4	p2	(GT)8	Class II	304502	304517	AAGAACCTGCAAGCCCAACCCA	TGGAATGCCCTGCAGTTGGA	337
FMgSSR-31448	scaffold14351	p2	(GT)8	Class II	9926	9941	AAGCAGTGGTGGTGGCACAAGA	ATACTCCCATCCTCCCTACTCCTC	334
FMgSSR-31449	scaffold23871	p2	(GT)8	Class II	1271	1286	AAGCCAGTCTCCAGATGCACCA	ACCTGAAAACGGGCTGCCTCAA	293
FMgSSR-31451	scaffold56266	p2	(GT)8	Class II	1919	1934	AATCACTGCGGAGGCTGCAA	TCAAGGGACACGCAACGCAA	295
FMgSSR-31455	scaffold148	p2	(GT)8	Class II	40304	40319	ACAGCAACACCCGCATCTCCAT	TTCACCATAAGGCAGGGCAGCA	258

SSR_ID	Scaffold	SSR_Type	SSR_Motif	SSR Class	SSR_Start	SSR_End	Forward sequence	Reverse sequence	Product_size
FMgSSR-31458	scaffold20293	p2	(GT)8	Class II	2243	2258	ACAGTGGAACTCTAGCGCCCTT	AGCTCCATCCCTTGCACAACCT	237
FMgSSR-31466	scaffold1883	p2	(GT)8	Class II	61391	61406	ACGCCTTCCACAAGCCAACA	AGGTGAAAGTTGAGGCTGCCCT	225
FMgSSR-31468	scaffold31869	p2	(GT)8	Class II	1847	1862	ACGTACAGCCCACAACAAGC	TACCTCGCTTCCGGCAATCA	205
FMgSSR-31469	scaffold7168	p2	(GT)8	Class II	23783	23798	AGAAGTGCGGCAGAAGCGAA	AAAAGTGAGGCGCTTCGAGCTG	339
FMgSSR-31473	scaffold6486	p2	(GT)8	Class II	1661	1676	AGCAATTGGAGCCGGCACTCTT	TAAAAAGCGACAGTGGGCGGGA	299
FMgSSR-31478	scaffold222	p2	(GT)8	Class II	43905	43920	AGCTGCATGCAAGGCGTCTA	ACACCGTGCGATGACACGTACT	247
FMgSSR-31479	scaffold2636	p2	(GT)8	Class II	20652	20667	AGCTGCCGTACCAGATGCAGTT	TGAATGGGTTTGGTGCGCGT	267
FMgSSR-31480	scaffold5156	p2	(GT)8	Class II	35042	35057	AGCTGGCGCAAGAGGAATGGTT	TCGCGCTTTGCTTTGGCACT	224
FMgSSR-31481	scaffold13489	p2	(GT)8	Class II	19865	19880	AGCTTCCCCATGTGTTTGTGG	AGGCACGAGAAACGCATGCAAC	323
FMgSSR-31482	scaffold13295	p2	(GT)8	Class II	12563	12578	AGCTTGCTTGTCGACGTGGAGT	ATCTCAGTGAAGACACTCGCACCC	209
FMgSSR-31484	scaffold2214	p2	(GT)8	Class II	31425	31440	AGGCGAGAGAGAAACGTGTAGTGC	GCGCTCAGCATCAATCACAGCA	293
FMgSSR-31489	scaffold124244	p2	(GT)8	Class II	689	704	AGTAGGTGGTTTACTCCCGCGT	ACCAACGCGTGTGCTAGTGA	345
FMgSSR-31490	scaffold2700	p2	(GT)8	Class II	23949	23964	AGTCAACGCGTGGCGTACAT	AAAAAGCAGCCAAACGGGACGG	212
FMgSSR-31493	scaffold2357	p2	(GT)8	Class II	44491	44506	ATCACATTGTCCGTCGCTCGCT	TTCCAGGCACCCGAACAAACGA	274
FMgSSR-31494	scaffold2387	p2	(GT)8	Class II	40587	40602	ATCACGGCGTCGAGCAAACA	ACATGGTCATGGTGCGGACA	317
FMgSSR-31495	scaffold6045	p2	(GT)8	Class II	7923	7938	ATCAGATGTGTCGCGAGTGCGT	ATTTTGCGCCGGTGCGTTTC	290
FMgSSR-31496	scaffold12607	p2	(GT)8	Class II	9847	9862	ATCATCGGCCAACGCAACATGC	ACACATCAACCGGATCGGAGCA	306
FMgSSR-31497	scaffold567	p2	(GT)8	Class II	73606	73621	ATCGAGTGGCACAGGCGAAGAA	TACCGCAAGCTCAGCCCACAAA	292
FMgSSR-31509	scaffold352	p2	(GT)8	Class II	59349	59364	CCCGCTTTCGCAGTTTTCAGGT	AGCGAGAGTGACGGATGGAA	229
FMgSSR-31511	scaffold1354	p2	(GT)8	Class II	87998	88013	CCGGCCTTGCAAAAGATCGCAT	GCTCTCCTGCGCGTTTGAGAAA	323
FMgSSR-31514	scaffold2111	p2	(GT)8	Class II	61503	61518	CGCTCCATCAAACAGAGGTCAA	TCGTTGTGCCACCGAAGTGGAT	350
FMgSSR-31517	scaffold3006	p2	(GT)8	Class II	30581	30596	GCACTCCGGCAGCTGCATATTT	TCGAGTACATCGACGACGCCTT	266
FMgSSR-31518	scaffold28604	p2	(GT)8	Class II	899	914	GCGACCTTTGACCAAGTAGCAAGT	ACAGGAGTGGTCTTAGGTCCCA	308
FMgSSR-31524	scaffold22859	p2	(GT)8	Class II	760	775	GGGGGACCTTTGGGTGGAACATTT	TCAAGTCTGCTTCGCGTGCT	245
FMgSSR-31525	scaffold1552	p2	(GT)8	Class II	38934	38949	GGGTGCGCCAATTTGCCATT	AGCTGCAGACCTCAGTGTCAGT	247
FMgSSR-31526	scaffold5156	p2	(GT)8	Class II	37719	37734	GGTGCGTGCCACATGTGTTT	TGTTACCTCACCTGCTCACCA	349
FMgSSR-31536	scaffold14436	p2	(GT)8	Class II	1001	1016	TCAAACTCGGTGCTTGGGGTGT	AATGGCAGGAGGAGCAGCGAAT	232
FMgSSR-31539	scaffold796	p2	(GT)8	Class II	85452	85467	TCACCCATCTTGAGGGGGAGTA	TGCCGCTGCATGATTGGACGAT	348

SSR_ID	Scaffold	SSR_Type	SSR_Motif	SSR Class	SSR_Start	SSR_End	Forward sequence	Reverse sequence	Product_size
FMgSSR-31540	scaffold7846	p2	(GT)8	Class II	24818	24833	TCAGCGGACCCACAGCTGTAAT	ATAAGGCGTGCGAAAGCGAGGT	231
FMgSSR-31541	scaffold14734	p2	(GT)8	Class II	19252	19267	TCATCTAGCTGGGGAACCGCAA	TGGCTCCAACAGAGACTGACCT	339
FMgSSR-31543	scaffold7642	p2	(GT)8	Class II	22790	22805	TCCAAGGGTCATGCGTTTGC	TCACCTCCTATGCGTCCGGTTT	348
FMgSSR-31545	scaffold512	p2	(GT)8	Class II	58000	58015	TCCCGCTTTCGCAGTTCAGGTT	AAGTGGAATCCCGCTCTTGGCT	255
FMgSSR-31547	scaffold4728	p2	(GT)8	Class II	50285	50300	TCCGAGCATGAGCTGTGACGTA	TGCAGTGTCGCTCTACACACACA	312
FMgSSR-31548	scaffold161	p2	(GT)8	Class II	108393	108408	TCCTCGATCGCCGACTACAAACT	TGCAGCTTGCCCATACACACCT	321
FMgSSR-31551	scaffold440	p2	(GT)8	Class II	115859	115874	TCGCGACCCGTGAAGTCTTT	ACGGAGGAGGTTAGCTTTCGGA	279
FMgSSR-31554	scaffold2458	p2	(GT)8	Class II	32900	32915	TCTGATCTGACGAGGACGGATG	TGGTGGTGGTCCTGCATCTTCT	253
FMgSSR-31561	scaffold1712	p2	(GT)8	Class II	77808	77823	TGCATCATGCGACACCTGACCA	TGGCACACAGCACACAGCAA	218
FMgSSR-31562	scaffold20283	p2	(GT)8	Class II	331	346	TGCCTGATGCCATTTCGCCA	TGCTCAATTTGGTGGCTCGC	223
FMgSSR-31563	scaffold201640	p2	(GT)8	Class II	157	172	TGCCTTGTGAGAGCCAAGGT	AGCGTCCAACACCAGCTCAACA	205
FMgSSR-31571	scaffold2075	p2	(GT)8	Class II	35493	35508	TGGTGCAGGTCGATCACGTT	TACGCGGGCGTAAAACAGCA	315
FMgSSR-31576	scaffold146	p2	(GT)8	Class II	119571	119586	TGTGGATCGTGTCGGCTTGACT	ACGACGCAGGCAGATTTAGCGA	350
FMgSSR-31577	scaffold4728	p2	(GT)8	Class II	50412	50427	TGTGTGTGTAGAGCGACACTGC	ACAGGTGCAAGAGAATGTAGGGCT	333
FMgSSR-31578	scaffold14173	p2	(GT)8	Class II	5074	5089	TGTGTTCTGCGACTGCGAGGTT	TGAGCAGAGCAACAAGGGATCA	253
FMgSSR-31580	scaffold210232	p2	(GT)8	Class II	528	543	TGTTGGCATGTAGCGCATCCT	TTTGATGTCCCTGGGGCGTTGT	201
FMgSSR-31581	scaffold612	p2	(GT)8	Class II	88775	88790	TGTTGTGTGTGCAGCGCCAAA	ACTTGGCGAAGGTGTCCAGT	314
FMgSSR-31584	scaffold1095	p2	(GT)8	Class II	798	813	TTCGCGTGCGATACACTGCT	AAAGCGCGGTGTGACCATCA	294
FMgSSR-31590	scaffold34600	p2	(GT)8	Class II	3806	3821	TTTGAACGACGCGTGGGCTT	TGTGTTCAAGTCAGCCCTTTGCCT	331
FMgSSR-31591	scaffold11188	p2	(GT)8	Class II	5171	5186	TTTGCGCCATTTGGTGGTGC	TGTGAAGGACGACGCGCATT	231
FMgSSR-31592	scaffold6277	p2	(GT)8	Class II	290	305	TTTTGGCGCAGCTGGTGGAA	ACACAAACAGCCAGCCCGTTCT	236
FMgSSR-31593	scaffold657	p2	(GT)8	Class II	60771	60786	TTTTGGGAGCCGAACTGGAGGA	TGCTTCTGGTGGTGCTGTCA	341
FMgSSR-31595	scaffold520	p2	(GT)9	Class II	82252	82269	AAACGAAGCAGCACACGGCA	TGTACACGTTTCCGCAGGCA	268
FMgSSR-31597	scaffold23965	p2	(GT)9	Class II	4903	4920	ACAAACCCGACGCGCTTAGTGA	ACATCTTCCGCGATTCGCACT	317
FMgSSR-31616	scaffold5084	p2	(GT)9	Class II	26946	26963	AGGCGCAATGCTTGCTTCGT	ACCGTAAGGCACTATGCGGTGA	259
FMgSSR-31618	scaffold4867	p2	(GT)9	Class II	20008	20025	AGTGCACACACTGCACATGGAC	AGCGCTCGGCATGATCAGGAAA	276
FMgSSR-31619	scaffold12960	p2	(GT)9	Class II	4411	4428	AGTGCAGCAGCAGGAAACTGT	TCTGCATTGGTAAAGGCGGCTG	209
FMgSSR-31621	scaffold9678	p2	(GT)9	Class II	22906	22923	ATAACGCACACTGCTGACCCGT	TGAAGCAGTCGACGGCCTTGTT	258

SSR_ID	Scaffold	SSR_Type	SSR_Motif	SSR Class	SSR_Start	SSR_End	Forward sequence	Reverse sequence	Product_size
FMgSSR-31623	scaffold78642	p2	(GT)9	Class II	932	949	ATGCCGCAGCAAAAGAGGCA	TTGTGTACTGGCAAGGCTGGCT	202
FMgSSR-31624	scaffold2767	p2	(GT)9	Class II	36058	36075	ATGGCTGCGTTGTGGAGGAA	TCCGCACAGCACCAACCAACTA	350
FMgSSR-31625	scaffold24482	p2	(GT)9	Class II	2016	2033	ATTCGACTGCCTGTGGTGGT	TGCAGCTGAGGATGACTTGCCA	343
FMgSSR-31626	scaffold294	p2	(GT)9	Class II	25620	25637	ATTGGTTGCACGTTGCACGG	TGATGAGCCCCACACAAGCA	271
FMgSSR-31627	scaffold665	p2	(GT)9	Class II	107489	107506	CGCGCGTCGTGTGTATGTGTAT	AGCGAGCGCCTACCACAAAA	328
FMgSSR-31630	scaffold14099	p2	(GT)9	Class II	17675	17692	CGTTCCTCAAAAGTACTTGGTCCC	AAGCCTCAAGGAGCGCACAA	260
FMgSSR-31632	scaffold56662	p2	(GT)9	Class II	97	114	GCAAACCAGCACGAACGAATGC	ACTGCCGCTCTAAATCGCACCA	229
FMgSSR-31633	scaffold14436	p2	(GT)9	Class II	611	628	GCACATCCATGCGTGCAAACAA	TTGCAAAGTTTGGGTGTGCCCG	234
FMgSSR-31634	scaffold9678	p2	(GT)9	Class II	23820	23837	GCGTTCGTGTTCAGCGACAA	AGCTTGCTCATGCTCTCGGT	296
FMgSSR-31635	scaffold17609	p2	(GT)9	Class II	13836	13853	GCTTGTGGCCGGTCTGCAAAAT	ATGCATGCGGATGCGGAAGA	235
FMgSSR-31637	scaffold2775	p2	(GT)9	Class II	2810	2827	TAAGCCCCAATAGCCCCGGTTT	TTGCCGTTCTAGTGAGTGGGCT	335
FMgSSR-31638	scaffold772	p2	(GT)9	Class II	68980	68997	TAGGCGTTCTTGTCGCGTGTGA	TGTGCTGCGAGAGCTGATGA	278
FMgSSR-31640	scaffold4036	p2	(GT)9	Class II	10326	10343	TCAGCGCAAGCCGCATCAAA	CCTCGCCCTTCTCCAGCTTATTCA	278
FMgSSR-31644	scaffold402	p2	(GT)9	Class II	63892	63909	TCCATGTGGCCAGCGTGAAGAA	ACCAAGGACACCGCACCATCTT	293
FMgSSR-31647	scaffold450	p2	(GT)9	Class II	14248	14265	TCCTCGTCTACCTCCACGATCA	CAGATGCTGCTGGTGACCAAGT	350
FMgSSR-31648	scaffold8931	p2	(GT)9	Class II	17118	17135	TCGAAGCGTGCCTGCCATTT	ATAGTCCAGGTCGCGTGCCAAT	257
FMgSSR-31653	scaffold3287	p2	(GT)9	Class II	53273	53290	TCGTCGTTCCGTCAACTGTGCT	ATTCAGACCAAACCGCGCCCTA	334
FMgSSR-31666	scaffold39109	p2	(GT)9	Class II	1753	1770	TGGTCTCCTTGTGGGAGCAA	ACAAGACATCAAGCACCCGCCT	275
FMgSSR-31668	scaffold665	p2	(GT)9	Class II	113374	113391	TGTCCGTGCCTATTGCTGGT	TGAGCTGGGCCATTTCCTGT	306
FMgSSR-31672	scaffold4938	p2	(GT)9	Class II	12226	12243	TTGTAGCAGGGGCCAAGCAT	TGCAAACAGACAGTGCCGGAGT	233
FMgSSR-31677	scaffold9513	p2	(TA)10	Class I	1911	1930	AAAAGCCCACCAGATCCGCACA	AGCCAGGCAGACCAGTTTTCGT	243
FMgSSR-31680	scaffold161	p2	(TA)10	Class I	30312	30331	AACGAAGCAGCTACGACCCCAT	GCGTTGCCGTGCCAAAGTCTAA	266
FMgSSR-31681	scaffold8760	p2	(TA)10	Class I	27333	27352	AAGCCAACAGCAGCCTAGCACT	AGCAGACACGACATGGTGGGTA	333
FMgSSR-31685	scaffold928	p2	(TA)10	Class I	84880	84899	AATCCGTAACCAAGCTCGGAGGTG	TCGATCCTCGCTCTCAGGCATT	316
FMgSSR-31688	scaffold486	p2	(TA)10	Class I	60094	60113	ACAAGGCTTGGGCGTGCATT	TATTGCACAGCCAGGCTACACG	342
FMgSSR-31692	scaffold30634	p2	(TA)10	Class I	2228	2247	ACCACGCTGCAGCAAATCCA	ATCACCGCAGCACTCACAGGTT	339
FMgSSR-31695	scaffold14751	p2	(TA)10	Class I	6986	7005	ACCGGCACCGAAAACGATTCTG	ATTATGGATGGGCTGGCTCGGT	334
FMgSSR-31699	scaffold1638	p2	(TA)10	Class I	9959	9978	ACGCACACTCCATCCGTTCGTT	AGGACCTGCACAACGGCAAGTA	268

SSR_ID	Scaffold	SSR_Type	SSR_Motif	SSR Class	SSR_Start	SSR_End	Forward sequence	Reverse sequence	Product_size
FMgSSR-31700	scaffold4950	p2	(TA)10	Class I	18793	18812	ACGCCGCAACCTGGCTAACATA	TTCGATGCGCAGCGTGATTG	339
FMgSSR-31702	scaffold2666	p2	(TA)10	Class I	42741	42760	ACGTAGCACGGACTTGACCCAA	AGCCCGTAAAGCGCTCAAGT	314
FMgSSR-31710	scaffold2633	p2	(TA)10	Class I	25350	25369	AGAGGCGGAAACTCTGTTCCGT	AACCAGCTGCGTGCTTACCA	300
FMgSSR-31720	scaffold2440	p2	(TA)10	Class I	46034	46053	AGTGGTCGATGAAAACCAAGCTGC	CCCGCTGCTCCCATGGTTTATT	326
FMgSSR-31721	scaffold6896	p2	(TA)10	Class I	9867	9886	ATCTGGTTGGACCAAACGACCG	TGAATGAAGCAGCTCTACCGCC	205
FMgSSR-31723	scaffold4862	p2	(TA)10	Class I	9143	9162	ATGCAAATGCGGCTCTCGCT	TCATTAGCAGCAGCAGCGGTGA	221
FMgSSR-31725	scaffold1374	p2	(TA)10	Class I	37219	37238	ATGCTTGTGGGCATGCACTGGT	ATGGCACCACATACTCCCTCCTGT	213
FMgSSR-31726	scaffold303	p2	(TA)10	Class I	121917	121936	ATGGCAACACGCAACCGACA	TGCACATGCAGAGCGTTCGT	264
FMgSSR-31727	scaffold1025	p2	(TA)10	Class I	36874	36893	ATGTTGCGCCGCTATGTGCT	TTTCAGGCAGGGTTGACACGGA	348
FMgSSR-31731	scaffold1833	p2	(TA)10	Class I	30754	30773	CCAATGCTAATTGGACGGGGCA	CGCTCGTCCAGCACATGGAAAA	209
FMgSSR-31733	scaffold114	p2	(TA)10	Class I	192846	192865	CCAGCACCCGCCAAGTAATCTT	TGTGTGTTCAGGTTCCGAGAGC	272
FMgSSR-31736	scaffold460	p2	(TA)10	Class I	55204	55223	CCCCGGCCAGGAAGTACTTTTGAA	TGGAGCACATGCACATCCGACT	347
FMgSSR-31737	scaffold34287	p2	(TA)10	Class I	3391	3410	CCCGCTCAGAAGCAGCTGAAAT	TAACCACCGCGCGTCACTTT	225
FMgSSR-31739	scaffold282	p2	(TA)10	Class I	27683	27702	CCGGTCTTGTATGCATAGCCTGGA	TGGCTGGTCTCTTCCTTCCCTGTA	348
FMgSSR-31743	scaffold8549	p2	(TA)10	Class I	14735	14754	CGCCTGATCCCCACGTTTACAA	ACGTGACCAACAACATTCCCCG	346
FMgSSR-31744	scaffold10946	p2	(TA)10	Class I	5043	5062	CGGCTCGCGCATGTAATTCT	ACCAACCTCTGCGACGTTACAA	336
FMgSSR-31745	scaffold2399	p2	(TA)10	Class I	11304	11323	CTCCAACCAGTTTAGAGGTCTCCT	ATGCCTGCACTTCTCTGCCAGT	317
FMgSSR-31748	scaffold3	p2	(TA)10	Class I	46138	46157	GCCAATTGGGTCACATTCGGCA	TCTCACCAGCACGACAATCCT	292
FMgSSR-31751	scaffold2277	p2	(TA)10	Class I	53441	53460	GCGCTTCAACGTCAAGGCAA	ACACCAACGACCATCACACGAC	340
FMgSSR-31752	scaffold4218	p2	(TA)10	Class I	37221	37240	GCTTCAACGTCAAGGCAAGGCA	AGCTGTCAACCTCCACCAGCTT	334
FMgSSR-31753	scaffold472	p2	(TA)10	Class I	100566	100585	GCTTGGAGTGACGCGATCAACA	AGCAGCAGTCCTGACGACAA	295
FMgSSR-31754	scaffold1311	p2	(TA)10	Class I	60465	60484	GCTTTCAGTTGAAGACCACACGAC	TTCCTGCCCATGTGGATTGCCT	235
FMgSSR-31761	scaffold6797	p2	(TA)10	Class I	5531	5550	TACGTACAACTCGCTTTCGGCG	GCTCGAGCAGCAACCATGCAAA	344
FMgSSR-31767	scaffold2158	p2	(TA)10	Class I	34724	34743	TCCGATGTCGATGAATGAGGACG	TGTTCTGTCAAGGTGGGCTTGC	280
FMgSSR-31779	scaffold1009	p2	(TA)10	Class I	90256	90275	TCGTTGGTGCACTATCGCGTCA	TTTCGGTGTGTGAGCCAGGGTT	347
FMgSSR-31782	scaffold2953	p2	(TA)10	Class I	47571	47590	TCTGCCGTACGCCCACTTTT	ACGCCTCTTTCGCTAACTCGT	305
FMgSSR-31783	scaffold1080	p2	(TA)10	Class I	35047	35066	TGAACACCGGATGGTTGACGTG	ACCGCGGTAGAAGCGACAAGAT	329
FMgSSR-31789	scaffold1712	p2	(TA)10	Class I	9315	9334	TGAGTGCACGAGTTAATGCGGA	AGCGGCGTAGCCGGAGATTTTT	274

SSR_ID	Scaffold	SSR_Type	SSR_Motif	SSR Class	SSR_Start	SSR_End	Forward sequence	Reverse sequence	Product_size
FMgSSR-31791	scaffold7556	p2	(TA)10	Class I	25457	25476	TGATGCGCGGGCACAAGAAA	TGGCGGCAATAGCAGGCAGTAA	234
FMgSSR-31793	scaffold637	p2	(TA)10	Class I	55379	55398	TGCAAGGTTTGCTTGGGCTCT	ATAGCCGAGCGAGCGAGGATTT	291
FMgSSR-31795	scaffold722	p2	(TA)10	Class I	52521	52540	TGCAGGCTCTGTGCATCGTT	AAGTTGGCACCGCTAGAGTAGT	350
FMgSSR-31796	scaffold775	p2	(TA)10	Class I	47299	47318	TGCAGGTCAAGGCTGCGTACAA	ACACGGGGAAAACGGAAAACAG	328
FMgSSR-31797	scaffold261	p2	(TA)10	Class I	91624	91643	TGCATGCCTGCATTGTCTGCT	AGCAGTGATAAGCACAGGGCA	312
FMgSSR-31799	scaffold2004	p2	(TA)10	Class I	5369	5388	TGCCATGGTGCTCTGTAGGACT	TCGCCCAGGACACATGTCATCA	344
FMgSSR-31802	scaffold6227	p2	(TA)10	Class I	22855	22874	TGCGGTCCTGCCATTGCTTACA	CCGTGTGCTTCATTCGTAGCGT	220
FMgSSR-31803	scaffold20	p2	(TA)10	Class I	114999	115018	TGCGGTTCGCAAGCTTATGCAG	CGAGTCACCATAAAGTGGCACTGA	344
FMgSSR-31804	scaffold3873	p2	(TA)10	Class I	5947	5966	TGCTACAACAGAGAAGCACACG	AAGGCCCAATCAGCGCAACT	209
FMgSSR-31805	scaffold5445	p2	(TA)10	Class I	21168	21187	TGCTCATGTTGCCTTTGGCCT	TGGCCTTCATGCCTTCATGCTGT	344
FMgSSR-31808	scaffold13737	p2	(TA)10	Class I	2021	2040	TGGGCACATGCCTTATTTGGGC	TGTGACGCCAAATGTCCTGC	329
FMgSSR-31809	scaffold318	p2	(TA)10	Class I	123303	123322	TGGGCCCAACATGGAAACAAAC	TCCCCAACACAGGTCAGTTGTCA	205
FMgSSR-31817	scaffold1953	p2	(TA)10	Class I	17770	17789	TTCAGTCCGGCCATCATGCACA	ATGGACGTTGTCCACCACACGA	276
FMgSSR-31820	scaffold13335	p2	(TA)10	Class I	10784	10803	TTCTGCGACGCAACCTTCCT	TGCACTCAAAGAAACGCCCTCG	323
FMgSSR-31826	scaffold1377	p2	(TA)10	Class I	75385	75404	TTTGCTGAATGTGCTGCGCT	TCGCCTATGTATGCGGACTGGA	284
FMgSSR-31828	scaffold2034	p2	(TA)10	Class I	47945	47964	TTTGTGAAGACCTACGTGGCCG	ACCGATGCTTAATCAGCTGGCA	264
FMgSSR-31829	scaffold1723	p2	(TA)11	Class I	12257	12278	AAACTGCGACAAGGGTTGCTGC	CACAGCGTGTCACGAGGCTTTT	304
FMgSSR-31831	scaffold714	p2	(TA)11	Class I	56017	56038	AACCTGAAAAGGTGCAGCGACG	GCGCGGTTCTGTTCAACGTA	263
FMgSSR-31832	scaffold14524	p2	(TA)11	Class I	12569	12590	AACGTGGTCAAGTAGCACGCCA	AGCTACCGTGTTCCCAACACCA	280
FMgSSR-31833	scaffold22405	p2	(TA)11	Class I	7943	7964	AAGCGCGGAGGAAAGGAAACGA	ACCCGACAACGAAACTTGCCCA	294
FMgSSR-31836	scaffold1115	p2	(TA)11	Class I	53066	53087	ACACACACACAGTTCGCGGAT	TCACCACGCGCGCACATAAA	242
FMgSSR-31841	scaffold313775	p2	(TA)11	Class I	91	112	ACCACCCGACCTGGCTACAGAATA	TGCACGTGCTTCCACTACGA	308
FMgSSR-31842	scaffold197	p2	(TA)11	Class I	140930	140951	ACCCGCTGGTCACCGTAGATTT	GCGTGCTTCCTTTTGGCTGGTT	349
FMgSSR-31843	scaffold2737	p2	(TA)11	Class I	31132	31153	ACCGAGCCTAGCAGATTCCTCA	CGTGGTAGAAGCAGTTGATCTGTC	328
FMgSSR-31846	scaffold3	p2	(TA)11	Class I	64555	64576	ACGCCACTCTTGCGAAGAACA	GCACGACTCTTGCTTAATTTCCCC	280
FMgSSR-31848	scaffold950	p2	(TA)11	Class I	61413	61434	ACGGCGCCCTTTGTCTGTTT	AACCAAGTGGCGAGTGTCGT	268
FMgSSR-31852	scaffold1398	p2	(TA)11	Class I	6309	6330	ACTGTCACCTGAAATCCGTCATGG	CCTTCATTGCACGAACAGGCCA	301
FMgSSR-31857	scaffold1364	p2	(TA)11	Class I	21871	21892	AGCAGATGTCTCTGGTACCGCAT	TGCCATGGTTTGCTGCCATGA	278

SSR_ID	Scaffold	SSR_Type	SSR_Motif	SSR Class	SSR_Start	SSR_End	Forward sequence	Reverse sequence	Product_size
FMgSSR-31861	scaffold149598	p2	(TA)11	Class I	987	1008	AGCCGCCCTCATCCAACAATGA	AGCCGGCTTCCGGTCAAAAA	221
FMgSSR-31862	scaffold96	p2	(TA)11	Class I	185537	185558	AGCCGTATAAGCTGCCGTGCTT	TGCTGAAGCAGATGACGGAGGA	246
FMgSSR-31863	scaffold4093	p2	(TA)11	Class I	2833	2854	AGCGCGCCATTTAACGCTGT	ATGCGATGGCGAGCTTCCTT	335
FMgSSR-31866	scaffold11710	p2	(TA)11	Class I	9062	9083	AGCTCGAAGCGTACGCCTATT	AAGAACCTGCGCGCAATCCA	346
FMgSSR-31867	scaffold628	p2	(TA)11	Class I	27203	27224	AGCTGTGGCAGCCTGTGAACTT	CGCTTGTGCTTGGCGCATTT	332
FMgSSR-31868	scaffold184	p2	(TA)11	Class I	96457	96478	AGCTTCTGCTCATTTGGGCTGGT	GCACTGTACCTGGAAACCTGGTGA	269
FMgSSR-31870	scaffold22683	p2	(TA)11	Class I	291	312	AGGAGTCGTTGTGCTCTCCGTA	TGAAGGCGTTCCATAGCCGA	284
FMgSSR-31873	scaffold24012	p2	(TA)11	Class I	9773	9794	AGGGTGCCCAATCAGGTTTCTGT	TGGGAATCTCTCCCGGTGTGTT	283
FMgSSR-31875	scaffold15261	p2	(TA)11	Class I	4150	4171	AGTCTCGGCAAGCACTGCTGTA	AACTGCCGTGTTGAGTGGGA	267
FMgSSR-31876	scaffold5042	p2	(TA)11	Class I	30478	30499	AGTTAAGGTCTGTCTCCGAAGCCT	TCCATCGATGCGATTTGGCTGA	217
FMgSSR-31877	scaffold365	p2	(TA)11	Class I	70396	70417	ATCGACAACCTGGGTCTCACAAGC	ACAGCACATCAGGATTCTGCCACA	264
FMgSSR-31883	scaffold598	p2	(TA)11	Class I	75937	75958	ATGGTCTGATGGCATGGCGTGT	ACACAGTTCAGGGCATGTCACG	345
FMgSSR-31884	scaffold156	p2	(TA)11	Class I	65698	65719	ATGTTCTTCTCAGCAGCCACGC	ATGACAACACCAGGCAGGCAGA	213
FMgSSR-31890	scaffold7331	p2	(TA)11	Class I	35572	35593	CGGTCCCCGAAACATTCACACA	ACGTGGCCAGTTGGGTTGACTA	239
FMgSSR-31891	scaffold23471	p2	(TA)11	Class I	1150	1171	CGTAGATCGGGTCTGCCTACAGAA	CACACCATCCTCCCTTTTATGCTG	345
FMgSSR-31893	scaffold123	p2	(TA)11	Class I	121306	121327	CTAGAAGCAATTCTGTGGCGGT	TCTGATGTGTTTGGATCCTCCACC	324
FMgSSR-31896	scaffold3208	p2	(TA)11	Class I	48506	48527	GCACCAATGCACGACTACTTTGGC	ATGCAGTAGAGCCACGCTGCTT	249
FMgSSR-31897	scaffold11140	p2	(TA)11	Class I	15588	15609	GCCAAACCTGTCTGAGCCCACTTA	TGCGTGCCAAGAGAGGAATGT	348
FMgSSR-31898	scaffold1760	p2	(TA)11	Class I	18659	18680	GCCATTTTGCCAGTCAGATCACCA	AGCCATGTAGGTGTGGGTTGA	331
FMgSSR-31899	scaffold772	p2	(TA)11	Class I	93389	93410	GCCCTACACTCAGTTGCTTGTTGC	ACGTGCTTTTGCCAACGCCAA	254
FMgSSR-31901	scaffold2386	p2	(TA)11	Class I	14253	14274	GGCATGGTGGTACAAATTATGGCG	TGCCAAGGATCACAAAGGCCA	314
FMgSSR-31902	scaffold4228	p2	(TA)11	Class I	5429	5450	GTGGCCACATTTCGCTGTTGT	TGGTGCATCAGGATCGACCAGT	346
FMgSSR-31903	scaffold287	p2	(TA)11	Class I	141376	141397	GTGTGGATCAACAGTGTACTAGGC	TGCAGCTGCGAGACAAGGTT	350
FMgSSR-31907	scaffold165176	p2	(TA)11	Class I	334	355	TAGTCGGGCAGGGTTGGTTT	AAAACAGCCGTCCAATACCGCC	327
FMgSSR-31908	scaffold2135	p2	(TA)11	Class I	24426	24447	TATCCACGCTGCACCGCTTT	TGAGGGCATGACTTGGATGTCCTT	239
FMgSSR-31909	scaffold485	p2	(TA)11	Class I	83397	83418	TCAAGCTCAGACGTGCACATGG	TGTCGACCGACACACACGAT	301
FMgSSR-31911	scaffold7492	p2	(TA)11	Class I	17787	17808	TCAGGCCACAAGCAAATCACGA	TGGACTGCACTAACCGAGCA	331
FMgSSR-31913	scaffold1680	p2	(TA)11	Class I	68798	68819	TCCACGAGCCCATCTCAAGGAT	TGAGTGGCGGAGGACAGAAAGA	328

SSR_ID	Scaffold	SSR_Type	SSR_Motif	SSR Class	SSR_Start	SSR_End	Forward sequence	Reverse sequence	Product_size
FMgSSR-31916	scaffold1816	p2	(TA)11	Class I	4585	4606	TCCTTCTCTGCATGCCCAAGT	ACAGAAGCTAAGCAAGAGGCAGCA	308
FMgSSR-31917	scaffold3513	p2	(TA)11	Class I	41685	41706	TCGGTCATCACAACGTGGCAGT	TGTGGCAGCAGATCACGAACGA	318
FMgSSR-31920	scaffold22163	p2	(TA)11	Class I	5578	5599	TGCAACCAGACACATGCGGT	AGAGCCGTGGCCGACATTTT	279
FMgSSR-31922	scaffold16117	p2	(TA)11	Class I	7124	7145	TGCACCTGGACGTTTGCTGT	TGCTCCCGTGTTAACCACCACA	307
FMgSSR-31926	scaffold2260	p2	(TA)11	Class I	46404	46425	TGCATGAATGGGCACACCTCGT	GCGTCGAGGCGTTCATCAACAA	285
FMgSSR-31930	scaffold1996	p2	(TA)11	Class I	31865	31886	TGCGTGAGTGTTGCACTCGCTA	TGCAGGAGAGCAGATGAGTTTCCA	267
FMgSSR-31931	scaffold9648	p2	(TA)11	Class I	5717	5738	TGCTCTTGCACCTCCTGCACAT	TCGGCTGCAACAGAACGAAGGA	210
FMgSSR-31932	scaffold1	p2	(TA)11	Class I	218299	218320	TGGAAGCGATCATTTTCTGGAGCC	TTGCCTGCTTTCAGGTGCAACG	332
FMgSSR-31940	scaffold6896	p2	(TA)11	Class I	13999	14020	TGGGTGCGTAATCCACGGCAAT	TTGGTCGTCGCATACGGGTTCA	272
FMgSSR-31945	scaffold15707	p2	(TA)11	Class I	4059	4080	TGTTTCCGCTCCAAACGAGTGC	TGGGAGCGTTACAACTTGGGAGT	344
FMgSSR-31946	scaffold854	p2	(TA)11	Class I	11450	11471	TTAACCACCCGCCCGTTCCAAA	ATTGCCATTCTTGCAGCAGCGG	233
FMgSSR-31948	scaffold3317	p2	(TA)11	Class I	46948	46969	TTCCGCTGGGTACTCCTCGTTT	ACTGGAAGCCACAAGCACGA	291
FMgSSR-31950	scaffold4460	p2	(TA)11	Class I	41200	41221	TTCGCAGGTAGCCTCACCTAGT	ACTGACAGAGCCACAGCAGTCA	347
FMgSSR-31951	scaffold10633	p2	(TA)11	Class I	7329	7350	TTCTGGCTGGGGTGTGGAAT	ATGCATCTACACCGACATGCAGGG	337
FMgSSR-31952	scaffold72	p2	(TA)11	Class I	82962	82983	TTGCACGTATGAACGGCCGGAT	AGCCGCTTCTAGTGTGTTGC	331
FMgSSR-31957	scaffold6692	p2	(TA)11	Class I	27720	27741	TTGTGGATTGCTGGCGTGGT	ACACCTGCCAACGGACATGGAA	292
FMgSSR-31958	scaffold8193	p2	(TA)12	Class I	28577	28600	AAACGTGGGCGCATCTTCGGAT	CGTGTCTTGTCCGGCACATTCT	263
FMgSSR-31960	scaffold5898	p2	(TA)12	Class I	19342	19365	AAGCAGCGACGGACTTGACT	ACGTCCAGGCGTTCAAGCAGAT	232
FMgSSR-31962	scaffold236704	p2	(TA)12	Class I	185	208	ACAACCGGCCAATGATGCCA	TGCGGTGTATCTCCCGCCATTT	289
FMgSSR-31963	scaffold6324	p2	(TA)12	Class I	10545	10568	ACAACTTGCGGCTCGGCTTT	AGCTGAGGTAATAGGCCGTTGCT	348
FMgSSR-31966	scaffold4116	p2	(TA)12	Class I	9724	9747	ACCACGCCTCCGTGACCATAAA	TGCATGCGTGAGTGGTTAGTGC	350
FMgSSR-31972	scaffold4163	p2	(TA)12	Class I	33385	33408	ACGCGCGTACGTTCTCCCAATA	TGCCATGTCAACTAGCACCACC	330
FMgSSR-31973	scaffold2489	p2	(TA)12	Class I	19574	19597	ACGTGAGCAATGGCCAACCA	TCTCCCGGTGTATTTTGTCACTGG	204
FMgSSR-31977	scaffold1267	p2	(TA)12	Class I	63135	63158	AGAGCCCCTCTGATTTCGTGGT	AAAAGCATCTTGGCTCAGCCGC	332
FMgSSR-31978	scaffold9303	p2	(TA)12	Class I	5674	5697	AGATTGTGGGCGGGGTACTGAA	CAGCATGCACCATTGCCTGA	329
FMgSSR-31980	scaffold2138	p2	(TA)12	Class I	34663	34686	AGCAGCGCTGGTGAATTATCCCT	TGCAGCAGCAAGTGACGCAT	292
FMgSSR-31983	scaffold11519	p2	(TA)12	Class I	22068	22091	AGCGCACTAGGCTGCTGTTT	AGGAGGGCCAGATGCTCAAA	347
FMgSSR-31984	scaffold463	p2	(TA)12	Class I	59261	59284	AGCTGCCGTCTGTTTCACTGCT	CGGTGCCTTTGCTTTGCTGT	298

SSR_ID	Scaffold	SSR_Type	SSR_Motif	SSR Class	SSR_Start	SSR_End	Forward sequence	Reverse sequence	Product_size
FMgSSR-31986	scaffold733	p2	(TA)12	Class I	69557	69580	AGGAGCAGCCTAATGACGGGTT	ACGCTACGCAATGTTGCCCT	217
FMgSSR-31987	scaffold26978	p2	(TA)12	Class I	2653	2676	AGGATTTTCCCCTGCGTGGCTT	TGGACGTCGGTAATGGCATGGA	340
FMgSSR-31990	scaffold223	p2	(TA)12	Class I	18987	19010	AGGCGGCTTGTTGCAAGTTGA	TGGCTGCAAGGTGCTAAGTCGT	315
FMgSSR-31992	scaffold14928	p2	(TA)12	Class I	12950	12973	AGGTTGGGTTCAGTCGGTGCTT	TGGGCCCGGTGACATTTGTA	277
FMgSSR-31993	scaffold10462	p2	(TA)12	Class I	6416	6439	AGTGGGGTTGGTTGGTTCATGCT	AACTCGAGCCAGGCAATGGAGA	329
FMgSSR-31995	scaffold1139	p2	(TA)12	Class I	46753	46776	ATACACGGCAGGGGTGTTGA	TATGGATGAAGCGACAGCGAGC	210
FMgSSR-31996	scaffold2971	p2	(TA)12	Class I	55547	55570	ATCAATGCTAGCAGCGCACCAG	TTTTCGGGTGTTGTCCTGCG	326
FMgSSR-31998	scaffold2067	p2	(TA)12	Class I	19411	19434	ATGCTTGCAGGCAGAGTGCT	GTGCAACGACAAGTTAAAAGCCCC	213
FMgSSR-32001	scaffold1055	p2	(TA)12	Class I	38195	38218	ATTACCCGCTTCGTCGCTTCGT	TGGTCACTTTGCAGCTTTTCGCC	308
FMgSSR-32008	scaffold2545	p2	(TA)12	Class I	19454	19477	CGGCTTTGTACCGCATCTATTGCC	GCAAGTTTGCAAAACCTGTCCG	347
FMgSSR-32010	scaffold5336	p2	(TA)12	Class I	30398	30421	GCAGCGCACCATTTAGCACTGT	GGCATTCTGAGATCAAGGCATCGC	346
FMgSSR-32012	scaffold75150	p2	(TA)12	Class I	927	950	GGTCCTCATCATCAAGAGTTGCTC	TCCTGCCGCATCTATGCAGATCA	327
FMgSSR-32018	scaffold15883	p2	(TA)12	Class I	2244	2267	TCCATTCCCAGTAAACAGCCCAGC	TGGAGCTGTTGCGGAAGTCCAT	329
FMgSSR-32020	scaffold1557	p2	(TA)12	Class I	17701	17724	TCCCTCCATTCCCCCTCCCAATTT	CACGCGCGCTGCATTATGTT	332
FMgSSR-32022	scaffold50164	p2	(TA)12	Class I	963	986	TCGGACTATGTGGAACGTGGCT	ATCGCTCGGCGATTCGTGTCAA	291
FMgSSR-32025	scaffold6842	p2	(TA)12	Class I	23669	23692	TGACGTTAGAACGGTTGGCCT	AGGTGCGGCACATGACACAT	278
FMgSSR-32027	scaffold6641	p2	(TA)12	Class I	11320	11343	TGCAATCGCACTGGTCCACA	AGATGACAATGGTGCAGTGGGT	348
FMgSSR-32028	scaffold7119	p2	(TA)12	Class I	1673	1696	TGCATCCAGCCCGTAATGGTGT	CGGTTTGCTGGCTGGTTTTGGT	200
FMgSSR-32034	scaffold1440	p2	(TA)12	Class I	63319	63342	TGCTGGTGATTGGGGTGTCGAT	AAAAGGCTAAGGTCGGCCGTGA	249
FMgSSR-32036	scaffold918	p2	(TA)12	Class I	41512	41535	TGGAGAAAAGCGAGCGGAGCAA	ACATATGGAGGTCAGGAGGTGGT	279
FMgSSR-32037	scaffold2491	p2	(TA)12	Class I	23954	23977	TGGAGGGGAGACAGGAGGCTTTTA	TTGGGGCGATTAGGCAGCTGAA	350
FMgSSR-32038	scaffold6331	p2	(TA)12	Class I	40795	40818	TGGCATGCTTGGTCAAAGTGGC	AAGATACGGCACACGCAGCA	287
FMgSSR-32040	scaffold7893	p2	(TA)12	Class I	8420	8443	TGGTGTGACGTGGATGCTGA	ACCTATTGCCCGAAGCAGTGT	334
FMgSSR-32042	scaffold3404	p2	(TA)12	Class I	10296	10319	TGTCCACCGCTTCACAACCACA	TCATCCAAAGGATGTCTGCCGT	220
FMgSSR-32047	scaffold11057	p2	(TA)12	Class I	19391	19414	TTATGGCCGTGTGGTGCTTGGA	TTTACGAATGGGGCAGCGCA	349
FMgSSR-32048	scaffold22559	p2	(TA)12	Class I	4619	4642	TTCGCGCCGTCTTAAGCCAA	AGCAAGACCCAAAAGCCTGGT	309
FMgSSR-32049	scaffold1060	p2	(TA)12	Class I	56231	56254	TTGCACTAGGTGCATGCGGA	ACACGCCCTGCCGGATAAAACA	306
FMgSSR-32050	scaffold1155	p2	(TA)12	Class I	64797	64820	TTGCGCCGTTCGTGCAAAGA	TGGAGTTGCGATAGTGAACCGTGA	273

SSR_ID	Scaffold	SSR_Type	SSR_Motif	SSR Class	SSR_Start	SSR_End	Forward sequence	Reverse sequence	Product_size
FMgSSR-32053	scaffold1184	p2	(TA)13	Class I	71219	71244	AAACCTCCGTGCGTGTGTGT	GTGTTCTTCTGCACGAACGTGGT	200
FMgSSR-32056	scaffold12503	p2	(TA)13	Class I	11010	11035	AACGGCCAACTAGGCGACTGAA	CGCGCCGCTGGACTTGATAAAA	308
FMgSSR-32057	scaffold2227	p2	(TA)13	Class I	56381	56406	AAGAGTTTTGGCGTGGTGGGGA	AGCGAGAATCGGCGAGAGAA	324
FMgSSR-32062	scaffold2462	p2	(TA)13	Class I	52655	52680	AATTGGCTCCGCAACTCCGACA	TCGTGGCTGCATCACGGTTT	280
FMgSSR-32064	scaffold8895	p2	(TA)13	Class I	7662	7687	ACACCGTATGGACGTGTTCACT	CTGAGTGGACGGATGCTTTCTTCA	350
FMgSSR-32065	scaffold6362	p2	(TA)13	Class I	9775	9800	ACAGCGTGCCATTTACCGCT	GGATAGCTGCTAAAGTCGATCAGCC	337
FMgSSR-32067	scaffold6218	p2	(TA)13	Class I	7545	7570	ACCCTACCATCACTCGTTCCTGGT	TTCCCTGGGCACTCGCAAAA	350
FMgSSR-32069	scaffold24664	p2	(TA)13	Class I	2546	2571	ACCGGCCGGCCAAATTAACTGA	TTCACCATGTTCCCCATCGAGC	349
FMgSSR-32073	scaffold519	p2	(TA)13	Class I	9659	9684	AGCAAATGTCTGACGGAGCCA	TGCCAGCAGTCTCCAGACATT	336
FMgSSR-32078	scaffold340	p2	(TA)13	Class I	106500	106525	AGTTTTGAGGGCCTGCTGCT	TGCCCAATCATGTCGCGTCT	220
FMgSSR-32080	scaffold5764	p2	(TA)13	Class I	29899	29924	CCCCAGGAAACGATCAAATAGCAC	ATTCAGCGTGCTGCGCTCCATA	328
FMgSSR-32082	scaffold5390	p2	(TA)13	Class I	23187	23212	CCTTCAGCAAATGGGGTGTCGT	AGGTGGGTTCATCGATCTAGAGGGT	350
FMgSSR-32085	scaffold11404	p2	(TA)13	Class I	17315	17340	GCAAATTAACCAGCGGCGCA	ACGCACATCCTCTCCCAGTTGT	350
FMgSSR-32089	scaffold4096	p2	(TA)13	Class I	8248	8273	GGACACCTAGGACTGTTGTTGCCT	TGGTCAAAGCATTTGGGGACGC	349
FMgSSR-32090	scaffold348	p2	(TA)13	Class I	54699	54724	GGGGGTTTGCTTGCATTACGTGT	TCGATGCTTGCTGCTAGGTCA	335
FMgSSR-32092	scaffold675	p2	(TA)13	Class I	43106	43131	TAGCCAGGCGTGCATGATGAGT	AATGCGAAGAGCGCCGGAAA	322
FMgSSR-32094	scaffold12244	p2	(TA)13	Class I	7602	7627	TCACAGCCCGCCACATCTAA	AACCGACCAGCACGCTGAAT	312
FMgSSR-32097	scaffold71	p2	(TA)13	Class I	116768	116793	TCCCAACTGCTTGCTGGTCGAT	GCGCGACTTGCAGCATCAAT	308
FMgSSR-32099	scaffold8250	p2	(TA)13	Class I	30382	30407	TCGGCCGATCACTGAGTTGCTT	AGTGACACAGTCACACCAAGCTG	216
FMgSSR-32104	scaffold4605	p2	(TA)13	Class I	15274	15299	TGCCAGACCAAATCCCCTGCAA	ACCTAGGCTGCTACTACGCCTT	317
FMgSSR-32105	scaffold9	p2	(TA)13	Class I	229915	229940	TGCCTGCAAACGCATGGTCA	AACGTTTCCTCGCACATGCACC	273
FMgSSR-32107	scaffold8199	p2	(TA)13	Class I	660	685	TGCGCACGCGTCAAAAACCA	TCTGCGACACCAACAACACCA	234
FMgSSR-32108	scaffold4843	p2	(TA)13	Class I	38792	38817	TGGCCACGTCCAACGTAATGA	CCGCCATACCATGCTGGCATTT	342
FMgSSR-32117	scaffold2244	p2	(TA)13	Class I	51778	51803	TTGCCAGTGACCTTTTCCGGGT	TGCACTACATATGGCACTGGTCCT	342
FMgSSR-32121	scaffold6639	p2	(TA)14	Class I	25192	25219	ACATATCGTGGGCCGCAATGGA	ACCGTGGCGAAATGGCATCA	227
FMgSSR-32123	scaffold1489	p2	(TA)14	Class I	63704	63731	ACCAGCATGCGTTGCGTCAT	AGCGGACACATAAACAGGCGCT	286
FMgSSR-32125	scaffold692	p2	(TA)14	Class I	57750	57777	ACCAGGATGCGTAATGTGCATGG	TGTGAACTCGTTTCGCCGAGGA	239
FMgSSR-32126	scaffold1619	p2	(TA)14	Class I	33418	33445	ACCCATTCACGTCATGGGCTT	TCCACTAGCAGCTTGACTAACAGC	320

SSR_ID	Scaffold	SSR_Type	SSR_Motif	SSR Class	SSR_Start	SSR_End	Forward sequence	Reverse sequence	Product_size
FMgSSR-32128	scaffold5583	p2	(TA)14	Class I	4299	4326	ACCGCGTCCAATAGCTGCATGT	TGCTCATGTCCGATGGTCAGCA	227
FMgSSR-32130	scaffold574	p2	(TA)14	Class I	53446	53473	ACGTGCCACATGCTTAAGGCGA	TTCAGCTGCTCCCAGTTTGGCA	324
FMgSSR-32133	scaffold3420	p2	(TA)14	Class I	39568	39595	AGCACAGTGCGATCACCTGCAT	ATCAGGCTGCAAGCCATGGGAA	305
FMgSSR-32134	scaffold12798	p2	(TA)14	Class I	15406	15433	AGCATTCAACCACTGGCCACACT	ATGAGTGGCGACCGGGTGTTTT	211
FMgSSR-32137	scaffold10591	p2	(TA)14	Class I	17283	17310	AGCGCCAGAGTGTAGCATTGGA	TACGTGTGACCCCAACGCAT	336
FMgSSR-32141	scaffold492	p2	(TA)14	Class I	43420	43447	AGGCTTGGAGCAAAGCCACTGA	TCCAGAGCTTACTCCAGTCGATAGC	285
FMgSSR-32146	scaffold9108	p2	(TA)14	Class I	7802	7829	ATAGTCACTGCAGCATGCCCAC	AGAGCTCGGCTATAACATGTCGTG	328
FMgSSR-32148	scaffold361	p2	(TA)14	Class I	14152	14179	ATGGCAAAAGCCCGGACGAA	TGGGTGCCAATGTGCCATCA	344
FMgSSR-32150	scaffold30687	p2	(TA)14	Class I	4442	4469	CACCGGCGTAAGCTGCATTTGT	AGGCGCCAGTCATCGAGATCAT	347
FMgSSR-32151	scaffold20888	p2	(TA)14	Class I	4037	4064	CCGACCTGAATGACTTATGTTGGG	CGTCTACATCGTCACTAAAGCCC	350
FMgSSR-32152	scaffold2441	p2	(TA)14	Class I	29118	29145	CCTTTTTAAGTGGGTCCCATGTCC	GCGCGTTTCCTTCTACTCTTCAGT	348
FMgSSR-32157	scaffold256	p2	(TA)14	Class I	7180	7207	GTGCGTTGCTTGCACGGTTT	ACGTGACAATCCACTGCTGCCA	243
FMgSSR-32159	scaffold2298	p2	(TA)14	Class I	15590	15617	TCACAACCACCACAACAGCTCTCA	ACCCAGCAAGCCATGCTCATCT	314
FMgSSR-32161	scaffold891	p2	(TA)14	Class I	34053	34080	TCCCGGTTTCATGGCATCGGTT	ACAGGTCCGCCGTGCAAATA	346
FMgSSR-32162	scaffold26863	p2	(TA)14	Class I	3744	3771	TCGGTGACCAGAGCGCTACTTT	ACGCGTTAAATAGCGGGACCA	336
FMgSSR-32165	scaffold2180	p2	(TA)14	Class I	47213	47240	TGACAAACTCGCGACAGCGT	ACGTAATTGCGTGCAGGCGT	263
FMgSSR-32167	scaffold5281	p2	(TA)14	Class I	3384	3411	TGCACCAATTACGGGAGCAGGT	TGGGTGGCTTGAAACGTGCAT	327
FMgSSR-32170	scaffold1501	p2	(TA)14	Class I	12486	12513	TGCTAGACCTTGCGTGTTTCGC	AGCGTTCCGCCATTGGTTGT	215
FMgSSR-32171	scaffold2925	p2	(TA)14	Class I	31959	31986	TGCTCTAGTCGTGTCAGTCGCA	ATGATTCCGTGGTTCCCAGAGG	204
FMgSSR-32174	scaffold186	p2	(TA)14	Class I	25336	25363	TGGCCTGCATGCCTGTCATA	ACGTGCGTGACCCATGAAGT	281
FMgSSR-32176	scaffold79458	p2	(TA)14	Class I	353	380	TGTTCGAGGCCGACGTGCTAAT	TTTTGCGTGTCACGTGGGGA	338
FMgSSR-32177	scaffold361	p2	(TA)14	Class I	16624	16651	TTCACTCCCAGCTGGTGCAA	AGGCTCAGCAGCATCCACCAAT	241
FMgSSR-32178	scaffold582	p2	(TA)14	Class I	113586	113613	TTGGTGGGCGGCTAACATTGGA	TGCTTGGACGCAACGCTTTCA	253
FMgSSR-32179	scaffold65	p2	(TA)14	Class I	65510	65537	TTTGCCGTGACCGAACCCAA	CTGCCCATAAAAGGGCAAACTCTG	307
FMgSSR-32180	scaffold65	p2	(TA)14	Class I	64839	64866	TTTTGCCGTGACCGAACCCA	TCCGGCTCTTTTGCTCCGTT	275
FMgSSR-32184	scaffold12559	p2	(TA)15	Class I	18083	18112	AACGATCAAGCAGCAGATCGGG	TCGCGATCATGGCGTCAACT	203
FMgSSR-32188	scaffold17301	p2	(TA)15	Class I	12161	12190	ACATGCGCGTGCTCTACGAT	TCCAGAGCGCCATCAGGGATTT	340
FMgSSR-32192	scaffold5197	p2	(TA)15	Class I	7286	7315	ACTCTGACTTGCCAACCCTCT	CGGATTTTGTTCTTGACACGCCAC	350

SSR_ID	Scaffold	SSR_Type	SSR_Motif	SSR Class	SSR_Start	SSR_End	Forward sequence	Reverse sequence	Product_size
FMgSSR-32193	scaffold25904	p2	(TA)15	Class I	7585	7614	AGCGCCCTGAAGATACGCTGAA	TTTGGTGGTGTGTGCGACGA	270
FMgSSR-32194	scaffold12786	p2	(TA)15	Class I	12482	12511	AGGCAGTCCTTGGGCAAAAC	TCAGTGCGGCATGAACGAAGT	346
FMgSSR-32195	scaffold4467	p2	(TA)15	Class I	35979	36008	AGGCGGACGCCTGTTACACTAA	ACTGCACCAACCGACCAGCTAT	277
FMgSSR-32199	scaffold30687	p2	(TA)15	Class I	4266	4295	ATCGGTGCCACGCTGCATTT	GCCGGTGCAAATGAAGCACCAA	339
FMgSSR-32200	scaffold2144	p2	(TA)15	Class I	37227	37256	ATGCCCTCACATGCTTTGCTGC	TGGCAAACCACTTTCCCCCT	349
FMgSSR-32202	scaffold21093	p2	(TA)15	Class I	11600	11629	ATGTGGAAAGTGGCTGGGGTGT	TGGGAATGTGATCCATGCGCCTT	280
FMgSSR-32203	scaffold17159	p2	(TA)15	Class I	616	645	ATTGCACGACCGTGGCGACTAA	AAAGTTAGCGCGTGTGGGCA	287
FMgSSR-32205	scaffold5042	p2	(TA)15	Class I	30842	30871	ATTTACTCGGCGACCTCTGCCT	AAACGCCGCCCTCCATGTTGAT	313
FMgSSR-32209	scaffold1	p2	(TA)15	Class I	101670	101699	CGTGATGACCCTGAACGGTGGAAA	AATGGCTATGCGCGGCAACA	349
FMgSSR-32211	scaffold200	p2	(TA)15	Class I	35306	35335	GCGTACCTAGCGTTTGGTAAAGGT	CCCTGGTTCAAGCATCTCGTCT	350
FMgSSR-32214	scaffold3269	p2	(TA)15	Class I	21585	21614	TACCACCAAAACTTGGGGCCGT	GCTGGAGCATGTTTCCGCGAAT	263
FMgSSR-32217	scaffold8716	p2	(TA)15	Class I	17859	17888	TCACGACGCACACACTCACACA	TGCCCGTCTGCTTCTTCACTGT	301
FMgSSR-32220	scaffold7075	p2	(TA)15	Class I	27822	27851	TCGTGCCACAATTTCCCTGCT	ACCAAGCATAGAACGGGCGCAT	334
FMgSSR-32223	scaffold2444	p2	(TA)15	Class I	26461	26490	TGACATGCATGCGTGGCTCAAC	ACATGCCAACGAAAAGCCAGCC	346
FMgSSR-32224	scaffold6	p2	(TA)15	Class I	273067	273096	TGACTGCTTCGTGTGCATCTGGA	TCGCGATGCCAAACACTGGA	258
FMgSSR-32227	scaffold9560	p2	(TA)15	Class I	18045	18074	TGCAGAACGCAGCAAAGGGT	AACGGAATGTGGCAGGCAGACA	298
FMgSSR-32229	scaffold133	p2	(TA)15	Class I	121764	121793	TGCGAGCTGGTGGTAGCCTTTT	ACCGAGCAAGTTGGCCACACAA	324
FMgSSR-32231	scaffold3269	p2	(TA)15	Class I	21440	21469	TGCTTTCTTTCCCTCTGCCCCT	ACGGCCCCAAGTTTTGGTGGTA	317
FMgSSR-32233	scaffold10745	p2	(TA)15	Class I	22163	22192	TGTGGCACCGCACGTATGAA	CCGTAGGTGCATCCTCCGATTA	348
FMgSSR-32234	scaffold14764	p2	(TA)15	Class I	9646	9675	TGTGTTCGTAGTGCGTTCCACCA	TCCCAGCAACAAGTGCGACCAT	257
FMgSSR-32235	scaffold16375	p2	(TA)15	Class I	1754	1783	TTACGCGAGCCTGTTTGGCA	TGCATGCTTGAGTGCTCCTGGT	201
FMgSSR-32236	scaffold6226	p2	(TA)15	Class I	8908	8937	TTCACAAGGCAGGTCAAGCCGT	TATGCTTGATGTGCGGGAGGGA	304
FMgSSR-32239	scaffold2043	p2	(TA)16	Class I	59919	59950	AAATCTCCGCACGCATGCCA	TGTCACCTGTGCTGTTTCGCT	305
FMgSSR-32241	scaffold6496	p2	(TA)16	Class I	27225	27256	ACCAGGAACTAGCCTTGGGCAT	CACGCCCACGAATGACGAATGT	325
FMgSSR-32242	scaffold7814	p2	(TA)16	Class I	30563	30594	ACCCACCTTGCCACTATAAGCA	AGAGGTTGGTTTGCCTGGACA	201
FMgSSR-32243	scaffold4205	p2	(TA)16	Class I	18293	18324	ACCGCGGCAGCTGTTTACTT	TGCTATGGCGCGAGATGACCTT	350
FMgSSR-32245	scaffold6850	p2	(TA)16	Class I	16009	16040	ACCTCGGGTGAATCCAAGCAGA	GCGCCGTTGATGTACTGCCAAT	266
FMgSSR-32247	scaffold11946	p2	(TA)16	Class I	9383	9414	ACGGCTTTTTGAAGGACGCTGC	AAGCGTCAGATTGGTTCGGCCT	244

SSR_ID	Scaffold	SSR_Type	SSR_Motif	SSR Class	SSR_Start	SSR_End	Forward sequence	Reverse sequence	Product_size
FMgSSR-32248	scaffold1825	p2	(TA)16	Class I	68643	68674	ACGTCCAAAATCTCCGTGCCCA	TTCTTGTCGCTCAGACCGAAGC	334
FMgSSR-32250	scaffold886	p2	(TA)16	Class I	89112	89143	ACTGCTGTCGATGAAATGTGCAGC	TGACAAGCACTTGGGCCGTT	348
FMgSSR-32251	scaffold11232	p2	(TA)16	Class I	3941	3972	AGCCTGCCCAGCACAAAGGAAA	AACAACGGTCTTCAGCGCCA	243
FMgSSR-32253	scaffold1489	p2	(TA)16	Class I	23673	23704	AGTGGTTGGAGCTTCGTTCCCT	ACCGTGGCGCATGTTTCACA	263
FMgSSR-32255	scaffold8477	p2	(TA)16	Class I	32887	32918	ATTTCGGAGCCGTTGCGGAGAT	TTTTCTTCCGGCGACCATGC	346
FMgSSR-32256	scaffold37	p2	(TA)16	Class I	147687	147718	GCAGACGGCTACCAAATCTCCCAT	AGGACCACAATCCGGACCGTTA	348
FMgSSR-32261	scaffold12659	p2	(TA)16	Class I	14822	14853	TCTGCTCAGCCAGGCCTTTTCA	TGCTGGTAAGACTGCGTGTTTG	278
FMgSSR-32265	scaffold15001	p2	(TA)16	Class I	7365	7396	TGCTCCACTCCATGGTGACA	TCAGGGCGAGTTAAGCTGCAA	343
FMgSSR-32266	scaffold447	p2	(TA)16	Class I	110182	110213	TGGGATGCCAGCTCGACATCATT	ACACCTGTAGCAACCACGGGTA	332
FMgSSR-32270	scaffold74	p2	(TA)17	Class I	154946	154979	AACATGCACTGCCGCACGAT	GCTCCCGATGCTGCATGTCAAA	288
FMgSSR-32273	scaffold2957	p2	(TA)17	Class I	33605	33638	ACATGGCACGATGGCAGTGA	CCGGAGGGTGTACACAGCAAAGTT	309
FMgSSR-32274	scaffold5068	p2	(TA)17	Class I	1448	1481	ACGTTCGCCATGTCCACGGATT	ATGGGCGTATGCACCAACAAGC	337
FMgSSR-32275	scaffold3769	p2	(TA)17	Class I	6140	6173	AGACCCTGCTGGACCAAGAGAA	ACACCGATCTGCAATCCATGCCT	314
FMgSSR-32278	scaffold2700	p2	(TA)17	Class I	29473	29506	AGCCTGTCCTGATGGCAACAA	ACGATAAGCTGTAGCAAGGGCGT	309
FMgSSR-32279	scaffold19905	p2	(TA)17	Class I	11543	11576	AGGTCGTGGCGGGATCAGAAAA	TAAAATGGCCAACTGCCGGTGC	293
FMgSSR-32285	scaffold12023	p2	(TA)17	Class I	3905	3938	CTACACCGATAAGCATGCCGAG	AAGGTGAAAGGACCGCGCAGAT	294
FMgSSR-32290	scaffold1718	p2	(TA)17	Class I	48913	48946	TCCAGTGGCGGCACATACCTTT	TGCTGAGGATGAGCTGCCTGTT	273
FMgSSR-32303	scaffold424	p2	(TA)17	Class I	108218	108251	TGCTACCGTAGAAGCACGAGCA	TGGTGCTCTACAAGCTGATGCG	251
FMgSSR-32306	scaffold33	p2	(TA)17	Class I	194180	194213	TGGTGACCTGAATATGGGCTGA	ACCAATCTTTCATCCCTCCTGCGA	350
FMgSSR-32307	scaffold673	p2	(TA)17	Class I	67350	67383	TGGTGGTATCTCTTGGAGGACTGT	AGCATACCCAGAGGGAGCGTTT	305
FMgSSR-32309	scaffold14148	p2	(TA)17	Class I	13896	13929	TTACCAATGGGGTGCGGTCTGT	TTTAACGTGGGGTGCTGCCT	328
FMgSSR-32310	scaffold125	p2	(TA)17	Class I	69204	69237	TTGCGAACTTCCGTGCTCGT	ATGCGAGCTACTCGTGGTCA	290
FMgSSR-32311	scaffold14242	p2	(TA)17	Class I	8444	8477	TTGTTGCACTGGATGCAGGCGT	GCACGAGCACATGGATGCTTCA	336
FMgSSR-32316	scaffold17072	p2	(TA)18	Class I	10029	10064	ACGGAAGTGTCGCCTTTCCA	TGTGATTCCGACGACGTGTCCA	350
FMgSSR-32319	scaffold3071	p2	(TA)18	Class I	3547	3582	ACGTGGTGCCATGGATGTGA	TCCCGGGGGAATTTTACGGT	296
FMgSSR-32322	scaffold2611	p2	(TA)18	Class I	49288	49323	AGGGTTACCGCTACATTCCCCA	TGCATGTCCGAAGCAGCACA	332
FMgSSR-32324	scaffold7497	p2	(TA)18	Class I	35300	35335	ATCCAATGACGCGGCCATGA	ACCTGTTGATGTCCTGCCTACT	345
FMgSSR-32326	scaffold711	p2	(TA)18	Class I	2040	2075	ATGCAGACACACGAAAGCAGCG	ACGGTACCAACAGCGTCGATGA	298

SSR_ID	Scaffold	SSR_Type	SSR_Motif	SSR Class	SSR_Start	SSR_End	Forward sequence	Reverse sequence	Product_size
FMgSSR-32327	scaffold11648	p2	(TA)18	Class I	15392	15427	ATGCCTGCCACACGGATCGAAT	TCGAACTGGCCGGAATCACGTT	344
FMgSSR-32329	scaffold937	p2	(TA)18	Class I	20081	20116	ATGGGCGCTTGCGTTGCATA	ACCAAGCAAAGCCTACCAGC	238
FMgSSR-32330	scaffold3715	p2	(TA)18	Class I	45155	45190	CACTCAAGACCAAAGTCAGCGT	GTGTGTTGGCATGATCGAACGGT	350
FMgSSR-32331	scaffold6466	p2	(TA)18	Class I	22377	22412	CCATCAGTTTGCTGCCGTGACA	AGACTGCAGAACGTTACCCCGA	276
FMgSSR-32335	scaffold986	p2	(TA)18	Class I	31793	31828	GCTAATGGCTATAAGGATGGGGCA	ACAACCATGATCTGGCGACCCA	307
FMgSSR-32339	scaffold68	p2	(TA)18	Class I	182188	182223	TGGCTTTTGCGCCTGCTTCT	AACACCTGTAGTGGCAGCGGTA	242
FMgSSR-32343	scaffold387	p2	(TA)18	Class I	64372	64407	TTTAGGAATGGTCCCGCTCACG	AGGCGGACACAGTTAAGCAGTT	308
FMgSSR-32346	scaffold1968	p2	(TA)19	Class I	3891	3928	ACACGCCCTGGTGTAGTGCATAA	TGTCAGTCCAAAGCAAGGCCCA	225
FMgSSR-32348	scaffold2091	p2	(TA)19	Class I	26081	26118	ACCGTGTTGGCTGTGGACATGA	ACCCCAATGCACCACATGCT	306
FMgSSR-32351	scaffold5056	p2	(TA)19	Class I	34841	34878	ACTCCTTGCCCACATCATGGGT	AGTGTGTTCCCATCGTGGTTGC	319
FMgSSR-32354	scaffold10936	p2	(TA)19	Class I	2094	2131	AGTCCCATACGGACACGCGTTA	ACCTCATGTCGAGTGCGTCGAT	345
FMgSSR-32355	scaffold13346	p2	(TA)19	Class I	12446	12483	AGTGAACCTACACCGGGTCCAA	CGCCCGCACCCATTCACATATT	327
FMgSSR-32357	scaffold11571	p2	(TA)19	Class I	13781	13818	ATGTTGATCCAAGGGACGCGCT	TGCGTGCGTTTGTCATTCGG	329
FMgSSR-32360	scaffold1337	p2	(TA)19	Class I	31778	31815	TCCTTCCTGCTGCACCATCT	TGTGGCACCACGGTGAAAGT	347
FMgSSR-32361	scaffold12076	p2	(TA)19	Class I	12248	12285	TCGAGACTTGTGGTTGGCGCAT	AATGCCTAGCTGGCAGGAGAGT	339
FMgSSR-32362	scaffold10180	p2	(TA)19	Class I	7506	7543	TGCATGCTGGCATCCATGGAGAT	ACCCAGATAATCCGACTGATTGGCG	318
FMgSSR-32363	scaffold2004	p2	(TA)19	Class I	11537	11574	TGCCTAGCAGCTTCAGGTCATCA	AGCTTCTGCCGCAACCAACT	318
FMgSSR-32366	scaffold1326	p2	(TA)19	Class I	2566	2603	TTTTCGCAGCAAGCCACGGA	ACGTACGTTCCTGTTGTAGGGT	305
FMgSSR-32367	scaffold1101	p2	(TA)20	Class I	43372	43411	ACACAGCGATCCGGTTTCCTT	CCGTTGCACCCATTTGGTTGGT	346
FMgSSR-32370	scaffold7303	p2	(TA)20	Class I	25864	25903	ACGGATCAAATCGTCGGCACT	AGGTGCGGTCTGCTGGTTTT	341
FMgSSR-32371	scaffold1000	p2	(TA)20	Class I	39006	39045	ACGTGCATGTCACACGGACCAA	TTTGTGCTTCGGCGTTCGCT	333
FMgSSR-32373	scaffold3382	p2	(TA)20	Class I	22535	22574	AGCACGCCACTGCAAATGACAC	TGCAGCAATGTGGAGGATGGCA	327
FMgSSR-32375	scaffold2271	p2	(TA)20	Class I	39082	39121	AGGCACGAATTCCGGCACAA	ACCATTTGATGTCCCGTCGCGT	300
FMgSSR-32377	scaffold14751	p2	(TA)20	Class I	9724	9763	ATGGTTGTGGATGAGCGGTGGT	AATGGTGGCCGCACAAATGC	328
FMgSSR-32380	scaffold16460	p2	(TA)20	Class I	11940	11979	GTGCCTCGACATCTGTGCACTATT	TCGGTTCACATCTGCGCTCT	316
FMgSSR-32383	scaffold5151	p2	(TA)20	Class I	12116	12155	TGATCAGGCCGGGAATCACT	ATGTGAGGCAACTCCCGGTT	341
FMgSSR-32386	scaffold3616	p2	(TA)20	Class I	40624	40663	TGCGTTCGTTTTCACGCCGA	GCCCGGTCTGTTCAGATTTGGT	350
FMgSSR-32390	scaffold570	p2	(TA)20	Class I	42811	42850	TGTGCGATACCTAGATGTTGGGAC	TCGACTAGGCTACCTTTCGGCA	320

SSR_ID	Scaffold	SSR_Type	SSR_Motif	SSR Class	SSR_Start	SSR_End	Forward sequence	Reverse sequence	Product_size
FMgSSR-32394	scaffold9671	p2	(TA)21	Class I	10251	10292	ACTTGTCCTGTTGCAGCCCTGA	TGACCTCCCTCGAACATGCTGAA	347
FMgSSR-32395	scaffold7724	p2	(TA)21	Class I	26902	26943	AGCTCGATCACGCCTTCGAT	GGTGGCGTCTGCACAAAACT	349
FMgSSR-32399	scaffold4133	p2	(TA)21	Class I	18362	18403	CGCACACACATGGCCTTCCAAA	CGTTTGCTGCTTTGGGCCTTGT	347
FMgSSR-32402	scaffold7969	p2	(TA)21	Class I	30532	30573	GCAGCCTTTTGAGGCCCATATCA	GTGACCGGGACTCTCTGTCTATT	350
FMgSSR-32405	scaffold3614	p2	(TA)21	Class I	16390	16431	TAACGTCAAGCTCAGCGCCA	AAATGCGTGCGGTGCAGTGA	350
FMgSSR-32411	scaffold3379	p2	(TA)21	Class I	37785	37826	TCGCATGTAAGTGGACTTGTGGC	ACCTGGGTTTTCGGCACACCTT	346
FMgSSR-32413	scaffold15535	p2	(TA)21	Class I	11720	11761	TGCTGCAGTTTTTGCACCCAC	TGCAGTGGTGGCATTCGTGA	285
FMgSSR-32414	scaffold943	p2	(TA)21	Class I	73141	73182	TGCTGCATGCTGGTATAGAAAGGG	TTGGGACGAGAGAGCGCAGTTT	320
FMgSSR-32416	scaffold48252	p2	(TA)21	Class I	4288	4329	TGGATCAGTGTCAGAACCGGCA	TGTGTGAAGCCTGCCAAACCA	255
FMgSSR-32417	scaffold6216	p2	(TA)21	Class I	22648	22689	TGTGCTTGTGCCTCTAGCCAGATT	AGGCTTCCTTCGAGCACACT	210
FMgSSR-32419	scaffold3590	p2	(TA)22	Class I	50357	50400	ACACTCCGTCTTTGCCACTTGT	AACCAGCGTGTTGCGTTCCA	306
FMgSSR-32427	scaffold594	p2	(TA)22	Class I	86104	86147	CGGTGCTAGGACTGTGTACGTT	TCCACGGCCGATTCCCTGTACATA	344
FMgSSR-32428	scaffold562	p2	(TA)22	Class I	116526	116569	TCGAGTGCAGGCTAGTTCGGTT	TGTCTGCAGGGATGTGACATGACG	281
FMgSSR-32430	scaffold2470	p2	(TA)22	Class I	32457	32500	TGCGCAAAGAGTGGCGAACA	AAATGACCGACACGCACAACGC	214
FMgSSR-32432	scaffold4329	p2	(TA)22	Class I	40901	40944	TGGAGCTTCTGTCGACAAGCCA	AACGACCCGGCACGTTGAAT	332
FMgSSR-32441	scaffold4116	p2	(TA)23	Class I	13057	13102	ACATGCAACATGGCTGGGGAGT	GCAAACTGGACCGATCCGAGAA	291
FMgSSR-32442	scaffold10026	p2	(TA)23	Class I	2162	2207	ACGAGTAGTCAGGTCGTTGGGT	ACCGATGTCAACGCACGGAA	298
FMgSSR-32445	scaffold35861	p2	(TA)23	Class I	3321	3366	AGCAGGAGGTGGTGGTTGAT	TGCGCGACATGATGAGATGAGC	224
FMgSSR-32446	scaffold9464	p2	(TA)23	Class I	691	736	AGCCTGGCGTGTACAAAGACT	TCGTTAAGCAACACTGGCACGA	346
FMgSSR-32448	scaffold1421	p2	(TA)23	Class I	12437	12482	ATTTCCTCGTCTGCGTCAGCCT	TCTGACGTGTCATGGTTCCTCT	270
FMgSSR-32450	scaffold5678	p2	(TA)23	Class I	2656	2701	CCTCTTCCAGTGCTCACCTCTTA	GAGAGGAGGAAGAGGAACGAGATT	350
FMgSSR-32454	scaffold3045	p2	(TA)23	Class I	34041	34086	TGGAGCTGTGGAAGTGCAGCAT	CCACAATACGCGGAGTGCTGTAA	338
FMgSSR-32457	scaffold8850	p2	(TA)23	Class I	14083	14128	TTCCTTCCCAGTTGGTTCCCCT	ACCGAATTCCGGGGCAAGATCA	248
FMgSSR-32464	scaffold83943	p2	(TA)24	Class I	1243	1290	ATCCCAGAAGGGTGTGTGTGT	TCATTCTCATCGCCACCCCA	285
FMgSSR-32468	scaffold983	p2	(TA)24	Class I	21274	21321	GGGAAGCACCACCTGCAAGTTA	TCAGGCACTGCTTCGCACAA	335
FMgSSR-32469	scaffold7903	p2	(TA)24	Class I	24167	24214	GGTCTGACTGAGTGTGATGGCA	ACGTGTGACATGGCTACGTGGT	350
FMgSSR-32474	scaffold483	p2	(TA)24	Class I	82344	82391	TGCATGATGGCTACTGGCTGGT	TGCGTGTCAGCAGCATGTGT	235
FMgSSR-32476	scaffold707	p2	(TA)24	Class I	7502	7549	TGGAGGGAAGAACTCCATCAGCGT	TCTCTCTCTCAGGTCTGTGGCA	295

SSR_ID	Scaffold	SSR_Type	SSR_Motif	SSR Class	SSR_Start	SSR_End	Forward sequence	Reverse sequence	Product_size
FMgSSR-32478	scaffold3012	p2	(TA)24	Class I	43910	43957	TTGCGTACCAAGGCCTGAGCTT	TCCACGGCTCACTGTGACAA	349
FMgSSR-32483	scaffold10242	p2	(TA)25	Class I	9126	9175	AGCATGAACTGGTCTTCTCGCT	CACCCTGGGTATGATTTAGCGTTG	349
FMgSSR-32484	scaffold5546	p2	(TA)25	Class I	40475	40524	ATCTCCATGAGCACGTCGTGTG	TCCTGAGCGCGCACAACTGTAT	280
FMgSSR-32488	scaffold19767	p2	(TA)25	Class I	1356	1405	CGCGCGTTGCGCTCCATATAAA	TGCGCATGTATAGCCAGTGGT	223
FMgSSR-32494	scaffold1301	p2	(TA)25	Class I	9318	9367	TGGGTGATCGTCTTTTTGTGGTGC	TGGGTTGCACCAATCGACCA	295
FMgSSR-32500	scaffold4247	p2	(TA)26	Class I	39665	39716	ATGTGACGTGGCTGGTTCAGGT	TACGCCTTGGTCGCAGATGACT	281
FMgSSR-32501	scaffold1462	p2	(TA)26	Class I	69654	69705	CGCCATTTAGCGCTGTCGTGTA	TGGCCTTCAAACGCCCTCTT	337
FMgSSR-32505	scaffold6954	p2	(TA)26	Class I	25334	25385	TGCACACGAGAGGAGCTGAA	ACGGCCACCATCGAAAACGA	256
FMgSSR-32510	scaffold1039	p2	(TA)27	Class I	32531	32584	AGTGCCGAAAACCGCACGTA	AGCCAATCAAAATCCCGGACATCG	323
FMgSSR-32512	scaffold150	p2	(TA)27	Class I	33444	33497	ATGGGGGTGTTCGTTTCCTAAGGC	AGCGTCAATACTCGCCCTGAGA	277
FMgSSR-32514	scaffold8780	p2	(TA)27	Class I	11460	11513	GTCTCCGTCCCATTAAAACTGTGC	CCACCTTCTTCTTGGACATCTGCT	350
FMgSSR-32515	scaffold7678	p2	(TA)27	Class I	7820	7873	TCATGCACCAGTTGGGCGAA	AGCTGGTACACGCACGATCCAA	207
FMgSSR-32521	scaffold14220	p2	(TA)28	Class I	7166	7221	TGCAGACAGTGAATGTGACACTGC	CCCCAGTCAAAGCGAATGCTGA	328
FMgSSR-32525	scaffold8284	p2	(TA)30	Class I	6861	6920	ACACGTGGCCATAGCTGAGA	AAGATTATGCCGGTGGCAGCGA	324
FMgSSR-32529	scaffold574	p2	(TA)30	Class I	62885	62944	TCTCCGTCGTCTCCAACTTCTCA	TCCAGAGCGCTACCCATTCCAA	323
FMgSSR-32534	scaffold3453	p2	(TA)31	Class I	50140	50201	AATGCAGGTCGGCTTTCATGCG	ACGTTCGGCCACATAGACAAACCA	349
FMgSSR-32535	scaffold11365	p2	(TA)31	Class I	17577	17638	ACGAGGAATAGGGCATGCCAAC	TTGTTCTTCTTGCGGCGTCA	324
FMgSSR-32538	scaffold3987	p2	(TA)31	Class I	23582	23643	ATTCCGTTCGGCTCTGCGTT	TGAGCCTGCGAGAAAACGATGC	275
FMgSSR-32540	scaffold1250	p2	(TA)31	Class I	39893	39954	TGCGAGCTGTTCCTGCACAT	CCATTCAAGCCCCAGCATTACA	348
FMgSSR-32541	scaffold589	p2	(TA)32	Class I	52997	53060	TGTGCAAGCCGGTGGAGTAT	TGAGCGTCTGGCTAAATCCGTGA	315
FMgSSR-32542	scaffold11762	p2	(TA)32	Class I	23253	23316	TTATGGTTGCGGGTCAGTCGCT	TGGCTGATGATTAGGCAGGGTGT	347
FMgSSR-32546	scaffold3478	p2	(TA)34	Class I	33725	33792	ACCGGGTTTGAACATCGGTGAGT	GCCTTGCAAAGCTGGGCTTCAT	324
FMgSSR-32547	scaffold13751	p2	(TA)34	Class I	15105	15172	GCATGGGGCTCCGAGCATTTTA	TCTCCAATCAGGTGGTGTCCGT	344
FMgSSR-32548	scaffold13751	p2	(TA)34	Class I	16281	16348	GTGGAGGAAGACGTACAGCCAA	AGGCTGTCTCCAATCAGGTGGT	346
FMgSSR-32551	scaffold1225	p2	(TA)35	Class I	66615	66684	ACAGCTTACGCTTCGCTGAGGT	TGAGGACGAGCTGTGCTCTTT	266
FMgSSR-32552	scaffold13	p2	(TA)35	Class I	42510	42579	AGCTTGCCTTGAGTTCAGCAGC	ACATCGCACACCTCTCAACACT	335
FMgSSR-32554	scaffold12425	p2	(TA)37	Class I	229	302	TTGTCACTGGCAGGCAACCACT	TTGACATTGCCGTAAGGGCCAC	311
FMgSSR-32555	scaffold1529	p2	(TA)37	Class I	49069	49142	TTGTGGAATCGCAGTGGTGGCT	TAAGAAGCCGCAGTCGAGGA	346

SSR_ID	Scaffold	SSR_Type	SSR_Motif	SSR Class	SSR_Start	SSR_End	Forward sequence	Reverse sequence	Product_size
FMgSSR-32557	scaffold4513	p2	(TA)38	Class I	6469	6544	TGGCCTGGCCGCATAAGAAA	ATCCCACAATGACTCCCCTGTCCA	227
FMgSSR-32558	scaffold62	p2	(TA)43	Class I	209450	209535	TGCATTGCTGCAGCGCATAAGT	TCAGCTAGGATGTGGTCTAGGTGC	278
FMgSSR-32563	scaffold5016	p2	(TA)6	Class II	39649	39660	AAACGAGCAACGCCCACAGT	TCGTGTCATCCACGCCACTT	300
FMgSSR-32564	scaffold118	p2	(TA)6	Class II	106689	106700	AAACGTGTAGCACGTGGGGAGA	ATTGCTATGCTGGCATGGGCCT	286
FMgSSR-32565	scaffold3128	p2	(TA)6	Class II	58486	58497	AAACTGCAACCAGGCAGGAGCA	AAAGCACGCATTCCGTTCTCCC	236
FMgSSR-32567	scaffold7414	p2	(TA)6	Class II	4054	4065	AAACTGGCAAGGTGGTCCGTGA	AACGACAACGGCCGAACGAA	295
FMgSSR-32568	scaffold535	p2	(TA)6	Class II	113282	113293	AAACTGGGGAGGCAGCACTTGT	GTGCATTGGCCTCATGTTAGGT	273
FMgSSR-32572	scaffold4940	p2	(TA)6	Class II	44547	44558	AAAGCACGCATTCCGTTCTCCC	ACCGCGAAAGGTGCAGAGTT	256
FMgSSR-32573	scaffold4189	p2	(TA)6	Class II	7975	7986	AAAGCAGGTGCAGGGCTATGGA	GGTGCTTATTCTCCACGCTATGCC	320
FMgSSR-32576	scaffold1722	p2	(TA)6	Class II	27830	27841	AAATCGGCTGGCCCACAACT	ACTGGCCTTAAACATCCCTGGCT	306
FMgSSR-32577	scaffold265	p2	(TA)6	Class II	89841	89852	AAATCTTGCGCCTCCAAGAGCC	ACTACTGCGTGCTTGCCCCTTT	301
FMgSSR-32580	scaffold7524	p2	(TA)6	Class II	10708	10719	AAATGGCAACGTGCGTGTCTCG	TCCATGGCCGGTTTGTCTGA	271
FMgSSR-32581	scaffold422	p2	(TA)6	Class II	13748	13759	AACAAAGCAGCTGTCAACGGCG	TTTTTCCTGCCGCAAGCACT	288
FMgSSR-32585	scaffold6474	p2	(TA)6	Class II	31242	31253	AACACGTCGCATTCACCGGA	TGCCCAATGACACCGAAAGATG	219
FMgSSR-32590	scaffold8882	p2	(TA)6	Class II	8737	8748	AACCTTCATGGGTTTCAGAGGGGG	TCAAGCTGTTAGGGCCCACCTT	301
FMgSSR-32592	scaffold1943	p2	(TA)6	Class II	1624	1635	AACGTGAAGAGTGCCTGATCGC	TGCTCTGCCATCCCTTCATCCA	334
FMgSSR-32594	scaffold30584	p2	(TA)6	Class II	4677	4688	AACTAGCAAATTGGCCCGCCCT	GCCAGCGTGAAGAGCTTGCATT	322
FMgSSR-32597	scaffold23386	p2	(TA)6	Class II	4468	4479	AACTCGGAGGGAGATGGCTGGTTA	TTTTGTGGTGCCCGAGTCGT	244
FMgSSR-32600	scaffold12036	p2	(TA)6	Class II	4791	4802	AAGAAGGGCTGCACGAGAGGAT	AACAAGGTTAACTCGCGGCCCA	280
FMgSSR-32602	scaffold383444	p2	(TA)6	Class II	82	93	AAGACGTCGTGCTCCCCTGTTT	GCTGCATGCGCGTGTCTGTAAA	202
FMgSSR-32603	scaffold6489	p2	(TA)6	Class II	7818	7829	AAGCACGCATCCAGCACCTT	ACGTTCACCACGCCAAAAACGA	308
FMgSSR-32604	scaffold7592	p2	(TA)6	Class II	32241	32252	AAGCATGCACCCTGGGTTGTGA	TGCCCTTTCCCAGGCTAAATCGT	223
FMgSSR-32611	scaffold1563	p2	(TA)6	Class II	37878	37889	AAGGGTCAGCATGCGTGCGATA	GCTGCTCGATCGACGACAACAA	328
FMgSSR-32613	scaffold14841	p2	(TA)6	Class II	3524	3535	AAGTCACTGCACGCTGGGAA	ACCGTCCGATCCAACGTCATCA	213
FMgSSR-32615	scaffold69671	p2	(TA)6	Class II	931	942	AAGTGACGCTGTTCTTGTGCCG	AACGAGGCTTCTTGCCACCCTT	288
FMgSSR-32616	scaffold55442	p2	(TA)6	Class II	3210	3221	AAGTGGAGTCGCAGACAGGCTT	ACACTGAAACTGAACCACCCCC	232
FMgSSR-32618	scaffold613	p2	(TA)6	Class II	27769	27780	AATCCAGCCGCAACTATCGGCA	AAATTGGGCTGTCCGGCTGT	298
FMgSSR-32626	scaffold18212	p2	(TA)6	Class II	7769	7780	AATGTTCCGGATACAGGGCGGT	CATGGAGCTAAGTAGCAGATGTGG	287

SSR_ID	Scaffold	SSR_Type	SSR_Motif	SSR Class	SSR_Start	SSR_End	Forward sequence	Reverse sequence	Product_size
FMgSSR-32629	scaffold26331	p2	(TA)6	Class II	2218	2229	AATTTGCATGCGACTGTCGGCG	AGCATCTGCTGGGATGCTCT	347
FMgSSR-32631	scaffold3182	p2	(TA)6	Class II	33144	33155	AATTTTGGCCCACGCCTGTGGA	AGACCGCTGCCTTTTTGCAT	290
FMgSSR-32632	scaffold38	p2	(TA)6	Class II	24992	25003	AATTTTTGCGGGGAGCTGTGGC	ATTTTGCAGCAGCAGTGGCTCG	209
FMgSSR-32635	scaffold10543	p2	(TA)6	Class II	27108	27119	ACAACACCCACATTCAGCAGCA	TGTTTAGTGCCCTGACCCCTGA	233
FMgSSR-32636	scaffold4893	p2	(TA)6	Class II	3482	3493	ACAACAGGCATCTCGCAGTCCT	CCGGCTCGTTGCCCTTCAAAAT	314
FMgSSR-32637	scaffold6861	p2	(TA)6	Class II	19772	19783	ACAACTCAGCAAACCAGGGGCA	CGAGGTGTCAAACAGTAACGCA	334
FMgSSR-32638	scaffold218531	p2	(TA)6	Class II	806	817	ACAACTCCAATGACAGAAGCCTCG	TCACGTTTTGGGGGCACGTACA	318
FMgSSR-32640	scaffold14549	p2	(TA)6	Class II	13624	13635	ACACAAACCGAGCACCTTGC	AGCTCGAAAAGAGCTAGCCGA	302
FMgSSR-32643	scaffold10029	p2	(TA)6	Class II	24256	24267	ACACACAGGGAGCAGGTTGT	TGCTGACTTTCCTGGTGCTGT	334
FMgSSR-32644	scaffold64	p2	(TA)6	Class II	130083	130094	ACACACCCATTCCGGTTGCT	AGCAGGCCAGTTGCCTTAGCTT	321
FMgSSR-32645	scaffold73307	p2	(TA)6	Class II	412	423	ACACAGCCATAGACCTGAGATGTGG	AGGATGCCGGTCCCATGTTGTT	218
FMgSSR-32650	scaffold8196	p2	(TA)6	Class II	26548	26559	ACACTGAGCAGCTAGGGTGTAGA	GCACCACCTCCATCTCTATTCAAC	334
FMgSSR-32655	scaffold675	p2	(TA)6	Class II	15137	15148	ACAGCGGCCAACAAGCAGTAGT	TGCCTCGCGTGAATTGGTGT	319
FMgSSR-32657	scaffold454	p2	(TA)6	Class II	26124	26135	ACAGGGCCTAAGATTCGTCACTCA	ACGCCCCAAGTCCAAAAACCCT	308
FMgSSR-32658	scaffold5998	p2	(TA)6	Class II	7983	7994	ACAGGTCGCATGGCCTAATGGA	TCCTCTACTTTCAGAGCAGCGGGT	297
FMgSSR-32664	scaffold4109	p2	(TA)6	Class II	7085	7096	ACATGACGTGTCCGGCCATA	ACTCACTCCATCGCTTCCACCA	281
FMgSSR-32665	scaffold8902	p2	(TA)6	Class II	32652	32663	ACATGAGCCTCGTAAGCGAACA	TGGCCGATAGAGGGGTGTTTGA	242
FMgSSR-32668	scaffold1981	p2	(TA)6	Class II	27250	27261	ACCAACACGATGCACTACTTGC	AGCGATGAAACGATGCCGCT	257
FMgSSR-32670	scaffold7614	p2	(TA)6	Class II	28804	28815	ACCAAGACAAACCGACAGAGCG	ACGTCCAACATTGCGGCCAA	274
FMgSSR-32673	scaffold2562	p2	(TA)6	Class II	34553	34564	ACCAGCATGCGCTTCAACCA	CCATGGGACGATGTTTGGATCTTC	302
FMgSSR-32675	scaffold1667	p2	(TA)6	Class II	41370	41381	ACCAGCGTCCATACAACGACA	ACACTCAAGTGACTGCCAACGACA	333
FMgSSR-32681	scaffold94552	p2	(TA)6	Class II	247	258	ACCCCCACCCGTGACCATAAAT	TCCGCGCATATGATTGAAGCCT	270
FMgSSR-32684	scaffold5022	p2	(TA)6	Class II	37479	37490	ACCCTGGCACCTAACAAATGGCT	ACAAATCAGCCGCCACCTTCCT	315
FMgSSR-32685	scaffold19275	p2	(TA)6	Class II	7617	7628	ACCCTGGCTCAATATGCCTGGT	AGGCACTGGAGGCTATAAGTGTC	343
FMgSSR-32686	scaffold5475	p2	(TA)6	Class II	27078	27089	ACCGAACAGGTCCTACAATAAGCC	CCACCGGAGGCATCTCTTCTTCTT	334
FMgSSR-32687	scaffold15986	p2	(TA)6	Class II	8921	8932	ACCGAGACGTGGATGTCCGAAA	AGGAAAGCAACGCGCTGAGA	299
FMgSSR-32700	scaffold1530	p2	(TA)6	Class II	61084	61095	ACCGTTTGGTCCACAAGTTCTGC	TGCCATGGGGTGGATCGCTAAT	307
FMgSSR-32706	scaffold2664	p2	(TA)6	Class II	45141	45152	ACGAAACTCGCCTATCTGGCCT	GGCCATGTTTCGATTGACTAGGAC	257

SSR_ID	Scaffold	SSR_Type	SSR_Motif	SSR Class	SSR_Start	SSR_End	Forward sequence	Reverse sequence	Product_size
FMgSSR-32708	scaffold1496	p2	(TA)6	Class II	2841	2852	ACGAACAGGCAACGCCGAAA	ATCCAGCCGCACAATCGACA	288
FMgSSR-32709	scaffold1621	p2	(TA)6	Class II	32224	32235	ACGAACGTGTCCACAGTCACA	TGTGGGCGCACGTTCATTGT	254
FMgSSR-32714	scaffold2513	p2	(TA)6	Class II	56048	56059	ACGACGCGCTGAACGAAGTACA	AGGCAGGAACGCGAGGAAAAGT	312
FMgSSR-32715	scaffold10	p2	(TA)6	Class II	66081	66092	ACGACGGCGCCAAAAGTTCA	TCTTTGACTGGGCCTGGTTGCT	301
FMgSSR-32721	scaffold10342	p2	(TA)6	Class II	10010	10021	ACGAGTTTGGCTTTGCTGCACG	TGGGCGTGGGAAGCTCCAAATA	332
FMgSSR-32722	scaffold6271	p2	(TA)6	Class II	25847	25858	ACGATAAAGCCGATCGTGGCCT	TGCTGCTCCTCGAAACCGTTGA	277
FMgSSR-32726	scaffold3960	p2	(TA)6	Class II	33160	33171	ACGCACTCTTCGGTTGTGCAGT	AGCGATGTTAGCCTTCGGGCAA	295
FMgSSR-32727	scaffold6216	p2	(TA)6	Class II	16689	16700	ACGCCCACATTGGGCTGCATTA	TTGTCCACGTTGCGAGCCAT	254
FMgSSR-32728	scaffold11029	p2	(TA)6	Class II	15731	15742	ACGCCGGTGATGCGAATTGA	ATGCATGCGTGGTGGCACTT	332
FMgSSR-32731	scaffold5393	p2	(TA)6	Class II	22229	22240	ACGCGACCACATGCCAACAA	AATTGGGCTGGTGGAGACTGCT	222
FMgSSR-32738	scaffold26029	p2	(TA)6	Class II	1449	1460	ACGTACTGCAGCCAAACCGT	AGGGTAGCGGTGGTCACAACTT	349
FMgSSR-32739	scaffold2360	p2	(TA)6	Class II	2235	2246	ACGTAGTGTTGGAGCTTCCTGC	AATGCTGAAGACTCGTGCGGCT	323
FMgSSR-32741	scaffold25470	p2	(TA)6	Class II	6072	6083	ACGTCGTTTGGCGGACTGTT	ACCGTAACGCCGAGAAGCCAAT	345
FMgSSR-32743	scaffold1433	p2	(TA)6	Class II	12970	12981	ACGTGCACTGATGTGGCCTT	TGTCGAGCACACGGACTTGCTA	267
FMgSSR-32745	scaffold6087	p2	(TA)6	Class II	35920	35931	ACGTGCTCAGCTGATTTCGTGC	AGGACGGACATGCAAGTGACCT	330
FMgSSR-32750	scaffold20782	p2	(TA)6	Class II	2120	2131	ACGTTAGTCCTTGCTACAGGAGG	TGTTCCGCAGAGGGTCCATGTT	311
FMgSSR-32752	scaffold510	p2	(TA)6	Class II	25881	25892	ACTAACCCGGCAGTTTGGACGA	ACCTCGTGCTTGGTGTTGGTCA	328
FMgSSR-32763	scaffold24	p2	(TA)6	Class II	217581	217592	ACTCGACAAAGAACCGGTTTCCGA	GCGCACTTGTCGAACGAACGAA	342
FMgSSR-32766	scaffold561	p2	(TA)6	Class II	81471	81482	ACTGACTGGGCGTGTGTTCGAT	ATGCCTTGTCGATGGTGGGTGA	346
FMgSSR-32767	scaffold51056	p2	(TA)6	Class II	3239	3250	ACTGCAGTGCGGAAGGTTGT	AACAGGCAGGTTCGCTGTGA	214
FMgSSR-32768	scaffold194	p2	(TA)6	Class II	84294	84305	ACTGGCACGCTTTGGCGTAT	TGTGATCGACGTCTTCACCAGA	329
FMgSSR-32769	scaffold1292	p2	(TA)6	Class II	52936	52947	ACTGTGAGCAGAAGCAACACCCA	TCCGTGGTGGTAATGGACGACA	320
FMgSSR-32774	scaffold1978	p2	(TA)6	Class II	35070	35081	ACTTCGCCGTCGTCGCATTT	ATGCTTGTTTGGACGACCAGCG	312
FMgSSR-32776	scaffold4580	p2	(TA)6	Class II	16442	16453	ACTTGCCGGCTTCAGCTTGT	AGTTGTTGAGTGGCCGAGCGAT	345
FMgSSR-32782	scaffold233	p2	(TA)6	Class II	129745	129756	AGAGGTGAAAGCATGGGCCTGA	AGAAGGTCGGGGCATAGAGACA	258
FMgSSR-32789	scaffold18919	p2	(TA)6	Class II	14610	14621	AGCAAAGAAGAGCTGCAGCGGA	ACGGACGAAGGTTCTTGGCACA	295
FMgSSR-32796	scaffold29015	p2	(TA)6	Class II	3963	3974	AGCACGTTGCTGCTGTTGACAC	AAGCACTGGACGGAGCAGCAAA	223
FMgSSR-32798	scaffold21999	p2	(TA)6	Class II	10519	10530	AGCAGAGAAGGGACGACGGTTT	TTTTCCGCGGTGCTGCTGTT	302

SSR_ID	Scaffold	SSR_Type	SSR_Motif	SSR Class	SSR_Start	SSR_End	Forward sequence	Reverse sequence	Product_size
FMgSSR-32799	scaffold31016	p2	(TA)6	Class II	6967	6978	AGCAGCAACAAGAGCGCCAA	AGCCAACCCAACTTGTGCGA	251
FMgSSR-32801	scaffold1136	p2	(TA)6	Class II	74477	74488	AGCAGGAACGACGAGCCAACAA	AGAGGGCCTCGGTCCATAACAT	318
FMgSSR-32805	scaffold19727	p2	(TA)6	Class II	12950	12961	AGCATGCAAGGCCGGAACGAAT	TGGGTGTGATTTAGCGCTGC	326
FMgSSR-32814	scaffold26715	p2	(TA)6	Class II	9547	9558	AGCCCACCGTACCAACATCA	ACAGCTCCAGGCCAGGCAATTT	232
FMgSSR-32819	scaffold3349	p2	(TA)6	Class II	9031	9042	AGCCGTGCAGAAAAACGCGA	CCGACAACATGATGCCAACGCA	335
FMgSSR-32824	scaffold3990	p2	(TA)6	Class II	23357	23368	AGCGAGCGAGAGTAGCATGTGA	AGTGGCGCGCGATTGCATTT	334
FMgSSR-32825	scaffold1448	p2	(TA)6	Class II	15933	15944	AGCGATGTTGCGGATGCTGA	AGCGAGCGTCCGTGTTTCAT	320
FMgSSR-32827	scaffold13317	p2	(TA)6	Class II	840	851	AGCGCGCAGCACACCATTTA	TTAGTCCTATGGGTGGGCCCCTTT	245
FMgSSR-32830	scaffold928	p2	(TA)6	Class II	59875	59886	AGCGGCGGAACCAGGATCAAAA	AGCGTATGCAGGCAAGGCAA	300
FMgSSR-32833	scaffold43	p2	(TA)6	Class II	31669	31680	AGCGGTCTAAGGCGCCAGATTA	AATTTGTCCTTGCTGGCTGCCG	286
FMgSSR-32834	scaffold23057	p2	(TA)6	Class II	3306	3317	AGCGTGCAGCGCGTCATTTA	ACCAGCGTGACATCGTGGCAAT	200
FMgSSR-32835	scaffold6553	p2	(TA)6	Class II	7155	7166	AGCGTTTTGTGGCCCGCATT	TTTCCTCCGCTGCTGCTTGAGT	292
FMgSSR-32837	scaffold375	p2	(TA)6	Class II	79744	79755	AGCTCATCTTGCACGGAGCGTT	TGTTCTTTGGCGCGCGACTT	276
FMgSSR-32840	scaffold1684	p2	(TA)6	Class II	65815	65826	AGCTTGCAGCGTTGTGCAGT	TTGTCGTCGCCCACTAGCAT	268
FMgSSR-32842	scaffold10036	p2	(TA)6	Class II	5515	5526	AGCTTTTTCCCCGCTCATGGGA	TGAAAGAAGGGGCAGGGTCA	338
FMgSSR-32846	scaffold973	p2	(TA)6	Class II	60558	60569	AGGAGCGAGTGAGCCACACAAT	GCAGACGGACGCAGCAGAAAAA	340
FMgSSR-32848	scaffold10	p2	(TA)6	Class II	36974	36985	AGGAGTGGGAGCAAATGCTGGT	AAGCCACAAACCAGTGCACCAC	297
FMgSSR-32849	scaffold2298	p2	(TA)6	Class II	14440	14451	AGGATGTGGATGCACGTGCTGA	TTTCGATGTGTGGCCTGGGGAA	242
FMgSSR-32851	scaffold2638	p2	(TA)6	Class II	3765	3776	AGGCAAACTGTCGAAGACCGA	AGCTGTCCCCCATGCTACTACTGT	201
FMgSSR-32855	scaffold299606	p2	(TA)6	Class II	109	120	AGGCAGCTCAATCCAGATAACGCA	TGCATGGCGCAAAGATGAGC	203
FMgSSR-32857	scaffold5704	p2	(TA)6	Class II	11855	11866	AGGCCCTAAGCGGCGATTTTGA	TTGGTTGCCGCTCATGTCTCGT	303
FMgSSR-32858	scaffold523	p2	(TA)6	Class II	84561	84572	AGGCCGCTGATTGCTGGAAGTT	ACCTCGAGTGAACAATGGTGGA	270
FMgSSR-32860	scaffold16211	p2	(TA)6	Class II	5307	5318	AGGCGATCCTGTAGCTCTGACCAT	ACTCTGAAGCGTCCACAGCA	214
FMgSSR-32861	scaffold851	p2	(TA)6	Class II	10246	10257	AGGCGCTCCTGCTGTGTTGAAT	ACACAGCCAAAGCCAATCCCAGT	273
FMgSSR-32862	scaffold1927	p2	(TA)6	Class II	4095	4106	AGGCGGGAGAAGAAAGCAACGA	ACCTTGATGGAAGCAGAAACGGGA	302
FMgSSR-32864	scaffold17112	p2	(TA)6	Class II	6754	6765	AGGCGTTCAAAGCCAAGCTTCA	ACGACCTTAACCGCATGCAACG	321
FMgSSR-32865	scaffold4933	p2	(TA)6	Class II	9176	9187	AGGCTCAACAGAGTGACATGCG	TGCGTGTGATGGAATGCGTGT	348
FMgSSR-32868	scaffold1853	p2	(TA)6	Class II	56714	56725	AGGGAATCCACGTTGCTGACT	ACACAGCGCCATGAAACGAG	344

SSR_ID	Scaffold	SSR_Type	SSR_Motif	SSR Class	SSR_Start	SSR_End	Forward sequence	Reverse sequence	Product_size
FMgSSR-32870	scaffold7052	p2	(TA)6	Class II	20639	20650	AGGGACATTGTCGTTGGCTGCT	AGTGTTGTTCTCTGGGCGAGGA	229
FMgSSR-32871	scaffold14524	p2	(TA)6	Class II	6804	6815	AGGGAGTTTGCATGCAGGAGGA	ACGTGTGTTGTGGAAGGCCA	225
FMgSSR-32875	scaffold84	p2	(TA)6	Class II	89611	89622	AGGGGGCAGGGCATTTGTTCTT	TGTTGGCCACCAAAACCGTGA	247
FMgSSR-32877	scaffold77	p2	(TA)6	Class II	154811	154822	AGGGTGCTACCCTTCATGCTGT	ACACGGTTGATGCACAGTGGGA	261
FMgSSR-32880	scaffold20765	p2	(TA)6	Class II	2482	2493	AGGTAGCTTGTCCAGCCCCAAA	ACAGGGCGTGATTGTTCCGACA	329
FMgSSR-32882	scaffold14968	p2	(TA)6	Class II	8656	8667	AGGTGCCAGTGCTCACTGTTGA	AGACACCTTCATCGCTGCCT	316
FMgSSR-32884	scaffold12150	p2	(TA)6	Class II	4432	4443	AGGTGCGAAGCACGTGGTAA	ACGCTGCGTAGAAGTGGCTGAA	349
FMgSSR-32891	scaffold288	p2	(TA)6	Class II	106741	106752	AGTCCACCCATGTGCAGTCTT	TGCAAGCACAGGTTGCAGGT	335
FMgSSR-32894	scaffold873	p2	(TA)6	Class II	957	968	AGTCCTGTGGCGGCCATAAA	CCGTCGGGACAAGTTGTGCATT	349
FMgSSR-32895	scaffold2432	p2	(TA)6	Class II	46852	46863	AGTCGACACGGTCTGCCTGTTA	TGCACACCTCCCCTTTTGTGT	277
FMgSSR-32905	scaffold14453	p2	(TA)6	Class II	9085	9096	AGTGCGGCAAGAGCATTGGT	TTTGGTAACGCAGCCACGCA	231
FMgSSR-32910	scaffold5766	p2	(TA)6	Class II	40468	40479	AGTGTAACAGGCCTCCGAAGAT	ACAGCTACAGTCTGGCTGCACA	350
FMgSSR-32915	scaffold419	p2	(TA)6	Class II	100008	100019	AGTTTCTGGAAGGCACGTGAGG	AGCGTGCATCTGCATCTGTCCT	270
FMgSSR-32917	scaffold3496	p2	(TA)6	Class II	5342	5353	ATAAAATGGGACAGGGCCTGGG	CCCTTGCGTTGCTTGACATGGT	244
FMgSSR-32918	scaffold72506	p2	(TA)6	Class II	1684	1695	ATACGCATCACGCGTTTCCTGC	TTGCTGCCGTGATCGCCATT	322
FMgSSR-32919	scaffold900	p2	(TA)6	Class II	97125	97136	ATAGCCACCTCATGCCCCCAAA	TGGCCGCGAGGAAGAAGATT	205
FMgSSR-32921	scaffold71933	p2	(TA)6	Class II	1580	1591	ATATCTTGGCGCGGCATCTCGT	AGCGCTCAAGTCGCTCTCAT	343
FMgSSR-32928	scaffold5225	p2	(TA)6	Class II	141	152	ATCCGCGAGAGACATGAACCCT	AGAGCAAACGTGTCTGTGGCA	341
FMgSSR-32933	scaffold688	p2	(TA)6	Class II	77632	77643	ATCGGGCGCAATTCACTCATCG	TATCGCCTCGCCTCGTGATT	200
FMgSSR-32935	scaffold70789	p2	(TA)6	Class II	504	515	ATCGTTGGCGCAATCCAGCA	GCTGGCGTTGGACATCCATCAA	267
FMgSSR-32936	scaffold985	p2	(TA)6	Class II	25507	25518	ATCTCGGCACCGCAGTTGAA	TGACTTGAATGAGTCCGTGCCA	339
FMgSSR-32937	scaffold12088	p2	(TA)6	Class II	389	400	ATCTGCATGCATCAGGGGCACA	ACCGGTCGAGCTTGCAAGAAA	344
FMgSSR-32938	scaffold73882	p2	(TA)6	Class II	697	708	ATGAAACGGAGCCGAACGCA	AGGCAGAATAACCCGTGAGGCA	231
FMgSSR-32944	scaffold446	p2	(TA)6	Class II	8046	8057	ATGCAGAAAGCAGTGGCGCA	TTTCTATCGTGCCAGCTGTGGAGC	229
FMgSSR-32945	scaffold10129	p2	(TA)6	Class II	13101	13112	ATGCATCGGTGTGTGGGTGT	ATCCCCTGCCTAACTCTTGCCT	335
FMgSSR-32951	scaffold1288	p2	(TA)6	Class II	39092	39103	ATGCGAAGTGCGAAGCGCAA	TGCCGGTCGCAAGCCATTTT	303
FMgSSR-32952	scaffold13813	p2	(TA)6	Class II	21680	21691	ATGCGTTGGAAGCAGTGTGC	TGTTCTCCATGTACGGGTTGGGT	342
FMgSSR-32953	scaffold8276	p2	(TA)6	Class II	29498	29509	ATGCTCAGGCAAGAGGACCCAA	ATCGAGCATGTGCGGCTCAGAA	325

SSR_ID	Scaffold	SSR_Type	SSR_Motif	SSR Class	SSR_Start	SSR_End	Forward sequence	Reverse sequence	Product_size
FMgSSR-32956	scaffold557	p2	(TA)6	Class II	115248	115259	ATGCTTAGCGGCGCAAACTCAG	ACGGATTTTTAGACCGGCCCATCA	217
FMgSSR-32957	scaffold4110	p2	(TA)6	Class II	47707	47718	ATGGCAAACCAGGTCACCATGC	CCCTGATGCTTGGAGACAGATCCA	341
FMgSSR-32959	scaffold5034	p2	(TA)6	Class II	11863	11874	ATGGTCACAGGATGGGTGCT	TGACCTTGCAGGCAGCTGAA	333
FMgSSR-32960	scaffold1260	p2	(TA)6	Class II	7735	7746	ATGGTGAAACGATGCGACGGGA	TGCGACTCCATGGCTCAGTCAA	333
FMgSSR-32961	scaffold24708	p2	(TA)6	Class II	1529	1540	ATGGTGTCGAACCGAAACGGGA	GCCGTCGATCGATTTGAACGCA	241
FMgSSR-32969	scaffold1737	p2	(TA)6	Class II	52049	52060	ATGTGCTGCGCCAACCGAAA	TGCTGCACCTTCATCACCTTCG	348
FMgSSR-32972	scaffold12056	p2	(TA)6	Class II	9143	9154	ATGTTGGCAACGGGCAGATACC	GGCTACGATAGCAGCATCACGTCT	272
FMgSSR-32975	scaffold2098	p2	(TA)6	Class II	21145	21156	ATTCATAGGCCGAGGCTGCTGT	TGCAAAGCAAGGCGGCTCAA	241
FMgSSR-32977	scaffold2017	p2	(TA)6	Class II	2464	2475	ATTCGTGCCGAGTTGACGCCAT	TGCGTGAGGACTTGCACGATCA	324
FMgSSR-32981	scaffold541	p2	(TA)6	Class II	47699	47710	ATTGGGTCAGGCATCTCAGGGT	TATAGCGCGGCTGCTGCATGAT	274
FMgSSR-32982	scaffold3241	p2	(TA)6	Class II	41026	41037	ATTGGTCTCGTGCATGTCTGGC	AAGCGGTGGTAGCATGGTGGAA	333
FMgSSR-32983	scaffold1657	p2	(TA)6	Class II	53817	53828	ATTGTGGGCCGAAGCTGGTT	TACTCATCTGTGCGTGCCGT	213
FMgSSR-32984	scaffold12194	p2	(TA)6	Class II	4573	4584	ATTGTTGGCTGGGTTCGCGT	ATCGAGCAGTCCTGGAACGA	312
FMgSSR-32985	scaffold113	p2	(TA)6	Class II	39276	39287	ATTGTTTCCACCGCCGCTGA	AGCACACTCTCCCGTGCTTACA	300
FMgSSR-32986	scaffold34192	p2	(TA)6	Class II	1347	1358	ATTTATGGTCACGGGTGGGGGT	TTGGGTCGCTGGGCTGAAAA	275
FMgSSR-32991	scaffold263	p2	(TA)6	Class II	139284	139295	CAGCACAACGTTTGCCCGTT	AAGCAGAGCTGCAGCACGAA	209
FMgSSR-32992	scaffold46	p2	(TA)6	Class II	112718	112729	CAGTTCGGCTGTATACTGAGTGAC	TCCCACCAATTGTCCGCCACTA	350
FMgSSR-32993	scaffold1124	p2	(TA)6	Class II	27337	27348	CCAAGCAGAGAGCCCATCTGAACT	AGCATGTTGCTCGACGGCAGTA	256
FMgSSR-32995	scaffold6136	p2	(TA)6	Class II	25875	25886	CCACGTGAATTTGGTCCATCGAGT	AGTTTCAAACCCGCCACGCA	329
FMgSSR-32997	scaffold333	p2	(TA)6	Class II	122897	122908	CCAGCCCGTGACAACTGACAAT	AATCAGAGTCCTTGCCTGGGGT	310
FMgSSR-32999	scaffold7554	p2	(TA)6	Class II	11674	11685	CCAGGCTGTTGCAAACCTACA	GTTGTTGGTGAAGACCTTGCTCTC	301
FMgSSR-33001	scaffold3944	p2	(TA)6	Class II	23528	23539	CCATGGCTCGAGCATTTACGATGG	ACCGCAGCCCCAACTTATGCAA	338
FMgSSR-33003	scaffold9938	p2	(TA)6	Class II	14200	14211	CCCTCCGTCTCAAAATAAGTGTCG	CATCGTGCTCATCGTCCATGT	350
FMgSSR-33004	scaffold23	p2	(TA)6	Class II	166938	166949	CCGACCTCCCTGCCTGTGAAATTA	TGCATGTGGGCTGCTTGCAT	315
FMgSSR-33007	scaffold4290	p2	(TA)6	Class II	48982	48993	CCGCGCGAAACGTTTTGTTGA	TGCCATCACGCTCACCCTGAAA	268
FMgSSR-33008	scaffold9459	p2	(TA)6	Class II	3976	3987	CCGTGGTTTCTCAAAACAGCGT	AAGCGCTACTGCGACATGCT	249
FMgSSR-33013	scaffold8288	p2	(TA)6	Class II	10250	10261	CCTTCGTTTCACACGTTCAGAAGC	TGCCTAGTCGCTACATCCAACTGA	262
FMgSSR-33016	scaffold13187	p2	(TA)6	Class II	5961	5972	CGAGACACACGTTTTGCTCCTTGC	TGCCTTGCTGACGACGACCAAT	248

SSR_ID	Scaffold	SSR_Type	SSR_Motif	SSR Class	SSR_Start	SSR_End	Forward sequence	Reverse sequence	Product_size
FMgSSR-33024	scaffold3285	p2	(TA)6	Class II	17140	17151	CGATTTGATTTCCTGCGGCGCT	TTGGTGTGTGTGTGCCGTGT	273
FMgSSR-33030	scaffold44248	p2	(TA)6	Class II	194	205	CGCCCCACCTGTTCATCACAAA	TCACGATCCGTCTGGATCTTTGCT	334
FMgSSR-33031	scaffold38617	p2	(TA)6	Class II	2412	2423	CGCCCTTGGTTTTCTGCCTGTT	GGTCACAGGTAGGAAGGCGAGTTT	317
FMgSSR-33040	scaffold1576	p2	(TA)6	Class II	9567	9578	CGGTGGCAAAATCAAGGAACCCA	TGTGTACGTGCCCGGAAACAAC	294
FMgSSR-33041	scaffold183	p2	(TA)6	Class II	79338	79349	CGGTTGGTTGACGTGCAAGGTT	TGCGCGCTCTTCTGTTGATGT	259
FMgSSR-33043	scaffold1804	p2	(TA)6	Class II	19297	19308	CGTCGTGCAGTGCGGTTAAA	GGTGATGAGCGCGTGCATTTGA	346
FMgSSR-33044	scaffold18998	p2	(TA)6	Class II	3201	3212	CGTGAAGTTCAGTGTCATAGAGGG	AGACGGCAAACAGTGCGGAT	313
FMgSSR-33046	scaffold2346	p2	(TA)6	Class II	53651	53662	CGTGGCCAACAGATCCGATGTT	AGCGTTGTGTGATGCGTCGT	225
FMgSSR-33047	scaffold18275	p2	(TA)6	Class II	10890	10901	CGTGGTGGTACGACCCTCTTTGAA	TGCGTTGCCACGGGCTAAAA	283
FMgSSR-33048	scaffold4721	p2	(TA)6	Class II	4139	4150	CGTGTGGGTGTGCCAAAACGAA	AGGTGCACAAAGTTGCCCCA	262
FMgSSR-33053	scaffold11155	p2	(TA)6	Class II	14690	14701	CTAGCCAATCGAGTTGCGTTCA	AGTGGGCAGGTTGTTTATGGTGGA	350
FMgSSR-33057	scaffold5574	p2	(TA)6	Class II	8223	8234	CTTGCCACGCGCATGTTGAA	TGCATTTGGTCGTGAGGGCA	319
FMgSSR-33060	scaffold14470	p2	(TA)6	Class II	17963	17974	GAGCTTTGGTTGCATAGCGTGA	CTTGCGACGCGGCAGAAAAA	270
FMgSSR-33070	scaffold5351	p2	(TA)6	Class II	20395	20406	GCACGAGCGCTATCGGTTAACAA	GTGTTGTCTTGCACTCTTGCCT	268
FMgSSR-33074	scaffold806	p2	(TA)6	Class II	108573	108584	GCACGTTGCTTCCTTTTGGCCT	TGTGTTTCTAGCGGGGTGTCCA	272
FMgSSR-33078	scaffold456	p2	(TA)6	Class II	108502	108513	GCATGAAGCCTGCCGTGATGTA	TGTCGTTCTGGCCGTTTTTCCA	346
FMgSSR-33083	scaffold857	p2	(TA)6	Class II	83058	83069	GCCACATCAAGCTCAGCAAAGCA	ATTCCCCATCCAAGTTGCGCCT	222
FMgSSR-33084	scaffold18038	p2	(TA)6	Class II	86	97	GCCATGGTAATGGAGAGCAGCA	TGCGACATATGGGGAACGCA	270
FMgSSR-33086	scaffold150	p2	(TA)6	Class II	67386	67397	GCCGCACTGAGTTGAAAAGGCA	ACCAAGCGACTGTGACCAACTG	304
FMgSSR-33097	scaffold6060	p2	(TA)6	Class II	25836	25847	GCGCGTCACACGCAATGATA	ATATGCTCACCGTACCTCGCCT	244
FMgSSR-33100	scaffold6029	p2	(TA)6	Class II	14424	14435	GCGTGGCAAGTAACAGCCGAAA	TGGCCCCAATGCAAACAACCAC	232
FMgSSR-33102	scaffold2811	p2	(TA)6	Class II	66172	66183	GCTGATGAAACAATGCCGGGCT	AAGGGCGCTGCCACATGACATA	271
FMgSSR-33107	scaffold6059	p2	(TA)6	Class II	34247	34258	GCTTGAGCAGCGCCACTTTACT	GCTGCGAAACTGCGAAAGCA	256
FMgSSR-33108	scaffold3856	p2	(TA)6	Class II	13259	13270	GGAATGTGTCAAGGTTGTGTTCCC	ACACCATTGGCTCCCACTGACA	336
FMgSSR-33110	scaffold3276	p2	(TA)6	Class II	29298	29309	GGACAAGCAAGGTATGATGTCTGC	TGGACCCTTTCTTTAGCGGCCA	299
FMgSSR-33117	scaffold3356	p2	(TA)6	Class II	52840	52851	GGCCAGGAATGACTTCACGATGGA	ACGAGGTGGCGTTTGATCCACA	242
FMgSSR-33119	scaffold14902	p2	(TA)6	Class II	7629	7640	GGCGGCAAGGGCAATTTTCTCA	TCTCGCAGCGTGGCTCATTT	261
FMgSSR-33125	scaffold3207	p2	(TA)6	Class II	18839	18850	GGTCCTTGTGTTATCCCTTGTAGC	ATGCTTGAGAGCATGTTGGCCC	261

SSR_ID	Scaffold	SSR_Type	SSR_Motif	SSR Class	SSR_Start	SSR_End	Forward sequence	Reverse sequence	Product_size
FMgSSR-33126	scaffold289	p2	(TA)6	Class II	13796	13807	GGTCTGCCCAATACATCTGCACCA	TGAACAAGGTCAGCGGAGCACA	300
FMgSSR-33130	scaffold5203	p2	(TA)6	Class II	7677	7688	GGTGGGTGCATCGATCTAGAAGGT	ATGGTTGAGGCAACCGATGACG	207
FMgSSR-33134	scaffold29810	p2	(TA)6	Class II	2727	2738	GGTTGTTCTGCAATTCCCATGC	AGTGGACTCATGCTTCGGTAGTGT	342
FMgSSR-33135	scaffold8452	p2	(TA)6	Class II	3959	3970	GGTTTAGCGTCTCTAAGTGACCCAG	TTCATTATGGTTGCGGGCCGGT	273
FMgSSR-33137	scaffold321	p2	(TA)6	Class II	91295	91306	GTCAAGGCCTCTATCACAGTAGGA	TGTTTGCGTGTGAACGGCCT	301
FMgSSR-33142	scaffold938	p2	(TA)6	Class II	52531	52542	GTGACCCTGAGCATCTATGCCTAT	TTTCAGAGAAGGAGCGCTGCAC	312
FMgSSR-33146	scaffold8355	p2	(TA)6	Class II	12509	12520	GTGGTACAACAATCCACGGAAGAC	CGTGCAAGTGTAACACGCGA	350
FMgSSR-33153	scaffold2625	p2	(TA)6	Class II	6090	6101	TAACTTGCCGGCTTCGGTCTGT	TTCGTGCGAGCGTCTGACAA	341
FMgSSR-33155	scaffold11333	p2	(TA)6	Class II	4007	4018	TAATGCCGCGCTTTCTTTCGGC	ATGCGTTGCGATTTCCGGCT	295
FMgSSR-33156	scaffold3459	p2	(TA)6	Class II	44694	44705	TACCACTTCCCACGTTCCCGTT	GTGCATTCCACAGGCTTTGCTGA	303
FMgSSR-33157	scaffold7043	p2	(TA)6	Class II	10885	10896	TACCGGAAACGCGAAACGAGGA	ACGTTCAAGGCCACCAAGCTCA	305
FMgSSR-33158	scaffold23585	p2	(TA)6	Class II	11458	11469	TACGCTTTACGCGGGTGGGAAA	CGGAGGGTATTTTGCAAGAACAGG	350
FMgSSR-33161	scaffold12706	p2	(TA)6	Class II	13260	13271	TAGCAGCCTGAGTGGCCGTAAT	TGACACTAGCACCGCGCAAT	274
FMgSSR-33165	scaffold20888	p2	(TA)6	Class II	4971	4982	TAGCCGCGAGACAGCATTTCGT	AGGGATGACAGCATGCAGCAGA	262
FMgSSR-33166	scaffold3060	p2	(TA)6	Class II	53167	53178	TAGCGACGCTGACAACCGACAA	TTCTCACCGGGTGGTGTGGTTT	223
FMgSSR-33176	scaffold126	p2	(TA)6	Class II	172774	172785	TAGTGGTTGGCGCAGAACGTGT	ACCATCCGCATGCCGTAACA	244
FMgSSR-33177	scaffold3723	p2	(TA)6	Class II	7216	7227	TATGACGCGCGAGCTACAGACCTA	TGTACAGCAGCCTTGCTTGGGT	326
FMgSSR-33180	scaffold4687	p2	(TA)6	Class II	22750	22761	TCAACGATGCTGGACGCAGA	GCCCTGCGCTGGTGTCAATAAT	286
FMgSSR-33181	scaffold4467	p2	(TA)6	Class II	39075	39086	TCAACGTGCAGCGCACCATTT	AGGGTTTCAGAGCTGCCAACTGA	202
FMgSSR-33183	scaffold5546	p2	(TA)6	Class II	20443	20454	TCAAGCACACCAGCATCCCACA	TGCCAGCTTAGTTTCCGCAACA	347
FMgSSR-33185	scaffold2818	p2	(TA)6	Class II	14965	14976	TCAAGTGTCACCACACGGTCA	AGTTGGCAATCAAACCCTGGCT	235
FMgSSR-33186	scaffold2325	p2	(TA)6	Class II	15435	15446	TCACAACGGCAAACGGCCAA	ATGCCATTTAGCTCCCAGCCGT	297
FMgSSR-33190	scaffold100603	p2	(TA)6	Class II	708	719	TCACGGTCCTCAACGTGTTTGG	ACCAAGGATATTGCGAGCTGCGT	328
FMgSSR-33191	scaffold214	p2	(TA)6	Class II	145765	145776	TCACGTGAAACCTGCAGTGCCT	AGCCTGGCCCAGCTGTGATTTT	303
FMgSSR-33192	scaffold21967	p2	(TA)6	Class II	11686	11697	TCACTCTCCGACGTCCACATCA	TGTCACGTACACCCATCAGGCA	335
FMgSSR-33194	scaffold3007	p2	(TA)6	Class II	5932	5943	TCAGCCCAACCACATTGCGT	AACATGCACCTGCACCTTCCCT	236
FMgSSR-33196	scaffold8670	p2	(TA)6	Class II	10497	10508	TCAGCCTAGCGGCAACATGGAA	AACCGATGGAGCCATTGTGC	283
FMgSSR-33200	scaffold8709	p2	(TA)6	Class II	13203	13214	TCAGGACGTAGCTGTTCCTGTATC	ATCGGCCCATCATAACGCCT	283

SSR_ID	Scaffold	SSR_Type	SSR_Motif	SSR Class	SSR_Start	SSR_End	Forward sequence	Reverse sequence	Product_size
FMgSSR-33201	scaffold2778	p2	(TA)6	Class II	50210	50221	TCAGGAGGAGCAAAACCAGCA	GGACGTGCCATAACAGCGCATT	260
FMgSSR-33202	scaffold31869	p2	(TA)6	Class II	3936	3947	TCAGGCAGCAACAATGCCGA	CGTGCTGCACATGTTAAAACCGGA	297
FMgSSR-33206	scaffold3911	p2	(TA)6	Class II	46838	46849	TCATGACAACTGCGCGGGCTAT	ACTCTTCAGGGCGGTGAGCTTT	226
FMgSSR-33207	scaffold418	p2	(TA)6	Class II	126832	126843	TCATGCAATTGGAGCCGAGGGA	GCTTGTCTGCTCAATCGCCGAA	219
FMgSSR-33209	scaffold112	p2	(TA)6	Class II	70483	70494	TCATTCAGCACCCACCTCGGTA	GGTGTTGGTGTCCGTTCGATCT	348
FMgSSR-33215	scaffold11384	p2	(TA)6	Class II	10272	10283	TCCACGCAGCTATAGCAACCCT	ACACGTACTCGGCAGCCATT	314
FMgSSR-33216	scaffold766	p2	(TA)6	Class II	32551	32562	TCCAGCAATGGCACGGTTCTCA	AATGGCAAAGGGCGGAATGAGC	337
FMgSSR-33221	scaffold2129	p2	(TA)6	Class II	66315	66326	TCCATGCATTGGCAAGCCGT	GGGTCATCGTCGATCGCGTTTT	231
FMgSSR-33222	scaffold4204	p2	(TA)6	Class II	21372	21383	TCCCACCAGGTACAGCCAGTAT	TTGATGCGTGGCAGCGAACT	277
FMgSSR-33227	scaffold4206	p2	(TA)6	Class II	22891	22902	TCCCTTCCCTTTTGCTAGCCGT	TCAGCTGGCAGCATATGAGGCAT	314
FMgSSR-33235	scaffold16464	p2	(TA)6	Class II	6361	6372	TCCGGACACAGACACGTGCAAA	ATGGCGCGCCTACTGAATGA	245
FMgSSR-33240	scaffold25011	p2	(TA)6	Class II	1814	1825	TCCGTGTACCATCATGCCCTTGT	AGCACGTAGACGATGGTGTTGCT	328
FMgSSR-33242	scaffold4	p2	(TA)6	Class II	129092	129103	TCCTCAAGGAAGCTGCAGATGGA	TGTTTGACGAGAGGGGTGTCT	225
FMgSSR-33262	scaffold1833	p2	(TA)6	Class II	13624	13635	TCGCGGCAATGTCATCCACA	AGCTATGGCACCGTGCTTCT	314
FMgSSR-33264	scaffold1462	p2	(TA)6	Class II	39378	39389	TCGCTCACTCGGGTGTGTTACT	AAATAGGTTGTCGCACGCGCAG	258
FMgSSR-33265	scaffold11338	p2	(TA)6	Class II	15569	15580	TCGCTTGTTGCTTCACTGTGGT	GCAGCAGCAGTGTGCTCAAA	317
FMgSSR-33267	scaffold8468	p2	(TA)6	Class II	20144	20155	TCGGCAAGGTTGTGTTGGGT	TCACTATGCAACGTGCGAGCCT	326
FMgSSR-33268	scaffold3837	p2	(TA)6	Class II	9956	9967	TCGGCCTGGCCCATTTGAGTTT	CCAGCAGCAATAAGCAGCGT	345
FMgSSR-33269	scaffold6823	p2	(TA)6	Class II	8253	8264	TCGGCTTTGAATCCCACTGATCG	TGCATACACCGGCCACAGAA	323
FMgSSR-33270	scaffold28	p2	(TA)6	Class II	67863	67874	TCGGGACCAACAACAGCACGAT	TGCAGCATGTGCGCCAACAA	347
FMgSSR-33272	scaffold164563	p2	(TA)6	Class II	502	513	TCGGTTTTATGCGCCGCGTT	AGTCGTCGTGTCAGAATCACGGA	257
FMgSSR-33274	scaffold14994	p2	(TA)6	Class II	3551	3562	TCGTAGAGGTTGTGCGTGGA	TTTGCTCCTTGCACGAGTGGCT	211
FMgSSR-33275	scaffold145	p2	(TA)6	Class II	19066	19077	TCGTCACGCTACTGCCATTGGT	TTGAATGGGCGACGTGCCTT	281
FMgSSR-33276	scaffold6573	p2	(TA)6	Class II	22661	22672	TCGTCCCATGCGTTGGAGAAGAT	TGCCTGCCCCAAATAAGCCTGT	301
FMgSSR-33278	scaffold2195	p2	(TA)6	Class II	1064	1075	TCGTCGGAGCACAAGTGGGTTT	GGGCTAAGGCAATTTTCGGCGT	315
FMgSSR-33280	scaffold2267	p2	(TA)6	Class II	69924	69935	TCGTCTGCTCTGTTGCGTTGT	TTTGCGATGCATCAGGTCGGGT	293
FMgSSR-33283	scaffold11750	p2	(TA)6	Class II	14558	14569	TCGTTCTCTGCTTGCCACACCA	TGCTGCGGGCATTTGTTCCA	337
FMgSSR-33294	scaffold1945	p2	(TA)6	Class II	44469	44480	TGAAATCCAGCCGGTTTAGGCG	ACGGTTGGGTGCATGACGAT	205

SSR_ID	Scaffold	SSR_Type	SSR_Motif	SSR Class	SSR_Start	SSR_End	Forward sequence	Reverse sequence	Product_size
FMgSSR-33295	scaffold436	p2	(TA)6	Class II	36937	36948	TGAACACGAGGATCGGAGGTGT	TGCCGCTCACTTGGGTCGTTAT	334
FMgSSR-33296	scaffold5411	p2	(TA)6	Class II	20209	20220	TGAACCGCTGCTGATGACCAA	AACACAGCCCTGGAAGGCTACA	243
FMgSSR-33299	scaffold11629	p2	(TA)6	Class II	24709	24720	TGAACTTCACTCGGCAGCAGCA	AAGCGAGGCAGCGCCATATT	323
FMgSSR-33306	scaffold4412	p2	(TA)6	Class II	4483	4494	TGACCTGCGGATTTAGCAGGGA	GTGTGTGTTGCACGTGCCTAGT	343
FMgSSR-33308	scaffold437	p2	(TA)6	Class II	101320	101331	TGACGATTTGGGTCCTGTTCGGA	ATTGGACTGCTGTGCGGAGT	238
FMgSSR-33310	scaffold10920	p2	(TA)6	Class II	28042	28053	TGACGTCACACGCCTAACTCGT	GAGCAAGGGAGACAATGTGCTAGT	318
FMgSSR-33311	scaffold16726	p2	(TA)6	Class II	6377	6388	TGAGAAGCGACATACTCTCTGGTG	ACTGGCTGGGGTAACGTTTGACA	320
FMgSSR-33316	scaffold12903	p2	(TA)6	Class II	21090	21101	TGAGCTCAAAGCTAGGCCAACA	AGCGTTGTGGATTTCCTGGAGT	292
FMgSSR-33332	scaffold11565	p2	(TA)6	Class II	15370	15381	TGCAAAATGCTCCGGCTGCT	AAGAACCCCATCCTGCCAGCAA	340
FMgSSR-33333	scaffold197	p2	(TA)6	Class II	4507	4518	TGCAAAGCCTGGCGATCAAGGA	ATGCCGTGCTGTTGAAACTCGC	341
FMgSSR-33337	scaffold1373	p2	(TA)6	Class II	4299	4310	TGCAAGCAACGATGAGGCGA	AAGCCGAGAGCGCCAAGTTT	341
FMgSSR-33340	scaffold71304	p2	(TA)6	Class II	2065	2076	TGCAAGTTGGCTGGGGTGTGAA	AAAGGCACTCCATGCACCGGAT	211
FMgSSR-33342	scaffold661	p2	(TA)6	Class II	13673	13684	TGCACCCCATAACCTGTGGCTT	AGGTCGTGTCAACCAGCAAGA	202
FMgSSR-33347	scaffold98	p2	(TA)6	Class II	183597	183608	TGCACGTCTCGCCCCCTAATAA	TGCAGTTGACACCACTCGCGTT	314
FMgSSR-33348	scaffold3835	p2	(TA)6	Class II	52903	52914	TGCACGTGGCGTTCACGATT	GGCTCCCCATCTGGCTATATGTACT	339
FMgSSR-33349	scaffold110	p2	(TA)6	Class II	86961	86972	TGCACGTGTGTTCTGCTCTGCT	AGGTGTCAAGCAACGCACACT	330
FMgSSR-33350	scaffold349812	p2	(TA)6	Class II	208	219	TGCACGTTTGTAGACCTGTGGT	GGCGTCTTGTGCATGTTTTCAGTG	202
FMgSSR-33351	scaffold6153	p2	(TA)6	Class II	26646	26657	TGCACTCCTTGATGGTATCGGT	TATGCGAGGATGAGCTGGGCTT	340
FMgSSR-33352	scaffold2277	p2	(TA)6	Class II	38541	38552	TGCACTGCATGTGTGCTGCT	AGGAGCATGGCAGGGACGAATA	309
FMgSSR-33358	scaffold2547	p2	(TA)6	Class II	57299	57310	TGCAGTGGACAGCAATGATCGTG	GCATGCAGCGTGTATTCGGT	282
FMgSSR-33366	scaffold47216	p2	(TA)6	Class II	715	726	TGCATTGAAACTCGGGCGCT	AAGGTGGCAGGGGCATTTGTGA	243
FMgSSR-33367	scaffold2376	p2	(TA)6	Class II	22121	22132	TGCATTGCCGCAGCGCATAA	TGCCTGGTGGTGGGGTGAATTA	313
FMgSSR-33368	scaffold7542	p2	(TA)6	Class II	27024	27035	TGCATTTCCTGTGCGGCTGT	AGGTGCTTAGCCACAGTCAGGT	299
FMgSSR-33371	scaffold4435	p2	(TA)6	Class II	16045	16056	TGCCCACTTTCTGCTCCGTT	GGAGCGTTCGAAGAAAGGTCTCCT	313
FMgSSR-33372	scaffold1275	p2	(TA)6	Class II	19835	19846	TGCCCAGCAGATTGTTGCTCCA	TCAACCAAACAACAGCACGCGG	333
FMgSSR-33374	scaffold19273	p2	(TA)6	Class II	13981	13992	TGCCCATTAGCAGGCCATGGAT	AGCGTAACGTCGAGGCAACACA	293
FMgSSR-33376	scaffold725	p2	(TA)6	Class II	95026	95037	TGCCGCTGCAAATGAAGCCA	AGGATGACGATGACCGAGACGA	315
FMgSSR-33378	scaffold16276	p2	(TA)6	Class II	10729	10740	TGCCGTTCCGGAAACTCGACAT	ACCAAGACTGAAGAGGCAGGCA	234

SSR_ID	Scaffold	SSR_Type	SSR_Motif	SSR Class	SSR_Start	SSR_End	Forward sequence	Reverse sequence	Product_size
FMgSSR-33388	scaffold1452	p2	(TA)6	Class II	27744	27755	TGCCTTGCACGCCTTTCGTT	ACGTTGATTGAGCCAGGCGT	289
FMgSSR-33389	scaffold215954	p2	(TA)6	Class II	309	320	TGCCTTTTTGGGGTCACCGT	TGTCTTCGCCCTGAGAGCTT	346
FMgSSR-33392	scaffold380	p2	(TA)6	Class II	31125	31136	TGCGACAGGGGGCAAAAACT	ATCGCATAGGCCACGACCACAT	297
FMgSSR-33394	scaffold5602	p2	(TA)6	Class II	10417	10428	TGCGAGATGGCGGTGGCTTATT	AGGGAAAAGCACCTAGGGCTACA	238
FMgSSR-33397	scaffold1155	p2	(TA)6	Class II	29133	29144	TGCGCGTATTTGAAGAGCCACT	AGCTGTGCATGTGAGAGAGCGT	344
FMgSSR-33400	scaffold10090	p2	(TA)6	Class II	287	298	TGCGGCCAAACACAAGTGGGTA	TGCCGCCTTGCTCATCGAAA	308
FMgSSR-33404	scaffold2183	p2	(TA)6	Class II	62280	62291	TGCGTGTGTCTGCTTGCATTGG	GCGCTTGTGTGCTGCTTGAT	246
FMgSSR-33406	scaffold2396	p2	(TA)6	Class II	8735	8746	TGCTACGTACAGCGCACTCA	ATCGCCGGTCGTCGCTTATTGT	277
FMgSSR-33407	scaffold3083	p2	(TA)6	Class II	23880	23891	TGCTACTGCGACCACGAGAACT	TGCCAATCGGTCCGCATGTT	265
FMgSSR-33418	scaffold16301	p2	(TA)6	Class II	3700	3711	TGCTGCCCCCAAAAGTCGAT	CCGTGCAAATGCACAGGCTA	319
FMgSSR-33421	scaffold12287	p2	(TA)6	Class II	5892	5903	TGCTGCTCCAAGCTGATGTGT	ACGAGCACTAAGATGTATGGCGCA	296
FMgSSR-33422	scaffold4375	p2	(TA)6	Class II	36865	36876	TGCTGCTGCCTGCTGGTCTATT	TCGTGATGCCTGAAACTGGCCT	335
FMgSSR-33423	scaffold287	p2	(TA)6	Class II	105043	105054	TGCTGGCACGGTCGATTGAT	TGTAGGCGCTAGAGCACCAAGT	219
FMgSSR-33426	scaffold711	p2	(TA)6	Class II	12875	12886	TGCTGTCTCGCCTTGGCGTTTA	TTTATTGGGCCTGGCCACCT	332
FMgSSR-33428	scaffold6666	p2	(TA)6	Class II	8408	8419	TGCTTCCGCTCTTGCGGTGTTA	AGGCTGTGCTCCTGCCCATTTT	305
FMgSSR-33429	scaffold17865	p2	(TA)6	Class II	6762	6773	TGCTTCGGTGCAATGACGAC	CCGGTGCAATCGCCTTTGACAT	349
FMgSSR-33431	scaffold17180	p2	(TA)6	Class II	9795	9806	TGCTTCTTTAAAAGGGGACACGGC	TCCACTCTAAGCTACCTCAGCCA	314
FMgSSR-33438	scaffold1563	p2	(TA)6	Class II	20945	20956	TGGAAAGGGAAGACGGCGTGAT	TGCACCGTACCCAAGTCCAAGT	330
FMgSSR-33439	scaffold3260	p2	(TA)6	Class II	30722	30733	TGGAAGCAACCGGCTGTTAGGA	ATCTACCAGGCGTCGCAAGTGT	284
FMgSSR-33440	scaffold3485	p2	(TA)6	Class II	36585	36596	TGGAAGGGGAAGATTGTGCGGT	AAACGGCTTGAACTCGGACGGT	347
FMgSSR-33444	scaffold716	p2	(TA)6	Class II	31045	31056	TGGAGGAGAGGGTTGCTCCAAA	AGGCCAGTTGCACCTACATGCT	333
FMgSSR-33447	scaffold5704	p2	(TA)6	Class II	11665	11676	TGGAGGCACTAAGCGGCGATTT	TCACTTTCCAGTCGTCCCAACG	341
FMgSSR-33451	scaffold192	p2	(TA)6	Class II	9344	9355	TGGCACGACGTGATTTTGCCT	GGCACTCATGTACACACAGTAGAG	290
FMgSSR-33456	scaffold2433	p2	(TA)6	Class II	1825	1836	TGGCAGCTCTGAGTGGCGATAA	TGGTTCTTGTATGGCGGGCT	234
FMgSSR-33458	scaffold26775	p2	(TA)6	Class II	2885	2896	TGGCCAAGCAAACACACGCT	TCCGAGCCAAACCGACCATGAA	313
FMgSSR-33460	scaffold4202	p2	(TA)6	Class II	46701	46712	TGGCCATGAACCCGTGACGAAA	CGCCTGCTTTATTTGCCACGCT	330
FMgSSR-33463	scaffold2438	p2	(TA)6	Class II	22752	22763	TGGCCGACCTTTTTGTTTGCCT	TGTTTGACGCGGAAGCAGCA	349
FMgSSR-33465	scaffold1109	p2	(TA)6	Class II	4702	4713	TGGCGAGTACACAGACGACA	TTGCGGTGGCCTTGGGTAAA	260

SSR_ID	Scaffold	SSR_Type	SSR_Motif	SSR Class	SSR_Start	SSR_End	Forward sequence	Reverse sequence	Product_size
FMgSSR-33468	scaffold1324	p2	(TA)6	Class II	33137	33148	TGGCGTGACATCACTTGCATGG	AGCTGGCATAAGGGCACCAA	277
FMgSSR-33471	scaffold4277	p2	(TA)6	Class II	11446	11457	TGGCTCCAGTTGCCATCATCGT	TCCACAGTGGTTTGACGTGC	332
FMgSSR-33472	scaffold4181	p2	(TA)6	Class II	40894	40905	TGGCTGATCGCAGCTTCTCCTT	ACCTCGCAGCCACAAACAGT	200
FMgSSR-33476	scaffold164067	p2	(TA)6	Class II	394	405	TGGGACATTCGCCAGGCCAAAA	TGTCCCTAGAAGGCACACTCGT	247
FMgSSR-33484	scaffold2653	p2	(TA)6	Class II	52710	52721	TGGGGAATTACTAAGCCGCGCA	CGGCAAGCCAAGCAGGGAATAA	245
FMgSSR-33496	scaffold25839	p2	(TA)6	Class II	4478	4489	TGGTAACCGGTTCACTGCAGTTTC	TTTGCTAGCTTTCTGGGCGGCT	274
FMgSSR-33499	scaffold125816	p2	(TA)6	Class II	201	212	TGGTCCTTGGCAGCTGGTTGTT	TGTACTGTGCGAGCGTGCAA	245
FMgSSR-33500	scaffold4016	p2	(TA)6	Class II	29183	29194	TGGTCTGCACACATGATCGGCT	TCTCAACACCAGCAATTGGGGA	264
FMgSSR-33504	scaffold3413	p2	(TA)6	Class II	21953	21964	TGGTGCCATTCGCCTTTCTCGT	CGCGTGTTTCGCAACGTTCT	300
FMgSSR-33510	scaffold2585	p2	(TA)6	Class II	54264	54275	TGGTTGGGCCGGACCATGTAAA	TCGGACCATGTGGAAAGTGGCT	244
FMgSSR-33515	scaffold4598	p2	(TA)6	Class II	8062	8073	TGTCCTGCATCCCTCCTCGTTT	ACAGCAGGTGGGTGTGTTTGCT	238
FMgSSR-33518	scaffold10272	p2	(TA)6	Class II	1794	1805	TGTCGGAAGCAAGAAACGGGGT	ACCACACGGATGATCGCTGTCT	293
FMgSSR-33520	scaffold39	p2	(TA)6	Class II	130659	130670	TGTGAGATGAGCACGGCGAT	AGGCTCAACTGGCCCTTTTCGT	268
FMgSSR-33521	scaffold3435	p2	(TA)6	Class II	55560	55571	TGTGAGCCCAACTCATGCAGTCA	AGTCTCGGTGGTTGCCGTTT	290
FMgSSR-33531	scaffold2803	p2	(TA)6	Class II	44946	44957	TGTGGGTGATTATGGCAGGCA	TTGCAGGATGGCAGTGGCAA	332
FMgSSR-33535	scaffold790	p2	(TA)6	Class II	14593	14604	TGTGTCTGTGTGGTTGCTTCTG	ACGTGTGTTGCCACGGACATT	298
FMgSSR-33536	scaffold2260	p2	(TA)6	Class II	49425	49436	TGTGTGCCCTGTCACAAACCCA	AGCTGTAACAAGCTGCTGCCCT	282
FMgSSR-33541	scaffold209119	p2	(TA)6	Class II	137	148	TGTGTTCTGGTTGCGTACGTGT	AAGAAGGGCGGCTTCATCGTCT	293
FMgSSR-33542	scaffold4817	p2	(TA)6	Class II	31213	31224	TGTTCATATACGTGCCCGGCGT	AGGTGCGGTTGCGCAGATAA	316
FMgSSR-33544	scaffold6152	p2	(TA)6	Class II	17019	17030	TGTTCTCTCGCCATGTCTCGT	AGCGAGCGAGTCACCAACAACA	350
FMgSSR-33546	scaffold2147	p2	(TA)6	Class II	41826	41837	TGTTGCAGCCCTGGTGCATT	ACTCCAATGCTGGGCCTGAGTT	266
FMgSSR-33547	scaffold1941	p2	(TA)6	Class II	23261	23272	TGTTGCTACTTGTCGCTCGGT	AGGCAACCACTAGCATGGTCA	286
FMgSSR-33549	scaffold2298	p2	(TA)6	Class II	51336	51347	TGTTGTTGGGTGGACAAGGACCA	GCAAGCAGGCGACCATGATGTTT	332
FMgSSR-33553	scaffold297263	p2	(TA)6	Class II	95	106	TTACGCCGGCCAATCCTTGT	TCGCCGTTACCGTGGCAAAT	342
FMgSSR-33554	scaffold8998	p2	(TA)6	Class II	28396	28407	TTACTGTGCACCACAGCCTCGT	TGGGCTTATGTCACAGGTCACAGC	322
FMgSSR-33556	scaffold171331	p2	(TA)6	Class II	109	120	TTATGGTCGCGGGGGAGTTTCA	AAGTCTGATGGCGGCACACGAA	210
FMgSSR-33559	scaffold69	p2	(TA)6	Class II	99050	99061	TTCATCGACGCGCACGTACACT	ACAGCGTCGTCAGCAAAACGTG	336
FMgSSR-33561	scaffold19618	p2	(TA)6	Class II	3113	3124	TTCCAACATGCGGTTGCGGT	TGATGTGGTCGTCACTCGGT	324

SSR_ID	Scaffold	SSR_Type	SSR_Motif	SSR Class	SSR_Start	SSR_End	Forward sequence	Reverse sequence	Product_size
FMgSSR-33563	scaffold248	p2	(TA)6	Class II	79202	79213	TTCCCAATGGCCACAACGCA	ATGTTCGTGACGTACGCCTG	227
FMgSSR-33564	scaffold817	p2	(TA)6	Class II	225	236	TTCCCACCTCGCGTGTTGTT	TGACATTGAGCGGCTGACGGAT	270
FMgSSR-33566	scaffold819	p2	(TA)6	Class II	20803	20814	TTCCCTGCATGGTGCGTTCGTA	AGCTGCTCTACACGGGGATT	306
FMgSSR-33567	scaffold37	p2	(TA)6	Class II	37034	37045	TTCGAGATCCCGGTCCGGTTTT	ACGGGGCATGCATCTTCAACGA	321
FMgSSR-33573	scaffold12660	p2	(TA)6	Class II	16088	16099	TTCGTGGGAGTTGCTCAGGCAT	AAACCGGACAGGGGCAAACT	309
FMgSSR-33574	scaffold343	p2	(TA)6	Class II	31879	31890	TTCTACCAGGCCACTGACCGTT	ACTGGAAACATGGGCCTTGC	303
FMgSSR-33583	scaffold5733	p2	(TA)6	Class II	8923	8934	TTGCAGCAACCAACGCCCAA	TCCGCGCAAGTCACCAAACT	341
FMgSSR-33584	scaffold856	p2	(TA)6	Class II	62607	62618	TTGCAGGTGGTGCAGCTGAT	GCAAGCCTTGGTGCTTTTTGGT	337
FMgSSR-33588	scaffold5083	p2	(TA)6	Class II	39985	39996	TTGCCAGGTCTCACGCGTTT	AAGAACCAAGCACGCACGGTGA	255
FMgSSR-33593	scaffold12947	p2	(TA)6	Class II	22271	22282	TTGCCTCCTCCCCTACGCTTAGAA	AGTGGCGGACCCAACATCAA	342
FMgSSR-33597	scaffold3283	p2	(TA)6	Class II	265	276	TTGCTTCGTGGGTTCACTCGCT	ACCATCTTCCTTTCCCGGCAACT	289
FMgSSR-33602	scaffold794	p2	(TA)6	Class II	12718	12729	TTGGCCCAGGCATGCGTAAT	GCCCTTCTTCTCAAAGGTTAGTGG	224
FMgSSR-33604	scaffold5166	p2	(TA)6	Class II	33742	33753	TTGGCGCTGGACAAATGAGC	TTTTCCCCGGCACCCGCATTTA	253
FMgSSR-33609	scaffold23872	p2	(TA)6	Class II	3261	3272	TTGGCTTGCTGAGGAGAGTCGT	AGCAAAATGAGTTGGCGGAGTGC	216
FMgSSR-33610	scaffold16364	p2	(TA)6	Class II	8157	8168	TTGGGAACGTTTGGAGGAGAGA	TAAGCTGGCTCGTGCAATGGCT	330
FMgSSR-33611	scaffold12617	p2	(TA)6	Class II	3172	3183	TTGGGGTTGATTTGGGCTCCGT	TCTCCTTGACAAGCCGACCA	219
FMgSSR-33616	scaffold2445	p2	(TA)6	Class II	15083	15094	TTGTCCAGAGTACCCCCTCGATCA	ACCGGTCGCCGCATTATTGA	306
FMgSSR-33620	scaffold24653	p2	(TA)6	Class II	3055	3066	TTGTGGATGACACGGCTGTTCG	AGTCCACCATGCGAGTTCAATTCC	329
FMgSSR-33621	scaffold241	p2	(TA)6	Class II	153817	153828	TTGTGGCAGACCACGTGTCACT	CCCATGAACGCGCGGACATATT	237
FMgSSR-33624	scaffold8921	p2	(TA)6	Class II	29451	29462	TTGTTGCCGGCCCATGCAAT	TGGTAGGCGGTGACGATGTT	315
FMgSSR-33633	scaffold401043	p2	(TA)6	Class II	166	177	TTTGAAAACCGCTGCTGCCACG	ACCGAGGTACGAAGCCATGA	212
FMgSSR-33638	scaffold92	p2	(TA)6	Class II	93074	93085	TTTGCTCTGATCGTCCACGGCT	ACGTAAGAAACACGGGCTGACA	273
FMgSSR-33639	scaffold13690	p2	(TA)6	Class II	9474	9485	TTTGCTTGCCTATGCTGCTGCG	TGCTTCAGCCCGGCCTGTTAAA	246
FMgSSR-33647	scaffold28498	p2	(TA)6	Class II	8026	8037	TTTTGCTTCGCCATCTGCCTGC	ACCACGCCGGCCAAAATACTGA	228
FMgSSR-33656	scaffold1297	p2	(TA)7	Class II	12367	12380	AACCCAACGCACGACTGACGAA	GCACGCACGACCAGCATTTT	265
FMgSSR-33659	scaffold4444	p2	(TA)7	Class II	4278	4291	AACGAAATCGCCGCGTCACT	TCGAGGACACAAGCGAAAGGGA	221
FMgSSR-33660	scaffold104	p2	(TA)7	Class II	168941	168954	AACGAAGTGCAGCGGGTGAT	AGCTACAACGACGCCGGCAATA	333
FMgSSR-33671	scaffold538	p2	(TA)7	Class II	79905	79918	AAGCACGCAAGGTTTGCACG	AAAACCCAGCAAGTGGCGCT	332

SSR_ID	Scaffold	SSR_Type	SSR_Motif	SSR Class	SSR_Start	SSR_End	Forward sequence	Reverse sequence	Product_size
FMgSSR-33672	scaffold116	p2	(TA)7	Class II	195427	195440	AAGCACGTCAGGTGGTTGGAGA	TGCCCTCATCCTGGACCAAA	278
FMgSSR-33674	scaffold1622	p2	(TA)7	Class II	69785	69798	AAGCGTACATCTCGCACACCCA	ATCTGAGCACAGCCCCTCTCAT	258
FMgSSR-33677	scaffold3150	p2	(TA)7	Class II	51351	51364	AAGGCGTCGTGCTCCCCTATTT	TGCCCACCGCATCGTCTTAACT	285
FMgSSR-33683	scaffold21645	p2	(TA)7	Class II	488	501	AATCGTCGTCGCCCATCCAGAA	AGTGATGCTCTAGTGAGGAGGCT	336
FMgSSR-33684	scaffold18669	p2	(TA)7	Class II	5364	5377	AATCTGCACCAAGCGCTCTCCT	TAGACCGGAGACCGGTGTCTTT	330
FMgSSR-33687	scaffold6539	p2	(TA)7	Class II	19284	19297	AATGCGCTGCCACATGGGAT	TGCAGGCCCAGGACCAACAAAA	248
FMgSSR-33688	scaffold235	p2	(TA)7	Class II	116183	116196	AATGGCATCGCCCACATCCACT	TCGCATTCTCTTAACGCGCGAC	200
FMgSSR-33689	scaffold2247	p2	(TA)7	Class II	38820	38833	ACAAACAGCACTGTCGGCAAGC	CAATTCGGCTGCCCTTTCCA	250
FMgSSR-33706	scaffold501	p2	(TA)7	Class II	7055	7068	ACCACTCAGAATCAGCGGCA	ACGCGGAGACGCCGAATAGTTT	249
FMgSSR-33710	scaffold7896	p2	(TA)7	Class II	18551	18564	ACCGCCAAGCTTTCAATCGCA	ACCGAGAACACACCAATAGGCGA	243
FMgSSR-33713	scaffold17221	p2	(TA)7	Class II	221	234	ACCTAAGTCATCCCCGTCGTGT	AGCCCAAGCCCGATGCTCTTTT	347
FMgSSR-33716	scaffold3631	p2	(TA)7	Class II	39219	39232	ACGAACCGTGCGTCAAGTCA	TTCATGAGCACCGTCCAACCGT	274
FMgSSR-33717	scaffold2331	p2	(TA)7	Class II	49595	49608	ACGAATCGATGCGACGCTGACT	AGCCGCCAGTGACGACTTTT	240
FMgSSR-33720	scaffold14462	p2	(TA)7	Class II	2684	2697	ACGAGCAGCATTTGCAGCCA	GCTTGTTTGAGGCGAGCGAGTT	245
FMgSSR-33721	scaffold3008	p2	(TA)7	Class II	39210	39223	ACGAGCCGGTGGTGGAAATGAA	TGTGTGTGCATGATGAGTGCGT	336
FMgSSR-33722	scaffold14193	p2	(TA)7	Class II	696	709	ACGATCCACGCACGGATCAACA	AACATCAAGGCCAGCGTGAGCA	280
FMgSSR-33726	scaffold3207	p2	(TA)7	Class II	7941	7954	ACGCACAATCCCAAGTACACAAGC	AGTCCGACCAACTCTTCGTGGT	294
FMgSSR-33729	scaffold1481	p2	(TA)7	Class II	52939	52952	ACGCGACACGGTAATGGACTCA	AAAAGTACCGCGTGCGCCTGAT	306
FMgSSR-33730	scaffold3345	p2	(TA)7	Class II	52101	52114	ACGCTGCGCTTTCAGATCTTGC	TCGTCTTCTTCGCCTGCAGCTT	270
FMgSSR-33731	scaffold9539	p2	(TA)7	Class II	29144	29157	ACGGAACAACTGAATCAAGGTGGC	TGTTGTCGTGTGCAGCTCCAGT	269
FMgSSR-33732	scaffold4728	p2	(TA)7	Class II	14247	14260	ACGGATGGGGCAGTTTCACT	TGCTGCTTTGGTGTTTTGGCGG	329
FMgSSR-33734	scaffold321	p2	(TA)7	Class II	26053	26066	ACGGCGGCTGTGAACTCAAGAT	TGCTTGCCTCTGCTGTCTCGTT	265
FMgSSR-33739	scaffold2497	p2	(TA)7	Class II	28475	28488	ACGTCGGCGAAGCAGCTAAA	AACAGTAGCCAGCGAGAGCA	341
FMgSSR-33740	scaffold2421	p2	(TA)7	Class II	18952	18965	ACGTGAGGAGAGGTGGGAAAGA	ACTGGACTTCGACGACAAGCGT	320
FMgSSR-33747	scaffold24899	p2	(TA)7	Class II	7038	7051	ACTCGGTGAGCACACAACACCA	AAACACGGGGGAATGGGGATGA	251
FMgSSR-33749	scaffold426	p2	(TA)7	Class II	40947	40960	ACTGAGCTTTTCGCGATCGCTTG	AGCGGTTCATGCGTAGGACA	338
FMgSSR-33753	scaffold680	p2	(TA)7	Class II	75664	75677	ACTGCGACGTGCAAAGCATGA	ACGCGCTAGCAGTGCTGTAT	346
FMgSSR-33757	scaffold2390	p2	(TA)7	Class II	19523	19536	ACTTGCTGCAACGCCCATGA	AACTTTGGCTGGGGATGACGGA	244

SSR_ID	Scaffold	SSR_Type	SSR_Motif	SSR Class	SSR_Start	SSR_End	Forward sequence	Reverse sequence	Product_size
FMgSSR-33765	scaffold4446	p2	(TA)7	Class II	6391	6404	AGAGCTTCTGCATCTGCGGAGT	AATCAGCAGCGTCATCGGCA	350
FMgSSR-33766	scaffold15327	p2	(TA)7	Class II	4394	4407	AGAGGAACGAAAAAGGCCCACG	TCCACTGGAGGCGTCCTTTGTT	224
FMgSSR-33767	scaffold588	p2	(TA)7	Class II	55040	55053	AGAGGAAGATGTGGGAAGGGCT	AACGACCAGTCCGGTCCAAGAA	261
FMgSSR-33778	scaffold568	p2	(TA)7	Class II	122256	122269	AGCGAAGCGATGCACTTGGA	TGTCGCCCTACTTAATCGCCA	209
FMgSSR-33782	scaffold13094	p2	(TA)7	Class II	21279	21292	AGCGCTCCAACTTCCCTAGTGT	TTGCCTGCGTGCCCTCCATATT	347
FMgSSR-33783	scaffold286	p2	(TA)7	Class II	89864	89877	AGCGGCAAGTGGAAGAAGTGGA	ATGTCGTGGCCTCACTCTGT	248
FMgSSR-33787	scaffold205	p2	(TA)7	Class II	74828	74841	AGCGTGCCACGACACATTCT	GGAGGCAGCGTGCAACATCAAA	335
FMgSSR-33790	scaffold884	p2	(TA)7	Class II	29729	29742	AGCGTGTAGCCACATGGTGAGT	CCCTACCACTGCCTTCATTGGTGT	289
FMgSSR-33793	scaffold2370	p2	(TA)7	Class II	18034	18047	AGCTAGAGCCCATGTGCAGCAA	ACGATTTTGGCAAGGGAGGGCA	316
FMgSSR-33795	scaffold911	p2	(TA)7	Class II	18253	18266	AGCTCCACGTCGGAACACGTAA	TATGGACGCATGCCGCCTATGT	316
FMgSSR-33798	scaffold2	p2	(TA)7	Class II	15674	15687	AGGATGCGGCAGAATCATCGGA	TAGCCTAGAACGGCAATGCGTG	343
FMgSSR-33801	scaffold128	p2	(TA)7	Class II	137865	137878	AGGCAACTAGAGTGACGGCT	TGACAGGGCTGGAGCTGAGATT	350
FMgSSR-33803	scaffold9330	p2	(TA)7	Class II	10985	10998	AGGCACCATGTGTTGTCTCGAAGG	AAATGGGCGTTCGTGGACATGC	200
FMgSSR-33806	scaffold778	p2	(TA)7	Class II	52118	52131	AGGCATTCGACCCAAGCAAGGA	ATGCAAAGCACCCAATGGCGTC	269
FMgSSR-33807	scaffold100	p2	(TA)7	Class II	26230	26243	AGGCCTAGATGTGTCCACTTTGCT	TGCATTTCCACAGGGCAAGGTCA	275
FMgSSR-33808	scaffold151815	p2	(TA)7	Class II	268	281	AGGCCTCTTAGTGGGTTGCACT	ATAAACACTGGCTGCAAGGCCC	302
FMgSSR-33810	scaffold13774	p2	(TA)7	Class II	16863	16876	AGGCTTTAGGCTTCTGGTCGGA	GGCTGGTTGGAAACTATGTTGCTG	343
FMgSSR-33811	scaffold162	p2	(TA)7	Class II	19114	19127	AGGGGACTGCAACATTCCTGGT	TGTGCAAACGCCTGAAGCACT	340
FMgSSR-33812	scaffold17664	p2	(TA)7	Class II	12048	12061	AGGGGGCAATGACATCAGCCTT	TTTTCCGTGGCAACTCACGGGT	267
FMgSSR-33813	scaffold7348	p2	(TA)7	Class II	5514	5527	AGGGTGGTGGTGGTAACCTTT	CGGTTCGATTCTGATCTAGTTCGC	275
FMgSSR-33814	scaffold1552	p2	(TA)7	Class II	15280	15293	AGGGTTTTGGCTCGTCGTGGAT	TTCCAGGAAAGGTCGGTAGCA	309
FMgSSR-33818	scaffold6533	p2	(TA)7	Class II	22196	22209	AGGTGTGCGCACGATTCACA	AGAGCAACGGTGCTGCAGAT	347
FMgSSR-33820	scaffold6470	p2	(TA)7	Class II	27790	27803	AGGTTGACATAGTCAGGCCACCA	TTAGTTACCGGTCTGGTGCGGA	208
FMgSSR-33822	scaffold5227	p2	(TA)7	Class II	6997	7010	AGTAGCGCGAGCTAACCTTACG	ACGTGGAGGGTTCTGAACTCCATT	347
FMgSSR-33825	scaffold50830	p2	(TA)7	Class II	481	494	AGTGAACTGCGGTCTGGTTTCG	TTTGCAGCCTTGCAGGCCAT	245
FMgSSR-33830	scaffold10166	p2	(TA)7	Class II	12940	12953	AGTGGCAACAACAAGAGGTCCTGA	AAACCAAGGGCCCACTCACT	332
FMgSSR-33835	scaffold18109	p2	(TA)7	Class II	7375	7388	ATAGCATGCGCACCCCCTTTCA	ACCGACAACGCACTGAGGAA	226
FMgSSR-33837	scaffold7437	p2	(TA)7	Class II	29146	29159	ATATGCACTGCGCTTGCCGT	TGCTGGGACCATACGTGAACGA	291

SSR_ID	Scaffold	SSR_Type	SSR_Motif	SSR Class	SSR_Start	SSR_End	Forward sequence	Reverse sequence	Product_size
FMgSSR-33838	scaffold5192	p2	(TA)7	Class II	5710	5723	ATATTGGCCTGGGTGCTGGAA	GCTTTTGCGCACCCAGTATGGT	336
FMgSSR-33839	scaffold157501	p2	(TA)7	Class II	271	284	ATCAAGATCGATCCACCCCGCT	ATGCAATGCGAAACGCCGCT	202
FMgSSR-33841	scaffold347629	p2	(TA)7	Class II	158	171	ATCACCGTTGGGCAGATAAGGA	CATCTTTTTGGGCATGGTCACCGC	200
FMgSSR-33848	scaffold224	p2	(TA)7	Class II	125126	125139	ATCGTGCGAGGTGAAAGGCA	ACAGGATGGGGACATGGGTGTT	284
FMgSSR-33850	scaffold975	p2	(TA)7	Class II	87371	87384	ATGCCCATGCCCATGCTTGA	AGCTCCCATCCAACTGCTGCAA	328
FMgSSR-33851	scaffold1539	p2	(TA)7	Class II	64784	64797	ATGCGCACACCACAATTGCC	TCCCATGAGACTTCCGATCCAGCA	230
FMgSSR-33854	scaffold394	p2	(TA)7	Class II	107979	107992	ATGTCCTGCTGCTTGATGAGC	AACACATGACACCCTGGCTGCT	341
FMgSSR-33859	scaffold3213	p2	(TA)7	Class II	42116	42129	CCAAACAGAGTCTGGAGCATTGTG	TGTGCTCGCCTTGTGTTTCCAC	343
FMgSSR-33860	scaffold10689	p2	(TA)7	Class II	17724	17737	CCAACAGCCACACACGCAGTTT	TGAGGTGATGTGGCTCAACATGG	320
FMgSSR-33861	scaffold36118	p2	(TA)7	Class II	911	924	CCACGCAGGCATGCATGTTTCT	TGCTTCAAAGGCACGCTACCCT	287
FMgSSR-33862	scaffold4817	p2	(TA)7	Class II	33556	33569	CCAGCGTGGCAGAAAACAGCTT	CGTGAGCACACGAGCATGAACA	342
FMgSSR-33873	scaffold154457	p2	(TA)7	Class II	595	608	CGCTAGTCTTCATTGAACCGCA	TGGCCCAGACGTGATAATCCGA	350
FMgSSR-33874	scaffold3960	p2	(TA)7	Class II	28487	28500	CGCTTGAAACTGCAACCGCA	CGCGTGCACCTGGAATCGAAAA	224
FMgSSR-33876	scaffold22233	p2	(TA)7	Class II	3636	3649	CGGTGGGAAGGGATATGAAGAAAC	TGCAGTGCACGCCGTAAGAA	350
FMgSSR-33878	scaffold22444	p2	(TA)7	Class II	8798	8811	CTCGACGTCATGCTCAAATTGC	CGTTGCTTTTCCTGTGCACCCA	312
FMgSSR-33881	scaffold1653	p2	(TA)7	Class II	22199	22212	GACAACCACCATTGACGAACAAGC	TGCACGAGCCACTAATGTGAAG	212
FMgSSR-33882	scaffold449	p2	(TA)7	Class II	2720	2733	GAGTTGTTGGCAAGGAAATACCCG	AGCCAACCGTCACACACAACCA	214
FMgSSR-33885	scaffold11168	p2	(TA)7	Class II	6172	6185	GCAAGACTTGAACTGGCGATCA	AGCAGAGCACTTGTCCTTGCCA	292
FMgSSR-33887	scaffold6064	p2	(TA)7	Class II	34103	34116	GCAAGGCATGCATCTCGCTTCA	AACGAAGCGGCTAGTGACCT	303
FMgSSR-33889	scaffold4296	p2	(TA)7	Class II	23991	24004	GCAGCGTCTTTCTCCTACACTGCT	ACAAGAGGCGGAGTGGCTGAAT	303
FMgSSR-33891	scaffold15756	p2	(TA)7	Class II	11304	11317	GCAGTGGTATAGATTCGGGCGCTA	ATCACACCTGTAGTAACGCACGGG	334
FMgSSR-33892	scaffold987	p2	(TA)7	Class II	58506	58519	GCATGAACAGAGGCCATATTGAGC	AGTGCTCTGTTGGTTTGGCTTGC	301
FMgSSR-33894	scaffold9116	p2	(TA)7	Class II	6038	6051	GCATGCTTCACCACAGCCAACA	TGGAATCATGCTGCGGTCGAGT	347
FMgSSR-33896	scaffold6490	p2	(TA)7	Class II	16107	16120	GCCCATTAACTGCTAGCAAAGAGC	TGTAATTCCTGACGGAGGCGCA	266
FMgSSR-33900	scaffold6088	p2	(TA)7	Class II	40449	40462	GCGCACGGATGACATTGCTGAT	TCGCCGGTCACTCATCCTCATT	323
FMgSSR-33901	scaffold141108	p2	(TA)7	Class II	818	831	GCGCCGAATTGGGGAAAAATCGT	AGGGTTTACCCCTAAGCCCCTA	272
FMgSSR-33903	scaffold4816	p2	(TA)7	Class II	41006	41019	GCGGCACAGGGCTTATGCTTTT	TCACACCCATGCGTTCAGGAGT	251
FMgSSR-33904	scaffold2923	p2	(TA)7	Class II	7514	7527	GCGTGATTCAAGCACACAGGAGT	AGGCCATCACGGGACGAACAAA	329

SSR_ID	Scaffold	SSR_Type	SSR_Motif	SSR Class	SSR_Start	SSR_End	Forward sequence	Reverse sequence	Product_size
FMgSSR-33906	scaffold2686	p2	(TA)7	Class II	44082	44095	GCTACCCGCTCCGAGTTTCTTTCA	GCCCGGTATGTTCCCCACAGAAAT	200
FMgSSR-33910	scaffold2607	p2	(TA)7	Class II	2333	2346	GCTGCACTGGAAGAAACAGCGT	TGCTGGAGCATGGACAGGAT	347
FMgSSR-33911	scaffold6323	p2	(TA)7	Class II	12862	12875	GCTGGCTGGGCCAACATAACAT	CCCACATTGTCCTGCAAACCA	350
FMgSSR-33917	scaffold510	p2	(TA)7	Class II	3052	3065	GGCAACCTGAGACTTTGTCTATGC	TTCAGGGACGGGACCTATTGCT	322
FMgSSR-33921	scaffold287	p2	(TA)7	Class II	75671	75684	GGCTTTCAAGTCCGGCAAACCA	TTAAGCACCGGCGAGCTGAT	326
FMgSSR-33922	scaffold3919	p2	(TA)7	Class II	20930	20943	GGGCCCCAACTACAATTCCTCGAT	ACTGGGGAGCAGGTTGGAAT	347
FMgSSR-33923	scaffold13958	p2	(TA)7	Class II	15042	15055	GGGCGCGCTGTGAAATGTAT	GCCCTGCCACACAAGATTACCT	266
FMgSSR-33925	scaffold6448	p2	(TA)7	Class II	21131	21144	GGTACCAATGAACCTGATGCACCT	AACACCCATCGGCTTCTGGCTT	326
FMgSSR-33934	scaffold5883	p2	(TA)7	Class II	4858	4871	GTGTACATAGGAGTAGCTAGGGCAC	ATGGGCCGTACATTGGCGTAGT	344
FMgSSR-33939	scaffold24664	p2	(TA)7	Class II	3588	3601	TAAGCAGGTCGGCCTGTGTTGT	ATCAGAGCGCCATTCACCCAGT	267
FMgSSR-33943	scaffold84089	p2	(TA)7	Class II	1639	1652	TAGCATGCGCACCCCCTTTCAT	AAGCTTCAGCGCCAGCTTTCCT	272
FMgSSR-33944	scaffold29	p2	(TA)7	Class II	164522	164535	TAGTGCCTCGATCACTGCCGTA	ACCTTCCGAGATTCATCAGCGT	332
FMgSSR-33946	scaffold8798	p2	(TA)7	Class II	26422	26435	TATTCCATGCATGCCACGCGAC	ACCAACGCCCAGCATTGAGACA	314
FMgSSR-33947	scaffold778	p2	(TA)7	Class II	15211	15224	TATTGCGGGCATGAGCCGTT	GGAAACGGACGTGCCATTTGGA	322
FMgSSR-33951	scaffold2832	p2	(TA)7	Class II	41072	41085	TCACCACGTTCCTCGACACGTT	TGCACAAGTGGTCGTCCCAA	322
FMgSSR-33953	scaffold341	p2	(TA)7	Class II	6818	6831	TCACTTTGTCTCGCGCGCTT	CCCATGCATGCGCGGTATTT	323
FMgSSR-33954	scaffold118	p2	(TA)7	Class II	80909	80922	TCAGCAATGCCTCGCCAACA	TTGGCTAACCCTGTGGTGCCTT	338
FMgSSR-33955	scaffold7437	p2	(TA)7	Class II	35209	35222	TCAGCACTTGCGAGTGGGTT	TGTGTGCGCGCGTGCTTTTT	252
FMgSSR-33956	scaffold852	p2	(TA)7	Class II	48114	48127	TCAGCGATCTGCATCCAACGGT	TTACATGGCCAACGCGGACGTA	230
FMgSSR-33959	scaffold27834	p2	(TA)7	Class II	3091	3104	TCATATGGTCGGGGTCATGCCT	TCAGGATTGATGTGCGGCCT	350
FMgSSR-33964	scaffold112	p2	(TA)7	Class II	25218	25231	TCCACGCCGGGTTCGTTTTT	CAAAGCGCCACCCAATCAGA	207
FMgSSR-33968	scaffold155081	p2	(TA)7	Class II	214	227	TCCCTTGCACGCATAAACGCA	ACCTAGGTCACGGTTGTTTGTGTG	349
FMgSSR-33971	scaffold5840	p2	(TA)7	Class II	28392	28405	TCCGGCGCCAACAATTTCCA	CCACAGACAGAGCCTGCAAGTCAA	278
FMgSSR-33973	scaffold2813	p2	(TA)7	Class II	13983	13996	TCCTCGAGGTGAAATCCGCCAA	AAGTTGATGTGCCGGAACGGGT	345
FMgSSR-33976	scaffold2009	p2	(TA)7	Class II	43348	43361	TCGACGTGTGCTGTCGGATT	TCCAACAGAGCGGCTAACTCA	232
FMgSSR-33979	scaffold14496	p2	(TA)7	Class II	13200	13213	TCGCTCCGTAAAAGCAGTGAGA	ACGACACATGCCTGAACAGCCT	278
FMgSSR-33980	scaffold6913	p2	(TA)7	Class II	6051	6064	TCGCTCGCTGTTGTGATAGCGT	AGAGCCGAGACACGCACTTT	276
FMgSSR-33982	scaffold622	p2	(TA)7	Class II	86141	86154	TCGGCGTGTGTGTGAGACAA	AAGAGATCAGCGCCGCATCGAA	337

SSR_ID	Scaffold	SSR_Type	SSR_Motif	SSR Class	SSR_Start	SSR_End	Forward sequence	Reverse sequence	Product_size
FMgSSR-33985	scaffold2472	p2	(TA)7	Class II	43146	43159	TCGTCGCTATCACGAGGAAACA	AGCTCACCACGCGAGCTGTAAA	323
FMgSSR-33990	scaffold3093	p2	(TA)7	Class II	16091	16104	TCTCTGCAACTGCAAGGCGT	TGCCGAATCTCTTTGCCGCAT	278
FMgSSR-33991	scaffold7269	p2	(TA)7	Class II	6520	6533	TCTTATGGTTGCGGGCTGGTGA	AGGCGTACTAGCTGGTTTGGGT	265
FMgSSR-33992	scaffold25919	p2	(TA)7	Class II	8276	8289	TCTTTCTTGCAAGTCGGTGCCT	TGGGCGTCGTGGTGTGTGAATA	293
FMgSSR-33993	scaffold3992	p2	(TA)7	Class II	24657	24670	TCTTTGGCCACAGGCCACGTAT	CCCTGGTGCGCATGAAGCAAAA	258
FMgSSR-33994	scaffold6795	p2	(TA)7	Class II	11354	11367	TGAACCATCAAGCCTCCAGCCA	TCAAGGCAACGGCGTCCAAA	346
FMgSSR-33997	scaffold3546	p2	(TA)7	Class II	8816	8829	TGACCAACGGACAACTGGGA	ACAAGGGTCTTAATGGCGGACA	339
FMgSSR-33999	scaffold4437	p2	(TA)7	Class II	40561	40574	TGACGGCGACCATAAAGACCGT	GCCGAGTTGGCTCAAATTCTGCAA	337
FMgSSR-34001	scaffold109819	p2	(TA)7	Class II	505	518	TGACTGACCCGAGCACTTTCGT	GGAAAGGTGTCATGTGTCGCCAA	337
FMgSSR-34003	scaffold2126	p2	(TA)7	Class II	19701	19714	TGAGCGTAGTTTGTTTGTCCCCGT	AGACGTGCTCAAGGCTCTCA	311
FMgSSR-34004	scaffold923	p2	(TA)7	Class II	34551	34564	TGAGGAGGCCGGAGCTTTTT	TTCCGTCCCAGCCACTTTGTGA	201
FMgSSR-34005	scaffold4578	p2	(TA)7	Class II	2700	2713	TGAGGCAACGCCGCACTATT	TCAGACTCTGCAGCAACTGCGT	251
FMgSSR-34007	scaffold1288	p2	(TA)7	Class II	69470	69483	TGAGGCCACCAGTACCCAAAGT	TCAAGTGCCACATGGCTTGG	320
FMgSSR-34011	scaffold80	p2	(TA)7	Class II	34451	34464	TGATCTGGTCAAGGAAGGGTGGT	AGCAAGTTGTACGGTCGCTCCT	326
FMgSSR-34013	scaffold3337	p2	(TA)7	Class II	46225	46238	TGCACAACAGAGTCCATGAATGCC	TGGTTCGGTGCATTGTCAGAGGT	293
FMgSSR-34014	scaffold4922	p2	(TA)7	Class II	11732	11745	TGCACACGGTGGATGAAGCAGT	TTGCTCGCGATGTCATCCCTCT	304
FMgSSR-34016	scaffold9506	p2	(TA)7	Class II	20999	21012	TGCACCGCGTTAGCTCGAAA	TGGTCTCACGGCACAAAGAAGC	271
FMgSSR-34018	scaffold180723	p2	(TA)7	Class II	221	234	TGCAGATTAGGCACAGCGACGA	AGCCGACAGGCCAAACAAGA	227
FMgSSR-34019	scaffold30534	p2	(TA)7	Class II	2248	2261	TGCAGCAGTGCGTGACCATT	TCCAAAGCCAATGTGTAGCGG	307
FMgSSR-34020	scaffold33381	p2	(TA)7	Class II	1722	1735	TGCAGCATGCTTTCAAGAGGCA	TGCTTTGGGCCTTTCTGGCCTT	276
FMgSSR-34021	scaffold1303	p2	(TA)7	Class II	82849	82862	TGCAGGGCCTTAAAAGGGACCA	AGTTGTCTTCGTGGGGCACA	326
FMgSSR-34022	scaffold10299	p2	(TA)7	Class II	3575	3588	TGCAGTCTACGGCTATATGCAGAGC	TGTTGCCCGGCCGTTTTAGT	304
FMgSSR-34023	scaffold742	p2	(TA)7	Class II	13420	13433	TGCATCCGCTGCATGACGAA	TTCGGCAACTCGTACGCTGA	320
FMgSSR-34025	scaffold49759	p2	(TA)7	Class II	3332	3345	TGCATGCGTCAAACCACTCCT	TGCGTTTGAGCATTGCTACGGT	255
FMgSSR-34028	scaffold11363	p2	(TA)7	Class II	25040	25053	TGCCATGCTTAGCACCCTGA	TGTTGGCACAAGCAGCAAGA	347
FMgSSR-34031	scaffold3012	p2	(TA)7	Class II	43515	43528	TGCCCTGCTTGTCTGACCTT	TGTCACGTAAAGCACCCTCCACA	203
FMgSSR-34032	scaffold40528	p2	(TA)7	Class II	263	276	TGCCGATACATGCCCCGTTA	TCGTGGTCAAAACCTGCGCT	312
FMgSSR-34033	scaffold1309	p2	(TA)7	Class II	29144	29157	TGCCGCTTGTGGTTGCCTTT	AGCCCACGTATCAACTTGGCGA	308

SSR_ID	Scaffold	SSR_Type	SSR_Motif	SSR Class	SSR_Start	SSR_End	Forward sequence	Reverse sequence	Product_size
FMgSSR-34035	scaffold3437	p2	(TA)7	Class II	11051	11064	TGCCTCTGTCCTCTGCTTGGAA	AGGAAGAAACGTGCAAGCCGGA	287
FMgSSR-34037	scaffold1486	p2	(TA)7	Class II	66687	66700	TGCGCCTATGGGATCAACGCTT	AGTTGGGCACAAACCATCCCGA	313
FMgSSR-34042	scaffold194673	p2	(TA)7	Class II	71	84	TGCGTCTCGATCAGCGCAAA	TATCGCGTGTGCCACATGGT	263
FMgSSR-34044	scaffold2201	p2	(TA)7	Class II	24549	24562	TGCTCAACAGCGGCGAATGT	TTCACCGGCATTTTCTTCCGGC	295
FMgSSR-34047	scaffold381	p2	(TA)7	Class II	133398	133411	TGCTGCACTGCTGCTGCTTT	AGCGGACGATTAGGGGCGATTA	206
FMgSSR-34051	scaffold5606	p2	(TA)7	Class II	14845	14858	TGCTTCATTTCCGAGCGCCT	AGCAAGAAACACCGCGGCAA	219
FMgSSR-34052	scaffold8051	p2	(TA)7	Class II	12581	12594	TGCTTCTTCGCAGGTGAATTGC	GGTCATGGCAACCAATCCAAAGGC	345
FMgSSR-34054	scaffold34658	p2	(TA)7	Class II	2713	2726	TGCTTTCCTCTCTCTCCTCCCA	AGCTCAGCTCCTACCGACCTTT	292
FMgSSR-34055	scaffold1995	p2	(TA)7	Class II	68516	68529	TGGAAGAAGTGATGGCATGGCG	AGCACACTACGGTCTGTTCAATGC	225
FMgSSR-34060	scaffold10495	p2	(TA)7	Class II	16439	16452	TGGCAATGCTCGGAAACTCGGA	GGCTGTGTCATCCTGAACTCGTCA	345
FMgSSR-34063	scaffold19129	p2	(TA)7	Class II	7807	7820	TGGCATAAAACGGCCAGGCT	GCCATGCAATCCGCAATGACGA	305
FMgSSR-34064	scaffold5905	p2	(TA)7	Class II	29475	29488	TGGCATCATGCACGTTGGGT	ACGTCGTGGTCTCAACAGATGCT	230
FMgSSR-34065	scaffold30618	p2	(TA)7	Class II	4886	4899	TGGCATGTTGGTCACGGTTGTG	GGATGGCAAAATGTTGCAGCCCA	322
FMgSSR-34067	scaffold3225	p2	(TA)7	Class II	2123	2136	TGGCCAACAAAAAGGGCGGA	GCTGCTGCGTCACCCAATCAAA	322
FMgSSR-34068	scaffold4264	p2	(TA)7	Class II	24989	25002	TGGCGATTGGTTGGTGCTGT	ACGTGAAATCACGACGGCCA	305
FMgSSR-34072	scaffold4087	p2	(TA)7	Class II	22552	22565	TGGCTCTGTTCGATCTGGCT	TGCTCTTCGTTTCTTCCCCTGCT	324
FMgSSR-34073	scaffold1325	p2	(TA)7	Class II	25376	25389	TGGGAGAATCGCGTGTGCAT	TCGTGGGCTGCTCATTATCGCA	326
FMgSSR-34075	scaffold8419	p2	(TA)7	Class II	1255	1268	TGGGCTTGTGGCTCCTGAATGT	AGCTGGCGCGTTATGCATCAGA	253
FMgSSR-34079	scaffold5978	p2	(TA)7	Class II	15272	15285	TGGTCACGGCCATTGTTGGT	TGCTTTGCTTTGCTCGGTTCGG	268
FMgSSR-34083	scaffold8127	p2	(TA)7	Class II	6591	6604	TGGTTGCGGGTGGTTGTAGGTT	TTGGCGTGAGCATACACGAGGA	314
FMgSSR-34084	scaffold1943	p2	(TA)7	Class II	35693	35706	TGGTTGGGTCGTCCTCCCAAAA	AACATGCAGGGACGACATGGGT	335
FMgSSR-34085	scaffold4122	p2	(TA)7	Class II	8056	8069	TGGTTTGAGGCGCTTGTGGT	GCGTTTGTGATGGCAGCAGTGT	293
FMgSSR-34086	scaffold9220	p2	(TA)7	Class II	17146	17159	TGTACCCGCATACCGTGACACA	TCCACGACTCGTCCATTGCT	213
FMgSSR-34087	scaffold2129	p2	(TA)7	Class II	55119	55132	TGTCCTCGCGCTTTTCTGCT	ACGAAGGTGAACTGGTGAAGGCT	287
FMgSSR-34093	scaffold4243	p2	(TA)7	Class II	11394	11407	TGTGCAAAGTTGTGCAGGCG	CCATCGTTTCATCATGGGTTTGCG	336
FMgSSR-34095	scaffold2124	p2	(TA)7	Class II	39298	39311	TGTGGCTGCATGGGAGTTGAGA	AAGAAAAGACGGCCCCTTCTGC	295
FMgSSR-34097	scaffold5668	p2	(TA)7	Class II	18583	18596	TGTTGAGCCCACAAACTTCAGC	AACGGCGATACGTGGATACGGT	347
FMgSSR-34099	scaffold32394	p2	(TA)7	Class II	1318	1331	TGTTGATGTTTCCGCGGTCG	GCACGCAGCTGAGCGTCATAAA	201

SSR_ID	Scaffold	SSR_Type	SSR_Motif	SSR Class	SSR_Start	SSR_End	Forward sequence	Reverse sequence	Product_size
FMgSSR-34101	scaffold1512	p2	(TA)7	Class II	30016	30029	TGTTGCTTCTGCCCCACAAAGC	ACTACTGCGCCTTCAGCCAT	337
FMgSSR-34102	scaffold423	p2	(TA)7	Class II	38869	38882	TGTTGGCGTTGTCCAGCACT	TCGCATGGGATTGCAGCCTT	283
FMgSSR-34104	scaffold3446	p2	(TA)7	Class II	24593	24606	TGTTGTGCCGCTGACTTCACT	AGTGATGACGATGATGCGTGCCT	344
FMgSSR-34105	scaffold397	p2	(TA)7	Class II	113639	113652	TTAACTGCCCCCACAAATGGCG	AGAGGCACACGAAACGCAACAC	201
FMgSSR-34110	scaffold818	p2	(TA)7	Class II	27777	27790	TTCCGCTGGACGCAGGTTTT	TTGGCACGTTCTCTCGGCAACT	346
FMgSSR-34111	scaffold308	p2	(TA)7	Class II	62638	62651	TTCCTTGCAGCTCACGTACTCG	TGTTGGTACGCCGTGTCCAGTT	341
FMgSSR-34112	scaffold228	p2	(TA)7	Class II	105098	105111	TTCGAGCAACCTTTCACCGCGA	TCTTTGGCCACAGGCACGTA	279
FMgSSR-34113	scaffold887	p2	(TA)7	Class II	59321	59334	TTCGCCCAATAGCCCTCTAGCA	TGCACCACCATTGCGGAACT	301
FMgSSR-34114	scaffold351	p2	(TA)7	Class II	137919	137932	TTCTTGAGAGCCGCCAACGTCA	AGCACCTCAACTGCGAGCACAT	334
FMgSSR-34118	scaffold16009	p2	(TA)7	Class II	8717	8730	TTGCAACCATGCGTACATGGCG	ATGCGAGTATGCACGCGAGA	207
FMgSSR-34121	scaffold2360	p2	(TA)7	Class II	52810	52823	TTGCCACGTGGTGAGGCTTT	TGGCACTGACTGTTTCTCACTTCG	273
FMgSSR-34122	scaffold16068	p2	(TA)7	Class II	3829	3842	TTGGCAGCACCTGTGGAGATGT	CCTGCGTTTTGCTGTATTGCTCCC	282
FMgSSR-34127	scaffold527	p2	(TA)7	Class II	74594	74607	TTGTGGAGAGAAGCTGGCGT	TCTGGCCATTTACCATCGCGT	331
FMgSSR-34128	scaffold18587	p2	(TA)7	Class II	10187	10200	TTGTTATCGCCCAGGAGCAGGT	TGGGGGACGCACAAGTAAGCTA	317
FMgSSR-34130	scaffold21984	p2	(TA)7	Class II	12537	12550	TTGTTGGTGCATGGCTGGCT	AAACATGGGGCAGACGGACTCA	350
FMgSSR-34131	scaffold9372	p2	(TA)7	Class II	19279	19292	TTTAAGCAGGCGGACGAGCA	AGCATGAAGCTCAACAAGCCGT	347
FMgSSR-34133	scaffold301	p2	(TA)7	Class II	1786	1799	TTTCCTCCCACTCCCAGGTTGA	TCCGCACGCTATTTTGCTCGT	212
FMgSSR-34137	scaffold6969	p2	(TA)7	Class II	16275	16288	TTTGGTCCTGCGGTGGACTACT	AGCTGCGCACGGAAGAACAT	262
FMgSSR-34141	scaffold18703	p2	(TA)7	Class II	10005	10018	TTTTCTCCGTCCGTCGTCTAGC	TGCTGTAAGAACTGGGAGGAGC	320
FMgSSR-34142	scaffold1290	p2	(TA)7	Class II	30373	30386	TTTTGGCAGCCGCATCCTCGAT	ACATCTGCTCCGGCGACATTGA	347
FMgSSR-34143	scaffold12140	p2	(TA)7	Class II	8817	8830	TTTTTCTGCCGGCCCTTCGT	AGTTGGGCCTTTGCCTGCTGTA	298
FMgSSR-34147	scaffold7922	p2	(TA)8	Class II	1476	1491	AAACGATGATCCAGCGCACACG	AATCGTGATGGCAGCGGCTT	259
FMgSSR-34150	scaffold10834	p2	(TA)8	Class II	20827	20842	AACAAACCAGGGGGCAGAACCA	TTGCCAATTTGAGCCGCTTGGG	305
FMgSSR-34151	scaffold872	p2	(TA)8	Class II	101846	101861	AACCAGGAGCAGAAGCAGAGCA	TGACCAACCCCAATTCCTTGACCT	216
FMgSSR-34152	scaffold6359	p2	(TA)8	Class II	11596	11611	AACCCGTACAAGCACCTTGC	GTCCGATGGTTGCGAAAATTGCTG	201
FMgSSR-34157	scaffold36256	p2	(TA)8	Class II	4300	4315	AAGGGCTCTGTCCAGCATCTCA	TGCCTACCTTACTGTGGACATCGT	245
FMgSSR-34161	scaffold1337	p2	(TA)8	Class II	73340	73355	AATGAAAAGGCAGGGCCCGCTA	AGACGCAACGTAGTGCGCAAA	269
FMgSSR-34164	scaffold59234	p2	(TA)8	Class II	50	65	ACACAGTAGTGGTCTGCCAGGT	ATGGCTGGCAGCAGAAAGCA	220

SSR_ID	Scaffold	SSR_Type	SSR_Motif	SSR Class	SSR_Start	SSR_End	Forward sequence	Reverse sequence	Product_size
FMgSSR-34167	scaffold4830	p2	(TA)8	Class II	28583	28598	ACAGCCACTTGGGAAGCCTA	AGCCCTGCGTTGATTCCTGTGA	338
FMgSSR-34170	scaffold22324	p2	(TA)8	Class II	8107	8122	ACAGTCCCCACGTTTGTCTCCT	TGCGTGCGTATCTCCAACGCTT	343
FMgSSR-34171	scaffold22389	p2	(TA)8	Class II	6885	6900	ACATTGCAGCGGAGGTCCAT	ATGGCAACCTTACACAGGCCGA	240
FMgSSR-34176	scaffold285	p2	(TA)8	Class II	4230	4245	ACCATGAAGGCGCTACAACTGC	TTGGAGGGTAGTGAACGCGACA	294
FMgSSR-34177	scaffold859	p2	(TA)8	Class II	53645	53660	ACCATGCAACTGAACGGGCCAT	TGCAGGCCTTCGTCTATGGT	317
FMgSSR-34181	scaffold1463	p2	(TA)8	Class II	83889	83904	ACCGATCGTTCCAGACATTCCGA	TTTGGGCCGTGCTTTTTCGC	344
FMgSSR-34183	scaffold2547	p2	(TA)8	Class II	1460	1475	ACCTCGAGTGAACATGGTGGA	ACAGGCCGCTGATTGTTGGA	275
FMgSSR-34184	scaffold5174	p2	(TA)8	Class II	13814	13829	ACCTGTCAGTGTCTGCACCA	TGACCAGGGGGCACACACATTT	215
FMgSSR-34187	scaffold3322	p2	(TA)8	Class II	12587	12602	ACGACGAGCAGGATGACAATGA	TGAAGCAAAGCGGCAGCAGA	335
FMgSSR-34191	scaffold4901	p2	(TA)8	Class II	7602	7617	ACGCCAGCTTCATCCATGGCAA	TGTCCCGTGCACCGTTTTCA	258
FMgSSR-34192	scaffold1382	p2	(TA)8	Class II	69545	69560	ACGCCATGCATGACTGGTTCT	TGAAACGTGGTTTCGGCCTGT	313
FMgSSR-34193	scaffold716	p2	(TA)8	Class II	77943	77958	ACGCCGAAGCAAAGCAGGAT	ACACAGGACACCTGGCTCGATT	252
FMgSSR-34194	scaffold1273	p2	(TA)8	Class II	46533	46548	ACGCCGCCAAACAACACCAA	TCGTTGACCACCCATGCCATCT	308
FMgSSR-34195	scaffold1556	p2	(TA)8	Class II	30818	30833	ACGCCTATTTTGCCGCTGCT	TTGGAACAGCCAAAGGGCGA	236
FMgSSR-34196	scaffold64	p2	(TA)8	Class II	173679	173694	ACGCGGGGAACGAACGAAAA	TAATCTGACCTGGCAGTGGGCA	332
FMgSSR-34197	scaffold1530	p2	(TA)8	Class II	67224	67239	ACGCTGAACCCTGCACACATGA	ACGATCTCGTGCTGCCCTTCAA	242
FMgSSR-34200	scaffold4234	p2	(TA)8	Class II	38868	38883	ACGTGCGTAGATGCGTAGGTCT	ACTGGATGTGTTCGTGTGGGCA	225
FMgSSR-34202	scaffold3008	p2	(TA)8	Class II	28749	28764	ACTAGAGGCCCTCAGACACACT	ACCATGACGCCCATGTTTCGT	210
FMgSSR-34208	scaffold4391	p2	(TA)8	Class II	28250	28265	ACTTGCTACCTCCGTGCCACAA	ACACCCGTGAACGAGTGCATGA	344
FMgSSR-34209	scaffold12679	p2	(TA)8	Class II	11067	11082	AGAACGAAATGCGTGCGTCCA	TACGCTGCTGAGAAACCGAGGA	294
FMgSSR-34211	scaffold1160	p2	(TA)8	Class II	49683	49698	AGACCCACCACGGTCACTGAAA	TCGCACAGTAGCAACCATGACCT	294
FMgSSR-34214	scaffold371	p2	(TA)8	Class II	53161	53176	AGATGCAATCCAGTGGCGGA	ACCAAGGCAGCGAGGAAGCTAA	339
FMgSSR-34217	scaffold65948	p2	(TA)8	Class II	2170	2185	AGCAGTGCCATGTCTCGTCTT	TGAAGCTACCTAACGCCACTAGGA	292
FMgSSR-34218	scaffold130874	p2	(TA)8	Class II	889	904	AGCCAGCAAAGACAGCGCCATA	CGATGTTGGTTTGGGTGACCGT	200
FMgSSR-34222	scaffold71147	p2	(TA)8	Class II	633	648	AGCCCTTGTTCCCCACAGTGAA	ACTCACCCCCTTTGAGGAACTGGA	308
FMgSSR-34223	scaffold450	p2	(TA)8	Class II	26422	26437	AGCCGTCCATCATGCATCCCAT	ACAAGGAAGTTTGCGCCAAGCG	310
FMgSSR-34224	scaffold12814	p2	(TA)8	Class II	14780	14795	AGCCTAAAAGCTGAGGCCCCTA	TCCGGCACGACAATTCGGCTTA	336
FMgSSR-34228	scaffold41906	p2	(TA)8	Class II	591	606	AGCGCTTGTGCTGAGAAGCTGA	TGCGCGATTAGGCGTCCTTT	305

SSR_ID	Scaffold	SSR_Type	SSR_Motif	SSR Class	SSR_Start	SSR_End	Forward sequence	Reverse sequence	Product_size
FMgSSR-34229	scaffold3361	p2	(TA)8	Class II	3948	3963	AGCGTACCATGGTTCAGCGGAT	ATGGATGGGTGCGGGTGTAGAA	256
FMgSSR-34231	scaffold22446	p2	(TA)8	Class II	9189	9204	AGCTTCCTTTAGGTGTTCCCGA	TTCAGTTAGCATGCGCCCGT	265
FMgSSR-34232	scaffold2004	p2	(TA)8	Class II	54459	54474	AGCTTCGTCTCTGTTCCGCA	TGCTGTTTGGTCTTCGCCGT	263
FMgSSR-34235	scaffold586	p2	(TA)8	Class II	90581	90596	AGGCACCGATTTCCTCCATTCCT	ACAGCAGAACTGCAGAAGCGT	321
FMgSSR-34237	scaffold56854	p2	(TA)8	Class II	2904	2919	AGGCGCCTCAGAACACTCAAGA	ACCCACGGTTGCAGTCGGTAAT	320
FMgSSR-34238	scaffold15107	p2	(TA)8	Class II	1040	1055	AGGCTGTTCTCCAGGCACCAAT	TGGCAAACTCGGACGAGCAA	290
FMgSSR-34246	scaffold10077	p2	(TA)8	Class II	3767	3782	AGTAGAACACCGTGGCCTTCCT	TGGGACAAATTTGCCGCTTGCC	207
FMgSSR-34252	scaffold6234	p2	(TA)8	Class II	29076	29091	AGTGGCTTTCCTTGTGCTGGA	GGGCTCGACCTCAGGTTAATGT	284
FMgSSR-34253	scaffold1191	p2	(TA)8	Class II	25957	25972	AGTGTGGGGGAGTTCTCTCTCT	AAAACGTTGGCCGCCGATGGAT	273
FMgSSR-34254	scaffold14439	p2	(TA)8	Class II	10871	10886	AGTGTGTGTCAAGGCCAGGT	ACGTCTGCGCAAGGTTATGTGC	347
FMgSSR-34256	scaffold5051	p2	(TA)8	Class II	34887	34902	AGTTGAATGAAGCGCCGCCA	ACCTGTGCCGAGTTGTGTGCAT	305
FMgSSR-34257	scaffold5593	p2	(TA)8	Class II	31778	31793	AGTTGATTGTGACCGGGCCAT	CGCATCACTTTGTTGTCGCCGT	275
FMgSSR-34258	scaffold1353	p2	(TA)8	Class II	27470	27485	ATAAGTGCGCGGCTGGTTCGTA	AATGGAAGCGGAAGCGGGTA	337
FMgSSR-34260	scaffold9585	p2	(TA)8	Class II	2925	2940	ATCCACAAATGAGCGGAGCGGA	ACATCGCCTTCGCTTGAGCA	281
FMgSSR-34262	scaffold7052	p2	(TA)8	Class II	20417	20432	ATCCGCCGGCACCAATGGTTAT	ACTGACAGCAGCCAACGACA	344
FMgSSR-34263	scaffold16590	p2	(TA)8	Class II	10182	10197	ATCGACTGATCGATTCTGGTCGCC	ACACGGTATTTGGTGCTGCGGA	241
FMgSSR-34266	scaffold3032	p2	(TA)8	Class II	43104	43119	ATGAGTGTTGCACATCGCCTGC	ATGCCAGTGCATTGTAGCGGGT	221
FMgSSR-34270	scaffold1	p2	(TA)8	Class II	335930	335945	ATGCGTTATCAGTTCCCCACGG	GCGTGGGTAGGAACATATTGGTGGT	314
FMgSSR-34278	scaffold4901	p2	(TA)8	Class II	47938	47953	ATTTGCATGCACGGGGTGGT	GCCGACGGATTATGCGTTTGGT	333
FMgSSR-34287	scaffold19129	p2	(TA)8	Class II	12827	12842	CGCAAATGCCGCCCAACAAA	AGAGGGTCTCCTCGTCTGATTCT	323
FMgSSR-34290	scaffold253415	p2	(TA)8	Class II	380	395	CGCTCAGGTGATTGAAACTTGGCG	AGGGTGAAAACCAAGGACAAGGCT	225
FMgSSR-34297	scaffold2228	p2	(TA)8	Class II	58805	58820	GAGTATGTGGACGCATACGCAA	AATACGGGGCCTGCAATACGCT	237
FMgSSR-34299	scaffold6002	p2	(TA)8	Class II	14254	14269	GCAAGCAAGAACCATCAGCGCA	TTCGGCATGGGCAGTTGCTAGT	301
FMgSSR-34301	scaffold160277	p2	(TA)8	Class II	754	769	GCAAGCCAGCCTATTTAGCTCACG	TGCAACGAGTCCATTCGCGT	207
FMgSSR-34303	scaffold10641	p2	(TA)8	Class II	23348	23363	GCACAGTAGCATGTTTGACCTCAG	ACCGTCGTTTGTACTTGGCCT	326
FMgSSR-34304	scaffold1288	p2	(TA)8	Class II	69798	69813	GCCATGTGGCACTTGACTGTGA	AACGGAAAAGGGGGAAGTGTCTCG	215
FMgSSR-34306	scaffold22055	p2	(TA)8	Class II	2926	2941	GCCGGGCCGAATTTGAGAACAA	CGTGTATGGTGAAACACACGGCA	261
FMgSSR-34309	scaffold104	p2	(TA)8	Class II	169128	169143	GCGCCATTATTATTGCCGGCGT	CACGCGACCGAGGTTTGTTTGT	271

SSR_ID	Scaffold	SSR_Type	SSR_Motif	SSR Class	SSR_Start	SSR_End	Forward sequence	Reverse sequence	Product_size
FMgSSR-34311	scaffold10980	p2	(TA)8	Class II	14267	14282	GCGGTGCCGGCATGAGTATTAT	TCCAAGAAATCCGGTCGGGCAA	300
FMgSSR-34312	scaffold1245	p2	(TA)8	Class II	8823	8838	GCTGCTTGCTTGCTTGATGGCT	TCACCCTCATGGAGTCGTGGTT	321
FMgSSR-34313	scaffold88598	p2	(TA)8	Class II	1433	1448	GCTGGCAGCAGGTATAACTAGAGGT	TGCTGGTGGTTGTGCAGCTT	265
FMgSSR-34314	scaffold3947	p2	(TA)8	Class II	33205	33220	GCTTGTGAACAGTGCAGGTTGC	AGGTGTCACAAGCAGATGAAGTGC	328
FMgSSR-34315	scaffold105308	p2	(TA)8	Class II	539	554	GCTTTCGCACAAAGAAGGGCCA	ATTACAAGCACCCGGGCACA	293
FMgSSR-34319	scaffold206	p2	(TA)8	Class II	83558	83573	GGCAGATGTTGATAGCACTCCATCG	TTCAAGACCGCACCTCTCGCAT	304
FMgSSR-34320	scaffold14618	p2	(TA)8	Class II	2186	2201	GGCTAGGTGCCAGTGCTCACTATT	CGCTGGTGCGTTTTGAGTGCAA	350
FMgSSR-34329	scaffold8017	p2	(TA)8	Class II	20804	20819	GTGCGTTTCAGCTTATTCACACGC	AGGGGCGGTGGCTATTGGATTT	341
FMgSSR-34335	scaffold341	p2	(TA)8	Class II	135255	135270	TACGCGTAGTGCGTAATGCGGA	AACCGGTGTGCTCCACGAAGTT	333
FMgSSR-34340	scaffold2010	p2	(TA)8	Class II	50698	50713	TATGCGTCGTCCTTGTCCGAGT	CCGGCCGGAATGCAAAAATCA	306
FMgSSR-34344	scaffold23211	p2	(TA)8	Class II	4311	4326	TCAGCACGGTTGCTGCGAAA	TGAAACGCTTTGATGGACAGGGGG	200
FMgSSR-34348	scaffold1814	p2	(TA)8	Class II	45964	45979	TCCGGCACCATGGGTAACAT	TTGCACCACCCGTTGTCGTT	346
FMgSSR-34353	scaffold16015	p2	(TA)8	Class II	8878	8893	TCGAGGGAAGCGGCATTGAT	ACGCGAGCTGCTTGGTCAAA	293
FMgSSR-34354	scaffold2653	p2	(TA)8	Class II	22727	22742	TCGCCAAGCACCTCCATGATCT	TGCAAGCTGCCATGGAAGGAGA	271
FMgSSR-34359	scaffold39633	p2	(TA)8	Class II	1327	1342	TCGTCGCGAAACAAGAGCCA	AGAGCAAAGCTGCAACGCCT	338
FMgSSR-34364	scaffold38185	p2	(TA)8	Class II	582	597	TCTGCGGTGAATTGACTGGAAC	GCCCACAAGCAGAAATGTGCGA	345
FMgSSR-34374	scaffold2729	p2	(TA)8	Class II	1683	1698	TGATGCGCAAGACGGGTTCA	AGTGTCCGTTTCACGCTGGT	319
FMgSSR-34375	scaffold11717	p2	(TA)8	Class II	2715	2730	TGATGGTCTTCCTCACCGCTCT	TCCGTTTTCCCTAGTGCTTCCCT	289
FMgSSR-34377	scaffold1415	p2	(TA)8	Class II	36356	36371	TGATTTTGTCGCGCCCTGAGGT	ACGCACGAGTCCTCTGCCATTT	349
FMgSSR-34378	scaffold1207	p2	(TA)8	Class II	82001	82016	TGCAGGCGTGCCAGTATACGTT	AGTTCTCGCGAGGCTCTAGGAT	314
FMgSSR-34381	scaffold62276	p2	(TA)8	Class II	1711	1726	TGCATTGCGCGCCATTTCAT	TTGATCGAAGACGACTGACGCC	329
FMgSSR-34384	scaffold161151	p2	(TA)8	Class II	515	530	TGCCCTCATCCTGGACCAAA	TGTGCCGCTCGGTTGAAACA	213
FMgSSR-34386	scaffold11571	p2	(TA)8	Class II	13525	13540	TGCCTTTCTGCATGCTCCACA	TGCATGGTTTGCACGGAGAGGT	266
FMgSSR-34391	scaffold2183	p2	(TA)8	Class II	65029	65044	TGCGTGCCCCATGCATGATA	TCCTCCCCACTACTCATCGGTCAA	275
FMgSSR-34392	scaffold25600	p2	(TA)8	Class II	2834	2849	TGCTGCACGAGCAATGCACA	TGTCTCCACTCGACTTCTCCCA	274
FMgSSR-34395	scaffold6488	p2	(TA)8	Class II	2835	2850	TGCTTTTGCGATGGGGAGTGGT	TTTGGGAGACGCTTGGCTGT	319
FMgSSR-34400	scaffold6	p2	(TA)8	Class II	130381	130396	TGGACTTGCAGCACGCACAA	ATCGCGCCTCTCTCTCCCAAAA	328
FMgSSR-34401	scaffold9341	p2	(TA)8	Class II	284	299	TGGCAATCTCTGTCCGCAGCAA	AGCACCGTGGGTGATGATTTGG	234

SSR_ID	Scaffold	SSR_Type	SSR_Motif	SSR Class	SSR_Start	SSR_End	Forward sequence	Reverse sequence	Product_size
FMgSSR-34407	scaffold3469	p2	(TA)8	Class II	13364	13379	TGGCTAATCGGCTCCGAACT	TCGTCACAGCGATCGACATCCA	337
FMgSSR-34415	scaffold1758	p2	(TA)8	Class II	7851	7866	TGGTTATCCGGCCACTAACAGCA	AAGGGTCATGCTCGTGGAAGGT	349
FMgSSR-34416	scaffold726	p2	(TA)8	Class II	44628	44643	TGGTTGCTTCGTTCTGCTCCCT	ATGGCACTGCCGACTCAGACTT	325
FMgSSR-34419	scaffold5990	p2	(TA)8	Class II	4784	4799	TGTCAACGCAACGCACTGCT	TCGAGGGTCACGACGAACTCTT	319
FMgSSR-34420	scaffold382	p2	(TA)8	Class II	85161	85176	TGTCCCTTCTTTTGGCCTTACCGT	AGGCAACAGAAAACCGCCCA	329
FMgSSR-34424	scaffold1009	p2	(TA)8	Class II	91738	91753	TGTCCTGCTCACACCACACGTA	ACCAACCGACCAGCGCTTTGAA	309
FMgSSR-34425	scaffold4852	p2	(TA)8	Class II	15124	15139	TGTCCTGGCTATGTGCTTCCAGT	TTCACGGGAAGGTGCAAGGA	325
FMgSSR-34426	scaffold483	p2	(TA)8	Class II	116621	116636	TGTGCAGCACCAACAAGCCA	TGCAGCTCACAGCTCACACACA	224
FMgSSR-34427	scaffold3918	p2	(TA)8	Class II	31683	31698	TGTGCAGGATCACAACCCAAAGGA	TGTCTGAGCATGCATAAGTGGGGA	235
FMgSSR-34430	scaffold210936	p2	(TA)8	Class II	255	270	TGTGGGATCAGGTTCGCCAT	TCAAGCGAGAGGGGTTGCAAGA	313
FMgSSR-34432	scaffold6262	p2	(TA)8	Class II	30104	30119	TGTGGTAGCCATCAGCACCCAA	AACCACGTGCGCAGCTTTCT	325
FMgSSR-34436	scaffold6423	p2	(TA)8	Class II	26995	27010	TGTTTCGCCGCTCCCAGTTT	TGCGCCACGTTGTAACGCAGTA	304
FMgSSR-34438	scaffold18053	p2	(TA)8	Class II	2915	2930	TTCAGCGTGCAACGCGTCAT	TTTTGGTGTGTGGGCCAGGGTA	207
FMgSSR-34439	scaffold8135	p2	(TA)8	Class II	5678	5693	TTCAGGTGGAAGAAGGCGCAGA	AACACCAATCGTGCACCCGT	311
FMgSSR-34442	scaffold7848	p2	(TA)8	Class II	4580	4595	TTCGCTTAGGTGGAAGTGCCGT	TTCCGCGTTACCAACAGCGA	319
FMgSSR-34444	scaffold387	p2	(TA)8	Class II	16443	16458	TTGATGGCGTCGCACACACA	CGCTATGCAATGCCAAGCACCA	334
FMgSSR-34450	scaffold6630	p2	(TA)8	Class II	29330	29345	TTGTATGGGGACCGGGACTTGGTA	TGCAGTTCTAGGTGGCCCTTGT	336
FMgSSR-34456	scaffold698	p2	(TA)8	Class II	21684	21699	TTTCTGCTGCTTGCACCGCT	ACCGCGGGTCAGTTCAGACTTT	332
FMgSSR-34457	scaffold824	p2	(TA)8	Class II	4993	5008	TTTGCCCAGCGGGTGCTATT	AACACTATTGGCCTGAGGCGGT	254
FMgSSR-34458	scaffold2211	p2	(TA)8	Class II	12658	12673	TTTGGAAGTGCGTGGACCCCAT	TGTACACAACGGGCTGTCACCA	293
FMgSSR-34460	scaffold2430	p2	(TA)8	Class II	5430	5445	TTTTTCTTCCGTCGCCCGTGGA	ACGCGCCATCCATTTCCGTT	350
FMgSSR-34461	scaffold30634	p2	(TA)9	Class II	1707	1724	AAAACCAGCCGTAACTGGGGCA	ACCAAGGCGCCTGACAAGTT	350
FMgSSR-34468	scaffold498	p2	(TA)9	Class II	44253	44270	AACCAAAGCCTCACACCAGCCA	CCCTAAGTGTGATTTAGCGATGCG	332
FMgSSR-34473	scaffold30394	p2	(TA)9	Class II	949	966	AACGTGGAAGGGAGGCATGA	TCGTTATGGGCTTTGCCCGGTT	333
FMgSSR-34476	scaffold3944	p2	(TA)9	Class II	9528	9545	AAGCCGACGACATGGATTGCCA	AGCCAAGCCTTTGCACCGAT	283
FMgSSR-34478	scaffold664	p2	(TA)9	Class II	51621	51638	AAGGCTAACGTCAAGGGAGGCA	AGCGTGCAAGCCATGCTGAT	271
FMgSSR-34480	scaffold16088	p2	(TA)9	Class II	17186	17203	AATGCTTGTGCGGCTCGGTT	TTGTCGAAGCCGTGCCAGAT	316
FMgSSR-34482	scaffold910	p2	(TA)9	Class II	65133	65150	ACAACCAATGGTACGGAGCGGT	ACGTCCGCCTGGAAACCGTAAA	313

SSR_ID	Scaffold	SSR_Type	SSR_Motif	SSR Class	SSR_Start	SSR_End	Forward sequence	Reverse sequence	Product_size
FMgSSR-34486	scaffold1744	p2	(TA)9	Class II	66870	66887	ACAGGCCGCCGGATTATTCCTT	TGGCATGCAGTGCTCGGTTT	230
FMgSSR-34490	scaffold8904	p2	(TA)9	Class II	4271	4288	ACCAGGCTTTTGGCCCAGTTCT	GGGTGAAGAAGCAATTTGGTGAGG	348
FMgSSR-34492	scaffold22127	p2	(TA)9	Class II	2298	2315	ACCATGTGCAACGGTCCCAA	ACAGACAAGCGCGCATCCTT	339
FMgSSR-34495	scaffold28604	p2	(TA)9	Class II	3155	3172	ACCTCTGTAGTGCAGCTTGTGC	ACGTGGTGATGTTATCCGCCGT	262
FMgSSR-34496	scaffold40762	p2	(TA)9	Class II	3675	3692	ACCTGCAGATATCAGAGACGCA	TAGCGCAAGGAGGGGCATGAAA	330
FMgSSR-34497	scaffold11551	p2	(TA)9	Class II	15436	15453	ACCTGCCGTTGATAAGCCACCA	ATTTATGGTCACGGGTGGGGGT	343
FMgSSR-34507	scaffold231	p2	(TA)9	Class II	129523	129540	ACGGTGGATGCAGAGCAACA	AGGACGTTCACAGTCGTTGGTG	350
FMgSSR-34508	scaffold30763	p2	(TA)9	Class II	1918	1935	ACGTACGGCAGTCTGTGTCA	TGCGCGTGCATGTTAGCTCAT	290
FMgSSR-34512	scaffold2310	p2	(TA)9	Class II	18602	18619	ACTCGTCACTCGTCAGGGGTGTAT	TTGCCCATTTTCTGGCCTGCAC	334
FMgSSR-34513	scaffold2659	p2	(TA)9	Class II	7460	7477	ACTGAACCATTGCCGCCGTT	ACTCTTGTGTGAGAACCGCCAC	350
FMgSSR-34515	scaffold18180	p2	(TA)9	Class II	9985	10002	ACTGCATGTCCCTGCAGCATGT	AACAGTGCCCAAGGCAATCGGA	245
FMgSSR-34518	scaffold11689	p2	(TA)9	Class II	22552	22569	AGAACAACGGATAGGGGCAAGA	CCCCCTTGCATGCGTGTTTT	286
FMgSSR-34522	scaffold6500	p2	(TA)9	Class II	7447	7464	AGCAGTGCCAGAGCCACTTT	GAAACAGAAGTGATGGTAGTCCCG	344
FMgSSR-34525	scaffold6912	p2	(TA)9	Class II	37017	37034	AGCCTGCAGTGTGCAAGGGAAT	TTAAACCGAGAGCAACGGCGGA	340
FMgSSR-34526	scaffold3453	p2	(TA)9	Class II	50341	50358	AGCGCGCTACGCGTCAAATA	TGCTTGACGAACGCACTGGCTA	324
FMgSSR-34527	scaffold2974	p2	(TA)9	Class II	53241	53258	AGCGTGCACTGACAACAACAGC	TTCATCGCATGCGCGTCACA	268
FMgSSR-34532	scaffold1334	p2	(TA)9	Class II	35602	35619	AGGCACGAGGCAGCAATCTT	TCGTCTCGCACGCTCATGGAAA	320
FMgSSR-34533	scaffold9240	p2	(TA)9	Class II	7763	7780	AGGCGATGGGCTCTGCTGTTAT	ACGCGACGCGAAAACACGAA	233
FMgSSR-34537	scaffold12806	p2	(TA)9	Class II	1952	1969	AGTTGGCAACGGCGAAACGA	ACCACGCAACCGGTCGAATA	325
FMgSSR-34541	scaffold3201	p2	(TA)9	Class II	25012	25029	ATGTCACGGGAGAGCTTGCAGA	ATCCCACTGTGTGCACCAGCAA	330
FMgSSR-34542	scaffold5203	p2	(TA)9	Class II	11515	11532	ATTATGGTCGCGGGATGGCGTT	GCAAAGGGGGTGTCGTTTTGGT	331
FMgSSR-34543	scaffold113	p2	(TA)9	Class II	73501	73518	ATTGCCGGCCAAGTAAAGGCGT	TGCAACTGGTGCATCATGGGAGT	306
FMgSSR-34544	scaffold2974	p2	(TA)9	Class II	30738	30755	ATTGGATTCCCACGCGCCAA	CGCACCATGCGCTTTTCGAT	278
FMgSSR-34545	scaffold7861	p2	(TA)9	Class II	6643	6660	ATTTCCATCGGAGCGACAGCGT	TTGAAACTGACGGCCACTCGCA	224
FMgSSR-34546	scaffold6542	p2	(TA)9	Class II	24972	24989	CACGCTAAAGCCAACTTAGCCA	TGCACACGACGACCCAACAA	310
FMgSSR-34549	scaffold7637	p2	(TA)9	Class II	20915	20932	CCACCCCACAACCATAAGACTTTC	AGCGCGCAACACGCCATTTA	333
FMgSSR-34550	scaffold606	p2	(TA)9	Class II	33032	33049	CCAGGATCTGTGATTGGACATGGGA	TTTCACCGACCGAGACGAGAGA	308
FMgSSR-34554	scaffold6500	p2	(TA)9	Class II	7655	7672	CGGGACTACCATCACTTCTGTTTC	TTCGCTAGGCTTGCCTTCTGGT	340

SSR_ID	Scaffold	SSR_Type	SSR_Motif	SSR Class	SSR_Start	SSR_End	Forward sequence	Reverse sequence	Product_size
FMgSSR-34556	scaffold3222	p2	(TA)9	Class II	31203	31220	CGGTTTAAGTGTAGGGACGACGTG	TGCGTTCTACACTCCACCAGCTT	314
FMgSSR-34557	scaffold29	p2	(TA)9	Class II	160368	160385	CGTGAATCCTTCCGCGAGCTAT	TCTTTGATGGGACGGGTGTGGA	340
FMgSSR-34560	scaffold16247	p2	(TA)9	Class II	6680	6697	GCACCTCAAGACAGCATCTATACC	TGAGGTGCAATGCGAGCAAGA	316
FMgSSR-34561	scaffold11531	p2	(TA)9	Class II	24666	24683	GCATGAAACGTAACCTGTGTGCAG	AGGAGGGGTGGTTTATCAGACGGA	347
FMgSSR-34564	scaffold6655	p2	(TA)9	Class II	33398	33415	GCGCCATTGAATCAGAACCTGC	TCCAACATTGCACAACACCTGCC	318
FMgSSR-34566	scaffold1437	p2	(TA)9	Class II	12503	12520	GCTGGTATCGTGAGAACAAGGTCA	TTCCAGCTGGCTGTCCTTTCCA	269
FMgSSR-34568	scaffold6103	p2	(TA)9	Class II	1047	1064	GGCGCGCCATTTAGCGTTTT	TGGTTGCAAGAGTTGGCTCGGT	272
FMgSSR-34569	scaffold1847	p2	(TA)9	Class II	68735	68752	GGCTGGGCAGCCTGTTATACAT	TCCTGCCTGCACAGACATCAA	263
FMgSSR-34574	scaffold4723	p2	(TA)9	Class II	44016	44033	GGGTCAATGCACATGGTGCAGAA	TGGGACAGTGGGTAAGGGAACA	261
FMgSSR-34577	scaffold4938	p2	(TA)9	Class II	41948	41965	GTGGCAGCTGAAACTGCTCCTT	AGGTTTGTTTCCGGAGCGCTGA	200
FMgSSR-34579	scaffold9238	p2	(TA)9	Class II	17931	17948	TAAACCATGTGGCCCTCGCT	TCCCATGCTTCAGAACAGCCA	332
FMgSSR-34583	scaffold12059	p2	(TA)9	Class II	22815	22832	TAGTGGGCTTCTTCCCCATGCT	AGCGGTCACCCCTTGTTGATGA	341
FMgSSR-34584	scaffold14066	p2	(TA)9	Class II	21291	21308	TATGGACAGTGCTGTTTCGGGC	TTGCCGTGAATCAATGGCGT	241
FMgSSR-34586	scaffold11081	p2	(TA)9	Class II	8492	8509	TCAACGCAGGCAGCACACTT	ACCTGCCCCTCTTTTGCTGGTT	223
FMgSSR-34587	scaffold158	p2	(TA)9	Class II	13892	13909	TCAAGTGCGCCTCGTGGCTATT	TGGCGACGGTGCTGTGTTTT	299
FMgSSR-34596	scaffold87384	p2	(TA)9	Class II	677	694	TCCACCACTTGCTTGAGTGTGT	TCAGGCCATGGCATACCTTTCT	323
FMgSSR-34598	scaffold980	p2	(TA)9	Class II	20484	20501	TCCCTTCTCAAACGCTGCTCCA	TTATAGCGAGAGGCTCCCTCCTGA	329
FMgSSR-34602	scaffold2400	p2	(TA)9	Class II	18690	18707	TCGAGATGAATGGAGCGCTGGT	TACATGGACATAAGGGGCGCGCTA	239
FMgSSR-34603	scaffold8244	p2	(TA)9	Class II	33832	33849	TCGATGGTTGTGTGGTGGGCTT	ATGCATGCGCGTGTGTGAGT	245
FMgSSR-34604	scaffold24932	p2	(TA)9	Class II	119	136	TCGCACTCCGGGCAACTTGATT	TGGCCTGCATCAGCTTGTTGTG	324
FMgSSR-34607	scaffold116159	p2	(TA)9	Class II	151	168	TCGCGGTCATAACGGAGAGCTT	TGGATTTCGTCGGAACGGGA	207
FMgSSR-34612	scaffold36476	p2	(TA)9	Class II	2599	2616	TGAACTGACCAGTCGCCCATGA	ACACGTACGCCACGCCAGTAAT	315
FMgSSR-34613	scaffold959	p2	(TA)9	Class II	8605	8622	TGACCCAGATGACACGTTCACGA	AGCGTAGCACCACGGATAGGTT	298
FMgSSR-34614	scaffold3901	p2	(TA)9	Class II	49035	49052	TGACCCGGCTGAACCAAAGT	AAGAGTGCCCGTGAGTGCAT	211
FMgSSR-34616	scaffold12671	p2	(TA)9	Class II	747	764	TGAGAGCTAGTGATTCCCACGGA	AGGTGGCAGGGACGTCATTT	269
FMgSSR-34624	scaffold15096	p2	(TA)9	Class II	17932	17949	TGCATGTGAGGCTCGGAAGT	AGTAGCCGCTGCAAGCATGA	227
FMgSSR-34626	scaffold3890	p2	(TA)9	Class II	20754	20771	TGCCATGCGTAGCAGTGCCTTT	ACCTTGGGCGAGGTTTGTGA	264
FMgSSR-34628	scaffold586	p2	(TA)9	Class II	86128	86145	TGCCTCTCACATCAGGGAAGCAT	AGACTAGTGAACGACAGAGGGACA	305

SSR_ID	Scaffold	SSR_Type	SSR_Motif	SSR Class	SSR_Start	SSR_End	Forward sequence	Reverse sequence	Product_size
FMgSSR-34630	scaffold889	p2	(TA)9	Class II	977	994	TGCGATGGCAACCGGTAAGT	AGGAGCTGCATTTGCCTCCCAA	254
FMgSSR-34632	scaffold30069	p2	(TA)9	Class II	6676	6693	TGCGTCCTCGGAACCTTATCCA	AACCATAGCAAGCTGGACCGGA	336
FMgSSR-34635	scaffold2511	p2	(TA)9	Class II	44223	44240	TGCGTTTGACCTCTTGTCGGGT	TCGCCCTGTGCATTTGTGCT	314
FMgSSR-34638	scaffold7973	p2	(TA)9	Class II	26300	26317	TGGACTTCCCCTAGCCAATGTCA	TTTGGCTCTCGTCCGTGTCT	225
FMgSSR-34644	scaffold341	p2	(TA)9	Class II	23871	23888	TGGCTTCAGAGGCATCTCAGT	ACGTGTAAAGCCTGCCCATCCA	302
FMgSSR-34645	scaffold2374	p2	(TA)9	Class II	3096	3113	TGGGCTGCCATGATTTCTAGGCT	TGCCATTGCCGGCATTTTTGC	325
FMgSSR-34646	scaffold12715	p2	(TA)9	Class II	2552	2569	TGGGGATCATTGTAGCACGGA	TGATTTGGTGCTCGGGTGCTGA	282
FMgSSR-34647	scaffold668	p2	(TA)9	Class II	106203	106220	TGGTCAAACGACCCGCACAA	CACCGAATAAACCTCCACCGCA	266
FMgSSR-34648	scaffold472	p2	(TA)9	Class II	99721	99738	TGTACGTGCGCAATAGCAAGCG	GCAATGCAAACGGAAGGCTGCT	287
FMgSSR-34652	scaffold716	p2	(TA)9	Class II	18718	18735	TGTCGTTGCAAATCACAGCCCG	TGCTCATCATTCCTGCGCGT	232
FMgSSR-34653	scaffold13462	p2	(TA)9	Class II	15860	15877	TGTGCAAATCCCTCCTGCATCCT	TTCTCGTAGATCGCGCGGAA	297
FMgSSR-34655	scaffold572	p2	(TA)9	Class II	41860	41877	TGTTCCCGTCGCTGTGTGTTGA	TGCCACCCATGTAATGTGCCA	344
FMgSSR-34657	scaffold8264	p2	(TA)9	Class II	14455	14472	TGTTGGCGTCCTTGTTCGCT	TGATTTGTGCGCCGGCATCT	340
FMgSSR-34665	scaffold34793	p2	(TA)9	Class II	4809	4826	TTGATGGTAGGGTACTGCAGGC	TCGTGCCGAGAAGTTCACCA	349
FMgSSR-34667	scaffold2659	p2	(TA)9	Class II	474	491	TTGGTGCCGCTCAAAGCTCA	TTAATCAACGGGCAGGCGGCAA	255
FMgSSR-34668	scaffold587	p2	(TA)9	Class II	76606	76623	TTGTGACGACTGAGGTGTCCTAGC	ATGCACGGGTGCGTGCTTTT	217
FMgSSR-34670	scaffold54830	p2	(TA)9	Class II	1347	1364	TTGTGCCGGTTTGGACTTGTGC	ACACCCCATGTTGATGATGGCCT	310
FMgSSR-34672	scaffold15746	p2	(TA)9	Class II	19422	19439	TTTGTGTGACCATGCGAGCAGC	CGCAGACGCCACTGTGATTA	255
FMgSSR-34674	scaffold8921	p2	(TC)10	Class I	22644	22663	AAAACGCATGGGGTGCTGGA	TTCCGCCGCAGCCTATTTCTGT	223
FMgSSR-34675	scaffold98	p2	(TC)10	Class I	158859	158878	AAACAGAGGTCCCCAACATCGC	TTGAGCAGCGTGCGGCATTA	253
FMgSSR-34678	scaffold2434	p2	(TC)10	Class I	4825	4844	AAACTCTCGGGCGGCATGACAA	AAAGAGAGCCCGATGCTGCT	319
FMgSSR-34680	scaffold4389	p2	(TC)10	Class I	50412	50431	AAAGCAAAGCGCGCGATGGA	AGGTGTGCGCTCTTCCTTTCCT	277
FMgSSR-34690	scaffold2499	p2	(TC)10	Class I	36875	36894	AACGTCATCAGGTTTCGCCCCA	TGCAAGAAAGTCGCCGCTGT	305
FMgSSR-34692	scaffold148	p2	(TC)10	Class I	100014	100033	AACTCCAGGTTGCTTTGCTGGC	TGGCAGGTGCTCTGCCTTTTCA	222
FMgSSR-34693	scaffold1219	p2	(TC)10	Class I	84118	84137	AACTGGTGCCCCACGGGAAAAT	ACCCATCTAGCTTCTGGCCCAT	336
FMgSSR-34694	scaffold1911	p2	(TC)10	Class I	58429	58448	AAGCAAGCAAGCAGCGGGTA	AGCGCCCTTTTGAGATTGAGCTG	267
FMgSSR-34697	scaffold2140	p2	(TC)10	Class I	28448	28467	AAGCGACACGAGCAAGATGC	TGCATCCAACAAAGCAGCACCA	298
FMgSSR-34699	scaffold17211	p2	(TC)10	Class I	603	622	AAGTGTTCCAAACTCGGGCGCT	ACGCCTTCGCATGGCAAGTT	223

SSR_ID	Scaffold	SSR_Type	SSR_Motif	SSR Class	SSR_Start	SSR_End	Forward sequence	Reverse sequence	Product_size
FMgSSR-34701	scaffold1243	p2	(TC)10	Class I	56647	56666	AATGCCCTTCAGCGAGCGTT	ACGTATTGGGCGCTGCTTGA	309
FMgSSR-34709	scaffold3412	p2	(TC)10	Class I	12073	12092	ACAACGACCGACTCCGATCACA	TGTTGCTAACCACTCGGCCACA	245
FMgSSR-34710	scaffold553	p2	(TC)10	Class I	88115	88134	ACAACGCCTGTGATAGTCGGCA	TGAGAGACCGATGCCCAAGCAA	349
FMgSSR-34717	scaffold2825	p2	(TC)10	Class I	50349	50368	ACACGTACCGCTTCAACGTGAC	TCGGGACCAAGGAACGGAGAAA	270
FMgSSR-34718	scaffold12335	p2	(TC)10	Class I	8652	8671	ACACGTGCTCTGCTCTGCAA	TTGTGCCGGATGGACAGTTGCT	235
FMgSSR-34720	scaffold28	p2	(TC)10	Class I	69785	69804	ACAGCGGTTGCCAAGAGACGAT	ATTGCATTGCCCGCATCGGA	243
FMgSSR-34724	scaffold12007	p2	(TC)10	Class I	5186	5205	ACCAAGTGCTTGCTAGGCTGCT	ATGGGCGTGCGAGAAGACAGAT	350
FMgSSR-34734	scaffold9	p2	(TC)10	Class I	104314	104333	ACGGCCAAGTAGCGAGCAAA	AGGCGCTTCAGGTAAGACGGTT	293
FMgSSR-34735	scaffold31786	p2	(TC)10	Class I	4776	4795	ACGGGGCATTGTCAGGTGCTTT	CGCTTTTGTTGCACCTTGCAGA	341
FMgSSR-34736	scaffold1725	p2	(TC)10	Class I	70244	70263	ACGTCTCCCAGCGTAAAAGGCA	TTGCTGGTCACCATTGCGCT	209
FMgSSR-34738	scaffold2605	p2	(TC)10	Class I	35306	35325	ACGTGCGCCTCACATTCTCACA	AAGTGTCGTCCGAGCTGTGGAT	319
FMgSSR-34740	scaffold1386	p2	(TC)10	Class I	61447	61466	ACGTGTCACTTGGGTGTCCAGA	ACAGTTCGAGGATAGGTGTGTTGG	329
FMgSSR-34742	scaffold1409	p2	(TC)10	Class I	67245	67264	ACGTTCGCAAAACGCGACTCA	ATGACGAAGCTCTCTGCTGGCT	265
FMgSSR-34746	scaffold15948	p2	(TC)10	Class I	5056	5075	ACTGCCCATCGGCTGCAAGTAT	AAGCATAGGCCATCGCAGTGGT	331
FMgSSR-34747	scaffold1557	p2	(TC)10	Class I	19264	19283	ACTGCGACTTCACGCCATGT	TGGCAATGGCGGTCCCTAACAA	326
FMgSSR-34748	scaffold6963	p2	(TC)10	Class I	24654	24673	ACTTGGCAACAGTCGCAGCA	CGTCGCAGCAACAGCGAAAT	319
FMgSSR-34751	scaffold44386	p2	(TC)10	Class I	991	1010	ACTTTCCACCGAGCGTGTTCGT	ACCCTCTGTCTTGCTTCACGCT	314
FMgSSR-34756	scaffold2580	p2	(TC)10	Class I	40465	40484	AGCAAGTGAGGGGTGGAAAGCA	TCGCTTTCTTTCGGGCTCGT	293
FMgSSR-34758	scaffold259	p2	(TC)10	Class I	142030	142049	AGCAAGTTGAGGAAAGATGGGCGT	AAGGCCACACACATGCTGCT	257
FMgSSR-34762	scaffold10302	p2	(TC)10	Class I	5816	5835	AGCAGCAGCAGCTAGTGTTCGT	TTCGGCACCAGTTACCATCGGA	348
FMgSSR-34763	scaffold2772	p2	(TC)10	Class I	33687	33706	AGCCAAGGCCAGACACTGTTGT	AGCCAATGGGCGATGGCATT	324
FMgSSR-34765	scaffold891	p2	(TC)10	Class I	24529	24548	AGCCCGTTGGGTTGTGTGTT	TCTGTCGCGTGTCGCTGTTA	346
FMgSSR-34767	scaffold3705	p2	(TC)10	Class I	43038	43057	AGCCTCTCCTTGTGGCTTGGAT	TAGCGTAAAAACGCCTGCACCG	257
FMgSSR-34768	scaffold4391	p2	(TC)10	Class I	3357	3376	AGCCTGCAGTGTTTGGGTTCCA	TTTTCGCCACGTCAACCCCA	320
FMgSSR-34769	scaffold2085	p2	(TC)10	Class I	71101	71120	AGCGCATGGATGAAGTGGGCAT	AGTTGTAGCTCGCGCTTCCT	346
FMgSSR-34774	scaffold94	p2	(TC)10	Class I	190354	190373	AGCTCACGGTTCCATCTGCTCA	AGGCGATGGATCGAATGCGACA	320
FMgSSR-34780	scaffold18	p2	(TC)10	Class I	40974	40993	AGGAAAACTTACCGGACGCACT	TTGCACCGACAGAGAGAGACGA	309
FMgSSR-34783	scaffold57753	p2	(TC)10	Class I	317	336	AGGCAGTGTCTGGCTGACCTTT	AACACCCGGCAGATCGAGCAAA	302

SSR_ID	Scaffold	SSR_Type	SSR_Motif	SSR Class	SSR_Start	SSR_End	Forward sequence	Reverse sequence	Product_size
FMgSSR-34787	scaffold5353	p2	(TC)10	Class I	27260	27279	AGGCTGTTTCGTGTGTCTGAGGA	ACCCGAATCCATGTTACGCGCT	242
FMgSSR-34797	scaffold19800	p2	(TC)10	Class I	3142	3161	ATAGTCCATCCCACAGCCCACT	TCAACGCATGGGGAGGAACA	345
FMgSSR-34810	scaffold13283	p2	(TC)10	Class I	2016	2035	ATGGGCGTGGCCCGTATCATTT	ACCACCACACCACGCTTTAGCA	304
FMgSSR-34811	scaffold352361	p2	(TC)10	Class I	130	149	ATTCTTCCACGTCCCGCTCT	TCTCGCGGCAGTAATTGGCA	285
FMgSSR-34812	scaffold22546	p2	(TC)10	Class I	8341	8360	ATTGCCTCTGTTTCCGCCCTGA	TGCTTGGTAGAGCCGTTGGGAA	347
FMgSSR-34813	scaffold691	p2	(TC)10	Class I	458	477	ATTGCTTTGCCCGGCCCTTT	TAATTTCTGGGGCCGCCGTTCA	238
FMgSSR-34819	scaffold3754	p2	(TC)10	Class I	26088	26107	CCGCAACTGCGGTTTTCACACA	AGATCCGGAGGGAAGGATGCAA	336
FMgSSR-34824	scaffold772	p2	(TC)10	Class I	108196	108215	CGCAGCAGGTGCGTTTTGTT	ATGAACCCGGCCTACGCGTTTA	224
FMgSSR-34825	scaffold11406	p2	(TC)10	Class I	3341	3360	CGCCAACCCGAGCCGAATTTTT	AACCAGCCAACGGACAGTCGAT	249
FMgSSR-34829	scaffold222	p2	(TC)10	Class I	64486	64505	CGGTGTGATCGACGTGCTGTTT	CGGCTTTGAAGCAGTGCACCTA	301
FMgSSR-34831	scaffold57753	p2	(TC)10	Class I	174	193	CGTTTGTCGACATGGCGGTGTT	TCTGGTGAGCCGACAAAGGTCA	322
FMgSSR-34834	scaffold7860	p2	(TC)10	Class I	14603	14622	GCAAGCAAGCAAGCGTCCCAAA	AGCTGAATGACACGGGGACACT	342
FMgSSR-34842	scaffold77194	p2	(TC)10	Class I	1274	1293	GCCCAATTTGCAAAGCACCCCA	AGACAGCCCCCTTTGTCACGTT	243
FMgSSR-34843	scaffold2404	p2	(TC)10	Class I	25974	25993	GCCCACAAAAGTCCCCTCTCAA	TTTGCCTCCCCCTCCCCTTAAT	330
FMgSSR-34844	scaffold57017	p2	(TC)10	Class I	1099	1118	GCCCATGAGCGAGGCCTAATTT	TGCACACAGGGACCAGTCAT	346
FMgSSR-34845	scaffold1	p2	(TC)10	Class I	28703	28722	GCGCGGTTGATTTTCTCCCGTT	ACTGTCGCGCCTTTCGTTGT	285
FMgSSR-34846	scaffold24184	p2	(TC)10	Class I	6969	6988	GCGCTTCTCATGAGCAAAGGGTCT	TGGTGCTCGCATGGAGTAGAGT	204
FMgSSR-34849	scaffold9133	p2	(TC)10	Class I	18232	18251	GGCCCAACTTGGCCATGTCATA	TCACGCCACCAAATAAGGGGTGA	213
FMgSSR-34851	scaffold1439	p2	(TC)10	Class I	81969	81988	GGCTCGCTGATGCTTTCCTTGA	CACCGCCTGGCTACCTAATCAT	325
FMgSSR-34852	scaffold7413	p2	(TC)10	Class I	10042	10061	GGGCAGACTTGCCAATGCATGTT	AGGTGGTGTGGTGGACAAACGA	294
FMgSSR-34853	scaffold19032	p2	(TC)10	Class I	5066	5085	GGGGACTGTTCGGAATCAAGGGTT	ATTGACAACCACCGCCACCACT	203
FMgSSR-34857	scaffold347	p2	(TC)10	Class I	131267	131286	GGTGTTTCTTCCGTCCACTGCT	GCGGCCTTAATTGCACTGTAGGAT	350
FMgSSR-34858	scaffold8750	p2	(TC)10	Class I	5180	5199	GTGACCATGAACGCGCTTTTGC	AGAATGTCGAGGCAAGCGCA	316
FMgSSR-34864	scaffold11036	p2	(TC)10	Class I	13697	13716	TACTCGTGGCAGTGTGCTGTGA	TGCTCCGAACGAAATCCACCGT	241
FMgSSR-34866	scaffold5341	p2	(TC)10	Class I	40132	40151	TAGGCACCAAAAGCTGCTCCGT	AGGTCCAGCTGATCCTCGCAAT	262
FMgSSR-34867	scaffold39990	p2	(TC)10	Class I	3238	3257	TATTGCTGCTGCGTCTGCGT	GGCAAGTGAAAATCCGGCGCAA	347
FMgSSR-34870	scaffold3247	p2	(TC)10	Class I	48677	48696	TCAACAACACATGCCTCTGCGG	ACGTCCTTTTCGTGCCAGCCAA	334
FMgSSR-34874	scaffold770	p2	(TC)10	Class I	9296	9315	TCACGTGCGAGCAGTTTTCCCT	ACACGCATGTCCGCATCAAG	321

SSR_ID	Scaffold	SSR_Type	SSR_Motif	SSR Class	SSR_Start	SSR_End	Forward sequence	Reverse sequence	Product_size
FMgSSR-34879	scaffold3758	p2	(TC)10	Class I	30810	30829	TCCGCCGATGATGCCGAAAGAA	ACATCTCATGAGCCGACGTCCA	340
FMgSSR-34880	scaffold23342	p2	(TC)10	Class I	5606	5625	TCCTTATCTCCGTCCCCTTGTGT	TCACCCAGCCATGCGGATTT	324
FMgSSR-34881	scaffold1978	p2	(TC)10	Class I	1216	1235	TCGCATGCACAATTCCCGAGGT	TATGCGAGCGAGGATGCCATGT	211
FMgSSR-34886	scaffold854	p2	(TC)10	Class I	24533	24552	TCGCTTGGTTCATCTGGTGGGT	ACTGGGCAACGAGAGCACAT	224
FMgSSR-34898	scaffold887	p2	(TC)10	Class I	57106	57125	TCTTCTACTGGGCGTCGCAACT	AAAGAAGAGCGCGCGAGAGT	237
FMgSSR-34901	scaffold6571	p2	(TC)10	Class I	1193	1212	TCTTGCAGCCGCACACCAAA	ACGGAAGGAACGTTGCGTGA	337
FMgSSR-34904	scaffold367	p2	(TC)10	Class I	15432	15451	TGACATGATCTCACCGCGCA	ACAACCTGATGCGCATGCCT	241
FMgSSR-34907	scaffold3063	p2	(TC)10	Class I	16935	16954	TGAGACCGGAGCGCATGTAA	AGCCGAAGCTGAAGTCCAGGTA	252
FMgSSR-34916	scaffold2659	p2	(TC)10	Class I	6982	7001	TGCAGCAGAGCCGAAAGACA	TCCGGTTGGTGTGACGTGTT	291
FMgSSR-34920	scaffold4375	p2	(TC)10	Class I	20747	20766	TGCAGTGTGGCGCTAAGCCTAA	GCAGTGGTTGCTCACCAGATGAA	346
FMgSSR-34921	scaffold6985	p2	(TC)10	Class I	28190	28209	TGCAGTTGCCATGACTGGTGGA	TCATTGCCGGCTACTGGCTACA	269
FMgSSR-34922	scaffold9084	p2	(TC)10	Class I	15627	15646	TGCATAGGAGAGCCGAGGCAAA	TGTTTATGGCGCCCTCCAGAGT	221
FMgSSR-34924	scaffold100937	p2	(TC)10	Class I	965	984	TGCATTGGCTGTCTTCGGCA	TGCACAGCCCTGAATCCTGA	283
FMgSSR-34925	scaffold24974	p2	(TC)10	Class I	1492	1511	TGCCAAACCTACTGCCCACA	GCTGCAATGCAAGCATGGCCTA	262
FMgSSR-34930	scaffold21576	p2	(TC)10	Class I	13798	13817	TGCCTCCTTCTCCTTGCTTGCT	AGCCCAAACAAGGCACCAACA	312
FMgSSR-34931	scaffold6	p2	(TC)10	Class I	258719	258738	TGCCTGCTTGCCGTGCTAAT	TGGTGATGCACATGCAGGGT	289
FMgSSR-34936	scaffold1105	p2	(TC)10	Class I	31168	31187	TGCGCGATAATGCTGATGTGCC	TTTCACATGTGGGTGCGTTGCC	290
FMgSSR-34938	scaffold19950	p2	(TC)10	Class I	859	878	TGCGGCCCATTTTTCTCCACCA	AAAGACAACAGCGTGGCCGT	344
FMgSSR-34939	scaffold2139	p2	(TC)10	Class I	30787	30806	TGCGTCCATGGCCTACCTGTAA	GCAAAACCATCTGGGCATGCAGT	350
FMgSSR-34944	scaffold6988	p2	(TC)10	Class I	9480	9499	TGCTCACTGCAAACCCGAACCA	TGCCTTTGCCCCAACCTTGCAT	235
FMgSSR-34955	scaffold4275	p2	(TC)10	Class I	42369	42388	TGCTTGTCTCCATTGCTGCCCA	AGCCATGGCGGAAGGCAAAA	254
FMgSSR-34960	scaffold9874	p2	(TC)10	Class I	941	960	TGGCAAAGATTGGGTCACGCCT	ATGCCTGGGACCATGAATCCT	239
FMgSSR-34962	scaffold178	p2	(TC)10	Class I	86420	86439	TGGCCGCCATGGAAGTACAAGT	AACACGACCAGAGCTTGTGGCT	309
FMgSSR-34963	scaffold2636	p2	(TC)10	Class I	54360	54379	TGGCCTTGTCAGGAGCAGCAAT	TGCAGGGTATTACGGATGTCCC	213
FMgSSR-34974	scaffold14587	p2	(TC)10	Class I	13924	13943	TGTACCACGCCCTTCAAAGCA	TTCTCGTCGCCCAACCCTTGTT	292
FMgSSR-34977	scaffold2125	p2	(TC)10	Class I	25439	25458	TGTCATGTAAGCACCGGGTGGA	ACAAGGATGCAGGAAGCGAGCA	225
FMgSSR-34984	scaffold5	p2	(TC)10	Class I	289534	289553	TGTTTGGGGCGCTTTGAGCA	AGCCCATGGCACTATGGCAGTA	285
FMgSSR-34986	scaffold12639	p2	(TC)10	Class I	9801	9820	TTATGTTGTCACGCGGTCGGCA	TCTGGTTGCGTTCTCTTGCGA	261

SSR_ID	Scaffold	SSR_Type	SSR_Motif	SSR Class	SSR_Start	SSR_End	Forward sequence	Reverse sequence	Product_size
FMgSSR-34988	scaffold12338	p2	(TC)10	Class I	21980	21999	TTCAGTCGGCATGTTGCAGGGT	TCGTGCATTTGTGCCCAACCA	288
FMgSSR-34990	scaffold7658	p2	(TC)10	Class I	4646	4665	TTCCTCATCACCTGAGCAAGCG	TGAACACGAGAAGAGGGCGCTA	310
FMgSSR-34992	scaffold2425	p2	(TC)10	Class I	4229	4248	TTCGCATCCGCTGTCTCCTTCT	TCGTCGTTGTGGATGGACAGCA	276
FMgSSR-34998	scaffold12570	p2	(TC)10	Class I	14581	14600	TTGCAGGGCATGATGCTGAGGT	TCCTGCGCACATGGTAACCCTT	268
FMgSSR-35000	scaffold447	p2	(TC)10	Class I	117153	117172	TTGCCGGACCCTTTCATGGAGA	TGCGTTCCATTCTGCAGGCACT	308
FMgSSR-35003	scaffold2442	p2	(TC)10	Class I	31798	31817	TTGCTGCACGAAGAACCCGT	AAGACGTGGCGATGCACACT	242
FMgSSR-35006	scaffold5034	p2	(TC)10	Class I	40003	40022	TTGGGAGTTCCGTTCCGTCCAA	AACAAAGCTGGTCGCCGTGT	276
FMgSSR-35010	scaffold1052	p2	(TC)10	Class I	9573	9592	TTTACTCTCCCCTCCCAGCCACTT	TTGGACATGGGTGTTGCCATCG	235
FMgSSR-35012	scaffold6498	p2	(TC)10	Class I	2830	2849	TTTCCTTGGTGCTGCGTGCT	AGCGAGCAATGCACTCGTCA	217
FMgSSR-35014	scaffold5302	p2	(TC)10	Class I	35669	35688	TTTGGTCGTCCCCACGCAGAAA	GCGCGGGAAAGCCAAAAAGCTA	214
FMgSSR-35015	scaffold5869	p2	(TC)10	Class I	4481	4500	TTTTGACCCGCGTTCGTCCTCA	TTTCCAGCATCCTCGCTTGTCG	326
FMgSSR-35017	scaffold6073	p2	(TC)11	Class I	35922	35943	AAAAGGACGAGCCCACATGACG	TGACGACGTCTCCAATGCGA	275
FMgSSR-35020	scaffold1578	p2	(TC)11	Class I	27978	27999	AAATAGTCGCGCCCTGGTCCAA	GGGTTCACGCCATGAGAAAGT	241
FMgSSR-35025	scaffold488	p2	(TC)11	Class I	102402	102423	AACGCCGTAGGGCACTGCTAAA	AACGGCGGGTTGGAATGGTT	289
FMgSSR-35031	scaffold113	p2	(TC)11	Class I	17073	17094	AAGCGGGTGTCTTTTGGCCT	AGTCCAGTCTGAGTTGTAACCGGC	348
FMgSSR-35032	scaffold1203	p2	(TC)11	Class I	69768	69789	AAGGCCAGTTACACTCACGGCA	AACATGGCTCGACCACAGCA	223
FMgSSR-35035	scaffold1204	p2	(TC)11	Class I	73605	73626	AATGCCACTTTGGTGCCATGCG	TGGCGCGTAGCACAGCATTT	297
FMgSSR-35038	scaffold6202	p2	(TC)11	Class I	2835	2856	ACACCGCGTGAACCACAACA	TGCCAAACAAAGCCCCCAGT	223
FMgSSR-35039	scaffold23470	p2	(TC)11	Class I	4115	4136	ACACTGCCACATCACCCCTCTT	ACACTCAGTTGCGAGCTTGGCT	223
FMgSSR-35041	scaffold20415	p2	(TC)11	Class I	8031	8052	ACAGGTTTGTTGCAGGTCTGGC	TGGAGCCGTGTTCTGCGTAA	314
FMgSSR-35043	scaffold3847	p2	(TC)11	Class I	4369	4390	ACCAGATCTCAGTGGTCACGCT	TGACAAGCGGAGCACGTTCA	332
FMgSSR-35051	scaffold630	p2	(TC)11	Class I	60009	60030	ACCTTGCTACGAACGGAGGCTT	AGCTCCTGCTTCCCTAGACACCAA	297
FMgSSR-35053	scaffold94	p2	(TC)11	Class I	140937	140958	ACGATGTGGGAGATGATGACTGCAC	TCTAGGTTGGGGAACAGTTTCTGC	327
FMgSSR-35054	scaffold20719	p2	(TC)11	Class I	12450	12471	ACGCCGAAGCGCATCCAAAA	GCGCCTCCGATCCTTTCTTTCT	308
FMgSSR-35061	scaffold3602	p2	(TC)11	Class I	29950	29971	ACGTCGCGGTGCGTAAACAT	TCGCAGGAGCTAGTGTTCGTCT	301
FMgSSR-35062	scaffold57271	p2	(TC)11	Class I	2522	2543	ACGTGCAACACGTCATGCGAT	AGAGAACCTCTCCAGCAATGCCA	208
FMgSSR-35064	scaffold6272	p2	(TC)11	Class I	11068	11089	ACTCGAAGGCCAGAACACATCC	TGGCCACGTTAGAGGCTGCTTT	220
FMgSSR-35067	scaffold4527	p2	(TC)11	Class I	40823	40844	ACTGCTGGCCGTAGATCCAT	AGGCAATGTGGGGGTAGGCAAA	273

SSR_ID	Scaffold	SSR_Type	SSR_Motif	SSR Class	SSR_Start	SSR_End	Forward sequence	Reverse sequence	Product_size
FMgSSR-35070	scaffold693	p2	(TC)11	Class I	4242	4263	ACTGTGGCACTTCACTGTCTCG	TGATACAAGTGCCGCGTCGAGT	252
FMgSSR-35072	scaffold1457	p2	(TC)11	Class I	28700	28721	AGACAAGCATGTGTGGACCCAA	TGCCGCACTTTGCTGGTGTA	285
FMgSSR-35075	scaffold458	p2	(TC)11	Class I	69688	69709	AGCACCAGGCGCTCATCTACAA	AGCCCCAGTACCCCACTGTGATTA	255
FMgSSR-35077	scaffold7175	p2	(TC)11	Class I	10547	10568	AGCACGGGCATCAAAGGAGTCT	TTCGCGTTGGTGCCTTGATCTG	350
FMgSSR-35078	scaffold10324	p2	(TC)11	Class I	26177	26198	AGCAGGATTGCAAGGCCATCAGA	AGCAACAGGGTTCCATGGAGGA	312
FMgSSR-35079	scaffold1111	p2	(TC)11	Class I	35262	35283	AGCAGGGGTTTCGCAGTACA	TTTTGCAGCAGCACACGGGA	253
FMgSSR-35080	scaffold120898	p2	(TC)11	Class I	679	700	AGCATGCATGGTGGAACCCACA	TTTGCCTTGGGCCTTGTGCT	266
FMgSSR-35081	scaffold950	p2	(TC)11	Class I	38140	38161	AGCCAACGGTGGAAGCTTGGAA	ACGGCTTCTTATCACAGCCACAGC	330
FMgSSR-35083	scaffold521	p2	(TC)11	Class I	7623	7644	AGCCGTATTTGCTGCTCCGA	AAAATGCGCCGTATCTGGGGGT	323
FMgSSR-35084	scaffold536	p2	(TC)11	Class I	103568	103589	AGCGCATGGATGAACTGGGCAT	TCGCCCTTTCGCGCTTCTTACT	339
FMgSSR-35086	scaffold5978	p2	(TC)11	Class I	7526	7547	AGCGCTCCAACAGTACCAGA	TGGTCGGGGTGGGCAATGTTAT	337
FMgSSR-35095	scaffold25014	p2	(TC)11	Class I	650	671	AGGCAGTACATGCTCCCGAT	ACGGCCATGATGCCACTACGTT	331
FMgSSR-35096	scaffold543	p2	(TC)11	Class I	88687	88708	AGGCAGTGCAACGACTCAGA	TGGGTGCACTATCCCGTTCCTT	336
FMgSSR-35097	scaffold426	p2	(TC)11	Class I	62274	62295	AGGCAGTTGGCGTTGTGGAA	AGTTACGAGTGCGGTGGTGCAA	341
FMgSSR-35104	scaffold234106	p2	(TC)11	Class I	311	332	AGTGGCGCCAAACACGTTCA	ATCAACACCGCCAAAACGAGCG	325
FMgSSR-35121	scaffold1656	p2	(TC)11	Class I	32295	32316	ATGGGGGAGAATCCACGCCAAA	TGCTCACCGTGGCATGTGAA	322
FMgSSR-35124	scaffold192	p2	(TC)11	Class I	85802	85823	ATGTTTGTCGCCGCCAATGC	AGCCTTGCAGCTTTGTTGCT	347
FMgSSR-35125	scaffold2745	p2	(TC)11	Class I	52087	52108	CATGTGCAGGGCTTTGATCAGT	TCCCTCTAATCTGCTCCGGTGT	304
FMgSSR-35126	scaffold2281	p2	(TC)11	Class I	61774	61795	CCCATCTCCCGTCTCATCGTCTTT	AGCCCCATCAGGTGAAGCAA	350
FMgSSR-35127	scaffold4440	p2	(TC)11	Class I	34042	34063	CCCCCAAAAGCTCCAGAAGTTAGG	TCCACAAGATCCGGCGAATGGT	334
FMgSSR-35134	scaffold2481	p2	(TC)11	Class I	35993	36014	GCAACGCACCCATAGCAAAGCA	TCGTTCAGGATGGCGAGCCTTT	321
FMgSSR-35135	scaffold2478	p2	(TC)11	Class I	4975	4996	GCAGCCCAATGCTACAACCACA	AGGGACATTTCGAAACGAAGGC	345
FMgSSR-35136	scaffold11407	p2	(TC)11	Class I	14917	14938	GCCAGCAACGCGTCAACAAA	AAGTCGGCCGTGTCAAAGCA	213
FMgSSR-35137	scaffold2760	p2	(TC)11	Class I	33581	33602	GCCGAATCGTTGGCGGATTTGT	TGCTGCAGGAAGCATGAGGAGT	234
FMgSSR-35140	scaffold716	p2	(TC)11	Class I	52497	52518	GCGGCCAGTTTGCTGCTAGAAT	ACTGCTCCGTCTGCTGCATT	350
FMgSSR-35141	scaffold2575	p2	(TC)11	Class I	10918	10939	GCGTACCTTTGCAAGCAAGCCA	ACGAAGCCAAGCGAAGCTGCTA	326
FMgSSR-35142	scaffold6278	p2	(TC)11	Class I	31438	31459	GCGTAGACAGCAACGCCATT	ACGCCGATCAAGCCGAATCA	272
FMgSSR-35144	scaffold305	p2	(TC)11	Class I	88821	88842	GCTGATTGAGTGCGAACCATGT	AGAGCGCTGTCCAGTTTCACA	339

SSR_ID	Scaffold	SSR_Type	SSR_Motif	SSR Class	SSR_Start	SSR_End	Forward sequence	Reverse sequence	Product_size
FMgSSR-35157	scaffold148	p2	(TC)11	Class I	111793	111814	TACTTGCGCCATGGCATCGT	CCGGCTTGCTCGGTTTTCGTTT	254
FMgSSR-35158	scaffold302	p2	(TC)11	Class I	55085	55106	TACTTGCTTTCGCCCCGCAA	CGTTCAACTTTGCGGTGGTCCT	260
FMgSSR-35163	scaffold9468	p2	(TC)11	Class I	18088	18109	TATTTTAGCCGGCGAGCACGGA	ATTTGCCAGCGGTCTCTCGTCA	279
FMgSSR-35166	scaffold29756	p2	(TC)11	Class I	2973	2994	TCAATGCCACCTTGCACCGACT	GGGGCGACGAGCCTTTTGATAA	202
FMgSSR-35173	scaffold961	p2	(TC)11	Class I	80086	80107	TCATCGGGACGATGGTGCTT	TCGGACACGAGCTGCAGTTTCA	204
FMgSSR-35174	scaffold141	p2	(TC)11	Class I	64561	64582	TCATTGCAACGCTGGTATGGCG	ATGATGCCCATCGAGCCCCATT	242
FMgSSR-35175	scaffold34287	p2	(TC)11	Class I	1901	1922	TCATTGCGTGCATGAGCTGC	TCGCAGGATCTGCCAAAGGACA	219
FMgSSR-35182	scaffold3822	p2	(TC)11	Class I	35464	35485	TCCCTCCCCACCTTTCTCCAAA	ACGCGTAGGCGGAAGAAAGT	313
FMgSSR-35184	scaffold65706	p2	(TC)11	Class I	1076	1097	TCCGATCGATCAGGCGTTGA	TGACGACTCTTCTGCGCGAT	304
FMgSSR-35188	scaffold21031	p2	(TC)11	Class I	10411	10432	TCCTGTTTTCGTCCTCACGGT	TCCTTGCTGTAGTCACATGCTTGC	274
FMgSSR-35190	scaffold8881	p2	(TC)11	Class I	23283	23304	TCGAGAGCGTGCTCTGCAAGTT	TCGTGGCCTCGTGTTTCAGCAT	324
FMgSSR-35196	scaffold934	p2	(TC)11	Class I	20574	20595	TCTCAATGCAGGCTGTGGGTCT	TGGGATGCGCCATGGAAGAA	294
FMgSSR-35197	scaffold925	p2	(TC)11	Class I	25253	25274	TCTCCAAACAGCCGCACACT	ACAGTTCGCATGGCGTGTAGT	350
FMgSSR-35200	scaffold20888	p2	(TC)11	Class I	1339	1360	TCTTTCTTTCCACCCACCCGCA	GTGACGACGATGGCATGGATCT	348
FMgSSR-35203	scaffold1424	p2	(TC)11	Class I	52942	52963	TGACGCCGTTCCTTCCATTTGT	AAACAGAAAGCCAACGCCAGCC	257
FMgSSR-35205	scaffold1935	p2	(TC)11	Class I	63576	63597	TGAGGGCAACTTGTAACGGGGT	GCAACGGGCCAACCTAAGAAGA	336
FMgSSR-35208	scaffold206240	p2	(TC)11	Class I	345	366	TGCAAAGCTGGAGCTGTCGAGT	ACAGAGGGCAGAGACTGAACGA	299
FMgSSR-35211	scaffold363	p2	(TC)11	Class I	10392	10413	TGCAGGTGCTGCCCCAAAAT	ATAGGGTTTGCGTCAGCGTG	320
FMgSSR-35212	scaffold3485	p2	(TC)11	Class I	26416	26437	TGCAGTGAGTGTTCCGGCATGA	TGTGCTGACCTGCACCTGACAT	213
FMgSSR-35214	scaffold4373	p2	(TC)11	Class I	15073	15094	TGCCACACCAAAATGCAAGGCA	AATCGGAAGAAGGCTCTCGTCG	345
FMgSSR-35215	scaffold171514	p2	(TC)11	Class I	83	104	TGCCACATGCACCTCTCTCTGT	AACAAACAAGGATGGGCCGGGA	294
FMgSSR-35216	scaffold1493	p2	(TC)11	Class I	41895	41916	TGCGAGCACGAAGCAACAACT	CGCAAACTCCTTTCCGCCACTT	338
FMgSSR-35217	scaffold34	p2	(TC)11	Class I	108484	108505	TGCTCATGCCTTCAGCCTTCCT	TGCATCTGCCGCATGCACAA	207
FMgSSR-35218	scaffold2446	p2	(TC)11	Class I	34173	34194	TGCTGCTGTTTTGCGGGGTA	ATCGCGTGCACCAAGAGCCTTA	287
FMgSSR-35226	scaffold220	p2	(TC)11	Class I	58846	58867	TGGCCGCGAATATCCTGTCAGT	TGCTTGCACATTTCAGACGGGC	340
FMgSSR-35244	scaffold88	p2	(TC)11	Class I	77713	77734	TGGTCAAGTTGGTAGGCCTGAA	CTCCGAAGCACTACAAGAAATGGG	239
FMgSSR-35247	scaffold2088	p2	(TC)11	Class I	50140	50161	TGTGCACCCTGCGGCCTATAAA	AGCACAGTGCAAATGGCAGCA	213
FMgSSR-35251	scaffold6530	p2	(TC)11	Class I	21203	21224	TTATCGCCGCGCATGATTGACG	AAAAGCGCCATCAAGTCGCCCT	341

SSR_ID	Scaffold	SSR_Type	SSR_Motif	SSR Class	SSR_Start	SSR_End	Forward sequence	Reverse sequence	Product_size
FMgSSR-35253	scaffold274	p2	(TC)11	Class I	18343	18364	TTCGTGGCGACTTCAGGACA	TCGTCGCGGAGGGTGAAAACAA	350
FMgSSR-35263	scaffold1657	p2	(TC)11	Class I	39236	39257	TTTAGGCCCACTGCACGAACGA	TTGCCGCACTGCCTACTGAA	340
FMgSSR-35264	scaffold4388	p2	(TC)11	Class I	40402	40423	TTTGCGCGTCCCTCTCGTTT	TGCAAACACCCCCACCATACCA	307
FMgSSR-35268	scaffold1091	p2	(TC)12	Class I	18525	18548	AAACGAGAACGGGAAAGGCGGT	CCTGCCTGTTGTTACATGCACT	350
FMgSSR-35269	scaffold598	p2	(TC)12	Class I	88561	88584	AAAGGGCGCCGGAATGGTTT	ACGGAATTGCAAGGTTGGGGGT	213
FMgSSR-35271	scaffold5913	p2	(TC)12	Class I	14667	14690	AACAACGCATGCCTACGTGC	AACTTGGGCCTTGCGAGAACGA	287
FMgSSR-35274	scaffold3093	p2	(TC)12	Class I	27516	27539	AACCGCCGTCTGAACCAAGT	AAGCAGGACGATGGCGGTGTAT	208
FMgSSR-35276	scaffold5083	p2	(TC)12	Class I	23027	23050	AACGGAGAGCATCGACACGCTT	AGCGACAACTGTCAGCTGGTT	330
FMgSSR-35277	scaffold2606	p2	(TC)12	Class I	8984	9007	AACTGCGCCGCTTCCTAATCCT	ATATTTTCAGGCCGGGCCCACA	242
FMgSSR-35278	scaffold13591	p2	(TC)12	Class I	10746	10769	AACTTGGCGCACAGCTCGAA	AGTGGGCACTGGCTGAGTTCTT	291
FMgSSR-35286	scaffold8572	p2	(TC)12	Class I	10638	10661	ACACCTGCTTCTTCGGCGTCAT	ACTGCATCATGCCCTCCACT	264
FMgSSR-35287	scaffold50109	p2	(TC)12	Class I	2413	2436	ACAGAAAACAGCTCGCGGTCCA	ACTGGCTGTGCTGATGGAGCAT	233
FMgSSR-35288	scaffold4797	p2	(TC)12	Class I	13787	13810	ACCAACGAGTCGTCGCATGTGT	ATCGCGGCATCACACACTGACT	350
FMgSSR-35293	scaffold2343	p2	(TC)12	Class I	52583	52606	ACCGACGGTAACCCAAACCACT	TGCAAGCGCTAATGATGGTGGTG	259
FMgSSR-35296	scaffold132522	p2	(TC)12	Class I	509	532	ACCGTGACACTTCGCCGTTT	AAGGCGCTTCAGTGCACGATCA	255
FMgSSR-35306	scaffold984	p2	(TC)12	Class I	55931	55954	ACGGACCGCATATGGACGGTAA	TTAGTGAGAAGGGCCAGTGCGT	306
FMgSSR-35307	scaffold1603	p2	(TC)12	Class I	59884	59907	ACGTATCAAACGCCCTCAGGTCA	AGACCAAAGCTGTACGCAGGA	298
FMgSSR-35308	scaffold148	p2	(TC)12	Class I	3753	3776	ACTGAAAGACCGTGACGTGCCA	TTGCGAGAGCGTATTGGGCCAT	328
FMgSSR-35309	scaffold1811	p2	(TC)12	Class I	63816	63839	ACTGAACGGCTTCTCTGACGCA	TGCCGCACAAATCAAGCCCT	347
FMgSSR-35313	scaffold297	p2	(TC)12	Class I	78203	78226	AGCAAGCCCCATGAAACGCA	TCCAGCCCATGCACACACAA	273
FMgSSR-35314	scaffold215	p2	(TC)12	Class I	19269	19292	AGCAAGCGCTAATGCACCAACA	AGCAACCAGGGGTTGATGGGAA	289
FMgSSR-35315	scaffold1234	p2	(TC)12	Class I	24398	24421	AGCACAAGGACGACGACAAGGA	TTAGCCCTCCTGCCGCCTTTTT	255
FMgSSR-35317	scaffold4138	p2	(TC)12	Class I	52845	52868	AGCGCCATTGGATTTGGACTGC	TCGCCTGGTAATCAGGAGCACA	306
FMgSSR-35319	scaffold9166	p2	(TC)12	Class I	23817	23840	AGCTCGTTACACCGCTACTGT	AGCAGTGCCCTGTTGGAGAAGT	204
FMgSSR-35320	scaffold18845	p2	(TC)12	Class I	1063	1086	AGCTGCCGTTTTCGTGATCCCT	AATTCCCACTGGTGTGTGGCGT	200
FMgSSR-35323	scaffold10295	p2	(TC)12	Class I	1325	1348	AGGCACATAGCAGCACGCTCAT	TGGACGAACAGGAATGAACCGGA	309
FMgSSR-35327	scaffold720	p2	(TC)12	Class I	20662	20685	AGGGAAGAGCAGAAGAAACCCTGC	AGGCTCGGTTTGTAGATTGGCA	349
FMgSSR-35333	scaffold5805	p2	(TC)12	Class I	3610	3633	ATCGGTGCCTCAACCGTCAA	ACTGTGGATGGTCCTGTGCGTA	247

SSR_ID	Scaffold	SSR_Type	SSR_Motif	SSR Class	SSR_Start	SSR_End	Forward sequence	Reverse sequence	Product_size
FMgSSR-35336	scaffold4234	p2	(TC)12	Class I	35032	35055	ATCGTCTTCTTCGCTCGCGT	TGGTGCAAAGCGCATCATCGT	266
FMgSSR-35340	scaffold4613	p2	(TC)12	Class I	18904	18927	ATGGCGATGCGTAACCACCACT	AGCAAACAAACCCACCCACCCA	350
FMgSSR-35342	scaffold20107	p2	(TC)12	Class I	4406	4429	ATGTGCGCCTGTGTTGTGCT	ATGTTCACTCCGCAACCAGCCA	299
FMgSSR-35344	scaffold2418	p2	(TC)12	Class I	41425	41448	ATGTTTGCCTTGCCTTGCCACC	AGATGAAAGAGCACGCGGCA	274
FMgSSR-35346	scaffold234	p2	(TC)12	Class I	64105	64128	ATTTCGCCGTCCCGGAAAACCT	AGCCGAACCCACGGCAATAACT	289
FMgSSR-35348	scaffold4312	p2	(TC)12	Class I	21797	21820	ATTTTCCGACAACCGCACGC	ACTCTTTCACGCACGAGGCA	285
FMgSSR-35351	scaffold7170	p2	(TC)12	Class I	21446	21469	CCCTGCACCCTTAGACTTGCAGTT	ACCGTTGCTTGCTGTTCCCT	335
FMgSSR-35353	scaffold9150	p2	(TC)12	Class I	18102	18125	CCTTCGCCGCGCTCATTAGTTT	ACTGCATGCGCCGTTAGGAA	295
FMgSSR-35356	scaffold11121	p2	(TC)12	Class I	18813	18836	CGTACATGTGTCCTCATATGCTGC	CTCCCGATAACTATGCACCAACCA	313
FMgSSR-35360	scaffold97	p2	(TC)12	Class I	59429	59452	GCACAGTTTCTATCGTGGCAGCA	AGCCGTCTTCATGTGTGTCCA	259
FMgSSR-35363	scaffold1557	p2	(TC)12	Class I	48047	48070	GCCGTGGGAATTGCTGGGATTT	TTGACAAGCAGCCTGGAGGGTT	243
FMgSSR-35368	scaffold938	p2	(TC)12	Class I	46726	46749	GTCTGTCATGTTTGCGCTGGCA	GACCCGTAATGTGTGTCAGCTT	338
FMgSSR-35373	scaffold418	p2	(TC)12	Class I	123931	123954	TCAAAGAGCCCGTCTGGGCTTA	TGGCCCGAAACTGTTGCGTT	247
FMgSSR-35374	scaffold392	p2	(TC)12	Class I	101950	101973	TCACCGGGAAGTGGTTGGTGTT	TGTTGTTGCCGCTACCGAGT	243
FMgSSR-35375	scaffold2983	p2	(TC)12	Class I	3417	3440	TCAGCCCAGCCCACGAAACAAT	TTCAGCGATCATGGGCCACTCA	318
FMgSSR-35378	scaffold28050	p2	(TC)12	Class I	4112	4135	TCGATCCGACGTCTCTCTGTCTGT	TTCAACCAAGGCCCAAGCCCAA	250
FMgSSR-35388	scaffold10398	p2	(TC)12	Class I	25139	25162	TGATTAGGCCCGCATTGCCA	AGTAGCAAAAGAGGGGGCATGT	295
FMgSSR-35390	scaffold2974	p2	(TC)12	Class I	12109	12132	TGCAATCCATGTTCTTCGCACGC	ACGCACACATGCGTCGCTTTTT	342
FMgSSR-35394	scaffold6726	p2	(TC)12	Class I	18850	18873	TGCAGATCCGCTTGGGGTCAAT	TTGCCAAGCGGTAGTGTGTCCT	229
FMgSSR-35396	scaffold1423	p2	(TC)12	Class I	20995	21018	TGCAGTCGCAGGAGCTTGGAAA	AAAACCGGATCCAACGTCACGC	218
FMgSSR-35404	scaffold1443	p2	(TC)12	Class I	37898	37921	TGCGCCACGTGCAGTACATT	GCATTCCCAGAAAATTTGCCGCC	273
FMgSSR-35407	scaffold9671	p2	(TC)12	Class I	24637	24660	TGCTCACGAGTAGAGCAGCCAA	TCGTTCCTACTGCGGTTCCAGT	268
FMgSSR-35408	scaffold4784	p2	(TC)12	Class I	26421	26444	TGCTCGTGATGCTTTGGAGGGA	AGGAGGTCGATCCTTCGATGCAA	266
FMgSSR-35413	scaffold580	p2	(TC)12	Class I	12135	12158	TGGCGCCAAAACATAACCCGA	AAGTGTGCGGGATGGAGAGCTT	322
FMgSSR-35423	scaffold3098	p2	(TC)12	Class I	37608	37631	TGTGGTCATGCACACAACCTGA	ACCTATTGGATTGCGGTTGCGGA	333
FMgSSR-35426	scaffold2655	p2	(TC)12	Class I	50359	50382	TGTTCCTTGCTTAGGTGGCGGA	TGGCCGCATGAGGTAGGCAAAA	294
FMgSSR-35427	scaffold950	p2	(TC)12	Class I	540	563	TTCAATCGCGCGACTCATCG	TGGATAAAACGCAGGCGGAGCA	274
FMgSSR-35432	scaffold23147	p2	(TC)12	Class I	4358	4381	TTGCACGCGTCCATGATCCA	TCACCCTGCAACGTGGCATTA	290

SSR_ID	Scaffold	SSR_Type	SSR_Motif	SSR Class	SSR_Start	SSR_End	Forward sequence	Reverse sequence	Product_size
FMgSSR-35435	scaffold22906	p2	(TC)12	Class I	4207	4230	TTGCGCTTCCCTCTCTCGATCA	AGCTAGCTCACACACTCCAGGT	210
FMgSSR-35438	scaffold241	p2	(TC)12	Class I	135532	135555	TTGGTAGGTGGCCTTTACGGCA	ACCCACCAGCATGTCAGTAGCA	287
FMgSSR-35439	scaffold6216	p2	(TC)12	Class I	24647	24670	TTGGTTGACTTGGGCTGGTGGT	TTCGGGCATTTCGCAGAGGA	221
FMgSSR-35441	scaffold1046	p2	(TC)12	Class I	57712	57735	TTTCAGTGCTCTCGCCCAGCTT	GCCGTTTGCCTCACTGTGTT	350
FMgSSR-35446	scaffold21420	p2	(TC)13	Class I	2715	2740	AAAGAGTTGCTGCCGCACGA	TGGGTTCACGGAAGAATGCACCA	234
FMgSSR-35452	scaffold156	p2	(TC)13	Class I	66831	66856	AAGACCGTCCCAGCGTCAGAAT	AGCGGGTTTTGTTGACCACCAC	350
FMgSSR-35454	scaffold63898	p2	(TC)13	Class I	998	1023	AAGCTATGCCTCCCGAAAGCGA	TACCCCGCCTACAGGTTTTGCT	331
FMgSSR-35465	scaffold11929	p2	(TC)13	Class I	14035	14060	ACACTCAGCGCAGGCAAAGA	AAGACGTCGCAAGAAGCGGAGT	278
FMgSSR-35466	scaffold6926	p2	(TC)13	Class I	15801	15826	ACACTCTTGCCCTAACTCCTCCCT	ACGGTTCACCGTCGCATTTT	228
FMgSSR-35471	scaffold3480	p2	(TC)13	Class I	12340	12365	ACCAACGGCTCTCTTCCGACAA	TTGAGCCATGCACCACCAACCT	311
FMgSSR-35473	scaffold7595	p2	(TC)13	Class I	23080	23105	ACCAGAGCAAGTTGCAGCTCTT	ACAGACCAACGGGGGACTCAAA	296
FMgSSR-35474	scaffold3242	p2	(TC)13	Class I	39519	39544	ACCCAACCACTGCAGCCTGAAT	TAACCCGCCACCGCAAGCATTA	221
FMgSSR-35481	scaffold276	p2	(TC)13	Class I	120707	120732	ACGCTCATGAGATCCACTACCTCC	TGCCGCTTTTGTGGCCGAAT	350
FMgSSR-35482	scaffold170	p2	(TC)13	Class I	11245	11270	ACGGCGACTGGTGCGCATATAA	AAAGTGGCACTGTGCTGGCT	336
FMgSSR-35483	scaffold940	p2	(TC)13	Class I	81151	81176	ACGTGCTGCGGATTCCTGACAA	TCCGGCGGTGTCTTGTTGTT	260
FMgSSR-35484	scaffold411	p2	(TC)13	Class I	106158	106183	ACGTGGACGTGACAAAGCGT	GCGGCAACTGGTTCAGCAACAA	286
FMgSSR-35499	scaffold721	p2	(TC)13	Class I	9161	9186	ATGCCAGCACCAGGTCGTAACT	AGGCAGTGAAGTGAGATCCCCA	324
FMgSSR-35500	scaffold1450	p2	(TC)13	Class I	73236	73261	ATGCGTTATGGTGCTTGGTGCC	CCGTCGCAAAAGCATTGGTGGT	217
FMgSSR-35503	scaffold15359	p2	(TC)13	Class I	10821	10846	ATGGACCACGGCGACGACATTT	TTGTTCGCTCGCTGCCTAATGC	235
FMgSSR-35504	scaffold585	p2	(TC)13	Class I	71834	71859	ATGTCAGCCCTTTTTGCACCCG	CCCTCATGCGTACGTGATACCT	200
FMgSSR-35506	scaffold13354	p2	(TC)13	Class I	9414	9439	ATGTGCGACACGTAGCGGAA	AGCTTGTAGCCGTCGTTCGT	312
FMgSSR-35515	scaffold6005	p2	(TC)13	Class I	38262	38287	GCACGATGTGCGGGTCAAACAA	GCGCTAGGCACGTTGTCCATTA	300
FMgSSR-35518	scaffold153	p2	(TC)13	Class I	45909	45934	GCCAACCTCGATGTGTTCGAT	TTGGACAAAACGCAGGCGGA	297
FMgSSR-35519	scaffold4610	p2	(TC)13	Class I	40817	40842	GCCACCGCGCATAAGCATTT	TTCGTGGTGCTGTCGTGCAT	261
FMgSSR-35520	scaffold968	p2	(TC)13	Class I	10401	10426	GCCTCGCTTGGCTGGAGATTTT	GCGTGTCGCTGGGAAAACGAAA	337
FMgSSR-35521	scaffold224	p2	(TC)13	Class I	44594	44619	GCGCGTGGTTAACGGAAAGGTT	TCCAAGACTGCAGCAACGCA	245
FMgSSR-35523	scaffold5174	p2	(TC)13	Class I	1054	1079	GCTCAAACTCACATTCATCAGCCG	ACGGTTGGTCCTTAACGGGT	345
FMgSSR-35525	scaffold2023	p2	(TC)13	Class I	72958	72983	GGACTTACTCCAATCCATTCCTGCC	TGAGATGAGCCCTAAGTGCGA	224

SSR_ID	Scaffold	SSR_Type	SSR_Motif	SSR Class	SSR_Start	SSR_End	Forward sequence	Reverse sequence	Product_size
FMgSSR-35527	scaffold16535	p2	(TC)13	Class I	9680	9705	GGCGGTTGAAAGGCGTTTCCTT	TCCTCAAACAAACACCCCGCGA	341
FMgSSR-35533	scaffold20013	p2	(TC)13	Class I	4668	4693	TATTCGAATCGCAGCAGGCGGT	ACGGACAACACGGCGGTTGATT	292
FMgSSR-35535	scaffold20454	p2	(TC)13	Class I	10386	10411	TCACTGAACGACCAGAGGTGTGA	ATGGTTGCAGGTGTCTGGGCAA	331
FMgSSR-35544	scaffold649	p2	(TC)13	Class I	60285	60310	TCTCCACCGCAATCCTGATCCA	TTGTGGAGCTCCCGCTCTTTGA	278
FMgSSR-35552	scaffold326508	p2	(TC)13	Class I	56	81	TGACACTGTTCTTCGTTTCCCG	TGCAGGCGACGCATGATTGA	300
FMgSSR-35555	scaffold229913	p2	(TC)13	Class I	170	195	TGCAAGGCATGGCGACGATT	ATGTGACTGCTCTTCGCACACG	218
FMgSSR-35558	scaffold20013	p2	(TC)13	Class I	6072	6097	TGCATCCGAGACATTCCGGT	TGATTGAGGGCACTCCGGTGAA	346
FMgSSR-35562	scaffold428	p2	(TC)13	Class I	41767	41792	TGCGACGCACGCATACAGGATA	TGAAAGTACAGGTACCCCCGGA	276
FMgSSR-35563	scaffold1552	p2	(TC)13	Class I	54695	54720	TGCGTCTGACTCTCGACCCTTT	TGGTCTGGCCTCTGCCTGTTAT	273
FMgSSR-35564	scaffold12909	p2	(TC)13	Class I	4249	4274	TGCGTGGCGCTTGGCAATTT	TGATCCGTTGCGGCTGCATT	327
FMgSSR-35565	scaffold18703	p2	(TC)13	Class I	10137	10162	TGCTCCTCCCAGTTCTTACAGCA	ACCGGTGATGATTGGCCAGCTA	290
FMgSSR-35568	scaffold1733	p2	(TC)13	Class I	14076	14101	TGCTTCCGTGTCACACACGCTT	AGCATCTAGCGAGGGTGTAAGCA	257
FMgSSR-35572	scaffold3288	p2	(TC)13	Class I	43142	43167	TGGCAGCATATGGTGATGCGGA	AGATCCTGCGTGACGTTGGAGA	254
FMgSSR-35573	scaffold1735	p2	(TC)13	Class I	64485	64510	TGGCTGCTGGATGACAGGTGAA	AGCAAGCATCCACCGCGTTT	323
FMgSSR-35574	scaffold75413	p2	(TC)13	Class I	381	406	TGGGATTTTCAGCCACCGTCCT	TGCATTAGGAGTTTGTGGCCGGA	308
FMgSSR-35579	scaffold5772	p2	(TC)13	Class I	34051	34076	TGTGGCGCCATGTTGTAGAC	ACGCATAGTTCAGTCGGTTCCA	300
FMgSSR-35585	scaffold158083	p2	(TC)13	Class I	203	228	TTCTGGCCCTTGATCACCCGTT	AATGCGTGACGTGCAGGGCTAT	256
FMgSSR-35587	scaffold2465	p2	(TC)13	Class I	52503	52528	TTGAGCCCAGCCTAGTTTCCCT	GGCAGGCTCAAGAAAGAACGAGGA	322
FMgSSR-35589	scaffold2774	p2	(TC)13	Class I	39204	39229	TTGCCGCTGTTGTCCTGCAT	TCCCATGATGTTGCTGCGGA	346
FMgSSR-35597	scaffold3830	p2	(TC)13	Class I	36913	36938	TTTGCGCATGTTGTGGCGCT	ACCGCCGCTGATTGAATTCCGA	302
FMgSSR-35601	scaffold3624	p2	(TC)14	Class I	8748	8775	AAACCCAAACCTTAACCCCGCC	AAACACACGCGTGCGGTAGA	268
FMgSSR-35603	scaffold67	p2	(TC)14	Class I	11966	11993	AACAAGCTCTTCGCAGCCAACG	TGCTGCGGTAACCAACCAACCA	231
FMgSSR-35605	scaffold18777	p2	(TC)14	Class I	11785	11812	AACGGCCTTTCCAGGTTCCACT	CGACGCTGGCAAAGTGGCAATA	219
FMgSSR-35608	scaffold3058	p2	(TC)14	Class I	31225	31252	AACTGAAACTGCCTGCGACGGT	TTGGCTCAGTGCGTCTGCAA	227
FMgSSR-35610	scaffold10891	p2	(TC)14	Class I	14946	14973	AAGCTGTGACGTGAAGGGCT	ACTGACACACGATCAGGAAGTCG	302
FMgSSR-35611	scaffold16598	p2	(TC)14	Class I	6145	6172	AATGCGGCGAGCATGGCATA	ACGACATGAGTGGCTCACGA	245
FMgSSR-35613	scaffold5905	p2	(TC)14	Class I	14931	14958	ACAACAATGTGCGGTTGCGG	TGGATCGGGGGAGTGGAATCAA	339
FMgSSR-35614	scaffold1121	p2	(TC)14	Class I	23168	23195	ACACATGAATGACCGGCCCACA	TGCAGCTTGACCAGAAGCCA	258

SSR_ID	Scaffold	SSR_Type	SSR_Motif	SSR Class	SSR_Start	SSR_End	Forward sequence	Reverse sequence	Product_size
FMgSSR-35630	scaffold8324	p2	(TC)14	Class I	15004	15031	ACGCTGCTAGAGCATTGGCT	AGTCCACACGATGCAGCTATGACC	281
FMgSSR-35633	scaffold1178	p2	(TC)14	Class I	50754	50781	AGCACCCAAGCCTCTAACTCATGC	TGTCGCAGATCCATTGGCGT	252
FMgSSR-35635	scaffold2583	p2	(TC)14	Class I	33561	33588	AGCATAACCTGCCCCGCTCAAA	TGACTGCCACGGAGGAAAGGAA	232
FMgSSR-35640	scaffold913	p2	(TC)14	Class I	13275	13302	AGGCAGGCAGGACAAACACA	TACAGACCCGACTAGAGGATCCCA	346
FMgSSR-35642	scaffold1699	p2	(TC)14	Class I	70189	70216	AGGCGGATAGGATGGTCGCAAT	TGTCCGAACATTGCCGGTAGA	304
FMgSSR-35644	scaffold24252	p2	(TC)14	Class I	5328	5355	AGGGCATTGGCTTCCAAGGT	TGCATGCGTTGCAGGTTGCT	331
FMgSSR-35646	scaffold106	p2	(TC)14	Class I	42212	42239	AGTCATGCTACTGCCTACTGCCT	ACAACTGGACGGAATCAGTCGGA	328
FMgSSR-35649	scaffold110068	p2	(TC)14	Class I	572	599	AGTGGTAAGCGATGCTGCTGCT	TGCACCGCATACATTCCTTTGCC	313
FMgSSR-35651	scaffold365	p2	(TC)14	Class I	91233	91260	ATCGACGCCACAAAGCAGCA	AGTTTCACGCTGCTCCGATCCT	267
FMgSSR-35655	scaffold13467	p2	(TC)14	Class I	12765	12792	ATGCGATCCTACTCCACCGCAT	TGTCGAGCTTCGACCAGAACA	330
FMgSSR-35659	scaffold110	p2	(TC)14	Class I	152718	152745	ATGTTTCTCTGCTGCCCTCCGT	GCTGCATGTCTTGCGCTTCACT	302
FMgSSR-35664	scaffold14914	p2	(TC)14	Class I	14947	14974	CCCCTTTGCACATTGCTTCCCT	AGTGTGTAGCCGGAAGGAACT	288
FMgSSR-35665	scaffold1059	p2	(TC)14	Class I	90271	90298	CCGGCGCCCGTGTTAAATTAGA	ACTGACGATGGCCGGCTGAAAA	350
FMgSSR-35669	scaffold2523	p2	(TC)14	Class I	4285	4312	GCCAAACGCCGGCATGTTCATT	TGCACTGACGCGCCTCTTTT	247
FMgSSR-35671	scaffold3611	p2	(TC)14	Class I	39200	39227	GCGAAATTGTGCGCCGTCAA	ATTAGCCGATCACGCCAGCA	336
FMgSSR-35673	scaffold5609	p2	(TC)14	Class I	11600	11627	GCGCACAGCACCGTGTACAAAT	TTGGACAACAGCATCAGCCCGT	320
FMgSSR-35678	scaffold3269	p2	(TC)14	Class I	34451	34478	GGTGACATAGATGTTCGGGTCACA	ATGTCAACTGGCAACGCGGA	290
FMgSSR-35679	scaffold5204	p2	(TC)14	Class I	13804	13831	TCACGCACAATGCCACATGC	TCCGTGCTTATTGGCCGGATCT	348
FMgSSR-35683	scaffold1081	p2	(TC)14	Class I	19999	20026	TCCAATGCCATCGTGCTCCA	GCATCACCAGTCAGAGACCGTT	273
FMgSSR-35687	scaffold109	p2	(TC)14	Class I	125319	125346	TCCTGCTTCAGCAGTGTGGCAA	ATGGTTGGCCATGCACCTCCTT	230
FMgSSR-35688	scaffold115	p2	(TC)14	Class I	117836	117863	TCGCAAGAGATACCTTGGGCCT	TGCGCCACGTTTCGCAAGTT	321
FMgSSR-35693	scaffold4053	p2	(TC)14	Class I	47233	47260	TCTCATCCAATCGACCTGCGCT	ATGCTCTCGCGCCGTTTGTT	322
FMgSSR-35694	scaffold262	p2	(TC)14	Class I	130388	130415	TCTGCTGCGCAAACCTGCAT	GCAGCTCTTCCCAACTCTCGAT	346
FMgSSR-35695	scaffold2082	p2	(TC)14	Class I	68292	68319	TGAAAAGTTGTCGCCGCCCA	AACGCAACGCGAAACGCAGT	314
FMgSSR-35706	scaffold5068	p2	(TC)14	Class I	40054	40081	TGGAGATGCCGTGCAGCAAA	AGATCGCTGGGTGAGGGTACAT	246
FMgSSR-35710	scaffold44183	p2	(TC)14	Class I	1082	1109	TGTAGCGGGGCCCAACAAATCA	ACAGGATCCAAGCGCCATCT	240
FMgSSR-35715	scaffold175	p2	(TC)14	Class I	6110	6137	TTCACGGTCCAATTCAAGGAGC	TGCCAAGCCAGCCAACTAAACC	217
FMgSSR-35718	scaffold8232	p2	(TC)14	Class I	8488	8515	TTCGCGCCTCCTCTCTCTCTTT	AAGGACAGCGCTCATCAACGGA	348

SSR_ID	Scaffold	SSR_Type	SSR_Motif	SSR Class	SSR_Start	SSR_End	Forward sequence	Reverse sequence	Product_size
FMgSSR-35720	scaffold934	p2	(TC)14	Class I	64498	64525	TTCTCCCACCACACATTGTCGC	ATGGAGAAGAGCCGCCAGAT	270
FMgSSR-35722	scaffold21709	p2	(TC)14	Class I	7570	7597	TTGGGGAACCAAACACCGACCA	TTGCATGCTAGCAACGTCGAGC	317
FMgSSR-35725	scaffold7547	p2	(TC)14	Class I	26649	26676	TTTTCCTTCACCCGCTGCCA	TGTAGTGCGTGCTCGCTTGGAT	323
FMgSSR-35727	scaffold21709	p2	(TC)15	Class I	4175	4204	AAACACCGACCACGACCTTGCT	TTTTGCGTACGGCGGGTGTT	319
FMgSSR-35729	scaffold1279	p2	(TC)15	Class I	61509	61538	AAAGACCCACTGCAACCCACGA	TCAACTAGATCTGCTGCCGCCT	342
FMgSSR-35738	scaffold4140	p2	(TC)15	Class I	30963	30992	AATGCCGGATCTGGCTGCATCA	TGCATGCCGTAGTCCCATCCAT	211
FMgSSR-35739	scaffold1388	p2	(TC)15	Class I	59177	59206	AATTTCCGTCGGTCTTCGACGC	GCTCCATTTTGTTGCGGCGAGT	332
FMgSSR-35741	scaffold14630	p2	(TC)15	Class I	1804	1833	ACACGCTTCCACTTTCGCCT	TGCTCTTTTGCGCACCACAACT	280
FMgSSR-35745	scaffold29598	p2	(TC)15	Class I	5970	5999	ACCAGTGCACGGGCATGATTGT	TGTAGTAGCGGGCATCGGTTGT	223
FMgSSR-35752	scaffold82	p2	(TC)15	Class I	39951	39980	AGAATCTGTTCGGACGCCCA	GCTAATAGCTGCGCCATGGAA	201
FMgSSR-35755	scaffold7930	p2	(TC)15	Class I	17884	17913	AGCGGCGTGATGGCAAGTTT	CGAGAGGGAGCGGAATTGAACT	302
FMgSSR-35757	scaffold550	p2	(TC)15	Class I	108223	108252	AGCTTTATGCCATCAGCAGCGG	TGTTGCGACGAACCCTGACA	326
FMgSSR-35761	scaffold787	p2	(TC)15	Class I	87946	87975	AGTCAAACGCCACCGTTCCTGA	TGTTCTGACCAATGGCCGCT	238
FMgSSR-35767	scaffold852	p2	(TC)15	Class I	55950	55979	ATCTGCGATGGTTGTCATGGCG	GTGGTCGGCGAGGAAACAAACA	303
FMgSSR-35768	scaffold1149	p2	(TC)15	Class I	72724	72753	ATGCACTGCAAGTGCCTGCT	AGGGAACACGAACAATGCACCCA	221
FMgSSR-35771	scaffold523	p2	(TC)15	Class I	104120	104149	ATTCAGCTGCTGCTGTGCGT	GATCCCTTCGCTTGCACACA	277
FMgSSR-35772	scaffold32	p2	(TC)15	Class I	197908	197937	ATTGCCTAAGCAAGCGCGTC	TGGTTTTCGCGCAAGCCTTCA	266
FMgSSR-35779	scaffold7547	p2	(TC)15	Class I	32853	32882	CGGATCGGAACTTTGCAAGGCA	ACATTAGAACTTCCCTGGCCCGT	215
FMgSSR-35780	scaffold1141	p2	(TC)15	Class I	46693	46722	GAAGTCACTCGCGTTCACAGT	TGTGCGTGCGACTGGTTTGT	274
FMgSSR-35783	scaffold4624	p2	(TC)15	Class I	5560	5589	GCTCGCGAAGCTTGTGAACT	ACATCTTGCGTGCGAAGCCA	341
FMgSSR-35784	scaffold25015	p2	(TC)15	Class I	5949	5978	TAAACTCCGCCGTGCATGTGGT	AAGCACCTTCGTGCGCCTTT	347
FMgSSR-35786	scaffold1683	p2	(TC)15	Class I	8875	8904	TCAAAGCCGCACGAGCACTACT	TTGTCCTGCCATGAAACGTGCG	344
FMgSSR-35791	scaffold8423	p2	(TC)15	Class I	1707	1736	TCGACACAACACAGGCACCGAA	AACCGGTCGTGATCTCCCTCAA	240
FMgSSR-35794	scaffold29409	p2	(TC)15	Class I	5462	5491	TCTATGCAAGGCGACACGGA	TCGCCTCTGGTCATCATGTCGT	227
FMgSSR-35795	scaffold65	p2	(TC)15	Class I	195562	195591	TCTTCTGCCTGTTGCTGTCCCT	CCCGGCCCACATTTCTTCGTTT	303
FMgSSR-35803	scaffold144581	p2	(TC)15	Class I	561	590	TGCCGTCCGCTCATTATTCTGC	ATGGCGCCAAAGGACAACAGGT	248
FMgSSR-35804	scaffold1283	p2	(TC)15	Class I	87934	87963	TGCGCTTATAAATGGGCACGCA	AAGGCTAGCGGTAGCACGCAAA	338
FMgSSR-35805	scaffold1087	p2	(TC)15	Class I	55005	55034	TGCGGTTCTTGCAAGTTGTTCGG	ACATGGCACACGCTGGTTTGA	214

SSR_ID	Scaffold	SSR_Type	SSR_Motif	SSR Class	SSR_Start	SSR_End	Forward sequence	Reverse sequence	Product_size
FMgSSR-35811	scaffold226	p2	(TC)15	Class I	59382	59411	TGGAGTCGTTGGAGATTGGACG	GCACAATGGTGCGGGTAGATTGA	219
FMgSSR-35814	scaffold1371	p2	(TC)15	Class I	36769	36798	TGGTTCGCCCATTTATGAGCGTTG	GCTTCCCCCTGACTTCGTCAAA	349
FMgSSR-35819	scaffold11198	p2	(TC)15	Class I	26506	26535	TGTGCCCATCTGCTCTCCGTTA	CCCAGCCTGCAATTTGGAAAGCA	305
FMgSSR-35820	scaffold2306	p2	(TC)15	Class I	15824	15853	TTCGGGATGTGCGCGATCTT	AAGCTCAAGTGCGCAGGCAA	343
FMgSSR-35828	scaffold53141	p2	(TC)15	Class I	2779	2808	TTGGCTTCGCCAACACCTCT	AAACACTGAGGCAAAGCGCGTC	342
FMgSSR-35829	scaffold175	p2	(TC)15	Class I	4912	4941	TTGTCGCTGTCCACGTCGATGA	CGGCGCCAACAGCCTAACATTT	312
FMgSSR-35832	scaffold28726	p2	(TC)15	Class I	4766	4795	TTTCTTGGTCCGTGCGAAGGCA	TGGATCACGCGTGCTGAATTTGC	212
FMgSSR-35837	scaffold3659	p2	(TC)16	Class I	47159	47190	AAAATGCCCGGGACTCTGCTGT	CGTCAAGGTCACGGCCCAGATAAT	325
FMgSSR-35839	scaffold3293	p2	(TC)16	Class I	16128	16159	AACAGTCCACCACCCAAAGCGT	AGACCTAGTAAGAAGGACCCTCCA	316
FMgSSR-35841	scaffold671	p2	(TC)16	Class I	77973	78004	AACGAACTCGACGCGTCCATCA	AACAAAGGGTGGCGGCGATT	326
FMgSSR-35852	scaffold1440	p2	(TC)16	Class I	64385	64416	ACAGCAGCCTCAATGACGGA	TGCGACTTCCCATGCTCCTT	348
FMgSSR-35860	scaffold8613	p2	(TC)16	Class I	21405	21436	ACTTCTTCCTCTCCGCCCTTCT	AAAAGCGGCTGTCGTTGGGA	264
FMgSSR-35861	scaffold5356	p2	(TC)16	Class I	14978	15009	ACTTGATACGGCCAGCCAGTGA	TGTCGATAACCTGCCACGCT	306
FMgSSR-35866	scaffold305	p2	(TC)16	Class I	122397	122428	AGCAATGAACAAGGCGGCGT	TTCCCAGGCAGGTTCACCATGT	206
FMgSSR-35870	scaffold1152	p2	(TC)16	Class I	11266	11297	AGCTTCAGGCTACCGGAATGGA	TAACGAGCCACCGCGCAAAA	226
FMgSSR-35882	scaffold755	p2	(TC)16	Class I	34432	34463	ATTCAGCAGCCACTTGGGGCAT	ATGCGTGTGTGGTGAGTGTGGT	246
FMgSSR-35886	scaffold549	p2	(TC)16	Class I	10962	10993	CCATTCAATGCTTTTTCCCCCGGT	ACGTGCATGGGTAATTGAGGCT	257
FMgSSR-35889	scaffold273	p2	(TC)16	Class I	88909	88940	GCAACACCTGCCGCTGTTTTCT	TTGCACGCACTTAGACACACGC	232
FMgSSR-35890	scaffold329	p2	(TC)16	Class I	104825	104856	GCAAGACTCACCACTGAACCGA	TCCCGCCCTTGACCAGATAAGT	350
FMgSSR-35893	scaffold5394	p2	(TC)16	Class I	20704	20735	GCGTGTCGCACGTGCATAGTAA	GGTCTATCACACGTTTTAAGCCCG	338
FMgSSR-35898	scaffold37058	p2	(TC)16	Class I	4621	4652	TACCGTGCAAGGATTGGGGTGT	TGAGTGCAATCTCATGGTGGGTGA	270
FMgSSR-35902	scaffold5660	p2	(TC)16	Class I	18632	18663	TAGCGGCGCGATTAAAGACCGT	ACGTGTGGAACAACCGCACA	345
FMgSSR-35908	scaffold42745	p2	(TC)16	Class I	1181	1212	TCCTGGTTGATGGTGGCATCGT	TGTTAGGACCGTGCGTGTGCAT	314
FMgSSR-35910	scaffold523	p2	(TC)16	Class I	104783	104814	TCGCAAGGTGGAAGACGAACCT	TGCGCAACAACCAGCAGAGA	291
FMgSSR-35912	scaffold142	p2	(TC)16	Class I	157499	157530	TCTCCTACCGAACAGGCGAACA	AATCGCCCCTTGTTGTGCCCAT	226
FMgSSR-35913	scaffold1172	p2	(TC)16	Class I	35061	35092	TCTGCACCGTAGAGCAGCAA	AATGCGGCAGCGAAGATTGAGC	331
FMgSSR-35918	scaffold2189	p2	(TC)16	Class I	35832	35863	TGATGTGTACGTGCACGGGT	ATCGAGCTGCGTGCTGCATT	291
FMgSSR-35919	scaffold20097	p2	(TC)16	Class I	8131	8162	TGCACTTGAACTGGAATGCACGC	TGTGTCCACCAGCATGGTCA	317

SSR_ID	Scaffold	SSR_Type	SSR_Motif	SSR Class	SSR_Start	SSR_End	Forward sequence	Reverse sequence	Product_size
FMgSSR-35927	scaffold1411	p2	(TC)16	Class I	72125	72156	TGGACTTGGCTCTTGTGACCA	AAGGCACTGGCATGGAGACT	298
FMgSSR-35928	scaffold5998	p2	(TC)16	Class I	24365	24396	TGGAGAGACGTGCGTGTTGT	GCATTCTGCAAGCCTCGTGT	292
FMgSSR-35931	scaffold10282	p2	(TC)16	Class I	2965	2996	TGGTCACTGTCCACCACTGTCT	TCATGTGAGCCTGCGGCAAT	348
FMgSSR-35937	scaffold21033	p2	(TC)16	Class I	3659	3690	TTCCACACATGCCGGCTTCA	TGCGCACGGCATAGACACAA	319
FMgSSR-35943	scaffold221	p2	(TC)16	Class I	132605	132636	TTGGGCGCGCACATTCGTTA	ATTTGCTGCGTGCATCCAGTCG	345
FMgSSR-35947	scaffold8489	p2	(TC)17	Class I	21065	21098	AACATAGCCAACGCCGATCGCA	AGGTTTCCGTGACACCGACT	207
FMgSSR-35949	scaffold167785	p2	(TC)17	Class I	48	81	AACCGCGATGCACCCAGTTTCA	TGGGAGTGGAGTGGTTTTGGGA	293
FMgSSR-35953	scaffold5218	p2	(TC)17	Class I	4946	4979	AAGGCGACAGGGACGTGTTT	ACCGATGGCGACAGTTTGCT	349
FMgSSR-35954	scaffold34236	p2	(TC)17	Class I	4611	4644	AAGTCAAGGCCAAACCGCCA	TCCCCCATGGTGCTCATCTCTCTT	344
FMgSSR-35962	scaffold7781	p2	(TC)17	Class I	2137	2170	ACACAATGGGCACCGGGATGTT	AGCCGAACGAAGGTCACAAGCA	318
FMgSSR-35963	scaffold17191	p2	(TC)17	Class I	7867	7900	ACATGCCAGACGCCCGTTTT	TTTATCACGTTGGCGGCGCA	259
FMgSSR-35971	scaffold24001	p2	(TC)17	Class I	2071	2104	ACTGATCCTTTGCTTCGCCCCA	AAACAGCAGCAGCAGGGGAACT	344
FMgSSR-35973	scaffold943	p2	(TC)17	Class I	31489	31522	ACTGCAGGGCAAGCGAGAGAAA	TGCCTGGCTCTGTGCAAACT	349
FMgSSR-35975	scaffold101939	p2	(TC)17	Class I	802	835	AGCAATGATCGGCGAACCCACA	TTGGGTTTTCGCAGCCAGCA	246
FMgSSR-35981	scaffold113	p2	(TC)17	Class I	12840	12873	AGCGACATCCCCAAAAGTGGCT	ACAAAAGCAGCGGTGATGGTGC	322
FMgSSR-35986	scaffold1694	p2	(TC)17	Class I	38202	38235	AGGAGCCAGCTACATGAGACTAGA	TCACGATTTGCTGGTTGCTGCG	287
FMgSSR-35989	scaffold4623	p2	(TC)17	Class I	29083	29116	AGGTCTGGTCCATGTCGCCTTT	ACCTGCGACTGCGAAGGAACAA	349
FMgSSR-35996	scaffold10958	p2	(TC)17	Class I	23083	23116	ATCTTTGCGCTCCCTCTCGCTT	AGCAGCACCGTACCACACAA	307
FMgSSR-35998	scaffold233097	p2	(TC)17	Class I	196	229	ATGAGCCCCACTTGCATGGTCA	AAAAGCACCAATTCAGCGGCCC	253
FMgSSR-35999	scaffold9556	p2	(TC)17	Class I	753	786	ATGCAACTTGACCGCCCCAT	TCCTACACGCTGGAACTGACCA	236
FMgSSR-36003	scaffold5834	p2	(TC)17	Class I	27633	27666	ATGGGCCGTACGGTTGAACACA	TGCAGCAAAAGGAGCAGCGA	261
FMgSSR-36008	scaffold1497	p2	(TC)17	Class I	52993	53026	CGCTCCATCTTTAGGGGGAGTGTT	ACCACCAATGCTAGCAAGGAGCA	279
FMgSSR-36009	scaffold15061	p2	(TC)17	Class I	19597	19630	GCCTTCACTCTGTTGCTTAGTGACC	AGGTAGCTCAAATCCAGATACGGG	202
FMgSSR-36010	scaffold6413	p2	(TC)17	Class I	28740	28773	GCTACGCACGACAGCTTTGCAT	CGTGCGCGAGCTTTTCAGTT	308
FMgSSR-36013	scaffold21130	p2	(TC)17	Class I	8830	8863	GGCAACACGAAGCAAAGGACGA	TCCTTCTCGAGGGGTGTGAAGGAT	329
FMgSSR-36015	scaffold537	p2	(TC)17	Class I	20125	20158	GGCCTCAAATAGATGTCCAGTGGGT	ACAGGGCCTTAAAGCTCAACTGC	330
FMgSSR-36016	scaffold4091	p2	(TC)17	Class I	3638	3671	GGGGCCGTGCAATTGAGTTAGA	GCTCATGTGCTCCCATAACGCA	348
FMgSSR-36019	scaffold7626	p2	(TC)17	Class I	19229	19262	TCCACTGCCACATGAGCCTT	AGGCCACATGCAGAAAACCGGA	280

SSR_ID	Scaffold	SSR_Type	SSR_Motif	SSR Class	SSR_Start	SSR_End	Forward sequence	Reverse sequence	Product_size
FMgSSR-36024	scaffold5932	p2	(TC)17	Class I	4729	4762	TCGGCTAGGGATTCAACCATTGGC	TGGTGCCAGTGCCCTATTGCAT	202
FMgSSR-36030	scaffold7201	p2	(TC)17	Class I	10098	10131	TGCATGGATCAGCAGCAGCA	TCGCCAACGCAAAGATCCCA	239
FMgSSR-36033	scaffold751	p2	(TC)17	Class I	66869	66902	TGCGGTTTTGGCCTCGTGTT	ATGCCTACTTGTTGCAGGCCGT	297
FMgSSR-36034	scaffold628	p2	(TC)17	Class I	66127	66160	TGCTCGTTGTTCCCTTCGCA	TGCCCACCAGGCTTTTGTGT	323
FMgSSR-36035	scaffold100	p2	(TC)17	Class I	170008	170041	TGCTGGGTGTGGTTGAACGGAA	TGCTCCCAAGTCCCAACCAA	329
FMgSSR-36038	scaffold292	p2	(TC)17	Class I	123610	123643	TGGCCATTAATTCGACCAGCCC	AGTTCCATGGTGCTTGCGCT	329
FMgSSR-36041	scaffold6352	p2	(TC)17	Class I	3140	3173	TGGTTGCAGACCGCGCAAAA	AGGTGTCTCTAGACATGCGGCT	310
FMgSSR-36042	scaffold825	p2	(TC)17	Class I	52659	52692	TGTAGCTGCCCGTGTACCTT	AGCTGCCCGATGATGCTCTGTT	262
FMgSSR-36045	scaffold14260	p2	(TC)17	Class I	20052	20085	TTGAAAGCCACCTTGGGGGTGA	ATACGGGCCAGTTTCCCTGCAT	250
FMgSSR-36046	scaffold24477	p2	(TC)17	Class I	1076	1109	TTGCATGTTCATCCGCCCGT	TTGGGCACGCAAGTCCAACA	336
FMgSSR-36051	scaffold8154	p2	(TC)17	Class I	19224	19257	TTTGTTACACCCCCTCAGGCCA	TGCGTGGGAAGGCAGACAAA	328
FMgSSR-36053	scaffold3312	p2	(TC)18	Class I	18475	18510	AAACCCAACTCACTCAGCAGCC	TGTCCACAGTTGCACCGCGATA	290
FMgSSR-36054	scaffold586	p2	(TC)18	Class I	28414	28449	AAAGACTCGTGCCCTTGGCTCT	GCCTTGATAGCAGAGTGCGGAA	269
FMgSSR-36056	scaffold733	p2	(TC)18	Class I	102432	102467	AACAACGGAACGGAGGTGGCAA	AGCCACCTTTTGCAGCAGGGTT	312
FMgSSR-36057	scaffold3365	p2	(TC)18	Class I	34586	34621	AACAATGCGTGTAGAGCGCACG	AGGCATGCGTGCTCCTGATT	300
FMgSSR-36058	scaffold3587	p2	(TC)18	Class I	25014	25049	AACGCAAACCCTGTTGCACG	TCCATTTATCCGGCCCGCTCTT	237
FMgSSR-36059	scaffold21	p2	(TC)18	Class I	173287	173322	AACTCATCATAGCTCGCCCGGT	ACCGGCACACATTTAACCAGCA	309
FMgSSR-36062	scaffold8537	p2	(TC)18	Class I	13035	13070	AAGCTTGGAGGAGCAAGCAGCA	AGAATTGCGCGCGTTGGGAA	210
FMgSSR-36069	scaffold1943	p2	(TC)18	Class I	11252	11287	ACCAGGGCAAACAAGGACAGCA	TGCATCCACACGCACATCGT	338
FMgSSR-36078	scaffold5321	p2	(TC)18	Class I	39721	39756	AGGCAATGCTTGACACCCCTGA	TGCACGAAACAAGCCGGACA	333
FMgSSR-36079	scaffold11849	p2	(TC)18	Class I	3814	3849	AGGCCACTTTGTGGGTAGGAACA	TAGGGGAGGAGGCCAACGTAAT	343
FMgSSR-36082	scaffold355	p2	(TC)18	Class I	55288	55323	AGGGGACATCACCCTAAAGCTC	AACATATGTGAAGGGCAGCGG	349
FMgSSR-36083	scaffold784	p2	(TC)18	Class I	35697	35732	AGGTTACTTAGGGGTTCTGGTGC	AGACTTGCTAGTATGTCCCTCCCA	345
FMgSSR-36090	scaffold471	p2	(TC)18	Class I	73822	73857	CACAACACACCAGTTGCCACCA	AATGTTAGTGAGGGGCCATGCG	274
FMgSSR-36100	scaffold9493	p2	(TC)18	Class I	1471	1506	GCTTTTGTCCTTTCCTGTGCGA	ATGACGTCACGCCGACAACA	311
FMgSSR-36109	scaffold1943	p2	(TC)18	Class I	2410	2445	TCGGCGAGCTGATGTGCTTCAA	TGTGTTGACTGACCAGCTGACCT	240
FMgSSR-36115	scaffold4918	p2	(TC)18	Class I	4740	4775	TGCAGCTGGAAGCGTTGGAA	TGGTTGCCGCTGAACCAGAT	334
FMgSSR-36122	scaffold14998	p2	(TC)18	Class I	4131	4166	TGCGCGGAGAGAAAGGCTTCAT	CGCGGCGATGCTGAAAGGATTT	286

SSR_ID	Scaffold	SSR_Type	SSR_Motif	SSR Class	SSR_Start	SSR_End	Forward sequence	Reverse sequence	Product_size
FMgSSR-36123	scaffold10367	p2	(TC)18	Class I	18311	18346	TGCGTCGCGTAGTTCGTTTCA	AAGCCTCGTCTGCGCTTCCATT	314
FMgSSR-36124	scaffold11968	p2	(TC)18	Class I	5897	5932	TGCTGCTGCTGTTTAGGCAGGT	AGCAAAGAAGGAGCAGCAGGGA	338
FMgSSR-36126	scaffold1392	p2	(TC)18	Class I	6171	6206	TGCTTTTCGGACTGCTCGCT	AAGGACAACATGTGGCACGC	243
FMgSSR-36128	scaffold20598	p2	(TC)18	Class I	1115	1150	TGGCAAGTACAGCAGCTCCTCA	TGCCACAAGTGTGGTCAGGT	292
FMgSSR-36129	scaffold56	p2	(TC)18	Class I	177045	177080	TGGCCATGGATGCTGGTCTTCA	TTGCCAAGTCTGCGGCTTCT	349
FMgSSR-36130	scaffold7653	p2	(TC)18	Class I	20597	20632	TGGTGGCAAGCAACCCTTCTCT	TGAGCTGTGGGCGATGAATGCT	341
FMgSSR-36135	scaffold2872	p2	(TC)18	Class I	37348	37383	TTAGTGACGGGCCTCTTCAATGGC	ACAATGTTGTGTGCCGGGGGAA	297
FMgSSR-36136	scaffold10592	p2	(TC)18	Class I	22171	22206	TTCACATGCGCTCGATTGCTGC	AACCGCCACCAACATCCCGTTA	297
FMgSSR-36137	scaffold18	p2	(TC)18	Class I	76093	76128	TTCACCGTGAGCCTCTTGCTGT	TGGCTTGCCTACACCTTCCCAA	294
FMgSSR-36138	scaffold71092	p2	(TC)18	Class I	714	749	TTCTCTCCCCACTCTCTCTTTCCC	AACTCTGAGCGAGCACAAAGGG	332
FMgSSR-36141	scaffold169	p2	(TC)18	Class I	145479	145514	TTGGCAGGTGGGCTCTTCCAAA	ACAGGCTATGCTGCTGCCTACA	329
FMgSSR-36144	scaffold23724	p2	(TC)19	Class I	4169	4206	AAACCCTTGCTGGGTTTTGCGG	TGCCTTTGTTGCCTGTGCGT	346
FMgSSR-36147	scaffold6610	p2	(TC)19	Class I	20529	20566	AAGAAAGATGCCAGCAGGGC	AAATGGCACGCACGACAGATCG	260
FMgSSR-36149	scaffold3201	p2	(TC)19	Class I	21560	21597	AAGCGCCGTACGTGTAGGTCAT	TTCAGACACTGGGCTGGGGTTT	317
FMgSSR-36156	scaffold20275	p2	(TC)19	Class I	11630	11667	ACCGGCACGTGAACCTGTTT	AGCTGGCCGGCGTCAAGATTAT	328
FMgSSR-36163	scaffold7457	p2	(TC)19	Class I	31320	31357	ACGGGTGCTGCTGCATTTCA	ACAAATTCTGCCGTGCCTGCT	268
FMgSSR-36167	scaffold449	p2	(TC)19	Class I	39017	39054	AGACACGCGAGAGCAAGACA	ACAGCAAAAGATAGGCCAGCCGT	334
FMgSSR-36170	scaffold12370	p2	(TC)19	Class I	3705	3742	AGCAACCGTTACCGAAGGCA	CCCTTGGGTTTTTGTTGCCGCT	224
FMgSSR-36171	scaffold3015	p2	(TC)19	Class I	42229	42266	AGCACAACTTCCCGCCAGAT	AGCGCTGCTGCCTCTTCTTCTT	317
FMgSSR-36172	scaffold5241	p2	(TC)19	Class I	4244	4281	AGCCTTTGGTGCGGTTTCGT	TCAGAACGCGTGAACTGGGT	321
FMgSSR-36177	scaffold2020	p2	(TC)19	Class I	18253	18290	AGTTCTGTGGCAGGTGCATTGG	ATACGCAGCTTTGATCCCGCCA	288
FMgSSR-36178	scaffold23747	p2	(TC)19	Class I	11192	11229	ATCACACCCTCGCTTGTTCCGT	TGGCTGCAGGTTTCGTGGTCAA	328
FMgSSR-36182	scaffold14285	p2	(TC)19	Class I	1881	1918	ATCGTGTGCCCGAGTGGAGAAA	ATTGTCACTGTCGTGCCTTGCC	257
FMgSSR-36184	scaffold22093	p2	(TC)19	Class I	2901	2938	CCACTGACGGTAACCCGAATCACT	ATCGATCGCAGGTGGGGAACAA	262
FMgSSR-36187	scaffold433	p2	(TC)19	Class I	20072	20109	CGGCTCAAACCCCGTTATGCTT	ACCAGGTGCGCAAAAGTGACCT	204
FMgSSR-36196	scaffold521	p2	(TC)19	Class I	72831	72868	TAATTGTGCGCTCATCACCCCG	TGCTGCGTGAAGCGAGGTTT	330
FMgSSR-36199	scaffold10878	p2	(TC)19	Class I	19058	19095	TCCAACTTCCAAGCCCGTTTCCA	TTTGCGCCTTTGCCTGGCTT	209
FMgSSR-36200	scaffold1648	p2	(TC)19	Class I	27526	27563	TCCAATCCCCGCAAGGGAACAA	TTTGCTTGTCGTGGCGCTCT	350

SSR_ID	Scaffold	SSR_Type	SSR_Motif	SSR Class	SSR_Start	SSR_End	Forward sequence	Reverse sequence	Product_size
FMgSSR-36201	scaffold4610	p2	(TC)19	Class I	20625	20662	TCCCAAGCGAACGCCAATGGAA	AGCTCTGGCAGTTTCAGGCACA	214
FMgSSR-36206	scaffold5800	p2	(TC)19	Class I	28490	28527	TCGGTCGTGGTGTTGAAGGA	CCTTGCAGCGCCTAATGCAA	305
FMgSSR-36219	scaffold10557	p2	(TC)19	Class I	21679	21716	TGCTGGTCCTTGTGCCTTCT	TGCTCATTCAGCACAAGGGGCT	256
FMgSSR-36222	scaffold1846	p2	(TC)19	Class I	18225	18262	TGTCGCGGCCTCAGTTGCTTTA	TCAGTGGCGGAATTGGACCAGA	344
FMgSSR-36224	scaffold1147	p2	(TC)19	Class I	43915	43952	TGTTGGTTACGCCAAGCCGA	CGGGATGCATGCGTTGAACTGA	293
FMgSSR-36227	scaffold2034	p2	(TC)19	Class I	35012	35049	TTCGACCGCCGCCGTAATTT	AGCACGCAAATGCAGCGGAA	218
FMgSSR-36231	scaffold5865	p2	(TC)19	Class I	13025	13062	TTGCCGAGCATCAGGTTGTCGT	GGCGAGCGTTGTGTCAGCAAAA	339
FMgSSR-36232	scaffold4425	p2	(TC)19	Class I	42649	42686	TTTGCTGGTGGATGGCACAGGA	AGCTGGCGTTGCTTGGTTGA	317
FMgSSR-36233	scaffold11661	p2	(TC)19	Class I	6434	6471	TTTTAGGGCGGCGGCTTGCTAT	ACGCAAAGCAGTGGGGTTGT	324
FMgSSR-36236	scaffold35467	p2	(TC)20	Class I	3437	3476	AACACGGATCCGGCAGCAAT	AGAGCCATGAACTAAAGGGGGCA	236
FMgSSR-36238	scaffold1334	p2	(TC)20	Class I	53755	53794	AACTACGACAACAGCGGGAGGT	ACAGTGGCAGTAGTGACGGCTT	347
FMgSSR-36249	scaffold190	p2	(TC)20	Class I	125483	125522	ACGAGAGCCTTTCCCTTGGT	ATCCACTGCAGCCTCCAAGGTT	286
FMgSSR-36257	scaffold6149	p2	(TC)20	Class I	28402	28441	AGCGCGTCCAACCTCAAGAT	AGCTGCACACAAACCGCAGA	252
FMgSSR-36258	scaffold73569	p2	(TC)20	Class I	1087	1126	AGCGGTGGCCCAATAAACCA	ATCTGGCGCTGTCGATGTGT	334
FMgSSR-36262	scaffold900	p2	(TC)20	Class I	95952	95991	AGTGTGGTGCATGAGCAAGCCT	AGGCAGCTTGCACGGTACACTT	345
FMgSSR-36263	scaffold10255	p2	(TC)20	Class I	3320	3359	AGTGTTGGGCCCCACTTCAA	GCAAGAAACAGGGAGCCAGTCTGA	305
FMgSSR-36264	scaffold248446	p2	(TC)20	Class I	388	427	ATCGGCACAAGCGCAAAGCA	GCACCACATGAGTTATACATGCCC	293
FMgSSR-36267	scaffold8570	p2	(TC)20	Class I	26374	26413	ATGATCACCGCGCGCAACAA	ACGCCCGAATCCACAGCTTTCT	278
FMgSSR-36269	scaffold147	p2	(TC)20	Class I	109841	109880	ATGCAGAAGCCTGTTGCTCCCA	ATCGGGGCACCCAATACCATGT	300
FMgSSR-36271	scaffold10306	p2	(TC)20	Class I	10589	10628	ATTCGCTCGGCCCCTCTTCTTT	ACAGCCGGGATGTGTCTTCA	273
FMgSSR-36272	scaffold5206	p2	(TC)20	Class I	19804	19843	ATTGGTTGCTCATCGCCCCA	ACCAACAAGTAGGCAAGGCGGT	251
FMgSSR-36279	scaffold1486	p2	(TC)20	Class I	14513	14552	GCATGCGTCATATCACTGTGGTG	ACCGGTGAAAGGTCGTCTGCTT	339
FMgSSR-36282	scaffold14057	p2	(TC)20	Class I	10821	10860	TCAACCTTGAGCCAGCCACGAA	TGTTGGCACCTGCTTGTCCT	304
FMgSSR-36286	scaffold31024	p2	(TC)20	Class I	5452	5491	TCCGGCTTTCATGATGTGGC	ACCTCGGGCAATGACTGAGTTTGA	340
FMgSSR-36289	scaffold44350	p2	(TC)20	Class I	130	169	TGAGTCGTGACCACATAGCCACA	AGGGATCGAGCTTTTGTCACAC	207
FMgSSR-36290	scaffold431	p2	(TC)20	Class I	50911	50950	TGCAAGTCTCCCTCATGCACT	TGGGTTGAAGGCCGAATGAGGT	295
FMgSSR-36292	scaffold181	p2	(TC)20	Class I	120427	120466	TGCAGCAGCAGAGGCAGAAGAT	TGAAACGGAACGCTCTCGCA	267
FMgSSR-36295	scaffold4485	p2	(TC)20	Class I	10868	10907	TGCCAAGCAGAAGCTCTCTCTCTC	TGAGGTTAGGGCATTGATGAGGCT	243

SSR_ID	Scaffold	SSR_Type	SSR_Motif	SSR Class	SSR_Start	SSR_End	Forward sequence	Reverse sequence	Product_size
FMgSSR-36296	scaffold834	p2	(TC)20	Class I	25145	25184	TGCCATCATTTCTACCGCTGAGCC	ACCCACCAAAAGGATCTAGCAAGC	231
FMgSSR-36297	scaffold12731	p2	(TC)20	Class I	9795	9834	TGCCGGCAAATGATGCTGCAA	AGTAACGGGCAGGACTAGAGCA	254
FMgSSR-36302	scaffold301	p2	(TC)20	Class I	941	980	TGCTTGCGCGCCATGTGAAT	ACTCATGCAAAGCTACCGCGTG	205
FMgSSR-36305	scaffold105	p2	(TC)20	Class I	150310	150349	TGGCAGACCAGCCACCAAACAT	ACAGGAGCTTGGGCCGAAAT	264
FMgSSR-36306	scaffold2	p2	(TC)20	Class I	181089	181128	TGGTCCAATTTCACCGGTCCGA	ACGGAGATGCAGCAAGCCAA	283
FMgSSR-36307	scaffold16743	p2	(TC)20	Class I	9544	9583	TGTCCAGTTATTCTGCCGCGA	TTTGGGCATGCAGCATCGGA	253
FMgSSR-36311	scaffold9536	p2	(TC)20	Class I	11996	12035	TTCCTCATCCTCTCTCCGAGCA	AATGGCACACACGTACCGCA	333
FMgSSR-36316	scaffold2237	p2	(TC)20	Class I	25644	25683	TTTGGCCCATGTCTTCTCAGCC	AAGAGGGGGCAACAATACCGCT	274
FMgSSR-36318	scaffold5017	p2	(TC)21	Class I	14476	14517	AACTTGGGACTATGCGGAGCCT	TGGTGACAGGATGAACTGGACTGA	240
FMgSSR-36322	scaffold9054	p2	(TC)21	Class I	9864	9905	ACGCAGCACCCCTCACGAATAA	AGCACAGCTGACTGCACAAAGA	282
FMgSSR-36334	scaffold635	p2	(TC)21	Class I	65383	65424	TCATAGCTTGCGACCTTGCGT	AGGAAACCGCGTAGACAGCAGA	345
FMgSSR-36335	scaffold17618	p2	(TC)21	Class I	12029	12070	TCCACATCAGCAAAGCGCCA	GTGACCGCGTCAACGCAAAA	212
FMgSSR-36337	scaffold843	p2	(TC)21	Class I	57546	57587	TCTGCGGTTCAGTCAGTGCT	TGGAACAATGAGTGTGCATGGGC	300
FMgSSR-36338	scaffold3447	p2	(TC)21	Class I	18417	18458	TGAAAAAGGGGAGGCTTCTCCGAC	ATCGGGGAAGCAAACCGCAA	349
FMgSSR-36340	scaffold4704	p2	(TC)21	Class I	27644	27685	TGGACGAGCCTTCCTCCAACAA	AGGCTGCAGCAAAGACGACA	338
FMgSSR-36351	scaffold11014	p2	(TC)22	Class I	10523	10566	AAAGGCCCACCAGTGAAGCA	CGCCCTTGGCTGTTGTTTGT	323
FMgSSR-36357	scaffold10421	p2	(TC)22	Class I	4724	4767	ACGCGCCTATGCCTCTTGTGTT	TGCCACCTTTTTGGCCTGACA	273
FMgSSR-36363	scaffold2905	p2	(TC)22	Class I	46785	46828	AGCATCGGAGTGCGCTAGCTTT	AATCATGGAGGTAGCCGGGACA	231
FMgSSR-36366	scaffold5305	p2	(TC)22	Class I	37843	37886	AGTAATGTGCACCCTGTGGCCT	CACAGCGCAAATGGCAGCAA	220
FMgSSR-36376	scaffold7397	p2	(TC)22	Class I	21017	21060	TCGTCACCTTGCTTGCCACT	AATGGCGGCTCGTTCGTTGA	344
FMgSSR-36381	scaffold5134	p2	(TC)22	Class I	15346	15389	TGACAGGGGTATACCACCACCTA	ATCCTGCCGAGCATGGTGTTGA	339
FMgSSR-36382	scaffold3245	p2	(TC)22	Class I	29778	29821	TGATGGCGGGGCTCCATTTT	ACAGTGCTCGACATTCCGACGA	289
FMgSSR-36384	scaffold3159	p2	(TC)22	Class I	55802	55845	TGCCAAAAGGCAGGCTAGCAA	ACGCAACACAAAACCGAGCAGG	220
FMgSSR-36385	scaffold1041	p2	(TC)22	Class I	16752	16795	TGCCATCTTCTCGCGGCTTT	AATTTCTACTCCCGCTGTCCCA	307
FMgSSR-36387	scaffold19878	p2	(TC)22	Class I	9837	9880	TGTCGCTTTTGAAGGTTTTCCGCC	ACCCATGATTGCTGGTTCGCT	266
FMgSSR-36392	scaffold10650	p2	(TC)22	Class I	6339	6382	TTTGCCCCTTGTTGCCATGC	AACCGATCAGAAACCCGCCGAT	346
FMgSSR-36394	scaffold2070	p2	(TC)22	Class I	8019	8062	TTTTTCCTCCGCCGCCACAT	TGGTTGGATCCACCGTTGTTGC	269
FMgSSR-36402	scaffold6103	p2	(TC)23	Class I	38294	38339	ACCTGCAACAGGTGGGCAACTT	TTAAGCTACGGCAGCGTCAGCA	324

SSR_ID	Scaffold	SSR_Type	SSR_Motif	SSR Class	SSR_Start	SSR_End	Forward sequence	Reverse sequence	Product_size
FMgSSR-36408	scaffold308	p2	(TC)23	Class I	143402	143447	AGGAAAAAGACGCATGCAGCCC	ACGAACACCACACGCTCTCA	316
FMgSSR-36409	scaffold4034	p2	(TC)23	Class I	41965	42010	AGGCGCAGATGGGTTGCAGTAA	ACACGGGCAAACCGCAAACA	253
FMgSSR-36415	scaffold4259	p2	(TC)23	Class I	42291	42336	TCACACGGCCCAGTTTTGCAT	TATCTGCCGCCCAAAGCAGCTA	241
FMgSSR-36418	scaffold22741	p2	(TC)23	Class I	292	337	TCCCGCTGGCAGTTTCCATT	ACATGCTACGCACACACGCA	274
FMgSSR-36419	scaffold19957	p2	(TC)23	Class I	12026	12071	TCCTCCAGCAAAAACACCACCTGT	ATGTGACTTTCGCCTCTGGGCT	259
FMgSSR-36427	scaffold7511	p2	(TC)23	Class I	13998	14043	TTCCAGCTGAGTCTCCCCCTTT	TGCACGTGGATGAGGGCAGATA	312
FMgSSR-36429	scaffold548	p2	(TC)23	Class I	71248	71293	TTGCCTCCTCTTCGCATCGCAT	TCGCGTGAAGGACAGTTGCACA	201
FMgSSR-36434	scaffold195612	p2	(TC)24	Class I	495	542	ACGGGCTGCCTGATTTGCTT	ATGGAGACCACCAGGGAGAAGTCA	329
FMgSSR-36442	scaffold2898	p2	(TC)24	Class I	25909	25956	TGAGTAAACGATGACGTTGTGGGC	ACTTCCACCGTTCCAGGTCACA	315
FMgSSR-36443	scaffold5604	p2	(TC)24	Class I	22659	22706	TGCACTATCCAGACCTCTCTCTGA	ACAAAGTCCGCACACAAGCCA	312
FMgSSR-36446	scaffold1318	p2	(TC)24	Class I	65856	65903	TGCTTATGAGAAGTGGCGCCGT	AGCAGGGCAACAACACAGAACC	284
FMgSSR-36447	scaffold13987	p2	(TC)24	Class I	10507	10554	TGGCCATCCAACTGCTTGGAGA	ATGCACATGCGCACGAAGTAGC	300
FMgSSR-36453	scaffold62876	p2	(TC)25	Class I	1835	1884	ACGTTGCTTCCGTGCTCGAT	GCGGCCGCCATTTTTGTGTT	301
FMgSSR-36456	scaffold1698	p2	(TC)25	Class I	25615	25664	AGCGTACATGTTTGGTCCGCA	ATACCCCGTGCGTCAGAGAAGT	305
FMgSSR-36458	scaffold13706	p2	(TC)25	Class I	8291	8340	TGCAATCCGCAACGCTTTCGT	ATCTCGGCTGGTGCTTGATGGA	337
FMgSSR-36460	scaffold4547	p2	(TC)25	Class I	32813	32862	TGCCTCAGCCTGATATTGGCA	ATAGGCGGCCAGCCTTTTGA	336
FMgSSR-36461	scaffold634	p2	(TC)25	Class I	63767	63816	TGGTGACGGCTGAAAAGGAGCA	AGAATGCGCGGCATGCTTGT	241
FMgSSR-36466	scaffold1757	p2	(TC)26	Class I	8578	8629	ACTCGTTTGGCAAGCCCACA	AGGTGTGAGGCTGCTTGCTT	243
FMgSSR-36468	scaffold2848	p2	(TC)26	Class I	18133	18184	TCCTCTAGCCTTGCTCGACAACT	TTGCTCGTCTGGTCCGTTCA	313
FMgSSR-36470	scaffold9134	p2	(TC)26	Class I	15024	15075	TCGCATCAACATTCGACGGC	AGACAGCCTCAGACCTGCCATT	343
FMgSSR-36472	scaffold13965	p2	(TC)26	Class I	9800	9851	TGCCCGGATTGTCACCAACGAA	GGCCAAGATGCATATACACCCCGT	262
FMgSSR-36478	scaffold16186	p2	(TC)27	Class I	10855	10908	TAACCCACAATCTGCGGCCCAT	ATGGCTGTGGCTAGGGTTGAGA	234
FMgSSR-36479	scaffold12055	p2	(TC)27	Class I	6632	6685	TCTTGTGGGCGATCCCTGGAAA	ACCTCTCCCACACATCCTATGGCT	337
FMgSSR-36486	scaffold597	p2	(TC)28	Class I	80428	80483	TATGGTAACCTACGTGGGTGCTGC	TGGGCAGCAATGGTATTGGCCT	201
FMgSSR-36489	scaffold389	p2	(TC)28	Class I	106991	107046	TGGTCAGAACACGTACTTGCCG	GCCGCAGCTCTCACCAACTAAA	307
FMgSSR-36490	scaffold6338	p2	(TC)29	Class I	47807	47864	ACCCTGACTCGGACCCGAATTT	ATTGCACCTAACATGCCGCGCT	318
FMgSSR-36493	scaffold1428	p2	(TC)29	Class I	30511	30568	TCTCCTCTCTAGCTACCTGCCT	TCGCTCCCTTGCTTCTGCTT	329
FMgSSR-36502	scaffold1063	p2	(TC)31	Class I	2063	2124	TTGCGTGATGGCGAGGTGATGA	TAAAGCTCGATCGGGCCATGCT	291

SSR_ID	Scaffold	SSR_Type	SSR_Motif	SSR Class	SSR_Start	SSR_End	Forward sequence	Reverse sequence	Product_size
FMgSSR-36503	scaffold763	p2	(TC)32	Class I	72346	72409	ACGCAGTGCACTTGTCAACCCT	AATTGTCACGGCGTTGCCCT	227
FMgSSR-36504	scaffold1578	p2	(TC)32	Class I	3191	3254	CGGTTCAACAGCCTTTCACAGA	AAGCACCAGAAACCCCTGCTGT	305
FMgSSR-36510	scaffold7148	p2	(TC)36	Class I	29719	29790	ATGCCGCTGGATAGTAAGGACT	AAAAGGTCGGGAGGCACACGTT	212
FMgSSR-36511	scaffold1383	p2	(TC)36	Class I	53857	53928	TTCTTCCCCTCGGGCTGAAACT	TCCATGCAAGGTTGACGGTTGGT	298
FMgSSR-36513	scaffold6937	p2	(TC)43	Class I	27476	27561	TCCCCAACCTCTCTTCCCTCTT	AGCAACACGTTCGCTCATCTCG	302
FMgSSR-36519	scaffold61	p2	(TC)6	Class II	188262	188273	AAAACGGTTGAGGAAGGCCAGC	TGCTGCAAAGTGCAAGCCGA	201
FMgSSR-36522	scaffold20717	p2	(TC)6	Class II	9086	9097	AAAAGCCAGGCCGCTTTGAACC	GCCCGCGCCCAAACTTTTCTTT	237
FMgSSR-36524	scaffold5	p2	(TC)6	Class II	280611	280622	AAAAGCCGCACCTTGCACCT	ACAATTCCTGGGCCGAAGTGGT	284
FMgSSR-36525	scaffold771	p2	(TC)6	Class II	41634	41645	AAAAGCGTGTGCTGTTCGGC	TGCTCCTCGTTGCGGCCATAAA	221
FMgSSR-36526	scaffold2002	p2	(TC)6	Class II	50797	50808	AAAATACTCTCCCCGCCCCTCT	CTGCACGCTTGATCATTGTACTGC	252
FMgSSR-36542	scaffold66	p2	(TC)6	Class II	164882	164893	AAACGGACGTTTTGTCGCCACC	ATCAATCAGCCAGCCGTGTGGT	338
FMgSSR-36545	scaffold87	p2	(TC)6	Class II	95388	95399	AAACTGGACACCGGACACCTCT	TGCTCTGGTAGGAGAGGGAAAGTGA	216
FMgSSR-36547	scaffold67	p2	(TC)6	Class II	33724	33735	AAAGCACACGAAGCGCCAGT	GCTGCAGTGAAGGAAATGGGCA	350
FMgSSR-36553	scaffold4728	p2	(TC)6	Class II	29616	29627	AAAGGCTAGCGGACCGACGAAA	TGGTGTTTGGGTGCTCGCAGTT	337
FMgSSR-36556	scaffold676	p2	(TC)6	Class II	79439	79450	AAATCGCCGGACCATGTGGTGT	ACGCCATGCAAGCACGAAACT	314
FMgSSR-36557	scaffold1653	p2	(TC)6	Class II	64801	64812	AAATGACGAGTTGGATCGGGGC	ATTCGTGCTCTCGCGCACAT	333
FMgSSR-36558	scaffold302	p2	(TC)6	Class II	79390	79401	AAATGAGGCGCAGGGTTGGA	CGCGACGCAACACAACACAA	216
FMgSSR-36559	scaffold3943	p2	(TC)6	Class II	34614	34625	AAATGAGTGGCACGGCCCTT	GGGCATCAACCAGCCACAAGTT	330
FMgSSR-36561	scaffold226	p2	(TC)6	Class II	62242	62253	AAATTGCAGCCACGCCGAAG	AGACAACGCCTGGAAAGCCA	272
FMgSSR-36571	scaffold4211	p2	(TC)6	Class II	19114	19125	AACCACTCCAACCACCACCGTA	ACAATGGAGCAGGTGGGTCTCA	319
FMgSSR-36574	scaffold1866	p2	(TC)6	Class II	15570	15581	AACCCACCCATGACAAGCAGGA	AATGTGGCAAAGCACGCAGT	321
FMgSSR-36575	scaffold655	p2	(TC)6	Class II	59393	59404	AACCCACTATCACCCACCAGCA	ATCCTCATGCAGATCCGTCGAG	295
FMgSSR-36577	scaffold54	p2	(TC)6	Class II	112937	112948	AACCCAGCCACATCGCCATT	TTGTTGCCTCGAGTTGCTCCCT	324
FMgSSR-36581	scaffold635	p2	(TC)6	Class II	79729	79740	AACCTAGCAGCCGTTGAGCACT	CGCAGTGGCAGAGGAAGAAAGA	288
FMgSSR-36588	scaffold11907	p2	(TC)6	Class II	20726	20737	AACGCAACCAACGGCTCGTA	TCGCGCGAATAAGGCAAGGT	312
FMgSSR-36589	scaffold3073	p2	(TC)6	Class II	39343	39354	AACGCACGCACAAACACACG	ACAGGGTAATCGCCACCGCAAA	307
FMgSSR-36591	scaffold7403	p2	(TC)6	Class II	20319	20330	AACGCAGGGGCAATATGGGACA	AGGGCAGCGACAACAATCGTGA	257
FMgSSR-36608	scaffold7161	p2	(TC)6	Class II	21573	21584	AAGAAGAAGCCGCGTTGGGA	AATCGGTGGGCAAGGCAGGAAA	347

SSR_ID	Scaffold	SSR_Type	SSR_Motif	SSR Class	SSR_Start	SSR_End	Forward sequence	Reverse sequence	Product_size
FMgSSR-36610	scaffold8086	p2	(TC)6	Class II	17622	17633	AAGAAGTGGGAGCACGGAAGCA	TTCCCAGCGCATAGGGTTCAGT	335
FMgSSR-36613	scaffold1183	p2	(TC)6	Class II	81043	81054	AAGATCACGTCGGACTCTGCCT	TTCAGATGAGCCCCAAGTGCGA	302
FMgSSR-36614	scaffold1669	p2	(TC)6	Class II	37601	37612	AAGATGCAGCAACCGGCCTT	AATGGAAGCGGAGTCGGAACCT	270
FMgSSR-36616	scaffold1789	p2	(TC)6	Class II	12371	12382	AAGCCGCAGAAAAGCGCCAT	AGACGCTTTGCTCACCAGGA	281
FMgSSR-36618	scaffold2051	p2	(TC)6	Class II	62294	62305	AAGCCTCCTTGGCTGCGAAT	ACGTGTGTTCCGTGCGCTTA	265
FMgSSR-36621	scaffold6911	p2	(TC)6	Class II	9471	9482	AAGCGAATGGGAATCGACGGCA	ATCGGCAGCGGCGACAAAAA	295
FMgSSR-36622	scaffold117	p2	(TC)6	Class II	110643	110654	AAGCGCCGTCTCTGGACTGTTT	TGCGTACGACCCAGCCACAAAA	332
FMgSSR-36633	scaffold17685	p2	(TC)6	Class II	2553	2564	AAGTACATCACGCAGGGGCT	ACCTGCACAGTGCGTGGACTAA	341
FMgSSR-36636	scaffold14340	p2	(TC)6	Class II	11213	11224	AAGTCTCCAACCCCAACCCCAA	TTGACCCGTTCTTGGCTGGCAT	250
FMgSSR-36641	scaffold13839	p2	(TC)6	Class II	13868	13879	AATACGCGCCATGAGTCCGAGT	AGCTTCAGAAGGAGGCAGAGGA	290
FMgSSR-36642	scaffold1582	p2	(TC)6	Class II	56085	56096	AATCACCACACAGTAGCGCCCA	TGGAAACTGGACACCCCTTGGT	333
FMgSSR-36647	scaffold19232	p2	(TC)6	Class II	9791	9802	AATCGAAACGTCATGGGGCAGG	TGCCATGTCTGGTAACAGGGCT	302
FMgSSR-36649	scaffold4806	p2	(TC)6	Class II	1222	1233	AATCTTTCCTCCGCCGCTTTGC	TTGCCTCTTGGTTTTGGGGGCT	338
FMgSSR-36656	scaffold1264	p2	(TC)6	Class II	22612	22623	AATGGACGCCAGCCATCCAACA	ACTCAACCACAGGTCGGTGT	217
FMgSSR-36665	scaffold11046	p2	(TC)6	Class II	13935	13946	ACAAAGCCCCACGTATCACCA	ATCGTGTGGGAGGTCGCATT	294
FMgSSR-36668	scaffold307	p2	(TC)6	Class II	131760	131771	ACAAAGGCGAGAGAGGCCAA	TGTGAGCTTGGCGTGCCATT	301
FMgSSR-36669	scaffold3788	p2	(TC)6	Class II	17112	17123	ACAACCAATCCAAGCTGCCCCT	TTCCAGCCATCGACAAGCTCCT	309
FMgSSR-36672	scaffold4159	p2	(TC)6	Class II	23751	23762	ACAAGAAACACACGCGGAACGA	ATGCGAAAGCCTCCTGTGGT	247
FMgSSR-36673	scaffold512	p2	(TC)6	Class II	35135	35146	ACAAGGCGACAATAGGCGGTGA	TCGCATGCGCATACCAACCT	325
FMgSSR-36674	scaffold124744	p2	(TC)6	Class II	1077	1088	ACAAGGCTGTTCTCGTCCCACA	ACAAACTTCAGCAGACGCGG	224
FMgSSR-36676	scaffold28733	p2	(TC)6	Class II	8730	8741	ACAAGGTTGGTAAGGCAGGGCT	ACCTTCGCCGTTGTTCATCCGT	296
FMgSSR-36685	scaffold38599	p2	(TC)6	Class II	724	735	ACACCTCTCCCATTTGACCACC	ACGCTGCAACGTTTTCCGGT	235
FMgSSR-36691	scaffold3294	p2	(TC)6	Class II	33688	33699	ACACGGGGGCGGAGTGTAAAAA	AATGCTGCCACACGCTGACA	278
FMgSSR-36695	scaffold654	p2	(TC)6	Class II	46128	46139	ACACTTTTGCGATGTGTCCCGC	TGCTCAACGCACTGGCATGT	308
FMgSSR-36700	scaffold17685	p2	(TC)6	Class II	2712	2723	ACAGCAATGCAGTGGAGACGA	ACAATACCTGCATGCGGCTGGA	231
FMgSSR-36704	scaffold1840	p2	(TC)6	Class II	42490	42501	ACAGCCAGAGCAACCATCCAGT	TGAAGTGAGGCGGCGAGAAACA	313
FMgSSR-36708	scaffold25721	p2	(TC)6	Class II	3363	3374	ACAGGACGCTAGCTGCATTGGT	AGCGGTTTGCAGCGGTAAGA	205
FMgSSR-36709	scaffold331	p2	(TC)6	Class II	124675	124686	ACAGGCAAACACCACGTAGGCA	TGCGAGGCGAAAAGCGACAT	303

SSR_ID	Scaffold	SSR_Type	SSR_Motif	SSR Class	SSR_Start	SSR_End	Forward sequence	Reverse sequence	Product_size
FMgSSR-36713	scaffold2636	p2	(TC)6	Class II	54523	54534	ACAGTCTAGGCGAACATGGGCT	TCCAAGACAGTGTGCAGGAAAGCA	320
FMgSSR-36719	scaffold2884	p2	(TC)6	Class II	15651	15662	ACATGCACTGTGGGCGCTAT	TGCAGCCACACGTTTGCACTT	350
FMgSSR-36724	scaffold49	p2	(TC)6	Class II	170221	170232	ACATGCGGGCCGTCAAACTT	ATGCACGCACAGTTGCTGGT	294
FMgSSR-36736	scaffold17359	p2	(TC)6	Class II	10130	10141	ACCACCGCTGCCCATATCAT	ACCGCGCATAGATCCACTGA	299
FMgSSR-36737	scaffold9586	p2	(TC)6	Class II	6264	6275	ACCACGAAAAACACGCGACG	TTCGCGTCGTCATCAACCGT	292
FMgSSR-36746	scaffold4327	p2	(TC)6	Class II	1346	1357	ACCAGCGATGTTCGCAAGGA	TTGCAGTTGCCAGGCGTGAA	323
FMgSSR-36748	scaffold44	p2	(TC)6	Class II	144481	144492	ACCAGGGCAAGACATGGCAA	TCCTTCATGGGGCTCAAGCCAT	301
FMgSSR-36755	scaffold8175	p2	(TC)6	Class II	15893	15904	ACCCAATCGCAGACGGTCCATT	TGAGCAACGCCAGTGGAAGT	332
FMgSSR-36757	scaffold858	p2	(TC)6	Class II	89279	89290	ACCCATCACCAGTCGGTGAGTT	TGATCAAGGCGCAGAAGCTCGT	273
FMgSSR-36766	scaffold2261	p2	(TC)6	Class II	44479	44490	ACCCGCATCGATGGATTGAGCA	TCGTGCAGGGGAAGAGGAAGAA	315
FMgSSR-36767	scaffold680	p2	(TC)6	Class II	65156	65167	ACCCGCTGTGATAGTCCTGGTT	TCGGCAATGGTGCCTGGTTCTA	345
FMgSSR-36776	scaffold2767	p2	(TC)6	Class II	29741	29752	ACCGCAAACAAAACCGTGCTCC	TGGTGCCGCGCAATGTATGT	347
FMgSSR-36777	scaffold5346	p2	(TC)6	Class II	7221	7232	ACCGCAAAGGCGAATGACAAGG	ATAGCCCGCAAGATGTCCAGGT	212
FMgSSR-36779	scaffold3026	p2	(TC)6	Class II	23847	23858	ACCGCCGAGAAGAATGCTTGTGA	TCACCAAAAGCTGCCTGCTTCA	349
FMgSSR-36780	scaffold19646	p2	(TC)6	Class II	15956	15967	ACCGCCTGCACTCTTCCAACAA	TGACGTCAGGCGTGTGTCTTGA	328
FMgSSR-36782	scaffold1651	p2	(TC)6	Class II	12089	12100	ACCGCTCAGATGTTGGTCGT	TTGGCCGCAGCTTCCTTTTTGC	323
FMgSSR-36787	scaffold7972	p2	(TC)6	Class II	14524	14535	ACCGTGCAAGCTCCAAATGAGT	TTTCACCATTGCCGGCACCA	338
FMgSSR-36789	scaffold661	p2	(TC)6	Class II	58914	58925	ACCGTGTGGGTGCACTTGTT	ATGACGTTGGCTCGTGCGAGAT	282
FMgSSR-36791	scaffold9468	p2	(TC)6	Class II	17162	17173	ACCTCATGCACTTGCCCCTT	ATTGACGACCACGGAACGGT	242
FMgSSR-36797	scaffold968	p2	(TC)6	Class II	56878	56889	ACCTTGCTTACGTCGCACCA	AGCGCAAACCTACGCAGCAT	309
FMgSSR-36798	scaffold12299	p2	(TC)6	Class II	19962	19973	ACCTTTAACCGTGGGACTCGCT	GTCGGAAAAACGGCCTTGCT	224
FMgSSR-36805	scaffold6220	p2	(TC)6	Class II	32594	32605	ACGACGCGAAACGACGTGAA	AGCAGACGCGACACCAAACA	314
FMgSSR-36807	scaffold78072	p2	(TC)6	Class II	351	362	ACGAGATGTGGGCGGTGTGTTT	ACACGCCGCTCTTGGACTAT	272
FMgSSR-36808	scaffold18263	p2	(TC)6	Class II	3090	3101	ACGAGGTGCGAAGCAAGGGAAA	TAATGCTTGGCTCCGCCAGT	321
FMgSSR-36809	scaffold94	p2	(TC)6	Class II	145936	145947	ACGATCCAGTGGCACGTCTTCT	GGGCGCGCAAGCCAATTTTT	310
FMgSSR-36810	scaffold76717	p2	(TC)6	Class II	336	347	ACGATCGTGGGTTGAGTTGGT	TCAACATGGCGGCACCATCA	273
FMgSSR-36814	scaffold1620	p2	(TC)6	Class II	27581	27592	ACGCATTGTATGCAGGCTAGGT	TGTTGCTTTTGCGGGTCGCT	342
FMgSSR-36817	scaffold10513	p2	(TC)6	Class II	14456	14467	ACGCCCACCGTATTCCACTTCT	TGCCATTGCGCGTTGAAGGT	248

SSR_ID	Scaffold	SSR_Type	SSR_Motif	SSR Class	SSR_Start	SSR_End	Forward sequence	Reverse sequence	Product_size
FMgSSR-36818	scaffold6681	p2	(TC)6	Class II	7621	7632	ACGCCCCGACACATTTTCCA	ATGCAAGTGGAGGAGGATTGCC	256
FMgSSR-36820	scaffold2066	p2	(TC)6	Class II	58294	58305	ACGCGCATGTCTTTCCTGGT	TTCGGGACGGTGGTCGAATCAA	264
FMgSSR-36823	scaffold9498	p2	(TC)6	Class II	7842	7853	ACGCTCATCGTCCACCACATGA	TTCGTGCGAGCGCCTCTTTT	307
FMgSSR-36824	scaffold2049	p2	(TC)6	Class II	37280	37291	ACGCTCTCTTCCACCGTCGATT	AGTCTCACGCGCTCTGCTACAT	257
FMgSSR-36826	scaffold4396	p2	(TC)6	Class II	11785	11796	ACGCTGTCTTGTCACGCACACA	ACGCGACACAACTCAGAGAGCA	317
FMgSSR-36830	scaffold194	p2	(TC)6	Class II	151152	151163	ACGGAGGACAGCGTGCAAAA	ACGGTGCTAGACGGCAACAA	349
FMgSSR-36832	scaffold329	p2	(TC)6	Class II	39676	39687	ACGGATGCAGACAATGGCGT	AACGGCGATCGTTCTCAGGCTT	326
FMgSSR-36843	scaffold1721	p2	(TC)6	Class II	47592	47603	ACGGGGCAGGCTTTTCTGAT	ACGCTTGTGTGACATGGTGGA	305
FMgSSR-36845	scaffold16276	p2	(TC)6	Class II	9187	9198	ACGGTCAACGTGCTATCGTTCG	CGGATGATCCACACACACAACA	284
FMgSSR-36848	scaffold6868	p2	(TC)6	Class II	9961	9972	ACGGTTCGATTCGTGCGGTT	ACGCAGAGCTGCAGGAAAGGTT	267
FMgSSR-36849	scaffold12363	p2	(TC)6	Class II	3872	3883	ACGGTTGGAAAGCGCCACAT	TTCTGGACTAGAGCAGTGAGCAGC	315
FMgSSR-36854	scaffold4849	p2	(TC)6	Class II	38101	38112	ACGTGAAATGCGCCGTGCTT	TGATCTGCAAGGCAGCAAGGGT	248
FMgSSR-36858	scaffold10772	p2	(TC)6	Class II	4196	4207	ACGTTAGCAGCTCGAATGCCGA	TGGTGGGAAGCAAGCACTAGCA	299
FMgSSR-36863	scaffold206	p2	(TC)6	Class II	28384	28395	ACTCTTCCTGTCACCAGTCGCA	ACACACGCGCGCCAAAAAGT	326
FMgSSR-36864	scaffold1197	p2	(TC)6	Class II	7483	7494	ACTCTTGTGTAACCCCCACTCTCT	TAGTGCCCCCTGATGATGGATGGA	307
FMgSSR-36869	scaffold1970	p2	(TC)6	Class II	53115	53126	ACTGCACTGCACCACAGTGA	AAAGCGACGTCGTACGGTAGCA	346
FMgSSR-36871	scaffold10176	p2	(TC)6	Class II	19120	19131	ACTGGATTACAGTGGCAGACGCA	AAGGCCACATGCTCATGGCT	335
FMgSSR-36874	scaffold176	p2	(TC)6	Class II	29963	29974	ACTGTCACGCTGCCTTCGTT	TGCTGCTCTGCATGTACCGT	271
FMgSSR-36875	scaffold65	p2	(TC)6	Class II	201572	201583	ACTGTTGCAGCGTGCGCATT	TGGGAATTCGATCGGCAAGGA	350
FMgSSR-36879	scaffold1133	p2	(TC)6	Class II	32845	32856	ACTTGCGGCTTTCACGCAGT	ACCTCCTCCTTGCTGACTTGGT	249
FMgSSR-36882	scaffold3699	p2	(TC)6	Class II	33347	33358	AGACACCAATGCCCGTCACT	CGCGTTGCTTTGGCAGTGTT	275
FMgSSR-36884	scaffold239	p2	(TC)6	Class II	108859	108870	AGACCTAACACTCCCCTCACCT	AACATTACCGCCCTGCCTGCTT	344
FMgSSR-36892	scaffold20270	p2	(TC)6	Class II	3121	3132	AGAGGCCAGCTAGCCCGTTTTA	ATTCAAGCCCCTGCCAGACTGT	284
FMgSSR-36897	scaffold28531	p2	(TC)6	Class II	756	767	AGCAACAACCCGAAAGTAGCCCA	TCGTTTGTCGGGGTGGCTTAGA	311
FMgSSR-36898	scaffold326866	p2	(TC)6	Class II	166	177	AGCAACCCGAGCCAACCCATTA	TGCGGTAGCATGAGAGTTGAGA	273
FMgSSR-36900	scaffold5342	p2	(TC)6	Class II	4533	4544	AGCAACGACAAACAGGGGCA	ACCACGATAACACTGTAGGGGCT	301
FMgSSR-36905	scaffold25839	p2	(TC)6	Class II	7849	7860	AGCAATCCGTGTCTTACCCCCT	ATCGACCATCCGCGTCCTTTGT	311
FMgSSR-36906	scaffold886	p2	(TC)6	Class II	39995	40006	AGCAATGCATTTCGCCCGCT	TGATCCACAGTGAGCAGCCACA	336

SSR_ID	Scaffold	SSR_Type	SSR_Motif	SSR Class	SSR_Start	SSR_End	Forward sequence	Reverse sequence	Product_size
FMgSSR-36910	scaffold4080	p2	(TC)6	Class II	3832	3843	AGCACCACCGGAAAATCGTCCA	CGTGCAACCGTGTGTTTGTGCT	210
FMgSSR-36914	scaffold9621	p2	(TC)6	Class II	20650	20661	AGCACTGTGCAGATCGCAAGGA	AGCACGAGGTGGATGGTTGA	209
FMgSSR-36916	scaffold2694	p2	(TC)6	Class II	16022	16033	AGCAGCAACCCCTTCGATGCTT	GCGTGTGCGCGTCTTCAAAA	281
FMgSSR-36917	scaffold8686	p2	(TC)6	Class II	17955	17966	AGCAGCACCGAATCTACACTCA	GCCAAAAGCAGCGGCAATGA	337
FMgSSR-36918	scaffold3679	p2	(TC)6	Class II	48748	48759	AGCAGCACCGTCGCCATTTT	AACGAGGGGAAGTGCGGAGAAT	350
FMgSSR-36919	scaffold131	p2	(TC)6	Class II	50816	50827	AGCAGCAGCCGACTTGCATT	ACTCGTCCATTCCACGGGCAAA	349
FMgSSR-36921	scaffold18845	p2	(TC)6	Class II	7593	7604	AGCAGCTGCCTTGCTGATGT	GCACGAAGACCAGTGCAAGA	350
FMgSSR-36922	scaffold2359	p2	(TC)6	Class II	19659	19670	AGCAGCTGCGGCAAAATGCTT	AGCTCCGTCCTCCTGATTCTACT	349
FMgSSR-36926	scaffold320	p2	(TC)6	Class II	119581	119592	AGCCAACGCGTTTCTGCTCT	GAGAGAATGGGGAGGATTGGAGAA	343
FMgSSR-36927	scaffold1678	p2	(TC)6	Class II	30091	30102	AGCCAGCACAAACGCACCAA	AGGTCACATGCAGGGTTCTGAGT	283
FMgSSR-36931	scaffold568	p2	(TC)6	Class II	79305	79316	AGCCGGGCAACTTTTCCCAA	TAGCTTGATCCCCATGCGCCAA	215
FMgSSR-36939	scaffold4723	p2	(TC)6	Class II	51480	51491	AGCGAGCTCACACAATCAGCA	ATCACCTCCACCTGCACACCAT	320
FMgSSR-36943	scaffold4728	p2	(TC)6	Class II	31675	31686	AGCGGACCGACGAAATGGAA	TTGCCTGACCGATGGCATGA	349
FMgSSR-36944	scaffold7511	p2	(TC)6	Class II	33940	33951	AGCGGATACCGGCGAAACCAAA	AGTGATGCCATGCCAACTCCCA	253
FMgSSR-36946	scaffold105967	p2	(TC)6	Class II	1334	1345	AGCGGGGCATAATCTGTGCTGA	ACCCTGCATCAGACAAGGTCCA	320
FMgSSR-36950	scaffold19472	p2	(TC)6	Class II	7631	7642	AGCGTTACATTTGGCCGCAAGC	TGTGTGGTTGGTGCAGCCTT	279
FMgSSR-36952	scaffold3782	p2	(TC)6	Class II	21404	21415	AGCTCAAGGGTGCGGCATACAA	AAGCCGTGATTGCAAGGCGA	236
FMgSSR-36954	scaffold845	p2	(TC)6	Class II	70484	70495	AGCTCCAGATGAGGTGAAGAAGCA	TGTCGTTGCTGGCTAGGTTCCA	224
FMgSSR-36962	scaffold7700	p2	(TC)6	Class II	27506	27517	AGCTTCTAGCTGCATGGGCAGT	AGCACGTGCAGTCGCTGAAA	289
FMgSSR-36966	scaffold12	p2	(TC)6	Class II	212590	212601	AGCTTGCTCTGGGCACACCATT	ACGAATGATTGCGACGCGGA	341
FMgSSR-36974	scaffold1585	p2	(TC)6	Class II	26992	27003	AGGAGGCAAAAACCAGCGGA	CGGTGGCGGCAATTTCTCCTTT	329
FMgSSR-36981	scaffold27375	p2	(TC)6	Class II	7412	7423	AGGCAGGCAAGGCAGCAAAT	AATGGAGATCCTGGTAGTCGAGGC	261
FMgSSR-36982	scaffold342	p2	(TC)6	Class II	83861	83872	AGGCAGGCAGAGAGCAAGCAAT	AGGAAGGCCCAAACACACACACA	262
FMgSSR-36983	scaffold161226	p2	(TC)6	Class II	105	116	AGGCATCATATTAGCGGGGCA	TTGTTAACACGTGCGCTCTCGC	229
FMgSSR-36985	scaffold25	p2	(TC)6	Class II	155662	155673	AGGCCTGGGCCACAATTCAT	TAGGCTTAGCTCAGCGGGATCT	316
FMgSSR-36988	scaffold7861	p2	(TC)6	Class II	574	585	AGGCGATTCATCAGGAGCGAGT	TGGCGCCCAGTTTCCCATTGAA	268
FMgSSR-36989	scaffold6165	p2	(TC)6	Class II	2379	2390	AGGCGTCATCTCTGCGCTTT	TGATGAGTTGGCATTCCCGGCT	208
FMgSSR-36990	scaffold286	p2	(TC)6	Class II	67937	67948	AGGCTTCAGTTGGAGCGCTGTT	TTTACGCCTTTACGCGGCCT	299

SSR_ID	Scaffold	SSR_Type	SSR_Motif	SSR Class	SSR_Start	SSR_End	Forward sequence	Reverse sequence	Product_size
FMgSSR-37001	scaffold10676	p2	(TC)6	Class II	9518	9529	AGGTCAATGCAAGCCGACGTGT	TGCGTCCACAAGTTGGACATCCT	349
FMgSSR-37003	scaffold30333	p2	(TC)6	Class II	1769	1780	AGGTCCAGAGTGGCCACAAGAT	TATTCGTCGAGGATCGGGCGTA	270
FMgSSR-37005	scaffold476	p2	(TC)6	Class II	26283	26294	AGGTGCAATCCCGAGGGAACTT	TGTGGATCAGCCGGTTGCACAT	238
FMgSSR-37006	scaffold19986	p2	(TC)6	Class II	10219	10230	AGGTGCATTGCTGTTGGTGTGAG	ACCCAACACACCCGAGCAAAA	282
FMgSSR-37009	scaffold21461	p2	(TC)6	Class II	3506	3517	AGGTGCTTTCGAACCTGCCGTA	TGCAGCCAAATGACGTGCCT	266
FMgSSR-37010	scaffold7579	p2	(TC)6	Class II	1929	1940	AGGTGGCGCAGTGTCGTTGTAT	ACCTCCATTGCACGCCATCA	218
FMgSSR-37011	scaffold1255	p2	(TC)6	Class II	46479	46490	AGGTGGCTTCGCCACAATAGCA	AGATCAGTGAGGTCGAGCGGAT	332
FMgSSR-37016	scaffold39174	p2	(TC)6	Class II	1201	1212	AGGTTGTAGGTCAACAGGCACT	ATGGTGTTGGTGAGGACAAGTGGG	312
FMgSSR-37021	scaffold5052	p2	(TC)6	Class II	31677	31688	AGTCAGCGCAACTTGCCGTA	AATCAGTCGCCAGTCGGCCAAA	323
FMgSSR-37024	scaffold5606	p2	(TC)6	Class II	26008	26019	AGTCCTCTCCCGGCTAAGGGTTAAT	TGGGCTTTTGGATCCGTCGT	349
FMgSSR-37029	scaffold51232	p2	(TC)6	Class II	3717	3728	AGTGCAAACACCCTTGTGCGA	TGCCTACCCAGTACGCACGAAA	343
FMgSSR-37034	scaffold605	p2	(TC)6	Class II	66501	66512	AGTGGCTCTAGACAGATCGCCA	TGCCACGATGGGGCTTAGCTTT	322
FMgSSR-37036	scaffold1351	p2	(TC)6	Class II	48174	48185	AGTTCACCGCTGCCTCTCTCAT	TCCTTGCCGCACCGAAATTGA	278
FMgSSR-37038	scaffold770	p2	(TC)6	Class II	79248	79259	AGTTGTTGTGTGCGGTGGAGGT	ACTGTTTGGTGTAGCAGCAGTCCA	233
FMgSSR-37041	scaffold3891	p2	(TC)6	Class II	12745	12756	ATATGTGAGTCCGCCTGCCTCT	TATGGGCCACACCACGAAGA	291
FMgSSR-37046	scaffold9234	p2	(TC)6	Class II	16340	16351	ATCACCGTCGGTTGCTAAGGCT	CTCGCACCAATGCGCATCAA	341
FMgSSR-37055	scaffold19355	p2	(TC)6	Class II	1702	1713	ATCCGCCGCCGAATTGACTCAT	ACGGTGTTGCTGCAGCTTGT	239
FMgSSR-37058	scaffold55	p2	(TC)6	Class II	138027	138038	ATCGCACGAACATCGTACACGC	TGCGTCGGTGTCCACGTAAA	229
FMgSSR-37062	scaffold1232	p2	(TC)6	Class II	75077	75088	ATCGTCATGCTGCGGCCATT	AGCGAAGCAATTGGGAGGTGCT	223
FMgSSR-37064	scaffold8617	p2	(TC)6	Class II	5110	5121	ATCGTGAACTGGGTGGTCCAGA	TAGCGTTGGTGGTTGGCCACAT	334
FMgSSR-37065	scaffold9186	p2	(TC)6	Class II	8278	8289	ATCGTTGCCGCAAGCCTGCTAT	ACTCGCGCAGTCCTCACAAT	260
FMgSSR-37068	scaffold78	p2	(TC)6	Class II	98836	98847	ATCTGTAGGACTGTCGTGCGAAGC	TGGCGCCAAAAACAGGGCAT	308
FMgSSR-37070	scaffold2635	p2	(TC)6	Class II	17407	17418	ATCTTTTGGCGCCTCGGTGT	GGCACAACGAGTAAAGCAGCGT	344
FMgSSR-37071	scaffold2697	p2	(TC)6	Class II	34502	34513	ATGAATTGCCGTTGGATGCGGC	AAAAGCGCCATGGGATGCGT	243
FMgSSR-37081	scaffold1233	p2	(TC)6	Class II	76032	76043	ATGCAGCACTGGAACCCACTCT	GTGCATGCATTGTTCAGCTGGC	288
FMgSSR-37087	scaffold750	p2	(TC)6	Class II	60004	60015	ATGCGAGAGTCGCCGTGGAAAT	TCCTAGTTGTCGAGCTTCCGCA	348
FMgSSR-37088	scaffold876	p2	(TC)6	Class II	61584	61595	ATGCGGGCGTAGAAGTTGGCAT	AGCAGCGTTTTTCATCCCACGC	252
FMgSSR-37089	scaffold110711	p2	(TC)6	Class II	58	69	ATGCTCAATTCCTGGCTCTGCC	ATTGCAAGCACGCGGTGGAA	278

SSR_ID	Scaffold	SSR_Type	SSR_Motif	SSR Class	SSR_Start	SSR_End	Forward sequence	Reverse sequence	Product_size
FMgSSR-37095	scaffold174631	p2	(TC)6	Class II	191	202	ATGGAGTGGGCCAACAGGAA	AAGGTGTCCATGCTTGGTGCGA	319
FMgSSR-37098	scaffold4759	p2	(TC)6	Class II	14835	14846	ATGGCCACGTGAAGTTTGCG	GTTTCAGGTTGCTTGGCCGT	274
FMgSSR-37099	scaffold13774	p2	(TC)6	Class II	17942	17953	ATGGCCATAGCACTAGCACCGA	AGTGCACCTCGTATCTGGGCAA	315
FMgSSR-37103	scaffold9459	p2	(TC)6	Class II	6277	6288	ATGGGTGCCGTGTGCTTTCA	TGCAGCTCAGCACATGCCAA	349
FMgSSR-37106	scaffold5439	p2	(TC)6	Class II	26104	26115	ATGTCGAATCACGAGCGGCA	TGGATTGGTGGCGCAATGGA	329
FMgSSR-37107	scaffold5315	p2	(TC)6	Class II	23535	23546	ATGTGAGCCACTGACGGTGT	TGTGGTTGGCGATGGTGTCA	345
FMgSSR-37115	scaffold6877	p2	(TC)6	Class II	16542	16553	ATTCGGCTGTTCTCGTCCCA	AAAGAGCGGAGGCGAAGACGTT	350
FMgSSR-37119	scaffold67812	p2	(TC)6	Class II	1295	1306	ATTGTGCAGCGCCAGCTTCT	AACGCCGCTCGCTCTCTGTTTT	317
FMgSSR-37121	scaffold6151	p2	(TC)6	Class II	14441	14452	ATTTCGAATGGCGTGCGAGC	TGGTGCACGTTGTTCATCAGGC	315
FMgSSR-37126	scaffold13335	p2	(TC)6	Class II	11900	11911	ATTTGGCCGGCTACGAGCAA	AGCATGCAGGTTGGGTGGACAA	318
FMgSSR-37133	scaffold37650	p2	(TC)6	Class II	3309	3320	CAGTTCCTGAGGTAACCCCTGCAT	GCGAGCAACCTCAAATGCAACAC	290
FMgSSR-37141	scaffold18877	p2	(TC)6	Class II	14069	14080	CCAGCAGCCCACCTGTCAAAAA	AGAAACGGCCCAGAAGAGCAGA	348
FMgSSR-37142	scaffold5252	p2	(TC)6	Class II	24022	24033	CCATACAAAGCGCCGCGAAACA	TGCCCAGCGTAGCCGTCATTTT	335
FMgSSR-37164	scaffold1202	p2	(TC)6	Class II	74052	74063	CGACGCAGGAAGCCAAGTAAGA	GGAGATGACGTTGTTGATGAGTGCC	302
FMgSSR-37165	scaffold30608	p2	(TC)6	Class II	5002	5013	CGACGCCCGAAAGCCATCAAAA	GCACGAAGTTTCAGTGGCCCTT	328
FMgSSR-37166	scaffold5898	p2	(TC)6	Class II	32415	32426	CGAGTGTCGTCCGACGTTCTTT	CGCAAGAAAACATGGCAACGGC	291
FMgSSR-37171	scaffold877	p2	(TC)6	Class II	35499	35510	CGCAGCGAAGCAGCCTACAAAA	ACAAGTGCCTGCTGTGTGGT	273
FMgSSR-37173	scaffold10254	p2	(TC)6	Class II	7971	7982	CGCATGTCATCAGCAACGAGCA	AGGTCAGGAGCTACTAGGCCATT	299
FMgSSR-37174	scaffold2671	p2	(TC)6	Class II	1467	1478	CGCCATGGAAGATTGGAACGCA	TGGAGCAGCCAATGCCAATCA	348
FMgSSR-37175	scaffold16080	p2	(TC)6	Class II	15433	15444	CGCCGTCTTCTTCCTTGCAACA	TGAATCCGTGGCCAAGCTGA	226
FMgSSR-37180	scaffold2271	p2	(TC)6	Class II	56336	56347	CGCTCTAGCTCGTTGATGTCACTG	ATTCCGGCGACAAAGGTGCCAT	348
FMgSSR-37181	scaffold6	p2	(TC)6	Class II	89772	89783	CGCTGGATAAACATATGCTGGGCT	GCCCGAACCGTGGTTTTGGAAT	350
FMgSSR-37187	scaffold3189	p2	(TC)6	Class II	15128	15139	CGGGGTTTGGCGCTCAATTT	ACGATATGCGTGGCCGGTTT	347
FMgSSR-37195	scaffold3775	p2	(TC)6	Class II	37848	37859	CTAGCAAGCATGTGGAACGAGT	CCGTGGTTCCAAAAACTGCAACAC	283
FMgSSR-37202	scaffold1609	p2	(TC)6	Class II	9793	9804	GCAAACCAAAGGTCAAAGGGGTGA	TGTCGATTTTGCTTGCGCTCG	235
FMgSSR-37206	scaffold30608	p2	(TC)6	Class II	4814	4825	GCAATTTCCGCTGCTGCCAT	TTTTGATGGCTTTCGGGCGTCG	274
FMgSSR-37210	scaffold183	p2	(TC)6	Class II	134225	134236	GCATCGCTGTAGCAGCCTATCT	TTGCCAGCCCAACGCTCTGAAA	349
FMgSSR-37211	scaffold10944	p2	(TC)6	Class II	10890	10901	GCATGGAATCAGTATTCGAGCACC	TTGTTACCGGCGGAGGTAGT	222

SSR_ID	Scaffold	SSR_Type	SSR_Motif	SSR Class	SSR_Start	SSR_End	Forward sequence	Reverse sequence	Product_size
FMgSSR-37213	scaffold685	p2	(TC)6	Class II	79441	79452	GCATTGGTCGGCCTTCCATTCA	ATGCGAAGCCACCGTGAACT	349
FMgSSR-37215	scaffold6866	p2	(TC)6	Class II	19401	19412	GCCCAACGCTGTCTTCGTTGTT	ATTGACGGACGCAGCGTGAT	298
FMgSSR-37218	scaffold5	p2	(TC)6	Class II	145149	145160	GCCCTTCGATGCACACAGCAAA	TCCCCACTGTGAAGTGTTAAGCCA	323
FMgSSR-37220	scaffold2105	p2	(TC)6	Class II	1766	1777	GCCTGGAAATGACGTGCTTGGA	GCCAGCGTGTGACAGGTTTTGT	236
FMgSSR-37222	scaffold14492	p2	(TC)6	Class II	15514	15525	GCGCAAAAACTTGCACGCCA	TTATTCGCCGGGAGCAGAGGAA	313
FMgSSR-37224	scaffold319950	p2	(TC)6	Class II	170	181	GCGCAGCTTGCTGGTCTTCAAA	GGGGCTCTCCATAGGGATTTGT	246
FMgSSR-37225	scaffold3029	p2	(TC)6	Class II	5524	5535	GCGCGCAGCTGCAATAATGA	TACAGCGCAGCAAGCAGTGT	321
FMgSSR-37226	scaffold1929	p2	(TC)6	Class II	67206	67217	GCGCGCGGTTGCTTTATTCA	ACCTTGAGGCTTGAACCACCCA	251
FMgSSR-37229	scaffold13915	p2	(TC)6	Class II	9963	9974	GCGGGATTTGCATTGGTGATGCT	TGCTACATGCATGCCAACGTC	342
FMgSSR-37234	scaffold85148	p2	(TC)6	Class II	1142	1153	GCGTAAGGTGCACCACGAACAA	CCATGCCGATGGCTGCAGATTT	276
FMgSSR-37239	scaffold2326	p2	(TC)6	Class II	38034	38045	GCTGCAGTTGGTCATCTCCCTT	TGCATGGTGCCAGTGCCCTATT	297
FMgSSR-37242	scaffold8	p2	(TC)6	Class II	173956	173967	GCTGCTGCTACCTGTTGCATCTT	ACGCTTGCAGCAACGAGAGGTT	321
FMgSSR-37245	scaffold1471	p2	(TC)6	Class II	2941	2952	GCTTTTTCGGCGAGCAATGCAA	GCACTGACGCCGCCATTGTTTT	324
FMgSSR-37248	scaffold1090	p2	(TC)6	Class II	43702	43713	GGACCGGGGCTGAGAATGTTTT	TCCTTCTGTGCTCAAGTGCGT	291
FMgSSR-37249	scaffold3158	p2	(TC)6	Class II	34860	34871	GGACGAAAGCGCGTCGACAAAT	TGCCGCATCGCAAACAAACG	294
FMgSSR-37250	scaffold221	p2	(TC)6	Class II	114411	114422	GGAGCACCACGGTGGCAATTTT	ACAGCTTGAGCGGCACCAAA	275
FMgSSR-37255	scaffold3601	p2	(TC)6	Class II	31629	31640	GGCACATGGATGCAGAAACTGCT	TGCAGCTTGCCCTAGCCAAA	247
FMgSSR-37257	scaffold12370	p2	(TC)6	Class II	12302	12313	GGCCGCATGGCGATTTTCAA	TGCAAGGCTAAGCAGCGTGA	340
FMgSSR-37258	scaffold10858	p2	(TC)6	Class II	6424	6435	GGCGCCCATTCTTTTTCACGCA	ATCGCTGCACGACGCCAAAA	318
FMgSSR-37260	scaffold1148	p2	(TC)6	Class II	38877	38888	GGCGTGCAGTGTGTTGGCAAAT	TTGGTCCAAACCGTGACCCACA	266
FMgSSR-37263	scaffold401	p2	(TC)6	Class II	125665	125676	GGGCAGAATAAGATGGGTGGACGA	CCAAAGAGTGTGTACTCCAAAGGC	281
FMgSSR-37266	scaffold14121	p2	(TC)6	Class II	11444	11455	GGGTGCATCTTACTTGGAGCAAGC	CGGCTCCAACCCATATGTAGCTCT	349
FMgSSR-37276	scaffold13817	p2	(TC)6	Class II	10953	10964	GTCACACATGCAACAGTGCCGT	ACATACGCGCGCTGCCTATACA	312
FMgSSR-37277	scaffold7515	p2	(TC)6	Class II	32243	32254	GTGGTCGCGCTCTTATTGACGA	TGCTGTGGTCAACTCCTGCGAT	308
FMgSSR-37285	scaffold26	p2	(TC)6	Class II	20090	20101	TAACGCGAAGGTCGCAGGTT	TGGCCCATTTGCTTTGGGCT	230
FMgSSR-37286	scaffold2430	p2	(TC)6	Class II	66710	66721	TAACGGCATTGTTGCTGCCG	TGACCACCAGCCACCACTTCTT	273
FMgSSR-37290	scaffold16967	p2	(TC)6	Class II	3231	3242	TAATCAATAGCGCCGCCGTTCG	GCCAGCGGGCCTTGTTCAATTT	334
FMgSSR-37296	scaffold2109	p2	(TC)6	Class II	36618	36629	TACATGCACGTACTGCGCCA	ATTGCCGGTATGCCGCCGTTAT	293

SSR_ID	Scaffold	SSR_Type	SSR_Motif	SSR Class	SSR_Start	SSR_End	Forward sequence	Reverse sequence	Product_size
FMgSSR-37298	scaffold186132	p2	(TC)6	Class II	87	98	TACCCCTCGTCTTCAACCTTGAGC	AGTTGAGCATCACCACCGTTGC	287
FMgSSR-37302	scaffold5496	p2	(TC)6	Class II	13869	13880	TACGCTCCCAAGGGCAAGGAAA	TCGAGAGGTGGGGTGGTCTTTT	203
FMgSSR-37303	scaffold754	p2	(TC)6	Class II	45953	45964	TACGGCGGCGTTGCCTTTAT	TTGCACCGAACTCGAACCGTCA	313
FMgSSR-37312	scaffold5656	p2	(TC)6	Class II	23152	23163	TAGGAGGCGTCTTCAATGGCGT	CGCACCCTTAATCTTTGGTGTCAG	252
FMgSSR-37317	scaffold7413	p2	(TC)6	Class II	14929	14940	TATTGCACCCCGGCCTCAAA	TCCGCCTCCCCCAAACCAATTA	235
FMgSSR-37319	scaffold2056	p2	(TC)6	Class II	12569	12580	TATTGTGGCCCGTGACATGCGA	ACTTTGCCCTCGCTACCGTT	348
FMgSSR-37326	scaffold9051	p2	(TC)6	Class II	380	391	TCAAATGTGGCTGGCTGGTGGA	TGCGCGTACAACATGGAGCA	299
FMgSSR-37336	scaffold33283	p2	(TC)6	Class II	5388	5399	TCAATGCAGTGAGCGGAGGT	AGATTCATGGGACGCAAGGCCA	259
FMgSSR-37339	scaffold16920	p2	(TC)6	Class II	1662	1673	TCACACACGCCATCGCTCATCA	AAGCTAAGGTGATGGTGGGGCA	274
FMgSSR-37340	scaffold6820	p2	(TC)6	Class II	8548	8559	TCACAGTCGCCGATGCTAGT	TGCATTTGGATGCACTGACGAGGA	301
FMgSSR-37342	scaffold16907	p2	(TC)6	Class II	16056	16067	TCACCTCCTCCTTGCTCCCTCTTT	GCGCCGCGAAACCCTATCAAAT	211
FMgSSR-37346	scaffold2089	p2	(TC)6	Class II	42193	42204	TCACGTGCTTCGCTATGCCA	TGAAGGGTCAGGGCCGATTAGA	316
FMgSSR-37350	scaffold228	p2	(TC)6	Class II	172315	172326	TCAGACGACGACACTGACACCA	TCGGACGCAACAAACACGCA	338
FMgSSR-37354	scaffold2809	p2	(TC)6	Class II	10870	10881	TCAGCGCGGCGAGTTGATAA	TCGCCAGCCAGTTGGCCTAAAA	325
FMgSSR-37356	scaffold2054	p2	(TC)6	Class II	44930	44941	TCAGCGTCGAGATGCGTTGTCA	TGGTGATGCAGGCAAAAGGCA	201
FMgSSR-37359	scaffold455139	p2	(TC)6	Class II	75	86	TCAGGCAAGTGGACACTGGAA	TTTGCTCGGTCGTCAGAGATGC	203
FMgSSR-37363	scaffold914	p2	(TC)6	Class II	103557	103568	TCATACACGACGCACACGCA	ACAGAGATCACTTCGACCGGCA	281
FMgSSR-37368	scaffold1865	p2	(TC)6	Class II	71680	71691	TCATTTGCGCCGCATTCGTCT	AAAAGGAATCGTCGATGGCGGG	200
FMgSSR-37374	scaffold1628	p2	(TC)6	Class II	76962	76973	TCCACCACCAACTCGTCTCTGT	TTTGCTCACCTAGTCACCCGCA	349
FMgSSR-37380	scaffold1269	p2	(TC)6	Class II	11454	11465	TCCCAAGATGCAGAGCAAGTGGA	ATCCGCTGAATTCCGGCACA	342
FMgSSR-37387	scaffold46	p2	(TC)6	Class II	12391	12402	TCCCCGACCTTCTCAAGCTTCT	TCGATCCCAAGCCAATCGAGA	316
FMgSSR-37388	scaffold7580	p2	(TC)6	Class II	6853	6864	TCCCCGTACACGCTTCTTGACT	GCGTGCAGTGGAGGCTATATCGTA	346
FMgSSR-37401	scaffold12197	p2	(TC)6	Class II	10433	10444	TCCGTTGTTCGTCCATCCCTGT	TACCACTTGGCCATGCCGTCAA	267
FMgSSR-37402	scaffold4584	p2	(TC)6	Class II	18865	18876	TCCTCAGCCATTCCTAGTCATCCA	TGTGGCCTTGAACGAGCAAACT	261
FMgSSR-37405	scaffold1386	p2	(TC)6	Class II	79909	79920	TCCTTCCTCTCCCTCCCTTTCT	ACTATCGGATGACACGCCCA	342
FMgSSR-37418	scaffold404	p2	(TC)6	Class II	32108	32119	TCGATAACCCGCCGTGCAAA	ACCGATAGCGCGGACCTTTCAT	329
FMgSSR-37423	scaffold10958	p2	(TC)6	Class II	19819	19830	TCGATGGCCAGCTGCTACTT	TAAGCACGCACCATCCGGAACT	335
FMgSSR-37430	scaffold334	p2	(TC)6	Class II	128621	128632	TCGCATCCGCATCGTCTCTT	TTACCCGGGTGTTCGTTGCCAT	202

SSR_ID	Scaffold	SSR_Type	SSR_Motif	SSR Class	SSR_Start	SSR_End	Forward sequence	Reverse sequence	Product_size
FMgSSR-37431	scaffold147	p2	(TC)6	Class II	26040	26051	TCGCATGTTTGACGCTGCCT	AAGCTCGATGCAAGCTGGGGAA	217
FMgSSR-37437	scaffold4025	p2	(TC)6	Class II	37418	37429	TCGCGACTGACGTTGTGCAT	AACAACGAAAGGTCTCGGCACG	288
FMgSSR-37442	scaffold2841	p2	(TC)6	Class II	34650	34661	TCGCTGCCGTGAGTGTTTGT	TGTGGACGCATTGAGCGTAGCA	243
FMgSSR-37443	scaffold6029	p2	(TC)6	Class II	15125	15136	TCGCTGCTAGCCGCTTTGTT	ATCGCGATTTCCACTGCGCT	245
FMgSSR-37444	scaffold773	p2	(TC)6	Class II	53473	53484	TCGGCAACAGCGACAGCATT	AGCATGAGCGACGACCGATT	314
FMgSSR-37445	scaffold19844	p2	(TC)6	Class II	4603	4614	TCGGCCAGAAGGTCGTCATCAA	TGATCATGCTCGACTTGCGGCT	300
FMgSSR-37447	scaffold10728	p2	(TC)6	Class II	13970	13981	TCGGCCTACATTTCTGCTTTGC	ACAAGCGCAGCAATCGGCTT	316
FMgSSR-37448	scaffold4875	p2	(TC)6	Class II	14799	14810	TCGGCGTCTTTCTGGTAGCTGT	ATGGCCACCGAATTGCCGAA	236
FMgSSR-37452	scaffold2032	p2	(TC)6	Class II	66008	66019	TCGGTGGCAAGCACCAGAAA	ACGTCATGCGCAGCATCAGTTG	338
FMgSSR-37454	scaffold7236	p2	(TC)6	Class II	6400	6411	TCGTAGTCGTCCAAGATCCGCT	TTTCACCCACTCCATCTCTCCCCT	328
FMgSSR-37460	scaffold31577	p2	(TC)6	Class II	1952	1963	TCGTGCCATGTGTGGCCAAT	GGAAGTCAAAAAGGCAGCCGCA	225
FMgSSR-37461	scaffold6443	p2	(TC)6	Class II	40863	40874	TCGTGGAAGCCCCAGTTTGCTT	AGTGTGACATATTGCTCCCGCA	321
FMgSSR-37463	scaffold4204	p2	(TC)6	Class II	48032	48043	TCGTTGCGAACCCAACCCAA	TGCTAACGCACGCGCCTTTT	308
FMgSSR-37467	scaffold1287	p2	(TC)6	Class II	39694	39705	TCTATTGCGGATCACACGCCA	AGGCCCAACTGAAGCAAAGCA	331
FMgSSR-37469	scaffold3299	p2	(TC)6	Class II	54110	54121	TCTCACATGCCATACACGCAGG	ACTCGCTCGCCACTTGGGAAAA	289
FMgSSR-37470	scaffold4053	p2	(TC)6	Class II	44331	44342	TCTCAGCATCTCTCGGCACCAT	TGCATGCATGGACACGGCAA	272
FMgSSR-37476	scaffold28272	p2	(TC)6	Class II	2135	2146	TCTGCCAAAGCTTGGCCTCT	TTACGCCGCGTCAACTCCTTGT	345
FMgSSR-37478	scaffold6827	p2	(TC)6	Class II	16285	16296	TCTGGAGCGAGCATTCAGCA	TACACTAGCATGCCGTCGGTCT	316
FMgSSR-37480	scaffold1715	p2	(TC)6	Class II	21693	21704	TCTGTCGGCAACGCTTTCCA	ACGGATTTGTAGCGGCCGAA	269
FMgSSR-37483	scaffold19039	p2	(TC)6	Class II	7876	7887	TGAAAAATGGGTCCACACCGCT	TCTCAAGATCACGCCAATGCCC	235
FMgSSR-37484	scaffold10504	p2	(TC)6	Class II	23763	23774	TGAAACATGGCCCTGTCGGT	AACAACGGAGCGAGCTAAGCGA	226
FMgSSR-37485	scaffold129	p2	(TC)6	Class II	119350	119361	TGAAAGCGCGATCGGTGGAA	TTGTCGCCAACTTGTGTGCTGC	326
FMgSSR-37489	scaffold2830	p2	(TC)6	Class II	49077	49088	TGAACTCGAGAGCTCCACAGCA	ACGGGCGAATGATGGCTCGAAA	349
FMgSSR-37493	scaffold140	p2	(TC)6	Class II	176809	176820	TGAAGCAAGCAAGCGCGGAT	AGGTTCCAGCCACAGTCCAA	346
FMgSSR-37497	scaffold10590	p2	(TC)6	Class II	15701	15712	TGAATCACGCCCAGCTTCCT	CCCGTCGCCTGCGCATTTTATT	298
FMgSSR-37499	scaffold444	p2	(TC)6	Class II	63760	63771	TGACACGTGGCCCCATCAAACA	TAAGAAACGTGAGGCAGGCGGA	303
FMgSSR-37507	scaffold1797	p2	(TC)6	Class II	7809	7820	TGAGGAAGCAAGCGACTCACA	GCCGCGTGAACCAACCGATTTA	331
FMgSSR-37508	scaffold2591	p2	(TC)6	Class II	17549	17560	TGAGGCAGTCCGTCTAACAGCA	AGTCTTCCCACCACCCCAAGAT	346

SSR_ID	Scaffold	SSR_Type	SSR_Motif	SSR Class	SSR_Start	SSR_End	Forward sequence	Reverse sequence	Product_size
FMgSSR-37511	scaffold381	p2	(TC)6	Class II	61594	61605	TGAGTGCGTGGGTACGGTTTGT	CGCCATGACAACAGCATCGACA	316
FMgSSR-37512	scaffold139	p2	(TC)6	Class II	171727	171738	TGATCGGCCCACATGCGTAGAA	TCGTGCACACGCACTTTCCT	262
FMgSSR-37517	scaffold2097	p2	(TC)6	Class II	3595	3606	TGCAAAAGCGTCTGGGAGGT	TGCAAGTAGGAGCAGCGCTATGT	342
FMgSSR-37521	scaffold6829	p2	(TC)6	Class II	37267	37278	TGCAAGTACCACCAACAAGGCGA	TGCACCATCTGAACCTGGCT	216
FMgSSR-37523	scaffold1593	p2	(TC)6	Class II	45133	45144	TGCAATGGCGGTTGTGAGCA	GCTCCATGACCACATAGATGACTG	269
FMgSSR-37524	scaffold100	p2	(TC)6	Class II	123792	123803	TGCACCATCAGCACCTGCAT	ACGACTCAACGTTCTCTGCGCT	323
FMgSSR-37528	scaffold4153	p2	(TC)6	Class II	36030	36041	TGCACGCCACCACTTGGTAGTT	TGCTCGCTCAACGGTTTGAGGT	281
FMgSSR-37529	scaffold466	p2	(TC)6	Class II	25345	25356	TGCACGGCGGGTTCAAAGAT	AAGCGTGGGGTGGCAAACAA	308
FMgSSR-37533	scaffold7878	p2	(TC)6	Class II	1312	1323	TGCAGCATGCACAATCCACACC	ATGCCGTCCAGTAGAGGGAGAGAT	244
FMgSSR-37534	scaffold244	p2	(TC)6	Class II	61675	61686	TGCAGCGGCGTGAATTTTGGT	TCGAGCACCTTGCTTTGCCA	303
FMgSSR-37535	scaffold1957	p2	(TC)6	Class II	40277	40288	TGCAGGAAACGGCATCGTCA	TCGACTCTTGTGCCATCGAGCA	329
FMgSSR-37538	scaffold10408	p2	(TC)6	Class II	19077	19088	TGCAGGGGTCACATGCAGTTCT	TTCACGCATGCCTGCCCTTT	238
FMgSSR-37543	scaffold736	p2	(TC)6	Class II	31234	31245	TGCATCTGCGCATTGCACACT	TGTTGCGCAGGAGGACAAGTCA	228
FMgSSR-37545	scaffold1559	p2	(TC)6	Class II	81334	81345	TGCATGCTGGTGCGTGGTTT	TGTATGCGTGCGTGCAAGTGT	277
FMgSSR-37550	scaffold146	p2	(TC)6	Class II	119307	119318	TGCCAAAGCAGCACATGGCCTA	AGTCAAGCCGACACGATCCACA	305
FMgSSR-37551	scaffold10473	p2	(TC)6	Class II	7847	7858	TGCCAAAGCATTGGGAGTTGCT	CCCGCTTAAACCGTAGCCCATT	350
FMgSSR-37567	scaffold5135	p2	(TC)6	Class II	20639	20650	TGCCGCACACGCTTCAGTAA	AGGAGGGAGGCGATGACCATTT	330
FMgSSR-37569	scaffold1428	p2	(TC)6	Class II	4801	4812	TGCCGCTTTCACGTCGTCAT	TCGATCGTGCGCTTGACTCGTT	294
FMgSSR-37577	scaffold26667	p2	(TC)6	Class II	3443	3454	TGCGATCGACAGAGCAGGTT	TGCTTGCTGCGCTCGTTACT	338
FMgSSR-37578	scaffold376	p2	(TC)6	Class II	122916	122927	TGCGCAGTCTCCACCAACAA	AACAAAGAGCGAAGCGCACACG	300
FMgSSR-37579	scaffold81	p2	(TC)6	Class II	95180	95191	TGCGCATGCATGTCACACAGT	TGGGCAGGATATGTCTTCGTCTCA	261
FMgSSR-37580	scaffold5135	p2	(TC)6	Class II	2609	2620	TGCGCCGCCGTTCTTATCTT	TCGATGCATGTCGGAACGGA	325
FMgSSR-37581	scaffold487	p2	(TC)6	Class II	50232	50243	TGCGCCGTACCGTTAGAGTT	TAGAGGTTGCATCGTGCCGT	272
FMgSSR-37583	scaffold2460	p2	(TC)6	Class II	59605	59616	TGCGCTAACGCAACCGGTAA	AATGTCGTGTGGGGTGACCA	334
FMgSSR-37588	scaffold2388	p2	(TC)6	Class II	33366	33377	TGCGGCTTCTTGTTTCGAGGCT	TGCAGACCGCCCGGACATAAAA	244
FMgSSR-37589	scaffold11304	p2	(TC)6	Class II	23312	23323	TGCGGTGTGACTGTGTGAGGTT	AGCCCAGCCCACTCCATCATTT	324
FMgSSR-37591	scaffold12150	p2	(TC)6	Class II	13464	13475	TGCGGTTGAACGTTGGAGTGCT	TGGCACGCAAGCTTTTCGTCA	330
FMgSSR-37592	scaffold75	p2	(TC)6	Class II	164600	164611	TGCGGTTGCGAGTGATGTTG	ACAGCTCCGAGCACCTACACAT	317

SSR_ID	Scaffold	SSR_Type	SSR_Motif	SSR Class	SSR_Start	SSR_End	Forward sequence	Reverse sequence	Product_size
FMgSSR-37598	scaffold6891	p2	(TC)6	Class II	10539	10550	TGCGTGTAACCATCGTGCTCTGT	AGCCTGCCAGCTGAACTCAA	317
FMgSSR-37600	scaffold8352	p2	(TC)6	Class II	12311	12322	TGCGTTCCTTGCTCGTCTGACT	ACCGACCGAAAAAGCAGCCGTA	338
FMgSSR-37604	scaffold3636	p2	(TC)6	Class II	38682	38693	TGCTACGAGTGGCATGTGGA	AGCAAGCGCGCAAAGAATGG	239
FMgSSR-37610	scaffold73725	p2	(TC)6	Class II	127	138	TGCTCGTGTGTACCACTCTGCT	AGCGCTGTTGCAGCTCTCTCAT	236
FMgSSR-37617	scaffold87	p2	(TC)6	Class II	121280	121291	TGCTGCCTGCTACACGTTGGTT	AGCTTGCGCGCCTTTTCTCT	293
FMgSSR-37619	scaffold336	p2	(TC)6	Class II	11329	11340	TGCTGCTGCCTGCATTCCTT	AAAGCGGCGAGAGGACGAAA	321
FMgSSR-37620	scaffold18	p2	(TC)6	Class II	193511	193522	TGCTGCTTGTGGCTGGTTCA	ACAACGGTAAGCCCGCATGCTA	234
FMgSSR-37621	scaffold18130	p2	(TC)6	Class II	9036	9047	TGCTGGAGGAGGGATTTGAGGT	TGCCGACCATGTCAGGAGTGAA	289
FMgSSR-37628	scaffold210763	p2	(TC)6	Class II	423	434	TGCTTGCCTTGGGCAGCTCTAT	TTGGGTGCCTTGTGTGCCTT	298
FMgSSR-37630	scaffold1139	p2	(TC)6	Class II	18854	18865	TGCTTTGCCACGGATCCCAA	AGGGTCAAGATGGCTGAGTTGA	336
FMgSSR-37631	scaffold9843	p2	(TC)6	Class II	13597	13608	TGGAAATGGTTAGGTGCGGCAG	TGGCTGGTGCATGCTGCAAT	347
FMgSSR-37633	scaffold21036	p2	(TC)6	Class II	2981	2992	TGGAACGGCACTGCATCACA	ACCAAAGAACCCATGGCCACCA	319
FMgSSR-37649	scaffold9281	p2	(TC)6	Class II	5829	5840	TGGCATAACATGAACGGCTCGC	TGCCAAAGCACCAGACACACA	348
FMgSSR-37650	scaffold25076	p2	(TC)6	Class II	4406	4417	TGGCATAGCGCCGTTGCATT	GCACCGAATGCGCTGAGAAGAA	205
FMgSSR-37655	scaffold11997	p2	(TC)6	Class II	3007	3018	TGGCCCAGCTAAATGCACCT	TCATGCACCGCAGATGAGGA	341
FMgSSR-37658	scaffold26184	p2	(TC)6	Class II	6651	6662	TGGCGGGACCTTGACGAAAACT	AGCCTCCTTTGTAGCTGCCAGA	303
FMgSSR-37660	scaffold4053	p2	(TC)6	Class II	48755	48766	TGGCGTTTCATGGCCCCTTT	AAACGCGGCCACAATGGCTT	342
FMgSSR-37671	scaffold2438	p2	(TC)6	Class II	43560	43571	TGGGCCATTTTGCCGTTGATCG	TCTCTGTGCTTCTTGGTCGGGT	223
FMgSSR-37673	scaffold2607	p2	(TC)6	Class II	36537	36548	TGGGCCTTTTCATTTGGGCTGAC	AGCAATGCTTCCGATGGCGT	318
FMgSSR-37675	scaffold4029	p2	(TC)6	Class II	51566	51577	TGGGCTCGTTCGTGCTCACATT	AGCGCGGAAGGAAACGGAGATT	242
FMgSSR-37678	scaffold31205	p2	(TC)6	Class II	3673	3684	TGGGCTTGCGAATGGACTGTCA	TGGGGAAAAGGAGGGACCAACA	323
FMgSSR-37680	scaffold10465	p2	(TC)6	Class II	17733	17744	TGGGGCAATCAATCCCGAATCGT	TTCATTGTGCTGCGCCAGAAGC	314
FMgSSR-37692	scaffold24117	p2	(TC)6	Class II	2056	2067	TGGTTTGAGCAGCAACGGCT	AGCTCAGCAAGGTGCAGACA	334
FMgSSR-37696	scaffold660	p2	(TC)6	Class II	45096	45107	TGTCAATGGCTGCTCCCTCA	TCGACCGTGGACGTGATTAGCA	342
FMgSSR-37705	scaffold463	p2	(TC)6	Class II	70130	70141	TGTCCTGGCATTCGCAGCTT	CACCGTTTCACTTTCGTCATCGC	294
FMgSSR-37711	scaffold4445	p2	(TC)6	Class II	49399	49410	TGTCGCCAATCCGCAACAAGA	AAGAACCTGACGCCATCGACCT	337
FMgSSR-37717	scaffold971	p2	(TC)6	Class II	46856	46867	TGTGCAAAGTTCCGGGCTTCTGA	TGCTGTTGGCGCTGCAGAAT	341
FMgSSR-37718	scaffold3790	p2	(TC)6	Class II	32121	32132	TGTGCATGCGTGTGCGTAGT	AGCAGCAGCAAGCAAGACCA	345

SSR_ID	Scaffold	SSR_Type	SSR_Motif	SSR Class	SSR_Start	SSR_End	Forward sequence	Reverse sequence	Product_size
FMgSSR-37723	scaffold8747	p2	(TC)6	Class II	25412	25423	TGTGCCTTGCTTCATGCGCT	TGGGGCACACATGTCCCTTGTT	252
FMgSSR-37724	scaffold3338	p2	(TC)6	Class II	36831	36842	TGTGCGCCCAACAAGGCAAT	TGGACGTTGTTGTTGGGTCCGT	297
FMgSSR-37730	scaffold6975	p2	(TC)6	Class II	19291	19302	TGTGGAGCGCGCGACAATAA	AGTTTTCAGGGCGCCACAGT	264
FMgSSR-37733	scaffold1951	p2	(TC)6	Class II	59385	59396	TGTGGCCAACGTAGCAGTGT	AGTTTGGCGGCCTTTGCTGA	255
FMgSSR-37735	scaffold4348	p2	(TC)6	Class II	23593	23604	TGTGGGAAGCTGCCACCATAAGT	TTCCTGGCACATCCCTGCAAGA	226
FMgSSR-37743	scaffold1591	p2	(TC)6	Class II	81841	81852	TGTGTGCGTCCCCATTGTTACTGT	TGGTACATGCCACCACACCCTT	294
FMgSSR-37749	scaffold4396	p2	(TC)6	Class II	45024	45035	TGTTAACCAGGCGGTGATGCT	AGCAAATTGGGCTCCCGCAT	311
FMgSSR-37760	scaffold3355	p2	(TC)6	Class II	9261	9272	TTAACTAACTTCTCCCCGTGCCGC	ATTTCGCGCCGCCGTCTTTT	311
FMgSSR-37761	scaffold567	p2	(TC)6	Class II	2222	2233	TTAAGCCGAGAGTCCGAGGTCA	TGTGCGGGCTGTTGTGTTCT	338
FMgSSR-37764	scaffold7649	p2	(TC)6	Class II	27340	27351	TTACAACGTTGGCGTGGCGT	TGTGGCAAGGCAAGGTGCAGTA	245
FMgSSR-37774	scaffold5045	p2	(TC)6	Class II	11836	11847	TTCAATGTCCGCAAGGCGCT	TGTGCAACCTCGCTAACTGC	336
FMgSSR-37776	scaffold7879	p2	(TC)6	Class II	12055	12066	TTCACATGGCAAACGGGGACCA	TGCAAACGTGTCGCACAGACAG	298
FMgSSR-37777	scaffold1558	p2	(TC)6	Class II	54826	54837	TTCACCGTGCTGACGGTTTCGT	ACGCCAGTGCGAGATTGTTTGC	332
FMgSSR-37778	scaffold3672	p2	(TC)6	Class II	52190	52201	TTCACGCCCTCGCAGCATTT	TCTAGTTCTGCAAGCACCGTGG	213
FMgSSR-37784	scaffold1601	p2	(TC)6	Class II	44855	44866	TTCATGCAGCCGCTTACCCT	ACACCACCTGCCATTGCCAGAA	330
FMgSSR-37788	scaffold3499	p2	(TC)6	Class II	22054	22065	TTCCATCCATCCCAAGCAGCGA	TACAGAACACACTGGGCAGGCA	268
FMgSSR-37794	scaffold181	p2	(TC)6	Class II	109296	109307	TTCCTCTCTCAACGCTGCTGCT	GCGGCCGTCTTTTAACGACCAT	331
FMgSSR-37801	scaffold10376	p2	(TC)6	Class II	14629	14640	TTCGATTGGGCAGGCTGTTGGA	AATTTCACCCCGTCACGGCT	288
FMgSSR-37805	scaffold5292	p2	(TC)6	Class II	15071	15082	TTCGGCTTCCACGGAACCTTCA	AGTGTGAACGGAGCAAGTGAGC	285
FMgSSR-37810	scaffold3779	p2	(TC)6	Class II	29310	29321	TTCGTTGTTAGGGCGAGACGCT	TGCCGGACGGAAGTCTCATCAA	241
FMgSSR-37813	scaffold2815	p2	(TC)6	Class II	61871	61882	TTCTCCCTCTAGATCCAGGCGT	TTCGATCCTTCAACCGGGCT	328
FMgSSR-37817	scaffold2937	p2	(TC)6	Class II	55661	55672	TTCTGGCCTCCCCTTACCAATGGA	AACGCGGCTAAAAGCTAGGCCA	258
FMgSSR-37819	scaffold4843	p2	(TC)6	Class II	12801	12812	TTCTTCCCCACCCAACCATTCC	TTGACGGCACTACACGCAGA	243
FMgSSR-37826	scaffold1356	p2	(TC)6	Class II	36585	36596	TTGATGCCGTACGGGTCCAACT	ATTGGGACATCGGGCATCGCTT	219
FMgSSR-37829	scaffold2623	p2	(TC)6	Class II	47858	47869	TTGCCACATCATCGATCCATCCCC	AGCCAGAACAGCACTGCGACAT	214
FMgSSR-37835	scaffold140	p2	(TC)6	Class II	3766	3777	TTGCTAGGCGCGTCCTTTTTGG	TCGCCGTTGCGAATTTGGGA	266
FMgSSR-37838	scaffold9191	p2	(TC)6	Class II	9157	9168	TTGCTGCATGGAGTGGAGGCTT	TGCTTGCCCCCATGTCAATGGT	290
FMgSSR-37847	scaffold115	p2	(TC)6	Class II	35228	35239	TTGGCTGGCTCCTCCAATCA	GAGCAACTTTAGTCTCGAGGATCG	246

SSR_ID	Scaffold	SSR_Type	SSR_Motif	SSR Class	SSR_Start	SSR_End	Forward sequence	Reverse sequence	Product_size
FMgSSR-37852	scaffold1145	p2	(TC)6	Class II	77937	77948	TTGGGTTTCAGATAAGCGCGGG	TTCACACTCTCACCGAGCCA	208
FMgSSR-37862	scaffold1549	p2	(TC)6	Class II	45045	45056	TTGTGTGTGCTGGCTGCGTT	AGCCCAGATGCAACTTGTGGTCA	278
FMgSSR-37866	scaffold21	p2	(TC)6	Class II	56906	56917	TTTAACAGCTGATGCCCGCTGC	AGGTCCAACAGTGAGGTGGACA	313
FMgSSR-37873	scaffold28307	p2	(TC)6	Class II	1395	1406	TTTCCGTTCGGGTCATGCGT	ATCAGCTTTGGCACTCGGTCGT	318
FMgSSR-37876	scaffold7558	p2	(TC)6	Class II	2112	2123	TTTCGCCGACAATGAACGTGCC	ATTAGCGGGCGCTTGTGGTGAT	325
FMgSSR-37883	scaffold413	p2	(TC)6	Class II	78580	78591	TTTGACAACTCCGCGTCGTCCT	TTAGTCAGCCACACGCCAGGAA	316
FMgSSR-37887	scaffold5058	p2	(TC)6	Class II	10844	10855	TTTGGCACTTGCCCTGCTCT	ACCCAGGCCGCGATTCGTTAAT	349
FMgSSR-37898	scaffold437	p2	(TC)6	Class II	76432	76443	TTTTCCTGTGTGTTTCGCCCGC	GCGTCAAGTGAATTGCAGGGCA	314
FMgSSR-37904	scaffold5064	p2	(TC)6	Class II	40525	40536	TTTTTGGGTCCGGCTGGGTA	TGGTATGCGCGTACTGTGAGCA	213
FMgSSR-37909	scaffold30535	p2	(TC)7	Class II	2901	2914	AAAACGGTAGCGCCGCCATA	ACTAACTGATCCTGGCTGCCGT	272
FMgSSR-37915	scaffold5128	p2	(TC)7	Class II	27795	27808	AAACGCAGCAGGTGTGGTGA	ACATGGCGGATCACATGCGT	340
FMgSSR-37917	scaffold1785	p2	(TC)7	Class II	41904	41917	AAACGGACAATGCCGGTGCT	GCCATGCACATGCGCACACATA	265
FMgSSR-37927	scaffold2793	p2	(TC)7	Class II	12350	12363	AAATCCCCACCCGTTAGCCT	AACTGGCATGGTGGGGAAGA	292
FMgSSR-37928	scaffold3346	p2	(TC)7	Class II	30512	30525	AAATCTCTCTCAGTCGGCGGCA	AGGACATGCAACAAGGCGCT	337
FMgSSR-37931	scaffold1440	p2	(TC)7	Class II	61760	61773	AACACCAGCGTCTCGTTCCAGT	ATAAATCGACGGTGGCGGTGCT	299
FMgSSR-37936	scaffold2038	p2	(TC)7	Class II	26682	26695	AACCGCTGACTTGGTCGTGA	AGCTGCTTTCCTACGACGCT	328
FMgSSR-37937	scaffold104	p2	(TC)7	Class II	79331	79344	AACCGTAGCACTTGCACCACCT	TGGCAACCGCGTAAGCTTCT	342
FMgSSR-37939	scaffold8919	p2	(TC)7	Class II	2808	2821	AACCTGCGATGCGCTCTTCA	ATTGGACCACCCAGCAGAGCAT	208
FMgSSR-37940	scaffold5878	p2	(TC)7	Class II	2596	2609	AACGAACAACGAGGCGCCAA	ACCGTGATTGATGAGTGGCCGA	297
FMgSSR-37942	scaffold2218	p2	(TC)7	Class II	55582	55595	AACGATGTGCGCGTCAAAAGGG	TCGATTGGATGCTTGGGCTGGA	329
FMgSSR-37947	scaffold1182	p2	(TC)7	Class II	79622	79635	AACTCCCAAAACACCGGCGACT	TCGCCTTGTTTCTCCGCCTTGT	207
FMgSSR-37952	scaffold65	p2	(TC)7	Class II	54481	54494	AAGAAACCGAGCCTCGCACT	CGGAAAACAAGTGGAAGCGGCA	288
FMgSSR-37956	scaffold2057	p2	(TC)7	Class II	33565	33578	AAGCAAACGCTCCGCCATGA	TTTCACACGACGCACTCAGGCA	336
FMgSSR-37963	scaffold1449	p2	(TC)7	Class II	47242	47255	AAGCTACCGGCTGAAAGCCGAA	AAGCGATGCTGACTCACTGGCA	309
FMgSSR-37967	scaffold19650	p2	(TC)7	Class II	4092	4105	AAGCTGTCATGCACGAGCGA	TTATTCACCTCCCCTGTGCGCT	334
FMgSSR-37976	scaffold3785	p2	(TC)7	Class II	42596	42609	AATACCCAAGCGCTATCCTTGCCC	TGTCCATGTGTCAACGTAGCTTGG	320
FMgSSR-37977	scaffold141	p2	(TC)7	Class II	12389	12402	AATCACTGCCACCCTTCGAGGA	ATCAGCCGGCCGTATGTTGT	269
FMgSSR-37979	scaffold1759	p2	(TC)7	Class II	73885	73898	AATCGGAATGGCCGCGAGTT	TTCCCTCAATCCCTCTCCTTCCCA	348

SSR_ID	Scaffold	SSR_Type	SSR_Motif	SSR Class	SSR_Start	SSR_End	Forward sequence	Reverse sequence	Product_size
FMgSSR-37982	scaffold2722	p2	(TC)7	Class II	15857	15870	AATGGCGCCCGCGGAATTTA	TCGTTGGGGAGGAGCAGCAAAA	314
FMgSSR-37983	scaffold13212	p2	(TC)7	Class II	11292	11305	AATGGCGTGTACGGACACAGGT	AGGGGCATGTCGATGACACA	251
FMgSSR-37987	scaffold4145	p2	(TC)7	Class II	20153	20166	ACAAAGTCCAGACAGCGTGCGA	ATTCGATCGATTCCTTCGGAGGCG	341
FMgSSR-37995	scaffold1916	p2	(TC)7	Class II	50201	50214	ACAATTCCTTGCCGCCGGTT	ACACAGCCACGTGCAGACGATA	339
FMgSSR-37999	scaffold99862	p2	(TC)7	Class II	660	673	ACACATGAAGTCGGCCAGCA	TGCCCCAAAGAACACCCCAT	285
FMgSSR-38002	scaffold1651	p2	(TC)7	Class II	37404	37417	ACACCTGCTGCTAATGTGCTGA	ACCATGTCCCAATGACGGCGAA	287
FMgSSR-38005	scaffold682	p2	(TC)7	Class II	114302	114315	ACACGGAGCACTCGTACCCTTT	TCTCTGTGGTCAACTCCTGCGA	341
FMgSSR-38012	scaffold6117	p2	(TC)7	Class II	3520	3533	ACATCAACACGTGCAACGGC	TGCCTGGGTTGATTCTTGCGGT	278
FMgSSR-38014	scaffold38483	p2	(TC)7	Class II	1232	1245	ACATGCACACCTCACACCAACG	TCCGGCAACGATTTGCTCAGTG	336
FMgSSR-38017	scaffold7224	p2	(TC)7	Class II	33127	33140	ACATTCACGACACGGCAAGAGT	AGCTCGCGTGGGGAAGGATAAA	268
FMgSSR-38019	scaffold19177	p2	(TC)7	Class II	12857	12870	ACCACAGCATCGGTTGCACA	TGCCAACACACACACACACACA	225
FMgSSR-38022	scaffold614	p2	(TC)7	Class II	99643	99656	ACCACCGCGCCTCTACACAAAA	CGGCAACTTGTGCGTGCTTT	224
FMgSSR-38026	scaffold1040	p2	(TC)7	Class II	16079	16092	ACCAGCTGGGAGTGACTTGGTACT	GCTGTGTGAAACTATAGCAGGCAGG	329
FMgSSR-38027	scaffold1235	p2	(TC)7	Class II	71359	71372	ACCATCGCACATCGCACGTACT	TGGCGTGGGCGAAGAAAAGGTA	218
FMgSSR-38033	scaffold614	p2	(TC)7	Class II	84792	84805	ACCGAACTTGTGAGGCCACGTA	ACAACGGCGCAAGTCCATGA	318
FMgSSR-38041	scaffold4714	p2	(TC)7	Class II	23283	23296	ACCGCATGGCATTTGCATGTCC	AGGCGGGAGTAGAAGGAGTCAA	318
FMgSSR-38043	scaffold13794	p2	(TC)7	Class II	9057	9070	ACCGCGAAGGCGGATAATGACA	CGCAGGATGTCCAGGTGGAGAAAA	232
FMgSSR-38044	scaffold11936	p2	(TC)7	Class II	21039	21052	ACCGCTGCATGCTAGCACAA	TGCATGCTTGCGACTGATCCA	255
FMgSSR-38054	scaffold17	p2	(TC)7	Class II	98194	98207	ACCGTATGTCTTCTCCAGCCGT	AGCAAGGAGCACCATGTCTGCAT	212
FMgSSR-38056	scaffold13850	p2	(TC)7	Class II	5276	5289	ACCTTGCTTCCAAAATCAGCCGTG	TTGAAATTGGCGAGGCCGCA	263
FMgSSR-38060	scaffold437	p2	(TC)7	Class II	10432	10445	ACGACAAAAAGCGAGGAGGGCT	GGGCAGCCAAAACAAGGCAACA	214
FMgSSR-38064	scaffold27374	p2	(TC)7	Class II	10789	10802	ACGCAATCATCGCAGTCGCA	AGCACGAACGCAGCCTGGATTA	286
FMgSSR-38067	scaffold2456	p2	(TC)7	Class II	8071	8084	ACGCCTGTCTCGGTGGAAAA	ATCGCGAGTGCCGAAACGAA	322
FMgSSR-38069	scaffold3348	p2	(TC)7	Class II	21462	21475	ACGCGCAGAAGAGCCAAGAA	ACATCCTGCGTGACGATCTCCT	306
FMgSSR-38074	scaffold950	p2	(TC)7	Class II	66708	66721	ACGGCGCTGTTCGTCGATTCTT	AGCAAACTCTGCCTGTCGGT	342
FMgSSR-38082	scaffold1497	p2	(TC)7	Class II	23680	23693	ACGTGAGTTCAACGCCCCACTA	TGGCGCCTCAAAACCAAGCA	347
FMgSSR-38084	scaffold5142	p2	(TC)7	Class II	7397	7410	ACGTGGAGCGCTTACACTTG	TGTCCGTGTGTCCTATATTGCTGCC	230
FMgSSR-38085	scaffold9433	p2	(TC)7	Class II	6825	6838	ACTCAGGCACATCTTCCTAGCCA	TTGGGCGCATGCGGATTCAT	346

SSR_ID	Scaffold	SSR_Type	SSR_Motif	SSR Class	SSR_Start	SSR_End	Forward sequence	Reverse sequence	Product_size
FMgSSR-38087	scaffold67053	p2	(TC)7	Class II	2120	2133	ACTCGCGGGGAGTTCTTTGT	CAGGCTCACAGAAACTCTCATAGC	342
FMgSSR-38094	scaffold54772	p2	(TC)7	Class II	2240	2253	ACTGCGATGCCGTTGGGATCAT	AACAGCGTCGCCTGGCAATGAA	329
FMgSSR-38097	scaffold4914	p2	(TC)7	Class II	13104	13117	ACTGTTGTCGGGGCATCGGTTT	ACGGCAACAGCTGACAACGA	307
FMgSSR-38103	scaffold59919	p2	(TC)7	Class II	2514	2527	ACTTGGCACTTTCGCAGCCA	GGCGCCCATGCATGTATCAACT	265
FMgSSR-38104	scaffold1984	p2	(TC)7	Class II	45511	45524	ACTTGGGCTGAACGGCGAAA	ACGGCAAAAAGGCGTGGACA	345
FMgSSR-38106	scaffold4767	p2	(TC)7	Class II	30673	30686	AGACACTGGGAGCACGGTTTCA	TCCCTTCCAGTTGCCGCTTTGT	301
FMgSSR-38110	scaffold6939	p2	(TC)7	Class II	4288	4301	AGAGAGTAATACATGCAGTCCCCC	ATGGCGGTGTAGTCACCAGGAA	340
FMgSSR-38114	scaffold16465	p2	(TC)7	Class II	12503	12516	AGCAAAAGCACCAACAGAGCCC	GTGCTTGCTACGTGGCCAGTTT	347
FMgSSR-38119	scaffold6146	p2	(TC)7	Class II	7077	7090	AGCACCAGAAAGCCCAGTAGCA	GGTGCTTGTTTTTGCCGTCGGA	318
FMgSSR-38122	scaffold5558	p2	(TC)7	Class II	1628	1641	AGCACTCCTCCATCTCAGCCTT	ATCGCGAGCTATGCGGTGTCAA	343
FMgSSR-38124	scaffold3030	p2	(TC)7	Class II	15906	15919	AGCAGCACGAGAGAGGCTTGTT	TTCGCGCGGACGTTTGAGAA	255
FMgSSR-38125	scaffold4905	p2	(TC)7	Class II	44188	44201	AGCAGCGACTCGTACTGGTTCA	ACGGCTCCTGACGTTGCGTTAT	205
FMgSSR-38127	scaffold34640	p2	(TC)7	Class II	2978	2991	AGCAGGCACGCTTGGAAAGT	AGTTTGTCCTTGAGGGCGGA	312
FMgSSR-38132	scaffold748	p2	(TC)7	Class II	61543	61556	AGCCAAATTCGGCCCAGAGTGA	AGCTCGCACGAGAGAGAGAGAGAT	245
FMgSSR-38133	scaffold241	p2	(TC)7	Class II	50776	50789	AGCCAAGCCAAACGTCAGGTGT	ACGAGTCACGACAACCCAACCT	268
FMgSSR-38134	scaffold9140	p2	(TC)7	Class II	2114	2127	AGCCAAGTTTCATGAGGGGCCA	AATGAAGGGAAGAGAGGGGCGA	292
FMgSSR-38139	scaffold239	p2	(TC)7	Class II	98849	98862	AGCCGAGTGTCTACGTGTTGCT	TTCATTGCGTTGGCCGAGGA	332
FMgSSR-38144	scaffold6067	p2	(TC)7	Class II	2031	2044	AGCCTTCGGTAACCAGTGATAGGC	TTCCATCGTGGAGAGGTGCTCA	214
FMgSSR-38146	scaffold1270	p2	(TC)7	Class II	78693	78706	AGCGCAAGCTGCAAAGAGCA	AGCCATGGGTGCAGTGGAAT	271
FMgSSR-38147	scaffold7275	p2	(TC)7	Class II	36774	36787	AGCGCCACTAGCCGATCGATTT	TGTTGGGCCGAAAGGAAGGT	297
FMgSSR-38149	scaffold9239	p2	(TC)7	Class II	11833	11846	AGCGTGCCGTGATAATTTCCCT	AGCACACGAGGCAGCACAAT	300
FMgSSR-38150	scaffold70	p2	(TC)7	Class II	172788	172801	AGCGTGGGCAGGACAAGGAAAA	TTTGCAAACACTGGCGGTGC	302
FMgSSR-38162	scaffold305	p2	(TC)7	Class II	16658	16671	AGGAAGCCGTTCATGCTCTCCA	ACGCTTGCTCCTCTAATGGCGT	247
FMgSSR-38167	scaffold15105	p2	(TC)7	Class II	8906	8919	AGGAGAGTACGTTTGGGCCAT	TGCCGTTGGCTTTGTGCTGT	231
FMgSSR-38176	scaffold2512	p2	(TC)7	Class II	62984	62997	AGGGTATTGCTCTGCGTCTGGT	AGCCAGCAGCAACCTGAACA	343
FMgSSR-38183	scaffold1015	p2	(TC)7	Class II	30600	30613	AGTAAGGCTGCACTCTCCGT	TTGCATTGCATTGGGCGCGT	302
FMgSSR-38184	scaffold5757	p2	(TC)7	Class II	28292	28305	AGTAGAAACGCCCACTGCTACA	TGGCTCCAACAAGCTGGGAA	277
FMgSSR-38186	scaffold9985	p2	(TC)7	Class II	20625	20638	AGTCACGCGCCAAAAGTTCGT	AGAACGCCACGTACACCGTA	278

SSR_ID	Scaffold	SSR_Type	SSR_Motif	SSR Class	SSR_Start	SSR_End	Forward sequence	Reverse sequence	Product_size
FMgSSR-38188	scaffold477	p2	(TC)7	Class II	86167	86180	AGTCGGCCCATTGCGTTACT	AAGCTCCACTTCCTGACAGGCT	334
FMgSSR-38191	scaffold1137	p2	(TC)7	Class II	82406	82419	AGTGACACCCACCTGATTTCCA	TAAGGTGGGAATGGAAGGCCGA	318
FMgSSR-38192	scaffold49	p2	(TC)7	Class II	186426	186439	AGTGCAGCCCTTTCTTCACCA	TGCATGCGTGCATTGGCATGA	325
FMgSSR-38193	scaffold360	p2	(TC)7	Class II	108369	108382	AGTGCCCACAAAGTCAACGGGT	TGCATCTTGTGCTATGCGCGGT	203
FMgSSR-38199	scaffold6237	p2	(TC)7	Class II	22896	22909	AGTGTGAGTGCGTTGTGACTGG	ACGCTAGGAAAGTGCAGGCT	259
FMgSSR-38205	scaffold552	p2	(TC)7	Class II	87926	87939	ATACCCCGGTGCGGGAGAAAAA	GGCAGCAAACTTCATCTTTCCGGG	312
FMgSSR-38206	scaffold2811	p2	(TC)7	Class II	30742	30755	ATACGCCTTCGTGTTGCTCGCT	GCACAACGTTTCACTGCTGATGC	342
FMgSSR-38214	scaffold332	p2	(TC)7	Class II	33266	33279	ATCCCACATAATCTGGCGGCGA	CCAGCGCAAGCAAGGAGAAAGA	266
FMgSSR-38220	scaffold1407	p2	(TC)7	Class II	42225	42238	ATCGCGCACTGCAAGGAGTCAT	ACAAGGGAGCTTCCGTCGACTT	303
FMgSSR-38223	scaffold150	p2	(TC)7	Class II	86529	86542	ATCGTGCATCTCCTGGTTGCGA	AGTGTAGGGAACCCCATGATGCT	214
FMgSSR-38225	scaffold338326	p2	(TC)7	Class II	120	133	ATCTCTCCCCAAACCCTACCCA	TTGTTGGAGGACGACATGAGCG	261
FMgSSR-38241	scaffold684	p2	(TC)7	Class II	13226	13239	ATGGCCGCCTCAGAGAAAGGAT	ATGCATCGCACGCTCCAACT	342
FMgSSR-38243	scaffold9482	p2	(TC)7	Class II	15396	15409	ATGGGAATCGAAAGAAGGGCCG	ATCACCCCCACCAAACCGTTGT	274
FMgSSR-38247	scaffold6883	p2	(TC)7	Class II	29667	29680	ATGTTTGGCTGCCGAAGGGT	GTGGGCGGCATTCAAGTTCA	200
FMgSSR-38248	scaffold4613	p2	(TC)7	Class II	27843	27856	ATTCCCTTCGCCACGTTTGC	ATGTTCCCGCCACAAGAAGCCA	305
FMgSSR-38253	scaffold57894	p2	(TC)7	Class II	3120	3133	ATTCTCGGCACCGCAAGCAA	AAGTGCCGCCGGTCCATTTT	260
FMgSSR-38254	scaffold110	p2	(TC)7	Class II	99663	99676	ATTCTCTCTCAGCCGATGCCGT	AGTGGAGAGCTTGGAGTGGAGA	270
FMgSSR-38257	scaffold1580	p2	(TC)7	Class II	17965	17978	ATTGGCCATTGCCGTCTTCG	ATTCGCGGCATCCAAGCGAT	346
FMgSSR-38261	scaffold167734	p2	(TC)7	Class II	267	280	ATTTGCCCCCTGATTTCGGACG	TGTCCTGGTTATCGAGGCCAGT	245
FMgSSR-38264	scaffold18317	p2	(TC)7	Class II	11108	11121	CAAGCTTCGCCAACGCCAAT	ATGTGTCCGACGCCGTTTCA	225
FMgSSR-38273	scaffold25603	p2	(TC)7	Class II	357	370	CCCGCACGCCTCTTGAAGAAAA	TGCCTCTTGCTTGCGGGAAT	313
FMgSSR-38276	scaffold8420	p2	(TC)7	Class II	24303	24316	CCGGCACACAGCAAAAACCACA	TGCATGGTTGGTTAGCAAGCCG	274
FMgSSR-38283	scaffold2275	p2	(TC)7	Class II	16403	16416	CGCAAGGTTATCAATCGTGCGGT	AGCAAGCAAGCAGAGCGGAA	297
FMgSSR-38284	scaffold3332	p2	(TC)7	Class II	51753	51766	CGCACGCCACGTCGATCAATTT	CAGATCCACCGATGGTCTAGGT	318
FMgSSR-38288	scaffold15156	p2	(TC)7	Class II	9486	9499	CGCCGTTGATGCTGATGATGCT	TAACAACGCCTGCCGATCTCGT	290
FMgSSR-38289	scaffold362736	p2	(TC)7	Class II	124	137	CGCCGTTTGCTCGCAAGTTT	ACAGGACGCGACAGGACGATTA	212
FMgSSR-38291	scaffold3076	p2	(TC)7	Class II	17632	17645	CGCGGCATGTGAGCTGAGAAAA	AGCTGTGATAACCATGGGCGGT	328
FMgSSR-38292	scaffold250	p2	(TC)7	Class II	99288	99301	CGCTGCAAATGGACGGGAAT	AATGAGCTCCTGAGGCGTGT	340

SSR_ID	Scaffold	SSR_Type	SSR_Motif	SSR Class	SSR_Start	SSR_End	Forward sequence	Reverse sequence	Product_size
FMgSSR-38293	scaffold11733	p2	(TC)7	Class II	15813	15826	CGGCTGCACTGGTTCTCTCTTT	TTTTTCCCACGGTGCTGCCA	273
FMgSSR-38300	scaffold358	p2	(TC)7	Class II	3484	3497	GCACGCACGTACGAACTCTTGA	AAGCACGACCGACCGACAAA	338
FMgSSR-38304	scaffold17506	p2	(TC)7	Class II	6612	6625	GCATCAGGCATGCGCCTCTTTT	TGCTTGCACGCAGATGCTAA	215
FMgSSR-38309	scaffold1639	p2	(TC)7	Class II	72065	72078	GCCATTCGTGGCACATGACGAA	GCCCAAAACTCACGGCCCAAAA	348
FMgSSR-38314	scaffold20827	p2	(TC)7	Class II	1066	1079	GCCTAAACAACACACACGCGCA	ACACACAACAGGCTTAGAGCAC	339
FMgSSR-38316	scaffold96	p2	(TC)7	Class II	12079	12092	GCCTTCTCTGTGTTGCACGCAT	TCAAGCCCTCAGCATTCGCACA	333
FMgSSR-38319	scaffold294	p2	(TC)7	Class II	19242	19255	GCGCGCAGCGAGTGAATACATA	ATGTCGTCGTCGTCGGTGGTTT	305
FMgSSR-38326	scaffold1252	p2	(TC)7	Class II	69270	69283	GCTCTCTCGCCAACTTCCCATT	TTTGTCGCCCGAGTCCAGAT	334
FMgSSR-38328	scaffold4795	p2	(TC)7	Class II	28659	28672	GCTGAGCTGTTTTGTGTCCGCA	AAGTTGTGGCCCGTGTGCAT	227
FMgSSR-38330	scaffold166508	p2	(TC)7	Class II	966	979	GCTGCCGCCTTGCTTTGCTATT	CATGGCGTGTGTTACTCTTCACTC	314
FMgSSR-38336	scaffold18430	p2	(TC)7	Class II	3810	3823	GCTTGGCTGAGACAAACGAGA	ACCTACCTGGCTTCGATTCGCT	310
FMgSSR-38337	scaffold325139	p2	(TC)7	Class II	83	96	GCTTTGGGTCGTGTCGACAACTT	TGCGTTTCCGTACCTTGCCA	262
FMgSSR-38340	scaffold2981	p2	(TC)7	Class II	27050	27063	GGATATCCGGTGCTACAAGCATGA	AGTCGATTGCACCGAGCACCAT	310
FMgSSR-38350	scaffold5478	p2	(TC)7	Class II	16073	16086	GTAGCCAGTTTCTGAAGCGTGA	TCCGTCAGTTCCTGTCGCTGTT	245
FMgSSR-38351	scaffold6810	p2	(TC)7	Class II	26348	26361	GTCACTTGAACTTCACCGATCAGC	CGTGGAGCCATGGACCAAGAAA	338
FMgSSR-38353	scaffold77	p2	(TC)7	Class II	10706	10719	GTGCTCACAATCATAATGCGGTGC	CCTCATCGATCTGTTGCTTGCCCT	285
FMgSSR-38357	scaffold27559	p2	(TC)7	Class II	10997	11010	TAAAGAGGCTGAAGGCTGCGCT	GGCAAAAGCATGACACACGGCA	298
FMgSSR-38363	scaffold696	p2	(TC)7	Class II	13206	13219	TACAAAATCGTCCCTCCGCGCT	ACGATGGCCAATTGCAGGCT	332
FMgSSR-38364	scaffold1197	p2	(TC)7	Class II	22634	22647	TACAAGGCAACAGCAGGCAGCA	TGGAACTCGATCCGGCGTTTGT	346
FMgSSR-38366	scaffold13390	p2	(TC)7	Class II	5643	5656	TACCAGTGGCACACACCACACA	TGCCTCCTGTAGCTTGCCTT	262
FMgSSR-38371	scaffold92	p2	(TC)7	Class II	65890	65903	TAGTTGGGCCGCAACCAACCAT	TCGCGAACTCTCTTGGTTGCCA	287
FMgSSR-38378	scaffold27815	p2	(TC)7	Class II	8372	8385	TCAAAGCAGCTGCCGTCCTCAT	AGAAAAGAGTTGGCGGTGGC	221
FMgSSR-38385	scaffold2842	p2	(TC)7	Class II	33747	33760	TCACACCTGCCACTCGCACATA	AAGCAGCTGGGCAACGAACA	292
FMgSSR-38386	scaffold7643	p2	(TC)7	Class II	27271	27284	TCACAGCGCGCAGCACAAAT	ACGGGTTACATGGCAGCACCTT	297
FMgSSR-38388	scaffold9014	p2	(TC)7	Class II	15032	15045	TCACCAGGCCATCCATCGTGAA	AGGCCTCGGAGAGAGAAAGGAGAT	310
FMgSSR-38393	scaffold662	p2	(TC)7	Class II	94567	94580	TCACGTACCGGGAAGCAGTGTT	AACCAGGCCCGTACCGTAGTTT	350
FMgSSR-38394	scaffold2619	p2	(TC)7	Class II	1348	1361	TCACTAGGAGGAGTCCATCGACCA	ACGTGTTAATGGGACGCGGA	296
FMgSSR-38398	scaffold2505	p2	(TC)7	Class II	55755	55768	TCAGCGCGTGAGATGCACAA	ACGCTTCTCGACTGCTGCTACA	221

SSR_ID	Scaffold	SSR_Type	SSR_Motif	SSR Class	SSR_Start	SSR_End	Forward sequence	Reverse sequence	Product_size
FMgSSR-38399	scaffold1629	p2	(TC)7	Class II	14810	14823	TCAGCTGTCTGGTCGCTCTTCT	TCGCGCATGTTTTGGTGGCT	286
FMgSSR-38402	scaffold11349	p2	(TC)7	Class II	1150	1163	TCAGTATGGAGTTAGGAGGCACCG	TGGCTCAAGCCACAACGAAGA	346
FMgSSR-38403	scaffold4719	p2	(TC)7	Class II	4752	4765	TCATAACCGGTGGGAAGTGGGT	TGCATGCACCACGACAACTCT	345
FMgSSR-38421	scaffold261008	p2	(TC)7	Class II	210	223	TCCATTGCAGCTGGCGCCTTAT	ATCGCTACGTCTCGACGCTGAA	200
FMgSSR-38422	scaffold20737	p2	(TC)7	Class II	10023	10036	TCCCAAAACCCCCACACTTGCAT	GCAACCTTTATGGGATTCCTGCCG	259
FMgSSR-38425	scaffold29469	p2	(TC)7	Class II	1243	1256	TCCGACGACGTATGTAGGGTTTGC	ACGTGCGGATCAACGTTTCCCT	215
FMgSSR-38426	scaffold1463	p2	(TC)7	Class II	45704	45717	TCCGATTCGATCCGATCCGCTT	TTGCCCCTTTGGACTTCGCA	321
FMgSSR-38433	scaffold16803	p2	(TC)7	Class II	13886	13899	TCCGTCTTGCTCGACACGAAA	ACTCCAGCTAAGACACGGTCAAGT	348
FMgSSR-38434	scaffold72065	p2	(TC)7	Class II	1501	1514	TCCGTGAGGCCCACATGTCAAT	CGCAGTCGTGCGGAAAAAGA	207
FMgSSR-38435	scaffold3801	p2	(TC)7	Class II	13683	13696	TCCTCCCTGCAAAACGGGTTCA	CGCTGTCACCGCTGTTTTGTGA	278
FMgSSR-38436	scaffold15753	p2	(TC)7	Class II	17811	17824	TCCTGAATCTTCGCGCGCTT	ATCACCTGCACGTTCGTTCGGA	237
FMgSSR-38440	scaffold2477	p2	(TC)7	Class II	14212	14225	TCGAGTGTGCTCTACATGCGT	TCCCGCTGCAAACATTCCCGAT	203
FMgSSR-38448	scaffold1296	p2	(TC)7	Class II	35638	35651	TCGCGCGCACTACCTCTTACAT	GCTTGTTTTCCTTGGGTACCCCCT	226
FMgSSR-38449	scaffold4419	p2	(TC)7	Class II	47160	47173	TCGCGCTTTTGCAAGTTGGC	ACGTCGCGTCATGAATCAGGCT	292
FMgSSR-38450	scaffold758	p2	(TC)7	Class II	19716	19729	TCGCGGCACGATTCTTCAACT	TGCAATCTTGTCCCCAGAGCCA	260
FMgSSR-38452	scaffold10324	p2	(TC)7	Class II	5886	5899	TCGGAATGTGGGATTGAGCAGAGC	AATAGGTGTGCCACGTCGGCTT	266
FMgSSR-38455	scaffold3436	p2	(TC)7	Class II	22840	22853	TCGGCAACGACAAAAGCCCT	GTGCAACGGGCCTGATTCAACA	311
FMgSSR-38459	scaffold93259	p2	(TC)7	Class II	885	898	TCGGCGATCCACCCCTTCAAAA	TCGCGGAGTTTGGTTTCCTGT	319
FMgSSR-38468	scaffold2954	p2	(TC)7	Class II	52806	52819	TCTCCTCTGCTTCGAGGCTTGT	AGCTGTGCGTTTTGTCCCGA	222
FMgSSR-38469	scaffold2612	p2	(TC)7	Class II	44253	44266	TCTCCTGGAGCAGTGCCATTCA	TACGTTGACCGCACCCCAACAA	342
FMgSSR-38471	scaffold1802	p2	(TC)7	Class II	29452	29465	TCTCTTTGGTGGCCAGTGGTGA	TGCATTGATGAGGAGGCGCT	345
FMgSSR-38472	scaffold190103	p2	(TC)7	Class II	204	217	TCTGCCGAACTGCCTTTTCCA	ATCGCGCAGAGATCGCAAGA	239
FMgSSR-38474	scaffold3569	p2	(TC)7	Class II	44452	44465	TCTGCTGCATGCACTGCGTT	AGCCACACGAGGCACCAAGATT	208
FMgSSR-38476	scaffold169	p2	(TC)7	Class II	111600	111613	TCTGTCGCCGGATGCAAACA	GCTGCGGCTGCCTTTCATCAAT	285
FMgSSR-38478	scaffold15	p2	(TC)7	Class II	67686	67699	TCTTCCCCCTCTCCTGCATCAT	TCTCGGGCAATCTCCTCTGTGT	346
FMgSSR-38482	scaffold763	p2	(TC)7	Class II	52768	52781	TCTTTCTCTCTCCCCCTCTCCTCA	AGGCGTGTTGCTGCTCTTCCAA	337
FMgSSR-38489	scaffold73	p2	(TC)7	Class II	25983	25996	TGACAGAGCATGGAAACCGGCA	AGCGCCGTCATTTCTCCCTTGT	264
FMgSSR-38491	scaffold1021	p2	(TC)7	Class II	69693	69706	TGACCCAATAGGCCAGCACCAT	ACAAGGAAAGTGGTGCCGACGA	296

SSR_ID	Scaffold	SSR_Type	SSR_Motif	SSR Class	SSR_Start	SSR_End	Forward sequence	Reverse sequence	Product_size
FMgSSR-38497	scaffold13996	p2	(TC)7	Class II	5483	5496	TGAGTGCTTGCAGTGAGGATCCAG	ATTCCAAGCCAACAACCTCCGC	248
FMgSSR-38499	scaffold2377	p2	(TC)7	Class II	59995	60008	TGATTCGGCTGTTCTCGTCCCA	AAGAGCGGAGGCAAAGGAGTCA	272
FMgSSR-38502	scaffold2027	p2	(TC)7	Class II	19116	19129	TGCAAAGACGTGGCAAGAGTGC	TCGGTGCACAATGGACAGCA	263
FMgSSR-38505	scaffold24463	p2	(TC)7	Class II	3977	3990	TGCAAGCGAACAGGCAACGA	TGACGAGATCGCCCTGGAGTTT	343
FMgSSR-38508	scaffold2133	p2	(TC)7	Class II	19737	19750	TGCACGCGTGTGGGTGAAAA	AGATCCACCAAAGGGCTTGTCC	293
FMgSSR-38512	scaffold27317	p2	(TC)7	Class II	5619	5632	TGCACTGACTGCAACTGCCA	AGCGTGGATCTAGCAACAGCGA	268
FMgSSR-38514	scaffold8540	p2	(TC)7	Class II	23022	23035	TGCAGCAATTAACCGAGGGGA	AAGCGTTGCTTGCCTCTGCT	267
FMgSSR-38515	scaffold18711	p2	(TC)7	Class II	6110	6123	TGCAGGTACACCATGACGACGA	TGACCAATGCGGTTTGCGGT	215
FMgSSR-38519	scaffold3897	p2	(TC)7	Class II	43432	43445	TGCATGCGCAGCCTACTCAA	TTGCCGCCGATGAATGGTCA	283
FMgSSR-38520	scaffold1030	p2	(TC)7	Class II	24887	24900	TGCATGGTTGGTGCATGATGGC	AAAGGGCAAGGTGGCGAAGT	229
FMgSSR-38521	scaffold408	p2	(TC)7	Class II	36290	36303	TGCATTGTGGGCTTGTGGCA	ACATTTTTGGGCGGCCTGCT	297
FMgSSR-38522	scaffold1414	p2	(TC)7	Class II	17579	17592	TGCCACCTGGTCCACAACTT	AAAAATAGACGCCGCGGTTGCC	307
FMgSSR-38523	scaffold3175	p2	(TC)7	Class II	59628	59641	TGCCACGTCCTTTCTGCATCCT	TTGTCGGCATGGTGGCATGT	268
FMgSSR-38530	scaffold780	p2	(TC)7	Class II	23006	23019	TGCCGCTATCTCTCTCTCGGCTAT	AATTGACGGGCCCAACGCAA	207
FMgSSR-38531	scaffold2330	p2	(TC)7	Class II	62309	62322	TGCCGGATCTGAGCTGTCAT	ACAGAGCAAACCCCACCACGAA	323
FMgSSR-38534	scaffold389	p2	(TC)7	Class II	23422	23435	TGCCGTGATGTACTAGTGTGCG	TGGTGTCACTTTGATCGGTGGCT	331
FMgSSR-38539	scaffold4613	p2	(TC)7	Class II	5816	5829	TGCCTGGCTAGTGGCTACTTCA	ACTCCCACATCGCCCTATATCGAC	314
FMgSSR-38541	scaffold5439	p2	(TC)7	Class II	44428	44441	TGCGAATGCTCTGCCGACTT	AGCCAGTTCCTGTTGATCCCGT	338
FMgSSR-38542	scaffold5973	p2	(TC)7	Class II	22390	22403	TGCGACAAGCGGCTGTCCTAAA	TCGTCTTCCGAGGAGAGCGAAA	344
FMgSSR-38544	scaffold4965	p2	(TC)7	Class II	27580	27593	TGCGATGTGAGAAGCGCAGA	AGGTCATCACGGTGCGCAAGAT	346
FMgSSR-38546	scaffold37082	p2	(TC)7	Class II	1998	2011	TGCGCATTGAAAGCTGGGCA	ACACCGTTGCCACTCACTACCA	296
FMgSSR-38548	scaffold15844	p2	(TC)7	Class II	4751	4764	TGCGCCACTCTGCCACTATTCA	CGGCGTTGAGCGCATAGTGTTT	237
FMgSSR-38549	scaffold21832	p2	(TC)7	Class II	1688	1701	TGCGCCCGGAAGTAGTTGTT	AACACTGGAAGCAGAGCTGCTGAG	310
FMgSSR-38561	scaffold2153	p2	(TC)7	Class II	5323	5336	TGCTAAACCACCGTCGTTGCCT	GGTGGCTTGCGATGTGCAACAA	344
FMgSSR-38562	scaffold23342	p2	(TC)7	Class II	5837	5850	TGCTCAAGCAGAAGTTGTGCCA	TTGGATCGCGCCTGATGCTCTT	349
FMgSSR-38565	scaffold1094	p2	(TC)7	Class II	32411	32424	TGCTCGCACGAGTTCCATGA	TTTCGAACCACTGCAGAAGGGC	306
FMgSSR-38568	scaffold5286	p2	(TC)7	Class II	37472	37485	TGCTGCTTTCGCGTTTGCGT	ACGAGCGACAACATTCGCGT	238
FMgSSR-38569	scaffold6669	p2	(TC)7	Class II	24288	24301	TGCTGGCCGTGTGGTGTTTAGT	ACGACATTATGTGGACACGCGGA	217

SSR_ID	Scaffold	SSR_Type	SSR_Motif	SSR Class	SSR_Start	SSR_End	Forward sequence	Reverse sequence	Product_size
FMgSSR-38572	scaffold29667	p2	(TC)7	Class II	5663	5676	TGCTTCAGGCCTTGGGCTTCTT	ATGAGGCGGATGATGTTGGGGT	348
FMgSSR-38576	scaffold864	p2	(TC)7	Class II	66456	66469	TGGAACAGCCTGCTCGCTGAAA	TCTGCTGAGGATCTTTGCCGGA	293
FMgSSR-38578	scaffold4166	p2	(TC)7	Class II	9178	9191	TGGACCGAGGGCTAAAGTGCAA	TCCCAAATCCGCCGTCGTTT	238
FMgSSR-38579	scaffold1287	p2	(TC)7	Class II	33346	33359	TGGACGAGGCCTTTGGATGT	GCCTCATGTGTGCATCTATCGTGG	323
FMgSSR-38580	scaffold1421	p2	(TC)7	Class II	11008	11021	TGGAGTGGCAAGAAACGCGA	AAGTTGCTCGTCGTCCCGTTGA	265
FMgSSR-38583	scaffold5692	p2	(TC)7	Class II	17321	17334	TGGATGCAGGTGGATGATGCGA	AGGAGCGCGCATGCTTTTCT	281
FMgSSR-38586	scaffold166508	p2	(TC)7	Class II	823	836	TGGCAAGGCATGCACTGAGAA	ATAGCAAAGCAAGGGGGCAGCA	338
FMgSSR-38592	scaffold2892	p2	(TC)7	Class II	29277	29290	TGGCCAACAGCTGGAAAGCTCA	TTCATGAACGCGCTCCTCTGCT	234
FMgSSR-38594	scaffold4929	p2	(TC)7	Class II	4090	4103	TGGCCCATATGGCTGAGGTTGT	AAGCATTGGTGGTGTGGAGCGA	335
FMgSSR-38600	scaffold948	p2	(TC)7	Class II	76935	76948	TGGCCTTCCCGCTGAATGACTT	TGTTGCCCACCAAAACGCGA	229
FMgSSR-38601	scaffold1583	p2	(TC)7	Class II	61855	61868	TGGCCTTGGTTGCTCCATCCAT	TCGCACTACACACTGGAAGCTG	221
FMgSSR-38602	scaffold4328	p2	(TC)7	Class II	12678	12691	TGGCGAGTGGCAGCAAGTTA	TGTGTTGAGGGCAAGTGCTGA	348
FMgSSR-38606	scaffold171616	p2	(TC)7	Class II	704	717	TGGCTGGTTGCTCAGCTTGACT	ACGTTTGGCCTCAGCACCTGTT	279
FMgSSR-38610	scaffold1830	p2	(TC)7	Class II	68615	68628	TGGGAGCGCATGCACATTGTTG	AGCTTCTGGCAGCTTGGTAGT	302
FMgSSR-38614	scaffold127	p2	(TC)7	Class II	101197	101210	TGGGCCTGTTCACTGTTCGGTT	CACACAAGCACACGGAGGTT	219
FMgSSR-38617	scaffold251	p2	(TC)7	Class II	138558	138571	TGGTCTACGTGCTCTACAGCGT	ACTTCACAAGCGGTGGTAACCT	346
FMgSSR-38621	scaffold69	p2	(TC)7	Class II	162265	162278	TGGTGCTGCCACTCTCAAGCAA	TAACACGGCAGCGCGTTCAT	324
FMgSSR-38622	scaffold4606	p2	(TC)7	Class II	40878	40891	TGGTGGACGGTGGTATGGTT	ATTTGACATCGTGTGCGCGG	322
FMgSSR-38642	scaffold270271	p2	(TC)7	Class II	108	121	TGTGCTACGCGTGTTGTTGCT	TCGATCTGCCGAGCAAAGCCAA	282
FMgSSR-38644	scaffold1819	p2	(TC)7	Class II	44957	44970	TGTGGCAACCTGGCAGCAAA	ATTCGCTGTTGCCTGAAGGGGA	264
FMgSSR-38650	scaffold166841	p2	(TC)7	Class II	778	791	TGTGTGTGTTTGGGCCTCCCTT	TTAAACAGGTCCAGCGCAGCAG	206
FMgSSR-38651	scaffold1615	p2	(TC)7	Class II	70656	70669	TGTGTTGCGTCCGCCTCTTT	ACAGGTGCGATGGAGCGTGATT	201
FMgSSR-38652	scaffold13453	p2	(TC)7	Class II	18747	18760	TGTTCAACTGGAGGCGGTCT	TTGGCAGATAGACACACGCAGC	348
FMgSSR-38653	scaffold1234	p2	(TC)7	Class II	23654	23667	TGTTGCGTTAACACTTGCGGCT	AACCAGGAGCTCGTCAAGTCCA	266
FMgSSR-38654	scaffold69	p2	(TC)7	Class II	109991	110004	TGTTGCTCTGCGCCACTTGAGA	AACATGGGGCTCAAGCCATAAC	300
FMgSSR-38655	scaffold3773	p2	(TC)7	Class II	29245	29258	TGTTTCGCGGATACACCCCT	TACGCCCGACGAATGCCAAT	269
FMgSSR-38657	scaffold60268	p2	(TC)7	Class II	959	972	TTACCGCACGCTTTTGTGCTGG	AAAATGACGTCGGCGCTGCT	200
FMgSSR-38658	scaffold4005	p2	(TC)7	Class II	42302	42315	TTACTACTGTGTGGGCGCGTGT	GCGTGGTCCACTGAAAACTACA	297

SSR_ID	Scaffold	SSR_Type	SSR_Motif	SSR Class	SSR_Start	SSR_End	Forward sequence	Reverse sequence	Product_size
FMgSSR-38662	scaffold239	p2	(TC)7	Class II	41676	41689	TTCAGTGCCAGCTCCCCTGTTT	TGATGATGCCAATGCCGCGA	301
FMgSSR-38665	scaffold1799	p2	(TC)7	Class II	22367	22380	TTCATTCTCCATCCATCGCCGC	ACCCGGCTTGCACACACAAA	339
FMgSSR-38669	scaffold31125	p2	(TC)7	Class II	4344	4357	TTCCCCAAAACCGGTGGTCACT	TGGACAACACACACAGCGGCAT	321
FMgSSR-38670	scaffold6466	p2	(TC)7	Class II	12422	12435	TTCCTGGGTCCCTGCTGTCTTT	TCGAGCTACCAAGACCACCTGA	293
FMgSSR-38674	scaffold61021	p2	(TC)7	Class II	948	961	TTCGCGCGCAGTTGAGAAGA	CGCACTGTGAGACACCGTTGAT	242
FMgSSR-38675	scaffold402	p2	(TC)7	Class II	85054	85067	TTCGGTCAGTTCTCATCCGCCA	ACGTCGGTTTGTGTGGCACT	335
FMgSSR-38684	scaffold92	p2	(TC)7	Class II	58461	58474	TTGAGCTACAAGCACGGCGACT	AGTGCGAACAACCGAACCGA	244
FMgSSR-38686	scaffold1513	p2	(TC)7	Class II	30616	30629	TTGAGGTCCACAGCCTTTCGCT	AGCTAGGCTTCACCAAAACCGCT	324
FMgSSR-38689	scaffold89	p2	(TC)7	Class II	161642	161655	TTGCACCAGGGTACCACTACCT	GGTGCTCATTACCAATAGGGCTGT	284
FMgSSR-38690	scaffold63	p2	(TC)7	Class II	63606	63619	TTGCATCCCTCCCGCAATCA	AGCTGTGGATGACTGGATGCCAA	267
FMgSSR-38692	scaffold629	p2	(TC)7	Class II	41379	41392	TTGCCAGCATCAGCATGCCA	TTCAGAAGCGGCACGACTTGGA	295
FMgSSR-38702	scaffold4654	p2	(TC)7	Class II	37997	38010	TTGCTTTTCCCCACTCACCCCA	AGCCATCGTGACGCTTCCTTGA	222
FMgSSR-38705	scaffold6963	p2	(TC)7	Class II	11918	11931	TTGGCGGGAGAGCTTCGGTTTT	GCCTTTTTCCGTCGCTGCGATT	298
FMgSSR-38706	scaffold161	p2	(TC)7	Class II	90776	90789	TTGGCTTCTCCTCGGCAGCTTA	TGCTGGCTGGCTCCAGTAAAGA	218
FMgSSR-38708	scaffold1436	p2	(TC)7	Class II	67359	67372	TTGGGCCTGCAGTGGCAGTAAA	AAGGCAATGCAGCCAATCGC	324
FMgSSR-38709	scaffold778	p2	(TC)7	Class II	108223	108236	TTGGTGATATGCCGACTGCCGA	ACTTTTGTCCCCCGGCTTGT	272
FMgSSR-38711	scaffold10808	p2	(TC)7	Class II	6767	6780	TTGTGAACGCGACACGCTGA	AACCTCGTCAACCCTTGGCT	262
FMgSSR-38713	scaffold136	p2	(TC)7	Class II	34292	34305	TTGTTCCTGCACAACAGCGTCC	ACCGTGGCAACTTGCTTGCAT	263
FMgSSR-38714	scaffold549	p2	(TC)7	Class II	77682	77695	TTGTTCGTACGCTGCTTGCTCC	AACCGCAAGGACGATGCAAA	200
FMgSSR-38716	scaffold16473	p2	(TC)7	Class II	12302	12315	TTTAGCCCGTCGGTGTAGCGAA	TCGTTGTCGTGCATAGCGCA	263
FMgSSR-38720	scaffold76	p2	(TC)7	Class II	20164	20177	TTTCCGCCCCTGTAAGATCCGT	TTGCACTTCTGCAGCGTCGT	337
FMgSSR-38722	scaffold5000	p2	(TC)7	Class II	29979	29992	TTTCCTTTCCGCTCCGCTGCTT	TGCATCGCAGCGAACTACTCT	233
FMgSSR-38731	scaffold32569	p2	(TC)7	Class II	2485	2498	TTTGCCACGCTTCTTGCCCT	TGGGAGGTTGTACAGGTAGGAGGA	340
FMgSSR-38735	scaffold1512	p2	(TC)7	Class II	46989	47002	TTTGGCACATGCGAGCGTCA	ACTGGTGGATTTGTCCCCGCAA	294
FMgSSR-38736	scaffold3971	p2	(TC)7	Class II	24249	24262	TTTGGCACGCGGAATTGCCA	ACAGGACAGCAAAGCAGGAGGA	320
FMgSSR-38745	scaffold12451	p2	(TC)7	Class II	5533	5546	TTTTGGCCGAAGCTTGTCCCGT	TTCCGCATGACACAGGTGCAGT	328
FMgSSR-38750	scaffold681	p2	(TC)8	Class II	108754	108769	AAAAGCCACACCCACAGCCGAA	TGCACTCCATTCTGGTCCGA	272
FMgSSR-38753	scaffold1146	p2	(TC)8	Class II	75596	75611	AAAAGTCAACTGCCACCCGTGC	ATGCATGCTTCTCCGAGCGA	208

SSR_ID	Scaffold	SSR_Type	SSR_Motif	SSR Class	SSR_Start	SSR_End	Forward sequence	Reverse sequence	Product_size
FMgSSR-38759	scaffold6095	p2	(TC)8	Class II	16247	16262	AAACGAGTTGAGAGCCGCAG	ACGCCGATCAGCAACGAGAA	346
FMgSSR-38761	scaffold17531	p2	(TC)8	Class II	10292	10307	AAACTCGGATCTTAACCCTGCCCC	AAGTCTTGGCTGCGCACGTT	332
FMgSSR-38762	scaffold493	p2	(TC)8	Class II	35237	35252	AAAGATCTACAGGGGTCAGGTCC	TGCGTGCAAACTGGTACGGT	219
FMgSSR-38765	scaffold2147	p2	(TC)8	Class II	54794	54809	AAAGCTGCAGCCAATAGAGGCG	TTGACCCTTTGGTTGGCCCT	253
FMgSSR-38768	scaffold2466	p2	(TC)8	Class II	4067	4082	AAAGTCATGTGCCCAGGTTGCG	AGCAAAGCAGCACCGTAGCA	329
FMgSSR-38773	scaffold18441	p2	(TC)8	Class II	3710	3725	AACAGAAGGACGACCAAGCGGT	GCAAGCGGTGATGAACGCAACA	300
FMgSSR-38778	scaffold9460	p2	(TC)8	Class II	19117	19132	AACCCCCTCTGAGCCCAAAGTT	AACTCGTAACCCATGTCGGCCA	246
FMgSSR-38783	scaffold1528	p2	(TC)8	Class II	33782	33797	AACGGTTACTGTCCCTCGCAGT	TCCAGGACAGTGTGCAAGAAAGCA	325
FMgSSR-38786	scaffold2761	p2	(TC)8	Class II	8824	8839	AACTTACCCATAGCTCTGGCCC	ACCCCACCGATGAAGAGAGAGA	293
FMgSSR-38788	scaffold2914	p2	(TC)8	Class II	51665	51680	AAGAAGAAGAGGTGCGGTGCGT	TTCGAAATCCAAGCGGGCCA	287
FMgSSR-38791	scaffold2203	p2	(TC)8	Class II	46296	46311	AAGAGATGGGTCCCTTTGGTAGGG	TGGTGGCTGGAGAGGAACGAAA	298
FMgSSR-38792	scaffold3294	p2	(TC)8	Class II	33414	33429	AAGAGGAGGAAGAAGGGAGGCGAT	AAATCCCAACGAACCCGACCGA	331
FMgSSR-38793	scaffold45054	p2	(TC)8	Class II	4270	4285	AAGATTGCACTGGGCTCTGCAC	AGCGCTGTACCTGGAATTCAGCA	288
FMgSSR-38794	scaffold4771	p2	(TC)8	Class II	19537	19552	AAGCACTTCTTCCTCCCCACCA	AGCAACCGCAGCAGCTCATT	286
FMgSSR-38795	scaffold41	p2	(TC)8	Class II	136505	136520	AAGCGCCTCGAAGAAGGAAGCA	TTCGTTTAACGCCCGGCACA	293
FMgSSR-38797	scaffold10169	p2	(TC)8	Class II	14904	14919	AAGCTACGTGGTGCTTCCTCCT	AAATTGACGGCCTCCCGTGACA	333
FMgSSR-38798	scaffold1551	p2	(TC)8	Class II	9075	9090	AAGCTCAGGGCCAGCTGATT	GTGTCACTAGCAACAAGAGCGA	349
FMgSSR-38804	scaffold1929	p2	(TC)8	Class II	2101	2116	AAGGCCAAACCATCGCGACA	TCGGTTGCGTCGCCTTGAAA	329
FMgSSR-38806	scaffold19967	p2	(TC)8	Class II	10577	10592	AAGGGCAGCCGATAGCTTGA	AGTCCTCGTGGCCCCATTTT	325
FMgSSR-38807	scaffold66	p2	(TC)8	Class II	105724	105739	AATAGTTCATGTCCGCGGCGTC	ACTCGCCTGGATCTGTGTCGAA	256
FMgSSR-38814	scaffold22238	p2	(TC)8	Class II	7643	7658	AATGGGCCTGAGCAGACTTGGT	GCAGAATCTCCGTGCGCAAACA	263
FMgSSR-38823	scaffold6841	p2	(TC)8	Class II	11301	11316	ACAAGGACGTGTTCTCCGCCAA	CGCGTAGTCAAGTCGTGATGCT	350
FMgSSR-38826	scaffold2789	p2	(TC)8	Class II	58173	58188	ACACACTTGCTTGTGGCCGA	ACTGTTGCCGCGCAACGAAA	302
FMgSSR-38830	scaffold327	p2	(TC)8	Class II	133193	133208	ACACGAATGGGCCACCAGACAA	GTGTCCAAGGGTACCTTCTCGT	324
FMgSSR-38832	scaffold10478	p2	(TC)8	Class II	13415	13430	ACACGTCGTCTGCTTCTCTGT	AGTAAGAGAATGCCGGGCCA	350
FMgSSR-38835	scaffold12337	p2	(TC)8	Class II	2536	2551	ACAGAGAGAAACCCGACCTTCCCA	AAAAGCGACAGCCGCCGATA	312
FMgSSR-38839	scaffold653	p2	(TC)8	Class II	103546	103561	ACATCAACGCGCTTGCTTGC	CCGACCGGCATGAAACAGGAAA	347
FMgSSR-38840	scaffold5826	p2	(TC)8	Class II	35938	35953	ACATCACCAAACGGTGAGACCA	TCATGGCGTATACCGAGTCCCT	337

SSR_ID	Scaffold	SSR_Type	SSR_Motif	SSR Class	SSR_Start	SSR_End	Forward sequence	Reverse sequence	Product_size
FMgSSR-38843	scaffold3886	p2	(TC)8	Class II	13961	13976	ACATGTTGGAACTCGACCGCCT	TGTTTTCTGCCCCTGCTGTGGT	257
FMgSSR-38845	scaffold24027	p2	(TC)8	Class II	8708	8723	ACCAAGGCCAAAGGCTGGTCAA	ACACCAACCGAACCGCATCA	314
FMgSSR-38850	scaffold110807	p2	(TC)8	Class II	577	592	ACCACGTTCCTTGCAGGGTCAA	TAAGGCTGCGCGAGAGCATTGT	242
FMgSSR-38855	scaffold4493	p2	(TC)8	Class II	29719	29734	ACCAGCGCGACACATACCTCAA	TTTCCCGCGAGTAAAATGGCCG	219
FMgSSR-38856	scaffold7030	p2	(TC)8	Class II	14202	14217	ACCAGGGGTGTCACAGTAAGATTGG	AGCAAGGCACAACGGATCAAGC	261
FMgSSR-38857	scaffold81755	p2	(TC)8	Class II	1659	1674	ACCAGTGACGGCCTATGCTGAA	TTGGGTCAGACGTGGGGTGTTT	336
FMgSSR-38861	scaffold13304	p2	(TC)8	Class II	14602	14617	ACCCGCCAAAACTGTCTGCCTT	ACGGCACGTGGTATCGTTGT	291
FMgSSR-38862	scaffold8118	p2	(TC)8	Class II	17210	17225	ACCCTGGGGTGCAAGACATTGA	TGCCGCATTGCCATCGAAGA	301
FMgSSR-38866	scaffold9063	p2	(TC)8	Class II	23244	23259	ACCGCAATGGCCGTAGAGTGTA	AGTTTGTGCGGCGTGTGTGT	273
FMgSSR-38871	scaffold10551	p2	(TC)8	Class II	5150	5165	ACCGGTGTCGAACCCCTTTGAA	AGGGTGATGGTGTCGTGTCA	322
FMgSSR-38874	scaffold1874	p2	(TC)8	Class II	5740	5755	ACCGTTTCCGCGGCTTCATT	TCGATCGTCGAGACGGTGTGTT	262
FMgSSR-38875	scaffold6101	p2	(TC)8	Class II	32231	32246	ACCTGCTCAACTCAGCATGAACCC	ACCAAATCAGCCCGCAACAA	329
FMgSSR-38876	scaffold1952	p2	(TC)8	Class II	45626	45641	ACCTGGCAACCTGCAAGCAA	TTGCGTGGCAGAGATTTGGTGC	288
FMgSSR-38877	scaffold77671	p2	(TC)8	Class II	1867	1882	ACGAATGTGGCATCTGGACCCT	AGGGAAGGAAAGTCTCGTCGCT	306
FMgSSR-38880	scaffold615	p2	(TC)8	Class II	78329	78344	ACGACTGCAACAACCGACTGGA	AATTGCACGCCAAGTGCGAC	211
FMgSSR-38884	scaffold5337	p2	(TC)8	Class II	21200	21215	ACGAGTGTAATCACTGCCGCT	ATCACATGCTCTTGCGCCCT	305
FMgSSR-38885	scaffold5554	p2	(TC)8	Class II	29053	29068	ACGATAAGTGTTCGTGCGGGT	TTGTCCATGTCACCGTGCCT	318
FMgSSR-38888	scaffold2797	p2	(TC)8	Class II	54247	54262	ACGCAGCATGCCAACAACTG	ATGCTCCGCCGCACACAAAA	328
FMgSSR-38889	scaffold2267	p2	(TC)8	Class II	56923	56938	ACGCAGCTAGCGTGACATTTGC	AAGCGGTGGTGCACTGCATAGT	288
FMgSSR-38892	scaffold9238	p2	(TC)8	Class II	3195	3210	ACGCGGGAAGTGAGTGCTTT	AAGACAAGTTTGGTGCGGGTGC	257
FMgSSR-38895	scaffold795	p2	(TC)8	Class II	81180	81195	ACGGATCATGTCTCGTAGGCGT	CGCAACGCTCGTAATGCAGCTT	287
FMgSSR-38897	scaffold11773	p2	(TC)8	Class II	5587	5602	ACGGCAATGGGCGCTTTCAT	TCGAGCCATATGTGTGCAACGGA	315
FMgSSR-38898	scaffold655	p2	(TC)8	Class II	43526	43541	ACGGCGAAACCGACCATTCT	TGTTGCAGCGCCAGTGAAGT	309
FMgSSR-38899	scaffold15050	p2	(TC)8	Class II	7907	7922	ACGGGATGCTCCATTGTTGCGA	TGATGGTCTGAACCTCGCGTGT	218
FMgSSR-38901	scaffold1333	p2	(TC)8	Class II	9164	9179	ACGTCGCCACAAAGGCTGCTAT	ACGCACCCAACGTTTCCTCA	343
FMgSSR-38903	scaffold111	p2	(TC)8	Class II	31937	31952	ACGTGTTTCCGTCGCCGTACAT	TGGCTGGCAGTTTCCGCTTT	331
FMgSSR-38907	scaffold36852	p2	(TC)8	Class II	1732	1747	ACTCGGCTCAATTCAGCACCT	TGCGAGCTCACAAGAACAGCCA	317
FMgSSR-38915	scaffold6194	p2	(TC)8	Class II	27865	27880	AGAACAGCTCACGTGTTGCCT	AGGAGGAAGGTGTTGTCGGTGA	285

SSR_ID	Scaffold	SSR_Type	SSR_Motif	SSR Class	SSR_Start	SSR_End	Forward sequence	Reverse sequence	Product_size
FMgSSR-38924	scaffold3779	p2	(TC)8	Class II	24972	24987	AGCAACCAACCCCGATGAGA	TGCGTGCGTCGCCTCTTTTT	341
FMgSSR-38925	scaffold8548	p2	(TC)8	Class II	17684	17699	AGCAACCGCCGCTTCAGAAA	TCGAATTTGACCCGAGTCGGAGA	202
FMgSSR-38928	scaffold5130	p2	(TC)8	Class II	33167	33182	AGCACATGGGAATGTCCCACCA	TCGCGTGTGCATGGTCTTGT	224
FMgSSR-38931	scaffold3905	p2	(TC)8	Class II	30606	30621	AGCAGTCATGCAATGGGCGA	TGGATGGCAAGAGAACCCGACT	332
FMgSSR-38932	scaffold587	p2	(TC)8	Class II	56529	56544	AGCAGTGTTCCTGCTGGTGCAA	TGTGCATGCAGCAAGAGTCCGA	307
FMgSSR-38936	scaffold16114	p2	(TC)8	Class II	372	387	AGCCCAGGGAACAAGCAATGGA	TTCTGCAGAGCTCCTGCGTAGT	250
FMgSSR-38940	scaffold3729	p2	(TC)8	Class II	23902	23917	AGCGACTGGGCGTAGAAGCAAA	TGCTTTGGGCTGGCTCTTTGGA	312
FMgSSR-38943	scaffold2012	p2	(TC)8	Class II	31312	31327	AGCGCCGCATTTGCACTTGT	GCTCCATTCCTGATAGCACGCAGA	294
FMgSSR-38944	scaffold7805	p2	(TC)8	Class II	32611	32626	AGCGCGGGCATTTGTCTAGT	ACAGCATATCAGCGACGGAGCA	250
FMgSSR-38949	scaffold8240	p2	(TC)8	Class II	19308	19323	AGCTGACCTGAACGCAGGAA	TGCTCACGCATCTCGCTCAT	281
FMgSSR-38950	scaffold2624	p2	(TC)8	Class II	43009	43024	AGCTGATGGCAAAGGCCGAA	ATATTCCGCATCCTGACGCGCT	337
FMgSSR-38952	scaffold7422	p2	(TC)8	Class II	30726	30741	AGCTGTGCGCAGCAAAAGTGA	AAGCGTCAGAATGTGAGCCGGT	257
FMgSSR-38953	scaffold2994	p2	(TC)8	Class II	31900	31915	AGCTTCAGACTTGTGTGGATGGCT	TCGCCATTTCCAAAGCTTGGCT	291
FMgSSR-38958	scaffold4213	p2	(TC)8	Class II	6562	6577	AGGAAACCGCTATGGCCCAA	AGGAGGGATGAGCACATGCGAA	325
FMgSSR-38959	scaffold7069	p2	(TC)8	Class II	17050	17065	AGGAAGCGAGCCACGTCAACAT	TGCAGCAGCAGGTAGCTGTAGT	313
FMgSSR-38964	scaffold1068	p2	(TC)8	Class II	41931	41946	AGGCCAAAGTCCTGCAGTTCCA	TTGGGCTGGGACACAAAAGCCT	346
FMgSSR-38965	scaffold3360	p2	(TC)8	Class II	46844	46859	AGGCCATGGACCTTGTTGGT	ACAACACAAGCACCCGCAGT	310
FMgSSR-38966	scaffold922	p2	(TC)8	Class II	94318	94333	AGGCGCAAAGCCAAGATGCT	ACATTGCGCTAGCCGTCGATA	294
FMgSSR-38968	scaffold308	p2	(TC)8	Class II	121525	121540	AGGCGTTTTGGCTCACTCGT	ACAAACACGCACGCGCAACT	329
FMgSSR-38971	scaffold1159	p2	(TC)8	Class II	4246	4261	AGGCTTGCCACGGCTGTACAAT	ACGGCACCTTTCCTGGCATCAT	337
FMgSSR-38972	scaffold117831	p2	(TC)8	Class II	610	625	AGGGCGTGCTGTTCGTTCTT	TGGGTGGCTGCGAACCACTTTT	257
FMgSSR-38975	scaffold4014	p2	(TC)8	Class II	30523	30538	AGGGTGTGTGATCAGCGAGGTT	AGCAGCAGCTTGGGCTTTGA	346
FMgSSR-38977	scaffold2106	p2	(TC)8	Class II	15568	15583	AGGGTTGCGTCACATGGCAA	TCGCCGTGATAAAGAGCTGCGT	228
FMgSSR-38983	scaffold2120	p2	(TC)8	Class II	55434	55449	AGGTTGCCCCTCCACTACAAACA	TGGGCAAGAATGCGACTGAAGC	246
FMgSSR-38984	scaffold2319	p2	(TC)8	Class II	45539	45554	AGGTTGTGGGTGGGCCAATGAT	TAGTACCGTGAATGGACCCGCT	280
FMgSSR-38985	scaffold398	p2	(TC)8	Class II	104021	104036	AGTAACCGTACGCCTGGCCAAA	TGTACTTCGGCTGCTGGTCA	328
FMgSSR-38989	scaffold8004	p2	(TC)8	Class II	7407	7422	AGTGCGCATGCTCTGACCAA	ACGGGAATTGACTGTGGTGCGA	238
FMgSSR-38993	scaffold6940	p2	(TC)8	Class II	7560	7575	AGTGGACGCGAAGCAACGAA	AGGTAGCTGGAGTAGTGGGAATGT	216

SSR_ID	Scaffold	SSR_Type	SSR_Motif	SSR Class	SSR_Start	SSR_End	Forward sequence	Reverse sequence	Product_size
FMgSSR-38994	scaffold35238	p2	(TC)8	Class II	706	721	AGTGGGGGACATGGCCTAGTTT	AAGAAACAAGCCAGCCGTGC	322
FMgSSR-38997	scaffold3971	p2	(TC)8	Class II	36606	36621	ATAATTCGCTGGTGCAGCCGGT	TGGCGCCATGCATCTCTGAT	294
FMgSSR-39000	scaffold3914	p2	(TC)8	Class II	2439	2454	ATCATCGGGTGCATGCTCGTCA	ATCCACGTGCCCCAAGCAAA	282
FMgSSR-39001	scaffold5438	p2	(TC)8	Class II	23217	23232	ATCCCTTTCCTCTGTTGCCCCT	ATTGGCCACCGTCGCACCTAAA	242
FMgSSR-39003	scaffold43	p2	(TC)8	Class II	175439	175454	ATCGCGCATTCCAAGGTCGT	TGCATGCCCGTACGCTTTGT	240
FMgSSR-39004	scaffold1966	p2	(TC)8	Class II	10458	10473	ATCGGTACGGCGTGTAATTGCG	AGCTAATAAACCGGGCGGCGAA	322
FMgSSR-39010	scaffold40690	p2	(TC)8	Class II	430	445	ATGCACCAGGCATCACAGCA	ATTGTTCGGGGTGGGCTAAGGA	313
FMgSSR-39011	scaffold60	p2	(TC)8	Class II	36986	37001	ATGCAGTGGTGTGATGCGCT	CCTCCCGTAGTTGTGTACTGTGCAT	322
FMgSSR-39030	scaffold4613	p2	(TC)8	Class II	22725	22740	ATTGCCACCAGCCCAGCAAT	TAGCCCGCTATCGTTCCGCAAA	237
FMgSSR-39032	scaffold12564	p2	(TC)8	Class II	17568	17583	ATTGTTTCGGCTTCCGGCAACG	GCGAACAGTTCCAATTGTTGAGCG	252
FMgSSR-39033	scaffold2214	p2	(TC)8	Class II	41556	41571	ATTTAGTGGTTGGCGGCGCGTA	TTGGGCTGGCGTTTTGGACA	260
FMgSSR-39034	scaffold37881	p2	(TC)8	Class II	4784	4799	ATTTCCCGCATGGCAGCACT	TGTGGCTTCGGAGCAGCATCTT	246
FMgSSR-39035	scaffold243993	p2	(TC)8	Class II	397	412	ATTTTCGAGTCGGAGGCGCA	ACCCGGGTGATGGTGAGGAAAA	218
FMgSSR-39037	scaffold10526	p2	(TC)8	Class II	24936	24951	CAAGATGAAATTCAAGGGGGCTGG	AATTAACGGTGGAGCAGGGGGT	339
FMgSSR-39045	scaffold5391	p2	(TC)8	Class II	32329	32344	CCATCCATCATGTGCGTGCGAT	ACAGTGTAGCTCACGACTTGGCA	236
FMgSSR-39046	scaffold6734	p2	(TC)8	Class II	4366	4381	CCATCTTGGAAAAGCGCACGGT	AACCGGCTCTCGTGCTTGAA	297
FMgSSR-39047	scaffold10547	p2	(TC)8	Class II	23481	23496	CCGCACGCCCGAAAACAAGTTA	TCGCTTGCGAACACCTCCACTA	253
FMgSSR-39048	scaffold11442	p2	(TC)8	Class II	13912	13927	CCGCTGAAACAAGGACATGGCA	GAGCTGAGCTTGAGGACCAGAT	235
FMgSSR-39050	scaffold2432	p2	(TC)8	Class II	21113	21128	CCTCTGGAGAGTCAACGTGGAAGT	ACTTCTGCGTGTGAGCGAGACT	243
FMgSSR-39055	scaffold223	p2	(TC)8	Class II	57667	57682	CGCCAAACCGGCGATCACATTT	ATGGCTCGCAAAGCCGCAAT	285
FMgSSR-39056	scaffold69957	p2	(TC)8	Class II	230	245	CGCGAATCCTTTGCTTCCTTCGT	AAAGGCGTGTGGATCATGGCGA	322
FMgSSR-39057	scaffold678	p2	(TC)8	Class II	68381	68396	CGCTGGCCTTCTCTCTCTTTCTCT	TCGTCGAAGAACCGTGAAAGGCA	220
FMgSSR-39060	scaffold3631	p2	(TC)8	Class II	8560	8575	CGTACACGCGCGCACTACAAAA	AAAATGCCTGCCGCACCATTCG	207
FMgSSR-39062	scaffold11744	p2	(TC)8	Class II	8495	8510	CGTCACGCAAATGCGCTAGGTT	GGGGAAGAATGGAGCCAAGCAA	334
FMgSSR-39063	scaffold1410	p2	(TC)8	Class II	9105	9120	CGTGCTACGGCAGAATGTGCAA	GGCACGAGCGCTTGAAATCT	297
FMgSSR-39066	scaffold11484	p2	(TC)8	Class II	7219	7234	GAAACACGCACATAGGCGAAAC	AAGGTGCACGTATGTTGGCACG	279
FMgSSR-39070	scaffold129458	p2	(TC)8	Class II	247	262	GCACACGCCCCAAAGTGTCAAA	TCTTGCTTGCTTGGCTTGCGA	206
FMgSSR-39072	scaffold2083	p2	(TC)8	Class II	9371	9386	GCACTTTCATGAGCGAAACAGGTG	TGATTTACCGCCCGCATCCCAA	286

SSR_ID	Scaffold	SSR_Type	SSR_Motif	SSR Class	SSR_Start	SSR_End	Forward sequence	Reverse sequence	Product_size
FMgSSR-39073	scaffold6033	p2	(TC)8	Class II	14869	14884	GCAGCACACCCAGCATCCAATA	ATCGGCGCCCACTTCACTTT	306
FMgSSR-39074	scaffold10428	p2	(TC)8	Class II	28476	28491	GCAGCGACGGCGAATCAAAA	TCCAGCTCAGGAGAGGGAAAGA	348
FMgSSR-39080	scaffold309	p2	(TC)8	Class II	88714	88729	GCCATGCCAAATTGCCAAGCCA	ACGCCGCACCCATTTCCAAT	308
FMgSSR-39081	scaffold13205	p2	(TC)8	Class II	11847	11862	GCCCAAGGCCCGTTTAGATGTT	TCTAAGGGCAGCACGAGCAT	231
FMgSSR-39084	scaffold3556	p2	(TC)8	Class II	55269	55284	GCCGGCCCAAAAACCAACGTAA	AAGGAGCAAAAGCCGCCCCTAT	343
FMgSSR-39085	scaffold2153	p2	(TC)8	Class II	43755	43770	GCCTTCTTTCCTACTTTGTCCCGC	TCGGGCATGTAGCGTTTTACCA	289
FMgSSR-39087	scaffold3113	p2	(TC)8	Class II	41462	41477	GCGCTTGAATGGCTTGAAACGGA	TGGCATGCTAAGAAAGTGCAGGC	321
FMgSSR-39089	scaffold458	p2	(TC)8	Class II	38273	38288	GCTGCTGCTGCGGCTAAAAA	AGCCCAAACGCCAAACGCAA	258
FMgSSR-39092	scaffold952	p2	(TC)8	Class II	2355	2370	GGCAGCCAACCAACGCTTTT	GCACGGGAGGATTGTCGTTGTT	247
FMgSSR-39093	scaffold11853	p2	(TC)8	Class II	12015	12030	GGCATTGCAAGTCGGCATCGTT	CACGCGGAGAGAGAGAAGCTAT	318
FMgSSR-39094	scaffold36900	p2	(TC)8	Class II	1923	1938	GGCCCGGTTTTTGTTGCGTT	TACTGTGCCAACGGGGTGTCAT	336
FMgSSR-39097	scaffold1232	p2	(TC)8	Class II	52717	52732	GGCTTGCACATCCTTGCACCAA	CCGTCTTGTTCCACAAGTGCCA	281
FMgSSR-39102	scaffold547	p2	(TC)8	Class II	50843	50858	GTAAGCCAAAGTGTTGGTGGGT	CACACACTAGCAAAGTCACAGACG	275
FMgSSR-39110	scaffold2108	p2	(TC)8	Class II	17144	17159	TAATGATTCCGGCGCTACCTGC	TTGCGCTCAGTTGGCTTTGA	302
FMgSSR-39116	scaffold40365	p2	(TC)8	Class II	2625	2640	TATGCCGATCCGTGTGCCTTCA	TGAAGCTTGCTACCGCCCAAGT	292
FMgSSR-39118	scaffold10986	p2	(TC)8	Class II	2002	2017	TCAAAGCCCATATCCCAGGCCA	ACGATGTGGAGCGTTGGAAGCA	257
FMgSSR-39119	scaffold2523	p2	(TC)8	Class II	58315	58330	TCAACAGCCACGCACAGCCATA	TGTACGTGCGTTCCACCGTT	312
FMgSSR-39120	scaffold701	p2	(TC)8	Class II	90616	90631	TCAAGCACCGGCAAAAAGCGA	ACCGACCATGGCTTCCCACAAA	298
FMgSSR-39122	scaffold9478	p2	(TC)8	Class II	30416	30431	TCACACACGATCCCGCAACT	AAACCTCTGCGCGGCCATTTT	200
FMgSSR-39124	scaffold12153	p2	(TC)8	Class II	1882	1897	TCACGCCGTGGTAAAGACCAGT	ATTTGCGCATCAACGGCAGC	345
FMgSSR-39126	scaffold23733	p2	(TC)8	Class II	4956	4971	TCACGCGATTGCAGGTCAGGAT	AGGGACGCAAGGAAAACTCCGA	298
FMgSSR-39128	scaffold3329	p2	(TC)8	Class II	32672	32687	TCAGAGGGATGTAAGACGGTGGA	TGCGCCGGATCATTCTCGATCA	311
FMgSSR-39129	scaffold1753	p2	(TC)8	Class II	27471	27486	TCAGCCGGTGCTGTCGTTTT	AGCTGCACAGCAGTCGTTCA	330
FMgSSR-39130	scaffold4373	p2	(TC)8	Class II	35106	35121	TCAGCGTCCGTTCATCATGAGTC	TTGGGCTAGGCCCCATGAACTT	348
FMgSSR-39134	scaffold4664	p2	(TC)8	Class II	20250	20265	TCCAATGCCTCCAGCAAGCTCT	TGACTTGCACTTTGCGTGCG	284
FMgSSR-39136	scaffold16592	p2	(TC)8	Class II	4771	4786	TCCACCAACCCACAGTACGCAT	ATAGCACCGAACACAGCGCA	251
FMgSSR-39137	scaffold10337	p2	(TC)8	Class II	20578	20593	TCCACGAGCGAGCATTGGACTT	TGCATGTTTCAGGATCAGGCCCA	316
FMgSSR-39138	scaffold25132	p2	(TC)8	Class II	1004	1019	TCCAGCTTCGTGGGCCAAAT	GTGACCTTTCGGTCAGATTTGAGG	274

SSR_ID	Scaffold	SSR_Type	SSR_Motif	SSR Class	SSR_Start	SSR_End	Forward sequence	Reverse sequence	Product_size
FMgSSR-39141	scaffold21422	p2	(TC)8	Class II	618	633	TCCCAGCAGCAAAACGGCAA	TTGCCCTAGGGTTAGGGTTCTGGA	320
FMgSSR-39142	scaffold12	p2	(TC)8	Class II	110143	110158	TCCCATGCTGCAGCTCCTGAAA	ATCGGCATTCTGTCCGGGTTCA	314
FMgSSR-39144	scaffold23815	p2	(TC)8	Class II	7906	7921	TCCCGACTTTGAGCAACGCCAT	TGCAAGCACGGGAAGACACA	307
FMgSSR-39156	scaffold2203	p2	(TC)8	Class II	55262	55277	TCGAGCTCAAGAACGTGACCGT	TGCACGAGCCACACGAAGAACT	218
FMgSSR-39157	scaffold549	p2	(TC)8	Class II	85055	85070	TCGATCGGCCACTTGATCACCA	ACGCGTGCGTCAAGGCTTTT	204
FMgSSR-39158	scaffold23932	p2	(TC)8	Class II	5264	5279	TCGATCTCCATCGCCTTGTGCT	ACGTTGTGGCTCCGCTGATT	288
FMgSSR-39159	scaffold12663	p2	(TC)8	Class II	10334	10349	TCGCACGCGCAGCGTATAAT	ACAGCGGATCAACCGCGAAA	200
FMgSSR-39163	scaffold36575	p2	(TC)8	Class II	1692	1707	TCGCCATGTCCTCCCAACAA	TGGCACAGTGCGTGCAGTAA	343
FMgSSR-39167	scaffold715	p2	(TC)8	Class II	4002	4017	TCGCCGCTGGGTTGTGAAAT	TGGGGACTGGAGTATCTTTGCGA	344
FMgSSR-39169	scaffold9488	p2	(TC)8	Class II	12930	12945	TCGCCTTTCCCGTGCTTGTT	CGCGCCATCCATTTTGTTCCGT	293
FMgSSR-39171	scaffold11989	p2	(TC)8	Class II	6140	6155	TCGGAACCCAGCGGCAAAAA	AACCTTGGGCAGTAGCTTGGCT	211
FMgSSR-39172	scaffold12021	p2	(TC)8	Class II	6386	6401	TCGGACAACATAGGCGCTGCAT	AGCGATGGCGTTCGTTCTCT	317
FMgSSR-39181	scaffold4444	p2	(TC)8	Class II	11275	11290	TCTGATCTGCAATGAGCGGCCT	AGATGTATGGGACCCGGAGACA	337
FMgSSR-39182	scaffold3240	p2	(TC)8	Class II	353	368	TCTGCACCGCTGCAAGTCAA	TGCGAGCCGTACCTGTTTGT	302
FMgSSR-39187	scaffold251607	p2	(TC)8	Class II	148	163	TCTGTCTGTCTGCTTAGCGGGT	ACCCAACAGCCGTGCATGAT	308
FMgSSR-39188	scaffold2659	p2	(TC)8	Class II	60221	60236	TCTTCAACCTCCAGCCATCGT	TGGAGCAGCTAGGCACAACACA	211
FMgSSR-39192	scaffold29693	p2	(TC)8	Class II	2871	2886	TGAACAGAGGCAGAGGTGCT	TGGTTGTGTGTTGACCAGACCCAG	218
FMgSSR-39197	scaffold465	p2	(TC)8	Class II	35267	35282	TGACGACGCCGCTTGTTTCA	AGCCGTTGAAAGGTTCGCCA	253
FMgSSR-39201	scaffold8493	p2	(TC)8	Class II	3007	3022	TGATCAACATGGCAGCGCGT	TTGGGGAGGCGATCTGGAGAAA	246
FMgSSR-39206	scaffold14098	p2	(TC)8	Class II	4446	4461	TGATCGATCGGCAAGGGGTAT	TCGAGCTTCGACCACAGCAACA	281
FMgSSR-39208	scaffold4514	p2	(TC)8	Class II	40549	40564	TGATTTCCGATGGCGCGCTT	ACCATCCATCGGCCACATGACA	337
FMgSSR-39212	scaffold2117	p2	(TC)8	Class II	34215	34230	TGCAAAGGTGGGGAGCCAACAT	ACCACCAGTGATCCAAAGCGGA	220
FMgSSR-39213	scaffold24658	p2	(TC)8	Class II	1586	1601	TGCAAGGCGAGATGTTGCTGT	TTTCAGCTGCGACTGATCCACG	329
FMgSSR-39216	scaffold14230	p2	(TC)8	Class II	4713	4728	TGCACAGTACACAACTACGGGAGG	ATCCAGGCTCGTTTCTTCCGCA	307
FMgSSR-39218	scaffold6913	p2	(TC)8	Class II	31053	31068	TGCACCAACATCAGGCAGCA	AGTCGCCGCAGTCAGTTGATGA	256
FMgSSR-39223	scaffold57	p2	(TC)8	Class II	129143	129158	TGCATCCATAGCAAAAGGGCGGA	TCAGGAGGGTGGCTCAAGAAACA	281
FMgSSR-39228	scaffold3636	p2	(TC)8	Class II	38270	38285	TGCCAAATGGGTCAATGGGCTG	AATACTACGGCAAAACGCCGGG	244
FMgSSR-39234	scaffold1112	p2	(TC)8	Class II	80500	80515	TGCCATGTTGGTCTCGTCTCAC	GCCACAGGCGCATGCACATAAT	297

SSR_ID	Scaffold	SSR_Type	SSR_Motif	SSR Class	SSR_Start	SSR_End	Forward sequence	Reverse sequence	Product_size
FMgSSR-39242	scaffold3056	p2	(TC)8	Class II	41355	41370	TGCGCGCCTCGCAATGAAAA	ACGAGGCACGCGTTTGGTTT	258
FMgSSR-39243	scaffold212	p2	(TC)8	Class II	116123	116138	TGCGGCGCGCCAAATAGAAA	GCCAAAGCGTCCTTTCGTTCGT	291
FMgSSR-39244	scaffold7320	p2	(TC)8	Class II	3978	3993	TGCGGTGGAAGCGTGGAAAT	AGCCCGTAAGACAGCGAAAACC	250
FMgSSR-39250	scaffold4656	p2	(TC)8	Class II	33482	33497	TGCTGCTCTCATGATCTGGGGAA	TTGTTCGCCGCCCTGTTGAA	287
FMgSSR-39251	scaffold7331	p2	(TC)8	Class II	2510	2525	TGCTGCTGAGTATCGCCGTTGT	TCGCTCAGGGCTTGTTGTGGTT	341
FMgSSR-39252	scaffold235	p2	(TC)8	Class II	113472	113487	TGCTGTAACCAACCGATCCAAGCA	ACCTCCCCCTAGCCAACTGGAAAA	226
FMgSSR-39253	scaffold2645	p2	(TC)8	Class II	36051	36066	TGCTGTAGCTTGCTGCTGCTCT	CCGCAATTCATCGACAAAGGCCA	243
FMgSSR-39254	scaffold33705	p2	(TC)8	Class II	3819	3834	TGCTGTATCCACCAGCCAACGA	CCAAATGGTATGGTCACAGACTGC	335
FMgSSR-39261	scaffold6259	p2	(TC)8	Class II	9419	9434	TGGAGGCGAAGCTGTTGCACAT	AGCATGCGCCACTCTGTTGT	256
FMgSSR-39264	scaffold8639	p2	(TC)8	Class II	14094	14109	TGGCACAAGGTGGCGAGATTGA	TGGGTTTGGTTTGGTCGGGT	217
FMgSSR-39267	scaffold935	p2	(TC)8	Class II	77663	77678	TGGCCAAAATGGAAGGCGCA	TGACGATTGTGCCTTGGGGA	326
FMgSSR-39268	scaffold8916	p2	(TC)8	Class II	26210	26225	TGGCCAAGGCCCAATATAGACAGC	TTGGTGCTCATGGCTGAACAGG	331
FMgSSR-39269	scaffold186980	p2	(TC)8	Class II	273	288	TGGCCATCCCGCATTGGGAAAA	ACGAGGAAATCCTTTGCCGGA	298
FMgSSR-39278	scaffold10190	p2	(TC)8	Class II	24535	24550	TGGCTCCTTGATCCTTACATGGGG	TGCGCATGGTGGCAAAAGTC	343
FMgSSR-39280	scaffold2118	p2	(TC)8	Class II	16579	16594	TGGCTGTCATGTGCCCTGTTGA	ACTTAGTCGGTTAGGAGGGAGGCA	261
FMgSSR-39281	scaffold6430	p2	(TC)8	Class II	31749	31764	TGGCTGTGGTTGTACGACGAGT	ACTGTCATCGCTCATTGGCCT	322
FMgSSR-39282	scaffold587	p2	(TC)8	Class II	55176	55191	TGGGCCCATTGGGATTGCTCAT	TCACAACCCAGGGTGCATGCTT	294
FMgSSR-39283	scaffold9441	p2	(TC)8	Class II	9311	9326	TGGGCCGAAACGATGAGCAA	GGCAGTGCCATGAGCATTCCTT	217
FMgSSR-39284	scaffold86340	p2	(TC)8	Class II	1404	1419	TGGGCTCACATGATTGCCACT	AGACTGGTGGTGCGTGTGAGAA	247
FMgSSR-39285	scaffold12376	p2	(TC)8	Class II	21778	21793	TGGGGCACCTCTTCGACAGATT	AAATAGAGGCGGTGCGAGGAGA	332
FMgSSR-39286	scaffold6578	p2	(TC)8	Class II	15692	15707	TGGGGGAAGTTATGCTGCGTCT	TCCAATACCACCACTGCCATCGT	324
FMgSSR-39288	scaffold2751	p2	(TC)8	Class II	39787	39802	TGGGGTGGCCTTTCATCTCA	ACAGGCATCGGTCGAACTCACA	259
FMgSSR-39289	scaffold262	p2	(TC)8	Class II	55982	55997	TGGGTGGTTGTGCTTCCTGA	AACAAAGTGGTGTCGTCCGCCT	346
FMgSSR-39291	scaffold13609	p2	(TC)8	Class II	18917	18932	TGGTACGCTTTCAACGGCTTCC	CGCAAGGCCAGCATTTTTCCCA	277
FMgSSR-39293	scaffold410	p2	(TC)8	Class II	16587	16602	TGGTCCCTAGGCGAGGTTTTGT	TCGTCAGTGGCTCTGGTCACTT	340
FMgSSR-39297	scaffold11188	p2	(TC)8	Class II	10742	10757	TGGTGCCATCTTATCTACCGCTGA	TGCCAACTTGAAGCGACGCA	314
FMgSSR-39298	scaffold3010	p2	(TC)8	Class II	22070	22085	TGGTGGAAGCTGCAAAGCTGGA	AGCTCTCATGCTGCTGCTGATGT	206
FMgSSR-39302	scaffold20717	p2	(TC)8	Class II	4004	4019	TGGTTTGGAGCATGGGCGTT	ACCTCTGGCTACTTGCTGCCTT	264

SSR_ID	Scaffold	SSR_Type	SSR_Motif	SSR Class	SSR_Start	SSR_End	Forward sequence	Reverse sequence	Product_size
FMgSSR-39303	scaffold2277	p2	(TC)8	Class II	38772	38787	TGTCACGTGCCTATAACGACCA	GAAAAACGTGCAGCGCATGA	216
FMgSSR-39306	scaffold92	p2	(TC)8	Class II	163757	163772	TGTCTTTCCCTGTGCGTGTGT	AGGCGTTCCACATCGTGCAT	314
FMgSSR-39311	scaffold2145	p2	(TC)8	Class II	22777	22792	TGTGGCACGCCATGTGTCAA	ATGCCGTGTTCGAGCTTGTTGC	304
FMgSSR-39312	scaffold10217	p2	(TC)8	Class II	9499	9514	TGTGGGGAATCCACGCCAAACA	CAGCCGCATGCATCATGACAGA	349
FMgSSR-39316	scaffold21822	p2	(TC)8	Class II	5418	5433	TGTTAGGCAGTGGCGTGTCCTT	TGCTCTTTCCAGCCAATGCTTCCA	204
FMgSSR-39320	scaffold113	p2	(TC)8	Class II	48621	48636	TTAATACCGCAGGGCCACTCT	TTTATGCCGCCCATGCTCGT	343
FMgSSR-39323	scaffold270	p2	(TC)8	Class II	134716	134731	TTCAACCATTGCCGTCCCGT	AGCTGTGCACGCGTGAGAGTAA	250
FMgSSR-39326	scaffold23679	p2	(TC)8	Class II	1577	1592	TTCACCTTGTTGTCGTTGCCGC	AAACACTCGGGGGTGGACAGTA	339
FMgSSR-39328	scaffold13657	p2	(TC)8	Class II	14102	14117	TTCAGCGTCTTCAACGGCGTCT	TCAGCCACCTCTCCAAAAGTGC	334
FMgSSR-39330	scaffold868	p2	(TC)8	Class II	23932	23947	TTCATTTGTGTGGCCGCAGGGA	TCCATTTTCTTCGGCAGCCCCT	231
FMgSSR-39331	scaffold4490	p2	(TC)8	Class II	40654	40669	TTCCCATGCCAAGGCGAAGGAT	AAAGATTCCAAGGCGCCGCA	243
FMgSSR-39335	scaffold5063	p2	(TC)8	Class II	45443	45458	TTCGACATGACGGCGATGGT	TGCGCCACATGCAAATTCTCCG	311
FMgSSR-39344	scaffold28233	p2	(TC)8	Class II	5729	5744	TTGAGACAACTTCCCTCCCTCCCT	TTTGAAGCAGAGGAGGGCTTGC	268
FMgSSR-39345	scaffold80	p2	(TC)8	Class II	165821	165836	TTGCATCTGTGCCGGTTGGT	TGTTGCTTGTCTGCGGTCCA	345
FMgSSR-39347	scaffold8603	p2	(TC)8	Class II	16695	16710	TTGCCTGATGTTGGCTGCCACT	TCCAGCAGCCATGCACCATTCA	321
FMgSSR-39350	scaffold1015	p2	(TC)8	Class II	35664	35679	TTGCTGCTGGAGAGTGGAGACA	TGTCGACGAATGGACCACCACT	346
FMgSSR-39351	scaffold694	p2	(TC)8	Class II	64537	64552	TTGCTGCTTCTGACCGGCTGAT	ATCGCGACTGAGATTTGGGCGT	296
FMgSSR-39354	scaffold24974	p2	(TC)8	Class II	2609	2624	TTGGACTGGCCCGTTTAGGTGT	AGTAGCTACACTCGGCGTGCAT	240
FMgSSR-39360	scaffold13430	p2	(TC)8	Class II	12193	12208	TTGTAGCGGCCCAGCACATT	ACCGATGCACCACCAACCCATT	251
FMgSSR-39361	scaffold6203	p2	(TC)8	Class II	30960	30975	TTGTCCATGTGCGGCTGAGGTT	TCCTGCCAACATCCTCTGTGCAA	200
FMgSSR-39362	scaffold7426	p2	(TC)8	Class II	18003	18018	TTGTCGGCATCACCGACACCAA	TGGCGGCATGGCTTGATGAT	260
FMgSSR-39376	scaffold4493	p2	(TC)8	Class II	29924	29939	TTTTACTCGCGGGAAAGGCACC	ATCGCGCAACCAGGTCAACA	348
FMgSSR-39378	scaffold19383	p2	(TC)8	Class II	2638	2653	TTTTGTGCGTGCGTGCGAGT	ACGATGGCGCGAGAATGGAAGT	292
FMgSSR-39384	scaffold10889	p2	(TC)9	Class II	5686	5703	AAACAGTAGCGTGCCGTGCT	ATCTGTCGCTTTGCTGCCGA	290
FMgSSR-39389	scaffold455	p2	(TC)9	Class II	25706	25723	AAAGACCCTCTTGCCCCGTCTT	TCCTTCACTCCCTTTCTCGGGT	231
FMgSSR-39393	scaffold5066	p2	(TC)9	Class II	43370	43387	AAAGGTTAGTGCGGCTCCGTCT	TCAGGCCAAAACGACAGCGA	215
FMgSSR-39395	scaffold5296	p2	(TC)9	Class II	20062	20079	AAATCCGCCACGCCGATGAA	ACCAATCGTGAGCCCTGCTTGA	268
FMgSSR-39398	scaffold59557	p2	(TC)9	Class II	317	334	AACAACCAGTGCCCGTCTTGGT	ACCTGTTTTGCGACTCTGCCCA	310

SSR_ID	Scaffold	SSR_Type	SSR_Motif	SSR Class	SSR_Start	SSR_End	Forward sequence	Reverse sequence	Product_size
FMgSSR-39400	scaffold2516	p2	(TC)9	Class II	58149	58166	AACACGTCCATCCCTACTGCCA	TTGCAGTCCCCGTTGCAACACA	254
FMgSSR-39402	scaffold13901	p2	(TC)9	Class II	6149	6166	AACAGGGGCTTTGGTTCGGA	TGTCTTCATTGCCTTGGGGC	273
FMgSSR-39404	scaffold132	p2	(TC)9	Class II	89168	89185	AACGCCTACACCCAGATGTTGC	CACGCGTGGCAACAAAAAGCA	299
FMgSSR-39414	scaffold6995	p2	(TC)9	Class II	38498	38515	AAGCAACCGACGGAAGGCAA	TGCTTTGAGAAGGTGAATGCCGC	333
FMgSSR-39416	scaffold5511	p2	(TC)9	Class II	12988	13005	AAGCACGGTCAAGGTCGTCT	TGAGGTTAGGGCATTGATCAGGCT	232
FMgSSR-39421	scaffold2558	p2	(TC)9	Class II	43957	43974	AAGGCGATGCCAAAGGGACACA	AGAAATTTCGGGTCCGCCGT	271
FMgSSR-39422	scaffold558	p2	(TC)9	Class II	74358	74375	AAGGGGCTGGGTCAGCATCATT	TGACACTCGTTCAGCATCTTGTGC	219
FMgSSR-39429	scaffold10361	p2	(TC)9	Class II	3003	3020	AATCGTTGATGGGCGTGCCT	TGTTGCATGGTGGTCCCCTTCA	327
FMgSSR-39435	scaffold5529	p2	(TC)9	Class II	35839	35856	ACAACAATAGCCCCCTGTGGCA	TGCCCTTGTCCGCCAACATT	342
FMgSSR-39439	scaffold9155	p2	(TC)9	Class II	8275	8292	ACACGAGGCCCTCAAACAACCT	TGACTACTCACGACGGGCAA	332
FMgSSR-39440	scaffold128	p2	(TC)9	Class II	180238	180255	ACACGCATAACGACAACCGAACC	AAGGAAGACCTCACAGCACACG	266
FMgSSR-39447	scaffold1206	p2	(TC)9	Class II	60781	60798	ACAGCGCAAGAGCAGAGCAA	ACGAGTGCAACATTCACCAGCG	347
FMgSSR-39448	scaffold1078	p2	(TC)9	Class II	42490	42507	ACAGGTTGAAGCCTCGTCGT	TGATCCGGCGGCAAAGTTGT	237
FMgSSR-39449	scaffold14524	p2	(TC)9	Class II	12041	12058	ACATCAGCAGCAGACAGCGGAA	TTGGCCCAGAGTGTACTTGCGA	231
FMgSSR-39451	scaffold1564	p2	(TC)9	Class II	1939	1956	ACATGCAGCGACCCGATTGA	TGGAGTTGGCACTTTGGCACA	213
FMgSSR-39453	scaffold6286	p2	(TC)9	Class II	37586	37603	ACCAAAAGGAGCTGCCTTGCAT	AGCTGCCAACACTGAGAGGT	321
FMgSSR-39455	scaffold187	p2	(TC)9	Class II	63236	63253	ACCACCAGCAACAGGAACCCTT	TGTTTGTGCATCTCCGGGTGCT	287
FMgSSR-39461	scaffold172861	p2	(TC)9	Class II	770	787	ACCCCAGGGTTGCATTTTGGCT	AGGGAGCGTGATCAACCAGT	211
FMgSSR-39462	scaffold8884	p2	(TC)9	Class II	24444	24461	ACCCGCGGCTAGTGGAATTT	ACGACAATGGCGGCGATGTT	304
FMgSSR-39464	scaffold95	p2	(TC)9	Class II	60013	60030	ACCCTTGCTTTCCAGTCAGGCA	TTTGGCAATGCGCAGGGTGA	206
FMgSSR-39466	scaffold28420	p2	(TC)9	Class II	1222	1239	ACCGCGCGAGGAAGAAAGGAAT	ACCCCACTGGCACTGTAGCAAT	229
FMgSSR-39469	scaffold1598	p2	(TC)9	Class II	59866	59883	ACCTAACTTCACTCCCCCACCCTT	TTGTCGCAAGGTCACCACTGGA	212
FMgSSR-39473	scaffold4224	p2	(TC)9	Class II	5279	5296	ACGAACAACACGCAAAACGTGC	TTCGACGTCGCTGTCGAGTA	329
FMgSSR-39475	scaffold5609	p2	(TC)9	Class II	12647	12664	ACGACGAGGCTGCAAAAGCA	TTCATCAGTGGGCGTCTCTGGA	239
FMgSSR-39480	scaffold237	p2	(TC)9	Class II	92238	92255	ACGCAAACCTCTGCAGCTCT	ATTGGGTGGAGTTTGCTCCGCT	343
FMgSSR-39483	scaffold8137	p2	(TC)9	Class II	18439	18456	ACGCAGCTATGCAGCATGGACA	AGCCTGAATCTGGCGTCGAA	350
FMgSSR-39484	scaffold1854	p2	(TC)9	Class II	62151	62168	ACGCATCAACGCCTTCTCTCGT	AGCCCTGCGGCAATTTGTTTGG	216
FMgSSR-39487	scaffold41461	p2	(TC)9	Class II	5021	5038	ACGCTGGAACGGAGCCTGTAAT	AGATCCGCGTCCACCATCGTAA	331

SSR_ID	Scaffold	SSR_Type	SSR_Motif	SSR Class	SSR_Start	SSR_End	Forward sequence	Reverse sequence	Product_size
FMgSSR-39490	scaffold8747	p2	(TC)9	Class II	23195	23212	ACGGGCCCACACATGGGTTTAT	GGCAACGACGTGAGCAATGCAA	344
FMgSSR-39491	scaffold8270	p2	(TC)9	Class II	8694	8711	ACGTCCAAAAGCGAAAGCGCA	TCGCGCTTGCATCGTTGTTGT	338
FMgSSR-39492	scaffold5914	p2	(TC)9	Class II	5049	5066	ACGTCGCAATCAATTCGGGGA	ACCGCGAGGGTAACTTCGTCTT	340
FMgSSR-39498	scaffold28341	p2	(TC)9	Class II	7731	7748	ACTCCCACCCTCTCCCATGTTT	TTGCACTGCACCTCCTCCATGA	264
FMgSSR-39510	scaffold2117	p2	(TC)9	Class II	32793	32810	AGAGCTGTGTTGGAAAGGCGGA	AACGGGCCATTTGTCTCAGCGT	299
FMgSSR-39513	scaffold13612	p2	(TC)9	Class II	4777	4794	AGCAGCGACGAACATATGCGGT	TGCACAAGACAGCTGAGGATGGA	329
FMgSSR-39514	scaffold244	p2	(TC)9	Class II	37099	37116	AGCAGGCCACACAAAAAGCAGC	TGCTCCGACAGCTGCTTGAAA	219
FMgSSR-39517	scaffold4744	p2	(TC)9	Class II	11242	11259	AGCCCACATACACTATCCTAGAGC	CGCCTTGAAGCAACTCGGTCAA	335
FMgSSR-39523	scaffold2386	p2	(TC)9	Class II	7651	7668	AGCGCATTCACTTCGCTGCT	AGCCGCAAAAGGCACAGCTT	312
FMgSSR-39529	scaffold2918	p2	(TC)9	Class II	13997	14014	AGCTCCATTTGGTGGCTGTCCA	AGCGATGCACGGTGCCAAAA	294
FMgSSR-39533	scaffold1551	p2	(TC)9	Class II	12841	12858	AGCTGCAGTGACGTTCGCAA	TCCAACGGCTTCTTTCCAATTGCC	325
FMgSSR-39535	scaffold1055	p2	(TC)9	Class II	1712	1729	AGCTTCAACCAGCAGCACGA	TTGTTGACCCAGTCGGCCGTTA	336
FMgSSR-39536	scaffold17097	p2	(TC)9	Class II	9181	9198	AGCTTGCAGTTGTAGCCGCTGT	TGAAGAAGGCCCCGTCAACAA	328
FMgSSR-39540	scaffold13736	p2	(TC)9	Class II	16324	16341	AGCTTTTTGCTTAGGCGGCG	TTGCTCGACGCCAGCTTCTT	348
FMgSSR-39551	scaffold4212	p2	(TC)9	Class II	37437	37454	AGTCGCCACCACCCTCATATCT	TTACACCGCCGCACCAAACT	349
FMgSSR-39559	scaffold17276	p2	(TC)9	Class II	2016	2033	ATAATGCGGCGACATGCTGGGA	TGTTTGGGGATGAAGTGGCGGT	312
FMgSSR-39563	scaffold2299	p2	(TC)9	Class II	28007	28024	ATATTGGCGCGCACCCAACAAC	TGCGAAGTGGGGCAAAGACA	310
FMgSSR-39568	scaffold21554	p2	(TC)9	Class II	6463	6480	ATCGCTCGTGCATAAGGCGACA	TAAGCAGAGCTGATGGCGCTGT	319
FMgSSR-39569	scaffold322	p2	(TC)9	Class II	22212	22229	ATCGTCCACTCTTTCGCCCCTT	AAGGCCAGCCCAAACAACCT	210
FMgSSR-39570	scaffold15277	p2	(TC)9	Class II	8088	8105	ATCGTGTGTTCGCGTTGGGT	TTGATGCCGCGAGCATTGGT	315
FMgSSR-39573	scaffold2	p2	(TC)9	Class II	305630	305647	ATGACACGCACGCCTCCTTT	TGACGGCGTTGCTGGGATTGAA	201
FMgSSR-39574	scaffold280	p2	(TC)9	Class II	110349	110366	ATGACATGGTGACGTCGTGGCT	TTGCACCGTTCATCCGAGGGAA	293
FMgSSR-39575	scaffold616	p2	(TC)9	Class II	32961	32978	ATGACCCGACTGAGCACACACA	AGGAGCCCAGGTGCCTTTTT	345
FMgSSR-39577	scaffold5	p2	(TC)9	Class II	255278	255295	ATGATGCTGGGCGATTGACCGT	TGCTCCAGGTTTTCCCGGTT	338
FMgSSR-39578	scaffold931	p2	(TC)9	Class II	69261	69278	ATGCAGTTCCGCAACCTGAGCA	ATGGCCATGGTCGTCCTGTGTA	211
FMgSSR-39581	scaffold8896	p2	(TC)9	Class II	23716	23733	ATGCCCACCAAGCGATGACA	TGACCACTCCTTTCACATCGCCA	342
FMgSSR-39589	scaffold418	p2	(TC)9	Class II	41380	41397	ATGGGCCAGTATCCGCAACA	ACAGCGCTCAGTAACTGGTGT	346
FMgSSR-39592	scaffold9074	p2	(TC)9	Class II	29219	29236	ATTCCCTGCTCCGCCGATCAAT	AGGGCAGGGCTGCAATGATA	236

SSR_ID	Scaffold	SSR_Type	SSR_Motif	SSR Class	SSR_Start	SSR_End	Forward sequence	Reverse sequence	Product_size
FMgSSR-39595	scaffold4004	p2	(TC)9	Class II	15238	15255	ATTGTGCCACCGGCTGTTCT	AGGTGCACGCTGACAACCTAGA	328
FMgSSR-39601	scaffold690	p2	(TC)9	Class II	82347	82364	CAGCGCGCATTTGCCTTCTT	TGCAGACGTGACGAGGGACTAA	231
FMgSSR-39602	scaffold5423	p2	(TC)9	Class II	30093	30110	CCAAAATCAGCGATGGCCGTGA	TTGAAATTGGCGAGGCCGGA	280
FMgSSR-39604	scaffold23	p2	(TC)9	Class II	46236	46253	CCCGTGTTGTGTTGTGTCGTGT	AATGGTGATGGCGGCTTTTGCC	327
FMgSSR-39607	scaffold72	p2	(TC)9	Class II	90879	90896	CCGGCGGTGACAACACAAAAGT	AGTGCTGCTGCACGATGGTT	212
FMgSSR-39616	scaffold8799	p2	(TC)9	Class II	15019	15036	CGCTCAGTGTGCCCTAGCATTT	TTGCATGTCCTGGAAGGCGGTT	349
FMgSSR-39620	scaffold19	p2	(TC)9	Class II	208145	208162	CGGAGGACGCCTTTGTGGAATTA	TGCAGCTTGATCGCAGCACA	350
FMgSSR-39621	scaffold14063	p2	(TC)9	Class II	4270	4287	CTGCAGCAACAAAGAGAAAGGCCA	ATGGGGAGTGGCTACGTACCGAAA	330
FMgSSR-39624	scaffold166	p2	(TC)9	Class II	51171	51188	GCCAGTTGGTGGCAAAACAAGGA	AGAAGATGCAACCTTCGCGG	324
FMgSSR-39627	scaffold4445	p2	(TC)9	Class II	8317	8334	GCCGCGTAGGGAACAGATTTGA	TGGCAAGAGAAAGCTCGGCACA	299
FMgSSR-39629	scaffold2421	p2	(TC)9	Class II	56792	56809	GCGACAATACTGGAGCAATGGGGA	AGTGCAAGCTGCAGAGGTCCAT	312
FMgSSR-39634	scaffold527	p2	(TC)9	Class II	38672	38689	GCTTGCACAAATAAGCGAGGTGC	TGCAGAGCGCATCCATGTCT	345
FMgSSR-39640	scaffold296	p2	(TC)9	Class II	106800	106817	GGCGGCGGCATGTTTCTTTT	TGCGCTGGTGGGTTTTGTAGAC	282
FMgSSR-39643	scaffold11389	p2	(TC)9	Class II	3147	3164	GGGCCTTCTGCTCCCTTTCATT	AAGCTTTGTTTCCTGGGCAGCG	310
FMgSSR-39652	scaffold4570	p2	(TC)9	Class II	4719	4736	TAATAGCGGGTGACGTGGGCAA	TGCGCCTGCTTGTTCAGGTT	320
FMgSSR-39654	scaffold7331	p2	(TC)9	Class II	17388	17405	TAGGCTCTCAGGTTTTGCAGGC	TGAAATCAGATCGGTCACCGCC	312
FMgSSR-39665	scaffold273895	p2	(TC)9	Class II	38	55	TCACCCTCTCTCTCTTGGTCTTCCT	TAGCGCCAAATCTACCGCTGCT	339
FMgSSR-39666	scaffold488	p2	(TC)9	Class II	101504	101521	TCACCGAGGCCTCCTCCATAATGT	TCCAAACCCTAGGGGAGAACCA	239
FMgSSR-39669	scaffold4546	p2	(TC)9	Class II	4640	4657	TCACTGGAGCACTTGGCAGCAT	GCTGGCTGTCCACAAGTGTAAGT	306
FMgSSR-39672	scaffold12182	p2	(TC)9	Class II	12101	12118	TCAGGGCTTGCCTTGTCTTCGT	TTGCAGACGGCCATCCTAACCA	335
FMgSSR-39673	scaffold3337	p2	(TC)9	Class II	28004	28021	TCAGGGGACATCACCCAAGAGGTT	ATATGTGAAGGGCAGGCGCA	317
FMgSSR-39674	scaffold14467	p2	(TC)9	Class II	17045	17062	TCATCACGCGGCCCATTTGA	ACGACGAGAAATCCCATGCCGA	270
FMgSSR-39677	scaffold237	p2	(TC)9	Class II	90200	90217	TCCACGCAGTCAGTGGTTCAGA	TCCCTATCGTGCAACACGGTCA	302
FMgSSR-39678	scaffold2978	p2	(TC)9	Class II	22672	22689	TCCACGCTTTATCCGCCTGCTT	ACTAACGGCGGATGCTCCATGT	306
FMgSSR-39681	scaffold1387	p2	(TC)9	Class II	54644	54661	TCCCCACAAACGCAACAGCA	ACAACAACACACAGCCACGCA	342
FMgSSR-39689	scaffold6427	p2	(TC)9	Class II	25027	25044	TCCTCCTCGTTTTCGCTCGCAT	AAACCCGCCCAGTTCACTCCTT	223
FMgSSR-39693	scaffold4814	p2	(TC)9	Class II	15367	15384	TCGCAATCACAAGCCTCGCT	AATGACGCGTTGGCCACGTT	213
FMgSSR-39695	scaffold9191	p2	(TC)9	Class II	18943	18960	TCGCGCGCGTGTACATTGAA	TTAATCATTGCTGCCGCCTGCC	206

SSR_ID	Scaffold	SSR_Type	SSR_Motif	SSR Class	SSR_Start	SSR_End	Forward sequence	Reverse sequence	Product_size
FMgSSR-39696	scaffold6808	p2	(TC)9	Class II	36676	36693	TCGCTGCCGACGCTTCAAAA	TGGACTCATGGTGGCCAAACCT	336
FMgSSR-39701	scaffold97585	p2	(TC)9	Class II	1607	1624	TCGTGTGGGGTCAGAATTTCGCA	AAATATGGACCGGACACCGGAGGA	212
FMgSSR-39706	scaffold7253	p2	(TC)9	Class II	10917	10934	TCTGGGCGTTCACCTTTCCTCT	TGGCGTCAGGTTTCTCCCAA	331
FMgSSR-39708	scaffold14063	p2	(TC)9	Class II	3230	3247	TCTTTCTGCTGCCACTCTGTCA	ACTTCCCCCGTGAATGTTGC	347
FMgSSR-39711	scaffold20363	p2	(TC)9	Class II	1918	1935	TGACAAACACGCCAGGCCAA	TGGTGCCGTCGTCCATTTGTGA	298
FMgSSR-39735	scaffold6950	p2	(TC)9	Class II	20119	20136	TGCCAAAAGGAGTCCACGTGCT	AGCATTAGGGCGACTGCGAGAT	224
FMgSSR-39739	scaffold3436	p2	(TC)9	Class II	52403	52420	TGCCATATGCGTGGCTGGCTTA	ATGGCCGCCGATCCATCAATGT	349
FMgSSR-39742	scaffold1797	p2	(TC)9	Class II	55081	55098	TGCCGGCGTTTACCATTGCT	AGAAGTCGCGTCGAAACCGTGT	200
FMgSSR-39743	scaffold13145	p2	(TC)9	Class II	10737	10754	TGCCGGTTAGATCGGCATGT	ACAGGTGCATCTCAGCATCTCA	280
FMgSSR-39746	scaffold4259	p2	(TC)9	Class II	33371	33388	TGCGCAACATCCTTTTCGTCCA	TGCCTACTGATGCTGCCATTGCT	348
FMgSSR-39747	scaffold767	p2	(TC)9	Class II	44760	44777	TGCGTGCGTGCGTGTGTTTA	ACACGCGCCATGGAAACGAT	270
FMgSSR-39748	scaffold426	p2	(TC)9	Class II	42432	42449	TGCGTTGTACTGTCTGCGTCGT	AGATTGCGTCGCCCGCTTTT	240
FMgSSR-39749	scaffold436	p2	(TC)9	Class II	77278	77295	TGCGTTTGGGGGACAGAAGT	AGCACTCAGTTTTGGGGCCCTT	244
FMgSSR-39750	scaffold4810	p2	(TC)9	Class II	990	1007	TGCGTTTGGTTGAGCGGCAA	AGGCCAAGGCATGTTGTGACAGT	349
FMgSSR-39754	scaffold5379	p2	(TC)9	Class II	24957	24974	TGCTGCAGGGACATGACAGA	AACTGTGCATGCCACGTTTGGG	257
FMgSSR-39757	scaffold1744	p2	(TC)9	Class II	64889	64906	TGCTTCACAGACCACCTGGA	TGATCTCGTGCGTGCGTGTT	297
FMgSSR-39758	scaffold11717	p2	(TC)9	Class II	17519	17536	TGCTTCGAAGATGCACCAGGTCT	AGGGGAAGCATGTGCACGAA	282
FMgSSR-39761	scaffold3	p2	(TC)9	Class II	256986	257003	TGGAGCGTCTCGTTTCGTCTGT	CCGTGGCGCAAGCCAATTTT	237
FMgSSR-39767	scaffold3226	p2	(TC)9	Class II	45548	45565	TGGCGAAGAGTGGTCCAAGAGT	TGGTTTGGCCATGGCTTCCT	283
FMgSSR-39768	scaffold64481	p2	(TC)9	Class II	1247	1264	TGGCGGTCGCAAATCGACTGAA	CGCTCGTGCGTGTGGTTTTT	239
FMgSSR-39772	scaffold222376	p2	(TC)9	Class II	381	398	TGGGCGTCCGAATCCAACAA	AGTGCGCCTGGAGTGCAAAA	269
FMgSSR-39777	scaffold56978	p2	(TC)9	Class II	764	781	TGGGCTTGGGCACGTTCAAA	AAGCTGACCGAATCGTCGTGGA	306
FMgSSR-39779	scaffold29904	p2	(TC)9	Class II	1971	1988	TGGGGCCCTGCATTTCTTTCGT	TTGGCACGAGTGGGAAAGGAGT	277
FMgSSR-39780	scaffold6938	p2	(TC)9	Class II	26961	26978	TGGGTTCGTGTTTCGAGCCCAT	AGGGGATCGTGATTTGCACACG	297
FMgSSR-39781	scaffold33841	p2	(TC)9	Class II	1241	1258	TGGTAACACGAAGCCGACCA	TAATTTGGCAGCAAGGGGCAGC	318
FMgSSR-39784	scaffold559	p2	(TC)9	Class II	90628	90645	TGGTCGGCTGTGGACATGTTT	CCGTTCTTTAGACACGCACGCA	266
FMgSSR-39785	scaffold162604	p2	(TC)9	Class II	36	53	TGGTGAGATCTCCGACGACCTT	TGAATCCGGTGCTCAACTGGGA	288
FMgSSR-39788	scaffold3502	p2	(TC)9	Class II	29457	29474	TGGTGGTAGTGAGAAAGCTGCAC	AAACATGACACGCGTAGGGCCA	313

SSR_ID	Scaffold	SSR_Type	SSR_Motif	SSR Class	SSR_Start	SSR_End	Forward sequence	Reverse sequence	Product_size
FMgSSR-39792	scaffold4018	p2	(TC)9	Class II	9025	9042	TGTCACACGCGGCGTCTTTT	AGCGCACGCTTCCACTCAAA	321
FMgSSR-39793	scaffold2290	p2	(TC)9	Class II	2998	3015	TGTCGCTGTTATCCCTTCCCCA	TGGCAAAGGATGCAACATGCCG	242
FMgSSR-39794	scaffold1288	p2	(TC)9	Class II	28352	28369	TGTCGTGTTATGCGGTGAGCGA	TTAACCGTGAAGAGCCCAGCCA	201
FMgSSR-39795	scaffold3387	p2	(TC)9	Class II	37147	37164	TGTGCACCATGTTGATCCGACGA	TGGATCAGGGCTGTCTCTGCAT	311
FMgSSR-39798	scaffold9262	p2	(TC)9	Class II	31582	31599	TGTTCTGGGCGAAAAGCGGT	TGGAAAAGCGGTGGATGCTCCT	319
FMgSSR-39800	scaffold16146	p2	(TC)9	Class II	4076	4093	TGTTGTTGCCGTTGTCGTCGT	ACGCCGACATCTGCTCTTGT	278
FMgSSR-39802	scaffold5035	p2	(TC)9	Class II	40060	40077	TTATTAGGTGGCGCCGGACGTT	ACACGACGCACTCACGTCAGAA	245
FMgSSR-39805	scaffold32	p2	(TC)9	Class II	101535	101552	TTCCAATCTTCTGGACACGCCC	TGCCACATGGCATTGACTTCCAGA	340
FMgSSR-39808	scaffold50596	p2	(TC)9	Class II	2763	2780	TTCCTCCCTGTGCTGTTCGCTT	ACGCTGCGCAACCAAACGAT	279
FMgSSR-39812	scaffold2181	p2	(TC)9	Class II	36749	36766	TTCGTCCCTGTTCTGCCACCTCAT	TTGGCGGATCCACCCACATCAA	280
FMgSSR-39813	scaffold6993	p2	(TC)9	Class II	5586	5603	TTCGTGGCGTCATCAGAGCA	CGCGCGCGAGTGAAATTTAGGT	215
FMgSSR-39814	scaffold7741	p2	(TC)9	Class II	28132	28149	TTCTGATGATGCAGGACTGCCG	AAGGGGATGAGCACGAGAACGA	225
FMgSSR-39817	scaffold5076	p2	(TC)9	Class II	29715	29732	TTGCATGAAGCCGTATGGGGCA	AGCGACTCAAGTTTCCACCCGT	262
FMgSSR-39822	scaffold4384	p2	(TC)9	Class II	32823	32840	TTGCGCTTGCTTGAACTGGTCG	GTGGCTTGGCTTGCTTGTCACT	243
FMgSSR-39824	scaffold76	p2	(TC)9	Class II	1923	1940	TTGGATGGGGACATGGGGCATT	TGCAGTGGCTGCGTGAGCTAAA	349
FMgSSR-39827	scaffold63	p2	(TC)9	Class II	118222	118239	TTGGGCACAGCCAGTAGCAA	ATCTGTTGTCCATGATGGCCGC	257
FMgSSR-39828	scaffold165408	p2	(TC)9	Class II	205	222	TTGGGGGTGGCATCTTCCAT	TGTTGATTGCGGATCGGACGGA	214
FMgSSR-39835	scaffold448	p2	(TC)9	Class II	68459	68476	TTTGATGGCAGCGACGGTGA	ACGCCAAAACTTTGGACGGGGT	247
FMgSSR-39836	scaffold65	p2	(TC)9	Class II	11412	11429	TTTGCCCGCTTCCGCAAGAT	ACCTCCTTGTCAGCAGCAGCAT	272
FMgSSR-39838	scaffold1655	p2	(TC)9	Class II	44012	44029	TTTGGTCACGGTTTGTGCGTGC	TGCTCGGCGGCTTGCTTTTT	259
FMgSSR-39840	scaffold631	p2	(TG)10	Class I	6671	6690	AAAGGTGGTGGCTCAGCCGTTA	ATATGCGACCACGCACGCAA	281
FMgSSR-39841	scaffold131	p2	(TG)10	Class I	103060	103079	AACCCAGGTTTTGCCGTCCA	TCGGTTGCTGCATGCTTCGT	329
FMgSSR-39844	scaffold3126	p2	(TG)10	Class I	49740	49759	AATTGGTCCCAGTAGTCCTGTCCG	AGACAACAAGACGCGCGGTA	291
FMgSSR-39845	scaffold9433	p2	(TG)10	Class I	14446	14465	ACAACTTCCTGGTGGCAACGGT	AAGGACGCGCAGCCAAACAT	244
FMgSSR-39846	scaffold4477	p2	(TG)10	Class I	7176	7195	ACACAGTTTCTGTTGCTCCCAG	TTCGATTCGCGCCTTGCTCA	321
FMgSSR-39849	scaffold3294	p2	(TG)10	Class I	41736	41755	ACAGGCCTGACTCTATTTCGCCA	TGGCCTGGTGGTTTGTGGAT	270
FMgSSR-39850	scaffold5084	p2	(TG)10	Class I	19242	19261	ACCGTTTCGAGCAGCAGCAT	TGCCAAAAGTTACATCGGGGCT	252
FMgSSR-39851	scaffold9676	p2	(TG)10	Class I	18339	18358	ACGCTGCTGCTTTGTCCTCCTT	TGGCCATGCGCCTCTCAAAA	228

SSR_ID	Scaffold	SSR_Type	SSR_Motif	SSR Class	SSR_Start	SSR_End	Forward sequence	Reverse sequence	Product_size
FMgSSR-39855	scaffold2027	p2	(TG)10	Class I	58942	58961	AGAATGCGCGCAAGCGTAAAC	AGGAAGCTTGTTGTTGCGGGGA	258
FMgSSR-39861	scaffold8071	p2	(TG)10	Class I	22879	22898	AGCTGCAAGGGGAAGTGCTTGA	TCAGACAGTTTTCACGGGCGGA	267
FMgSSR-39862	scaffold1690	p2	(TG)10	Class I	2359	2378	AGGCCGATGTAGATGTTGGCGA	TGGCGCTGTACGGCTTCATT	293
FMgSSR-39863	scaffold457	p2	(TG)10	Class I	106954	106973	AGGGAAAGCTTTGTCGAGGCCA	CGTGAAACCCTGCTTTTGTTGACC	316
FMgSSR-39865	scaffold4405	p2	(TG)10	Class I	5042	5061	AGGTCCTTGTTTGACAGTCGCA	TGGTTCCATCGTGCTTGGCA	231
FMgSSR-39866	scaffold903	p2	(TG)10	Class I	18628	18647	AGTGCTTCGGTCGTTGCATTG	TGCTGTGATCGCCCTTTGGA	347
FMgSSR-39872	scaffold10086	p2	(TG)10	Class I	11315	11334	ATTTGCTGGCCTCTCTGGGTCT	TGATTTGGGCAACAGCCGCA	323
FMgSSR-39874	scaffold299	p2	(TG)10	Class I	144656	144675	CGCACTCAAAGCCACGCAAGAA	ACAGCACAAAACGAGGGCGT	259
FMgSSR-39875	scaffold927	p2	(TG)10	Class I	77093	77112	CGCGATGCATTTGGAGTTTGGGT	TTGTTTTCGGCGGGCAGGTT	340
FMgSSR-39876	scaffold563	p2	(TG)10	Class I	67867	67886	CGCTATGCTCTTGAGGGTAGTGGT	TGCAGGCAAAACAATCATGCCCC	336
FMgSSR-39877	scaffold1250	p2	(TG)10	Class I	33127	33146	GCAGCAGCAGTTTCCTCAATGT	AGGGGCCTGTAGTGATTGTGCT	287
FMgSSR-39878	scaffold1125	p2	(TG)10	Class I	27736	27755	GGATTGATGCCAGAAACCGTGA	ACTTGCTGTCCGCAATACCAGC	241
FMgSSR-39879	scaffold9898	p2	(TG)10	Class I	8468	8487	GGTACCACGCTGCTTTTAATGTGC	GGCGAAAGCCATGGTTGCCAAA	225
FMgSSR-39882	scaffold648	p2	(TG)10	Class I	82403	82422	TATGTTGCTTGCTGCCGTCCGA	AAAACGCACCAAAGAGGTGGCG	243
FMgSSR-39883	scaffold104685	p2	(TG)10	Class I	323	342	TCGAGCCAATCGGCACAAGT	AGCCTGCCTACCTACTTTGCAACC	242
FMgSSR-39887	scaffold891	p2	(TG)10	Class I	48209	48228	TGCAGTTCCAGTGTGAGCCA	TGAAGGCACATAGGCGGCCAAA	203
FMgSSR-39888	scaffold6056	p2	(TG)10	Class I	40717	40736	TGCGCGGGCCAATTTGTGAT	TGGATAACGCAAGGCTGCCA	344
FMgSSR-39893	scaffold131	p2	(TG)10	Class I	30524	30543	TGTCCGTGTTCTTCCACTCGCA	TTTTTGGGTTGGGCAGCCGT	346
FMgSSR-39896	scaffold5387	p2	(TG)10	Class I	18387	18406	TGTGTGTGACGCAGCGTTCT	GCATTGATCGTGCAGTGTGCGT	258
FMgSSR-39897	scaffold888	p2	(TG)10	Class I	37840	37859	TTCGTGTGCACGTTGGCTGT	TCGGTCGACCATACCACTGCAT	321
FMgSSR-39905	scaffold1273	p2	(TG)11	Class I	47590	47611	AACTGCGAGGCAACAGCGAT	AAGCGCGAAACTGAAGGCGA	272
FMgSSR-39907	scaffold163	p2	(TG)11	Class I	27907	27928	ACGAGGTGTCCCGTGGTGTTTA	AACTACGATTGCCGCTGCCCAA	265
FMgSSR-39908	scaffold7905	p2	(TG)11	Class I	16905	16926	ACGCAGCTGAAACCAGATCGCA	ACCATGTGCACCACTTAACAGGCA	345
FMgSSR-39909	scaffold3259	p2	(TG)11	Class I	36991	37012	ACGCTTGCATTCAGTGACGGGA	AAACCGCGCCAAAGTACACACG	331
FMgSSR-39913	scaffold1476	p2	(TG)11	Class I	34928	34949	AGCACCATCCACTCTTTCCCGT	ATTTCCAGTGCAGTCGGCGT	332
FMgSSR-39918	scaffold209	p2	(TG)11	Class I	150166	150187	AGGGCCACCATACAAGCCACTA	ACGACAACGAGGCCCAACTCTT	313
FMgSSR-39919	scaffold2335	p2	(TG)11	Class I	8463	8484	ATCTGCCGAACGCGCGAAAA	ATGCGCGTTCTTGCATGCGT	284
FMgSSR-39920	scaffold146	p2	(TG)11	Class I	7901	7922	ATTCAACGCTCGCCCTGATCCT	AATCAGAATTTCGGCCGGGGCA	339

SSR_ID	Scaffold	SSR_Type	SSR_Motif	SSR Class	SSR_Start	SSR_End	Forward sequence	Reverse sequence	Product_size
FMgSSR-39922	scaffold13395	p2	(TG)11	Class I	17201	17222	CCTAATCACAGGTTCACCACCACA	ACCCAGCTCCCTAATCACTTGC	343
FMgSSR-39924	scaffold3206	p2	(TG)11	Class I	33163	33184	GCGCCGACACGCGGATTAAAAA	GGAACGTGCGGTGCATGCATTA	337
FMgSSR-39927	scaffold8847	p2	(TG)11	Class I	3820	3841	GGGGAGAAGAGGGCCAAACAAA	TCCGTGCTTCTTTCTGCCCA	288
FMgSSR-39928	scaffold1175	p2	(TG)11	Class I	41171	41192	TACAGTCGCAAACAGAGCGTGC	TGCTTGTTCACGAACAGGGTGC	322
FMgSSR-39932	scaffold52011	p2	(TG)11	Class I	2154	2175	TCGGCACGGAATCGAGTCAT	TGGACTGGACTGCGGAGAACAA	318
FMgSSR-39936	scaffold2125	p2	(TG)11	Class I	20914	20935	TGCCCTTGTGCTATGGTGCCTT	TCGGCAACCTGCAATACCGAGT	336
FMgSSR-39937	scaffold1506	p2	(TG)11	Class I	36117	36138	TGCCTGCTGTGCTGGTTGTT	GGTGATATGCATAGGAGAGCGTGA	291
FMgSSR-39938	scaffold2414	p2	(TG)11	Class I	32001	32022	TGGCGGCGTTGGACAAAACA	TGCGAGCGCCTACAGCTATCTA	254
FMgSSR-39940	scaffold6939	p2	(TG)11	Class I	5336	5357	TGGTGGGTTCACGCATTGCT	AGGAGCATTGGCACGTCGTT	281
FMgSSR-39941	scaffold2027	p2	(TG)11	Class I	37902	37923	TGTGTGCGCTTGCTTGCTGT	TCACAAGTCCGCCTTTGGTGCT	300
FMgSSR-39942	scaffold12609	p2	(TG)11	Class I	7086	7107	TTGCTATCCTGGCAAATCCAGC	GCGCAATGTGGTTTTTGCATCGC	242
FMgSSR-39945	scaffold9910	p2	(TG)12	Class I	15753	15776	AACCCATGCAGCAGCACGAT	TTTGCGCCGGCCTGTTCTTA	231
FMgSSR-39950	scaffold87	p2	(TG)12	Class I	101314	101337	ACAGAGCAGTGTGTGAAGCGA	TCACGGCCGCGCAATTTACA	340
FMgSSR-39952	scaffold1	p2	(TG)12	Class I	135901	135924	ACCTGCTCTGCTTCACTGCT	CGCATGCACGCTCTCTCTCTTT	214
FMgSSR-39953	scaffold260132	p2	(TG)12	Class I	284	307	AGGGGTTTATACGGTGGGGTTT	TCGTGGGCTGCACTGGAAGAAA	312
FMgSSR-39954	scaffold6648	p2	(TG)12	Class I	22438	22461	AGGGTCGCACATTGAGTGAGGA	AAAATCACTGCCCCGTCAAGCC	321
FMgSSR-39957	scaffold12597	p2	(TG)12	Class I	3755	3778	CGCGTTGTATGTACCTCAGCA	ACCACGCTCTCCAGTTTGGGTT	350
FMgSSR-39958	scaffold23890	p2	(TG)12	Class I	5750	5773	GCATTGCTCAGTGCGAGAAGCA	TTCGGCATGCTTTCAGGCTCCA	286
FMgSSR-39963	scaffold3923	p2	(TG)12	Class I	35574	35597	TCGGCTGCACCCAAAACCTT	AGCCATTGGCACCATCAATGCG	229
FMgSSR-39964	scaffold5546	p2	(TG)12	Class I	5688	5711	TGATGACGCCATGGCACTGT	TCCCGTACGCACCCTGCAATTT	205
FMgSSR-39967	scaffold3819	p2	(TG)12	Class I	40585	40608	TGTTAACCGCCGGCACACATT	TGGCATGACCTGATGAGAGCAG	280
FMgSSR-39972	scaffold10	p2	(TG)13	Class I	254684	254709	AACGCTGGACGGACCGTTGTTA	TTTGTAACGGGCCGGAAGCGAA	341
FMgSSR-39976	scaffold5411	p2	(TG)13	Class I	20559	20584	AGCCTTCCAGGGCTGTGTTCTT	AACGTGAAGCGGAGAGCTCGAA	286
FMgSSR-39977	scaffold128	p2	(TG)13	Class I	114941	114966	AGCTCGGCGCCAAAACGAAA	ACAACCTCTCACATTCCCCCTC	309
FMgSSR-39980	scaffold1296	p2	(TG)13	Class I	41590	41615	ATACGTACCACATGACGGCGCA	TTACTGGGCCAACAACGCCT	260
FMgSSR-39981	scaffold1180	p2	(TG)13	Class I	2221	2246	CGAAAACACCAAAACGGGGCTGT	TGGTGCGCATATGTTGCCGT	294
FMgSSR-39984	scaffold98714	p2	(TG)13	Class I	813	838	TCGAGCTGGCTGTGCTCAACAT	TTTGTTTGTCGCCAGCCGCA	344
FMgSSR-39986	scaffold550	p2	(TG)13	Class I	73009	73034	TGCTTGCCATGCGAGCTGTT	TGTCGCACAGGTGTCACAGGTT	210

SSR_ID	Scaffold	SSR_Type	SSR_Motif	SSR Class	SSR_Start	SSR_End	Forward sequence	Reverse sequence	Product_size
FMgSSR-39988	scaffold19953	p2	(TG)13	Class I	5923	5948	TGTCACTCAGATTCGGCCTGGA	TGGCCAATACGCCATCTACGACA	207
FMgSSR-39989	scaffold2394	p2	(TG)13	Class I	11487	11512	TGTCAGGTTCGTGCTGTTGTCAC	TATCGCTGCCCGGGAACTACCATA	314
FMgSSR-39990	scaffold1081	p2	(TG)13	Class I	2529	2554	TGTGACCTTTCTGGCGACCT	TGCCTTTCACTGACGCTAGCACT	293
FMgSSR-39992	scaffold7398	p2	(TG)13	Class I	12980	13005	TTCGCCACATCCTCGTTTGCCT	TGCAAGCTCGGTCCATGTCA	286
FMgSSR-39998	scaffold1883	p2	(TG)14	Class I	62546	62573	AACGGAGCAAGCCTTCCAACCT	TGCTTGCAACGCAGACTTGCT	252
FMgSSR-39999	scaffold574	p2	(TG)14	Class I	11162	11189	ACCAGCAAACAGGCAAGCTCCA	AGCAGGAGCGCGGATTTGAT	247
FMgSSR-40001	scaffold21872	p2	(TG)14	Class I	6091	6118	ACGCGTTGCTTGATGCCTTGA	AGCTGCAAGCGGAGAGTCACTT	304
FMgSSR-40002	scaffold9417	p2	(TG)14	Class I	10439	10466	AGCGTCAGCATCACAAGGCA	TCGATCGTGCGTTTCAAGCTCC	252
FMgSSR-40004	scaffold2523	p2	(TG)14	Class I	10547	10574	ATCCATCGGTCCCGTACGTGTT	CCGCCCTAGATGCCGATGAGAAAA	208
FMgSSR-40006	scaffold5257	p2	(TG)14	Class I	14600	14627	TATGGTGGTGGCGCTGTTGT	AGCTCGGGACGGATGTCATCTT	334
FMgSSR-40007	scaffold3388	p2	(TG)14	Class I	53386	53413	TGATTGGACCCATCGTGTCGGA	TCAGCGGTCATCATCAGCGT	205
FMgSSR-40009	scaffold2758	p2	(TG)14	Class I	14193	14220	TGGCATCATGACCACCCTGACT	TGTGAGTCACCCGGTTGAAGGAA	277
FMgSSR-40013	scaffold11646	p2	(TG)15	Class I	14058	14087	ACTGCAACATGGAACCCCCTCA	TTGGCATCCATACCCTCGCCTT	207
FMgSSR-40014	scaffold13429	p2	(TG)15	Class I	2319	2348	ACTGTCTCCATTCCGGCGTT	TGCTGTGCACCGAGATTTTCGC	350
FMgSSR-40016	scaffold72500	p2	(TG)15	Class I	650	679	AGCTAGTGATTGGCAGAGGGCA	TTGGTCTCACTCTTCCGCTGCT	343
FMgSSR-40018	scaffold6626	p2	(TG)15	Class I	14410	14439	ATCCAACGCAGTGTCTGAACGC	AGTCAACAGCGCGAAAGGCA	243
FMgSSR-40023	scaffold100	p2	(TG)15	Class I	121766	121795	TGCGGCGCGCTCATCTTTTT	CCGTGCAAGCACTCTCCTTCAT	333
FMgSSR-40025	scaffold34222	p2	(TG)16	Class I	3069	3100	ACCTATGCCACGTGGAACCCAT	GCCGCGCTTGTGCAAATGAT	225
FMgSSR-40030	scaffold3670	p2	(TG)16	Class I	48736	48767	ATCGCGGGCACCATGTCTTT	TGATCGTGTCACGCCGTGCTAA	249
FMgSSR-40031	scaffold1579	p2	(TG)16	Class I	30253	30284	ATGCTACGGTCCAACTCCCACA	TGCTGCCACAATGTCGTGCT	270
FMgSSR-40032	scaffold2847	p2	(TG)16	Class I	55676	55707	CCTTGTCAACCGGCGTGAGTTT	TCGGCGAAACCTCCTGAAACAGT	220
FMgSSR-40033	scaffold6939	p2	(TG)16	Class I	7262	7293	GCATGATGACAACAAGCAGCAC	AGCTCGCAGGCACAACACTGAA	347
FMgSSR-40034	scaffold1781	p2	(TG)16	Class I	64207	64238	GTCAGCTGGAGTACCAGCCTGTAA	AGTGCAACGCCGATGTGGGTAA	344
FMgSSR-40038	scaffold4	p2	(TG)16	Class I	134107	134138	TGCCAAGAGGTACAACCCGT	CCAAATGTAGGCACGCGAGGTT	272
FMgSSR-40040	scaffold11323	p2	(TG)16	Class I	13575	13606	TGTTTCGTGACCCATTCCGTGG	TGAGTGGTCACCCACGCAAGAA	309
FMgSSR-40044	scaffold7920	p2	(TG)17	Class I	29354	29387	TCCAGCTGTGAGTCCTTTCTTGGC	TCCTTGAAGTGCCATCCAGTCG	327
FMgSSR-40049	scaffold18	p2	(TG)17	Class I	96385	96418	TGTCCCATGAAGTGGTGCCAA	GCATTTGGGAGTGATTGGTGCCT	306
FMgSSR-40050	scaffold586	p2	(TG)17	Class I	45481	45514	TGTGGCTCTCTCCCACAAGTGA	ACAGCCCCTTTTGCGCCTAGTT	337

SSR_ID	Scaffold	SSR_Type	SSR_Motif	SSR Class	SSR_Start	SSR_End	Forward sequence	Reverse sequence	Product_size
FMgSSR-40053	scaffold17207	p2	(TG)18	Class I	7839	7874	AGCAATGGTGCCTCCCATGT	TAGCTAGCGTTGGGGCAAGCAA	320
FMgSSR-40054	scaffold11329	p2	(TG)18	Class I	18232	18267	ATCAGCCGCAGTGAAAGCGT	AAGATGCGGCCCATGAGCTA	204
FMgSSR-40056	scaffold172	p2	(TG)18	Class I	65952	65987	GCGCGCCGCCTAATTTACTTCA	AAGCCCGAACCAACAAGGCA	290
FMgSSR-40060	scaffold10955	p2	(TG)19	Class I	14658	14695	ACGACGACCACGACAATGGCAA	AGGCCCAGCTCATCTTGTGGTT	234
FMgSSR-40063	scaffold103942	p2	(TG)19	Class I	1288	1325	TGCATGTGCATGAGTGGCCT	TGGCGCATGACACCAAAGTT	216
FMgSSR-40064	scaffold9574	p2	(TG)19	Class I	17632	17669	TGCGAGAAGATCAGTCGCAATG	TCATGCAACGCAACCTCCTGT	343
FMgSSR-40065	scaffold1467	p2	(TG)20	Class I	45377	45416	ATGACACTTGGCAAGCCCACTC	TCCACTGACCACAAGATGGCACA	281
FMgSSR-40066	scaffold5704	p2	(TG)20	Class I	17787	17826	GCCGGAAGCCAATGCAGTATGA	ACCTGTCATCCCGTTGCACACA	305
FMgSSR-40067	scaffold76	p2	(TG)20	Class I	170126	170165	GCTCGGCTGTTTCCTCCGTAAT	TCGAGGCACTTGACCTTGTAGCA	350
FMgSSR-40070	scaffold2469	p2	(TG)21	Class I	11624	11665	AAAACGCAAGATGGCGACACGG	ATTTTTCCGAGCGACCAGGGGA	201
FMgSSR-40074	scaffold11753	p2	(TG)22	Class I	10018	10061	ACAGCGCAACTCGTAGGGAAGT	ACTGAGCTGGGCCATTTCCTGT	344
FMgSSR-40085	scaffold2027	p2	(TG)24	Class I	3470	3517	TGCATGCCAACCCAAAAGCAGC	TCGTTCACACACGCCGAACT	272
FMgSSR-40090	scaffold1531	p2	(TG)27	Class I	61010	61063	ACCGGGTCGACCAAACCTATATCT	AGCACGCGTGAACACACACA	294
FMgSSR-40091	scaffold120	p2	(TG)27	Class I	175870	175923	TCACCATGAATCACGCCGGCTA	AGGGGTGCGATTGGTTGTGTGT	327
FMgSSR-40092	scaffold1531	p2	(TG)27	Class I	61197	61250	TGCTGCCCTCATCATTGTGTGTGT	TCAGGGCAGAGGCTAAAGCA	284
FMgSSR-40096	scaffold2612	p2	(TG)35	Class I	43678	43747	CGGTGCCATTGTTGGGCTTCTT	TGTGGGAGAAACACTAGCCACCT	343
FMgSSR-40099	scaffold54830	p2	(TG)6	Class II	598	609	AAAACCTCGGCGTTGCGTTG	ACCCAGGAACACACACACACACA	289
FMgSSR-40101	scaffold20494	p2	(TG)6	Class II	6157	6168	AAAAGGGCGTTTGGCGAAGCTG	TGGCAAGACCGCAAGAGAGA	298
FMgSSR-40102	scaffold5552	p2	(TG)6	Class II	22274	22285	AAAAGGTGCAACGGAGACGTGC	TGGATCCGATTTCACCCCTTCCA	252
FMgSSR-40103	scaffold622	p2	(TG)6	Class II	77884	77895	AAAATGACAGCGCCGGACGA	TTTGGCTGGGCGTTTGCGTT	275
FMgSSR-40104	scaffold2890	p2	(TG)6	Class II	46453	46464	AAACAAGGGGCCGGTGCAAT	TGCCGGCGCACAGTAGTAAA	306
FMgSSR-40105	scaffold305	p2	(TG)6	Class II	41649	41660	AAACAAGGTCATGGCGCTGCAC	AGAGGATCCAAACCGGCTCTT	252
FMgSSR-40106	scaffold51260	p2	(TG)6	Class II	3578	3589	AAACATGCGGCGGTGACTGA	TCGCCCTGTGTGACATCCGTTA	328
FMgSSR-40108	scaffold21514	p2	(TG)6	Class II	490	501	AAAGAAATGCGCGCCGGCTA	TTGCATTGACCATGTGGCACGC	228
FMgSSR-40113	scaffold15373	p2	(TG)6	Class II	5540	5551	AAAGGCAAAGCCAGGCGAGA	ACCCCTGCTTGACCCAAACACA	348
FMgSSR-40116	scaffold13782	p2	(TG)6	Class II	6508	6519	AACAAAAGGCGGCACCGAAA	ATGCAGGATGCGCTTTGCAG	283
FMgSSR-40120	scaffold302	p2	(TG)6	Class II	16793	16804	AACAGCAGGCCCCGTGAAGATT	TTTTGCCCGGTCCTTTCAGCCT	229
FMgSSR-40122	scaffold693	p2	(TG)6	Class II	20059	20070	AACCGCACCAAAACGCTGCT	TCAGTGAGCGGGGTGCACTTAT	310

SSR_ID	Scaffold	SSR_Type	SSR_Motif	SSR Class	SSR_Start	SSR_End	Forward sequence	Reverse sequence	Product_size
FMgSSR-40124	scaffold4997	p2	(TG)6	Class II	24149	24160	AACCTGCAAGCACCATCCCACT	TCAGCAAGATGGAATGCCCTGC	346
FMgSSR-40126	scaffold39832	p2	(TG)6	Class II	1099	1110	AACGCGACGGTACGGCTAAA	TTGCGTCCTCCAAGCTGTTGCT	224
FMgSSR-40131	scaffold14701	p2	(TG)6	Class II	1257	1268	AAGATGATGAGCGCGTGCAGGT	GCACGGCGAAACAAAGTGGGAA	215
FMgSSR-40134	scaffold372	p2	(TG)6	Class II	115883	115894	AAGCGCGCAGCCATCAACAT	ACGCCTTGGCGCAATTGAAGT	300
FMgSSR-40135	scaffold1368	p2	(TG)6	Class II	84788	84799	AAGCTGGTCTGGTGCCACAGTT	TGCTAAACGCATGCACACGCA	344
FMgSSR-40149	scaffold4360	p2	(TG)6	Class II	8913	8924	AAGTGCCCGCAGAACCAGACAA	AGCAGTGGCGAATCAAGTCCACA	338
FMgSSR-40153	scaffold7073	p2	(TG)6	Class II	14007	14018	AATCGGCCAACGCGTCGATGTA	TCCGACTCCGACGAACAACACA	329
FMgSSR-40160	scaffold590	p2	(TG)6	Class II	62105	62116	ACAAGACTCGAGTTGCGACGA	TTGTTGCGACGGATTGCAGC	203
FMgSSR-40165	scaffold772	p2	(TG)6	Class II	17936	17947	ACAGCACCGCTTGGAAGTCA	TACGTGTGCCGGTTGGTGAT	350
FMgSSR-40168	scaffold2225	p2	(TG)6	Class II	29114	29125	ACAGCCTCTAGTGCCCTTTGT	GTCTTCGGCAATCTATAGCCTGGT	236
FMgSSR-40169	scaffold8567	p2	(TG)6	Class II	7804	7815	ACAGGAATGATCCCAGTCCGCA	ACCACTGGCAACGTGCATGA	305
FMgSSR-40173	scaffold2271	p2	(TG)6	Class II	2269	2280	ACATGTGGCCACTTCCTAGGGGTA	AAGGCCTGCTCCGGTTCATA	283
FMgSSR-40175	scaffold126168	p2	(TG)6	Class II	473	484	ACCAATAGCAGGCGAGCAGCAT	AAACCGACCGACTCCTCCTTGA	239
FMgSSR-40176	scaffold14809	p2	(TG)6	Class II	19627	19638	ACCACCGATGGTGCACAGACAA	TGACGCATCTTTGGTTCGGGGT	219
FMgSSR-40177	scaffold293	p2	(TG)6	Class II	14891	14902	ACCAGCTTGCAATGCAGTCGGA	AGTTGGTGAAGCACCGACCTGA	295
FMgSSR-40178	scaffold3017	p2	(TG)6	Class II	9086	9097	ACCATGCCAAAGCGGGAGAAGA	AACATGTTCGGAGGAAGGGCGA	255
FMgSSR-40179	scaffold19246	p2	(TG)6	Class II	9645	9656	ACCCATTCTCCACACAGTTCTCGT	AGGAACCCCTAACCCTAACCTAGC	223
FMgSSR-40180	scaffold5608	p2	(TG)6	Class II	22945	22956	ACCCCAAATGCACCAAGCCA	AGGCCATGCGCACATCAACTCT	228
FMgSSR-40192	scaffold4153	p2	(TG)6	Class II	36308	36319	ACCTCAAACCGTTGAGCGAGCA	AACAGACGTGGACCACACGGAA	326
FMgSSR-40193	scaffold182445	p2	(TG)6	Class II	181	192	ACCTGATGCCAACCCAATGACC	CCCTGCTGCCTTTTTGCAGCTT	240
FMgSSR-40197	scaffold170572	p2	(TG)6	Class II	228	239	ACGACCCATCATGAGGACGCTT	AGTAGCGGCAACTCATTCAGGT	270
FMgSSR-40198	scaffold35139	p2	(TG)6	Class II	449	460	ACGAGGCTGCAGAGCTTAGTCTT	TTTCCGACAGCTAGCGGCTT	243
FMgSSR-40199	scaffold1342	p2	(TG)6	Class II	65419	65430	ACGAGTCGTTCACTCACAGCTT	TGCAAAAGGGGGCTATCCTCTGA	312
FMgSSR-40204	scaffold2914	p2	(TG)6	Class II	5103	5114	ACGCTGCGGTGAGTCGATTT	AGCATACCCTTGTGGCGCTTGT	332
FMgSSR-40206	scaffold2184	p2	(TG)6	Class II	9321	9332	ACGGAGCCGTTTCCGCTTTT	ATGGGCGTTTCGTGGCCCAATA	327
FMgSSR-40207	scaffold523	p2	(TG)6	Class II	103689	103700	ACGGATTTACGCCGCGTTTG	ATGCCCGAGAACACACCGAAGT	317
FMgSSR-40208	scaffold1475	p2	(TG)6	Class II	5438	5449	ACGGCCACTCGCACTATTGGAA	AGACCCCATGCATGCTCTGCTT	307
FMgSSR-40209	scaffold14177	p2	(TG)6	Class II	16345	16356	ACGGCTAAACATCGACACTTGC	AAACGACGCGCTGGTCTGAT	300

SSR_ID	Scaffold	SSR_Type	SSR_Motif	SSR Class	SSR_Start	SSR_End	Forward sequence	Reverse sequence	Product_size
FMgSSR-40212	scaffold2934	p2	(TG)6	Class II	53702	53713	ACGTCATCAACGCCTCACCA	TTTTCGCTGCACGCGTCACT	272
FMgSSR-40213	scaffold2451	p2	(TG)6	Class II	13815	13826	ACGTGTCGAGATGGAACTTGGC	GTGTGGGTCATCCAAGTGATCTTC	308
FMgSSR-40216	scaffold221	p2	(TG)6	Class II	83329	83340	ACTCAGCTGCACTGTTCACTTGC	AGCAGCAGCGAAGCACCAAA	261
FMgSSR-40217	scaffold33873	p2	(TG)6	Class II	4169	4180	ACTCCCTTCGACAACCAAACGCA	ACACAGCGCGCACTGATGAAT	227
FMgSSR-40218	scaffold10077	p2	(TG)6	Class II	17368	17379	ACTCTGTGCCTGCCCAAAACCT	TGCCAAAAAGCCACCGACCA	305
FMgSSR-40220	scaffold2571	p2	(TG)6	Class II	10619	10630	ACTGGAGCTTGGTGCTGGAA	AGGAGCTAAAAGGCCATGCCACA	345
FMgSSR-40226	scaffold8446	p2	(TG)6	Class II	19659	19670	AGAAAAGGTTCGATCCGACGCC	AACCTCTGTATGCCTGTGGCCT	254
FMgSSR-40236	scaffold6876	p2	(TG)6	Class II	19169	19180	AGCAGCAGGGCATGCAAAGA	TGCTGCATTCTAGCTGCGGTGA	315
FMgSSR-40238	scaffold5849	p2	(TG)6	Class II	8236	8247	AGCAGTTGGCTTGCAGAGTTGT	ACCCCAACTGTAGCCATGGAGT	333
FMgSSR-40239	scaffold10567	p2	(TG)6	Class II	1188	1199	AGCATGGATGCACACTGCGA	TTTGTCCGTGCTGGTGTCGT	318
FMgSSR-40240	scaffold245660	p2	(TG)6	Class II	281	292	AGCCACCTTGCACAACATGACA	TTCAACGCGCTCCATGCCAA	265
FMgSSR-40244	scaffold1941	p2	(TG)6	Class II	23088	23099	AGCCCACCAGCATGTTATGGCA	ACCGAGCGACAAGTAGCAACA	318
FMgSSR-40245	scaffold1709	p2	(TG)6	Class II	61806	61817	AGCCGCAAGGTTGATGTGTTGA	TGCGGTGGCGAGAAACTGCTAT	328
FMgSSR-40246	scaffold172941	p2	(TG)6	Class II	135	146	AGCCGTCTCGTCGAGGGTATTT	TGCCGGTCGGCTAGTTGACAAT	221
FMgSSR-40247	scaffold35971	p2	(TG)6	Class II	2963	2974	AGCCGTGAGCTCTAACATGCGT	AACGAGACGAACATCACCGGCA	250
FMgSSR-40250	scaffold3	p2	(TG)6	Class II	80713	80724	AGCGACTCGACCTGTTGTGAGT	TGAAGGACTGGGCCGTCGATTT	336
FMgSSR-40251	scaffold158	p2	(TG)6	Class II	140422	140433	AGCGAGCGGTCCGTGTCAAAAA	ACCACGTCGTCGTTCTCATCGT	290
FMgSSR-40261	scaffold4183	p2	(TG)6	Class II	43176	43187	AGCTGCTCACGCATTTGGCA	TTTTCATGGCAGGCGTGCGGTT	253
FMgSSR-40262	scaffold2950	p2	(TG)6	Class II	4856	4867	AGCTGCTGGAGCTCCTTGATGT	CGAGGATCGCCGATAGAGGCTAAA	253
FMgSSR-40263	scaffold26376	p2	(TG)6	Class II	6801	6812	AGCTGTTAGCGGCGTATGAGCA	TTGTGTTTTCCCCACTCCGGCA	284
FMgSSR-40264	scaffold9576	p2	(TG)6	Class II	16379	16390	AGCTTGGGTGGTTGAGTGGT	CACGTTTCATGCCAACCACAGTGA	313
FMgSSR-40268	scaffold750	p2	(TG)6	Class II	85945	85956	AGGATCGACCGTGTAACACCA	ATGCTGTTCGGCAGCCAGTT	343
FMgSSR-40269	scaffold157	p2	(TG)6	Class II	17936	17947	AGGCACCATGGTTGATGACCCT	TTCCGCTTCTTCTTGCCCCGAT	271
FMgSSR-40270	scaffold32435	p2	(TG)6	Class II	2245	2256	AGGCAGTGGAGCATATCCAAGT	TGCTAGCTAAAAACGCGGGTGC	214
FMgSSR-40273	scaffold377	p2	(TG)6	Class II	121759	121770	AGGCGGAAGAATGTTGGTGGCA	TTCACGTCTCCAGACTCCAGCA	244
FMgSSR-40275	scaffold12788	p2	(TG)6	Class II	10402	10413	AGGGACGTAGACGTTTGGAAGCA	AGCGAATGACCCGCTCAAACA	219
FMgSSR-40276	scaffold1541	p2	(TG)6	Class II	58487	58498	AGGGCAGAGCATCTACCACACA	AAACCAGCACGCAGCAAGCA	324
FMgSSR-40281	scaffold1844	p2	(TG)6	Class II	53564	53575	AGGTCGACAAAGACGGCTCACA	GCAGCTAAGGTTGCTGATGGCA	330

SSR_ID	Scaffold	SSR_Type	SSR_Motif	SSR Class	SSR_Start	SSR_End	Forward sequence	Reverse sequence	Product_size
FMgSSR-40282	scaffold5867	p2	(TG)6	Class II	25115	25126	AGGTCGGATTGGTGGTGCCTAT	AGCAGCAATGTTTCGTGGGCT	297
FMgSSR-40284	scaffold14200	p2	(TG)6	Class II	15718	15729	AGGTGCTGCGAGGCATCATGTA	ATGGCGCGTGCATGGGTAAA	301
FMgSSR-40285	scaffold7574	p2	(TG)6	Class II	1847	1858	AGGTGGAGGGGTCAACATGTTCCT	ACCCACACCCACAACACACACA	314
FMgSSR-40286	scaffold3751	p2	(TG)6	Class II	44372	44383	AGGTGGATTGCTGCACGCTGAT	CGGAGACGCGCTTGTGAAGTTT	331
FMgSSR-40290	scaffold2039	p2	(TG)6	Class II	55159	55170	AGTACTGAGTGGTCCTTCGGCT	ACAGGGCATAAGGGCACAAAGA	207
FMgSSR-40291	scaffold153	p2	(TG)6	Class II	116168	116179	AGTCCAGCATGCACCAACGA	TTGGAAACGAACGCCGTAACGC	292
FMgSSR-40297	scaffold222	p2	(TG)6	Class II	53068	53079	AGTGTTGTCCGAACAGTGGGT	TGCGACACACACACACACACA	252
FMgSSR-40298	scaffold634	p2	(TG)6	Class II	71644	71655	AGTTAGTTCGCTCGCGGCTT	GGAACCACGAGTTTCAGCGT	350
FMgSSR-40300	scaffold5852	p2	(TG)6	Class II	3214	3225	AGTTCTGGTTGGTTGGCACGGT	CCAGGGCATGGTTACGAGCATT	287
FMgSSR-40312	scaffold539	p2	(TG)6	Class II	113797	113808	ATAGGCCTTGGCTCGCTGGAAT	ACAATGACCGCAGGGCTCCAAA	239
FMgSSR-40314	scaffold12808	p2	(TG)6	Class II	15986	15997	ATCCGAACGCAACCCCAACCAT	TGTGCATTGGCAGATCCAGCCT	297
FMgSSR-40315	scaffold15096	p2	(TG)6	Class II	9857	9868	ATCCGGGCTGCTCTGCTCTTTT	TCCATCGTCGGCAGCTTTCA	318
FMgSSR-40317	scaffold4444	p2	(TG)6	Class II	27977	27988	ATCCGTGTTTGAGCGCCAGT	ATGAGTGGATGGAGGTCGTCCTGT	289
FMgSSR-40320	scaffold1201	p2	(TG)6	Class II	21608	21619	ATCTACCGCTTCGTAGTGGCCT	TGCGCCTAAGTGGGTCAAACCT	322
FMgSSR-40324	scaffold515	p2	(TG)6	Class II	12961	12972	ATGCAGGCATGGGCAAAGGT	TCTGCGGCAAATGTGGGTCA	302
FMgSSR-40329	scaffold818	p2	(TG)6	Class II	108007	108018	ATGCCGTTGCACTCCGATGGAT	ACACAGCGGGATGTGCCTTT	344
FMgSSR-40331	scaffold3944	p2	(TG)6	Class II	39717	39728	ATGCGGAATCCAAGGCTGGGTA	TCAGTGGGGTTACAACCAATCACG	311
FMgSSR-40333	scaffold5032	p2	(TG)6	Class II	8753	8764	ATGCTTGTGGCGTGTCATGTGC	TGCCAGAAAGCCTCTCCTACACA	218
FMgSSR-40337	scaffold2108	p2	(TG)6	Class II	2276	2287	ATGGTGGTGCACGCCAGTGATT	TACATGTGTCCGTCCGGTGACT	346
FMgSSR-40338	scaffold3294	p2	(TG)6	Class II	15080	15091	ATGTACGCTTGCACTGCGTGGT	ATACGTGCGCGTTGGCGTTT	212
FMgSSR-40340	scaffold518	p2	(TG)6	Class II	28454	28465	ATGTGTGCGGTTCGTGTCGT	AGGTTTCACTTTCACGCCCTCCT	202
FMgSSR-40346	scaffold70807	p2	(TG)6	Class II	492	503	ATTTGGCGAAAACGCCGCAG	AATTCGCGTGTAGGCAGCGT	303
FMgSSR-40347	scaffold131	p2	(TG)6	Class II	102423	102434	ATTTTGTCCGGTGCGGCTGT	TTTTCACACACGCTCGCAGG	267
FMgSSR-40348	scaffold1116	p2	(TG)6	Class II	58319	58330	CACCAGTTTCGTCACACAGAGACA	TTTTCTGGTGCGCGGCACAT	216
FMgSSR-40350	scaffold2566	p2	(TG)6	Class II	11313	11324	CACGGCCTCTTTTACATGACCA	GCCTGGCAAGCATGACCAACAA	330
FMgSSR-40353	scaffold2090	p2	(TG)6	Class II	7409	7420	CCAGTACATACACTGCTACTGCTG	ACATGCACAGCTGCCCCATT	330
FMgSSR-40356	scaffold2386	p2	(TG)6	Class II	13756	13767	CCCTGCCATCTCTTCATGTGGGTT	TGCCGCTTTGTTTGGCCCAT	254
FMgSSR-40357	scaffold3212	p2	(TG)6	Class II	32717	32728	CCGAATTGCACCCAGCCGTTTT	ACTGCTCAGTGAGGACTACAGGA	307

SSR_ID	Scaffold	SSR_Type	SSR_Motif	SSR Class	SSR_Start	SSR_End	Forward sequence	Reverse sequence	Product_size
FMgSSR-40358	scaffold2830	p2	(TG)6	Class II	16512	16523	CCGAGTTGCCTTGTGCCTTGTT	ACGTGTGTCTGTCTTGTCGCGT	214
FMgSSR-40361	scaffold2126	p2	(TG)6	Class II	16946	16957	CCTGTTTTACCACTTGTGCCTTGC	CGTGCGCGCATGAGAATCATTG	293
FMgSSR-40362	scaffold182	p2	(TG)6	Class II	140113	140124	CCTTGTGCGCTTGTGTGCTTGT	ACCAAATGCGCCCATGCATCA	270
FMgSSR-40363	scaffold670	p2	(TG)6	Class II	37254	37265	CGAACGCGCCTAATGGAAGAGA	AAAAACTGCGCGGTGGAGAC	257
FMgSSR-40367	scaffold10462	p2	(TG)6	Class II	1007	1018	CGCATGCTTGCGGCCTTCTAAT	TGACGTGGCGTGATTTGTGAGGA	330
FMgSSR-40369	scaffold5410	p2	(TG)6	Class II	10405	10416	CGCCAACTTGGGCGTCCTTTTT	ACATTTGCACGTCGCCCTGA	204
FMgSSR-40371	scaffold3231	p2	(TG)6	Class II	34290	34301	CGCGGCAGATGAATGGAAAGCA	AGTTGGGCGCATGGATGCAA	323
FMgSSR-40384	scaffold44	p2	(TG)6	Class II	40786	40797	GCAAGGCAAGTGAGAGAAGCGA	ACCATGTGCTTGCTGTGCTCCA	312
FMgSSR-40389	scaffold309	p2	(TG)6	Class II	57792	57803	GCACGAGAAACATCCATTCGGAGG	GTGTGCTGCAAACCAGACATTGC	283
FMgSSR-40390	scaffold15399	p2	(TG)6	Class II	15629	15640	GCAGTGGTGGCTTTTACTCGCT	TCACTCGTCAGCAGGTGGCAAA	350
FMgSSR-40394	scaffold6440	p2	(TG)6	Class II	4372	4383	GCCCCGATTTTGTGCCGCATTA	GCGGGCAGTTCATGTGGATT	246
FMgSSR-40399	scaffold787	p2	(TG)6	Class II	6649	6660	GCCTTGGAGAATAGTGTGTATGCG	AGCTTGCACGTAACTGGGGCAA	331
FMgSSR-40401	scaffold4072	p2	(TG)6	Class II	30218	30229	GCGCATAGCGCTGTGTTGTT	CGTTCCATCTGCTATCTGGCACAA	282
FMgSSR-40403	scaffold11329	p2	(TG)6	Class II	20625	20636	GCGGACATCCAATCCATTGCT	ACGCATTCCACGATGTCAGACT	350
FMgSSR-40404	scaffold13566	p2	(TG)6	Class II	20436	20447	GCGTCGGTGTTTAATGCCGCTT	TGAGCCATCAGCTGAGCGAGTT	270
FMgSSR-40408	scaffold939	p2	(TG)6	Class II	56496	56507	GCTGGAGAATGACTGTAAGCATCGC	ATCAACAGCAGCCGAGCCACCTAA	321
FMgSSR-40411	scaffold4454	p2	(TG)6	Class II	24337	24348	GGAACGTCGCAGCCCTGTTTTT	TGCACGGATGCAGCACACAA	219
FMgSSR-40416	scaffold2449	p2	(TG)6	Class II	63574	63585	GGGCTTGCCGAAGTCAATCGAA	TCCCACTAACAACCTTCACGGA	300
FMgSSR-40418	scaffold7747	p2	(TG)6	Class II	14472	14483	GGGTGCACGCGTGTGTGTATTT	TGTCCCTGCAGTTTGCAGTCAG	340
FMgSSR-40419	scaffold1480	p2	(TG)6	Class II	24963	24974	GGGTGGTTGTGTTCTTGCTGGT	TGGCATGGTAGCCTGCCTATT	334
FMgSSR-40421	scaffold2987	p2	(TG)6	Class II	40812	40823	GTCCAGCTGCAAAACGTGGTCA	TGGAGAACGCGCGACTCAAAGA	296
FMgSSR-40423	scaffold7328	p2	(TG)6	Class II	29457	29468	TAACCCGCAGCCTGCTGTCTTT	AATCGCCGGCACTTGAGACT	341
FMgSSR-40427	scaffold409	p2	(TG)6	Class II	75704	75715	TAATGGCAGTTCCGCCCATGCT	AAAAGCCAACGCGCTGCTCA	321
FMgSSR-40428	scaffold25683	p2	(TG)6	Class II	1802	1813	TACCACTGCGCCACTCTTGCTT	CACCAGAGCTCTTCTAACGCCT	346
FMgSSR-40436	scaffold641	p2	(TG)6	Class II	39672	39683	TATGGCGGGTTCAGTAGTGGGT	TCAGATCTGGCCGCGCAAGTAA	217
FMgSSR-40437	scaffold5095	p2	(TG)6	Class II	16478	16489	TATGGTTTCTGTGCCGCGCT	TTGTCTCGATGCCTTCGCGGAT	235
FMgSSR-40440	scaffold5071	p2	(TG)6	Class II	26354	26365	TCAAAAAGCGGCGACGAGGA	ATTGAGCTTCAGCACGGCCA	284
FMgSSR-40441	scaffold121	p2	(TG)6	Class II	57755	57766	TCAAGCAAAGGCGCAAAGCGT	TGCCTAATGCACAGGGCAGCTT	279

SSR_ID	Scaffold	SSR_Type	SSR_Motif	SSR Class	SSR_Start	SSR_End	Forward sequence	Reverse sequence	Product_size
FMgSSR-40443	scaffold2571	p2	(TG)6	Class II	9417	9428	TCACATTCCCCGTGCCACTTCT	TAGAAACCGCAAGCGTCGGCAT	335
FMgSSR-40445	scaffold78	p2	(TG)6	Class II	153495	153506	TCACCATTGGCGAGAGCAAGCA	ACGCAGAGGCATGCCGAATA	317
FMgSSR-40446	scaffold55	p2	(TG)6	Class II	151838	151849	TCACGAACGTCGGGTGGTTCAA	AACATCACAGCGGCCACAGCTT	254
FMgSSR-40448	scaffold1191	p2	(TG)6	Class II	62032	62043	TCACGGCCATGAGGAGATGTCA	CCCGCTTTGGAGGTGATCCATT	325
FMgSSR-40450	scaffold20067	p2	(TG)6	Class II	8621	8632	TCACTGAAGCATCCCTGGCCTT	AGCTTCACCAGCAGCAGCAA	247
FMgSSR-40455	scaffold939	p2	(TG)6	Class II	58260	58271	TCAGTGGAGCTACTTGCTGGAGA	TGGCAGAGCTTCGTACAAGCCAA	345
FMgSSR-40458	scaffold29390	p2	(TG)6	Class II	8742	8753	TCATGCTGGCATGCTGCTGT	ACGTGTGTGGAGCTTCAGCAGTT	272
FMgSSR-40461	scaffold13876	p2	(TG)6	Class II	10971	10982	TCCAAGCGAAAGGAGCGCAA	TCCATCCCCTGCACACAGGTTA	344
FMgSSR-40462	scaffold1115	p2	(TG)6	Class II	74003	74014	TCCACGAGTTTTGGGGCATCA	TGAATACAGGAGGGATCTAGGGGGT	345
FMgSSR-40463	scaffold127	p2	(TG)6	Class II	165613	165624	TCCAGTCCACCGGCATTGATGT	CAGCCAGTTCTTTTGCACTCAGCA	314
FMgSSR-40465	scaffold1174	p2	(TG)6	Class II	30636	30647	TCCGAATTGTCCGGGCCTGTTT	TGCAACAACGGTCCCTGAATCG	218
FMgSSR-40467	scaffold12253	p2	(TG)6	Class II	13271	13282	TCCGATCCAAGGCTGCTACCAT	TCCGTGCACAACACATGCCT	321
FMgSSR-40472	scaffold13218	p2	(TG)6	Class II	18820	18831	TCGACCGTTAATGCGTGGCA	ATGGCTGTGCTTCTCCGTGTGT	316
FMgSSR-40474	scaffold7542	p2	(TG)6	Class II	17500	17511	TCGAGATTGAGGTAGGGGACTGA	TGAGGCGTACGACACGATCA	214
FMgSSR-40477	scaffold36553	p2	(TG)6	Class II	4308	4319	TCGCAGCGATTTTCTGCCCA	GGGGTGCAACCATTAACGCACA	238
FMgSSR-40478	scaffold14536	p2	(TG)6	Class II	10894	10905	TCGCAGGAATCACACCCACA	TCGCCAAGGAACACTCAGCCAT	310
FMgSSR-40479	scaffold2779	p2	(TG)6	Class II	4059	4070	TCGCCAGTGACACGCCATTT	TGTGTTCCCGCATTTTCGTGGC	290
FMgSSR-40481	scaffold500	p2	(TG)6	Class II	121895	121906	TCGCCGTTACTTGCCTGCTT	TGCACGCGCACATCTTACAA	265
FMgSSR-40483	scaffold1791	p2	(TG)6	Class II	437	448	TCGGCCAAAAGGCGTGTCAA	TGCCCGTGCAAACCCTTCAA	341
FMgSSR-40487	scaffold201054	p2	(TG)6	Class II	560	571	TCGGTCGGTATTGACTAGGCGT	AAGAGGGAGAGAGACGAGAGCACA	287
FMgSSR-40488	scaffold1478	p2	(TG)6	Class II	60895	60906	TCGTCACCAGGCAAGCTCCATT	GCGGGACATTAACGAGGATGTGCT	204
FMgSSR-40491	scaffold37	p2	(TG)6	Class II	156389	156400	TCGTTCCGTATCGTCGTGCT	AACGTGCGGGTGACCTTGTT	298
FMgSSR-40493	scaffold7231	p2	(TG)6	Class II	6087	6098	TCGTTTGGTGTTTTGCGCTCGG	TAGCTGCCGTGCCATCTGAACT	329
FMgSSR-40494	scaffold436	p2	(TG)6	Class II	28104	28115	TCGTTTTTCCGCGTGTGCGT	CGGCATTGGCAAATCGCCACAA	294
FMgSSR-40506	scaffold12051	p2	(TG)6	Class II	17099	17110	TGAAAGTATGCTGCCGCTCCCA	ACAAGTGCAACTCCATGGGCA	334
FMgSSR-40508	scaffold2225	p2	(TG)6	Class II	36324	36335	TGAACATGTCCTGGGTGCCT	TCCTTTGCTTCACCTCCACGGT	329
FMgSSR-40509	scaffold9290	p2	(TG)6	Class II	8483	8494	TGAACCCCCTTTGCAGCACACT	ATGGCACCAGAGACAGCCTACA	336
FMgSSR-40513	scaffold494	p2	(TG)6	Class II	92620	92631	TGAATCTCCCAACTGAGAGGGGT	TAGCTGCAGGAAGCATGTCGCT	260

SSR_ID	Scaffold	SSR_Type	SSR_Motif	SSR Class	SSR_Start	SSR_End	Forward sequence	Reverse sequence	Product_size
FMgSSR-40514	scaffold11464	p2	(TG)6	Class II	12019	12030	TGAATGCAGTGCGGGGTCTT	TTGCTCCCCACTGCCCAAACAA	326
FMgSSR-40515	scaffold1185	p2	(TG)6	Class II	67214	67225	TGACCACACGCTTGCCATGT	TGGCACACTAGCCCGTTGTT	347
FMgSSR-40518	scaffold18912	p2	(TG)6	Class II	7481	7492	TGAGGAGAGTTCTGCGCTGT	ATGGCGTTGGCTGTTGTGCT	237
FMgSSR-40520	scaffold8270	p2	(TG)6	Class II	5619	5630	TGAGTTGGTGACCACGCGTCAA	AGCTCACCGTGCCATGAAGA	307
FMgSSR-40521	scaffold4178	p2	(TG)6	Class II	17466	17477	TGATGCAGCTGCTAGCTTGGCT	ACTTTGGAGGCTGCAGTTAGGA	279
FMgSSR-40523	scaffold6335	p2	(TG)6	Class II	35235	35246	TGCAAAGGCACGATCGCCAA	CGTGCGATGCTACGGGTTTT	293
FMgSSR-40528	scaffold5034	p2	(TG)6	Class II	24123	24134	TGCACATGCCATTGAAGGCAGC	AAGGGTCAAGCACACACGCA	326
FMgSSR-40534	scaffold102	p2	(TG)6	Class II	66824	66835	TGCACTTCGTGCGGATGGTT	ATGGCTGCGCAGTGATGAGT	253
FMgSSR-40535	scaffold1251	p2	(TG)6	Class II	81964	81975	TGCAGAGAGACACTGTACCGACCA	TGCTGACTTCCGTGACTGCTGA	317
FMgSSR-40537	scaffold8082	p2	(TG)6	Class II	7714	7725	TGCAGATGCAGCTGTCGATGTCT	ACCACCAGAAGCAGTTACCATCG	251
FMgSSR-40538	scaffold8797	p2	(TG)6	Class II	22461	22472	TGCAGCACAATGAGCCCAGAGA	AGCAGCGTTAGAGTGTGGGCAT	290
FMgSSR-40539	scaffold2955	p2	(TG)6	Class II	31220	31231	TGCAGCCAACAAAACAGCCCA	ATGCGCGGTGGTGCTTTTGA	269
FMgSSR-40540	scaffold6303	p2	(TG)6	Class II	30601	30612	TGCAGCTTTCACAATGCAGCAC	AGCAGCACAACGCTTCAGGT	274
FMgSSR-40543	scaffold164627	p2	(TG)6	Class II	709	720	TGCATGCCTGGAGTGCAAGA	AACTGCAGAGAACCCTGAGTCCCT	238
FMgSSR-40544	scaffold84268	p2	(TG)6	Class II	1082	1093	TGCATGCTAACTGCCGTGTAGGT	AACCAGGTAGCCATCCTGACGA	350
FMgSSR-40552	scaffold2579	p2	(TG)6	Class II	11546	11557	TGCCTGAGCGAACATATGGGTCA	AATCGGCAGCCTGCGTTCTT	267
FMgSSR-40555	scaffold5966	p2	(TG)6	Class II	36212	36223	TGCCTTGTCGCTGTGAAGCA	TTTCAGGGAATACCTCGGCAGT	303
FMgSSR-40558	scaffold1396	p2	(TG)6	Class II	14525	14536	TGCGAGCCTGCGACTACTGAAT	TGTCGATCTGTTGCGGCCAT	277
FMgSSR-40559	scaffold8796	p2	(TG)6	Class II	11481	11492	TGCGATGCGTGCGTTGCTAA	TCCGATCGCATCACCACACACA	279
FMgSSR-40561	scaffold1829	p2	(TG)6	Class II	58193	58204	TGCGCACCTGAACTGCATCT	AAATTTCAGGCTGCGGAACGGG	328
FMgSSR-40562	scaffold3075	p2	(TG)6	Class II	38263	38274	TGCGCAGCGAGCATGTTTCT	TCGCTGTGGACAAAAGCGGGTT	338
FMgSSR-40563	scaffold1839	p2	(TG)6	Class II	29065	29076	TGCGCAGGCATCTCTTGCAATC	GCGGAGCCGGGAATCAACATTT	350
FMgSSR-40566	scaffold5802	p2	(TG)6	Class II	23841	23852	TGCGGCAAGATCATTCCCGGTT	CCTTGCCGCCCTCGTTTCTTTT	328
FMgSSR-40570	scaffold3277	p2	(TG)6	Class II	25013	25024	TGCGGGGTTATGGCATGGAACT	ACCCCTCTGCTGCGACCATAAA	331
FMgSSR-40571	scaffold2444	p2	(TG)6	Class II	47391	47402	TGCGTCACCAGTCATTGGCACT	TGAGATCGCGCGTCATCAGCAA	200
FMgSSR-40572	scaffold45	p2	(TG)6	Class II	95238	95249	TGCGTGTCTTGTGTGCGCTT	ACACACCATGTGTATGGCGCA	257
FMgSSR-40573	scaffold516	p2	(TG)6	Class II	26388	26399	TGCTCGCTGGTTTGGATCTCCT	TGCTCACATGGTGCTTGCAG	301
FMgSSR-40574	scaffold2663	p2	(TG)6	Class II	16433	16444	TGCTCGCTTCTTTGTTGCCCTG	ACGCACCCCATACTCTGCAA	243

SSR_ID	Scaffold	SSR_Type	SSR_Motif	SSR Class	SSR_Start	SSR_End	Forward sequence	Reverse sequence	Product_size
FMgSSR-40578	scaffold4737	p2	(TG)6	Class II	8551	8562	TGCTCTTCACGGAAAACCACGC	TTGGCGGTTGCTTGCTCCAT	219
FMgSSR-40583	scaffold2271	p2	(TG)6	Class II	11683	11694	TGCTTCGCTACGTACCAAACTGC	TGCACAAGCTTTTTGGCCCCTG	344
FMgSSR-40588	scaffold4826	p2	(TG)6	Class II	3990	4001	TGGAACCAACGGTGGTGAAGGA	TGGCACCTAGACATGCGAGCAA	278
FMgSSR-40591	scaffold898	p2	(TG)6	Class II	75978	75989	TGGAGGGCAGTCCAAGTTCACA	ACACGCACAATCCCCCGCATAA	252
FMgSSR-40592	scaffold15299	p2	(TG)6	Class II	12893	12904	TGGATCATCAGGTGGTGGCACA	AAGGCCTGCTACCCGGTTCATA	251
FMgSSR-40597	scaffold147677	p2	(TG)6	Class II	426	437	TGGCCTGTGTTTCGGTACTCTCA	AAAGGCATCTGCGGACAACCCA	320
FMgSSR-40602	scaffold5173	p2	(TG)6	Class II	5891	5902	TGGGCATGGGATTTGGCAGGAA	CCGATCCATAAACAGAGCACACTC	278
FMgSSR-40603	scaffold118451	p2	(TG)6	Class II	488	499	TGGGCGAAGGTTTGGAGGTT	TGTGCCAACGTGGTTGACGA	276
FMgSSR-40604	scaffold36	p2	(TG)6	Class II	117998	118009	TGGGCTGCAACTCACACTGCAA	ACAAGTTACCAGGGAGCGAGGA	345
FMgSSR-40611	scaffold5341	p2	(TG)6	Class II	42671	42682	TGGTGAGTGGCTGCGACGATTA	ATTTGGCGCGCCGCATATCT	321
FMgSSR-40618	scaffold2596	p2	(TG)6	Class II	37366	37377	TGTCGGTTGCCATTGACCATGT	GGACGGTTGCAATAACTCTGGT	263
FMgSSR-40620	scaffold8	p2	(TG)6	Class II	144697	144708	TGTGAATCCGGTGCTGCTGGAA	AGTATATGCAGGCAAAGCGCCG	207
FMgSSR-40621	scaffold68	p2	(TG)6	Class II	76249	76260	TGTGACCGATCGACGCACAT	AACGGTTTCTCGCCAGCTCT	307
FMgSSR-40624	scaffold5500	p2	(TG)6	Class II	41246	41257	TGTGCAAGCGTCAACCGCAT	ACGCCCAAGCTAACGTGCAA	323
FMgSSR-40628	scaffold1814	p2	(TG)6	Class II	51399	51410	TGTGGAGATACCCTGCGTTCCT	TCTTGTCGGAGCTTCTCGCCTT	260
FMgSSR-40630	scaffold1694	p2	(TG)6	Class II	26000	26011	TGTGGCATGCATTTGTGGCACT	AATAGCTGTGAGCAGAGGCGCT	323
FMgSSR-40633	scaffold19010	p2	(TG)6	Class II	3074	3085	TGTGTGCGCGCTACCTACTT	TGCGAGCAGCAAACATGGCA	287
FMgSSR-40636	scaffold16972	p2	(TG)6	Class II	7268	7279	TGTGTTGGCATTGACGGGCA	TCCGGTGCACCCTCTCATATACT	301
FMgSSR-40638	scaffold518	p2	(TG)6	Class II	111286	111297	TGTTGCCACATGCTTGGGATCG	TGATCAGGTAAGCGGCTCCCAT	316
FMgSSR-40641	scaffold7742	p2	(TG)6	Class II	33628	33639	TGTTTGGGTTTCGCGTGGAGGA	ACCGTGCGTACAGGTCTTTGA	346
FMgSSR-40644	scaffold429	p2	(TG)6	Class II	67416	67427	TTAGCTGCACCAAGACGGGACA	ACGACCGCTGATGAAACGCA	205
FMgSSR-40645	scaffold13415	p2	(TG)6	Class II	4016	4027	TTCCAACCAAAGTGCAGCACGC	AAACCGGTGCATCACATTCCGC	308
FMgSSR-40647	scaffold138	p2	(TG)6	Class II	45939	45950	TTCCCCGTCTTCTCCGATGGAA	TGGAGCCAAACCAGGTGACGAT	330
FMgSSR-40654	scaffold529	p2	(TG)6	Class II	90803	90814	TTGCAAGTGCTCAGGCAACG	TCGATGGCAGCACACCAAAGGA	327
FMgSSR-40656	scaffold7326	p2	(TG)6	Class II	14105	14116	TTGCCCATTGCTGGGTGCCATA	ACGGCACCACATGCGTACAT	348
FMgSSR-40657	scaffold24613	p2	(TG)6	Class II	2924	2935	TTGCCGTCTACACCACAGAGT	AAAAGGGATCAGGCGGCTGGAA	347
FMgSSR-40660	scaffold1405	p2	(TG)6	Class II	27321	27332	TTGCTCGGCTGGCTAGTGTT	AGCCAAGCCACATCGCTGTCAA	315
FMgSSR-40661	scaffold16919	p2	(TG)6	Class II	13550	13561	TTGCTGCTGCGTGCGTGTTT	ACCTGCTTGCCAAAGTGCTGAC	299

SSR_ID	Scaffold	SSR_Type	SSR_Motif	SSR Class	SSR_Start	SSR_End	Forward sequence	Reverse sequence	Product_size
FMgSSR-40664	scaffold90	p2	(TG)6	Class II	157050	157061	TTGGGTTGAGCTGCTTGCTGGT	AACGCGGCCGTCTTGTACTT	328
FMgSSR-40668	scaffold2741	p2	(TG)6	Class II	52276	52287	TTGTGCGTGCGTCTGCGTTT	TACGTTGGGTTTTGGGGCGGAA	302
FMgSSR-40671	scaffold16416	p2	(TG)6	Class II	7916	7927	TTGTTCACCACCGGCAGCAT	ACCCCCAAACACACGTACGACA	308
FMgSSR-40674	scaffold2074	p2	(TG)6	Class II	69934	69945	TTTCACACCACCTCAGCACCCA	TGGCGCGTCTGGATGGAAAT	339
FMgSSR-40677	scaffold251	p2	(TG)6	Class II	112705	112716	TTTCTCGTCTGCCCGTTCGT	AACGGCGGCGGAAAAATGAG	315
FMgSSR-40679	scaffold255095	p2	(TG)6	Class II	90	101	TTTCTGCCACGCGACGTAGT	GCTCACAACACACGCACACACA	231
FMgSSR-40685	scaffold1087	p2	(TG)7	Class II	76128	76141	AAAACAGGGACCGGCAGATGCT	GCAGTGGCAAAGCAAACAAGGA	309
FMgSSR-40686	scaffold182743	p2	(TG)7	Class II	505	518	AAAACGAACAGCGGGCAGCA	ACACACACAAACAGGGGTCGGT	303
FMgSSR-40688	scaffold6203	p2	(TG)7	Class II	643	656	AAACACAGTTCCGTCCGCGT	TCAGCATCCCCTTTCGGCCTTT	259
FMgSSR-40690	scaffold1819	p2	(TG)7	Class II	61111	61124	AAATCATGTGGGTTTCGCCCGC	ACCTGGTAGCCCAAAAGCGA	272
FMgSSR-40691	scaffold25	p2	(TG)7	Class II	145167	145180	AACCAAAGCCCATGCCTGGTGA	TTGGGCAGGGGAGCGAACAAAA	273
FMgSSR-40693	scaffold14239	p2	(TG)7	Class II	17100	17113	AACCCCCTTTGCAAGCACACCT	TGACACCAGTGACAGCCTACACA	324
FMgSSR-40698	scaffold7809	p2	(TG)7	Class II	16681	16694	AACGCGTGGGTCCGTCACTAAT	AACATAGGCAGAGCCGCAGT	327
FMgSSR-40700	scaffold4145	p2	(TG)7	Class II	27450	27463	AACTACGGCGGCGTACATGCAA	TCGCCATCGACATCGTGAGA	323
FMgSSR-40703	scaffold4160	p2	(TG)7	Class II	35055	35068	AAGCTGCACACACTTGCCGT	AGTTCGTCACGGGGCATAACCT	221
FMgSSR-40707	scaffold1772	p2	(TG)7	Class II	74142	74155	AAGGTGACCGCCGATGTGGAAA	TTGGGACAAGCCAAGCATCC	341
FMgSSR-40708	scaffold3337	p2	(TG)7	Class II	7817	7830	AAGGTGCGTCAATGTGGCGT	TCCAACTATTAGCCGCAGGTGCT	215
FMgSSR-40709	scaffold2282	p2	(TG)7	Class II	32952	32965	AATGCCGGTGGTCGTCATGT	AGCCTGAAGGGATGCTTGCGAT	257
FMgSSR-40710	scaffold1066	p2	(TG)7	Class II	40680	40693	AATTCGGCACGCTGGGGTTT	ACGCGCGCCAGTAGATCAATCA	219
FMgSSR-40716	scaffold15754	p2	(TG)7	Class II	6262	6275	ACACAACGTCGTCTTCGCCT	AAGAGAGGGAGGGAGCGAGAAA	279
FMgSSR-40720	scaffold11865	p2	(TG)7	Class II	16901	16914	ACACCATGTGAGCTGTGACGCA	TGCGCGCCTCCATTCTTCAA	281
FMgSSR-40723	scaffold391	p2	(TG)7	Class II	50669	50682	ACACCGGAGCACAACCAACA	ATCGAAACGGAACCGCGCAA	317
FMgSSR-40724	scaffold2548	p2	(TG)7	Class II	8240	8253	ACACGGTGTTCCCATCAAGAGCA	ACCAAAGCACAAGCAGGCGA	208
FMgSSR-40726	scaffold2807	p2	(TG)7	Class II	19700	19713	ACAGGGCAGGGGACAGAGAAAT	GTGGCCGTTCTTTTAGATAGCGGA	289
FMgSSR-40728	scaffold55	p2	(TG)7	Class II	108097	108110	ACATGGGCGAGCTTCCTGACAA	ATTTCACGTGACGGCCCATCCT	222
FMgSSR-40732	scaffold11198	p2	(TG)7	Class II	2630	2643	ACCCCGTTGTGATAGCTCCTCTGT	TGGACCCTTAGGGGCAATAAGCA	231
FMgSSR-40734	scaffold2448	p2	(TG)7	Class II	35765	35778	ACCGAAGTACCGGTAGGCTCAT	ACTTTGGGAGCAAGCAATCGGT	336
FMgSSR-40736	scaffold1	p2	(TG)7	Class II	262087	262100	ACCGTGACAGTGTGTGTGGT	TGGCACCCATTCACACCGAAGT	272

SSR_ID	Scaffold	SSR_Type	SSR_Motif	SSR Class	SSR_Start	SSR_End	Forward sequence	Reverse sequence	Product_size
FMgSSR-40743	scaffold660	p2	(TG)7	Class II	76429	76442	ACGCCCGCTTCTTATTCGCA	TCACCCGACACATGTACGCTCA	307
FMgSSR-40745	scaffold336615	p2	(TG)7	Class II	247	260	ACGCGTACTCCACGGTAACAGA	TCGATGGATGCACACGCGAA	277
FMgSSR-40746	scaffold4995	p2	(TG)7	Class II	32704	32717	ACGGCGAGGTTTCAGTGTTGGA	ACGTTTTGTGGCACCGCTGA	314
FMgSSR-40749	scaffold625	p2	(TG)7	Class II	69985	69998	ACGGGCGGGGTCATGAAAAGAA	ACGCTTCACCTTTCTCGTCGCA	344
FMgSSR-40751	scaffold14137	p2	(TG)7	Class II	2065	2078	ACGGTGCTCAACGCCAACAA	TTCGGATTCAGCTCAGCCCA	295
FMgSSR-40756	scaffold6165	p2	(TG)7	Class II	35214	35227	ACTACTAGCACATAGAGGAGGGGG	ACATCCATTATACCTCCGCGACCC	283
FMgSSR-40757	scaffold15050	p2	(TG)7	Class II	13012	13025	ACTCAAAACCCGCCCCTGAACT	TGATGCGCCACCCTTACCTTGT	303
FMgSSR-40758	scaffold200795	p2	(TG)7	Class II	688	701	ACTCTGCCACGGGTTTTCGTCA	TGGTACGGCCAGCAAGAGTGAT	336
FMgSSR-40759	scaffold10693	p2	(TG)7	Class II	28349	28362	ACTGCCGCCTTGTTGTCTCT	TGACTACCGAACGGCACTTGCT	315
FMgSSR-40763	scaffold6149	p2	(TG)7	Class II	6645	6658	AGCAAAGTGCGCGACTTCCA	TCGACATGTGTGTGCCGCGTAT	279
FMgSSR-40765	scaffold883	p2	(TG)7	Class II	90318	90331	AGCAACGTTGGCCACGTCAA	TTCGACGTGTTTCTGCCGCT	235
FMgSSR-40768	scaffold277	p2	(TG)7	Class II	65667	65680	AGCAGAATGCGCAAGCGAGT	TCCTGGACACCGTCAACACCAA	266
FMgSSR-40772	scaffold7427	p2	(TG)7	Class II	10472	10485	AGCAGGAGGCAGCGCTTAAA	ACACAACAAAGAGTGCACGCCA	329
FMgSSR-40775	scaffold49166	p2	(TG)7	Class II	1647	1660	AGCCGGCAAACAACGGAGAA	CGTTTGGTTGGTGCGATGCGAT	340
FMgSSR-40782	scaffold37194	p2	(TG)7	Class II	2165	2178	AGCTAGGGCCACTAGATGGATACC	GGAGCCACAAAACACACAACAC	348
FMgSSR-40785	scaffold2817	p2	(TG)7	Class II	49087	49100	AGCTGGGCTGCACCAAAACA	ACCTGACCCCCATGCCATTTCA	318
FMgSSR-40788	scaffold4598	p2	(TG)7	Class II	4304	4317	AGGACACCAGCGATCTGCAA	TCAAATAGACCTGACCCAACCCCC	310
FMgSSR-40791	scaffold858	p2	(TG)7	Class II	97715	97728	AGGCTAAGCCAGCGAAGGTGAA	TCACGCGCGTAACATCGTCT	297
FMgSSR-40792	scaffold17250	p2	(TG)7	Class II	13471	13484	AGGCTGCATCACACACCTCT	TCATGGCATAAGACAGCGCCA	257
FMgSSR-40793	scaffold1577	p2	(TG)7	Class II	30873	30886	AGGGCACAACGTTTCCGTGCTA	AATGCCCTCCCACTTCACCTGT	307
FMgSSR-40797	scaffold7070	p2	(TG)7	Class II	1817	1830	AGTCCTGTTTCGACACGTGCAT	ACACAACTTGCATCCAGCCGT	275
FMgSSR-40798	scaffold12335	p2	(TG)7	Class II	7536	7549	AGTCGAGCAGCAAACGAACCCA	TGCCAGGCTTGCATCTCTCTCT	313
FMgSSR-40802	scaffold211	p2	(TG)7	Class II	45865	45878	AGTGGGTGCTAATGGCCTTCT	GCACCAAGCAGGTGTTTGCAT	350
FMgSSR-40803	scaffold40661	p2	(TG)7	Class II	1489	1502	AGTTCCGGTTCCGGTTCCTTGT	TGCGCAAAGGCTACCACCAA	257
FMgSSR-40804	scaffold24065	p2	(TG)7	Class II	6483	6496	AGTTCGAGCCCAGCACGTTTGA	TCGGCAGAAATGGACGACGGTA	249
FMgSSR-40806	scaffold3555	p2	(TG)7	Class II	16985	16998	AGTTTGCGCAGCTCACTGCT	ACCCAAGCACAGGACGTGAA	248
FMgSSR-40807	scaffold536	p2	(TG)7	Class II	19704	19717	ATACCAACTTCCGCCCAGTGGT	TAGTGGCGTTTTTCGGCCCTCA	331
FMgSSR-40811	scaffold2830	p2	(TG)7	Class II	50300	50313	ATCGTGTGCGACGCCATTGT	ATGATGCGTCACGCCATGGACA	296

SSR_ID	Scaffold	SSR_Type	SSR_Motif	SSR Class	SSR_Start	SSR_End	Forward sequence	Reverse sequence	Product_size
FMgSSR-40815	scaffold42886	p2	(TG)7	Class II	595	608	ATGCGTGGAGCACGGAATGACA	TCATGGCAGGCTCCGGAACAAA	221
FMgSSR-40816	scaffold1896	p2	(TG)7	Class II	53006	53019	ATGGCGACAACATGACGGTTGC	AGCTTGTCGTTCTATTGGCTCGGT	258
FMgSSR-40817	scaffold660	p2	(TG)7	Class II	315	328	ATGGGCCATATTGGGCCGAGTT	AGTGTCACCACGACACGAACGA	312
FMgSSR-40820	scaffold836	p2	(TG)7	Class II	28791	28804	ATGTGGCAAGATGCGAGGCA	ACCCGACCGACTTTGTTTGGCT	340
FMgSSR-40823	scaffold623	p2	(TG)7	Class II	50633	50646	ATTGGGGATCACCCGAGCAA	ACCAGGCACAGAATGGCAAGGT	218
FMgSSR-40825	scaffold4082	p2	(TG)7	Class II	22327	22340	CAGCTGCAGCGGCATTAGTA	AAACCATGGCGCACAGCACA	209
FMgSSR-40826	scaffold1417	p2	(TG)7	Class II	40042	40055	CAGTGTTTCCCCATCCTATCACAC	AGCGGCCCAAGTACAAGGAT	350
FMgSSR-40828	scaffold3518	p2	(TG)7	Class II	45220	45233	CCAGCGAAAGCGGTGATGATGA	AGCGGTCGGGATGAGAAAGT	229
FMgSSR-40829	scaffold19966	p2	(TG)7	Class II	1134	1147	CCAGTATCGTCAAACAAAACCCGC	ACCTGGAAACTTCCGTGCGTCA	329
FMgSSR-40830	scaffold3011	p2	(TG)7	Class II	57649	57662	CCATGCATGTCGTTGCAGGGTT	ACAAACAAGCAGGCGGGTCTGA	336
FMgSSR-40831	scaffold2828	p2	(TG)7	Class II	18342	18355	CCCAGAAGTTGTCGTTTCTCTTCG	TGGTTTCCATCAGTGCTTGCCT	290
FMgSSR-40839	scaffold5151	p2	(TG)7	Class II	31242	31255	CGCTTCATGGCCAGCAGCTTTT	TTTGCCGTGACCTACTAGCCT	297
FMgSSR-40840	scaffold3207	p2	(TG)7	Class II	53087	53100	CGCTTCTGTTTCTGCATTGCACC	AAGCTGCCTCGTAACGCACA	316
FMgSSR-40842	scaffold983	p2	(TG)7	Class II	2929	2942	CGTGATGGCAGCAATGCGAGTT	AGGAGACCCGAAAAGACGGCAA	260
FMgSSR-40846	scaffold3819	p2	(TG)7	Class II	29682	29695	GCAGTCCAAGGCTTGTCTTCAGCA	AGCTCCCCCTGCTAGGATTTGACA	350
FMgSSR-40849	scaffold895	p2	(TG)7	Class II	37569	37582	GCTCATGCTTCCGAGCATTGGT	TAGGCCCGAACCCCATGACAAA	330
FMgSSR-40851	scaffold4549	p2	(TG)7	Class II	36181	36194	GCTGCATACACAGAATAGGCTGCT	TAGCAACGCGCAGGCAAAGT	234
FMgSSR-40852	scaffold15991	p2	(TG)7	Class II	15514	15527	GCTGTTGCGAGATTTGGTCGCT	TCGCTCCACGCCAACCATTT	209
FMgSSR-40856	scaffold14802	p2	(TG)7	Class II	3487	3500	GGCCTCGGAAAAAGCGCAAA	ATGCGTGCCACCAACCGAAA	300
FMgSSR-40860	scaffold18104	p2	(TG)7	Class II	8597	8610	GGCTCTTTGCACAAGGCAGCAA	CGCGGCTCGGATTGAATGAGTT	347
FMgSSR-40864	scaffold1842	p2	(TG)7	Class II	42672	42685	TAAGGCACCTGCCTCTAGAACCCT	TTTGCGGGACACCTTTGGCT	343
FMgSSR-40868	scaffold3508	p2	(TG)7	Class II	57206	57219	TCAAAGCGCAGTTTGCTGCTG	AGGCCCAACACACACACACACA	251
FMgSSR-40870	scaffold995	p2	(TG)7	Class II	77003	77016	TCACAGGTCTATCTGGCGCA	TGTGGTGCCGCAGCTTTCTT	264
FMgSSR-40882	scaffold110206	p2	(TG)7	Class II	1259	1272	TCCGCTTGGATGAGCACCAACA	ACGGCCGAACCAAGGTTCCATT	301
FMgSSR-40885	scaffold111	p2	(TG)7	Class II	110398	110411	TCGCAAGCCCAATGGCATGA	AGTGGGGAGCCGATCGTTTCAA	338
FMgSSR-40888	scaffold921	p2	(TG)7	Class II	25995	26008	TCGCCTCCTTTACTGCACCA	AGGCACGTTGACTTGACGGA	249
FMgSSR-40889	scaffold4864	p2	(TG)7	Class II	10821	10834	TCGCTGCCATTGCCAAGATGGA	TCCTAACGCACGCTACACAGCA	228
FMgSSR-40893	scaffold8655	p2	(TG)7	Class II	25157	25170	TCGTGCCCGTGTGTGTGTTT	TCGCAAGCATACTACGGCCCAT	210

SSR_ID	Scaffold	SSR_Type	SSR_Motif	SSR Class	SSR_Start	SSR_End	Forward sequence	Reverse sequence	Product_size
FMgSSR-40900	scaffold3073	p2	(TG)7	Class II	27666	27679	TGACGAGGAAAGCCTGGAGAGA	TCAGTTGGGCGAATGGGAACA	334
FMgSSR-40901	scaffold78766	p2	(TG)7	Class II	658	671	TGACGAGTTGACGACGGGGAAA	TGGGCGGACGACTCTACTTCAA	317
FMgSSR-40902	scaffold161	p2	(TG)7	Class II	150376	150389	TGACTGCACTTTGGGGTCGT	AGCAATGTGTTCGCGAGTGC	350
FMgSSR-40907	scaffold168	p2	(TG)7	Class II	17995	18008	TGATGCCGTCGCATGAGTTGGA	ACAGAAATGGAAGCCGACGCCA	336
FMgSSR-40908	scaffold1128	p2	(TG)7	Class II	24310	24323	TGCAACACGGCTGCTGGAAT	ACTATGGCCGACGACCCATTCA	320
FMgSSR-40911	scaffold202344	p2	(TG)7	Class II	293	306	TGCACTGGTGCCTCGAATCCAT	ATTCCATCATTCCGCGCCCT	308
FMgSSR-40912	scaffold1389	p2	(TG)7	Class II	39398	39411	TGCAGAAGAAGCAGAGCGCA	ATTCAGTTCCGTTCGGTCCCGT	219
FMgSSR-40913	scaffold87	p2	(TG)7	Class II	36356	36369	TGCAGTTGTGGAGGCATGGGAA	TGCGATCAAGAGCAATGAGGCA	342
FMgSSR-40919	scaffold16321	p2	(TG)7	Class II	5398	5411	TGCCGCTCGTTGTTGGAGTT	TGGTCGGGCTTGAAGGGGATTT	326
FMgSSR-40920	scaffold5673	p2	(TG)7	Class II	25928	25941	TGCCTTGATCTGGGGCTGCAAT	ACTTCAGCATCTGTGCCACCCA	332
FMgSSR-40921	scaffold13567	p2	(TG)7	Class II	6051	6064	TGCGCCAGTGTTCGGTTCTT	TGCTGTAACCTTCGCATCAGGA	337
FMgSSR-40926	scaffold7725	p2	(TG)7	Class II	8823	8836	TGCTGCTCGCAGGTTTTGGT	ACTCCCTTGTCGTTGCTGTCGT	350
FMgSSR-40927	scaffold16598	p2	(TG)7	Class II	17793	17806	TGCTGGCCGTCCAAACACTT	TGGCAAAACACAGGCCAGGGAA	275
FMgSSR-40931	scaffold22747	p2	(TG)7	Class II	7010	7023	TGCTTTGCTTGTGCCAAAGCCT	AAACTCCGGTCTAGGGGAAGAC	241
FMgSSR-40934	scaffold358	p2	(TG)7	Class II	99780	99793	TGGACGGTTGGCCAGTTTGTCA	AGTGCAGCCATGTGCGGTAGTT	341
FMgSSR-40938	scaffold11950	p2	(TG)7	Class II	1436	1449	TGGCTGTGCACCGCTTACGAAT	TCTCACGGGGCAAACGAAACT	228
FMgSSR-40939	scaffold1782	p2	(TG)7	Class II	65117	65130	TGGGCAGACTATGGTCGCACTT	AAGCACATTGGTTACACGCGGC	307
FMgSSR-40940	scaffold118451	p2	(TG)7	Class II	57	70	TGGGCCGGGATTTGTTCAGA	TTTTCCTGGACGAGCGATGCCT	286
FMgSSR-40941	scaffold32154	p2	(TG)7	Class II	6522	6535	TGGGGATAAGAGGGCCAAACA	AAATTGGCGTCCCGGGTACAGA	243
FMgSSR-40942	scaffold39583	p2	(TG)7	Class II	4623	4636	TGGGGCACCATTGTTGTTGGA	GCTGCCAAAAGTTGCTGCGA	302
FMgSSR-40946	scaffold4206	p2	(TG)7	Class II	721	734	TGGTAGTGGCCAGCAAGTGGAA	TTGCAACACACCGGCGAACT	263
FMgSSR-40953	scaffold1109	p2	(TG)7	Class II	90024	90037	TGGTTTTGCTCCCTGGACTCGT	AGCAGCACACAGCTTGCGAA	294
FMgSSR-40954	scaffold21	p2	(TG)7	Class II	97202	97215	TGTCACGAACCGGCCCAAAA	TATGCGACGCCTGAAACGCT	327
FMgSSR-40955	scaffold5517	p2	(TG)7	Class II	20912	20925	TGTCACGCATGTCAAAGCAGTG	TGACAGGCTGCGCGTCAAAT	313
FMgSSR-40956	scaffold3704	p2	(TG)7	Class II	11041	11054	TGTGAGAAGTGTGGCGTGGAGA	TTCGCCTGCATGGCTGCATA	276
FMgSSR-40958	scaffold11323	p2	(TG)7	Class II	13957	13970	TGTGCACGGTTGCCACAAGT	GGCTTTCCAAATGCAAACACCG	202
FMgSSR-40960	scaffold296	p2	(TG)7	Class II	99544	99557	TGTGTGCCTCCAAAGCAGAGCA	ACCATGACGCCACTGCCATGAA	282
FMgSSR-40961	scaffold276881	p2	(TG)7	Class II	383	396	TGTGTGTGCGTGCGTGCAAT	CAAATGGAAGAACCTCCCCCATGC	200

SSR_ID	Scaffold	SSR_Type	SSR_Motif	SSR Class	SSR_Start	SSR_End	Forward sequence	Reverse sequence	Product_size
FMgSSR-40963	scaffold2409	p2	(TG)7	Class II	35094	35107	TGTGTGTGTGTGTGGGTTCACCA	TCCAGGACAACTGGATGGTGCT	252
FMgSSR-40967	scaffold2095	p2	(TG)7	Class II	54177	54190	TGTTGCAGAACGGCAAAGGTGC	TACAGTGTGCGCCCTGTTGT	330
FMgSSR-40968	scaffold1989	p2	(TG)7	Class II	49659	49672	TTACGCAAGGGCCGCACATA	CAGCAAACGTCAGTTCGGTGGT	223
FMgSSR-40969	scaffold30753	p2	(TG)7	Class II	5736	5749	TTAGGGCCTAGTGGGCAGACTT	TAACCACAAACTCCTCGCCCCA	230
FMgSSR-40970	scaffold5316	p2	(TG)7	Class II	18220	18233	TTAGGGCGATTCGTTGCTGCAC	GCTACAGCACAACAAGCCGACA	345
FMgSSR-40974	scaffold225	p2	(TG)7	Class II	121704	121717	TTGCTTGGTGGGGATGCACT	TGTGAGTGCCTTTTGCCCCCTT	340
FMgSSR-40976	scaffold44103	p2	(TG)7	Class II	376	389	TTGGTCTTGGTTTTTCGGCGGC	TTGTACACGTGTGGGATGCGGA	205
FMgSSR-40977	scaffold3935	p2	(TG)7	Class II	33123	33136	TTGTTCGTGTGCGCCTTCTC	ATCAGTGAGGCCGTGAGAGGTT	283
FMgSSR-40981	scaffold25167	p2	(TG)7	Class II	1188	1201	TTTGGTCCACAGGATGTGCCGA	TGCGGTCTTGGCCTTTGCTT	221
FMgSSR-40985	scaffold540	p2	(TG)7	Class II	50925	50938	TTTTGTGATGTACACGGGCGGC	AATCATCCCCTTTGCCGTCGTC	322
FMgSSR-40990	scaffold10614	p2	(TG)8	Class II	14272	14287	AACCGTCCGGCCCAAAACAA	GCCATGCATGTGCGGCTTTT	347
FMgSSR-40992	scaffold289	p2	(TG)8	Class II	125551	125566	AAGACGAGCGCAAGGAAGACGA	AGTTCACGACAGCGGCAGTT	327
FMgSSR-40993	scaffold92	p2	(TG)8	Class II	151333	151348	AAGGAGCACAATTGCGGCGT	TATCAGCAGGCCAGCACACACT	320
FMgSSR-40995	scaffold10408	p2	(TG)8	Class II	19273	19288	AAGGGCAGGCATGCGTGAAA	TCAGCACACAGCAGGGAGCTTT	219
FMgSSR-40998	scaffold1251	p2	(TG)8	Class II	19708	19723	AAGTTCGGCAGTGGCCTTGT	AGCGACCAAGCCGCCTAAAGAA	282
FMgSSR-40999	scaffold2387	p2	(TG)8	Class II	64185	64200	AATAACAAAGCGCAGCCCCACG	TTGGCACTGGCTCGTGTCTT	319
FMgSSR-41000	scaffold10421	p2	(TG)8	Class II	25958	25973	ACAAGGGAGCACGACCAACT	AGCCAATGTGTTTTTCCGGGG	226
FMgSSR-41001	scaffold4935	p2	(TG)8	Class II	18293	18308	ACACAAGCACGGAGCCGTTT	AGCCCTTGAGCATTTCTGGGGT	312
FMgSSR-41002	scaffold625	p2	(TG)8	Class II	11203	11218	ACAGCGCTGCTTGTTTACTGGA	CGGCAGAAATCACCCTGGACTTCA	333
FMgSSR-41004	scaffold780	p2	(TG)8	Class II	57573	57588	ACCAAGAGTGTTGAATGCTTCCCC	AGCAGAGCAGAGCGGAGCTTTT	309
FMgSSR-41006	scaffold318	p2	(TG)8	Class II	61740	61755	ACCCACTTACAAGGAACGCATGAC	TGACGCGCTCTCATTCAGTTCTCA	228
FMgSSR-41007	scaffold1233	p2	(TG)8	Class II	70605	70620	ACCGCACAATCGTGGGGAACAA	ACCTGCTGATGCAACATGCTC	226
FMgSSR-41009	scaffold822	p2	(TG)8	Class II	85398	85413	ACCTGCGAATGTTGCCCTCT	ACCGAGCGTGCACAACAACT	265
FMgSSR-41011	scaffold1528	p2	(TG)8	Class II	21491	21506	ACGCGTACGGACAAAACGCA	ACGTGATTGGCAGAAAACCCCAC	232
FMgSSR-41018	scaffold156	p2	(TG)8	Class II	98063	98078	ACGGCTGACTTGCGCCATTT	TCAACACTGGCCACTGAGCGTT	298
FMgSSR-41019	scaffold841	p2	(TG)8	Class II	83716	83731	ACGTGGTGGACGGCTGTATCTT	TGGTCGTGGCGCCGGTATTTAT	272
FMgSSR-41024	scaffold6689	p2	(TG)8	Class II	20399	20414	ACTGAGCAAAGGGGAAGGCCAA	TGTCTTGGAAGAGGTGAGTTCCCA	200
FMgSSR-41025	scaffold1533	p2	(TG)8	Class II	58404	58419	ACTGGCACCCTTGTTGCGAA	AGCCCCGTTCTAAACGCAGA	240

SSR_ID	Scaffold	SSR_Type	SSR_Motif	SSR Class	SSR_Start	SSR_End	Forward sequence	Reverse sequence	Product_size
FMgSSR-41028	scaffold2806	p2	(TG)8	Class II	45377	45392	ACTGTCAGTCAACACAGCTGACAC	TTACAGTTATTCGCGGCGGGGT	262
FMgSSR-41029	scaffold4626	p2	(TG)8	Class II	9372	9387	ACTTGATCCCCATTGCTGCTGT	AGACGAACCAAAGTGCTGGGT	335
FMgSSR-41032	scaffold1614	p2	(TG)8	Class II	38002	38017	ACTTTGCCTTGGCGAGCATGA	ACTGGAGTAGTGGCCATGCAGT	212
FMgSSR-41036	scaffold7069	p2	(TG)8	Class II	33047	33062	AGAGCTTGGTGCCAAAGCCA	AGCAGCATCATACCTGCACCT	350
FMgSSR-41038	scaffold324	p2	(TG)8	Class II	101051	101066	AGCACCCGAGCTCAGCAAAT	AAAAAGCACCGGGACCCAGCAA	236
FMgSSR-41040	scaffold11440	p2	(TG)8	Class II	6291	6306	AGCAGTATCTCTCTGGCAAGCGT	TTCATTCTTCCCGTCGCCCCAA	243
FMgSSR-41050	scaffold915	p2	(TG)8	Class II	18157	18172	AGGGCGAGAGCCTTTTTGCT	TCTGCCCACAGACGCTGTTT	332
FMgSSR-41053	scaffold4172	p2	(TG)8	Class II	7873	7888	AGTGGACTGGGTTGTTGGTTGC	ACGGCGGCCGCATTTATTCA	345
FMgSSR-41055	scaffold5221	p2	(TG)8	Class II	6961	6976	ATGCTGCATGGGGATGTGTTCC	TCAGGCTGGTCTTGTGAAGCGT	223
FMgSSR-41063	scaffold4023	p2	(TG)8	Class II	8022	8037	CAGGACAATTCCGTTGTGCCGA	ACACCAGCGGTAGACCACATGA	268
FMgSSR-41065	scaffold7038	p2	(TG)8	Class II	25378	25393	CCAAATGCCTCCGCCCTTGTTT	TCGGGATGTTCGCCATGCTCAA	281
FMgSSR-41066	scaffold13022	p2	(TG)8	Class II	2430	2445	CCACTTCGGCGCAAATGTTGT	TCCACGCGCAACAAAAAGCC	251
FMgSSR-41068	scaffold251523	p2	(TG)8	Class II	217	232	CCATGTGGGCGCCGATGAATTT	CGCAAGTGACGCTTTGACTCCA	275
FMgSSR-41069	scaffold435	p2	(TG)8	Class II	16339	16354	CCGTGCTCCAAGGCCAAAAT	TGACTGTCTAGCAGGGCACAAGT	285
FMgSSR-41072	scaffold2066	p2	(TG)8	Class II	2919	2934	CGCCAGGAATTCTTTGGGAAACGA	CACAGCAAAAACGCAAGTGCCA	211
FMgSSR-41073	scaffold58267	p2	(TG)8	Class II	2205	2220	CGCTACTAAAATGGAACGGCAGCG	AGCTTCGCCCAACACGCTTCTA	303
FMgSSR-41074	scaffold9148	p2	(TG)8	Class II	7916	7931	CGTCATGTGTAGACGTGAGGTAGT	AGCTCTGTTTTGCACGGTGG	257
FMgSSR-41075	scaffold5044	p2	(TG)8	Class II	16206	16221	CGTGTGCATGAGCTTTGTCGCA	TCCATGGTGCCCACATGAAGGT	220
FMgSSR-41076	scaffold738	p2	(TG)8	Class II	12148	12163	GAGCATTGCCCATTGCGTCTGT	AGCGGGTTTTACGTATCAGGCTCA	200
FMgSSR-41078	scaffold1330	p2	(TG)8	Class II	64392	64407	GCACAAGCGTTTGCTGCTCCAT	TGTGCACCATGGCCGTTGAA	326
FMgSSR-41080	scaffold6505	p2	(TG)8	Class II	25696	25711	GCACAGGAACCTTCCACCTCTCTT	CGCATTTGGGATCAGAAACGTGAC	350
FMgSSR-41081	scaffold13961	p2	(TG)8	Class II	19291	19306	GCACCCAATCGAATCGAACGCA	AGTGAGAGCGGGTTGCTTGT	327
FMgSSR-41082	scaffold36169	p2	(TG)8	Class II	609	624	GCCTTTAGGAGTTGTATGCTCCTGC	CCCGCAAAGTGACTAGTAGAGGAC	350
FMgSSR-41085	scaffold367	p2	(TG)8	Class II	16684	16699	GCTTTGCTGCCCACCGATTTGA	TGCCAAGAAGGTGTCGTACTCGT	278
FMgSSR-41086	scaffold330718	p2	(TG)8	Class II	292	307	GGGATGTTGTATTGCTTGGGCA	TCCAGTCAGGCAGTCACGTCAA	300
FMgSSR-41088	scaffold3073	p2	(TG)8	Class II	4742	4757	GTGACGCGTCCAGCCAAAAACA	AACCCGCGTTTGAGAGCACA	331
FMgSSR-41090	scaffold3181	p2	(TG)8	Class II	56403	56418	TAACGAGGAAGCCACCTGCACT	TGGCGAGTCTCTCAAAAGAGGG	245
FMgSSR-41094	scaffold75	p2	(TG)8	Class II	174945	174960	TATTACGCGTGGTGGGCCGTAT	GCTCACGGGCGAAAACCAAACA	203

SSR_ID	Scaffold	SSR_Type	SSR_Motif	SSR Class	SSR_Start	SSR_End	Forward sequence	Reverse sequence	Product_size
FMgSSR-41097	scaffold5871	p2	(TG)8	Class II	24837	24852	TCAGCTTGTCGGCATGTCAGGT	TATGCCTGCGTGGCCAATTGCT	332
FMgSSR-41103	scaffold1770	p2	(TG)8	Class II	15174	15189	TCCGAAGTGACAACCTATCCCTGG	TGTCCATAGGACACCGCTTAGTGT	328
FMgSSR-41105	scaffold13395	p2	(TG)8	Class II	18693	18708	TCCTCCACGTTTCTTGGGGTCT	TTGCCGCATTTCCACTCGGA	350
FMgSSR-41106	scaffold26392	p2	(TG)8	Class II	1827	1842	TCCTTGCTTTTCCCTCCTCTCTCCT	ACGTGTGATGCCAACCAGCA	260
FMgSSR-41107	scaffold1805	p2	(TG)8	Class II	46087	46102	TCGAGCGCGGAGACAAGATT	TCAACGCAACACTCCTCCTCCT	319
FMgSSR-41109	scaffold14683	p2	(TG)8	Class II	4299	4314	TCGCCTGTCAACAGCATAGCCA	ACAAGCCGCCCAATTACTCGCT	299
FMgSSR-41111	scaffold15900	p2	(TG)8	Class II	17257	17272	TCGGAGTTAGTGTTCGTGGCCT	TCGAGCCCCATTGCAAGACAGT	285
FMgSSR-41112	scaffold13	p2	(TG)8	Class II	267346	267361	TCGTCATCAACTTGGGCCACCT	TCCACGCTATGAAGCAACCCGT	311
FMgSSR-41113	scaffold17188	p2	(TG)8	Class II	6054	6069	TCTCAGACGGATGTACCATGCTG	TGCTATTTCCCTTGACCAATCCCCC	228
FMgSSR-41119	scaffold5517	p2	(TG)8	Class II	33623	33638	TGCAAGGTTCGCGCACAGAAA	ACTGCTGCCGACGATCCAAA	280
FMgSSR-41121	scaffold3969	p2	(TG)8	Class II	41081	41096	TGCAATTGGGACAGTGAAGGCA	TCAAGGGGGAGAATGTTGGTGTGA	342
FMgSSR-41129	scaffold4444	p2	(TG)8	Class II	12758	12773	TGCGTACTTGTTTGGGCACTGA	TCGCCTCCATTTCGTCCTCGAT	274
FMgSSR-41132	scaffold14228	p2	(TG)8	Class II	11971	11986	TGCTGTACATCTGGTCGTGCT	TCGACGCTCAAGCCTGCAAA	266
FMgSSR-41134	scaffold115	p2	(TG)8	Class II	39007	39022	TGCTTTCCACGTCCTTGTCCCT	ATAGCAAAGAGGTGGCGCGTGT	348
FMgSSR-41135	scaffold719	p2	(TG)8	Class II	47636	47651	TGGAAGCGTGTCGAGACGAACT	CCCTTTGTTAAGTGCACGCACGA	305
FMgSSR-41138	scaffold4848	p2	(TG)8	Class II	32075	32090	TGGCGGAGCCAGCCACAAAATA	ACGCCTACACGATGCATCAACCA	236
FMgSSR-41140	scaffold19190	p2	(TG)8	Class II	16406	16421	TGGGGAAGCCACAACTGGAA	AGGGTTGTGTGATGGACCGA	331
FMgSSR-41142	scaffold19254	p2	(TG)8	Class II	11320	11335	TGTAGCGATGCGACCGTCCAAA	AGTGTGCAGCCAGCTCTCGATT	304
FMgSSR-41143	scaffold214	p2	(TG)8	Class II	132528	132543	TGTATTGGTGTGCAGCGCCT	AATGCATCGCGACCACTCTTGC	250
FMgSSR-41144	scaffold9515	p2	(TG)8	Class II	13628	13643	TGTCCTGTGCCCTTGGCTTTCT	GCAAAGCTTTTGCGTGTTCGC	333
FMgSSR-41145	scaffold349	p2	(TG)8	Class II	109182	109197	TGTCTCCTTGTGTGCTAGGTGGT	GCATGGAAGTCAAAAGGAGCTGACG	349
FMgSSR-41147	scaffold784	p2	(TG)8	Class II	98548	98563	TGTGTCGAGTAGACGTTGGTGTGT	TCGTCGCCACACATGAGGTTGT	296
FMgSSR-41148	scaffold215	p2	(TG)8	Class II	74079	74094	TGTTCTGCCCAGACAGCAGCAA	TCTCCACTCACTGTAGCAGCGT	289
FMgSSR-41149	scaffold13996	p2	(TG)8	Class II	18003	18018	TTATGAGAAGGCGCGTGCAGGA	GCTGCCACTTGTTTGCTGTGGA	338
FMgSSR-41150	scaffold1168	p2	(TG)8	Class II	20466	20481	TTCACTGACCGTCCGTTTGGGT	CCGCAGCGGATTCAAATGCAAC	321
FMgSSR-41151	scaffold2195	p2	(TG)8	Class II	67591	67606	TTCAGTGACGGGGAAAGCGA	AAACCGCGCTGAAGTACACGGT	348
FMgSSR-41153	scaffold6380	p2	(TG)8	Class II	14966	14981	TTCCTGCAAGGCAAGACCTCCA	ACTGAAGGTGCCAACCCACACA	333
FMgSSR-41155	scaffold5440	p2	(TG)8	Class II	34674	34689	TTCTTGCTAGTGCCACCGCCAT	TGTGCGGCAACAGTTCTGGA	216

SSR_ID	Scaffold	SSR_Type	SSR_Motif	SSR Class	SSR_Start	SSR_End	Forward sequence	Reverse sequence	Product_size
FMgSSR-41156	scaffold277	p2	(TG)8	Class II	78842	78857	TTCTTGGTGCCGCATTGCCA	AGTGGCGTGCCTTTCTCTTGCT	323
FMgSSR-41157	scaffold551	p2	(TG)8	Class II	41242	41257	TTGCATGCAGCTCGCTCTCA	TGCAAATGGCCAAAGCGCCA	265
FMgSSR-41158	scaffold168	p2	(TG)8	Class II	734	749	TTGCCGACCTGAAACGGTCA	TGCAAAGCATTGCTCGCCGAA	254
FMgSSR-41164	scaffold15249	p2	(TG)8	Class II	2841	2856	TTGTGGCCTCTGCCCTTCACTT	GCACAGCACACGGCCACATAAT	340
FMgSSR-41167	scaffold20645	p2	(TG)8	Class II	9297	9312	TTTCAACCGGCAGCTCCTAGTG	TACCCCATCCCGGTGTAGGTTGAA	320
FMgSSR-41178	scaffold8	p2	(TG)9	Class II	60052	60069	AAGTGGCGTGGGACAGAAGACA	TGAAATGAGGGATGCGCATTGG	350
FMgSSR-41180	scaffold3621	p2	(TG)9	Class II	12637	12654	ACCACCACGACTGTTGGCATCT	TGCATTGGCGCATGGACACA	207
FMgSSR-41181	scaffold815	p2	(TG)9	Class II	8774	8791	ACCAGATTCTTTGGTGGGTGGGT	ACGAAACAAACCCCTCCACTCCT	308
FMgSSR-41183	scaffold179672	p2	(TG)9	Class II	162	179	ACCCAACTGTAGGGCTGCAA	AGGATTGCGTGGAATGGCAGA	224
FMgSSR-41187	scaffold3123	p2	(TG)9	Class II	38502	38519	ACGCACACCAACAACCGCAA	TTCAGATCCCGCGCAAACGA	235
FMgSSR-41189	scaffold214	p2	(TG)9	Class II	80911	80928	ACGTGCAGTCATGCAGAGACA	AAAGCTGGGCTTTTGCGTGC	278
FMgSSR-41190	scaffold465	p2	(TG)9	Class II	67429	67446	AGACGACGAAGCAGCGTTGAA	ACGTGAAACCACCGCGTCAA	278
FMgSSR-41193	scaffold2117	p2	(TG)9	Class II	12640	12657	AGCCGGAAAAAGCAGCGTGA	ATCGTGTCAGGTGGTTTGGGCA	309
FMgSSR-41197	scaffold1575	p2	(TG)9	Class II	65142	65159	AGCGGACAAGCAACGGGTATGT	TGCCGGCTCTGCTGCTTTTT	334
FMgSSR-41200	scaffold7069	p2	(TG)9	Class II	12403	12420	AGCTCGGTTGCACCGTTGTT	ACGTTACGTATTGCCCGTCCCA	341
FMgSSR-41202	scaffold19177	p2	(TG)9	Class II	13033	13050	AGCTTCAACGATCGCATCTACAGC	TAGTCCCCATATGCCGCAGAGT	337
FMgSSR-41204	scaffold9262	p2	(TG)9	Class II	20614	20631	AGGACGGGACACACGGAACAAA	AACCCGCGTCTTGCGTAGATGT	297
FMgSSR-41206	scaffold6726	p2	(TG)9	Class II	12578	12595	AGGGTGCAGTCGAAGTTGCT	AGATCAACGCCTCCATGCCT	209
FMgSSR-41208	scaffold235	p2	(TG)9	Class II	114025	114042	AGTCTACGGATCTAGGGGCCTT	CGCGAGAATTTAGTGGCCATCGT	246
FMgSSR-41209	scaffold9027	p2	(TG)9	Class II	6197	6214	AGTCTTTGGTGGCGGAGCTCTT	ATGCCGCCTCTGTGTTGCAT	288
FMgSSR-41210	scaffold37745	p2	(TG)9	Class II	511	528	AGTGCATGCTTACTTCCTGGCCT	ACCACCATCACCAGTTCACCACT	350
FMgSSR-41217	scaffold1526	p2	(TG)9	Class II	77062	77079	ATGTGCTCCCACGCAGACAA	TCGTTTGACCTGTCTGCCGCAT	276
FMgSSR-41223	scaffold40	p2	(TG)9	Class II	48648	48665	CGCAACAGTCATCGACTGCAGTATC	TCATCGCCTATCGCGTGCAA	278
FMgSSR-41224	scaffold43478	p2	(TG)9	Class II	5153	5170	GCAGCCTCTCCACGCAAAAT	GCAGCGTTGCGGAATGCGAATA	287
FMgSSR-41225	scaffold2636	p2	(TG)9	Class II	46573	46590	GCATGTGAAGGGCGCCTGTTTT	TGGCGTTGCTGTGAACCTCAGT	313
FMgSSR-41235	scaffold3395	p2	(TG)9	Class II	29798	29815	TATCCTTGTCAGTGCGGCTGCT	AGGCACCAATCGTCCAAGTCGT	253
FMgSSR-41236	scaffold7073	p2	(TG)9	Class II	10379	10396	TCACTCACTGCGACCTTTCGGA	GCAGTGTCATTCAACGCATGCCAA	243
FMgSSR-41238	scaffold9311	p2	(TG)9	Class II	24227	24244	TCATCAGGGTCTGCAACCGACA	TACACCGGATGGGCGTAGCTTT	280

SSR_ID	Scaffold	SSR_Type	SSR_Motif	SSR Class	SSR_Start	SSR_End	Forward sequence	Reverse sequence	Product_size
FMgSSR-41240	scaffold1636	p2	(TG)9	Class II	5093	5110	TCGACGACGGCAAGCTCTCAAA	TCCATTGCCCACACACACGTCA	294
FMgSSR-41241	scaffold4200	p2	(TG)9	Class II	30628	30645	TCGATTGTTGCGTCTCTGCCCT	TCCATCGAACAGGTTCCTTGCCA	224
FMgSSR-41243	scaffold93	p2	(TG)9	Class II	176974	176991	TGAAGGCTGGAGGTAGCCTGAT	GGGTGCCGATCGGAGATTGTAT	335
FMgSSR-41244	scaffold262	p2	(TG)9	Class II	119583	119600	TGAATTGTGCTCGTGCGGCT	ACGAAGAATGCAGGGAACACGC	206
FMgSSR-41246	scaffold21949	p2	(TG)9	Class II	11070	11087	TGACGCGCGCTGGATTTGTT	TGGCGGTTGAAGAAGGAACGGA	333
FMgSSR-41251	scaffold23348	p2	(TG)9	Class II	402	419	TGATGGCAGTTGGCAGCAGT	TCGATCGAGCGTGCACAACA	344
FMgSSR-41256	scaffold5679	p2	(TG)9	Class II	17746	17763	TGCCAGAGCAAGCTGAACGTGA	AGGGTGTGCTACGTGTCTGCAA	218
FMgSSR-41258	scaffold1749	p2	(TG)9	Class II	13572	13589	TGCCCCTATTTGATGCATTGAGCC	TGCTGTCATCATTTGGCCCCT	227
FMgSSR-41259	scaffold1293	p2	(TG)9	Class II	62042	62059	TGCCTAGCAGTGTCATGGATCA	TGTTCTTCTTCCCCTCTACCCCCT	281
FMgSSR-41263	scaffold1474	p2	(TG)9	Class II	87678	87695	TGCGTAAGCCGGTAACCGAAGT	ACACTTGCTCCTGGATGTGGGT	268
FMgSSR-41264	scaffold67679	p2	(TG)9	Class II	1363	1380	TGGAGTTTGTCCCCGTCGAGTT	ACGTGTGGCAGCAACGTGAT	274
FMgSSR-41265	scaffold5000	p2	(TG)9	Class II	32885	32902	TGGCTTCAGAACCTCCACGA	ATCTCCTTGACTCCTTCCTCGGGT	270
FMgSSR-41266	scaffold8731	p2	(TG)9	Class II	25703	25720	TGGTAGCGTGTTTGCGCAGT	ACCCAGCTGCCGCATGATAA	337
FMgSSR-41272	scaffold1349	p2	(TG)9	Class II	48689	48706	TGTTTGCACGTCCACGCAGT	AGACGCTGAGCAACAGCACA	312
FMgSSR-41273	scaffold30474	p2	(TG)9	Class II	3776	3793	TTAGCCACCAACCCTTGCCGTT	TGTGGGTTGCTCTCGTGTTGCT	319
FMgSSR-41274	scaffold925	p2	(TG)9	Class II	39083	39100	TTCACCGAGCAACCCAGCTGAT	TCCATTGCGGGCTGTGCTAT	242
FMgSSR-41276	scaffold6046	p2	(TG)9	Class II	9654	9671	TTGCAGCACGATGAGGTGCTGAT	AGGCATGCCAGCAACGATTT	313
FMgSSR-41277	scaffold2775	p2	(TG)9	Class II	3953	3970	TTGGCCATATGAGCATCGCCGT	TAGCGGACTGCATGGTCTGGTT	303
FMgSSR-41278	scaffold2817	p2	(TG)9	Class II	44926	44943	TTGGGCAGCAAGGGTCAGTCAA	CGCTGCTGCTGCTGATCAACTT	295
FMgSSR-41297	scaffold5299	p3	(AAC)11	Class I	3935	3967	AGCGGTTGGCAGACATTAGGCA	ACGTGGTGTTGGGAATGGTTTGC	239
FMgSSR-41337	scaffold5393	p3	(AAC)5	Class II	22706	22720	AAGAAGCGGTGCATGCAGGA	TGGCTGCGACAGGGTAACAACA	349
FMgSSR-41350	scaffold4713	p3	(AAC)5	Class II	21046	21060	AAGGCAGCATCTTCACCAGCGT	ACCTTCACCAGACACTTGGCCT	322
FMgSSR-41387	scaffold2390	p3	(AAC)5	Class II	55600	55614	ACCCGGACCATGTTAGCAGT	ACTGCGTTTTTGGCGGTGGT	265
FMgSSR-41394	scaffold5709	p3	(AAC)5	Class II	17058	17072	ACCTTCATCACGGCAAACAGC	ACATGGTAGGCATTGGGCACGA	347
FMgSSR-41395	scaffold7303	p3	(AAC)5	Class II	2182	2196	ACGAAAGCGGTTTTAGGCACTCCT	AGGCTGCTGCTGCTGCATTT	278
FMgSSR-41401	scaffold5914	p3	(AAC)5	Class II	28166	28180	ACGTACATTTGGCCTCGGCTCT	TGAATGTGCTTGTCGCGCCT	346
FMgSSR-41402	scaffold264	p3	(AAC)5	Class II	126298	126312	ACGTACTGCTGCTGTTTGGCTGA	ACCCAGACAAGCGCAATCCCTA	299
FMgSSR-41405	scaffold943	p3	(AAC)5	Class II	32130	32144	ACGTTGATGTGCCAACCGCT	TTTGCGTCGTCGTCTGTTTGCG	341

SSR_ID	Scaffold	SSR_Type	SSR_Motif	SSR Class	SSR_Start	SSR_End	Forward sequence	Reverse sequence	Product_size
FMgSSR-41419	scaffold2447	p3	(AAC)5	Class II	25941	25955	AGAGGACGTGCCAGTTGGTCTA	ACTTGGCAGTGTCCGAGGTT	305
FMgSSR-41420	scaffold5392	p3	(AAC)5	Class II	29292	29306	AGCAGCTTCACACGTTCACGAC	ATCATCGTCTCGAAGTCCGGCA	217
FMgSSR-41428	scaffold6732	p3	(AAC)5	Class II	4279	4293	AGCCATGGTTCCATCAACAGCGA	GTACTTGAACACACACCCAGCA	221
FMgSSR-41439	scaffold32948	p3	(AAC)5	Class II	5349	5363	AGCTTCATCCCCACACTTCCACA	TCTTGCATCAATGGGGTGGGCA	336
FMgSSR-41440	scaffold6530	p3	(AAC)5	Class II	17731	17745	AGGACAGTGGGCATACGACTGA	TGGTTGGGTGCTCCACAATGGAA	267
FMgSSR-41455	scaffold4686	p3	(AAC)5	Class II	25561	25575	AGTTTCCCGACGGTCAAGCTCA	TCAAGTTGAGGCTGCCAGGTGT	320
FMgSSR-41467	scaffold5465	p3	(AAC)5	Class II	34623	34637	ATGGTCGGCAGCAGGCTTCTTT	TGCCTCTCCTCCGTTCCAACTCTT	299
FMgSSR-41469	scaffold3761	p3	(AAC)5	Class II	25436	25450	ATGGTGTGTGACCAAGCGCA	ACAGCTACATGACGCCACTGCT	314
FMgSSR-41471	scaffold200	p3	(AAC)5	Class II	133529	133543	ATGTGTTACGCTGGCACGCT	ACACGCGTAAGCTTTTCCGCA	289
FMgSSR-41477	scaffold10324	p3	(AAC)5	Class II	22525	22539	CACGAAATGCACCACCACCACT	TTGAATGCGCTTCGAACTCGGC	350
FMgSSR-41510	scaffold6749	p3	(AAC)5	Class II	8551	8565	GCAGTTACGCACATTCAGCAACG	ACAGAGCAACAGGAGGGGCAAA	266
FMgSSR-41516	scaffold4349	p3	(AAC)5	Class II	20137	20151	GCTGTCTTGTCCAGTGCTTTCA	GGGGTCATAGTCTACACCCTCACT	350
FMgSSR-41526	scaffold15264	p3	(AAC)5	Class II	14776	14790	TACAACATAGGCCCGCTCCTGA	AGCCGTAGCCCCATGAGTTACA	349
FMgSSR-41527	scaffold127488	p3	(AAC)5	Class II	291	305	TACCATCAACACCGCAGCCACT	TGGCCCTGCGTGTCAAACAA	234
FMgSSR-41556	scaffold74192	p3	(AAC)5	Class II	677	691	TCCGCAACTGCAGAGCATGA	TGCTACGCACCTTTGTGAGTC	313
FMgSSR-41560	scaffold1087	p3	(AAC)5	Class II	79339	79353	TCCGGCTTTGCCGTTTGGTA	GCGCGTCTCATTCATGGGCATT	307
FMgSSR-41562	scaffold736	p3	(AAC)5	Class II	69170	69184	TCCTGGCGCGCAAGTTCTTT	TGGTCAGCTCGTGCGCAAAT	263
FMgSSR-41579	scaffold2224	p3	(AAC)5	Class II	18884	18898	TCGTTCCATCCGTGCTTGTCAC	AGGCGAATTGGCGGGAACTT	209
FMgSSR-41584	scaffold513	p3	(AAC)5	Class II	76728	76742	TCTCTCGCGCTTGCTTGGAA	CGACGACAACAACGAAGGGGAA	303
FMgSSR-41591	scaffold34	p3	(AAC)5	Class II	25718	25732	TGACGGGTATGGTCTGGGTCAA	TGAAAGGAGGCATGAGCTCGCA	246
FMgSSR-41592	scaffold52880	p3	(AAC)5	Class II	1552	1566	TGAGACCCTGCCTTGCTGCATA	AAGCACACAGCTCTGGGCACAT	350
FMgSSR-41593	scaffold56762	p3	(AAC)5	Class II	673	687	TGAGACGACATGCGCAAGCA	TCAAGTAGACCAGTGCACGCGA	288
FMgSSR-41596	scaffold10275	p3	(AAC)5	Class II	7353	7367	TGCAACCAACGGTATGATCGGGA	TGCGTGCCGATACGAGAAACCA	304
FMgSSR-41605	scaffold15722	p3	(AAC)5	Class II	3791	3805	TGCATGCCCACTGCGATACTGT	TTCCGCCTGCACATGGACATCA	321
FMgSSR-41620	scaffold3544	p3	(AAC)5	Class II	9382	9396	TGCTGTACGTGTGTGTGGCT	AAGGCATCGTGGGTGACGTGAA	341
FMgSSR-41627	scaffold1175	p3	(AAC)5	Class II	74550	74564	TGGGCAGCATACACACTGCT	AGCGCTCAATGCTGCAGGAAA	346
FMgSSR-41639	scaffold7967	p3	(AAC)5	Class II	14371	14385	TGTCAGCTCGCTTGCTGCCTAA	GGCGCCAAGAAACCAACCAACA	218
FMgSSR-41644	scaffold432	p3	(AAC)5	Class II	12502	12516	TGTGCGTGGTGAACCCACTGAA	AAGGCGTTGTGGGTGACGTGAA	349

SSR_ID	Scaffold	SSR_Type	SSR_Motif	SSR Class	SSR_Start	SSR_End	Forward sequence	Reverse sequence	Product_size
FMgSSR-41647	scaffold340	p3	(AAC)5	Class II	124292	124306	TGTGGGAGCGAGCGATCCATAA	AAGCAGAAGAGCAAGATGGCCC	320
FMgSSR-41648	scaffold18745	p3	(AAC)5	Class II	4519	4533	TGTGGGGCCTTGAAACTTCGTG	TGCTTTCGAGGGGACCAAGTGA	345
FMgSSR-41654	scaffold35155	p3	(AAC)5	Class II	6068	6082	TTCAACACGTACGGCCACACCA	GCATGCACGTTTGAACAGCCCA	301
FMgSSR-41672	scaffold21	p3	(AAC)5	Class II	2239	2253	TTGTCTTCCGTTCCCTGGCTGT	TGGCCAGCAGTTGCAACAAA	237
FMgSSR-41680	scaffold733	p3	(AAC)6	Class II	62125	62142	AAAACGACGCGGCACACACA	CGGCCGCAAGGTCCAATACAAT	337
FMgSSR-41683	scaffold3693	p3	(AAC)6	Class II	6984	7001	AACACGACATGGCGCTGACGAA	TCCGGTCGGTCGGTTTTTCT	302
FMgSSR-41716	scaffold848	p3	(AAC)6	Class II	85077	85094	ACCGACTCCAGAAACGCCTACT	ACGCGTCGGAAACGAACCAA	259
FMgSSR-41723	scaffold2567	p3	(AAC)6	Class II	6910	6927	AGCAGGAGCGGACACCAAAA	TCGTTGTCCCTGCAGCATGT	326
FMgSSR-41739	scaffold313	p3	(AAC)6	Class II	103249	103266	AGTTGTGTGTGTGCGGTTGGTG	AGGCTGGAATTGGTCGTTCACCT	348
FMgSSR-41753	scaffold210	p3	(AAC)6	Class II	32094	32111	ATTTGGCGAGCGCATGCAAC	TGCATGCGATGCGACTGTGA	350
FMgSSR-41761	scaffold42903	p3	(AAC)6	Class II	500	517	GCAACCAGAACAAAAGGTTGGCCT	ACCGTAACTTCCCTGTCCGTT	255
FMgSSR-41770	scaffold7630	p3	(AAC)6	Class II	23923	23940	GGCGGCGGCTAGGGTTTTTATT	TGTTTGGCTTCGTGCATGCGT	273
FMgSSR-41777	scaffold170	p3	(AAC)6	Class II	42052	42069	TCCATCGAGCCTGGCTGCTATATT	AGCTGCATGTCATTTTCCCTGGC	337
FMgSSR-41787	scaffold5456	p3	(AAC)6	Class II	33481	33498	TGCAATCAGTTGGGGGCTCTGA	AGTTCATGCCTCTTGCCTGC	212
FMgSSR-41793	scaffold7959	p3	(AAC)6	Class II	24896	24913	TGCATGCTCTTTTCGCCGCA	AGCGGATCGAGAACTCCACCAA	306
FMgSSR-41795	scaffold425	p3	(AAC)6	Class II	126857	126874	TGCCCTCCAACACTTGGATCACT	TCCGAATGAACCATGCACGC	336
FMgSSR-41823	scaffold5913	p3	(AAC)6	Class II	16306	16323	TGTTGACACACTGCCACTGCT	ATGCAAGCGCTCAATGCTGC	318
FMgSSR-41829	scaffold419	p3	(AAC)6	Class II	7552	7569	TTGAAGGCAGCGCGGAACAA	TGTCACTGACCGTTGAGGCGTT	213
FMgSSR-41858	scaffold18637	p3	(AAC)7	Class I	1112	1132	ATCACCCTCCTTCGCCATTGCT	TCCAGGCGTAGGGCGATTGTTT	253
FMgSSR-41890	scaffold2579	p3	(AAC)7	Class I	25410	25430	TGCATCAAGAAAGATGCCGACGC	ACCATGTACGTGTGCAACGGGT	204
FMgSSR-41893	scaffold8220	p3	(AAC)7	Class I	22826	22846	TGTACACATGTCCGCCCAACT	TGTGCCATGTGACAGAATCAAGCC	332
FMgSSR-41894	scaffold4849	p3	(AAC)7	Class I	20691	20711	TGTCTTGTCCTGTGCTTCCGGT	ACACCCTACGAAAGGCCAGTGT	342
FMgSSR-41899	scaffold6505	p3	(AAC)7	Class I	6670	6690	TTGCTCATCACTCGTCGTCGCA	TTGTGGTGGTCGGTTACGCT	265
FMgSSR-41905	scaffold7332	p3	(AAC)7	Class I	19596	19616	TTTCCCTGTGGCAAAGCACGGT	TTGGCCCCACTGTCAAAGACGA	271
FMgSSR-41926	scaffold6626	p3	(AAC)8	Class I	17757	17780	TCGCCTGCCTAGCATCGTCAAA	CTCTCCAGATACAGGCAAACTTCG	347
FMgSSR-41935	scaffold3787	p3	(AAC)8	Class I	7903	7926	TGGTAACCTGCAACCCGCTTGT	TGCGCACGAACCACAAAGCA	302
FMgSSR-41963	scaffold1016	p3	(AAG)11	Class I	28155	28187	AGCATCTTCGTGCTGCCAGTGA	TGCGGCCATCCCATTTCTTGGA	251
FMgSSR-41968	scaffold3050	p3	(AAG)12	Class I	34436	34471	CGTTGCATTTGCGCAGACCT	AGACTTGACGCCAACATCGCA	301

SSR_ID	Scaffold	SSR_Type	SSR_Motif	SSR Class	SSR_Start	SSR_End	Forward sequence	Reverse sequence	Product_size
FMgSSR-41969	scaffold1365	p3	(AAG)12	Class I	71390	71425	TGGCTTTCCTCTGCGTAGCTCA	AGTACACAAAAGCCCGCACCGA	345
FMgSSR-41971	scaffold11994	p3	(AAG)14	Class I	3709	3750	CGCTTGCCAATTTGCCATTGCT	ATAGGCACGAAGGCAAGCGTGA	331
FMgSSR-41972	scaffold4918	p3	(AAG)14	Class I	42220	42261	TGAACGTCTCTGCGCCACAA	GTCACGGTGGACGTCATGAGAA	303
FMgSSR-41976	scaffold320	p3	(AAG)18	Class I	66671	66724	GAGCAGGCGGCTTCCAAAAA	TGCGTTGGCCTGGAGCATTA	332
FMgSSR-41977	scaffold162	p3	(AAG)18	Class I	72648	72701	TTTTTCCCCGGCGGAACTGTGT	AATCCGAAACGGCCGCAAGT	327
FMgSSR-41978	scaffold11835	p3	(AAG)19	Class I	2045	2101	AGCGCCCCAACAATCCCAAT	TGGTTCACCGCCTGGAAGAA	250
FMgSSR-41981	scaffold4918	p3	(AAG)19	Class I	42012	42068	TGCTCTGCTTGGGTTCCTTCCA	TCACGGTGGACGTCATGAGA	350
FMgSSR-41986	scaffold1517	p3	(AAG)22	Class I	59377	59442	ACCTGCACCACCACAACCACTT	TGTTTCAGAAAGGGGCAGGGCT	248
FMgSSR-41987	scaffold9017	p3	(AAG)22	Class I	19962	20027	AGAATCGTGGCCAAGCGCAA	TCCGTCGTCCTTATTGTGCTGC	350
FMgSSR-41989	scaffold4234	p3	(AAG)22	Class I	4269	4334	CGCGCATCGCGATCAAGAAA	TGCCTTGTTCTCCAACTCCGCT	289
FMgSSR-41990	scaffold10940	p3	(AAG)22	Class I	19117	19182	TGGCCCCGAGATGATGCTAGAGTA	TCGACTGCTAACACCTGGCGAT	237
FMgSSR-42005	scaffold5195	p3	(AAG)5	Class II	39987	40001	AACAGTGTCAGGGGGAGCAA	ATGGCCAATGTGTGTCGGCT	321
FMgSSR-42008	scaffold5921	p3	(AAG)5	Class II	20170	20184	AACCATCACAACAGCGAGGGCA	TCTGCTTGGCGTTCGCGATT	304
FMgSSR-42051	scaffold613	p3	(AAG)5	Class II	18969	18983	AACTCAGGTGAACAGCCTGCCA	ATGCTTCGCTCCAACCCCAACA	248
FMgSSR-42080	scaffold1156	p3	(AAG)5	Class II	52660	52674	AAGAACTCTGTGCATTGCCGCC	TGCCGACGGTCCACATGAAGAT	335
FMgSSR-42084	scaffold1356	p3	(AAG)5	Class II	69177	69191	AAGAGCACGCTCTGTTTCGACG	TCCAACAATGTGGGCGCTGT	339
FMgSSR-42090	scaffold4100	p3	(AAG)5	Class II	21624	21638	AAGCAGAAGAAGGCGTGGTGGT	TCGTGCTAATGGCGGCAGGATT	251
FMgSSR-42092	scaffold295	p3	(AAG)5	Class II	125367	125381	AAGCAGCAGCTCCAGTCCATCA	AGTCCAGCCAACGCCTAACCAA	273
FMgSSR-42093	scaffold8998	p3	(AAG)5	Class II	27739	27753	AAGCAGCAGGAAGAGGAAGGT	TCCTCGAATGGTGAACCTAGAGCC	273
FMgSSR-42098	scaffold60953	p3	(AAG)5	Class II	691	705	AAGCGACAGTGTGCAATCCGGT	TTTCGCCTTGCACTCGCCTT	317
FMgSSR-42099	scaffold2306	p3	(AAG)5	Class II	23451	23465	AAGCGATCCGCTTGTCTCTGGT	GCTTGCGAGTGCAATCATGCTCA	344
FMgSSR-42101	scaffold29104	p3	(AAG)5	Class II	6395	6409	AAGCTGAGCTCCGTGAACGTGT	AGGTTCTCGTCGCGCAAACA	268
FMgSSR-42103	scaffold4862	p3	(AAG)5	Class II	35950	35964	AAGGACAACGAGGGGCACGATA	TCCGATGCGATGCAGTCGATGA	314
FMgSSR-42116	scaffold122110	p3	(AAG)5	Class II	458	472	AAGGAGGCAACACATGAGGCGT	TTGCCAGCCCACCCTGTTCATT	303
FMgSSR-42120	scaffold82502	p3	(AAG)5	Class II	1055	1069	AAGGCTCGTGATCCGTGTTGCT	AGCACCAGAGGAGGAACTTCTTGA	314
FMgSSR-42142	scaffold6884	p3	(AAG)5	Class II	30589	30603	ACAATGCCTGCGCTGCTGTA	ACCTGACGCGTGTGAAAGCGTA	334
FMgSSR-42146	scaffold2041	p3	(AAG)5	Class II	13829	13843	ACACCACGCACCAACACCAA	ATTATCCACCGCCACCGCAA	223
FMgSSR-42187	scaffold6422	p3	(AAG)5	Class II	28585	28599	ACCACGGTCAACCGCGAACATA	TGCCCATGGCAATCTCTCTCCT	340

SSR_ID	Scaffold	SSR_Type	SSR_Motif	SSR Class	SSR_Start	SSR_End	Forward sequence	Reverse sequence	Product_size
FMgSSR-42188	scaffold10704	p3	(AAG)5	Class II	5839	5853	ACCAGACCAGAGCCACCGAAAA	ATTGGTTCACGGGTTGGCGT	289
FMgSSR-42357	scaffold3908	p3	(AAG)5	Class II	14302	14316	ACGAACGCCAGTACAAACCGCT	GGTCACAATTTTCCGCCGCA	231
FMgSSR-42361	scaffold6628	p3	(AAG)5	Class II	8765	8779	ACGACGAGTGCACCAACACCAA	AACCCAGCGCGTGGCATATCTT	295
FMgSSR-42396	scaffold19884	p3	(AAG)5	Class II	4933	4947	ACGCGAACAACCTGCAACGA	TTCAATGCCGTCGTGTGCCT	201
FMgSSR-42403	scaffold12875	p3	(AAG)5	Class II	10930	10944	ACGGGTTACGTGTGGGGACATT	AGGCTGGAGCCTGAAACACT	306
FMgSSR-42435	scaffold7377	p3	(AAG)5	Class II	9938	9952	AGAAGATGGCACAGCACGCA	TTCAGCTCCATGGTGTCGAGGT	276
FMgSSR-42436	scaffold1110	p3	(AAG)5	Class II	89234	89248	AGAAGCACTTGGCACGTTGGA	AAAAACCGCCGCCGCAACTT	296
FMgSSR-42440	scaffold968	p3	(AAG)5	Class II	24723	24737	AGAGATGTGAGCAACCAGCCGA	AGGCACCATCCTACTAACTCGGT	277
FMgSSR-42441	scaffold9761	p3	(AAG)5	Class II	12052	12066	AGAGCCGGGAAACAGAGGAAGA	TTCAGCAGCATCACTCCCCT	325
FMgSSR-42469	scaffold14717	p3	(AAG)5	Class II	7435	7449	AGCGACCTGCTCCAAAGCAT	GCCAACCTTTTGGCGCTCTTGT	227
FMgSSR-42472	scaffold3019	p3	(AAG)5	Class II	19985	19999	AGCGCCATCATTCAGCCACA	TTCCTGCGTCAGCTTCACATGG	206
FMgSSR-42474	scaffold1058	p3	(AAG)5	Class II	8727	8741	AGCGTGTCGTCAACCCTCTT	TGTCATTATCTGCTGCCGCTGG	338
FMgSSR-42476	scaffold163102	p3	(AAG)5	Class II	534	548	AGCTCGGGGACAGTCAGATTCA	AAGCGCATTCGCCTTCTCGT	259
FMgSSR-42494	scaffold626	p3	(AAG)5	Class II	58731	58745	AGGAAGCAGATGCTACTGAGCCT	CGGCAGTAGCTCGGAGGTTCTTTT	296
FMgSSR-42495	scaffold67	p3	(AAG)5	Class II	43963	43977	AGGAAGCGGACATTCACGCA	AACGCGTCTTGTGTTCGGGT	285
FMgSSR-42523	scaffold1831	p3	(AAG)5	Class II	5678	5692	AGGCCGGGGGTAACAATTGCAT	TCAGTTGACGAGCTGGTGGAT	249
FMgSSR-42546	scaffold2061	p3	(AAG)5	Class II	70365	70379	AGTCCCCTCCCTCCTCACTAAT	TTCGATTTGACGCGATGCGG	207
FMgSSR-42548	scaffold3509	p3	(AAG)5	Class II	4137	4151	AGTCGTCACCAGTCGCCTCAAA	TGCTGGGCTGCAAATGTCCCAT	293
FMgSSR-42549	scaffold20888	p3	(AAG)5	Class II	3204	3218	AGTCTGTCGCGTGCATGGAA	GGCACCAGCTTTTTCTTCTGACCC	350
FMgSSR-42550	scaffold120908	p3	(AAG)5	Class II	747	761	AGTGACGATGTGGGGTCTGGAA	ACAAGCACGGATTGGCTGGT	255
FMgSSR-42577	scaffold8165	p3	(AAG)5	Class II	17489	17503	ATATAAACCGCCTCACCCCGCT	TGGCAAGGAAGCGGTTGACT	299
FMgSSR-42578	scaffold1060	p3	(AAG)5	Class II	75783	75797	ATATGCGCGTGTGGTCGGCAAA	TGCATTGCGCTGCAGACAGTT	224
FMgSSR-42580	scaffold1463	p3	(AAG)5	Class II	69563	69577	ATCAAGCTGCACGCCATGCT	TCGGTGCCTGACGATGTCTTGA	285
FMgSSR-42585	scaffold25665	p3	(AAG)5	Class II	1682	1696	ATGATCATGTGGCGCTGCTGGA	TCCACACAATCGCTGGACGGTA	342
FMgSSR-42589	scaffold391	p3	(AAG)5	Class II	59478	59492	ATGCCAGAGGGTGAGAGCACTT	TTCCTGGCCATGCCACCAACAA	290
FMgSSR-42592	scaffold4559	p3	(AAG)5	Class II	32769	32783	ATGCTGTGCTTGTGCAACGACG	TTTTGACGTGTCGGGGCAGTGA	278
FMgSSR-42613	scaffold2130	p3	(AAG)5	Class II	8209	8223	CAGCTCACACACAAACGACAAAGC	TGGTCAGCGGACATGCGAATCA	222
FMgSSR-42617	scaffold14878	p3	(AAG)5	Class II	11925	11939	CCCCATCGATTGCACTCCAGGAAA	ACGCGGAGACGAAACCTCTTCA	275

SSR_ID	Scaffold	SSR_Type	SSR_Motif	SSR Class	SSR_Start	SSR_End	Forward sequence	Reverse sequence	Product_size
FMgSSR-42641	scaffold1533	p3	(AAG)5	Class II	52061	52075	CGCATCCCCAAAAGAATGCCCA	TCGCCGACAGCCTAGACAAGAT	242
FMgSSR-42651	scaffold182	p3	(AAG)5	Class II	11978	11992	GATCATTGACTCCTGCTACAAGGG	TGATTATGGGGTGTGTTGGCCT	246
FMgSSR-42652	scaffold3694	p3	(AAG)5	Class II	12149	12163	GCAAGTTGCCTGCAATCGGTGA	TCCTTTCTGGCACCACCTTGTGA	265
FMgSSR-42657	scaffold2543	p3	(AAG)5	Class II	45370	45384	GCCGCACACGCGGATATAACAA	TGTGCAGATCGGCAGCACAAGA	296
FMgSSR-42663	scaffold18372	p3	(AAG)5	Class II	5356	5370	GCTGCATGCTCGTGTGCAAT	TTGTGCGGCAGCCTAAACCT	250
FMgSSR-42676	scaffold109	p3	(AAG)5	Class II	33915	33929	GGTGAAGTGCAAAAGGTGTGGTGG	AGCAGCAGCTCGGAGGTTCTTT	325
FMgSSR-42684	scaffold7756	p3	(AAG)5	Class II	27591	27605	GTGCATATGCTGAATCACCCAGAC	AGCTCAGTTGGTAGAGCAGAGGAC	305
FMgSSR-42705	scaffold273	p3	(AAG)5	Class II	118726	118740	TAACAACCCGTTCGCGGCAA	TGAAGTCGTCGCAGCACTCGAA	248
FMgSSR-42719	scaffold2714	p3	(AAG)5	Class II	13948	13962	TCAAAGCAATCGGTGGCGTCCT	AGCAGCAGAGCAGCAACAGGAA	279
FMgSSR-42770	scaffold9563	p3	(AAG)5	Class II	2676	2690	TCACCGGGGGCTTTCTGTTT	TGAACTGCCAGTGGTGCCAA	330
FMgSSR-42794	scaffold950	p3	(AAG)5	Class II	28714	28728	TCCAGAAGCAACCGCGTCAA	ATTGACCAAACCCGCCACGA	346
FMgSSR-42797	scaffold956	p3	(AAG)5	Class II	805	819	TCCCCGGCTGATTCTCTTGCAT	TTGCCACAACCACGTCACCA	294
FMgSSR-42807	scaffold66	p3	(AAG)5	Class II	162793	162807	TCCGGTGCTATTGTCACTTCTCGT	AGGGGACGATGAATTGCTGCCA	344
FMgSSR-42829	scaffold2142	p3	(AAG)5	Class II	27446	27460	TCGCATCAACCGGCATGCAA	CAGCTGATTAGCAGCAGCACGA	281
FMgSSR-42833	scaffold1186	p3	(AAG)5	Class II	24893	24907	TCGTCATCCGGCTGCAGATT	TCCTCCACGCGGTGCATAAA	312
FMgSSR-42834	scaffold8350	p3	(AAG)5	Class II	33162	33176	TCGTCGTCTCGCAAGTCGTGAT	TCAGCAGCTGGCTGTCGAACTT	313
FMgSSR-42836	scaffold822	p3	(AAG)5	Class II	74485	74499	TCGTTGCAGGCGTGAAGGAAGT	TCAACAATGCGTAGCCGCGA	310
FMgSSR-42844	scaffold231	p3	(AAG)5	Class II	65442	65456	TGAACCAAACACCCGGCCTTGA	TCTGAATCCACTGCGCTGGACA	299
FMgSSR-42845	scaffold6752	p3	(AAG)5	Class II	28123	28137	TGAACTCCATCAACGCGGCT	AAGACTGCATCACGCACGCA	329
FMgSSR-42914	scaffold7273	p3	(AAG)5	Class II	9635	9649	TGAGACGCAGCTTTACACCGGA	TGCAAATGTCGCTGCCAACT	308
FMgSSR-42919	scaffold753	p3	(AAG)5	Class II	83102	83116	TGAGGACGTCACCGCGGAAAAA	TGCCTACTTCGACTGCGTGACA	220
FMgSSR-42968	scaffold170	p3	(AAG)5	Class II	20480	20494	TGCAATGCAAGCGGCACACT	GCGAGAACGGCGCAGTCAATTA	209
FMgSSR-42970	scaffold2341	p3	(AAG)5	Class II	38243	38257	TGCACGCCTGAAGTTGCTGA	TGCACCGAGTTTGTTTCCGCT	284
FMgSSR-42971	scaffold571	p3	(AAG)5	Class II	79445	79459	TGCACTCGGCAATGGAAGCA	TTGCAACATCACAAGGCCGC	257
FMgSSR-43091	scaffold12081	p3	(AAG)5	Class II	12124	12138	TGCCATGAAGCAAAGAGGCACG	ATGGGGCTGCTACACAACTCGT	348
FMgSSR-43095	scaffold220	p3	(AAG)5	Class II	74801	74815	TGCCGCCCTGTGTTTGAATACCT	GCACGCGAACTGGCAACTTCAA	225
FMgSSR-43102	scaffold16303	p3	(AAG)5	Class II	16616	16630	TGCGCGTGAATTGTGGCAGA	TGCGAGTGACGTGACACCACAT	210
FMgSSR-43105	scaffold4375	p3	(AAG)5	Class II	42927	42941	TGCGGCGATGATGATGAGTCGT	CACTGCGCTCAAAACCTCAGCA	342

SSR_ID	Scaffold	SSR_Type	SSR_Motif	SSR Class	SSR_Start	SSR_End	Forward sequence	Reverse sequence	Product_size
FMgSSR-43118	scaffold10543	p3	(AAG)5	Class II	14258	14272	TGCGTGCCGCACCTCTAAAA	ATGGGCTCGTTGAACTTGGGCA	241
FMgSSR-43126	scaffold17412	p3	(AAG)5	Class II	3027	3041	TGCTCGCGCAGCTGATTTTCA	TGCAAACCGGTAAGCTGCCA	240
FMgSSR-43160	scaffold7203	p3	(AAG)5	Class II	31446	31460	TGGACGGCGGTGATCTGTTGAA	TGCACGTTCGCCGTCTCAAA	332
FMgSSR-43178	scaffold744	p3	(AAG)5	Class II	42706	42720	TGGCACATGCCGCAAACAGA	AGGACAAGCTCCACTGCCCTTT	298
FMgSSR-43201	scaffold22508	p3	(AAG)5	Class II	8433	8447	TGGGGCCCTTACGTCAAAAGGA	AGACGCCTAGGCCCATTACAT	284
FMgSSR-43211	scaffold9546	p3	(AAG)5	Class II	30015	30029	TGTCAAGAAGCCCAGTTCGCGT	AGCAGCCTGCGGGTTACAACAT	232
FMgSSR-43220	scaffold148	p3	(AAG)5	Class II	40997	41011	TGTGCAGGCTGGATGTGCAA	ACTTGCGTGCCATTGTCGGA	350
FMgSSR-43237	scaffold3376	p3	(AAG)5	Class II	36781	36795	TTATCACTGGAAGCGTGCGGGA	GCGCGCGTGTGTACAGAAATGA	347
FMgSSR-43240	scaffold8592	p3	(AAG)5	Class II	5306	5320	TTCACACCGCGCACCTTCAA	GCACTGTAGTGGTCTTCGTCAGGA	335
FMgSSR-43241	scaffold28000	p3	(AAG)5	Class II	8422	8436	TTCACCGGGTACAAACAGCCCA	TGGTTGCACTGTTGCTGGCT	323
FMgSSR-43245	scaffold176282	p3	(AAG)5	Class II	486	500	TTCATGCTCTGCCGCTGATCCA	TCAGCGGTACTTCAGCAAGGT	347
FMgSSR-43249	scaffold6641	p3	(AAG)5	Class II	21557	21571	TTCCTCCGATCTGCCTCCTCTTCA	AGCTTGTTGCTGCTGCTGCT	293
FMgSSR-43250	scaffold17870	p3	(AAG)5	Class II	11172	11186	TTCCTCTTGTGATGCGGCCT	TGCGCTGCTTGCTCTGGTTT	212
FMgSSR-43255	scaffold8509	p3	(AAG)5	Class II	21422	21436	TTCGGCGAAAATGGAGCCCT	TGGGTCCGCCAATGAATGCGAT	319
FMgSSR-43275	scaffold5971	p3	(AAG)5	Class II	29325	29339	TTGCTTGTGCCTTGTGCCTT	CGCCGATGCCGGGAAACAAAAT	350
FMgSSR-43282	scaffold11907	p3	(AAG)5	Class II	14340	14354	TTTGAAGCGCGCGTGGTCAT	AGCCACAGCCACGAAAGCGATA	322
FMgSSR-43301	scaffold8496	p3	(AAG)6	Class II	24878	24895	ACAAGCAAGTAGCCATCGCAGC	GCCACAAGCTGAAGCTCGATCA	259
FMgSSR-43315	scaffold2075	p3	(AAG)6	Class II	6504	6521	ACGCCGCAGGCACTTATCTTGT	TGTGCAACGTCTCGTCAGCCTT	239
FMgSSR-43326	scaffold10268	p3	(AAG)6	Class II	24584	24601	AGCCATTCTTTTTACCCCGCCA	TGTCGCGCCACAAGATTCAC	350
FMgSSR-43328	scaffold2208	p3	(AAG)6	Class II	10040	10057	AGCTAACCCCACCCAGCTCCAATA	TGCAGCTTGCATTACGCGGT	230
FMgSSR-43331	scaffold3473	p3	(AAG)6	Class II	40030	40047	AGGCCGCGAATGTGCATCAA	CCTTACTCATCCGTTCCAGTAGGT	217
FMgSSR-43332	scaffold8650	p3	(AAG)6	Class II	18042	18059	AGGCGCGTACTCGTAAACAGGT	TGCACGTACAAATCGAGGGGCA	294
FMgSSR-43339	scaffold2140	p3	(AAG)6	Class II	22315	22332	ATCCTTTGCTCCCCACGCAA	AACCGCGGCTTGTCCGTTTT	217
FMgSSR-43343	scaffold5187	p3	(AAG)6	Class II	36848	36865	ATTTTCTGTCGCGCCCAACGAC	ACAGGTGATGCAGCAGGGTT	348
FMgSSR-43346	scaffold50902	p3	(AAG)6	Class II	1915	1932	CGCGGTGCTGTTGTTCGTTT	TAGACCACGTTGATCCGCCT	350
FMgSSR-43348	scaffold126	p3	(AAG)6	Class II	42678	42695	GCCCATCAGCAAAACGTCCACT	TTTGAACCCACGCCCTTTCGGA	313
FMgSSR-43349	scaffold5018	p3	(AAG)6	Class II	31035	31052	GCCGGCGTTTAAGCGAAAACCA	TGCATCCCAAGCTTACCCGCAA	209
FMgSSR-43358	scaffold15210	p3	(AAG)6	Class II	14676	14693	TACCGTTGCCCGTTTCAGCA	GCGGGCGGCAAAGTTCGTTAAT	257

SSR_ID	Scaffold	SSR_Type	SSR_Motif	SSR Class	SSR_Start	SSR_End	Forward sequence	Reverse sequence	Product_size
FMgSSR-43369	scaffold4140	p3	(AAG)6	Class II	18234	18251	TCCCCGCGAAGAAGGGAAAT	TCAACACGCCGCATACCACA	302
FMgSSR-43372	scaffold60269	p3	(AAG)6	Class II	33	50	TCGATGCCGTCTCCAAAGAAGGA	TGTTTTGGCTCAGGCTCCGGTT	275
FMgSSR-43373	scaffold15712	p3	(AAG)6	Class II	10789	10806	TCGCAAGAAGCTCCGCAACTGT	TCGTGAGAAACATCTTGGCGGC	216
FMgSSR-43374	scaffold4520	p3	(AAG)6	Class II	8117	8134	TCGGGAGACGCCGTCAAAAA	TTGTGCGGGATGACGCCAAT	246
FMgSSR-43375	scaffold7211	p3	(AAG)6	Class II	9818	9835	TCGGTGCAGAGCCGCCATTTAT	ATGTACGCCACAAGCTTCTCGTCC	223
FMgSSR-43376	scaffold2831	p3	(AAG)6	Class II	28277	28294	TCGGTGTTTTCGACTTTGGCCG	GCGTGCGCGTCGTCCTATTTTT	328
FMgSSR-43380	scaffold392	p3	(AAG)6	Class II	71950	71967	TCTGCGCTGCCAGATCGATAGT	AATACTGGCTTCACCGGCTCCA	215
FMgSSR-43389	scaffold2001	p3	(AAG)6	Class II	36244	36261	TGATGGACACTGGTGGTGCT	ACGTGTCAATCAGCCGGTCGAA	311
FMgSSR-43400	scaffold15023	p3	(AAG)6	Class II	9193	9210	TGCTGATGGAGAAGCAGAGCCT	AGTGAGTTCAAACTCGGCACCT	305
FMgSSR-43412	scaffold14673	p3	(AAG)6	Class II	14606	14623	TGTGCTGCCACTGTCATGCT	GCCGCGAGCCAAGATGTGTTTT	321
FMgSSR-43418	scaffold7473	p3	(AAG)6	Class II	2681	2698	TTGGCAATGGCAGCCACGTA	TCACACACCTTCGTTCTCCCCT	206
FMgSSR-43420	scaffold804	p3	(AAG)6	Class II	81997	82014	TTGGTGATGCGCTGCTCGAAGT	CGCCACCTTCGTGTACGTTT	249
FMgSSR-43424	scaffold2430	p3	(AAG)7	Class I	53176	53196	AACGAGGCTCATTCCCTGAACG	TCGCCGACTCCTTGTGAACTGT	322
FMgSSR-43425	scaffold1718	p3	(AAG)7	Class I	13447	13467	AAGCGCACATTCTGCAGCGA	AGATGAAGGCGACGCTCAAAGG	348
FMgSSR-43427	scaffold574	p3	(AAG)7	Class I	56908	56928	ACACCGAGGTTTGAGCCAACTGA	AAGCCGCCTTGTGAGTCCATCA	327
FMgSSR-43430	scaffold6963	p3	(AAG)7	Class I	1342	1362	ACCACCGTCCGAGCATTTGT	TTCTTGTTGTGCTGGCCCACGA	330
FMgSSR-43431	scaffold1532	p3	(AAG)7	Class I	4880	4900	ACCCGCTTGCCTCATCCATGTT	ATTGCCACGTATAGCGGCACCA	343
FMgSSR-43437	scaffold877	p3	(AAG)7	Class I	36084	36104	AGCAGCAGCAAGGACAGCATGA	AGTTGACCACGCTGGTGTTCTC	342
FMgSSR-43440	scaffold2438	p3	(AAG)7	Class I	6779	6799	ATCCTTGCCATTTCCACCGGCT	ATGCGCCATCGATCTGCTTCCT	317
FMgSSR-43442	scaffold8614	p3	(AAG)7	Class I	12265	12285	ATTGTCCCTGTTGTGCCCGT	TTCCTGCTGCGTGTCGTTCT	260
FMgSSR-43458	scaffold671	p3	(AAG)7	Class I	97712	97732	TGGGTGCACGAAAATGATGACGG	TGCTGGTGGCAACAATGAGGGT	289
FMgSSR-43459	scaffold23272	p3	(AAG)7	Class I	2565	2585	TGGTTATTTGCGGCGGCGAT	AAGGCGACGCAAATCATGCGA	286
FMgSSR-43460	scaffold2094	p3	(AAG)7	Class I	63068	63088	TGTCGTGGCAGCTGTTCAACCT	AATCAAGGGCCAGCAGACGA	340
FMgSSR-43468	scaffold291	p3	(AAG)7	Class I	19987	20007	TTTGAAGCAAACAGGAGGGGCG	TGCACGTGCGATTCGCCAAA	228
FMgSSR-43470	scaffold28517	p3	(AAG)8	Class I	6613	6636	AACGCGCTGGTGAAATGTGACG	TTCATGCGACGCAGACACAC	290
FMgSSR-43471	scaffold7239	p3	(AAG)8	Class I	1647	1670	AAGGGGTCCAAAAGTCACGCCA	ACAAATCGCGGCAGGCCAAT	211
FMgSSR-43474	scaffold47870	p3	(AAG)8	Class I	909	932	ACGAGGTAGCCTGCACAAGA	TCGTGGTGCCTTTGCCCTTT	215
FMgSSR-43475	scaffold1151	p3	(AAG)8	Class I	11914	11937	ACTCGCACTGCCGCAAGAAA	AGCAGCGGAGAAGCAGCAAGAA	346

SSR_ID	Scaffold	SSR_Type	SSR_Motif	SSR Class	SSR_Start	SSR_End	Forward sequence	Reverse sequence	Product_size
FMgSSR-43477	scaffold15011	p3	(AAG)8	Class I	6234	6257	AGGCCAGCAATGCAAGTGGA	TCGCATGTGTGCAGCAGCTA	323
FMgSSR-43479	scaffold8572	p3	(AAG)8	Class I	16735	16758	AGTTGGCGTTCGGCCATCAA	TGCGGATTCGTTTCCTTGCTCG	233
FMgSSR-43480	scaffold835	p3	(AAG)8	Class I	31540	31563	TACTGCCGCACAGCAAGCAT	AGCGTCCAGTGTCATTCCCT	307
FMgSSR-43481	scaffold2191	p3	(AAG)8	Class I	51602	51625	TAGAGTGTTCGTCCGGGTTCTCGT	ACGATGCGTGTGAGGCAGAGAT	334
FMgSSR-43486	scaffold4827	p3	(AAG)9	Class I	27868	27894	TCGTTTCGGTCCAGGGTTCA	TGTTGTCGCTCGCTTCCCCATT	338
FMgSSR-43495	scaffold12357	p3	(AAT)10	Class I	15552	15581	AGCGTGAAGCAGCCGAAGAGTT	ACCTTCAGCAGGTTGCTGTCCA	308
FMgSSR-43497	scaffold6858	p3	(AAT)10	Class I	26850	26879	ATAGGGAAGATGGGCTGGACCT	ACTGAATTGGCACTCAGCAGCA	260
FMgSSR-43499	scaffold1045	p3	(AAT)10	Class I	7841	7870	TAAACTTGCGTCTTCCCCGGCA	ATTGCATTGCATGCCTCCCG	261
FMgSSR-43501	scaffold9519	p3	(AAT)10	Class I	14866	14895	TGGACGCCTACTTCCGTCTTCT	ACCATCGAGAAACGCAGCCA	341
FMgSSR-43502	scaffold389	p3	(AAT)11	Class I	19992	20024	AATGAAGCGCCCAGCATCCA	ACTGGCCATGCTTGTGCCTT	316
FMgSSR-43510	scaffold3531	p3	(AAT)12	Class I	38984	39019	AGGAAGAACCAGCAAGCCCCAT	TAGGGCCCTGCAGTTTTGAGCA	311
FMgSSR-43513	scaffold9791	p3	(AAT)12	Class I	14371	14406	TATTTGCTGAGTTGGGCGCACG	AGTACGACCACTTTCTCGGGTCCT	295
FMgSSR-43518	scaffold9760	p3	(AAT)14	Class I	27427	27468	ACACATGCCAACTTGCCGCT	AGCAGGGCGCGAATTCCAAA	299
FMgSSR-43519	scaffold1703	p3	(AAT)14	Class I	8377	8418	ACAGCTGAAGAACAGCAGGGCT	AGGCTAGTTGGGCGTGGATCAAA	290
FMgSSR-43523	scaffold10797	p3	(AAT)14	Class I	4337	4378	TCCCTTCAAGCGGAAGCCCATA	TGGGCTTCGAAGATGTAAGTGTGG	286
FMgSSR-43524	scaffold4	p3	(AAT)15	Class I	201077	201121	CGCAGGTAGTTGAGACTGACAGCA	AATGGCGGGAAGGTGCACATGA	332
FMgSSR-43530	scaffold2095	p3	(AAT)17	Class I	75074	75124	ACGATGCGAATTGGACGGCT	ATTGCCGTTGAAGCCACGGT	248
FMgSSR-43532	scaffold20294	p3	(AAT)17	Class I	4918	4968	TGACACGACGGTCGAAATGACTGA	AGGCTCAAGCCTTCTGCTGACA	319
FMgSSR-43535	scaffold1255	p3	(AAT)19	Class I	73713	73769	TGCATGGAGCGCAGTGACAA	ACAAGCATCGCAGCCAATAGCA	263
FMgSSR-43538	scaffold10808	p3	(AAT)20	Class I	7132	7191	ACAACAACTAAGACGACCGCGA	ACGGCGGGCCCATATTCATCTT	288
FMgSSR-43540	scaffold2686	p3	(AAT)21	Class I	48851	48913	TCGCTCGGCCCAGAGTTTGTTA	AGCACATCGCATGGTAGCACT	289
FMgSSR-43542	scaffold1571	p3	(AAT)22	Class I	75891	75956	TGCCAGCAGGTTGGACACAT	TGCGTGGACGTGCTGCAAAA	303
FMgSSR-43544	scaffold15029	p3	(AAT)5	Class II	13714	13728	AAAACACACGGGTCACCGCA	TGGGGGTAACTTCGTCAGGACT	206
FMgSSR-43546	scaffold2430	p3	(AAT)5	Class II	39488	39502	AAAATGACGCCGCCGATCCA	TTCCGACCGCTTTCACCTGTCA	222
FMgSSR-43547	scaffold301	p3	(AAT)5	Class II	77885	77899	AAAATGCTAGCGTGGCATGCGG	GGCACACGCGTGAAAAGGTGAT	338
FMgSSR-43550	scaffold5581	p3	(AAT)5	Class II	28321	28335	AACACGCAGGGCGACAACTT	AAGACCAGGCCGATGTGTGGAT	337
FMgSSR-43551	scaffold5583	p3	(AAT)5	Class II	14083	14097	AACCAGCCAGTCCTTGGTGCAA	TGTGCCGTCGTGTTGTTTGGTG	236
FMgSSR-43560	scaffold18565	p3	(AAT)5	Class II	14579	14593	AAGGTGAGCATCCCGAAGCAGT	TGTGTGCGCGTGTGTGTGTT	330

SSR_ID	Scaffold	SSR_Type	SSR_Motif	SSR Class	SSR_Start	SSR_End	Forward sequence	Reverse sequence	Product_size
FMgSSR-43563	scaffold14197	p3	(AAT)5	Class II	10919	10933	AATCCGTGGCCAAGCTCGACAT	TTGTCCCGTTCTGCAGCGATCA	236
FMgSSR-43564	scaffold29105	p3	(AAT)5	Class II	4878	4892	AATCGGGCGGCGTTTCGTTT	TAGTGCCATCAAGCCGGCCAAA	301
FMgSSR-43567	scaffold263	p3	(AAT)5	Class II	59656	59670	AATGCCCACGCCACGAGTACAA	TGACCAGGAATACGCGGCATCT	225
FMgSSR-43568	scaffold2266	p3	(AAT)5	Class II	16588	16602	AATGCGACCTCTGTCGTCGT	ATGGGCACGCATCACTCACT	348
FMgSSR-43570	scaffold1332	p3	(AAT)5	Class II	17804	17818	ACAAACCTTGTCCAGCGTCTCCT	TGCATGGTGTTGTTGCTAGGC	344
FMgSSR-43577	scaffold30238	p3	(AAT)5	Class II	7695	7709	ACACGGCAGAACACCTGCTT	TTCCGCAAACAAGCACCGCA	349
FMgSSR-43578	scaffold6903	p3	(AAT)5	Class II	7446	7460	ACAGGCAAGGAGGCAAAGCTGA	TTGGTGGGCGTTGGAGTTGT	324
FMgSSR-43587	scaffold19363	p3	(AAT)5	Class II	7363	7377	ACCGCAGCGATGTGTGTGAA	GCTCCCAGCAGCAGCAGTAGTAAT	348
FMgSSR-43588	scaffold11821	p3	(AAT)5	Class II	2376	2390	ACCTAACCAGACGTCTCCTCCCTT	AATTTGACGCGGCCAATGCG	303
FMgSSR-43590	scaffold15391	p3	(AAT)5	Class II	15906	15920	ACGAAACTGCTGCGTGCCTT	AGTGCACAAACGTGGCCCAT	316
FMgSSR-43591	scaffold23636	p3	(AAT)5	Class II	10893	10907	ACGAAATGGATGCCTCGGCT	TGTAGTTTCGCGTCCCAACCT	281
FMgSSR-43596	scaffold8839	p3	(AAT)5	Class II	18307	18321	ACGGAGCAGAAAGAAGCTCAACCA	CCGATGGCGCCGATGTTTGTTT	237
FMgSSR-43597	scaffold1506	p3	(AAT)5	Class II	61915	61929	ACGGTCACAAAGGCTGGGAT	AGCACGCAGGCCAGCAAAAA	349
FMgSSR-43598	scaffold58133	p3	(AAT)5	Class II	614	628	ACGTGCCATCTGGTAGAGTCCT	TTTTAATGGCGCCCACCCGA	344
FMgSSR-43599	scaffold167	p3	(AAT)5	Class II	76778	76792	ACGTTCAAGCCAAGCGCAGT	AACAGCAGCAGCAGCTCCATCA	350
FMgSSR-43601	scaffold1485	p3	(AAT)5	Class II	34891	34905	ACTCCCGCCAGCAACCACTAAA	AGCTCTGAGGTGAGTTGTGCCT	312
FMgSSR-43605	scaffold4120	p3	(AAT)5	Class II	18734	18748	ACTGGCAGGGTTGTATTGCCA	TGCACAGCACGCTCAAGCATT	303
FMgSSR-43608	scaffold2127	p3	(AAT)5	Class II	27551	27565	ACTGTGTCAGTGGTGGTGCT	ACAGTCACAGGTGTGTTTGGCCT	258
FMgSSR-43610	scaffold138	p3	(AAT)5	Class II	25019	25033	AGACGCCGGCCTTTTCACTT	AGCAGAGCCAGCCTGCCAATAA	340
FMgSSR-43612	scaffold178	p3	(AAT)5	Class II	41427	41441	AGATGAACGTCTGCGGGCAA	TCCATCGGGCTCATCACGCAAA	348
FMgSSR-43633	scaffold2984	p3	(AAT)5	Class II	35217	35231	AGCACAGGTAGGAGGAACTGGGAA	TGGAAGATGCCAAATAGGTCGGGT	298
FMgSSR-43642	scaffold493	p3	(AAT)5	Class II	26708	26722	AGCCTGTTCACGGTGCTTCA	TTCCGGCCCGTGAAGGAAAT	228
FMgSSR-43644	scaffold18274	p3	(AAT)5	Class II	3811	3825	AGCCTTTTGTGCAGGGTTCAGC	TTGAACTGCCCCAAACCGACCT	237
FMgSSR-43647	scaffold4425	p3	(AAT)5	Class II	18996	19010	AGCGTTTAGACGGGCACAAGGA	TGTGTGGCTGTGCGGCAAAT	332
FMgSSR-43649	scaffold22766	p3	(AAT)5	Class II	7220	7234	AGCTTGTGGCCTGTGACTTGA	AACACGCCCTACTCCGGTTGAT	323
FMgSSR-43650	scaffold8121	p3	(AAT)5	Class II	3389	3403	AGGAAGGCGGCCTAAAATGTGA	AGCAGCCGCCATCTACCTTTGT	253
FMgSSR-43664	scaffold36424	p3	(AAT)5	Class II	351	365	AGGGCATACAGGTGAGAACCGT	AGTGGCGTTGTAGAGGTCAGGA	291
FMgSSR-43667	scaffold327	p3	(AAT)5	Class II	146708	146722	AGTGTACGTGCACCCTGCAGAA	ATAGCTCAGTTGGCCAGAGCGT	225

SSR_ID	Scaffold	SSR_Type	SSR_Motif	SSR Class	SSR_Start	SSR_End	Forward sequence	Reverse sequence	Product_size
FMgSSR-43675	scaffold473	p3	(AAT)5	Class II	120645	120659	ATCTCTCCACGGCTCGTTGGTT	ACAGATGACACGGCACACAGT	232
FMgSSR-43676	scaffold186	p3	(AAT)5	Class II	64815	64829	ATGCAGTGCCGTCTGTGAGTGT	CGCACCACACGCCATCAAAA	276
FMgSSR-43677	scaffold1081	p3	(AAT)5	Class II	12513	12527	ATGCATGGCCCCACTTCAGACA	GCGTGTGTTCTCGAAAGCATGGA	343
FMgSSR-43687	scaffold1648	p3	(AAT)5	Class II	45913	45927	CACAGGCACATACTTCCTCTGTTC	GGGGTGGTTATGGATGTGAGTCGT	323
FMgSSR-43693	scaffold9435	p3	(AAT)5	Class II	19576	19590	CGATTCCAGGCAACTTGTAATGGC	TGCTCAGAGGGCGTTTTGGA	342
FMgSSR-43694	scaffold716	p3	(AAT)5	Class II	70771	70785	CGCATATGCATGCAGGTTCCTC	TCGCCAGCGGTCTTTGTTCA	203
FMgSSR-43695	scaffold150	p3	(AAT)5	Class II	107233	107247	CGCTTGCAAAGGGTGCAGCATA	AATCCGGTGGGTCAGTTTGGGT	291
FMgSSR-43700	scaffold29164	p3	(AAT)5	Class II	706	720	CTCACAATATGTTGAATCCGGCCC	ACCCACTAACTTTCCCTAAGCCGC	211
FMgSSR-43702	scaffold2586	p3	(AAT)5	Class II	44037	44051	GCAAAAGCGACCTGGCAATGCT	GCAGGATGATGACCTTGCGGTT	350
FMgSSR-43707	scaffold895	p3	(AAT)5	Class II	85387	85401	GCATATGGCGCACACTGGCTTT	TGAAACCAACCGCGCACTCT	213
FMgSSR-43709	scaffold1778	p3	(AAT)5	Class II	58402	58416	GCATGTTGCAGCAGGCGTCTAT	TCAGCAGCTAAGAAACATGCCGTG	303
FMgSSR-43710	scaffold3	p3	(AAT)5	Class II	264181	264195	GCCATGGTTATCCACAGAGACTGC	TGTAATCACTGATGGGGGAGGGCT	272
FMgSSR-43712	scaffold22004	p3	(AAT)5	Class II	9325	9339	GCTGTCTAAACCTCTGTACATGCCC	AGTTCGCGAGGAGAGGACCCATTA	302
FMgSSR-43716	scaffold1522	p3	(AAT)5	Class II	50925	50939	GGCCTATATATTGTCGCCAACCCA	CGCCGCATAAATTGTTAGGCCA	341
FMgSSR-43717	scaffold521	p3	(AAT)5	Class II	128247	128261	GGCTCAATGATGCTGCCATGCT	TTCCTTTCGTGACCGCAGGAGT	305
FMgSSR-43719	scaffold253	p3	(AAT)5	Class II	78634	78648	GGCTGCTCTGCACCAGTAGTTA	GTGCGTTCACTGTGGCAACT	350
FMgSSR-43722	scaffold3346	p3	(AAT)5	Class II	47674	47688	GGGGTAAAGTCTAGTTTTGGCCCAG	TCCCGTGAAAGGTGCTCAACA	349
FMgSSR-43726	scaffold140	p3	(AAT)5	Class II	3259	3273	GTCCAACCAAGACATAATGCGCGA	GCTGGGCGGATCAAAACGTGAT	302
FMgSSR-43727	scaffold9153	p3	(AAT)5	Class II	23360	23374	GTCCATTTGTGGCCAAAGCAGC	AGGAAACGAAAGCTGCCCCCAA	214
FMgSSR-43728	scaffold3914	p3	(AAT)5	Class II	53194	53208	GTGAAACAGGGGATGATGAGCAAC	TGCCTTGGCCTCCTTACATTGC	253
FMgSSR-43732	scaffold6927	p3	(AAT)5	Class II	14734	14748	TACCAGTGGCATCACGCTCA	TCCATTGCACTTGGGAGGAGCA	344
FMgSSR-43740	scaffold11614	p3	(AAT)5	Class II	13745	13759	TCATGTGCACGCTTGTGTGG	TGCGCCACCTTTGGAAGCTTTG	328
FMgSSR-43742	scaffold3362	p3	(AAT)5	Class II	45230	45244	TCCATCCGAACCGTTGTTGCCA	TCGACTCTGCTCGACTTACCAGGA	201
FMgSSR-43745	scaffold719	p3	(AAT)5	Class II	65331	65345	TCCGCTTACCTCTGCTCTGCAT	CGCCGCGAATCAACAAATCCCA	348
FMgSSR-43747	scaffold2603	p3	(AAT)5	Class II	38363	38377	TCCTTCAGAATTCGGGCCTGT	TGTTTGTGAAGCTCACCGTGCT	349
FMgSSR-43748	scaffold329	p3	(AAT)5	Class II	14088	14102	TCGACTAGCAATGGTGAAGCTTGG	TCCCAGGACCCTTTCTTGTTGT	310
FMgSSR-43749	scaffold7967	p3	(AAT)5	Class II	13672	13686	TCGCGGACAGTAATGCGAGAGA	TTGGCGAGATCGACAACCGA	288
FMgSSR-43750	scaffold2445	p3	(AAT)5	Class II	32829	32843	TCGCTGTACAATGGAAGCCTGC	TGCTCTGTTCGTTGGACCGAT	337

SSR_ID	Scaffold	SSR_Type	SSR_Motif	SSR Class	SSR_Start	SSR_End	Forward sequence	Reverse sequence	Product_size
FMgSSR-43756	scaffold4903	p3	(AAT)5	Class II	3507	3521	TCGTCCAACCAAGACACTGCAC	CGTGGTCAGTTGTATCACAGTGGG	343
FMgSSR-43757	scaffold3847	p3	(AAT)5	Class II	35631	35645	TCGTCGGATGTTGTCTCTGTCGT	TGCCTTGCGTAGCACACGTT	317
FMgSSR-43758	scaffold528	p3	(AAT)5	Class II	114118	114132	TCGTGAACTCCTGAGTCTTGGTCA	ATCCTTACCGCCACTTCGCACT	304
FMgSSR-43760	scaffold24254	p3	(AAT)5	Class II	5736	5750	TGAAGCTGAGGGTTCTGGACTCA	AGGAAGTGCCACGCTTGAGGTT	285
FMgSSR-43761	scaffold51462	p3	(AAT)5	Class II	3642	3656	TGACCTGAGACAACGGGGGAAA	AAACGGCTACACGGCGTCCAAA	243
FMgSSR-43765	scaffold228	p3	(AAT)5	Class II	34337	34351	TGAGGGCCCAGGAAAACAGA	AGGGAGTTCCTTTTGGACCGT	333
FMgSSR-43772	scaffold4986	p3	(AAT)5	Class II	7331	7345	TGCAAGTGCCCAAGTGCCAA	GGGTCTTGTGCCGAACAGGAAT	229
FMgSSR-43776	scaffold3556	p3	(AAT)5	Class II	54160	54174	TGCAGACGACAAGAGAGCGT	CCCCCGTCCAACACCAAGATTCAA	275
FMgSSR-43779	scaffold1892	p3	(AAT)5	Class II	47885	47899	TGCCACCGCCGTCAAATCAA	TTTGTAGAGGCTGCGGAACCGA	273
FMgSSR-43782	scaffold6443	p3	(AAT)5	Class II	13090	13104	TGCCATGTCGTCAGGAAGCAGA	AAAGCTCGCAAGGCAAGCAACC	314
FMgSSR-43784	scaffold2853	p3	(AAT)5	Class II	48198	48212	TGCCTATTTGCCTCTGCCACCA	ATTTGCCGTTGCATGGAGTCGC	312
FMgSSR-43786	scaffold1709	p3	(AAT)5	Class II	49120	49134	TGCGCTTTTGTGACGGTGGA	CAAGGCGCAAAAGTTGACATGG	326
FMgSSR-43787	scaffold803	p3	(AAT)5	Class II	361	375	TGCGTAGGCGATAGATTGTGGC	ACATGTTGCTTGATGCTGAGCC	330
FMgSSR-43788	scaffold4214	p3	(AAT)5	Class II	28145	28159	TGCGTCACTCAGACGCTCCAAA	CCGGCCAACAACCACCAAACTT	338
FMgSSR-43792	scaffold11472	p3	(AAT)5	Class II	17206	17220	TGCTTGCCGGCACGGTAATCTT	TGTTCTCTGATCCCCACCGTGT	337
FMgSSR-43798	scaffold1797	p3	(AAT)5	Class II	67683	67697	TGGCATGGCTTTCAGCTCTCT	ATGCTAGGGCCCCTTTGATGCT	349
FMgSSR-43799	scaffold14714	p3	(AAT)5	Class II	3360	3374	TGGCCAACACCCGCAACAAT	TTGCTGCTGCTACGTGCCAT	326
FMgSSR-43807	scaffold1733	p3	(AAT)5	Class II	28674	28688	TGGTGCTGACACCATGTCGCTT	TGCAGATGGCAACGCTGTGA	315
FMgSSR-43808	scaffold6573	p3	(AAT)5	Class II	5256	5270	TGGTTGCATGACGGCACTTGT	AGCAAAAGTGCAGGTTGCTCTG	344
FMgSSR-43812	scaffold12553	p3	(AAT)5	Class II	7398	7412	TGTCACGTTGGCACACACCA	TTGCCGCCGGCTTTTTCAGA	305
FMgSSR-43817	scaffold525	p3	(AAT)5	Class II	121240	121254	TGTGCGGTGCGCTCTCAAAT	TAACCGATCCGTGCGTTGCT	300
FMgSSR-43821	scaffold1295	p3	(AAT)5	Class II	23060	23074	TTATGCGCGCAAGCACCCTGAA	AAAGCCGATGACAACGCACA	219
FMgSSR-43822	scaffold11739	p3	(AAT)5	Class II	3029	3043	TTCACCTGCCGCAACACCTCAA	TGCTTGTCCCAATTACGTGGCCT	336
FMgSSR-43829	scaffold764	p3	(AAT)5	Class II	87308	87322	TTGTCGGACCACGCGAATGT	TGCCGTGGTTCCGTTCAACCAA	346
FMgSSR-43833	scaffold30371	p3	(AAT)6	Class II	498	515	AAAGAGGCCTTTAGCAGCGGCA	TCGCATCGTGCCCCGAAACATT	271
FMgSSR-43834	scaffold4697	p3	(AAT)6	Class II	48909	48926	AAAGGCCCCTGAAAACTGGAGC	ACAAGCAATGGTGCTTGGTGCG	260
FMgSSR-43836	scaffold34	p3	(AAT)6	Class II	5367	5384	AAGAGGACACGAGCCAACGCTT	TGCCTTTGGTTGCGACTGGT	224
FMgSSR-43837	scaffold13554	p3	(AAT)6	Class II	10441	10458	AAGCATCCCAATCGTCTCCCCA	ACAGGCAGTATGAGATTGGCCG	343

SSR_ID	Scaffold	SSR_Type	SSR_Motif	SSR Class	SSR_Start	SSR_End	Forward sequence	Reverse sequence	Product_size
FMgSSR-43838	scaffold302	p3	(AAT)6	Class II	62424	62441	AATGAAGAACGCGGCCCACT	AACGGCCGGGGAAATCAAACCT	296
FMgSSR-43839	scaffold8872	p3	(AAT)6	Class II	2040	2057	ACAACTCGTCACTCGTGCTCGT	ATGCCTAGCTGTTGGAAGGGGT	301
FMgSSR-43840	scaffold4181	p3	(AAT)6	Class II	44923	44940	ACACACATCCGCTCCAACACCT	CGGTGGCTGTCACAATGCAA	259
FMgSSR-43845	scaffold723	p3	(AAT)6	Class II	1809	1826	ACCCATTGGATACCGATACCCACCA	TCTCCAAGCAGTCTCCCTGCAA	317
FMgSSR-43852	scaffold2530	p3	(AAT)6	Class II	35987	36004	AGAGCCACAGGTAGTGTGTGT	TGGGTATCCAAGGTCATCAAACCC	311
FMgSSR-43854	scaffold855	p3	(AAT)6	Class II	104372	104389	AGCAACCCATGAGGCACCCAAA	GCGTGACTTCAATCATCGGACACC	252
FMgSSR-43858	scaffold715	p3	(AAT)6	Class II	35183	35200	AGGAGAGCAGCGCTTTCGTTGT	AAATTCTGCCGCCGTACGCA	345
FMgSSR-43859	scaffold115570	p3	(AAT)6	Class II	602	619	AGGCAGCAGCGTTAGAGGCAAA	TAGCCAGGCTTGTGCTATGCAG	215
FMgSSR-43861	scaffold48019	p3	(AAT)6	Class II	820	837	AGGCTCTCGTGCAAGCTGTTGA	AGCACGCACGCCTTCTGTTT	348
FMgSSR-43862	scaffold148816	p3	(AAT)6	Class II	876	893	AGGCTTGGCATACGAAATGGCCT	AGGACCCCTCTCTGCACATTGA	313
FMgSSR-43864	scaffold21524	p3	(AAT)6	Class II	11128	11145	AGGGCAGAATAAGCACGGACGA	TTATCCCTCCGGCTGACGTTGT	274
FMgSSR-43868	scaffold240	p3	(AAT)6	Class II	80297	80314	ATACGAGCCCATGTGTTCCGGT	ACCGTTTTGTCAGCCGTGCAA	316
FMgSSR-43870	scaffold7075	p3	(AAT)6	Class II	34152	34169	ATGTGCCAGCGTCAATGCGA	AGCGGTCAGACGCGTTGTACTT	327
FMgSSR-43871	scaffold4302	p3	(AAT)6	Class II	41609	41626	CCAAAGCCTGCACACATTGGGA	ACCACGGAGCCACGTGAATTGT	348
FMgSSR-43881	scaffold341	p3	(AAT)6	Class II	29041	29058	GGCCAGCCGGCAATGAAATGAA	TGCGTCCGTTCAACGCTCTGAT	346
FMgSSR-43891	scaffold20709	p3	(AAT)6	Class II	1898	1915	TCCCACATAGGACATCCCTTGAGA	ATGCTGGAATCGGGCAATGG	273
FMgSSR-43893	scaffold950	p3	(AAT)6	Class II	96403	96420	TCCGCGTCGTTCGTGCAAAA	TGTACGTCCCTGTGGCTTGA	273
FMgSSR-43895	scaffold189322	p3	(AAT)6	Class II	585	602	TCGCGTATGCAGGCAGATGA	TCCCCTTTAGCTGATGGCTGCT	346
FMgSSR-43897	scaffold995	p3	(AAT)6	Class II	8586	8603	TCGTATGGAACTCTCGACGCCT	ACTGCTTTGTTCCGTGCCCT	232
FMgSSR-43898	scaffold10811	p3	(AAT)6	Class II	1034	1051	TCGTCAGGCCTCGTTTGACA	GGAGAAACGTCAGACTTCCGGATT	325
FMgSSR-43899	scaffold133	p3	(AAT)6	Class II	92623	92640	TGAACCAACGACGAGGTACGCA	CGCGCTGCTTGCAGTATGCTTT	228
FMgSSR-43900	scaffold1935	p3	(AAT)6	Class II	79906	79923	TGACGGCATAAGCCTGCACT	TGCCCAACAGCGTCACCATT	347
FMgSSR-43903	scaffold68764	p3	(AAT)6	Class II	2312	2329	TGCAACAGTAAGCAAGGAGGCCA	GGGTCTGGTGATCTCATGGTCCAA	268
FMgSSR-43914	scaffold318	p3	(AAT)6	Class II	109761	109778	TGGCCCGCATGCTCTAGTTT	ACGGACCATGCTGGTGCAAA	315
FMgSSR-43916	scaffold4501	p3	(AAT)6	Class II	16557	16574	TGGGACGTGTATGTGCCGTT	CTCTGTGAACAGTGCTGAACTCAC	273
FMgSSR-43917	scaffold10102	p3	(AAT)6	Class II	4995	5012	TGGGCATCAACAGGGGATGCAA	GGCCATGTGTTTTGTACCTGCCA	299
FMgSSR-43920	scaffold183	p3	(AAT)6	Class II	131893	131910	TGTGGAGTATCCCTCTTCGGATCA	ACGTGTTCCGATATTCCCCGCT	313
FMgSSR-43924	scaffold5222	p3	(AAT)6	Class II	33743	33760	TGTGTGTCACGCCTTAGAGATCC	ACTCATGTCGATGCGACGGT	238

SSR_ID	Scaffold	SSR_Type	SSR_Motif	SSR Class	SSR_Start	SSR_End	Forward sequence	Reverse sequence	Product_size
FMgSSR-43925	scaffold6048	p3	(AAT)6	Class II	40633	40650	TGTTCTGGTGTTGTACCTCGCT	TTTCTCCTTCGGACGTCCTCTCGT	345
FMgSSR-43927	scaffold4594	p3	(AAT)6	Class II	42224	42241	TTGAGCGCGTTCTGGACGTT	TTCAGCCAAGTCTGGTGGCA	325
FMgSSR-43928	scaffold3097	p3	(AAT)6	Class II	17769	17786	TTGGAGCCAAAGTCAGCAGTCTCC	TGCAGCTTTGGCGTTTCAGC	269
FMgSSR-43929	scaffold19450	p3	(AAT)6	Class II	1903	1920	TTGGGACTGCTGAGCTTCTGCT	TCTCAGTGACCTCTTGCACCA	337
FMgSSR-43930	scaffold160	p3	(AAT)7	Class I	173662	173682	AAATCATCACCCCGTCCACCGT	ACTGTGGTCACTGCGTGCAT	336
FMgSSR-43931	scaffold8972	p3	(AAT)7	Class I	23106	23126	AAGATGATGGCGATCGGTGGCA	GGCACAACAAATGCCTGCGT	269
FMgSSR-43932	scaffold7715	p3	(AAT)7	Class I	25929	25949	AAGCGAGCTTCTTCCTTCGTCG	TCAGTGCCTCGTTGTCGTTCCA	306
FMgSSR-43933	scaffold10558	p3	(AAT)7	Class I	19030	19050	AAGGGTGGCAAGACGACGAA	ACGGTGGTGACAGCTGTTTTGC	216
FMgSSR-43935	scaffold1491	p3	(AAT)7	Class I	21002	21022	ACAATGGCTCGCCGTCAGTT	TCAGGACGTCAAGTCAACCCACA	350
FMgSSR-43939	scaffold9578	p3	(AAT)7	Class I	7966	7986	ACGTCCTCACATCCGACGTAGT	TGGGTTCTAAGAATGGAGTGGTGC	297
FMgSSR-43940	scaffold22258	p3	(AAT)7	Class I	80	100	AGAACAAGGAACAGCCTCCCTCA	TTGGGCTGTTGTGATGCTTGGG	291
FMgSSR-43942	scaffold33	p3	(AAT)7	Class I	126773	126793	AGCCATGTTGGCCTGCAAGT	AGCACACTACGCTGTGCCAT	276
FMgSSR-43954	scaffold2985	p3	(AAT)7	Class I	29403	29423	TCGTGAAACGAGCGAACCGT	AGCGGCGGTGACTTTGGATGAT	297
FMgSSR-43958	scaffold1306	p3	(AAT)7	Class I	13797	13817	TGCCCGTGCAAAAATCAGTCGT	TGTCTAATCAAGCGCGTTGCG	274
FMgSSR-43965	scaffold1565	p3	(AAT)7	Class I	47381	47401	TGGTGGGACAAGCAAGCACT	TCATCAGGCTGCGGGCAAAA	292
FMgSSR-43967	scaffold9695	p3	(AAT)7	Class I	5423	5443	TTGTGCTGCTCCGATAGCACCT	CCCGGTCAAACAGCCCGTTATT	339
FMgSSR-43973	scaffold479	p3	(AAT)8	Class I	5908	5931	AGGGGTAGGGTTTAGGACCACGAA	CAGGAACAGCAGCTAGCCTTGA	340
FMgSSR-43977	scaffold5648	p3	(AAT)8	Class I	21318	21341	CGAACACCCTCTAAATAGCACACC	TGCAGTGCTGCTGCCAACTT	292
FMgSSR-43978	scaffold17432	p3	(AAT)8	Class I	5081	5104	GAGTCATCAAGGGTAAGAGTGCCA	GCTGAACATTCGATGGCTTGGAGT	322
FMgSSR-43979	scaffold1035	p3	(AAT)8	Class I	11143	11166	GCTGCTCATGCTCCTCTTCTCCAA	TTGTGCACGCAGTAGCTCACCA	216
FMgSSR-43981	scaffold10648	p3	(AAT)8	Class I	17583	17606	TCAACCTCGCGCTCTGTCCAAT	ACCGACCACCCCTGGTTTGTTA	262
FMgSSR-43984	scaffold10467	p3	(AAT)8	Class I	26852	26875	TGCCAGCCGACGATGGAGAAAA	AACCAGATGATTGCCGGAGCCA	239
FMgSSR-43985	scaffold13322	p3	(AAT)8	Class I	3910	3933	TGCTGAAACTCGCACCGTCCTT	ACGTTAGGTTGGGACCATCTGC	317
FMgSSR-43988	scaffold42821	p3	(AAT)9	Class I	654	680	AGCCGATGGCGAGGTGAACAAT	AAGTCCCCTAGCTTCGAACGGTCA	314
FMgSSR-43992	scaffold1846	p3	(AAT)9	Class I	29799	29825	GCTGCAGCAAAACAGCAGCA	AGATCAAGCGGGAGCAGCAGTA	323
FMgSSR-43994	scaffold17296	p3	(AAT)9	Class I	219	245	TATGCAGTACCGCCGTGCAGTT	AGTCGCGCATCATCTACGCA	246
FMgSSR-43997	scaffold3822	p3	(AAT)9	Class I	9257	9283	TGCCGGCGCGTACTAGTCAAAA	ACAAGTCCGGCATGCCCAAAA	297
FMgSSR-43999	scaffold75772	p3	(AAT)9	Class I	1076	1102	TGCGCGCAACAGCAACTCTA	TGAGGCAGAGCATTGAACGACA	291

SSR_ID	Scaffold	SSR_Type	SSR_Motif	SSR Class	SSR_Start	SSR_End	Forward sequence	Reverse sequence	Product_size
FMgSSR-44002	scaffold7715	p3	(AAT)9	Class I	31293	31319	TTGCCGCGATTCATAGCCCTCA	TACGGGAGGCGACGAAGATGAT	318
FMgSSR-44004	scaffold9437	p3	(ACA)10	Class I	10393	10422	AGCGACGTAGGAGCATTCCA	TCGTCGGCAACCAAGTCCACAA	294
FMgSSR-44008	scaffold285	p3	(ACA)10	Class I	119797	119826	TCCCAAGCATGTTCAGAGGTGC	TGAGTCATTCCCGCTGAGCCTT	276
FMgSSR-44009	scaffold9286	p3	(ACA)10	Class I	13084	13113	TCTGATGGAATCAGGCCGTGT	TTGAGCTGTTCACGTGGTGAC	288
FMgSSR-44020	scaffold21629	p3	(ACA)14	Class I	10013	10054	TGAGGCAGGTGCATGCTCAAA	CGGTCAGAACCTGATGCCATGT	314
FMgSSR-44021	scaffold65	p3	(ACA)15	Class I	207741	207785	AGGCTGCAAGAACTTCCTGTACGA	TGTCCTGTCTCTCCTGCTCTGA	318
FMgSSR-44026	scaffold9815	p3	(ACA)5	Class II	21508	21522	AAAATGCACCACCACCAGTAGG	TTCGGGCTTGATGATCCAAGTGGC	315
FMgSSR-44027	scaffold1175	p3	(ACA)5	Class II	41516	41530	AAACACCTCCTCTGGCTCAGCA	ATTTCCCCACCAGCCACGTCTT	246
FMgSSR-44030	scaffold4313	p3	(ACA)5	Class II	32724	32738	AAAGGCGCATTTGGTCGAGG	TTGGTTGGGAGGGTACGGCAAA	305
FMgSSR-44036	scaffold2418	p3	(ACA)5	Class II	55083	55097	AACGACAACGTCGACCGCAT	AGCGCCGTCGTAATAGCACT	251
FMgSSR-44039	scaffold6891	p3	(ACA)5	Class II	12691	12705	AAGAGAAGCAGCAGCCGCACTA	AAAACGAGTCGTCGTGGCAGGT	330
FMgSSR-44044	scaffold5511	p3	(ACA)5	Class II	26118	26132	AAGGCCCCAGCCCATGTACAAA	GGTGCCATTGTTGTATGCTTTGCC	344
FMgSSR-44055	scaffold1677	p3	(ACA)5	Class II	54900	54914	ACATGCGACCTGTCGAACACCT	AGATGGTGAAGCATTCCCGCCA	350
FMgSSR-44058	scaffold11128	p3	(ACA)5	Class II	4310	4324	ACCAGCTGCTGTGGTTGCTT	CGACAGTCAGGCATACAGGACACA	261
FMgSSR-44062	scaffold3993	p3	(ACA)5	Class II	2487	2501	ACCCATCGCGCGTTAGCATA	AAGCACGCGACATGCGTTGT	312
FMgSSR-44072	scaffold1486	p3	(ACA)5	Class II	33493	33507	ACTGCTTGTTCGCTGCACTTGA	CGGAACGCACTTGGTTCAGT	347
FMgSSR-44076	scaffold4196	p3	(ACA)5	Class II	31409	31423	AGCAGTAGTCGCACTTATTGCCCC	TGTCACTTTGAACTCGGCCAGACC	207
FMgSSR-44079	scaffold3376	p3	(ACA)5	Class II	37099	37113	AGCGAATGAGCCTGACCGCTTT	TTTTTGCAGGCGCCAAAGGTCC	348
FMgSSR-44084	scaffold5415	p3	(ACA)5	Class II	38431	38445	AGCTGCGCCGCCATTGAAAA	GCAACTGCATGCTTGAGGTGGT	282
FMgSSR-44086	scaffold19374	p3	(ACA)5	Class II	3004	3018	AGGACCGAGCCGAGGTTGAAAA	AAGGCGGTCAGGAAAGCTTGGT	332
FMgSSR-44093	scaffold15228	p3	(ACA)5	Class II	14814	14828	AGGGGCCTGAACTGTTGTGA	TGCCTCCCCCTTTTTGGGTTCA	297
FMgSSR-44105	scaffold14178	p3	(ACA)5	Class II	9028	9042	ATGGAGGTCATTCCTTGGCCCT	TAGGCCACACCACAGCATCACT	326
FMgSSR-44110	scaffold4767	p3	(ACA)5	Class II	46096	46110	ATTATTAGTGGGGGTGGCGGAGGA	TTGCTCGCAAAGGTTCGGCA	349
FMgSSR-44112	scaffold1291	p3	(ACA)5	Class II	62217	62231	ATTCGTGGCTGGTGGAATCGCT	TTTTAAACGCCACCCGGGCA	245
FMgSSR-44118	scaffold922	p3	(ACA)5	Class II	26500	26514	CCGCTAGCACAATGTTGGATGTGC	TCTTCGTGGAGTGGTGTCCTCT	294
FMgSSR-44129	scaffold1185	p3	(ACA)5	Class II	8086	8100	GCTTGCCGACGTTAACACCT	ACTTGTACAGCCCGTCTCGCTT	348
FMgSSR-44135	scaffold20900	p3	(ACA)5	Class II	14120	14134	TAGTTGGCCCTTCGTCAGGGTT	ATGGGCGTATGGCGACCTAACA	253
FMgSSR-44137	scaffold3801	p3	(ACA)5	Class II	7710	7724	TCAAGGTTCACAGCCGCCTCAA	TCGCCAAATGCTCCAGCTGACT	338

SSR_ID	Scaffold	SSR_Type	SSR_Motif	SSR Class	SSR_Start	SSR_End	Forward sequence	Reverse sequence	Product_size
FMgSSR-44151	scaffold3466	p3	(ACA)5	Class II	42323	42337	TCGCCCATTTGCTTCCTTCCCT	TCCTTGATTGCGTTTGGTGCCT	323
FMgSSR-44152	scaffold657	p3	(ACA)5	Class II	110842	110856	TCGCCGAATCCCACCAGAAA	ACGTTGAGTACCGCTGCTTCGT	316
FMgSSR-44160	scaffold2401	p3	(ACA)5	Class II	66012	66026	TCTGTACTCAGCAGCCAGCA	AGGCCCAAATCTGAGCTGGCAA	343
FMgSSR-44171	scaffold200	p3	(ACA)5	Class II	13808	13822	TGCACGCCTTTTGGCTTTGGT	AAAGGGCGCAACCTAACCTGCT	243
FMgSSR-44181	scaffold14492	p3	(ACA)5	Class II	20828	20842	TGCCATACTGGTGGCTTCGCTA	AGGGAACGTGCTGCTGCATT	302
FMgSSR-44184	scaffold17798	p3	(ACA)5	Class II	13079	13093	TGCCTGGTCTTGGTATGTAGCC	TGTTCTGCAGCTCTACTCCCCA	332
FMgSSR-44189	scaffold639	p3	(ACA)5	Class II	8241	8255	TGCTGCGAGGTTGCATTCCA	TCGATGTAGCACGAGATGGCGA	340
FMgSSR-44193	scaffold575	p3	(ACA)5	Class II	22124	22138	TGGATGCCAACTGCAACTCGGT	AGGCTTGCTTGGGAAAAGGCT	236
FMgSSR-44197	scaffold2430	p3	(ACA)5	Class II	2318	2332	TGGCGCCTGAAAATCCGTGA	AGCTCGGCACATCGCACAAA	335
FMgSSR-44212	scaffold188	p3	(ACA)5	Class II	118761	118775	TGTGGGAGGACAAAGGGGAGAA	AGCTTGGGCTTCAGTGTTGCGA	331
FMgSSR-44214	scaffold1164	p3	(ACA)5	Class II	55833	55847	TGTTCCCAAGGGCAGCTCTGTT	TGGAAGCGCACCGACAAGAAGT	277
FMgSSR-44223	scaffold7156	p3	(ACA)5	Class II	18702	18716	TTGCATGCAGGACCACACCT	AGCGTTGGAGAACATCGTGTC	313
FMgSSR-44225	scaffold19392	p3	(ACA)5	Class II	9779	9793	TTGCGCTACCGCCTCAATCT	AAGGGGCACCGTTACAACCA	294
FMgSSR-44231	scaffold4371	p3	(ACA)6	Class II	29652	29669	AAGAACCTTGAGGGCATGGGAC	ACGCTGTGCATTCAGCTCACA	226
FMgSSR-44238	scaffold7124	p3	(ACA)6	Class II	26642	26659	ACATCGCCAGGAAGCTTTGGGA	AGGCGATCTGGTGATTTCGCGT	285
FMgSSR-44245	scaffold1644	p3	(ACA)6	Class II	3032	3049	ACGCTCTTGCTGTTGCAGCATT	TGCGAAACGTGTGGGACTCT	276
FMgSSR-44248	scaffold29707	p3	(ACA)6	Class II	3267	3284	ACGTGGGTTTTCGAGGTGGACA	TGCAGCGCGATCCAGAGAAT	242
FMgSSR-44252	scaffold9394	p3	(ACA)6	Class II	5145	5162	ACTCTGATCTCCTGCTGCTTTCG	GCCATCAATTGCGGCATTGCAAGA	220
FMgSSR-44255	scaffold246791	p3	(ACA)6	Class II	286	303	AGCAAGGCAGTTCAGCTCCGAT	TTTAGCGCCTGCTGCCTTGA	252
FMgSSR-44260	scaffold280078	p3	(ACA)6	Class II	277	294	AGGCCGTGCGATTACTCACCAA	TTCTGTCGCTGTTGTGTCGCGT	267
FMgSSR-44270	scaffold55	p3	(ACA)6	Class II	39686	39703	ATTGCTGCGGATGGAGCACTGA	CCCTTGACAGGGTTAAGTGTCAGC	230
FMgSSR-44289	scaffold15995	p3	(ACA)6	Class II	9588	9605	TCCAGCTGCATTGCAAAAGCGT	ACGGAGCTGTAGGGAAACAGAAGC	319
FMgSSR-44296	scaffold61887	p3	(ACA)6	Class II	621	638	TGATTGCGCACAAAGCGGATGG	TGTTCGATGAAGACGGCGAGGA	252
FMgSSR-44317	scaffold18418	p3	(ACA)6	Class II	2276	2293	TGGCGAACATATCCGTCCCGAA	CTCTTGTTGTTGTCAGGCAGTCAG	260
FMgSSR-44319	scaffold267434	p3	(ACA)6	Class II	268	285	TGGCTGGCATAGGAGTAGGAGA	CTGGTGGTTCAATTGCATCTGCTC	265
FMgSSR-44324	scaffold11046	p3	(ACA)6	Class II	22079	22096	TGTGGATGATGCTTGATACCCGCA	TTCTCGATCCCCCAAAGCTCGT	338
FMgSSR-44332	scaffold119333	p3	(ACA)6	Class II	712	729	TTTGGCGCCAGCAAAACCGA	TTCTTGTGGCTGCCGACATGGT	257
FMgSSR-44333	scaffold24560	p3	(ACA)7	Class I	8820	8840	AAAGGAGGTCCGCTTATGCCCA	CGCTGGCATGATTTGAGAAGATGG	290

SSR_ID	Scaffold	SSR_Type	SSR_Motif	SSR Class	SSR_Start	SSR_End	Forward sequence	Reverse sequence	Product_size
FMgSSR-44351	scaffold1980	p3	(ACA)7	Class I	46212	46232	ACTCTGACAAGCTCAGAGAGTGCT	GCCGCTTTTCTGGCTTTTGCCT	204
FMgSSR-44361	scaffold18130	p3	(ACA)7	Class I	6355	6375	ATGGCGAGCCTTGACTTGCACT	ACGAGTGGCCTTGGATTGCGTT	222
FMgSSR-44367	scaffold17627	p3	(ACA)7	Class I	11890	11910	CGTTGTACATTTGCGGCGGTGA	AAATTTGGTGCGCGGATGCC	349
FMgSSR-44371	scaffold6242	p3	(ACA)7	Class I	16757	16777	GCTGCCTGTCCGTTGTTTCGTT	ATTCATCAACCCCAGCGCACGA	294
FMgSSR-44380	scaffold364	p3	(ACA)7	Class I	67874	67894	TCGAGCCATTGAGTCGGCCATT	TGGGTTTGCTGCAGCCATGA	285
FMgSSR-44381	scaffold49825	p3	(ACA)7	Class I	526	546	TCGAGTCACCTACATCAAGGGGCA	GCCCGTCAGCATTTGTTCGGTT	348
FMgSSR-44393	scaffold287	p3	(ACA)7	Class I	94126	94146	TGCCGGCAGTGAATCCATCCAT	AACGGCCAAAGCGAAGGCAA	226
FMgSSR-44395	scaffold4910	p3	(ACA)7	Class I	25345	25365	TGCTGATACATATGCTCCCTCTGG	TGGACAAGACAGCCTCACGTGTT	266
FMgSSR-44396	scaffold9626	p3	(ACA)7	Class I	5655	5675	TGCTGTACTGGTTCCCATGCT	TGGCAGGTACGTTGACATGATTGC	347
FMgSSR-44404	scaffold3695	p3	(ACA)7	Class I	14556	14576	TGTGCAGCACCACTTTGGATTGC	TTGACGCTACCCATGCGTGACA	337
FMgSSR-44425	scaffold12714	p3	(ACA)8	Class I	7168	7191	ACCCTTGTTATGTGGCACGCCT	TCGAGCGAGGCCATAGTGAA	224
FMgSSR-44430	scaffold23461	p3	(ACA)8	Class I	1840	1863	AGGATGGACGGCGGCTTCTTTA	ATGATTCAGCAGCTGGGACTGC	334
FMgSSR-44451	scaffold632	p3	(ACA)8	Class I	6470	6493	TGCTGCCACGATGAGCCATT	CTCCTGAACTGTTAACTGGTTGCC	310
FMgSSR-44453	scaffold139	p3	(ACA)8	Class I	138725	138748	TGGCACAGAGGATCAACAGCA	GCCAAACTGTTTCCCCACAGCA	342
FMgSSR-44461	scaffold10372	p3	(ACA)8	Class I	8852	8875	TTGCGAAATTGCTCTGCGTCCC	ACCAGGGAAGCTCAACATGGCA	241
FMgSSR-44476	scaffold8236	p3	(ACA)9	Class I	12235	12261	TCAGCGGAAAAGGGTTCAGCACA	GCTGTCCAGCGCAATATACAGTCC	240
FMgSSR-44481	scaffold9356	p3	(ACA)9	Class I	17855	17881	TGTGTGCAAATGTACCTGCTCC	TGGCGTTAATTGAGGGGACCCA	289
FMgSSR-44484	scaffold16910	p3	(ACC)5	Class II	13798	13812	AAAAGGGAGGGGAGGGTCGAAA	AACGTGGTGGCAGAGGAGGATA	340
FMgSSR-44486	scaffold5795	p3	(ACC)5	Class II	42521	42535	AACAAGCTTAGCCCCCTGCT	TGCCACAATGGACATGCAGC	328
FMgSSR-44488	scaffold9641	p3	(ACC)5	Class II	11456	11470	AACGCCCCGATGCTTAGGTGAA	AAAGCAGCGTGTCGAGGCTACA	274
FMgSSR-44489	scaffold1428	p3	(ACC)5	Class II	75887	75901	AACGTGCTCATCTTCGACTCGC	AGCTCACTCACATGAAGGCGCA	249
FMgSSR-44493	scaffold2401	p3	(ACC)5	Class II	44102	44116	ACACCGACGCCGTTTTACATGC	TGCCTGCATGGTGTGGTGAA	232
FMgSSR-44494	scaffold5031	p3	(ACC)5	Class II	31521	31535	ACATATGGACTGAGGGGTGCAT	TGACCCCGCATCACAGAGCAAA	344
FMgSSR-44501	scaffold267	p3	(ACC)5	Class II	41279	41293	ACGCGCAAAGCCCATCCAAA	AGATCGGGGAGGTAGTCAGACA	338
FMgSSR-44502	scaffold5874	p3	(ACC)5	Class II	31929	31943	ACGGCAAAGCTGCGAAGATG	TCCCGTAAGACTTGCTACGCT	251
FMgSSR-44508	scaffold181	p3	(ACC)5	Class II	161148	161162	AGAACGTGCTCGTCTTCGACT	ATGCATGCAAGCACCGCCAT	316
FMgSSR-44510	scaffold6226	p3	(ACC)5	Class II	4449	4463	AGCAAAGCAAAGCCGACGCT	AACCATCATCCGCAACCGCGAA	245
FMgSSR-44511	scaffold17296	p3	(ACC)5	Class II	4541	4555	AGCGGTGCTTGTGCTTTTGGTG	ACGCAGTGTAGGAAAGGCAACA	337

SSR_ID	Scaffold	SSR_Type	SSR_Motif	SSR Class	SSR_Start	SSR_End	Forward sequence	Reverse sequence	Product_size
FMgSSR-44512	scaffold12914	p3	(ACC)5	Class II	625	639	AGCTCGTCGGAGAATTCGAGGT	AAATGGCGGTGGTGTTGGCT	337
FMgSSR-44514	scaffold148730	p3	(ACC)5	Class II	197	211	AGGACGGTCGTCATTTTCGGGT	TCACCTCCTTTTCGTACCGGGAT	289
FMgSSR-44517	scaffold8584	p3	(ACC)5	Class II	18434	18448	ATACTCCACGGCCAAAAGCGGT	TGGACGCTTGCCCGGGTTATTT	285
FMgSSR-44519	scaffold357	p3	(ACC)5	Class II	82277	82291	ATGGCCGTGCATACTGCAACCT	TGCTAGAACGTGTGGCCATTTC	299
FMgSSR-44520	scaffold450	p3	(ACC)5	Class II	55721	55735	ATGTGTGCACGTGAAGCAAGGC	TGGTGCCTGCAGAGCGCTAATA	318
FMgSSR-44521	scaffold12796	p3	(ACC)5	Class II	12326	12340	CACGTCCAAAAGTTAGCCGCCA	AGCCTACTTCGAGCTTGTGCGT	205
FMgSSR-44522	scaffold2474	p3	(ACC)5	Class II	4703	4717	CCAAAGAGGCAACTTCACCAAGCA	ACAGGCATGGTGGTGGCAAA	309
FMgSSR-44528	scaffold24940	p3	(ACC)5	Class II	913	927	TAATCCACGTCGTCTTCCGCCA	ATCACAGGGGCGAATTGGTGGT	255
FMgSSR-44529	scaffold43	p3	(ACC)5	Class II	118242	118256	TACATGTAGGCTCGGAGCAGAAGC	TTTTCGCAGCCGTGAACGCA	330
FMgSSR-44531	scaffold34954	p3	(ACC)5	Class II	1642	1656	TCAAAGCTGCGTGCGTCCTT	TTGCTAGTTGCCGGGTTACCGA	312
FMgSSR-44532	scaffold1058	p3	(ACC)5	Class II	54419	54433	TCAACCGTCGTCGTTTCAGAGC	AGCGAATGTGCGGCTGTTTG	252
FMgSSR-44536	scaffold89	p3	(ACC)5	Class II	94678	94692	TCAAGCTCGTCCGCGCATTT	TGCCAGGCGTGAGCCGTAATTT	248
FMgSSR-44538	scaffold1849	p3	(ACC)5	Class II	10534	10548	TCCACCCTACCCGCTTTCCTTT	ACGAAGGGCGTGCGTCTTTT	202
FMgSSR-44542	scaffold4613	p3	(ACC)5	Class II	38685	38699	TCGCCAGCCACCTGCAAAAGTA	TGCGCTGCAGGATATGGAGGAT	320
FMgSSR-44543	scaffold3203	p3	(ACC)5	Class II	46299	46313	TCGGTTGTTTGCCCTTCACCCT	ACACCGCATTTCTGCCCGAT	305
FMgSSR-44544	scaffold2591	p3	(ACC)5	Class II	20841	20855	TCGTTTTCCACCACCACCACCA	TTGCAGACAGCACGTTCCGA	320
FMgSSR-44547	scaffold7655	p3	(ACC)5	Class II	26937	26951	TCTCGCTAAACCTGCCTGTGGA	TTCCAGTGCAGGTCCTGGTTGA	340
FMgSSR-44548	scaffold8385	p3	(ACC)5	Class II	9334	9348	TCTCGTGTGGAGCGGAGCATAA	ATGCTGCCGTCGTTGTCGAA	349
FMgSSR-44551	scaffold43	p3	(ACC)5	Class II	142778	142792	TGCAAGCGATAAGCAGGTAAGGGA	GTGGAGGGAAATGGAGAATTGGAG	342
FMgSSR-44552	scaffold5944	p3	(ACC)5	Class II	19342	19356	TGCATCAGGCGTCAAAGCGT	AGGTTTGTTGGAGGACGCAGCA	301
FMgSSR-44555	scaffold3115	p3	(ACC)5	Class II	7308	7322	TGCATTGATGACTGCCGCAC	TGGGCGAGTGCGTTGAACTGAA	268
FMgSSR-44557	scaffold47	p3	(ACC)5	Class II	14531	14545	TGCGCTGTCGGGTTTCGATT	TGTCGATCTCCAGCGTCTTGGT	247
FMgSSR-44560	scaffold20688	p3	(ACC)5	Class II	12001	12015	TGTTCCAATCAGCGCCATCC	AGGACGTCGGTTGTTGATGC	237
FMgSSR-44564	scaffold2091	p3	(ACC)5	Class II	37189	37203	TTGATGTGTGCCGACGTTGC	ATGCGGCCTCACACACACTTGA	345
FMgSSR-44568	scaffold136	p3	(ACC)5	Class II	12563	12577	TTGCCCGAGCTCCACAACAACT	TGAGCAGCTCGGTGAAAGAGGA	310
FMgSSR-44574	scaffold2256	p3	(ACC)6	Class II	18844	18861	AAAAATCCCCCAAATCCCGCCC	TGCGGGAATCAAGGTTCCGT	279
FMgSSR-44575	scaffold87	p3	(ACC)6	Class II	141574	141591	AACAGCAGCCAGAGCTACGTGA	GGCGTTGACGTTGCGGAAAT	336
FMgSSR-44576	scaffold445	p3	(ACC)6	Class II	20306	20323	AAGCACAACCACGCCCTCAT	ATCAAGGGAGCACGACGCATCA	309

SSR_ID	Scaffold	SSR_Type	SSR_Motif	SSR Class	SSR_Start	SSR_End	Forward sequence	Reverse sequence	Product_size
FMgSSR-44585	scaffold136	p3	(ACC)6	Class II	12906	12923	AGCAGCTTCAGCAGCACCACTT	CGGAGATGTGCGGTGAATTGGA	270
FMgSSR-44586	scaffold10982	p3	(ACC)6	Class II	12206	12223	AGGGCGCCGTCGAGAAACTAAA	ATGTGGTCCGGGTGCATGGTTT	334
FMgSSR-44587	scaffold742	p3	(ACC)6	Class II	80930	80947	AGTTGCGGTTGCGGCAATCAA	AGTTGTCGTCCGCTGGTCTT	279
FMgSSR-44591	scaffold1956	p3	(ACC)6	Class II	45536	45553	ATGCACGGCGACATCTGCATCT	TTGTGCTTTGACATGAGCGCCG	317
FMgSSR-44593	scaffold11584	p3	(ACC)6	Class II	15598	15615	ATTTCGCCTCGCCGTGTTGA	AAACGGGTGGCCTGTCGGAAAT	313
FMgSSR-44594	scaffold5795	p3	(ACC)6	Class II	42832	42849	CCCTTCTGGTGCATTTTGTGCCA	ACAGTGGTGAAGGCTGCTGACA	296
FMgSSR-44601	scaffold6830	p3	(ACC)6	Class II	8864	8881	TCATCCTGCTGCCGTTCCACAA	TGTGCTGGTGGGTGGTGGTTTT	287
FMgSSR-44603	scaffold2936	p3	(ACC)6	Class II	17703	17720	TCTGCTTCCAGATGCAGCGA	ATGCGATGTTGACTTGGGGCGT	270
FMgSSR-44604	scaffold3362	p3	(ACC)6	Class II	6415	6432	TGACGCAACTGAACAAGCAGCG	TGCAGCGCATGCACGCATTT	203
FMgSSR-44607	scaffold745	p3	(ACC)6	Class II	37849	37866	TTCTACTTCTGCGCCGAGTTCC	TTCATCTGGAACCGCGGACT	279
FMgSSR-44608	scaffold34066	p3	(ACC)6	Class II	6836	6853	TTTGGACACTTGGTCGGGCCTT	AGAGGTGTTGGCCGTCAAAGGA	243
FMgSSR-44609	scaffold34564	p3	(ACC)7	Class I	1188	1208	AAGCGTGCGTGCAAAATGGC	ACTCGCCGAACCACCAGAACTT	267
FMgSSR-44612	scaffold135430	p3	(ACC)7	Class I	493	513	AGAGACCACGCGTGCAACAA	AACGAACGGTCTCGCGACTT	324
FMgSSR-44615	scaffold51757	p3	(ACC)7	Class I	887	907	TGCCGCCCATAATTTGCCTCCT	ACGTCGTCGCTGGTGAGATT	220
FMgSSR-44617	scaffold3008	p3	(ACC)8	Class I	8893	8916	AATCGCGCTTGCCTTGCCTT	GCTTGCTTCAACGCCTTGGT	350
FMgSSR-44619	scaffold14224	p3	(ACC)8	Class I	4332	4355	CGCGTTGCCGAAATTGAAGGGA	ACAACTCGTAACGCCAACGC	344
FMgSSR-44626	scaffold473	p3	(ACG)5	Class II	76642	76656	ACACCACCGGCGGTCTTTTT	ACAAGCTGGGTGGATGAGGAGT	268
FMgSSR-44628	scaffold12481	p3	(ACG)5	Class II	15179	15193	ACGAGGGTAATGACGCAAAGGCA	TTCGTCCTCAACGCCTTCCGAT	271
FMgSSR-44634	scaffold11568	p3	(ACG)5	Class II	15659	15673	AGCAGGGACATCACGGCACAAT	TTCTCCGGCAAGCGGTCGATTT	203
FMgSSR-44635	scaffold855	p3	(ACG)5	Class II	50532	50546	AGCAGGTATGCTTACACGCACG	AAGGGTGCGGCAGCGAAAAA	323
FMgSSR-44640	scaffold549	p3	(ACG)5	Class II	102650	102664	AGGGGCAATCACCGTGAAGA	AGGGCCACGGCAACTGAAAA	327
FMgSSR-44641	scaffold3076	p3	(ACG)5	Class II	6332	6346	AGGTAGGCCTTGATCTCGTCGAA	ACCAGAGACCAGCTGAAGACGA	272
FMgSSR-44643	scaffold225	p3	(ACG)5	Class II	37897	37911	AGTGGATGCGGAGGAGGATGAA	ACGCCACCAGCATCGTGTAT	341
FMgSSR-44644	scaffold1001	p3	(ACG)5	Class II	17621	17635	AGTTCGTCCCATCTCAAGGTGC	TCGCGAGCGAGCAAAGGAAA	278
FMgSSR-44654	scaffold383	p3	(ACG)5	Class II	23570	23584	TATGCGCACCCCTTTGATCCGA	TTATCCGCGCAGCACACCAA	236
FMgSSR-44657	scaffold5301	p3	(ACG)5	Class II	1038	1052	TCGTCCATGGCGTTTTGTGGCT	TGAAGCACATGCACCACGCA	349
FMgSSR-44660	scaffold194	p3	(ACG)5	Class II	6996	7010	TGACCACACGCTCACGCTTT	ACGTACGCCGCAGAAGTGTT	287
FMgSSR-44662	scaffold225	p3	(ACG)5	Class II	36923	36937	TGAGGAATGGCGTTGTTGCG	TGGGCCGACAAACAAGCTGA	342

SSR_ID	Scaffold	SSR_Type	SSR_Motif	SSR Class	SSR_Start	SSR_End	Forward sequence	Reverse sequence	Product_size
FMgSSR-44664	scaffold3902	p3	(ACG)5	Class II	44248	44262	TGCAAGAGGTTCGCGTGCAGTA	ACTTGCAGCATGATGAACGGCG	294
FMgSSR-44674	scaffold6053	p3	(ACG)5	Class II	22401	22415	TTCTACGCGCGCCTTCCTCATT	TTCGCTGGCCATGACGACCTTT	261
FMgSSR-44680	scaffold3004	p3	(ACG)5	Class II	62466	62480	TTTCCTGTTGGGCTCACCCTCA	GGCACGTTCCCGTTTGTCTTGT	325
FMgSSR-44688	scaffold5355	p3	(ACG)6	Class II	2797	2814	TGCACTGCATGCAACGACGA	AGAGGGCACAAGTGGCTTGACT	336
FMgSSR-44693	scaffold1255	p3	(ACG)7	Class I	7872	7892	ACAACCCAAACGCGCCTAGT	ACGGCGGGTTTCTTCTTGGA	280
FMgSSR-44694	scaffold1334	p3	(ACG)7	Class I	34766	34786	AGCTCATCATGGAGATCGCGGA	AGAAGCACACACCCAACGCA	344
FMgSSR-44696	scaffold7885	p3	(ACG)8	Class I	3299	3322	ATTGATGCATTGCCGCGACG	TCAATTTACCCGCGCCGACT	346
FMgSSR-44698	scaffold6300	p3	(ACT)10	Class I	29472	29501	ATAAACGCCGAAGGTGCCGA	TGCTTCAGAAAGCTGCACCGAC	295
FMgSSR-44700	scaffold10676	p3	(ACT)13	Class I	12618	12656	TTCTTTGCCGGGAAGCTCCTGA	TCGCTGCATGCGTCAGACAA	349
FMgSSR-44703	scaffold268319	p3	(ACT)17	Class I	166	216	TAGCGTGCCGTAATGCTCCA	GCAGCAGCCCAGCTTTTTCGTT	224
FMgSSR-44706	scaffold272392	p3	(ACT)5	Class II	317	331	AAAATGCCAGCTGCGTCTGC	TTCGTGTGGGGATGATGGCGAA	299
FMgSSR-44709	scaffold26898	p3	(ACT)5	Class II	9216	9230	AACACGGTAAGGAGCGAGCGAT	TGACTGCACTGCTTGCCACA	203
FMgSSR-44710	scaffold26775	p3	(ACT)5	Class II	9368	9382	AAGGCGCAGCATGCGGAAAT	AAGAAGAGCGAGGAGGACGACA	327
FMgSSR-44711	scaffold5388	p3	(ACT)5	Class II	42621	42635	ACAAAGCGATGGCGGACACA	TGGAGGGGTTGGCCAAAAAGA	201
FMgSSR-44712	scaffold11629	p3	(ACT)5	Class II	10048	10062	ACAAGTTGCACTAGCGCATGG	ACGCTGTACAAGTTTTGGCGCT	224
FMgSSR-44715	scaffold40	p3	(ACT)5	Class II	96184	96198	ACCAAGAGCAACAACAGCAGCC	AAGGCATGCTGCAATGGAAGGC	202
FMgSSR-44718	scaffold221	p3	(ACT)5	Class II	125774	125788	ACGGCACTGGAGAAAGAGAGAGGA	ATGTGTCAGCTGTCTGCTTGCG	314
FMgSSR-44719	scaffold17349	p3	(ACT)5	Class II	12090	12104	ACGGCCAGCACAAACGTGAT	ACCCGTACCGGAGAAGAAGTGA	312
FMgSSR-44720	scaffold3730	p3	(ACT)5	Class II	20437	20451	ACGTCTGCACCTGTAAGCGA	TGCTACCGGCACACAGTGGAAA	305
FMgSSR-44724	scaffold4936	p3	(ACT)5	Class II	7094	7108	AGCCAGCACAGCGGTGATCTTT	AAACAGGGCAATGGCTTGACGC	204
FMgSSR-44725	scaffold211256	p3	(ACT)5	Class II	341	355	AGCGAGCCGATCAAAGCGAT	TCAATGAGGCGAGGGTTGCCTA	287
FMgSSR-44736	scaffold4887	p3	(ACT)5	Class II	2660	2674	ATTAGGAGCGTCGATAGTGGCGGA	GCGTCCAGCAAAGTGCCAAGAT	336
FMgSSR-44737	scaffold2575	p3	(ACT)5	Class II	55735	55749	ATTGCAGCCCTTCGATTGCAGC	ACGAAGCAAGCAGCTCTCAGCA	246
FMgSSR-44738	scaffold7717	p3	(ACT)5	Class II	11987	12001	CCCTCGAAACGTTTTGCCGCTT	TCTGTTTGAGTGCGGGTCGAGA	318
FMgSSR-44743	scaffold29493	p3	(ACT)5	Class II	4254	4268	GGGCGCTTGCAAGAAAGTTTGGT	TCCGTTCTCAAACCTGCGCATC	343
FMgSSR-44757	scaffold19814	p3	(ACT)5	Class II	8456	8470	TGCCGCTTGCAAGAACAAGCA	GCGCGTGTTGCTCAATGGATGT	335
FMgSSR-44762	scaffold196	p3	(ACT)5	Class II	142558	142572	TGGTTTGCCACAGAACGTCCCA	ACCAGAGACCACTGCTTGCGAT	325
FMgSSR-44764	scaffold2273	p3	(ACT)6	Class II	43637	43654	AAAGCACGCAGACACGACGA	AGAGCATGGTGCAGATGGTGGT	339

SSR_ID	Scaffold	SSR_Type	SSR_Motif	SSR Class	SSR_Start	SSR_End	Forward sequence	Reverse sequence	Product_size
FMgSSR-44772	scaffold366	p3	(ACT)6	Class II	74063	74080	TCCCTGCCGCGCTTCTTATT	ATTCACGTCGCCTTCTCGGCTT	247
FMgSSR-44773	scaffold688	p3	(ACT)6	Class II	21723	21740	TGCAGCCGGAGATTGTTCGT	AGCAGTAACATGGTGTCTTGGGCA	215
FMgSSR-44775	scaffold1983	p3	(ACT)7	Class I	25278	25298	AACCACTTGTGTGTGGCCGT	TGCCATCGGTCATTGCTTCAGT	251
FMgSSR-44779	scaffold456	p3	(ACT)9	Class I	97280	97306	AGGCGAGATTAAACCGGTCGAG	TGCACGTGACAGCGGAAGAAGT	340
FMgSSR-44781	scaffold7093	p3	(AGA)10	Class I	22574	22603	ACCGGCGACCTAGATCAGTCAGTA	TGGGCTGAAGCAGTCCAAGT	300
FMgSSR-44782	scaffold45	p3	(AGA)10	Class I	193997	194026	GCGCGCGTATAAAGCAGCAA	TGATGTCCCGCTTGCACAGGAA	200
FMgSSR-44784	scaffold1794	p3	(AGA)10	Class I	46882	46911	TGGTGCTCCAACTCCAAGCACA	TCGCGCATCATCTCCAACGA	313
FMgSSR-44786	scaffold4752	p3	(AGA)11	Class I	16549	16581	TGTGACCACTCATGGGCTTCCT	TGCTGCTGCTCGAGTTGTGAGT	254
FMgSSR-44787	scaffold583	p3	(AGA)11	Class I	106275	106307	TGTGTGATGGCGAAGGCATGGA	TCGATGCCCATCGCAAGGTTGT	287
FMgSSR-44789	scaffold598	p3	(AGA)12	Class I	88355	88390	TAGCTAGCGTTGGCTTGGCTGT	AAACCATTCCGGCGCCCTTT	302
FMgSSR-44792	scaffold8796	p3	(AGA)12	Class I	19090	19125	TGCTCTGCTTACGGAACATGCCT	AGCCACAAAGGAAAGGGCGTGA	255
FMgSSR-44793	scaffold3828	p3	(AGA)12	Class I	9761	9796	TTCCGTGTTGAAGCTTTGCGGC	AACAGACAAGCGTGCCAGTTGC	313
FMgSSR-44795	scaffold7255	p3	(AGA)14	Class I	2831	2872	ACATGGGCGCAGTGGATGAA	CCACCTAACACATGGACAACGCCT	277
FMgSSR-44802	scaffold9926	p3	(AGA)19	Class I	7966	8022	ACATCCCGGAAGTGGTCATCA	AGCACCACCACCGCCTTGTTAT	232
FMgSSR-44804	scaffold856	p3	(AGA)20	Class I	102267	102326	CCGATGACGACGCAGGAATAGGAT	AGCAGCTTCACTGGAAGCGACA	205
FMgSSR-44805	scaffold1029	p3	(AGA)21	Class I	56082	56144	TTCCACCAGTGGAGCGTGTGTT	TCATACTCCGCCGTCGTGAA	319
FMgSSR-44808	scaffold4224	p3	(AGA)25	Class I	17484	17558	TGATGCGAAGGTGCAGAGCA	TCGTTGCTGCCGCCTTTGTT	255
FMgSSR-44815	scaffold2818	p3	(AGA)5	Class II	39397	39411	AAATCGGAGAGGGGTTAGGGCA	AGCTTCTGATACGCCCGCTTGA	341
FMgSSR-44816	scaffold4038	p3	(AGA)5	Class II	50158	50172	AACACAACATGGGATGGCCGGA	TCGTCTGAGGAAAGCGCAACGA	340
FMgSSR-44822	scaffold10270	p3	(AGA)5	Class II	25685	25699	AAGAGAAACTCGGCGAGACGCA	AGGAGCGCCATGACACACACAA	309
FMgSSR-44824	scaffold29392	p3	(AGA)5	Class II	4451	4465	AAGCGATCACTCGGCATCAGCA	ATTGCAAGGCAAGCGACCATGC	280
FMgSSR-44825	scaffold1018	p3	(AGA)5	Class II	85426	85440	AAGGCAACAACGACCAGCCA	AAGAGGCATTGGGGCTGCATGA	284
FMgSSR-44827	scaffold26331	p3	(AGA)5	Class II	8680	8694	AATCGAAAAGGAGCTCGCCGCT	TTGCGCCATTTCGTCGAGCA	250
FMgSSR-44828	scaffold12570	p3	(AGA)5	Class II	2918	2932	AATGAAAGCTCGGTGGGGCGAT	ACACCTTCTCCGCCAGTCGTTT	244
FMgSSR-44829	scaffold12107	p3	(AGA)5	Class II	2667	2681	ACAACTGCGTGAATTTCCGGGG	ATGTCATCGTGAGGGCCTTGCT	281
FMgSSR-44831	scaffold251	p3	(AGA)5	Class II	65026	65040	ACACGAACTGTCCACGACGCAT	TGCGAATCGACTCATGCCAGGAA	349
FMgSSR-44832	scaffold3447	p3	(AGA)5	Class II	27693	27707	ACACGACCTCATTTGCCTCGCA	GGGTCACGCATCATCTGCAA	223
FMgSSR-44833	scaffold13	p3	(AGA)5	Class II	89213	89227	ACACGTCACAACGGATCAGCCA	ATGGAGGCGAGGCAACAAACCT	205

SSR_ID	Scaffold	SSR_Type	SSR_Motif	SSR Class	SSR_Start	SSR_End	Forward sequence	Reverse sequence	Product_size
FMgSSR-44835	scaffold1058	p3	(AGA)5	Class II	23544	23558	ACCCAAAACACATGACTCGCCA	TCCTTACGCCTGCCATGCTTCT	344
FMgSSR-44841	scaffold453	p3	(AGA)5	Class II	116043	116057	ACGAAGACGCCTGCGAATGT	TGCCTTGTTTTGGGCCTCGT	332
FMgSSR-44842	scaffold19647	p3	(AGA)5	Class II	14329	14343	ACGACCGCCACCTTCAAATGCT	AACGGCAGCGACGAAGAAGA	229
FMgSSR-44846	scaffold1123	p3	(AGA)5	Class II	50939	50953	ACGTTGCAGTAGAGTCGACGGA	AGCTTCCGTTTCCACCCAGCTT	236
FMgSSR-44849	scaffold4194	p3	(AGA)5	Class II	43493	43507	ACTGACGGGCCCGGTTTTAGAT	ATGGGTTGGGTTGGGTTGGGTT	286
FMgSSR-44850	scaffold85	p3	(AGA)5	Class II	169471	169485	ACTGGATCACCCAGAGTAGCGT	TAGAGGCGGGCAAGAAACACCT	265
FMgSSR-44853	scaffold3093	p3	(AGA)5	Class II	8985	8999	AGATTGAGGTGCAGGTGGAGCA	TGTCCGTCGCAGCACTTGTT	328
FMgSSR-44860	scaffold867	p3	(AGA)5	Class II	24672	24686	AGCAGAAGGCGCATTTGCCGTA	AGCAGCATCGCCATGACGAGAA	339
FMgSSR-44863	scaffold87	p3	(AGA)5	Class II	96205	96219	AGCGCCGGAGGAGTCTTTTT	TCGCACTTCGCCTCCATCAT	322
FMgSSR-44864	scaffold2528	p3	(AGA)5	Class II	25793	25807	AGCGGCCTTGGGAGTGACTAAA	AGCGATAGGTTGTGCCAACACAC	343
FMgSSR-44876	scaffold2331	p3	(AGA)5	Class II	47981	47995	AGGTGCGAACAGAAGGTGTGCT	TCACGCAACCAAGCAGTGTCA	241
FMgSSR-44877	scaffold946	p3	(AGA)5	Class II	2070	2084	AGTGGCACTGTTAAGGCAGGCA	GCCCTGCAACAGCCAAACTGTA	284
FMgSSR-44892	scaffold384	p3	(AGA)5	Class II	55112	55126	CCGTGGATTTGAAGGGGCGAAT	TCCACAGATGCCAAGTGCCAACA	301
FMgSSR-44894	scaffold818	p3	(AGA)5	Class II	15936	15950	CGCGATACGCGAATGCCGAAAA	AGGCGCTGCATTGGACGTAGTT	279
FMgSSR-44900	scaffold11463	p3	(AGA)5	Class II	8061	8075	CGTGCGTGCGGTTTCTGTTT	TCCGTTGTCACACACGCACA	291
FMgSSR-44901	scaffold7438	p3	(AGA)5	Class II	26666	26680	GCAAAAATGAAGCCCCAACACC	TGGGAAATGGCACCCACAAGGA	269
FMgSSR-44908	scaffold3158	p3	(AGA)5	Class II	28891	28905	GCTGCCTTTGGAGGTTAACGTTGC	ACGCTTCTCAGCTCAACGCCAT	349
FMgSSR-44913	scaffold5465	p3	(AGA)5	Class II	37816	37830	TACATCGTGTGCAACCCGGCAA	AGCTGCTCCCATGCCGTAAT	201
FMgSSR-44926	scaffold24410	p3	(AGA)5	Class II	9582	9596	TCCCAAAACAAAGAAGCCCCCGT	ACCCAGAGCATATGCAGGAGATGG	283
FMgSSR-44928	scaffold2271	p3	(AGA)5	Class II	15330	15344	TCCGATGTGCTGGTGGTTTCCT	ACGCCATCGTCATCAGCCACAA	340
FMgSSR-44933	scaffold298298	p3	(AGA)5	Class II	105	119	TCGAGGCGGAGCTGAAAAAGCA	TGCAGTTGATCCCAAACTCGCTGT	200
FMgSSR-44934	scaffold9492	p3	(AGA)5	Class II	4505	4519	TCGATCCGTTTGTCTCCGCCAA	AGCAGCACCTGCATCAGCAT	223
FMgSSR-44939	scaffold1944	p3	(AGA)5	Class II	29721	29735	TGAGTAACGAAAACCGGGGCGT	GCGCAATGGGGTGGTTGTTAGT	215
FMgSSR-44940	scaffold4528	p3	(AGA)5	Class II	5031	5045	TGATGGCGGCCGAAGAAGAA	GGCAATTGCGTCACTCACCCAT	249
FMgSSR-44945	scaffold6767	p3	(AGA)5	Class II	59420	59434	TGCCCAGCAAGGAAGCATGT	TGCAATGTCGGTTCACCTGCAA	260
FMgSSR-44947	scaffold730	p3	(AGA)5	Class II	17422	17436	TGCTGCTCTGTTTCAGAGGCCA	TGTTCGACGAAATGCGCGAC	310
FMgSSR-44948	scaffold13743	p3	(AGA)5	Class II	5360	5374	TGCTGGCGCAAATTTAGCCGT	ACGTTCGGGTCGATGGTGACTT	335
FMgSSR-44950	scaffold3822	p3	(AGA)5	Class II	34236	34250	TGGAAGAAAGCGCGTCGACAA	AAGCGCCGTGTGCGTTTGTT	212

SSR_ID	Scaffold	SSR_Type	SSR_Motif	SSR Class	SSR_Start	SSR_End	Forward sequence	Reverse sequence	Product_size
FMgSSR-44953	scaffold13866	p3	(AGA)5	Class II	6536	6550	TGGCAAAACAGTCGCCAAATCG	TCGTCAATCGCGCGCCAAAA	299
FMgSSR-44966	scaffold22508	p3	(AGA)5	Class II	10753	10767	TGTGCCATGCTCTGCTTCCA	ATGTGACGCTCTCGTTTGCCCT	260
FMgSSR-44967	scaffold104337	p3	(AGA)5	Class II	584	598	TGTGCGATTAACACGGTTGGCG	AGCGCCGCGATAAACTTCCA	247
FMgSSR-44968	scaffold161	p3	(AGA)5	Class II	30739	30753	TTAACAGCACGCAGCCAACGTC	ATTTTCGCGCATGACTGCCG	227
FMgSSR-44969	scaffold6558	p3	(AGA)5	Class II	10044	10058	TTCGGTCTCGTGCTGTGCAA	TGGGCACTGTTTGCTGGGTT	314
FMgSSR-44970	scaffold7305	p3	(AGA)5	Class II	26960	26974	TTGCAACTGCTGGCCCAAGT	TGTCAGCACCTGGTGGACAAT	268
FMgSSR-44980	scaffold3247	p3	(AGA)6	Class II	48201	48218	ACACAACCGTCTCACCGGATCA	AAGCTGAATGCGTTGCGGGA	294
FMgSSR-44986	scaffold6977	p3	(AGA)6	Class II	4414	4431	AGCAAACCCAATTGCGACCCA	ACCGAGGACTTTTGGTGATGCGT	213
FMgSSR-44990	scaffold189	p3	(AGA)6	Class II	90576	90593	ATCCACCCCAAGCAAGGCACAA	TCCACGCGCACCTTGAAGGAAT	266
FMgSSR-44992	scaffold243	p3	(AGA)6	Class II	91153	91170	ATGCACTACTTCCACGGCGAGT	AAGCGAGACAGAGCAAGCAGCA	331
FMgSSR-44994	scaffold2095	p3	(AGA)6	Class II	65092	65109	CACCGGCGGCGAAATTCAAA	AGCATGAACGGCCAATGCGT	271
FMgSSR-44998	scaffold5794	p3	(AGA)6	Class II	37615	37632	CGATCGGCATGCATGAACGTGT	GCGACGGCTTTGCTTTTGCT	295
FMgSSR-44999	scaffold1474	p3	(AGA)6	Class II	61958	61975	GCACGTTCTCATCAAGCCAACGA	TTTACCAGCCGCCACTCCTTCT	230
FMgSSR-45005	scaffold38	p3	(AGA)6	Class II	114552	114569	TCCATTGGGCTTTTGCCTCGGA	AGGTGCCACTGCAGAGATAGACA	347
FMgSSR-45010	scaffold480	p3	(AGA)6	Class II	51396	51413	TCGTCGTCGGGATGCTTGCTTT	TTTGACGGGCGCATGCATCA	294
FMgSSR-45019	scaffold1410	p3	(AGA)6	Class II	3712	3729	TTGGACAGCGCACAACACGA	AATTCTAACCCGCCGTGCGT	231
FMgSSR-45021	scaffold183	p3	(AGA)7	Class I	123782	123802	AACCCACGATGTGTGAGCAGCA	ACTCAGTGATCACGGTGCGA	212
FMgSSR-45022	scaffold14235	p3	(AGA)7	Class I	16226	16246	ACAATGTAGCAGCTCGGCAGT	AAAGGTCGCCGTGCATCCAT	341
FMgSSR-45023	scaffold8358	p3	(AGA)7	Class I	24393	24413	ACGACGACTCCTGTGATGCGTT	TTCGGCTGCACCAACGTGAA	346
FMgSSR-45029	scaffold463	p3	(AGA)7	Class I	103441	103461	AGGTGAGTCGTCGTCCAACACA	AGCTGCAGCATCTTCCACCGAT	274
FMgSSR-45030	scaffold9189	p3	(AGA)7	Class I	28430	28450	ATAAAGCGCGTCGCAAGCCA	AGCACACGTCAACAATCGCC	319
FMgSSR-45033	scaffold557	p3	(AGA)7	Class I	106958	106978	ATTTTGCTCCGCTGGCTCAGGT	TGACCACGGGGTACGTCTTCAT	271
FMgSSR-45034	scaffold3208	p3	(AGA)7	Class I	38637	38657	CCGTGCGTGCGGTTTTTGTT	AATCCGTTGTGACGCACGCA	310
FMgSSR-45036	scaffold14254	p3	(AGA)7	Class I	15219	15239	CGCAGCGGCAGCTTGTGTTATT	TGCGATGCCGAAGAACTGGTCA	326
FMgSSR-45040	scaffold4813	p3	(AGA)7	Class I	2308	2328	TCGTGTTGCAGGCGCATGAT	ACGCGTTGCACAGCGTCTTT	262
FMgSSR-45041	scaffold5623	p3	(AGA)7	Class I	12010	12030	TGCAAACCGTTGCACGGAACA	TCAGCGTTGGAGCAGAGTGCTT	307
FMgSSR-45042	scaffold6088	p3	(AGA)7	Class I	40040	40060	TGCAAGATTGTGCAGGCGCT	TCCGTGCGTTTCAACGACCTCA	269
FMgSSR-45043	scaffold1517	p3	(AGA)7	Class I	79631	79651	TGCATGCTCGAAAGGCGATGA	TGCATGGTGTGGTCATGGGAGT	286

SSR_ID	Scaffold	SSR_Type	SSR_Motif	SSR Class	SSR_Start	SSR_End	Forward sequence	Reverse sequence	Product_size
FMgSSR-45044	scaffold4018	p3	(AGA)7	Class I	26060	26080	TGGAACGCAACCACAACACACC	TGTGTGCATCCTCCGTGATCGT	224
FMgSSR-45046	scaffold825	p3	(AGA)7	Class I	31667	31687	TGGCTGCCAAGCAAGGCAAA	AGGCAGCGACAAGACCAATGCT	336
FMgSSR-45048	scaffold286	p3	(AGA)8	Class I	74681	74704	AAGGAAGCAAAGGAAACGGCGG	TCCAACAAGAGCACCAGCACCA	256
FMgSSR-45049	scaffold1898	p3	(AGA)8	Class I	39907	39930	ACGAACTTGGACTCGTCGCAGA	TGCAAGCTTGAGCGCTGGAT	203
FMgSSR-45050	scaffold7630	p3	(AGA)8	Class I	3244	3267	AGCAAATCAGCAGGTGGTGGCA	ACACCTCGCTCATCACTGGCTT	346
FMgSSR-45052	scaffold1557	p3	(AGA)8	Class I	18096	18119	ATGAAGAAGCTGGTCGGCGTGT	ATTGGCCACCACGCACAGTT	303
FMgSSR-45054	scaffold9200	p3	(AGA)8	Class I	24452	24475	TCAGTGCGAGCAACATTGAAGC	TGGTTGCCAGTATCCCACTCCT	257
FMgSSR-45055	scaffold3922	p3	(AGA)8	Class I	22538	22561	TCCCAGTGGTGATGCCGGATTT	ACGATGGGCAAATCCACGACCT	312
FMgSSR-45058	scaffold3101	p3	(AGA)9	Class I	7086	7112	AAAGGTGAAGGCGACGCAGACA	CTTTAATTCGTCGCCGCGCA	239
FMgSSR-45060	scaffold37649	p3	(AGA)9	Class I	5180	5206	ACCATGGCGAGCGCAAGAAA	ACCGCATATCATCACCCATCCCT	306
FMgSSR-45064	scaffold6081	p3	(AGA)9	Class I	2340	2366	TCGCGGCCTACGTTTGTCTTCA	TTTGCGTACGCCCGCTTCCTTT	347
FMgSSR-45065	scaffold559	p3	(AGA)9	Class I	100701	100727	TGCAGGGTTGGTTTTTGCTGCC	TGAATCAGTGCGTCGATGGGGT	249
FMgSSR-45066	scaffold138823	p3	(AGC)10	Class I	321	350	AGCCACCAGAATTTGCACACGC	TGCTTCTGTTGCGTGCTCACT	339
FMgSSR-45067	scaffold60931	p3	(AGC)12	Class I	245	280	TGCAGTTCCGTGCAGTTGCT	TTTGGTTGCTCGGGAGCGTT	255
FMgSSR-45069	scaffold25210	p3	(AGC)5	Class II	5114	5128	AAAAGGGCGCGGCTTGTGTA	ACCGGTGCCAATAAGCAGAGCA	326
FMgSSR-45071	scaffold603	p3	(AGC)5	Class II	123439	123453	AAACCCAAGCCCGCTCCCTAAA	TCGCTTGCCCTTGTCTTCGTCA	259
FMgSSR-45072	scaffold3828	p3	(AGC)5	Class II	20691	20705	AAAGAACAGGGAGCCAGCCAGA	AAGCACTCGCGTTCAGCGTT	238
FMgSSR-45073	scaffold4320	p3	(AGC)5	Class II	22462	22476	AAAGTCGAACCAGAGCTGCACG	AGCAACAGCATCACCACCAGCA	290
FMgSSR-45076	scaffold4902	p3	(AGC)5	Class II	25365	25379	AAATGCGTGCGTCCGAGTGA	TCACACCACACGCCATCCCAAT	280
FMgSSR-45078	scaffold19173	p3	(AGC)5	Class II	14774	14788	AACAACCACCGCTCTCGTCCAA	TGCTTGCCGCTTGAGAGTCCAT	308
FMgSSR-45080	scaffold20087	p3	(AGC)5	Class II	4399	4413	AACCAGTTGCAGGCACGGAA	ACGCTGCACGCACTGATGAT	338
FMgSSR-45081	scaffold29394	p3	(AGC)5	Class II	1382	1396	AACCCGTGATTATTGCGCGGTC	TGCTTTTGCAACGGTGATGGGC	314
FMgSSR-45083	scaffold17308	p3	(AGC)5	Class II	10014	10028	AACGCCAGCAGCAGCAGTAA	TGGTGGAGTGCATCTTGCCTGT	350
FMgSSR-45088	scaffold1342	p3	(AGC)5	Class II	96227	96241	AAGCGCGCAGGAACAAGAGCTA	ACGCATCATCCGGTTCGTCA	212
FMgSSR-45090	scaffold7850	p3	(AGC)5	Class II	11011	11025	AAGGGCAAGCCGTCCAAGAACA	TGCATCATCTGCAGGTCCTCGT	200
FMgSSR-45097	scaffold1428	p3	(AGC)5	Class II	7532	7546	AATTTCCCGTGCTCTCACCGCT	TTTGGGGCTTGGAGGCATGGAA	331
FMgSSR-45098	scaffold14998	p3	(AGC)5	Class II	3896	3910	ACAACAGCAACAGCCAGCCT	AATGAAGCCTTTCTCTCCGCGCAC	224
FMgSSR-45099	scaffold100	p3	(AGC)5	Class II	6097	6111	ACAAGGGCCGTCATCAGTCGTA	TAACGTGTTCGGCCGTGTCCTT	216

SSR_ID	Scaffold	SSR_Type	SSR_Motif	SSR Class	SSR_Start	SSR_End	Forward sequence	Reverse sequence	Product_size
FMgSSR-45101	scaffold3469	p3	(AGC)5	Class II	3013	3027	ACACCGTGCCGTACGTCTTT	TCGCAACTGCGAGTACGAAGCA	259
FMgSSR-45105	scaffold1202	p3	(AGC)5	Class II	52793	52807	ACAGACCAGGCCAAAACGCA	TTCCAACTCGGCCTTGCCAACT	269
FMgSSR-45109	scaffold9135	p3	(AGC)5	Class II	27464	27478	ACATGCACCAGCAGCAGTAGA	AGGAGAGCACGGGTCTTTTTGCT	347
FMgSSR-45110	scaffold1852	p3	(AGC)5	Class II	9964	9978	ACCAATCTGCCACAAGCACCGA	TTCGTCAGCGCGATGTTGGT	323
FMgSSR-45112	scaffold35155	p3	(AGC)5	Class II	4948	4962	ACCACAGGGCACGCAGTTTT	AAAAAGGGGAGCGTCCATCCGT	232
FMgSSR-45113	scaffold147	p3	(AGC)5	Class II	133040	133054	ACCACAGGTTTTGACAGCGCCA	AAATGAGCAACGATCCGGCG	265
FMgSSR-45117	scaffold6939	p3	(AGC)5	Class II	8668	8682	ACCATTTCACGGGCGCAGAA	ACGACGACGTTGTTGCTCCT	324
FMgSSR-45118	scaffold301	p3	(AGC)5	Class II	6266	6280	ACCCAGCGTCCAGTTGTCTTGA	ACGGGAAGCGGAAGTTCGGTAT	207
FMgSSR-45119	scaffold1974	p3	(AGC)5	Class II	1566	1580	ACCCTTCAAAGACGACGGGCTT	TCCGTCAGGTGTGCCTACACTCTT	309
FMgSSR-45123	scaffold13052	p3	(AGC)5	Class II	7297	7311	ACCGGTGCGTGCTTTGCATT	TGCCGCGCGGACAAAAGAAT	213
FMgSSR-45130	scaffold9771	p3	(AGC)5	Class II	4571	4585	ACGCGATGCTGCAAAGGTGT	TGGCGCCTTGAAGTTTGCTGA	242
FMgSSR-45136	scaffold520	p3	(AGC)5	Class II	27496	27510	ACTGATTTCATGGCGTGTGCGT	ACGTAGTTGAGCGTGCCGAT	344
FMgSSR-45141	scaffold12652	p3	(AGC)5	Class II	4579	4593	AGAAAACGACCACGCTGCCA	AGGTGCGGCAACGAAACTCA	348
FMgSSR-45143	scaffold106376	p3	(AGC)5	Class II	618	632	AGAGTGTTTGTGCTGGCGCT	TCTGTTCGTGCTCGCCACAA	230
FMgSSR-45145	scaffold27499	p3	(AGC)5	Class II	1954	1968	AGCAAGAAGCGAAGCAGCCT	AATTCCCAGCTTGCCAGCCA	339
FMgSSR-45146	scaffold200	p3	(AGC)5	Class II	30675	30689	AGCACAGCACATCCTTCGCACT	TGTTCGTCGTGCTCGTTGGT	287
FMgSSR-45147	scaffold225	p3	(AGC)5	Class II	35154	35168	AGCACGAACAACAACGACGGGA	TCCTTGTTCACCGTGCGCATCT	332
FMgSSR-45153	scaffold1098	p3	(AGC)5	Class II	26707	26721	AGCCCATAGCTCATCGCGCAAA	AAGGTAGCCATCGACCGCGTTA	300
FMgSSR-45156	scaffold1157	p3	(AGC)5	Class II	40198	40212	AGCTAGCACTAGCAGCGGTGAA	TGCCATGCATGCGCGATCTT	313
FMgSSR-45160	scaffold1995	p3	(AGC)5	Class II	58254	58268	AGCTTCTTCGTGTGGCGACGAT	AGCCCGTGTCATTCCATTCCGT	343
FMgSSR-45163	scaffold152616	p3	(AGC)5	Class II	680	694	AGGCACTCGGTTGCAAATCAGC	TTTGCCGAGGGGGAGTTTTGCT	310
FMgSSR-45165	scaffold11619	p3	(AGC)5	Class II	4026	4040	AGGCGCGGATCAAGAGAGTGAT	CCAGTGTTGCCGCCAAACCAAA	322
FMgSSR-45166	scaffold1024	p3	(AGC)5	Class II	47575	47589	AGGCGGAGCAATTGCGCTTT	CGCCGGCTTTGAAGTTTCAGCA	339
FMgSSR-45171	scaffold1684	p3	(AGC)5	Class II	63053	63067	AGTCCGATGAACCCAACCCGAA	AGCTGTCGTTGTTGATCCGCGA	254
FMgSSR-45172	scaffold745	p3	(AGC)5	Class II	38173	38187	AGTCCGCGGTTCCAGATGAA	TTCCGGACTTGCGATGCCTGTT	249
FMgSSR-45175	scaffold830	p3	(AGC)5	Class II	64930	64944	ATAAGCGCACCACCGTGTCT	TGCATTGGCTGCGCGTTGTT	280
FMgSSR-45178	scaffold1719	p3	(AGC)5	Class II	125155	125169	ATCAACCTCGCGTCCTCTTGCT	ATGGTTTCCGTCGGTTGTGGCT	299
FMgSSR-45186	scaffold4	p3	(AGC)5	Class II	56359	56373	ATGAATGGAGTTGGGGGCTGGT	TGCAGGATGGAGGGCAGAGATT	271

SSR_ID	Scaffold	SSR_Type	SSR_Motif	SSR Class	SSR_Start	SSR_End	Forward sequence	Reverse sequence	Product_size
FMgSSR-45188	scaffold70292	p3	(AGC)5	Class II	981	995	ATGATCGAGACGACGAAGGCGT	TGATCCGAGCGGAAGGAACT	281
FMgSSR-45189	scaffold6594	p3	(AGC)5	Class II	32062	32076	ATGCCTTGCTGTTCCTCGTGCT	TCGCGTCCGTACTTTGCAGT	223
FMgSSR-45191	scaffold3006	p3	(AGC)5	Class II	11972	11986	ATGCTCGACCACCCTTCGTA	ATCGCGTACTCGCACGATGA	347
FMgSSR-45192	scaffold286	p3	(AGC)5	Class II	8375	8389	ATGCTTTCCACGGCCTGGTT	CCTGGTCAACAAAGCCGCAACA	299
FMgSSR-45204	scaffold10038	p3	(AGC)5	Class II	6766	6780	CCTTGCGCAATCGCCTTCTGAT	TGGGATTGGGTTGCTCTGTCCA	295
FMgSSR-45206	scaffold5296	p3	(AGC)5	Class II	3509	3523	CGCCGCGTTGAAGTAATTGGCA	GCTCAACGCCTCACATTGCTGT	253
FMgSSR-45209	scaffold6466	p3	(AGC)5	Class II	22794	22808	CGTCTGGTGGAGACAAAGGCAA	TTCGCACGCCAGGCACAAAT	348
FMgSSR-45211	scaffold21729	p3	(AGC)5	Class II	4969	4983	CGTGTCGTCTTCGTCGGTGTTT	TTGGCGCACCGCTCGAAAAA	241
FMgSSR-45214	scaffold581	p3	(AGC)5	Class II	19028	19042	GCAGTCCGGCACACTGATTT	AGCTGACCTCCTTCTGTCCTGCTT	266
FMgSSR-45217	scaffold1774	p3	(AGC)5	Class II	8312	8326	GGAGAGAAACCCGCATTGCGTT	ATCCGCAATGGCGTCAGAAGGT	212
FMgSSR-45218	scaffold730	p3	(AGC)5	Class II	18268	18282	GGCAGCAGCGTCTTCATGGAGTAT	TCCACACACACCGACTGCTCAA	330
FMgSSR-45219	scaffold12205	p3	(AGC)5	Class II	3143	3157	GGCGCGAGCGATTTCACATT	ACGATGGTGGTGGACAGGTTGA	314
FMgSSR-45225	scaffold3516	p3	(AGC)5	Class II	13437	13451	TAACGAAGGGTCTGCGTGTGCT	TGACGCGTACAACGGGGACAAA	312
FMgSSR-45227	scaffold437	p3	(AGC)5	Class II	70681	70695	TACTGCGCCAGGGAAACCATGA	TGGCAACACCAAGCAGCCAA	215
FMgSSR-45228	scaffold14558	p3	(AGC)5	Class II	10120	10134	TACTGTGCGGACGGTTTCGT	TCGAAAGCAGGACGCCGAAT	332
FMgSSR-45229	scaffold1684	p3	(AGC)5	Class II	65492	65506	TATACCGGTGGCGCATGGCTTT	CACAACGCTGCAAGCTCGATCA	349
FMgSSR-45231	scaffold4654	p3	(AGC)5	Class II	9191	9205	TATGCCGGCCGATCAAAATGGC	TGCCGGTGCTGGTGAAGAAGAT	290
FMgSSR-45232	scaffold14393	p3	(AGC)5	Class II	18838	18852	TCAATCCGCTGCTGCTCCTCTT	AGCGGCGTCTTCATGCTGTT	340
FMgSSR-45236	scaffold2597	p3	(AGC)5	Class II	9034	9048	TCCATCCACCATTTTGCCCCCA	AGCCAACGGATGCAGCATGT	272
FMgSSR-45239	scaffold15746	p3	(AGC)5	Class II	18520	18534	TCCGGGCTTCTGGTTGTTGTGT	TTGCTTGGCGTCGAGCTCTT	239
FMgSSR-45241	scaffold4594	p3	(AGC)5	Class II	35171	35185	TCGAAAGGGAACGGAGCAAGCA	TGTTGAGCTTTGGCGGCACT	241
FMgSSR-45244	scaffold3217	p3	(AGC)5	Class II	40666	40680	TCGCGTCGGTTGCATCACAA	TGAAGGCGTCGCCGTACTTGAT	229
FMgSSR-45245	scaffold10423	p3	(AGC)5	Class II	8502	8516	TCGCTCACGCCTGCACCTAAAT	TGCATCGAGATTCCGTTGGCCT	326
FMgSSR-45246	scaffold1364	p3	(AGC)5	Class II	62608	62622	TCGGAATCCATCTGCGACCT	ACCGCGCAGGTTAGCATAGCAA	218
FMgSSR-45248	scaffold71739	p3	(AGC)5	Class II	366	380	TCGTCGATCGAGAGGTAGAGCA	AGCAACTATCGCGACGGACA	293
FMgSSR-45249	scaffold1928	p3	(AGC)5	Class II	77386	77400	TCTCATCGTCAGCACCCACA	TGACCAGGCGAGCCAAACCAAT	260
FMgSSR-45253	scaffold31302	p3	(AGC)5	Class II	4525	4539	TGACCAAGGAGCACACTGGCAA	AGGTGTGGGAACTCATCGCCAA	348
FMgSSR-45255	scaffold174	p3	(AGC)5	Class II	139182	139196	TGACGCCCACGATCTTCGAT	AGGAAGAAGGCGCTGTACCTGA	226

SSR_ID	Scaffold	SSR_Type	SSR_Motif	SSR Class	SSR_Start	SSR_End	Forward sequence	Reverse sequence	Product_size
FMgSSR-45257	scaffold3754	p3	(AGC)5	Class II	44701	44715	TGAGACAACCGTGACGCCTGAA	AGGACGAACAACCCGATTCGCT	284
FMgSSR-45260	scaffold870	p3	(AGC)5	Class II	31520	31534	TGAGCGTGTCAACCGTGGAT	ACGGTTGTTCTTCCCTGCTGCT	295
FMgSSR-45261	scaffold3203	p3	(AGC)5	Class II	2925	2939	TGATGCCCAACACTCGAGCAGA	ACGGCCGTTTCAAGTACGACGA	295
FMgSSR-45262	scaffold9248	p3	(AGC)5	Class II	8622	8636	TGATGCTGTCCCCGGAGATGTT	ACGCGTCGTCTTCTTCGCTT	260
FMgSSR-45266	scaffold1485	p3	(AGC)5	Class II	20089	20103	TGCACTGCAGCAGCTTGTGT	TGCTGCTTTCTGCCTCCGTTCA	289
FMgSSR-45268	scaffold265	p3	(AGC)5	Class II	109991	110005	TGCAGACATGCGGACACGAA	ACAGCTTCGTCAATGCGCCA	222
FMgSSR-45271	scaffold6115	p3	(AGC)5	Class II	28405	28419	TGCATCGCATGTGGCGAGATT	ACTCGATGATCGCTGCCAAGCA	343
FMgSSR-45274	scaffold17404	p3	(AGC)5	Class II	13996	14010	TGCGAGAAGAAATGGCGACGGA	TGCTGCCAAAGCCAAACCACA	310
FMgSSR-45276	scaffold6115	p3	(AGC)5	Class II	28253	28267	TGCGCACACGTCATATGGAGGA	TGCATGTGCGAAGGGAAGTTGC	324
FMgSSR-45278	scaffold1713	p3	(AGC)5	Class II	52756	52770	TGCGCGCGATCCCAAATTCA	GCGCAGGTTACAAGTCGGAAGA	298
FMgSSR-45279	scaffold142000	p3	(AGC)5	Class II	555	569	TGCGGAGTTGGCGTTACGTT	TGCAAAGGCGTTCTGACGCA	252
FMgSSR-45280	scaffold11081	p3	(AGC)5	Class II	12583	12597	TGCGGTAGCGCCCTTCTCTTTT	TGGTCGTGGTTGTTGGTCGTGT	334
FMgSSR-45283	scaffold5244	p3	(AGC)5	Class II	24463	24477	TGCTTGCATCGGCAGGCTAA	ACGGGCATATCTTGTGCACGGT	264
FMgSSR-45284	scaffold44529	p3	(AGC)5	Class II	2887	2901	TGCTTTGCTCGTTCATCCCCGA	AGTGTCGAAACACGGACAGGCA	221
FMgSSR-45286	scaffold1746	p3	(AGC)5	Class II	17618	17632	TGGAATGGAAGGCAGCGGAA	TCAAGTCAACCACACCACCGCT	332
FMgSSR-45290	scaffold6383	p3	(AGC)5	Class II	35161	35175	TGGCCATGGATGGTCAGCAACA	ATCCGCCAGGGTTAGAATTCGG	235
FMgSSR-45294	scaffold133841	p3	(AGC)5	Class II	609	623	TGGTGGAACGACGTGCACTTGA	ATTGGCACCGGTAGCAGCACAT	306
FMgSSR-45296	scaffold3760	p3	(AGC)5	Class II	24620	24634	TGTCTCACGAGACCTTGGCGAT	ACAATCTTGCGCTGGCGCTT	259
FMgSSR-45297	scaffold2535	p3	(AGC)5	Class II	20374	20388	TGTGCACTGCAGCAGCTTGT	ATGAGGAGGACGCCCATGATCT	272
FMgSSR-45298	scaffold11287	p3	(AGC)5	Class II	24215	24229	TGTTCCACCTCAAGCCGCAGAT	TGGCGTCGTTGTTGCTGCAT	228
FMgSSR-45299	scaffold75840	p3	(AGC)5	Class II	516	530	TGTTCGTCACAGCGGTTGCT	TTGCGGTGCAAGGAGTCATGGA	260
FMgSSR-45311	scaffold526	p3	(AGC)5	Class II	101332	101346	TTCAATCAGGCAGCTGGTGACG	AGCACAAGTCTTGCCTTGCC	306
FMgSSR-45313	scaffold1865	p3	(AGC)5	Class II	50304	50318	TTCATGCAGGTGTGGGCGTT	ATCCGTCGGCTCGCATTTTCCT	330
FMgSSR-45315	scaffold19648	p3	(AGC)5	Class II	7508	7522	TTCCGCCGCGCAAGAAACAT	AAGCACTCACGTCCCTGCACTA	214
FMgSSR-45319	scaffold2799	p3	(AGC)5	Class II	45704	45718	TTCTTTTCCGCACCGGAGGT	ACACGGATCCACTGCTGCTGTT	225
FMgSSR-45320	scaffold6115	p3	(AGC)5	Class II	28071	28085	TTGAGCGCATGTTCCGTGCT	ATATGACGTGTGCGCACCAACC	267
FMgSSR-45322	scaffold7573	p3	(AGC)5	Class II	31069	31083	TTGCCACCACCGAACATCCCAT	GCTCTTGGTTGCAGGTCATGGT	279
FMgSSR-45329	scaffold5599	p3	(AGC)5	Class II	22578	22592	TTGTTGTTGCTGCCGTCCGT	AACAATGATCTCCGGGCCGTGT	221

SSR_ID	Scaffold	SSR_Type	SSR_Motif	SSR Class	SSR_Start	SSR_End	Forward sequence	Reverse sequence	Product_size
FMgSSR-45331	scaffold6994	p3	(AGC)5	Class II	6251	6265	TTTGCGGCATGGGTTCAGCA	ACCAGAACCTTGGTCTTTGCCCT	344
FMgSSR-45333	scaffold240248	p3	(AGC)5	Class II	230	244	TTTTTGCGATGGAGGCAGCCAG	TGGAAACCGACGCGTGGATGAT	254
FMgSSR-45334	scaffold9528	p3	(AGC)6	Class II	7245	7262	AACCACGCAGCTCCAAGGAA	AGCTCTGCCTGCGTTTCGTCAT	273
FMgSSR-45342	scaffold12463	p3	(AGC)6	Class II	6293	6310	AATCGTTCCCGAGCGGCTTCAT	TTGGTGTGCTTGGGGAAATGGC	296
FMgSSR-45345	scaffold6362	p3	(AGC)6	Class II	6645	6662	ACCTCGCTCACGAGCAACAGAT	ACGCGGGATCTGCAAGGAGAAA	254
FMgSSR-45350	scaffold6505	p3	(AGC)6	Class II	38966	38983	AGCACTCGCTATGCGCTTCA	TGCCGAGTAGAGGGGCTACAAA	335
FMgSSR-45351	scaffold861	p3	(AGC)6	Class II	78304	78321	AGCGCTGCTTCATTCGCTCA	AGCACCTCGGTGTAGACCTCAA	338
FMgSSR-45354	scaffold9011	p3	(AGC)6	Class II	19422	19439	AGTCCTTGCTGCATCCCCACAT	ACGCGCTGCTAGTGCTAGTT	303
FMgSSR-45361	scaffold491	p3	(AGC)6	Class II	122333	122350	ATCTGCAACTGCGACAACCTCC	ATGAACGGAATCCACCGACCGA	257
FMgSSR-45366	scaffold4918	p3	(AGC)6	Class II	42482	42499	CCATCGTGGTGTCGCTCAAA	AGCAACCAGGCAACCACTCACA	299
FMgSSR-45369	scaffold52117	p3	(AGC)6	Class II	1259	1276	CGCTTCGCGCTCAAGTGAAT	TTGCAACTCTTCGCAGCCCCAT	325
FMgSSR-45371	scaffold10940	p3	(AGC)6	Class II	18235	18252	GCCGCACAAAGCAATAGGCT	TCAGGGGTCGCCAATTCGATCT	232
FMgSSR-45376	scaffold2976	p3	(AGC)6	Class II	20968	20985	TAAGCTGCGTGTGATCTGGTGC	TGTTGGTGTTGGTGGAGCAGAG	269
FMgSSR-45379	scaffold43	p3	(AGC)6	Class II	147160	147177	TCACAAACTGCGCTGCTCCA	ACGACACCATGACAGACAGGCT	305
FMgSSR-45381	scaffold3890	p3	(AGC)6	Class II	27899	27916	TCCCCCTCTTCCATCATCTCCA	AGCCGCGTTGCTGTTGTTGT	249
FMgSSR-45383	scaffold6062	p3	(AGC)6	Class II	28827	28844	TCGCGCGTTGCTTTGCTTTG	ACCAAACGAGCAGCGTCTTCTGA	318
FMgSSR-45391	scaffold920	p3	(AGC)6	Class II	89893	89910	TGCGTGTTCAGCAGCAGCAA	GCAAGTCGTGGAACCCGATCAT	300
FMgSSR-45393	scaffold1628	p3	(AGC)6	Class II	64687	64704	TGGCTGTCCAATTACAAGGGCGA	ACGAACATGCGTGACGTCCAGT	251
FMgSSR-45397	scaffold70581	p3	(AGC)6	Class II	1226	1243	TTCGGCAAGTCGGAGTCCTGAT	TTTTCTTTCCCGTCGGAGCCCA	331
FMgSSR-45402	scaffold14216	p3	(AGC)6	Class II	3133	3150	TTGCTGGGACCGCATACTCGTT	ATCGCGACGCCAGGTTTCAA	343
FMgSSR-45403	scaffold28	p3	(AGC)6	Class II	240268	240285	TTGGCCGTGAGGACAAACGTGA	ACCCCGCCAATGCCAACAAT	205
FMgSSR-45407	scaffold13820	p3	(AGC)6	Class II	18172	18189	TTTGGTAAACCTGCCCTGCGGT	TGTACTGCTGGATTGGAGAGGTCG	259
FMgSSR-45410	scaffold6603	p3	(AGC)7	Class I	11575	11595	AAGACCCCATCGGACGCACAAT	ACAGAGCGTGGCCTGGATTTCA	203
FMgSSR-45411	scaffold3225	p3	(AGC)7	Class I	20875	20895	AAGCACGTTGTTGTGAAGCCCG	ACAGCGTTAGGCGTCGTTGT	251
FMgSSR-45421	scaffold6372	p3	(AGC)7	Class I	32091	32111	AGAGCGCAATGCAAAAGCGG	CAAGGCTCGGAGCATGTGATT	309
FMgSSR-45426	scaffold2855	p3	(AGC)7	Class I	52847	52867	ATAGGCAGCGCGAGGAGAACAA	CATGCCATGCTACGCACTGT	237
FMgSSR-45427	scaffold734	p3	(AGC)7	Class I	57962	57982	ATCCTTCGAATTCGTACCCCGACG	CCATGTCGTCACGCTGCTTGTT	324
FMgSSR-45430	scaffold905	p3	(AGC)7	Class I	83540	83560	TCCTTTGCGCCTGCTTGTCCTT	TTTTCTCGTGCGGTCGCGGATA	318

SSR_ID	Scaffold	SSR_Type	SSR_Motif	SSR Class	SSR_Start	SSR_End	Forward sequence	Reverse sequence	Product_size
FMgSSR-45432	scaffold1582	p3	(AGC)7	Class I	23674	23694	TCGCGTCGCCAAAAAGACCA	TTGCCGTAGTCGTGCACCTTGA	286
FMgSSR-45439	scaffold224892	p3	(AGC)7	Class I	517	537	TGCTGCCGACGATTCACCTTGT	ACCAACAACAAGTGGCCGAGCA	314
FMgSSR-45445	scaffold48	p3	(AGC)8	Class I	14074	14097	ACCCTTCTCTGTTCCGGCCTTT	ATGCAATCGTTCGCCCCCAT	295
FMgSSR-45447	scaffold272772	p3	(AGC)8	Class I	250	273	AGTTCCTCCGCATGTTTGGGCT	TCCACAATGTCGCGCGTGTT	274
FMgSSR-45448	scaffold71648	p3	(AGC)8	Class I	936	959	GTGGTGGTGCTGCACTGAAT	AAGGTGTTGGTGCGCATGAC	346
FMgSSR-45453	scaffold1146	p3	(AGC)8	Class I	37307	37330	TTCATGGCATGGAGCGAGGT	AGGGAGCAGAGCTCACTGAATGGA	339
FMgSSR-45455	scaffold1814	p3	(AGC)9	Class I	33175	33201	AGTGGCTGCTTGTTCTCTGCCT	TGTCAAACAGTGGCGACTGGCA	271
FMgSSR-45456	scaffold208	p3	(AGC)9	Class I	12635	12661	TCGTTCCCAATTTTCGCCGTGT	ACGAAGCAAACCCGTCAAGCAC	346
FMgSSR-45457	scaffold56163	p3	(AGG)10	Class I	1689	1718	AAGAGCGCCGCGTAGTAGTTGA	AAAGGTGGAGACGTTCCTGTGC	346
FMgSSR-45459	scaffold56163	p3	(AGG)10	Class I	1897	1926	GCACAGGAACGTCTCCACCTTT	TCCTTGCCACCTCCTCCTCTTCAT	321
FMgSSR-45460	scaffold1906	p3	(AGG)10	Class I	187	216	TCAGCATCAGGTCCTCAGGTCA	TTCGCCGACTTCAACCTCCT	335
FMgSSR-45462	scaffold349	p3	(AGG)11	Class I	121166	121198	AGCAAAAGCGTTCGACGCATGG	GCGCCCTTGTTTTGGCCATT	228
FMgSSR-45464	scaffold631	p3	(AGG)11	Class I	55868	55900	TTCACGCCGTGCTCGTCCTATT	AAGGAAGGGGAGCGGCATTT	304
FMgSSR-45476	scaffold6865	p3	(AGG)5	Class II	29816	29830	AACACGAGAAGCAGCAGCGTGA	TTGAAGCTAATCCCGCCGCCAT	251
FMgSSR-45486	scaffold9153	p3	(AGG)5	Class II	6714	6728	AACGTGTGGCAGCGTTGCTT	AAAATGGCAGAGGCAACGGTAGGG	200
FMgSSR-45487	scaffold1034	p3	(AGG)5	Class II	82711	82725	AACTCTCGCGTGACGTCGTT	GCAAGGACACGCCAGCATTGAT	344
FMgSSR-45492	scaffold38535	p3	(AGG)5	Class II	1446	1460	AAGAGGTAGCAAGTGGCGGT	ATTTTCTCTCCCTCCCATCCCCCA	205
FMgSSR-45497	scaffold7989	p3	(AGG)5	Class II	5637	5651	AATGACGCGCCATCGGATCA	TGGGAAGTTTTTGGCAGCCCGA	330
FMgSSR-45499	scaffold874	p3	(AGG)5	Class II	37768	37782	AATGCTGGAGGAGGATGGTGCT	AACCATTTCACGCGGAGCCT	290
FMgSSR-45500	scaffold657	p3	(AGG)5	Class II	10803	10817	ACAACAACACCGACGACCACCA	TGCGAGTACTTGGCAAGCGT	337
FMgSSR-45501	scaffold15133	p3	(AGG)5	Class II	12608	12622	ACAACCCACCGCTCGTTGTA	TTCCAGCATGCGAGGCAACA	340
FMgSSR-45503	scaffold6539	p3	(AGG)5	Class II	17640	17654	ACAAGCGACGATTGCAACAGCA	TAAGCGGGCAGCGTTCCATT	243
FMgSSR-45507	scaffold11853	p3	(AGG)5	Class II	13857	13871	ACATCGACTTCGACTACGGCT	AGCGGCACCAGTGGTTGATT	275
FMgSSR-45508	scaffold10322	p3	(AGG)5	Class II	23722	23736	ACATGCCCGTGGGATTCCCTTT	AAACGGCATCGTGTGCTGGT	253
FMgSSR-45510	scaffold10502	p3	(AGG)5	Class II	2440	2454	ACCAAGTCGGGCCTTGGAAA	TGGGCTGCTGTAACCGTCATCA	348
FMgSSR-45512	scaffold5289	p3	(AGG)5	Class II	40975	40989	ACCATTGCCTGCATCTGTCGCT	ACCTTTTGAGCACCGCACCA	293
FMgSSR-45516	scaffold23558	p3	(AGG)5	Class II	2272	2286	ACCGCCGCCAATGCTAACTCAT	ACGTCACCACGCGGTCTATT	338
FMgSSR-45523	scaffold108074	p3	(AGG)5	Class II	55	69	ACGATTCGCATAAGCCGGAGGA	TTTTGATGCGTGGGCAGCGT	307

SSR_ID	Scaffold	SSR_Type	SSR_Motif	SSR Class	SSR_Start	SSR_End	Forward sequence	Reverse sequence	Product_size
FMgSSR-45525	scaffold8657	p3	(AGG)5	Class II	16711	16725	ACGCCATGGCAACGTCAGAA	TCATGAGCAGCACCCACGTT	297
FMgSSR-45527	scaffold5296	p3	(AGG)5	Class II	2910	2924	ACGCGCCCGTACCCTTCTAATA	AGTACACGTGCCTTGACGGGTT	238
FMgSSR-45530	scaffold7718	p3	(AGG)5	Class II	18403	18417	ACGCGTGATGTAGACCGTCGAT	TGGTGCACCTCGTTGACGTT	229
FMgSSR-45531	scaffold6628	p3	(AGG)5	Class II	19379	19393	ACGTCGTCGGATCCTGCTTT	TTGAGCCGGTGTTCCACGTT	237
FMgSSR-45537	scaffold3	p3	(AGG)5	Class II	31961	31975	ACTGCATGCCGCTACTGCAA	TGCTCACACCTCGTTTTCGCCT	229
FMgSSR-45539	scaffold3545	p3	(AGG)5	Class II	13084	13098	ACTTCGAGTGCAGGGAGGCTAA	AGTTTCAGAGGCTACTGCGCT	342
FMgSSR-45543	scaffold1735	p3	(AGG)5	Class II	24955	24969	AGCACGGAGCTCATGGTGTAGA	ATCGCCACCGCCGATGATAA	245
FMgSSR-45544	scaffold302	p3	(AGG)5	Class II	64160	64174	AGCAGCACCAGCGCCTTTTT	TTGCACGTCGTCGAGCAGGTAA	345
FMgSSR-45547	scaffold11958	p3	(AGG)5	Class II	17944	17958	AGCATCTCATCCGCGGCAAA	AAACATCAGCAACCACCGCTGC	330
FMgSSR-45548	scaffold9038	p3	(AGG)5	Class II	23209	23223	AGCATGGTTGTCGTCTCGAGGT	ACGGCGCCACAGCTGCTAATAA	287
FMgSSR-45550	scaffold1776	p3	(AGG)5	Class II	64901	64915	AGCCATGGAACCAGGAGACGAA	TCCCGCGCCCGTTTTTCTTT	302
FMgSSR-45551	scaffold2619	p3	(AGG)5	Class II	25303	25317	AGCCGTCCAGGAGGCAGTTATT	TGCGAGCGTGCCGTGTCATTAT	236
FMgSSR-45555	scaffold9087	p3	(AGG)5	Class II	30464	30478	AGCTGCGGCTGCTGATTGAA	ATTGATCCGACGTCTCGCGCTT	344
FMgSSR-45557	scaffold13564	p3	(AGG)5	Class II	2142	2156	AGGACGAGGCAATGCAGAGT	ATCTTTGGTCTGCTCCAGACGTGC	291
FMgSSR-45568	scaffold17995	p3	(AGG)5	Class II	7489	7503	ATAAAGTTGGCGCCTCCACG	ATGACAGGCCTTTGGGCAGCAA	244
FMgSSR-45584	scaffold703	p3	(AGG)5	Class II	42491	42505	CCGCCTCGTCTGTGCGATTATT	TCCGCTTCAGCTGCACCTTT	217
FMgSSR-45596	scaffold48	p3	(AGG)5	Class II	129229	129243	GCCGCTTGATGATCGTGATCTCCT	TTCGACTTCACGCTCGGGTTCA	206
FMgSSR-45598	scaffold6048	p3	(AGG)5	Class II	5275	5289	GCGCTTGCTCATCGGCTCATTT	TGGCACCCAAGGCCCTTAAT	288
FMgSSR-45599	scaffold3010	p3	(AGG)5	Class II	23057	23071	GGCGCCATTGGCCTTTTGTT	CGTGCTGCTGCCCATTGTTGTT	315
FMgSSR-45604	scaffold7474	p3	(AGG)5	Class II	29470	29484	TCAAACACACAAGCACGGCAGC	ACGTTGTAGCCTGCTCGCAAA	200
FMgSSR-45607	scaffold2906	p3	(AGG)5	Class II	62960	62974	TCACATACGCGTACCGGCTT	TGCAGCTGGACCGAAAGATCGAA	221
FMgSSR-45608	scaffold5623	p3	(AGG)5	Class II	24846	24860	TCACATGCCCGTGGATTCCCTT	ATGCTCCACAGCAATCAACCGC	284
FMgSSR-45615	scaffold6816	p3	(AGG)5	Class II	8064	8078	TCCAGTTCCAGCGTGTGGTTCA	ATGGCGGTGACGTTGTGGAA	313
FMgSSR-45616	scaffold3240	p3	(AGG)5	Class II	12264	12278	TCCATTCGCTTGATCCGTGGTGT	TGCCTGCCGTGCACAATCAA	258
FMgSSR-45630	scaffold21100	p3	(AGG)5	Class II	3905	3919	TGATTTCGCGCAACAGGGCA	TTGTTCCCGAACCGCACCAAGA	309
FMgSSR-45635	scaffold549	p3	(AGG)5	Class II	83701	83715	TGCATCCCATTCGCGCAAGTT	TGCTCCTCGTGCAGAAATGGT	270
FMgSSR-45636	scaffold614	p3	(AGG)5	Class II	36725	36739	TGCGCAAGTGCAAGAACTGCT	AACAAAGATGCGGGCTTCGC	333
FMgSSR-45637	scaffold1554	p3	(AGG)5	Class II	74682	74696	TGCGCCTAGAGCCAAAAACCGT	ACACTGTGACCAGTGCGTGGAT	329

SSR_ID	Scaffold	SSR_Type	SSR_Motif	SSR Class	SSR_Start	SSR_End	Forward sequence	Reverse sequence	Product_size
FMgSSR-45638	scaffold4490	p3	(AGG)5	Class II	39248	39262	TGCGCTGGTGTTCTGTAGCTT	TGCTCTTGCTAGGTCACCGT	317
FMgSSR-45640	scaffold4051	p3	(AGG)5	Class II	48948	48962	TGCGTTCGTGATTTTTGGGCGT	ACCTGGTCCATCTTGAAGGTGTGG	346
FMgSSR-45641	scaffold5107	p3	(AGG)5	Class II	4820	4834	TGCTGCTCGATGAGGTCCATGA	TGCTTTGCTTGGCGTGGACA	347
FMgSSR-45643	scaffold708	p3	(AGG)5	Class II	93169	93183	TGGAAGTCGTCTTCGCTGCT	TTGTTGCCTGCGGTTGTCGT	260
FMgSSR-45647	scaffold262	p3	(AGG)5	Class II	17986	18000	TGGCCAAGCTGAAACGAGCA	AAGCCCAGCCAAACACCTTGGA	300
FMgSSR-45648	scaffold96950	p3	(AGG)5	Class II	899	913	TGGCGATGAAGGCAGCGATT	TGTGCCATGGCCTCCTTTTCCT	347
FMgSSR-45650	scaffold12740	p3	(AGG)5	Class II	14210	14224	TGGCTGCCCTTGCCCATCTATT	ATGTCGTCGTTGCTGCTCTGCT	268
FMgSSR-45651	scaffold17537	p3	(AGG)5	Class II	9281	9295	TGGGCGTTTTACAGGGGAGA	ATTACAACACGCGCTGGAGGGT	273
FMgSSR-45666	scaffold13706	p3	(AGG)5	Class II	7826	7840	TGTTCGTGAACAATCGCGTGCT	TACTTGCGGAAGAGCGCGTA	283
FMgSSR-45669	scaffold2633	p3	(AGG)5	Class II	13443	13457	TGTTTCACTTGCAGCCGCCAT	TCCTTGACCGGCTTATTGTCGCA	310
FMgSSR-45670	scaffold2502	p3	(AGG)5	Class II	36231	36245	TTACACGCGCAGGAAATGCG	ACGTACCGCTGCTTTCGTTCA	240
FMgSSR-45677	scaffold1128	p3	(AGG)5	Class II	35690	35704	TTCCTGTTGCTGCTTCCGCA	AACTTGAGGCTCCCGCACTTCT	292
FMgSSR-45679	scaffold2544	p3	(AGG)5	Class II	43070	43084	TTGCAACCACCTAGCAGCAGGA	AATGGGCACCGCGTCAAATTCC	347
FMgSSR-45682	scaffold4683	p3	(AGG)5	Class II	9515	9529	TTGGCGACGACGGGTTCGAAAT	AACCGAAATCGCCCGCAAGA	227
FMgSSR-45695	scaffold295	p3	(AGG)6	Class II	102004	102021	AACGCCGGCCACACTTTGAA	TGAAAACGAAACGCAGCGGGT	260
FMgSSR-45696	scaffold6155	p3	(AGG)6	Class II	24727	24744	AACGGTAGCACGCAAGACGA	TGGCCGCCATATAAACGCCACT	323
FMgSSR-45699	scaffold3189	p3	(AGG)6	Class II	50893	50910	AACTAGTACGCCGCTCAGCAGT	TTGTTTGGGGAAAGGCCGCA	294
FMgSSR-45700	scaffold558	p3	(AGG)6	Class II	92497	92514	AACTGGTGGTGGTGCAGGTCAA	AGTGGTGCCATGTCGTCGTT	210
FMgSSR-45701	scaffold450	p3	(AGG)6	Class II	15428	15445	AAGAATGAGCCAGCAGCGAAGC	GCAGCAAAGAACATGGCGGTGA	210
FMgSSR-45708	scaffold13766	p3	(AGG)6	Class II	8095	8112	ACAAACAAGCGCCGTGGTCA	ACGTGTCGTCGTCATTGCGT	275
FMgSSR-45713	scaffold169	p3	(AGG)6	Class II	60431	60448	ACCGCAAGCTGTTTGTCCGA	TCGCAAGCTGCACGCAGAAA	330
FMgSSR-45722	scaffold11771	p3	(AGG)6	Class II	6423	6440	AGAGAAATGATGAGCCACGGCG	TCGACGCCGGGTAACGTATGAA	341
FMgSSR-45725	scaffold1863	p3	(AGG)6	Class II	5105	5122	AGCAGGTGGCATCACAGTCT	GGGCGTCTAAATCATCCTTCACTGC	334
FMgSSR-45726	scaffold11383	p3	(AGG)6	Class II	14328	14345	AGCCGCCCAATTTGGCAACAA	TCAAGCAGCGAGTTGAGCGA	327
FMgSSR-45739	scaffold7678	p3	(AGG)6	Class II	6385	6402	ATGAAGACGTTGTTCACGCGCC	GTGCGCCTCACTTCAACTCACA	306
FMgSSR-45742	scaffold194	p3	(AGG)6	Class II	152463	152480	GCAGGCCATGCGTCGTTTTT	TACCGTGACCGAAGCAATGCGA	348
FMgSSR-45743	scaffold187871	p3	(AGG)6	Class II	404	421	GCATCATCATGGAGACCCGAGACA	ATGGCATTGCTCCACGGGAA	337
FMgSSR-45744	scaffold3152	p3	(AGG)6	Class II	58244	58261	GCGTTGGTTTTGCGTTCCGT	TCACGATGAAGAGCGCGGTGAT	298

SSR_ID	Scaffold	SSR_Type	SSR_Motif	SSR Class	SSR_Start	SSR_End	Forward sequence	Reverse sequence	Product_size
FMgSSR-45745	scaffold14619	p3	(AGG)6	Class II	3100	3117	GGTGACGGCCAAAAACGCATGA	TGGTAGTGTTGCCCTCCTCCTT	350
FMgSSR-45749	scaffold814	p3	(AGG)6	Class II	27370	27387	TAGTGCTGCAAAGGTGCGCT	ACTTGTGCTGCCGAGAATGCCT	342
FMgSSR-45753	scaffold8639	p3	(AGG)6	Class II	14327	14344	TCGACGTGGGTGCACATGGATA	ACGACACCTGCGACATCCTCCTAT	347
FMgSSR-45763	scaffold1216	p3	(AGG)6	Class II	34084	34101	TGCGTCGTCATCGTCGTGAA	TGCGGGCCACCATTAACACA	278
FMgSSR-45766	scaffold3276	p3	(AGG)6	Class II	19235	19252	TGGCGGTTATGACGAAGCCA	TCGTGCCCCCATGCAATGAA	272
FMgSSR-45775	scaffold320	p3	(AGG)6	Class II	53107	53124	TTCGCTCGGAGGAGGAGGAAAA	GCCACGATGTGGCAGTTGAAGT	228
FMgSSR-45776	scaffold3521	p3	(AGG)6	Class II	33887	33904	TTCTGTAGAAGCTCCAGCCGT	TCAATGTCCATCGGCAAGCG	350
FMgSSR-45784	scaffold1296	p3	(AGG)7	Class I	27906	27926	AATGAGGAGGTGGTCTCCTTCGGA	AAAGGCAACAAAGCGGCAGCA	200
FMgSSR-45787	scaffold227	p3	(AGG)7	Class I	62822	62842	ACGAGGTTGGTGATCTGGCGTA	AATAGCTAGACGGGCTAGGCGACA	328
FMgSSR-45793	scaffold4330	p3	(AGG)7	Class I	32186	32206	AGGAGCCGCCGTGGCTTATTAT	AAAGCATGGACTGGCACGCT	280
FMgSSR-45795	scaffold3783	p3	(AGG)7	Class I	39746	39766	AGTCAACGGCACCATCGTCA	TGTGCGTGCAGTGTTCCAGT	325
FMgSSR-45804	scaffold2199	p3	(AGG)7	Class I	69041	69061	TCTCTCCCCAGCAAGCAAGCAA	ACGCCGAAGCAACAGCTGAA	219
FMgSSR-45807	scaffold4015	p3	(AGG)7	Class I	1228	1248	TGCGCGCCACGTTCTTGAAA	ACTCCGTCGTCCAACTGCTCAA	207
FMgSSR-45809	scaffold13256	p3	(AGG)7	Class I	5659	5679	TGCTTATCTGCCCCACGTCACT	TGGTTGACGGCAGTGAACAAGT	333
FMgSSR-45814	scaffold12840	p3	(AGG)8	Class I	10874	10897	AACACCACCAACAGGCACCACA	AGCCTCACCCTGCCATTCCATT	296
FMgSSR-45816	scaffold5879	p3	(AGG)8	Class I	3237	3260	AATGCCGTGGCCGACAAGTT	TTGCCGATGGGTTCGTGCTT	302
FMgSSR-45822	scaffold65	p3	(AGG)8	Class I	57405	57428	GCGTTTCGCGCGCTTTTCTA	TCTGCGACGGCGAGAAATTGT	228
FMgSSR-45831	scaffold3506	p3	(AGG)8	Class I	42613	42636	TGTTGACGATGCGCTAGCCTGT	TGTGTGACGCGGCAGAGTTT	214
FMgSSR-45832	scaffold436	p3	(AGG)8	Class I	37996	38019	TTGGAGATGTTCCCCCGCTTGA	ACATTGCAAAGCACGGCGAG	272
FMgSSR-45837	scaffold30	p3	(AGG)9	Class I	134047	134073	TCATCAGGCACAAGCCGCAA	AAGCAGCAGCCTTGTTCCGT	311
FMgSSR-45840	scaffold1486	p3	(AGG)9	Class I	48906	48932	TGCTCAAAGCACACCCAGCA	TCCACACGAACAACGTTCCCGA	222
FMgSSR-45842	scaffold174306	p3	(AGT)15	Class I	341	385	TGATGAGGGCCAAAGGCAGCAA	TTCGGCGCCATCGTGTTTGA	260
FMgSSR-45845	scaffold4586	p3	(AGT)5	Class II	29243	29257	AAAGGCGAAGCCACACGGTT	TCGCGATCATCCAAAGGCTTGC	328
FMgSSR-45854	scaffold86	p3	(AGT)5	Class II	68299	68313	ACGACCAAGAAACGCCGCAT	TGCTTGCTCCCCTGGAATGCTT	287
FMgSSR-45855	scaffold1935	p3	(AGT)5	Class II	26062	26076	ACGAGATGCCGAGCGTTGTT	AGGGGCATGTGCGTGTTTCT	345
FMgSSR-45856	scaffold392	p3	(AGT)5	Class II	84442	84456	ACGCCCAACAACAACTCCCAGT	TGCCTTTGTGCAAGACCATGCC	286
FMgSSR-45858	scaffold2595	p3	(AGT)5	Class II	12150	12164	ACGGAGGTAGCAGGCATGAGAT	CGGAATGTTGGATGCGAGCGTA	244
FMgSSR-45861	scaffold214	p3	(AGT)5	Class II	82465	82479	ACTGATGCCCAATTGACCCCCT	TGGTCCCTGCGTGTACTGCAAA	308

SSR_ID	Scaffold	SSR_Type	SSR_Motif	SSR Class	SSR_Start	SSR_End	Forward sequence	Reverse sequence	Product_size
FMgSSR-45862	scaffold15415	p3	(AGT)5	Class II	1096	1110	ACTTGCCGCAGATTGGTGCT	TCACCGGGCTAAACGAACCACT	310
FMgSSR-45864	scaffold207	p3	(AGT)5	Class II	75352	75366	AGCTCATCACGTGCCCGTTT	AAAGAGCGACCCATCCTTGTGC	265
FMgSSR-45866	scaffold558	p3	(AGT)5	Class II	91713	91727	AGGATGCAACACCTTGGCTGA	ATCGAATCTGTGCCCTGCCTGT	328
FMgSSR-45869	scaffold7515	p3	(AGT)5	Class II	24530	24544	ATGGCGCGGAATCTTTTACGGC	TGGTGGTGGTGGGTGCTTGTTT	340
FMgSSR-45870	scaffold2874	p3	(AGT)5	Class II	29174	29188	ATTGCCACGCAGGCATGCAGAA	CGGAACGTCGTATCGGCATGTA	306
FMgSSR-45875	scaffold5797	p3	(AGT)5	Class II	11205	11219	CTGTGTTTGCAACGCACGCA	TCCGTGCACCAACCCACTCAAT	349
FMgSSR-45881	scaffold3566	p3	(AGT)5	Class II	15950	15964	GCTTTGAAGAGCCTGACTACCAAGG	CGAGCGTTAAGATGTGGTGAGTCC	299
FMgSSR-45883	scaffold1367	p3	(AGT)5	Class II	70122	70136	TAAGCAGCGGCCGGGTGATAAT	ATCTTGCATTGGTCGCGGCA	332
FMgSSR-45884	scaffold4605	p3	(AGT)5	Class II	12463	12477	TCAGCTTGGTGCCTCACGTCAA	ATGGACGCGCTTGCAGAGTT	310
FMgSSR-45885	scaffold1996	p3	(AGT)5	Class II	32823	32837	TCATGAGCGTGCAACGGGAA	AGCAACCAAGCAGCAGCAGT	276
FMgSSR-45887	scaffold2047	p3	(AGT)5	Class II	6167	6181	TCGGAGCTTTCCTTCCTTCCGA	ACGCCTCGAGTTTCCCTTTGGA	280
FMgSSR-45889	scaffold1068	p3	(AGT)5	Class II	81465	81479	TCTTGCCTTTTGCCTGGCGT	TGTGCAACGCATCACACGCA	216
FMgSSR-45890	scaffold4532	p3	(AGT)5	Class II	23149	23163	TGAGGGTTGCCTAGACCAAAGGGA	TGTGAGCACACGAGGGTGAA	213
FMgSSR-45891	scaffold440	p3	(AGT)5	Class II	68878	68892	TGATAACATGAGCGGCGGCA	TGGGTGGACAACAACGGGTTCA	254
FMgSSR-45895	scaffold4237	p3	(AGT)5	Class II	5831	5845	TGCGGGACGCATGCAAATTCA	GAAGCCGGCGGCATTAAGAT	266
FMgSSR-45896	scaffold785	p3	(AGT)5	Class II	68924	68938	TGCGGGTCATTCACGCAGTA	GCTTGATGTTAGCGAAGCCTGTCA	350
FMgSSR-45900	scaffold4623	p3	(AGT)5	Class II	17279	17293	TGGTTGGGCGCGGAATCTTT	ACGAGCGCTGGATTCCGTTT	289
FMgSSR-45902	scaffold287	p3	(AGT)5	Class II	115679	115693	TGTGGGATCGGAGCTCCATGAT	TCCTGCATACACGACTCTGCCA	227
FMgSSR-45905	scaffold433	p3	(AGT)5	Class II	41903	41917	TTCTGGCGCAGTTTTGCCCT	TCCGGCCTCTCGGCAAAGTAAA	326
FMgSSR-45906	scaffold1842	p3	(AGT)5	Class II	48995	49009	TTGCGTGGTCACTGGACAGGAA	TTCCCGTGCTAGTTGTGTGCCT	331
FMgSSR-45908	scaffold5912	p3	(AGT)5	Class II	38293	38307	TTGGCGAGTGGCAGCAGTGTAA	TGCGCGGCAACACACCAAAA	244
FMgSSR-45911	scaffold7239	p3	(AGT)5	Class II	30821	30835	TTGTTCCTGGCTGCTCCACA	TGTCACCATGTTGCCGCTGT	350
FMgSSR-45912	scaffold204	p3	(AGT)6	Class II	78717	78734	AAAGAAAGGGAAGGCGAGGCGA	CGTGTGTGCAAGTGTGTGTGCT	274
FMgSSR-45914	scaffold1486	p3	(AGT)6	Class II	62751	62768	AACTGGCCGTTCCACCATGTCA	AACAACAACCTGGCGTCGCT	210
FMgSSR-45916	scaffold8	p3	(AGT)6	Class II	122981	122998	ACCAACGGCAGCTGTGCTGATA	TGCCATCGTTCGCGATTCGT	272
FMgSSR-45921	scaffold4257	p3	(AGT)6	Class II	44678	44695	AGGCACGTTGTCACGTCAACCA	CGACCAAGGCAAACGCCAAACA	339
FMgSSR-45922	scaffold4944	p3	(AGT)6	Class II	26592	26609	AGTAGACGACGTACACACGCA	ATCATGGGTGGCACCGACAACT	343
FMgSSR-45924	scaffold109	p3	(AGT)6	Class II	113994	114011	ATGCGTACACAAGAGCGGCTGA	ACTTGCACGACGTTCCGTGT	298

SSR_ID	Scaffold	SSR_Type	SSR_Motif	SSR Class	SSR_Start	SSR_End	Forward sequence	Reverse sequence	Product_size
FMgSSR-45925	scaffold162222	p3	(AGT)6	Class II	375	392	GAATCGCTGCACACGCACAA	CCATCACTGGCCATTTCACCCT	350
FMgSSR-45928	scaffold140	p3	(AGT)6	Class II	131022	131039	GGGCCATTGCAAGGAGGAAAGA	AATTTGAGCCACTCGCCGTCCA	290
FMgSSR-45929	scaffold2459	p3	(AGT)6	Class II	28024	28041	TCTAGTTGTTGACCCGCCGT	GGTGAGCATGCGTGCCAAAA	257
FMgSSR-45930	scaffold140	p3	(AGT)6	Class II	173265	173282	TGCAGTAGACGCGCAGCTGAAT	TAAAACGGGAACGGCTGCAACG	311
FMgSSR-45936	scaffold346	p3	(AGT)8	Class I	55422	55445	TGCTCTGTTTCGCACGAACCCT	ACGTGTGCTGGTCAATCACCGA	267
FMgSSR-45939	scaffold1549	p3	(ATA)10	Class I	68955	68984	GCGACGGCTGCGCTTAAGTTTT	ACCAGTCTCGTTGCTTGTCGAA	222
FMgSSR-45943	scaffold4225	p3	(ATA)12	Class I	37518	37553	ACCGGCAGGCAAAATGCCAA	ACATCGGGTGTTGCCATCACGA	279
FMgSSR-45944	scaffold40389	p3	(ATA)12	Class I	1023	1058	CCGCAGCATAGTAGGAAAGAGGCA	TTGCCCATGAGTCTCGCAAGGT	252
FMgSSR-45946	scaffold17207	p3	(ATA)13	Class I	15469	15507	ACGAGAAACCCACAGCAGGCAT	ATGGCGCCGCCTCTTATCATCA	264
FMgSSR-45953	scaffold31697	p3	(ATA)16	Class I	3698	3745	TCCCAACAGCAAGCAGACGA	TGCAGACACTGGCTTTCGCT	347
FMgSSR-45954	scaffold4549	p3	(ATA)16	Class I	18199	18246	TTTGTGAACACGGAGGAGGGGT	TCCTCCCCTCGCCGCTTATTAT	283
FMgSSR-45956	scaffold17112	p3	(ATA)19	Class I	1991	2047	ACGTGAGTCAGGACGACAGCTT	TCTGTGAGAACAGCTGGGCA	350
FMgSSR-45958	scaffold2295	p3	(ATA)20	Class I	18090	18149	AAAGACGGGAGGGAGTACAAGC	CCAGGTTTCCTTCAGTTGCTGGGT	214
FMgSSR-45963	scaffold8812	p3	(ATA)20	Class I	15720	15779	TCGTGAAGCCTCCATCAACCGA	AGCGCCTAAGCAATTCTGCAAGG	315
FMgSSR-45965	scaffold1312	p3	(ATA)20	Class I	20043	20102	TTGAAGACGACGATGACCCCCA	AGTGGGATGGCCTGCACATTT	314
FMgSSR-45966	scaffold6489	p3	(ATA)22	Class I	5855	5920	TCGCTGAGGGCATTGCATCT	TGGGCAAAACGTGACAAGTGC	238
FMgSSR-45968	scaffold11144	p3	(ATA)5	Class II	14442	14456	AACTTGCCTCACTGCGCTCT	ATTTGCGCTGGAAGGCGTGA	346
FMgSSR-45980	scaffold2818	p3	(ATA)5	Class II	64752	64766	ACCGAGTGCATGGGAAGTCTGA	AGCTGAGCCTGTGTTGTTTGCT	243
FMgSSR-45981	scaffold7263	p3	(ATA)5	Class II	33610	33624	ACCGTAACGATGAAGCTCGCGT	TTACGGGCTACTGCAGTCCTCT	306
FMgSSR-45987	scaffold314	p3	(ATA)5	Class II	3879	3893	AGCAATGAGCGATCACCACCTG	AACATGCATGGCGTTGGCGT	272
FMgSSR-45989	scaffold1659	p3	(ATA)5	Class II	62469	62483	AGCCAAAACACGACAACCCCCA	TCCGTAATGCTCTACCCAGGCA	342
FMgSSR-45999	scaffold29364	p3	(ATA)5	Class II	4997	5011	ATCGAACGACGCCTGCACTT	TGCCCAACAGTCACTCTGTAACTC	346
FMgSSR-46005	scaffold157	p3	(ATA)5	Class II	30279	30293	CCAGCTGGTAATGACCCAACCTGT	AGCGGCACTGTAATACCAACACCA	230
FMgSSR-46009	scaffold533	p3	(ATA)5	Class II	22006	22020	GCAACGTGCAGCAACAAGTGTC	ACGACGGTGGCATCTCTTTTCAC	349
FMgSSR-46017	scaffold8389	p3	(ATA)5	Class II	26438	26452	GCGATCTTGTTCTACGTGCACACC	ACCACCATCACGACAGTACCGA	334
FMgSSR-46018	scaffold1258	p3	(ATA)5	Class II	52274	52288	GGAGCCAACCAGCGTCAAAT	TGGAGGTGACAGCGATGCAA	232
FMgSSR-46024	scaffold84373	p3	(ATA)5	Class II	1297	1311	TCACCACCACCTGCTGTTTCA	ACCCTCCACTATCAGGAGTGCAA	295
FMgSSR-46029	scaffold118946	p3	(ATA)5	Class II	823	837	TCCGCTGTGCTTGACCTGAACT	ACTGACGGCCTGCCAGAAAGAA	304

SSR_ID	Scaffold	SSR_Type	SSR_Motif	SSR Class	SSR_Start	SSR_End	Forward sequence	Reverse sequence	Product_size
FMgSSR-46030	scaffold10811	p3	(ATA)5	Class II	1180	1194	TCCGGAAGTCTGACGTTTCTCCA	TTCATCTCGCCACACCGTCACT	305
FMgSSR-46035	scaffold7171	p3	(ATA)5	Class II	13621	13635	TGACATGGTCGTGATGGGCTGA	TGCGTCTTCTCCCCTTCCTTGT	240
FMgSSR-46037	scaffold2918	p3	(ATA)5	Class II	12351	12365	TGCAGGATGAACGTGCCCAA	AATTATTCGAAACGCCGCCGCC	331
FMgSSR-46038	scaffold1374	p3	(ATA)5	Class II	17720	17734	TGCATGCTGCTGCTGCCATT	ATGTACGCGCTCAACTGCTTCG	200
FMgSSR-46043	scaffold504	p3	(ATA)5	Class II	111828	111842	TGCGTGTGCTTCTGGAGGTCAA	AAATGGCCTTCAGCGCTTGG	319
FMgSSR-46050	scaffold32805	p3	(ATA)5	Class II	1589	1603	TGGGGGAGAGAAATATGGACACCC	AGTTGGCTAACAGTGTGGCCT	336
FMgSSR-46052	scaffold10430	p3	(ATA)5	Class II	11585	11599	TGTATCTGGCTTTGGGGCGA	AGCGTTCAGGAAATGCCCGT	347
FMgSSR-46053	scaffold1588	p3	(ATA)5	Class II	34784	34798	TGTCCGCTCAGCTCGGCAAATA	ACCTCTAGCTACCTCGCAACCA	218
FMgSSR-46054	scaffold7443	p3	(ATA)5	Class II	22837	22851	TGTGGCACACATCGAGCCTT	AGGCTGGGATTTCATGAGCACCT	267
FMgSSR-46055	scaffold190	p3	(ATA)5	Class II	97286	97300	TGTGTGGATCTGTCACGTTGC	GTCCCAGATAACTGTGAACCGTCT	280
FMgSSR-46058	scaffold1301	p3	(ATA)5	Class II	1588	1602	TTGCATTGCCCTCGGTGGTT	TGAAGCTCTCAGTCTCAGTCCG	347
FMgSSR-46059	scaffold5095	p3	(ATA)5	Class II	22957	22971	TTGGTGTGCATCGTCGCTCT	TGTACCCCGTGGTGCAAGAGAA	236
FMgSSR-46061	scaffold103039	p3	(ATA)6	Class II	773	790	AAGCAAACCGGCTGACCGAA	ATTGCTGCCCTGGTATGTCGGA	249
FMgSSR-46062	scaffold752	p3	(ATA)6	Class II	30855	30872	AAGCTGTACGGTTTCCCTGCGT	TTCGGTTGGTCGTCTCCCTTGA	341
FMgSSR-46065	scaffold94	p3	(ATA)6	Class II	170514	170531	ACGTTGTTGCAGATGTTGGCGG	CGTGATTTTCGGGCGGGTAT	319
FMgSSR-46069	scaffold4296	p3	(ATA)6	Class II	3371	3388	AGCGTGTGCAGAAACGGCAA	AGCTCCACATGGCCGGATCAAA	298
FMgSSR-46074	scaffold10512	p3	(ATA)6	Class II	15229	15246	ATTTTCTGTCGACCACCACGG	TGGATGATTCCCACACGAGTGCT	324
FMgSSR-46075	scaffold370220	p3	(ATA)6	Class II	46	63	CCTCCGTGCCGTGATTTTCCAA	CAGCCGGCCAATAAAGGCAA	252
FMgSSR-46089	scaffold9010	p3	(ATA)7	Class I	24512	24532	ACCACCCGTTGCAGTGGAACAT	ACTGCCGCCAGTAGCAAACTGT	322
FMgSSR-46090	scaffold26743	p3	(ATA)7	Class I	3436	3456	AGCTCGTCTGGACGCGTAACAT	ACGTCCAGTGGCTGCTCAAT	272
FMgSSR-46092	scaffold341	p3	(ATA)7	Class I	36784	36804	GGGCAATTTCTCTTTTCCAGGCCG	CCGCCATCACCAAGGACTTCAA	348
FMgSSR-46101	scaffold19646	p3	(ATA)8	Class I	15518	15541	CGAGTCATTTTAAGGCGCCAGT	TTCCGAGTCCACTTGCCAGACA	335
FMgSSR-46105	scaffold2601	p3	(ATA)8	Class I	56279	56302	TGTGTGTTGCACTCTTGCCGA	ACACTTGCCTCTATGGCTCTATCC	246
FMgSSR-46107	scaffold350	p3	(ATA)9	Class I	36793	36819	AGAACGGAGGCTCCAGATCGTT	GCAGCGCACGCTCTTCAAAA	256
FMgSSR-46109	scaffold16903	p3	(ATA)9	Class I	10583	10609	CGTGGCGTCGTCAGTATACGAAA	TGGACGTGCTGCAAAAACCA	350
FMgSSR-46115	scaffold340	p3	(ATA)9	Class I	78051	78077	TGGTGCATGTGGATCAGGGT	TGCATGCATCATGTTGCCCA	244
FMgSSR-46133	scaffold134	p3	(ATC)11	Class I	179705	179737	GCACACAATCAAGGTCCAAGCCA	AAGCAAGAGCCTAGTGCACGGA	275
FMgSSR-46140	scaffold314074	p3	(ATC)12	Class I	128	163	TGGCACCTCCTATGCTATGCCAGT	TTTGGACTGCTGGCGTCCTT	324

SSR_ID	Scaffold	SSR_Type	SSR_Motif	SSR Class	SSR_Start	SSR_End	Forward sequence	Reverse sequence	Product_size
FMgSSR-46142	scaffold504	p3	(ATC)12	Class I	88283	88318	TTGTTCGTCCGCAGCTAGGTCA	TTTAGGCAGGCTGGGTCTACGA	282
FMgSSR-46148	scaffold1247	p3	(ATC)5	Class II	92332	92346	AAAAGCATCCCAATCGTCCCCC	AGGACATGGTCCGAGCTTCAGT	209
FMgSSR-46159	scaffold13716	p3	(ATC)5	Class II	14862	14876	AAGTCAGCGACTTTGAGCACCC	TGACCGGTACTTCACGGACGAA	254
FMgSSR-46161	scaffold18146	p3	(ATC)5	Class II	11804	11818	AATGCAACACCGCAAAGCCC	AGAGAGCGCAGTGAGCAAGT	221
FMgSSR-46162	scaffold662	p3	(ATC)5	Class II	78361	78375	AATGCACTTACGGGAGGCGA	TTGCAGAACGCCGACGCAAT	227
FMgSSR-46171	scaffold5717	p3	(ATC)5	Class II	2281	2295	ACATGCCGTTCTGCGGTTCT	ATCACCGGCGGCAAAATGGT	283
FMgSSR-46173	scaffold8233	p3	(ATC)5	Class II	8273	8287	ACCAATGTCATGGACCACGGCT	TGTGGTAGAACCTCGTATGCCTCA	342
FMgSSR-46175	scaffold354	p3	(ATC)5	Class II	112345	112359	ACCACGTCGTCAATAAGGCCCA	ACTTGCCGTAGAAACTGCTGCTG	201
FMgSSR-46189	scaffold49340	p3	(ATC)5	Class II	1445	1459	ACGGACGCACGAAGTTGTAGCA	TGGAGCCATGCGCTGATAAGGT	319
FMgSSR-46190	scaffold367	p3	(ATC)5	Class II	22225	22239	ACGGCAGCAACAAGTGCGAA	TCGAAACGGCGCAAGTTGGT	290
FMgSSR-46198	scaffold4856	p3	(ATC)5	Class II	43010	43024	ACGTGACGCAACACGGGAACTA	TCGCGTGAAATCGCCACGAA	322
FMgSSR-46199	scaffold1201	p3	(ATC)5	Class II	84604	84618	ACTCCAAGCAACAAGCGGCA	AGTTCTTCGCCGGTGGCATT	224
FMgSSR-46202	scaffold8522	p3	(ATC)5	Class II	27615	27629	ACTTGCAACCCTTGTCCGCT	AGGGTGGACACTCTGACCCAAA	339
FMgSSR-46236	scaffold18753	p3	(ATC)5	Class II	8346	8360	AGGAACGGATCAGGCATGCAA	ACAAGTAGCGAGAGCCGAGT	288
FMgSSR-46238	scaffold5344	p3	(ATC)5	Class II	21205	21219	AGGAGCATTGTGGTCTTGGTCG	AGGAGGCAAAGGAGTGGAGCAT	340
FMgSSR-46271	scaffold862	p3	(ATC)5	Class II	51311	51325	ATCGCTTGCACCGCCAAACA	ACGCGTCGTAGATGGCCACATT	299
FMgSSR-46273	scaffold10931	p3	(ATC)5	Class II	14219	14233	ATGCATGGGTCGTAGGTGTGGA	TGGGAAGACACATGAGTTGGGCA	344
FMgSSR-46283	scaffold2437	p3	(ATC)5	Class II	15549	15563	CACGCACGTCGCTGCAAAAT	AGTTGCTCAACAACCCAGCCCA	294
FMgSSR-46286	scaffold22006	p3	(ATC)5	Class II	10932	10946	CCATTTGTTGCTGGCACCGTCA	AATGCTCCATACGGGATTGGGACG	350
FMgSSR-46289	scaffold1045	p3	(ATC)5	Class II	52893	52907	CCGTGCGTAATCAGCCTCTGTT	TGCAAGGCGTTCTTCGGCAA	252
FMgSSR-46290	scaffold117111	p3	(ATC)5	Class II	210	224	CGAAGCAAGTTCCCTAGACTCAGCA	TGAAGAGACTGCTGCCTTGTGA	264
FMgSSR-46292	scaffold4641	p3	(ATC)5	Class II	40927	40941	CGCCGGCAAAATGAGGAGAT	ACAAGCAGCAAACAACGCGACA	265
FMgSSR-46331	scaffold221	p3	(ATC)5	Class II	25932	25946	TCAAAAGCAGCCCGCTCGAT	TTTGGGCGTGCTTGGGTTGT	305
FMgSSR-46335	scaffold284	p3	(ATC)5	Class II	95916	95930	TCAGCTTCTTGCGTGCCAGAGT	ACGGGTGCTGGAGCTAACATGA	322
FMgSSR-46337	scaffold95	p3	(ATC)5	Class II	15243	15257	TCCACCAAGCACCAACGTCACA	ACGCTTACGGCGATGTCGTT	322
FMgSSR-46347	scaffold1117	p3	(ATC)5	Class II	22874	22888	TCCTCTGCGCGTTGCTTTGT	TGCGAGTGTGCGTCAGGAAT	277
FMgSSR-46361	scaffold575	p3	(ATC)5	Class II	87614	87628	TGAAGCTTTGTCCTCCTGGCCT	GCCAGCATGGAAAACAACAAGG	335
FMgSSR-46362	scaffold453	p3	(ATC)5	Class II	86740	86754	TGAAGTGGCGACGCTGCAAA	AGCGTTCGTCGGCAAGTTCAGA	247

SSR_ID	Scaffold	SSR_Type	SSR_Motif	SSR Class	SSR_Start	SSR_End	Forward sequence	Reverse sequence	Product_size
FMgSSR-46363	scaffold10366	p3	(ATC)5	Class II	13328	13342	TGACACCACACGCCATAGCACA	ATTGCCCATTGGTGGTGCGA	339
FMgSSR-46365	scaffold216	p3	(ATC)5	Class II	129728	129742	TGACCCTTGGTTCTGCTCGTCT	GCAAGCAAGTCATTGCACAGGC	284
FMgSSR-46380	scaffold503	p3	(ATC)5	Class II	117015	117029	TGCCAGTGCCAGGCAAAACA	TAGTCGACGAGACGGAGATCCA	255
FMgSSR-46387	scaffold758	p3	(ATC)5	Class II	13334	13348	TGCGCGCAACAGCAGTAGTA	AGGTGATGACGCAACCCAAGCA	332
FMgSSR-46406	scaffold181	p3	(ATC)5	Class II	108845	108859	TGGCAGCATTGTCGGGTAAGCA	AGCCCGGTTCCGGAGCAAATTA	236
FMgSSR-46422	scaffold10804	p3	(ATC)5	Class II	26554	26568	TGTCTTGCCTCGCCTCAACA	CGCTGGTTTTTCGTGTCTGCCA	320
FMgSSR-46423	scaffold548	p3	(ATC)5	Class II	24389	24403	TGTGCGCGTGCCTTCTTTTCT	ATGTCGGCATGCTAGTGCGA	257
FMgSSR-46424	scaffold1983	p3	(ATC)5	Class II	65100	65114	TGTGTGCGTGGGACTTGGATGA	TGTCGAAGGCCCATCCGTTGTA	314
FMgSSR-46425	scaffold2463	p3	(ATC)5	Class II	56980	56994	TGTGTGGATTGCCATCCGGCTT	ACCTTTGGGCAATTGGCTCG	291
FMgSSR-46442	scaffold1709	p3	(ATC)5	Class II	55962	55976	TTTCCCACGCAACGACACGA	GCACGGCCAATAAACTGCGTCA	322
FMgSSR-46458	scaffold49649	p3	(ATC)6	Class II	2933	2950	AATCCTCGACGCGGCTTCCAAT	ACAGCTGCAAGGGTTGCTCCAA	290
FMgSSR-46460	scaffold1428	p3	(ATC)6	Class II	7086	7103	AATTCCGCGAACGGCACTGT	AATAATTGGGGCCGAGCCTGGT	303
FMgSSR-46476	scaffold5955	p3	(ATC)6	Class II	9265	9282	ACGAACGAACGATTTCCGGTGC	TATGCGCGATTGGGATTGGGGA	231
FMgSSR-46479	scaffold1074	p3	(ATC)6	Class II	35220	35237	ACGCACTCATGGGATTCGGCAA	TGGAGGGCAGTTCACAGGAA	349
FMgSSR-46481	scaffold1360	p3	(ATC)6	Class II	81828	81845	ACGGTGAACTCACCATAAGGTCCTG	TGCATGAGTAGTGCACGCAAAGC	295
FMgSSR-46520	scaffold2620	p3	(ATC)6	Class II	27520	27537	AGGACTTCCGCAACCGCATCTT	TTGCAACGGCAGCAGCATCT	293
FMgSSR-46556	scaffold414171	p3	(ATC)6	Class II	62	79	CGCTCTCCTTTTCTGATTGGGAAC	AGATTGGGGCGACGTCTTGT	230
FMgSSR-46578	scaffold5882	p3	(ATC)6	Class II	14283	14300	TACCGCGTGTCCAGATGCTT	GCATCGGTTTAAGGATGCGCGA	329
FMgSSR-46581	scaffold1826	p3	(ATC)6	Class II	36297	36314	TCAACAGATGCCCAGCCAACGA	TGGGTGTGCAAGTAGGCAAGGA	330
FMgSSR-46585	scaffold3433	p3	(ATC)6	Class II	19837	19854	TCATGATGGACCACGACGACGA	ATGGCGAACGGCATCGCATT	298
FMgSSR-46587	scaffold326	p3	(ATC)6	Class II	84767	84784	TCGAAGTCTCTGTGCAGGGCAT	AATTGAGAGCCACAGGCCGGAT	268
FMgSSR-46592	scaffold26816	p3	(ATC)6	Class II	7493	7510	TCGTGCTAAACCCGCCACTGAT	TGTGCAAATGCTGCCGTGCT	317
FMgSSR-46593	scaffold115735	p3	(ATC)6	Class II	585	602	TCTGCGGGCGTTTTGGATGT	TCGTGGTTTTCCTCACCCGGAA	336
FMgSSR-46634	scaffold26816	p3	(ATC)6	Class II	10306	10323	TGGACGGCAAGCAACAGCAA	ACCTTGCACGCACGCGAAAT	201
FMgSSR-46650	scaffold574	p3	(ATC)6	Class II	55551	55568	TGGCGAAGCAAACCGCAAACA	TGAGCGAATGCTGCAGGTCA	263
FMgSSR-46697	scaffold2134	p3	(ATC)7	Class I	49542	49562	ACAACGCCGATGATGACCCCAA	TGCTGAATGTCTGCGACACGGA	249
FMgSSR-46705	scaffold12197	p3	(ATC)7	Class I	11496	11516	ACGCGTGCCTAACTGCTTGA	AAGCAGAGGACGTCGATCGGAA	218
FMgSSR-46734	scaffold1945	p3	(ATC)7	Class I	29053	29073	TCACCAACTCGCAAACCGCT	AGCGCGTGTTCTGCAGGATCTA	336

SSR_ID	Scaffold	SSR_Type	SSR_Motif	SSR Class	SSR_Start	SSR_End	Forward sequence	Reverse sequence	Product_size
FMgSSR-46765	scaffold1532	p3	(ATC)8	Class I	23361	23384	CGCATGCACAACCAAAACACGC	AGCGGACAAGCGAACCCGATAA	235
FMgSSR-46766	scaffold19027	p3	(ATC)8	Class I	11095	11118	GCGCTGAAGTTCCAAATGTGGGT	TGTGCATGCCCAAACCCGAT	247
FMgSSR-46779	scaffold804	p3	(ATC)8	Class I	11144	11167	TGGATGGCATTGGCGAGCAT	ACGCTGAATCGGCACTACAGCA	262
FMgSSR-46787	scaffold2591	p3	(ATC)9	Class I	46226	46252	ACGCTTCTTAAGGCGCTCCTTCTC	GCTGCACAATGCTTTAACCACC	314
FMgSSR-46803	scaffold7023	p3	(ATC)9	Class I	35002	35028	TTGTGTGGATGTGGCCCTCAGA	GCCTATGCTTTATGGTAGAGCGTGC	321
FMgSSR-46807	scaffold21764	p3	(ATG)11	Class I	5694	5726	ACACATTCTTCCTATCGCGCCC	TTGCTGGTCGATGCATGGCT	200
FMgSSR-46819	scaffold3729	p3	(ATG)5	Class II	15216	15230	AACCTGGACGCTACCTTGATCTGC	AAAGCTGCACCACCACCATCCA	335
FMgSSR-46826	scaffold6628	p3	(ATG)5	Class II	18908	18922	AAGCAGATCACGCACGACGA	ACATGCATGGACGTCGGGGAAA	266
FMgSSR-46828	scaffold10643	p3	(ATG)5	Class II	28253	28267	AAGGAAGGCCTCTGCCAGCATT	TGCATGACGCCGTGGACTTT	348
FMgSSR-46831	scaffold14921	p3	(ATG)5	Class II	10577	10591	AATGTGCCCTTCCCTTGGCA	AGGCCATGTCAACTTCGGGCAA	298
FMgSSR-46841	scaffold1865	p3	(ATG)5	Class II	14562	14576	ACCCACACCTGCACCAACAT	AAGAACAATCGCGTGGCGCT	267
FMgSSR-46845	scaffold1696	p3	(ATG)5	Class II	22129	22143	ACCGTGTTGCAATTCCCGTT	ACCACTACCCACCATCATGCCT	330
FMgSSR-46848	scaffold1892	p3	(ATG)5	Class II	5977	5991	ACGACTGTTGTGGGCTGTCA	TTTGGTAGCAGTAGCAGCAGCC	200
FMgSSR-46851	scaffold4682	p3	(ATG)5	Class II	43100	43114	ACGCTACCTCGGTCCGGATATT	AAGCCGAGGGAGGTCAAAAGCA	341
FMgSSR-46874	scaffold160	p3	(ATG)5	Class II	68501	68515	AGCTGTTCACGGTCGTCGTGTT	TGCGCAACAGCATCATCCGA	203
FMgSSR-46881	scaffold17933	p3	(ATG)5	Class II	4028	4042	AGGCAACCATCCACAAAGCCCT	AGTGGCGCAGTCGTGATCTT	346
FMgSSR-46882	scaffold26891	p3	(ATG)5	Class II	6240	6254	AGGCAGATGCCGATGTCACCTT	ACCACTTCCCTCCGAAGCCATT	339
FMgSSR-46899	scaffold12289	p3	(ATG)5	Class II	16919	16933	ATGACGCAGGCCAAAAGCCA	AGTGGGAGGCTACCAGCAGATT	344
FMgSSR-46906	scaffold383126	p3	(ATG)5	Class II	126	140	CAGAAACTGACACAGAAGGTGGCA	ACACGCTTTCTACCAACAGAGTGG	335
FMgSSR-46909	scaffold977	p3	(ATG)5	Class II	13069	13083	CCCTCTCGGTGGGAGACAAATGAT	AGCAACTACCTCACCTGCTCAGA	326
FMgSSR-46929	scaffold7300	p3	(ATG)5	Class II	5749	5763	GCACGCCAGCAACCACACAAAT	TGCCGTCAACGACATTGGCA	253
FMgSSR-46940	scaffold11856	p3	(ATG)5	Class II	17107	17121	GGCCGGTTCCCCTTGATTGTAA	ACCCATGGATCCGCAAGGAA	230
FMgSSR-46949	scaffold450	p3	(ATG)5	Class II	27232	27246	TAGCTGTGCGCCGAGTTCACAA	AGCTGCCGGGATTCTTCTTCCA	300
FMgSSR-46951	scaffold3486	p3	(ATG)5	Class II	21726	21740	TATGCACGCGCACGACTTGA	TGGCCATGTACTCCGCAATCA	316
FMgSSR-46960	scaffold12	p3	(ATG)5	Class II	232574	232588	TCCATTCAGCGGAAAGACCGGA	TGTTCTTGGCCACGACTCGGTT	344
FMgSSR-46965	scaffold425	p3	(ATG)5	Class II	37763	37777	TCGCTGCCGCCATTCTTGTT	AACTCTCGCGCCACTTTCTCCT	249
FMgSSR-46973	scaffold450	p3	(ATG)5	Class II	94057	94071	TGACCAGAGAGCCATTTGTGC	CGGTGTTCCACTGATGACAATGCG	323
FMgSSR-46981	scaffold558	p3	(ATG)5	Class II	28979	28993	TGCAAGGGATGACAGATCGCACT	AGCGCAGCAAGCCACCATTT	337

SSR_ID	Scaffold	SSR_Type	SSR_Motif	SSR Class	SSR_Start	SSR_End	Forward sequence	Reverse sequence	Product_size
FMgSSR-46983	scaffold7843	p3	(ATG)5	Class II	34816	34830	TGCACGCAACAAGCGAACAC	AGAGCGGTTCGGCGGTAAAT	296
FMgSSR-47020	scaffold1878	p3	(ATG)5	Class II	53033	53047	TGGTCGTCGAGTCAAGTCGAAA	ATGCCGTGAGAGTCTCTTTGTCGG	291
FMgSSR-47023	scaffold22001	p3	(ATG)5	Class II	10272	10286	TGTCTTGAGTCGCACGGCTAGT	TGCAATTTGTTGGGGCGGCA	333
FMgSSR-47033	scaffold214	p3	(ATG)5	Class II	109701	109715	TGTGGTTTCCGTTCGCGAGT	TGCATGCAATGCAACCTGGC	302
FMgSSR-47034	scaffold1457	p3	(ATG)5	Class II	60391	60405	TGTGTTATCCACCCCACCCGTT	AGGCAGGCAGGCCATCATAA	296
FMgSSR-47035	scaffold10129	p3	(ATG)5	Class II	21085	21099	TTAACCGGCGACGCAACAAG	TCGCGTTCCGTTGCGTTTGT	349
FMgSSR-47036	scaffold2284	p3	(ATG)5	Class II	40744	40758	TTCAGCTGCATATCGGGGCACA	TATTCTGGCCATCAAGCTCTGGGC	302
FMgSSR-47038	scaffold502	p3	(ATG)5	Class II	79216	79230	TTGCATTGGCCTCGCCTTCT	AGCAGTCTCGTCGTGGTTCCTT	345
FMgSSR-47039	scaffold1416	p3	(ATG)5	Class II	53249	53263	TTGCCACAAAATAACCCGCCGC	TGCCGCACCTTGGTAACGTA	341
FMgSSR-47049	scaffold949	p3	(ATG)5	Class II	51565	51579	TTTTCGGCCCTTCCGCTTCT	ACGCATGCTGAAGTTGCTGC	348
FMgSSR-47050	scaffold5455	p3	(ATG)6	Class II	36599	36616	AAAAGGTGTCACGCTGGGCA	ACAGACTGCTTACTGTCCTCCCTGT	279
FMgSSR-47053	scaffold2255	p3	(ATG)6	Class II	59628	59645	AACGCCTGTCTTGTGCTGGAGT	ATCGCCAGCGGACAATGAACCA	299
FMgSSR-47055	scaffold4985	p3	(ATG)6	Class II	24208	24225	ACCAAATGCCGGTTGCACCCTA	TGTGTTGAAGTGTCCACCCTCCT	210
FMgSSR-47060	scaffold3971	p3	(ATG)6	Class II	38273	38290	ACGGTGCGAATACGGCAGTT	TGCACCTACATCACCGTCGTCA	333
FMgSSR-47073	scaffold12080	p3	(ATG)6	Class II	13086	13103	AGGCAACGACGACACGTCTGAA	TCCTACCAGCAAAAGTCACGCCA	308
FMgSSR-47074	scaffold4774	p3	(ATG)6	Class II	7865	7882	AGGTCGACGACGACTTCCTGTT	ACCACCGCCTGAAAATGGGA	239
FMgSSR-47089	scaffold1802	p3	(ATG)6	Class II	27057	27074	GCGCCATAGCAGCAGTAGTGAA	AGCGGTTCCTCCGTTTCCTTGT	326
FMgSSR-47091	scaffold20290	p3	(ATG)6	Class II	5660	5677	TATGCACAGGAAGCGGTGGAGT	AGTTGTTGTTGCCGCTGTCCAC	253
FMgSSR-47096	scaffold10512	p3	(ATG)6	Class II	9975	9992	TCCGTATGGCTCACGCATGT	CACCAGAAGTTGCCGCCACAAT	223
FMgSSR-47098	scaffold7949	p3	(ATG)6	Class II	22164	22181	TCGCGTAACCCACCAAGTCCAT	TTGCGGAGCAGAGTGTCGTTGA	306
FMgSSR-47106	scaffold183043	p3	(ATG)6	Class II	343	360	TGCCAGCACCTGAGATTCCAGA	AGTCCGAGGCTGTAGCAAAAGGA	290
FMgSSR-47109	scaffold35034	p3	(ATG)6	Class II	2750	2767	TGCTCCGTCATATGGTCGCT	TTATCCGCATGCCTCCGTCGAA	251
FMgSSR-47111	scaffold11520	p3	(ATG)6	Class II	23278	23295	TGCTGCTGCTCTGCTCATCGTA	AAGCCCAGCAACAAAACGGTGC	285
FMgSSR-47128	scaffold6722	p3	(ATG)6	Class II	27880	27897	TTCCGTGCAGCTAATAAGGGGTGG	AGCAAACGGGCAGTAGCAGTGT	286
FMgSSR-47129	scaffold3452	p3	(ATG)6	Class II	54746	54763	TTGAACCGCAAGCGCCAACA	TCCATGTGCACCCTGAACTGTCA	233
FMgSSR-47133	scaffold622	p3	(ATG)6	Class II	80354	80371	TTGTGGAAACGGAATCTGCCCG	CGGCAGGAAAAGGCAAAGGCAA	257
FMgSSR-47140	scaffold2504	p3	(ATG)7	Class I	40241	40261	AGCGCGATGTCCAGAACAACCA	AAAGGTCGCAGATTGGCTTGCG	258
FMgSSR-47157	scaffold77821	p3	(ATG)7	Class I	728	748	TGAGCTGTTGGGCACTCTTGCT	TTGTCCGTTTGGTTTGGCCAGC	287

SSR_ID	Scaffold	SSR_Type	SSR_Motif	SSR Class	SSR_Start	SSR_End	Forward sequence	Reverse sequence	Product_size
FMgSSR-47163	scaffold736	p3	(ATG)7	Class I	38611	38631	TGGGTGCAAGTTGCGCTTGT	ACAGCGTGCAAACTTTGAGGTGC	216
FMgSSR-47166	scaffold26959	p3	(ATG)8	Class I	3580	3603	AGCCTTCCCAGCTGGTCTTT	ACGTGGGTGCGTGTTTACGCTA	334
FMgSSR-47172	scaffold2193	p3	(ATG)8	Class I	19933	19956	TCGGCAGCAACCAAAACGCA	GCACAGATTGGGGCAACAAACCA	269
FMgSSR-47191	scaffold5826	p3	(ATT)10	Class I	36254	36283	TATATGTGTGGGTCACGTGCAGGG	GGCGACCGCCTGCTTCAAATAA	210
FMgSSR-47195	scaffold185	p3	(ATT)12	Class I	145110	145145	AACCCATCGAGTGCGCGATACA	TGGGCCATCCCGTTTGCATT	201
FMgSSR-47197	scaffold781	p3	(ATT)13	Class I	51523	51561	GAGAGAGAGGAGAGAGAGAGACTCA	TCGAAGGTGGTGCTCCAACCAA	331
FMgSSR-47201	scaffold21914	p3	(ATT)13	Class I	353	391	TGCTCAATGCACTGCAAGCCA	TGCGCCTTGGAGGCGTATTT	305
FMgSSR-47202	scaffold8572	p3	(ATT)14	Class I	16074	16115	AAAAGGTACGATGCGCCGCT	ACCGTGGGATCCATGTAAGTCG	344
FMgSSR-47203	scaffold19567	p3	(ATT)15	Class I	11347	11391	AGGTGATTCCGAGTTTTGGAGGG	CCTCACTGACTTACCATTTCCCAC	341
FMgSSR-47204	scaffold1787	p3	(ATT)16	Class I	25358	25405	ACTCTCCGGCATCAAAAGCGT	TGACAGGCATTGCAGCCGTT	315
FMgSSR-47206	scaffold14271	p3	(ATT)16	Class I	10647	10694	GTGCGTTCATCGTTTGACCTGA	ACAGCACATCTTACAGCAGGCA	290
FMgSSR-47216	scaffold165	p3	(ATT)19	Class I	118720	118776	AGTCGTGGCCACTATAGGTTGGT	TCGTGCGGCGCTCCTTGTAAAT	274
FMgSSR-47218	scaffold977	p3	(ATT)20	Class I	16765	16824	AAATTCTGTCGGGCCAGCCCAT	TTTTCAGGGGTGGCCCAGCTTT	271
FMgSSR-47223	scaffold140	p3	(ATT)23	Class I	110771	110839	ATCAGCATGAGAAGGCTTGCTCCC	ATGGCGATGACCCAGGCCAAAA	310
FMgSSR-47226	scaffold16688	p3	(ATT)5	Class II	12259	12273	AAAAAGGGATGGGCAGCGGACA	TCCACAGTTCCAGCGGGCAATA	296
FMgSSR-47229	scaffold272	p3	(ATT)5	Class II	89866	89880	AACAGCATCAGTTTGGCGAGCG	TTGTGCGATGCACCAAGACCGT	208
FMgSSR-47230	scaffold97506	p3	(ATT)5	Class II	837	851	AACATGCAATCCCGCCTTTGCC	CGCTGGGCATTGCCGAAGAAAA	248
FMgSSR-47231	scaffold209	p3	(ATT)5	Class II	22957	22971	AACGCAGCGAGTCCGGTGTTAT	TCGCCAGTAGCAGGAGTGTT	350
FMgSSR-47236	scaffold807	p3	(ATT)5	Class II	83433	83447	AAGAACGCCGCAGCTATACGGT	AAGGTGCAACAGCGGCAGAT	245
FMgSSR-47238	scaffold243073	p3	(ATT)5	Class II	282	296	AAGCAGGGACCAAAGCTGGA	TGAGCGGTCGAGAGGCCTTTTA	320
FMgSSR-47240	scaffold856	p3	(ATT)5	Class II	112717	112731	AAGGCGCATCAGTGGCTGGTAA	CGAAGTCATGCTTTTGCCACTGTC	206
FMgSSR-47247	scaffold7	p3	(ATT)5	Class II	61239	61253	ACACAGCCACACACGTTGGACA	GACGCGTGTGGTATCTCTTTCTTC	312
FMgSSR-47248	scaffold18587	p3	(ATT)5	Class II	16359	16373	ACACGGGAGGAAAGGGAATGGA	TCGTAGCTACTCTCGTGTGCAT	325
FMgSSR-47250	scaffold5874	p3	(ATT)5	Class II	22895	22909	ACCAAGGCACCAGTGATGGAGT	ACTCAGATCGTCCTCCTCAGCAGT	333
FMgSSR-47252	scaffold2049	p3	(ATT)5	Class II	29047	29061	ACCAATGAGCCGTGGTTCTGA	AGCAGGAAGCCAACTCGTGTGT	340
FMgSSR-47260	scaffold3741	p3	(ATT)5	Class II	1918	1932	ACCCCTGCTACCAGTTTAAGCA	TGCGCCAACGTTGCCTGTTT	330
FMgSSR-47261	scaffold483	p3	(ATT)5	Class II	8267	8281	ACCGAAGCGTGGACTTTGGA	TGTTGCACGACGCAGCATGA	314
FMgSSR-47263	scaffold880	p3	(ATT)5	Class II	17981	17995	ACCGACGACATCTGTTTTGCGT	TTTCTGCGTGCAGGGCTCTT	281

SSR_ID	Scaffold	SSR_Type	SSR_Motif	SSR Class	SSR_Start	SSR_End	Forward sequence	Reverse sequence	Product_size
FMgSSR-47274	scaffold654	p3	(ATT)5	Class II	48749	48763	ACCGTGGAACACGGACATGTGA	AGTGTCATGCAAGCGGCAGAGT	339
FMgSSR-47279	scaffold365	p3	(ATT)5	Class II	56804	56818	ACGCATGGGCGAGAAATAGCCT	TAACGCCTTCGGAAGCCACCAA	307
FMgSSR-47281	scaffold6	p3	(ATT)5	Class II	22230	22244	ACGCCGCCGGAGATCATTTT	TCAGATGTCCCCAGCACAGAGA	207
FMgSSR-47282	scaffold11284	p3	(ATT)5	Class II	9423	9437	ACGGCAGTGTGCCTCGAAAT	CCAGTGTGGATGCGGGTTATGA	350
FMgSSR-47285	scaffold284	p3	(ATT)5	Class II	14327	14341	ACGTACTGCTCATCGGCTCTGA	TACGCAAGGCGCATGTTCCA	307
FMgSSR-47288	scaffold3779	p3	(ATT)5	Class II	6720	6734	ACTGCACGTTTCCCTCCAAAGA	TCGTTCTGGTACTCCATTCACAGC	300
FMgSSR-47289	scaffold9062	p3	(ATT)5	Class II	853	867	ACTTGCCTCTATCTCTCGCGTGT	TGTGTTGCACTGTTGCCTGTCG	241
FMgSSR-47295	scaffold4302	p3	(ATT)5	Class II	24459	24473	AGCTAGTGTCCGTGTGTTGGCA	ACGTTGCTCTGCCCATTTGC	275
FMgSSR-47301	scaffold2767	p3	(ATT)5	Class II	27729	27743	AGCTTGGAGCTGAGTGAGAAACCA	CGATCGCCTGGCCTGATTGATT	336
FMgSSR-47302	scaffold1904	p3	(ATT)5	Class II	68522	68536	AGGACCGTTTTGCTGCTCCCAT	TCGCGCTCGATAATCATGGGGT	277
FMgSSR-47311	scaffold10231	p3	(ATT)5	Class II	10479	10493	AGTCGGCAAACGCGCAACTT	ACACAGTAGCGCGCGTGAGTAA	345
FMgSSR-47317	scaffold131044	p3	(ATT)5	Class II	894	908	AGTTTCAGCAAGACCCTGCCCT	ATCAAACAGCCGCAGCCACA	329
FMgSSR-47336	scaffold5089	p3	(ATT)5	Class II	45942	45956	ATTCAAGCGCGGCACAACGA	GCGTGCCAACGATTGCAAGTT	247
FMgSSR-47349	scaffold1697	p3	(ATT)5	Class II	14382	14396	CGACCCAGCGAACTGTTCTTCA	TTGGGTTTGGGTTTCGCGGT	329
FMgSSR-47351	scaffold4667	p3	(ATT)5	Class II	3072	3086	CGAGTAGTTCCCGATGATCTCTTGC	TGGTGTTTCCAACCACCTACGTCA	230
FMgSSR-47359	scaffold18357	p3	(ATT)5	Class II	9735	9749	CGGGTACACATATGTTCGAAGCTC	TGGTTGATGAACATGGGCGTCTGA	295
FMgSSR-47361	scaffold496	p3	(ATT)5	Class II	89316	89330	CGTGATAACCAACAAACCGCAAGC	ATTGAAGGTTGCAGGCGTGG	289
FMgSSR-47362	scaffold324	p3	(ATT)5	Class II	113003	113017	CGTGCATGCAGATAGTGGAGGA	TGGGCTAACGAAACGTTCACGC	349
FMgSSR-47365	scaffold30180	p3	(ATT)5	Class II	5058	5072	GAGAAGGCATGCATTGTGCTGA	TGCACGGCACACTGGAACAA	343
FMgSSR-47375	scaffold309938	p3	(ATT)5	Class II	346	360	GCGGTATGTCGTGGAAGCTGTT	AGAAAAAGGACCACCAGCAACG	235
FMgSSR-47377	scaffold2962	p3	(ATT)5	Class II	27260	27274	GCTCATACTAGCCTCCAGTAGCA	AGCAGAACCCATCATGGAAAGC	350
FMgSSR-47386	scaffold11330	p3	(ATT)5	Class II	12188	12202	TATGCCCAAGCGCCGTTTCA	TGCAATTGGGCGATGGTCAGTG	255
FMgSSR-47388	scaffold6699	p3	(ATT)5	Class II	11023	11037	TCACCCTCTCATGCACCGGTTT	TGCAATGGTAGCTGGTTGAGCG	345
FMgSSR-47393	scaffold187	p3	(ATT)5	Class II	167064	167078	TCCAGGGACAGAAGGTATGTGC	AGCAAGGGATGGAAGCACCA	280
FMgSSR-47400	scaffold1614	p3	(ATT)5	Class II	50754	50768	TCCCTCGTTTCCCTTCAGGCTT	TGCCCATTACATGGCATCACGA	298
FMgSSR-47401	scaffold2438	p3	(ATT)5	Class II	40267	40281	TCCGATCGTGACGCTTAGGGTT	TGCACTTGGAGTCAGTGTGCGT	290
FMgSSR-47406	scaffold10296	p3	(ATT)5	Class II	13173	13187	TCGGTCTTGCTGCTGTCATGCT	TGACATCCGCAGCTCGTCTT	307
FMgSSR-47407	scaffold5632	p3	(ATT)5	Class II	22971	22985	TCGGTTTTTCCGCATGGCAACC	ACTTCCAATCCACCGCCCAACA	304

SSR_ID	Scaffold	SSR_Type	SSR_Motif	SSR Class	SSR_Start	SSR_End	Forward sequence	Reverse sequence	Product_size
FMgSSR-47413	scaffold1130	p3	(ATT)5	Class II	1744	1758	TCTCAACCCGCCTAAACCCTGA	AATTGTTACGTGGAGCGGTGCC	248
FMgSSR-47414	scaffold5223	p3	(ATT)5	Class II	43379	43393	TGAATCGCCCTGGTTACCTTGA	CAGGATCCCCTAACGATGTGATGT	343
FMgSSR-47417	scaffold853	p3	(ATT)5	Class II	29275	29289	TGAGGCGCAACTCGTTAGCGTA	TTGCACAGTGATCTGCCGCT	279
FMgSSR-47418	scaffold15118	p3	(ATT)5	Class II	8472	8486	TGATCCGTGGAATGGGGTGCAT	TCCTGCACCAAGAAAGGGTGGA	283
FMgSSR-47438	scaffold27	p3	(ATT)5	Class II	180809	180823	TGCCGGCACGAATAACTCGAA	TGTCTTGTTCGCTGGTGGCAGT	347
FMgSSR-47445	scaffold3150	p3	(ATT)5	Class II	9546	9560	TGGCCAAAGCCACGACCATCAT	CAGAATCACAAACTGCAAGGAGCC	224
FMgSSR-47446	scaffold5381	p3	(ATT)5	Class II	32908	32922	TGGCCATGTCAATCGGCTTGT	TGCTCCAACGCAACATGAGCCT	210
FMgSSR-47456	scaffold6498	p3	(ATT)5	Class II	29546	29560	TGGTTCGGCTTCTCCATCCACA	ACACGGCAAAAGCCTGGTCA	274
FMgSSR-47457	scaffold82476	p3	(ATT)5	Class II	250	264	TGTCAGAATCAGACGCCGCGTAA	TCGCTCGCTGCATGATGCTT	343
FMgSSR-47467	scaffold22213	p3	(ATT)5	Class II	386	400	TTCGTGTCAGCGCGTGCAAA	TCTCTGCCCTGTTTCAGATGCT	275
FMgSSR-47470	scaffold12548	p3	(ATT)5	Class II	13243	13257	TTGCTTCAGCGGGTGGAACACT	ACGGGTCATCTTTTGCCTCACC	322
FMgSSR-47477	scaffold7147	p3	(ATT)5	Class II	26441	26455	TTGGTGGGTGTCGGCATTGTGA	ACAGGCAAGGAGGCAAAGCTGA	340
FMgSSR-47488	scaffold975	p3	(ATT)6	Class II	54502	54519	ACGGAGATGTCGCTCGAAAGGA	TGGTTGGCGAACGACCTCAACT	293
FMgSSR-47491	scaffold200	p3	(ATT)6	Class II	98936	98953	AGGATTGGCTCGTGAGCATCAGT	TGGAGGTTGTTGCTCAGCTTGT	335
FMgSSR-47493	scaffold314	p3	(ATT)6	Class II	117111	117128	AGGGGAGTTTTTACGGTAGTCCG	TGTCACGTTTTGGGTCTGTCACG	279
FMgSSR-47496	scaffold18030	p3	(ATT)6	Class II	475	492	AGTGAGCCTAATAACCCCCGTGA	TCGGTTTGGTTCATTAGCTCGG	308
FMgSSR-47515	scaffold43	p3	(ATT)6	Class II	53869	53886	TACATCGACGCGTTGGCCGATT	ACCAGCTCCACCAATTCTACGC	304
FMgSSR-47525	scaffold9953	p3	(ATT)6	Class II	4591	4608	TGCATACCACTTTCCGGCACT	AGGGTAATGATGCCCGTTCGT	307
FMgSSR-47527	scaffold1729	p3	(ATT)6	Class II	54403	54420	TGCCGTTGAGCTGTCGCAAA	ATGACCCAGAGGTGCAGTCCTCTT	266
FMgSSR-47529	scaffold4610	p3	(ATT)6	Class II	46636	46653	TGCGGATTCGTCAACATGCCCT	GGTGTTTTGTTGCCAACGACGGA	265
FMgSSR-47536	scaffold207	p3	(ATT)6	Class II	144488	144505	TGTCCTCTTGGTGGCACAATAACG	AAACCGCTCCAAATGCGCCA	335
FMgSSR-47537	scaffold528	p3	(ATT)6	Class II	88754	88771	TGTGGCCTCAAGCGCACATATT	TGCGTGTCCGTTCAGTTTCCAGT	247
FMgSSR-47542	scaffold13304	p3	(ATT)6	Class II	9589	9606	TTGGCGTGCCAAATTCCTGAGC	TGGCGGGAAATGCTGAGAGT	205
FMgSSR-47548	scaffold3	p3	(ATT)7	Class I	109328	109348	AACAGGCAGCGGCAAAATGC	TGGTGGGACAAGCAAGCACT	289
FMgSSR-47551	scaffold21607	p3	(ATT)7	Class I	11728	11748	ACCCTTCCCAACCAACAGCACT	TGTCCCAAGAACCTCCGCACAA	312
FMgSSR-47558	scaffold23949	p3	(ATT)7	Class I	7614	7634	AGCCTGTGCGAGAATACCTGCT	TGGGTGCCGTACGTGTGTTTGA	240
FMgSSR-47561	scaffold6210	p3	(ATT)7	Class I	41282	41302	AGTGATGGCCTGTGGCTGTT	AACGCGGAGCTCGGAGCAAAAA	263
FMgSSR-47564	scaffold462	p3	(ATT)7	Class I	49354	49374	ATGCTGCGGAGAAGACTGTGGA	TTGTTCTCGGGCCGCCTATT	267

SSR_ID	Scaffold	SSR_Type	SSR_Motif	SSR Class	SSR_Start	SSR_End	Forward sequence	Reverse sequence	Product_size
FMgSSR-47568	scaffold4548	p3	(ATT)7	Class I	31317	31337	TCATTGAGGCTGCGCCATGT	TGGCAGCAGCATTAGGCAGA	259
FMgSSR-47571	scaffold6274	p3	(ATT)7	Class I	32726	32746	TGAGAATGCCACGGCCTGAA	TCGGAGGCTGCAGAACTTCACT	336
FMgSSR-47574	scaffold6950	p3	(ATT)7	Class I	23132	23152	TGTTAAGCAAGAGAGGTGGGCA	GGTTGAGTCTGGACAAACTAGTGG	282
FMgSSR-47578	scaffold3287	p3	(ATT)7	Class I	27514	27534	TTTCGACAGCGTGTGGGGATGA	AAAGCTTTGCCATAGCGGCAGG	331
FMgSSR-47581	scaffold12485	p3	(ATT)8	Class I	4812	4835	ACCACAATCACGGTGACGACGA	GCGTCGTTCGCCCCTAACTTTT	343
FMgSSR-47583	scaffold3841	p3	(ATT)8	Class I	39667	39690	ACTCGCGATTTGCATGGCTTCA	AAGGTGCGGTGTACGAGAGTGA	256
FMgSSR-47585	scaffold1155	p3	(ATT)8	Class I	22575	22598	ATTTGCCGCCTCCGTTGCAT	CCAGTGAGCTCCAAGGGCTCAAAA	249
FMgSSR-47588	scaffold7869	p3	(ATT)8	Class I	16421	16444	TCCCCGTTTTGAGGCTCGTCTT	TCATGGCCGCCGTTAATTCGGA	342
FMgSSR-47591	scaffold20578	p3	(ATT)8	Class I	4137	4160	TGGTCACTTGACACCTTGGGT	AGGCACAATCATTGCAGGCAGC	286
FMgSSR-47592	scaffold151	p3	(ATT)8	Class I	131216	131239	TTAACGGTGCCTTCGCCTCCTT	TGGCAGAGCAGGAGGATTCCAT	331
FMgSSR-47596	scaffold7372	p3	(ATT)9	Class I	22517	22543	ACGTTCAGGTTACATCAGGTGGGA	GCACGTTTCCAACCAGGCACTA	342
FMgSSR-47597	scaffold281	p3	(ATT)9	Class I	33127	33153	AGCACAGGAGCACCAGAGTTGT	CGCAGTCCAGTTTGGCTAGCAT	332
FMgSSR-47598	scaffold1521	p3	(ATT)9	Class I	70134	70160	AGCACTGCATCAGCGACCAA	AACCCAAAGCGGTCGCCAAA	296
FMgSSR-47604	scaffold3328	p3	(ATT)9	Class I	59555	59581	TTAAACAGGCCCACATGCAGCC	TGCCCACACGGCTTCTACTCTT	242
FMgSSR-47609	scaffold13124	p3	(CAA)10	Class I	4187	4216	ACCCATTGCGCATTCACCTCCT	AAGGGCGTTGTCACCTTGCTCA	248
FMgSSR-47610	scaffold14938	p3	(CAA)10	Class I	702	731	ACCTTGTGCCCCAAGTTGGAA	AATGCTGGACGCAGGCTTGA	309
FMgSSR-47622	scaffold795	p3	(CAA)10	Class I	89315	89344	GGTTGACACTTTGACACAGCTTCC	TGCCCTCAACCATGCAGCAA	325
FMgSSR-47651	scaffold9789	p3	(CAA)13	Class I	4417	4455	AGCAACTTCCTCACTTGCAGCAG	TATCGGCTCGCGCGTTGTACTT	328
FMgSSR-47675	scaffold4617	p3	(CAA)5	Class II	31156	31170	AAAAACCATGGCGGGCGTGA	TTTGTTGACGCTGACGCGGT	226
FMgSSR-47676	scaffold4243	p3	(CAA)5	Class II	9051	9065	AAAAAGGAAACCCCCGAGGCGA	TTCAGCGGCATGTCTGGCTT	287
FMgSSR-47687	scaffold1558	p3	(CAA)5	Class II	37804	37818	AACAAGCGTTGCCGCACGTA	TCCCCAACTGCAAGAAATGCTGC	347
FMgSSR-47690	scaffold2026	p3	(CAA)5	Class II	39504	39518	AACAGCTCCGGCTTCTTGCT	TGAATGTCTCTCTGTCTCCCCCGT	292
FMgSSR-47692	scaffold5820	p3	(CAA)5	Class II	34603	34617	AACAGGTCCAGCAGCAGCAACA	ACTGGATGTGAGCGACGTTTGC	283
FMgSSR-47701	scaffold758	p3	(CAA)5	Class II	24610	24624	AACCATGCCGACCGCAACAA	TGCTGCTGCTGCTGCTGAGATT	232
FMgSSR-47705	scaffold6471	p3	(CAA)5	Class II	4625	4639	AACCTCGGCTCAAGCGAATCGT	TGCCGAGTCTGTTAGTTCCACT	341
FMgSSR-47707	scaffold636	p3	(CAA)5	Class II	20175	20189	AACGCGGTTGGTTGACCGTA	AAGCTGGCATTGCGTGCAGA	247
FMgSSR-47722	scaffold20	p3	(CAA)5	Class II	149062	149076	AAGCTGACTACGCAACGCCT	AGCTGATGGTGGTGATGCTAGGT	292
FMgSSR-47732	scaffold8279	p3	(CAA)5	Class II	3482	3496	AATCCAGTGGCAGCGACCTCTT	TCGCTGCTGCTACTCGTGTTCA	339

SSR_ID	Scaffold	SSR_Type	SSR_Motif	SSR Class	SSR_Start	SSR_End	Forward sequence	Reverse sequence	Product_size
FMgSSR-47733	scaffold16457	p3	(CAA)5	Class II	152	166	AATCCCAGCTCTGCTTTGCTCG	TGGTCCATCCAAAACCGTCGCA	244
FMgSSR-47736	scaffold145188	p3	(CAA)5	Class II	272	286	AATCTGACCAGCGCACAGGA	TTGTATGCGATGCCGACGCT	308
FMgSSR-47750	scaffold417	p3	(CAA)5	Class II	131087	131101	ACAACGAAGCTACGACCACGCA	TAGGCATGATTCTGCACCCCGT	300
FMgSSR-47755	scaffold8755	p3	(CAA)5	Class II	9352	9366	ACACCGTCGGCCCAAAGATCAA	TGACCACCTTGTTGCAGGGCAT	300
FMgSSR-47772	scaffold4902	p3	(CAA)5	Class II	25714	25728	ACCACGCGGCGAATACCAAA	GCGGCCATGCATGCACAAAT	246
FMgSSR-47805	scaffold6865	p3	(CAA)5	Class II	11656	11670	ACCTACGCAGGCCCAGAAAGTT	ACACGTGCGGTGTTTGCATT	247
FMgSSR-47809	scaffold77	p3	(CAA)5	Class II	138432	138446	ACGAGGCATGAACATGGGTGGA	TGCCGATCATCGCCTCGGTATT	321
FMgSSR-47812	scaffold215318	p3	(CAA)5	Class II	104	118	ACGCCAATCCACCAACGTGT	ACCAAAACCAGGAGGAGGGCAT	206
FMgSSR-47814	scaffold1238	p3	(CAA)5	Class II	43961	43975	ACGCCTCCTTCAGTCTTCCCAA	GCTGCATGTTCTGCACAACCGA	347
FMgSSR-47831	scaffold4544	p3	(CAA)5	Class II	27744	27758	ACTTGTAGCATGCACGGAGCCA	GGCTGCTACGCTCTTCAACACT	210
FMgSSR-47835	scaffold13631	p3	(CAA)5	Class II	10928	10942	AGACATGGCAAGACACGGGAGT	TCCAGCCAGCCATTCAAGACCA	312
FMgSSR-47838	scaffold4218	p3	(CAA)5	Class II	3338	3352	AGAGAGTGCCACATGCCCACAT	ACGGGTTTGATGCATGGCGT	294
FMgSSR-47847	scaffold22451	p3	(CAA)5	Class II	6398	6412	AGCAGCACCCCTTTGATATTGGCA	AGGTGCACTAGAGACTGGAGACCA	204
FMgSSR-47887	scaffold9417	p3	(CAA)5	Class II	12095	12109	AGGGCAACAACCCACCAAGA	TCAATGACGTGATGATGCGGGG	329
FMgSSR-47898	scaffold172480	p3	(CAA)5	Class II	551	565	AGTGGGATGTGTTCAGGTTGGA	AACTGCGCCCAGGCCTATCAAT	241
FMgSSR-47905	scaffold9370	p3	(CAA)5	Class II	26981	26995	ATCAAAACGCGCCTTCCGTTCC	AGCGACAAGAAATCGGCCCTGA	227
FMgSSR-47911	scaffold177113	p3	(CAA)5	Class II	227	241	ATGACAACCAAGAGTGAGCCGC	ATGCAGACTGCCACGTCCAACA	267
FMgSSR-47923	scaffold539	p3	(CAA)5	Class II	123831	123845	ATTGGGCTGCGTTTGTGACG	ACACCAAGGCAGCACGAACTGA	339
FMgSSR-47924	scaffold6538	p3	(CAA)5	Class II	24222	24236	ATTGTCAAGGGTTGCACGGCAG	CCAACAAGGAATGCTGGTTACGGG	348
FMgSSR-47925	scaffold49096	p3	(CAA)5	Class II	3233	3247	ATTGTGGATGCCCTGCCTGT	AACAGGAGGGACTGGAGTTGGA	271
FMgSSR-47932	scaffold6300	p3	(CAA)5	Class II	12657	12671	CCAAGTTGCTGGCACGAGAGAA	TGGCGCCGGTGATTCAACTT	203
FMgSSR-47939	scaffold5286	p3	(CAA)5	Class II	38466	38480	CGAACCAACCAACGCACACGAA	GAGGACGACGATCTCCTCATCAA	205
FMgSSR-47956	scaffold1906	p3	(CAA)5	Class II	40075	40089	GCGGGTTGCAACGCTTTGTT	TCCCTGCTTACGTTTCGGCA	256
FMgSSR-47963	scaffold207408	p3	(CAA)5	Class II	101	115	TACTCTGCCTGCACCGTCATCT	TGACCGGGCATAGCGAGGTTTT	243
FMgSSR-47967	scaffold3216	p3	(CAA)5	Class II	47337	47351	TCACAACGCAAAGCGAACCCAC	TCGAATCGCTGAATTTCGCCGC	203
FMgSSR-47968	scaffold86535	p3	(CAA)5	Class II	506	520	TCACAAGCTCGCAGACCATCA	TTGCCGGTCATGTTGTCGCT	317
FMgSSR-47972	scaffold9005	p3	(CAA)5	Class II	4264	4278	TCAGAGCACGCGCATCATCA	ACGAGTGGGTGACCTTGTTGCT	201
FMgSSR-47978	scaffold49694	p3	(CAA)5	Class II	3536	3550	TCCAGACTATGACACTAGCCGC	ATGGAGACCGCACAAGGAGT	340

SSR_ID	Scaffold	SSR_Type	SSR_Motif	SSR Class	SSR_Start	SSR_End	Forward sequence	Reverse sequence	Product_size
FMgSSR-47979	scaffold475	p3	(CAA)5	Class II	42138	42152	TCCGCTGATGTTTCCGTCCT	ACCCTGGGCCCACTCGTAAAAA	237
FMgSSR-47985	scaffold5005	p3	(CAA)5	Class II	10660	10674	TCGAGCTGTAGGGTGCTAAACGA	AAAGCAAGCCCGCCTTTGGT	295
FMgSSR-47994	scaffold5978	p3	(CAA)5	Class II	20806	20820	TCGTGATGCAGGCACAACTGGA	GCCATGTTGGGTCGCACCTTTT	335
FMgSSR-47996	scaffold351	p3	(CAA)5	Class II	71604	71618	TCTGTGATGCGCCTGCCATT	TGGATTGCTGCCCTCGTGTT	338
FMgSSR-48003	scaffold349	p3	(CAA)5	Class II	43163	43177	TGCACAGCCTTTCATGCCGT	TGGATGGCACTGCCCGATCTAT	248
FMgSSR-48009	scaffold29535	p3	(CAA)5	Class II	6311	6325	TGCAGCTCCCGCATTCTTTGGT	TGCAGCATATGTTCCATCGGCG	282
FMgSSR-48013	scaffold12345	p3	(CAA)5	Class II	13985	13999	TGCGAGGTCAATGTGCTGCT	TGATCAAGGCGCTCGTCAAGGA	339
FMgSSR-48016	scaffold1247	p3	(CAA)5	Class II	35202	35216	TGCGGTGGAATGTCCAAGCAGA	AGACTGTTGTGGTGGGCAGGAA	299
FMgSSR-48021	scaffold7296	p3	(CAA)5	Class II	27611	27625	TGCTCCTTGTTGCTTGGCGA	AGAGAGCACACCGTCAGCTTGT	324
FMgSSR-48024	scaffold8013	p3	(CAA)5	Class II	8366	8380	TGCTTGCCACTTTGCTCCTTGC	ACACATGCATTAGCCCCCTGCT	224
FMgSSR-48026	scaffold3284	p3	(CAA)5	Class II	34963	34977	TGGAAGGGGATGAGACAACCCAGT	AGCAAGTAGGGACAGCCTTGACA	341
FMgSSR-48033	scaffold10297	p3	(CAA)5	Class II	22651	22665	TGGCTGAACGAGGTTCCGTTGT	TGCTCTGTGACTGACTGTGTGC	345
FMgSSR-48051	scaffold21300	p3	(CAA)5	Class II	13029	13043	TGTGTAGTAGGCGGTGTGAGGGAA	TGATGCTGTCGGTGTAGTGGGA	200
FMgSSR-48055	scaffold19768	p3	(CAA)5	Class II	3597	3611	TGTTCTGACCTGCGGTGGAAA	TGTGGAGCGAAAGAATTGCAGG	329
FMgSSR-48069	scaffold31334	p3	(CAA)5	Class II	2949	2963	TTGCCGGCGTTGGAGAAGATGA	TGCTCGTCGCATCGCATTGT	329
FMgSSR-48070	scaffold2152	p3	(CAA)5	Class II	12474	12488	TTGCGTGTGCAACCCGATGA	TTGAGACGTGGCCAGTAGACGA	334
FMgSSR-48075	scaffold7282	p3	(CAA)5	Class II	27327	27341	TTGTGTTGGTGGGGCCGTTT	TTCAGTTGATGGGAGGCAGGCA	232
FMgSSR-48078	scaffold8051	p3	(CAA)5	Class II	14354	14368	TTTCAGCGGCAGCCTTCTCA	TCTTGCTCTCCACATGCAGCCT	214
FMgSSR-48109	scaffold28044	p3	(CAA)6	Class II	2142	2159	AAGAGCACGAGAGGCAGAGCAT	GCGGGCAACAAAGTGATGCTGA	350
FMgSSR-48132	scaffold196	p3	(CAA)6	Class II	79708	79725	ACAGCCGGAGCAGTTCACAGTT	AGCTAATACCAGTGGGTACACGGC	301
FMgSSR-48138	scaffold54	p3	(CAA)6	Class II	220040	220057	ACATAGCCTCTCTCGCCAGTCCAT	AGGATTTTTGGCGCTGCTCA	349
FMgSSR-48144	scaffold2342	p3	(CAA)6	Class II	42779	42796	ACCCCAAGCGTTTGGTCTTCCA	CCCGCTGCCTGTTTCTTCTTCT	265
FMgSSR-48166	scaffold698	p3	(CAA)6	Class II	47076	47093	ACTGGCAACTTCAATGCCGAGC	TAAAGGCCGCTCCCTACGAACA	273
FMgSSR-48177	scaffold3124	p3	(CAA)6	Class II	56737	56754	AGCACCACGACTGCATCGTCAA	AAACGCGTGGAAATCAGCGTCC	325
FMgSSR-48193	scaffold997	p3	(CAA)6	Class II	75896	75913	AGGCAGAACGGAATCGGGTT	TGCAAGCCAACACATGGTGC	349
FMgSSR-48198	scaffold3058	p3	(CAA)6	Class II	27441	27458	AGTAGCGCAAGTGGCGAAGA	TGCTGCGAATGCTGCTTGCT	215
FMgSSR-48200	scaffold1237	p3	(CAA)6	Class II	59003	59020	AGTGCAGCGAGCAACACAGT	TCACCGAGGATCACCCTTTGGA	306
FMgSSR-48206	scaffold453	p3	(CAA)6	Class II	110566	110583	ATCTCATCGACGACGTGCTCCT	TGTCACTGTCGCCAGCACTGTT	338

SSR_ID	Scaffold	SSR_Type	SSR_Motif	SSR Class	SSR_Start	SSR_End	Forward sequence	Reverse sequence	Product_size
FMgSSR-48209	scaffold950	p3	(CAA)6	Class II	58165	58182	ATGATGAAGCCATCGCCCCCAT	TTGCCGCAGTTGCAGTCCTT	227
FMgSSR-48223	scaffold6537	p3	(CAA)6	Class II	11348	11365	CACGTGCCATGCGCGAAAAT	TGTCGGGTCGTTAGGATGAGTT	349
FMgSSR-48228	scaffold387	p3	(CAA)6	Class II	29439	29456	CGCATTTCAGCTGGCAACACCA	TTGGAACTGCAAAGGGGAGGGA	330
FMgSSR-48242	scaffold276315	p3	(CAA)6	Class II	241	258	GCGCTCGTTGTTCGCGAATTGA	TTGGCAAAAGGAGGCCATGCAC	254
FMgSSR-48243	scaffold1008	p3	(CAA)6	Class II	46384	46401	GCGGGCGACAAAACTCCAACTT	ATCGCCCGGTGCCCATAAATGA	200
FMgSSR-48244	scaffold3022	p3	(CAA)6	Class II	18301	18318	GGTGTGACCTTTTCCCTGTGCT	AACTCGAAGCGGTACTGGGTGT	297
FMgSSR-48258	scaffold9560	p3	(CAA)6	Class II	12012	12029	TCGTTCTGACCAACAGTCCCA	TTAGGCGATGCACGTAGAGAGC	341
FMgSSR-48260	scaffold437	p3	(CAA)6	Class II	22589	22606	TGACGGGGTGGCATAGCTAACA	TGCATAAGTTCCCGGCTGGT	345
FMgSSR-48281	scaffold5593	p3	(CAA)6	Class II	2407	2424	TGGAACATGGTTCTGACCAACGGT	TGGGCAATGCACGTAGAGAGCTT	347
FMgSSR-48298	scaffold3036	p3	(CAA)6	Class II	14774	14791	TGTCCGCGAAGGAAAACCACCT	TGTCGTGCAATGCGAAGCCAT	220
FMgSSR-48314	scaffold1002	p3	(CAA)7	Class I	15035	15055	AACAGCTGGACGAACTTGCC	AGAGCAGCTACTGCACGGAA	292
FMgSSR-48320	scaffold8245	p3	(CAA)7	Class I	4826	4846	AAGGCGACTGCAGCATCATCGT	TTGGTGCGCGCGTATCTGTT	339
FMgSSR-48365	scaffold17100	p3	(CAA)7	Class I	15928	15948	AGGGTCTACCGTCATGGGCTAA	AGGCCAGGCCAATGCCGATAAT	278
FMgSSR-48372	scaffold16825	p3	(CAA)7	Class I	5165	5185	ATCACCAATCGCAGGACCAGCA	CGCTCGCGGCACACATATAACA	245
FMgSSR-48375	scaffold102	p3	(CAA)7	Class I	3376	3396	ATCAGTTGGACGACTGGGTGCT	TGCTGTTGTGGCTCGTCAGT	331
FMgSSR-48405	scaffold31395	p3	(CAA)7	Class I	2654	2674	TCAGCTGCCCATGCTGTTCT	AACGTGCACTGTGACCGTGAGA	272
FMgSSR-48420	scaffold8	p3	(CAA)7	Class I	258294	258314	TGCAGTACCAGTCCCACACCAA	GGTCTGCCACTGCCCAGATAAACA	266
FMgSSR-48424	scaffold25648	p3	(CAA)7	Class I	9571	9591	TGCGGTGGCCATGACATCCTAA	GGCCATTCCCAAGGAAGCTTGT	331
FMgSSR-48437	scaffold13447	p3	(CAA)7	Class I	19869	19889	TTAAACGCCCAAAACCCACCGC	AACCCGAGGAGGACGATGATCT	306
FMgSSR-48450	scaffold28251	p3	(CAA)8	Class I	10485	10508	AACTGCTTGCGGGACAACATGG	TGCATCAGAACCACCACAGAGCA	224
FMgSSR-48479	scaffold55851	p3	(CAA)8	Class I	3318	3341	AGTGCTGCTTCCAGTGCCTT	TTGTTGCCAGCGCCCATAGT	346
FMgSSR-48491	scaffold23468	p3	(CAA)8	Class I	5394	5417	GGACCAAAGCATGTGATGCAAACG	ACAAACCTGCAGCATCCTTGGC	308
FMgSSR-48502	scaffold14140	p3	(CAA)8	Class I	6751	6774	TGCAGCATGTCAGCAAGGAAGA	TGAGCATCAGAAGGCAGGACCA	346
FMgSSR-48526	scaffold8058	p3	(CAA)9	Class I	16553	16579	ACCGGAGCAGATAGGCTTAGCA	TTGTTCGGATCGCAAGGGCA	241
FMgSSR-48529	scaffold1500	p3	(CAA)9	Class I	69667	69693	ACGGAACAGAGGCGACCACAAA	ACGACGGCATGGCTGTTGAT	314
FMgSSR-48533	scaffold68234	p3	(CAA)9	Class I	1162	1188	AGCAATAACCTGGGCAGCAGCA	TGGGGAAGTTGCTCAACAGCGT	244
FMgSSR-48551	scaffold5986	p3	(CAA)9	Class I	7016	7042	GGCAACAGATTGCCTGGACAGT	ACCATGGCTGGTGGGAGTGATT	337
FMgSSR-48564	scaffold9421	p3	(CAA)9	Class I	12905	12931	TGGCTTGGGATCATCGGTGCTT	TTGCCGTGATCCGGGCAGTTTT	232

SSR_ID	Scaffold	SSR_Type	SSR_Motif	SSR Class	SSR_Start	SSR_End	Forward sequence	Reverse sequence	Product_size
FMgSSR-48569	scaffold17108	p3	(CAC)5	Class II	11970	11984	AAACAGCTTGCCGAGAAGGGGA	TGGATGCGGCGGCGATTATT	288
FMgSSR-48573	scaffold2017	p3	(CAC)5	Class II	10332	10346	AACAGCTGGTGGCATGGGTTCA	AACGGGATCGTCGAGGCAATGT	284
FMgSSR-48576	scaffold38311	p3	(CAC)5	Class II	5915	5929	AAGGGGATGATGAAGGCGCT	AGCAGCAGCAGGGTCGGTTATT	305
FMgSSR-48579	scaffold745	p3	(CAC)5	Class II	48884	48898	AATTCTGCTGCCGTCCAACGTG	TTGGTTGCCTGACGCTTCGT	281
FMgSSR-48580	scaffold7467	p3	(CAC)5	Class II	20452	20466	AATTGCGAGAGAGCAAGCCACG	ATCGAGAACATGGCCGCGTTGA	317
FMgSSR-48582	scaffold680	p3	(CAC)5	Class II	101337	101351	ACACTCTACCTGGCGCACAT	TGACTGCCTGAGTGTGTCCTT	329
FMgSSR-48583	scaffold6056	p3	(CAC)5	Class II	11720	11734	ACACTGCCGCAACATCTGCT	AGCGTCGATCCAACCAGTCCTA	291
FMgSSR-48587	scaffold18268	p3	(CAC)5	Class II	5448	5462	ACCACGGCCATGAACACCACAA	GCGGAGTTTAGCAGCGCAACAT	241
FMgSSR-48593	scaffold161493	p3	(CAC)5	Class II	984	998	ACCGCTGCAGCACTTCAACT	ATCCGAAGGCCTCGATGTCCAA	298
FMgSSR-48594	scaffold258	p3	(CAC)5	Class II	73933	73947	ACGACGACCTGCTCCATATGCT	GGCTGGAAGTGGTTGTTGTGCT	226
FMgSSR-48596	scaffold57347	p3	(CAC)5	Class II	1499	1513	ACGCCCCATTTGTCTTGCAGTG	ATTGCGGCGCCTACAAACAC	312
FMgSSR-48598	scaffold34352	p3	(CAC)5	Class II	4629	4643	ACGCTTCATCCACCCCAGCATA	TGATGCTGATGATGGTGGCCT	280
FMgSSR-48602	scaffold10939	p3	(CAC)5	Class II	26447	26461	AGCAATAGCTGCATGGATCCCG	ACCTTGGTCTTGCCGAACTGGT	338
FMgSSR-48604	scaffold18363	p3	(CAC)5	Class II	12582	12596	AGCAGCACGTTCACGACGTA	TCGCAATCATCGCGGCTTCT	313
FMgSSR-48605	scaffold10837	p3	(CAC)5	Class II	18212	18226	AGCATGTGGTCGAACGAACGGT	ACGCACACGCACACAGTACA	208
FMgSSR-48606	scaffold644	p3	(CAC)5	Class II	86189	86203	AGCGAAACCCGTGGATGTGA	ACGAAACACGACGGAGCTGGTT	285
FMgSSR-48608	scaffold1519	p3	(CAC)5	Class II	34854	34868	AGCTCGCGCAGAATCAACGA	ATTTTCCGCACGCGCTTCCA	276
FMgSSR-48615	scaffold8729	p3	(CAC)5	Class II	10224	10238	ATGCACGCGTCGGTTTCCAT	ACGAACGTGCTTGTTCCGTTGC	238
FMgSSR-48617	scaffold9417	p3	(CAC)5	Class II	22401	22415	ATGGGAAACCTCGCCGACAACT	AGTCGCAAGGCATGGCATCA	228
FMgSSR-48619	scaffold499	p3	(CAC)5	Class II	87947	87961	ATGGGAGCCTCGCCGACAATTA	AATCGCGAGTCAAACGAGGGCA	264
FMgSSR-48626	scaffold118277	p3	(CAC)5	Class II	69	83	CCAGCTGCGTTCCAAGCTTCTT	TTTCTCCTCGCGTGTTTGTCGC	321
FMgSSR-48629	scaffold4334	p3	(CAC)5	Class II	36760	36774	CGGTTGCAGAGGCATGCTCAAA	TCGACGAGATGTGCATGGCAGT	334
FMgSSR-48631	scaffold90384	p3	(CAC)5	Class II	146	160	GCACATCGCAAGATGCCGTT	TGTTCTCGCTGCTGTCCGTCTT	306
FMgSSR-48637	scaffold6988	p3	(CAC)5	Class II	32996	33010	TCAAGCGGTGGAGGAAGGAGAT	ATCATGATTGCGGTGGCGGT	326
FMgSSR-48638	scaffold2549	p3	(CAC)5	Class II	26695	26709	TCACCCTCCAGTCAAAGTGGCT	TTGCTTGAGCACCAGGCAAGGA	276
FMgSSR-48639	scaffold480	p3	(CAC)5	Class II	89504	89518	TCCAAAGCTTGCCACAGGACCA	AGACACCCGCAACGTGACAA	234
FMgSSR-48640	scaffold50350	p3	(CAC)5	Class II	3603	3617	TCCCCAATCCCCATCTCCTCCATT	TTGAGCCGTTTCACGCTGTCCT	347
FMgSSR-48643	scaffold11968	p3	(CAC)5	Class II	4781	4795	TCCTCGCTCTGCTATGACGACT	GCGGCGCCGATAATATCCCTTT	256

SSR_ID	Scaffold	SSR_Type	SSR_Motif	SSR Class	SSR_Start	SSR_End	Forward sequence	Reverse sequence	Product_size
FMgSSR-48644	scaffold3840	p3	(CAC)5	Class II	30086	30100	TCCTCTGTAGCTGCGGCTTTGT	CGCGTGGGTTTGCGCATATGAT	320
FMgSSR-48648	scaffold157644	p3	(CAC)5	Class II	166	180	TCGCGTAAAACTCTCTCGGCGT	ATCCGGTGACACCAATGACGCA	291
FMgSSR-48649	scaffold1789	p3	(CAC)5	Class II	56480	56494	TCGGGGCACGTTTTCGTCAA	TCGTGCGGGGTTTCGTTTCTCA	245
FMgSSR-48650	scaffold716	p3	(CAC)5	Class II	53645	53659	TCGGTGACATTCAGCAGAGCCA	AGCTAGTCGAGCTTCCATGCGT	218
FMgSSR-48653	scaffold34352	p3	(CAC)5	Class II	4465	4479	TCTCCGGGTTCAGACGCTTCAT	TCGTTCGACCATATGCTGGGGT	210
FMgSSR-48654	scaffold296	p3	(CAC)5	Class II	86240	86254	TGACGCTGCTCGTCATCGTT	TTTGCTACGCTTGACGCCGA	251
FMgSSR-48656	scaffold3205	p3	(CAC)5	Class II	54876	54890	TGATGTTCACGACCTGCTCCCA	TTGATCCACGAGGCGTAGTGCT	268
FMgSSR-48660	scaffold7372	p3	(CAC)5	Class II	13496	13510	TGCGGTTTCGACATGATGGGCT	TCAAGACGACAAGCGAGCGACT	244
FMgSSR-48662	scaffold68234	p3	(CAC)5	Class II	391	405	TGCTTCTTCCCAACAGCCCACT	TCGTCCAAAGACAGCCGCAA	313
FMgSSR-48666	scaffold23882	p3	(CAC)5	Class II	6314	6328	TGTAGGCATCCATGCGCGTT	AGCGACAAGGTTCGTGGTCA	283
FMgSSR-48674	scaffold13513	p3	(CAC)5	Class II	16736	16750	TTGCAGCAACTGCAGCACCA	ACACCATCCCCCTTTGCAGCAT	340
FMgSSR-48677	scaffold9218	p3	(CAC)5	Class II	17193	17207	TTGTGTGTGCTCTCTCGCGT	TGTTGGGTTGCTGCTGGTGT	287
FMgSSR-48678	scaffold968	p3	(CAC)5	Class II	1713	1727	TTGTTGGTGGTGCAGAGGTGGA	TGGTGGAACAGGAAGTGGCGTT	301
FMgSSR-48680	scaffold24073	p3	(CAC)5	Class II	2997	3011	TTTTCCAGGAGCTATGGGGGTGCT	ACAGTTGTTCCCGAGCTGGT	214
FMgSSR-48681	scaffold1611	p3	(CAC)5	Class II	14716	14730	TTTTTGGTAGTCGGGGAGGAAGGG	TTTAAGCAACGGCGTGCGAC	298
FMgSSR-48685	scaffold5318	p3	(CAC)6	Class II	36623	36640	AAGAAGAAACGCAACGGCAGCG	GGCTCTTGCTGCCCTTCTTGAAT	292
FMgSSR-48687	scaffold566	p3	(CAC)6	Class II	61375	61392	ACAACGTCGTCGTCTTCCGT	TACCTCCACAGCAACAAGGGGT	290
FMgSSR-48691	scaffold202235	p3	(CAC)6	Class II	259	276	ACCGGGCTTTAATCCGTCCGTT	ATTGGGAATTGGGGGCGCTCTT	244
FMgSSR-48696	scaffold8161	p3	(CAC)6	Class II	26300	26317	AGGGCAGGGCAAACAAAGCA	AAAAAGGGCGAGGGAGTGGT	315
FMgSSR-48698	scaffold6281	p3	(CAC)6	Class II	32574	32591	ATGCTGGCAAGAAGGCAGAGCA	TTCAAGACCACGTTTGCCGC	215
FMgSSR-48703	scaffold2962	p3	(CAC)6	Class II	18779	18796	TCATGCCGGCAATGCACTCA	ACTGGTGTGTGATGCAGCTCCT	340
FMgSSR-48706	scaffold580	p3	(CAC)6	Class II	113569	113586	TGACCAACGAAGCACACCACCT	ACTCACACTCTCTGCTGCGTGA	238
FMgSSR-48707	scaffold3672	p3	(CAC)6	Class II	19046	19063	TGCACGGTGGCAATGAGACA	TGCGCCTGGTGATGTTGATGGT	260
FMgSSR-48709	scaffold7955	p3	(CAC)6	Class II	14695	14712	TGCCTCCAAACCAAACGTCCGA	ATAGTTGCTGCGTTCCGCGA	288
FMgSSR-48715	scaffold1487	p3	(CAC)6	Class II	11936	11953	TGTCACACACAAACCGCACTTG	TCTTCCACAGCAACAGCCGA	328
FMgSSR-48716	scaffold3841	p3	(CAC)6	Class II	6179	6196	TTCAACCCGCTCATGCTGCT	AGGGCATGGGCTTGCAGATCAA	337
FMgSSR-48718	scaffold147911	p3	(CAC)7	Class I	208	228	AACGCCATCATGGGCAGCAA	AGGCAGTTTGGCGTCACAGA	225
FMgSSR-48721	scaffold386593	p3	(CAC)7	Class I	106	126	ACCTTCACCTTGGCGCTCTT	TTTGACAAATAGCAGGGCGGCG	210

SSR_ID	Scaffold	SSR_Type	SSR_Motif	SSR Class	SSR_Start	SSR_End	Forward sequence	Reverse sequence	Product_size
FMgSSR-48725	scaffold5030	p3	(CAC)7	Class I	29977	29997	CAAACCGCAACAGCGAAGCA	ACTCCGCGTTTTGTTCGGGT	258
FMgSSR-48727	scaffold12083	p3	(CAC)7	Class I	21310	21330	TCACGAGGTCAAACAGGCCCTT	CGTGCCAGCAGCCATGTTTCTA	246
FMgSSR-48733	scaffold3191	p3	(CAC)8	Class I	7265	7288	ACGCTTCCTTGCCGTTCACT	AACCGTGCCGGGAGAAGAAGAA	325
FMgSSR-48739	scaffold5547	p3	(CAC)8	Class I	3309	3332	TGCAGCAGGTGCTCGATTCT	ACGTTGGAGCCGGAGATCTTGA	250
FMgSSR-48741	scaffold332	p3	(CAC)9	Class I	109940	109966	AACATTGAGCCCAGCAGTAGCC	ATCCCAGCCGCCGAGATGAAAT	272
FMgSSR-48743	scaffold179417	p3	(CAG)10	Class I	128	157	ACTTTCGCGCCTCATCAGCA	TCACCGACGTGAAGAAGCCA	238
FMgSSR-48746	scaffold2129	p3	(CAG)5	Class II	44205	44219	AAATAGGGCCGTTCCCGCCAAA	AGGCGTTCACGGTTCTTGTGCT	345
FMgSSR-48748	scaffold147278	p3	(CAG)5	Class II	485	499	AACAGCAGCAGCGAGAACAGGT	TGAAGTGGAACTTCGGCGCCAT	298
FMgSSR-48749	scaffold152175	p3	(CAG)5	Class II	784	798	AACAGCAGTCGCAGCAGCCTTA	TGACTGCCAAGCATGCCAACT	254
FMgSSR-48755	scaffold92197	p3	(CAG)5	Class II	545	559	AACGGCGGCAAGCTCAAGAT	TCATAGCCAGACTGGTGGTGATGC	206
FMgSSR-48760	scaffold768	p3	(CAG)5	Class II	62832	62846	AAGAGCAGGGTTGGCAATCGGT	AGAGAAAAACTGAGGTGGCGGC	286
FMgSSR-48763	scaffold41	p3	(CAG)5	Class II	1463	1477	AAGCACGCGAACCAAGCGAA	ATTCCAGCGCGGAGCACTTACT	347
FMgSSR-48768	scaffold3650	p3	(CAG)5	Class II	54506	54520	AAGCGAGGAGGCATGGCATCTA	AGCACCGTCTGCATGCATCATC	330
FMgSSR-48769	scaffold71	p3	(CAG)5	Class II	136601	136615	AAGCGAGTGGTTGCATGACG	TGTGTTCTCTTTGCTGGTTGGG	203
FMgSSR-48774	scaffold1621	p3	(CAG)5	Class II	26965	26979	AATATGCGCGCAACGCATGG	TGCATGGACGACGCCTGAAT	315
FMgSSR-48775	scaffold30025	p3	(CAG)5	Class II	6142	6156	AATCAGCGCAATCGCGGCAA	ATTCTGGGCAGCGTGCTGTT	303
FMgSSR-48777	scaffold5460	p3	(CAG)5	Class II	30023	30037	AATTCCCAATCCCGTGACGTGC	TCCGTATGCCGCATGCAACT	330
FMgSSR-48784	scaffold92	p3	(CAG)5	Class II	164586	164600	ACAATGAATCGGGCGGCGAA	AGTTGGATTTCGGGTGGGGCAA	267
FMgSSR-48785	scaffold1312	p3	(CAG)5	Class II	62282	62296	ACACACTCACACCCGCTCGTAT	AAGCAGGCAAACCCTGTCACCA	215
FMgSSR-48799	scaffold12723	p3	(CAG)5	Class II	21475	21489	ACCCGTTTCCGCATCTTCAGCA	TGAGCGACATGGAACTGCCACA	305
FMgSSR-48815	scaffold12482	p3	(CAG)5	Class II	21640	21654	ACGAAGCAGTCGTGGAAGAGCA	AGGGTGCGCTGGTTTGATGA	212
FMgSSR-48816	scaffold9621	p3	(CAG)5	Class II	17707	17721	ACGCATCCAAAGGTACTGCGA	TGCACACATGCACGCGTTTT	340
FMgSSR-48817	scaffold2835	p3	(CAG)5	Class II	34289	34303	ACGCCAAGCTGTTGAGCACT	AGCCACACGCGGATCTTTTGGA	299
FMgSSR-48820	scaffold15973	p3	(CAG)5	Class II	9783	9797	ACGGCATATTGGCTCGGGTTCT	AACGTGAACAGGCTGCACCA	342
FMgSSR-48821	scaffold15876	p3	(CAG)5	Class II	18221	18235	ACGGCGACGTACGCATGTAGTAT	TGACCACGGACGACGGATGAAT	292
FMgSSR-48829	scaffold8733	p3	(CAG)5	Class II	23150	23164	AGAAATCCCGCCGCATCGTT	TCGCGAATCGCAGCCATCTT	344
FMgSSR-48832	scaffold59199	p3	(CAG)5	Class II	1020	1034	AGACGGCAACAGCAGCAACA	CCGACATTGCCGCTGACATTGA	206
FMgSSR-48834	scaffold5336	p3	(CAG)5	Class II	15925	15939	AGCAAACCTGCTGCCGAGAA	TGCGGCGGTTCATCATGTCA	298

SSR_ID	Scaffold	SSR_Type	SSR_Motif	SSR Class	SSR_Start	SSR_End	Forward sequence	Reverse sequence	Product_size
FMgSSR-48848	scaffold223845	p3	(CAG)5	Class II	386	400	AGCGAGTTCTCCATGCCCCTTT	AAGCTGCTGCTGCTGTTGATCG	251
FMgSSR-48858	scaffold47900	p3	(CAG)5	Class II	1844	1858	AGGCCAGATTGCGTGGTGTTCA	ACGTAAGCCAGTGCCATTCGAT	350
FMgSSR-48859	scaffold252	p3	(CAG)5	Class II	39510	39524	AGGCCGGCTGCTTCAACTTT	TGTCGTCGTAGTTGGCCACGAA	345
FMgSSR-48863	scaffold16	p3	(CAG)5	Class II	35396	35410	AGGTCAAGCAGTATGACGCGCT	TCTCTGCGTTCTTGTCCCCTGT	235
FMgSSR-48871	scaffold6139	p3	(CAG)5	Class II	6855	6869	AGTTGTCCTGCTTCGGCGAT	TTGGTGGAGGTGTACTTGGCCT	340
FMgSSR-48879	scaffold537	p3	(CAG)5	Class II	67078	67092	ATCGGATCGCAAGAACGAGGGA	ATCCAAAATCGCGGGGCGAA	328
FMgSSR-48880	scaffold19248	p3	(CAG)5	Class II	583	597	ATCTGTCCCTGGCCACCTTTGA	TCACGCGGGGTATGCAAGGAAA	235
FMgSSR-48892	scaffold5068	p3	(CAG)5	Class II	26289	26303	CCATGCAGTCAGGCGTCAAGAT	TGGTGCTTGTCGATGTCGCTGA	310
FMgSSR-48893	scaffold28	p3	(CAG)5	Class II	88475	88489	CGCACGCTTTTGCACCGAAA	TCGAGGCAACCCTGTCACGAAA	241
FMgSSR-48894	scaffold79533	p3	(CAG)5	Class II	232	246	CGCTCGTTGCGAGCAACAACAA	TCGACGACGACGGGACAATGAA	304
FMgSSR-48902	scaffold110213	p3	(CAG)5	Class II	450	464	CGTCCAGCACGCTTTTGCGAAT	TGGCCAAATGGATTGGCACG	324
FMgSSR-48903	scaffold336525	p3	(CAG)5	Class II	153	167	CGTCTGTTTGTTGCTGCACGGT	ATCGCCATCAGGCTCGCAAA	229
FMgSSR-48904	scaffold390518	p3	(CAG)5	Class II	72	86	CTCGATCGTTTTGCCCTTCTATGC	TCAGTGGAAACAATGTGCACCACC	200
FMgSSR-48911	scaffold58	p3	(CAG)5	Class II	144569	144583	GCACGCCACAAACCTTCCTT	TGTTCCTTCCTTCGAGCTCCTTCC	239
FMgSSR-48916	scaffold73083	p3	(CAG)5	Class II	392	406	GCGATGATGTTGAAAGCGGCCA	ATGCAACGCCTCAACGTGGT	320
FMgSSR-48920	scaffold808	p3	(CAG)5	Class II	18953	18967	GTCGTCCATGGCTTTGCAGCAT	TCCTGCAAGCGTTAATTGCGGC	336
FMgSSR-48928	scaffold1023	p3	(CAG)5	Class II	23909	23923	TACGGCCTCTAGGAATTCACGCCT	GCAGTTTGTCCGTCGTTTACAAGC	347
FMgSSR-48932	scaffold9614	p3	(CAG)5	Class II	8240	8254	TCAGCGACTCAAGCCACCAA	CAGTTGCTGTGTCGGCATCTGA	344
FMgSSR-48933	scaffold1691	p3	(CAG)5	Class II	23662	23676	TCATCGGCGTCGAGGAAGAA	AGAAAAGCCGTCAGAGCCGA	285
FMgSSR-48938	scaffold9667	p3	(CAG)5	Class II	2374	2388	TCCAGGTCGGCGGACTAAAT	ACGGCAGTGGGCAAAGTGTT	350
FMgSSR-48940	scaffold10074	p3	(CAG)5	Class II	10031	10045	TCCATTCCAGTGCATTGCCCGT	AGCGCAACCTGCTTCTGGAAGAT	280
FMgSSR-48943	scaffold349	p3	(CAG)5	Class II	126345	126359	TCCCTTTGCTTTCGCTGCCA	TGCAGCTGGTGGTGATGCTGAT	349
FMgSSR-48944	scaffold2331	p3	(CAG)5	Class II	44013	44027	TCCTACTCTCCTCCTCTCCACCAA	AAATCGTTGCCCTCCTCCTCGT	298
FMgSSR-48949	scaffold6088	p3	(CAG)5	Class II	9485	9499	TCGAGCCGGAACATGTGGAA	TCAATCGCCCCTTGTTCGCA	333
FMgSSR-48950	scaffold2748	p3	(CAG)5	Class II	51874	51888	TCGCAGATCGCGTTGCACTT	AGCACCGGCTGCTTCTGATGAT	230
FMgSSR-48951	scaffold3121	p3	(CAG)5	Class II	40533	40547	TCGCGCTGCAGATGCAACAA	TGCTCGTGCTCCTCATGGTGAT	202
FMgSSR-48956	scaffold9023	p3	(CAG)5	Class II	11787	11801	TGACTCGTTGTTGCACCGGGAA	AGCAGCACCACCACCAACAA	313
FMgSSR-48957	scaffold3050	p3	(CAG)5	Class II	49619	49633	TGAGGCCAGCCGGGGAAAATAA	ACCTCGCATCAGCCCAAGGAAA	231

SSR_ID	Scaffold	SSR_Type	SSR_Motif	SSR Class	SSR_Start	SSR_End	Forward sequence	Reverse sequence	Product_size
FMgSSR-48960	scaffold68	p3	(CAG)5	Class II	67426	67440	TGATACCCCACAGCTGCCTCAA	TGCTGTGCCTGCATGCTGTT	233
FMgSSR-48962	scaffold16981	p3	(CAG)5	Class II	6690	6704	TGCAAGATTTGGTTGCGAGCCC	AGGTTGTTGCCCCTGCATCAGT	303
FMgSSR-48965	scaffold910	p3	(CAG)5	Class II	66770	66784	TGCAAGGGACGATTTGCGGT	AGTCGGCGTTAGCGGACAAA	287
FMgSSR-48966	scaffold5656	p3	(CAG)5	Class II	37279	37293	TGCACGGGCGTGAGGAATCAAT	TGAGTCCAGCAGGAACTGGTGA	322
FMgSSR-48968	scaffold1839	p3	(CAG)5	Class II	19660	19674	TGCAGCAGCAGCAGGAACAAAA	AAGGCAAAGGTGTTGCTGGTGG	209
FMgSSR-48969	scaffold32881	p3	(CAG)5	Class II	3594	3608	TGCCAGGCCAGCAAACCAAT	CGGTAACACGCCGCAAAGTGAA	326
FMgSSR-48972	scaffold9402	p3	(CAG)5	Class II	18352	18366	TGCCGCGAACAAAGCAACGA	CGCACGTGTTCGTGAACCTA	329
FMgSSR-48973	scaffold30025	p3	(CAG)5	Class II	6367	6381	TGCGCCACGCTCTGCATAAA	GCGCAATCCAGCGGCAATTT	230
FMgSSR-48974	scaffold5924	p3	(CAG)5	Class II	25180	25194	TGCGCGAGTTGATTTGGCGA	TCTGCTTCCTTCGGTTCAGGGA	305
FMgSSR-48981	scaffold84729	p3	(CAG)5	Class II	1199	1213	TGGACCGGACCAAAAAGGGT	GGCGCTGGTGGATGCTTTTCAT	302
FMgSSR-48982	scaffold17871	p3	(CAG)5	Class II	918	932	TGGCAGCCTGGCTAGTGGAAAA	CGTGTGTTTGTCTGCACCGCAT	300
FMgSSR-48987	scaffold688	p3	(CAG)5	Class II	82349	82363	TGGGCCGAATGGACTTTGAACCT	AAGCAGCCAGTGCTTCGGTT	280
FMgSSR-48988	scaffold4830	p3	(CAG)5	Class II	20014	20028	TGGGCTTGATTCTTGGTGGGCT	TCGACTCGACCAAGCAACTCGT	326
FMgSSR-48989	scaffold5170	p3	(CAG)5	Class II	42450	42464	TGGGGCACAAGCTTTGGCAT	AGGTCGGTGCTTTTGGAACCCT	242
FMgSSR-48993	scaffold576	p3	(CAG)5	Class II	82963	82977	TGGTGCAGAGTTGCAAGTCCCT	TCGACACGACCATCTTCCTCTGA	265
FMgSSR-48997	scaffold1269	p3	(CAG)5	Class II	85911	85925	TGTGTTCGGGACCGTGGACTTT	AGGAATGCGGCGTTCAGTTCCA	248
FMgSSR-49003	scaffold1621	p3	(CAG)5	Class II	37309	37323	TTCACTGATCGATCTGCGCGAC	ATGGCAGGGCTTGCTTGCTT	327
FMgSSR-49004	scaffold58183	p3	(CAG)5	Class II	743	757	TTCCACGAGCACAGGAGGCTTT	TGTTGGTCCTGGAGCTGCTGTT	202
FMgSSR-49007	scaffold430386	p3	(CAG)5	Class II	94	108	TTCGCGAGACAAAGTATCGGC	TCTATAGCCTCGCGATCCTTGC	202
FMgSSR-49019	scaffold5599	p3	(CAG)5	Class II	25231	25245	TTGAAGAGTTGGTTGGGCGACG	ACCAGTACTACCACGGCTTCCT	253
FMgSSR-49020	scaffold130	p3	(CAG)5	Class II	79002	79016	TTGACGTGTTGGCCTTCGCT	ACCGCTCGCTGGTTTCTTCT	348
FMgSSR-49022	scaffold12845	p3	(CAG)5	Class II	11934	11948	TTGCAACTGTACGCAGCTTGGG	TGCCGACATGAGCTGTGTGT	271
FMgSSR-49023	scaffold2477	p3	(CAG)5	Class II	59670	59684	TTGCACGCGCCGGAAATACT	TGCACAAAGGCGCCGCAAAT	260
FMgSSR-49024	scaffold85	p3	(CAG)5	Class II	145783	145797	TTGCCGTGGTACATGCCCTTGA	TGCGCGCTCACCTTCTTGTACT	233
FMgSSR-49025	scaffold23591	p3	(CAG)5	Class II	3746	3760	TTGCGGTCGATGCGGGATACTT	ACCCGCGTTGTTGTAGTGCT	302
FMgSSR-49026	scaffold3060	p3	(CAG)5	Class II	4514	4528	TTGGACTGGAAACCCGTGCCTT	TGATGACGGCTTCATGGGCA	264
FMgSSR-49029	scaffold5945	p3	(CAG)5	Class II	18270	18284	TTTCCGCCGCTGCCCAAAAT	AAGGCAGGGCCGGTTCCTTTTT	247
FMgSSR-49030	scaffold2082	p3	(CAG)5	Class II	48958	48972	TTTCGCTACAAAAGGGCGCACG	ACGGCACTGGATGCTGTTGA	275

SSR_ID	Scaffold	SSR_Type	SSR_Motif	SSR Class	SSR_Start	SSR_End	Forward sequence	Reverse sequence	Product_size
FMgSSR-49038	scaffold17683	p3	(CAG)5	Class II	12711	12725	TTTTGCGTACGACGACCCGA	TTGGCGGAGCCGGCATTGTTAT	219
FMgSSR-49058	scaffold6088	p3	(CAG)6	Class II	8981	8998	ACAGCATCTCGGCGGGCAATAA	TGCAGCGTTACCATCGTGGGTT	338
FMgSSR-49059	scaffold357216	p3	(CAG)6	Class II	260	277	ACCATGGCATTGCAGGTCCTGTT	TCCCCCTTCTTATTGGTGGGCT	248
FMgSSR-49062	scaffold14636	p3	(CAG)6	Class II	18982	18999	ACGTGCCAGCAGCAGCAAAA	TCGCAAAAGCTATTGGGCACGC	298
FMgSSR-49064	scaffold7843	p3	(CAG)6	Class II	19228	19245	AGAAATGGACCGGCAGCTCTGT	AACCCGTTCCTGCCCTACTT	220
FMgSSR-49065	scaffold5425	p3	(CAG)6	Class II	35519	35536	AGCACAGACTCGGAAAGCAGCA	TTGTGGGCTTGTGGCCTTGT	321
FMgSSR-49070	scaffold383860	p3	(CAG)6	Class II	106	123	AGCCTCACTGGCAGGGTTTTGA	TCCATGCTGCTCGCAAACGA	205
FMgSSR-49072	scaffold458	p3	(CAG)6	Class II	130800	130817	AGCGCAAGTTCGAGTGCCAT	AGTGATGATGCGGTGCGAGAGT	228
FMgSSR-49074	scaffold15	p3	(CAG)6	Class II	150032	150049	AGCTGCTGCCAAACAACGGT	AACTTGCCGCTGCTTCTGCT	221
FMgSSR-49079	scaffold130395	p3	(CAG)6	Class II	760	777	AGGTGGAGAGGTCCCAAGTCAA	ATGCGATGCAGCCTCCAACA	323
FMgSSR-49089	scaffold347	p3	(CAG)6	Class II	77430	77447	ATGGCGCAGCGATAGCAGGATT	AACGCGGCTGTGCACAAGTA	277
FMgSSR-49090	scaffold4497	p3	(CAG)6	Class II	2518	2535	ATGTCCTCGAACAGCAGCAGCA	AATGACGGGAGCGAAGCGAT	272
FMgSSR-49092	scaffold660	p3	(CAG)6	Class II	36477	36494	GCAGCTCGTGCAACAGAGCAAA	AAACTAGCACGGCTGCGACT	252
FMgSSR-49097	scaffold22591	p3	(CAG)6	Class II	1004	1021	TATGCCCTTGCACCAGCCACAA	ACCCATTGATGCCTGCTGGGTA	208
FMgSSR-49098	scaffold7496	p3	(CAG)6	Class II	6299	6316	TCAAAGCGCCAGCGGAAACT	AGGAATCAAAGCAGGCGACGGT	238
FMgSSR-49106	scaffold135901	p3	(CAG)6	Class II	97	114	TCCTTCGACACCGACAAGCA	ATGGCAAGCGCACCACCAAT	248
FMgSSR-49107	scaffold730	p3	(CAG)6	Class II	79329	79346	TCGCATTCGCACAAGCTACTCG	ATTTTTGGACGACCTCGGCG	268
FMgSSR-49108	scaffold21260	p3	(CAG)6	Class II	6488	6505	TCGTTCTCCGGCCTTGTGCATT	AGCTTGATGGAGGCGCCAAAGT	285
FMgSSR-49119	scaffold6149	p3	(CAG)6	Class II	13742	13759	TGCTGCCGAGTGGTGCTAAT	AGGACGGCGCGCAATTATCT	331
FMgSSR-49120	scaffold4559	p3	(CAG)6	Class II	3286	3303	TGCTTCTTGCTCCTGCTGCTCA	AGGCGATGTTAATGCGGAGCCT	284
FMgSSR-49125	scaffold622	p3	(CAG)6	Class II	111253	111270	TTACCGCTTGGTGCAGAGTTGC	AGGATAGGGTGATGCTGCGGAT	297
FMgSSR-49127	scaffold6459	p3	(CAG)6	Class II	9861	9878	TTGCCTAGCTGCCGCATGTT	ACAGCCGGAAGTTGAAGCCGAT	268
FMgSSR-49129	scaffold10077	p3	(CAG)7	Class I	15837	15857	AAGCAACGTCGCTGCCTTTTCC	AAAGCGTCAGCAGCACACCA	255
FMgSSR-49131	scaffold299	p3	(CAG)7	Class I	59049	59069	AAGGTTCAAACACTTGCGGCGG	AGTTGGTCAGCTTTGGCGGT	305
FMgSSR-49132	scaffold1544	p3	(CAG)7	Class I	48300	48320	ACAACAACCACCACCACCACCA	GCGTGAAGCACCGCGACATTTA	347
FMgSSR-49137	scaffold15114	p3	(CAG)7	Class I	16582	16602	AGCAGAGAACACGGGAAACGCA	AACCAGAGCACGAGCAAGAGCA	282
FMgSSR-49138	scaffold129168	p3	(CAG)7	Class I	152	172	AGCAGATCAACGGGCGGTAT	ACGACGGCGAAAGCATCTGT	237
FMgSSR-49139	scaffold3492	p3	(CAG)7	Class I	9228	9248	AGTCCGACTGCAATGACCCGTT	AGCGTGCCGAAGATGCTGAA	311

SSR_ID	Scaffold	SSR_Type	SSR_Motif	SSR Class	SSR_Start	SSR_End	Forward sequence	Reverse sequence	Product_size
FMgSSR-49142	scaffold8025	p3	(CAG)7	Class I	29982	30002	ATTCGATTCGATTGGGGCTGCG	AAACGGGAAGCAGCGAGACA	341
FMgSSR-49143	scaffold8805	p3	(CAG)7	Class I	29333	29353	GCATCTGCAGCGGCATGTTCAA	AACAAGCGTTCCGGTTCCGT	341
FMgSSR-49144	scaffold4877	p3	(CAG)7	Class I	6935	6955	GCTGAGACTGCGAGCGATCAAA	TCCAACAGCACGAGACGCAA	270
FMgSSR-49145	scaffold12911	p3	(CAG)7	Class I	23375	23395	TAGAGCGCCAGCCAGTCAAACT	TCACCTACATATACGCGGCGGT	343
FMgSSR-49152	scaffold240	p3	(CAG)7	Class I	114445	114465	TGCGAACCAGTACACCAACGCT	AGAAGACAGGTGTGCGTCAGGT	335
FMgSSR-49156	scaffold74	p3	(CAG)7	Class I	107134	107154	TTCAATGCGAGCAGAGGCCA	AATGAATGCGCGGCATGTAGCC	341
FMgSSR-49160	scaffold791	p3	(CAG)7	Class I	98171	98191	TTTCCGCCGCTGCTCAATGCAA	TCATGGAAACGCACCAAGGC	278
FMgSSR-49162	scaffold169	p3	(CAG)8	Class I	1248	1271	ACCAGTCGACGATTTTGGCCGT	TCAACACCGCCCCCAAAGTT	270
FMgSSR-49165	scaffold55441	p3	(CAG)8	Class I	1814	1837	AGTTCTGCGTCTACACCAACGC	AACAAGTTGTCGGTCGCAAGGG	280
FMgSSR-49167	scaffold1298	p3	(CAG)8	Class I	22197	22220	TGTTGGAAAAGGCGAGGTCGGT	CGCGGGTACAAGCACTTCAA	325
FMgSSR-49168	scaffold214179	p3	(CAG)9	Class I	603	629	AAATTGAGCTTCGACCTCCGGC	TCGACATCTCCTTCTCCATCTGC	213
FMgSSR-49174	scaffold177	p3	(CAT)15	Class I	126540	126584	GGTCCCCTCATTGTCAAGCACACA	TGGGAAGTCTTGGCATCAAGGACG	208
FMgSSR-49175	scaffold4306	p3	(CAT)17	Class I	37492	37542	ACAACGACCCGTTTTGATGGGA	TCCACCAGGGCTACTCATGCTGAT	343
FMgSSR-49177	scaffold1292	p3	(CAT)5	Class II	46013	46027	AAACGATAAGCTTGCGCGGC	TTTGGCTTGCAGCCTCTGCT	256
FMgSSR-49186	scaffold2357	p3	(CAT)5	Class II	16197	16211	AAGACGTTGAGGAACACCGACG	TCACGCTGCTCTGGCATTTCCT	214
FMgSSR-49190	scaffold2404	p3	(CAT)5	Class II	17540	17554	AAGGGACGGGCGAAACAGGAAT	AGCGAGCATGCCATGGATGTGA	316
FMgSSR-49194	scaffold2304	p3	(CAT)5	Class II	13443	13457	ACAGGGCTATCAAGCGCAGCAT	AAATGATGCCCCGGTGCTTGGA	310
FMgSSR-49199	scaffold41559	p3	(CAT)5	Class II	1438	1452	ACCCAGCTGGTTATGTGGTCGT	ACGGCAACCTGAAGCCGTTCAA	286
FMgSSR-49201	scaffold200505	p3	(CAT)5	Class II	545	559	ACCTGAAAACGATGCGGGGTGT	TGAGGCGGGGATTCAGATTCGT	219
FMgSSR-49202	scaffold2694	p3	(CAT)5	Class II	15571	15585	ACGACGAGGCAGCATTGCAT	TTTTACTGCCCAGAGGCCGACA	262
FMgSSR-49210	scaffold1455	p3	(CAT)5	Class II	29760	29774	ACGTCGTCGTACTGTTCTCTCT	ACTTTGGCGTTGCCCCTGTT	303
FMgSSR-49211	scaffold6394	p3	(CAT)5	Class II	21999	22013	ACTAAGCCCAAGTGCAGCGT	TGCGAGCACCGAAACATGTCA	229
FMgSSR-49227	scaffold9225	p3	(CAT)5	Class II	26578	26592	AGCAATCTTTCCCGTTCCTGTCCT	TGGCTCCAGTCAAAATGCGGAC	313
FMgSSR-49228	scaffold9342	p3	(CAT)5	Class II	26718	26732	AGCACCCGTAGTCAACGTGTCA	TGGGGCGTGACGTAGTCTGTTT	295
FMgSSR-49230	scaffold40	p3	(CAT)5	Class II	109982	109996	AGCAGCTCGTGCAACACAACA	AGGGCGGCTGCATCTAATGT	343
FMgSSR-49245	scaffold3104	p3	(CAT)5	Class II	14365	14379	AGGCGTGCATGGAGCACATGAT	TGTTTACGCGCGTTCCACGA	350
FMgSSR-49248	scaffold1298	p3	(CAT)5	Class II	35100	35114	AGGGCGTTGACTGGATTGGT	AGGAGGCGCGTATGACAACA	305
FMgSSR-49261	scaffold828	p3	(CAT)5	Class II	59972	59986	ATCAACAGCAGGCAAGGATGGC	AGCCCGTGGCAACATACAGT	221

SSR_ID	Scaffold	SSR_Type	SSR_Motif	SSR Class	SSR_Start	SSR_End	Forward sequence	Reverse sequence	Product_size
FMgSSR-49264	scaffold8685	p3	(CAT)5	Class II	6647	6661	ATCGCACCAAGTGCAGCAACCT	TGGCACCTGTTGTGCATGTGT	252
FMgSSR-49270	scaffold346	p3	(CAT)5	Class II	54697	54711	ATGGCACGTTCTGTTGCCGCTA	TTGCTTTGCTTGCTGCGCGA	326
FMgSSR-49271	scaffold6719	p3	(CAT)5	Class II	22697	22711	ATTAAAGGAAGCCGGTGGCGCA	AAATGGCGTACGACGAGGGGAA	265
FMgSSR-49274	scaffold483	p3	(CAT)5	Class II	113100	113114	CATGCATGCAGGCAAAGGCA	ATGCCCAAGAGCCAACAAGC	239
FMgSSR-49280	scaffold3705	p3	(CAT)5	Class II	5249	5263	CGGGTTGTCACAAAGCCGTTGA	ACAACCACCAAGCCCTGCCATT	341
FMgSSR-49286	scaffold5329	p3	(CAT)5	Class II	4670	4684	GCGACCCGTAACAAACGGTGAA	TGCGTGCCGTGTTTTGACCA	237
FMgSSR-49290	scaffold2001	p3	(CAT)5	Class II	66169	66183	GGAACCATGGTAGCAAACTGAGCC	TGAGCGCCAACCCCGTTTTA	304
FMgSSR-49299	scaffold359	p3	(CAT)5	Class II	66918	66932	TATCGCGCGCCCCTTTAACA	TGCCAATTGCCAACAAGAGGCG	285
FMgSSR-49301	scaffold2908	p3	(CAT)5	Class II	20847	20861	TCACAAACAAAAGCCCCCGCT	TGCCATTGGCCAAGCACACA	333
FMgSSR-49302	scaffold1614	p3	(CAT)5	Class II	33182	33196	TCACGGTGGCCGTTGATTTCCT	GCACGCCATGATGGGCTAGTTT	279
FMgSSR-49308	scaffold56163	p3	(CAT)5	Class II	2131	2145	TCCTCCGTCTCATCCTCTCCTT	TCGACGTGCCGTTCCAGTTCAA	210
FMgSSR-49310	scaffold1013	p3	(CAT)5	Class II	9396	9410	TCCTTGTGGATGCAACGGGAA	ATCCACCCGTTCATGTGCTCGT	250
FMgSSR-49319	scaffold10275	p3	(CAT)5	Class II	7218	7232	TCGGATGGCCGGTGAGATTGTT	AGCTTGGAGAATCGGGCACA	302
FMgSSR-49320	scaffold686	p3	(CAT)5	Class II	58390	58404	TCGTCCTCTTCGCCGTTGTGTT	ACGGAGCGGCGAAGCTTTTT	288
FMgSSR-49321	scaffold17627	p3	(CAT)5	Class II	12952	12966	TCGTCGCGTGTGTTTGGGTT	TGTCATCATCAACGTGCGTGCG	344
FMgSSR-49324	scaffold45	p3	(CAT)5	Class II	205006	205020	TCTTCTTCCTTGTCCGGCGA	TAGGCAGGCACACATGCCACAT	292
FMgSSR-49340	scaffold20410	p3	(CAT)5	Class II	10802	10816	TGCCGTGCATGTCGCACTTT	AGGATGGTCGCTGTCAACTGCT	258
FMgSSR-49343	scaffold15589	p3	(CAT)5	Class II	8315	8329	TGCGCGCTCAAAGAAAACGCA	TTTGTGGACATGGGAGCGGCTA	322
FMgSSR-49358	scaffold4190	p3	(CAT)5	Class II	12563	12577	TGGCATCTGAAACGTTGGGAGCA	TGTCAGAGCTATGGAGGACGGTGA	328
FMgSSR-49365	scaffold14071	p3	(CAT)5	Class II	558	572	TGGTCCGCAGCTCATTGTCAGA	AACTGGGTGAGTGAGGCTGCAT	226
FMgSSR-49366	scaffold76	p3	(CAT)5	Class II	99952	99966	TGGTTCTGACCCCATCCATAGCGA	TGAGCCTTGAAGCTTCTTGGTGGT	268
FMgSSR-49373	scaffold13513	p3	(CAT)5	Class II	12940	12954	TTCAGTGAACGTCGGTGGCA	GCGTACAAACAGTAACGGCGCA	343
FMgSSR-49377	scaffold7968	p3	(CAT)5	Class II	23246	23260	TTGAACGCGTAGGGGCAGTCAA	TCGGCCGAGACTGATGACAA	266
FMgSSR-49381	scaffold6691	p3	(CAT)5	Class II	26511	26525	TTGGCATGCACATGGCCTCA	ACGTGGCGGCGAATTGGTAA	324
FMgSSR-49383	scaffold3011	p3	(CAT)5	Class II	56179	56193	TTTGCCTGGTTGCCGAGGGTTT	CACACTGCCATCATCGTGTTCGT	269
FMgSSR-49385	scaffold4687	p3	(CAT)5	Class II	23178	23192	TTTGGGCGGCTTCACAACCA	AAGACTGAAAGGGTGCCGACCA	345
FMgSSR-49389	scaffold12680	p3	(CAT)6	Class II	21287	21304	AAACTAGTGCAAGGGCAAGTCC	ATGGCGGTTGAGATGGTGGA	350
FMgSSR-49391	scaffold2143	p3	(CAT)6	Class II	2285	2302	ACAACGTGGCCTGCATGCTT	TGAGGCACACGGAAACTGCT	316

SSR_ID	Scaffold	SSR_Type	SSR_Motif	SSR Class	SSR_Start	SSR_End	Forward sequence	Reverse sequence	Product_size
FMgSSR-49397	scaffold787	p3	(CAT)6	Class II	7443	7460	AGCAGCGTGCTGAATTGCTGA	GCGTCGGCATGCAACAACAA	340
FMgSSR-49400	scaffold396	p3	(CAT)6	Class II	1723	1740	AGTGCCATGCCGTTGCATCA	TGGCTCCTGTTGTTGACAGCGA	346
FMgSSR-49415	scaffold276553	p3	(CAT)6	Class II	413	430	TGATGTCCGGCACGTTGAGA	AGCATGATGAGGCTCCGGTTGT	247
FMgSSR-49420	scaffold42411	p3	(CAT)6	Class II	3842	3859	TGCTCATGAGGCGAACGAACGTA	TGCTCGTCGGCAGCAAGAACAA	317
FMgSSR-49421	scaffold24566	p3	(CAT)6	Class II	8917	8934	TGGACTACAACACACGTGGCGA	ACGCCAATAGGAGGAAGACGGA	205
FMgSSR-49429	scaffold12613	p3	(CAT)6	Class II	18777	18794	TTCCGGCTCTTCGCTTCGAT	AGAGAAAAGTGGCGCTGCGT	259
FMgSSR-49432	scaffold543	p3	(CAT)6	Class II	117001	117018	TTGGTTGTTGCTCCTGACGC	AGAAACTGCCGTACACCTTCGC	333
FMgSSR-49435	scaffold71709	p3	(CAT)7	Class I	1793	1813	ACAGCAACACCGCCAAGCAT	GCCAGGCACGTCTTGTTCATCT	245
FMgSSR-49436	scaffold52962	p3	(CAT)7	Class I	3192	3212	ACAGCAACGGGGGAATGAACGA	CGACCGCGACGTCTGAATTCTT	346
FMgSSR-49440	scaffold1155	p3	(CAT)7	Class I	41144	41164	ACCAGCATTACATCGGCGGT	ATGGCCCCCATTGAACAAGGCA	344
FMgSSR-49444	scaffold1191	p3	(CAT)7	Class I	34833	34853	AGAAGCCCATACCCAACCACCA	AGCTGCACCTGCAACTTGGACT	268
FMgSSR-49448	scaffold6383	p3	(CAT)7	Class I	32465	32485	CAAAAACGCGCACGCATGGA	AGCGCGGAGCAAACACACTT	350
FMgSSR-49457	scaffold7171	p3	(CAT)7	Class I	4398	4418	TCCGTCCTAGCAATCCGACCAA	AGGCGTTGGCGATGCTCTTGTA	313
FMgSSR-49460	scaffold2622	p3	(CAT)7	Class I	52433	52453	TGTCCACACCAACACACGCA	GCTTCTCCGCCGTTTGGCTTTA	346
FMgSSR-49465	scaffold2767	p3	(CAT)7	Class I	1811	1831	TTGCTGGCATGGCATCACTCCA	AAAGCCCGCCTACCCTGTAA	337
FMgSSR-49467	scaffold988	p3	(CAT)8	Class I	56505	56528	AGCAACAAGAGGTGGGACCGAA	ATTAAACGCCCAAGCAACGCCC	330
FMgSSR-49469	scaffold15458	p3	(CAT)8	Class I	736	759	GCACGCCACCGCATTATCATCA	ACGACTTCTACGACGTCAGCCT	227
FMgSSR-49474	scaffold125	p3	(CAT)8	Class I	123254	123277	TTCTTGTCCGGCGCCCCTTTTT	TGGCGGCCACACAAATGTTGA	333
FMgSSR-49478	scaffold16119	p3	(CAT)9	Class I	2097	2123	TTCCAGTGTCACTTGCCCCACA	CGCGGTGCACTGCAATTTGT	217
FMgSSR-49480	scaffold2898	p3	(CCA)10	Class I	24871	24900	TGGTTGGCGAACAGAAACTGCG	TGGCAAGCAGACCGATCGACAA	266
FMgSSR-49482	scaffold1821	p3	(CCA)5	Class II	10264	10278	AAAAAGGGCCCTGCATCGCT	AAAGCACTGCCACCATGCCA	206
FMgSSR-49485	scaffold594	p3	(CCA)5	Class II	27364	27378	AAAAGCCCAGTAACCCGCACCA	AGAAAGAAAGCGGCGTGCGT	348
FMgSSR-49509	scaffold4328	p3	(CCA)5	Class II	14402	14416	AACGATGCACTGCTGTCTGCGA	ACGCTTGTGCGTGTGACCTT	302
FMgSSR-49559	scaffold33	p3	(CCA)5	Class II	19697	19711	AATGCAGAGGCAGCTGAGCA	TCCAACGGCCAAGCGACAAA	319
FMgSSR-49584	scaffold19905	p3	(CCA)5	Class II	2329	2343	ACAAGAGCCAAGGACGCCAAGT	TTCGGTCACGCCACGAACAA	243
FMgSSR-49587	scaffold3413	p3	(CCA)5	Class II	39053	39067	ACAATTAATCCCCGGCCGCA	AGGCAACCGCGTAGGGTGTTTT	265
FMgSSR-49609	scaffold13447	p3	(CCA)5	Class II	19571	19585	ACAGAAGCACACGAGCCCGTAA	TGCGGTGGGTTTTGGGCGTTTA	213
FMgSSR-49629	scaffold264	p3	(CCA)5	Class II	63979	63993	ACCAGGCGAACGAAACAGCA	AACCATGTGGTGTCTCCCGTCT	277

SSR_ID	Scaffold	SSR_Type	SSR_Motif	SSR Class	SSR_Start	SSR_End	Forward sequence	Reverse sequence	Product_size
FMgSSR-49695	scaffold6106	p3	(CCA)5	Class II	14141	14155	ACCTTTGGTTGGCGAGCAGA	TGGGTTTGGCAAGCAGACCGAT	266
FMgSSR-49706	scaffold3838	p3	(CCA)5	Class II	2523	2537	ACGGACATGAGAGGGAAGGGTT	ACTGCTACGAACACAGCAGCGA	221
FMgSSR-49707	scaffold2342	p3	(CCA)5	Class II	51244	51258	ACGGCACCAGAATCACAGCA	ATGGTAGTGGCGTTCTGCACCT	337
FMgSSR-49711	scaffold158	p3	(CCA)5	Class II	9540	9554	ACGTCTTCCTCCACGTCGTTGT	AAGGATTCCCCCTGGAACGACA	200
FMgSSR-49715	scaffold4182	p3	(CCA)5	Class II	10067	10081	ACTGGTCGGCTGTGGTGACATA	AGTGTGCTGATGCGGCATAGAT	328
FMgSSR-49719	scaffold595	p3	(CCA)5	Class II	87976	87990	AGAAGCAGCAGCAACCGCAA	TCCCGACGACAGTTGTTCCA	341
FMgSSR-49724	scaffold4654	p3	(CCA)5	Class II	43971	43985	AGCAGCAACTCAACCGCACT	TTCGAATCCCGACGCTGACTGA	307
FMgSSR-49735	scaffold150997	p3	(CCA)5	Class II	764	778	AGCCACCACGAACGAAACGA	CGGTTTTGCCTTTTCCGCCTGT	289
FMgSSR-49747	scaffold303570	p3	(CCA)5	Class II	172	186	AGCTCCACGAACTCCGCAAT	CGCTGTTCGACCGCTTCTTCTT	229
FMgSSR-49748	scaffold9798	p3	(CCA)5	Class II	3871	3885	AGGAATCGAGCTGGGCCAACAA	TGGATCCATCCAATGCGCTTCT	309
FMgSSR-49750	scaffold11464	p3	(CCA)5	Class II	10239	10253	AGGAGGACGACGAGTTCGACAA	ATCATCTGTCGGCGCTGCTT	304
FMgSSR-49753	scaffold3399	p3	(CCA)5	Class II	29256	29270	AGGGTTCTTGCGGAAGCGTT	AGCTGTCATCGGCAATCACCTG	350
FMgSSR-49797	scaffold119316	p3	(CCA)5	Class II	118	132	ATCCTACAAACGCATCTGCCGC	AACGAGTTGGAGAGGTTGGCGT	257
FMgSSR-49798	scaffold1272	p3	(CCA)5	Class II	32827	32841	ATCGTTGTCGCCGTTGTTCACG	ACGACGACGTGGTTGTTGTGGT	248
FMgSSR-49821	scaffold483	p3	(CCA)5	Class II	82012	82026	CACCGCCCATGCACGTTTTT	TCGGATGTGTGCAACCACCA	286
FMgSSR-49838	scaffold5440	p3	(CCA)5	Class II	25852	25866	CGACGCAGAGGCAGCACAAAAT	TGAATCGCAACACGCCGACT	234
FMgSSR-49840	scaffold9156	p3	(CCA)5	Class II	29783	29797	CGCGGCCTCGTCTCAAGAAAAA	AAGGCCAAGGAGCTCATGCTGT	332
FMgSSR-49842	scaffold33955	p3	(CCA)5	Class II	4777	4791	CGTGGCGTGCACACGTATTA	ACGAAACCGGAAGAGACGAGCA	313
FMgSSR-49860	scaffold2905	p3	(CCA)5	Class II	23536	23550	GCCAACCTCACACCTTCTTCCT	ATCTGAGGGTTTCGGTGGCA	339
FMgSSR-49861	scaffold2233	p3	(CCA)5	Class II	43044	43058	GCCGTGTTGCGAGCATGTGTTT	AGTGTGACAAGCTTGGGCGT	202
FMgSSR-49863	scaffold773	p3	(CCA)5	Class II	62013	62027	GCGCGGGAACACGAAACAAA	TTACCTGGACGCCAAATCGGCA	348
FMgSSR-49898	scaffold6269	p3	(CCA)5	Class II	991	1005	TAAGCTGCCCCGTGAACAAGCA	AGGAGGCTTTGCATGCGGTT	263
FMgSSR-49916	scaffold4234	p3	(CCA)5	Class II	18912	18926	TCAATGGATAGGCCCCTGCCAA	ATTGGGTGGTGGTGATCCTGCT	221
FMgSSR-49927	scaffold7258	p3	(CCA)5	Class II	10445	10459	TCATGTTGCCTTCAGTGGCGGA	CGGAGTGTACACGACATGCATGGAA	207
FMgSSR-49928	scaffold21153	p3	(CCA)5	Class II	4523	4537	TCCACAACGCGCATCGCATA	TTGGGCGAATCGAGGCAGGAAT	326
FMgSSR-49955	scaffold532	p3	(CCA)5	Class II	17273	17287	TCCGCAGTTAATTCGGCCAGCA	CGTCGCACGCGAACAACAAA	344
FMgSSR-49960	scaffold8344	p3	(CCA)5	Class II	16580	16594	TCCTCTTCGACCTGCTCTCTGA	TTGACGAACCGGCCCTTGAT	331
FMgSSR-49967	scaffold4155	p3	(CCA)5	Class II	24549	24563	TCGTACGAGGAGCGCCTCTTTTT	TCTTGCTGAGGTAGCGCCTGAA	319

SSR_ID	Scaffold	SSR_Type	SSR_Motif	SSR Class	SSR_Start	SSR_End	Forward sequence	Reverse sequence	Product_size
FMgSSR-50209	scaffold66	p3	(CCA)5	Class II	75160	75174	TGATGAGCAGATGCGGAGACCA	TGTCGCGGAGACGAGCATGTTT	257
FMgSSR-50218	scaffold770	p3	(CCA)5	Class II	76427	76441	TGCCACATTGCCCATCGCTA	TGCGGCCGACAACTTTGTAACC	227
FMgSSR-50221	scaffold1905	p3	(CCA)5	Class II	5095	5109	TGCCCCACCACCGCTATTATCT	AATGGCAGCCGGTACAATGCCA	285
FMgSSR-50223	scaffold3201	p3	(CCA)5	Class II	4405	4419	TGCCGCCGTCGTCACTACTAAT	AAGAGCACGCTCGTTTCCTCCT	232
FMgSSR-50225	scaffold6329	p3	(CCA)5	Class II	5304	5318	TGCCGTCGTCAACAAACGGT	AGTCCCGGCGTTGTTTACCTGT	334
FMgSSR-50227	scaffold3381	p3	(CCA)5	Class II	47249	47263	TGCCGTTAAAACTGCCGCTTCG	TGACAAGGTGGTGGCCAAGT	333
FMgSSR-50234	scaffold12	p3	(CCA)5	Class II	171708	171722	TGCGATGCGATTCGTCAGCA	TTGGCGTCGAACACGGTGAA	347
FMgSSR-50235	scaffold9435	p3	(CCA)5	Class II	3648	3662	TGCGCTCATCTGCAGCCATCAT	ATCCGCGATCTTCGTCGTTGGA	284
FMgSSR-50242	scaffold1687	p3	(CCA)5	Class II	24270	24284	TGGACGGCGGTGAAACCAAA	ATTTTGTGGGGGCGTTTGCAGC	310
FMgSSR-50243	scaffold1412	p3	(CCA)5	Class II	79661	79675	TGGACTTGGGCAACCTCCATGA	CACTACTTTGGGAGACACGGCT	341
FMgSSR-50248	scaffold618	p3	(CCA)5	Class II	89436	89450	TGGCCATGCAACATGCCCTATCA	TGATCGTAAGTGGCAGCGGT	241
FMgSSR-50252	scaffold1657	p3	(CCA)5	Class II	47205	47219	TGGCGTCGTCCCTGGAATACAA	AAATGAAGCCGGGAGCAGGA	226
FMgSSR-50260	scaffold41130	p3	(CCA)5	Class II	3029	3043	TGGTGGCTGGAAGCTGCAAT	ATTCTAGGGTGCTGCTGTCGCT	248
FMgSSR-50277	scaffold961	p3	(CCA)5	Class II	36108	36122	TGTTGTGCCAATCACCCCCACT	TGACATAGTCGGCCATGGGGAA	245
FMgSSR-50284	scaffold78	p3	(CCA)5	Class II	152500	152514	TTCCTGTGCTGCGAATGCGA	GCCATGGCGATTCGGACTTTCT	342
FMgSSR-50285	scaffold4752	p3	(CCA)5	Class II	16892	16906	TTCGACAGCCATCATCCAGCCT	TGTTCATGGTGCTCCTCCGCTT	302
FMgSSR-50290	scaffold287	p3	(CCA)5	Class II	117928	117942	TTGCTGCTGTGTTGCAGCGT	AATGCCATCGCCTCCTGGGTAA	346
FMgSSR-50291	scaffold4258	p3	(CCA)5	Class II	4240	4254	TTGGAAGCACGCCCTCCATGTT	ACAAAGACACGAGCGAGCAGTG	311
FMgSSR-50297	scaffold1201	p3	(CCA)5	Class II	86326	86340	TTTAGCGCGCACAGAGAAGCCA	AATCAGTGCGAGGAAAGGGCGA	316
FMgSSR-50303	scaffold6269	p3	(CCA)6	Class II	1676	1693	AACGCCGGACTCGTCTACTT	TGTTGACCAGGTCGTAGCTGA	265
FMgSSR-50304	scaffold421037	p3	(CCA)6	Class II	163	180	AACTGCCACCACTGCCTACA	ACTTGTCGTACCTCCTGCCACT	203
FMgSSR-50307	scaffold765	p3	(CCA)6	Class II	51974	51991	AATAAACGGCCAAGATCGCGCC	TTGTCATTGCCGTACATCGCCG	202
FMgSSR-50308	scaffold136	p3	(CCA)6	Class II	13213	13230	AATTCACCGCACATCTCCGC	AGCGGCCAAAAAGCAAGCAGA	302
FMgSSR-50315	scaffold1983	p3	(CCA)6	Class II	17180	17197	ACACGCGAGTACCGTTGTGACT	TGCTGAACAAGGACGGCACCAT	319
FMgSSR-50324	scaffold8835	p3	(CCA)6	Class II	8131	8148	ACCGTCCACATAGGTGGGCATA	ACACCAGCCAATGCACCCTTGA	331
FMgSSR-50334	scaffold14158	p3	(CCA)6	Class II	2565	2582	AGCCCGGTTGATAGCCAACAAGT	CGCTGGATTTCTGTTTGTGTTGCG	298
FMgSSR-50337	scaffold1235	p3	(CCA)6	Class II	74560	74577	ATGAGGATTTGCGTGACGGTGG	ACAGAACAAACATGCGAGGACC	310
FMgSSR-50339	scaffold1312	p3	(CCA)6	Class II	63359	63376	ATGTGTAGCAGCACACGCCT	ACCCGTACGTCAGTGACTTGGT	253

SSR_ID	Scaffold	SSR_Type	SSR_Motif	SSR Class	SSR_Start	SSR_End	Forward sequence	Reverse sequence	Product_size
FMgSSR-50347	scaffold2591	p3	(CCA)6	Class II	20621	20638	GCGCTGCCACGAAATCAAGCAT	AGACAGGAGGACCAGCTGCATT	300
FMgSSR-50348	scaffold1583	p3	(CCA)6	Class II	56909	56926	GCGTTCACGAAATGGGGCAT	AGGACGTCGTCGTTGGCGATAA	278
FMgSSR-50350	scaffold1181	p3	(CCA)6	Class II	58240	58257	TAACGCCAATGCCGCTTCCA	AAGCACTAGCGTGGCAGCAT	261
FMgSSR-50352	scaffold3595	p3	(CCA)6	Class II	35215	35232	TAGCAGGCCAAAAAGGTGCCCA	ATACGTCGACCTCACGCACA	347
FMgSSR-50353	scaffold120	p3	(CCA)6	Class II	4478	4495	TCAGCTGCTTTGCCTCCCATCT	TGGCCAATGCGAAGGAAGTGGT	324
FMgSSR-50358	scaffold4974	p3	(CCA)6	Class II	23327	23344	TCCATTTCACGCTCTCGACGCA	AAGGGCATGGCATGTACGGT	258
FMgSSR-50379	scaffold3133	p3	(CCA)6	Class II	43987	44004	TGCACGCAACGCAACTTCCT	ATGCACAGCACTGCCACGTA	308
FMgSSR-50381	scaffold446	p3	(CCA)6	Class II	128352	128369	TGCGCACAACAACACAGGCA	TGATTTGCACGCGCTGTCCT	207
FMgSSR-50383	scaffold7655	p3	(CCA)6	Class II	22237	22254	TGGTGTTCGGCAGCAAGTCA	ACCGCGCGCCTAATCAAACT	235
FMgSSR-50389	scaffold11765	p3	(CCA)6	Class II	29338	29355	TGTGGCCCCCGTACAACAAACA	ACGGAATTTGCATCGTCCCACC	262
FMgSSR-50393	scaffold4474	p3	(CCA)6	Class II	52487	52504	TTTGGTCGCGTCCGTTCAGT	ACAAGCCAAGGTGCGGACAA	339
FMgSSR-50399	scaffold1396	p3	(CCA)7	Class I	57369	57389	ACGTTCGTGCGGTTGGTTCT	TGCCCCTATGCCTGTAGTGCTT	349
FMgSSR-50400	scaffold90	p3	(CCA)7	Class I	14791	14811	AGCCAGGCGAACACCGATTT	AGAAAAGGGGAGGAGAAATGCCG	332
FMgSSR-50405	scaffold164689	p3	(CCA)7	Class I	521	541	ATCTCCTGCTCTCTGCCCGTTT	TGGGCATCAAAAGCACGCCA	260
FMgSSR-50410	scaffold174928	p3	(CCA)7	Class I	138	158	TCGTCTGCTTGCTGCAACGAT	TCCCAGCCTGAGCGTCAATCAA	254
FMgSSR-50415	scaffold1396	p3	(CCA)7	Class I	56806	56826	TGCAGGACCCAAACACTTGC	TCCCGAGGCATCTTGGTATCTGA	350
FMgSSR-50426	scaffold1458	p3	(CCA)8	Class I	20287	20310	TCAGAGCGGATAAACGCGCA	TGGTTCACGTCGTGCTCGGAAA	296
FMgSSR-50428	scaffold1125	p3	(CCA)9	Class I	23784	23810	AGCGCCTGGCTTCAGCTACAAA	TGGAGTATTGCAACGCACGGT	350
FMgSSR-50429	scaffold2537	p3	(CCA)9	Class I	41867	41893	TCATCTGCACCGTACCCCTATCT	TTGGAGTCGCGCGTTGGATT	298
FMgSSR-50434	scaffold1439	p3	(CCG)5	Class II	81536	81550	AAAACCGCGAGTCGCACAAC	ACCGGGGAAAAAGCGACGTT	202
FMgSSR-50435	scaffold8166	p3	(CCG)5	Class II	27277	27291	AAAACCTCGAACCCTAGCTTCGGC	TTCTTGTCCTTGCTCGACTGCG	333
FMgSSR-50438	scaffold12485	p3	(CCG)5	Class II	5178	5192	AAAAGTTAGGGGCGAACGACGC	AAGCCGGAACGAGTGTTGACCT	276
FMgSSR-50439	scaffold12024	p3	(CCG)5	Class II	6375	6389	AAACCAGGGGCTGCCTGCAAAT	TACTGGACGCAGGTCGCTCAAT	251
FMgSSR-50441	scaffold662	p3	(CCG)5	Class II	8576	8590	AAACCCACCCATCATCCCGT	TGAGAGTTTTGCGGCGGCTT	227
FMgSSR-50442	scaffold55517	p3	(CCG)5	Class II	2591	2605	AAACCTCGGCAGATGGGTCGAT	TGACCGAAAGCCTGCTGCAA	315
FMgSSR-50445	scaffold4610	p3	(CCG)5	Class II	24868	24882	AAACTCTTCTTCGCCGCCACCT	ACAAGAAGGAACGATCACGGCG	332
FMgSSR-50446	scaffold29627	p3	(CCG)5	Class II	2568	2582	AAAGAATTGCGGCTGCGCGT	AAGCAGCCAAACAAAGCCCG	232
FMgSSR-50454	scaffold3670	p3	(CCG)5	Class II	41223	41237	AACAACACCTCACGCACGGT	AGCAATGTTAGCGACACCCACG	204

SSR_ID	Scaffold	SSR_Type	SSR_Motif	SSR Class	SSR_Start	SSR_End	Forward sequence	Reverse sequence	Product_size
FMgSSR-50459	scaffold5244	p3	(CCG)5	Class II	14905	14919	AACATCGTGCTCTACGCCGA	AGGATCCTGAAGAGGAGATGCGT	278
FMgSSR-50460	scaffold10436	p3	(CCG)5	Class II	18277	18291	AACCACCAACGCACGTAGCA	TGCTACGGCCCAAGCCAAAA	350
FMgSSR-50462	scaffold1690	p3	(CCG)5	Class II	42455	42469	AACCCCAACCCCAACGCAAA	TTGTACAGCTCCGCGATGTCGT	279
FMgSSR-50464	scaffold404	p3	(CCG)5	Class II	12253	12267	AACGGCGAGAGAGAGAGAGAGA	TTGAGGATTGAGGAGGCCGTGT	349
FMgSSR-50473	scaffold4446	p3	(CCG)5	Class II	14932	14946	AAGAAGCGGTGCGCGTTGTA	GCCCGCATCCAGGTTCACATTA	306
FMgSSR-50474	scaffold157	p3	(CCG)5	Class II	82121	82135	AAGACGACCTTGAACCTGGCGT	TGCTCGTTGCCTCTCCTTCT	334
FMgSSR-50479	scaffold1272	p3	(CCG)5	Class II	85130	85144	AAGATGACCATGGCGGTGACGA	TTTTTCCCCCATCGGAAAGGCG	346
FMgSSR-50481	scaffold2821	p3	(CCG)5	Class II	45341	45355	AAGCACACTTGCCTCGTCGT	TTGGACTTTGGAAGTGGTGGCG	333
FMgSSR-50486	scaffold1757	p3	(CCG)5	Class II	34985	34999	AAGCGCGGAGGGACTTTTGT	TTCTTCTCATTCGAGTGCGGGC	323
FMgSSR-50491	scaffold8623	p3	(CCG)5	Class II	14463	14477	AAGCTGCTGCTAAGGCTGCT	TGGCCTGCATCGTCACTGCATA	215
FMgSSR-50492	scaffold3725	p3	(CCG)5	Class II	33709	33723	AAGCTTGGATGGGCCTCGCAAA	AGTGACCGGATTGCGGAGTA	267
FMgSSR-50494	scaffold1060	p3	(CCG)5	Class II	87978	87992	AAGCTTTATTCCGCGACCGCCT	AGCGGAACAATTCGGTGCAGT	320
FMgSSR-50496	scaffold39732	p3	(CCG)5	Class II	1246	1260	AAGGAAAGCAAAGCGAGCGAGG	TCAGCACCGCTTGTCAGTTCTCA	344
FMgSSR-50497	scaffold10597	p3	(CCG)5	Class II	3600	3614	AAGGCCCACCTTTGTTGCCA	AGCACCCGATCGAGAAACAGGA	350
FMgSSR-50498	scaffold6661	p3	(CCG)5	Class II	34521	34535	AAGGCGACGTAGGCGAGAAA	TGTTGCGGGGAAAAGGAGGA	283
FMgSSR-50499	scaffold1093	p3	(CCG)5	Class II	91605	91619	AAGTCGAACAGCTGCGCCTTCT	AGAGAGCATCGGCATCGTGT	309
FMgSSR-50501	scaffold17627	p3	(CCG)5	Class II	11467	11481	AAGTGCGCGCATTCCTTCCA	TTGACGTCACCGCCGCAAAT	325
FMgSSR-50506	scaffold4755	p3	(CCG)5	Class II	43304	43318	AATCAGATCGCGCTTCACCCCA	GGCGATACCACTTCGATAGCCA	322
FMgSSR-50507	scaffold5609	p3	(CCG)5	Class II	17931	17945	AATCCGTGGCAAAGGGGCTT	CCATGCGCCGCTTGACCAATAA	337
FMgSSR-50509	scaffold30	p3	(CCG)5	Class II	141421	141435	AATGCCGATCCCTGTGCCGATA	AGCGAGCTGCACGAGCAAGTAA	332
FMgSSR-50510	scaffold17024	p3	(CCG)5	Class II	1879	1893	AATGTTCTTCAAACCCGGCGGC	TGTTGAACTGCGGCGAGCAA	345
FMgSSR-50511	scaffold5907	p3	(CCG)5	Class II	27749	27763	AATTCGCACGGTGTCGGCCTAT	TCGTACCGGCGCAAAAACCT	283
FMgSSR-50515	scaffold436	p3	(CCG)5	Class II	21137	21151	ACACCATTTCTGGCGTGCGT	TGGACCGGGGAACGAAACAAGT	257
FMgSSR-50516	scaffold19948	p3	(CCG)5	Class II	8554	8568	ACACGCTCTTGGACCCACAGTT	ACAAACGCCGAGCTCATTCCCA	250
FMgSSR-50520	scaffold3629	p3	(CCG)5	Class II	23610	23624	ACAGCGGCTTCTTGGTGTTGGA	AGGAGACGGCAAAGCAGCAA	290
FMgSSR-50524	scaffold1937	p3	(CCG)5	Class II	67266	67280	ACATGGTCGGTGAGCTTGTCCA	ACAGCACGATTGAACGGTCGGA	295
FMgSSR-50525	scaffold80296	p3	(CCG)5	Class II	616	630	ACCAAACGACGCTGCCAGAA	AACGGCATCATAACGTCCGCGA	206
FMgSSR-50530	scaffold9398	p3	(CCG)5	Class II	4694	4708	ACCACGCCACGAAAGCAAACA	AAAACTTGCCCAGCCCGTGT	301

SSR_ID	Scaffold	SSR_Type	SSR_Motif	SSR Class	SSR_Start	SSR_End	Forward sequence	Reverse sequence	Product_size
FMgSSR-50533	scaffold367	p3	(CCG)5	Class II	30918	30932	ACCATTGTTGCCGTTGCCCT	AAGGCCGTCAAGTTCAGCCT	270
FMgSSR-50541	scaffold834	p3	(CCG)5	Class II	97481	97495	ACCGCGAACTCCTCTTCGTCTT	ACGCCGCCGAATCTTCCTCAAT	278
FMgSSR-50542	scaffold10342	p3	(CCG)5	Class II	10417	10431	ACCGCTTTGGGCTCGTTGAA	TTGGCATTTTGGCAAGCCCG	251
FMgSSR-50543	scaffold17181	p3	(CCG)5	Class II	13560	13574	ACCGGGCGTGCAATCGAATA	AGGGGGAAAGGAGGAGATGGGAAA	294
FMgSSR-50545	scaffold981	p3	(CCG)5	Class II	101774	101788	ACCGTGTTTGACTCTGGCTCGT	TTCCTCGACTCCCCGTTCAGTT	342
FMgSSR-50548	scaffold4446	p3	(CCG)5	Class II	14462	14476	ACCTGCCGTTCACCTCAACACA	TGCACATGGAGCAGGCCTTCAA	325
FMgSSR-50549	scaffold6516	p3	(CCG)5	Class II	24686	24700	ACCTGCTCTCGCCTTGTTGT	TCGTAGAAGCCGTGGTGCATGT	301
FMgSSR-50550	scaffold1202	p3	(CCG)5	Class II	28920	28934	ACCTGCTGAAACCGAAGCTGTGA	ATCCCGCACACGAACACGAA	281
FMgSSR-50554	scaffold946	p3	(CCG)5	Class II	6022	6036	ACCTTGAAATGGGCCGTGTCGT	AGCAACGGCAGGAAAAGCTGGA	259
FMgSSR-50560	scaffold223	p3	(CCG)5	Class II	104140	104154	ACGCACTCACGCATCATTCC	TTAATCCTTCCGACGGCTTGGC	304
FMgSSR-50563	scaffold5084	p3	(CCG)5	Class II	30214	30228	ACGCGCGCATGAAGAGTTGA	AAAGAAGGGGCTGTCCCGATGA	266
FMgSSR-50565	scaffold327	p3	(CCG)5	Class II	126019	126033	ACGCGTGCCGTGTCTTTGTA	AGACGATGATCCCGTACGCT	286
FMgSSR-50570	scaffold59208	p3	(CCG)5	Class II	2075	2089	ACGGCATTGGCTTACCGCAT	TGTTGAAGTCGCAGTAGCCGCA	255
FMgSSR-50574	scaffold3117	p3	(CCG)5	Class II	49833	49847	ACGTACGACGAAGTTGCGCT	ACTGCGACACCACCACCTTT	333
FMgSSR-50576	scaffold25076	p3	(CCG)5	Class II	11818	11832	ACGTGTCGCACGACGATTCA	AAGACCGTGGATCTGGAGGCTT	237
FMgSSR-50586	scaffold633	p3	(CCG)5	Class II	12114	12128	ACTGCAACCTCGACTTCCTGCT	GCCGTGCAGGATGAAGAACA	317
FMgSSR-50591	scaffold6816	p3	(CCG)5	Class II	8735	8749	ACTTCAACTCCATCTCCGCCGT	AAGTAGCAGAGCGCGACGAT	307
FMgSSR-50595	scaffold11836	p3	(CCG)5	Class II	11264	11278	ACTTGTAGCAGGCACCAGGA	ACACCAAGGGCTTCGGCTTCAT	327
FMgSSR-50598	scaffold10658	p3	(CCG)5	Class II	3674	3688	AGAAGCAACAGGCGACCCAA	ATCATCTCCGCCATGGTGCCTT	339
FMgSSR-50600	scaffold1361	p3	(CCG)5	Class II	70303	70317	AGATGGCGCCTGCGGTTTTT	CGCATCATGCACGCCTCAGAAA	330
FMgSSR-50602	scaffold145499	p3	(CCG)5	Class II	149	163	AGCAAGACCTTCCGCAACCTCA	TTCGACCTTGGCGGAGTTGA	210
FMgSSR-50603	scaffold2047	p3	(CCG)5	Class II	32790	32804	AGCAAGTGCAAGTCTGCCGT	CAGGGACTGGTAGAACTCCACGAT	344
FMgSSR-50609	scaffold12832	p3	(CCG)5	Class II	7566	7580	AGCACTAACCTCGGCAACCTGT	AGCCCGTTCAAGTACGTGGTGT	231
FMgSSR-50612	scaffold443	p3	(CCG)5	Class II	45930	45944	AGCAGGTTGGTGGCGAAGAAGA	TCGAGTTCTCGGACGGCATTGT	253
FMgSSR-50616	scaffold5296	p3	(CCG)5	Class II	6838	6852	AGCCCAGCAAAACAGCCAAAGC	TGCCTTTTGGAAGCGTAGCCT	340
FMgSSR-50623	scaffold200	p3	(CCG)5	Class II	89957	89971	AGCGACTTGTTGCTGTCCTCCA	ATCTCGTGAAGAGCGCAGGCAA	343
FMgSSR-50625	scaffold29985	p3	(CCG)5	Class II	305	319	AGCGCATCGAGGACGTGTTT	TACTCGATCTTGATCGGCGACTGC	253
FMgSSR-50628	scaffold895	p3	(CCG)5	Class II	76703	76717	AGCGTTAGTGCGTTACTGCCCA	TTTCATCGCCAAGGATGCGG	268

SSR_ID	Scaffold	SSR_Type	SSR_Motif	SSR Class	SSR_Start	SSR_End	Forward sequence	Reverse sequence	Product_size
FMgSSR-50630	scaffold4310	p3	(CCG)5	Class II	38459	38473	AGCTCGTCTTCACCTTCACCGT	AGCTCAAGTGACCAGGCAACCA	343
FMgSSR-50636	scaffold319	p3	(CCG)5	Class II	105415	105429	AGCTTTGCATCTCCCGCTCCTT	AAACAGAACAGGCCGCGCTT	226
FMgSSR-50638	scaffold4312	p3	(CCG)5	Class II	37664	37678	AGGAAGAAGCCGGCCATGATGT	TCTGATTGCTGCGCCGTTCA	321
FMgSSR-50641	scaffold296673	p3	(CCG)5	Class II	335	349	AGGACTAGACTGGATCCGCGTT	TTGGCCGCATCCAGTGATGA	238
FMgSSR-50642	scaffold351	p3	(CCG)5	Class II	23985	23999	AGGAGGAAGAACGCGAAGCAGA	AAGCATCCGAGGCTCTGGACAA	349
FMgSSR-50647	scaffold360	p3	(CCG)5	Class II	53265	53279	AGGCTTTGAGTATTGGCCGCA	AGCCGACGGCGAAGGTAAAA	216
FMgSSR-50649	scaffold1116	p3	(CCG)5	Class II	83844	83858	AGGGGATGTCAACGGCATTGGT	ATGATGGCGGTGTTCGAAGGGA	279
FMgSSR-50651	scaffold4172	p3	(CCG)5	Class II	27762	27776	AGTGCCAATCCGGGAGCAAA	CCACGCACGTGTTGATCTGGAA	315
FMgSSR-50653	scaffold1140	p3	(CCG)5	Class II	37248	37262	AGTGTGCCTGACCTATGTGTCCT	TGGAAGTGGAGCAGAATCGCCT	346
FMgSSR-50655	scaffold4813	p3	(CCG)5	Class II	2126	2140	AGTTGGGCGAGCCCGAAAAA	TTTTCGCCGTGCTTGGCCTT	323
FMgSSR-50656	scaffold26495	p3	(CCG)5	Class II	3686	3700	ATAATGTGTTCGCTCGCTCGCC	AAACTGCAGGCGCTTGAGATCC	347
FMgSSR-50658	scaffold5470	p3	(CCG)5	Class II	591	605	ATATACCGTCAGGGGAAGTCGG	TCGTGGGACACAGCAGGAGAAA	200
FMgSSR-50660	scaffold215	p3	(CCG)5	Class II	76171	76185	ATCAACGCGGCGCAAATCAC	GCTTTTCGGCCAAAGCCGGTTA	297
FMgSSR-50662	scaffold13459	p3	(CCG)5	Class II	21566	21580	ATCACCATCCGCGACTGCAT	TGGTGCTGCTAGGTAGGGTTCGAT	313
FMgSSR-50663	scaffold1701	p3	(CCG)5	Class II	32249	32263	ATCAGCAGTCAGGTATGCCCGT	AAGCGGGTGTTGCTTAGCCT	263
FMgSSR-50665	scaffold3109	p3	(CCG)5	Class II	48848	48862	ATCATCTTGTGCCTGCCGGCTT	AGTCAAACTCGCCCCCACAT	302
FMgSSR-50667	scaffold301	p3	(CCG)5	Class II	95145	95159	ATCCTGCACCACGTGTCCGTTT	ATGATTTCGCCAGCGCCGTT	253
FMgSSR-50668	scaffold82	p3	(CCG)5	Class II	95492	95506	ATCGACGACATGGGATCCGTCT	TCGTACATGGAGCACGCGAA	350
FMgSSR-50669	scaffold18013	p3	(CCG)5	Class II	13660	13674	ATCGACTACGCCTCCCCTGTTT	AGCGCAGAAAGGAACCGACA	337
FMgSSR-50670	scaffold1158	p3	(CCG)5	Class II	33343	33357	ATCGATACGATGGCAAGACGGC	AAATATAGCGGCTCACGTCCCG	349
FMgSSR-50671	scaffold2469	p3	(CCG)5	Class II	30670	30684	ATCGATCGGCCAACACCCAACA	TGAGCGGCTGGAACGAGAAT	298
FMgSSR-50673	scaffold1168	p3	(CCG)5	Class II	94939	94953	ATCGCGATCCTGGGTCATTTGC	TTGTTGTGCCGCGACGATGA	301
FMgSSR-50674	scaffold66	p3	(CCG)5	Class II	119731	119745	ATCGCGGACTCGGGGTTAAT	TGTGGATGGACATGCGCGTT	320
FMgSSR-50679	scaffold1418	p3	(CCG)5	Class II	14635	14649	ATGAAAATGCGCTGCCAGACGG	TTGGCGCTTTGCGTTGCTTG	254
FMgSSR-50681	scaffold424915	p3	(CCG)5	Class II	34	48	ATGAATGGCACCTACGGCACCCAT	ACATCGACGCCAGTCTCGAA	215
FMgSSR-50682	scaffold298901	p3	(CCG)5	Class II	199	213	ATGAGGGAAACGAAAACGCGGC	ACCTTCTTGCGGCCGATCTTGT	335
FMgSSR-50687	scaffold3689	p3	(CCG)5	Class II	47983	47997	ATGGCGCCCGCGGTTTTTAT	GCGCGTGCACGATTGAGGAAAT	309
FMgSSR-50690	scaffold4886	p3	(CCG)5	Class II	45100	45114	ATGGCGTCCTTGGCGTTCTTGA	TCACTGCGCAGGCAACATCT	343

SSR_ID	Scaffold	SSR_Type	SSR_Motif	SSR Class	SSR_Start	SSR_End	Forward sequence	Reverse sequence	Product_size
FMgSSR-50695	scaffold8722	p3	(CCG)5	Class II	6502	6516	ATTAGGAGACCGCCGATGCGTT	AGATGTCGTGGCCGTGCTTT	306
FMgSSR-50698	scaffold423	p3	(CCG)5	Class II	88309	88323	ATTGCCGGCTAACGCTTGCT	TGAGGGGGCCTTCTGCAAATCA	350
FMgSSR-50700	scaffold317574	p3	(CCG)5	Class II	22	36	ATTTCTCGCTTGCGAGGTCC	AGCAATGGAAGCTGGCCACTCA	245
FMgSSR-50709	scaffold521	p3	(CCG)5	Class II	83705	83719	CCAAAGCAACCTTGCGAGCAGA	ACAGCACCGTGTGGAAGAGAGA	349
FMgSSR-50711	scaffold229582	p3	(CCG)5	Class II	428	442	CCAATCGATTCGGCGGTGAAA	ACTAGTCCGCGCTTGCGTTT	204
FMgSSR-50715	scaffold1648	p3	(CCG)5	Class II	3566	3580	CCGCGTCGATAACAAACCGCAA	ATGGTCTTGCCCTTGTTGGGCT	341
FMgSSR-50716	scaffold299	p3	(CCG)5	Class II	60264	60278	CCTTGTCGTTGGCCATGCACTT	TGCAGCAGCGATCGTCATCA	304
FMgSSR-50719	scaffold260028	p3	(CCG)5	Class II	301	315	CGATGAAGTGGCTGCTGGAGAA	TCGCGCACAAAGAACGGCAA	314
FMgSSR-50720	scaffold5690	p3	(CCG)5	Class II	34920	34934	CGCAAATCTGCCACGATCGCTT	AAGGCAGACCAAGAAACCCCGA	250
FMgSSR-50721	scaffold18	p3	(CCG)5	Class II	109228	109242	CGCACGCACAACGTAAGCAA	AGCGCCGGCTGTAGTCAAAA	316
FMgSSR-50723	scaffold222	p3	(CCG)5	Class II	32954	32968	CGCATCGCTGATTGAAACGGCA	TAGACGAGAATGGGAAGGCGCT	325
FMgSSR-50725	scaffold2821	p3	(CCG)5	Class II	45606	45620	CGCCACCACTTCCAAAGTCCAA	AAGAGGATGGTGCCGGTTTGGT	342
FMgSSR-50727	scaffold7397	p3	(CCG)5	Class II	19352	19366	CGCCGCGAAGAAGGAAGTAA	AGCGGCGACGGATGACAAAA	289
FMgSSR-50730	scaffold17700	p3	(CCG)5	Class II	15747	15761	CGCGCGTGTTCGAAAAGAAACG	ACACGGCCGCTTATACATGGAG	326
FMgSSR-50732	scaffold5622	p3	(CCG)5	Class II	24637	24651	CGGTGCAACTCGACCAACACAA	TGGTGCGTCATCAGTTTCGGGT	284
FMgSSR-50733	scaffold28431	p3	(CCG)5	Class II	3787	3801	CGTTTGAAGTGCGAAGGCAACCA	AGCATGGGAGAATGTGGAGACG	315
FMgSSR-50734	scaffold6878	p3	(CCG)5	Class II	13095	13109	GAAAAGGCGCCGCGAAGATT	AGGACGAGCTGGACGAAGAGTT	206
FMgSSR-50735	scaffold2322	p3	(CCG)5	Class II	41918	41932	GCAAAAACGCCCCTTTTCCGGT	TCACCATGAGCACTCGAGCA	301
FMgSSR-50736	scaffold18	p3	(CCG)5	Class II	136809	136823	GCAAACATGACGACGGAAGCGA	TCAGTACCAGCTGTTGTGGGCA	248
FMgSSR-50739	scaffold6746	p3	(CCG)5	Class II	35428	35442	GCCAGCCGCGCTTTGAGTTATT	ACACGCCGAAGGTGTCGATGAA	313
FMgSSR-50740	scaffold21	p3	(CCG)5	Class II	236826	236840	GCCCACATCGTCTGACACAGTCAA	ACGCCACTGCTTGTGCTTGT	347
FMgSSR-50745	scaffold7668	p3	(CCG)5	Class II	8529	8543	GCCGCCGCACTTCAATACAA	ACTGAAGACGAGGACAAGCCGA	204
FMgSSR-50746	scaffold6815	p3	(CCG)5	Class II	35899	35913	GCCGCGAACAAGCCGAAAAT	AGGCTCGATCTCTGGTGGTTTCCA	201
FMgSSR-50747	scaffold2794	p3	(CCG)5	Class II	39881	39895	GCCTGCCAAAAACCAGCACACAA	TCGTGCAGGTGCTGACAGGAAA	324
FMgSSR-50751	scaffold1111	p3	(CCG)5	Class II	39779	39793	GCGCCAACAACCTTGACCACTT	AATTCGATGCGCGCAAGCAG	332
FMgSSR-50754	scaffold92226	p3	(CCG)5	Class II	1116	1130	GCGTTGCGTCGATTGGCATT	TTCTCGTCTTCCTCCACGGGTT	349
FMgSSR-50758	scaffold2726	p3	(CCG)5	Class II	34980	34994	GCTTGCTTCGCCTGAACTCTGA	TGTCCAGCGCGAACTTTGCCTA	296
FMgSSR-50761	scaffold5971	p3	(CCG)5	Class II	15537	15551	GGCCTGCCACCATTGTTGTTGT	TTGTTCGCGTGCAGGTGGAT	256

SSR_ID	Scaffold	SSR_Type	SSR_Motif	SSR Class	SSR_Start	SSR_End	Forward sequence	Reverse sequence	Product_size
FMgSSR-50762	scaffold2206	p3	(CCG)5	Class II	65510	65524	GGCGGCATGATTGGCTTGTTGA	TCTCGCAAAACCCAGTGCCT	212
FMgSSR-50765	scaffold41899	p3	(CCG)5	Class II	4825	4839	GTGTCGGGCGCATCAAATGTGT	AGGGAGACCAAAGCGCACGAAA	341
FMgSSR-50766	scaffold73337	p3	(CCG)5	Class II	909	923	GTTGAACGTGCCACGGCATT	TTTGAAGTGCTGCTCGACCC	218
FMgSSR-50773	scaffold188	p3	(CCG)5	Class II	110226	110240	TACACGACACGAACCTGGCAGA	ATTCGGTTCCTGCCGTTGGA	287
FMgSSR-50780	scaffold30811	p3	(CCG)5	Class II	6188	6202	TACTCATGGCAGGATGTGCAGC	TGACTTCGTAAACGCCAGCCGA	255
FMgSSR-50782	scaffold548	p3	(CCG)5	Class II	34619	34633	TATACCTGCACGTAGGGCGTGA	TTCCGTCCCCTTCGATCGGTTT	344
FMgSSR-50787	scaffold21061	p3	(CCG)5	Class II	9420	9434	TATTTTGCACGCAGCCCCCTGA	TCTTCGCGAGTCGGGACATT	226
FMgSSR-50791	scaffold445	p3	(CCG)5	Class II	19698	19712	TCAACGCGCTCCTCAATCCA	AGTAGCTCACGCAGTCGTCGTA	350
FMgSSR-50796	scaffold346959	p3	(CCG)5	Class II	31	45	TCAGCTGATAGGCGCTGACGAT	ACCAGCATGTGAAGGTCGAGGA	215
FMgSSR-50797	scaffold7057	p3	(CCG)5	Class II	5762	5776	TCAGGCGCCCACAAAGCAAA	GGAGTACCTGAGTTGTTGCGCT	309
FMgSSR-50798	scaffold598	p3	(CCG)5	Class II	46098	46112	TCAGTTTGTCGCCAAGAGCCCA	CGGAACCATTAGGGACCGGGATTT	214
FMgSSR-50799	scaffold745	p3	(CCG)5	Class II	22303	22317	TCCAAACCAGGGGGCATTTTGC	AACGCTGCCTCCGTTGAACA	290
FMgSSR-50805	scaffold2021	p3	(CCG)5	Class II	17789	17803	TCCAGTGCAAGCCGTTCCTT	AATGGATGCACTGGCACGCA	313
FMgSSR-50807	scaffold20981	p3	(CCG)5	Class II	10277	10291	TCCCACCACCCTCCCAAAGATA	AGAAGGGAGGGCGAGGAAATCA	304
FMgSSR-50809	scaffold175	p3	(CCG)5	Class II	71039	71053	TCCCGAAAAACCCAAACACGGC	ATCGCGGTAGTGTTGCAGCAGA	236
FMgSSR-50810	scaffold15783	p3	(CCG)5	Class II	5554	5568	TCCCGGCACGTTGTTGTGTT	AGCCGTGCCGTCGTAACAAT	256
FMgSSR-50814	scaffold15754	p3	(CCG)5	Class II	4863	4877	TCCGCGCCTCAAATTGCTCA	TGCTGTTGAGGTTGCCCAGAGA	302
FMgSSR-50815	scaffold820	p3	(CCG)5	Class II	85223	85237	TCCGTCGAGCAGGTGAGGATTT	GCCTGCTTGTGGCTGTAGAAGT	343
FMgSSR-50817	scaffold401787	p3	(CCG)5	Class II	205	219	TCCGTCTCCACTCCCATAAGCAGT	TGCCCCAAGAAGTGCTTCTCCT	246
FMgSSR-50819	scaffold2141	p3	(CCG)5	Class II	17809	17823	TCCGTTCAACGAACTGGACGCA	TCACGGAAGGGAGCGAATTGGA	303
FMgSSR-50820	scaffold138	p3	(CCG)5	Class II	78381	78395	TCCTCGTCCTCTTCCTAATCGCCT	TTGCCGATCTTGTGCTCCACGA	346
FMgSSR-50821	scaffold302	p3	(CCG)5	Class II	80035	80049	TCCTCTGCGTCTGCCTCTTCAA	TGAACAACGCGTGGAACCCA	287
FMgSSR-50827	scaffold318	p3	(CCG)5	Class II	37310	37324	TCGAAGAATGTGGTCGGCGAGT	GCCCATGCCTTGTTGCGTTT	338
FMgSSR-50830	scaffold11999	p3	(CCG)5	Class II	16259	16273	TCGACAATCCACGCGGAATCA	AGCAGCCAGCAGCAGTTGTAGT	327
FMgSSR-50838	scaffold22044	p3	(CCG)5	Class II	8742	8756	TCGCGATCCAAATTCCCACCGA	TCAGGCCGTGAAGGCATCAA	337
FMgSSR-50840	scaffold603	p3	(CCG)5	Class II	53696	53710	TCGCGGCGCGCAAATTGAAA	CGGCGTTGAACGTGAGGAACTT	345
FMgSSR-50841	scaffold1494	p3	(CCG)5	Class II	9918	9932	TCGCGGCGCTGCTATTACAA	TGCAGAGAGGACAGCGGTCAAA	288
FMgSSR-50844	scaffold9818	p3	(CCG)5	Class II	6668	6682	TCGCTGAAGCTTCTCCTCCA	GCGGGTTGAGTTTGAGCTTGAGGT	297

SSR_ID	Scaffold	SSR_Type	SSR_Motif	SSR Class	SSR_Start	SSR_End	Forward sequence	Reverse sequence	Product_size
FMgSSR-50846	scaffold1295	p3	(CCG)5	Class II	36181	36195	TCGGCTTCGCTCGTGGATAGTA	TGGCCGGTGTAAATGAGCGA	219
FMgSSR-50848	scaffold4718	p3	(CCG)5	Class II	40249	40263	TCGTGATTGCGGTGAGCCAT	TGCTGGACTGTGAGTCGTTCCT	333
FMgSSR-50851	scaffold770	p3	(CCG)5	Class II	6852	6866	TCGTTCAGGTTGGCATCGCCTT	GGGGATTTTCGGCGGGAACTAT	291
FMgSSR-50857	scaffold3257	p3	(CCG)5	Class II	25621	25635	TGAAGAGGGTGAGCCATTCCACA	TGGAGTCCGCAAAAGCGTCGAA	303
FMgSSR-50862	scaffold357	p3	(CCG)5	Class II	40905	40919	TGAGCCAGCACGCATACGAT	ATGGAGGACGCCGCAATGTT	346
FMgSSR-50864	scaffold8776	p3	(CCG)5	Class II	5243	5257	TGAGGGGGTTCCACGCAAAACA	TGAGACACGGCTCTGGAATGGT	302
FMgSSR-50867	scaffold104	p3	(CCG)5	Class II	104109	104123	TGATGAACCACGTCAGCCTCGT	ATTGGTGAGCGCGAGCATGT	327
FMgSSR-50870	scaffold19851	p3	(CCG)5	Class II	2743	2757	TGCAAAAAGGCCCCTGGTTTCG	TCGGAAAATTGCCCGCACCA	343
FMgSSR-50874	scaffold5101	p3	(CCG)5	Class II	38915	38929	TGCACATGCAGCAAACCGATCA	TTCTGCTTCATCAGCGGCGA	265
FMgSSR-50886	scaffold2547	p3	(CCG)5	Class II	26870	26884	TGCCCTTAAGCTGGATCTGCGA	CAGTCATTGCCTCCCCTTGACT	337
FMgSSR-50890	scaffold6676	p3	(CCG)5	Class II	17734	17748	TGCGAAGCGATGACAAGCCA	TTCAAAAGGGTGCGACGCCA	231
FMgSSR-50894	scaffold30979	p3	(CCG)5	Class II	4181	4195	TGCGCCAACCTGCTTCCATT	AACCGAATGTGCTTCAGCGGGT	259
FMgSSR-50895	scaffold388	p3	(CCG)5	Class II	52233	52247	TGCGCCACCTGCAAAGAACA	TTCGGATCTTGATCTGGGCGGCTA	344
FMgSSR-50896	scaffold4690	p3	(CCG)5	Class II	7319	7333	TGCGCTCAAGAGGGGAAGAA	AAGCATCGCGCATGGAACCT	301
FMgSSR-50899	scaffold12140	p3	(CCG)5	Class II	14938	14952	TGCTAAATCAGCCGACCGGGAT	TGGTAGCAGCAGCAGCAGTTGT	298
FMgSSR-50900	scaffold5569	p3	(CCG)5	Class II	26480	26494	TGCTAGTCGAACAGACGCGA	TCCTTCCATTCCCTTGCTGCGT	325
FMgSSR-50903	scaffold93761	p3	(CCG)5	Class II	62	76	TGCTCGAACCGGAGCAGCAAAT	TTGCTGCAGTTCATCGCCCAGA	233
FMgSSR-50906	scaffold3589	p3	(CCG)5	Class II	27693	27707	TGGAACCTCCGACGCAAGAAGA	AAGATTCCGGCGAACTGGAACG	311
FMgSSR-50909	scaffold3296	p3	(CCG)5	Class II	31577	31591	TGGAGGTGGCGGTCTTGTCAAA	ATTGTGGAAGGGCTCAGCACCA	307
FMgSSR-50911	scaffold795	p3	(CCG)5	Class II	55481	55495	TGGCAGTTGTAGTGCACCGA	TGGGCGAACGCCTCTGTTTT	283
FMgSSR-50912	scaffold1766	p3	(CCG)5	Class II	38234	38248	TGGCCCAGTTGCCACGTTTA	AACTCCGGTGGCATACTCTGGT	294
FMgSSR-50917	scaffold59208	p3	(CCG)5	Class II	1935	1949	TGGCGCGATAACGATGAGGT	ATGCGGTAAGCCAATGCCGT	211
FMgSSR-50920	scaffold1060	p3	(CCG)5	Class II	4865	4879	TGGGCTTGACGGCATTCGTACT	TTCGGGTGTGACCTGCGAAT	264
FMgSSR-50921	scaffold6351	p3	(CCG)5	Class II	4630	4644	TGGGTGACTTCGTCACTTCGGA	TCATCTGCACCACTCCCACCTT	333
FMgSSR-50924	scaffold15020	p3	(CCG)5	Class II	3472	3486	TGGTGGTTGACGAGGTGGAAGA	AGGGCTGCAGATCAACCGCAAT	281
FMgSSR-50925	scaffold1840	p3	(CCG)5	Class II	68749	68763	TGGTTCTTGCCGAAGCCGTT	TCTTGCGTCGTGAGGGGAAGAA	284
FMgSSR-50926	scaffold8459	p3	(CCG)5	Class II	18957	18971	TGTAGGCGCTCCGTTTTGCT	AGGCGTCTTGCACCAAACGA	316
FMgSSR-50927	scaffold10202	p3	(CCG)5	Class II	19548	19562	TGTATGCAACGGAGAACGGCCT	ATGTAGCCAAGCGCCAGGAA	253

SSR_ID	Scaffold	SSR_Type	SSR_Motif	SSR Class	SSR_Start	SSR_End	Forward sequence	Reverse sequence	Product_size
FMgSSR-50932	scaffold4	p3	(CCG)5	Class II	303383	303397	TGTCTTTGCAAATGTGCGGCCC	TCAAATCTCCGCTTGTCCGCGA	346
FMgSSR-50933	scaffold1172	p3	(CCG)5	Class II	48790	48804	TGTGACGTCATCATGGCCGA	ACCCTGCAGCAAGCGAAACA	227
FMgSSR-50934	scaffold707	p3	(CCG)5	Class II	104902	104916	TGTGTGTGCAAGTGTGCAACGA	TCGAAGTCCGCAAATCGCCA	327
FMgSSR-50936	scaffold4091	p3	(CCG)5	Class II	23046	23060	TGTTCTTCGAACCCAGCGGA	ACCGCTGTTTGCCACCTCATCA	215
FMgSSR-50941	scaffold12476	p3	(CCG)5	Class II	16127	16141	TGTTGGTTGGGAGAGCAGCAGT	TTCGCGAGCTGTCGGAAGTTGA	299
FMgSSR-50943	scaffold2015	p3	(CCG)5	Class II	17381	17395	TTACCTTGTCGAGCAGGAGGCA	ACGCGTTCTGCATGTGGCAT	213
FMgSSR-50946	scaffold228	p3	(CCG)5	Class II	84449	84463	TTCAACGTCGCGCTCAAGTC	AGCTGGTTCGTGCGTAGCTT	249
FMgSSR-50950	scaffold13804	p3	(CCG)5	Class II	9910	9924	TTCAGGTCGCGGTCGAAGAT	TTCCTTTTTCCACGGGGCCCAA	285
FMgSSR-50955	scaffold4967	p3	(CCG)5	Class II	7441	7455	TTCCGCAGTTCTGCTCAGACGA	AACGCTGAGTTCTTCGCCTCCA	326
FMgSSR-50964	scaffold31124	p3	(CCG)5	Class II	8120	8134	TTCTCCACACTTCGACGCTCCA	ATTTGTGCAGCGCACGCAAC	223
FMgSSR-50965	scaffold4879	p3	(CCG)5	Class II	41730	41744	TTCTCCGAGTCCTTGACGTGGT	TCCCGAATCTCGACGCACGAAA	334
FMgSSR-50966	scaffold740	p3	(CCG)5	Class II	22622	22636	TTCTGCCTGCTCTTCTCAGGGT	TAGCAGCGGGGAAAGAAGCGAA	324
FMgSSR-50967	scaffold16735	p3	(CCG)5	Class II	10505	10519	TTGAACGGGACGACGATGCCAA	TTCGCGTAGCATCGACAGCA	312
FMgSSR-50972	scaffold7241	p3	(CCG)5	Class II	11387	11401	TTGCAGCTGCCTTCTCCTCCTT	ACGAAATTGCCACTCCCGACCA	350
FMgSSR-50973	scaffold3441	p3	(CCG)5	Class II	36208	36222	TTGCAGGCAGAACCGACCGAAT	ACTACGCCAGGAAGCGCAAGAT	292
FMgSSR-50975	scaffold90	p3	(CCG)5	Class II	141212	141226	TTGCGAGGGGGACATTCGAT	AGTACGTGGCGCGATGGACATA	316
FMgSSR-50976	scaffold21033	p3	(CCG)5	Class II	2984	2998	TTGCGGATGTGGCTGCTGTT	ACGCACGGCACGCGATATAA	314
FMgSSR-50978	scaffold209	p3	(CCG)5	Class II	138460	138474	TTGCGTACACCGAGCCCATCTT	AGGTGGCGGTTGCTCACTTA	333
FMgSSR-50980	scaffold281	p3	(CCG)5	Class II	102693	102707	TTGGATCGGACCGAAACGCA	AGTTCACCATCCGCTGGCCAAA	318
FMgSSR-50986	scaffold4473	p3	(CCG)5	Class II	44796	44810	TTTACACGCGACGCCTTCCA	ACGGCGAACAGGACGATGTT	347
FMgSSR-50992	scaffold2785	p3	(CCG)5	Class II	34670	34684	TTTCTCCGCAGCCTTCTTCGCT	TCAGCGTCAGCGCCACAAAA	317
FMgSSR-50995	scaffold1322	p3	(CCG)5	Class II	76802	76816	TTTGGGCCTGCCTATGATGGCT	ATGAGTGGTGCGCTCAGCTT	328
FMgSSR-50997	scaffold19189	p3	(CCG)5	Class II	7923	7937	TTTTCAAACGAGCCCCCGGT	AAGCAGAGACGAATGAGCGGCA	326
FMgSSR-51002	scaffold48718	p3	(CCG)6	Class II	974	991	AAAATCGACTCCTCATGCCCGC	GCCGCAGCATTGGGATTGGAAA	319
FMgSSR-51009	scaffold6630	p3	(CCG)6	Class II	34111	34128	AACAACGTCAAACGCCACGC	AGGTGAAGTTCATGACGCCGGA	265
FMgSSR-51010	scaffold315	p3	(CCG)6	Class II	69009	69026	AACAAGCCGGATGCGAGGAGGATT	AAGAAGCGCGAACGAACCTGGA	339
FMgSSR-51012	scaffold35590	p3	(CCG)6	Class II	2763	2780	AACCACTGAGCGAAGGCGGAAA	TTGCCACCGCCGAAGAAGAA	294
FMgSSR-51014	scaffold180691	p3	(CCG)6	Class II	304	321	AACCGAGGAAAAGCGCAGCA	TGAGCGCTAGCTTGAAGCGGAA	290

SSR_ID	Scaffold	SSR_Type	SSR_Motif	SSR Class	SSR_Start	SSR_End	Forward sequence	Reverse sequence	Product_size
FMgSSR-51022	scaffold325	p3	(CCG)6	Class II	84898	84915	AAGAACGCGGTTTCTGCCGT	TGCTGGGATCCCCAACCAAA	345
FMgSSR-51024	scaffold44	p3	(CCG)6	Class II	155828	155845	AAGACGGCCATGTCGTCGAT	TCGCTGTGGCTGTTCGGAAT	296
FMgSSR-51026	scaffold1542	p3	(CCG)6	Class II	77485	77502	AAGCCTAAGTGGTCTCGCTCGT	TGGCACCATCAGGCATCGAA	217
FMgSSR-51028	scaffold6412	p3	(CCG)6	Class II	22397	22414	AAGGCGGTTGAGCCGTGACAAT	TGTGTGCGTTGCACGTCAGT	295
FMgSSR-51037	scaffold6153	p3	(CCG)6	Class II	25525	25542	ACACTTGCCAAGCTTCCAGCCT	ACGCGAAGAACACTGGCGAA	271
FMgSSR-51040	scaffold445	p3	(CCG)6	Class II	66493	66510	ACCGAGAGATCGAAGATGCGCT	TGGTGGTGGTTGCTGTTGTGGT	347
FMgSSR-51041	scaffold1204	p3	(CCG)6	Class II	68062	68079	ACCGGTGACCATCTTGAGCA	ATGCCAACAATGCACGCCAC	342
FMgSSR-51048	scaffold103066	p3	(CCG)6	Class II	851	868	ACGGACCCATTATTTGCCCAGGA	AGCAGCACCCCGATGTTTGA	337
FMgSSR-51051	scaffold426	p3	(CCG)6	Class II	128128	128145	ACGTACGCGGGAGATGTTGA	TCGAGCCATGGGCTTCAAATGGA	345
FMgSSR-51052	scaffold43271	p3	(CCG)6	Class II	3796	3813	ACGTCCGGGCCATGAAAGATCA	ACGAGTACGACATCGGCAACCT	313
FMgSSR-51061	scaffold5588	p3	(CCG)6	Class II	27715	27732	AGATGTTGACGCGCTTGCTG	TCCTCCATTGACGAACGGCAACA	316
FMgSSR-51062	scaffold12733	p3	(CCG)6	Class II	7851	7868	AGCACAAGAGGAACAACGGCA	TCGCAGTGTTCGCTGTGTCCAA	341
FMgSSR-51067	scaffold8794	p3	(CCG)6	Class II	17607	17624	AGCCCAACCGTCGTTAGCATGA	AACCTGGAAGCAAAGCGGAGGA	311
FMgSSR-51069	scaffold74423	p3	(CCG)6	Class II	126	143	AGCGTCCACAGCTCAATGGTGA	AAACGTCGATCGCCGCTGCTAA	234
FMgSSR-51070	scaffold25755	p3	(CCG)6	Class II	3157	3174	AGCTAACGCGTCGAATGGCT	CGGAGCAAGAATTAAGCGGGCA	285
FMgSSR-51072	scaffold3875	p3	(CCG)6	Class II	5151	5168	AGGAGTCGGTCACCACGTAGAT	TTGCAGCAAGGTGTCGTCGT	212
FMgSSR-51080	scaffold1353	p3	(CCG)6	Class II	33712	33729	ATCCACAATTCCACACGCGGCA	TGTCAAGCGCGGGATGGTTT	345
FMgSSR-51083	scaffold11487	p3	(CCG)6	Class II	4216	4233	ATCGATACGACGGCAAGACGGT	CGTTCGTGGAAGACCAGCAA	330
FMgSSR-51085	scaffold392	p3	(CCG)6	Class II	86622	86639	ATCGTCAGGAACACCCACACGA	ATGGCCACTGGCTTCTTCTGCT	346
FMgSSR-51087	scaffold10376	p3	(CCG)6	Class II	788	805	ATGATTCGCGCGTCAGGGAT	GCGAGAGAAATCCAGCGCGAAA	271
FMgSSR-51088	scaffold50	p3	(CCG)6	Class II	115552	115569	ATGCCCGCAAAAGCCTTGGA	TCGAACCCGTCGTAGCAGAT	339
FMgSSR-51089	scaffold216344	p3	(CCG)6	Class II	245	262	ATGCGCTTGCCGATAGGCTT	ATTTCACGCAGGAAGACCGCCT	306
FMgSSR-51091	scaffold10020	p3	(CCG)6	Class II	4693	4710	ATGGCGCGAACCTTGTGGAA	AACGGCCCGTGCACGTAAAA	272
FMgSSR-51092	scaffold3082	p3	(CCG)6	Class II	19779	19796	ATGGTCCGTTCCAGCCCACAAA	TGAATTCAACGGAGGCGAGTGC	288
FMgSSR-51094	scaffold15576	p3	(CCG)6	Class II	16816	16833	ATTCCGGCGAGCATCGACTTGT	TGTTGTTGGCCGATGCCTCA	321
FMgSSR-51095	scaffold2143	p3	(CCG)6	Class II	65666	65683	ATTTTGTCACACTCGCCGGGCT	AAGCTTCCAGGCGGTGATCAGA	344
FMgSSR-51104	scaffold20765	p3	(CCG)6	Class II	3833	3850	CGTGTGCGGGCTGTTCTTTTGT	ATGATGGAGCAGCCGTTCTGGT	344
FMgSSR-51105	scaffold2210	p3	(CCG)6	Class II	51048	51065	GCAAAGCCACGTGCACAAACCT	ATGACGAAGGCGTTGCGGTT	301

SSR_ID	Scaffold	SSR_Type	SSR_Motif	SSR Class	SSR_Start	SSR_End	Forward sequence	Reverse sequence	Product_size
FMgSSR-51109	scaffold2162	p3	(CCG)6	Class II	25844	25861	GGCAAAACAGAGCGCGAGTACA	TGCCGTCGTGACTCGTGAAA	259
FMgSSR-51113	scaffold933	p3	(CCG)6	Class II	17909	17926	TAACCCTGCTTTGCTTCCGCCA	AGGCGTAGGAAGGCTTCTGTGT	306
FMgSSR-51114	scaffold8993	p3	(CCG)6	Class II	15096	15113	TACGCGGCACAAAATCGCGT	TCGACCGGCAATTTGGCCTT	302
FMgSSR-51117	scaffold6852	p3	(CCG)6	Class II	18341	18358	TCACACAAGCTCCGCTTTTGGC	TCCGCGATCCAATTTTCGCCAC	349
FMgSSR-51118	scaffold883	p3	(CCG)6	Class II	77981	77998	TCACCAGCTCGAACACCAGGAT	ACAGCCGACGACAGGAACAA	286
FMgSSR-51119	scaffold23117	p3	(CCG)6	Class II	8655	8672	TCAGCAACTCACTTGGCCAGCA	TTCGCGCCAATTGGGAGGGTAT	229
FMgSSR-51124	scaffold15384	p3	(CCG)6	Class II	17457	17474	TCCAGGCTAAACTTCCCAGGCT	GCGTACATGAGCATGGAAGGCA	326
FMgSSR-51128	scaffold321	p3	(CCG)6	Class II	19787	19804	TCCGCCGCAACAAAGTGGAA	TTCTTCCTGGTCGCCGCATCTA	254
FMgSSR-51134	scaffold3905	p3	(CCG)6	Class II	734	751	TCGGAAGGAGGCGAAGGCATAA	TCGATCTGGTGCTCGTTCCT	304
FMgSSR-51142	scaffold3257	p3	(CCG)6	Class II	28932	28949	TGAGAGGTGATGACAAGTGGGCA	AGCACCAAGGAAGCAGAGGT	272
FMgSSR-51143	scaffold1748	p3	(CCG)6	Class II	10531	10548	TGATGTCACGCAGCTGGCAA	GCTCGGTTCTTGATGAGCTGGA	206
FMgSSR-51146	scaffold810	p3	(CCG)6	Class II	89082	89099	TGCATGGTGCCCAAGTTCAGGT	ACGATGGCGAACATGAAGGGGT	312
FMgSSR-51147	scaffold8247	p3	(CCG)6	Class II	25716	25733	TGCCACTGCTCAATGGACCGTA	ACGTGGGCAAAGGCAACGAA	288
FMgSSR-51156	scaffold62	p3	(CCG)6	Class II	62581	62598	TGGCCTTGTCCTTGATGACGGT	AATTGCGCGTAACAGCAGGC	331
FMgSSR-51157	scaffold180	p3	(CCG)6	Class II	34230	34247	TGGCGAGGGGGTTTCTGCAAAT	AACAAAGCCACGGCGGAGAT	242
FMgSSR-51158	scaffold9056	p3	(CCG)6	Class II	6186	6203	TGGTGGAGACGACCTTTGACGA	TTGACGACGAGTCAACAACGCC	231
FMgSSR-51162	scaffold3984	p3	(CCG)6	Class II	22775	22792	TTCAGCCGGTTTCTTGGGTGGA	TCAAAGAAGCGAGCGCGAACA	284
FMgSSR-51166	scaffold763	p3	(CCG)6	Class II	86939	86956	TTCGTGCGGCCGTGATCAAT	TCTCGCGCAGCACAACCTTA	313
FMgSSR-51169	scaffold24932	p3	(CCG)6	Class II	2577	2594	TTGGGGGAAAAGGCGAAAAGCG	CGTCACAACGGCGTGAATCCAA	328
FMgSSR-51171	scaffold159	p3	(CCG)6	Class II	80846	80863	TTGGTGTCGTCGTCGTTGTCGT	GAAACGCGCGCAACAACTCA	350
FMgSSR-51178	scaffold19983	p3	(CCG)6	Class II	9316	9333	TTTGCCTCAGTGCCCGCTAACT	AGCCGGTCAGAATTGAGCGTGA	276
FMgSSR-51182	scaffold4225	p3	(CCG)7	Class I	28640	28660	AACCGCGTCGTTGTTCTCCT	AAGCAAGCAGGAAGGCCACA	291
FMgSSR-51186	scaffold1270	p3	(CCG)7	Class I	64186	64206	ACACAACGCATGCAACCGTCA	ACGAAGAAGATGACGGCCAGGA	244
FMgSSR-51187	scaffold22062	p3	(CCG)7	Class I	3814	3834	ACAGGCCCACTCGAATAACCCA	TCCGCTTCGCATGCTCAGAT	333
FMgSSR-51189	scaffold6304	p3	(CCG)7	Class I	33096	33116	ACCGCCGAGAAGAAGAAGCACA	AGTTGCAAAGCTGCGGCAAG	298
FMgSSR-51192	scaffold28209	p3	(CCG)7	Class I	4489	4509	AGCCCAAAATCGAGCGAGCGTT	GGCGGACACTTACGCCCAAAAA	266
FMgSSR-51199	scaffold730	p3	(CCG)7	Class I	24333	24353	ATTCGGTAAGAGAGCCGCAGCA	AAGCTCGCGCTGGCTGAAAT	277
FMgSSR-51204	scaffold1739	p3	(CCG)7	Class I	40523	40543	GGCACGAGGGACTTCTTGTTGT	GCATCCGCCTCGTCACTCAAAT	333

SSR_ID	Scaffold	SSR_Type	SSR_Motif	SSR Class	SSR_Start	SSR_End	Forward sequence	Reverse sequence	Product_size
FMgSSR-51205	scaffold21047	p3	(CCG)7	Class I	1744	1764	GGGCAAACCATGAAAATGGTGCGT	AGTTGACGAGGACGGCGTACAT	343
FMgSSR-51208	scaffold19	p3	(CCG)7	Class I	173047	173067	TCACGTCAAACCCACAGACCGA	TTGGGCCACACCCGGATTTTCT	245
FMgSSR-51209	scaffold776	p3	(CCG)7	Class I	33767	33787	TCATCAGCAGCAGCAGCACA	CGCGCGCCATTGTCTCATCATT	301
FMgSSR-51212	scaffold725	p3	(CCG)7	Class I	27240	27260	TCCCCAACGAACGCCTCTTCAT	TCGGCCTTCAGCTTCTTCTCCA	310
FMgSSR-51218	scaffold860	p3	(CCG)7	Class I	5010	5030	TGCCATGAGCTCTGGTGAACA	AGGGGCTTGCTGCAACAACACTA	319
FMgSSR-51220	scaffold4068	p3	(CCG)7	Class I	52712	52732	TGCGATGCGCAGCAAAAGGA	ATAACTGCGGAACCCGCGACAT	344
FMgSSR-51229	scaffold1272	p3	(CCG)8	Class I	69145	69168	ATTGGAGCCAAGCAGCCACTGA	ATTACCGCAATAGAGGGCTGCG	322
FMgSSR-51230	scaffold104	p3	(CCG)8	Class I	146538	146561	CGGCCCATGTGAAACCAACCAA	TGGTTAGTGCCTGGAAGCGA	322
FMgSSR-51231	scaffold75	p3	(CCG)8	Class I	105329	105352	TAGCAGAATCTCCTGGATCCCCCA	ATTGGGCGATTTGCTGGGCA	200
FMgSSR-51232	scaffold10058	p3	(CCG)8	Class I	1508	1531	TCGACGACGACAATGGAAGCGT	TCTCCAAAGCGGCACGCAAA	244
FMgSSR-51235	scaffold164	p3	(CCG)8	Class I	154238	154261	TCGCCGAAGTTGAACAGCACGA	TATAGCAGAGCTCGAGGAAGAGGC	315
FMgSSR-51236	scaffold3850	p3	(CCG)8	Class I	17267	17290	TGCGCAAGAAACGGCACACA	ACCGTCTCCACGCTCGATACAA	319
FMgSSR-51238	scaffold6043	p3	(CCG)9	Class I	5637	5663	AGCTCATGCCCATGGCTTGGATA	TCTGATGGGCGTTTTGGCACA	297
FMgSSR-51239	scaffold3106	p3	(CCG)9	Class I	28500	28526	TCACCTGCCAGTATCGCCAA	ATTCGTTCACATGACGGGGCGA	257
FMgSSR-51246	scaffold4447	p3	(CCT)10	Class I	35982	36011	TAACCATGGCCGCAATCTGCCT	AGGGAGTTTGGTGAATGGGGGA	216
FMgSSR-51252	scaffold16359	p3	(CCT)10	Class I	15081	15110	TGTTCCGTGGTGGTTGGTGT	AATCAAGAGAACGGCGCCCA	329
FMgSSR-51255	scaffold53813	p3	(CCT)11	Class I	2295	2327	TGCTGGTCCCTTTTGCCAGTGT	TCCACCCCGTCCACAACATCAT	294
FMgSSR-51257	scaffold54	p3	(CCT)12	Class I	67451	67486	ATAATGGAACGGCGGCGAACCT	TTGCGGCAGCTGATGTGGAA	226
FMgSSR-51258	scaffold800	p3	(CCT)12	Class I	50746	50781	TCCTGGATGAAGCCGTTGCAGA	AGGATGAGCACGATGAAGCCGA	349
FMgSSR-51265	scaffold2896	p3	(CCT)5	Class II	22523	22537	AAAATGTGGTCTGGTCGTCGCC	AATGGAGGACATGGCACACGGT	208
FMgSSR-51266	scaffold81	p3	(CCT)5	Class II	145082	145096	AAACACTTGGCGCGCGTTGA	TGAAGGTTTTCTTGCGCGAGGC	213
FMgSSR-51271	scaffold1889	p3	(CCT)5	Class II	47063	47077	AAAGCCGCTTCCATCGCCAA	AGCAACGACATTCGCACTGCAC	337
FMgSSR-51273	scaffold30557	p3	(CCT)5	Class II	4866	4880	AAAGCTCCACAGCCACCAGA	GGGGTAAATAATGCAGACCAAGGC	231
FMgSSR-51278	scaffold159	p3	(CCT)5	Class II	69942	69956	AACAACACCCACCACCGCAT	ACACGCCTAACACGTACGCA	314
FMgSSR-51279	scaffold4200	p3	(CCT)5	Class II	3993	4007	AACAAGCTTTCTCGTTGCCGCC	AGCCAGACCCCGATTGGAAACT	225
FMgSSR-51283	scaffold282	p3	(CCT)5	Class II	42388	42402	AACCACGCACTGTTGCTCTCCA	ACAACGAAGAAGACGACGCCCA	316
FMgSSR-51287	scaffold11286	p3	(CCT)5	Class II	20045	20059	AACCTTCGTCAGGGCCAGGTTT	AGAATTTCGGTGGGCCGGTT	298
FMgSSR-51294	scaffold10271	p3	(CCT)5	Class II	2096	2110	AACGTTTGCCTGGCCTGAGAGA	AGTGAGAACGCCGGTGTTGGTT	315

SSR_ID	Scaffold	SSR_Type	SSR_Motif	SSR Class	SSR_Start	SSR_End	Forward sequence	Reverse sequence	Product_size
FMgSSR-51299	scaffold8656	p3	(CCT)5	Class II	19678	19692	AACTCTTCGTGGACCCCACA	TGGTGGTGGCAGTGTCATGT	332
FMgSSR-51303	scaffold773	p3	(CCT)5	Class II	44172	44186	AAGCAAGAAGAACACGGGGGCA	TGGTCAACCTCGTCTCGTCTT	325
FMgSSR-51316	scaffold417	p3	(CCT)5	Class II	32990	33004	ACAAGCCTACAAGGCGAAGCCA	AGGACGATTTGAGCGAGGCCAA	314
FMgSSR-51317	scaffold143160	p3	(CCT)5	Class II	536	550	ACAATCACCCTTGGCGTCGT	AACGGTTGTTGCTGCTCCGA	301
FMgSSR-51321	scaffold20690	p3	(CCT)5	Class II	4993	5007	ACAGCAGCAATCGTCGTGGT	AACACGTTGTTGCGGAGGAGCA	225
FMgSSR-51326	scaffold30180	p3	(CCT)5	Class II	4101	4115	ACATGCTCTCGTAGCTCTCCATCC	ACATACCCAGGAGCGAAGACGA	259
FMgSSR-51327	scaffold23932	p3	(CCT)5	Class II	5655	5669	ACCAACTTCCCAGACGACGA	ACTCCCGTTTACGCCAGCATCT	278
FMgSSR-51333	scaffold30557	p3	(CCT)5	Class II	3668	3682	ACCATGGGCTCCTCCTTGACAT	TCACAAGTGGCGAGAACCAGCA	343
FMgSSR-51340	scaffold22670	p3	(CCT)5	Class II	6290	6304	ACCGCTTCTCTGACCCCACAAT	AGAGCGCGATGTGGTGTTCTT	245
FMgSSR-51341	scaffold5556	p3	(CCT)5	Class II	33911	33925	ACCGGCTTCGGATGATGACGTT	TCCTTGTTTGAGGCGGGCAA	328
FMgSSR-51343	scaffold9341	p3	(CCT)5	Class II	16038	16052	ACCTGCAGCGCAACCAATCA	TTTCACCCCTGCCTCGTTTGCT	328
FMgSSR-51345	scaffold7895	p3	(CCT)5	Class II	12149	12163	ACCTTTCCCGCCTCCCCTTAAT	ATGGAGAAGCTGGTGAGTGGGT	279
FMgSSR-51348	scaffold1977	p3	(CCT)5	Class II	19139	19153	ACGACGGTGGAGCTGGTTTT	TTGATCTTGGATGCCGCAGGGA	329
FMgSSR-51351	scaffold478	p3	(CCT)5	Class II	15540	15554	ACGCAGCCTATGTGCCTGTTGT	TGCCGGTTCATGCTGCTGTT	247
FMgSSR-51352	scaffold782	p3	(CCT)5	Class II	97625	97639	ACGCATGCAAATCGTGGCCT	AACAAAGCACGAGCCTCTCGGT	211
FMgSSR-51354	scaffold236578	p3	(CCT)5	Class II	110	124	ACGCTATGCGTCGGGTCAAA	TCGATCGGTGGCGAACAACT	246
FMgSSR-51358	scaffold4280	p3	(CCT)5	Class II	29054	29068	ACGGCTCGTTGCCTTTCTTGGA	AGTTTGAGGGAAGAGGCGGT	223
FMgSSR-51359	scaffold2175	p3	(CCT)5	Class II	47353	47367	ACGTACACCGTCGGCAATTCA	TGAATCGGTGCAGCTTCTCGAC	293
FMgSSR-51365	scaffold7152	p3	(CCT)5	Class II	3991	4005	ACTAGCTGTGATCATGCGCCGT	TGCTCGTTTTCAGCAGGTGGCT	299
FMgSSR-51366	scaffold18170	p3	(CCT)5	Class II	14504	14518	ACTCGACTTGCTGGGCTCTCTT	TGTGTGATGCCTGCTCCAACGA	305
FMgSSR-51378	scaffold4353	p3	(CCT)5	Class II	18036	18050	AGCAGCTGAGGAGGTGTTGT	TGCGGCGAAGACTGGACAAGAA	336
FMgSSR-51385	scaffold28685	p3	(CCT)5	Class II	1927	1941	AGCCTTGGTTCCTGCTTCCCAA	ATTGTTGCTGCGGCCCTCAT	234
FMgSSR-51387	scaffold3712	p3	(CCT)5	Class II	8491	8505	AGCGCTAACAAGTGGAGCCCAT	AAAATTTTGGGGGCCCTGTGCG	344
FMgSSR-51396	scaffold4419	p3	(CCT)5	Class II	46782	46796	AGGAACCGGTCGAGGTAGATGT	ATCAACAGATGACGACGGGGGA	286
FMgSSR-51398	scaffold1041	p3	(CCT)5	Class II	82647	82661	AGGAGAGGAACCATCCAAGCGT	TGTCGTCGAAGGGCACGGATTT	321
FMgSSR-51399	scaffold13513	p3	(CCT)5	Class II	11790	11804	AGGCCCTTGAATGCGAGAGCAA	GCTTGTTCGAGCTGTTGAGGCA	333
FMgSSR-51402	scaffold572	p3	(CCT)5	Class II	65400	65414	AGTCCCCGTCCTTTTTAACGCC	AGTTCGTCACCACTCCCAAGCA	349
FMgSSR-51405	scaffold299	p3	(CCT)5	Class II	107006	107020	AGTGCATGACGTGGCCCTTGAA	ATGTGCAGGCAGAACGTCGTCA	232

SSR_ID	Scaffold	SSR_Type	SSR_Motif	SSR Class	SSR_Start	SSR_End	Forward sequence	Reverse sequence	Product_size
FMgSSR-51406	scaffold27374	p3	(CCT)5	Class II	10024	10038	AGTGGCTGGAGCTTTCGGAT	AGGGGCTCTTCTTGCATTGGCT	335
FMgSSR-51408	scaffold15905	p3	(CCT)5	Class II	7219	7233	ATAAAAGCAGCCGAGCGCGT	AGCGCCTTGGTTGCCGAAAA	233
FMgSSR-51416	scaffold23301	p3	(CCT)5	Class II	1288	1302	ATCGCGATGAGGCGCTTGTACT	TGGCCAGGCACGAACTCTGATA	235
FMgSSR-51439	scaffold789	p3	(CCT)5	Class II	49305	49319	CACCGCTTCCAAACAAGTGGGT	TGTGTTACGTTTCCCTCGGCGT	346
FMgSSR-51440	scaffold9540	p3	(CCT)5	Class II	23178	23192	CCATCCTTGCACACGAAGCACA	AACACACTCCGCAAAGGCCA	344
FMgSSR-51441	scaffold7473	p3	(CCT)5	Class II	22206	22220	CCCACGAGAAAGATGATGGACGGA	ACCATGAACCGGACGTTGACA	345
FMgSSR-51442	scaffold3247	p3	(CCT)5	Class II	31416	31430	CCCGAAACCAGCTCCAACAACA	TTCCACGCGTGCGCGTTTTT	317
FMgSSR-51448	scaffold36	p3	(CCT)5	Class II	165890	165904	CGGGAGGCAGAAAAGAAGATTGCC	ACAAGAGAAGGTCGATCCGCCA	323
FMgSSR-51449	scaffold38879	p3	(CCT)5	Class II	1474	1488	CGGTCGACCTGCTGGTGTTAAA	ACAAGGCACGAGCTCAACGACA	238
FMgSSR-51452	scaffold39007	p3	(CCT)5	Class II	3535	3549	GCAGTGCACCTGCCCAAGTTTT	TGAAACACCGCTGTGCCTGGAT	303
FMgSSR-51453	scaffold15279	p3	(CCT)5	Class II	4950	4964	GCATCAGGCTGAGAAGTAGCAATC	TCCAGGCAATGTCTGACCTGA	304
FMgSSR-51455	scaffold468	p3	(CCT)5	Class II	40121	40135	GCCATGTCCAAGCTGCTCTT	TCGGCACAAAATCGGGAAGCCT	261
FMgSSR-51457	scaffold19415	p3	(CCT)5	Class II	7010	7024	GCGCAATGACGACCCATGATGA	TCTCTTCATGCCGCTGCTGT	350
FMgSSR-51459	scaffold8290	p3	(CCT)5	Class II	19963	19977	GCTCAATTCAGTGCAAGCCGCA	TGGGGAAGATAGGAAGGACCAGGA	332
FMgSSR-51464	scaffold4677	p3	(CCT)5	Class II	7050	7064	GGCGCGCCACAGCTGAAATTAT	ATGTCGCTGCTGATGGCGAA	322
FMgSSR-51468	scaffold16634	p3	(CCT)5	Class II	11844	11858	GGTAACATGTCGCTGGAGCGTT	TGTGCACGGCATCGTCATCA	232
FMgSSR-51469	scaffold6750	p3	(CCT)5	Class II	11439	11453	GGTCGTCCGATCTACATCCAATGGT	TGACGTGGATAGAGGGGTCACA	348
FMgSSR-51484	scaffold6763	p3	(CCT)5	Class II	25168	25182	TCAAGCCCCTCCAAATCCTCCA	AGGAGGGGCATCATGCAACTAGGA	303
FMgSSR-51487	scaffold2619	p3	(CCT)5	Class II	231	245	TCACTGCGCCTCTCATGGTT	TATCAGCAGGGTTCACGAGGAGGA	274
FMgSSR-51488	scaffold15640	p3	(CCT)5	Class II	16236	16250	TCAGCGACGAGCTGACGATT	AAAATCGAGGCAGCCACCACCA	322
FMgSSR-51505	scaffold13995	p3	(CCT)5	Class II	5825	5839	TCCCGCCGAACCAACCAATCAA	ATGCCGCACAGCACGATAGA	304
FMgSSR-51513	scaffold10804	p3	(CCT)5	Class II	15400	15414	TCCGCTTCCTCTTCGTCCTACAA	AGGAGCTGGGAATGAGAGGTGGAA	335
FMgSSR-51537	scaffold35593	p3	(CCT)5	Class II	5855	5869	TCGTTGAGCCGCTTGAGGTT	TCACGCGCACCAATGGCTAA	278
FMgSSR-51539	scaffold12197	p3	(CCT)5	Class II	15459	15473	TCTCCTGTGGACCGCTGTTTGA	TTACGCCTACGCATCCACCCTT	259
FMgSSR-51543	scaffold1609	p3	(CCT)5	Class II	45665	45679	TCTGGTGTCATGTCCCAGCAGT	TGCGGATCCATGGGGTTTGCAT	226
FMgSSR-51544	scaffold6767	p3	(CCT)5	Class II	3374	3388	TCTTCGCCGACGAATCTCCT	TGTTGCGTACGTCGGGTACGAT	346
FMgSSR-51546	scaffold3285	p3	(CCT)5	Class II	56009	56023	TCTTGGTCAACGTGGTGGGT	AAATCGCCGGGAGAGAGAGAGGAA	230
FMgSSR-51548	scaffold12947	p3	(CCT)5	Class II	12188	12202	TGACAGCAGCCTGATGCAGAGT	TGGCAACGTGCGCAAACGAA	335

SSR_ID	Scaffold	SSR_Type	SSR_Motif	SSR Class	SSR_Start	SSR_End	Forward sequence	Reverse sequence	Product_size
FMgSSR-51552	scaffold7799	p3	(CCT)5	Class II	2664	2678	TGATTCTCACACACCGCCCTCT	AATGGAAGGAGGAGGCGACGAT	263
FMgSSR-51555	scaffold569	p3	(CCT)5	Class II	34397	34411	TGCAAGGTGGTGGCCGAAAA	TCAAGGGCGTCGAAGAAGGTGT	270
FMgSSR-51559	scaffold669	p3	(CCT)5	Class II	93296	93310	TGCAGAATTGCAGACCGCGA	AGCGCTAACTCATCGGCATTGG	318
FMgSSR-51565	scaffold296	p3	(CCT)5	Class II	35439	35453	TGCGACACGAAACAGCCCAA	TCCAAGCAAGCACTCACCAGCA	338
FMgSSR-51575	scaffold3558	p3	(CCT)5	Class II	5275	5289	TGCTGCAGGTCAGACCCCAATTA	TGCACAGGGACCGGTTTTGGAA	316
FMgSSR-51578	scaffold1995	p3	(CCT)5	Class II	38191	38205	TGCTTCCTCTGCCTCGCCTTAT	TGTTGCTGAGGTCGAGCGTT	310
FMgSSR-51581	scaffold4409	p3	(CCT)5	Class II	42799	42813	TGCTTTAGCGGTGCAGCCAAT	AGCGCTAGCGTTCTAGTTGGGT	277
FMgSSR-51588	scaffold12094	p3	(CCT)5	Class II	23624	23638	TGGCAATTTGGCATGGCACG	TGCTGCACAGGATGAGGAGGAA	250
FMgSSR-51591	scaffold233	p3	(CCT)5	Class II	152995	153009	TGGCGCTTGCTTTCTCCTTCAC	CGCAGGACTTGGGGATCGATTT	320
FMgSSR-51592	scaffold1978	p3	(CCT)5	Class II	52600	52614	TGGCTGCGGTGCAAGAACAA	ACGAACATTGGAAGCGGCTG	318
FMgSSR-51595	scaffold328948	p3	(CCT)5	Class II	296	310	TGGGACAAGCGCGGTACTAA	TGGAAACGACTCCGACTTGTACG	338
FMgSSR-51609	scaffold5050	p3	(CCT)5	Class II	42025	42039	TGTGTGTGTGTGCGTGTGTCT	TGCTCAAAGGAGGGCAGCTCAA	324
FMgSSR-51611	scaffold6279	p3	(CCT)5	Class II	16959	16973	TGTTCGCACTGGACGCACTT	TGGCGCTTCCCAATTTGTCCCT	292
FMgSSR-51613	scaffold672	p3	(CCT)5	Class II	89894	89908	TGTTGCAGCTTGCAGGTGGT	AGATTGCGCATTTGTGCGGC	232
FMgSSR-51618	scaffold1274	p3	(CCT)5	Class II	67677	67691	TTAGCTTGCACTTGCACGGTGG	ATTGCCGGCACCATCATCTGCT	318
FMgSSR-51622	scaffold7350	p3	(CCT)5	Class II	30163	30177	TTCAACCCCCGTACACTGTCACAC	AGCTGGCGTCACATCCTCTT	235
FMgSSR-51626	scaffold73184	p3	(CCT)5	Class II	1845	1859	TTCCAATCCGACGCTCCCTGTT	AAAACGATACTGCGGCGGCT	322
FMgSSR-51627	scaffold2598	p3	(CCT)5	Class II	15212	15226	TTCCACGCTCATGTCCAACGGA	AGCTGGGACAATGCCACCTT	219
FMgSSR-51629	scaffold3835	p3	(CCT)5	Class II	2008	2022	TTCCTAGCCCTGCTGTCTTCCT	AGGCGGACTAGCATCTGTGCTT	203
FMgSSR-51631	scaffold2975	p3	(CCT)5	Class II	9253	9267	TTCGAAATGCGGCCAAGCCT	TGCCTACGGGACATGATTGCTG	238
FMgSSR-51634	scaffold12472	p3	(CCT)5	Class II	2860	2874	TTCGCCTTCTCATCATCGCCCT	GGCGGCGCTTCTTGGGATTTAT	230
FMgSSR-51637	scaffold16229	p3	(CCT)5	Class II	2373	2387	TTCTCCTCTCTGCCATTGCCCA	AGCTTGAGGGCCTCCATTGCTA	265
FMgSSR-51647	scaffold3962	p3	(CCT)5	Class II	21854	21868	TTGGGAGCCATGCTTTGCGT	TGGGATGAAGGCAGAGGCGTAT	350
FMgSSR-51654	scaffold8173	p3	(CCT)5	Class II	3107	3121	TTTCCGTCATTCCGCGTCGT	TTACAATGGCACCGGCAACGGA	290
FMgSSR-51666	scaffold45	p3	(CCT)6	Class II	50068	50085	AAAATCGCTTCCGTTCCCGC	ATGTGCCAGTGGCCAAGTGTGT	205
FMgSSR-51672	scaffold354	p3	(CCT)6	Class II	91129	91146	AACCCTGCCTCCGTCCGAAAAA	ACGAATGTCAGGCACCCGAT	265
FMgSSR-51674	scaffold15279	p3	(CCT)6	Class II	2770	2787	AACGGAGGACGGCAATGGAACT	TGCTCGTGGTAGAAAGCCTCGT	349
FMgSSR-51676	scaffold8083	p3	(CCT)6	Class II	9384	9401	AACTTGACTCGTCGTCGGTCGT	ACGCGCTCTTCTCCGTGAAGTT	346

SSR_ID	Scaffold	SSR_Type	SSR_Motif	SSR Class	SSR_Start	SSR_End	Forward sequence	Reverse sequence	Product_size
FMgSSR-51677	scaffold31431	p3	(CCT)6	Class II	2144	2161	AAGAACAGGGGTCGCCGTCAAA	ACCCAACCCAACGCCTACAA	341
FMgSSR-51679	scaffold2619	p3	(CCT)6	Class II	364	381	AAGCGGATCCCCCAATGCCTTT	GCCATTTGAAAGTAGGAAGGCTGC	312
FMgSSR-51693	scaffold7556	p3	(CCT)6	Class II	24973	24990	ACACGGGATGCGTCGTTGGATT	TGTCGTTGATGTTGCTGCTCGC	259
FMgSSR-51694	scaffold137012	p3	(CCT)6	Class II	897	914	ACAGATCCGCCGTGCATTTCCT	TGCCGAGTTGATGCGGTTTGT	324
FMgSSR-51695	scaffold3714	p3	(CCT)6	Class II	26279	26296	ACAGCCACCACAACCTCCTCAA	TCGCCGCTTGTTGTTGTTGGT	259
FMgSSR-51701	scaffold262	p3	(CCT)6	Class II	19699	19716	ACCCCATCCAACAACCACAGCA	GCGAAGGGGTGGAATTGCTCTT	225
FMgSSR-51702	scaffold568	p3	(CCT)6	Class II	28685	28702	ACCCCGCGCAATGCAAACAA	TCTGCTGCTTCTGCTTGGAGGA	341
FMgSSR-51708	scaffold5506	p3	(CCT)6	Class II	34465	34482	ACGAAGACCGAGTTCATCGGGT	AGAAGCAGAACAAGCGGTGC	278
FMgSSR-51710	scaffold69	p3	(CCT)6	Class II	148649	148666	ACGGCCACAGCCAATTCCAA	AAAAGGGAGCGAGGAACGGACA	238
FMgSSR-51711	scaffold9292	p3	(CCT)6	Class II	3753	3770	ACGTGGTTGAACTGGAACCCCA	AGCCGTCCAAAACCCCACGAAA	256
FMgSSR-51712	scaffold2775	p3	(CCT)6	Class II	21464	21481	AGCAACGCTGAATCCAATGCCG	TAGTGTGCGTGTGCTAACCTGC	333
FMgSSR-51713	scaffold7341	p3	(CCT)6	Class II	40733	40750	AGCAATTTTGCCCTCCGCACA	TCGAATTTGAACTCGCCCCCGT	313
FMgSSR-51715	scaffold281	p3	(CCT)6	Class II	2824	2841	AGCAGCGTGTCCGTCTTGTT	ATGCGATGCGAGCACAAGTTGC	286
FMgSSR-51726	scaffold752	p3	(CCT)6	Class II	92940	92957	AGGCAACAAACCAGCCAGCA	GCGCGGGGATTTGGGAAAAA	238
FMgSSR-51732	scaffold218	p3	(CCT)6	Class II	74334	74351	AGTCCAAAACGGCAAACGGC	ACCACTCTTCACATGCGTCGCT	349
FMgSSR-51739	scaffold804	p3	(CCT)6	Class II	98421	98438	ATGCGAACCACGCCCAACAA	TCATCATCGGCATGGTGCAGGT	256
FMgSSR-51742	scaffold386979	p3	(CCT)6	Class II	160	177	ATGTTGTCACTGCCATGTGGGC	TTACCGTGCAGGACGACGAA	208
FMgSSR-51749	scaffold9	p3	(CCT)6	Class II	260957	260974	CGGCTCACCTGCATGTTGTTGT	AGACAGCTGCTGCTGGAAGA	257
FMgSSR-51754	scaffold8791	p3	(CCT)6	Class II	15181	15198	GCCGGATGAAGGTCTTCCACTT	AAACCGCTCCAATTTCCCCCGA	339
FMgSSR-51756	scaffold35719	p3	(CCT)6	Class II	5685	5702	GGCGGCGCCTTTTTCACTTT	TGCTCGATCAGAACTGCTGGCT	315
FMgSSR-51761	scaffold1771	p3	(CCT)6	Class II	49285	49302	TATCGGCGGGGTCAAACAACGA	TCGATGACAACGGTTGCGGAGT	337
FMgSSR-51763	scaffold94	p3	(CCT)6	Class II	153955	153972	TATTCACGCCCTCACTCGTTGC	AGATCGTGATCCTTGGCGGT	338
FMgSSR-51766	scaffold12391	p3	(CCT)6	Class II	3503	3520	TCACCTTCTTCCCTGAGCAGCA	TCTTGGCCGTTGCCTCAGTT	292
FMgSSR-51772	scaffold14636	p3	(CCT)6	Class II	12014	12031	TCCAGCGCCTGCATCTTACTGA	TCAAGAGCTTCTCGTCGGAGCA	337
FMgSSR-51773	scaffold443	p3	(CCT)6	Class II	50819	50836	TCCCCTCCAATCTCTCCCCAAT	AGGCATACCTCTGTCCTTGTCCCT	304
FMgSSR-51780	scaffold14049	p3	(CCT)6	Class II	9677	9694	TCGCACCTTGTGCCAGTGTT	TCCGCCCAAGAACCTGTGAA	349
FMgSSR-51785	scaffold5396	p3	(CCT)6	Class II	23941	23958	TCGTCGTCTTTCGTTTGGCTGC	ACATGAACGCTACGCTGGAGCA	299
FMgSSR-51787	scaffold3016	p3	(CCT)6	Class II	59753	59770	TCGTCGTTGTCGCTGTCGTT	GCGACGACAAGTACGCCATT	295

SSR_ID	Scaffold	SSR_Type	SSR_Motif	SSR Class	SSR_Start	SSR_End	Forward sequence	Reverse sequence	Product_size
FMgSSR-51795	scaffold4343	p3	(CCT)6	Class II	37258	37275	TGAACCCAAATCTCGCGGCA	ACGCGCTTGCTGGGGTTATAGA	331
FMgSSR-51796	scaffold682	p3	(CCT)6	Class II	49877	49894	TGAATGTCGGCGTGGCTTCT	CGTTCCCTCGAAACGCATCA	335
FMgSSR-51804	scaffold25	p3	(CCT)6	Class II	205520	205537	TGCGGTGGCGATCTAGGAAGTT	TCCGCGGTGAATTGGACACA	288
FMgSSR-51812	scaffold365	p3	(CCT)6	Class II	64714	64731	TGGCGCCCTTAATGATGCCGAT	AAGAGTCGGTTGCAGCGAAAGC	295
FMgSSR-51825	scaffold1981	p3	(CCT)6	Class II	57270	57287	TTCGCCCACGCTTTTACGCT	ACGATCGACGATTGCCTGGA	274
FMgSSR-51830	scaffold4109	p3	(CCT)6	Class II	41144	41161	TTGCTCGTGGTGTGGTTGCT	ACCGCAGTTCCATGTGCCTCTA	327
FMgSSR-51838	scaffold1110	p3	(CCT)7	Class I	45935	45955	AAACCCACCACCAACGACTGCT	TCGGCGGTGAAGTTCCTGTTGA	218
FMgSSR-51839	scaffold116376	p3	(CCT)7	Class I	555	575	AAACCCACCGAATCGTCGCA	TCGAAATCGCGGAGGGAACGTA	291
FMgSSR-51841	scaffold347	p3	(CCT)7	Class I	94191	94211	AATGACCCGTTTCGTCGCGT	AAGCCAAAGCCAAGCTGCCA	306
FMgSSR-51842	scaffold94	p3	(CCT)7	Class I	138289	138309	ACAAAAACCGCCAGCGACCT	TCTGCCTGCCACGGAGAAAT	260
FMgSSR-51843	scaffold33447	p3	(CCT)7	Class I	3332	3352	ACAACCTCGGGCAAAAGCGA	TGAAGCTGCCACTGCCCAAA	338
FMgSSR-51845	scaffold2909	p3	(CCT)7	Class I	17194	17214	ACATCTGGTGGGATAGCTGCCA	TCCACCAAAAGGAAGGCGGA	273
FMgSSR-51847	scaffold7463	p3	(CCT)7	Class I	12922	12942	ACCAGCATATCCTCCTCCTCCT	TGCACACGCAATTCGCCTCTT	346
FMgSSR-51852	scaffold10068	p3	(CCT)7	Class I	25342	25362	ACGCCACGCATCTGTAAGACGA	AAGCGTTCTCGGCTTGGAAGGT	311
FMgSSR-51853	scaffold34	p3	(CCT)7	Class I	71296	71316	ACTTACTCCTGCGTGTCTCCTCA	CCCTTGGTGAAGAAGGTGAATGTG	320
FMgSSR-51857	scaffold1592	p3	(CCT)7	Class I	32936	32956	AGCACAGCACGGCAACCATT	AGGAAGAAAGAGGTGGGGCCCATA	338
FMgSSR-51860	scaffold9882	p3	(CCT)7	Class I	8501	8521	AGTGCAGAATTGCGGCTCCA	ATCGCCTTCGCCCAACACAA	346
FMgSSR-51861	scaffold4330	p3	(CCT)7	Class I	12023	12043	AGTTTGTGTCGCTCGCTCCA	AACCGAGGGTAGGAGGAACAAG	242
FMgSSR-51863	scaffold2365	p3	(CCT)7	Class I	28717	28737	ATAGCGTTCGCCCCTTAGTGCT	AAGCCACCCCATTCGCCTTGAA	284
FMgSSR-51868	scaffold7087	p3	(CCT)7	Class I	30611	30631	CCACGAATTTGTGCCGTGGGAA	AGCGCGGGAAACATCAGACAGT	349
FMgSSR-51869	scaffold7355	p3	(CCT)7	Class I	7379	7399	CGACGCGCACTCGAACTTTT	ATTGAACCGGCAAGAGCGCA	284
FMgSSR-51870	scaffold6137	p3	(CCT)7	Class I	20743	20763	CGCGCTCCACTGCAAACAAA	ATGAAGAGGAGGAGCAGGTACG	345
FMgSSR-51871	scaffold6117	p3	(CCT)7	Class I	970	990	GCGTGATGGAGATGGAGATGGA	GGGATTGCCGGATTGGGGAAAA	319
FMgSSR-51874	scaffold13664	p3	(CCT)7	Class I	3046	3066	TAGCACTGCCCCATCCCTTT	AGCCGCTGCTGCATTTTTCTCG	263
FMgSSR-51879	scaffold207	p3	(CCT)7	Class I	85075	85095	TCATCAACGCCGCACTCAACAC	TCGGCATGGCTGGGGAATTGTT	300
FMgSSR-51886	scaffold5888	p3	(CCT)7	Class I	17420	17440	TCGTGCAACCTCAACCGTTCAC	TTGCTCGGTGGCATGGAGTCAT	261
FMgSSR-51889	scaffold142	p3	(CCT)7	Class I	4447	4467	TGATGCCCTATCACCGCCAT	ATGGCAGCCGGTACAATGCT	338
FMgSSR-51902	scaffold7074	p3	(CCT)7	Class I	27219	27239	TTGCGCTGCACCCACTTCAT	AATCGTCGCAGACCCAAACCCT	304

SSR_ID	Scaffold	SSR_Type	SSR_Motif	SSR Class	SSR_Start	SSR_End	Forward sequence	Reverse sequence	Product_size
FMgSSR-51911	scaffold38098	p3	(CCT)8	Class I	3645	3668	AGAAGGCCAGAAGCCCAGAACT	AACATCCCGCCACACACACCAT	339
FMgSSR-51912	scaffold1691	p3	(CCT)8	Class I	23492	23515	AGCAGACACAGGCAGGAGCTTA	TGCTGCTGCTGCTGGCTCTTAT	346
FMgSSR-51913	scaffold477	p3	(CCT)8	Class I	97394	97417	AGCTCGTCCTTGAGCAGCAT	ACTTGACCGGTACCTGACGCAA	300
FMgSSR-51915	scaffold2010	p3	(CCT)8	Class I	25061	25084	ATCAGCAGCAGAGCAGGCTT	TTCAACGCCAAGGGCACCAT	320
FMgSSR-51918	scaffold455	p3	(CCT)8	Class I	71405	71428	CCAACTCTGCTCCCCAGCACTAAT	CGCCTCAGTTCGGTTAGGGTTT	296
FMgSSR-51925	scaffold4118	p3	(CCT)8	Class I	16096	16119	TCGTTTGGTGGGGGCGATCATT	AAACCGGCAGGCTCCCATTACA	346
FMgSSR-51926	scaffold6622	p3	(CCT)8	Class I	23575	23598	TGCAACAGGGTGAGGCACAA	GGTGGTGCTGGTGCATCCAAAT	325
FMgSSR-51934	scaffold2281	p3	(CCT)9	Class I	48847	48873	AAATATGTGTCGCGTCGGTGGC	ACCCGGAGTTCGACACGTTGAT	337
FMgSSR-51936	scaffold8121	p3	(CCT)9	Class I	14637	14663	ACTTCATCTGCTTCCAGCCCCT	AGCGATGCAGACAAGTCACGGT	284
FMgSSR-51939	scaffold8894	p3	(CCT)9	Class I	27466	27492	CGTCGTCTTCGCAAACACACCA	AACGAGAAACTGACGCGGGA	216
FMgSSR-51941	scaffold1266	p3	(CCT)9	Class I	18205	18231	TCACAGCCGCAGCTCATCAGAA	GCACCGTCGTCATCGTCAACAT	211
FMgSSR-51942	scaffold3236	p3	(CCT)9	Class I	7868	7894	TCCTCCTTTTCCACCCCAAGCA	AGCGAGCCGATGTGTTCGAAGT	305
FMgSSR-51946	scaffold11330	p3	(CGA)5	Class II	22210	22224	AAAAGGGAGCATCGGCAGCA	TTCCTGTGTTCATCCTCGGCGT	209
FMgSSR-51948	scaffold1073	p3	(CGA)5	Class II	67531	67545	AAATGCAGCGGCTCTTCTTCGC	TGCAATGCCAGTCGCGATCA	258
FMgSSR-51949	scaffold21027	p3	(CGA)5	Class II	2254	2268	AACAACCACGCCACAGCCAT	CGTGCGAGACGAAGCGTTTCAA	330
FMgSSR-51950	scaffold49452	p3	(CGA)5	Class II	3278	3292	AACGCCAAGAAGTGGGTCGT	ACGGAATCACCAAGCGGTCT	304
FMgSSR-51952	scaffold481	p3	(CGA)5	Class II	106129	106143	AACGGGTTCAGCCCGATCAAGT	CCGACGCGATGCAACAGTTTGA	319
FMgSSR-51953	scaffold1982	p3	(CGA)5	Class II	29588	29602	AACTGCTTCAACGGCCAGCA	ACCAGGCCCTGACGCGATTTTT	238
FMgSSR-51954	scaffold13106	p3	(CGA)5	Class II	13197	13211	AACTTCGAGTTCTCGGTGGGCA	ATGCCCTTGTCGCTCTGCTT	260
FMgSSR-51958	scaffold5355	p3	(CGA)5	Class II	39235	39249	AAGGCGGCATGGTCAAGGTGAA	TCAGATCCCCTCGCGAAGGAAAAA	301
FMgSSR-51961	scaffold1058	p3	(CGA)5	Class II	21967	21981	AATCGCTTGGAGCACGACGAGT	TGCCCCGTCGAACTTTTCCT	309
FMgSSR-51962	scaffold5233	p3	(CGA)5	Class II	24440	24454	ACAAGTACGTCGTCAGCGCA	TCTTCACCGGCACGTTCACT	340
FMgSSR-51963	scaffold2088	p3	(CGA)5	Class II	74317	74331	ACCACGAACAACGGCATCGT	AGGAGAGCATGCAGAGCATGGA	343
FMgSSR-51966	scaffold67	p3	(CGA)5	Class II	155250	155264	ACGACGGCATGTAATGTACCGC	ACCGAAGGCAAACGCCATGA	326
FMgSSR-51974	scaffold5260	p3	(CGA)5	Class II	4282	4296	ACTTCCAGTGCATCTCGGCGAA	TTGTGGCGGGCTCTGGTTTT	266
FMgSSR-51978	scaffold16191	p3	(CGA)5	Class II	9643	9657	AGATCCCGTGGCTTGATTGGCT	ATCGGCAAACACCGCAGCAA	312
FMgSSR-51983	scaffold1216	p3	(CGA)5	Class II	17633	17647	AGCCATGGAGCATCGCGTTGTA	AGGTGCTCCGCCTGTTCATT	285
FMgSSR-51985	scaffold4823	p3	(CGA)5	Class II	12965	12979	AGCGGCATAAATGCGCTCTC	TTTCTCCGCCCTGTCGTCGTTT	317

SSR_ID	Scaffold	SSR_Type	SSR_Motif	SSR Class	SSR_Start	SSR_End	Forward sequence	Reverse sequence	Product_size
FMgSSR-51986	scaffold1240	p3	(CGA)5	Class II	11310	11324	AGCGGCGACTTCGTCATCATCA	AAAGCGCCGCTGTTCATCATGG	259
FMgSSR-51992	scaffold2586	p3	(CGA)5	Class II	22059	22073	AGTGCTACCGCGTGTTCCTCTT	ATGCGGTAAGTGGTCGTGGACA	229
FMgSSR-51995	scaffold4052	p3	(CGA)5	Class II	18668	18682	AGTTCCTCAACGGCGGATTCGT	ACGATCAGTGCTGGTGCTGT	216
FMgSSR-51996	scaffold2326	p3	(CGA)5	Class II	52053	52067	ATATTCGCGAGCGGCTTCGT	AATCCCGTGCGACACCTCATCA	258
FMgSSR-51997	scaffold157490	p3	(CGA)5	Class II	399	413	ATCACAGCGACGATGACAGCGA	GCCAGAGCGCGATTTTCTCACA	311
FMgSSR-52001	scaffold23160	p3	(CGA)5	Class II	8842	8856	ATGCAGATTTGAAGGCCGCGCA	ATTCACTTGCTTCGGCCGGT	346
FMgSSR-52003	scaffold30617	p3	(CGA)5	Class II	3392	3406	ATGGAGCGGATCGAGGTCAACA	ACATGAGGTAGCCCCTCAACGA	308
FMgSSR-52006	scaffold3128	p3	(CGA)5	Class II	22281	22295	ATGGCCACGGAATTGCCAAGGT	TTTAGCGACCGCAACCGCAA	339
FMgSSR-52013	scaffold315	p3	(CGA)5	Class II	6806	6820	CGAGGAGGGAAAGAAGGAGAAGGA	CTCTGCTCCTTGCTCCTCGAAA	346
FMgSSR-52017	scaffold926	p3	(CGA)5	Class II	28493	28507	CGGGCATCAGCAGTTGCGAATTA	TGCTCGCCGGAGGAGATTACTA	343
FMgSSR-52018	scaffold1023	p3	(CGA)5	Class II	23779	23793	CGTCCGCCTCGGTGACAAAAAT	TGCTGTGGAGGCGTGAATTCCT	350
FMgSSR-52019	scaffold868	p3	(CGA)5	Class II	88544	88558	GCACGCGTTGCTGCTTTTGT	TCGGTGAAATTGAAACCGGCCT	287
FMgSSR-52021	scaffold14069	p3	(CGA)5	Class II	11893	11907	GGCTGCTCGAGGAAGCATTGAA	TTTCTTCTTGCTCCGCCGCT	261
FMgSSR-52025	scaffold68994	p3	(CGA)5	Class II	1727	1741	TCAAGGCCAAGCTGGTCGAGAT	TTGGTGATCAGCTTCTCGGCGT	339
FMgSSR-52027	scaffold441	p3	(CGA)5	Class II	34244	34258	TCAGCAGCTTGGCCCAATGAGA	TTGGCTTCTTGCCGAACACAGG	224
FMgSSR-52034	scaffold7482	p3	(CGA)5	Class II	8487	8501	TCGTGGACGCCGAAAATACGA	TCCCGCTTGAAACTACGCGACA	244
FMgSSR-52035	scaffold50541	p3	(CGA)5	Class II	1451	1465	TCTCCGTACTGTTCCACTGCAC	TCGAACTCGTCGTCGTCCTCAT	271
FMgSSR-52037	scaffold563	p3	(CGA)5	Class II	105563	105577	TGAATTCAGTCGCACGGCCT	TCGAGTGCTGGACCGTGCATTT	321
FMgSSR-52038	scaffold17970	p3	(CGA)5	Class II	7081	7095	TGCAACCAACCCGTCATCCA	TCAGCTGCTGCGCGTTATGA	309
FMgSSR-52040	scaffold555	p3	(CGA)5	Class II	70901	70915	TGCAGCTTGTCGATTCTCCGCA	GGCATCGTCACAAAACACGCCA	269
FMgSSR-52044	scaffold26977	p3	(CGA)5	Class II	4731	4745	TGCCGCCTCCTTTCTCACCAAA	ACCAAACAGCGCGTGCAGTAA	237
FMgSSR-52046	scaffold18603	p3	(CGA)5	Class II	9308	9322	TGCGAGTCCTCGACGAACAT	AACGGCAAGAAGGGGAACAGCA	242
FMgSSR-52051	scaffold11243	p3	(CGA)5	Class II	16229	16243	TGGAGCCTCGTTCGCATTGGTT	ATCGCAGAACGGCACAAGCA	207
FMgSSR-52053	scaffold157490	p3	(CGA)5	Class II	244	258	TGGCCCTTTTGGCCATCGAA	GCATCAGTCAACGTCCTCGATCCT	301
FMgSSR-52054	scaffold84099	p3	(CGA)5	Class II	396	410	TGGCTTCTGCGCACGTCAAA	TGGGAGCCAAATTCGCCACTGA	295
FMgSSR-52060	scaffold138	p3	(CGA)5	Class II	75335	75349	TTAACGGCTTGGGCCTTCTTGG	AAGATGCGGCTCCGTAGGTCTT	333
FMgSSR-52061	scaffold253	p3	(CGA)5	Class II	63065	63079	TTATTGGAGAGGCGGCAACCGA	AAGCCGAAACACGTGAGCGT	340
FMgSSR-52064	scaffold41024	p3	(CGA)5	Class II	1177	1191	TTCAACTGGGGAGCCTGCAT	ACCGTCGACGCCCATTTGTT	350

SSR_ID	Scaffold	SSR_Type	SSR_Motif	SSR Class	SSR_Start	SSR_End	Forward sequence	Reverse sequence	Product_size
FMgSSR-52068	scaffold478	p3	(CGA)5	Class II	28677	28691	TTCTGCCGCGCTCTGGCTAATA	TTCGCAACAACATGGCACGC	347
FMgSSR-52070	scaffold20075	p3	(CGA)5	Class II	8139	8153	TTGGCAGCGAACACGTGGAA	TCGCAGGTACGATGATGACGCA	229
FMgSSR-52071	scaffold46	p3	(CGA)5	Class II	101445	101459	TTGGTGGCCATGGAGATGCAGT	GCGCAGCTTGCGGAGTTTTT	204
FMgSSR-52076	scaffold200	p3	(CGA)5	Class II	12753	12767	TTTCGCAGGTGATCCGACGGCTAT	TCTTCTTCGCGCTGCTCTTC	347
FMgSSR-52083	scaffold75862	p3	(CGA)6	Class II	898	915	AAGCGGTCCGTCCAATTCGT	TTTGGTCCCTCAAGCGCAAGGA	325
FMgSSR-52087	scaffold3362	p3	(CGA)6	Class II	5913	5930	ACTGTTGTGTGTGCACGGGA	GCAAAGTCACAGCCCAAGCTCA	296
FMgSSR-52088	scaffold22527	p3	(CGA)6	Class II	5102	5119	AGAGAAACCACGCACACGCA	AGCGGAAGGCGTAATCGTAGCA	250
FMgSSR-52093	scaffold25122	p3	(CGA)6	Class II	8828	8845	AGTTGCATGGAGTGCGTGTGTG	AGCTAGCCATTGATCCGCCTCT	341
FMgSSR-52095	scaffold9818	p3	(CGA)6	Class II	18504	18521	ATCCGTTTCATGCGCCACCA	AAGAGGACGAACGCGACGAAGT	348
FMgSSR-52097	scaffold9882	p3	(CGA)6	Class II	9661	9678	ATGAAACGTTCCCGGCGGATGA	TCCAACACTCGCACGACAACGA	204
FMgSSR-52099	scaffold6698	p3	(CGA)6	Class II	29655	29672	CACGCCAAATTTCACTGCCCGT	AGCAACAGCAGCAGCATCAGGA	343
FMgSSR-52102	scaffold654	p3	(CGA)6	Class II	58005	58022	TCAAGCGCAAGGACCAAGGACA	AGTGCGAGGCACAGCTTGTT	281
FMgSSR-52104	scaffold13006	p3	(CGA)6	Class II	20108	20125	TCTTTTCGCGGGTGATCGCA	GCGCTATTAGCATTCGCACGCA	311
FMgSSR-52106	scaffold84099	p3	(CGA)6	Class II	144	161	TGGACGTGTTAAGCCTCGCA	TTGCCGCTGTCATTGTCGCT	303
FMgSSR-52107	scaffold1211	p3	(CGA)6	Class II	29478	29495	TGGCGCACCACGTTGTTCAT	TCGTGGCGTACCTTTCCTCCAA	331
FMgSSR-52116	scaffold10249	p3	(CGA)7	Class I	27373	27393	AACAGCGCGTTCTCTTCCCCAT	AGAAGTCGCGCATCTGGCTT	337
FMgSSR-52117	scaffold24881	p3	(CGA)7	Class I	4127	4147	ATGATTACCCCGGCCACCTGAA	TCCTGCACGTCGCCGAAAAA	304
FMgSSR-52118	scaffold1087	p3	(CGA)7	Class I	41615	41635	TTTCAACCCTGTCCCACAGCGA	GTGTTGCAGGCGCTTGTTGA	334
FMgSSR-52120	scaffold626	p3	(CGA)8	Class I	84295	84318	AGCGCTATTACGCTCGCCAT	TTGCTGGCTCTCGAGGACTCAT	226
FMgSSR-52122	scaffold1781	p3	(CGA)8	Class I	16209	16232	TTGAGGTGGATGACGGTGCTCT	ACCGGCATGAAGAAGAAGCCGA	347
FMgSSR-52124	scaffold6466	p3	(CGC)5	Class II	25705	25719	AAAAACCGAGAGGGCACCCA	TGAGGTTTCGGTGTTTGGGCGA	294
FMgSSR-52126	scaffold3462	p3	(CGC)5	Class II	38593	38607	AAAAATGGCCGGAGCTCTGCGT	TGCCGCTTGGACAGATGCAA	335
FMgSSR-52128	scaffold3630	p3	(CGC)5	Class II	49289	49303	AAAACGCACTGCAAGTCCAGCC	TAAGACAGCGTCTGCGACGGAA	340
FMgSSR-52129	scaffold5682	p3	(CGC)5	Class II	11609	11623	AAAAGGCAGCCTGCTCGCTA	AAGCAAGGCGTGCTAAGCGGAT	271
FMgSSR-52132	scaffold406	p3	(CGC)5	Class II	70925	70939	AAACCAGATCCGTGGTGCCTGT	TTCAAGGGAGATGCCGATGCGA	246
FMgSSR-52134	scaffold4856	p3	(CGC)5	Class II	43564	43578	AAACGTGTGTGCGTGGCTGT	AGAGGGCGTTCCGCAGAGATTT	336
FMgSSR-52139	scaffold7895	p3	(CGC)5	Class II	17929	17943	AAAGTCACGCTGCCGACGAA	ATGTACACGCAGCCAGCAGA	324
FMgSSR-52142	scaffold2605	p3	(CGC)5	Class II	24781	24795	AAATTTGTCGGCGACGGCCT	ACGTGCATATATAGGGCCTGCG	250

SSR_ID	Scaffold	SSR_Type	SSR_Motif	SSR Class	SSR_Start	SSR_End	Forward sequence	Reverse sequence	Product_size
FMgSSR-52144	scaffold131628	p3	(CGC)5	Class II	809	823	AACACCGTCAAGCTGGGCATCA	AGCACGTTGTTGCGCTCGTA	287
FMgSSR-52146	scaffold6283	p3	(CGC)5	Class II	7356	7370	AACATCCAGTGTCGTGGGCTGT	TGGCCTTCACGTTGTCGATGGT	344
FMgSSR-52147	scaffold10470	p3	(CGC)5	Class II	17464	17478	AACATCGGACTCACCTCCCCTT	TCGAGCCAACACGTGACCATGA	340
FMgSSR-52151	scaffold845	p3	(CGC)5	Class II	104978	104992	AACCGCGGATATGCATCTGGGA	AAGGAAACGTAGCGGCAGCA	327
FMgSSR-52153	scaffold19496	p3	(CGC)5	Class II	12570	12584	AACCTCCCGAACCCCGCAAAAA	TTTCGCGGAAACACAGCCGA	233
FMgSSR-52154	scaffold10299	p3	(CGC)5	Class II	22218	22232	AACCTTGTCCTCCAGAGTCCGA	AGCTCGCGGAGGAGAAATGGAA	269
FMgSSR-52156	scaffold19698	p3	(CGC)5	Class II	13069	13083	AACGAAAGCGGGAAGGCGAA	AGCGGCCATGAGCGAATCAA	333
FMgSSR-52159	scaffold15196	p3	(CGC)5	Class II	8702	8716	AACGCGGGTGTGTTGTGTCA	ATCATCATCACGGCGTTGGCGA	309
FMgSSR-52160	scaffold300	p3	(CGC)5	Class II	80165	80179	AACGTCGACGGCGATGTCTTCA	AGGAGCAACCGGCGTTATCTCT	240
FMgSSR-52163	scaffold16416	p3	(CGC)5	Class II	1989	2003	AACTGGCCTGCGGCCCATTATT	AAACGCGAAAGTAGGAGGCGGT	225
FMgSSR-52164	scaffold791	p3	(CGC)5	Class II	78897	78911	AAGAAAGCAGCAAGCTCCCGGT	ATGGCGAAGCATCGTGACAGGT	277
FMgSSR-52168	scaffold1092	p3	(CGC)5	Class II	33178	33192	AAGAAGCGCGGAGGGACTAA	GCCACACACCGCATGTTTCTCA	318
FMgSSR-52169	scaffold5130	p3	(CGC)5	Class II	26425	26439	AAGCACGAGCAGGAGGAGTACA	TGGAGATGGCGTTACGTACCTTGC	323
FMgSSR-52171	scaffold977	p3	(CGC)5	Class II	96491	96505	AAGCATCAGCCGAACCAATCCG	GCGCGAATAAAGAGCGGAGCA	330
FMgSSR-52172	scaffold5244	p3	(CGC)5	Class II	15947	15961	AAGCATGGATCAGCGCCTGT	TTCATGGTTGCGAATGCGGTGG	348
FMgSSR-52177	scaffold582	p3	(CGC)5	Class II	31007	31021	AAGCGCAGAAAAGGAAACCGGC	TCGAGTTCTATGCGCGTGACGA	206
FMgSSR-52181	scaffold3808	p3	(CGC)5	Class II	33561	33575	AAGCTCAACTTCCCCGACTCCT	ATGGCGGCCACTAACACTCGAA	346
FMgSSR-52184	scaffold3011	p3	(CGC)5	Class II	55055	55069	AAGGACGCAATCCAAGCGCA	GCGGGGATCGATGCGAGATTTT	319
FMgSSR-52185	scaffold610	p3	(CGC)5	Class II	115035	115049	AAGGCCCACTTGTGGCCAATCA	ACTGGGCAGGTGATGTATCGGT	281
FMgSSR-52186	scaffold22659	p3	(CGC)5	Class II	5827	5841	AAGGCCGACAACGACCTCAA	ACGACTGCCACACGGAACAGAA	206
FMgSSR-52192	scaffold2150	p3	(CGC)5	Class II	37724	37738	AAGTATGCGAAGCTGTCCTGCC	AACAACAAGCAGCAGCAGGC	349
FMgSSR-52200	scaffold5042	p3	(CGC)5	Class II	6067	6081	AATACTCACACGACGGCAGCGA	TGTACACCCCTATGTTCCGCCA	333
FMgSSR-52201	scaffold88543	p3	(CGC)5	Class II	121	135	AATCACGGGTTGGTTCCCGGTT	TGCAATGATATGTGCGCCACGG	302
FMgSSR-52204	scaffold2562	p3	(CGC)5	Class II	6664	6678	AATCGCGAATCAATCGGGGAGG	TGATGAAGCCGCTGAGGGCAAT	276
FMgSSR-52208	scaffold9796	p3	(CGC)5	Class II	17627	17641	AATGGACTCCACCGCTTCCTGA	AAACGGAGGAGGTGCTGGCAAA	329
FMgSSR-52214	scaffold16731	p3	(CGC)5	Class II	1708	1722	ACAAAGGGCACATCACGCCA	AACCAGGCGAGCAGATGCCAAT	295
FMgSSR-52222	scaffold1306	p3	(CGC)5	Class II	2841	2855	ACACTGACAGTCGCAGAGAGCA	GCCTTTTCATGCACCGGCAACA	228
FMgSSR-52225	scaffold23266	p3	(CGC)5	Class II	877	891	ACAGAACCGAGACAAGCGCA	AGCTTCTTGCCGATGCCGTT	262

SSR_ID	Scaffold	SSR_Type	SSR_Motif	SSR Class	SSR_Start	SSR_End	Forward sequence	Reverse sequence	Product_size
FMgSSR-52226	scaffold14636	p3	(CGC)5	Class II	18601	18615	ACAGGTTGGTCGCCGTTATGGT	TTTGCTGCTGCTGGCACGTA	249
FMgSSR-52227	scaffold5513	p3	(CGC)5	Class II	36110	36124	ACATCCCATCCATCACAGCAGC	AAGAAGGATCCAAACGCCGCCT	334
FMgSSR-52232	scaffold840	p3	(CGC)5	Class II	53154	53168	ACCACCACCATGTCGTTCACCA	GCCGTGGCTGCTGTTGATCTTA	303
FMgSSR-52233	scaffold289	p3	(CGC)5	Class II	81593	81607	ACCACCGTTTGGCGAACGAA	TCGGCATTGGGATTGTGCCAGT	257
FMgSSR-52236	scaffold8989	p3	(CGC)5	Class II	31377	31391	ACCCGAGCAAGGATGAATGCGT	TTGGTCAGCGCCGGAAATGGTA	248
FMgSSR-52239	scaffold877	p3	(CGC)5	Class II	32738	32752	ACCCTAACCAGACACTCGCGTT	ACGCACATCGGCAAGGTCAA	350
FMgSSR-52251	scaffold5945	p3	(CGC)5	Class II	1003	1017	ACGAAAGCAGCAAGCTCCCGAT	ACGTCGCCGTCGTCGTTATT	346
FMgSSR-52260	scaffold7739	p3	(CGC)5	Class II	12274	12288	ACGCCGTCAATGAAGCAGCA	ATAATGTGCCGGAACCGCGA	227
FMgSSR-52261	scaffold349	p3	(CGC)5	Class II	26342	26356	ACGCCGTCTTTTCGACGGTCTT	TCTTGCCTTTCTGCTCGGCT	310
FMgSSR-52262	scaffold297	p3	(CGC)5	Class II	135903	135917	ACGCGAGCATTGACCCGAAGAA	ATGTCGCGCTAGGGTTTCGT	349
FMgSSR-52265	scaffold26977	p3	(CGC)5	Class II	1145	1159	ACGGAAGGTCAAGCACTGACGA	AAGCTCCAATCTCTGCGCTCCA	272
FMgSSR-52266	scaffold364	p3	(CGC)5	Class II	102980	102994	ACGGACGACTTGGGTGGTGATT	TGAGACGGCGATGGTTCGATGT	293
FMgSSR-52267	scaffold5831	p3	(CGC)5	Class II	17959	17973	ACGGAGGCGCTAAAATCGCA	AGACGGACCACAAAGACCACCA	339
FMgSSR-52274	scaffold6184	p3	(CGC)5	Class II	35352	35366	ACGGCTGAGTTTAGCCAGCACT	TGGTGGACATAGAAGGGTTCCGGT	341
FMgSSR-52280	scaffold12573	p3	(CGC)5	Class II	3417	3431	ACTACAGCTCGTACATCCGCGT	ATTTTCCGCACTCCCGCAGCAA	226
FMgSSR-52282	scaffold1123	p3	(CGC)5	Class II	87965	87979	ACTCCGAGCAGAAGGCGTTT	AGGAACGTTGAATCGCGACGGA	280
FMgSSR-52283	scaffold633	p3	(CGC)5	Class II	11491	11505	ACTCGAACGCCATGCACCAT	GCAGCGAAGCTGTGAAGACGAA	303
FMgSSR-52287	scaffold2243	p3	(CGC)5	Class II	10471	10485	ACTGGAACAACAACAACGACGC	ACCTGAAGACGGCCTGGATCTT	349
FMgSSR-52288	scaffold6821	p3	(CGC)5	Class II	7874	7888	ACTTCCTAAACTTGGTGCGGCG	ACGAGCTGCGGCATCGAAAA	263
FMgSSR-52290	scaffold2864	p3	(CGC)5	Class II	39308	39322	ACTTGCCGATCCGAAGAAGC	CGGAAAGGATCGAAGCGCAAGA	346
FMgSSR-52295	scaffold75337	p3	(CGC)5	Class II	743	757	AGAAGAGCCGAGAGGGCATT	TCCATGACTCTAGCAAGTCGGC	316
FMgSSR-52300	scaffold12563	p3	(CGC)5	Class II	18140	18154	AGAGCCATGGAGGTGATCTTGC	ACGCAACGCAAGCCTCCAAA	336
FMgSSR-52313	scaffold29	p3	(CGC)5	Class II	187619	187633	AGCCATCCGCATTCACCTCGTT	TCGCACAGACTAAACACCGCGA	287
FMgSSR-52314	scaffold9119	p3	(CGC)5	Class II	14132	14146	AGCCATGCCAAGCTGGTGATGAT	TACGAGGGAAAGCACACGCA	324
FMgSSR-52316	scaffold3752	p3	(CGC)5	Class II	28285	28299	AGCCGTCAAACATGCGAAGCA	ACGAGCTCGACAACAAAGGCGT	324
FMgSSR-52319	scaffold4803	p3	(CGC)5	Class II	37342	37356	AGCGACAATGCTACGGCCATCA	AGCGCAAGAAGAGGAGGAACGA	317
FMgSSR-52320	scaffold938	p3	(CGC)5	Class II	37857	37871	AGCGACGAGGAAATGGCGATCA	GCAAGCACACATCATCACGGCA	308
FMgSSR-52321	scaffold655	p3	(CGC)5	Class II	25952	25966	AGCGCCTGCGATTTGAGGAA	TCGGTTGCAGCAGCATCATCCA	228

SSR_ID	Scaffold	SSR_Type	SSR_Motif	SSR Class	SSR_Start	SSR_End	Forward sequence	Reverse sequence	Product_size
FMgSSR-52322	scaffold1109	p3	(CGC)5	Class II	52562	52576	AGCTAGCTGTTCACGCGTCAGT	ACGACATCGTGTCCGTGGAT	288
FMgSSR-52323	scaffold18328	p3	(CGC)5	Class II	1768	1782	AGCTCCACCGCTTCGCTTTT	GGCGGCACTTGCTATGGAAT	332
FMgSSR-52324	scaffold3886	p3	(CGC)5	Class II	22068	22082	AGCTCGAAACGGCAGAGCACAT	TGATCGGCATCATCTCGCGT	301
FMgSSR-52326	scaffold9667	p3	(CGC)5	Class II	2739	2753	AGCTGAGGCAATCCAACGCA	TCACCATCGTCGTCTGAGCTGT	230
FMgSSR-52331	scaffold235693	p3	(CGC)5	Class II	239	253	AGGAAGGCAGGCAGCAAAAGGT	AGCTGATCGTGCAACCCACT	242
FMgSSR-52336	scaffold1406	p3	(CGC)5	Class II	27264	27278	AGGCAGCGTTGGATGCAAGA	TTAAGCCAACGCACGAGAGGCA	308
FMgSSR-52342	scaffold9843	p3	(CGC)5	Class II	21370	21384	AGGCTACACGTGTGATCTGCGT	TCCACACGAGCGCACTGAAA	315
FMgSSR-52350	scaffold6815	p3	(CGC)5	Class II	15694	15708	AGTTAATTTGCGTACGGCGGC	AATCAGCGCGGAGAGTGAGT	336
FMgSSR-52356	scaffold22681	p3	(CGC)5	Class II	5185	5199	ATACCGTACGCGGTGTCTGGAT	GGTGTGAGTGTGAAGAGCGCAA	331
FMgSSR-52357	scaffold2661	p3	(CGC)5	Class II	15782	15796	ATACGGATCGGTTTTGGGCTGC	TTGCCTCGCAAGCAGCAGAGTA	343
FMgSSR-52359	scaffold624	p3	(CGC)5	Class II	103969	103983	ATAGCGGCGACGCGAAAGTT	AGGCTCAAGCTGCCACGAAA	341
FMgSSR-52360	scaffold112	p3	(CGC)5	Class II	157773	157787	ATAGCGTTCCCGCTAACAGCGT	AAAGCCCGTGGGCCTAAATGGA	316
FMgSSR-52362	scaffold4737	p3	(CGC)5	Class II	34128	34142	ATCACCCTGCACACCAGCAA	AACGAGCTGCTCCACTTCCA	266
FMgSSR-52367	scaffold5510	p3	(CGC)5	Class II	22184	22198	ATCCAACATCGCGTTCCCGT	GGCGGAGTTGTTTCCTGCGATT	314
FMgSSR-52369	scaffold3075	p3	(CGC)5	Class II	40039	40053	ATCCCCTCCCTAAACCGCACAA	AGTGCAACGCTCAGGACGACAA	207
FMgSSR-52371	scaffold7132	p3	(CGC)5	Class II	17756	17770	ATCCTTTCCAACCCACGGCACA	ATGTGCGCGACACAGTGGTT	272
FMgSSR-52373	scaffold8638	p3	(CGC)5	Class II	32617	32631	ATCGCCAAGGGAATCGCTGT	TCGGCACAGCAACGCAATGA	216
FMgSSR-52376	scaffold1494	p3	(CGC)5	Class II	52631	52645	ATCGTGTCGGTGCGGATGAAGT	ACGACCCCATCCTGTTCGAT	280
FMgSSR-52378	scaffold1205	p3	(CGC)5	Class II	14093	14107	ATGACGATGCCCACGCACAT	AGCGGTGCAGAAGGACTTGGAA	316
FMgSSR-52379	scaffold4225	p3	(CGC)5	Class II	37755	37769	ATGACGCTGAAAGCAGTGCG	TCAAGTGCGAGTGCGCGTTT	227
FMgSSR-52380	scaffold2027	p3	(CGC)5	Class II	30520	30534	ATGAGGTGATGGTGCTGCCTGT	TGGCATGTTTGCTCGCACCA	299
FMgSSR-52382	scaffold2015	p3	(CGC)5	Class II	17642	17656	ATGCAGAACGCGTGTGACGA	AGCAGCAGCAAGAGCTTGGA	299
FMgSSR-52385	scaffold3327	p3	(CGC)5	Class II	29249	29263	ATGCAGATGGAGATGCAGGTCG	GCGGCGGCTGCTTTATTACT	341
FMgSSR-52390	scaffold6475	p3	(CGC)5	Class II	27978	27992	ATGCGGCCGATAACCTCAAC	TTCCGCACACCCCAAGCTCTTT	282
FMgSSR-52391	scaffold1572	p3	(CGC)5	Class II	22204	22218	ATGCTCGCCAGAAAGTCCTCCT	TGCAGCATCACCACCAGCTCAA	331
FMgSSR-52403	scaffold522	p3	(CGC)5	Class II	32559	32573	ATGGGGCAGAATAGCGAGCGTT	AGGGAAAACGGACAGCTGCGTA	271
FMgSSR-52404	scaffold13689	p3	(CGC)5	Class II	14556	14570	ATGGGGTTCGGCATGGGATT	AGCAAAGCCAGGACCGCATT	326
FMgSSR-52408	scaffold3712	p3	(CGC)5	Class II	49780	49794	ATGTCACGCTGGTGGCGACTTA	AAGAGCCATGCTCCGGTAACGA	311

SSR_ID	Scaffold	SSR_Type	SSR_Motif	SSR Class	SSR_Start	SSR_End	Forward sequence	Reverse sequence	Product_size
FMgSSR-52417	scaffold11268	p3	(CGC)5	Class II	12587	12601	ATTACCAGGGCACGTGCAAGGA	AGAAGCGTGGACCGGTTGAA	217
FMgSSR-52418	scaffold18607	p3	(CGC)5	Class II	10562	10576	ATTCACCAAATTCCCCCGCCCT	TAGTCGTTGTTGTCGACGCGGA	270
FMgSSR-52419	scaffold272533	p3	(CGC)5	Class II	220	234	ATTCAGTGCTCCACCGAACGCT	ACGCACACGGCGCAAACAAA	304
FMgSSR-52421	scaffold13102	p3	(CGC)5	Class II	4463	4477	ATTCATCAACCGCACGCTGCCT	TAAACCTCGCTGCAGACCACGA	339
FMgSSR-52423	scaffold4248	p3	(CGC)5	Class II	14452	14466	ATTCCCGACCGCTGTTGTCACT	ATGACGGCATGCAGGTCTCCTT	216
FMgSSR-52433	scaffold4583	p3	(CGC)5	Class II	3668	3682	ATTTTACGCCCAACGCGGAAGG	TGGTGGTGCAGCAAGGGGAAAA	233
FMgSSR-52437	scaffold6	p3	(CGC)5	Class II	287287	287301	CAGCATCATCACCTAGACCACCA	GCTCCTACTCCTACTCCTACTCCT	350
FMgSSR-52439	scaffold5624	p3	(CGC)5	Class II	22415	22429	CCCGCAAGCGCGTTCTACAAAT	TCGACGACGTGGTGGAAGATGA	214
FMgSSR-52442	scaffold162	p3	(CGC)5	Class II	122068	122082	CCGCTGCTGTTGTTGTTGCT	ATGACGACCAATAAGCCCGCGA	305
FMgSSR-52447	scaffold28897	p3	(CGC)5	Class II	3960	3974	CGCAGGTGATCCCCTTGTTGAA	TGCCACGGGTTTGTGAGTTCCT	304
FMgSSR-52449	scaffold22204	p3	(CGC)5	Class II	808	822	CGCGCGCACGATCTTGATGATT	TGCTCTGCGCCTGCAGGAATTT	314
FMgSSR-52450	scaffold409	p3	(CGC)5	Class II	121416	121430	CGCGCGGTGTGTTTGTTGTT	TTATAGACGACGACGAGGGCGA	273
FMgSSR-52451	scaffold7658	p3	(CGC)5	Class II	24788	24802	CGCTTCAGGCCTTCAGTTCCTA	ACGTCGGAGCAGAGGAAACA	349
FMgSSR-52452	scaffold31	p3	(CGC)5	Class II	171685	171699	CGGCAATCAGCAAGCAACACGA	TGCTGCTGCTTCTTTCAACGGC	259
FMgSSR-52457	scaffold22	p3	(CGC)5	Class II	101702	101716	CGTTGAGGGAGAAGAGCTGAGA	ATGACTCTAGCAAGTCGGCGAG	323
FMgSSR-52458	scaffold11234	p3	(CGC)5	Class II	24904	24918	CTGAACACGTCGTCGCAGAA	TGAGGACGTTCGTGGTGGAAGA	282
FMgSSR-52463	scaffold1715	p3	(CGC)5	Class II	34728	34742	GCAGTGACACCCCGCTTTTT	TCCATGACGATGAGCCAGTGCT	317
FMgSSR-52470	scaffold1212	p3	(CGC)5	Class II	9188	9202	GCGCACGCGTCATCAAAACT	ATGTCCGACTCGTCGAACTCCT	232
FMgSSR-52474	scaffold8391	p3	(CGC)5	Class II	9474	9488	GCTGCCACAACACTCGCAAACA	TCGACTGCGATTGGCCACAA	237
FMgSSR-52480	scaffold263	p3	(CGC)5	Class II	20011	20025	GGCGCATTTCGTCGAACACCTT	ATCTGGCTGGGGTTTGGAAGCA	297
FMgSSR-52481	scaffold1272	p3	(CGC)5	Class II	16480	16494	GGCGGGCGGGTGAATTGAATTTT	AAAAGGGAAATGGCCGCGCT	303
FMgSSR-52482	scaffold2210	p3	(CGC)5	Class II	1640	1654	GGGAAACGTCATCGCAACAGCA	TACTCAAGAACACCACGGCGCA	215
FMgSSR-52486	scaffold14882	p3	(CGC)5	Class II	17619	17633	GTCCTAGCATTTCGTTGCGCCT	TCAACAGCCGACATGGAGCA	317
FMgSSR-52490	scaffold19797	p3	(CGC)5	Class II	7250	7264	TAAAGCCCCAAAATCCGGCGCA	AAGTCGGGGTGTCATGGTGCTT	327
FMgSSR-52491	scaffold10043	p3	(CGC)5	Class II	20129	20143	TACAGGTACGCGATGGGGATCA	TGCGGCGTGCAAGAAGCAAA	295
FMgSSR-52493	scaffold5305	p3	(CGC)5	Class II	35128	35142	TACAGTCTGCTGGCACGCGTAT	AGTTGTCTGCGAGGACGGTGAA	221
FMgSSR-52494	scaffold2945	p3	(CGC)5	Class II	28011	28025	TACATACTTGCGCGGCCCAACA	TCGAGGAAGAAGAGCTGGCA	338
FMgSSR-52497	scaffold275	p3	(CGC)5	Class II	99238	99252	TACCTGTTTGGAGAGGGGACCT	TCTCTGCACGCTGGTTCCTT	343

SSR_ID	Scaffold	SSR_Type	SSR_Motif	SSR Class	SSR_Start	SSR_End	Forward sequence	Reverse sequence	Product_size
FMgSSR-52502	scaffold26361	p3	(CGC)5	Class II	2902	2916	TACGGTGAGCCTCGAAGCAA	TGCAACCAACACATCATGCGGA	221
FMgSSR-52503	scaffold16995	p3	(CGC)5	Class II	13975	13989	TACTGGTGCGGGTGCATGTAGT	ATGCTGCTTGGCACCTCTGCAT	347
FMgSSR-52504	scaffold6961	p3	(CGC)5	Class II	19722	19736	TAGACGACTGCGTCATGTCCGA	ACGTGAAGCAGCTGGAACAGCA	319
FMgSSR-52514	scaffold3	p3	(CGC)5	Class II	124437	124451	TCACCGTCAATGTCGTCGTCCT	GCAACAACAATGCCGATGCC	329
FMgSSR-52516	scaffold1804	p3	(CGC)5	Class II	5213	5227	TCACTTCACCCAGACACCCACA	GGCGTTAGGGTTTTCTCGCA	330
FMgSSR-52520	scaffold755	p3	(CGC)5	Class II	88807	88821	TCATGCTCCTCCACAACGGCAA	ATTCCCACGATCACGGTCACCA	333
FMgSSR-52524	scaffold3879	p3	(CGC)5	Class II	21945	21959	TCCACTTCTCCGTTCGGCAA	AGACGCCGACGATGCAAGTT	332
FMgSSR-52529	scaffold97	p3	(CGC)5	Class II	159564	159578	TCCCCGCTTACCGAATGCAACA	AAGTACTGCTTGCGCGCGTT	255
FMgSSR-52530	scaffold4	p3	(CGC)5	Class II	186100	186114	TCCCTCCGCCAAATCCGAACAT	TTGAGCTGCAAGACACTGGCGA	235
FMgSSR-52540	scaffold312963	p3	(CGC)5	Class II	235	249	TCCTGTTCTGCTGAAGCAGCCT	ACAATCGCAGCATGGCCCAA	274
FMgSSR-52541	scaffold16	p3	(CGC)5	Class II	125121	125135	TCCTTCCACCGTGCATTTGGCA	ACGACACCACCAAAGCACCA	240
FMgSSR-52543	scaffold57748	p3	(CGC)5	Class II	2502	2516	TCGAAGCGTCGCTGACACCAAT	ACGCCCCCAAACTGAAAGGGAA	254
FMgSSR-52553	scaffold10431	p3	(CGC)5	Class II	9427	9441	TCGCACGTAACGGAACGGAA	ATGATGCTTGCGACGACGAGGA	315
FMgSSR-52558	scaffold44	p3	(CGC)5	Class II	107409	107423	TCGCGAACTGACCAAACGACA	ACGCCTCGACCAAAAGACTGCT	346
FMgSSR-52559	scaffold819	p3	(CGC)5	Class II	31128	31142	TCGCGCGTGAAATCGTCTGT	TGGCTTGGCTGCGTTGCTTT	310
FMgSSR-52560	scaffold22987	p3	(CGC)5	Class II	9748	9762	TCGCGGAAAATCAGGTGCGA	TTTCGTGGAGCCTCGGCAGTTT	314
FMgSSR-52562	scaffold2757	p3	(CGC)5	Class II	29433	29447	TCGGCAGCAACTTCAACCGCTA	ACACGCAAAAACTCGCAAGGGG	294
FMgSSR-52563	scaffold640	p3	(CGC)5	Class II	70357	70371	TCGGCCGTCGTTGATGATGACA	ACTCACCATCCATGCCGTGA	271
FMgSSR-52564	scaffold25	p3	(CGC)5	Class II	67149	67163	TCGGGATGCCCGAGAAGTGTTT	AAGAAGCCAGCGTGCGACAT	300
FMgSSR-52566	scaffold1772	p3	(CGC)5	Class II	48284	48298	TCGTGATCGCCAAGGTAACGCT	GGGACCGTGGAGAAGTTCATGT	281
FMgSSR-52571	scaffold9882	p3	(CGC)5	Class II	9825	9839	TCGTTGTCGTGCGAGTGTTGGA	TCCCAGTGTACGCGTGCTTCAA	319
FMgSSR-52572	scaffold6283	p3	(CGC)5	Class II	3311	3325	TCTACCGTCCGCGTCAATCA	ATTAGGGAACGTGCCTTGCGCT	276
FMgSSR-52575	scaffold3876	p3	(CGC)5	Class II	12877	12891	TCTCCGAATCGGGGCAAGAT	TGCCATTCCCAAAACCCTACCT	226
FMgSSR-52577	scaffold9444	p3	(CGC)5	Class II	11767	11781	TCTGCTTCTGTTGCTGCCGT	TCCACCCACAAGCATCATCACCA	335
FMgSSR-52579	scaffold11256	p3	(CGC)5	Class II	24878	24892	TGAAAACGGGTCGCGCCAAA	AATGGCAAGCAGCAACGGCT	219
FMgSSR-52580	scaffold547	p3	(CGC)5	Class II	3261	3275	TGAACCTGTTCGCCAAGCAACG	ATGGCGCCGTCCATTCCAATCT	296
FMgSSR-52581	scaffold759	p3	(CGC)5	Class II	46742	46756	TGAACTGCGCCACGCAGAAT	AGCAGCTAGCTAAACCGGACACT	344
FMgSSR-52582	scaffold744	p3	(CGC)5	Class II	8747	8761	TGACACGCAAAACGCCGCAT	TTCCTCCATCATTTCACGCCGC	313

SSR_ID	Scaffold	SSR_Type	SSR_Motif	SSR Class	SSR_Start	SSR_End	Forward sequence	Reverse sequence	Product_size
FMgSSR-52585	scaffold18632	p3	(CGC)5	Class II	10659	10673	TGACGGAATGACTGCCGCAA	TGTTAGCGTCAGAGGTCGTGCT	290
FMgSSR-52596	scaffold6824	p3	(CGC)5	Class II	28182	28196	TGCAAAACTTTGCGTCGGCA	CGGGTAATTGATGCTGGGCGTT	269
FMgSSR-52597	scaffold468	p3	(CGC)5	Class II	125744	125758	TGCAACTGCAAACCCATCTCCC	ACCGCCGTTCCCGTTTCATACA	274
FMgSSR-52598	scaffold2698	p3	(CGC)5	Class II	1344	1358	TGCACAACTTCTTGGCGTCGT	GCGATCGTCAGCAAGGAAGACA	271
FMgSSR-52600	scaffold911	p3	(CGC)5	Class II	88270	88284	TGCACATCAGAACCCAGGCGTA	ATCTTGCGTTCGTCTCCGACCA	348
FMgSSR-52602	scaffold6630	p3	(CGC)5	Class II	35217	35231	TGCAGATGCAGATGCCGTTCAC	CACGAACCCGCATTGCAAGCTA	338
FMgSSR-52611	scaffold39735	p3	(CGC)5	Class II	2114	2128	TGCCCCGAGAAAGGAAAAGCCA	ATCTCGGAGAGGAGGTTGTCGT	350
FMgSSR-52612	scaffold45868	p3	(CGC)5	Class II	1929	1943	TGCCGGTACCCGTTGAACCTTA	TAAAAACCTTCACCACCCGGGGCA	208
FMgSSR-52615	scaffold903	p3	(CGC)5	Class II	85093	85107	TGCCTTGTGTGACGTTCCGT	AAACGCCAAGGTGCGGCTCTAT	267
FMgSSR-52619	scaffold583	p3	(CGC)5	Class II	59322	59336	TGCGCCTGATCGAGAAGCTGAA	TGTTGTACTGCAACCGCAACGG	273
FMgSSR-52624	scaffold18	p3	(CGC)5	Class II	193766	193780	TGCGGGCTTACCGTTGTGGTAT	ACCACCGGTACGCCAACAATCA	311
FMgSSR-52627	scaffold28420	p3	(CGC)5	Class II	8332	8346	TGCGTTTCGCACAGCAGGAA	TGTGACGTCCGTTGCGTTCA	204
FMgSSR-52633	scaffold17830	p3	(CGC)5	Class II	18866	18880	TGCTAGTTGGTGTTCCCGCT	TACAAGCGGATAAGCGCCGT	346
FMgSSR-52634	scaffold2413	p3	(CGC)5	Class II	5424	5438	TGCTCCTTGGAGAAGGCAGTGT	TGGCCAGCAAATCAACGGTCCA	333
FMgSSR-52639	scaffold1724	p3	(CGC)5	Class II	11826	11840	TGCTTCACCTGGCCGAAACAGA	ACATGACGAAGCAGAGCGTGGT	345
FMgSSR-52641	scaffold8373	p3	(CGC)5	Class II	13563	13577	TGCTTCTGCAGCTCCGTCAT	AAAACGCAGCCAGCGCACAA	246
FMgSSR-52643	scaffold781	p3	(CGC)5	Class II	96758	96772	TGCTTGCGCTCTTTGCGCTT	CACCACCAACTGGATGTTGACG	332
FMgSSR-52646	scaffold3966	p3	(CGC)5	Class II	22585	22599	TGCTTTGCGTTGTCAAACCGCT	TGAGTGAGTAGCGGAGAAGCTCA	331
FMgSSR-52649	scaffold771	p3	(CGC)5	Class II	43884	43898	TGGACCACAATTACAGGGGAGACC	CGCGAAAACCGAGAGCAAGCAT	336
FMgSSR-52650	scaffold11342	p3	(CGC)5	Class II	19922	19936	TGGCAAGCTTTGACTTGGCG	GGACACGGCAACACGGCAATTT	300
FMgSSR-52651	scaffold12803	p3	(CGC)5	Class II	11627	11641	TGGCAGCTGCGAAACACGAA	TGCGATGGCCATGGGTGTTCAT	325
FMgSSR-52653	scaffold70732	p3	(CGC)5	Class II	1440	1454	TGGCGCGATGCGTTTTGGTT	TCGTCGTCCTCGTCGTCTTCTT	294
FMgSSR-52655	scaffold216	p3	(CGC)5	Class II	59019	59033	TGGGAGCTGCACATCACCAACT	AATTCGCCTGCGTAGCAGCA	332
FMgSSR-52656	scaffold448	p3	(CGC)5	Class II	104342	104356	TGGGCCGGCGTACAAACAAA	TTGGCAAAAGCGGAGGGGAA	249
FMgSSR-52659	scaffold4524	p3	(CGC)5	Class II	30707	30721	TGGTACAGCTTCACCAACACCG	TCCTTAGTGCCGTCTGGTTCCT	340
FMgSSR-52660	scaffold3274	p3	(CGC)5	Class II	54839	54853	TGGTAGGACCAGTCACCTTCGTCA	TCAAGCCCAGCTGACTACGCAA	263
FMgSSR-52664	scaffold218	p3	(CGC)5	Class II	136078	136092	TGGTTGTCGAGATCCAGCGGAA	ATTACCCGATCGAGCAGCCA	347
FMgSSR-52675	scaffold518	p3	(CGC)5	Class II	83765	83779	TGTGTGCACGACACGAGCCTTA	AGTCGCACATGAAAGCGTTGCC	312

SSR_ID	Scaffold	SSR_Type	SSR_Motif	SSR Class	SSR_Start	SSR_End	Forward sequence	Reverse sequence	Product_size
FMgSSR-52680	scaffold856	p3	(CGC)5	Class II	35592	35606	TGTTGGGCTCCGCAACAGTA	TTCTCCAACTGCTCCGCACT	323
FMgSSR-52681	scaffold593	p3	(CGC)5	Class II	62000	62014	TGTTTAAGGGGCTCTCGCGT	AGCGCGTCATTTGGTGTCTTGC	333
FMgSSR-52682	scaffold7615	p3	(CGC)5	Class II	33927	33941	TGTTTGGCGGGCAACCTCTACA	AAGTCGTCTTCCTCGATGGCCT	286
FMgSSR-52683	scaffold10941	p3	(CGC)5	Class II	20880	20894	TGTTTGGGCTGTTGGTCTGGCA	ACCACCACTCCAAGGGAAGAAGA	294
FMgSSR-52684	scaffold18906	p3	(CGC)5	Class II	5238	5252	TTACCAGCTCCCGCTTCATGCT	CCTCCGCAACGCAATCCATCAA	281
FMgSSR-52685	scaffold558	p3	(CGC)5	Class II	39515	39529	TTACCGCAGTTGACTTGGGCGA	AGCGACAGCAGCATCAAGCA	346
FMgSSR-52686	scaffold1960	p3	(CGC)5	Class II	17146	17160	TTAGGCGCACGCTCGTCATCAT	TTGTTCTGCGTCGGCAGCTT	276
FMgSSR-52690	scaffold6453	p3	(CGC)5	Class II	8078	8092	TTATTAGGCGCAGCCGTCGT	TCGAGACCATCCTCTCGGGAAA	307
FMgSSR-52694	scaffold1572	p3	(CGC)5	Class II	58623	58637	TTCAGATGTTCACGGGCTTCGC	AAGCCGCGTCCTTTGGAAGT	332
FMgSSR-52700	scaffold13462	p3	(CGC)5	Class II	16101	16115	TTCCGCGCGATCTACGAGAA	AGCCGTCGGAACTGCCTTTTGA	304
FMgSSR-52707	scaffold58197	p3	(CGC)5	Class II	1416	1430	TTCGCGTTCGTCCTGTACCACT	ACCACGCTTTGTGATCCCCAGA	321
FMgSSR-52708	scaffold141934	p3	(CGC)5	Class II	955	969	TTCGGCGCAGCTAAAACCCA	TCGTGTATGCCGTCCTGCTCTT	278
FMgSSR-52709	scaffold3973	p3	(CGC)5	Class II	6325	6339	TTCGTCGCATGCAGGATTGCAG	TTCGACGGCGCTTTCCTCAA	340
FMgSSR-52710	scaffold8311	p3	(CGC)5	Class II	9422	9436	TTCTCCCAGTCGAAACCGCTCT	ACCTGGACGCTGAGCAGAAT	212
FMgSSR-52711	scaffold9978	p3	(CGC)5	Class II	23694	23708	TTCTGATCTCCGCTCTGACGCT	AACGAATGCCCACGCTGGAA	330
FMgSSR-52712	scaffold445	p3	(CGC)5	Class II	25883	25897	TTCTTCTTGCTCGTCACGGCCT	AGCCTGCCGTCCTTCCTCAAAT	254
FMgSSR-52718	scaffold3922	p3	(CGC)5	Class II	38165	38179	TTGAGAGAGCAAGGGGAGGAGGAA	AGTACTCACAGCAGCCGCAT	223
FMgSSR-52719	scaffold884	p3	(CGC)5	Class II	10632	10646	TTGATGGGGTGCGCGATTGA	AACCACCACGCCGCCATTGATA	305
FMgSSR-52721	scaffold16427	p3	(CGC)5	Class II	5597	5611	TTGCGCGAAATCGAGAGCGT	TGCGCGCAGAAACGAATCGAA	232
FMgSSR-52738	scaffold3398	p3	(CGC)5	Class II	27047	27061	TTTGCTGCTAACACCAGCCG	ACGCAAGACACGCTACATGC	292
FMgSSR-52739	scaffold17563	p3	(CGC)5	Class II	3625	3639	TTTGGCGTGCAATTGGTGACCG	TCGTGATGGCACCTATGCTCCT	287
FMgSSR-52745	scaffold1272	p3	(CGC)6	Class II	63435	63452	AAATCGGTACCTCTTCGCCGGA	TGCAGACGGATTTGCCTGACGA	263
FMgSSR-52746	scaffold7891	p3	(CGC)6	Class II	17155	17172	AACCCACTCGCGCGAAATCA	ACAAACCTCCTCTCGCGGAT	337
FMgSSR-52749	scaffold4	p3	(CGC)6	Class II	79012	79029	AACTCGATCCAACGGCCCAACT	ATTTTCCGAGCAATCCGCCACG	286
FMgSSR-52752	scaffold88	p3	(CGC)6	Class II	22654	22671	AACTCGCCCGTGCGTAACAA	TGCCTCAGCATCACCGGAGTTT	288
FMgSSR-52755	scaffold55	p3	(CGC)6	Class II	203755	203772	AAGCAGCACCCCAAAACCCT	AGCCACGCAGCCACCATTAGAA	245
FMgSSR-52759	scaffold6088	p3	(CGC)6	Class II	38225	38242	AAGGACGACGCCGCTTGTATGT	ACACACGCGTTTTCGGTGACT	220
FMgSSR-52761	scaffold6274	p3	(CGC)6	Class II	2420	2437	AATGGATGCCGCCGCATTTG	TAGCGCAGGAATGGCATGCAGA	260

SSR_ID	Scaffold	SSR_Type	SSR_Motif	SSR Class	SSR_Start	SSR_End	Forward sequence	Reverse sequence	Product_size
FMgSSR-52766	scaffold88	p3	(CGC)6	Class II	135626	135643	ACCAATCTTGCCGCCGGTTT	ATGAACAACAGGGCGGGAGCAA	267
FMgSSR-52769	scaffold7209	p3	(CGC)6	Class II	6777	6794	ACCGCTTCCCGCTCAACAAA	AAGCGGACCTGCCATTTGGA	338
FMgSSR-52771	scaffold279248	p3	(CGC)6	Class II	227	244	ACGACTTTAGGACGACGCCGAT	ATCACCTGCTCGTGCGGTATCT	200
FMgSSR-52772	scaffold12	p3	(CGC)6	Class II	234076	234093	ACGAGGCGTTGTTCCCTTCGAT	TTTCTTCTCCTTCGTCGTCGTGGC	212
FMgSSR-52775	scaffold18779	p3	(CGC)6	Class II	7589	7606	ACGCTAGACAGCAGCAGCAT	ACAGCCTTCGAAGCAGAGTGGA	250
FMgSSR-52776	scaffold1471	p3	(CGC)6	Class II	61690	61707	ACGGAAAGTGCAGCACACGA	AAGGGCAAGGTGCTCGACGAAA	256
FMgSSR-52787	scaffold4038	p3	(CGC)6	Class II	46256	46273	AGCCAAAGGAAGCCCGTCTTGT	ATTCGTCGAACAGCATGGACGC	241
FMgSSR-52790	scaffold1245	p3	(CGC)6	Class II	71223	71240	AGGAAGGAAGAAGGAGCAGACGGA	TGCAGTTCTGCTGCCTCACA	280
FMgSSR-52791	scaffold22	p3	(CGC)6	Class II	13879	13896	AGGAATTGGTGTCGCGTGCCTT	ATGGCTGCTTCTTCGTCCTCCT	203
FMgSSR-52793	scaffold18542	p3	(CGC)6	Class II	7892	7909	AGGCCGCTTTACAGCCCAAA	AAGACTAGCAGTGTTCCGGCCA	238
FMgSSR-52796	scaffold2365	p3	(CGC)6	Class II	12497	12514	AGTTTCTGGGGCAGGACGAAGA	TCGCTGCCGTTGAGGTTTGGTT	274
FMgSSR-52800	scaffold886	p3	(CGC)6	Class II	52334	52351	ATCGGTGTCCACGTGCAGTA	AACGTTTTAATTCGGGGCGGGC	330
FMgSSR-52801	scaffold258	p3	(CGC)6	Class II	23686	23703	ATCGGTTCTTACTGCTCCCCCA	ATTGCCCTGCGCTTCGTGTT	345
FMgSSR-52802	scaffold2364	p3	(CGC)6	Class II	68759	68776	ATGACTTCCGCCGCTCAGTCTT	AGCAGCCTCAGCTGATTGTGTCA	348
FMgSSR-52808	scaffold2954	p3	(CGC)6	Class II	41358	41375	ATGTCGTCCAGCCGCAGTTCAT	GCGATCCGGTTTAGGGGGCATATT	307
FMgSSR-52809	scaffold130	p3	(CGC)6	Class II	117298	117315	ATGTTGAGCACCGCGTTCATGC	AACAACGCCGCAAGTGCAGA	297
FMgSSR-52810	scaffold430	p3	(CGC)6	Class II	80849	80866	ATTCGCGGCCAGCAGTTAATCG	TTGCAGAACCACCACCAGCA	280
FMgSSR-52812	scaffold9033	p3	(CGC)6	Class II	21872	21889	CGATGCCCGCAAAAGCCTGAAT	ATCCCACGCATGATGGCGAA	253
FMgSSR-52814	scaffold1782	p3	(CGC)6	Class II	66741	66758	CGCGAGCTGCGCCCTTAATTTT	TCGACTTTGGCCTCGCTCATGT	308
FMgSSR-52820	scaffold6268	p3	(CGC)6	Class II	24292	24309	GCCCGCGAAACGTCCTAAAA	ACAGCGGCCGAAAGTTCCAGAT	334
FMgSSR-52821	scaffold14941	p3	(CGC)6	Class II	21554	21571	GCCGAGCGCACAAAAGGAAT	ATTGTACCGGAGAGGCCTGGTT	330
FMgSSR-52824	scaffold32	p3	(CGC)6	Class II	205065	205082	GCGCCGAAGTGGGTCAAAATGT	TCCTTCAACACCGTGAAGCCGT	350
FMgSSR-52825	scaffold17147	p3	(CGC)6	Class II	8428	8445	GGCACAGCCGTTGTTCCTCTTT	AGGTGCGAAGGACGCAGAAT	200
FMgSSR-52833	scaffold27438	p3	(CGC)6	Class II	5631	5648	TCACCATCAAACGCCACACG	TGGCCTTTGCGAAGGAGGATGT	314
FMgSSR-52834	scaffold7288	p3	(CGC)6	Class II	10685	10702	TCAGCGCCACGTTGAAACACA	TGTGGGGGTTGTTGGTGAGTGA	340
FMgSSR-52835	scaffold585	p3	(CGC)6	Class II	41522	41539	TCAGGAAGAGGAACGCCACCTT	CCCCTGAATTTCGTTCGGGGAA	345
FMgSSR-52837	scaffold26971	p3	(CGC)6	Class II	618	635	TCATGCCAATCTCACTCGCGTC	TGCATCGTCACCTCCAACACTG	343
FMgSSR-52839	scaffold6543	p3	(CGC)6	Class II	23384	23401	TCCAACCTCCAACCTCCAACCA	GCGAAATTTCAGCGGGCTCAGT	314

SSR_ID	Scaffold	SSR_Type	SSR_Motif	SSR Class	SSR_Start	SSR_End	Forward sequence	Reverse sequence	Product_size
FMgSSR-52840	scaffold837	p3	(CGC)6	Class II	26034	26051	TCCACCTTGCCGCACAGATCAT	TGCCGGCCTTCACTTACGTT	220
FMgSSR-52842	scaffold1316	p3	(CGC)6	Class II	64521	64538	TCGATGCTCTCATCATGGCGGT	TGGCTACCGTTGCACTTCGT	220
FMgSSR-52850	scaffold554	p3	(CGC)6	Class II	69665	69682	TCGGTTGCTGCGAAATCGGT	ACATTCGGCGCCCTTGAACA	347
FMgSSR-52851	scaffold266	p3	(CGC)6	Class II	85944	85961	TCGTCCTAGCATTTCGTTGCACCT	TGGATCAGCACAGTACCACGCA	256
FMgSSR-52853	scaffold4081	p3	(CGC)6	Class II	37254	37271	TCTTTTTGCTGCCGCCGAGA	TGGCGGTGGAATACGCAACT	263
FMgSSR-52857	scaffold130546	p3	(CGC)6	Class II	361	378	TGAGAAGATGCGTTTTGCGCCC	TTTGCCGTCGCTTGGCCTTT	287
FMgSSR-52860	scaffold11962	p3	(CGC)6	Class II	15536	15553	TGCAAAACGTTGCGCCGACA	AAGACGAACGGAGCCGAAAGCA	284
FMgSSR-52863	scaffold6656	p3	(CGC)6	Class II	22703	22720	TGCAAGCCCGACGAGTTCAA	AACGCGCATCCCGTCACATT	295
FMgSSR-52864	scaffold768	p3	(CGC)6	Class II	38556	38573	TGCAGACTTGCAGCTACGAGCA	TCGAGTCCACGTGCACCTTT	298
FMgSSR-52867	scaffold39122	p3	(CGC)6	Class II	1746	1763	TGCCGCTTTTCGCGTTTCACT	AAGCTCGGCAATGCACTCACCA	316
FMgSSR-52871	scaffold4229	p3	(CGC)6	Class II	10238	10255	TGCTCGTGGCAGTGACGAAA	TCTTCGTTTGGCGGCGAAGT	200
FMgSSR-52873	scaffold1147	p3	(CGC)6	Class II	60996	61013	TGGCGCTGCCGTTACAGTACAA	ATCCCAGCTGGCGTGGAAACAA	327
FMgSSR-52874	scaffold4846	p3	(CGC)6	Class II	32268	32285	TGGTTCCAGCCACCGTCGAAAT	TGGTGCGTCGCTGATTCTGT	274
FMgSSR-52876	scaffold126	p3	(CGC)6	Class II	171343	171360	TGTCGCCGAACGATTGCGTA	TTCGACTTGAGCGATAGCGGCA	331
FMgSSR-52877	scaffold1007	p3	(CGC)6	Class II	11168	11185	TGTCGTTACGGCTCCTCGGTTT	TGCGTTACCGTCGTCCCTTT	310
FMgSSR-52881	scaffold208	p3	(CGC)6	Class II	44548	44565	TTCGAAGCGCCACATCGGTT	ACGGTGCGAGACTGTGCTTGAA	333
FMgSSR-52882	scaffold12425	p3	(CGC)6	Class II	22165	22182	TTCGGCCCATTTGCCGTACCTT	AAAGGCGGTGTAAGAGACCACG	325
FMgSSR-52885	scaffold11083	p3	(CGC)6	Class II	15005	15022	TTCTTGTGATCGCCGCCCAAGT	ACGACGGGAGGCAACGATTT	242
FMgSSR-52889	scaffold203	p3	(CGC)6	Class II	131758	131775	TTGTAGACGGCGATGGCGTT	GCTTCCGCCGCAACCATTTCAA	277
FMgSSR-52891	scaffold63	p3	(CGC)6	Class II	82649	82666	TTGTTTGGGATGCCGCCGAAGA	ACAGCCGCACCACACCAAAA	311
FMgSSR-52893	scaffold13312	p3	(CGC)6	Class II	11837	11854	TTTCGGCCGTTCCCGTTTGA	ACGCCATAATGACGCTCGAGGA	253
FMgSSR-52897	scaffold34136	p3	(CGC)6	Class II	2549	2566	TTTTGTTGGACGGACGCGCT	GCGAGCGGCGTACGGTATTTTT	328
FMgSSR-52899	scaffold500	p3	(CGC)7	Class I	62778	62798	AAATCGCGGGAGGAAGCAGCTA	CCAAGCGCAGGCCTAGTTTACTCA	343
FMgSSR-52903	scaffold448029	p3	(CGC)7	Class I	145	165	AATGGAAGTACCAGCCGGAGGA	CCTTCTCTCTGCCTCTGCGATT	232
FMgSSR-52904	scaffold7542	p3	(CGC)7	Class I	32518	32538	ACACTCGCATCGCATCCAGA	TCCGAGTTCTTCGTCACCAGCA	255
FMgSSR-52906	scaffold3516	p3	(CGC)7	Class I	59206	59226	ACCACCACCACGGCAACTACTA	TCCGGTGATCGGCTGTGAAA	200
FMgSSR-52908	scaffold121	p3	(CGC)7	Class I	23727	23747	ACCGGTGTGCGGATTCCAAA	ACTCGTGTGCTTCTTGTGGGCT	328
FMgSSR-52909	scaffold81	p3	(CGC)7	Class I	116966	116986	ACGCAGAGCTCCCACGAATCAA	TTTTCACGAGCGGGGGACGAAA	341

SSR_ID	Scaffold	SSR_Type	SSR_Motif	SSR Class	SSR_Start	SSR_End	Forward sequence	Reverse sequence	Product_size
FMgSSR-52911	scaffold9	p3	(CGC)7	Class I	301142	301162	ACGGACTCCAAGACCATTGCGA	AGCGCAGAGGATGAACTCCTTGA	333
FMgSSR-52916	scaffold428	p3	(CGC)7	Class I	83780	83800	AGCAGCAGCAGCTTACGCAT	AAGGAGGTGAGCTTGCAGTCGT	241
FMgSSR-52917	scaffold241	p3	(CGC)7	Class I	138049	138069	AGCCGGCTGTTGTGTGACTTGT	GGGACGTTGTAGTCGTTGTGGT	233
FMgSSR-52918	scaffold42	p3	(CGC)7	Class I	32515	32535	AGCTACCCATGCATGCCCAA	ACCTCGAGCCGCATCAGTTCAT	299
FMgSSR-52919	scaffold4060	p3	(CGC)7	Class I	37398	37418	AGCTGTCTGGTAATACGGATGCGG	TTGTCGCCGAATCGAAGCCGAA	312
FMgSSR-52922	scaffold3916	p3	(CGC)7	Class I	43062	43082	ATCCGTCGACGAAGTCGCAAGT	ACGATTTCCGCACCAGCACA	246
FMgSSR-52923	scaffold1686	p3	(CGC)7	Class I	48973	48993	ATGCAGGCTTCCGACGAACGAA	TATGGGTGGGACAAAGGCGT	328
FMgSSR-52925	scaffold5015	p3	(CGC)7	Class I	12204	12224	ATGGAGGTGGCTGCAATGGA	TCGCAGCCGCCGATATTGAA	343
FMgSSR-52929	scaffold9846	p3	(CGC)7	Class I	3243	3263	CCACCCTCCATTAGAGCTGGTCTT	TTCACGGCGGCATCTTCACCAT	212
FMgSSR-52931	scaffold543	p3	(CGC)7	Class I	3452	3472	GCCCATTTTTGCTTTCGGCCCT	ACCTTCATGGTTGCGTTGCG	245
FMgSSR-52932	scaffold212	p3	(CGC)7	Class I	140261	140281	GCGCCGAGTAGACGGGTTTTTA	ATGCGGAAGCATTGGGGGCTTA	251
FMgSSR-52934	scaffold3712	p3	(CGC)7	Class I	48859	48879	TCACGTGCGCGTACTCGATGAA	ATAATCCGAGTTGGCTCCGGCA	226
FMgSSR-52936	scaffold14082	p3	(CGC)7	Class I	3138	3158	TCCGCTTTTGTGTCCTCGTCGT	AATGACAGGTGGACCCAGCTCA	347
FMgSSR-52938	scaffold52	p3	(CGC)7	Class I	205300	205320	TCGGGCGAAGCCGTCACATTAT	ACGAAATCAGGATCACCAGCCCA	295
FMgSSR-52939	scaffold568	p3	(CGC)7	Class I	121679	121699	TGCACCAGTTCTCGTTGGGCTT	TTCGAGCAGGGCTACGGTCTTT	208
FMgSSR-52940	scaffold19178	p3	(CGC)7	Class I	2837	2857	TGCAGTAACGCAGGCTACCACA	ACTGTGCCGTCATCAAAGCAGGA	348
FMgSSR-52944	scaffold6301	p3	(CGC)7	Class I	41343	41363	TGTGTGCTCGCAGCAGTTCT	AATCGCGACCATCAGGCCGAAA	332
FMgSSR-52949	scaffold18646	p3	(CGC)7	Class I	7833	7853	TTGGGTCATGCGGTCGAAGA	TATCGCCGGACGCCTATACGAT	329
FMgSSR-52954	scaffold477	p3	(CGC)8	Class I	38270	38293	AACACATGCAAGCTCTGCTCCC	TTCTTGAACCCCGCCTCCATGT	218
FMgSSR-52959	scaffold624	p3	(CGC)8	Class I	67898	67921	TAAACGAAACGGCCCAACAGCG	ATGCGTCCCGCGATTGAACT	346
FMgSSR-52961	scaffold7547	p3	(CGC)8	Class I	21093	21116	TTCAGCGCCACGTTGAAGCA	TGGGGGTTGTTGGTGAGTGA	342
FMgSSR-52964	scaffold352	p3	(CGC)9	Class I	142675	142701	ATTTGGGTCCACGCAACGGT	ATTCAGTCAGAAGGGCGGTGGA	275
FMgSSR-52969	scaffold2391	p3	(CGG)5	Class II	60068	60082	AAAAAGGTCGCTGTCACGTGCG	TTCGCCTGCATCTGCGTTGT	285
FMgSSR-52973	scaffold2358	p3	(CGG)5	Class II	55491	55505	AAAAGGTTGGACGCGAAGGGGA	ACGGTTCACACGTTTGCGGT	337
FMgSSR-52977	scaffold933	p3	(CGG)5	Class II	31286	31300	AAACCACCGCTTCGTGCAGT	AAAAGCCCTCGCGTTTCTTGCC	320
FMgSSR-52983	scaffold986	p3	(CGG)5	Class II	84943	84957	AAACTGATGCGTCGGCTGCT	ACCCGTTCTTCCGGTTGCACTT	298
FMgSSR-52993	scaffold4347	p3	(CGG)5	Class II	16199	16213	AACAGGCCCGGTCCCAAAAA	GCAAGCGAGCAAAGATTGACGC	350
FMgSSR-53005	scaffold364256	p3	(CGG)5	Class II	189	203	AACTGGGCCATGAAGATGCCGT	TGCAGATCGTGGTGTTCTCGGT	226

SSR_ID	Scaffold	SSR_Type	SSR_Motif	SSR Class	SSR_Start	SSR_End	Forward sequence	Reverse sequence	Product_size
FMgSSR-53006	scaffold519	p3	(CGG)5	Class II	84111	84125	AAGAACGCCTTGCCCTGGAA	TGCTCTTCATGGCGCGTAGGTT	220
FMgSSR-53009	scaffold51	p3	(CGG)5	Class II	142567	142581	AAGAGATGAGAGCGACCTGTGC	TCCGTCGCCTACAAGAACGTCA	349
FMgSSR-53013	scaffold1407	p3	(CGG)5	Class II	38859	38873	AAGCACGAATGCGAGCAGAGGA	TTTCCCTGCGGCGAGGTTTT	263
FMgSSR-53014	scaffold24459	p3	(CGG)5	Class II	11702	11716	AAGCAGCAGCAGGATAGCAGGA	AGGCAGTGCATGTTCCCGAT	242
FMgSSR-53015	scaffold1410	p3	(CGG)5	Class II	23120	23134	AAGCAGCTCGTCGTCCGACATT	TATGCTTACTGCCAGCCGCCAT	311
FMgSSR-53016	scaffold35377	p3	(CGG)5	Class II	470	484	AAGCCCACTTCGAAGCCCAT	TCGGACGTTTTACTGCTGGCGA	303
FMgSSR-53020	scaffold344	p3	(CGG)5	Class II	26192	26206	AAGCGGGCAAATGGTGACGA	ACAAATAAGCCCTTCGGCGGCT	323
FMgSSR-53028	scaffold14114	p3	(CGG)5	Class II	12161	12175	AAGGAACGGGCTTACAACGGCA	AGTGAGTATCGACCCTGCCAGA	346
FMgSSR-53031	scaffold3113	p3	(CGG)5	Class II	37232	37246	AAGGCGGCGTTATTGTCGCT	ACTGCCTGTCGTCGATCGCATT	268
FMgSSR-53032	scaffold19884	p3	(CGG)5	Class II	4658	4672	AAGGGCCGGGGGAATTGAAAGA	TCGTTGCAGGTTGTTCGCGT	274
FMgSSR-53036	scaffold1756	p3	(CGG)5	Class II	51046	51060	AAGTCGTTGGATCGAGCAGGGA	TGCCCGAAGTTGACGACGAA	346
FMgSSR-53039	scaffold2744	p3	(CGG)5	Class II	24078	24092	AATCGAGCTACAGCTTGGACGC	CGCTTCCTCTGGCAGTACAACA	346
FMgSSR-53040	scaffold6824	p3	(CGG)5	Class II	1745	1759	AATCGCGATCCAAACCAGGGGT	ATTTGCAGCGCGTCAGCACT	310
FMgSSR-53042	scaffold4237	p3	(CGG)5	Class II	32887	32901	AATCTACACGTGCGGGTTGGCA	ACGCTCCACCACAAGGAGAA	234
FMgSSR-53044	scaffold2182	p3	(CGG)5	Class II	49367	49381	AATGCTACTCTGCGACGGCT	AGCGCAAACACGCAGGCATA	324
FMgSSR-53045	scaffold3783	p3	(CGG)5	Class II	10689	10703	AATGCTGTCGCGAAGCTGGGAA	TTGGCGAAGTGCTTAGGTGCGA	316
FMgSSR-53046	scaffold749	p3	(CGG)5	Class II	69330	69344	AATGGAGGCGCTGGTGCTTGAT	ACCAGGCCAATGTGTTTGCACG	327
FMgSSR-53048	scaffold1115	p3	(CGG)5	Class II	52872	52886	AATTAGGCCACTTCACGTCCCG	TGTTGGCATGGACGCAACGA	279
FMgSSR-53055	scaffold16198	p3	(CGG)5	Class II	16028	16042	ACAACTGATCGAGGCGGCAACA	AAGGTGCGTGGAAGTGGCAT	267
FMgSSR-53056	scaffold1781	p3	(CGG)5	Class II	44171	44185	ACAAGCGGAACGAGGACGTTGA	TTCGTTTCCGGTGTGCCTGACA	248
FMgSSR-53057	scaffold6392	p3	(CGG)5	Class II	11688	11702	ACAATGAGGCACCACGAAGCAC	TGCAACGGAACAGAGCACCACA	303
FMgSSR-53062	scaffold3744	p3	(CGG)5	Class II	5974	5988	ACACGAACCTTGCCCGAGGAAT	ACGGCCTACCTGAACTTGCTGA	329
FMgSSR-53067	scaffold29691	p3	(CGG)5	Class II	1921	1935	ACAGCCTTGTGCAGCGTGAT	TGGCCTTCTGTTTCGCGGTA	335
FMgSSR-53070	scaffold576	p3	(CGG)5	Class II	6492	6506	ACATTGCAGGCGCAAACCGA	CCGGAAGCACAAAATCCGGGAA	257
FMgSSR-53079	scaffold33651	p3	(CGG)5	Class II	1881	1895	ACCATCCCGGCGATCAATGT	TTCCCTCCGCATAAGCCGCAAA	205
FMgSSR-53082	scaffold6622	p3	(CGG)5	Class II	15469	15483	ACCCTGCGAACGCAGACTTT	AGCTTGATCAACGTAGCGCTGG	251
FMgSSR-53083	scaffold3490	p3	(CGG)5	Class II	9030	9044	ACCGCCGGTGGTTACCTTTA	AAGACGTTGCACGACGCGAT	301
FMgSSR-53084	scaffold3616	p3	(CGG)5	Class II	38546	38560	ACCGCGCAAGGATGCCAAAT	TCCTCGAACTCGCTCAGACACA	228

SSR_ID	Scaffold	SSR_Type	SSR_Motif	SSR Class	SSR_Start	SSR_End	Forward sequence	Reverse sequence	Product_size
FMgSSR-53085	scaffold2234	p3	(CGG)5	Class II	19861	19875	ACCTGAAGCGCGTCATCTACCT	ATGTCGTAGGGCGATTGGTGGA	244
FMgSSR-53086	scaffold7952	p3	(CGG)5	Class II	18821	18835	ACCTTCGTTCTCGCTTTGCAGC	TTCCGAGACAAAAAGCGCCG	267
FMgSSR-53087	scaffold9421	p3	(CGG)5	Class II	21695	21709	ACGAAATGTAAACCAGGGACCGACC	ATTACGCCAACACGCGCACCAA	350
FMgSSR-53092	scaffold513	p3	(CGG)5	Class II	6999	7013	ACGACCTCGTTGTCGAACACCT	AACCCTAGGCTCCAATGCAGGA	247
FMgSSR-53093	scaffold3800	p3	(CGG)5	Class II	14354	14368	ACGACGACCTCGTGCATGATT	ACTGCAACCTCGACTTCCTCCT	256
FMgSSR-53097	scaffold28503	p3	(CGG)5	Class II	3072	3086	ACGATTGCGATTTGGGAGGGCA	AAAGTTGCCGACGCAGGAGT	241
FMgSSR-53102	scaffold279	p3	(CGG)5	Class II	2674	2688	ACGCGCGACAAAGAGAGTGT	ACATGTAGGTGTTGAACGACCCCG	343
FMgSSR-53103	scaffold1233	p3	(CGG)5	Class II	32315	32329	ACGCGCTCGCTCGATAAAAGGA	ATCGCCTCTGCCTCATCGAA	303
FMgSSR-53105	scaffold2288	p3	(CGG)5	Class II	40092	40106	ACGGCGAGTGCAAGCAGAAT	TGCAATAATCTCGGGCTGGCGT	350
FMgSSR-53110	scaffold118379	p3	(CGG)5	Class II	668	682	ACGGTTTGCGCATCACCATCCA	TGGCCATTGCCATTCTCGCCAT	310
FMgSSR-53112	scaffold1838	p3	(CGG)5	Class II	64368	64382	ACGTGCCGCAGGTAATCGGTAT	GCCGCTGACCAATTCCACGATT	330
FMgSSR-53115	scaffold27	p3	(CGG)5	Class II	172439	172453	ACGTGGGCAAAGGCAACGAA	TCTTTCTTCTTCCCGGTGGCGT	326
FMgSSR-53116	scaffold1485	p3	(CGG)5	Class II	50293	50307	ACTAACCCAGCACCACGGCAAA	GCAAGCTCTTCGTCTGGACCAT	298
FMgSSR-53117	scaffold3040	p3	(CGG)5	Class II	52659	52673	ACTAGGGCTTGCATCGGCGAAT	TCGCGATCCAAATTAGGGGGCA	319
FMgSSR-53119	scaffold1095	p3	(CGG)5	Class II	6074	6088	ACTGAGGCTGAATTTGGCGCTG	TCCGGCGAAGCAAAATCCGA	321
FMgSSR-53123	scaffold13730	p3	(CGG)5	Class II	5310	5324	ACTGCCATGAAGCGAGCCAT	TTACGTACCGTGAGCTTGTGCG	338
FMgSSR-53127	scaffold8704	p3	(CGG)5	Class II	3659	3673	ACTTCCAACCCCGCGACATCAT	AGGGGGCGCGTAGTATTGAT	290
FMgSSR-53128	scaffold5526	p3	(CGG)5	Class II	6199	6213	ACTTCCTGCTCAAGCGGTCCTA	AGCGACATGAAGAAGAACCCGC	304
FMgSSR-53129	scaffold551	p3	(CGG)5	Class II	77015	77029	ACTTCGCTCCATGAGGCTCGTT	TGAAACGAGGGCACCAACCCAA	242
FMgSSR-53130	scaffold4997	p3	(CGG)5	Class II	10479	10493	ACTTCTCCGACTTCCACACCGT	TTGTCCACGAACACGTTGGC	304
FMgSSR-53131	scaffold7533	p3	(CGG)5	Class II	26482	26496	ACTTGCGCTTGGGCTTGACT	ATCAGGCCTCTTCAGCCCAACA	253
FMgSSR-53134	scaffold5339	p3	(CGG)5	Class II	5658	5672	AGAAGAGCTTCCACTGGTTGCG	AAATCGCTCGCCGTCTGCAA	289
FMgSSR-53138	scaffold10153	p3	(CGG)5	Class II	18634	18648	AGAGCTGCAGCAACCAACCA	AGCTCAAATCGTTGCGGCCT	318
FMgSSR-53140	scaffold1068	p3	(CGG)5	Class II	12653	12667	AGATCACACGCGCACACACA	GCAAACCCGGAGGCCAACAAAT	336
FMgSSR-53144	scaffold458	p3	(CGG)5	Class II	69415	69429	AGCACCTCGGCCTGCTGATAAA	GCCGATCGAGCAGCGAAAGAAT	323
FMgSSR-53146	scaffold208	p3	(CGG)5	Class II	11887	11901	AGCATCGCCTCAAGAACGCA	AAAGAAGGCGCTGATGTCCACG	300
FMgSSR-53147	scaffold4317	p3	(CGG)5	Class II	4704	4718	AGCCACAGACTCGCGAAGAA	TCAAGCGAGACCTTTGCTCCCA	205
FMgSSR-53153	scaffold3779	p3	(CGG)5	Class II	49335	49349	AGCGCTTTCGCCGCCTCTAAAT	TGAGCCTGGGCCAAGTTGTT	313

SSR_ID	Scaffold	SSR_Type	SSR_Motif	SSR Class	SSR_Start	SSR_End	Forward sequence	Reverse sequence	Product_size
FMgSSR-53157	scaffold6213	p3	(CGG)5	Class II	24578	24592	AGCGTCGAGTAAGTGGTGCT	TTTCAGGCCACAGAGCCCAA	263
FMgSSR-53158	scaffold50752	p3	(CGG)5	Class II	3346	3360	AGCGTCGCCGGAATTATGGTGT	TGTCCCTGACGGTCATCTTCCTCA	256
FMgSSR-53163	scaffold22757	p3	(CGG)5	Class II	5849	5863	AGCTGCAGGTACTTCGTGGTGT	CGAGGGACCTTTTCCGATGACCAA	349
FMgSSR-53165	scaffold225	p3	(CGG)5	Class II	119163	119177	AGCTGGTGCAACTGCGGAAA	TTATTCCAAGCCGCCTCCATCG	307
FMgSSR-53171	scaffold2306	p3	(CGG)5	Class II	69212	69226	AGCTTTCGTGTGGCGATGGACT	ATCACCCAAGCAGCAGCACA	336
FMgSSR-53173	scaffold6672	p3	(CGG)5	Class II	38049	38063	AGGAAGCATCTTGCTGGGCA	AAAAGGCACAGGAGGTGACGCA	225
FMgSSR-53177	scaffold7241	p3	(CGG)5	Class II	25944	25958	AGGGAGTTCGGAAGCTCAGACA	AATCGAATTTCTCCCCCGCACC	327
FMgSSR-53179	scaffold19	p3	(CGG)5	Class II	214052	214066	AGGGCTTGGAAATGCCATCGGA	AAAGGCCAAAGCCAGCCAGACT	349
FMgSSR-53185	scaffold6452	p3	(CGG)5	Class II	12512	12526	AGTCCGCAGAACGTAAATCGGC	ACTCTCACAGAGCGAGGAAAGCA	316
FMgSSR-53186	scaffold305	p3	(CGG)5	Class II	76480	76494	AGTCCGCGACTGAGCTGAAA	AGGGGGCTGCCTGCAAATTTCT	206
FMgSSR-53187	scaffold8206	p3	(CGG)5	Class II	145	159	AGTCGTACACCACGCAGCAA	CGCCGCCATGTGGAAGCAATTT	248
FMgSSR-53190	scaffold3703	p3	(CGG)5	Class II	4688	4702	AGTGCCGCCCAAATCCAACT	TGGTGTCGCTGAGATGCACCAT	263
FMgSSR-53191	scaffold5858	p3	(CGG)5	Class II	8122	8136	AGTGGAGGCTGGAGATAACTGCT	TCCGTCGACACTTCACACCA	284
FMgSSR-53194	scaffold1832	p3	(CGG)5	Class II	41401	41415	AGTGGCTGCAAGGGCAATGAGT	AGTCGCGATCTAAAGTAGGGTGGT	258
FMgSSR-53195	scaffold170379	p3	(CGG)5	Class II	488	502	AGTGGGACGTAGTGAGGAGGTTGT	ACCGTGCCTGTCCTCTTCAA	332
FMgSSR-53202	scaffold9429	p3	(CGG)5	Class II	20577	20591	ATACCAGCGCTTGCAGACGA	ACCGGCCCAAATTCCTTCCCAA	214
FMgSSR-53203	scaffold74	p3	(CGG)5	Class II	156278	156292	ATACTCCAGTGTCGCATGCTCG	AAAACGGGGAAGCGGTGTGT	339
FMgSSR-53205	scaffold2325	p3	(CGG)5	Class II	32593	32607	ATAGTTCAGTGCTGCTCGCCGT	TTCGTCAAGCGCGTGGATGT	256
FMgSSR-53206	scaffold1074	p3	(CGG)5	Class II	5055	5069	ATATACAGAGGCAGCGCCGCAA	ACACGCTCCACACCATGAGCAA	248
FMgSSR-53207	scaffold2597	p3	(CGG)5	Class II	25823	25837	ATATTAATCACACCCGCCCGCC	TTTTCGGGCTGACGACAGAGCA	304
FMgSSR-53208	scaffold4202	p3	(CGG)5	Class II	16073	16087	ATCACGGCCTCGCAAGTCACAA	TCCTTACCGAAGTGCCCGATGA	289
FMgSSR-53214	scaffold61	p3	(CGG)5	Class II	49427	49441	ATCGAGTGGCGCGATGGACATA	TGCACTCATCGTATGGCTCTCC	304
FMgSSR-53218	scaffold1146	p3	(CGG)5	Class II	23740	23754	ATCGGCCCTGCCCAAATCCAAA	ACAGGTCCAAACCTGTCCCGAA	320
FMgSSR-53224	scaffold5703	p3	(CGG)5	Class II	28263	28277	ATCTGGGTCGTCGGCATCTT	AGCAATGGGCACGCGGTAAT	317
FMgSSR-53227	scaffold2102	p3	(CGG)5	Class II	68181	68195	ATCTTTGCCCACCGAAGCGT	GCACAAATCACCCGCCGAAACA	207
FMgSSR-53228	scaffold1579	p3	(CGG)5	Class II	73835	73849	ATGAACAGCTCGAACCGCGA	ACCGCGATCCTGTTAAGTCCGT	326
FMgSSR-53230	scaffold2027	p3	(CGG)5	Class II	30808	30822	ATGACAAGAGCGCGGAGGAGAA	ATCAAGCTGCTGCCAAGGCT	219
FMgSSR-53231	scaffold76	p3	(CGG)5	Class II	165680	165694	ATGACCTCGACATACGGCAGCA	ACCCCCGAAAGTCCGAAACA	339

SSR_ID	Scaffold	SSR_Type	SSR_Motif	SSR Class	SSR_Start	SSR_End	Forward sequence	Reverse sequence	Product_size
FMgSSR-53232	scaffold4489	p3	(CGG)5	Class II	21011	21025	ATGACCTTCATAGTGGCGGGGA	ATGCGGCGAATTGACGGAGGAT	326
FMgSSR-53233	scaffold305	p3	(CGG)5	Class II	67865	67879	ATGACGACGAACGGAGCCGAAA	AATTTCTGAGCCGGCGGCCATT	289
FMgSSR-53234	scaffold26455	p3	(CGG)5	Class II	6980	6994	ATGAGATGCCGGCAGAGAACGA	TTGAGTGGACCAAGAACGCGGA	318
FMgSSR-53238	scaffold11104	p3	(CGG)5	Class II	19719	19733	ATGCGCCCATCATGCCCAAT	AAGAACGCGGCAAAGCAGCA	330
FMgSSR-53240	scaffold6345	p3	(CGG)5	Class II	8047	8061	ATGGAGTTTGGTCGGCGCAAGA	ACGAAGAAGGAGGGAAACGGAC	295
FMgSSR-53248	scaffold11999	p3	(CGG)5	Class II	645	659	ATTCCTCGCCTTCAACCGCCAT	TGCTGCTGGTTCTTCTGGGT	243
FMgSSR-53251	scaffold2540	p3	(CGG)5	Class II	60795	60809	ATTCGCAACCTTTGGAGGGCGT	TGTTCTGCGCCAGCAATCCA	335
FMgSSR-53254	scaffold759	p3	(CGG)5	Class II	1512	1526	ATTCGTGGACGTTCGGCGTT	AAGCAGCGCCGGTTTTTGGA	258
FMgSSR-53255	scaffold735	p3	(CGG)5	Class II	82738	82752	ATTCTGACGGGCGTTTGGCA	TCATGCCCATGGCTTGGATGT	285
FMgSSR-53259	scaffold7614	p3	(CGG)5	Class II	13369	13383	CAAACAACACACGACGCGCA	AAACCCATTTTCCCCCACCCCT	202
FMgSSR-53262	scaffold63113	p3	(CGG)5	Class II	1460	1474	CATTGCGCACTGCAAGAGGT	ACAGCACATTGCTCGCCCAT	268
FMgSSR-53267	scaffold214	p3	(CGG)5	Class II	67366	67380	CGCCATCAGCAAAACCAGCACA	ATCCCGCACGCGCATTTCTT	283
FMgSSR-53270	scaffold94274	p3	(CGG)5	Class II	305	319	CGCTATCTGAACGTCGCACCTA	ATGCGGTTGATGTACCCCAGCA	245
FMgSSR-53272	scaffold7021	p3	(CGG)5	Class II	21314	21328	CGGCGGCAGAAACAAAACCA	ACACGCGTAGGTTCGCTGACTT	231
FMgSSR-53273	scaffold1554	p3	(CGG)5	Class II	67870	67884	CGGGTGAGAGCTGAGAAAGAGA	TCTTTCTCCTCCCCTTCGTTCCT	238
FMgSSR-53275	scaffold179570	p3	(CGG)5	Class II	170	184	CGTCTCGCCCTGGATGATGTTGAT	ATGTTCCACAGCGTCGCCAA	292
FMgSSR-53277	scaffold2535	p3	(CGG)5	Class II	52640	52654	GCACCACGGCGAAGACCAAAAA	ACCAGGGCCGCAAGAAGAAGAA	342
FMgSSR-53281	scaffold3000	p3	(CGG)5	Class II	11862	11876	GCAGTTGGCGACGAGGTAAT	TCAGGCCTTCAGCTTTCCCAGT	310
FMgSSR-53284	scaffold3464	p3	(CGG)5	Class II	23580	23594	GCCGGTCCGCCTTGTTCATTTT	ATCTCTCGCATATCCGCGTCCA	218
FMgSSR-53293	scaffold93	p3	(CGG)5	Class II	2743	2757	GCTCCGGCTAGTGCCTTTGTAA	ACTTCGGTTTGCTTTGGCGGT	305
FMgSSR-53297	scaffold2176	p3	(CGG)5	Class II	66655	66669	GGAGCGCGGGCTTTGTTTGATT	TTGGACAGCCGCAGCTTCTT	306
FMgSSR-53299	scaffold154	p3	(CGG)5	Class II	135088	135102	GGCAACAGCGGCAACAACAT	TTCGCTCAGGGAAGCGCGATAA	288
FMgSSR-53302	scaffold2368	p3	(CGG)5	Class II	30775	30789	GGCGGCAATGGTTTGTCGTT	ATCGTCCGTGTTATTGCCTCGC	338
FMgSSR-53303	scaffold2804	p3	(CGG)5	Class II	50627	50641	GGCTCCGATTCACGTGTCGATT	CGCGACAAAAAGGTGCTCTGCT	346
FMgSSR-53308	scaffold279	p3	(CGG)5	Class II	71102	71116	TAAAACGACTCGAGGGCGGACA	CGTCGTGTTCAACGGCATGTCA	309
FMgSSR-53311	scaffold50169	p3	(CGG)5	Class II	1009	1023	TAAGGTTCGTACCCGTGGGCGTAA	AGGAAGGAACTGCGGAGGTTGT	263
FMgSSR-53315	scaffold77	p3	(CGG)5	Class II	28461	28475	TACAAGCATGAGGCGGCGTA	TGCACCGTGCTTGTTCTTCACG	258
FMgSSR-53318	scaffold259	p3	(CGG)5	Class II	140199	140213	TACCACCGGATGCTCTCCAACA	AAGGCGGAGAAACTCACGCT	261

SSR_ID	Scaffold	SSR_Type	SSR_Motif	SSR Class	SSR_Start	SSR_End	Forward sequence	Reverse sequence	Product_size
FMgSSR-53319	scaffold9044	p3	(CGG)5	Class II	1895	1909	TACCATCCCGGCGATCGATGTT	TGGCAAAAAGCCCAACCCCT	240
FMgSSR-53320	scaffold275	p3	(CGG)5	Class II	15317	15331	TACCCGGGAAGAAAAAGCGGCA	TCGCATGTTCCGCGGTCTTT	285
FMgSSR-53322	scaffold5252	p3	(CGG)5	Class II	9895	9909	TACTCGCTGTCCACCTGCTTGA	TACCCGCAGCACCTTTCGTA	272
FMgSSR-53324	scaffold3828	p3	(CGG)5	Class II	15740	15754	TAGCCCAACCCAATGGAAGCCA	AAGAGGATCTTCGTGCGGGT	276
FMgSSR-53326	scaffold8391	p3	(CGG)5	Class II	31112	31126	TAGCTGGACACTTGCTTGGCGA	ACGATTCACCAGCGGGCATT	222
FMgSSR-53327	scaffold345	p3	(CGG)5	Class II	46707	46721	TAGGCACAGCGAGAGCAAGCTA	AGCGGACGAAAGGTCGGTGAAA	291
FMgSSR-53330	scaffold574	p3	(CGG)5	Class II	66662	66676	TATCTGCAGCAGCAACGGTCCA	ACCATCGCTTCTACCGCCGAAT	301
FMgSSR-53331	scaffold696	p3	(CGG)5	Class II	12790	12804	TATGGATAAGCAGAGCTCCTGGGC	AGCGCGGAGGGACGATTTTGTA	327
FMgSSR-53332	scaffold19785	p3	(CGG)5	Class II	3474	3488	TCAAGCAGATGGCGTGGTCT	AAGCATGAAGCAGGGGCTCGAT	298
FMgSSR-53333	scaffold1852	p3	(CGG)5	Class II	14302	14316	TCACCATGCTCCCCTTACTCGT	AGCCCATCCTTTCCTTGCGA	340
FMgSSR-53341	scaffold3902	p3	(CGG)5	Class II	44431	44445	TCATGCTGCAAGTTCGTTCGCC	ATGCATTGCACACGCTGCGA	279
FMgSSR-53348	scaffold3388	p3	(CGG)5	Class II	12371	12385	TCCATGAATTGAGCTGCGCC	TCAGTCTCCTCAGCAAGCTCGT	237
FMgSSR-53351	scaffold783	p3	(CGG)5	Class II	53818	53832	TCCCGGCGCCATGAATGAATGA	ATAAACCAAGAAGGTGCGCGGC	344
FMgSSR-53352	scaffold3313	p3	(CGG)5	Class II	29507	29521	TCCGAGCAAAACCAGCACAAGC	TGCAGGTGGTCCTTGCTGTTGT	238
FMgSSR-53355	scaffold1294	p3	(CGG)5	Class II	48547	48561	TCCGCAAAAACCGAGAACACGC	TTGTTCCGGCAACGAATCGC	263
FMgSSR-53360	scaffold4950	p3	(CGG)5	Class II	12961	12975	TCCTTGGTCTTGGTCACGGCAA	AGCCGCTTGTGTGTTCGTGA	298
FMgSSR-53367	scaffold691	p3	(CGG)5	Class II	111960	111974	TCGCGCGTCAAGCATAAGCA	AAGCAACGCAAGGCAGGCAT	350
FMgSSR-53369	scaffold1202	p3	(CGG)5	Class II	37357	37371	TCGCGTCGCTGCTCTCCTTTTT	ACGTAACCTGTTGCCGCAGT	255
FMgSSR-53370	scaffold4430	p3	(CGG)5	Class II	29250	29264	TCGGAAACCTTCCAGTCCCTCT	TCCAGTCACCAGACGTGGGTTT	347
FMgSSR-53371	scaffold673	p3	(CGG)5	Class II	41099	41113	TCGGCGCGCTAACTTGATGA	ACCCAAAGCCCAAGGGGACTAA	278
FMgSSR-53372	scaffold4902	p3	(CGG)5	Class II	43377	43391	TCGGCTAAATCACCGCCATCCA	TTCCGGATGCGGCCGATAACTT	215
FMgSSR-53375	scaffold41129	p3	(CGG)5	Class II	5926	5940	TCGTCGAAGGAGTAGGAGGCAA	AACCCCATCAACGGCCATGT	332
FMgSSR-53380	scaffold4748	p3	(CGG)5	Class II	41015	41029	TCGTTGCTAACCTCCTCTGCGT	ACGAGTACGACGACATGCCT	217
FMgSSR-53383	scaffold75	p3	(CGG)5	Class II	128185	128199	TCTGCAATCGTGGGCGAACA	ACGCCTGCCTTCGATGTTTGGT	340
FMgSSR-53384	scaffold7397	p3	(CGG)5	Class II	30010	30024	TCTGCCGTCCTTCGTTTGCT	ACCTTGCGCTCGTTTCGTCA	227
FMgSSR-53386	scaffold3522	p3	(CGG)5	Class II	45759	45773	TGAAAGGACGCGACTGGAGTT	TGGAGGAGCTGCAAAGCAGACA	322
FMgSSR-53387	scaffold707	p3	(CGG)5	Class II	33794	33808	TGAAATCCGGCCTTGGCGAGAA	AGTCATATGGTTGCGGGCGT	346
FMgSSR-53395	scaffold67313	p3	(CGG)5	Class II	276	290	TGAGGAAGCTCTCGCTCATCGT	ATGAACGCCGCCTATTTCCCGA	282

SSR_ID	Scaffold	SSR_Type	SSR_Motif	SSR Class	SSR_Start	SSR_End	Forward sequence	Reverse sequence	Product_size
FMgSSR-53396	scaffold1797	p3	(CGG)5	Class II	11057	11071	TGAGGCGCCGTTGTTGTTGT	TGCAAAGGAGACCAGCAGGCTT	204
FMgSSR-53397	scaffold12509	p3	(CGG)5	Class II	12889	12903	TGAGTTCACATGGCGCGAGA	AGAGAAGCAGAATAGGCCCGCA	315
FMgSSR-53398	scaffold1967	p3	(CGG)5	Class II	20933	20947	TGATCGGCTGCGAGATGGAT	TTCTTCCGCGGATGCAACGA	339
FMgSSR-53400	scaffold14683	p3	(CGG)5	Class II	13737	13751	TGCAAATTAGTCCCTCCGTGCGA	TGGCGCGTCCATTGGATTTTGG	204
FMgSSR-53401	scaffold1638	p3	(CGG)5	Class II	78010	78024	TGCAACAGCTCGAGAGTGCCTT	GGGCGTCCAACCCACAGAATTT	282
FMgSSR-53402	scaffold787	p3	(CGG)5	Class II	111192	111206	TGCAATGTCGGAGCAACCGT	AAGCTGTAGAGGTACCAGCCGA	339
FMgSSR-53406	scaffold332	p3	(CGG)5	Class II	133547	133561	TGCACTTCACCTGCGGGAAA	TTTTGTTCCAGGTGGCGCGT	287
FMgSSR-53409	scaffold11463	p3	(CGG)5	Class II	7110	7124	TGCCCCAAGAAGTGCTTCTCCT	CAACGCACTGTGGCCGTTTT	269
FMgSSR-53417	scaffold64431	p3	(CGG)5	Class II	425	439	TGCGCGAGGTGGAAGAGGAAAA	GCCGCCCCTTCCAAAATCCAAA	343
FMgSSR-53418	scaffold1901	p3	(CGG)5	Class II	59997	60011	TGCGCGGTTATTCCGAGTCCAT	ACAACCGTCCACTCTCCACACA	338
FMgSSR-53425	scaffold3050	p3	(CGG)5	Class II	10860	10874	TGCTGCTTGTTGGAGGAGGA	TGTATCAGCCGGTGCAGACCTT	229
FMgSSR-53426	scaffold2380	p3	(CGG)5	Class II	56827	56841	TGCTGTTCGGGTACCAGCCTTT	TGTCAGCGACGTGAACGTGA	317
FMgSSR-53428	scaffold477	p3	(CGG)5	Class II	51348	51362	TGCTTCGCTCAGAAACCCAGCA	AACGCACGCGTCATCCTCAA	274
FMgSSR-53429	scaffold16585	p3	(CGG)5	Class II	4987	5001	TGCTTGCTTACTTCCAGGCCGA	ACGATCACACGATCACACGCGA	290
FMgSSR-53435	scaffold9882	p3	(CGG)5	Class II	12593	12607	TGGCATGGGCGCAATCAATG	TCCGGCTCCTACTTCTTCATCAGC	341
FMgSSR-53440	scaffold20149	p3	(CGG)5	Class II	4384	4398	TGGCGCGCATCAGTTTCAGT	AAACGCTTCTCCCAGTCTCCCA	250
FMgSSR-53441	scaffold2242	p3	(CGG)5	Class II	29743	29757	TGGCGGCAACTACGTTGTCAGA	TTCGAAGGCCACCATCAACCGT	236
FMgSSR-53444	scaffold21153	p3	(CGG)5	Class II	1603	1617	TGGCTGTTGGAAATTCTGCGACG	AGAAGGCCGCAGCAACTACTGA	328
FMgSSR-53445	scaffold5942	p3	(CGG)5	Class II	40836	40850	TGGCTTGGCTGCGTTGCTTT	TCGCGCGTGAAATCGTCTGT	201
FMgSSR-53453	scaffold43271	p3	(CGG)5	Class II	4042	4056	TGTCGTACTCGTGCCTGATCCA	TCCGGGGAACGAGAAGAACAACA	203
FMgSSR-53455	scaffold951	p3	(CGG)5	Class II	86437	86451	TGTGAACGGACATACGCGGT	TAAACACCCCTGGTTTCGGTGG	320
FMgSSR-53458	scaffold5355	p3	(CGG)5	Class II	32285	32299	TGTTCAGCTGTTCTTGGCGTGC	ACTTCTTGCTCAGCACCGCT	269
FMgSSR-53464	scaffold19563	p3	(CGG)5	Class II	2531	2545	TTACAGCAGCAGGGGCTACGAA	ATCGCACCGCATCCCCAAAT	307
FMgSSR-53467	scaffold2195	p3	(CGG)5	Class II	73501	73515	TTCATCGAGCACGTCACTGCCA	TTGGCAATGCGGAGGATGTCGT	215
FMgSSR-53468	scaffold1864	p3	(CGG)5	Class II	43849	43863	TTCCAAGAAGTCGTCGTCCTCACG	TGCTGCATCTGCAGGTAGTGGT	240
FMgSSR-53469	scaffold1679	p3	(CGG)5	Class II	35758	35772	TTCCCGTTGTTGTCGCCGAT	ACGCGGTCCCGTATTTCAGA	240
FMgSSR-53471	scaffold6493	p3	(CGG)5	Class II	26969	26983	TTCCGACCCCGAGCTGAAAGAT	AAGGCGACAAGGAAACCAAGCG	305
FMgSSR-53480	scaffold1349	p3	(CGG)5	Class II	53882	53896	TTGAATCAAGCGCGACGGAGCA	GCCAAATTTCGGCCCAGTCA	205

SSR_ID	Scaffold	SSR_Type	SSR_Motif	SSR Class	SSR_Start	SSR_End	Forward sequence	Reverse sequence	Product_size
FMgSSR-53486	scaffold1047	p3	(CGG)5	Class II	28311	28325	TTGCGGTTGCAGGAGTGGAT	ATCGGGCATGCATGGACAACCA	309
FMgSSR-53487	scaffold43847	p3	(CGG)5	Class II	1374	1388	TTGCGTCTCAGCTGACGTGTGA	ACACAGACGAAGAATGGCCCTCA	269
FMgSSR-53489	scaffold6417	p3	(CGG)5	Class II	12455	12469	TTGGCGGGATCAAATGCCGT	TGCTTTCTTGCCTTGCCGCT	306
FMgSSR-53491	scaffold4811	p3	(CGG)5	Class II	12764	12778	TTGGGCTGCTGCTTCTTCTTCG	AACCACGCGCAGAAAGCCAT	269
FMgSSR-53502	scaffold199517	p3	(CGG)5	Class II	172	186	TTTCCCAGCACCCGGCTCTTTT	AGGATGTCAGTGGCACAGCACT	296
FMgSSR-53505	scaffold3161	p3	(CGG)5	Class II	30432	30446	TTTGGAAGCGGAGGCAAGAGCA	AGGGAAAAGGGCGACGGGAAAT	295
FMgSSR-53506	scaffold447	p3	(CGG)5	Class II	29581	29595	TTTGGCCTCCGGCTGTGGATTT	ATAAGCAGCGGCACCTTACGTC	312
FMgSSR-53509	scaffold6276	p3	(CGG)5	Class II	4807	4821	TTTTGTGGTTTGAGGTGCGGGC	TGCCTCATTGAGCGTGTAGCGT	350
FMgSSR-53512	scaffold989	p3	(CGG)6	Class II	55439	55456	AACACAAGCGCCTGTACGCT	TTCTTTCCTTCGCCGGAGCTGA	346
FMgSSR-53513	scaffold6660	p3	(CGG)6	Class II	17496	17513	AACATGCAGCCACATGGACCGA	TTTTCTGACCCGGCCTCGAT	350
FMgSSR-53518	scaffold19564	p3	(CGG)6	Class II	8401	8418	AATTGCAAGCATCCCCGCAC	TGGCTTCTTGGGCCTGTTCGTT	348
FMgSSR-53521	scaffold20086	p3	(CGG)6	Class II	11416	11433	ACAAGGAAAACCGGGGGTGT	AGCACAAACCCTAACACGAGCC	350
FMgSSR-53522	scaffold3213	p3	(CGG)6	Class II	25981	25998	ACAATCACCGCCGCCAAAAGT	GCGTTCGTCGCGAGAGGAAAAA	342
FMgSSR-53523	scaffold637	p3	(CGG)6	Class II	107648	107665	ACCAAAACCAACCACCGTTCCCT	AGGTCGTACATGAAGACCCGCA	350
FMgSSR-53525	scaffold3301	p3	(CGG)6	Class II	29469	29486	ACCATCAACGGCCACCTGTTCA	TCAGTTCTTTCACGGCGCGTT	207
FMgSSR-53526	scaffold3142	p3	(CGG)6	Class II	14054	14071	ACCGCTCTTCGCAACCGAAA	CGCAGCCACTGCTCGATACAAT	278
FMgSSR-53529	scaffold1598	p3	(CGG)6	Class II	19610	19627	ACGAGGATGAGACAGTTGGGGAGT	ATTCCCCACCCCGCAACGAAAT	208
FMgSSR-53540	scaffold169513	p3	(CGG)6	Class II	534	551	AGCCAGAGCAAGGATAGCAGCA	ATCACCAGCCGGTTGAGCTT	338
FMgSSR-53542	scaffold5287	p3	(CGG)6	Class II	21631	21648	AGCCCCAAACAAAGCAGAGCCT	ACCGTTTTAGGGTGGTTCCTGC	330
FMgSSR-53543	scaffold5872	p3	(CGG)6	Class II	24644	24661	AGCCGTCGGTGAGTAAGCACAA	AGCTGATGAGCGCGCCTAAA	251
FMgSSR-53549	scaffold6023	p3	(CGG)6	Class II	911	928	AGGAGAGCAAGCCGTCAATCGT	AGCCTACAAGCAGGCGCAAA	349
FMgSSR-53551	scaffold307	p3	(CGG)6	Class II	48387	48404	AGGCATTCATGTTCACGGCGA	AGATGATGTCTCGCGACGGT	233
FMgSSR-53554	scaffold374	p3	(CGG)6	Class II	30842	30859	AGGCTGCGATTGCCGAGAAA	TTGCCTCACTCGCACGTTGA	294
FMgSSR-53555	scaffold3455	p3	(CGG)6	Class II	17070	17087	AGGGCAGAGGTGCCGATTTT	ACAAAGTCGCCGATGTTGCG	291
FMgSSR-53556	scaffold14848	p3	(CGG)6	Class II	22487	22504	AGGGGCGCTTCCACAGAAAGAT	AAGCCCCTGGTTTCGACCAACT	325
FMgSSR-53557	scaffold3990	p3	(CGG)6	Class II	19370	19387	AGGTCCATGTAGGCGTTGACGA	TCTTCTTCTCCCTCGACTCGCT	304
FMgSSR-53559	scaffold13845	p3	(CGG)6	Class II	17862	17879	AGTGGATTGTGAGGGCGATGGA	AAACAGATCGGCGCAGTCAGGA	216
FMgSSR-53564	scaffold180319	p3	(CGG)6	Class II	316	333	ATGCACGCGACTACGTGGAGTT	GCACGTTCCCACCGCCTTATTA	281

SSR_ID	Scaffold	SSR_Type	SSR_Motif	SSR Class	SSR_Start	SSR_End	Forward sequence	Reverse sequence	Product_size
FMgSSR-53567	scaffold223	p3	(CGG)6	Class II	86783	86800	ATGGTGCTGATTACGAGCGCGA	ACGGCGAGCTTCTTGGGTAA	343
FMgSSR-53569	scaffold561	p3	(CGG)6	Class II	30329	30346	ATTGATGGCCCCGCTGTTCA	AGGTGCGTTGAATCGCCACA	305
FMgSSR-53570	scaffold23076	p3	(CGG)6	Class II	3512	3529	ATTGCGATTTGCGAGGGCGA	TTGTGCGCCGTGACAACGTA	227
FMgSSR-53571	scaffold3022	p3	(CGG)6	Class II	20906	20923	ATTGGTGCCTGGTGCTCGAA	TGTGGCTTGCGGTGTTTGCT	303
FMgSSR-53572	scaffold692	p3	(CGG)6	Class II	39748	39765	CCGCCATGATCCAGTTGAGGAA	TCCACATCGACGCTGTCTTCGT	292
FMgSSR-53573	scaffold17883	p3	(CGG)6	Class II	10424	10441	CGCAATGATGACGCAGGCGATT	TCGCAAACGGGTTTGTCCCA	263
FMgSSR-53575	scaffold6314	p3	(CGG)6	Class II	36447	36464	CGGCAAAACAGAATCGGACGACA	TGCCTACCGCGACCTTGTAA	314
FMgSSR-53579	scaffold2320	p3	(CGG)6	Class II	48477	48494	GCAAAGTCGGGCCTTGCGTAAA	AACGAGATCGCTGGAGGTGGAA	337
FMgSSR-53582	scaffold1072	p3	(CGG)6	Class II	67543	67560	GCGATGGCCACAACTTCGTT	TGGTGGTTGCCTACTGCCAACT	342
FMgSSR-53589	scaffold192	p3	(CGG)6	Class II	149090	149107	TCAACGGCCGAAAGATCGGGTA	AGCTCCGGTCCATTGTTGCCAT	268
FMgSSR-53590	scaffold18222	p3	(CGG)6	Class II	945	962	TCAAGGACACGAACCCGTTGCT	TTGTAGTTGCCCAGACGGACCT	250
FMgSSR-53593	scaffold653	p3	(CGG)6	Class II	57392	57409	TCCATTGATGGCCCCGTTGT	TTGGTGCGTTGAATCGCCCT	309
FMgSSR-53595	scaffold266	p3	(CGG)6	Class II	105810	105827	TCCGGCCATAGAACACGGATGA	ATGCAGTACCACCACCGCAA	325
FMgSSR-53597	scaffold9118	p3	(CGG)6	Class II	18781	18798	TCGACGCGGTTCTTACACTGGT	TCCCCCTTCCGAACCAACTT	309
FMgSSR-53604	scaffold189	p3	(CGG)6	Class II	11239	11256	TCTGGCGAACCAAAGAAGGGGA	TGCGCAAGCACGGTCAAACT	346
FMgSSR-53607	scaffold320	p3	(CGG)6	Class II	27438	27455	TGAAGAGGAAGGAAGTGGGGGA	AAAGCATGAAGCAGGGGCTGGA	221
FMgSSR-53614	scaffold9321	p3	(CGG)6	Class II	31247	31264	TGCCAAATCACGCGCAGAGT	TCGCGTTTTGCAGGAATCCCCT	267
FMgSSR-53616	scaffold6946	p3	(CGG)6	Class II	16086	16103	TGCGGAACAGCCAATGGCACTA	AGCTCGCGTTTCAGAGTGGT	349
FMgSSR-53617	scaffold26	p3	(CGG)6	Class II	104786	104803	TGCGGGATGACGTTGGCAAGAA	TTGCCGTCGTTGCTCGTCTT	248
FMgSSR-53622	scaffold170470	p3	(CGG)6	Class II	500	517	TGGCAAACCCAACACCACCA	ATGTGCGTGCGCTTCATGCT	234
FMgSSR-53626	scaffold8309	p3	(CGG)6	Class II	11626	11643	TGGGCCGTTGTTCTGTGTGTCA	AAGGGGCCAATCCGAGTCTTGA	324
FMgSSR-53630	scaffold23045	p3	(CGG)6	Class II	4176	4193	TGGTTCTCCTTCACGGCGTT	TGCTCAAGTGCGAGGTCTGGAT	325
FMgSSR-53640	scaffold434	p3	(CGG)6	Class II	114172	114189	TTGCACGGCAAGGTGATCGT	TAGCCGTGCACGTGGTAGTTGT	314
FMgSSR-53643	scaffold12733	p3	(CGG)6	Class II	5200	5217	TTGGTGACGACATGGCCCTT	TTTCTCACCAACGCGGCTCA	274
FMgSSR-53649	scaffold338	p3	(CGG)6	Class II	123181	123198	TTTTACGGCTGTGGCGCGAA	AGAACCTCGTAGCGGGATGTGA	285
FMgSSR-53655	scaffold64604	p3	(CGG)7	Class I	824	844	AACCCGGCCAAGCAGGAAAA	ACTGCTTCTGCTGCTGCTGACT	238
FMgSSR-53661	scaffold6868	p3	(CGG)7	Class I	9682	9702	ACCGACGATGTCGCCATAGCTT	TCAGCCTGAAAAAGGTGCGGGT	341
FMgSSR-53662	scaffold780	p3	(CGG)7	Class I	78617	78637	ACGACGACGATGGATGCGAA	TGGCTGTGGCGCTAAAATCGGA	323

SSR_ID	Scaffold	SSR_Type	SSR_Motif	SSR Class	SSR_Start	SSR_End	Forward sequence	Reverse sequence	Product_size
FMgSSR-53666	scaffold2006	p3	(CGG)7	Class I	12284	12304	AGCAACAGCGTGCCGAGTTT	AACCCCGATCTCAACGCGAACT	206
FMgSSR-53668	scaffold613	p3	(CGG)7	Class I	22044	22064	AGGACGTCTCTTTGGCTACTGGCT	TGCGGATCTCCCCGGTGAATTT	232
FMgSSR-53671	scaffold5527	p3	(CGG)7	Class I	38823	38843	ATCCGTGCGTGCAGGTGATGAT	ACACCGTCCGGTACTGGAACAA	317
FMgSSR-53673	scaffold4302	p3	(CGG)7	Class I	46389	46409	CAGCGGCGCTAAGTTCACAA	ACTCCGCGGATGCAAAACCA	261
FMgSSR-53674	scaffold6073	p3	(CGG)7	Class I	24942	24962	CCAAATTCCGCGCCACGTCAAA	GCGTGGAAGGATTGCTTCTGCT	291
FMgSSR-53683	scaffold145	p3	(CGG)7	Class I	148764	148784	TGAAACAACGCGGCCAAGGT	TCGCATCCGCATCCGAGAAACT	238
FMgSSR-53685	scaffold790	p3	(CGG)7	Class I	1411	1431	TGAAGGGGATCCTCCTCCTGAT	AAGCGGTCCATCGCGAAGAT	327
FMgSSR-53686	scaffold1186	p3	(CGG)7	Class I	7337	7357	TGCCACAACGCAAAGGCCAAA	ACTACTTGCTGGGCTGGGCATT	317
FMgSSR-53687	scaffold15011	p3	(CGG)7	Class I	22022	22042	TTACGTGCGACTGCGTGCAT	ACGCTATAATGCGCGTGGCT	287
FMgSSR-53689	scaffold4140	p3	(CGG)7	Class I	3423	3443	TTGCGTCCGCGCGAAGATTA	ACGACGGACCACAAGCAGGAAT	200
FMgSSR-53690	scaffold7580	p3	(CGG)7	Class I	16053	16073	TTGGGAGCGGGCGTTTGTTT	ATTGGAGCCAACGAACTCGGCA	332
FMgSSR-53691	scaffold827	p3	(CGG)7	Class I	88548	88568	TTTGCCTAAACCGACATGCGCC	TGAAGCTGAGCAGGAAGCAGGA	349
FMgSSR-53692	scaffold15149	p3	(CGG)8	Class I	7490	7513	AATATCGCCAGCCACCAAACCC	AAGATGCCCCCTTGCACTTGCT	310
FMgSSR-53694	scaffold1365	p3	(CGG)8	Class I	55918	55941	ACATCGACCTGCGGCTGATT	TGGAAGAGCAAGAAGCAGGGCT	225
FMgSSR-53700	scaffold48836	p3	(CGG)9	Class I	102	128	TCGGCCTGCTACTTGATGCTGA	AAGCTGCCCACCGCAAGATGTA	216
FMgSSR-53702	scaffold4922	p3	(CGG)9	Class I	25609	25635	TTCTTGCCGCAGTGGTTGAGGA	ATGTTGGCGACAATGGCATCCG	329
FMgSSR-53704	scaffold7775	p3	(CGT)5	Class II	31057	31071	AAAGGCGCGCGTGAGTTACA	AATAAGGTGCGGCGTGTGGT	300
FMgSSR-53705	scaffold121	p3	(CGT)5	Class II	50089	50103	AAATCGCACGCAGGCCAACA	TGGTGCCCACGAATCTCGCTTT	290
FMgSSR-53714	scaffold9218	p3	(CGT)5	Class II	29735	29749	AATTTTGCTGAATCGGGCGCGG	TGGACGCAAGAAGGCCATGTGA	263
FMgSSR-53717	scaffold3229	p3	(CGT)5	Class II	39195	39209	ACACCGTAATGTCGCCCTCCAA	AGAAGCAGGGCGGGTGAGAAAA	232
FMgSSR-53722	scaffold4609	p3	(CGT)5	Class II	4726	4740	ACGCACCACGAGATCCACAT	AGTAGACCCTCATGTTCCGCGT	303
FMgSSR-53723	scaffold2679	p3	(CGT)5	Class II	52475	52489	ACGCGTAGCATTTGCGCTTG	TGCTGCATTTCGGTGGCGAAT	207
FMgSSR-53725	scaffold6749	p3	(CGT)5	Class II	15840	15854	ACTTGGCTTCCTCGCAGCAGAT	TTTCGCCGCGCTTCTTGCTT	270
FMgSSR-53726	scaffold4396	p3	(CGT)5	Class II	9696	9710	AGACGACGACGGGCTGATCTAT	ACTCTCGCGCACAAGCCAAA	274
FMgSSR-53730	scaffold70471	p3	(CGT)5	Class II	1327	1341	AGCAGCAGCTAGCAGCAATGGA	ACGACATCATGGAGGAGCAGCA	335
FMgSSR-53731	scaffold2112	p3	(CGT)5	Class II	28311	28325	AGCAGGTGATGGGTTTGAGGGA	TGTCCCGCATCGACTACGACAA	252
FMgSSR-53734	scaffold3705	p3	(CGT)5	Class II	13930	13944	AGCTATGAACAACGACACGCGG	AAGAAGGCGTTCGCGCAGAT	298
FMgSSR-53737	scaffold3496	p3	(CGT)5	Class II	7949	7963	AGGCATGACAACAGGACCGAGA	ACAGCCTGCGATGAACCTCCTT	283

SSR_ID	Scaffold	SSR_Type	SSR_Motif	SSR Class	SSR_Start	SSR_End	Forward sequence	Reverse sequence	Product_size
FMgSSR-53740	scaffold32	p3	(CGT)5	Class II	114473	114487	AGGGACAACCTCTGGCCAACAT	TGAGGAATGGCGTTGTTGCG	288
FMgSSR-53741	scaffold2420	p3	(CGT)5	Class II	64324	64338	AGGTTGAGCAAGACGATCCCGA	TTGCCTTGCTCGTCTGGGAA	262
FMgSSR-53744	scaffold1802	p3	(CGT)5	Class II	35113	35127	ATCCCATCCGCAAAACGGCA	TGTTCATCCCACGCACTCTCCT	281
FMgSSR-53748	scaffold1506	p3	(CGT)5	Class II	46821	46835	ATCGCGGAGACCCAAGTCAAGT	TTCTTCCTCGACTGCTCCTTCGGT	345
FMgSSR-53751	scaffold434336	p3	(CGT)5	Class II	103	117	ATTCCGGAAAGACAGGACTCGC	TGAAGTCGTCACGGAAGAGGGT	217
FMgSSR-53752	scaffold1506	p3	(CGT)5	Class II	45992	46006	CACAGGTGATGGGCTTGATGGA	TCGCGCATCGACTATGACAACCT	250
FMgSSR-53754	scaffold1185	p3	(CGT)5	Class II	27207	27221	CGGCTTGGGCTTGTTCTTGGAT	TTCGAGTTTCGTTCCCCCGA	331
FMgSSR-53758	scaffold1342	p3	(CGT)5	Class II	16749	16763	CGTTGCAACAACTCGCAACCCA	GCATGCCCTGCATACATTCGCA	343
FMgSSR-53760	scaffold1709	p3	(CGT)5	Class II	24084	24098	GCGAACCGCCGTTTGAAGAA	ATCCTTGCCCACTGCCACTGAA	212
FMgSSR-53761	scaffold17002	p3	(CGT)5	Class II	14472	14486	GCGCTTCTCGCTTTTGGAGA	CAACAAGCGCCACGTCATCA	350
FMgSSR-53765	scaffold1655	p3	(CGT)5	Class II	35993	36007	TACGCCGATCACCGGCAAGTTT	TTTATGCAACGACTGCTCGCCG	342
FMgSSR-53768	scaffold9512	p3	(CGT)5	Class II	28300	28314	TAGTTCCGGGCAAATAGGCCGT	CGAGCTGCTGAGGATGAGGATGAT	285
FMgSSR-53770	scaffold1045	p3	(CGT)5	Class II	52665	52679	TCATCACCGCCGTCTCGTTCTT	ATGAACGACCTCGTCACGCA	290
FMgSSR-53772	scaffold994	p3	(CGT)5	Class II	24039	24053	TCCGAAATCTTCTTCGCCCCCA	AGCAAAGCCACAGCGACACA	342
FMgSSR-53773	scaffold1978	p3	(CGT)5	Class II	37470	37484	TCGACGGCAACGATGGTGATGA	ATGTTGCCGAGGCTCTCCTT	313
FMgSSR-53775	scaffold21167	p3	(CGT)5	Class II	13665	13679	TCGAGCTGTTCATCGTCGAGGT	ACCGCTTGCATAAACCGGCA	253
FMgSSR-53777	scaffold971	p3	(CGT)5	Class II	13670	13684	TCGGCGAACGAACTCTGCAA	ACCTGATGGTGCGTGTGGTGTT	294
FMgSSR-53780	scaffold2106	p3	(CGT)5	Class II	20579	20593	TGAAGCCGTGCCGTGCTTAT	ACGCTTTGCTCTGTTCCGCA	225
FMgSSR-53781	scaffold10585	p3	(CGT)5	Class II	19095	19109	TGAGTGACTGCACGACGCTGTT	TTCTGCGCCGACAATTCGCT	229
FMgSSR-53787	scaffold15721	p3	(CGT)5	Class II	2447	2461	TGGCCCAAGAGGCGGACTATTT	AGGACGACACGGGCATCATT	292
FMgSSR-53788	scaffold1946	p3	(CGT)5	Class II	35188	35202	TGTAGAGCGGCGCGAACAAA	TTCAACGGCGCTCTGCACTT	319
FMgSSR-53789	scaffold2085	p3	(CGT)5	Class II	36217	36231	TGTCTCACCCGCGTCTGTGTTT	GCTCCTCGCAAAAGAGCAGCAA	293
FMgSSR-53792	scaffold31431	p3	(CGT)5	Class II	3813	3827	TTCGCCGTTCCTGGCAAACT	AATCTCGTTTTGGGGCCGGA	314
FMgSSR-53796	scaffold2460	p3	(CGT)5	Class II	61579	61593	TTGCAGAGCAAAGCCTGGCA	TTTGCTGCTGCGTGTCCCTT	337
FMgSSR-53797	scaffold3634	p3	(CGT)5	Class II	38839	38853	TTGTGCCCGTCACCATTCACCA	AGCCGTGCATACACGCCAAA	305
FMgSSR-53798	scaffold5172	p3	(CGT)5	Class II	16363	16377	TTGTTCTTGAACGCGCGGAGGA	ACGAACCTGCTGTCAACGACAC	307
FMgSSR-53800	scaffold661	p3	(CGT)6	Class II	79450	79467	AAACAGGCCCGCAGTTCAAAGC	TCCAACCAAGCGAACGTGCT	346
FMgSSR-53803	scaffold37248	p3	(CGT)6	Class II	1859	1876	AACCGCCCGTTTCCCAATGGAT	AGCCGTCGCTTTCACGAGTT	312

SSR_ID	Scaffold	SSR_Type	SSR_Motif	SSR Class	SSR_Start	SSR_End	Forward sequence	Reverse sequence	Product_size
FMgSSR-53811	scaffold1489	p3	(CGT)6	Class II	68740	68757	AGTCAATGCGCGTGGCACAA	TGCTCTGGTTCGCGTGTTCAGT	239
FMgSSR-53814	scaffold4903	p3	(CGT)6	Class II	21909	21926	CGGCGAAGAAGACAAGCACCAA	TAAATCAGCGCCTTTCCCCGCA	306
FMgSSR-53819	scaffold182	p3	(CGT)6	Class II	138886	138903	TGACGTGCCTGCTGTTTCCA	CCGCAATCGAGGCCCTGTTTTT	283
FMgSSR-53822	scaffold900	p3	(CGT)6	Class II	102449	102466	TGATCGCGGCCTTCTTGATGGA	AACCGTGCGTTCTTTGTCGC	335
FMgSSR-53835	scaffold986	p3	(CGT)8	Class I	5685	5708	AGTGGCGGATGTGACGTGAA	TTTCTGCGGCGGTGATAAGCCT	250
FMgSSR-53836	scaffold7580	p3	(CGT)8	Class I	10307	10330	TGCCCAACGCAACAAACGGA	TCGGCCTGCGAGACTTTTGAGT	225
FMgSSR-53839	scaffold4665	p3	(CTA)5	Class II	5476	5490	AAACGGGGGAACAATGGAGGCA	GCATTCTGCAGCATTGGGAGCA	207
FMgSSR-53845	scaffold2633	p3	(CTA)5	Class II	11628	11642	AAGCTGCAGGCGTGGAAACA	GGTGCACAGATCACCGCACAAA	253
FMgSSR-53857	scaffold1590	p3	(CTA)5	Class II	21209	21223	ACCTCCTTTTCTGCCCCCACAA	TGCAGCAGCAGCAACAACAACC	307
FMgSSR-53863	scaffold7	p3	(CTA)5	Class II	135882	135896	ACGTGCAGGCCCGTAGTTTT	ACCGCGGTTGGGCAAGAAAT	276
FMgSSR-53867	scaffold7590	p3	(CTA)5	Class II	2391	2405	AGAGGAACGCCGCAGGTATTGT	AGAGTTGTAGTGGCGCAGGCAA	200
FMgSSR-53868	scaffold1300	p3	(CTA)5	Class II	20014	20028	AGCAAAACCTCGCCAGTGCT	GCTTGTTCTGTGTTGCCGCGAT	270
FMgSSR-53874	scaffold999	p3	(CTA)5	Class II	28048	28062	ATACTGCGGTTGCAGGTCCCAA	ACGCACGCAAGCACTACTGT	318
FMgSSR-53875	scaffold392	p3	(CTA)5	Class II	95046	95060	ATGGAGCGTGGCCTGTTGTGTT	TAGGTGGCACGCACGTGAGAAA	335
FMgSSR-53877	scaffold1312	p3	(CTA)5	Class II	64670	64684	CGCTCGCTTTTTGTTCGCCA	ACCGAATTCCGCAGTGCCAA	261
FMgSSR-53878	scaffold257321	p3	(CTA)5	Class II	304	318	CGTCTTCGCGTGAGGCATTCTT	AACCAGCCACAGCCAGTGTTCA	231
FMgSSR-53884	scaffold6688	p3	(CTA)5	Class II	2049	2063	TCCTGACTATCGACGACCACACA	TGCTTTCTGTCGCGGGAACT	339
FMgSSR-53886	scaffold2075	p3	(CTA)5	Class II	33785	33799	TGAGAGCGCACTGTCCGTGAAT	TACGCGGTGCTGCCGGTATAAA	230
FMgSSR-53888	scaffold10495	p3	(CTA)5	Class II	3526	3540	TGCAAGCAGTTTCCCTATCCTGGG	ACTGCTGCTGGAGCAAATGGGT	278
FMgSSR-53889	scaffold11680	p3	(CTA)5	Class II	9457	9471	TGCACAAACTGCCCACCCAA	GCGTTGTGTAGTGAGTGCCCTA	345
FMgSSR-53892	scaffold14685	p3	(CTA)5	Class II	7007	7021	TGGCAGGTTCGTTGGCTCACAT	ATCATGTCCGGCTCGGGTTT	302
FMgSSR-53897	scaffold16933	p3	(CTA)5	Class II	9476	9490	TTATTGCACTCGCTCCGTCGCT	AGCTCGTCCGCGTGTCAAAA	260
FMgSSR-53898	scaffold76	p3	(CTA)5	Class II	27742	27756	TTCGTGTCAGCTGAAGCCCCTT	TGGCTGGCTAGCTTAGCTTCTGGA	229
FMgSSR-53899	scaffold804	p3	(CTA)5	Class II	89909	89923	TTGCCGCGTATGGACACACA	TGCTTCAAGGACAGACAGGTGGA	332
FMgSSR-53904	scaffold3855	p3	(CTA)6	Class II	11019	11036	AGGTGCGTCGCCAATACTGTCA	AAACAGAGCCGCCGCAAGTT	262
FMgSSR-53908	scaffold4120	p3	(CTA)6	Class II	29389	29406	TTGGTTTGTGCCTTGTCCGC	AGCCGCATGTTTGTTGCCGA	259
FMgSSR-53909	scaffold145660	p3	(CTA)8	Class I	307	330	CGGCGGCAATGAAAATGGCA	ACAAAGCCAGCGGTAGCAGT	277
FMgSSR-53911	scaffold15787	p3	(CTC)10	Class I	15916	15945	AAGTCGTCCTTGCGTGCGAA	AGCTTGTTGTGCGTTCCCGA	344

SSR_ID	Scaffold	SSR_Type	SSR_Motif	SSR Class	SSR_Start	SSR_End	Forward sequence	Reverse sequence	Product_size
FMgSSR-53913	scaffold1186	p3	(CTC)10	Class I	52868	52897	AGAAGCGGGGGTTTGTTCCTCT	TTGTTCATGCGACCGTGCGT	348
FMgSSR-53915	scaffold1999	p3	(CTC)10	Class I	36309	36338	ATTCCACGGTCTGGTGGAGCAA	AAATGGCGTGAAGCTCCGCA	317
FMgSSR-53919	scaffold588	p3	(CTC)11	Class I	49760	49792	TCGAGGTAGGTTGCGGACAACA	ACTTGTCTGAAGCCCCTTCGCA	304
FMgSSR-53921	scaffold26631	p3	(CTC)12	Class I	3469	3504	AACACCGACCGCGACAACTT	GGCGGATTGCTTGGTTTTGCGT	320
FMgSSR-53923	scaffold8148	p3	(CTC)12	Class I	6956	6991	ATACAGGACGTTTGGTGCGTGG	TCTCGGAGCAGTTGATGGCTCT	341
FMgSSR-53924	scaffold9404	p3	(CTC)13	Class I	18031	18069	AACTGCGTTTGGCAGCGACA	ACGGCGCATATGCAAGACACT	342
FMgSSR-53930	scaffold562	p3	(CTC)5	Class II	58149	58163	AAACACTTCCAGCTTCAGCCGC	GCTTGGAAGAAAAGGAGCAGGCA	241
FMgSSR-53933	scaffold317	p3	(CTC)5	Class II	32824	32838	AAACCGCAGCACGGAAAGGA	TCCGCGTCGAAATAGAAGGCGA	299
FMgSSR-53937	scaffold2498	p3	(CTC)5	Class II	58400	58414	AAATGCGCAGGAGATCGAGCCA	AATGCGCCTGCGTCCGTAAA	314
FMgSSR-53940	scaffold182	p3	(CTC)5	Class II	153038	153052	AACAACAACTTGGGCGGAGACG	AAAGCCAAAAGCGTGCGTGC	289
FMgSSR-53944	scaffold7166	p3	(CTC)5	Class II	26416	26430	AACATGCTTGGCTGCGGTGT	TGCAGCAACTCACGACGCAA	205
FMgSSR-53945	scaffold8051	p3	(CTC)5	Class II	14039	14053	AACCAAGGCTTCGTGTGGCA	TGCTGGACGTCAGCGGTAAGAT	337
FMgSSR-53950	scaffold4563	p3	(CTC)5	Class II	32565	32579	AACGACAGCGAGACGGTTGCTA	ATTTGGGATGCCGACCATCGGA	255
FMgSSR-53951	scaffold1466	p3	(CTC)5	Class II	67203	67217	AACGGTGACACTCTGCTCTGCT	TGAGCGCGTTGAGGTTGAGGAA	333
FMgSSR-53954	scaffold1889	p3	(CTC)5	Class II	23331	23345	AAGAAAAGCTCTTCCCGGCGGT	ATGCGCCCAGCCTTTTGGTT	291
FMgSSR-53960	scaffold4966	p3	(CTC)5	Class II	33956	33970	AAGTAACAGCCCTGAATACCGCCG	AGGAGCCGTTCAGAGAAGAGGGAA	308
FMgSSR-53961	scaffold8650	p3	(CTC)5	Class II	14919	14933	AAGTCCTGCTGCGCGATGTT	TCGAACCCCTCCAGCATCAT	350
FMgSSR-53962	scaffold4204	p3	(CTC)5	Class II	3580	3594	AATATTCGTCGCCCTCTTGCTCCC	CGCCGATCTTGCACCGAAACAA	280
FMgSSR-53965	scaffold2681	p3	(CTC)5	Class II	2077	2091	AATCCAACGCTCCACCTTCCGT	TCATGGCCTCTTGCGAGCAT	342
FMgSSR-53969	scaffold1284	p3	(CTC)5	Class II	18776	18790	AATGGCTTCGAACCAAGGGGCT	ACGACCTCATCAACGCTGCCAA	214
FMgSSR-53970	scaffold18956	p3	(CTC)5	Class II	5774	5788	AATGTCAAGCACTGCCGCCT	AACTCGCTTGTGCACCTCAACG	317
FMgSSR-53974	scaffold27422	p3	(CTC)5	Class II	1711	1725	ACAAGGACACTGACACACGCCT	AATGCTTACCTGCTTCTGGCGG	333
FMgSSR-53979	scaffold297	p3	(CTC)5	Class II	62320	62334	ACACGCGACAAAGACTTGGCA	TTGGCAACTGCTGCTGGGAT	309
FMgSSR-53980	scaffold5395	p3	(CTC)5	Class II	7902	7916	ACACGGCAGTGACTTTCGCA	TGTTCCGGCGCACAATGGTT	289
FMgSSR-53985	scaffold21758	p3	(CTC)5	Class II	6594	6608	ACATGCTGCCTCGATGACTGGT	TCAAGTCGTCAAGACAAGCGCC	232
FMgSSR-53992	scaffold61	p3	(CTC)5	Class II	121450	121464	ACCACAAGCAGCCCAAGCCTAA	TGTTGGGGAGTCGAGCTTTGCT	217
FMgSSR-53993	scaffold223	p3	(CTC)5	Class II	97621	97635	ACCACCAGCCATCGAGTTCCAA	AACAGAGCAGTCCGCGTGAACA	341
FMgSSR-53994	scaffold768	p3	(CTC)5	Class II	21862	21876	ACCAGGCCTAACGGCGTTTTCA	ACACCATCGGAGTTTCTTCGC	324

SSR_ID	Scaffold	SSR_Type	SSR_Motif	SSR Class	SSR_Start	SSR_End	Forward sequence	Reverse sequence	Product_size
FMgSSR-53995	scaffold404	p3	(CTC)5	Class II	106036	106050	ACCATTGTTGGCTTGGCTCACG	ATAGTCACGCGCGGAAGGAA	334
FMgSSR-53999	scaffold22045	p3	(CTC)5	Class II	1180	1194	ACCTCCGCTTTGCTCTTCGCTT	TGTTTCCCCGTACGCCATCGTT	213
FMgSSR-54001	scaffold55	p3	(CTC)5	Class II	49936	49950	ACCTGCTGCATGGGAATCCA	TCGAGGGTCTTGTTGAGCTGGT	320
FMgSSR-54002	scaffold17748	p3	(CTC)5	Class II	10129	10143	ACCTTCTTGCGCGAATCACCGT	TAAGCGCTTCAAGAATCCCGGC	293
FMgSSR-54010	scaffold71	p3	(CTC)5	Class II	167902	167916	ACGTTCGTGATTCCGCCGAT	TGCAGGTCCACGAAGAATCCGT	316
FMgSSR-54011	scaffold1374	p3	(CTC)5	Class II	28120	28134	ACTAATCATGGAGCGGCGCA	GCCATTGTTGCTGGTTGCGGTT	342
FMgSSR-54014	scaffold23471	p3	(CTC)5	Class II	6595	6609	ACTGCCAGCATCGTCGCTTT	ACGTTCTTTATCGGGCGTGCCT	308
FMgSSR-54024	scaffold2875	p3	(CTC)5	Class II	16837	16851	AGAAGGCGCCACGTGCATTT	TCGACCGCACACTTACCGTGTT	339
FMgSSR-54027	scaffold2619	p3	(CTC)5	Class II	48236	48250	AGCAAGCAACGACACAAACGCA	ACCTGACGGCAATGAAGCCA	296
FMgSSR-54043	scaffold856	p3	(CTC)5	Class II	95054	95068	AGCGCCAACAATGGGTCCAA	AGGAAGAGCTGAAGGAGGGCAT	322
FMgSSR-54044	scaffold28527	p3	(CTC)5	Class II	6858	6872	AGCGCGAGTTCCACAACGAGAT	AGTTGACTCCACGGCATTCGCT	340
FMgSSR-54052	scaffold2954	p3	(CTC)5	Class II	28412	28426	AGGACTCGCACCAACAGCAACA	TCACTTGTCAGCAGACAGCAGC	349
FMgSSR-54059	scaffold602	p3	(CTC)5	Class II	112948	112962	AGGGCTGAGCTTCCATGCCTTT	AGACGAAAGCACGAGGCAGA	259
FMgSSR-54063	scaffold1991	p3	(CTC)5	Class II	16403	16417	AGTGTACCTGCCGCCACTAA	TGACTGGGCACAGACCGTAGTAGT	348
FMgSSR-54064	scaffold4569	p3	(CTC)5	Class II	37588	37602	AGTGTGTGAACCCCAACCCACA	AATGGGCGAGGGGATTGTTCGT	303
FMgSSR-54065	scaffold4072	p3	(CTC)5	Class II	451	465	AGTTCACGTGCAGCACCATCCA	TGCGCACTAAAGAATCTCCCGGAC	314
FMgSSR-54069	scaffold5056	p3	(CTC)5	Class II	4361	4375	ATATTTCACCGCGGCGTGCT	ATCCGCGTCATCGAGAACCTGT	264
FMgSSR-54070	scaffold1953	p3	(CTC)5	Class II	6580	6594	ATCATCAGCAGCAGCAGCGT	TGCCAACACGCAGGAGCATT	253
FMgSSR-54074	scaffold5973	p3	(CTC)5	Class II	2051	2065	ATCGGCATCGCCATCTGCTT	TTGAGCACGCACTTGTACCCCT	227
FMgSSR-54076	scaffold2322	p3	(CTC)5	Class II	36132	36146	ATCTCACCGTAACTCCGTGATGCG	ATGCTCATGTTGCAGCGGGT	350
FMgSSR-54078	scaffold1844	p3	(CTC)5	Class II	51710	51724	ATGAACTCCATGACCAGCAGCG	AAGAACCTAGTGGGCAAGGGCA	317
FMgSSR-54083	scaffold804	p3	(CTC)5	Class II	22713	22727	ATGATGGCGATGTGCGTCGT	GCAGTGCAAGCATCGGATGGAA	310
FMgSSR-54085	scaffold579	p3	(CTC)5	Class II	95952	95966	ATGCTGCCGAAGCAGAGGAAGA	GTCATCGTCAACACGTTCCGCT	236
FMgSSR-54086	scaffold394	p3	(CTC)5	Class II	126296	126310	ATGCTGGAGAAGTGGGTCGGAA	TGCGACGCCTACGCAATTCA	319
FMgSSR-54087	scaffold6485	p3	(CTC)5	Class II	30298	30312	ATGGCAGCAGCATCGTTGACCT	AGGGCAGCAGCTAGAGGAAAT	345
FMgSSR-54091	scaffold3531	p3	(CTC)5	Class II	12113	12127	ATGTGTGCAGGCACTCGGTGAT	AAGAGGAGAGATCGAAGCAGCG	278
FMgSSR-54093	scaffold22044	p3	(CTC)5	Class II	3867	3881	ATTCCAGGGGAGCCGATGAACA	TTCGTGCCGGTGAACTTCTCGT	293
FMgSSR-54094	scaffold8986	p3	(CTC)5	Class II	10423	10437	ATTCGTGCCTGACCTCGACT	AGCACGATGACCGGGTTGAA	341

SSR_ID	Scaffold	SSR_Type	SSR_Motif	SSR Class	SSR_Start	SSR_End	Forward sequence	Reverse sequence	Product_size
FMgSSR-54104	scaffold393	p3	(CTC)5	Class II	109408	109422	CGCCGCAGATGGGGTACGTAATAA	AAGTTTCTCCGCCGCTGCAT	295
FMgSSR-54105	scaffold10594	p3	(CTC)5	Class II	27891	27905	CGCCGGCCACCAGTTTACAAAA	ATGGTGGCGCTGCACTACTT	271
FMgSSR-54106	scaffold8148	p3	(CTC)5	Class II	6040	6054	CGCTGCACGCCATCAACAAT	TGGCTGTAAGTGACGTGGCGAT	301
FMgSSR-54107	scaffold4354	p3	(CTC)5	Class II	4881	4895	CGGCTGTTGGAATGGGCGAAAA	TCCTGGGCAGACAATCTGCTGT	320
FMgSSR-54111	scaffold1733	p3	(CTC)5	Class II	60846	60860	GCCCCTTGATTGCCTTGTCGAT	ACAAGCCAACCGATGAGCCT	291
FMgSSR-54114	scaffold13353	p3	(CTC)5	Class II	137	151	GCCTCCTTCGTCTCCATCTACGTT	AATGCAGAGCTCTCCTGCTGCT	208
FMgSSR-54115	scaffold999	p3	(CTC)5	Class II	28973	28987	GCGAAGACGTGGCAACCAAT	TCGCTGTCTGTTGCAGCCAT	306
FMgSSR-54116	scaffold852	p3	(CTC)5	Class II	2611	2625	GCGAAGCCATGACGGACATT	AAGAATATCGGCGCCCGCAA	335
FMgSSR-54121	scaffold189520	p3	(CTC)5	Class II	103	117	GCTCACAGCATCCCCAATTCCT	TACAACGACTTCCGCGACCT	350
FMgSSR-54123	scaffold20276	p3	(CTC)5	Class II	1210	1224	GGCACTCGGACAACTCCTCCTTTT	TAGTGGATGTGGTCGGGAGCAA	273
FMgSSR-54125	scaffold995	p3	(CTC)5	Class II	20394	20408	GGTGCAACTCCACCAACACCAA	TCGTGGTGGTGCGTCATCAT	289
FMgSSR-54136	scaffold107	p3	(CTC)5	Class II	4455	4469	TCAAACACCACGCGACAGGA	ATTCCATGGATGGCGTCGCT	283
FMgSSR-54139	scaffold7252	p3	(CTC)5	Class II	29141	29155	TCACTGACAAGGGTAACCCGCA	AGAAAAGCTCGCACCCGCAA	264
FMgSSR-54140	scaffold11499	p3	(CTC)5	Class II	16825	16839	TCACTGACCTCGAGTTTGCGGA	ATCTATTTCGTGCTCCCCGCGA	283
FMgSSR-54149	scaffold1	p3	(CTC)5	Class II	177120	177134	TCCAGCGGCGTTCGCTTTCATA	AAGCGGGTCCAGCAAACGAA	246
FMgSSR-54151	scaffold6101	p3	(CTC)5	Class II	31020	31034	TCCATTCCCGAACCCAAGCA	ATGTCTCGCGGAGGAAGAAGAC	287
FMgSSR-54154	scaffold3744	p3	(CTC)5	Class II	19200	19214	TCCCCTTCGCGCTCAACAGTTT	GCGCCGGAAAGCTTCTTCCTTT	328
FMgSSR-54156	scaffold3473	p3	(CTC)5	Class II	13694	13708	TCCCTGCCGAATCACAACCA	CAGTTGCGAATGTCGGCGAA	283
FMgSSR-54176	scaffold3158	p3	(CTC)5	Class II	30667	30681	TCGAGCAGCACAAGCAAAGCA	ATGCTGCTGCTTCAGGTGGGAT	226
FMgSSR-54177	scaffold17	p3	(CTC)5	Class II	227426	227440	TCGCAACAGACATGAACCGGCA	TGGGGGCTTGCCTTCATGCTTA	322
FMgSSR-54180	scaffold21799	p3	(CTC)5	Class II	5561	5575	TCGCTCTTGTCTCTCTGCGA	TTGCGTTCCTTGTGGAGGCT	276
FMgSSR-54186	scaffold14224	p3	(CTC)5	Class II	14997	15011	TCGTCGGCCTCCCCTTATTCTT	AACAACAACGGCACCCTGCT	271
FMgSSR-54187	scaffold311	p3	(CTC)5	Class II	24568	24582	TCGTGGTGCACGTCGTTTGA	ATGCCGTGGTGTCGTCGTCTTT	237
FMgSSR-54189	scaffold21034	p3	(CTC)5	Class II	4818	4832	TCGTTTGTTCCCACTCCCGT	AAGCCCGACGTGCATTCCTAGT	298
FMgSSR-54196	scaffold28	p3	(CTC)5	Class II	95740	95754	TGACAAAGCCCATGCGGCAA	ACGATGGCTGCAAATGGTCGTG	330
FMgSSR-54198	scaffold173	p3	(CTC)5	Class II	55976	55990	TGACACCAGCAAGTAGGCGCAA	TGTGCAACGTCGTCCTGGAA	280
FMgSSR-54202	scaffold588	p3	(CTC)5	Class II	77182	77196	TGAGAACAGCACGCTACGCT	TTGCACAGCTTCACCAGTCCCT	345
FMgSSR-54207	scaffold380	p3	(CTC)5	Class II	31909	31923	TGATCGCCTCGTAGTTGAGCGT	TCAAGGTGGAGGCCGAGAACAA	225

SSR_ID	Scaffold	SSR_Type	SSR_Motif	SSR Class	SSR_Start	SSR_End	Forward sequence	Reverse sequence	Product_size
FMgSSR-54208	scaffold1098	p3	(CTC)5	Class II	49480	49494	TGATTCGGCTCCTCCGCTTTCA	TGCAAAGGGTGCTAGTTTGGCG	348
FMgSSR-54209	scaffold53350	p3	(CTC)5	Class II	2693	2707	TGCAACAAGGACACTGACACGC	TGCTAGACGCCTTGATTTGTGCG	298
FMgSSR-54214	scaffold2660	p3	(CTC)5	Class II	8049	8063	TGCACCGGAAACGGAAAGCA	TGAATGCCACGGCAAGCTCA	245
FMgSSR-54226	scaffold51	p3	(CTC)5	Class II	158023	158037	TGCTCGCCCTTGATCTGCTTGT	ACGAAGCCCAGTTCGTCACGTT	276
FMgSSR-54239	scaffold7518	p3	(CTC)5	Class II	17531	17545	TGGACAAGCTCTTCGGCTGCTT	TCGTGAGTCATGGCCGTTTCCA	343
FMgSSR-54248	scaffold21130	p3	(CTC)5	Class II	8413	8427	TGGGTCCATAGCACTCACCAGT	TCGTCCTTTGCTTCGTGTTGCC	327
FMgSSR-54251	scaffold682	p3	(CTC)5	Class II	89939	89953	TGGTGCTTCAGAAAGGTGGGCT	CGGCGGAAACACCCACAACAAA	295
FMgSSR-54252	scaffold72	p3	(CTC)5	Class II	116672	116686	TGGTGTGGTGCTCCAGCATTGA	TGCCTCTCTCGATCAGGACGAT	276
FMgSSR-54263	scaffold566	p3	(CTC)5	Class II	62036	62050	TGTTGTTGGAGCAGGCGTTGT	TCGCCGTCCAGCAATCTTGT	238
FMgSSR-54264	scaffold2463	p3	(CTC)5	Class II	41511	41525	TGTTTCGGGTGCCTGCTGTCTT	ACTTCGAGTGCAGGGAGGCTAA	318
FMgSSR-54283	scaffold12077	p3	(CTC)5	Class II	13130	13144	TTGATGACCCACTGGTGAAGGC	TGGTCGAGGCTTGCTCAGTT	283
FMgSSR-54292	scaffold11321	p3	(CTC)5	Class II	24493	24507	TTGGGTGCTCCAATCAGCGT	AACATGGGCTCACCACACCCAT	301
FMgSSR-54295	scaffold5254	p3	(CTC)5	Class II	11008	11022	TTGGTTGCTGCCGCCTTCTT	ACAGGAGTCTTCCACCGAATCCA	327
FMgSSR-54296	scaffold2088	p3	(CTC)5	Class II	47477	47491	TTGTCCCAGCAGCCCGTTTCTT	ACAACACAGACGAGCGAGAGCA	304
FMgSSR-54297	scaffold12387	p3	(CTC)5	Class II	14085	14099	TTGTCGCCATGACCACCGCTAA	GCAATGCGTCGCACATTGACT	215
FMgSSR-54301	scaffold1063	p3	(CTC)5	Class II	55992	56006	TTGTGGCTGTTGAGCACCGT	TTGCAGCATTTTCGGCTCGC	221
FMgSSR-54315	scaffold83192	p3	(CTC)5	Class II	1071	1085	TTTTCGGCATCTCCAGCCGT	ATACCCATCCCCGTCGCCTTTT	245
FMgSSR-54318	scaffold2534	p3	(CTC)6	Class II	31802	31819	AAAACCTCACCAACACCGCCCT	TGCGCAGCAACAAAACAGCG	314
FMgSSR-54320	scaffold5099	p3	(CTC)6	Class II	27797	27814	AACCACCTGTCCCTTTGCGT	TCTTGGTGGTGAGGTGGTGGTT	279
FMgSSR-54327	scaffold3551	p3	(CTC)6	Class II	20697	20714	ACAATGGATCGAGGGCGCTT	TCCGTGTCACCGGAGCATTTCA	263
FMgSSR-54328	scaffold112	p3	(CTC)6	Class II	103109	103126	ACAGCAGAGCACGAGCGAAA	ATCATGTTCGCGCCGTCGAT	293
FMgSSR-54329	scaffold38963	p3	(CTC)6	Class II	94	111	ACAGCTTGGACTTGATGCCGGA	ACACCACGCAACGAAACAGAGC	222
FMgSSR-54332	scaffold510	p3	(CTC)6	Class II	106980	106997	ACCACCATCGTCATCTTACCCG	ACGATGTGACGTTGTTGCCG	330
FMgSSR-54335	scaffold3050	p3	(CTC)6	Class II	51071	51088	ACGAACGCCATTGACGCAGT	CTGATGCACCAGCACGCAAA	349
FMgSSR-54339	scaffold12937	p3	(CTC)6	Class II	16219	16236	ACGCTGGCAGTAGTTGCACTTG	ATCGAGATCCGCCATGGACA	334
FMgSSR-54348	scaffold540	p3	(CTC)6	Class II	99252	99269	AGATCTAGGGGAGCTCCAGTGT	TGTTCATGTTGTCGCGCCCAT	230
FMgSSR-54351	scaffold4355	p3	(CTC)6	Class II	36367	36384	AGCCCAGCAGCCACCATTTA	AATCACTGCTTGTCGCCCGT	334
FMgSSR-54352	scaffold12150	p3	(CTC)6	Class II	9332	9349	AGCCGCATGACTGAGTGCAA	TCGCCGCAACCAGGATGAAGAT	326

SSR_ID	Scaffold	SSR_Type	SSR_Motif	SSR Class	SSR_Start	SSR_End	Forward sequence	Reverse sequence	Product_size
FMgSSR-54362	scaffold67	p3	(CTC)6	Class II	9542	9559	AGTGTGCGTGTCCCAATGCT	TGCTGCGCCGATCAGAGCTAAA	215
FMgSSR-54363	scaffold1597	p3	(CTC)6	Class II	46625	46642	ATCGGCCTGCCGCATAACACAA	ATTCGTTTCTCGGCACCGCA	283
FMgSSR-54366	scaffold3193	p3	(CTC)6	Class II	34695	34712	ATGCCGTTGGCCTTCTCCTT	ACGAGCTGCAGCGTTTTGCT	289
FMgSSR-54368	scaffold8921	p3	(CTC)6	Class II	4464	4481	ATGCTGTTCTTCCCTCGCGT	TCGCGGAGCCAGTAGTAGTTGT	221
FMgSSR-54369	scaffold128373	p3	(CTC)6	Class II	569	586	ATGGATACGCGTGGTGGTGCTA	AGCTTCGATCGCTGGTCTGCAT	230
FMgSSR-54377	scaffold2306	p3	(CTC)6	Class II	57861	57878	CGCAAGTCCAACAACAACGCCA	AGGAACGGCCACTTGTCGTCTT	203
FMgSSR-54380	scaffold18569	p3	(CTC)6	Class II	6864	6881	GCACAACCACATGTTCACGCCA	AAACGCGTGCCCTTCACGTA	332
FMgSSR-54381	scaffold7707	p3	(CTC)6	Class II	23618	23635	GCACTGCATTGCACCGGTATGT	AGTCTTGCCCCACAGCGAAA	262
FMgSSR-54384	scaffold8389	p3	(CTC)6	Class II	20804	20821	GCGAGCGAAAGAGGCGAACAAA	TGCGCGCAGAAATTACATGGGG	242
FMgSSR-54397	scaffold6300	p3	(CTC)6	Class II	15843	15860	TCCGGCGGATTTGCCAAGAA	TGCTCCATGTCCCAAAGCTCACA	261
FMgSSR-54402	scaffold33565	p3	(CTC)6	Class II	2128	2145	TCGCAGCTCGGTCTCGAAATGT	AGCGGTGGAAGTTGCGGATT	241
FMgSSR-54403	scaffold1156	p3	(CTC)6	Class II	6489	6506	TCGCCGATCTTCTCGATGACCT	TCTTGATCACTGCCGACCGA	327
FMgSSR-54405	scaffold13486	p3	(CTC)6	Class II	1947	1964	TCGGCAACTTGCTGGCCAAT	ACGCCTGTCTGCGTGAAACTGA	341
FMgSSR-54407	scaffold2034	p3	(CTC)6	Class II	52642	52659	TCGTTCAAGGCGACGGAGATGT	AAAAAGCGTAGTTCCCGTCCTCCC	217
FMgSSR-54408	scaffold14544	p3	(CTC)6	Class II	14911	14928	TCGTTCTTCTCGTCCTCTCGCT	ATCCATGCCGGGTGCATGTT	345
FMgSSR-54426	scaffold929	p3	(CTC)6	Class II	11816	11833	TGCTGCTCCAAAGGCAGTTGT	ACACCGATGTGCCGCTGTTT	344
FMgSSR-54428	scaffold19894	p3	(CTC)6	Class II	3488	3505	TGGCATGGATGCTCGTCCAGAT	AGCTCCACGCTATTGCCAGCTT	332
FMgSSR-54430	scaffold177592	p3	(CTC)6	Class II	317	334	TGTTGCGCCAGCCTCTTCTT	CAGGTCATCACCTCCTCCACAT	219
FMgSSR-54433	scaffold8923	p3	(CTC)6	Class II	19591	19608	TTCAGACTTCCAGCCACCACCT	AACAAGGCCTGAGATAGCGGGA	263
FMgSSR-54439	scaffold5151	p3	(CTC)6	Class II	10552	10569	TTTCAACGGCCCCGATTCCTCT	AAAATCGACGGACGGAGGCT	289
FMgSSR-54440	scaffold27192	p3	(CTC)6	Class II	2185	2202	TTTCGGCTGCAGGAGCCATCTT	AGTCCACCAACGGCGAATTG	339
FMgSSR-54447	scaffold1230	p3	(CTC)7	Class I	81672	81692	AACGGTGCCAAAATCCCCCGAT	AGGCAGTCGTGGCTCCGAAAAA	312
FMgSSR-54448	scaffold10648	p3	(CTC)7	Class I	21189	21209	AAGTGAAGCCGCAAGTCATCGC	AGCTGCTGCCCCAAGGAAAT	296
FMgSSR-54455	scaffold9020	p3	(CTC)7	Class I	13695	13715	ACCATCATGGGCAACCCCAACT	AGCCAAGCGTAGGGAAATCGCA	295
FMgSSR-54457	scaffold270	p3	(CTC)7	Class I	106136	106156	ACCGGCATCGGTCATCTTGA	ACCACGCATTCTTCTTCGGCCT	320
FMgSSR-54468	scaffold4549	p3	(CTC)7	Class I	44062	44082	CCAGGTTACCTCCGTTCGTCGATT	AGTAGCAGATGAGGAGGTGCAGGA	302
FMgSSR-54469	scaffold1216	p3	(CTC)7	Class I	68112	68132	CGCCGGGAACCTACATCATCCAAA	GCGGTGAGGTTGTTGGTGAA	286
FMgSSR-54476	scaffold65	p3	(CTC)7	Class I	27523	27543	TCAACTGAGGTTGGGCCAGA	TGCGATCAACCATCAGTCCAGC	348

SSR_ID	Scaffold	SSR_Type	SSR_Motif	SSR Class	SSR_Start	SSR_End	Forward sequence	Reverse sequence	Product_size
FMgSSR-54483	scaffold161	p3	(CTC)7	Class I	91471	91491	TCGCTTTCACGACCGGGTCAAA	TCGCACAGCGGATGATTCACGA	214
FMgSSR-54490	scaffold7117	p3	(CTC)7	Class I	19419	19439	TGGTGCTTGGCTGCTTGCTT	AGCGGATCGAAGGTGTTTGGCT	267
FMgSSR-54492	scaffold7928	p3	(CTC)7	Class I	7845	7865	TTCCCAGCCATGACATGACGCA	ATCTGTCTCCACCACCACGACA	251
FMgSSR-54497	scaffold1041	p3	(CTC)7	Class I	44806	44826	TTGGCTCAACACGGCCCATT	TTGGGGCAGCAATGTTGCGA	315
FMgSSR-54510	scaffold4	p3	(CTC)8	Class I	83087	83110	ACAGCTCGCGCTTCTTGACGTA	ACCCGTGTGCAGTGACACCATT	347
FMgSSR-54511	scaffold7917	p3	(CTC)8	Class I	13310	13333	ACATGGTTTTCCCAGCTCCCCA	TCTTTGTAGGTTGGCGGCGA	244
FMgSSR-54515	scaffold4468	p3	(CTC)8	Class I	40792	40815	AGCCAAGGTTTGCGCATGGT	TTCTGGATGGTGGCGTTGAGCA	223
FMgSSR-54517	scaffold13042	p3	(CTC)8	Class I	2733	2756	AGGGCGAAAGGAACGACCAACA	GCGAAGGAGTTCTGCGTGCATT	293
FMgSSR-54518	scaffold213	p3	(CTC)8	Class I	46325	46348	ATCCTGCAGAGCGTCGTGAACA	GCCATGCCCGTCTGAAAGGAATTA	283
FMgSSR-54524	scaffold3955	p3	(CTC)8	Class I	11115	11138	TGCCATTGCAAGCAGAGCCT	ACGCAACGGAACGCCATACT	339
FMgSSR-54527	scaffold2170	p3	(CTC)8	Class I	56768	56791	TGCTCTTCATGGCGCTCTTCGT	ACAAATCAACGCGACCTGCCTG	326
FMgSSR-54532	scaffold65	p3	(CTC)8	Class I	26815	26838	TTGCTCCTCCGCAATCCGCTTA	TTCTTACGAGACGGGTGAGGAGGT	308
FMgSSR-54538	scaffold394	p3	(CTC)9	Class I	101101	101127	ATTGTGGTGCAGCAACAGCAGC	AGCAAGTGCAGGGCAGACAA	293
FMgSSR-54540	scaffold1040	p3	(CTC)9	Class I	14434	14460	GCGAGTGGAAGTTCCGTCCTAA	TCCACCTCTACGCTCACGAGTT	263
FMgSSR-54542	scaffold3113	p3	(CTC)9	Class I	39622	39648	TCCCGTCGAAGCACGATCAA	AGAATCCTACGGGAACACCGCA	344
FMgSSR-54543	scaffold9697	p3	(CTC)9	Class I	25266	25292	TCCCTTTCGAACCAGGCCGAAT	ACGCTGGGCTCAAGCCAGATTT	287
FMgSSR-54544	scaffold14628	p3	(CTC)9	Class I	2799	2825	TCGCACGAACCGAACACGGATA	ACGACGACGAGCTTGTACTCCA	313
FMgSSR-54545	scaffold508	p3	(CTC)9	Class I	111037	111063	TCGCGAAGAACGCGAGTAGT	ACCGATCCGCACCGCATTTT	328
FMgSSR-54546	scaffold14276	p3	(CTC)9	Class I	1710	1736	TGCGTGCCCCGTGCTTAAAA	TCATTCGTCCACGCGACGTT	257
FMgSSR-54547	scaffold14033	p3	(CTC)9	Class I	5200	5226	TGCGTTGCGTTGCACACTTGA	CCACCAACGTGCAGCCTATGAT	235
FMgSSR-54548	scaffold583	p3	(CTC)9	Class I	17793	17819	TTGCAGCACGCGATGACGAA	AGCTCTGTTAGCACCGGGCATT	308
FMgSSR-54551	scaffold61943	p3	(CTG)10	Class I	692	721	AAATGGGCGTGGTGTTGACGTG	AATGACAGAGCCTTGCCGGGTT	331
FMgSSR-54553	scaffold235685	p3	(CTG)10	Class I	156	185	GTGCAGCAGGAACAGATCCACA	AAATGTACCGCAACGAGGCCGA	218
FMgSSR-54556	scaffold87	p3	(CTG)5	Class II	31634	31648	AAAGAAGGCCAGCCAGCTCTCA	TTGGTGTGCAGTCGGAAGCA	245
FMgSSR-54560	scaffold10092	p3	(CTG)5	Class II	17486	17500	AAATTGCGGTGGGTTGGTGGGT	TCGCGGCGCTTCTTCTTCTT	289
FMgSSR-54568	scaffold63919	p3	(CTG)5	Class II	368	382	AACGGCACCGTTCCGATGAA	AGCTGTTACTGCCTGCTGCAT	325
FMgSSR-54571	scaffold992	p3	(CTG)5	Class II	35390	35404	AAGAACATCTCTGCCTCTGGTGCG	TAACTGCTACTCCCAAAGCAGCGG	276
FMgSSR-54574	scaffold7331	p3	(CTG)5	Class II	2281	2295	AAGATGGCGAAGGTTCTCCCGA	AACCTGCTGCATGGGAAAGGCA	330

SSR_ID	Scaffold	SSR_Type	SSR_Motif	SSR Class	SSR_Start	SSR_End	Forward sequence	Reverse sequence	Product_size
FMgSSR-54576	scaffold81	p3	(CTG)5	Class II	64892	64906	AAGCATGGACATCGGACGCA	ATTTGGCTCCTGCCGCAACA	337
FMgSSR-54578	scaffold8266	p3	(CTG)5	Class II	33743	33757	AAGCGGTGGTTCAGGTCGTT	CCACACCACCACAGCACACAAA	347
FMgSSR-54579	scaffold1064	p3	(CTG)5	Class II	34736	34750	AAGCGTTTGGCGGTTTGCAG	ACGCATACGCATGAGCGCAA	235
FMgSSR-54590	scaffold15334	p3	(CTG)5	Class II	5070	5084	ACATCCATTGCTGCCGCTGT	AGGCAGGGGGTTGAACTCGTAGAT	342
FMgSSR-54598	scaffold1157	p3	(CTG)5	Class II	18906	18920	ACCTTCTTCTTCGCGCAGTCGT	TTGCCACTTGCCACCACAGA	338
FMgSSR-54611	scaffold102	p3	(CTG)5	Class II	64443	64457	ACTGAGCAGCATCCGTCAGACA	TCAGCCACCCTTTGCTGTCCTT	226
FMgSSR-54612	scaffold2410	p3	(CTG)5	Class II	51109	51123	ACTGCGAGCACGTTGCTGTT	AACACACACAGCGACGCCAA	277
FMgSSR-54617	scaffold8029	p3	(CTG)5	Class II	291	305	AGAAAGCACTCAGCAGCGCA	TTTTGAGGCAAGAAGCCGGCGA	323
FMgSSR-54618	scaffold7010	p3	(CTG)5	Class II	18273	18287	AGCAAGGCGCATAGTGGCAA	AGCTCGTGCGCGCCACTAATAA	283
FMgSSR-54623	scaffold1710	p3	(CTG)5	Class II	5232	5246	AGCATTTACGCCCGTATCCTCAC	ACGGGCATACAAGGTACGTCT	306
FMgSSR-54625	scaffold969	p3	(CTG)5	Class II	102791	102805	AGCCGAAGCGAACAAACGCA	TCACGCAGCTGCAAGAGGAA	220
FMgSSR-54632	scaffold27639	p3	(CTG)5	Class II	1070	1084	AGCGGTTTGGTACCTGGCTTCA	AGCAAGCTTCTGGTTGTCTGCCA	320
FMgSSR-54635	scaffold784	p3	(CTG)5	Class II	52150	52164	AGCTCGATGCGGTACGGCTTAT	TTCCAAAGTGCGCCACGTCT	342
FMgSSR-54637	scaffold1572	p3	(CTG)5	Class II	22436	22450	AGCTTGAGCTGGTGGTGATGCT	AGAAGCTGCGAGTCCAACATCG	347
FMgSSR-54639	scaffold2409	p3	(CTG)5	Class II	2213	2227	AGGAAACCTGTGTCGTGCGT	TGGACACAATCGACCACCGCAA	285
FMgSSR-54640	scaffold2669	p3	(CTG)5	Class II	45959	45973	AGGAGGAAGCTTCACGTCCACA	TCGCCAGCATGAACACCACCAT	207
FMgSSR-54641	scaffold359	p3	(CTG)5	Class II	55623	55637	AGGCAATCCGACCGGTTATTCGT	ACTGGCAGCAAGCTGTGTGA	343
FMgSSR-54643	scaffold4123	p3	(CTG)5	Class II	42603	42617	AGGCCCCCACAGTGGAAAATCT	AGCTGTACATGCCCTCCCATGA	350
FMgSSR-54644	scaffold305	p3	(CTG)5	Class II	1362	1376	AGGCCCTGAGCTGGTATTTGCT	ACAGGCGTGTTAGGCTTGGGAT	339
FMgSSR-54652	scaffold2294	p3	(CTG)5	Class II	50551	50565	AGTGACAGTGCCGCGATCATCA	AACACCGGCAACTGAGCTCCAA	256
FMgSSR-54654	scaffold6015	p3	(CTG)5	Class II	9534	9548	AGTGCAGCGGCATCCGAAAT	CCGGAGCACGCAACAACAAT	240
FMgSSR-54657	scaffold53600	p3	(CTG)5	Class II	240	254	AGTTAGTGCTTGCTCCGCCT	ACGCAACAGCGCAATCAGCA	346
FMgSSR-54659	scaffold234	p3	(CTG)5	Class II	61850	61864	ATAGAAGGCGTGACTGCTGCGT	AGGCGTTCATCCGAAGCGTGTT	324
FMgSSR-54660	scaffold88356	p3	(CTG)5	Class II	133	147	ATCCGTGCTGCGCTTCTTCTGT	GCTGCTGCAGAAACGGGGAAAA	226
FMgSSR-54662	scaffold3801	p3	(CTG)5	Class II	44132	44146	ATCGGCACAGCGCACGTAAA	TGGCGTTCAAGATCCCTCCCTACT	300
FMgSSR-54663	scaffold3021	p3	(CTG)5	Class II	41476	41490	ATCGTGTCCAAGCAGAGCACCA	AAAGCGTTTTCGTGCCGTGG	296
FMgSSR-54667	scaffold8466	p3	(CTG)5	Class II	3200	3214	ATGCACTGCTGGAAGGTGGGTT	ATTTGCACCGCCAACAACGC	247
FMgSSR-54668	scaffold7467	p3	(CTG)5	Class II	21710	21724	ATGGCAGCTGTTGCCGTGTT	TGACAAGGGCTGGTGCAGCTAA	292

SSR_ID	Scaffold	SSR_Type	SSR_Motif	SSR Class	SSR_Start	SSR_End	Forward sequence	Reverse sequence	Product_size
FMgSSR-54673	scaffold1272	p3	(CTG)5	Class II	39866	39880	CCTGCGCAGCCGAAAAACAGAA	CGCGTTCCGCCGCAAGAAATAA	349
FMgSSR-54674	scaffold13047	p3	(CTG)5	Class II	7938	7952	CGAAGCGAAGGCAGCGAACAAA	AGTTACCGTTACCGTAGGCCGT	344
FMgSSR-54675	scaffold2299	p3	(CTG)5	Class II	38905	38919	CGGCGGCACAAAGTTGAGGTTA	AGCAGCAGCAGCAATGGAGA	343
FMgSSR-54676	scaffold64663	p3	(CTG)5	Class II	150	164	CGTCGGAACTCATCACCTGGAA	TGGCAACGTCGTGTTTGTGC	239
FMgSSR-54677	scaffold131760	p3	(CTG)5	Class II	199	213	CGTTGATCGTGATCTTGCCCGA	ATGAAGCGCAGAACCCGGAAGT	317
FMgSSR-54679	scaffold54	p3	(CTG)5	Class II	66669	66683	GCAGGATGCAGCTGGTTCAA	AGCAGCAGCAGCACGTTGTT	276
FMgSSR-54686	scaffold48145	p3	(CTG)5	Class II	2453	2467	GCTGCGTATTCAGCGTTTCGGT	TGCAACAGCAGCGTTCCCAT	204
FMgSSR-54695	scaffold80	p3	(CTG)5	Class II	158162	158176	TAGATCGCTGACGGTTGCTGGT	TATCCACCGTGCTGCTGTTGCT	259
FMgSSR-54697	scaffold13545	p3	(CTG)5	Class II	7424	7438	TCACAAACAGCAGGAGGGAGGA	ATCGCAGCAACCGCAATGATGG	309
FMgSSR-54699	scaffold208	p3	(CTG)5	Class II	73104	73118	TCAGTTAGGGTTTTGCGCCG	AATGCAACGCCAAACTCGTCGG	338
FMgSSR-54704	scaffold5035	p3	(CTG)5	Class II	38486	38500	TCCTCACGCGCTTCTTCCCTTT	TTGTAGTCGTTGCCGCCGAA	301
FMgSSR-54720	scaffold21978	p3	(CTG)5	Class II	7459	7473	TGAAACCCTGCACGCTCACCAT	TGCGCGTTCGTTTACTTGCCT	296
FMgSSR-54727	scaffold4425	p3	(CTG)5	Class II	47943	47957	TGATCCAGTGTAGTGCCGCT	TGCGCGCCTTGCAACTTTGA	345
FMgSSR-54733	scaffold781	p3	(CTG)5	Class II	3648	3662	TGCCTTCTCTGCTTGTCGCT	CGGCTACGCGCCTGAAACAAAT	258
FMgSSR-54736	scaffold293	p3	(CTG)5	Class II	39212	39226	TGCGGACGATTGTGGCATCA	TCAAAATCCGGTGAGCGCTGGT	283
FMgSSR-54739	scaffold2749	p3	(CTG)5	Class II	34978	34992	TGCGGCTGCCAACGAAGTAA	ATCAGCGGCTGCTGCAACTACA	245
FMgSSR-54745	scaffold9114	p3	(CTG)5	Class II	17553	17567	TGCTGCCTCAACTTCTTGCCCT	AACACACAGCTGCACGAGCGTA	221
FMgSSR-54746	scaffold188	p3	(CTG)5	Class II	62134	62148	TGCTGCTATCATCGCTGCTGGT	AGGAACCCGTGCAAAGCATGGA	341
FMgSSR-54750	scaffold2159	p3	(CTG)5	Class II	54084	54098	TGCTTCATCCTCCTCTCCGCAA	TGCCAGCGCTCGTGATTTGA	231
FMgSSR-54754	scaffold8140	p3	(CTG)5	Class II	17453	17467	TGGCAAGATGAAGGTGGCCGAT	TGCAACCTGTAGCCCGCTTT	298
FMgSSR-54756	scaffold5914	p3	(CTG)5	Class II	37185	37199	TGGCACACGTGTTACCGCAA	AGTCGTTGGCACTCTGTGCAGT	257
FMgSSR-54757	scaffold6220	p3	(CTG)5	Class II	26155	26169	TGGCAGTTGGTTGGGACATGGA	TGTTTTGTTCCGCACCGCCA	281
FMgSSR-54758	scaffold358085	p3	(CTG)5	Class II	338	352	TGGCCCAGGCACACTCATTT	TGGTTGGTGGTCAGGTGCACAA	225
FMgSSR-54761	scaffold2075	p3	(CTG)5	Class II	33978	33992	TGGGCGTTGAATTGGCCTCA	TTGAACGAAAGCAACAGCCGGG	261
FMgSSR-54762	scaffold331	p3	(CTG)5	Class II	127150	127164	TGGGCTCAAAAAGCAGGGCA	TGCATCGAATCCTGTGCCGA	316
FMgSSR-54767	scaffold6003	p3	(CTG)5	Class II	32287	32301	TGTAACTGTTGCGCCGCCAT	TTCATCAGGCGCAGATGACGCA	220
FMgSSR-54771	scaffold906	p3	(CTG)5	Class II	80970	80984	TGTGCGGCAAAGGGGATGTA	TGGATGCCGGAGTTGAAGCA	220
FMgSSR-54772	scaffold1073	p3	(CTG)5	Class II	32631	32645	TGTGCTCGCGCTTTGCTTCA	TACCATGGCTGGTGCAGCTT	310

SSR_ID	Scaffold	SSR_Type	SSR_Motif	SSR Class	SSR_Start	SSR_End	Forward sequence	Reverse sequence	Product_size
FMgSSR-54779	scaffold53600	p3	(CTG)5	Class II	445	459	TGTTCAGCCATCGCCTGCTTCA	AGGAAAACGCCCGTCAGCAA	305
FMgSSR-54784	scaffold4173	p3	(CTG)5	Class II	12948	12962	TTCCGCTGCTTCTGCGCTAA	CCGCAACAGGTCCGTTTTGGTT	346
FMgSSR-54785	scaffold661	p3	(CTG)5	Class II	63689	63703	TTCGCCGACGACATCCTTCT	ACACCTACGCCTGCTGCATT	239
FMgSSR-54789	scaffold1378	p3	(CTG)5	Class II	17695	17709	TTCTTTCGGTTTTGGGCGCACG	AAACAGGCATCCCAGCGGTGAA	234
FMgSSR-54792	scaffold1828	p3	(CTG)5	Class II	34250	34264	TTGAGCTCCTGCACATCCCACA	TTTCAGGCGTTTCTTGGCGTCG	328
FMgSSR-54795	scaffold182	p3	(CTG)5	Class II	39751	39765	TTGATTGCCCGCCTGCATGA	TCGACCTGGTTTGGTTGGCTCT	235
FMgSSR-54799	scaffold63446	p3	(CTG)5	Class II	886	900	TTGCTCCTGGTTGTGCTCGT	TCGAAGCGTTACTATGGCCGCT	237
FMgSSR-54803	scaffold3602	p3	(CTG)5	Class II	12381	12395	TTGGCGCGGCGCTATTTTGT	ATCTTCCTCATCGGCGGCTTCA	234
FMgSSR-54805	scaffold1160	p3	(CTG)5	Class II	38144	38158	TTGGGTTCCGTGCTGGTTGACA	TGCATCACAAGTGACATGCACCG	320
FMgSSR-54808	scaffold9996	p3	(CTG)5	Class II	1413	1427	TTGTGGACGCTGCCAAGCAA	GCTGCTTCAGCCGGTGATCATCTA	224
FMgSSR-54811	scaffold12	p3	(CTG)5	Class II	83305	83319	TTTTGGAGCGCGCAAGCATC	ACGTCCCCCACGAACACGAAAT	223
FMgSSR-54815	scaffold1789	p3	(CTG)6	Class II	4883	4900	AACATGCCGGCGTTCATCATGC	ACCGAAGGCTTTGCATTGCT	330
FMgSSR-54817	scaffold238	p3	(CTG)6	Class II	77688	77705	AAGACCGATGGGGGACCAGAAT	TTCCTGATCGGTGGCAAGGT	292
FMgSSR-54818	scaffold237	p3	(CTG)6	Class II	7199	7216	AAGATACGCCCGCTGTGACTGT	TTGTCCGCCCACACGAACTT	349
FMgSSR-54820	scaffold1999	p3	(CTG)6	Class II	70128	70145	AAGTCGACGAACGGCATCTCCA	AACCTGCGCCTCTACAGCCTTT	281
FMgSSR-54823	scaffold10603	p3	(CTG)6	Class II	16503	16520	AATTGCATGGGAACCATGGGCG	ATGGGCTCCGGAGACAACTTCA	334
FMgSSR-54827	scaffold3682	p3	(CTG)6	Class II	27648	27665	ACAGAACGCTGAACGCATCGTG	AACACGGCGGCGCTCAATTA	324
FMgSSR-54835	scaffold3749	p3	(CTG)6	Class II	21637	21654	ACGAAGCAGCCGAGGAACAT	AGATCGAGCGGTGTTCTGACGA	318
FMgSSR-54840	scaffold1620	p3	(CTG)6	Class II	27193	27210	ACGTGGAACGATCCTCCGTCTT	TGCAAGCAGACGACCACAGT	344
FMgSSR-54846	scaffold4940	p3	(CTG)6	Class II	18276	18293	AGCAAGTCCACACGTGAACCA	AAAGAGGGGCAACCTGCTGGAA	344
FMgSSR-54853	scaffold10459	p3	(CTG)6	Class II	26664	26681	ATACGAGGCGCGGGCATAAA	TGGCTTGTTGGGTTTGGCGT	249
FMgSSR-54856	scaffold860	p3	(CTG)6	Class II	77565	77582	ATGAGCAGCAAGGCGTAGGTGA	ATCTGGCGCCAAGCAATGGT	311
FMgSSR-54858	scaffold7442	p3	(CTG)6	Class II	31334	31351	ATGCATCGCACGCGAGAGGATT	AGCTGACTTTGTAACCGCGGCA	233
FMgSSR-54859	scaffold81	p3	(CTG)6	Class II	18337	18354	ATGTCGTGCCCCTTGTCCTCTT	TTCTTGGCTGCGAACTGCGA	323
FMgSSR-54860	scaffold607	p3	(CTG)6	Class II	71243	71260	ATGTGCAAAGAAGCCCGCCA	AGCTCAGCCCTGCAACAACA	279
FMgSSR-54861	scaffold1259	p3	(CTG)6	Class II	23513	23530	CCCTGATTTGCTTTGGTGTGGCA	TCAACAAGCGCAGCAGACAACG	343
FMgSSR-54862	scaffold87813	p3	(CTG)6	Class II	1445	1462	CGAACGGACATTTTAGCGGCGT	ATCATGCTGACCCGCTTGTGGT	247
FMgSSR-54865	scaffold2660	p3	(CTG)6	Class II	21017	21034	CGTCCTGCAACAAAACGGACGA	TTTTGCGATGCAGGGTGATGCG	335

SSR_ID	Scaffold	SSR_Type	SSR_Motif	SSR Class	SSR_Start	SSR_End	Forward sequence	Reverse sequence	Product_size
FMgSSR-54866	scaffold25309	p3	(CTG)6	Class II	6708	6725	GCAGCAGCTTCGTGTTGAAGCA	ACTGCTGGATTGGGGAGGTCAA	316
FMgSSR-54867	scaffold139262	p3	(CTG)6	Class II	496	513	TAAGAACATCTCTGCCTCTGCGCC	AACTGCTACTCCCAAAGCAGCG	273
FMgSSR-54871	scaffold7029	p3	(CTG)6	Class II	32366	32383	TAGCCGCACTCTGCGTGAATGA	TGGGTGCAGCGCAATCACAT	339
FMgSSR-54874	scaffold68	p3	(CTG)6	Class II	19950	19967	TCACCACTTGTTTCCCCTGCCT	ACCACCGCACACACACTCACAT	213
FMgSSR-54876	scaffold9035	p3	(CTG)6	Class II	30712	30729	TCATCGCCTCTGGTGCGTTTGA	GGCGGCGACCAAAACAACAA	228
FMgSSR-54877	scaffold3617	p3	(CTG)6	Class II	25570	25587	TCATGCAAGAACCGAGGCGACA	TCACGCACGCAGGTTGCTTT	219
FMgSSR-54880	scaffold8466	p3	(CTG)6	Class II	1301	1318	TCCGCTCTGCTGTGGATGTT	ACAGCACTGAACGAGGCACTGA	201
FMgSSR-54883	scaffold11520	p3	(CTG)6	Class II	23029	23046	TGAAGATTGGCGCAGCCAGTGA	TTGCTTTTGGAGACAGGTGCCG	271
FMgSSR-54884	scaffold586	p3	(CTG)6	Class II	42511	42528	TGATCGGCCCTGTCTGCTTT	AGATGCCGATGGAAGAGGAGGT	349
FMgSSR-54888	scaffold27648	p3	(CTG)6	Class II	3307	3324	TGCGCTAACACAAGCTGAGGA	AGAACGCAAGAGGCCGAAGT	200
FMgSSR-54898	scaffold5807	p3	(CTG)6	Class II	9278	9295	TGTTGCAAACTCTTCGCAGCCC	CGCTTCGCGCTCAAGTGAAT	336
FMgSSR-54900	scaffold831	p3	(CTG)6	Class II	86881	86898	TTCCAGTTGCTGCTGTTGCTGC	ACCACCCACAAGCCCATCATCT	302
FMgSSR-54901	scaffold25309	p3	(CTG)6	Class II	6463	6480	TTCCCTGATCGCAACAGCAGCA	TCAGGCCATGCTTCAACACGA	343
FMgSSR-54902	scaffold31304	p3	(CTG)6	Class II	6772	6789	TTCCGGAACACACGAGCAAGCA	AGCGTGCTTTTTCTGCGGCT	311
FMgSSR-54903	scaffold110950	p3	(CTG)6	Class II	372	389	TTGAGATCCATGGGCTGCACTG	AGAGCAGCAAGAGCATCACCCT	349
FMgSSR-54911	scaffold1164	p3	(CTG)7	Class I	40215	40235	AAGGCCCCCACAGTGGAAAA	AGCACTGCAACTAGCCAAGCCT	330
FMgSSR-54914	scaffold4850	p3	(CTG)7	Class I	16268	16288	ACGTTCGCGATCGGCATCAA	TGCATGCCTTCGCTTCGCTT	343
FMgSSR-54935	scaffold470620	p3	(CTG)8	Class I	118	141	AATTGCTGGCCCATTCCTGTGC	CGGTGCATGTGCATGACATCTT	221
FMgSSR-54941	scaffold1296	p3	(CTG)8	Class I	17491	17514	TCGCTGCTCATCATCGCACA	ATGAGGTTGTCAGGACGACGGT	254
FMgSSR-54943	scaffold1024	p3	(CTG)8	Class I	20526	20549	TTCCGAAGAAACCGCAGCCCTT	AAATTTCAACCCCCGGGCCCAA	244
FMgSSR-54944	scaffold3480	p3	(CTG)8	Class I	25449	25472	TTGCAAGGGAACCATGGGCA	ACAACTTCACGAACCCGGCA	346
FMgSSR-54948	scaffold631	p3	(CTT)10	Class I	12438	12467	AACAACGAATCCCACCCGGCAT	ATGGACCGGAGTTGGTTACGGA	247
FMgSSR-54951	scaffold15551	p3	(CTT)11	Class I	5809	5841	TCTGGGATTACTGGCCCCCATT	AGCTTGTTCAAAGAGACGAGGGGA	277
FMgSSR-54955	scaffold744	p3	(CTT)12	Class I	53580	53615	AAGATGCCCAAGCGTGCGTT	TGGAACGTTGGTGAAGGGGCTT	307
FMgSSR-54956	scaffold219	p3	(CTT)12	Class I	47838	47873	ATCTTGTGCCTGGTTTCCGCGA	AGCAGGCGAAACGAAACCGT	350
FMgSSR-54957	scaffold21377	p3	(CTT)12	Class I	2025	2060	ATGCTAACGGAGGCCCATGT	ACAGTGATCGATTCCCCATCCCCT	241
FMgSSR-54958	scaffold12806	p3	(CTT)12	Class I	11604	11639	TACGCGTACAGCGCCATGAA	TCGCCAACAAACACCACCCA	278
FMgSSR-54959	scaffold804	p3	(CTT)13	Class I	10567	10605	AGCAACGAAGTCGACAAGGGGT	TCGTGCACTTGCCCGGTTTT	252

SSR_ID	Scaffold	SSR_Type	SSR_Motif	SSR Class	SSR_Start	SSR_End	Forward sequence	Reverse sequence	Product_size
FMgSSR-54966	scaffold855	p3	(CTT)15	Class I	33482	33526	TGCTCATCGTCGTCTTGCTCTCA	AATCAGAGCGCCCCAACACCAA	261
FMgSSR-54971	scaffold2087	p3	(CTT)21	Class I	55698	55760	TCGAGTGCAAACGAGCGAGGAT	TCGCCCTTCGCTCTTGGTAGTT	342
FMgSSR-54972	scaffold661	p3	(CTT)21	Class I	54633	54695	TGCCGTGATGTCAGTTTCGGTGT	TGGGTTGGGCCCGAGAAATGTT	257
FMgSSR-54974	scaffold17193	p3	(CTT)26	Class I	8066	8143	ACGAGAAACTTGCAACTCCGAAGG	ACAGGCGCATGTGATTAGGCCA	288
FMgSSR-54977	scaffold447103	p3	(CTT)5	Class II	41	55	AAAAGGATTGCCGCTAGTGCCG	TCAGCATCCCTTCTTTCAGCGGT	201
FMgSSR-54979	scaffold10077	p3	(CTT)5	Class II	9387	9401	AAACGGCCGGAGTTCCATCACA	TGGAGGAGCCTTAACGGCGAAA	255
FMgSSR-54980	scaffold1183	p3	(CTT)5	Class II	48218	48232	AAAGATCAGTTGCGGCAGGGCA	CGCGCGAACGCAAGAAAGTT	350
FMgSSR-54992	scaffold187	p3	(CTT)5	Class II	107230	107244	AAATCGCCTCGGTTTCGCCT	ATGTGCGCGTCCCATCTTCACA	201
FMgSSR-54994	scaffold13083	p3	(CTT)5	Class II	8730	8744	AACAACAAGTGCTGCCACTGCC	ACCATCCGTACCAGCGACGTTT	244
FMgSSR-55000	scaffold50916	p3	(CTT)5	Class II	767	781	AACAGCATGCTCCCCTCCAACA	ACTGATGACAACCGGCAGGCAA	283
FMgSSR-55046	scaffold26883	p3	(CTT)5	Class II	1363	1377	AACCCTAGCAGCCACACACGTT	TTGCTCCCGCCCTTCATCAT	294
FMgSSR-55047	scaffold68	p3	(CTT)5	Class II	167619	167633	AACCTGCGCATTCCGTCCAA	ACGACAGCAAAGACGGCTCCAA	302
FMgSSR-55061	scaffold153	p3	(CTT)5	Class II	63393	63407	AAGATGGATGTACCTTGCAGGCGG	ATCGACGCACACTTCCACACGA	260
FMgSSR-55100	scaffold6210	p3	(CTT)5	Class II	20992	21006	AAGTGAAACGACGCGCATCTCG	TTTTGCGCCGCTCGTCAAGT	314
FMgSSR-55102	scaffold1166	p3	(CTT)5	Class II	55293	55307	AATAAACCGGCCGTCGTCACCA	AATCGTTGACGCTCTGCTGGGA	338
FMgSSR-55109	scaffold8178	p3	(CTT)5	Class II	6109	6123	AATGCCAGAAAGGGGAAGGCGA	TGCGGAATGAAAGCGCCCTA	314
FMgSSR-55115	scaffold23461	p3	(CTT)5	Class II	8271	8285	AATTTGGGCGGCGGGTACTT	TCGGAAGGCACAACTGCACA	322
FMgSSR-55123	scaffold161	p3	(CTT)5	Class II	163465	163479	ACACAACGTGGAGATGGTCCCT	TGCAACAAGCATTGGAAGCCCG	221
FMgSSR-55153	scaffold4598	p3	(CTT)5	Class II	34242	34256	ACCAGCAACGGTAGCAACACCA	ACCGAGCAAAAGTGTGCAGC	268
FMgSSR-55158	scaffold1609	p3	(CTT)5	Class II	22345	22359	ACCCACCACAGCCATGGTCATT	TTGGCTAGCACAAAGCCTCACC	338
FMgSSR-55181	scaffold1567	p3	(CTT)5	Class II	42399	42413	ACGGCGATCGATGTTGCCTCTT	TCGCAGCACACGCAAACTGA	347
FMgSSR-55183	scaffold11759	p3	(CTT)5	Class II	10482	10496	ACGTCATCACACGCGTCAACCT	ACCAACCACATAGATGTGCCGT	341
FMgSSR-55192	scaffold18901	p3	(CTT)5	Class II	7668	7682	ACGTGTTGAGTCTGGCGTCT	ACCTCAAGGCCACGGAGGAAAA	291
FMgSSR-55205	scaffold3	p3	(CTT)5	Class II	310635	310649	ACTTCCGACCGCGTGACAAT	TGGCAACTTTGTTTGGCCTCGC	313
FMgSSR-55236	scaffold957	p3	(CTT)5	Class II	84724	84738	AGCACCGCCGAAGAGTAAAGCA	AAACACGTCACCAAGCGCGA	278
FMgSSR-55237	scaffold12	p3	(CTT)5	Class II	73732	73746	AGCACGCACTCACGGAAACA	TGGTCATCACCTCGCTGACGTT	277
FMgSSR-55239	scaffold975	p3	(CTT)5	Class II	32172	32186	AGCAGCCCTTGTTGTAGCGT	TAGCAGCAGAGCAAGAGGAGCA	348
FMgSSR-55240	scaffold1016	p3	(CTT)5	Class II	63905	63919	AGCAGCCGCAAGAAGCAGAGAT	TGGTTGGCGCGCGAAATTCT	221

SSR_ID	Scaffold	SSR_Type	SSR_Motif	SSR Class	SSR_Start	SSR_End	Forward sequence	Reverse sequence	Product_size
FMgSSR-55249	scaffold3602	p3	(CTT)5	Class II	3568	3582	AGCGCGTTGAGAACCTTGCT	ATAAAGGGCGTCGCAAGCCA	232
FMgSSR-55257	scaffold371	p3	(CTT)5	Class II	36220	36234	AGCTTCAAACTCCCTCCCTCCA	ACAGCGCAGCAGCGAAACAA	343
FMgSSR-55269	scaffold2523	p3	(CTT)5	Class II	45833	45847	AGGATCCACAGCGTGCCATGAT	GCGCTAACCAAGTCGCCAACAA	221
FMgSSR-55314	scaffold8	p3	(CTT)5	Class II	274476	274490	AGGCGACAGACTCATTCCGA	TCAACGAATCTTGTTGGACCGGG	349
FMgSSR-55327	scaffold20	p3	(CTT)5	Class II	215206	215220	AGGTTCTCGTGTTTCCGCTTCGT	AACGTCGCCGTGCGGATAAA	268
FMgSSR-55340	scaffold210	p3	(CTT)5	Class II	21027	21041	AGTTCGTTCTCTGGGTTCCGCA	TGCGTTGATGATCGAGGCTCACT	220
FMgSSR-55341	scaffold14760	p3	(CTT)5	Class II	13639	13653	AGTTGTTGACGCGGTTCCGT	TGCTGTCACGACGACCACCATT	211
FMgSSR-55342	scaffold773	p3	(CTT)5	Class II	70893	70907	ATAGCCGCTGCATTTCCGGTGT	CGTGGCCTCCACAGATTAGTGCTT	247
FMgSSR-55343	scaffold75844	p3	(CTT)5	Class II	395	409	ATATGGGTCGCCTGGCCATT	GGCGAACAAGAGGCCCATGAAA	274
FMgSSR-55358	scaffold261	p3	(CTT)5	Class II	27019	27033	ATCTCGAAGTGGTCGCGGAAGA	ACACGACGAGCTACGCGCTTTA	325
FMgSSR-55365	scaffold615	p3	(CTT)5	Class II	53029	53043	ATGCAGCTGACGATGCTGCT	ACTGTACACGGCTCTGCACT	311
FMgSSR-55370	scaffold682	p3	(CTT)5	Class II	50387	50401	ATGGTTGCGGCGATGATGGT	TTGAGCTTGCGACTTGGTTCGC	344
FMgSSR-55378	scaffold2234	p3	(CTT)5	Class II	55785	55799	CACGGCTACTCCTTTTGCAGGT	TCTGGCCACGTCTTTCCAGT	223
FMgSSR-55400	scaffold30	p3	(CTT)5	Class II	120284	120298	CGCAAGCAAAAGCAACCGCT	ATAATGCCTTGGTCTCTGGAGGCG	349
FMgSSR-55471	scaffold241410	p3	(CTT)5	Class II	511	525	GCCTGCGTGCACCAGCAATAAT	TCGTTCGCCATCCTGTTAGGCT	302
FMgSSR-55492	scaffold489	p3	(CTT)5	Class II	77677	77691	GGCACCACCTAAGATTCATCAACG	AACCGTGCGAAAGAGGCACA	211
FMgSSR-55495	scaffold3809	p3	(CTT)5	Class II	27340	27354	GGGCCAAAGCGCAACAACAA	AAGAATGCCGGGGTCACGAACT	307
FMgSSR-55537	scaffold8178	p3	(CTT)5	Class II	6475	6489	TAGGGCGCTTTCATTCCGCA	TGTGGGGCACGCACAAACAA	299
FMgSSR-55539	scaffold1073	p3	(CTT)5	Class II	21481	21495	TATCGCCGGTAGAAGATCCGCA	ACTCGCGTTCCGTTCGTGTT	310
FMgSSR-55564	scaffold3379	p3	(CTT)5	Class II	21904	21918	TCCAGCGGACGATGAATCAGGT	ACAAGGACAACGACGAGCACGA	309
FMgSSR-55577	scaffold439	p3	(CTT)5	Class II	25686	25700	TCCGCGTAGACCAAGGGTATGT	AACTGGGTGACGTCGTGGTGAT	310
FMgSSR-55611	scaffold25504	p3	(CTT)5	Class II	2140	2154	TCGCATCGGTTCCGCATAGCTT	TGGACTGCTCGTGTTGAGCCTA	322
FMgSSR-55658	scaffold7074	p3	(CTT)5	Class II	11020	11034	TCTTCCATGCGCATCCCTTCCT	TCACACACCTCAAAAGCTGCCAGT	323
FMgSSR-55660	scaffold18921	p3	(CTT)5	Class II	6111	6125	TGAAAGCGTTCGCCGGAACA	TGGCGTGGTAGCCATTTGATGC	305
FMgSSR-55668	scaffold77	p3	(CTT)5	Class II	106075	106089	TGACGGCAAACCAGTGTCGGAA	ACCGCGATCAAGACACAGAGGA	283
FMgSSR-55816	scaffold10127	p3	(CTT)5	Class II	19532	19546	TGCAGAAAGCGCGTCAAGGT	GCGGCGAGGAAGGGAAGAATAA	274
FMgSSR-55818	scaffold21627	p3	(CTT)5	Class II	1765	1779	TGCATCTTCGCCTTTCCGGTGA	AATGATCAGCCGTCCGCAGT	296
FMgSSR-55892	scaffold983	p3	(CTT)5	Class II	2098	2112	TGCCGACGGTCCACATGAAGAT	TGTTCTTCAACGCGCAGCGA	304

SSR_ID	Scaffold	SSR_Type	SSR_Motif	SSR Class	SSR_Start	SSR_End	Forward sequence	Reverse sequence	Product_size
FMgSSR-55909	scaffold2460	p3	(CTT)5	Class II	62977	62991	TGCGTAGCGTGGCGTGAAAA	ATGCCACTGTGAGACGGCAAA	221
FMgSSR-55915	scaffold975	p3	(CTT)5	Class II	32624	32638	TGGACAAGCGCCTGAGGATGTT	ATGGCTTCTGCCTTTGCCGCTA	291
FMgSSR-55916	scaffold479	p3	(CTT)5	Class II	63477	63491	TGGACGACATGGCTCACAGACA	TCCATGACGCCGTTGCGTTT	239
FMgSSR-55919	scaffold1838	p3	(CTT)5	Class II	65803	65817	TGGCACTGGTAGCGTCACGATT	TGGCTAAGCAGAAGTGACCTGT	229
FMgSSR-55936	scaffold184582	p3	(CTT)5	Class II	111	125	TGGGCTGGTTGGGCAGAAAGAA	TGCTTGTGCCTCCACTGCAA	269
FMgSSR-55938	scaffold8796	p3	(CTT)5	Class II	11909	11923	TGGTGTGTGTGGTGATGCGA	AGGCTAGGGCTGCCAAGGAAAT	316
FMgSSR-55939	scaffold98	p3	(CTT)5	Class II	134075	134089	TGGTTGGCACAACCGCACAA	AGGTGTTGATCTGCCGCCAT	313
FMgSSR-55950	scaffold16117	p3	(CTT)5	Class II	9300	9314	TGTCTGTGACCAAGTTTCCTCTGG	ACGAGGCTGAAAATCACGGGCA	301
FMgSSR-55954	scaffold30180	p3	(CTT)5	Class II	6703	6717	TGTGAGGCACAAAACCCGCA	AGCCTCGCGGGGAAGAAAACAA	219
FMgSSR-55979	scaffold2633	p3	(CTT)5	Class II	45571	45585	TTCAGCATCAGGAGGGTCATCAGC	TCATCATGCTGCAGTTCGCCA	348
FMgSSR-55980	scaffold133680	p3	(CTT)5	Class II	211	225	TTCAGTCCTGCTCGGTTTGGGA	TCACAGCCGATTCGGGAAGT	280
FMgSSR-55988	scaffold1956	p3	(CTT)5	Class II	13948	13962	TTCCTCCTTGGCCTTGGCATCA	ACATCCACGATGACGACCACCA	218
FMgSSR-55994	scaffold9783	p3	(CTT)5	Class II	8798	8812	TTCGCTTTGGCCGCGTAGAT	CGCTGGCAGCCGATTGATAA	321
FMgSSR-56006	scaffold6830	p3	(CTT)5	Class II	28338	28352	TTGACTTTGCCAGTTGCGGC	ATCGTGTTCGGCATCGTGCT	267
FMgSSR-56009	scaffold1773	p3	(CTT)5	Class II	34912	34926	TTGAGTGCCACCAACGCACT	GCTCACCACGCGACAGCTTTTT	258
FMgSSR-56014	scaffold860	p3	(CTT)5	Class II	8574	8588	TTGCAGTGTTGCAGAGGGCGTA	CCGGCAACAACAACAACCAGCA	325
FMgSSR-56105	scaffold2266	p3	(CTT)5	Class II	48627	48641	TTGTACCGGGGCGCATCATCTT	AAGAGGAGCGGCAATGGCACTT	294
FMgSSR-56109	scaffold28336	p3	(CTT)5	Class II	1130	1144	TTGTCTCTTGGTGCGTCGGCTT	TCAAAGCGCCGTCAAACCCA	343
FMgSSR-56122	scaffold874	p3	(CTT)5	Class II	101376	101390	TTTGCTGCTCAAGCTCCATCGC	ACTGGAAGGGGCACAAGTGCAA	291
FMgSSR-56138	scaffold657	p3	(CTT)6	Class II	44486	44503	ACAACACCTTGCCGCCAACA	ACCTGTCCGAGAACAACCTCGT	282
FMgSSR-56140	scaffold5149	p3	(CTT)6	Class II	32105	32122	ACACACACACATCACGCCCCTT	ACATCGCGTCCTTGGCACTT	340
FMgSSR-56147	scaffold13834	p3	(CTT)6	Class II	12605	12622	ACCGTCAACGTGGCTCTAGCTT	AGAACGCGTCCTCCTTGGACTT	209
FMgSSR-56149	scaffold319	p3	(CTT)6	Class II	31995	32012	ACCTGCCGGAACCCATTCGTTT	ACGCCGATGAAGTGAACGACGA	232
FMgSSR-56151	scaffold2043	p3	(CTT)6	Class II	66449	66466	ACGCCGTGATTCTCACCAAGCA	ATCGGTTCCTTCACGCAGCA	223
FMgSSR-56157	scaffold1005	p3	(CTT)6	Class II	34596	34613	ACGGTGCGGAGGAAACGGAAAA	TGGCTCTGTATTGTTGCCGCCT	338
FMgSSR-56164	scaffold26203	p3	(CTT)6	Class II	4831	4848	AGCCAGATGGGCTTCTGCTTGT	TGGATCGTTCACGGTGTCGCTT	342
FMgSSR-56177	scaffold4139	p3	(CTT)6	Class II	14231	14248	ATTCAACTTCCTCCTCGCCGGA	TCTGCAGGCCAACGTGTTCA	346
FMgSSR-56182	scaffold2023	p3	(CTT)6	Class II	42541	42558	CCGGCAGGCAAGTTCAAACGAT	TGGCGGCCAAGAGCAATCAA	283

SSR_ID	Scaffold	SSR_Type	SSR_Motif	SSR Class	SSR_Start	SSR_End	Forward sequence	Reverse sequence	Product_size
FMgSSR-56190	scaffold7644	p3	(CTT)6	Class II	390	407	GCCCACAATCCATTCTTGCTGC	AAGGCCTTCGAAACACGGGTGA	318
FMgSSR-56198	scaffold3572	p3	(CTT)6	Class II	21779	21796	TAGCCGCCTCGATCGCCTTTTT	AGGCATTGGCGAAGTGCATTG	337
FMgSSR-56199	scaffold1111	p3	(CTT)6	Class II	80719	80736	TATGCGGCCAGTTTGGGCTGAT	TTGCCGTTTAGCGCCGTTCA	293
FMgSSR-56200	scaffold5221	p3	(CTT)6	Class II	12183	12200	TCAACCGAACGGCGTGATGT	AGGCCGTCCGGGCTATATGAAT	305
FMgSSR-56207	scaffold11037	p3	(CTT)6	Class II	9591	9608	TCGCCGAGCTCAGCATCATCAA	TTGGCGTTTGCAGTGACGTG	202
FMgSSR-56210	scaffold4315	p3	(CTT)6	Class II	8738	8755	TCTGCAACGTCCTTTAGGGCCA	TTGTGGCCTCGTTTCGCAGT	249
FMgSSR-56233	scaffold4528	p3	(CTT)6	Class II	13630	13647	TGGCTGCGCACGGTTAGTTT	AGCGTGGCTTGTCGCAAACA	267
FMgSSR-56237	scaffold3533	p3	(CTT)6	Class II	2837	2854	TGTTGCTCTCCTGCTGCTGCTT	CGTTGCTGCAAGCTTGAGTGCT	341
FMgSSR-56240	scaffold24	p3	(CTT)6	Class II	163079	163096	TTAGCGTGCTCTGCTTGTGGCA	AACAAAGCTGCGCGGGACAT	335
FMgSSR-56241	scaffold37623	p3	(CTT)6	Class II	1739	1756	TTCGGCTTCCCAAACCCCACAA	TGCCGCGGGGAATCAAGAAA	286
FMgSSR-56243	scaffold4902	p3	(CTT)6	Class II	25046	25063	TTGCAGGCAGCAGCAGCTAT	TCACTCGGACGCACGCATTT	222
FMgSSR-56246	scaffold241	p3	(CTT)6	Class II	28233	28250	TTGGTGCCCTGCCAAAAGAAGC	AGTGAAGTACACACGCTTGGGG	257
FMgSSR-56253	scaffold323	p3	(CTT)7	Class I	20036	20056	ACACCGGTCGGCACTTGTAGTT	TGCGTGTAATGCCGCAGGAA	229
FMgSSR-56255	scaffold1564	p3	(CTT)7	Class I	59060	59080	ACCATCTGAGCCCATCATGGCA	AGCATTGCGGTATGTCCGTGT	293
FMgSSR-56258	scaffold3121	p3	(CTT)7	Class I	24595	24615	ACTGCATGATGGGCCAACACA	CCCTCCCACCCAGCAAAAACAT	334
FMgSSR-56261	scaffold1122	p3	(CTT)7	Class I	92771	92791	AGATGGGGCGTGCAGCAAAT	TCTGGTCCGGCAGATGCAAA	295
FMgSSR-56268	scaffold5410	p3	(CTT)7	Class I	28808	28828	TAGCGTTAGTGGGGGAAGCAGT	TCCCAGTTCCGCCAATGCATCA	327
FMgSSR-56271	scaffold5173	p3	(CTT)7	Class I	14058	14078	TCCCGCGTCATGCAATGCAAA	AAGCAGCATGGAGTTGCGGA	269
FMgSSR-56273	scaffold28516	p3	(CTT)7	Class I	159	179	TCGATGGTCACGTTGACGGGTT	TCCTCCGGCTCCACCAAGAAAA	218
FMgSSR-56282	scaffold3121	p3	(CTT)7	Class I	39726	39746	TGGCTCTGGCTGGGAAAAGA	TGGGCCTTGTCCATGCAACGAA	306
FMgSSR-56283	scaffold4066	p3	(CTT)7	Class I	21907	21927	TGGCTGCAGTGGCTATCTTGGT	ACGCCGTTTTTCCTCGGGAT	335
FMgSSR-56285	scaffold14724	p3	(CTT)7	Class I	14278	14298	TGTCACCAACGACAGCGACCAA	CGCCGATGGAACAAAAAGGCGT	318
FMgSSR-56295	scaffold28633	p3	(CTT)8	Class I	6270	6293	AGCTGTTCGATGCATTGCCCCT	TTGGACGCGAGGACCGAAAT	241
FMgSSR-56296	scaffold14235	p3	(CTT)8	Class I	14654	14677	ATGAAGATGGTCCTGCTGCCGA	AGCAAGAACACAAGTCGAGCGGA	342
FMgSSR-56299	scaffold4105	p3	(CTT)8	Class I	50700	50723	TGATTGCCTTACTGTCCGGTCT	TCAGTCCAAGCAATGTTAACCCCC	306
FMgSSR-56302	scaffold1	p3	(CTT)9	Class I	328347	328373	ACCACACCCACCCAACCAAA	TGTGCTTTGCTGTTGGGGGAGAA	318
FMgSSR-56305	scaffold155602	p3	(CTT)9	Class I	368	394	TCCACAGGCAGTGCCACGTAAA	TGCCGTGTCTGCATGTCGTA	341
FMgSSR-56307	scaffold103330	p3	(CTT)9	Class I	864	890	TGGCAGCAACAACACCCCTT	TTCGGTGCTTCATCGGGTCTGA	278

SSR_ID	Scaffold	SSR_Type	SSR_Motif	SSR Class	SSR_Start	SSR_End	Forward sequence	Reverse sequence	Product_size
FMgSSR-56308	scaffold22695	p3	(GAA)10	Class I	8208	8237	AGCTCAAATCTCCACGTCCCGT	TGCCATCCTCTCGCCATTTGCT	306
FMgSSR-56312	scaffold1943	p3	(GAA)10	Class I	47606	47635	TGCAGCGACGAACACATCCT	CGGCGAAGCAGCAGCCATTATT	249
FMgSSR-56313	scaffold228	p3	(GAA)11	Class I	136439	136471	ACCAACTTTGCAGCCGCCAT	ATGTGAACTCACCGCACGCT	257
FMgSSR-56316	scaffold574	p3	(GAA)11	Class I	62484	62516	GCAAAGACGCCAAGAACGGCAA	TCGCCCACAAACGCTTGGAT	338
FMgSSR-56320	scaffold9798	p3	(GAA)12	Class I	12787	12822	AACCATGGACGCCGGACAAA	ACGCGTAGAACCCGCACTTT	284
FMgSSR-56329	scaffold3015	p3	(GAA)15	Class I	37687	37731	ACACGCGTGCAGATGCAGAA	TCCCTCCCCCACCAATACCAAT	345
FMgSSR-56330	scaffold605	p3	(GAA)15	Class I	108360	108404	AGGAACTTCCATGAGGGCAGCA	TCCCCGTCATTCTCGAACGTCT	333
FMgSSR-56335	scaffold3860	p3	(GAA)19	Class I	47172	47228	ATGGCTGAATGGACGTCGGA	TTGAACACCCGCGTCAAGGA	286
FMgSSR-56338	scaffold365	p3	(GAA)20	Class I	38256	38315	AACGACGAAGCAGCTGGGATCA	TCACCGCACCAAGCTCAACT	346
FMgSSR-56339	scaffold145	p3	(GAA)20	Class I	30724	30783	ATCGACCAGCTGAAGTTGCCGT	AAGCTCAACTTCCCGGCTGA	253
FMgSSR-56340	scaffold21626	p3	(GAA)20	Class I	2112	2171	ATCGCCAACGACCAAACCCA	TGAAGACGGCATGCTGGCCATA	208
FMgSSR-56344	scaffold9969	p3	(GAA)23	Class I	14965	15033	TGTTGCACCATCCCATGGAAGC	TCCCTCCTTGCAGCTGTTGTCA	234
FMgSSR-56351	scaffold756	p3	(GAA)5	Class II	76964	76978	AAAGGACGACGCGCAGATGT	TTCCACGCCAGCCAGTTGAGTT	350
FMgSSR-56359	scaffold1807	p3	(GAA)5	Class II	19454	19468	AACGACCAACGACCACGACGAA	TCGTGCTGCTGCCTTGTTGA	264
FMgSSR-56369	scaffold182	p3	(GAA)5	Class II	129363	129377	AACTGGCACCACGGAGCATTGA	TCCCTTGAGAGCCACTACACATGA	324
FMgSSR-56387	scaffold30474	p3	(GAA)5	Class II	4185	4199	AAGCCGTACGCGCTGATGAT	ACGGCGACGATGCACATGAA	293
FMgSSR-56388	scaffold54	p3	(GAA)5	Class II	39975	39989	AAGCTCCAGCCATCCGCAAT	ATTTGAAGCGGCCGAAGCACAG	322
FMgSSR-56393	scaffold22986	p3	(GAA)5	Class II	7400	7414	AAGGGGAACCACATTGAGCACG	AATTTCTGCACCCTGCCCCA	273
FMgSSR-56412	scaffold3808	p3	(GAA)5	Class II	23593	23607	AATTGTTCGACGGGGTAGCGGA	ACAGCGCACCACACAACCTT	337
FMgSSR-56440	scaffold10109	p3	(GAA)5	Class II	6448	6462	ACCACGCATCCGATTGGTCTCT	AGATAAGCAGCTCCAGCACCCA	336
FMgSSR-56441	scaffold2270	p3	(GAA)5	Class II	57171	57185	ACCAGCACCCATAGCCACATGA	TCAGCCTCTGCAGTGTGGTCAA	334
FMgSSR-56443	scaffold6820	p3	(GAA)5	Class II	28456	28470	ACCAGGTTGTGCTTCTGCAGTCA	AGATTGACAACGGGCAGCCTCT	257
FMgSSR-56448	scaffold1718	p3	(GAA)5	Class II	35448	35462	ACCCACCACCACCTTCCCTTTT	TTGGGCCGAGCCAGATTCCTTT	220
FMgSSR-56480	scaffold961	p3	(GAA)5	Class II	97482	97496	ACGCTCCCAACCACCGAAAAGT	AACGTTTTTGGCAGGCCGACAG	343
FMgSSR-56487	scaffold13958	p3	(GAA)5	Class II	7306	7320	ACGTCCGTTGCGCCACCTATTT	ACCGTCCATGTGGAACGGCTAT	334
FMgSSR-56528	scaffold293	p3	(GAA)5	Class II	2243	2257	AGCACACCTCCATCGCGTTA	TTCGTCGTTGCGTGCTTCCT	292
FMgSSR-56530	scaffold21623	p3	(GAA)5	Class II	6524	6538	AGCACGACGGCAACAGCTACAT	TTCCGCTCATCGGAGAGCAT	205
FMgSSR-56535	scaffold847	p3	(GAA)5	Class II	63574	63588	AGCAGTCGCTCTTTGAAGCCCA	AGCATGTGGGAGAAGGTCGT	235

SSR_ID	Scaffold	SSR_Type	SSR_Motif	SSR Class	SSR_Start	SSR_End	Forward sequence	Reverse sequence	Product_size
FMgSSR-56536	scaffold1374	p3	(GAA)5	Class II	28294	28308	AGCATGCCGTGTCCTCCAAT	TTCCTTGTCGGCGTTCTTGCCT	228
FMgSSR-56547	scaffold1525	p3	(GAA)5	Class II	37478	37492	AGCGTGGCGAGAAGGAAAACCA	TAAGAGGCAGCAGCAGGCAACA	274
FMgSSR-56550	scaffold18785	p3	(GAA)5	Class II	8978	8992	AGCTGCAACAATGGGCTCGT	TCCTTGCAGTCCATGTGCCAGT	248
FMgSSR-56556	scaffold16516	p3	(GAA)5	Class II	12909	12923	AGGAGGAGCTGCATCGCAAACA	TGCCCAATGGCATGATCGCA	271
FMgSSR-56574	scaffold10016	p3	(GAA)5	Class II	5554	5568	AGGGCCTTCTACATCTGGTCCCAA	TTGTGCTCGGATCCCTTGTGCT	247
FMgSSR-56576	scaffold21332	p3	(GAA)5	Class II	10393	10407	AGGGGTGTGCTCGGCTTTGATT	TGCTCCCTTCCGCGTCATGAAT	260
FMgSSR-56582	scaffold40335	p3	(GAA)5	Class II	916	930	AGGTGGGTTGGGAAGCAGAGTT	GGTGCATACATTCTGGTTGTGTCC	247
FMgSSR-56592	scaffold3858	p3	(GAA)5	Class II	49711	49725	AGTGAGGAAGGCGTGGCAACAT	TTTGCAGCCCACACAGTCGT	308
FMgSSR-56633	scaffold4523	p3	(GAA)5	Class II	33399	33413	ATCAAGGAGGGCAGGGGACAAA	AGGCCAAACCAAGGGGCAAGAA	329
FMgSSR-56634	scaffold688	p3	(GAA)5	Class II	101910	101924	ATCACCACCGGCAATCCCAACA	TCACGCGTCACTGCCTGAAA	273
FMgSSR-56654	scaffold571	p3	(GAA)5	Class II	17329	17343	ATGCCGCCTCCGCTCATCTTTT	TGCTCTGTCCTCTTGCTCGCTT	278
FMgSSR-56662	scaffold853	p3	(GAA)5	Class II	30191	30205	ATGTGTGCATGCGTGCTGGA	TGCTGCAGCCACTACGTCTTGA	339
FMgSSR-56664	scaffold805	p3	(GAA)5	Class II	83467	83481	ATTGGGTGCATGGTGTGGACGA	ACACAACTCCAACGATGGGCGA	337
FMgSSR-56666	scaffold15554	p3	(GAA)5	Class II	2226	2240	ATTTGCGTGCTGGCCCACTT	CGCAATTGGTCGCTGTAACTCTGC	274
FMgSSR-56667	scaffold5398	p3	(GAA)5	Class II	28328	28342	ATTTGCGTGCTGGGCCACTT	TCGGTCGCTGTAACTCTGCTCT	275
FMgSSR-56669	scaffold369963	p3	(GAA)5	Class II	97	111	CAGCACTTGCGCAGGAGTTTATGG	AGGTAGAAGAAGCACCCGCA	200
FMgSSR-56672	scaffold75975	p3	(GAA)5	Class II	959	973	CAGTCGCACTGCGTGTGAAACA	TCAAGCGCGCAGGACTACAA	296
FMgSSR-56673	scaffold3744	p3	(GAA)5	Class II	31870	31884	CCACAGCGCGCAGTTCAAAA	TTAACCCCGACGAACTCCGCAA	294
FMgSSR-56683	scaffold9020	p3	(GAA)5	Class II	14343	14357	CGCAAAACTTGCTCGCGTGT	ACCGCGATCGGAATGAAGCA	316
FMgSSR-56692	scaffold1943	p3	(GAA)5	Class II	10497	10511	GCCAGGGCGTACACATTCTCAT	TCGTCGTCTGTGTGTTTGCTTGC	349
FMgSSR-56710	scaffold1763	p3	(GAA)5	Class II	72073	72087	GGCAGAACAAGCGATGAGCGTT	TGAATGTGGTCGCCAGCTTGGT	287
FMgSSR-56713	scaffold14376	p3	(GAA)5	Class II	1831	1845	GGCGTGTGGCAGTGATCAAA	ATCAGAGCGCTTCCTGCACT	290
FMgSSR-56714	scaffold14224	p3	(GAA)5	Class II	11632	11646	GGCTTGCGAGTGGACGAATGAA	TGAACAGACCAAGGGCACGACA	216
FMgSSR-56727	scaffold16602	p3	(GAA)5	Class II	543	557	TAATAATGGCGCCCGCAAGCTG	TCCACGCGTCTTCGATTCTCCT	239
FMgSSR-56729	scaffold614	p3	(GAA)5	Class II	56603	56617	TACTGTGCTCGGCATGCATCGT	ATATGCGGAGCTCGTGCGTT	249
FMgSSR-56743	scaffold803	p3	(GAA)5	Class II	36724	36738	TCACCCTTGTGCTGAGGTGT	TGCAAGGCTTTCGCAGGACTGA	216
FMgSSR-56745	scaffold8018	p3	(GAA)5	Class II	4081	4095	TCACGCGGTGCCTTGTTGAT	TGGAGCACCACCGCTGAAGAAA	205
FMgSSR-56775	scaffold115854	p3	(GAA)5	Class II	736	750	TCAGTCAGTGCGGGCGTAAA	TGGCAGCCTGGCAAACAGTA	279

SSR_ID	Scaffold	SSR_Type	SSR_Motif	SSR Class	SSR_Start	SSR_End	Forward sequence	Reverse sequence	Product_size
FMgSSR-56827	scaffold696	p3	(GAA)5	Class II	96769	96783	TCCCAGAACGTCAGACGGCAAA	TGAGCGTGAAGAACGCGACA	225
FMgSSR-56866	scaffold6234	p3	(GAA)5	Class II	25426	25440	TCGCACACGAGCCGATTTCT	ACGTCACCATCAATCCACGCCA	318
FMgSSR-56867	scaffold299	p3	(GAA)5	Class II	41821	41835	TCGCGAAGCAGAACAAGGACGA	GCTGGTTCAGCTGCTGGCTTTT	322
FMgSSR-56924	scaffold6151	p3	(GAA)5	Class II	9911	9925	TGCAAGTTGACAGGACTATCCGGG	ATGCCTTTGTGTCTGTCGCCCA	308
FMgSSR-56925	scaffold8654	p3	(GAA)5	Class II	10494	10508	TGCAATGTCAAGGCCGGCAA	AGCCACAAGCATGAGGCATGA	302
FMgSSR-56950	scaffold2638	p3	(GAA)5	Class II	55910	55924	TGCGCGTGCTGCTTTCTCTT	ACGAGTATGTCGTGTCGTGCGT	247
FMgSSR-56952	scaffold5465	p3	(GAA)5	Class II	6808	6822	TGCGGCATCACGAATCACATCA	TGACCTTTCCAGAGACAAGGCAGA	337
FMgSSR-56955	scaffold3176	p3	(GAA)5	Class II	38415	38429	TGCGTGCGTCATGACAGAGA	ACCATCCCTTCTAGCCCAGCCTTT	333
FMgSSR-56967	scaffold133	p3	(GAA)5	Class II	112924	112938	TGCTGCTGCATCATCGCGTTT	AGCTCGCGCGGAATCCATTA	207
FMgSSR-56989	scaffold988	p3	(GAA)5	Class II	94491	94505	TGGCCTTTGACGAGCAGGAACA	AGCCGATAAAGCCGTCGTCA	318
FMgSSR-56993	scaffold2790	p3	(GAA)5	Class II	45095	45109	TGGGAAGGCAGCACACTCACAT	AGCGCTGCTCTTGCGATTGA	216
FMgSSR-57021	scaffold4572	p3	(GAA)5	Class II	47801	47815	TGTGCGTGCTCTGTTCAGCA	ACGTGTGCGCCTGCTTTTCT	275
FMgSSR-57047	scaffold80722	p3	(GAA)5	Class II	632	646	TTCATTTGTCCCACGGCACCAG	TGGGCGGCTGTTTACCTTTGGT	236
FMgSSR-57049	scaffold128	p3	(GAA)5	Class II	126738	126752	TTCGAGAACATGTCTGAGCACG	TCGGAGTTATCCAGCGCAGT	202
FMgSSR-57074	scaffold971	p3	(GAA)5	Class II	81765	81779	TTCGCCGGCAACCATGGAAT	GCGGAGCAGACTGCGTTTGATT	333
FMgSSR-57103	scaffold1978	p3	(GAA)5	Class II	38077	38091	TTGCAGGAAACGCCACGTCA	ACGTACCCGAACGTGGAGAA	259
FMgSSR-57111	scaffold1233	p3	(GAA)5	Class II	67573	67587	TTGCGGTACTTGGACACCCACT	TTGCTTTTCGCGAGTGCCGT	330
FMgSSR-57131	scaffold4862	p3	(GAA)5	Class II	34929	34943	TTTCTGTGCTGTCCGCCTGT	TGCTGCGACAACACGAACAGT	295
FMgSSR-57149	scaffold35239	p3	(GAA)5	Class II	4402	4416	TTTTCACGAGCGGCCTTCCA	CGTCATCTACGGATGAGAATGCAC	330
FMgSSR-57151	scaffold65	p3	(GAA)6	Class II	184222	184239	AACAACGGCAATGGCGCATC	CGGCAGGCAGTCAACAAACACA	346
FMgSSR-57152	scaffold16591	p3	(GAA)6	Class II	7205	7222	AACAGCGAGGTGGGTGTGATGA	TGTCTTGTAGCGCTGTGCTGGA	334
FMgSSR-57153	scaffold68627	p3	(GAA)6	Class II	1772	1789	AACCAGAACCTGGCGTTGGACA	AACGTGCGAGCCAGCTATGA	293
FMgSSR-57162	scaffold1181	p3	(GAA)6	Class II	57860	57877	ACATGGCCTACTTCGCATTCGC	TGTTGCCGAGGTTGTACACG	219
FMgSSR-57164	scaffold1561	p3	(GAA)6	Class II	49917	49934	ACCGAAAACGCGCGAGGAAA	TCGGCGTCAGTCAGATCGTT	299
FMgSSR-57165	scaffold132204	p3	(GAA)6	Class II	108	125	ACCGAGAACAGCCACCTGGAAA	GCGCACGACGGTTCTGTTTCTT	230
FMgSSR-57166	scaffold68627	p3	(GAA)6	Class II	1340	1357	ACCGGCCCAACCCAAATTGA	TGTCCAACGCCAGGTTCTGGTT	312
FMgSSR-57169	scaffold733	p3	(GAA)6	Class II	32968	32985	ACGAGAGGCGACAAGCGAAA	AATCGAACCCCATGCTGCCTGT	314
FMgSSR-57172	scaffold8878	p3	(GAA)6	Class II	27922	27939	ACGTCTCTTGGACGCAACACGA	AGCAGTTCTAGCTCCTCGGCTT	349

SSR_ID	Scaffold	SSR_Type	SSR_Motif	SSR Class	SSR_Start	SSR_End	Forward sequence	Reverse sequence	Product_size
FMgSSR-57173	scaffold14385	p3	(GAA)6	Class II	9551	9568	ACGTGCCATCCAGTGGCATA	TTTTCCGACGCACGCAACGA	339
FMgSSR-57174	scaffold11174	p3	(GAA)6	Class II	5705	5722	ACTCACCAGCTCGGCTTGAT	AGCTCCTGTCTTTGCAGCACCA	290
FMgSSR-57192	scaffold4324	p3	(GAA)6	Class II	24209	24226	AGGTGTGCCCGCTTTGAGTTGA	TGCTCTGCCTTCTTCTCGGCAA	305
FMgSSR-57193	scaffold9231	p3	(GAA)6	Class II	26174	26191	AGGTTGGTTTTGTGGCGCCTT	TCCCTGTTGTCTGCTTCGCCTT	334
FMgSSR-57211	scaffold417	p3	(GAA)6	Class II	44992	45009	ATGAAAGAGACCGGACGCGGAA	GCAGGCAAGGCTGCACTGTTTT	261
FMgSSR-57212	scaffold4823	p3	(GAA)6	Class II	19098	19115	ATGGATGAAAGCGGCGCAGA	TGGAAAGCAGAAAGGCGGGACT	317
FMgSSR-57214	scaffold2942	p3	(GAA)6	Class II	54433	54450	ATTTTCTGAGGCTGGCACGACC	ACCAGCAACAGATCATGCGCGA	240
FMgSSR-57215	scaffold26108	p3	(GAA)6	Class II	9051	9068	CAATGCAACGCAAAGCCCGA	TGCTGTGCTGGTAGTCGCAA	264
FMgSSR-57218	scaffold10268	p3	(GAA)6	Class II	24372	24389	CCGGGCAGTAGTCGGCATTAAGAA	ACATGATCAGGCATCCCACGGA	332
FMgSSR-57219	scaffold10175	p3	(GAA)6	Class II	19148	19165	CGACGACGCCATTGTTGCCTTT	AACCATCGTCGGGTAGCAGCAA	278
FMgSSR-57220	scaffold19041	p3	(GAA)6	Class II	1331	1348	CGCATCACGTTTGCCGCTTT	ACACCTGGCACGCTTCGATT	266
FMgSSR-57221	scaffold37	p3	(GAA)6	Class II	76439	76456	CGCGCGACGAGAAAAGCAGAAA	TTGCGGAACGTGGCTTCGAT	333
FMgSSR-57222	scaffold387	p3	(GAA)6	Class II	75185	75202	CGGAGTTGCTGGTGCGTCTTTA	TAGTGCCTGCGCACACCAAA	248
FMgSSR-57223	scaffold2737	p3	(GAA)6	Class II	32375	32392	CGTGCTTCTTTGCTTTTCGTTGCG	GGAACTGACTAACCGGAACGTTTG	331
FMgSSR-57244	scaffold4922	p3	(GAA)6	Class II	40282	40299	TCCAGGAATTTGCCCGACGCTT	ACGAATCCACGCCTGTTCGGTT	313
FMgSSR-57250	scaffold8418	p3	(GAA)6	Class II	16191	16208	TCGAACCAGGTGCCAATTCCA	TTGGTGTGGCAGTTCGTCCA	310
FMgSSR-57254	scaffold15307	p3	(GAA)6	Class II	11227	11244	TCGCGCGCGTGTGCTTATTT	ACACAGGCTGTTTCGCTCGT	267
FMgSSR-57255	scaffold1654	p3	(GAA)6	Class II	62574	62591	TCGTCGTCGGCTTCTGGTTGTT	TGGGCAGCAACGACTACCTCAA	345
FMgSSR-57265	scaffold23850	p3	(GAA)6	Class II	6585	6602	TGCCATCTGCACGACTGCTCAT	TCCGGGCGTGGTTCAACAAA	276
FMgSSR-57267	scaffold5205	p3	(GAA)6	Class II	14675	14692	TGCCTCTTTAAGCACAGCATGTCG	ACCTTGCCTTCATTACTCTGGAGC	305
FMgSSR-57268	scaffold98	p3	(GAA)6	Class II	25104	25121	TGCCTGATTCTGCTGGATTGCCA	TCCATCTCTGAGACGTGTTGCTGT	272
FMgSSR-57271	scaffold149217	p3	(GAA)6	Class II	504	521	TGCTGCTGTTGAGCTTCCTGCT	TGCGCTTGAATCCCAGGTTTGTG	280
FMgSSR-57275	scaffold409	p3	(GAA)6	Class II	31339	31356	TGGCCCAATTGTCCCATGCT	TCATATTCCGCCGCCAACTTGC	207
FMgSSR-57278	scaffold1694	p3	(GAA)6	Class II	27505	27522	TGTCATGCCTTTGTGGCCGT	TGGCGCAGCTCAGAGTTCAA	290
FMgSSR-57279	scaffold367	p3	(GAA)6	Class II	14928	14945	TGTGAGGGGATCCAACCATCCA	TGGTTGGTGTGGGAGTAAGCGA	334
FMgSSR-57293	scaffold131	p3	(GAA)6	Class II	24529	24546	TTTCCACAGTGCAGTGCAGCCT	TCGCGTGTGCATAGAAGCCA	291
FMgSSR-57294	scaffold354	p3	(GAA)6	Class II	135200	135217	TTTGACCGCACCCTAACGCGAA	TCCACGCGGCAATCAGTAACCA	218
FMgSSR-57305	scaffold818	p3	(GAA)7	Class I	3709	3729	ACGACGGTCAAGCCCCAAAA	ATCAGCACCAAGATCCACCCGA	248

SSR_ID	Scaffold	SSR_Type	SSR_Motif	SSR Class	SSR_Start	SSR_End	Forward sequence	Reverse sequence	Product_size
FMgSSR-57309	scaffold2670	p3	(GAA)7	Class I	64149	64169	AGGAACTGCAACACCACCAGCA	ATCGATGCTTCACAGGGGCA	330
FMgSSR-57311	scaffold1407	p3	(GAA)7	Class I	43041	43061	AGGGGAGGCAACCAACAACTCA	ACAAAAGCAAAGGCTGACGCCG	278
FMgSSR-57325	scaffold12327	p3	(GAA)7	Class I	9757	9777	TCCTTTGTGCTTCACGCCAGACT	ATGCAGGATTGCAGGGGCATCT	321
FMgSSR-57329	scaffold4372	p3	(GAA)7	Class I	29632	29652	TCGGTGATCATCGTCGCGTT	TAGCCATTGTTGCTGCGCGA	345
FMgSSR-57330	scaffold8859	p3	(GAA)7	Class I	24893	24913	TGCAAAGCTAGCAAAGCCCGA	ATGCATCGATCAAACTGCCGCC	229
FMgSSR-57337	scaffold17498	p3	(GAA)7	Class I	2545	2565	TGGCCATCAGTCCTCTCACTCT	TGGGTTGACCTTCTCTCTTCCCCT	348
FMgSSR-57339	scaffold7861	p3	(GAA)7	Class I	27940	27960	TGGTGGTTGTCACGCGGTTT	AGCACTTCCCAACGGGTGAA	324
FMgSSR-57344	scaffold1296	p3	(GAA)8	Class I	27405	27428	AAGACCTTGTCGCTCCCAGCTT	TGTTCTCCGCCGTCGAGTTCAT	247
FMgSSR-57351	scaffold9884	p3	(GAA)8	Class I	13699	13722	CACACACGGCCGTCATGTAAT	TGCTTGCGCACGAACAGGAA	236
FMgSSR-57359	scaffold1697	p3	(GAA)8	Class I	4848	4871	TCGTTTCGTACCGGCTGACT	TCGCCAGCTGCTGCCTTTTT	342
FMgSSR-57362	scaffold36	p3	(GAA)8	Class I	132823	132846	TTCCCGGATGTCAGGTTGGT	AACAAACGACGGATTGGCTCGC	226
FMgSSR-57364	scaffold58554	p3	(GAA)8	Class I	771	794	TTGAGTAAGAGCAGCGGCGGAA	AACCGCGGGTTTCGTTTGGT	248
FMgSSR-57367	scaffold1230	p3	(GAA)9	Class I	62545	62571	AAGGATCACCAGCAGCAGCA	TTACGGGTGGCACGGATTTCCA	338
FMgSSR-57370	scaffold3233	p3	(GAA)9	Class I	17395	17421	ATACATGCACCCGGCCGTAA	ATTCTCCGAGAGCGCAACGACA	332
FMgSSR-57371	scaffold17277	p3	(GAA)9	Class I	7177	7203	ATTGGTGGGCACGCGGAAAA	TCGGCCGGGTCAAACAAATCCA	202
FMgSSR-57372	scaffold2280	p3	(GAA)9	Class I	62316	62342	CGCAGCTGCAAGATCATCACGA	TGGCAGCAACAACACCCCTT	298
FMgSSR-57375	scaffold8796	p3	(GAA)9	Class I	4981	5007	TGGTTGTTGGCAGCAGCAGAGA	ACCATCTTGGTGTGCGTGTGCT	308
FMgSSR-57383	scaffold7926	p3	(GAC)5	Class II	31250	31264	AAACCGAAACCCACACGCCA	TTCTTGTACCGCAACTCCGCCA	302
FMgSSR-57384	scaffold6476	p3	(GAC)5	Class II	18556	18570	AAAGCGATGTGATCGTTCCTCCCC	GCTTTGCTGTAGCCGCGTTCAA	274
FMgSSR-57387	scaffold14155	p3	(GAC)5	Class II	1212	1226	AACCGTTCCGTTCGGTGTTGGT	ACGCACAGTCGGTGGCAGTAAT	223
FMgSSR-57388	scaffold10323	p3	(GAC)5	Class II	14144	14158	AACGCCGACGACGAAGAAGA	TGATCTTGGTGGAGGTGGCAGA	268
FMgSSR-57391	scaffold2974	p3	(GAC)5	Class II	23972	23986	AAGACAAGGGCCCCGCTTTGAA	ACGTCGTCGACAGCTCATCA	252
FMgSSR-57393	scaffold1254	p3	(GAC)5	Class II	6736	6750	AAGCACAGGCGCCCAGAAAA	TGACCGCACGGGCTGCTTATTA	293
FMgSSR-57394	scaffold104	p3	(GAC)5	Class II	105589	105603	AAGTGGGATATCCTCTCCAACGGC	ATCATGTCGTTGCTGCTCTGGC	233
FMgSSR-57395	scaffold1562	p3	(GAC)5	Class II	43229	43243	AATGAATGGAGCGCGCCTGA	ACGCCGCCCACCTAACATAACA	266
FMgSSR-57397	scaffold5063	p3	(GAC)5	Class II	44455	44469	AATTTCTCGGCAAGCCGTCCCA	TGCTCGTTACTGTGCAGCGTT	233
FMgSSR-57401	scaffold2875	p3	(GAC)5	Class II	32433	32447	ACACGGTGCGACGTTTGTCA	TCGTAGCAATCGGCAAGGCTGT	257
FMgSSR-57402	scaffold16779	p3	(GAC)5	Class II	5095	5109	ACATGGCCAGGGACTCGATCTT	TGCAGGCGCTGAAGCTGATT	237

SSR_ID	Scaffold	SSR_Type	SSR_Motif	SSR Class	SSR_Start	SSR_End	Forward sequence	Reverse sequence	Product_size
FMgSSR-57403	scaffold30	p3	(GAC)5	Class II	102203	102217	ACATTTGGATCGGCAGGACGGT	TCGAACGAAATGGCGAAACGGC	252
FMgSSR-57404	scaffold4055	p3	(GAC)5	Class II	18894	18908	ACCACGTAGAGGAACAACGCCA	TCGCAGCAGCAAACAAACCACC	301
FMgSSR-57405	scaffold56	p3	(GAC)5	Class II	52180	52194	ACCAGCGGATCGCTCCTACAAA	ATGCATTCCAGGCATTTGCGGC	326
FMgSSR-57407	scaffold96	p3	(GAC)5	Class II	98414	98428	ACCGATCGGATGGCGAACAA	AGCACGCCATAGCCAGGAAA	310
FMgSSR-57408	scaffold204	p3	(GAC)5	Class II	151513	151527	ACCGATTCTACCGCAACGTGCT	TTCCGCCACGTGAAACCCAA	213
FMgSSR-57409	scaffold213	p3	(GAC)5	Class II	159345	159359	ACCGCATCTACGGGCCAACAAA	TGATGGATCGTCGTCGCGTTGT	231
FMgSSR-57411	scaffold52865	p3	(GAC)5	Class II	2153	2167	ACGAGCTTGACGTTGTACTGCGA	AGGAAAGACGACATGACCACCGT	347
FMgSSR-57418	scaffold387	p3	(GAC)5	Class II	136569	136583	ACTAACGAGGACTCGGCGACTA	AAGGCCTCGCTACTGTCCTCAT	288
FMgSSR-57421	scaffold271	p3	(GAC)5	Class II	84006	84020	AGCACCGGTTGCGATGATGA	AACCACTTGGCGTGCGACTT	280
FMgSSR-57422	scaffold8236	p3	(GAC)5	Class II	17619	17633	AGCACGCTCCAGATCTTCACCT	ACGCGAAGGCGATACTGTGCTT	322
FMgSSR-57423	scaffold528	p3	(GAC)5	Class II	87422	87436	AGCAGGAGCGAATCAGATCCGTA	ACAACGCGCAGAGAGGGATT	350
FMgSSR-57424	scaffold2999	p3	(GAC)5	Class II	27003	27017	AGCAGGCATTGCAGAGCAGCTA	TGCCCAGCTCGCAGTTTAGCAT	333
FMgSSR-57425	scaffold3152	p3	(GAC)5	Class II	4554	4568	AGCCAAGAAACCCACCAACCCA	TAGAGCGTACCTGGGCAATGGT	346
FMgSSR-57428	scaffold610	p3	(GAC)5	Class II	114591	114605	AGCGAGTTCTTGCCGCAGTA	AACGGCTCCGACTCCGATATGT	320
FMgSSR-57429	scaffold1412	p3	(GAC)5	Class II	17791	17805	AGCTCGGTTGCAGGAGAAGCAA	TTTCCCAGTCCAATCGAGCCCA	203
FMgSSR-57431	scaffold30719	p3	(GAC)5	Class II	4833	4847	AGGTCGGCGCAGCTTTTCTT	AAGTTGAGTGGTGGTGGTGCGT	233
FMgSSR-57432	scaffold5921	p3	(GAC)5	Class II	29283	29297	AGTACTGAACGACGCGCTCT	TGAATCTGCGCGTTCTGCGT	265
FMgSSR-57433	scaffold2164	p3	(GAC)5	Class II	14856	14870	AGTGCTGTGTGACGACGAGA	TTGCGGCCGCGTTCTCTAACAA	250
FMgSSR-57435	scaffold2830	p3	(GAC)5	Class II	5813	5827	AGTTGTTTGGCGCACAAAGCGT	TCGTCCAACACGATGCAGAGGT	333
FMgSSR-57436	scaffold296	p3	(GAC)5	Class II	121163	121177	ATAAGATGAAGGCGGCGCGTGA	ATCTGCTGCTTCAGCGACCTCA	206
FMgSSR-57443	scaffold15301	p3	(GAC)5	Class II	8977	8991	ATCGCGTTCGAAGGGGTTAGGT	AACGAACGGCAAAACGTGGC	299
FMgSSR-57449	scaffold3681	p3	(GAC)5	Class II	3883	3897	ATTACAAGCGCGACGTTGGGGA	AAGAATGCGTCGGTTCGCGGTT	213
FMgSSR-57450	scaffold166	p3	(GAC)5	Class II	167153	167167	ATTTCCTCGGCTTCGTGCGT	TCCTGCTTTCCTTGCTGCCTGT	249
FMgSSR-57451	scaffold470	p3	(GAC)5	Class II	70109	70123	ATTTGAACCCGACGAGTTACCCGC	TTCTTCTACGCCTCCGTCGTCA	294
FMgSSR-57453	scaffold554	p3	(GAC)5	Class II	80496	80510	CGATGAACATACCGGACTGGCAGA	GGCAAAGCGTCAGTCCTCGAAT	269
FMgSSR-57455	scaffold4176	p3	(GAC)5	Class II	15832	15846	GCCAAACCCAAAACCTGCTCCA	ATCTCTGCGTCACAGGCTGCAT	329
FMgSSR-57458	scaffold3133	p3	(GAC)5	Class II	51617	51631	GGCGCAGCCGCAGATTATTACT	CGACGCGAAACGTCGTCTTCTT	332
FMgSSR-57459	scaffold25221	p3	(GAC)5	Class II	3846	3860	GGGCATACTAGCATCCACCGATGT	TGCGTCTTGCCCGCATCAAT	211

SSR_ID	Scaffold	SSR_Type	SSR_Motif	SSR Class	SSR_Start	SSR_End	Forward sequence	Reverse sequence	Product_size
FMgSSR-57463	scaffold61	p3	(GAC)5	Class II	118280	118294	TCAAGTACGAGGAGCTTGGCTTGG	TTAGCACGCGCAAGGCTGTT	347
FMgSSR-57471	scaffold63278	p3	(GAC)5	Class II	252	266	TCCTTCAACGGCATCTACGCCT	AGGTCTGCGGCCTTTTGCAT	281
FMgSSR-57472	scaffold112	p3	(GAC)5	Class II	39524	39538	TCGAAAACCGGTGGTGCCAA	TGCAAGCGGTAATTAAGCGGCG	282
FMgSSR-57476	scaffold1682	p3	(GAC)5	Class II	29600	29614	TCGTCGGCTGCGTGACAAAA	TGCGTTTCACGTGCTGCTTCA	213
FMgSSR-57480	scaffold1369	p3	(GAC)5	Class II	21607	21621	TGCCGCCTTCTTCCTCTGTTGT	TCGCAGCAGCAAACGAACCA	346
FMgSSR-57481	scaffold1604	p3	(GAC)5	Class II	47752	47766	TGCGCATCCAAGAACCCGAA	ATTTGGCCCAGACAGAGGCA	281
FMgSSR-57485	scaffold3864	p3	(GAC)5	Class II	35309	35323	TGCTTGGCGTCCATTACGGGTT	GCAGCCGGCAAACATCATCA	345
FMgSSR-57487	scaffold1281	p3	(GAC)5	Class II	59209	59223	TGGATTCCGTTGTGCTAGGCGT	ACTTGAAGACCATGCCGTCCCT	336
FMgSSR-57488	scaffold1460	p3	(GAC)5	Class II	18000	18014	TGGCGCTTTGGCAGCATTGT	AGTGGTTCTTCACGCACACCGT	257
FMgSSR-57494	scaffold7071	p3	(GAC)5	Class II	22703	22717	TTAGCTTGATTCGGGCGCGT	ATTTGCGCCCAGGCACATCA	289
FMgSSR-57497	scaffold8073	p3	(GAC)5	Class II	20842	20856	TTCTTGATGGCGGCGTCGTT	TTTGCGGTTGGAGACGACACGA	307
FMgSSR-57505	scaffold19651	p3	(GAC)6	Class II	13697	13714	AAAGGCAGTCACGGCCCAGTTT	AATTGGGGTGGACTCCTCGGAA	313
FMgSSR-57506	scaffold1797	p3	(GAC)6	Class II	15538	15555	AACCAGCCCCACATCAGTCACA	AACGCAAATCGAGACGGGCT	207
FMgSSR-57512	scaffold148521	p3	(GAC)6	Class II	1031	1048	ACGAAGAACGGAGAGGCGCATT	AGGCTCTTCAGAGACTGGATCAGC	328
FMgSSR-57515	scaffold183	p3	(GAC)6	Class II	91519	91536	ACGCCGATGAAGGCGACAAT	AGCGGCAAAGGAGGTTTTCG	209
FMgSSR-57516	scaffold6195	p3	(GAC)6	Class II	17565	17582	ACGCCGTGGCTTTGTTCGAT	ACGCGGAGCAAGAGTTGATGGA	209
FMgSSR-57526	scaffold5327	p3	(GAC)6	Class II	39879	39896	GCGCGGCAAAATCGGCATTA	CGAAGCGCGTCTCCATGCTTTT	328
FMgSSR-57530	scaffold159	p3	(GAC)6	Class II	160427	160444	TCCCGCAACGCCAATCTGATCT	TCATCTCGACGCACCGGATCTT	334
FMgSSR-57533	scaffold8881	p3	(GAC)6	Class II	17315	17332	TCGTTGGTCACAAACCAGCAGC	ACCTGCGATGTGGTGTTGGAGT	327
FMgSSR-57534	scaffold10175	p3	(GAC)6	Class II	21253	21270	TGAAGAATCGGGCGCCATGT	AGCAGCTGCGGCTCTTGTTCTT	288
FMgSSR-57537	scaffold11458	p3	(GAC)6	Class II	4319	4336	TGCGCTGACAAGTGTCTTTCCG	TGCCGAGTGCCGTCACTTTT	311
FMgSSR-57541	scaffold4594	p3	(GAC)6	Class II	18796	18813	TTTGTAAAAGTCGCCGCTGGGG	TTCCGAGCATTCATGGTCGCCT	280
FMgSSR-57543	scaffold96	p3	(GAC)7	Class I	57633	57653	AAGCTCGCGAGGAAGAGGAA	ATGGCAAATAGAGCGAGACCCC	350
FMgSSR-57544	scaffold975	p3	(GAC)7	Class I	72414	72434	ACAGCAGTGTTGGGGCACAGAA	TCGATGGCGGAAAGCTCACT	287
FMgSSR-57545	scaffold50927	p3	(GAC)7	Class I	518	538	ACGCGTCGTCGGAGGATATT	AGCCGCCAGTTCCGTCTCATTT	290
FMgSSR-57546	scaffold6002	p3	(GAC)7	Class I	3749	3769	AGCGGCGGCTCGTCATTTTT	TGCCTTGGTTGGCTGGGTGATT	317
FMgSSR-57547	scaffold10149	p3	(GAC)7	Class I	23294	23314	GCGCGCGCAACATGTCAAAA	CATGTCCATGCAAACGGCGA	276
FMgSSR-57551	scaffold4974	p3	(GAG)10	Class I	27154	27183	AACTAACCCGAGCGGGCAGAAA	AAGCGGCGGCGTTAACGAAT	254

SSR_ID	Scaffold	SSR_Type	SSR_Motif	SSR Class	SSR_Start	SSR_End	Forward sequence	Reverse sequence	Product_size
FMgSSR-57555	scaffold31843	p3	(GAG)10	Class I	3027	3056	AGCGCCAAGGCAGCATGTTT	AGGATTGAGGTCCGTGCTGT	344
FMgSSR-57557	scaffold3514	p3	(GAG)10	Class I	28514	28543	ATGGCTTGGCCTTGGGGTTGAT	TCTCATGTAAGCGCGCATCCTC	285
FMgSSR-57561	scaffold8161	p3	(GAG)11	Class I	22776	22808	TGAGCAGCAGGCACATGACA	AAGCAGCCATCCGTGCCATT	210
FMgSSR-57564	scaffold81	p3	(GAG)12	Class I	140445	140480	AGCTGCTGCTTTTTGGGAGGA	ACCGGTGACGCTTGCCAAAT	208
FMgSSR-57565	scaffold4697	p3	(GAG)12	Class I	24248	24283	ATCTGCCTCCACCACCACGATA	AGCAACGCAATCTGCTTACGCC	342
FMgSSR-57568	scaffold5526	p3	(GAG)5	Class II	6555	6569	AAAAGGGGGCGGGCAGTAAT	AGGTTTGAAATTCCGGACGCGG	211
FMgSSR-57582	scaffold50927	p3	(GAG)5	Class II	722	736	AAATGAGACGGAACTGGCGGCT	TGTTGAAACGTGAGCGGGACCT	240
FMgSSR-57586	scaffold243344	p3	(GAG)5	Class II	318	332	AACCAACCGTGTCGCGCTTT	GCGGCATGCTCCTCATGTTCTT	350
FMgSSR-57591	scaffold62467	p3	(GAG)5	Class II	554	568	AACGCTCGATCACCTTGCCCTT	TCGCCTTGCCTTTTTGCCCA	338
FMgSSR-57592	scaffold497	p3	(GAG)5	Class II	81838	81852	AACGGTGCGTGCGTGATTCT	TGGCAGCATGTGTCTCACGACT	350
FMgSSR-57596	scaffold127740	p3	(GAG)5	Class II	582	596	AACTTGCCACTGTCACAGCCGA	ATGCGCATCTCGTCCACATCGT	227
FMgSSR-57614	scaffold5609	p3	(GAG)5	Class II	31263	31277	AAGGTCGGTGCTGAGGCAACAA	GGCGTTTTCCAAGCGCAGAA	308
FMgSSR-57616	scaffold2002	p3	(GAG)5	Class II	37878	37892	AATCCACCACGCACTAGGCACT	ACGTCCAGGAAACAAGGCACCA	340
FMgSSR-57617	scaffold2036	p3	(GAG)5	Class II	22010	22024	AATCCAGCGTGGATGCCGTT	GCGCCGCGGTGGTATAAAAT	269
FMgSSR-57618	scaffold4901	p3	(GAG)5	Class II	11072	11086	AATCGGTTCTTCGGCGATTGGG	CCGCTGGCCTACAAATGCATCA	263
FMgSSR-57627	scaffold13774	p3	(GAG)5	Class II	11247	11261	ACACAGCACCAGCAAGCAAGGA	AGCTAGCTGTGCCTTACTGCCA	347
FMgSSR-57634	scaffold61	p3	(GAG)5	Class II	123691	123705	ACACGGCGCGGTTGTTTGTA	TGCGAACCGTTGGGGTTGAT	343
FMgSSR-57636	scaffold6930	p3	(GAG)5	Class II	35963	35977	ACACTGCTCGCCCAAAACCT	ATTCGGACTCCTCGCGCTTT	317
FMgSSR-57646	scaffold1127	p3	(GAG)5	Class II	37376	37390	ACATGCATCGGCCGTGAAGCTA	TCCCCTCTATCGGTGAACTCCTGT	218
FMgSSR-57651	scaffold110022	p3	(GAG)5	Class II	1181	1195	ACCAGACGGCGTTGACGATGAT	TCGTTTGGCTCCCCTACCAAGT	289
FMgSSR-57653	scaffold694	p3	(GAG)5	Class II	32527	32541	ACCAGGACGACAGCCGAACTTT	TGTTTGTCGCGATGCAGCCT	265
FMgSSR-57656	scaffold10842	p3	(GAG)5	Class II	2446	2460	ACCCTTGCGCTCCAAACGATCA	TGCTCGCCGGAATCCAAATCGT	252
FMgSSR-57659	scaffold1286	p3	(GAG)5	Class II	9750	9764	ACCGGTCGGCTAATCGTCAGTT	TTTCGTACAGGGTTCACGACGG	298
FMgSSR-57660	scaffold6364	p3	(GAG)5	Class II	25380	25394	ACCGTGTCGTTCGAGGACTACT	ACACGCCGCAAACGAATCAA	271
FMgSSR-57661	scaffold8264	p3	(GAG)5	Class II	15625	15639	ACCTCGCTCGACAGCCTTGAAT	AGCGCTTCGTTTCTGGGCAT	289
FMgSSR-57663	scaffold265	p3	(GAG)5	Class II	37080	37094	ACGACCGAGCATGAGCACAA	AATCGCCAATCGGACTCGGTCT	332
FMgSSR-57667	scaffold257	p3	(GAG)5	Class II	126849	126863	ACGAGTACGAGGAAGCCGATGA	TACCGCTACCACCTTTCTGCGA	342
FMgSSR-57668	scaffold12345	p3	(GAG)5	Class II	14964	14978	ACGAGTGCACGGAAATTGCGA	ACGAGTCACACACAGGCCACAA	277

SSR_ID	Scaffold	SSR_Type	SSR_Motif	SSR Class	SSR_Start	SSR_End	Forward sequence	Reverse sequence	Product_size
FMgSSR-57669	scaffold22466	p3	(GAG)5	Class II	12185	12199	ACGATGATGGCGCACAGTCT	CCTCCGTCGATGAGCTCCTATT	339
FMgSSR-57670	scaffold3034	p3	(GAG)5	Class II	14721	14735	ACGATGTTGCGGTTGCCGTT	TCGTTGAGAAGCAGATGGCGCA	216
FMgSSR-57671	scaffold2816	p3	(GAG)5	Class II	25626	25640	ACGCACAATGCCTGAACGGA	TGACACAGCGGCGGAATCTT	315
FMgSSR-57674	scaffold3491	p3	(GAG)5	Class II	48129	48143	ACGCGACAACGACGACATCA	AGCAAGCGAGAGCCATGGAGAA	307
FMgSSR-57675	scaffold7932	p3	(GAG)5	Class II	29693	29707	ACGCGTCCATGAACACGATG	TTGTGCGTCTTCGTCACGGT	273
FMgSSR-57677	scaffold12804	p3	(GAG)5	Class II	11445	11459	ACGGCATCATGCGTTGTTCCCT	ACGTGTGTTCGCAACCCAAAGC	340
FMgSSR-57679	scaffold14802	p3	(GAG)5	Class II	6503	6517	ACGGTGAATGCGAACTGGGT	ATGCGGTGCTGCTGATGTGT	290
FMgSSR-57681	scaffold78	p3	(GAG)5	Class II	130172	130186	ACGGTGGATTGGTGCGACAT	AGCTGCTCGCACTCTTTCTCCA	335
FMgSSR-57683	scaffold464	p3	(GAG)5	Class II	67755	67769	ACGTCGACATCCCGATGCTGTT	TCTTCTTACACCACGGGCAGCA	250
FMgSSR-57685	scaffold5609	p3	(GAG)5	Class II	11093	11107	ACGTGGACGGCCGAGATAACAA	ACACGCTGCAGAAGGTCATGGT	343
FMgSSR-57693	scaffold10430	p3	(GAG)5	Class II	13897	13911	ACTTCCCGTGCGGGACTTTGTT	ACTCAATACCCCAGCGCCCTTT	303
FMgSSR-57697	scaffold6212	p3	(GAG)5	Class II	12311	12325	AGACGATGACGCGTTGGATG	TGTCGTGGATCATCGGCTGT	300
FMgSSR-57702	scaffold13839	p3	(GAG)5	Class II	12473	12487	AGATTAGGAAAGCCTCGTCGGC	TGCCTCCGCTAACCTCGATT	211
FMgSSR-57705	scaffold1891	p3	(GAG)5	Class II	35528	35542	AGCAAAACACCCAAGCCCGA	TGCAACACACAACACCGGCA	337
FMgSSR-57706	scaffold16499	p3	(GAG)5	Class II	13059	13073	AGCAAAGGCAGCAATGGCGA	ACGTTTGCACGGAAGTCTTGCT	258
FMgSSR-57710	scaffold880	p3	(GAG)5	Class II	51923	51937	AGCACGCGACGTGGTTGAAA	CGTGTGGTGGTGCGAATCGTAA	273
FMgSSR-57727	scaffold148	p3	(GAG)5	Class II	2224	2238	AGCGAGGTGAAGATGATGCCGT	AATGTCCTTGCCGCTCTCGT	330
FMgSSR-57729	scaffold30824	p3	(GAG)5	Class II	4262	4276	AGCGCACGTAGTTGCGAATGA	ACCCAAGCCGCAACAACAGTCA	289
FMgSSR-57731	scaffold5218	p3	(GAG)5	Class II	35458	35472	AGCGGGGCGGATGTTTTTGA	TGTCTGCAGCGAGAGCGAGTAA	327
FMgSSR-57738	scaffold2579	p3	(GAG)5	Class II	41809	41823	AGGAAGAAGGTGGAGGCAAGGT	AGCGACTAACACTAACCCGCCA	287
FMgSSR-57739	scaffold1006	p3	(GAG)5	Class II	81000	81014	AGGAAGCGAGTAGGGTCAATGGGA	ACGGGCAACCCAACAATAGGCA	215
FMgSSR-57744	scaffold15821	p3	(GAG)5	Class II	11122	11136	AGGAGGAGAAGAACGCGGTACA	TCGCCCGTCAGACACCATTTGA	310
FMgSSR-57751	scaffold8361	p3	(GAG)5	Class II	23407	23421	AGGCAGCTTGCTCTTTTCCCCA	TCAGCAGCAAGGACGACATCCA	213
FMgSSR-57753	scaffold2108	p3	(GAG)5	Class II	64386	64400	AGGCGCATAGATTTGTGGGGACA	ACCAACGGAATCACTTCCCGCT	344
FMgSSR-57754	scaffold1090	p3	(GAG)5	Class II	25158	25172	AGGCGCGTAGTTACTGGCTCTT	TCCGGCAAGAAATCCCGCTT	293
FMgSSR-57762	scaffold5758	p3	(GAG)5	Class II	36191	36205	AGGTCCAAACAAAGCGTCCGT	ATAAAGAGCGACGGCAACGGGT	345
FMgSSR-57764	scaffold37	p3	(GAG)5	Class II	97831	97845	AGTACTCGGGCCGGAGCAAAAT	GGTTTGTTGCGTGATGGCGAGT	233
FMgSSR-57773	scaffold792	p3	(GAG)5	Class II	45257	45271	ATAGGCTGGCGACGACGATGAT	TTGTTGAGCCATCGCCGGTACA	309

SSR_ID	Scaffold	SSR_Type	SSR_Motif	SSR Class	SSR_Start	SSR_End	Forward sequence	Reverse sequence	Product_size
FMgSSR-57774	scaffold3381	p3	(GAG)5	Class II	46240	46254	ATCAACCGAACGCCGAACGA	CGTCGCCGTCATCCTCCATTACAT	338
FMgSSR-57775	scaffold3435	p3	(GAG)5	Class II	51919	51933	ATCAAGCAGAGCATGCGTGGGA	TTCTCCGCCGTCTCCATCAACA	202
FMgSSR-57776	scaffold243	p3	(GAG)5	Class II	90759	90773	ATCACGAGCACGAGCAATCGGT	GTACACCGCCATTGCAGCTT	311
FMgSSR-57777	scaffold12731	p3	(GAG)5	Class II	5412	5426	ATCAGAGGCCAACAAGCAGGCA	TTGGCAGCAGGCGATTCAAGGA	212
FMgSSR-57779	scaffold22020	p3	(GAG)5	Class II	7732	7746	ATCCCAGGCAAACGATGGCA	ACGTTGAAACGCCTCACGCT	325
FMgSSR-57780	scaffold16805	p3	(GAG)5	Class II	11244	11258	ATCCCCACAGTCAATGAGGCCA	TAAGTGGCGCCGCAAGCAAA	323
FMgSSR-57783	scaffold2344	p3	(GAG)5	Class II	29471	29485	ATGAAGCTGTCGGTCTTCGGGT	TCCGATGCAATCTCGCCACCAT	311
FMgSSR-57784	scaffold3852	p3	(GAG)5	Class II	41225	41239	ATGAGAAAGGCACGGCGGAGTA	AGAGAACAGAGCAATGCCCAGC	293
FMgSSR-57793	scaffold5562	p3	(GAG)5	Class II	28468	28482	ATGGCGGCATCCTATGTTCGCT	ATTCCTCCTCTCCGCTTGAGGCTT	276
FMgSSR-57799	scaffold4052	p3	(GAG)5	Class II	38874	38888	ATGTTGTACGTCTTCACCGCCG	AAGTAGTCCCACCTCAATGGCG	348
FMgSSR-57800	scaffold2907	p3	(GAG)5	Class II	11320	11334	ATTCGACCCGAGCACGAACCTA	ACGAAGGCAGTTCTTGCAGCCT	332
FMgSSR-57802	scaffold10666	p3	(GAG)5	Class II	21252	21266	ATTCTGCCGGACCGAGAACT	GGCAACGCGCGAGTAAACGTAA	339
FMgSSR-57806	scaffold5869	p3	(GAG)5	Class II	19952	19966	CAGCAAGGCTTCGTTCTTGCCA	AATGACGTTGGCGTCGGACA	264
FMgSSR-57808	scaffold1003	p3	(GAG)5	Class II	5024	5038	CCACCGGCGAAAATCGTGCAAT	TCCCACGTTTGCATCTCGCA	237
FMgSSR-57811	scaffold304	p3	(GAG)5	Class II	114973	114987	CGCTGCCTTGTCTTGCTGTT	ACTGCCCACCTCTTCGTTTGCT	210
FMgSSR-57819	scaffold2150	p3	(GAG)5	Class II	37933	37947	GCCTGCTGCTGCTTGTTGTTGT	TTTGTTGACCGCCATCAGGC	275
FMgSSR-57823	scaffold811	p3	(GAG)5	Class II	86245	86259	GCGTCAAGGCAGAGGATGTGTT	AGCACTTCGATCCAGCCTTCGT	202
FMgSSR-57828	scaffold5789	p3	(GAG)5	Class II	30948	30962	GGCCAGCAAAAGTACGTGGT	TCCAGCACGCCTTTGACAGT	227
FMgSSR-57834	scaffold223148	p3	(GAG)5	Class II	546	560	TAATCGAAGGCCCTCGCCATCA	TGCCGTGGAGCAAGATCTGA	313
FMgSSR-57844	scaffold8372	p3	(GAG)5	Class II	16922	16936	TATCGGTGGATCATGCGAGCGT	TGTGCCCTGACGAGAATTGCCA	323
FMgSSR-57845	scaffold2472	p3	(GAG)5	Class II	63272	63286	TCAAACCAACGTGCGGCTCA	ACACTGGAAAGTGTTGCGGC	237
FMgSSR-57849	scaffold957	p3	(GAG)5	Class II	15465	15479	TCACGCTGCTCGTTCAAGTCCA	TGCTCAAAGCCGGTGATGACGA	252
FMgSSR-57855	scaffold1031	p3	(GAG)5	Class II	23416	23430	TCAGCTCCTGGTCCACAAAGCA	ACGACAGTTGCAGATCCAGGCT	310
FMgSSR-57860	scaffold296	p3	(GAG)5	Class II	113843	113857	TCCAAAGCTGCCGTGCATCA	ACGGCGAAATCTGGGTGTTGGT	275
FMgSSR-57863	scaffold4604	p3	(GAG)5	Class II	50425	50439	TCCAGAAAGTGGCGGGCAAA	TCAGTGTACCTCCTTGTCTGGGCT	267
FMgSSR-57870	scaffold296	p3	(GAG)5	Class II	80337	80351	TCCTCGGCCACTTCATCTCTCT	ATTCGCAGCGGGTCATCTCGTT	329
FMgSSR-57871	scaffold1589	p3	(GAG)5	Class II	19151	19165	TCCTGCATCGAGTCCTCCTCTT	AACATGCGCAACTTCCCGCT	239
FMgSSR-57872	scaffold154	p3	(GAG)5	Class II	124968	124982	TCCTGTACAAAACGCCGGCTGA	TTCGCGCGTGTTCTCCTCAA	283

SSR_ID	Scaffold	SSR_Type	SSR_Motif	SSR Class	SSR_Start	SSR_End	Forward sequence	Reverse sequence	Product_size
FMgSSR-57876	scaffold6533	p3	(GAG)5	Class II	20967	20981	TCGAGCAAGACCAGCTGCAA	TGGCCATGGTCGCCATAGGATT	268
FMgSSR-57878	scaffold4050	p3	(GAG)5	Class II	25843	25857	TCGCAGCTCCCAACTTGTCGTT	GCATGCTCATCTCCGGCTGAAA	240
FMgSSR-57880	scaffold1439	p3	(GAG)5	Class II	68538	68552	TCGCCAACGCGGTCTACTTCAA	ACGACCGCCTTATGCGCTAACT	334
FMgSSR-57882	scaffold3421	p3	(GAG)5	Class II	27876	27890	TCGCTCTCGCCGGCTTTTACTT	ACATGCGCTACGTGCGTTTCA	264
FMgSSR-57883	scaffold1420	p3	(GAG)5	Class II	58318	58332	TCGCTGACGAACATGACCTCGT	ACACTCTTCAAGCACACCCCACA	326
FMgSSR-57884	scaffold18901	p3	(GAG)5	Class II	9511	9525	TCGGAGAGCGAATTGCAGGCAT	AGGAAAAACACCGGATCGGGCA	245
FMgSSR-57886	scaffold16689	p3	(GAG)5	Class II	17225	17239	TCGGTTTGGCTAGGGTTCGACT	TCGCCATAACCCGTTGCTCCAT	278
FMgSSR-57887	scaffold662	p3	(GAG)5	Class II	50404	50418	TCGTAGCTCCTAACACCTCCGT	TTGAGGGTTTTGCGGCGGCTTT	334
FMgSSR-57892	scaffold2447	p3	(GAG)5	Class II	22698	22712	TCGTTGCTTCCTCGGTTCGCAA	TATCACCACCTGCTCATGCTGC	339
FMgSSR-57895	scaffold967	p3	(GAG)5	Class II	46200	46214	TCTGCTCCGTGTGAAAGAGGCA	GGACGTGTTTGATTGGTCCGCA	282
FMgSSR-57899	scaffold3103	p3	(GAG)5	Class II	28352	28366	TGAAGCGGCGGTACAAGATGGA	AACAAGTCCCCGCCAACCCTTT	228
FMgSSR-57901	scaffold1376	p3	(GAG)5	Class II	5994	6008	TGACCGGGGTTTTCTTTTCGCC	ACGGCCGTCTGATGAAGACCAA	240
FMgSSR-57905	scaffold108491	p3	(GAG)5	Class II	722	736	TGACGTTCTCTGCCACGTGCTT	TTCTCTCTCGCGCTATCATGCC	244
FMgSSR-57914	scaffold6767	p3	(GAG)5	Class II	6326	6340	TGCAGCTTCGCATGAAAGGCA	TTCGCTGCTGGATTCGCTCT	336
FMgSSR-57915	scaffold5577	p3	(GAG)5	Class II	24971	24985	TGCAGGAACGCCCTAATGACCA	ATCCTGAGACGCGTCAGCTCTT	343
FMgSSR-57916	scaffold5110	p3	(GAG)5	Class II	8740	8754	TGCAGGGACGCTTCGAGAATCA	TACCCCCTGAGTATTGCGTCCCTT	209
FMgSSR-57921	scaffold9314	p3	(GAG)5	Class II	17870	17884	TGCATGGCGCATGGAGTTGGTA	TGTGTGTGTGCGTGCGTGTT	282
FMgSSR-57922	scaffold4787	p3	(GAG)5	Class II	45436	45450	TGCATTCTGAACCAGGGCCTCA	AGCAGAAACGGACGCCACAA	242
FMgSSR-57929	scaffold100	p3	(GAG)5	Class II	111480	111494	TGCGAAGCTGTGTCTTGGGA	AGCAGAAAACGAACGGGCCA	234
FMgSSR-57932	scaffold1022	p3	(GAG)5	Class II	33122	33136	TGCGCGCACTTGGTCTTGAA	TCCTCGCCAAGCAAAACCACCT	292
FMgSSR-57934	scaffold11773	p3	(GAG)5	Class II	16212	16226	TGCGCTTCGATTTTGGGGGT	AAGCGAACAGGCGAACAGCA	279
FMgSSR-57936	scaffold17877	p3	(GAG)5	Class II	12003	12017	TGCTACATGGAGCGTGCGTT	AAGGGCAAACCCTAGGAAGCCA	249
FMgSSR-57942	scaffold34945	p3	(GAG)5	Class II	407	421	TGCTGCTGTGCTGTGCTGTT	AACTCCTCTTCTCGGAGCACCA	347
FMgSSR-57943	scaffold3245	p3	(GAG)5	Class II	4248	4262	TGCTGTGTGTGATGTCGCCACT	AGGCATGGCTTTCTCGGCAAGT	270
FMgSSR-57944	scaffold235061	p3	(GAG)5	Class II	402	416	TGCTTCCATCCTACCGAGCA	AGCAATAACAGCAGCAGCACCG	282
FMgSSR-57952	scaffold10666	p3	(GAG)5	Class II	9007	9021	TGGCAGCGAGCAAGGACAGAAA	ACGCACTTGCACGGCTTTCA	333
FMgSSR-57956	scaffold5547	p3	(GAG)5	Class II	1716	1730	TGGCTCCGGTCTGCATCTCATT	TCTTGGCTGCTCTTCTTCGGCT	255
FMgSSR-57960	scaffold5440	p3	(GAG)5	Class II	16336	16350	TGGGGGAGAACCAGAGAATGAGAG	AATATCTCTTCCCTTCCCCCACCC	230

SSR_ID	Scaffold	SSR_Type	SSR_Motif	SSR Class	SSR_Start	SSR_End	Forward sequence	Reverse sequence	Product_size
FMgSSR-57978	scaffold10862	p3	(GAG)5	Class II	17205	17219	TGTTATTCGGGAACCCGGCCAT	GCATACGTGCGTTGCGTTGA	300
FMgSSR-57991	scaffold2121	p3	(GAG)5	Class II	33582	33596	TTCAGTGGGTACAGCACGGT	ACAAGGAGTTGATGGCGGCACT	308
FMgSSR-57994	scaffold244	p3	(GAG)5	Class II	52217	52231	TTCGAGAACCTCGCGGAACA	GCTCGGCTCCGGTGTTTCAAAT	346
FMgSSR-57996	scaffold54	p3	(GAG)5	Class II	16851	16865	TTCGCCTTCGTCGATGTCCTCA	TGCGACATGTACTCCTCGTCGT	323
FMgSSR-58002	scaffold10166	p3	(GAG)5	Class II	14813	14827	TTGAGATGAAGAAGGTGGAGGCGG	AGCAGCACGCGCTTTCTCTTCA	238
FMgSSR-58013	scaffold4513	p3	(GAG)5	Class II	20647	20661	TTGTGCTGTTGGTGGTGGCT	CGTGTCGTCATCGCAACAGA	268
FMgSSR-58017	scaffold4140	p3	(GAG)5	Class II	3071	3085	TTTCCGCTGTATGCGCGTCT	TAATCTTCGCGCGGACGCAA	324
FMgSSR-58018	scaffold4829	p3	(GAG)5	Class II	8302	8316	TTTCCTCCGCTTCCACCGTCAA	AGCAGCAGAAGCTGGTCTCCTT	316
FMgSSR-58020	scaffold1953	p3	(GAG)5	Class II	39113	39127	TTTGCAACTGCGTGGTGGGT	GCCGGTCACGATGCAAACCTTA	340
FMgSSR-58031	scaffold2207	p3	(GAG)6	Class II	39166	39183	AACGCCAACCGCCAGAATCA	AGCGCATTGAGGTCTGGTCCAA	300
FMgSSR-58041	scaffold682	p3	(GAG)6	Class II	101933	101950	AAGTCACCACGAACGGCGTCTT	AACGCGAGGGCATTCGTTCA	279
FMgSSR-58044	scaffold22020	p3	(GAG)6	Class II	13251	13268	AAGTTGGAGGCGGTGAGGACAA	ATTTCATCACCATCGGCGGCCA	296
FMgSSR-58048	scaffold4917	p3	(GAG)6	Class II	21287	21304	AATGGCGTGCCCACTGTTGA	TGTTCACCGTGCTGCTGTCA	220
FMgSSR-58052	scaffold2714	p3	(GAG)6	Class II	14611	14628	ACCGGAAGCTCATGTTCCACCT	ACCGCCGGATTATGCCTTGT	253
FMgSSR-58057	scaffold137287	p3	(GAG)6	Class II	218	235	ACGCTGGAGAAGAGGAAGTCGT	AACTTCGTCTACACGCACCACG	299
FMgSSR-58065	scaffold25812	p3	(GAG)6	Class II	1709	1726	AGCAGCAAACGTGTGTCCTCTCT	TGGGCCGACATGATGACGATGA	341
FMgSSR-58076	scaffold24242	p3	(GAG)6	Class II	5178	5195	AGGCAGGCCAACTGCAAACT	ACGACAAGAAGGACGCCCAA	221
FMgSSR-58079	scaffold3828	p3	(GAG)6	Class II	10917	10934	AGGTGAAGGAGGGGATTTGCGT	TTCGAAGCGGAAGCTCCACCAA	318
FMgSSR-58080	scaffold179884	p3	(GAG)6	Class II	724	741	AGTAGAGATCGCGTCGTTCCGT	TTTCCCTCTCTCTCCTCTCTGCAC	291
FMgSSR-58091	scaffold1624	p3	(GAG)6	Class II	46031	46048	ATGCAATGAGGGAGCCACCTGT	AGCCAAACGCACGCAGCAAA	287
FMgSSR-58092	scaffold980	p3	(GAG)6	Class II	74694	74711	ATGCAGACGATGCGCTCCTTGA	AGCCCAAGCCAAAGCCAAATCC	244
FMgSSR-58093	scaffold16512	p3	(GAG)6	Class II	16745	16762	ATGCAGTTGATGAGGTGCGCGT	AAGGCGAAATCGCCGAACCA	230
FMgSSR-58101	scaffold201	p3	(GAG)6	Class II	7420	7437	ATGTCGAACTCGAAGTCGTCGG	TCCATATAGGCCACAACGCGCA	257
FMgSSR-58104	scaffold148	p3	(GAG)6	Class II	39256	39273	ATTCCGAGGAGGATTTGCCTCCAG	GCTGCACTGCTCGTCAACAACA	292
FMgSSR-58105	scaffold657	p3	(GAG)6	Class II	46376	46393	CACGGAATTCGAAACGACGCCA	ACCATCCAAGCGTCCTTCCT	296
FMgSSR-58108	scaffold15088	p3	(GAG)6	Class II	5350	5367	CCTCGGTGCTTGCGCTTAACAT	GCAATGTGGAACACTGCAAGCCT	345
FMgSSR-58109	scaffold22906	p3	(GAG)6	Class II	3285	3302	CCTGCATCTGCATTTGTGGGCT	CGCACGGGTGGTGCTTGATTTT	309
FMgSSR-58115	scaffold354	p3	(GAG)6	Class II	22205	22222	GCGTGAAGCAATAGGCGTCGTT	TTCGTGGTGGAGTGACGATGCT	306

SSR_ID	Scaffold	SSR_Type	SSR_Motif	SSR Class	SSR_Start	SSR_End	Forward sequence	Reverse sequence	Product_size
FMgSSR-58116	scaffold320	p3	(GAG)6	Class II	10986	11003	GCTCGATTTGCGGCTTCGAT	ACGCTTGGCCACACACTTTCT	350
FMgSSR-58117	scaffold1109	p3	(GAG)6	Class II	39624	39641	GGCTTACGACGTGGAAAAGCGT	AAACGGCAATTAACCGGCGTCC	276
FMgSSR-58119	scaffold1179	p3	(GAG)6	Class II	21163	21180	GTGTACCCGCAGCTGTTGTTGT	ATGAGCAGCAGCAAGTCCGT	332
FMgSSR-58132	scaffold26685	p3	(GAG)6	Class II	2471	2488	TCCGGCAAGCTTGAGTGCTT	ACGCAACCATCCCACCTCTT	339
FMgSSR-58138	scaffold65994	p3	(GAG)6	Class II	2366	2383	TCGCAGCAGATGGGGAAACGAA	AGCGGCGGAGCAAAAGAAAAGC	230
FMgSSR-58139	scaffold1508	p3	(GAG)6	Class II	41414	41431	TCGCTACAAATACCCCGCGCAA	TGGACGACGAGATCGCCTTCTT	313
FMgSSR-58143	scaffold113598	p3	(GAG)6	Class II	81	98	TCGTCACGACAGGACCCATT	AGTGCGCAAGTTGCCGTCTT	327
FMgSSR-58146	scaffold7126	p3	(GAG)6	Class II	35530	35547	TCTTCGCGGAAGACGATGACGA	AAGAGGATCAGGACGAAGGCGA	201
FMgSSR-58151	scaffold27935	p3	(GAG)6	Class II	1916	1933	TGATTTGGCTCGGCTGTGCT	ACGATGCCATTAAAGCCCGACG	259
FMgSSR-58154	scaffold977	p3	(GAG)6	Class II	55017	55034	TGCAGAAATACCGGCTGCACAC	GCCTCACTAGCTGTTCACGACGAA	295
FMgSSR-58159	scaffold31389	p3	(GAG)6	Class II	5586	5603	TGGAATTGGAAAGGGCGGGA	TTGCGGGTTGATTCGTCTCCCT	272
FMgSSR-58161	scaffold243	p3	(GAG)6	Class II	73932	73949	TGGCAGAGGAACGAATAGGCGA	TCGTGGTGGAATTACCCCTGCT	315
FMgSSR-58163	scaffold3564	p3	(GAG)6	Class II	27227	27244	TGGCTTGCGAATCTCGTCGT	TTCCCCTTTCTTGCTCTCGCCT	246
FMgSSR-58165	scaffold119208	p3	(GAG)6	Class II	613	630	TGGTGATCAGCAACAACCGCTG	TCCACCGCTTCGCTTTCAGCTA	240
FMgSSR-58166	scaffold17	p3	(GAG)6	Class II	189101	189118	TGGTGATGCTGATGAGGCGT	ACGCGGACTACGAAACTGGA	244
FMgSSR-58169	scaffold2855	p3	(GAG)6	Class II	377	394	TGTCGCTGGCCTGGCTTATT	TTGCGCTACGATTGGGGCAAGT	329
FMgSSR-58179	scaffold20290	p3	(GAG)6	Class II	45	62	TTCTTAGCGCTGGGAGATTTGGGG	TTACTCCGGAGCTCTGCGACAT	202
FMgSSR-58197	scaffold12	p3	(GAG)7	Class I	234510	234530	ACCACCAAGAACAGCAGCAGCA	TCCATGACGCTGCACACCTTCT	349
FMgSSR-58201	scaffold1331	p3	(GAG)7	Class I	15719	15739	ACGGCGGCGACTGTTGAAAA	TTCAAAGCCAAATGCGCCGACC	291
FMgSSR-58204	scaffold2410	p3	(GAG)7	Class I	38235	38255	AGAAACGCGTACGCTGCGAT	TCCGGAAAGACGTCGCTGTTGA	292
FMgSSR-58211	scaffold25440	p3	(GAG)7	Class I	4296	4316	AGGACCAACCAAACATGCGACT	TCCAAGCCATTGAGGTCCACTCAG	234
FMgSSR-58216	scaffold578	p3	(GAG)7	Class I	71141	71161	AGTTGCGGGAGGATGCAAGTGA	TGCCCTTAGCGAACAGACCA	271
FMgSSR-58217	scaffold10410	p3	(GAG)7	Class I	22816	22836	ATCAACACTGAGACCACGCCGA	TCAAAACGGCAGCAGCAGCA	238
FMgSSR-58226	scaffold3733	p3	(GAG)7	Class I	27643	27663	GCTGGCTTGCTCGAAAGGAGAA	TCAACAGCCTGCGCTGCAAA	350
FMgSSR-58235	scaffold18001	p3	(GAG)7	Class I	11148	11168	TCCAGATCGTCTGCTTCGAGGT	TTGTCCTGTCGCTTGCTCCGAT	332
FMgSSR-58236	scaffold16168	p3	(GAG)7	Class I	15806	15826	TCCATCCATGTGGCTTGCGA	TTTTGATCCACCGCGAGGGT	330
FMgSSR-58241	scaffold1428	p3	(GAG)7	Class I	6747	6767	TCCGTCCGGGGTTATTATGCAG	ATGCAGCCGACGCTTGCTTT	347
FMgSSR-58244	scaffold7851	p3	(GAG)7	Class I	19773	19793	TCGACCACGTCAGTTAGGCGAT	ACGACGCCCATTGTTGCCTT	308

SSR_ID	Scaffold	SSR_Type	SSR_Motif	SSR Class	SSR_Start	SSR_End	Forward sequence	Reverse sequence	Product_size
FMgSSR-58246	scaffold20726	p3	(GAG)7	Class I	12858	12878	TCGTCCTCTTCTGCACCAGCAT	TGGAGCAACAACGGCCAACA	326
FMgSSR-58249	scaffold19536	p3	(GAG)7	Class I	6958	6978	TCGTGGCGCACCAAGGAAGAAA	TCGTGCATGCGTTCCACGTA	269
FMgSSR-58257	scaffold5126	p3	(GAG)7	Class I	15101	15121	TGGTTTGCACACAGACGCGA	TCACCCCAAACTGCACGGATCT	320
FMgSSR-58264	scaffold3111	p3	(GAG)8	Class I	36287	36310	AAAGGATCGCATGATGGCCGCT	AAAAGGCAGGCAGGTTGTGTGG	300
FMgSSR-58265	scaffold98	p3	(GAG)8	Class I	148538	148561	AACAGGTGGGAGGACAACCAGA	TTCCCTCCTGAATGCTGCCTCT	326
FMgSSR-58266	scaffold35639	p3	(GAG)8	Class I	1305	1328	AACATCCCGCCACACACACCAT	AGAAGGCCAGAAGCCCAGAACT	269
FMgSSR-58273	scaffold15755	p3	(GAG)8	Class I	14153	14176	ATGTCCAGTGGTGGTGGAGGAA	AGTTGCTCGCTAACCCGCAT	330
FMgSSR-58274	scaffold1718	p3	(GAG)8	Class I	634	657	CACCGACCACGACGAATCATCA	AGTAGCAGTGAAGGCACCACCT	214
FMgSSR-58276	scaffold9687	p3	(GAG)8	Class I	5854	5877	GCGAATCCAAGAAGCCGCACAT	ATGCAGTTGGGAGAGTTGGCGT	302
FMgSSR-58277	scaffold10847	p3	(GAG)8	Class I	23195	23218	GCGGCGGAAACGAATGGAAT	TCATTTCGCCGCGTCTTCGT	277
FMgSSR-58280	scaffold4006	p3	(GAG)8	Class I	6141	6164	TAGCGACATGTTGCAGGTGTGC	ATAAAGCCGGTGCAGATAGGTGGG	237
FMgSSR-58281	scaffold15754	p3	(GAG)8	Class I	16144	16167	TATGGCGTCGGAGATTGCGCTT	AGCTACAGATTCAGCAGCGCCT	217
FMgSSR-58282	scaffold22389	p3	(GAG)8	Class I	9892	9915	TCAAAGTGGAGGTGGCGCTT	ACCCGCCGCTCACAACAAAT	271
FMgSSR-58283	scaffold4789	p3	(GAG)8	Class I	26919	26942	TCAATGGAGGAGGAGGAGGCGTTA	TCCTCTTCGAGGTCCCTTCCAT	236
FMgSSR-58285	scaffold36981	p3	(GAG)8	Class I	3862	3885	TGCAGTGGCACTTGAACCCA	CCGCAATCACCACTTCGCAT	276
FMgSSR-58286	scaffold14082	p3	(GAG)8	Class I	2936	2959	TGGCCGCTCCCCTTGATTAGAA	ACGACGAGGACACAAAAGCGGA	262
FMgSSR-58287	scaffold3922	p3	(GAG)8	Class I	27480	27503	TGGCGTTTTGCTCTCGACCCAT	ACAACACCGCACCACATGGA	349
FMgSSR-58290	scaffold2778	p3	(GAG)8	Class I	58002	58025	TTTCAACGCTGGCACCGGAA	TCGGATTGCAATTGGCCCGA	341
FMgSSR-58291	scaffold691	p3	(GAG)9	Class I	19843	19869	AAAAGGTCGACGCAGACGGT	ATCGGAAGGCCGACTGGATCTT	219
FMgSSR-58293	scaffold4187	p3	(GAG)9	Class I	46708	46734	AAGTTCGCAAGGCAAACGGC	AACGCGTCCTTGTTCGAAAGCC	296
FMgSSR-58295	scaffold9153	p3	(GAG)9	Class I	5446	5472	AATTTGCGCAGCATCGGACG	AGGCAGAAGAAGCTGCTGGCAA	265
FMgSSR-58304	scaffold322	p3	(GAG)9	Class I	26459	26485	GCGCGCGAGCATTCAGAAAT	TTGCTTCACGACAGCAGCTCCA	288
FMgSSR-58352	scaffold253151	p3	(GAT)5	Class II	171	185	AACAAGAGCACGATGAGCGCGA	AGCCGTTTCCCAGGAAGGTCAA	243
FMgSSR-58359	scaffold1318	p3	(GAT)5	Class II	62453	62467	AACGACAAGGACGCCACCCATT	ATCATTCCTTGGTGGCGGTGGT	321
FMgSSR-58379	scaffold1206	p3	(GAT)5	Class II	45264	45278	ACAAAAGATCTCGCTCCGGGGT	TCCCGCTCCGAACACAACAA	214
FMgSSR-58380	scaffold1388	p3	(GAT)5	Class II	62615	62629	ACAAAGAGAGCTCCAAGCACCA	AGCACTAACACCAACCCCGA	274
FMgSSR-58417	scaffold1794	p3	(GAT)5	Class II	41225	41239	ACCGCCAGACAATTGAGGCA	AATAGTGAAGAAGCGGCGGGCA	328
FMgSSR-58422	scaffold2271	p3	(GAT)5	Class II	44004	44018	ACGACGAAGGGCATCGATGGTT	ACAGCACGTCTTCTTCGACAGC	278

SSR_ID	Scaffold	SSR_Type	SSR_Motif	SSR Class	SSR_Start	SSR_End	Forward sequence	Reverse sequence	Product_size
FMgSSR-58427	scaffold9017	p3	(GAT)5	Class II	21188	21202	ACGCAGCACAAGAAGCAGCA	TTGGTTGACGCAGCCGACAT	306
FMgSSR-58429	scaffold725	p3	(GAT)5	Class II	48614	48628	ACGCTCCTGCACTTGCCATGAT	ACCTCAAGAAGCAGCTCAAGCC	235
FMgSSR-58463	scaffold4680	p3	(GAT)5	Class II	25991	26005	AGCAACGTCGCCACACCAAGAA	ATTGAGAGGTCCGCACGCAA	267
FMgSSR-58478	scaffold1296	p3	(GAT)5	Class II	39882	39896	AGCCGCACAGAGCAGTCAACTT	CGTTTTGAAAAGAGCCCGCAGT	241
FMgSSR-58482	scaffold12088	p3	(GAT)5	Class II	10528	10542	AGCGCGGCAGGTCTTTTGTT	TGGCTTTGTCCCCAACTTCCT	210
FMgSSR-58483	scaffold12781	p3	(GAT)5	Class II	4599	4613	AGCGCGTGTTGTATCCGCTGTA	AACCCCAAGTTGCGTTGCGA	329
FMgSSR-58494	scaffold3696	p3	(GAT)5	Class II	46524	46538	AGGAAAGCGTCGGTGCAAGT	TCTTCGCATGCGTCAATTGGGT	296
FMgSSR-58510	scaffold2164	p3	(GAT)5	Class II	68249	68263	AGGTGCCGATGCCTTGTGTT	TACGTGTTTCACAACGCGCC	238
FMgSSR-58512	scaffold25261	p3	(GAT)5	Class II	6311	6325	AGTGCACCTGTCTGAGGAGGAA	ACGTGGGAAAGTGGGACATGACA	338
FMgSSR-58519	scaffold8540	p3	(GAT)5	Class II	11780	11794	AGTTGATGAGGAGGGCTCGTGT	TCAGCTTGCGCACACTTTGCT	327
FMgSSR-58520	scaffold5769	p3	(GAT)5	Class II	11335	11349	ATAAGCGGCGCAACCGATGA	AAGCGCGGATGTCACGTACCTT	318
FMgSSR-58521	scaffold811	p3	(GAT)5	Class II	79648	79662	ATCAAGTCGTTGGCGTGCTCGT	AGCAGCAGCATCCTCTGTTTCCA	225
FMgSSR-58528	scaffold20256	p3	(GAT)5	Class II	8899	8913	ATGCGGTCTTCGCCACAAGA	AGGTGACGTTGTCTGCTGCT	212
FMgSSR-58534	scaffold2778	p3	(GAT)5	Class II	57142	57156	ATTACCAGACGGCCCTCAGACT	TGCTTTTGTGTTGGCTTCCCCA	278
FMgSSR-58535	scaffold3435	p3	(GAT)5	Class II	38725	38739	ATTCAAGATTGGCGCCTCCCCT	TCCGCAGGCTCTTGTTGTGTGA	260
FMgSSR-58542	scaffold3245	p3	(GAT)5	Class II	6345	6359	CCCTAGGGTGGTATTTGAGGTGGT	ACACTTAGGCAATGAGATGGCACC	331
FMgSSR-58548	scaffold25361	p3	(GAT)5	Class II	2008	2022	CGGATCAGATCGCCAATCCGACAT	AGCGTGAAGGCAGCCGAAAT	330
FMgSSR-58566	scaffold13701	p3	(GAT)5	Class II	19582	19596	GCAGCGCAGAGCAAGTTTCGTT	AGCAATGGGCGCATGGGAAA	307
FMgSSR-58575	scaffold4084	p3	(GAT)5	Class II	41620	41634	GCGCAATCGCACATGCAGAA	ACCTGAAAACGATGCGGGGTGT	345
FMgSSR-58595	scaffold6417	p3	(GAT)5	Class II	16359	16373	GGCTGTTGCATCGTGGACAACT	TGCCGAATTTTTCGGTGGGCTG	336
FMgSSR-58605	scaffold8894	p3	(GAT)5	Class II	4065	4079	TAAACGGCAGCACCGTGGTAGT	TTCATGGCCGCAATCTGCCT	343
FMgSSR-58612	scaffold622	p3	(GAT)5	Class II	63406	63420	TAGGGGAGCGTGAAGAGGAGTT	TGCTGCCGGACGAGATCTTGAA	292
FMgSSR-58631	scaffold797	p3	(GAT)5	Class II	11587	11601	TCACCACATGACTGCATCCCCA	AAGCTGGGGCATCGTTTGTTCG	296
FMgSSR-58678	scaffold342694	p3	(GAT)5	Class II	61	75	TCGCTGGGCATGATGGTCTT	TGGCCGAAGTCGTTGTCGTT	323
FMgSSR-58726	scaffold4058	p3	(GAT)5	Class II	38428	38442	TGATGAGCAAACGCACCGCT	AGTCGTACAACTTCGCCGGCAT	306
FMgSSR-58755	scaffold5035	p3	(GAT)5	Class II	39840	39854	TGCCTCAGCGGAAAACGTGA	ATGAACGCGCATGTGTCGGTTG	292
FMgSSR-58798	scaffold28341	p3	(GAT)5	Class II	8153	8167	TGCTGCGTGGTTACCAGCTT	ACCAAGGCTCTTAGCCGTCACT	306
FMgSSR-58802	scaffold3830	p3	(GAT)5	Class II	23928	23942	TGCTTGTGCATGGAGCGTGCTA	GAGGACACAGAACCCTCAAGAATG	303

SSR_ID	Scaffold	SSR_Type	SSR_Motif	SSR Class	SSR_Start	SSR_End	Forward sequence	Reverse sequence	Product_size
FMgSSR-58803	scaffold1897	p3	(GAT)5	Class II	51949	51963	TGCTTTGCTCCTGTTGCGCT	TCATTCGCCAACGGAAGCCTCA	286
FMgSSR-58804	scaffold203	p3	(GAT)5	Class II	87204	87218	TGGAAGTAGAAGACGACGGGGA	AGCAAGAAATGGCCACCTCCGA	307
FMgSSR-58823	scaffold450779	p3	(GAT)5	Class II	46	60	TGTCTATGGCAAATGCAGGCG	TAACAACGAGTCCACGGCCACA	224
FMgSSR-58824	scaffold35249	p3	(GAT)5	Class II	3417	3431	TGTGCAACACCATGTACGGACG	TTTGGCACTCGGCAAAGCCA	322
FMgSSR-58846	scaffold64	p3	(GAT)5	Class II	174055	174069	TTGAGCAGCATGTACGGAGGCA	AAATGCGCTGAGCCGTTTTCCC	262
FMgSSR-58859	scaffold803	p3	(GAT)5	Class II	58916	58930	TTGGCTGCAACACGGCTTCCTA	CAGTGCAAACGGCTGCTGAT	320
FMgSSR-58881	scaffold15	p3	(GAT)6	Class II	216785	216802	ACAAGGGGCAAAAAGGCGCT	AGTCTCGCAACAGTGGATGGCT	275
FMgSSR-58887	scaffold3837	p3	(GAT)6	Class II	5268	5285	ACCCAACAATGAGCTGGGCCTT	TTGCTGAGCACCGCCTTGTA	283
FMgSSR-58893	scaffold36745	p3	(GAT)6	Class II	4873	4890	ACGCGTCTGCTCAAATGGAGTG	ACCATCTGTTGGTGGCGGTA	341
FMgSSR-58902	scaffold298	p3	(GAT)6	Class II	70504	70521	AGCAACGGATGGAGCTGAGCAA	GCGCATCATCATAACGCGGCAA	215
FMgSSR-58912	scaffold347	p3	(GAT)6	Class II	122505	122522	AGGCCGGCAATTACCTGTCGTA	TACAGAGCCGCCCACGAAAACA	280
FMgSSR-58922	scaffold18635	p3	(GAT)6	Class II	9095	9112	AGTCGTCAATGGGCGCTAGCTT	ACTGTGGAGGTGATCATGGCGT	265
FMgSSR-58926	scaffold5734	p3	(GAT)6	Class II	15228	15245	AGTTGCCGAGTGTGGCACGTAT	AGCGTTGCAACAAGGGATGGGT	325
FMgSSR-58930	scaffold2687	p3	(GAT)6	Class II	49160	49177	ATCACACACGGCATCCTCCAGA	TGCTACACCGCATCAGGCCTTT	341
FMgSSR-58937	scaffold210734	p3	(GAT)6	Class II	152	169	CAGTGACAGCGACAACGATAACGA	TGCACACAATTAGGTCCGAGCCA	330
FMgSSR-58947	scaffold9692	p3	(GAT)6	Class II	16679	16696	GCCGTCGGGTTTGGTTTGGTTT	ACATGCGTTCAGGCGAACAACG	337
FMgSSR-58950	scaffold2796	p3	(GAT)6	Class II	28439	28456	GCGCTAGGTACCATGCACTTGA	TTCCTCCTCTGTCATTGGCGCA	338
FMgSSR-58972	scaffold3991	p3	(GAT)6	Class II	28723	28740	TCAGGCAGCGGATAAAGACTCGT	AGCTGTCGAGAGATGCCAAGGT	290
FMgSSR-58977	scaffold1522	p3	(GAT)6	Class II	45019	45036	TCGACTGCTCCAGGGTCAGCTAAA	TCGCACGAACACACACACTGA	232
FMgSSR-59004	scaffold1677	p3	(GAT)6	Class II	62566	62583	TGCCGGCCGTTCTCTCAAAACT	AACCGATCGACGAGAGGCGTTA	246
FMgSSR-59033	scaffold793	p3	(GAT)6	Class II	8517	8534	TGTCCTGCCAACAAGATGCGGT	TGACGCCTTTCCACGCTGTA	227
FMgSSR-59061	scaffold2355	p3	(GAT)7	Class I	11035	11055	AGGTGGCAACGCAACACCTT	ATCACCTTCTGCCCTGCGACTT	304
FMgSSR-59065	scaffold186820	p3	(GAT)7	Class I	677	697	ATGCGTGCTTTGGGGGACAA	TCATCGGTGCAGAAGACGCA	316
FMgSSR-59076	scaffold631	p3	(GAT)7	Class I	8187	8207	TCGCGAGATGCGATGACGATGA	AGCTCCTCAAACCCGCAACACA	347
FMgSSR-59101	scaffold128094	p3	(GAT)8	Class I	644	667	AATTGCGGCTGTGCCAGAGGAA	ACCACGCTGTAAGCAGTGTGTCA	304
FMgSSR-59105	scaffold2992	p3	(GAT)8	Class I	43339	43362	AGCAAGCCCAGCTGAACACCAT	TTCTTCGACAGCTACCACCCCA	334
FMgSSR-59114	scaffold1345	p3	(GAT)8	Class I	22178	22201	CACATGCGCAACCGCTCAAA	CAGCCTGCACACAGTTTTCGCA	205
FMgSSR-59118	scaffold541	p3	(GAT)8	Class I	85278	85301	TCATGTCCTGCAGCTCGTTGGT	TCGGTGACTTCTTCCCTGCTGA	345

SSR_ID	Scaffold	SSR_Type	SSR_Motif	SSR Class	SSR_Start	SSR_End	Forward sequence	Reverse sequence	Product_size
FMgSSR-59122	scaffold37657	p3	(GAT)8	Class I	899	922	TCCACGTCGAACCCGACTGATT	ATCGTCGGCACACGATTCCA	296
FMgSSR-59153	scaffold758	p3	(GCA)10	Class I	32618	32647	ATGCTCGCAAGTCGCAGTGA	TTGGAGTCGATGAACACCGCCA	287
FMgSSR-59154	scaffold68748	p3	(GCA)11	Class I	2206	2238	AACAAATGACCAGCCGGAGCAG	ATCACCGTCACGTTGGTGTGCT	244
FMgSSR-59155	scaffold1157	p3	(GCA)5	Class II	41568	41582	AAAATCATCCGACGTGGCCGAG	TGGATGCGGGTCATCTGCAA	307
FMgSSR-59156	scaffold4068	p3	(GCA)5	Class II	26481	26495	AAAATGGCAGAGGCAACGGTAGGG	AACGTGTGGCAGCGTTGCTT	316
FMgSSR-59159	scaffold713	p3	(GCA)5	Class II	48007	48021	AAAGGCAGCGATTGACCGGGAA	TTGCGGCAGCGGCTGAAATA	316
FMgSSR-59162	scaffold5503	p3	(GCA)5	Class II	6184	6198	AACACGGTGCCTCCATCGATCA	GGAAGAGGTTGTTGCCTGTGGT	312
FMgSSR-59163	scaffold24065	p3	(GCA)5	Class II	5141	5155	AACAGGCTGAAGCCCATGTCGT	CTCTGCTGGTTCATAGCATGACTG	244
FMgSSR-59165	scaffold5209	p3	(GCA)5	Class II	29657	29671	AACGAAGTGCCGTAGGCTGT	TCGACGCTCCGGTTGAAACA	347
FMgSSR-59169	scaffold67	p3	(GCA)5	Class II	50004	50018	AACGTCACCAACGCCTTCGTCT	TCGAGTTTGCGCATTACCCCT	325
FMgSSR-59170	scaffold2878	p3	(GCA)5	Class II	50908	50922	AACGTGTACGCGCCCGATAA	TGTTGGGTCATCAGGTGCCA	313
FMgSSR-59175	scaffold27	p3	(GCA)5	Class II	181356	181370	AAGATGTCGAACCGCACGCA	CGCCGCTCGGTGGTTAATTTGT	290
FMgSSR-59194	scaffold17469	p3	(GCA)5	Class II	1626	1640	ACAACCACAGCGACAGCGAT	TGCTGCTGCTGCGAGCTAAGAT	221
FMgSSR-59195	scaffold125307	p3	(GCA)5	Class II	840	854	ACAAGGCCATGACTGCGAGGAA	ACCACTCCCAACCAACGGCAAA	234
FMgSSR-59197	scaffold36403	p3	(GCA)5	Class II	1203	1217	ACACGGCACATCACAGCCAA	ATCGCTCGCATGTCGCGTTA	224
FMgSSR-59198	scaffold10220	p3	(GCA)5	Class II	14363	14377	ACACTGCAAGGGTGTGTGCT	AGTCCCAGCTCGTGTTGACCTT	277
FMgSSR-59204	scaffold473	p3	(GCA)5	Class II	76328	76342	ACCAAAACGTTCGTCCGCGT	TTGCTTTTCTGCGACGAGCTGC	245
FMgSSR-59211	scaffold568	p3	(GCA)5	Class II	88737	88751	ACGACGGTTCGACATCGACAA	GGCGTGCCAATTGGAAAACGGA	214
FMgSSR-59212	scaffold11280	p3	(GCA)5	Class II	22480	22494	ACGCACACGTCCTTTTGCACTG	TCCATCATTTCCGTGCCGCTCA	338
FMgSSR-59214	scaffold7275	p3	(GCA)5	Class II	32065	32079	ACGCCGGAGGGACATTGAGATT	TATCAACGGCTGCCGACCATCA	332
FMgSSR-59215	scaffold30669	p3	(GCA)5	Class II	7077	7091	ACGCCTGCTGATAAAAGGGCGA	ACGGTGCCGCTGTTTGTCTT	327
FMgSSR-59216	scaffold6445	p3	(GCA)5	Class II	5566	5580	ACGCGGCTCGTGCAAAAACA	AGGGCCAGTCTTGGTATGGAAGGA	233
FMgSSR-59218	scaffold11565	p3	(GCA)5	Class II	10916	10930	ACGGCCAAGACGCTCATTGT	AGAAATTCGTGCGAGCGCCT	346
FMgSSR-59221	scaffold2858	p3	(GCA)5	Class II	33750	33764	ACGGGCATATGCTGCACACGTA	TCATGCTGACTGTATGGCCGCA	329
FMgSSR-59224	scaffold2928	p3	(GCA)5	Class II	23212	23226	ACGTTGCCCACTTCACCACCAA	AGGCAGTAGTCGTAGCCGATGT	257
FMgSSR-59228	scaffold102599	p3	(GCA)5	Class II	1179	1193	ACTCTCGTGCTGGTCATTGCGA	TTGGGCAGGTTGCAGACGTT	296
FMgSSR-59230	scaffold5622	p3	(GCA)5	Class II	30591	30605	ACTGCGAGCGCTGGAAAAGA	TCCGCATTCAAGTCGCAGCA	329
FMgSSR-59231	scaffold274	p3	(GCA)5	Class II	133625	133639	AGAAAAGCTTAAAAGGGCGCGG	TCAGCGCTCGTGTCGTTCTT	325

SSR_ID	Scaffold	SSR_Type	SSR_Motif	SSR Class	SSR_Start	SSR_End	Forward sequence	Reverse sequence	Product_size
FMgSSR-59232	scaffold10231	p3	(GCA)5	Class II	19384	19398	AGAAACGACGTGCGACGGAA	TAGCGACTTGCGAGCCATTGGT	236
FMgSSR-59233	scaffold30	p3	(GCA)5	Class II	102980	102994	AGACGGGGCAGGTGATGATGTT	TGCCATCCGAGAAGGGCAAT	224
FMgSSR-59237	scaffold11506	p3	(GCA)5	Class II	16852	16866	AGCACAGCAGCAATCAGAGGCA	ACTGGCGCTGGTAAACGACA	348
FMgSSR-59243	scaffold5016	p3	(GCA)5	Class II	21318	21332	AGCAGCCTTGGAGCAAACCAGT	ACAGGACGCAGGCTGCAATGAT	267
FMgSSR-59247	scaffold4389	p3	(GCA)5	Class II	26287	26301	AGCGAGACGCGAAACATCCA	TCGTCAGGGTAGTTGACAGTAGCG	295
FMgSSR-59248	scaffold3922	p3	(GCA)5	Class II	25970	25984	AGCGAGCGACAGTGCAGTTGTA	TTTTAGCATGCCGCTCGATGCC	324
FMgSSR-59250	scaffold11342	p3	(GCA)5	Class II	23452	23466	AGCGCAAGGCTTCCTGCAAT	TGCAGGCGTCGATCTTACTCTTGG	335
FMgSSR-59261	scaffold868	p3	(GCA)5	Class II	27010	27024	AGGCTCAAGCCAAACCCTTCCT	TTCTTGCCCTCGATCTTGCCGT	269
FMgSSR-59262	scaffold306689	p3	(GCA)5	Class II	279	293	AGGCTGTTGGCTCCGTACATGA	ACCAACTACGACCGCAAGCACA	320
FMgSSR-59263	scaffold120410	p3	(GCA)5	Class II	1197	1211	AGGGGAAGATCGCGCCTAAACA	ACCGCTCACCCATTTGCTTGGT	251
FMgSSR-59278	scaffold8247	p3	(GCA)5	Class II	25578	25592	ATCTGGCAGTTGAACTCCGCGT	ACGGTCCATTGAGCAGTGGCAT	350
FMgSSR-59281	scaffold5945	p3	(GCA)5	Class II	264	278	ATGGTGCTTGCACTGCTCGT	ACGCGGTTGAAAAACGTCGCT	228
FMgSSR-59288	scaffold11103	p3	(GCA)5	Class II	8191	8205	ATTGCGCGGATTGCTTCGGT	AGCAGGTCGAACCGCATGTA	256
FMgSSR-59289	scaffold24942	p3	(GCA)5	Class II	10567	10581	ATTGCGCGGCAAAGTCGTCA	TGGTGGCCATGAACGAGAGGTA	214
FMgSSR-59291	scaffold452112	p3	(GCA)5	Class II	112	126	CGATGATCACCGGCATCGACAT	TTTCCAGCTACCTGGTGCGCTT	207
FMgSSR-59295	scaffold5460	p3	(GCA)5	Class II	20715	20729	CGGCGACGAAAAAGGCTTTGCT	ATTCGGGAGATGCGTGCAGA	313
FMgSSR-59296	scaffold158	p3	(GCA)5	Class II	121418	121432	CGGCGGCAGAAGGAGCAAAATA	AAAAGAAGCGAGCGGAAAGCCG	220
FMgSSR-59297	scaffold2722	p3	(GCA)5	Class II	15413	15427	CGTCGGTCATTCAGCTGAGTCCAT	TCATGAAGCTGCCGTCGTTCCT	348
FMgSSR-59298	scaffold149525	p3	(GCA)5	Class II	620	634	CGTGCATCGGCCATCATCAACA	ACAGCCATGGTGGCTATGCT	311
FMgSSR-59302	scaffold517	p3	(GCA)5	Class II	42329	42343	GCCAAAATCCCGTCGCAGCAAT	TGGTGCAGATGCAGCGTTGA	328
FMgSSR-59305	scaffold3764	p3	(GCA)5	Class II	35523	35537	GCGCGCCATCAAGTCAATCGAA	GCGTGGTGGCGTGGTGATTTTT	294
FMgSSR-59307	scaffold28656	p3	(GCA)5	Class II	5536	5550	GCGTCGCGTTGACCATTGAA	CCCTCCCTCTTGCATATGAACCCA	202
FMgSSR-59314	scaffold42232	p3	(GCA)5	Class II	1011	1025	TAACGCGCATCTCCACAGCA	CGGGTGAACACCGCAACAACAA	215
FMgSSR-59319	scaffold194	p3	(GCA)5	Class II	111273	111287	TCACGGTCAAATGCTGGCCACT	AGGTGCTCTGCACAAAGGGCAT	257
FMgSSR-59330	scaffold292232	p3	(GCA)5	Class II	117	131	TCCGCCGACTTCCCAAACGAAA	ATGGGCACGGGAACAAAGGAGA	201
FMgSSR-59331	scaffold42468	p3	(GCA)5	Class II	1526	1540	TCCGCGTACATCACCACGTCTT	AGCCGAAGCGAACAAACGCA	342
FMgSSR-59332	scaffold36884	p3	(GCA)5	Class II	826	840	TCCGGGCAACGCCATTGATT	ATACGGCACCACGACCACATGA	263
FMgSSR-59335	scaffold13973	p3	(GCA)5	Class II	19535	19549	TCCTCTTCCAAGGCGCAACA	TTCCAAGTCCGGCAGGTTGCTT	236

SSR_ID	Scaffold	SSR_Type	SSR_Motif	SSR Class	SSR_Start	SSR_End	Forward sequence	Reverse sequence	Product_size
FMgSSR-59346	scaffold3974	p3	(GCA)5	Class II	25329	25343	TCGTGCGTACCTTTCCTTTGGCT	AGAGCATGTGCGGTTGCAGT	305
FMgSSR-59352	scaffold26404	p3	(GCA)5	Class II	648	662	TGACACACCCAGATGTTCGGCA	GCGAAGGCCGCATTCCCAAAAA	320
FMgSSR-59354	scaffold12564	p3	(GCA)5	Class II	3566	3580	TGATGCAAAGGAACGACGGGGT	ACGTACGCGCAAGTCAATCGGA	339
FMgSSR-59355	scaffold1600	p3	(GCA)5	Class II	29103	29117	TGATGCGTCGTGTCTACAGGGT	AGAGCCGGTGGATCAAAGCAGT	346
FMgSSR-59362	scaffold2387	p3	(GCA)5	Class II	10860	10874	TGCACACACTCGCACTGCTT	ACGCAGCTCCAACACGGAAA	201
FMgSSR-59363	scaffold1475	p3	(GCA)5	Class II	1276	1290	TGCACAGTCCCGGCTGATTT	ACAGCGGCATGCTGGACTTT	289
FMgSSR-59369	scaffold686	p3	(GCA)5	Class II	40614	40628	TGCCCATGCAAATCCTCCACCT	AGTGGGCAGCTGTGAGCACAAT	205
FMgSSR-59382	scaffold348	p3	(GCA)5	Class II	24337	24351	TGGATGCACAACCGATACCCCA	TGACACCTGCGCGACATGAA	333
FMgSSR-59386	scaffold2624	p3	(GCA)5	Class II	37155	37169	TGGCCACCAACAGTAGCCAA	GCGGCAGCGGCAGCAATAATAA	316
FMgSSR-59387	scaffold4088	p3	(GCA)5	Class II	29126	29140	TGGCCACGTTCATGTACCCCTT	TGTTCGGGCTGCTGCTTCTT	316
FMgSSR-59392	scaffold51552	p3	(GCA)5	Class II	940	954	TGGTAGAGGAGGAGGGAAAGGA	AAAGCGCTGGCTCATGGAGT	335
FMgSSR-59394	scaffold35317	p3	(GCA)5	Class II	2428	2442	TGTACAAGAAGATCCGCCGCCA	TGGAAGAAGGGGTTGTTGCGCT	328
FMgSSR-59395	scaffold13914	p3	(GCA)5	Class II	11919	11933	TGTCATGTCGTCGCACCGGATA	TGGTCGTGAGTGGCTGCTTGTT	291
FMgSSR-59399	scaffold4745	p3	(GCA)5	Class II	39585	39599	TGTTTAGTTTAGCACGCGCCG	AATCCACTATGGAGCCAGCCGA	320
FMgSSR-59400	scaffold3489	p3	(GCA)5	Class II	799	813	TGTTTAGTTTAGCACGGGCCGC	AGAAGAAGCAACAACGTGGCGT	259
FMgSSR-59401	scaffold29628	p3	(GCA)5	Class II	1802	1816	TTACGCTCAAACTCGCCGTCCA	AGAAGCTGGCGCGTAACGAA	238
FMgSSR-59406	scaffold3590	p3	(GCA)5	Class II	40656	40670	TTCCATCGAGCGGTTACGTGCT	TGGTTGCGGTTTGCGAACGA	251
FMgSSR-59407	scaffold1971	p3	(GCA)5	Class II	50530	50544	TTCCTTCGATGGCGGTGACCAT	TGGTGAAGGTGAGCAACTCCGT	215
FMgSSR-59409	scaffold2894	p3	(GCA)5	Class II	8873	8887	TTGCAAGTGCGAGGTGTCCA	AGTTAGGAAGCCAAAGCCCGCA	325
FMgSSR-59411	scaffold1686	p3	(GCA)5	Class II	45414	45428	TTGCAGCACGCGGAAACGAA	AGCCGTGATGATGACTGTCCGT	315
FMgSSR-59412	scaffold9265	p3	(GCA)5	Class II	7458	7472	TTGCATGTTCTGCATCCGCA	TGCATCCACAGGATCTGGTTCA	314
FMgSSR-59413	scaffold233	p3	(GCA)5	Class II	78512	78526	TTGCCAACCTCTGCTGGATGGA	CGTCCTGAATCTGGCTTGTGCT	302
FMgSSR-59415	scaffold110808	p3	(GCA)5	Class II	500	514	TTGGGTTGCTGATGGGGTGGTT	TCACCAACCCCCTGCTGTTTCT	210
FMgSSR-59416	scaffold1189	p3	(GCA)5	Class II	75007	75021	TTGTACGTGCAGTTGGGTGCCT	TGTTGGCACGTCACCAGGTT	236
FMgSSR-59418	scaffold10489	p3	(GCA)5	Class II	1647	1661	TTTCTTCCTGTGCGTGGTGGCT	ATCACCGGCGACTGTGACTGTT	268
FMgSSR-59421	scaffold4977	p3	(GCA)5	Class II	22658	22672	TTTGCCACTTTGCTGCGCGT	TTCGGCGTGGGCAGAAGAAAGT	231
FMgSSR-59423	scaffold3166	p3	(GCA)5	Class II	55574	55588	TTTGTTTGTGGGGAACAGCCCG	CGCACATTGCCGTGGCAAAAA	314
FMgSSR-59427	scaffold421724	p3	(GCA)6	Class II	187	204	AATCTCGCCGGAAGATCTGGCA	TGCTGATATTGGCCCTGCTGA	231

SSR_ID	Scaffold	SSR_Type	SSR_Motif	SSR Class	SSR_Start	SSR_End	Forward sequence	Reverse sequence	Product_size
FMgSSR-59428	scaffold2377	p3	(GCA)6	Class II	32656	32673	AATCTGACGCCTCCAAGGGGTT	TCGACGAGATCGACCACCCATA	343
FMgSSR-59434	scaffold11629	p3	(GCA)6	Class II	24488	24505	ACGCGGCTGGTTGGGATAAA	ATGCGCCGAGGGTAGCAATGAT	337
FMgSSR-59435	scaffold10636	p3	(GCA)6	Class II	17698	17715	ACGCGTTCACCCCCAAACTCTT	AACCTGGTTGTGAATTGCGGGC	247
FMgSSR-59438	scaffold3294	p3	(GCA)6	Class II	39689	39706	ACTGGGGTTTGGTCAAGGCAGT	ACACTAAAAGGGGCCGCACT	333
FMgSSR-59444	scaffold192103	p3	(GCA)6	Class II	63	80	AGCAGGCGTCGATGATGCAGAT	GGTTTGCGGCATCACCTTCT	221
FMgSSR-59448	scaffold1490	p3	(GCA)6	Class II	63182	63199	AGGCACGCCCACTGATCTGATT	ATGCTCTCGCACGACGACTT	271
FMgSSR-59460	scaffold714	p3	(GCA)6	Class II	111221	111238	ATCGCCACCTGGCACAGAGTTT	ATGAGCTGATGGTCCGCCATGT	272
FMgSSR-59464	scaffold5016	p3	(GCA)6	Class II	23349	23366	GAGCACCGCCATTTGCATTG	ATGCGCACACATGCAGGACA	350
FMgSSR-59465	scaffold1233	p3	(GCA)6	Class II	44612	44629	GCAGGTGCATCGTGGAACAGAA	TGCCGGTCTCGATCCGTGAAAT	239
FMgSSR-59470	scaffold3083	p3	(GCA)6	Class II	36108	36125	TAAGCCACAGCACACCCACA	ACGGGAGCGTCACGAATGGAAA	346
FMgSSR-59475	scaffold17618	p3	(GCA)6	Class II	12890	12907	TCAACTGCGCACACATCACCT	TGTACGCACGTACGCATCAG	246
FMgSSR-59477	scaffold349606	p3	(GCA)6	Class II	71	88	TCAGTCGGCGTACCAAGAGT	AGCTCATACTCAGCGCCACGTT	302
FMgSSR-59478	scaffold2170	p3	(GCA)6	Class II	53673	53690	TCCAATCCAGTTCCGCCAATCCA	TTCCACATTGCTGGCGGCTT	249
FMgSSR-59480	scaffold56	p3	(GCA)6	Class II	83979	83996	TCCTCCGTCGTCTCTGATTGCT	AGGCTGCATCCTTCGCTCAT	293
FMgSSR-59484	scaffold7385	p3	(GCA)6	Class II	32462	32479	TCGGTTCCTTCGAGTTCGCT	GCGCTCATGTTCATGTTGGGCA	289
FMgSSR-59485	scaffold7405	p3	(GCA)6	Class II	15490	15507	TCGTCGGCGGCAACAAGAAGAT	AGGGTCTTGCCACGGACACAAA	275
FMgSSR-59489	scaffold51	p3	(GCA)6	Class II	198226	198243	TGAGGGTGCTTGGCACGAAA	TGCTGTGTCTGTGTCGTTGGCT	266
FMgSSR-59493	scaffold12044	p3	(GCA)6	Class II	24256	24273	TGCAGATCAACCGCTTCGCT	ACCATCGTCGCTGCTGCTTACA	332
FMgSSR-59498	scaffold9218	p3	(GCA)6	Class II	12547	12564	TGGTCGCGCTCGTGCAGATAAT	TGCAATCGCGTGGAACGACA	329
FMgSSR-59499	scaffold105	p3	(GCA)6	Class II	14753	14770	TGTGTGTTGTACCGATCGGCGT	AACATCAGGGGTCTTGGCGT	312
FMgSSR-59502	scaffold106572	p3	(GCA)6	Class II	881	898	TTCAGAAACGCCCTGGCGAA	TGTTTATGGCGTAGCCGCGT	209
FMgSSR-59503	scaffold7415	p3	(GCA)6	Class II	4134	4151	TTCGCTTGGAGCTGCGGAAT	TGCTACGGCTTTGCCAGTCT	207
FMgSSR-59508	scaffold4774	p3	(GCA)6	Class II	23581	23598	TTTGGTGCATGATCGGGTCGCT	TGGTGTTCCTACACCACGCA	342
FMgSSR-59510	scaffold76810	p3	(GCA)7	Class I	113	133	AAGGTAACCCCGGCCTTGTTGA	TGGGCCAAACCCAATGGGAACA	342
FMgSSR-59511	scaffold5794	p3	(GCA)7	Class I	39735	39755	AAGTATGGGGGCGGATGTCGTT	TTGCTGCGAAAGACCAGCCA	219
FMgSSR-59517	scaffold16210	p3	(GCA)7	Class I	3207	3227	ACCTCTTTCACTGTCTCGCGCT	TAACGCCACGGCGATGAAGACT	333
FMgSSR-59519	scaffold30404	p3	(GCA)7	Class I	6735	6755	ACGCTATCCAGCTCGATGCAAA	TCCGTGAGCATGGTGGTGAAGT	240
FMgSSR-59521	scaffold681	p3	(GCA)7	Class I	3347	3367	ACGGTGCGTGCCAACTTTATCC	GCGATGATGCCGCCATGGTTTT	216

SSR_ID	Scaffold	SSR_Type	SSR_Motif	SSR Class	SSR_Start	SSR_End	Forward sequence	Reverse sequence	Product_size
FMgSSR-59524	scaffold1133	p3	(GCA)7	Class I	54673	54693	AGCTGGTTCCTGCTTCCATGGTT	ATCCGCGCTGGTCAGTTGAT	257
FMgSSR-59529	scaffold2865	p3	(GCA)7	Class I	31549	31569	ATGTTGTGTAATGCACGGCGGC	TCTCGGCCGCTTCTGTTTGT	204
FMgSSR-59531	scaffold6243	p3	(GCA)7	Class I	17825	17845	CGGCCACGCGTACAAACAAGAA	AGCGAGCCTCGGGTAAAGAAGA	240
FMgSSR-59535	scaffold9885	p3	(GCA)7	Class I	10449	10469	TCAGACGGCAGGCAACTGGTTT	TGGGCTCAGCTCAAATCTCACCA	315
FMgSSR-59536	scaffold4654	p3	(GCA)7	Class I	8119	8139	TCAGGCTGCCTTCGTCATCAGT	TGTGCCCTTATTCCGTTGGCT	279
FMgSSR-59537	scaffold2758	p3	(GCA)7	Class I	66982	67002	TCCAAGGCTTTTTCGCGCTGT	AGCGCTAAAGCCCAGAACCCAT	222
FMgSSR-59539	scaffold658	p3	(GCA)7	Class I	6593	6613	TCGTGACCAAGACAAGCACCCA	CGCGCCACGCACAAAAAGTA	310
FMgSSR-59541	scaffold2271	p3	(GCA)7	Class I	24829	24849	TCTGGAAGATTGGTTGGCCCGT	ATCTTCTCTTGCTGGGCGGTGT	209
FMgSSR-59554	scaffold230510	p3	(GCA)7	Class I	263	283	TTGAGTCTCGGGGCCATTTCCA	TTTCCGTTGGTTGCGGCCTT	334
FMgSSR-59559	scaffold2135	p3	(GCA)8	Class I	63765	63788	AGGCCGTTAGGGGCATGAAGAA	TGCAGCATCGAGTGTTGGCT	254
FMgSSR-59570	scaffold2299	p3	(GCC)5	Class II	45799	45813	AAAAACCACGGGACGCGCAA	TCCATGCGATGCGAGCACAA	341
FMgSSR-59573	scaffold8115	p3	(GCC)5	Class II	16377	16391	AAAACTAACAACCGCCGCGCTC	TCGGAAACCCCCAAAACCCACA	262
FMgSSR-59578	scaffold13343	p3	(GCC)5	Class II	11308	11322	AAACCTTTCCTGGAGGAGGCCA	AGATTGGAGCGGCGGTGAAA	339
FMgSSR-59580	scaffold5358	p3	(GCC)5	Class II	30784	30798	AAACGCGGCACCGAAGCAAA	TGGATTTCGGATGAGACGGGGT	319
FMgSSR-59582	scaffold5107	p3	(GCC)5	Class II	14433	14447	AAAGATCCGTCAACGACTGCCG	AATCCTAGTCGCGTCCTCTGCT	349
FMgSSR-59583	scaffold12597	p3	(GCC)5	Class II	2337	2351	AAAGGCGTTGCGCGAGATGA	AAGAACGAAGCGACTTGGCG	332
FMgSSR-59585	scaffold1994	p3	(GCC)5	Class II	14000	14014	AAAGGGTGCCGGCGATTTGT	TTTTGGATGGAGAGGCCTGGCT	265
FMgSSR-59586	scaffold3753	p3	(GCC)5	Class II	41625	41639	AAATACATCGCGCGTGGCAGTG	TCCTTCATCGCCGTCTCGACAT	286
FMgSSR-59588	scaffold7092	p3	(GCC)5	Class II	16368	16382	AACAACCACCGCGCTTACGA	TGAGCTCGGAAACCCCCAAA	285
FMgSSR-59592	scaffold55	p3	(GCC)5	Class II	55587	55601	AACATCCGGTTCCGTCGCTT	CGGCCATCGAAAACGTGAGCAA	223
FMgSSR-59593	scaffold1075	p3	(GCC)5	Class II	42019	42033	AACCACTTCTTCGCGCTGCT	TCTCCGGCGAGGCCAGTAAAAA	343
FMgSSR-59598	scaffold2043	p3	(GCC)5	Class II	33502	33516	AACCGGCAAATCGCTAGGCT	AGTTCGCCGTTTTGGTGACTGC	346
FMgSSR-59599	scaffold927	p3	(GCC)5	Class II	10786	10800	AACGGCGAACGAGCAAGCAA	TGCAACAAGGGTTTGCCCGA	319
FMgSSR-59605	scaffold4155	p3	(GCC)5	Class II	40867	40881	AAGCAAAGAGAGGGAGGCAACC	TGTCCTTTCCGTCTGGTACGCT	297
FMgSSR-59613	scaffold16730	p3	(GCC)5	Class II	5274	5288	AAGCTGCTCACGACCAGGTTCA	TAGGCGCCATGGATGGTGTT	241
FMgSSR-59614	scaffold359	p3	(GCC)5	Class II	17467	17481	AAGGAAAAGCCACCGCAGCA	ATGGGACGCTGGTACATCTTCG	343
FMgSSR-59623	scaffold58360	p3	(GCC)5	Class II	1167	1181	AATTCAAGGCCGCCCTGCAACA	TTGATGCCGTCGATGCTGCT	337
FMgSSR-59626	scaffold7746	p3	(GCC)5	Class II	24429	24443	ACAAGCTGCAGGGACGTACA	TCCGGCCCTAACAACTGCATGA	201

SSR_ID	Scaffold	SSR_Type	SSR_Motif	SSR Class	SSR_Start	SSR_End	Forward sequence	Reverse sequence	Product_size
FMgSSR-59634	scaffold13211	p3	(GCC)5	Class II	23158	23172	ACACGCCAAATCTGAGCCAACG	AAGACGTCCACGGCTTGTGCTA	338
FMgSSR-59637	scaffold805	p3	(GCC)5	Class II	30690	30704	ACAGGGCATACAGGCAAGGCAA	ACTGTGCGAGTGGCCCATTT	326
FMgSSR-59642	scaffold14673	p3	(GCC)5	Class II	11039	11053	ACCAAACCCAACCAGAGCAGCA	TTCGCCTTGTACGCCTCCAACT	314
FMgSSR-59649	scaffold455	p3	(GCC)5	Class II	87404	87418	ACCGCCACCACAACACTCTT	AGAAGCGCTCTTGAGAAGCTCG	244
FMgSSR-59651	scaffold2435	p3	(GCC)5	Class II	11723	11737	ACCTCTGACGAACCGAGCTCTT	TTTCCACCGCGTCCACGAAT	332
FMgSSR-59653	scaffold128	p3	(GCC)5	Class II	6784	6798	ACGAAGCAGATGTGGCACGGAA	TGAGCTGAGCTGATTTCGGCGT	221
FMgSSR-59654	scaffold25882	p3	(GCC)5	Class II	5482	5496	ACGACAAGCAACGCCTGTTC	TGGATTACTGTGTGTGGCCGCT	335
FMgSSR-59655	scaffold1049	p3	(GCC)5	Class II	50001	50015	ACGACGAAGAGACGGCATGT	TGGGAGCACGGTAGCAACAA	328
FMgSSR-59664	scaffold1657	p3	(GCC)5	Class II	44628	44642	ACGCAGCATCTGCTTGGTCA	TCTTTGGGCGCTGCAACGAT	255
FMgSSR-59667	scaffold6530	p3	(GCC)5	Class II	27459	27473	ACGCCTCAGCATGGAAGATGAC	ACCTGGAGAACAGCTCGCTT	270
FMgSSR-59669	scaffold1848	p3	(GCC)5	Class II	57681	57695	ACGCGATGAAGAAGAGGGCTGT	TCGTGACGACATCGTGCGTT	285
FMgSSR-59674	scaffold2861	p3	(GCC)5	Class II	22369	22383	ACGGACAGCTGCTTGAGGAACA	TGTGCTGTTTGCTTGCACCGT	291
FMgSSR-59678	scaffold13077	p3	(GCC)5	Class II	18663	18677	ACGGGAAGTTGTAGGCTTGGCA	ATTCTCCCCCGTGGAAGCGTGATT	270
FMgSSR-59683	scaffold8410	p3	(GCC)5	Class II	17060	17074	ACGTACGAGGGCAAGCACAA	ACGCCCCGAAACCAGAGTAA	220
FMgSSR-59684	scaffold6291	p3	(GCC)5	Class II	27845	27859	ACGTCATCGTCGACGCCAAA	AACAACGGCGGCATGATGGT	283
FMgSSR-59686	scaffold2077	p3	(GCC)5	Class II	33294	33308	ACGTGCTTGCGCCATGGATA	TGTCGCGGGAAGAAGAAGCCAT	243
FMgSSR-59690	scaffold19685	p3	(GCC)5	Class II	10709	10723	ACTGCACCACGCATGGGAAA	AATGCCCCCACCCTCAGCTAAA	334
FMgSSR-59694	scaffold13624	p3	(GCC)5	Class II	10862	10876	AGAATGCGACTGCCGCGAAA	TTGTGTCCAGGGTTCGGCTGAT	300
FMgSSR-59700	scaffold81908	p3	(GCC)5	Class II	1469	1483	AGCAAACGAGGAGGAACGGA	TTTGCTGCGCAGGTGGAAGT	249
FMgSSR-59701	scaffold7057	p3	(GCC)5	Class II	12750	12764	AGCAACAAGCGAAGCACGCA	ATTGCGGAGCGATTCGCCTACT	316
FMgSSR-59704	scaffold3175	p3	(GCC)5	Class II	55459	55473	AGCAGAGGAACGCAATCGCA	AGCGATACATTGCTGCGCGT	291
FMgSSR-59707	scaffold12723	p3	(GCC)5	Class II	24147	24161	AGCAGGCCACAGCAAAGCAA	ATTTTATCCTGGCGCGTGGAGG	346
FMgSSR-59708	scaffold12128	p3	(GCC)5	Class II	14990	15004	AGCAGGGGAAAAAGGCCTGGAA	TGCAGCTCCGCCTTGTTCTT	342
FMgSSR-59710	scaffold11052	p3	(GCC)5	Class II	7864	7878	AGCATGCAGTTGATGTGGCCCT	AGCCGGCAATGCAGCGATAA	245
FMgSSR-59716	scaffold13766	p3	(GCC)5	Class II	2713	2727	AGCGAAAAGGCAGCAGCTGAGA	ACCAGATACGTGAGGTGTGCCA	339
FMgSSR-59717	scaffold1024	p3	(GCC)5	Class II	879	893	AGCGTGTTGTGGGCGTTGTT	AGCACCTAGTTGCGGAGAAGCA	255
FMgSSR-59718	scaffold19564	p3	(GCC)5	Class II	14622	14636	AGCGTTCGATTCCCCGCATT	TCGAACCGTGTCGAAGACGA	342
FMgSSR-59719	scaffold528	p3	(GCC)5	Class II	47000	47014	AGCTACCAAAGCACAATGCAGC	TCGCTCTTTTGTCGCGCAGT	309

SSR_ID	Scaffold	SSR_Type	SSR_Motif	SSR Class	SSR_Start	SSR_End	Forward sequence	Reverse sequence	Product_size
FMgSSR-59723	scaffold706	p3	(GCC)5	Class II	22415	22429	AGCTGGAAAGGGTACACGCTGA	AGTGTCACACAGTCCTGCCGAT	320
FMgSSR-59733	scaffold25	p3	(GCC)5	Class II	135467	135481	AGGCCGATGCTGTAGTCGATGT	AGCTCGTGAACGAGGAGATCGT	262
FMgSSR-59736	scaffold4290	p3	(GCC)5	Class II	10055	10069	AGGCTTCTCCGCAACGTTCA	ACCTGGGCTGTGAGCCTTCAAA	345
FMgSSR-59744	scaffold791	p3	(GCC)5	Class II	87187	87201	AGGTAGGCGCTGCAGATACGAA	AAGGAGTGTTCCTGGACGTGCT	343
FMgSSR-59747	scaffold4917	p3	(GCC)5	Class II	11133	11147	AGGTTGCACGAACCGCACTT	ACTTCCAAGGCGAAGGCCAACA	205
FMgSSR-59751	scaffold1808	p3	(GCC)5	Class II	17293	17307	AGTCCCGATGACATCTCCCTGA	AGCATGGTGGTCACACACGTCA	343
FMgSSR-59755	scaffold186474	p3	(GCC)5	Class II	515	529	AGTGCCTTCCACTGGTCGTT	TTCGGCTCCAATTCCTCCTCCT	276
FMgSSR-59757	scaffold2088	p3	(GCC)5	Class II	68059	68073	AGTTCTCCAAGTTTGGGTCCGC	TTAGCGCCGCGATTGCATCA	226
FMgSSR-59758	scaffold9	p3	(GCC)5	Class II	119978	119992	AGTTGCGTGCGTACATTGCG	TTTGCGACAATCTGGTCGCTCG	340
FMgSSR-59759	scaffold450	p3	(GCC)5	Class II	122102	122116	AGTTGTTGCCGACCTCGCTT	TTGACTCCTTCCTTCCCGCA	345
FMgSSR-59763	scaffold2020	p3	(GCC)5	Class II	47579	47593	ATCACCCGCCGGTTCAGCATTT	TGCCGCACAACAGGACCTTTCA	203
FMgSSR-59766	scaffold32238	p3	(GCC)5	Class II	5193	5207	ATCCGCATGCCTCGCATCAT	AGCCTGCTCTATCCATTCGCCT	220
FMgSSR-59768	scaffold5490	p3	(GCC)5	Class II	1758	1772	ATCGGACAAGCTGGTGTTGC	AGCCACGTGCGCTGCATTTA	276
FMgSSR-59769	scaffold1100	p3	(GCC)5	Class II	27654	27668	ATCGGAGGCGCAACCAACAT	AACGGCATTGGCCAGCAACT	232
FMgSSR-59770	scaffold1604	p3	(GCC)5	Class II	32556	32570	ATCGTCATTGTCGCCCTCGT	TGCATCTGCTGCTGGTACTGCT	324
FMgSSR-59773	scaffold7799	p3	(GCC)5	Class II	5671	5685	ATCTGGTGTTGTGTTCGCCCGT	TGGCGAAGATGTTGGAGCGA	281
FMgSSR-59774	scaffold116	p3	(GCC)5	Class II	82543	82557	ATCTTCCAGAAAGCGCTGCCGT	TCCACAACCTTCTCCATCGCGT	271
FMgSSR-59776	scaffold4294	p3	(GCC)5	Class II	4521	4535	ATGAAGCGACGGGCTAATCGCA	AGTTGAGGCTCCTTCCTCAAGGGT	282
FMgSSR-59777	scaffold270886	p3	(GCC)5	Class II	308	322	ATGACGCTGGCCGAAAGGATGA	TCGACCCGCAATTTCTGCGA	292
FMgSSR-59779	scaffold1574	p3	(GCC)5	Class II	17926	17940	ATGCACGCAACGCAAGCACA	TGATGAGCGGCAGCAACCTT	295
FMgSSR-59781	scaffold715	p3	(GCC)5	Class II	32440	32454	ATGCAGCACATGTGGCCAGT	ACGTGTCTCGGCAAGGACAT	241
FMgSSR-59784	scaffold10240	p3	(GCC)5	Class II	17233	17247	ATGCGTGACCATGCTAGAGTCG	TGGACTTGGCGCTCATGCAA	311
FMgSSR-59787	scaffold8650	p3	(GCC)5	Class II	25234	25248	ATGGGCAAGCTGTCGTGGAAGT	ACATGAGCACCTGCCGTTTCCT	337
FMgSSR-59789	scaffold1357	p3	(GCC)5	Class II	56479	56493	ATGGTCTCGAACCGGACTCCTT	ATCGCGTGAGGGATATCGAGGT	296
FMgSSR-59790	scaffold9219	p3	(GCC)5	Class II	18598	18612	ATGGTTGCTGTACCAGGCGTCA	AGCAACAACGAGAAACGCAGCC	224
FMgSSR-59791	scaffold8752	p3	(GCC)5	Class II	21263	21277	ATGTCCTGCCCCTCAAGCACAA	ACGTACACGCAGCAAACGGA	241
FMgSSR-59792	scaffold22940	p3	(GCC)5	Class II	1653	1667	ATGTCGCACGCCTCCTTCTT	AAGAATTGCGGCCCGCTGTA	237
FMgSSR-59793	scaffold423	p3	(GCC)5	Class II	53228	53242	ATGTCGCAGCCCATCAACGGAA	TTGTGCCCCAATTCGCACCT	254

SSR_ID	Scaffold	SSR_Type	SSR_Motif	SSR Class	SSR_Start	SSR_End	Forward sequence	Reverse sequence	Product_size
FMgSSR-59796	scaffold6153	p3	(GCC)5	Class II	3416	3430	ATTCGGACACCCATTTGCCCGT	GGAAAGCACCTTTTCGTCGGCA	258
FMgSSR-59797	scaffold3430	p3	(GCC)5	Class II	45840	45854	ATTCGTGGCTGCTCTGGACCAT	AGATCTTCTCCAGGCGCGAT	306
FMgSSR-59798	scaffold1630	p3	(GCC)5	Class II	57126	57140	ATTGGCGTCATGGCATGCGAAC	TTGAATCAAGCGCGACGGAGCA	342
FMgSSR-59802	scaffold18570	p3	(GCC)5	Class II	8961	8975	CACAGTCCGCCAACAGCAAT	CGCCGAGCCTTTTCAAAAACCG	286
FMgSSR-59803	scaffold4986	p3	(GCC)5	Class II	8504	8518	CACCCGTTCTTTGAAGGCGTGA	TCCAGACTCCACATCTGCACCA	201
FMgSSR-59804	scaffold1486	p3	(GCC)5	Class II	6698	6712	CACGCCGCCATAATGTCCTT	ACAAGTATAGGAAGACGAGGTCCCC	339
FMgSSR-59805	scaffold750	p3	(GCC)5	Class II	39758	39772	CAGCGAAGCGAAAATGCACCCA	ATTTCGGGCGGAAAGGGCAT	306
FMgSSR-59807	scaffold2774	p3	(GCC)5	Class II	49639	49653	CCAAAACGCCTCGTACGCCATT	TTTCCGGATTGCTCTTGCTCGG	334
FMgSSR-59815	scaffold770	p3	(GCC)5	Class II	7280	7294	CGAAGTCCGCGTTGTTGAGGAA	TGCATTGTGCAGGAGGGACA	350
FMgSSR-59816	scaffold15149	p3	(GCC)5	Class II	6383	6397	CGCAACATGAGTGCGCCATTGA	TGTGACCTTCTTCCTCACCGCA	243
FMgSSR-59817	scaffold6281	p3	(GCC)5	Class II	883	897	CGCACATCGCCTTGATGCTCTT	TTCTCGGACTTCGAACCGCTCA	265
FMgSSR-59819	scaffold36221	p3	(GCC)5	Class II	5724	5738	CGCATTGCTTGGCCACTCGTTT	TCGCAAAAAGCGCAGCACCA	350
FMgSSR-59821	scaffold3289	p3	(GCC)5	Class II	46371	46385	CGCCAGCACAACGCAGAAAT	ACGACGACAACGCCAAAGGT	238
FMgSSR-59823	scaffold6939	p3	(GCC)5	Class II	7845	7859	CGCCGACCAACCTGCCATTTTT	ATGCAGCAATGCCGCCTGAA	318
FMgSSR-59825	scaffold7443	p3	(GCC)5	Class II	16672	16686	CGCCGCTTGCATTTGGAAAGGT	AGCTTGGCCTTCTCTTGCACA	243
FMgSSR-59826	scaffold1439	p3	(GCC)5	Class II	67641	67655	CGCGCCTTCATCGAAACACGAT	TTGCTGAAGCTCACGGTCCT	314
FMgSSR-59827	scaffold1281	p3	(GCC)5	Class II	15046	15060	CGCTCAGCGTCGCCTGATTTTT	AGCAGCGGTGCTGGACTTTT	250
FMgSSR-59829	scaffold55	p3	(GCC)5	Class II	172238	172252	CGGCGCCATAGTCGTTGCTATT	ACGCCGTTGCTGCGAGTTTT	346
FMgSSR-59831	scaffold943	p3	(GCC)5	Class II	17292	17306	CGTTGTTCGCCTCCACCATCAT	GCTGCCAGCAAGCTCCAACAAT	225
FMgSSR-59833	scaffold745	p3	(GCC)5	Class II	13290	13304	GCAGGCAAACCAGGGACCTTTT	GCTGCGTTGGGAGCGAAAAT	343
FMgSSR-59834	scaffold23304	p3	(GCC)5	Class II	10944	10958	GCATGCAAACGCACAACGCA	ATAGCGAGAGGCCTGTGAGGAA	338
FMgSSR-59842	scaffold112408	p3	(GCC)5	Class II	309	323	GGCCAGCCAACGGCTACTTTTT	AAGAGCAGCACGCAAAGGCA	255
FMgSSR-59848	scaffold657	p3	(GCC)5	Class II	110451	110465	TAACTTTGGCCTTTGCTGGCCC	ACATCACCGTCAACGAGGCTCA	282
FMgSSR-59849	scaffold2135	p3	(GCC)5	Class II	64178	64192	TAAGCGCCGCCGGAATAATCGT	TGTACATCACGAGCCCGAAGAACG	252
FMgSSR-59850	scaffold447	p3	(GCC)5	Class II	82023	82037	TAATCGCGGGTTCTGAGCGA	AATTGTCGCGCAACTCATGGGC	308
FMgSSR-59856	scaffold4296	p3	(GCC)5	Class II	6350	6364	TACGTAGCAACACGCCAGCCAA	TATTGCAGGTCATGTCGCGGCT	315
FMgSSR-59859	scaffold8118	p3	(GCC)5	Class II	31277	31291	TAGCATGGTGCCGGACAGCAAA	ATTACCGATGCGGCCTGTGT	321
FMgSSR-59860	scaffold6643	p3	(GCC)5	Class II	14626	14640	TAGCCCAAACAGCGGCATCCAT	AGAAACCTAGTGGGGAGGCAGT	340

SSR_ID	Scaffold	SSR_Type	SSR_Motif	SSR Class	SSR_Start	SSR_End	Forward sequence	Reverse sequence	Product_size
FMgSSR-59861	scaffold1123	p3	(GCC)5	Class II	57012	57026	TAGCGAAGAGGAGTCGACGAGA	TACACCAAGTTGCCGCCACA	293
FMgSSR-59865	scaffold9739	p3	(GCC)5	Class II	14710	14724	TATGGTGCCTTCGCCTTGCT	TGGCAACTCACCACCTCATGGA	344
FMgSSR-59867	scaffold3078	p3	(GCC)5	Class II	35235	35249	TCAACAGCAACGGCGAAGCA	ACTCCAACACAAGCACGGCA	327
FMgSSR-59868	scaffold343	p3	(GCC)5	Class II	141198	141212	TCAAGATGGGCTCGGCCCAAAA	TTGACACCCGCAGCACCATA	333
FMgSSR-59869	scaffold3058	p3	(GCC)5	Class II	28185	28199	TCAAGCCCGACCTTTCCTCA	TGCATGCAGTATTAGCCAGCCC	290
FMgSSR-59871	scaffold178	p3	(GCC)5	Class II	74008	74022	TCAATGGCAGGTGATTGTCGGA	ACGGTGAACAACTGGACGCTGT	327
FMgSSR-59873	scaffold1911	p3	(GCC)5	Class II	38893	38907	TCACGAACCTCCGGTAATGGCT	TCCACAAGGAGTTCACCGTCGT	201
FMgSSR-59874	scaffold21832	p3	(GCC)5	Class II	1213	1227	TCACGCACACACGGGACTTT	TGAAACTTCGCTTGCTCGTGGC	301
FMgSSR-59876	scaffold25520	p3	(GCC)5	Class II	942	956	TCACTTGTTACCGCGTGCCT	AGTAAGCCATGTACCGTCCCGA	288
FMgSSR-59879	scaffold318	p3	(GCC)5	Class II	56363	56377	TCAGGTTCGAGGTGCGGAATGA	AAGCAGGAGCTGGAACCAAGCA	343
FMgSSR-59880	scaffold795	p3	(GCC)5	Class II	73397	73411	TCAGTCATTCCACAGCGCGA	AGCAAGTACGACGTGAAGCGCA	339
FMgSSR-59882	scaffold38729	p3	(GCC)5	Class II	5280	5294	TCATCATGCTTGCGTAAGCGGC	AAGACGAAGGAAAACGGGGGCT	263
FMgSSR-59889	scaffold8468	p3	(GCC)5	Class II	28234	28248	TCCCCAATTCCTTCAGCACCGA	AGGCACCCATGTGGATCTGGAA	262
FMgSSR-59891	scaffold545	p3	(GCC)5	Class II	87736	87750	TCCGAACCAGCGCTCATACGTT	AATGCGACCGTGCCAGATGT	346
FMgSSR-59894	scaffold4982	p3	(GCC)5	Class II	39592	39606	TCCGCCTCCTCCAATACCGATACA	TGGACGAACATCTCGCGCAACT	316
FMgSSR-59899	scaffold20928	p3	(GCC)5	Class II	1437	1451	TCCGTTTCTTCCGGTCGCTGAT	ACCTTGGGCAAGTGCTGACGAA	283
FMgSSR-59902	scaffold4691	p3	(GCC)5	Class II	23371	23385	TCGACGGGAAACCAAAAACGGA	GCAGGCCGAGAACTTCATCAACA	308
FMgSSR-59906	scaffold493	p3	(GCC)5	Class II	68746	68760	TCGCCGAAGTTGAACAGGACCA	TATCAGAGCTCGAGGAAGAGGC	313
FMgSSR-59908	scaffold1168	p3	(GCC)5	Class II	51870	51884	TCGCCTTTCCCCAACCAAACCA	AGCGACATGGGGATTGCTTCGT	238
FMgSSR-59912	scaffold1991	p3	(GCC)5	Class II	46732	46746	TCGCGGTTTCACCATCCACACA	AATGGCCAGCGAAACCCGAA	299
FMgSSR-59923	scaffold1467	p3	(GCC)5	Class II	8888	8902	TCGTCTACCAGTGGCTTCGACT	TGACGTAGGGGCACTTGTCGTT	277
FMgSSR-59925	scaffold11096	p3	(GCC)5	Class II	20083	20097	TCGTTGTAGTCGTCATCCGCCA	GCAACCAGGGACGCTAACAA	350
FMgSSR-59931	scaffold4375	p3	(GCC)5	Class II	25036	25050	TCTCCGGCCAACTACACCCAAA	AACGGCTATTGGATGGCGGAGA	332
FMgSSR-59932	scaffold4253	p3	(GCC)5	Class II	46461	46475	TCTCGCGCCACCATTCCAAA	TGCAGTTCGACGATGGCGATGA	295
FMgSSR-59934	scaffold2737	p3	(GCC)5	Class II	18436	18450	TCTGCCTACGAAACGGCACA	TGCATACCGCCAAGCAGAGTGT	301
FMgSSR-59938	scaffold1627	p3	(GCC)5	Class II	6985	6999	TGAACCGCTGGTTCTGCTTGA	TCGTTCTCACGGGCAGCTGTTT	293
FMgSSR-59941	scaffold6595	p3	(GCC)5	Class II	33002	33016	TGACGCAAACACGTACGCCA	TGCACCACGATTTTGCCCCT	243
FMgSSR-59945	scaffold4538	p3	(GCC)5	Class II	44649	44663	TGCAAGCGCACGAGGCAAAT	TGGCAAGCACCAAACCAGACA	208

SSR_ID	Scaffold	SSR_Type	SSR_Motif	SSR Class	SSR_Start	SSR_End	Forward sequence	Reverse sequence	Product_size
FMgSSR-59948	scaffold328	p3	(GCC)5	Class II	23510	23524	TGCACGCTCGCCAAAACCTT	AAACGCGATCTCGCAGGATGGT	208
FMgSSR-59956	scaffold4055	p3	(GCC)5	Class II	45658	45672	TGCCTCGCGAACTCTAACCACT	ATGCCCTCACCCACCAGCAAAA	282
FMgSSR-59959	scaffold11065	p3	(GCC)5	Class II	9413	9427	TGCGCTCATACTTGTGGGCGTT	TGTTCAGCTGTGGCTGTGCTGT	307
FMgSSR-59960	scaffold674	p3	(GCC)5	Class II	30791	30805	TGCGGAAGAAACGCTCTCCA	ATCCGCAGCCGTATCCTGCATT	212
FMgSSR-59968	scaffold75719	p3	(GCC)5	Class II	1734	1748	TGCTCGCCGCAGTTGAAGAA	CGTCGCTGGCAAACAAGCCAAA	310
FMgSSR-59978	scaffold3620	p3	(GCC)5	Class II	21843	21857	TGGAAGGTGAAACGCGCACA	TGCTGATCATGCCGCACTTCCT	347
FMgSSR-59979	scaffold299637	p3	(GCC)5	Class II	214	228	TGGAATCAGGCGGCGTTACA	TTGATTGGGATGCGGACGCT	293
FMgSSR-59980	scaffold3186	p3	(GCC)5	Class II	27786	27800	TGGCAACGTGCCGGTCTCATTA	TGCGTCTCCCGAAACCATCTGA	287
FMgSSR-59983	scaffold4921	p3	(GCC)5	Class II	4692	4706	TGGCGGCACTAAATCGAGCA	TTGCTGTTGTCCTCGCCCTTGT	237
FMgSSR-59984	scaffold270	p3	(GCC)5	Class II	155337	155351	TGGCTCGAGGTTGACAAGGTCT	TGCACGCCAGTGGAAGGATT	206
FMgSSR-59987	scaffold1326	p3	(GCC)5	Class II	20485	20499	TGGGCTTGTTTGGCAGGGAAGA	TTATGCAAAGCGCGATTCCGCC	264
FMgSSR-59988	scaffold28759	p3	(GCC)5	Class II	8952	8966	TGGGTCGAGTTGACCGTCCTTT	AGCACAAGCAGAAACGGGTGA	334
FMgSSR-59989	scaffold5588	p3	(GCC)5	Class II	17160	17174	TGGTGGTGTCGATCCTTGACGA	TTTGGCGGTGCTTGGATTTGGC	224
FMgSSR-59995	scaffold3226	p3	(GCC)5	Class II	24211	24225	TGTCCGACTCATCGCCAACACT	CGCGATTGGGGTTGAGAGGTTT	271
FMgSSR-59996	scaffold10570	p3	(GCC)5	Class II	5541	5555	TGTCTCCCTCATCGCTGCAA	AAGAGGGAGAAGGGAGTACGCT	219
FMgSSR-59999	scaffold25	p3	(GCC)5	Class II	194429	194443	TGTTGCGATCCACCGGCAAT	ATGAGGGTGAAGGACCTGCTGT	216
FMgSSR-60000	scaffold980	p3	(GCC)5	Class II	52128	52142	TTAGGCGTGAACCACGAAGGCT	AGCGTGTCGTGGTGGTAGTT	304
FMgSSR-60002	scaffold3307	p3	(GCC)5	Class II	17561	17575	TTCACGGCGATGCAGGAGTTGA	AGTCGACTCGAAGCCGATGAGA	281
FMgSSR-60006	scaffold9115	p3	(GCC)5	Class II	29633	29647	TTCCACCTCCTCGATTTCCCGA	TTGTCGGAGCGCATGAACAG	344
FMgSSR-60008	scaffold21469	p3	(GCC)5	Class II	5794	5808	TTCCGGAAGAAGCTGTACTCCACG	AACTGCGGTGCAAAGCGAGA	349
FMgSSR-60009	scaffold7083	p3	(GCC)5	Class II	29579	29593	TTCCTCTGTTCGCCGAAGTCGT	TCACCAGCCACTTCCTCCCAAA	325
FMgSSR-60010	scaffold30322	p3	(GCC)5	Class II	3259	3273	TTCCTGCTGACGGCTTCGATCA	TGCAGCGCAATGCCTCCTTT	312
FMgSSR-60011	scaffold2387	p3	(GCC)5	Class II	58988	59002	TTCCTTCTCCTCGTCATCGGCT	ACCCCGCCACGGATTAAACACA	337
FMgSSR-60019	scaffold33	p3	(GCC)5	Class II	74986	75000	TTCTTGGCGCAGATCAGGCT	ATTAGATTGCACGGCGCAGGGA	317
FMgSSR-60020	scaffold57248	p3	(GCC)5	Class II	2680	2694	TTGACCAGCCTTCTGCTGCGTT	ACCTGCAGTCGTGCAGCATA	212
FMgSSR-60022	scaffold7176	p3	(GCC)5	Class II	14546	14560	TTGATCAGCGCGTCCGACAT	TGCGGATGGGGAACCGAGTTTT	329
FMgSSR-60024	scaffold2327	p3	(GCC)5	Class II	7163	7177	TTGCCCAGCAAACGAACGCT	AGTTGGGACGAACGACACAGGA	306
FMgSSR-60031	scaffold6973	p3	(GCC)5	Class II	9958	9972	TTGGCATCAGGTAGCCAGGT	TGGACACACCAGTGGAAGAAACCA	337

SSR_ID	Scaffold	SSR_Type	SSR_Motif	SSR Class	SSR_Start	SSR_End	Forward sequence	Reverse sequence	Product_size
FMgSSR-60032	scaffold5529	p3	(GCC)5	Class II	883	897	TTGGTCGGCGTTTGGACAGT	CGTCAGACGAACGAAGTTCACA	308
FMgSSR-60035	scaffold1503	p3	(GCC)5	Class II	37678	37692	TTGTTACGCTCGCTTCCGGGAT	AGTGGAAGTTGCCGCCTCTGTT	277
FMgSSR-60041	scaffold1856	p3	(GCC)5	Class II	46771	46785	TTTAGCTGGCGCGACGCAAT	ACACTCCGCCACCACTTGGTAA	332
FMgSSR-60042	scaffold7735	p3	(GCC)5	Class II	18034	18048	TTTCAACCCTAACCGCCCACCA	ACGACCAAACGGCACCATGT	283
FMgSSR-60046	scaffold8986	p3	(GCC)5	Class II	10110	10124	TTTCCGTTGCCACTTGCGATGC	TTGAACAGCGGCTCAAGCGA	307
FMgSSR-60047	scaffold390	p3	(GCC)5	Class II	109794	109808	TTTCTGGAGCGAGGTGCGGTTT	TGCTTTTGATGCGGCGAGGA	348
FMgSSR-60050	scaffold8264	p3	(GCC)5	Class II	12689	12703	TTTGACGATGCTTGCTGCTGCG	TGTGGCGATCCATGGAGACTGT	322
FMgSSR-60052	scaffold18999	p3	(GCC)5	Class II	7688	7702	TTTGGCGAACTTCTCCGCGT	AACAAGAAGGCAGGCTCGCA	204
FMgSSR-60057	scaffold6648	p3	(GCC)5	Class II	9188	9202	TTTGTGAGGCCTGTCTCTGTCG	ACGCGGGTAGCCCAACCTTTTT	242
FMgSSR-60059	scaffold301	p3	(GCC)5	Class II	144246	144260	TTTGTTTTTATGCCCCCGCCGC	AGCCTCAACGTGCACCTCTT	297
FMgSSR-60063	scaffold9132	p3	(GCC)6	Class II	19593	19610	AAAGCTTAAGGTGCCGGCGT	ACTGCCATATCCTCGTCGGTCT	204
FMgSSR-60065	scaffold60	p3	(GCC)6	Class II	184753	184770	AAGAAGGCCAAGCGCAAGCA	ATCCGGAAATTGGCGAGCGAGT	302
FMgSSR-60066	scaffold65	p3	(GCC)6	Class II	38664	38681	AAGCAAGGCCGTTGGATGGGAA	TTGCGGATGGTGGACTTGAGGT	289
FMgSSR-60067	scaffold22057	p3	(GCC)6	Class II	4051	4068	AAGCGCGCGCACGCAATAAA	CGGGTTTCCTCTCGTCGCTTTT	320
FMgSSR-60068	scaffold7678	p3	(GCC)6	Class II	35021	35038	AATGAAGACACGATCGGCCTGG	AGGTTAGGAAGGAGGAGAGCGT	305
FMgSSR-60069	scaffold57248	p3	(GCC)6	Class II	2425	2442	AATGCGTGCGCAGAGCTACA	TGGCATCGACGCAGCAGAAA	286
FMgSSR-60072	scaffold1269	p3	(GCC)6	Class II	9952	9969	ACACGTACAGGAACGCGCAGAA	GCTCAGCGGCGCAATATTCACA	218
FMgSSR-60076	scaffold90079	p3	(GCC)6	Class II	1352	1369	ACCATCGCTTCTACCGCCGAAT	AGCCCGGCTAAAACCCTGAA	330
FMgSSR-60077	scaffold373	p3	(GCC)6	Class II	105504	105521	ACCATGCCGAACGTTGACGA	TGTTCTGCATCCGTCTCGCA	337
FMgSSR-60078	scaffold377	p3	(GCC)6	Class II	91545	91562	ACCCGAAATTTACCGTCGCCCA	TTCGAAGCAGGCGACGTTCCTT	348
FMgSSR-60081	scaffold5387	p3	(GCC)6	Class II	28048	28065	ACCGGAGGTAGGAGAGAAACGGAA	TTGTTCAGACGCCGCAGGTT	265
FMgSSR-60084	scaffold7160	p3	(GCC)6	Class II	3504	3521	ACCTGCACAAGCACGAACCA	TCCACACGCCGCATCACGAATA	212
FMgSSR-60087	scaffold4433	p3	(GCC)6	Class II	41012	41029	ACCTGTTACTTGCGTGCTGCTG	ACAGTGCACGGTTTCTTGTGTGC	322
FMgSSR-60091	scaffold4131	p3	(GCC)6	Class II	17191	17208	ACGTCGGTTGCTGCCACATT	TCCATGCTTTGCCGTTAGCCGT	255
FMgSSR-60092	scaffold5795	p3	(GCC)6	Class II	19268	19285	ACGTGCTGTGAATGCAGGCT	TGCAGATCACCACCGCAAACCA	336
FMgSSR-60103	scaffold2148	p3	(GCC)6	Class II	43792	43809	AGGCAACTGCAAGCTACGGCTA	AGGGGAGAGAGAAATGGGGAAGGA	241
FMgSSR-60104	scaffold49744	p3	(GCC)6	Class II	1303	1320	AGGCGGCCATCCAACTCAACAA	ATCGCCTGCTTTTGCCTCCT	311
FMgSSR-60107	scaffold526	p3	(GCC)6	Class II	17498	17515	AGGTATTCAACGAGGCGCCGTA	TGCACCCCATCGGACCAAAGAA	331

SSR_ID	Scaffold	SSR_Type	SSR_Motif	SSR Class	SSR_Start	SSR_End	Forward sequence	Reverse sequence	Product_size
FMgSSR-60111	scaffold24870	p3	(GCC)6	Class II	6291	6308	ATACGGAACGATGCACTGCTGC	ACGGCAGCAACAAGCAGACT	324
FMgSSR-60112	scaffold590	p3	(GCC)6	Class II	29784	29801	ATACTGCGCCCATCGCCATT	TCATCTTGTCCATGCCGCGT	348
FMgSSR-60115	scaffold268	p3	(GCC)6	Class II	24687	24704	ATGCTCAGCGAGATGGCGTA	TCAGCAGCAGCAAGGACGTTCA	316
FMgSSR-60116	scaffold5921	p3	(GCC)6	Class II	31160	31177	ATTGCGCTGTCCACTGGGTGAT	TGGGTACAGCTACAACCCCTCCTA	314
FMgSSR-60118	scaffold2620	p3	(GCC)6	Class II	24655	24672	GCCAACAAGACGCACACGAA	ATGACGATGATAGGTGCCGCGA	223
FMgSSR-60123	scaffold17141	p3	(GCC)6	Class II	13591	13608	GCGTACAGGCGGTTGTTGTCTT	TGAGTTTGCAGAGCGTGCCA	279
FMgSSR-60124	scaffold16730	p3	(GCC)6	Class II	5886	5903	GCGTCCAAAAATTCGAGGCCGA	TTGAGCGCAACTGCCGATGT	322
FMgSSR-60125	scaffold2216	p3	(GCC)6	Class II	30358	30375	GCGTTCATCATGATCTCGGCGT	TTCGCGTACGGCCCAAACTA	329
FMgSSR-60126	scaffold4184	p3	(GCC)6	Class II	25493	25510	GCTCGCTCGCCATTGACGATTT	TAACAAGCAGCAGGCAGGCA	347
FMgSSR-60128	scaffold7532	p3	(GCC)6	Class II	13125	13142	TACCACCACATCCTTGCGCT	TCTGCACTTCTCCGGCAACA	291
FMgSSR-60129	scaffold657	p3	(GCC)6	Class II	8940	8957	TAGAGCGCGCAGATGACTTGGT	TCACGCTAGCGAGCTGCAAT	287
FMgSSR-60131	scaffold2738	p3	(GCC)6	Class II	66551	66568	TATCTATGCCTGCGCCACCACA	TACGCTTGAGGTTCTCGCGGTA	234
FMgSSR-60132	scaffold159	p3	(GCC)6	Class II	73197	73214	TATTGCATGCTTTTCGGGCGGC	AAGGAGGTGAGCTTCCAGTCGT	288
FMgSSR-60134	scaffold4591	p3	(GCC)6	Class II	29119	29136	TCACGTTGTGGTTCGCTGTGT	AAAACGGCGGCGTTGTGGTA	331
FMgSSR-60135	scaffold65423	p3	(GCC)6	Class II	1352	1369	TCAGTTGTGGATGCACTGGC	CGCGCCTGTGAAACGGAATTGT	334
FMgSSR-60137	scaffold570	p3	(GCC)6	Class II	64270	64287	TCCACAACAGCACCCATCACCA	ATACTGTCGGGGAAGACGAAGG	241
FMgSSR-60138	scaffold2297	p3	(GCC)6	Class II	35672	35689	TCCACAACGTCCAAGCCCAT	TCGACCGGCCTGTGAAACAA	258
FMgSSR-60140	scaffold5449	p3	(GCC)6	Class II	32576	32593	TCGCATCACGAGGACAGCAACA	TGCAGCGAGGAGAAGCAACA	201
FMgSSR-60148	scaffold26423	p3	(GCC)6	Class II	855	872	TGCCAAGCTGCCTCGTGAAA	AGCGCTTTGAATTGGCCGGA	236
FMgSSR-60151	scaffold4694	p3	(GCC)6	Class II	17583	17600	TGCCTTGAAGCCGACGGAGAAA	TTTGAGTGCGGTGGTGCTTTGC	257
FMgSSR-60152	scaffold1943	p3	(GCC)6	Class II	63579	63596	TGCGATCAAGCACACAACGCA	AACCGAACGGAGTCGTCGTGAA	293
FMgSSR-60155	scaffold5761	p3	(GCC)6	Class II	39741	39758	TGCTGCCTCCTTTGTTGGCT	AAGCAAGGCAGCCAAAAGCACC	319
FMgSSR-60157	scaffold8672	p3	(GCC)6	Class II	14670	14687	TGCTTCTTCGGCTTCGCCTT	AGCCCACATCATCAGACCGCAA	251
FMgSSR-60158	scaffold787	p3	(GCC)6	Class II	32867	32884	TGGAATACGGGTTGCGTCCT	AAGATCGTGCGCACCTGGTGAA	330
FMgSSR-60162	scaffold32	p3	(GCC)6	Class II	57552	57569	TGGTACAGCGACTGCTGCAT	AGTAATTTCGCGTGCGGTCGT	341
FMgSSR-60167	scaffold12018	p3	(GCC)6	Class II	8681	8698	TGTGCCTACCGCGACCTTGTAA	ACCGTTTCCGTTTGCCTGCT	279
FMgSSR-60171	scaffold1107	p3	(GCC)6	Class II	28416	28433	TTCCCGAGTCAAACCGACGCAA	CGGCTTGCTCCTCCTCAACTTCTT	341
FMgSSR-60174	scaffold87327	p3	(GCC)6	Class II	602	619	TTCTCCACCATCTTGAGCGCGT	AAACCAGAGCGTCCAGCCAT	243

SSR_ID	Scaffold	SSR_Type	SSR_Motif	SSR Class	SSR_Start	SSR_End	Forward sequence	Reverse sequence	Product_size
FMgSSR-60175	scaffold12494	p3	(GCC)6	Class II	3418	3435	TTCTCTTGCCACCACACCGT	TGCGTGTGCGGGTTTTCACA	289
FMgSSR-60178	scaffold19730	p3	(GCC)6	Class II	11095	11112	TTGTACACCGGCTGACATGTGG	TGCGAATCAAGGAGGAGGGAACA	229
FMgSSR-60181	scaffold19344	p3	(GCC)7	Class I	228	248	AAGGGCGTGACTCTTGCGAT	ATGAGCACCTCGTACTTGCGCT	306
FMgSSR-60186	scaffold35538	p3	(GCC)7	Class I	893	913	AGCAGTGCAACTGCCGACGATT	ATGTACTTCGCCGCATTCCCCT	313
FMgSSR-60191	scaffold10840	p3	(GCC)7	Class I	23107	23127	ATGTGACCCACGGGCCCAAATA	TGGAGACTTGCATTGAGCGGGA	304
FMgSSR-60194	scaffold2206	p3	(GCC)7	Class I	54957	54977	CCGTTAGCACGTCACTCTGAGACA	AGGCGGCAACAAAGGTGAGTGA	330
FMgSSR-60196	scaffold24940	p3	(GCC)7	Class I	270	290	TCGACCATGAGCTCTTCGCGTT	TCCAGCCTTGAAAAGCCCACCA	267
FMgSSR-60197	scaffold11280	p3	(GCC)7	Class I	7708	7728	TCGTTGCAGCCCTTGAAGCA	AGTGCGTTTTTGGCCATGGAGC	246
FMgSSR-60198	scaffold3092	p3	(GCC)7	Class I	19433	19453	TGCATGCATGGACCCAACACCA	TTGTCCCTTTGAACAGACGGCG	306
FMgSSR-60200	scaffold4879	p3	(GCC)8	Class I	37347	37370	AATGGCCTCCATCTCCGCCTTA	TGCGGGCCACTGATCTGCTTTT	312
FMgSSR-60201	scaffold1090	p3	(GCC)8	Class I	87388	87411	ACACAACGCCGAACCGTTGA	AAGCGAATCCAGCCATGCGA	343
FMgSSR-60212	scaffold6237	p3	(GCC)9	Class I	4581	4607	GCGGCTGGAAGTAGCCATTCTT	TCCCCAATGGATAGCAGCAGCA	208
FMgSSR-60213	scaffold7590	p3	(GCC)9	Class I	20639	20665	TCTGCTGGCGTCCAACTTCTCT	GCCCCTCCATTGATCCGCCTAAAA	297
FMgSSR-60217	scaffold1725	p3	(GCG)5	Class II	2989	3003	AAAAGCGGCGTTTTGCCGGT	TCCTTTTGCGCCCTCGTTCCTT	349
FMgSSR-60219	scaffold340	p3	(GCG)5	Class II	17312	17326	AAAATTCAGTTCACCCGCCCGC	AGCCTCGTTGGGGTTCGTCAAA	211
FMgSSR-60221	scaffold1203	p3	(GCG)5	Class II	76574	76588	AAACAGCCCACAGTGGCACA	TTGCGCGGGCGAACGAAAAA	333
FMgSSR-60225	scaffold1992	p3	(GCG)5	Class II	48821	48835	AAACCCTAGAGCACCGGCTT	ACCTGTAATGCGGCACAGAGCA	342
FMgSSR-60226	scaffold113295	p3	(GCG)5	Class II	317	331	AAACCGATCGTCTGCTGGCT	TCGACGCTCTTCATCGTGCT	346
FMgSSR-60227	scaffold232	p3	(GCG)5	Class II	97521	97535	AAACGAGGAGAACCTTCCGCCA	GCCGGGAAATTGAGCAACGCAA	278
FMgSSR-60228	scaffold2226	p3	(GCG)5	Class II	69055	69069	AAAGCGGTGATGGTCTTGCCCT	TCCGCGTCGATAACATACCGCA	335
FMgSSR-60229	scaffold1066	p3	(GCG)5	Class II	26018	26032	AAAGTGAAGCGGCACAGCGA	AAGTGCAGACACGAAGCAGAGC	338
FMgSSR-60231	scaffold5329	p3	(GCG)5	Class II	4113	4127	AAATGCACACGCGCCGACAA	TGCCGCAACCAATTGCGAAGT	295
FMgSSR-60235	scaffold1372	p3	(GCG)5	Class II	64432	64446	AACAACGGCGGCAGACGTAA	ACCGTGGGTGTGTCGCATTT	311
FMgSSR-60240	scaffold25442	p3	(GCG)5	Class II	6904	6918	AACAATCACCGCCGCCAACA	AAATCCCTACCCCGCAATCCGT	309
FMgSSR-60241	scaffold5052	p3	(GCG)5	Class II	32328	32342	AACACAGACGGCCTGTAGCA	AACTCTAAAGCACGCGGCGGAA	331
FMgSSR-60245	scaffold211	p3	(GCG)5	Class II	143336	143350	AACATCCCAAACCGTCACCCGA	AGCGCCGAATCGAGAAGGAA	278
FMgSSR-60247	scaffold9882	p3	(GCG)5	Class II	17984	17998	AACATGGGCCACTTCTTCGGCA	AGCGTGTGCACCACCACTGTAT	289
FMgSSR-60250	scaffold547	p3	(GCG)5	Class II	102237	102251	AACCACCAACCTCGACCCAA	ATTCAGCCCCCAAGCAGCAA	316

SSR_ID	Scaffold	SSR_Type	SSR_Motif	SSR Class	SSR_Start	SSR_End	Forward sequence	Reverse sequence	Product_size
FMgSSR-60252	scaffold5225	p3	(GCG)5	Class II	44986	45000	AACCCACCACCGCTTCTCTCTT	AGAAGGCTTCGTTCAGCGCA	288
FMgSSR-60253	scaffold2430	p3	(GCG)5	Class II	16109	16123	AACCCAGCATCTTCTCACCCGA	CCCCACATCTTGCAACCGCAAT	205
FMgSSR-60256	scaffold2182	p3	(GCG)5	Class II	42263	42277	AACCGCTAATCTGCGACGGCTA	AATCATGCCGGCAGTCCAACCA	291
FMgSSR-60258	scaffold1745	p3	(GCG)5	Class II	10211	10225	AACGCACCCCTGCATCGAAA	TCAAGTACCAACCTGCGGGA	328
FMgSSR-60261	scaffold1013	p3	(GCG)5	Class II	41420	41434	AACGCTTCAGGCTTCACGAC	CGCGTCGGCAGAAGAAGAAGAA	350
FMgSSR-60263	scaffold1915	p3	(GCG)5	Class II	16443	16457	AACGTGACGCTCCACTCTGA	ACTGCGCCAGCACGTACAAT	218
FMgSSR-60265	scaffold6983	p3	(GCG)5	Class II	19240	19254	AACTCAATTCGGTTCACGCCCC	AGGTTCGTGATCACCGTCGTCT	281
FMgSSR-60272	scaffold33275	p3	(GCG)5	Class II	4855	4869	AAGAATGTACTGGCCGGCGGTT	AAAATTTTCGCGGAGGCGAGGC	243
FMgSSR-60276	scaffold9109	p3	(GCG)5	Class II	4692	4706	AAGATCAAGCGGCGGAAGCA	AGCGGATCGGCGAAGCTGAATA	317
FMgSSR-60278	scaffold296	p3	(GCG)5	Class II	81993	82007	AAGCCGCGCTACAACAACCT	TTCTTCCGCTTCTGCCCTCTGT	286
FMgSSR-60282	scaffold899	p3	(GCG)5	Class II	57405	57419	AAGCTTCGGCATCCGACGATGA	ACGTGGACCCGGAAATTGCAGA	275
FMgSSR-60283	scaffold446	p3	(GCG)5	Class II	29855	29869	AAGGAACATGAGGTCGCGCA	GGCAACGCAACATGGCAACA	316
FMgSSR-60292	scaffold30	p3	(GCG)5	Class II	177964	177978	AAGGCGGCACGGAGTGGAAAAA	ACAAGTGGAATCAAGCGTGCCC	306
FMgSSR-60295	scaffold2377	p3	(GCG)5	Class II	54854	54868	AAGGGTCCTTGAAGTAGCCGGT	GGAAGGAGAAGAGAGTGACGGTGA	339
FMgSSR-60296	scaffold15797	p3	(GCG)5	Class II	9318	9332	AAGGTAACGGCCAAGAGCAGCA	GCTGCGAAAATTCCACCGCT	318
FMgSSR-60298	scaffold3379	p3	(GCG)5	Class II	23114	23128	AAGTGCCCTGCCACAACAGAGT	TCACGTGGGCAACGCAAAGA	350
FMgSSR-60299	scaffold38730	p3	(GCG)5	Class II	694	708	AAGTTGCCTGCTCTGTTACGGC	AACGAGAAGAAACGCGGCGA	332
FMgSSR-60301	scaffold780	p3	(GCG)5	Class II	8031	8045	AATCGTGTGATAACGGCGGCCT	AGGAGGACCTTGTAGTTCTCGGGT	321
FMgSSR-60302	scaffold5002	p3	(GCG)5	Class II	44147	44161	AATCTCTGGTGCGGGTGCAGAA	ACGCGCACCTCAACTACCAGAA	332
FMgSSR-60304	scaffold9213	p3	(GCG)5	Class II	320	334	AATGCTCGCGGACACAGACCAA	TGCTGTCCGCTCCCCATTTCAA	334
FMgSSR-60306	scaffold1951	p3	(GCG)5	Class II	66238	66252	AATGGCGAGAAACTTCGGCGGT	TGGCGACCTCGGCGTATACTTT	345
FMgSSR-60307	scaffold1295	p3	(GCG)5	Class II	57272	57286	AATGGCGCAGTATAGGCAGTCC	TGCGCCGAGTACGCCATTCTTT	345
FMgSSR-60314	scaffold5790	p3	(GCG)5	Class II	27046	27060	AATTTTGCTGCCCCCGCTCA	ATGCAGAAGCTGGGCATCGT	308
FMgSSR-60318	scaffold496	p3	(GCG)5	Class II	120039	120053	ACAAGCACCGGAAGCTACAGCA	TTGTACAGCGGCAGCAGCAT	325
FMgSSR-60321	scaffold84	p3	(GCG)5	Class II	52357	52371	ACACATCACAGCAGCTTCCGCT	AGCGTGAGAAGGATGCCAAGCA	258
FMgSSR-60322	scaffold71047	p3	(GCG)5	Class II	727	741	ACACCACCACCAGCCGATTTGT	TAGTGCATCACCAGCTCCTCGT	258
FMgSSR-60324	scaffold579	p3	(GCG)5	Class II	31793	31807	ACACCATCAAGAAGCTGCCGGT	TTGTCCTTGTGTTCGCCACCGA	346
FMgSSR-60325	scaffold383	p3	(GCG)5	Class II	53931	53945	ACACGAGATTTGCAGAGGCGTCA	TGCGTGCAAAATGTCTCCGC	315

SSR_ID	Scaffold	SSR_Type	SSR_Motif	SSR Class	SSR_Start	SSR_End	Forward sequence	Reverse sequence	Product_size
FMgSSR-60327	scaffold21208	p3	(GCG)5	Class II	13343	13357	ACACGCTTTCAGCCCCATCCAT	TCGCTCTCGCCGGACATAAT	342
FMgSSR-60329	scaffold18219	p3	(GCG)5	Class II	12205	12219	ACAGCCGCGTTGCTTCATCA	TTCGACACGTTGGAGGCGTT	300
FMgSSR-60330	scaffold16416	p3	(GCG)5	Class II	14140	14154	ACAGCTCTGCGTCGGAAAGA	ACGGTGGAAGGAAAACCCGCAT	302
FMgSSR-60334	scaffold20807	p3	(GCG)5	Class II	5945	5959	ACATGTGGCATGCGGTGAGGAA	ACGCCATTAACGCCCCAACA	336
FMgSSR-60335	scaffold11342	p3	(GCG)5	Class II	22065	22079	ACCAAGAACCAAAGAGGGCGCA	AAAAACGCGAAACGCTCCCC	332
FMgSSR-60338	scaffold3452	p3	(GCG)5	Class II	9810	9824	ACCAGCAGCGAACCGACAAA	AGACCGGCCCGTCATGAAAT	332
FMgSSR-60342	scaffold8266	p3	(GCG)5	Class II	21163	21177	ACCCACCAATGGCAGCCAAA	TGCAAAGGAGCAGCGAAAGCA	346
FMgSSR-60343	scaffold4160	p3	(GCG)5	Class II	39003	39017	ACCCAGTCAGCCAACGACAACA	AACACCGTCTTCTTCCTCGCCT	350
FMgSSR-60345	scaffold1016	p3	(GCG)5	Class II	73043	73057	ACCCGCACGAAGTCATCGTATGT	ACCCGGCATTTGTCCCGTTT	281
FMgSSR-60346	scaffold11371	p3	(GCG)5	Class II	5742	5756	ACCGACGATGTCATCCACGCAT	AAGGTTTCCCTTGCCGACCGTT	339
FMgSSR-60348	scaffold186	p3	(GCG)5	Class II	62048	62062	ACCGGCGTTCGTTTCCAACA	TTGTGCTGCTGGTGCTGGTT	334
FMgSSR-60351	scaffold1202	p3	(GCG)5	Class II	33750	33764	ACCTCTATTCCCAGCACGAACCGA	TCCATCACAGCGTTGGCCTTGT	325
FMgSSR-60356	scaffold10126	p3	(GCG)5	Class II	15568	15582	ACCTTTCATTCGCTCGCGGACT	ACGACGAAGATGCCTCAACCGT	288
FMgSSR-60359	scaffold4739	p3	(GCG)5	Class II	17797	17811	ACGAAGCCGCAGGAGAGGAAAA	ACTGTGATGTGGGTCCATGGCT	337
FMgSSR-60360	scaffold6193	p3	(GCG)5	Class II	17092	17106	ACGACACTGTTTGCCGACGACT	TCATGGTTGAGGCAACCGCA	278
FMgSSR-60364	scaffold1683	p3	(GCG)5	Class II	59831	59845	ACGCAAACTGCCTCCCCTTTGT	CGCACTTAATCGCCTGGATTCG	345
FMgSSR-60370	scaffold127291	p3	(GCG)5	Class II	961	975	ACGCCGGCTTCTTGCTCTTCTT	AGGCGCACAAGTACTTCGACCA	323
FMgSSR-60375	scaffold1725	p3	(GCG)5	Class II	44492	44506	ACGCGACGTGGGTTACATGA	TTCCACCCGGCGCACAAAAA	248
FMgSSR-60381	scaffold935	p3	(GCG)5	Class II	19987	20001	ACGCTGTTGACGAGCTCAACGTA	AGCTCGTTCCCTTTCCTTCCCT	322
FMgSSR-60392	scaffold306830	p3	(GCG)5	Class II	328	342	ACGGGCTGCCGACGATAACAAA	TCCCCCTCCACACCAACCTTTT	235
FMgSSR-60394	scaffold360	p3	(GCG)5	Class II	33651	33665	ACGGGTAGGTGATGTTGTGGCT	ACAGTCAACACGTCCGGCAA	323
FMgSSR-60405	scaffold1828	p3	(GCG)5	Class II	72278	72292	ACTGATTGCATGCGGCGTGA	TTTCGGTTTCGAGCTCGCGT	261
FMgSSR-60408	scaffold2038	p3	(GCG)5	Class II	20931	20945	ACTGCGAGCAGCATCCGAAA	TCTGCACCGTGTGTGAAGGAGA	318
FMgSSR-60409	scaffold3473	p3	(GCG)5	Class II	13538	13552	ACTGGCTGCTGTTGCTGACA	GGCGTGGTTGAGGAGGTGTTTT	298
FMgSSR-60410	scaffold38945	p3	(GCG)5	Class II	4077	4091	ACTGTGACGGGCTTGTGTGT	TTGACGGCGTTGCTGTGGTA	300
FMgSSR-60412	scaffold4320	p3	(GCG)5	Class II	32918	32932	ACTTGAACACAGACCCCGCA	ATGTACACCGGCCAGTTCGTCT	349
FMgSSR-60414	scaffold19336	p3	(GCG)5	Class II	14428	14442	ACTTGCAAATTCGCCCGTTCCC	TCTCCCTGCAAGCCCTGAACAA	303
FMgSSR-60415	scaffold5682	p3	(GCG)5	Class II	37116	37130	AGAACAGGGGTCGCGTCAAA	CGTTGCAGCGGCAAGAGAAGAA	348

SSR_ID	Scaffold	SSR_Type	SSR_Motif	SSR Class	SSR_Start	SSR_End	Forward sequence	Reverse sequence	Product_size
FMgSSR-60417	scaffold328618	p3	(GCG)5	Class II	244	258	AGAACGGCACGTACGACATGA	TCGCCTGGTTCGCGATCTTCTT	204
FMgSSR-60418	scaffold16678	p3	(GCG)5	Class II	12677	12691	AGAAGATTGGCGGCAGAGGA	ACGTCTTGGCTTGCCTTAGTCTCC	322
FMgSSR-60419	scaffold584	p3	(GCG)5	Class II	110318	110332	AGAAGCACCGCCATCTACTCCT	TCCTGCCGCGAAGAAAAGGAA	305
FMgSSR-60420	scaffold767	p3	(GCG)5	Class II	49763	49777	AGACGACGTAGGCGACAAGGAA	CAGCGCACGAGTTCATCCTCTT	252
FMgSSR-60423	scaffold9111	p3	(GCG)5	Class II	14278	14292	AGAGGAATCGCCGGAACGAT	TCCCACTCGTCGGAAAACCCAA	213
FMgSSR-60425	scaffold2914	p3	(GCG)5	Class II	51492	51506	AGATACCGCCGCACAGCAAA	TAAAAGTCACGCAACCGCCCCA	313
FMgSSR-60426	scaffold4283	p3	(GCG)5	Class II	6060	6074	AGCAAGAAGCACCGCTGGAAGT	ACGACGTCAAGAACATGCCACCA	315
FMgSSR-60429	scaffold16226	p3	(GCG)5	Class II	14031	14045	AGCACTGAGGAGGGAGGTGATA	TGCCATGGGCTTTATCGTCGTG	205
FMgSSR-60430	scaffold1460	p3	(GCG)5	Class II	61722	61736	AGCAGACACCAATCGCACCT	TTCTTTCCCCAACGCGGACA	307
FMgSSR-60431	scaffold6272	p3	(GCG)5	Class II	14695	14709	AGCAGCATGATTCCGCAGCA	TGGACAATACGGAAGCGGGTGT	335
FMgSSR-60433	scaffold5914	p3	(GCG)5	Class II	27873	27887	AGCAGCGATTCATGGCCTTCCT	TGTACATGCGTTCCAGCCGT	279
FMgSSR-60434	scaffold2859	p3	(GCG)5	Class II	21795	21809	AGCAGCTGCACATTCCGTGT	TGATCCCGAGGCAGCATCTTGT	330
FMgSSR-60440	scaffold2919	p3	(GCG)5	Class II	38647	38661	AGCGCCTGTGCTGTGCAAAA	TTTGCGGCGTTACCTGTGTTCG	338
FMgSSR-60443	scaffold6338	p3	(GCG)5	Class II	45111	45125	AGCGCGTTGACGGAGGATTT	ATGGGCCATGGGCACACAAT	276
FMgSSR-60448	scaffold15222	p3	(GCG)5	Class II	4637	4651	AGCGGTACAGGTCGATGTTGGA	TCAGGGCTACAGATCAACGGCA	313
FMgSSR-60451	scaffold10510	p3	(GCG)5	Class II	23327	23341	AGCTCCGAGATGGTGTCAACGA	GGAGCCGAAGCGTTTGCACTTT	303
FMgSSR-60454	scaffold125	p3	(GCG)5	Class II	107142	107156	AGCTTCATCGTCGCCTTGTCGT	ACCGCCGCCACTAAGAAAACCA	329
FMgSSR-60456	scaffold2409	p3	(GCG)5	Class II	25624	25638	AGGAAGCAGCAGCAAATGAGCG	GCGTGTAAGGGTGCGAGTTGTT	310
FMgSSR-60463	scaffold7405	p3	(GCG)5	Class II	9529	9543	AGGAGGTGCTAGGTTTCTGCGT	ACTGCAATGGCGCAGGAGAA	326
FMgSSR-60471	scaffold17207	p3	(GCG)5	Class II	6796	6810	AGGCGCAATAAAAAGGCCTCGC	AAGATGACCATGGCGGTGACGA	304
FMgSSR-60473	scaffold762	p3	(GCG)5	Class II	65482	65496	AGGGCCAGGCAACAACCGAAAT	ACTACTTACCGTCGCAGGCT	306
FMgSSR-60474	scaffold32589	p3	(GCG)5	Class II	2133	2147	AGGTTAGGAGCAGGACGCGAAT	ACCGCCGACCAAAGCAAACA	314
FMgSSR-60476	scaffold14995	p3	(GCG)5	Class II	3279	3293	AGTCATCATAGTCATGGGGTCGCC	AAATCCCCAAATCGCCCTCCGA	288
FMgSSR-60477	scaffold1673	p3	(GCG)5	Class II	85363	85377	AGTCGTCGTTGTGTCCGTCCAA	ACGCAACGCAAGGCAGCTAA	228
FMgSSR-60478	scaffold3966	p3	(GCG)5	Class II	33819	33833	AGTGCGGCCCCAGTAATTGAGA	ACAAGACCGTCTGCGAGAAGCA	339
FMgSSR-60480	scaffold13865	p3	(GCG)5	Class II	7308	7322	AGTTTGACACGCGGAAGCTGA	AAAGTGCCGCAAACAGCGGA	257
FMgSSR-60482	scaffold8584	p3	(GCG)5	Class II	8465	8479	ATAAAAACCCACTGCCCGGCGA	AGTTGCGATGGCAGCTCGAA	320
FMgSSR-60485	scaffold1082	p3	(GCG)5	Class II	32781	32795	ATCACATCGGACCAGTTCAACCCG	ACGTGCGTGCACAACAAACCT	349

SSR_ID	Scaffold	SSR_Type	SSR_Motif	SSR Class	SSR_Start	SSR_End	Forward sequence	Reverse sequence	Product_size
FMgSSR-60486	scaffold3986	p3	(GCG)5	Class II	42034	42048	ATCAGACACAGACAGCGCCCTT	CGCCGCCGCATTCAATACAA	228
FMgSSR-60489	scaffold1733	p3	(GCG)5	Class II	50311	50325	ATCATCCATCAGCAGCCGCACA	AGTCCCCGAAATTGAACACCGC	345
FMgSSR-60495	scaffold74	p3	(GCG)5	Class II	169456	169470	ATCGATCGGTGCCCGAAGAA	GTGGCACGCGAAGAAGAGAA	309
FMgSSR-60496	scaffold975	p3	(GCG)5	Class II	3173	3187	ATCGCGACCTTGATCATCGCGT	TGCACTGACGACGAGTACGA	285
FMgSSR-60497	scaffold1045	p3	(GCG)5	Class II	57679	57693	ATCGCGATGCAAACCAGGGGAT	TGGGGAAAGGGCGTTTCAGA	311
FMgSSR-60501	scaffold1594	p3	(GCG)5	Class II	51255	51269	ATCGTCGTCGTCGGAACACACA	TTCACCCGACAACAAGGACGGA	344
FMgSSR-60502	scaffold94382	p3	(GCG)5	Class II	1025	1039	ATCGTGCTGATTGCGCTGCT	ACGCTGACTGTCGCTTTGCT	348
FMgSSR-60504	scaffold5258	p3	(GCG)5	Class II	37472	37486	ATCTCGACCACCCCAACCTCAA	AGCTCGTGCGTGGGTCTTGAAA	286
FMgSSR-60508	scaffold17188	p3	(GCG)5	Class II	4675	4689	ATGAACGGCTGGGATTGCTCGT	GCCACAGGTTGAACTCCTGGAT	342
FMgSSR-60510	scaffold8548	p3	(GCG)5	Class II	13242	13256	ATGACGACCGTGGTGGAAGT	TTCAGCCCAGTTTCGCCTAGCA	257
FMgSSR-60513	scaffold167	p3	(GCG)5	Class II	7237	7251	ATGCAGTGAAGGCGAAGCGT	TGGGGTGAACAAGACTAGTCGCA	221
FMgSSR-60515	scaffold115319	p3	(GCG)5	Class II	902	916	ATGCGCTGCAGGATCAGGTTGT	ATCGGCAACCTGCTGATCGTCT	305
FMgSSR-60519	scaffold347	p3	(GCG)5	Class II	129170	129184	ATGGCGCCAATGCTTGGGAA	TCCGCTCCGGCTCAAGCTTATT	217
FMgSSR-60522	scaffold22948	p3	(GCG)5	Class II	1669	1683	ATTGCGGACACCCTGCGAAA	TGTCGTCGGCGCTCGCTATAAA	271
FMgSSR-60524	scaffold2298	p3	(GCG)5	Class II	26312	26326	ATTGGAGAAGGCCGCAACCACA	ATGAGCTCCCACACCATCCACA	290
FMgSSR-60532	scaffold3065	p3	(GCG)5	Class II	13957	13971	CATCGCTACCGAGGGAAATGGA	ACATGGACGATCAGCTGCGA	264
FMgSSR-60533	scaffold134	p3	(GCG)5	Class II	5031	5045	CCCGTCGTTTTTGATTTGCCCGT	TGTCGGGCGTCCACGAATTGAA	300
FMgSSR-60535	scaffold4274	p3	(GCG)5	Class II	37096	37110	CCTCATCATCGCCTTGTTGCGA	ATGCACGACCGTGACTTGACCA	272
FMgSSR-60538	scaffold572	p3	(GCG)5	Class II	38996	39010	CGCCACTTGCGTGGGTTTTT	TTCGGTTGCCGTGCAGAACT	250
FMgSSR-60539	scaffold5717	p3	(GCG)5	Class II	12929	12943	CGCGCGTGGCGCCAATATTTTA	ACATGACCACCAGCAAGCACGA	288
FMgSSR-60540	scaffold15856	p3	(GCG)5	Class II	2545	2559	CGGCAATCTCGTGCGCGATAAA	ACAGCGGCGGAAGCATCAAA	348
FMgSSR-60542	scaffold14581	p3	(GCG)5	Class II	5740	5754	CGGTGGTGGCGCTGCTATAAAT	ACGTCGTCGAAGAGGTACTCTGA	308
FMgSSR-60543	scaffold2582	p3	(GCG)5	Class II	59325	59339	CGTCCCCAGCATGGAATCAAGGAA	AAGCGATTGCAGGAATGGCAGC	335
FMgSSR-60544	scaffold4656	p3	(GCG)5	Class II	37522	37536	CGTCGGCATCTTTGCTCACA	TGCCGGCTGATGTCTGTTTCCA	332
FMgSSR-60551	scaffold14495	p3	(GCG)5	Class II	1936	1950	CTCCAAGCGCAAGCAACCAA	TTCCAGTTGCGGCGCTTCTT	337
FMgSSR-60555	scaffold827	p3	(GCG)5	Class II	33876	33890	GCAAAAGCTAATCGCCGTGCGT	TCGCCGATATGCAGTCGAAGGA	326
FMgSSR-60557	scaffold550	p3	(GCG)5	Class II	71025	71039	GCAAACGCACCGAACCTCCTAA	TCGCGTCTTCTGTTTCGTTGCG	346
FMgSSR-60568	scaffold5336	p3	(GCG)5	Class II	9726	9740	GCGCATTCGCATCATCGCAA	TTGTGGTACTGCAGCATCCCGT	348

SSR_ID	Scaffold	SSR_Type	SSR_Motif	SSR Class	SSR_Start	SSR_End	Forward sequence	Reverse sequence	Product_size
FMgSSR-60573	scaffold9010	p3	(GCG)5	Class II	24016	24030	GCGTCCTTGTAATACCCGCTCA	GTCGGAGAGGAAGAAGCCGATT	338
FMgSSR-60575	scaffold1680	p3	(GCG)5	Class II	37034	37048	GCGTTGGTGCTGCAGCTTTT	TGCGCGGAAAAGGACCGATT	342
FMgSSR-60576	scaffold332	p3	(GCG)5	Class II	98514	98528	GCTCACGCTGTTACGCTTTGCT	TACTGCTACCGCGTGTTCTCGT	306
FMgSSR-60586	scaffold215	p3	(GCG)5	Class II	27638	27652	GGCCGCGATTTCACACAAATGGT	AAAACCCCGAGCCCCGCTAAAA	318
FMgSSR-60587	scaffold6911	p3	(GCG)5	Class II	25878	25892	GGCCGGTTGTTTGTTGCGAA	TCATCGACTGGTCGGAGCACTT	235
FMgSSR-60588	scaffold1182	p3	(GCG)5	Class II	88295	88309	GGCGGCAACCCAACAAACCAAT	TGTCGTGCTTCCCCTTTTGCCT	210
FMgSSR-60589	scaffold8245	p3	(GCG)5	Class II	4455	4469	GGCGTCGGAAGGAAACACTCAA	ACGATGATGCTGCAGTCGCCTT	325
FMgSSR-60594	scaffold12483	p3	(GCG)5	Class II	10501	10515	GTGGGCAAGAAGATGGGCAA	TGCACGGGCGATTGGGATTT	291
FMgSSR-60597	scaffold2446	p3	(GCG)5	Class II	11900	11914	TACAGCGGCGAGAAGAAGCA	TGGCGCCGAAGATCACGATT	337
FMgSSR-60598	scaffold424332	p3	(GCG)5	Class II	120	134	TACCATGCCGCGATGATCCAGT	CCTCGCAGCTTCTGGTCAATCA	214
FMgSSR-60599	scaffold11155	p3	(GCG)5	Class II	17909	17923	TACTTCCGGTGTCGGCACAA	ACCGGCATTGGCATCTGCAT	284
FMgSSR-60601	scaffold309	p3	(GCG)5	Class II	31289	31303	TACTTCGCCGCCAAGACCAACA	AACCCAAGCGTGCACTCGAT	338
FMgSSR-60602	scaffold10853	p3	(GCG)5	Class II	1715	1729	TAGAAAGCCCGTGGGCCCAAAT	TTGCTTCGTGTTAGCTGGGCGT	260
FMgSSR-60603	scaffold517	p3	(GCG)5	Class II	14980	14994	TAGCGCGTCGCTTGTTCTCT	ACCATGCCGAACGTTGACGA	322
FMgSSR-60604	scaffold3061	p3	(GCG)5	Class II	36037	36051	TAGCTCAAGCTGAACCCGCA	TGGCGCGTTGTTGATGAAGC	293
FMgSSR-60605	scaffold249	p3	(GCG)5	Class II	21310	21324	TAGTTGCGGAGCACGAAGCTCT	ATCGCCTCGGCATCCAAGAACA	221
FMgSSR-60606	scaffold11973	p3	(GCG)5	Class II	23800	23814	TATAGTGACACGGCGGCAAGCA	GCTGCAATCCCATGCTTCGAGA	296
FMgSSR-60607	scaffold1550	p3	(GCG)5	Class II	38715	38729	TATATACGCGGGTTGCGGCTGT	TCGTGGGTGTGCTCGCTAGAAA	266
FMgSSR-60609	scaffold1925	p3	(GCG)5	Class II	38257	38271	TATTTCTGCGCCCGCGAATCCT	ATCGTCAGCGCCAAACCCAA	251
FMgSSR-60610	scaffold774	p3	(GCG)5	Class II	44337	44351	TCAAAGCCCATCCTCGGCTTCA	AGGCTTCTCCGCAACGTTCA	235
FMgSSR-60611	scaffold909	p3	(GCG)5	Class II	27292	27306	TCAAATCTGACGCTGTGCCGGA	AGCACATCACCGCCCTCTATCT	301
FMgSSR-60612	scaffold1614	p3	(GCG)5	Class II	3433	3447	TCAACACCACACTGTCACGCCA	AGTAGCTGTAGTAGACGCCAGCAG	341
FMgSSR-60617	scaffold3496	p3	(GCG)5	Class II	39140	39154	TCAGAAAGCAGCGAGTGGACGA	TTCGTTCTTGCGGCACAAGCTG	339
FMgSSR-60618	scaffold2499	p3	(GCG)5	Class II	36072	36086	TCAGCCGAGAGCACGCAGAAAT	TGTTGTGCCTGAGCTGAAAGCC	343
FMgSSR-60622	scaffold468	p3	(GCG)5	Class II	54381	54395	TCCAGCGTGTTCACGAAGCTCA	ATCCTGCAGCAACACGGAGACA	267
FMgSSR-60623	scaffold10848	p3	(GCG)5	Class II	6962	6976	TCCATCCTCCTCGACCTGAAACGA	ACCTGTAGCAGGGGTTCGACTT	292
FMgSSR-60630	scaffold928	p3	(GCG)5	Class II	68431	68445	TCCCGTGATCCGCATCGCAAAT	AAATCGCCGTCGATCTCCTCGT	334
FMgSSR-60632	scaffold809	p3	(GCG)5	Class II	34094	34108	TCCGAGGAAAGCGTCTCGTT	AACAACAGCCACGGGCATCA	328

SSR_ID	Scaffold	SSR_Type	SSR_Motif	SSR Class	SSR_Start	SSR_End	Forward sequence	Reverse sequence	Product_size
FMgSSR-60634	scaffold9445	p3	(GCG)5	Class II	7848	7862	TCCGTCAGCGGGCGTTTTTA	AATCGAACGCCGGCTCTGAACT	266
FMgSSR-60636	scaffold60	p3	(GCG)5	Class II	190064	190078	TCCGTGAACGTGTTCATGCCGA	TGTGCCCAACAGAATCGCGT	298
FMgSSR-60637	scaffold7643	p3	(GCG)5	Class II	24911	24925	TCCTCCCGCTTGAACCTGTTGT	AACACCAACAGCACCAGCCA	302
FMgSSR-60644	scaffold2121	p3	(GCG)5	Class II	21708	21722	TCGACATCGTCGCCCAAGTA	GCCGCCGCAAAATCTTCGAT	301
FMgSSR-60645	scaffold90447	p3	(GCG)5	Class II	878	892	TCGACGAGATCATCAAGCGCAC	TTTGCACGCGACGAGCAGTT	348
FMgSSR-60649	scaffold15452	p3	(GCG)5	Class II	1318	1332	TCGAGCCAACACGTGACGAT	GGCGTGGATGAAACCCTCATCA	235
FMgSSR-60653	scaffold1214	p3	(GCG)5	Class II	70448	70462	TCGATGCACCGGCAATCGGTTA	TTTTGCCGTGGTCGTCGTCGTA	321
FMgSSR-60656	scaffold8199	p3	(GCG)5	Class II	18300	18314	TCGCATCGCATGCACCGAAT	AGTGAGATTCATCAGCCGCGT	346
FMgSSR-60658	scaffold5440	p3	(GCG)5	Class II	15411	15425	TCGCCAAAACGTCATCGGCA	ATGTCAGCATCTTCCGGCGCTT	320
FMgSSR-60660	scaffold343394	p3	(GCG)5	Class II	102	116	TCGCCCGAAGCCTATTTCCGTT	AAGTGAAAACCCTTCACCCCACCC	263
FMgSSR-60661	scaffold37547	p3	(GCG)5	Class II	2969	2983	TCGCCCGCAGCTATTAGCAA	TCCATGGCAGCATGCTTGTGAA	338
FMgSSR-60663	scaffold994	p3	(GCG)5	Class II	41302	41316	TCGCGTTGGTGCGACGAAAA	TCTGCCCTGCCGTGAAACAA	319
FMgSSR-60665	scaffold19336	p3	(GCG)5	Class II	14212	14226	TCGCTTTCAGGACCCACTCGAT	CCCTATTGCGGATCGCGAAGTT	284
FMgSSR-60667	scaffold1115	p3	(GCG)5	Class II	70973	70987	TCGGGATCCTTGCAGTAGGAGT	ATTCCCACGCCTCCCCAAAA	341
FMgSSR-60668	scaffold6752	p3	(GCG)5	Class II	26436	26450	TCGGGCCAAAGCAAATCCTCCA	ATTACCCGCGATCAACACCAGC	232
FMgSSR-60670	scaffold792	p3	(GCG)5	Class II	69993	70007	TCGGTTCGGTTGCTCCGATTCA	TGCCAGTGCCTTGACGCTTT	325
FMgSSR-60673	scaffold1357	p3	(GCG)5	Class II	45280	45294	TCGTCGAAGAAGCAGCAGCA	ATCGCGGGTTGATTTCGCCA	297
FMgSSR-60685	scaffold476	p3	(GCG)5	Class II	9414	9428	TCTCAGAAGGCAAGGTTTCCGC	TGATTGCTCCCCACTCGCTT	295
FMgSSR-60686	scaffold3379	p3	(GCG)5	Class II	16143	16157	TCTGCCACGCTCCATCGTTT	TTTCCGCTTCTGCTTTGCGAGC	271
FMgSSR-60687	scaffold4652	p3	(GCG)5	Class II	38619	38633	TCTTCAGATCACGGCGACGA	TTGACGTCTTGCAGCTTGCCGA	322
FMgSSR-60690	scaffold16286	p3	(GCG)5	Class II	10910	10924	TCTTGCACCTCAGCTTCTCCGT	TATAAGGCAAGCCGCACGCA	297
FMgSSR-60691	scaffold1262	p3	(GCG)5	Class II	4198	4212	TCTTGGTGCCATCGAGCAGT	TGCCACGAAATGCCTGTCAGA	203
FMgSSR-60692	scaffold8352	p3	(GCG)5	Class II	21411	21425	TCTTTGCGGCCGCGATTCTT	CGTTCCGCAAGCCCAACAACAA	274
FMgSSR-60693	scaffold4374	p3	(GCG)5	Class II	13911	13925	TGAAGAAACCCGGCGGCAAA	AAAACCACCGTGCGCTGAAAGG	282
FMgSSR-60696	scaffold12153	p3	(GCG)5	Class II	9637	9651	TGACAGACTCCATGACCACCGT	ATTACCAGGGCACGTGCAAGGA	350
FMgSSR-60698	scaffold2975	p3	(GCG)5	Class II	17801	17815	TGACGATGCGAGAGCGAACA	AACGGGTGAACAATGGCGCA	326
FMgSSR-60699	scaffold31124	p3	(GCG)5	Class II	7727	7741	TGAGACGCTGAAGCCTCGGAAA	ATGCAAAACGCCCCCTCGTT	309
FMgSSR-60700	scaffold777	p3	(GCG)5	Class II	85987	86001	TGAGCTTTTGGAATGTGCGGCG	AGTGTTCCTGGCGGCAAACA	332

SSR_ID	Scaffold	SSR_Type	SSR_Motif	SSR Class	SSR_Start	SSR_End	Forward sequence	Reverse sequence	Product_size
FMgSSR-60703	scaffold21417	p3	(GCG)5	Class II	1548	1562	TGATTACGATGTCTGCCGGCGT	TCGCGCAGCACAAGTCACAA	313
FMgSSR-60709	scaffold6423	p3	(GCG)5	Class II	23570	23584	TGCAGCTGATCGGCTTCTTCCT	GGCACAATGCCCAGGTTGTGTT	322
FMgSSR-60711	scaffold10761	p3	(GCG)5	Class II	9177	9191	TGCAGGTTGTTTGTGGTTCGTGG	TGGCACCGTCGCCATTTCTT	207
FMgSSR-60720	scaffold133	p3	(GCG)5	Class II	111946	111960	TGCCGGAGACACAACAGAGAGA	ACCAGCAGCAGCAGCAACAA	212
FMgSSR-60724	scaffold14917	p3	(GCG)5	Class II	19747	19761	TGCGACTGTGGCCAAGGATGAA	AGCAGCAGCCCGAGCTATTT	205
FMgSSR-60725	scaffold222	p3	(GCG)5	Class II	43631	43645	TGCGAGAAGGTGATGGTGTGCAT	CACACACGTAGACGCCTTGCAT	344
FMgSSR-60726	scaffold2382	p3	(GCG)5	Class II	56774	56788	TGCGCGCAAGGATGGATTTACG	ACTTATCAACGCGCACAGCGA	336
FMgSSR-60728	scaffold2956	p3	(GCG)5	Class II	8573	8587	TGCGGCAGAGGGATTTGGATGT	TGGCTCTCGCTGGTCTCTTT	220
FMgSSR-60729	scaffold782	p3	(GCG)5	Class II	86523	86537	TGCGTGGAAAAGCAAGCAGCA	TCGCTGGACACTGGCTGGAAAT	269
FMgSSR-60730	scaffold575	p3	(GCG)5	Class II	70120	70134	TGCGTGTCATCTACGCCGACTT	CGGAATTGCATTGGTGCGTTGC	324
FMgSSR-60732	scaffold3183	p3	(GCG)5	Class II	35549	35563	TGCTGCATGCGCTCACACAA	AAAGCTGCGACGCAACAAGC	291
FMgSSR-60736	scaffold298	p3	(GCG)5	Class II	90324	90338	TGCTGCTTCTGTGGGATGGA	ACATGGAACCAGCCCAGAACGA	232
FMgSSR-60748	scaffold2605	p3	(GCG)5	Class II	32057	32071	TGGAATCAACCAACTCCGGCA	TAAGCGAACGAAGACCGCGT	348
FMgSSR-60750	scaffold2095	p3	(GCG)5	Class II	32646	32660	TGGAGAGCGACTTTATGGCGGA	ATGGCGCCTGCCTTATCCGAAA	207
FMgSSR-60752	scaffold915	p3	(GCG)5	Class II	30614	30628	TGGCAGACTCGAGTGCTTCATCA	TTCATGTGCTCGGCCTCGAA	331
FMgSSR-60753	scaffold15458	p3	(GCG)5	Class II	2455	2469	TGGCAGCCATGGTGGTTGAT	TTCGTTGGCATCGACGACGGTT	329
FMgSSR-60754	scaffold62412	p3	(GCG)5	Class II	890	904	TGGCGACAACAACCTGGAGA	TCCGGTGGCGAGCTTCTTGTTA	328
FMgSSR-60755	scaffold27360	p3	(GCG)5	Class II	11247	11261	TGGCGCTGAATGATGTCCCTGA	TGCTGCTTCATCAGGCGAGT	336
FMgSSR-60756	scaffold13218	p3	(GCG)5	Class II	5037	5051	TGGCGGATGCGGATGTGGAATA	TTCCTGGCCCAATAGCCACT	231
FMgSSR-60757	scaffold320	p3	(GCG)5	Class II	86922	86936	TGGCTGATGCCATTGGTGCTGA	TCTCCCCAGCAGCACCCAAAAT	214
FMgSSR-60761	scaffold8132	p3	(GCG)5	Class II	2143	2157	TGGGTCAGGCGGCGAAGAAAAA	TGGGCTGAACAAACATGCGTGG	332
FMgSSR-60762	scaffold354	p3	(GCG)5	Class II	112099	112113	TGGGTCATCTGCAGGGTGTTCA	GCAGCAGCAGCGAAGTGATGAT	332
FMgSSR-60763	scaffold567	p3	(GCG)5	Class II	79159	79173	TGGTCCCCGTTATTGAGCACGA	TCGTCACATCCAAGCCCCCTTT	286
FMgSSR-60764	scaffold127291	p3	(GCG)5	Class II	1128	1142	TGGTCGAAGTACTTGTGCGCCT	ACCCAGCCATGCAAGGAAGCAA	305
FMgSSR-60765	scaffold11850	p3	(GCG)5	Class II	10227	10241	TGGTCTGGGACAAAAGCGACCA	TCTTTGCGGGTGTGCTGCAT	225
FMgSSR-60766	scaffold107355	p3	(GCG)5	Class II	601	615	TGGTGCGCCGCTTTTTCGAT	ACGACGACCATCACGCCAATCA	292
FMgSSR-60769	scaffold8859	p3	(GCG)5	Class II	22180	22194	TGGTGGGTGCGATTCCACTT	AAGGCGGTCACGGACTTGAA	342
FMgSSR-60770	scaffold15020	p3	(GCG)5	Class II	15986	16000	TGGTGGTTGACGAGGTGGAAGAA	AACTCCCAACTGTAGCGCCCAT	325

SSR_ID	Scaffold	SSR_Type	SSR_Motif	SSR Class	SSR_Start	SSR_End	Forward sequence	Reverse sequence	Product_size
FMgSSR-60772	scaffold833	p3	(GCG)5	Class II	27518	27532	TGTACGACGCCGGCTTCTTGTT	TGGTTGCACGCACCTTCTTTGC	302
FMgSSR-60773	scaffold7799	p3	(GCG)5	Class II	26891	26905	TGTAGTGCTGCTCCGACGAA	AGCCTCCGCTTCGCTTCTTCTT	299
FMgSSR-60779	scaffold1560	p3	(GCG)5	Class II	49654	49668	TGTCGTGTGGTGGCCATGAA	GCGACTTCTTCATGATGCGCGA	217
FMgSSR-60782	scaffold9714	p3	(GCG)5	Class II	14964	14978	TGTGCACGAGGATCGAGGTTGT	AACATCGCATGAAGCGGTGCAG	310
FMgSSR-60783	scaffold42	p3	(GCG)5	Class II	217348	217362	TGTGCATCCAGACCTTGAAGACGG	ACTCGGACTACTACGACGACGA	345
FMgSSR-60785	scaffold216	p3	(GCG)5	Class II	118422	118436	TGTGCGAGCTTCCGTTCCAGAAA	ACGCGCGTTAAAAACCGTGC	349
FMgSSR-60787	scaffold7185	p3	(GCG)5	Class II	19878	19892	TGTTCGCCACCGGTGAGATT	AGTGCTCGTCGCTGATGTTCCA	234
FMgSSR-60788	scaffold2173	p3	(GCG)5	Class II	41343	41357	TGTTCGTCGTGCCGTCTGTT	ACGTGCATCGTCATCGCCAA	346
FMgSSR-60789	scaffold15883	p3	(GCG)5	Class II	7876	7890	TGTTCGTTGGCAGTGCGAGA	ACAACTCTGGAGCCAAACGGGT	208
FMgSSR-60791	scaffold4948	p3	(GCG)5	Class II	36483	36497	TGTTCTTCCTCGCGCACCTT	GCGACCTTCAAAAGCAGCAGCA	311
FMgSSR-60795	scaffold9049	p3	(GCG)5	Class II	2313	2327	TGTTTGATGCTTTGCCTGAGCC	TCGCACACCCTGCGAACAAA	212
FMgSSR-60798	scaffold500	p3	(GCG)5	Class II	63661	63675	TTAAATCCCGCGGCGAACGA	AAACACGTACGCCGTCCTCT	321
FMgSSR-60799	scaffold969	p3	(GCG)5	Class II	66874	66888	TTCAAAGTGCGGTGGCGGAA	TATGCAGTTTGGCAGCGGCA	294
FMgSSR-60801	scaffold429	p3	(GCG)5	Class II	87047	87061	TTCAGCAAGGTGTCAGCACCGA	TGCGTACGCGGACAAGCTAA	314
FMgSSR-60804	scaffold3010	p3	(GCG)5	Class II	23716	23730	TTCCAGTTCCTGAACGCCATGC	GCGAGGGTGTGCTTGTTGTTGT	305
FMgSSR-60808	scaffold56291	p3	(GCG)5	Class II	1864	1878	TTCCGGTTCTTGTTGCTCAGCG	AGCCTGGAATGGCATGGCGTAA	295
FMgSSR-60810	scaffold785	p3	(GCG)5	Class II	22254	22268	TTCCTGTCGCTGCGCTTTACCT	TTTACTTTCCGCTCGCCCCTGT	315
FMgSSR-60813	scaffold136	p3	(GCG)5	Class II	11946	11960	TTCGTCGTCGTCACCACCAACA	ACACCTTGCTGTGCCGGTCTTT	235
FMgSSR-60814	scaffold2247	p3	(GCG)5	Class II	16928	16942	TTCGTGAGGTAGCGGAAGAGCA	ATGCAGCCAGACATCGAAGACG	337
FMgSSR-60816	scaffold855	p3	(GCG)5	Class II	3293	3307	TTCTGACGGCGGCTTGAATCGT	ATTGCTTGGGTGGCTGTGGCTT	281
FMgSSR-60818	scaffold17550	p3	(GCG)5	Class II	11758	11772	TTGACGCGAGCCTCCATCGAAA	TGGAGCAGTAGCGAGGAAGAAG	314
FMgSSR-60826	scaffold10	p3	(GCG)5	Class II	98712	98726	TTGCGGGTCTTGAAGGACGAGA	ACGAAGGCGACTTTGCTGACGA	258
FMgSSR-60827	scaffold1073	p3	(GCG)5	Class II	60922	60936	TTGCGTGGCCGTGAAGAAGT	AACCGGTCCGAAACCCTAGGAA	345
FMgSSR-60831	scaffold4938	p3	(GCG)5	Class II	25756	25770	TTGGACAAGGCAACACGGCA	AAGCTTTGACTTGGCGTGCG	304
FMgSSR-60832	scaffold814	p3	(GCG)5	Class II	85504	85518	TTGGCAGTGGAGACGATGTGGA	TCGCTCGATTCACCGTCCAT	330
FMgSSR-60833	scaffold2878	p3	(GCG)5	Class II	55308	55322	TTGGCCGTGTACACCTTGCT	TTCTCTCCCTCTCCTTCTCGCA	343
FMgSSR-60837	scaffold106206	p3	(GCG)5	Class II	611	625	TTGGCGTCCTTGGTGGACATCT	AACAAGGACGGCTTCATCGCGT	350
FMgSSR-60838	scaffold3875	p3	(GCG)5	Class II	23288	23302	TTGGCGTTGCCTGTCACGAA	ACCATGTGCCCTGTGCCGATAA	228

SSR_ID	Scaffold	SSR_Type	SSR_Motif	SSR Class	SSR_Start	SSR_End	Forward sequence	Reverse sequence	Product_size
FMgSSR-60840	scaffold1830	p3	(GCG)5	Class II	21259	21273	TTGTCGTTGCGGCGGCATTA	ACTGCGAACACCGCCACCTTAT	329
FMgSSR-60842	scaffold5879	p3	(GCG)5	Class II	13444	13458	TTGTGCTTCCTGCCCACCATCA	TATCGATCGCGCCACACCAA	313
FMgSSR-60843	scaffold3338	p3	(GCG)5	Class II	29258	29272	TTGTGTGTCTCCGTCGTCGTCA	TGCGCGGTAACGGCATTTTGA	251
FMgSSR-60849	scaffold682	p3	(GCG)5	Class II	13407	13421	TTTCCTCCACGTTGGTCGTCGT	TCGACCGCCTCAACACAAGTCA	210
FMgSSR-60851	scaffold5002	p3	(GCG)5	Class II	8790	8804	TTTCTTAATCGCGCTCCCCCGT	ACAAGCGAAAAGTCGAGCAGGC	350
FMgSSR-60855	scaffold1087	p3	(GCG)5	Class II	67361	67375	TTTGCGGGCGCGCGATTATT	GCAAGCAAAACGTGCGTCTCCT	205
FMgSSR-60863	scaffold2446	p3	(GCG)6	Class II	11612	11629	AAAGAAACCCAGTAACGCGACG	AAGATGGCGCCGAAGATCACGA	200
FMgSSR-60865	scaffold11690	p3	(GCG)6	Class II	19343	19360	AACATGCAAACTGGTGGTCGGC	ACGGTGACCACAGCACAACCAA	299
FMgSSR-60866	scaffold11103	p3	(GCG)6	Class II	20084	20101	AACCAACGCCCTGAAGGTCT	ATGAGTAACAGTCCGTGGGCCT	283
FMgSSR-60867	scaffold2992	p3	(GCG)6	Class II	4081	4098	AACCCTAGAAGCAGCGACCGTT	TTGAGGGGGCTGCGTGCATTTA	224
FMgSSR-60870	scaffold14569	p3	(GCG)6	Class II	9086	9103	AACCGCCACGCCTTCTTACA	ACACCTGGACCTGGTTGTTGCT	342
FMgSSR-60872	scaffold51955	p3	(GCG)6	Class II	3196	3213	AACGCGAACCCGTTCGTCAA	ATGATGTCGGCCAGTGCGTT	323
FMgSSR-60874	scaffold3001	p3	(GCG)6	Class II	28338	28355	AAGAAGCGCGAACGAACCTGGA	TCACATCAGCTGCAGGGCGAAT	295
FMgSSR-60877	scaffold10224	p3	(GCG)6	Class II	28294	28311	AAGCAACGCCACCACATCGT	AAAGCATGGGGCGCCTAGAA	346
FMgSSR-60878	scaffold22895	p3	(GCG)6	Class II	456	473	AAGCTCGCGGAAGACTGCAA	TCAGTCGATCACAGCCGCAGAA	317
FMgSSR-60887	scaffold1401	p3	(GCG)6	Class II	27404	27421	ACAAAGATGGCCGCTACCAGGA	TGATGAAGCCGCTGTTGCAGA	211
FMgSSR-60888	scaffold141	p3	(GCG)6	Class II	161857	161874	ACAAGCAGAGGAAGCTCTCGCA	AACATGGAGCACGATGGCGT	292
FMgSSR-60890	scaffold81	p3	(GCG)6	Class II	132871	132888	ACACCGGCAGAGAATTGCGT	AAAGGCCAGCTAGCACTGCGAA	295
FMgSSR-60893	scaffold2095	p3	(GCG)6	Class II	30613	30630	ACAGCAAGAACGTGGAGGACGA	ACTACTAGGCGTGGCCGTTGTT	297
FMgSSR-60894	scaffold3881	p3	(GCG)6	Class II	4501	4518	ACAGGCCTCACCTTGCTGAA	AAGTTGTCGACGAGGGTGGAGA	341
FMgSSR-60895	scaffold8608	p3	(GCG)6	Class II	11169	11186	ACCACCTTCCCCTGCATCCAAA	ACCACCGGAAACACGCACTA	302
FMgSSR-60897	scaffold39739	p3	(GCG)6	Class II	2818	2835	ACCAGCAGCACCGTCAGTTT	ATGCCCAACGTGCCAGCTAT	295
FMgSSR-60909	scaffold347	p3	(GCG)6	Class II	50585	50602	ACGGCGAGGGCGAAATGTTA	TCGGCAGCCAAGTCCGTTTT	341
FMgSSR-60919	scaffold22489	p3	(GCG)6	Class II	6752	6769	AGCAGCACCCCGATGTTTGA	CAGGTGCTTTTCTCGCAACTCGT	343
FMgSSR-60921	scaffold11955	p3	(GCG)6	Class II	10793	10810	AGCATCGCCTCCATGGCATT	TTTTTGCTCCGTGTCCCATCCC	344
FMgSSR-60924	scaffold2709	p3	(GCG)6	Class II	5169	5186	AGCTCCTGGATCGCATCATCTC	AGGTGAACCTGGTGGAGTGGTT	341
FMgSSR-60925	scaffold5310	p3	(GCG)6	Class II	28303	28320	AGGATGGGTGGTGCGCAGTAAA	ATGCCCCTGCTACAACCTTTCG	349
FMgSSR-60928	scaffold2175	p3	(GCG)6	Class II	42481	42498	ATCGTTGCGGCGATGACGAA	TGGGATCTTTGTGCTCGGGTGT	348

SSR_ID	Scaffold	SSR_Type	SSR_Motif	SSR Class	SSR_Start	SSR_End	Forward sequence	Reverse sequence	Product_size
FMgSSR-60930	scaffold6412	p3	(GCG)6	Class II	35515	35532	ATGCGGCTTTTCCGGTCGTCAT	TTGGCCGCTGGGATCCAAAT	333
FMgSSR-60935	scaffold2015	p3	(GCG)6	Class II	52285	52302	ATTGCCGTTGCCACTCCTCACA	CGTCGCATAACGAGCACAGGAA	202
FMgSSR-60941	scaffold601	p3	(GCG)6	Class II	35590	35607	CCATGCAAGATGCAGCCAAGCA	TCGTCATCAAGGGCAGACGACA	336
FMgSSR-60943	scaffold301	p3	(GCG)6	Class II	99303	99320	CGCAATCCAAAATAGGGGGCGT	TGGAAGCTCGCGGCATTGAA	236
FMgSSR-60945	scaffold3980	p3	(GCG)6	Class II	9151	9168	CGCGAGTGCACCAGCATGAAAA	TCCCGCCATCAAAGAACCCT	316
FMgSSR-60946	scaffold13572	p3	(GCG)6	Class II	20476	20493	CGGCAGCAACTGCGAATGAGAT	ATCTCAGCGCACAGCAAAGC	245
FMgSSR-60950	scaffold11223	p3	(GCG)6	Class II	20480	20497	GCACTCCCCACTTGAACGCAAT	ATCGCAGCTAGCTTCTGCCACA	239
FMgSSR-60952	scaffold58	p3	(GCG)6	Class II	157623	157640	GCCAGAATTGAAAGCGGCGACA	AAAACAAACCCCGGCCACGA	316
FMgSSR-60957	scaffold6095	p3	(GCG)6	Class II	21830	21847	GGTGCGGCTGGACGATTTAT	TGGACCTGCGTAGATGTGCT	209
FMgSSR-60959	scaffold122	p3	(GCG)6	Class II	63125	63142	GTGCCTGCCAAAGGCGAAAT	AACAGCGAGTCAGCGAGTCCAA	339
FMgSSR-60960	scaffold7152	p3	(GCG)6	Class II	38903	38920	TAATTGACGCCTCTCAGCACGC	TGAAAGCCCGGTCGTGTCAT	224
FMgSSR-60961	scaffold6595	p3	(GCG)6	Class II	33724	33741	TACAGCGTGCGGCCACTATT	AAATCGCGGGAGGAAGCAGA	317
FMgSSR-60963	scaffold346	p3	(GCG)6	Class II	85548	85565	TAGAGCCCGTTGCTTCGTTCGT	TCAGGAATGCTCATGGCACGCT	340
FMgSSR-60971	scaffold5697	p3	(GCG)6	Class II	539	556	TCAGTACCCGCATCGATGTCCA	ACCTCCAACCCTTTTCCCGA	326
FMgSSR-60982	scaffold1885	p3	(GCG)6	Class II	48602	48619	TCGCACTCGCGGCATTATCA	ATCCACGCGATGGAACGAACCT	300
FMgSSR-60987	scaffold13031	p3	(GCG)6	Class II	12318	12335	TCTTCCGAACGCAACTCGCT	TTGGGCTGGGCTGTAGATCGTT	267
FMgSSR-60988	scaffold24457	p3	(GCG)6	Class II	9731	9748	TGAAGCTTCGGATGCAGGGTCA	GCCAGCACCACAGAAGACACAT	288
FMgSSR-60990	scaffold1572	p3	(GCG)6	Class II	39476	39493	TGATGATGGTGATGGCGACGGT	CGCGAACCGCACCTTCTTCTTA	208
FMgSSR-61000	scaffold977	p3	(GCG)6	Class II	53278	53295	TGGCTCAAAGAGGAGCTCGCAA	TGCTGGTTGGTTTCTTGGCG	205
FMgSSR-61003	scaffold40291	p3	(GCG)6	Class II	2621	2638	TGTCGGATCTCTTGCGCCTCTT	TCCCCAACTCGGCATCATCTCT	350
FMgSSR-61007	scaffold64	p3	(GCG)6	Class II	83493	83510	TTCACCCAACAACGGACGCA	AGAGATGAAGGCGAGGCGTGAT	242
FMgSSR-61008	scaffold1029	p3	(GCG)6	Class II	56314	56331	TTCACGACGGCGGAGTATGA	TGTTGGTTGCGGAGGATGCAGT	265
FMgSSR-61012	scaffold7743	p3	(GCG)6	Class II	837	854	TTCTCCGAGGAGGAGGCCATAA	AAGCATAATCCACGACGGCCCA	233
FMgSSR-61017	scaffold8408	p3	(GCG)6	Class II	23369	23386	TTGATTGTTGCGGCTCCGGT	TGCCGCCGTCAATTTACCCA	348
FMgSSR-61020	scaffold5715	p3	(GCG)6	Class II	3809	3826	TTGCGAATGGCCTCGACGTT	AGGAGCCCAGTCCAAATCCCAA	335
FMgSSR-61026	scaffold2271	p3	(GCG)6	Class II	11159	11176	TTGGCAGGACCGGGGAAAAGTT	AGCGAAGCAACACTGCAACAGC	320
FMgSSR-61032	scaffold1659	p3	(GCG)6	Class II	29294	29311	TTTGCTGCTGTTGCCGCCTT	TGAGCCTGACACACACACGACA	292
FMgSSR-61033	scaffold92	p3	(GCG)6	Class II	67370	67387	TTTGGGCCCCTTTCATCGACCA	ACGCGAAGTCGAAGCAGTTGT	346

SSR_ID	Scaffold	SSR_Type	SSR_Motif	SSR Class	SSR_Start	SSR_End	Forward sequence	Reverse sequence	Product_size
FMgSSR-61035	scaffold474	p3	(GCG)7	Class I	9307	9327	AAGCTCCGGCAGCTCAAACT	AGCAAGCCGCCTTCCTTCTTCA	237
FMgSSR-61037	scaffold6439	p3	(GCG)7	Class I	6556	6576	AATCGGAACGGGGTCAGTCACA	AGGCCGCAGCGTTTGTTGTT	313
FMgSSR-61044	scaffold2266	p3	(GCG)7	Class I	49037	49057	ACGCGGACGATGGAACTTGGAA	AACATCCAGGCCCGCGTAACAT	251
FMgSSR-61047	scaffold2124	p3	(GCG)7	Class I	14809	14829	ACTTGCAGTTGCCGGCCATA	AGCACAGCCGAGTGAGTTGTGT	319
FMgSSR-61052	scaffold825	p3	(GCG)7	Class I	19030	19050	AGCGGCAGGCTTCTTGTTGT	CCAATGGACCTTACCGCACACA	324
FMgSSR-61054	scaffold5644	p3	(GCG)7	Class I	24841	24861	AGGACTCCTCGCATTGCATGT	AAGATTTCGAGGCGGCTGAGGA	325
FMgSSR-61055	scaffold8543	p3	(GCG)7	Class I	5116	5136	AGGGGTCCCAGCTGCAAATTGT	AGAGTGATGAGTGCGCGGTT	277
FMgSSR-61056	scaffold113	p3	(GCG)7	Class I	29786	29806	ATCACGGTGGCTGGATTTGGGT	ACATAAAAACCCCGCACCCCAG	215
FMgSSR-61057	scaffold4319	p3	(GCG)7	Class I	25617	25637	ATCAGCGAGCAATGGAGTGGGA	TGGAGTTAGCCACGCGCAAA	211
FMgSSR-61058	scaffold1738	p3	(GCG)7	Class I	3858	3878	ATCCTCAACCGTCACGTCCTCT	CACGCGCAAAGCCTGTCATT	350
FMgSSR-61059	scaffold4791	p3	(GCG)7	Class I	30061	30081	ATCGACATGTTGACGAGCACGC	TGGCGAAACAGCATCATCCCCT	292
FMgSSR-61065	scaffold299	p3	(GCG)7	Class I	96069	96089	CGGCACATCCTGAAAACGGA	TTCCGAAAACAGTCCACCACGC	308
FMgSSR-61067	scaffold5892	p3	(GCG)7	Class I	23187	23207	GCCGCCGTGGAAGTAGATGATT	AACTTCCTACCCAGCAGCAGCA	340
FMgSSR-61068	scaffold315	p3	(GCG)7	Class I	76313	76333	GCCGTTGGCCCTGTCTGAAAAA	TGATGCATCCTGGCCAAGCAGA	278
FMgSSR-61070	scaffold9987	p3	(GCG)7	Class I	4878	4898	GGCATATGGCGGACTGCTGAAA	ATGGAGAACACAGCTGGTGCGA	313
FMgSSR-61072	scaffold28335	p3	(GCG)7	Class I	4810	4830	TATCCGCCACCCAGTTGTCACT	CGACTTCGTCTTTTCGCGCCAT	274
FMgSSR-61073	scaffold4227	p3	(GCG)7	Class I	4522	4542	TCCTCCATGGTCCTTTCGACCT	TCGCGCATCTAAACCCTTGCTCA	315
FMgSSR-61074	scaffold24172	p3	(GCG)7	Class I	4382	4402	TCGACGGAAGCGCCAAGATTGT	GCGATCCAAACTAGGGGGTGCTTT	293
FMgSSR-61077	scaffold645	p3	(GCG)7	Class I	80580	80600	TCTGACATCGTGCAGGGGTT	TTGACGAGGAAGGCGAGCATCT	314
FMgSSR-61087	scaffold1457	p3	(GCG)7	Class I	292	312	TTGCTTGCTCCGCCGGAAAT	ACCAAAGAGCCACAGCACAGGA	297
FMgSSR-61091	scaffold1703	p3	(GCG)7	Class I	6452	6472	TTGGCGGCGCGAAAAGAACA	ATGCCTGCGCAGCCCAATTT	348
FMgSSR-61092	scaffold3203	p3	(GCG)8	Class I	21361	21384	AAAGCCCGAACCGATGAACCGA	TCGGCGTCAAGCTTGTGGAT	299
FMgSSR-61095	scaffold17207	p3	(GCG)8	Class I	5501	5524	ACGCAACACAACAGCAGCACA	TTGTTGGTTGAGAGCCCGTCCA	210
FMgSSR-61098	scaffold458	p3	(GCG)8	Class I	122388	122411	ATCTGCAACACGCCCCAACT	CCGCGCAACAAAGAGGCAAA	275
FMgSSR-61099	scaffold528	p3	(GCG)8	Class I	96617	96640	ATGCCGCTGTAGAACTCGAGCA	CAGCCGCAGCCGCAATTTTT	286
FMgSSR-61101	scaffold3256	p3	(GCG)8	Class I	23574	23597	CGGATCGAGAAAATTTCGCCGC	ACTTCGGGTGGCTTTGAACCGT	301
FMgSSR-61102	scaffold16926	p3	(GCG)8	Class I	9631	9654	GCAGATTCGCCATTTGCGAGGA	ATTGCTGCTCCGGGTGTTTCCA	348
FMgSSR-61103	scaffold8893	p3	(GCG)8	Class I	24296	24319	TCCATTTGCAGCAACCGTCG	CAACAAGCACACGCACGCAT	297

SSR_ID	Scaffold	SSR_Type	SSR_Motif	SSR Class	SSR_Start	SSR_End	Forward sequence	Reverse sequence	Product_size
FMgSSR-61105	scaffold2173	p3	(GCG)8	Class I	35322	35345	TGAGAATGTCTGCGCCGCTT	TGCAGCAAGAGGCGTTCGAT	302
FMgSSR-61109	scaffold5355	p3	(GCG)9	Class I	25648	25674	AGAGCCTGGAATCAAGCCCGTT	ATGCGGCCCATCGAAACCAA	319
FMgSSR-61112	scaffold113737	p3	(GCT)10	Class I	357	386	TGCTTGTTTTACGTCGGCCACC	AGCTACTTGTACGGCAACGGCA	267
FMgSSR-61117	scaffold1515	p3	(GCT)5	Class II	3554	3568	AAAAGAAGGCGCGCGCAAGT	ATCAGCCCTTGATGTACGCTGC	348
FMgSSR-61126	scaffold8373	p3	(GCT)5	Class II	19450	19464	AACAACCACTGCTGCTGCCA	TGATCACTGGGTGGGTGCACAA	243
FMgSSR-61130	scaffold1799	p3	(GCT)5	Class II	36906	36920	AACGGGGAACAAATTGGCCAGC	GGCACATGCAAAATGGCAGTCT	329
FMgSSR-61134	scaffold225	p3	(GCT)5	Class II	134011	134025	AACTCAGCACGAATGCCGCCAA	ATCGATACGCTCGCGGCAAA	325
FMgSSR-61140	scaffold1406	p3	(GCT)5	Class II	39848	39862	AAGATGGCAGCTGCTGAGGCAA	AGAGTCACCACCATGTGCCA	326
FMgSSR-61145	scaffold1304	p3	(GCT)5	Class II	21158	21172	AAGTTCGCAGGCCATTAGGCCA	TGGTGGAGGCAAAGGCAACA	303
FMgSSR-61148	scaffold6778	p3	(GCT)5	Class II	25338	25352	AATAAGTGGCAACGCCGCCT	ACCCATCAGCCACCATCCTCTT	348
FMgSSR-61149	scaffold1455	p3	(GCT)5	Class II	13226	13240	AATCCGCAACCAACGGACTGGA	AAAGCAGCGAGGAATGCGGA	284
FMgSSR-61150	scaffold19647	p3	(GCT)5	Class II	6837	6851	AATGAAGCCCAGCTAAGGCCCA	CGACTTTCACCCGCCGCAAAAT	206
FMgSSR-61152	scaffold822	p3	(GCT)5	Class II	84702	84716	AATTCCCGCCAAAAGGCACACG	TGCGTTTGCACGAGCACTGA	311
FMgSSR-61156	scaffold9038	p3	(GCT)5	Class II	29348	29362	ACAAGGCCCTCGCCAAACTCTT	TCACCGTTGCTTCCTGCACT	349
FMgSSR-61157	scaffold2420	p3	(GCT)5	Class II	31086	31100	ACACGCAAGCAAGCACTTGGA	GCGTGTTTGTTTCTCGGCGT	337
FMgSSR-61159	scaffold21385	p3	(GCT)5	Class II	635	649	ACAGGCTCTTCTTGCTCGCCAA	TTGGTGATCAGCCCACTGGCAT	216
FMgSSR-61160	scaffold144	p3	(GCT)5	Class II	161320	161334	ACAGGTGACTGCACAACGCT	TCACGAGCTGTGCGTGAAATGC	340
FMgSSR-61161	scaffold5475	p3	(GCT)5	Class II	7933	7947	ACAGGTTGTGGCTGCTGGTGTT	GCGACGATTAATATGCCGCCGA	323
FMgSSR-61163	scaffold18023	p3	(GCT)5	Class II	4688	4702	ACCAAAAGTGCAGGTAGCCCCA	TGCAGGAGCGACCATTGCAAGA	283
FMgSSR-61166	scaffold8805	p3	(GCT)5	Class II	20712	20726	ACCACCACCGCGTCAGGAATTT	AGCACGCTGGATTCCGCATT	263
FMgSSR-61168	scaffold4819	p3	(GCT)5	Class II	27166	27180	ACCACTTCCGCGCTCACAAA	ACCATCTCACTAGTGCTGGCGT	283
FMgSSR-61180	scaffold2999	p3	(GCT)5	Class II	23982	23996	ACGCCTGTCCCAACACAAGACA	TGGTCACCTGTCAGTACCTGCT	345
FMgSSR-61181	scaffold476	p3	(GCT)5	Class II	96684	96698	ACGGCACGCAGTTAAAACGGA	TCCGCGCCTTAGCAATTTCGGA	343
FMgSSR-61183	scaffold3980	p3	(GCT)5	Class II	42670	42684	ACGGTTCGTAGAGCGTGTCGAT	CGGTGTTCGCGGAATATTGGCA	277
FMgSSR-61184	scaffold27574	p3	(GCT)5	Class II	1079	1093	ACGGTTGTCATCAACGCGGA	AGCGTGAAGAGCAGCAGCAA	326
FMgSSR-61185	scaffold1180	p3	(GCT)5	Class II	45882	45896	ACTCGATGTTAACGCGGCCA	TGGAAAAAGCGGTCGACAACGC	320
FMgSSR-61190	scaffold3799	p3	(GCT)5	Class II	10868	10882	AGACACGAACAGCGGCAACA	TTTGGCTGGCATCGGCATCA	318
FMgSSR-61193	scaffold2515	p3	(GCT)5	Class II	31073	31087	AGAGCCGTGTGTCGACATTCAA	AAGCAAAGCGGAAGCGCTCA	324

SSR_ID	Scaffold	SSR_Type	SSR_Motif	SSR Class	SSR_Start	SSR_End	Forward sequence	Reverse sequence	Product_size
FMgSSR-61198	scaffold78	p3	(GCT)5	Class II	68103	68117	AGCACAGCACAACTGCAGGA	TGTTGTTTGGTCCGGCGTCA	231
FMgSSR-61200	scaffold4618	p3	(GCT)5	Class II	37643	37657	AGCACGAGGTTGCCGAAGTTGA	TGCTCCCCGGAATGCTGGAAAA	235
FMgSSR-61201	scaffold16015	p3	(GCT)5	Class II	4071	4085	AGCAGGAAACAACAGCCGCA	ACGCGTCCGTAGGAACAAGCAT	236
FMgSSR-61202	scaffold16762	p3	(GCT)5	Class II	1820	1834	AGCATCATCAACAGCCGCGGTA	TGGACACCAACAACCAGGCA	278
FMgSSR-61205	scaffold6845	p3	(GCT)5	Class II	4540	4554	AGCTCACAAGTGGGCCGTAA	TCCCGTGCATGCAAAAGGGT	202
FMgSSR-61218	scaffold10741	p3	(GCT)5	Class II	23130	23144	AGTGCCTCCTATTCCCACTTGAG	ACAGTGGTTGCCTGTCCCTT	350
FMgSSR-61219	scaffold2855	p3	(GCT)5	Class II	14430	14444	AGTTGGTACGGTGGTGGTGCAA	AGCTCGTACACGGTCCGTAA	243
FMgSSR-61220	scaffold6453	p3	(GCT)5	Class II	35213	35227	ATATGGCTCGGCAGCTTTGCCT	AAGGCGCAACAGAAGCGGAA	221
FMgSSR-61233	scaffold30407	p3	(GCT)5	Class II	937	951	ATGGCTTCATCCTGCGGCCTTT	TGCGGCGTAAATCGGCGTAA	262
FMgSSR-61235	scaffold8826	p3	(GCT)5	Class II	11350	11364	ATTAGCATGGGTGCAGCGGT	ACCGCATGCAGGTCTTTGGA	260
FMgSSR-61236	scaffold2914	p3	(GCT)5	Class II	814	828	ATTCGGCTGGGGTGTCTGTTGT	TCAGGCAAACGGCTTTGGCA	329
FMgSSR-61241	scaffold743	p3	(GCT)5	Class II	25090	25104	ATTTTTATGCCGCTGCTGGCCG	ATTGCAGCGCGGAGGATTAC	322
FMgSSR-61242	scaffold1213	p3	(GCT)5	Class II	10788	10802	CAACCGCGGTGCGATTTGAT	TTCAAGGCCCATGCCTGCAGAT	260
FMgSSR-61244	scaffold269773	p3	(GCT)5	Class II	261	275	CATCCAAAGCTGTTGGCGCT	AGCAGGATCAATCTGGCGGCTA	211
FMgSSR-61245	scaffold7931	p3	(GCT)5	Class II	9347	9361	CCAACCCGTTCCTGCCTTACTT	TCACTGAAAGCCAGCCCAAAGC	204
FMgSSR-61246	scaffold178312	p3	(GCT)5	Class II	472	486	CCCCAAAGCGCCTCAAACGAAA	AGTTGCGTGTCGGTGAGACA	261
FMgSSR-61247	scaffold20731	p3	(GCT)5	Class II	7091	7105	CCCGCTCGCTTTTTCTCCGTTT	AGCCGTCCGTACAGAACGCAAA	255
FMgSSR-61249	scaffold57862	p3	(GCT)5	Class II	104	118	CCGCCGCGATCAAAGAAGCAAA	TACTCGCTGTCATTGCCGTCGT	317
FMgSSR-61253	scaffold3202	p3	(GCT)5	Class II	46076	46090	CGCCGCACCACGAAACTATGAT	AATGCAGAAGACGCACGCCA	200
FMgSSR-61255	scaffold380385	p3	(GCT)5	Class II	161	175	CGGCTTTTGTACACGCTTTTGCG	TCGCGCCTCCTCTCTCTGTTTT	225
FMgSSR-61259	scaffold2077	p3	(GCT)5	Class II	33940	33954	GCACGCCCTTGTACTTCTTGCT	ACGTCGCCACAGTTCCCAAA	321
FMgSSR-61260	scaffold255963	p3	(GCT)5	Class II	232	246	GCACGGGAACCTGCACCAAAAA	AGCTGCTGCTGCCAGAGAAA	215
FMgSSR-61262	scaffold847	p3	(GCT)5	Class II	55330	55344	GCACTGTTTTGCTGGCTTGCCT	CGTACTTCGCCGCCAGTTTGAA	305
FMgSSR-61264	scaffold4976	p3	(GCT)5	Class II	8763	8777	GCATGCTGCCAGAACGTGGTTT	TTGGCAACACTCGCGGTTCA	266
FMgSSR-61265	scaffold7463	p3	(GCT)5	Class II	17030	17044	GCCATGTCCGGCGTCATTTGTT	ATCTCGAGCTTATGCGGTGCGT	235
FMgSSR-61266	scaffold11177	p3	(GCT)5	Class II	6498	6512	GCCGCAGACATGCTTGCTTTGT	AAATACGCCCCGGCTCTCAA	304
FMgSSR-61270	scaffold2858	p3	(GCT)5	Class II	27658	27672	GGATCCAACGACGCAACGAACT	ACGCGTTTTTCTCTCGCCCA	241
FMgSSR-61271	scaffold5296	p3	(GCT)5	Class II	8196	8210	GGCGCTCGATGTATGTGGTTGT	TCTGGCAGTACAACAAGGACGC	344

SSR_ID	Scaffold	SSR_Type	SSR_Motif	SSR Class	SSR_Start	SSR_End	Forward sequence	Reverse sequence	Product_size
FMgSSR-61277	scaffold6878	p3	(GCT)5	Class II	16850	16864	GTCGCAACGGCAATGCCAAA	ACATACGCTTGCGGTTTGGTGC	256
FMgSSR-61282	scaffold298	p3	(GCT)5	Class II	27646	27660	TACGGCTTCGTGCCTTCGTT	ATTGCTCCGTGGACCTTGCT	211
FMgSSR-61287	scaffold7707	p3	(GCT)5	Class II	24537	24551	TCACCCATCTCTCTGCGAGCAA	GCACGCTCGCTCTCTTTTGGAA	345
FMgSSR-61289	scaffold2368	p3	(GCT)5	Class II	43814	43828	TCAGCGACCTCACAGTGCAT	AATACGGCAATCAGGCGACGCT	322
FMgSSR-61290	scaffold115762	p3	(GCT)5	Class II	695	709	TCAGCGCAAAAGCCTGCTGA	AACTTGAACCGATGCTGGCGGT	286
FMgSSR-61292	scaffold8	p3	(GCT)5	Class II	169759	169773	TCATCTCTCACTCGCTTGCCCA	TGCACGCACGTACCGATGAA	325
FMgSSR-61302	scaffold11599	p3	(GCT)5	Class II	20571	20585	TCCTCAGCATCGCCATTACCGT	AAGGGCAGCTGCAGAACGAA	254
FMgSSR-61304	scaffold434	p3	(GCT)5	Class II	30726	30740	TCCTTCACGAACCGCATCTCCT	TCATCCGATGCCAAATCCAGCTC	221
FMgSSR-61306	scaffold232086	p3	(GCT)5	Class II	182	196	TCGAACCCGAGAAACAGCAGGA	AGCAAGAAGCGAAGCAGCCT	223
FMgSSR-61309	scaffold1557	p3	(GCT)5	Class II	30942	30956	TCGCCGATGCTCCACAAGAA	TGCACCATGCTAGACTTCTGCG	218
FMgSSR-61310	scaffold326114	p3	(GCT)5	Class II	236	250	TCGCTGCTGCTAGGACACAA	TCTCCGTGGATCTGTGGAGCAA	206
FMgSSR-61311	scaffold325	p3	(GCT)5	Class II	322	336	TCGGTTGCTTTTGTGCGCGT	GGTCGCCGATCTGTCACAAGAA	293
FMgSSR-61313	scaffold2948	p3	(GCT)5	Class II	46544	46558	TCGTCCATGAATCGCTGGCTCA	ACCGGCGTGGTAGATGAGGAAA	347
FMgSSR-61318	scaffold691	p3	(GCT)5	Class II	96132	96146	TGAAGGACCAGCGCGTGTTT	CCGCATTGAGGTGCAACCGTTTT	211
FMgSSR-61319	scaffold2403	p3	(GCT)5	Class II	14865	14879	TGACGCCTCGTTCATGCCGTAA	TTTGCCTACGTCGTCAAGCACG	329
FMgSSR-61321	scaffold692	p3	(GCT)5	Class II	38956	38970	TGAGCCGCGCCAATATCTCT	ACTCCATGCCCGACAACCTCTT	255
FMgSSR-61324	scaffold38462	p3	(GCT)5	Class II	3044	3058	TGATCAGTCGATTCGCTCGCT	AGCCGATTCCCCAACTCCACAT	267
FMgSSR-61326	scaffold571	p3	(GCT)5	Class II	20764	20778	TGCAAATCCGTCGCGTTCGT	CGCCTGCAACACGCATCATT	217
FMgSSR-61329	scaffold7878	p3	(GCT)5	Class II	8137	8151	TGCACTGGTCACCCTTCGTGTT	GCAAGGCACCCTGCAAAAGCAA	294
FMgSSR-61336	scaffold2719	p3	(GCT)5	Class II	6618	6632	TGCTCAATCTTGCTTCGCACGC	TGCATCGCGTGTCGTCCAAA	280
FMgSSR-61341	scaffold25932	p3	(GCT)5	Class II	3085	3099	TGCTGCTACAGATGGTGCTGCT	CGAGTGGCACGAATTGGCCTTT	345
FMgSSR-61343	scaffold52420	p3	(GCT)5	Class II	1101	1115	TGCTGCTGCTGCTGCTTATCT	ACACCGACTGTGCTTGTGAGAGT	247
FMgSSR-61344	scaffold381131	p3	(GCT)5	Class II	180	194	TGCTGGCCACCACGATCTTCTA	TCGAGCTGGAAGTAGCCCTTGA	211
FMgSSR-61346	scaffold3405	p3	(GCT)5	Class II	29751	29765	TGCTTGCGTCGTCGTCTTCT	GCGCTGCTGATGTGAACGTGAA	225
FMgSSR-61349	scaffold2296	p3	(GCT)5	Class II	44161	44175	TGGAAAGCCCTGATAGCGCA	AGTGAAGATTGGAGATGGCCGCA	296
FMgSSR-61350	scaffold214	p3	(GCT)5	Class II	157655	157669	TGGCAGCTTTGCAGCTGTTGT	TCAAACCCCGGCTTCCAGTCAT	338
FMgSSR-61351	scaffold21440	p3	(GCT)5	Class II	3794	3808	TGGCCAACCGATCTCCATCCTT	AGGCGACGACACGGTTTGTT	348
FMgSSR-61352	scaffold5138	p3	(GCT)5	Class II	10684	10698	TGGCCCAAACAGCCTATCGTCA	TCCTCGCCAAGTTCGACGAGTT	292

SSR_ID	Scaffold	SSR_Type	SSR_Motif	SSR Class	SSR_Start	SSR_End	Forward sequence	Reverse sequence	Product_size
FMgSSR-61355	scaffold2221	p3	(GCT)5	Class II	60405	60419	TGGCGCACACGGTGAATTGAA	AATGATTGTGCGAACCGCCG	314
FMgSSR-61356	scaffold12731	p3	(GCT)5	Class II	21249	21263	TGGCGCCTTGAAGTTTGCTGA	TCGTCAACAATTGGGCTGGGCT	279
FMgSSR-61357	scaffold981	p3	(GCT)5	Class II	34438	34452	TGGCGGCTTTGCCTTATGCT	ACATGCAAAAGCAGACACGGGG	263
FMgSSR-61359	scaffold4396	p3	(GCT)5	Class II	19541	19555	TGGGCTCCATTGTGCTCTGCTT	AGTTGAACACGGCGGGGAACTT	343
FMgSSR-61366	scaffold282050	p3	(GCT)5	Class II	643	657	TGGTTTTGGGCTGGCCACAT	TTTCTTCCCCTTGCCAACGCCA	274
FMgSSR-61368	scaffold8466	p3	(GCT)5	Class II	6070	6084	TGTCGGTTCATCACGTCGCT	AGCGAAGCCGCATCCCATTT	306
FMgSSR-61369	scaffold78	p3	(GCT)5	Class II	19318	19332	TGTCTCAGCGGCGTACCTTGAA	AACTGTGCCGTGCAACAGGA	232
FMgSSR-61374	scaffold27	p3	(GCT)5	Class II	223180	223194	TGTGTACGCACGCAACGCAA	TTCGCAGAAGCAAACGGGGT	314
FMgSSR-61377	scaffold30407	p3	(GCT)5	Class II	1091	1105	TTACGCCGCAGTAAACGCCT	ACGTCGCGCGAAATCCGAAA	344
FMgSSR-61382	scaffold869	p3	(GCT)5	Class II	101940	101954	TTCTTCCATCGCGGTTGGTCGT	TGCGATGCACACTCGCACAT	347
FMgSSR-61386	scaffold23636	p3	(GCT)5	Class II	1958	1972	TTGCACCTGGCCCATTGTGA	ACAATGTTGCCCGGACCATACG	229
FMgSSR-61388	scaffold189836	p3	(GCT)5	Class II	435	449	TTGCAGCCACAGCTGCATCA	AGCCCACTGCAGTTGCACTTCT	306
FMgSSR-61389	scaffold163239	p3	(GCT)5	Class II	728	742	TTGCAGGGTAACGAATCGCCCA	TGGCTCTCAAAGCCCGTACGTT	222
FMgSSR-61391	scaffold14678	p3	(GCT)5	Class II	4739	4753	TTGCCGTGGTGCACGCTAAT	TCAAACGGCTTTTGGCCAGC	288
FMgSSR-61397	scaffold19768	p3	(GCT)5	Class II	1527	1541	TTGTTGTTCTGCAGTGCTGGCG	TGCTACCATGGCTTCGCGTT	235
FMgSSR-61398	scaffold568	p3	(GCT)5	Class II	45796	45810	TTTCATCTGCCTCGCCTCAGCA	ATTGCAAGCCGGAGGTGCAT	347
FMgSSR-61399	scaffold524	p3	(GCT)5	Class II	48212	48226	TTTCTCTTTCCTCTTGCCCTCCGC	ACCATCAGGAGCAAGAGCAGCA	247
FMgSSR-61401	scaffold1367	p3	(GCT)5	Class II	47240	47254	TTTGGCTTCTTGGCGCGTGT	AAGGAAGCGCATGTAGCCACCA	325
FMgSSR-61403	scaffold100	p3	(GCT)5	Class II	170400	170414	TTTGGTTGGACCAGGGGTGT	TGCCGACGCTGCCCAATAAT	297
FMgSSR-61406	scaffold90	p3	(GCT)6	Class II	33882	33899	AACGAAACGGAGCAGGTTTGCG	ACACCCGTTTCTGCATCAGCGA	267
FMgSSR-61408	scaffold7346	p3	(GCT)6	Class II	39434	39451	AAGCGTATCAACTGCAGCGCCA	CCATTGCATCGAATCCGCGTCA	337
FMgSSR-61422	scaffold111109	p3	(GCT)6	Class II	520	537	ACGGTCTTTGGCGTCAGCTTCT	ACCTTCTTCCGCGAGCCAAAGT	277
FMgSSR-61426	scaffold424	p3	(GCT)6	Class II	56960	56977	AGCAACAGGCAAGTGCGACA	ACCAAGACCCCAGCACTCAACA	280
FMgSSR-61430	scaffold54660	p3	(GCT)6	Class II	1477	1494	AGCAGCAACAGTACAGAGGCGT	TGTGCAGTGCGTGATCTGAGGT	266
FMgSSR-61446	scaffold18	p3	(GCT)6	Class II	215998	216015	ATGCGTGCAGCAGCAACTGA	ATGCGATGCGTAGTGTCGTGGA	307
FMgSSR-61449	scaffold1651	p3	(GCT)6	Class II	30468	30485	ATGTTGTCACGGAAGCCGTCCA	AACAAGCACAGCGGTGGCAA	323
FMgSSR-61453	scaffold8308	p3	(GCT)6	Class II	3723	3740	GAAGTAGAGCGGGACAGAGGAA	AGCATCAGCAGCCACCACCATT	245
FMgSSR-61454	scaffold12620	p3	(GCT)6	Class II	9424	9441	GACCGCCAAAATGACAGTTGCT	ACGGTTCCTCCTGCTCAACA	276

SSR_ID	Scaffold	SSR_Type	SSR_Motif	SSR Class	SSR_Start	SSR_End	Forward sequence	Reverse sequence	Product_size
FMgSSR-61455	scaffold11553	p3	(GCT)6	Class II	5280	5297	GCTTCCTTTAATTCGCCGCGCT	CCGTCGTGCATTATCGGCATGT	217
FMgSSR-61459	scaffold4602	p3	(GCT)6	Class II	21382	21399	TCACCGTCTTCACCTTGCTGTG	ACTGGGGGTTGCATTTCGCA	278
FMgSSR-61462	scaffold1389	p3	(GCT)6	Class II	33461	33478	TCCAACACGCATTCCGGTACACT	TACTTCCGCAACTTCCGCGT	332
FMgSSR-61463	scaffold2458	p3	(GCT)6	Class II	4883	4900	TCGATGCTAAAGCGGGCTTCCT	CGGTGTGCGCGTTTCGGTTAAT	283
FMgSSR-61466	scaffold494	p3	(GCT)6	Class II	31562	31579	TCGTCCAACGAGACGACGGTTT	ACCAAATGTTCGGCCCCAGTCA	226
FMgSSR-61467	scaffold2828	p3	(GCT)6	Class II	59490	59507	TCGTCGCAGCATGAGATTGGCA	ACGAAGCCTTCCTTGGCTGT	249
FMgSSR-61468	scaffold692	p3	(GCT)6	Class II	39511	39528	TCGTGGAAAGGCATGGTCGT	TTCCTCAACTGGATCATGGCGG	287
FMgSSR-61470	scaffold6117	p3	(GCT)6	Class II	21162	21179	TGAACTTAGGTGCGAGGAGCA	TGGGAGTAAGTGGTCCCTGACA	334
FMgSSR-61471	scaffold52325	p3	(GCT)6	Class II	1752	1769	TGAATGCCATCGGGGAGAAGGT	ACAGGCAGCGCTCCCCTTAATA	345
FMgSSR-61472	scaffold3661	p3	(GCT)6	Class II	29855	29872	TGATGAGCTGCTGGATCCGT	CGCGACACTGCGACAGTCAAAA	324
FMgSSR-61473	scaffold447	p3	(GCT)6	Class II	101384	101401	TGCACGGCCAACGATTCCAT	AGTGCTGCTCCACGTGACTT	288
FMgSSR-61474	scaffold800	p3	(GCT)6	Class II	69903	69920	TGCATCAGGAAAGCCAGCGAGT	TACCAACACAGAGATGGGCACG	318
FMgSSR-61479	scaffold1854	p3	(GCT)6	Class II	61171	61188	TGCGCTTCAGAGTCGTTGAGCA	ACTGAGGCTTGCTTGGTCCACA	344
FMgSSR-61480	scaffold1696	p3	(GCT)6	Class II	29741	29758	TGCTAAAGCGGGCTTCCTGCAT	AACAGAGCGAGATGGCAAGCGA	328
FMgSSR-61482	scaffold3065	p3	(GCT)6	Class II	1411	1428	TGCTGGCTTCCCACTGGAGTTT	ACACAAATCTGTGCCCGCGT	330
FMgSSR-61487	scaffold297	p3	(GCT)6	Class II	70447	70464	TGGTCGTGGGCATGGTGTACTT	GCCGTGGCAAAGAGTTGGAGTT	236
FMgSSR-61493	scaffold496	p3	(GCT)6	Class II	48174	48191	TTAGCCACAACGCCTTCTCCGA	ATCGATGGGAATGCGAGCAGCA	277
FMgSSR-61494	scaffold7796	p3	(GCT)6	Class II	1268	1285	TTCCTCCACCAGATCGCGTGTT	TGAGCAGCAGCGTCAGATACGA	211
FMgSSR-61498	scaffold11128	p3	(GCT)6	Class II	24756	24773	TTGGCAAAGTAGCAGGCGCA	TGCAAGCCCTGCACCAAAGT	263
FMgSSR-61499	scaffold12738	p3	(GCT)6	Class II	12774	12791	TTTACCGCGACAAGCCGACA	AGCGCGCCCAAATAAAGACGA	304
FMgSSR-61503	scaffold13837	p3	(GCT)7	Class I	16060	16080	AACGCTTGTGTCTTGTGCCACG	TTGCCGTCAACTTTCTCGTGCG	337
FMgSSR-61504	scaffold1830	p3	(GCT)7	Class I	54902	54922	ACCTGCTGTCCAGACACAATGTAG	TGTCTAGCAAACAGGCGGGCAA	211
FMgSSR-61505	scaffold16384	p3	(GCT)7	Class I	1666	1686	ACGGCGACCTTAGCCTTACCTT	ACGGACCCCTTCAAATCACCCT	283
FMgSSR-61506	scaffold5336	p3	(GCT)7	Class I	10017	10037	ACGGGATGCTGCAGTACCACAA	TTAACGACGGTCGAGACGGGAT	209
FMgSSR-61507	scaffold138	p3	(GCT)7	Class I	15034	15054	AGCATCGTCCTCTCCTCTCCTCTT	TTTACCACAGGCAAGGCGCA	320
FMgSSR-61508	scaffold196	p3	(GCT)7	Class I	56957	56977	AGCGCATCCAGCTCTTGCTT	AATAAAGCCGCAAGTGCGAGGC	203
FMgSSR-61509	scaffold21695	p3	(GCT)7	Class I	13147	13167	AGCTAGCATCTTCCCTGTGGCT	TGGCAGCATCTGCAGCAGTGTA	308
FMgSSR-61510	scaffold548	p3	(GCT)7	Class I	76611	76631	GCCACGTGATTAACGCGGTTGT	TCGTTTTTGCGATCTGCCACCG	270

SSR_ID	Scaffold	SSR_Type	SSR_Motif	SSR Class	SSR_Start	SSR_End	Forward sequence	Reverse sequence	Product_size
FMgSSR-61512	scaffold140	p3	(GCT)7	Class I	44223	44243	TGAACAGAACTGCGCCGTGA	ATGCGTCGTCCGCACATTCA	258
FMgSSR-61514	scaffold77537	p3	(GCT)7	Class I	1157	1177	TGTCTTTCCATCGCCGCCAA	AACTGTGCCAACGCCAGCAT	259
FMgSSR-61518	scaffold2951	p3	(GCT)7	Class I	22750	22770	TTGCGCTTCTTGGACGACGA	ATGTCCATGCACGGCTCTTCCT	272
FMgSSR-61519	scaffold9187	p3	(GCT)7	Class I	2655	2675	TTGCTGTGCTTGGCATGGCT	AGGCAAAGAAACGTGTGCGACC	280
FMgSSR-61521	scaffold10779	p3	(GCT)8	Class I	18304	18327	ATGTTGCGCCTGCACAAGAAGG	TTCGCAGATGCTGACCGTGACA	218
FMgSSR-61525	scaffold15440	p3	(GCT)8	Class I	14276	14299	TCGCGACTGCTTCGCTCTTT	GCTCGGTGCGTGCCTTTTCTAA	267
FMgSSR-61526	scaffold2003	p3	(GCT)8	Class I	36530	36553	TGACAGGCGGCGAGGATGTTTT	ACATGTGGCCGTGAGAACTGGA	337
FMgSSR-61529	scaffold1201	p3	(GCT)8	Class I	77850	77873	TTGCGCAGCTGCTCGTAGAATC	TCCTCAACAACAGCGAGCAGGT	274
FMgSSR-61538	scaffold23846	p3	(GGA)11	Class I	8892	8924	GCAGCAGAATTCAGCGCAAAGC	TTTGTACGGCCACGCCAGTT	303
FMgSSR-61549	scaffold1063	p3	(GGA)5	Class II	16239	16253	AAACCATTGGAGGCAGCACGGA	CCGTTGTCTCTGGCGCTTTTGT	331
FMgSSR-61552	scaffold4084	p3	(GGA)5	Class II	17963	17977	AAAGTCGGCGACAAGTGCGA	TGCCCGCCCTCAAAAGCAAA	222
FMgSSR-61554	scaffold5327	p3	(GGA)5	Class II	12185	12199	AACAACGACGGCAACGACGA	TAAATCAAGCCCGCGGCCAGAA	327
FMgSSR-61559	scaffold34409	p3	(GGA)5	Class II	2885	2899	AACGATGGAGATGGCCGCTGTT	TCGGCTCGGCGGAAATGGTAAA	238
FMgSSR-61562	scaffold8034	p3	(GGA)5	Class II	17886	17900	AACGTTCATCAGCGCGAACAGC	ATCAACGCCCCTAGCGAGTGAA	230
FMgSSR-61569	scaffold10445	p3	(GGA)5	Class II	11189	11203	AAGCAGGAGCAGTTCTTCCGCA	AGACCAGAACCCTAGCCCCAAT	286
FMgSSR-61570	scaffold2351	p3	(GGA)5	Class II	16348	16362	AAGCTGCAGCAGACGGTCAA	AGGTGGTCCGCCTGCAATAACA	314
FMgSSR-61571	scaffold3973	p3	(GGA)5	Class II	7589	7603	AAGGACGCCCAGCTTCAGGATT	CGCATTCGACATAAACGCTGCCT	247
FMgSSR-61572	scaffold189	p3	(GGA)5	Class II	109078	109092	AAGGAGACCTTGTCCATGCCCAGA	TTCGAGAACAACGCCACGGGAA	344
FMgSSR-61580	scaffold4	p3	(GGA)5	Class II	242215	242229	AATGACACCGCCGTCAGGAA	ACTGAAGTGCCAGCCATCGTGT	237
FMgSSR-61583	scaffold5025	p3	(GGA)5	Class II	8552	8566	AATGCATCGGTATCCTCGGCGT	ACAAGCCCGCCCCACAAAAT	267
FMgSSR-61584	scaffold1556	p3	(GGA)5	Class II	63393	63407	AATGGGCGAGGGGTTTGTTCGT	AGTGTGTGAACCCAACCCACAC	296
FMgSSR-61591	scaffold2430	p3	(GGA)5	Class II	14301	14315	ACAGCGGTTTATGAGGCGACCA	TAAAGCCAACAAGCCTCCGCGT	206
FMgSSR-61595	scaffold24742	p3	(GGA)5	Class II	1137	1151	ACATCGCTGTGTTGCTAGGGGT	AGCGAACATTCCAACGGGCA	335
FMgSSR-61605	scaffold190925	p3	(GGA)5	Class II	325	339	ACCCGTTGCGTTTCCCCAAT	TGCCCGGCCCATCATTGCTTTA	241
FMgSSR-61606	scaffold2839	p3	(GGA)5	Class II	39009	39023	ACCCTTGGTGAAGGACACCAGGTA	CGCAAGCGCAGAAGAGCATT	319
FMgSSR-61610	scaffold312	p3	(GGA)5	Class II	92158	92172	ACCGCCATGGCTTGATCTGGTT	TGTTGAAGGAGGCTGCTGCT	334
FMgSSR-61611	scaffold3663	p3	(GGA)5	Class II	7016	7030	ACCGGTGTCCATGTCGTTCA	TAAAAGCGTGCACCCACCTG	275
FMgSSR-61614	scaffold6368	p3	(GGA)5	Class II	4251	4265	ACGATTAGGGTTTGGCGGTGCT	TCTGTTGGCTGGTGGCTCAT	206

SSR_ID	Scaffold	SSR_Type	SSR_Motif	SSR Class	SSR_Start	SSR_End	Forward sequence	Reverse sequence	Product_size
FMgSSR-61615	scaffold558	p3	(GGA)5	Class II	113938	113952	ACGCACAACAGCAACTCCGA	ACAACACTGGGCCTGGGTTACA	332
FMgSSR-61619	scaffold922	p3	(GGA)5	Class II	95347	95361	ACGGGAGCAGGAGCATTTTTGC	AGTCCGGCGCCATTTGCATT	315
FMgSSR-61625	scaffold5957	p3	(GGA)5	Class II	10184	10198	ACTCAGAAGGAGGAGGAGCAGGTA	TGACAGCGCAGCTAGGAAAGCA	238
FMgSSR-61626	scaffold950	p3	(GGA)5	Class II	26126	26140	ACTGGCTTCGGCTTCAGCTT	AACACCTCATCCACCTCAGCGA	274
FMgSSR-61633	scaffold3509	p3	(GGA)5	Class II	28528	28542	AGATAGATGGCCACGACGACGA	ATTACAAGGGAGAGCACGCCGA	332
FMgSSR-61637	scaffold76033	p3	(GGA)5	Class II	1130	1144	AGCAAGATGGCTTCCGAGCGTT	AACCACGTGAACCCCAGAGA	274
FMgSSR-61640	scaffold36692	p3	(GGA)5	Class II	765	779	AGCAGCAACACGTGCCATGA	TTTTTCCAGCAAGTGGTCGGGG	345
FMgSSR-61643	scaffold26335	p3	(GGA)5	Class II	7462	7476	AGCAGCATGGCGAAGACACA	ACTCAACCCAAGCTTCGCCACT	261
FMgSSR-61655	scaffold1995	p3	(GGA)5	Class II	40192	40206	AGCGACGGAAGAGGAAACTTGC	TTCACGATGTTGGTGTGGCGGA	307
FMgSSR-61658	scaffold2478	p3	(GGA)5	Class II	69365	69379	AGCGGACGAGCCTGTTCAAT	AGTAGAATTCCTCGTCGCCGGA	333
FMgSSR-61661	scaffold2875	p3	(GGA)5	Class II	35113	35127	AGCTCAAGAGCGTGGCCATT	ACGCTGCGGTTGCATGTTCA	256
FMgSSR-61671	scaffold692	p3	(GGA)5	Class II	34740	34754	AGGCGAGACAACCATGTTGCGA	GTCACTTGCAAAAATGCGCGAC	345
FMgSSR-61672	scaffold492	p3	(GGA)5	Class II	70855	70869	AGGTAGGAAGACGGTAGAGGCACA	AAAACATGGTGCTGGAGCTGCG	349
FMgSSR-61674	scaffold2138	p3	(GGA)5	Class II	15035	15049	AGGTGGTGAAGAAGTGGTGCGT	TTGCTTCACTCGCTCGGACCAA	318
FMgSSR-61678	scaffold22645	p3	(GGA)5	Class II	10367	10381	AGTTCGCCACTGCCTCTGTAGT	TCTTCCTCTGTCTCTCTCCCGA	303
FMgSSR-61679	scaffold102395	p3	(GGA)5	Class II	399	413	AGTTGATCCGCCACACCACGTT	ATCTTCGCCGACGAGTATCTGC	346
FMgSSR-61680	scaffold15132	p3	(GGA)5	Class II	11929	11943	AGTTGTTGCTCTCCGCGCAT	TTCTTCCTCTGCTACAACGCGAGC	201
FMgSSR-61683	scaffold459	p3	(GGA)5	Class II	55677	55691	ATATCACCGCGCCTCCAACCTT	TTTCGCACCCAAAGCGACGA	336
FMgSSR-61685	scaffold8358	p3	(GGA)5	Class II	160	174	ATCATCGTTGCTTTTGCGCGGG	ACTAAGATCAACGACGGCGCGA	294
FMgSSR-61687	scaffold9074	p3	(GGA)5	Class II	15112	15126	ATCGATCTCTGCCCCGAACTCA	AGGAGGACATCGACGACAGGTT	340
FMgSSR-61693	scaffold1045	p3	(GGA)5	Class II	25512	25526	ATCTGTTGAGCGCGGTGGAA	AGAGCAGCTGGCCCTTGAAA	333
FMgSSR-61694	scaffold212	p3	(GGA)5	Class II	148629	148643	ATGAACCTCGCTGACCGGAACA	TCCTTGTTTGCTCCCGCTTGGT	349
FMgSSR-61695	scaffold1	p3	(GGA)5	Class II	43268	43282	ATGACCTTGCTGCTGGTGGCTT	AACCCGTCGTTCCGCTACTA	322
FMgSSR-61696	scaffold1005	p3	(GGA)5	Class II	77745	77759	ATGAGCGAGTTCCAGGACACGA	AAAAAGTTAGCGGTCCTCCCGC	264
FMgSSR-61701	scaffold450	p3	(GGA)5	Class II	60158	60172	ATGCGGCAGCTCGAATAAGCCA	ATGACTGTGTAGTGGCAGCGGA	214
FMgSSR-61703	scaffold613	p3	(GGA)5	Class II	9011	9025	ATGGATGCGGTTTGGGCCAT	TGCGCGTACAACGTTCCAGT	251
FMgSSR-61711	scaffold6	p3	(GGA)5	Class II	21631	21645	ATGTGAGGGAAACGCCAAGGGT	AGTGCGCAGATGAGGATGCAGA	348
FMgSSR-61712	scaffold6630	p3	(GGA)5	Class II	35386	35400	ATGTTCGGGATGCCCTTCCCTT	TCAGGACCGTTGCAACACCGAA	303

SSR_ID	Scaffold	SSR_Type	SSR_Motif	SSR Class	SSR_Start	SSR_End	Forward sequence	Reverse sequence	Product_size
FMgSSR-61715	scaffold69	p3	(GGA)5	Class II	144126	144140	ATTCGGTCGGAGCCGAGCATTT	ACGCAATGCAGCCCACGATT	205
FMgSSR-61718	scaffold4771	p3	(GGA)5	Class II	24066	24080	ATTTGGGGGCACTTTGGCCGTT	AGCCCGATCGCCATCTTTTCCA	303
FMgSSR-61720	scaffold4626	p3	(GGA)5	Class II	31787	31801	CCATGCGGTGTTGCCACAAA	AGGAAAGCTTCGACCTTGCAG	346
FMgSSR-61723	scaffold1557	p3	(GGA)5	Class II	17287	17301	CGAATGCGGAGGAAGACCTTGA	AGGTTCGAAATTGGGAGGGGGA	258
FMgSSR-61724	scaffold225	p3	(GGA)5	Class II	118864	118878	CGACGTGGCTGTGGATCGAAAA	TGCAAAATATGTGCCACCGCCG	234
FMgSSR-61729	scaffold2880	p3	(GGA)5	Class II	27067	27081	GCAGCCGGCAGTAAAACAGCTT	TTCTGGTGCCTCCTCTTCAGCA	280
FMgSSR-61739	scaffold33565	p3	(GGA)5	Class II	3182	3196	GGCGACGACGATCTGATGATGA	ATACTCCATCTCCTTCCCGAGGCA	345
FMgSSR-61746	scaffold95325	p3	(GGA)5	Class II	1098	1112	TACGTTGCGCCCCACACTAT	AGGCGCAGGTTGACCATCGAAT	278
FMgSSR-61759	scaffold2114	p3	(GGA)5	Class II	38743	38757	TCACGCACACTTGAGAACGCCT	TGCTTGTATCCCATGCCTCGCA	273
FMgSSR-61760	scaffold1536	p3	(GGA)5	Class II	35016	35030	TCACGCCGGAAGAACCTGAA	TTCGTACTCCTCCTCGAACCCT	305
FMgSSR-61762	scaffold213	p3	(GGA)5	Class II	150457	150471	TCAGCTCACCTGCAGCAATCCA	ATCCGCGTGAAGGTCAAGCA	211
FMgSSR-61773	scaffold39348	p3	(GGA)5	Class II	2383	2397	TCCGTGTATCCCTGCTTGGTCT	AAAACGGCGGCTTCCTTCCT	339
FMgSSR-61779	scaffold4752	p3	(GGA)5	Class II	17569	17583	TCGACGAGGCCATACGCTACAT	AGTGCATTCCATGGCTGCTCTGT	333
FMgSSR-61780	scaffold194	p3	(GGA)5	Class II	53158	53172	TCGACGCATGGTTTACCAGCCA	TCCCGTTCGCTTTTGGGAACCA	235
FMgSSR-61782	scaffold5588	p3	(GGA)5	Class II	18347	18361	TCGAGCTCGCCACCAACAACAA	AGCTGAGCACAACAATGGCGT	297
FMgSSR-61786	scaffold8798	p3	(GGA)5	Class II	20254	20268	TCGATGGCAATGAGCGCCAA	CGCAGTTGCAGCGTTGTCAGTT	304
FMgSSR-61788	scaffold275	p3	(GGA)5	Class II	92152	92166	TCGCCGTGATGAGGATCAGAGT	ACAGCCAGCAGCATTGCTCA	331
FMgSSR-61792	scaffold259	p3	(GGA)5	Class II	121523	121537	TCGTCATCGGAAGTGGCGTTGT	TGGCCTCAATGACTGCCACA	204
[truncated: 463,010 more chars]
