# Supplementary material for: Genome and Transcriptome sequence of Finger millet (Eleusine coracana (L.) Gaertn.) provides insights into drought tolerance and nutraceutical properties
Source: BMC Genomics. 2017 Jun 15;18:465. doi: 10.1186/s12864-017-3850-z (PMC5472924; doi:10.1186/s12864-017-3850-z)
Supplement: Supplementary file 5 — List of SSRs used for fingerprinting of finger millet germplasm accessions. (PDF 525 kb) [file 12864_2017_3850_MOESM5_ESM.pdf]

Supplement File 5: List of SSRs used for fingerprinting of finger millet germplasm accessions.

Sl #	SSR_ID	SSR_Type	SSR_Motif	Forward sequence	Reverse sequence	Annealing Temperature (° C)	Product_size (bp)
1	FMgSSR-39840	di- type	(TG)10	AAAGGTGGTGGCTCAGCCGTTA	ATATGCGACCACGCACGCAA	55	281
2	FMgSSR-7062	di- type	(AC)10	AAAGCGGAACCGTCAGGGGAAA	AACTGCAGCAGCAAGTGCGT	55	342
3	FMgSSR-46807	tri- type	(ATG)11	ACACATTCTTCCTATCGCGCCC	TTGCTGGTCGATGCATGGCT	55	200
4	FMgSSR-69389	tri- type	(TGT)5	AAGCGTTGTTGTTGCGCACG	TCCATGGCCCTACCGGAAAGAA	55	200
5	FMgSSR-73210	tetra- type	(TTGC)5	AGCATCAGGGGCAAGCCTGTTT	ACGCGTATCTGCCTCTCACA	55	256
6	FMgSSR-72878	tetra- type	(GTTG)5	TTTGGCAAGCTTCTTCGGTCGC	AGCAGGTGGCCTGAGCAAAA	55	246
7	FMgSSR-73212	tetra- type	(TTGT)5	ACACGACGACACGTACGCAGAT	TGCCATCGCAAAACCTCCCCAT	55	217
8	FMgSSR-73213	tetra- type	(TTGT)5	AGGTGTGCGTGTGCGACTTT	TGGCGTTTTCACGCTTCTTGGG	55	304
9	FMgSSR-54772	tri- type	(CTG)5	TGTGCTCGCGCTTTGCTTCA	TACCATGGCTGGTGCAGCTT	55	310
10	FMgSSR-70729	tri- type	(TTG)11	TCTGATGGCCACGCAGGAAT	AGGAGGGGATCCAAGAGCAATGA	55	240
11	FMgSSR-73406	penta- type	(AAAAG)6	TTTGCCTGGGTTCTCCCTCAGA	TCCGGTCATCCTCAGGTCAAACA	55	350
12	FMgSSR-10490	di- type	(AG)54	ATTTGCACCATCTCCGGCCGTT	ATAACCGACGCCTGGGTGTCTT	55	248
13	FMgSSR-72875	tetra- type	(GTTG)5	AAGTGGGCACGCATCTTCGCTT	AACCAACGTCCCGTCACGATTC	55	299
14	FMgSSR-72622	tetra- type	(CTTG)5	ACGAGATGCCACTTGGACCACT	ACGATGCGTGAGGTGTTCCT	55	227
15	FMgSSR-13778	di- type	(AT)10	AAGGAATATGCGCCAGCCAGGA	GCAAAACGTGAGCGGAGCAACT	55	213
16	FMgSSR-74663	penta- type	(GAGAG)5	AGGTCGAGCGGATGACACGTTA	GCGGCATTTGTTTCGGGCAA	55	286
17	FMgSSR-18258	di- type	(CG)6	AAAAATCCGCGGTCAAGAGGCG	ACGCGCCGAAACGAAAAACG	55	293
18	FMgSSR-75582	penta- type	(TTTCT)4	ATTCTTTCAGCTGGTGCGGGCT	TTGCTCAACGCCTCTTAGCCGA	55	333
19	FMgSSR-75994	hexa- type	(CACCAA)7	GGACAGGGTGCCGCTTCTTTTT	TGGGATTCGTCTTGCCGTCCTT	55	341
20	FMgSSR-76557	hexa- type	(TCCTCT)4	TGCATCTTCCTCGTCGCAACCT	TGTTCGTGGCTGCCGCTTTT	55	311
21	FMgSSR-26243	di- type	(GA)6	AAACACAACCAGCAGCGAACCC	TGCAAATGGCTTCACCTTGCCG	55	339
22	FMgSSR-73524	penta- type	(AAGAA)4	AGTGGCACACCACCAGTTCCTT	ACTTTGAGGCTGTGCGGGAT	55	226
23	FMgSSR-74392	penta- type	(CTCGC)4	ACAACGGTTGATGTGATCCCCAGG	AGCAATCTCTCGCTTGTTGCCT	55	346
24	FMgSSR-67291	tri- type	(TGA)11	TCGTCGTCAGGGAAAAGCACCA	ACAACAAGCCACTTCACGGGGA	55	228

25	FMgSSR-36100	di- type	(TC)18	GCTTTTGTCCTTTCCTGTGCGA	ATGACGTCACGCCGACAACA	55	311
26	FMgSSR-73465	penta- type	(AAAAT)6	TTGCTCATTGCTGGGCCGAA	TCGACATGTGTGTGGACCCGAT	55	335
27	FMgSSR-35931	di- type	(TC)16	TGGTCACTGTCCACCACTGTCT	TCATGTGAGCCTGCGGCAAT	55	348
28	FMgSSR-54818	tri- type	(CTG)6	AAGATACGCCCGCTGTGACTGT	TTGTCCGCCCACACGAACTT	55	349
29	FMgSSR-59580	tri- type	(GCC)5	AAACGCGGCACCGAAGCAAA	TGGATTTCGGATGAGACGGGGT	55	319
30	FMgSSR-60219	tri- type	(GCG)5	AAAATTCAGTTCACCCGCCCGC	AGCCTCGTTGGGGTTCGTCAAA	55	211
31	FMgSSR-63455	tri- type	(GTG)9	TGTTCTGGTCCGACGTCATCGT	TTGTGATGCCCGTGACACCTGA	55	292
32	FMgSSR-73351	tetra- type	(TTTG)5	TGGTCACGCCGGTGGATTCATT	GCGCAACCATTGCTGTGAGCTA	55	343
33	FMgSSR-75635	penta- type	(TTTTA)5	TCCAGTTTCTCGCTGCCGAA	AATGGCGTGATCTGGTGGCACA	55	327
34	FMgSSR-14754	di- type	(AT)6	AAATCTTCTCCCCAGGAACAGCGG	GTCGACGCTTTTGGCTTGCTGA	55	243
35	FMgSSR-18886	di- type	(CT)10	AAACAAGCCCAACCGTGAACCG	TGGAGCAAAGCAGATCCGGACA	55	320

List of germplasm accessions used for fingerprinting

Sl no.	Accession number	origin	State/ Country	Comments
1	GEC 301	Africa	Kenya	
2	GEC 298	Africa	Kenya	
3	GEC 273	India	Uttar Pradesh	
4	GEC 288	India	Uttar Pradesh	
5	GEC 65	India	Kerala	
6	GEC 1	India	Karnataka	
7	GEC 290	India	Uttar Pradesh	
8	GEC 59	India	Orissa	
9	IE 942			
10	GEC 270	India	Uttar Pradesh	
11	IE 2780			

12	IE 2008			
13	IE 2568			
14	IE 2093			
15	IE 886			
16	GEC 255	India	Tamil Nadu	
17	IE 2921			
18	IE 510			
19	IE 1023			
20	GEC 35	India	Uttar Pradesh	
21	GEC 71	India	Tamil Nadu	
22	IE 2030			
23	IE 518			
24	GEC 518	Africa	Zambia	
25	GE 7123	*Eleusine africana*		Wild species
26	GE 7124	*Eleusine africana*		Wild species
27	GE 7125	*Eleusine africana*		Wild species
28	GE 7126	*Eleusine africana*		Wild species
29	GE 7127	*Eleusine africana*		Wild species
30	GE 7128	*Eleusine africana*		Wild species
31	GE 7129	*Eleusine africana*		Wild species
32	GE 7130	*Eleusine africana*		Wild species
33	GE 7131	*Eleusine africana*		Wild species
34	GE 7134	*Eleusine indica*		Wild species
35	GE 7135	*Eleusine indica*		Wild species
36	GE 7138	*Eleusine indica*		Wild species
37	GE 7140	*Eleusine tristachya*		Wild species
38	GE 7141	*Eleusine jaegeri*		Wild species

Fingerprint profile of two SSRs resolved on PAGE and agarose gels.

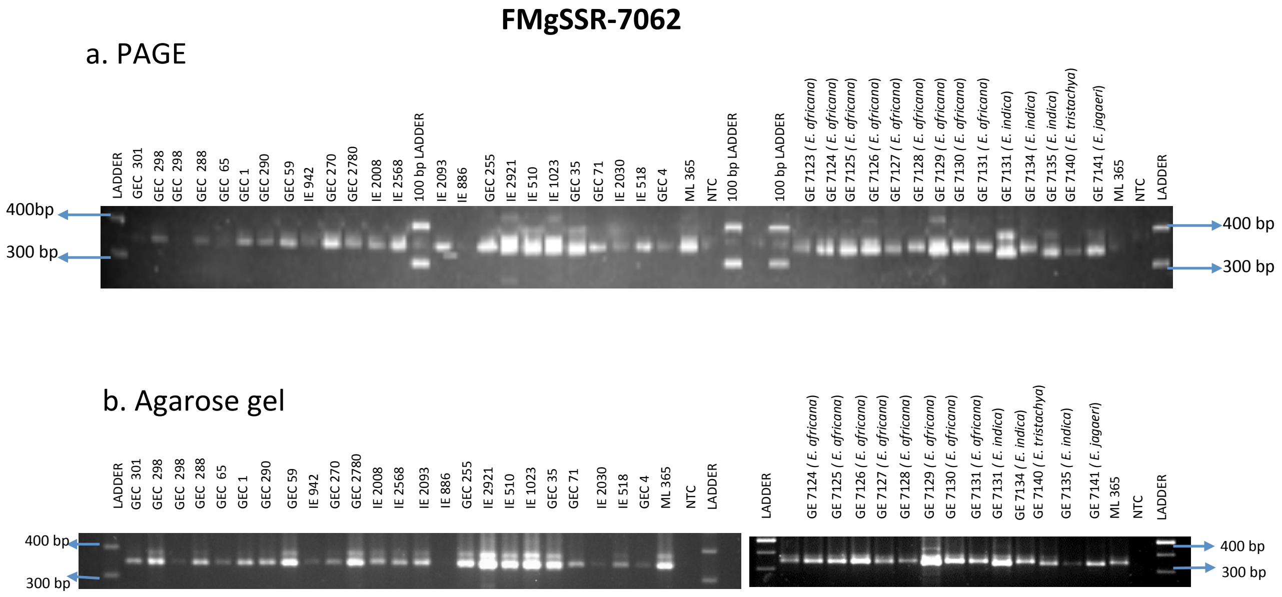

NTC is No Template Control

FMgSSR-39840

a. PAGE gel

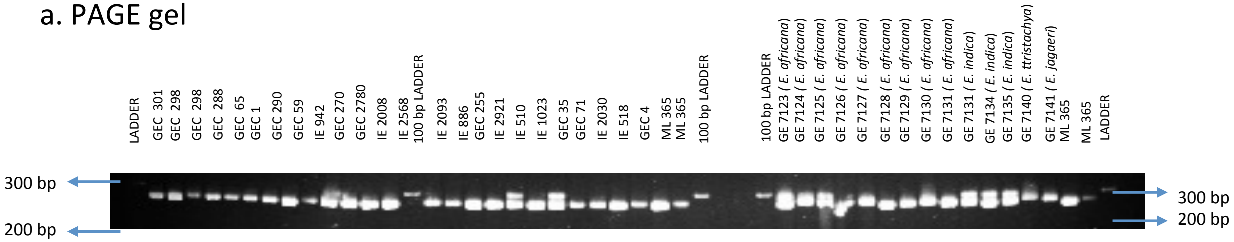

b. Agarose gel

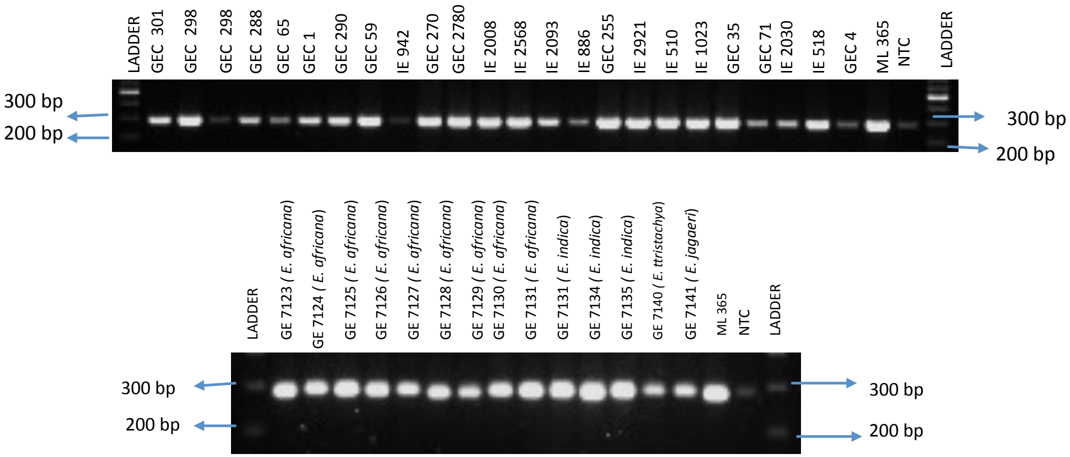

NTC is No Template Control
