# Supplementary material for: Genome and Transcriptome sequence of Finger millet (Eleusine coracana (L.) Gaertn.) provides insights into drought tolerance and nutraceutical properties
Source: BMC Genomics. 2017 Jun 15;18:465. doi: 10.1186/s12864-017-3850-z (PMC5472924; doi:10.1186/s12864-017-3850-z)
Supplement: Supplementary file 6 — Validation of differentially expressed drought responsive genes through qRT-PCR. (PDF 116 kb) [file 12864_2017_3850_MOESM6_ESM.pdf]

Supplement File 6: Validation of differentially expressed drought responsive genes through qRT-PCR.

Gene	Function	Pfam Domain	Uniprot Entry	RNA-seq Expression		qRT-PCR		
				WW	LMS	Normalized gene expression (fold change)*	WW	LMS
g161426.t1	Uncharacterized protein	late embryogenesis abundant (LEA) proteins	K3Z9P0	Down regulated	Up regulated	55.33	Higher Ct value	Lower Ct value
g77173.t1	Uncharacterized protein	Probable lipid transfer (LTP_2)	K3ZD63	Down regulated	Up regulated	64.22	Higher Ct value	Lower Ct value
g86441.t1	Uncharacterized protein	Potato inhibitor I family	J3M5N4	Down regulated	Up regulated	7.61	Higher Ct value	Lower Ct value
g134601.t1	Uncharacterized protein	NA	A0A1E5WJT2	Up regulated	Down regulated	0.90	Lower Ct value	Higher Ct value
g40229.t1	Uncharacterized protein	AhpC/TSA antioxidant enzyme	K4ACR1	Up regulated	Down regulated	0.66	Lower Ct value	Higher Ct value

NA- Not Available
Ct- Threshold Cycle
WW- Well-watered
LMS- Low moisture stress

* Ct values were normalized based on expression of Elongation factor (EF) housekeeping gene and gene expression was calculated based on 2-ΔΔCt method

Primer sequences used for qRT-PCR

Gene ID	Forward primer sequence (5'—>3')	Reverse primer sequence (5'—>3')	Product size (bp)

g161426.t1	AGCTACCTGGGCCAGAAGAC	GGAGTCCTTGGCGTACTCG	204
g77173.t1	ACAACAAGCAGGCGTCTCTC	TTGGGGTCCCTCTTGTACTG	220
g86441.t1	CTCCGCTTCCAAGACAGAGT	ACCGTCGCCACAGTATCAAC	177
g134601.t1	TGGTCTTCCTCGTCGTCTCT	CGCACCTGTTCTTGTAGGTG	156
g40229.t1	GCTGGCTTCTGTTCTCAAGG	ATATTTTGGCGCTAGCTGGA	224
Elongation Factor	TTTCACTCTTGGTGTGAAGCAGAT	GACTTCCTTCACGATTTCATCGTAA	103

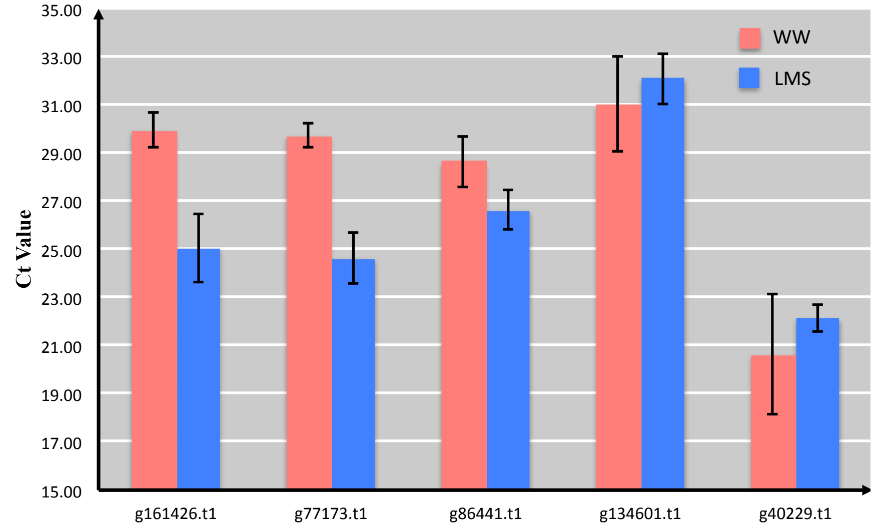

Graph showing the 'Ct' values of differentially expressed genes under well-watered (WW) and low moisture stress (LMS) samples. Lower and Higher 'Ct' values represent up and down regulation of a gene, respectively. Bars represent the standard errors of the mean values.
