# Supplementary material for: Genome and Transcriptome sequence of Finger millet (Eleusine coracana (L.) Gaertn.) provides insights into drought tolerance and nutraceutical properties
Source: BMC Genomics. 2017 Jun 15;18:465. doi: 10.1186/s12864-017-3850-z (PMC5472924; doi:10.1186/s12864-017-3850-z)
Supplement: Supplementary file 7 — Gene counts of ML-365 genome shared across other species of plants. (PDF 66 kb) [file 12864_2017_3850_MOESM7_ESM.pdf]

**Supplement File 7:** Gene counts of ML-365 genome shared across other species of plants

| Botanical name | Common name | No. of genes shared |
|---|---|---|
| Setaria italica | Foxtail millet | 1206 |
| Malus domestica | Apple | 1085 |
| Phyllostachys heterocycla | Moso bamboo | 716 |
| Aegilops tauschii | | 697 |
| Fragaria vesca | Wild strawberry | 694 |
| Sorghum bicolor | sorghum | 579 |
| Zea mays | Maize | 529 |
| Phoenix dactylifera | Date palm | 489 |
| Oryza sativa subsp. Japonica | Rice | 483 |
| Oryza punctata | Wild species of rice | 381 |
| Oryza barthii | Wild species of rice | 362 |
| Jatropha curcas | Jatropha | 350 |
| Linum usitatissimum | Flax | 327 |
| Musa acuminata | Banana | 311 |
| Oryza sativa subsp. indica | Rice | 292 |
| Brassica rapa | Rape mustard | 270 |
| Cannabis sativa | | 242 |
| Pyrus bretschneideri | Chinese white pear | 235 |
| Triticum urartu | | 159 |
| Oryza brachyantha | Wild rice species | 139 |
| Brachypodium distachyon | Brachypodium | 137 |
| Utricularia gibba | | 135 |
| Vitis vinifera | Grape vine | 127 |
| Theobroma cacao | Cacao tree | 124 |
| C.cajan | Pigeon pea | 76 |
| C. annum | Chilly | 65 |
| Gossypium raimondii | Diploid cotton | 64 |
| Nelumbo nucifera | Indian lotus | 62 |
| Triticum aestivum | Bread wheat | 58 |
| Azadirachta indica | Neem | 50 |
| Solanum tuberosum | Potato | 50 |
| Ricinus communis | Castor | 49 |
| Hordeum vulgare | Barley | 48 |
| Selaginella moellendorffii | Spike moss | 44 |
| Oryza glaberrima | African rice | 43 |
| Saccharum officinarum | Sugar cane | 42 |
| Arabidopsis lyrata | Arabidopsis | 40 |
| Carica papaya | Papaya | 33 |
| Artemisia annua | sweet wormwood | 29 |
| Glycine max | Soybean | 22 |
| Cicer arietinum | Chickpea | 21 |
| Picea abies | Norway spruce | 21 |

| | | |
|---|---|---|
| Thellungiella parvula | | 20 |
| Medicago truncatula | Medicago | 19 |
| Nicotiana tabacum | Tobacco | 19 |
| Physcomitrella patens subsp. patens | Moss | 17 |
| Solanum lycopersicum | Tomato | 14 |
| Arabidopsis thaliana | Arabidopsis | 11 |
| Capsella rubella | | 11 |
| Gossypium hirsutum | Cultivated cotton | 10 |
| Aquilegia coerulea | | 9 |
| Citrullus lanatus | Water melon | 9 |
| Helianthus annuus | Sunflower | 9 |
| Micromonas sp. RCC299 | | 9 |
| Lactuca sativa | Lettuce | 8 |
| Manihot esculenta | Cassava | 7 |
| Picea glauca | White spruce | 7 |
| Amborella trichopoda | | 6 |
| Brassica napus | Rapeseed | 6 |
| Mimulus guttatus | | 5 |
| Populus trichocarpa | California poplar | 5 |
| Raphanus sativus | Radish | 5 |
| Chlorella sp. NC64A | | 4 |
| Citrus clementina | Clementine | 4 |
| Citrus sinensis | Orange | 3 |
| Eucalyptus grandis | Eucalyptus | 3 |
| Lotus japonicus | Lotus | 3 |
| Ostreococcus sp. RCC809 | | 3 |
| Prunus persica | Peach | 3 |
| Vigna unguiculata | Cow pea | 3 |
| Arachis hypogaea | Pea nut | 2 |
| Cucumis sativus | Cucumber | 2 |
| Coccomyxa sp. C-169 | | 1 |
| Picea sitchensis | Sitka spruce | 1 |
| Volvox carteri | Green algae | 1 |
