# Supplementary material for: Genome and Transcriptome sequence of Finger millet (Eleusine coracana (L.) Gaertn.) provides insights into drought tolerance and nutraceutical properties
Source: BMC Genomics. 2017 Jun 15;18:465. doi: 10.1186/s12864-017-3850-z (PMC5472924; doi:10.1186/s12864-017-3850-z)
Supplement: Supplementary file 9 — Nucleotide sequences of calcium accumulation and transportation genes identified in ML-365 genome. (PDF 485 kb) [file 12864_2017_3850_MOESM9_ESM.pdf]

Supplement File 9: Nucleotide sequences of calcium accumulation and transportation genes identified in ML-365 genome

CaM ATPase genes

>g2583.t1
atgaccctccatcacggcgcggccgcaccttgccagtccgacgacgagtcaagcaatgggtggtccggatcggggtggtgcggtgtcatctctcctgaggaa
gctgctgcagcggcgatggccggggtgcggaaccctgaggcgtcgcccggacgctacgtgcggcgccgcgacgagatcgacgacggctgcgacgacgt
gctcggcgtcgacgtcgggggcgcgggggccgaccccttcgacatccccgccaagcgcgcctccgtcgagcggctgcggcgctggaggcaagctgctctt
gtcctcaatgcttctcgacgattcagatatacgcttgacctaaaaaaagaggaggagaaggaacatatcaggaggaaaatcagggctcatgctcaagtcatacg
ggcggcactgctttttaaagaggctggagaaaagaagaatgatgacagggaattgccagctgtcagtgattataaacagtctctgcagttccaacacctaaacga
ggagaagcaaaacatccaagtggaggttattaggggtggtagaaggattcaagtgtcaatatttgatattgtagttggcgatgtagtggctctaaaaattggtgatc
aggtaacggcagtcggcttaaacactgaatggggtttattaatggccagtatttcagaagacaataatgaagaaactccattgcaggtgagattgaatggagtag
caacactcataggcatcttaggactttctgttgctgccctggtcctcatagtcatgtttgcaagatattttacaggacatactacagactcaaatggaacagttcagttt
gttaaagggcagacaagtgcgaaatctgcaatatttggatcaataaagatactaactgttgcggtaactattgttgttgttgctgtgcctgagggcctaccactggct
gtaacattgaccctggcatattctatgcggaaaatgatggctgacaaagcactggtccggaggctttctgcctgtgaaacaatgggttctgctactacaatttgcag
tgacaagacaggcacattaactttgaaccagggagattcaaatgttcatgtacactggaaaggagctgctgaaatagttcttgctttatgtacaagttggcttgaca
cggatggttcaattcatgaaatgacaactgataaggctgatcagttcaaagaatacattgagtatatggctgagcaaagtcttcggtgtgttgcttttgcatacagaa
ctatcgagaacgatgtcccgagtgaagagcaaagaatcaactggcaattgccagataatgaccttactctaattggaatagtgggaatgaaggatccttgtcgcc
ctggagtaagagaggctgttgaattgtgcatcaatgctggtgtaaaggtacggatggtaacaggagataatctgaagacagctagagcgatagcacttgagtgt
ggaatactcactgatcatgaggcatctgccccgattataatagaaggaagagttttccgtgcttacaatgatgctgaaagggaggcaattgccgaaaagatttctg
tgatggcaagatcttctcctaatgacaagcttctccttgtaaaagctcttaagaaaagaggccatgtcgttgctgttactggtgatggtacaaatgatgctcctgcatt
gcatgaggctgatattggtctttctatgggcatccaaggaacagaagtagctaaagaaagctcagatattattattctcgacgacaatttttcgtcagttgtgaaggt
ggtccgctggggtcgttctgtttatgcaaatatccaaaagtttattcagttccaacttactgtaaatgtcgcggctcttgtcatcaatgtggttgctgccatttcatcggg
aaatgttcctctaaatgctgttcagctgctctgggtaaacctcatcatggacacacttggagctcttgcattggctaccgaaccacctacagatcagcttatgatgcg
gccacctgtcgggcgaagggaacctcttgtgactaatatcatgtggagaaacttattcattcaggtgtttaatgagttcaattctcgtaaaccagaagaactaaacat
cttcgatggggtttcaagaaaccatcttttcttgggagtagtgagcataactgtcattctgcaggtgataattatcgagttccttggaaagttcacatcaacagtgaga
ctcagctggaagctatggcttgtttctgtagctattgcatttgtcagttggcccttggctttcgttggaaaattcattccagttccaaagacaccattgaaggattttctc
atgagatgttggcccaaacgaaggaagcaaggtgacgaaggagcaacaccaccggtgtga

>g5694.t1
atggcgtacctccggaacaagtcgatggagttcctgaagaggttcgaggtgccggcgaagaacccgtcggaggacgcgcagcgccggtggcgggacgcc
gtcggcacgctcgtcaagaaccgccggcgccgcttccgcatggtccctgacctcgaaaagcgatcgcaggtcgagacccagcgccgccagatccaggaga
agcttcgtgttgcactctacgtgcagaaggccgcactgcagttcatcgacgccgcccgcaagaaggagcaccctctaccggagctggcgcggcaatgcggct
tctccatcagctccgaggagcttgcctccgtggtgcgtggccacgacagcaagagcctgaggctccacaagggcgtggacggcgtggcgcggaaggtgaa
cgtctccctcgcggacggcgtcaagtcggacgacgccggcgtccgcgcggaggtgtacggcttgaaccagtacgccgagaagccggcgcgcacgttctgg
atgttcctctgggacgcgagccaggacatgacgctgatgctgctggcgttgtgcgcgctcatctccgtgtgcatcgggctcgccacggaaggttggcccggcg
gcatgtacgacggcgtgggcatcatgcttaccatcttcctggtggtgatgatcaccgccgccagcgactacaagcagtcgctgcagttccgggacctcgacaa
ggagaagaagaagatcgacatgcacgtcacccgcgacgggtaccggcagaaggtgtccatctacgacattgtcgtcggcgacatcgtgcacctgtccatcgg
cgaccagccggcgcgcacgttctggatgttcctctgggacgcgagccaggacatgacgctgatgctgctggcgttgtgcgcgctcatctccgtgtgcatcggg
ctcgccacggagggttggcccggcggcatgtacgacggcgtgggcatcatgcttaccatcttcctggtggtgatgatcaccgccgccagcgactacaagcagt
cgctgcagttccgggacctcgacaaggagaagaagaagatcgacatgcacgtcacccgcgacgggtaccggcagaaggtgtccatctacgacattgtcgtc
ggcgacatcgtgcacctgtccatcggcgaccaggtccctgccgacgggctgtacatcgacggctactcctttttggtggacgagtcgagcctgtcgggcgaga
gcgagccggtgcacgtgtcgacggcgaacccgttcctgcttggtgggaccaaggtgcaggacgggtcggctcggatgctggtgacggcggtggggatgcg
caccgagtgggggaacctgatggagacgctgagccagggcggcgaggacgagacgccgctgcaggtgaagctcaacggcgtggccaccatcatcggca
agatcgggctggcgttcgcggtgctcaccttcgccgtgctcatggcgcggttcctcgtcgccaagaaggagctgatgcggtggagcatggcggacgcgctgt
cggtgctcaacttcttcgccgtggcggtgaccatcatcgtggtggccgtgcccgagggcctcccactcgccgtcacgctcagcctcgccttcgccatgaagaa
gctgatgcaggagcgcgccctcgtgcgacacctctcggcgtgcgagaccatgggatccgcaagctgcatctgcaccgacaagaccggcacgctcaccacca
accacatggtcgtcgagaaggtctgggcggcgggggccacgcagacggtcaccacatccaagggctacgacgacctcatcatcaagtccgcctcagagac
cttctcgaagcttctactggagggcgtcttccactgctctggctccgaggtggtgcgcgacaaggacgggaagaccaccgtcatgggcacgcccaccgagac
cgccatcctcgagttcgggctcgaggtggagaagcacgccaagctcgagcacgccgccgccgccaagaagctcaaggtggagcccttcaactcggtgaag
aagaccatggcggtggtcgtggcgtccccgaacgccgccggccggccacgcgcgttcctcaagggcgcgtccgaggtcgtcctgcagcggtgcagctgcg
tcgtcgacggcgccggcgccgtcgagaagctcacggaggccaagaccaagcgggtcgccgccgccatcgacgcgttcgcgtgcgaggcgctgcgcacg
ctatgcttggcgtaccaggacgtcggcaccggcaacgacgtccccgccgacgggtacacgctcattgccgtctttgggatcaaggacccgctccgtccgggc
gtcagggaggccgtcaagacgtgccatgacgcggggatcaatgtacgcatggtcaccggagacaacatcaacacggcgaaagccattgcgagggagtgcg
gcattctgactgatgatggcattgccatcgaggggcctgagtttcgggtgatggctcggtcgctgccgttggacaagcacacactggtgaccaacttgaggga
atgttcaacgaggttgtggcggtgactggcgatggcacgaacgacgcgccggcgctacacgaggctgatattggcctcgccatgggaatcgctggaacaga
ggttgccaaggaaaacgctgacgtgatcatcatggacgacaatttctccaccatcatcaacgttgctaaatggggccgttccgtctacatcaacatccaaaaattc
gtgcagttccagctcacggtgaacatcgtcgccctgatggtgaacttcgtttctgcgtctttcacaggaagtgctccattgactattgtgcaactactttgggtgaac
ctgatcatggacacactcggtgctctggcgctagcaacagagccccccaacgattcgatgatgcagaggccgccggtaggccgaggcgacaacttcatcaca

aaggttatgtggaggaacatcatcggacaaagcatctaccagctcatcgtgctaggcgtcctcctcttcaaagggaagaccctgttgctgttgaatggcactcaat
ctgacgcccagctcaataccttcatattcaacacctttgtcttctgtcaggttttcaacgaggtgaacagcagggagatggaaaagattaatgtcttcgccggcattt
tcaacagctggatattctcggcagtagccggcgccaccgccgcgttccaggtgattattgtagagctcctggggacctttgctagcacggtgcatctgagcggc
aggctctggctcatcagtgtactaatcggatcagtcagcctagtgattggtgccatcttgaagtgcatccctgtcgactccagcagcgactcctctgaccgccaag
atggttaccagcccatccccaccggccccaacgccgtgtga
>g5801.t1
atgcctcttgagctgaggaatcgtcagatgaaaaaggattctagatcacaaaagtggtcagacatgtgcaagcttgaagcaatgccatcaaagaattttgtcttcct
gcaattgtttgaactgaaggatgactttattcaagctgaaataagaaaaccttcttatcaatcagtctgttcgaatgatgatagaaacaagatgtctgtgctgctgcaa
tcagctgttgttttctga
>g8064.t1
atgtttgccaaggccattgcacgcgcctgcgttgccgccttgagcgatgtcgacctcacatcaagcgaaggccttacaagcttggaggaggtggttgaaaagtc
acgagtcaagaggaaggatgacttcactgggctttgcttcgtggcaaagaacgaccacgacactgactctggattcgatttcgacacaagcgagtgctgttcgtg
a
>g16824.t1
atggagaagtacctgcaggagcacttcgatctgccgcacaagaacccctccgaagaggcgcagcggcgatggcgctccgccgtcggcaccatcgtcaaga
accgccgccgccggttccgcatggtgcctgacctcgaccgccgccagaaggacgaggaggcgcgtcgctccatccaggaaaaaattcgggttgctctctatg
tgcagaaggctgcgatcattttcagtgatggtgccagcaagaaagagtacaagctgacagaggacattatgaatgctggattttcgatcaatccagatgaactgg
catcaatcacaagcaagcatgatatgaaagctttcaagatgcatggtggtgttgatgggatatccaaaaagatccgttcaactttcgaccgtggcatatcaactagc
gatctggataaaagacagatgatctacggtgaaaatcgttatgccgagaaacctgcaagaagcttctgggcatttgtttgggatgcgttgcaggacatgaccctta
tcattcttatgttcaaggagctagacaatgagaagaaaaagatattcatccatgtaaccagagacggtgagagtgaccctgtgtacatttcggaggataagccctt
catcttggcaggaactaaagttcaagatggatctgctaaaatgttggtaactgctgtgggtatgcgtactgaatggggaaggttgatgagcacattgagtgaggg
aggagaggatgagacaccattgcaggttaaactaaatggagttgcaaccatcattgggaagattgtgaactactttgcgacagcggtcactattattgttgttgctg
ttcctgaagcatgtgagacaatgggatctgctggtacaatctgcacagataagacaggaactttgacaactaaccatatgggtatttttgagaacacgagtgcaga
agtagtcaaggataaggatggtaaacaaactgttttaggaactccaactgaaagggcgatattagaatttggcttgatgttggaaggacatgataccgaggatag
aacctgcacgaaggtcaaggttgagcctttcaattcggtgaagaagaagatggcagtgttggtctcagtacctaacggcacataccgctggttcagcaaagaaa
gtcctacaagtggttttactctgatatgcatctttggcatcaaggaccctgtgcgcccaggagtcaaggatgctgttaaggcctgcatatctgctggcatcattgtga
gaatggtgactggtgataacatcaatacagctaaagctattgccaaggagtgtggcatactgactgatgacggcatagcaatagaaggaccagagttccgcaac
aagagcccagaagaaatgagggacttgatacctaagattcaggtcatggctcgttcgttaccattggacaaacatacccttgtgacaaacttgcgaggtatgttcc
gtgaagtggtcgctgtgacaggtgatggcacaaatgatgctccagcattgcatgaatcagatattgggcttgctatgggcatagctggcacagaggtagcaaag
gagagtgctgacgtgatagtgcttgatgacaattttactactataataaatgttgcaaggtggggtcgggcagtttacctcaacatccaaaagtttgtacagtttcagt
tgactgtcaatattgttgctttagtcatcaactttgtctcagcatgcattacagggagtgctcctctcacagcagtgcagttgttgtgggtcaatatgattatggataca
ctaggagctttggctctagctaccgagcctccaaatgatgacatgatgaagagaccacctgttgggcgaggggagagtttcattaccaagatcatgtggaggaa
tatcattggccaaagtttatatcagttggttgtacttggagctctaatgtttggtggggaacgacttctgaacatcaagggtgcagattccaaatctgtaatcaatactc
tcatattcaactcctttgtgttctgccaggtattcaatgaaataaacagcagggaaatgcagaagatcaatgttttccatggaatgtttagcaactggatcttccttgga
atcattgctgtgacagtagcatttcaagtggtgatcatagagtttnnnnnnnnnnnnnnnnnnnnnnnnnnatactgactgatgatggcatagcaatagaa
ggaccagaattccgcaacaagagccccgaagagatgagggacttgatacctaagattcaggtcatggctcgttcgttaccattggacaagcatacccttgtgac
aaatttgcgaggtatgttccgtgaagtggtcgctgtgacaggtgagtgccctatagctgttatcaatacatccagtcctaatgcccatggtgatgcttcagtattcaat
gaaataaacagcagggaaatgcagaagatcaatgttttccatggaatgtttagcaactggatcttccttggaatcattgctgtgacagtagcatttcaagtggtgatc
atagagtttcttggcacttttgccagcaccgttccacttgattggcagctttggttggttagcatcggcctgggatccatcagcttgattgttggcgccatcttgaagt
gcataccagtggaatcaggtgggacttctgctagtccaaatggctacgccccactccccagcggccccgataacatttaa
>g17111.t1
atggaggcgggcaggagctggagcatcgacggctacctcaacgagcacttcgacatccccgccaagaacccgcccagcgaggcccgcctccgatggcgc
cgcgccgtcggcctggtccgcaaccaccgccgccgcttcggcatgttcacccccctacacgccctcgacgacacccagcgccgcaagatcctgatgtttgtttt
tgttacccagggaaaagttcaggttgtgatcaatgtgcacaaggcagcgcttcattttatcgatggtgtaaaacgctaccaactatcccctgagcttattcaagaag
gattttgcatcagtccggatgaactggcagcaattactggctttggtgcagattcaacaatcttcaaaacgcatggtggagtcagtggaatatctaggaaaatcaaa
gcctcattggaggatggcgtcagggaagctgaaattacaacgaggcagaaactgtatggcaccaacaagcatgcagagaagcaacctagaagcttcttgatgt
ttgtgtgggatgcattacatgacacaactctgattattctaatgatatgtgctatggtatctctagtggttggccttgcaaccgaggggtggccgaagggaatctatg
atggccttggcattatactcagcattctattagtagtactagttacagcatcaagtgattacaagcagtcacggaaattcatggagctggaccgtgagaagaagaa
gatctatgtccttgtcactagagataagaaaaccaagaaggttctgattcatgacttggttgttggggacatattgcacctttcaataggtgatgttattcctgcagatg
gcttgttcatctccggctacagcttagtgatagacgaatctagcttatcaggtgagagtgaaccagtcaatgtttctgaagaaaagccctttcttcatgctggaagta
aagtggtggatgggaccgccaagatgcttgttactgctgttggtatccgtactgagtgggcgttaagaacattgtgcatcgcgtttaaggatctgaatgaaatttctg
atgatgaaaccataccagaagacagctacactctaatagcactttttggtataaaggatccagtccgtcctggtgtcaaggatgcagtgatgacatgcatgaatgc
tggaatcacagtaagaatggtaactggggacaacatcaacactgctaaagctatcgccaaggaatgtggaatactaactgaggacggaatagcaatagaaggg
cgagagcttcataataaaagctcagatgaactgaaggagctcctgcctaaaattcaggtaatggctcggtccttgcccatggataaatacaaactggtaacaagc
ctagaagatatgtatcaagaagttgtagcagttactggtgatggaaccaatgatgccccagcattgtgtgagtcagacattggcctggccatgggcattgcaggc
actgaggtcgctaaagagagtgctgatgttgtaataatggatgacaacttcagcaccattgtaaatgttgctagatggggtcgtgcagtttacctgaacattcagaa
gtttgtgcagttccagcttacagtaaatatagtggctctgatagtgaatttcggctcagcttgtattgtcggtactgcaccacttactgccgtccagttactatgggtga
acatgatcatggacacattaggagccttggccttagctacagaaccacccaatgatgaaatgatgaagcgaccacctgtatga
>g23346.t1

atggaccccgtggagaggttcctgcaggagcacttcgatctgccgcccaagaacccctccgaggaggcgcagcggcgatggcgctccgccgttggcaccat
cgtcaagaaccgccgccgccgattccgcatggtgcccgacctcgaccgccgccagaaggacgaagagacgcgccgctccatccaggaaaaaattcgggtt
gctctttatgtgcagaaggctgcgatcattttcagtgatggcgccagcaagaaagagtacaatctgacagaggacattatgaatgctggattttcaatcaatccaga
tgaactggcatcaatgacaagtaaacatgatatgaaagctttcaagatgcatggtggtgtcgatgggatatccaaaaaggtccgttcaactttcgaccatggcatat
caactaacgatttggataaaagacagataatctacggtgaaaatcgctacaccgagaaacctgcaagaagcttctggtcatttgtttgggatgcgctgcaggacat
gacccttatcattcttatggtatgtgctgtgctctctgctgtagtgggtctggcatcggaaggttggcctaagggcatgtatgacggcttgggaatcatactcagcat
tttattggttgtgatggttactgctatcagtgactataggcagtcgcttcagttcaaggagctagacaatgagaagaaaaagatattcatccatgtaactagagacgg
ttctcgacagaagatctctgtatatgacttggtagttggtgatatcgtgcatctatcaattggagaccaggtacctgctgatggactgttcattcatggatattctctttt
gcttgatgagtccagcttgtcagtgaactactttgcgacagcagtcactattattgttgttgctgttcctgaaggtctgccgttagctgttactttgagccttgcatttgc
gatgaaacagttaatgaaggataaagcacttgttagacatctttcagcatgtgagacgatgggatctgctggtacgatctgcacagataaaacaggaactttgaca
actaaccatatgggtatttttgagaacacgagtgcagaagtagtcaaagataaggatggtaaacaaactgttttaggaactccaactgaaagggcgatattagaat
ttggcttgatgttggaaggacatgataccgaggataggacctgcacgaaggttaaggttgagcccttcaattcggtgaagaagaagatggcagtgttggtgtcat
tacctaacggcacacatcgctggttcagcaaagaaagtcctacaagtggttttactctgatatgcatttttggcatcaaggatcctgtacgcccgggagtcaaggat
gctgttaaggcctgcatatctgctggcatcattgttagaatggtgactggtgataacatcaatacagctaaagctattgccaaagagtgtggaatactgactgatgat
ggcatagcaatagaaggaccagaattccgcaacaagagccccgaagagatgagggacttgatacctaagattcaggtcatggctcgttctttacccttggacaa
gcataccettgtgacaaacttgcgaggtatgttccgtgaagtggttgctgtgacaggtgacggcacaaatgatgctccagcattgcatgaatcagatattgggctt
gctatgggcatagctggcacagaggtattcaatgaaataaacagcagggaaatgcagaagattaatgttttccatggaatgttcagcaactggatctttcttggaat
cattgctgtgacagtggcatttcaagtggtgatcatagagtttcttggcacatttgccagcaccgttccacttgattggcagctttggttggttagcatcggccttggg
tccatcagcttgattgttggtgccatcttgaagtgcatacctgtggaatcaggtggaacttctgcaagtccaaatggctacgcgccactccccagcggtcctgataa
catttaa
>g34383.t1
atgctccccgcgccgacgcacgacgcccctgcttggtgttccgtgaggccaccaccctgcttccccacgccgtcggtctctggatcgatcgaccccgtcgcctt
gacaacccagcgtcccagaccggatctccccacctctcactcctgcgccgccacacgacgcacgtgctcggcgttgcgcgaggccgccgtcctccttcccca
cgctgccgacctctag
>g36319.t1
atggcttctcgccgtcccatcctcgtccatggcggcgacgctaggctccgattgaatgcggtgaccatcggcgcctgcacggggccggagaaaattggatctg
gtgggaatgatggtgctggattcgttcttttgtcggtggacggcgcgggacgagcggcgatggaaggcggcggcgtcaattcggtaaagtttaatgaatacaat
gctgttgtagtgtcggctggttatgacctgcggacaattgatacttttcaagatagtgttatgtcagtcatcctaaccaaaacagaaattattgctggtagtgttgatgg
tactgttagcacatttgacatccgcattggtagggagactgttgataatctggggcatcctgtcaactgtatatctttgtcaaatgacagccattgtcttttagcaaact
gtttagattctactgtgaggcttcttgacaagtaa
>g37783.t1
atggctgctactggccgggaggagatcgtcagccagcagcagcagcggcaggagtacggcggcgtggacggtaaggactacacggacccgccgccgga
gccagtggtcgccacgtcggagctgcgggggtggtcgctgtaccgcgccgcgatcgccgagttcgtggccacgctgctgtttctctacgtcacggtggccac
ggtcatcgggcacaagcgcagccagtccgactcctcgtcggacgcgtgcggcggcgtcggcatctccggtgagtga
>g39065.t1
atggagagctacctggaggagaacttcggtggcgtcaaggccaagaactcctcggaggaggcgctccggcggtggcgcccgcctctgcagcgtcgtcaaga
accccaagcgacggttccgcttcaccgccaacctcgacaagcgaggggaggcgcaggccatcaagcacgccaaccacgtccgtacacatctcctcttcctct
ctcatccatcatcccctttcctccgcaccgggatccgcctcgctcgcggcggcgggatctggactggagccgcgccgccggcgggggcgggaggagctcat
cctgcggttgtgttgcaggagaagctgcgtgttgccgtgctggtctcgaaggccgcgcttcagttcttacatggtctctcacttcgaagcgaatatgttgtccctga
ggaagtcaaggctgcagggtttcagatttgtgccgatgagcttgggtccattgtggagggccacgatagcaaaaagctgatcatacatggcggagtcgatgga
attgctgccaagcttgcgacatcacaaatggatgggctgaggacagctgaggaggacaacattaagcgcaggcaagagatttatggactcaacaagttcacag
aaagcgaggtccgcagtttctgggtgtttgtatgggaagcgcttcaagatacaactcttataattcttgccgtctgcgcatttgtatctctggtcgttggcattgcgat
ggaaggatggccaaaaggtgcccatgacggtcttggaattgttgcgagtatcctcctggtagtgtttgtgactgcaacaagtgattaccgacaatcgctgcagttc
aaaaacctggacaaggagaaaaagaaaattcgggtgcaagttacaagggacggatttaggcagtggatatcgatatatgaccttcttcctggagatgtcgtccat
ttacaaattggagaccaggttcctgcagacgggctcttcatttcagggttttctctgttgatcaatgaatccagcctaacgggtgaaagtgaacccgtcgttgtaaat
gaagataatcctttcctttgtcgggaaccaaagtgcaggatgggtcctgcaagatgctggttacgaccgttggtatgcgcacccaatggggaaaactgatggcc
actctcagtgagggcggcgatgatgaaactccactgcaggtcaaactcaatggtgttgcaactatcattggaaagattgggctattctttgctgttataactttcattg
tcttgtcccaagggttatttggcaacaaataccatgacggacagcttttgagctggtcaggagatgatgcactggaacttctggagcattttgctattgcagttacca
ttgttgttgtggctgttcctgagggattgccattagcagtcacgctgagccttgcatttgccatgaagaaaatgatgaatgacaaggcacttgttcgcaacttagctg
catgtgaaactatgggctcagcgaccaccatctgcagtgacaagacagggacattgacaaccaatcatatgactgtcgttaaggcctgcatctgtgggaaaatc
aaggaggttaacagtcctcagaatgcatccaagttatgctctgaatttccggaaactgtcgtcaaaacactcctggagtctatatttaacaatacaggtgttgaggtt
gtgattaaccaagatggtaaacgtcagatcctagagaaatggaagaaggttttcctggcacagagcaaataccattacaagggtacacatgcattggtattgtag
>g50728.t1
atgtgcaantcgtacccggtgccgccggggctgccgttcgcgcagctccccgacgactggcggtgcccgacgtgcggcgcggcgcagtccttcttcgagag
caagagcgtcgagatcgcggggttcgcggagaaccagcagttcgggctcggtggcaactcgctcacctccgggcagaagggcttcgtattctgcagcattgtt
agatgcgcaaaacatgatctggaggacgtattgtag
>g54563.t1
atggatctccgcatcgccgtgctcgtggccgcggcgctcgccgcgggcggcgggtggtggggaggaggctgcggcgtggaggcgtccatccacacttacg
accgggagcccttccgagaggtgggcaacgcgttcctcctctccggcggcagcgagggcatcgtcgccgacgggaccgaccccgcagcgcccgcctcgtc
cttcatcaagtaa

>g64531.t1
atgggatcagctactacaatatgcagtgataagacaggaactcttaccttaaataagatgacagttgtggaagcatactttggaggaacaaagttggatccctgct
atgatatgagccaaatgtcagccaatgtagcatcgcttattattgaaggaattgcacagaacacaacaggaactgtattcctgccagaggatgggggagaagca
gaacttacaggttcaccaactgaaaaggccattctttcttggggccttaaggcagggatggatttccatggtgtgagatcaaaatcttcagttcttcatgtttttccattt
aattcggagaagaaacgtggagctgttgcaatacagtatgatgaattctggaaatctattgaagacatggcagcaacctcattacgttgtgttgcttttgcatatcgt
ccctgtgagcctgaaaagataccagaggaagacatagctaattggaaattgcctgaggatgacctgactctgcttggcatcataggaataaaggccgatatagg
tctttcaatgggcatctcaggcacagaagttgctaaggaaagttctgacattataatcttggatgatgacttcacatcagttgtcaaggttgttcgttgggggcgatct
gtctatgcgaatattcagaaattcatccagttccagctcacggttaatgttgccgcccttgtaataaatgtggttgctgctgtgtcatctggtgatgtgcccctgaatgc
cgtagaggttgggaccttttcttga
>g73960.t1
atggcgtacctccggaacaagtcgatggagttcctgaagaggttcgaggtgccggcgaagaacccgtcggaggacgcgcagcgccggtggcgggacgcc
gtcggcacgctcgtcaagaaccgccggcgccgcttccgcatggtccctgacctcgaaaagcgatcgcaggtcgagacccagcgccgccagatccaggaga
agcttcgtgttgcactgtacgtgcagaaggccgcactgcagttcatcgacgccgcccgcaagaaggagcaccctctaccggagctcgcgcggcaatgcggct
tctccatcagcgccgaggagctggcctccgtggtgcgcggccacgacagcaagagcctgaggctccacaagggcgtggacggcgtggcgcgcaaggtga
acgtctccctggcggacggcgtcaagtcggacgacgccggcgtccgcgcggaggtgtacggcttgaaccagtacccggagaagccggcgcgcacgttctg
gatgttcctctgggacgcgagccaggacatgacgctgatgctgctggcgttgtgcgcgctcatctccgtgggcatcgggctcgccacggagggttggcccgg
cggcatgtacgacggcgtgggcatcatgcttaccatcttcctggtggtgatgatcaccgccgccagcgactacaagcagtcgctgcagttccgggacctcgac
aaggagaagaagaagatcgacatgcacgtcacccgcgacgggtaccggcagaaggtgtccatctacgacattgtcgtcggcgacatcgtgcacctgtccatc
ggcgaccagggcggcgaggacgagacgccgctgcaggtgaagctcaacggcgtggccaccatcatcggcaagatcgggctggcgttcgcggtgctcacc
ttcgccgtgctcatggcgcggttcctcgtcgccaagaaggagctgctgcggtggagcatggcggacgcgctgtcggtgctcaacttcttcgccgtggcggtga
ccatcatcgtggtggccgtgcccgagggcctgccgctcgccgtcacgctcagcctcgccttcgccatgaagaagctcatgcaggagcgggccctcgtgcggc
acctctcggcgtgcgagaccatgggatccgcaagctgcatctgcaccgacaagaccggcacgctcaccaccaaccacatggtcgtcgagaaggtctgggcg
gcgggggccacgcagacggtcaccacctccaagggcttcgacgagctcatcaagtcagagaccttcttctcgaagcttctactcgagggcgtcttccactgctc
tggctccgaggtggtgcgcgacaaggacgggaagatcaccgtcatgggcacgcccaccgagaccgccatcctcgagttcgggctccaggtggagaagcac
gccaagatcgagcacgccgccgccgccgccaagaaactcaaggtggagcccttcaactcggtgaagaagaccatggcggtggtcgtggcgtccccgaacg
ccgccggccggccccgcgcgttcctcaagggcgcgtccgaggtcgtcctgaagcggtgtagctgcgtcgtcgacggcgccggcaccgtcgagaagctcac
ggaggccaagaccaagcgggtcgccagcgccatcgacgcgttcgcgtgcgaggcgctgcgcacgctatgcttggcgtaccaggacatcggcgccggcaa
cgacgtccccgccgacgggtacacgctcattgccgtcttcgggatcaaggacccgctccgtccgggcgtcagggaggccgtcaagacgtgccatgacgcgg
ggatcaatgtacgcatggtcaccggagacaacatcaacacggcgaaagccatcgcgagggagtgcggcattctgactgacgatggcattgccatcgaggga
cccgagtttcgggtgatggctcggtcgctgccgttggacaagcacaccctggtgaccaacttgaggggaatgttcaacgaggtcgtggcagtgaccggcgac
ggcacgaacgacgcgccggcgctacacgaggctgacattggcctcgccatgggaatcgctggaacagaggttgccaaggaaaacgctgacgtcatcatcat
ggacgacaatttctccaccatcatcaacgttgctaaatggggccgttccgtctacatcaacatccaaaaatttgtgcagttccagctcacggtgaacatcgtcgccc
tgatggtgaacttcgtctctgcgtctttcacaggaagcgctccattgactattgtgcaactattgtgggtgaacttgatcatggacacactcggtgctctggcgctag
caacggagccccccaacgattccatgatgcagaggccgccggtaggccgaggcgacaacttcatcacaaaggttatgtggaggaacatcatcggacaaagc
atctaccagctcatcgtgctaggcgtcctcctcttcaaagggaagaccctgttgctgttgaatggcactcaatctgacgcccagctcaataccttcatattcaacac
ctttgtcttctgtcaggttttcaacgaggtgaacagcagggagatggaaaagattaatgtcttcgccggcattttcaacagctggatattctcggcagtagccggcg
ccaccgccgcgttccaggtgattattgtagagcttatggggacctttgctagcacggtgcatctgagcggcaggctctggctcatcagtgtactaatcggatcagt
cagcctagtgattggtgccatcttgaagtgcatccctgtcgactccagcagcgactcctctgaccgccaagatggttaccagcccatccccaccggccccaacg
ccgtgtga
>g76043.t1
atggagaagagaactacactagagacagatgcatctacccatgtcaaaggacacaagatatcgacgcagatgagagatagcttgaagaagttttcaggcttgat
tgaagaggggggtgacccagaagccctgtcattagctgtaaccgagatggaaggcatccagagcgagcaggaagtgtacattcaacagctcgagcgggaga
aggcagtactgttgcgtaaaatacatgagcttggttctgaaattatcaggacttcgtccggacctccaaggacaccttactag
>g88536.t1
atggagtcctcgtcgtcgggcggcgggagggcgcggcggcgcagcagcggcggtagctggggcagcgtcggtgacgccttcgacatccccgccaaggg
agcgcccgtggagcggctaaaaaagtggcggcaagcagcccttgtgctgaatgcgtcaaggcgctttaggctgcctttcgtttcaaagaagctggtcgagtac
atgttcagccaaaggaggcaccagtaccacatgctgatggagcacttggctttggaatcaaagaggaacagattactgcgttcacaagagatcacaactactct
gcgcttcaacaatatggagggttttgcagtaccacatgctgatggagcacttggctttggaatcaaagaggaacagattactgcgttcacaagagatcacaacta
ctctgcgcttcaacaatatggagggatttcaggtgtagcaagtatgctgaagactgatacagagaaaggaattaatggcgatgattctgacttgacggcaaggcg
aaatgcatttgggtcaaatacatatcctcgtaagaaaggaaggagctttttggcttttgtatgggatgcttgtaaagacatgacactgataatcctcatggttgcggct
gctgtttcacttgccttgggcataacaactgaggttgcccgaggcggaaggcgaataatggtatcaatatatgatttggttgttggagatgttgtgcctctgaagatc
ggtgatcaggtccctgctgatgggatcctcatcagcggtcactcgctttccatagatgaatcaagcatgacaggagaaagtaaaatcgtacacaaagaccagaa
atcacctttcctaatgtcaggttgcaaagttgcagatggttatggcacaatgctggtgactgctgttggtattaacactgaatggggattactaatggcaagcatatct
gaagattctggtgaagagacacctctgcaggttcgcctgaatggtgttgctactttcattggaatggttgggctttctgttgcatttgcagttttggttgtccttctggcc
agatacttcactggtcatacatataatcctgatggatcggcgcaatatgtgaaaggaaagatgggtgtgggccagacgatacgtggagtagttcgaatcttcactg
tggcggtcactatcgtggtcgtggctgttcctgaagggttaccgttggcagtcacattgacgcttgccttttcaatgcgcaagatgatgagggacaaggcactgga
attgcacagaacacttctggaagcatatttgagccagaggcatctgcttttgtatggtatgattactgacttattttcaatgtctcagcagggtggtcaagaaccaga
ggtgactgctgttggtattaacactgaatggggattactaatggcaagcatatctgaagattctggtgaagagacacctctgcaggttcgcctgaatggtgttgcta
ctttcattggaatggttgggctttctgttgcatttgcagttttggttgtccttctggccagatacttcactggtcatacatataatcctgatggatcggcgcaatatgtgaa
aggaaagatgggtgtgggccagacgatacgtggagtagttcgaatcttcactgtggcggtcactatcgtggtcgtggctgttcctgaagggctaccgttggcagt

cacattgacgcttgccttttcaatgcgcaagatgatgagggacaaggcactggaattgcacagaacacttctggaagcatatttgagccagaggcatctgcttttg
tatggtatgattactgacttattttcaatgtctcagcagggtggtcaagaaccagaggttgctgaattcaagagattcatcgaagatatggctgcttctagcctccgct
gtgttgcctttgcatatagacctcatgacatgggtgaggttccaaaggaggatcagaggaacgactggaagttgcctgaagataacctgattatgcttggaattgt
gggaataaagcaaaactattag
>g89161.t1
atggagagctacctgaacgagaactttgggggcgtcaagcccaagcactcgtccgacgaggcgctggggcgatggcgcaaggtcgtcggcgtcgtcaaga
accccaagcgccgcttccgattcacggccaacctcagcaagcgctcagaggccgcgcagatgaagcgatccaaccaggagaagctgcgtgttgctgtgcttg
tttccaaggctgcacttcagttcctccacggccttgcaccgcaaagcgagtacacggtccctgacaacgtcaagaccaagggcttcggcatctgcgccgagga
gctgagctccatcgtggagggccacgacctcaagaagctcaagtcacatggcggcgtcgagggcctcttgtcgaagctgtccacctcggagtccgacggtgt
cgacacgtcccgcaagaggctgtcgagaagggaggccatcttcggcgtgaacaagttcatcgaggcagagtcccgcggcttctgggtcttcgtctgggaggc
gctccaggacatgacgctcatgatcctcgcggcgtgcgcgttcgtctcgctcatcgtcggcattgccaccgaagggtggcccaagggcgcgcacgacggcct
cggcatcgtggccagcatcctgctcgtcgtgttcgtcaccgcgaccagcgactaccgccagtccctgcagttcaaggacctcgacaaggagaagaagaagat
caccgtgcaggtcacccggagcgggtacaggcagaagctctccatatacgagctcctcgccggcgacgttgtccacctctccattggtgatcaggtgccggcc
gacgggctgttcctgtcgggattctcgctgctgatcaacgagtcaagcttgaccggggagagcgagccggtcgcggtcaacgccgagaacccgttcctcctat
cggggacaaaggtgcaggacggttcgtgcaagatgctcatcaccacggtcggcatgaggactcagtggggcaagctcatggcaactctcagcgagggtggt
gacgacgagacgccgttgcaggtcaagctgaacggcgtggccaccatcattggtaagatcggactcatcttcgccgtggtcacgttcgcggtgctcaccgaaa
gcctgttccgtcggaagatcatggacgggtcgtacttgagctggaccggagatgacgcgttggagctgctcgagttcttcgccattgccgtcaccattgtggttgt
tgctgtacctgaaggactgccgcttgcagtgacgctgagccttgcttttgccatgaagaagatgatgaacgacaaggcgctcgtcaggcaccttgctgcttgcga
gaccatgggctcggccacctccatctgcagcgacaagaccggcacgctgacgacaaaccatatgaccgtggtcaaggcctgcgtctgcggcaaggtcaaag
agctggacggtgtttcagagacaaagaccttgttctctgagttgccggactctgtcatgacgatgctcatgcagtccatattcaacaacaccggcggtgacgttgt
cattaaccaggatggcaagcgggaaatactgggcacaccaactgagacagcgattctcgagttcggcctgtcactcggaggggacttctcagctgtgcgaaaa
gaaagcaccatgatcaaggtggagccgttcaactcggcaaagaagagaatgggcgtggtcattcaactcccagggggcagtgccgttcctctcgatgaagcg
accgtcaagcacttgaatgccacgatcgagagctttgcaaacgaggcacttcgcacgctgtgccttgcctacgcggaagtccctgatgggttctcagccaatgat
cagattccgatggatgggtacacttgcattggcattgtggggatcaaggaccccgtccggcctggcgtcaaggaatcagttgccatctgcaggtctgctggtatt
actgttaggatggtcacaggtgacaacatcaacactgccaaggcaatcgcccgggaatgtggcattttaactgaagatggcattgccattgaaggcccggagtt
cagaaccaagactccagaagaaatgaccgaattgatacccaagatacaggtgatggcaagatcttcgccacttgacaagcacacccttgtgaagcatcttcgga
cttcacttgatgaggttgtcgcggtgactggcgacgggacaaatgatgcacctgcgctacatgaggctgacattgggcttgcaatgggcattgctggaactgag
gtattcaatgaggtgagttcaagagagatggagaagataaatgtatttgaaggcattctagacaacaatgtgtttgtcttcgtcctaagtagcaccgtcatcttccag
ttcatcataatacaatttctgggcgagtttgcaaacactacacctctctcattcatgcagtggatatactgcattttcatcggtttcataggcatgccaatcgctgctatt
gtcaagatgatccccgttggttcttggtag
>g93962.t1
atggcgacgacgaggccggcgcgcagcgacccgcacctgccgccggaggaggcggcgcgtgtggaggccgaggtgcggggctactttgacagcgtcgc
gccgaggcgacccgccaagccgccgcgcagcgacccgtcggaggacgtcgccgccgcggaggctggcgatggggacagcgacctgccggagctccgc
aagctccaggtcctcgccgcggagccccagaagctggtgctggacggagcaggggacgtggacggcggggaggagtacgttgagacgcgctactacgat
gggctcaacggcatcgacaagcagcatcacacgctacccatgtga
>g112607.t1
atgaatgctgagaagtatgatgaattctggaaatctattgaagacatggcagcaacctcattacgctgtgttgcttttgcatatcgtccctgtgagcctgaaatgatac
cagaggacgacatagctaactggaaattgcctgaggatgacctgactctgcttggcatcataggaataaaggctgatataggtctttcaatgggcatctcaggga
cagaagttgctaaggaaagttctgacattataatcttggatgatgactttacatcagttgtcaaggttgttcgttggggccgatctgtctatgcgaatattcagaaattc
atccagttccagctcacggttaatgttgccgcccttgtaataaatgtggttgctgctgtgtcatctggtgatgtgcctctgaatgccgtagaggttgggaccttttcttg
a
>g120143.t1
atgggtcggaggaatcatgacctcatgttgaccgctcgggagtgcttcaagattgctgataagttcgcgttgggcaagttggcgctggatgacatccacggcaa
aggcaaggagaagcgctcggacaagccggagtcctccaagaaggacaagaaaaggaggtctgacaacatggttgctacggtagaccgatccctgaagaac
cctcggacgaaccagcagaccatggatgacttgctaagtggcccatgcctatggcacccaaaggggaaccacaaagcaaaggactattaccgactgaaggg
ctttgtgactgccacgctcaaggtggccaaggatctgccaagacacaatggcaaagacaaggctaaagacggtgacaacaaggacaaagaaggagagttcc
aggagccccggaaggaagtcaactttatctttggaggaccggatgcctacgtgagatcaactccgtag
>g121814.t1
atggaggagatgggggcggggccggtgagcgaaacctctgctcgcgctggtagctcgccgcggggtaggacctcagccttcttggctcggagccgggcct
caacaccggcgacagtggagatcccgagcttgtccggccatgaaagatggcaagctcgccgcccttctcaagcccgcgtcggcaccgccgccccgttggcc
cctggtctcgccctgcccgccccgagcgccgcccgcgcccaggcctcgtccatggcgagctcgttgcccgtgccaagcctcccgagcttgcctctcggcgcc
gccctccgccaccccgcctcccggtgccgccctccgccgcccctcccacgcccgcgctctcgtttgcgccccgcgctctcgtttgcgccccgcgccgccgct
gtcgagttccggtgcgagcaggagatggagaagccgagatcggggtggtgcggtgtcatctctcctgaggaagctgctgcggcggcgatggccggggtgc
ggaaccctgaggcgtcgcccgggcgctacgtgcggcgccgggacgagatcgacgacggctgcgacgacgtgctcggtgtcgacgtcgggggcgcgggg
gccgaccccttcgacatccccgccaagcgcgcctccgtcgagcggctgcggcgctggaggcaagctgctcttgtcctcaatgcttctcgacgattcagatatac
tcttgacctaaaaaaggaggaggagaaggaacatataaggaggaaaatcagggctcatgctcaagtcatacgggcggcactgctttttaaagaggctggagaa
aagaagaatggtgacagggaattgccagctgtcagtgattataaacagtccctgcagttccaacacctaaatgaggagaagcaaaacatccaagtggaggttat
taggggtggtagaaggattccagtgtcaatattcgatattgtagttggtgatgtagtggctctaaaaattggtgatcaggtcccagctgatggtgttttaattaatggt
cattctcttgccatcgacgagtccagtatgactggggaaagcaagattgtaacggcagtcggcttaaacactgaatggggtttattaatggccagcatttcagaag
acaataatgaagaaactccattgcaggtgcgattgaatggagtagcaacactcataggcatcttaggactttctgttgctgccctggtccttatagtcttgtttgcaag

atattttacaggacatactacagactcgaatggaacagttcagtttattaaagggcagacaagtgcaaaatctgcaatatttggttcaataaagatactaactgttgc
ggtaactattgttgttgttgctgtgcctgagggcctaccactggctgtaacattgaccctggcctattctatgcggaaaatgatggctgacaaagcactggtccgga
ggctttctgcctgtgaaacaatgggttctgctactacaatttgcagtgacaagacaggcacattaactttgaaccagggagattcaaatgttcatgtacactggaaa
ggagctgctgaaatagttcttgctttatgtacaaattggcttgacacggatggttcaattcatgaaatgacaactgataaggctgatcagttcaaagaatacattgagt
atatggccgagcaaagtcttcggtgtgttgcttttgcatacagaactatcgagaacgatgtcccgagtgaagagcaaagaatcaactggcaattgccagataatg
accttactctaattggaatagtgggaatgaaggatccttgtcgccctggagtaagagaggctgttgaattgtgcatcaatgctggtgtaaaggtgatggcaagatct
tctcctaatgacaagcttctactcgtaaaagctcttaagaaaagaggccatgtcgttgctgttactggtgatggtacaaatgatgctcctgcattgcatgaggctgat
attggtctttctatgggcatccaaggaacagaagtagctaaagaaagctcagatattattattcttgatgacaatttttcgtcagttgtgaaggtggtccgctggggtc
gttctgtttatgcaaatatccaaaagtttattcagttccaacttactgtaaatgtcgcggctcttgtcatcaatgtggttgctgccatttcatcgggaaatgttcctctaaat
gctgttcagctgctctgggtaaacctcatcatggacacacttggagctcttgcattggctaccgaaccacctacagatcagcttatgatgcggccacctgtcgggc
gaagagaacctcttgtgactaatatcatgtggagaaacttattcattcaggctgtctttcaagttgctgttcttctgaccctcaactttaggggccgagatctcttgcac
ttgacccaagacaccctggatcactccagtaaagtgaaaaatacagttatattcaatacatttgtcttgtgccaggtgtttaacgagttcaattctcgtaaaccagaag
aactgaacatctttgatggggtttcaagaaaccatcttttcttgggagtagtgagcataactgtcattctgcaggtgataattattgagttccttggaaagttcacgtca
acagtgagactcagctggaagctgtggcttgtttctgtagttattgcatttgtcagttggcccttggctttagttggaaaattcattccagttccaaagacaccattgaa
ggattttctcatgagatgttggcccaaacgaaggaagcaaggtgacgacggagcaacaccaccggtgtga
>g122894.t1
atgtcatcgcctaacaggagagaagatccgacggagcaatttgagtcgctgtcaccggtggagtccacgtggatgatcgtggacatccaagggtccgacgtag
tcgaagaaggggcggcgcagccaaagaatgggaggcggcgcagggggaggaaggagaggcgcggccagggggtggcggcgctggttgttaccgtgcc
ctggatcagacggcggcgtttggtgggtggagaggcagcggaagggaaggcacgaaatcagggataccctggactggagaagcttcatgttcttgtgctggt
atcaaaatccaccctgcagtatgaacatggtgtttcgcttcagagtgagtatgttgctcctgaaagtgttaaagctgctgggtttcagatttgtgctgacgaactggc
atcaattgttgaaactcgtgacaatgaaaagttgtctatgcatggccagttggatggaattgcaggcaaattggtgacatcattaactgatggaattagtacagatga
gtatatcctgaatctaagacgagacatatatggagtgaacaagtttaccgagggtgaggagaagcttcatgttcttgtgctggtatcaaaatccaccctgcagtatg
aacatggtgtttcgcttcagagtgagtatgttgttcctgaaagtgttaaagctgctgggtttcagatttgtgctgacgaactggcatcaattgttgaaactcgtgacaat
gaaaagttgtctatgcatggccagttggatggaattgcaggcaaattggtgacatcattaactgatggaattagtacagatgagtatatcctgaatctaagacgaga
catatatggagtgaacaagtttaccgaggttacaatcgttgttgttgctgttcctgaggggcttcctttagcagttacattgagccttgcatttgcaatgaagaagatg
atgaatgacaaggctctggtccgacaattagctgcctgtgaaacaatgggctccgccacagtcatttgtagtgacaagacaggaacactaacaaccaattgcatg
tctgtcgtgaaggcctgttctgtggcaacatgccgatctgctggcatttcagttcgaatggttactggagacaacataaatacagcaaaggcaattgctcgtgaatg
tggtatacttacagaggatggcctttcaattgaaggtgctgaattcagggagaaaagtcgtaaagaaatccttgagttgattccaaagatgcaggtactggcccga
tcttcaccaattgataagcatacactggtgaaacacttgcgtacaacgttcaatgaggttgttgctgtgactggtgatggcacaaacgatgcacctgctctgcgcga
ggcagatgtcggacttgccatgggcattgcagggactgaggtgttcaatgagataagctcaagagacatggaagctatcaacgttctcaagggcctgccagata
actcaattttcatgggcatcctcgctggcaccattggttttcagttcatccttgtccaatttcttggcgactttgccaatacggcaccgctcacccaactacagtggttt
gtcagcgttttgtttggactccttgggatgcccatagcggctgccattaaacttattcctgtggaacgtcatgaagaagatacacatccgtcatag
>g135510.t1
atggagagctacctgaacgagaactttgggggcgtcaagcccaagcactcgtccgacgaggcgctggggcgatggcgcaaggtcgtcggcgtcgtcaaga
accccaagcgccgcttccgattcacggccaacctcagcaagcgctcagaggctgcgcagatgaagcgatccaaccaggagaagctgcgtgttgctgtgcttg
tttccaaggctgcacttcagttcctccacggccttgcaccgcagagcgagtacacggtccctgacaacgtcaaggccaagggcttcggcatctgcgccgaaga
gctgagctccatcgtggagggccatgacctgaagaagctgaaatcacatggcggcgtcgagagcctcgtgtcgaagctgtccacctcggagtccgacggcgt
cgacacgtcgtccaggaagaggctggcgagaagggaggccatcttcggcgtcaacaagttcgtggaggcagagtcccgcggcttctgggtgttcgtctggga
ggcgctccaggacatgacgctcatgatcctcgcggcgtgcgcgctcgtctcgctcatcgtcggcattgccaccgaagggtggcccaagggcgcgcacgacg
gcctcggcnnnnnnnnnnnnnnnnnnnnnnnnnnnnnnnnnnnnnnnnnnnnnnnnnnnnnnnnnnnnnnnnnnnnnnnnnttgcac
cgcagagcgagtacacggtccctgacaacgtcaaggccaagggcttcggcatctgcgccgaagagctgagctccatcgtggagggccatgacctgaagaa
gctgaaatcacatggcggcgtcgagagcctcgtgtcgaagctgtccacctcggagtccgacggcgtcgacacgtcgtccaggaagaggctggcgagaagg
gaggccatcttcggcgtcaacaagttcgtggaggcagagtcccgcggcttctgggtcttcgtctgggaggcgctccaggacatgacgctcatgatcctcgcgg
cgtgcgcgctcgtctcgctcatcgtcggcattgccaccgaagggtggcccaagggcgcgcacgacggcctcggcatcgtggccagcatcctgctcgtcgtgtt
cgtcaccgcgaccagcgactaccgccagtccctgcagttcaaggacctcgacaaggagaagaagaagatcaccgtgcaggtcatccggagcgggtacagg
cagaagctctccatatacgagctcctcgccggcgacgttgtccacctctccattggtgatcaggtgccggccgacgggctcttcctgtcaggattctcgctgctga
tcaacgagtcgagcttgaccggggagagcgagccggtcgcggtcaacgcagagaacccgttcctcctgtcgggcacaaaggtgcaggacggttcgtgcaa
gatgctcatcaccacggtcggcatgaggactcagtggggcaagctcatggcaactctcagcgagggtggtgacgacgagacgccgttgcaggtcaagctga
acggcgtcgccaccatcattggtaagatcggcctcatcttcgccgtcgtcacgttcgcggtgctcaccgaaagcctgttccggcggaagatcatggacgggtcg
tacttgagctggaccggagatgacgcgttggagctgctcgagttcttcgccattgctgtaaccattgtggttgttgcggtgcctgaaggactgccgcttgcagtca
cactgagccttgctttcgccatgaagaaaatgatgaacgacaaggcgctcgtccggcaccttgcggcttgcgagaccatgggctcggctacctccatctgcagc
gacaagaccggcacgctcacgacgaaccatatgaccgtggtcaaggcctgtgtctgcggtaagtccatattcaacaacacgggtggcgacgttgtcattaacc
aggatggcaagcgggaaatactgggcacaccaactgagacggcgattctggagttcggcctgtcgctcggcggggacttctcggctgtgcggaaacaaagc
accatgatcaaggtggagccgttcaactcggcaaagaagagaatgggcgtggtcatccagctcccagggggtgcgctgcgtgcacactgcaaaggcgcctc
agagatcatactggcgtcttgcaacaaatacctgaatgatgaaggcagtgtcgttcctctcgataaagcgaccgtcaagcacttgaatgccacgatcgagagcttt
gcaaacgaggcacttcggacgctgtgccttgcctacgcggaagtccctgaggggttctcagccaatgatcagattccgatggatgggtacacttgcattggcatt
gtggggatcaaagaccccgtccggcctggcgtgaaggaatcagttgccatctgcaggtctgctggtattactgttaggatggtcacaggtgacaacatcaacac
cgccaaggcaatcgcccgggaatgtggcatcttaactgaagatggcattgccattgaaggcccggagttcagaaccaagactccagaagaaatgaccaaattg
atacccaagatacaggtgatggcaagatcttcgccacttgacaagcacacccttgtgaagcatcttcggactgcactcgacgaggttgtcgctgtgactggcgac
gggacaaatgatgcacctgcgctacatgaggctgacattgggctcgcaatgggcattgctggaactgaggttgcaaaagagagtgccgatgtcattattcttgat

gacaacttctccactattgtcactgtcgcaaaatggggtcggtctgtgtacatcaacattcagaagtttgtgcagtttcagctgacagtcaatgtggttgctctcgttgt
gaacttctcctcagcctgcttgacagggagtgctcctcttactgctgtgcaattgctctgggttaacatgatcatggatacacttggagcactggcattggccaccg
aacctccaaataatgaactgatgaagagaactcctgttggaaggaaaggaaatttcatcagcaacattatgtggaggaacatcctgggacaggccttgtatcagtt
cattgtgatttggtacctacagaccgaagggaagcgggtatttggacttgtaggcgacaattcggatcttgtcttgaacacactcatcttcaattgcttcgtgttctgt
caggtattcaatgaggtgagctcaagagagatggagaagataaatgtatttgaaggcattctagacaacaatgtgtttgtcgccgtcctaagtagcaccgtcatctt
ccagttcatcataatacaatttctgggcgagtttgcaaacactactcctctctcattcatgcagtggatatactgcattttcatcggtttcataggcatgccaatcgctg
ctattgtcaagatgatccccgttggttcttggtag
>g138947.t1
atggagctccgcctcctcctccctcgcgtgtcgccggggcccgccctcctccgatcggatccggcgagggccttcctcctccggccccccctcccctgctctccc
cgccagcgagcacgacatgctggccttgcacggaggcggcctcagcttcgacgacccggacccgccgccgccagccgagctcagattcggtgggatccgg
ccccgctggatgctgccgacaccatcgatggagcagcggcggccgacgctggcgcgcgaagagcaacggctgtag
>g146910.t1
atggagagctacctggaggagaacttcgggggcgtcaaggccaagaactcctcggaggaggcgctgcggcggtggcgccgcctctgcagcgtcgtcaaga
atcccaagcgacggttccgcttcaccgccaacctcgacaagcgcggcgaggcgcaggccatcaagcacgccaaccacgtccgtacacatctcctcttccctc
atccatcccctttccttcctttcctccgcgccgggatccgcctcgctcgcggcggcgggatctggactggagccgcgccgccggcgggggcgggagattccg
agaaactgcgtgttgccgtgctggtctccaaggccgcgcttcagttcttacatggtctctcacttcggagcgagtatgttgtccctgaggaagtcaaggctgcagg
gtttgagatctgtgccaatgagcttgggtccattgtggagggccacgatagcaaaaagctgattatacatggaggagtcgatggaattgctgcaaagcttgcgac
gtcacaaacggatggtctgagtacagctgaggacaacattaagcgcaggcaagagatttatggactcaacaagttcacagaaagcgaggtccgaagtttctgg
gtgtttgtatgggaagcacttcaagatacaactctcataattcttgctgtgtgcgcatttgtatctctggtcgttggcattgcgatggaagggtggccaaaaggtgcc
catgacggtcttggaattgttgcgagtatcctcctggtagtgtttgtgactgcaacaagcgattaccgacagtcgctgcagttcaaggacctggacaaggagaaa
aagaaaattcgagtgcaagttacaagggatgggtttaggcagtggatatcgatatatgaccttcttcctggagatgttgtccatttacaaatcggagaccaggttcc
tgcagacgggctcttcatttcagggttttctctgttgatcaatgaatccagcctaacaggtgaaagtgaacctgtcgctgtaaatgaagataacccttttcttttgtcgg
ggaccaaagtgcaggatgggtcctgcaagatgctggttacaacagttggcatgcgcacccaatggggaaaactgatggccacactcagtgagggtggtgatg
acgaaactccactgcaggtcaaactcaatggtgttgcaactatcattggaaagattgggctattctttgctgttataactttcattgtcttgtcccaagggttattcggc
aataaatatcatgacggacagcttttgagctggtcaggagatgatgcactggagcttctggagcattttgctattgcagtgaccattgttgttgtggctgttcctgag
ggattgccattagcagtcacgctgagccttgcattcgccatgaagaaaatgatgaatgacaaggcactggttcgcaacttagctgcatgtgaaactatgggttcag
caaccaccatctgcagtgacaagacagggacattgacaaccaatcatatgactgtcgttaaggcctgcatctgtgggaaaatcaaggaggttaacggtcctcag
aatgcatccaagttatgctctgaatttccggaaactgtcgtcaaaacactcctggagtctatatttaataatacaggtggtgaggttgtgattaaccaagatggtaaa
cgtcagatcctagatgagacaggcagtgttgttcccatggataaaacaactttggaaaagctcaatggtattatcgacaattttgctggtgaagctcttaggacact
atgccttgcttacagggaaatggaagaaggtgtgagggagtctgttgcaacttgccggtctgctggaattatggtgagaatggtcacaggagacaacataaatac
ggcaaaggcgattgcacgtgaatgtggtatactaactgaagatggtgtggctattgaaggacctgaattcagagagaaaaaacttgaagaactccttgagctggt
tccaaaaatccaggttatggcccggtcatcgccacttgacaagcatacactagtaaagcatttgcgcacgacattcaatgatgttgttgcagttactggtgatggca
caaatgatgcacctgcattgcatgaagcagatatcggacttgcgatgggcattgcaggaactgaggtggcaaaagagagtgctgatgttataattctggatgaca
acttctctacaattgtgactgttgcaaagtggggacgctctgtttacatcaatatccaaaagtttgtgcagtttcagcttactgtcaatgtagtagcactgctggttaact
ttcttctgcttgctttacgggaaatgcaccattgacggctgttcagctcctttgggtcaacatgatcatggacacccttggtgcactagcattggccacggaaccac
cgaacgatgagttgatgaagagagagccagtaggaagaacagggaaattcattacaaatgtaatgtggaggaacatcatgggacagtctttctaccaatttttgt
tatgtggtatctccagacgcaagggaaatcctttttggtcttgatgaaagctctgatactgatattgtgctgaacacaataattttcaactcattcgtcttctgtcaggtt
atggcccggtcatcgccacttgacaagcatacactagtaaagcatttgcgcacgacattcaatgatgttgttgctgttactggtgatggcacaaatgatgcacctgc
attgcatgaagcagatatcggacttgcgatgggcattgcaggaactgaggtggcaaaagagagtgctgatgttataattctggatgacaacttctctacaattgtg
actgttgcaaagtggggacgctctgtttacatcaatatccaaaagtttgtgcagtttcagcttactgtcaatgtagtagcactgctggttaacttttcttctgcttgcttta
cgggaaatgcaccattgacggctgttcagctcctttgggtcaacatgatcatggacacccttggtgcactagcattggccacggaaccaccgaacgatgagttg
atgaagagagagccagtaggaagaacagggaaattcattacaaatgtgttcaatgagatcagttcaagggagatggagagggtgaacgtgatcaagggcatc
ctaaagaattatgtcttcatggccgtccttaccagtacggtcatcttccagttcatcatggtacagttcctgggcgagttcgccaacacgacaccgctcaccaggct
gcagtggctcgccagcgtgctccttggcctcgtagggatgcccatcgctgctgctgtcaagatgatccccgtcggctcctcatga
>g167054.t1
atgatgacctgcaccagctgcatcgcctccgaccgcatcagatccatcgacgggcagcacccgcctccgccgctgcgcgccatggcgtgcgcgcgtgcccc
gcaccggttagcggccggcgcacagctcctccgtcagatcccgggagatccgacgagggattcgggatcgatttcgctcgggcggagcggatctccggcgg
gcgcggcggtggcctga
>g167600.t1
atggatgattgtttttccattgatgagcaaataccgctgcaaggatacatatgcattggtattgttggcatcaaagatcctgtgcgtccaggggtcatgcagtctgtg
gcaacatgccgatctgctggcatttcagttcgaatggttacaggagacaacataaatacagcaaaggcaattgctcgtgaatgtggtatacttacagaggacggc
ctttcaattgaaggtgctgaattcagggagaaaagtcctaaagaaatccttgagttgattccaaagatgcaggtactggcccgatcttcaccaattgataagaatac
attggtgaaacatttgcgtacaacgttcaatgaggttgttgctgtgactggtgatggcacaaacgacgcacctgctctgcgcgaggcagatatcggacttgccatg
ggcattgcagggactgaggtggctaaagagaatgctgatgttgtagttctggatgacaacttctccaccattgtaacagttgtcaaatgggggtgttctgtttatgtc
aatatccaaaagtttgtgcagttccagctgactgttaatatagttgcattgctagttaatttttcttttgcatgctttacaggtgttcaatga

CaMK1 genes

>g636.t1

atggggctcggggcggtgatgatggggctggccgacccgccggcgaactacggctcgatctccgcggtggggctgttcgtcgcgctcatgtgcgtctgcatc
atcgtcggacacctcctcgaggagaaccggtggatgaacgagtccatcaccgcgctcttcatcgggctcgggaccggcgccgtgatcctgatggcgtccagc
gggaagcactcccgcgtgctggtcttcagcgaggacctcttcttcatttacctgctcccgccaattatattcaacgcagggttccaagttaagaagaaacaattctt
ccgcaatttcatgactatcacactcttcggcgcagttgggactttgatctctttcactgtaatatcccttggtgctctaggactagtatcaaagcttaacattggtgagc
tggagctaggagattatcttgcacttggggcaatattctcggcgacggactctgtgtgcaccttgcaggtgttaaaccaagatgacacccctttcttatacagcttg
gtgtttggtgaaggtgttgtcaatgatgcaacatccgttgtgctgttcaacgcaatccagagtttttctaccgggagtttcagtggcgttaatttacttaaatttggtggc
agcttcctctatctgttctgcaccagcacctttcttggagtagcatctggacttatcagtgcctatgtcatcaagaaactgtactttggcaggcattccactgatcgtga
agtcgcgattatgatgctcatggcttatttatcttacatgatagctgaaaaagttcgagggtcacaaccaaaaagttcgagggtcacaaccaagtaa
>g701.t1
atgggcaactgctgcgtggcccggccgtcgcccggcaagcggcgtgccggcggcggcgggaaccaccgcggcggccgcctcggtggcgccaacatgca
gtcctgctccgcgatctcctccatcaccgaccccggaggtgcctcccgcgggtgggcccccgccaccgtgctggggaaaggcctggcccccgacgccacc
gccgcggagctgctccggcggtacgagctcggcgaggagctcgggcgcggcgagttcggcgtgacgcggcggtgcacagacacggccacgggcgagtc
gctggcgtgcaagtccatcagcaagcgccggctgcgcagcagcgtggacatcgaggacgtgcggcgggaggtggccatcatgcggtcgatgcccgcgca
cccgaacgtcgtgcgcctgcgcgaggcgttcgaggataacgaggccgtgcacctcgtcatggaggtttgcgagggcggggagctcttcgaccgcatcgtctc
cagggggcactataccgagcgcgccgccgctgccgtcatgcgcaccatcgtcgaggtcgtgcagcattgccataagaacggtgtcatgcatcgggatctcaa
gcctgaaaactttctgtacgccaacacatctgagaactccccccctcaaggtcatcgactttggtctctccgtgtgcttccaaccaggtgataggttcagtgagattgt
cggatccccatactacatggctcctgaagtcctcaagaggaactacggacaagaaatagacatatggagtgccggagtcatactctatatcttgctatgtggtgtc
ccacccttctgggctgaaactgatgaagggattgcacaggctatcatccggtcaaaacttgatttcgagagggagccttggcctaaggtttctgagaatgcaaaa
gatcttgtcaggaagatgcttgatccgagcccttatggacgattgacagctcagcaggttttagaacatccttggatacagaatgcgaactcagctcccaacatcc
ctcttggagaagcagtaaggtccaggcttaagcaatttactgtcatgaacaagttcaagaagaaggccctacttattgtggcagaatacctaccaactgaagagct
ggaggcaattagggagctgttccacatgttggacaccaacaaggacgggcacttgactattgacgaacttcgcaagggattgcgagtgatagggcagaatgca
gacattgacggcaacggtaccctggacttgaaggagtttgtgacagtctccattcatttgaagaaaatcagaagcgaggatcacttgccaaaggtattcagctact
ttgacaagaatggaagcggttacattgaaattgaagaattgaaggaggcgctctctccacgaggaggccagaaggcaatcgatgacataatcctggatgtcga
caaagacaaggcagacattgacggcaacggtaccctggacttgaaggagtttgtgacagtctccattcatttgaagaaaatcagaagcgaggatcacttgccaa
aggtattcagctactttgacaagaatggaagcggttacattgagattgaagaattgaaggaggcgctctctccacgaggaggccagaaggcaatcgatgacata
atcctggatgtcgacaaagacaaggatgggaagataagctacgaggagtttgagctgatgatgaaagtgggccacttgctggagctgcaaaagaacaacgag
aggttgactgatgagcgatgtgtttcgtacagtggatgtatcgaaacagaaagtgtgtga
>g1997.t1
atgggcggccgcgcctcccgccaccgccaatcgcccgaggaccgaaactcgcagcctcccaagcagaagcagcagcagcatcagtcgacccgcccatca
cccaagcagcagcacaggccacagccgccgccgccgccgaaacagctgcagcagcctcctcctccgccgcgcgcttcccctcttccgccggcagcaggg
ccggacgatggcgtgggccgggtgctgggccgcccactggaggacgtgcgcgcgagctacaccttcgggcgcgagctgggccggggccagttcggcgtc
acctacctggcgacccacaagcccacgggccgccgccacgcctgcaagtccatcgccacacgcntcacctacctggcgacccacaagcccacgggccgc
cgccatgcctgcaagtccatcgccacacgaaagctcgcgcgcgccgacgacgccgacgacgtccgccgggaggtgcacatcatgcaccacctcacgggcc
accgcaacatcgtcgagctccgcggcgcctacgaggacaggcactccgtcaacctcgtcatggagctctgcgagggcggggagctcttcgacaggatcatc
gccaggggacactactccgagcgcgccgccgccgcgctgtgcagggagatcgtctccgtcgtgcacacctgccattccatgggggtcatgcacagggacct
caagcctgagaacttcctgttcctcaacaaatcagaagactcgccgctcaaagcgacagactttggactatccgtattcttcaagccaggtgagcagttcaggga
ccttgttgggagtgcctattacgtggccccggaagtgctcaaacggcgatatggggctgaggccgacatctggagcgccggagttatcctgtacatccttctatc
cggcgtccctcctttctgggccggacatccatggattcgagaggatggagaggccccagacaaaccactcgatattgcagtaatcggcagaatgaagcaattc
agggcaatgaacaagcttaagaaggttgctttgaaggttgttgcagagaacttgtcagaggaagagatcgtcagattaaaagaaatgttcaaatccctggatact
gacaacagtgggacaataactctggaagaactaagaactggcttaccaaagcttgggaccaaagttactgaatcagaaataagacagttgatggaggcggctg
atgttgatggaaatggtatcattgattatgctgaattcatatcagcaacaatgcatatgaatagactagagaaggaggatcacatatataaagcatttgagtattttgat
aaggatcacagcgggtacataacagttgatgagttggaagaagctttaaagaagtatgatatgggcgatgaggcaacgataaaagaaattattgccgaagtgga
tacagaccatttcgcaaaatag
>g2451.t1
atgcaaccggacccgagcgggaacgccaatgcgaaggggaagctgccgcagccggtgacggcgccggcgccgtcgtccggccggccggcgtccgtgtt
gccgtacaaaacggcgaacgtgcgcgacttctaccgcatcggtaagaagctggggcaggggcagttcggcaccacgtaccagtgcgtggacaaggccgat
ggcgcagagtacgcgtgcaagtccatccccaagcgcaagctgctgtgccgggaggactacgaggacgtgtaccgcgagatccagatcatgcaccacctttc
ggagcaccccaacgtcgtgcgcatccgcggcgcttatgaggatgcgctcttcgtgcacatcgtcatggaaatctgcgccggtggtgagctctttgatcgcatcgt
cgcaaagggacactacagcgagcgtgcggcggcgaagctcatcaagaccatcgtcggggttgtggaggggtgccactcgcttggtgtcatgcaccgggacc
tcaagccggagaacttcctctttgccagcacagcagaggacgcacctctcaaggctactgactttgggctctccatgttctacaagcccggtgataaattctctgat
gtcgttgggagcccctattatgtcgcaccagaggtgcttcagaaatgctatggtccagaagctgatgtctggagtgctggggttatcctgtacattttgctttgtgga
gttcccccattttgggctgaaactgaagcaggaatcttcaggcagattctacggggaaaacttgattttgaatctgaaccctggcctagcatctctgatagtgccaa
agatctggtccgtagtatgcttactcgtgatcctagaaagagatatagtgctcatgaagttctctgtcacccatggatcattgatgatgccgttgcacctgataagcct
attgattctgctgttttgtcaaggctgaaacatttctcagcaatgaacaagctcaagaagatggcattgagggttattgctgaaagcttgtcagaggaagagattgg
aggcctcaaggagctgttcaaaatgattgatactgacaatagtgggactataacatttgatgagctgaaaaatggcttgaaaagggttggctcagagttaacaga
gaatgagatccaagctttgatggaagcagctgatattgacaacagtggaaccatcgactacggtgaatttatcgcagctacattgcacatgaataaactagagag
ggaggaaaacttggtgtcagcattctcgtttttcgacaaagatggaagtggctttataaccatagatgagctgtcacaagcatgccgtgaatttggtcttgatgatgt
tcaccttgaggatatgattaaagatatcgatcaaaacaatgatgggcaaattgactacagcgagttcacggcaatgatgaggaagggcaacgctggttgtgcaa
caggtaggaggaccatgaggaccaatttgaatcttggtgaactcttgaatcccagcaacagctag
>g3293.t1

atggaaacagagaagaatggtaacattctgctgcaaaggtatgaggttggtagattacttgggcagggcacttttgctaaggtttaccatgctaggaacatagtga
catcacagagtgttgctatcaaggtgattgataaagacaaaattttcaaggttggtctaatggagcagatcaagcgggagatatctgtgatgaaattagtgaggca
ccccaacattgtacagctttatgaggtcatggctaccaaatcaaagatatattttgttcttgaatatgtcaagggtggtgaattgtttaacaaaatagctaaaggtaagt
taagagaagatgcggctaggaagtacttccagcagttgataagtgctgttgatttctgccatagtagaggtgtataccatcgtgatctgaagccagaaaacctactt
gttgatgagaatgggaatctgaaaatttcagattttggattgagtgctcttgctgagtcaagacgccaagatggccttctacacacaacttgtggtactccagcatat
gtggcgcctgaggttatcagccgaaaagggtatgatggtgccaaggttgacacatggtcttgtggggtgatcctgttcgttctcatggctgggtatctgccttttca
agattccaatttggtggagatgtatagaaaaattgggaaagcggagttcaagtgcccgccttggtttccatctgatgatctgtggaacatggaaacagagaagaat
ggtaacattctgctgcaaaggtatgaggttggtagattacttgggcagggcacttttgctaaggtttaccatgctaggaacatagtgacatcacagagtgttgctatc
aaggtgattgataaagacaaaattttcaaggttggtctaatggagcagatcaagcgggagatatctgtgatgaaactagtgaggcaccccaacattgtacagcttt
atgaggtcatggctaccaaatcaaagatatattttgttcttgaatatgtcaagggtggtgaattgtttaacaaaatagctaaaggtaaattaagagaagatgcggcta
ggaagtacttccagcagttgataagtgccgttgatttctgccatagtagaggtgtataccatcgtgatctgaagccagaaaacctacttgttgatgagaatgggaat
ctgaaaatttcagattttggattgagtgctcttgctgagtcaagacgccaagatggccttctacacacaacttgtggtactccagcatatgtggcgcctgaggttatc
agccgaaaagggtatgatggtgccaaggttgacacatggtcttgtggggtgatcctgttcgttctcatggctgggtatctgccttttcaagattccaatttggtggag
atgtatagaaaaattgggaaagcggagttcaagtgcccgccttggtttccatctgatcatcatgttcttaagttgcacctgccatcaattgtagttgattaa
>g4006.t1
atggcggatcagctcaccgacgaccagatcgctgagttcaaggaggccttcagcctcttcgacaaggacggtgatggctgcatcaccaccaaggaacttggta
ctgtaatgcgctctttggggcagaaccccactgaggctgagcttcaggatatgatcaatgaagtggatgctgatggcaatggaactatagacttccccgagttcct
caacctgatggctcgtaagatgaaggacacagactctgaagaggagcttaaggaggctttccgtgtgtttgacaaggaccagaacggcttcatttctgcagctga
gctccgccatgtcatgaccaaccttggctgcatcaccaccaaggaacttggtactgtaatgcgctctttggggcagaaccccactgaggctgagcttcaggatat
gatcaatgaagtggatgctgatggcaatggaactatagacttccccgagttcctcaacctgatggctcgtaagatgaaggacacagactctgaagaggagctta
aggaggctttccgtgtgtttgacaaggaccagaatggcttcatttctgcagctgagctccgccatgtcatgaccaaccttggtgagaagctgacagacgaggaa
gtggatgagatgattcgtgaagctgatgtggacggtgatggccagatcaattacgaggaatttgtgaaggtgatgatggccaagtga
>g7072.t1
atgaaccacntgcaccatgaggctctccaggaggccctccctggtgacaatgttggtttcaacgtgaagaatgttgctgtgaaggatctgaagcgtgggtacgtg
gcctccaactccaaggatgaccctgccaaggaggctgccagcttcacctcccaggtcatcatcatgaaccatcctggacagattggcaatggttatgccccagtt
ctggactgccacacctcccacattgctgttaagtttgctgagctgcttaccaagattgataggcgatctggtaaggagcttgagaaggagcctaagttcctaaaga
acggtgatgctggtatggtgaagatgattcccaccaaacccatggtggtggagaccttctctcagtaccctcctcttggtcgttttgctgtccgtgacatgaggcag
acggttgctgttggagtcatcaagagcgttgagaagaaagaccccactggtgccaaggtgaccaaggctgctgtcaagaagaaatga
>g8189.t1
atggggaaggagaaggttcacatcaacattgtggtgatcggccatgttgattctggcaaatcgaccaccactggtcacctcatctataagcttggaggaatcgac
aagcgtgtcattgagaggttcgagaaagaggccgctgagatgaacaagaggtccttcaagtacgcctgggtgcttgacaagctcaaggctgagcgtgagagg
ggtatcaccattgacatcgccctctggaagtttgagaccaacaagtactactgcactgtgattgatgctcctgggcaccgtgacttcatcaagaacatgatcaccg
ggacctcccaggctgattgcgccgtccttatcatcgactccaccaccggaggctttgaggctggtatctccaaggatgggcagacccgtgagcacgcgctgctt
gctttcactcttggtgtcaggcagatgatctgctgctgcaacaagatggacgccaccactcccaagtactccaaagctcggtatgatgaaattgtcaaggaagtg
ggctcgtacctgaagaaggttgggtacaacccggagaagatccccttcgtgcccatctctgggtttgagggtgacaacatgattgagaggtccacgaacctgga
ctggtacaagggcccgaccctccttgaggccctggaccagatcaacgagcctaagaggccctcagacaagcccctccgccttcccctgcaggacgtgtacaa
gatcggcggcattggcacggttccggtgggccgtgtggagacgggtaccctgaagcccggcatggtggtcacctttgggcccagcggcctgaccaccgagg
tgaagtcggtggagatgcaccacgaggccatgcaggaggctctgcccggcgacaacgtgggcttcaacgtgaagaatgttgccgtcaaggacctgaagcgt
ggctatgtggcctccaactccaaggatgaccctgccaaggaggctgccagcttcacggcgcaggtgatcatcatgaaccaccctggtcagatcggcaacggc
tacgcccccgtgctggactgccacacttcccacatcgccgtcaagttctctgagctgctgaccaagatcgacaggcgatccggcaaggagctggannnnnnnn
nnnnnnnnnnnnnnnnnnnnnnnnnnnnnnnnnnnnnnnnnnnnnnnnccggtgggccgtgtggagacgggtaccctga
>g8190.t1
atgcaccacgaggccatgcaggaggctctgcccggcgacaacgtgggcttcaacgtgaagaatgttgccgtcaaggacctgaagcgtggctatgtggcctcc
aactccaaggatgaccctgccaaggaggctgccagcttcacggcgcaggtgatcatcatgaaccaccctggtcagatcggcaacggctacgcccccgtgctg
gactgccacacttcccacatcgccgtcaagttctctgagctgctgaccaagatcgacaggcgatccggcaaggagctggatagcgcccccaagttcctcaaga
acggtgatgccgggttcgtgaagatgatccccaccaagcccatggtggtggagaccttctcagagtaccctccgctggggcggtttgcggtgcgcgacatgag
gcagacggtggccgtgggcgtgatcaagagcgtggagaagaaggacccgacgggggccaaggtcaccaaggctgcagccaagaagaagtga
>g9985.t1
atgggcaactgctgcccgggctccggggatgcggagcccgccgccggcgacgcctcgtcccgcctccacgccgcctccctcaaggccggcggcagcgnn
nnnnnnnnnnnnnnnnnnnnnnnnnnnnnnnnnnnnnnnnnnnnnnnnnnnnnnnnnnnnnnnnnnnnnnnnnnnnnnnnnnnnnnnnnnnnnnnnnnnnnnnnnnnnn
nnnnnnnnnnnnnnnnnnnnnnnnnnnnnnnnnnnnnnnnntcccccatccgccgccgccgccccgcgcgccatgggcaactgctgcccgggctccggg
gatgcggagcccgccgccggcgacgcctcgtcccgcctccacgccgcctccctcaaggccggcggcagcggcagcggcagcgggccgacgtccgcgcc
cacgccgaccaagccgccggcgccgatcgggcccgtcctgggccggcccatggaggacgtccgcagcatctacaccgtgggcaaggagctgggccgcg
gccagttcggcgtgaccagcctgtgcacgcacaaggccacgggcgagcggttcgcgtgcaagaccatcgccaagcggaagctgtccaccaaggaggacgt
ggaggacgtgcggcgcgaggtgcagatcatgtaccacctggcgggccagcccaacatcgtcgagctcaagggcgcctacgaggacaagcagtccgtgca
cctcgtcatggagctctgcgcgggaggggagctcttcgaccggatcatcgccaaaggcaagtacacggagcgcgccgccgccgcgctgttgcgcaccatcg
tcgagatcgtgcacacgtgccactcgctcggcgtcatccaccgcgacctcaagccagagaacttcctcctcctcagcaaggacgagcacgcgccgctcaagg
ccaccgatttcgggctctccgtattcttcaagcagggggaggtgttcaaggacatcgtgggaagcgcctactacatcgcgccggaggtgctcaagaggagcta
cggaccagaggcggatatctggagcgtcggtgtcatcctctacatcctactctgcggagtgcccccgttctgggcaggcaagttcatccctgttcaatcggagca
cggcatcttcaacgccatcctccggggacaggtcgacttctccagcgacccatggccgcgcatttcctccggcgccaaggatctcgtcaggaagatgctcacct

ctgaccccaagaagaggatctcggcctacgacgtcctcaatcatccttggatcaaggaagacggcgaggcgcctgacacgccgctggacaacgctgtcatga
acagactcaagcagttcagggcaatgaaccagttcaagaaagccgcgctcagggtcattgccgggtgcctgtcggaggaagagatcaaggggctcaaggag
atgttcaagggcatggacgctgacaacagcggcaccatcaccgtggacgagctgcggcgagggctggccaagcagggcaccaagctctcggaggccgag
gtggagcagctcatggaggccgccgacgcggacgggaacggcacgatcgactacgaggagttcatcaccgcgacgatgcacatgaaccggatggacagg
gacgagcacctctacgccgcgttccagtacttcgacaaggacggcagcggctgcatctccaaggaggagctggagcaggcgctcagggagaagggcctgc
tcgacggccgggacatcaaggacatcatctccgaggtcgacgcagacaacgacgggaggatcgactacagcgagttcgtggcgatgatgaggaaagggaa
ccccgaccccaaccccaagaagcggcgcgacgtcgtgctgtag
>g11720.t1
atggcgatgccgcaacagccgcgaactgtggcctacggacgctgcctccgccggtcgacgaccaccatcgcggcgtcacccccctgcccacgttcttcgggtc
agcggcgagggcccggttctgccgtgccggcagccgttccacaggagaggagaggagaggagagcgcgcaccaccggaatggagggagctgacaagg
atggcaacccgttgaggaattaccgaattggtaagactctggggattggttcattcggtaaggtgaaaatcgcggagcatataaccactggtcacaaggtggcta
ttaagatcctcaatcgtcgcaagatcagaggcatggaaatggaagagaaagattgtgagccccgaatgttctgccccagcannnnnnnnnnnnnnnnnnata
accactggtcacaaggtggctattaagatcctcaatcgtcgcaagatcagaggcatggaaatggaagagaaagttaaaagagagatcaagatattgaggttgttt
atgcacccacatattattcgcctctatgaggtgatagatacgccagctgacatttatgtggttatggaatatgttaagtgtggggagttgtttgattacattgttgaaaa
aggtagactgcaggaggaagaggctcgccgttttttccagcagattatatctggagttgagtattgccatcgaaacatggtggtccaccgtgacctaaaaccaga
gaacctcctattggattcgaaatggaatattaagattgcagattttggcttaagtaatgttatgcgggatggccatttccttaagacaagttgtggtagcccaaattatg
ctgctcctgagattgatgacgagactcttcgtgaggttataagtatgggttatgacaagaacgttttggtggaatcaatacaaaacagattacaaaatgaggcaact
gttgcgtattatttacttttggacaataggcttcgtacaactagtggctatcttggagctgagtgtcaagaagctatgctttacaaaacaagagacgagaaataccttc
ttgacttgcaaagagtcaccggaccgcagctcctctttctggacctgtgtgcggccttttttagctcagctgagagttctttga
>g11944.t1
atgaaccaccctggacagatcggcaacggctatgccccagtgctggactgccacacctcccacattgctgtcaagtttgctgagctgctgaccaagattgacag
gcgatctggcaaggagcttgagaaggagcctaagttcctaaagaatggtgatgctggtatggtgaagatgattcccaccaagcccatggtggtggagaccttct
ctcagtaccctcctcttggtcgttttgctgtccgtgantaccctcctcttggtcgttttgctgtccgtgacatgaggcaaacagttgctgttggtgtcatcaagagtgtt
gagaagaaggacccaaccggtgctaaggtgaccaaggctgctgtcaagaagaaatga
>g12550.t1
atggagctcgtcgccgtcaaatacatcgagcgcggcgagaaggtcattttaacgccaacccatcttgctattgtgatggaatatgcatctggtggtgagctttttga
gagaatatgtaaaaatgtgcgattcagtgaagatgaggcacgctatttcttccagcagcttatctcaggagtaagctactgccattcaatgcaagtatgtcaccgtg
atctgaagctggagaatacactgctggatgggagtgatgctcctcgcttgaagatatgtgacttcggctattccaagtcttcagttcttcattcacagccaaagtcaa
ctgttggaacaccggcttatattgcacctgaagttcttttgaaaaaagaatatgatggcaagatagctgatgtatggtcttgtggtgtaaccctctatgttatggtagtt
ggcgcatatccttttgaagaccctgaagagcctaagaacttccgcaagacaattcagcgaatcttgaatgttcagtactcaattccagataatgtgaacatatctcc
agagtgcaggcatctaatttcgaggatatttgttggtgatcctgctatgcggatttcaatccctgaaatccgaaaccatagttggttcttgaagaaccttcctgctgat
ctgatggatgatgatagcatgagcaaccagtacgaggagcctgatcagccgatgcagaccatggatcagatcatgcaaattttgacagaggccaccataccac
ctgcttgttctcgcagcataaacgccctagctgatggactggacatggacatggacgatgacatggatgacctcgagtccgactctgatcttgatgttgatagcag
tggtgagatcgtatcgtgcatgcttaaaggggaggagatgaagcgagagcgctggaacgcgtacagtgcagagcctgacagcctgatcgaggagaggaag
gaggttctggacatggtcaggaacgagtttgaacaagaaaaataa
>g13082.t1
atgcgccacctccccaagcaccccaacatcgtcaccctcagggacacctacgaggacaacaatgccgtccacctcgtcatggagctctgcgagggcgggga
gctctttgatcggatcgttgcccgtggacactacacggagcgcgctgccgccttggtcactaagaccattgtcgaggtcgtgcaggtgagattggcttcattgttc
gtgcaggttcattga
>g13118.t1
atgggcaactgctgcgtgacgcctaacggcgccgatggcggcggcaagaagccgaagcagcccaagcagaagaagggcaagaagccgaacccgttctc
gatcgactacaaccggtcggcgccgccgggctcgaggctggtggtgctgcgggagcccacggggcgggacatcgccgcgcggtacgagctgggcgggg
agctcggccgcggcgagttcggggtcacctacctctgcaccgaccgcgccacgggggaggccctcgcctgcaagtccatctccaagaagaagctccgcac
ccccgtcgacgtcgaggacgtgcgacgggaggtcgagatcatgcgccacctccccaagcaccccaacatcgtcacgctcagggacacctacgaggacgac
aatgccgtccacctcgtcatggagctctgcgagggcggggagctctttgatcggatcgttgcccgtggacactacacggagcgcgctgctgccttggtcactaa
gaccattgtcgaagtcgtgcagatgtgccataagcatggagtgatgcacagagatctcaaaccagagaacttcttgtttgcaaacaagaaggaaacagcggctc
tcaaggcaattgattttggtctgtccgtatttttcactccaggtgaaaggttcactgagattgttggaagtccttattatatggctccagaggtactgaagagaaattat
ggcccggaagttgatgtttggagtgcaggagtgattctttacattcttctttgtggggttcctccattttgggcagaaactgaacagggtgttgctcaagctattattcg
ttcggtcattgattttaaaagagacccatggccaagggtctccgataacgccaaagatcttgtcaggggaatgcttaatccagatccgaaacggagattgacagc
tcagcaagtgcttgatcatccttggctgcagaacataaagaaggctccaaatgtcaatttgggtgaaactgttaaggccaggcttcaacaattttctgtgatgaaca
agttcaagaagcatgcgctcagggtcatagctgatcatctttcagtagaagaggctgctgacataaaagatatgtttgagaaggtagatcttaacaaagatcagat
gattagttttgatgagctgaagcttggtctgcataagtttgggcaccaaatgcctgatgcagatgtccaaatacttatggaagctgctgatgccgatggaaatggaa
ccttggactatggagagtttgttactctatctgttcacctaagaaagattggcaatgacgaacatctgcataaggcgtttgcttactttgatcggaatgagactggata
tatcgaaatcgatgaactccctgatgacctgggacaaaatcctgaagatgttatcaatgccatcatacgtgacgtggacactgataaggatggcaagataagcta
cgatgaatttgcggcaatgatgaaggctggaacagactggaggaaagcatcaagacagtattcaagagaacggttcaccaaccttagcctaaagctgcaaaag
gacggatcgttgcagatgacgagtacccggtag
>g14573.t1
atggaagggctgacgggggagcagatagttgctttccaggaggctttctccctcttcgacaagaacggcgacggatgcatcaccatggaagagctggctgca
gtgactcgctccctaggccttgatcctagtgatcaggagctcatcgatatgatgagcgaagttgatactgatggcaatgggataattgattttcaggagtttctgagc
cttattgccaggaagatgaaggatggtgatggcgatgaggagctgaaggaggcttttgaggtcctagacaaggaccagaatggatttatctcccctactgagct

gaggacggtgatgaccagccttggggagaagatgactgatgaagaggttgagcagatgatcaaagaagcggatactgatggtgatggacaggtgaactacg
atgagtttgtacttatgatgaaaaatgctgaacggaagatatctgggtga
>g16078.t1
atggaggagaggtacgaggcgctcaaggagctgggcgccggcaacttcggggtggcgaggctggtcagggacaaacggaccaaggagctcgtcgccgtc
aagtacatcgagaggggaaaaaagatcgatgagaatgtgcagagagagattatcaatcaccggtcacttcgacatcctaatatcgtacggttcaaggaggtaatt
cagaaatcattaactctatcttttgtttga
>g16714.t1
atgggtggccgcgcctcccgccaccgccaatcgcccgaggaccgaaactcgcagccccccaagcacaagcagcagcagcatcagtctacccgcccggcg
gcacccaagcagcagcagcagcacaggcacccgccgccgccgccgaagcagcagcagcctcctcctccgccgccgcacgcttcccctcctccggctccg
ccggaagcagggccggacgcgggcgtgggccgggtgctgggccgcccactggaggacgtgcgcgcgagctacaccttcgggcgggagctgggccggg
gccagttcggcgtgacctacctggcgacccacaagcccacgggccgccgccatgcctgcaagtccatcgccacacgaaagctcgcgcgcgccgacgacgc
cgacgacgtccgccgggaggtgcacatcatgcaccacctcacgggccaccgcaacatcgtcgagctccgcggcgcctacgaggacaggcactccgtcaac
ctcgtcatggagctctgcgagggcggggagctcttcgacaggatcatcgccaggggacactactccgagcgcgccgccgcagcgctgtgcagggagatcgt
ctccgtcgtgcatacctgccattccatgggggtcatgcacagggacctcaagcctgagaacttcctcttcctcaacaaagcagaagactcgccgctcaaagcaa
cagactttggactatccgtattcttcaagccaggtgagcagttcagggatcttgttgggagtgcctattacgtggccccggaggtgctcaaacggcgatatgggg
ctgaggccgacatctggagtgccggagttatcctgtacatcctgctatccggcgtccctcctttctgggccggacatccatggattcgagaggacggagaggcc
ccagacaaaccactcgatattgcagtaattggcaggatgaagcaattcagggcaatgaacaagcttaagaaggttgctttgaaggttgttgcagagaacttgtca
gaggaagagatagtcggattaaaagaaatgttcaaatccctggatgctgacaacagtgggacaataactctggaagaactaagaactggcttaccaaagcttgg
gaccaaagttactgaatcagaaataagacagttgatggaggcggctgatgttgatggaaatggtaccatcgattatgctgaattcatatcagcaacaatgcatatg
aatagactagagaaggaggatcacatatataaagcattcgagtattttgataaggatcacagcgggtacataacagttgatgagttggaagaagctttaaagaagt
atgatatgggcgatgaggcaacaataaaagaaattattgccgaagtggatacagatcataacatccttgcaaaggtgaaaataaaggggcaagcagagaatgc
tccaagatcagagatcttactgtttgactga
>g16824.t1
atggagaagtacctgcaggagcacttcgatctgccgcacaagaacccctccgaagaggcgcagcggcgatggcgctccgccgtcggcaccatcgtcaaga
accgccgccgccggttccgcatggtgcctgacctcgaccgccgccagaaggacgaggaggcgcgtcgctccatccaggaaaaaattcgggttgctctctatg
tgcagaaggctgcgatcattttcagtgatggtgccagcaagaaagagtacaagctgacagaggacattatgaatgctggattttcgatcaatccagatgaactgg
catcaatcacaagcaagcatgatatgaaagctttcaagatgcatggtggtgttgatgggatatccaaaaagatccgttcaactttcgaccgtggcatatcaactagc
gatctggataaaagacagatgatctacggtgaaaatcgttatgccgagaaacctgcaagaagcttctgggcatttgtttgggatgcgttgcaggacatgacccttа
tcattcttatgttcaaggagctagacaatgagaagaaaaagatattcatccatgtaaccagagacggtgagagtgaccctgtgtacatttcggaggataagcccctt
catcttggcaggaactaaagttcaagatggatctgctaaaatgttggtaactgctgtgggtatgcgtactgaatggggaaggttgatgagcacattgagtgaggg
aggagaggatgagacaccattgcaggttaaactaaatggagttgcaaccatcattgggaagattgtgaactactttgcgacagcggtcactattattgttgttgctg
ttcctgaagcatgtgagacaatgggatctgctggtacaatctgcacagataagacaggaactttgacaactaaccatatgggtatttttgagaacacgagtgcaga
agtagtcaaggataaggatggtaaacaaactgttttaggaactccaactgaaagggcgatattagaatttggcttgatgttggaaggacatgataccgaggatag
aacctgcacgaaggtcaaggttgagcctttcaattcggtgaagaagaagatggcagtgttggtctcagtacctaacggcacataccgctggttcagcaaagaaa
gtcctacaagtggttttactctgatatgcatctttggcatcaaggaccctgtgcgcccaggagtcaaggatgctgttaaggcctgcatatctgctggcatcattgtga
gaatggtgactggtgataacatcaatacagctaaagctattgccaaggagtgtggcatactgactgatgacggcatagcaatagaaggaccagagttccgcaac
aagagcccagaagaaatgagggacttgatacctaagattcaggtcatggctcgttcgttaccattggacaaacatacccttgtgacaaacttgcgaggtatgttcc
gtgaagtggtcgctgtgacaggtgatggcacaaatgatgctccagcattgcatgaatcagatattgggcttgctatgggcatagctggcacagaggtagcaaag
gagagtgctgacgtgatagtgcttgatgacaattttactactataataaatgttgcaaggtggggtcgggcagtttacctcaacatccaaaagtttgtacagtttcagt
tgactgtcaatattgttgctttagtcatcaactttgtctcagcatgcattacagggagtgctcctctcacagcagtgcagttgttgtgggtcaatatgattatggataca
ctaggagctttggctctagctaccgagcctccaaatgatgacatgatgaagagaccacctgttgggcgaggggagagtttcattaccaagatcatgtggaggaa
tatcattggccaaagtttatatcagttggttgtacttggagctctaatgtttggtggggaacgacttctgaacatcaagggtgcagattccaaatctgtaatcaatactc
tcatattcaactcctttgtgttctgccaggtattcaatgaaataaacagcagggaaatgcagaagatcaatgttttccatggaatgtttagcaactggatcttccttgga
atcattgctgtgacagtagcatttcaagtggtgatcatagagtttnnnnnnnnnnnnnnnnnnnnnnnnnnnnnatactgactgatgatggcatagcaatagaa
ggaccagaattccgcaacaagagccccgaagagatgagggacttgatacctaagattcaggtcatggctcgttcgttaccattggacaagcatacccttgtgac
aaatttgcgaggtatgttccgtgaagtggtcgctgtgacaggtgagtgccctatagctgttatcaatacatccagtcctaatgcccatggtgatgcttcagtattcaat
gaaataaacagcagggaaatgcagaagatcaatgttttccatggaatgtttagcaactggatcttccttggaatcattgctgtgacagtagcatttcaagtggtgatc
atagagtttcttggcacttttgccagcaccgttccacttgattggcagctttggttggttagcatcggcctgggatccatcagcttgattgttggcgccatcttgaagt
gcataccagtggaatcaggtgggacttctgctagtccaaatggctacgccccactccccagcggccccgataacatttaa
>g19529.t1
atgggccaatgctgcgccaagggcggcgacccggccgccgccgccggcgacgacaaggaccgccccgcgtcgtcgtcgtcggagcccaagccggagg
cctccagccgcggcggcgcgaacgccgccccgccgcgctcatcgtcgtcttcctccaaccacccggcggtgggcccggtgctgggccggcccatggagga
cgtgcgcgcgacgcactcggtggggaaggagctgggccggggccagttcggcgtgacccacctgtgcacacagaagcccacgggggagaagctggcct
gcaagaccatcgccaagcggaagctctccacccgcgaggacgtggacgacgtgcgccgcgaggtccagatcatgcaccacctctccggccagcccaacgt
cgtcgtcctcaagggcgcctacgaggacaagcacaacgtacacctcgtcatggagctctgcgccggcggcgagctcttcgaccgggtcatcgccaagggac
agtacacggagcgcgccgcggcggcgctgctccgcaccatcgtgcagatcgtgcatacctgccacgccatgggagtcatgcacagggatatcaagccgga
gaatttcttgctgcttagcaaggacgaggacgcgccgctcaaggccacggatttcgggctatccgtcttcttcaaggaaggggaggtgttcagggacatcgtag
gaagtgcctactacatcgcgccggaggtgcttaagaggaagtacgggccagaggcggatatttggagtgttggagtcatgctatacatcttccttgctggagtgc
cgccccttctgggcggagaatgagaatggcatcttcaccgccatcctgcgtggccagattgatctcgccagcgacccgtggccaaagatttcgtcgggggcaaa
ggatcttgtcaagaagatgctcaccatcaaccccaaggagcggcttacggcgttccaggtcctcaatcacccgtggatcaaagaagatggagatgcacctgata

cgccacttgacaacgttgttctcaacagactcaaacagttcagggccatgaaccagttcaagaaagcggcactaaggatcatagctgggtgcctatcggaagag
gagatcaccgggctgaaggagatgtttaagaacatcgacaaggataacagtgggaccattacgcttgaagagctcaagaacgggttggcaaagcacggaac
aaagctgtcggacggtgaaattcagcaactcatggatgcagctgatgctgatggcaacggattgatcgactacgatgaatttgtcaccgcgacggtgcacatga
acaaactggacagagacgaccacctctacactgcattccagtatttcgacaaggataacagcgggtacattacaaaagaagagcttgagcaagctttgaagga
gcaaggattatatgacgccgaggagattaaggacgttatctcagatgctgatgctaacaatgacgggaggatagattattcagagtttgtggcgatgatgaggaa
aggtactgctggcgctgagacggcgaaccccaagaaaaggcgcgacctagtactgtag
>g20054.t1
atgggtctctgccatggcaagccaacgcaaatcccggagcctgaggcggaagaagatccccatgtagcctccggcgccggcgacgccgccggtgcccgga
acggcgcctccgcgccgtcgccagcgcctgcgacgaagccgggcacgccgaagcagcccaagttcccgttctacctaccgagcccgctcccgccgtcgag
ctacaagggctcgccggcgaactcgagtgtggcatcgacgccggcgcgcggagggttcaagcggccgttcccaccgccatcgccggcgaagcacatccgc
gcgctcctggcgcggcggcacggctcggtgaagcctaatgaggcgtccatccctgagggcggcgagccggagctcggcctggacaagagcttcggcttctc
aaagcacttcttcgccaagtacgagctcggcgaggaggtcggccgcggccacttcggctacacctgctctgcnnnnnnnnnnnnnnnnnnnnnnnnnn
nnnnnnnnnnnnnnnnnnnnnnnnnnnnnnnnnnnnnnnnncacccccattcccctctgacccaagcctttcgctcaaagcttccagctttttcaattgtttg
gtcacttcaagaatcaaggaatatgttcttgcttcttgggcgatagtctga
>g22121.t1
atggggctcggggcggtgatgatggggctggccgacccgccggcgaactacggctcgatctccgcggtggggctgttcgtcgcgctcatgtgcgtctgcatc
atcgtcggacacctcctcgaggagaaccggtggatgaacgagtccatcaccgcgctcttcatcgggctcgggaccggcgcggtgatcctgatggcgtccagc
gggaagcactcccgcgtgctggtgttcagcgaggacctcttcttcatttacctgctcccgccaattatattcaacgcagggttccaagtgaagaagaaacaattctt
ccgcaatttcatgactatcacactcttcggtgcagtcgggactttgatctctttcactgtaatatcctttggtgctctaggactagtatcaaagcttaacattggtgagct
ggagctaggagattatcttgcacttggggcaatattctctgcaacggactctgtatgcaccttacaggtgttaaaccaagatgataccccccttcttgtacagcttggt
gtttggtgaaggtgttgtcaatgatgcaacatccgttgtgctgttcaacgcaatccaaggtttttctaccgggagtttcagtggcgttaatttacttaaatttggtggca
gcttcctctatctgttctgcaccagcacctttcttggagtagcatctggacttctcagtgcctatgtcatcaagaaactgtactttggcaggcattccactgatcgtgaa
gtcgcgattatgatgctcatggcttatttatcttacatgatagctgaagttattatatggtgggcggggctcatgagaggtgctgtatccattgcgctggcctacaata
agttcgcaaggtcaggccacactcagcttcctagcaatgccatcatgatcaccagcacgattagtgttgttcttttcagcacaattgtttttgggatactgacgaagc
cgttgatcagactcttgaatccgccaaggtacctatccagggaacagagcgccctctctgaaccatccagccccaagaccttccttgagcggctgatcgagaac
ggtcctgatgctgatcttgagaatggagtgagcctacgccgcccttcgagtctccggctcctcctggcaagcccaacacggtctgtccactattactggcgcaag
tttgacaattccttcatgcggccggtgtttgggggccgaggctttgttcccttcgtcccggggtcaccaactgagagcagtgtgccgttgcttgctacaactgagg
acaactga
>g22370.t1
atgggcaacacctgcgtcggccccagcgcggcgggccgcagcggatttctcgcctccgtcaacctgtggcggcccgcgcagcccgccgccgccctcccgc
cgccctcctcccccgcctccgacaaggcgcccgagcccgtcacaatccccgcctccgagcactcctcccaccactcctcccgatcctccgaccagccgcaga
cgccctccgcggagccgcagacgcaggacaataatcctccggcgaagaggcccgtgcccaaggtgaagcgcgtccagagcgcgggcctcctcgcggact
ccgtcctcaagcgcgacgtcaacaccgcccggctcaaggatctctacaccatcggcaagaagctagggcaggggcagttcggcaccacctacctctgcgtcg
agaaggccaccggccgggagttcgcctgcaagtccatcgccaagcggaagctgctcacggaggaggacgtcgaggacgtgcgcccgcgagatccagatca
tgcaccaccttgcgggtcacgccaacgtcgtctccatcgtcggcgcctacgaggacgcggtcgccgtgcagctcgtcatggagctctgcgccggcggggaa
ctattcgacaggattatccaaaggggacattactccgagaaggccgccgcgcagctggcgcgggtgatcgtcggcgtcatcgaagcctgccactcgctcggt
gtgatgcacagggatctcaagccggagaacttcctgttcgtcaaccagaaggaggactcgccgctcaaggccattgatttcggtctctccatctttttcaaaccag
aacaggattcccccccgcctatctggtgaatatatactga
>g22373.t1
atgggtaacacatgcgtgggccccagcatcaccaagaacggcttcttccagtccgtgtccacgctgatgtggaagacgccgcaggacggcgacgcggcgct
gcccgccgccgccgccaatggctccgaaggcggcagcccgggccggtcgcagcccaccaagcccgaagtgcagagcaaggccccggagcccatgaag
atcgccgcagcccaacaaccggatgagtcgccgccgccgcccagcaataagcagcagcaggacacgaagccaaacgccggcgccggcgaggcgccca
gcaacaagccgcggcccaaggttcctcaggtgaagcgcgtgtcgagcgccgggcttctggtgggctccgtgctgaagcgcaagacggagaacctcaagga
gaagtacagcctgggccgccggctcgggcagggccagttcgggacgacgtacctgtgcgtggagcgcggcacggggaaggagctggcgtgcaagtccat
cctgaagcggaagctggtgaccgacgacgacgtggaggacgtgcgccgtgagatccagataatgtaccacctggcgggccacccgaacgttatctccatcc
gcggcgcgtacgaggacgccgtcgccgtgcacctcgtcatggagctctgcgccggcggggagctcttcgaccggatcgtgcagaggggacactacaccga
gaggaaagccgccgagctcgcaagggtcatcgtcggcgtcgtcgaggtgtgccactccatgggcgtcatgcatcgggatctcaagcctgagaacttcctcttc
gttgacaacaaggaggaggccgcgctcaagaccattgactttggtctctccatcttcttccgcccaggtcaagtattcacggatgttgtcggtagcccatactacgt
cgcgccagaggtcttgaaaaagaaatatggtcccgaggcagatgtctggagtgctggtgtgatcatctacattttgctgtgcggtgtgccaccattttggcagag
aacgagcagggtatatttgaagaggttttacatggaaaacttgactttcagtcagagccgtggcctagcatctccgaaggtgccaaagatcttgtgaggagaatg
cttgttagggaccccaaaaagagattgacggctcatgaagttctacggcatccatgggttcaggttggcggtttggctcctgacaagccgctggattctgctgttct
gtctcgcatgaaacaattctcggctatgaataagctgaaaaagatggctcttagggtcattgcagagaacttatccgaggacgagatcgccggcctgaaagaaat
gttcaagatgatagacacggacaacagtgggcacatcacctacgaggaactgaaggtcgggttgaaaaaagttggtgccaacctccaggaatccgagatcca
ggcactcatggaagctgcggacgtggataacagcggcacgatagactacggcgagttcatcgcggccactctgcacctgaacaaggtggagagggaggac
cacctgttcgctgcgttccagtacttcgacaaggacggcagcgggtacatcacgcccgacgagctgcagctggcgtgcgaggagttcggcctgggcggaga
cgtccagctcgaggagatgatccgagaagtggaccaagacaacgacgggcgcatcgattacaacgagttcgtggcgatgatgcagaagcccacgctgggg
ctcccgaagaagtccgggggtctgcagaacagcttcagcatcggcttcagggaggcactgaggatttcgtaa
>g27338.t1

atgcatagacacaagcttgaaagagatgagcatctgtttaaggcattccaatactttgataaagataacagcggcttcatcacaagggatgaactagaatctgcttt
gattgaacatgaaatgggtgatgcaagtaccatcaaagatatcatttcagaagttgacactgacaatgatggaaggatcaactacgaggaattttgtgctatgatga
gaggaggaatgcagcagccaatgaggctcaagtag
>g27641.t1
atggcgattgctactgagtcgtgcggcggcagtgaggacaaggctgcatcacatgcaaatgaggagaagcgttgggtgctgccagattttgaggttggaaagc
cacttggcaggggaaagtttggccatgtttacttggcaagagaaaaaaggagcaatcagattgtggctttgaaagttcttttcaagagccaactgaaacagtctca
agtcgagcatcagcttcggcgtgaagttgaaatccagagtcacctaaggcaccccaatattctacgcctgtatggttacttctatgaccaggtacataatagataa
>g28784.t1
atgggcaacacgtgcggcgtcacgcttagatccaagtacttcaccagcttccgtggctcgcagcgccacgacaccgcgggatacgcgcccgtcgccgtcgcc
gcctccgacgaccccgcgccgcgcgggaaaggcaagcgggcctcgccccgcacggccgatgcggcggccgccgcggcggacgcggacgcggccgct
ccgccccccgcccccggcatgcggcgcggggcgcccgcgcccgcggagctcacggccaacgtgctcggccacccgacgccgagcctccgggagcact
acgcggtcgggcggaagctcgggcagggtcagttcggcaccacgtacctctgcaccgaccttgccacgggggtcgactacgcctgcaagtccatctccaag
cgcaagctcatctcgcgggaggacgtcgaggacgtgcgccgcgagatccagatcatgcaccacctcgccggccaccgtaacgtcgtcgccatcaagggcg
catacgaggaccagcagtacgtccacatcgtcatggagctctgcgcgggcggggagctgttcgaccgcatcatccagcgcgggcattacagcgagcgcaag
gcggccgacctcacgcgcatcgttgtgggggtcgtcgaggcgtgccactcgttaggggtgatgcacagggatctcaagcccgagaacttcttgcttgctaaca
aggatgacgatatgtctctcaaggcaatcgattttggactctctgtgttcttcaagcctggtcaaatttttaccgatgttgttggaagcccctactatgtagctccagaa
gtgctgcgcaaacgttatggaccagaagctgatgtgtggacggccggagtgattctatatatattgctaagtggcgttccaccattttgggcaggtcatccctggat
ttgtgaccagggagtggctcctgatcggcctcttgatccagctgtcctctctcgcattaagcagttctcagcaatgaataagttgaagaagatggctttgagagtca
ttgctgagagtctttcagaggaggagatagcaggattgaaggaaatgttcatggcaatggatacagataacagcggggccattacatatgatgagctaaaagaa
ggcttgaaaaaatatggttctacactaaaggacactgaaattcgtgatcttatggaggcggatggccgcattgattacggtgaatttgttgctatgatgacaaaggg
caacttgggagttgggagaagaacaatgagaaacagcttgaatataagcttgagggacacacctgctgctctctga
>g32373.t1
atggagaagtacgagctgctcaaggacatcggctccggcaacttcggggtggcgcggctgatgcggaacaaggagaccaaggagctcgtcgctatgaagta
catcccgcgggggctcaagattgacgagaatgtggcgagggagatcatcaaccaccgctcgctgcggcaccccaacataatccggttcaaagaggttgtgct
cacgcccacgcacctggccatcgtcatggagtatgccgctggcggcgagctgttcgaccggatctgcggtgccgggaggttcagcgaggatgaggcgaggt
atttcttccagcaactgatttgcggtgtcagctactgccacttcatgcaaatctgtcaccgggacttgaagctcgaaaacacgctgctggatggcagcccggcacc
gcgccttaagatttgtgacttcggctactccaagcaaatctgtcaccgggacttgaagctcgaaaacacgctgctggatggcagcccggcaccgcgccttaaga
tttgtgacttcggctactccaagatggcagatgtttggtcctgtggagtgaccctttatgtgatgctggttggtgcatacccttttgaggatcccgacgatcccaaga
atttcagaaagacaattgggagaatagtatctattcaataccaaataccagaatatgtccacatatcccaagattgcaggcagctcctctcgaggatcttcgttgcg
aatcctgcaaagagaataacaattagggagattagaaatcacccctggttccttaagaacttgcctagagagcttacagaagctgcacaagcgaagtactacaa
gaaggacaacagcacacccacctactctgatcagactgttgaagagatcatgaagattgtagaggaggctcgcacaccacccaaattatctactcctgtggctg
gtttcggttgggctgaggaagaagagcaagaagatggcaagaaacctgatgatgaagaacatgatgaagaggatgaggaatatgatggtgaggacgagtatg
acaagcaggtgaaggcagtacatgccagcggtgattttcaacatctgataaaaggaagcaactag
>g34089.t1
atgggaaacgcatgcggcggctcccttagatccaggtatctgccgagcttcaagacctccgcctcgcagcgccacgactccgaccacagcgccgccgccga
ctcgccgaagaagccctcccgccccgccacgccccccgccgccgcgacggacgcccacgcggcacccgcgccgggcctgccgccgccgccgcccggc
atgaggcgcggcggcgcggcgcccccggacctcggttccgtgctgggccaccccaccccgaacctccgtgacctctacgccgtgggccgcaagctcgggc
agggccagttcgggaccacctacctctgcaccgacctcgccacgggcgcccagtacgcctgcaagtccatctccaagcgcaagctcatcacccgggaggac
gtcgacgacgtgcgccgcgagatccagatcatgcaccacctctcgggacaccgcaacgtcgtcgccatcaagggcgcctacgaggaccagctctacgtcca
catcgtcatggagctctgcgccggcggggagctcttcgaccgcatcatacagcgcggacactacagcgagcgaaaggccgcagagctcacgcggatcatcg
tcggggtcgtcgaggcgtgccactcgctcggggtcatgcaccgggacctcaagccagagaacttcctgctcgccaacaaggacgatgacctctcgctcaagg
ctatcgatttcggcctctccgtcttcttcaagccgggtgagctgctgctcattactgattcatattccgacttgattactgtttcggtcaacgattgtgctgtcaatgatat
gcgactaannnnnnnnnnnnnnnnnnnnnnnnnnnnnnnnnnnnnnnnnnnnnnnnnnnnnnnnnnnnnnnnnnnnnnnnnnngtggg
ccgcaagctcgggcagggccagttcgggaccacctacctctgcaccgacctcgccacgggcgcccagtacgcctgcaagtccatctccaagcgcaagctca
tcacccgggaggacgtcgacgacgtgcgccgcgagatccagatcatgcaccacctctccggacaccgcaacgtcgtcgcaatcaagggcgcctacgagga
ccagctctacgtccacatcgtcatggagctctgcgccggcggggagctcttcgaccgcatcatacagcgcggacactacagcgagcgaaaggccgcagagc
tcacgcggatcatcgtcggggtcgtcgaggcgtgccactcgctcggggtcatgcaccgggacctcaagccagagaacttcctgctcgccaacaaggacgatg
acctctcgctcaaggctatcgatttcggcctctccgtcttcttcaagccggaaacgcagcaaggaatatttgatgctgtattgaaaggtgacattgatttcgattctga
cccgtggcctgtgatatctgatagtgccaaagacctgataaaaagaatgctcaatcctcgccctaatgaacgcttaacagcacatgaagttctatgccatccatgg
attcgtgatcatggagtagcacctgatcgtccacttgatcctgctgtcctatctcgcatcaagcaattctctgcaatgaacaagttgaagaagatggctttgcgagta
atagctgagagtctctcagaggaggaaattgcagggttgaaagaaatgttccagaccatggacactgacaacagtggcgcgattacatatgatgagctaaaaga
aggattgagaagatatggctccacactaaaggatactgaaattcgtgatcttatggacgcggctgatattgacaacagtgggacgattgactacatagaattcatt
gctgcaacattgcatctcaataaactggagcgtgaggaacatctggtggcagcatttacatattttgacaaagatggtagtggttacatcactgtggatgagctgca
acaagcttgcaaagagcataacatgccagatgcttttcttgatgatgtcattaaagaagctgatcaggacaatgacggacgcattgactatggagagtttgttgcca
tgatgaccaagggcaatatgggagttggtcgaagaacaatgagaaacagtttgaatatcagcatgagggacgcacctggtgcattttag
>g34176.t1
atgggcaactgcttcaccaagacgtacgagatacccatcaccacggagccgtcggatcggcggcgcgcgccgtcgtcgtacggctaccagccgccgagga
gccccgatcagcgcaagtcccgcgacgtgccggtgatgacctcgcgccggccgtccttccgtccgccgccgcccttccggccgtcgagcacgacgggcac
cgggtcgtcgtcggcgtcgtcgatgccccgtcgcgcgccctcctccggcgaggtgggcccggtcctgcagcggccgatggtgaacgtgcgcacgctgttcc
atctggagcggaagctggggagcgggcagttcgggaccacgtacctgtgcacggagcgcgagacggggctccggtacgcgtgcaagtccgtgtccaaacg

caagctggtgcgccgcgccgacgtggaggacatgcgccgggagatcaccatcctgcagcacctcagcgggcaggccaacgtggccgagttcaagggcgc
cttcgaggacgccgactacgtgcacctcgtcatggagctctgctccggcggggagctcttcgaccgcatcaccgccaagggcacctactccgagcgccaggc
cgccgccgtgtgccgcgacatcgtcaccgtcgtgcacgtctgtcacttcatgggggtcatgcaccgcgacctcaagccagagaatttcctgcatgccagcccc
gcggaggacgcgccgctcaaggccatcgatttcggactctccgtcttcatcgaagaagaacatccatggctcaaagaaggtggtgcatcagacagacctatcg
acagtgcagtcctatcaagaatgaagcaattcaaggcaatgaacaagttaaagcaattagcactcaaggtaatagcggagaacctatcaccagaagaaatcaa
gggattgaagcaaatgttcaataacatggatactgacaagagtggcacaatcacagttgaagaactgaaggaaggactgacgaaactagggtcgaagattagt
gaagcagaggttcagaaacttatggaagcagttgatgtagacaagagtggcagcattgattatacagagttccttactgacatgatgaacaaacataaagtggaa
aaggaggaggatttgctccgtgcatttcagcacttcgacaaagatagcagcgggtacataacaagagatgaactggaacaagccatggaagagtatggaatgg
gtgatgaggcaagcattaaacaagtactggatgaagttgataaagataaggatgggaaaattgactatgaagagtttgtggaaatgatgaggaaaggaagctat
acctga
>g36737.t1
atgggtggctgctactccgtcatcgcggtcaccaggctgaagatgcttcgccgcaaccgtggcgccgcggccgtcctccccatcaccaacggcgacgacgac
acgtgctgccgctctcccgacaacaacaacaacgccatcgacgacgacaataagggcatgggctgctgcagggcgaagaggaggtgcaggaaggacaag
tcgaagcacgcctcgatcctggggcccgacgccgccggcgagccggacttcgctcggcggtacaggctgggcgcggagctcgggcgtggcgagttcggc
gtgacgcggcggtgcgaggacgtcgccacgggggaggcgctggcgtgcaagaccatccgccgccggcgcctgctgctgcgccgcgggggggcccgacg
ccgtcgacgtgcgccgggaggtggagatcacgcggcgcatgtcggagctcgggggccccgtggtgcggctccgcgaggcctgcgaggacgccgacggg
gtccacctcgtcatggagctctgcgagggcggcgagctcttcgaccgcatcttcgcgcgagggcactacaccgagcgcgccgccgccaagctcgctcacac
catcgtcgaggtcgtgcagctgtgccacgagaacggggtgatgcaccgggacctgaagccggagaatttcctgttcctgaacaagtcggaagactcacctctc
aaggccatcgacttcggcctctcggtgtacttcaaaccaggggatcggttcacggaagtggttggcagcgggtgttacatggctccagaggttctcaacagaag
ctatgggccagagatagacgtgtggagcgctggtgtcatcctgcacatccttctctgcggcttccctcctttctggggagattccgatgagaaaattgcacaggca
atactacgtggaggaattaacttacaaagagacccatggcacaaggtctcccagagtgcaaaggatcttatcaggaagatgcttgacccgaatccttctaacaga
ttgacagcgaagcaagtccttgaacatccttggctcaagaacgctgataaggctcccaatgtgtcacttggagaggttgttcgatccaggctgaagcaattctcgg
ccatgaacaggtttaaaaagaaggcacttggagtcgttgccatgaatttaccggcagaagagatcgacaaatataatgagatgttccaaaagatggacaaagat
aaggatggtaatttgacacttgaggagctgaaggagggtctccagattaatggccatcctgtcccagaggcagagataaagatgctattagaagctggtgacat
agaaggaactggaatgttagactgtgaggagtttataacaatcttacttcacataaaaaagatgagtaacgaggagtacctacccaaagcttttaaattctttgacaa
agatggcaatggctttattgatatggaggaattgatggaggctttaggtgatgatgaattaggacccaatgaacaagtgattcatgatattgttcgtgacgtcgaca
aggataaggtggataatgctgttaagtttacaaaggagggacacatcgacatttaa
>g37557.t1
atgggtaaggagaagagtcacatcaacattgtggtcattggtcatgtcgactctggaaagtcgaccaccaccggccacctgatctacaagcttggtggtattgac
aaacgtgtgatcgagaggtttgagaaggaggccgctgaaatgaacaagaggtccttcaagtatgcgtgggtgctggacaagctcaaggctgagcgtgagaga
ggtatcaccattgatatcgccttgtggaagtttgagaccaccaagtactactgcactaatgttgctgtgaaggatctgaagcgtgggtacgtggcctccaactcca
aggacgaccctgccaaggaggctgccagcttcacctcccaggtcatcatcatgaaccatcctggacagattggcaatggttatgccccagttctggactgccac
acctcccacattgctgttaagtttgctgagctgcttaccaagattgataggcgatctggtaaggagcttgagaaggagcctaagttcctaaagaacggtgatgctg
gtatggtgaagatgattcccaccaaacccatggtggtggagaccttctctcagtaccctcctcttggtcgttttgctgtccgtgacatgaggcagacggttgctgttg
gagtcatcaagagcgttgagaagaaagaccccactggtgccaaggtgaccaaggctgctgtcaagaagaaatga
>g39065.t1
atggagagctacctggaggagaacttcggtggcgtcaaggccaagaactcctcggaggaggcgctccggcggtggcgccgcctctgcagcgtcgtcaaga
accccaagcgacggttccgcttcaccgccaacctcgacaagcgaggggaggcgcaggccatcaagcacgccaaccacgtccgtacacatctcctcttcctct
ctcatccatcatcccctttcctccgcaccgggatccgcctcgctcgcggcggcgggatctggactggagccgcgccgccggcgggggcgggaggagctcat
cctgcggttgtgttgcaggagaagctgcgtgttgccgtgctggtctcgaaggccgcgcttcagttcttacatggtctctcacttcgaagcgaatatgttgtccctga
ggaagtcaaggctgcagggtttcagatttgtgccgatgagcttgggtccattgtggagggccacgatagcaaaaagctgatcatacatggcggagtcgatgga
attgctgccaagcttgcgacatcacaaatggatgggctgaggacagctgaggaggacaacattaagcgcaggcaagagatttatggactcaacaagttcacag
aaagcgaggtccgcagtttctgggtgtttgtatgggaagcgcttcaagatacaactcttataattcttgccgtctgcgcatttgtatctctggtcgttggcattgcgat
ggaaggatggccaaaaggtgcccatgacggtcttggaattgttgcgagtatcctcctggtagtgtttgtgactgcaacaagtgattaccgacaatcgctgcagttc
aaaaacctggacaaggagaaaaagaaaattcgggtgcaagttacaagggacggatttaggcagtggatatcgatatatgaccttcttcctggagatgtcgtccat
ttacaaattggagaccaggttcctgcagacgggctcttcatttcagggttttctctgttgatcaatgaatccagcctaacgggtgaaagtgaacccgtcgttgtaaat
gaagataatcctttccttttgtcgggaaccaaagtgcaggatgggtcctgcaagatgctggttacgaccgttggtatgcgcacccaatggggaaaactgatggcc
actctcagtgagggcggcgatgatgaaactccactgcaggtcaaactcaatggtgttgcaactatcattggaaagattgggctattctttgctgttataactttcattg
tcttgtcccaagggttatttggcaacaaataccatgacggacagcttttgagctggtcaggagatgatgcactggaacttctggagcattttgctattgcagttacca
ttgttgttgtggctgttcctgagggattgccattagcagtcacgctgagccttgcatttgccatgaagaaaatgatgaatgacaaggcacttgttcgcaacttagctg
catgtgaaactatgggctcagcgaccaccatctgcagtgacaagacagggacattgacaaccaatcatatgactgtcgttaaggcctgcatctgtgggaaaatc
aaggaggttaacagtcctcagaatgcatccaagttatgctctgaatttccggaaactgtcgtcaaaacactcctggagtctatatttaacaatacaggtgttgaggtt
gtgattaaccaagatggtaaacgtcagatcctagagaaatggaagaaggttttcctggcacagagcaaataccattacaagggtacacatgcattggtattgtag
>g39068.t1
atggataggacggcggcgacgacggggccgctggggatggagatgccgataatgcacgacggggaccggtacgagcacgtcaaggacatcgggtccgg
caacttcggcgtcgcgcgcctcatgcgcaaccgcgcctccggcgagctcgtcgccgtcaagtacatcgaccgcggcgagaagatcgacgagaacgtgcag
cgggagatcatcaaccacaggtcgctgcggcatcccaacatcatccgattcaaggaggttatcctgacgccgactcacctcgccatcgtcatggagtacgcctc
tggaggagagctcttcgagcgcatctgcagtgccggaagattcaacgaggacgaggcccggttcttcttccagcagctgatttctggggtcagctactgccact
ccatgcaagtatgtcatcgtgacctgaagctcgagaatacattgctggatggaagtaccgctccacggcttaagatctgcgactttggttattcaaagtcatccgtt
cttcattctcaaccaaaatcaacagttggtactccagcttatattgcccctgaagttttgctcaagaaagaatatgatggcaagattgctgatgtgtggtcatgtggtg

taaccctctacgtcatgttggttggcgggtacccattcgaggacccggaagatcctaggaatttcaaaaagacaattcagaaaatattgggtgttcagtatgtgatt
ccagattacaggatcaccatgcctgaaataaaaagtcatccttggttcgttaagaatctcccagcagacctcatggatgatgccacaatgagcagccagtatgag
gagcctgaccagcctatgcagaacatgaacgagatcatgcagatactagcagaggcaactataccagccgctggtacccgtggaatgaaccagtttttggttga
cagccttgaccttgatgatgatatggaggatctagactcagaccttgacatcgacattgagagcagtggagagatagtgtatgccatgtga
>g39207.t1
atggatgaggtggagcagacgctgacaaaggagcagatcaaggagttccgggaagccttcagcctcttcgacaaagatggcgatgggacgatcacgagcaa
ggagcttggcacggtgatgcgttcgctggggcagagccctacggaggcggagctgcaggagatggtggcggaggtggacgccgatggcagcggcgccat
tgatttccacgagttcctcgtcctcctcgggcgcaagatgcgcgacgccggcgccgacgacgagctccgggaggccttccacgtgttcgaccaggaccagaa
cggatacatctcccgcgacgagctgcgccacgtcctcgagaacctcggcgagaagctctccgacgaggagctcgtcgagatgctgcgcgaggccgacgtc
gacggggacggccagatcaactacaacgagttcgcgaaggtcatgatggccaaacgaagaaaccagatgatggacgacgacgatagtggttcgcactccca
cgggagcagcccctgctgtacaatcctctga
>g44056.t1
atgggaggttgccacgcgaagccgctcacccacgacccggacaactcgccgccgcacgcggcgccggcgacgccgctcccggagggccgcacctccgc
gtccgccacgcccgcgaagaagcagcactgggcgtcgtcgcccttcttcccgttctccacgccgagcccgagtccggcgcaccacctcttcggctcgtccgc
ggcgtccccgcgcaagtcccctgcaccgcccggcgcgggctccgcgcccaccacaccggccagacggctgctgcggctgcccttcccgccgccgtcgcc
cgccaagcacatcagggcggcgctcgcgcggcggcacgggccgtcgcggccgtccatccccgaggaaggtggcggcgagggggacggcagcggcag
ggggctcgacaaggggttcgggttcagtaaggggttcgcgaccaagtacgagatgggcgatgaggtcgggaggggacacttcggctacacctgtgccgcca
aggtgaagaagggggcgcgcaagggcgagtctgttgccgtcaaggtcatccccaaagccaagaatttcctttttacttcgaaagatgagaattcccaacttaag
gctatcgactttggcttgtcggattttgtaaaaccagatgaacgcctaaatgatattgttggaagtgcttactatgttgctccagaagttctgcatagtcgtccattttgg
gcacgcactgaatctggcatattccgttctgttctcaaagctgaccccagctataatgaggcaccatggccttctctgactccggaagcaatggattttgttaagcgt
ttgctgtgcaaggatccacgtagaaggatgactgcagcacaggctttaagtcatccatggatcagaaattataatgacattaagctgccgttggacgtccttatatt
ccgacttatcaaagcttatatccgttcctcatctttacgaaaagctgctttgagggctctatcgaagactttaactgttgatgaacttttctatctgaaagcacagttttcc
ttgttggaaccagacagaaatggatgcattactcttgataatatcagaacggcattaacaagggaggccactgatgcaatgaaagaatcacgagttcaagagatt
cttgtttcgttgagcgctcttcagtacagacgaatggacttccaagagttctgtgcagcagcagttagtgtgcaccagctcgaagcattggatagatgggagcaac
atgcccgatctgcttatgaatattttgagaaggatggcaatcgtgctattgtaattgatgaactggcttctgaattgggtctcagcccttccgtgccattgcatgttgttc
tgcaagattggatcagacatactgacgggaaactgagctttcttggttttgtcaagctattgcatggcatgtccagcaggtccctgtcaaaggtgaggtag
>g46612.t1
atgcagcacgatcaacgcaagaagagctccgcggaggcagagtttttcacggagtatggggacgcaagtcgatacaggattcaggaagtcattggtaaagga
agctatggggttgtatgctctgcaatagatgtccacaccggagagaaagtagccatcaagaagatacatgacatctttgagcacatatctgatgctgcacggattc
tccgtgagatcaagcttctgagactcctaagacatcctgacattgttgagatcaaacatattatgctgcctccatcaagaagggacttcaaagatatttatgttgtttt
gaacttatggagtctgaccttcatcaggattatgttgcaacaagatggtatagagctccagagctctgtggatccttcttttcaaagtatacaccggccattgatattt
ggagcattggatgcatctttgctgaggtgctaacaggaaagccgttgtttcctggtaaaaatgttgtccatcagttagatttgatgactgatcttctgggaaccccatc
cttggataccatttctcgggtctgcaatgagaaagcaaggagatatctgagcagcatgagaaagaaagaaccaatctcattttcacagaagtttccaaatgcaggt
cctttggccttggatcttttgcaaaggctgttagcatttgatccaaaggaccgtccaactgcagaagaggcattggctcatccatacttcaaagggctagccaaggt
tgagagagaaccatcttgtcaaccaatcacaaagatggagtttgagtttgaacgtagaagagtgacaaaggaagacataagggagctgatattccgtgagatac
tggaataccatccacatgctgttgatcaattcaggaagcaatttgctcatctcgaagagaataatggaaatggccctgtgattccgatggaaagaaaacatgcttct
cttcctagatctacaattgttcactctactccaatccctgtcaaggaacaaccccgtattggctcgtcaagagacaaggcttcacctgatgagtcctacagaaatcct
cgtgagacagacagattttccggcaatgccccgagaacctcacaagctccccatagagtgccagcagcgagaccgggaagggttgttggtccagtaatgcca
tatgaaaatgggagcaccaaagacccctatgacagacgaaagtttgcattgaattcaggatatcctccccaacaacaaatccaacaaacgtatggttattatcaga
catccggcaaggtagcttgctctgagcagtcgcaggctgaaagcagggcctgcaagtga
>g47095.t1
atggagcggtacgaggtgatccgggacattgggtctgggaactttggtgtggccaagctcgtccgcgacgtcgggaccaaggagctctacgccgtcaagttca
tcgagagggggctgaagcaagtgtgccacagggatctgaagctagaaaacacccttttagatggaagtgttgcacctcggcttaagatttgtgatttcggttactc
caagtcttctgtgctgcactctcaaccaaaatcaactgttggcacacctgcgtacattgctccagaggtcctctctagaaaagaatatgatgggaaggttgctgatg
tttggtcatgcggagtaacactatatgtgatgcttgttggtgcatatccttttgaggaccctgatgatccaaggaacttccgcaagacaatcaccagaatactcagtg
tacagtactcaattccagactatgttagaatttcaatggaatgcaggcatctgctgtcccggatatttgttggtaatcctgagcagcgaattaccattccagagatcaa
gaaccacccatggttcctgaagaacctgcccatcgagatgactgacgagtaccaacagcagatgaagttggcagacatgaacaccccggagcaaagcctag
aggaggtcatggcgatcatccaggaggcgcggaaaccaggggatgacctgaggcttgccgggcaggtaccctgccccctaggaagcatggaccttgatga
cactgatttggacgatattgacgacattgacatagagaatagcggcgatttcgtttgtgcaatgtga
>g47734.t1
atgctcccgccctcgcgccgcgacttccgcgacatctacgtcgtcttcgagctcatggagtccgacctccaccaggtcatcaaggccaacgacgaactctcgg
ccgagcaccagcagttcttcctctaccagctactccgcggcatgaagtacatccacgccgccaatgtcttccacagggacctcaagcccaggaacatccttgcc
aatggcgattgcaaactcaagatttgtgacttcggccttgctcgggtgtcgttcaatgataccccttctgcgatattctggacggtaacctga
>g48661.t1
atgggcaacacgtgcggcgtcacgcttagatccaagtacttcaccagcttccgtggctcgcagcgccacgacaccgcggggtacgcgcccgtcgccgccgc
cgccgcctccgacgaccccgcgccgcgcgggaaagggaagcgggcctcgccccgcacggccgatgcggcagctgccgcggcggacgcggccgctcc
gccccccgcccccggcatgcggcgcggggctcccgcgcccgcggagctcacggccaacgtgctcggccacccgacgccgagcctccgggagcactacg
cggtcgggcgcaaactcgggcagggccagttcggcaccacctacctctgcaccgacctcgccacgggggtcgactacgcttgcaagtccatctccaagcgc
aagctcatctcgcgggaggacgtcgaggacgtgcgccgcgagatccagatcatgcaccacctcgccggccaccgcaacgtcgtcgccatcaagggcgcat
acgaggaccagcagtacgtccacatcgtcatggagctctgcgcgggcggcgagctgttcgaccgcatcattcagcgcgggcattacagcgagcgcaaggcg

gccgacctcacgcgcatcgttgtgggggtcgtcgaggcgtgccactcgttaggggtgatgcacagggatctcaagcccgagaacttcttgcttgccaataagg
atgacgatatgtcgctcaaggcaatcgattttggactctctgtgttcttcaagcctggtcaaatttttaccgatgttgttggaagtccctactatgtagctccagaagtg
ctgcgcaaacgttatggaccagaagccgatgtgtggacagctggagtgattctttatatattgctaagtggtgttccaccattttgggcagagacacagcaaggaa
tatttgatgctgttttaaaaggtggcattgattttgactcggatccctggcctgtaatctctgatagtgcaaaggatcttataagaaaaatgttgaaccctcgaccagc
agagcgcctaacagcacatgaagttctatgtcatccctggatttgtgaccagggagtggctcctgatcggcctcttgatccagctgtcctttctcgcattaagcagtt
ctcagcaatgaataagttgaagaagatggctttgagggtcattgctgagagtctttcagaggaggagatagcaggattgaaggaaatgttcacggcaatggatac
agataacagcggggccattacatatgatgagctaaaagaaggcttgaaaaaatatggttctacgctaaaggatactgaaattcgtgatcttatggaggcggcaga
tgtagacaacagtggaactattgattatatagaattcattgcggccactttgcacctcaataaacttgagcgtgaggaacatctagtggcagccttttcgtattttgac
aaggatggcagtggttatatcacagtggatgagcttcagcaagcttgcaaagagcataacatgccagatgcttttctggatgacgtcattaacgaagctgaccaa
gataatgatggccgcattgattacggtgaatttgttgctatgatgacgaagggcaacttgggagttgggagaagaacaatgagaaacagcttgaatataagcttg
agggacacacctgctgctctctga
>g48899.t1
atgggcctctgctcctcctccaccgcgtccgcgcaccgtgccggtgccgcgccggggaagaaggagaacaagggcgccggaggcggcagccgggggat
cgtggcgtgtgggaagcggacaaacttcggctacgacaaggacttcgaggcccggtactcgctcggcaagctgctgggccacggacagtttggctacacctа
cgccgccgtcgaccgcaactccggtgagcgcgtcgccgtcaagcgcatcgacaagaacaagggattaggagtcatagttgcctctcatttctga
>g49337.t1
atgagtacaaccaaggtgaagagacgtgtggggaagtatgagcttggccggaccataggcgagggaacatttgcaaaagtcaggttcgcacgggacaccga
gactggcgacccggtggccatcaagattctggacaaggagaaagttctcaagcacaagatggttgagcaggtgatgggaagtaaaacaaagatctacattgtg
ttagagtatgtaaccggcggcgagctctttgatacaatcgttcttgaggatcaaggttatgatggtgcgatggctgatctgtggtcatgtggagttatcctgtttgttct
actagcagggtatttgccttttgaggactctaatcttatgacgttgtataagaaaatatcaaatgcagtgtttacatttcctccttggacatcgtttcctgccaagaggtt
gttaacaagaatccttgatccaaatccaataacgttgaggctttcaaaggtcaaagcagggaggaagggaaacctcaatgtagccaccgagatattgcaagttg
cacccctctcttcacatggtagaagtccgaaaagcaaaaggtgacactctggaatttcacaagttctacaagaacctttccaagaccttaaaggacgttgtctggaa
atccagtgacatgcaaatgcagcccacttcttag
>g49339.t1
atggatgaggtggagcagacgctgacgaaggagcagatcgaggagttccgggaagccttcagcctcttcgacaaagatggcgacgggacgatcacgagca
aggagcttggcacggtgatgcgttcgctgggtcagagcccgacggaggcggagctgcaggagatggtggcggaggtggacgccgacggcagcggcgcc
attgatttccacgagttcctggtcctcctcgcccgcaagatgcgcgacgccggcgccgacgacgagctccgggaggccttccacgtgttcgaccaggaccag
aacggatacatctcccgcgacgagctgcgccacgtcctcgagaacctcggcgagaagctctccgacgaggagctcgtcgagatgctgcgcgaggccgacgt
cgacggggacggccagatcaactacaacgagttcgcaaaggtcatgatggccaaacgaagaaaccagatgatggacgacgacgacagtggttcgcactccc
acaggagcagcccctgctgtacaatcctctga
>g50524.t1
atgctcaaggttgcagctgagtgccacctgcatggtttggttcatcgggacatgaaaccagagaatttcctactcaaatcaacaaaagaggactcaccccttaag
gctacagattttggtctttcagacttcataagaccagggaaacaattccgtgacattgttgggagtgcctactatgtagcaccagaagtactcaagccacatgattg
ggtcagagaaggaggtaaggcatctgaaataccattggatatatcagtattacacaatatgcgacaatttgtgaaatacagtcgcttcaagcaatttgctttaaggg
cattggcatcaacactaaatgcagaagagctttctgatcttcgtgaccagtttgatgccatcgatgttgacaaaaacggcacaatcagtctggaggaactgaagca
gattgacagtaacacagatgggttggtcgattttgaagagtttgttgcagcgacactacatgtgcatcagctggcggagcatgatactgagaagtggagatcgttg
tctcaagctgcatttgataaatttgacgttgatggagatggttatattacatctgatgaattgagaatggtatcttaa
>g50691.t1
atggcggaccagctcaccgacgagcagatcgcggagttcaaggaggccttcagcctcttcgacaaggatggcgacggttgcatcactacaaaggagcttgga
actgtgatgcggtctcttggccagaaccctaccgaggcagagctgcaggacatgatcaacgaggttgacgctgatggcaatggtactattgacttcccggagtt
cctgaacctgatggcaaggaagatgaaggacactgactctgaggaggagctcaaggaggccttccgtgtcttcgacaaggaccagaacggtttcatctcagct
gcagagctccgccatgtcatgaccaaccttggggagaagctgactgacgaggaagtcgacgagatgatccgcgaggctgatgttgatggtgatggccagatc
aactacgaggagtttgtcaaggtcatgatggccaaagcccatgcattattcaatgttctcaggcatcgaccatatgcagcatgtcagatcgccaaagacgacctc
gttacggccctccgctcgctgggcctgaacgtcgacgaggccgaggcgcagcgcctcctcggcgacgccgcctccggcggcgcgatcgagttcccggcgt
tcctgtccgtcgcggcgcgcaagatggccagcgacgggaagcagacggcggagaggctggcggaatgcttcgacgtgttcgacgacggccggagcgggt
cgatcccggcggagcagctgcggcaggtgatgacgagccacggcgaccggctcacggaggaggaggccgacgccatggtccgcgaggccgacccgcg
cggcgagggccgcgtcgagtacaaggagtacgtcaaggtgctgaccaagaacaagtga
>g53597.t1
atgggcaactcgtgccgcggctacaccacaagcttgtacgattgcgactaccagcacagcagcacctcctattcgtcgtcgtcgtcgaatttctccagcaggcg
cagcagcagcagcatcgtctgtggaggcggcggcatgcgcgggcaccagcaacagctttcgtccccgacggccgtgctcgggcacgtgaccccaccgctg
cgggagctctactcgctggggcggaagctcgggcagggtcagttcggcaccacgtacctgtgcacggatctggccacgggcgcgtccctggcctgcaagtc
catcgccaagcgcaagctgctcaccccggaggacgtggacgacgttcgccgcgagatccagatcatgcaccacctcgccggccacggcagcgtcgtcacc
atcaagggcgcctacgaggacccgctctacgtgcacatcgtcatggagctctgcgagggcggcgagctcttcgaccgcatcgtcaaccgcggctacttctccg
agcgcaaggccgccgagatcgcgaggatccaatag
>g54372.t1
atgggcaactgctgcggcgcgccggcgacgccgggcggccgcaagaaccggcggaagcagaaggcgaacccctacaacgtctcgtacaaccgtggggc
ggcgccgccgccggcgcgtccggggctgatggtgctgcgggacccgacggggcgggacatgaacgcgcggtacgagctcggcggcgagctggggcgc
ggcgagttcggcgtcacgtacctgtgcacggaggtggccacgggggcgcggttcgcgtgcaagtcgatatcgaagaggaagctgcggacgccggtggacg
tggaggacgtgcgccgggaggtggacatcatgcgccacatgccgtcgcaccccaacatcgtcagcctcagcgccgcctacgaggacgaggacgccgtgca
cctagtcatggagctctgcgagggaggggagctcttcgacaggatcgtcgcgcggggccactacaccgagcgcgccgccgccgccgtcacgcgcaccatc

gtggaggtggtccagatgtgccacaggcatggtgtcatgcacagggaccttaaaccagagaacttcttatatgcaaacaagaaggagagttcccctctgaaagc
aattgattttgggctgtctgtgttcttcaggcctggtgagcgctttacagaaattgtaggcagtccatactacatggctccagaggttttaaagcgaaactacggtcct
gaagttgatgtctggagtgcaggagtgatactttatatacttctttgtggcgtgccaccattttgggcagagactgaacagggagtagcacaggcaattatacgctc
agttgttgatttcaaaagagatccatggccaagagtatctgaacctgctaaagatcttgtcaggcgtatgctggacccaaatccaaacacaaggctttccgcagca
caagtacttgaacatccatggctgcatgactctaaaaagatgccggacatccctcttggtgatacagtccgagcgagactgcagcaatttgcagcaatgaacaaa
ttaaagaagaaagctcttagggtgattgctgaacatctctccgtagaggaagtagctgatataaagcaaatgtttgatggcatggatgtgaacaaaaatggcaagc
taacctatgatgaattcaaagctggtcttcgtaaacttggaaaccaaatgcctgattcagacattaggatattgatggatgctgctgatgtcgataaaaatgggaccc
tagactatggagaatttgttgctgtgtctatccatgtccgaaaaattggcaatgatgaacatatccagaaagctttctcgtactttgaccaaaataacactgggtacat
agaaattgaagaacttagacaggctctggctgatgaattggagggaaatgatgatgatattattaatggcatcatccgtgatgtggacacagataaggacggaaa
aataagcttcgacgagtttgcgaccatgatgaaggctggcactgactggaggaaggcgtctaggcagtactcacggcaaaggttcagcaacctcagcctgaag
cttcaaaaggatgggtccatcggcgctgatacaaagtag
>g54373.t1
atgggcaactgctgcggcgcgccggcgacgccgggcggccgcaagaaccggcggaagcagaaggcgaacccctacaacgtctcgtacaaccgtggggc
ggcgccgccgccggcgcgtccggggctgatggtgctgcgggacccgacggggcgggacatgaacgcgcggtacgagctcggcggcgagctggggcgc
ggcgagttcggcgtcacgtacctgtgcacggaggtggccacgggggcgcggttcgcgtgcaagtcgatatcgaagaggaagctgcggacgccggtggacg
tggaggacgtgcgccgggaggtggacatcatgcgccacatgccgtcgcaccccaacatcgtcagcctcagcgccgcctacgaggacgaggacgccgtgca
cctagtcatggagctctgcgagggaggggagctcttcgacaggatcgtcgcgcggggccactacaccgagcgcgccgccgccgccgtcacgcgcaccatc
gtggaggtggtccagatgtgccacaggcatggtgtcatgcacagggaccttaaaccagagaacttcttatatgcaaacaagaaggagagttcccctctgaaagc
aattgattttgggctgtctgtgttcttcaggcctggtgagcgctttacagaaattgtaggcagtccatactacatggctccagagagactgaacagggagtag
>g54450.t1
atggcggaccagctcaccgacgaccagatcgccgagttcaaggaggccttcagcctcttcgacaaggacggcgatggttgcatcacaaccaaggagctggg
aactgtcatgcgttcattgggtcagaacccaaccgaggctgagcttcaggacatgatcaatgaggttgatgctgacggcaatggcaccatcgacttccctgagtt
cctgaacctcatggctcgcaagatgaaggacactgactctgaggaagagctcaaggaggcgttcagggtgttcgacaaggaccagaacggcttcatctctgct
gcggagctccgccacgtcatgaccaaccttggagagaagctaaccgatgaggaggtcgatgagatgattcgcgaggctgatgtcgacggcgatggccagat
caactatgaggagttcgtcaaggtgatgatggccaagtga
>g57551.t1
atggcgatcgccactgagtcgtgcggcggcagcgaggacaaggctgcatcacatgcaaatgaggagaagcgttgggtgctgccagattttgaggttggaaag
ccacttggcaggggaaaatttggccatgtttacttggcaagagaaaaaggattgtggctttgaaagttcttttcaagagccaactgaaacaatctcaagtcgagc
atcagcttcggcgtgaagttgaaatccagagtcacctaaggcaccccaatattctacgcctgtatggttacttctatgaccagactcgtgtttatttgatcctggaata
tgctgccaaaggagagctgtacaaggagctgacaagatgcaaacatttcaatgagagacgttcagctacttacatcgcatcactggcaagagcactcatttacct
tcatgggaagcatgtcatccatagagacattaaaccagagaatcttctaattggagctcagggcgagctgaaaattgcagactttggctggtctgttcatacattca
atcgaagacgaacaatgtgcggaacgctagattacctgccacctgaaatggttgagaagactgagcatgattaccatgttgacatctggagcttgggaatactct
gttatgagttcctctatggagccccgccttttgaagcgaaagaacattccgagacatatcgaaggatagtgaacgtggacctgaagttcccattgaaaccgtatgtt
tctgctgccgcgaaggacctaatttcacaggatagtgaacgtggacctgaagttcccattgaaaccgtatgtttctgctgccgcgaaggacctaatttcacagatg
cttgtgaaaaactctgcgaaccggcttcctctgtataa
>g57892.t1
atggcggatcagctcaccgacgaccagatcgctgagttcaaggaggccttcagcctcttcgacaaggacggtgatggctgcatcaccaccaaggaacttggta
ctgtaatgcgctctttggggcagaaccccactgaggctgagcttcaggatatgatcaatgaagtggatgctgatggcaatggaactatagacttccccgagttcct
caacctgatggctcgtaagatgaaggacacagactctgaagaggagcttaaggaggctttccgtgtgtttgacaaggaccagaacggcttcatttctgcggctg
agctccgtcatgtcatgaccaaccttggtgagaagctgacagatgaggaggtggatgagatgatccgtgaagccgatgtggatggctgcatcaccaccaagga
acttggtactgtaatgcgctctttggggcagaaccccactgaggctgagcttcaggatatgatcaatgaagtggatgctgatggcaatggaactatagacttcccc
gagttcctcaacctgatggctcgtaagatgaaggacacagactctgaagaggagcttaaggaggctttccgtgtgtttgacaaggaccagaacggcttcatttct
gcggctgagctccgtcatgtcatgaccaaccttggtgagaagctgacagatgaggaggtggatgagatgatccgtgaagccgatgtggatggtgatggccag
atcaattacgaggaatttgtgaaggtgatgatggccaagtga
>g59931.t1
atgagcgtgtccgtcgggagaacgcgggtggggaggtacgagctgggccggacgctcggggaaggcaccttcgccaaggtcaagttcgccaggaacgtc
gagaccggcgagaatgtcgccatcaagatcctcgacaaggagaaggtgctcaggcacaagatgatcggacagataaagcgtgagatctcgaccatgaagct
catcaggcacccgaacgtcataaagatgcacgaggtgatggccagcaagacaaagatttacatcgtgatggaacttgtcactggtggtgaacttttcgacaaaat
tgcttcacgtgggaggctgaaagaggatgatgccaggaagtatttccagcaactggtcaatgctgttgattactgccacagcagaggcgtgtaccaccgtgatct
gaagcctgaaaatcttctgcttgatgctagtggcactctcaaggtgtcagatttcggactgagtgcactttctcaacaagtccgagaggatggtcttctgcacacaa
cctgtggaactcccaactatgttgcccccgaggtccttcgnaatatttttccatatctttctgtcaacttgcaggaggatggtcttctgcacacaacctgtggaactcc
caactatgttgcccccgagccaaggctgatctttggtcatgtggagtga
>g61469.t1
atggaggcggctgatgttgatggaaatgggaccattgattatgcagagttcatatcagccacaatgcacttgaatagattggagaaggaagaccacatactcaaa
gcattcgagtattttgataaagaccacagcgggtanttggagaaggaagaccacatactcaaagcattcgagtattttgataaagaccacagcggatacataact
gtagatgagctggaagaagctctgaagaagtatgatatgggagatgataaaacaattaaagaaatcattgcggaagtagattcagataatgatcacacacgtaca
agtgaatccatgttggattaa
>g62237.t1
atggggaacacctgctccggtccaacgccccccctcggaccgccatggtttcttcgactccgtctccctcgccgtgctctggcgcccacccgccgccgccaggg
ccgagccccccttctccatcggactccaggccctccgcctcctcgacgacttcctccaggcctcccgagtccgtcaccttcaccgatgccgagcactccccccac

gcctccccctccgcgccggaccctaacggcaagcccaggcctaaggtgaagcgcgtgcagagcgccggcctccttgtcggctccgtcctgaaacgcaactc
cgagcggctcaaggacctctacaccctgggcaagaagctgggacaggggcagtttggcaccacctaccagtgcgtcgagaaggccaccgggaaggtattc
gcctgcaagtccatcgccaagcggaagctcgtcagcgaggaagacgtcgaggacgtgcgccgcgagatacagatcatgcatcacctctccggccacccgaa
tgttatctccatcgttggggcttatgaggacgccgttgcggtgcaccttgtcatggagctgtgtgcgggaggggaactgttcgatagaatcatacagcgggggca
ctactctgagaaagctgctgcacaattggcacgggtgatcattggagtggtcgaggcatgccattctctcggggtgatgcacagagacctcaagccagagaatt
tcttgtttgtgaaccacaaggaggatgcaccgctaaaggcgatcgattttgggctctccatattcttcaaaccaggtgaaacattttttgatgtcgttggaagtccgta
ctatgttgcacctgaagttctgttgaaacactatggctgtgaggtggatgtgtggagtgctggtgtaataatttatatcttgctgagcggagtccctccattttgggat
gaatctgaacaaggaatatttgaacaagtcctgaaaggtgatcttgacttttcttctgagccctggcccagcatctcaaggagtgcaaaggatttggtcaggaaga
tgttaatccgtgatcccaaaaagagattgactgcacatgaagctctatgtcatccatgggtttgtgttgatggagttgctcctgacaaacctcttgattctgctgtctta
accagattgaaacagttttctgcaatgaacaaattaaagaagatggcccttagggtcattgctgagaatttgtctgaagatgaaattgccgggttgcgagaaatgtt
caaaatgctggacactgacaacagtggccaaattacattggaagaactaaaaagtggcttgaagagagttggtgctaacttaaaggactctgaaattacaacgtt
aatggaggcggctgatattgataacagtggttcaattgattatggagagttccttgctgctactttgcatctgaacaaagttgagcgagaagataatctctttgctgc
gttctcatattttgataaagacggcagcggttacattactcaagatgagctacagaaagcatgtgaggagtttggtataggagatgaacaccttgaagatattatca
gagacattgatcaagataataacttcattctgccatttgagagagagagagagagagagagagagcagccgccggcactggacgtggagccgctgcttgttcctc
cagctggaccagcagcaatactgcgctgcggttccccatcgaagcagtggccgcccaacagcagcagagcctccgcctcctcttcaccttcggagcagcggc
aacctgccagcagcagagccttcgcctccgcctcctcttcaccttcggagcagcggcaacctaccaggagcagagcctccgcctctgcctcctcttcaccttcc
gagcagcggcaacctgccagcagcgcctcaacctccgcctcctcttcttatgctccatgtccagctcgtaa
>g62898.t1
atgggccccggcgtggtggcgcacctggtgcgcctgggcgtgctgagctcgacctccgactacacctcggtggtgtccatcaacctgttcgtggcgctaatctg
cgcttgcatcgtcctcggccacctcctcgaggagaaccggtggatgaacgagtccatcaccgcgctcatcattgggctgtgcactggcgtggtgatcttgctga
cgaccaaaggaaagagctcgcatatcctagtgttcagcgaggacctcttcttcatctacctcctccctccaatcatcttcaatgccggttttcaggtaaagaagaaa
caattcttccggaatttcatgacaatcacgttgtttggtgctgttgggacaatgatatccttcgtcacaatatcagttggcgccatagcaatattcagcaggatgaaca
ttgggacacttgatgttggggattttcttgcaattggagccatcttttctgcaacagattctgtctgcacattacaggtcctccatcaggatgaaactcctcttctataca
gtcttgtgtttggtgaaggagttgtgaatgacgctacgtcagttgtgctcttcaacgcgctccagaactttgatctcaacaagattgacgtagccgttgtacttaaatt
cttgggaaacttctgctatttatttttgtcaagcaccttccttggagtgttttctggattgctcagtgcttatataatcaagaaattatatataggaaggcattccactgatc
gcgaggttgcgcttatgatgctcatggcttacctctcttacatgttagctgagctatcagatttgagtggcattctcactgtgttcttctgcggcatcgtgatgtcacatt
acacttggcataatgtgacagagagctcaagagttacaaccaaacatgcttttgcaactttgtccttcatcgctgagacatttctcttcctctatgttggtatggatgca
ctcgatatcgaaaagtggaagtttgccagtgacagccctggaaagtccattggaataagctcaattttgctaggattggttctggtgggaagagctgcgtttgttttc
cctctctcattcctgtctaacttgacaaaaaagggtcctttggaaaaaataacctggagacaacaaatcgtaatatggtgggctggactgatgagaggggctgtgt
cgatcgcccttgcttacaataagtttactagatctggacacactgagctccacggcaatgcgataatgataaccagcacaatcactgtagttctgtttagcaccatg
gtgttcgggatgatgacaaagccattaatccagttcttgctcccggcgtcaagccacacggtcacctccgagccgaattcgcccaagtctctccactcccctctcc
tcaggagcatgcaggggtctgacctcgagacggcctcgcagtcgcatattgtcaggccttccagtctccgcatgctcctcagcaagccgacccacaccgtcca
ctactactggcgcaagttcgatgatgcgctgatgcggcccatgtttggcggacgcgggttcgttcctttctctcctggatcacccaccgagcagagcgttcatggt
ggacagtga
>g64531.t1
atgggatcagctactacaatatgcagtgataagacaggaactcttaccttaaataagatgacagttgtggaagcatactttggaggaacaaagttggatccctgct
atgatatgagccaaatgtcagccaatgtagcatcgcttattattgaaggaattgcacagaacacaacaggaactgtattcctgccagaggatggggggagaagca
gaacttacaggttcaccaactgaaaaggccattctttcttggggccttaaggcagggatggatttccatggtgtgagatcaaaatcttcagttcttcatgtttttccattt
aattcggagaagaaacgtggagctgttgcaatacagtatgatgaattctggaaatctattgaagacatggcagcaacctcattacgttgtgttgcttttgcatatcgt
ccctgtgagcctgaaaagataccagaggaagacatagctaattggaaattgcctgaggatgacctgactctgcttggcatcataggaataaaggccgatatagg
tctttcaatgggcatctcaggcacagaagttgctaaggaaagttctgacattataatcttggatgatgacttcacatcagttgtcaaggttgttcgttgggggcgatct
gtctatgcgaatattcagaaattcatccagttccagctcacggttaatgttgccgcccttgtaataaatgtggttgctgctgtgtcatctggtgatgtgcccctgaatgc
cgtagaggttgggaccttttcttga
>g65772.t1
atggggaaggagaaggttcacatcaacattgtggtgatcggccatgttgattctggcaaatcgaccaccactggtcacctcatctacaagctcgggggaatcga
caagcgtgtcattgagaggttcgagaaagaggccgctgagatgaacaagaggtccttcaagtacgcctgggtgcttgacaagctcaaggctgaacgtgagag
gggtatcaccattgacatcgccctctggaagtttgagaccaacaagtattactgcactgtgattgatgctcctgggcatcgtgacttcatcaagaacatgatcaccg
gaacctcccaggctgattgcgccgtccttatcatcgactccaccaccggaggctttgaggctggtatctccaaggatgggcagacccgtgagcacgcgctgctt
gctttcactcttggtgtcaggcagatgatctgctgctgcaacaagatggacgccaccactcccaagtactccaaggctcggtatgatgaaattgtcaaggaagtg
ggctcgtacctgaagaaggttgggtacaacccggagaagatccccttcgtgcccatctctgggttcgagggtgacaatatgattgagaggtccacgaacctgga
ctggacgtgtacaagatcggtggcatcggcacggtcccggtgggccgtgtggagacgggtaccctga
>g66239.t1
atgcacaggagaggtgggaaatattctgaagaagatgccaaggttgtcattcatcaaattctaagtgttgcttcattttgccatcttcagggtgttgttcaccgggatc
tgaaaccagagaatttcctttcatgtccaaggatgagaattctgccttgaaagtcatagactttggtttgtctgactttgttaggccagatgaaagacttaacgatatt
gttggaagtgcatactatgttgctccggaggtgctccaccgatcttacggcaccgaggcagatatgtggagcattggagtgattgcgtatattttgctttgtggaag
ccgcccttctgggcacggaccgagtcaggaatatttcgggctgtcctaaaggcagagcctagttttgatgaatccccatggcctaacctttctgctgaagcaaaa
gactttgtaagaaggctgctcaataaggattaccggaaaaggatgactgctgcacaggcccctctgccatccatggatctgtggcggccaagaagttaagattaa
cttggacatgattatttacaggcttatgagggcttacataagttcatcctctcttcggaagtctgctttaagggctatgcacaggagaggtggaaagtattctgaaga
agatgccaaggttgtcattcatcaaattctaagtgttgcttcattttgccatcttcagggtgttgttcaccgggatctgaaaccagagaatttcctttcatgtccaagga
tgagaattctgccttgaaagtcatagactttggtttgtctgactttgttaggccagatgaaagacttaacgatattgttggaagtgcatactatgttgctccggaggtgc

tccaccgatcttacggcaccgaggcagatatgtggagcattggagtaattgcgtatattttgctttgtggaagccgccctttctgggcacggaccgagtcaggaat
atttcgggctgtcctaaaggcagagcctagttttgatgaatccccatggcctaacctttctgctgaagcaaaagactttgtaagaaggctgctcaataaggattacc
ggaaaaggatgactgctgcacaggccctttgtcatccatggatctgtggcggccaagaagttaagattaacttggacatgattatttacaggcttatgagggcttac
ataagttcgtcctctcttcggaagtctgctttaagggcattgacgaagaattccacaagtgcaatgaatgattcaagggttcttgatttcgttaacacaatatgcaatgt
ccagtaccgaaagcttgattttgaggagttctgtgcttcggctattagtgtttatcagatggaaggtctggagacctgggaacaacatgctcgagaagcatatgaat
tatttgacaaggagggaaatcgaccaattctaatggaagaacttgcatcggtatag
>g66771.t1
atgtataaaactaagagatctgcttcacttaaagtgaagcgccgtgttgggaagtatgagcttgggcgcacaattggggaaggaacctttgctaaggttcggattg
caaagaacacggagactggggaacatgttgctatcaagatccttgacaaggcaaaggttctgaagcacaaattggttgagcagttagagaacttgctactggat
attgctggaacccttaaaatttctgattttgggttaagtgctatatctgagcaattgaaggctgatggattgctgcacactacatgtggaacggctaactatgttgctcc
agaggttatcaatgacaagggctatgatggtgccactgcagacctttggtcttgtggggtaatcctttttgtgttgcttgcaggatatctgcctttcgaggatgacaat
actgtctccctctataaaaagatctcaggagctcaatttacttgcccctcttggttttctgctggagcaaagaagctgatctgtagaattctggatcctaatccttcaac
tgtaagtgacccttga
>g68626.t1
atggcggcggacgcgtctgcgtcggggaggaagacgcgggtggggccgtacgagctggggaagacgatcggggagggcagcttcgccaaggtcaagct
cgcccgaaactcgcgcaccggcgacgtctgcgccatcaaggtgctcgaccgcaaccacgttctccgccacaagatggtcgagcagataaagcgggagattt
cgacaatgaagctaatcaaacatccgaatgtagtccagttgcatgaggttatggctagcaaatcaaagatatacatggttcttgaatttgttgatggaggcgagcta
tttgataagatctttgtagatgctgctgtttacgtgttctga
>g70664.t1
atgcagatctacaaagcggacttcagttgtccttcttggttctccacaagtgcaaagaagctcatcaagaaaattctagatcctaaccctagcactagaataactatt
gcagatctcatcaacaatgagtggttcaagaaagggtatcagcctcctaggtttgagacagcagatgttaatctggatgatgtgaactccatttttaacgaatctgg
ggaccccgctcagcttgttgttgagaggcgagaagaaaggccatcagtgatgaatgcttttgagctgatatccacatctcagggtctcaatcttggcacgctctttg
agaagcaaacgggttctgttaagcgagaaacaagatttgcgtcaaggcttcctgcaaatgagatattgtcaaaaattgaagcagcagcaggacccatgggcttc
aatgtacagaagcgcaactacaagctgaagctgcaaggggagaatcctggaaggaaaggtcagctggccattgcaacggaggttttcgaagtcacgccttcg
ctgtacatggttgagctacgcaagtcgaacggtgacaccctcgagttccacaagttctatcacaatatctcaaatggcctgaaagacgtgatgtggaagccggag
agtggcatcgtcgaaggtgccgagatcaagctccgaagttcgccatga
>g71498.t1
atgtacgggtggaggaaaggaggttctggggttttcttgatctgtgtgagagatcttctctatcagtcaatgcccacgggcttcttacccccgcaggtcattgctgat
catttgtcggctgaggaagttgaagatataaaggagatgttcaaggctatggatactgacaatgatgggattgtatcttatgaagaactaaagagtggaatagcaa
aatttggttctcatcttgcggaatcagaagtacaaatgctcattgaagctgtggatacaaatggcaggggagcactagattacggtgaatttttggctgtctcacttc
atttgcaaaggatggcaaatgatgagcacttgcggcgagccttcctatttttgacaaggatggcaatggttttattgagcccgaggagctccgagaggctctaat
ggatgaagggggcactgatagcatggaagtggtgaatgacatattgcatgaagtcgacactgataaggacggcaagattagttatgatgaatttgtagcgatgat
gaagaccggcacagattggagaaaggcatcccggcactattcgagaggaagattcaatagccttagcatgaagcttataaaggatgggtctgtaaaattgggtg
ttgagtga
>g72393.t1
atggaccgggcggcggtgacggtggggccggggatggacatgccgataatgcacgacggcgaccggtacgagctcgtccgtgacatcggctccggcaact
tcggcgtcgcgcgcctcatgcgcagccgcgccgacgggcagctcgtcgccgtcaagtacatcgagcgcggcgagaagattgacgagaacgtgcagcgcg
agatcatcaaccaccggtcgctgcgccaccccaacatcatccgcttcaaggaggtcatccttacgcccacgcacctcgccatcgtcatggagtacgcctcagg
aggggaactcttcgagcgcatctgcaacgccggcaggttcagcgaggacgaggtccagaccctttctgccttcttcctgtcacctctgcagtgctcactgaatca
aacatcagtgctgccctctgttgacgttccttcgtcggttcttcattcacagccgaagtctactgtaggaactccagcatacattgctcctgaggtccttctgaagaaa
gaatacgatggaaaggttgctgacgtgtggtcatgtggggtaacactttatgtgatgctggtcggtgcatatcctttcgaggatcctgatgagcccaagaatttcag
gaagacaattcagagaatactgggtgtgcagtactcaattccagattatgtccacatatctcccgagtgccgagatcttatttcaaggatatttgttgcggacccatc
tactagaatcaccatacctgagatcacaaaccatccatggttcacgaagaacctcccagctgacttaatggatgagaacacaacgagcaaacagtatgaagagc
ctgatcagccagtgcagagcatggatgagatcatgcagatactggccgaggcgacaataccggcagctggttctcgaatcaaccagttcttaaatgatggtcttg
acctcgatgacgacatggacgatctagattcagatgccgatctcgacttggaaagcagcggggagattagaatactgggtgtgcagtactcaattccagattatg
tccacatatctcccgagtgccgagatcttatttcaaggatatttgttgcggacccatctactagaatcaccatacctgagatcacaaaccatccatggttcacgaaga
acctcccagctgacttaatggatgagaacacaacgagcaaacagtatgaagagcctgatcagccagtacagagcatggatgagatcatgcagatactggcgg
aggcgacaataccggcagctggttctcaaatcaaccagttcttaaatgatggtcttgacctcgatgatgacatggatgatctagattcagatgccgatctcgacttg
gaaagcagcggggagattgtatatgcgatgtga
>g73227.t1
atgcagccggacccacaaggcccgggcagggagaagcccggcggcaccaatgtgaacgtccggctgccgccgccggtgacggtggggtcgggtggccg
gccggcgtccgtgctgccgcacaagacggccaacgtgcgcgaccactaccgcatcgggaagaagctgggacaggggcagttcggcaccacgtaccagtg
cgtcggcaaggccgacggcgccgagtacgcgtgcaagtccatccccaagcgcaagctgctgtgccgcgaggactacgaggacgtgtggcgcgagatcca
gatcatgcaccacctctccgagcacccaaacgtcgtccgcatccgcggcgcatacgaagacgccctcttcgtgcacctcgtcatggagctctgcgccggcgg
cgagctcttcgaccgcatcgtcgccaagggccattactccgagcgcgccgcagcgcagctcatcaggacgatcgtgggcgtggtcgaaggctgtcactcgct
gggcgtcatgcaccgggacctcaagccggagaacttcctcttcgcgagcaccgctgaggatgcgccactcaaggccactgactttgggctctctgtgttctaca
agcctggtgataaattttctgacgttgtcgggagcccctattatgttgcaccggaggtgcttcaaaaatgctatggcccagaagctgatgtctggagtgctggagt
gattctgtacattttgctatgtggtgtgcccccattctgggcaggtgataaattttctgacgttgtcgggagcccctattatgttgcaccggaggtgcttcaaaaatgct
atggcccagaagctgatgtctggagtgctggagtgattctgtacattttgctatgtggtgtgcccccattctgggcagaaagtgaagcaggaatctttaggcagatt
ttgcgaggcaaacttgacttggaatctgaaccttggcctaatatctctgatagtgctaaagatctggtccgtaagatgcttacccgggatcctacaaaaagactgac

tgctcatgaagttctatgtcatccctggattgtggatgatgctgttgcacctgataagcctattgattctgctgtgttgtcaaggctgaaaaacttttctgcaatgaaca
agcttaagaagatggcattgagggtgatagccgagagtctgtctgaggaggagattggtggcttgaaggagctgttcaaaatgatcgatactgacaatagtggc
acaataacttatgatgaactgaaaaacggcctgaaaagggtaggttctgatctaatggaacctgaaatcaaggctttaatggatgcggccgatattgacaacagt
ggaaccattgactatggagagttcctagcagctacattgcacatgaataaactggagagggaggaaagcttggatggacaaattgattatagcgagtttgcagca
atgatgagaaagggcaatgctggtggtgcagggaggagaaccatgaggaacagcttgcatgtgaaccttggtgaaatcttcaaacccgctgagaagtaa
>g73323.t1
atggcggaccagctcaccgacgaccagatcgccgagttcaaggaggccttcagcctcttcgacaaggacggcgatggttgcatcacaaccaaggagctggg
aactgtcatgcgttcattgggtcagaacccaaccgaggctgagcttcaggacatgatcaacgaggttgatgctgacggcaatggcaccatcgacttccctgagtt
cctgaacctcatggctcgcaagatgaaggacaccgactctgaggaagagctcaaggaggccttcagggtgtttgacaaagaccagaatggcttcatctctgctg
cggagctccgccacgtcatgaccaaccttggagagaagctaaccgatgaggaggtcgatgagatgatccgtgaggctgatgtcgacggcgatggccagatc
aactacgaggagttcgtcaaggtgatgatggccaagtga
>g73726.t1
atgggcaactgctgctgcggggggtccaagcaggatcccgccgcagagccggcgtcctccggctccagccgccccgtgggcagcacgacgacctcggtct
cgccgtcctcggctccggcgcccatgaagcccccgccgcggtgggccctgtcctggggcggcccatggaggacgttcggagcacgtacagcatcggcaa
ggagcttgggcgcgggcagttcggcgtgacgtcgctgtgcacgcacaaggcgacggggcagaagctggcgtgcaagacgatcagcaagcggaagctgtc
gaccaaggaggacatcgaggacgtgcggcgcgaggtgcagatcatgtaccacctctccggccagcccggcgtcgtggagctcaacggcgcgtacgagga
caaggcctccgtccacctcgtcatggagctctgcgccggcggcgagctcttcgaccggatcatcgccaagggccactatacggagcgcgcggcggcggcg
ctgctccgcaccatcgtcgagatcgtgcacacgtgccacgccatgggcgtcatccaccgcgaccttaagcccgagaacttcctcctcctcagcaaggacgaga
gcgcgccgctcaaggccaccgacttcggcctctccgtcttcttcaaagaaggggaggtgttcagggacatcgtcggcagcgcatactacatcgcgccggagg
tgctgaagcggagctacgggccggaggccgacatatggagcatcggcgtcatcgtctacatcctgctctccggcgtcccccccgttctgggcaggcaagcgat
ctgttgtcttcagagagacgcacggcatcttcaattccatcctgagggggcaagtcgacttcaccagcgacccctggccgcgtatctcgtctggtgccaaggac
cttgtcaggaagatgctcaactcggaccccaagcagcggatatctgcgtatgacgtcctcaaccatccgtggatcaaagaagacggcgacgcaccggacaca
ccgctggacaacgcggtcctcggccggctcaagcaattcagagctatgaaccagttcaagaaagcagcgctaagggtcatcgccggatgcctgtcagagga
ggagatcaaggggctgaaggagatgttcaggagcatggacgcggacaacagcggcaccataaccgtggacgagctgcggcgcgggctggccaacaagg
gcaccaagctcagcgaagccgaggtccagcagctgatggaagctgccgacgcggacgggaacgggacgatcgactacgacgagttcatcacggcgacga
tgcacatgaacaggatggaccgggacgagcacctgtacacggcgttccagtacttcgacaaggacagcagcgggtacatcaccatcgaggagctggagcag
gctttgagggagaagggcctgctcgacgacggccgggacatcaaggacatcatatcggaagtcgacgccgacaacgacgggaggatcaactacacggagt
tcgtggcgatgatgaggaaaggggacccggaggtggccaaccccaagaagcgccgcgacgtcgtgctatag
>g78519.t1
atgggcaacacctgcgtcggccccagcgcggcgggccgcagcggatttctcgcctccgtcaacctgtggcggcccgcgcagcccgccgccgccctcccgc
cgccctcctcccccgcctccgacaaggcgcccgagcccgtcacaatccccgcctccgagcactcctcccaccactcctcccgatcctccgaccagccgcaga
cgccctccgcggagccgcagacgcaggacaataatcctccggcgaagaagcccgtgcccaaggtgaagcgcgtccagagcgcgggcctcctcgcggact
ccgtcctcaagcgtgacgtcaacacagcccggctcaaggatctctacaccatcgacaagaagctagggcaggggcagttcggcaccacctacctctgcgtcg
agaaggccaccggccgggagttcgcctgcaagtctatcgccaagcggaagctgctcacggaggaggacgtcgaggacgtgcgccgcgagatccagatcat
gcaccacctcgccggccacgcaaacgtcgtctccatcgtcggcgcctacgaggacgcggtcgccgtgcagctcgtcatggagctctgcgccggcggggag
ctattcgacaggatcattcaaaggggacattactccgagaaggccgctgcgcagctggcgcgggtgatcgtcggtgtcatcgaagcctgccactcgctcggtg
tgatgcacagggatctcaagccggagaacttcctgttcgtcaaccagaaggaggactcgccgctcaaggccattgatttcggtctctccatctttttcaagccagt
ggaatcatttactgctcatatgggtgttgcagaacaggttattgctgagagtttatccgaggaagaaattgcagggttgaaagaaatgtttaaaatgcttgacagtga
caatagtggccatataacattggaggaattgaaaactggcctgcagagagttggagctactttgatggactcagaaattgatgctttgatgcaagcagcagatatc
gacaatagtggcacaattgattatggggagttcattgctgcaactttgcatataaacaaagttcagaaggaggataagctctttgcagctttctcatattttgacaaag
acggcagcggttacataactcaagatgagctccaaaaggcatgtgaggagtttggtataggagatacccggcttgaggatattattggggacatcgatcaggat
aatgatggacggatcgactacaatgagtttgtcgcaatgatgcagaaaggagataatccactagggagaaagggtcaaagtgcggattttggtcttggtgaagc
actgaagcttcgataa
>g78522.t1
atggggaacacgtgcgtgggccccagcatcaccaagaacggcttcttccagtccgtctccacgctgatgtggaagacgccgcaggacggcgacgcgctgcc
cgccgccgccaatggccccgaaggcggcagcccgggccggtcgcagcccgccaagcccgaagtgcagagcaaggccccggagcccatgaagatcgcc
gcagcccaacaaccggatgagttgccgccgcccaaaaccaagcagcagcaagacgcgaagcccaacgccggcggcgaggcgcccagcaccaagccgc
ggcccaaggttcctccggtgaagcgcgtgtcaagcgccgggcttctggtgggctcggtgctgaagcgcaagacggagaacctcaaggagaagtacagcctg
ggccgtcggctagggcagggccagttcgggacgacgtacctgtgcgtggagcgcggcacggggaaggagctggcgtgcaagtccatcctgaagcggaag
ctggtgaccgacgacgacgtggaggacgtgcgccgggagatccagataatgtaccacctggcgggccacccgaacgtgatctccatccgcggcgcgtacg
aggacgccgtcgccgtgcacctcgtcatggagctctgcgccggcggggagctgttcgaccggatcgtgcagaggggacactacacggagaggaaggcgg
ccgagctcgcaagggtcatcgtcggggtcgtcgaggtgtgccactccatgggcgtcatgcatcgggatctcaagcctgagaacttcctcttcgttgacaacaag
gaggaggccgcgctcaagaccattgactttggtctctccattttctttcgccctggtcaagtattcacggatgttgtcggtagcccatactacgtcgctccagaggtc
ctgaaaaagaaatatggtcccgaggcagatgtctggagtgctggtgtgatcatctacatcttgctgtgcggtgtgccaccattttgggcagagaacgagcagggt
atatttgaagaggttttacatggaaaacttgatttccagtcagagccgtggcctagcatctccgaaggtgccaaagatcttgtgaggagaatgcttgttagggaccc
caaaaagagattgacagctcatgaagttctacggcatccatgggttcaggttggtggtttggcgcctgataagccgctggactcggctgttctgtctcgcatgaaa
caattctcggctatgaataagctgaaaaagatggctcttagggtcattgcagagaacttatccgaggacgagatcgccggcctgaaagaaatgttcaagatgata
gacacggacaacagtgggcacatcacctacgaagaactcaaggtcgggttgaaaaaagttggtgccaacctccaggaatccgagatccaggcactcatggaa
gctgcggacgtggataacagcggcacgatagactacggcgagttcatcgcggccactctgcacctgaacaaggtggagagggaggaccacctgttcgcggc
gttccagtacttcgacaaggacggcagcgggtacatcacgcccgacgagctgcagctggcgtgcgaggagttcggcctaggaggggacgtgcagctcgag

gagatgatccgcgaagtggaccaagacaacgacgggcgcatcgattacaacgagttcgtggcgatgatgcagaagccgacactggggctcccgaagaagt
ccggcggtctgcagaacagcttcagcgtcggattcagggaggcgctgaggatcgcgtaa
>g79282.t1
atgttggacaccaacaaggacgggcacttgactattgacgaacttcgcaagggattgcgagtgatagggcagaatgttagtgaaactgatgtggatatgcttatg
gaagctgcagacattgacggcaacggtaccctggacttgaaggagtttgtgacagtctccattcatttgaagaaaatcagaagcgaggatcacttgccaaaggt
attcagctactttgacaagaatggaagcggttacattgaaattgaagaattgaaggaggcgctctctccacgaggaggccagaaggcaatcgatgacataatcct
ggatgtcgacaaagacaaggttagtcgcacgaccattctgaaggaaactcaaaagtgttag
>g79424.t1
atgttcaaggccatggacaccgacgccagcggagccatcaccttcgacgagctcaaggaagggctaaggagatacggatcaaacctcaaggagagcgaga
tcagggacctcatggacgccgccgacgttgacaagagcggcaccatcgactacgacgagttcatcgccgccaccgtgcacatgagcaagctcgagcgcga
ggagcacctcctcgccgccttcgcctacttcgacaaggacgccagcggatacatcaccgtcgacgagctcgagcaggcctgcagggaccacaacatggcag
acgtcggcatcgacgaaatcatcagggaggtggaccaggacaacgatgggcgcatagactacggcgagtttgtggccatgatgaagaagggcatcattggg
aacggaaggctcaccatgaggcacacctctgacggcagcgttctccatggcgccggccacctctgctag
>g80828.t1
atggggctcggcgtggtggcgcacctggtgcgcctgggcgttctgagctcgacctccgaccacgcctcggtggtgtccatcaacctgttcgtggcgctaatctg
cgcctgcatcgtcctcggccacctcctcgaggagaaccggtggatgaacgagtccatcaccgcgctcatcattgggctgtgcactggcgtggtgatcttgctga
cgacaaaaggaaagagctcgcacatcttagtgttcagcgaggacctcttcttcatctacctcctccctccgatcatcttcaatgccgggcattccactgatcgcga
ggttgcgcttatgatgctcatggcttacctctcttacatgttagctgagctatcagatttgagtggcattctcactgtgttcttctgcggcatcgtgatgtcacattacact
tggcataatgtgacagagagctcaagagttacaaccaagcatgcttttgcaactttgtccttcatcgctgagacatttctcttcctctatgttggtatggatgcacttga
tatcgaaaagtggaagtttgctagtgacagccctggaaaatccattggaataagctcaattttgctaggattggttctggtgggaagagctgcgtttgtttttcctctct
cattcctgtctaacttgacaaaaaagggacctttggaaaaaataacctggagacaacaaatcgtaatatggtgggctggactgatgagaggggctgtgtcgattg
cgcttgcttacaataagtttactagatctggacacactcagcttcatggcaatgcgataatgataaccagcacaatcactgtagttctgtttagcaccatggtgttcgg
gatgatgacaaagccattaatccggttcttgctcccggcttcaagccacacggtcacctccgagccgaattcgcccaagtctcttcactcccctctcctcaggagc
atgcaggggtctgacctggagacggcctcgcactcgcacattgtcaggccttctagcctccgcatgctcctcaccaagccgacccacaccgtccactactactg
gcgcaagttcgatgatgcgctgatgcggcccatgtttggcggacgcgggttcgttcctttctcccctggatcacccaccgagcagagcgttcatggaggacagt
ga
>g84684.t1
atgggctccgccccgtcgtcgcgtgtccagagctcgtcgggggaggcgccgttcttcacggacttcggcgaggcgagccggtacgaggtcacggaggtggt
cggcaagggcagctacggcgtggtcgccgccgccgtcgacacgcacaccggcgagcgcgtcgccatcaagaagatcaacgatgtcttcgagcacatctcc
gacgccacccgcatcctccgcgagatcaagctgctccgcctcctgcgccacccggacatcgtccagatcaagcacatcatgctcccgccctcgcgccgcgac
ttccgcgacatctacgtccagttcttcctctaccagctactccgcggcatgaagtacatccacgccgccaatgtcttccacagggacctcaagcccaggaacatc
cttgccaatggcgattgcaaactcaagatttgtgacttcggccttgctcgggtgtcgttcaatgataccccttctgcgatattctggacgtatactcctgcgattgatat
ttggagcataggatgtatatttgctgaaatgcttacagggaagcctctctttcctggcaagaatgtggtacaccaattagatctcatgactgatcttcttggcactcctt
cagcagaaacactttccaagattttggagtaccatcctcagatgttgcaggagtatctttgcggaggggatatggcaaactttgtgtacccaagggaaaaagtaaa
tggcaacgggaatgagcttgagaagccaaatgcagactacgtcataaaattgcatgagtttcgctacgtgctcgcccctctccttgcagcttgtgctgatgagcaa
tga
>g85619.t1
atgggcaacgtgtgcttctgcggcacgacgtccacgtcccctgacgagcctccatctgatcccaaaaaatccgtagcgccgccgcaacagcaaggcaagagg
ccggcgacgcctccaagcagcagccatgaaaagagccccactcccacccccaggccgaagcagaagcagcagcaacagcaacccgagccccgtacgcg
cgccaagcccataaagcccaacccgtacgactgggcatcgccgtcgtcgtcgcgcgtgctggacggcgtggtcccgcaccacccgcgtctccgcgtgacgg
acaagtacacgctgggccgcgagctgggccgcggcgagttcggcgtgacgcgtctggcgacggaccgggccacgcgtgagcggctggcgtgcaagtcca
tccccaagcgccgcctccgcacggccgtggacgtgggcgacgtgcggcgcgaggtggccatcatggcgtcgctccccgaccacccgtccctggtgcgtctc
cgcgcggcgtacgaggacgccgacgccgtgcacctggtcatggagctctgcgacggcggggagctgttcgaccgcatcgtggcccgcgggcggtacacg
gagcgggccgcggcggcggcggcccggacggtggccgaggtggtccgggcctgccacgcgcacggggtgatgcaccgggacctcaagcccgagaact
ttctgtacgaggggcggggtgatgacgcgcggctcaaggccatcgactttgggctgtcggtgttcttccggcccggggagaggttcacggagatcgtgggca
gcccgtattacatggcgcccgaggtgttgcgacggagttacgggcccgaggtggacgtgtggagcgccggggtgatactgtatatcctgctgtgcggggtgc
cgccgttctgggccgagacggagcagggcgtggcgagggctatactgcgagggagcctggacctggaccgcgagccctggccccggatctcccacgggg
ccaaggacctcgtcaggcagatgctgcagatggaccccaagaagcggccaaccgcgcagcaagtcctcgagcacccgtggcttcagaacgcgcggaagg
cgccgaacgtgccgctgggcgacgtggtgcgcgcgcggctgcagcagttctcggccatgaacaagttgaagaagaaggccatgcgggtcatcgcggagca
cctgtccgtggaggaggtggaggtgatccgggacatgttcgcgctcatggacacggacaatgacggcaaggtcacgctgcaggagctcaaggcggggctc
aacaaggtcggatccaagctcgccgacccggagatggagctgctcatggaggccgccgacgtgaatggcaacgggtacctggactacggcgagttcgtgg
ccatcaccatccacctgcagcgcctctccaacgacgaccacctccgcacggcgttcctcttcttcgacaaggacagcagcggctacatcgagcgcgacgagct
ggccgacgcgctcgccgacgactccggccaaaccgacgaagccgcgctcaacaacgtcctccgagaagtagacacggacaaggacggtcggataagtttc
gacgagttcgttgccatgatgaaagccggcacggactggaggaaggcgtcacgacagtactcaagggaacggttcaagactctgagcaacagcctcatcaa
ggacggctcgctcgccatggcgcactga
>g86042.t1
atggaagggctgacgggagagcagatagttgctttccaggaggccttctccctcttcgacaagaacggcgacggatgcatcaccatggaagagctggctgca
gtgactcgctccctaggccttgatcctagtgatcaggagctcatcgatatgatgagcgaagttgatactgatggcaatgggataattgattttcaggagtttctgagc
ctcattgccaggaagatgaaggatggtgatggcgatgaggagctgaaggaggcttttgaggtgctagacaaggaccagaatggatttatctcccctactgagct

gaggacggtgatgaccagccttggggagaagatgactgatgaagaggttgaacagatgatcaaagaagcggataccgatggtgacgggcaggtgaactacg
atgagtttgtgcttatgatgaaaaatgctgaacggaagatatctggttga
>g86873.t1
atgggccagtgctacggcaaggcgggcgggggctcctcgtcgcgggccgaccacgacgacatcggggtcgtcgcgccgccgtccccgctgcccgccaac
ggcgcgccgcagcagccggccacgccgaggcgtaggaagtccggatcgaccacgccggtgcaccacacgcccggggtcgcctggccgagtccctaccc
cgccggcggaacgagcccgctgccggcgggggtgtcgccgtcgccggcgaggtcgacgcccaggaggttcttcaagcgccccttcccgccgccctcgcc
ggccaagcacataaaggccacgctcgctaagaggctgggcgggggggaagcccaaggaggggaccataccggaggaaggcggtgtcggggctgccggt
gctggaggggggtgcggatggagccgaggcggagcggccgttggacaagacgtttggcttcgccaagaacttcggggccaagtacgagctggggaaggag
gtgggaaggggccacttcggacacacctgctccgccgtcgtcaagaagggagagtacaagggccacaccgtcgccgtcaagatcatttccaaagctaagatg
acgacggccatatccatcgaagatgttcgtagggaagtcaagattctgaaagctctctcagggcacaataatcttgtcagattctatgatgcatgtgaggacgccc
tcaatgtctacattgtcatggagtatgttttttatga
>g88536.t1
atggagtcctcgtcgtcgggcggcgggagggcgcggcggcgcagcagcggcggtagctggggcagcgtcggtgacgccttcgacatccccgccaaggg
agcgcccgtggagcggctaaaaaagtggcggcaagcagcccttgtgctgaatgcgtcaaggcgctttaggctgcctttcgtttcaaagaagctggtcgagtac
atgttcagccaaaggaggcaccagtaccacatgctgatggagcacttggctttggaatcaaagaggaacagattactgcgttcacaagagatcacaactactct
gcgcttcaacaatatggagggttttgcagtaccacatgctgatggagcacttggctttggaatcaaagaggaacagattactgcgttcacaagagatcacaacta
ctctgcgcttcaacaatatggagggatttcaggtgtagcaagtatgctgaagactgatacagagaaaggaattaatggcgatgattctgacttgacggcaaggcg
aaatgcatttgggtcaaatacatatcctcgtaagaaaggaaggagctttttggcttttgtatgggatgcttgtaaagacatgacactgataatcctcatggttgcggct
gctgtttcacttgccttgggcataacaactgaggttgcccgaggcggaaggcgaataatggtatcaatatatgatttggttgttggagatgttgtgcctctgaagatc
ggtgatcaggtccctgctgatgggatcctcatcagcggtcactcgctttccatagatgaatcaagcatgacaggagaaagtaaaatcgtacacaaagaccagaa
atcacctttcctaatgtcaggttgcaaagttgcagatggttatggcacaatgctggtgactgctgttggtattaacactgaatggggattactaatggcaagcatatct
gaagattctggtgaagagacacctctgcaggttcgcctgaatggtgttgctactttcattggaatggttgggctttctgttgcatttgcagttttggttgtccttctggcc
agatacttcactggtcatacatataatcctgatggatcggcgcaatatgtgaaaggaaagatgggtgtgggccagacgatacgtggagtagttcgaatcttcactg
tggcggtcactatcgtggtcgtggctgttcctgaagggttaccgttggcagtcacattgacgcttgccttttcaatgcgcaagatgatgagggacaaggcactgga
attgcacagaacacttctggaagcatatttgagccagaggcatctgcttttgtatggtatgattactgacttattttcaatgtctcagcagggtggtcaagaaccaga
ggtgactgctgttggtattaacactgaatggggattactaatggcaagcatatctgaagattctggtgaagagacacctctgcaggttcgcctgaatggtgttgcta
ctttcattggaatggttgggctttctgttgcatttgcagttttggttgtccttctggccagatacttcactggtcatacatataatcctgatggatcggcgcaatatgtgaa
aggaaagatgggtgtgggccagacgatacgtggagtagttcgaatcttcactgtggcggtcactatcgtggtcgtggctgttcctgaagggctaccgttggcagt
cacattgacgcttgccttttcaatgcgcaagatgatgagggacaaggcactggaattgcacagaacacttctggaagcatatttgagccagaggcatctgcttttg
tatggtatgattactgacttattttcaatgtctcagcagggtggtcaagaaccagaggttgctgaattcaagagattcatcgaagatatggctgcttctagcctccgct
gtgttgcctttgcatatagacctcatgacatgggtgaggttccaaaggaggatcagaggaacgactggaagttgcctgaagataacctgattatgcttggaattgt
gggaataaagcaaaactattag
>g89054.t1
atgggcaactgctgcgtggcccggccgtcgtccggcaagcggcgtgccggcggcggcgggaaccaccgcggcggccgcctcggcggcgccaacatgcg
gtcctgctccgcgatctcctccatcaccgaccccggaggcgcctcccgcgggtgggcccccgccaccgtgctggggaaaagcctggcccccgacgccacc
gccgaggagctgctccggcggtacgacctcggcgaggagctcgggcgcggtgagttcggcgttacgcggcggtgcacagacacggccaccggcgagttg
ctggcgtgcaagtccatcagcaagcgccggctgcgcagcagcgtggacatcgaggacctgcagcgggaggtggccatcatgcggtcgatgccggcgcacc
cgaacgtcgtgcgcctgcgcgaggcgttcgaggacaacgaggccgtgcacctcgtcatggaggtctgcgagggcggggagctcttcgaccgcatcgtctcc
agggggcactacaccgagcgagccgccgccgccgttatgcgcaccatcgtcgaggtcgtgcagcattgccataagaacggtgtcatgcatcgggatctaaag
cctgaaaactttctgtacgccaacacatctgagaactccccccctcaaggtcatcgactttggtctctccgtgtgcttccaaccaggtgataggttcagtgagatcgt
cggatccccatactacatggctcctgaagtcctcaagaggaactacggacaagaaatagacatatggagcgccggagtcatactctatatcttgctatgtggtgtc
ccacccttctgggctgaaactgatgaagggattgcacaggctatcatccggtcaaaacttgatttcgagagggagccttggcctaaggtttctgagaatgcaaaa
gatcttgtcaggaagatgcttgatccgagcccttatggacgattgacagctcagcaggttttagaacatccttggatacagaatgcgaactcagctcccaacatcc
ctcttggagaagcagtaaggtccaggcttaagcaatttactgtcatgaacaagttcaagaagaaggccctacttattgtggcagaatacctaccaactgaagagct
ggaggcaattagggagctgttccacatgttggacaccaacaaggacgggcacttgactattgacgaacttcgcaagggattgcgagtgatagggcagaatgtc
agtgaaactgatgtggatatgcttatggaagctgcagacattgacggcaacggtaccctggacttgaaggagtttgtgacagtctccattcatttgaagaaaatca
gaagcgaggatcacttgccaaaggtattcagctactttgacaagaatggaagcggttacattgaaattgaagaattgaaggaggcgctctctccacgaggaggc
cagaaggcaatcgatgacataatcctggatgtcgacaaagacaaggatgggaagataagctacgaggagtttgagctgatgatgaaaggtggaatggactgg
agaaatacatctcgacagtattcgagagcagtttacaacacccttagccgaaagatatttaaggacatttccttgaagcttgatccctgtagtgggccacttgctgc
agctgcaaaagaacaacgagaggttgactga
>g89161.t1
atggagagctacctgaacgagaactttgggggcgtcaagcccaagcactcgtccgacgaggcgctggggcgatggcgcaaggtcgtcggcgtcgtcaaga
accccaagcgccgcttccgattcacggccaacctcagcaagcgctcagaggccgcgcagatgaagcgatccaaccaggagaagctgcgtgttgctgtgcttg
tttccaaggctgcacttcagttcctccacggccttgcaccgcaaagcgagtacacggtccctgacaacgtcaagaccaagggcttcggcatctgcgccgagga
gctgagctccatcgtggagggccacgacctcaagaagctcaagtcacatggcggcgtcgagggcctcttgtcgaagctgtccacctcggagtccgacggtgt
cgacacgtcccgcaagaggctgtcgagaagggaggccatcttcggcgtgaacaagttcatcgaggcagagtcccgcggcttctgggtcttcgtctgggaggc
gctccaggacatgacgctcatgatcctcgcggcgtgcgcgttcgtctcgctcatcgtcggcattgccaccgaagggtggcccaagggcgcgcacgacggcct
cggcatcgtggccagcatcctgctcgtcgtgttcgtcaccgcgaccagcgactaccgccagtccctgcagttcaaggacctcgacaaggagaagaagaagat
caccgtgcaggtcacccggagcgggtacaggcagaagctctccatatacgagctcctcgccggcgacgttgtccacctctccattggtgatcaggtgccggcc
gacgggctgttcctgtcgggattctcgctgctgatcaacgagtcaagcttgaccggggagagcgagccggtcgcggtcaacgccgagaacccgttcctcctat

cggggacaaaggtgcaggacggttcgtgcaagatgctcatcaccacggtcggcatgaggactcagtggggcaagctcatggcaactctcagcgagggtggt
gacgacgagacgccgttgcaggtcaagctgaacggcgtggccaccatcattggtaagatcggactcatcttcgccgtggtcacgttcgcggtgctcaccgaaa
gcctgttccgtcggaagatcatggacgggtcgtacttgagctggaccggagatgacgcgttggagctgctcgagttcttcgccattgccgtcaccattgtggttgt
tgctgtacctgaaggactgccgcttgcagtgacgctgagccttgcttttgccatgaagaagatgatgaacgacaaggcgctcgtcaggcaccttgctgcttgcga
gaccatgggctcggccacctccatctgcagcgacaagaccggcacgctgacgacaaaccatatgaccgtggtcaaggcctgcgtctgcggcaaggtcaaag
agctggacggtgtttcagagacaaagaccttgttctctgagttgccggactctgtcatgacgatgctcatgcagtccatattcaacaacaccggcggtgacgttgt
cattaaccaggatggcaagcgggaaatactgggcacaccaactgagacagcgattctcgagttcggcctgtcactcggaggggacttctcagctgtgcgaaaa
gaaagcaccatgatcaaggtggagccgttcaactcggcaaagaagagaatgggcgtggtcattcaactcccagggggcagtgccgttcctctcgatgaagcg
accgtcaagcacttgaatgccacgatcgagagctttgcaaacgaggcacttcgcacgctgtgccttgcctacgcggaagtccctgatgggttctcagccaatgat
cagattccgatggatgggtacacttgcattggcattgtggggatcaaggaccccgtccggcctggcgtcaaggaatcagttgccatctgcaggtctgctggtatt
actgttaggatggtcacaggtgacaacatcaacactgccaaggcaatcgcccgggaatgtggcattttaactgaagatggcattgccattgaaggcccggagtt
cagaaccaagactccagaagaaatgaccgaattgatacccaagatacaggtgatggcaagatcttcgccacttgacaagcacacccttgtgaagcatcttcgga
cttcacttgatgaggttgtcgcggtgactggcgacgggacaaatgatgcacctgcgctacatgaggctgacattgggcttgcaatgggcattgctggaactgag
gtattcaatgaggtgagttcaagagagatggagaagataaatgtatttgaaggcattctagacaacaatgtgtttgtcttcgtcctaagtagcaccgtcatcttccag
ttcatcataatacaatttctgggcgagtttgcaaacactacacctctctcattcatgcagtggatatactgcattttcatcggtttcataggcatgccaatcgctgctatt
gtcaagatgatccccgttggttcttggtag
>g90096.t1
atgggcaactcgtgccgcggctacaccataagcttgtacgattgcgactaccagtaccagcacagcagcagcacctcctattcgtcgtcgtcgaatttctccagc
aggcggagcagcatctgtggaggaggcggcggcatgcgcgggcaccagcagcagctttcgtccccgacggccgtgctcgggcacgtgaccccaccgctg
cgggagctctactcgctggggcggaagctcgggcaggggcagttcggcaccacgtacctgtgcacggatctggccacgggcgcgtccctggcctgcaagtc
cattgccaagcgcaagctgctcaccccggaggacgtggacgacgttcgccgcgagatccagatcatgcaccacctcgccggccatggcagcgtcgtcacca
tcaagggcgcgtacgaggacccgctctacgtgcacatcgtcatggagctctgcgagggcggcgagctcttcgaccgcatcgtcaaccgcggctacttctccga
gcgaaaggccgccgagatcgccagggtcatcgtcggcgtcgtcgaggcgtgccactcgctgggcgcgtgccactcgctgggcgtcatgcaccgggacctc
aagcccgagaacttcctgctgctcaagccccccctcaagatccaataccaagaatcagcagcatgaggacgacgacgacgaggcgtccctcaaggccatcga
cttcggcctctccgtcttcttcaagcccggccagatcttcaccgacgtcgtgggctccccctactacgtggcacccgaggtgctctgcaagcactacggcccgg
aggccgacgtgtggaccgccggcgtcatcatctacatcctcctcagcggcgtgccacccttctgggcagagacgcagcagggcatctttgacgccgtgctcaa
gggcgccatcgacttcgactccgatccctggcccaccatctccgacagcgccaaggacctcatccgccgtatgctacgctcgccgcccgccgaccggctcac
ggcgcaccaggtgctttgccacccatggatctgcgagaacggcgtcgcccccgacgcgccgcttgacccggcggtgctgtcgcggctcaagcagttctcgg
ccatgaacaagctcaagaagatggcgctgcgggtcatcgcgcagaacctttccgaggaggagctggcggggctcaaggagatgttcaaggccatggatacg
gacgccagcggagccatcaccttcgacgagctcaaggaagggctaaggagatacggatcaaacctcaaggagagcgagatcagggacctcatggacgcc
gccgacgttgacaagagcggcaccatcgactacgacgagttcatcgccgccaccgtgcacatgagcaagctcgaccgggaggagcacctcctcaccgcctt
cgcctacttcgacaaggacgccagcggatacatcaccgtcgacgagctcgagcaggcctgcagggaccacaacatggcagacgtcggcatcgacgaaatc
atcagggaggtggaccaggacaacgatgggcgcatagactacggcgagtttgtggctatgatgaagaagggcatcattgggaacggaaggctcaccatgag
gcacacctctgacggcagcgttctccatggcgccggccacctctgctag
>g91314.t1
atgatcgacaaggacaaggtcatgaagattggtctcacggatcagatcaagagggagatctcgataatgagactggtcaggcacccgaacgtcctgcagctttt
tgaggtgatggcgaccaggaacaaaatctactttgtcctggagtacgcgaaaggcggtgagcttttcaacaagatcgccaaggggaagctcactgaggatgct
gcgaggaagtattttcagcagttgatcagcgctgtggagtactgccacagccggggtgtttatcaccgtgacctgaagcctgagaacctgctactggacgagaa
tgagacactcaaggtctccgattttgggttaaagctcaaggagctcctttatggaatcctagatccagatcctaatactaggatgtccatctcaaggctaaagagaa
gtgcttggtacaagaagcctgttggaataacagcaccgaaaactgaaacatgcttcaaaacttgtccaagcgatggatttgatatgtccaagttgtttgaggaaag
atatggccgaagggaggcacgctttacttcgaaacagtcggcaacaacggtctttgcgaagctggaggaaatggccagacgtttgaagctcaaactgacaaat
aaagacaacggattgctgaaattggccacagccaaggaaggcaagaagggtgttcttgagcttgatgcagagatatttcagattgctccttctttcctgttagtcga
gttgaagaagacaaatggtgacacgttggagtaccagaaactgatgaaagatgacattaagccatcacttagagacattgtttggacatgggaaggtgatcacc
agcagtcacatctgttgcggcatgcagagcaacagcagcctgcaatgacaccagttccaccactaacgtaa
>g92472.t1
atgcagccggacccgagcgggaacgccaatgcgaaggcgaagctgccgcagccggtgacggcgccggcgccgtcgtccggccggccggcgtccgtgct
gccgtacaagacggcgaacgtgcgcgacttctaccgcatcggcaagaagctggggcaggggcagttcggcaccacgtaccagtgcgtggacaaggccga
cggcgcagagtacgcgtgcaagtccatccccaagcgcaagctgctgtgccgcgaggactacgaggacgtgtaccgcgagatccagatcatgcaccacctct
ccgagcaccccaacgtcgtgcgcatccgcggcgcttatgaggatgcactcttcgtgcacatcgtcatggaaatctgtgctggcggtgagctgtttgatcgcatcg
ttgcaaagggacactacagcgagcgcgcggccgcgaagctcatcaagaccatcgtcggggttgtggagggggtgccactcgcttggtgtcatgcaccgggac
ctcaagccggagaacttcctctttgccagcacagcagaggacgcacctctcaaggctactgactttgggctctccatgttctacaagcccggtgataaattctctg
atgtcgttgggagcccctattatgtcgcaccagaggtgcttcagaaatgctatggtccagaagctgatgtctggagtgctggggtgatcctgtacattttgctttgtg
gagttcccccattttgggctgaaactgaagcaggaatcttcaggcagattctacggggcaaacttgattttgaatctgaaccctggcctagcatctctgatagtgcc
aaagatctggtccgtagtatgcttactcgtgatcctagaaagagatatagtgctcatgaggttctctgtcacccatggatcattgatgatgccgttgcacctgataag
cctattgattctgctgttttgtcaaggctgaaacatttctcagcaatgaacaagctcaagaagatggcattgagggttattgctgaaagcttgtcagaggaagagatt
ggaggcctcaaggagctgttcaaaatgattgatactgacaatagtgggactataacatttgatgagctgaaaaatggcttgaaaagggttggctcagagttaaca
gagaatgagatccaagctttgatggaagcagacgggcaaattgactacagcgagttcacggcaatgatgaggaagggcaacgctggttgtgcaacaggtagg
aggaccatgaggaccaatttgaatcttggtgaactcttgaatcccagcaacagctag
>g92837.t1

atggcggccggcggggtggggccggcgcagcaccagacgcccaacggcagcagcaacggcgttgctggggcggcggcggcggccacgccgctccac
ggctccgcgacatccaccgtcaacggcgccgccgatgggtacgacagcgacggctacagcttcgcgccgcctacgccatcaactttatcaatgtccatacctc
cggagcttgcaggagcaattccactgattgatagattccaggttgagggatttcttaaggcaatgcagaaacagattcattcagctggaaaacgtggattttttcaa
agaaatcagttgggccccaagctcgtgagaagttcactttggaagatatgttgtgcttccaaaaggatcctattcctacatcgttactgaaaataagtagcgaccttg
taagccgctcaattaagttgttccatgtcatactaaagtacatgggcattgattcgcctgcaataataagtatggatgaaagaatagaacttgttgcgaagctttacaa
gcatacattgaagcgttctgaacttcgagatgaactctttgcacagatttcaaagcaaacgcgtaacaatcccgacaggggttggtcaataagagcctgggagct
tatgtatctttgcgcgtcgtccatgccaccaagcaaggatattggggcatacctgtctgaatatgttcactatattgctcatggagccacaactgattctgatattcgc
gttttagcgttgaacacactaaatgcgttgaaacgttcagttaaggcaggccctagggttacaatccctgcacgtgaagagattgaagctcttttaaccagccgga
agcttacaacaattgtatttttcttggatgaaacttttgaggaaatcacctatgacatggcaacaactgttgctgatgctgttgagctatcatatgtccagttgcaacat
gattatattttgggaaactatccagtaggaagagacgatgctgcacaactctctgccctacagatattagttgagattggtttcattgataatcctgagtcttgtgtgta
a
>g96026.t1
atgcgagcgcactccgaactcggccttgcagcgagacctagcaccgtctacaccaggcacaagtcctctgtattgcactctcagcccaaatcaactgttagcac
accagcttacattgctccagaggtccttgctagaaaggagtatgatggaaaggttgctgatgtttggtcctgtggagtaactctttacgtgatgcttgtcggggctta
tccctttgaagatcctgatgaaccgaggaatttttgcaagacacttactcggattctcagtgtacaatatgcagtccctgattttgttcgagtttcaatggagtgcaga
catttgctatcccggatcttcgtggcaaaccctgagcaacgaataaccatcccggagatcaagaaccacccatggttcctgaagaacctccccatcaagatgac
cgacgagtaccagacgaacctgcagatcatcaacatgaacgttccctcgcaatgcttggagatcatggccgtcatacaggaggccatcatggccgtcatacata
caggaggcccggaagcctggcaaatgggctga
>g96774.t1
atggacaagtacgagctgctcaaggacatcggcgccggcaacttcggcgtcgcaaggctcatgcggcacaaggagaccaaggagctcgtcgccatgaagta
catcccgcgaggccagaagattgacgagaatgtggcgagggagatcatcaaccaccgctcgctgcggcaccccaatatcatccggttcaaggaggtgctggt
cacgccgacgcacctggcgatcgtgatggagtacgccgccggaggcgagctgttcgaccggatctgcaacgccgggaggttcagcgaagacgaggccag
gtacttcttccagcagctaatttgcggtgtgagctactgccacttcatgcaaatttgccaccgagacctgaagctggagaacacgctgctggacggcagcccgg
cgcctcgactcaagatctgcgacttcggttactccaagtcgtcgctgctgcactcgaagcccaagtcgacggtgggcacgccggcgtacatcgccccggaggt
gctctcacgccgggaatacgacggcaagaggataatgtctatccagtacaaaataccggagtatgtccatgtatctcaggactgcaaggatatgcttgcaaaaat
tttcgtcgcaaaccctgcaaagagaattacaatcagggaaatcaggaaccacccctggttcctaaagaacttgcccagagaactcacagaagccgcacaggca
atgtactacaagaaggataacagtgccccaacttattccgtccagtcagtcgaggagatcatgaagatcgtcgagcaggcacggacgccacctccttcctccac
ccctgtggctggctttggttgggtagaggaggatgaagagaacagcaagaaaccagaggagaaacaggaggaagaagaggatgctgaagatgaatatgag
aaacaagtgaatgaagtccgcgccagcggtgaatttcagatcagctga
>g97633.t1
atgggcgcttgcttctcctccgcctccgctgccaccgccgccgccgggccgcccaaggcggagaagaggaagcgccgcggcgccgcctcgccggacgga
caggggaaggcggtgcgggtggagttcggctacgacagggatttcgaggcgcggtacgaggtcggcaggctcctcggccatggacagttcgggtacacctt
cgccgccaccgaccgcggctccggggaccgcgtcgccgtcaagcgcatcgacaaggccaagatggaccgccctgttgccgtggaggatgtgaaaagaga
agtgaagattcttaaagcacttcaaggacatgagaatattgttaacttctacaatgcatttgaggatgattcgtatgtgtatattgtgatggagtaa
>g98523.t1
atgggtctctgctatggcaagtcagcggcggctccggagccggcgggggtggaggaagcctgcgtagccaacggcgccggtgacccggtcccggacagc
gtcgcgtcgccggcgaagccccggacaccgaagcagcccaagttctcgttctacctgccgagcccgctcccgccttcgagctacaagggttcgccggcgaa
ctcgagcgtggcttccacgcccgcgcgcggcgggttcaagcggccattcccgccgccgtctcccgccaagcacatccgcgccctgctggcgcggcggcac
ggctcggtgaagccgaacgaggcttccatccccgagggcggggagccggagctgggcctggacaagagcttcggctactcgaagcacttctcggctaagta
cgagctcggccgcgaggtggggcgcggccacttcggctacacctgcgccgccaaggccaagaaaggcgagctcaagggcgaggacgtcgccgtcaagg
tcattcccaaggctaagatgacaactgctattgccatagaagatgtcagaagagaagtcaaaatattgagttctttgacaggtcacagcaacctagtgcaattttat
gatgcttttgaggatgaagaaaatgtatacgttgttatggagctatgcacaggaggtgaactgctggacaggatcttggctagaggtggaaagtattctgaagaa
gatgccaaggttgtcattcatcaaattctaagtgttgcttcattttgccatcttcagggtgttgttcaccgggatctgaaaccagagaatttccttttcatgtccaaggat
gagaattctgccttgaaagtcatagactttggtttgtctgactttgttaggccaggttaa
>g100730.t1
atgcagcaggagcagcccaagaagagttctgcagaggccgagtttttcacagagtatggcgatgcaaaccggtacaagatccaagaagtcatcggtaaagga
agctatggtgttgtgtgctctgccattgatttgcacactcgacagagagtggcgatcaagaagatacacaatatctttgagcacgtctctgatgctgcaaggatcct
ccgtgagattaaacttctgagactcctaaggcatcctgacattgttgagatcaagcatattatgttacctccttcgagaaaggacttcaaggatatttatgttgtttttga
gctgatggagtctgatctccatcaagttataaaggccaacgatgacttgacaaaagagcattaccaattctttctctatcaattgctccgtgccctcaaatacattcat
actgtgaacttaactgcagctaatgtttatcaccgtgacctgaagcccaagaacattttagcgaattctaactgcaaactgaaaatatgtgactttggactagcacga
gttgcattcaatgatacaccaacaacagtcttctggacggattatgtggcaacaagatggtacagagctccagagctctgtggatccttcttctcaaaatatacacc
ggctattgacatttggagcattggatgcatctttgctgaggtattgactgggaagcctctgtttcctgatggattaggatttgctgagtttgttgttccctccatcctcgg
caggttcggaatgagaaagcaagaaggcattgcgtga
>g100925.t1
atgggtaaggagaagagtcacatcaacattgtggtcattggacatgttgactctggcaagtccaccaccactggccacctgatctacaagcttggaggaattgac
aagcgtgtgatcgagaggttcgaaaaagaggcagctgaaatgaacaagaggtccttcaagtatgcctgggttcttgacaagctcaaggctgagcgtgagagag
gctga
>g102361.t1
atgggcaactgctgcccgggctccggggatgcggagcccgccgccggcgacgcctcgtcccgcctccacgccgcctccctcaaggccggcggcagcggc
agcggcgccgggccgacgtccgcgcccacgcccaccaagccgccggcgcccatcgggcccgtcctgggccggcccatggaggacgtccgcagcatcta

caccgtgggcaaggagctgggccggggccagttcggcgtgaccagcctgtgcacgcacaaggccacgggggagcggttcgcgtgcaagaccatcgccaa
gcggaagctgtccaccaaggaggacgtggaggacgtgcggcgcgaggtgcagatcatgtaccacctggcgggccagcccaacatcgtcgagctcaaggg
cgcctacgaggacaagcagtccgtgcacctcgtcatggagctctgcgcgggaggggagctcttcgacaggatcatcgccaagggcaagtacacggagcgc
gaagccgccgcgctgctgcgcaccatcgtcgagatcgtgcacacgtgccactcgctcggcgtcatccaccgtgacctcaagccagagaatttcctcctcctca
gcaaggacgagcacgcgccgctcaaggccaccgatttcggcctctccgtattcttcaagcagggggaggtgttcaaggacatcgtgggaagcgcctactacat
cgcgccggaggtgctcaagaggagctacgggccagaggcggatatctggagcgtcggagtcatcctctacatcctactctgcggagtgcccccgttctgggc
aggcaagtcgttcatctctgtcttcatccatcacggcatcttcaacgccatcctccggggccaggtcgacttctccagcgacccgtggccgcgcatttcctccggc
gccaaggacctcgtcaggaagatgctcacctctgaccccaagaagaggatctcggcttacgacgtcctcaatcatccctggatcaaggaagacggtgaggcg
cctgacacgctgctggacaacgctgtcatgagcaggctcaagcagttcagggctatgaaccagttcaagaaagccgcgctcagggttatcgccgggtgcctgt
cggaggaagagatcagggggctcaaggagatgttcaagggcatggacgccgacaacagcggcaccatcaccgtggacgagctgcggcgagggctggcc
aagcagggcaccaagctctcggaggccgaggtggagcagctcatggaggccgccgacgcggacgggaacggcaccatcgactacgaggagttcatcacc
gcgacgatgcacatgaaccggatggacagggacgagcacctctacgccgcgttccagtacttcgacaaggacggcagcggctgcatctccaaggaggagct
ggagcaggcgctcagggagaagggcctgctcgacggccgggacatcaaggacatcatctccgaggtcgacgcagacaacgacgggaggatcgactacag
cgagttcgtggccatgatgaggaaagggaaccccgaccccaaccccaagaagcggcgcgacgtcgtgctgtag
>g104248.t1
atggagcggtatgagttggggagactgttggggaaaggcacatttggcaaggtgcactatgcaaggaaccttgagtccaatgagagcgtcgccataaagatga
ttgataaggacaaagcactgaaggttgggctttcagagcagataaaacgtgagatcacaacaatgcggttggtggcacataagaacattgtcgagcttcatgag
gtcatggctacacggaacaagatctactttgttatggagtatgtcaaaggtggagagctctttgacaagattgagaaaagtggccggctcacagaggaaatagca
cataagtatttccagcagcttattagtgcagtggatcactgccacagtcgaggtgtgtttcaccgagacttgaagcctgagaacctgctgctggatgagaatgaga
acctgaaggtctcagactttgggttgagtgcactttcagaatcaaggagacaagatggattgctccatacgacctgtggaaccccggcatatgtagctccagagg
tgatcagcaagacaggctatgacggtgcgaaatcagatatatggtcttgtggtgctgtcctatttgttcttgttgctggttatctccctttccaaggatcaaatttgatg
gagatgtatcggaagatccagcaaggtgatttcaggtgccccagttggttttcacacaaactcaaaaagctgttgtacaagatcctggaccccaacccaaacacc
agaatttcaatccagaagataaaagagtctacatggttccggaaaggtcctagntcttgtggtgctgtcctatttgttcttgttgctggttatctccctttccaaggatc
aaatttgatggagatgtatcggaagatccagcaaggtgatttcaggtgccccagttggttttcacacaaactcaaaaagctgttgtacaagatcctggaccccaac
ccaaacaccagaatttcaatccagaagataaaagagtctacatggttccggaaaggtcctagtgagatccgtgcagtaaaggagagaactcctagtgaaaatgc
catcactaatactacttcaatacatggtgccgcaaggcgcaagaagattgctcttgaagatgtgaagcccctgactgtggcaaacctaaatgcctttgaaattatct
ctctctccacagggtttgatctgtctggcctattcattgaaaaggactgcagaaaggagacaagatttacatcagataagtctgcctcagcaatcatctcaaagctt
gaagatgttgcaaagagcctgaatcttagggttaggaagaaagataatggtatagtcaagattcaagggaggaaggntagggttaggaagaaggataatggta
tagtcaagattcaagggaggaaggaaggaaggaatggtgtcatgcagtttgatgcagagatttttgagatcacaccttcccatcatcttgttgagctgaagcaaac
aagtggtgattccatcgagtatcggaaactattggaagaaggcatccggccagcgctgaaagaaatagtctgggtctggcatggggatgatcagcaacagaag
caagagtag
>g107035.t1
atggaccgcggcacgcgggagctgctggcctgcaagtccatctcgaagcggaagctgcggacgcccgtggacgtggaggacgtgcgccgcgaggtggcc
atcatgcggcacctgcccaagagccccagcatcgtgtcgctgcgggaggcgtgcgaggacgacggcgccgtgcacctcgtcatggagctctgcgagggag
gggagctgttcgaccgcatcgcgtgcgaggacgacggcgccgtgcacctcgtcatggagctctgcgagggaggggagctgttcgaccgcatcgtcgccag
gggacactacacggagcgcgcggcggccgccgtcgtgcgcaccatcgtcgaggtcgtgcagctctgccaccgccacggcgtgatccaccgcgacctcaag
cccgagaacttcctcttcgcaaacaagaaggagaactccccgctcaaggccatcgattttggcctctccatcttcttcaagcctggtgaaaaattttcagaaatagt
gggaagtccctactacatggctcctgaagtgctgaagaggaattatggtcctgaaatagacatctggagtgctggtgttatcctgtatatattgctatgtggtgttcct
ccattttgggctgaggttgaggacataaaggaaatgttcaaagtgatggacacagacaatgatggtatagtttcctatgaagaattgaagagtgggatcgcgaag
tttggttcccatcttgcagaatctgaagtccaaatgcttattgaagctgtggatacaaagggtaggggagcactagattatggtgaatttttggctgtctcacttcattt
acaaaggatggcaaatgacgagcaccttcggcgggccttcctgtttttcgacaaggatggaaatggttacattgagcctgaagagcttcaggaagcccttgtgg
aggatggaggagctgatagcatggacgtggtcaatgacatattgcaagaagttgacactgataagaactga
>g107749.t1
atggatgaggtggagcagacgctgacaaaggagcagatcgaggagttccgggaagccttcagcctcttcgacaaagatggcgatgggacgatcacgagcaa
ggagcttggcacggtgatgcgttcgctgggtcagagcccgacggaggcggagctgcaggagatggtggcggaggtggacgccgacggcagcggcgcca
tcgatttccacgagttcctcgtcctcctcgcccgcaagatgcgcgacgccggcgccgacgacgagctccgggaggccttccacgtcttcgaccaggaccaga
acggatacatctcccgcgacgagctgcgccacgtcctcgagaacctcggcgagaagctctccgacgaggagctcgccgagatgctgcgcgaggccgacgt
cgacggagacggccagatcaactacaacgagttcgctaaggtcatgatggccaaacgaagaaaccagatgatggacgacgaggacagtggatcgcattccc
acgggaggagcccctgctgtacaatcctctga
>g107750.t1
atgagtacaaccaaggtgaagagacgtgtggggaagtatgagctgggccgaaccataggcgagggaacattcgcaaaggtcaggtttgcacgggacactga
gacaggcgacccggtggccatcaaaatcctggataaggagaaggttctcaagcacaagatggttgagcaggtgatgggaagtaaaacaaagatctacattgtg
ttagagtatgtaaccggtggtgagctctttgacataattgttaaccatggcagaatgagggaagatgaggcaaggagatactttcaacagttaatcaatgcagttga
ttattgtcatagcaggggcgtgtaccaccgggatttaaaaagaataacaatccctgaaatactagaggatgagtggttcaaaaaaggctacaagcgcccagagtt
tgatgaaaaatatgacacaacgttggatgatgtggatgctgtcttcaatgattcagaagagcaccatgtgacagaaaggaaagaagaagaaccagcaagtctga
atgcgtttgaactaatttcaatgtcagaaggtctaaaccttggtaacttattcgactcggagaaggaatacaaaagagaaacaaggttcacatcaaaatgtccaccc
caagaaattgtccgcaagattgaggaagcagcaaagcctctaggatttgatgttcaaaagaaaaattacaaggttccttga
>g112607.t1
atgaatgctgagaagtatgatgaattctggaaatctattgaagacatggcagcaacctcattacgctgtgttgcttttgcatatcgtccctgtgagcctgaaatgatac
cagaggacgacatagctaactggaaattgcctgaggatgacctgactctgcttggcatcataggaataaaggctgatataggtctttcaatgggcatctcaggga

cagaagttgctaaggaaagttctgacattataatcttggatgatgactttacatcagttgtcaaggttgttcgttggggccgatctgtctatgcgaatattcagaaattc
atccagttccagctcacggttaatgttgccgcccttgtaataaatgtggttgctgctgtgtcatctggtgatgtgcctctgaatgccgtagaggttgggaccttttcttg
a
>g113382.t1
atgggggtcttccaccgggatctcaagcccgagaacttcctcttcctcaacaacaaggaggactcgccgctcaaggccacggactttggcctctccgtcttcttc
aagcccggccatccatggattagagaagacggagaggccccagataagccacttgacattacggtcatcggtagaatgaaacagttcagggcaatgaacaag
cttaagaaagttgcattgaaggttgttgccgagaacttatcagatgaggagattatgggcttgaaagagatgtttagatccttggataccgataacagtgggacaat
tacgcttgatgagctaagatctggtttaccaaagcttgggactaaaatttctgaatcagaaattagacagataatggaggcggctgatgttgatggaaatgggacc
attgattatgcagagttcatatcagccacaatgcacttgaatagattggagaaggaagaccacatactcaaagcattcgagtattttgataaagaccacagcggat
acataactgtagatgagctggaagaagctctcaagaagtatgatatgggagatgataaaacaattaaagaaatcattgcggaagtagattcagataatgatggaa
gaattaactaccaggagtttgttgccatgatgaggaacaacagccctgagattgttccaaaccggaagcgcatgttttaa
>g113452.t1
atgggcaacgtgtgcttctgcggcacgacgtccacctcccctgacgaccctccatctgatcccaaaaaacccggagcgcctccgcaacagcaaggcaagag
gccggcgacgcctccaagcagccgccaggagaagagccccacccccaggtcgaagcagcagcccaagccccggacgcgcgccaagcccaagcccaag
cccaccccctacgagtacgactgggcgtcgccgccgtcgcgcgtgctggacggcgtggtcccgcaccacccgcgtctccgcgtgacggacaagtacacgct
gggccgtgagctgggccgcggcgagttcggcgtcacgcggctggcgacggaccggggccacgcgggagcggctggcgtgcaagtccattcccaagcgcc
gcctccgcacggccgtggacgtgggcgacgtccggcgcgaggtggccatcatggcgtcgctccccgaccacccgtcgctggtgcggctccgcgcggcgta
cgaggacgccgacgccgtgcacctggtcatggagctctgcgacggcggggagctcttcgaccgcatcgtggcccgcggccggtacacggagcgggccgc
ggcggcggcggcccgcacggtcgccgaggtggtgcgggcctgccacgcgcacggggtgatgcacagggacctcaagcccgagaactttctgtatgaggg
gaggggcgacgacgcaaggctcaaggccatcgactttgggctgtcggtgttcttccggcccggagagcggttcacggagatcgtgggcagcccgtattacat
ggcgcccgaggtgctgcgacggagctacgggcccgaggtggacgtgtggagcgcgggcgtgatactgtatatcctgctgtgtggggtgccgccgttctggg
ccgagacggagcagggcgtggcgagggccatcctgcgggggagcctggacctggaccgcgagccctggcccaggatctcccacggggccaaggacctc
gtcaggcagatgctgcagatggacccaaagaagcggccaaccgcgcagcaagtcctcgagcacccgtggctgcagaacgcgcggaaggcaccgaacgtg
ccgctgggcgacgtggtgcgtgcgcggctgcagcagttctcggccatgaacaagttgaagaagaaggccatgcgggtcatcgcggagcacctatccgtgga
ggaggtggaggtgatcagggacatgttcgcgctcatggacacggacaaggacggcaaggtcacgctgcaggagctcaaggcggggctcaagaaggtcgg
atccaagctcgccgacccagagatggagctgctcatggaggccgccgacgtgaatggcaacgggtacctggactacggcgagttcgttgcaatcaccatcca
cctgcagcgcctctccaacgatgaccacctccgcacggcgttcctcttcttcgacaaggacagcagcggttacatcgagcgcgccgagctggccgacgcgct
cgccgacgactccggccagaccgacgacgccgcgctcaacaacgtcctccgagaagacggtcggataagtttcgacgagtttgttgccatgatgaaagccgg
cacggactggagaaaggcgtcacgacagtactcaagggagcggttcaagactctgagctacagcctcatcaaggacggctcgctcgccatggcgcactga
>g114194.t1
atgggcgcctgcttctcctccgcctccgctgccaccgccgccgccgggccgcccaaggcggagaagaggaagcgccccggcgccgcctcgaccgacgga
caggggaaggcggtgcgggtggagttcggctacgacagggatttcgaggcgcggtacgaggtcgggaggctcctcggccatggacagttcgggtacacctt
cgccgccaccgaccgcggctccggggaccgcgtcgccgtcaagcgcatcgacaaggccaagatggaccgccctgttgccgtggaggatgtgaaaagaga
agtgaagattcttaaagcacttcaaggacatgagaatattgttaacttctacaatgcatttgaggatgattcatatgtgtatattgtgatggagtaa
>g116892.t1
atgggcaacatctgcgccgggtcgcggtttcccaagctcggctccatctggccgtcgcgctcctccacgccgtcccagcccaccaccacctcccgctccgtcc
ccgtcgtccaggtccagcccagcgaatccacctccaccgccgactccaagccgcttccggtcccggcagccgcgcaaacccacacctcctgctcccatcgtca
tttccgaaccagcacgacactcatcatcacaatcagagcccccacggccacaaccaaaaagagaggacgcgacgtcccaacaaccagcgccttcctcgtcgt
cgcagccgcaggcgaagaagaagcccgcgcacatcaagcgcatctccagcgcgggtctgcaggtggaatccgtgctgcgtcgcaagaccgagaatctcaa
agacaagtacagcctgggacgaaaactcgggcagggccagttcggcacgacgtacctgtgcgtggacaaggccacggggcgtgagcacgcgtgcaagtc
catcgccaagcggaagctggtcaccgacgaggacgtggaggacgtgcgccgcgagatccagatcatgcaccacctcgccggccacccaaacatcatctcc
atcgtcggggcgtacgaggacgccgtggccgtgcacgtggtgatggagctgtgtgcgggtggggagctgttcgatcggattgtgaggaggggccattactcg
gagcggcaggcggcggcgctggcgcgggtcatcgttgcggttgttgagtcgtgtcattcgttgggggtcatgcaccgcgacctcaagccggagaatttcctgtt
tgttgggaatgaggaggatgcgccgctcaagaccatcgatttcggactctccatgttcttccggccaggcgaggcgttcactgacgtggtgggaagtccgtact
acgtggcgccggaggtgttgaagaagaattacgggcaggaggcggacgtgtggagcgccggcgtcataatctatatcctgctatgcggcgtgccgccgttct
gggcggagacggagcagggcatcttcgagcaggtgctgcacggctcgctggacttcgagtccgacccatggcccaacgtgtcggacaacgccaaggacct
gcttaggaggatgctcgtcagggaccccaagaaacggctcactgcgcaccaagtcctctgccacccgtggcttcagatgatcggctcggcgcccgataagcc
gctagactcagcggtgctctcgcgactgaagcagttctcagcgatgaacaagctaaagaagatggccttgagggtgattgcggagaacctttcagaagaggag
attgctggtctgaaggagatgttcaagatgatggataccgacaacagtgggcagattaacttcgaggagctcaaggcggggctccatagagtcggcgccaata
tgaaagagcccgagatacatcagctaatgcaggctgccgatattgacaacagtggtaccatagattatggtgagttcatagctgctactttgcacctcaataaagtt
gaaagggaggatcatctgttcgctgccttccaatacttcgacaaagatggaagtgggtacatcacagctgatgagctccagcaggcatgcgatgaattcggcatt
gaagatgtccgattggaagacatgatcggtgaagtagatcaagacaatgatgggcggattgactacaacgagttcgtcgccatgatgcagaaatcaactagtgg
ttttgggaagaaagggagcagatcagaccaggacggggatcataaacgaggttgcttgaggatcctgtggatcttcttgccagggcggaggggttgggtcacc
ctgcgcaattccagctcaggcagcgtgatcaggggcatcctcggcagggcggcaccactcactggactccgcaatatattccaagcagcggcagtttctggat
ctatagaatcagtagtctccggcagcaggtag
>g118827.t1
atgggtaaggagaagagcaagagtcacatcaacattgtggtcattggtcatgtcgactcaggcaagtcgaccaccactggccacctgatctataagcttggtttta
ttgataagtatgagattgagaggctcgagaaggaggctgctaaaatgaacagaagctccttcaagtatgcatgggtgctggacaagctcagggccgagcgtga
gagaggtattaccattgatatcaccatgtggaagttcgagactgccaagtaccgctggactgtaattgatgcccctggacaccgtgacttcataaaaaatatgatc
accggtacctcccaggctgactgtgctattcttgtcattgattcttgttgttttgaggctggcatgtccaagaagggtcaaacccgtgagcatgcactccttgctttca

ctcttggtgtgaagcaaatgatttgttgctgcaacaagatggatgccaccacacccaagtactcaaaggtccgttatgatgtgattgtgaaggatatctcttcttatct
caagaaggtcggctacaaccttgacatgattccctttgttcccatctctggttttaatggcgataatatgattgagaggtccaccaaccttgactggtacaagggccc
gacattgcttgaggctcttgaccagatcaacgagcccaagaggccctcagacaagcccctgcgtctccccttcaggatgtatacaagattggtggtattggaac
tgtccctgtcggtcgtgttgagactggtgccctcaagcctggtatggttgtcacctttggtcccagcggcctgagtactgaggccaaatctattgagatgggcaac
aagactctccaggaggcctttcctggtgacaatgtagcattcaacgtgaagaacgttgctgtgaaaaatctccagcgtgggtatgtggcctccaactccaaggat
gaccccgccaaggaggctgccagcttcaactccctggtcatcatcttgaaccaccctggaaagatttgcaatggctacaccccaattatggactgccacacctcc
cacattgctgtgaagtttgctaaactgctaagcaagattgacagacaatctggcaaggatattgagtggaagcccaaatttctcacgaatgattcccaccaaaccc
atggtggtggaggccttctctaa
>g119568.t1
atgggccaatgctgcgccaagggcggcgaccaggccgccggcgacgacaaggaccaccaccccgccgcgtcgtcgtcgtcggatcccaagccggatgcc
tccacccgcggcggcggcgccaacaccaacgccccgccgccgcgcccgtcgtcgtcctcctccaaccacccggctgcggcggcgggcgcgtcggtgggc
ccggtgctgggccggcccatggaggacgtgcgggcgacgcactcggtggggaaggagctgggccggggccagttcggcgtgacccacctgtgcacacac
aagcccacgggggagaagctggcctgcaagaccatcgccaagcggaagctctcctcccgggaggacgtggacgacgtccgccgcgaggtccagatcatg
caccacctctccggccagcccaacgtcgtctccctgaaaggcgcctacgaggataagcacaacgtacacctcgtcatggagctctgcgccggcggcgagctc
ttcgacagggtcatcgccaaagggcagtacacggagcgcgccgccgcggcgctgctccgcaccatcgtgcagatcgtgcacacctgccacgccatgggagt
catgcacagggatatcaagccggagaacttcttgttgcttagcaaggacgaggacgcgccgctcaaggcaacggatttcgggttatccgtcttcttcaaggaag
gggaggtgttcagggacattgttgggagtgcgtattacatcgcgccggaggtgcttaagaggaagtatgggccggaggcggatatatggagtgttggagtcat
gctatacatcttccttgctggagtgcctcccttctgggcggagaatgagaatggtatcttcactgccatcctgcgtggccagattgacctcgccagcgacccctgg
cccaagatttcgtcgggagcaaaggatcttgtcaagaagatgctcaacatcaaccccaaggagcggcttaccgcgttccaggtcctcaatcacccgtggatcaa
agaagatggagatgcccctgatacgccacttgacaatgttgtcctcaacagactcaaacagttcagggccatgaaccaattcaagaaagcggcactaaggatca
tagctgggtgtctatctgaagaggagatcaccgggctaaaggagatgtttaagaacatcgacaaggataacagtgggaccattacgcttgaagagctcaagaat
ggcttggcaaagcacggaaccaagctgtcggacggtgaaattcagcaactcatggatgcagctgatgctgatggcaacggattgatcgactacgacgaatttgt
caccgcgacggtgcacatgaacaaactggacagagacgaccacctctacactgcattccagtatttcgacaaggataacagtgggtacatcacaaaagaaga
gcttgagcaagctttgaaggagcaaggattatatgacgccgaggagattaaggacgttatttcagatgctgatgctaacaatgacgggaggatagattattcaga
gtttgtggcgatgatgaggaaaggtactgctggcgctgagacggcgaaccccaagaaaaggcgcgatctagtactgtag
>g119576.t1
atggatgaggtggagcagacgctgacaaaggagcagatcaaggagttccgggaagccttcagcctcttcgacaaagatggcgatgggacgatcacgagcaa
ggagcttggcacggtgatgcgttcgctgggtcagagcccgacggaggcggagctgcaggagatggtggcggaggtggacgccgacggcagcggcgcca
tcgatttccacgagttcctcgtcctcctcgcccgcaagatgcacgatgccggcgccgacgacgagctccgggaggccttccacgtcttcgaccaggaccagaa
cggatacatctcccgcgacgagctgcgccacgtcctcgagaacctcggcgagaaactctccgacgaggagctcgctgagatgctgcgcgaggccgacgtcg
acggagatgggcagatcaactacaacgagttcgcaaaggtcatgatggccaagtga
>g120444.t1
atggattttgttaagcgtttgctgtgcaaggatccacgtagaaggatgactgcagcacaggctttaagtcatccatggatcagaaattataatgacattaagctgcc
attggacgtccttatattccgacttatcaaagcatatatccgttcctcatctttacgaaaagctgctttgagggttgttcttaaactggctctatcaaagactttaactgtt
gatgaacttttctatctgaaagcacaattttccttgttggaaccagacagaaatggatgcattactcttgataatatcagaacggcattaacaagggaagctactgat
gcaatgaaagaatcacgagttcaagagattcttgtttcgttgagcgctcttcagtacagaagaatggacttccaagaattctgtgcagcagcagttagtgtgcacca
gcttgaagcattggatagatgggagcaacatgcccgatctgcttatgaatattttgagaaggatggcaatcgtgctattgtaattgatgaactggcttctgaattggg
cctcagcccttccgtgccgttgcatgttgttctgcaagattggatcagacatactgacgggaaactgagctttcttggttttgtcaagttattgcatggtatgtccagc
aggtccatgtcaaagatgaggtag
>g121814.t1
atggaggagatgggggcggggccggtgagcgaaacctctgctcgcgctggtagctcgccgcggggtaggacctcagccttcttggctcggagccgggcct
caacaccggcgacagtggagatcccgagcttgtccggccatgaaagatggcaagctcgccgcccttctcaagcccgcgtcggcaccgccgccccgttggcc
cctggtctcgccctgcccgccccgagcgccgcccgcgcccaggcctcgtccatggcgagctcgttgcccgtgccaagcctcccgagcttgcctctcggcgcc
gccctccgccaccccgcctcccggtgccgccctccgccgcccctcccacgcccgcgctctcgtttgcgccccgcgctctcgtttgcgccccgcgccgccgct
gtcgagttccggtgcgagcaggagatggagaagccgagatcggggtggtgcggtgtcatctctcctgaggaagctgctgcggcggcgatggccggggtgc
ggaaccctgaggcgtcgcccgggcgctacgtgcggcgccgggacgagatcgacgacggctgcgacgacgtgctcggtgtcgacgtcgggggcgcgggg
gccgaccccttcgacatccccgccaagcgcgcctccgtcgagcggctgcggcgctggaggcaagctgctcttgtcctcaatgcttctcgacgattcagatatac
tcttgacctaaaaaaggaggaggagaaggaacatataaggaggaaaatcagggctcatgctcaagtcatacgggcggcactgctttttaaagaggctggagaa
aagaagaatggtgacagggaattgccagctgtcagtgattataaacagtccctgcagttccaacacctaaatgaggagaagcaaaacatccaagtggaggttat
taggggtggtagaaggattccagtgtcaatattcgatattgtagttggtgatgtagtggctctaaaaattggtgatcaggtcccagctgatggtgttttaattaatggt
cattctcttgccatcgacgagtccagtatgactggggaaagcaagattgtaacggcagtcggcttaaacactgaatggggtttattaatggccagcatttcagaag
acaataatgaagaaactccattgcaggtgcgattgaatggagtagcaacactcataggcatcttaggactttctgttgctgccctggtccttatagtcttgtttgcaag
atattttacaggacatactacagactcgaatggaacagttcagtttattaaagggcagacaagtgcaaaatctgcaatatttggttcaataaagatactaactgttgc
ggtaactattgttgttgttgctgtgcctgagggcctaccactggctgtaacattgaccctggcctattctatgcggaaaatgatggctgacaaagcactggtccgga
ggctttctgcctgtgaaacaatgggttctgctactacaatttgcagtgacaagacaggcacattaactttgaaccagggagattcaaatgttcatgtacactggaaa
ggagctgctgaaatagttcttgctttatgtacaaattggcttgacacggatggttcaattcatgaaatgacaactgataaggctgatcagttcaaagaatacattgagt
atatggccgagcaaagtcttcggtgtgttgcttttgcatacagaactatcgagaacgatgtcccgagtgaagagcaaagaatcaactggcaattgccagataatg
accttactctaattggaatagtgggaatgaaggatccttgtcgccctggagtaagagaggctgttgaattgtgcatcaatgctggtgtaaaggtgatggcaagatct
tctcctaatgacaagcttctactcgtaaaagctcttaagaaaagaggccatgtcgttgctgttactggtgatggtacaaatgatgctcctgcattgcatgaggctgat
attggtctttctatgggcatccaaggaacagaagtagctaaagaaagctcagatattattattcttgatgacaatttttcgtcagttgtgaaggtggtccgctggggtc

gttctgtttatgcaaatatccaaaagtttattcagttccaacttactgtaaatgtcgcggctcttgtcatcaatgtggttgctgccatttcatcgggaaatgttcctctaaat
gctgttcagctgctctgggtaaacctcatcatggacacacttggagctcttgcattggctaccgaaccacctacagatcagcttatgatgcggccacctgtcgggc
gaagagaacctcttgtgactaatatcatgtggagaaacttattcattcaggctgtctttcaagttgctgttcttctgaccctcaactttaggggccgagatctcttgcac
ttgacccaagacaccctggatcactccagtaaagtgaaaaatacagttatattcaatacatttgtcttgtgccaggtgtttaacgagttcaattctcgtaaaccagaag
aactgaacatctttgatggggtttcaagaaaccatcttttcttgggagtagtgagcataactgtcattctgcaggtgataattattgagttccttggaaagttcacgtca
acagtgagactcagctggaagctgtggcttgtttctgtagttattgcatttgtcagttggcccttggctttagttggaaaattcattccagttccaaagacaccattgaa
ggattttctcatgagatgttggcccaaacgaaggaagcaaggtgacgacggagcaacaccaccggtgtga
>g122894.t1
atgtcatcgcctaacaggagagaagatccgacggagcaatttgagtcgctgtcaccggtggagtccacgtggatgatcgtggacatccaagggtccgacgtag
tcgaagaaggggcggcgcagccaaagaatgggaggcggcgcagggggaggaaggagaggcgcggccagggggtggcggcgctggttgttaccgtgcc
ctggatcagacggcggcgtttggtgggtggagaggcagcggaagggaaggcacgaaatcagggataccctggactggagaagcttcatgttcttgtgctggt
atcaaaatccaccctgcagtatgaacatggtgtttcgcttcagagtgagtatgttgctcctgaaagtgttaaagctgctgggtttcagatttgtgctgacgaactggc
atcaattgttgaaactcgtgacaatgaaaagttgtctatgcatggccagttggatggaattgcaggcaaattggtgacatcattaactgatggaattagtacagatga
gtatatcctgaatctaagacgagacatatatggagtgaacaagtttaccgagggtgaggagaagcttcatgttcttgtgctggtatcaaaatccaccctgcagtatg
aacatggtgtttcgcttcagagtgagtatgttgttcctgaaagtgttaaagctgctgggtttcagatttgtgctgacgaactggcatcaattgttgaaactcgtgacaat
gaaaagttgtctatgcatggccagttggatggaattgcaggcaaattggtgacatcattaactgatggaattagtacagatgagtatatcctgaatctaagacgaga
catatatggagtgaacaagtttaccgaggttacaatcgttgttgttgctgttcctgaggggcttcctttagcagttacattgagccttgcatttgcaatgaagaagatg
atgaatgacaaggctctggtccgacaattagctgcctgtgaaacaatgggctccgccacagtcatttgtagtgacaagacaggaacactaacaaccaattgcatg
tctgtcgtgaaggcctgttctgtggcaacatgccgatctgctggcatttcagttcgaatggttactggagacaacataaatacagcaaaggcaattgctcgtgaatg
tggtatacttacagaggatggcctttcaattgaaggtgctgaattcagggagaaaagtcgtaaagaaatccttgagttgattccaaagatgcaggtactggcccga
tcttcaccaattgataagcatacactggtgaaacacttgcgtacaacgttcaatgaggttgttgctgtgactggtgatggcacaaacgatgcacctgctctgcgcga
ggcagatgtcggacttgccatgggcattgcagggactgaggtgttcaatgagataagctcaagagacatggaagctatcaacgttctcaagggcctgccagata
actcaattttcatgggcatcctcgctggcaccattggttttcagttcatccttgtccaatttcttggcgactttgccaatacggcaccgctcacccaactacagtggttt
gtcagcgttttgtttggactccttgggatgcccatagcggctgccattaaacttattcctgtggaacgtcatgaagaagatacacatccgtcatag
>g123258.t1
atggagatgcttaatcaggagcagatctccgagttccgcgaggcgttctccttcttcgacaaggacggcgacgggtgcatcacggtggaggagctggcgacg
gtgatggggtcgctgcaggggcagcggccgagcgcggaggagctccgcgagatgatccgtgacgccgacgccgacggcaacggcaccatcgagttcgc
cgagttcctgacactcatggcgcgcaacaagaccgccgacaaggacgaccatgacgccgacgaggagctccgcgaggccttcaaggtcttcgacaaggac
cagaacggatacatctccgccaccgagctgcggcacgtgatgatcaacctcggcgagaagctgacggacgaggaggtggagcagatgatccgggaggcc
gacctcgacggcgacggccaggtcaactacgacgagttcgtcaggatgatgatgctctccgacggcggccacgcgccggccaccaaacactaccaatga
>g124260.t1
atgggcctctgctcctcctccaccgcgtccgcgcaccgtgccggtgccgcgccggggaataaggagaacaagggcgccggaggcggcagccgggggatc
gtggcgtgtgggaagcggacagatttcggctacgacaaggacttcgaggcccgttactcgctcggcaagctgctgggccacggacagtttggctacacctac
gccgccgtcgaccgcaactccggtgagcgcgtcgccgtcaagcgcatcgacaaaaacaaggtgcctcatttctccgtttgttttttgtttcgtttttcttacttgctg
gatcggatcgcgttagtggctga
>g135275.t1
atgtataaaactaagagatctgcttcacttaaagtgaagcgccgtgttgggaagtatgagcttgggcgcacaattggggaaggaacatttgctaaggttcggattg
caaagaactcggagactggggaacatgttgctatcaagatccttgacaaggcaaaggttctgaagcacaaattggttgagcaggctgatggattgctgcacact
acatgtggaacggctaactatgttgctccagaggttatcaatgacaagggctatgatggtgccactgcagatctttggtcttgtggggtaatcctttttgtgttgcttg
caggatatctgcctttcgaggatgacaatactgtctccctctataaaaagatctcaggagctcaatttacttgcccctcttggttttctgctggagcaaagaagctgat
ctgtagaattctggatcctaatccttcaactcggatttcagttcctcaaatacaaaaggatccctggttcaaaacaggctacaagccacctgtttttgacaagaaatgt
gaaggaagtttagatgatgttgatgctgcttttggagactcagaggaacatcatgtgacagaggaaatggaaggccagccaccctcgatgaatgcatttgagttg
atatcattaaacaaggggctgaatctggagaatttgttcgactctaataagatgcaaatggagaatccaaaagcaggaaggaagggcaatcttcatgttgcgact
gagggcaggagacacggtgaatattcaatattggaagcaaataaatga
>g135393.t1
atgggtctctgccatggcaagccaacgcaaatcccggagcctgaggcggaagaagatccccatgtagcctccggcgccggcgacgccgccggtgcccgga
acggcgcctccgcgccgtcgccagcgcctgcgacgaagccgggcacgccgaagcagcccaagttcccgttctacctaccgagcccgctcccgccgtcgag
ctacaagggctcgccggcgaactcgagtgtggcatcgacgccggcgcgcggagggttcaagcggccgttcccaccgccatcgccggcgaagcacatccgc
gcgctcctggcgcggcggcacggctcggtgaagcctaatgaggcgtccatccctgagggcggcgagccggagctcggcctggacaagagcttcggcttctc
aaagcacttcttcgccaagtacgagctcggcgaggaggtcggccgcggccacttcggctacacctgctctgctagggccaagaaaggcgagcacaagggg
caggacgtcgccgtcaaggtcatccccaaggccaagatgacaactgctattgcaattgaagatgtcagaagagaagttagaatattgagttctctgtcaggccac
aacaacctagtgcagttctatgacgcttttgaggatgaagataacgtgtatatagttatggagttatgtaaaggaggcgaactccttgatagaatattggcgagagg
tggaaagtattctgaagaggatgcaaaggttgttatggtacaaattttgagtgttgtatcattttgccatcttcaaggtgttgtccatcgcgatctgaaaccagagaatt
tccttttctcttcaaaggatgaaacctctgccttgaaggtcatagattttggcttgtctgacttcgtgaagccagtttatcatgttctcatcatgtga
>g135394.t1
atgggtctctgccatggcaagccaacgcaaatcccggagcctgaggcggaagaagatccccatgtagcctccggcgccggcgacgccgccggtgcccgga
acggcgcctccgcgccgtcgccagcgcctgcgacgaagccgggcacgccgaagcagcccaagttcccgttctacctaccgagcccgctcccgccgtcgag
ctacaagggctcgccggcgaactcgagtgtggcatcgacgccggcgcgcggagggttcaagcggccgttcccaccgccatcgccggcgaagcacatccgc
gcgctcctggcgcggcggcacggctcggtgaagcctaatgaggcgtccatccctgagggcggcgagccggagctcggcctggacaagagcttcggcttctc
aaagcacttcttcgccaagtacgagctcggcgaggaggtcggccgcggccacttcggctacacctgctctgcnnnnnnnnnnnnnnnnnnnnnnnnn

nnnnnnnnnnnnnnnnnnnnnnnnnnnnnnnnncacccccattcccctctgacccaagcctttcgctcaaagcttc
cagctttttcaattgtttggtcacttcaagaatcaaggaatatgttcttgcttcttgggcgatag
>g135510.t1
atggagagctacctgaacgagaactttgggggcgtcaagcccaagcactcgtccgacgaggcgctggggcgatggcgcaaggtcgtcggcgtcgtcaaga
accccaagcgccgcttccgattcacggccaacctcagcaagcgctcagaggctgcgcagatgaagcgatccaaccaggagaagctgcgtgttgctgtgcttg
tttccaaggctgcacttcagttcctccacggccttgcaccgcagagcgagtacacggtccctgacaacgtcaaggccaagggcttcggcatctgcgccgaaga
gctgagctccatcgtggagggccatgacctgaagaagctgaaatcacatggcggcgtcgagagcctcgtgtcgaagctgtccacctcggagtccgacggcgt
cgacacgtcgtccaggaagaggctggcgagaagggaggccatcttcggcgtcaacaagttcgtggaggcagagtcccgcggcttctgggtgttcgtctggga
ggcgctccaggacatgacgctcatgatcctcgcggcgtgcgcgctcgtctcgctcatcgtcggcattgccaccgaagggtggcccaagggcgcgcacgacg
gcctcggcnnnnnnnnnnnnnnnnnnnnnnnnnnnnnnnnnnnnnnnnnnnnnnnnnnnnnnnnnttgcac
cgcagagcgagtacacggtccctgacaacgtcaaggccaagggcttcggcatctgcgccgaagagctgagctccatcgtggagggccatgacctgaagaa
gctgaaatcacatggcggcgtcgagagcctcgtgtcgaagctgtccacctcggagtccgacggcgtcgacacgtcgtccaggaagaggctggcgagaagg
gaggccatcttcggcgtcaacaagttcgtggaggcagagtcccgcggcttctgggtcttcgtctgggaggcgctccaggacatgacgctcatgatcctcgcgg
cgtgcgcgctcgtctcgctcatcgtcggcattgccaccgaagggtggcccaagggcgcgcacgacggcctcggcatcgtggccagcatcctgctcgtcgtgtt
cgtcaccgcgaccagcgactaccgccagtccctgcagttcaaggacctcgacaaggagaagaagaagatcaccgtgcaggtcatccggagcgggtacagg
cagaagctctccatatacgagctcctcgccggcgacgttgtccacctctccattggtgatcaggtgccggccgacgggctcttcctgtcaggattctcgctgctga
tcaacgagtcgagcttgaccggggagagcgagccggtcgcggtcaacgcagagaacccgttcctcctgtcgggcacaaaggtgcaggacggttcgtgcaa
gatgctcatcaccacggtcggcatgaggactcagtggggcaagctcatggcaactctcagcgagggtggtgacgacgagacgccgttgcaggtcaagctga
acggcgtcgccaccatcattggtaagatcggcctcatcttcgccgtcgtcacgttcgcggtgctcaccgaaagcctgttccggcggaagatcatggacgggtcg
tacttgagctggaccggagatgacgcgttggagctgctcgagttcttcgccattgctgtaaccattgtggttgttgcggtgcctgaaggactgccgcttgcagtca
cactgagccttgctttcgccatgaagaaaatgatgaacgacaaggcgctcgtccggcaccttgcggcttgcgagaccatgggctcggctacctccatctgcagc
gacaagaccggcacgctcacgacgaaccatatgaccgtggtcaaggcctgtgtctgcggtaagtccatattcaacaacacgggtggcgacgttgtcattaacc
aggatggcaagcgggaaatactgggcacaccaactgagacggcgattctggagttcggcctgtcgctcggcggggacttctcggctgtgcggaaacaaagc
accatgatcaaggtggagccgttcaactcggcaaagaagagaatgggcgtggtcatccagctcccagggggtgcgctgcgtgcacactgcaaaggcgcctc
agagatcatactggcgtcttgcaacaaatacctgaatgatgaaggcagtgtcgttcctctcgataaagcgaccgtcaagcacttgaatgccacgatcgagagcttt
gcaaacgaggcacttcggacgctgtgccttgcctacgcggaagtccctgaggggttctcagccaatgatcagattccgatggatgggtacacttgcattggcatt
gtggggatcaaagaccccgtccggcctggcgtgaaggaatcagttgccatctgcaggtctgctggtattactgttaggatggtcacaggtgacaacatcaacac
cgccaaggcaatcgcccgggaatgtggcatcttaactgaagatggcattgccattgaaggcccggagttcagaaccaagactccagaagaaatgaccaaattg
atacccaagatacaggtgatggcaagatcttcgccacttgacaagcacacccttgtgaagcatcttcggactgcactcgacgaggttgtcgctgtgactggcgac
gggacaaatgatgcacctgcgctacatgaggctgacattgggctcgcaatgggcattgctggaactgaggttgcaaaagagagtgccgatgtcattattcttgat
gacaacttctccactattgtcactgtcgcaaaatggggtcggtctgtgtacatcaacattcagaagtttgtgcagtttcagctgacagtcaatgtggttgctctcgttgt
gaacttctcctcagcctgcttgacagggagtgctcctcttactgctgtgcaattgctctgggttaacatgatcatggatacacttggagcactggcattggccaccg
aacctccaaataatgaactgatgaagagaactcctgttggaaggaaaggaaatttcatcagcaacattatgtggaggaacatcctgggacaggccttgtatcagtt
cattgtgatttggtacctacagaccgaagggaagcgggtatttggacttgtaggcgacaattcggatcttgtcttgaacacactcatcttcaattgcttcgtgttctgt
caggtattcaatgaggtgagctcaagagagatggagaagataaatgtatttgaaggcattctagacaacaatgtgtttgtcgccgtcctaagtagcaccgtcatctt
ccagttcatcataatacaatttctgggcgagtttgcaaacactactcctctctcattcatgcagtggatatactgcattttcatcggtttcataggcatgccaatcgctg
ctattgtcaagatgatccccgttggttcttggtag
>g136047.t1
atggagacgctgaaagggccaaagggtggcctcccgatgtggaagcagatatccatctctgacgcgctgctcaccaatgagattcttgtaatgaggaggatagt
tgagaacgtctcgcctcatcccaatgtcatcggcttgcatgacgtttatgaggatgcacacggcgtgcacttgatccttgagctgtgctctggtggtgaactgtttga
tagaatagtggggcgtgaccggtactcggagttcgatgctgcgtgtgttattcgccagattgctagaggcctagaggctcttcataaggcaaacatcatccacag
ggacttgaagccggagaattgtttgttctctgacaaaaatgaagattccacattgaagatcatggattttggtctgagttctgttgaggacttcagtgatccaattgtg
gccttgtttggctcgatagattatgtttcaccagaagctctctcaaggcaagatgtttcagctgcaagtgatatgtggtctgttgggatgccctccatttcatgctgca
actaa
>g140089.t1
atgtttacttggcaagagaaaaaaagggacaatcatattgtggctttgaaagttcttttcaagagccaactgaaaaagtctcaagtcgaacatcagcttcagtgtga
agttgaaatccagagtcacctaaggcatcctaatattctatgcctatacggttacttctatgaccagactcgcgtttacttgatcctggactatgctgccaaaggaga
gctgtacaaggagctgacaagatgcaaacttttcaatgagagacgttcagctatgtacatcgcatcactggcaagatcactcatttaccttcatgggaagcatgtca
tccatagaaacattaaaccagagaatcttttaattggagctcaggacaatcatattgtggctttgaaagttcttttcaagagccaactgaaaaagtctcaagtcgaac
atcagcttcagtgtgaagttgaaatccagagtcacctaaggcatcctaatattctatgcctatacggttacttctatgaccagactcgcgtttacttgatcctggactat
gctgccaaaggagagctgtacaaggagctgacaagatgcaaacttttcaatgagagacgttcagctatgtacatcgcatcactggcaagatcactcatttaccttc
atgggaagcatgtcatccatagaaacattaaaccagagaatcttttaattggagctcaggatagtgaatgtggacctgaagttcctattgaaaccgtgaggcccca
gcagcgggcagcaacaccatcagcctccgccctcccccgcatccggtgccggcctcctcccctctgctccgccatgatggacctggatccccgcctttacga
gaacgtggttacggtggtttctatattatgtccgaaaagcttggtttccaggatttttggagttgttga
>g141673.t1
atgggtccgccccccgccgcgcaggactccgcgtcgtcgtcgccgtccggctcctccaggcgccgcttccgccggctcgaccgccgcaacgcgtccaaga
acatcgcctacgacgccgcaaacttctgcagctacccgccgtccccgacgccctcctcctcctccgcgccggcatccgcggcctgctccctcgacctcctctcc
agcttccgcatcggcggcagcggcgacgggggcggggacctgcagctcctctgccagagcctagggctctccggccccgacgacttcgccatcccggtcg
ccgactgggaggcgcacaaggccgtccgcaactcctcggcctccagctccccgtcctccgcgcgccacaaccccgacgcccccgcgcgggactccccgct
ccgccgcgagggtgcggaggagccggcccagcccgcggacgccgcccccgagctgccggcggcgtggaacgccccaatcgaagctctggagcggccc

caacagctggatcggatagagcccgtccgtctggaggtgaagagggcagccggcgagggaggaatcaagggcgtgcggccgccgccggtgctcaagcc
gccgccctcgatggcgatgccggctgtctgcggggcggggtccacgtgggacatcctggcttcgttcgcgcctgatgagaaggagcgcgccccggcgagc
agatctggcggtggtcttgtacgccaggaggaggacgaggacgaagacgcggcggtagcgttggcgttggaggagctcaggctggggagacttccgagg
atttcacgggcacatcttccatgtcgacagtaaacgacgacgagacgtccagcaccaccaccgagtccatgttctacatctctcccaacgggaggttccggagg
aagatccgatcatggaaccgaggggtgctcctggggagcggctccttcgggacggtatatgagggcatcagcgaagacataaaatgtgcaaatatactggtgc
atgctaatggatctgtgaaacttgcagactttgggcttgctaaggagatattaattcttggtaatcatgcggagctggctgaaaaggaggcggtttacctgcagctg
gtaatgtcaacactcaacagcattctcatcgtcatcgtctgtttcattgattga
>g142373.t1
atgggcaactgctgctgcgcggggtccaagcaggagcccgccgcagagccggcgtcctccggctccagccgccccgtgggcagcacgacgacctcggtc
tcgccttcctcggcgccggcgcccatgaagcccccccccgccgtgggccctgttctaggccgacccatggaagatgttcggagcacgtacagcatcggcaag
gagcttggccgcggccagttcggcgtgacgtcgctgtgcacgcacaaggccaccgggcagaaattcgcgtgcaagaccatcagcaagcggaagctgtcga
ccaaggaggacatcgaggacgtgcgtcgcgaggtgcagatcatgtaccacctctccggccaaccgggcgtcgtggagctcaagggcgcgtacgaggacaa
ggcctccgtccacctcgtcatggagctatgcgccggcggcgagctcttcgaccggatcatcgccaagggccactacacggagcgcgcggcggcggagctg
ctccgcaccatcgtcgagatcgtccacacgtgccacgccatgggcgtcatccaccgcgacctcaagcccgagaacttcctcctccttagcaaggacgagacc
gcgccgctcaaggccaccgactttggcctctccgtcttcttcaaagaaggggaggtgttcagggacatcgtcggcagcgcctactacatcgcgccggaggtgc
tgaagcggagctacgggccggaggccgacatatggagcatcggcgtcatcgtgtacatcctgctctccggcgtccccccgttctgggcaggcaagcgatctgt
tgtcttcagagacacggtcgacttcaccagtgacccatggccgcgtatctcgtcgggtgcaaaggaccttgtcaggaagatgctcaactcggatcccaagcagc
ggatatctgcgtatgacgtcctcaaccatccgtggatcaaagaagacggcgacgcgcctgatacaccgctggacaacgcggtcctcggccggctcaagcaatt
cagagctatgaaccagttcaaaaaagcagcactaagggtcatcgccggatgcctttcagaggaggagatcaaggggctgaaggagatgttcaggagcatgga
cgtggacaacagcggcacgataaccgtggatgagctgcggcgcgggctggccaacaagggcaccaagctcagcgaagccgaggtccagcagctgatgga
agctgccgacgcggacgggaacgggacgatcgactacgacgagttcatcacggcgacgatgcacatgaacaggatggaccgggacgagcacctctacac
agcgttccagtacttcgacaaggacagcagcgggtacatcaccatcgaggagctggagcaggctctcagggagaagggcctgctcgacgacggccgggac
atcaaggaaatcatatcggaagtcgacgccgacaacgacgggaggatcaattacacggagttcgtggcgatgatgaggaaaggggacccggaggtggcca
accccaagaagcgccgcgacgtcgtgctatag
>g142379.t1
atggcggaccagctcaccgacgagcagatcgcggagttcaaggaggccttcagcctcttcgacaaggatggcgacggttgcatcactacaaaggagcttgga
actgtgatgcggtcccttggtcagaaccctaccgaggcagagctgcaggacatgatcaacgaggttgacgctgatggcaatgggactattgacttcccggagtt
ccttaacctgatggcaaggaagatgaaggacactgactccgaggaggagctcaaggaggccttccgtgtcttcgacaaggaccagaacggtttcatctcagct
gctgagctccgccatgtcatgaccaaccttggggagaagctgactgatgaggaagtcgacgagatgatccgcgaggctgatgttgatggtgatggccagatca
actacgaggagtttgtcaaggtcatgatggccaaggcccatgcattattcaatgttctcaggcatcgaccatatgcagcatgccagatcgccacggaggagctg
gtcacagcgctccgctcgctgggcctgaacgtcgacgaggccgaggcgcagcgcctcctcagcgacgccactgacgtctcctccgccggcggcgcgattg
acttcacggcgttcttgtccgtcgcggcgcgcaagatggccggcggcggtaagcagacggcggagcggctggcggggtgcttcgacgtgttcgacgacgg
ccggagcgggtcgatcccggcggagcagctgcggcaggtgatgacgagccacggcgaccggctcacggaggaggaggccgacgccatggtccgcgag
gccgacccgcgcggcgagggccgcgtcgagtacagggagtacgtcaaggtgctgaccaagaacaagtga
>g142424.t1
atggggaaccgcgggtcgcgccaccaccgccaccccgcggaccagcaggcggccccgccttcgcagcccaagccgcagtcctccccaccgccccagcc
caagccgcatgcctccccgccgccgaagcccaagccccagccgccggcgcccgctccggcggcgtcgggggacgtggggcgcgtgctgggtcggccga
tggaggacgtccgggccacctacaccttcggccgcgagctcggccggggccagttcggggtcacctacctcgtcacgcaccgcgagacggggcagcgatt
cgcctgcaagtccatcgccacgcggaagctcgtccaccgcgacgacatcgaggacgtccgccgggaggtgcagatcatgcaccacctcacgggccaccgc
aacatcgtcgagctccggggcgcctacgaggaccgccactcggtcaacctcgtcatggagatttgcgagggcggtgagctcttcgaccgcatcatcgccaag
ggacactacaccgagcgcgccgccgccgcgctctgccgcgagatcgtcgcagtcgtgcacagctgccactccatgggggtcttccaccgggatctcaagcc
cgagaacttcctcttcctcaacaacaaggaggactcgccgctcaaggccacggactttggcctctctgtcttcttcaagcccggtgagtgcttgatttccgccgca
tctgtgggatcctga
>g142425.t1
atggggaaccgcgggtcgcgccaccaccgccaccccgcggaccagcaggcggccccgccttcgcagcccaagccgcagtcctccccaccgccccagcc
caagccgcatgcctccccgccgccgaagcccaagccccagccgccggcgcccgctccggcggcgtcgggggacgtggggcgcgtgctgggtcggccga
tggaggacgtccgggccacctacaccttcggccgcgagctcggccggggccagttcggggtcacctacctcgtcacgcaccgcgagacggggcagcgatt
cgcctgcaagtccatcgccacgcggaagctcgtccaccgcgacgacatcgaggacgtccgccgggaggtgcagatcatgcaccacctcacgggccaccgc
aacatcgtcgagctccggggcgcctacgaggaccgccactcggtcaacctcgtcatggagatttgcgagggcggtgagctcttcgaccgcatcatcgccaag
ggacactacaccgagcgcgccgccgccgcgctctgccgcgagatcgtcgccgtcgtgcacagctgccactccatgggggtcttccaccgggatctcaagcc
cgagaacttcctcttcctcaacaacaaggaggactcgccgctcaaggccacggactttggcctctccgtcttcttcaagcccggtgagtgcttgatttccgctgca
tctgtgggatcctga
>g144903.t1
atggagctctgcgccggcggggagctcttcgaccgcatcatacagcgcggacactacagcgagcgaaaggccgcagagctcacgcggatcatcgtcgggg
tcgtcgaggcgtgccactcgctcggggtcatgcaccgggacctcaagccagagaacttcctgctcgccaacaaggacgatgacctctcgctcaaggctatcga
tttcggcctctccgtcttcttcaagccgggtgagctgctgctcattactgattcatattccgacttgattactgtttcggtcaacgagtcctgtcccattggatgtactta
ctaa
>g145423.t1
atgcagccggacccgcaaggcccgggaagggagaagcccggcggcaccaatgtgaacgtccggctgccgccgccggtgacggtggggtcgggtggcc
ggccggcgtccgtgctgtcgcacaagacggcgaacgtgcgcgaccattaccgcatcgggaagaagctggggcaggggcagttcggcaccacgtaccagtg

cgtgggcaaggccgacggcgccgagtacgcgtgcaagtccatccccaagcgcnnnnnnnnnnnnnnnnnnnnnnnnnaaaaaaattctgtatactctc
atttctag
>g145424.t1
atgcagccggacccgcaaggcccgggaagggagaagcccggcggcaccaatgtgaacgtccggctgccgccgccggtgacggtggggtcgggtggcc
ggccggcgtccgtgctgtcgcacaagacggcgaacgtgcgcgaccattaccgcatcgggaagaagctggggcaggggcagttcggcaccacgtaccagtg
cgtgggcaaggccgacggcgccgagtacgcgtgcaagtccatccccaagcgcaagctgctgtgccgcgaggactacgaggacgtgtggcgcgagatcca
gatcatgcaccacctctccgagcacccaaacgtggtccgcatccgcggcgcatacgaggacgccctcttcgtgcacctcgtcatggagctctgcgccggcgg
cgagctcttcgaccgcatcgtcgccaagggccattactccgagcgcgccgcggcgcagctcatcaggacgatcgttggcgtggtcgaaggttgccactcgct
cggcgtcatgcatcgggacctcaagccggagaacttcctcttcgcgagcaccgctgaggatgcgccgctcaaggccactgactttgggctctctgtgttctaca
agcctggtgataaattttctgacgttgtcgggagcccctattatgttgcaccggaggtgcttcaaaaatgctatggcccagaagctgatgtctggagtgctggagt
gattctgtacattttgctatgtggtgtgcccccattctgggcagaaagtgaagctggaatcttcaggcagattttgcgaggcaaacttgacttggaatcggagccat
ggcctaatatctctgatagtgctaaagatctggtccgtaagatgcttacccgggatcctacaaaaagactgactgctcatgaggttctatgccatccctggattgttg
atgatgctgttgcacccgataagcctgttgattctgctgtattgtcaaggctgaaaaacttttctgcaatgaacaagctcaagaagatggcattgagggtgatagct
gaaagtctgtctgaggaggaaattggtggcttaaaggagctgttcaaaatgatcgacactgacaatagtggcacaataacttatgatgaactgaaaaacggcctg
aaaagggtgggttctgatctaatggaacctgaaatccaggctttaatggatgcgattttgcgaggcaaacttgacttggaatcggagccatggcctaatatctctga
tagtgctaaagatctggtccgtaagatgcttacccgggatcctacaaaaagactgactgctcatgaggttctatgccatccctggattgttgatgatgctgttgcacc
cgataagcctgttgattctgctgtattgtcaaggctgaaaaacttttctgcaatgaacaagctcaagaagatggcattgagggtgatagctgaaagtctgtctgagg
aggaaattggtggcttaaaggagctgttcaaaatgatcgacactgacaatagtggcacaataacttatgatgaactgaaaaacggcctgaaaagggtgggttctg
atctaatggaacctgaaatccaggctttaatggatgcggccgatattgacaacagtggaaccattgactatggagagttcctagcagctacattgcacatgaataa
actggagagggaggaaagcttggtatcagcattttcattttttgataaggatggaagtggcttcataacaattgatgagctctcacaagcatgcaaacagtttggcc
tttctgatgttcatcttgaggatatgatcaaagatgtggatcaaaacaatgatggacaaattgattatagcgagtttgcagcaatgatgagaaagggcaatgctggt
ggagcagggaggagaaccatgaggaacagcttgcatgtgaaccttggtgaaatcttcaaacccgctgagaattaa
>g145738.t1
atgggtaaggagaagagtcacatcaacattgtggtcattggccacgtcgactctggaaagtcgaccaccactggccacctgatctacaagcttggaggaattga
caagcgtgtgatcgagaggtttgagaaggaggcagctgaaatgaacaagcggtcattcaagtacgcgtgggtgctggacaagctcaaggctgagcgtgaga
gaggtatcaccattgatatcgccctgtggaagtttgagaccaccaagtactactgcactgtcattgatgcccctggacaccgtgacttcatcaagaacatgatcac
cggtacctcacaggctgattgtgctgtccttatcattgactccaccactggtggtttcgaggctggtatctccaaggatggccagacccgtgagcatgctctccttg
ctttcactcttggagtgaagcagatgatttgctgttgcaataaggtataa
>g146170.t1
atgggtaaggagaagaatcacatcaacattgtggtcattggccacgtcgactctggaaagtcgaccaccactggtcacctgatctacaagcttggaggaattgac
aagcgtgtgatcgagaggtttgagaaggaggctgctgaaatgaacaagcggtcattcaagtacgcgtgggtgctggacaagctcaaggctgagcgtgagaga
ggtatcaccattgatatcgccctgtggaagtttgagaccaccaagtactactgcacttttgagaccaccaagtactactgcactgtcattgatgcccctggacaccg
tgactttatcaagaacatgatcaccggtacctcccaggctgactgtgctgtccttatcattgactccaccactggtggtttcgaggctggtatctccaaggatggcc
aaacccgtgagcatgctctccttgctttcactcttggagtgaagcagatgatttgctgctgcaacaagatggatgcaaccacaccgaagtactcaaaggctcgtta
cgatgagattgtgaaggaagtctcttcctacctgaagaaagttggctacaaccctgacaagattcccttcgttcccatctctggtttcgagggtgacaacatgattg
agaggtccactaaccttgactggtacaagggtccaaccctgcttgaggctctcgaccagatcaatgagcccaagaggccctcagacaagcccctgcgtctccc
ccttcaggatgtgtacaagattggcggtattggaactgtccctgtcggtcgtgttgagactggtgtcctcaagcctggtatggttgtcacctttggtcccagcggcct
gactactgaggtcaagtctgttgagatgcaccacgaggctctccaggaggcccttcctggtgataatgtgggattcaacgtgaagaatgttgctgtgaaggatct
caagcgtgggtacgtggcctctaactccaaggatgaccctgccaaggaggctgccagtttcacctcccaggtcatcatcatgaaccaccctggacagatcggc
aatggctacgccccagtgttggactgccacacctcccacattgctgtcaagtttgctgagctgctgaccaagattgacaggcgatctggcaaggagcttgagaa
ggagcctaagttcctgaagaatggtgatgctggtatggtgaagatgattcctaccaagcccatggtagtggagaccttctctcagtaccctcctcttggtcgttttgc
tgtccgtgacatgaggcaaacggttgctgttggtgtcatcaaaagtgttgagaagaaggatccaaccggtgctaaggatgtatacaagattggtggtattggaact
gtccctgtcggtcgtgttgagactggtgtcctcaagcctggtatggttgtgacctttggtcccagcggcctgactactgaggtcaagtctgttgagatgcaccacg
aggctctccaggaggcccttcctggtgataatgtgggattcaacgtgaagaatgttgctgtgaaggatctcaagcgtgggtatgtggcctctaactccaaggatg
accctgccaaggaggctgccagtttcacctcccaggtcatcatcatgaaccaccctggacagatcggcaatggctacgccccagtgttggactgccacacctcc
cacattgctgtcaagtttgctgagctgctgaccaagattgacaggcgatctggcaaggagcttgagaaggagcctaagttcctgaagaatggtgatgctggtatg
gtgaagatgattcctaccaagcccatggtagtggagaccttctctcagtaccctcctcttggtcgttttgctgtccgtgacatgaggcaaacggttgctgttggtgtc
atcaaaagtgttgagaagaaggatccaaccggtgctaaggtgaccaaggctgctgtcaagaagaaatga
>g146173.t1
atgggtaaggagaagagtcacatcaacatcgtggtcattggccacgtcgactctggaaagtcgaccaccactggtcacctgatctacaagcttggaggaattga
caagcgtgtgatcgagaggtttgagaaggaggctgctgaaatgaacaagcggtcattcaagtacgcgtggtttgagaccaccaagtattactgcactgtcattga
tgcccctggacaccgtgactttatcaagaacatgatcaccggtacctcccaggctgactgtgctgtccttatcattgactccaccactggtggttttgaggctggtat
ctctaaggacggccagacccgtgagcatgctctccttgctttcactcttggagtgaagcagatgatttgctgctgcaacaagatggatgccaccacacccaagta
ctcaaaggctcgttatgatgagattgtgaaggaagtctcttcctacctgaagaaagttggctacaaccctgacaagattcccttcgttcccatctctggtttcgaggg
tgacaacatgattgagaggtcaaccaaccttgactggtacaagggcccaactctgcttgaggctcttgaccagatcaacgagcccaagaggccctcagacaag
cccctgcgtctccccccttcaggatgtatacaagattggcggtattggaactgtccctgtcggtcgtgttgagactggtgtcctcaagcctggtatggttgtcaccttt
ggtcccagcggcctgactactgaggtcaagtctgttgagatgcaccacgaggctctccaggaggcccttcctggtgataatgtgggattcaacgtgaagaatgtt
gccgtgaaggatctcaagcgtgggtatgtggcctctaactccaaggatgaccctgccaaggaggctgccagtttcacctcccaggtcatcatcatgaaccaccc
tggacagatcggcaacggctacgccccagtgnnnnnnnnnnnnnnnnnnnnnnnnnnnnnnnnnnnnnnnnnnnnnnnnnnnnnnnnnnnnnnn
nnnnnnnnnnnnnnnnnnnnnnnnnnnnnnnnnnnnnnnnnnnnnnnnnnnnnnnnnnnnnnnnntggactgccacacctcccacattgctg

tcaagtttgctgagctgctgaccaagattgacaggcgatctggcaaggagcttgagaaggagcctaagttcctgaagaatggtgatgctggtatggtgaagatg
attcctaccaagcccatggtggtggagaccttctctcagtaccctcctcttggtcgttttgctgtccgtgacatgaggcaaacggttgctgttggggtcatcaaaagt
gttgagaagaaggatccaaccggtgctaaggtgaccaaggctgctgtcaagaagaaatga
>g146358.t1
atgggtaaggagaagagtcacatcaacattgtggtcattggccatgtcgactctggaaagtcgaccaccactggccacctgatctacaagcttggagggatcga
caaacgtgtgatcgagaggttcgagaaggaggccgctgaaatgaacaagcggtcattcaagtacgcgtgggtgctggacaagctcaaggctgagcgtgaga
gaggtatcaccattgatatcgccttgtggaagtttgaaaccaccaagtactactgcactgtcattgatgcccctggacaccgtgacttcatcaagaacatgatcacc
ggtacctcccaggctgactgtgctgtccttatcattgactccaccactggtggttttgaggctggtatctccaaggacggccagacccgtgagcatgctctccttgc
tttcactcttggagtgaagcagatgatttgctgctgcaacaagtcgatgtaa
>g146823.t1
atggggaactgctgtcgctcgccggccgccgcggcgcgggaggacgtcaagtcgtcgcacttcccggcctcggccggcgggaagaagaagccgcaccag
gcgcggaacggcgggggaccgggcggcggggagaagaagcggctgtcggtgctcggggaggatgggtgcgacgtgagcggcggcggcgggatcgac
gagaaatacgcgctggaccgggagctcgggcgcggcgagttcggggtgacgtacctgtgcatggaccgcggcacgcgggagctgctggcctgcaagtcc
atctcgaagcggaagcttcggacgcccgtggacgtggaggacgtgcgccgcgaggtggccatcatgcggcacctgcccaagagccccagcatcgtgtccct
gcgggaggcgtgcgaggacgacggcgccgtgcacctcgtcatggagctgtgcgagggaggggagctgttcgaccgcatcgtcgccaggggacactacac
ggagcgcgcggcggccgcggtcgtgcgcaccatcgtcgaggtcgtgcagctctgccaccgccacggcgtgatccaccgcgacctcaagcccgagaacttc
ctcttcgcaaacaagaaggagaactccccgctcaaggccattgattttggcctctccatcttcttcaagcctggtaagctctcggcgccgagatctgtccctgattt
gggcgaactgaagaagcggctgtcggtgctcggggaggatgggtgcgacgtgagcggcggcggcgggatcgacgagaaatacgcgctggaccgggagc
tcgggcgcggcgagttcggggtgacgtacctgtgcatggaccgcggcacgcgggagctgctggcctgcaagtccatctcgaagcggaagcttcggacgcc
cgtggacgtggaggacgtgcgccgcgaggtggccatcatgcggcacctgcccaagagccccagcatcgtgtccctgcgggaggcgtgcgaggacgacgg
cgccgtgcacctcgtcatggagctgtgcgagggaggggagctgttcgaccgcatcgtcgccaggggacactacacggagcgcgcggcggccgcggtcgt
gcgcaccatcgtcgaggtcgtgcagctctgccaccgccacggcgtgatccaccgcgacctcaagcccgagaacttcctcttcgcaaacaagaaggagaactc
cccgctcaaggccattgattttggcctctccatcttcttcaagcctggtgaaaaattttcagaaatagtgggaagtccctactacatggctcctgaagtgctaaagag
gaattatggtcctgaaatagacatctggagtgctggtgttatcctgcaaaatttgcagagactgaacaaggagttgcacaagctatccttcggggaaatatag
>g146907.t1
atggataggacggcggcgacgacggggccgctggggatggagatgccgataatgcacgacggggaccggtacgagcacgtcaaggacatcgggtccgg
caacttcggcgtcgcgcgcctcatgcgcaaccgcgcctccggcgagctcgtcgccgtcaagtacatcgaccgcggcgagaagatcgacgagaacgtgcag
cgggagatcatcaaccacaggtcgctgcggcaccccaacatcatccgattcaaggaggttatcctgacgccgactcacctcgccatcgtcatggagtacgcttc
tggaggggagctcttcgagcgcatctgcagtgccggaagattcaacgaggacgaggcccggttcttcttccagcagctgatttctggggttagctactgtcactc
catgcaagtatgtcatcgtgacctgaagctcgagaatacattgttggatggaagtaccgctccacggctcaagatctgcgactttggttattcaaagtcgtctgttct
tcattctcaaccaaaatcaacagttggtactccagcttatattgctcctgaagttttgctcaagaaagaatacgatggcaagaaaatattgggtgttcagtatgtgatt
ccagattacgtgcacatatctccagagtgccggcacctcatctcaaggatttttgacgccaacccagaaactaggattaccatgcctgagataaaaagtcatcctt
ggttcgttaagaatctcccagcagacctcatggatgatgccacaatgagcagccagtatgaggagcctgaccagcctatgcagaacatgaacgagatcatgca
gatactggcagaggcaactataccagcagctggtacccgtggaatgaaccagttcttggttgacagccttgaccttgatgatgatatggaggatctagactcaga
ccttgacatcgacattgagagcagtggagaaatagtgtatgccatgtga
>g146910.t1
atggagagctacctggaggagaacttcgggggcgtcaaggccaagaactcctcggaggaggcgctgcggcggtggcgccgcctctgcagcgtcgtcaaga
atcccaagcgacggttccgcttcaccgccaacctcgacaagcgcggcgaggcgcaggccatcaagcacgccaaccacgtccgtacacatctcctcttccctc
atccatcccctttccttcctttcctccgcgccgggatccgcctcgctcgcggcggcgggatctggactggagccgcgccgccggcgggggcgggagattccg
agaaactgcgtgttgccgtgctggtctccaaggccgcgcttcagttcttacatggtctctcacttcggagcgagtatgttgtccctgaggaagtcaaggctgcagg
gtttgagatctgtgccaatgagcttgggtccattgtggagggccacgatagcaaaaagctgattatacatggaggagtcgatggaattgctgcaaagcttgcgac
gtcacaaacggatggtctgagtacagctgaggacaacattaagcgcaggcaagagatttatggactcaacaagttcacagaaagcgaggtccgaagtttctgg
gtgtttgtatgggaagcacttcaagatacaactctcataattcttgctgtgtgcgcatttgtatctctggtcgttggcattgcgatggaagggtggccaaaaggtgcc
catgacggtcttggaattgttgcgagtatcctcctggtagtgtttgtgactgcaacaagcgattaccgacagtcgctgcagttcaaggacctggacaaggagaaa
aagaaaattcgagtgcaagttacaagggatgggtttaggcagtggatatcgatatatgaccttcttcctggagatgttgtccatttacaaatcggagaccaggttcc
tgcagacgggctcttcatttcagggttttctctgttgatcaatgaatccagcctaacaggtgaaagtgaacctgtcgctgtaaatgaagataaccctttttcttttgtcgg
ggaccaaagtgcaggatgggtcctgcaagatgctggttacaacagttggcatgcgcacccaatggggaaaactgatggccacactcagtgagggtggtgatg
acgaaactccactgcaggtcaaactcaatggtgttgcaactatcattggaaagattgggctattctttgctgttataactttcattgtcttgtcccaagggttattcggc
aataaatatcatgacggacagcttttgagctggtcaggagatgatgcactggagcttctggagcattttgctattgcagtgaccattgttgttgtggctgttcctgag
ggattgccattagcagtcacgctgagccttgcattcgccatgaagaaaatgatgaatgacaaggcactggttcgcaacttagctgcatgtgaaactatgggttcag
caaccaccatctgcagtgacaagacagggacattgacaaccaatcatatgactgtcgttaaggcctgcatctgtgggaaaatcaaggaggttaacggtcctcag
aatgcatccaagttatgctctgaatttccggaaactgtcgtcaaaacactcctggagtctatatttaataatacaggtggtgaggttgtgattaaccaagatggtaaa
cgtcagatcctagatgagacaggcagtgttgttcccatggataaaacaactttggaaaagctcaatggtattatcgacaattttgctggtgaagctcttaggacact
atgccttgcttacagggaaatggaagaaggtgtgagggagtctgttgcaacttgccggtctgctggaattatggtgagaatggtcacaggagacaacataaatac
ggcaaaggcgattgcacgtgaatgtggtatactaactgaagatggtgtggctattgaaggacctgaattcagagagaaaaaacttgaagaactccttgagctggt
tccaaaaatccaggttatggcccggtcatcgccacttgacaagcatacactagtaaagcatttgcgcacgacattcaatgatgttgttgcagttactggtgatggca
caaatgatgcacctgcattgcatgaagcagatatcggacttgcgatgggcattgcaggaactgaggtggcaaaagagagtgctgatgttataattctggatgaca
acttctctacaattgtgactgttgcaaagtggggacgctctgtttacatcaatatccaaaagtttgtgcagtttcagcttactgtcaatgtagtagcactgctggttaact
tttcttctgcttgctttacgggaaatgcaccattgacggctgttcagctcctttgggtcaacatgatcatggacacccttggtgcactagcattggccacggaaccac
cgaacgatgagttgatgaagagagagccagtaggaagaacagggaaattcattacaaatgtaatgtggaggaacatcatgggacagtctttctaccaatttttgt

tatgtggtatctccagacgcaagggaaatcctttttggtcttgatgaaagctctgatactgatattgtgctgaacacaataattttcaactcattcgtcttctgtcaggtt
atggcccggtcatcgccacttgacaagcatacactagtaaagcatttgcgcacgacattcaatgatgttgttgctgttactggtgatggcacaaatgatgcacctgc
attgcatgaagcagatatcggacttgcgatgggcattgcaggaactgaggtggcaaaagagagtgctgatgttataattctggatgacaacttctctacaattgtg
actgttgcaaagtggggacgctctgtttacatcaatatccaaaagtttgtgcagtttcagcttactgtcaatgtagtagcactgctggttaacttttcttctgcttgcttta
cgggaaatgcaccattgacggctgttcagctcctttgggtcaacatgatcatggacacccttggtgcactagcattggccacggaaccaccgaacgatgagttg
atgaagagagagagccagtaggaagaacagggaaattcattacaaatgtgttcaatgagatcagttcaagggagatggagagggtgaacgtgatcaagggcatc
ctaaagaattatgtcttcatggccgtccttaccagtacggtcatcttccagttcatcatggtacagttcctgggcgagttcgccaacacgacaccgctcaccaggct
gcagtggctcgccagcgtgctccttggcctcgtagggatgcccatcgctgctgctgtcaagatgatccccgtcggctcctcatga
>g147245.t1
atgcaatcgtgggttcgtgaagccgagcggccattcccgccgccgtctcccgccaagcacatccgcgccctgctggcgcggcggcacggctcagtgaagcc
gaacgaggcttccatccccgagggcggcgagccggagctgggcttggacaagagcttcggctactcgaagcacttctcggctaagtacgagctcggccgcg
aggtggggcgcggccacttcggctacacctgcgccgccaaggccaagaaaggcgagctcaagggcgaggacgtcgccgtcaaggtcattcccaaggctaa
gttttgctcttga
>g147906.t1
atgaaccccccctgtagtccctcctgtggttgtcccaccaaagccaacaccagacacgattcttggcaaaccatatgaagatgtgcgttccgtgtactccattggta
aggagcttggccgtggtcagtttggggtgacatacctctgtactgaaattgcctctggtaggcagtatgcctgcaagtccatttccaagcgcaaactcaccagcaa
ggcggacagggaggacattcgcagggagatccagatcatgcagcactnnnnnnnnnnnnnnnnnnnnnnnnnnnnnnnnnnnnnnnnnn
nnnnnnnnnnnnnnnnnnnnnngattctggtcatggaggaaccaatggttatggctattcacaccagcaaaaaccagcacagacacctcccagctaca
acccttctcaaccacagtcaacggccgaagtgaggtacacaccaccagccatgaaccccccctgtagtccctcctgtggttgtcccaccaaagccaacaccaga
cacgattcttggcaaaccatatgaagatgtgcgttccgtgtactccattggtaaggagcttggccgtggtcagtttggggtgacatacctctgtactgaaattgcctc
tggtaggcagtatgcctgcaagtccatttccaagcgcaaactcaccagcaaggcggacagggaggacattcgcagggagatccagatcatgcagcacttgtct
ggacaaccaaatatagtcgagtttcggggagcatacgaggacaagagcaatgtgcatgtggtgatggagctctgcgctggtggggagctgttcgatcggatca
tcgcaaaggggcactacacggagcgggcagctgccaccatctgcagggctgttgtgaatgttgtcaacatttgccatttcatgggagtgatgcatcgtgatctaa
aaccagagaatttcttgcttgcgaccaaggaggagaatgcaatgctcaaggccactgattttggactttcagtcttcatcgaagaaggaaaaatgtacagggaca
ttgttggaagtgcttattatgttgctccagaagttcttaggcgaagctatggaaaagagatagatgtgtggagtgctggtgtcattttgtacattcttcttagtggtgtgc
ctccattttgggctgaaactgaaaagggcatatttgatgctattctgcatgaggaaattgactttgaaagtcagccatggccatcaatttctgagagtgctaaagacc
tggttaggaagatgttgacacgggatccaaagaaaagacttacttcagctcaagttcttcggagggtggtgaggcatcagacaagcctattgacagtgctgtactt
tccagaatga
>g149417.t1
atgggcaaggagaagagtcacattaacatcgtggtcattggacatgtcgactcaggcaagtccaccaccactggccacctgatctacaagcttggaggaattga
caagcgtgtgattgaaaggttcgagaaagaagctgcagaaatgaacaagaggtccttcaagtatgcctgggttcttgacaagctcaaggctgagcgtgagaga
ggtattaccattgatattgctctgtggaagtttgagaccaccaagtactactgcacagtcattgatgcccctggacaccgtgacttcatcaagaacatgattactggt
acctcccaggctgactgtgctgttcttatcattgactccaccactggtggttttgaggctggtatctccaaggatggccagacccgtgagcatgctcttcttgctttca
ctcttggagtgaagcagatgatctgctgctgcaacaagccaactaaagctatatttcttccttgcttgtcagtctaa
>g149418.t1
atgggcaaggagaagagtcacattaacatcgtggtcattggacatgtcgactcaggcaagtccaccaccactggccacctgatctacaagcttggaggaattga
caagcgtgtgatcgaaaggttcgagaaagaagctgcagaaatgaacaagaggtccttcaagtatgcctgggttcttgacaagctcaaggctgagcgtgagaga
ggtattaccattgatattgctctgtggaagtttgagaccaccaagtactactgcacagtcattgatgcccctggacaccgtgacttcatcaagaacatgattactggt
acctcccaggctgactgtgctgttcttatcattgactccaccactggtggttttgaggctggtatctccaaggatggccagacccgcgagcatgctcttcttgctttc
actcttggggtgaagcagatgatctgctgctgcaacaagatggatgccaccactcccaagtactcgaaggcccgttatgatgagattgtgaaggaagtctcttcat
acctcaagaaggaagtctcttcatacctcaagaaggttggctacaaccctgacaagatccccttcgttcccatctctgggttcgagggtgacaacatgattgagag
atccaccaaccttgactggtacaagggccccaccctgctggaggctcttgaccagatcaatgagccgaagaggccctcagacaagcccctgcgtgttcccctt
caggacgtgtacaagattggtggtattggaactgtgcctgtgggccgtgtggagactggtgtcctcaagcctggtatggttgttacctttggtcccactggactga
ccactgaggtcaagtctgtggagatgcaccacgaggctctcctggaggccctccctggtgacaatgttgggttcaacgtgaagaacgttgctgtgaaggatctg
aagcgtgggtatgtggcttccaactccaaagatgaccctgccaaggaggctgccagcttcacctcccaggtcatcatcatgaaccaccctggccagatcggca
atggctatgccccagtgctggactgccacacttcccacattgctgtgaagttcgctgagcttctgaccaagattgacagacgatctggtaaggagctcgagaagg
agcccaagttcctgaagaatggtgatgctggtatggtgaagatgattcccaccaagcccatggttgtggagaccttctcccagtaccctcctctcggtcgttttgct
gtccgtgacatgaggcaaacggttgctgttggtgtcatcaagagcgtggagaagaaggaccctactggtgccaaggttaccaaggctgctgccaagaagaaat
ga
>g150172.t1
atgggccagtgctacggcaaggcgggcggctcgtcgtcgcgggccgaccacgacgacatcggggtcgtcgcgccgccgtccccgctgccggccaacggc
gcgccgcagcagccggccacgccgaggcgtaggaagtccggatcgaccacgccggtgcaccacacgcccggggtcgcctggcccagcccctaccccgc
cggcggaacgagcccgctgccggcgggggtgtcgccgtcgccggcgaggtcgacgcccaggaggtttttcaagcggcctttcccgccgccctcgccggcc
aagcacataaaggccacgctcgctaagaggctgggcgggggggaagcccaaggaggggaccataccggaggaaggcggcgtcggggctggcggtggcg
gaggaggtgcggatggagccgaggcggagcggccgttggacaagacgtttggcttcgccancgtcggggctggcggtggcggaggaggtgcggatgga
gccgaggcggagcggccgttggacaagacgtttggcttcgccaagaacttcggggccaagtacgagctggggaaggaggtgggaagggggcacttcgga
cacacctgctccgccgtcgtcaaaaagggcgagtacaagggccagaccgtcgccgtcaagatcatttccaaagctaagcaactgatatgcgtcgcgccaattc
tgtgctag
>g151365.t1

atgatggggtcagcgcccgataagccgctagactcggcggtgctctcgcggctgaagcagttctccgcgatgaacaagctaaaaaagatggccttgagggtg
attgcggagaacctttcagaagaggagattgctggtctgaaggagatgttcaagatgatggataccgacaacagtgggcagatcaacttcgaggagctcaagg
ccggcctccacagagttggcgccaatatgaaggagccggagatacatcagctaatgcaggctgccgatattgacaacagtggtaccatagattatggtgagttc
atagccgctactttgcacctcaacaaagttgaaagggaggatcatctgttcgctgccttccaatacttcgacaaggatggaagtgggtacatcacagctgatgag
ctccagcaggcatgcgatgaattcggcattgaagatgtccgattggaagacatgatcggcgaagtagatcaagacaatgatgggcggattgactacaacgagtt
cgtcgccatgatgcagaaatcaactagtggttttgggaagaaaggccaccggaacaatcttagcattggactaatagacgcactgaaaccacatagttga
>g155326.t1
atgggcaactgctgccgttccccggcggccgtcgcgcgggaggacgtcagctcctcccacttccccgcatccaacgcgaagaagaagccgcaccagccgc
ggaacggcgccgccgggggaggcggcggcggcggggcgcagaagcggctcacggtgctgggcgaggaggggcgcgaggtgggcgggatcgacga
gaggtacgccttggacagggagctcggccgcggggagttcggggtcacgtacctgtgcatggatcgggacaccaaggagctgctcgcctgcaagtccatct
ccaagcgcaagctgcggaccgccgtcgacgtcgaggacgtgcgccgggaggtcgccatcatgcgccacctgcccaagagccccagcatcgtgtccctgcg
cgaggcctacgaggacgagggcgccgtgcacctcgtcatggagctctgcgagggcggcgagctcttcgaccgcatcgtcgcgcgggggcactacacgga
gcgggccgccgccaacgtcacccgcaccatcgtcgaggtcgtgcagctctgccaccgtcacggcgtgatccaccgcgacctcaagcccgagaacttcctctt
cgctaacaaaaaggagaactcgccgctcaaggccatcgacttcggcctttccatcttcttcaagcccgaacattttggtggtctgttagctgcagtggtggagcca
gacctcaacattaggggagctgagcggaaacttacatcagccttggccatcgctgactcccctgttcctactcctaccgcttga
>g159122.t1
atggctcttcacttgcttaagcgtcttctttcttttgatcctaaggagcgtccaagtgctgcagaggtaagagtggacccatactttaatggattagcaaacaatgaac
gagagcccatagcacagcctatctcaaaacttgaatttgagttcgagaagagaaaactgggcaaagatgatgtccgagaattaatttaccgagagattttggagt
accatcctcaaatgttgcaagaatacctacgtggaggagaccagaaccagatgagcttcatgtaccccagtggggttgatcgctttaaacggcaatttgttcatttg
gaagaaggtaaaccaaagggtgagaagaatagtccacagctgcggcagaatgtttccttaccaagggaacgagtaatgggcaataaacatggagacagtga
gtactgcataaatattttggagtaccatcctcaaatgttgcaagaatacctacgtggaggagaccagaaccagatgagcttcatgtaccccagtggggttgatcgc
tttaaacggcaatttgttcatttggaagaaggtaaaccaaagggtgagaagaatagtccacagctgcggcagaatgtttccttaccaagggaacgagtaatgggc
aataaacatggagacagtattaatacgggttctaatgtcgagaataagagagagagcttgccctcctgaacacgttcaaccttccagtcagtggtgacggagaggac
gttgatgtgcctatcatcagcaacatggagacagagggtgaagaagataagaaacctgttggtgggtcatccttccaacgacgtaaatataatcggttctccgag
gagcagtctgacatgcttgaagcaagctttcagcaaaaccactccccagacaaggtgactttcacactctag
>g162572.t1
atggccgtccagcttagcgacgagcagattaccgaattcaaggaggccttcagcctcttcgacaaggacggcgacggttgcatcacggccaaggagctgggt
acggtgatgcgttcgctgggtcagaacccgacggagcgtgagctgcaggacatgatcaacgatgtggacgcggacgggaacggcaccatcgacttccagg
agtttctggggctgatggcccggaagatgaaggacaaggacaccgaggaggagctcatggaggcgttccgcgtgttcgacaaggaccagaacgggctcatc
tccgcggcggagctccggcacgtgatgaccaacctcggcgagaagctgagcgacggcgaggtcgacgagatggtccgcgaggccgacgtcgacggcga
cgggcacatcaactaccaggagttcgtcaaggtcatgatggccaaacggaggaagcaggcgaagacagaggagaagacggcggtgcggggcaagaaga
acaaggcggtggcggcaccgtccgacgccgccggcaagcgttcccagaagtgcgtgatcctgtga
>g162952.t1
atgggtaaggagaagagtcacatcaacattgtggtcattggccatgtcgactctggaaagtccaccaccaccggccacctgatctacaagcttggtggtattgac
aagcgtgtgatcgagaggtttgagaaggaggccgctgaaatgaacaagaggtccttcaagtatgcgtgggtgctggacaagctcaaggctgagcgtgagaga
ggtatcaccatcgatatcgccttgtggaagttcgagaccaccaagtactactgcactgtcattgatgctcctggacaccgtgacttcatcaagaacatgatcaccg
gtacctcccaggctgactgtgctgtcctcatcattgactccaccactggtggttttgaggctggtatctccaaggatggccagacccgtgagcatgctctccttgctt
ttactcttggagtgaagcagatgatttgctgctgcaacaagatggatgccaccacacccaagtactcgaaggcccgctatgatgagattgtgaaggaagtctcttc
ctacctcaagaagatggatgccaccacacccaagtactcgaaggcccgctatgatgagattgtgaaggaagtctcttcctacctcaagaaggtcggctacaacc
ctgacaagattcccttcgttcccatctctggtttcgagggtgacaacatgattgagaggtccaccaaccttgactggtacaagggccccacccttcttgaggccctt
gaccagatcaatgagcccaagaggccctcggacaagcccctgcgtctccccttcaggatgtgtacaagattggtggtattggtactgtccctgtcggtcgtgtt
gagactggtgtcctcaagcctggtatggttgtgacctttggtcccagtggcctgactactgaggtcaagtctgttgagatgcaccatgaggctctccaggaggcc
ctccctggtgacaatgttggtttcaacgtgaagaatgttgctgtgaaggatctgaagcgtgggtacgtggcctccaactccaaggatgaccctgccaaggaggct
gccagcttcacctcccaggtcatcatcatgaaccatcctggacagattggcaatggttatgccccagttctggactgccacacctcccacattgctgttaagtttgct
gagctgctgaccaagattgacaggcgatctggtaaggagcttgagaaggagcctaagttcctaaagaacggtgatgctggtatggtgaagatgattcccaccaa
gcccatggtggtggagaccttctctcagtaccctcctcttggtcgtttcgctgtccgtgacatgaggcagacggttgctgttggagtcatcaagagcgttgagaag
aaagacccgaccggtgccaaggtgaccaaggctgctgtcaagaagaaatga
>g163673.t1
atggagaagtacgagctgctcaaggacatcggctccggcaacttcggggtggcgcggctgatgcggaacaaggagaccaaggagctcgtcgccatgaagt
acatcccgcgggggctcaagattgacgagaatgtggcgagggagatcatcaaccaccgctcgctgcggcaccccaacataatccggttcaaagaggttgtcc
tcacgcccacgcacctggccatcgtcatggagtatgccgctggcggcgagctgttcgaccggatctgcggcgctgggaggttcagcgaggatgaggcgagg
tatttcttccagcaactgatttgcggtgtcagctactgccacttcatgcaaatctgtcaccgggacttgaagctcgaaaacacgctgctggatggcagcccggcac
cgcgccttaagatttgtgacttcggctactccaagatggcagatgtttggtcctgtggagtgacccttatgtgatgctggttggtgcatacccttttgaggaccccg
acgatcccaagaatttcagaaagacaattgggagaatagtatctattcaataccaaataccagaatatgtccacatatcccaagattgcaggcagctcctctcgag
gatcttcgttgcgaatcctgcaaagagaataacaattagggagattagaaatcacccctggttccttaagaacttgcctagagagcttacagaagctgcacaagc
gaagtactacaagaaggacaacagcacacccacctactccgatcagactgttgaagagatcatgaagattgttgaggaggctcgcacaccacccaaattatcta
ctcctgtggctggcttcggttgggctgaggaagaagagcaagacgatggcaagaaacctgatgatgaagaacatgatgaagaggatgaggaatatgatggtg
aggatgagtatgacaagcaggtgaaggcagtacatgccagcggtgattttcaacatctgataaaaggaagcaactag
>g165869.t1

atggggacgctgaaagggccaaagggtggcctcccgatgtggaagcagatatccatctctgacgcgctgctcaccaatgagattcttgtaatgaggaggatag
ttgagaacgtctcgcctcatcccaatgtcatcggcttgcatgacgtttatgaggatgcacacggcgtgcacttgatccttgagctgtgctctggtggtgaactgtttg
atagaatagtggggcgtgaccggtactcggagttcgatgctgcgtgtgttattcgccagattgctagaggcctagaggctcttcataaggcaaacatcatccaca
gggacttgaagccggagaattgtttgttctctgacaaaaatgatgattccacattgaagatcatggattttggtctgagttctgttgaggacttcagtgatccaattgtg
gccttgtttggctcgatagattatgtttcaccagaagctctctcaaggcaagatgtttcagctgcaagtgatatgtgttgcgcagatggagagaacgccacattggc
ggagtttgaggaagtactaaaagcaatgaaaatggactcactgattcctctcgccccacgtgtattcgacttgtttgacaacaaccgtgacggcaccgtcgacatg
agggagattctttgcgggctttccagcctccggaactcgcgaggggatgatgctcttcgcctctgcttccagatgtatgacgcggatcggtcaggctgtatcagc
aaggaagagctggcatcaatgctgcgagcgttgcccgaagactgcctcccgggcgacatcacggagccgggaaagctggacgagatattcgaccaaatgg
acgcgaacagcgacggaaaggtcaccttcgatgagttcaaggctgccatgcagaaggacagctccctccaggacgtcgtcctctcctccttgcgcccggtgca
atag
>g167600.t1
atggatgattgtttttccattgatgagcaaataccgctgcaaggatacatatgcattggtattgttggcatcaaagatcctgtgcgtccaggggtcatgcagtctgtg
gcaacatgccgatctgctggcatttcagttcgaatggttacaggagacaacataaatacagcaaaggcaattgctcgtgaatgtggtatacttacagaggacggc
ctttcaattgaaggtgctgaattcagggagaaaagtcctaaagaaatccttgagttgattccaaagatgcaggtactggcccgatcttcaccaattgataagaatac
attggtgaaacatttgcgtacaacgttcaatgaggttgttgctgtgactggtgatggcacaaacgacgcacctgctctgcgcgaggcagatatcggacttgccatg
ggcattgcagggactgaggtggctaaagagaatgctgatgttgtagttctggatgacaacttctccaccattgtaacagttgtcaaatgggggtgttctgtttatgtc
aatatccaaaagtttgtgcagttccagctgactgttaatatagttgcattgctagttaatttttcttttgcatgctttacaggtgttcaatga
>g168322.t1
atgggaaacgcatgcggcgggtcccttagatccaggtacctgccgagcttcaagtcagccgcgtcgcagcgccacgactccgaccacagcgccgccgccg
actcgcccaagaagccctcccgccccgccacgccccccgccgccgcgacggacgcccacgcggcacccgcgccggggctgccgccgccgccccggcat
gaggcgcggcggcgcggcgcccccggacctcggctccgtgctgggccaccccaccccgaacctccgcgacctctacgccgtgggccgcaagctcgggc
agggccagttcgggaccacctacctctgcaccgacctcgccacgggcgcccagtacgcctgcaagtccatctccaagcgcaagctcatcacccgggaggac
gtcgacgacgtgcgccgcgagatccagatcatgcaccacctctccggacaccgcaacgtcgtcgcaatcaagggcgcctacgaggaccagctctacgtcca
catcggccagttcgggaccacctacctctgcaccgacctcgccacgggcgcccagtacgcctgcaagtccatctccaagcgcaagctcatcacccgggagg
acgtcgacgacgtgcgccgcgagatccagatcatgcaccacctctccggacaccgcaacgtcgtcgcaatcaagggcgcctacgaggaccagctctacgtc
cacatcgtcatggagctctgcgccggcggggagctcttcgaccgcatcatacagcgcggacactacagcgagcgaaaggccgcagagctcacgcggatcat
cgtcggggtcgtcgaggcgtgccactcgctcggggtcatgcaccgggacctcaagccagagaacttcctgctcgccaacaaggacgatgacctctcgctcaa
ggctatcgatttcggcctctccgtcttcttcaagccggaaacgcagcaaggaatatttgatgctgtattgaaaggcgacattgattttgattctgacccgtggcctgt
gatatctgatagtgcgaaagacctgataaaaagaatgctcaatcctcgccctaatgaacgcttaacagcacatgaagttctatgccatccatggattcgtgatcatg
gagtagcacctgatcgtccacttgatcctgctgtcctatctcgcatcaagcaattctctgcaatgaataagttgaagaagatggctttgcgagtaatagctgagagt
ctctcagaggaggaaattgcagggttgaaagaaatgttccagaccatggacactgacaacagtggtgcaattacatatgatgagctaaaagaaggattgagaag
atatggctccacactaaaggatactgaaatccgtgatcttatggatgcggctgatattgacaacagtggcacgattgactacatagaattcattgctgcaacattgc
atctcaataaactggagcgtgaggaacatctggtggcagcatttacatattttgacaaagatggtagtggttacatcactgtggatgagctgcaacaagcttgcaa
agaacataacatgccagatgcttttcttgacgatgtcattaaagaagctgatcaggacaatgacggacgcattgactatggagagtttgttgccatgatgaccaag
ggcaatatgggagttggtcgaagaacaatgagaaacagtttgaatatcagcatgagggacgcacctggtgcattttag
>g168792.t1
atgttcaataacatggatactgacaagagtggcacaatcacagttgaagaactgaaggaaggactgacgaaactaggatcgaagattagtgaagcagaggttc
agaaacttatggaagcagttgatgtagacaagagtggcagcattgattatacagagttccttactgccatgatgaacaaacataaagtggaaaaggaggaggatt
tgctctgtgcatttcagcacttcgacaaagatagcagcgggtacataacaagagatgaactggaacaagccatggcagagtatggaatgggtgatgaggcaag
cattaaacaagtactggatgaagttgataaagataaggatgggaaaattgactatgaagagtttgtggaaatgatgaggaaaggaagctatacctga
>g168793.t1
atgggcaactgcttcaccaagacgtacgagatacccatcaccacggagccgtcggatcggcggcgcgcgccgtcatcgtacggctaccagcagccgccga
ggagccccgatcagcgcaagtcccgcgacgtgccggtgacgacctcgcgccggccgtccttccgtccgccgccgcccttccggccgtcgagcacgacgac
gggcaccggctcgtcgtcgatgatgccccgtcgcgcggcctcctccggcgaggtgggcccggtcctgcagcggccgatggtgaacgtgcgcacgctgtacc
agctggagcggaagctggggagcgggcagttcgggacgacgtacctgtgcacggagcgcgcgacggggctgcggtacgcgtgcaagtccgtgtcgaagc
gcaagctggtgcgccgcgccgacgtggaggacatgcgccgggagatcaccatcctgcagcacctcagcgggcaggccaacgtggccgagttcaagggcg
ccttcgaggacgccgactgcgtgcacctcgtcatggagctctgctccggcggggagctcttcgaccgcatcaccgccaagggcacctactccgagcgccag
gccgccgccgtgtgccgcgacatcgtcaccgtcgtgcacgtctgccacttcatgggggtcatgcaccgggacctcaagccagagaatttcctgcatgccagcc
ccgcggaggacgcgccgctcaaggccatcgatttcggactctccgtcttcatcgaagaaggttcgtcgttctga
>g168970.t1
atgggcaaggagaagagtcacattaacatcgtggtcattggacatgtcgactcaggcaagtccaccaccactggccacctgatctacaagcttggaggaattga
caagcgtgtgatcgaaaggttcgagaaagaagctgcagaaatgaacaagaggtctttcaagtatgcctgggttcttgacaagctcaaggctgagcgtgagaga
ggtattaccattgatattgctctgtggaagtttgagaccaccaagtactactgcacagttattgatgcccctggacaccgcgacttcatcaagaacatgattactggt
acctcccaggctgactgtgctgttcttatcattgactccaccactggtggttttgaggctggtatctccaaggatggccagacccgtgagcatgctcttcttgctttca
ctcttggagtgaagcagatgatctgctgctgcaacaagatggatgccaccactcccaagtactcaaaggcccgttatgatgagattgtgaaggaagtctcttcata
cctcaagaaggttggctacaaccctgacaagatcccсttcgttcccatctctgggttcgagggtgacaacatgattgagagatccaccaaccttgactggtacaa
gggccccaccctgctggaggctcttgaccagatcaatgagccgaagaggccctcagacaagcccctgcgtgttccccttcaggacgtgtacaagattggtggt
attggaactgtgcctgtgggccgtgtggagactggtgtcctcaagcctggtatggttgttacctttggtcccactggactgaccactgaggtcaagtctgtggagat
gcaccacgaggctctcctggaggccctccctggtgacaatgttgggttcaacgtgaagaacgttgctgtgaaggatctgaagcgtgggtatgtggcttccaactc
caaagatgaccctgccaaggaggctgccagcttcacctcccaggtcatcatcatgaaccaccctggccagatcggcaacggctatgccccagtgctggactgc

cacacctcccacatcgctgtgaagtttgctgagcttctgaccaagattgacagacgatctggtaaggagctcgagaaggagcccaagttcctgaagaacggtga
tgctggtatggtgaagatgattcccaccaagcccatggttgtggagaccttctcccagtaccctcctctcggtcgttttgctgtccgtgacatgaggcaaacggttg
ctgttggtgtcatcaagagcgtggagaagaaggaccctaccggtgccaaggttaccaaggctgctgctaaaaagaaatga
>g171444.t1
atggggaacacctgctccggtccaacgccccctcggaccgccatggtttcttcgactccgtctccctcgccgtgctctggcgcccacccgccgccgccaggg
ccgagccccttctccatcggactccaggccctccgcctcctcgacgacttcctccaggcctcccgagtccgtcaccttcaccgatgccgagcactccccccac
gcctcccctccgcgccggaccctaacggcaagcccaggcctaaggnnnnnnnnnnnnnnnnnnnnnnnnnnnnnnnnnnnnnnnnnnnnnnnnnnn
nnnnnnnnnnnnnnnnnnnnnnnnnnnnnnnnngacgacttcctccaggcctcccgagtccgtcaccttcaccgatgccgagcactcccccacgcctcccc
ctccgcgccggaccctaacggcaagcccaggcctaaggtgaagcgcgtgcagagcgccggcctccttgtcggctccgtcctgaaacgtaactccgagcggc
tcaaggacctctacaccctgggcaagaagctgggacaggggcagtttggcaccacctaccagtgcgtagagaaggccaccggtaaggtattcgcctgcaagt
ccatcgccaagcggaagctcgtcagcgaggaagacgtcgaggacgtgcgccgcgagatacagattatgcatcacctctccggcaaccccaatgttatctccat
cgttggtgcttatgaggacgctgtcgcggtgcaccttgtcatggagctgtgtgcgggaggggaactgttcgatagaatcatacagcgggggcactactctgaga
aagctgctgcgcaactggcacgggtgatcattggagtggtagaggcatgccattctctcggggtgatgcacagagacctcaagccggagaatttcttgtttgtga
accacaaggaggatgcactgctaaaggcgatcgattttgggctctccatcttcttcaaaccagcgttcatgacatatggtgaaacattttcggatgtcgttggaagt
ccgtactatgttgcacctgaggttctgatgaaacactatggctgtgaggtggatgtgtggagtgctggtgtaataatttatatcttgctgagcggagtccctccatttt
gggatgaatctgaacaaggaatatttgaacaagtcctgaaaggtgatcttgacttttcatctgagccctggcccagtatctcaaggagtgcaaaggatttggtcag
gaagatgttaatccgtgatcccaaaaagagattgactgcgcatgaagctctatgtcatccatgggtttgtgttgatggagttgctcctgacaaacctcttgattctgct
gtcttaaccagattgaaacagttttctgcaatgaacaaattaaagaagatggcccttagggtcattgctgagaatttatctgaagatgagattgcagggttgcgaga
aatgttcaaaatgctggacactgacaacagtggccaaattacattggaagaactaaaaagtggcttgaagagagttggtgctaacttaaaggactctgaaattaca
atattaatggaggcggctgatattgataacagtggttcaattgattatggagagttccttgctgctactttgcatctgaacaaagttgagcgagaagataatctctttg
ctgcgttctcatattttgataaagacggcagcggttacattactcaagatgagctacagaaagcatgtgaggagtttggtataggagatgaacaccttgaagatgtt
atcagagacattgatcaagacaatgatggccggatcgactacaatgagtttgtaacgatgatgcagaagggaaataatattttaggtaaaaagggacaaggcca
gatgagttttggtcttagggaagcattgaagcttggttaa

CaMK2 genes

>g636.t1
atggggctcggggcggtgatgatggggctggccgacccgccggcgaactacggctcgatctccgcggtggggctgttcgtcgcgctcatgtgcgtctgcatc
atcgtcggacacctcctcgaggagaaccggtggatgaacgagtccatcaccgcgctcttcatcgggctcgggaccggcgccgtgatcctgatggcgtccagc
gggaagcactcccgcgtgctggtcttcagcgaggacctcttcttcatttacctgctcccgccaattatattcaacgcagggttccaagttaagaagaaacaattctt
ccgcaatttcatgactatcacactcttcggcgcagttgggactttgatctctttcactgtaatatcccttggtgctctaggactagtatcaaagcttaacattggtgagc
tggagctaggagattatcttgcacttggggcaatattctcggcgacggactctgtgtgcaccttgcaggtgttaaaccaagatgacacccctttcttatacagcttg
gtgtttggtgaaggtgttgtcaatgatgcaacatccgttgtgctgttcaacgcaatccagagtttttctaccgggagtttcagtggcgttaatttacttaaatttggtggc
agcttcctctatctgttctgcaccagcacctttcttggagtagcatctggacttatcagtgcctatgtcatcaagaaactgtactttggcaggcattccactgatcgtga
agtcgcgattatgatgctcatggcttatttatcttacatgatagctgaaaaagttcgagggtcacaaccaaaaagttcgagggtcacaaccaagtaa
>g701.t1
atgggcaactgctgcgtggcccggccgtcgcccggcaagcggcgtgccggcggcggcgggaaccaccgcggcggccgcctcggtggcgccaacatgca
gtcctgctccgcgatctcctccatcaccgaccccggaggtgcctcccgcgggtgggcccccgccaccgtgctggggaaaggcctggcccccgacgccacc
gccgcggagctgctccggcggtacgagctcggcgaggagctcgggcgcggcgagttcggcgtgacgcggcggtgcacagacacggccacgggcgagtc
gctggcgtgcaagtccatcagcaagcgccggctgcgcagcagcgtggacatcgaggacgtgcggcgggaggtggccatcatgcggtcgatgcccgcgca
cccgaacgtcgtgcgcctgcgcgaggcgttcgaggataacgaggccgtgcacctcgtcatggaggtttgcgagggcggggagctcttcgaccgcatcgtctc
cagggggcactataccgagcgcgccgccgctgccgtcatgcgcaccatcgtcgaggtcgtgcagcattgccataagaacggtgtcatgcatcgggatctcaa
gcctgaaaactttctgtacgccaacacatctgagaactccccctcaaggtcatcgactttggtctctccgtgtgcttccaaccaggtgataggttcagtgagattgt
cggatccccatactacatggctcctgaagtcctcaagaggaactacggacaagaaatagacatatggagtgccggagtcatactctatatcttgctatgtggtgtc
ccacccttctgggctgaaactgatgaagggattgcacaggctatcatccggtcaaaacttgatttcgagagggagccttggcctaaggtttctgagaatgcaaaa
gatcttgtcaggaagatgcttgatccgagcccttatggacgattgacagctcagcaggttttagaacatccttggatacagaatgcgaactcagctcccaacatcc
ctcttggagaagcagtaaggtccaggcttaagcaatttactgtcatgaacaagttcaagaagaaggccctacttattgtggcagaatacctaccaactgaagagct
ggaggcaattagggagctgttccacatgttggacaccaacaaggacgggcacttgactattgacgaacttcgcaagggattgcgagtgatagggcagaatgca
gacattgacggcaacggtaccctggacttgaaggagtttgtgacagtctccattcatttgaagaaaatcagaagcgaggatcacttgccaaaggtattcagctact
ttgacaagaatggaagcggttacattgaaattgaagaattgaaggaggcgctctctccacgaggaggccagaaggcaatcgatgacataatcctggatgtcga
caaagacaaggcagacattgacggcaacggtaccctggacttgaaggagtttgtgacagtctccattcatttgaagaaaatcagaagcgaggatcacttgccaa
aggtattcagctactttgacaagaatggaagcggttacattgagattgaagaattgaaggaggcgctctctccacgaggaggccagaaggcaatcgatgacata
atcctggatgtcgacaaagacaaggatgggaagataagctacgaggagtttgagctgatgatgaaagtgggccacttgctggagctgcaaaagaacaacgag
aggttgactgatgagcgatgtgtttcgtacagtggatgtatcgaaacagaaagtgtgtga
>g1997.t1
atgggcggccgcgcctcccgccaccgccaatcgcccgaggaccgaaactcgcagcctcccaagcagaagcagcagcagcatcagtcgacccgcccatca
cccaagcagcagcacaggccacagccgccgccgccgccgaaacagctgcagcagcctcctcctccgccgcgcgcttcccctcttccgccggcagcaggg
ccggacgatggcgtgggccgggtgctgggccgcccactggaggacgtgcgcgcgagctacaccttcgggcgcgagctgggccggggccagttcggcgtc
acctacctggcgacccacaagcccacgggccgccgccacgcctgcaagtccatcgccacacgcntcacctacctggcgacccacaagcccacgggccgc
cgccatgcctgcaagtccatcgccacacgaaagctcgcgcgcgccgacgacgccgacgacgtccgccgggaggtgcacatcatgcaccacctcacgggcc

accgcaacatcgtcgagctccgcggcgcctacgaggacaggcactccgtcaacctcgtcatggagctctgcgagggcggggagctcttcgacaggatcatc
gccaggggacactactccgagcgcgccgccgccgcgctgtgcagggagatcgtctccgtcgtgcacacctgccattccatgggggtcatgcacagggacct
caagcctgagaacttcctgttcctcaacaaatcagaagactcgccgctcaaagcgacagactttggactatccgtattcttcaagccaggtgagcagttcaggga
ccttgttgggagtgcctattacgtggccccggaagtgctcaaacggcgatatggggctgaggccgacatctggagcgccggagttatcctgtacatccttctatc
cggcgtccctcctttctgggccggacatccatggattcgagaggatggagaggccccagacaaaccactcgatattgcagtaatcggcagaatgaagcaattc
agggcaatgaacaagcttaagaaggttgctttgaaggttgttgcagagaacttgtcagaggaagagatcgtcagattaaaagaaatgttcaaatccctggatact
gacaacagtgggacaataactctggaagaactaagaactggcttaccaaagcttgggaccaaagttactgaatcagaaataagacagttgatggaggcggctg
atgttgatggaaatggtatcattgattatgctgaattcatatcagcaacaatgcatatgaatagactagagaaggaggatcacatatataaagcatttgagtattttgat
aaggatcacagcgggtacataacagttgatgagttggaagaagctttaaagaagtatgatatgggcgatgaggcaacgataaaagaaattattgccgaagtgga
tacagaccatttcgcaaaatag
>g2451.t1
atgcaaccggacccgagcgggaacgccaatgcgaaggggaagctgccgcagccggtgacggcgccggcgccgtcgtccggccggccggcgtccgtgtt
gccgtacaaaacggcgaacgtgcgcgacttctaccgcatcggtaagaagctggggcaggggcagttcggcaccacgtaccagtgcgtggacaaggccgat
ggcgcagagtacgcgtgcaagtccatccccaagcgcaagctgctgtgccgggaggactacgaggacgtgtaccgcgagatccagatcatgcaccacctttc
ggagcaccccaacgtcgtgcgcatccgcggcgcttatgaggatgcgctcttcgtgcacatcgtcatggaaatctgcgccggtggtgagctctttgatcgcatcgt
cgcaaagggacactacagcgagcgtgcggcggcgaagctcatcaagaccatcgtcggggttgtggaggggtgccactcgcttggtgtcatgcaccgggacc
tcaagccggagaacttcctctttgccagcacagcagaggacgcacctctcaaggctactgactttgggctctccatgttctacaagcccggtgataaattctctgat
gtcgttgggagcccctattatgtcgcaccagaggtgcttcagaaatgctatggtccagaagctgatgtctggagtgctggggttatcctgtacattttgctttgtgga
gttcccccattttgggctgaaactgaagcaggaatcttcaggcagattctacggggaaaacttgattttgaatctgaaccctggcctagcatctctgatagtgccaa
agatctggtccgtagtatgcttactcgtgatcctagaaagagatatagtgctcatgaagttctctgtcacccatggatcattgatgatgccgttgcacctgataagcct
attgattctgctgttttgtcaaggctgaaacatttctcagcaatgaacaagctcaagaagatggcattgagggttattgctgaaagcttgtcagaggaagagattgg
aggcctcaaggagctgttcaaaatgattgatactgacaatagtgggactataacatttgatgagctgaaaaatggcttgaaaagggttggctcagagttaacaga
gaatgagatccaagctttgatggaagcagctgatattgacaacagtggaaccatcgactacggtgaatttatcgcagctacattgcacatgaataaactagagag
ggaggaaaacttggtgtcagcattctcgtttttcgacaaagatggaagtggctttataaccatagatgagctgtcacaagcatgccgtgaatttggtcttgatgatgt
tcaccttgaggatatgattaaagatatcgatcaaaacaatgatgggcaaattgactacagcgagttcacggcaatgatgaggaagggcaacgctggttgtgcaa
caggtaggaggaccatgaggaccaatttgaatcttggtgaactcttgaatcccagcaacagctag
>g3293.t1
atggaaacagagaagaatggtaacattctgctgcaaaggtatgaggttggtagattacttgggcagggcacttttgctaaggtttaccatgctaggaacatagtga
catcacagagtgttgctatcaaggtgattgataaagacaaaattttcaaggttggtctaatggagcagatcaagcgggagatatctgtgatgaaattagtgaggca
ccccaacattgtacagctttatgaggtcatggctaccaaatcaaagatatattttgttcttgaatatgtcaagggtggtgaattgtttaacaaaatagctaaaggtaagt
taagagaagatgcggctaggaagtacttccagcagttgataagtgctgttgatttctgccatagtagaggtgtataccatcgtgatctgaagccagaaaacctactt
gttgatgagaatgggaatctgaaaatttcagattttggattgagtgctcttgctgagtcaagacgccaagatggccttctacacacaacttgtggtactccagcatat
gtggcgcctgaggttatcagccgaaaagggtatgatggtgccaaggttgacacatggtcttgtggggtgatcctgttcgttctcatggctgggtatctgccttttca
agattccaatttggtggagatgtatagaaaaattgggaaagcggagttcaagtgcccgccttggtttccatctgatgatctgtggaacatggaaacagagaagaat
ggtaacattctgctgcaaaggtatgaggttggtagattacttgggcagggcacttttgctaaggtttaccatgctaggaacatagtgacatcacagagtgttgctatc
aaggtgattgataaagacaaaattttcaaggttggtctaatggagcagatcaagcgggagatatctgtgatgaaactagtgaggcaccccaacattgtacagcttt
atgaggtcatggctaccaaatcaaagatatattttgttcttgaatatgtcaagggtggtgaattgtttaacaaaatagctaaaggtaaattaagagaagatgcggcta
ggaagtacttccagcagttgataagtgccgttgatttctgccatagtagaggtgtataccatcgtgatctgaagccagaaaacctacttgttgatgagaatgggaat
ctgaaaatttcagattttggattgagtgctcttgctgagtcaagacgccaagatggccttctacacacaacttgtggtactccagcatatgtggcgcctgaggttatc
agccgaaaagggtatgatggtgccaaggttgacacatggtcttgtggggtgatcctgttcgttctcatggctgggtatctgccttttcaagattccaatttggtggag
atgtatagaaaaattgggaaagcggagttcaagtgcccgccttggtttccatctgatcatcatgttcttaagttgcacctgccatcaattgtagttgattaa
>g4006.t1
atggcggatcagctcaccgacgaccagatcgctgagttcaaggaggccttcagcctcttcgacaaggacggtgatggctgcatcaccaccaaggaacttggta
ctgtaatgcgctctttggggcagaaccccactgaggctgagcttcaggatatgatcaatgaagtggatgctgatggcaatggaactatagacttccccgagttcct
caacctgatggctcgtaagatgaaggacacagactctgaagaggagcttaaggaggctttccgtgtgtttgacaaggaccagaacggcttcatttctgcagctga
gctccgccatgtcatgaccaaccttggctgcatcaccaccaaggaacttggtactgtaatgcgctctttggggcagaaccccactgaggctgagcttcaggatat
gatcaatgaagtggatgctgatggcaatggaactatagacttccccgagttcctcaacctgatggctcgtaagatgaaggacacagactctgaagaggagctta
aggaggctttccgtgtgtttgacaaggaccagaatggcttcatttctgcagctgagctccgccatgtcatgaccaaccttggtgagaagctgacagacgaggaa
gtggatgagatgattcgtgaagctgatgtggacggtgatggccagatcaattacgaggaatttgtgaaggtgatgatggccaagtga
>g9985.t1
atgggcaactgctgcccgggctccggggatgcggagcccgccgccggcgacgcctcgtcccgcctccacgccgcctccctcaaggccggcggcagcgnn
nnnnnnnnnnnnnnnnnnnnnnnnnnnnnnnnnnnnnnnnnnnnnnnnnnnnnnnnnnnnnnnnnnnnnnnnnnnnn
nnnnnnnnnnnnnnnnnnnnnnnnnnnnnnnnnnnntcccccatccgccgccgccgccccgcgcgccatgggcaactgctgcccgggctccggg
gatgcggagcccgccgccggcgacgcctcgtcccgcctccacgccgcctccctcaaggccggcggcagcggcagcggcagcgggccgacgtccgcgcc
cacgccgaccaagccgccggcgccgatcgggcccgtcctgggccggcccatggaggacgtccgcagcatctacaccgtgggcaaggagctgggccgcg
gccagttcggcgtgaccagcctgtgcacgcacaaggccacgggcgagcggttcgcgtgcaagaccatcgccaagcggaagctgtccaccaaggaggacgt
ggaggacgtgcggcgcgaggtgcagatcatgtaccacctggcgggccagcccaacatcgtcgagctcaagggcgcctacgaggacaagcagtccgtgca
cctcgtcatggagctctgcgcgggaggggagctcttcgaccggatcatcgccaaaggcaagtacacggagcgcgccgccgccgcgctgttgcgcaccatcg
tcgagatcgtgcacacgtgccactcgctcggcgtcatccaccgcgacctcaagccagagaacttcctcctcctcagcaaggacgagcacgcgccgctcaagg
ccaccgatttcgggctctccgtattcttcaagcagggggaggtgttcaaggacatcgtgggaagcgcctactacatcgcgccggaggtgctcaagaggagcta

cggaccagaggcggatatctggagcgtcggtgtcatcctctacatcctactctgcggagtgcccccgttctgggcaggcaagttcatccctgttcaatcggagca
cggcatcttcaacgccatcctccggggacaggtcgacttctccagcgacccatggccgcgcatttcctccggcgccaaggatctcgtcaggaagatgctcacct
ctgaccccaagaagaggatctcggcctacgacgtcctcaatcatccttggatcaaggaagacggcgaggcgcctgacacgccgctggacaacgctgtcatga
acagactcaagcagttcagggcaatgaaccagttcaagaaagccgcgctcagggtcattgccgggtgcctgtcggaggaagagatcaaggggctcaaggag
atgttcaagggcatggacgctgacaacagcggcaccatcaccgtggacgagctgcggcgagggctggccaagcagggcaccaagctctcggaggccgag
gtggagcagctcatggaggccgccgacgcggacgggaacggcacgatcgactacgaggagttcatcaccgcgacgatgcacatgaaccggatggacagg
gacgagcacctctacgccgcgttccagtacttcgacaaggacggcagcggctgcatctccaaggaggagctggagcaggcgctcagggagaagggcctgc
tcgacggccgggacatcaaggacatcatctccgaggtcgacgcagacaacgacgggaggatcgactacagcgagttcgtggcgatgatgaggaaagggaa
ccccgaccccaaccccaagaagcggcgcgacgtcgtgctgtag
>g11720.t1
atggcgatgccgcaacagccgcgaactgtggcctacggacgctgcctccgccggtcgacgaccaccatcgcggcgtcacccccctgcccacgttcttcgggtc
agcggcgagggcccggttctgccgtgccggcagccgttccacaggagaggagaggagaggagagcgcgcaccaccggaatggagggagctgacaagg
atggcaacccgttgaggaattaccgaattggtaagactctggggattggttcattcggtaaggtgaaaatcgcggagcatataaccactggtcacaaggtggcta
ttaagatcctcaatcgtcgcaagatcagaggcatggaaatggaagagaaagattgtgagccccgaatgttctgccccagcannnnnnnnnnnnnnnnnnata
accactggtcacaaggtggctattaagatcctcaatcgtcgcaagatcagaggcatggaaatggaagagaaagttaaaagagagatcaagatattgaggttgttt
atgcacccacatattattcgcctctatgaggtgatagatacgccagctgacatttatgtggttatggaatatgttaagtgtggggagttgtttgattacattgttgaaaa
aggtagactgcaggaggaagaggctcgccgttttttccagcagattatatctggagttgagtattgccatcgaaacatggtggtccaccgtgacctaaaaccaga
gaacctcctattggattcgaaatggaatattaagattgcagattttggcttaagtaatgttatgcgggatggccatttccttaagacaagttgtggtagcccaaattatg
ctgctcctgagattgatgacgagactcttcgtgaggttataagtatgggttatgacaagaacgttttggtggaatcaatacaaaacagattacaaaatgaggcaact
gttgcgtattatttacttttggacaataggcttcgtacaactagtggctatcttggagctgagtgtcaagaagctatgctttacaaaacaagagacgagaaataccttc
ttgacttgcaaagagtcaccggaccgcagctcctctttctggacctgtgtgcggccttttttagctcagctgagagttctttga
>g12550.t1
atggagctcgtcgccgtcaaatacatcgagcgcggcgagaaggtcattttaacgccaacccatcttgctattgtgatggaatatgcatctggtggtgagctttttga
gagaatatgtaaaaatgtgcgattcagtgaagatgaggcacgctatttcttccagcagcttatctcaggagtaagctactgccattcaatgcaagtatgtcaccgtg
atctgaagctggagaatacactgctggatgggagtgatgctcctcgcttgaagatatgtgacttcggctattccaagtcttcagttcttcattcacagccaaagtcaa
ctgttggaacaccggcttatattgcacctgaagttcttttgaaaaaagaatatgatggcaagatagctgatgtatggtcttgtggtgtaaccctctatgttatggtagtt
ggcgcatatccttttgaagaccctgaagagcctaagaacttccgcaagacaattcagcgaatcttgaatgttcagtactcaattccagataatgtgaacatatctcc
agagtgcaggcatctaatttcgaggatatttgttggtgatcctgctatgcggatttcaatccctgaaatccgaaaccatagttggttcttgaagaaccttcctgctgat
ctgatggatgatgatagcatgagcaaccagtacgaggagcctgatcagccgatgcagaccatggatcagatcatgcaaattttgacagaggccaccataccac
ctgcttgttctcgcagcataaacgccctagctgatggactggacatggacatggacgatgacatggatgacctcgagtccgactctgatcttgatgttgatagcag
tggtgagatcgtatcgtgcatgcttaaaggggaggagatgaagcgagagcgctggaacgcgtacagtgcagagcctgacagcctgatcgaggagaggaag
gaggttctggacatggtcaggaacgagtttgaacaagaaaaataa
>g13082.t1
atgcgccacctccccaagcaccccaacatcgtcaccctcagggacacctacgaggacaacaatgccgtccacctcgtcatggagctctgcgagggcgggga
gctctttgatcggatcgttgcccgtggacactacacggagcgcgctgccgccttggtcactaagaccattgtcgaggtcgtgcaggtgagattggcttcattgttc
gtgcaggttcattga
>g13117.t1
atgggcaactgctgcgtgacgcctaacggcgccgatggcggcggcaagaagccgaagcagcccaagcagaagaagggcaagaagccgaacccgttctc
gatcgactacaaccggtcggcgccgccgggctcgaggctggtggtgctgcgggagcccacggggcgggacatcgccgcgcggtacgagctgggcgggg
agctcggccgcggcgagttcggggtcacctacctctgcaccgaccgcgccacgggggaggccctcgcctgcaagtccatctccaagaagaagctccgcac
ccccgtcgacatcgaggacgtgcgacgggaggtcgagatcatgcgccacctccccaagcaccccaacatcgtcaccctcagggacacctacgaggacaac
aatgccgtccacctcgtcatggagctctgcgagggcggggagctctttgatcggatcgttgcccgtggacactacacggagcgcgctgctgccttggtcactaa
gaccattgtcgaggtcgtgcaggtgagattggcttcattgtngatgatttcatgtaagttttga
>g13118.t1
atgggcaactgctgcgtgacgcctaacggcgccgatggcggcggcaagaagccgaagcagcccaagcagaagaagggcaagaagccgaacccgttctc
gatcgactacaaccggtcggcgccgccgggctcgaggctggtggtgctgcgggagcccacggggcgggacatcgccgcgcggtacgagctgggcgggg
agctcggccgcggcgagttcggggtcacctacctctgcaccgaccgcgccacgggggaggccctcgcctgcaagtccatctccaagaagaagctccgcac
ccccgtcgacgtcgaggacgtgcgacgggaggtcgagatcatgcgccacctccccaagcaccccaacatcgtcacgctcagggacacctacgaggacgac
aatgccgtccacctcgtcatggagctctgcgagggcggggagctctttgatcggatcgttgcccgtggacactacacggagcgcgctgctgccttggtcactaa
gaccattgtcgaagtcgtgcagatgtgccataagcatggagtgatgcacagagatctcaaaccagagaacttcttgtttgcaaacaagaaggaaacagcggctc
tcaaggcaattgattttggtctgtccgtatttttcactccaggtgaaaggttcactgagattgttggaagtccttattatatggctccagaggtactgaagagaaattat
ggcccggaagttgatgtttggagtgcaggagtgattctttacattcttctttgtggggttcctccattttgggcagaaactgaacagggtgttgctcaagctattattcg
ttcggtcattgattttaaaagagacccatggccaagggtctccgataacgccaaagatcttgtcaggggaatgcttaatccagatccgaaacggagattgacagc
tcagcaagtgcttgatcatccttggctgcagaacataaagaaggctccaaatgtcaatttgggtgaaactgttaaggccaggcttcaacaattttctgtgatgaaca
agttcaagaagcatgcgctcagggtcatagctgatcatctttcagtagaagaggctgctgacataaaagatatgtttgagaaggtagatcttaacaaagatcagat
gattagttttgatgagctgaagcttggtctgcataagtttgggcaccaaatgcctgatgcagatgtccaaatacttatggaagctgctgatgccgatggaaatggaa
ccttggactatggagagtttgttactctatctgttcacctaagaaagattggcaatgacgaacatctgcataaggcgtttgcttactttgatcggaatgagactggata
tatcgaaatcgatgaactccctgatgacctgggacaaaatcctgaagatgttatcaatgccatcatacgtgacgtggacactgataaggatggcaagataagcta
cgatgaatttgcggcaatgatgaaggctggaacagactggaggaaagcatcaagacagtattcaagagaacggttcaccaaccttagcctaaagctgcaaaag
gacggatcgttgcagatgacgagtacccggtag

>g14573.t1
atggaagggctgacgggggagcagatagttgctttccaggaggctttctccctcttcgacaagaacggcgacggatgcatcaccatggaagagctggctgca
gtgactcgctccctaggccttgatcctagtgatcaggagctcatcgatatgatgagcgaagttgatactgatggcaatgggataattgattttcaggagtttctgagc
cttattgccaggaagatgaaggatggtgatggcgatgaggagctgaaggaggcttttgaggtcctagacaaggaccagaatggatttatctcccctactgagct
gaggacggtgatgaccagccttggggagaagatgactgatgaagaggttgagcagatgatcaaagaagcggatactgatggtgatggacaggtgaactacg
atgagtttgtacttatgatgaaaaatgctgaacggaagatatctgggtga
>g16078.t1
atggaggagaggtacgaggcgctcaaggagctgggcgccggcaacttcggggtggcgaggctggtcagggacaaacggaccaaggagctcgtcgccgtc
aagtacatcgagaggggaaaaaagatcgatgagaatgtgcagagagagattatcaatcaccggtcacttcgacatcctaatatcgtacggttcaaggaggtaatt
cagaaatcattaactctatcttttgtttga
>g16714.t1
atgggtggccgcgcctcccgccaccgccaatcgcccgaggaccgaaactcgcagcccccaagcacaagcagcagcagcatcagtctacccgcccggcg
gcacccaagcagcagcagcagcacaggcacccgccgccgccgccgaagcagcagcagcctcctcctccgccgccgcacgcttcccctcctccggctccg
ccggaagcagggccggacgcgggcgtgggccgggtgctgggccgcccactggaggacgtgcgcgcgagctacaccttcgggcgggagctgggccggg
gccagttcggcgtgacctacctggcgacccacaagcccacgggccgccgccatgcctgcaagtccatcgccacacgaaagctcgcgcgcgccgacgacgc
cgacgacgtccgccgggaggtgcacatcatgcaccacctcacgggccaccgcaacatcgtcgagctccgcggcgcctacgaggacaggcactccgtcaac
ctcgtcatggagctctgcgagggcggggagctcttcgacaggatcatcgccaggggacactactccgagcgcgccgccgcagcgctgtgcagggagatcgt
ctccgtcgtgcatacctgccattccatggggggtcatgcacagggacctcaagcctgagaacttcctcttcctcaacaaagcagaagactcgccgctcaaagcaa
cagactttggactatccgtattcttcaagccaggtgagcagttcagggatcttgttgggagtgcctattacgtggccccggaggtgctcaaacggcgatatgggg
ctgaggccgacatctggagtgccggagttatcctgtacatcctgctatccggcgtccctcctttctgggccggacatccatggattcgagaggacggagaggcc
ccagacaaaccactcgatattgcagtaattggcaggatgaagcaattcagggcaatgaacaagcttaagaaggttgctttgaaggttgttgcagagaacttgtca
gaggaagagatagtcggattaaaagaaatgttcaaatccctggatgctgacaacagtgggacaataactctggaagaactaagaactggcttaccaaagcttgg
gaccaaagttactgaatcagaaataagacagttgatggaggcggctgatgttgatggaaatggtaccatcgattatgctgaattcatatcagcaacaatgcatatg
aatagactagagaaggaggatcacatatataaagcattcgagtattttgataaggatcacagcgggtacataacagttgatgagttggaagaagctttaaagaagt
atgatatgggcgatgaggcaacaataaaagaaattattgccgaagtggatacagatcataacatccttgcaaaggtgaaaataaaggggcaagcagagaatgc
tccaagatcagagatcttactgtttgactga
>g16824.t1
atggagaagtacctgcaggagcacttcgatctgccgcacaagaacccctccgaagaggcgcagcggcgatggcgctccgccgtcggcaccatcgtcaaga
accgccgccgccggttccgcatggtgcctgacctcgaccgccgccagaaggacgaggaggcgcgtcgctccatccaggaaaaaattcgggttgctctctatg
tgcagaaggctgcgatcattttcagtgatggtgccagcaagaaagagtacaagctgacagaggacattatgaatgctggattttcgatcaatccagatgaactgg
catcaatcacaagcaagcatgatatgaaagctttcaagatgcatggtggtgttgatgggatatccaaaaagatccgttcaactttcgaccgtggcatatcaactagc
gatctggataaaagacagatgatctacggtgaaaatcgttatgccgagaaacctgcaagaagcttctgggcatttgtttgggatgcgttgcaggacatgacccta
tcattcttatgttcaaggagctagacaatgagaagaaaaagatattcatccatgtaaccagagacggtgagagtgaccctgtgtacatttcggaggataagcccctt
catcttggcaggaactaaagttcaagatggatctgctaaaatgttggtaactgctgtgggtatgcgtactgaatggggaaggttgatgagcacattgagtgaggg
aggagaggatgagacaccattgcaggttaaactaaatggagttgcaaccatcattgggaagattgtgaactactttgcgacagcggtcactattattgttgttgctg
ttcctgaagcatgtgagacaatgggatctgctggtacaatctgcacagataagacaggaactttgacaactaaccatatgggtatttttgagaacacgagtgcaga
agtagtcaaggataaggatggtaaacaaactgttttaggaactccaactgaaagggcgatattagaatttggcttgatgttggaaggacatgataccgaggatag
aacctgcacgaaggtcaaggttgagcctttcaattcggtgaagaagaagatggcagtgttggtctcagtacctaacggcacataccgctggttcagcaaagaaa
gtcctacaagtggttttactctgatatgcatctttggcatcaaggaccctgtgcgcccaggagtcaaggatgctgttaaggcctgcatatctgctggcatcattgtga
gaatggtgactggtgataacatcaatacagctaaagctattgccaaggagtgtggcatactgactgatgacggcatagcaatagaaggaccagagttccgcaac
aagagcccagaagaaatgagggacttgatacctaagattcaggtcatggctcgttcgttaccattggacaaacataccccttgtgacaaacttgcgaggtatgttcc
gtgaagtggtcgctgtgacaggtgatggcacaaatgatgctccagcattgcatgaatcagatattgggcttgctatgggcatagctggcacagaggtagcaaag
gagagtgctgacgtgatagtgcttgatgacaattttactactataataaatgttgcaaggtggggtcgggcagtttacctcaacatccaaaagtttgtacagtttcagt
tgactgtcaatattgttgctttagtcatcaactttgtctcagcatgcattacagggagtgctcctctcacagcagtgcagttgttgtgggtcaatatgattatggataca
ctaggagctttggctctagctaccgagcctccaaatgatgacatgatgaagagaccacctgttgggcgaggggagagtttcattaccaagatcatgtggaggaa
tatcattggccaaagtttatatcagttggttgtacttggagctctaatgtttggtggggaacgacttctgaacatcaagggtgcagattccaaatctgtaatcaatactc
tcatattcaactcctttgtgttctgccaggtattcaatgaaataaacagcagggaaatgcagaagatcaatgttttccatggaatgtttagcaactggatcttccttgga
atcattgctgtgacagtagcatttcaagtggtgatcatagagtttnnnnnnnnnnnnnnnnnnnnnnnnnnnatactgactgatgatggcatagcaatagaa
ggaccagaattccgcaacaagagccccgaagagatgagggacttgatacctaagattcaggtcatggctcgttcgttaccattggacaagcataccccttgtgac
aaatttgcgaggtatgttccgtgaagtggtcgctgtgacaggtgagtgccctatagctgttatcaatacatccagtcctaatgcccatggtgatgcttcagtattcaat
gaaataaacagcagggaaatgcagaagatcaatgttttccatggaatgtttagcaactggatcttccttggaatcattgctgtgacagtagcatttcaagtggtgatc
atagagtttcttggcacttttgccagcaccgttccacttgattggcagctttggttggttagcatcggcctgggatccatcagcttgattgttggcgccatcttgaagt
gcataccagtggaatcaggtgggacttctgctagtccaaatggctacgccccactccccagcggccccgataacatttaa
>g19529.t1
atgggccaatgctgcgccaagggcggcgacccggccgccgccgccggcgacgacaaggaccgccccgcgtcgtcgtcgtcggagcccaagccggagg
cctccagccgcggcggcgcgaacgccgccccgccgcgctcatcgtcgtcttcctccaaccacccggcggtgggcccggtgctgggccggcccatggagga
cgtgcgcgcgacgcactcggtggggaaggagctgggccggggccagttcggcgtgacccacctgtgcacacagaagcccacgggggagaagctggcct
gcaagaccatcgccaagcggaagctctccacccgcgaggacgtggacgacgtgcgccgcgaggtccagatcatgcaccacctctccggccagcccaacgt
cgtcgtcctcaagggcgcctacgaggacaagcacaacgtacacctcgtcatggagctctgcgccggcggcgagctcttcgaccgggtcatcgccaagggac
agtacacggagcgcgccgcggcggcgctgctccgcaccatcgtgcagatcgtgcatacctgccacgccatgggagtcatgcacagggatatcaagccgga

gaatttcttgctgcttagcaaggacgaggacgcgccgctcaaggccacggatttcgggctatccgtcttcttcaaggaaggggaggtgttcagggacatcgtag
gaagtgcctactacatcgcgccggaggtgcttaagaggaagtacgggccagaggcggatatttggagtgttggagtcatgctatacatcttccttgctggagtgc
cgcccttctgggcggagaatgagaatggcatcttcaccgccatcctgcgtggccagattgatctcgccagcgacccgtggccaaagatttcgtcgggggcaaa
ggatcttgtcaagaagatgctcaccatcaaccccaaggagcggcttacggcgttccaggtcctcaatcacccgtggatcaaagaagatggagatgcacctgata
cgccacttgacaacgttgttctcaacagactcaaacagttcagggccatgaaccagttcaagaaagcggcactaaggatcatagctgggtgcctatcggaagag
gagatcaccgggctgaaggagatgtttaagaacatcgacaaggataacagtgggaccattacgcttgaagagctcaagaacgggttggcaaagcacggaac
aaagctgtcggacggtgaaattcagcaactcatggatgcagctgatgctgatggcaacggattgatcgactacgatgaatttgtcaccgcgacggtgcacatga
acaaactggacagagacgaccacctctacactgcattccagtatttcgacaaggataacagcgggtacattacaaaagaagagcttgagcaagctttgaagga
gcaaggattatatgacgccgaggagattaaggacgttatctcagatgctgatgctaacaatgacgggaggatagattattcagagtttgtggcgatgatgaggaa
aggtactgctggcgctgagacggcgaaccccaagaaaaggcgcgacctagtactgtag
>g20054.t1
atgggtctctgccatggcaagccaacgcaaatcccggagcctgaggcggaagaagatccccatgtagcctccggcgccggcgacgccgccggtgcccgga
acggcgcctccgcgccgtcgccagcgcctgcgacgaagccgggcacgccgaagcagcccaagttcccgttctacctaccgagcccgctcccgccgtcgag
ctacaagggctcgccggcgaactcgagtgtggcatcgacgccggcgcgcggagggttcaagcggccgttcccaccgccatcgccggcgaagcacatccgc
gcgctcctggcgcggcggcacggctcggtgaagcctaatgaggcgtccatccctgagggcggcgagccggagctcggcctggacaagagcttcggcttctc
aaagcacttcttcgccaagtacgagctcggcgaggaggtcggccgcggccacttcggctacacctgctctgcnnnnnnnnnnnnnnnnnnnnnnnnn
nnnnnnnnnnnnnnnnnnnnnnnnnnnnnnnnnnnnnnnnnnnnnnnnnnncacccccattcccctctgacccaagcctttcgctcaaagcttccagctttttcaattgtttg
gtcacttcaagaatcaaggaatatgttcttgcttcttgggcgatagtctga
>g22121.t1
atggggctcggggcggtgatgatggggctggccgacccgccggcgaactacggctcgatctccgcggtggggctgttcgtcgcgctcatgtgcgtctgcatc
atcgtcggacacctcctcgaggagaaccggtggatgaacgagtccatcaccgcgctcttcatcgggctcgggaccggcgcggtgatcctgatggcgtccagc
gggaagcactcccgcgtgctggtgttcagcgaggacctcttcttcatttacctgctcccgccaattatattcaacgcagggttccaagtgaagaagaaacaattctt
ccgcaatttcatgactatcacactcttcggtgcagtcgggactttgatctctttcactgtaatatcctttggtgctctaggactagtatcaaagcttaacattggtgagct
ggagctaggagattatcttgcacttggggcaatattctctgcaacggactctgtatgcaccttacaggtgttaaaccaagatgataccccccttcttgtacagcttggt
gtttggtgaaggtgttgtcaatgatgcaacatccgttgtgctgttcaacgcaatccaaggtttttctaccgggagtttcagtggcgttaatttacttaaatttggtggca
gcttcctctatctgttctgcaccagcacctttcttggagtagcatctggacttctcagtgcctatgtcatcaagaaactgtactttggcaggcattccactgatcgtgaa
gtcgcgattatgatgctcatggcttatttatcttacatgatagctgaagttattatatggtgggcggggctcatgagaggtgctgtatccattgcgctggcctacaata
agttcgcaaggtcaggccacactcagcttcctagcaatgccatcatgatcaccagcacgattagtgttgttcttttcagcacaattgtttttgggatactgacgaagc
cgttgatcagactcttgaatccgccaaggtacctatccagggaacagagcgccctctctgaaccatccagccccaagaccttccttgagcggctgatcgagaac
ggtcctgatgctgatcttgagaatggagtgagcctacgccgcccttcgagtctccggctcctcctggcaagcccaacacggtctgtccactattactggcgcaag
tttgacaattccttcatgcggccggtgtttgggggccgaggctttgttcccttcgtcccggggtcaccaactgagagcagtgtgccgttgcttgctacaactgagg
acaactga
>g22370.t1
atgggcaacacctgcgtcggccccagcgcggcgggccgcagcggatttctcgcctccgtcaacctgtggcggcccgcgcagcccgccgccgccctcccgc
cgccctcctcccccgcctccgacaaggcgcccgagcccgtcacaatccccgcctccgagcactcctcccaccactcctcccgatcctccgaccagccgcaga
cgccctccgcggagccgcagacgcaggacaataatcctccggcgaagaggcccgtgcccaaggtgaagcgcgtccagagcgcgggcctcctcgcggact
ccgtcctcaagcgcgacgtcaacaccgcccggctcaaggatctctacaccatcggcaagaagctagggcaggggcagttcggcaccacctacctctgcgtcg
agaaggccaccggccgggagttcgcctgcaagtccatcgccaagcggaagctgctcacggaggaggacgtcgaggacgtgcgccgcgagatccagatca
tgcaccaccttgcgggtcacgccaacgtcgtctccatcgtcggcgcctacgaggacgcggtcgccgtgcagctcgtcatggagctctgcgccggcggggaa
ctattcgacaggattatccaaaggggacattactccgagaaggccgccgcgcagctggcgcgggtgatcgtcggcgtcatcgaagcctgccactcgctcggt
gtgatgcacagggatctcaagccggagaacttcctgttcgtcaaccagaaggaggactcgccgctcaaggccattgatttcggtctctccatctttttcaaaccag
aacaggattcccccccgcctatctggtgaatatatactga
>g22373.t1
atgggtaacacatgcgtgggccccagcatcaccaagaacggcttcttccagtccgtgtccacgctgatgtggaagacgccgcaggacggcgacgcggcgct
gcccgccgccgccgccaatggctccgaaggcggcagcccgggccggtcgcagcccaccaagcccgaagtgcagagcaaggccccggagcccatgaag
atcgccgcagcccaacaaccggatgagtcgccgccgccgcccagcaataagcagcagcaggacacgaagccaaacgccggcgccggcgaggcgccca
gcaacaagccgcggcccaaggttcctcaggtgaagcgcgtgtcgagcgccgggcttctggtgggctccgtgctgaagcgcaagacggagaacctcaagga
gaagtacagcctgggccgccggctcgggcagggccagttcgggacgacgtacctgtgcgtggagcgcggcacggggaaggagctggcgtgcaagtccat
cctgaagcggaagctggtgaccgacgacgacgtggaggacgtgcgccgtgagatccagataatgtaccacctggcgggccacccgaacgttatctccatcc
gcggcgcgtacgaggacgccgtcgccgtgcacctcgtcatggagctctgcgccggcggggagctcttcgaccggatcgtgcagaggggacactacaccga
gaggaaagccgccgagctcgcaagggtcatcgtcggcgtcgtcgaggtgtgccactccatgggcgtcatgcatcgggatctcaagcctgagaacttcctcttc
gttgacaacaaggaggaggccgcgctcaagaccattgactttggtctctccatcttcttccgcccaggtcaagtattcacggatgttgtcggtagcccatactacgt
cgcgccagaggtcttgaaaaagaaatatggtcccgaggcagatgtctggagtgctggtgtgatcatctacattttgctgtgcggtgtgccaccattttgggcagag
aacgagcagggtatatttgaagaggttttacatggaaaacttgactttcagtcagagccgtggcctagcatctccgaaggtgccaaagatcttgtgaggagaatg
cttgttagggaccccaaaaagagattgacggctcatgaagttctacggcatccatgggttcaggttggcggtttggctcctgacaagccgctggattctgctgttct
gtctcgcatgaaacaattctcggctatgaataagctgaaaaagatggctcttagggtcattgcagagaacttatccgaggacgagatcgccggcctgaaagaaat
gttcaagatgatagacacggacaacagtgggcacatcacctacgaggaactgaaggtcgggttgaaaaaagttggtgccaacctccaggaatccgagatcca
ggcactcatggaagctgcggacgtggataacagcggcacgatagactacggcgagttcatcgcggccactctgcacctgaacaaggtggagagggaggac
cacctgttcgctgcgttccagtacttcgacaaggacggcagcgggtacatcacgcccgacgagctgcagctggcgtgcgaggagttcggcctgggcggaga

cgtccagctcgaggagatgatccgagaagtggaccaagacaacgacgggcgcatcgattacaacgagttcgtggcgatgatgcagaagcccacgctgggg
ctcccgaagaagtccgggggtctgcagaacagcttcagcatcggcttcagggaggcactgaggatttcgtaa
>g27338.t1
atgcatagacacaagcttgaaagagatgagcatctgtttaaggcattccaatactttgataaagataacagcggcttcatcacaagggatgaactagaatctgcttt
gattgaacatgaaatgggtgatgcaagtaccatcaaagatatcatttcagaagttgacactgacaatgatggaaggatcaactacgaggaattttgtgctatgatga
gaggaggaatgcagcagccaatgaggctcaagtag
>g27641.t1
atggcgattgctactgagtcgtgcggcggcagtgaggacaaggctgcatcacatgcaaatgaggagaagcgttgggtgctgccagattttgaggttggaaagc
cacttggcaggggaaagtttggccatgtttacttggcaagagaaaaaaggagcaatcagattgtggctttgaaagttcttttcaagagccaactgaaacagtctca
agtcgagcatcagcttcggcgtgaagttgaaatccagagtcacctaaggcaccccaatattctacgcctgtatggttacttctatgaccaggtacataatagataa
>g28784.t1
atgggcaacacgtgcggcgtcacgcttagatccaagtacttcaccagcttccgtggctcgcagcgccacgacaccgcgggatacgcgcccgtcgccgtcgcc
gcctccgacgaccccgcgccgcgcgggaaaggcaagcgggcctcgccccgcacggccgatgcggcggccgccgcggcggacgcggacgcggccgct
ccgccccccgcccccggcatgcggcgcggggcgcccgcgcccgcggagctcacggccaacgtgctcggccacccgacgccgagcctccgggagcact
acgcggtcgggcggaagctcgggcagggtcagttcggcaccacgtacctctgcaccgaccttgccacgggggtcgactacgcctgcaagtccatctccaag
cgcaagctcatctcgcgggaggacgtcgaggacgtgcgccgcgagatccagatcatgcaccacctcgccggccaccgtaacgtcgtcgccatcaagggcg
catacgaggaccagcagtacgtccacatcgtcatggagctctgcgcgggcggggagctgttcgaccgcatcatccagcgcgggcattacagcgagcgcaag
gcggccgacctcacgcgcatcgttgtgggggtcgtcgaggcgtgccactcgttaggggtgatgcacagggatctcaagcccgagaacttcttgcttgctaaca
aggatgacgatatgtctctcaaggcaatcgattttggactctctgtgttcttcaagcctggtcaaatttttaccgatgttgttggaagcccctactatgtagctccagaa
gtgctgcgcaaacgttatggaccagaagctgatgtgtggacggccggagtgattctatatatattgctaagtggcgttccaccattttgggcaggtcatccctggat
ttgtgaccagggagtggctcctgatcggcctcttgatccagctgtcctctctcgcattaagcagttctcagcaatgaataagttgaagaagatggctttgagagtca
ttgctgagagtctttcagaggaggagatagcaggattgaaggaaatgttcatggcaatggatacagataacagcggggccattacatatgatgagctaaaagaa
ggcttgaaaaaatatggttctacactaaaggacactgaaattcgtgatcttatggaggcggatggccgcattgattacggtgaatttgttgctatgatgacaaaggg
caacttgggagttgggagaagaacaatgagaaacagcttgaatataagcttgagggacacacctgctgctctctga
>g32373.t1
atggagaagtacgagctgctcaaggacatcggctccggcaacttcggggtggcgcggctgatgcggaacaaggagaccaaggagctcgtcgctatgaagta
catcccgcgggggctcaagattgacgagaatgtggcgagggagatcatcaaccaccgctcgctgcggcaccccaacataatccggttcaaagaggttgtgct
cacgcccacgcacctggccatcgtcatggagtatgccgctggcggcgagctgttcgaccggatctgcggtgccgggaggttcagcgaggatgaggcgaggt
atttcttccagcaactgatttgcggtgtcagctactgccacttcatgcaaatctgtcaccgggacttgaagctcgaaaacacgctgctggatggcagcccggcacc
gcgccttaagatttgtgacttcggctactccaagcaaatctgtcaccgggacttgaagctcgaaaacacgctgctggatggcagcccggcaccgcgccttaaga
tttgtgacttcggctactccaagatggcagatgtttggtcctgtggagtgaccctttatgtgatgctggttggtgcatacccttttgaggatcccgacgatcccaaga
atttcagaaagacaattgggagaatagtatctattcaataccaaataccagaatatgtccacatatcccaagattgcaggcagctcctctcgaggatcttcgttgcg
aatcctgcaaagagaataacaattagggagattagaaatcacccctggttccttaagaacttgcctagagagcttacagaagctgcacaagcgaagtactacaa
gaaggacaacagcacacccacctactctgatcagactgttgaagagatcatgaagattgtagaggaggctcgcacaccacccaaattatctactcctgtggctg
gtttcggttgggctgaggaagaagagcaagaagatggcaagaaacctgatgatgaagaacatgatgaagaggatgaggaatatgatggtgaggacgagtatg
acaagcaggtgaaggcagtacatgccagcggtgattttcaacatctgataaaaggaagcaactag
>g34089.t1
atgggaaacgcatgcggcggctcccttagatccaggtatctgccgagcttcaagacctccgcctcgcagcgccacgactccgaccacagcgccgccgccga
ctcgccgaagaagccctcccgccccgccacgccccccgccgccgcgacggacgcccacgcggcacccgcgccgggcctgccgccgccgccgcccggc
atgaggcgcggcggcgcggcgcccccggacctcggttccgtgctgggccaccccaccccgaacctccgtgacctctacgccgtgggccgcaagctcgggc
agggccagttcgggaccacctacctctgcaccgacctcgccacgggcgcccagtacgcctgcaagtccatctccaagcgcaagctcatcacccgggaggac
gtcgacgacgtgcgccgcgagatccagatcatgcaccacctctcgggacaccgcaacgtcgtcgccatcaagggcgcctacgaggaccagctctacgtcca
catcgtcatggagctctgcgccggcggggagctcttcgaccgcatcatacagcgcggacactacagcgagcgaaaggccgcagagctcacgcggatcatcg
tcggggtcgtcgaggcgtgccactcgctcggggtcatgcaccgggacctcaagccagagaacttcctgctcgccaacaaggacgatgacctctcgctcaagg
ctatcgatttcggcctctccgtcttcttcaagccgggtgagctgctgctcattactgattcatattccgacttgattactgtttcggtcaacgattgtgctgtcaatgatat
gcgactaannnnnnnnnnnnnnnnnnnnnnnnnnnnnnnnnnnnnnnnnnnnnnnnnnnnnnnnnnnnnnnnnnnnnnnnnnnnngtggg
ccgcaagctcgggcagggccagttcgggaccacctacctctgcaccgacctcgccacgggcgcccagtacgcctgcaagtccatctccaagcgcaagctca
tcacccgggaggacgtcgacgacgtgcgccgcgagatccagatcatgcaccacctctccggacaccgcaacgtcgtcgcaatcaagggcgcctacgagga
ccagctctacgtccacatcgtcatggagctctgcgccggcggggagctcttcgaccgcatcatacagcgcggacactacagcgagcgaaaggccgcagagc
tcacgcggatcatcgtcggggtcgtcgaggcgtgccactcgctcggggtcatgcaccgggacctcaagccagagaacttcctgctcgccaacaaggacgatg
acctctcgctcaaggctatcgatttcggcctctccgtcttcttcaagccggaaacgcagcaaggaatatttgatgctgtattgaaaggtgacattgatttcgattctga
cccgtggcctgtgatatctgatagtgccaaagacctgataaaaagaatgctcaatcctcgccctaatgaacgcttaacagcacatgaagttctatgccatccatgg
attcgtgatcatggagtagcacctgatcgtccacttgatcctgctgtcctatctcgcatcaagcaattctctgcaatgaacaagttgaagaagatggctttgcgagta
atagctgagagtctctcagaggaggaaattgcagggttgaaagaaatgttccagaccatggacactgacaacagtggcgcgattacatatgatgagctaaaaga
aggattgagaagatatggctccacactaaaggatactgaaattcgtgatcttatggacgcggctgatattgacaacagtgggacgattgactacatagaattcatt
gctgcaacattgcatctcaataaactggagcgtgaggaacatctggtggcagcatttacatattttgacaaagatggtagtggttacatcactgtggatgagctgca
acaagcttgcaaagagcataacatgccagatgcttttcttgatgatgtcattaaagaagctgatcaggacaatgacggacgcattgactatggagagtttgttgcca
tgatgaccaagggcaatatgggagttggtcgaagaacaatgagaaacagtttgaatatcagcatgagggacgcacctggtgcattttag
>g34176.t1

atgggcaactgcttcaccaagacgtacgagatacccatcaccacggagccgtcggatcggcggcgcgcgccgtcgtcgtacggctaccagccgccgagga
gccccgatcagcgcaagtcccgcgacgtgccggtgatgacctcgcgccggccgtccttccgtccgccgccgcccttccggccgtcgagcacgacgggcac
cgggtcgtcgtcggcgtcgtcgatgccccgtcgcgcgccctcctccggcgaggtgggcccggtcctgcagcggccgatggtgaacgtgcgcacgctgttcc
atctggagcggaagctggggagcgggcagttcgggaccacgtacctgtgcacggagcgcgagacggggctccggtacgcgtgcaagtccgtgtccaaacg
caagctggtgcgccgcgccgacgtggaggacatgcgccgggagatcaccatcctgcagcacctcagcgggcaggccaacgtggccgagttcaagggcgc
cttcgaggacgccgactacgtgcacctcgtcatggagctctgctccggcggggagctcttcgaccgcatcaccgccaagggcacctactccgagcgccaggc
cgccgccgtgtgccgcgacatcgtcaccgtcgtgcacgtctgtcacttcatgggggtcatgcaccgcgacctcaagccagagaatttcctgcatgccagcccc
gcggaggacgcgccgctcaaggccatcgatttcggactctccgtcttcatcgaagaagaacatccatggctcaaagaaggtggtgcatcagacagacctatcg
acagtgcagtcctatcaagaatgaagcaattcaaggcaatgaacaagttaaagcaattagcactcaaggtaatagcggagaacctatcaccagaagaaatcaa
gggattgaagcaaatgttcaataacatggatactgacaagagtggcacaatcacagttgaagaactgaaggaaggactgacgaaactagggtcgaagattagt
gaagcagaggttcagaaacttatggaagcagttgatgtagacaagagtggcagcattgattatacagagttccttactgacatgatgaacaaacataaagtggaa
aaggaggaggatttgctccgtgcatttcagcacttcgacaaagatagcagcgggtacataacaagagatgaactggaacaagccatggaagagtatggaatgg
gtgatgaggcaagcattaaacaagtactggatgaagttgataaagataaggatgggaaaattgactatgaagagtttgtggaaatgatgaggaaaggaagctat
acctga
>g36737.t1
atgggtggctgctactccgtcatcgcggtcaccaggctgaagatgcttcgccgcaaccgtggcgccgcggccgtcctccccatcaccaacggcgacgacgac
acgtgctgccgctctcccgacaacaacaacaacgccatcgacgacgacaataagggcatgggctgctgcagggcgaagaggaggtgcaggaaggacaag
tcgaagcacgcctcgatcctggggcccgacgccgccggcgagccggacttcgctcggcggtacaggctgggcgcggagctcgggcgtggcgagttcggc
gtgacgcggcggtgcgaggacgtcgccacgggggaggcgctggcgtgcaagaccatccgccgccggcgcctgctgctgcgccgcgggggggcccgacg
ccgtcgacgtgcgccgggaggtggagatcacgcggcgcatgtcggagctcgggggccccgtggtgcggctccgcgaggcctgcgaggacgccgacggg
gtccacctcgtcatggagctctgcgagggcggcgagctcttcgaccgcatcttcgcgcgagggcactacaccgagcgcgccgccgccaagctcgctcacac
catcgtcgaggtcgtgcagctgtgccacgagaacggggtgatgcaccgggacctgaagccggagaatttcctgttcctgaacaagtcggaagactcacctctc
aaggccatcgacttcggcctctcggtgtacttcaaaccaggggatcggttcacggaagtggttggcagcgggtgttacatggctccagaggttctcaacagaag
ctatgggccagagatagacgtgtggagcgctggtgtcatcctgcacatccttctctgcggcttccctcctttctggggagattccgatgagaaaattgcacaggca
atactacgtggaggaattaacttacaaagagacccatggcacaaggtctcccagagtgcaaaggatcttatcaggaagatgcttgacccgaatccttctaacaga
ttgacagcgaagcaagtccttgaacatccttggctcaagaacgctgataaggctcccaatgtgtcacttggagaggttgttcgatccaggctgaagcaattctcgg
ccatgaacaggtttaaaaagaaggcacttggagtcgttgccatgaatttaccggcagaagagatcgacaaatataatgagatgttccaaaagatggacaaagat
aaggatggtaatttgacacttgaggagctgaaggagggtctccagattaatggccatcctgtcccagaggcagagataaagatgctattagaagctggtgacat
agaaggaactggaatgttagactgtgaggagtttataacaatcttacttcacataaaaaagatgagtaacgaggagtacctacccaaagcttttaaattctttgacaa
agatggcaatggctttattgatatggaggaattgatggaggctttaggtgatgatgaattaggacccaatgaacaagtgattcatgatattgttcgtgacgtcgaca
aggataaggtggataatgctgttaagtttacaaaggagggacacatcgacatttaa
>g39065.t1
atggagagctacctggaggagaacttcggtggcgtcaaggccaagaactcctcggaggaggcgctccggcggtggcgccgcctctgcagcgtcgtcaaga
accccaagcgacggttccgcttcaccgccaacctcgacaagcgaggggaggcgcaggccatcaagcacgccaaccacgtccgtacacatctcctcttcctct
ctcatccatcatcccctttcctccgcaccgggatccgcctcgctcgcggcggcgggatctggactggagccgcgccgccggcgggggcgggaggagctcat
cctgcggttgtgttgcaggagaagctgcgtgttgccgtgctggtctcgaaggccgcgcttcagttcttacatggtctctcacttcgaagcgaatatgttgtccctga
ggaagtcaaggctgcagggtttcagatttgtgccgatgagcttgggtccattgtggagggccacgatagcaaaaagctgatcatacatggcggagtcgatgga
attgctgccaagcttgcgacatcacaaatggatgggctgaggacagctgaggaggacaacattaagcgcaggcaagagatttatggactcaacaagttcacag
aaagcgaggtccgcagtttctgggtgtttgtatgggaagcgcttcaagatacaactcttataattcttgccgtctgcgcatttgtatctctggtcgttggcattgcgat
ggaaggatggccaaaaggtgcccatgacggtcttggaattgttgcgagtatcctcctggtagtgtttgtgactgcaacaagtgattaccgacaatcgctgcagttc
aaaaacctggacaaggagaaaaagaaaattcgggtgcaagttacaagggacggatttaggcagtggatatcgatatatgaccttcttcctggagatgtcgtccat
ttacaaattggagaccaggttcctgcagacgggctcttcatttcagggttttctctgttgatcaatgaatccagcctaacgggtgaaagtgaacccgtcgttgtaaat
gaagataatcctttccttttgtcgggaaccaaagtgcaggatgggtcctgcaagatgctggttacgaccgttggtatgcgcacccaatggggaaaactgatggcc
actctcagtgagggcggcgatgatgaaactccactgcaggtcaaactcaatggtgttgcaactatcattggaaagattgggctattctttgctgttataactttcattg
tcttgtcccaagggttatttggcaacaaataccatgacggacagcttttgagctggtcaggagatgatgcactggaacttctggagcattttgctattgcagttacca
ttgttgttgtggctgttcctgagggattgccattagcagtcacgctgagccttgcatttgccatgaagaaaatgatgaatgacaaggcacttgttcgcaacttagctg
catgtgaaactatgggctcagcgaccaccatctgcagtgacaagacagggacattgacaaccaatcatatgactgtcgttaaggcctgcatctgtgggaaaatc
aaggaggttaacagtcctcagaatgcatccaagttatgctctgaatttccggaaactgtcgtcaaaacactcctggagtctatatttaacaatacaggtgttgaggtt
gtgattaaccaagatggtaaacgtcagatcctagagaaatggaagaaggttttcctggcacagagcaaataccattacaagggtacacatgcattggtattgtag
>g39068.t1
atggataggacggcggcgacgacggggccgctggggatggagatgccgataatgcacgacggggaccggtacgagcacgtcaaggacatcgggtccgg
caacttcggcgtcgcgcgcctcatgcgcaaccgcgcctccggcgagctcgtcgccgtcaagtacatcgaccgcggcgagaagatcgacgagaacgtgcag
cgggagatcatcaaccacaggtcgctgcggcatcccaacatcatccgattcaaggaggttatcctgacgccgactcacctcgccatcgtcatggagtacgcctc
tggaggagagctcttcgagcgcatctgcagtgccggaagattcaacgaggacgaggcccggttcttcttccagcagctgatttctggggtcagctactgccact
ccatgcaagtatgtcatcgtgacctgaagctcgagaatacattgctggatggaagtaccgctccacggcttaagatctgcgactttggttattcaaagtcatccgtt
cttcattctcaaccaaaatcaacagttggtactccagcttatattgcccctgaagttttgctcaagaaagaatatgatggcaagattgctgatgtgtggtcatgtggtg
taaccctctacgtcatgttggttggcgggtacccattcgaggacccggaagatcctaggaatttcaaaaagacaattcagaaaatattgggtgttcagtatgtgatt
ccagattacaggatcaccatgcctgaaataaaaagtcatccttggttcgttaagaatctcccagcagacctcatggatgatgccacaatgagcagccagtatgag
gagcctgaccagcctatgcagaacatgaacgagatcatgcagatactagcagaggcaactataccagccgctggtacccgtggaatgaaccagtttttggttga
cagccttgaccttgatgatgatatggaggatctagactcagaccttgacatcgacattgagagcagtggagagatagtgtatgccatgtga

>g39207.t1
atggatgaggtggagcagacgctgacaaaggagcagatcaaggagttccgggaagccttcagcctcttcgacaaagatggcgatgggacgatcacgagcaa
ggagcttggcacggtgatgcgttcgctggggcagagccctacggaggcggagctgcaggagatggtggcggaggtggacgccgatggcagcggcgccat
tgatttccacgagttcctcgtcctcctcgggcgcaagatgcgcgacgccggcgccgacgacgagctccgggaggccttccacgtgttcgaccaggaccagaa
cggatacatctcccgcgacgagctgcgccacgtcctcgagaacctcggcgagaagctctccgacgaggagctcgtcgagatgctgcgcgaggccgacgtc
gacggggacggccagatcaactacaacgagttcgcgaaggtcatgatggccaaacgaagaaaccagatgatggacgacgacgatagtggttcgcactccca
cgggagcagcccctgctgtacaatcctctga
>g44056.t1
atgggaggttgccacgcgaagccgctcacccacgacccggacaactcgccgccgcacgcggcgccggcgacgccgctcccggagggccgcacctccgc
gtccgccacgcccgcgaagaagcagcactgggcgtcgtcgcccttcttcccgttctccacgccgagcccgagtccggcgcaccacctcttcggctcgtccgc
ggcgtccccgcgcaagtcccctgcaccgcccggcgcgggctccgcgcccaccacaccggccagacggctgctgcggctgcccttcccgccgccgtcgcc
cgccaagcacatcagggcggcgctcgcgcggcggcacgggccgtcgcggccgtccatccccgaggaaggtggcggcgagggggacggcagcggcag
ggggctcgacaaggggttcgggttcagtaaggggttcgcgaccaagtacgagatgggcgatgaggtcgggaggggacacttcggctacacctgtgccgcca
aggtgaagaagggggcgcgcaagggcgagtctgttgccgtcaaggtcatccccaaagccaagaatttcctttttacttcgaaagatgagaattcccaacttaag
gctatcgactttggcttgtcggattttgtaaaaccagatgaacgcctaaatgatattgttggaagtgcttactatgttgctccagaagttctgcatagtcgtccattttgg
gcacgcactgaatctggcatattccgttctgttctcaaagctgaccccagctataatgaggcaccatggccttctctgactccggaagcaatggattttgttaagcgt
ttgctgtgcaaggatccacgtagaaggatgactgcagcacaggctttaagtcatccatggatcagaaattataatgacattaagctgccgttggacgtccttatatt
ccgacttatcaaagcttatatccgttcctcatctttacgaaaagctgctttgagggctctatcgaagactttaactgttgatgaacttttctatctgaaagcacagttttcc
ttgttggaaccagacagaaatggatgcattactcttgataatatcagaacggcattaacaagggaggccactgatgcaatgaaagaatcacgagttcaagagatt
cttgtttcgttgagcgctcttcagtacagacgaatggacttccaagagttctgtgcagcagcagttagtgtgcaccagctcgaagcattggatagatgggagcaac
atgcccgatctgcttatgaatattttgagaaggatggcaatcgtgctattgtaattgatgaactggcttctgaattgggtctcagcccttccgtgccattgcatgttgttc
tgcaagattggatcagacatactgacgggaaactgagctttcttggttttgtcaagctattgcatggcatgtccagcaggtccctgtcaaaggtgaggtag
>g46612.t1
atgcagcacgatcaacgcaagaagagctccgcggaggcagagtttttcacggagtatggggacgcaagtcgatacaggattcaggaagtcattggtaaagga
agctatggggttgtatgctctgcaatagatgtccacaccggagagaaagtagccatcaagaagatacatgacatctttgagcacatatctgatgctgcacggattc
tccgtgagatcaagcttctgagactcctaagacatcctgacattgttgagatcaaacatattatgctgcctccatcaagaagggacttcaaagatatttatgttgtttt
gaacttatggagtctgaccttcatcaggattatgttgcaacaagatggtatagagctccagagctctgtggatccttcttttcaaagtatacaccggccattgatattt
ggagcattggatgcatctttgctgaggtgctaacaggaaagccgttgtttcctggtaaaaatgttgtccatcagttagatttgatgactgatcttctgggaaccccatc
cttggataccatttctcgggtctgcaatgagaaagcaaggagatatctgagcagcatgagaaagaaagaaccaatctcattttcacagaagtttccaaatgcaggt
cctttggccttggatcttttgcaaaggctgttagcatttgatccaaaggaccgtccaactgcagaagaggcattggctcatccatacttcaaagggctagccaaggt
tgagagagaaccatcttgtcaaccaatcacaaagatggagtttgagtttgaacgtagaagagtgacaaaggaagacataagggagctgatattccgtgagatac
tggaataccatccacatgctgttgatcaattcaggaagcaatttgctcatctcgaagagaataatggaaatggccctgtgattccgatggaaagaaaacatgcttct
cttcctagatctacaattgttcactctactccaatccctgtcaaggaacaaccccgtattggctcgtcaagagacaaggcttcacctgatgagtcctacagaaatcct
cgtgagacagacagattttccggcaatgccccgagaacctcacaagctccccatagagtgccagcagcgagaccgggaagggttgttggtccagtaatgcca
tatgaaaatgggagcaccaaagacccctatgacagacgaaagtttgcattgaattcaggatatcctccccaacaacaaatccaacaaacgtatggttattatcaga
catccggcaaggtagcttgctctgagcagtcgcaggctgaaagcagggcctgcaagtga
>g47095.t1
atggagcggtacgaggtgatccgggacattgggtctgggaactttggtgtggccaagctcgtccgcgacgtcgggaccaaggagctctacgccgtcaagttca
tcgagagggggctgaagcaagtgtgccacagggatctgaagctagaaaacacccttttagatggaagtgttgcacctcggcttaagatttgtgatttcggttactc
caagtcttctgtgctgcactctcaaccaaaatcaactgttggcacacctgcgtacattgctccagaggtcctctctagaaaagaatatgatgggaaggttgctgatg
tttggtcatgcggagtaacactatatgtgatgcttgttggtgcatatccttttgaggaccctgatgatccaaggaacttccgcaagacaatcaccagaatactcagtg
tacagtactcaattccagactatgttagaatttcaatggaatgcaggcatctgctgtcccggatatttgttggtaatcctgagcagcgaattaccattccagagatcaa
gaaccacccatggttcctgaagaacctgcccatcgagatgactgacgagtaccaacagcagatgaagttggcagacatgaacaccccggagcaaagcctag
aggaggtcatggcgatcatccaggaggcgcggaaaccaggggatgacctgaggcttgccgggcaggtaccctgccccctaggaagcatggaccttgatga
cactgatttggacgatattgacgacattgacatagagaatagcggcgatttcgtttgtgcaatgtga
>g47734.t1
atgctcccgccctcgcgccgcgacttccgcgacatctacgtcgtcttcgagctcatggagtccgacctccaccaggtcatcaaggccaacgacgaactctcgg
ccgagcaccagcagttcttcctctaccagctactccgcggcatgaagtacatccacgccgccaatgtcttccacagggacctcaagcccaggaacatccttgcc
aatggcgattgcaaactcaagatttgtgacttcggccttgctcgggtgtcgttcaatgataccccttctgcgatattctggacggtaacctga
>g48661.t1
atgggcaacacgtgcggcgtcacgcttagatccaagtacttcaccagcttccgtggctcgcagcgccacgacaccgcggggtacgcgcccgtcgccgccgc
cgccgcctccgacgaccccgcgccgcgcgggaaagggaagcgggcctcgccccgcacggccgatgcggcagctgccgcggcggacgcggccgctcc
gccccccgcccccggcatgcggcgcggggctcccgcgcccgcggagctcacggccaacgtgctcggccacccgacgccgagcctccgggagcactacg
cggtcgggcgcaaactcgggcagggccagttcggcaccacctacctctgcaccgacctcgccacgggggtcgactacgcttgcaagtccatctccaagcgc
aagctcatctcgcgggaggacgtcgaggacgtgcgccgcgagatccagatcatgcaccacctcgccggccaccgcaacgtcgtcgccatcaagggcgcat
acgaggaccagcagtacgtccacatcgtcatggagctctgcgcgggcggcgagctgttcgaccgcatcattcagcgcgggcattacagcgagcgcaaggcg
gccgacctcacgcgcatcgttgtgggggtcgtcgaggcgtgccactcgttaggggtgatgcacagggatctcaagcccgagaacttcttgcttgccaataagg
atgacgatatgtcgctcaaggcaatcgattttggactctctgtgttcttcaagcctggtcaaatttttaccgatgttgttggaagtccctactatgtagctccagaagtg
ctgcgcaaacgttatggaccagaagccgatgtgtggacagctggagtgattctttatatattgctaagtggtgttccaccattttgggcagagacacagcaaggaa
tatttgatgctgttttaaaaggtggcattgattttgactcggatccctggcctgtaatctctgatagtgcaaaggatcttataagaaaaatgttgaaccctcgaccagc

agagcgcctaacagcacatgaagttctatgtcatccctggatttgtgaccagggagtggctcctgatcggcctcttgatccagctgtcctttctcgcattaagcagtt
ctcagcaatgaataagttgaagaagatggctttgagggtcattgctgagagtctttcagaggaggagatagcaggattgaaggaaatgttcacggcaatggatac
agataacagcggggccattacatatgatgagctaaaagaaggcttgaaaaaatatggttctacgctaaaggatactgaaattcgtgatcttatggaggcggcaga
tgtagacaacagtggaactattgattatatagaattcattgcggccactttgcacctcaataaacttgagcgtgaggaacatctagtggcagccttttcgtattttgac
aaggatggcagtggttatatcacagtggatgagcttcagcaagcttgcaaagagcataacatgccagatgcttttctggatgacgtcattaacgaagctgaccaa
gataatgatggccgcattgattacggtgaatttgttgctatgatgacgaagggcaacttgggagttgggagaagaacaatgagaaacagcttgaatataagcttg
agggacacacctgctgctctctga
>g48899.t1
atgggcctctgctcctcctccaccgcgtccgcgcaccgtgccggtgccgcgccggggaagaaggagaacaagggcgccggaggcggcagccgggggat
cgtggcgtgtgggaagcggacaaacttcggctacgacaaggacttcgaggcccggtactcgctcggcaagctgctgggccacggacagtttggctacaccta
cgccgccgtcgaccgcaactccggtgagcgcgtcgccgtcaagcgcatcgacaagaacaagggattaggagtcatagttgcctctcatttctga
>g49337.t1
atgagtacaaccaaggtgaagagacgtgtggggaagtatgagcttggccggaccataggcgagggaacatttgcaaaagtcaggttcgcacgggacaccga
gactggcgacccggtggccatcaagattctggacaaggagaaagttctcaagcacaagatggttgagcaggtgatgggaagtaaaacaaagatctacattgtg
ttagagtatgtaaccggcggcgagctctttgatacaatcgttcttgaggatcaaggttatgatggtgcgatggctgatctgtggtcatgtggagttatcctgtttgttct
actagcagggtatttgccttttgaggactctaatcttatgacgttgtataagaaaatatcaaatgcagtgtttacatttcctccttggacatcgtttcctgccaagaggtt
gttaacaagaatccttgatccaaatccaataacgttgaggctttcaaaggtcaaagcagggaggaagggaaacctcaatgtagccaccgagatattgcaagttg
caccctctcttcacatggtagaagtccgaaaagcaaaaggtgacactctggaatttcacaagttctacaagaacctttccaagaccttaaaggacgttgtctggaa
atccagtgacatgcaaatgcagcccacttcttag
>g49339.t1
atggatgaggtggagcagacgctgacgaaggagcagatcgaggagttccgggaagccttcagcctcttcgacaaagatggcgacgggacgatcacgagca
aggagcttggcacggtgatgcgttcgctgggtcagagcccgacggaggcggagctgcaggagatggtggcggaggtggacgccgacggcagcggcgcc
attgatttccacgagttcctggtcctcctcgcccgcaagatgcgcgacgccggcgccgacgacgagctccgggaggccttccacgtgttcgaccaggaccag
aacggatacatctcccgcgacgagctgcgccacgtcctcgagaacctcggcgagaagctctccgacgaggagctcgtcgagatgctgcgcgaggccgacgt
cgacggggacggccagatcaactacaacgagttcgcaaaggtcatgatggccaaacgaagaaaccagatgatggacgacgacgacagtggttcgcactccc
acaggagcagcccctgctgtacaatcctctga
>g50524.t1
atgctcaaggttgcagctgagtgccacctgcatggtttggttcatcgggacatgaaaccagagaatttcctactcaaatcaacaaaagaggactcaccccttaag
gctacagattttggtctttcagacttcataagaccagggaaacaattccgtgacattgttgggagtgcctactatgtagcaccagaagtactcaagccacatgattg
ggtcagagaaggaggtaaggcatctgaaataccattggatatatcagtattacacaatatgcgacaatttgtgaaatacagtcgcttcaagcaatttgctttaaggg
cattggcatcaacactaaatgcagaagagctttctgatcttcgtgaccagtttgatgccatcgatgttgacaaaaacggcacaatcagtctggaggaactgaagca
gattgacagtaacacagatgggttggtcgattttgaagagtttgttgcagcgacactacatgtgcatcagctggcggagcatgatactgagaagtggagatcgttg
tctcaagctgcatttgataaatttgacgttgatggagatggttatattacatctgatgaattgagaatggtatcttaa
>g50691.t1
atggcggaccagctcaccgacgagcagatcgcggagttcaaggaggccttcagcctcttcgacaaggatggcgacggttgcatcactacaaaggagcttgga
actgtgatgcggtctcttggccagaaccctaccgaggcagagctgcaggacatgatcaacgaggttgacgctgatggcaatggtactattgacttcccggagtt
cctgaacctgatggcaaggaagatgaaggacactgactctgaggaggagctcaaggaggccttccgtgtcttcgacaaggaccagaacggtttcatctcagct
gcagagctccgccatgtcatgaccaaccttggggagaagctgactgacgaggaagtcgacgagatgatccgcgaggctgatgttgatggtgatggccagatc
aactacgaggagtttgtcaaggtcatgatggccaaagcccatgcattattcaatgttctcaggcatcgaccatatgcagcatgtcagatcgccaaagacgacctc
gttacggccctccgctcgctgggcctgaacgtcgacgaggccgaggcgcagcgcctcctcggcgacgccgcctccggcggcgcgatcgagttcccggcgt
tcctgtccgtcgcggcgcgcaagatggccagcgacgggaagcagacggcggagaggctggcggaatgcttcgacgtgttcgacgacggccggagcgggt
cgatcccggcggagcagctgcggcaggtgatgacgagccacggcgaccggctcacggaggaggaggccgacgccatggtccgcgaggccgacccgcg
cggcgagggccgcgtcgagtacaaggagtacgtcaaggtgctgaccaagaacaagtga
>g53597.t1
atgggcaactcgtgccgcggctacaccacaagcttgtacgattgcgactaccagcacagcagcacctcctattcgtcgtcgtcgtcgaatttctccagcaggcg
cagcagcagcagcatcgtctgtggaggcggcggcatgcgcgggcaccagcaacagctttcgtccccgacggccgtgctcgggcacgtgaccccaccgctg
cgggagctctactcgctggggcggaagctcgggcagggtcagttcggcaccacgtacctgtgcacggatctggccacgggcgcgtccctggcctgcaagtc
catcgccaagcgcaagctgctcaccccggaggacgtggacgacgttcgccgcgagatccagatcatgcaccacctcgccggccacggcagcgtcgtcacc
atcaagggcgcctacgaggacccgctctacgtgcacatcgtcatggagctctgcgagggcggcgagctcttcgaccgcatcgtcaaccgcggctacttctccg
agcgcaaggccgccgagatcgcgaggatccaatag
>g54372.t1
atgggcaactgctgcggcgcgccggcgacgccgggcggccgcaagaaccggcggaagcagaaggcgaacccctacaacgtctcgtacaaccgtggggc
ggcgccgccgccggcgcgtccggggctgatggtgctgcgggacccgacggggcgggacatgaacgcgcggtacgagctcggcggcgagctggggcgc
ggcgagttcggcgtcacgtacctgtgcacggaggtggccacgggggcgcggttcgcgtgcaagtcgatatcgaagaggaagctgcggacgccggtggacg
tggaggacgtgcgccgggaggtggacatcatgcgccacatgccgtcgcaccccaacatcgtcagcctcagcgccgcctacgaggacgaggacgccgtgca
cctagtcatggagctctgcgagggaggggagctcttcgacaggatcgtcgcgcggggccactacaccgagcgcgccgccgccgccgtcacgcgcaccatc
gtggaggtggtccagatgtgccacaggcatggtgtcatgcacagggaccttaaaccagagaacttcttatatgcaaacaagaaggagagttcccctctgaaagc
aattgattttgggctgtctgtgttcttcaggcctggtgagcgctttacagaaattgtaggcagtccatactacatggctccagaggttttaaagcgaaactacggtcct
gaagttgatgtctggagtgcaggagtgatactttatatacttctttgtggcgtgccaccattttgggcagagactgaacagggagtagcacaggcaattatacgctc
agttgttgatttcaaaagagatccatggccaagagtatctgaacctgctaaagatcttgtcaggcgtatgctggacccaaatccaaacacaaggctttccgcagca

caagtacttgaacatccatggctgcatgactctaaaaagatgccggacatccctcttggtgatacagtccgagcgagactgcagcaatttgcagcaatgaacaaa
ttaaagaagaaagctcttagggtgattgctgaacatctctccgtagaggaagtagctgatataaagcaaatgtttgatggcatggatgtgaacaaaaatggcaagc
taacctatgatgaattcaaagctggtcttcgtaaacttggaaaccaaatgcctgattcagacattaggatattgatggatgctgctgatgtcgataaaaatgggaccc
tagactatggagaatttgttgctgtgtctatccatgtccgaaaaattggcaatgatgaacatatccagaaagctttctcgtactttgaccaaaataacactgggtacat
agaaattgaagaacttagacaggctctggctgatgaattggagggaaatgatgatgatattattaatggcatcatccgtgatgtggacacagataaggacggaaa
aataagcttcgacgagtttgcgaccatgatgaaggctggcactgactggaggaaggcgtctaggcagtactcacggcaaaggttcagcaacctcagcctgaag
cttcaaaaggatgggtccatcggcgctgatacaaagtag
>g54450.t1
atggcggaccagctcaccgacgaccagatcgccgagttcaaggaggccttcagcctcttcgacaaggacggcgatggttgcatcacaaccaaggagctggg
aactgtcatgcgttcattgggtcagaacccaaccgaggctgagcttcaggacatgatcaatgaggttgatgctgacggcaatggcaccatcgacttccctgagtt
cctgaacctcatggctcgcaagatgaaggacactgactctgaggaagagctcaaggaggcgttcagggtgttcgacaaggaccagaacggcttcatctctgct
gcggagctccgccacgtcatgaccaaccttggagagaagctaaccgatgaggaggtcgatgagatgattcgcgaggctgatgtcgacggcgatggccagat
caactatgaggagttcgtcaaggtgatgatggccaagtga
>g57551.t1
atggcgatcgccactgagtcgtgcggcggcagcgaggacaaggctgcatcacatgcaaatgaggagaagcgttgggtgctgccagatttgaggttggaaag
ccacttggcaggggaaaatttggccatgtttacttggcaagagaaaaaaggattgtggctttgaaagttcttttcaagagccaactgaaacaatctcaagtcgagc
atcagcttcggcgtgaagttgaaatccagagtcacctaaggcaccccaatattctacgcctgtatggttacttctatgaccagactcgtgtttatttgatcctggaata
tgctgccaaaggagagctgtacaaggagctgacaagatgcaaacatttcaatgagagacgttcagctacttacatcgcatcactggcaagagcactcatttacct
tcatgggaagcatgtcatccatagagacattaaaccagagaatcttctaattggagctcagggcgagctgaaaattgcagactttggctggtctgttcatacattca
atcgaagacgaacaatgtgcggaacgctagattacctgccacctgaaatggttgagaagactgagcatgattaccatgttgacatctggagcttgggaatactct
gttatgagttcctctatggagccccgccttttgaagcgaaagaacattccgagacatatcgaaggatagtgaacgtggacctgaagttcccattgaaaccgtatgtt
tctgctgccgcgaaggacctaatttcacaggatagtgaacgtggacctgaagttcccattgaaaccgtatgtttctgctgccgcgaaggacctaatttcacagatg
cttgtgaaaaactctgcgaaccggcttcctctgtataa
>g57892.t1
atggcggatcagctcaccgacgaccagatcgctgagttcaaggaggccttcagcctcttcgacaaggacggtgatggctgcatcaccaccaaggaacttggta
ctgtaatgcgctctttggggcagaaccccactgaggctgagcttcaggatatgatcaatgaagtggatgctgatggcaatggaactatagacttccccgagttcct
caacctgatggctcgtaagatgaaggacacagactctgaagaggagcttaaggaggctttccgtgtgtttgacaaggaccagaacggcttcatttctgcggctg
agctccgtcatgtcatgaccaaccttggtgagaagctgacagatgaggaggtggatgagatgatccgtgaagccgatgtggatggctgcatcaccaccaagga
acttggtactgtaatgcgctctttggggcagaaccccactgaggctgagcttcaggatatgatcaatgaagtggatgctgatggcaatggaactatagacttcccc
gagttcctcaacctgatggctcgtaagatgaaggacacagactctgaagaggagcttaaggaggctttccgtgtgtttgacaaggaccagaacggcttcatttct
gcggctgagctccgtcatgtcatgaccaaccttggtgagaagctgacagatgaggaggtggatgagatgatccgtgaagccgatgtggatggtgatggccag
atcaattacgaggaatttgtgaaggtgatgatggccaagtga
>g59931.t1
atgagcgtgtccgtcgggagaacgcgggtggggaggtacgagctgggccggacgctcggggaaggcaccttcgccaaggtcaagttcgccaggaacgtc
gagaccggcgagaatgtcgccatcaagatcctcgacaaggagaaggtgctcaggcacaagatgatcggacagataaagcgtgagatctcgaccatgaagct
catcaggcacccgaacgtcataaagatgcacgaggtgatggccagcaagacaaagatttacatcgtgatggaacttgtcactggtggtgaacttttcgacaaaat
tgcttcacgtgggaggctgaaagaggatgatgccaggaagtatttccagcaactggtcaatgctgttgattactgccacagcagaggcgtgtaccaccgtgatct
gaagcctgaaaatcttctgcttgatgctagtggcactctcaaggtgtcagatttcggactgagtgcactttctcaacaagtccgagaggatggtcttctgcacacaa
cctgtggaactcccaactatgttgcccccgaggtccttcgnaatatttttccatatctttctgtcaacttgcaggaggatggtcttctgcacacaacctgtggaactcc
caactatgttgcccccgagccaaggctgatctttggtcatgtggagtga
>g61469.t1
atggaggcggctgatgttgatggaaatgggaccattgattatgcagagttcatatcagccacaatgcacttgaatagattggagaaggaagaccacatactcaaa
gcattcgagtattttgataaagaccacagcgggtanttggagaaggaagaccacatactcaaagcattcgagtattttgataaagaccacagcggatacataact
gtagatgagctggaagaagctctgaagaagtatgatatgggagatgataaaacaattaaagaaatcattgcggaagtagattcagataatgatcacacacgtaca
agtgaatccatgttggattaa
>g62237.t1
atggggaacacctgctccggtccaacgcccccctcggaccgccatggtttcttcgactccgtctccctcgccgtgctctggcgcccacccgccgccgccaggg
ccgagccccccttctccatcggactccaggccctccgcctcctcgacgacttcctccaggcctcccgagtccgtcaccttcaccgatgccgagcactccccccac
gcctccccctccgcgccggaccctaacggcaagcccaggcctaaggtgaagcgcgtgcagagcgccggcctccttgtcggctccgtcctgaaacgcaactc
cgagcggctcaaggacctctacaccctgggcaagaagctgggacaggggcagtttggcaccacctaccagtgcgtcgagaaggccaccgggaaggtattc
gcctgcaagtccatcgccaagcggaagctcgtcagcgaggaagacgtcgaggacgtgcgccgcgagatacagatcatgcatcacctctccggccacccgaa
tgttatctccatcgttggggcttatgaggacgccgttgcggtgcaccttgtcatggagctgtgtgcgggagggggaactgttcgatagaatcatacagcgggggca
ctactctgagaaagctgctgcacaattggcacgggtgatcattggagtggtcgaggcatgccattctctcggggtgatgcacagagacctcaagccagagaatt
tcttgtttgtgaaccacaaggaggatgcaccgctaaaggcgatcgattttgggctctccatattcttcaaaccaggtgaaacatttttttgatgtcgttggaagtccgta
ctatgttgcacctgaagttctgttgaaacactatggctgtgaggtggatgtgtggagtgctggtgtaataatttatatcttgctgagcggagtccctccattttgggat
gaatctgaacaaggaatatttgaacaagtcctgaaaggtgatcttgacttttcttctgagccctggcccagcatctcaaggagtgcaaaggatttggtcaggaaga
tgttaatccgtgatcccaaaaagagattgactgcacatgaagctctatgtcatccatgggtttgtgttgatggagttgctcctgacaaacctcttgattctgctgtctta
accagattgaaacagttttctgcaatgaacaaattaaagaagatggcccttagggtcattgctgagaatttgtctgaagatgaaattgccgggttgcgagaaatgtt
caaaatgctggacactgacaacagtggccaaattacattggaagaactaaaaagtggcttgaagagagttggtgctaacttaaaggactctgaaattacaacgtt
aatggaggcggctgatattgataacagtggttcaattgattatggagagttccttgctgctactttgcatctgaacaaagttgagcgagaagataatctctttgctgc

gttctcatattttgataaagacggcagcggttacattactcaagatgagctacagaaagcatgtgaggagtttggtataggagatgaacaccttgaagatattatca
gagacattgatcaagataataacttcattctgccatttgagagagagagagagagagagagagagcagccgccggcactggacgtggagccgctgcttgttcctc
cagctggaccagcagcaatactgcgctgcggttccccatcgaagcagtggccgcccaacagcagcagagcctccgcctcctcttcaccttcggagcagcggc
aacctgccagcagcagagccttcgcctccgcctcctcttcaccttcggagcagcggcaacctaccaggagcagagcctccgcctctgcctcctcttcaccttcc
gagcagcggcaacctgccagcagcgcctcaacctccgcctcctcttcttatgctccatgtccagctcgtaa
>g62898.t1
atgggccccggcgtggtggcgcacctggtgcgcctgggcgtgctgagctcgacctccgactacacctcggtggtgtccatcaacctgttcgtggcgctaatctg
cgcttgcatcgtcctcggccacctcctcgaggagaaccggtggatgaacgagtccatcaccgcgctcatcattgggctgtgcactggcgtggtgatcttgctga
cgaccaaaggaaagagctcgcatatcctagtgttcagcgaggacctcttcttcatctacctcctccctccaatcatcttcaatgccggttttcaggtaaagaagaaa
caattcttccggaatttcatgacaatcacgttgtttggtgctgttgggacaatgatatccttcgtcacaatatcagttggcgccatagcaatattcagcaggatgaaca
ttgggacacttgatgttggggattttcttgcaattggagccatcttttctgcaacagattctgtctgcacattacaggtcctccatcaggatgaaactcctcttctataca
gtcttgtgtttggtgaaggagttgtgaatgacgctacgtcagttgtgctcttcaacgcgctccagaactttgatctcaacaagattgacgtagccgttgtacttaaatt
cttgggaaacttctgctatttatttttgtcaagcaccttccttggagtgttttctggattgctcagtgcttatataatcaagaaattatatataggaaggcattccactgatc
gcgaggttgcgcttatgatgctcatggcttacctctcttacatgttagctgagctatcagatttgagtggcattctcactgtgttcttctgcggcatcgtgatgtcacatt
acacttggcataatgtgacagagagctcaagagttacaaccaaacatgcttttgcaactttgtccttcatcgctgagacatttctcttcctctatgttggtatggatgca
ctcgatatcgaaaagtggaagtttgccagtgacagccctggaaagtccattggaataagctcaattttgctaggattggttctggtgggaagagctgcgtttgttttc
cctctctcattcctgtctaacttgacaaaaaagggtcctttggaaaaaataacctggagacaacaaatcgtaatatggtgggctggactgatgagaggggctgtgt
cgatcgcccttgcttacaataagtttactagatctggacacactgagctccacggcaatgcgataatgataaccagcacaatcactgtagttctgtttagcaccatg
gtgttcgggatgatgacaaagccattaatccagttcttgctcccggcgtcaagccacacggtcacctccgagccgaattcgcccaagtctctccactcccctctcc
tcaggagcatgcaggggtctgacctcgagacggcctcgcagtcgcatattgtcaggccttccagtctccgcatgctcctcagcaagccgacccacaccgtcca
ctactactggcgcaagttcgatgatgcgctgatgcggcccatgtttggcggacgcgggttcgttcctttctctcctggatcacccaccgagcagagcgttcatggt
ggacagtga
>g64531.t1
atgggatcagctactacaatatgcagtgataagacaggaactcttaccttaaataagatgacagttgtggaagcatactttggaggaacaaagttggatccctgct
atgatatgagccaaatgtcagccaatgtagcatcgcttattattgaaggaattgcacagaacacaacaggaactgtattcctgccagaggatgggggagaagca
gaacttacaggttcaccaactgaaaaggccattctttcttggggccttaaggcagggatggatttccatggtgtgagatcaaaatcttcagttcttcatgtttttccattt
aattcggagaagaaacgtggagctgttgcaatacagtatgatgaattctggaaatctattgaagacatggcagcaacctcattacgttgtgttgcttttgcatatcgt
ccctgtgagcctgaaaagataccagaggaagacatagctaattggaaattgcctgaggatgacctgactctgcttggcatcataggaataaaggccgatatagg
tctttcaatgggcatctcaggcacagaagttgctaaggaaagttctgacattataatcttggatgatgacttcacatcagttgtcaaggttgttcgttgggggcgatct
gtctatgcgaatattcagaaattcatccagttccagctcacggttaatgttgccgcccttgtaataaatgtggttgctgctgtgtcatctggtgatgtgcccctgaatgc
cgtagaggttgggaccttttcttga
>g66239.t1
atgcacaggagaggtgggaaatattctgaagaagatgccaaggttgtcattcatcaaattctaagtgttgcttcattttgccatcttcagggtgttgttcaccgggatc
tgaaaccagagaatttcctttcatgtccaaggatgagaattctgccttgaaagtcatagactttggtttgtctgactttgttaggccagatgaaagacttaacgatatt
gttggaagtgcatactatgttgctccggaggtgctccaccgatcttacggcaccgaggcagatatgtggagcattggagtgattgcgtatattttgctttgtggaag
ccgccctttctgggcacggaccgagtcaggaatatttcgggctgtcctaaaggcagagcctagttttgatgaatccccatggcctaacctttctgctgaagcaaaa
gactttgtaagaaggctgctcaataaggattaccggaaaaggatgactgctgcacaggccctctgccatccatggatctgtggcggccaagaagttaagattaa
cttggacatgattatttacaggcttatgagggcttacataagttcatcctctcttcggaagtctgctttaagggctatgcacaggagaggtggaaagtattctgaaga
agatgccaaggttgtcattcatcaaattctaagtgttgcttcattttgccatcttcagggtgttgttcaccgggatctgaaaccagagaatttcctttcatgtccaagga
tgagaattctgccttgaaagtcatagactttggtttgtctgactttgttaggccagatgaaagacttaacgatattgttggaagtgcatactatgttgctccggaggtgc
tccaccgatcttacggcaccgaggcagatatgtggagcattggagtaattgcgtatattttgctttgtggaagccgccctttctgggcacggaccgagtcaggaat
atttcgggctgtcctaaaggcagagcctagttttgatgaatccccatggcctaacctttctgctgaagcaaaagactttgtaagaaggctgctcaataaggattacc
ggaaaaggatgactgctgcacaggccctttgtcatccatggatctgtggcggccaagaagttaagattaacttggacatgattatttacaggcttatgagggcttac
ataagttcgtcctctcttcggaagtctgctttaagggcattgacgaagaattccacaagtgcaatgaatgattcaagggttcttgatttcgttaacacaatatgcaatgt
ccagtaccgaaagcttgattttgaggagttctgtgcttcggctattagtgtttatcagatggaaggtctggagacctgggaacaacatgctcgagaagcatatgaat
tatttgacaaggagggaaatcgaccaattctaatggaagaacttgcatcggtatag
>g66771.t1
atgtataaaactaagagatctgcttcacttaaagtgaagcgccgtgttgggaagtatgagcttgggcgcacaattggggaaggaacctttgctaaggttcggattg
caaagaacacggagactggggaacatgttgctatcaagatccttgacaaggcaaaggttctgaagcacaaattggttgagcagttagagaacttgctactggat
attgctggaacccttaaaatttctgattttgggttaagtgctatatctgagcaattgaaggctgatggattgctgcacactacatgtggaacggctaactatgttgctcc
agaggttatcaatgacaagggctatgatggtgccactgcagacctttggtcttgtggggtaatcctttttgtgttgcttgcaggatatctgcctttcgaggatgacaat
actgtctccctctataaaagatctcaggagctcaatttacttgcccctcttggttttctgctggagcaaagaagctgatctgtagaattctggatcctaatccttcaac
tgtaagtgacccttga
>g68626.t1
atggcggcggacgcgtctgcgtcggggaggaagacgcgggtggggccgtacgagctggggaagacgatcggggagggcagcttcgccaaggtcaagct
cgcccgaaactcgcgcaccggcgacgtctgcgccatcaaggtgctcgaccgcaaccacgttctccgccacaagatggtcgagcagataaagcgggagattt
cgacaatgaagctaatcaaacatccgaatgtagtccagttgcatgaggttatggctagcaaatcaaagatatacatggttcttgaatttgttgatggaggcgagcta
tttgataagatctttgtagatgctgctgtttacgtgttctga
>g71498.t1

atgtacgggtggaggaaaggaggttctggggttttcttgatctgtgtgagagatcttctctatcagtcaatgcccacgggcttcttacccccgcaggtcattgctgat
catttgtcggctgaggaagttgaagatataaaggagatgttcaaggctatggatactgacaatgatgggattgtatcttatgaagaactaaagagtggaatagcaa
aatttggttctcatcttgcggaatcagaagtacaaatgctcattgaagctgtggatacaaatggcaggggagcactagattacggtgaatttttggctgtctcacttc
atttgcaaaggatggcaaatgatgagcacttgcggcgagccttcctattttttgacaaggatggcaatggttttattgagcccgaggagctccgagaggctctaat
ggatgaaggggggcactgatagcatggaagtggtgaatgacatattgcatgaagtcgacactgataaggacggcaagattagttatgatgaatttgtagcgatgat
gaagaccggcacagattggagaaaggcatcccggcactattcgagaggaagattcaatagccttagcatgaagcttataaaggatgggtctgtaaaattgggtg
ttgagtga
>g72393.t1
atggaccgggcggcggtgacggtggggccggggatggacatgccgataatgcacgacggcgaccggtacgagctcgtccgtgacatcggctccggcaact
tcggcgtcgcgcgcctcatgcgcagccgcgccgacgggcagctcgtcgccgtcaagtacatcgagcgcggcgagaagattgacgagaacgtgcagcgcg
agatcatcaaccaccggtcgctgcgccaccccaacatcatccgcttcaaggaggtcatccttacgcccacgcacctcgccatcgtcatggagtacgcctcagg
aggggaactcttcgagcgcatctgcaacgccggcaggttcagcgaggacgaggtccagaccctttctgccttcttcctgtcacctctgcagtgctcactgaatca
aacatcagtgctgccctctgttgacgttccttcgtcggttcttcattcacagccgaagtctactgtaggaactccagcatacattgctcctgaggtccttctgaagaaa
gaatacgatggaaaggttgctgacgtgtggtcatgtggggtaacactttatgtgatgctggtcggtgcatatcctttcgaggatcctgatgagcccaagaatttcag
gaagacaattcagagaatactgggtgtgcagtactcaattccagattatgtccacatatctcccgagtgccgagatcttatttcaaggatatttgttgcggacccatc
tactagaatcaccatacctgagatcacaaaccatccatggttcacgaagaacctcccagctgacttaatggatgagaacacaacgagcaaacagtatgaagagc
ctgatcagccagtgcagagcatggatgagatcatgcagatactggccgaggcgacaataccggcagctggttctcgaatcaaccagttcttaaatgatggtcttg
acctcgatgacgacatggacgatctagattcagatgccgatctcgacttggaaagcagcggggagattagaatactgggtgtgcagtactcaattccagattatg
tccacatatctcccgagtgccgagatcttatttcaaggatatttgttgcggacccatctactagaatcaccatacctgagatcacaaaccatccatggttcacgaaga
acctcccagctgacttaatggatgagaacacaacgagcaaacagtatgaagagcctgatcagccagtacagagcatggatgagatcatgcagatactggcgg
aggcgacaataccggcagctggttctcaaatcaaccagttcttaaatgatggtcttgacctcgatgatgacatggatgatctagattcagatgccgatctcgacttg
gaaagcagcggggagattgtatatgcgatgtga
>g73227.t1
atgcagccggacccacaaggcccgggcagggagaagcccggcggcaccaatgtgaacgtccggctgccgccgccggtgacggtggggtcgggtggccg
gccggcgtccgtgctgccgcacaagacggccaacgtgcgcgaccactaccgcatcgggaagaagctgggacaggggcagttcggcaccacgtaccagtg
cgtcggcaaggccgacggcgccgagtacgcgtgcaagtccatccccaagcgcaagctgctgtgccgcgaggactacgaggacgtgtggcgcgagatcca
gatcatgcaccacctctccgagcacccaaacgtcgtccgcatccgcggcgcatacgaagacgccctcttcgtgcacctcgtcatggagctctgcgccggcgg
cgagctcttcgaccgcatcgtcgccaagggccattactccgagcgcgccgcagcgcagctcatcaggacgatcgtgggcgtggtcgaaggctgtcactcgct
gggcgtcatgcaccgggacctcaagccggagaacttcctcttcgcgagcaccgctgaggatgcgccactcaaggccactgactttgggctctctgtgttctaca
agcctggtgataaattttctgacgttgtcgggagcccctattatgttgcaccggaggtgcttcaaaaatgctatggcccagaagctgatgtctggagtgctggagt
gattctgtacattttgctatgtggtgtgcccccattctgggcaggtgataaattttctgacgttgtcgggagcccctattatgttgcaccggaggtgcttcaaaaatgct
atggcccagaagctgatgtctggagtgctggagtgattctgtacattttgctatgtggtgtgcccccattctgggcagaaagtgaagcaggaatctttaggcagatt
ttgcgaggcaaacttgacttggaatctgaaccttggcctaatatctctgatagtgctaaagatctggtccgtaagatgcttacccgggatcctacaaaaagactgac
tgctcatgaagttctatgtcatccctggattgtggatgatgctgttgcacctgataagcctattgattctgctgtgttgtcaaggctgaaaaacttttctgcaatgaaca
agcttaagaagatggcattgagggtgatagccgagagtctgtctgaggaggagattggtggcttgaaggagctgttcaaaatgatcgatactgacaatagtggc
acaataacttatgatgaactgaaaaacggcctgaaaagggtaggttctgatctaatggaacctgaaatcaaggctttaatggatgcggccgatattgacaacagt
ggaaccattgactatggagagttcctagcagctacattgcacatgaataaactggagagggaggaaagcttggatggacaaattgattatagcgagtttgcagca
atgatgagaaagggcaatgctggtggtgcagggaggagaaccatgaggaacagcttgcatgtgaaccttggtgaaatcttcaaacccgctgagaagtaa
>g73323.t1
atggcggaccagctcaccgacgaccagatcgccgagttcaaggaggccttcagcctcttcgacaaggacggcgatggttgcatcacaaccaaggagctggg
aactgtcatgcgttcattgggtcagaacccaaccgaggctgagcttcaggacatgatcaacgaggttgatgctgacggcaatggcaccatcgacttccctgagtt
cctgaacctcatggctcgcaagatgaaggacaccgactctgaggaagagctcaaggaggccttcagggtgtttgacaaagaccagaatggcttcatctctgctg
cggagctccgccacgtcatgaccaaccttggagagaagctaaccgatgaggaggtcgatgagatgatccgtgaggctgatgtcgacggcgatggccagatc
aactacgaggagttcgtcaaggtgatgatggccaagtga
>g73726.t1
atgggcaactgctgctgcgggggggtccaagcaggatcccgccgcagagccggcgtcctccggctccagccgccccgtgggcagcacgacgacctcggtct
cgccgtcctcggctccggcgcccatgaagccccccgccgcggtgggccctgtcctggggcggcccatggaggacgttcggagcacgtacagcatcggcaa
ggagcttgggcgcgggcagttcggcgtgacgtcgctgtgcacgcacaaggcgacggggcagaagctggcgtgcaagacgatcagcaagcggaagctgtc
gaccaaggaggacatcgaggacgtgcggcgcgaggtgcagatcatgtaccacctctccggccagcccggcgtcgtggagctcaacggcgcgtacgagga
caaggcctccgtccacctcgtcatggagctctgcgccggcggcgagctcttcgaccggatcatcgccaagggccactatacggagcgcgcggcggcggcg
ctgctccgcaccatcgtcgagatcgtgcacacgtgccacgccatgggcgtcatccaccgcgaccttaagcccgagaacttcctcctcctcagcaaggacgaga
gcgcgccgctcaaggccaccgacttcggcctctccgtcttcttcaaagaaggggaggtgttcagggacatcgtcggcagcgcatactacatcgcgccggagg
tgctgaagcggagctacgggccggaggccgacatatggagcatcggcgtcatcgtctacatcctgctctccggcgtcccccgttctgggcaggcaagcgat
ctgttgtcttcagagagacgcacggcatcttcaattccatcctgagggggcaagtcgacttcaccagcgacccctggccgcgtatctcgtctggtgccaaggac
cttgtcaggaagatgctcaactcggaccccaagcagcggatatctgcgtatgacgtcctcaaccatccgtggatcaaagaagacggcgacgcaccggacaca
ccgctggacaacgcggtcctcggccggctcaagcaattcagagctatgaaccagttcaagaaagcagcgctaagggtcatcgccggatgcctgtcagagga
ggagatcaaggggctgaaggagatgttcaggagcatggacgcggacaacagcggcaccataaccgtggacgagctgcggcgcgggctggccaacaagg
gcaccaagctcagcgaagccgaggtccagcagctgatggaagctgccgacgcggacgggaacgggacgatcgactacgacgagttcatcacggcgacga
tgcacatgaacaggatggaccgggacgagcacctgtacacggcgttccagtacttcgacaaggacagcagcgggtacatcaccatcgaggagctggagcag

gctttgagggagaagggcctgctcgacgacggccgggacatcaaggacatcatatcggaagtcgacgccgacaacgacgggaggatcaactacacggagt
tcgtggcgatgatgaggaaaggggacccggaggtggccaaccccaagaagcgccgcgacgtcgtgctatag
>g78519.t1
atgggcaacacctgcgtcggccccagcgcggcgggccgcagcggatttctcgcctccgtcaacctgtggcggcccgcgcagcccgccgccgccctcccgc
cgccctcctcccccgcctccgacaaggcgcccgagcccgtcacaatccccgcctccgagcactcctcccaccactcctcccgatcctccgaccagccgcaga
cgccctccgcggagccgcagacgcaggacaataatcctccggcgaagaagcccgtgcccaaggtgaagcgcgtccagagcgcgggcctcctcgcggact
ccgtcctcaagcgtgacgtcaacacagcccggctcaaggatctctacaccatcgacaagaagctagggcaggggcagttcggcaccacctacctctgcgtcg
agaaggccaccggccgggagttcgcctgcaagtctatcgccaagcggaagctgctcacggaggaggacgtcgaggacgtgcgccgcgagatccagatcat
gcaccacctcgccggccacgcaaacgtcgtctccatcgtcggcgcctacgaggacgcggtcgccgtgcagctcgtcatggagctctgcgccggcggggag
ctattcgacaggatcattcaaaggggacattactccgagaaggccgctgcgcagctggcgcgggtgatcgtcggtgtcatcgaagcctgccactcgctcggtg
tgatgcacagggatctcaagccggagaacttcctgttcgtcaaccagaaggaggactcgccgctcaaggccattgatttcggtctctccatctttttcaagccagt
ggaatcatttactgctcatatgggtgttgcagaacaggttattgctgagagtttatccgaggaagaaattgcagggttgaaagaaatgtttaaaatgcttgacagtga
caatagtggccatataacattggaggaattgaaaactggcctgcagagagttggagctactttgatggactcagaaattgatgctttgatgcaagcagcagatatc
gacaatagtggcacaattgattatggggagttcattgctgcaactttgcatataaacaaagttcagaaggaggataagctctttgcagctttctcatattttgacaaag
acggcagcggttacataactcaagatgagctccaaaaggcatgtgaggagtttggtataggagatacccggcttgaggatattattggggacatcgatcaggat
aatgatggacggatcgactacaatgagtttgtcgcaatgatgcagaaaggagataatccactagggagaaagggtcaaagtgcggattttggtcttggtgaagc
actgaagcttcgataa
>g78522.t1
atggggaacacgtgcgtgggccccagcatcaccaagaacggcttcttccagtccgtctccacgctgatgtggaagacgccgcaggacggcgacgcgctgcc
cgccgccgccaatggccccgaaggcggcagcccgggccggtcgcagcccgccaagcccgaagtgcagagcaaggccccggagcccatgaagatcgcc
gcagcccaacaaccggatgagttgccgccgcccaaaaccaagcagcagcaagacgcgaagcccaacgccggcggcgaggcgcccagcaccaagccgc
ggcccaaggttcctccggtgaagcgcgtgtcaagcgccgggcttctggtgggctcggtgctgaagcgcaagacggagaacctcaaggagaagtacagcctg
ggccgtcggctagggcagggccagttcgggacgacgtacctgtgcgtggagcgcggcacggggaaggagctggcgtgcaagtccatcctgaagcggaag
ctggtgaccgacgacgacgtggaggacgtgcgccgggagatccagataatgtaccacctggcgggccacccgaacgtgatctccatccgcggcgcgtacg
aggacgccgtcgccgtgcacctcgtcatggagctctgcgccggcggggagctgttcgaccggatcgtgcagaggggacactacacggagaggaaggcgg
ccgagctcgcaagggtcatcgtcggggtcgtcgaggtgtgccactccatgggcgtcatgcatcgggatctcaagcctgagaacttcctcttcgttgacaacaag
gaggaggccgcgctcaagaccattgactttggtctctccattttctttcgccctggtcaagtattcacggatgttgtcggtagcccatactacgtcgctccagaggtc
ctgaaaaagaaatatggtcccgaggcagatgtctggagtgctggtgtgatcatctacatcttgctgtgcggtgtgccaccattttgggcagagaacgagcagggt
atatttgaagaggttttacatggaaaacttgatttccagtcagagccgtggcctagcatctccgaaggtgccaaagatcttgtgaggagaatgcttgttagggaccc
caaaaagagattgacagctcatgaagttctacggcatccatgggttcaggttggtggtttggcgcctgataagccgctggactcggctgttctgtctcgcatgaaa
caattctcggctatgaataagctgaaaaagatggctcttagggtcattgcagagaacttatccgaggacgagatcgccggcctgaaagaaatgttcaagatgata
gacacggacaacagtgggcacatcacctacgaagaactcaaggtcgggttgaaaaaagttggtgccaacctccaggaatccgagatccaggcactcatggaa
gctgcggacgtggataacagcggcacgatagactacggcgagttcatcgcggccactctgcacctgaacaaggtggagagggaggaccacctgttcgcggc
gttccagtacttcgacaaggacggcagcgggtacatcacgcccgacgagctgcagctggcgtgcgaggagttcggcctaggaggggacgtgcagctcgag
gagatgatccgcgaagtggaccaagacaacgacgggcgcatcgattacaacgagttcgtggcgatgatgcagaagccgacactggggctcccgaagaagt
ccggcggtctgcagaacagcttcagcgtcggattcagggaggcgctgaggatcgcgtaa
>g79282.t1
atgttggacaccaacaaggacgggcacttgactattgacgaacttcgcaagggattgcgagtgatagggcagaatgttagtgaaactgatgtggatatgcttatg
gaagctgcagacattgacggcaacggtaccctggacttgaaggagtttgtgacagtctccattcatttgaagaaaatcagaagcgaggatcacttgccaaaggt
attcagctactttgacaagaatggaagcggttacattgaaattgaagaattgaaggaggcgctctctccacgaggaggccagaaggcaatcgatgacataatcct
ggatgtcgacaaagacaaggttagtcgcacgaccattctgaaggaaactcaaaagtgttag
>g79424.t1
atgttcaaggccatggacaccgacgccagcggagccatcaccttcgacgagctcaaggaagggctaaggagatacggatcaaacctcaaggagagcgaga
tcagggacctcatggacgccgccgacgttgacaagagcggcaccatcgactacgacgagttcatcgccgccaccgtgcacatgagcaagctcgagcgcga
ggagcacctcctcgccgccttcgcctacttcgacaaggacgccagcggatacatcaccgtcgacgagctcgagcaggcctgcagggaccacaacatggcag
acgtcggcatcgacgaaatcatcagggaggtggaccaggacaacgatgggcgcatagactacggcgagtttgtggccatgatgaagaagggcatcattggg
aacggaaggctcaccatgaggcacacctctgacggcagcgttctccatggcgccggccacctctgctag
>g80828.t1
atggggctcggcgtggtggcgcacctggtgcgcctgggcgttctgagctcgacctccgaccacgcctcggtggtgtccatcaacctgttcgtggcgctaatctg
cgcctgcatcgtcctcggccacctcctcgaggagaaccggtggatgaacgagtccatcaccgcgctcatcattgggctgtgcactggcgtggtgatcttgctga
cgacaaaaggaaagagctcgcacatcttagtgttcagcgaggacctcttcttcatctacctcctccctccgatcatcttcaatgccgggcattccactgatcgcga
ggttgcgcttatgatgctcatggcttacctctcttacatgttagctgagctatcagatttgagtggcattctcactgtgttcttctgcggcatcgtgatgtcacattacact
tggcataatgtgacagagagctcaagagttacaaccaagcatgcttttgcaactttgtccttcatcgctgagacatttctcttcctctatgttggtatggatgcacttga
tatcgaaaagtggaagtttgctagtgacagccctggaaaatccattggaataagctcaattttgctaggattggttctggtgggaagagctgcgtttgtttttcctctct
cattcctgtctaacttgacaaaaaagggacctttggaaaaaataacctggagacaacaaatcgtaatatggtgggctggactgatgagaggggctgtgtcgattg
cgcttgcttacaataagtttactagatctggacacactcagcttcatggcaatgcgataatgataaccagcacaatcactgtagttctgtttagcaccatggtgttcgg
gatgatgacaaagccattaatccggttcttgctcccggcttcaagccacacggtcacctccgagccgaattcgcccaagtctcttcactcccctctcctcaggagc
atgcaggggtctgacctggagacggcctcgcactcgcacattgtcaggccttctagcctccgcatgctcctcaccaagccgacccacaccgtccactactactg
gcgcaagttcgatgatgcgctgatgcggcccatgtttggcggacgcgggttcgttcctttctcccctggatcacccaccgagcagagcgttcatggaggacagt
ga

>g84684.t1
atgggctccgccccgtcgtcgcgtgtccagagctcgtcgggggaggcgccgttcttcacggacttcggcgaggcgagccggtacgaggtcacggaggtggt
cggcaagggcagctacggcgtggtcgccgccgccgtcgacacgcacaccggcgagcgcgtcgccatcaagaagatcaacgatgtcttcgagcacatctcc
gacgccacccgcatcctccgcgagatcaagctgctccgcctcctgcgccacccggacatcgtccagatcaagcacatcatgctcccgccctcgcgccgcgac
ttccgcgacatctacgtccagttcttcctctaccagctactccgcggcatgaagtacatccacgccgccaatgtcttccacagggacctcaagcccaggaacatc
cttgccaatggcgattgcaaactcaagatttgtgacttcggccttgctcgggtgtcgttcaatgataccccttctgcgatattctggacgtatactcctgcgattgatat
ttggagcataggatgtatatttgctgaaatgcttacagggaagcctctctttcctggcaagaatgtggtacaccaattagatctcatgactgatcttcttggcactcctt
cagcagaaacactttccaagattttggagtaccatcctcagatgttgcaggagtatctttgcggaggggatatggcaaactttgtgtacccaagggaaaaagtaaa
tggcaacgggaatgagcttgagaagccaaatgcagactacgtcataaaattgcatgagtttcgctacgtgctcgcccctctccttgcagcttgtgctgatgagcaa
tga
>g85619.t1
atgggcaacgtgtgcttctgcggcacgacgtccacgtcccctgacgagcctccatctgatcccaaaaaatccgtagcgccgccgcaacagcaaggcaagagg
ccggcgacgcctccaagcagcagccatgaaaagagccccactcccacccccaggccgaagcagaagcagcagcaacagcaacccgagccccgtacgcg
cgccaagcccataaagcccaacccgtacgactgggcatcgccgtcgtcgtcgcgcgtgctggacggcgtggtcccgcaccacccgcgtctccgcgtgacgg
acaagtacacgctgggccgcgagctgggccgcggcgagttcggcgtgacgcgtctggcgacggaccgggccacgcgtgagcggctggcgtgcaagtcca
tccccaagcgccgcctccgcacggccgtggacgtgggcgacgtgcggcgcgaggtggccatcatggcgtcgctccccgaccacccgtccctggtgcgtctc
cgcgcggcgtacgaggacgccgacgccgtgcacctggtcatggagctctgcgacggcggggagctgttcgaccgcatcgtggcccgcgggcggtacacg
gagcgggccgcggcggcggcggcccggacggtggccgaggtggtccgggcctgccacgcgcacggggtgatgcaccgggacctcaagcccgagaact
ttctgtacgaggggcggggtgatgacgcgcggctcaaggccatcgactttgggctgtcggtgttcttccggcccggggagaggttcacggagatcgtgggca
gcccgtattacatggcgcccgaggtgttgcgacggagttacgggcccgaggtggacgtgtggagcgccggggtgatactgtatatcctgctgtgcggggtgc
cgccgttctgggccgagacggagcagggcgtggcgagggctatactgcgagggagcctggacctggaccgcgagccctggccccggatctcccacgggg
ccaaggacctcgtcaggcagatgctgcagatggaccccaagaagcggccaaccgcgcagcaagtcctcgagcacccgtggcttcagaacgcgcggaagg
cgccgaacgtgccgctgggcgacgtggtgcgcgcgcggctgcagcagttctcggccatgaacaagttgaagaagaaggccatgcgggtcatcgcggagca
cctgtccgtggaggaggtggaggtgatccgggacatgttcgcgctcatggacacggacaatgacggcaaggtcacgctgcaggagctcaaggcggggctc
aacaaggtcggatccaagctcgccgacccggagatggagctgctcatggaggccgccgacgtgaatggcaacgggtacctggactacggcgagttcgtgg
ccatcaccatccacctgcagcgcctctccaacgacgaccacctccgcacggcgttcctcttcttcgacaaggacagcagcggctacatcgagcgcgacgagct
ggccgacgcgctcgccgacgactccggccaaaccgacgaagccgcgctcaacaacgtcctccgagaagtagacacggacaaggacggtcggataagtttc
gacgagttcgttgccatgatgaaagccggcacggactggaggaaggcgtcacgacagtactcaagggaacggttcaagactctgagcaacagcctcatcaa
ggacggctcgctcgccatggcgcactga
>g86042.t1
atggaagggctgacgggagagcagatagttgctttccaggaggccttctccctcttcgacaagaacggcgacggatgcatcaccatggaagagctggctgca
gtgactcgctccctaggccttgatcctagtgatcaggagctcatcgatatgatgagcgaagttgatactgatggcaatgggataattgattttcaggagtttctgagc
ctcattgccaggaagatgaaggatggtgatggcgatgaggagctgaaggaggcttttgaggtgctagacaaggaccagaatggatttatctcccctactgagct
gaggacggtgatgaccagccttggggagaagatgactgatgaagaggttgaacagatgatcaaagaagcggataccgatggtgacgggcaggtgaactacg
atgagtttgtgcttatgatgaaaaatgctgaacggaagatatctggttga
>g88536.t1
atggagtcctcgtcgtcgggcggcgggagggcgcggcggcgcagcagcggcggtagctggggcagcgtcggtgacgccttcgacatccccgccaaggg
agcgcccgtggagcggctaaaaaagtggcggcaagcagcccttgtgctgaatgcgtcaaggcgctttaggctgcctttcgtttcaaagaagctggtcgagtac
atgttcagccaaaggaggcaccagtaccacatgctgatggagcacttggctttggaatcaaagaggaacagattactgcgttcacaagagatcacaactactct
gcgcttcaacaatatggagggttttgcagtaccacatgctgatggagcacttggctttggaatcaaagaggaacagattactgcgttcacaagagatcacaacta
ctctgcgcttcaacaatatggagggatttcaggtgtagcaagtatgctgaagactgatacagagaaaggaattaatggcgatgattctgacttgacggcaaggcg
aaatgcatttgggtcaaatacatatcctcgtaagaaaggaaggagctttttggcttttgtatgggatgcttgtaaagacatgacactgataatcctcatggttgcggct
gctgtttcacttgccttgggcataacaactgaggttgcccgaggcggaaggcgaataatggtatcaatatatgatttggttgttggagatgttgtgcctctgaagatc
ggtgatcaggtccctgctgatgggatcctcatcagcggtcactcgctttccatagatgaatcaagcatgacaggagaaagtaaaatcgtacacaaagaccagaa
atcacctttcctaatgtcaggttgcaaagttgcagatggttatggcacaatgctggtgactgctgttggtattaacactgaatggggattactaatggcaagcatatct
gaagattctggtgaagagacacctctgcaggttcgcctgaatggtgttgctactttcattggaatggttgggctttctgttgcatttgcagttttggttgtccttctggcc
agatacttcactggtcatacatataatcctgatggatcggcgcaatatgtgaaaggaaagatgggtgtgggccagacgatacgtggagtagttcgaatcttcactg
tggcggtcactatcgtggtcgtggctgttcctgaagggttaccgttggcagtcacattgacgcttgccttttcaatgcgcaagatgatgagggacaaggcactgga
attgcacagaacacttctggaagcatatttgagccagaggcatctgcttttgtatggtatgattactgacttattttcaatgtctcagcagggtggtcaagaaccaga
ggtgactgctgttggtattaacactgaatggggattactaatggcaagcatatctgaagattctggtgaagagacacctctgcaggttcgcctgaatggtgttgcta
ctttcattggaatggttgggctttctgttgcatttgcagttttggttgtccttctggccagatacttcactggtcatacatataatcctgatggatcggcgcaatatgtgaa
aggaaagatgggtgtgggccagacgatacgtggagtagttcgaatcttcactgtggcggtcactatcgtggtcgtggctgttcctgaagggctaccgttggcagt
cacattgacgcttgccttttcaatgcgcaagatgatgagggacaaggcactggaattgcacagaacacttctggaagcatatttgagccagaggcatctgcttttg
tatggtatgattactgacttattttcaatgtctcagcagggtggtcaagaaccagaggttgctgaattcaagagattcatcgaagatatggctgcttctagcctccgct
gtgttgcctttgcatatagacctcatgacatgggtgaggttccaaaggaggatcagaggaacgactggaagttgcctgaagataacctgattatgcttggaattgt
gggaataaagcaaaactattag
>g89054.t1
atgggcaactgctgcgtggcccggccgtcgtccggcaagcggcgtgccggcggcggcgggaaccaccgcggcggccgcctcggcggcgccaacatgcg
gtcctgctccgcgatctcctccatcaccgaccccggaggcgcctcccgcgggtgggcccccgccaccgtgctggggaaaagcctggcccccgacgccacc
gccgaggagctgctccggcggtacgacctcggcgaggagctcgggcgcggtgagttcggcgttacgcggcggtgcacagacacggccaccggcgagttg

ctggcgtgcaagtccatcagcaagcgccggctgcgcagcagcgtggacatcgaggacctgcagcgggaggtggccatcatgcggtcgatgccggcgcacc
cgaacgtcgtgcgcctgcgcgaggcgttcgaggacaacgaggccgtgcacctcgtcatggaggtctgcgagggcggggagctcttcgaccgcatcgtctcc
agggggcactacaccgagcgagccgccgccgccgttatgcgcaccatcgtcgaggtcgtgcagcattgccataagaacggtgtcatgcatcgggatctaaag
cctgaaaactttctgtacgccaacacatctgagaactccccccctcaaggtcatcgactttggtctctccgtgtgcttccaaccaggtgataggttcagtgagatcgt
cggatccccatactacatggctcctgaagtcctcaagaggaactacggacaagaaatagacatatggagcgccggagtcatactctatatcttgctatgtggtgtc
ccacccttctgggctgaaactgatgaagggattgcacaggctatcatccggtcaaaacttgatttcgagagggagccttggcctaaggtttctgagaatgcaaaa
gatcttgtcaggaagatgcttgatccgagcccttatggacgattgacagctcagcaggttttagaacatccttggatacagaatgcgaactcagctcccaacatcc
ctcttggagaagcagtaaggtccaggcttaagcaatttactgtcatgaacaagttcaagaagaaggccctacttattgtggcagaatacctaccaactgaagagct
ggaggcaattagggagctgttccacatgttggacaccaacaaggacgggcacttgactattgacgaacttcgcaagggattgcgagtgatagggcagaatgtc
agtgaaactgatgtggatatgcttatggaagctgcagacattgacggcaacggtaccctggacttgaaggagtttgtgacagtctccattcatttgaagaaaatca
gaagcgaggatcacttgccaaaggtattcagctactttgacaagaatggaagcggttacattgaaattgaagaattgaaggaggcgctctctccacgaggaggc
cagaaggcaatcgatgacataatcctggatgtcgacaaagacaaggatgggaagataagctacgaggagtttgagctgatgatgaaaggtggaatggactgg
agaaatacatctcgacagtattcgagagcagtttacaacacccttagccgaaagatatttaaggacatttccttgaagcttgatccctgtagtgggccacttgctgc
agctgcaaaagaacaacgagaggttgactga
>g89161.t1
atggagagctacctgaacgagaactttgggggcgtcaagcccaagcactcgtccgacgaggcgctggggcgatggcgcaaggtcgtcggcgtcgtcaaga
accccaagcgccgcttccgattcacggccaacctcagcaagcgctcagaggccgcgcagatgaagcgatccaaccaggagaagctgcgtgttgctgtgcttg
tttccaaggctgcacttcagttcctccacggccttgcaccgcaaagcgagtacacggtccctgacaacgtcaagaccaagggcttcggcatctgcgccgagga
gctgagctccatcgtggagggccacgacctcaagaagctcaagtcacatggcggcgtcgagggcctcttgtcgaagctgtccacctcggagtccgacggtgt
cgacacgtcccgcaagaggctgtcgagaagggaggccatcttcggcgtgaacaagttcatcgaggcagagtcccgcggcttctgggtcttcgtctgggaggc
gctccaggacatgacgctcatgatcctcgcggcgtgcgcgttcgtctcgctcatcgtcggcattgccaccgaagggtggcccaagggcgcgcacgacggcct
cggcatcgtggccagcatcctgctcgtcgtgttcgtcaccgcgaccagcgactaccgccagtccctgcagttcaaggacctcgacaaggagaagaagaagat
caccgtgcaggtcacccggagcgggtacaggcagaagctctccatatacgagctcctcgccggcgacgttgtccacctctccattggtgatcaggtgccggcc
gacgggctgttcctgtcgggattctcgctgctgatcaacgagtcaagcttgaccggggagagcgagccggtcgcggtcaacgccgagaacccgttcctcctat
cggggacaaaggtgcaggacggttcgtgcaagatgctcatcaccacggtcggcatgaggactcagtggggcaagctcatggcaactctcagcgagggtggt
gacgacgagacgccgttgcaggtcaagctgaacggcgtggccaccatcattggtaagatcggactcatcttcgccgtggtcacgttcgcggtgctcaccgaaa
gcctgttccgtcggaagatcatggacgggtcgtacttgagctggaccggagatgacgcgttggagctgctcgagttcttcgccattgccgtcaccattgtggttgt
tgctgtacctgaaggactgccgcttgcagtgacgctgagccttgcttttgccatgaagaagatgatgaacgacaaggcgctcgtcaggcaccttgctgcttgcga
gaccatgggctcggccacctccatctgcagcgacaagaccggcacgctgacgacaaaccatatgaccgtggtcaaggcctgcgtctgcggcaaggtcaaag
agctggacggtgtttcagagacaaagaccttgttctctgagttgccggactctgtcatgacgatgctcatgcagtccatattcaacaacaccggcggtgacgttgt
cattaaccaggatggcaagcgggaaatactgggcacaccaactgagacagcgattctcgagttcggcctgtcactcggaggggacttctcagctgtgcgaaaa
gaaagcaccatgatcaaggtggagccgttcaactcggcaaagaagagaatgggcgtggtcattcaactcccagggggcagtgccgttcctctcgatgaagcg
accgtcaagcacttgaatgccacgatcgagagctttgcaaacgaggcacttcgcacgctgtgccttgcctacgcggaagtccctgatgggttctcagccaatgat
cagattccgatggatgggtacacttgcattggcattgtggggatcaaggaccccgtccggcctggcgtcaaggaatcagttgccatctgcaggtctgctggtatt
actgttaggatggtcacaggtgacaacatcaacactgccaaggcaatcgcccgggaatgtggcattttaactgaagatggcattgccattgaaggcccggagtt
cagaaccaagactccagaagaaatgaccgaattgatacccaagatacaggtgatggcaagatcttcgccacttgacaagcacacccttgtgaagcatcttcgga
cttcacttgatgaggttgtcgcggtgactggcgacgggacaaatgatgcacctgcgctacatgaggctgacattgggcttgcaatgggcattgctggaactgag
gtattcaatgaggtgagttcaagagagatggagaagataaatgtatttgaaggcattctagacaacaatgtgtttgtcttcgtcctaagtagcaccgtcatcttccag
ttcatcataatacaatttctgggcgagtttgcaaacactacacctctctcattcatgcagtggatatactgcattttcatcggtttcataggcatgccaatcgctgctatt
gtcaagatgatccccgttggttcttggtag
>g90096.t1
atgggcaactcgtgccgcggctacaccataagcttgtacgattgcgactaccagtaccagcacagcagcagcacctcctattcgtcgtcgtcgaatttctccagc
aggcggagcagcatctgtggaggaggcggcggcatgcgcgggcaccagcagcagctttcgtccccgacggccgtgctcgggcacgtgaccccaccgctg
cgggagctctactcgctggggcggaagctcgggcaggggcagttcggcaccacgtacctgtgcacggatctggccacgggcgcgtccctggcctgcaagtc
cattgccaagcgcaagctgctcaccccggaggacgtggacgacgttcgccgcgagatccagatcatgcaccacctcgccggccatggcagcgtcgtcacca
tcaagggcgcgtacgaggacccgctctacgtgcacatcgtcatggagctctgcgagggcggcgagctcttcgaccgcatcgtcaaccgcggctacttctccga
gcgaaaggccgccgagatcgccagggtcatcgtcggcgtcgtcgaggcgtgccactcgctgggcgcgtgccactcgctgggcgtcatgcaccgggacctc
aagcccgagaacttcctgctgctcaagccccccctcaagatccaataccaagaatcagcagcatgaggacgacgacgacgaggcgtccctcaaggccatcga
cttcggcctctccgtcttcttcaagcccggccagatcttcaccgacgtcgtgggctccccctactacgtggcacccgaggtgctctgcaagcactacggcccgg
aggccgacgtgtggaccgccggcgtcatcatctacatcctcctcagcggcgtgccacccttctgggcagagacgcagcagggcatctttgacgccgtgctcaa
gggcgccatcgacttcgactccgatccctggcccaccatctccgacagcgccaaggacctcatccgccgtatgctacgctcgccgcccgccgaccggctcac
ggcgcaccaggtgctttgccacccatggatctgcgagaacggcgtcgcccccgacgcgccgcttgacccggcggtgctgtcgcggctcaagcagttctcgg
ccatgaacaagctcaagaagatggcgctgcgggtcatcgcgcagaacctttccgaggaggagctggcggggctcaaggagatgttcaaggccatggatacg
gacgccagcggagccatcaccttcgacgagctcaaggaagggctaaggagatacggatcaaacctcaaggagagcgagatcagggacctcatggacgcc
gccgacgttgacaagagcggcaccatcgactacgacgagttcatcgccgccaccgtgcacatgagcaagctcgaccgggaggagcacctcctcaccgcctt
cgcctacttcgacaaggacgccagcggatacatcaccgtcgacgagctcgagcaggcctgcagggaccacaacatggcagacgtcggcatcgacgaaatc
atcagggaggtggaccaggacaacgatgggcgcatagactacggcgagtttgtggctatgatgaagaagggcatcattgggaacggaaggctcaccatgag
gcacacctctgacggcagcgttctccatggcgccggccacctctgctag
>g91314.t1

atgatcgacaaggacaaggtcatgaagattggtctcacggatcagatcaagagggagatctcgataatgagactggtcaggcacccgaacgtcctgcagctttt
tgaggtgatggcgaccaggaacaaaatctactttgtcctggagtacgcgaaaggcggtgagcttttcaacaagatcgccaaggggaagctcactgaggatgct
gcgaggaagtattttcagcagttgatcagcgctgtggagtactgccacagccggggtgtttatcaccgtgacctgaagcctgagaacctgctactggacgagaa
tgagacactcaaggtctccgattttgggttaaagctcaaggagctcctttatggaatcctagatccagatcctaatactaggatgtccatctcaaggctaaagagaa
gtgcttggtacaagaagcctgttggaataacagcaccgaaaactgaaacatgcttcaaaacttgtccaagcgatggatttgatatgtccaagttgtttgaggaaag
atatggccgaagggaggcacgctttacttcgaaacagtcggcaacaacggtctttgcgaagctggaggaaatggccagacgtttgaagctcaaactgacaaat
aaagacaacggattgctgaaattggccacagccaaggaaggcaagaagggtgttcttgagcttgatgcagagatatttcagattgctccttctttcctgttagtcga
gttgaagaagacaaatggtgacacgttggagtaccagaaactgatgaaagatgacattaagccatcacttagagacattgtttggacatgggaaggtgatcacc
agcagtcacatctgttgcggcatgcagagcaacagcagcctgcaatgacaccagttccaccactaacgtaa
>g92472.t1
atgcagccggacccgagcgggaacgccaatgcgaaggcgaagctgccgcagccggtgacggcgccggcgccgtcgtccggccggccggcgtccgtgct
gccgtacaagacggcgaacgtgcgcgacttctaccgcatcggcaagaagctggggcaggggcagttcggcaccacgtaccagtgcgtggacaaggccga
cggcgcagagtacgcgtgcaagtccatccccaagcgcaagctgctgtgccgcgaggactacgaggacgtgtaccgcgagatccagatcatgcaccacctct
ccgagcaccccaacgtcgtgcgcatccgcggcgcttatgaggatgcactcttcgtgcacatcgtcatggaaatctgtgctggcggtgagctgtttgatcgcatcg
ttgcaaagggacactacagcgagcgcgcggccgcgaagctcatcaagaccatcgtcggggttgtggaggggtgccactcgcttggtgtcatgcaccgggac
ctcaagccggagaacttcctctttgccagcacagcagaggacgcacctctcaaggctactgactttgggctctccatgttctacaagcccggtgataaattctctg
atgtcgttgggagcccctattatgtcgcaccagaggtgcttcagaaatgctatggtccagaagctgatgtctggagtgctggggtgatcctgtacattttgctttgtg
gagttcccccatttgggctgaaactgaagcaggaatcttcaggcagattctacggggcaaacttgattttgaatctgaaccctggcctagcatctctgatagtgcc
aaagatctggtccgtagtatgcttactcgtgatcctagaaagagatatagtgctcatgaggttctctgtcacccatggatcattgatgatgccgttgcacctgataag
cctattgattctgctgttttgtcaaggctgaaacatttctcagcaatgaacaagctcaagaagatggcattgagggttattgctgaaagcttgtcagaggaagagatt
ggaggcctcaaggagctgttcaaaatgattgatactgacaatagtgggactataacatttgatgagctgaaaaatggcttgaaaagggttggctcagagttaaca
gagaatgagatccaagctttgatggaagcagacgggcaaattgactacagcgagttcacggcaatgatgaggaagggcaacgctggttgtgcaacaggtagg
aggaccatgaggaccaatttgaatcttggtgaactcttgaatcccagcaacagctag
>g92837.t1
atggcggccggcggggtggggccggcgcagcaccagacgcccaacggcagcagcaacggcgttgctggggcggcggcggccacgccgctccac
ggctccgcgacatccaccgtcaacggcgccgccgatgggtacgacagcgacggctacagcttcgcgccgcctacgccatcaactttatcaatgtccatacctc
cggagcttgcaggagcaattccactgattgatagattccaggttgagggatttcttaaggcaatgcagaaacagattcattcagctggaaaacgtggattttttcaa
agaaatcagttgggccccaagctcgtgagaagttcactttggaagatatgttgtgcttccaaaaggatcctattcctacatcgttactgaaaataagtagcgaccttg
taagccgctcaattaagttgttccatgtcatactaaagtacatgggcattgattcgcctgcaataataagtatggatgaaagaatagaacttgttgcgaagctttacaa
gcatacattgaagcgttctgaacttcgagatgaactctttgcacagatttcaaagcaaacgcgtaacaatcccgacaggggttggtcaataagagcctgggagct
tatgtatctttgcgcgtcgtccatgccaccaagcaaggatattggggcatacctgtctgaatatgttcactatattgctcatggagccacaactgattctgatattcgc
gttttagcgttgaacacactaaatgcgttgaaacgttcagttaaggcaggccctagggttacaatccctgcacgtgaagagattgaagctcttttaaccagccgga
agcttacaacaattgtatttttcttggatgaaacttttgaggaaatcacctatgacatggcaacaactgttgctgatgctgttgagctatcatatgtccagttgcaacat
gattatattttgggaaactatccagtaggaagagacgatgctgcacaactctctgccctacagatattagttgagattggtttcattgataatcctgagtcttgtgtgta
a
>g96026.t1
atgcgagcgcactccgaactcggccttgcagcgagacctagcaccgtctacaccaggcacaagtcctctgtattgcactctcagcccaaatcaactgttagcac
accagcttacattgctccagaggtccttgctagaaaggagtatgatggaaaggttgctgatgtttggtcctgtggagtaactctttacgtgatgcttgtcggggctta
tccctttgaagatcctgatgaaccgaggaatttttgcaagacacttactcggattctcagtgtacaatatgcagtccctgattttgttcgagtttcaatggagtgcaga
catttgctatcccggatcttcgtggcaaaccctgagcaacgaataaccatcccggagatcaagaaccacccatggttcctgaagaacctccccatcaagatgac
cgacgagtaccagacgaacctgcagatcatcaacatgaacgttccctcgcaatgcttggagatcatggccgtcatacaggaggccatcatggccgtcatacata
caggaggcccggaagcctggcaaatgggctga
>g96774.t1
atggacaagtacgagctgctcaaggacatcggcgccggcaacttcggcgtcgcaaggctcatgcggcacaaggagaccaaggagctcgtcgccatgaagta
catcccgcgaggccagaagattgacgagaatgtggcgagggagatcatcaaccaccgctcgctgcggcaccccaatatcatccggttcaaggaggtgctggt
cacgccgacgcacctggcgatcgtgatggagtacgccgccggaggcgagctgttcgaccggatctgcaacgccgggaggttcagcgaagacgaggccag
gtacttcttccagcagctaatttgcggtgtgagctactgccacttcatgcaaatttgccaccgagacctgaagctggagaacacgctgctggacggcagcccgg
cgcctcgactcaagatctgcgacttcggttactccaagtcgtcgctgctgcactcgaagcccaagtcgacggtgggcacgccggcgtacatcgccccggaggt
gctctcacgccgggaatacgacggcaagaggataatgtctatccagtacaaaataccggagtatgtccatgtatctcaggactgcaaggatatgcttgcaaaaat
tttcgtcgcaaaccctgcaaagagaattacaatcagggaaatcaggaaccacccctggttcctaaagaacttgcccagagaactcacagaagccgcacaggca
atgtactacaagaaggataacagtgccccaacttattccgtccagtcagtcgaggagatcatgaagatcgtcgagcaggcacggacgccacctccttcctccac
ccctgtggctggctttggttgggtagaggaggatgaagagaacagcaagaaaccagaggagaaacaggaggaagaagaggatgctgaagatgaatatgag
aaacaagtgaatgaagtccgcgccagcggtgaatttcagatcagctga
>g97633.t1
atgggcgcttgcttctcctccgcctccgctgccaccgccgccgccgggccgcccaaggcggagaagaggaagcgccgcggcgccgcctcgccggacgga
caggggaaggcggtgcgggtggagttcggctacgacagggatttcgaggcgcggtacgaggtcggcaggctcctcggccatggacagttcgggtacacctt
cgccgccaccgaccgcggctccggggaccgcgtcgccgtcaagcgcatcgacaaggccaagatggaccgccctgttgccgtggaggatgtgaaaagaga
agtgaagattcttaaagcacttcaaggacatgagaatattgttaacttctacaatgcatttgaggatgattcgtatgtgtatattgtgatggagtaa
>g98523.t1

atgggtctctgctatggcaagtcagcggcggctccggagccggcgggggtggaggaagcctgcgtagccaacggcgccggtgacccggtcccggacagc
gtcgcgtcgccggcgaagccccggacaccgaagcagcccaagttctcgttctacctgccgagcccgctcccgccttcgagctacaagggttcgccggcgaa
ctcgagcgtggcttccacgcccgcgcgcggcgggttcaagcggccattcccgccgccgtctcccgccaagcacatccgcgccctgctggcgcggcggcac
ggctcggtgaagccgaacgaggcttccatccccgagggcggggagccggagctgggcctggacaagagcttcggctactcgaagcacttctcggctaagta
cgagctcggccgcgaggtggggcgcggccacttcggctacacctgcgccgccaaggccaagaaaggcgagctcaagggcgaggacgtcgccgtcaagg
tcattcccaaggctaagatgacaactgctattgccatagaagatgtcagaagagaagtcaaaatattgagttctttgacaggtcacagcaacctagtgcaattttat
gatgcttttgaggatgaagaaaatgtatacgttgttatggagctatgcacaggaggtgaactgctggacaggatcttggctagaggtggaaagtattctgaagaa
gatgccaaggttgtcattcatcaaattctaagtgttgcttcattttgccatcttcagggtgttgttcaccgggatctgaaaccagagaatttccttttcatgtccaaggat
gagaattctgccttgaaagtcatagactttggtttgtctgactttgttaggccaggttaa
>g100730.t1
atgcagcaggagcagcccaagaagagttctgcagaggccgagtttttcacagagtatggcgatgcaaaccggtacaagatccaagaagtcatcggtaaagga
agctatggtgttgtgtgctctgccattgatttgcacactcgacagagagtggcgatcaagaagatacacaatatctttgagcacgtctctgatgctgcaaggatcct
ccgtgagattaaacttctgagactcctaaggcatcctgacattgttgagatcaagcatattatgttacctccttcgagaaaggacttcaaggatatttatgttgtttttga
gctgatggagtctgatctccatcaagttataaaggccaacgatgacttgacaaaagagcattaccaattctttctctatcaattgctccgtgccctcaaatacattcat
actgtgaacttaactgcagctaatgtttatcaccgtgacctgaagcccaagaacattttagcgaattctaactgcaaactgaaaatatgtgactttggactagcacga
gttgcattcaatgatacaccaacaacagtcttctggacggattatgtggcaacaagatggtacagagctccagagctctgtggatccttcttctcaaaatatacacc
ggctattgacatttggagcattggatgcatctttgctgaggtattgactgggaagcctctgtttcctgatggattaggatttgctgagtttgttgttccctccatcctcgg
caggttcggaatgagaaagcaagaaggcattgcgtga
>g102361.t1
atgggcaactgctgcccgggctccggggatgcggagcccgccgccggcgacgcctcgtcccgcctccacgccgcctccctcaaggccggcggcagcggc
agcggcgccgggccgacgtccgcgcccacgcccaccaagccgccggcgcccatcgggcccgtcctgggccggcccatggaggacgtccgcagcatcta
caccgtgggcaaggagctgggccggggccagttcggcgtgaccagcctgtgcacgcacaaggccacgggggagcggttcgcgtgcaagaccatcgccaa
gcggaagctgtccaccaaggaggacgtggaggacgtgcggcgcgaggtgcagatcatgtaccacctggcgggccagcccaacatcgtcgagctcaaggg
cgcctacgaggacaagcagtccgtgcacctcgtcatggagctctgcgcgggaggggagctcttcgacaggatcatcgccaagggcaagtacacggagcgc
gaagccgccgcgctgctgcgcaccatcgtcgagatcgtgcacacgtgccactcgctcggcgtcatccaccgtgacctcaagccagagaatttcctcctcctca
gcaaggacgagcacgcgccgctcaaggccaccgatttcggcctctccgtattcttcaagcaggggggaggtgttcaaggacatcgtgggaagcgcctactacat
cgcgccggaggtgctcaagaggagctacgggccagaggcggatatctggagcgtcggagtcatcctctacatcctactctgcggagtgcccccgttctgggc
aggcaagtcgttcatctctgtcttcatccatcacggcatcttcaacgccatcctccggggccaggtcgacttctccagcgacccgtggccgcgcatttcctccggc
gccaaggacctcgtcaggaagatgctcacctctgaccccaagaagaggatctcggcttacgacgtcctcaatcatccctggatcaaggaagacggtgaggcg
cctgacacgctgctggacaacgctgtcatgagcaggctcaagcagttcagggctatgaaccagttcaagaaagccgcgctcagggttatcgccgggtgcctgt
cggaggaagagatcaggggggctcaaggagatgttcaagggcatggacgccgacaacagcggcaccatcaccgtggacgagctgcggcgagggctggcc
aagcagggcaccaagctctcggaggccgaggtggagcagctcatggaggccgccgacgcggacgggaacggcaccatcgactacgaggagttcatcacc
gcgacgatgcacatgaaccggatggacagggacgagcacctctacgccgcgttccagtacttcgacaaggacggcagcggctgcatctccaaggaggagct
ggagcaggcgctcagggagaagggcctgctcgacggccgggacatcaaggacatcatctccgaggtcgacgcagacaacgacgggaggatcgactacag
cgagttcgtggccatgatgaggaaagggaaccccgaccccaaccccaagaagcggcgcgacgtcgtgctgtag
>g107035.t1
atggaccgcggcacgcgggagctgctggcctgcaagtccatctcgaagcggaagctgcggacgcccgtggacgtggaggacgtgcgccgcgaggtggcc
atcatgcggcacctgcccaagagccccagcatcgtgtcgctgcgggaggcgtgcgaggacgacggcgccgtgcacctcgtcatggagctctgcgagggag
gggagctgttcgaccgcatcgcgtgcgaggacgacggcgccgtgcacctcgtcatggagctctgcgagggaggggagctgttcgaccgcatcgtcgccag
gggacactacacggagcgcgcggcggccgccgtcgtgcgcaccatcgtcgaggtcgtgcagctctgccaccgccacggcgtgatccaccgcgacctcaag
cccgagaacttcctcttcgcaaacaagaaggagaactccccgctcaaggccatcgattttggcctctccatcttcttcaagcctggtgaaaaattttcagaaatagt
gggaagtccctactacatggctcctgaagtgctgaagaggaattatggtcctgaaatagacatctggagtgctggtgttatcctgtatatattgctatgtggtgttcct
ccattttgggctgaggttgaggacataaaggaaatgttcaaagtgatggacacagacaatgatggtatagtttcctatgaagaattgaagagtgggatcgcgaag
tttggttcccatcttgcagaatctgaagtccaaatgcttattgaagctgtggatacaaagggtaggggagcactagattatggtgaatttttggctgtctcacttcattt
acaaaggatggcaaatgacgagcaccttcggcgggccttcctgtttttcgacaaggatggaaatggttacattgagcctgaagagcttcaggaagcccttgtgg
aggatggaggagctgatagcatggacgtggtcaatgacatattgcaagaagttgacactgataagaactga
>g107749.t1
atggatgaggtggagcagacgctgacaaaggagcagatcgaggagttccgggaagccttcagcctcttcgacaaagatggcgatgggacgatcacgagcaa
ggagcttggcacggtgatgcgttcgctgggtcagagcccgacggaggcggagctgcaggagatggtggcggaggtggacgccgacggcagcggcgcca
tcgatttccacgagttcctcgtcctcctcgcccgcaagatgcgcgacgccggcgccgacgacgagctccgggaggccttccacgtcttcgaccaggaccaga
acggatacatctcccgcgacgagctgcgccacgtcctcgagaacctcggcgagaagctctccgacgaggagctcgccgagatgctgcgcgaggccgacgt
cgacggagacggccagatcaactacaacgagttcgctaaggtcatgatggccaaacgaagaaaccagatgatggacgacgaggacagtggatcgcattccc
acgggaggagcccctgctgtacaatcctctga
>g107750.t1
atgagtacaaccaaggtgaagagacgtgtggggaagtatgagctgggccgaaccataggcgagggaacattcgcaaaggtcaggtttgcacgggacactga
gacaggcgacccggtggccatcaaaatcctggataaggagaaggttctcaagcacaagatggttgagcaggtgatgggaagtaaaacaaagatctacattgtg
ttagagtatgtaaccggtggtgagctctttgacataattgttaaccatggcagaatgagggaagatgaggcaaggagatactttcaacagttaatcaatgcagttga
ttattgtcatagcaggggcgtgtaccaccgggatttaaaaagaataacaatccctgaaatactagaggatgagtggttcaaaaaaggctacaagcgcccagagtt
tgatgaaaaatatgacacaacgttggatgatgtggatgctgtcttcaatgattcagaagagcaccatgtgacagaaaggaaagaagaagaaccagcaagtctga

atgcgtttgaactaatttcaatgtcagaaggtctaaaccttggtaacttattcgactcggagaaggaatacaaaagagaaacaaggttcacatcaaaatgtccaccc
caagaaattgtccgcaagattgaggaagcagcaaagcctctaggatttgatgttcaaaagaaaaattacaaggttccttga
>g112607.t1
atgaatgctgagaagtatgatgaattctggaaatctattgaagacatggcagcaacctcattacgctgtgttgcttttgcatatcgtccctgtgagcctgaaatgatac
cagaggacgacatagctaactggaaattgcctgaggatgacctgactctgcttggcatcataggaataaaggctgatataggtctttcaatgggcatctcaggga
cagaagttgctaaggaaagttctgacattataatcttggatgatgactttacatcagttgtcaaggttgttcgttggggccgatctgtctatgcgaatattcagaaattc
atccagttccagctcacggttaatgttgccgcccttgtaataaatgtggttgctgctgtgtcatctggtgatgtgcctctgaatgccgtagaggttgggaccttttcttg
a
>g113382.t1
atgggggtcttccaccgggatctcaagcccgagaacttcctcttcctcaacaacaaggaggactcgccgctcaaggccacggactttggcctctccgtcttcttc
aagcccggccatccatggattagagaagacggagaggccccagataagccacttgacattacggtcatcggtagaatgaaacagttcagggcaatgaacaag
cttaagaaagttgcattgaaggttgttgccgagaacttatcagatgaggagattatgggcttgaaagagatgtttagatccttggataccgataacagtgggacaat
tacgcttgatgagctaagatctggtttaccaaagcttgggactaaaatttctgaatcagaaattagacagataatggaggcggctgatgttgatggaaatgggacc
attgattatgcagagttcatatcagccacaatgcacttgaatagattggagaaggaagaccacatactcaaagcattcgagtattttgataaagaccacagcggat
acataactgtagatgagctggaagaagctctcaagaagtatgatatgggagatgataaaacaattaaagaaatcattgcggaagtagattcagataatgatggaa
gaattaactaccaggagtttgttgccatgatgaggaacaacagccctgagattgttccaaaccggaagcgcatgttttaa
>g113452.t1
atgggcaacgtgtgcttctgcggcacgacgtccacctcccctgacgaccctccatctgatcccaaaaaacccggagcgcctccgcaacagcaaggcaagag
gccggcgacgcctccaagcagccgccaggagaagagccccacccccaggtcgaagcagcagcccaagccccggacgcgcgccaagcccaagcccaag
cccaccccctacgagtacgactgggcgtcgccgccgtcgcgcgtgctggacggcgtggtcccgcaccacccgcgtctccgcgtgacggacaagtacacgct
gggccgtgagctgggccgcggcgagttcggcgtcacgcggctggcgacggaccgggccacgcgggagcggctggcgtgcaagtccattcccaagcgcc
gcctccgcacggccgtggacgtgggcgacgtccggcgcgaggtggccatcatggcgtcgctccccgaccacccgtcgctggtgcggctccgcgcggcgta
cgaggacgccgacgccgtgcacctggtcatggagctctgcgacggcggggagctcttcgaccgcatcgtggcccgcggccggtacacggagcgggccgc
ggcggcggcggcccgcacggtcgccgaggtggtgcgggcctgccacgcgcacggggtgatgcacagggacctcaagcccgagaactttctgtatgaggg
gaggggcgacgacgcaaggctcaaggccatcgactttgggctgtcggtgttcttccggcccggagagcggttcacggagatcgtgggcagcccgtattacat
ggcgcccgaggtgctgcgacggagctacgggcccgaggtggacgtgtggagcgcgggcgtgatactgtatatcctgctgtgtggggtgccgccgttctggg
ccgagacggagcagggcgtggcgagggccatcctgcgggggagcctggacctggaccgcgagccctggcccaggatctcccacggggccaaggacctc
gtcaggcagatgctgcagatggacccaaagaagcggccaaccgcgcagcaagtcctcgagcacccgtggctgcagaacgcgcggaaggcaccgaacgtg
ccgctgggcgacgtggtgcgtgcgcggctgcagcagttctcggccatgaacaagttgaagaagaaggccatgcgggtcatcgcggagcacctatccgtgga
ggaggtggaggtgatcagggacatgttcgcgctcatggacacggacaaggacggcaaggtcacgctgcaggagctcaaggcggggctcaagaaggtcgg
atccaagctcgccgacccagagatggagctgctcatggaggccgccgacgtgaatggcaacgggtacctggactacggcgagttcgttgcaatcaccatcca
cctgcagcgcctctccaacgatgaccacctccgcacggcgttcctcttcttcgacaaggacagcagcggttacatcgagcgcgccgagctggccgacgcgct
cgccgacgactccggccagaccgacgacgccgcgctcaacaacgtcctccgagaagacggtcggataagtttcgacgagtttgttgccatgatgaaagccgg
cacggactggagaaaggcgtcacgacagtactcaagggagcggttcaagactctgagctacagcctcatcaaggacggctcgctcgccatggcgcactga
>g114194.t1
atgggcgcctgcttctcctccgcctccgctgccaccgccgccgccgggccgcccaaggcggagaagaggaagcgccccggcgccgcctcgaccgacgga
caggggaaggcggtgcgggtggagttcggctacgacagggatttcgaggcgcggtacgaggtcgggaggctcctcggccatggacagttcgggtacacctt
cgccgccaccgaccgcggctccggggaccgcgtcgccgtcaagcgcatcgacaaggccaagatggaccgccctgttgccgtggaggatgtgaaaagaga
agtgaagattcttaaagcacttcaaggacatgagaatattgttaacttctacaatgcatttgaggatgattcatatgtgtatattgtgatggagtaa
>g116892.t1
atgggcaacatctgcgccgggtcgcggtttcccaagctcggctccatctggccgtcgcgctcctccacgccgtcccagcccaccaccacctcccgctccgtcc
ccgtcgtccaggtccagcccagcgaatccacctccaccgccgactccaagccgcttccggtcccggcagccgcgcaaaccacacctcctgctcccatcgtca
tttccgaaccagcacgacactcatcatcacaatcagagcccccacggccacaaccaaaaagagaggacgcgacgtcccaacaaccagcgccttcctcgtcgt
cgcagccgcaggcgaagaagaagcccgcgcacatcaagcgcatctccagcgcgggtctgcaggtggaatccgtgctgcgtcgcaagaccgagaatctcaa
agacaagtacagcctgggacgaaaactcgggcagggccagttcggcacgacgtacctgtgcgtggacaaggccacggggcgtgagcacgcgtgcaagtc
catcgccaagcggaagctggtcaccgacgaggacgtggaggacgtgcgccgcgagatccagatcatgcaccacctcgccggccacccaaacatcatctcc
atcgtcggggcgtacgaggacgccgtggccgtgcacgtggtgatggagctgtgtgcgggtggggagctgttcgatcggattgtgaggagggccattactcg
gagcggcaggcggcggcgctggcgcgggtcatcgttgcggttgttgagtcgtgtcattcgttgggggtcatgcaccgcgacctcaagccggagaatttcctgtt
tgttgggaatgaggaggatgcgccgctcaagaccatcgatttcggactctccatgttcttccggccaggcgaggcgttcactgacgtggtgggaagtccgtact
acgtggcgccggaggtgttgaagaagaattacgggcaggaggcggacgtgtggagcgccggcgtcataatctatatcctgctatgcggcgtgccgccgttct
gggcggagacggagcagggcatcttcgagcaggtgctgcacggctcgctggacttcgagtccgacccatggcccaacgtgtcggacaacgccaaggacct
gcttaggaggatgctcgtcagggaccccaagaaacggctcactgcgcaccaagtcctctgccacccgtggcttcagatgatcggctcggcgcccgataagcc
gctagactcagcggtgctctcgcgactgaagcagttctcagcgatgaacaagctaaagaagatggccttgagggtgattgcggagaacctttcagaagaggag
attgctggtctgaaggagatgttcaagatgatggataccgacaacagtgggcagattaacttcgaggagctcaaggcggggctccatagagtcggcgccaata
tgaaagagcccgagatacatcagctaatgcaggctgccgatattgacaacagtggtaccatagattatggtgagttcatagctgctactttgcacctcaataaagtt
gaaagggaggatcatctgttcgctgccttccaatacttcgacaaagatggaagtgggtacatcacagctgatgagctccagcaggcatgcgatgaattcggcatt
gaagatgtccgattggaagacatgatcggtgaagtagatcaagacaatgatgggcggattgactacaacgagttcgtcgccatgatgcagaaatcaactagtgg
ttttgggaagaaagggagcagatcagaccaggacggggatcataaacgaggttgcttgaggatcctgtggatcttcttgccagggcggagggggttgggtcacc
ctgcgcaattccagctcaggcagcgtgatcaggggcatcctcggcagggcggcaccactcactggactccgcaatatattccaagcagcggcagtttctggat
ctatagaatcagtagtctccggcagcaggtag

>g119568.t1
atgggccaatgctgcgccaagggcggcgaccaggccgccggcgacgacaaggaccaccaccccgccgcgtcgtcgtcgtcggatcccaagccggatgcc
tccacccgcggcggcggcgccaacaccaacgccccgccgccgcgcccgtcgtcgtcctcctccaaccacccggctgcggcggcgggcgcgtcggtgggc
ccggtgctgggccggcccatggaggacgtgcgggcgacgcactcggtggggaaggagctgggccggggccagttcggcgtgacccacctgtgcacacac
aagcccacgggggagaagctggcctgcaagaccatcgccaagcggaagctctcctcccgggaggacgtggacgacgtccgccgcgaggtccagatcatg
caccacctctccggccagcccaacgtcgtctccctgaaaggcgcctacgaggataagcacaacgtacacctcgtcatggagctctgcgccggcggcgagctc
ttcgacagggtcatcgccaaagggcagtacacggagcgcgccgccgcggcgctgctccgcaccatcgtgcagatcgtgcacacctgccacgccatgggagt
catgcacagggatatcaagccggagaacttcttgttgcttagcaaggacgaggacgcgccgctcaaggcaacggatttcgggttatccgtcttcttcaaggaag
gggaggtgttcagggacattgttgggagtgcgtattacatcgcgccggaggtgcttaagaggaagtatgggccggaggcggatatatggagtgttggagtcat
gctatacatcttccttgctggagtgcctcccttctgggcggagaatgagaatggtatcttcactgccatcctgcgtggccagattgacctcgccagcgacccctgg
cccaagatttcgtcgggagcaaaggatcttgtcaagaagatgctcaacatcaaccccaaggagcggcttaccgcgttccaggtcctcaatcacccgtggatcaa
agaagatggagatgcccctgatacgccacttgacaatgttgtcctcaacagactcaaacagttcagggccatgaaccaattcaagaaagcggcactaaggatca
tagctgggtgtctatctgaagaggagatcaccgggctaaaggagatgtttaagaacatcgacaaggataacagtgggaccattacgcttgaagagctcaagaat
ggcttggcaaagcacggaaccaagctgtcggacggtgaaattcagcaactcatggatgcagctgatgctgatggcaacggattgatcgactacgacgaatttgt
caccgcgacggtgcacatgaacaaactggacagagacgaccacctctacactgcattccagtatttcgacaaggataacagtgggtacatcacaaaagaaga
gcttgagcaagctttgaaggagcaaggattatatgacgccgaggagattaaggacgttatttcagatgctgatgctaacaatgacgggaggatagattattcaga
gtttgtggcgatgatgaggaaaggtactgctggcgctgagacggcgaaccccaagaaaaggcgcgatctagtactgtag
>g119576.t1
atggatgaggtggagcagacgctgacaaaggagcagatcaaggagttccgggaagccttcagcctcttcgacaaagatggcgatgggacgatcacgagcaa
ggagcttggcacggtgatgcgttcgctgggtcagagcccgacggaggcggagctgcaggagatggtggcggaggtggacgccgacggcagcggcgcca
tcgatttccacgagttcctcgtcctcctcgcccgcaagatgcacgatgccggcgccgacgacgagctccgggaggccttccacgtcttcgaccaggaccagaa
cggatacatctcccgcgacgagctgcgccacgtcctcgagaacctcggcgagaaactctccgacgaggagctcgctgagatgctgcgcgaggccgacgtcg
acggagatgggcagatcaactacaacgagttcgcaaaggtcatgatggccaagtga
>g120444.t1
atggattttgttaagcgtttgctgtgcaaggatccacgtagaaggatgactgcagcacaggctttaagtcatccatggatcagaaattataatgacattaagctgcc
attggacgtccttatattccgacttatcaaagcatatatccgttcctcatctttacgaaaagctgctttgagggttgttcttaaactggctctatcaaagactttaactgtt
gatgaacttttctatctgaaagcacaattttccttgttggaaccagacagaaatggatgcattactcttgataatatcagaacggcattaacaagggaagctactgat
gcaatgaaagaatcacgagttcaagagattcttgtttcgttgagcgctcttcagtacagaagaatggacttccaagaattctgtgcagcagcagttagtgtgcacca
gcttgaagcattggatagatgggagcaacatgcccgatctgcttatgaatattttgagaaggatggcaatcgtgctattgtaattgatgaactggcttctgaattggg
cctcagcccttccgtgccgttgcatgttgttctgcaagattggatcagacatactgacgggaaactgagctttcttggttttgtcaagttattgcatggtatgtccagc
aggtccatgtcaaagatgaggtag
>g121814.t1
atggaggagatgggggcggggccggtgagcgaaacctctgctcgcgctggtagctcgccgcggggtaggacctcagccttcttggctcggagccgggcct
caacaccggcgacagtggagatcccgagcttgtccggccatgaaagatggcaagctcgccgcccttctcaagcccgcgtcggcaccgccgccccgttggcc
cctggtctcgccctgcccgccccgagcgccgcccgcgcccaggcctcgtccatggcgagctcgttgcccgtgccaagcctcccgagcttgcctctcggcgcc
gccctccgccaccccgcctcccggtgccgccctccgccgcccctcccacgcccgcgctctcgtttgcgccccgcgctctcgtttgcgccccgcgccgccgct
gtcgagttccggtgcgagcaggagatggagaagccgagatcggggtggtgcggtgtcatctctcctgaggaagctgctgcggcggcgatggccggggtgc
ggaaccctgaggcgtcgcccgggcgctacgtgcggcgccgggacgagatcgacgacggctgcgacgacgtgctcggtgtcgacgtcgggggcgcgggg
gccgaccccttcgacatccccgccaagcgcgcctccgtcgagcggctgcggcgctggaggcaagctgctcttgtcctcaatgcttctcgacgattcagatatac
tcttgacctaaaaaaggaggaggagaaggaacatataaggaggaaaatcagggctcatgctcaagtcatacgggcggcactgctttttaaagaggctggagaa
aagaagaatggtgacagggaattgccagctgtcagtgattataaacagtccctgcagttccaacacctaaatgaggagaagcaaaacatccaagtggaggttat
taggggtggtagaaggattccagtgtcaatattcgatattgtagttggtgatgtagtggctctaaaaattggtgatcaggtcccagctgatggtgttttaattaatggt
cattctcttgccatcgacgagtccagtatgactggggaaagcaagattgtaacggcagtcggcttaaacactgaatggggtttattaatggccagcatttcagaag
acaataatgaagaaactccattgcaggtgcgattgaatggagtagcaacactcataggcatcttaggactttctgttgctgccctggtccttatagtcttgtttgcaag
atattttacaggacatactacagactcgaatggaacagttcagtttattaaagggcagacaagtgcaaaatctgcaatatttggttcaataaagatactaactgttgc
ggtaactattgttgttgttgctgtgcctgagggcctaccactggctgtaacattgaccctggcctattctatgcggaaaatgatggctgacaaagcactggtccgga
ggctttctgcctgtgaaacaatgggttctgctactacaatttgcagtgacaagacaggcacattaactttgaaccagggagattcaaatgttcatgtacactggaaa
ggagctgctgaaatagttcttgctttatgtacaaattggcttgacacggatggttcaattcatgaaatgacaactgataaggctgatcagttcaaagaatacattgagt
atatggccgagcaaagtcttcggtgtgttgcttttgcatacagaactatcgagaacgatgtcccgagtgaagagcaaagaatcaactggcaattgccagataatg
accttactctaattggaatagtgggaatgaaggatccttgtcgccctggagtaagagaggctgttgaattgtgcatcaatgctggtgtaaaggtgatggcaagatct
tctcctaatgacaagcttctactcgtaaaagctcttaagaaaagaggccatgtcgttgctgttactggtgatggtacaaatgatgctcctgcattgcatgaggctgat
attggtctttctatgggcatccaaggaacagaagtagctaaagaaagctcagatattattattcttgatgacaatttttcgtcagttgtgaaggtggtccgctggggtc
gttctgtttatgcaaatatccaaaagtttattcagttccaacttactgtaaatgtcgcggctcttgtcatcaatgtggttgctgccatttcatcgggaaatgttcctctaaat
gctgttcagctgctctgggtaaacctcatcatggacacacttggagctcttgcattggctaccgaaccacctacagatcagcttatgatgcggccacctgtcgggc
gaagagaacctcttgtgactaatatcatgtggagaaacttattcattcaggctgtctttcaagttgctgttcttctgaccctcaactttaggggccgagatctcttgcac
ttgacccaagacaccctggatcactccagtaaagtgaaaaatacagttatattcaatacatttgtcttgtgccaggtgtttaacgagttcaattctcgtaaaccagaag
aactgaacatctttgatggggtttcaagaaaccatcttttcttgggagtagtgagcataactgtcattctgcaggtgataattattgagttccttggaaagttcacgtca
acagtgagactcagctggaagctgtggcttgtttctgtagttattgcatttgtcagttggcccttggctttagttggaaaattcattccagttccaaagacaccattgaa
ggattttctcatgagatgttggcccaaacgaaggaagcaaggtgacgacggagcaacaccaccggtgtga
>g122894.t1

atgtcatcgcctaacaggagagaagatccgacggagcaatttgagtcgctgtcaccggtggagtccacgtggatgatcgtggacatccaagggtccgacgtag
tcgaagaaggggcggcgcagccaaagaatgggaggcggcgcagggggaggaaggagaggcgcggccagggggtggcggcgctggttgttaccgtgcc
ctggatcagacggcggcgtttggtgggtggagaggcagcggaagggaaggcacgaaatcagggataccctggactggagaagcttcatgttcttgtgctggt
atcaaaatccaccctgcagtatgaacatggtgtttcgcttcagagtgagtatgttgctcctgaaagtgttaaagctgctgggtttcagatttgtgctgacgaactggc
atcaattgttgaaactcgtgacaatgaaaagttgtctatgcatggccagttggatggaattgcaggcaaattggtgacatcattaactgatggaattagtacagatga
gtatatcctgaatctaagacgagacatatatggagtgaacaagtttaccgagggtgaggagaagcttcatgttcttgtgctggtatcaaaatccaccctgcagtatg
aacatggtgtttcgcttcagagtgagtatgttgttcctgaaagtgttaaagctgctgggtttcagatttgtgctgacgaactggcatcaattgttgaaactcgtgacaat
gaaaagttgtctatgcatggccagttggatggaattgcaggcaaattggtgacatcattaactgatggaattagtacagatgagtatatcctgaatctaagacgaga
catatatggagtgaacaagtttaccgaggttacaatcgttgttgttgctgttcctgaggggcttcctttagcagttacattgagccttgcatttgcaatgaagaagatg
atgaatgacaaggctctggtccgacaattagctgcctgtgaaacaatgggctccgccacagtcatttgtagtgacaagacaggaacactaacaaccaattgcatg
tctgtcgtgaaggcctgttctgtggcaacatgccgatctgctggcatttcagttcgaatggttactggagacaacataaatacagcaaaggcaattgctcgtgaatg
tggtatacttacagaggatggcctttcaattgaaggtgctgaattcagggagaaaagtcgtaaagaaatccttgagttgattccaaagatgcaggtactggcccga
tcttcaccaattgataagcatacactggtgaaacacttgcgtacaacgttcaatgaggttgttgctgtgactggtgatggcacaaacgatgcacctgctctgcgcga
ggcagatgtcggacttgccatgggcattgcagggactgaggtgttcaatgagataagctcaagagacatggaagctatcaacgttctcaagggcctgccagata
actcaattttcatgggcatcctcgctggcaccattggttttcagttcatccttgtccaatttcttggcgactttgccaatacggcaccgctcacccaactacagtggttt
gtcagcgttttgtttggactccttgggatgcccatagcggctgccattaaacttattcctgtggaacgtcatgaagaagatacacatccgtcatag
>g123258.t1
atggagatgcttaatcaggagcagatctccgagttccgcgaggcgttctccttcttcgacaaggacggcgacgggtgcatcacggtggaggagctggcgacg
gtgatggggtcgctgcaggggcagcggccgagcgcggaggagctccgcgagatgatccgtgacgccgacgccgacggcaacggcaccatcgagttcgc
cgagttcctgacactcatggcgcgcaacaagaccgccgacaaggacgaccatgacgccgacgaggagctccgcgaggccttcaaggtcttcgacaaggac
cagaacggatacatctccgccaccgagctgcggcacgtgatgatcaacctcggcgagaagctgacggacgaggaggtggagcagatgatccgggaggcc
gacctcgacggcgacggccaggtcaactacgacgagttcgtcaggatgatgatgctctccgacggcggccacgcgccggccaccaaacactaccaatga
>g124260.t1
atgggcctctgctcctcctccaccgcgtccgcgcaccgtgccggtgccgcgccggggaataaggagaacaagggcgccggaggcggcagccgggggatc
gtggcgtgtgggaagcggacagatttcggctacgacaaggacttcgaggcccgttactcgctcggcaagctgctgggccacggacagtttggctacacctac
gccgccgtcgaccgcaactccggtgagcgcgtcgccgtcaagcgcatcgacaaaaacaaggtgcctcatttctccgtttgttttttgtttcgtttttcttacttgctg
gatcggatcgcgttagtggctga
>g135275.t1
atgtataaaactaagagatctgcttcacttaaagtgaagcgccgtgttgggaagtatgagcttgggcgcacaattggggaaggaacatttgctaaggttcggattg
caaagaactcggagactggggaacatgttgctatcaagatccttgacaaggcaaaggttctgaagcacaaattggttgagcaggctgatggattgctgcacact
acatgtggaacggctaactatgttgctccagaggttatcaatgacaagggctatgatggtgccactgcagatctttggtcttgtggggtaatcctttttgtgttgcttg
caggatatctgcctttcgaggatgacaatactgtctccctctataaaaagatctcaggagctcaatttacttgccccctcttggttttctgctggagcaaagaagctgat
ctgtagaattctggatcctaatccttcaactcggatttcagttcctcaaatacaaaaggatccctggttcaaaacaggctacaagccacctgtttttgacaagaaatgt
gaaggaagtttagatgatgttgatgctgcttttggagactcagaggaacatcatgtgacagaggaaatggaaggccagccaccctcgatgaatgcatttgagttg
atatcattaaacaaggggctgaatctggagaatttgttcgactctaataagatgcaaatggagaatccaaaagcaggaaggaagggcaatcttcatgttgcgact
gagggcaggagacacggtgaatattcaatattggaagcaaataaatga
>g135393.t1
atgggtctctgccatggcaagccaacgcaaatcccggagcctgaggcggaagaagatccccatgtagcctccggcgccggcgacgccgccggtgcccgga
acggcgcctccgcgccgtcgccagcgcctgcgacgaagccgggcacgccgaagcagcccaagttcccgttctacctaccgagcccgctcccgccgtcgag
ctacaagggctcgccggcgaactcgagtgtggcatcgacgccggcgcgcggagggttcaagcggccgttcccaccgccatcgccggcgaagcacatccgc
gcgctcctggcgcggcggcacggctcggtgaagcctaatgaggcgtccatccctgagggcggcgagccggagctcggcctggacaagagcttcggcttctc
aaagcacttcttcgccaagtacgagctcggcgaggaggtcggccgcggccacttcggctacacctgctctgctagggccaagaaaggcgagcacaagggg
caggacgtcgccgtcaaggtcatccccaaggccaagatgacaactgctattgcaattgaagatgtcagaagagaagttagaatattgagttctctgtcaggccac
aacaacctagtgcagttctatgacgcttttgaggatgaagataacgtgtatatagttatggagttatgtaaaggaggcgaactccttgatagaatattggcgagagg
tggaaagtattctgaagaggatgcaaaggttgttatggtacaaattttgagtgttgtatcattttgccatcttcaaggtgttgtccatcgcgatctgaaaccagagaatt
tccttttctcttcaaaggatgaaacctctgccttgaaggtcatagattttggcttgtctgacttcgtgaagccagtttatcatgttctcatcatgtga
>g135394.t1
atgggtctctgccatggcaagccaacgcaaatcccggagcctgaggcggaagaagatccccatgtagcctccggcgccggcgacgccgccggtgcccgga
acggcgcctccgcgccgtcgccagcgcctgcgacgaagccgggcacgccgaagcagcccaagttcccgttctacctaccgagcccgctcccgccgtcgag
ctacaagggctcgccggcgaactcgagtgtggcatcgacgccggcgcgcggagggttcaagcggccgttcccaccgccatcgccggcgaagcacatccgc
gcgctcctggcgcggcggcacggctcggtgaagcctaatgaggcgtccatccctgagggcggcgagccggagctcggcctggacaagagcttcggcttctc
aaagcacttcttcgccaagtacgagctcggcgaggaggtcggccgcggccacttcggctacacctgctctgcnnnnnnnnnnnnnnnnnnnnnnnnnn
nnnnnnnnnnnnnnnnnnnnnnnnnnnnnnnnnnnnnnnnnnnnnnnnnncacccccattcccctctgacccaagcctttcgctcaaagcttc
cagctttttcaattgtttggtcacttcaagaatcaaggaatatgttcttgcttcttgggcgatag
>g135510.t1
atggagagctacctgaacgagaactttgggggcgtcaagcccaagcactcgtccgacgaggcgctggggcgatggcgcaaggtcgtcggcgtcgtcaaga
accccaagcgccgcttccgattcacggccaacctcagcaagcgctcagaggctgcgcagatgaagcgatccaaccaggagaagctgcgtgttgctgtgcttg
tttccaaggctgcacttcagttcctccacggccttgcaccgcagagcgagtacacggtccctgacaacgtcaaggccaagggcttcggcatctgcgccgaaga
gctgagctccatcgtggagggccatgacctgaagaagctgaaatcacatggcggcgtcgagagcctcgtgtcgaagctgtccacctcggagtccgacggcgt
cgacacgtcgtccaggaagaggctggcgagaagggaggccatcttcggcgtcaacaagttcgtggaggcagagtcccgcggcttctgggtgttcgtctggga

ggcgctccaggacatgacgctcatgatcctcgcggcgtgcgcgctcgtctcgctcatcgtcggcattgccaccgaagggtggcccaagggcgcgcacgacg
gcctcggcnnnnnnnnnnnnnnnnnnnnnnnnnnnnnnnnnnnnnnnnnnnnnnnnnnnnnnnnnnnnnnnnttgcac
cgcagagcgagtacacggtccctgacaacgtcaaggccaagggcttcggcatctgcgccgaagagctgagctccatcgtggagggccatgacctgaagaa
gctgaaatcacatggcggcgtcgagagcctcgtgtcgaagctgtccacctcggagtccgacggcgtcgacacgtcgtccaggaagaggctggcgagaagg
gaggccatcttcggcgtcaacaagttcgtggaggcagagtcccgcggcttctgggtcttcgtctgggaggcgctccaggacatgacgctcatgatcctcgcgg
cgtgcgcgctcgtctcgctcatcgtcggcattgccaccgaagggtggcccaagggcgcgcacgacggcctcggcatcgtggccagcatcctgctcgtcgtgtt
cgtcaccgcgaccagcgactaccgccagtccctgcagttcaaggacctcgacaaggagaagaagaagatcaccgtgcaggtcatccggagcgggtacagg
cagaagctctccatatacgagctcctcgccggcgacgttgtccacctctccattggtgatcaggtgccggccgacgggctcttcctgtcaggattctcgctgctga
tcaacgagtcgagcttgaccggggagagcgagccggtcgcggtcaacgcagagaacccgttcctcctgtcgggcacaaaggtgcaggacggttcgtgcaa
gatgctcatcaccacggtcggcatgaggactcagtggggcaagctcatggcaactctcagcgagggtggtgacgacgagacgccgttgcaggtcaagctga
acggcgtcgccaccatcattggtaagatcggcctcatcttcgccgtcgtcacgttcgcggtgctcaccgaaagcctgttccggcggaagatcatggacgggtcg
tacttgagctggaccggagatgacgcgttggagctgctcgagttcttcgccattgctgtaaccattgtggttgttgcggtgcctgaaggactgccgcttgcagtca
cactgagccttgctttcgccatgaagaaaatgatgaacgacaaggcgctcgtccggcaccttgcggcttgcgagaccatgggctcggctacctccatctgcagc
gacaagaccggcacgctcacgacgaaccatatgaccgtggtcaaggcctgtgtctgcggtaagtccatattcaacaacacgggtggcgacgttgtcattaacc
aggatggcaagcgggaaatactgggcacaccaactgagacggcgattctggagttcggcctgtcgctcggcggggacttctcggctgtgcggaaacaaagc
accatgatcaaggtggagccgttcaactcggcaaagaagagaatgggcgtggtcatccagctcccagggggtgcgctgcgtgcacactgcaaaggcgcctc
agagatcatactggcgtcttgcaacaaatacctgaatgatgaaggcagtgtcgttcctctcgataaagcgaccgtcaagcacttgaatgccacgatcgagagcttt
gcaaacgaggcacttcggacgctgtgccttgcctacgcggaagtccctgaggggttctcagccaatgatcagattccgatggatgggtacacttgcattggcatt
gtggggatcaaagaccccgtccggcctggcgtgaaggaatcagttgccatctgcaggtctgctggtattactgttaggatggtcacaggtgacaacatcaacac
cgccaaggcaatcgcccgggaatgtggcatcttaactgaagatggcattgccattgaaggcccggagttcagaaccaagactccagaagaaatgaccaaattg
atacccaagatacaggtgatggcaagatcttcgccacttgacaagcacacccttgtgaagcatcttcggactgcactcgacgaggttgtcgctgtgactggcgac
gggacaaatgatgcacctgcgctacatgaggctgacattgggctcgcaatgggcattgctggaactgaggttgcaaaagagagtgccgatgtcattattcttgat
gacaacttctccactattgtcactgtcgcaaaatggggtcggtctgtgtacatcaacattcagaagtttgtgcagtttcagctgacagtcaatgtggttgctctcgttgt
gaacttctcctcagcctgcttgacagggagtgctcctcttactgctgtgcaattgctctgggttaacatgatcatggatacacttggagcactggcattggccaccg
aacctccaaataatgaactgatgaagagaactcctgttggaaggaaaggaaatttcatcagcaacattatgtggaggaacatcctgggacaggccttgtatcagtt
cattgtgatttggtacctacagaccgaagggaagcgggtatttggacttgtaggcgacaattcggatcttgtcttgaacacactcatcttcaattgcttcgtgttctgt
caggtattcaatgaggtgagctcaagagagatggagaagataaatgtatttgaaggcattctagacaacaatgtgtttgtcgccgtcctaagtagcaccgtcatctt
ccagttcatcataatacaatttctgggcgagtttgcaaacactactcctctctcattcatgcagtggatatactgcattttcatcggtttcataggcatgccaatcgctg
ctattgtcaagatgatccccgttggttcttggtag
>g136047.t1
atggagacgctgaaagggccaaagggtggcctcccgatgtggaagcagatatccatctctgacgcgctgctcaccaatgagattcttgtaatgaggaggatagt
tgagaacgtctcgcctcatcccaatgtcatcggcttgcatgacgtttatgaggatgcacacggcgtgcacttgatccttgagctgtgctctggtggtgaactgtttga
tagaatagtggggcgtgaccggtactcggagttcgatgctgcgtgtgttattcgccagattgctagaggcctagaggctcttcataaggcaaacatcatccacag
ggacttgaagccggagaattgtttgttctctgacaaaaatgaagattccacattgaagatcatggattttggtctgagttctgttgaggacttcagtgatccaattgtg
gccttgtttggctcgatagattatgtttcaccagaagctctctcaaggcaagatgtttcagctgcaagtgatatgtggtctgttgggatgccctccatttcatgctgca
actaa
>g140089.t1
atgtttacttggcaagagaaaaaaagggacaatcatattgtggctttgaaagttcttttcaagagccaactgaaaaagtctcaagtcgaacatcagcttcagtgtga
agttgaaatccagagtcacctaaggcatcctaatattctatgcctatacggttacttctatgaccagactcgcgtttacttgatcctggactatgctgccaaaggaga
gctgtacaaggagctgacaagatgcaaacttttcaatgagagacgttcagctatgtacatcgcatcactggcaagatcactcatttaccttcatgggaagcatgtca
tccatagaaacattaaaccagagaatcttttaattggagctcaggacaatcatattgtggctttgaaagttcttttcaagagccaactgaaaaagtctcaagtcgaac
atcagcttcagtgtgaagttgaaatccagagtcacctaaggcatcctaatattctatgcctatacggttacttctatgaccagactcgcgtttacttgatcctggactat
gctgccaaaggagagctgtacaaggagctgacaagatgcaaacttttcaatgagagacgttcagctatgtacatcgcatcactggcaagatcactcatttaccttc
atgggaagcatgtcatccatagaaacattaaaccagagaatcttttaattggagctcaggatagtgaatgtggacctgaagttcctattgaaaccgtgaggcccca
gcagcgggcagcaacaccatcagcctccgccctccccccgcatccggtgccggcctcctcccctctgctccgccatgatggacctggatccccgcctttacga
gaacgtggttacggtggtttctatattatgtccgaaaagcttggtttccaggatttttggagttgttga
>g141673.t1
atgggtccgcccccgccgcgcaggactccgcgtcgtcgtcgccgtccggctcctccaggcgccgcttccgccggctcgaccgccgcaacgcgtccaaga
acatcgcctacgacgccgcaaacttctgcagctacccgccgtccccgacgccctcctcctcctccgcgccggcatccgcggcctgctccctcgacctcctctcc
agcttccgcatcggcggcagcggcgacgggggcggggacctgcagctcctctgccagagcctagggctctccggccccgacgacttcgccatcccggtcg
ccgactgggaggcgcacaaggccgtccgcaactcctcggcctccagctccccgtcctccgcgcgccacaaccccgacgcccccgcgcgggactccccgct
ccgccgcgagggtgcggaggagccggcccagcccgcggacgccgcccccgagctgccggcggcgtggaacgccccaatcgaagctctggagcggccc
caacagctggatcggatagagcccgtccgtctggaggtgaagagggcagccggcgagggaggaatcaagggcgtgcggccgccgccggtgctcaagcc
gccgccctcgatggcgatgccggctgtctgcggggcggggtccacgtgggacatcctggcttcgttcgcgcctgatgagaaggagcgcgccccggcgagc
agatctggcggtggtcttgtacgccaggaggaggacgaggacgaagacgcggcggtagcgttggcgttggaggagctcaggctgggggagacttccgagg
atttcacgggcacatcttccatgtcgacagtaaacgacgacgagacgtccagcaccaccaccgagtccatgttctacatctctcccaacgggaggttccggagg
aagatccgatcatggaaccgaggggtgctcctggggagcggctccttcgggacggtatatgagggcatcagcgaagacataaaatgtgcaaatatactggtgc
atgctaatggatctgtgaaacttgcagactttgggcttgctaaggagatattaattcttggtaatcatgcggagctggctgaaaaggaggcggtttacctgcagctg
gtaatgtcaacactcaacagcattctcatcgtcatcgtctgtttcattgattga
>g142373.t1

atgggcaactgctgctgcgcggggtccaagcaggagcccgccgcagagccggcgtcctccggctccagccgccccgtgggcagcacgacgacctcggtc
tcgccttcctcggcgccggcgcccatgaagcccccccccgccgtgggccctgttctaggccgacccatggaagatgttcggagcacgtacagcatcggcaag
gagcttggccgcggccagttcggcgtgacgtcgctgtgcacgcacaaggccaccgggcagaaattcgcgtgcaagaccatcagcaagcggaagctgtcga
ccaaggaggacatcgaggacgtgcgtcgcgaggtgcagatcatgtaccacctctccggccaaccgggcgtcgtggagctcaagggcgcgtacgaggacaa
ggcctccgtccacctcgtcatggagctatgcgccggcggcgagctcttcgaccggatcatcgccaagggccactacacggagcgcgcggcggcggagctg
ctccgcaccatcgtcgagatcgtccacacgtgccacgccatgggcgtcatccaccgcgacctcaagcccgagaacttcctcctccttagcaaggacgagacc
gcgccgctcaaggccaccgactttggcctctccgtcttcttcaaagaaggggaggtgttcagggacatcgtcggcagcgcctactacatcgcgccggaggtgc
tgaagcggagctacgggccggaggccgacatatggagcatcggcgtcatcgtgtacatcctgctctccggcgtcccccgttctgggcaggcaagcgatctgt
tgtcttcagagacacggtcgacttcaccagtgacccatggccgcgtatctcgtcgggtgcaaaggaccttgtcaggaagatgctcaactcggatcccaagcagc
ggatatctgcgtatgacgtcctcaaccatccgtggatcaaagaagacggcgacgcgcctgatacaccgctggacaacgcggtcctcggccggctcaagcaatt
cagagctatgaaccagttcaaaaaagcagcactaagggtcatcgccggatgcctttcagaggaggagatcaaggggctgaaggagatgttcaggagcatgga
cgtggacaacagcggcacgataaccgtggatgagctgcggcgcgggctggccaacaagggcaccaagctcagcgaagccgaggtccagcagctgatgga
agctgccgacgcggacgggaacgggacgatcgactacgacgagttcatcacggcgacgatgcacatgaacaggatggaccgggacgagcacctctacac
agcgttccagtacttcgacaaggacagcagcgggtacatcaccatcgaggagctggagcaggctctcagggagaagggcctgctcgacgacggccgggac
atcaaggaaatcatatcggaagtcgacgccgacaacgacgggaggatcaattacacggagttcgtggcgatgatgaggaaaggggacccggaggtggcca
accccaagaagcgccgcgacgtcgtgctatag
>g142379.t1
atggcggaccagctcaccgacgagcagatcgcggagttcaaggaggccttcagcctcttcgacaaggatggcgacggttgcatcactacaaaggagcttgga
actgtgatgcggtcccttggtcagaaccctaccgaggcagagctgcaggacatgatcaacgaggttgacgctgatggcaatgggactattgacttcccggagtt
ccttaacctgatggcaaggaagatgaaggacactgactccgaggaggagctcaaggaggccttccgtgtcttcgacaaggaccagaacggtttcatctcagct
gctgagctccgccatgtcatgaccaaccttggggagaagctgactgatgaggaagtcgacgagatgatccgcgaggctgatgttgatggtgatggccagatca
actacgaggagtttgtcaaggtcatgatggccaaggcccatgcattattcaatgttctcaggcatcgaccatatgcagcatgccagatcgccacggaggagctg
gtcacagcgctccgctcgctgggcctgaacgtcgacgaggccgaggcgcagcgcctcctcagcgacgccactgacgtctcctccgccggcggcgcgattg
acttcacggcgttcttgtccgtcgcggcgcgcaagatggccggcggcggtaagcagacggcggagcggctggcggggtgcttcgacgtgttcgacgacgg
ccggagcgggtcgatcccggcggagcagctgcggcaggtgatgacgagccacggcgaccggctcacggaggaggaggccgacgccatggtccgcgag
gccgacccgcgcggcgagggccgcgtcgagtacagggagtacgtcaaggtgctgaccaagaacaagtga
>g142424.t1
atggggaaccgcgggtcgcgccaccaccgccaccccgcggaccagcaggcggccccgccttcgcagcccaagccgcagtcctccccaccgccccagcc
caagccgcatgcctccccgccgccgaagcccaagccccagccgccggcgcccgctccggcggcgtcgggggacgtggggcgcgtgctgggtcggccga
tggaggacgtccgggccacctacaccttcggccgcgagctcggccggggccagttcggggtcacctacctcgtcacgcaccgcgagacggggcagcgatt
cgcctgcaagtccatcgccacgcggaagctcgtccaccgcgacgacatcgaggacgtccgccgggaggtgcagatcatgcaccacctcacgggccaccgc
aacatcgtcgagctccggggcgcctacgaggaccgccactcggtcaacctcgtcatggagatttgcgagggcggtgagctcttcgaccgcatcatcgccaag
ggacactacaccgagcgcgccgccgccgcgctctgccgcgagatcgtcgcagtcgtgcacagctgccactccatgggggtcttccaccgggatctcaagcc
cgagaacttcctcttcctcaacaacaaggaggactcgccgctcaaggccacggactttggcctctctgtcttcttcaagcccggtgagtgcttgatttccgccgca
tctgtgggatcctga
>g142425.t1
atggggaaccgcgggtcgcgccaccaccgccaccccgcggaccagcaggcggccccgccttcgcagcccaagccgcagtcctccccaccgccccagcc
caagccgcatgcctccccgccgccgaagcccaagccccagccgccggcgcccgctccggcggcgtcgggggacgtggggcgcgtgctgggtcggccga
tggaggacgtccgggccacctacaccttcggccgcgagctcggccggggccagttcggggtcacctacctcgtcacgcaccgcgagacggggcagcgatt
cgcctgcaagtccatcgccacgcggaagctcgtccaccgcgacgacatcgaggacgtccgccgggaggtgcagatcatgcaccacctcacgggccaccgc
aacatcgtcgagctccggggcgcctacgaggaccgccactcggtcaacctcgtcatggagatttgcgagggcggtgagctcttcgaccgcatcatcgccaag
ggacactacaccgagcgcgccgccgccgcgctctgccgcgagatcgtcgccgtcgtgcacagctgccactccatgggggtcttccaccgggatctcaagcc
cgagaacttcctcttcctcaacaacaaggaggactcgccgctcaaggccacggactttggcctctccgtcttcttcaagcccggtgagtgcttgatttccgctgca
tctgtgggatcctga
>g144903.t1
atggagctctgcgccggcggggagctcttcgaccgcatcatacagcgcggacactacagcgagcgaaaggccgcagagctcacgcggatcatcgtcgggg
tcgtcgaggcgtgccactcgctcggggtcatgcaccgggacctcaagccagagaacttcctgctcgccaacaaggacgatgacctctcgctcaaggctatcga
tttcggcctctccgtcttcttcaagccgggtgagctgctgctcattactgattcatattccgacttgattactgtttcggtcaacgagtcctgtcccattggatgtactta
ctaa
>g145423.t1
atgcagccggacccgcaaggcccgggaagggagaagcccggcggcaccaatgtgaacgtccggctgccgccgccggtgacggtggggtcgggtggcc
ggccggcgtccgtgctgtcgcacaagacggcgaacgtgcgcgaccattaccgcatcgggaagaagctggggcaggggcagttcggcaccacgtaccagtg
cgtgggcaaggccgacggcgccgagtacgcgtgcaagtccatccccaagcgcnnnnnnnnnnnnnnnnnnnnnnnnnaaaaaaattctgtatactctc
atttctag
>g145424.t1
atgcagccggacccgcaaggcccgggaagggagaagcccggcggcaccaatgtgaacgtccggctgccgccgccggtgacggtggggtcgggtggcc
ggccggcgtccgtgctgtcgcacaagacggcgaacgtgcgcgaccattaccgcatcgggaagaagctggggcaggggcagttcggcaccacgtaccagtg
cgtgggcaaggccgacggcgccgagtacgcgtgcaagtccatccccaagcgcaagctgctgtgccgcgaggactacgaggacgtgtggcgcgagatcca
gatcatgcaccacctctccgagcacccaaacgtggtccgcatccgcggcgcatacgaggacgccctcttcgtgcacctcgtcatggagctctgcgccggcgg
cgagctcttcgaccgcatcgtcgccaagggccattactccgagcgcgccgcggcgcagctcatcaggacgatcgttggcgtggtcgaaggttgccactcgct

cggcgtcatgcatcgggacctcaagccggagaacttcctcttcgcgagcaccgctgaggatgcgccgctcaaggccactgactttgggctctctgtgttctaca
agcctggtgataaattttctgacgttgtcgggagcccctattatgttgcaccggaggtgcttcaaaaatgctatggcccagaagctgatgtctggagtgctggagt
gattctgtacattttgctatgtggtgtgcccccattctgggcagaaagtgaagctggaatcttcaggcagattttgcgaggcaaacttgacttggaatcggagccat
ggcctaatatctctgatagtgctaaagatctggtccgtaagatgcttacccgggatcctacaaaaagactgactgctcatgaggttctatgccatccctggattgttg
atgatgctgttgcacccgataagcctgttgattctgctgtattgtcaaggctgaaaaacttttctgcaatgaacaagctcaagaagatggcattgagggtgatagct
gaaagtctgtctgaggaggaaattggtggcttaaaggagctgttcaaaatgatcgacactgacaatagtggcacaataacttatgatgaactgaaaaacggcctg
aaaagggtgggttctgatctaatggaacctgaaatccaggctttaatggatgcgattttgcgaggcaaacttgacttggaatcggagccatggcctaatatctctga
tagtgctaaagatctggtccgtaagatgcttacccgggatcctacaaaaagactgactgctcatgaggttctatgccatccctggattgttgatgatgctgttgcacc
cgataagcctgttgattctgctgtattgtcaaggctgaaaaacttttctgcaatgaacaagctcaagaagatggcattgagggtgatagctgaaagtctgtctgagg
aggaaattggtggcttaaaggagctgttcaaaatgatcgacactgacaatagtggcacaataacttatgatgaactgaaaaacggcctgaaaagggtgggttctg
atctaatggaacctgaaatccaggctttaatggatgcggccgatattgacaacagtggaaccattgactatggagagttcctagcagctacattgcacatgaataa
actggagagggaggaaagcttggtatcagcattttcatttttgataaggatggaagtggcttcataacaattgatgagctctcacaagcatgcaaacagtttggcc
tttctgatgttcatcttgaggatatgatcaaagatgtggatcaaaacaatgatggacaaattgattatagcgagtttgcagcaatgatgagaaagggcaatgctggt
ggagcagggaggagaaccatgaggaacagcttgcatgtgaaccttggtgaaatcttcaaacccgctgagaattaa
>g146823.t1
atggggaactgctgtcgctcgccggccgccgcggcgcgggaggacgtcaagtcgtcgcacttcccggcctcggccggcgggaagaagaagccgcaccag
gcgcggaacggcgggggaccgggcggcggggagaagaagcggctgtcggtgctcggggaggatgggtgcgacgtgagcggcggcggcgggatcgac
gagaaatacgcgctggaccgggagctcgggcgcggcgagttcggggtgacgtacctgtgcatggaccgcggcacgcgggagctgctggcctgcaagtcc
atctcgaagcggaagcttcggacgcccgtggacgtggaggacgtgcgccgcgaggtggccatcatgcggcacctgcccaagagccccagcatcgtgtccct
gcgggaggcgtgcgaggacgacggcgccgtgcacctcgtcatggagctgtgcgagggaggggagctgttcgaccgcatcgtcgccaggggacactacac
ggagcgcgcggcggccgcggtcgtgcgcaccatcgtcgaggtcgtgcagctctgccaccgccacggcgtgatccaccgcgacctcaagcccgagaacttc
ctcttcgcaaacaagaaggagaactccccgctcaaggccattgattttggcctctccatcttcttcaagcctggtaagctctcggcgccgagatctgtccctgattt
gggcgaactgaagaagcggctgtcggtgctcggggaggatgggtgcgacgtgagcggcggcggcgggatcgacgagaaatacgcgctggaccgggagc
tcgggcgcggcgagttcggggtgacgtacctgtgcatggaccgcggcacgcgggagctgctggcctgcaagtccatctcgaagcggaagcttcggacgcc
cgtggacgtggaggacgtgcgccgcgaggtggccatcatgcggcacctgcccaagagccccagcatcgtgtccctgcgggaggcgtgcgaggacgacgg
cgccgtgcacctcgtcatggagctgtgcgagggaggggagctgttcgaccgcatcgtcgccaggggacactacacggagcgcgcggcggccgcggtcgt
gcgcaccatcgtcgaggtcgtgcagctctgccaccgccacggcgtgatccaccgcgacctcaagcccgagaacttcctcttcgcaaacaagaaggagaactc
cccgctcaaggccattgattttggcctctccatcttcttcaagcctggtgaaaaattttcagaaatagtgggaagtccctactacatggctcctgaagtgctaaagag
gaattatggtcctgaaatagacatctggagtgctggtgttatcctgcaaaatttgcagagactgaacaaggagttgcacaagctatccttcggggaaatatag
>g146907.t1
atggataggacggcggcgacgacggggccgctggggatggagatgccgataatgcacgacggggaccggtacgagcacgtcaaggacatcgggtccgg
caacttcggcgtcgcgcgcctcatgcgcaaccgcgcctccggcgagctcgtcgccgtcaagtacatcgaccgcggcgagaagatcgacgagaacgtgcag
cgggagatcatcaaccacaggtcgctgcggcaccccaacatcatccgattcaaggaggttatcctgacgccgactcacctcgccatcgtcatggagtacgcttc
tggaggggagctcttcgagcgcatctgcagtgccggaagattcaacgaggacgaggcccggttcttcttccagcagctgatttctggggttagctactgtcactc
catgcaagtatgtcatcgtgacctgaagctcgagaatacattgttggatggaagtaccgctccacggctcaagatctgcgactttggttattcaaagtcgtctgttct
tcattctcaaccaaaatcaacagttggtactccagcttatattgctcctgaagttttgctcaagaaagaatacgatggcaagaaaatattgggtgttcagtatgtgatt
ccagattacgtgcacatatctccagagtgccggcacctcatctcaaggatttttgacgccaacccagaaactaggattaccatgcctgagataaaaagtcatcctt
ggttcgttaagaatctcccagcagacctcatggatgatgccacaatgagcagccagtatgaggagcctgaccagcctatgcagaacatgaacgagatcatgca
gatactggcagaggcaactataccagcagctggtacccgtggaatgaaccagttcttggttgacagccttgaccttgatgatgatatggaggatctagactcaga
ccttgacatcgacattgagagcagtggagaaatagtgtatgccatgtga
>g146910.t1
atggagagctacctggaggagaacttcgggggcgtcaaggccaagaactcctcggaggaggcgctgcggcggtggcgccgcctctgcagcgtcgtcaaga
atcccaagcgacggttccgcttcaccgccaacctcgacaagcgcggcgaggcgcaggccatcaagcacgccaaccacgtccgtacacatctcctcttccctc
atccatcccctttccttcctttcctccgcgccgggatccgcctcgctcgcggcggcgggatctggactggagccgcgccgccggcgggggcgggagattccg
agaaactgcgtgttgccgtgctggtctccaaggccgcgcttcagttcttacatggtctctcacttcggagcgagtatgttgtccctgaggaagtcaaggctgcagg
gtttgagatctgtgccaatgagcttgggtccattgtggagggccacgatagcaaaaagctgattatacatggaggagtcgatggaattgctgcaaagcttgcgac
gtcacaaacggatggtctgagtacagctgaggacaacattaagcgcaggcaagagatttatggactcaacaagttcacagaaagcgaggtccgaagtttctgg
gtgtttgtatgggaagcacttcaagatacaactctcataattcttgctgtgtgcgcatttgtatctctggtcgttggcattgcgatggaagggtggccaaaaggtgcc
catgacggtcttggaattgttgcgagtatcctcctggtagtgtttgtgactgcaacaagcgattaccgacagtcgctgcagttcaaggacctggacaaggagaaa
aagaaaattcgagtgcaagttacaagggatgggtttaggcagtggatatcgatatatgaccttcttcctggagatgttgtccatttacaaatcggagaccaggttcc
tgcagacgggctcttcatttcagggttttctctgttgatcaatgaatccagcctaacaggtgaaagtgaacctgtcgctgtaaatgaagataacccttttcttttgtcgg
ggaccaaagtgcaggatgggtcctgcaagatgctggttacaacagttggcatgcgcacccaatggggaaaactgatggccacactcagtgagggtggtgatg
acgaaactccactgcaggtcaaactcaatggtgttgcaactatcattggaaagattgggctattctttgctgttataactttcattgtcttgtcccaagggttattcggc
aataaatatcatgacggacagcttttgagctggtcaggagatgatgcactggagcttctggagcattttgctattgcagtgaccattgttgttgtggctgttcctgag
ggattgccattagcagtcacgctgagccttgcattcgccatgaagaaaatgatgaatgacaaggcactggttcgcaacttagctgcatgtgaaactatgggttcag
caaccaccatctgcagtgacaagacagggacattgacaaccaatcatatgactgtcgttaaggcctgcatctgtgggaaaatcaaggaggttaacggtcctcag
aatgcatccaagttatgctctgaatttccggaaactgtcgtcaaaacactcctggagtctatatttaataatacaggtggtgaggttgtgattaaccaagatggtaaa
cgtcagatcctagatgagacaggcagtgttgttcccatggataaaacaactttggaaaagctcaatggtattatcgacaattttgctggtgaagctcttaggacact
atgccttgcttacagggaaatggaagaaggtgtgagggagtctgttgcaacttgccggtctgctggaattatggtgagaatggtcacaggagacaacataaatac
ggcaaaggcgattgcacgtgaatgtggtatactaactgaagatggtgtggctattgaaggacctgaattcagagagaaaaaacttgaagaactccttgagctggt

tccaaaaatccaggttatggcccggtcatcgccacttgacaagcatacactagtaaagcatttgcgcacgacattcaatgatgttgttgcagttactggtgatggca
caaatgatgcacctgcattgcatgaagcagatatcggacttgcgatgggcattgcaggaactgaggtggcaaaagagagtgctgatgttataattctggatgaca
acttctctacaattgtgactgttgcaaagtggggacgctctgtttacatcaatatccaaaagtttgtgcagtttcagcttactgtcaatgtagtagcactgctggttaact
tttcttctgcttgctttacgggaaatgcaccattgacggctgttcagctcctttgggtcaacatgatcatggacacccttggtgcactagcattggccacggaaccac
cgaacgatgagttgatgaagagagagccagtaggaagaacagggaaattcattacaaatgtaatgtggaggaacatcatgggacagtctttctaccaattttttgt
tatgtggtatctccagacgcaagggaaatcctttttggtcttgatgaaagctctgatactgatattgtgctgaacacaataattttcaactcattcgtcttctgtcaggtt
atggcccggtcatcgccacttgacaagcatacactagtaaagcatttgcgcacgacattcaatgatgttgttgctgttactggtgatggcacaaatgatgcacctgc
attgcatgaagcagatatcggacttgcgatgggcattgcaggaactgaggtggcaaaagagagtgctgatgttataattctggatgacaacttctctacaattgtg
actgttgcaaagtggggacgctctgtttacatcaatatccaaaagtttgtgcagtttcagcttactgtcaatgtagtagcactgctggttaacttttcttctgcttgcttta
cgggaaatgcaccattgacggctgttcagctcctttgggtcaacatgatcatggacacccttggtgcactagcattggccacggaaccaccgaacgatgagttg
atgaagagagagccagtaggaagaacagggaaattcattacaaatgtgttcaatgagatcagttcaagggagatggagagggtgaacgtgatcaagggcatc
ctaaagaattatgtcttcatggccgtccttaccagtacggtcatcttccagttcatcatggtacagttcctgggcgagttcgccaacacgacaccgctcaccaggct
gcagtggctcgccagcgtgctccttggcctcgtagggatgcccatcgctgctgctgtcaagatgatccccgtcggctcctcatga
>g147245.t1
atgcaatcgtgggttcgtgaagccgagcggccattcccgccgccgtctcccgccaagcacatccgcgccctgctggcgcggcggcacggctcagtgaagcc
gaacgaggcttccatccccgagggcggcgagccggagctgggcttggacaagagcttcggctactcgaagcacttctcggctaagtacgagctcggccgcg
aggtggggcgcggccacttcggctacacctgcgccgccaaggccaagaaaggcgagctcaagggcgaggacgtcgccgtcaaggtcattcccaaggctaa
gttttgctcttga
>g147906.t1
atgaaccccccctgtagtccctcctgtggttgtcccaccaaagccaacaccagacacgattcttggcaaaccatatgaagatgtgcgttccgtgtactccattggta
aggagcttggccgtggtcagtttggggtgacatacctctgtactgaaattgcctctggtaggcagtatgcctgcaagtccatttccaagcgcaaactcaccagcaa
ggcggacagggaggacattcgcagggagatccagatcatgcagcactnnnnnnnnnnnnnnnnnnnnnnnnnnnnnnnnnnnnnnnnnnnnnnnnnnn
nnnnnnnnnnnnnnnnnnnnnnnngattctggtcatggaggaaccaatggttatggctattcacaccagcaaaaaccagcacagacacctcccagctaca
acccttctcaaccacagtcaacggccgaagtgaggtacacaccaccagccatgaaccccccctgtagtccctcctgtggttgtcccaccaaagccaacaccaga
cacgattcttggcaaaccatatgaagatgtgcgttccgtgtactccattggtaaggagcttggccgtggtcagtttggggtgacatacctctgtactgaaattgcctc
tggtaggcagtatgcctgcaagtccatttccaagcgcaaactcaccagcaaggcggacagggaggacattcgcagggagatccagatcatgcagcacttgtct
ggacaaccaaatatagtcgagtttcggggagcatacgaggacaagagcaatgtgcatgtggtgatggagctctgcgctggtggggagctgttcgatcggatca
tcgcaaaggggcactacacggagcgggcagctgccaccatctgcagggctgttgtgaatgttgtcaacatttgccatttcatgggagtgatgcatcgtgatctaa
aaccagagaatttcttgcttgcgaccaaggaggagaatgcaatgctcaaggccactgattttggactttcagtcttcatcgaagaaggaaaaatgtacagggaca
ttgttggaagtgcttattatgttgctccagaagttcttaggcgaagctatggaaaagagatagatgtgtggagtgctggtgtcattttgtacattcttcttagtggtgtgc
ctccattttgggctgaaactgaaaagggcatatttgatgctattctgcatgaggaaattgactttgaaagtcagccatggccatcaatttctgagagtgctaaagacc
tggttaggaagatgttgacacgggatccaaagaaaagacttacttcagctcaagttcttcggagggtggtgaggcatcagacaagcctattgacagtgctgtactt
tccagaatga
>g151365.t1
atgatggggtcagcgcccgataagccgctagactcggcggtgctctcgcggctgaagcagttctccgcgatgaacaagctaaaaaagatggccttgagggtg
attgcggagaacctttcagaagaggagattgctggtctgaaggagatgttcaagatgatggataccgacaacagtgggcagatcaacttcgaggagctcaagg
ccggcctccacagagttggcgccaatatgaaggagccggagatacatcagctaatgcaggctgccgatattgacaacagtggtaccatagattatggtgagttc
atagccgctactttgcacctcaacaaagttgaaagggaggatcatctgttcgctgccttccaatacttcgacaaggatggaagtgggtacatcacagctgatgag
ctccagcaggcatgcgatgaattcggcattgaagatgtccgattggaagacatgatcggcgaagtagatcaagacaatgatgggcggattgactacaacgagtt
cgtcgccatgatgcagaaatcaactagtggttttgggaagaaaggccaccggaacaatcttagcattggactaatagacgcactgaaaccacatagttga
>g155326.t1
atgggcaactgctgccgttccccggcggccgtcgcgcgggaggacgtcagctcctcccacttccccgcatccaacgcgaagaagaagccgcaccagccgc
ggaacggcgccgccgggggaggcggcggcggcggggcgcagaagcggctcacggtgctgggcgaggaggggcgcgaggtgggcgggatcgacga
gaggtacgccttggacagggagctcggccgcggggagttcggggtcacgtacctgtgcatggatcgggacaccaaggagctgctcgcctgcaagtccatct
ccaagcgcaagctgcggaccgccgtcgacgtcgaggacgtgcgccgggaggtcgccatcatgcgccacctgcccaagagccccagcatcgtgtccctgcg
cgaggcctacgaggacgagggcgccgtgcacctcgtcatggagctctgcgagggcggcgagctcttcgaccgcatcgtcgcgcgggggcactacacgga
gcgggccgccgccaacgtcacccgcaccatcgtcgaggtcgtgcagctctgccaccgtcacggcgtgatccaccgcgacctcaagcccgagaacttcctctt
cgctaacaaaaaggagaactcgccgctcaaggccatcgacttcggcctttccatcttcttcaagcccgaacattttggtggtctgttagctgcagtggtggagcca
gacctcaacattaggggagctgagcggaaacttacatcagccttggccatcgctgactcccctgttcctactcctaccgcttga
>g159122.t1
atggctcttcacttgcttaagcgtcttctttcttttgatcctaaggagcgtccaagtgctgcagaggtaagagtggacccatactttaatggattagcaaacaatgaac
gagagcccatagcacagcctatctcaaaacttgaatttgagttcgagaagagaaaactgggcaaagatgatgtccgagaattaatttaccgagagattttggagt
accatcctcaaatgttgcaagaatacctacgtggaggagaccagaaccagatgagcttcatgtaccccagtggggttgatcgctttaaacggcaatttgttcatttg
gaagaaggtaaaccaaagggtgagaagaatagtccacagctgcggcagaatgtttccttaccaagggaacgagtaatgggcaataaacatggagacagtga
gtactgcataaatattttggagtaccatcctcaaatgttgcaagaatacctacgtggaggagaccagaaccagatgagcttcatgtaccccagtggggttgatcgc
tttaaacggcaatttgttcatttggaagaaggtaaaccaaagggtgagaagaatagtccacagctgcggcagaatgtttccttaccaagggaacgagtaatgggc
aataaacatggagacagtattaatacgggttctaatgtcgagaataagagagagcttgccctcctgaacacgttcaaccttccagtcagtggtgacggagaggac
gttgatgtgcctatcatcagcaacatggagacagagggtgaagaagataagaaacctgttggtgggtcatccttccaacgacgtaaatataatcggttctccgag
gagcagtctgacatgcttgaagcaagctttcagcaaaaccactccccagacaaggtgactttcacactctag
>g162572.t1

atggccgtccagcttagcgacgagcagattaccgaattcaaggaggccttcagcctcttcgacaaggacggcgacggttgcatcacggccaaggagctgggt
acggtgatgcgttcgctgggtcagaacccgacggagcgtgagctgcaggacatgatcaacgatgtggacgcggacgggaacggcaccatcgacttccagg
agtttctggggctgatggcccggaagatgaaggacaaggacaccgaggaggagctcatggaggcgttccgcgtgttcgacaaggaccagaacgggctcatc
tccgcggcggagctccggcacgtgatgaccaacctcggcgagaagctgagcgacggcgaggtcgacgagatggtccgcgaggccgacgtcgacggcga
cgggcacatcaactaccaggagttcgtcaaggtcatgatggccaaacggaggaagcaggcgaagacagaggagaagacggcggtgcggggcaagaaga
acaaggcggtggcggcaccgtccgacgccgccggcaagcgttcccagaagtgcgtgatcctgtga
>g163673.t1
atggagaagtacgagctgctcaaggacatcggctccggcaacttcggggtggcgcggctgatgcggaacaaggagaccaaggagctcgtcgccatgaagt
acatcccgcgggggctcaagattgacgagaatgtggcgagggagatcatcaaccaccgctcgctgcggcaccccaacataatccggttcaaagaggttgtcc
tcacgcccacgcacctggccatcgtcatggagtatgccgctggcggcgagctgttcgaccggatctgcggcgctgggaggttcagcgaggatgaggcgagg
tatttcttccagcaactgatttgcggtgtcagctactgccacttcatgcaaatctgtcaccgggacttgaagctcgaaaacacgctgctggatggcagcccggcac
cgcgccttaagatttgtgacttcggctactccaagatggcagatgtttggtcctgtggagtgaccctttatgtgatgctggttggtgcatacccttttgaggaccccg
acgatcccaagaatttcagaaagacaattgggagaatagtatctattcaataccaaataccagaatatgtccacatatcccaagattgcaggcagctcctctcgag
gatcttcgttgcgaatcctgcaaagagaataacaattagggagattagaaatcacccctggttccttaagaacttgcctagagagcttacagaagctgcacaagc
gaagtactacaagaaggacaacagcacacccacctactccgatcagactgttgaagagatcatgaagattgttgaggaggctcgcacaccacccaaattatcta
ctcctgtggctggcttcggttgggctgaggaagaagagcaagacgatggcaagaaacctgatgatgaagaacatgatgaagaggatgaggaatatgatggtg
aggatgagtatgacaagcaggtgaaggcagtacatgccagcggtgattttcaacatctgataaaaggaagcaactag
>g165869.t1
atggggacgctgaaagggccaaagggtggcctcccgatgtggaagcagatatccatctctgacgcgctgctcaccaatgagattcttgtaatgaggaggatag
ttgagaacgtctcgcctcatcccaatgtcatcggcttgcatgacgtttatgaggatgcacacggcgtgcacttgatccttgagctgtgctctggtggtgaactgtttg
atagaatagtggggcgtgaccggtactcggagttcgatgctgcgtgtgttattcgccagattgctagaggcctagaggctcttcataaggcaaacatcatccaca
gggacttgaagccggagaattgtttgttctctgacaaaaatgatgattccacattgaagatcatggattttggtctgagttctgttgaggacttcagtgatccaattgtg
gccttgtttggctcgatagattatgtttcaccagaagctctctcaaggcaagatgtttcagctgcaagtgatatgtgttgcgcagatggagagaacgccacattggc
ggagtttgaggaagtactaaaagcaatgaaaatggactcactgattcctctcgccccacgtgtattcgacttgtttgacaacaaccgtgacggcaccgtcgacatg
agggagattctttgcgggctttccagcctccggaactcgcgaggggatgatgctcttcgcctctgcttccagatgtatgacgcggatcggtcaggctgtatcagc
aaggaagagctggcatcaatgctgcgagcgttgcccgaagactgcctcccgggcgacatcacggagccgggaaagctggacgagatattcgaccaaatgg
acgcgaacagcgacggaaaggtcaccttcgatgagttcaaggctgccatgcagaaggacagctccctccaggacgtcgtcctctcctccttgcgcccggtgca
atag
>g167600.t1
atggatgattgtttttccattgatgagcaaataccgctgcaaggatacatatgcattggtattgttggcatcaaagatcctgtgcgtccaggggtcatgcagtctgtg
gcaacatgccgatctgctggcatttcagttcgaatggttacaggagacaacataaatacagcaaaggcaattgctcgtgaatgtggtatacttacagaggacggc
ctttcaattgaaggtgctgaattcagggagaaaagtcctaaagaaatccttgagttgattccaaagatgcaggtactggcccgatcttcaccaattgataagaatac
attggtgaaacatttgcgtacaacgttcaatgaggttgttgctgtgactggtgatggcacaaacgacgcacctgctctgcgcgaggcagatatcggacttgccatg
ggcattgcagggactgaggtggctaaagagaatgctgatgttgtagttctggatgacaacttctccaccattgtaacagttgtcaaatgggggtgttctgtttatgtc
aatatccaaaagtttgtgcagttccagctgactgttaatatagttgcattgctagttaatttttcttttgcatgctttacaggtgttcaatga
>g168322.t1
atgggaaacgcatgcggcgggtcccttagatccaggtacctgccgagcttcaagtcagccgcgtcgcagcgccacgactccgaccacagcgccgccgccg
actcgcccaagaagccctcccgccccgccacgccccccgccgccgcgacggacgcccacgcggcacccgcgccggggctgccgccgccgcccggcat
gaggcgcggcggcgcggcgcccccggacctcggctccgtgctgggccaccccaccccgaacctccgcgacctctacgccgtgggccgcaagctcgggc
agggccagttcgggaccacctacctctgcaccgacctcgccacgggcgcccagtacgcctgcaagtccatctccaagcgcaagctcatcacccgggaggac
gtcgacgacgtgcgccgcgagatccagatcatgcaccacctctccggacaccgcaacgtcgtcgcaatcaagggcgcctacgaggaccagctctacgtcca
catcggccagttcgggaccacctacctctgcaccgacctcgccacgggcgcccagtacgcctgcaagtccatctccaagcgcaagctcatcacccgggagg
acgtcgacgacgtgcgccgcgagatccagatcatgcaccacctctccggacaccgcaacgtcgtcgcaatcaagggcgcctacgaggaccagctctacgtc
cacatcgtcatggagctctgcgccggcggggagctcttcgaccgcatcatacagcgcggacactacagcgagcgaaaggccgcagagctcacgcggatcat
cgtcggggtcgtcgaggcgtgccactcgctcggggtcatgcaccgggacctcaagccagagaacttcctgctcgccaacaaggacgatgacctctcgctcaa
ggctatcgatttcggcctctccgtcttcttcaagccggaaacgcagcaaggaatatttgatgctgtattgaaaggcgacattgattttgattctgacccgtggcctgt
gatatctgatagtgcgaaagacctgataaaaagaatgctcaatcctcgccctaatgaacgcttaacagcacatgaagttctatgccatccatggattcgtgatcatg
gagtagcacctgatcgtccacttgatcctgctgtcctatctcgcatcaagcaattctctgcaatgaataagttgaagaagatggctttgcgagtaatagctgagagt
ctctcagaggaggaaattgcagggttgaaagaaatgttccagaccatggacactgacaacagtggtgcaattacatatgatgagctaaaagaaggattgagaag
atatggctccacactaaaggatactgaaatccgtgatcttatggatgcggctgatattgacaacagtggcacgattgactacatagaattcattgctgcaacattgc
atctcaataaactggagcgtgaggaacatctggtggcagcatttacatattttgacaaagatggtagtggttacatcactgtggatgagctgcaacaagcttgcaa
agaacataacatgccagatgcttttcttgacgatgtcattaaagaagctgatcaggacaatgacggacgcattgactatggagagtttgttgccatgatgaccaag
ggcaatatgggagttggtcgaagaacaatgagaaacagtttgaatatcagcatgagggacgcacctggtgcattttag
>g168792.t1
atgttcaataacatggatactgacaagagtggcacaatcacagttgaagaactgaaggaaggactgacgaaactaggatcgaagattagtgaagcagaggttc
agaaacttatggaagcagttgatgtagacaagagtggcagcattgattatacagagttccttactgccatgatgaacaaacataaagtggaaaaggaggaggatt
tgctctgtgcatttcagcacttcgacaaagatagcagcgggtacataacaagagatgaactggaacaagccatggcagagtatggaatgggtgatgaggcaag
cattaaacaagtactggatgaagttgataaagataaggatgggaaaattgactatgaagagtttgtggaaatgatgaggaaaggaagctatacctga
>g168793.t1

atgggcaactgcttcaccaagacgtacgagatacccatcaccacggagccgtcggatcggcggcgcgcgccgtcatcgtacggctaccagcagccgccga
ggagccccgatcagcgcaagtcccgcgacgtgccggtgacgacctcgcgccggccgtccttccgtccgccgccgcccttccggccgtcgagcacgacgac
gggcaccggctcgtcgtcgatgatgccccgtcgcgcggcctcctccggcgaggtgggcccggtcctgcagcggccgatggtgaacgtgcgcacgctgtacc
agctggagcggaagctggggagcgggcagttcgggacgacgtacctgtgcacggagcgcgcgacggggctgcggtacgcgtgcaagtccgtgtcgaagc
gcaagctggtgcgccgcgccgacgtggaggacatgcgccgggagatcaccatcctgcagcacctcagcgggcaggccaacgtggccgagttcaagggcg
ccttcgaggacgccgactgcgtgcacctcgtcatggagctctgctccggcggggagctcttcgaccgcatcaccgccaagggcacctactccgagcgccag
gccgccgccgtgtgccgcgacatcgtcaccgtcgtgcacgtctgccacttcatgggggtcatgcaccgggacctcaagccagagaatttcctgcatgccagcc
ccgcggaggacgcgccgctcaaggccatcgatttcggactctccgtcttcatcgaagaaggttcgtcgttctga
>g171444.t1
atggggaacacctgctccggtccaacgccccccctcggaccgccatggtttcttcgactccgtctccctcgccgtgctctggcgcccacccgccgccgccaggg
ccgagccccccttctccatcggactccaggccctccgcctcctcgacgacttcctccaggcctcccgagtccgtcaccttcaccgatgccgagcactccccccac
gcctcccccctccgcgccggaccctaacggcaagcccaggcctaaggnnnnnnnnnnnnnnnnnnnnnnnnnnnnnnnnnnnnnnnnnnnnnnnnnn
nnnnnnnnnnnnnnnnnnnnnnnnnnnnngacgacttcctccaggcctcccgagtccgtcaccttcaccgatgccgagcactccccccacgcctcccc
ctccgcgccggaccctaacggcaagcccaggcctaaggtgaagcgcgtgcagagcgccggcctccttgtcggctccgtcctgaaacgtaactccgagcggc
tcaaggacctctacaccctgggcaagaagctgggacaggggcagtttggcaccacctaccagtgcgtagagaaggccaccggtaaggtattcgcctgcaagt
ccatcgccaagcggaagctcgtcagcgaggaagacgtcgaggacgtgcgccgcgagatacagattatgcatcacctctccggcaaccccaatgttatctccat
cgttggtgcttatgaggacgctgtcgcggtgcaccttgtcatggagctgtgtgcgggaggggaactgttcgatagaatcatacagcgggggcactactctgaga
aagctgctgcgcaactggcacgggtgatcattggagtggtagaggcatgccattctctcggggtgatgcacagagacctcaagccggagaatttcttgtttgtga
accacaaggaggatgcactgctaaaggcgatcgattttgggctctccatcttcttcaaaccagcgttcatgacatatggtgaaacattttcggatgtcgttggaagt
ccgtactatgttgcacctgaggttctgatgaaacactatggctgtgaggtggatgtgtggagtgctggtgtaataatttatatcttgctgagcggagtccctccatttt
gggatgaatctgaacaaggaatatttgaacaagtcctgaaaggtgatcttgacttttcatctgagccctggcccagtatctcaaggagtgcaaaggatttggtcag
gaagatgttaatccgtgatcccaaaaagagattgactgcgcatgaagctctatgtcatccatgggtttgtgttgatggagttgctcctgacaaacctcttgattctgct
gtcttaaccagattgaaacagttttctgcaatgaacaaattaaagaagatggcccttagggtcattgctgagaatttatctgaagatgagattgcagggttgcgaga
aatgttcaaaatgctggacactgacaacagtggccaaattacattggaagaactaaaaagtggcttgaagagagttggtgctaacttaaaggactctgaaattaca
atattaatggaggcggctgatattgataacagtggttcaattgattatggagagttccttgctgctactttgcatctgaacaaagttgagcgagaagataatctcttg
ctgcgttctcatattttgataaagacggcagcggttacattactcaagatgagctacagaaagcatgtgaggagtttggtataggagatgaacaccttgaagatgtt
atcagagacattgatcaagacaatgatggccggatcgactacaatgagtttgtaacgatgatgcagaagggaaataatattttaggtaaaaagggacaaggcca
gatgagttttggtcttagggaagcattgaagcttggttaa

CAX1 genes

>g20617.t1
atggcgggcgggcagctcaaggtgctcacgacgctggaccaggcgaagacgcaatggtaccacttcatggccatcgtgatcgccggcatgggcttcttcacg
gatgcctatgatctgttctgcatctcccttgtctccaagctactcggccgcctctactacagcgagcctaacagccccaatcccggcagcctgccacccaacgtgt
cggcggccgtgaacggtgtcgccctatgcggtacactggctggtcagctctttttggctggctcggcgacaagctcggccgcaagagcgtctacggcttcacg
ctcattctcatggtggtgtgctccgtggcgtccgggctctcgtttgggcacacggcgaagggggtcatcgccactctctgcttcttccggttctggctgggattcg
gcatcggtggcgactacccgctgtcggcgaccatcatgtctgagtacgccaacaagagaacccgcggtgcgttcatagctgccgtgttcgccatgcagggctt
cggtatcctcttcggcgccatcgtcgcgctcgtcgtgtcctccggcttccgcaactcgtatccggcgccgtcctacgagcagaatgccaccgcgtctctggtccc
ggaggccgacttcgtctggcgcatcatcctcatgttcggcactatcccggcagctctcacctactactggcgcatgaagatgccagagacggcacgttacacgg
cactcatcgcgcgcaacgcgaagcaggctgcagcagacatgtccaaggttctcaacaccgagattgtagaggacaaagaccaggccgagctcgccagcgc
cagtgccggcaacaatgagtgggggctcttctcgccacagtttttgcgtcggcacgggctccacctcctgggcaccaccagcacatggttcctactggacatcg
ccttctacagccagaacctcttccagaaggacatcttctccaaggtgggatggatcccgccgcccaagaccatgaacgccattgaggaggtgttccgcatcgca
cgggcacaggcgctcatcgcactttgcggcaccatcccgggctactggttcaccgtgttcttcatcgacatcgtcggccgttttgccatccagctgatggggttctt
tatgatgaccgtcttcatgctcggcctcgccgtgccgtaccaccactggaccactgcaggacaccacaccggcttcgtggtcatgtacggcttcactttcttcttttg
ccaactttggtcccaacagcacctccttcatcgtgccggccgagatcttcccagcgcggctgcggtctacgtgccacggcatctcggccgccgcaggcaaggc
tggcgcgatcatcggtgcctttggtttcctgtacgccgcgcaggatccccacaagccggaggccggctattcgccaggcatcggcatccgcaacgcgctgttc
gtgctcgccggcactaactttcttggaatgatcatgactcttctcgttccagaatccaagggcctgtcgctcgaggaaatatccaaggagaccgtcgacgacgaa
gaggcgacttga
>g20618.t1
atggcgcgcgggggaggagataatctccaggtgctaagcgcgcttgacgcggccaagacgcagtggtaccacttcacggccatcgtggtggccgggatgg
gcttcttcacggacgcctacgacctgttctgcatctccctggtgaccaagctcctgggccgcatctactacaccgacaccagcaagcccgacccgggctccctc
cctcccaacgttgccgcggcggtgaacggcgtggccttctgcggcacgctggccggtcagctcttcttcgggtggctgggcgacaagctgggtcgcaagagc
gtgtacggcatgacgctgatgctgatggtgatctgctccatcgcgtcgggcctctccttcgggcacaccccgacgggcgtcatcgccacgctctgcttcttccgc
ttctggctcgggttcggcatcggcggcgattatccactatccgcgaccatcatgtccgagtacgccaacaagaagacccgcggcgccttcatcgccgccgtgtt
cgcgatgcaggggttcggcatcctcgccggcggcattgtcacgctcatcatctcggccgcgttccggaccgcgtacccggcacccgcgtaccaggtggacgc
cgcggcctccaccgtgacgcaggccgacttcgtgtggcggatcatcctcatgctcggcgccttacccgcgctgctcacctactactggcggatgaagatgccg
gaaactgcgaggtacaccgccctcgtggccaagaacgccaagcaggccgcggcggacatgtccaaggtgctgcagaccgagatcgtggacgagcaggag
aagctggacgatctcgtcacgcgcagcaccaacagcttcggcctcttctccagggagttcgcgcgccgccacgggatgcacctcatcggcacggcctccacg
tggttcctcctcgacatcgccttctacagccagaacctgttccagaaggacatcttcacggccatcaactggatccccaaggcgcgcaccatgagcgcgctcga
ggaggtgttccgcatctcccgggcgcagacgctcatcgccctgtgcgggaccgtcccgggatactggttcaccgtcgcgctcatcgacatcgtgggacgattc

gccatccagctcatggggttcttcatgatgaccgtcttcatgctcggcctcgccgtgccgtaccaccactggaccaccgccggcaaccacatcggcttcgtcgtc
atgtacggcttcaccttcttcttcgccaacttcgggccaaactccacgaccttcatcgtgccggccgagatcttcccggcgcggctgaggtcaacgtgccatgga
atctccgctgcggcgggcaaggcaggagcaatcatcgggtcgttcgggttcctgtacgcggcgcaggacccggaccatcccgaccacgggtacaagggcg
ggatcggcgtgcggaactcgctgttcgtgctcgccgcgtgcaacatgctcggattcgtactcaccttcctcgtgccggagtccaaggggaagtcgctcgagga
ggtgtccggcgaggccgacgacgccgaggaggaagccggcagcgccgtccggccggccgatggggcccacatggcctag
>g25744.t1
atgatgggggcggagaagttcgggctggggatggaagcagaggaggaggtggagctggccacgccgaccgcgagctcgccgctcccgccgcggaaaat
gcagtcgctggacttcgagcacatcggctcgctggcggcggtggcggagtctctctcgccaggtagcaggtggcggagggtgctcaccagcgtgcacatcgt
catcttccaggcgaagatcaacgtgctcctccccttcggcccgctcgccgtcttgcttcattacctcaccggcaagcaccaaggatgggttttccttttcagcttaat
cggcataacaccgttggccgagagattgggatatgcaaccgagcaacttgcttgctacactggcccaactgttgggggactcctgaacgcaacatttggaaatg
cgactgaaatgattatctcgatatatgcgctgaaaaatgggatgattcgtgttgtccaacagtcgctactaggctcaatattgtcaaatatgctgctggttcttgggtg
tgctttccttgctggtggtattgtccattctgatagggaccaggtcttcaacaaggcatcagctgttgtaaactcagggctgttattgatggctgtcttaggtctactgtt
cccagcagtgcttcacttcacacactcagaagcgcaatatggcaaatctgaagtagctctttcaaggtttagtagctgcattatgcttgtggcttatgctagctatctg
ttttttcaactaaagacccaccgcagtgtgtacagcccaatcggtgaagatgatgaagcctctgaggatgaggaggatgaaaaggagataacacaaggggaag
caatctgctggctttttgtattgactatttggatttccgtactctctggatacctggtagatgctatacagggtgcttctgaatcattaagcttgccagtggcctttatcag
tgttattctgcttcctattgtggggaatgctgctgaacatgcaagtgccattatgtttgccatgaaaaacaaattggacattactttaggagttgctattgggtcatcaa
cacagatatccatgtttgtgattccattctgtgtagtaattggttggatgatggggcaggaaatggacttaaattttcaactgtttgagacagcgactctcttcataaca
gttttagtggttgcatttatgctacaggaaggaacatctaactactttaagggccttatgctcatcttatgttaccttattgttgctgcaagcttctttgttcatgttgatccc
accgcaaatgataattaa
>g31482.t1
atggctggcggcgagcctttgcatgttcttagcgcccttgacgccgccaagacgcaatggtaccacttcactgcgatcgtgatcgccggcatgggtttcttcacg
gacgcgtacgacctgttctgcatctcgctggtgacgaagctgctgggtcgcatctactactaccgcgacggcgccgccgaccctggttcgcttcctccgagagt
ggccgccgccgtgaacggggtggccttctgcggcaccctctccggtcagctcttcttcggctggctcggtgacaagatgggtcgcaagagggtctacggcatg
acgctcatgtgcatggtgctctgctctctcgcctctggcttatccttgtcggacaagccaagctctgtcatggccacgctgtgcttcttccgtttctggctcggcttcg
gcatcggcggcgactaccccttgtcggcgacgatcatgtcggagtacgccaacaagaagacccgtgggggattcatcgccgccgtgttcgccatgcagggat
tcggcatcctcaccggcggcgtggtcacgctcgtcgtctccgccgccttcagatcggcgttcccggctccctcgtaccagaacggggcactggcttccacgcc
ggcgcaagcggatttcgtgtggcgcctcatcctcatgttcggtgcaattccggcgctgctcacctactactggaggatgaagatgcccgagacggccaggtaca
cggcgctcgtcgccaagaacgccaagcaggcagcagcagacatgtcaaaggtcctccacatggacatcactcctccttgtcaagctgcttctagtaatggaca
aggacaaggagaagcatttggtctcttctcgaagcagtttctgcgtcgccatgggcttcatctccttggcactgcaacgacatggttcctgctggacatcgccttct
acagccagaacctgttccagaaggacatcttcactgcaatcaactggatcccaaaggccaagaccatgagcgcactcgaggaggtgtaccgcatcgcgcgcg
cgcagacgctcatcgcgctctgcggcaccgtcccgggctactggttcaccgtcttcctcatcgacgtcatcggtcggttctggatccagatggtagggttcgcna
tcatcggcaggttctggatccagatggtggggttcgccatgatgtcggtcttcatgctcggactcgcgttcccctaccaccactggaccacgccaggcaaccac
atcgggttcgtcgtcatgtacgcattcacattcttcttcgcaaacttcgggcccaactccaccacattcatcgtccccgccgagatcttcccggcgagactgcgatc
cacgtgtcacggaatctccgccgccgcggggaaggcaggcgccatcatcggatccttcggattcctgtacgcggcgcagaaccaggacaaggccaaggcg
gaccacggatacccagcaggcatcggcgtacgcaactccctcttcgtcctcgctgcctgcaacgtcttgggactcctcttcacactcctcgtcccagagtccaag
ggaaagtcgctcgaggagctctccggcgaagataaccatgacgacgacgccctcgcagtcgcacacgcaagaacagtacccgtcgtctaa
>g33161.t1
atggcgggcgagcagatgcacgttctctccgcgctggacggcgccaagatgcagtggtaccacttcactgccatcatcgtctccggcatgggcttcttcaccga
cgcgtacgacctcttctgcatctccctcgtcaccaagctcatcggccgcgtctactacaccgtcgacggctcgcccaaccccggcagcctcccacccgacgtct
ccgcggccgtgaacggcgtggctttcgtcggcacgctctccggtcagctcttcttcggctggctcggcgacaaggtcggccggaagagcgtctatgggatgac
gctcctcataatgatcatctgttccctggcgtccgggctctcgttcggccactcgccgacctgcgtcatggccacgctctgcttcttccgcttctggcttggcttcgg
catcggcggcgactacccgttgagcgccaccatcatgtccgagtacgccaacaagaagacccgcggcgccttcatcgccgccgtgttcgcgatgcaggggtt
cggcatcctcgccggcggggcggtggcgatcggggtcaccgctttgttcaagaaaaaattccccgcgccgccgtacgccgtcgacccggcggcgtccacgc
cgccggaggctgatctcgtgtggcgcatcatcctcatgttcggggccgtccccgccgcgctcacattctactggcggatgaagatgccggagacggcgcgtta
cacggcgctcgtggccaagaacgccgaacgtgccgctgcggacatgtccaaggtgctccacgtcgagatcgcgaagccacaggcagaggaggaggacac
cgccgcgccgatgaaaagcagcgagcgatccttcggcctcttctcgtcggagttcgtgcggcggcacggggcccacctcgtgggcacgacgtcgacgtggc
tcctcctggacgtggcctactactcgcagaacctgttccagaaggacatcttcagcgcgatcgggtggatcccggcggcgcgcacgatgagcggccttgacg
agctgtaccacatcgcgcgtgcgcagacgctgatcgcgttgtgcggaaccgtacccgggtactggttcaccgtcgcgttcatcgacgtgttcgggcggttcaag
atccagctcgtcgggtttctcatgatgacggccttcatgctgggcctggccgtgccgtacgagcactggaagaccgcggagaaccatatctggttcgtcgtcatg
tacgccttcacattcttcttcgctaacttcgggcccaacgccaccacgttcatcgtgccggccgagatctacccggccaggctccgcgcgacgtgtcacgggatt
tcggcggcggcggggaaggtgggcgcgatcatcgggtcgttcgggttcctgtacctggcgcagagcccggacgcggccaagacggcgcacgggtaccta
cccggcatcggggtgcggaactcgctcttcgcgctcgccggatgcagcttcgcggggttcatgctcaccttcctcgtgccggagcccaagggaaagtcgctcg
aggaaatgtcgcgtgagaacgagcacgccgagccgtag
>g54928.t1
atgcctgtccctggtagtggcagtgaatccctcgactcttattggggcggagacggaggcgctgtcgccggagaagagaggagggggaggtggcgcatgga
gcccctcctcgacacctccaaggagatggccgtccgccacctcggccgcaccgcccacaacctgtcgtcctcctccctccgcaagaagtccgacgtggccct
ggtccgcaaggtcccctgcgccgccctccgccgattcctcgtcaatctccaggaggtcctcctcggcaccaagctcgtcctcctcttccccgccgtcatcctcgc
gctcgcggcacgattcttcaacttcggacaggagtggatatttgttcttagcttaatcggtctcattcctcttgctgagcgactaagcttcctaactgagcaggttgca
ttttacactggacctactgtgggtggactgctgaacgcgacatttggaaatgtgacggaggtcatcattgcgctctttgcccttcatgaaggccaagtcgtggtggt
gaaatgctccctacttggttccatattgtccaacctgctgcttgttcttggaacctcccttttctgtggtggccttgccaaccttggaaaggatcagccattcgacaga

atgcaagcagatgtcagtacaggacttctgattctgggtgtactatgtcactctcttccgctgatgctgagcgtagtcatgctcctggcatatgtggcctaccttttctt
ccaactgaagacccatcgccagctctttgagccccaagaggatgaagatgatggcgatgattcggttgctcaggatgaggcagttataggattttcaagtgcgat
gatctggctgggtatcgtgaccctgatgactgctgtattatcagagtttgttgtgagtacaattgaggtacggagttcaacgtcctatgcttaa
>g58152.t1
atggcaggcgagcagatgcacgttctctcggcgctggacggcgccaagacgcagtggtaccacttcacggccatcatcatctccggcatgggtttcttcaccg
atgcgtacgacctcttctgcatctccctcgtcaccaagctcatcggccgcgtctactacaccgtcgccggctcgccccaccccgggagcctgccgcccgacgtc
tccgcggccgtgaacggcgtggctttcgtcggcactctcttgggccagctcttcttcggctggctcggcgacaaggtcggccggaagagcgtgtacggcatga
cgctgctgctcatggtcatctgctccatagcgtcgggcctctcgttcgggcacacgcccaacagcgtcatggccacgctctgcttcttccgcttctggctcggctt
cggcatcggcggcgactacccgttgagcgccacaatcatgtccgagtacgccaacaagaagacccgcggtgccttcatcgccgccgtgttcgcgatgcagg
ggttcggcattctcgccggcggcgcggtggcgatcgggatcaccgctttgttcaagaaaaaattccccgcaccgccgtacgccgtcgacccggcggcgtcca
cgccgccagaggctgatttcgtgtggcgcatcatcctcatgttcggggccgtccccgccgctctaacattctactggcggatgaagatgccggagacggcgcg
gtacacggcgctcgtcgccaagaacgcggagcgtgccgcggcggacatgtccaaggtgctccacgtcgagatcgcgaagccacatgcggaggaggagac
cgctgcgccgatgaagagtgaccaatccttcggcctcttctcgtcggagttcgtgcggcgtcacggggttcacctcatgggcacgacgtcgacgtggctcctcc
tggacgtggcctactactcgcagaacctgttccagaaggacatcttcagcgcgatcgggtggatccccgcggcgcgcacgatgagcggcctggacgagctgt
tccacatcgcgcgggcgcagacgctgatcgcgctgtgcggcacggtgcccgggtactggttcacggtcgcgttcatcgacgtgttcggccggttcaagatcca
gctggtcgggttcctcatgatgacggccttcatgctgggcctggccgtgccgtacgagcactggaagaccgtggacaaccacatctggttcgtcgtcatgtacg
ccttcacattcttcttcgccaacttcgggcccaacgccaccacgttcatcgtgcccgccgagatctacccggccaggctccgcgcgacgtgtcacgggatatcg
gcggcggcggggaaggtgggcgccatcatcgggtcgttcgggttcctgtacctggcgcagagcccggacgcggccaagacggcgcacgggtacctgccc
gggatcggcgtgaggaactcgctcttcgcgctcgccggatgcagcttcgcggggttcctgctcaccttcctcgtgccggagcccaagggaaagtcgctcgag
gaaatgtcgcgtgagaatgagcatgccgagccgtag
>g59931.t1
atgagcgtgtccgtcgggagaacgcgggtggggaggtacgagctgggccggacgctcggggaaggcaccttcgccaaggtcaagttcgccaggaacgtc
gagaccggcgagaatgtcgccatcaagatcctcgacaaggagaaggtgctcaggcacaagatgatcggacagataaagcgtgagatctcgaccatgaagct
catcaggcacccgaacgtcataaagatgcacgaggtgatggccagcaagacaaagatttacatcgtgatggaacttgtcactggtggtgaacttttcgacaaaat
tgcttcacgtgggaggctgaaagaggatgatgccaggaagtatttccagcaactggtcaatgctgttgattactgccacagcagaggcgtgtaccaccgtgatct
gaagcctgaaaatcttctgcttgatgctagtggcactctcaaggtgtcagatttcggactgagtgcactttctcaacaagtccgagaggatggtcttctgcacacaa
cctgtggaactcccaactatgttgcccccgaggtccttcgnaatatttttccatatctttctgtcaacttgcaggaggatggtcttctgcacacaacctgtggaactcc
caactatgttgcccccgagccaaggctgatctttggtcatgtggagtga
>g66576.t1
atggcccacgatcacaaggtgctggacgccttggacgcggcgaagacgcagtggtaccatttcacggcggtggtgatcgccggcatgggcttcttcaccgac
gcctacgacctcttctccatctccctcgtcaccaagctcctcggtcgtatctactacttcgatccaagctccaggacccccggcaccctcccgcccagcgtctccg
ccgccgtcaacggcgtggccttctgcggcacgctcgccgggcagctcttcttcggctggctcggctacaagatggggcgcaangggccgcatctactacttcg
atccaagctccaggaccccgggcaccctccctcccagcacaagatggggcgcaagaaggtctacggcatgacgctcatgctgatggtcatctgctgcctggc
ctccggcctctcgttcggcgtctccgccgccgtcaacggcgtggccttctgcggcacgctcgccgggcagctcttcttcggctggctcggctacaagatgggg
cgcaagaaggtctacggcatgacgctcatgctgatggtcatctgctgcctggcctccggcctctcgttcgggtccacacccaaggccgtcatggccacgctctg
cttcttccgattctggctcggcgtcggcatcggcggcgattacccgctgtccgccaccatcatgtcagagtacgccaacaagaagacccgcggcgcgttcatcg
cggcggtcttcgccatgcagggcttcggcaacctcaccggcggcatcgttgccatcatcatctcggccacgctcaaggcacgattcgatgctccggcgtacaa
agatgacccggccggttccaccgtgtcacaggctgactatgcgtggcgcatcgtcctcatgttcggcgccatcccggctctgctcacctactactggcgcatga
nctacaagatggggcgcaagaaggtctacggcatgacgctcatgctgatggtcatctgctgcctcgcctcgggcctctccttcgggtccacgcccaaggccgt
catggccacgctctgcttcttccggttctggctcggcgtcggcatcggcggcgattacccgctgtccgccaccatcatgtctacggcatga
>g100219.t1
atggctgggggtcagctgaacgtcctgaccactcttgaccaggccaagacgcaatggtaccacttcatgaccatcgtgatcgccggcatggggttcttcacgga
cgcgtacgacctgttctgcatctcgctggtgaccaagcttcttggccgcctgtactacacagacccaagcagcaaggaccccggcagcctcccgcccaacgtg
tctgccgccgtgaacggcgtggccctctgcggcacgcttgccgggcagctcttcttcgggtggctcggcgacaagctggggcgcaagagcgtgtacggcttc
actctcatcctcatggtgctctgctccgtggcgtcgggcctctcgttcgggcacaccccaaaaggggtgatcggcacgctctgcttcttccggttctggctcgggt
tcggcatcggcggagactatccattatcagcgacgatcatgtcagagtacgccaacaagaagacccggggcgcattcatcgccgccgtgttcgcgatgcagg
gattcggcatcctcttcggcaccatcgtcgcgctcgtcgtctccgccgcgttccggaacgcgtacccggcgcctccttactacgtggacgccaaggcgtcgctg
gtgcccgaggcggactacctgtggcgggtcgtgctcatgttcggcactgtgcccgctgcgctcacttactactggcgcatgaagatgcccgagacggcacggt
acacggccctcatcgcgcgcaacacgaaacaggccacggcagacatggccaaggtgctcaaaaaggatatccaggaagacgatgagcaggtggagcgac
aggtcgttgctggtggtgacacatggggactcttctccatgcagttcctgaggcgacacgggctccacctcctggccaccacatcgacatggttcctattggaca
ttgcgttctactcccagaacctgttccagaaggacatcttcagcaaggtcggctggatcccgcccgccaggaccatgaacgcgatcgaggaggtcttccgcatc
tcccgcgcccaggcgttcatcgcactctgtggaaccatcccgggatactggttcaccgtcgcactcatcgatgtcatcggaaggttctggatccagatcatggga
ttcctcatgatgacagtattcatgatcgccctcggggcgccatacgagcactggacgcagccggcaaaccacaccgggttcgtcgtgctctacggactcacgtt
cttcttcgcaaacttcgggccaaacagcaccacgttcantttgccattcttgaactctctacaccatagctatctaacaacgccttacctgaaatag
>g100220.t1
atggctgggggtcagctgaacgtcctgaccactcttgaccaggccaagacgcaatggtaccacttcatgaccatcgtgatcgccggcatggggttcttcacgga
cgcgtacgacctgttctgcatctcgctggtgaccaagcttcttggccgcctgtactacacagacccaagcagcaaggaccccggcagcctcccgcccaacgtg
tctgccgccgtgaacggcgtggccctctgcggcacgcttgccgggcagctcttcttcgggtggctcggcgacaagctggggcgcaagagcgtgtacggcttc
actctcatcctcatggtgctctgctccgtggcgtcgggcctctcgttcgggcacaccccaaaaggggtgatcggcacgctctgcttcttccggttctggctcgggt
tcggcatcggcggagactatccattatcagcgacgatcatgtcagagtacgccaacaagaagacccggggcgcattcatcgccgccgtgttcgcgatgcagg

gattcggcatcctcttcggcaccatcgtcgcgctcgtcgtctccgccgcgttccggaacgcgtacccggcgcctccttactacgtggacgccaaggcgtcgctg
gtgcccgaggcggactacctgtggcgggtcgtgctcatgttcggcactgtgcccgctgcgctcacttactactggcgcatgaagatgcccgagacggcacggt
acacggccctcatcgcgcgcaacacgaaacaggccacggcagacatggccaaggtgctcaaaaaggatatccaggaagacgatgagcaggtggagcgac
aggtcgttgctggtggtgacacatggggactcttctccatgcagttcctgaggcgacacgggctccacctcctggccaccacatcgacatggttcctattggaca
ttgcgttctactcccagaacctgttccagaaggacatcttcagcaaggtcggctggatcccgcccgccaggaccatgaacgcgatcgaggaggtcttccgcatc
tcccgcgcccaggcgttcatcgcactctgtggaaccatcccgggatactggttcaccgtcgcactcatcgatgtcatcggaaggttctggatccagatcatggga
ttcctcatgatgacagtattcatgatcgccctcggggcgccatacgagcactggacgcagccggcaaaccacaccgggttcgtcgtgctctacggactcacgtt
cttcttcgcaaacttcgggccaaacagcaccacgttcatcgtgcccgcggagatcttccccgcccggctgcggtccacgtgccacggaatctcagccgcggcg
ggaaaggccggagccatcatcggagccttcgggttcctctacgccgcgcaggacccaaaaaacccagaccacggctactcgcccgggatcggcatacgca
acgcgctattcctactcgcaggaacaaacttcctaggaatgctcatgtccctattcgtcccggagtcaaagggaaagtcgctagaggaactgtcaaaggaaaac
gtcgtcgcacaagacgcttaa
>g104638.t1
atgagccgatcggcgcaagcgttgcgccgatcggcgcgacctcccttctgggtgatctcaccagtgttgagtgtggatggcacgtttttgactgggaaatacaa
gggtactttaatgattgttgttggaattgatacggaggatcagcttattcctattgcatttgcactcaccgaggctttggttgatcggtggcggcctgaaactcacacg
ttccatctaccttgtggtgagctcacagttactctacaggacgtggccatgataacagccctctcgattgatggcatggctatctgtgacacggtgaatcctgaggc
gtggctggatgttgtggaggagtggcttggcctacggacgcccgaactggatgaagatgagcgggacaagaaggctgttggtgtctcgtgcaagtggcagctt
tttgttcctagatggtag
>g106245.t1
atgcgcccctgtcaccccgtgttggggaggtcggcagggctgcaatctcatcgggagggctggccgattctcgagaggagtcactggctgacggcagccgtg
ctcttcaccagtggttggctgctccctaggcacatcggcctcgtgcaggctgccatcgtggaggattgcatcgcagcatattctccctcctccaacaatcttctgttc
tcgcactcagcggcggaggaagaggaagctatttattcagttgttggtgacggcggccccaaaccaaaaccaaaacaacaacacacagacacagaaagaaa
gaaagaaaggaaggaagaaagaaaggaagacacggccatggctggcgagcctttgcatgttctgagcgcccttgacgctgccaagacgcaatggtaccact
tcaccgccatcgtgatcgccggcatgggtttcttcacggacgcgtacgacctgttctgcatctcgctggtgacgaagctgctgggtcgcatctactactaccgcg
acggcgccgccgacccgggctcccttcctccgagggtggccgccgccgtgaacggggtggccttctgcggcaccctctccgggcagctcttcttcggctggc
tcggagacaagatgggccgcaagagggtctacggcatgacgctcatgtgcatggtcatctgctctctggcttccggcctctcctttgggcacacacccaacagc
gtcatggccacgctgtgcttcttccggttctggctcggcttcggcatcggcggcgactaccccttgtcggcgacgatcatgtcggagtacgccaacaagaagac
ccggggcggcttcatcgccgccgtgttcgccatgcagggattcggcatcctcaccggcggcgtcgtcacgctcatcgtctccgccgccttcagatcggcgttcc
cggcgccggcctaccaaaacggggcactggcttcgacgccggcgcaggcggatttcgtgtggcgcctcatcctcatgttcggtgcaattccggcgctgctcac
ctactactggaggatgaagatgcccgagacggccaggtacacggcgctggtggccaagaacgccaagcaggcagcggcagacatgtcaaaggtcctccac
atggacatcagtgctcctactccttctgaagctcctgctgctggaggaggagacgaagcatcaaacttcggtatcttttcgaagcagtttctgcatcgccatgggct
tcatctccttggcactgcaacgacatggttcctactggacatcgccttctacagccagaacctgttccagaaggacatcttcactgcaatcaactggatcccaaag
gccaagaccatgagcgccctcgaggaggtgtaccgcatcgcgcgcgcgcagacgctcatcgcgctctgcggcaccgtcccgggctactggttcaccgtcttc
ctcatcgacgtcatcggtcggttctggatccagatggtagggttcgccatgatgtcagtcttcatgctcggcctcgcgttcccctaccaccactggaccacgcccg
gcaaccacatcggattcgtcgtcatgtacgcattcacattcttcttcgcaaacttcggccccaactccaccacgttcatcgtccccgccgagatcttcccggcgag
actgcggtccacgtgccacggaatctccgccgccgcggggaaagcaggcgccatcatcggatccttcggattcctctacgcggcgcagaaccaggacaagg
ccaaggcggaccacggataccccgcaggcatcggcgtacgcaactcactcttcgtcctcgccgcctgcaacgtcttgggcctcctcttcaccctcctcgtacca
gagtccaagggaaagtcgctcgaggagctatccggcgaaaataacgatgacgacgcgcccgcagtcgcacacgcaagaacagtacccgtctaa
>g106246.t1
atgagagtgtaccgcatcgcgcgcgcgcagacgctcatcgcgctctgcggcaccgtcccgggctactggttcaccgtcttcctcatcgacgtcatcggtcggtt
ctggatccagatggtagggttcgccatgatgtcagtcttcatgctcggcctcgcgttcccctaccaccactggaccacgcccggcaaccacatcggattcgtcgt
catgtacgcattcacattcttcttcgcaaacttcggccccaactccaccacgttcatcgtccccgccgagatcttcccggcgagactgcggtccacgtgccacgg
aatctccgccgccgcggggaaagcaggcgccatcatcggatccttcggattcctctacgcggcgcagaaccaggacaaggccaaggcggaccacggatac
cccgcaggcatcggcgtacgcaactcactcttcgtcctcgccgcctgcaacgtcttgggcctcctcttcaccctcctcgtaccagagtccaagggaaagtcgctc
gaggagctatccggcgaaaataacgatgacgacgcgcccgcagtcgcacacgcaagaacagtacccgtctaa
>g124496.t1
atgcttgccgccgccgcctccgtcaagccctccgccgctgccgccgccgcggccacatccaagccggcggccttcaagcccctccacctccctcccctcccc
gccgccgtcccccggcgcccgctctccctcgccgcgcggcccctgtaccgccagcaggacccgctcttcgtcgtctccgccgcccgcaacgaccgcggcg
ccgcgccgccgtccgccaccgccgacggctcccggcccgtggaggtcgcggcgcccgcggagaccgcgcggcgcgccaagatcggtgtttacttcgcca
cgtggtgggcgctgaacgttatcttcaacatctacaacaaaaaagtgctcaacgcgttcccatacccgtggctcacctccacgctggcgctcgccgccggctcc
gccatcatgctcgcgtcctgggccaccaggatcgccgaggcgccagataccgacgtcgatttctggaagtcgctcgcgccggtggcgatcgcgcacaccatc
gggcacgtcgccgcgacggtgagcatggccaaggtggccgtctcgttcacgcacatcatcaagagcagtgagccggcgttcagcgtgctcgtctcaaggttct
tccttggcgagcactttccggcgccggtctacttctccctcctccccatcatcggtggatgcgccctcgccgccgtcaccgagctcaacttcaacatgattggattc
atgggggcaatgatctcaaacctcgctttcgtcttccgcaacattttctccaagaaggggatgaagggcaagtcggtcagcgggatgaactactacgcctgcctc
tccatactctcactggtgatcctcctccccttcgccttcgccatggaggggcccaaggtgtgggctgcaggttggcagaaagcagtcgccgagatcggtcccaa
cttcgtctggtgggtggcggcgcagagtgtgttctaccacctgtacaaccaagtgtcctacatgtcgttggatgagatctcgccgctgacattcagcgtcggcaac
accatgaagaggatttctgtcattgtcgcctccatcatcatcttccacacaccggtccaacccatcaacgcgctcggagccgccatcgccatccttggaactttcat
ctactcccaggctaagcagtaa
>g130090.t1
atggcgcgcgggggaggagataatcttcaggttcttagcgcgcttgacgcggccaagacgcagtggtaccacttcacggccatcgtggttgccgggatgggc
ttcttcacggacgcctacgacctgttctgcatctccctggtgaccaagctccttggccgcatctactacaccgacaccagcaagcccgacccgggttccctccct

cccaacgtggccgcggcggtgaacggcgtggccttctgcggcacgctggcgggtcagctcttcttcgggtggctgggcgacaagctgggtcgcaagagcgt
gtacggcatgacgctgatgctcatggtgatctgctccatcgcgtcgggcctctccttcgggcacaccccgacgggcgtcatcgccacgctctgcttcttccgcttc
tggctcggcttcggcatcggcggcgactatccattatccgcgaccatcatgtcagagtacgccaacaagaagacccgcggcgccttcatcgcggccgtgttcg
cgatgcaggggttcggcatcctcgccggcggcattgtcacgctcatcatctcagccgcgttccgggccgcgtacccggcccccgcgtaccaggtggacgccg
cgacctccaccgtgtcgcaggccgacttcgtgtggcggatcatcctcatgctgggcgccttacccgcgctgctcacctactactggcggatgaagatgccgga
aactgcgcgatacaccgccctcgtggccaagaacgccaagcaggccgcggcggacatgtccaaggtgctgcagaccgagatcgtggacgagcaggagaa
gctggacgatctcgtcacgcgcagcaccaacagcttcggcctcttctccagggagttcgcgcgccgccacgggatgcacctcatcggcacggcctccacgtg
gttcctcctggacatcgccttctacagccagaacctgttccagaaggacatcttcacggccatcaactggatccccaaggcgcgcaccatgagcgcgctcgag
gaggtgttccgcatctcccgcgcgcagacgctcatcgccctgtgcgggaccgtcccgggatactggttcaccgtcgcgctcatcgacatcgtgggacgattcg
ccatccagctcatggggttcttcatgatgaccgtcttcatgctcggcctcgccgtgccgtaccaccactggaccaccgccggcaaccacatcggcttcgtcgtca
tgtacggcttcaccttcttcttcgccaacttcgggccaaactccacgaccttcatcgtgccggccgagatcttcccggcgcggctgaggtcaacgtgccatggaa
tctccgctgcggcgggcaaggcaggagcaatcatcgggtcgttcgggttcctgtacgcggcgcaggacccggaccatcccgaccacgggtacaagggcgg
gatcggcgtgcggaactcgctgttcgtgctcgccgcgtgcaacatgctcggattcgccgacttcgtgtggcggatcatcctcatgctgggcgccttacccgcgct
gctcacctactactggcggatgaagatgccggaaactgcgcgatacaccgccctcgtggccaagaacgccaagcaggccgcggcggacatgtccaaggtg
ctgcagaccgagatcgtggacgagcaggagaagctggacgatctcgtcacgcgcagcaccaacagcttcggcctcttctccagggagttcgcgcgccgcca
cgggatgcacctcatcggcacggcctccacgtggttcctcctggacatcgccttctacagccagaacctgttccagaaggacatcttcacggccatcaactggat
ccccaaggcgcgcaccatgagcgcgctggaggaggtgttccgcatctcccgcgcgcagacgctcatcgccctgtgcgggaccgtcccgggatactggttca
ccgtcgcgctcatcgacgtcgtgggacgattcgccatccagctcatggggttcttcatgatgaccgtcttcatgctcggcctcgccgtgccgtaccaccactgga
ccaccgccggcaaccacatcggcttcgtcgtcatgtacggcttcaccttcttcttcgccaacttcgggccgaactccacgaccttcatcgtgccggccgagatctt
cccggcgcggctgaggtcaacgtgccatggaatctccgctgcggcgggcaaggcaggagcaatcatcgggtcgttcgggttcctgtacgcggcacaggacc
cggaccatcccgaccacgggtacaagggcgggatcggcgtgcggaactcgctgttcgtgctcgccgcgtgcaacatgctcggattcgtactcaccttcctcgt
gccggagtccaaggggaagtcgctcgaggaggtgtccggcgaggccgaggacgccgaggaagaacccagcagcgccgtccggccggccgatggggct
cacatggcgtag
>g130093.t1
atggcgggcgggcagctcaaggtgctcacgacgctggaccaggcgaagacgcaatggtaccacttcatggccatcgtgatcgccggcatgggcttcttcacg
gatgcttatgatctcttctgcatctccctcgtctccaagctgctcggccgcctctactacaccgagcctaacagccccaatcccggcagcctaccgcccaacgtgt
cggcggccgtgaacggtgtcgccctgtgcggtacgctggccggtcagctctttttggctggctcggcgacaagctcggccgtaagagcgtctacggcttcac
gctcatcctcatggtggtgtgctccgtggcgtccgggctctcgtttgggcacacggcgaagggggtcgtcaccacgctctgcttctttcggttctggctaggattc
ggcatcggtggcgactacccgctgtcggcgaccatcatgtctgagtacgccaacaagaggacccgcggtgcgttcatagctgccgtgttcgccatgcagggct
ttggtatcctcttcggcgccatcgtcgcgcttgtcgtgtccggcggcttccgcaactcgtacccggcgccgtcctacgagcagaacgccgccgcgtctctggtcc
cagaggccgacttcgtctggcgcatcatcctcatgttcggcaccatcccggcagctctcacctactactggcgcatgaagatgccagagacggcacgttacacg
gcactcatcgcgcgcaacgcgaagcaggctgcagccgacatgtccaaggttctcaacaccgagattgtagaggacaaagaccaggtcgagctcgccagtgc
cagtgccagtgccggcagcaatgagtgggggctcttctcgccacagtttttgcgccggcacgggctccacctcctgggcaccaccagcacctggttcctactg
gacatcgccttctacagccagaatctcttccagaaggatatcttctccaaggtgggatggatcccgccacccaagaccatgaacgccattgaggaggtgttccg
catcgcacgggcgcaggcgctcatcgcactttgcggcaccatcccgggctactggttcaccgtgttcttcatcgacatcgtcggccgctttgccatccagctgat
ggggttctttatgatgaccgtcttcatgctcggcctcgccgtgccgtaccaccactggaccactgccggacaccacaccggcttcgtggtcatgtacggcttcact
ttcttctttgccaactttggtcccaacagcaccaccttcatcgtgccggccgagatcttcccagcgcggctgcggtctacgtgccacggcatctcggctgccgca
ggcaaggctggcgcgatcatcggtgcctttgggttcctgtacgccgcgcaggatccccacaagccagaggccggctattcgccaggcatcggcatccgcaat
gcgctgttcgtgctcgccggcactaacttccttggaatgatcatgactcttctcgtgccagaatccaagggcctgtcgctcgaggaaatatccaaggagaccgtc
gacgacgaagaggcggcttga
>g130228.t1
atgtacggtctcaccttcttcttcgccaacttcgggcccaacagcaccaccttcatcgtgcccgcagagatcttcccggccaggctccgttccacgtgccacggc
gtctccgccgcggcaggcaaggccggcgccatcatcggcgcgttcgggttcctgtacgccgcgcaggaccccaagaaacccgaccacgggtattcaccgg
gtatcggtatccgcaactcgctcttcttgctcgccggcaccaacttcctcggcatgctcatgacgctgttcgtgccggaggccaagggcaagtcgctcgaggag
gtctccaaggagaacgtcggggaggagcaggcataa
>g131764.t1
atggggcttccgctcctcctccggctctctcctctccatcgccctccgaatatacctcgacgcacttcgttccactccgtgatgaagcggtggctgcgcttcggctc
agagccaaggagctccaacgacggcgccggcgggcggcgcaaggaaaggcttgatgccggcgatggcgaaggaatggatctcctagacagcggcctgc
cggatgagcaccggacgctgtgccggcggctccagaatgttgacgaggaaggcaacatctgccaagccatgataccttctgtgaggctctctcctacccctgt
ggcctttgctggctccaatctacgctccaaatctgcatcagttccatcagtctctagtctcaaaccctccaaatatgtggtctcctccctcagaccactctaccttgca
ccactaaatggttcacaccctgctgagctgaagcttcagaggcagccgcttgagttccgttgtgcagcttctgcagctgatgacaatgagcccaaggctgaggtg
gcaccagctcgctcagaaggtgcacagaggttaaagatctccatctatttcgcaacatggtgggcgcttaatgtgatatttaacatttacaacaagaaggttctcaa
tgctttcccgtatccctggctcacctctacactctcccttgcctgcggctctgcgatgatgcttttctcatgggccactcgccttgttgaagcccccaagactgactta
gatttctggaaagtgcttttcccggttgctgttgcgcacacaattgggcatgttgctgcgaccgtgagcatgtctaaggtggcagtatcattcacacatatcattaaa
agtgctgagcctgcattcagtgttttggtgtcgaggttcctccttggggagacattcccagttcctgtctatctttcccttctcccaatcattggtggttgtgctctagct
gctgtcacagaactgaacttcaatatggttggatttatgggtgccatgatttccaatcttgcatttgttttccgcaacatcttctccaagagaggcatgaaggggacgt
ctgtcagtggcatgaattactatgcttgcttgtcgataatgtccctggtcatactcactccatttgctattgctatggagggtccccaaatgtgggctgctggttggca
aaaggctcttactgaagttggccctaatgttatctggtgggttgctgctcagagcgtgttctaccacttgtataaccaagtatcgtacatgtctcttgatcaaatttctcc
attgacgtttagcattggcaatacaatgaagcgcatatcagtgattgtttcatcaataattatcttccacacacctgtacgccctgtcaatgcactaggagctgccatt
gctatccttggcacattcctgtattcccaggcaaaggcataa

>g135558.t1
atgctgcagaatgacaccttttcagaaagcacaggtttgccatatgctcctactcttgttgggatcctaaagtctgttggttacctgactctagcaattggagatggtg
gtaatgatgttaggatgattcaagaagctaatattggagtagggattagtggtagggaaggactgcaagctgcaagagctgctgattacagcattggaagtaagt
atcttttgcttgttgggatcctaaagtctgttggttacctgactctagcaattggagatggtggtaatgatgttaggatgattcaagaagctaatattggagtagggatt
agtggtagggaaggactgcaagctgcaagagctgctgattacagcattggaagtgagtgcgtgcttactcatgtgcaacagtaa
>g136350.t1
atgcttgccgccgccgcctccgtcaagccctccgccgccgcggccgcggccgccacatccaagccggcggccttcaagcccctccacctccctcccctccc
cgccgccgtcccccggcgcccgctctccgtcgccgcgcgccccctgtaccgccagcaggacccgctcttcgtcgtcgccgccgcccgcaacgaccgcggc
gcagcgccgccgtccgccaccgccgacggcgcccggcccgtggaggtcgcggcgccagcggagaccgcgcggcgcgccaagatcggtgtttacttcgc
cacgtggtgggcgctgaacgttatcttcaacatctacaacaaaaaagtgctcaacgcgttcccctacccgtggctcacctccacgctggcgctcgccgccggct
ccgccatcatgctcgcgtcctgggccaccaagatcgcagaggcgccagacaccgacctcgatttctggaagtcgctctcgccggtggcgatcgcgcacacca
tcgggcacgtcgccgcgacggtgagcatggccaaggtggccgtgtcgttcacgcacatcatcaagagcggtgagccggcgttcagcgtgctcgtctcaaggt
tcttcctcggcgagcacttcccggcgccggtctacttctccctcctccccatcatcggtggatgcgccctcgccgccgtcaccgagctcaatttcaacatgaccg
gattcatgggggcgatgatctcaaacctcgctttcgtcttccgcaacattttctccaagaaggggatgaagggcaagtcggtcagcgggatgaactactacgcct
gcctctccatactctcactggtgatcctcctccccttcgccttcgccatggaggggcccaaggtgtgggctgcaggttggcagaaagcagtcgccgagatcggt
cccaacttcgtctggtgggtggcggcgcagagtgtgttctaccacctgtacaaccaagtgtcctacatgtcgttggatgagatctcgccgctgacattcagcgtcg
gcaacaccatgaagaggatttctgtcattgtcgcctccatcatcatcttccacacgccggtccaacccatcaacgcgctcggagccgccatcgccatccttggaa
ctttcatctactcccagagtgtgttctaccacctgtacaaccaagtgtcctacatgtcgttggatgagatctcgccgctgacattcagcgtcggcaacaccatgaag
aggatttctgtcattgtcgcctccatcatcatcttccacacgccggtccaacccatcaacgcgctcggagccgccatcgccatccttggaactttcatctactccca
ggctaagcagtaa
>g141536.t1
atggcgcctccacagaacggcggcggcggcgacctcctggaggccgggctgctggcagccgcccccaacggcggcggcggcaagcaggagacggcgt
cctcgccgaagaataagaggccggcgtggcggcgcgcggcggcgcagctgcgggaggtgttcctgggcacgaggctattcccgctcttctccgccgtgcc
gctcgccgtcgccgccgagcacctccgactcggacgggcttgggtgttcgttttcagcttgataggtctggcgccccttgcagaacgtgtgagcttcctgagcg
agcatattgccgatactgcagggccaacagctggtggcctactgaatgcgacctgtggaaatgtacctgagctaatcattgcactgttcgctctgcataaggaga
agctggaaatcctcaagtggtcacttttaggttccatattatccaatttgctccttgtccttggttcatcgctccttctcggaggtcttgctaatgcgggcaaggaacat
ccgtttgacagggatagttcagagagtagtagtacaagcaatgatgacgatgctgcagataatacagctataggatttgccaatgcagtgatatggctgattggga
tggctgcagtcatggcaatgctttctaattacgttgtcacaacaattgaggaagcatcagaatcattgggtatccctgtcaggcatattgccgatactgcagggcca
acagctggtggcctactgaatgcgacctgtggaaatgtacctgagctaatcattgcactgttcgctctgcataaggagaagctggaaatcctcaagtggtcactttt
aggttccatattatccaatttgctccttgtccttggttcatcgctccttctcggaggtcttgctaatgcgggcaaggaacatccgtttgacagggatagttcagagagt
agtagtacaagcaatgatgacgatgctgcagataatacagctataggatttgccaatgcagtgatatggctgattgggatggctgcagtcattgcaatgctttctaa
ttacgttgtcacaacaattgaggaagcatcagaatcattgggtatccctgtcaggtttgttagtattatcctgcttcctattgttggaaatgctgcagagcatgcaggc
gctatcatatttgctttcaagaacaaaattgatatcacccttggaattgccctcggctcagcgactcaaatatctttgcttgtgcaacatcaaagaaggtacatgctgc
ctgagctgctcaaggtgatactgtcccatgtttcagatcagacctacgcgtctaatggtgtggagtgctcagtgatacaccgcaccagtcagatcaagctactcaa
tgtgctaaactaa
>g151141.t1
atggcgcggcaggagctgcaggtgctgaacgcgctcgactccgccaagacgcaatggtaccacttcacggcgatcgtggtcgcaggcatgggtttcttcacc
gacgcctacgacctgttctgcatctcgctggtgaccaagctgctgggccgcatctactacaccgaccccaccagtcccaacccgggttcgctgcctcccaacgt
ggccgccgcggtgaacggcgtcgcgctgtgcggcaccctcgcgggtcagctcttcttcgggtggctcggcgacaagctcgggcgcaagagcgtctacggc
atgacgctgctgctcatggtcatctgctccatcgcgtcgggcctctcgttcgggcacacgcccaacagcgtcatggccacgctctgcttcttccgcttctggctcg
gcttcggcatcggcggcgactacccgttgagcgccaccatcatgtcggagtacgccaacaagaagacccgcggcgccttcatcgccgccgtgttcgcgatgc
agggcttcggcatcctcgccggcggcatcgtcacgctggccatctcctccgtgttccgcgcggcgttcccggcgccggcgtaccagatcagcgccgcggcgt
ccaccgtgccgcaggccgactacgtgtggcgcatcatcctcatgctcggcgccgtgcccgccatgctcacctactactggcggatgaagatgcccgagacgg
cgcggtacacggcgctggtggccaagaacgcgacgcaggccgcgtccgacatgtccaaggttctgcaagtcgagatcgaggcggaggccaagaagctgg
acgagatcatcacccggaacaaggactacggcctcttctcctcacaattcctgaagcgccacgggctgcacctcctcggcacggccacgacgtggttcctcgt
cgacgtggcctactacagccagaacctgttccagaaggacattttcacgagcatccactggatccccaaggcgcgcaccatgagcgcgctggaggaggtgtt
ccgcgtctcccgcgcgcagacgctcatcgcgctctttggcaccgtgccgggctactggttcaccgtcttcctcatcgacgtcatcggccgcttcgcaatccagct
cctgggcttcgcgatgatgaccgtcttcatgctgggcctcgccatcccgtaccaccactggaccacgccgggcaaccagatcgggttcgccatcatgtacgggt
tcacattcttcttcgccaacttcgggcccaacgcgaccacgttcatcgtgccggccgagatttttccggcccggctccggtccacctgccacggcatctccgccg
ctgcgggcaaggcgggcgccatcatcggagcgttcggattcctctacgcggcgcagccgcaggacaaggcgcacgttgacgccgggtacaggccgggga
ttggcgtgcagaaggcgctgtacgtgctggccgtgtgcaatctgctcggcttcctctgcaccttcctcgtgccggaatcgaaagggaagtcgctcgaggagatg
tccggcgaggctgacaacgaagacgaagccgccggtaacagcaccagcaccgtgcagccttcctccggaattgagatggtctag
>g151142.t1
atggcgggaggccagctgaacgtcttatccaccctcgaccaggccaagacgcaatggtaccatttcctggcgattgtcatcgccggcatgggcttcttcaccga
tgcatacgatctcttctgcatcgccctcgtcaccaggctgctcggccgcatctactacactgatcctaccaagcccgacccgggttctctgccacccaacgtgtcg
gccgcagtgaccggtgtcgccctctgcggcacgcttgcagggcagctgttcttcggatggctcggcgacaagctcgggcgcaagagcgtctacggcttcacc
ctgatcctcatggtgctatgctccatggcgtcgggcctttcgttcgggcacacgcctaagagcgtcattggcacgctctgcttcttcaggttctggctcggcttcgg
cataggcggtgactaccctctcagcgccaccatcatgtctgagtacgccaacaagaagacccgtggtgccttcatcgccgcggtgtttgccatgcagggtttcg
gcatcctcttcggcacaattgtggcactcgtcgtctccggggcattccggaacgcgtacccggctccggcatattacattgatgccaaggcgtccctcgtgccgc
aggctgatttcatgtggcgtgtcatcctcatgttcggcaccgttccggccgctctcacctactactggcgcatgaagatgcccgagacggcgcgttacacggcgc

tcattgcccggaacacgaagcaggctgcagctgacatgtccaaggtgctccacaaggacatccaggaggacgagcaggtggaacgtgaggtggtcgccac
cggtgacacttggggcctcttctcgaagcagttcctgcgtcgccatggcctccacctcctggccactaccaccacgtggttcttgctagacatcgcgttctacagc
cagaacctgttccagaaggacatcttcaccaaggtcgggtggatcccggcgggcaggaccatgaacgccatcgaggaggtgttccgcatctcgcgtgcccag
gctctcatcgcgctctgcggaaccgtgcccgggtactggttcaccgtggccttcatcgacatcatcggcaggttctggatccagctcatgggcttcctcatgatga
ccgtgtttatgatcgcgctggccgttccatatgagcactggaccgagccggcacaccacaccgggttcctcgtcatgtacggtctcaccttcttcttcgccaacttc
gggcccaacagcaccaccttcatagtgcccgcagagatcttcccggccaggcttcgttccacgtgccatggcatctctgccgcagcaggcaaggccggtgcc
atcatcggtgcgtttgggttcctgtacgcggcccaggaccccaagaagcccgaccatggctactcgccgggaattggtatccgcaactcgctcttcttgctcgct
ggcaccaacttcctcggcatgctcatgaccctgttcgtgccggaggccaagggcaagtcgcttgaggaggtctccaaggagaacgttggcgacgaggaggc
ataa
>g151143.t1
atggcgggaggccagctgaacgtcttatccaccctcgaccaggccaagacgcaatggtaccatttcctggcgattgtcatcgccggcatgggcttcttcaccga
tgcatacgatctcttctgcatcgccctcgtcaccaggctgctcggccgcatctactacactgatcctaccaagcccgacccgggttctctgccacccaacgtgtcg
gccgcagtgaccggtgtcgccctctgcggcacgcttgcagggcagctgttcttcggatggctcggcgacaagctcgggcgcaagagcgtctacggcttcacc
ctgatcctcatggtgctatgctccatggcgtcgggcctttcgttcgggcacacgcctaagagcgtcattggcacgctctgcttcttcaggttctggctcggcttcgg
cataggcggtgactaccctctcagcgccaccatcatgtctgagtacgccaacaagaagacccgtggtgccttcatcgccgcggtgtttgccatgcagggtttcg
gcatcctcttcggcacaattgtggcactcgtnnnnnnnnnnnnnnnnnnnnnnnnnnnnnnnnnnnnnnnnnnnnnnnnnnnnnnnnnnnnnnnnnnnn
nnnnnnnnnnnnnnnnnnnnnnnnnnnnnnnnnnnnnnnnnnnnnnnnnnnnnnnnnnnnnnnnnnnnnnnnnnnnnnnnnnnnnnnnnnnn
atggcgggaggccagctgaacgtcttatccaccctcgaccaggccaagacgcaatggtaccatttcctggcgattgtcatcgccggcatgggcttcttcaccga
tgcatacgatctcttctgcatcgccctcgtcaccaggctgctcggccgcatctactacactgatcctaccaagcccgacccgggttctctgccacccaacgtgtcg
gccgcagtgaccggtgtcgccctctgcggcacgcttgcagggcagctgttcttcggatggctcggcgacaagctcgggcgcaagagcgtctacggcttcacc
ctgatcctcatggtgctatgctccatggcgtcgggcctttcgttcgggcacacgcctaagagcgtcattggcacgctctgcttcttcaggttctggctcggcttcgg
cataggcggtgactaccctctcagcgccaccatcatgtctgagtacgccaacaagaagacccgtggtgccttcatcgccgcggtgtttgccatgcagggtttcg
gcatcctcttcggcacaattgtggcactcgtcgtctccggggcattccggaacgcgtacccggctccggcatattacattgatgccaaggcgtccctcgtgccgc
aggctgatttcatgtggcgtgtcatcctcatgttcggcaccgttccggccgctctcacctactactggcgcatgaagatgcccgagacggcgcgttacacggcgc
tcattgcccggaacacgaagcaggctgcagctgacatgtccaaggtgctccacaaggacatccaggaggacgagcaggtggaacgtgaggtggtcgccac
cggtgacacttggggcctcttctcgaagcagttcctgcgtcgccatggcctccacctcctggccactaccaccacgtggttcttgctagacatcgcgttctacagc
cagaacctgttccagaaggacatcttcaccaaggtcgggtggatcccggcgggcaggaccatgaacgccatcgaggaggtgttccgcatctcgcgtgcccag
gctctcatcgcgctctgcggaaccgtgcccgggtactggttcaccgtggccttcatcgacatcatcggcaggttctggatccagctcatgggcttcctcatgatga
ccgtgtttatgatcgcgctggccgttccatatgagcactggaccgagccggcacaccacaccgggttcctcgtcatgtacggtctcaccttcttcttcgccaacttc
gggcccaacagcaccaccttcatagtgcccgcagagatcttcccggccaggcttcgttccacgtgccatggcatctctgccgcagcaggcaaggccggtgcc
atcatcggtgcgtttgggttcctgtacgcggcccaggaccccaagaagcccgaccatggctactcgccgggaattggtatccgcaactcgctcttcttgctcgct
ggcaccaacttcctcggcatgctcatgaccctgttcgtgccggaggccaagggcaagtcgcttgaggaggtctccaaggagaacgttggcgacgaggaggc
ataa
>g151145.t1
atgggcttcttcaccgatgcatacgatctcttctgcatcgccctcgtcaccaggctgctcggccgcatctactacactgatcctaccaagcccgacccgggttctct
gccacccaacgtgtcggccgcagtgaccggtgtcgccctctgcggcacgcttgcagggcagctgttcttcggatggctcggcgacaagctcgggcgcaaga
gcgtctacggcttcaccctgatcctcatggtgctatgctccatggcgtcgggcctttcgttcgggcacacgcctaagagcgtcattggcacgctctgcttcttcagg
ttctggctcggcttcggcataggcggtgactaccctctcagcgccaccatcatgtctgagtacgccaacaagaagacccgtggtgccttcatcgccgcggtgttt
gccatgcagggtttcggcatcctcttcggcacaattgtggcactcgtcgtctccggggcattccggaacgcgtacccggctccggcatattacattgatgccaag
gcgtccctcgtgccgcaggctgatttcatgtggcgtgtcatcctcatgttcggcaccgttccggccgctctcacctactactggcgcatgaagatgcccgagacg
gcgcgttacacggcgctcattgcccggaacacgaagcaggctgcagctgacatgtccaaggtgctccacaaggacatccaggaggacgagcaggtggaac
gtgaggtggtcgccaccggtgacacttggggcctcttctcgaagcagttcctgcgtcgccatggcctccacctcctggccactaccaccacgtggttcttgctag
acatcgcgttctacagccagaacctgttccagaaggacatcttcaccaaggtcgggtggatcccggcgggcaggaccatgaacgccatcgaggaggtgttcc
gcatctcgcgtgcccaggctctcatcgcgctctgcggaaccgtgcccgggtactggttcaccgtggccttcatcgacatcatcggcaggttctggatccagctca
tgggcttcctcatgatgaccgtgtttatgatcgcgctggccgttccatatgagcactggaccgagccggcacaccacaccgggttcctcgtcatgtacggtctcac
cttcttcttcgccaacttcgggcccaacagcaccaccttcatagtgcccgcagagatcttcccggccaggcttcgttccacgtgccatggcatctctgccgcagca
ggcaaggccggtgccatcatcggtgcgtttgggttcctgtacgcggcccaggaccccaagaagcccgaccatggctactcgccgggaattggtatccgcaac
tcgctcttcttgctcgctggcaccaacttcctcggcatgctcatgaccctgttcgtgccggaggccaagggcaagtcgcttgaggaggtctccaaggagaacgtt
ggcgacgaggaggcataa
>g151146.t1
atggcgggaggtcagctgaacgtcttatccactctcgaccaggccaagacgcaatggtaccatttcctggcgattgtcatcgccggcatgggcttcttcaccgat
gcatacgatctcttctgcatcgccctcgtcaccaggctgctcggccgcatctactacactgatcctaccaagcccgacccgggttctctgccacccaacgtgtcg
gccgcagtgaccggtgtcgccctctgcggcacgcttgcagggcagctgttcttcggatggctcggcgacaagctcgggcgcaagagcgtctacggcttcacc
ctgatcctcatggtgctatgctccatggcgtcgggcctttcgttcgggcacacgcctaagagcgtcattggcacgctctgcttcttcaggttctggctcggcttcgg
cataggcggtgactaccctctcagcgccaccatcatgtctgagtacgccaacaagaagacccgtggtgccttcatcgccgcggtgtttgccatgcagggtttcg
gcatcctcttcggcacaattgtggcactcgtnnnnnnnnnnnnnnnnnnnnnnnnnnnnnnnnnnnnnnnnnnnnnnnnnnnnnnnnnnnnnnnnnnnn
nnnnnnnnnnnnnnnnnnnnnnnnnnnnnnnnnnnnnnnnnnnnatggcgggaggccagctga
>g151167.t1
atggcgggaggccagctgaacgtcttatccactctcgaccaggccaagacgcaatggtaccatttcctggcgattgtcatagccggcatgggcttcttcaccgat
gcatacgatctcttctgcatcgccctcgtcaccaggctgctcggccgcatctactacactgatcctaccaagcccgacccgggttctctgccacccaacgtgtcg

gccgcagtgaccggtgtcgccctctgcggcacgcttgcagggcagctgttcttcggatggctcggcgacaagctcgggcgcaagagcgtctacggcttcact
ctgattctcatggtgctctgctccattgcgtcgggcctctcgttcgggcacacgcccaagagcgtcattggcacgctctgcttcttccggttctggctcggcttcgg
catcggcggcgactaccctctcagcgccaccatcatgtctgagtacgccaacaagaagacccgtggcgccttcatcgcggcggtgtttgccatgcagggcttc
ggcatcctgttcggcacaatcgtcgcgctcgtcgtctccggggcattccggaacgcgtacccggctccggcatattacatcgacgccaaggcgtccctcgtgcc
ccaggccgatttcatgtggcgtgtcatcctcatgttcggcaccgttccggccgctctcacctactactggcgcatgaagatgcccgagacggcgcgttacacgg
cgctcatcgcccggaacacgaagcaggctgcagctgacatgtccaaggtgctccacaaggacatccaggaggacgagcaggtggaacgggaggtgatcgc
caccggcgacacttggggcctcttctcgaagcagttcctgcgtcgccatggcctccacctcctggccactaccagcacgtggttcttgctagacatcgcgttctac
agccagaacctgttccagaaggacatcttcaccaaggtcgggtggatcccggcgggcaggaccatgaacgccatcgaggaggtgttccgcatctcgcgtgcc
caggctctcatcgcgctctgcggaaccgtgcccgggtactggttcaccgtggccttcatcgacatcatcggcaggttctggatccagctcatgggcttcctcatg
atgaccgtgttcatgatcgcgctggccgttccatatgagcactggaccgagccggcacaccacaccgggttcctcgtcatgtacggtctcaccttcttcttcgcca
acttcgggcccaacagcaccaccttcatagtgcccgcagagatcttcccggccaggcttcgttccacgtgccatggcatctctgccgcagcaggcaaggccgg
tgccatcatcggtgcgtttgggttcctgtacgcggcccaggaccccaagaagnnnnnnnnnnnnnnnnnnnnnnnnnnnnnnnnnnnnnnnnnnnnnnnnnnnn
nnnnnnnnnnnnnnnnnnnnnnnnnnnnnnnnnnnnnnnnnnnnnnnnnnnnnnnnnnnnnnnnnnnnnnnnnnnnnnnnnnnnnnnnnnnnnnnnnn
nnnnnnnnnnnnnnnnnnnnnnnnnnnnnnnnnnnnnatataa
>g151168.t1
atggcgcggcaggagctgcaggtgctgagcgcgctcgactccgccaagacgcaatggtaccacttcacggcgatcgtggtggcaggcatgggcttcttcacc
gacgcttacgacctgttctgcatctcgctggtgaccaagctgctgggccgcatctactacaccgaccccaccagtcccaaaccggctcttcttcgggtggctcgg
cgacaagnnnnnnnnnnnnnnnnnnnnnnnnnnnnnnnnnnnnnnnnnnnnnnnnnnnnnnnnnnnnnnnnnnnnnnnnnnnnnnnnnnnnn
nnnnnnnnnnnnnnnnnnnnnnnnnnnnnnnnnnnnnnnnnnnnnnnnnnnnnnnnnnnnnnnnnnnnnnnnnnnnnncttcggcatcctcgccggc
ggcatcgtcacgctggccatctcctccgtgttccgcgcggcgttcccggcgccggcgtaccagatcagcgccgcggcgtccaccgtgccgcaggccgacta
cgtgtggcgcatcatcctcatgctcggcgccgtgcccgcgatgctcacctactactggcggatgaagatgcccgagacggcgcggtacacggcgctggtggc
caagaacgcgacgcaggccgcgtcggacatgtccaaggtgctgcaggtagagatcaaggcggaggccaagaagctggacgagatcatcgcccggaacaa
ggactacggcctcttgtcgtcgcaattcctaaaacgccacgggctgcacctcctcggcacggccacgacgtggttcctcgtcgacgtggcctactacagccag
aacctgttccagaaggacatcttcacgagcatccactggattcccaagtcgcgcaccatgagcgcgctcgaggaggtgttccgcgtctcccgcgcgcagacgc
tcatcgcgctcttcggcaccgtgccgggctactggttcaccgtctttctcatcgacgtcatcggccgcttcgccatccagctctttggcttcgcgatgatgaccgtat
tcatgctgagcctcgccatcccgtaccaccactggaccacgccgggcaaccagatcggcttcgccatcatgtacgggttcacattcttcttcgccaacttcgggc
ccaacgcgaccacgttcatcgtgccggccgagatcttcccggcccggctgcggtccacctgccacggcatctccgccgctgcgggcaaggcgggcgccatc
atcggagcgttcgggtttctctacgcggcgcagccgcgggacaaggcgcacgtcgacgccgggtacaggccggggattggcgtgcagaaggcgctgtacg
tgctcgccgtgtgcaatctgctcgggttcctctgcaccttcctcgtgccggaatcgaaagggaaatcgctcgaggagatgtccggcgaggctgacaaccaaga
cgaagccgccggtaacagcaccagcaccgtccagccttcctccggaattgagatggtctag

TPC1 genes

>g2435.t1
atggttgtttgtcattcgttgccaagcgagctggtgacgctgttcgcggcgttcgacaaggactccgacggccggatctccccgtcggagcttcgcgtgtgcatg
aagacgacgctgggcgaggaggtctcggcggcggaggcggaggcgctggtggcctcggtggacgcggacggcgacgggcttctcgacggcgacgagtt
cgtcaggctggtctcggcggcggaggtggccgagcacgacgacgagcgccgccgcggtctcagggaggcgttcggcatgtacgagatggaggggcaag
gctgcatcacgccgacgagcctcaagcggatgctggcccgcctcggctcccaccagggcatcgacgactgccgcgccatgatctgcaggttcgacctcaac
ggcgacggcgtcctcacattcgacgagttcatcgtcatgatgaacgcctcctga
>g54470.t1
atgcgcatcgcagacatggaggcgcaggatttcgaggcattcctgtattatgtgtacaccgacacgttgccggagatgaaggggacgcggcagcgatgcta
cccgaccttgttgccgcggcgaacaggtataagatggagaggctaaggctgctctgtgaagacaagctatgcgagtttgtgaacgtgaggaccgtggtagcca
tgctggagttcgccggcgagcaccattgccatgggcttaaggaggcgtgcttgcaattcctccaggatcctgggaatctgagggagatagtgaaggtcaatgg
cctggagcatctcagcgaaagctgcccatctgtgtttaaggatctggttgccaagcttgctagcgacgcaattagatag
>g120964.t1
atggttgtttgccattcgttgccgagcgagctggtgacgctgttcgcggcgttcgacaaggactccgacggccggatctccccgtcggagcttcgcgtgtgcatg
aagacgacgctgggcgaggacgtgtcggcggcggaggcggaggcgctggtggcctcggtggacgcggacggcgacgggcttctcgacggcgacgagtt
cgtgaggctggtctcggcggcggaggtaatggccgaggacgagcgccgccgcgggctcagggaggcgttcggcatgtacgagatggaggggcaaggct
gcatcacgccgacgagcctgaaacggatgctgggacgcctcggctccgaccagggaatcgacgactgccgcgccatgatctgcaggttcgacctcaacggc
gacggcgtcctcacattcgacgagttcatcgtcatgatgaacgcctcatga
