# Supplementary material for: Genome and Transcriptome sequence of Finger millet (Eleusine coracana (L.) Gaertn.) provides insights into drought tolerance and nutraceutical properties
Source: BMC Genomics. 2017 Jun 15;18:465. doi: 10.1186/s12864-017-3850-z (PMC5472924; doi:10.1186/s12864-017-3850-z)
Supplement: Supplementary file 10 — Protein Sequences of C4 photosynthetic genes identified in ML-365 genome. (PDF 119 kb) [file 12864_2017_3850_MOESM10_ESM.pdf]

Supplement File 10: Protein Sequences of C4 photosynthetic genes identified in ML-365 genome

Carbonic anhydrase (CA)

>g6613.t1
MAALCSTPARPPRANLGTRSRRPARMVVVAAGARVSGAEARASLVLALASQALATSQRRAADLAAETVKY
AFPSRRFEPRTLEEALMSVPDLETIPFRVLKREADYEIREVESYFIAETTMPGTSGFDFSGSSQSFNLLA
SYLFGKNTTSEQMEMTTPVITWKGESAGEKMDMTTPVITKKSASENKWKMSFVLPSKYGSNLPL
PKDPSVTVKEVPSKIVAVAAFSGPLRYNDTAETVMVGGTRYKLKQLHWHSPSEHTINGHRFAVELHMVHY
SEDGDITVVAILYRYGRPDPFLFQIKDKLAELYGEGCKAEKGDPLPVGVVDMTELRQGADRYFRYVGSLT
APPCTEKVIWNILDEVREMTKEQAADLMAPLEGSYRHNCRPTQPLNGRTVHFYDRSLKIRNMQ
>g10252.t1
MDPVEHLKSGFDKFKADVYDTKPDLFEPLKAHQSPKYMVFSCADSRVCPSVTLGLQPGEAFTVRNIANMV
PSYDKARYASVGSAIEYAVCALKVEVIVVIGHSRCGGIKALLSLKDGTPDTFHFVEDWVRIGCPAKEKVL
AEHAEAPFADQCTLLEKEAVNMSLENLKSYPFVKEGLEKGTIKLVGAHYDFVDGKFETWDH
>g100950.t1
MMLPPMKVTQSPAPQAVAKERRCVSFAAATAPPDRPPRTASGAKRTPAARKDTYTAVRNRAAHPIFWPPR
PA
>g31422.t1
MVRGKTEMKRIENATSRQVTFSKRRNGLLKKAFELSVLCDAEVALIVFSPRGKVYEFASATRIKKSVSSS
SHFLEEPAHEGSS
>g34871.t1
MEKRAVKLWMFNGEDFPYWKSRTNGYLLSQGHAIWDIVKREYAIPEDLNTTVVASDEQMECIPDEELSLL
TRKFRKF
>g10253.t1
MDMDPVARLKSGFEDFKVNVYNKKPELFEPLKEGQAPTDMDPVARLKSGFEDFKVNVYNKKPELFEPLKE
GQAPTYMVFGCADSRCCPSVTLGLQPGEAFTMRNIASMVGPYDKNKYTGTGSAIEYAVXLFLQDMDPVAR
LKSGFEDFKVNVYNKKPELFEPLKEGQAPTYMVFGCADSRCCPSVTLGLQPGEAFTMRNIASMV
GPYDKNKYTGTGSAIEYAVCALKVSVIVVIGHSRCGGIKALLSMEEGAPDNFHFVEDWVRIGLPAKRKVQ
TECKALPFDAQCSVLEKEAVNVSLDNLKTYPFVKEGVENGSLKLVGAHYDFVNGKFETWDAPTPPPSKAV
DAVARLKTGFQNFKIDVYDKKPELFEPLKEGQAPTFMVFACADSRCCPSVTLRLQPGEAFTIRN
IASMVGPYDKTKYTGIGSAIEYAVCALKVSCIVVIGHSRCGGIRALLSMQDGAPNNFHFVEDWVKICYAA
KKKVQIECKALPFDDQCSVLEREAVNVSLDNLMTYPFVKEGVASGKLKLVGGHYDFVNGKFETWTR
>g10254.t1
MSSTFASVVATRGAAATTIAGTTAPAKSVRRRRSSPVLAAPTNTTAIVNMDAVERLKSGFQKFKTDVYDK
KPELFEPLKEGQAPTYMVFACSDSRCCPSVTLGLQPGEAFTVRNIASMIGPYDKTKYTGTGSAIEYAVCA
LKVSCIVVIGHSRCGGIKALLSMQDGSADNLICHPAKKKVHAECKMLPFDEQCSILERAAVNVS
LDNLKTYPFVKEAVANGKLKLVGGHYDFVNGKFETWVSS
>g31580.t1
MAPALLRAASQCLHPASPCAGADSGRSHGAVTIGGSRPRDVALRAGGSGRENISNCQSLAEQQTPKFMVV
ACADSRVCPTSILGFQPGEAFTVRNVANLVPPYQHGTSEVSAALEFAVNTLEA
>g39692.t1
MPAMAMSSRAAVVLLVLAGSVALSYGSGPKLFGYTAGSLNGPENWAKLSPENKICGDGKRQSPVDIVTKQ
AISAPNLDTLTRTYAATNATLINNGHDVSMTFQGKVGSITVNGKVYNFEKLHWHLPSEHTINGQRFPIEL
HLVHKNDAGNLAVIAILYQFGAPDSFYFQLKNKLAELAKEKDSCNLEEEVSQVPAGVIHMRSLQ
KRTGSYFRYEGSLATPPCTENVVWNVLGKVRQISKEQVALIETLLPAKHTARPAQQLNGRVIQFYNPPNS
TISFQM
>g46700.t1
MSQSNHHAVWAALLAAALLLLATSVPTARAQYETGGTTYSKNEKEFSYVRGAKNGPERWGKINKNWTKCG
TGEMQSPIDLSDKRVKLVRSLGYLNHSYRPAEASIVNRGHDIMVKFGGDAGSLVINGTTYNLHQIHWHSP
SEHTVNGRRYDMELHMVHETVPNKTAVIGILYKVGNPDEFLRKLEPFLKLIADRKDEAEPIGVV
DPRDARGKANAYYRYMGSLTTPPCTEGVIWTLVTEVRSVSKRQLQLLREAVHDGMEDNARPLQELNNRNI
SLFKPYPLKYN
>g46704.t1
MRQSGHLAVLAAVLLLLAAAVPGARAQEETEHEEGFSYVPGAKNGPERWGEIKEEWATCSTGKMQSPIDL
SDKRVKLVPSLGYLDHSYRAAEASIINRGHDIMVKFNGDAGSLVINSTAYNLHQVHWHTPSEHTINDRRY
DMELHMVHQSTANKTAVIGVLYVVGSPDEFLRKLEPFLKLIADKKGREEPIGVVDPHDALGRAN
AYYRYMGSLTTPPCTEGVIWTVVREVHTVSKYQLQLLREAVHDGFENNARPLQELNNRDISLFKPCLRKH
N
>g46706.t1
MAAIYATHRDGPLFPDHSGCCSLNMSQSRHLTVSAAATFAAALLLLAGVVPTARAQEETKREEEFSYVRG

AKNGPEHWGAIKQEWAACGTGRMQSPIDLSDQRVSLMCSLGYLKHSYRAAEASIVNRGHDIMVKFKGNTG
SLVINGTAYNLLQVHWHTPSEHTINGRRYDLELHMVHQSATNKTAVIGVLYKVGSPDEFLCKLE
PFLKLIADQKDREEPIGQVDPRDALGRSYAYYRYMGSLTTPPCTEGVVWTVVREVRTVSKYQLQLLREAV
HDGFENNARPLQELNNRDISLFKPYPHKHN
>g46707.t1
MRQSRHLTVSSAAILLLLLAAAAPAARAQEETEHEEEFSYVPGEENGPEHWGAIKEEWAACGTGKMQSPI
DLSHHRVSLVRSLGYLNHSYRSAEASIVNRGHDIMVKFNGDAGSLVINGTAYNLHQIHWHSPSEHTVNGR
RYDMELHLVHQSAENKAAVVGLLYELGSSDGFLRKMEPFLKRIADKKDREERVGVVNPAGARGR
ASVYYYRYMGSLTTPPCKEGVIWTIVRRVRTVSKYQLQLLREAVHDEMENNARPTQDSNNRDVSLFRPIPR
KQY
>g51447.t1
MAERSGPRRPPSSCFCVVLLLVLLIGQPYCLFVRQLGFLSLSQLVSRLQIKLWXSTSSGIKFASNSDELL
AVADQVVVRADGVSVGINYGQIADNLPSPRRVSWLLRSMQVSKVKLYDADPYVLSAFLDTDVEFVVGIGN
ENVSAMVDPAAAQAWIQRHVQPYLPSTRITCITVGNERHVQPYLPSTRITCITVGNEVFKGNDT
QLKADLLPAMQSVYQALTALGLQGRVNVTTAHSLDIMGNSYPPSAGAFRPDVVPYMQPILGFLSMARSSF
LINCYPFFAYKADPGSVPLDYALFQPNPGVTDPNTKLNYDNMLYAQIDSVYAAIQALGHTDIDVKISETG
WPSRGDPDEVGATPENAGTYIGNLLQRIEMKQGTPLRPSVPIDVYVFALFNENLKPGPASERNY
GLFYPDGRPVYNVGLRGYLPPIDDSRGTRKDGRSRSRSAGRQRGSRVDSARLRVQAGSEFFGQRGGRAGK
MTTSSGNAVFVLLLSACCLATLACDPNGAKFGYVGSMGPDHWGSLNPNFTRCAMGTNQSPIDIATDEAVY
DPSMKALHRNYTVANASIVDNVFNIGLRMEGGDAPGCVNVDGKHYRLKQIHWHSPSEHTFNGQR
FPLELHMVHTSDDGNVTVVAILYRIGRPDPFFWQIQDKLARLYAEGCEAEKGAPVPAGFVNMLSLRRHAY
MYYRYVGSFTTPPCTENVVWNILAQVREMTPDQAAALMAPLEEEYRHNNRPTQPTNGRTVRLYHRFWKKN
KRSP
>g63388.t1
MSERVRSIRSATPPAAGKQSADEALQTLLDGFQRFRSDVFPEQRELFSKLANQQSPRAMFITCADSRIVP
ELITQSDPGSLFVTRNVGNVVPPYGQMNGGVSTAIEYAVMGLGVQHIIICGHSDCGAMKAVLNPQSIERM
PTVKAWLRHAEVAKTVTQQSCSCAEHQELGVXDDWGRAAEQQGGALLIERHGLNRRLEGRALNM
RRAMA
>g66376.t1
MYAVRSSGKPAAAAIVIVILLLCSGAIHRARAQQESDDERGFSYLPDAANGPSQWGSVRGEWAACAVGRL
QSPIVLSAGVPGLDDGRAGRLGRSYRRAAAASLVNRGHDITVRFYSDPGGVAYRLRQMHWHAPSEHAIDG
KGFDMELQMLHQSEATNRSAVVAQLYRISRRRDGTIRRLERYIRRIARREDHEELIDEPVNPR
RPVGGSTVYYKYMGSFTTPPCTEGVTWLVANRVRSVTWRQVRLLRNAVHDGARRNARPLQEANGRNISFY
YTSPAQNRGTTTRS
>g66377.t1
MHAAAAAVVVFVASFSLPLAFPFGGRALVPRAAAQELDDEGEFSYRRDAGNGPARWGVMRRDWCACAYGH
LQSPIGLSDTVAALADRPGRLARAYRPAAASLVNRGHDIMVRFNSDAGGVVIDGVAYRLRQMHWHTPSEH
AVNGKRYDMELHMLHQSEATNRFAVVSQLYRIGRRRDKTISRIERYIARIARKRDHEELIDEVV
DPRRQVRRSTVYYRYTGSFTTPPCTEGVTWLVAKKVRRVRRRQVRMLRNAVHDVISNGPFFFALVLSYVL
FKLINRALVQGARRNARPLQEANGRAVGFYYASPEHGQVTSTMGT
>g85868.t1
MKDFPDYLLNGYKNFMSGRYAGERDRYRVLADTGQRPQTLFIACCDSRSAPETIFDCGPGELFVVRNVAN
MVPPFEPDGQYHATSAAIEYAVQVLKVKDIVVMGHGRCGGIQAALDPNFESLSPGDFIGKWMNLVKSAAE
QIQSNDVMTSGERQTALERVSIRNSIHNLRGFPFVKAQETAGKTALERVSIRNSIHNLRGFPFV
KAQETAGKVKLHGAWFDISTGELWVMDSKTGDFRRPEV
>g85917.t1
MAPALLRAASQCLHPASPCAGADSARSHGAVTFMVVACADSRVCPTSILGFQPGEAFTVRNVANLVPPYQ
HGTSEFMVVACADSRVCPTSILGFQPGEAFTVRNVANLVPPYQHGTSEVSAALEFAVNTLEA
>g95716.t1
MAWQRNGPRDSPTHAPLASLVIFAFCAGYASAEANTPQSTFNYTKGTGDPSKWATLQKDWALCGNGTKQS
PINITKVEASKDLGPLDQTYKVGAATIQNRGHDFMLNFTGGNGNLTIEGKEYRLQQVHWHTPAEHTINGT
DLDAEMHMVHEGSSKARAVVSLLFSTKAGRPSKLLSDLEPYFKRLAGKENEEEKVKGTIDPAAW
IDKASGYYRYEGSLTTPPCTEGVIWTIMSKVADASTEQVNLLKSALEPDARPIQKINNRVVRYYEAAPGP
TKAPAPAPGPTKAAAR
>g108872.t1
MRQSRHLTVPSAAILLLLLAAAVPAARAQEETEHEEEFSYVPGEENGPEHWGAIKEEWAACGTGKMQSPI
DLSHHRVSLVRSMGYLNHSYRSAEASIVNRGHDIMVKFNGDAGSLVINGTAYNLHQIHWHSPSEHTVNGR
RYDMELHLVHQSAENKAAVVGLLYEVGSSDGFLRKMEPFLKRIADKKDREERVGVINPAGARGR
ASVYYYRYMGSLTTPPCTEGVIWTIVRRVRTVSKYQLDLLREAVHDEMEKNARPTQDLNNRDVSLFRPIPR
KQY

>g108873.t1
MNQSRHLTVSSSAILAAALLLLAAAVPDARAQEETEREEEFSYVHGKENGPEHWGAIKQEWAACGTGRMQ
SPINLSDQRVSLVCSLGYLKHSYRAAEASIINRGHDIMVKFKGNTESLVINGTAYNLLQVHWHTPSEHTI
NGRRYDLELHMVHQSAANKTAVIGILYKVGGPDEFLCKMEPFLKLIANQKDREEPIGIVDPCNA
LGRAYAYYRYMGSLTTPPCTEGVIWTVVREVRSVSKYQLQLLREAVHDGFENNARPLQELNSRDTSLFKP
YPRRHD
>g108874.t1
MRQSGHLTVSAAILLLAAATAVPGARAQEETEHEEEFSYVPGAKNGPERWGEIKEEWATCSTGKMQSPID
LSDKRVKLVPSLGYLDHSYRAAEASIINRGHDIMVKFNGDAGSLVINSTAYNLDQVHWHTPSEHTINGRR
YDMELHMVHQSAANKTAVIGVLYVVGNPDEFLRKLEPFLKLIADKKGREEQIGIVDPRDALGRA
NAYYRYMGSLTTPPCTEGVIWTVVKEVHTVSKYQLQLLREAVHDGFENNARPLQELNNRDISLFRPCPRK
HN
>g108875.t1
MELHMVHETVPNKTAVIGILYKVGNPDEFLRKLEPFLKLIADRKDEKPEPIGVVDPRDARGKANAYYRYM
GSLTTPPCTEGVIWTLVTEVRSVSKHQLQLLRDAVHDGMEDNARPLQELNNRNISLFKPYPLKYN
>g108876.t1
MELHMVHETVPNKTAVIGILYKVGNPDEFLRKLEPFLKLIADRKDEKPEPIGVVDPRDARGKANAYYRYM
GSLTTPPCTEGVIWTLVTEVRSVSKHQLQLLRDAVHDGMEDNARPLQELNNRNISLFKPYPLKYN
>g108877.t1
MSQCSHHAVSAALLATALLLLATSVSTARAQYETENEKKFSYVRGAKNGPEHWGEINENWTKCGTGDMQS
PIDLSDKRVKLVRSLGYLNHSYRPAEASIVNRGHDIMIHWHSPSEHTVNGRRYDMELHMVHETVPNKTAV
IGILYKVGNPDEFLRKLEPFLKLIADRKDEKPEPIGVVDPRDARGKANAYYRYMGSLTTPPCTE
GVIWTLVTEVRSVSKHQLQLLRDAVHDGMEDNARPLQELNNRNISLFKPYPLKYN
>g137467.t1
MAMSSRAAVVLLVLAGSVALSYADGSGPKMFGYTAGSLNGPENWAKLSPENKMCGDGKRQSPVDIVTKQA
ISAPNLDSLTRTYAATNATLINNGHDVSMTFQGKVGSITVNGKVYNFEKLHWHLPSEHTINGQRFPIELH
LVHKNDAGNLAVIAILYQFGAPDSFYFQLKNKLADLAKEKDSCNLEEEVSQVPAGVIHMRSLQK
RTGSYFRYEGSLTTPPCTENVVWNILGKVRQISKEQVALIETLLPAKHTARPAQQLNGRVIQFYNPPNST
ISFQM
>g145182.t1
MATVELHMVHYSEHGDITVVAILYRYGRPDPFLFQIKDKLAELYVEGCKAEKGDPLPVGVVDMTELRQGA
DRYFRYVGSLTAPPCTEKVIWNILDEVREMTKEQAADLMAPLEGSYRHNCSPTQPLNGRTVQFYDRSPKI
RNTR
>g160385.t1
MATEFFHGHLMASPVPWFLAPRAVSDVEESFALASGEGVQDGSAGGGSTRLVSAYRLKSYGSADGSPYGW
IYSVIPSFLHECSPLPGSEFSGQRGGRAEKMATSSGNAVFVLLLSACCLATLACDPNGAKFGYVGSMGPD
HWGSLNPNFTRCATGTNQSPIDIATDEAVFDPSMKALHRNYTVANASIVDNVFNIGLRMEGGDA
PGCVNVDGKHYRLKQIHWHSPSEHTFNGQRFPLELHMVHTSDDGNVTVVAILYRIGRPDPFFWQIQDKLA
RLYAEGCDAEKGVPLPAGFVNMLSLRRHAYMYYRYVGSFTTPPCTENVVWNILAQVREMTLDQAAALMAP
LEEEYRHNNRPTQPTNGRTVRLYHRFWKKNKRSP
>g160851.t1
MSSTFASVATRGAACSTIAGTTAPAKSVRRRRSSPVLAAPTNTTAIVNMDAVERLKSGFQKFKTDVYDKK
PELFEPLKEGQAPTYMVFACSDSRCCPSVTLGLQPGEAFTVRNIASMIGPYDKTKYTGTGSAIEYAVCAL
KVSCIVVIGHSRCGGIKALLSMQDGSADNLICHPAKKKVQAECKMLPFDEQCSILERAAVNVSL
DNLKTYPFVKEAVANGKLKLVGGHYDFVNGKFETWVSS
>g160853.t1
MDMDPVARLKSGFEDFKVNVYNKKPELFEPLKEGQAPTYMVFGCADSRCCPSVTLGLQPGEAFTMRNIAS
MVGPYDKNKYTGTGSAIEYAVCALKVSVIVVIGHSRCGGIKALLSMEEGAPDNFHFVEDWVRIGLPAKKK
VQTECKALPFDAQCSVLEKEAVNVSLDNLKTYPFVKEGVENGSLKLVGAHYDFVNGKFETWDAP
TPPPSKYMVFGCADSRCCPSVTLGLQPGEAFTMRNIASMVGPYDKNKYTGTGSAIEYAVCALKVSVIVVI
GHSRCGGIKALLSMEEGAPDNFHFVEDWVRIGLPAKKKVQTECKALPFDAQCSVLEKEAVNVSLDNLKTY
PFVKEGVENGSLKLVGAHYDFVNGKFETWDAPTPPPSKAVDAVARLKTGFQNFKIDVYDKKPEL
FEPLKEGQAPTFMVFACADSRCCPSVTLRLQPGEAFTIRNIASMVGPYDKTKYTGIGSAIEYAVCALKVS
CIVVIGHSRCGGIRALLSMQDGAPNNFHFVEDWVKICYAAKKKVQIECKALPFDDQCSVLEREAVNVSLD
NLMTYPFVKEGVASGKLKLVGGHYDFVNGKFETWSR
>g160855.t1
MVFSCADSRVCPSVTLGLQPGEAFTVRNIANMVPSYDKSRYASVGSAIEYAVCALKVEVIVVIGHSRCGG
IKALLSLKDGTPDTFHFVEDWVRIGCPAKEKVLAEHAEAPFADQCTLLEKEAVNMSLENLKSYPFVKEGL
EKGTIKLVGAHYDFVDGKFETWDH
>g160856.t1

MGGCCCCFPAHKPPRENPMHPSREPLIRHGPDFTTLHHPPQMITYSEGLSAVGRLKAGFRTFKRTIYDQN
PKLFGPLKTGQFPKYMVFACSDSRVCPSVTLNLKPGEAFTVRNIAGLVPAYSQRIFSMGSAIEFAVTVLK
VEYIVVIGHSCCGGIRELLSLKENKPRTFHFIDDWVKTALATKKKVEQENSLMSFDEQCTVLEQ
EVVNLSLSNLKTYPFVMDQIARGKLKLIGAHYDFVNGRTATVLATLSVSLSAELRLRNKDPSSVTELAAT
SGSCPVQLCFTARFSWWPAAAVRWMRAGQPAVMLDDRRSLSLPSSSGFLSSPADASISEGAAASAGSTRP
VVDGVPVFPRVTTMHPRIRSAVEDLSRVDTSRAPWPRVRRRLGSQNSSGS
>g165962.t1
MHAAAPSRRSSGRVAAAVVVFVASFSLPLAFPFVPRAAAQELDDEGEFSYRRDAGNGPARWGVMRRDWSA
CAYGHMQSPIGLSDTVAALAYRPGRLARAYRPAAASLVNGGHDIMVRFNSDAGGVVIDGVAYRLRQMHWH
TPSEHAVNGKRYDMELHMLHQSEATNRFAVVSQLYRIGRRRDKTISRIERYIARIARKRDHEEL
IDEVVDPRRPVRRSTVYYRYTGSFTTPPCTEGVTWLVAKKVRRVRKRQVSMLRNAVHDQANMIRDSGVQG
ARRNARPLQEANGRAVGFYYASPEHGQTDRATNGQVDMYAVPSSGKPAAAAVVIVILLCSGAIHRARAQQ
ESDDERGFSYLPDAANGPSQWGSVRSDWAACAVGRLQSPIVLSAGVPGLDDGRAGRLGRSYRRA
AAASLVNRGHDIMVRFYSDPGGVVIDGVAYRLRQMHWHAPSEHAIDGKGFDMELQMLHQSEATNRSAVVA
QLYRISRRRRDGTIRRLERYIRRIARREDHEELIDEPVNPRRPIGGSTVYYRYTGSFTTPPCTEGVTWLV
ANRVRSVTRRQVRLLRNAVHDGARRNARPLQEANGRNVSFYYTSPAQNRGATTRS

phosphoenolpyruvate carboxylase (PEPC)

>g7297.t1
MLNLANLAEEVQIAHRRRIKLKRGDFADEASAPTESDIEETLKRLVSQLGKSREEVFDALKNQTVDLVFT
AHPTQSVRRSLLQKHGRIRNCLRQLYAKDITADDKQELDEALQREYHGMPSCIPNKALC
>g91710.t1
MASSRPPPASSSVSTSAAARGSYSVFVYGPLMVDEVVSIFLGRVPPSQRFCTEGRIYPAVVPVAGKIVTG
KVFRGITTKELSVLDRLKVGHGEFERKAVEISIPGMLERSLAYTYIWFKKNDPELFGVWDFEVLAQEFML
QLPFLL

>g12019.t1
MASTTKSMERHQSIDAQLRLLAPGKVSEDDKLVEYDALLVDRFLDILQDLHGPDLREFVQECYELSAEYE
GKRDASKLDELGARLTSLNPADAIVVASSFSHMLNLANLAEEVQIAHRRRNKLKRGDFADEASATTESDI
EETLKRLVSELGKTREEVFDALKNQTVDLVLTAHPTQSIRRSLLQKHARIRNCLTQLYAKDITA
DDKQELDEALQREIQAAFRTDEIRRTQPTPQDEMRAGMSYFHETIWKGVPKFLRRVDTALKNIGINERLP
YNAPLIQFSSWMGGDRDGNPRVTPEVTRDVCLLARMMAANLYFSQIEDLMFEFLEPLEVCYRSLCDCGDK
TIADGSLLDFLRQVSTFGLSLVKLDIRQESDRHTDALDAITTHLGIGSYRDWPEEKRQEWLLSE
LRGKRPLLGSDLAQSEEVADVLGTFRVLAELPADSFGAYIISMATAPSDVLAVELLQRECQVRHPLRVVP
LFEKLADLEAAPAAVARLFSVDWYMDRIGGKQEVMIGYSDSGRLSAAWQLYKAQEEMVQVAKRYGVKLTM
FHGRGGTVGRGGGPTHLAILSQPPDTINGSLRVTVQGEVIEHSFGEEHLCFRTLQRFTAATLEH
GMHPPVSPKPEWRGLMDEMAVVATQAYRSIVFKEPRFVEYFRSATPETEYGRMNIGSRPSKRKPSGGIES
LRAIPWIFAWTQTRFHLPVWLGFGAAFKHVMNKDIRNVQMLREMYNEWPFFRVTLDLLEMVFAKGDPGIA
GLYDQLLVADDLKPFGDKLRNNYLDTRQLLLQVAGHKDILEGDPYLKQRLRLRDPYITTLNVCQ
AYTLKRIRDPSFQVTAQPPLSKEFADENQPAGLVKLNPASEYAPGLEDTLILTMKGIAAGMQNTG
>g12020.t1
MASATKHHSIDAQLRLLAPGKVSEDDKLVEYDALLIDRFLDILQDLHGADLREFVQECYETSAEYESKRD
SSKLEELGSKLTSLNPADAIVVASSFSHMLNLANLAEEVQIAHRRRNKLKRGDLSDESNATTESDIEETL
KRLVSELGKTPEEVFEALKSQTVELVLTAHPTQSLRRSLLQKHTKIRNCLTQLYAKDITEDEKK
ELDEALQAEIQAAFRTDEIRRAQPTPQDEMRYGMSYFHETIWKGVPKFLRRVDTALKSIGINERLPYNAP
LIKFSSWMGGDRDGNPRVTPDVTRDVCMLARMIAANLYVAQIEDVMFELSMWRCNDELRAEAEKHLATRE
TKKHYIEFWRQIPATEPYRVLLGHVRDKLYNTRERALHMLTKGFSEIPLDTTIRSVEEFMAPLE
LCYKSLCDCGDKTIADGVLLDFMRQVSTFGLSLAKLDIRQESERHTDAIDAITTHLGIGSYKEWPEEKRQ
EWLLSELQGKRPLMVPDMPVSDEVADVLGCFKVLAELPSDSFGPYIISMATAPSDVLAVELLQRECKVRN
PLPVVPLFERLADLQVEKIDELVDPAAECPCLRGAPLLH
>g13877.t1
MSQPYGSLTGPIKITEQGEVISDKYALPGLGRRNLESALAAVIEASVLHRSAQLPADTLAEWDATMDRVA
AAGQEAYRALVRDEALVPFFVAATPVDELGKMNIGSRPAKRPGGAGGLDDLRAIPWVFGWTQSRIILPGW
YGVGSGLAAAAAEGRGDALRAMYAEWDFFRTFLSNVQMTLAKTDLDIAAEYVEALVPAEHRHLF
DTIRDEHARTLEQVLAISGQGELLQDAPVLRGTLQLRDTYLAPLHALQVALLRRARDGADETPPADDQRA
LLLTINGIAAGLRNTG
>g39847.t1
MRAAGMEDTAAVVERQLEAELAAMSLEDALCLARAFSHYLNLVGIAETHHRVRKAREVEHLSKSCDDIFD

KLIQSSVPPEQLHDTVCKQEVEIVLTAHPTQINRRTLQYKHLRIAHLLEYNERPDLSHEDKEMLIEDLVR
EITAIWQTDELRRHKPTPVDEARAGLHIVEQSLWKAVPHYLRRVSNALKKQSCGSSVLADGRLA
DLIRRVATFGMVLMKLDVRQESGRHTEALDDVTSYLDLGVYSEWDEERKLDFLTRELKGKRPLVPPNMEV
AADVKEVLDTFRVAAELGSDSLGAYVISMASNASDVLAVELLQKDARLAVSGDLGRPCPGGTLRVVPLFE
TVKDLREAGSAIRKLLSIDWYREHVIKNHNGHQEVMVGYSDSGKDAGRFTAAWELYKAQEDVVA
ACTEFGIKVTLFHGRGGSIGRGGGPTHLAIQSQPPGSVMGTLRSTEQGEMVQAKFGLPQTAVRQLEIYTT
AVLLATLRPPHPPRDATWRRVMEEISRASCAHYRRTVYEDPEFITYFHEATPQAELGHLNIGSRPVKRRP
AGGIASLRAIPWVFAWTQTRLVLPAWLGVGTGLQAALDAGRGAELRAMYAEWPFFQSTVDLIEM
VVAKADANMAKHYEEMLVPEGARRAVGAELRRELATTERCVLAVSGHSKLSAHNRSLRRLIESRLAYLNP
MNMLQVEVLRRLRRDDDNRKLRDALLITINGIAAGMRNTG
>g42715.t1
MAKTPSDVLAVHLLLKEAGIPFAMPVAPLFETLDDLNNANDVMSKLLNIDWYRGFIQGKQMVMIGYSDSA
KDAGVMAASWAQYQAQDALIKTCEKAGVSLTLFHGRGGTIGRGGAPAHAALLSQPPGSLKGGLRVTEQGE
MIRFKYGLPEVTIASLSLYTGAILEANLMPPPXXXXARQPQRRPARDGTGRNDSLQVRLAGSHY
RQPVALYRRDSGSQPDAAAGTQTGMA
>g52416.t1
MMAANLYFSQIEDLMFELSMWRCSDELRVRADELHRSSRRAAKHYIEFWKQVPQNEPYRVILGDVRDKLY
YTRERSRHLLTTGISDIPEEATFTNVEQFLEPLELCYRSLCACGDKPIADGSLLDFLRQVSTFGLALVKL
DIRQESDRHTDVLDAITTHLGIGSYAEWSEEKRQDWLLSELRGKRPLFGSDLPQTEEVADVLGT
FHVLAELPADCFGAYIISMATAPSDVLAVELLQRECHVKQPLRVVPLFEKLADLEAAPAAVARLFSIDWD
KLYYTRERSRHLLTTGISDIPEEATFTNVEQFLEPLELCYRSLCACGDKPIADGSLLDFLRQVSTFGLAL
VKLDIRQESDRHTDVLDAITTHLGIGSYAEWSEEKRQDWLLSELRGKRPLFGSDLPQTEEVADV
LGTFHVLAELPADCFGAYIISMATAPSDVLAVELLQRECHVKQPLRVVPLFEKLADLEAAPAAVARLFSI
DWYMNRISGKQEVMIGYSDSGKDAGRLSAAWQMYKAQEELIKVAKHYGVKLTMFHGRGGTVGRGGGPTHL
AILSQPPDTIHGSLRVTVQATLEHGMHPPISPKPEWRAMMDEMAVVATKEYRSIVFQEPRFVEY
FRSATPETEYGRMNIGSRPSKRKPSGGIESLRAIPWIFAWTQTRFHLPVWLGFGAAFKHIMQKDIRNIHT
LKEMYNEWPFFRVTLDLLEMVFAKGDPGIAAVYDKLLVTDDLQSFGEQLRKNYEETKELLLQVAGHKDVL
EGDPYLKQRLRLRESYITTLNVCQAYTLKRIRDPNFQVSPQPALSKEFADEKQPAELVQLNTES
EYAPGLEDTLILTMKGIAAGMQNTG
>g61742.t1
MLGYSDSAKDGGLLASRWALQRTQVELTALAREAGVRIVFFHGRGGSVSRGGGKTGRAINAAPRGSVDGS
LRVTEQGEVIHRNRGGGKTGRAINAAPRGSVDGSLRVTEQGEVIHRKYGIRALALRNLEQSTAAVLQATL
RPRAPDPREDSWRGIATELAEVSRTHYRALVHERADFPDXXXYRALVHERADFPDYFRAATPID
VIERLQIGSRPSRRRDGGINNXXXXXXXXPPRGGGSSPGRRTAPA
>g85032.t1
MLGKLLGDTIKEALGENILDQVEAIRKLSKSSRAGNDTHRKELLNTLQNLSNEELLPVARAFSQFLNLTN
VAEQYQTISQSGEGANHPELLKKTFETLKQQKDIRESDILAAIESLSLELVLTAHPTEITRRTLIHKLVE
VNSCLKQLDHSDVSDYERNQIMRRLRQLVAQAWHTDEIRKYRPTPIDEAKWGFAVVENSLWEGV
PAFLRELNEQVEEAFGIKLPVDFVPVRFTSWMGGDRDGNPNVTASITRHAMQLSRWKATDLFLRDIGVLI
SELSMSECSDEIRELSGDPEAIEPYRVILKRLRSQLMSTQSFLEHRLKGERLPRPADLLVSNDQLWDPLF
AIYQSLQQCGMGIIANGQLLDTLRRVKCFGVPLVRIDLRQESTRHTEAIAEVTRYLGLGDYESW
SEADKQAFLIRELNSKRPLLPRSWEPSDETREVLETCRVAAEAPQGSIAAYVISMAKTPSDVLAVHLLLK
EAGISYAMPVAPLFETLDDLNNANDVMSQLLSIDWYRGFIQGKQMVMIGYSDSAKDAGVMAASWAQYQAQ
DALIKTCEKAGISLTLFHGRGGSIGRGGAPAHAALLSQPPGSLRGGLRVTEQGEMIRFKYGLPA
VTIASLSLYTGAILEANLMPPPEPKREWRDIMNQLSADSCAMYRGYVRENADFVPYFRSATPEQELGKLP
LGSRPAKRRPTGGVESLRAIPWIFAWTQNRLMLPAWLGAGAALQQAMAAGHQDQLEAMCRDWPFFSTRLG
MLEMVFAKADLWLAEYYDQRLVDKSLWPLGKQLRDQLDADXXXLPAWLGAGAALQQAMAAGHQD
QLEAMCRDWPFFSTRLGMLEMVFAKADLWLAEYYDQRLVDKSLWPLGKQLRDQLDADIKAVLTIANDSHL
MADQPWIAESIALRNVXXXXXXXTTRT
>g94976.t1
MRAAGMEDTAAVVERQLEAELAAMSLEDALCLARAFSHYLNLVGIAETHHRRSTDDCRLLGSLLHDVLLR
ELGPRFIHILERNRILAQSAVSMRAAGMEDTAAVVERQLEAELAAMSLEDALCLARAFSHYLNLVGIAET
HHRVRKAREVEHLSNSCDDIFDKLIQSGVPPEQLHDTVCKQEVEIVLTAHPTQINRRTLQYKHL
RIAHLLEFNERPDLSHEDKEMLIEDLVREITAIWQTDELRRHKPTPVDEARAGLHIVEQSLWKAVPHYLR
RVSNALKKQSCGSSVLADGRLADLIRRVATFGMVLMKLDVRQESGRHTEALDAVTSYLDLGVYSEWDEER
KLDFLTRELKGKRPLVPTNMEVAADVKEVLDTFRVAAELGSDSLGAYVISMASNASDVLAVELL
QKDARLAVSGDLGRPCPGGTLRVVPLFETVKDLREAGSAIRKLLSIDWYKEHIIKNHNGRQEVMVGYSDS
GKDAGRFTAAWELYKAQEXKQSCGSSVLADGRLADLIRRVATFGMVLMKLDVRQESGRHTEALDAVTSYL
DLGVYSEWDEERKLDFLTRELKGKRPLVPTNMEVAADVKEVLDTFRVAAELGSDSLGAYVISMA
SNASDVLAVELLQKDARLAVSGDLGRPCPGGTLRVVPLFETVKDLREAGSAIRKLLSIDWYKEHIIKNHN

GRQEVMVGYSDSGKDAGRFTAAWELYKAQEXXXXXXXKKKTQNITLIKKTAKQSCGSSVLADGRLADLIR
RVATFGMVLMKLDVRQESGRHTEALDAVTSYLDLGVYSEWDEERKLDFLTRELKGKRPLVPTNM
EVAADVKEVLDTFRVAAELGSDSLGAYVISMASNASDVLAVELLQKDARLAVSGDLGRPCPGGTLRVVPL
FETVKDLREAGSAIRKLLSIDWYKEHIIKNHNGRQEVMVGYSDSGKDAGRFTAAWELYKAQEDVVAACTE
FGIKQGEMVQAKFGLPQTAVRRLEIYTTAVLLATLRPPHPPRDATWRRVMEEISRASCAHYRRT
VYEDPEFITYFHEATPQAELGHLNIGSRPAKRRPAGGIASLRAIPWVFAWTQTRLVLPAWLGVGTGLQAA
LDAGRGAELRAMYAEWPFFQSTVDLIEMVVAKADANMAKHYEEMLVPEGARRAVGAELRRELARTERCVL
AVSRHSKLSAHNRSLRRLIESRLAYLNPMNMLQVEVLRRLRRDDDNRKLRDALLITINGIAAGM
RNTG
>g104762.t1
MASATKHHSIDAQLRLLAPGKNAPASVERLFSIDWYLKRIAGKQQIMVGYSDSGKDAGRLSAAWQLYQAQ
EEVAKVAKKYNVQLTFFHGRGGTVGRGGGPTHLAILSQPPDTINGSLRVTIQGEVIEHSFGEEHLCFRTL
QRFTAATLEHGMHPPISPKPEWRKLMDDMAVVATEAYRSVVVKEPRFVEYFRSATPETEYGRMN
IGSRPAKRRPGGGITTLRAIPWIFSWTQTRFHLPVWLGVGTAFKSAIDKDIKNFQVLKDMYNEWPFFRVT
LDLLEMVFAKGDPGIAALYDKLLVAEELKPFGEQLRSKYLETEDLLLKIAGHSEILAGDPYLKQRLRLRD
PYITTLNVCQAYTLKQIRDPNFKVTTNPPLNKEPADLVKLNPASEYAPGLEDTLIITMKGIAAG
GICHSAIDKDIKNFQVLKDMYNEWPFFRVTLDLLEMVFAKGDPGIAALYDKLLVAEELKPFGEQLRSKYL
ETEDLLLKIAGHSEILAGDPYLKQRLRLRDPYITTLNVCQAYTLKQIRDPNFKVTTNPPLNKEPADLVKL
NPASEYAPGLEDTLIITMKGIAAGMQNTG
>g111535.t1
MKKPKSFFFRDLLEGDPYLKQRLRLRDAYITTLNVCQAYTLKRIRDPDYHVALRPHLSKEILDSSKPAAE
LVKLNPGSEYAPGLEDTLILTMKGIAAGLQNTG
>g116785.t1
MAAAAPWGAAAARPTRRSCRSRPXXXXXXXXXGRGGTVGRGGGPAHEAILSQPPGSVNGRFRTTEQGEMIR
FKFGMPDIAEQNLNLYLAAVLEATLLPPPAPRQAWREQMDKLAADGVATYRGVVREHPQFVEYFRQATPE
QELGRLPLGSRPAKRREGGVESLRAIPWIFAWTQTRLMLPAWLGWEAALRNALQRGEGQLLSEM
REQWPFFRTRIDMLEMVLAKADESIARLRARWCWA
>g118845.t1
MAAPLGGGKVERLSSIDAQLRMLVPGKLSEDDKLIEYDALLLDRFLDILQDLHGDDLRELVQECYEVAAE
YETKHDHQKLDELGKMITSLDPGDSIVIAKSFSHMLNLANLAEEVQIAYRRRIKLKKGDFADENSAMTES
DIEETLKRLVVDLKKSPAEVFDALKSQTVDLVLTAHPTQSVRRSLLQKHSRIRNCLVQLYSKDI
TPDDKQELDEALQREIQAAFRTDEIRRMQPTPQDEMRAGNT
>g119294.t1
MDKLAADGVATYRGVVREHPQFVEYFRQATPEQELGRLPLGSRPAKRREGGVESLRAIPWIFAWTQTRLM
LPAWLGWEAALRNALERGEGEVLREMREQWPFFRTXXXIFAWTQTRLMLPAWLGWEAALRNALERGEGEV
LREMREQWPFFPFGXPWIFAWTQTRLMLPAWLGWEAALRNALERGEGEVLREMREQWPFFRTRI
DMLEMVLAKADESIARLYDERLVTAELQPLGAHLRDLLSQASEVVLGLTGQSQLLVHSPETLEFITVRNT
YLDPLHLLQAELLARSRQREQEPGSALEQALLVSVAGIAAGLRNTG
>g119295.t1
MSSLARQVAELDIELVLTAHPTEVSRRTLIQKYDAISAQLAEQDHSDLSGAEREAIASRLQRLIAEAWHT
EEIRRVRPTPVDEAKWGFAVIENSLWYALPTVMRKADQALQRETGQRLPLDAAPIRFASWMGGDRDGNPN
VTARITREVLLLARWMAADLYLRDIDSLAAELSMQRANDELRARAGDSAEPYRAVLKQLRERLR
ATRTWAHESLAGPVNPPAQVLHDNAELREPLLLCYRSLHDCGMGVIADGPLLDFLRRVSTFGLFLVRLDV
RQDATRHASAMAEITEYLGLGRYDSWDEEQRLAFLQDELSGRRPLLPADFNPSDDTAEVLATCREVAAAP
AASLGSYVISMAGAASDVLAVQLLLKEAGLRRPMRVVPLFETLADLDNAAVAIERLLGLPDYRA
GLQGPQEVMIGYSDSAKDAGTTAAAWAQYRAQESLVRICREHDVELLLFHGRGGTVGRGGGPAHEAILSQ
PPGSVNGRFRTTEQGEMIRFKFGMPDIAEQNLNLYLAAVLEATLLPPXSAPGAPPSRAR
>g126792.t1
MPMTGKMERHQSIDAQLRLLAPGKVSEDDKLVEYDALLVDRFLDILQDLHGPHLREFVQECYELSAEYEN
DRDEARLGELGSKLTSLPPADSIVVASSFSHMLNLANLAEEVQIAHRRRIKLKRGDFADEASAPTESDIE
ETLKRLVSQLGKSREEVFDALKNQTVDLVFTAHPTQSVRRSLLQKHGRIRNCLRQLYAKDITAD
DKQELDEALQRESIGISALLLLVLLLETN
>g126793.t1
MPMTGKMERHQSIDAQLRLLAPGKVSEDDKLVEYDALLVDRFLDILQDLHGPHLREFVQECYELSAEYEN
DRDEARLGELGSKLTSLPPADSIVVASSFSHMLNLANLAEEVQIAHRRRIKLKRGDFADEASAPTESDIE
ETLKRLVSQLGKSREEVFDALKNQTVDLVFTAHPTQSVRRSLLQKHGRCGRWVTVPSIDLLCYQ
V
>g138303.t1
MRADVRLLGELLGRVLRESGSPGLFEDVERLRTATIDAYTDESGEAFDRAVAIADALPSARAEEVARAFT
VYFHLANLAEEHQRVRVLRERARTDVDATRTDTVPGAYAQLAAELGPEAARERLQSLRFHPVFTAHPTEA

RRRAISDSIRRLAGLLDELDGGGSGAAVEGPEGGRIHRSMLEEVDTLWRTAPLRSEKPTPVDEV
RSVMAAFDETLYTAVPEVYRRIEAILHGDEAGAVPPVVRPFVRVGTWVGGDRDGNPFVTAKVTRKAAGIA
SEHVLIGLERSAQRIGRSLTLHVGTTPASAALQQLFQRMADADGAAAEILERSPGELHRATVLLIGRRIG
ATRSRDADLAYREPAELLADLRTVQDSLAAAGARRQAFGGLQELVWQVETFGFHLAELEVRQHS
AVHAKVLAELSAGGALSEQAEEALETVRTIAHIQHRFGPAAAGRYIVSFTQSAEDLANVHRLARAAVGPD
GVPPVLDVVPLFETFADLQAAPAILAEIVEHPEFASRLEATGRKLEVMLGYSDSSKDVGPVAATLALYEA
QQRIADWARESGIELTLFHGRGGALGRGGGPANSAILGQPPHSVDGRFKLTEQGEVIFARYGDS
DIAMRHMDQVAAAVLLASAPSIEQRNEGAATRYAEVAATMDAASRERFFALVKAEGFAPWFATVTPMEEI
GQLALGSRPARRGLSVSSLEDLRAIPWVFSWTQARINLTGWFGLGTALDAVGDLPLLQQAYRDWPLLRTM
IDNVAMSLAKADERIARHYLALGDRDDLAQLVLDEMALTRDWVVRIAGGGEVLSNKPVLQRAVK
MRSPYVDALSLLQLRALRALRDAPEGAEVDPDLQRLLLLSVSGVAAGLQNTG
>g138732.t1
MAAPLGGGKVERLSSIDAQLRMLVPGKLSEDDKLIEYDALLLDRFLDILQDLHGDDLRELMAAPLGGGKV
ERLSSIDAQLRMLVPGKLSEDDKLIEYDALLLDRFLDILQDLHGDDLRELVQECYEVAAEYETKHDHQKL
DELGKMITSLDPGDSIVIAKSFSHMLNLANLAEEVQIAYRRRIKLKKGDFVDENSAMTESDIEE
TLKRLVVDLKKSPAEVFDALKSQTVDLVLTAHPTQSVRRSLLQKHSRIRNCLVQLYSKDITPDDKQELDE
ALQREVHIANLSCSVDAKA
>g143563.t1
MTQDSRSSAVPAMPDELRADVRLLGELLGTVLTESGGADLLADVEALRALTIEAYASDEDSLARAEELVE
SFTPERAAEVARAFTCYFHLVNLAEEYHRVRTLRGRDSSAPGAMLPAVDTIPGAISQLRGEVGEAETYAR
LAKLEFRPVLTAHPTEARRRAVSAAIRRISENLVLLDDPRANDAERAAIRRALLAGIDVMWRTA
PVRTEKPSPLDEVRTAMSVFDTTLFEQIPLIYRALDAALQPETSGTVPPKAPAFVRLGSWIGGDRDGNPN
VTAKITREAAIIAAEHVLLGLEKHTLRIGRELTLDASTTPPSRELLDLRERHRDLSEDLTGTVEERSPGE
PHRQVLLVMAARVRATRERNADLAYPDPEEATRERNADLAYPDPEEFLADLRTVQTSLAAAGDV
RSAYGELQDLIWQVETFGFHLAELEVRQHSQVHRETLEEIDRLGIDGALSERSREVLETYRILGRIQRRF
GPKASRRYIVSFTQEAADIAAVYRLAELAAGGAKPPVIDAVPLFETFEDLHNSVDILTAALEQPAVQARL
AATGRKIEVMLGYSDSSKDVGPVSATLALYDAQARIAEWARANDLELTLFHGRGGALGRGGGPA
NRAVLAQPPHSVDGRFKLTEQGEVIFARYGDKAIATRHIDQVGAATLMASAPSNEQRNAGAAERFAQVAE
TMDRVSRERFYELVRADGFPQWFASVTPLEEVGLLALGSRPAKRGLSVNSLEDLRAIPWVFSWTQARINL
TGWFGLGSALAAVGDVETLHAAYREWPLLRTMLDNVEMSLSKADERIARRYLALGDRDDLAQLV
LDEMQLTREWVLRTTQHTRPLESSRVLGRAVQLRSPYVDALSLIQLRALKRLRAGASEAEVPELRQLLLL
SVNGVAAGLQNTG
>g150385.t1
MSVFLTMLLLFNSLLGWEEIVTLSMWRCNDELRSRADELHRSSKKDAKHYIEFWKKVPPNEPYRVILSDV
RDKLYNTRERSRELLSSGNSDIPEEATLTNVEQATPETEYGRMNIGSRPSKRKPSGGIESLRAIPWIFAW
TQTRFHLPVWLGFGAAFKHALQKDIRNLHMLQEMYNQWPFFRVTIDLIEMVFAKGNPDIAALYD
NLLVSEELRPLGEKLRANYEETQKLLLQVAGHRDLLEGDPYLKQRLRLRDAYITTLNVCQAYTLKRIRDP
DYHVALRPHLSKEIMDSSKPAAELVKLNPGSEYAPGLEDTLILTMKGIAAGLQNTG
>g153502.t1
MMDEMAVVATKEYRSIVFQEPRFVEYFRSATPETEYGRMNIGSRPSKRKPSGGIESLRAIPWIFAWTQTR
FHLPVWLGFGAAFKHIMQKDIRNIHTLKEMYNEWPFFRVTLDLLEMVFAKGDPGIAAVYDKLLVTDDLQS
FGEQLRKNYEETKELLLQVAGHKDVLEGDPYLKQRLRLRESYITTLNVCQAYTLKRIRDPNFQV
SPQPALSKEFADEKQPAELVQLNTESEYAPGLEDTLILTMKGIAAGMQNTG
>g162058.t1
MTPVSKRGRACQYFGGMRELTRTEAIDLVGRYEAEQEIPEAMRADVRMLGSMLGQVLRESGSPPPLXXXX
XXXDDSPEAFERAAAIAESFSVSRADEVARAFTVYFHLVNLAEEHQRVRLLRERAGISDQDSASDSIPAA
YAQLSSEVGEDEAARRLRELRFHPVFTAHPTEARRRAVSQSIRRLVTLLETLENSAAGGAEHRR
ARRRMLEEIDTLWRTSPLRAEKPTPTDEVRAVMAVFDDTLYTTIPLVYRRLDDALQGEVAGSRPPVVAPF
VRLGTWVGGDRDGNPFVTASVTKKAAGIAAEHHCGIASPRPTTRAPPTP
>g166856.t1
MEERHQSIDAQLRLLVPGKVSEDDKLVEYDALLVDRFLDILQDLHGPDLREFVQECYEVSAEYEGKRDAS
KLDELGARLTSLNPADAIVVASSFFHMLNLANLAEEVQIAHRRRNKLKRGDFADEASATTESDIEETLKR
LVSELGKTREEVFDALKNQTVDLVLTAHPTQSIRRSLLQKHARIRNCLTQLYAKDITADDKQEL
DEALQREIQAAFRTDEIRRTQPTPQDEMRAGMSYFHETIWKGVPKFLRRVDTALKNIGINERNPRVTPEV
TRDVCLLARMMAANLYFSQIEDLMFEFLEPLEVCYISLCDCGDKTIADGSLLDFLRQVSTFGLSLVKLDI
RQESDRHTDALDAITTHLGIGSYREWSEERRQDWLLSELRGKRPLLGSDLPQTSEEVADVLGTF
RVLAELPADSFGAYIISMATAPSDVLAVELLQRECQVRHPLRVVPLFEKLADLEAAPAAVARLFSVDWYM
DRIGGKQEVMIGYSDSGKDAGRLSAAWQLYKAQEEMVQVAKRYGVKLTMFHGRGGTVGRGGGPTHLAILS
QPPDTINGSLRVTVQGEVIEHSFGEEHLCFRTLQRFTAATLEHGMHPPVSPKPEWRKLMDEMAV
VATEAYRSIVFKEPRFVEYFRSATPETEYGRMNIGSRPSKRKPSGGIESLRAIPWIFAWTQTRFHLPVWL

GFGAAFKHVMNRDIKNVQMLREMYNEWPFFRVTLDLLEMVFAKGDPGIAGLYDQLLVADDLKPFGEQLRN
NYLDTRQLLLQVAGHKDILEGDPYLKQRLRLRDPYITTLNVCQAYTLKRIRDPSFQVTAQRPLS
KEFADENQPAGLVKLNPASEYAPGLEDTLILTMKGIAAGMQNTG
>g166857.t1
MASATKHHSIDAQLRLLAPGKVSEDDKLVEYDALLIDRFLDILESLHGADLREFVQECYETSAEYESKRD
SSKLEELGTKLTSLNPADAIVVASSFSHMLNLANLAEEVQIAHRRRNKLKRGDLSDESNATTESDIEETL
KRLVSELGKTPEEVFEALKSQTVELVLTAHPTQSLRRSLLQKHTKIRDCLTQLYAKDITEDEKK
ELDEALQAEIQAAFRTDEIRRAQPTPQDEMRYGMSYFHETIWKGVPKFLRRVDTALKSIGINERLPYNAP
LIKFSSWMGGDRDGNPRVTPDVTRDVCMLARMIAANLYVAQIEDVMFELSMWRCNDELRAEAEKHLATRE
IKKHYIEFWRQIPATEPYRVLLGHVRDKLYNTRERALHMLTKGFSEIPLDTTIRSVEEFMAPLE
LCYKSLCDCGDKTIADGVLLDFMRQVSTFGLSLAKLDIRQESERHTDAIDAITTHLGIGSYKEWDEEKRQ
EWLLSELQGKRPLMVPDMPVSDEVADVLGCFKVLAELPSDSFGPYIISMATAPSDVLAVELLQRECKVRN
PLPVVPLFERLADLQNAPASVERLFSIDWYLKRIAGKQQIMVGYSDSGKDAGRLSAAWQLYQAQ
EEVAKVAKKYNVQLTFFHGRGGTVGRGGGPTHLAILSQPPDTINGSLRVTIQGEDAGRLSAAWQLYQAQE
EVAKVAKKYNVQLTFFHGRGGTVGRGGGPTHLAILSQPPDTINGSLRVTIQGEVIEHSFGEEHLCFRTLQ
RFTAATLEHGMHPPISPKPEWRKLMDDMAVVATEAYRSVVVKEPRFVEYFRSATPETEYGRMNI
GSRPAKRRPGGGITTLRAIPWIFSWTQTRFHLPVWLGVGAAFKSAIDKDIKNFQVLKDMYNEWPFFRVTL
DLLEMVFAKGDPGIAALYDKLLVADELKPFGEQLRSKYLETEDLLLKIAGHSEILAGDPYLKQRLRLRDP
YITTLNVCQAYTLKQIRDPNFKVTTNPPLNKEPADLPADLVKLNPASEYAPGLEDTLIITMKGI
AAGMQNTG

NADP-malate dehydrogenase (NADP-MDH)
>g7737.t1
MAKEPMRVLVTGAAGQIGYALVPMIARGIMLGVDQPVILHMLDIPPAAEALNGVKMELVDAAFPLLKGVV
ATTDVVEACTGVNVAVMVGGFPRKEGMERKDVMSKNVSIYKAQASALEAHAAPNCKVLVVANPANTNALI
LKEFAPSIPEKNITCLTRLDHNRALGQISERLNVQVLVVANPANTNALILKEFAPSIPEKNITC
LTRLDHNRALGQISERLNVQVSDVKNVIIWGNHSSTQYPDVNHATVKTASGEKPVRELVADDEWLNGEFI
KTVQQRGAAIIKARKLSSALSAASSACDHIRDWVLGTPEGTFVSMGVYSDGSYGVPAGLIYSFPVTCSGG
EWKIVQGLPIDELSRQKMDATAQELSEEKTLAYSCLE
>g54516.t1
MGKVKYVVELARALAATTGVHRVDLLTRQITSPEVDWTYGEPVEMITRASDVSDDGGDSAGAYIVRLPCG
PRDKYIPKESLWPHIPEFVDRALAHITNVARALSEELLGSGGNGVDQPPQPVWPC
>g157580.t1
MSGKKAGDTSGDGVMIQRVIREVSSGVSYPVLTKTNYSDWALLIKVKLKATALWNVIENDGADAQEEMMA
LDALCSVVPPEMVPTIAKKETAKEARDAIATMRIGDDRVKRSMA
>g47863.t1
MLNYAVEYRGVKTKEEKGMEQIEEQRVIEAQIRMRQQELQDDAERMKNKQTAVSMDTVATAHVEHCDTGV
TSTTGLYIVCCPF
>g23958.t1
MAAAAIATLSFISSVPRKKLLGHGASTSNPQQLSGCNPHWHSVISLKPRTPSRSRAATRAIVARAGGTYK
VAVLGAAGAVGQPLSLLIKMSPLVSTLHLYDVSDSDVKGVAADLGHCNTPARVAGFSGAAELASCLAGAD
LVVIIAAAGGDYTSNPSATTAPDDLTFAAAAGVVRELAEAVADHAPAGALLLVVAGEVVDSAVP
VAAETLKRKGAYDPRRLLGVTTLDVVRANTLVAGRKGLPLADVDVPVVGGPAGPAALPLLSKARPKAAFT
EEEVEVITARVRERAGTDEAPLSAAYAAARLVEAALRGLGGEGGVYECAYVQSQVVPELPFFACRVRLGK
EGVEEVVGSELRGVSDYEARALEELKPLLKASIDRGVAYVQQQPTEAALN
>g26130.t1
MASAAIATLSFISTVPRKKQLRASISNAQQFSGSNTHWHSVTSLKPRTPASAPTSRSRAATRAIVAQAGG
TYKVAILGAAGAVGQPLSLLIKMSPLVSTLHLYDVSDSDVKGVAADLGHCNTPALVAGFSGAAELASCLA
GADLVVVIAAGGGGDDPINPSATTAPDDLAFAASADVVRELAEAVADHAPAGALVLVVAGEVVD
SAVPVAAETMKRKGAYDPRRLLGVTTLDVVRANTLVAGRKGLPLADVDVPVVGGHAGPAALPLLSKARPK
AAFTEEEVQEITARVRERAGAPLAAAYAAARLVEAALRGMGGEGGVYECAYVQSQVVPELPFFACRVRLG
REGVEEVVGSELRGISDYEARAMEELKPLLKASIDRGVAYVQQQPTEAALN
>g44889.t1
MAKEPMRVLVTGAAGQIGYALVPMIARGIMLGADQPVILHMLDIPPAAEALNGVKMELVDAAFPLLKGVV
ATTDVVEACTGVNVAVMVGGFPRKEGMERKDVMSKNVSIYKAQASALEAHAAPNCKVLVVANPANTNALI
LKEFAPSIPEKNITCLTRLDHNRALGQISERLNVQVSDVKNVIIWGNHSSTQYPDVNHATVKTA
SGEKPVRELVADDEWLNGEFIKTVQQRGAAIIKARKLSSALSAASSACDHIRDWVLGTPEGTYVSMGVYS
DGSYGVPAGLIYSFPVTCSGGEWKIVQAICLGIMVTPMHNDLNNKQLATAGTVASQRPELPHPPSPRRGS
RHKTGVCTPNTGKDERGSTCLPAAKRGKTASGEKPVRELVADDEWLNGEFIKTVQQRGAAIIKA

RKLSSALSAASSACDHIRDWVLGTPEGTYVSMGVYSDGSYGVPAGLIYSFPVTCSGGEWKIVQAICLGIM
VTPMHNDLNNKQLATAGTVASQRPELPHPPSPRRGSRHKTGVCTYARTARRVSVRCAADGLVSDAN
>g48942.t1
MEVMELEEADAISPSSEARMASTVSISSVTGQAVLIPKARSHGVTSYAGLKASSSVSSVSQSAFLGRNVS
LRASVAPRIVPKAKSGSQISPQASYKVAVLGAAGGIGQPLGLLIKMSPLVSELHLYDIANVKGVAADLSH
CNTPAQVLDFTGPSELANCLKGVDVVVIPAGVPRKPGMTRDDLFNINAGIVKTLVEAVADNCPE
AFIHIISNPVNSTVPIAAEVLKQKGVYNPKKLFGVTTLDVVRANTFVAQKKNLKLIDVDVPVVGGHAGIT
ILPLLSKTRPSVTFTDEETEELTKRIQNAGTEVVEAKAGAGSATLSMAYAAARFVESSLRALAGDADVYE
CTFIQSEVTELPFFASRVKLGKNGVESIISADLEGVTEYEAKALEALKPELKASIEKGIAFAHK
PKEAAASV
>g49420.t1
MSLPPATSPSFARPVSTVAIVGAGSVGATLAYAALVRGAARRVVLYDVNAAKVKAEAADIGHGIEFISQA
TVEGSDDIEICRGADIVVFTAGAKQHPGQSRMELAATTVGLVKKVLPTLVDIAPRAVHMMVTNPVDVVTY
AAQKITGLPPNQIFGSGTVLDSSRLRFLISRECGVAVQNVHAYVLGEHGDSEVPMWSSASIGGV
PLLEWLGPDGSPMFTEGVRDRLGHDVVHAAYSIIEGKGATNYAVGLAVTRIIEAILRNEHRVLPVSTRID
DDFLGISDVCLSMPTIVDAMGARERLGLPMSQREMEMLRESADHIRDAARTLGF
>g53752.t1
MRPILKSASQLLRRRNYSSAANPERKVAILGAAGGIGQPLALLMKLNPLVSSLSLYDIAGTPGVAADCSH
INSPALAKGFMGDDQLGEALEGSDVVIIPAGVPRKPGMTRDDLFNINAGIVKALCTAIAKHCPNALVNMI
SNPVNSTVPIAAEVFKKAGTYDEKKLFGVTTLDVVRARTFYAGKAGVPVNEVNVPVVGGHAGIT
ILPLFSQATPASNSLSDEDIKALTKRTQDGGTEVVEAKAGKGSATLSMAYAGAVFADACLKGLNGAPDIV
ECSYVQSTVTELPFFASKVRIGKNGVEEVLGLGELSAFEKEGLEKLKGELKSSIEKGIKFAQSN
>g58461.t1
MELEEADAISLSSEARMASTVSISSVTGQAVLIPKARSHGVTSYAGLKASSSVSSVSQSAFLGRNVSLRA
SVAPRIVPKAKSGSQISPQASYKVAVLGAAGGIGQPLGLLIKMSPLVSELHLYDIANVKGVAADLSHCNT
PAQVLDFTGPSELANCLKGVDVVVIPAGVPRKPGMTRDDLFNINAGIVKTLVEAVADNCPEAFI
HIISNPVNSTVPIAAEVLKQKGVYNPKKLFGVTTLDVVRANTFVAQKKNLKLIDVDVPVVGGHAGITILP
LLSKTRPSVTFTDEETEELTKRIQNAGTEVVEAKAGAGSATLSMAYAAARFVESTLRALAGDPDVYECTF
IQSEITELPFFASRVKLGKNGVESIISADLEGVTEYEAKALEALKPELKASIEKGIAFAHKPQE
AAASV
>g67107.t1
MTRDDLFNINAGIVKNLATAIAKYCPNALVNMISNPVNSTVPIAAKVFKKAGTYDEKRLFGVTTLDVVRA
KTFYAGKANVPVTDVNVPVVGGHAGITILPLFSQATPATNALSHEDIKALTKRTQDGGTEVVEAKAGKGS
ATLSMAYAGAVFADACLKGLNGVPDIVECSYVQSTVTELPFFASKVKLGKNGVEEVLGLGELSD
FEKEGLENLKGELKSSIEKGIKFANEN
>g79345.t1
MLDLQHAAAFLPRTRLVSDTDIAAVTKGSDLAIVTAGARQIPVETRLNLLQRNVALFRKIIPALAENSPD
ALLLIVSNPVDILTYVAWKLSGFPVSRVIGSGTNLDSSRFRFLLDVNAQDVQAYMVGEHGDSSVAVWSSV
SIAGMPVLKSLQQSHATASSASDASMISKEKKEEALEGR
>g79346.t1
MKKAASLSELGFDSDGASSGFFRAVADGVAACTPTAHRQRLTKVSVIGVGNVGMAIAQTILTRDLADEIA
LVDALPDKLRGEMLDLQHAAAFLPRTRLVSDTDMAVTKGSDLAIVTAGARQIPGETRLNLLQRNVSLFRK
IIPALAEHSPEALLLIVSNPVDILTYVAWKLSGFPASRVIGSGTNLDSSRFRFLLAEHLDVNAQ
DVQAYMVGEHGDSSVAVWSSVSVAGMPALKTLQQSHSSFNEDALEGIRKAVVDSAYEVISLKGYTSWAIG
YSVANLVATLLRDQRRIHPVSVLATGFHGIADDHEVFLSLPARLGRGGVLGVAEMELTEEEAKRLRRSAK
TLWENCQLLGL
>g92876.t1
MKVAVLGAAGGIGQALALLLKTQLPAGSALSLYDIAPVTPGVAVDLSHIPTAVTIEGFSGEDATPALKGA
DVVLISAGVARKPGMDRADLFNVNAGIVRNLIEQVAATXAVTIEGFSGEDATPALKGADVVLISAGVARK
PGMDRADLFNVNAGIVRNLIEQVAATAPKALIGVITNPVNTTVAIAAEVLKKHGVYDKNRLFGV
TTLDIIRANTFVAALKGKQPDQVEVPVIGGHSGVTILPLLSQVKGVSFSDQEVKGVSFSDQEVADLTKRI
QNAGTEVVEAKAGGGSATLSMGQAAARFGLSLVRALNGEANVVECAYVEGEGEHARFFSQPLLLGKNGIA
ERKPIGTLSAYEQQALSGMLETLKKDIEQGEAFVKQ
>g96607.t1
MAYLLLFVGGDPGQIVRNPSTPKQYQKFIMLPNSVFQRLVKWCPWLLRVRDEPKQPVKVLVTGAAGQIGY
AIVPMIARGLMLGPDQPVVLHMLDLPRSADALKGVRMELIDAALPLLRGVVVTSDEAEAFKGVNFAVLIG
GWPRKEGMERNDLIAKNVTIYRSQASALQKHAAPNCKVLVVANPANTNALVLKEFASAIPTKNI
TCLTRLDHNRALGQISEKLGVHVGDVKNAIVWGNHSSTQFPDASHATVRTEQGEKPVRELVADEKWLREE
FVSIVQQRGAAVIKARKQSSSLSAASSACDHMRDWILGTPKGSWVSMGVHSDGSYGVPEGIFYSFPVTCE
KGEWAIVQGLQVDEFARSKMELSVNELEEEKVIAYELVSS

>g97529.t1
MTAVRAAAVLAAAAALALLLPGWAAAEWKLTKKGSVVTYDERSLLIDGKRDLFFSGAIHYPRSPPEMWPK
LMKLAKEGGLNTIETYVFWNAHEPEPGKFNFEGRLDLIKFVKLIQEHDMYAVVRIGPFIQAEWNHGGLPY
WLREIDRIIFRANNDPFKKEMEKFVRFIVQKLKDAELFASQGGPIILSQIENEYGNIKKDHVIE
GDKYLEWAAQMALSTQTGVPWAHTFELPEDNLCLSFLSNNNTGEDGTVVFRGEKLYIPSRSVSILAGCKN
VVYNTKRVLVVANPANTNALILKEFAPSIPEKNITCLTRLDHNRALGQISERLNVQVSDVKNVIIWGNHS
STQYPDVNHATVKTASGEKPVRELVADDEWLNGEFIKTVQQRGAAIIKARKLSSRENSHLRNWV
MGTHL
>g101033.t1
MEQQQQHAAARRMAALASHLRPHPTSQEVSLLRGSNCRAKGAAPGFKVAILGASGGIGQPLALLMKMNPL
VSVLHLYDVVNTPGVTADISHMNTGAVVRGFVGQPQLEDALTGMDLVIIPAGVPRKPGMTRDDLFNINAG
IVRTLCEGIAKCCPKAIVNVISNPVNSTVPIAAEYRHGEFLNIPLHFSVYREKFLGLTRERSVF
L
>g104616.t1
MNTKARKVMIIGTGNVGASAAYALLNQNICEELILVDVNAPRVEGHAQDLADAAAFMPGMMSISTRSIEA
CADVDIAVITVSGGALKPGQTRLDELGNTARIVKNIVPTMMANGFNGIFLVATNPCDIITWQVWSLSGLP
RNQVIGTGVWLDTTRLRRLLAQALDIGAQSIDAFIMGEHGDTQFPVWSHSSIYGSPIADVYQQK
TGQPLDADALADRVRRHGFEIFARKGCTEYGIAGTIAEICRNIFTGSHRALAISCILDGEYGAHNVAIGV
PAVLTHSGVEQIIELHLTEDEVAKFNHSVEVIKANIARLP
>g109848.t1
MTPPVTLTLSGAAGQIGYALLFRVASGEMFGPDTPVRLRMLEVEGALAAADGVALELQDVASPLLTDVEV
TADPVRAFDGVDVALLVGARPRTAGMERADLLRANGAIFGPQGRALNEHAADGVRVLVVGNPANTNALIT
RQHAPDVPADRFTALTRLDHNRAVAQVALRAGVRTDEVRRVAIWGNHSRTQYPDLRHAVVAGRP
ALEVLPADWVEGEFVDTVAQRGAAIIAARGGSSVASTATAIIDQVRLRREGVPEGDWTSTGLVSDGSYGV
PAGLVCSFPVTARDAAWEIVPGLSLDAFSRARIDASVAELVQEREAVRALGLVPTLRSGSEFES
>g114780.t1
MTMKKPVRVAVTGAAGQIGYALLFRIASGEMLGKDQPVILQLLELPIDKAQAALKGVIMELEDCAFPLLA
GVIGTDDAEVAFKDADIALLVGARPRGPGMERKDLLLENAKIFTAQGAALNKVASRDVKVLVVGNPANTN
AYIAMKSAPDLNPRNFTAMLRLDHNRALSQLATKADVAVGDIEGLIVWGNHSPTMYPDYRFXXX
XXXVWGNHSPTMYPDYRFATANGVNLKDKVNDADWNANTFIPQVGKRGAAIIEARGLSSAASAANAAIDH
IRDWVLGSNGKWVTMGVPSDGSYGIPEGVIFGFAVTTQNGEYTLVKDIEVDDFSQKAIDKTLAELEEERS
GVAHLLA
>g117654.t1
MEQQQQHAAARRMAALASHLRPHPTSQEVSLLTGSNCRAKGAAPGFKVAILGASGGIGQPLALLMKMNPL
VSVLHLYDVVNTPGVTADISHMNTGAVVRGFVGQPQLEDALTGMDVVIIPAGVPRKPGMTRDDLFNINAG
IVRILCEGIAKCCPKAIVNVISNPVNSTVPIAAEVFKKAGTYDPKRLLGVTTLDVVRANTFVGE
VLGLDPREISVPVVGGHAGVTILPLLSQVNPPCSFTSEEVSYLTSRIQNGGTEVVEAKAGAGSATLSMAY
AAAKFADACLKGLRGDVGIVECSYVASQVTELPFFASKVRLGRYGIEEILPLGPLNEFERAGLEKAKREL
AESIQKGVSFVNK
>g119734.t1
MATPASNSLSDEDIKALTKRTQDGGTEVVEAKAGKGSATLSMAYAGAVFADACLKGLNGAPDIVECSYVQ
STVTELPFFASKVRLGKNGVEEVLGLGELSAFEKEGLEKLKGELKSSIEKGIKFAQSN
>g124977.t1
MVPVAAAGQIGYAIVPMIARGLMLGPDQPVVLHMLDLPRSADALNGVRMELIDAALPLLRGVVVTSDEAE
AFKGVNFAVLIGGWPRKEGMERNDLIAKNVTIYRSQASALQKHAAPNCKVLVVANPANTNALVLKEFASA
IPAKNITCLTRLDHNRALGQISEKLGVHVGDVKNAIVWGNHSSTQFPDASHATVKTEQGEKPVR
ELVTDEKWLREEFVSIVQQRGAAVIKARKQSSSLSAASAACDHMRDWILGTPKGTWVSMGVYSDGSYGVP
EGIFYSFPVTCEKGEWTIVQGLQIDEFARSKMELSANELEEEKAIAYELVSS
>g153703.t1
MARKKIALIGAGNIGGTLAHLAALKGLGDIVLFDVVEGVPQGKALDLSQCGPVEGFDANIKGSNDYADIA
GADVIIVTAGVARKPGMSRDDLLGINLKVMKAVGEGIRDNAPDAFVICITNPLDAMVWALREFSGLPANK
VVGMAGVLDSARFSHFLAEEFGVSVKDVNTFVLGGHGDTMVPVLEYSTVSGIPKRILPAAAHLS
GEYGIDNLYVGVPVVIGAGGVEKVVEVKLSDEAKANLQVSVDAVKELLVACKGXXXXXXXXQGHRREPGV
I
>g153997.t1
MKKACSLSELGFDADSASSGLFRAVAANGGDHHHDSSGTSMHRLTKVSVIGAGNVGMAIAQTILTRGLAD
EIALVDALPDKLRGEMLDLQHAAAFLPRTRLVSDTDIAAVTRGSDLAIVTAGARQIPGETRLNLLQRNVS
LFRKIIPALAQNSPEALLLVVSNPVDILTYVAWKLSGFPVSRVIGSGTNLDSSRFRFLLAEHLD
VNAQDVQAYMVGEHGDSSVAVWSSVSVAGMPVLKTLQDSHTTSASASMIMKEEALEGIRRAVVNSAYEVI
GLKGYTSWAIGYSVANLVATLLRDQRRIHPVSVLATGFHGIADDHEVFLSLPARLGRAGVLGVVDMELTH

EETKRLRHSAKTIWENCNQLGL
>g153998.t1
MKKAASLSELGFDSDGASSGFFRPVSDGVAACTPTAHRQRLTKVSVIGVGNVGMAIAQTILTRDLADEIA
LVDALPDKLRGEMLDLQHAAAFLPRTRLVSDTDMAVTKGSDLAIVTAGARQIPGETRLNLLQRNVSLFRK
IIPALAEHSPEALLLIVSNPVDILTYVAWKLSGFPASRVIGSGTNLDSSRFRFLLAEHLDVNAQ
DVQAYMVGEHGDSSVAVWSSVSVAGMPALKTLQQSHSSFNEDALEGIRKAVVDSAYEVISLKGYTSWAIG
YSVANLVATLLRDQRRIHPVSVLATGFHGIADDHEVFLSLPARLGRGGVLGVAEMELTEEEAKRLRRSAK
TLWENCNLLGL
>g155412.t1
MFARDGFRSAVPGVDVNVPVVGGHAGITILPLFSQATPATNALSHEDIKALTKRTQDGGTEVVEAKAGKG
SATLSMAYAGAVFADACLKGLNGVPDIVECSYVQSTVTELPFFASKGDCVADVGPERSPGQSKPSSSQNH
FISLSKSTVSPMSKSLARKKSGPSAVAPPAAKLAPNAKSSALLIAWLGARLRLACSVGWSPQKL
PDSGI
>g160502.t1
MQPDAGSPAHRIARIAAHLNPQMEESASSALRPAACRAKGGAPGFKMAILGAAGGIGQPLSMLMKMNPLV
SVLHLYDVVNTPGVTADISHMDTSTVVRGFLGSQQLDAALTGMDLVIIPAGLPRKPGMTRDDLFNKNAGI
VRVLCEGVARCCPDAIVNLISNPVNSTVPIAAEVFKKAGTYNPKRLLGVTTLDVVRANTFVAEV
LGVDPRDVSVPVVGGHAGVTILPLLSQVTPPCSFTPDEIKYLTNRIQNGGTEVVEAKAGAGSATLSMAFA
AAKFGDACLRAMKGEAGIVECSYVASQVTELPFFATKVRMGLEMAKKELNESIQKGIAFMNK
>g170179.t1
MLPDAGSPADRIARIAAHLNPQMEEGASSALRPAACRGKGGAPGFKVAILGAAGGIGQPLSMLMKMNPLV
SVLHLYDVVNTPGVTADISHMDTSTVVRGFLGPQQLDAALTGMDLVIIPAGLPRKPGMTRDDLFNKNAGI
VRVLCEGVARCCPDTIVNLISNPVNSTVPIAAEVFKKAGTYNPKRLLGVTTLDVVRANTFVAEV
LGVDPRDVSVPVVGGHAGVTILPLLSQVTPPCSFTLDEIKYLTNRIQNGGTEVVEAKAGAGSATLSMAFA
AAKFGDACLRAMKGEAGIVECSYVASQVTELPFFATKVRMGLEMAKKELNESIQKGIAFMNK
>g171764.t1
MRPMLKSASQLLRRRNYSSAANPERKVAILGAAGGIGQPLALLMKLNPLVSSLSLYDIAGTPGVAADCSH
INSPALAKGFMGDDQLGEALEGSDVVIIPAGVPRKPGMTRDDLFNINAGIVKALCTAIAKHCPNVSVALL
PL

NADP-malic enzyme (NADP-ME)
>g2007.t1
MELDYESKRPLYIPYAGPILLEFPLLNKGSAFTLEERNEFNLNGLLPEAVESIEEQAKRAWRQFQDFKNN
NDKHVYLRNIQDTNETLFYRLLDNHLEEMMPIIYTPTVGAACEHFSEIYRRARGVFISYPNRDRIEDMLQ
NATKQNVKVIVVTDGERILGLGDQGIGGMGIPIGKLSLYTACGGISPAYTLPVVLDVGTNNQQL
LNDPLYMGWRHPRITGEEYDAFVNEFIQAVKRRWPKVLLQFEDFAQKNAMPLLNRYREEVCCFNDDIQGT
AAVTVGTLIAASRAAGSRLCEQKVVFLGAGSAGCGIAEQIIAQMKSEGLSDDEARSRVMMVDRFGLLTDK
LPNLLDFQSKLVQKSENLQNWDVASDAISLLDVVRNAQPNILIGVSGQPGLFTEEIIREMHKHC
KRPIVMPLSNPTSRVEATPADIIAWTDGAALVATGSPFSPVSWNGKTYPIAQCNNSXFRSAGSVHRRDHP
>g4203.t1
MSSEMEMAGGGVEDAYGEDRATEEQLVTPWSFSVASGYTLLRDPRHNKGLAFSEAERDAHYLRGLLPPAY
ASQELQEKKIMHNLRQYTVPLQRYIAMMDLQERNERLFYKLLIDNVEELLPVVYTPTVGEACQKYGSIYR
RPQGLYISLKDKGKILEVLKNWPERSIQVIVVTDGERILGLGDLGCQGMGIPVGKLSLYTALGG
VRPSACLPITIDVGTNNETLLNDEFYIGLKQRRATGEEYHELLEEFMTAVKQNYGEKVLIQFEDFANHNA
FDLLAKYSKSHLVFNDDIQAERDAHYLRGLLPPAYASQELQEKKIMHNLRQYTVPLQRYIAMMDLQERNE
RLFYKLLIDNVEELLPVVYTPTVGEACQKYGSIYRRPQGLYISLKDKGKILEVLKNWPERSIQV
IVVTDGERILGLGDLGCQGMGIPVGKLSLYTALGGVRPSACLPITIDVGTNNETLLNDEFYIGLKQRRAT
GEEYHELLEEFMTAVKQNYGEKVLIQFEDFANHNAFDLLAKYSKSHLVFNDDIQGTASVVLAGLLAALKV
VGGTLADHTYLFLGAGEAGTGIADLIALEMSKHTEMPIDECRKKIWLVDSKGLIVESRKESLQH
FKKPWAHDHEPLKTLLEAVESIKPTVLIGTSGVGRTFTKEVIEAMASFNEKPVIFSLSNPTSHSECTAEE
AYTWSQGRAVFASGSPFDPVEYEGKVYVPGQSNNAYIFPGFGLGVVISGAIRVHDDMLLAASEALAEQVT
EEHFGKGLIFPPFTNIRGISARIAAKVAAKAYELGLASRLPRPDDLVKYAESCMYTPAYRSYR
>g12712.t1
MASLSRNLRRSLAADRVRRLLAPAPRGYVTAECHRPVVLHKRGPDILHDPWYNRGTGFSMTERDRLGLRG
LLPPSVISSQQQIDRFMLDLHRLQKYAKDGPSDTYPLAKWRILNRLHDRNETMYYKVDMIVVTDGSRILG
LGDLGVHGIGIAIGKLDLYVSAAGINPQRVDMIVVTDGSRILGLGDLGVHGIGIAIGKLDLYVS
AAGINPQRVLPVMIDVGTNNEKLLKDPLSYENGLAFYSVLGDEDSIAFLDS
>g73306.t1
MHVEEFDNFMESEDNMVFNDYDERSKFTAIQDREFMFVRKFDHNFLEQIGVLADFEYALNTAGWTLA
>g14619.t1

MELEYESKRPLYIPYAGPILLEFPLLNKGSAFSIEERNEFNLNGLLPEAVETIEEQAKRAWRQFQDFKNN
NDKHVYLRNIQDTNETLFYRLLDNHLEEMMPIIYTPTVGAACEHFSEIYRRARGVFISYPNREHIEDMLQ
NATKQNVKVIVVTDGERILGLGDQGIGGMGIPIGKLSLYTACGGISPAYTLPVVLDVGTNNQQL
LNDPLYMGWRHPRITGEEYDEFVNEFIQAVKRRWPKVLLQFEDFAQKNAMPLLERYRDEVCCFNDDIQGT
AAVTVGTLIAASRAAGSRLCEQKVVFLGAGSAGCGIAEQIIAQMKSEGLSDEEARSRVMMVDRFGLLTDK
LPNLLDFQSKLVQKSDNLKDWDTASDAISLLDVVRNAKPDILIGVSGQPGLFTEEIIREMHKHC
KRPIVMPLSNPTSRVEATPADIIAWTDGAALVATGSPFSPE
>g16331.t1
MSVTNVFDEMSEDKLTEHKVALSESASGMVARWDRAASREGVAGKGAAMRARMEAWTVRLGELAAGVGSA
RGRGHQWQHWEAGSARRARSGAGEREAEAARSLRWCIGRRWASSAGETMESTMKEIRGDGAPCVLDMDDA
ATVGGGVEDTYGEDRATEEQLVTPWTVSVASGYNLLRDPRYNKGLAFTERERETHYLRGLLPPQ
IVTQELQERKIMNNIRQYQLPLQKYMALMDLQEGNERLFYKLLIDNVEELLPIVYTPTVGEACQKYGSIF
SRPQGLYISLKEKGKILEVLKNWPERSIQVIVVTDGERILGLGDLGCQGMGIPVGKLALYTALGGVRPSA
EYAEFLQEFMSAVKQNYGEKVLIQFEDFANHNAFDLLAKYGTTHLVFNDDIQGTASVVLAGLVA
AQKLISGTLADHKYLFLGAGEAGTGIAELIALEISRQTKAPIEECRKKIWLVDSKGLIVSSRKESLQHFK
KPWAHDHEPVGSLLDAVHAIKPTVLIGTSGKGQTFTQDVVEAMSSFNEKPIILALSNPTSQSECTAEQAY
SWSKGRAVFATGSPFDPVEYDGKIHVPGQANNAYIFPGFGLGVVMSGAIRVHDNMLLAASEALA
QQVTEENFEKGLIYPPFSNIRKISAHIAANVAAKAYELGQHPTCIFVI
>g22250.t1
MRFRLSPELPRRWAQDTAFPMTERDRLGLRGLLPPRVMSFEQQYERFIDSFRSLENNTRGEPEAIVALAK
WRILNRLHDRNETLYYRVLIDNIKDFAPIIYTPTVGLVCENYSGLFRRPRGMYFSAKDKGEMMSMIYNWP
QEKVDMIVVTDGSRILGLGDLGVQVIGIPIGKLDVYVAAAGINPQKFEDFQMKWAFETLQRYRH
RFCMFNDDVQGLITKDRKGLDPAVARFARGHGPDEIEDLHEGASLVEVFGTDHQVKKVKPHVLLGLSGVG
GIFHEEVLKAMKESDSPRPAIFAMSNPTTKAECTPDHVFKYVGENAIFASGSPFTNVSLGNGRTGYANQA
NNMYLFPGIGLGALLSGARHISDGMLQGAAECLASYITDDEIRKGVLFPSVSRHITARVGAAVV
RAAVAEDLAESSCDVGPKELGSMSEVLY
>g22500.t1
MLRSAVARRSRELRRLLSSSAAAAGAAVPGPCIVHKRGNDILNDPWYNKDTAFPLTERDRLGLRGLLPPR
VMTFKEQYERFMNSFKSLENNTRGEPDSIVSLAKWRILNRLHDRNETLYYRVLIDNIKDFAPIIYTPTVG
LVCENYSGLFRRPRGMYFSAKDKGEMMSMIYNWPQENVDMIVVTDGSRILGLGDLGVQGIGIPI
GKLDLYVAAAGHGN
>g22584.t1
MMPVIYTPTVGWACERFSDIYRRARGVFISWQNRGSMDDILQNVPNHNIKVIVVTDGERILGLGDQGIGG
MGIPIGKLSLYTSCGGISPAYTLPVVLDVGTNNQQLLNDPLYMGSRHPRITGDEYYAFVDEFIQAVKQRW
PDVLLQARRRSPSVR
>g72403.t1
MRFRLSPELSRRWAQDTAFPMTERDRLGLRGLLPPRVMSFEQQYERFIDSFRSLENNTRGEPDAIVALAK
WRILNRLHDRNETLYYRVLIDNIKDFAPIIYTPTVGLVCENYSGLFRRPRGMYFSAKDKGEMMSMIYNWP
QEKVDMIVVTDGSRILGLGDLGVQGIGIPIGKLDVYVAAAGINPQKVLPVMLDVGTNNQKLLED
KLYLGLRQPRLEGEEYLSVVDEFMEAVRARWPKAVVQFEDFQMKWAFETLQRYRHRFCMFNDDVQGLITK
DRKGLDPAVARFARGHGPDEIEDLHEGASLVEVFGTDHQVKKVKPHVLLGLSGVGGIFHEEVLKAMKESD
SPRPAIFAMSNPTTKAECTPDHVFKYVGENAIFASGSPFTNVSLGKVLKAMKESDSPRPAIFAM
SNPTTKAECTPDDVFKYVGENAIFASGSPFTNVSLGNGRTGYANQANNMYLFPGIGLGALLSGARHISDG
MLQGAAECLASYITDDEIRKGILFPSVSRHITARVGAAVVRAAVAEDLAEGSCDVGPRELXITARVGAAV
VRAAVAEDLAEGSCDVGPKELGSMSEVLY
>g74021.t1
MLSAHAATASASPVSLWKRGGSEGGSCDGCRSCRDVVRKRAAAVRVRATAPRRVEAVAMGSAAETEKEEV
EVEVVATEKEELAAAGGGVKDPYGEDRATEDLPVTPWAFSVASGYTLLRDPHHNKGLAFTEKERDAHYLR
GLLPPGVVSQETQIKKFMHNLRQYQLPLQRYMAMMDLQERNERLFYKLLIDNVEELLPVVYTPT
VGEACQKYGSIFRQPQGLYISLKDKGRVLEVLRNWPQRNIQVIVVTDGNGNSCG
>g95382.t1
MASLSRNLWRSLAVDRVRRLLAPAPRGYVTAECHRPVVLHKRGPDILHDPWYNRVRTPASPPLALSPPPF
ARGGCSNVASACRRWRVLQGTGFSMTERDRLGLRGLLPPSVISSQQQIDRFMLDLHRLQKYAKDGPSDTY
PLAKWRILNRLHDRNETMYYKVDMIVVTDGSRILGLGDLGVHGIGIAIGKLDLYVSAAGINPQR
VLPVMIDVGTNNEKLLKDPLCSSSYD
>g107840.t1
MSSEMEMAGGGVEDAYGEDRATEEQLVTPWSFSVASKKLATTMDIFLIVSSITLPVSTSDALKQSKERNR
NKARCSQFSNQNLFFFLLLLVVVVVGTNSGYTLLRDPRHNKGLAFSEAERDAHYLRGLLPPAYASQELQE
KKLMHNLRQYTVPLQRYIAMMDLQERNERLFYKLLIDNVEELLPVVYTPTVGEACQKYGSIYRR
PQGLYISLKDKGKILEVLKNWPERSIQVIVVTDGERILGLGDLGCQGMGIPVGKLSLYTALGGVRPSACL

PITIDVGTNNETLLNDEFYIGLKQRRATGEEYHELLEEFMTAVKQNYGEKVLIQFEDFANHNAFDLLAKY
SKSHLVFNDDIQGTASVVLAGLLAALKVVGGTLADHTYLFLGAGEAGTGIADLIALEMSKHNEM
PIDECRKKIWLVDSKGLAFSEAERDAHYLRGLLPPAYASQELQEKKLMHNLRQYTVPLQRYIAMMDLQER
NERLFYKLLIDNVEELLPVVYTPTVGEACQKYGSIYRRPQGLYISLKDKGKILEVLKNWPERSIQVIVVT
DGERILGLGDLGCQGMGIPVGKLSLYTALGGVRPSACLPITIDVGTNNETLLNDEFYIGLKQRR
ATGEEYHELLEEFMTAVKQNYGEKVLIQFEDFANHNAFDLLAKYSKSHLVFNDDIQGTASVVLAGLLAAL
KVVGGTLADHTYLFLGAGEAGTGIADLIALEMSKHNEMPIDECRKKIWLVDSKGLIVESRKESLQHFKKP
WAHDHEPLKTLLEAVESIKPTVLIGTSGVGRTFTKEVIEAMASFNEKPVIFSLSNPTSHSECTA
EEAYTWSQGRAVFASGSPFDPVEYEGKVYVPGQSNNAYIFPGFGLGVVISGAIRVHDDMLLAASEALAEQ
VTEEHFGKGLIFPPFTNIRGISARIAAKVAAKAYELGLASRLPRPDDLVKYAESCMYTPAYRSYR
>g124469.t1
MESTMKGIRGDGAPCVLDMDDAATVGGGVEDTYGEDRATEEQLVTPWTVSVASGYNLLRDPRYNKGLAFT
ERERETHYLRGLLPPQIVTQELQERKIMNNIRQYQLPLQKYMALMDLQEGNERLFYKLLIDNVEELLPIV
YTPTVGEACQKYGSIFSRPQGLYISLKEKGKILEVLKNWPERSIQVIVVTDGERILGLGDLGCQ
GMGIPVGKLALYTALGGVRPSACLPITLDVGTNNEELLNDEFYIGLRQRRATGQEYAEFLQEFMSAVKQN
YGEKVLIQFEDFANHNAFDLLAKYGTTHLVFNDDIQGTASVVLAGLVAAQKLISGTLADHKYLFLGAGEA
GTGIAELIALEISRQTKAPIEECREKIWLVDSKFEDFANHNAFDLLAKYGTTHLVFNDDIQGTA
SVVLAGLVAAQKLISGTLADHKYLFLGAGEAGTGIAELIALEISRQTKAPIEECREKIWLVDSKAIKPTV
LIGTSGKGQTFTQDVVEAMSSFNEKPIILALSNPTSQSECTAEQAYTWSKGRAVFATGSPFDPVEYDGKI
HVPGQANNAYIFPGFGLGVVMSGAIRVHDDMLLAACLATRRPRPKDLVKYAESCMYSPVYRNYR
>g136528.t1
MAGSGEEKNGGVAAVSGGVEDAYGEDRATEDQPVTPWAVCVASGHSLLRDPRHNKGLSFTEKERDAHYLR
GLLPPIVLYQELQEKRLLQNVRQFEVPLQRYMALMDLQARTLISSDAYTFILLNDPSGLQERNERLFYKL
LVDNVEELLPVVYTPTVGEACQKYGSIFRRPQGLYISLKEKGRILEVLRNWPEKSIQVIVVTDG
ERILGLGDLGCQGMGIPVGKLALYSALGGVRPSANYGQKVLVQFEDFANHNAFALLEKYRENHLVFNDDI
QGTAAVVLAGLIAALKSVGGSLGDHTFLFFGAGEAGTGIAELVALAISRQSKISIDEARNKIWLVDSKGL
VVRSREGTLQPFKKRYAHEHEPIKDLLGAVKAIRPTALIGSAGVARSFTKEVVEAMSSINKRPI
ILALSNPTSQSECTAEQAYSWSQGHAIFGSGSPFDPVKYNNKVFVPAQAGTGIAELVALAISRQSKFSID
EARNKIWLVDSKGLVVRSREGTLQPFKKRYAHEHEPIKDLLGAVKAIRPTALIGSAGVARSFTKEVVEAM
SSINKRPIILALSNPTSQSECTAEQAYSWSQGHAIFGSGSPFDPVKYNNKVFVPAQAGTGIAEL
VALAISRQSKFSIDEARNKIWLVDSKGLVVRSREGTLQPFKKRYAHEHEPIKDLLGAVKAIRPTALIGSA
GVARSFTKEVVEAMSSINKRPIILALSNPTSQSECTAEQAYSWSQGHAIFGSGSPFDPVKYNNKVFVPAQ
ANNAYIFPGFGLGVVIAGAIRVTDDMVLAAAEGLAEQVTLEHIDKGLIYPPFSIIRKISANIAA
RVAEKAYDLGLASHLPRPKDLVKYAESCMYSPIYRSYR
>g136530.t1
MAGSGERNGADMPVGVVTGGVEDAYGEDRATVDQPIITPWAVCIASGRSLLRDPRHNKGLAFTEKERDAH
YLRGLLPPAVLSQELQEKRVLQNLRQFKVPLQRYMALIDLQERNERLFYKLLIDNVEELLPIVYTPTEGE
ACQKGRVLETLRNWPEKNIQVIVVTDGERILGLGDLGCQGMGIPVGKLALYTALGGVRPSACLP
VTIDVGTNNEDLLKDEFYIGLRQRRVTGQEYSELLDEFMAAVRQNYGQNVLVQFEDFANHNAFTLLEKYR
ENHLVFNDDIQGTAAVVLAGLIAALKSTGGTLADHTFLFFGAGEAGTGIAELLALAISRQGLIVTSRNET
LEPFNKRYAHKHEPIKDLLEAVKAIKPTALIGSSGVGQSFTKEVIEAMSSINERPIILALSNPT
SKSECTAEQAYSWSQGRAIFGSGSPFDPVKYNGKLFVSAQANNAYIFPGFGLGLVIAGAIRVKDDMVLAA
AEGLAEQITPEQIDRGQIYPPFSNIRKISANIAARVAAKAYDLGLASQLPRPKDLVKYAESCMYSPIYRS
YR
>g138018.t1
MAGAEERNDADMPMRDDATGVVTGGVEDAYGEDRATEDQPIITPGHSLLRDPRHNKGLAFTEKERDAHYL
RGLLPPAVLSQELQEKRVLQNLRQFKVPLQRYMALTDLQERNERLFYKLLIDNVEELLPIVYTPTEGEAC
QKGRVLETLRNWPEKNIQVIVVTDGERILGLGDLACQGMGIPVGKLALYTALGGVRPSACLPVT
IDVGSNNEDLIKDEFYIGLRQRRVTGQEYSELLDEFMAAVRQNYGQDVLVQDVLVQFEDFANHSAFTLLE
KYRENHLVSNDDIQGTAAVVLAGLIAALKSTGGTLADHTFLFFGAGEAGTGIAELLALAISRQSKVSVEE
ARKKIWLDDAKGLIVTSRNETLEPFNKRYAHKHEPIKDLLEAVKAIKPTALIGSSGVGQSFTKE
VIEAMSSINERPIILALSNPTSKSECTAEQAYSWSQGRAIFGSGSPFDPVKYNDKLFVPAQANNAYIFPG
FGLGLVISGAIRVKDDMVLAAAEGLAEQITPEQIDKGQIYPPFSNTRKISANIAARVAAKAYDLGLASQL
PRPKDLVKYAESCMYNPVYRSYR
>g138020.t1
MAGSGEEKNGGVAAVSGGVEDAYGEDRATEDQPITPWAVCVASGHSLLRDPRHNKGLSFTEKERDAHYLR
GLLPPVVLSQELQEKRLLQNVRQFEVPLQRYMALMDLQERNERLFYKLLVDHVEELLPVVYTPTVGEACQ
KYGSIFRRPQGLYISLKEKGRILEVLRNWPEKSIQVIVVTDGERILGLGDLGCQGMGIPVGKLA
LYSALGGVRPSANYGQKVLVQFEDFANHNAFALLEKYREIHLVFNDDIQGTAAVVLAGLIAALKSVGGSL
GDHTFLFFGAGEAGTGIAELVALAISRQSKISIDEARNKIWLVDSKGLVVRSREETLQPFKKRYAHEHEP

IKDLLGVVQAIRPTALIGSAGVARSFTKEVVEAMSSINKRPIILALSNPTSQSECTAEQAYSWS
QGHAIFGSGSPFDPVKYNNKVFVPAQGHAIFGSGSPFDPVKYNNKVFVPAQANNAYIFPGLGLGVVISGA
IRVTDEMVLAAAEGLAEQVTPEHIDKGLIYPPFFIIRKISANIAARVAEKAYDLGIFAAWLVISLAPKIS
>g139643.t1
MLSAHAATASASPVSLWKRGGSEGGSCDGCRSCRDVVRRRAATVRVRATAPRRVEAVAMGSAAETEKEEV
EVEVVATEKEELAAAGGGVKDPYGEDRATEDLPVTPWAFSVASGYTLLRDPHHNKGLAFTEKERDAHYLR
GLLPPGVVSQETQIKKFMHNLRQYQLPLQRYMAMMDLQERNERLFYKLLIDNVEELLPVVYTPT
VGEACQKYGSIFRQPQGLYISLKDKGRVLEVLRNWPQRNIQGMGIPVGKLALYTALGGVRPSACLPITID
VGTNNEKLLNDEFYIGIRQRRARGQEYHELIEEFMSAVKQIYGEKVLIQFEDFANHNAFDLLAKYRKSHL
VFNDDIQGTASVVLAGLLAALKVVGGTLAEHTYLFLGAGEAGTGIAELIALEISRQTKAPIEEC
RKRVWLVDSKSIKPTVLIGTSGVGKTFTKEVVEAMASFNERPIIFSLSNPTSHSECTAEEAYNWTQGRAV
FASGSPFAPVEYNGKLHVSGQANNAYIFPGFGLGVVISGAIRVHEDMLLAASEALAAQATQENFDKGSIF
PPFTNIRKISAHIAAAVAEKAYELGLATRLPPPKDLVKYAESCMYTPVYRNYR
>g157120.t1
MEPPALSSPQVPAPRADGPLQISLEFHSPATTRSVPSRAVPSPPSRTSSSSSRPRARDVAFGGGAAFEGA
PASPLVVRTGGRGRRAGPLHRAQARQRHPQRPMVQQGKAKPSRHPPPRCPRPAXAPAAGAAVPGPCIVHK
RGNDILNDPWYNKVRLSPAAIPLLDARDLLDRDSGSSLGNFCIDGAQDTAFPLTERDRLGLRGL
LPPRVMTFKEQYERFMNSFKSLENNTRGEPDSIVSLAKWRILNRLHDRNETLYYRVLIDNIKDFAPIIYT
PTVGLVCENYSGLFRRPRGMYFSAKDKGEMMSMIYNWPQENVDMIVVTDGSRILGLGDLGVQGIGIPIGK
LDLYVAAAGHGN
>g163541.t1
MSAVKQIYGEKVLIQFEDFANHNAFDLLAKYSKSHLVFNDDIQGTASVVLAGLLAALKVVGGTLAEHTYL
FLGAGEAGTGIAELIALEISRQTKAPIEECRKRVWLVDSKSIKPTVLIGTSGAGKTFTKEVVEAMASFNE
RPIIFSLSNPTSHSECTAEEAYNWTQGRAVFASGSPFAPVEYNGKLHVPGQVLPFVFKSLLDKA
MQRCADLNVFH

pyruvate orthophosphate dikinase (PPDK)
>g823.t1
MEKLMPEAFQQFLAICNRLETHYRDMQDMEFTIERGQLWMLQTRCGKRTTRAAMKIAVDMVEEGLITAEE
AVCRIDPSSLDQLLHPTIDSSIERPVIGSGLPASPGAASGEIVFSAEEAVAAKAEGRSVILVRMETSPED
IHGMHAAEGILTTRGGMTSHAAVVARGMGIPCVTGAGSMRVDLRNGVLIGIGCMLRKGDVITID
GSSGRVLRGEVPMTQPELSGDFGKLMEWADGARRMTXXXALWRTSHDG
>g63587.t1
MYDKYTNRSRRFGFVTMSSAEEANAAIEALNGTADPNMCVLDMYQEVEGRKIKVNVTESFLPNIDRSAPE
PEPVFVDSQYKVYVGNLAKTVTTEVLKNFFSEKGQILSATVSRVPGTSKSKGYGFVTFSSEEEVEAAVAT
FNNAG
>g104072.t1
MLLMNRWSAFLTRGCPCSQGSSGSSTTTGRREEEFSRTCFECGDPGHFIADCPKKNTKAGYGDNNIEEFK
QKKNHFYKKGKNSKKLGKAIACACIAALSDVDLTSSKGLTSLEEEVEKP
>g6353.t1
MAVLVQEIVNADYAFVIHTTNPSSGDSSEIYAEVVKGLGYPSKPIGLFIRRSIIFRSDSNGEDLEGYAGA
GLYDSVPMDEEDEVVLDYTTDPLIVDGGFRNSILSSIARAGHAIEELYGSPQDVEGVVKDGKIFVVQTRP
QMYSARGRTRDYYYQVLGVTVHSTPQEIKEAYRKLQKQHHPDIAGYQGHDYTLLLNEAYKVLMR
NMSRHADGHVQSTGGSGSGYTGDGYSSWNGPVRSQALFVDENKCIARTFAMDDVLGSARVTVQFGDLEQQ
IQVAVESCPVNCIHWVESQELPVLEFLARPQPKEGHGVFGGGWERPRNLFAAAKNFAKKLEREEQELERE
QYSRSSNGKMNRWDADGEAETAAQAEARRRAGQELRRAQRSVTFS
>g28460.t1
MAGSYAATATPLETSVPGRRAPLAAGATRSRQCALLSPLRLGAGGHHRLAVSSRHRRRLGAVYAQNSGRF
SLESNSELQVAVNPAREGSLVEVELVATNTGSSSLSLHWGALQQGRREWVLPSRRPDGTRTPLSRLPSKA
FNIAADLAEQAKDAGHLGLSGLLVWMRFMATRQLVWNKNYNVKPREISQAQDRFTDILQNLYKT
HPQYRETIRMIMSAVGRGGEGCSFWSRSRIFYRNLYRSEGFMVGVKVNPVKELPPGFQELLKFVMEHIDD
KSVEALVEALLEARAEIRPLLCGSSSRLKDLIFLDIALDSTVRTAVERSYEQLNNAAPEKIMYFISLVLE
NLALSTEDNENLLCCLKVKELKEKMQGCGMPWPGDEGEQRWEQAWMAIKKVWASKWNERAYFST
RKVKLDHDYLSMAVLVQEIVSADYAFVIHTTNPSSGDDREIYAEVVKGLGETLVGAYPGRALSFVCKKDD
LNSPKVLGYPSKPIGLFIKQSIIFRSDSNGEDLEGYAGAGLYDSVPMDKEEEVVLDYTTDPLIIDCSFRN
SILSSIARTGYAIEELYGSPQDIEGVVKDGKIYIVQTRPQM
>g46883.t1
MKIEKGLRNDTELSVRDLEELVSQYKNVYVVAKGEQFPSDPKRQLHLAVLAIFDSWNSARAKKYRSINQI
TGLKGTAMNVQCMVFGNMGDTSGTGVLFTRNPSTGEKKLYGEFLTNAQGEDVVSGIRTPQDLDAMKKCMP

EPYAELVENCVVLERHYKEMMVMVNADTPADALTGRNNGAEGIGLCRTEHMFFSSDARIKAMRQ
MIMADTAEQRQKALDLLLPYQRTDFEGIFRAMNGLPVTIRLLDPPLHEFLPEGSIEDMLPMLSFDIKSTN
DEILARIEKLSEVNPMLGFRGCRLGISYPELTAIQARAIFEAAIAINDQGFQELDQQVRVIRQVADKVFA
NAEKTISYKVGSMIEVPRAALIADEIAELAEFFSGTNDLTQMTFGYSRDDVGKFLPTYLSQGIL
QHDPFEVLDRKGVGELLKIATERGRRSRPGLEVGVCGEHGGEPSSVAFFAKAGLDYVSCSPFRVPIARLA
AAQAVLSDKSEYM
>g52745.t1
MKIEKGLRNDTELSVRDLEELVSQYKNVYVVAKGEQFPSDPKRQLHLAVLAVFDSWNSARAKKYRSINQI
TGLKGTAVNVQCMGEDVVAGIRTPQDLDAMKECMPEPYAELVENCVILERHSKEMMDIEFTVQENKLWML
QCRTGKRAGKGAVKIAVDMVNEGLIDHGSAIKMVEPRHLDQILHPQFERPSSYEEKVITTGLPA
SPGAAVGQIVFTADDAETWHAQGESVILVRTETSAEDVGGMHAAAGILTARGGMTSHAAVVARGWGKCCV
AGCSSIHVNDIEKVVVVGSRVLREGDWLSLNGSTGEVILGKVPLSPPALSGDLGTLMSWVDELKQLKVMV
NADTPADALTGRNNGAEGIGLCRTEHMFFSSDARIKAMRQMIMADTVEQRQKALDLLLPYQRTD
FEGILRAMDGLPVTIRLLDPPLHEFLPEGSIEDMLDMLSFDIKSTNDEILARIEKLSEVNPMLGFRGCRL
GISYPELTAMQARAIFEAAIAMDDQGFEVFPEIMVPLVGTRQELGQQLRVIRQVADKVFANAEKTISYKV
GSMIEVPRAALIADEIAELAEFFSFGTNDLTQMTFGYSRDDVGKFLPTYLSQGILQHDPFEVSI
YRTSIHVVVLHASVCAGARVAWVPIARLAAAQAVLSDKSEYM
>g99058.t1
MAGSSYAATAMLPETSVLGRRAPLAAGATRSRQCALLPPPRLGAGGHRLAVSSRHRRRLGAVSAQNSGRF
SLESNSELQVAVNPAREGSLVEVELVATNTGGSSLSLHWGALQQGRREWVLPSRRPDGTRTCEDAALRTP
FKSCGTNSTIKIEIDDPAVESIEFVIVDEAQNKWFKNNDQNFQIHLRKTDVHQGQHSATAETRR
HSAERIVRKNRDAMQLLSKPATSHALFLEEQGRAKVQLATNYAEQLILHWALAQKAGEWKAPPAGIVPPG
STLLEMACESSFSEATFDGLGYQAMEIELDDDSYKGMPFVLRSNETWIKNNTSDFYLDFSRKLTKSKEDG
TEGGKGTAKALLETIADLEEDAQRSLMHRFNIAADLAEQAKDAGHLGLSGLLVWMRFMATRQLV
WNKNYNVKPREISQAQDRFTDILQNLYKTHPQYRETIRMIMSAVGRGGEGIVPPGSTLLEMACESSFSEA
TFDGLGYQAMEIELDDDSYKGMPFVLRSNETWIKNNTSDFYLDFSRKLTKSKEDGTEGGKGTAKALLETI
ADLEEDAQRSLMHRFNIAADLAEQAKDAGHLGLSGLLVWMRFMATRQLVWNKNYNVKPREISQA
QDRFTDILQNLYKTYPQYRETIRMIMSAVGRGGEEWHQKLHNNTSPDDVVICQALMDYLNSNLDIKVYWD
TLNKNGITKERLLSYDHPIHSEPNLKIEQKDGLLRDLTNYMRSLKAVHSGADLESSIGTCTGYRAESEGF
MVGVKVNPVKELPPGFQELLKFVMEHIEDKSVEALVEALLEARAEIRPLLCGSSARLKDLIFLD
IALDSTVRTAVERSYEQLNNAEPEVKELKEKMQGCGMPWPGDEGEQRWEQAWMAIKKVWASKWNERAYFS
TRKVKLDHEYLSMAVLVQEIVSADYAFVIHTTNPSSGDDLEIYAEVVKGLGETLVGAYPGRALSFVCKKD
DLNSPKVLGYPSKPIGLFIKQSIIFRSDSNGEDLEGYAGAGLYDSVPMDKEEEVVLDYTTDPLI
IDCSFRNSILSSIARTGYVIEELYGSPQDIEGVVKDGKIYVVQTRPQM
>g112156.t1
MAPAAHHQRGGAADETAQRVFHFGKGRNDGNKAMKDLLGGKGANLAEMATIGLSVPPGFTVSTEACRQYQ
LNGGTMPPGLWDEILDALRWVEGAMDAGLGDCSSPCAPGPPSLCPA
>g112172.t1
MSGLSAAAAAERCALGIRGRPAAASSPAASLRRAKVPRRPTALVASRRGPVSPRAIATTPADRASPDLAG
RFTLDSNSELQVAVNPAPQGSLVVVDLVATNTSGSLILHWGALRPENWLLPSRRPDGTAVYKNRALRTPF
VKSGNNSTLRIEIDDPAVQAIEFLVFDEAQNKCESDSPASESTVNEVPEDLVQVQAYIRWEKAG
KPNYPPEKQLLEFEEARKELQAELDKGISVDQLKKKILKGNIESKVSKQLKNKKYFSVERIQRKKRDIMQ
LLNKHKHDVIEEKVETAPMQPTVLDLFTQSLQDKVGCEILSRKLFKFGDKEILAPPSNIVPSGSKLLDMA
CETSFTESQLDGLHYQVVEIEFDDGGYKGMPFVLRSGESWIKNNGSDFYLDFSAHVTNTNNTKA
LIDYIKSDFDISVYWDTLNKEGITKERLLSYDRAIHSEPNFRSEQKEGLLRDLGNYMRSLKAVHSGADLE
SAIATCMGYKSEGEGFMELLEFVLDHVEDKSAEPLLEGLLEARVELRPLLLNSPERMKDLIFLDIALDST
FRTATERSYEELNDAAPEKIMYFISLVLENLALSIDDNEDILYCLKGWNQALEMAKQKHDQWAL
YAKAFLDRIRLALASKGEQYYNMMQPSAEYLGSLLRVDQWAVNIFTEEIIRGGSAATLSALLNRFDPVLR
NIAHLGSWQVISPVDVSGYVVVVDELLAVQNKSYDKPTVLIAKSVKGEEEIPDGVVGVITPDMPDVLSHV
SVRARNCKVLFATCFDPSILSELEGHDGKLFFFKTTSADVTYREISESELQQSGSSNAEADNAV
PAVSLVKKKFLGKYAISAEEFSEEMVGAKSRNIAYLKGKLPSWVGIPTSVAIPFGTFEKVLSDDLNKEVA
QDIEKLKGRLAQEDFSALAEIRKVVLNLTAPSQLVNELKEKMLGSGMPWPGDEGNQRWEQAWMAIKKVWA
SKWNERAYFSTRKVKLDHDYLSMAVLVQEIVNADYAFVIHTTNPSSGDSSEIYAEVVKGLGETL
VGAYPGRAMSFICKKEDLDSPKVNELKEKMLGSGMPWPGDEGNQRWEQAWMAIKKVWASKWNERAYFSTR
KVKLDHDYLSMAVLVQEIVNADYAFVIHTTNPSSGDSSEIYAEVVKGLGYPSKPIGLFIRRSIIFRSDSN
GEDLEGYAGAGLYDSVPMDEEDEVVLDYTTDPLIVDGGFRNSILSSIARAGHAIEELYGSPQDV
EGVVKDGKIFVVQTRPQM
>g113712.t1
MAPAQCVHAQRVFHFGKGKSDGNKSMKELLGGKGANLAEMASIGLSVPPGFTVSTEACQQYQAAGQALPP
GLWDEIVDALRWVEEYMGARLGDPDSPLLLSVRSGAAVSMPGMMDTVLNLGLNDAVAEGLAAKSGDRFAY

DSYRRFLDMFGNVVMDIPHALFEEKLEAMKAAKGLKNDTDLSASDLKELVGQYKDVYVEAKGEQ
FPSDPRKQLELAVLAVFDSWDSPRAKKYRSINQITGLRGTAVNVQCMVFGNMGNTSGTGVLFTRNPSTGE
KKLYGEYLVNAQGEDVVAGIRTPEDIDTMKDNMPGAYEELVENCKILESHYKDMMDIEFTVQENRLWMLQ
CRTGKRTGKGAVKIAVDMVNEGLVDHNAAIKMVEPGHLDQLLHPQFENPSAYKDKVIATGLPAS
PGAAVGQIVFTAEDAEAWHAQGNSAILVRTETSPEDVGGMHAAAGILTARGGMTSHAAVVARGWGKCCVS
GCSSIRVNDAEKAVVIGEMVLHEGEWLSLNGSTGEVILGKQPLSPPALSGDLGNFMSWVDAVRQLKVMAN
ADTPEDALAARNNGAEGIGLCRTEHMFFASDERIRAVRQMIMAPTLELRQKALDRLLPYQRSDF
EGIFRAMDGLPVTIRLLDPPLHEFLPEGNIEEIVHELCSETGSNQEEALARIEKLSEVNPMLGFRGCRLG
ISYPELTEMQARAIFEAAIAMTNQGVQVFPEIMVPLVGTPQELGHQVTLIRQIADKVFAATNKTVGYKVG
TMIEIPRAALVADEIAEHAEFFSFGTNDLTQMTFGYSRDDVGKFIPIYLAQGILQHDPFEVLDQ
RGVGELVKFGTERGRKARPNLKVGICGEHGGEPSSVAFFAKTGLDYVSCSPFRVPIARLAAAQVVL
>g113714.t1
MKQLLGGKGANLAEMSSIGLSVPPGFTVTTEACEQYQKAGKELPAGLWEEIVDGLQWVEEYMGARLGDPE
KPLLLSVRSGAAVSMPGMMDTVLNLGLNDEVAAGLAAKSGDRFAYDSYRRFLDMFGNVVMDIPHEKFEEK
LEHMKEAKGVKADTDLTAADLKELVGQYKECYIEAKGEPFPSDPKKQLELAVRAVFNSWDSPRA
NKYRAINQITGLKGTAVNVQTMVFGNMGNTSGTGVLFTRNPSTGEKKLYGEYLINAQGEDVVAGIRTPED
LDTMKENMPEAYAELVENCNILESHYKDMMDIEFTVQENRLWMLQCRSGKRTGQGAVKIAVDLFENPSAY
KDQVIATGLPASPGAAVGQIVFTAEDAEAWQAQGKAAILVRTETSPEDVGGMHAAAGILTARGG
MTSHAAVVARGWGKCCVSGCSSVRVNDAEKTVTIGDKLLQEGDWLSLNGSTGEVVLGKQPLAPPALSGDL
GTFMSWVDAVRQLKVMANADTPEDALAARNNGAEGIGLCRTEHMFFASDERIKTVRQMIMAPTLELRQKA
LDRLLPYQRSDFEGIFRAMDGLPVTIRLLDPPLHEFLPEGNIEDIVQELCSETGADQEEALARI
EKLSEVNPMLGFRGCRLGISYPELTEMQARAIFEAAIAMSNQGVQVNPEIMVPLVGTPQELNNQVNLIHA
TANKVFSATGKTVVYKVGTMIEIPRAALVADEIAEHAEFFSFGTNDLTQMTFGYSRDDVGKFLPIYLGQG
ILQHDPFEVLDQRGVGELIKFATERGRKARPNLKVGICGEHGGEPSSVAFCAKVGLNYVSCSPF
RVPIARLAAAQVLV
>g114689.t1
MAPAAHHRRGGPAETAQRVFHFGKGRSDGNKAMKDTLGGKGANLAEMATIGLSVPPGFTVYTEACXXXXX
XXXXXXXXXXXXXXXXXXXXXXXXXXXXGGTMPPGLWDEILDALRWVEGAMDAGLGDSRRPLLLSVRSG
AAVSMPGMMDTVLNLGLNDHVVAGLAERSGRRFAYDSYRRFLDMFGDVVMGIPHGLFEEKLEAM
KIEKGLRNDTELSVRDLEELVSQYKNVYVVVKGEQFPSDPKRQLHLAVLAVFD
>g127123.t1
MRRSCGDVSMQRVFHFGKGKSDGNKSMKELLGGKGANLAEMASIGLSVPPGFTVSTEACQQYQAAGQALP
PGLWDEIIDALRWVEEYMGARLGDPESPLLLSVRSGAAVSMPGMMDTVLNLGLNDAVAEGLAAKSGDRFA
YDSYRRFLDMFGNVVMDIPHALFEEKLEAMKAAKGLKNDTDLSASDLKELVGQYKDVYVEAKGE
QFPSDPRKQLELAVLAVFYSWDSPRAKKYRSINQITGLRGTAVNVQCMVFGNMGNTSGTGVLFTRNPSTG
EKKLYGEYLVNAQGEDVVAGIRTPEDIDTMKDNMPGAYEELVENCKILESHYKDMMDIEFTVQENRLWML
QCRTGKRTGKGAVKIAVDMVNEGLVDHNAAIKMVEPGHLDQLLHPQFENPSAYKDKVIATGLPA
SPGAAVGQIVFTAEDAEAWHAQGNSAILVRTETSPEDVGGMHAAAGILTARGGMTSHAAVVARGWGKCCV
SGCSSIRVNDAEKAVVIGEMVLHEGEWLSLNGSTGEVILGKQPLSPPALSGDLGNFMSWVDAVRQLKVMA
NADTPEDALAARNNGAEGIGLCRTEHMFFASDERIKAVRQMIMAPTLELRQKALDRLLPYQRSD
FEGIFRAMDGLPVTIRLLDPPLHEFLPEGNIEEIVHELCSETGSNQEEALARIEKLSEVNPMLGFRGCRL
GISYPELTEMQARAIFEAAIAMTNQGVQVFPEIMVPLVGTPQELGHQVTLIRQIADKVFAAMNKTVGYKV
GTMIEIPRAALVADEIAEHAEFFSFGTNDLTQMTFGYSRDDVGKFIPIYLAQGILQHDPFEVLD
QRGVGELVKFGTERGRKARPNLKVGICGEHGGEPSSVAFFAKTGLDYVSCSPFRVPIARLAAAQVVL
>g127125.t1
MASVTRAVCCVQSPTSNGRRSSELASGRRSVAASRTRLTKATVIRASGRGEHCAPAKAIADAAPVTATKR
VYPFGKGKSEGDKSMKQLLGGKGANLAEMSSIGLSVPPGFTVTTEACEQYQKAGKELPAGLWEEIVDGLQ
WVEEYMGARLGDPEKPLLLSVRSGAAVSMPGMMDTVLNLGLNDEVAAGLAAKSGDRFAYDSYRR
FLDMFGNVVMDIPHEKFEEKLEHMKEAKGVKADTDLTAADLKELVGQYKECYIEAKGEPFPSDPKKQLEL
AVRAVFNSWDSPRANKYRAINQITGLKGTAVNVQTMVFGNMGNTSGTGVLFTRNPSTGEKKLYGEYLINA
QGEDVVAGIRTPEDLDTMKENMPEAYAELVENCNILESHYKDMMDIEFTVQENRLWMLQCRSGK
RTGQGAVKIAVDLVSEGLVDRDTAIKMVEPGHLDQLLHPQFENPSAYKDQVIATGLPASPGAAVGQIVFT
AEDAEAWQAQGKAAILVRTETSPEDVGGMHAAAGILTARGGMTSHAAVVARGWGKCCVSGCSSVRVNDAE
KTVTIGDKLLQEGDWLSLNGSTGEVILGKQPLAPPALSGDLGTFMSWVDAVRQLKVMANADTPE
DALAARNNGAEGIGLCRTEHMFFASDERIKAVRQMIMAPTLELRQKALDRLLPYQRSDFEGIFRAMDGLP
VTIRLLDPPLHEFLPEGNIEEIVQELCSETGADQEEALARIEKLSEVNPMLGFRGCRLGISYPELTEMQA
RAIFEAAIAMSNQGVQVHPEIMVPLVGTPQELNNQVNLIHATANKVFSATGKTVVYKVGTMIEI
PRAALVADEIAEHAEFFSFGTNDLTQMTFGYSRDDVGKFLPIYLGQGILQHDPFEVLDQRGVGELIKFAT
ERGRKARPNLKVGICGEHGGEPSSVAFCAKTGLNYVSCSPFRVPIARLAAAQVLV
>g156383.t1

MAILVQEMLQPDLSFVLHTISPADHDPKFVEAEVAPGLGETLASGTRGTPWRFSCDKFDGKVTTLAFANF
SEEMVVLNSGPANGEVVRLTVDYSKKPLSVDSTFRKQLGQRLAAIGQFLEQKFGSAQDVEGCLVGKDIFI
VQSRPQP
>g158840.t1
MLPPSIKEPVVLIVNKADGDEEVKAAGDNIVGVILLQELPHLSHLGVRARQLKSYTSGVNGTSGVLELSE
ASVESSGAKAAACGTLSVLASLSNKVYSDQGIPAAFGVPTGAVIPFGFMEHALKNTGSLDSYTSLLERIE
TAKIENSELDSLSSELQSIVSLLAPSEKTIESLKNIFDPNARLIVRSSANVEDLAGMSAAGLYD
SIPNVSLCDPSAFGAAVGQVWASLYTRRAVLSRRAAGVPQRDAKMAILVQEMLQPDLSFVLHTISPADHD
PKFVEAEVAPGLGETLASGTRGTPWRLSCDKFDGKVTTLAFANFSEEMVVLNSXSHVTSLMAKSPL

PPDK-regulatory protein (PPDK-RP)
>g3032.t1
MTTDRSVFYISDGTAITAEVLGHAVLSQFPVNITSLTLPFVENVQRALAVKAQINALYQQSGVRPLVFFS
IVTPEVRDIIVQSEGFCQDIVQALVAPLQQELGLAPAPVAHRTHGLDASNLGKYDARIAAIDYALAHDDG
ISLRGLEDAQVILVGVSRCGKTPTSLYLAMQFGIRAANYPFIADDMDNLKLPPALRAHQNKLFG
LTIDPERLAAIRQERAENTRYASMRQCRLEVGEVEALFRTHQIRYLNSTNYSVEEIATKILDIMGLTRRM
Y
>g114612.t1
MLVRAIHSCAVEYPEVAGSVVHLLMDFLGDTNVAAAVDVVLFVREIIETNPKLRVSMIQRLIDTFYQIRA
SPVCACALWILGEYSLSLSEVESAIATIKQCLGELPFYTLSEEGEATDSAKPAQPVVNSVTVSXQPSQW

>g26333.t1
MIGGGATAAESLAEETAPRASAQLSRWSRARALRPGRRTGRAALSAPAIKPPPLLAEEESFSAAASKEEE
DDDVSPPASRDATAPGKAIYMVSDGTGWTAEHSVNAALGQFEHCLGDRRCAVSTHLFSGVDDMDRLIEII
KQAAKEGALVLYTLADPSTAEAIKKACDFWGVPSTDVLRPTVEAIASHIGVAPSGIPRSSPSRQ
GQLTEDYFQRIDAIEFTIKQDDGAQPQNLNRAHIALVGVSRTGKTPLSIYLAQKGYKVANVPIVMGVDLP
KVLFEIDQDKIFGLTINPAVLQAIRKTRAKTLGFDEHQSNYAEMEHVDDMDRLIEIIKQAAKEGALVLYT
LADPSTAEAIKKACDFWGVPSTDVLRPTVEAIASHIGVAPSGIPRSSPSRQGQLTEDYFQRIDA
IEFTIKQDDGAQPQNLNRAHIALVGVSRTGKTPLSIYLAQKGYKVANVPIVMGVDLPKVLFEIDQDKIFG
LTINPAVLQAIRKTRAKTLGFDEHQSNYAEMEHVRHELDHANKIFSQNPVWPVIEITGKAIEETAAVVMR
IYHDRKQKCSMPRISRRESRKSVLFDACRLAKSLLELPPHKRCRMIHVVWVEMLCYAANK
>g26334.t1
MFSSSRPNPRRATDKQPARHAKSLRKRREILVATRHRLQASRHRHKGLIISTLPSHARTARVPALLRSQL
ALDSAMIGGATNPFAAPPLLPPSPGRRVAAGPGPLACVADPAALSPAAESPPAAHEDSTGSAQPPEQASP
RSPTSPSSSPTPTLRASSQLTRFSRARALRSGRRLTRTALSSAPVTTTPPPPAPMPSLPDGLPM
AGDDEDDDVCIAETDAATGKSIYLVSDGTGETAEHSVKAALGQFEHCLVDQRCTVNTHLFSGVDDTDRLV
EIIKQAAKEGAVVLYTLVDPSMAEAMKKACNIWGVPSTDVLRPTVDAIASHIGVAPSGIPRSSPSRKAQL
SEDYFRRIEAIDFTIKQDDGAQPENLNRAHIVLVGVSRTGKTPLSIYLAQKGYKVANVPIVMGV
NLPKSLFEINQDKIFALTINPVVLQAIRKTRAKTLGFDGYQSNYAEMDHVRHELVHANKIFAQNPGWPVI
EVTGKAIEETAAIVVRIHHDRKQKCSMPRISKRVAPVLVYDYMSHVRDA
>g83920.t1
MIGGGATANPLAEETAPRPHPSSSPAPRASAQLSRWSPARALRPGRRTGRAALSAPATKPPPPPLAEEKS
FSAAASTEFDDDDDDVSPPASRDAAAPGKAIYMVSDGTGWTAEHSVNAALGQFEHYLGDRRCAVSTHLFS
GVDDMDRLIEIIKQAAKEGALVLYTLADPSTAEAIKKACDFWGVPSTDVLRPTVEAIASHIGVA
PSGIPRSSPS
>g83921.t1
MGVDLPKALFEIDQDKIFGLTINPAVLQAIRKTRAKTLGFDEHQSNYAEMEHVDDMDRLIEIIKQAAKEG
ALVLYTLADPSTAEAIKKACDFWGVPSTDVLRPTVEAIASHIGVAPSGIPRSSPSRQGQLTEDYFQRIDA
IEFTIKQDDGAQPQNLNRAHIALVGVSRTGKTPLSIYLAQKGYKVANVPIVMGVDLPKALFEID
QDKIFGLTINPAVLQAIRKTRAKTLGFDEHQSNYAEMEHVRHELDHANKIFSQNPVWSVIEITGKAIEET
AAVVMRIYHDRKQKFSMPRISKRY
>g105292.t1
MIGGATNPFAAPPLLPPSPGRRVAAGPGPLACVADPAALSPAAESPPPAHEDATGPAQAAEQXXXXQASP
RSPTSPSSSPTPTLRASSQLTRFSRARALRSGRRLTRAALSSAPVTTPPPPPAPMPSLPDGLPMAGNDED
DDVCIAETDAATGKSIYLVSDGTGETAEHSVKAALGQFEHCLVDQRCTVNTHLFSGVDDTDRLV
EIIKQAAKEGAVVLYTLVDPSMAEAMKKACNIWGVPSTDVLRPTVDAIASHIGVAPSGIPRSSPSRKAQL
SEDYFRRIEAIDFTIKQDDGAQPENLNRAHIVLVGVSRTGKTPLSIYLAQKGYKVANVPIVMGVNLPKSL
FEINQDKIFALTINPVILQAIRKTRAKTLGFDGYQSNYAEMDHVRHELVHANKIFAQNPGWPVI
EVTGKAIEETAAIVVRIHHDRKQKCSMPRISKRY

PEPC kinase (PPCK)
>g7805.t1
MGREERFPVWEAALGAGVATAFATGLVGVYLSMPDSDYSFLKLPRNLEELQILTKDQEVSYGLNWSIAGR
GVVVKDKVFYNLETSELQKGGATYIECLSGIPLHVRGNVVSGVPDVSKGQFAKLLKLVTFHLSSISSLYV
QDGAIGSSTECNAKVRVISDNPSAAMLLSNVLWKVSDRAISHDTSPLTIYAASSISSNVKTVLVSGAQYA
NGFAVADIDRSSLILCGKAFADLTVVKNALTALTAPIFSARGGLPVPGWFLSFGSSVVLLFAPVDLIRSC
SEIQNVLLKEDDIPAYMINAKRSGKQINGNIN
>g30872.t1
MGREERFPVWEAALGAGVAAAFAVGLAGVYLSMPDSDYSFLKLPHNLEELQILTPIAKHREPRRGHVTEN
LIEPKIGRAHLLGSILPYTPFTAAVDLWTSSACRRDGRRPTWYVTPDSPPSPPAAAVSRGEQPPTATAPR
RWAGKWKQITCWAFSDVLPAPPRHRLRQRRAYGRKDQEVSYGLNWSIAGRGVVVKDKVFYNLETSELQKG
GATYIECLSGIPLHVRGNVVSGVPDVSKGQFAKLLKLVTFHLSSISSLYVQDGAIGSSTELADIERSSLI
LCGKAFADLTVVKDALTALTAPIFSARGGLPVPGWFLSFGGSVVLLFAPVDIIRSCSEIQNVLLSLDCGV
VISSKESTVLFPIKARREPKLSSRATSVIIVSSDSTDAIPSVSKLSPGQAAYHFLAGYHDGRFVPAYSNG
PSPAEPLALASSLFSHLKEDDIPAYMINAKRSGKQINGKEFIKLLELALSDNLPSIKTDNIRVRELKQKY
RSFLSSKFGECLPKDFSF
>g32108.t1
MVGSEPNHDHKATPRRGKMATPNGLARIETNGKKKHENGVCHDDSSAPVRAQTIDELHSLQRKRSAPTTP
IKEGAASAFAAALSEEQRQKQQLQSISASLASLTRETGPKVVRGDPARKGEAAAKSAATTPVPVHHHHHP
AAPTIAVSDSSLKFTHVLYNLSPAELYEQAIKYEKGSFITSTGALATLSGAKTGRSPRDKRIVKDEAATQ
ELWWGKGSPNIEMDEHTFLTNRERAVDYLNSLDKVFVNDQFLNWDPENRIKVRIISARAYHSLFMHNMCI
RPTDEELESFGTPDFTIYNAGQFPCNRYTHYMTSSTSVDINLARREMVILGTHASLASLTRETGPKVVRG
DPARKGEAAAKSAATTPVPVHHHHHPAAPTIAVSDSSLKFTHVLYNLSPAELYEQAIKYEKGSFITSTGA
LATLSGAKTGRSPRDKRIVKDEAATQELWWGKGSPNIEMDEHTFLTNRERAVDYLNSLDKVFVNDQFLNW
DPENRIKVRIISARAYHSLFMHNMCIRPTDEELESFGTPDFTIYNAGQFPCNRYTHYMTSSTSVDINLAR
REMVILGTHASLASLTRETGPKVVRGDPARKGEAAAKSAATTPVPVHHHHHPAAPTIAVSDSSLKFTHVL
YNLSPAELYEQAIKYEKGSFITSTGALATLSGAKTGRSPRDKRIVKDEAATQELWWGKGSPNIEMDEHTF
LTNRERAVDYLNSLDKVFVNDQFLNWDPENRIKVRIISARAYHSLFMHNMCIRPTDEELESFGTPDFTIY
NAGQFPCNRYTHYMTSSTSVDINLARREMVILGTQYAGEMKKGLFGVMHYLMPKRRILSLHSGCNMGKHG
DVALFFGLSGTGKTTLSTDHNRLLIGDDEHCWSDNGVSNIEGGCYAKCIDLSKEKEPDIWNAIKFGTVLE
NIVFDEHTREVDYTDKSVTENTRAAYPIEYIPNAKIPCVGPHPKNVILLACDAFGVLPPVSKLNLAQTMY
HFISGYTALVAGTEDGIKEPQATFSACFGAAFIMLHPTKYAAMLAEKMQKYGATGWLVNTGWSGGRYGVG
KRIKLAYTRKIIDAIHSGELLTANYKKTEVFGLEIPTEIDGVPSEILDPINTWTDKAAYKETLLKLAGLF
KNNFEVFASYKIGNDSSLTDEILAAGPSF
>g71461.t1
MKIAAVDSWPRSRTLKALRGFLGLTGYYRKFIAGYGAVAAPLTTLLKEAFAWSDVAEEAFVNLKRALTTA
PLLQMPDFAKWFIVDCDA
>g119907.t1
MRYAGEMKKAMFSVQNFLLPEKDVLPMHCAANVGEEGDVTLFFGLSGTGKTTLSADQSRYLIERAGDLEG
HPXKTTLSADQSRYLIGDDEHGWGVGTVFNMEGGCYAKCIDLSEKNEPVIWKAIQFGAVLENVVLDPQTR
QPNYADDSLTQNSRAAYPRELIDKRAEHNLGGEPNAVIFLTCDLTGVLPPVSILNNEQAAYHFLTPIC
>g123970.t1
MATPNGLARIETNGKKKHENGVCHDDSAAPVRAQTIDELHSLQRKRSAPTTPIKEGAASAFAAALSEEQR
QKQQLQSISASLASLTRETGPKVVRGDPARKGEATAKSAATTPAPVHHHHHHPAAPTIAVSDSSLKFTHV
LYNLSPAELYEQAIKYEKGSFITSTGALATLSGAKTGRSPRDKRIVKDEAAAQELWWGKGSPNIEMDEHT
FLTNRERAVDYLNSLDKVFVNDQFLNWDPENRIKVRIISARAYHSLFMHNMCIRPTDEELESFGTPDFTI
YNAGQFPCNRYTHYMTSSTSVDINLARREMVILGTQYAGEMKKGLFGVMHYLMPKRRILSLHSGCNMGKH
GDVALFFGLSGTGKTTLSTDHNRLLIGDDEHCWSDNGVSNIEGGCYAKCIDLSKEKEPDIWNAIKFGTVL
ENIVFDEHTREVDYTDKSVTENTRAAYPIEYIPNAKIPCVGPHPKNVILLACDAFGVLPPVSKLNLAQTM
YHFISGYTALVAGTEDGIKEPQATFSACFGAAFIMLHPTKYAAMLAEKMQKYGATGWLVNTGWSGGRYGV
GKRIKLAYTRKIIDAIHSGELLTANYKKTEVFGLEIPTEIDGVPSEILDPINTWTDKAAYKETLLKLAGL
FKNNFEVFASYKIGNDSSLTDEILAAGPSF
>g123971.t1
MGKDGDVALFFGLSGTGKTTLSTDHNRLLIGDDEHCWSDNGVSNIEGGCYAKCIDLSKEKEPDIWNAIKF
GTVLENVVFDEHTREVDYTDKSVTENTRAAYPIEYIPNAKIPCVGPHPKNVILLACDAFGVLPPVSKLNL
AQTMYHFISGYTALVAGTEDGIKEPQATFSACFGAAFIMLHPTKYAAMLAEKMQKYGATGWLVNTGWSGG
RYGVGKRIKLAYTRKIIDAIHSGELLTANYKKTEVFGLEIPTEIDGVPSEILDPINTWTDKAAYKETLLK
LAGLFKNNFEVFASYKIGNDSSLTDEILAAGPSF
>g138099.t1
MPRHPTEYGNLLRDLIAQHGVDCWLVNTGWTGGAYGIGKRMPIKATRALLTAALTGELKNAQFRTDAHFG

```
FAVPTSLEGIDNSILDPRSTWADGAAYDAQAKKLVNMFVTNFTKFEDHVDSTVRDAAPGLLAAAE
>g139091.t1
MRVNGLTSQDLAALGIVDTTEVVYNPDYDTLFQEETRPDLEGFARGTLTQSGAIAVDTGIFTGRSPKDKY
IVRDDTTRDTLWWNDQGTGKNDNQPLSQETWNALKSCVTRQLSGKRLFVVDAFCGANADSRLSVRFVTEV
AWQAHFVKNMFIQPTDAELADFTPDFVVMNGAKCTNPDWQAQGLHSENFVAFNLTERMQLIGGTWYGGEM
KKGLFAIMNYLLPLKGIASMHCSANVGKAGDVAKGLFAIMNYLLPLKGIASMHCSANVGKAGDVAVFFGL
SGTGKTTLSTDPDRQLIGDDEHGWDDDGVFNFEGGCYAKTINLSEQAEPEIYRAIRRNALLENVVVREDG
SVDYADGSKTENTRVSYPINHIDNIVQPVSKAGHAKKVIFLTADAFGVLPPVSRLTPEQTQYHFLSGFTA
KLAGTERGVTEPTPTFSACFGAAFLTLHPTQYAEVLVKRMEASXPEQTQYHFLSGFTAKLAGTERGVTEP
TPTFSACFGAAFLTLHPTQYAEVLVKRMEASGAQAYLVNTGWNGSGKRISLKNTRAIINAILAGELDDAP
VETLPIFNLQMPVALGXEVLVKRMEASGAQAYLVNTGWNGSGKRISLKNTRAIINAILAGELDDAPVETL
PIFNLQMPVTLGELDSETLDPRRSWESEEKWTAAAEGLAQRFIDNFDKYTDNAAGAALVKAGPQR
```
