# Supplementary material for: Genome and Transcriptome sequence of Finger millet (Eleusine coracana (L.) Gaertn.) provides insights into drought tolerance and nutraceutical properties
Source: BMC Genomics. 2017 Jun 15;18:465. doi: 10.1186/s12864-017-3850-z (PMC5472924; doi:10.1186/s12864-017-3850-z)

**Supplement File 11**: Phylogenetic tree of C4 pathway depicting the sharing of common ancestry among different cereals (a) PEPC, phosphoenolpyruvate carboxylase; (b)PPCK, PEPC kinase; (c)NADP-MDH, NADP-malate dehydrogenase; (d)NADP-ME, NADP-malic enzyme; (e) PPDK, pyruvate orthophosphate dikinase, (f) PPDK-RP, PPDK-regulatory protein; and (g) CA- carboxylating anhydrase. The gene IDs are denoted as- Sb, *Sorghum bicolor*; ZE, *Zea mays; Si, Setaria italica;* BR, *Brachypodium distachyon*, Os, *Oryza sativa,* and g, *Eleusine coracana*.

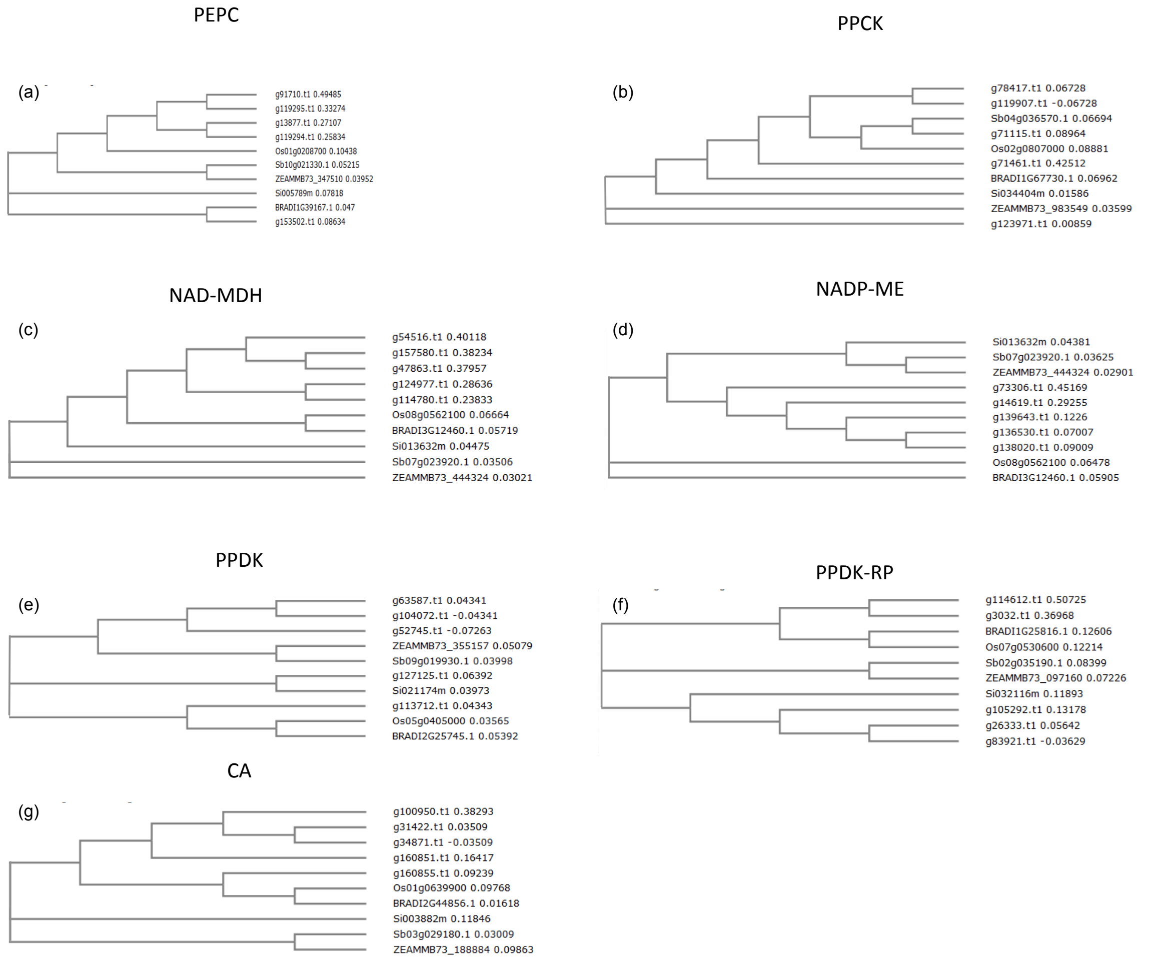

Supplement: Supplementary file 11 — Phylogenetic tree of C4 pathway depicting the sharing of common ancestry among different cereals. (PDF 1716 kb) [file 12864_2017_3850_MOESM11_ESM.pdf]
