# Supplementary material for: Genome and Transcriptome sequence of Finger millet (Eleusine coracana (L.) Gaertn.) provides insights into drought tolerance and nutraceutical properties
Source: BMC Genomics. 2017 Jun 15;18:465. doi: 10.1186/s12864-017-3850-z (PMC5472924; doi:10.1186/s12864-017-3850-z)
Supplement: Supplementary file 8 — Nucleotide sequences of resistance genes identified in ML-365 genome. (PDF 3136 kb) [file 12864_2017_3850_MOESM8_ESM.pdf]

Supplement File 8: Nucleotide sequences of resistance genes identified in ML-365 genome

Coiled-coil domain containing genes
>g48727.t1
atgaagagatacgggctgcagctacgcgcgaagccggcggcgtcctcgtcgcgtgcgccaccgccgccggtgcggccgttggctgcgttcgccgacg
acggcgacgacgatgtggaggccgagatcctccgccaggccgccaagtcgcgcgccctccaaaaggtagaggagcagcagaagaaggcaatggag
gaggacccttcagtgttcgcctacgacgaagtgtacgatgatatgaaggagaaagccgcccggcccaagatgcaggacaaggtcgtccgcgagtcaa
aatatattgcacaacttaaggaaaaggcagaacaacgtaagcgtgaacaggacataatatatgagaggaagcttcagaaagagaggagcaaggag
gatcacctctttggtgacaaggaaaagtttgtaacatctgcctacaggaaaaaacttgaggaacagcagaagtggctggaggaagagaaaaggcggc
agcgtcaagaagaaaaggaagataaagagaggagcaaggaggatcacctctttggtgacaaggaaaagtttgtaacatctgcctacaggaaaaaac
ttgaggaacagcagaagtggctggaggaagagaaaaggcggcagcgtcaagaagaaaaggaagatgttactaagaagaaggaccttagtgattttt
actttgggcttcagaataatgttgcttttggtgcacagacgcatgacagtgcaaaacatgctaagcctcaaaagacggacaagaaaccagatgatacca
aaaccaacagttttggtccggaaatctctgatcgttctccaaagcgtagaagagaatccagtgtagaatcagagcgggctaaaagtgtggaaaagccc
tcagcagatctacctgagcggggtaaaagtgtggaacgaccttcagcaaaccaatcaaaagattcaacttctgctggatctactgagaagaatgctgat
gttccggcaaatgcttcacagaccccaccgaacactgagccggcaaaggttacagacgatcactacaaaagaactgatgatgcacttgctgccgctag
agcacgagctctggcccgcaagagagcgaaagagcagcaattactcgagggctaa
>g56817.t1
atggagcccgcggcgatggaccgcatcgccacccgcctctccaccgtggagggcctctacttcccctcctccttcctcggcacctcccccctcctccggcg
ccgccgacgcccccgaccacgcgccccccctccccgccgcggcgccaggaggagctccggtccctccttgcccgcgacgcgccgctcttcctcgagcgc
tacggctcgtccctctgcgccgacgagctcgccgccttcgacgcgctgagcccggactacgaggtggactggcacctccgccgcctgcgcgccgccgc
ggcaggggagccgcggcccgcgtggcaggtgcgcaaccggcgccgcgcgtacctcgaccgcctcgtgcgccaggggactacttctccgaggaggc
catgcgggagcgggagccctacctgcaccacgagtacctcgggaggttccaggacccgctcggcagggccatggcgcaccccggggagaggtggtc
cgacacgctgatgcgccgcgcggaggaggccgtcattgtggagaagatccgtggggagcagattaagagaggggttcatcccagcgagtgggtgggt
ggtggtggagctgatgaggcgatgcaagaggaagaagaggaagaggaggaggaggaggaggaagaagaagacgacgacgacgatgtcatgaag
gaaaaggaggcagaggtcgaaaaggccactgaggtggttgcaaatggggatgcccccgaggaatccaacaacgttggaggttctcaagcagggact
ttcaagcaaactttatcttctgaagaaatgcaaggttctcaagcagggactttcaagcaaactttatcttctgaagaaatgcaggatcagctggaacagt
tcacatacctgatgcaagaaaggttcctatctggtcaagacactgagcatatggactactctcagatcgataatgatgagatgctggatgaccattggtc
cagagaagccaactatgatgcagaggagaagtatttcgaagaagattaa
>g76246.t1
atggaaccctcagcgatggaccgcatcgccacccgcctctccaccgtggaaggcctctacttcccctcctccttcctcggcacctcccccctcctcccgcgc
cgccgacggcccctcccacgcccacacgccaccctccccgccgcgccgcaaggaggagctccggtccctcctggcccgcgacgcgccgctcttcctcg
agcgctacggctcgtccctctgcgccgacgagctcgccgccttcgacgcgctcagcccggactacgaggtggactggcacctccgccgcctgcgcgcc
gccgcggcgggggagccgcggcccgcgtggcaggtgcgcaaccgccgccgcgcctacctcgaccgcctcgtgcgccaggggactacttctccgagg
aggccatgcgggagcgggagccctacctgcaccacgagtacctcgggaggttccaggacccgctcggcagggccatggcgcgacccggggagaggt
ggtccgacacgctgatgcgccgtgctgaggaggccgtcatcgtggagaagatccgtggggagcagattaggagaggggttcaccccagcgagtgggt
gggtggtggtggagctgatgaggcgatgcaagaggaagaagaggaagaggaagaggaggaggaagaagaagacgatgtcatgaaggaaaagca
ggcagaggtcgaaaaggccactgaggtggttgcaaatggggatgcccccaaggaatccaacaacgttggaggttctcaagcagggactttcaagcaa
actttatcttctgaagaaatgcaggatcagctggaacagttcacatacctgatgcaagaaagagaagccaactatgatgcagaggagaagtattttgaa
gaagattaa
>g80567.t1
atgaagagatacgggctgcagctacgcacgaagccggcggcgtcctcgtcgcgtgcgccaccgccgccggtgcggccgttggcagcgttcgccgacg
acggcgacgacgatgtggaggccgagatcctccgccaggccgccaagtcgcgcgccctccaaaaggtagaggagcagcagaagaaggcaatggag
gaggacccttcagtgttcgcctatgacgaagtgtacgatgatatgaaggagaaagccgcccggcccaagatgcaggacaaggtcgtccgcgagtcaa
aatatattgcacaacttaaggaaaaggcagaacaacgtaagcgtgaacaggacataatatatgagaggaagcttcagaaagagaggagcaaggag
gatcacctctttggtgacaaggaaaagtttgtaacatctgcctacaggaaaaaacttgaggaacagcagaagtggctggaggaagagaaaaggcggc
agcgtcaagaagaaaaggaagatgttactaagaagaaagaccttagtgatttttactttgggcttcagaataatgttgcttttggtgcacagacgcatga
cagtgcaaagcatgacagtgcaaaacatgctaagcctcaaaagacggacaagaaaccagatgataccaaaaccaacagttgtggtccggaaatctct
gatcgttctccgaagcgtagaagagaatccagtgtagaatcagagcggcctaaaagtgtggaaaagccctcagcagatctacctgagcgggctaaaa
gtgtggaacgaccttcagcaaaccaattgaaagattcaacttctgctggatctactgagaagaatgctgatgttccagcaaatgcttcacataccccgcc
gaacactgagccggcaaaggttacagacgatcactacaaaagaactgatgatgcacttgctgctgctagagcacgagctctggcccgcaagagagcg
aaagagcagcaattactcgagggctaa
>g127266.t1
atggaagcaagcggtagccgtgcaacggaaggctcgccggcgacgcgcctcccggaggcggactcgctccccgacggcttcgtcgacagcttcgcag
ctgatcaggcccttcctctctcctcaagccccgtggcgaatgacctcccgcgaaccgccgtcgactccgatcgccctgcggccagcgtctctgatgtcggt
gaaaccctaggcgctccctcttcccgcacctctggcgttgcctccggagaaatcctagatgcctcctccgccgccgatgccctggggaccgtcgccttgg
acgcctctgcggagcaggatcgcgctcacgaacagcaagagaccaccggcgacacagcagatgctaaagggtccttgaaggagagcagtggttcag
aaccagcagaatctactgctggtcaaaaggagactggcgagctgaaacgcaaggtcactaagcgcagtaaactagagaaggacagagagttgttgc
agctcgcccagggataccagcaagtgctggcagaaagagatgcagccatcgcagtcaaagaaaaactagaatctctttgcagggagtttcagcttcaa
aacaaaatgctaaaggatgagtgtcgaaaggtgtcaacagaggggcataacatgcgtatggaactgtctgaaaaattcaataatgctataaaggatgt

gagtatcaagcttgaggagcagaaaaatgaatgcattgctcagctggaagagaacaatatgttaagaaacaaacttaaagaccttgccgatcagtatg
acattacccagcagaaatatgctcaccagttgaaagagaagatgctggaacttgagcttgctgatctgaagattcaacatcatcaagaaaaggctgcta
aagaacatactcaaatgcagttatatgctgagcaagtttctcaacttatcactactgaaaagaacctgcgagcacaactagctgccgatggagagaggt
ttcaacagtttcaggaaactctgtcaaaaagcaatgaagtctttgaaacttataagcaggagatggaaaagatggtgaaggtgatcaaaactctgaag
aaggagaatgaatttttgaagggaaaatgtgagaattcagatattgctcttgtgaagctcattgaagaggtaatgtgcatcgcaaaagctgattgttctg
aaacagggacacatgcaactatcgcctggacaggccttgcttga
>g145233.t1
atgccgccaaagcgggcgaacccgccggccggtgccgccgcggcctcgaccgagttcgagttctgcgagctgtgccgccgcaaccacgaccagggcc
ggcgccaccgctatttccccgctcaccgcgctgccctcgccgccgcgctcactcgcttccgctccaagctctccgaccttcgcggtgcctcctcctcgcag
catcagcagccgcagccccgactctggtgcaccttctgcgccatcgatctcgtcggcctcgatagccgctccgcttgcactgagcacctgaagggcgtca
aggatttcctgcggaagcatgggggagggatggatcaggtggattcacttaggatttcagaggacgaacttgctaagctgagtgtgtcagtttgtgccgt
ggcagaatga
>g149422.t1
atggaagcaagcggtagccgtgcaatggaaggctcgccggcgacgcgcctcccggaggcggactcgctccccgacggcttcgtcgacagcttcgcag
ctgatcaggcccctcctctctcctccagccccgtggcggatgacccccgcgaaccgccgtcgactccgatcgccctgcggccagcgtctctgatgtcgg
ggaaaccctaggcgctccctctccccgcacctctggcgttgccgccggagaaatcctagatgcctcctccgctgccgatgccctggggaccgtcgccttg
gacgcctctgcggagcaggagcgcgctcacgaacagcaagagaccaccggcgacacagcagatgctaaagggtccttgaaggagagcagtgcttca
gaaccagcagaatctactgctggtcaaaaggagactggcgagctgaaacgcaaggtcactaagcgcagtaaactagagaaggacagagagttgttg
cagctcgcccagggataccagcaagtgctggcagaaagagatgcagccattgcagtcaaagaaaaactagaatctctttgcagggagtttcagcgtca
aaacaaaatgctaaaggatgtgagcatcaagcttgaggagcagaaaaatgaatgcattgctcagctggaagagaacaatatgttaagaaacaaactt
aaagaccttgctgatcagtatgacattacccagcagaaatatgctcaccagttgaaagagaagatgctggaacttgagcttgctgatctgaagattcaa
catcatcaagaaaaggctgctaaagaacatactcaaatgcagttatatgctgagcaagtttctcaacttatcactactgaaaagaacctgcgagcacaa
ctagctgctgatggagagaggtttcaacagtttcaggaaactctgtcaaaaagcaatgaagtctttgaaacttataagcaggagatggaaaagatggtg
aaggtgataaaaactctgaagaaggagaatgaatttttgaagggaaaatgtgagaattcagatattgctcttgtgaagctcattgaagaggtaatgtgc
attgcaaaagctggttgttctgaaacagggtactgttga

Leucine-rich repeats domain containing genes

>g843.t1
atggcgctgctaccgtggtggtgggcggcggtactgctggttcttgcggcggcggccgaggcaggagcagatggaggcgacggggacgggcaggcg
ctgatggcgttgaaggccgggttcgggaacgcggccaacgcgctggtggactgggacggcggccgcgaccactgcgcctggcgcggggtcgcctgcg
acagcgcctccttcgcagtcgtcgccctgaacctgtcaaatctaaacctcggaggggagatctcgccagctataggggcgctcaagagcctacagtccg
tggatttgtctttcaatttgctgtatggagacatccccttctccgtctccaagctcaagcagctcgaggaactgattttgaagaataaccagctcactggac
ccattccttccacattgtcccagattccaaatctcaagatcttaggcttgaggggtaattcactgactggaacactatcacctgacatgtgccagcttaca
ggcctgtggtactttgatgtaaggggaaacaatctcactggaaccattccagagggcattgggaactgcactagctttgagattctggacatttcttacaa
ccaaatctcaggagaaataccttacaatattggttaccttcaagtagccacgctgtcacttcaaggaaatagactgactggaaaaatcccagaagtgatt
ggcctcatgcaggctcttgctgtcctagacctgagtgagaatgaactggttgggccaattcctccaatactaggcaacctatcctacacaggcaaactct
atttacatggcaacaaacttagtgggcacataccaccggaattggggaacatgagtaaactgagctacctgcagctgaatgacaatgaactagtgggc
acaattccatctgagcttggcaagctcacagaactatttgaattaaatcttgccaacaacaaccttgaaggtcatattcctgccaacatcagttcttgcac
agcactaaacaaattggatctttcctacaatgaattctatggaccagttcctcctaccattggtgatcttgagcatcttcttgaattgaatttgagtaaaaat
catcttactgggtctgtgcctgctgaatttggaaacttgaggagcgtccaagtaatcgacatatcctacaataatttgtctggtcatctccctgaagaactt
ggccagttgcagaaccttgatagtctgattcttaacaacaacaatttggctggagagatccctgctcagttggctaactgcttcagcctaaataccttgaa
tttgtcaaataacaacctttctggacgtgttccgtctggaaagaatttctcaaaatttccaatggatagctttgtgggtaatccaatgctacatgtgtactgc
caaaactctgcctgtggacactctcatggaactaaagttatttctcggacggtggttgcttgcatcatcttaggcttcataatattgctctgccttatgctatt
ggcaatatacaaaacaaaccggccagaaccacctgagaaaggatctgacaagccaatgcaagggccaccaaagctagtaagccttcaaatggacat
ggctatccatacttatgaagatattatgaggttgactgagaatttgagtgagaagtacatcattggttatggtgcgtcaagtaccgtgtacaagtgcctcc
tgaaaagtggcaaggccattgcagtcaagcggctttacagtcagtataaccacagccttcgtccatcaaagaaggtgaagcttgattgggacacgcgg
ctaaagattgcagttggtgctgcacaagggctggcatatctccaccatgactgcaacccacgtattgttcacagggatgtcaagtcctctaacatcctcct
tgatgagaactttgaagcgcatctttcagatttcggtatcgccaagtgtgtcccggctgccaagtcccatgcgtccacttacgttctaggaaccatcggct
acattgatccagagtatgcccggacgtcaaggctcaatgagaaatcagatgtctacagctttggcattgttcttttggagttgcttacagggaagaaggc
tgtcgacaacgaatcaaacttgcatcaattgatactctcaaaagctgatgacaacacggtgatggaggctgtggactcggaggtgtcagtgacgtgcac
agacatgaacctcgtccggaaggcattccagctcgcactgctgtgcaccaagaggcacccctccgaccggccaaccatgcacgaggtcgctagggttc
ttctctccctcctgccagcccctcctgtgaagccaaccacaacaaagacggttgactacactcgcttcctcgcgacaacagcagctgaaatgtcgcacga
catatctgacatcggagacaacagctcctctgacgagcagtggttcgtgaagtttggcgaggtcatatccaagcacacaatatcatga
>g1028.t1
atgcagcagctcatgaagaatccccgagatctcgtgaccatgctcctcaccatcgtcctatccctcgccgccgttgcccacgccggcgacgcagtagtag
gctgcgacaagtccgacggcgcggcattgctggccgtgaagaaggccctgggcgacccccgggtctctcgggctggaactccaccctcccctgctgc
gcgtggaagggcgtctcctgcaacgccaccacgggccgcgtgacggagctgaccatcttcgcgctcaacatctcggcgccgatccccatggccatggc
caacctcaccaagctgcagtccgtgaacctcgcctacaaccgcctgtacgggcccatcccgtccttcctgggcccgctggccctcccgggcctcaactac

ctccgcctcgacggcaaccgcctgacgggcaccatcccgcccacgctcacggtcctagacctcagcctcgtgggcaacctcctggtgggcccgatccct
gccaccttcggcgccgcgcagttcaacagcatggacctcgccgacaaccagctctccggcgacgcgtcgctgctgttcggcgccaagaagcagatcaa
cgcgctgcggctgtcgcgcaaccagttcaagttcgacctcggaagggtggagctgccggagccgctcgacatcctcgtcatcgaccacaacctcgtgta
cgggaccatcccgccggccgcggcggcgaggtccaggaagtggctggccttcgacgtcagcttcaaccagctgtgcgggcccataccgcagggacgc
ctcacgcggcggtttgggcccagacacttcgccgggaacaagtgcctctgcgcccgcccgctgccgccctgcagctaa
>g1832.t1
atggagcatttcttgatgcaacgtaccaccacatggccactccgtttcttcctctgctcgcctatcctgctgctcttcttggcaccagccaactccctgaatc
agagctcctgcgaccagcgcgatcttgacgccctgcaggggttctccaaaggtctcgctggaggaggcgtcagcggctggacgtttccaaactcgagct
ccgacaaggccaactgctgtgcatggcttggtgtcacctgcgacgatggtggacgagtcatcgggttggaccttcaaggcaggaggctcaggggcaag
ctgtcgcactcgctcggnnnnnnnnnnnnnnnnnnnnnnnnnnnnnnnnnnnnnnnnnnnnnnnnnnnnnnnnnnnnnnnnnn
nnnnnnnnnnnnnnnnnnnnnnnnnnnnnnnnnnnnnnnnnnnnagctgtcgcactcgctcggacagctggcccagctccagtggctc
aacctctccgacaacaatttccgcggcaccattccggcgcctgtgctccagctacaaaagctgcagcggttcgacctcagctataacgagctcaccggc
gcgttcccagccaacatatctctccctctgatcgaggtcttcaatatatcttacaacctcatcaatggctcccatcctacactccgtggctcgccacagctc
gtgttgttcgacgcaggatacaatttgtttgccgggcaaatcgacacaagcatctgcgagtcgtctggtatgatccgcgtgctgcagttcacatccaacct
cttcgctggggacttccccgcaggttttgggaactgcaccaagctcgaggagttataccttgacatcaacagcatctccggaatcttaccagatgacctg
ttcaggataccatctctgaggaaccttttctgcaggagaatcagctctctggcaggatgagccaaagattcagcaacctgtctagccttgcaaagctgg
acatatctttcaattcgttctctggataccttccaaatatttttggtagccttgacaagctggagtacttctccgcgcagtccaacttcttcaggggcccattg
ccttcctctttgtcccactcaccatcactgaagatgttgtacctgagaaacaattcgctgcatggagagatcagtctcaattgctcagcgatgacacaact
gacatcgcttgaccttgggacaaacaagttcattggcacaattgatagcattatctgtccttcaagactgttcaagcctaacgagcctagtgctgaccaa
gaacttccatga
>g1874.t1
atggtcgccagatgttattcatcatcatcagagtcagtagtagcactagtaccacttcgagtggtggtggttttggggttattagtggtgctcgtgtgtggc
agctcgtcgcagcaatgccacgaggaggacgaggcagcgctactggcgttgaacgccggtctgggcagcccataccacttcgagtcatggagccccg
acacgtggtgctgcgactggtacgacgtggactgcgacaacgtcaccggccgcgtcgtgggtctctccgtgttccaggacagcaacctcaccgccggc
gccatccccgacgccgtcgccaacctcacgcacctccgcagcctcactctgcaccacctcccggggctcaccggcgccatcccggagtcccttgctctcc
tcgccaacctctccgtcctcaccatctcctacaccggcatgtcgggacccgtgccgtcatttctgtcgcggctcacctccctgactttgctcgacctctctta
caactccttcaccggcgccatcccggcatccctcgccgatctccctaacctcgccggaatcaacctcagccgtaaccgtctcaccggcacaatcccgcac
atgctctttagcaaattatcatcctcatcttcttcctctgattctgaggagcagaagcagcagcagcagcagcagggaagaattgttagtctgtggctgtc
gcacaacgagctctccggcagcatcccggcagagttcgccgccgtcaacttcgcgtacgtcgacctgtcaaggaacgcattcaccggcgacccgtcgg
aggcgttcttcggcgcggcggcgaggccatcgatacagcacctggacctgtcaaggaaccgcttcgcgttcagcctcaccggcgccgagctgccggag
cagctcagcttcttggacctcagccacaacggcatccggggccggatcccggagcagctggcaaatctcaccaatctcaaggcgttcaacgtcagctac
aacaagctgtgcggccaggtgcccggtggtgggatcatgtccaggttcgacgcatacagcttccagcacaacaagtgcctctgcggagcgccgctcca
ggcatgccccaaaatgagctaa
>g1875.t1
atgatgctcctgctctgcttgttcgtcgccgtcattgcagcctcaacatcatcctcgtcgtcgtccagcagtagcagcagctgccaccccgacgacaaggc
cgccttgctggccatgaaagctgcgtggggcgatcccaagcagctggcctcctggacgcccgactcctcctcctgctgctgcgacggcgactggcacca
cgtcgagtgcgacgactccacctccaccaccaccggacaacggcgagtcaccagcctctccatcgtcgtctcccaacaacaagacgacgccgacgatg
acagtggcagcagcggtggtctcgtcgccggcgccatccccgacgccatcgcggggctgactcacctggagaaccttgacctgcgccgcctcccgggt
ctctccggccccatccctccggccatcgccaagctgtcgaagctcgcattcctcagcatctccaggacggggatctccggccccgtgccgtcgttcctcg
gcgacctaacctcgctgacggagctcgacctctcctacaactccctctccggctccatcccgccgtcttcgctcccctgctgcaacctctggagcatcgac
ctccgacacaaccgtctcaccggatccctcccaaagctcctcttctccaatcatcctactacaagcattaaccgagaacaacaacaagaagaagatgac
gatgacgatgaggaagaagaagatgagtataattacctgatcgatctgaggctgtcgcacaacaggctatccgggcccatcccggcggagtggtcgac
ggtgagcttcgggacggtctcgctctcccacaacgccctcacgggggacccgtcggcggcgctgctcggccgggacaggccgctgcagctgctcgaca
tctcccgcaacgccttcagcttcaacctctccgccgtggagctgcccgaggacctcgggttcatggacctcagccacaacgccgtctacggcgccgtccc
cgcgcaggcggctaacctgcagtacctcaacgtcagctataaccgcctctgcggcgagctgcccgccggcatggtaaacttgttcgacgcagacagctt
ccagaacaacacatgcctctgcggcgcccctctcgacccctgccggtag
>g1876.t1
atggcagccagcctggcgcttgacttgtcgctgagactgagagtcctctcttgtggcctcctcgatatatatctcgcaaccgcattgctcggttgtctacta
aataaaatcctcaaagaacacaagacgatggcaatgacgacgcgcttcctcctcctcctcgtcgtactagtactagtagtaccgctgcagctggccgcc
gcttcagcagcatccgggaagaagacgacaaagaagggagaccgtcgttgccactccggcgacaaggcggcgcttctggcggtgaaggcggcgctg
ggcaacccgtaccatttcgcgtcatggacgcccgacacgtcctgctgcgacgactggtacgacgtggactgcgacgccgccacgggacgcgtcgtcgg
cctcgccgtgttccaggacgccaacctcaccggcgccatccccgacgccatcgccggcctcgtccacctccagaacctcatgctgcaccacctcccggg
catctcgggtcccatcccttccgccatagccaagctctccaacctctcccagctcaccatctcatggaccggggtgtcaggtcccgtgccatcattccttgg
ctcgctcaccaagctcaccttgctcgacctctccttcaactccctctccggcgccatccctgcgagtctcggcaacctccctaacctcgccggcatcaacct
cagccggaaccgcctcacaggcaccatcccgcccctcctcttcagcaccaaatcttggtcgtcttcctcatcatcacccgacaacaatcagcaggcctac
ctgtggctgtcgcacaacaacctctccggcaccatcccggcagagttggccgccgtcaatttcgcgcacatcgacctctcgcgcaacgcattcaccggc
gacgccacacccattctcttctccggcgccggcgtcaacagcaagcagctgcagtacgtggacctttcccggaacggattcagcttcaacctctccgccg
tagccgccatgccggagcagctctacttcatggacgtcggccacaacgccatctacggcggcatcccggcgcaggtggcaaacctcaccaacctgcag

ttcttcaacgtcagctacaaccggctctgcggccacatccccgacggtggcaacatgcccagatttgatgtcttttccttccagcacaacaagtgcctctg
tggaaccccccttcctccctgcaactag
>g2272.t1
atgtcgtcgtcgcgtctgagcttccaaacgctcgagccggcgctcgccgcggcggcggagacgctgttccactcccgctccccgccgcgcgcggtcctc
cggcgcgcgcgccccccttcacgcgctcctcgtcgtctcctccctgtcctccgcgcccacgcgcaccaccttccttgtcaaccagctgctcgcgctctactcc
cgcctcgccgccgtccccgacgccctcgcgctcctccgatccacgccgcacccctccgtcgtctcctacaacaccgtgctctccgctctctcccgctccgc
gaggcacgcgccgcaggcgtttccgctcttccgggacctgcacgcgtcgggtctccgtcccacggctcccagcctctgcgccatcctccgcgccgtcggt
gtgctgcactacggtcgggcaggggccgccgcgcactctcaagccgcggtgctgggcttcctggccagcgacattgtgccgacggcgctgctgcagat
gtactcggcctgcgggtcgccgcgggacgccaaccaggtgttcgacgaaatgacaacgcgggatgtggtggcgtggaactgcgtcatgcactgcaac
gtgaggtatggctaccttgatcgcgctctcaggaaattctgcgggatgatgaggattgggctggcgcccacggagagcacgctttcatcagtgctgagt
gggtgtgcgcgcactggagcctcgcgccatggacgtgcacttcatggatgggtggtgaaatcagaagagcttgatcctgacctgccgctgcagaatgct
ctgctcgacatgtactgcagctgtggtgatatggactctgcacggcgtgtgtttcagaggattgaaacgcctgacttggtgtcatggaataccataattgc
tgggttttcaggcgttggggatggatggaatgctatggatacctttgttcagctcaaagccgtggtctccagtgaacaactcgctcctgatgagtacacgt
ttgcagctttggtgtctgctactgctgcattaccagcagtgtgtagtggcaagccgcttcacgcccaagtgatcaaagctgggttggagatcagcgttttt
gtagggaatacactgatcaatatgtacttcacgaacgaggagccggagtctgctcggattttgtttaataagcttatggtgaaagatgtaatcatgtgga
ccgagatggttgctggccactctgtactaggtgagggagaattggccctaaagtactttactaggatgctggaggaggggcacaagagcctattccatt
ctcgcctcatagcctcactgaagatgggagactaccactgtttgctatacatcttggttgcctcagtattgctccacgtccatggtgctggtggtcagttcct
gagccagacttgcgattccgctgacctggaggtgcttatatctttttccaatggcttggacaagaaggacgctgggcttgttggatggggccccgacaac
gcgtcctgctgctcctggactggtgttgcctgcgatcttgggagggtggtcgaggtggatctcttcaacaagagcctccatggcgtcatttcctcatcgatc
gcctccctcgacaggctcgtgactctaaacctctcccggaactcgctccatggccggacgcctgtagagcttggccgcctcgcgaggctgcaggtgctc
gacctcagtgcaaactggctatctggagagttttactctaccgaagggagcttcccggctatcaaagtggtgaatatctcctgcaaccaattcacgggcc
cgcatcctaggtttcctggcgctgtgaatctgacaattcttgatatctccagcaacaccttctctggtggcatcaacgccagcgcactctgtcttgcaccga
tcaagatcatatggttctcagggaatgaattcgcaggtcagatcccgtctgacttcagcgtctgcaagacacttgaagagctctgtctagatggcaatgg
cctcacagggagcctacccagcgaactgtacacgctgttggaattgatgaggctaatcttatgggaaaatcagctctctggtagcatcagcaaggacct
gggtaacctctctcagcttttgcagcttaacctatccttcaacaggttcaccggccccatccctgaagtgtttggaggtatgatgaggctggagtccataa
acttggccacaaatgggttcactggtgaattgcctgcttccctgtcaaggtgcccaacgctgagggtaatcaatctaaggaacaactcgctgtctggtgt
gattgctattgacttcaacttgctgccgaggctgaatacttttgatgctggaagcaacaatttgagtggtgttatacctcctggcttcgtgaggtgcaccaa
gttgaggacgctgaaccttgcaaggaacaagtttattggggagataccagagacctttaaggacttgaggtccctgtcgtacctctcactaacagggaa
tggcttcaccaatctttcgttggcattgcaagtcttgcagcacctgcccaacctaacgagcttggtgcttaccacaaatttccgtggggggagacaatgc
ctgtggctggcatcaatgggttcaagagcttgaagcactgctccacgtccatggtgctagtgggcggtggccagttcctgaaccagacttgcgacctctc
cgacctggaggcgcttatatctttctccaatggcttggacaaggacgccgggcttgccagatag
>g2274.t1
atgggaggattccgctggctgttcctcttctcgcttgtcgccgttctgctccacgttcatggcgcccagtcccagaaccagacgtgccacccggcagacct
gaaggcgcttcttgccttctccaatggcttggacaggaagggcgaggggcttgtcgggtggggctccggcgacgccgcctgctgttcctggaccggcgt
cgcctgcgatcttgggagggtggtcgggttggatctctacaacaagagcctacatggcggcatctcctcctccgtcgcctccctcgatggccttgtgacgc
taaacctctccggcaactcccttcgcggctcgccgccggcggagctcggcagcctgacgaggctgcgggtgctcgacctcagcaggaacgggctctcc
ggcccgttcgcgactagcgacaacggcttcccggcgatcgaggtggtcaacatctcctccaacaatttcaccgggccgcacccttcgttccccggcgcg
gcgaacctgacggttcttgatatctccggcaacagcttcaccggtggcatcaacgcctctgcgctctgtgttgcgccggtcgaaactctgcggttctcgga
gaatgcgttctccggtgaggtccccggcggcttcggcctgtgcaaggcgctggttgagctctctctcgacggcaatggcttaactgggaacctacctagc
gacctgtacacgatgccgaagttgacgaggctcagcttacaggacaatcagctctccggcaacctcggcaatgggcttggtaacctctctcagcttgtgc
agcttgacttatcctacaacaggttcaccggtcccatccctgatgtgtttggaggtatgaggaggctggagtccataaacttggccacaaataggttcttt
ggtgaattgcctccttccttgtcaagttgcccaacactgagggtaatcagtctaaggaacaacacactgtttggtgctattgatatcaacttcgacatgctg
ccaaggctgagcacttttgatgctggaagcaacaatttgactggtcatatacctcctggcttcgcatcgtgcactgagttgaggacgctgaaccttgcaa
ggaacaagcttgatgggaagataccagagaccttcaaggatttgaggtccctgtcgtacctctcactaacagggaatggcttcactgatctgtcatcagc
attacaaatcttgcagcacctgcccaacctggcgagcttggtgctcaccaggaatttccgtggtggtgagataatgccagntcttgcagcacctgcccaa
cctggcgagcttggtgctcaccaggaatttccgtggtggtga
>g2540.t1
atggccgccaaagctcttccgctcctcctcgccctcgccgccttgctcatcaccgccccgtcgacgcacgcgtcgtcctgctcggccgcggaccgcgacg
cgctgctctccatccgggcggcgctctccgacgacgcgcacctgggcgtcttctccacctggacagcaaacaccgactgctgcgccaactggtacggtg
tggcctgcgaccccaccaccggccgcgtggggtacctctccctccgcggcgaggccgaggacgccgtgatggcccccgccggccgccccacctccggc
gtcatgtcaggctacgtctccgacgcggtctgccacctcgaccgcctctccatgctcgtgctcgccgactggaaacgcatcacgggtcccgtccccacct
gcatccccacctccctcccttccctccgcgtcctcgagctcccgggcaactccctcaccggcgacatcccggccgccatcggaaccctctcgcgtctcac
ggtgctcaacctcgccgacaaccacctcacgggctccatcccggcctccatcacatccctcgggtcctcgctcaagcacctcgacctcgccaacaaccg
gctcacgggccgcgtcccgacggacttcgggaacctcgccatgctcagccgggccctgctgggccggaacaggctgtccgggcccatcccggcgtcgg
tggcctccatgccccggctcgccgacctcgacctctccgagaaccagctcacgggcccggtcccggccgccctcggctccagccgcgtgctcgcgtcgc
tctacctcggcgcgaaccggatctcggggaatattccggcgagcctgctcgggaacagcgggctggggatcctcaacctcagccgcaacgccgtcgac
ggcgccatccccgacgcgttcacgccaaggtcctacttcacgatgctcgacctctcgaggaaccggctcacgggcgccgtgccgcggtcgctcgccgac
gccgcgtacgtgggacacctcgacctcagccataaccggctctgcgggaccatccccgccggcgcgccgttcgaccacctcgacgccgcgtcgttcgc
aagcaacagctgcctctgcggcggacccctcggcaagtgcacgtga

>g2580.t1
atgcgggccggaggcggacggcgcctcgggccgctcgggattgaggtcctcgccttcctgctgctgatcgccgcggcggcattggtgccgcgttcgcgt
gcggccaccgacccctcagatgttgctgcgataaatgagctttatgttgctcttggatcaccaactctgcccggatggacggcgagtgctggagatccct
gtggtgagaagtggcaaggtgtcacatgtatcggcacaagtataaactcaatagtttttaatgctgcaaatttggggggacagctaggcggcctggaga
agttcacgacaatagctgaaatctttctctcagataaccaactcactggaatcttagtgttgctggtaatattttgcctgtccaagtaccaagagagagag
tcaagacgtgattatgctacaagtcagttggggagggtgcgtcagagagttgaggagccgaaaatcaagcaagttccagtacagtctacaaatgatgc
taagaaaggttcagctgagattcttgaacgaaagaagcctcgtgaaataaatttatcggtaccaggttcagctgagattcctggaaagaagcctcgtga
aataaatttatctgtaccagctgcccttgagaaacctcctgaaaagagaaaggagcatgttatcaatttggaaagaacagaatcggagatctttgcgac
aacacctccaccacctccacctccgccaccaatgccaccatcacaaccaccgccgccaccaccatcacagtcagcaccagcgccgccaccaccaccat
cgccgccactaccatcacaaccatcaccgccacagccgccgccacctcctcctcctgttgaaaaattattggaggttatgaaaatcgataatgctaatga
tagaataccagtagatgactttctggagctggttgcacgtatatcagatattaggcatcctaacattcttgagcttgttggatactgtgctgaatatggaca
acggttactggtctataaccacttcagtagagaaacactacatgatgtactacatgagggcgagggtctggatgatactttatcctggaacgatcgcctc
catgttgctcttgatgctgcaaaagcgttagagtatctccatgacacttgtgaaccgccagttgtgcatcagaattttgaaccagccaatgtgctccttgat
gatggatgctcagtacgagttgctgaatgtggactggcagaactgatggggtcaggttctgttacacagacatcatcagtcgctgtattcagatcctcctc
gtccttgaggccaaagtgcctctctattagacttctggctgcatacaagtacttttcaactgctttccttgttcttcatgcttatactggtaaaagaaattga
>g3162.t1
atgcacgtccgggcgcgatggcagcagcaggaggccgcggcgctcctccagctgaagcagtccttctcttttattggcccttcgttctgcagcaacagcg
aggcatcagacgacacgaccattttattgccgtcgtggagggccggcaacgactgctgccgctgggaaggcattagctgtgaccgtgttgctgtcaggg
ttaccgcactcgacctttcgaatcaacatatgcagatcagaggctttcacccggccctgttcaacctcacctcactcagatatctcaacctggagcacaac
gagttctgtggctcccacctgccagattttggttttgataggttgacacagcttcaagtccttaacttaagggaaagcaacctctctgaaattcttgacctt
gcttataatccattcgaacacggaacattacctcttggcatcactcatctgaaaaacctcaagtatcaggtggcagtcgatgtcgccatgaaacagcagt
acatgagggtctctaaaatctttagcgacctggtggtgattgacctgtcgaacaacagattcagcggctccattcccaggacggtaggcaaccttacgtc
cttgctcgtgctaaacatgtctcataacgcattgaccggcccaatccccggcgagcttggccgtcttgctcagattgagtccctcgacctctcgtggaacc
gtctcacaggggagatcccccggcagcttgccgcattgacggccctcgaatggctcaacctctcacataatgacctcagcgggagtataccatcgggaa
gccagttctcgacgttcaccagcagctcgttccaaggcggcaacccagggctgtacggatgcccgcttcctgtgagatgcaatctgacgcagccatcac
caggcaccaaggtcctgccgccgccgtcaccgcaacagcaggttcccgatggggcatcggctgaccgcaggttcgagcttatcgtgctctggctcttag
tcggttctggctatggtatgggcttcgcactggcagttgtcttgcaggtggtcctcacccgcaggcgcaagaaaatactagcacggcaaagtgtttaa
>g3164.t1
atgctagaattcaaggtcacagttcacatcaacctggccaacaataaactcagcgggcctttcctatatgcggggtgccattctaatgatcagctgcaaa
tacttgccctatccggcaacaacttaaatgggtcgatccctcctcatctgctcaaaggctgcactggtcttgcagtgctaaatcttaggggcaatcgcctc
agtggaacatggcctgatgagatggacaagttttgcggcctgacgcttgtagatttacacgggaaccagttcgaaggtcctctgccaaggactcttgtaa
attgcacagagttgcagttccttgatgttggtgggaacaattttgtggatgtattcccaacatggttgggtaaccttcgagatcttcgtatacttgttctgag
atccaataaattttacggcccattaagcatgccgcttgcgcttgggaggaaccatacaaccaccagtggttacttccccagtctccaagtcattgaccttg
caggaaacagcttcaccggagtcataccttcagaactcttcgacagtttcatgtccatggtgcgcggcccactccacaatactcgaagcggttataattat
agcacactgtatgttgactctgcgacatcaacttacggtgtggaggtggaggtcgccctgaagcaacaatacatgaagatgcttaaagtcttcagcgat
ctggttgttattgacctatccaacaacagattcagcggccccattcccaatacggtcggcaacctaatggccttggttgtgctcaatatgtcgcataatgc
cttcactggtggtatcccaggtgagctcggccgcctggctcagattgagtcactcgacctctcatggaacaatttcactggcgagatcccgcgagaattg
ctcgcattgacggccctcgagtggctcaacctatcacacaacggtctgagcgggagcatacctttgggcggccagttatcgacgttccccagcagctcgt
tccaaggcaaccgaggactctatggatgcccgcttcttgtgcgatgcaatctgacgcagccaccgccgggcaccaatattctgcctccactgcaagtgcc
tgatgaatcggctggccataggttcgaacttatagtgctatggctcttggttgtggctatggtgtgggctttgcactggcagttgtcttgcagttggtcttca
cccgcaggcgcaacaaaaacaacacacatgtaa
>g3906.t1
atgctccggcgcagcgctgccgccgcggcggcggcggtgctctgtgcgctgctactgctactgcaccccgccgcgtcgcaggagggcgacgtgccgga
atcctacgccgcctcgttcgccgcccggttcgccgcgccgccgtcctggtcgtttcccaacccgcgcctccgcgccgcgtacgccgcgctgcaggcgtgg
aagcgcacggccatcttctccgacccgtccaacttcaccggcaactgggtgggccccaacgtctgcgcctacaacggcgtcttctgcgccccgctcccc
ggatcctacggccacgacctggtcgtcgcggggatcgacctcaaccacgccgacatcgccggctacctcccggcgtcgctgccgctgggcgtccctgat
ctggcgctgctccacctcaactccaaccgcttctgcggcgtggtgcccaccaccttccgccgcctgcacctcctccacgagctcgacctcagcaacaacc
gcttcgtcggcgccttcccggaagtcgtcctctccctgccgtccctgaaatacctcgacctccggttcaacgagttcgagggctccctcccgccggcgctg
ttcgatcggcccctcgacgccatcttcgtcaactcgaaccggctgcgcaacccgatcccagccaacctcggcaactccccggcctccgtggtggtgctcg
cgcacaaccgcctcggcggatgcatcccgccgtccatcggccggatggcaggcacgctcaacgagatcgtcctcatcgccgacgagctcgtcgggtgc
gtcccgccgcaggtcgggttgttgaagaaggtcaccgtgttcgacgtcagcgacaaccacctccagggccagatcccggcgagtgttgcggggatggt
cgccgtcgaggagctcgacttcgccgggaacaggttcgagggcgcggtcccggcgggcgtgtgcgggctcgtggccttgaagaacctgacgtatacca
acaacttcatcagctccaggcccagctgcgccaaggccacggccgacggcgcgtggaactgtattcccggcgccccggcccagcgcccgccggccca
gtgcgctgccgccgccgcacgcccattcgactgcagcaaggcgcagtgtcaggctgcggcgtacacgcccaccacgccggcgggatcggtgcctcctt
ccaagggacccaccacgccgtcctacccgtctccgccgtcgagcgcgaccacgccgtcgcacccctcgccgccgactcaggggcccagcaccccgtcg
cacccctcgccgccgacccaggggcccaccaccccgtcgtacccgccgccgccgtccagcgcgacgacaccgtcttaccacatgccgccgacgggatc
cccactcccgcgccgtcgacatacccgtcgccgccgtcaagctccagcacgccgtcctaccaccactcgtcgccgccgccgcagggcacgcccaccc
cgtcccacccgtcgccatcacctccagtgacaacgcacacgccgcccccgccgcccacgtccgccgacgagccggacgtgcggtacgcgccgccgcc
gcgctcgcacaggccccacccgtccacgccgccgtctcacggtggcgcgtcttccccgcctccctcatcaacgggctaccaccagccacccaccgagca

cccaggctacgtcttgccgccaccgtcccacccgggctcccacgcgccgccgacgacgccgtcgtccccgccgccgcaccactgcgcgccgccgcctc
agagcggcagcgccggcggtgaccaccaacccggcgggaagctgccgttcccgcccgtctacggcgtgtcgtacgcgtcgccgccgccgccggtgaa
gccgtacaactag
>g4195.t1
atgaggcggcggccaccaccacctttcgtcgcccgtgccgccgccgtgttcctgcttcttctgctgtcccccgccccgacctcccagctcggcctcggcgc
cgccattggcgcctggatcaacggcgcgccgccaccttctgctgctgctgatcccacctccggcagccagcagcagggtcagcagccgagcccggagt
acacctcgctgcaggcattgaagtccgccgtcaccgacgacccccgcggcgtgctgtccacctggcacgggcccaacgtctgcgcctacaagggcgtc
tactgctccgcctcccccgacgccgacgccgacgccacgacggtggtcgtcgccggcgtggacctcaaccggttcaacctccgcggcacgctccccga
cgcggcggtcgccggcctggcccacctcacgttcctccacctcaacagcaaccgcctctcgggcgccgtcccggacgccctccgcgacctggcctacct
caccgagctcgacctgagcaacaacctcttctccggcgcgttccccacctcccctctcctcgcgctctccctcgtctacctcgacctccgcttcaacgcctt
ctccggcgccctcccgccggacctcttcaccaaacccaacctcgacgccctcttcctcaacaacaaccagttcgacggcgagattcccgacacgctctg
gtcctccccggccaccgtcatcaccctcgccaacaaccgcttcaccggccagctcccctccacctacggctacaccggcggtggtagagtccgggaagc
gctgttcctcaacaacaacctgaccgggtgcgtgccggaggcgctgggattcatgcccagcatccaggtgctcgatttaagctacaacgcgctcaccgg
ccacctccccggaaccatctcctgcctctccggcatcgaggtgctcaacgtcgcgcacaaccagctcacgggggagctacccgacctgctctgcgacct
gcgccggatcaccaacctctccgtcgcgttcaacttcttctccgggatcagccagcgctgcgacaggctcgccgggcgcagcatgttcgacttcgtcggg
aactgcgtgcccggaagagacatgcagcggccgcaacccgagtgtgatgggtttgggggtgatggcgggctgagctgcctccgtattcccggggcaa
ggccggttgggtgcgccgaggcggcggtgtcggtcggcgttggagttggagtcagtgttgggggactgccgttcgggttgcccggcgcgtcggccggc
ggcgtggtcaccgtctccgtgccatga
>g4246.t1
atggaggcgcctccgtcttcttcttccccgtccctgttgctcctcctcctcctgctcgtctccccttcatcttcgtctgccgtcctctcccccaagggcgtcaac
tacgaagtgcaagctctgatggcgatcaaggacctcctcaaagatccccatggcgtgttcaaggcctgggacataaactctgtggatccctgcagctgg
gctatggtcacttgctcaccagatagttctgtcactggactggaagccccaagtaagaatttctccggcacgctctccccaagcataggaaacctgacca
atattgagactgttctcctgcagaacaacgatataaccgggccaataccaacagagattggcaagcttgcaaagctcaagacccttgatctctccagca
accacctgtatggcgaaatccccacctctgtgggccaccttgaaagcctccagtacttgaggctcaacaacaacaccctgtctggtccattcccctcggc
atcagctaatttatcccaacttgttttcctagacttatcatataataaccttagtggtccaatactaggatctttggcaagaacattcaacatagttgggaat
cctctcatatgtggaacgagtatggagaaagattgttacggcactgcgccaatgccagtgtcctacaatctgactagctcacagggtgctgtaccgccgg
caaaatccaaaagccacaagtttgcagttgcatttggttctgccattggttgcatcagcttccttttccttgctgctggatttctcttctggtggaggaaccg
tcgaaaccgccagatccttttccatgtcgacgaccaacacatggaaaacgttagccttggaaacgtgaagcggttccagttcagggagcttcagtccgc
aacagacaatttcagcagcaaaaacatactaggtaaaggcggctttggaaacgtttacagagggcagctccctgatggaactcttgtggccgtcaagc
gactgaaggatggcaatgctgctggcggggaggcacagttccagactgaagttgagatgatcagcttggcactgcacaggaacctcctcaggctctat
ggattttgcatgactgctacagagaggctactggtctatccatacatgtcaaatggaagcgtcgcatcgcgtctgaaagggaagccacctttggactgg
atgaccaggaagaggatagcccttggggcagggagagggttactgtacctgcacgagcagtgtgaccccaagatcattcatagggacgtcaaggcag
ccaacatactcctggatgaatactgtgaggcgatcgtcggcgacttcggtcttgccaagcttctcgatcaccgggattcacatgtcaccactgcagtgag
gggcacagttggccacattgcgcctgagtacctcagcactggccagtcttctgagaagactgatgttttcggcttcggcatcctgctgctggaactgatca
ctgggcagaccgcgctggagttcggtaaatcagcaaaccagaagggagccatgctggattgggtgaaaaaaatgcaccaggagaagaagctggacg
tgctggtggacaccggcctgaagggcggctacgacaggatcgagctggaggagatggtgcaggttgcgctgctgtgcacgcagtacctccctgtccac
cggccgaagatgtcggaggtggtacggatgctggagggcgatgggctcgcggagcggtgggaggcgtcgcagcgcgcagactcgcacaagttcaag
gtgccggacttcaccttcagccggtgctattccgacctcaccgacgactcgtcgctgctggtgcaggccgtcgagctctccgggccgagatga
>g4711.t1
atgaacaactacgatcaaggcgagacgcccgcaagcatcggaaacctcaggaacctcacgtacctgtacttggcggcttgcagcttgacaggggagat
accttcctccatcttccagctcactgccctggaaacactggacatatcaatgaacaacctcgccggcaggatccccggagccattggcaacctccggaa
cctgtggaagatcgagctgtacaagaataacctctccggggagctgccgccggagctcgggaagctgacggggctgcgggaaatcgacgtgtcccag
aaccagctcagcggcgggatcccggcggccatggccgagctcancaacctctccggggagctgccgccggagctcgggaagctgacgaggctgcag
gagatcgacgtgtcccagaaccagctcagtggtgggatcccggcggccatggccgagctcaagggaacctga
>g5308.t1
atgggaaagggtgcagctttgccggagatgcaagggcgggaggagaagtgccggaggccggtacatgcagcgaagggggcgccgcccaaccgagg
tcgccctgcggagcccgatgcggcggcgccggcggcggtctcgtgcaggggagcgagaaaaccagcagggggaagggcgggagaaggagggccg
atggctggtacctgtgacaaaggagggccggagggggcggtgatggcagcggcggtgacgccctccgttccgtctgctccgttccagcgctcggggct
tagcgattcttttttttttttggcgaggtgttctgaaccggctatcgcctttaactgtggtcagctgcaatcgttaaacctgggttggtgcgacagtattactg
acaagggggtgaccagcttggcatcaggatgccctgaacttagggctctggacttgtgtggttgtgttctgataacagatgagagcgtggttgccctcgc
gaacgggtgcccgcacctgcgttccctggggctgtactactgccagaacatcaccgaccgcgccatgtactccctggccgcgaacagccgcgtgcgga
gcaacaagggccgtggctgggacgcggtgaagagcgacaaggacgggctggcgagcctgaacatcagccagtgcacggcgctgacgccgccggcg
gtgcaggccgtgtgcgactcgttcccggcgctgcacacgtgcccggaacggcactcgctcatcatcagcggctgcctcagcctcacctccgtccactgc
gcctgcgcgctccacccgcaccgcgccggcggcagggccatcctctccaaccagcacgcctattga
>g5583.t1
atggctctccagctccttcccatcgtgatactggccgtcgtgctgccggcggctgcccagagcaacgacaccgacctcgacgcactgctcgggttccag
gcacagctctcggaccctctcggcgttctgcgtggcaactggacgcccgacacgtccttctgcaactggctgggcgtctcgtgcagccgacgccgtcag
cgcgtcacggccctggagctaacaaacactcccctccgtgggagcatttctccccacatcggtaacctctctttcctctctgtcctgatcctcgccaacgcc
aatatcacaggatcaattccagctgagcttggaaggctgcgccgtctcaaggtccttgctcttcctgaaaacagcctgttaggttccatccctccagccat

cggaaacctcacggagctccagcaacttgttctgtacaagaacagtctgtcagggcagatcccacctgagctgcaaaacctgcgaaatcttaagtatttt
gatctgcatgggaatttcttaactggagaaataccacatgaactcttcaacaacacgcctcacctaagccatctgatccttgccaacaacagcctgttcg
gacatataccgggcgaattccacctgaattggggcagttga
>g5744.t1
atggccaagttgctaatcccaatcatcatcatctttcatgtcggcctcgtgccgatcgctgtcgcggaagcagtcgccgcaccagcaccagtgatcgacg
acgaccgctccgcgctcctcgcgttcctctccaacgtgtcatccgaccccggcggcgccctcgcggactggggccgctcgccggagttctgcaactgga
cgggcgtcacgtgcggcggcccgggccggcggcgcgtcgtcacgcagctggtgctcagcgggaagggcatccgcggcgtggtctcgccggcgttggg
ccggctgtctttcctctccgtgctcgacctctcaagcaacgctctctccggcgcgatcccgcgagaggttgcaaatctggccatgctgacgcagctgagct
tgacgaacaacctcctcgagggcgccatcccggccggcctcggcctcctccagaagctctactacctcgacctcagcagcaaccgtctctccggcggca
ttccccagacgctcttctgcaactgctccgcgctgcagtacttggacctcgcaaacaactccctcgccggcgacatcccgtacgccgacgagtgccgtct
ccccaacctccgtttcctcctcctctggtccaacaccctctccggcacgatcccgccggcgctggccaactcctccatcctcgagtgggtcgacttcgagt
cgaactacctctccggcgaactcccgtcgcaggtgttcgacaagcttccgcggctcgtattcctctacctctcctacaacaacctgtcgagccacaacgg
caacaccgacctggaccccttcttccgctccctgagcaactgcactcgcctgcaggagctggagctcgccggaaacaacctcggcgggcgtctccctcc
ctcctccaccggccgcctcccgcgcggcctccggcagctccacctcgaggacaacgccatcacgggccccatccacccaaacatctccggcctcgtcaa
cctcacctacctcaacctctccaacaacctgctcaacggctccatcccgccggccatctcccgcatgcagcgcctcgagcggctgtatttatccaacaac
ctcctctccggcgagatcccccggtccatcggcgagaccctccgcctcggcctcgtggacctctccgggaaccgcctttccggaccgatccccgacacgt
tctccaacctgacgcagctcaggcggctgatgctccaccacaaccggctctccggcgccatcccccgagcctcggcgactgcctcaaccttgaaatcct
ggacctctcctacaacggcttgcaaggccccatcccggcctacgtcgctgccctgaacagcctcaaactctacctcaacctctccaacaaccaccttgac
gggcccctccccatcgagctgagcaagatggacatgatcctcgctctagatttgtcagtgaacaggttatccggcacgatcccgtcgcagctcggcggg
tgcgtcgcgctcgagtaccttaacctgtccgggaacgcgctgcacggcgcgctcccgtcgtccgtcgccgcgctgccgttcctgcggtccatcgacgtgt
cgcacaacgcgctgtcgggacccctccccgcgtcgctgcaggcgtccacgtcgatccgggaggccgacttctcctacaacaacttctccggcgtcatcc
ccgacgccggcggcggcggcgtcctcgggagcctctcggccgacgcgttccgtggcaacccgggcctgtgcgggcggcacggcattattgccgcgtgc
gaagcgaacagcaagcgcgggcggcgccaccgccgccgtcggccactggtctccgtcgccgtgggcatcgtggcgtgcgtctcgctcatgctctgcgc
ggtcgggtaccggtccgggcggcgcaacgggtcgtcgatgatgcggctcgtggacgccgaaaatacgaacccgacgacgacgacgacggagcggga
ggagtacccgaggatatcgtaccgggagctctccgacgcgacggccgggttcgcgccagagaggcttatcgggtcgggtcggttcgggcgggtgtacg
aggggacgctccgcgacggcgccacgcgcgtggcggtgaaggtgcttgacccgaaaggcgggtgcggcggggaggtgtcgcgtagtttcaagcggg
agtgcgaggtgctgcgtcgcacgcggcacaggaacctcgtcagggtgatcaccacgtgcagcacggacagcttcaacgcgctcgtgctgccgctcatg
cgcaacggcagcctcgaggaccgcctccacccgccgctcggggacgacgacgaaggcggcggaggagggctgcgtttcgggcagatcatggccgtc
gtgggcgacgtcgcggaggggatggcgtacatgcaccactacgcgccggtgaaggtcgtgcactgcgacctcaagcccagcaacgtcctcctcgacg
acgggatgcgcgccgtcatctcggacttcggcatcgccaagctcgtcctcgccggtggcgtcggaggaggncatctcggacttcggcatcgccaagctc
gtcctcgccggtggcgtcggaggaggggaagccagcagcgcaagcgacgaggccgcgccgtgcaactccatcaccgggctgttgcagggctcggttg
ggtacatcgcacctggtactatggattaggagggcacccttcgacgcagggagacgtgtacagcttcggcgtgatgcttctggaactgatcaccgggaa
gaggccgacggacgtgatcttccaggagggcctcacgctgcacgactgggtcaggcggcactacccgcacgacgtcgcggccgtggtcgcgcacgcg
ccgtggcgagggcgcgagctgccgcagccggcggccgacgtcgcggtcgtcgagctgatcgagctcgggctcgtgtgcacgcagcactcgccggcgc
tgcggcccacgatggtcgacgtatgccacgagatcacgctgctcaaggaggatctcgcgaggcacggcgccgacgaagatgacggcggacgccggt
cgttctcgaccaaggactcgctgttctcgaactga
>g5745.t1
atggaggttgcaccaatgtccgctatcatcttcacctactttttctcttcttctccaacggtccaagcccgatttttcagctggctggtcgcacgagcgctc
tgcacttctatccttcaagtctggtgtgtctaatgaccccaagggagctcttgctggttggggttcatctaatgtgtgcaactggaccggtgttgcttgcga
catggcgaggagacatgtcgtgaagctggagctaagagaccaaaagctctccggtgaggtctctcctgcacttggcaacctctcccacctcaacatact
caatctatcgggaaatctcttcaccgggaaggtcccgtcggagttggggaacctctttcacctcaatttactagacatatcggcgaattcatttgccggga
ggattccgccggagcttggcaacctctccagcctcaattcacttgacctctccggaaacttgttcaccggagaagtgctgccggagctcgggaacctctc
caaactgacgcaactgagcattggcagcaacaatctggaagggtccatccccaccgagctcacacacattcggaatctcatttacctcaacctcggcga
gaacaacctctccgggcacatcccggaggccatcttctgcaacttctctgcgttgcagtacattgacctctcctcgaattcgctcgacggcacgattccca
tccgtggtgactgcccgctccctgacctgatgttcctggtcctgtggtcgaacaacctcgtgggcggcatccccagctcgatatccaactccactaagctc
aaatggctgctgctggagtccaatttccttaccggcgagctgccatccgacatgttcagcaacacgagagacttggagctcctttacctctcatacaacta
cttggagagcccggagaacaacaccaacctcgcgccgttcttcgcgtccctcacaaactgcaccaagctcaaggagctcggcgtcgccgggaacgag
atcgccgggacgatcccgccggtcgtgggccaactctccacggaactcaggcagctccacctcgagtacaacaagatcttcggcccggtcccggccaa
cctctccgacctcgccaaccttaccgcgctgaacctctcccacaacctcctcaacggctccatcccgccgggcatcgcggggatgcagcgcctcgagcg
gctgtacctctcgaacaacctgctctccggcgagatcccgccgtccgtcggcgcgatcccacggctggggctcgtcgacctctcccacaaccggctcgc
cggcgccgtccccgccgcgctctccaacctcacgcagctccgcgtgctcgtgctctctcacaaccgcctctccggctccatacctccgacgctctcccagt
gcgtgaacctgcagaatttcgatctctctcgcaacgcgttgcacgggaagatccccgcggacctgtccgagctgagcagcctcctctacctgaacctctc
cagcaacatgctcgaaggacccatcccggtgaccgtcagccagatgacgatgctgcaggtgctcaacctgtcgtccaacaggctgtcaggcccgatac
cgccgcagctcggcagctgcgtcgcgctcgagtacgtcgacgtctccggaaacgcgctcgccggcggcctcccggactccgtcgccgccctgccgttcc
tgcagctgctcgacgtgtcgcgcaacgcgctgaccggcgcgctgccgctgtcgctggaggcgtcggcgtcgctgcgacgcgtcaacttctcgtacaacg
agttctccggtgaggtgccgggagccggcgcgttcgcggggttccctcccgacgcgttcctcggcaacccgggcctgtgcggcggtcacgacgtggcg
gggttggcgccgtgcggtaaccacgcgacgcgccgcgtcgcgtggctccgtgaccggcgcgtcgtgctgcccgtggtgatcacggtggccgtgttcatt
ctcgccatcgtcatcggcgccgtcgcgcggcgcgcgacggcgcgcgcggaggtgaggcgcgactcgcggcggtcgacgctgctggccgacggcgcc
gacgagccgcagccgccgatggagcgggaccacccgcgcgtgtcgcaccgcgagctcgccgaggcgacccgcggcttcgagccggcgcggctgatc

ggcgcggggcgattcgggcgcgtgtacgagggcacgctccgcgacggcacgcgcgtggcggtgaaggtgctggacccgaagggcggcggggaggtt
tcgaggagcttcaagcgggagtgcgaggtgctgcggcgcacgcggcacagaaacctcgtcagggtggtcaccgcgtgcagccagccggatttccacg
cgctcgtgctcccgctcatgcgaaacggcagcctcgagagccggctgtacccgcccgacggcgggccgggtcgcgggctcgacctcgcgcgggtggtc
gccattgctggcgacgtcgcggaggggctcgcgtacctgcaccactacgcgccggtgcgcgtcgtgcactgcgacctcaagccgagtaacgtgctgctc
gacgatgacatgaccgccgtcgtggcggactttggtatcgccaagctggtgaaggacgtcggcgacggcgacttcggtagctcggctgatccctgcaa
ctccattaccgggttgttacagggttctgtcggctacatcgcgccagagtacggattaggaggccacccttcgacacaaggcgacgtgtacagcttcggc
gtcatgcttctcgagctgattaccgggaagaggccgacggacgtgatcttccaggagggcctcacgctgcacgactgggtcaagcagcactacccgca
cgacgtcggcgcgatcgtcgcgcggtcgtggctgacggacgcggcgacggcggtggcggacgagacgctcagggaggacgtcatggtcgtgctaatc
gacctggggctccagtgcacgcaacactcgccaacggcgcggccgacgatggtggaggtgtgccacgagatcacactcctcaaggaagacctcgcca
agcaccggggctctgtggtcgcagtggcaagaggggcggggtcggtgacgatcacagcaagcgagcggtcatgctccagctcggattcgtctttatga
>g6133.t1
atggcgatgggcgttgccggagtggccgtggtgctattggccgccgccttcgtggggactaccgccgacaccaactccgacgatgtgaccgccctcaac
accttctacacaaccctgaattcgccatcggagctgacaaactgggtgtcccagaatggcgacccttgtggccagtcgtggttcgggattgcttgctctg
gttcaagagtgacaacaataaatcttgaaaacaacagcttcacagggacattaccttactccatttcccagatggctgcacttaaatatctaaatcttggt
cataatcagctgtcagacatcaatgctgagttcaaccaactcaccaacttaacaacactaaatgttgcaaataaccaattcagcggatcgattcctgata
agctgaagagcataagcaatctccagacaaatggcaactcctttagtaatagtcctgcagcagcagcccctgcacctcaaccacacactccaccattgc
aaccaatccaaccatcgcctactggagttcctagtcatagtaacaaaagtccgtccagtggaagccacacagacaatggtggcaaagatgctcgtggt
agcacaaacggtggtcgcaaaagctccaaagtaggaggtggcactgttgcgggaattgtggtatctctggtggctgttggtgcattggtttccttctgtgt
gatcaagaggaaatctgcgatgcgtcaattgggggatgatcctgaaaagaatggacatctcagtcctcttgcttctggaaaaattaaacagctgaggcc
catctgcgctgtctcactctcaccaactggaaaggaaagattggaaaagagtctctcaatgaacttgagaccgccatcgaaaatcgagttacacaagtc
cttcgatgaaaatgatcccacaagcaagcctgtggccaaggaaataaacatttcttccatcagggcaactgtgtatactgtagcagacctgcaagtggc
aacggagagctttaacgccgacaatctggtcggtgaaggttctttcggatgtgtctacagagcaaggttgccatatcagaaggtatctacatga
>g6264.t1
atggccgtacaactggaggcattgctggagttcaagaagggcatcgcatctggagagacccgaaaggcgtgctactatccaactggaccgtcgggga
aggcagcggcgtcgttggtggtctcccgcggcactgccactgggaatacgccaacggccacgtcaccgtccatcatgatgcaggggacctggttcgag
ggcgcgttgtcgcctttcctaggcaacatctcgacgctcaaggtcctctatctcacgtggaaccgcttcgtgggtggcatcccaccgcagttcggtcgcct
cggtgagctccagcagctactcctctacgccaacaacttcgacggcaccatcccgccggaggcgctctcgatcgaacacaacaacctcaccggcacga
ttcccgactgcaatggcaatctgtccaatctgaagatcttccaggcctactcaaacaacctccagggatggtgcgccttcgccgccgttgtcagttcgccc
cggcgagagcgagcctgcctttcggagcagatcgtcggtcagcaacgacacccggcaaagttcctgtgctctccactcgcgtcgctctcagtgatagttt
ggtgcgtttctgccgtagcgctgctctcggcggctgccgcacctgactcgtactggtccatgcacctcgaggcgttgctggcgttgaagaagtgcgtcac
ggcggacccaagcggcgcgctatccaactggatcgggcacgtcgtcatcattgtcgaccatggcccccgccgcactgcaactggaccggcgtcgcgt
gcgacggcgccggccacgtcacgtcgatccagctccaggccgcccgtctccgtggcaggctcacccccttcctcggcaacgtctccacgctccagatcc
tcgacctgtcgtcgaacaggttcgtcggcgcgataccgccgcagctcggccgcctctcgtcatcctatacgacaacagcttcaccggcggagctcggca
acctgagccggagtctcgaaaccctcgacctgaacaggaacaggctcaacggcgtcgtcgtcccgcgccgcctctgcaactgctcggcgttgtggtggc
tcggcatgcactacaacaacctcacgcggctgcagacgttggacctcagcgacaacaacttttccggctcgatcccctcgggtatcggcaacttctccaa
cctcaaaatcctgcagctgtttgagaaccagttctccggcgcgattccgcgggagattggcgggtgcaagaacctgatcatgctcaacgtatacagcaa
ccggttcaccggcgacatcccaagcgagctcggggagctatggaaactgcaacagctgcgcttgtacgacaacgcgctgtcgtcggggatccctcgct
cgttcggacgactaacgtcgctttctgtgcttgaaatgtctggcaaccagctcaccgggtcgattccgctggagctcggaatgctacggagcctcagacg
gttggctttatgttataacaagctgtctggcgagataccggaggaactgtacgactgcggcgatctgtacgtgctgagcctcgcggagaacaacttcagt
ggtggactgagccgccgcgtcggccggctcagcgagctcagatggttggacctgcaggggaacgctctgtcggggatgataccggaggagatcggca
accttacggagctcacactactgctgctccggggaaaccgcttctttgggccgattccgtaa
>g6265.t1
atggcgtcgttctcgaagaagactcgttacgaaccattattgccagcagccctagtcgcgctgctagtggcagcagcagcattcgttgccgctccagcgg
cggcggcggccgcggtcgtcccagactcgtcgacggcggtggccgcgcacctcgaggcgctgctggcgttcaagaagggcgtcacggcggacccgct
cggcgcgctgtccaactggacggtggggccagcaggcgtcgtcccgcggcactgcagctggaacggcgtcgtgtgcgacgacgccggccacgtcacg
tccatccagctcctggagacccagctccgcggcatgctgacccccttcctcggcaacatctccacgctccaactcctcgacctgtctgcgaacagtttcgt
cggcgccatcccgccgcagctcggccgcctcgccgcgctcgaggcgctcggcctcggcggcaacagcttcaccggcggcatcccgccggagctcggc
gacctcgggagcctccaaatcctggacctcagcgagaacacgctccgcggcgggatcccgcgccgcctctgcaactgctcggcgctgtgggcggtcgg
catggagtacaacaacctcaccggctcaatcccagattgcatcggtgacttgtccaacctcaagatattccagacctactacaaccagcttgagggcga
cttgccaccgtcgttcgcaaagctcacgaagatggagacgctggacctcagcaacaaccagttctccggccctatcccgccggctatcggcaacttctcc
cacctcaacatcctccaactgcaggacaacatgttctccggtgctattccatcggagctcggccggtgcacgaacctgacgcggttgaatctatacaaca
accagttcaccggctcgatccccgcacgctcgggcggctgacgtcgctgctgaacttgggtctctccatgaaccggctgacgggctcgatccccaccg
agctcggcgcgctccggtcggtccgcaagctgacactccacaacaaccggctcacaggaacagtgccggcgtcgctgacgagtctcgtgaacctgacg
tacctctccttcagctacaactccctgtccggccctctcccggaacaactcggctcgctgcggaacctccaaatgctggtgatccaaggcaactcgctctc
cggcccgatcccggcgagcatcgccaactgcacgtcgctggtcaacacgagcatggcttccaaccagttcaccgggccgctgcctgccggcctcggcc
ggctaccgaacctcaccttcttctcagtaccgtacaacaacctctccggcgagataccggaggacatcttcgactccgcgagcctccgtacgctgacgct
ggcctggaacggcttcaccggctcgctgagccgccgcgtcggccggctcagcgacctcacgctgctgcagctgcaggggaacgcgctgtcgggaccg
atcccctcggagatcggcaacctgacgaagctcgtcggtctgaacctcgggcggaaccgtttcgtgggccgcgtcccggcttccatctccaacatgtcat
cgtcgctgcaagagctcgacctaagcgagaaccgcctccacgggcccctccccggcgcggtgttcaacctgcggcgactcaccatcctcgacctcgcgt

cgaacaggttcgccggccccgtcccggacgccatctccaaccttggcgcgctgtccctcctcgacctctccaacaacgcgctcagcggcacgctccccg
ccgcgctgtccggtgacgggctcctggcgctggacctctcccgcaaccggttcaccggcgccgtccccggcgcggtgctcgccaacctcagcaacctgc
agacgttcctcaacctgtcgcacaacgcgttcgacggcgacatcccgccggagatcgggaagctcgcgatggtccaggccgtcgacgtatctgacaac
cggctgtccggcggcgtccccgcggcgctcgccgggtgcttgaacctcgccctgctcgacctgtcggcgaacagcctcaccggcacgctgccgccggg
gctgttcccgcgtctcgacgtgctcaccagcctcaacgtctccggcaacaagctcgacggggagatcccttccaacatcgcggaactgaagcgtgtaca
gacactggacctgtcgaggaacgcgttcacgggcggcataccgcctgggctggggaatttgaccagcctcaagtttctcaatttgtcgtggaaccatttg
gaggggcccgtgccggacgccggcgtgttcaggaacatgagcgcgtcgagcctgcagggcaacgacgggctctgcgggtggaagctcctcgcggcat
gccagcgtcacagcggcaacaagcgtgtgttctcgaggacgggactggtgatcctggtcgtgctgctggtcctgtccttgctgcttcttgtgctcctcgcg
acgattacatgcgtcgcgtacaagcgctacaagaagaaaaagcgaggaggaggcaacgacggcggcgcgaggttctcggagacggtcatcgtgccg
gagctgaggaagttcacgtacagcgagctggaccacgccacgggctcgttcgaggaaggcaacgtcatcgggcgcagcgccctgagcacggtgtaca
agggcgtgctcgtcggatcaggcaaggcggtggccgtgaagcggctcaacaacctggagcggttcccgtccagcagctccgccgacaagtgcttccta
acggagctcgcggcgctgagccgcctgcggcacaagaacctggcgcgcgtggtcgggtacgcgtgggagcccgggaagctcaaggccctcgtcctcg
agttcatggtcaacggcgacctcgacgccgtgttgcacgggcgcgacgcggcggcgccaaggtggacggtgcgggaccggctgcgcgtgtgcgtctc
ggtggcgcacgggctcgtgtacctgcacaccgggtacgacttccccgtcgtgcactgcgacgtcaagccctccaacgtgcttctcgacgccgagtggga
ggcgcgcgtcagcgacttcggcacggccaggatgctcggcgtccacctcacggacgcagccgccgcgcagtccgcgacgtcgtcggcgttcaggggc
accgtcggctacatggcaccagagttcgcgtacatgaggaccgtctcgccgaaggccgacgtgttcagcttcggcgtcctggtgatggagctcttcacg
aagcgccgccccaccggcatcatcgaggaggacggcgttccgctgacgctgcagcagtacgtcgacaacgcgctctcgaggggcctcgacggggtcg
ccgacgtcctcgaccccgacatgaaggtcaacaccgagggcgacctgtccacggcggcggacgttctcaacctggccgtgtcctgcgccgcgttcgag
ccggcggagcggcctgacatggacgccgtgctgtcgtccttgctcaagatgacaaagatctgtggagaaaactag
>g7171.t1
atggccgccgccggagaggacgacgcggtgtgggaccgcgccatctccgcggcctccaagaccgccgcccctccgccgcagcgcccaagaccctca
ccctcgacggcaccgtcaagtccacaacgggccgcctcccctcgccggcggtcctcgagcgcgtcgccgcatccctcgaagagctttccgtcgcgggcg
cgcgcctctcctcgctcgcgggcctccctcgactcccggcgctgcgccgcctctcgctccccgacaaccgcctctacggcgccgccgcgctctccgcggt
cgcggaggcctgcggcgccacactgcgatacctcgacctgggaaacaatcggttcgccgatgttgaggagctcgttccgctcgccaaggtcgggatcg
aggcgctcgacttgtaccagtgccccgtcaccaaggtcaaggggtatagagagaaggtcttcgcgctggttccaagcttgaagtacctcgatggcgtcg
acgccgagggtaacgagcgattcgagacggatgacgaagaggacgatgaggaggaggaagacgaggaaggagatgaggaggatgaaggtgggg
aagaagaggaaggagaagaggatgatggggacgacggggaaggggatgaagaagatggagaaggtgatgatgaggaagaagaaggcgatgagg
cggtgaggattttgctctaa
>g8442.t1
atggcgccggccttcgccgctgccgcgtcctcctccgtatgcggtgcttgcgtccccgcggcgtgccatttcgaagcgcggagtatggttgccgcctcca
gatttcttcgtaatcgggtggaagttatagatttctacaagaatctgggatttgaagtggaccctcaaggcatcaaaggcatgttctgtcttgttgacaata
acaatctgtctggacctcttcctccagaatttgcggcggcacctgcattgaaaatatttcttgttgacaataacaatctgtctggacctcttcctccagaatt
tgcggcggcacctgcattgaaaatattggatcttggctggaaccagctcacaggatcaataccaactaataagcttgcttcaaatatcactacaattgatt
tgtcgcacaacatgcttaatggaactatcccgttgaacttctcggggctgcctaatcttcagatattggacttccaaaacaattctctcaagacaattccgg
ctgcatttgatcctccagcaaatgttactgtcatgcacgtacttgctagttgggaaattactctttcggatgtgtttggtccatatgagcttctcaacttcaca
ctaggttcctatgctgatgaacttccaactgcagcttccgtaggtttaagcaaggttgtgctgggcagtattttggcaggcacaatagctggtgctattgcg
ctttctgtggtggccaccacccttatactgaaaaggcgttcaagatataaaacagtctcaaaatcaaaccggtcattgtcgagattttctgtcaaaattga
tggagtaagatgcttcacatttgaagaaatggctaccgccacaaataatttcgacctatcagctcaagttggtcaaggaggttatggaaaagtctacaa
aggtattctagctaatggagcagttgtagcgatcaaaagggcacatgaagattctcttcagggttctacagagttctgcacagaaatagagttgttatca
agactgcatcatcggaatttggtgtcattagttggctactgtgatgaagaagatgaacagatgctggtttatgaattcatgccaaatggcactttacgtga
tcatctttctgccaagtccaaaagagcactaagctttggtttgaggttacaaattgcattgggtgctgcaaagggaattttatatctacacgctgaagcaa
accctcctatatttcaccgtgatgttaaagccagcaatattctgttagactccaaattcattgcaaaggtagctgattttggtctctcgaggcttgctccagt
gcctgatgttgaagggacgttaccagctcatgtgtcaactgtcgttaaaggtaccccgggttaccttgatccagaatacttcctaactcataaactgacgg
ataagagcgatgtttacagccttggtgttgtgtttcttgaaatgttaactgggatgaaacctattgagcacggtaaaaacatcgtgagagaggtgaactc
agcttgccaatcaggcaacatttcggaaactatcgacaaccggatgggtctgtacccttcagaatgcgtcaggaggttcctctccctagcaaccaagtgc
tgccaggatgagaccgacgccaggccttccatgtgggagatcgttagggaacttgagaccatcttgagaatgatgccggaagaggaccttgttctgctc
gaaaccccggatacagactcaaccgacgtgagcaaatcgctgacagtttcaagaagcgggactctctttgtctcgtcgcaaacctccgatagcattgat
gcaagcggtggcactctttctggaaggctaactcctcggtga
>g8885.t1
atgctagggtctcgctgcagatctgttgatggtctacgttctctgtttgatgagaaggtagactacctgagtgttaatgaaatacatcgctctaagtcaact
ttggatctgtattctggaccttctcctgatacttaccaaacactaaacatggaggacaatggttcacttggttgctctgatgctgctgatgagggtcaacgg
tcttctgcaagcgcagaagaaaattttgtcagggatgggatgctagttggtcatgaatactgggatgtcaaatacatcagtggtgatcactcagtcgatc
gaattgcccctttctccgtggattcaagggatgtctgtcaccattctgcccatgatggtggcatcagtgatgccatggatcaagagatagaggagaagct
atttaacagagataatactcttcacaaccaatcacttgttgttgaagtgcctgacaccaggaacatgtcgaacacaaaggatattggtgaagaacctga
acgtggtaaagaagttattgatgaggacccaaatgagctttctccaaaggcttatagcattaaaagaatcgaggagtggatcaaccaaatcaatattga
tgatctcactgtgaatgagcaaggagaaagctcattttctgctttggcggaatctcgtgagccagcagctggtgttcctgctgtgtggcctgatgccaaaa
gtccacttggcatggagatagcctacacatatatttcaaagttaactcctgtttcatcctcggcacagttggcaaaccttggtttggttgcgatcccacgac
tgagtgcattctcaggcttacgcctcttgaacttatcagggaactcaattggtctggcctcctgttcttcattgaaggagttatatctggctggtaacaagat
tagtgaggttgatggtcttcaccgccttctgaagctgaaagttctggacttgcgccacaacaagatttctacatccaagggcctcggccagctagctgca

aattacaattctctagaagccattaaccttgatggcaaccctgctcagaagaatgttggtgatgagcacctgaagaaatacctcttaggtctgctgccaa
accttgtcgtctacaacaagcaccctataagagcagctggatcaaaagatgtctctgatcgtcactcccgcaaaatctcctcttcccatcgttctgaccgt
ggtgttagatcggatcgcaaatcttccaggctggtgggtggctcatcttctcacaaggcacagagctcacggcatgcccatgctgggtatgcttcaggct
cagcctctttgctgaagcattcaagggtgagaaacactccaatgccattgttggggtcaagaccagcaagtgctgtagatctgacgaagctagcccaaa
tggaaggaaaggcgcagtaa
>g8890.t1
atggccaccaacaaccgcgccatactcccgctccaactcctcctcttcctcgcgctcaccgccctggcgcgcgccgccgccccgtgccacccggacgac
ctccgcgcgctgcgggccttcgcggcgaacctcatcgccgcccccaccgcgtggccctccggcgacgacgccgccgcctgctgctcctggcccggcgtc
ggatgcgacgccgacggccgcgtcacggcgctgcgcctcccgcggtggggcctggagggtcccctcccggccgccgccctcgccggcctcgcgcgcct
cgaggacctcgacctcagccacaacgccctctccggcgacctcgccccgctcctcgtctcctcctcctccgccgccggcggcgggaggaacctccgcgc
cgccaacctctcctccaacctcctctccggcgcgctccccgacctctcctccaacccgtccctcgccgtcctcgacgccagcaacaactccctctccggct
tcctcgcccccgacctttgcgccggcgcgccggccctgcgagtgctcgacctctccgccaaccgcctcgccggcgcgctcccctccgattcctcctcctcc
tcctccgcgtcgccgccgccctgcgccgccacgctccgggacctctccctcgcctccaacgccttctccggcgccctcccgcccgcgctcttcgccctcgc
cgccctccaaaccctctccctcgcctccaacgcgctcgccggccagctcaccacccgcctccgcgacctcaaaaacctcacctttctcgatttctccggaa
acgccttctccggccacctccccgacgtcttcgccgacctcacctcgctcgagaatttggccgcacactccaacaacttatccggcccccctcccgccgtcg
ctgtctctcctctcgtccctccgtgtccttgacctccgcaacaattcgctgtctggtccagtctccctggtcaatttctcagccatgccgttcctcgcttcggtt
gaccttgccaccaaccacttcaacggcacgctccctgtcaccctagcggattgccaggagctcaaatcactcagcctcgccaaaaacaagctcaccggc
caactacccgaggattacagccggctcgcctcgctgtccatgctgtccctgtccaacaacagcctgcacaacatctcaggggcgctcaccgtgctccgtg
gctgtaagaacctcaccacgctgatcctcaccaagaatttcgacggtgaggagctaccggccgacggcatcgccgggtttgacgccatcgaggtgctg
gcactaggtaattgtgctctcaggggggagggttcctgattggctgactcagtgcaagaagttggaggtgcttgatctgtcctggaaccggttggttggaa
ccatcccacaatggatcggtgactttgagtacttgagctacctggatctctcaaacaactccttggttggtgagataccaaagagcttgacgcggctcaa
gagcctcgtcactgctagtgctaggcagtcgcagggtatagcgtttacgagcatgccgctctatgtgaagcataacaggagcataaatggccgtcagta
taaccagctctcgaatttcccgccgtcgttgttcctgaacgacaacaggctgaatgggactatctggcctgagtttgggaacttgagggagctacatgtg
cttgatctgagcaacaactccatatctgggagcattcctgattcactatccaggatggataatctggaggtccttgacctttcgtccaataacctgagtgg
attgattccgtcatccctcacggacctcaccttcttgtcaaagttcagtgttgctcacaatcacttggtggggcagatcccaaatggagggcagttcttcac
cttctccaacaccagtttcgaggggaacccaggtttgtgcagatcaacatcctgcaacctgaatctgtcgctgtcgaggcaagcttctagtggcaagaag
atgcagccttcagctagtgtgaggaacaataaaaacaaaatacttggggtggcgatctgcgttggattggctctcgcggtacttttgactcttattttggtt
aacatgtcaaagagggatgtcagcgccatcgactacgaagatactgagggctcctgtcaggaattgtatgattcttactcgaagccggtgctcttctttca
gaattctgcggtgacggaactaacagtcagtgacctcgtcagatcgacaaacaactttgatcaagccaacattatagggtgtggtggatttggcctggtg
tacaaggcctaccttcctgatggaacaaaggcagcagtgaagaggctctctggtgattgcggacagatggagagggagttccgggctgaggtagaag
cactgtcacaggctcaacacaagaatcttgtgacgctcagagggtactgccgctatggaaatgataggctgctgatttactcgtacatggagaatggta
gccttgattactggctgcatgagaggtcggatgcaggttacatgctgaaatgggagtcaaggctcagaattgctcagggctctgccaggggggctggctt
acctgcacaaggaatgtgagcccaacatcatccaccgagacgtgaagtccagcaacattcttcttaatgaaaatttcgatgcatgcctggccgatttcgg
tctagcaaggctgatacagccctatgacactcatgtgaccaccgatttggttggtacgttggggtacataccaccagagtacagtcaatcggtgatcgcc
accccaaaaggagatgtattcagcttcggtgtagtcttgcttgaactgctcactggtaaacggcccttggatgtgtcaaagatgaagggatcaagggact
tggtctcctgggttcttcagatgaaatctgaaaagaaggaagagcagattttcgacagaccgatatggagtaaagtgcatgagaagcaactattgtcag
tccttgagactgcatgcaagtgtattagcactgatccgcggcagaggcctacaatagaagaagttgtcagttggcttgacagtttgtaa
>g9043.t1
atggcgtctcggtcctcccgtcgcgtcctcctgctagccttgctgcccctcctcctcctcctcatcgcgccccggtcgtcgtccacaccgacgaccaccttc
ccgggcgacgccgccgcattggcctcgctcaaatcgtcctccatcccgccgcaatcctgcctcgcgacgtgggacttcgcacgccgcgacccctgcgcc
gcgttcccctgcggcctccgctgcgtcgcgccgcccaactccacccacctccgcgtcgccggcgtcgcgctcgacccggccggctactccggcgcgttcc
cgacccggctcctcacttccctcccctacctcacgaccctctccctcgccggcaaccgcttccacggcccccctcccggatggcgtcccgctcccaccaacc
ctccgcgtcctcgacctctccgggaacgccttcacgggccccatcccgggctccctctttaccacccgtgcgtcggaactgcaagagctctacctctcccg
caacgcgttctccggcgtcgtcccgccggagctcgcgtcgctggcgtcgctcacgcggctcgagctccagcacaacgcgctcgcggggagcccgagcc
tccctcccgggatgcgcgccatgcgcgcgctcgcacacctcgacctcacgggcaacaagctctccggcccgccgccgctcgacgcgctgcctccctcgc
tcgtctcgttcgtcgcccgcgacaacgccttctcgggcgccgtacccgacgcgccgctcgccgctctcggctcgatccgggtgctggacctcaccggcaa
cgcgctgtccggcgcggtgcccgacgccgcgctcgcgcacccgtcgctgcagcagctgcggctcggctccaaccggctcgacgcggtcgcggagccc
gacggcggcggctgcgcgtcgagccagctcgcggagctggacctgagcgggaaccagatcgcgggacggctcccgacgtgcctcggttcgatgccgc
ggctcgtggcggtggcgctggaccggaaccggttcacggggccggtcccccacgcgtacgccgcgcgggtcgcggcgcaggaggccaccgacgagt
gggtcccgttcgccaggctcatgctgcaggggaacttcctctgcggggcgctgccgggccggtggacgcagctcagggaggaggacggcgccaaggt
cagcgtcgcggacaactgcctgccgaggtgcccgcgcaagttcttcttctgccagggcgcgccgcagaagaaccaggccgcgtgcccgaccaagtgc
gatggcgccgccatacgccatgtccgcgagcacgaggatttgctgctggaaatgccgtag
>g9158.t1
atggcttcttcactcatctcggtcgtcctcttgctctacttccacgtcgccgtggcaaccgcgcaggtcggggacgcgcctgtgccggagcttccgcggtc
ggagctggcggccgtgttccgcgtgatggcggacctcctcggcgacccgacgtgggcgcaggcccacccgcgtccctgcacggacacgccgtggcctg
gtctgcagtgcgaggtggcccccgacgacgcccgccgcctccgcgccacgcgcctccacttcggccccgacgtcgccaccccgccctgccgtcccggc
gccgcgctcgccgccgacgcgctgcgcggcctgccccacctcaagacgctctccctcttctcctgcttcaccagcgactccggcgtggcgaccgtcgag
ctgccgcctgcgctgttcgcgaacgcgtcgtcgtcgctggagcaggtcgtgctcaagtccaacccggggctgaccggcgcgatccccgccacgctggg
cggcgtgcgcggcctgcgcgtgctcagcctgtcgcagaacgggttccgcggcggcatccccagggagctcggcggcctcgccgcgctgcagcagctcg

acctcagctacaacaacatcaccggcgagatacccgaggagatcggagggatggcgagcctgaccatcctcgacctgagctggaacggcatcaccgg
cgcggtgcccagcgccatcgggaagctgcacaacctccagaaggcggacctcagctacaaccgactcaccggccgcgtcccgccggagctcggctcg
ctgcagaacctcgtgttcctggacctgggccacaacgctctcgcgggcccgcttccctccacgctggccgggctgggcaggctgcagtacctgctcctgc
aggacaacccgctgggcacggcggtgccggcggtggtgggctcgatgcggcggctgcaggtgctgggcctgtcgggctgcggcctcacgggcccggt
cccgttcttcgcgggcctgcgcagcctcaccgcgctgtcgctggaccggaaccgcctcgacgggcccatcccgtcgggactcgccgcgctgccgcacct
gggacagcttaacctcagccagaaccgcctcgcgggggagatcgccctgcccggggacttcgtcgcgcggctcgggcggcgcctcgacgtgcgcggg
aacacacagctttgcgtcggaaaggggctgcaggggacgaccggcggatacctcgtcggcgcgagcccgtgcgagagggacgatggcgacgcgttg
ccggcgcgggcggaggggtcgggctccgccgcccgtcgttggggacggggttacgccgtccctgtcgtgttgttgtttctggtgtaccgtttctga
>g9423.t1
atgaagcagctttctgctccaacaaggcgcgtgatccttcttcccctcctccttgtcgccctgctgtcgagccaaacaaatgcagcgcatgacgacgagg
acaggccgtcgccgccgtgctcaccggccgaccgcgcggcgctgctgggcttcaagtccggcatcgccgcggacacgacgggcatcctcgccacgtg
gtccggcgccgactgctgcggcgcgtgggatggcgtgtcctgcgacgccgccacggggcgggtcgtggcgctgcagctggaggccccaccgcccaag
gagcgccactacatggagggcaccatttccccgtccctcggcaacctcgagttcatggagacactggtcatccgcgacatggggaaaatcggaggcgc
catcccggcggcggcgttgtcgcggctcacgcgtctcaagcagctctacctcgagggcaacctgctcgtggggcccatacccgggagcctcagcaagc
tcaggtcactccagtacctgtccctcgccggcaacaggctggaggggcatctcccgccggaaattagcttcgtctccggcctgcaacagatcaacgtcg
cacggaaccgcctcgccggcgcgttgccgccgagctacaagaatctctccaaactggcgtacctcgacatgagcaacaacttgctgtccggcgccattc
ccgggttcctcggccagttcaagaacctggcgttgctggacctcagcaacaacagcttttcaggtgacattccggcgtcgctctgcgccttgcgcagcct
gacagacctctcgctgagccataacaagctcaccggccagatcccatcgaaactgggcagcctccggtcactgagctccctggccatcgctgacaacat
gctcgtcggctccatcccgtcactgctcgggttgcagaagctgtggaacctgaacctgtcaagaaacgcgctctcgggccccccttcccgccgtcggcttg
tcgtccttgatgtccatggacctctccgacaaccgtctcaccggcgaaatcgacgatttcctgaaaagcttatccttatccatacccgtgcgcgccacgct
ccccaacaacaagaagaagaacttattgatcgttcttccccagaaactggagcacctggacgtgtcggggaacagggtcaccggcgcgctgcctgagt
tcgccggcggcggactcaagtggctcgacgtctcgagcaacgccatcggcgggcagataccgagcacggtgtcgaagctccgagacctcgagaggct
ggacgtgtcgaggaacagagttcggggtgtcatccctgcgtccatggccgggatggcgcggttgcagtggcttgatctgtcgagcaacgagatcgtcgg
gaggatacctgagaatttcacgaggttggggagcgtccggcacgcgagttttaggggcaataagctctgcggacagataccgcagggccggccgttca
atctgttcccggccgccgcgtacgcgcataacctgtgcctgtgtggcaagccgctgccaccgtgcaggaagatactgtaa
>g9980.t1
atggctgcgaaggccgcgggggccctggcgttggcgctcgtcctcgcgcttgcgggggccaactccgagggcgacgcgctctcggcgctgcgccgca
gcctgcgagaccccggcggcgtgctgcagagctgggaccccacgctcgtcaacccctgcacctggttccacgtcacttgcgaccgcgacaaccgcgtc
acccgcttgtga
>g10414.t1
atgacgtggctgcagaggcttgacctgtcctggaacggcatcaactcctcaacagtggacgcctggttctggaatgtgcccacccttccctacctggacc
tctccttcaacttgctgtccggcccgttccccgacgccctccaggagctctacctcgacggcaacagcatggtcggcatgatccccgcaactctgcagcg
cctctgtagcctgcaggtggtgcacgtgtcctggaaccagatcaacggcgacatgtcagagttcatggaccggctcccgcgctgcgcgttgagccacgt
gcaggtgctgcatctgtcggcgaccaacatgtctggggaacttcctcagtggattggcgacatgtcggagctgacgtccctcgacctgtcctacaacaat
cttgcaggggacattcctctagggatcggcaggctctccaagctcacgaaactgttcctgttcatgaataatatgaatggacgcttgacagaggagcact
tttccaatatggtgagcttggagtggatcgatctgtcgcagaactccatgtccatggaggtcaggccgagttggaggcctccatgcaaactgatccgcgc
ttacttctctgacgtcaagatgggtcctctatgcagtatacctcaacatctctgtcaaccagatcagtggcaggctgccatcttctctgagattcatgacct
cagctaa
>g10417.t1
atgcacgtggcatcggcgatcctgttcctgttggcgctgtccgtgctcggcggcgccggcggctgcgtccctgcagagcgcgatgcgctgctcgccgtga
agcgtggcttctccagcgacccggacggcgtcctcgcgtcgtgggccgccagcaagttcccggactgctgccggtgggccggcgtcgtctgcgacacc
aacggcaccgaccgcgtcgttgagctccgtctccgcaacgcctttgccgacgccggcagccccgggctcagcggcgagataagcacggcaatcacca
gcctctcgcggcttgagtacctggacctcagccggaacagcatcggcggcatggcaaacttgccgatcccacggttcctcggcgcgctctccggcctcc
gctacctcaacctctccggcacgggcctcgtcggcgagatcccccacagctcggaaacctctcccgtctcgtctccctcgacctgagttcctacggcctc
tactccggcgacctcttctggctcgccggcttgtcctcgctcgagcacctcgacatgaccagcgtcaacctcaacgcatccattcactggcagagcgacg
tcaacatactcccttccctacgcgtcatctccctctccgactgcgggctaataacgacgtcgtcgcaggtgccacccgcacacaccaacctgacacgtctt
cagcgtctggacctgtcatccaacggcatcaacgcctcgaccgtgaacgcctggttctggaacgtgccaacacttcgttatcttgacctctccggcaacg
cgctgtccggcccgttccccgacgccctcggcaacatgacgcgtctccaggagctctacctctacggcaacaacatggtggggatgatcccggcgactc
tgaagcgcctctgcagcctgcaggtggtggacgtgtccgtgaaccagatcaacggcgacatgtcggagttcatgaaccggctcccgcgatgcgcgctg
aggcgcgtgcaagtgctgcagctctcggcgaccaacatgtccgggcacgtgccggaacagatcggtgacatgtctgaactcacatacctggatctgtcc
tccaacaaacttgcaggggacattcctcaggggatcggcaggctctcgaatctcacgaggctgttcctgctcaagaacaacctgaatggcagcttgaca
gaggagcatttttccaatatggtgagcttggagtggatcgacttgtcacagaactccttgtcaatggaggtcacgccattctggaagcctccatgcaaatt
ggtgtatgcgtatttccctgacgtcaagatgggtcctcggttccctgcgtggatcaggcaccagccggacatcaagtaccttgacatttctcatgcaggca
tagtggacaccctgccacactggttctgggagtcgttcacagatgcggtttacctgaacatctcggtgaaccagatcagtggaaggctgccaccttctct
gagatttatgacctccgctctagcgatatatctcgggtcgaacaacctcaccggctccatgccgttgttaccggagcagctgcttgtgttggatctctccag
aaactctttatctggcccaattccaatagaatttggtggtcctgagttagtggagctggacgtctcctcaaacagaatcaacggagcagtgccagcatcc
ctatgccagtttccaaacttgttgcatttggacctgtcaaacaacaatctgagcggccacctccctcggtgcccaaacatgtcatcaggtctcgggctcac
cactctcatcttgtacaagaacaatctttcaggcgactttcctgtgttgttgagacactgtcaagctatgacattccttgatcttgcccagaacatgttctcg
gggatgctaccggagtggatcgggaggaagatgccatccttgacacatcttcggctgcgatcaaacatgttcactggaatcatcccgaatcagctggca

cagcttggtgacctgcaactcttggacttagctggcaacaggatatccggttccataccccattctctagggaacatgaaaggaatgacaaaggagcac
acgccacttgctctgaacccattgacaggctacggtgcgtcagggaatgatagaattgtggacagtcttcccatcgtcaccaaaggacaggatcgtggc
tatactagtggagttatctacatggtgagccttgatctgtccaacaacattatcagtggagagatacctgaagaggtttcatctcttactggcctggtgaa
cttaaatttgtcctggaatcagttgaccggaacaattcctcaagacattggtcatatacaaaagctggagtctctcgacttgtccatgaatctgctttcggg
tgcgattccatcgagtctgtcagacttgacttcattaggccacctgaacttgtcctacaacaacctgtcaggaaaaataccttcaggcaaccagctccag
gcacttgccaattcagcatacatctacattggcaatgctggtctctgtgggccacctctgttgaagaactgttcatctggagatgataataacagtagcca
tgctcctcgccatggagataaagatttatccaagatgtccttctaccttggtctagccgtaggtaaggttggggaaacccggggataa
>g10458.t1
atggacactcccatcaacctcttgctcctcctctcctcgattttggtgtcgtcgtcgtcaacattcacgacgtcggtggtggctgcggcggcggaggagac
cgacgcacaggcgctcctgcggttcaaggagtccatcaagagagacccacgaggggtcctctcgtcttggcagccggaccagatcggcaacttatgca
gctggtatggcgtgtcgtgcaacggcgaggagcgtgtgacgcggatcaacctcgccggctgcggcctcgtaggccgcgcggcgatcgccgagctgtcc
gccgtctacgggctgtgccacctcaacctctccggcaacggcgcgctccacgcggacgccgccgacctcttgagcctcccacgcgcactgcagacgctc
gacttctccgacggcggcctcactggcagactccccagcgacctgctcgcgcactacccgaacctcaccgacgtaggtctcgcccggaacaacctcacc
ggtacgtttccggtgtcgcttctctccaacgcgtcgtcgtcgtcgagcaggattcaactgttcgacgtctccagcaacaacctgtctggtgacatctctggc
gcgtcatcgttcccagacacgctgaccgtgctcgacctctccggcaacgcattcaccggcgcgatcccggcggctttgtcggggtgcacggggctcagg
acgctgaacctgtcgtacaacgggttctcccggccgatcccggcttcggtcaccggcatcggagggagcctcgaggtgttggacgtctcgtcgaatcac
ctcacggggcagatccctgacacaatcgggagcgcgtgctcgtcgctgcgggttctcaaggtgtcgagcaacaacatctcggggccggtcccggagtc
gctgtcatcatgccgcgcgctccaggtgctggacgccgccaacaacaagctgtcaggccccatcccggccgccgtgctggggaacctgacgtcgctgg
agagcctccttctcagcaacaacttcatcaacgggacgctgccgggcaccatttcctcatgtgccagtctgaggattgctgacctcagcagcaataagat
ctctggagcgctgccggaggaaatctgttcctcgtccggcgatgcggcgctggaggagctccggttgccggacaacatgctcacgggtaccatcccgcc
cgggctggccaactgctcgaaattgagggtgatcgacttgagcatcaactacctgcgcggcgcaatcccgcgggagctgggccgactccgcggcctcg
agcagctggtgatgtggttcaacgggctggaaggccggatcccgtccgagcttggtcagtgccggcgtctccgaacgctgatcctgaacaacaacttca
tcggcggcgacatccccgcggagctcttcaactgcaccaacctcgagtgggtgtcgttcacgagcaaccggctgaccggcacgatccgtccggatttcg
ggcgtctgacccgtctcgcggtgctgcagctggccaacaacagcctggagggcgccatcccgaccgagctcggcaactgcagcagcctcatgtggctt
gacctcaacagcaaccggctcaccggcccgatcccgcgccggctgggccggcagctgggctccacgcccctgagcgggatcctggccgggaacacgc
tggcgttcgtgaggaacgtgggaaactcgtgcaagggcgtgggcgggcttctagagttcgcgggcatccggccggagcggctgctgcaggtgcccac
gctcaagagctgcgacttcacaaggctctactccggcgcggcggtcagcgggtggacacggtaccagacgctggagtatttggatctctcgtacaacgc
gctgtccgggagcatcccggaggagctgggcgacatggtggtgctccaggtgctggacctggccagtaacaacttcaccggtgagatcccctggtcgct
cggcaaattgcgtaatctcggcgtgttcgatgtgtcgcacaacatgctcagcggtgggatcccctattccttctccaacctgtcgttccttgtccagatcga
cgtgtcggataacaaccagacgggagagatcccccagctcgggcagctcagcacgctgccggccacccagtacgccggcaacccgggcctctgcggt
atgccgctgctcccgtgcagtgctcgaccacgtgccaccgcatccgtgctcgcggatccgccggataacgacaggcgcgccaccagaacgggaaccgt
ggtcctcgccgtcctggtcgcggccgggctggcgtgtctggtggcagcgtccgcgctgatcatcgttgttgtggcgcgcgcgcggcggagggaggcgcg
ggaggcgatgatgctgagcagcctgcaggacgggacccgcacggccaccacctggaagctgggcaaggtcgcggagaaggaagccttgagcatca
acgtggccacgttccagcggcagctgcgtcggctgaccttcacgcagctgatcgaggccaccaacggattctccgcaggcagcctggttgggtccggc
gggttcggcgaggtgttcaaggccacgctcaaggacggctcctgcgtggccatcaagaagctcatccacctcagctaccagggcgaccgcgagttcac
ggcggagatggagacgctgggcaagatcaagcactgcaacctcgttcccctactggggtactgcaaggtcggggaggagcgcctgctcgtctacgagt
acatggcccacggcagcctcgaggacgcgctgcacggccgccagcttctacttccgtggcaacggaggaagaggatcgctcgcggggccgccaagg
gcctctgctttctgcaccacaactgcatcccgcacatcatccaccgggacatgaagtccagcaacgtgctcctggacggggacatggaggcccgcgtgg
cggacttcggaatggcgcgcctcatcagcgcgctcgacacgcacctcagcgtcagcacgctggccggcacgcccgggtacgtgccgcccgagtattac
cagagcttccggtgcacggccaagggcgacgtgtactcgctcggcgtcgtgctgctcgagctcatcaccgggaggaggcccaccgacaaggaggattt
cggggacaccaacctcgtcggctggatcaagatgaaggtcagggagggcaatggcaaggaggtggttgacccggagctcgtcgttgccgccgtcgat
gatgaggggacggagatggccaggttcctcgagctggcgctgcagtgcgtcgacgacttcccatctaagcgccccaacttgctgcaggttgtggccacg
ctcagggagatcgacgacgcgccgccgtcccatcaggcgccaacagcaccatcaccggtcactcctgctattgcctga
>g11013.t1
atgaccaagttgctcatcccaatcatcatcatcatcatctttcatgtcggcctcgtgccgatcgctgtcgcggcagcagtcgccgcaccagcaccagtgat
cgacgacgaccgctcctcactcctcgcgttcctctccaacgtgtcagccgaccccggcggcgccctcgcggactgggaccgctcgccggacttctgcaa
ctggacgggcgtcacgtgcgtcggcccgggccggcggcgcgtcgcgcagctggtgctcaccggaaagggcatccgcggcgtgatctcgccggcgttg
ggccggctgtctttcctctccgtgctcgacctctcaagcaacgctctctccggcccgatcccgcgagaggttgccgcgctgtcgatgctgacgcagctga
gcttgacgaacaacctcctcgagggccccatcccgcccagcctcggcctcctccaggagctctactacctcgacctcagcggcaaccggctctccggcg
tcattccccagactctcttctgcaactgctccgcgctgcagtacttggacctcgccaacaactccctcgccggcgacatcccgtacgccgacgagtgccgt
ctccccaacctccgtttcctcctcctctggtccaacaccctctccggcgcgatcccgctggcgctggccaactcctccatcctcgagtgggtcgacttcgag
tcgaactacctctccggcgagctcccgtcgcaggtgttcgacaagcttccgcagctcgtattcctctacctctcctacaacaacctgtcgagccacaacgg
caacaccgacctggaccccttcttccgctccctgagcaactgcactcgcctgcaggagctggagctcgccgggaaccacctcggcgggcgtctccctcc
ctccaccggccgcctgccgcgcggcctccggcagctccacctcgaggacaacgccatcacgggccccatccacccaaacatctccggcctcgtcaacc
tcacctacctcaacctctccaacaacctgctcaacggttccatcccgccggccatctcccgcatgcagcgcctcgagcggctgtatttatccaacaacctc
ctctccggcgagatcccgcggtccgtcggcgagaccctccgcctcggcctcgtcgacctctccgggaaccgattcgtgggaccgatccccgacacgttct
ccaacctcacgcagctcagacggctgatgctccaccacaaccggctctccggcaccattcccccgagcctcggcgactgcctcaacctggaaatcctgg
acctctcctacaacggcttgcaaggcggcatcccggcctacgtcgccgccctgagcagcctcaaactctacctcaacctctccaacaaccacctggacg
ggcccctccccatcgagctcagcaagatggacatgatcctcgctctagatttatcagccaacaggttatccggcacgatcccgtcgcagctcggcggctg

cgtcgcgctcgagtacctcaacctgtccgggaacgcgctgcgtggcgcgctcccgtcgtccgtcgctgcgctgccgttcctgcggtccatcgacgtgtcg
cgcaacgcgctgtcgggacccctacccgcgtcgctgcaggcgtccacgtcgatccgggaggccgacttctcgtacaacaacttctccggcgtcgtcccc
gacgccggcggcgtcctcgggaacctctccgccgacgcgttccgtggcaacccgggcctgtgcgggcacggcattgccgggtgcgaagcgaacaagc
agcgtcggcgccaccgccgccggccactggtttctgtcgccgtcggcatcgtggcgtccgtgtcgctcatgctctgcgcggtcgggtgccggtccgggcg
caacgggtcgtcggtgcggctcgtggacgccggggagaacccgacgacgacgacgacggagcgggagtacccgaggatatcgtaccgggagctctc
cgacgcgacgggcgggttcgcgccggagaggcttatcgggtcggggcggttcgggcgggtgtacgaggggacgctccgcggcggcgcgcgcgtggc
ggtgaaggtgctggacccgaagaagggcgggtgcggcggggaggtgtcgggcagcttcaagcgggagtgcgaggtgctgcggcgcacgcggcaca
ggaacctcgtcagggtcatcaccacgtgcagcaccgacagcttcaacgcgctcgtgctgccgctcatgcgcaacggcagcctcgaggaccgcctccac
ccgccgctcggggacgacgaaggcctggatttcgggcagatcatggccgtcgtgggcgacgttgcggaggggatggcgtacatgcaccactacgcgc
cggtgaaggtcgtgcactgcgacctcaagcccagcaacgtcctcctcgacgacgggatgcgcgccgtgatatcggacttcggcatcgccaagctcgtc
gccggtggcgtcggaggggaaaccagcagcgccaacgacgaggccgcgccgtgcaactccatcaccggactgttgcagggctcggttgggtacatcg
cacctgagtatggattaggagggcacccttcgacgcagggagacgtgtacagcttcggcgtgatgattctcgagctgatcaccgggaagaggccgacg
gacgtgatcttccaggagggcctcacgctgcacgactgggtcaggcggcactacccgcacgacgtcgcggccgtggtcgcgcacgcgccgtggcgag
ggcgcgagctgccgcaggcggcggccgacgtcgcggtcgtcgagctgatcgagctcgggctcgtgtgcacgcaatactcgccggcgctgcggcccac
gatggccgacatctgccacgagatcacgcttctcaaggaggatctcgtgaggcacggcgccgacgaggagggcggcggacgccggtcgttctcgacc
aaggactcgctgttctccaactga
>g11014.t1
atgtctgctatcatcttcacctattttttctcttcttctccaacggtccaagccctattctttcagctggcttgtcgcacgagcgctctgcacttctatccttca
agtctggtgtgtctaatgaccccaagggagctcttgctagttggggttcacctaatgtgtgcaactggaccggtgttgcctgcgacatggcgaggagaca
tgttgtgaagctggagctaagagatcaaaagctctccggtgaggtctctcctacacttggcaacctctcccacctcaacatactcaatctctcgggaaac
ctcttcaccgggaaggtcccgtcggagttgggaaacctctttcgcctcaatttactagacatatcggcgaattcatttgccgggaggattccgccggagct
tggcaacctctccagcctcaattcacttgacctctccggaaacttcttcaccggagaagtgccgccggagctcgggaacctcttcaaactgacgcaactg
agcattggcaacaacaatttggaagggtcaatcccgaccgagctcacgcacattcggaacctcatttacctcaacctcggcgagaacaatctctccggg
cacatcccggaggccatcttctgcaacttctccgccttgcagtacattgacctctcctccaactcactcgacggcacgatccccatcagagaaggtgactg
cccgctccctgacctgatgttcctggtcctgtggtcgaacaacctcgtcggcggcatccccagctcgatatccaactccactaagctcaaatggctgctgc
tggagtccaatttccttaccggcgagctgccgtcaaacatgttcagcaacatgagagacttggagctcctttacctctcctacaactacttggagagtcct
gagaacaacaccaacctcgcgccgttcttcgcgtccctcacaaactgcaccaagttgaaggagctcggcgtcgccgggaacgagatcgccgggacga
tcccgccggtcgtgggccgcctgtccactgaactcaggcagctccatctcgagtacaacaagatcttcggcccggtcccgggcaacctctccgacctcgc
taacctcaccgcgctgaacctctcccacaacctcctcaacggctccgtcccgccgggcatcgcggggatgcagcgcctcgaacggctgtacctctcgaa
caacctgttctccggcgagatcccgccgtcgctcggcgcgatcccacggctgggcctcgtcgacctctcccacaaccggctcgccggcgccgtccccgc
cgcgctctccaacctcacgcagctccgcgtgctcgtgctctctcacaaccacctctccggcgccatacctccgaccctctcccagtgcgtgaacctccaaa
acttggacctctctcacaacgcgttgcgcgggagcatccctgcggacctttcccagctgagcagcctgctgtacctgaacctctccagcaacatgctcga
agggcccatcccggtgaccgtcagccagatgacgatgctgcaggtgctcaacctgtcgtccaacaggctgtccggcccgatcccgccgcagctcggcc
gctgcgtcgcgctcgagtacgtcaacgtctcgggaaacgcgctcgccggcggtctcccggactccgtcgccgccctgccgttcctgcaggtgctcgacgt
gtcgcgcaacgcgctgaccggcgcgcttccgctgtcgttggagacttcgcccttgttgcaacgtgtcaacttctcgtacaacgagttttccggggaggtgc
ccgtggcgggcgcgttcgcggggttccctcccgacgcgttcctcggtaacccgggcctgtgcggcggtcacgacgtggcgggattggctccgtgcggcg
accacgcgacgcgccgcgtgtggctccgtgaccggcgcgtggtgcttcccgtggtgatcacggtggccgggttcattctcgccatcgttatcggcgccgt
cgcgcgccgcgcggcggcgcgcgcggaggctcggcgcgactcgcggcggtcgacgctgctggccgacggcgccgacgagcagcagccgatggagc
gggaccacccgcgcgtgtcgcaccgggagctcgccgaggcgacgcgcggcttcgagccggcgcggctgatcggggccggccggttcgggcgcgtgt
acgagggcacgctccgcgacggcacgcgcgtagcggtgaaggtgctggacccgaagggcgggtgcggcggggaggtgtcgcgcagcttcaagcgg
gagtgcgaggtgctgcggcgcacgcggcaccggaaccttgtgcgcgtggtcaccgcgtgcagccagccggacttccacgcgctcgtgctcccgctgat
gcgaaacggcagcctcgagagccggctgtacccgcccgacggcgggccgggacgcgggcttgacctcgtgcgggtggtcgctattgctggagacgtc
gcggaggggctcgcgtacctgcaccactacgcgccggtgcgcgtcgtgcactgcgacctcaagcccagcaacgtcctcctcgacgatgacatgaccgc
cgtcgtggcggactttggtatcgccaagctggtgaaggacgtcggcgacggcgacttcggcagctcggcagatccgtgcaactccatcaccgggttgtt
acagggttctgtcggctacatcgcgccagagtacggattaggaggccacccttcgacacaaggcgacgtgtacagcttcggcgtcatgcttctcgagct
cctcaccggaaacaggccgaccgatgtgatcttccaggagggcctcacgctgcacgattgggtcaagcagcactacccgcacgacgtcggcacgatcg
tcgcgcggtcgtggctgacggacgccgccacggcggtggcggacgagacgctcagggaggacgtcatggtcgtgctcatcgacctcgggctccagtg
cacgcagcactcgccgacggcgcgaccgacgatggtggaagtgtgccacgagattacactcctcaaggaagacctcgccaagcaccggggctctgtg
gtcgcagtggcaagaggggcggggtcggtgacgatcacagcaagcgagcgctcattttccacctcggattcgtctttctga
>g11015.t1
atgcaggctcttgacctgtcggcaaacaagctctcagggacaataccggcagaaattagaggttgtgtagggcttgactatgtcaatctctccagaaac
atgcttcagggcagccttccttcttccactggcactttgccaaatctccaggtccttgacgtttctttcaatagccttaccggcgtgcttcctcagtctttgca
ggcagcgaaggtgctctggttcgccaacttctcgtacaacatcttctccggtgaggtttccaaggaaggggcgtttgcgaaactcacatatgactcatttc
tgggcaatcctgacctgtgcgggtcaattcccggcatggccccatgcaaaactggaaataggcggcatgctcgtttcctgcacatagtcatcgctatcgt
tgtcatcgttcttgccggtttattggccaccatgtgctttgttgatcgtggactgatgacgactcgtttgaggttgacagccccatcagttggtcaattgtca
cgtttcccgacaggatctgtcagtgccgcggtcgaaatggagagcgatcatccaaggatctcatatcgtgagcttgtggatgctactgatggcttttcgg
aagtgaatttgattggcaaaggaggatatggacatgtctacaagggcatcctcgctgaagggacagcagtcgctgtcaaggtcctgcaccaggaccat
gccggtgaggtgatcgctggcagcttcgtgagggaatgccgggtgctcaggagcatccggcaccggaacctcatccgcgtcgtgacggcgtgcagcac
gccggatttcaaggccgtcgtgctcccgttcatgccgaacggcaacctcgacagcctcgtccacgggcttcccggaggaggtgataagccggcagcac

ggcggcgcctagaccttgacctgctgctcagcgtcgccagcgacgttgcagagggcgtggcgtacttacaccaccacgcccccgtcaaggtcgtgcact
gcgacctcaagccgagcaacgtccttctcgatggcgacatgaccgccatcgtctccgatttcggcatctcgaaactgttcaaggatgcaagagatccag
agatgagctacgcgtccatgacgtccgcatgcagcagctccattactcggctgttacaaggctctgttggttacattgcacccgagtatgggttgggagg
gcggccatccacgcaaggtgatgtgtacagcttcggggtgatgctcctggagatgatctccgggaagaggccgacggacgtgatctccgaggagggg
cacagcctgcacgaatgggtcaggatctgctgcctgcgacagcacgaagaggacgccgtcgtcgagcgctcgctgctgcctggtgatccggttcccgg
gcacggcgaggttatggaggtcatcgtggagcttctcgagctcggcgttgcatgcactcagcatgtgcctgccatgaggcccagcatggacgacgtggc
gcacgagatcgcttgcctaagggatgatacatggagaaagtacagggtggcagatttcaatactcacggcaaatccacactctaa
>g11016.t1
atgcgtacatttctcgcacttcttattaccatcaccattgctactatcagctccaactatgcttctccggtggattcacatgaaggcgaccacactgcccttc
tctccttcaagtccggcgtttggggcaacctatctgactggagctcccttggatcaatgtgcaattggactggtgttgaatgtgattcaagagggcgcgtg
attcatctctttctctctcactctaacctcaccggcgtcatctccccggcaatcggcaacctttctgtgctcgagaggcttgagctcgacaacaaccaactc
tcgggaatcctcccaccagagctcggaatgctgtcatcgttgttagagctgagcctttacaacaacttcctggaagggccgattcctgaaacgcttggcc
ttctgagaaacctgacctacctctccctcgagtctaacaatatcaatggtagcatccctgaggctgtcgtatgtaactgctcctccttgacttacattggcct
aagcagcaactccctcaccggtgaaattcctttcaccgcacactgccggctcccaggcctgaacgaactcgttctctacgagaacaagcttgttggagcc
atcccgccgtcgatattgaacttcacaagcttagagtgggtgcttttgtatgataatttactcagtggtgcattgccatctcagatgttcagcaagatgccg
aacctcacatacctttacttgtcatacaacaatttctcaagtgatggcgggaacaccaactttgagcctttccttgcttctctagtgaactgcaccagcttgc
aagagcttgggttggcctcaaacggcatcgaaggaaaaataccatccatcatcggcaacttatctgccaatctttacaggctcatgttgcatgacaacaa
aatnnnnnnnnnnnnnnnnnnnnnnnnnnnnnnnngtgtag
>g11721.t1
atgtcgccatcctccgttgccgcgctcctgtccctgctcctgcttgtagtaccacccatgtccccggcggcggcggcggccatggacccggccgagcgtg
agacgctattccgcgtgatggaggccgtctcctccgaccgcgactggcgcgccgagagccccgacccctgcggcgcgccgtggcccgggctggagtgc
aagccatcggcggggaccccggcgcgcatgcacgtcacgcggctggacttgggcgtggagcccaacccgacgtgcagggacgccgcgacgttcccg
cccgacgcgttcgccctgccccgcctccagtccctcttcttcgtcgactgcttcaccaacccggccgctgtcaccacgctcgccctcccccgctcgcaaa
cctgtccgcggcgtccaccctgcagcagctcagcatccgtgccaacccggcgctgtccggcacgctgcccccgagctcgccagcctccgctccctcca
ggtgctcaccgtctcccagaacgcgctggtccgcggcggcatcccgccgggcatcggcgcgctcacctcgctcgtgcgcctcgacctcagctacaactc
gctcaccggtccagtaccaaacgaggtcggcgagctgaagagcctggtcgggctggacctcagctacaactccctttcaggcgccgtccccacccgga
tcggcgagctgcggcagctgcagaagctggacctcagcgcgaacaacctcaccggcgccatcccggaaaccgtcgccaacctgaccgcgctcacgtt
cttcgcgctcagcagcaacggcctcactggccgcttccctccgggcatctccgggctgcggaacctgcagtacctgatcatggacaacaacccgatggg
cgtcccgctgccgtcggagctcggcggcctcgcgcgcctccaggagctgcggctgtcgggatccgggtactcgggcccgatcccggcggcgttcggcc
ggctcgccagcctgaccacgctgtcgctgcaggacaacaacctgacgggacccatcccggcggcgctcagccggctgggaaggatgtaccacctcaa
cctcagcaacaacgcgctcggcggggccgtcccgttcgacggcgcgttcctgcgccagctcgggcggaacctcgacctcagcggcaactcggggctgt
gcatggaggaccggagcggcgtcctgcggggcgtcggcgtcggggtcggctcgtgccgcggcggtggtgatggcagcggcgccggcggtgacacgt
ctgcggcggcggcgcgtcggggtacgggacgaacggcgtggggttccggtcccagagacggtttcccgttcgggctgctctgcccggcgtgcgtgctc
gtgttgcccgtcgtttttgccctgaacgagcacgggccgaattga
>g12870.t1
atgccatcttggtggctgatagtactgctcttctttgtaacggctgatgctgcggggacggtgctggacgatctacatctgctggagcagttcaaggctgc
cctgcccaacgtcaaggttctagatggctggactagccgcaatggttcttgtagcttttcgggtgtaggctgcaagaatggaagggtcacgtcagtttccc
tcactggtatcaaccttagtgccagctttagtgctgtggccagcagtctgctgcagctgaaaagtgtggaggcgatcacaatccgtgatgccaacattac
cggcatgctctctgcggccgcggcctgcggggagaagttgtctcttcttgatttgtcaggtaattctggccttaatggctccgtggccgatatagctgcaat
gatcagtgtttgtcgtgggttaagggtgctgaatttctctggtaatggagttggtgctgagaaagctgctagagaaggtagcatgacgtttgctgggctgg
aggttcttgacctttccaataataggattgataataatggtgatctacaatggatgattagtgtgggtgctggtgcagtgcggtggctggatctctctagg
aacatgatttccggggaacttccggtattcatcaactgctctggattgcagtatcttgacctctctagcaaccttatatccagcagcgtggaagcaggggt
cctatctggatgtgtcagcttgagcgtcctcaacctctcctacaacaggtttgttggcatgttcccaccagatatttcacgcctcatgtctcttactgtcctca
acctatcctgcaacaacttctcaggcgttatttctggcaaggcttttatcaatcttcctactatcaggacagttgccctctccttcaactatttcaatggctcc
atcccagatgtggtcacaatgcttccggagcttgagataatggacctaagctccaatgcactctccggcaccatcccagccagcctatgctccttaaaaa
cgatcaccagactccaagttctatacctgcagaacaactacctcactggtggcatccctgagaccatctccaattgtaccggccttgtttcacttgacctc
agtctcaactatctcaacgggcacattcctttatccctaggcatgctctctatccttcgtgacctgttgttgtggcaaaatgagctttcaggtgagataccat
cctcgctgtccaatatgcgtgctcttgagactcttattcttgattacaataatcttgttggcagcatcccagcggggttagaactcgcatatacaacgaagg
tgactgaaaaatgcgacgtctacagctttggcgtgattgttcttgagctgttcatgggatctcatcctggtgaccttctttcgatgctcttttgcacacacag
gaaaagcacgtcgctgaaggatttgctggacgcccgtctcccgctccctgaagctgagactgcaagggaaatctttgggtttgttacagtcgccctccac
tgcttggatcccaacccggcaagtcgtccgacaatgcagtctgcaattcggaagctttctgaagttcttagaacgggggatgctgattacctgcacactg
acatcaccaataccagcacccatggttag
>g13115.t1
atggcgtcgagagcatccttcctcctcctcgtcctgctggtcgtggcggccgccgccgcatcgccggcgctgtcccacccgccgccgtcgtgcaacgcgg
cggacctccacgcgctgctggccgtgaagcagtcgctgggcaacccgaagacgctgtcgacgtggtcccccctcctcccccgactgctgcaagtgggac
cacgtccgttgcgacgacgcgtccggccgcgtgaacaacgtcttcatcgacggcgcggacgacgtgcgcggccagatcccgtctgccgtggcgggtct
cacctcgctcatgtcgctctccctcttccgcctcccgggcctctccggcaccatcccgccctgcctcgccacgctccccaacctgcagttcctcaccctctc
ccacaccaacgtgtccgggcccatcccggaccacctggcccggatccgcaccctcgactccgtcgacctctccaacaaccgcctcacgggctgcatccc
ggccgccttcgccgacctgcccaacctccggtccctcgacctccgccacaaccagctgacggggcccatcccggcgggactcgtgcagggccagttcc

ggtcactcatcctctcctacaaccagctgtccggcccgatcccgcgggacgacgcgcaggacgagatcaacaccgtcgacctgtcgcacaaccggctc
accggcgacgcctcgcacctgttcgtccccggcaggcccatcgggaaggtggacctctcgtggaacgacctcgacttcgacctcagcaaggccgtcttc
ccgcccgagctcacgtacctggacctctcacacaaccgcatccacggcaccgtgccgccgtcgctggggaggctgtccacgctgcagaagctcgacct
cagctacaaccgcctgtgcgggccgctgcccaggttccacggcgtcatcaagcacggctgccaggcgtacgagcacaaccagtgcctgcgcggggcg
cccatcgagacgtgccaccggtactcgtag
>g13116.t1
atgatgggcccctgtatgttcagaaacgtggtccagggccagttccggtcactcatcctctcctacaaccagctgtccggcccgatcccgcgggacgacg
cgcaggacgagatcaacaccgtcgacctgtcgcacaaccggctcaccggcgacgcctcgcacctgttcgtccccggcaggcccatcgggaaggtgga
cctctcgtggaacgacctcgacttcgacctcagcaaggccgtcttcccgcccgagctcacgtacctggacctctcacacaaccgcatccacggcaccgt
gccgccgtcgctggggaagctgtccacgctgcagaagctcgacctcagctacaaccgcctgtgcgggccgctgcccaggttccacggcgtcatcaagc
acggctgccaggcgtacgagcacaaccagtgcctgcgcggggcgcccatcgagacgtgccaccagttctcgtag
>g13731.t1
atgctccaccgctcagccaccagcttcctgctgctgcttctctcagctaccaccttcagccatggcgtctcgcttaccacccacgcgctgtggcagcctca
accaccagctggcagtaatactgcaagctgcatagggaaggagagggacgcgctgctagccttcaagcaaggcatcacaagtgacccagaaggaga
tctcgcctcatggaacgaagaccaagaagattgctgccggtggagaggcgtccagtgcagcaacgcgacaggccacgtcctcaagcttcgccttcgaa
acaagcacgctgacccctacggcaacccaattacacctctcgtcggacagataagtcagtctttgcttgctttggaacgtttggagtaccttgacctgagc
tcgaatcaccttgagggaacaactggtcgtattccggagttcctgggctctctcaagaacctaaagtaccttaacttatctggttga
>g14379.t1
atggcggcgtcgcgcgctgatgtcatcctctcgatcctcttggccctcgccgcgacgagctccgcctcccccgtcccggagagccccgacgccgcggcg
ctgctcgcgttcaagtcgtcctccccgccccgcgtccgccgcgtggtcctcgagggcctccgtctcggcgggcacgccgcggcgctggagctcctcgcg
ggcggcctcccccctgctctcgtccctcagcctgaagaacaacacgttcacggggtccctccacgacgtcgacttctcccgcctggcgccgcacctcaagc
tcctccacctctccgggaacgcgttctcggggccgttcccggagtccgtcctgcggctccgccacctgcgccgcctcgacctctccggtaaccggctcac
gggcgccgtcccgcgggacatcgggaaccgcctccgcgctctcctgacgctgaacctctcgcgcaattcgttctccgggcccgtgccggactcgctgga
aacgttgaccgggctcgcagagctcgacgtctccggcaaccactttgagggccgaatccccgggcgccttgcggccgtgttcccggggtcgtctttcgc
gggcaatccgggactctgtggcgcgccgctgccccgccggtgcaataatggacagcagcagatcgtgcacggcaacggcagcacagcagaaggcgg
gtcgtgggcggggaggaggaggaggaggaagcgagaccgttggatggtcgtgctgatcatgtcagccgtgggcgcggcggtcgcgtcgctgatcctc
gcggccctgtgcgccgcgctgtggctcaagaaccggaagccaacgagacgcccgcgcggcaccaccaagtcgacctcacgctccgtggtggtggcgc
cgcgggaggagacgatgcgcttcgacgggtgctgcgacgagttcgacgtggccgcgctcatgcgcggcgcggcggagatgctggggaaaggcgcgg
cggcgacgacgtaccgcgtggtcatgaccggaggcaccgacgacgaggaggaggccgttggtggtagtaagggcgaggcggtggtggtgaagcgg
atgcgacggcgggacggcgccctgaagaacaacacgttcacggggtccctccacgacgtcgacttctcccgcctggcgccgcacctcaagctcctcca
cctctccgggaacgcgttctcggggccgttcccggagtccgtcctgcggctccgccacctgcgccgcctcgacctctccggtaaccggctcacgggcgcc
gtcccgcgggacatcgggaaccgcctccgcgctctcctgacgctgaacctctcgcgcaattcgttctccgggcccgtgccggactcgctggaaacgttg
accgggctcgcagagctcgacgtctccggcaaccactttgagggccgaatccccgggcgccttgcggccgtgttcccggggtcgtctttcgcgggcaat
ccgggactctgtggcgcgccgctgccccgccggtgcaataatggacagcagcagatcgtgcacggcaacggcagcacagcagaaggcgggtcgtgg
gcggggaggaggaggaggaggaagcgagaccgttggatggtcgtgctgatcatgtcagccgtgggcgcggcggtcgcgtcgctgatcctcgcggccc
tgtgcgccgcgctgtggctcaagaaccggaagccaacgagacgcccgcgcggcaccaccaagtcgacctcacgctccgtggtggtggcgccgcggga
ggagacgatgcgcttcgacgggtgctgcgacgagttcgacgtggccgcgctcatgcgcggcgcggcggagatgctggggaaaggcgcggcggcgac
gacgtaccgcgtggtcatgaccggaggcaccgacgacgaggaggaggccgttggtggtagtaagggcgaggcggtggtggtgaagcggatgcgacg
gcgggacggcgcgtcgcgggaggacgagcggcggcgcagggagctggtgcggcagatgggcgcgtggcggcacgccaacgtcgtggcgctgcgcg
cgttctacgcgtccgccgacgagctcctcctcgtcttcgactacgtccccaacggcagcctgcacggcctcctgcacggtgagcacgcacgcacgcactt
ttcccccccctcgcgagcgttacacttaccgttcgaacgttccaactgacgtagccttctctgacgccacgcgcgcgtgcgacaaatttgctttgtcttccac
agagaacaggggaccggcaagagtgcctctcgactggcccaccaggctgaagctggcgcaggacgcggcgcacggcctggcctacctccacggcgc
gtgcggcgggaccctctcccacggccacctcacctcctccaacatcctcgtcgacggctccggcgccgcgcgcgtctccgacttcgccctcctccacctg
ctcgcgcccgcgacacagaccggggcggcggccctgcagcagaagcaggacgtgcacggcttcggcgtgatcctgctcgagatcctcacgggccggg
cgcccggggccgacgacctggtgcggtgggcccgcgcggtggcgaaggaggagtggacgccgcgcgacgcgttcgacgtggagctgccaaggagc
aaggccgccgaggacgagatggtggcgctcctgcaggtggcgatgctgtgcgtggccgacgacccgagggagcggcccaggatggcggtggtggcc
aagatgatcgaggatattagggaccgcgggagcaggaggaacggcaagtgctccgcgtcgccgtcgcaggctgggcgctcgtacgagacatcgccta
gcgtctcggaggacaccacgaggtccactcctgcttcaggctcttga
>g14607.t1
atgccaaccaccatctaccgcaaaatgtccatggccttcctcatctcatgctgctgctttgcggcggtggcagcgcacgaacgctcctccgatgccgacg
ccatcgccgacttgggtcgatccctcgtgaagccgccctcttcctggaggatcgctggtgacgacgtctgctcattcaagggcatctcgtgctcctcctct
ggccgcgtcactgccatcgacctcgccgcgatgggcctggccgggaccctgccctcctccctctcctccctcaccgcgctcgagtccctccagctccggg
gcaacgccctctccggcgccatcccgccactgcgattcccctccctcaccaatctctcgctcgacggcaatgccttcacttcccttcccgaggatttcccgc
gggggatgcccgcgctgcagcacgtcaccatggacaacctccccttcttgccgccatggtccttccccgaacaagccattcttgccggctgcccctctctc
cgcaccttctccgcctccaacgcctccatcgccggcccccctcaccaatgctatcgcaaacctcaagtcgattaccacgctgaggctctcgcacaacctgc
tcaccggggtggtgccgccggctctgggcaagagcgccagcctcagagacatctctctatccggcaactacctccagggcccggtgccacagttcgcc
gccggcgtgaatgccgacgtggtcgccgggaacggtttctgcttggacaaacccgggccgtgtgacgcgctcgtggccactcttctccgggtggcagag
gggtttggctacccgctttaccttgccaggtcatggaagggtaacgacccgtgcggtgactactggcttggcgtggaatgctacttgtcatcgcgccacgt
atccaccatcttcttgtatagcgtcgacttgtcagggacaatctcgccggccatcggggatctcaccaagctcaaaatgcttaacctggcaaacaacagt

ctcaccgggggataccggaatctctcaccacattgcccagactcgaagtccttgatgtcaccaacaataagctcaccggacaagtgcccaagtttaag
ccatatgtccgtgtcttgacagatggcaacgcgtttagcccgccgggctcaaaataa
>g14823.t1
atgggcagcctcatgtcatcgaagcaacgaacagagtacgtctggaatcaagtgtacaatcagtttcgaagtgagctcttgaaaacagacgatgtgca
ggcaattctgaagctaagctacaacgacttaccagggaaccttagaaattgcttcctatactgcagtctgtttcctgaagattgcactatagcgcgggag
agccttgttaggcagtgggttgctgaaggctttgcaatggccactaagaacaacacaccggaggatgttgctgagttaaatctcatggaattcatcacca
gaaacatgctgcaagtagtggactatgatgagcttgatagggtgagcacctttctcgagctgcaagattctgcaatcacagaagtgccagcatctattca
gaacttgttcaatctacgttacattggcttgcggcgcacaatggtcaaatcactgccagattgtattgagaaattgtcaaacctacagaccctggatatca
agcaaacaaaaatagaaaagttaccacgaggcatagctaagctcaagaagctgcggcatctattagctgacagagtcatcgacgagaagcaaagaa
gtttccagtacttcgtcggggtacagccacccaaggatctttcgaacttgttggaattgcagacactcgagacggtggaagctagtgatgaccttgcaga
ccaactggagaaaatgaacaggctgcagagcgtgtggattggcaatgtaaatgctacccattctgcaaaactttttgtagcctgtcgaaggtgccactt
ctttctagcttgcttctaaatgcatgtgatgtggatcagcccctttccttggaagctctcaacccacagtccagacagctccacagactcattgtcagagga
cgctgggctgctgggatgctgcagtgcccaatgtttcagtttgatggtcatgggaagaatctcaagtatcttgctctaagctggtcttgtcttgtggaagac
ccactgctactgcttgcaccacaggtgccgaacctcacttatctcagccttaacaaggcgaacagtgtagagaccttggttgtttctgatgggtgcttccc
aaagctgaagacacttgttttgaagaacttggagaatgtcaacaagattacaattggaaaaggtgcccttcaaagtatccaaggattgtatgttgtgacg
ctgcccaatctcgacaaggttcctcaagatatagaataccttacctcactgaagaagctgtggctgctttatcttcacggtaacttcaaagttcagtggtg
cgagaatgggatgcacaagaagatggcacatgttctcgagcttcgagtctag
>g15028.t1
atggccacgctccaccacatcgctctcctcctcctcctcgccgccgccgccgccgacgcgctcacgccggacgggcaggcgctgctgtccttcaagacg
gcagttctgcgcgaccccgacggcgcgcttgccgactggaacgcgagcgacgcggacccctgcgcgtggaacggcgtctcatgctcctcctcctcctcc
tccgccgccgcccgccgcgtcgtcgcgctctccctcccgcggaagcgcctggtcgcctcgctccccgcctcggcggcgctcccctcctcgctccgccacc
tcaacctccgcgacaaccgcctgttcggccccgtcccccgcagctcctggcgggtgcgcccgcgctgcagagcctcgtcctctacggcaacgcgctgc
gcgggcccctccccgccgacgcgctcgccggcctcgcctacctccagatcctcgacctctcctccaacgcactcgacggcgccctccctgactcgatcct
caaatgccaccgcctgcggacgctcgcgctcgcgaggaacaacctcacgggtccgctccccgcggggctgggcattcaactgccggggctggagcgg
ctcgatctgtcatacaatcgcttctccggaggcctccccgaggacctcggtaacctcaccaggctccaggggaccgtcgacctctcgcacaaccagttct
ccggcccgattccggtgagcctcggccggctgccggagaaggtgtacatcgatctcacatacaacaacctctccggcccgattccgcagaacggggcg
ctggagaaccggggccccacggcgttcgtcggcaacccggggctctgcggcccgccactcaagaacccgtgctcaccggacgccgcgccctcgtccg
acccgttggtgcccaaggacggggactcctccggggctggcagcggcaaagggaagaagagcaaggggttggggaaggtcgccattgtggccatcg
tgctgagcgatgtggtggggatcctcatcatcgcgctggtcttcttctactgctactggagggcggtttcgtccaaggacaagaccggggctaccggttcc
aaaggatccaaatgtggtggcagggattgcggctgcttcagccgggacgagtccgagactccctctgagcagtacgaccttgtggcgttggaccagga
actgaggtttgatctcgatgagctcctcaaggcatcggcattcgtgttggggaagagcggcatcgggattgtgtataaggttgttctcgaggatgggctc
atcatggcggtcaggcgactcggggaaggggggattgcagaggtttaaggaattccagactgaggttgaggcaatcggcaaggtccgccatccaaaca
ttgttaccttgagggcctactactggtctttcgatgagaagttgctcgtctatgattacattcctaatggcagcctctctgcagcaattcatggtagtaaacc
tgggacaatgacattcacaccattgccatgggaagcccgtctaaacatcatgaaaggagttgccaagggaatgtcattcctgcatgaattcagccccaa
aaagtatgttcatggggacttgagaccaaacaatgtgctccttggaacaaacatggaggcatatatttcagattttggcctcgggcgacttgcaaacatt
gctggagggtcgccttatgtgcaatcaaatcgagttggtttagaaaagggtcagagccagccatcagatgcctcagtgagccctctggtccgccatcca
aacattgttaccttgagggcctactactggtcttttgatgagaaattgctcatctatgattacattcctaatggaagcctctctgcagcaattcttggtagta
aacctgggacaatgacattcacaccattgccatgggaagcccgtctaaacatcatgaaaggagttgccaagggaatgtcattcctgcatgaattcagcc
ccaaaaagtatgttcatggggacttgagaccaaacaatgtgctccttggaacaaacatggaggcatatatttcagattttggccttgggcgacttgcaaa
cattgctggagggtcgccttatgtgcaatcaaatcgagttggtttagaaaagggtcagagccagccatcagatgcctcagtgagccctctggtgagcag
agggtcatgctatcaagcaccagaagcgctcaaaacgctaaaaccatcacagaaatgggatgtctactcttatggagtggtcttgcttgaaattattact
ggcagatcacctgctgttctcctggaaaccatgcagatggatcttgttcaatgggtccagttctgcattgaggagaagaagccatccgctgatgtgctgg
accctttccttgcccaagactcggaacgggaagacgagatgatcacggtactcaaagtggctctagcctgtgttcaggcaaaccccgagtggagaccgt
cgatgagacatgtcgtagagaccctggagcgcctcaatggttcaagctag
>g15694.t1
atgactagcaagcagtgcagcttcgtcctgctcgttcttgctctatccttgttcttgtgcattctggttgcacctgtatgcttagccacagcagccacagctc
ctgccagtctcctgcaagtgaagtccagcctcgttgaccctgaaggcgtcctctccggctggtcgccggaggccgacgtgtgctcatggcacggcattac
gtgctcgctacccggagagggcattgtgaccggcctcaacttttcggggtacggcttgtcaggcgtgataccgccggcgatcggtggcctcgtctccgtc
gagttcattgacctgtcctccaactctctctgggtcgatcccgccggagctgggcatgctggagaacctgaaggttttgctgctcttctccaactccctc
accggcacagtcccgccggagctcggcctcctaaagaacctgaaggttctcaggatcggtgacaacaggctgcacggcgagatcccgccgcagctcg
gcaactgcaccgagctggagacgcttggcctggcgtatggccagctgaacggcaccattcctgctgagctcggcaacctgaagcacctgcagcagctt
gtcctggacaacaacacccttaccggcggtatcccggagcagcttgccggttgtgcaagtttgcaactactgtcagtggctgataatatgctgcaaggca
gcattccttcgttcattggcagcctcggtgatctccggtctctgaatttagcaaataaccagttgaccggtgacattccggcagagattgggaacctttccg
gtctgacatacctcaacctgctcggcaacagcctgaccggctccatctcgggagaactgaaccggttgagccagctgcaggttctggacttgtccggca
acaacatttccgggaagatcatcatctctgcgtcacagctgaagaacctcaagtaccttgtgctgtctggcaatcttcttgaaggcgccatccctgacgac
ttgtgcgctgaggagtcgagcctggagaacctgttcctggccgacaacaaactcggaggcggcatcgaggggttgctgaactgcgtcactctgcagtcc
atcgacgtgtcaaacaacagcttcaccggagccataccgccgggcatcgacaaactgtcgagcctcgtcaaccttgcgctgcacaacaacagcctcac
cggcgctttgccgcctcagatagggaacctcagcaacctggagatcctgtctctgttccacaatggccttaccggcacgatcccgccggagatcgggag
gcttcagaagctgaagctgctgttcctctacgagaaccagatgtcaggaaccatccctgacgagatcaccaactgcacgagtttggaagaggtggactt

cttcggcaaccacttccatggttccatccctgacaagattgggaacctcaagaacctcgccgtgctccagctccggcagaacgacctgtcgggccccatc
ccggcgagcctcggcgactgcaggagcctccaggcgctggcgttggccgataaccggttttccggcgagctgccggacacgttcggccagctcacgga
gatcagcgtgatcactctgtacaacaactccctcgaaggcccccctgccggaatcactgtccgggctcaagaacctgacggtgatcaacttctcgcacaa
ccggttcaccggcagcgtcgtccctctcctcggctccagctccctctccgtgctggccctgacgggaaacagcttctccggggtgatccctgcggcggta
gcccagtcgagtgacatggtccggctccagctcggcggcaaccggtttaccggtgcaataccggcccagctgggaaacctcaagcacctccacatgctt
aacctctcatccaacgacctgtccggaaacatccccgtcgagctctccaactgcacggcgctcacccacctgaagctcgacgggaacggcctgaccgg
cagcgtccccgcatggctcggcagcctccggtccctcggcgagctcgacctctcgtcgaacgcgttaaccggtgccataccggtggacctcggcaactg
ctcgggactgctcaaactgtccctcagcgacaaccacctctccggcagcatcccacaagagataggcagcctcacatctctcaacgttctcaacctgga
caagaacaggctcaccggcgccataccgccggcgctccggcagtgcaacaagctgtacgagctgaggctgtctgagaactcgctggagggcccagtc
ccgccggagctcgggcagctgccggagctgcaggtcattctggacttgagccggaaccggctctccggcgagataccggagtccctcggcaacctcgt
gaagctggagcggctcaacctgtcgtccaaccagctccatggaccaattccatcttcacttctgcagctgacgagcctgcacatcctgaacctctccgac
aacctcctctccggctcggtccccgccgcgctgtcgaccttcccggccgagtccttcaccggcaacaacgagctctgcggcgcgccgctgccgacgtgc
cggggctcgtcgtccggccggcttccggggaccgaggtggccgccatcgtggcgggcatcgccgtcgtgtcggcggtggtgtgcgtgacgctgctgtac
actatgctcagggtgtggagcaactggagggcggtgtcagtgtgcagctccgacaacgaggaggactcgcatggcaacaagcgcggcggcaagtgg
ggcgccgccgacggcaaatactggaaggtcgggtggccgccggcgtcatcggcggaggagaagcacagctcggggtcggagaccagcgtgcttcac
gggaactccaaggaggttggcgctggcgccgcgaagagctag
>g15696.t1
atgtggatgcgtctgctgctcggcgtcgtctgcctgaccacggtagccatggcagcgagcggcggcgacgaggccgcgctgctcgctttcaaagccgg
gctcagcaacggcggcagcgcgctggcctcgtggaacggcagcaccgggttctgcagctgggagggcgtggcgtgcacccgcggtcggaacccgcc
gcgcgtggtggtgctgagcctgcccaagatggggctcgccgggacgctctcggcggccatcggaaacctcacgttcatgcaagcgatcgagctgacat
tcaactggctgcacggagacgtcccggcgagcctcggccgcctccggcgcctccgctacatgagcctaggctacaactccctctccggcgagatccctg
ccaacctgacctcctgcgtggccctggagcagctgttcctcgacagcaacaagctcagcggccgcgtgcccgccgagctcgggaacgcgctgagccgg
ctttggctgctccgactcaagaacaacagcttcaccggacccgtcccagagtcgctggccaacgcgtcgctgctaagctacctctccctcgccaacaacc
agttcgacggcgccatcccgccggggctcgccggcctcgccggcctctggcacctcgacctctccgtgaacaagctgcgcggcgctctgccgctgtccc
tgtacaacctgtcatcgctgaggatcttccacgtggaagggaaccggctgcacggcaccattcccgcggacatcggcagcaagttcccggccatgaag
gacttgagcttcgccaacaacaggttcaccggcggcatccctgccaacctgacctcctgcgtggccctggagcagctgttcctcgacagcaacaagctc
agcggccgcgtgcccgccgagctcgggaacgcgctgagccggctttggctgctccgactcaagaacaacagcttcaccggacccgtcccagagtcgct
ggccaacgcgtcgctgctaagctacctctccctcgccaacaaccagttcgacggcgccatcccgccggggctcgccggcctcgccggcctctggcatct
cgacctctccgtgaacaagctgcgcggcgctctgccgctgtccctgtacaacctgtcatcgctgaggatgttccacgtggaagggaaccggctgcacgg
caccattcccgcggacatcggcagcaagttcccggccatgaaggacttgagcttcgccaacaacaggttcaccggcggcgtccctccttctatctcgaa
cctcaccaacctcacaagcctccagctctccatcaacgggttcacggggctcgtgccgcacgatctcggcaggctgtatcgtctccagtacctgtatatg
ccttacaatatgcttcaggcagatgacacaaagggctgggagttcatcacttcactggcgaactgcagccagctggtgcagctgagcctcagtgacaac
gccttcgccgggcagctgcctagctcactggtgaacctgtcatccacactccagtatctctatctgggagactgtggtatctctggcacaatcccccagga
catcagcaatttggctggactaagcatacttgatttctcaaacacctccatatccggggtgatccctgacaccattggcaagctagcaaacctggtcgag
cttgtcttgtacaggaccagtttgcgcaatgcatggctcatcctctacttcgcgatcacgccaggattggcgaaccaacggcgtgccgcgtcgccgcggg
actag
>g15956.t1
atggccatcaacaaacgcgccctactcccgctccaactcctcctcttcctcgcgctcaccgccccggcgcgcgccgccccgtgccacccggacgacctcc
gcgcgctccgggccttcgcggcgaacctcaccgccaccccgaccgcgtggccctccggcgacgacgacgccgactgctgcgcctggcccggcgtcgg
atgcgacgccgacggccgcgtcacggcgctgcgcctcccgcggtggggcctcgaggggcccctcccggccgccgccctcgccggcctcgcgcgcctc
gaggacctcgacctcagccacaacgccctctccggggacctcgccccgctcctcgtctcctccgccaacctccgcgccgccaacctctcctccaacatcc
tcaccggcgcgctccccgacctctcctccaacccgtccctcgccgtcctcgacgccagcaacaactccctctccggcttcctcgcccccgacctctgcgcc
ggcgcgccggccctaaggctgctcgacctctccgccaaccgcctcgccggcgcgctcccctcctcctcctcctcctcctcctcctcctcctcctccgcg
ccgccctgcgccgccacgctccgggacctctccctcgcctccaacgccttctccggcgccctcccacccgcgctcttcgccctcgccggtctacacaagct
ctccctcgcctccaacgcgctcgccggccagctcacctcccgcctccgcgacctcaaaaacctcacatttctcgatctctccgggaacgccttctccggcc
acctccccgccgacgtcttcgccgacctcacctcgctccagaatttggccgcccactccaacaacttatccgggcccctgccgccgtcgctgtctctcctc
acatctctccgtgtgcttgacctccgcaacaattccctgtcgggtcctgtcactctcgtcaatttctcagtgatgccgttcctggcttcggttgacctcgcca
ccaaccacttcaacggcacgctccctgtcaccctagccgactgccaggagctcaaatccctaagcctcgccaaaaacaagctcaccggccaactaccc
gaggattacagccggctcgcctcgctgaccatgctgtccctctccaacaacagcctgcacaacatctcaggggcgctcaccgtgctccgtggctgtaag
aacctcaccacgctgatccttaccaagaatttcgacggcgaggagctaccggccgacagcatcgctgggttcgacgccatcgaggtgctggcactagg
taattgtgctctcagggggagggttcctgattggctgactcactgcaagaaactggaggtgcttgatctgtcctggaaccagttggttggaaccatccca
caatggatcggtgactttgagtacttgagctacctggatctctcaaacaactccttggttggtgagataccagagagcttgacgcagctcaagagccttgt
cactgctagtgctaggcagtcgccgggtatagcgtttacgagcatgccgctctatgtgaagcataacaggagcataaatggccgtcattataaccagct
ctcgaatttcccgccgtcgttgttcctgaacgacaacaggctgaatgggactatctggcctgagtttgggaacttgagggagctacatgtgcttgatctga
gcaacaactccatatctgggagcattcctgattcactatccagcatggataatctggaggtccttgacctttcgtccaataacctcagtggatcgattccgt
catccctcaccgacctcaccttcttgtcaaagttcagtgtggctcacaatcacttggtggggcagatcccaaatggagggcagttcttcaccttctccaac
accagtttcgaggggaacccagggttgtgcagatcaacatcctgcaacctgaatctgtcgctgtcgaggcaagcttctagtggcaagaaggtgccacct
tcagcaagtgtgaggaacaacaaaaacaaaatacttggggtggcgatctgcattggattggcccttgcagtattgttaaccattattttggttaacatgtc
aaagagggatgtcagtgccatcgactatgaagatactgaaggttcctgtcaggaattgtatgattcttactcgaagccggtactcttctttcagaattctg

cggtgacggagttaacagtcagtgacctcgtcagatcgacaaacaactttgatcaagccaacattatagggtgtggtggatttggcctggtgtacaagg
cctaccttcctgatggaacaaaggcagcagtgaagaggctctctggtgattgcgggcagatggagagggagttccgcgctgaggtagaagcactgtca
caggctcaacacaagaatcttgtgacgctcagagggtactgccgctatggaaatgataggctgctgatttactcgtacatggagaatggtagccttgatt
actggctgcacgagaggtcggatgcaggttacatgctgaaatgggagtcaaggctcagaattgctcagggctctgccaggggggctggcttacctgcac
aaggaatatgaagggatcaagggacttggtctcctgggttcttcagatgaaatctga
>g16134.t1
atgaagggaaaggtcgcggcgctgaacacgattatggagatatggggcctgcaagcgtcgccgaggtggaacatcagcggcgagccatgcagcggc
atcgccgttgacaacaccgacgtcgacaatgatcccaacatcaaactgggcatcaagtgcgactgctccttcaacgcctccaccgtctgccatatcacca
agctatacttgggtagctctggattcagcggtccattcccttcaacgttctcaaaacttacaaacttgaggatcctgtgggcaccagacaacgattttact
ggaaaaatacctgattatcttgggagcttaactaagctggaagatttaatattgaggaactgtaggatatctgataatcttgcaacagtaaatttttctaa
gcttgaaggattaacgttgctggatctgagttttaacaatatcactggtcaaattcctgaatccatcttgaatctgcaaaatcttggattcttgtaa
>g16424.t1
atggcggctcgcgcaagcttcttcgccgtcgtagcgctccttgccctgctcgccggcgccgcgcgggcgtccaacgaggagggagacgcgctctacgc
gctgcgccagcgccttacggaccccaacggcgtgctgcagagctgggatcccaccctcgtgaacccctgcacctggttccatgtcacctgtgaccaggc
cagccgcatcgtccgcctggatttaggcaactccaacatctctggctccatcggccctgagctcggtcgccttgtgaacctcaaatacctggagctgtacc
ggaacaatcttattggcgatatcccacaagaactgggcaacctaaaggatttgatcagcttggatttgtatgctaacaaactcactggaggaatccccaa
gtcactttccaagctcaactcactgagattcatgcgcttgaacaacaacaagcttacaggatcgattccgagggagtttgctaaactctccaacctgaaa
gtcattgacttgtctaacaatgacctctgtggaacaattcctgttgatggccctttctcaaccttccctcttccaagctttgagaacaacggtaggctcaatg
gaccggaactgcaaggattggttccttatgactttggatgctag
>g16703.t1
atgactcccctgctcatcctctgcttcctcccgctcgccggcgcagccgccgccagtgaggtctccggtgacatcaccgccctgctttccgtcaagtccgc
gctctctgaccccgacgccgctctctccggatgggacccgcgcgcctccccgtccccttgcaactggccgcacatcctctgctccaacaaccgttccgat
gcctccgacgccatcgtctcctccctcctcctctccaacctctccctcgccggcgagttccccaccccgctctgctccctgagatccctcacccatctcgtcc
tctcctacaattccctgtcaggcccgctccccgcctgcctcgccaccgcgctaccctcgctcacgcacctcgacctcgccggcaacgcgttctccggcgcc
gtcccggctagctacggcgcgttcccctccctctccaccctcagcctcgccggaaacgagctgtccggccccttcccgagcttccttgtcaccaacgccac
caataagctcgaggagctcctcctcgcgtacaaccccttcgacccatccccgctgcccccggatgactccttctcctccttcagcctccgcgtgctgtggc
tcgccggctgcaacctcgtcggcgcgatcccggagtccgtgggcaccctcccgcgcctcgtcaacctcgacctctccaccaacaacctcaccggcccga
tccccgccaccattatacagctaaaaggcatcgtgcagatcgagctctactccaaccagctcaccggctcagtaccgccgggcctgggcgcgctgcggc
atctgcggttcctcgacgcgtccatgaaccgcctctccggcccgctccctgccgacctgttcctggccccggcgctcgagagcctgcacctctacgacaa
cgacctctccggcgcgctgccggccacgctgggccgctgcgcgccggcgctcgccgacctgcgcctcttcagcaaccgcctcgcgggggcgctgccgc
cggagctgggacacaactccccgctcgagttcgtcgacctctccgacaaccgcatctccggcccgatcccggtggggctgtgccgcgcggggaggctc
gagcagctgctgatcctcaacaaccagctggtgggccccatcccggcggagctcggtgagtgccggacgctcacgcgggtgcggctacccaacaacc
agctgtcgggggacgtgccgccgggcgtgtggggcctgccgcacctgtacctcctcgacctcgccgggaacgcgctgtccgggaccgtggccccgacc
atcggtatggccaacaacctgtcgcagctgatcgtttccggcaaccggttcaccggcgcgctgccgcgtgagctcggcggcctgccgcgcctgttcgaa
atcgcagccgcggataatcggttctccggtccggtgccggcgtcgctcacggaggtctccacgctcggccggctcgacctgaggaacaactcgctgtcg
ggcgagatcccgcggggcgttgttggagcgtggcgggcgctcacgcagctcgatctctccaacaaccggctcaccgggagcgtgccggccgagctcg
gcgagttacccgtgctcaactcccttgatttatcgaacaacgagctaaccggcggcgtgccggtggagttggagagcctcaagctcagccggctcaacc
tgtccaacaaccggctcaccggcgcgctgccgcctctcttttctggtggcatttacgagaccagcttcctgggcaacccgggtctgtgccgcggcgacgc
gtgcgccactggcagcggaggcagcgccatcgccggcggccgcgggttggtcgggagcgtcgcgtcggtcctcgccgtcgccggcgtcgtgttcgtgc
tcggcgtcgcctggttctgccaccagtaccgtaagaatcgtcgtgccatcgcggaggaggtggttaacgacattgataaaagcggcgggaagccgagg
tgggcgctgacgtcgttccacaaggcggagttcgacggcgacgacatcctccgctgcctcgacgacgacaacgtcatcggcgtcggcgccgcgggga
aggtgtaccgcgccgtcctccgccatcacggcggcattgaaggcgccgttaccgtcgtcgccgtcaagaagctctggtctaccggcggcggcggcaag
gcggccgtcaacgacacgttcgacgcggaggtggccacgctgggcagggtgcgccacaagaacatcgtcaagctctggtgctgcctccggagtggag
actgccgcctgctcgtgtacgagcacatggccggtggcagcctcggcgacctcctccacggcgacatcaagggtggaatcctgctcgactggccggcg
aggtggcggatcatggtggacgcggcggaggggctcgcgtacctgcaccacgactgcgcgccgcccatcgtgcaccgcgacgtcaagtcgaacaaca
tcctcctcgacgccgacctccgcgccaaggtcgccgacttcggcgtcgccagggtcgtcggcggcgccggagccgccgccgtgaccgccatcgccgga
tcatgcgggtacatcgcgccagagtactcgtacacgctgcgcgtcacggagaagagcgacgtgtacagcttcggcgtggtgatgctggagctgctcac
ggggaagaggcccgtggggccggaccttggggacaaggacctgccgcggtggacgcgcggcgccgtcgagcgcgacggcgtggacgccgtgctgg
acccgaggctcgcgtgcagggacgacatggccagggcgctgcacgtcgcgctgctctgcacggccagcctacccatcaaccgcccgtccatgagggc
cgtcgtcaagctgctactcgaggccgcaccaatgccgacatccacagagagcaagccggcggcgaccgaggagaaacctcttgatgtctga
>g18116.t1
atgcagcatcgcctccacagcgtccggctgtgtggctgtgctgtgctgtgctggtgcgtccgggcggggcatctgtctcgcgctagaaaccaggcggcc
gtcaacgaagcgaagctggcgagcccggcccacgcacgcttgtaccctcggcaggcatgtgaccccccctgacacttcctcgaggggagctgcccacct
gcctcccctccttcacttccattccaccacccttctctcactcctcttccccaccttccctaaacctcaagagagcccctcttcctcctccgccactaccaccg
ccgccgcattgctgtcgtcaccagtaccaatgatgaccggtggggggaggcgcctccgatgggtggcggctctgctcgtcctcgtggcgctggcgctgc
gggcttcagcgcagcagccgaacccgcgtctccgcacggcgttcctggctctccagacgtggaagcgcacggccatcttctccgaccccttcaacttcac
ggccaactgggtgggccccaacgtgtgcgcgtacacgggcatcttctgcgcgccgcacccgtcggacggtcagctcgcggtggccggtctggacctga
accacgccgacatcgcgggctacatcccggcggacctcccgcgcggcctccccgacctggcgctgctccacctcaactcgaaccgcttctgcggcgtgc
tcccggacaccttcctccacctccgcctcctccacgagctggacctcagcaacaaccgcttcgtgggccccttccccgccgtcgtcctcgccctcccggag

ctccggtacctcgacctccgcttcaacgacttcgagggccccatcccgccgcggctcttcgacctgcccctcgacgccatcatcctcaacgacaaccgcc
tcacccgccccatcccttccaacctcggcaactcccccgcctccgtcgtcgtcctcgccaacaaccgcctcggcggatgcatcccgccgtccatcgggcg
gatggggaccacgctcaacgagatcgtcctcatcaacgacgggctcacgggctgcatcccgccgcaggtggggatgctcagccaggtcaccgtgttcg
acgtcagcgacaacaggttgcagggtcggctccccgggagcgtcgcggggatggcggccgtgcaggtgctcaacgtcgcggggaacctgctcaaggg
acccgtgccaggtggggtgtgcgggttgcagcgcaccctcaggaacttcacctacgaggacaacttcttcacggcgcggcctgggtgtcctgtggcaac
ggcggacgggaggtggaactgtatccctggggcgcccgcgcagcgcccgccgccgcagtgcgctgccgccggggcgccgttcgactgcagcacggc
acagtgccaggccgcgccgcccaatgggcccagcccgcctcctgggtctgactctgggcctggcagcccttcctcctcgccgcccgggtccccacctgg
agggagctccaatgcgccgccgtacccgtctccgcctggaggaagctcttctggtcaacccacggcgccgccacccgctgagcaccctggcggcgtat
ggccgccgaacactcccggagcaccaggatccccattgactcccggagcaccaagatcgccgggatccactccgtccgttcccggcgcatcaggatca
ccagggtctgctcccggcgcaccaggttcaccaccagggtcgccattgactcccggcgcacctggctcgccgggatccactccgtcggctcccggaaca
ccaggagggtcacccactactcctggggcaccaggcggatcaccaggagggtcgccgtcaactccaggaactcccggcgcaccaggaggttcgccat
cgacgcccggtggcacaccaggctcccaccctccggctcccggaacaccaggttctccaggttcatggtcgccgcccggaggtggtgaccacggcaac
cagcactccccgcccggaggcctgccattcccgcccgtccacggcctgccatactcctcgccgccgccgccgcctcgcgccacgctgccgttcccgccg
gtccgcggcgtgtcctacgcctcgccgccacctccgatgtcggtccagggtgtgtcctacgcctcgccgcctccgccgcacacggtccatggggtggcat
actcgtcgccgccgccgccgccatcgcatacggttcacgggatggcatactcgtcgccgccgccgccacaacacatgtag
>g18757.t1
atgccacaccaccgcttgcactccggccgcctcgccgctctgctcctcgtcgtggccgccgccgccgcctcgccgacggaagcatcgtcagcagaagca
gactcatcgccatcgtcgaactgcaccgcgctgttcccggacgccgccgccgtccgcgcggcgttcctgcgcgtcggcaacttcccccctgcggcaacttg
gcgcgtgccgacccgtgcggcgcctccgcttcccgttgcacaacctcacgggcaccgtcgactgggcggagctggccaacctgaccagcctcctctccg
tcgacctcgccggtaacgctctcgacggcggcgtcgaccccgcgttctggcacgcgccggcgctccgcatcgcggacgtctccggcaaccacctcgccg
gcacgctccggtttgactttgaccgccgcgcgaatagcacgcgcctggcgtcgctgaacgtgtcaggtaaccggttcgactccgttgagggcgtggcgg
cgctcgttggtctggaggacctcgacgtgtccagaaacatgatcagaaaggtgcctgacgggttgcgcctattgacccgggttcagcggctcgacttgtc
cgggaacgcgatgaccgggaggttccccgacgacttgcccccgcttgagggactcgtgttcttgaacatctccaacaacaacttctccggaatcgtgcg
cgccgacgccgtccggaagttcggccgcgccgctttcctcaacgccggcgaagcgtcctctctggtgatcgaagacgacaccgccccgccgccaccag
cgccatcttcgccgccgtcgagaagcgaaaacaaagggcgcaggcgcgtggtcatcgtaaccattgccagcgcggtggcggtggccgcgctggcgct
ggcgctcgtggcggcacggttacggataggagtggatagaagtagtttgctgttataa
>g18854.t1
atgcagctcctccgcggctccgcaccggacaacatggcccgcctcctcctcccggccctcgccgccctccacctcctcctcctcgcggccgtcgccgtcg
ccgaggacgggccaacgctgctggagatcaagaagtcgttccgcaacggcgacagcgcactgcacgattggtccggcgacggcgcgtcgccgggcta
ctgctcgtggcgcggcgtgctatgcgacaatgtcaccttcgctgttgcggcgctggatttgtcaaacaatcagcttactggagaaatcccattcaatattg
gtttcctgcaagtggcaacattatctttacaagggaacaagttctctggccctataccatctgtgattggactaatgcaggcacttgcagtgctggatctg
agtttcaacgagttatctggcactataccatctatactgggcaatttaacatacacagaaaaattggaactgaatgacaaccagctgactgggtctattcc
gcccgatcttggaaagctcacagaattgtttgacttgaacctgtcatcaaatcatctcagtggagcaattccaaatgaagtagcaagaatgagaaattta
gacacattggacctatcatgtaacaagatgtctggttcgattccctcagctttcgggaaactagaacatcttttgagacttaacttgagcaataataatctg
gttggacacattcctgctgagtttggaaacttgaggagtatcatggagattgatttatccaacaatcacctgggtggcttgattcctaaagaggttggaat
gctacaaaatctgatactattaagattagaaagtaacaatattactggggatgtctcttcactgactaactgcttcagtctcaatgccttaaatgtatcgtt
caacaaccttgctggtattgtacctacagacaacaacttttctcgattttcaccggacagtttcttgggtaaccctggactttgtggctattggctcggttctt
catgctactcgtcaagtcacgcacagagatatacctttgcctcagcatcaagcattgttcctcccaagcttgtgatcctccacatgaacatgtccctccatg
gatatgatgatataatgaggatgactgaaaatttgagtgaaaaatatattataggatatggagcatcgagcacagtctacaagtgtattctgaaaaactc
caagccagttgcaataaaaaagctatatgctcactacccgcagagcttgaaggaatttgagactgaacttgagactgttggtagtatcaaacaccggaa
tcttgtcagccttcaggggtactccttatcacctgctgggaatcttctcttttatgattacatggaaaatggcagcctctgggatgttttgcatggttcctcca
agaaggagaaactggattggaaggctcgcctccggattgcacttggtgctgctcaaggcctggcttaccttcaccatgaatgcaacccacgtataattca
cagggatgtaaaatcaaagaatattcttctagacaaggacaatgaagcacatattgctgactttggcatcgccaagagcctgtgcgtctcaaagacaca
cacatcaacctacgtgatgggcaccattggctacattgaccctgatttcttgggtaaccctggactttgtggctattggctcggttcttcatgctactcgtca
agtcacgcacagagatatacctttgcctcagcatcaagcattgttcctcccaagcttgtgatcctccacatgaacatgtccctccatggatatgatgatata
atgaggatgactgaaaatttgagtgaaaaatatattataggatatggagcatcgagcacagtctacaagtgtattctgaaaaactccaagccagttgca
ataaaaaagctatatgctcactacccgcagagcttgaaggaatttgagactgaacttgagactgttggtagtatcaaacaccggaatcttgtcagccttc
aggggtactccttatcacctgctgggaatcttctcttttatgattacatggaaaatggcagcctctgggatgttttgcatggttcctccaagaaggagaaac
tggattggaaggctcgcctccggattgcacttggtgctgctcaaggcctggcttaccttcaccatgaatgcaacccacgtataattcacagggatgtaaa
atcaaagaatattcttctagacaaggacaatgaagcacatattgctgactttggcatcgccaagagcctgtgcgtctcaaagacacacacatcaaccta
cgtgatgggcaccattggctacattgaccctgagtatgcacgaacatcccggctcaatgagaaatccgacgtatacagctatggcattgtcttgctggag
ttgctgactggcaaaaagccggttgatgatgagtgcaatcttcatcacttgatgctaaccaaagccgcagacaacacggtcatggagatggttgatccg
gagatctctgacacgtgcaaagacctcggtgaggtcaagaaggttttccaattggcgctcctttgcagcaagcggcaaccttcggatcggccaacgatg
catgaggtcgtgcgtgttttggacagcctggtctccccagacccgctcccgaagccggcaaaaccaccagcatcgcagcagtcatccacaactccgagc
tacgtcagtgaatatgttagcctacgaggcggcagctcgctatcctgcgccaattcatcaagcacgtcagacgcagaactctttatgaagtttggcgagg
tgatatcacggggcacggaatag
>g19124.t1
atgacggcgcacagcaccctcctcccattcctcctcctcgccctcctcctctcccctcagctcctcgcctccgcggcccacatccgccgccgccagctcctc
cagtacgacaatggcggcgatagcaacgtagtggacccgtcgtacacgttcccgaacccgcgcctccgcgacgcgtacatcgcgctgcaggcatgga

agcgcgccatcctctcggacccctacaacgtgacgggctcctggtcggggcccgacgtgtgctcctaccacggcgtcttctgcgcgccctcccccctccga
cccctacctcaccgtggtggcctccatcgacctcaaccacgcggacctggcgggccacctccccgacgagctgggcctcctgggcccggacctggccgt
gctccacctcaactccaaccgcttctgcggcctggtcccgcgctccctcgcccacctccacttgctccacgagctcgacctcagcaacaaccgcctcgtg
ggcgggttcccgggggccgtgctggagatgccggcgctgcggtacctcgacctccgctacaacgagttcgagggccccgtcccgccggagctcttcga
ccgcccgctcgacgccatcttcctcaactccaatcggttcaggttcaggatccccgacaacgtgggaaactcgccggcatccgtgctcgtgctcgccaac
aacgacttcggcgggtgcctcccggcctccgtcgccaacatgtccgccacgctcaacgagatcatcctcctcaacacggggctcaagtcctgcgtgccc
agagagttcgggatgctcacggaactcaccgtgctcgacctcagcttcaacagcctcatgggaccggttcctgaggagctggcgaacctgcgcagcat
cgagcagctcaacctcgcgcacaacaggctcgtcggcgacgtgccggagggcatctgccacctgcccagcctccagaacttcacctacagctacaact
atatctccggcgagccgccggtgtgcttgcacgtgaagaacgtcgatgatacgaggaactgtatcccggaacgcccggagcagaggtccccggaaca
gtgcgagttcttcaagagtcatcaccccgtcaactgcgacgcattcggatgcaagaagttcgtgctaccctcgccgcctccgccgccgccttcacctccg
ccgccgtcgccgtcgccgccccctccctcgccgccaccaccatctcctccgccaccttcgccatctcctcccccgccatcgcctccgccaccgtctcctcc
accaccttcgccgtctcctccgccaccttcacctcccccaccgtctcctccacctccatccccgtcgcctcctcctccttctccaccaccaccttccccacct
ccnnnnnnnnnnnnnnnnnnnnccccccgccatccccatccccgccgccgccttccccaccaccaccttccccacctccgccatccccatccc
cgccaccgccgtctccggtctactacaactcgccaccgccaccttactacgaggtgtcaccggaggaccggtacctctcgccgccgcctcctgcctacca
ggagtcaccgccgccaccatcctacgaggtctcacctga
>g19434.t1
atgtcacgctcgggcgcccgctaccctctcttccccttctccccgccttgctcctgctgctggtcgccggcgggtgccgcgcgcagccctccggcgaccg
cgacacgctcctcgcggcgaagaaggattggggcagccccgcgcagctcaattcatggaattcctcttcctccgaccactgcagctggcacggcgtcac
ctgcgccgccgggtcagtgacggagctatcactccagagcctgaacctgagcggctcggtgccggcgtccgtgtgctcgctcaagagcctcgcccgcct
cgacctctcctacaacaacctcaccggcgcgttccccgccgccgcgctctacgcctgctcgaacctcacctacctcgacctctccaacaacctcttctcgg
gacccctccccgccgacatcgacgggctctcgccggcgatggagcacctcaacctctccacgaaccggttcgcgggtccggtgccgccgcccgtggcg
cggctgccggcgctcaagtccctgatgctcgacaccaaccgcttcgccggcgcgtacccggcggcggggatcagccggctcgccgggctggagcggct
cacgctggctgacaacgagttcgcgccggggcccgtgccaccggagttcgccaagctgaccaaactcacctacctgtggatgtcaaatatgaacctcac
cggcgagattccggacgcgctcggcagcctcacggagctgaggacgatggccatgtcggggaacatgctcaccggcgccgtcccggcgtgggtgctg
cagcacgagaagctcgaggtggtgtacctctacgacaacaggctctccggtccgctgccgcgcaacgtgtcggcgccgaacgtggtggagctggacct
gtccacgaaccagctcaccggaaccataccggacagcttcggcaacctcaagagcctgtacctgctgttcctctacaagaaccagctcaccggcacgat
tccggcgagcatcggcctcctgccgcggctcggcgacatccgtctcttcttcaaccagctctccggcgagctcccccggagctgggcaagcactcgcc
gctcggcaatctggaggtgtccaacaacaacctctccggcccgctgcgggagtcgctgtgcgccaacgggaagctctacgacatcgtcgtcttcaacaa
cagcttctccggcgcgctcccggcgaacctgcgcgactgcgtcctgctcaacaacctcatgctctacaacaaccggttcacgggcgagttcccggcgga
gatctggtcgttccccaagctcaccaccgtgatgatccagaacaacagcttcaccggcacgctgccgcccgtgatatccttcaacatgtcacggatcgag
atgggggacaacagcttcaccggctcggtgccaacgtcggccaccaagcttcacgtgtttaaggccgggaacaaccggttcaccggcgagttgccggc
cgacatgagcaagctcgcgagcctgacggatttcctggtgcccgggaacaggataaccggctccataccgggttcgatcaagctgctgcagaagctga
attcgctcgacctgagcggcaacctgctttccgggccgatcccgccggggagcatcgggcagctcccggtgctcaacatcctcgacctatccagcaacg
agctcaccggcgacattccgtcgaatattcacaacgcgttcatctcgctgaacctgtcgtcgaaccggctgaccggcgaggtgccggccgcgctgcaga
ccgcggcgtacgaccgcagcttcctcggcaacgaccgtgggctctgcgcgcgggccgggtccgggacgggcctcccgacgtgctccggcgggccagg
cgggcacgacgcgctgtccaagggcctcatcgtcctgttctcgatcctcgccggcgtggtcctggtcggcatcgtgggcatcgcgtggctgctgctccgg
cggcgcaaggacatccgcgaggtgaccgactggaagatgacggcgttcacgcagctggacttcacggagtcggacgtgctgagcaacctccgcgagg
agaacgtgatcggcagcggcgggtccgggaaggtgtaccggatccacctcgccggcggcggcaacggaggaggcaggatggtggcggtgaagagg
atctggaactcgaggaagctggacgcgaagctggacaaggagttcgagtcggaggtgaaggtgctgggcagcatccggcacaacaacatcgtcaag
ctgctgtgctgcgtctccagccaggacgcaaagctgctggtgtacgagtacatggagaaccgcagcctcgaccggtggctgcatcatcgggaccgtga
cggcgcccccgtaccgcccgactggcccaccaggctcgccatcgccatcgacgccgcgaaggggctcagctacatgcaccacgactgcgcgcagccc
atcgtgcaccgggacgtcaagtccagcaacatcctgctggaccttgacttccaggccaagatcgccgatttcggtctcgcgaggatcctcgtcaagtccg
gcgagcccgagtcggtgtcagcgatcggtggaacgttcgggtacatggcgccggagtacgggtacaggcccaaggtgaacgagaaggtggacgtgt
acagcttcggcgtcgtgctgctggagctcaccacaggcaaggtcgccaacgacagcggcgccgacctgtgcctggcggaatgggcgtggaagcggta
ccagaagggcgcgccgttcgaggacgtggtcgacgaggccatccgggggagccggcgttgatgcaggacatcttgtcggtgttcactctcggggtga
tctgcacgggggagaatcctatgacacggccgtcaatgaaggaggtcctgttccacctcatccgctgcgacaggatgtccgccgaggcgcaggcgtgc
caggtggactacgatggcggcggcggcgcgcagctaccggaggtcaagaagaaaggcagcgggcggcggagcacgtcgagctccggcaggtggga
tgaggacgacgaggacagcggcaacttcgtcgtgcacgtggtctag
>g19435.t1
atgtcgtggccgggcacctgcaacctctcattatcaccactactgcacgtgctagtcctgctgctgctcatcgccggcgagggtattgcccagccagcca
ctggcgaccgcaacacattgcttgtcatcaagaaagactggggcagcccgccgcagctcacgtcctgggatgccaccaccgacgccgatgccgatcac
tgtcgctggaaaggcgtcacctgcgccggcgccggcagtggagtcgtcacggagctgtccctcccgttcctcaacctcaccggttcagtcccggcgtcc
gtgtgcacgctcaagaatctcacccacctcgatctctcatacaacaacctcgccggagcattccccgccgccacgctcttcgcgtgcacccacctccgca
cccttaacctctccaacaacctcttctccggtcccctcccagccgacatcgacggcctctcgccggcgatggagcacctcaacctctccagcaaccggttt
gccggcgaggtgccgccggccgtggcgcggctgccggcgctcaagtccctgctgctcgacaccaaccgcttcaccggcgcgtacccggcggccgaga
taggcaggctcgccgggctggacgtgctcacactggccaacaacatgttcgctccggcgccggtgcctacggagttcgcaaagctgaccaagctcacc
tacctctggatgttcaacatgagcctcaccggggaaatccctgaagcgttctcaaatctcaccgagctgaggacaatggacttgtcatggaacaacctta
ccggcacgatcccggcgtgggtgctgcagcacaggaagctcgagcaggtttacctgtattacaacgtcctctccggtgagctgccgcgcaacgtcacgg
cggtgaacttgatcgagcttgacgtgtcatcgaatcagctcaccggaaagatacctgaaagctttgggaatctcaagaacctgagtttgatgtaccttta

ccagaaccagttcaccggcacgatcccagcgagcattgtgctgctaccaatgctcagtgacatcaagatatttgacaaccagctatccggtgagctgcc
acctgagctcggtaagcattccccgctcagcaacctcgagatggatgacaacctcctctccgggccgctgccggagactctctgcgccaacggcttcctc
tatggcctcgtcgccttcaacaacagcttctccggcgtgatcccggccagcctggggaactgcgtcacactgaagaacctgatgctccaggacaacaat
ttttcggacgaattcccagcgaagatatggtcgttgccacgactgaccagggtgaggattcagaacaacaagttcactggtgctctgccggctcagatat
cctctaacatcacacgaattgagatgggcaacaacatgttctctggctccatcccgacctcggcgacagggctgcaagcgttccatgccgaaagtaacc
ggcttggcggcgaattgccttctgacatgagcaagcttgccaaccttacttatttctcggtgcccggcaataggattactggttccataccaacgtcaatc
aagctgctgcagaagctcacgtcactgaacttgagcggcaatcacttatccggtgcgataccgccagggagtgtcgggttgcttccaacgctgacaacg
cttgatctgtccaacaatgagctcactggcaccataccgtctgatataagcagcatggttaacccgctcaacctgtcatcaaaccagctcaccggcgagg
tgccgatggcactgcagagtgcggcatatggccgcagcttcctcggcaaccagctctgtgccagggcgaactccggcacgaacctcccaccatgtcca
ggcggaggccagggcgcccatgtcaagctatccacgggtttcatcatcctcttcgcgatgctcgccagtgtagtccttgtcagctgcatctgcatcatctg
cctgcttttccggcgccgcaaggacaacgacaatgtgatcgactggaagatgacggttttcaaccaactgaatttcaccgagttggatgtgctcaacaac
atccgagaggagaatgtgatcggcagtggcgggtctgggaaggtgtaccgcatcagagtcggctcctctgcacacaatgtggagggctgcaggatggt
ggctgtcaagaggatttggaactcgagaaagctggatgccaagctggacaaggagttcgagtccgaggtgaaggtgctgggcaacatccggcacaac
aacatcgtcaagctgctctgctgcatctccagccaggaagctaagctgctcgtctacgagtacatggagaatggcagtctcgatcgttggctacaccatc
gagaccgcgacggcgcccctgtacctctggattggccgacgaggctggccatcgccatcgacgcggtgagggggctcagctatatgcaccatgagtgt
gtgcagccgatcgtgcacagggacatcaagtccagcaacatcctgctcgacccggacttccaggccaagatcgctgacttcggtctcgctcggatactc
gtcaagcagtccagcgagcctgagtccgtgtcagcaatcggcgggacatttggctacatggctccagaatacgggcacaggcgaaaggtgaacgaga
aggtggacgtgtacagcttcggcgtcgtcctgctggagctcactaccggcagggtcgccaacgacagcaacgccgacctgtgcctggcggaatgggcg
tggcagcggtaccagaaaggtgttccgttcaacgatgttgtggacgaggccatccgggagccggcattgatgcaggacatcttgtcggttttcactctcg
gggtgatctgcacaggggagaaccctgtgacacggccatcgatgaaggaggtcttgcactaccttatccggtgtgaccagatgtctggagatgcaggg
ttgtgccagatggaatgcaatggtgacctcgaggcgaataagaatatcagctga
>g19580.t1
atggccggcccccgcgcgcccgtggcggcggcggtggcggccgtcctggcactcctctccctcgcgttcgcggagccgccggcgagcgagcggtcgg
cgctgctggccttccagaccgccaccccgcaccagcgccggctcggctggaccacctccacctccgcctgcagctggttcggcgtgacctgcgacccca
ccaactccacggtcgtcgacgtccgcctcccgggcgtcggcatcgtcggcgccgtcccgcccggcaccttcggccgcctccccaacctccgcgtgctctc
cctccgctccaaccgcgtcctcggcgcgctcccctccgacatcctcgccctccccaacctcaaatccctcttcctccagcgcaacgacatctccggcccca
tccccgccgggatcacggggctggcctccctggagcgcctcgtcctcgcgcacaacaatttgtcaggatccatccccttcgcgctcaacaacctcacgtc
cctccgcgtgctgcgcctcgacggcaaccgcctgtcgggaagcctccccagcatcagcatcccggggctcgacgtcttcaacgtctccgacaacagcct
caacgggtccatccccaggtcgctctcgcggttccccaaggattccttcgcgggcaaccggctcctctgcggcgcgccgctggacccgtgcggcagccc
gttcttcccccggcggcgtcgcccggggctccacccgggggcgagcccgtcccggcggggaataagaagcggaagctctccggcgcggccatcgcc
ggaatcgtcgtcggcgcggtggtggcggcgctgctcctcctcatcgtctgcgtgctctgcgccgtgcaccaccgccgcggcgccaccgcacgtcaggag
gggcccaagggcgccgtggcctccgcggcgcctgcgccggcgcgagggccgacatccggcgagggcggcgggatgacgtcctcgtccaaggaggac
ctcggcggcggcggcacgtccgggtcgacaacggccgcggcagcagctgctgccgccgcggctggggccggcgcgggtggggagcagagccggctt
gtgttcgtggggaagggcgcagggtacagcttcgacctggaagacctgctgcgcgcgtcggcggaagtgctgggcaagggcagcgtgggcaccagct
acaaggccgtgctggaggaaggcaccaccgtcgtcgtcaagcggctcaaggacgtcgccgcgcagcgccgcgagttcgacgcgcacatggaggcgc
tcggcagggtggagcaccgcaacgtgctgcccgtgcgcgcctactactactccaaggacgagaagctgctcgtctacgactacgtgcccaacggcagc
ctctccgcaatgctacacggaagccgtgggtccgggcggacgccgctggactgggaggcgcgcatgcgttcggcgctgtccgccgcccgcggtctggc
gcacctgcacaccgcgcacaacctcgtgcacggcaacgtgaaggcctccaacgtgctgctccgccctgaccaggacgccgccgcgctgtcggacttct
gcctgcacccgctcttcgccccgtcgtcgacgcgcgccggcgggtaccgcgcgccggaggtggtggacacgcggcgtctgtcgttcaaggccgacgtg
tactcgcttggggtgctgctgctggagctcctgacgggcaagtcccccctcgcacgcgtcgctggagggcgacggcacgctggacctgccccggtgggtg
cagtccgtggtgcgggaggagtggaccgccgaggtgttcgacgtggagctggtgcggctgggcgccagcgccgaggaggagatggtggcgctgctgc
aggtggccatggcgtgcgtggccaccgtgcccgacgcgcgtcccgacgcgcccaacgtggtcaggatgatcgaggagatcggcgccgggcacgggc
ggaccaccacggaggagagcgaggaggggcatcgcggcacgtcggaggaggggcgctccagggacacgccgccggccgacccgacgcggtga
>g20696.t1
atgatcaccgggacgattcccgccagttacggcggcatggtcgcgttgcaggacctgagcctcgcgtcgaaccgtctcgtcggcacggtacccccgga
gctcggcggactgccgttactgatcaacctggatttgagcggcaacatgttggaaggccggatcccggtcgcggtcggcaacgccaaggggatgctgc
ggctggacctgtccgggaaccgcctcgaaagtggcgtgccggtggagctgaccaggctatcgcacatgtggcacctcaacctgagccggaacaacctc
agcggcgaagtgccggcgctgctcgggaagatggaggcgctgcaggagctggacctcagcggcaaccccggtctgtgcgggcagattgccgggctg
agaccgtgcagcttggattccgttggcgcaggccggtccaaacgcagcaacgtgaagctcgtcgttgctctggcttccgccgcggttctgctggtctgca
tcgtcgcggtgagctgcgtcgtgctggcccgcatcaaccggcggcgccaaaccggcaaggacacgagcggatacacgacggggagcggcagcgaga
cgaccctgacggcgtcgatctggagcaaggacgcggatttcacgttcggcgacatcctcgcggccacagagcacttcaacgacgcctactgcatcggc
aagggcagcttcgggagcgtgtaccgcgccgacctccccacgggcgcccgcctcgccgtgaagcgcctcgacgcgtgctggggcgcgagtgagcgga
gcttcgagaacgaggtgcgcgcgctgacgctggtgcggcaccgcaacatcgtcaagctgcacgggttctgcgcgtcggcggggcacatgtacctggcc
tacgagctggtggagcgcggcagcctggggaaggtcctgtacggcggcgggagcagccgcgagcggttcgactgggcggcgcgggtgcgcgcggtc
cgtggcatggcgcacgcgctggcgtacctgcaccacgactgctcgccgccgatggtccaccgcgacgtgtcggtcaacaacgtgctgctgggcccgga
cttcgagccccgggtttcggacttcggcacggctaggttcctcgacccgggccggtccgagtgcgccagcgtggcggggtcgtacgggtacatggcgcc
ggagctggcgtacatgagggtcaccaccaagtgcgacgtgtacagcttcggcgtcgtggcgctggagatcctcatggggcagtaccccggcggactca
tcagctccctgcacgcgcggctcccaagcggcgacctcaaggacgcggtggaccagaggctggacccgcccaccgggcacctggcggggcaagtcgt

gctcgccttcctcgtcgcgctgtcctgcgttcgggcggacccggacgcgcggccgaccatgcggcacgtcgcacaagagctctcggagcggaggcgga
cctccctcgacaggccgttcgccgtgatcaggatcggcgacctgacaacttcgaacaaatga
>g21255.t1
atggcgtctgcggcgtcggcgcggttcctgctcccggcggtgatcctcctcgccgtcgtccatccgacgacaccggcgaacgacggcgcagcggcggc
ggcagcgctgctggagttcaagtctgcgctgcaagacccagacgggcgcctcatctcgtggaccaccaccaccgccaccgccgcgggcagcgaccac
ccgtgctcatgggccggcattgcctgctccaccaccggcgaggtcaccggcatcaccctccccaaccacaacctccacggcaccctctccacctcctccc
tctgccattccctcccgcacctcgccgtcctcaacctctccaccaacgcgctctccgggccggtccccaccaccctctccctctgcggcgcgctacacacg
ctcgacctcagcaccaacgccttccactccggcatcccgtccgccctctgctccctcccgcgcctccgccgcctcttcctcagcgagaacctcctcaccgg
ccccatcccgccgtccatcggcaacctctcctccctcgaagagctcgagatctacagcaacaacctcacgggcgcgatccccgccacggtgcgcgccct
ccgccgcctccgcgtcctccgggccgggctcaacgcgctctccggcccggtccccgcggccctgggccggtgcgagggcctccaggtcctcggcctcgc
gcagaaccgcctctccggcccgctcccccgcgccgcgctggcccgcctccaaaacctcaccaccctcatcctctggcagaacgcgctgtccggcgaaat
ccccccgagctcggccgctgcacgaacctccagatgctcgccttgaacgacaacgcgttcacggggagcgtccccgcggagctcggaaatctcccca
acctcgccaagctctacatctaccgcaaccgcctcgacggcgtcgtcccgccggagctgggcaacctgcgggccgcgctcgagatagacctctccgag
aaccgtttgcggggcaccatcccgcgggagctcggccggatccgcacgctgcggctgctctacctcttcgagaaccggttgcaggggaccatcccgcc
ggagctcggccggctccgccgcgtccggagaatcgacctctccatcaacagcctcaccggcgcgatcccgccggagctccaggacctgcccagcctcg
agtaccttgagctcttcgacaaccagctccacggtgccatccctcctttactcggcgccaacagcaacctctccgtgctggacttatcggacaaccacctc
accggcaccattccgccacacctctgcaagcaccagaagctcatcttcctcagcctcggctccaaccgcctcgccggcaacatccccgaaggcgtcaag
gcgtgccggacgcttacgcagctccgcctcggcggcaacttgctcaccgggagcctccccgtcgagctcaccctcctgcagaacctctcctcgctcgag
atgaaccagaaccggttctccggcccgatcccgccggagatcggcgagttcaggaccgtcgagcggctgattttatccaacaactacttcctcggacac
atccccgccgccattggcaacctgaccgagctcgtcgccttcaacatctcatccaaccaattaaccggaccgatccctcgggagctggcgcggtgcacg
aagctccagaggcttgacctcagcaggaactccttcaccggagcgatcccacaggagctcggttctctggtgaacctggagcagctcaagctatctgat
aacggactgaacgggactgttccgggcagtctcggaggcctgtcgcggctcacagagctgcagatgggggggaaccgtctgtccggtcgaatcccggt
cgagctcggcgagctcgcctccctccagatcgctctcaatgtcagctacaacatgctctccggcgagatcccctcgcagctcggcaacctgcgcatgctt
gagtacctgtacctgaacaacaatgagcttcaagggaaggtcccttcctcgtttggggagctctcgagcctcatggagtgcaacctctcgtacaacaacc
tcgtcgggccgctccccagcacgcccttgtttgagcacctggaccgcagcaatttccttggcaacaatggcctctgtttcatgaaagggagtacatgtca
gggttcatcaccattttcaagcaacgaagaggaagcacagaagaagcgcttcctcagggagaagatcatcagcgtcgtctccatagtcatcgcattggt
ttcgttggtgctgatcgccgttgtttgctgggcattaagagccaagataccggagcttgtatcaagcgaagagctgcgcagaaccgggttctctggcccg
caatacttcatcaaggaacgggtgaactatcaggagcttatgaaagcaactgagagcttttcagagagtgctgtgattggaaggggtgcgtgtggcacg
gtttataaagctgtcatgcctgacggccggaaaatcgcggtcaagaagctgaaaactcaaggagaaggctcaaatatcgacagaagcttccgagcag
agataaccatgcttggaaatgtcaggcaccgcaacatagtcaagctctatggtttctgctctcatcaggactcccatcttatcctctatgagtacatggca
aatggtagccttggagagttgcttcatggcagcaaggatgcctgcctactggactgggacacacgttaccggatcgcgataggtgccgctgaaggcctg
cggtaccttcacagtgactgcaagccgcaggtcattcaccgtgatatcaaatcgaacaacatacttctggatgagatgatggaggctcatgtgggggac
tttggtttggccaaacttattgacatttccaactccaaaacaatgtctgctattgctggttcatatggctacattgcgccagagtacgctttcaccatgaagg
tcactgaaaaatgtgatgtgtacagttttggagttgttttgttggaactattgaccgggcagtctccaatccaacctcttgaaaaaggctga
>g21541.t1
atggccgggacgctcaacgagctgattctcctcaacagtggcatctcctcttgtatcccgccggagatcggcagactggacaagctcacagttctcgacc
tcagcttcaacagcatcgccggcacgctgccagacaccatcggcaacatgcgcgcgctggaacagctcaacgtcgcgcacaacatgcttgccggaga
gatacccgagagcatctgcgcactgcctaacctaaagaacttcacctactcgtacaacttcttctgcggcgagccacaccggtgcttcgaggtgccacgc
atcgacgacaggcagaattgcatcgccggacgcccagaccagcggtccggcgaggagtgcatcgcgttcctgcaccggccaccggtgcactgcgatg
cgcatgggtgcatcgcgccgccaacggcacatgcgcctccgccgccggtatactga
>g22697.t1
atgcgctcctcatcacttatcaagcactgcgccatcgtggtagtcgccgtgctcctcgccgcagtggcggctgcggcagcacggacgaagaacgactgc
cattccggcgacaaggcggcgctgctggccatcaagtccgccttcggcaacgcctcctacttcaactcgtggacgctcaacacccccctgctgcgagtgg
gccgatgtcacctgcgaccgcgctgcctccccaaacaccacgcgccgcgtcatcggcgtgaccctgcagaacgacgccagcctcgccggcccgctgcc
cggcgccgccatcgcccgcctcaccgcgctgcagacgctcgagctgaactacttgcccggcgtgaacggcaccatcccccgcgacctgacccgtctca
acgccaccctcggcttgctccagatcgtctccacgggcatcttcggccccgtgccgccgttcctgtccgagatcacggcgctcggctacctcaggctctcg
gccaacaagctcatgggccccatcccgaactcgctcgtcgatatgcccaacctctccttcctcgacctcagctacaaccgcatcacgggcacgctcccac
cgctgctcttcagcaaggcctccggctccgacgcgtacctctacctgtcccacaataaggtcaccggccgcgtccccgccgagttcgccgccgtgaactt
ttggaccatcgacctctcgcacaacgccctggctggcgacgcctcattcctgctcggcgctaacaagtcgttgaagaatctggacctatcgcacaacgca
ttcagcttcaacctctccgccatgccgctgccggaggacctcatcagtttggagatcagccagaacgacatctacggcgacctcccggaaaccatgccg
gaggacctcagctatttgaacgtcagctacaatcgcctcagcggcatagtgccctcctatggcaacctcgactcgtttgatgcgtcatgcttcgatcacaa
caaggggctctgtggaagtccacttccaccctgcaagcactga
>g23311.t1
atgtcgctcatcaatctgctgctcacaattctagcagtattagtggcactcacgccggctgcctcggagccgagcccaggcaacgccgccgaccttgcgg
cgctgctttccttcaaggccgagctccccgacccgcttggtgtgctccgccacaactggtccaacagcacgaccccttgcagctggattggcgtatcatg
cagcaaacgacacccagggcgcgtaaaatctctggtgctccccgacgtgccgctccaaggaaaaattgctcccagccttggtaacctctctttcctctcc
atccttaacctcaccaatgctagcctcgcagggccgatcccgcctgcccttggcctcctacgccgtctacgcaatgtcctgcttgacgaggagtttaccgc
gcatctggcggattttggtattgcaaaattgttgctaggggatgacacctccattatcactgctagtatgcctgggacaatcggctacatggctcctgagt
atggttcgatgggacgagcatcgcggaagagcgatgtttttagctatgggatcatgttactcgaggttatcaccgggaaaaggccaacagaccccgctt

ttgttgcagagcgaagcctaagacagcaggtcaatgatgcatttcgtacccaaatctttcatgttattgaccccaacctgctgatgcaagagaagctagg
tggctttggcgacatcggcacaacttcaaggtccagttctgatacgctgaatagctgcctcgtgtcagtggtggagttgggtctgctgtgcaccagcgag
ctgcctgagaaaaggatttcgatgacagatgtggtcaaagggttgaccaagatcaagacggagtacactcgcatgcgtaatgaataa
>g23398.t1
atgcagctcattcgattcgacgaggggcggcggaagctgcgtgtttgtcgagctgtggcggtagagctcgccgatggctttggaaaacgttatagtcttg
gcaagcgtattgctgagctgctggaggatctagctgggctaattgaggaagggaaccaattcaaaacatttgcatccaagcgtttaccagaatttgtcg
aggagcgaccacggacacaaacctttggcattgaaccggtcctgagagatttaaggaaatcttttgacagcagtgatgtgagcatcattggagtttggg
gtccagggggtgtggggaagaccacactcctcaacaccttcaacaacgagctcaagacatggggcagggattaccaggtggccatcatggtcgaggt
gtccaactcgggaactctaaacaaagcagccatccaacgcacaattacagaccgccttggtttgccatggaatgacacagagacagaggaggctcgtg
ccaggttcttggcaaaagcactgagcagaaaaaagtttgtgattttgctggatgatatcaggagcaaattccaactagaagatgttgggattccaactcc
ggatacagggagtaaaagcaagttgattctcacatcacgttatgagaatgtgtgctatcaaatgggagcaaaccagagcttgatcaagatggagtacct
cgaaaaagaagcagcatgggagctcttccgaagcaatttgagcgccaaggcaattaaggccattgagtcacctgagccaaacaatgttgtaaaagaa
tatgctaatgccattgtccaaagctgtggtggtttgccactagcactcaaggtggccatcatggtcgaggtgtccaactcgggaactctaaacaaagcag
ccatccaacgcacaattacagaccgccttggtttgccatggaatgacacagagacagaggaggctcgtgccaggttcttggcaaaagcactgagcaga
aaaaagtttgtgattttgctggatgatatcaggagcaaattccaactagaagatgttgggattccaactccggatacagggagtaaaagcaagttgattc
tcacatcacgttatgagaatgtgtgctatcaaatgggagcaaaccagagcttgatcaagatggagtacctcgaaaaagaagcagcatgggagctcttc
cgaagcaatttgagcgccaaggcaattaaggccattgagtcacctgagccaaacaatgttgtaaaagaatatgctaatgccattgtccaaagctgtggt
ggtttgccactagcactcaaggtaatagggagcgctgtggcagggctaacaaaaccaaaggaatggagtttggcaatgcaagcaacaaaagatgaca
tcaaggacttggatggaatcccagaaatgttccataaactgaaatacagttatgaaaagttaacagcaacacagcaggactgcttcctgtactgcaccc
tcttcccagaatatggctcaattaacagagatcagcttgtagattattggatggcacaagacttgatacctcaggatcccaataagggtcattggataatc
cgtagcctggtatccgcttgcttgctggagagttgtggatctgacttagaggtgaaaatgcaccacataattcgtcaattggggctatcacttgcagtgca
gcaaaatattgttgtgaaggtaggaatgagcttggcgaaggcaccaccacacagagagtggcgaacagcaataatgatttcactcatgttcaatgatat
aaaggacctggacatgtcgcctgaatgcaaggatcttgtaactttgctggttcagaataatcctaacttggacaggctgagtcaaaccttcttccagtcca
tgcattgtttgaaggttcttgatctttcacacaccaacattacagcacttccgctctgtagcacattggccaagctcaagtatctcaatttgtcccacacact
aattgagaggcttcctgcagatttctggatgctaaaaaacctaaggcatctagatttaagcgttaccaaggcattgaaggagaccttcgacaactgttcc
aagctatacaagctaagggtgctgaacatcttccgaagcaactatgggattcgtgatgtcaacgacctgaatattgattccctgacggaactgaaattcc
ttggcatcacaatctatgctgaggatgtattaaagaagttaacaaagacgcatcctctagcaaagtcaacacagcgccttagcctcatgcactgcgaac
aaatgcagttaatccaaatatcagacttcacacatatggtgcaacttcgagaactctatgttgaatcgtgtcttgacttgatagaactcattgccgaccttg
acaagcaaaaagcttcttgcctgcaggttctcacccttgctaaacttcctgccctccaaactattcttattggatcttcacctcatcacttccgcaacctccg
agaaattacaatatctcactgccaaaaattgaatgacatcacctgggttctgaagctggaagcacttgagaagctttctatccatcattgccacgaattg
gagcaagttgttcacgaaacagtcaacaaggtggaaaatatgagcaggaggaatgagccgagcagtgttcagagatgtgggagaaataatggtctgt
ctgaagaacaacaagtacatggtatggcagaagatgcatataatgaatatatgaagggttatcagaacatggccaaatggggacaggtcaatggaac
agtgcgtcatgtggattttccaaaacttagattcctggtgctgacttctctaccaaagctggctgagatatgctatccaagagacttccctagtcttgagat
tattagagtggaaggttgcccactcttgaaaacacttccagtgggtcaaatgtacgaatgcccaaaactaaagcagatttgtggatcttatgaatggtgg
gaaaggctagaatggaatggcagagagataatggagaataagtacttcatccccatccaggatgagggatag
>g23523.t1
atggtgctcctcctcctcgagtatctcctgctcgcttttatctttctttcatgtcctgccacatacatgacacatgcctcactcgcatctcgtggaccatccac
cccaaacattgtcgaccagcttgcgctcatgtcgttcaagtcacgcataagaagtgacccgtcacgagccctggcgacatggggcaatctgtcccttccg
atctgccggtggcgcggagtggcgtgcggcttgaggggacggagccgtggccgtgtggtggcgctggacctcgccgagctcaacctacttggcaccat
cacccctgcgctgggcaacctcactcacttgagacagctcgacctttcatcgaaccgcttccacggtatcctgccgccagagctcggaagtgacatggtt
gctcatgttggtgattttggactcgcgagatttctacatcaagtcactgacaaatcaagtggatgggcagcaatgagaggatctattggttatgcagctcc
aggtggcatccggaggaggggaggttaccttccgcatagatttgaggaagcaggaaacgcagcggtgaaggttgccgcggggcgcgggaactacgc
caagcaggacctggactccgacggcagtttcactagctacgagagagcagtcactggagtgggggcggggaggcgcttggcagaggagctcatcggt
gtcggatga
>g23782.t1
atggccatggacacaactgaccctgaatattggctcaactggaggttcttattgtgtgggatctgggtctactcctgcatggccttggcgtgctacttaatt
tggaaatatgagggggcgcgttcgagggatggcaatggcaatgatggtggagacagcgaggaagcaaggccctggttcaggcctggagttgtttacct
tgatgattgctggaagccatgcatcgaggagatccaccctgcctggctgctggcttatcgccttgtggccttctttgttatggcttcactgctcgcggttga
ccttattactgatggatggagcatcttcctatactacactcaatggacgttcttgcttgtcaccttgtattttgggattctgatcgggacgcattcaatcaatg
ttgttttcataattggtgatgttgctctgaataacatgcgattcccgtggtttagaatcgcatatttcctgctatggacaggcatttttgttaatgttcagtgg
atcatccatgctaacgtttcaatgtggtggccttacccatttctagacttgaaattccctgcagcacctgtatggtatttggtagtggcagtgctgcacttcc
cctgttatgcgttgttcgctctggtcctgaggctgaagcattccctgctggaaagcggcgctgctgctgccgtcgcgcggcggaattgtggtgtggagctg
tggagtggcctgctccgcttcttctccttgttcgccgaattgaatctgctggctgtggcgatgcgggtgctggtgtggatgcggtggtgggtggcggttgcc
ggcctgctcgccgtgatcctgccgccgggcaccgcgacgctctccccggccggaatcaactacgaagttgtggccctgatggccatcaagatggagctg
gaggacccccacaacgtgctcgacaactgggacatcaactcggtcgacccctgcagctggaggatggtcacctgctccgccgatggctacgtctctgcc
ttaggcttacccagccagagcttgactggaaagttatcgcccggcattggcaacctcaccagactgcaatccgtgctgttacagaacaatgccatctctg
gacctattcctggtaccataggaagactgggaatgctaaggactcttgacatgtcagacaatcaactcactgggagcatcccaaattcaattgggaatct
caagaatctcgactacctgaaattgaacaacaatagcttgtctggagttctacctgattcacttgccaccattgatggtcttgctctcgtcattgctggaaa
tccaatgatttgtggtcttcagtctggagacaactgctcttctgtgtcattggacccactctcttatccaccagatgatctgaagatgcgagggactgttgg

gcatatagcgcccgagtatttgtcaactggccagtcatctgagaagactgatgtttttggatttggagtcttattggttgagttgatcactggtcagaaggc
attagattttggaagagtagcaaatcagaagggcagagtgcttgactgggttaagaagcttcaacaagaaaagcagctgagcatgatggttgataagg
acctaggtaggaactacgacagggttgaactggaagaaatggttcaggtggctttgctatgcacacagtactacccgtctcatcgccccagaatgtccg
agtttatcaggatgcttgaaggggacgggctcgcagagaaatgggaagcatcacagaatgtggacacaccaaagtccgtatcatcagagatcctacct
ccaaagtacatgaattttgccgcagacgaatcttcacttggcctagaagacatggagctatctggaccaaggtga
>g23885.t1
atggccgcgagcggcggcgacgaggccgcactgctcgctttcaaagccgggctcagcaacggcggcagcgcgctggcctcgtggaacagcagcacc
gggttctgcagctgggaaggcgtggcgtgcacccgcggtaggagtaggaacccgccgcgcgtggtggccctgagcctgcccaagatggggctcgccg
ggacgctctcggcggccatcgggaacctcacgttcctgcaggcgatcgagctgaccttcaactggctgcacggcgacgtcccggcgagcctcggccgg
ctccggcgcctccggtacatgagcctaggctacaactccctctccggcgagatcccttccaacctgacctcctgcgtggccctggagcagctgttcctcg
acagcaacaacctcagcggccgcgtgcccctcgagctcgggaacgcactgacccggctttggctgctccgactcaagaacaacagcttcaccggacct
gtgccagagtcgctggccaacgcgtcgctgctaagctacctctccctcgccaacaaccagttcgacggcgccatcccgccggggctcgccggcctcgcc
ggcctctggcatctcgacctctccgtgaacaagctgcgcggcgctctgccgctgtccctgtacaacctgtcatcgctgaggatgttccacgtggaaggga
accggctgcacggcaccattcccgcggacatcggcagcaagttcccggccatgaaggacttgagcttcgccaacaacagcctcggccggctccggcgc
ctccggtacatgagcctaggctacaactccctctccggcgagatcccttccaacctgacctcctgcgtggccctggagcagctgttcctcgacagcaaca
acctcagcggccgcgtgcccctcgagctcgggaacgcactgacccggctttggctgctccgactcaagaacaacagcttcaccggacctgtgccagag
tcgctggccaacgcgtcgctgctaagctacctctccctcgccaacaaccagttcgacggcgccatcccgccggggctcgccggcctcgccggcctctgg
catctcgacctctccgtgaacaagctgcgcggcgctctgccgctgtccctgtacaacctgtcatcgctgaggatgttccacgtggaagggaaccggctgc
acggcaccattcccgcggacatcggcagcaagttcccggccatgaaggacttgagcttcgccaacaacaggttcaccggcggcgtccctccttctatct
cgaacctcaccaacctcacaagcctccagctctccatcaacaggttcaccgggctcgtgccgcgtgatctcgggaggatgtatcgtctccagtacctgta
catgcctcacaacatgcttcaggcagatgacacgcagggctgggagttcatcacttcactggcgaactgcagccagctgctgcagctgagcctcagtga
caacgccttcgccgggcagctgcccagctcactggtgaacctgtcatccacgctccagtatctctatctgggagactgtggtatctctggcagaatcccc
aggacatcagcaatttggctggactaagcatacttgatttcgcaaacacctccatatgcggggtgatccctgacagcattggcaagcttgcaaacctggt
tgagcttggcttgtacaggaccaggttatcaggcctcataccaggggcactcggaaaccttacacggctgaataggattattgcatacagtagcaacttg
gaaggacccatcccagcaagccttggcaacctaaggaatctataccttcttgatctagcaggaaactaccggctaaatggttcaattccaaaggaaata
ttactgacttcgctttcttctctcctcgatttgtcatacaattcactctctggaccccttccatcggaagttggcaacttggttaacctcaaccagttattccta
tcaggaaaccgcttgtctggccgcatacctgacaccattgggaactgcctagttctggagtcattgatgctggataacaacagtttgcgcaatgcatggc
tcatcctctacttcgcgatcacgccaggattggcgaaccaacggcgtgccgcgttgctgtgggactag
>g23886.t1
atgactagcaagcagtgcagcttcgtcctgttcgttcttgctctatccttgttcttgtgcattctggttgcacctgtatgcttagccacaccaaccacagatcc
tgctactctcctgcaagtgaagtccagcctcaccgaccctgaaggcgtcctctccggctggtcgccggaggccgacgtgtgctcatggcacggcattac
gtgctccttacccggagagggcattgttactggcctcaacttgtcggggtacggcttgtcaggcgtgataccgccggcgatcagcggcctcgtctccgtc
gagttcattgacctgtcctccaactccctctccgggccaatcccgccggagttgggcatgctggagaacctgaaggtcttgcttctcttctccaactccctc
accggcacagtcccgccggagctcggcctcctcaagaacctgagagttctcaggatcggcgacaataggctgcacggcgagatcccaccgcagctcg
gcaactgcaccgagctggagacgcttggcctggcgtacggccagctgaacggcgccattcctgctgagctcggcaacctgaagcgcctgcagcagctg
gtcctggacaacaacaccctcaccggcggcatcccggagcagctcgccggttgctcaagtttgcaacttctgtcagtggctgataatatgctgcaaggc
agcattccttcgttcattggcagcctcggtgatctccggtctctgaatctagcaaataaccagttgtccggtgaaattccggcagagattggtaacctttcc
ggtctgacatacctcaacctgctcggcaacagcctgaccggctccatcccgggagaactgaaccggctgagccagctgcaggttctggacttgtccggc
aacaacatttccgggaagatcagcatctctgcgtcacggctgaagaacctgaactaccttgtgctgtctggcaatcttcttgaaggcgacatcccagacg
acttgtgctctgaggagtcgagcctggagaacctgttcctggccgacaacaaactcggaggcagcatcgatgggttgctgaactgcgtcactctgcagt
ccatcgacgtgtcgaacaacagcttcaccggagccataccgtcgggcatcgacaagctgtcgagcctcgtcaaccttgcgctgcacaacaacagcctc
accggcgctttgccgcctcagattgggaatctcagcaacctggagatcctgtctctgttccacaatggccttaccggcacgatcccgcaggagatcgga
ggcttcagaagctgaagctgctgttcctctacgagaaccagatgtcaggaaccatccctgacgagatcaccaactgcacgagcttggaagaagtggac
ttcttcggcaaccatttccatggttccatccctgacaagattgggaacctcaagaacctcgccgtgctccagctccggcagaacgacctgtcgggccccg
tcccagcgagcctcggcgactgcaggagcctccaggcgctggcgttggccgacaaccggttctccggcatgctgcctgacacgttcggtcagttcacgg
agatcagtgtgatcactctgtacaacaactccctggaaggcccccctaccggaatcactgtccgggctcaagaacctgacggtgatcaacttctcacaca
accggttcaccggcagcatcgtcccactcctcggctccagctccctctccgtgctggccctgacgggaaacagcttctccggcgtgatcccggcgtcgat
cgcgcggtcgagtgacatgatccggctccagctcggcggcaaccggtttaccggtgcaataccggccgagctgggaaacctcaagcaccttcacatgc
tcgacctctcatccaacgacctctccggtgacatccccgtcgagctttgcaactgcgcggcgctcacccacctgaacctcgacgggaacggcctgaccg
gcggcgtcccggcatggctcggcggcctccggtccctcggcgagctcgacctctcgtcgaacgcgttaaccggcaacataccggtggacctcggaaac
tgttccggcctgctcaagctgtccctcagcgacaaccacctctacggcagcatcccacaagagatcggcaggctgacatctctgaacgtcctcaacctg
gacaagaaccgcctcaccggcgccataccgccggcgctccggcagtgcaacaagttgtacgagctgaggctgtctgagaactcgttggagggcccgat
cccgccggagctggggcagctgccggagctgcaggtcattctggacctgagccggaaccggctctccggcgagataccggagtccctcggcaacctcg
tgaagctggagtggctcaacctgtcgtccaaccagctccatggaacaataccatcttcattgctgcagctgacgagcctgcacattctgaacctctccaa
caacctcctctccggctcagtacccgccgcgctatcgaccttcccggccgagtccttcaccggcaacgacgagctctgcggcgcgccattgccgacctgc
cgggggtcatcgtccgggcggcttccggggaccgaggtggccgccatcgtggcgggcatcgccgtcgtgtcggcggtggtgtgcgtgacgctgctgta
caccatgctcagggtgtggaacaactggagggcggtgtccgtgtgcagctccgataacgaggaggactcgcatggcaagaagcgtggcggcaagtgg
ggcgccgccgacggcaagtactggaaggtcgggtcgccggcgtcgtcggcggaggagaagcacagcacggcgacggagaccagcgtgcttcacgg
gaactccaaggaggctggcgctggcgccgcgaagagctag

>g24745.t1
atgccgccgtggctgccgctctgcgtggcggccctgctcctcaccaccttcctcttgccctgctcctccgagccggacgccgacaagtccgcgctgctcg
acttcctcgccgcggtgggccacagcaactcccgcatcaactggcccaccacccctctcgcctgcggcggcggcggctggacgggcgtcacctgc
agcgccgacgggtcccacgtcgtcgcgctccacctcccgggcctgggcctgtcgggccgcatcccggcgcgcacgctgggccgcctcgccgcgctaca
ggtgctcagcctccgcgccaacaacctctccggcccgctcccggccgacctgctcgccctgcccaacctcacgggcctccacctccagcgcaacgccct
cgccggcccgctcccggcccacctcttcggcaccaccaccaccaccaccctccagacgctcgacctctccttcaacggcttctccggcgccctcctcccc
gccgccgccgcgctctccaacctcacccacctcgtcgccctcaacctctccaacaactccctctccggccgcgtccctgacctccgcctcccccagctccg
cttcctcaacctctccaacaaccacttcgacgggcccatccccgattccctcctcaggttccccgacgccgcgttcgcagggaacaacctcacgcgcccc
ccactctctcctccgctctcgccgtcgccttcttccgcgactggcactcccaccaccaccgagagcagtggacacgcacgcaccccgcgcctcggcgag
gcggccatcctcgccatcgcggtcggcggctgcgcgctcctcttcgccgtcgccgcggtgctgctcatcaccttctgcgccaccagagacaccaccaccg
ccagcagcaacaacatcgtcgtgtccaaggcggcgggtggcgggaaggggaaggagtcgcccgagtccaaggcggtcatcatcggcggtggcaggg
ccggcgacggcaaccggatggtcttcttcgagggcgcctcgctcgcgttcgacctcgaggacctgctccgcgcctccgccgaggtgctcgggaaaggc
gccttcgggaccgcctaccgcgcggtcctcgaggacgccaccaccgtcgtcgtcaagcggctcaaggacgtcagcgccgggcgacgtgacttcgagc
agcagatggagctcgtggggcggattcgacacgacaatgtcgtcgagctgcgcgcatactactactccaaggacgagaagctgctcgtctacgattact
acagcagtggaagcatatccaacatgcttcacgggaagaggggagaggacaggatgcctttggattggcaaaccagggtaaagatagcacttggagc
agcacgagggattgcacacattcacactgagaacaatgggaaatttgttcatggcaacatcaaggcctcaaatgttttcataaacaagcatcaatacgg
ctgcatctctgatctcggcttggcggcactgatgaacccaatcaccggcaggtcccgtactctagggtactgcgcaccagaggtcactgacacgaggaa
agcatcacagtgcgcagatgtatacagcttcggcgtcttcatattggagctcctcaccggcaagtcgcctgttcaaataaccggcggaggcaatgaggt
cgtccacctagtgagatgggtgcagtctgttgtccgggaagagtggaccgccgaggtgtttgactccgagctgctgagataccccaacattgaggagga
gatggtggagatgctccagatcgccatggcctgcgtctcaaggaccccggagcggcggcccaagatcccagacgtggtgaggatgatcgaggaggtg
cggaggagcgacaccgggacgcgaccttccaccgaggcctccactccggctgtagaagctcagaacagagcagagagctcatctccggccccttact
ga
>g25116.t1
atgccgccgcccgccgcatcgaggctcgcgcttctcgtacggcccctcgccttcttcgtgctacttcttctagtctccccgtgccactgcctgaacgagcag
ggccaggcgctgctgcgatggaaggcgtcgctgcggccggtggacggcggcgcgctgtcgtcgtggcgcgcgtcggaccccaacccgtgcgaatggt
ccggcgtgatgtgcgacgcgcgcggcggcgtggccgagctgagcatcacgtcggtggacctgggcggcccgctcccgtccaacctacagccgctggc
gccgtcgctcaagacgctggtgctgtcgggcaccaacctcaccggcacgatcccgccgcagctcggcgacctcgccgcgctgaccgagcttgacgtca
gcaagaaccagctcaccggcgcgatcccgccggagctctgccgcctcgcgaagctcgagtcactggcgctcaactccaactccctgcgcggcgccatc
cccgacggcatcggcaacctcaccggcctgacttccctcacgctctacgacaacgagctgagcgggcccatcccgcccggcatcggcgacctcaagaa
tttacaggtgctccgcgccggcgggaacccgggaatgaagggcccggtgccggcggagatcggcgggtgcagcagcctcaccatgctcgggctcgcc
gagaccggcgtctccggcagcctcccggacacgatcgggcagctcaagaaggtccagaccatcgccatctacaccaccctgctctccggccgcatccc
ggagtccatcggcaactgcaccgagctcaccagcctgtacctctaccagaactcgctctccgggccgataccggcgtcgctcggccagctcagaaagct
ccagacgctgctcctgtggcagaaccagctcgtcggcgcgatcccgcccgagctcgggcagtgcaaggagctcacgctgatcgacctgtcgctgaattc
tcttaccggtccgatcccggcgagtctcggggaattgcccaacctccagcagctgcagctgagcacgaaccagctgacgggcgccatccctccggagc
tctccaactgcacgtcgctgacggacatcgaggtcgacaacaaccagctgtcaggggagatccgcatcgatttttcgcggcttcacaacctgactctctt
ctacgcgtggaagaaccggcttaccggccgcgtgccggccggcctcgccgagtgccccggcatgcaggcggtcgacctgtcctacaacaacctcaccg
ggtccatccccaaggagctcttcggcctgcagaacctcaccaagctgctcctgctcaacaacgagctgtccgggctcgtcccgccggagatcggcgact
gcaccagcctctaccgcctccggctcaacggcaaccgcctctccggcacggtgccggccgagatcggcaacctcaagagcctcaacttcctcgacatg
agcgacaaccacctcgtgggccccatacccgccgccatctccgggtgcgccagcctccagttcctcgacctccactccaacgcgctgtccggctcactcc
ccgacacgctcccccgcagcctccagctcatcgacgtctccgacaaccagctcgccggcccgctgagccccggcatcgggtcaatgccggagctgacc
aagctcctctaccgcctccggctcaacggcaaccgcctctccggcacggtgccggccgagatcggcaacctcaagagcctcaacttcctcgacatgagc
gacaaccacctcgtgggccccatacccgccgccatctccgggtgcgccagcctccagttcctcgacctccactccaacgcgctgtccggctcactccccg
acacgctcccccgcagcctccagctcatcgacgtctccgacaaccagctcgccggcccgctgagccccggcatcgggtcaatgccggagctgaccaag
ctgtaccttggccggaaccggctcaccggcggcatcccgccggagctcgggtcctgcgcgaagctccagctgctggacctcggcggcaacggcttctcc
ggcgccatcccgcccgagctcggggagctcccgtcgctggagatcgcgctcaacctcagcagcaaccggctctccggcgagatcccgccgcggttcgc
cgcgctcgacaagctcgccagcctcgacctgtcgcgcaatcagctgtcggggactctcgccccgctcgccgcgctgcagaacctcgtcacgctcaacgt
ctcctacaacggcttctccggcgagctccccgacacgcccttcttccagaagctccctctcagcgacctcgccggcaaccggcacctcgtcgtcggcgac
ggctcctcccggcacggcggcgcgctctcgtcgctgaagatcgccatgtcagtgctcgccgtgttcagcgcggtcctcctggcggcggcgacatacatg
ctggcacgcacccaccgcaatagcggcgccgggggaaagatggtccacggcgagggcggcccgtgggaggtgacgctgtaccagaagctggacatc
gccatggacgacgtgctgcgggagctgacgtcgtcgaacgtgatcgggacgggcagctcgggggtggtgtaccgcgtggagacgcccaacgggtaca
ccttcgcagtgaagaagatgtggtcggcgtccgacgagnnnnnnnnnnnnnnnnnnnnggcgtccgggctcgtcccgccgga
gatcggcgactgcaccagcctctaccgcctccggctcaacggcaaccgcctctccggcacggtgccggccgagatcggcaacctcaagagcctcaact
tcctcgacatgagcgacaaccacctcgtgggccccatacccgccgccatctccgggtgcgccagcctccagttcctcgacctccactccaacgcgctgtc
cggctcactccccgacacgctcccccgcagcctccagctcatcgacgtctccgacaaccagctcgccggcccgctgagccccggcatcgggtcaatgcc
ggagctgaccaagctgtaccttggccggaaccggctcaccggcggcatcccgccggagctcgggtcctgcgcgaagctccagctgctggacctcggcg
gcaacggcttctccggcgccatcccgcccgagctcggggagctcccgtcgctggagatcgcgctcaacctcagcagcaaccggctctccggcgagatc
ccgccgcggttcgccgcgctcgacaagctcgccagcctcgacctgtcgcgcaatcagctgtcggggactctcgccccgctcgccgcgctgcagaacctc
gtcacgctcaacgtctcctacaacggcttctccggcgagctccccgacacgcccttcttccagaagctccctctcagcgacctcgccggcaaccggcacc
tcgtcgtcggcgacggctcctcccggcacggcggcgcgctctcgtcgctgaagatcgccatgtcagtgctcgccgtgttcagcgcggtcctcctggcggc

ggcgacatacatgctggcacgcacccaccgcaatagcggcgccgggggaaagatggtccacggcgagggcggcccgtgggaggtgacgctgtacca
gaagctggacatcgccatggacgacgtgctgcgggagctgacgtcgtcgaacgtgatcgggacgggcagctcgggggtggtgtaccgcgtggagacg
cccaacgggtacaccttcgcggtgaagaagatgtggtcggcgtccgacgagacgttccgcagcgagatcgccgcgctgggatctatccgccaccgcaa
catcgtgaggctgctgggctgggccgccaacggcggcaccaggctgctgttctacacctacctgcccaacggcagcctcagcggcctgctccacggcg
gcggtgggtcggggaagggcagcgccaacgacgagtgggccgcgcggtaccacgtggcgctcggcgtggcccacgccgtggcctacctccaccacg
actgcgtgccggctatcctacacggggacatcaaggcaatgaacgtgttgctgggccccgcgtacgagccgtacctcgccgacttcggactcgcgcgc
gtcctgtcatcggcgagccccaagcagcagctcgacgacaccatcaggcagccacgcatcgccggctcatacggctacatcgcgccagagtacgcgtc
gatgcagcggatcagcgagaagagcgacgtgtacagcttcggcgtggtggtgctggagatcctgacgggcaggcacccgctggaccccacgctcccg
ggcggcgcacacctggtgcagtgggtgcgggcccaccttcaggccacgcgcgacgccgccgagctcctggacccgcgcctcaccgcgcccggcgccg
ccgccgccgacgtgcaggagatgcgccaggtgctgtccgtggccgcgctctgcgtcgcgcaccgcgccgacgaccgccccgccatgaccgacgtcgtc
gcgctgctcaaggagatccgacgacccgccgccgccgcaggcgacgacgccacgaagccggcacccacgcagccggtgtcgccggtgcggagcccg
cactcgagggaccactcgtccagctgctccttcgcctga
>g25176.t1
atgggcaccgcggtggagccggcggcgttcggctcggtggacggcgtggtgggggagatcatgcggctgcaccggtcgctgccggcgcggccgtcgc
tggaggacgtggaggcggcggagtcgctggtgcgcgcggcggaccgggaggagcgggcgcggctcgacgccgtcgagcgcctccgccgccctcccg
ccgtgcccgacgagctgttcggcgtcgcgctggagatgcaccgcgcgctcgccggcttccactgccgcgagcagaagcgcgacgccgcgcggctgctc
gagctcgacgcgctccacgcgctcttcgacgacctcgtcacgcgcgcctcgcagtgcgtgccgtccacctcctcgcgcgccgcgccgcgcatcccgtct
acctcctcctcggcggcggcctcctcgtcctccgctgcctcatcgtcgtcggcggcggcggaccggtactcgtccatgaacaccaacgggttcagtgcgc
agaggccggcggcgcgcgtctccatggatgacagctacgtgaagaagtccaaggcgcctgtatgggacggcggagctgtggcgacgagctcgcatat
gccgcgaggaggagccgttgcagccaactccgtggttgttcagatggatggtggttacggtgatgacaacgagaaattgagcctaattaagctggcta
gcacgattgaagtggctgcaaagaaaggcacccgggatctgaacctccaaggcaagctcatgaaccagatagagtggctgcctgattcaattggaaa
gctcactggacttgttacccttgatatttcggagaaccggatattggccttgccagaagaaattgggaggctttcttccttgaccaaaatggaccttcatgc
taaccgaattgctcagctccctgacacagttgctgatcttagcaacttgatttttctcgatctgagaggtaatcagctttcatcgttacccgctggtcttggc
aggctggtgaatcttgaggagcttgatgtgagtgcaaaccagctcacctcactccctgattcaattggaagccttgcacggttgaagaaattaatcatcg
agacaaacaacattgacgagttgccgtacactattggtaattgtgtttctctggtagaactgcgggcaggttataatcatctcagggccctgccagaggc
tgtcggaaagctagattctctggagatcctatctgtgcgatacaacagtatcagaggatttccgacaacaatggcatctctcacaaagctgaaggaggtt
gatgcaagcttcaacgaacttgagtctattcctgagaacttttgctttgttacttctcttgtgaagctgaatgttggtaacaatttcgctgacatgcaagctc
tgccgcgttctattggtaaccttgagatgcttgaagagttggatataagcaataatcagattagggtccttccagattcttttggaaacctgcagcgtctcc
gtgtgcttcgtgcagaagagaaccctctacaagtgccaccaagagacatagctttgaagggagctcaggcagaaccgaccacctga
>g25199.t1
atggcggttccgcggggcgttgtggcagcggcggcggttctggtgctggcggtgttggcttcgtccgcggcggcgctgaacacggacgggctggcgct
gcttgccctcaagttcgcggtgtccgacgaccccggcggggtcctcggctcatggcaggacgccgacgcggacccctgcgcgtggtcgggcgtgacgt
gcgccgacggcggcgggggccgcgtcgccgccgtggagctcgggaacgcctcgctggcggggtacctcccctcggagctgtccctgctctcggagctc
cagacgctctccctcccctacaaccgcctctccggccagatcccggtggccgtcgccgcgctgcagaagctggccgcgctcgacctcgcgcacaacca
gctgtccggcccgatccccgagggcatcgtgcggctgctctccctctcccgcctcgacctctcctccaaccagctcaacgggacgctgccgccggcgatc
gcggggctgccccgcctctccggcgtgctcaacctcagctacaaccacttcaccggcgggatcccgccggagttcggcgggattccggtggcggtgag
cctggatttgcgggggaacgacctcgagggcgagatcccgcaggtggggtccctcgtcaaccagggccccaccgcgttcgacggcaacccgaggctg
tgcgggttcccgctcaaggtcgagtgcgcgggggagagggacgacgagccgaggatgccggagtcagggaccgggccgagcgaccccggcgccgc
ggcggaggtgcggcggccgcccccgaagcggcacgcgtcgtccccgacggtgccgattctcgcggccattgtggtggtcgtcatcgtggcgggagtcg
tgttgcagtggcagtgccgcaggcggtgcgcggcggcggccggggacgaggaggagaaggggtcgtcggcgtcggggaaggagaggagcgcggc
ggtgacgctcgcggggagcgaggagcggcggagtgggggaggcggcggtggagaggagggggaggtgttcgtggccgtggacgacgggttcggg
atggagctggaggagctgctccgcgcgtcggcctacgtcgtcggcaagagccgcggcggcatcgtgtaccgggtcgtgcctgtacgcggcgaggcagt
cgctgtgcgacggctcagcgagcccgacgacggcggtgatagcggcggtgggtccgagtccgggtggcgccgccgacgcgcggcgttcgaggccga
ggccgccgccatcggccgcgcgcgccaccccaacgtggcccggctccgcgcctactactacgcacccgacgagaaactgctcatctacgactacctcc
ccaacggctcactccactccgctctccacggtgggccgacggcgtcgccgacgccattgccgtggtcgacgagactgtccatcgtgcagggcgcggcg
cgtgggctggcgtacctgcacgagtgcagcccgcggcggtacgtgcacggctgcatcaagtcatccaagatcctgctcgacgaggagctccgcgcgca
cgtgtccgggttcggcctggcccgcctcgtcgccggcgccgcgggacactcctacaacaagaagcgcgccggcgtcgcgctccgctccggcgccggcg
cggtgacctacgtcgcgccggagctgcgcgcgccgggcggcgcgacggcggcgacgcagaagggggacgtgttcgcgttcggggtcgtgctgctgga
ggccgtgacgggccgggagcccgcggagggggagggcggcctcgacctggaggcgtgggtgcgccgcgcgttcaaggaggagcgcccgctgtcgg
aggtggtggaccccacgctgctcggggaggtgcacgccaagaagcaggtgctcgcggtcttccacgtcgcgctcgggtgcaccgaggccgacccgga
gatgcggccgcgcatgcgcgccgtcgccgagagcctcgaccggatcggttgctga
>g25628.t1
atggccaaccggccagttctcctgatgaccgtactactgtctttccagctgctcatcacgacgtcactttgtcagacgtctcaggtgtccgaccgtgacctc
gcggcgctgatcaaggtcagggagcacttcggcaacccgaacctgttcccggggtggcggctcggtacgacctgcatctcgtggtggcccgcggcgtg
caacgagcagggccgcgtaaccaagctcttcctcgacaaccttctcggcatcaaggaacttctaagtgtcaacaccacgctcccgccggccatcagcga
actcgagatgctagagacgctgagcattatgagcattcccgacctgcacggccccatcccagaatcctacggcaacctcccgcacctctccctcttcaac
atcataggcaccaatgtctccggctcgataccggcgtccctctccctcaccaatctcacctccgtcagcttatccaagaacaagctcaccggcccgatcc
cgaggtcgctgcggaagctgccgtacctttcctacttcgacgcctcgtacaacgacctcatcgggaccattccgccgggtctggtgcgcagcacgcctga
cagcccggccgccctgcagctggatggcaaccggctgtcagggaccatacccagatcgtacagattcgagaggaactcgatggagttcagggtggcc

aacaaccggctcaccggagacgcgtcgttcctgttcgggcggcacaagacggtgtcagggaccatcgatttgtccgggaacaatttccggttcaacctg
acaggggtggagatgccgatgcatcttgggttcctgaacctgagccacaaccggatctacggcggcgtgcccgtgtcgctgagggagtccagggtctc
ggtgctggatctcagctataacgagctctgcggggagatacccaccggcgggcacatggtgcagttcaaggcagcggcgtacgaacacaacaaatgc
ctctgtggtacgccgctgccaccgtgcgccaacggccgctga
>g25629.t1
atggccaaccggccagttctcctgatgaccgtactactgtctttccagctgctcatcacgacgtcactttgtcagacgtctcaggtgtccgaccgtgacctc
gcggcgctgatcaaggtcagggagcacttcggcaacccgaacctgttcccggggtggcggctcggtacgacctgcatctcgtggtggcccgcggcgtg
caacgagcagggccgcgtaaccaagctcttcctcgacaaccttctcggcatcaaggaacttctaagtgtcaacaccacgctcccgccggccatcagcga
actcgagatgctagagacgctgagcattatgagcattcccgacctgcacggccccatcccagaatcctacggcaacctcccgcacctctccctcttcaac
atcataggcaccaatgtctccggctcgataccggcgtccctctccctcaccaatctcacctccgtcagcttatccaagaacaagctcaccggcccgatcc
cgaggtcgctgcggaagctgccgtacctttcctacttcgacgcctcgtacaacgacctcatcgggaccattccgccgggtctggtgcgcagcacgcctga
cagcccggccgccctgcagctggatggcaaccggctgtcagggaccatacccagatcgtacagattcgagaggaactcgatggagttcagggtggcc
aacaaccggctcaccggagacgcgtcgttcctgttcgggcggcacaagacggtgtcagggaccatcgatttgtccgggaacaatttccggttcaacctg
acaggggtggagatgccgatgcatcttgggttcctgaacctgagccacaaccggatctacggcggcgtgcccgtgtcgctgagggagtccagggtctc
ggtgctggatctcagctataacgagctctgcggggagatacccaccggcgggcacatggtnnnnnnnnnnnnnnnnnnnnnnnnnnnnnn
nnnnnnnnnnnnnnnnnnnnnnnnnnnnnnntgcatcacgcatatttgatcgcactgcagctcaataa
>g25630.t1
atggatccggcgccgcagacccatccgatcctctcctacgtgctcgcccgcatccccaccctctccaagctcaagtcccccgccgccggcagcggcgac
ttcgacatcgagcagccgcccgtgcacacgccatcccctcggacgccgtctaccgtgggggagttcgagctcgtggagcgtatgccgggtctgcgccac
ccgtccgtgctccgcgccatgacccgcgccgtcgccgacgtctccgccgcgcgcgccgcgctcgaggtgctcggcccgcggcccgaccacgagctcgt
agactcctcccgcgccatcattgcggccgccgaggccggggactccgaggtccccgaggccgacctccagggctgccgcgccgtggtgcggctcgag
gagacgcacgacgcctacgaggccctgctgcaggatgccgagggcaggctcgagaaagtgtaccggtccgcgatggaggggaccgacctggatgac
gaggcggaggggaaggaggacgagcttgcggcgggaggcgccgagggcggggatgcggcggtgcaggaggaggtggtggcggtgctcaagcagg
ccgaggaggggaagtctgtggagagcgtgcgcctcgtggatcggcagctgcggcaactgcccgaggcgttcggcaggatccaggggctccgggtgct
ggacgtctcccgcaaccagctcgagcctgttacggtagaactactaggtgcatgnctgcgccacccgtccgtgctccgcgccatgacccgcgccgtcgc
cgacgtctccgccgcgcgcgccgcgctcgaggtgctcggcccgcggcccgaccacgagctcgtagactcctcccgcgccatcattgcggccgccgagg
ccggggactccgaggtccccgaggccgacctccagggctgccgcgccgtggtgcggctcgaggagacgcacgacgcctacgaggccctgctgcagg
atgccgagggcaggctcgagaaagtgtaccggtccgcgatggaggggaccgacctggatgacgaggcggaggggaaggaggacgagcttgcggcg
ggaggcgccgagggcggggatgcggcggtgcaggaggaggtggtggcggtgctcaagcaggccgaggaggggaagtctgtggagagcgtgcgcct
cgtggatcggcagctgcggcaactgcccgaggcgttcggcaggatccaggggctccgggtgctggacgtctcccgcaaccagctcgaggttattccag
atgctataggaggacttgatcaacttgaggagcttcgtctctctaccaatgctttggtttctctgccagattctattggacttctatccaatctgaagattttg
aatgttgcaaataacaggctgagggcacttcctgatagcatatcaaagtgcagcaacttcagtgacctgaaagagctccctgcttcctttggtgacctgc
tgaatctgcgtgagctttacctgagcaacaaccagatccatgctcttcccgacagttttggtcgtctggataaactggagaggcttaacctggagcagaa
tcccctgggcatgccaccatctgacattgtgaacaagggcgtggatgcagtgaaagagtacatgtcaaaaaggtggttggacatcttgcttgaggaaga
acagaagcgcattgctgcagaaaccccacaggcatcatcaacccccaaagcttggctggaccgcagcgtctcttgggtctcaggcgtttctgggagcct
tgtagggtaccttagcggaaatgagaagtcagagaaagatgcgtatctgaaccagcagttatga
>g25636.t1
atgagccaggagcaccaccaccaccagcaccccgtcaccaccggcgtggacgcgctgtcgctggaccttctcgcccacgccctctccaccctgaccga
cccacacgaccgcaagtcctgccgcctcgcctcccgcgccttcacccgcgccgaatccctctcccgccgcaccgcgcgcccgctcacccgcgacgccct
cccacacgtcctccgcctcttcccctccctcaccgccctcgacctctccgcctgcgcgggcctcgacgccgcctccctcgccctcctcccccgctcccccct
cctccgccgcgtcaacctcgcgcgggccagcggggtgggcccgcgcgggctggacgcgctggtgacggcctgcccgcggctcgaggccctcgacctg
tcacactgcgtcgccgccggggaccgcgaggtggccgcggtcgcgggcgccgaggggatcacggacctcgtgctggataagtgtctcgaggtgacgg
atgttgggttggctaaggtcgccgtcgggtgccccgggttggaacggctcagcgtcaggtggtgccgcgagatctccgatattggggtccatttgctggc
gaacaagtgccggaacctccgcaccgtcgacgtctcgtacctcaaggtcagcaatgaatccctcaggtctctttccactcttgagaagctacaacactta
gcaatggttagctgctcgttcatagatgatgatggcctgcaactgctgagtattggaggcagctcattacagagcattgatgtatcgagctttgcagcact
tgtcaaggtgctcagaactgctaaccttaaagcttggcctctgcccaattgcggtgccatcactgatgatgggctggcagctttagctggtggctgtaaga
agatgaggatgctcaacctacgttattgtgacaaaatcactgatgctggtttgaagcatctaagctcactagaagaactgacgaacctggagatgagat
gcctggtgcgtgtcacaggcatcggaatcgcttccattgccactggctgccctaacctgaaagagctggacttgaagcgctgttactctgtagatgatgct
agcgtctgggctgttgcaagatactctcagaacctcagacagctcgacatatcctactgccaaatcacaggcttgggcctgtgccacctgttgagctccc
tgaggtgccttcaggacttgaagatggtccacctctcctgggtctccattgaagggtttgagatggcgctgcgagccgcctgtgggaggctgaagaaag
tgaagatgttgagtgggatgcggtccgtgctctccccggagctgctccaaatgctgcaggcctgcggctgccgtgtccggtggatcgacaagccgctcg
tgtacaagtga
>g26001.t1
atggccggcccccgcgcgcccgtggcggcggcggcggcggtcgtcctggcgcttctctccctcgcgttcgcggagccgccggcgagcgagcggtc
ggcgttgctggccttccagaccgccaccccgcaccagcgccggctcggctggaccacctccacctccgcctgcagctggttcggcgtgacctgcgaccc
caccaactccacggtcgtcgacgtccgcctcccgggcgtcggcatcgtcggcgccgtcccacccggcaccttcggccggctccccaacctccgcgtcct
ctccctccgctccaaccgcgtcctcggcgggctcccctccgacctcctcgccctccccaacctcaaatccctcttcctccagcgcaacgagctctccggcc
ccatccccaccgggatcacggggctggcctccctcgagcgcctcgtcctcgcgcacaacaatctctccggccccatccccttcgcgctcaacaacctcac
gtccctccgcgtgctgcgcctcgacgggaaccatctatctggaagcatccccagcatcagcatccaggggctcgacgtcttcaacgtctccgacaacag

cctcaacgggtccatccccaggtcgctctcgcggttccccaaggattccttcgcgggcaaccggctcctctgcggcgcgccgctggacccgtgcggcag
cccgttcttcccccggcggcgtcgcccggggccggcccgcccgggggcgagcccgtgccggcggggaataagaagcggaagctctccggcgcggc
catcgccggcatcgtcgtcggcgcggtggtggcggcgctgctcctcctcatcgtctgcgtgctctgcgccgtgcaccaccgccgcggcgcggccgcccg
tcaggaagggcccaagggcgccgtggcctccgcggcggccgcgccggcgcgagggccgacatccggcgagggcgccgggatgacgtcctcgtccaa
ggaggacctgggcggcggcggcacgtccgggtcaacaacggccgcggctgctgctgctgctgccgcggcggcgggggccggcgcgggtggggagc
agagccggctggtgttcgtggggaagggcgccgggtacagcttcgacctggaggacctgctccgcgcgtcggcggaggtgctgggcaagggcagcgt
gggtaccagctacaaggccgtgctggaggaaggcaccaccgtcgtcgtcaagcggctcaaggacgtcgccgcgcagcgccgcgagttcgacgcgca
catggaggcgctcggcagggtggagcaccgcaacgtgctgcccgtgcgcgcctactactactccaaggacgagaagctgctcgtctacgactacctgc
ccaacggcagcctctccgcaatgctacacggaagccgtgggtccgggcggacgccgctggactgggaggcgcgcatgcgttcggcgctgtccgccgc
ccgcggtctggcgcacctgcacaccgcgcacaacctcgtgcacggcaacgtgaaggcctccaacgtgctgctccgccctgaccaggacgccgccgcgc
tgtcggacttctgcctgcacccgctcttcgcgccgtcgtcgacgcgcgccggcgggtaccgcgcgccggaggtggtggacacgcggcgtctctcgttca
aggccgacgtgtactcgctgggcgtgctgctgcttgagctcctgacgggcaagtccccctcgcacgcgtccctggagggcgacggcacgctggacctg
ccccggtgggtgcagtccgtggtgcgcgaggagtggaccgccgaggtgttcgacgtggagctggtgcggctgggcgccagcgccgaggaggagatg
gtggcgctgctgcaggtggccatggcgtgcgtggccaccgtgcccgacgcgcggcccgacgcgcccgacgtggtcaggatgatcgaggagatcggcg
ccgggcacgggcgcaccaccacggaggagagcgaggaggggcatcgcggcacgtcggaggaggagcggtccaggggcacgccgccggccgaccc
gacgccgtga
>g26305.t1
atggagttcgtggtgtccatcgtggacacagtgttccggcctctcaaggactacttcgcgcggaccttcggctacgtgatgtcctgcggcgagtacatcg
acgcgctgggcaacgctatgaacgagctcaagagcaagcgcgacgacgtgaagcgcatggtggactccgccgagcggcagggcatggaggccacc
agccaggtcatgtggtggctggagtgtgtcgccgcgctggaggtcgccgccgcgcggatcgtggacgagtaccaggcgcgcctgcagctcccgccgg
accaggcccccgggatgaaggccacctaccacctcagcaagaaggccgacgagacgctcgccgaggccaccagcctcaaggagaagggcgacttcc
acaaggtcgccgacgagctcgtgcaggtccgcttcgaggagatgcccagcgcgcccgtggtcggcagggacgcgctgctccaggagctccacgcctg
cgtccgcgacggcgacgtcggcatcgtcggcatctacggcatggccggggtcggcaagaccgcgttgctcaacaagttcaacaacgagttcctcgtca
acacccccgacatcaatgtcgccatctacatcgaggtcggcaaggagtacaacctcgacgacatccagaggatcgtcggcgaccggctcggcgtgtcc
tgggagaacaggacgcccaaggagcgcgccggggtcctctacagggtgctcagcaagatgaatttcgtgctgcttctggacgatgtctgggagcccct
caatttcaggatgctcggcatccccgtgccgaagcagaattccatgagcaagatcgtcttgaccacgaggattgaagacgtgtgcgaccgcatggacgt
ccgccgaaagctcaagatggagtgcctgccatgggcgccctcctgggagctcttccgtgacaaggttggggagcacatcatgagtgcgacccggaga
ttcggcagcaggcgcaggcgctggccatgaagtgcggcgggctgcccctcgcgctcatcactgtcggccgggcgatggccagcaagcgcactgcaaa
agagtggaagcacgccatcactgtgctcaagatcgccccatggcagctccttggcatggagatggatgtccttgtgcctctcaaggacagttacgacaa
cttgcccagcgacaagctaaggctctgcctcctgtattgctctctgttcccagaggaattctccatttctaaggattggatcattgcctactgcatcggtga
aggtttcatagatgaattgtacactgagatggatgagatatacaacaaggggcatgaccttcttggtgatctcaagattgcctctttgttggaaaaaggtg
atgatgaggatcatatcagaatgcatcccatggtccgtgccatggccctatggatagcgtcagagtttggccaaaaagagaccaaatggctcgtccgtg
ccggagtcatgctgaaggaggcaccaggtgcagaaaaatggagtgatgctgagcggatttccttcatgcggaacaacattcttgaactgtatgagaag
cctaattgcccttttgctgaagactctgatgctacaaggtaatcctgcactggacaagatatgtgacggattcttccagtacatgccatctctcagagtttta
gatctgtcccatacttctattagtgaattaccttcaggcttcagttccttggttgagttgcagtacctggatttgtacaacaccaacatcagggcactgcca
agggaactcgggtcgctggtgactctgcgattcttgcttctctcaaatatgccactggaaatgatcccgagtggcgttatatgcagtctcactaggctgca
agtcttgtacatggacctcagctatggagattggaaggttggtgagagtggaaatggtgttgattttcaggaacttgagagcctgcgtaggctcaaggct
ctcgacatcactttacagtcgcttgatgccctggagcggttgtcaaggtcatttcgccttgctggttccacaagatacatactgataaagacttgttccagc
ctaacaaagattgagcttcctggaagcaacctctggaagaacatgactgggctgaagagggtgtggatcgctagctgcagcaacttggaggaggtcat
cattgatggtagccaagaaactgttcggggcaatgcgctcccccatgccatcttgcaggtacaggctcgagctggacttgtggatgacgagcagcccat
ccttccaaacctgcatgatgtcattcttcagggccttcataaggtaaagatcatatacaaaggcggatgcgtccagaatctttcatcgctgttcatctggta
ttgccatggactggaggagctgattactcttagtgatgaagaacaagaaacgactgccggtggaggcgaacaagctgcaggattcaagatcatcacac
cattccctaacctcaaggacctgtatctccatggattggcgaaattcaggaggatgggcagcagtacatgcacgctgcgattcccttcactggagagcct
gaagattgtcgagtgcccgaatctgaagcagctgaaactttctgctggagggctgaatgtggtacaatgcacgagagaatggtgggatgggctggagt
gggacgacgaggaagtcaaagcgtcttacgagccgctcttccgtccaatgcactga
>g27941.t1
atgcgccgccgcctccttgagcttcttcccctcctctcactccacctcctcttctccgccgcctcctatgcctcctcttcatcggaggttgcgttcctgacgtc
gtggctgaacacgacgtcgtcgcggccggcggactggtctccgtcggccgcgtcgccctgcaaatggtcccacatcacctgtgacggaggagtgagag
tgacttccgtgaccttccagtccgtccacctcgcggtcccctccctcctggcctctgcgccgcgctcccttctctgacgtcgttggtcgcctccgacgcga
acctgaccggctccctccctgacgacctccacctgtgccgccgcctcgccgtcctcgacctgagcaacaacgcgctgacgggccccatcccggcgtcgc
tcggcaacgcgtcggcgctggagacgctggcgctcaactcgaaccagctctccggcgccatcccccggacctcggccaccacctgaacctgctcaag
aacctgctcctcttcgacaaccagctctccggcgagctccctccctcgctgggcgacctgaccctcctggagtccctccgcgcgggtggcaaccgcgacc
tctccggctcgattccggattccatctccaagctgtccaacctcgtcgtgctgggcctcgccgacaccaagatctccggcgcgctccctgcgtcgctgggc
cagctccagaacctcgagacgctctccgtgtacaccacctccctctccggctccatccctccggagctcggcaactgctcgtccctcgccttcgtgtacct
ctacgagaaccagctcaccggcccgctcccttcgtcgctgggtaataataacctccctcggttgcagaagctgcttctatggcagaacgcgctgacggg
gcccatcccggactcgttcgggaacttgacctcgttggtctccctcgacctctccatcaactccatctccggcgccatccccggtacgcttgggcgtctgcc
atcgttgcaggacctgatgctcagcgacaacaacatcacgggcaccatcccgacctctctcgccaacgccacgtccctcgtgcagctgcaggtggacac
caacgagatctccgggctcatccctccggagatcgggagcaggttatccaacctccaggtgctgttcgcgtggcagaaccagctggaaggcttgatccc
gcccacgctcgcgtcgctcaccaacctccaagcactggacctctcccacaaccacctgaccggccccatcccacccggactgttcctcctccgcaacctc

accaagctcctcctcctctccaacgacctgtcgggccccttgccgccggagacgatgggctccgcggcgaacctcgtccgtctccggctcggcgggaac
cggctcgcggggaccattccgggtaccatctccgggctgaggagcatcagcttcctggacctcgccaacaaccgtctcgcgggcacgattcccccgga
gatgggcaactgctcccagctccagatgctggacctgagcaacaacgcgctcacggggaccatcccggagtctctcgcggacgcccgcggcctccagg
agatggacgcgtcgcacaaccggctcacgggcgccatccccgacaagctgtttgttcagttgagccggctggtcctgcgcggcaactcgttgtccggcc
cgatccccgcgtcgctcgggaagtgccgcaacctggagctcctcgacctcagcgacaacgcgttcacgggtcccatcccggacgcgctgtgcggcctc
gacgggctcgacatcgcgctcaacctcagccggaacgcgctcaccggccccatcccgcgcaacatctccgcgctctcgaagctcagcgtgttggacgt
gtcgtacaataaccttgacggcggactggcgccgctggcggggctgtccgacagcctcgtggcgctcaacgtctcccacaacaacttgtcggggtacct
ccccgacaccaagctgttccggcagatgtccgcgtcggggctcgcggggaacgcggggctgtgcacgaaaggcggcgacgtgtgcttcgtcgccgtca
acaatggcgacgcacgcacggcgagcagctccgacgacgaggcggcgcgcgtgcggaggctcaagctggccgtcgcgctgctggtgaccgccacgg
tggccatggtgctgggcatggtggggatcctccgcgcgcgacggatggggatcgggaagggctgtggaagcagtaatggagatagccagctggcgtg
gccgtggcagttcacgccgttccagaagctgagcttcagcgtggaccaggtggtgcgtagcctcacggacgccaacgtcatcgggaaagggtgttccg
gcgtggtgtaccgcgtcagcatcgacaccggcgaggtcatcgccgtcaagaagctctggcccaacggcaacacgcacccgaccaaggaccacgacgt
gatccgcgactcgttctcggcggaggtgcgcacgctggggtccatccgacacaagaacatcgtcaggttcctcggctgctgctggaacaagaccacgc
ggctgctcatgtacgactacatgcccaacgggagcctcggcgccgtgctgcacgagcggaggtgtggtggtggggcgcagcagctggagtgggacgt
caggtacaggatcgtgctgggcgcggcgcaggggctcgcgtacctccaccatgactgcgtcccgcccatcgtgcacagggacatcaaggccaacaac
atcctcatcggcctcgatttcgaggcctacatcgcggatttcggactcgcaaagctcgtcgaggacggcgatttcgggaggtcgtcaaacaccgtcgcc
ggatcgtacggttacatcgcacctgagtacgggtacatgatgaagatcacccagaagagcgacgtgtacagctacggcgtggtggtgctggaggtgct
gacggggaagcagcccatcgacccgaccatccccgacggcctgcacgtcgtcgactgggtgcgccggtgcaagggcggcgccgacgtgctggaccc
ggcactcaaaggccggaacagctccgaggtggaggagatgctgcaggtcatgggcgtcgcgctgctctgcgtcagcaccacgcccgacgacaggccg
accatgaaggacgtcgcggcaatgctcagggagatcaagctcgagcgggaggactttgcgagtaatgtcgatctgctgctcaagggcggcgatcatca
ggctcctaacaataacaacaacacggcggcggtacccaaggggacggcaatgtcgtcgtccacgtccagcacgccgccgtcgtacaggcaaggtccg
agcaacagcaacagctgcagtagcagcagcttcgtctctgctgccaccatctactcatccaaggaaaagacaccgtttggctcaagtagtgctcaactc
aacaaaacagagtcttag
>g28172.t1
atggcgccgcagtcatcgccacgcggccaccatgccctgccctgcttcctctgcctcgccttatcctgcctcctcctcctcccgcgccacgccgcggg
gcagagcgtcgtcgtcgacgagggccagctcctcctgcagatcaagcgcgcgtgggggggacccggcccagctcggctcatggaccgcggggaccaca
tcgtcacgctgcaccaactggacctacgtgtcctgcgacgccacgggccgggtgacctcgctcgcgctgcccaacgtcacgctcccggccggcgccgcc
gtccccgccgccatcggcagcctggcgtccctcgcaacgctcgacctcagcaacaccagcgtcgccggcgggttccccgcctccctctacaacctcacc
ggtctcacccacctcgacctctcgtcgaacagactcggcggcgagctacctaccgatatcgaccggctcgggtccagcctcacatacctcgccctggac
cacaacaatttcaccggcgagatcccaccatcggtggggaagctcaggaacctcacgtacctcgccctcaacgagaacttgctcaccggcacggtccc
gccggagctgggcgagctgatcaacctcgaggcgctcaagctggaggcgaacccgttcagtcccggccgctgccggcgtcattcaagaacttgaag
aagctgaggacggtctggtttgcaggttgcaacctcaccggcgacttcccgggctacttcgcggagatggctgacatggaattcctggacctatcggtg
aacgggctcaccgggagcataccgcccgggatatggagcctcccgaagctgcagatcatgtacctgtacagcaacagaatctccggcgacgtcgtcgt
cggtaacaacggcacgatgggcgcgacgagtctgatcgagctcgacatttcggacaaccagctgacgggaaccatcccggagagctttgggagcttg
atggacgtcagggtcctcaacctcttctcgaacaacttcacaggcgagataccggcgagcctggcgcagctgaggtcggtggtgatcatgaagctgttc
tacaacaagctcacgggaccgctcccggccgagctcgggaagcactcgcccgtgctccgggacatcgagatcgacggcaacgacatctccggaccga
taccggccgggatctgcgacaaccgcgctctgtggatcctatccgcttcgggaaaccggttgaacggctccatcccggccagtctcgccacctgcccgg
ccctcatcagcctccagctccaggacaaccggctgtccggcgaggtcccgccagagctctggacggagacgaagctcatcactctgctcctgcagaac
aacggggaacttaccggcaccttaccggagaagctgttctggaacctgacgcgtctgttcctcgacagcaaccggttcaccggcccggttccggcgtcg
gccacccggctccagaagttccacgccgcgaacaactcgttctcgggcgacatacccgcggcgttccccgcggggatgtccatgctgcaggagctgga
cctgtcgtcgaaccggctctccggggccatcccgccgagcatcgcgtcgctcaacgacctcacgcagatgaacttcagcgggaaccggctcaccggcg
agataccggcgacgatcgggtccatgccggtgctcaccctgctcgacctctcgtccaaccagctctccggcgccatccctccagcactaggatcgttgag
ggtcaaccagctcaacttatcctcgaaccagctcaccggagagatccccgccgcgttcgccaccgccgcctacgaagagagcttcctgggcaacccga
acctgtgcacgtccacgcggcagctcctcacgggcgtgcacgcgtgcgggggggccacgtcatcgcccgaccgcgtctcccctggcctccgcgcgggc
ctgctcgccgccggcgccgcgctgctggtgctcatcgccgggctcgcgttcctcgccgcgcgcgacatcaagcgacggaagaggaaacttgccgccgc
cgcggaggaggaggaggagtggaagctgaacccgttccagccgctggagttcagcgaggcggcgctgctgcgtgggctctccgacgacaacctcgtc
gggaagggcggctccggccgcgtgtaccgcgtcgcgtacgcgaaccgtgctcgcggcggaacgagcggcaccgtggcggtgaagcgggtgtggacg
gggcgaagctggacacgagcctggagcgggagttcgaggcggaggtgggcatcctcggccacgtccgccacaccaacatcgtgaagctgctgtgct
gcctgtcacgcgcggagacgaagctgctcgtgtacgagtacatggaatacggcagcctcgacaagtggctgcacgggcacaagtgggtggcgcggga
cggcgtcgtgttgacgacgacgacgacgagggtcccgtcggtcaggcgcgcgccgctggattggccggcgagggttagggtggctgttggggctgca
agggggctctgctacatgcaccacgagtgctcgccgccgatcgtgcaccgcgatgtcaagtcgagtaacatcttgctcgactcggagctcaacgccaag
gttgccgacttcgggctcgccaggatgttggctcaggccggcaagcccgacaccgtgtccgccgtcgccggaacgttcggatacatggctcctgagtgc
gggtacacgaggaaggtgaacgagaagctcaacgccaaggttgccgacttcgggctcgccaggatgttggctcaggccggcaagcccgacaccgtgt
ccgccgtcgccggaacgttcggatacatggctcctgagtgcgggtacacgaggaaggtgaacgagaaggtggacgtgtacagcttcggcgtcgttctc
ctggagctgaccacgggtagggaggccaacgacggcggggagcacgggtctctggccgactgggcgtggcggtacctgcagtcggggaagagcatc
gccgacgcggcggacaagtgcatcaggaacgccgggtacggcgacgagatcgaggccgtgttcaagctcggcatcatctgcacgggcgcgcagccg
gccacgcggccgaccatgaaggacgtgctgcagatactgctgcggtgcgagcaggcgtaccagaaaagcttggacgagaaggtcgccgagtttgacg
cggcgccgctcatccaggtgcgaggaggcagccgacggaagcagttctcggacgctaaagtggtagatgatgccgaggcctgctttgactccatcgtc
tga

>g28337.t1
atggctccctccgattcctctccgaaggccgccgctcttttcctcctcttcctcccgctcctctgctccaccatcgccacccccgccgtagcacaacaacta
gcctcctcgcaggcgaaggcgctcctccgcgtgcgccgcctcctgggcaaccctccggcgctcgagcccctccgccacgtgccggaccccctgcgcgctc
ccgccgacgccgacgctcaccgtcgcttgttcgggcgggcaggtcaccgccctctccgtcctcggcgaccgcgacccatcgtccgcgctcccctccacct
tctcctccgacgcgctcttcaccacgctcacccgcctcccgtccctctcccgcctctccctcgtccggctcggcatgcggggcccgctcccgggcgccaag
ctccgccgcctccaggcgctgcaggtcctcaacctcaccggcaaccacctctccggcgccgtccccgccgacctcgcccgcatgtactcgctccagagc
ctcgttctgtcccgcaaccggctcaacggctccgtgccctcgctcgccgcgctgcagttcctcgaggagctcgacctgtcgcacaacgcgctgggcccgc
ccgccgcgttcccggaggtgggcaacgcggtggagagattagtcctcgcggagaacaacttcacggggaagattccggcgtgggtgggcaagctggg
ccagctccggttcatggacgtgtcggggaacaagctgcaggggtggatcccctccgccatcttcgcgctcccgtcgctgcgccacgtcgacctgtcgcg
gaaccggctctccgggcagctgccgccggccacggcgtgcggcggcgcgctggcgttcgtcgacgtgtcgtccaacctgctcacgggcgcgcggccgg
cgtgcatgcgggggaacaactcggcggcggcgcggacggtgctcgtcgcggggaactgcttctccgacgccggcgccgagcggcagcggcccagct
cgtactgcgcacccggcgcgctggccgcagtgctgccgccgccgcagggggggcgggaagggcggggaggggaaggggaagggcggcgaggtcgg
gatggtcctcgctgttgctggcagtgtcgtgggcggcgcgttgctgatcgcgctcgtgctggtggtcgtgctgagagtggtcaggaagcagcacccgga
ggtgagcagcgtcttgccgacctccccggcggccacgccggcgaagaaacctccggttaaggccacccacaagattattgctcctgccgataagagac
atgcatcacaagccgcaagggtgaatacactggaagtaccagcgtaccgtgtgtatacgctggaggagctgcaagaggcaaccaacaacttcagcgc
ctccaaattgatcaagagcagtccccatgcacagatttcctcagagatcccatttgctattgatgacgatagtggcagcggcagtgctcataaccaggaa
caaggggaaaagcaagatatctatcagtttggcctaattctcctggaagtgatcaccggcaaaacaacagagtcacaaagacaactcgagtccctaaa
agcacagataagggagacactcgctgaagatccagaattgctgaaagacatagctgacccagcgatccatggcactttcgcggtggactctctgagca
cagtcgctgaggtagccctcaactgcacggctagtgccccgggcgagcggccttccattgacgacgtcctctggaacctgcagtactcgatgcaggtgc
aggatggatgggcgagcagcgagagcttaagcatgagcatcaaatcacaggcctga
>g28594.t1
atgaggatgaagccaaggctgagtcgctgcagcagcttcgtccatggcgtccctttgcttctgctgttcttgctgccacttctgtgccattcttggagggcg
gctgcgcaggctccgcagactgatccggttgaaacggcggcggtgaacgcgatcctgagcaagctggggctgagcgcgccggcgtcgtggaacatca
gcggcaacccgtgcagcggcgcggctacggacgacacgtcgctggacgacaacccggcgttcaacccggccatcaaatgcgactgctccgaccagaa
caacaccgtctgccatgtcatcagattgaagataaacatgctggacgcggttggtccgattccagaagagctgcggaacctcactcacctcaaaaagtt
ggacttcagaagaaattacttcactggaccattaccagcattcataggagaattgactgctctagagactgtgggaatcaatgcattatcggggcctctc
ccaaaggagcttgggaatcttacaaatctcaaatcactggcactgggctcaaatcattttaacggctcacttcctgatgagctgggcaaattgacgaaac
ttcaacagatgtacattgatagtaatgacttcagtggtccactaccatcaacattgtcccaactcaagaacctctcagtcttgtgggcatcagacaataat
ttgagtgggcagattcctgattatcttgggagcttgactaacttgactcaagtgtaa
>g28615.t1
atggttgcggcggccattggtgtggtggtgctgttcttggctgtaacttcatttggggctaatgccagcaccgactctaacgacgttgatgcactcaatgtc
ttctacacgaccatgaattcaccaacccagctcacaaactgggtgtcccaaaacggggatccctgtggacaatattggcttggggtcgcctgctcaggtt
ccagggtgaaaacaataaagttatctggaatgcggcttaacgggaccttggggtacaatatgaatcacctgactgcgctagttgagctcgatatgagca
acaataatcttggagggaatgacattccttataatctcccaccaaatcttgagagcctaaatcttggaggaaataattttactgggaccgtaccatactcc
atctcccagatggttgcacttagacatttaaatctcggtaataatcatctttcgaacatcaatgatatgtttaatcagctcacaaatttaacactaatggac
atatcatacaacgcattttctggtgacatcccacaaagcttccagacattgactaatttgaaaaaactaaatgtggagaacaatcaattcactgggtgga
taccagataaactaaagggaatcaggagtatccagctaaatcatgcaaaccttgctgagctatctggttactgctcagagcatggacagtgcttgctagc
atatgaactctaccggaatggttctctccacgacttccttcatgtgaaggatgaatacagcaaaccgttgtcttggaacaatcgtgtcaagattgccctag
gatctgcaagggcattagaatacctacatgaaacttgttcgccgcctatagtccacaagaacttcaagtcatcgaatattttgctggatagtgaactgaat
ccccacgtgtcagattcagggtttgcaggccttattcccaaccaagaatttcaggaatcagatgagaactcaggaggatacagggcaccagaggtgac
catgtctggtcagtactccctgaagagcgacgtgtatagcttcggcgtcgtcatgctagagctcctgactggccggagaccgttcgacagatcacggccg
cggccggagcagtcgctggtccgatgggccacgccgcagctgcacgacatcgacgcgctggaccggatggtcgacccggcgctgcagggcctgtacc
cttccaaatccctctcccgcttcgccgacgcgatcgccctttgcgtccagcccgagccggagttccggccgccaatgtcggaggtggtgcagtcgctggt
gcggctcgtgcagagggcgaacatgacgagggcgcacgagaatcagtcgcggagatccggcgagtctggggactacgagttctaa
>g29008.t1
atggaagcaagagcgtcagtgctggtcctcgtagcagcagtgacactactgtcatcattgcccaccaccggaggagccgtcgatgagccggcggcgct
gcttgcgctgaaggcggggttcagtgacccgttgggcgcgctcgccgactggaagaaagaggacgcctcggcgccgcattgcaggtgggccggcgtc
gggtgcaacgccggcgggcgcgtggacagcctggacctctcagggaagaacctgagcggcaaggtctccgacgacgtgttccggctggcgtcgctcc
ggttgctcaacctgtcgtacaacgccttcgactcggtgctgcccaagtcgctggcgccgctgtccaacctgcaggtgctggacgtgagccagaactcctt
cgaaggcgcgttcccggcgggcctcggctcctgcgccgacctggccaccgtcaacgcctccggcaacaacttcgtgggcccacttcccgtggacctcgc
gaatgccacgtcgctggaggtgctcgaccttcgcggcagcttcttcgaaggcgccatcccggcggcgtaccggagcctgacgaagctcaagtttctggg
gctctccggcaacaacatcaccggcaagatcccgccggagctcggcgagctggagtcgctcgagagcctcatcatcgggtacaatgtgctggacggcg
gcatcccgccggagctggggaacctggggagcctgcagtacctggacctcgccgtcggcaacctcgacgggcccatcccggcggagctgggcaagct
tccgtcgcttacatcgctgtacctgtacaagaacaatcttgaagggaagatcccggctgagctgggcaacatctcgacgctggtgttcctggacctgtcg
gacaacctgctctccggcccgatccccgaggaggtgtcgcagctgagccacctccgtctcctgaacctgatgtgcaaccacctggacggcacggtgcct
tcgtccatcggcgacaagatgacgagcctggaggtgctggagctgtggaacaactcgttgactggcccgctcccggcgagcctgggccggaacgcgc
cgctgcagtgggtggacgtgtcgtcgaatctgttcacgggtcccgtcccactcgggatctgcgacggcaaggcgctggtgaagctcatcatgttcaaca
acgggttcacgggcggcatccccgccggtctggcgtcgtcgtgcgcgtcgctggtgcgcgtcaggatgcaagggaaccggctcaacggcacggtgccc
gtcgggttcgggaagctgccgctgctgcagcgcctcgagctcgccgggaacgacctgtccggcgagatcccggtcgacctcgcgacgtccacttcgttg

tcgttcatcgacgtgtcgcgcaaccgtctgcagtactcgctgccttccaacatattcacgatcccgacactgcagagcttcctggcctccgacaacctcat
ctccgggtcgatccccgacgacttccaggactgcccggccctggcggcgctggacctgtcgaataaccggttggtaggcacggtcccgtccagcctcgc
gtcgtgtcagaggctcgtcaagctgaacctccggcgcaaccggctggaaggggagatccccggcgcgctggcgaagatgcccgccatggccatcctc
gacctctcggggaatgcgttgaccggcaccataccggagagtttcgggagctcgccggcgctggagacgctcaacctggcgtacaacaacctgagtgg
tctcgtccccgggaacggcgtcctccgcaccatcaaccccgnnnnnnnnnnnnnnnnnnnnnnnnnnnnnnnnnnnnnnnnnnnnnnnn
nnnnnnnnnnnnnnnnnnnnnnnnnnnnnnnnnnnnnnnnnnnnnnnnnnnnnnnnnngtggaacaactcgttgactggccc
gctcccggcgagcctgggccggaacgcgccgctgcagtgggtggacgtgtcgtcgaatctgttcacgggtcccgtcccactcgggatctgcgacggca
aggcgctggtgaagctcatcatgttcaacaacgggttcacgggcggcatccccgccggtctggcgtcgtcgtgcgcgtcgctggtgcgcgtcaggatgc
aagggaaccggctcaacggcacggtgcccgtcgggttcgggaagctgccgctgctgcagcgcctcgagctcgccgggaacgacctgtccggcgagat
cccggtcgacctcgcgacgtccacttcgttgtcgttcatcgacgtgtcgcgcaaccgtctgcagtactcgctgccttccaacatattcacgatcccgacact
gcagagcttcctggcctccgacaacctcatctccgggtcgatccccgacgacttccaggactgcccggccctggcggcgctggacctgtcgaataaccg
gttggtaggcacggtcccgtccagcctcgcgtcgtgtcagaggctcgtcaagctgaacctccggcgcaaccggctggaaggggagatccccggcgcgc
tggcgaagatgcccgccatggccatcctcgacctctcggggaatgcgttgaccggcaccataccggagagtttcgggagctcgccggcgctggagacg
ctcaacctggcgtacaacaacctgagtggtctcgtccccgggaacggcgtcctccgcaccatcaaccccgacgagctcgccggcaacccggggctctg
cggcggcgtgctcccgccctgcaactcccgtggttccaacaacatggtggccaacacgcggccgcgtgtcacggcggcgcgtctgaagcgcgtggcgg
tggggtggctcgtggggatgctcgtggtcttcgccctggtagctgcgatgctcgccggacggtacgtgtaccggcgctggtacatgtacactgatggcga
ggaggagatctccagtggtggcgcgtggccgtggcggctgacggcgttccagcgcgtcgggttcacgagcgcggacgtggtggcgtgcgtcaaggaa
gccaacgtggtgggcatgggcgccacgggcgtggtgtacaaggcccagctcccgcgcgccaccatcgccgtcaagaagctctggaggccttcttctca
gtctcaggcggaggagacgacggagactaggacggaggaggtgctcaaggaggtgaaccttctgggtcggctccgtcaccggaacatcgtgcggatg
ctggggtacgtgcacaacgacagggacgccatgatgctctacgagttcatgcccaacggcagcctctgggaagcgctgcacggccactcgccggatga
ccaccaccccaggcttttggaggactgggtgtctcgctacgacgtggccgccggcgtcgcgcagggcctggcgtacctccaccacgactgccacccgcc
ggtcatccaccgcgacatcaagtccaacaacatcctgctcgatgccaacatggaggcgcgcatcgccgatnnnnnnnnnnnnnnnnnnnnnn
nnnnnnnnnnnnnnnnnnnnnnnnnnnnnnnnnnngtggaacaactcgttgactggcccgctcccggcgagcctgggccggaacgcgc
cgctgcagtgggtggacgtgtcgtcgaatctgttcacgggtcccgtcccactcgggatctgcgacggcaaggcgctggtgaagctcatcatgttcaaca
acgggttcacgggcggcatccccgccggtctggcgtcgtcgtgcgcgtcgctggtgcgcgtcaggatgcaagggaaccggctcaacggcacggtgccc
gtcgggttcgggaagctgccgctgctgcagcgcctcgagctcgccgggaacgacctgtccggcgagatcccggtcgacctcgcgacgtccacttcgttg
tcgttcatcgacgtgtcgcgcaaccgtctgcagtactcgctgccttccaacatattcacgatcccgacactgcagagcttcctggcctccgacaacctcat
ctccgggtcgatccccgacgacttccaggactgcccggccctggcggcgctggacctgtcgaataaccggttggtaggcacggtcccgtccagcctcgc
gtcgtgtcagaggctcgtcaagctgaacctccggcgcaaccggctggaaggggagatccccggcgcgctggcgaagatgcccgccatggccatcctc
gacctctcggggaatgcgttgaccggcaccataccggagagtttcgggagctcgccggcgctggagacgctcaacctggcgtacaacaacctgagtgg
tctcgtccccgggaacggcgtcctccgcaccatcaaccccgacgagctcgccggcaacccggggctctgcggcggcgtgctcccgccctgcaactccc
gtggttccaacaacatggtggccaacacgcggccgcgtgtcacggcggcgcgtctgaagcgcgtggcggtggggtggctcgtggggatgctcgtggtc
ttcgccctggtagctgcgatgctcgccggacggtacgtgtaccggcgctggtacatgtacactgatggcgaggaggagatctccagtggtggcgcgtgg
ccgtggcggctgacggcgttccagcgcgtcgggttcacgagcgcggacgtggtggcgtgcgtcaaggaagccaacgtggtgggcatgggcgccacgg
gcgtggtgtacaaggcccagctcccgcgcgccaccatcgccgtcaagaagctctggaggccttcttctcagtctcaggcggaggagacgacggagact
aggacggaggaggtgctcaaggaggtgaaccttctgggtcggctccgtcaccggaacatcgtgcggatgctggggtacgtgcacaacgacagggacg
ccatgatgctctacgagttcatgcccaacggcagcctctgggaagcgctgcacggccactcgccggatgaccaccaccccaggcttttggaggactgg
gtgtctcgctacgacgtggccgccggcgtcgcgcagggcctggcgtacctccaccacgactgccacccgccggtcatccaccgcgacatcaagtccaa
caacatcctgctcgatgccaacatggaggcgcgcatcgccgatttcggactggcacgcgcgctggccaggaccaatgaatcagtctccgtcgtcgcag
gatcatacggatacatcgcgccagagtacgggtacactctgaaggtggaccagaagagcgacatctacagctacggcgtggtgctcatggagctcatc
acggggcgccgcgcggtggagccggagttcggggaaggccaggacatcgtgggctgggtcagggacaagatcaggacaaacacggtggaggagtt
tctcgacccaaacgtcggcggccggtgcgcgcacgtcagggaggagatgctgctcgtcctgcgcatcgccgtcctctgcaccgcaagggcccccaggg
acaggccatccatgcgcgacgtcatcaccatgctcggcgaagccaagccaagaagaaagagcgccaccaacaacaacacaaccgtcgtggacaagg
acaaaccagtcttcaccaccacgccggacgcagactatgcctag
>g29523.t1
atgaacagccatttcaacaagaaccagttatctggagcacttacgggcctcttcaactctaatatgactctcatacacatattgtttgatagcaatcagttt
tccggcccaatcccagctgagatagggggcattgaaacactccaagttcttcgattagacaggaataaatttacgggagcacttcctcctaacattagca
acctggtcaacttaaacgagctaaacttggcaagcaaccagctcactggagcactgccagatctaagccgcatgaccaagttaaatgttgtgtaa
>g30165.t1
atgcggatactgngcagcaacaagtttgagggaggtgtaccaccagagattgggaaatgccgactcctcacttacttggacttgagccagaacaacct
ctctgggaagataccaccggccatctctggtatgcggatactgaactacctcaacctgtcacggaaccatcttgatggagagataccgccatccattgcg
acgatgcagagcttgacggcagtcgatttctcatataacaacctgtccgggcttgtgccggggactggccaattcagctacttcaacgccacttcttttgtt
ggcaacccaggcctgtgcggaccataccttggtccatgcctcccgggtgttgctggtaccgaccaccctgcccatggccatggtgggctgtccaatactg
tcaagctgctcattgtgcttggctntgctggcccgctcgttgaagaaagctag
>g30849.t1
atggcgcaaaatacgggaccgcgtcctgcgctcctcctcctcctgtccaccctcctcctctcctcctcctcctccatcctcgccgccgggcagcaggggta
cgaccagcagcagcagcagcagccaccgtcgtcgccatcaccggaggaggtgccgaagcagcagacggcggcgaccaacccgcggttgcagaaag
cctacgtggcgctacaggccctgaagcgcgccatcacggatgacccacacaacctgacctccaactggtgcgggcccgacgtgtgcgcctacacgggc
gtcttctgcgccgccgcccccgacgacccgcacgccctcaccgtcgccggtctcgacctcaaccacggcgacctcgccgccaccttcccggaggagctc

ggcctcctctccgacctcgccctcctccacctcaactccaaccgcttctccggcacgctcccggaatccctccccaaactccacctcctacacgagctcga
cgtcagcaacaaccgcctctccggcgggttcccgcagcacatcctctgcctccccaacatcaagtacgtcgacatacggttcaacaacctctgcggcac
cgtccccgccgccatcttcgacaacaacctcgacgcgatcttcctcaacgacaaccacttcgactttgagctaccgcacaacttcggaaactccccggct
tcggtcatcgtgttggccaacatcaagctgcgcgggtgtatcccgcctagtgtcgggaagatggcggccacgctgaacgagcttgtcgtgctcaactcc
gggttacgctcgtgtttgccgcaggaactcgggtggctccgggagctcaccgtgctcgacctcagctcgaaccagctgcaaggacagttgccggagtcg
ttggcggggttgcacaagctggagcagctcgacgtcgcgcataacgagctgtcgggtcacgtgccggaagggatctgcgcgctgccgagcttgaaga
atttcacatactcgtacaactacttctgcagcgagccgcagcggtgcttggatatacgcagcgtcgacgaccggcagaactgtattgccggacggcctg
accagcggcccgtcgaccagtgcatggcgttcctccaccgcccgccggtacactgcgacgaccacggatgcttcgccacgcccacgcccaagccgcag
cccaagccacagccgccgcactactga
>g31226.t1
atgcctccacgctcgcgcgccactaccagagcgaggaggccgcttcttcttgctctgctcccgtgcgtcttcctgttcgcgccgtgccagtgcgtcaacga
gcagggccaggcgctgctgcgatggaaggactcgatgaaagccacggccggcggcgccgcgctggcctcgtggaaggcgtcggacgcgtccccgtg
ccggtggtccggcgtgtcgtgcgacgcccgcggcgacgtcgagtcgctgagcatcaagtcggttgacctcggcagcgcgctgccgaccgacctccggc
cgctgcggtcgtcgctgaagacgctggtgctctccggcgccaacctcaccggcgcgatccccaaggagctcggcgacctcgccgagctgaccacactt
gacctcagcaagaaccagctctccggcgccatcccgcccgagctctgccggctgggcaagctccagtcgctggcgctgaacaccaactcgctgcgcgg
cgccatccccgacgccatcggcaacctcacgagcctcacgcacctcacactctacgacaacgagctgagcggcgtcatcccggggagcatcggcaact
tgaagaagctccaggtgttgcgcgccggcgggaaccaggcgttgaagggcccgctgccgcgggagatcggcgggtgcacggagctcaccatgctcgg
gctcgccgagaccggcatgtccagcagcctccccgacacgatcgggcagctgaagaaggtccagaccatcgccatctacacggccatgctcaccggct
ccatcccggagagcatcggcaactgcaccgagctcaccagcctctacctgtaccagaacgccctcaccggcccgatcccgccgcagctcggtcggctg
cgcaagctccagaccgtgctcctctggcagaaccagctggtcggcacgatcccgcccgagatcagcaactgcaaggacctcgtgctcatcgacctgtc
gctcaacatgctgaccggccccatcccgcgcagcttcggcacgctgcccaacctgcagcagctgcagctgagcaccaacaagctgaccggcacgatcc
cgagggagctatccaactgcacgtcgctcaccgacgtcgaggtggacaacaacgagctcaccggggagatcgacctcgacttcccgcggctggggaa
cctcacgctcttctacgcgtggcagaaccggctcactggccccgtgccacccggcctcgcgcggtgcgaggggttgcagtcgctggacctctcgtacaa
caacctcaccggccctgttccacgggagctattcgcgctgcagaacctcaccaagctgctcctcctcagcaacgagctctccgggttcatcccgccggag
atcggcaactgcaccaacctgtaccggctccggctcaacgataaccggttgtccggcacgataccggccgagatcggcaacttgaagaacctcaatttc
cttgacctgggcaacaaccgcctcgtcgggcccttaccagtggcactttccggctgcgacaacctcgagttcatggacctgcattccaacgcaatgtctg
gcacgttgccggacgagctcccccgcagtctgcaattcgtcgacatctccgacaacaagctgaccgggctcctcggccccggcatcgggtcgctgccgg
agctgacgaagctgaacctgggcaagaaccggatctcgggcggcataccgccggagctcgggtcctgcgagaagctccagctgctggacctgggcgg
caacgcgctgtccggcggcatcccgccggagctcgggaagcttccgtcgctggagatcgcgctcaacctcagctgcaaccgcctctccggcgagctccc
cccgcagctcggcgagctagggaagctcggcgcgctcgacctctcctacaaccagctctccggcagcctcgcgccgctcgccaggctggaggccctcg
tcacgctcaacgtttcgttcaattcgttctcgggcgagctcccggacacgcccttcttccagaagctcccgctcagcgacatcgccggcaaccacaacct
gctcgtcgtcggcgaccacggcgacgcggccgcccgccgcgcgtcgtccgtctcggcgctcaagctggcgatgacggtcctcgccgttgccagcgcgc
tcctcctcgtcgccgccacattcgcgctcgcgcgcgcctcccgccgccgcgaccggagcacctttcacggtgccggcggcgaggggggcgtgggaggtg
acgctgtaccagaagctggacttgtcggtggacgaggtggttcggtcgctgacgtcagcgaacgtgatcgggacgggcagctctggcgtggtgtaccg
ggtgtccctgcccaacggagaccacctcgccgtgaagaagatgtggtcgtcgaacgaggccggcgcgttccgcaacgagatcgcggcgctgggctcg
atccgccaccgcaacatcgtgcgcctcctgggctgggccgccaaccgcagcacgaagctgctcttctacgtctacctccccaacggcagcctcagcggg
ttcctccaccgcggcggcgtcaagggcgccgccgactgggccccgcgctacgacgtcgcgctcggggtggcccacgccgtggcatacctacaccacga
ctgcctccccgccatcctgcacggggacatcaaggccatgaacgtcctgctcgggcccaggaacgagccgtacttggctgatttcgggctcgcccgcgt
gctctccggcgccgtcgcggccggatccgccaagatcgactcgtccaagccaaggatcgcagggtcgtacggctacttttgttccaatcatgttcgtgac
agcgaacaacaaaaaacagaacgtttttttttttttgctgataattttggttatgacgatgtgacgacagagtacgcgtccatgcaacggatcacggaga
agagcgacgtgtacagcttcggcgtggtgctgcttgagatcctgacggggcggcacccgctggacccgacgctgccgggcggcacgcacctggtgca
gtgggtgcgggaccacgtgcaggccaagcgcgccacggcggagatgctggacccgcggctgcgcgggaagcccgaggcgcaggtgcaggagatgc
tgcagctcttcgcagtggcgctgctctgcatcgcgcaccgcgccgacgaccggcccgcgatgaaggacgtcgtcgcgctgctcaaggaggtcaggcgg
ccggcggagggcgtcggcgaagaggggaaggagcaggcggcgtgcaatggcgccgccgcgccgccgtctgcgcagcaggcgccggaccggagcc
cgctgcccaagggcggctcgtcgagttgctcgtttgccatgtccgactactccagctag
>g31956.t1
atggcccgaaataagctcctggcgacgacgatggtgctggtactgacgacagtgctactgatggccggcgctgagccgccggcgagcgagcgttcgg
cgctgcaggcgttcgtgtccgggacggcgcacgagcgcgcgctgggctggaacgcgtccgtgccggcgtgcgcgtggaccggcgtgacgtgcgacaa
agccaccaactccaccgtcgtcgagctccggctccccggcgtcggcctcatcggccgcctcccatcgggcacgctgggccggctgaagaacctgcagg
tcatctcgctgcggtccaaccgcctctccggctccatccccgacgacctcttctccctcccgcggctccggtcgctcttcctccaggggaacctgctcaccg
gcgctctccccgccgagatcgccaggctgtcgtcatcgctcgagcacctcgcgttgtcacataacaacctctccggcccggtcccctttcgcgctcaacag
tttaacgaagctccggtcgctgatgctcgacggcaacagcttctccggcagcctcccgagcatcaccatccgccgcctcgaggtgttcaacgtctccgat
aacaacctcaacggctccatcccatcttctctcgcgcggttcccgccggaatccttcgccggcaacctccacctctgcggcgcgccgctcctcgaccgcc
cgtgccacccattcttcccgccctctcctcctcctcccgctgctgctcctcctcctcctcctggatcacctgatgatgagtctaacaaaaggaagaagctttc
cggcgcggcggtggtggccattgcggtgggcgctggcgcggctgctctgctcgcgctggtgctcctcgtcctctgcttggtccaccaccaccggcgccgg
aaatccgttgatcaggacggaagcaaggcggcgaccaccaccagtaggggaggcgtgacgccgtcgtcgggagagatgggcatgggtggcggcga
gttcacctcctcgtccaaggagatcagcgcggcggcggccacggcggcggggcttattaccaagctcgtcttcgtcggcggcaacaagcacggcggcc
agcagcagagcttcgacctggaggacctgctgcgcgcgtcggcggaggtgctggggaagggcagcctcggcacgtcgtacaaggcgacattggagg
acgggaccaccgtcgtcgtgaagcgcctccgcgacgtggcggcggcgaggagggagttcgccgcctgcgtcgaggcggccgccgccgtcgagcacc

gaaacctggcgccgatgagagggtactactactccaaggacgagaagctgctcgtcggagactacctgcccgccggaagcctatccgccagactaca
cggtggccgtggcaccggccggacggcgatggactgggaggcccggatgcgcgccgcgctgtgcgccgcccgcggcgtggcgcatttgcacaccgcc
cacggcctcccgcacggcaacatcaagtcctccaacctccttctccggccagaccccgacgcggcggcgctctccgactacggcctcaaccagctcttc
acatcatctgcaactccggcgcggcctattagcgccgccggcagcagcggctaccgcgcgccggagctcgtggacgcgcggcggccgacgttcaagt
cggacgtgtactcggtcggggtgctgttcctggagatgctgacgggcaagtccccgtcgtcgggcgaggtggacctgccgcggtgggtgcagtcggtg
gtgcgggaggagtggacggcggaggtgttcgacggcgagctggtggggatggcggcggaggaggagatggtggcgctgctgcaggtggccatggcg
tccgtcgccaccgcgcccgacgcgcgacccgacgccggcgatgtcgtcaggatggtcgaggagatcggcgtctcctcctccaggggcgccacgccctg
a
>g32778.t1
atgagagcacttgcaaatctaatcaatttggtaaaccttgatcttgaaggccttgtgctattacaaagcttggtgctttctgatactgaagttggtaccaat
ggactccgccatctgtctggtcttcgcaatttgcagagccttaatatctcatttaccttgatcacggatgatagtatgaagaagattgccacattgacttcg
ctgaagtcaattaatctagataatcgccagattactgatataggcttggcagcacttacaggtcttagcgggttgactcatcttgaccttttggagctcgc
ataaccgactacggaacaaactgcttgagatgccttgtgctattacaaagcttggtgctttctgatactgaagttggtaccaatggactccggcatctgtc
tggtcttcgcaatttgcagagccttaatatctcatttaccttgatcacggatgatagtatgaagaagattgccacattgacttggctgaagtcaattaatct
agataatcgccagattactgatataggcttggcagcacttacaggtcttagcgggttgactcatcttgaccttttggagctcgcataaccgactacggaa
caaactgcttgagattttttaagaaccttcaatcccttgaaatctgtggtgggtcaatcaccgacgctggagtgaaaaatatcaaggatctgaaagctttg
acgttcctgaatctttcgcaaaatgttaacctgacagacaaaactttggagctgatttctggtttgactgctttggtctcgttgaacgtctcaaactcgcgg
gtatcgaatgcaggtctcaaacacctaaaggacctgcagaacctgcggtcgttgtctcttgattccagcagggtaacagctgccgagatgaagaagctc
caggcaacgacgcttcctaatctgataagcatgcgacccgagtag
>g33993.t1
atgtttttggtgctcgccaccgtcgtcagctggccgctcctggcatcggcccagccggcgccgagcatgccgccgccttccccgccggcggctgccacga
acaactcgcggctggagaaggcctacgtggcgctgcaggcgctgaagcgcgccatcactgacgaccccaagaagctgacgcacaactggtgcgggc
ccgacgtctgcaactacttcggtgtgttctgcgcgccagcgcccgacgacccgtgccagcgcacggtggccggcgtggacctcaatcacggcgacctcg
tcggcacgctcccggaggagctgggcctcctctccgacctggccgtcttccacctcaactccaaccgcttctgcggcactctccccgagtccctccgctcc
ctccacctcttgcacgagatcgatgtcagcaacaaccagctctccggaggatttcccacccagcttctctgcctccccaacctccagtacgtcgacatcag
gttcaacaacttctgcggtgaggtgccggcagccatctttgacaaaaagatcgacgctctgttcatcaacaacaaccacttcgagttctcgctgccgaac
agcttcagcaactcaacagcgtcggtgatcgtgctggcgaacctgccgcgtctggggggctgcctgccgagcagcatcggcgacatggccgggacgct
caacgagctgattctcctcaacagtggcatctcctcttgtatcccgccggagatcggcagactggacaagctcacagttctcgacctcagcttcaacagc
atcgccggcacgctgccagacaccatcggcaacatgcgcgcgctggaacagctcaacgtcgcgcacaacatgcttgccggagagatacccgagagca
tctgcgcactgcctaacctaaagaacttcacctactcgtacaacttcttctgcggcgagccacaccggtgcttcgaggtgccacgcatcgacgacaggca
gaattgcatcgccggacgcccagaccagcggtccggcgaggagtgcatcgcgttcctgcaccggccaccggtgcactgcgatgcgcatgggtgcatcg
cgccgccaacggcacatgcgcctccgccgccggtatactga
>g33994.t1
atgtttttggtgctcgccaccgtcgtcagctggccgctcctggcatcggcccagccggcgccgagcatgccgccgccttccccgccggcggctgccacga
acaactcgcggctggagaaggcctacgtggcgctgcaggcgctgaagcgcgccatcactgacgaccccaagaagctgacgcacaactggtgcgggc
ccgacgtctgcaactacttcggtgtgttctgcgcgccagcgcccgacgacccgtgccagcgcacggtggccggcgtggacctcaatcacggcgacctcg
tcggcacgctcccggaggagctgggcctcctctccgacctggccgtcttccacctcaactccaaccgcttctgcggcactctccccgagtccctccgctcc
ctccacctcttgcacgagatcgatgtcagcaacaaccagctctccggaggatttcccacccagcttctctgcctccccaacctccagtacgtcgacatcag
gttcaacaacttctgcggtgaggtgccgtcagccatctttgacaaaaagatcgacgctctgttcatcaacaacaaccacttcgagttctcgctgccgaag
agcttcagcaactcaacggcgtcggtgatcgtgctggcgaacctgccgcatctggggggctgcctgccgagcagcatcggcgacatggccgggacgct
caacgagctgattctcctcaacagtggcatctcctcttgcatcccgccggagatcggcaagcnnnnnnnnnnnnnnnnnnnnnnnnnntatcagt
gggagagatttcaagatcagtgagaaaaggtactaa
>g34226.t1
atgcctcctcgtctgctcatgctcgtgctcctcctcgccgccgcttcggcgtcgccggagcgtgacgtgtacgcgctgggcaagctgaaggctgccctgg
ccccgtccacacccacccacgcgctcatcgactgggaccccgccgccacgccgccggcgcactgctacttctccggcgtgacctgcgacgacgccggc
acaggccgcgtggtggcgatcaacatcaccgggattccgctccacggcgggttcctccctcccgaggtagccctcctggacgcgctgaccaacctgacc
gtggcggcgtgctcgctccctggccacatcccgccgaccatcgcgtccatgccggagctccgattcctcaacctctccaacaacaacctcaccggcccct
tccccatacccgtcgacggaatcgatgactacttcccttcgctggaggtcatcgacgtctacaacaacaacctctccggcgtgcttcctcccttccgcagc
aataataaactcctccggtacctccatctgggcggcaactacttctcggggaccatcccggagagcttctcggagctggcgtcgctcgagtacctgggcc
tcaacggcaacgctctctctggtcgcgttccctcttcattatcgcgcctcacgaggctgcgcgagatgtacctgggctacttcaaccagtacgacggcgg
cgtgccacccgagttcagcaacctctcgtcgcttgtccggctcgacatgagcagctgcaacctcacgggtcccatcccgccggagctcgggaacctggc
caacctcgacacgctcttcctcctcatgaaccgcctctcgggggagataccttcccagctaggcgacctcaggagcctcaagtcgctggacctctccatc
aacgacctcggcggcgagatcccggccagcttcgccaacctgaccaacctcgagctgctcaacctcttccgcaaccacctccgcggcaccatcccgga
ctttatcgccgacgggttccccaacctggaggtgctccaaatctgggagaacaacttcaccggcactcttcctcagggtcttggaaagaacggccggctc
aagaagctggacgtgacggggaaccacctgactggcccgatcccgccggacctctgcgcgggtcgcaagctggagctgctcgtgctcatggagaacg
gcctgttcgggcccattcctcagtcgctcggggaatgcaagacactcacgagggtccgactcggcaagaatttcctcagcggcccgatccccgccgggc
tcttcgacctcccccaggccgacatgctggagctcacggacaacctgctcaccggcgagctccccgacgtcatcggcggtgacaagatcggcatgttgt
tactagcgaacaacgcaattaccgggcgcatcccgccgggcatcggcaacctccccgcgctgcagacgctgtcgctcgagtccaacaacttcaccggt
ccgttgcctgcagagatcgggaaactccggaccctcagccggctgaacgtgagcgggaacgcgctcaccgggaccatcccaccggagctcttcacgg

gatgcggctcactcgccgccgtcgacctcagccgcaacgccatctccggcgctataccggacgccatcacgtcgctcaagatcctgtgcacgctcaacg
tgtcgcggaacatgctgtcgggccagctccccgccgagatgtccaacatgaccagcctcaccacgctcgacgtctcctacaacatgctgtccggacccg
tgccgatgcagggccagttcctcgtcttcaaccagagctccttcgtcggcaacccaggcctatgccgcggcgggcccattgacgactgctcatcgctatc
atcaacagacggcggaggacctgctagtcgcctctaccaaaacaacaagaagaagatgcttcttctgtggctggggatcatcattgtgttcgctgctgc
gataatactcggcttcttcctcgtcggaggagggtggacaaagttgcacctactactagtagagaaaacccggcggcggcaagggtggaagatgacg
gcgttccagaagctggacttctcggcggaggacgtggtggagtggctcaaggaggacaacatcatcggcaagggcggcgccgggatcgtgtaccacg
gcgtcacgcgcgccggcgtcgagctcgcaatcaagcgcctcgtcggacgcgggtgcggccacgaccacgacaggggggttcacggccgaggtcacca
cgctcggccgcatccgacaccgcaacatcgtccggctcctcggattcgtctccaaccgcgagaccaacctcctgctctacgagtacatgcccaacggat
cgctaggggagatgctccatggcggaaagggaggccatctcggatgggacgcgagggcgcgcgtcgccgtcgaggccgcccgcgggctgtgctacct
ccaccatgactgcgcgccaaggatcattcaccgcgacgtcaagtcaaacaacatcctcctcgactccgcgttcgaggcccacgtcgctgatttcggact
agcaaagttcctcggcggagcaacctcagagtgcatgtccgccatcgcaggatcatacggctacatcgcgccagagtacgcttacaccctccgcgtgg
acgagaagagcgacgtgtacagcttcggcgtggtgctgctggagctgatcaccggccgccgccccgtcggcagcttcggcgacggcgtggacatcgtg
cactgggtccggaaggtgannnnnnnnnnnnnnnnnnnnnnnnnnnnnnnnnnnnnnnnnnnnnnnnnnnggcgcgcgtcgccgtcga
ggccgcaagggggctctgctacctccaccatgactgcgcgccaaggatcattcaccgcgacgtcaagtccaacaacatcctcctcgactccgcgttcga
ggcccacgtcgctgatttcggactcgcaaagttcctcggcgtcggtgcggccgccggagccacgtcagagtgcatgtccgccatcgccgggtcatacgg
ctacatcgcgccagagtacgcttacaccctccgcgtggacgagaagagcgacgtgtacagcttcggcgtggtgctgctggagctgatcaccggccgcc
gccccgtcggcagcttcggcgacggcgtggacatcgtgcactgggtccggaaggtgactgccgagctgccggacacgtcggacacagcggcggtgct
cgcggtggcggaccgccgcctgacgccggagccggtggcgctgatcgtggacctgtacaaggtggcgatggcgtgcgtggaggaggccagcaccgc
ccggcccaccatgcgcgaggtcgtccacatgctctccaactccgctgcggcccagcccaacgacaccttctga
>g35592.t1
atgtcgtggctgggcacctgcagcttctcactcgactcactgtcaccactactgcacgtgttagtcctgctgctgctcgtcgccggcgagggcattgccca
gccagccactggcgaccgcgacacattgcttgtcatcaagaaggactggggcagcccgccgcagcttacgtcatgggatgccgccagcgccgccgatc
actgccgctggaaaggcgtcacttgcgccggcgccggcagtggagttgtcacggagctgtccctcccgttcctgaacctcaccggttcagtcccggcgtc
catgtgctcgctcaagaacctcacccgtctcgatctctcgtacaacagcttcgccggagcattcccggccgccgcgctcttcgcgtgcacccacctccgc
accctcaacctctccaacaacttcttctccgggcccctcccggccgacatcgacggcctctcaccggcgatggagcacctcaacctctccagcaacctat
tcaccggcgacgtgccgccggccgtggcgcgtctgccggcgctcaagttcctgctgctcgacaccaaccgcttcaccggcgcgtacccggcggccgag
ataggcaagctctctgggctcgaggtgctcacactggccaacaacatgttcgctccagcgcccgtgccgccggagttcgcgaagctgaccaagctcacc
tacctctggatgttcaacatgagcctcatcggggagatccctgaagcgttctccaatctcaccaagctgaggaccatggacttgtcatggaacaacctta
ccggcacgatcccggagtgggtgctgcagcacaggaagctcgagcaggtttacctgtattacaacatcctctccggtgagctgccgcacaacgtcacg
gcgatgaacttgatcgagcttgacgtgtcgtcaaatcagctcaccggaaggatacctgaaagctttgggaatctaaagaacctgagtttgatgtaccttt
accaaaaccagttcactggtacgatcccggcgagcattgtgctgctaccgaagctcagtgacataaagatatttgacaaccagctatccggcaagctgt
catcagagctcggtaagtattcgccgctcagcaacctcgagatggatgacaacaacctctccgggccgctgccggagactctctgcgccaacggcttcc
tgtatggcctcgtcgccttcaacaacagcttctccggcgcgattccggccagcctggggaactgcgtcacactgaagaacctgatgctccaggacaaca
gtttttcgggcgatttcccagcgaagatatggtcgttgccacggctgaccacggtgaagatccagaacaacaacttcaccggtgctctgccggctcagat
atcctctaacatcgcacgacttgaaatgggcaacaacatgttctctggctccatcccgacctcggcgacagggctgcaagcgttccatgcggaaagtaa
ccggtttgatggggaattgccttctgacatgagaaagctagccagccttacttatttgtcactggccggcaacaggattactggttctataccgacatcaa
tcaagctcctgcagaagcttacttcactgaacgtgagcggcaatcacttgtccggtgcgataccgccagggagtgtcgggctgcttccaacgctgacaa
cgcttgatctgtccaacaatgagctcgctggcggcataccgtctgatataagcagcatggttaacccgctcaacctgtcatcaaacaagctcaccggcg
aggtaccggctgcactgcagagtgcagcgtatggccgcagcttcctcggcaaccagctatgtgctaggcccagctccggcacgaaccttccaccatgtc
caggcggaggccagggcgcccatgtcaagctatccacgggcttcatcatcctcttcgcgatgctcgccggcactgtccttttcagcagcctcggcatcat
ctgcctgcttctctggcgccgcaaggacaacgacgatgtgatcgactggaagatgacggcgttcaaccaactgaatttcaccgagttggatgtgctcaa
caacatccgagaggagaacgtgatcggcagtggcgggtctgggaaggtgtaccgcatcagagtcggctcctctgcccacaatgtggagggctgcagg
atggtggctgtcaagaggatttggaactcgaggaagctggacgccaagctggacaaagagttcgagtccgaggtgaaggtgctgggcaacatccggc
acaacaacatcgtgaagctgctctgctgcatctccagccaggaagctaagctgctcgtctacgagtacatggagaacggcagtcttgatcggtggctgc
accgccgcaaccgcgacggcgcccctgtaccgctggactggcccaccaggctggccatcgccatcgatgcggcgagggggctgagctacatgcacca
tgagtgtgcgcagccaatcgtgcacagggacatcaagtccagcaacatcttgctcgacctggacttccaggccaagatcgccgacttcggtcttgctcg
gatactcgtcaagcagtccagcgagcctgagtccgtgtcagcaatcggcgggacatttggctacatggctccagaatacgggcacaggcgaaaggtga
acgagaaggtggacgtgtacagcttcggcgtcgtgctgctggagctcacaaccggcaaggtcgccaacgacagcaacgccgacctgtgcctggcgga
atgggcgtggaagcggcaccagaagggcgcgccgttcgaggacgtggtcgacgaggcgatccgtggggagccggcgttgatgcaggacatcctgtc
agtgttcacgctgggcgtgatctgcacgggggagaaccctctgacacggccgtcaatgaaggaggtcttgaattaccttatccggtgtgaccagatgtct
gcagatgcagggttgtgccagctggaatgcaatggtgacctcgaggcgaaggagaatggcagctga
>g35789.t1
atggcagccacctccgtaccccatctcgtcgtcctcctcttcctcctctcgccactgattgacgcctcaacctcgccgtcgactgaagaggcgcagacacc
gcaagaccttctcctcgacttcaggtccaccctgcacgacccgtccggcgcgctctcctcctggtcgcgcgccacccccctactgcaactgggcccacgtg
acctgcaccagcgccacggccgccgccaacgccaccgtctccgtctccctctccctcgacggcctcggtctctccggcgagctctccgccgcctcgctct
gccgcgtgcccggcctcgcctccctcagcctcgcctccaacgccttcaaccagaccgtcccgctggagctcgcgcgctgcgcctcgctcgcgtcgctcaa
cctcagcgccgccgggttctggggcccgctccccgaccagctcgccgctctcccctttctcgtctcgctcgacctcagcggcaacagcatcgagggccag
gtgccgcccggtcttgccgcgctgaggagcctcgagacgctcgacctcggccgcaaccagctctccggcgtgctccacccggctctgttccgcaacctc
accagcctgcacttgctcgacctctccaggaaccagttcctggagtccgagctgccgtctgagatcggcaagatgagcggcctccggtggctgttcctgc

aggggtcagggttcatcggaacgatccccgaatccttcctcgatctggagcagctcgaggtccttgacctgtccatgaacaacctcaccggagtcgtgcc
gcccgggttcggcctcaagcttcagaagctgatgaccctggatttgtcccagaacgggctgtccggtccgttcccggaggacatcgacaagtgcttgat
gctgcagaggttcgaggtgcacgacaacgcgttcaccggaacgctccccgccgggctctggctgctgccggacctgcgcgtgatccgcgcccagaaca
accggttcaccggccggttcgaggtgcacgacaacgcgttcaccggaacgctccccgccgggctctggctgctgccggacctgcgcgtgatccgcgcc
caaaacaaccggttcaccggccggttacgggatttacccggcgggcagtctcggctcgagcaggtccagctagacaacaacagcttctccggcgggat
acctcgaagcatcggccaggttcgcaccttgtaccagttctccgcctccctgaacgagctgagcggcggcttgccggacaacctctgcgactccccggt
gatgagcatcgtaaacatctcgcggaactcgctctccggcacgatcccggagttcaggaactgcaagaggctggtgtctctgtacctgtccggcaacgg
cttcaccgggccgataccggcgtcgctaggggacttgccggttctgacgtacatcgacctgtccagcaacaacctcaccggtggcattcctgtggagcta
cagaacctgaagcttgcgctgctgaacgtgtcgttcaatcagctctctggccgtgtgccggcgtctctgatctccggccttcctgctgtgtttctccaaggg
aacccggggctctgcggtcctgggttgccaaacgattgcgatgcgccgttgaagaagcatcagggtctagtattggctgcaaccgtggtgttgttggtca
ccggagtgatgctgctcgctctaggagcatttgcagcgtggagaagaagaatgtcattggcaggggagcatttggaaaggtttatctga
>g35902.t1
atgagaaggcacatccttgcttgcctccttctggtcatattcctcgccctctctgtaaataccagtggccaaatcgatccacaaacacaagcccttcttcaa
ttcaaggccagcctgaaagaccctctgaaccacctccagtcatggacgaacaccaactcgccgtgccgcttcttcggcgtccggtgcaacgaggattcc
agcacggtcacggagatctcgctgtcaaacatgaacctctccggcgatatctcgccgtccatctccaccttgcacggcctgacactactcgagctcgact
caaactcgttgtcagggcccgtgccacctgagctcagcaggtgcacccagctcaggcaccttaacctttcagtcaactacctcaccggagaattgccgg
acctgtcagtgctgacagtcctcgagaccctcgacgtcgccaacaatgaattctctggccggtttccggagtgggtcggcaaggtttccggcctcagctg
gctcagcatcggtctgaacaactacgatcaaggcgagacgccagcaagcatcggaaacctcaggaacctcacgtacctgtacttggcggcctgcagct
tgacaggggagataccttcctccatcttccagcttacttccctggaaacactggacatatcaatgaacaacctcgccggcaggatccccggagccattgg
caacctccggaacctgtggaagatcgagctgtacaagaataacctctccggggagctgccgccggagctcgggaagctgacggggctgcgggaaatc
gacgtgtcccagaaccagctcagcggcgggatcccggcggccatggccgagctcaagggaatctga
>g36764.t1
atgtgcagatgtgtcatcaaagtcaataccgaggcattgggggtaaccacgaacaacccagcgagctttgcagcgagccagggagaccacattgaca
ccaagagggcacggaacaactatgtaggtgttattgtccacgagcacttggaatttgtcctgcatagcagcccgccagatgggatcagcgagatctacc
ttatgggaaggagagacatcagctggggaagtctgagcgacaaggttgagacgatcaaccggttggatgatcttatgtttgcggcgagtcatcatgtgg
tatacaaggtgttcgtattggacgactctactcttgactctccatcagccctggagtgcggggacgacttccgaacaagaaggaacgcttcacctggact
ctgcacaaaattgcagtgggtgagaagttgcatcaccatggcaaccaggcgaacgagcacccagatcactctcctcctcgccgtcctctccgtcgttctc
atcccgttccagctaccggtgagcacgtgcaccgctatcatctcaacaccaggcaccgaatatcaggcgctgctgaggtggaagtccacgctgcaatgg
acgcccaacggcagcagcagcagctcctcgctctcctcatggtcagcctccaccgcgagcacggcctgccattcatgggctggcgtctcgtgcgacact
gtgggcagcgtgtctgggatcgcgctccccggggccgggctcaccggcacgcttgcggccctcgacctcagggttgcagtgggcaccgaatatcaggc
gctgctgaggtggaagtccacgctgcaatggacgcccaacggcagcagcagcagctcctcgctctcctcatggtcagcctccaccgcgagcacggcct
gccattcatgggctggcgtctcgtgcgacactgtgggcagcgtgtctgggatcgcgctccccggggccgggctcaccggcacgcttgcggccctcgacc
tcagggttgcagtgggcaccgaatatcaggcgctgctgaggtggaagtccacgctgcaatggacgcccaacggcagcagcagcagctcctcgctctcc
tcatggtcagcctccaccgcgagcacggcctgccattcatgggctggcgtctcgtgcgacactgtgggcagcgtgtctgggatcgcgctccccggggcc
gggctcaccggcacgcttgcggccctcgacctcagggttgcagtgggcaccgaatatcaggcgctgctgaggtggaagtccacgctgcaatggacgcc
caacggcagcagcagcagctcctcgctctcctcatggtcagcctccaccgcgagcacggcctgccattcatgggctggcgtctcgtgcgacactgtggg
cagcgtgtctgggatcgcgctccccggggccgggctcaccggcacgcttgcggccctcgacctcagggttgcagtgggcaccgaatatcaggcgctgc
tgaggtggaagtccacgctgcaatggacgcccaacggcagcagcagcagctcctcgctctcctcatggtcagcctccaccgcgagcacggcctgccat
tcatgggctggcgtctcgtgcgacactgtgggcagcgtgtctgggatcgcgctccccggggccgggctcaccggcacgcttgcggccctcgacctcagg
gttgcagtgggcaccgaatatcaggcgctgctgaggtggaagtccacgctgcaatggacgcccaacggcagcagcagcagctcctcgctctcctcatg
gtcagcctccaccgcgagcacggcctgccattcatgggctggcgtctcgtgcgacactgtgggcagcgtgtctgggatcgcgctccccggggccgggct
caccggcacgcttgcggccctcgacctcacggccttcccggcgctcacgaacctcaacctcagcggcaaccgcctcgctgacgccatcccaaccaccat
ttccaacctatcctggctggtattgatcgatctggccgacaacgacctcagcagcggcgtcccggcgtcactcggttggctgccagcgttgcgagtcctc
gtgctgcgcaacaactcgctcggcggctccatcccggcatcgctcggccagctgcggaaactggaacagctcgaccttcatgccacggggctggtctcc
acgctgccgccggagatggggcacatgacgagcctacgcttcctcgacctctcagtgaacaacctcaccggcgtgctgccgaaatccttcgtcgggatg
agcaagatgagggagttctacctgtcaaggaacaggctctcgggtaagatcccacctgacgtgtttaaaagctggcctcagcttactctgttctacctgc
actacaactccttcaccggaagaattccgccggaaatcgccgaggcgaagacgctgcagcggctgtctcttttcagcaacaacctcactggcacaattc
cggaagaaatcgaaggcttggcaagcctgcagatgctgcacttgggacagaattgtctcagtgggcaaacccctcgttcgttggggaacttgactaagc
ttgtgatactgattttgtccttcaacaacctcacaggcagagttccaactgaaattggcaatttaggagcactgcaaaaccttgacctcaacaacaatcag
ttggagggtgagctgccagcaacacttacattgctcaaagaacttaatgatctttccttgagcatcaacaagttcaccggagctgtcccaaacgcatgtg
tttaa
>g37426.t1
atgggtgtacatgataatgaccttgaggagatcggaaagctgctaccccgccttaagttcctctccctacgaggatgcacacagatcaaacaactgccg
gattcatttggtggcttgaggcagctgcagactttggatgtcaggcatacatccatagtcatgctgccaccgtccatcttccagatagagaagttgcagta
catccgtgctggaacttctgcatggcatgcagatgatgacatgctcaaaatcgtaccagtggtatatgagaaccagacatcagcactgccactgttaga
agacagtgacagtgacggcagctcagcgccaccaatagcaacacagtcgcatgcccattttgatgcaactccagccccatctggaatggatggtgatc
atacatcatcagcacaacagggtggcaatggcagcttaccacagcaaggccgtgatgaaactgccccaatactgccagcggggaatagggacggtcg
agcatcaccaccgcaagaagatcgtggtcatgccagcacacatggccaagcagatcctccacaagcagcacaaaacaacaagacgtggtcatcctgg
ttggggttctcaaagaagcccaagccgcatgctaacgctcataatgaaggtgttgaacttcctgctgctgcagggattggaaagctgacggccttacata

ctattggtgttatcaatgtcaatggcgcaggttgtagtaaagccatcctggaggagctcaaaaaacttacacaattgcgcaagctcggggcgtctggcat
caataggggaaacatcgaggatttcatttctgccatttcaaaacaccgccatctggagtcgttgtcactgcaacttgatgaagatagtttcctggatggca
tctccctgccaaagccagtagcagagactctgaagagccttaagctatatgggaacatgaagaaattgcctgtcagcttgatcaagcaacttggcaatc
tcaagaagttgaagcttgagttaaccatatcagggcaagacgacatcaatgctctttcagatttgccacgcatcgaagttgaaaaaaacgaaatcctaa
ctcgtctttgtgtcaagctgactcaaggtggcaaggtcagttttggatatgatgattcgttattttttaacttcagaagccttgcagttcttgagattgattgc
atctccagttcagaggttacttttaaatnggagaatgctgcaaatttattttccgagcttgtcaacctaagcataatccagcaatcaagaagcaagctcat
aagccaagtcaacactttttccatgaatacatcatctcacagcccatggaggataaccttgtgtttgcattggaagggagttgcagcccaaacacccaa
cgtgcaggacggcacctcaccataaggagctcttgggacggagatatcaatgtgttcatgagcattgatttctcacggctacgatcattgacagtgcttg
gggagtggagttcattcttcatctctaacaagatcatgatgagagtgcttcgggtgcttgatttggagagcaatatgggtgtacatgataatgaccttgag
gagatcggaaagctgctaccccgccttaagttcctctccctacgaggatgcacacagatcaaacaactgccggattcatttggtggcttgaggcagctgc
agactttggatgtcaggcatacatccatagtcatgctgccaccgtccatcttccagatagagaagttgcagtacatccgtgctggaacttctgcatggcat
gcagatgatgacatgctcaaaatcgtaccagtggtatatgagaaccagacatcagcactgccactgttagaagactgtgacagtgacggcagctcagc
gccaccaatagcaacaccgtcgcatgcccatattgatgcaactccagccccatctggaatggatggtgatcatacatcatcagcacaacagggtggca
atggcagcttaccacagcaaggccgtgatgaaactgccccaatactgccagcggggaatagggacggtcgagcatcaccaccgcaagaagatcgtgg
tcatgccagcacacatggccaagcagatcctccacaagcagcacaaaacaacaagacgtggtcatcctggttggggttctcaaagaagcccaagccg
catgctaacgctcataatgaaggtgttgaacttcctgctgctgcagggattggaaagctgacggccttacatactattggtgttatcaatgtcaatggcgc
aggttgtagtaaagccatcctggaggagctcaaaaaacttacacaattgcgcaagctcggggcgtctggcatcaataggggaaacatcgaggatttca
tttctgccatttcaaaacaccgccatctggagtcgttgtcactgcaacttgatgaagatagtttcctggatggcatctccctgccaaagccagtagcagag
actctgaagagccttaagctatatgggaacatgaagaaattgcctgtcagcttgatcaagcaacttggcaatctcaagaagttgaagcttgagttaacca
tatcagggcaagacgacatcaatgctctttcagatttgccacgcatcgaagttgaaaaaaacgaaatcctaactcgtctttgtgtcaagctgactcaagg
tggcaaggtcagttttggatatgatgattcgttattttttaacttcagaagccttgcagttcttgagattgattgcatctccagttcagaggttacttttaaat
actggatgattaatgttgagctactgaaggtttgttgctctaataagaaggcgtcgctgaccttcttcgagctggataaactcttaagtcttaaggaagtct
ggctcatgggttcatttgatgacaaattcaacaaagaaatgcagcagcagcttgccaagcatataaataagcctgtcttgaagcaggtagtgaagcaac
gttcctcctga
>g38091.t1
atggaggccgccggccgcctcacgctcgccgcgtcgtcgctcctcctcctcctcgtcctctcctccttcgccagccatggagcctgggccttatccga
cttggaggcctccgacctcgcgcggcggcagcgtcacctcctctccctccgctccccgtccggcgacctccccgacgacttcgagttcgacatccgcgtc
gacgtgaccttcgcgaacgcgcgcctccgccgcgcctacatcgccctccagtccttccgccgcgccgtccactccgaccccaagaacttcacggccaac
tggcgcggcgccgacgtgtgcgcctacttcggcgtgacctgcgcggcggcgctcgacgacccgtcgctcaccgtggtcgccggcgtcgacctcaacgg
cggcgacatcgcggcgtacctgcccccgagctgggcctcctcgtcgacgccgcgctcttccacgccaactccaaccgcctctgcggcgtcgtcccgga
gacgttcgcgaagctcgcgttgatgcacgagctcgacctcagcaacaaccgcctcgtggggcccttccccgacgtcgtgttgaagatgcccgtgctcaa
gtacctcgacctgcggtacaacgagttcgaaggcaccgtcccgcgggggctcttcgagaaggacctcgacgcgatcttcctcaacagtaaccggttcgc
cgggtccctgccggagaatttcggcaactccacagcctcggtggtcgtgctcgcggacaacgcgttcgtggggtgtatcccgaggagcgtgggccggat
gggcgccacgctggaccagctcatgctcaaggggaaccgcctcgacgggtgcatcccgaacgaactcgctcatctccgcaacaccacggtggtggac
gtgagcgagaacctcctcgtgggcacgctgcccgaccggctccacgagatggccgggctccagcagctggacgtgtccaggaaccggctcgcgggcg
aggtgggcgacaaggtctgcacgattcccgcgctcgtcaacttcagcttcgccgataacttcttcacccacgaggcgccggcgtgcgtgccgtcgtcca
acgcgcacgtcaccctcgacgacgcggggaactgcatccgaggagaaaggccggggcagaagacacccgtggagtgcgcgcccgtgttatcgcacc
cggtggactgccggaccaacgtgtgttctggtgggggtggtggtggtcccgtaccgccggtgccgaagaagaaggagcctgaggaggcggacccgcc
ggtggaggctccggagcccgtacctcccactagagccgatgaggtcgccgccgccactagtgaaggctccatcaccgatggcgccggtcagttccccg
ccaccaccggtgaaggctccaccacctccggcaccggtcagctcacctcctccaccggtgaaatcgcctccaccaccagctccagtgacctctcctcctc
caccaccggcgccagtgagctcacctccccctccggtaaaatcacctccaccaccagcaccagtgagctctcctcctccaccggtgaaatcgcccccac
cgccaccactggtgcgctcaccaccaccgcctgtaagatcacctcctccaacgagttcaccgccgccaccggtaaaatctccacctccaccgactccag
tgagctcaccacctcctccggaaaaatctccgccaccaccggctccggtgagctctccacctcctcctgtaaaatcaccgccaccgccagcaccaccgg
tgacctctacgcctcctcctctaaaatcaccgccaccaccagcaccggtgagctctccacccccaccagtaaaatccccaccacccccatctccggtgag
ctcaccgcctccaccacccgtcccatcacctccgccaccggcaccgtcgttgcctccaccaccggcagaggaggtcatcctgccaccgatcatatcaca
aaattacgcgtcaccaccgccgccagtgttccaaggatactaagtggacaggacacaccagcaaggtcaatcagtgggaacgacgacggatag
>g38221.t1
atgcctagtgtcgttcatgaaccctctcaacttcctcttgtgtcatccttgctcgtcttctccttcatgtgcctgcagcttcctcatggcgccctgacgtgcgct
ctggctgaccggattgcggtctcagccttctctaagcatcttgacatcagtgccatcaatggatggccaagagcagaaaactactccagcgactgttgtc
gatggccaggcgtcagttgcagctgggtcggagcaaaggagctccggattgtcagcctggacctcgcttggcatggcctcaccggcgtcctaacgagc
gggctggcaggcctggacgagctgcgcgtcctcaacctctccgggaactcgttctatggggccgtgccgtccgagctcctgcgcatgcaaaatcttagg
gtgcttgatctgagccagaacggctttaccggcgagctcggcggagacttgccgccggcgccagcacccgacgcatcggggatccgaatcctcgatgt
ctcgttcaatagcctgtccaccctacatgccggaatattcgtccatctcccggaactgcgcagtttctcgggtgaatccaaccttctcactggaacacttcc
taccccgctttcctccggctctgagctggagtgcctgaacatggccaacaactccctgtacggcaatcttggtttgttcgacttctcccggctgccgaggct
ccgtgccctccatctcgactggaacaagttgagcggcaaccttccagagaccctgtcgcgctgccgggaacttagagttgtgaaccttcgccggaacag
cttctccgggacagtcccgtcctccttccaacagcttcagtcactggctttttcgacatcggcgacaacaacatcaccggcatagtgcaggcgctccggg
tgctccaggagtgccgtgcccttgtcgtccttatcctcaccaccaacttccacggcgaagcgatgcccgacgacgatgctggtgagggcaaccgagggt
tcccaagcatgcgcctcctcggcatcgccaactgcgacctcaccggcgccgtaccaacgtggctgcttgctagcgcacggttgagcgtgctggacctgt
catggaacaggttgagcggccgcttgccgccatggctcggcaacttcgacgcgctgttccggatagacctctcgggcaactcgctgaccggggacatcc

cggtctccctggcattcctgaaatcgcttgcaagcaacgcatcggttgtccaactgtcactgtccgacgagtacggtgtaaggctgtacaattggcacgt
cgaacgtggccagctctggtacaacagttacatcccgccatcactggacctgagccggaacggcctcaccggcacgatcccggaggagcttggcaacc
tccgggctctgaacctgctgaacctgagctggaattcgttgtccgagatgatccctagttcgttggcgtctctgacatccttgcagacgctggacctgtca
aacaacgagctcactggagaaattccttggtcgctcgcgatgctcacctttctctcgtgcttcgatgtctcctacaaccggctgaacggcaccatcccgga
ccgtggccagttctccacgttcccctgctccaactttgcaggcaaccctgggctttatgtcaagtactgcgacggccaagttgatgcaggaacaccgggg
accgacggggacttgtcttctatgattgtgatcgaggatttgtggctgccattttggcttggaatggtcaccggcgtcttggccacgctgttcgctcatgtg
ctagcgctagagcatgcttgcaacagctga
>g38325.t1
atggaaacttcatcagaggcctcgaagcaggcagatggtaaaccggtgaagccgcggcaagcggtttccaccggcaggttcgcgttggggactgcttc
atcagacaagaagcggactgagggcagtgcttcagtgggtgtgtcccggtcttccttgacgaagtctacttcgtcggtgaatgccggttcggtgcagag
aagaagtagcactggaagtgctggaaagcagcagtacaatggggtctcagatgttggtaatgctaagaaggccagtcctacgatgtcggatgggacg
aagaagagtaagccgctgtcagcctccactgttagttcaaagccttcactggaaaagaaaacatctttgtcagagagaaccaaggtggatgtgacgaa
gaaacctgctgctaaggcatcaccaactattacgctgaagaaggtccagtccaaaacagagagctccaatggaagcagtgccagcgctaggagagtg
gcctcgaacacctctttgccctcaccgcgttcatcttctgtttcaagtaatgtttcaaagaagttgggttctctggcatcatcagcagacaaagggtctgtg
tctagtcggaggaagagctcaacggctgacagtcgcgattccaggttcatgatgctgccacaggtggatttcaaagccagcgatgaagtgagactgga
cgcaagaggtcacagggttcgtagtctgaagcagttgagattaacaaatgctcttgaggttcttgacttgagctttaatgattttaagcttcctgggtttga
ggctcttgaaaattgcaaagtcctccaggttcttgacttgagctttaatgattttaagcttcctgggtttgaggctcttgaaaattgcaaagtcctccagcat
ttgcgtgtcgaggataacccactgctcgagatgccgcatttggaagctgctactattctgcttataggtcctacattgaaaaagttcaatgaccgagatct
gaaccccagggaggctgaggtagcaaagcagtatcctgctcacacagctatttgcattcgtgacggctgggaattttgctctcctgaacttgctcctgatt
caacattttccttcctgcttgagcaatggaagaataagttacctcaagattacacagtgaagaaagcttatgttgactacccgtttgaagaagatttgtgc
cgttgccattttagcttcacaaatcttggtagcaagggcgaggtgtactggcctaagcgtgaggatgccggaagatgtttgaaagttgagtgcactctga
tactgaatgatgccgaatttccacctatttttgctgtatctttacctgtttctccaggaactggatgtccaaaggtcatcaatctggcagtcaatggtgaact
agtggaaggaaacatactcaccggagttcctgaaatcgcctggtgtggtgggacaccaggcaaaggagttgcaagttggttaaggcggagatggaac
gataacgctgtagtaatagatggagccgacaagatggaatatcagctgacaattgatgatatcaattcaagtttggtgtttatgtacacccctgttacaa
atgaaggtgtcaagggagagccacattgtacaatgacagattttgtgaaggctgcaacaccatcagtcagtgatgtacatgtcctgggagatgttgttg
aagataacattatcaaagggaacgggaagtacttcggtggcaaagaggggctaagcaaactccagtggtttagagaaaaggaaaatggtgaatatct
tctagtgttgtcaaatagcatggaatatacactaaccatggatgacgtggggaggcacctcaaatttgtatacacccccataaatcttgaaggtctggaa
ggtgaacctgcttgcgcaataacagatgtggttaaaaaagcccctccaaaagtcttcaacttaaagatttctggggaagcaagggaaggaagcaaaat
atcagtgagtgctactgttacaggtggaactgaagggtccagcagggttcagtggtacaaggcatcttcctctgaatttgagaacaagcatgagcttga
agccctgagtacatcaaaagtttctaagacattccgcattcctcttggtgctgttggacactatattgttgcaaaatttactcctgtggcacctgatggtga
aattggcgaaccagcatatgctctatccgataatattgttgaaaaatgtctagatcttagtcttgctgatgttgattgccggattgaacttgtgtatacacct
gtacgagatgatggagtgcaggggctacccagacgtgtcatttctgatacaattgtctctggcgctgggaaatatatatggtttcgaaataaagagaagt
ttgataacttggaatatgatttggtagctgcatcttcagaagttgtcggtgaaaccttaaagtacaagcctccattggatgatgttggctcctatctgatttt
tgtttgggttcctacacgatgtgatggaaagactggaaatccattgatgactatcactgatgccccagtcatggcagcgtttccatctgttttggatgttca
cttggagcagaaaagttcagatgtctattgtggagtaggcatctattatggtggatatgagggatcaagcttatataggtggtacagggaatctaatgat
ggaaccaggcttcatatagatggtgctaattcagatacacatgaagtgactgatgcagattacagctgccgtctagtgtttggctatactcctgttcgttca
gatgggattgttggtgaagaaaagctgtctgaaccatctagtgttatttttcctgaactactcaaaatcgagacacttattttcaagggcaatcaggttga
gagagaaacactgactgctgttgaacaaattccaagcagtgagattcaacaacatatatggaacaactataagaaagagatgaaatatcaatggtttg
tttccactggatcaggagtggatcagtccctttgaaccattggctacccagtgttctcgctcgtacaaagtacgcttcgaggatgttggtcattgtgtgaaat
gtgaatgctttgtcactgacatatttgggcgatccagtgaagtggtatcagctgtgactgctcccatcttaccaggaatacctaaaatttcaaaactggaa
attgaaggaagaggtttccatactaacctgtatgctgttgggggtacctacagtggaggcaaagaaggcaagagtaaaatccagtggcttagatcgat
ggttggaagccctgatcttatctccattcctgagcctgaaatttaccgggaagttaaacaaaaacttgatgatggtttagttaaatttgaggttctgtgtga
caaggatcgtacacccaaaaaggcacaagtaatgggacatttggaacggcggattttggaagtcaataaaaagagaattaaggttgtaaaacctgga
tccaagacatctttcccaactactgaagtgcgaggaacatatgctccgcccttccatgtcgagctgtatcacaacgatcagcatcgcttcaagattgtcgt
tgatggagacaatgaagttgatttaatggtgcaaacccggcatatgcgagatgtcatcattctcgtgatcagaggtctcgcccagaaattcaacagcacc
tccctcaacacgcttctcaagatcgaagcatga
>g38456.t1
atggcgtttcgctttgaagcagcagcaatactccttgctggttttctagcacttgcaacactggcaagctgcaatactgaaggtgacattctgtatgcgca
aaggatggagtggtatgacccaaataatgtgttggagagttgggatcctaccctcgtcaatccctgtacctggtttcatgtcacctgcaataatgataact
ccgtcatccgtgtggatttgggcaatgcaggcctgggggggttctctaattccagatctgggaagactgaagaaccttcagtacctgtaa
>g38602.t1
atggctctccagctccttcccatcgtgatactggccgtcgtgctgccggcggctgcccagagcaacgacaccgacctcgacgcactgctcgagttcaag
gcacagctctcggaccctcttggcgttctgcgcggcaactggacgcccgacacgtccttctgcaactggctgggcgtatcgtgcagccgacgccgtcag
cgcgtcacggccctggatctaacaaacactcccctccgtgggagcatttctccccatatcggtaacctctctttcctctctgtcctgatcctcgccaacgcc
aatatcacaggatcaattccagctgagcttggaaggctgcgccgtctcaaggtcctcgctcttcctgaaaacagcctgttaggtttcatccctccaaccat
cggaaacctcacggagctccagcaacttgttctgtacaagaacagtctatcagggcagatcccacctgagctacaaaacctgcgaaatctcaagtatttt
gatctgcatgggaatttcttaattggagaaataccacatgaactcttcaacaacacgcctcacttgagccatctgatccttgccaacaacagcctgttcgg
acatataccggaagtgtaccagaagggggtgttttcacaaacatcagtttgcagtccttgctggggaatgacgcactttgtggtgctccacgcctaa
>g38779.t1

atgcaggccgctatcagtgcggcgatctgggtggtgggcaaggcgctgtcccctgtggttgatggcttcctggaggcgtgggcggcaagcgtcgggctg
ggcccaaacgtggaggagctcaagctgcagctgctctacgcgcaggccatgctcaacaacacccggggcaaggagatcgagaacccggccctcaac
gagctgctgcacaagctgcgggacacggcgtacaacgccgacgacgtgctcgacgagctcgagtacttccgcatccaggacgcgctcctcggcaccta
ccacgccgccaccgaggtcgatgccgccgccggtgggtgcgtctgtggccaccttctaaatgctgggcaggcttgcagagctgtttctactagtaagctc
aatctctgctgctcggcctcgcgcgacaccgcaagttcagatgaagtactgatgatcaaccaggaagaccaggtggatggcggtggatgcatcccgaa
ggtcacttctgcagccaggaacactgccctatcgtcagttggtaaacgcctcttatgttgctttcctcaatctagatctgctaatgatggtgctcagtctggc
atgactgaggatgaggatcctgacatgccagaaaatggaccacgactttactgttgtgcatggacatccaaggcacaacagagaaaacgtgatgtcgt
ggaaacaccacggctcaagtttgatagggtggaaatatctaggaagatgaaagatatcgtagagcagctcaaaccaatctgtggcatggtctccacca
ttctgaacctagagctcttgggctccaaccttaagactaccagggatattgctatgagtaatcgacccaaaacatcaccgcaaattatagagcccaaatt
atatgggagggatgaccagttcaagagcattgtagagagaattaccgatgatcacatcaccggagcccttactgtgcccatgtgcagcttcttcaggtcc
tcccttaccaccatagtctttcattggaatgatgagctggaacgcttttcaatggagcaagaggaggcccttcagctcctcacctccctcaagcaccttaa
acttcacgggtgctccaagctgcagtgcctccctgcgagcttggaaagacttcccaacctcaagaaactagagatcatctactgtgaagccatccagtca
ctacccatggaaggtttcccaagttctctggaagttttacatatcatcttatgcagaggcatctcgtccctgccaaaggcgggcctcccaagttcattaca
agaattatacatcgaagactgcccagccatccggttgcgcttgccggctgctgctagccaccgggaaacgtgctactgctccttgccgccgggaaagctt
cactgcctcctcggcgttgtcgagctgctcaccacggagaaggtgttgtgcgacgtcccggaggacgacgtgcacgccagtgtcacgagtgggtttcat
ggccgtgcctatgccgactacatggtggacaccaccggccgcccctactggcacaaccacaacatcgcgatcccgcaggcgtttctttacaaggaacta
ttagatcaaagaacgttatttatctga
>g39849.t1
atgaagcgggctggcggcggcggcaggctcctcttggctcttggcctgctcctgctcttgctccatggctgctcggggcaggcggaggaggacgacggc
tccgccgcctcgggagcgacggcgcccatggacgagaaggagaagcgggccctgttcgcggccatcgagagcttcgtcgggaaagggtggaacggc
tccgggctctaccccgacccatgcggctggtctcctatccagggtgtgtcatgtgacatcttcaatggcgtgtggtacccgacggtgatcagcatcggtcc
agtgctggacaactcgctgcagtgcgccccagacgcccggttcagcccgcagctgttggacctgcggcacctcaagtccctctccttgtacagctgcttc
ccggcgagcaacccgacctccatcccgtccacgagctgggagaagctcgccggcagcctggagacgctcgagttccgcacgaacccgggcctggcgg
gcccgattccgccctccctgggccggctcgccagcctgcagtccctcgtcctcgtcgagaacaacctcacggggcccgtgccgcccgagctgggcttgc
tgctgcggctgaagcggctcgtgctgtccgggaacgggttgtcgggtccgttacccgcggcgctcggtaacgcccgccacgacgcgctgctgatcctgg
acgccagcaacaactatctaaccgggtctctcccgtcttcgctaggtggcttcaccgggctgctcaagctcgacctcagcaacaaccggctgcaaggga
gcatcccggccgagctcgccgagctgaagagcctcacgctgctcgacctcaggaacaacagcctcaccggcggcgtgccgcggttcgtgcaggggat
ggcggcgctgcaggacctgctgctctcgaacaacccgttgttgggcgggagcctggtggagtccgggtgggagaaactgggtagtaacctggccaccc
tcgacctgtccaacgttggtctggtcgggacgattccggagtccatggcggcgctcaccgggctgcggttcctcgcgcttgatcacaaccggctcaccgg
gaaggtgccggcgaagctcgccgagctgcccaacatcggcgcgctgtatctgaacgggaacaacctcaccgggacgctggatttcgcgccggggttct
accagaaaatgggcaggaggttcgcgtcctgggacaacccggggctgtgctacgacctcgccgccgtcgacgccgcgaacgcgccgtcgggagtggt
ggtgtgcaaggatttgcaggagcccagtgttggtccgacggataataaggacggtgggaagaagcctgaggccagctccagcctcgtggcctcttcttc
ttgttcgattaccaccagtaaggtcgtcaatggattttggcacctggtgatggttcagggaatatggttggctgctggacgatcgcttgggtcgttgatgct
gctatag
>g39865.t1
atggcgctgctgccgtggtggtggtgggcggcggtgctgctggttcttgcggcggcggcggcggcggctgaggcaggagcagcagatggaggcg
acggggatgggcaggcgctgatggcgttgaaggccgagttcgggaacgcggccaacgcgctggtggactgggacggcggcagcgaccactgcgcct
ggcgcggcgtcgcctgcaacaccgcctccttcgccgtcgtcgccctgaacctgtcaaatctaaacctcggaggggagatctcgccagctataggggagc
tcaagagcctacagtccgtggatttgtctttcaatttgctgtatggagacatccccttctccatctccaagctcaagcagctcgaggaactgattttgaaga
ataaccagctcactggacccattccttccacattgtcccagattccaaatctcaagatcttaggcttgaggggtaattcactgactggaatactatcacctg
acatgtgccagcttactggcctgtggtactttgatgtaaggggaaacaatctcactggaaccattccagagggcattgggaactgcactagctttgagat
tctggacatttcatacaaccaaatatcaggagaaataccttacaatattggttaccttcaagtagccacactgtcacttcaaggaaatagactgactgga
aaaatcccagaagtgattggcctcatgcaggctcttgctgtcctagacctgagtgagaatgagctggttgggccaattcctccgatactaggcaacctat
cctacacaggcaaactaaatcttgccaacaacaaccttgaaggtcatattcctgccaacatcagttcttgcactgcactgaacaaattcaatgtgtatggc
aataaattaaatggatctatccctgtgggtttccagaagctggagagtttgacattcctgaacctttcttcaaataatttcaaaggtcaaattccttctgaa
cttggtcacatcgtcaacttggatactctggatctttcctacaatgaattctatggaccagttcctcctactattggtgatcttgagcatctccttgaattgaa
tttgagtaaaaaccatcttactgggtctgtgcctgctgaatttggaaacttgaggagcgtccaagtaatgatccttaataacaacaatttggccggagag
atccctgctcagttggctaactgcttcagcctaaataccttgaatttgtcaaacaacaacttatctggacgcattccatctggaaagaatttctcaaaattt
ccaatggatagctttgtgggtaatccaatgttacatgtgtactgccaaaactctgcctgtgggcgttctcatggaactaaagttatttctcggattgcttgc
atcatcttaggcttcataatattgctctgccttatgctattggcaatatacaaaacaaaccggccagaaccacctgagaaaggatctgacaagccagtgc
aagggccaccaaagctagtaagccttcaaatggacatggctatccatacttatgaagatattatgaggttgactgagaatttgagtgagaagtacatcat
tggttatggtgcgtcaagtaccgtgtacaagtgcctcctgaaaagtggcaaggccattgcagtcaagcggctttacagtcagtataaccacagccttcgt
ccatcaaagaaggtgaagcttgattgggacacgcggctaaagattgcagttggtgctgcacaagggctggcatatctccaccacgactgcaacccacg
cattgttcacagggatgtcaagtcctctaacatccttctcgatgagaactttgaagctcatctctcagattttggtatcgccaaatgtgtcccggctgccaa
gtcccatgcgtccacttatgttctaggaaccatcggctacattgatccagagtatgcccggacgtcaaggcttaatgagaaatcagatgtctacagctttg
gcattgttcttttggagttgcttacagggaagaaggctgtcgacaacgaatcaaacttgcatcaattgatactctcaaaagctgatgacaacacagtgat
ggaggctgtggactcggaggtgtcagtgacttgcacagacatgaacctcgtccggaaggccttccagctcgctctgctgtgcaccaagaggcacccctc
tgaccggccaaccatgcatgaggtcgctagggttcttctctccctcctgccggcccctcctgtgaagccaaccacaacaaagacggttgactacactcgc

ttcctcgcgataacagcagctgaaatgtcgcacgacatatctgacatcggagacaacagctcctctgacgagcaatggttcgtgaagttcggcgaggtc
atatccaagcacacaatatcatga
>g40158.t1
atggcctccggagagggcgccatcgatccgcaggctgcggcctctggcgctgaggcagacgccgagatggagacggaggagcaggagggaccggtt
gtgtgcctggacctgacgagctaccagctgcacgatctgaacgaggtggagattccacccacgctggaagagctcgacctcaccgccaaccgcctctc
ctccgtcgacccccgcataggccgcctcaccggactccgcaagctctccttccgccagaatctcctggatgacgccgcagtcgcaccgctctcgtcctgg
gaaacaattgctggtctgcaggagttggtccttcgagataacaaacttacaagaattcctgatgccagcatcttcaagggtcttcttgtgttcgatgtatct
ttcaatgagatatcttccttgagtgggctatccaaggtttccagcacactgaaagaactttatgtttcaaaaaatgaagttggaaagatggaagaacttg
aacatttccatgcgctagaaattcttgaacttggcagcaacagattaagggactgtattgcattagaagaactgtacctcagccataatggaatccaaaa
gatggaagggctctctaccttgcagaacctccgtgttctagatgtttcatctaacaagataacagctatagaaaatattgaaagcctaaccaggttggag
gacctgtggttgaatgacaaccaaataccgtcactcgatgggatagaggctgcattagctggttcccgggagacgctgacaacaatttatcttgagcgc
aatccttgtgcaaaaactccaaactactcagccacattgaagagaacatttccaaatcttgagcaaattgattcagatatcatagcatga
>g40555.t1
atgtcaacgtncgcgtggggcgacccgccggtgctgctggcatggaacgccacggcatcggccgcacactgcgggtggcccttcgtccggtgcaacag
agccgggcgcgtctccggcctctacctcgctcatgccagagtctccggcccgttcccagatgccatcgccagcctctcccgcctcagacacctcgacctc
cggtacaacaacatcgctggcgtgttcccgacctcgctgtaccgctgccgttctctgaagtacctcagcctgtcatggaacaacttcatcggggagctcc
cggctgacatcggcgttggtctttcagcgagcctgaccacgctgtccctcagcagaaacaggttcaacggcaccatacagccttcgctctccaggctcca
taaccttcggtttctcaggctgtccaacaacttcttcgtcggcaccatcccagatgccatcgccagcctctcccgcctcagacacctcgacctccggaaca
acaacatcgctggcgtgttcccgacctcgctgtaccgctgccgttctctggagtacctcgacctgtccatgaaccacttcgccggggagctcccggctga
catcggccttggtctttcagcgagcctgaccacgctgtacctcagcagaaacaggttcaacggcaccatcccagcagagctcggcgagctgacaagcct
ccaggacctgtaccttgggaataattcattcgctgctggcgagcttccgccatcgttcaggaagctgaccaacctgactgcccttggagtgtggagctgc
aaccttgtcggagattttccgacctttctagtggagatgccggagctggagattctggacctctccaacaactcgttgaccggaagcatacctccgggga
tttggagactcaacaagctgcaagttttgtttttgtccgagaacaacttctctggtgaaataccggcaagcatcgcccggttgtcaacactgattgatttgg
agctggacaacaacaggtttaatggaacacttccaccagagctcgggaagcactctttggacctggctagtgtttcagtggataacaatgacctcaccg
gtgcaattccagagtggttatgcaatggaggtaatctctattccttctctgcctcgagcaaccgattgaacggctccataccagcaggactcgccaactgc
cccagtctttcagaactgaggctggacaataaccagttgtcaggacaagtgccctag
>g40910.t1
atggcggctcgcgcgagcttcatcgccgtcgtagcgctccttgccctggtcgctggcgccgcgcgggcgtccaacgaggagggagacgcactctacgc
gctgcgccagcgccttacggaccccaacggcgtgctgcagagctgggatcccaccctcgtgaacccctgcacctggttccatgtcacctgtgaccaggc
cagccgcgtggtccgcctggatttaggcaactccaacatctctggctccattggccctgagctcggtcgccttgtgaacctcaaatacctggagctgtacc
ggaacaatctcattggcgatatcccacaagaactgggcaacctaaagaatttgatcagcttggatttgtatgctaacaaactcactggaggaatcccca
agtcactttccaagctcaactcactgagattcatgcgcttgaataacaacaagcttacaggatcgattccgagggagtttgctaaactctccaacttgaa
agtcattgatttgtctaacaatgacctctgtggaacaattcctgttgatggccctttctcaaccttccctcttcaagggtatgcctga
>g41013.t1
atggagttcagggtggccaacaaccggctcacgggcgacgcgtcgttcctgttcgggcggcacaagacggtgtccgggaccatcgatttgtccgggaa
caagttccggttcaacctgacaggagtggagatgcccatgcatcttgggttcctgaacctgagccacaaccggatctatggcggcgtgcccgtgtcgctg
agggagacgagggtctcggtgctggacctcagctataacgagctctgcggggagatacccaccggcgggcacatggtgcagttcaaggngtcgctga
gggagacgagggtctcggtgctggacctcagctataacgagctctgcggggagatacccaccggcgggcacatggtgcagttcaaggcggcggcgta
cgaacacaacaaatgcctctgtggaacgccgctgccaccgtgcaccaacggccgctga
>g41348.t1
atggagcgccatggctacaactacaagcaccacggttgctccgtcgtcctcgtcgtttcccttctctttgcattgccgtcttgggcttgggccgccacggga
aacgcagtagcagacagtgaccaccgcgccctcacgcagtttcggtccctcgtcacggacgacccacggggggccttggcgtcatggccggcggca
acgcgtccgggccgtgcgggtggcacggcgtgacgtgcggggcccgcgggcgccgccgcggccgcgtcacggcgctggatctccggggtctgggcct
ggccggctccgtcgggtcgtccctctccggcctcacctacctccggcggctcgacctcgccgagaaccgcctcagcggcggcgtgccgtcgccgctgcc
gccctccctcgagcacctcaacctcagccacaacgcgctgcacggcaccgtgccgcctgagctggggtcgctgcgccgccttcagatgttcaatctcgtc
cacaacaatctcaccggaacaatccccgcctcggttggcaacctcacctccctcattgtcctcagcctcaccaacaacaacctcgccggcgccatccccg
gcgccctcggggaaaggttatgctcgaagcaaatgatgatgcggattgggggttcatggatagcctga
>g41400.t1
atggcgtcgagcaaaccgctctcccccagctcgttgcttcctctgctttccctgctcctcctcgtcctctgctccaccaacatcgcctcctcgtccgccgcca
cggactgcgacccggacgaccgcgccgcgctgctccgcgtcaagtcccagctcggcgacccagtgcggctgtcctcgtggcagccgtccaccaactgc
tgcgcgtgggagcccgccgtcttctgcaacgccgcgggccgcgtcacgggcctggccctctacgccctcagcgagctcaaggcgccggtgccggcggc
cctcggcgacctcaccgcgctcgagatcctccagatcgagtccgtgccgggcctgtcgggcccaatcccaccctccttctccaacctcacgcgcctccgc
gacctcgacatcaacggcacctccatctccggcccggtccccgccttcctctccttcgccaacctcagcacgctcgtcatcgccaacagcaagctcgcgg
gcccgatcccggcggccctggccgccctccctaacctccggtacctcgacctcagcggcaacatgctcagcggcgccatcccgcccgggctgctgcacg
ggagcttcaggttcctcgtcctctccaacaaccggctcacggggaagatacccccgacgactacagcgacgtcgacaccgtcgacctctcgcgcaacc
ggctcaccggcgacccgacgccgttcctgttcgggatcaccaggcccgccgtcaagatcgacctgtcgtggaacgagctggagttcgacatgaccggg
gtgtcgtttccgcaccacctccggttcctggacctcagccacaacaagatcaccgggagcgtggccaagtcgctgatggacgtcaacctcgagcacttc
gacgtcagctacaacgagctctgcggcgagataccggcggggaggttgatggcggtgcacgggccggactgctacgcgcagaacaagtgcctgtgcg
gcacgccgctgcctccctgcgacacagccatgtag

>g41512.t1
atggccgctgccgcccgcctcgtccttgccctcgcgggcctcctcctcgccgcggccgcggcgcccaccagcgcgctcaccgacgacgtgctcgcgctg
gtggtcttcaagacgggcgtctcggacccgtcaggccgcctcgccgcgtggactgaggacgacgaccgcccctgcgcgtggcctggcgtcggctgcga
cccgcgcacgggccgcgtcacctcgctctccctccccgactcgtcgctctcgggccgcctcccccgcgcgctgctccgcctcgacgcgctcgcctccctct
ccctaccccgcaacaacctctcgggccccgtgcttcccaacctgctcgtggcgctcccgcgcctccgctccctcgacctctcctccaaccgcctcgccgcg
cccgtccccgacgagctcttcgcgcaatgccgctccgtccgcgcaatctccctcgcccacaaccagttctcgggatacatcccacccgccgtcgcctcct
gctcctccctcgtgtcactcaacctctcctccaaccgcctcgccgggcccatccccgacgggctctggtcgcttccttcgctccggtcgctcgatctctccg
gcaacgagctatccggaagcgtgcctggcgggttccccaggggcagctcgctgcgggagttggacttgagccgtaacctccttgcttgggagataccg
gcggacatcggccaggcggcgctgcttaagtcccttgacttgggccacaatttgttcaccggtgtcctgccggactcgctccggaggcttaccgggctgc
ggtctctcaacgccggcggcaatgcgctcacgggggacctgcccgcgtggatcggggaaatgtgggcgctcgagcggctcgacttatcgggcaaccg
cttcacgggcgccatcccgtacaccgtcgcgaactgcaagaaccttgtggaggtcgacctcagcagcaacgcgctcaccggcgagcttccctggtgggt
attcggcctgccgctgcagcgcgtctcggtcgccgggaacaagctgaacgggtgggtcaaggtgcccgacgacgcggcgattgcgctacgggtgctgg
acctctcgagcaacgggttctccggcgagatcccgccgcgcatcactgccttcgccggtttgcagtctctgaatctgtcgtcgaattccttctcgggccgtc
tgccggccggcattggcgggatgaggctgctcgaggtgctcgacgtcagctccaaccggctcgacggagccgtgccgtcggagattggcggcgccgtg
gcgctccgggacctgcggatggggaggaactcgctcaccggacacatcccggcccagattgggaactgcagctccctcattgccctggatttgtcgcgc
aatgggctcacaggttcaattcctcacaccatgggtaacctgaccagcctccaagtggcgaatctttctcaaaacaagctgaatggtacattaccggtgg
agctttctaatctgctcagcctccacatctttgacgtctcccacaacatgctgtcaggggacctgccaaatagccgcttcttcaaccacatccctcagccct
tcattgcagaaaatgctggtctctgtagctcgcggaggaacgattcatgcagcacagttatgccgaagccgattgtgctgaaccccaactcctcgtccaa
cccctcttcgctggacactcccacggcccctagcaacatgcatcacaagaaaatgatactgagcatatctactctcattgccattgccggtgctgctgcta
ttgcagttggagtgatcacaatatctgtgctcaaccgccgtgtacgtgcccgggcatcagctccccgctcagctcctgctattgcactatctgatgattatt
ttagccaatccccagagaataatgctacctctgggaagcttgtcatgtttggtaaaggtagccctgagttcagctccggtggacatgccttgttgaataag
gattgcgagcttggacgtggaggctttggtgcagtctacaagacagtgctgagagatggacagccggtagccatcaagaagctcactgtctcaagcct
ggtcaagtctaaggatgattttgagcgacaagtcaagttgcttagcaaggtgcggcaccgcaatgttgtgtcactcagaggattctactggacttcatca
ctgcagcttctcatctatgattacctgcctggagggaatttgcataagcacctgcacgagtgcactgaagataactcactctcttggatggagaggtttga
catcatcctcggtgttgcaaggggggttgacatacctgcaccagcatggtatcgtccactacaatctcaagtccagcaatgtgctgttggacagcaatggt
gagcccagggttggtgactatggtcttgccaagctgctgccaatgctagaccgctatgtcctaagcagtaaaattcagagtgcacttgggtacatggcgc
cagaatttgcatgcaagacagtgaagattactgaaaaatgcgatgtatatggctttggggtccttgtgcttgaggtcttgactggcagaagacctgttga
gtacttggaagatgatgttgtggtgctgtgtgatttggtcaggagcgctctggagggaggcagactggaggattgcatggatccacgattatgtggtgag
ttctccatggacgaggccctgccgatcatcaagttgggtctcgtgtgcacctcacaggtgccgtccaatcggcctgacatgggcgaggtggtgaacatac
ttgagctggtgaggagtccacaagatagtgccgaggatgagctggtttga
>g42045.t1
atgcagctcctccgcggctccgcaccggacatggcccgcctcctcttcccggccctcgccgccctctacctcctcctcctcgcggccgtcgccgtcgccga
cgacggggcaacgctgcttgagatcaagaagtcgttccgcaacggcgacagcgcactgcacgattggtccggcgacggcgcgtcgccgggctactgct
cgtggcgcggcgtgctatgcgacaatgtcaccttcgctgttgcggcgctggacttatcctctaacattctagaaggagacataccattttccatatctaag
ctgaagcaccttgagaatttgatcttgaagaacaaccagctggtgggtgtaataccatcgactctctctcaacttccaaatctgaagatattggatttgtc
aaacaatcagcttactggaggaatcccattcaatattggtttcctgcaagtggcaacattatctttacaagggaacaagttctctggccctataccatctgt
gattggactaatgcaagcacttgcagtgctggatctgagtttcaacgagttatctggcactataccatctatactgggcaatttaacatacactgaaaaat
tggaactgaatgacaaccagctgactgggtctattccgcctgatcttggaaagctcacagagttgtttgacttggacctatcatgtaacaagatgtctggt
ttgattccctcagctttcgggaaactagaacatcttttgagacttaacttgagcaataataatctggttggacacattcctgctgagtttggaaacttgagg
agtatcatggagattgatttatccaacaaccacctgggtggcttgattcctcaagaggttggaatgctacaaaatctgatactattaagattagaaagtaa
caatattaccgggaatgtctcttcactcactaactgcttcagtctcaatgccttaaatgtatcgttcaacaaccttgctggtattgtacctacagacaataac
ttttctcgattttcaccggacagcttcttgggtaaccctggactttgtggctattggctcggttcttcatgctactcgtcaagtcacacgcggagatatacctt
tgcctcagcatcaagcattgttcctcccaagcttgtgatcctccacatgaacatgtccctccatggatacgatgatataatgaggatgactgaaaatttga
gtgaaaaatatattataggatatggagcatcgagcacagtctacaaatgcattctgaagaactccaagccagttgcaataaaaaagctttatgctcacta
cccgcagagcttgaaggaatttgagactgaacttgagactgttggtagtatcaaacaccggaatcttgtcagccttcaggggtactccttatcacctactg
ggaatcttctcttctatgattacatggaaaatggcagcctctgggatgttttgcatggttcctccaagaaggagaagcttgattggaaggctcgcctccgg
attgcacttggtgctgctcaaggcctggcttaccttcaccatgaatgcaacccacgaataattcacagggatgtaaaatcaaagaatattcttctagaca
atgacaatgaggcacatattgctgacttcggcatcgccaagagcttaaatgtatcgttcaacaaccttgctggtattgtacctacagacaacaacttttct
cgattttcaccggacagtttcttgggtaaccctggactttgtggctattggctcggttcttcatgctactcgtcaagtcacgcacagagatatacctttgcct
cagcatcaagcattgttcctcccaagcttgtgatcctccacatgaacatgtccctccatggatatgatgatataatgaggatgactgaaaatttgagtgaa
aaatatattataggatatggagcatcgagcacagtctacaagtgtattctgaaaaactccaagccagttgcaataaaaaagctatatgctcactacccg
cagagcttgaaggaatttgagactgaacttgagactgttggtagtatcaaacaccggaatcttgtcagccttcaggggtactccttatcacctactggga
atcttctcttctatgattacatggaaaatggcagcctctgggatgttttgcatggttcctccaagaaggagaagcttgattggaaggctcgcctccggattg
cacttggtgctgctcaaggcctggcttaccttcaccatgaatgcaacccacgaataattcacagggatgtaaaatcaaagaatattcttctagacaatga
caatgaggcacatattgctgacttcggcatcgccaagagcttgtgcgtctcaaagacacacacatcaacctacgtgatgggcaccattggctacattgac
cctgagtatgcccgcacatcccggctcaatgagaaatccgacgtatacagctatggcattgtcttgctggagttgctgactggcaagaagcctgttgatg
atgagtgcaatcttcatcacttgatgctaaccaaagccgcagacaacacggtcatggagatggttgatccagagatctctgacacgtgcaaagacctcg
gcgaggtcaagaaggttttccaattggcgctcctttgcagcaagcggcaaccttcggatcggccaacaatgcatgaggtcgttcgtgttttggacagcct

ggtctccccggacccgctcccgaagtcggcaaaaccaccggcatcgcagcagtcatccacaactccgagctacgtcagtgaatatgttagcctacgag
gcggcagctcgctatcctgcgccaattcatcaagcacgtcagacgcagaactctttatgaagtttggcgaggtgatatcacggggcacggaatag
>g43072.t1
atggggttgaggagtggcggcgcggtggattctgcaggaatgttgcttcggctgctgctgctgctcgtgacggcgaccggcggcgcgtgggggcttaac
gccgacggtgagctgctcatgtcgttcaggaacgcggtgacctccgacccgctgggcgcgctctccggctggagctacggcaacgtggagccgtgcgc
gtggaacggtgtcgtctgcgccggcttcccttcgcctgacgcagtcaacgtgacgtcatcctccggcgaagtgaacgccaccgacgcgtcttcgggcct
gaacgcgtcgatggcggcggtggcgacggtgtcgcgcgtcatcagcctggtcctccccaacgcgcagctgtccgggtcgctggcccccgacctggccc
gcctcgaccgcctccgccacctcgacctctccgggaacgcgctcaacggcaccgccttccccgccgcgctgctcaacgcctccgacctccgcgtcctctc
gctcgcgggcaaccgcatcgccggctccctccccgactcagcgtccgcctactctcgcagcctccaggagctcaacctctcgggcaacgcgctcgccgg
ccgcctccccgccgcgctgtgccgtctgccgcgcctcgccgtgctgggcctcgccgacaaccgcctcgccggcgcgctccccatcggcgggctcggccc
cgcgctggagctcgtcgacctcagcaccaactccttcaacggctccgtcccgtccgacttcggcggggcccacctgcgtctcctgaacgtgtcctccaac
aacctcaccggcgcgctccccaccgagctcgccgccgtcgtgccggcgaacgccacgctggacatgtcccgcaacaacctcaccggcgcggtgccgc
aggcgggggcgctcgccgtgcaaccggcggcggcgttcgagggcaacccgggcctgtgcgggccgccgctggcgcaggcgtgctccatcccgtcgtc
gctgtccaacccgcccaacgccaccgactcgccgccggcgttcgccgccatccccaagaacaacccagctcgctccccgccgggggccccacctcagg
atccgtcacaagggcaggggaagctccggcccgccgcgattgtggccatcgtcgccggggacatcgccggcgtggggatcctcttcgcgctgttcttgt
acgcgttccatgtcaggaagaagaggcggcagcggcggcgggaggaggaggaggatgaccaccacccggcgccattacagatgggtgccaagagc
gtgcgagcgatcggcggcgacggggtgaagacgctggacatcggcagagaggagaaggcgtccacgtcgacggggtgctgtatcgggcgcggcaa
cgacagctcggacgaggagagctccgagtgctcggtctcgtcggacggcgagcccgacgacggcacgaagaagagggggagcctgatcggccgga
gcacgccgcagcacggtagcaagaagtacaacacccctgcgccgccgccaccgcagcagcaggcgccggcgacgctggtgacggtggacggcgac
ggcgagctggagatggagacgctgctcaaggcgtcggcctacatcctcggcgccacgggctccagcatcgtgtacaaggccgtgctcgccgacggca
cggcgctggccgtgcgccgcatcggcgagagcggcggcgccgagaagctcagggacttcgaggcgcaggtgcgcgtcgtcgcgaggttccggcacc
ccaacatcctccggctgcgagggttctactggggcgccgacgagaagttgctcatccacgagtacgcgcccaacggcagcctcgccaacatcgccttc
agcagacggtttggggcgtcgtcgccgctgcacctgagcctggaagcgcggctgcggatcgcgcgtggcgtggcgcgcggtctggccttcatccacga
gaagaagggnccgcgcctcgccgtgctgggcctcgccgacaaccgcctcgccggcgcgctccccatcggcgggctcggccccgcgctggagctcgtc
gacctcagcaccaactccttcaacggctccgtcccgtccgacttcggcggggcccacctgcgtctcctgaacgtgtcctccaacaacctcaccggcgcgc
tccccaccgagctcgccgccgtcgtgccggcgaacgccacgctggacatgtcccgcaacaacctcaccggcgcggtgccgcaggcgggggcgctcgc
cgtgcaaccggcggcggcgttcgagggcaacccgggcctgtgcgggccgccgctggcgcaggcgtgctccatcccgtcgtcgctgtccaacccgccca
acgccaccgactcgccgccggcgttcgccgccatccccaagaacaacccagctcgctccccgccgggggccccacctcaggatccgtcacaagggca
ggggaagctccggcccgccgcgattgtggccatcgtcgccggggacatcgccggcgtggggatcctcttcgcgctgttcttgtacgcgttccatgtcagg
aagaagaggcggcagcggcggcgggaggaggaggaggatgaccaccacccggcgccattacagatgggtgccaagagcgtgcgagcgatcggcg
gcgacggggtgaagacgctggacatcggcagagaggagaaggcgtccacgtcgacggggtgctgtatcgggcgcggcaacgacagctcggacgag
gagagctccgagtgctcggtctcgtcggacggcgagcccgacgacggcacgaagaagagggggagcctgatcggccggagcacgccgcagcacgg
tagcaagaagtacaacacccctgcgccgccgccaccgcagcagcaggcgccggcgacgctggtgacggtggacggcgacggcgagctggagatgg
agacgctgctcaaggcgtcggcctacatcctcggcgccacgggctccagcatcgtgtacaaggccgtgctcgccgacggcacggcgctggccgtgcgc
cgcatcggcgagagcggcggcgccgagaagctcagggacttcgaggcgcaggtgcgcgtcgtcgcgaggttccggcaccccaacatcctccggctgc
gagggttctactggggcgccgacgagaagttgctcatccacgagtacgcgcccaacggcagcctcgccaacatcgccttcagcagacggtttggggcg
tcgtcgccgctgcacctgagcctggaagcgcggctgcggatcgcgcgtggcgtggcgcgcggtctggccttcatccacgagaagaagggcgtgcacg
gcaacctgaagccgagcaacatcctgctgggcgccgagatggagcccatgatcggcgacctgggcctggaccggctcctctccggcgaggccgcggt
gcaccgcgccggcgcgtcggcgcggctgttcggcagcaagcggtccatgcactcgaccagcagccttcccgacctgtcccagatgcccgggcccggcg
cgagcccgtgcgggtccgcgtcggcggcggcgtcgtccggcccaaccaccggcccgtaccaggcccccgagtgcctcaagaacctccggcccaacgc
caagtgggacgtctactccttcggcatggtgctcctggagctgctctccgggcgggtctactccgaggtggagctgtgccagtggcacgccggcctcgtc
gccgaggagcgggcccgcgtgctgcggatggcggaccccaccctccgtggcgaggctgacggcagggaggacgcgctcgtcgcctgcttcaggctcg
cgttcgcctgctgcgccatggcgcccagcaagcggcccgccatgagggacgccgtggtcgtgctcgacaggacggcggcggcttccacgacgccggg
cgcgtcgtcgagcagcaacggcgccattccttga
>g43927.t1
atgagtgatgaagcagtgtctcggtatttgggagaatctggaggatgccttactgaattaatgctaaacagtatcgagaaggctggagaccttactgctc
ttgcaattgctcaaaagtgctctcttcgcttggaggttctggatctttccttctgccgtgaactgacaaatgaagctttggggctgatcgttgacaactgctc
atcattgagggttctcaaactatttggatgtacccaggtctctgatattttcctcaagggccactcaaacggattggtaacaattgtggggatggaaggga
acatattggagcaaatggataatcgagcgagtgaggaggggagaagattgctttcctcttnctctcttcgcttggaggttctggatctttccttctgccgtg
aactgacaaatgaagctttggggctgatcgttgacaactgctcatcattgagggttctcaaactatttggatgtacccaggtctctgatattttcctcaagg
gccactcaaacggattggtaacaattgtggggatggaagggaacatattggagcaaatggataatcgagcgagtgaggaggggagaagattgctttc
ctcttcagcacatgtggttccttttccgtcgcgcatgattgaaggatcgcgtgagaaaaggaaatcgccttcctatcccaaagtcgtgtgtctcctcctcct
cctcctctctctctctctctctctctctctcttcaccggcagcctccccttcctcgtcttctggctcttctggattgccaaccgcaccttcgccgccacccgctc
cacggccgtcagctccgtcgtctcgtccagcttccgcgcgggcgtcggtgccgcgggggcgtgcttgttcgtgctcgccgccgccttccgcaccatttcca
tggccgtgcccaaccacagtgcctggttcaccgccttcgtcgacttgaggtccgccgccttcgacttcgtgcgctgcagcaccacgggctccgagtcctg
ggtgatgttgcgacggcgctggctccatatctggagctga
>g44065.t1
atggctgcggcggccattggtgcggtgatgctgttcttggctgcaacttcatttggggctaatgccagcaccgactccaacgacgttgatgctctcagtgt
cttctacacgaccatgaattcaccaccccagctgacaaactgggtgtcccaaaatggggatccatgtggacaatcttggcttggggtcgcctgctcgggt

tccagggtgaaaacaataaagttatctggaatgcggcttaacgggaccttggggtacaatatgaatcagctgactgcgctagttgagctcgatatgagc
aacaatgaccttggagggaatgacattccttataatctcccaccaaatcttgagagcctaaatcttggaggaaataattttactgggaccgtaccatactc
catctcccagatggttgcacttagacatttaaatctcggtaataatcatctttcgaacatcaatgatatgtttaaccagctcacaaatttaacactaatgga
catatcatacaacgcattttctggtgacatcccacaaagcttccagtccttgacgaatttgaaaaaactaaatgtggagaacaatcaattcactgggtgg
ataccagataaactaaagggaatcaggagtatccaaacaagtggaaactcatttaacgatggtcctgctccaccacctccaccatctccaccatcaacg
tatacacctccacagactagtcgacctgctgttccaagtagtactggtggtgatgaaatgaaatctgttaaatctatcaaggtcatatcaacaattggcaa
ggaagaattacaaaagactgtttcaatgagcttgaagcctccaacgaagattgacctgcacaagtccttcgacgaaaatgatatcactaacaagttcat
cacaaggaaagtcttagctgtaaagaaaatcaatttttctgcatttccaagccatccttcagattcatttgttgagttggttgcaaacatttcaaggctaaa
tcatgcaaaccttgctgagctatgtggttactgctcagagcatggacagtgcttgctagcatatgagctctaccggaatggttctctccatgacttccttca
tgtgaaggatgaacacagcaaaccgttgtcttggaacaatcgtgtgaagattgccctaggatctgcaagggcattagaatacctacatgaaacttgttcg
ccgtctgtggtccacaagaatttcaagtcatccaatattttgctggctagcgaactgaatccccacatctcagattcagggtttgcaggccttattcccaac
caagaatctcaggaatcagatgagaactcaggatacagggcaccagaggtgaccatgtccggccagtactccctgaagagcgacgtgtatagcttcgg
tgtcgtcatgctagagctcctgactggccggagaccgttcgacagatcacggccgcggtcggagcagtcgctggtccgatgggccacgccgcagctgc
acgacatcgacgcgctggaccggatggtcgacccggcgctgcaggggctgtacccttccaaatcactctcccgcttcgccgacgcgatcgccctttgcg
tccagcccgagccagagttccggccgccgatgtcggaggtggtgcagtcgctggtgcggctcgtgcagagggcgagcatgacgagggcgcacgaga
atcagacgcggcgatcgggcgagtcttctggggactatgagttctga
>g44521.t1
atggattgtataaataatggtttatacgtgatcatcagtgccatcaacaccttctccttggacgccatgacaaggcagctgtactgtactggtggagagag
ggagaaaagcagcagtgccatcaacaccttctcctttgacgccatgacaaggcagctgtactcctacccgttcccctacaactccggtgacaaggcggc
gctgctagccatcaagtccgccttcggcaacgcctccttcttcaattcgtggacgcccgacaccccgtgctgcgagtgggccaacgtctcttgcgactcct
cctcctcctccacggcgcgccgcgtcgttgtcgtctccttcctcaacgacgccggcctcgccgggcccttacccggcgccgccatcgcccgcctcaccgc
gctgcagcagctcgtgctgaacaacgtgccaggcgtgaacggcaccatcccgcacgacctcacccgcctctccgctacactcagcttcctcaccatcac
ctccacgggcatctccggtccggtgccgccgttcctctccgagatcaccgcgctgggctacctcagcctctcctccaactcgctcacgggccccatcccg
gcgtcgctcggggacatgcccaacctctacttcctcgacctctcctccaacttgctcacgggcaccatcccgccgctgctcctcagcaaggcccccggcg
acgcgtacctatacctctcccacaacaggctcgtcggcggcgttcccgccgagttcgccgccgtgaacttctggacgcttgacgtctcacacaacggtct
ctccggcgacgccttgttcttgttaggggcgaacaagccgctggagaacctggacctgtctagcaacggcttcagctttaacctctccgccgtgcagctg
ccggagaacctcaacagtttggagctgagtcacaatgacatctacggggatataccggagcaggtggtcgacctgatgctggagtacttgaacgtcag
ctataatcgcctcagcggcacggtgcccgccggtggcaacatggcatggtttgatcagtcctgcttccagcacaacaaggggctctgtggaattccactt
cctccctgcaaggagtaa
>g44522.t1
atgctcgcctcgtcatcagtcaggcaccgtgccaccatggcagccgtcgtgctcctcgccgcagctgttgcggtttcggcagcacgggcgaagaatgag
tgccactccggcgacaaggcggccctgctggccatcacgtccaccttgggcagcaacgcctcctccttccagtcctggaattccgacaccccgtgctgcg
actggaccaacgtcttctgcagcaacgtctcctccacatcccccctacaccgcgcgccgcgtcatcggcttgtccttgcaggacgacgacatcctcgccgg
cccgtttcccggagccgccatcgccggcctcaccacgctgcagcagctcctgatgatcaacgtgcccggagtgaagggcaccatcccccacgacctca
cccgccttaacgccaccctttctttcgtcgacatcagctccacaggcatctccggcccggtgccgtcattcctatccgagatcacggcgctgggccacctc
aggctatccacgaacaagctcacgggtcccatcccggcgtcgctcggggacatgcccaacctctatttcctcgacctcagcaacaaccgcctcacgggc
acgctcccgcggctgctcttcagcaaggcctctgattattactccgacaagtacctctacctctcccacaacaagctcaccggcagcgtccccgccgagt
tcgccaccgtcaacttctggggcctcgacctctcgcacaacggattcaccggtgacgcgtcgttcctgttcggcgtgaacaagtcgctggactacctgaa
cctgtcgcacaacgtcttcgacttcaacttctccgccgtgcagctgccggtgaacctcgacggtttggacctcagccacaaccacatctacggcgacatc
ccggaccaggtggtcgacgtgacggtgacctattttaacgttagctacaatcgcctcaccggcgtagtgcccaccggtggcaccatgttctggtttgatg
catcctgcttccagcacaacaaggggctctgtggaactccacttccaccctgtattaagcactga
>g44717.t1
atgcctctgtaccgtggtcatgattttgattacagaacagcattgactgatgatagccttaaggctctatcacgctactgccccaagcttcaggttgttgag
ttcacctttacattttgtaatgctgcctatccatcagaaataggcttcacgcaggagggtattataatgctgattcagtcgtgcccaattcgtgttgtcatgc
taaatggtgccaccattttgatgatgagggaatgaaggccctctcatcctcccaattgttggaaaggctcgagcttgtggactgcaatggcattacaga
cgctgggataggtttcatgatgtgtgccccgtccctgatcagcttaactctccaacaatgcgaaagagtgactgaccatggaatggccgagttctttagc
actctgaacacctgctccatggaggatctcccagagccactgattgcagagatcatcaagaggactacctggacaagtgatcttaattctctctcccttgt
gtcaaagcggctgtacaaagttgaagcagatgaaaggggttttatctgtgttggatgtggcctcaaccctgcaacagaagctttgtcatctctgtgctccc
gattccccaacttgtggaaagtagagatcaattactctggttggtcgtctaaccaagggcagcagttagataaccagggccttcatgtgctttcatctcac
tgcccatcgctgatggatctcacgctgagcttctgctcacacataaatgactctggtcttggttatctggcacattgcaaaaagcttatggccctcaggctg
aacaatgctccagcaataagctcgaatgggcttctctcggtagcggttgggtgcaagagtttatctgttttctgttttgcatcttactga
>g45692.t1
atgccaagctccattgccgtcaccctcctcttcatcctcgtcgccgccgcattatttccctccgcgtcctcccaacccaccgccgccccacaaccacaacc
cagagggttctacataaactgcgggtcggacaaggaggtgcagatcgggagcgtgaagtggatccaggatgacggcttcgtctccgtgggcaacgtgt
cggccatcaacaagccccacctcctccccgtcctggccacgctgcgctacttcccggacaagaccgcgcgcaagtactgctaccaggtccccgtggtca
ggggcacgcgctacctcgtccgcaccacctacttctacggcggcttcgacggcggcgccgacccgcctgtcttcgaccagatcgtcgacggcacgcgct
ggagcgccgtcaacaccaccgacagcttccgccgcggcatgtccacctatttcgaggtggtcgcgccggcgcagggcaagaccatgagcgtgtgcctt
gcgcgccgccccgacacgacgtccagccccttcatctccgccatcgaggtcgtcgacctcgccgactccatgtacaacgccaccgatttcagcaggttc
gccatgaccaccgtcgcgcgcagccggttcggctccaagggcgagatcgtcagctatccagatgacccgtacaaccggtactgggcgccgttctccga

cgccaacccggccgtggagagccactcgccgatcgcgcccgacgacttctggaacctgccgccggcgaaggcgctcaaggccggggtcacggcgag
ccgggggaagaagctcgccgtgcagtggccgccggcggagctgcccgcggcgagctactacgtggcgctctacttccaggactcgcgcacggccagc
cccttcagctggcgcgtcttcgacgtcgccgtcaacgggcaggagttcttccgcgggctcaacgcgtccgccgccggcgtcatggtgtactccagcatga
tgcggctggccgggaagacggagatcctgctcacgcccaacgagacgtcgcctgtcggacccatgatcaacgccggcgagatatacatgattctacca
ctcggcggcaggacgaccaccagagatgctggtgcaatggaagatctcgcaagaagcttcaagaacccacctccagactgggccggagatccttgcc
tgccaaggaataactcatggaatggggtcgaatgctccgaggaacttcctgtgcgcgtcttgtctttggatctaaaaaatcatggtctttcaggatcactt
cccgacagctttggaaatttgactgcactgaaaaccatctatctcagtggcaacaaactctcaggctccatacctgacttgagcggcatgcgcaccctta
ctgctttgcatcttgatggcaatcagttcaacggcacgattaacccatcaatggaaaagctcatcaatcttaaggagctatacctgaacaacaataatctc
acgggccaaataccaatcggcctgaaaaacaaacctgggcttgatctcagggatctaaaaaatcatggtctttcaggatcacttcccgacagctttgga
aatttgactgcactgaaaaccatctatctcagtggcaacaaactctcaggctccatacctgacttgagcggcatgcgcacccttactgctttgcatcttgat
ggcaatcagttcaacggcacgattaacccatcaatggaaaagctcatcaatcttaaggagctatacctgaacaacaataatctcacgggccaaatacc
aatcggcctgaaaaacaaacctgggcttgatctcagaacggaggggaacaaatttggatga
>g45830.t1
atgaggtcgattgccgcggccgtcgccgtcgcctcggttctcctcgccgtggccggaggaagcgtcgtcaccaaccgcgacgacgagcatgttctgcac
gtggtcgccgccgcgctgggacccgccgggcggtccctcaactgggacaactgcacccggccgtgccaggactgggaaggggtgagctgcaacgcca
acggcagtgtggtcggcatctcggcgcggggtgccggcttcaacggcacgctactggaggacgcgcactacctccgggagttgcatcacctcgacctc
cgcgacaacgacatcaccggcccgctccccgacgtgccgttcctcaacctcaagaccctgcacctggactgcaacgcgttcaccgccgtgccagctgg
actctccgcggaacgggaagccgcctcgccaacctcaacacgatccaagccgacaacgcctcccatcaccggcacactctctgccttcctccgcaacg
gcaccgcctacgccaacctcgtccacgtctccctcgcgaggaaccgcctcacgggccccgtgccggccacgttcgtcagcaagaagctcgagcacctc
gacctcagcgacaatctcctcaccggcacgatcgacttcgtcatcaatttccccaatatcaaaattcttcgtctggacggcaactccttcgccgggcagct
tccagacttccagaggctcaaaaagctggagatccttcttctgggccagaaccagctcaccgggctggttccgccgtcgctgatgcagctcccaaatctc
gccgccgtgactctcgccgacaacctcttccaggggcccctgcccgagttcgggccgtgggtgcacagtgacaccgacggtgacgcgggaggaagct
ggtgccgcctcgcggcgagctggaagggcaacagcccgtgcgccggctggatgggcgttcactgcgacaagaccggcgcgacgatcaccggcatca
atttgtgccgccacgggctcaacggcacaatccacccggcgatcggcggcctgcgctcgctccgggccgtcctgctttccggcaacaacctcggcggcg
ctgttccggcaaccatctcgcgcctcgctgacctcctcctcgacgtatccgacaaccagatcaacggaagcattccgcgccttcccagaggcgcggt
cgtctgggctgatcagggcaacaccgccgtcgcactcagctcgccgccggcaagcctccccaccgccaccatcttcttcgtcctggcaacgttctgttac
agtttgtcatga
>g46200.t1
atgacaagtgctactccaaggcagagtagcacttcacacttgttcttccccttgtccttctccttggcgctcctgtgctgcattgccgcgtgcaatgcggct
ggcgatgaggccgcggcgctgctagccatcaaggagtcgctcgtcgaccccttgggcaagctccagggctggactgccgcgtcagcctcgtcgcattgt
acatgggaaggcgtgaagtgcgtcaagggcgtcgtcaccggcctcaacctcgccggcatgaacctgagcggcactttcccggaggacatcacccgcct
ctccgggctcacctcgatcatccttcagagcaacgcgttcgccgacgagctgcctcaggtgctcgtctccatcccgactctcgaggaggtcgacgtcagc
gacaacaacttcggcggccactttcccgccggcctcggcgcgtgcgccacactgacatacctcaacgcgtcgggcaacaacttcgccggcccgcttccg
gcggacctcggcactgccacggcgctcgagaccatcgacttcaggggcggcttcttctccggcacgatcccgaaatcctacggtaagctcaagaagctc
aagttcctgggcctctcaggcaacaatctcggcggcgcgctaccatcagagctgttcgagacgtcggcattggagcagctcatcattggctacaacgag
ttctccggcgcaattcctgccgccatcggcaacctcgccaatctccagtacctcgacctggcgatcggcaagctggaaggtcctctccccgcggagctcg
gccggctaccgtacctcaacaccatctacctctacaagaacaatatcgacggcccgatccccaaggagctcggcaacctctcctccctcgtcatgctcga
cctctccgacaacgcgctcaccggcaccatcccgtcggagctgtcgcagctcaccaacctgcagctgctcaacctcatgtgcaaccggctgaagggcg
gcgtcccgtcgggcatcggcgatctccccaagctcgaggtgctggagctctggaacaactccctcaccggcccgcttcctccctcgctgggccgcgcgc
agccgcttcagtgggtggacgtgtcgacgaacgcgctgtcgggccccgtgcccgcgggcctctgcgacagcggcaacctgacgaagctgatcctcttc
aacaacgcgttcacgggcccgatcccggcgggcctgaccacgtgcgcgacgctcgtccgcgtgcgggcccacaacaaccgtctcaacggcacggtgc
ccgcgggcctgggccggctcccgcggctgcagcggcttgagctcgcgggcaacgagctgtcgggagagattcccgacgacctggcgctgtccacgtcg
ctctccttcatcgacctctcccacaaccggctgcggtccgcgctgccgtccaacgtgctctccatcccggagctgcagacgttcgcggcggcggacaacg
agctcaccggcggcgtccccgacgagatcggcgggtgcccggcgctctccgcgctcgacctctccagcaaccgcctctccggcgcggtcccggccagc
ctcgcctcctgccagcgcctcgtctcgctcagcctccggaacaaccggttcacggggaacatccccgccgcgatcgccatgatgtcgacgctgtccgtgc
tcgacctctccaacaacctcctcaccggcgacatccccagcaacttcggcagctcccccgcgctcgagacgctcaacctcgccaacaacaacctctccg
gtcccgtgccgtcgacggggctgttgaggaccatcaaccccgacgagctcgccgggaacccgggattgtgcggcggcatcctacctccctgtggctcca
cctccctgcgcgcctcgtcgtctaatcaagcggctggtcactcacacgtgaagcacgtcgcggccgggtgggcgatcggcatctccgcggccctcgtgg
cgtgcgtcgccgcgttccttgggaagcagctttaccagcgatggtacgtcaacggcgggtgctgcgacgacgccggcatcgaggaagacgggacagg
cgcgtggccgtggcgcctgactgcattccaaaggctgagtttcaccagcgcggaagtgctcgcgtgcatcaaggaggacaacatcgtgggaatgggcg
gcacgggggtggtctaccgcgccgagatgccacgtcaccacgcggtggtcgcggtcaagaagctctggcacgcggcgaggtgccctgaccaggaggc
cgctgccaccgtaacagacatggagtccggcggcgagttcgcggcggaggtgaagctgctggggcggctgcggcaccgcaacgtggtgcggatgctc
gggtacgtcagtaacaacctggacacgatggtgctgtacgagtacatggtcaacggcagcctgtgggaggcgctgcacgggaaggggaaaggcaag
atgctgctggactgggtgtcgcgatacaacgtcgcggcgggcgtcgcggcagggctcgcatacctgcaccacgactgcaggccgccggtcatccaccg
cgacgtcaagtccagcaacgtgctcctcgacacaaacatggacgccaagatcgcggatttcgggctcgcacgcgtcatggcccgggctcacgagaccg
tctccatggtcgccggatcctacggctacatcgcgccagagtacgggtacacgctgaaggtggaccagaagagcgacatctacagcttcggggttgtg
ctgatggagctgctcacggggcgtcggccgatcgagccggattacggcgagacgcaggacatcgtgggctggatcagggagcggctgcgcagcaac
agcggagtggaggagctgctcgacgcgagcgtcggcggccgcgtggaccacgtcagggaggagatgctgttggtgctgcgcatcgcggtgctgtgca

cggcaaagtcgcccaaggaccggcccaccatgcgggacgtggtcaccatgctcggcgaggccaagccgcgccggaagagcagcagcgccaccgtg
gccgccaccgtcgtggacaaggacaagccggtgttcacgacgtcgccggactccgattacctatag
>g46284.t1
atgagcggcggcaccgacgagaagcgtgaccaccatcgcaggtcgcggccatcatggcgttattgtctcgcggttgcttggcttgtcgcggcgttggtta
ctgaaggaggcgcggaggtgacggagctggagcaggaggagctccgcgacgagcggcgcgggctgctgctcctccgcgacgcgctccgctcggcgc
tggacctgcactccaactggacgggcccgccctgccacggcgaccgcagccggtgggccggcgtcgcctgcgacgccgacggccgcgtggtgtccctc
gccctctcccacgcgcagctcacaggcaccctcccgcgcgacgcgctccgcggcgccacgcggctgtcggagctccgcctccgcgggaacgcgctccg
cggcgcgctgccaaggctggaagcgctgggcctgggccggctccgggtgctcgacctgtccagcaaccggttctccggccccatcccgcgtggctacg
cggggatgcgggcgctggaacgggtggagctgcaggacaacctgctcaccggcggcgtcccggcgttcgcccagcgcgggctggtgacgttcaacgt
gtcgtacaacttcctccgcggcgaggtgccccgcgcgatgtggcggttcccggccagcgcgttcgggcacaacctcgggctctgcggcgaggccgtac
gcgccgcgtgcccgcccccgtcttcgggctccgccggaagcagcagaagcgacaatactaatccggccgtgacgaagccgggtggggagggcagcg
aacaaggcgcagcgcggttcaggctcgcgacgtggagcgtcgtggcgatcgcgctcatcgccgcgctggtgccggtcgcggctgtgctcatcttcctgt
accacacgaggaggagccggagccggagccgggaggtccgcctcggtggtggccgcgccggcgccccaggcaacgtgacaggagcaggggagga
catcaaggacaaggcggcagagcagggcaagtcagggagcggcagccggagcacggactcgtcggccaagagcggcgtcgagctgcagttcttccg
ttgggacagggcgggcttcgacgtcgacgatctgttcaggtcgacggcggagacgctcggcaagggccggctggggatcacgtaccgcgtgacgctc
gaccacgccaaaagcggcgtggtggtcgtgaagcggctgcgcaacatggcgcgcgtgccgcgccgggagttcagccacaccatgcggctcctgggca
agctcccccggcacgacaacgtcttgggcctcatcgcgtgctactactcgaaggaggagaagctcgccgtgtacgagcacgtccccgggtgcagcctct
tccagcttctccacgagaacagaggcgaggggaggacgccgctgccgtggccggcgcggctggcgatcgcgaagggtgtggcgcgcgggctggccc
acctgcaccactcgctgccatacttccaccgcccgccgcacggcaacctcaagtcctccaacgtcatcgtcgccgcctcctccaccggaaaccatcatca
tcaccaccagcacaagcaggtgccgaagctgacggactacggcttccacccgctgctcccgcaccacgcgcaccgcctcgccgcggccaagtgcccg
gagttcgcgcggggcggcggcgggcggcggctgtcgtcgcgcgccgacgtgtactgcctcggcgtcgtgctgctggagctggtcacggggaaggtgc
ccgtggaggaggacggcgacctggcggagtgggcccgcctcgcgctcgcgcacgagtggtccacggacatcctcgacgtcgagatcgtcgccgacca
gccacgccacggggacatgctgcgcctcacggaggtcgcgctgctctgcgccgccgtcgacccggagcgccggcccaaggtgcacgacgtcgtcgtg
atgatcgacgcgattggcgatgctggggacggggaacagcagccgccagctgggcttcgttga
>g48163.t1
atgcgcttccactacctcgccgtcctcctcctcctcctgctcgccgccggcgccgccgcggcggacgacgcggacgcggacgcgctgctggcggtgaag
gcggcgctggtggacccggccggcgcgctcgcctcatggaagaagaacgccacctctccggcgagcccctgcacatggctgggcatcacctgcaacg
cccgcgcccacgtcatcgggctggacctcgtgggccgcaacctctccggcccggtcccaggcgcggccctctcccgtctggcccacctggcccgcctcg
acctcgccgccaacgccctgtccggccccatcccgccgccgctcgccgcgctccgcttcctcacccacctcaacctctccaacaacctcctcaactccac
cttcccttcctccctcttccacggcggcggtctccgcgcgctgcgcgtgctcgacctctacaacaacaacctcaccggcgcgctcccggcggagctcccc
gcggccgcccccttgctccgccacctccacctcggcggcaacttcttctccggctcgatcccgcgcgagttcgggcgctggtcccggcttcagtacctcgc
cgtctccgggaacgagctctcgggccccgtcccgccggagctgggcaacatcaccagcctcaacgagctctacatcggctacttcaacagctactccgg
cgatctccccccctgaactcggcaacctcacgcgcctcgtccgcctcgacgccgccaactgcggcctctccggcgcgatcccgccggacctcgggaagct
cgccaacctcgacacgctcttcctccaagtgaacaccctcgccggtgccatcccacccgagctcggccacctttccagcctcagctcgctcgacctctcc
aacaacgcgctgtccggcgagatcccggcgaccttcgcggacttaaaaaacctcactttactcaacctcttccgtaacaagctcaccggcaacatcccc
gacctcgtcggcgacctccccggtctcgaggtgctccagctctgggagaacaacttcaccggcggcgtcccccgccgtctcggccgcaacggccggctc
cggctcgtcgacctctcctccaaccgcctcaccgggactctcccacccgacctctgcgccggcggcaagctcgaaactttaatcgccctcggcaacttcc
tcttcggctccatcccggcgtctctcggcaaatgcgggtccctcactcgcgtccgcttaggggagaactacctcaacgggtccatcccagaggggctgtt
cgagcttcccaacctcacgcaggtcgagctccagggcaacctcctctccggcgccttcccgtcgcccaaatccgcggcggcgcagaacctcggcgccat
cagtctctccaacaaccagctcgccggcgcgctgccggccaccatcggcgacttctccgggcttcagaagctgctcctcgaccacaacaagttcaccgg
cgcggtcccgccggagctcggccgcctgcagcagctctccaaggccgacctgagcggcaacgcgttcgccggcgcggtcccgccggagatcgggaaa
tgcaggctgctcacctacctcgacctcagccagaacaacctctccggcgagatcccccggccgtctccgggatgcggatcctcaactacctcaacctgt
cacggaaccgcctcgccggggagataccagggaccatcgccggcatgcagagcctcacggcggtggacttctcgtataacaacctgtccgggctggtg
ccggcgacggggcagttcagctacttcaacgccacatccttcctcggcaaccccgggctgtgcgggccctacctcggaccctgccacgtcatcggcggg
gcccccggaacggaccacggtagacactctcgcgccggcgggatgtcaaacacgttgaagctgctcatcgtcctcggcttgctcctctgctcgatcgcgt
tcgcggccatggcgatcctcaaggcgcgggcgctgaagaaggccagcgaggcgcgcgcgtggaggatgacggcgttccagcggctcgagttcacctg
cgacgacgtgctcgacagcctgaaagaggagaacgtgatcggcaagggcggcgccggggtcgtgtacaaggccgccatgcccgacggcgaccacgt
cgccgtcaagcgcctctcggcgatgagcagccgcggcggcgggtcggcgcacgaccacggattctccgcggagatccagacgctcggcaggatccgg
caccggtacatcgtgcgattgctggggttctgctccaacaacgagaccaacctgcttgtgtacgagtacatgcccaatgggagcttaggggagctgctc
cacggcaagaagggtgggcacctgcattgggacactcggtacaagatcgccgtcgaggccgccaaggggctcagctacctccaccatgactgctcgc
cgccgatcctgcaccgcgacgtcaagtccaacaacattctgcttgactctgagtttgaagctcatgttgccgatttcgggctcgccaagttcttgcaggac
tccggcacgtcgcaatgtatgtctgccattgccggctcctatgggtacattgctccagagtatgcttatacgctcaaagtcgatgaaaagagcgatgtcta
cagctttggggttgtgctgcttgagctggtcaccggaaagaagccagtaggggagttcggtgacggcgtggacattgtccaatgggttaagacgatgac
ggactcgaacaaggagcaggtgatcaagatcatggaccctagactctcgacggtgccggtgcacgaggtcacgcacgtcttctacgtcgcgttgctctg
tgtcgaggagcagagcgtgcagcggcctacgatgagggaggtggtgcagatgctcagtgagctccccaagccaactgatcagaggcaaggagacga
gctccctggtgatggagatggttctgcatctgatcaggagcctccagtgtcgtctgaatctgttgaagcaccgacaaacgaagccaaagagcagcagca
aacgagctcgccgtcttcaccagctcctgatctcatcagcatctga
>g48577.t1

atgccgccgtcgcgggcgctgttcctgctgctcatgctttccctggcgccgttccacccggcggcgcaggcgccagggtctgtgccgccggtggcgcgg
acggccgaggtgcaggccgagatcgacgcgctcctcgcgttccgtcgcgggttgcgcgacccgtacggcgccatgtccggttgggacgcgtcctcgcc
gtcggcgccctgctcctggcgcggcgtcgcgtgcgcgcccggcggcgccggccacgtcgtggagctgcagctcccgcggctccgcctctcgggcccca
tctcgccggcgctgggctcgctcccttacctcgagcgacttagcctccggtccaactccctctccggcgccatcccggcgtcgctctcccgcgtcaccgct
ctccgcgccgtcttcctccagtccaactcgctctccggccccatcccccagtccttcctctccaacctcaccaacctcgaaaccttcgacgtctccggaaac
ctcctgtccggccccgttcccgcttcattcccttcgaccttgacatacatcgacctctcctccaatgccttctccggcatcatcccggccaacatcagcgcct
ccgcggcgaaccttcagttcctgaacctttcctga
>g48582.t1
atggcaacacacacacactcagtgagcttggtgttgttcgtgctatcttccgcacttgcggccggagccaacgacgaggctgcgctgcatgctttcaagg
tcgcagcgatcagcggcaggaatggtgaccctcttgcctcatggaacagcagcctaagcggctactgcagctgggagggggtgaggtgcaggggcag
gggcaggcaccagcgagtggtggccctcagccttccatcttaccagctcaccggcgtcctctcaccagccattggcaacctgacgtcgctacgaactctc
aacctgagcaagaacagcttcagcggggacatccctgccagcctcggccgtcttcaccacctccacgtcctcgacctgagccgaaactccttctccggc
gaggtccccacaaacctgacttcttgcatcaatctaacaattttgaacctcaacttcaaccagctccatgggtgtgtgtcctctgagatcggtgacaaact
ggcaggccttagatttctcggcctggcaaaaaataaccttacaggaaccatccccgcgtcccttggcaacttatcatcacttattatcctgcaactcggat
tcaatcaattagagggcatcatccctcccatattcggtgtcatgagaggcatccaatttctagacctcgccttcaacaacctctctgctgccaagcacaat
atcgaatctatcaacaaatctgcaattcctccgttttga
>g48595.t1
atggcgcggccgttcttagtggcgatgatggccatggctctgctttcgtgcgtggcgacggcggacgacggggccgtgctgctggaggtgaagcgcgc
gctcgtcggggacccatcgggccccctgtcgacctggtcctccggttcctcatcagacttctgctcgtgggccggcgtggcgtgcgacgacgcggggctc
cgcgtggtgggcctgaacctctccggcgcggggctgtcgggcccggtcccgcgcgcgctggcccgcctcgacgcgctccaagtcctcgacctgtcgga
gaacgcgctgaccggccccgtcccggcgtcgctcggcgcgctacagaatctcgagcgcctcctcctcttctccaaccacctcgccggcgagataccggc
gtcgctgggcgggctctcggctctccaggtgctccggctcggcgacaacccggcgctgtcgggcccgatccccgcgtccctcggcgggctcgccaacct
caccgtgctgggcctggcctcctgcaacctcaccggcccggtcccgaccaccctgggccggctcgcggccctcaccgcgctcaacctgcagcagaacg
cgctgtcagggcccgtcccgccggagctgggcaacttggggaatctcgaggtgctcgcgctcgccggcaaccagctcaccgggaagattcccccgag
atcgggaggctcgccgcgctgcagacgctgaacctggggaacaactcgctcgacggcggcgtcccgccggagctcggcgcgctgggcgggctcgagt
acctcaacctcatgaacaaccggctcaccggccgcatcccgcgcgagctcgccgcgctccaacgcgcgcgcaccatcgacctctccgggaacctcctct
ccggcgagctccccgcggagctgggccggctccccgagctcaacttcctcgtcctctccgacaaccgcctgtccggcaccgtccccggcgacctctgcg
gcaccggcgatgatgatcaagaagaggcagagtccattaccagcagcagtctggaacacctgatgctctccaccaacaacctctccggctcgatcccg
gcggggctgtcgcggtgccgcgcgctgacgcagctggacctggcgaacaacagcctgtcgggccccatccccgccgcgctgggcgagctcggcaacc
tgaccgacctgctgctcaacaacaacaccctctccggctccctgcccccgagctcttcaacctcaccgcgctccagacgctcgcattgtaccacaacaa
gctcaccggcgggctccccgactccatcggccgcctcggcgacctcgaggtgctgtacctctacgagaaccagttcacaggctccatcccagagtccat
cggccggtgcacgagcctgcagatgatcgacttgttcgggaacaagttcagcggcaccatcccgtcctccatcggcaacctgacccagctcgtgttcctc
gacctccggcagaacgagctgtccggtcacatcccgccgtccctcggcgcgtgcgcgcagctcgaggtgctcgacctcgccgacaacgcgctctccgg
cgccatcccggccaccttcggcaacctcctgtcgcttgaacagttcatgctctacaacaactccctgtcgggccccatccccgacgccatgttcgcgtgcc
gcaacatcacgcgggtcaacgtcgcgcacaacgcgctcaccggcaccctcctgcccctcacgggcacggcgcggctgctctccttcgacgccaccaac
aactccttctccggcgcgctccccgcccagctcggccggtcccgctccctccagcgcgtgcgcgtcggcagcaaccgcctcacggggcccatcccgccc
gccctcggcgccgcggcggagctcacgctgctcgacgcctccgacaacgcgctcaccggcgggatcccgggtacactcgcgcactgcgcccggctca
gcgccgtggtgctcggccgtaacaggctgtcgggtccggtgcccgactggctcggcgcgctgccgcagctcggggagctcacgctctcgggcaacgcg
ttcgccgggccggtccccgccgggctcagcaactgctccaagcttctcaagctctccctcgacgggaaccaaatcaatgggacagtgccggccgagct
gggcgggctggcgtcgctcaacgtgctcaacctagcgggcaaccagctctccggcccgatcccgaccacgctcgccaagctgaccaacctctacgagc
tcaacctgtcgcggaactacctgtccggcacgatcccaccggacctcggcaagttgcaggagctgcagagcttgctggacctgagcggcaacaacctc
agcggccgcatccctgcgtcactgggttcgctctccaagctggaggacctgaacctctcccacaacgcgctggtcggggaggtcccgtcgcagctcgcc
gggatgagcagcctggtgcagctggacctgtccagcaaccagctccaaggaaaactcggcgagcagttcgcgcggtggccgcaggacgcgttcctcg
gcaacgccgacctctgcggcagcccgttgcgaggctgcgacaacaccggaagcggcgaaaccaaccggtcggcgctgcactcggcgtcgatcgcgct
gatctccgcggccgtgacgctggcgatcgtccttgtaatcatcattcttgcattactggccgcgcggcgcagggggagacgccgttcaagggaggtgaa
ctgcgcggcgtcgacgggaacgagctcctcgggcggcacgcaccgcccccctggtggtgaagggctcggcgcgtcgcgagttccggtgggacgcgatc
atggaggcgacggcgaacctgagcgaccagttcgccgtgggctccggcgggtccgggaccgtgtaccgcgcggagctccccagcggcgagaccgtg
gcggtgaagcggatcgtcgtcgacgacagcgacgcgctgctggcccacgaccgcgggggggttcgcaagggaggtcaggatcctgggccgcgtgcgc
caccgccacctcgtcaagctgctcggcttcgtcgcgtgccacgaccgcggcggcggcatgctcgtctacgagttcatggagaacggcagcctctacgac
tggctgcacggcgacggcgaccgcaagaagcgggtgctcagctgggacgcaaggatcagggtcgccgccgggctcgcgcagggcgtcgagtacctg
caccacgactgcgtgcccagggtcgtgcaccgggacatcaagtcaagcaacgtgctcctcgacggcgacatggaggcgcacctcggggatttcgggct
cgccaaggccgtcgcagagaaccgccaggctgccaaggactgcaccgaatcggcttcatgctttgcaggatcatacggctacattgctccagagtgtg
cttactccctgaaggcgaccgagaagagcgacgtctacagcatgggcattgtgctcatggagctcgtcacggggctcatgcccacggacaaaaccttc
agcggcgacatggacatggtgaggtgggtgcagtccgggatcgacgcgccgcgcacggtgcgggaccaggtgttcgacccggccctgaagccgctcg
cgccgcgcgaggagtcgtccatgatggagatgctcgaggtggcgctgcggtgcaccaggaccgcgccgggggagaggcccacggcgaggcaggtct
ccgatctgctgcgacacgtctcgctcgattactatggagcaggggagcacaagcgttag
>g48747.t1
atggcggcgttggagaggctggatttatccggtaatcgcttcgtcggcgccatcccggacggcatttcaggctgtaagaacctagtcgaggtcgacctc
agccggaacgcgttcactggggagctcccgtggtggttgcttgggctgccgctgcagcgcgcttcggtcgccggcaatgcactgtcagggtgggtcaag

gttcccgatgatgctgccggaactttgcaaactctggacctgtcaggcaatgagttcaccggtgctatcccgcctgagattgggacctttgcagaaatgc
agtatctgaacttgtcctcaaattccatatcggggcagcttccggccagcattgaacagatgattctgctagaggtgttggatgttagtggaaaccagttg
gatgggaatctaccgccggagattggatccgtggtggcgctccggaagctgctgatggggaagaattcgcttaccggagtcatcccggcgcagattgg
cacttgcagctcactaattactttctcaaagaacaactcctgctatggagtcatgccaaagccaattgttttcaatcctaactcctcatctggtcccttgtca
gaggatgccccaagttcctcaagcaaccagcaccacaagaaaatgctattgagcatctccacactcatcgccattgtgggtggagcagtcattgttattg
gcgtggtcaccatcacattgctaaacctccgtgtccatgcaaaatcttctcgctctgcacttcccactgcattgtcggatgattaccatagccaatcaaccc
aatccccagagaacggcgccaactctggaaagcttgttatgttcggcagaggcagctcagatttcagtgctgatgggcatgcattgctgaatagggatt
gtgagcttgggcggggaggatttggcactatttacagggcagtcctcagagatggccagccagtggctatcaagaagctgacagtttcaagcttggtca
agtcagaggatgaattcaggcagcaggttaagcttctaggcaaggtgcggcaccataacattgttaccctcagaggcttctactggacttcctcgctgca
gctcctcatatatgaatttgtgcgtggcgggaacttgcatcagcacctgcacgagtgctcagaagagagctcgctctcatgggtggagaggtttgacata
atcacgggtgtagcccgggcacttgtccacttgcaccgccatggtataatccacttcaatctgaagtccagcaatgtcttgctagacacaaatggcgagc
caagggttggtgactatggtctagtgaacctgctgccgatgctggaccgctatgtgctgagcagcaagatccagagcgcgttgggttacatggcaccag
agttcacttgcagaacggtcaagatcacagagaagtgcgatgtctcaaagaacaactcctgctatggagtcatgccaaagccaattgttttcaatcctaa
ctcctcatctggtcccttgtcagaggatgccccaagttcctcaagcaaccagcaccacaagaaaatgctattgagcatctccacactcatcgccattgtg
ggtggagcagtcattgttattggcgtggtcaccatcacattgctaaacctccgtgtccatgcaaaatcttctcgctctgcacttcccactgcattgtcggat
gattaccatagccaatcaacccaatccccagagaacggcgccaactctggaaagcttgttatgttcggcagaggcagctcagatttcagtgctgatggg
catgcattgctgaatagggattgtgagcttgggcggggaggatttggcactatttacagggcagtcctcagagatggccagccagtggctatcaagaag
ctgacagtttcaagcttggtcaagtcagaggatgaattcaggcagcaggttaagcttctaggcaaggtgcggcaccataacattgttaccctcagaggc
ttctactggacttcctcgctgcagctcctcatatatgaatttgtgcgtggcgggaacttgcatcagcacctgcacgagtgctcagaagagagctcgctctc
atgggtggagaggtttgacataatcacgggtgtagcccgggcacttgtccacttgcaccgccatggtataatccacttcaatctgaagtccagcaatgtc
ttgctagacacaaatggcgagccaagggttggtgactatggtctagtgaacctgctgccgatgctggaccgctatgtgctgagcagcaagatccagagc
gcgttgggttacatggcaccagagttcacttgcagaacggtcaagatcacagagaagtgcgatgtgtatggttttggagtgctgatccttgagattttga
caggaaggagtccggtgcagtacttagaagacgatgtcattgtgctttcagattttgtcagagcagcacttgaggaaggccggctggaggactgcctgg
atccatggctgtcaggcgagttctcaatggaggaggcgtcggtgatcatcaagctggggctggtttgcacgtcacaggtgccatctcaacggccggaca
tggctgaggtggtcgctatgcttgagatgtgtggtagtagctcagaggatgaactggtttga
>g48932.t1
atggtaaatggcttcatatcatcgtggatagcgttcgtcgcgctggtgcttcagctcgcagtgtatggctgctcggcggtcaatcttgaaggtacgtaccg
gcggcgtgccaccgtacctgtgttcaattttancgtcgcgctggtgcttcagctcgcagtgtatggctgctcggcggtcaatcttgaagggtcggcgctgc
tcaagttccagtcaaaggtggaggaagacccgcatggcgccatggctggctggaagccccgtgacagggatccttgcagctggaacggcgtcagctg
cgttgatggcagggttgtgatgctgtga
>g49147.t1
atggccgccctccgcctcttcctcgcgctgttcctcctgctccgggtccacgccattgcggcggcctcctccgacggcgacctcctcctcgccgccaagtc
ggcgctctccgaccccaccggcgcgctcgccggatggcagtccggctccggcggcggctccccgtgccgctggaaccacgtgtcctgctccaacgact
ccgccggcgccgtcgtcgggctcgacctctcaaagctctccctctccggcgagttcccggccgcgctctgctccttgcggtccctggagcgcctcgacgt
ctccctgaacgacctggtgggccctctgccggcatgcctcgctgcgcttccggcgctcacgcacctcaatctcgccgggaacaacttctccggcgaggtc
ccgccggcgtggggcgcggggttccggtcgctcgtcgtgctcaacctgctccagaacctgatctccggcgagttcccggcgttcgtcgccaacctctcta
cgctccaggagctcgagctcgcctacaacccgctcgcgccgtcgccattgccagagaagctcggcgacctcaccgagctccgcgtactgttcgtcgcca
actgctccctgatcggtaacatcccttcttccattgagtggcttaggaatctcgtcaatttggacctctcgtcgaacaatcttaccggcgagataccgaaa
ggcatcaggaatttgatttctctggagcagattgagctctactccaaccatctctccggggaaattccggtgggattgggtcgcttcgagaagctccgac
gcctggacatgtccatgaatcatctcactgggcggataccggaggacatgttcagggcgccgagcctcgagagcgtgcacatatatcagaacaatctg
accgggcggttgccggagacgctggggacggcgccaagtctgtccgacctcaggattttcagcaaccagcttgaagggccgctgccaccggagttggg
gaagaattgcccctcgagttcctcgacacgtcggacaaccggctgtcgggtcctataccggcgaatctgtgcgcttccgggaagctgagtcagatcat
gctgttggataacgagtttgaaggtgccattccggccgaattggggcaatgccagacgctgttgagagtgcgtctgcagagcaacaggctgtccggccc
tgtgccgccggaactctggggactgccgggtgtcattctgcttgagcttcgagacaatgcattatcagggactattcatcctgccattgctggtgccaaga
acttatgcaagctgctcatacaggacaaccagttcactggtactctgcctacagagttgggggcattgtccagtctggcagagttggtggcttcgaacaa
tggcttctccgggccgctgcccacttcgctcgccgagctttctgctctttacaagcttgatctcagcaacaactccctctccggtgggatcctgcctggattt
tctaagttgaagaatcttgcctgtgcaattggaaaatcttag
>g50229.t1
atgccgcccgccgccgctccgcttccccaccacctcctcgccttcctcttactcctcctggcctccgcgccgcgccccgcggcgcccgcgtccgccgaccg
cgcggcgctcctcgccttccgcgccgccctctcccctccctcccgcgccgcgctcgcctcctggagcggcccgctctcgccgacctggctcggcgtctcgc
tccacccggacgcccccgcccccgccgccgcgccgccctccgtctccgcgctcgagctcccgggcctcaacctcacaggcccgctgcccgcgccggcg
ctcgcgctcctccgccgcctccgtacgctcgacctctccggcaacgcgctctccggcgagctcccctgctccctcccgcgctcgctccaggcgctcgacct
ctcccgcaacgcgctgtcgggcgccgtccccacctgcctgccctcctccctccccgcgctccgctccctcaacctctccgccaacctcctccgcctcccgc
tctccccgcgcctctccttcccctcccgcctcgccgcgctcgacctctcccgcaacgccatccccggcgcggtgcccccacggatcgtggccgaccccga
aaactccgcgctcctcctcctcgacctctcccacaaccgcttctccggcgagatccccgccggtgtcaccgcgattcggagcctgcaggggctgttcttcg
cgggcaaccagctgtccggggagatcccttccggaattgggaacctgacctacttgcaggtactggatttgtcgcataatcggttgtccggtgcagtgcc
agccggcctcgcgggctgcttccagcttctgtacctgcggctcgggggtaatcgactctcaggggcgctgcgaccggagcttgacgcgcttgatagtctc
atggttctggatgtgtccaacaacaagatatcaggggagattccgctgcccctggctggttgccgatctcttgaggtggtgaacttgtcaggaaatgaga
tctcaggggagctcagtagtgctgtggccaaatggcagagcttgaggtttctgtcactggccgggaaccagctctccggccaactacctgattggatgtt

ctcatttccagtgctccagtggcttgatttgtctggtaataaattcgtgggtttcatcccggatggtggattcaatgttagtgcagtgctcaatggtggaggt
ggtcaggggattccatcagaagaagtgctcccgcctaaattgtttgtttcagcatctgtggacacaatgtcatggcaattgaatctggggtacgatcttca
gtcaactactgggatagatctgtcaagaaatgagttccgtggggcgataccagaagggttggctgcaatgaaaggattggagtatttgaacctatcttgt
aattatttggccgggcagatccctgaggggcttggagaaatggggaagttgcggacacttgatttttcacataatgtgctatcaggagaggtgcctcctg
tgattgcttccatgacagagcttgaggtgcttaacctctcctacaatagcctgtctgggcctctaccaacaactgatggtttacagaaatttccaggagca
ttagccggaaacccagggatatgcagtggggaagggtgcccggaggacgcaatgtgcagtacatttcagtacaacgcctaa
>g50686.t1
atggtcgccggaacccagtccccgaccgcaacggccgccgccgccgccatcctcgtggcggtggcgctgacgtccctgtcgccggcggcggctaacga
cgagagcaaggcgctgctggctttccggaacggtttcgaggatcctcgtggcattctggattgggtgccaaacatggtggatccgtgtacctgggtccgg
gtcgaatgcaacgacgacaaccgcgtcacgcagttggagctggccgggaacaacttcgagggaccgatcccgccagagtacggtaacctgacgagcc
tggtagccatggacctgttcaacaacaagctttcggggcccatacctcgacgatcgggaacctgaagctgctggttttcctgcgcatcgatcacaaccg
gttgagtggacccatccctgcggagctggccgcactgctcgcccttgcagaagcggatttctccaacaacgacttgtgcggcaccatcccgaccggcgg
agcgttccagaactttcctgccaagagcttcgacaacaacccgcgcctgatacgccccgggatggagggcactgacgcggacggctccagctgctag
>g50904.t1
atgctcctcctcctcccgcggctgctgctgctgctgccgccgctccttctccttgccgccgcggtggacgcgcagcagccgcagccgctgtcgtcgggga
cggacctggcgggcctatacagcctgcgcgggtcgctgggcctgcgcgcccgggactggccggcccggtcggacccgtgctcggcctggaccggcgt
cacctgccgcgcgggccgggtcgtggccctcaccgtcgcgggcctccgccgcacccgccgcgcctccctcgccccgcgcctcgccctcgacggcctgc
gcaacctcaccgcgctcgaacgcttcaacgcatcgggcttcccgctgcccggcgagatccccgcctggttcgcctccgggtccggcctcccgccgccgc
tcgccgtgctcgacctcacctccgcgggggtcaacggcaccctccccgccggcctcggcgcggcgtcggggaacctcacgaccctgctcctcgccggg
aaccgcatctccggcccggtccccgcgccgctgctctcggtcaacggcattcgcgtcctcgacctctcccgcaacaatctcaccggcgagcttcccaaca
tctccggtcccgccccgtcatcctcactgttcaacatttccgggaactcgctgtacggcgtggccggcgacgccgtccgggcgctgaggagacggtttca
ggtggtggacgtgtcgagcaactacttggacggaccatggaccgggtcggcctccgatggaacagtagtgcttgccacgacgaactgcttctccggcg
cgccgccgggtcagcgcggccgtgcggaatgtgaggagttttacaggaggcagggcgtgaggctcgttgacgctccggcgccctcgccttcgctgcag
ccgttaccggtaccggtgaagggggaagaagcagaggatctccaggaacgtgctcatcggagtcattgtcgccgcgggagcgttgatgatcttgtttctg
gtcgcgttggcgttctgtttcatgggaaggagaaggagaagaaggggaggaagaggagcaggaagaggcatcgagccaaatgaggaaggcacacg
ctccgggcgtaggagggacagcagtgtgaacccggtggcatcgtcgccgtcagcaatgtcaccgagagcgaacgccgtatccaacaaggctgcgtctt
ccgtttcagacgatgacttcacctttgagcagctggttcatgccacggggggcttcggcgacgacaacctcctcaagcacggccactcaggggacattt
accatggcatcttggagaccggctcccacgtcgttgtgaagaaggttggcgccaagagcggaaacaagcacgcgggcgagctggacttctacaagcg
gtacacgcacgacaggatcgtccccttgctcgggcatttcgccatggacgacgacgtgtttctggcttacaagtacatgccgaaggggggacttgaccgg
tgcgttgcacaagaagcctgctgtggacactgaagatggcttgccctcgttggactggataactaggctcaaaattgctactggcgtggctgaagccatg
tgcttccttcatgatgagtgtagcccgccacttgttcacaggtcgctagagaagcacacatcagccccgccggccacatgctcgtacgacgtccactgct
tcggcaaggtgctcctggagctggtcaccggcaacttcagcatcagcggtcccttcgccgcctccgacgagtcgtcggagtggctgacgaccacgctga
gccacatcagcgccggcgacaagtcggccatcacgaacatcatcgacccgctgctggtggtggacgaggaccacctggaagaggtgtgggcggtggc
catcatcgccaagacgtgcctgggctccaagccgtcgcgccgcccgtcggcgcgctacgtgctccgcgcgctggagaacccgctgcgcgtagcgcgcg
cgtccagctcggcgcgccagttgcggagctgctcgtcgcggagctcgtggcagtccgtgttccaggggaacgccaatggcggccgcgtgcagagcttc
gacgtggcggcgctcgaccggaggcactcggcgaggtcgttcgggagcggcggaggaggagggggaggggcgtcgtcgttctcgttcaggaggact
ccggcggccaccacgccggagatcgtgccggagccgacggcaccgcgggaagaggaagaagacggtgccggagccgacagcggccaccgcaccg
agacaggatcacaccattga
>g51693.t1
atgggtttccaaaggccatcagggatttccccggcctgtttcccaggcgggcgggcttccccgagattgggccgggatccccgcggggaaggccgttcc
caaagaagccccaatgcgggccacatctccaggatcatgctccccggggccgggctcatcggtacactcgtggccctgaaccccacggcgttcccggt
gctcacgaacctcaaccttagtggcaaccgcctcgctggcgccatcccaaccaccatttccaacctatcctcgttgttgctggtattgcttgatttggccac
tcggtcaagtcctcatgctgagcaacaacttagtcaagtccgtgaaaatcaatatcacaagcttagtcaagtcctcgtgctgagcaacaactcgctcggc
ggctccattccgtcattgctcggtcggctatggaaactacaacagctcgaccttcatgccatgaggctggtctctaggctgccgctggagatggggcaca
tgacgagcctccacatcctcgacctctcgctaaacaacctcaccagctag
>g52169.t1
atggctcgtcgacggcgcgcactgccaccgccgccgctcgtcggcctggccgtcctcctctccgtggccgcagccacggtggcaaagaccgaccagtct
gacgtaactgcactcaacgttatgtttaatagtatgaacaaaccatctaagttatctggttggaaaggtagcggcggtgatccgtgtggtgacgacgatt
cgtggaagggaatagaatgcagcggctcatctgtcacagaaattaacttgtcggggcttggactaagcggcacgctaggctatcagttatcaagcttga
aatcagtaacgaaatttgatgtgagcaagaataatctcaatggtgagattccctaccagcttccacctaacgtggttcaattaaatctgtatgggaattcc
tttactggtggagttccatattcgatttcacaaatggttgatctggaaacattgtag
>g52456.t1
atggtgctgatcttgtccctgcaactgcaactgcaactggtgtcgtcggcaaccgacggggacgctctgctggcgttcaaggcccgcgtgaccgacccc
accggagcgctcgcctcttggaacgcctcgacgcacttctgccggcggtgggccggcgtgacgtgcaccgcgggccgcgtcacgtcgctggacctgtc
ggagcgccgcctcaccggaacgctccctccggccatcggcaacctcacgcacctctcatctctcaacctcacccaaaacggcatctccgggagcattcc
ggcgagcctcggccggctccatagcctctcgtacctcagcctctgcgacaacgccttctccggcgagattcccgacgcgctccgcaactgcaccgcgct
ctccgccctgtacctcaacgacaaccgcctcaccggcagcgtgccggactggctcggcggcggcggcgcgttcccggtcctcatgtacctcgggctcgg
caccaaccagctctccggccgcatcccgccgtcgctggccaacatcacgatgctcaggtggctcggactcgatcagaactttctggaaggcacggtccc
tgacgacttgtcgcgcctcaccaagctcatgtccttgaacgtctaccagagccacctcgccggcgagatcccgccgttcctcttcaacatgtcgtcgctgc

aaggcttgacactcgccaacaacgcattcgagggcgagctccccgccgacgccggcgtgggctggccgaaccttcagtacctctacctcggtgggaac
cggctcaccgggccgatcccggcgtcgctcgctgaggcgtccagtctgacggtcctcagcctggccaacaacagcttcaccgggcaggtgcccacggc
gatcggcaagctctgcttgcaggcgttgcagctgtcaaacaacgagctcacgacctccgacgccggcggctgggagttcttggagggcttgaccaactg
cagatcgctgtcggaaatctctctcgacggtaacaaatttactggagcaatgccgagctccattgcccacctctcgccgaacctccagacgctgaaactc
ggcggcaatggtggcatatccggagttataccgccgggcatcgggaacatcgtcgggctacagacgctggatttcgagtcgaatctcctatcgggcgtg
ataccggaaggcattgggaagctcaagaacctcaaagaactacggttgcagcacaacaagctaacaggccctattccatcctccattggcgacctcag
ccagctcctagtcatcgacctgagcaacaatttgctgaacggctcgatcccgcccagcctcggtgacctccagcagctcacactgcttaacctctccggt
aacgggcttaccggccacgtccccggagaactgttcaagctgtcgtcgctgtcctcggcgatggacctgtcagataaccggctggacgggacgcttccg
ggagacgtcggccagcttgcgaaactcgcacttttggccttgtccggaaaccgcttctccggcgaggtgccgtccgagctgggcttgtgcgcgagcttgg
agtttcttgatctggacagcaatttcttcaccggaagcatcccgtcatctttcaaaagtctcaaggggctccggaggctaaacctgacaagcaacaaatt
gttaggacgcatcccgccggagcttgggcagatgtcagggctgcaggagctgtacctgtcacggaacgaactgtccggcagaataccggcagaactg
gagaacgtgacgtccctgaaagagctcgacctgtcttacaacaacctcgacgggccagttccattgcgcggcgtgttcacgaacacgaccgggtttcag
atggccggcaacggcgcactctgcggcggtgcggcacggctccgcttgccgccatgtccgccatcagcgaattccacttccacccgacgcggtcacgtg
ttcctcaagatcgcgttgcccatcatccttggcggcgtggctctctgctttgggatgctgattgctggccagagccactga
>g52596.t1
atggcggtgcacgtctcgcggcggccggatccggggcctccgctcctctccacggtggcgcagtttcacgacggccagatccggggcctccacccctct
ccacggcgcaccacatcacgcggcggccggatccggggtggccgcccctccatggcggcgcacgacacgcggcagcccgattcatggcctccgcccc
tctccacggccccgcctcctctcttcgccctcttcgtcggatccattcgccttcatcagctactgaagtgtagcggcggaaggcgatacctgccgctacaa
caaaaagctagcacctccgttgtgggttgtggccgaaacataacgcccgtgtcccctttcctactcctcttcctgctcgttgccatcccgctcgccacctca
gctacgccgttgaacacctcatccggcatcctcctttccttcctcgccgccctgccgctggaatcacagcagctcctcctcccctcgtggcgagccaatgc
cggcaactcgacaccggatccacattgcgcgtttctcggcgtcacgtgctctgccgttggcgccgtagccgcgctcaacctctccggcgtggggctgtcc
ggcgagctatcagagtccgcgccgcacctctgcgcgctgccggagctctccctgctcgacctcagctccaacaatttcagcggcgccgtgcccgccgcg
ctcgccacttgctccggcgtcaccacggtcgtactcgccgacaacaggctctccggcggcatcccgccggagctgctctcctcgcgccagctccgcgat
gtggacctcagcttcaacgcgctcgccggcaagatcctaatggcgccttcctccgcagacaaatcggttctcgagttcgtagacctcaagaacaattccc
tctccggcgccattcccgcagagctcgccgcgctcccggagctcagacacctggacctgagcatgaaccaactgtccggtccaataccagacttcccaa
ctcaatgcaagatcgaatccctccagctctacaacaacgagatagctggtgagcttccccgcagcctcagcaactgcgccaatcttacagttctgctcct
acccttcaacaagatcaccggcgcagtgccggatttctttgcatcgctgccgttgctcaaaaagatttatctcgacgacaatgccttcactggggagttgc
ctgtgagcattggtgagcttctcaatttggagaaactggtggcttcgacaaactggttcactggaccggtccccgggtcaattgggaagtgtcagtcgttg
accacactaaggctgaacgacaaccagttcacagcattgtggcaaatgccggacatggagttggcgctctacaacaacagcctaagtggagaagttcc
tgcagaggttgctcttatgaggaatctaacagagttcaacttagcatttaacaacttcactgggagctcgccggaggcgatgggaagcgcgctgcacgg
gaacgtcgcgctccacgggaactag
>g53371.t1
atggggtctcagtttgcagcagcaactcttctgactggccttcttgcttttgccatgtctgcgaactgcaacactgaaggtgatatactgtatgcccaaaga
atggcgtggcagcagcctattgatgctctggagagctgggatcccacacttgagaatccctgtctatgggaggatttagggaatgcaggtatatcaggc
cctctagttccagaactgggagatcttgagaagcttcagtacctgaggttacatggaaacaacctgacgggagccataccatcgtctctgggcagtctca
cgaaccttgtggacttagagcttcagaataatgccctcagtggatctattcctgcctccctgggcaacatcaagacacttcggttgctgcgactgaatgac
aatatgctaactggcacggttccattggaaatcctctctcttgttaactctgggaacttgtctgatctgtga
>g53523.t1
atggccatggccatggcagcaccaccacaccgccagcccaagcgccgccgctcctctaccaccccggctcagtctccgctggactcgctggccgacga
gctcctgttcctggtcctggaccgcgtggccgccgcggacccgcgcgcgctcaagtccttctccctcgcctcgcgcgcctaccacgccgccgagtcgcgc
caccgccgcatcctccgcccgcgtcgcgccgacctcctcccctccttgctcgcgcgctaccccgccgccgccatcctcgacctcaccctctgcccgcgcct
cgccgacgccgccctcgccgccgtctcctcgtcgccgtgcctccgcgccgtcgacctttcccgctcctcggggttccgcgccgccggcctcgccgcgctc
gccacggcgtgccccgacctcgccgacctcgacctctccaatggggtcgacctcggcgacgccgcggccgccgaggtggcccggatgcgcaggctcc
gaaggctctcgctgtcgcggtgcaagccggtcactgacatggggctcggatgcgtcgccgtcgggtgccccgacctacgagacctgtctctcaagtggt
gcattggggtcacggatttggggatcaagctcctcgcacacaagtgcaggaacctgaggacgctggatctctcctacgccatgatcacaaaagactcct
ttccagccatcatgaagctacccaatcttgaggtgttgacactggtgggctgttctggaattgatgatgatgcccttagcagcattgagaaacattgcatc
aaatcactgaaggttactccttctatgggctga
>g54007.t1
atggagtccatggcgtgggtgatggacggcatcgtcgacgcgaccgaactctggcgaggcctcgacgtttacggcgttcctcgggtcaagccgttccat
ccggttgtaagtgtggacggctccaacgacgtcatctgcttcgtgacttgtcatggctccgtgacccgagcgacgcggaggtcgagtttcaggtttcttat
gcttcataccggcagctctgcatctggaaatgaaacagaccggctgtcactgctagacttcaagaaggccatcactcttgatccccagcaagccttggcc
tcatggaatgacagcacccacttctgcagctgggaaggcgtcctctgcacagcaaacaacccccgccgtgtcacctccatccgcctcgaaaggcgagg
cctagccgggcaaatctctccttcacttgcaaatttaacattcctacgaaacctttcacttgcaacaaaccagttcactggagaaatccctgcatcccttgg
ccacctgcaggttttgcgtgacatcaaggtccctctaggcgacatcaagatcctttcaatgtga
>g54239.t1
atggccatggcagcagcagcgcaccaccagcccaagcgccgccgcttctctacctcccccgcccccctacgccgccacgccgctcgactcgctggccga
cgagctcctgttcctggtcctggaccgcgtggccgccgccgacccgcgcgcgctcaagtccttctccctcgcctcccgcgcctgccacgccgccgagtcg
cgccaccgccgcgtcctccgcccgctccgcgccgacctcctcgccgccgcgctcgcgcgctaccccgccgccgccaccctcgacctctccctctgcccgc
gcgtcgccgacgccaccctcgccgccgtctccgccgcgtcgtcgtgcctccgcgccgtcgacctctcccgctcctcggggttccgcgccgccggcctcgc

cgcgctcgccagggcctgcccgggactcgccgacctcgacctgtccaatggggtcgatctcggcgacgccgcggccgccgaggtggcgcggatgcgg
cggctccggaggctgtcgctgtcgcggtgcaagccggtcaccgacatggggctcggctgcgtcgccgtcgggtgccccgacctccgcgacctctcgctc
aagtggtgcattgcggtcacggatttggggctcaagctcctcgcccacaagtgcaagaacctcaggacgctggatctctcctacggcatgatcacaaaa
gactcatttccagccatcatgaagctgcccaatcttgaggcgttgacactggtggggtgttctgggattgatgacgatttcctcaatagtattgagaaaga
ttgcaacaaatcattgaaggtgcttgatatatctcattgtccgaatgcgactgatgtgggggtttcatctgtagtgaagtcgatacccaatctatcagaatt
gaatctttcttactgctgtcctgttactccttctatggcaagcagcttccacaggattgctaatctgaagaccctcaagctggaaggctgcaaattgatggc
tggtgcacttagatccattggaaagtcttgtgtctctgttagggagttaagtctgagcaagtgttctggagtggcagatgcagaactttcttttgctgtgtca
agattaaagaatttgctgaagctggacattacttgttgtcgcaatattactgatgcttcactagcttccatcactacttcatgcacttctctcatctctctgag
aatggagtcctgcgcccatgtttctagtgaaggacttaaattgattggaaagtactgtcatcaattggaagagctggaccttactgacaatgatttggatg
atgaagggctgaaagctattgctggatgtagcaaactctcaagcctaaaaattggcatatgcctgaagataagtgatgaaggtctcactcagattggaa
agtcttgccctgaactccgagacattgatttgtacaggtgcggagacattagtgataatggtgttattcgtattgcgcaaggctgtccaaagctagagtct
ataaatctgtcctactgtacaggaataactgaccgttcactgatctccctctcaaaatgttcaaatctgaacacactggagattcgtggctgccctagagtt
tcatcctctgggatagcagaaattgcaatgggatgcaggctactttccaagcttgatattaagaaatgctttgagatcaatgacgtggggatgctttacat
ttcccagttctctcatagcctccgtcagataaacttgtcatattgttcagtcaccgatattggacttctttcgctttctagcatttgtggcttgcagaacatga
ccattatacatttagctggtatttcgcctaatggcttgatagctgctcttctggtctgtggtggtttgacaaaggttgagctagaaccatgtgatgtatggaa
gcaacagtcacaagatgtgctggttcgatga
>g54480.t1
atgagggacatgggagagaagaggagacatcatggccacggccatgccgttggatcgtttggagctggaagacatttagagcacgaggagaacaag
cacaaggaccagaagctagacatgagcggcatgaacatggaaaccatcccacatctcaccatgacactcggccacatcaccaccttggatctctccaa
caataacctacagaacatccctgaatcaataatcgcgaggctcctgaacgtagtcgtgctggacgtgcactccaaccagctcacgtcgctgcccaactc
catcggttgcctctccaagctcaaggtcctcaacgtctccggcaacctgctcgagtccctgcccgccaccatcgaggaatgccgtttgctggaggagctg
aacgccaacttcaaccagctgacgaagctgccggacacgctgggcttcgagctccacggcctccgccggttgtctgtcaactccaacaagctcacctcc
cttcccttctccacctcccacatgatagcgctgcgcgcgctcgacgcgcgcctcaactgcctccgcgcgctgccggacgggcttgaaaacctgggcaac
ctggagacgctcaacgtgagccagaactttcactacctgcgcgagcttccctatggcatcggcctgctcgtctcgctccgggagctggacatcagctaca
actccatctccgcgctcccggactccatgggctgcctcaccaagctcgtcaagttcagcgccacgggcaacccgctggtgtgcccgcccatggaggtcg
tggagcagagcctcgacgccatgcgcacctacctcagcgcgcggatcaacagcgccgacgacggcaagaagaagaagaggtcatggatgcccaaga
agctggtcaagtacagcaccttcacggcagggatgatgacgcccggccgcgccaaggtgcacggcaacggcgacggcctcctcatgtcggattaccg
gtcgatcgacggccacatcgcgtcaccggggttcctcagcatgctgtcgccccgaaggctcttctcgccacggaggaattcgcccaagcattag
>g55096.t1
atggggttgaggagtggcggcgcggaggattcagcagtaatgtcgctttcgctgctgctgctgctcgtgacggcgtgcggcggcgcgtgggcgctg
aacgccgacggggagctgctcatgtcgttcaagaacgcggtcacctccgacccgctgggcgcgctctccggctggaactacggcaacgtggagccgtg
cgcgtggaacggcgtcgtctgcgccggcttccctttgccggacgccgcggcagccaacgtgacgtcatcctccggcgaagaagtgaacgcgtcgatgg
cggcggcgacggtgtcgcgcgtcatcagcctggtcctccccaacgcgcagctgtccgggtcgctggcccctgacctggcccgcctcgagcacctccgcc
acctcgacctctccgggaacgcgctcaacggcaccgccttccccgccgcgctgctcaacgcctccgacctccgcgtcctctcgctcgcgggcaacggca
tcgccggctccctccccgactcagcgtccgcctactcgcgcagcctccaggagctcaacctctcgggcaacgccctctccggccgcctccccgccgcgct
gtgccggctgcagcgcctcgccgcgctgggcctcgccgacaaccgcctcgccggcgcgctccccatcggcgggctcggccccgcgctggagctcgtcg
acctcagctccaactccttcaacggctccgtcccgtcggacttcggcggggcccacctgcgggtgctgaacgtgtcctccaacaacctcaccggcgcgct
ccccaccgagctcgccgccgtcgtgccggcgaacgccaccctcgacctgtcacgcaacaacctcaccggcgcggtgccgcaggcgggggcgctcgcc
gtgcagcccgcggcggcgttcgagggtaacccgggcctgtgcgggccgccgctggcgcaggcgtgctccatcccgtcgtcgctgtccaacccgcccaa
cgccaccgactcgccgccggcgttcgccgccatccccaagaacaaccctgctcgctcaccgccgggggccccacctcaggatccggcgcaggggcag
gggaagctccggcccgccgcgattgtggccatcgtggccggggacatcgccggcgtggggatcctcttcgcgctgttcatgtacgcgttccatgtcagg
aagaagaggcggcagcggcggcgcgaggaggaggatgaccaccacccgtcgtcgttgcagatgggtaccaagagcgtgcgagcgatcggcggcga
cggggtgaagacgctggacatcggcagagaggagaaggcgtccacgtcgacggggtgctgtatcggccgcggcaacgacagctcggacgaggaga
gctccgagtgctcggtctcgtcggacggcgagcccgacgacggcacgaagaagagggggagcctgattggccggagcacgccgcagcacggcagca
agaagtacaacacccctgcgccgccgccgccgcagcagcaggcgccggcgacgctggtgacggtggacggcgacggcgagctggagatggagacg
ctgctcaaggcgtcggcctacatcctcggcgccacgggctccagcatcgtgtacaaggccgtgctcgccgatggcaccgcgctggccgtgcgccgtatc
ggcgagagcggcggcgccgagaagctcagggacttcgaggcgcaggtgcgcgtcgtggccaggttccggcaccccaacatcctccggctgcgaggat
tctactggggcgccgacgagaagctgctcatccacgagtacgcgcccaacggcagcctcgccaacatcgccttcagcaggcggttcggggcgtcgtcg
ccgctgcacctgagcctggaggcgcggctgcggatcgcgcgcggcgtggcgcgcgggctggccttcattcacgagaagaagggcgtgcacggcaacc
tgaagccgagcaacatcctgctgggcgccgacatggagcccatgatcggcgacctgggcctggaccgcctcctctccggcgaggccgcggtgcaccg
cgccggcgcttcggcgcggctgttcggcagcaagaggtccatgcactccaccagcagcctgccggacctgtcccagatgcccgggcccggcgcgagc
ccgtgcgggtcggcgtcggcggcggcggcgtccggcccaaccaccggcccgtaccaggcccccgagtgcctcaagaacctccggcccaacgccaagt
gggacgtctactccttcggcatggtgctcctggagctgctctccgggcgggtctactccgaggtggagctgtgccagtggcacgccggcctcgtcgccga
ggagcgggcccgcgtgctgcggatggcggaccccaccctccgtggcgaggcagacggcagggaggacgcgctggtggcctgcttcaggctcgcgttc
gcctgctgcgccatggcgcccagtaagcggcccgccatgagggacgccgtcgtcgtgctcgacaggacggcggcggcttccacgacgccgggcgcgt
cgtcgaccaccaacggcgccattccttga
>g55110.t1
atggacaagaccgagaagaagaaggaggagggcgccacggccacgcgagcggtgggtctgcgcgccgctgccttcgccattgcggccctcctctgct
ccacggcatcgctggcgctcagccctgatggcgaggctctgctggagctcaagctggccttcaacgccaccacccagcggctcaccacctggaggcctt

cggaccccaatccctgcggctgggagggcatctcctgctccctacccgacctcagagtccaatccataaatctgccctacatgcagcttggcggcattat
atcgcccagcattggcaagctggacaagctccagagactggctctgcaccagaacagcttgcacggctcaatccctccagagattaagaactgcaccg
agctcagagcaatttatatgagagctaactacctgcaaggaggcatcccttcagagattggcgagctcatccacctcactatcttggacttgtctagcaat
ctgttgaggggcacaattccagcatccattggaagcctcactcacctgcgttttctgaatctgtccaccaatttcttctccggtgaaataccgaatgttggt
gtccttggaaccttcaaaagcagctcgttcgttggaaatctagagctttgcgggctgtccatccagaaagcttgccggggaacccttgggtttcctgcagt
gctaccgcattctgatcccctctcctcatctggtaagtccttcatttcaatctga
>g55143.t1
atgcctcccagcggcggcactgctccgccgctcctcctctcgccaccgctcctgctgctgctgcttctctgccacgccaatgccggctcgctggagtc
cgacgtggccgcgctctcggacttccgcctcgccgcggaccgcacgtccgcgctgacgagctggaacctgtccgcgaacccgtcaccctgcgcatcctg
gcgcggcgtcacctgcgccggcggccgcgtgacgcgtctggtcctcgaggacctgtccctctccggcgacgcagcgctcccggcgctcgcgaggctgg
gcgggctccgcgtgctcagcctcaagcgcaacgccctctccggcagcatccccgacctctccccgctcgccgggctcaagctgctcttcctcgcgcaca
acgcgctgtccggcccggtcccaccctcgctcgggaagctctaccgcctgtaccgcctggacctatcctgcaacaacctctccggcgaggtcccgccgg
agctcaaccggctcgaccggctgctgacactcaggctggagtccaaccgcctcaccggcgggatcgacgccttaaatttaccgaggctgcaagagttc
aatgtgtcccacaattcaatgtcggggaagatcccgtccccgctggccgcgttccccgccgcggcgttcgacgggaacgccggcctgtgcggcgcgcc
gctcccgccgtgcaacgcgtccgaggcggcggcggggcggtgtcctccatccacggtggcgtcgtcgccctacgcgaagccggcgggcgcggagacg
ccgcctgaaagtgggaaagggaagatgagccgcgccgccgtggtggccatcgtggcgggggatttcgcggtggtggccctcgtcgccgcactcctctt
ctgctacttctacccgcggctctccaaccggcgccgcctccgacaaggggagaaaatcgtctactcctccagcccgtacggcgccgccggggtcgtggc
cgcggctgggggtggtgctgccggcggcggcggcacgttcgagcaccgggggaagatggtgtttctggaggacgttacgagcagcagcggcgggac
gaggcggttcgagctcgaggagctgctgcgcgcatcggcggagatgctggggaaaggcgggtccggcacggcgtacaaggccgtgctcgacgacgg
caccgtcgtcgccgtgaagcggctgcgcgagaccgctcccggcgcggtgggcggcgcgtcgtcgaggaaggagttcgagcaccacatggcggtcctt
ggtcgcctgcgccaccccaacgtcgtgccgctcaaggcgtattactatgcccgcgacgagaagctgctggtgtacgagtacatgcccaacggcagcctc
ttctccatcctccacggcaagtcctttgcttctcagttctcatgggcggcgcggctgcgcatcgcgtcgggcgcggcacggggtctggcgtacatccacc
acgcgggccggcggcggacgacggggtcgccgcacaagctggcccacggcaacgtgaagagcaccaacatcctcctcgacaagtccggcgcggccc
ggctggccgactgcggcctggcccacctggtcccgccgtcgccgcgctccgcggggtaccgcgcgccggaggcgccgccgccgcaccggccctgggc
gtcgcagaagggcgacgtgtacgcgctcggcgtcgtgctgctcgagctcctcacggggcggttccccggcagcgagctgcccaacggcggcgtggtgg
cggagctgccgcggtgggtgcagtccgtggtccgggaggagtggacctccgaggtgttcgacctggagctcatgaaggacaggggcatcgaggagga
gatggtcgccatgctgcagctcgcgctcggatgcgcggccgcggcgcccgagcagaggcccaagatcggctacgtcgtcaagatgatcgacgaggtc
cgcgcctgcggggaggcgtcgtcgcgggaggagtcgtccatggacgagtcgtccggcgtatctgactcgcccgccgtgtcggaaggcggcgcgattag
cggctaa
>g55674.t1
atggatccgacgccgcagtctcacccgatccttgcctacgtgctgtcccgcctgccgtcgctgtcggcgcggacgacgccgcggtcctcgtcgctctcctc
cccgcggtcgcgggacctcgagcagccgtccccgcgcgcgccgtcgggcgccgcggaggccgagctggtcgcgctcatgccggggctccgtcacctgt
ccgtgctctcctccatgacgcgcgccgtggccgatgtcgccaccgcgcgcgacgcgatccggctcctcgggccccgcccggaccacgagctcgtcgac
gactcccgcgccgtcatcgccgccgctgaatcctccgtggggaaggtcgaggaggaggagaaggtggcggcgagccgggaggtggtgcggctggag
gaggagcacgaggcgtacggcaccatgctgcgcgacgcggaggggaaactggagcgcgtgtaccggatggcgatgcacgggagggacatccagga
ggcgagcaaggaggaagaggaggagaaggaggggtccggggcggtagtggacgaggaggtggtgcgggtgctcagggaggcggaggaggggaa
gccggtggagcgggtgtgcctcgccgatcgccagctgcgcctcctgccggagcccgtgggccggatccgcggcctcctcgcgctcgacgtgtcccgca
accagctcaaggtggtgcctgatgctattggcggtctggaacagctggaagaactccgcctgaattccaacgccctggtttctctcccggactccatcgg
cctcctctccaacctcaagatcctcgacgtttccggcaaccggctgaaggtcctcccagatagcatctcgaaatgcaggtcactggtagagctggacgc
gagctacaacgcgctggtgtacctcccgaccggcatcggtcacgagctggtgaacctgcagacgctgcgcgtgcacctcaacaagctgcgctccctgc
cgtcgtccgtctgcgagatgcgttcgctgcgcctcctcgacgcccacttcaaccagctccacggcctgccgcccgccatcggccagctgtccgcgctgga
gacgctggatctgagcagcaacttcagcgacatgcgcgacctgccgccctccatcggcgacctagcgggcctgcgcgacctcgacctcagcaacaacc
agatccgcgcgctgccggactgcttcggccgcctcgccaggctggagcgcctgcgcctcgaccagaacccgctcgccgtgccgcccgcggaggtggtg
gccaggggcgtcgccgccgtgaaggagtacatggcgaagcggtgggccgaggccgtcgccgaggaggagcgtcggcgcgccggcgtggcggtgga
gagccccaaggcggccgccacgcccagggagtggctgacgcggagcgtgtcgtcgctgagcacctgggtgtcggacgtcaaggagaaggtcgtcgg
gcaggacaaggtggcggaggaggacgagtttctccagcagcagttctga
>g55882.t1
atgagccgggagggccggcggctggaccacggcggcggcggcggggacgacgtcgccggcggcgtcggggtcctcgtgctgtcgctggacctgctg
ggccaggtgctcgaccgcctgcgcgacccgcgggaccgcaaggcgtgccgcctcgtcagccgcgccttcgagcgcgccgaggcggcgcaccgccgc
gcgctgcgcgcgctccgccgggagccgctcccgcgcctgctccgcgcgttcccggcgctcgagcggctcgacctctccgcctgcgcctccctcgacgac
gcctgcctcgccgtcgccgtcgcgggcgccgcaggcgccggggacctgcgcatccgcagggtgtgcctcgcgcgggccagcggtgtcgggtggcgcg
ggcttgaggcgctcgtcgccgcctgccccaggctggaggccgtcgacctctcgcactgcgtcagcgccggggaccgcgaggccgccgcgctggcggc
agcggccgggctcagggatctcaagctggacaagtgcctcggcgtcacggacatggggctcgccaaggtggtggttgggtgccccaggctggagaag
ctcagtgtcaagtggtgccgcgagatctctgacatcggcatcgatctgctcgccaaaaagtgcccagagctccgcagcctcgacatctcctacctacag
gtgggcaatgagtcccttagatcaatatcctccctagagaagctagaggagttggcaatggttggttgctcatgtatagacgatgaaggcctggaactg
ctaagcaaggggagcaactcactgcagagtgttgatgtatcaagatgtgatcacgtgacctccaagggattagcttcactcatagatggtcacaattttt
ccagaagctatatgctgcagattgtttgcatgtgagttga
>g56061.t1

atggcgcaacaacggttgtccgtggcgctcgccgccgcgttcgtcgtcgttctaacgacgatggtccggccgggcgaggcgctgtcgccggacgggaa
ggctctgctctccctcctccccggggcgccatcgcccgtgctgccgtcgtgggaccccaagtcctccacgccgtgctcgtggcagggcgtgacgtgctcg
ccgcagagccgggtggtctccctctccctccccaacaccttcctcaacctctcctccctcccgccggacctcgcctccctctcctccctccagcttctcaac
ctctccacctgcaacatctccggcaccatcccgccctcctacgcctcgctctccgcgctccaagtgctggacctctcctcgaacgcgctgggcggcgaca
ttcccgccgagctcggcgcgctgtcgcagctccagttcctgctcctcaactcgaaccggctcaccggcggcatcccgcggtccctcgccaacctctccga
cctccaggtgctctgcgtccaggacaacctcctcaacggcaccatcccctcctccctcggcgcgctcgcggcgctgcagcagttccgggtgggggcaa
cccggacctgtcggggcccatcccgccgtccctcggcgcgctggccaacctcaccgtgttcggcgcggcggcgacggcgctgtcggggcccatcccgg
aggagctcggcaacctggtgaacctccagacgctggcgctgtacgacaccagcgtgtcgggtcccatccccgccgcgctgggcggctgcgtcgagctg
cgcaacctctacctccacatgaacaagctcacgggcccgatcccgccggagctgggccggctgcagaagctcaccagcctgctcctctggggcaacgc
gctgtccgggaccatcccgcccgagctctccaactgctccgcgctcgtcgtcctcgacctgtcggggaaccggctcagcggcgaggtccccggggagct
cggccgcctcgacgcgctcgagcagctccacctctccgacaaccagctgaccggtcgcatcccgcccgagctgagcaagctgagcagcctcaccgcgc
tgcagctcgacaagaacgggttcacgggcggcatcccgccgcagctcggggagctcaaggcgctgcaggtgctcttcctgtggggcaacgcgctgtcc
ggccccatcccgccgtcgcttggcaactgcacggagctctacgcgctcgacctctccaagaaccgcctcacggggaccatccccgacgaggtcttcgcg
ctccaaaagctcagcaagctgctcctcttgggcaacgcgctgtcgggcccgctcccgcccagcgtcgccgactgcgtctccctcgtccggctccggctcg
gcgagaaccggctcgcgggcgaggttcccagagagatcgggaagctgcagaacctggtgttcctcgacctctactccaacggcttcaccggcgcgctg
cccgccgagctcgccaacatcaccgtcctggagctgctcgacgtgcacaacaacagcttctccggcgccgtcccgccgcagttcggggacctcatgaac
ctcgagcagctcgacctcagcatgaacaacctcacgggggacatcccgcccagcttcggcaacttcagctacctcaacaagctcatcctcagcgccaac
aacctctccggcccgctgcccaactccatccgcaacctgcagaagctcaccatgctcgacctcagcaacaacagcttctccggcccgatccctcccgag
atcggcgacctgtccagcctcagcatcagcctcgacctcagctccaacagcttctctggcgagctccccgacgagatgtccggcctcacgcagctgcag
tcgctcaacctggccaacaataacctctacggcagcatcgccgtgctcagcaggctcaccagcctcacctccctcaacgtctcctacaacaacttctccg
gcgcaatcccggtcacggcattcttcaagacgctgtcgtccaactcctacacgggcaacgccaacctctgccagtcctacgacggccacacctgcacgt
cggacatggttcgaaggtcggcgctcaagaccgtcaagaccgtcatcctcgtctgcgccgtcctgggcgccgtcacgcttctgctcgtcgtggtctggat
gctcatcaacagaaccaggaggctcgccggcgacaagtccaccatgagcttgtcgggtgcgggcggcgacgacttctccaacccgtggacgttcaccc
cgttccagaagctcaacttcagcatcgaccacatcctagcgtgtctcaaggacgagaacgtgatcggcaaagggtgctccggggtagtgtacagagct
gagatgcccaacggcgagatcatcgccgtcaaaaagctctggaaggccggcaaggacgagccgatcgacgcgttcgcggctgagattcagatactgg
gccacatcaggcaccggaacatcgtgaagttgctcgggtactgctccaacaggtccaccaaactcctactctacaactacattcccaatggcaatctgct
acagctcctcaaggagaacaggaacctggactgggatactcgttacaagatcgccgtcgggacggcgcaagggctggcctatctgcaccatgactgcg
ttccggcgatactgcatcgtgacgtcaagtgcaacaacatactgctggattccaagtatgaggcctacttggctgattttggtctggccaagctgatgaac
tcgcccaactaccatcacgccatgtcacgcatcgccggctcctatggatacattgctccaggcaacatactagttgttaacatctctgcatttgtttcagag
tacggttacacaagcaacatcaccgagaagagcgacgtgtacagctacggcgtggtgctgctggagatcctgagcgggcgtagcgccatcgagccga
tggtcggcgacagcctccacatcgtggagtgggccaagaagaagatgggcagctacgagccaccagtgaacatccttgacccgaagctgcgcggcat
gccggaccagctcgtgcaggagatgctgcagacgctgggcatcgccatcttctgcgtgaacgcgtcgccggcggagcggccgaccatgaaggaggtg
gtggcgctgctcaaggaggtgaagagcccgccggaggagtgggccaagacgtcgcagcagccgctcatcaagcccggcagccagcaaggatga
>g56066.t1
atggctccctccaattcctctccgaacgccgccgctctgctcctcctcttcctcccgctcctctgctccaccatcgccacccccgtggcggcgcaacagctg
gcgtcctcgcaggcgaaggcgctcctccgcgtgcgccgcctcctgggcaaccctccggcgctcgaccccctccgccacgcgccggacccctgcgcgct
cccgccgacgccgacgctcgccgttgcctgctcgaacgggcaggtcaccgccctctccgtcctcggcgaccgcgacccatcgtccgcgctcccctccac
cttctcctccgacgcgctcttcaccacgctcacccgcctcccgtccctctcccgcctctccctcgtccggctcggcctgtggggcccgctcccgggcgcca
agctccgccgcctgcaggcgctgcaggtcctcaacctcaccggcaacctcctctccggcgccgtccccgccgacctcgcccgcatgtactcgctccaga
gcctcgtcctgtcccgcaaccggctcaacggctccatgccctcgctcgccgcgctccagttcctcgaggagctcgacctctcgcacaacgcgctgggccc
acccgccgcgttcccggaggtgggcaacgcggtggagatattagtcctcgcagagaacaacttcacgggcaagattccggcgtgggtgggcaagctg
ggccagctccggttcctggacgtgtcggggaacaaactccaggggtggatcccctcctccatcttcgcgctcccctcgctgcgccacgtcgacctgtcgc
ggaaccggctctccggacagctgccgccggccacggcgtgcggcggcgcgctggagttcgtcgacgtgtcgtccaacctgctcacgggcgcgcggcc
ggcgtgcatgcgggggaacaactcggcggcggcggcggcgcggacggtgctcgtcgcggggaactgcttctccgacgccggcgccgagcggcagcg
gcccagttcgtactgcgcccccggcgcgctggccgcagtgctgccgccgccgcagggggggcgggaagggcggggaggggaaggggaaggggcggcg
aggtcgggatggtcctcgccgtggccggcagcgtcgtgggcggcgcgttgctgatcgcgctcgtgctggtggtcgtgctgagagtcgtgaggaagcag
catccggaggtgaccagcgtcttgccgacctccccggcgaccacgccggcgaagaaggcgaaagctccggttaaggccacccagaagattattgctcc
tgccgataagaggcgtgcatcacaagccgcaagggtgaatacactggaagtaccagcgtaccgtgtgtatacgctggaggagctgcaagaggcaacc
aacaacttcagcgcctccaaattgatcaacagcagtccccatgcacagattttctcagagatcccatttgctgtcgatgacgatagtggcagtggcagtg
ctcatgacccggaaggaggggaaaagcaagatatctatcagtttggcttaattctcctggaagtgatcaccggcaaaacaacagagtcacaaagacag
ctcgagtccctaaaagctcagataagggagacactcgctgaagatccagaattgctgaaagacatagctgacccagcgatccacggcactttcgcggt
ggactctctgagcacagtcgctgaagtagccctcaactgcacggctagtgccccgggcgagcggccctccattgacgacgtcctctggaacctgcagta
ctcgatgcaggtgcaggatggctgggcgagcagcgagagcttaagcatgagcatcaaatcacaggcctga
>g57352.t1
atggcgttcctggcgatcttggcctgcctgagcctgacgctgccagccaccacagcggcggtgagccaaccacagccggagccgaaggtgaagccga
gcgacaccgacgcgctgaccatcttccgccacggcgccgacgcgcacggcatcctggcggcgaactggtccaccggcgacgcctgcggcggccgctg
gaccggcgtggtctgctccgccgacggccgccgcgtggtctccctctcactcccgtcgctggacctgcggggcccgctggacccgctctcccacctcacg
gagctccgcgcgctcgacctccgcggcaaccgcctcaacggcacgctggacgcgctcctccgcggggcccgcagcctcgcgctgctctacctctcccgc
aacgacgtctccggcgagatcccgaccgacgcgctcgcgcggctgccccgcctcgtccgcgtcgacctcgccgacaacagcctgagcgggcccgtccc

gtcggcgtccgcgcttgccgccggcctcgtcacgctcaggctccaggacaacctcctcaccggcctcgtacccgacctcgccgccgcactgcccaacct
cgccgacttcaacgcctccaacaaccagctctccggccgggtgcccgacgccatgcgcgccaggttcggggtgagctccttcgcggggaacgccgggc
tgtgcgggcccgagtcgccgctgccgccctgctcgtcgttcctgcccaacaagcccgccccgacgccgccttcttcttcctcgtcttcttcttcttccgtgcc
ggcgtcgtcttccgtagtgccgtccaacccggcggcgtcctcgtcgaactccgtcgcttcgtcgtcgccggcgttggccacgccggagtcggtcggtggt
ggtgcggggaatgggaggctgagccccggcgcgatcgcggggattgcggccggcaacgccctgttcttccttgctttggcgtcgctgctagtcgtgtgct
gctgctgccgccgtcaaggaaccgtcggcggcgagccagccaagaagaagaagaagaggaagagccgcgtcggcctcgaggacggagacgg
cgggctgttcggccaccaccacatcaagggggaccagcatccgcctcgtcaagacagcgacggcgccgacagtgacggggcgcgcagcaagctggt
attcttcggcgccgacgacgacgatgacggcagcgacggcgccgagagggacgggtcgacgctgacgtcgcacctgcggcagacccagcggcggcg
gagcggggtgttgttccagctggaggagctgctgcgcgcgtcggcggagatggtcggccgcgggagcctcggcaccgtgtaccgcgcggtggtcggc
gacgaccgcacggtcgccgtgaagcggctgcgcgacaccaacccgtgcgcgcgcgacgagttccaccgttacatggacctcatcggccggctccgcc
accgcaacctcgtcccgctcagggcgttctactacgccaagcaggagaagctcctcatctacgattacctccccaacggcaacctccacgatcgcctcc
acgggcaccgggtgtcaggagaatcgcagccattggactggacgacgcgggtaaggctgctgctcggcgcggcgcgcgggctggcttgcatccacag
agagcaccgcgcgtcgggcataccacacggcaacatcaaatcctccaacgtcctgctcgacacggccggcgccgcccgcgtcgcggacttcggcctcg
ccctcctcctcagcccggcgcacgccatcgcgcggctcggagggtacatggcgccggaacaagccgacgacaagcggctctcgcaggaggccgacgt
ctacagcttcggcgtgctcgtgctcgagacgctcaccggcaaatccccaacgcaacacctgcaactgcaccacgctgatgatcctcagagcagagaca
acactaacaacaagaagaaggagagcgcgacgacggtggtgtgtggcctcgcggagtgggtgcggtccgtggtgagggaggagtggacggcggag
gtgttcgacgtggagctgctgaggtacaggggcgtcgaggaggagatggtcgcgatgctgcacgtcgggctggcgtgcgtcgcgacggagcccgagc
agaggccggccatggatgacgtcgtgcggatgatcgagagcgtgccggtggatcagtcgcccgtgccggaggaggataggggtggtttctcggtgac
gtcgccgtccattggcgttaccacggacgatggtggagatggtcggctcggctactactag
>g57582.t1
atgaagcagctttctgcttcaacaaggctcgtgacccttcttcccctcgtcgtcctgctgtcttgccaagcaaatgcaggacacgacgacgacggcaggc
cgtcgtcgccgtgctcgccgaccgaccgcgcggcgctgctgggcttcaagtccggcatcgccacggacacgacgggcatacttgccacgtggtccggc
gccgactgctgcggcgcgtgggacggcgtgtcctgcgacgccgccacgggtcgggtcgtggcactgcagctggaggcgccaccgcccaaggagcgcc
actacatggcgggcaccatttcccagtccctcggcaacctcgagttcatggagacattggtgatccgcgacatggggaaaatcggcggtgccatcccgg
cggcggcgctgtcgcggctgacgcggctcaagcagctctacctcgaggacaacctgctcgtgggccccatacccgggagcctcgccaagctcaggtca
ctccagttcctgtccctcgccggcaacaggctggagggggcatctcccgccggagctcggcgccgtctccggcctgcaacagatcaacgtcgcacggaa
ccgactcgccggcgcggtgccgcagagctacaagaatctctccaaactggcgtacatcgacatgagcaacaatttcctgtccggtcccattcccgggttc
atcggccagttcaagaacctggcgttgctggacctcagcaacaacagcttttcaggtgacataccggcgtcgctctgtaccttgcgcagcctgacggacc
tctcgctgagccataacaagctcaccggccagatcccatcgaaactgggcagcctccggtctctgagctcactggccatcgctgacaacaagctcgtcg
gctccatcccgtcactgctcgggttgcagaagctgtggaacctgaacctgtcaagaaacctgctctcgggcccactgcctgccgtcggcttgtcgtccttg
gtgtccatggacctttcccacaaccgtctcacaggcggcatcgacgatttcctcagaagcttatccttatccatacccgtgcgcgccacaactgtccccca
aaacaagaagaacttattgatcgttcttcttccgcagaaactggagcacctggacctgtcggggaacagggtcgccggcgcgctgcctgagttcgccgg
cggcgggctcaagtggctcgacgtctcgagcaacgccattggcgggcagataccgagcacggtgtcgaagctccaagacctagagaggctggacgtg
tccaggaacagagttcggggtgtcatccctgcgtccatggccggcttggcgcggttgcagtggctcgatctgtcgagcaacgagatcgtcgggaggata
cctgagaacttcacgaggatggggagcatccggcacgggagttttaggaggaataagctgtgcgggcagataccgcaggccaggccgttcaacttgtt
cccggccgccgcgtacgcgcataacctgtgcctgtgtggcaagccgttgccaccgtgcaggaagatactgtaa
>g58146.t1
atgtctccgcctcccccgccagatccgatcgagccgtccccgccggcgatggacgtcgcgctccccggcgccgtcgtcgccaccatcctctcacgactcg
acgtccgctccctcgtcctcgccgccgccgcatgccgcggtctccgcacctgcgcctcccacgcactcgccttcctcccgtccttccacctcctcgaggtg
gccctgacgcgcgacctgctgcgcccgctgctgccaccgaaccagagcctgcagagtctgcgtctggactgcgcgcggctggaggacgccgccatcgg
ttgcctcgcgcgccccagcctacatgagctcctactcctccactgcgagaacatcagcggtcgcttgctctgcgagcttggcgccacatgcagagatctc
agggtgctttctctcgacactcttggtgagcgtaggggcctggttgtaaatttctctgatctacaggaattgctcaatggatgttctcagttggaggatgca
ccaattatagagcctacatcatcatcagatctcactaatgctggtcttcagcaaattaatccgcatgatgttggtattcagagattatcgttcaacaaagat
ctcaatgtactggatcttagggactgccgcagtttgggtgatgatgctgtaagagctctgagctgccttcccaagttgcaaacattgtcattggacgatgtt
ggtattcagagattatcgttcaacaaagatctcaatgtactggatcttagggactgccgcagtttgggtgatgatgctgtaagagctctgagctgccttcc
caagttgcaaacattgtcattggacggaattactggtagtttgaacagggttatggtggatgccctggctagaagccgccctttcttgcacatggcatgtc
gtggtgaagaattggggactaccgtgctctgggacacctcctctgactggtatagacacaatgatgacgatcttgatgagcttgagcagtggcttttgga
tggtgaacctgtgtcagatgatgacgccatcatggaagaatga
>g58276.t1
atgccttccgcctcctgccgcagctccctcctcttgcccatcttcttcctggctctcggcgtcggcgctgccggcgcggacggggatgccgccgcaatgca
ggccctgcttcatgagctgtcgccgccgggctggggcccgggcccggaccactgcgcgtggcgcggcgtcacctgcgcccctcccccggaccgcgccg
tcaccgccatcgacctgcctcgccggggcctccgtcccggcggcgacttctccgcggcggggtccctcccctccctgacccgcctcgacctctccggcaa
cgccctcctcggccgcatccctccctccctcggcgccctccgccgcctctccctcctcgacctctccaacaacgcgctctccggcgccatccccgcctccct
cggctccgccaccgcgctccgcttcctcaacctctccaacaacgcgctctccgaagcggccctcccctctgccttccgcgccctcacgcgcctcgaggag
ctccagatttccggcaacaacctgacgggcccccctcccgtcctggctcgcgtccctccccaacctccgcgtcctctccgcctacgagaacgcgctctccg
gcccgatcccgcccgggctgggccgggccgcgccgctccaggccctcaacctccactccaacgcgctctccgggcccatcccgtccaccctcttcgccc
gcgggacgctccaggtgctcgtcctcacgctcaaccgcctcaccggccccgtctccggcgccatcggccggtgcagggacctctccaacgtccgcatcg
gcgacaacgccctctccggcgcgattccttcctccatcggggacgccgccagcctcacctacttcgaggccaacgggaacctcctcaccggcggcatcc
ccgcggaattcgcgcgctgcgccaacctcaccctcctcaacctcgccttcaaccgcctgacaggagaggtgccggccgcgctcggcgggctgcggaac

ctgcaggagctgctggtggccgggaacgcgctcgtcggcgcgttcccgacgcccgtgctgcggtgccgcaacctcagcaagctcgacctcagctacaa
ctccttccacggcgcgctgcccgacgacgacctgatctgctcgccgcggatgcagttcctcgtgctcgaccacaaccgcttctccggccgcattccacct
ggaatcgagcgctgcacgcggctcctcgcgctgcagctcggcaacaacaacctcagcggggaggtaccgcccgagatcggcagggtcaagagcctg
cagatcgcgctcaacctcagcttcaaccacttggtcggcccgctcccccgcgagctcggccggctcgacaagctcgtcacgctcgacctgtccagcaac
cagatatcgggacagatccccggcgacatgagagggatgctcagcttgatcgaggtcaatctgtccaacaactggctcacgggcgccataccggactt
cgtgccgttccagaagagcgcggcgtccagcttctccggcaaccccgggctgtgcggcgacccgctagacatcgcctgcagaggatccgtttacgacg
gctcatccggttatgaactggaccgcaacaggatatcgtacagggtggcgctggccgtcgcgggctcctgcgtcctcatcttctcgctggtggcgctgct
ggtggcgctcttcatgtggcgcgagaggcaggagaaggaggctgccgaggccgacaaggcaggggaggtcgtggcggcagccgcggcgccgcagg
tcgcggcgtccgccgtgttcgtcgagagcctgcagcaggccgtcgacttccagagctgcgtcaaggccacgttcaaggacgccaacatcctcagcaac
ggcacgttcagcgtcacctacgaggcagtcatgccgtcgggcacggtggtgtgcgtcaagaagctcaagtcggtggaccgcgccgtcgtgcagcaccg
gtccaagatgatccgggagctggagcgactcgcccacataacccaccccaacctcgtgggcccgatcggctacgtcatctacgaggacgtcgcgctgc
tgctgcaccaccacatgcccaacggcactctgcttcagctcctgcacggtggtgatggtggcgacgctgccgatggtagtgaggaccggaaacccgact
ggccgaggctgctgtcgatcgccatcgacgtcgcgcaagggctggcgttcctgcaccaggtcgccacgattcacctcgacatctcttcggggaacgtctt
cctcgacccgcattacaatgcgcttctcggtgaagtggagatctccaagctgctggaccggtccaagggcacggccagcatcagtgcggtcgcgggca
cgtttggttacatacctccagttggtttcggtgcagagtacgcctacacgatgcaggtcacagtgccaggcaacgtgtacagctttggggtggttctgctg
gagatactgacatccaagctgccagcagatgaggggtttggggaaggtgtggaccttgtcaggtgggtgcacaccgccccggccagaggggagacgc
cggagcagatcatggacccgagactcagcaccgtgtcgttcgcgtggcgcaggcagatgctcgcggtgctccaggtcgcgatgctgtgcaccgagcat
tcgccggcgaagcggcccaagatgaagaaagtggtggagatgctgcaggatgccaggaagagctga
>g58575.t1
atgctcactggatccatgcctcctggattgctccacggaagctttcggttcctcatcctctccaacaaccggctcaccggagagatccccgaagattacg
gaaccggtgacattgacgccattgatctttcacacaaccagctcaatggcaacccatcgttcttgttcgacgccgcaaagccgatgacgaagatcgatct
ttcatggaacgagcttgggttcgacatggcaaaagtcaggtttccttaccacctgacctacttggacctgagtcataaccgtattacaggaagggtggca
aaggccctgaaggatataaagctccggcacttcaatgtcagctacaatgagctctgcgctcccacgaacaagcaaagcagtatgaagcttggagaatc
acagcagcagcagcagcagcagcagcaggatgaatag
>g58587.t1
atggtgggcaccgcggtggagccggaggcgttcggctcggtggacggcgtggtgggggagatcatgcggctgcaccggtcgctgccggcgcggccgt
cgctggaggacgtggaggcggcggagtcccttgcgcgcgcggcagaccgggaggagcgcgcgcggctcgacgccgtcgagcgcctccgccgcccgc
ccgccgtgcccgacgagctgttcggcgtcgcgctggagatgcaccgcgcgctcgccgccttccactgccgcgagcagaagcgcgacgccgcgcggct
gctcgagctcgacgcgctccacgcgctcttcgacgacctcgtcacgcgcgcctcgcagtgcgtcccgtccacctcctcgcgcgccgcgccgcgcatccc
gtccacctcggcggcggcggcatcctcctcgtcctctgctgcctcatcttcggcggcggcggaccgctactcgtccatgaacaccaacgggttcagtgcg
cagaggccggcggcgcgcgtctccatggacgacagctacgtcaagaagtccaaggcgcctgtatgggacggcggagctgtggcgacgagctcgcata
tgccgcgaggaggagccgttgcagcgaactccgtggttgttcagatggatggtggttacggtgatgacaacgagaaattgagcctaattaagctggcta
gcacgattgaagtggctgcaaagaaaggcacccgggatctgaacctccaaggcaaactcatgaaccagattgagtggctgcctgattcaattggaaag
ctcactggacttgttacccttgatatttcggagaatcggatattggccttgccagaagaaattgggaggctttcttctttgaccaaaatggaccttcatgct
aacagaattgctcagctccctgactcagttggtgatcttagcaacttggtatttctcgatctgagaggtaatcagctttcatcgttacctgctggtcttggca
ggctggtgaatcttgaggagcttgatgtcagtgcaaaccagctcacctcactccctgattcaattggaagccttgcacggttgaagaaattaatcatcga
gacaaacaacattgacgagttgccgtacactattggtaattgtgtttctctggtagaactgcgggcaggttataatcatctcagggccctgccagaggct
gtcggaaaactagagtctctggagatcctctctgtgcgatacaacagtatcagaggatttccgacaacaatggcgtctctcacaaagctgaaggaggtt
gatgcaagcttcaacgaacttgagtctattcctgagaacttttgctttgttacttctcttgtgaagctgaatgttgggaacaatttcgctgacatgcaagctc
tgccgcgttctattggcaaccttgagatgcttgaagagttggatataagcaataatcagattagggtccttccagattcttttggaaacctgcagcgacttc
gtgtgcttcgtgcagaagagaaccctctacaagtgccaccaagagacatagctttgaagggagctcaggctgctgtccaatacatgtctgagtatgttgc
caagaaagctacacggtcacaaccaacgacaaagacaaagaagacttgggctcagcagaaccgaccacctgaaggaatctga
>g58806.t1
atgtctcgtgccgccttcctgctcctcgtcacgctcgctgccaccgtcgcgccgccgtcggaggcgtcgtccgcggtcgccggcgaagcggctgtcctcc
actccttcctcgcctccctacctctggcctcccgccgcgtcctccgcccgttgtggaaggctagcgccgctgcaaccagcagccgcaacgggtccggcgg
ccactgcgcgtttctcggcgtcgaatgcgccgctgccggcgacgtcgccgcggtcaatctctccggggtgggtctctccggcgaactcgaggcgtccgc
accgcggctctgcgcgctccgggcgctcgccacgctcgacctcaccgggaacaggttcacgggcgccgtccccgccgcgctcgccgagtgttccggcg
tcacgaccctccttcttggtggcaaccttctcaacggcgcggtcccgccggagctcctctcctcgcggcagctccgtaaggtcgacctcagcgtcaacaa
tttcaccggcgagattcccgtcccccgctcgccagttctcgagtacctcgacctcagcgtcaacgccctctccggtgccatttcgccagagctcgccgcgc
tgccacagctcagccgcctggacctgaacagcaacaacctgtccgggccagtgccggagttcatggcgcaatgcaggcttacctacctcagcctcttct
ccaaccagatcttcggttacctcccccacagcctcagcaactgcgacaatctcaccgccttgtacctatccttcaatgggatcagtggcaaggtgccgga
tttctttgcctccatgccgaatttacaaagtctgtatcttgatgataaccaatttaccggacaactgccggcgagcattggtgagcttgtgaaattggaaga
attggtggtgtcaaataactggttcgctggacctgtaccagacgcaattgggaagtgtcagtccttgacgatgctatacttgaatggcaaccagttccctg
gcccaattccagcgttcattggaaatttaagtagattgcataagttctctatagcggacaatggcatttctggaagaattccaccagagattggaaaatg
cggggagttagttgagctccaacttcagaacaacagcctctatggggctgtaccatcggagatcagtaaccttacccagttgcagagactctatctattc
aataacttgcttcatggacctgttccttcaggattgtggcaattaccgaacatggtagaattgtacctcgacaataatagcttcagtggtgaggtgcatgc
acagatcactcaaatgaggaagctaagagagatctcattgttcagcaataatttcacaggcatgttgccacaagccctggggttgaacacaacacctgg
acttcttcgtgttgaactcactggcaaccacttccatgggaagattccactgggtctctgcactggaggccagctcagccttcttgatcttggagacaatc
acttcagtggtgggtttccagctgaaattgctaaatgttggctggactga

>g58811.t1
atgacgttcagaagaaacgcggctgtagttgttcttgtgctcgccggggttctgctgcacggcgttctgggcggctcctcgagccacaccgggttcgcca
ggaccggcaatgcgccgccgtccaccggcggcgtgaaaaacttcacggcgccggcgccatcgagcggtgcgctagaaggaggttgcggttgcggtcc
gacgccgccgccttgggactttctgaacgagaagcttgcggcgctgttcccggtgatccaggcgttcaagcgcagcatcacatgcgaccctctgggcgt
gacgtcgacatgggagggcaccaagctctgcgacagccactccggcggcaccgcgtacaagggcttctactgcgactaccctcccaccgcgaacacg
acgctgaccatcgcgtccatcgacttcaacggcttcggcctgtgcgccccttccctggccggcttcgtcgacgccttccctgacctagccctcttccacgcc
aactccaacaacttctccggcgccgtgcccgacctcaccgccctccccttcttctacgagctcgacctctccaacaacaacttctccggcgcgtttcccga
cacggtggtcccgctcggcggcctcctcttcctggacctccgcttcaaccggttcgcggggtccgtgccgccgcccgtgttcggcatcacgctggaggcg
ctcttcctcaacaacaacgggttcacgggccggatcccggacacgttggggagcaccacggcccagtaccttgtggtggccaacaaccggttcacggg
cccgatcccgcggtccatcttcaacacgtcggcccacctcgaggaggtgctcttcctcaacaacgacctctccgggtgcctcccctacgagatcgggctc
gtggaggggctcacgctcttcgacgcgggagggaaccagatcaccgggcccatcccgctctcgtttgggtgcctggcccacgccgaggagatcaacct
cgccgggaaccagctgtacggcaacgtccccgacgtgctctgcctgctggccaagaacgccaagctcaccaacctctcgctctccgacaactacttcca
ctccgtgggacaccactgcatggagctcgtaaggagcagggtgctggacgtcagacgcaactgcatcctgggattccccgatcagagacccctgctcg
agtgcacctggttctacgcagacccgccgcagcactgcccattcgtgccgcacatcccgtgcgacctgcccggctaccacaaaccgcatgccaacaag
cccgcccttcttccggccgcgcatcgccaaggcggcggcggcgggaactga
>g58835.t1
atgggaggattccgctggctgttcctcttctcgcttgtcgccgttctgctccacgttcatggcgcccagtcccagaaccagacgtgccacccggcagacct
gaaggcgcttctttccttctccaatggcttggataggaagggcgaggggcttgtcgggctcagcttacaggacaatcagctctccggcaacctcggcaat
gggcttggtaacctctctcagcttgtgcagcttgacttatcctacaacaggttcaccggtcccatccctgatgtgtttggaggtatgaggaggctggagtc
cataaacttggccacaaataggttctttggtgaattgcctccttccttgtcaagttgcccaacgctgagggtaatcagtctacggaataacacactgtttgg
tgcgattgatatcaacttcgacatgctgccaaggctgagcacttttgatgctggaagcaacaatttgactggtgatatacctcctggctttgcatcgtgcac
tgagttgaggacgctgaaccttgcaaggaacaagcttggtggcaagataccagagaccttcaaggatttgaggtccctgtcgtacctctcactaacagg
gaatggcttcactgatctgtcatcagcattacaaatcttgcagcacctgcccaacctggcgagcttggtgctcaccaggaatttccgtggtggtgagaca
atgccagtggatggcatcaatgggttcaagagcatccagagtggttcagctcctggttcagatgtcacaacagtgaagtctgcttcaaaattcatctatg
actga
>g59137.t1
atgtcgctcgctggccgcctgaacctggaaggcctcaccgagctgccccagctccagagcctcaacctcagcagcaacgggttctatgacaggctatgt
tccaatggtaccacattggagacatttgtgataagctgcaacaactttaatgggaggatccctccatctatcaccaagtgtgtcaatctcatctggatatct
cttgtgggcaatcacctcactgataaagtgccacctggcttcggaaacctcaagaagctcgccattttgcagcttaacaataattcgctctctggcttggtt
ccaaccgaactaggcagctgcagcaacctcatctggcttgacctcaacagcaactacttcacaggcacaataccagaagaattagcagctcgggcagg
gctcatcgatggggggaatagtatctgggaagcagtttgcgttcctccggaatatggcagggaacatctgtcctggcgcgggtgtgctgttcgagttccat
gacatccgcccagaacggttactcaagttacctgccatgaaatcatgcccatccacaaggatatatactggcacaacagtgtacacatttcgcaacaac
gggagcatgatcttccttgacctctcatacaatggtctgaccggtgcaatccccacaagcctgggtagcataatgtatcttcaggtcctcaatttaggcaa
caatgaactagctggcccaatcccagatacattcgcagggctcaagtcacttggcgcacttgtactcacccacaatcacctcactggtgccattccacat
gggcttggcaacctaaatttcctagttgacctggatgtctccaacaataacctgactggccagattcctacatcagggcagctcatgacatttcctccatc
cagctatggaaacaactcctacctttgtggcagcccaggttaa
>g59138.t1
atgggcgccgctcgcatggcggcgcctgcagctactctgctgctcctcctcctttacctggcgagcgtagcagccgtcgggcccggcgacgaggccgcg
gcgctgctcgcgttcaagcgcgcgtccgtggccaacgacccgcacggcacgctcgccgactgggccccggccaactccagctcagcagcgtcgccctg
cgcctgggccggcgtctcgtgcgcggacggccacgtcgaggcgctcaacctcagcggtatgtccctctccggccggctccacctcggcgccctgctcgc
gctcccggagctccggagcctcgacctccgcgggaacgccttccacggcgacctcacccaccggacctcgccgcggcggccggagccaccgtgcgct
ctcgaggacgtggacctctcgtcaaacgccttgaacgggacgctgcccaggacgttcttggcgtcctgcagcgggctacggttcctcaacttgtccagg
aacaccctcaccggcggcgggttccccttcccggcatctttacgccgcctggacatgtccaggaacaggctgtcagatgccggcttgctgaactactccc
tgaccggctgccacggggtccagtacctcaacctctcggccaaccaattcaccgggggactcccggggttcgcgccctgcagcggggtcgctattcttg
acttgtcatggaacctcttgtcgggcgccctgcctgcgaggcttgtggccgctgggccggtcaatttgacacacttgagcgttgccgggaacaatttctct
ggcgacatcttgaagcatcagtttggtggttgtgcaaatctcactattccaacagaagttttatttcttcctaaacttgttgatctggtcatgtgggcaaacc
atctctctggtgaaataccagacaagctatgctccaatggcacaacactggagacgctggtaataagctacaacaacttcactggaggcatccctccat
ccattaccagatgtgtgaatctcatatggttgtcacttgctggcaaccaactcatcgggagcgtgcctgcaggctttggcaacttccagaagcttgccatc
ctacagctgcacaagaattccctctctggtaaggtaccagccgagtttggcagctgcagcaacctcatctggcttgatctcaacagcaataacttcactg
gctcaataccaccgcagttagcagcacaggcgggactcatcactggaggtattgtttctggaaagcattttgcattccttaggaatgaagctggcaatat
ctgccctggtgctggagtattgttcgagtttctcgacatccgtccagagcggctggcccagttctcacctgtgaattcgtgcaccccaacaaggatataca
ctggcacaacggtctacacctttagcaataatggaagcatgatctttctggatctctcatacaacagcctcactggtacaattcctgagagcttggggaat
atgacatatcttgaggtccttaatttgnnnnnnnnnnnnnnnnnnnnnnnnnnnnnttaa
>g59139.t1
atgggcgccgctcgcatggcggcgcctgcagctactctgctgctcctcctcctttacctggcgagcgtagcagccgtcgggcccggcgacgaggccgcg
gcgctgctcgcgttcaagcgcgcgtccgtggccaacgacccgcacggcacgctcgccgactgggccccggccaactccagctcagcagcgtcgccctg
cgcctgggccggcgtctcgtgcgcggacggccacgtcgaggcgctcaacctcagcggtatgtccctctccggccggctccacctcggcgccctgctcgc
gctcccggagctccggagcctcgacctccgcgggaacgccttccacggcgacctcacccaccggacctcgccgcggcggccggagccaccgtgcgct
ctcgaggacgtggacctctcgtcaaacgccttgaacgggacgctgcccaggacgttcttggcgtcctgcagcgggctacggttcctcaacttgtccagg

aacaccctcaccggcggcgggttccccttcccggcatctttacgccgcctggacatgtccaggaacaggctgtcagatgccggcttgctgaactactccc
tgaccggctgccacggggtccagtacctcaacctctcggccaaccaattcaccgggggactcccggggttcgcgccctgcagcggggtcgctattcttg
acttgtcatggaacctcttgtcgggcgccctgcctgcgaggcttgtggccgctgggccggtcaatttgacacacttgagcgttgccgggaacaatttctct
ggcgacatcttgaagcatcagtttggtggttgtgcaaatctcactgtgctggattggtcgtacaacagattgagcggcatggggttaccacctagtcttgc
caattgccgccggttggagacgcttgacttgtcctag
>g59140.t1
atgtgggcaaaccatctctctggtgaaataccagacaagctatgctccaatggcacaacactggagacgctggtaataagctacaacaacttcactgga
ggcatccctccatccattaccagatgtgtgaatctcatatggttgtcacttgctggcaaccaactcatcgggagcgtgcctgcaggctttggcaacttcca
gaagcttgccatcctacagctgcacaagaattccctctctggtaaggtaccagccgagtttggcagctgcagcaacctcatctggcttgatctcaacagc
aataacttcactggctcaataccaccgcagttagcagcacaggcgggactcatcactggaggtattgtttctggaaagcattttgcattccttaggaatga
agctggcaatatctgccctggtgctggagtattgttcgagtttctcgacatccgtccagagcggctggcccagttctcacctgtgaattcgtgcaccccaa
caaggatatacactggcacaacggtctacacctttagcaataatggaagcatgatctttctggatctctcatacaacagcctcactggtacaattcctgag
agcttggggaatatgacatatcttgaggtccttaatttggggcataatgatcttacgggagcaattccagatgcattcacagggctcaagacgattggtgt
acttgatctctcacacaatcacctcactggcctcatccctgcagaacttgggtgtctgcatttccttgcagactttgatgtctccaacaacaatctgactggt
gaaatcccgacgtcagggcagctcattacatttccagcatcccgctatgaaaacaactctggcctctgtggcattcccttgattccttgtgtgcacaattcc
agttctggaggtctgcctcaatcttcccgtaatgtgcgaaagaagtttcttgaagaaagtgtgctcctcacagtggcattttcagttatcatagtgctctcg
cttatagtcactctgtacaaactcaggaggcccccctacaagtaaagctgacgagattcaaactgggtactgcgacagcctccacgcatccagcaattta
agttggaagttttctggttctcgtgagccactaagcatcaatttggccatttttgagaatcccataaggaagctaacttatgcccacctgcgtgaagctact
aatggcttcagctcagaggcgcttattggctcaggagggtttggggaggtttacaaggctaagctgataaatggcggtgttgtagccgtcaagaagctg
atgcattttacaggccagggtgaccgggagttcacagcagaaatggaaaccattggcaaaatcaagcatcgtaaccttgtaccattgctaggctactgc
aaaattggtgatgagcgtctccttgtttacaagtatatgaagcatggcagcttggatgttgtgcttcatgacaaggccaagaccgatgtgcaactcgattg
ggcatcaaggaaaaagattgcaattggttcagcaaggggcctagcctttctccaccatagctgcatcccacacatcatacaccgggacatgaagtcga
gcaatgtgctgcttgatgataacttcgacgcctatgtatcggatttcggaatggcacggctcatgaacgcactcgattcacatctgactgtgagtatgctct
caggaacacctggttatgtcccacctgagtattgccagtcttgcatgtgcacaaccaagggtgacgtatacagttatggtgttgtgctcttggagcttctct
caggaagaaaaccaattgatccaaccgcatttggtgatagtaaccttgttgattgggtaaagcagatggttaaggaggacagaggcaatgagatattc
gatcctatattgaccaccatgtcttgtgaattggagctgtatcagtatctgaagattgcttgtcagtgtttggatgatcagccaatccgtaggcctaccatg
atccaggtcatggcaatgttcaaagagctacagattgattcagacaataacttcgttgatggcttctcaataagttcaccaaatatagaggaatcatctga
gaaattagtgtaa
>g59402.t1
atggcgcagcaacggttgtccgtggcgttcgccgccgcgttcctcgtcgtgctactgtcgatggtccggccgtgcgcgtcgctgtcgccggacgggaag
gctctcctctccctcctccccggggcaccgtcgcccgtgcttccgtcgtgggaccccaagtcatcaacgccgtgctcgtggcagggcgtgacgtgctcgc
cgcagagccgggtggtgtccctctccctccccaacaccttcctcaacctctcctccctcccgccggacctcgcctccctctcctccctccagcttctcaacct
ctccacctgcaacatctccggcaccatccctccttcctatgcctccctctccgcgctccgcgtgctggacctctcctcgaacgcgctgggcggcgacatcc
cctccgacctcggcgcgctgtcacagctccagttcctcctcctcaactcgaaccggctcaccggcggcatcccgtggtccctcgccaacctctccgacctc
caggtgctctgcgtccaggacaacctcctcaacggcaccatcccctcctccctcggcgcgctcgccgcgctgcagcagttccgggtgggcggcaacccg
gagctgtcgggccccatcccttcgtccctcggcgcgctggccaacctcaccgtgttcggcgcggcggcgacggcgctgtcggggcccatcccggagga
gctcggcaacctggtgaacctccagacgctggcgctgtacgacaccagcgtgtcgggtcccatccccgccgcgctgggcggctgcgtcgagctgcgca
acctctacctccacatgaacaagctcaccggcccgatcccgcgtgagctgggccgcctgcagaagctcaccagcctcctcctctggggcaacgcgctgt
ccgggaccatcccgcccgagctctccaactgctcgtccctcgtcgtgctcgacctgtcggggaaccggctcggcggcgaggtgcccggggagctcggc
cgcctcgacgcgctcgagcagatgcacctctccgacaaccagctgaccggtcgcatccctccggaactcagcaagctgagcagcctcaccgcgctgca
gctggacaagaacgggttcacgggtggcatcccgccgcagctcggggaactcaaggctttacaggtgctcttcctctggggcaacgcgctgtccggccc
catcccgccgtcgctcggcaactgcacggagctgtacgcgctggacctctccaagaaccgtctcacggggaccatacccgacgaggtcttcgctctcca
gaagctcagcaagctcctcctcttggggaacgcgctgtccggcccgctcccgcccagcgtcgccgactgcgtctccctcgtccggctccggctcggcga
gaaccggctcgccggcgaggtgcccagagagatcgggaagctgcagaacctggtgttcctggacctctactccaacggcttcacgggcgcgctgcccg
ccgagctcgccaacatcaccgtcctcgagctgctcgacgtgcacaacaacagcttctccggcgccgtcccgccgcagttcggggacctcatgaacctcg
agcagctcgacctcagcatgaacaacctcacgggggacatcccgcccagcttcggaaacttcagctacctcaacaagctcatcctcagcgccaacaac
ctctccggcccgctgcccaggtccatccgcaacctgcagaagctcaccatgctcgacctcagcaacaacagcttctccggcccgattccgcccgagatc
ggcgacctgtccagcctcagcatcagcctcgacctcagctccaacagcttctccggcgagctccccgacgagatgtccggcctcacgcagctgcagtcg
ctcaacctggcaaacaacgacctctacggcagcatctccgtgctcagcaggctcaccagcctcacctccctcaacgtctcctacaacaacttctccggcg
ccatcccggtgacgccattcttcaagacgctgtcgtccaactcctacaccggcaacgccaacctgtgccagtcctacgacggccacacctgcgcgtcgg
acatggttcgaaggtcggcgctcaagaccgtcaagaccgtgatcctcgtctgcgccgtcctgggcgccgtcacgctgctgctcgtcgtggtatggatgct
catcaacaggaccaggaggctcgccggcgacaagtccgccatgagcctgtcgggtgcgggcggcgacgacttctccaacccgtggacgttcacgccgt
tccagaagctcaacttcagcatcgaccacatcctggcgtgtctcagggacgagaacgtgatcggcaaagggtgttccggggtggtgtacagggctgag
atgcccaacggcgagatcatcgccgtcaagaagctctggaaggcgggcaaggacgagccgatcgacgcgttcgcggctgagattcagattctcggcc
acatcaggcaccggaacatcgtcaagttgctcgggtactgctccaacaggtccaccaagctcctgctctacaactacatccccaatggcaatctcctgca
gctcctgaaggacaacaggagcctcgactgggatacacggtacaagatcgccgtcgggacggcgcaggggctggcctacctgcaccatgactgcgtt
ccgtcgatactacatcgtgacgtcaagtgcaacaacatactgctcgattccaagtatgaggcctacttggctgattttggctggccaagctcatgaactc
ccccaactatcatcatgccatgtcgcgcatcgccggctcctatggatacattgctccaggcaagtga
>g59486.t1

atgattactgccctcaacacgctgtttactagcatgaattcacctgggcagctacagggttggaaggtgaatggtggcgatccatgtggagaattgtggc
agggcatcacttgctctggctcctcagttaccgcaattaaattaccaagcttggggctctctggaacactgtcctacaacatgaacaacatggaatcact
agttgagcttgacatgagccaaaacaatctcggtaatggccagcagattccttacaatcttccaaataagaagcttgaaaggctcaatctggcaggaaa
ccagttcactggaactgtgccatactcgatatccactatgtcaaaacttaagtaccttgacctttccttcaattctctcactggtaatctaccgcaaagtttc
tcttcattgccgagcctgaaaactctgtacttgcagaacaatcaacttacaggatccatcaatgttcttgctaatctccccttgatgatttgtga
>g59876.t1
atgcatgctgcatgccacagcatcaccaccggattccatctcaagaatatctccatggctgtctcagtactgctgcagctagcagtagcggtcgagcttct
ctgcgccgtcgtcgcgtcggcgcttaaccaggacggcatcttgctcctctcgttcaagctgtcgctgggcggggacccgctaggttccctctccgactgg
gccccgtacacggacgccaccccgtgctcgtggaacggcatcgtgtgcgactccgactcgcgcgtggtgagcgtggtcctcccgaacgcgcagctggt
gggccccgtggcccgggagctgggcttcgtggagcgcctccgccacctggacctctccggcaacgcgctcaacggcagcgtccccggcgacctgttcc
gcgctccggagctccgcgtgctttccctcgccggcaacggcctcaccggcccgctcccggagccgcaagcgggtcagctccgcagcctccgcgcgctc
aacctcgccggcaacgcgctctccggcgccgtgccacgaaacctcgtgacgctgccgaacctcacggccgtgtccctcgcgggcaacttcttctccggc
gaggtccccggcggagaggtctcgccggcgctgcagatcctcgacgtcagcgccaacctgctaaacggcaccctgcccgcggacttgggcggcgccg
ggctgcggtacgtgaacctgtcgtcgaaccggatcgcggggggatccccgcggagatggcggcgcggctgccggcaaacgtcaccatcgacctcag
ctacaacaacctgaccggcgcggtccccgccgtggcgcccttctccacgcagcgggcggacgcgttcgaggggaacgcggagctgtgcggggcgccg
ctccagaggctctgctccttcgcgtcggcctctgcagtggaacccaagtcgccgccggccatcgcggcgattcccaagcaggaggaccccaccactga
agccgcgtcgtcttcgggaaggaagaagcgtcttgtggccatcgcggccatcgccgttggtgacgtggcgggcgttgcggtgctcttcttggtggcgctg
tatgtgtacaggttgaggaggaagcgtaagcagaggacgatgggcccagtcgtgtttaagaagcccgacccggaggaatcaccggacgctgtgggcc
ggagcctctcctgctgcctgcgcaacaacaacaaggccgacgacagcgaccgtgacgtgacggacacgtcgccctccttcgccgccaaggaagagaa
cactaaggccgccgacgtcgaagccgggcaggaggaggaggagaagaagaagaagaagaagaaggggggtggtggtgacggtggacggaga
aggggaggagctggagctggagacgctgctcaaggcgtcggcgtacatcctgggctccgccggcggcagcatcgtgtacaaggccgtgctggcggac
ggcgccgtgctggccgtacgcaggatcggcagcgacgacgccgccgtgcggaggttcgccgacctcgacgcgcacatgcgcgccgtcgccaagctcc
ggcacgggaacgtcctccggctgcgcgggttctactgggggccagacgagatgctaatcatccacgacttggcagtcaacggcagcctcgccaacctc
tccgtcaaaaggaagcctgggtcgtcgccggtgaacctcgggtggagcgcgcggctccgcatcgcacgcggcgtggcccgggccctcgcgttccttca
cgacaagaagtgggtgcacggcaacgtgaagcccagcaacgtgctcctcgacgccgacatggagcccctcctcgccgacctcggcatcgaccgcctc
gtgcgcaccaccgctgactccggcagccaccaacacatgtcgtcggtcacaactacagcnnnnnnnnnnnnnnnnnnnnnnnnnnnnnnnnn
nnnnnnnnnnnnnnnnnnnnnnnnnnnnnnnnnnnnnnnnnnnnnnnnnnnnnnnnnnnnnnnnnnnnnnnnnnnnnnnnnnnnnnn
nncacggcaacgtgaagcccagcaacgtgctcctcgacgccgacatggagcccctcctcgccgacctcggcatcgaccgcctcgtgcgcaccaccgct
gactccggcagccaccaacacatgtcgtcggtcacaactacagcattcaagttcgggaccaagcgttccgcgaagagcctccccgaccaagcaccggc
gagcccgcttccctccgacgaccacacggcggcgcactaccgggcgccggagaagacgaagccgagcgccaagtgggacgtgtacgcgttcggcgt
gctgctgctggaactgctggccggccgcgcgctcaccagcctcgagctgtgccagtgcgccgccgcgccggaagagaataaggcgcaggcgatgatg
cggatggcggacccggcgctgcgggcggagatgggaggcagggaggaggcggtggccagctgcctcaggctcggcgccgcgtgctgcaatttggcg
ccgaccaagaggccgtccatgagggacgcgctgcaggccatcgacaggataccggctcttgcctctgctgctgcttcctcttcctcctcctccagggagg
cggcaggagctcggtga
>g60015.t1
atggcgtgggcggcggcggtggcgctggtggttgcggcggcggcgctttcgccggcctccttggcgtcggcgtccaactcggagggcgatgcgctgta
cgcgctgcggcaggcgctcgcagaccccggggcgtgcttcagagctgggaccccacgcttgtcaacccctgcacctggttccatgtcacctgcaacgg
cgacggccgcgtcacccgcgtgtga
>g60093.t1
atggccgcggcgtcgttcgcccgtcccttgtccctcgtcttccttactctgcttcttgcgggcgctgcgcagggcaagacggtgaagagagacgtgaaag
cattgaatgagattaaggcttcactaggctggagggttgtatactcatgggtcggcgatgacccttgtggacatggtgatctaccccatggtctggtgtg
acatgttctcagcaaggagattatagagttgttacagaattgtacttaagcttcaataacttcaaaggtgaaattcctctcgagcttgcaaacctaccaga
actgcggtatctgtatcttcatgaaaataggttcactggtcggatccccctgaacttggaaccctcaagaacctacgtcacctgtaccttaacaataatg
aattgactggtgtgcttccagatcagattgcaaatttgacgaaccttgagattttccacttatccaataataagctaattggatcaatatcgccaaagcttg
ttcatattccgagattgacctatttgtatttggacaataacaacttcattgggagaatacctgaaggcatatacaagcatccatttttgaaggagctgtaca
ttgaaggaaaccattttagacccgggagcaaatccaaaggaacgcacaaggtgcttgaattgcctgatgctgacattttagtttag
>g60222.t1
atggagcatttcttgatgcaacgtaccaccacatggccactccgtttcttcctctgctcgcctatcctgctgctcttcttggcaccagccaactccctgaacc
agagctcttgcgaccagcacgatcttgacgccctgcaggggttctccaaaggtctcgctggaggaggcgtcagcggctggacgtttccaaactcgagtt
ccgacaaggccaactgctgtgcatggcttggtgtcacctgcgacgatggtggacgagtcatcgggttggaccttcaaggcaggaggctcaggggcgag
ctgtcgcactcgctcgggcagctggaccagctccagtggctcaacctctccgacaacaatttccgcggcaccattccggcgcctgtgctccagctacaaa
agctgcagcggttcgacctcagctataacgagctcaccggcgcgttcccagccaacatatctctcccgctgatcgaggtcttcaatatatcttacaacctc
atcaatggctcccatcctacactccgtggctcgccacagctcgtgttgttcgacgcaggatacaatttgtttgccgggcaaatcgacacaagcatctgcg
agtcgtctagtatgatccgtgtgctgcagttcacgtccaatctcttcgctggggacttccccgcaggttttgggaactgcaccaagctcgaggagttatat
cttgacatcaacagcatctccggaagcttaccagatgacctgttcaggatacaatctctgaggaaccttttctgcaggagaatcagctctctggcaggat
gagcccaagattcagtaacctgtctagccttgcaaagctggacatatctttcaattcgttctctggataccttccaaatatttttggtagccttggcaagctg
gagtacttctccgcgcagtccaacttcttcaggggcctattgccttcctctttgtcccactcaccatcactgaagatgttgtacctgagaaacaattcgcttc
atggagagatcagtctcaattgctcagccatgacacagctgacatcgcttgaccttgggacaaataagttcattggcacaattgataggcctaccccaat
gtcattcaactcatactccaatgatagctgcaagaaatag

>g60681.t1
atgactcgcgccgcgcgcgggctcctcctccgggcgctgccgctgctgctgctcctctcgctggcggcggcgcccgggccggtggtggcgcagacgca
gcagcctccctctatcctcgcgtcgcgcgcggatctcgccgcgctctacgcgctgcgtggctcgctcggcctgcgggcgcgggactggccgcgcaaggc
ggacccgtgcgcggcctgggcgggcgtcggctgccgcggcggccgcgtcgtctccctcacgctcgccggcctccgccgcacgcgcctgggccgcctcg
ccccgcgcttcgccgtcgacggcctgcgcaacctcacgcgcctcgaggacttcaacgcctccgggttcgccctgccggggcccatcccggcgtggctcg
gcgcgggcctcgcgcccaccttccgctccctcgacatctccgcctgcgacgtctccggggagatccccgcctcggcgctcgcggggctcgacaacctcg
ccacgctcaacctcgcggggaaccggctgtcggggccgctcccggctgccgcgctcgccaacctcacgcggctcagcgtcctcaacctgtccggcaat
gcgttctccggcgcgctgcctgacgcggtctggtcgctcccggggctcgccgttctcgacgtgtcccgggccaacctcaccggcgcgttgcccacggcg
gggcttgcgctgccttccaatgcgcaggtggtggatctgtctgggaacctcttgtacggcgccgtgccggactcgttcggccggctgtttagccgggtgg
cgctggccaatatctccgggaactacttcgacggcaagctcggtgcgtctgatggtggtgggaatgtgtcgtctgagttgaattgctttcaagactttccg
gggcagcggagccaggccgattgccagcagttctatgctacgcgtgggttgccttatgatggaccggttgccccaccggcgccttcaccttctgcaccag
cgaggaagaagaagcacaagaatttgaagtacatactgatcggggccattgttggaggcgtcctgctgatagccttggttgcggccctagtgttttgctt
tgtgtgctctgggaggaggaggaggagaaccggcactgaccagagggaaagtggagcaccaaatgcacaggcgggggtgtcaggtaccagcgcgg
ctgctgttggtggtggctcacagccttctgcattgtccgcaaacatggcgaaggttggtgattcgtttgcgtatgatcaacttgccagcatcacctctggat
tcagcgaggagaggcttatcaagcacgggcattcaggtgatctctaccatggtgtgctccaagatggaactgccgtggtggtgaagagggtcacttctc
gggtggctaagaaggatgcatatctggcagagttggatttgtttgcaaagggatgccatgaaaggctggttccgcttatggggcattgtcttgatcatga
agaagaaaaggctcttgtgtacagatttgtccgaaatggtgacttatcaagtgcactgcacagaaagccaagagaggatgaggaggggatgcaatctt
tggattggataaagaggttgaagattgcaacaggggtggctgaggctctttgctatctgcaccatgagtgtaatccaccaatggttcacaggatggcag
atcaaggctcttctggctttccatctgcaacctgctcatacgatgtttattgttttgggaaagttttaatggagctggtgacaggaagacttggaatgagtg
catcaaatgatgctgctacaagcgaatggcttgataatagtttgcggtatatcaatatttatgagaaggagctcatgagcaagatcattgatccaaccctt
atcattgatgaggaccacctggaggaagtctgggcaatggcaattgttgccaagtcttgcttgaatcctaggtcatctaaacgtccaccaatgaagtaca
tccttaaagcactagagaatcccttgaaggtcgtgagggaagataatggttctagctcagccaggttgagagctacatcttcacgaggttcatggaatgc
tgcattgtttggaagttggcggcatagctcctccgatataggtccttcaagggatgacaacattctgaaacgctcagagacaataaaatcgtctgggggg
agtaatggagatcattcttcctcccgccggaggcaatctaaggagatcttccctgagccatctgactctcgtgataccgaggattaa
>g61141.t1
atggaccggatcctcaagtccgccagggagtccggcttcctcaacctctccaatcgctccctcagggttgatttatcaaacaatcacctcactgagcttcc
aaatagccttgccaaatgcctggccttgtcagagatcaaggcatcaaacaacaacataaccagaataccagatgtgcttgctgattgttccaagctcag
caagttggatctggaggaattggacttgagtggccttggtgtggccagtgtaccaccggcagcatgggagacaagtgatgtggtgaaacttgatctttct
aaaaactcaatagaagatcttcccaatgagctgtcattatgttcatcacttcaagtatcatccattgatcttgtgtccctgtccaaacttgaagtgctcgatt
taagtggcaacgcgtctgcgcttcctgatccgtctgttgtttctgcattaccaaacctgcaggagctgtacctgagacgaatgaaactccatgaatttccc
aatgctttgctgggcttaaaacagctgaggattcttgacctgagtcagaacagccttacaactgttccggagatttga
>g61769.t1
atggcatctccgcggctggtggctctactggtgctcttcctcctcgtcgtcgcgtcagtggccgccgacgacgcccagctgctggagcagttcaaggcgg
ccgtgccgccgccaggccagccggacctccgccgctggaccgcagccgacggcgcctgcaagttcccaggcgcgcggtgccaggccggccgcctcac
gtcgctgtcgctcgcgggcgtcccgctcaatgccgacttccgcgccgtggccgcctcgctgctgcagctggcgagcctcgaggagctcagcctccgcgg
cgccaacgtcagcggcgcgcttgccgccccgccgcgctgcgccgccgccaagctgcgggcgctcgacctctccgggaatgccggcctgcgcggctccg
tcgccgacgtggacgcgctcgcggccgcctgcgccgggctgagggcgctcaacctctccggcgccgcggtcggcgcatccaaacccactagcaccaa
caccaacggcgtggggtcgggattcgccgcgctggactcgctcgacctctccggcaacaagatctccggcgacgccgacctgcggtggatggtggccg
cgggcgcgggcgcggtgcggcgcctggacctctccgacaacaagatctccggcgcgctgccggacctcgcaaactgctccggcctcgagcacctcga
cctctccggcaacctcatcgacggcgaggtcgccgcgggggcgctctccgactgcaagggcctcaagacgctcaacctctccggcaaccacctcgtcg
gcgccttcccgcccgacgtcgccgccctcccctcgctcctcgcgctcaacctctccaacaacaacttctccggggacctccccgccgacgccttcaccaa
cctgccgcacctcgccgtgctctccctctccttcaaccacttcaacggcaccgtaccggacacgctcgccgcgctccccgaactcgaggtgctcgaccta
agctccaacaccttctccggctcgatcccctcctccctctgccgggacccaaacgccgcgctccgcatgctctacctccagaacaactacctctccggcg
ccatccccgactccgtctccaactgcaccaacctcgagtcgctcgacctcagcctcaacctcatcaacggctcgctgcccgcgtcgctcggcgggctcgg
caacctgcgggacctcatcctgtggcagaacgagctggagggggagataccgccctccctcgccagcatgcacatgctcgagcatctcatcctcgacta
caacggcctcaccggcgccatcccgctggaactggccaagtgcaaggagctcaactggatatccctcgccagcaaccgcctctccggccccatcccgt
cctggttcggccagctcagcaacctcgccatcttgaagctcagcaacaactccttctccggtccgataccgcctgagcttggcgactgccagagcttggt
ctggctcgacctcaacagcaaccagctcaacgggtccattccgccggagctggccaagcagtccgggaagatgaatgtcggtctcgtcattgggcggc
cgtatgtgtatctccgcaacgacgagctcagcagtgagtgccgcggcacggggagtttgctggagtttaccagcatccggtccgaagacctcagccgg
atgcccagcaagaagctgtgcaacttcaccagaatgtacatgggcaacaccgagtacaccttcaacaagaatggatccatgatcttcctcgacttgtcct
tcaatcagctggactcggagatcccaaaggagcttgggaccatgtactacctcatgatcatgaatctcgggcacaacctcctgtctggcctcatcccgtc
ggagctagccgctgaatgggtcgattccggagcttggttcccttgccacgttccccaagagccagtatga
>g61779.t1
atggatctgcatctggcggccgtgctcggctcgctcgccctcggcggcgcgcttctggcgctgctcttcggcaagtggtggcagccgctggccgacgcg
gacaagcgcgtgaaggagctggccgacgcggtggacgcgctgctccgtcttcgcgccgacctgctcgggcgcgaggaccagccgccgtcggacccgg
tgcgcgcgtgggcgcgccgcgtgggggaggcgcaggacgaggcggcgtccgtccgcgcgcggcacggcgcgggcgggcagcagctctacgtggtg
cgcctggtgcagtacctcctcctctcctccgggcccgacgacgccgagctcgtccaccgccacctcaagtccgcgcgcgcgctgcacgagcagggcgc
cgcgctgctcgacgccgcgctcgccgcgccgcaggcgccgccgcccctgctccgccagcccgaggacctcgacgagagcgacctccccgcggaggcg
acgaatgcggggcccaccgttcgggcgcgcctcaacgacatgctccgcttcctcggcgactgcgacgcggcgctcggcgtgtggggcgcgggcgggg

tgggcaagtccacggcgctgcggctggtgcgcgaggtgtgcggccgcgtggcgcggttcgaccacgtcgtcgtcgtggccgcgtcaagggactgcacg
gtggcgaagctgcagaaggaggtggtggccgtgctcgggctgcgggacgcgcccacggagcaggcgcaggccgcgggcatcctcggcttcctcagg
gacaagagcttcctcctgctgctggacggcgtgtgggaccgcctcgacctcgagcgggtcggcatcccgcagccgctcggcgtggtcgccggaaaggt
caggaaggtgatagtggcgtccaggagcgaggccgtgtgcgccgacatggggtgccgcaagaagatcaggatggagtgctggaacgaggaggattc
ctggagcctgttccaagccagcgttggcgacgacgccatcggtcgccaccctcagatttccacacttgctagacaggttgctgcggaatgcagaggatt
gcctctgctcctcgttaccgtcggccgcgccatgtcaaagaagagcacaccggaggactgggccgaggcactcgacaccctgaaatcgtcgtcgcagc
ccatttcagacaagagcacacacgctcttgtgaagttctgcttcgacaacctggagagcgacacggcgagggagtgcttcctgaccaccgcgctgtggc
cggaggaccacaacatctccaaggacgagctggtgcagtgctggaccgggctgggccttctccccgagcacgccgacgtggacgaggcccaccgggc
cggccactcggtgctcgccgccctggagtccgcgcgcttgctggagcgcggcgacaaccaccggtacaacctgttcccgtccgacacgcacgtccggg
tgcacgacgccgtccgcgacgcggcgctccggttcgcccccggcaagtggctggtccgcgcgggcgccgggctccgcgatcccccgcgcgacgaggc
gctgtggcgcggcgcgcggcgcgtctccctgatgcacaacgccatcgaggacgcgccgccggcccgggccctcgcggtggcgcagccggcggaatcg
ctgatgctgcagggcaaccgcgcgctgccgaggcggatgctgcaggcggtccagcacttcagcaagctcacgtacctggacctggaggacacgggca
tcacggactcgttccccgtggagatctgctgcctggtcggcctggagtacctcaacctgtccaagaaccggatcctgtcgctgccggtggagctgggca
acctcacccagctcaagtacttctacctccgcgacaactactacatccaggtcaccatcccgccgggtctgatcgcgcggctcgggaagctgcgcgtgtt
cgaggtgttcaccgccagcatcgtctccgtcgcggacgactacgtcgcgccggtgatcgacgacctcgaggccagcgccggcgcgaccgtggcgtcgc
tcgggttatggctggacagcacccgcgacgtggagaggctggcccggctcgcgccgcgcgtgcgcgcccggtgcctccacctgcggaagctgaaggg
cgcgcggtcgctgccgctgctgtcggcgcggcacgcggcggagctcggcggcgtgcaggaggggctccgcgagctggtggtgtactcgtccgacgtg
gaggagctggtggccgacgcgcgcgcgccgaggctggaggtgatcaagttcgggttcctcaccaggctgcgcgtcatggcgtgggcgccagccgcgt
cggccagcctccgggaggtcgcgctcggcgcgtgccacagcctcacgcacctcacttgggtgcagcacctcccatgcctcgagacgctcaacctcagc
ggatgcaacgggctcaccaggctcatgggcgggcccgaggagggaggcagcgccgcggaggaggtcgtcgcgttcccacggctcaggctgctggcg
ctgctgggcctgccgaagctggagggcttacgcgccgacggggagtgcgcgttcccggagctgcggcgcgtgcagatgagggggtgcccgcggctga
aacggtttcagatgcagccggtccgcgggcaggagggacaggtgcggatcgagtgcgacaagcactggtgggaggcgctgcagtgggccaacgacg
acgtcaagtcctgcttcgtcaccgtcttctga
>g62004.t1
atggtaaatggcttcatgtcatcgtggatagcgttcgtcgcgctggtgcttcatctcgcagtgtatggctgctcggcggtcaatcttgaagggtcggcgct
gctcaagttccagtcaagggtggaggaggacccacatggcgccatggctggctggaacccccgcgatagggatccttgcagctggaacggcgtcagct
gcgttgatggcagggttgtgatgctattgctttccagtaacagctttcagtggcctctgatccggaattcctatgggaactttggtcaggaaaatggttttg
atatctatgataatctcgagagggataatatgaatcaaagaactgagcatgggttcggatctggttcttcatcagaagagaacaagaaggagagtagc
aatctttctgcaaaaaaccccgctgcacaatttagccagcgcagattgcttcaggatagcaatcttgctgcaccttcttctggaaatggtcctgctccagct
gctgttcctgttccttccacaggcagtggttcattctcagcatttagtcccaacaacgcacctgaaccagctgttgcacctccacctgttccatccatgtctc
ctgctactcctacagaagctctggaaggaaagtcattcaagttgttttacgtcatcgcgcttccattgatcgtgctgctacttatcagtatagtgtgcatggt
tatgctatgccgcaataagtcagggaaaacgataggcccatggaagaccggactcagtggccagctccaaaaggcgtttgtgacagtggagggatttg
atcctattgattggaatggtaggatgagggtaatcatgggagtagcatactgtatccagcacatgcatgagctgagccctcctatagcacacccgggcct
caagtcaagtgcgatattgctatctgaggatggagcagcaaagattgcagacatgagtgtttggcatgaagtatattccaaaggaaacatgcccaaga
atgatgaatttgttgatcaccatgagctggtagctgctgatccagctgggaatgtatgcagttttggtctactcatgctggaaatcatctcaggaaagcctt
catactccgaagacaaggtctcccttacgaacttgagatattctgagttgatgagaaagaaataa
>g62562.t1
atgaaggctggtgcagtaggaaaatttctgctggtgttcattgcttacagtatgctgcacaccggcagctctgcatctggaaatgaaacagatcggctgt
cactgctagacttcaagaaggccatcactcttgatccccagcaagtcttggcctcatggaatgacagcacccacttctgcagctgggaaggcatcctctg
cacagcaaacaacccgcgccgtgtcgcctccatccgcctcgaaagacgaagcctagtcgggcaaatctctccttcacttggaaatttaacattcctacga
aacctctcgcttgcgacaaaccagttcactggagaaatccctgcatcccttggccacctgcagtacctccaatacctctacctgagcaacaacacacttc
aggggcagataccaagctttgcaaactgttcaagtctcagggtgatgtggctgcatcgcaacggtcttgttgggcagttccctgcagacctgccctctgg
ccttcgccagctgcagatttcagttaataatcttagtggaactatccctgcatctctgtccaacataacaaaacttgctgtgattagctgtgcatataataa
cttcgtgggaaatgtccctgatgagtttgccaaattatctaacatgcagcagctgtatcttggtgccaatcagttgtcaggcaggtttccacgggccatcat
gaatttttctgccttaactgacctcagccttggtctcaatgagtttagtggagaggtgccaccggatctcggtagcgtgctgcgcaatctcctcctttttgag
ttgcctgtcaacttcttccatggtcctatcccaccttcactgacaaatgcttccaatctgtactatattgagatatcaaggaacaatttcactgggctgttcc
ctgcagacctgccctctggccttcgccagctgcagatttcagttaataatcttagtggaactatccctgcatctctgtccaacataacaacacttgctgtga
ttagctgtgcatataataacttcgtgggaaatgtccctgatgagtttgccaaattatctaacatgcagcagctgtatcttggtgccaatcagttgtcaggca
ggtttccacgggccatcatgaatttttctgccttaactgacctcagccttggtcttaatgagtttagtggagaggtgccaccggatctcggtagcgtgctgc
gcaatctcctcctttttgagttgcctgtcaacttcttccatggtcctatcccaccttcactgacaaatgcttccaatctgtactatattgagatatcaaggaac
aatttcactgggctgctttcatcaaataatctatctgctgaaatccctagcacattgggcaattgcgagagtttggaagaaatcgaacttgactacaatgc
tttcagtggaaacatccctgtttcactaggcaacataagaactctaaaagttctcaacttatcgcacaacaacatatctgggttgataccagtctctcttgg
aaacctacaacttcttgaacagctagatttgtcattcaacaatcttaaggaatgtgcgggaggtggtcaagtctcaactgcctcagacgtctacagcttcg
gagttgttctcctcgaagtgttcatccggaggaggccaacggatgacatgtttaaggaggaaatgagcatcacaaagtttacagagatcaacttccctg
agaatagatga
>g63045.t1
atggggtaccgggctttgcagctgggtgtgatgctggcctttctgctggcctatggatcccagggtgtgaaccacgaaggctggctcctcctggcgctga
agagccagatggttgacactctccaccaccttgataattggaacgcaagggatccaacaccatgcgcctggaggggcgtcaactgctcgttgccgccca
gcccagccgtggtgtctctcgacctcagcaacatgaacctgtcgggcatcgtcacgcccagcattggaggtctggctgaactgacctaccttgatctgtc

cttcaatgagttctatggtaccatccctgcagaaattgggaattgctcgaaactggaggtgcttaacttgttcaacaacaatttcatcggttcaatcccacc
tgagcttgggaagctgtctaagctggtcactttcaatctgtgcaacaacaggctccatggggcaatacctgatgaaattggtaacatggcatcacttgag
aacttggtggggtga
>g63310.t1
atgggcaccctcctattacggctcctcctgatcaccaccatttttcttccctcgtctttagcactgacggccgatgaggtggccaccattactcgccgtcag
ctactcgccttcgggcggaagagcaatgatggtgttcacgtcgacgtcggcgttgacatcaagatcagcaacccgcttctgctcacagcacacaaggcc
cttcaggcgctcaaggacgccttgtactccgaccccaacaatttcaccgaaaattgggtcggccccgacgtgtgtgcctacaacggcgtgttctgcgtcc
cgtcgatgtacaaccagtcggtaagcgccgtcgcgacgctggacatgaatggtgccgatgtcgcggggtacctgcccaaggagatcggccttatgaag
gaccttgccgtgctccatctcaactccaaccggttctgcgggatcatccccgaggagatcagaaatatgacggagttgtatgaattcgacgtcagcaac
aaccgtttcgtcggcccatttcctacggtggtacttggcgtccccaagcttagctacctcgacatcagattcaacgactttgacggcccgatcccgccaga
gctcttcctcaagccctatgacgctatcttccttaacaacaacaggttcagctctggcatcccggagactatcggcaaatcaaaggccacggtgattgtcc
ttgcaaacaataacctcggtggatgcattcctcggagcattggtgaggctgctgccacacttgaccagttcatcttcatcaacaacagcctcagcgggtg
ccttccggttgagacggggctcctgacgggcacgacagtgttcgacgtcagtggcaacgctctcactggctcaatcccactaacgctggctagattatcc
aaggtcgagcagctagacttgtcacgcaacatgttcaccggggaagtgccccggggtgtatgcaaggcccatgcagaggaccaaaggtga
>g63762.t1
atggtgctcctcctgctcgccttcatctgcctctttgcacttgttgctagcacccactcagccttgatatccaatgctgaaattgccacaagtaatgccagtg
accaccttgctctcctatccttcaagtctcacataaggactgacccatcgcaagctctggagtcatggggcaaccggtccatccccatgtgccagtggcg
cggcgtgacatgcggtagacgaggacgccctcatggccgtgtcatggctctggatctcgagaatctcagccttctgggcgccatctcctcttcggtagcc
aacctgacattcttgacgacgcttcacctccctagtaatcacctccatggtgcggtatcatcggtgcttggtcgcctgctatatctcaagcatgtggacctttt
ctggtaactttctcaacagtaagattccccgtcgctttcacagtgcaagcgtcttgagagcatcagcctcgaattcaacagcctgcaaggtgagatacc
gcgtgagcttggctctttgcacaatgccaagttgctgaatcttggccacacaagcttactggtgcaatccctctggagatcagcaaccttgtgagtcttg
agttcctgcacatcggaagaaacaagctaacaggagagataccagctgggatagagtgtgagactttaagatgtgcaaggcatcgtaaccttgtgaag
atcataacagcatgctcaagtgtggacaacacaggcactgatttcaaggccctcatatttgagttcttgccaaatggaaacctggagcagtggctacacc
agccaattttcagagatggagaatacaatgcgtttgatcttgttagaagtctagatatttcaatcgatgtggcttctgcccttgaatatctacaccaaaaca
agccattgccaattattcactgcgatctcaagcctagtaatattcttcttgaccatgacatggttgctcatgttggagatgtcgggctggcaaggttctacc
atgaggaccccaatgtcacttctgagaaatcaagtgcttgggttaaaatgagaggaacaaccggctatgttgccccagttgacacagataatggtagga
caggaggaggagaggatactacctccaacatggattatatgacgacttcaacgctgcggcgaaggttgccgccgaggatggagaaggcgcgagcca
agaaggggatggtggacctccacggtggcaggcggcttcactggcagcctagagtcatccctgcgatggtctttgtagtagcggaggcatacaacaga
gtactcactggcgtgggacgggacccggctggtagaggcgctcactggcgtcggaagagatcgccgacgggggcgcagggcgccggccaccgcgcg
gcggcaacgacaaccattgtagcagttgaagctataggcccctgggcttgggccctagaaactgtcgaacatgttatatatgaaacccaaaagaacct
aagcacaagataa
>g63763.t1
atggtgctcctcttcctcgagtatctcctgctcgcttttatcttcctttcatgtcctgccacatacatgacacatgcgtcactcgcatctcgtggatcatccac
cccaaatattgccgaccagctcgcgctcatgtcgttcaagtcacgcataagaagtgacccgtcacaagccctcgtgacatggggcaatctgtcccttccg
atctgccagtggcgcggggtggcttgcggcttgaggggacgaaactgtggccgtgtggtggcgctggacctcgccgagctcaacctgcatggcaccat
caccccctgcgctaggcaacctcactcacttgaggcagctcgacctttcatcgaatcgcttccacggtatcctgccggcagagctcgcacccgccccaaac
attgccgaccagcttgcactcatgtcgttcaagtcacgcataagtagtgacccatcgcaagccctcgcgacatggggcaatctatcccttccgatctgcc
ggtggcgcggagtggcgtgcggcttgaggggacggcgccgtggccgtgtggtagctctggacctggccgagctcaacatacttggcaccatcacccct
tcgctgggcaacctcacctacttgaggcgactcgacctttcatcgaatcgtttccatgacatcctgccaccacagctcggtaatctccgtgacctcaagat
actatatcttcactacaactccatcaatgggcaaattcccttgtcactctccaactctagccacctcgttgaaatttcattctatggaagcattccttccaac
attcggagccttgtgaatttgaagaagctcattctacaaagaaataacatgacaggagaaattcccaaagggataggaaaccttgtcaacctcgtccat
ctgggcctcgccagtgccacaaatggatttgcttctgagaacctcatcagagctgggagctttggctcagtctataaaggaagaatgagatgcaatgat
cagcatgatgtcatcgctgttaaagttctcaacctcatgcaacgtggcgcttctcaaagttttgttgcagaatgtgagactttacgatgtgttcggcatcgc
aacctggtgaagatattgacaatctgctcaagcattgattttcagggccgtgacttcaaggccctagtatatgagtttctaccaaatggaaatttagacca
atggctgcaccaacatataatgaaagatggtgaacggcaggccctagatctgattgcgaggctacgcattgcaattgacgtggcatcctcccttgattat
cttcatcaatataagcctgtgctgattgtccattgtgatcttaagccaagcaatgttctcctagacactgatatggttgctcatgttggtgattttggactgg
cgagatttctacatcaagacacggataaatcaagtggatgggcagcaatgagaggatcaattggttatgcagctccaggtctgcttctatttcctgtagtt
gtttcttctcctctaatatag
>g64095.t1
atgatgaccgggtggaggcgcctccaatgggtggcggctctgctcgtcctcctagcgctgcaggcatcagcgcagcagccatcgccgaacccgcgtctc
cgcacggcgttcctggctctccagacgtggaagcgcacggccatcttctccgacccgttcaacttcacggccaactgggtgggccccaacgtgtgctcgt
acacgggcatcttctgcgcgccgcacccgtcggacggccagctcgcggtggccggcctggacctgaaccacgccgacatcgcgggttacatcccggcg
gacctcccccgcggcctccccgacctggcgctgctccacctcaactcgaaccgcttctgcggcgtgctgcccgacaccttcctccacctccgtctcctcca
cgagctggacctcagcaacaaccgcttcgtggggcccttcccggcggtggtcctcaccctccccgagctccggtacctcgacctccgcttcaacgacttc
gagggccccatcccgccgcggctcttcgacctcccccttggacgccatcatcctcaacgacaaccgcctcacccgccccatcccgcccaacctcggcaac
tcccccgcctccgtcgtcgtcctcgccaacaaccgcctcggcgggtgcatcccgccgtcgataggccggatggggcccaccctcaacgagatcgtcctc
atcaacgacgggctcaccgggtgcatcccgccacaggtggggatgctcagccaggtcaccgtgttcgatgtcagcgggaacaggttgcagggccggct
ccccgggagtgtcgcggggatggcggccgtgcaggcctgggtgtcctgcggcaacggcggacgggaggtggaactgtatccctggagcacccgcgca
gcgcccgccgccgcagtgcgctgccgctggggcgccgttcgactgcagcacggcacagtgccaggccgcgccacctag

>g64649.t1
atggccccccttcgcacgccgcctcttcgccctcctcttcctcctcgccgccgccaccaccaccaccacggcgaccgacgtcgaggaggcttccgcgctgc
tggcgctcggcgcggcgctggacccgacgggccgcctgctcccgtcgtgggcccccggtcgcgacccgtgcgcgtcattcgagggcgtggcctgcgac
gcgcgcggggccgtggccaacgtctcgctgcaggggaaggggctcgccggcacgctgtcccccgccgtgtcgcggctccgcgcgctcacggggctcta
cctccactacaactccctgcgcggcggcgtgcccagggagctcgcggacctcgccaacctcaccgacctctacctcaacgtcaacaacctctccgggcc
catcccgccggagatcggcgccatgccgtccctgcaagtggttcagctgtgctacaaccagctgagcggcagcatccccacgcagctgggcaacctga
gccgcctcgccgtgctggccctgcagtcgaaccggctgaccggcgccatccccgccagcctcggcgacctcccgctgctcacgcgcctcgacctcagct
tcaacaacctcttcggctccatccccgtcaggctagcgcgcctgccacgcctcaccgcgctcgacgtcaggaacaactcgctcaccggcagcgtgcccg
cagagctggcgacgaagctgcgggccgggttccagtacggcaacaacagcgacctgtgcggcgcggggctccccgcgctccggccgtgcacgccgg
cggacctcatcgacccggacagaccgcagccattcagcgccggcatcacgccgggcggttcccctaattccgacgccggccacggccgctgcgtcggc
gcacactgcgcgccgtcgaccagggcgctcgcggccgtcgtcgtcgccgccgtcgtgctcctcgccgccacggcggccggcctgttcgcgttctcctgg
cgccgctggcgcagccagagggtcgcctcccgggcctcctcgccgccggccaccgactctgccgcgaaggtgccttcatcgcgcaagtcgtcggcgct
cgcgagcctcgagtactcgaccgcgtgggacccgctcgccgccgacgatcgcggcggcggcggcgggccgtcgtcggcgtcgtcgttcccgcaggag
gtgtcgcagctgagcacggaggaggtggagtccgcgacgcgctacttctccgagctcaacctgctgccgggcggccggaacaagaaggccgccggcg
gcggcgtcgcggccacgtacaggggcgcgctgcgggacggcacgcccgtggcggtgaagcggctggggaagacgtgctgccggcaggaggaggcc
gagttcgcgcgggggctgaggatgctcgccgagctccggcacgacaacgtggtggcgctcagggggttctgctgctccagggcgcgcggggagtgct
acctcgtctacgacttggtgcccaacggcagcctctcgcagttcctcgacgtcgacgcctccgcctctgccgccgccgccggacgggttctcgggtgggc
caccaggatctccatcatcaagggcgttgctaaaggaatcgagtatttgcacagcacgagaccgaacaagcccaggcctctggtgcaccagaacatct
ccgcagacaaggtcctgcttgactacacctacaagccgctcatctccgggtgcggcctgcacaagctcctcgtcgacgacctcgtgttctccacgctcaa
ggccagcgccgccatggggtacctcgcgccggagtacaccacggtcggccgcctctcggagaagagcgacgtctacgcattcggggtcatcgtgctcc
aggtgctcaccggcaagaggaagacgaggacgacgcagcctccgtcgccggcctcagataacagcgacgtcgacgagctcgtcgacgccaacctgc
ggggaagctactcccccgccgaggccgcgatgctggccaagatcgtggcggcttgcaccaacgaggacccggaccggaggccgacgatgacagagt
tgctccaagagctgggcaccatctga
>g65292.t1
atggtgctgctcctgtccctgcaactggtgtcgtcggaaaccgacggggacgctcttctggcgttcaaggcccgcgtgaccgaccccaccggagcgctc
gcctcttggaacgcctcgacgcccttctgccggcggtgggccggcgtgacgtgcaccgcgggccgcgtcacgtcgctggacctcattccggcgagcctc
ggccggctccgccgcctctcgtacctcagcctctgcgacaacgccttctccggcgagattcccgacgcgctccgcaactgcaccgcgctctccgccctgt
acctcaacgacaaccgcctcaccggcggcgtgccggaccggctcggcggcgcgttgatgccgtacctcatgtacctcgggctcggcaccaaccatctct
ccggccgcatcccgccgtcgctggccaacatcacgatgctcagtttgctcggactcgaccagaactttctggaaggcacggtccctgacgacttgtcgcg
cctcaccaacctcatgtccttgaacgtctaccagagccgcctcgccggcgagatcccgccgttcctcttcaacatgtcgtcgctgcaaggcttgaccctcg
ccaacaacgcgttcgagggcgagctccccgccgacgccggcgtgggctggccgaaccttcagttacttctcctcggcgggaaccagctcaccgggccg
atcccggtgtcgctcgctgaggcgtccagtttgaaggtcctcagcctagccaacaacagcttcaccgggcaggtgcccacggcgatcggcaagctctgc
ttgcaggcgttgcagctgtcaaacaacgagctcacgggcggctgggagttcttggagggcttgaccaactgcacgtcgctgtcggaaatctctctcgac
ggtaacagatttactggagcaatgccgagcgccattattgcccgcctctcgcccaacctccagacgctgaaactcggcggcaatggtggcatatccgga
gtcataccgccgggcatcgggaacatcgtcgggctacagacgctggatttcgagtcgaacctcctttccggcgcgataccggagggcatcgggaagct
caggaacctcaaagagttacggttgcagcagaacaagctaacaggccctattccatcctccattggcgacctcagccagctcctagtcatcgacctgag
caacaatttcctcaacggctcgatcccgcctagtctcggtgacctccagcagctcacactgcttaacctctccggtaaccagcttaccggccacgtccccg
gagaagtgttcaagctgtcgtcgctgtcctcggcgatggacctgtcaggtaaccggctggatgggacgcttccgggagacgtcggccagcttgcgaaac
tcgcgcttttggcagtgtcccggaaccgcttctccggcgaggggctccggaggctaaacctgacaagcaacgaattgtctggaagcatcccgccggag
cttgggcagatgtcggggctgcaggagctgtacctgtcacggaacgaactgtccggcagaataccggcagaactggagaacgtgacgtccctgaaag
agctcgacctgtcttacaacgatctcgacgggccagttccattgcgcggcgtgttcacgaacacgaccgggtttcagatggccggcaacggcgcgcttt
gcggcggtgcggcacggctccgcttgccgccatgtccgaattccatccgacgcggccacgtgttcctcaagatcgcgttgcccatcatcgttggcggcgt
ggctctctgcttggggatgctgattagtactctgctctgctgccgcaagaagaggaggaactcgccaactgcagataacaacacagctcgaagcgtgct
caacggcaacaactacccaagagtctcttacgccgagctggccagagccactgacagcttcgcggaagacaacctcatcggcgtggggaagtacggg
tccgtgtaccgggggacgctgtcactgaagacgaacgggagctgtgtgcacgaggacgtcgtggtggcagtgaaggtgttccatctgcgacaagtcgg
cgcttccaagaccttcgtgtcagaaatccaggcgctgcgaagcgtcaaacaccggaacctgatcagcatcgtcacctgctgctccagtatcgacgcgga
cgggaacgacttcagagctctcgtgtttgacttcatgccaaactacagcctggacaggtggctgcacccgagttctgctagtaatgaaagcaaggaagg
gcgtgtcctaagcgtaacccagaggttcaacgttgccgtggacattgccgacgctctcaactatctccacaacagctgcacgccaccgatcatacactgc
gatctcaagccgagcaatgttctccttggcgacgacatgacggcctacatcggcgattttggcctcgcgaagttgattcttgatccggggtgccatgatgc
ggcgaccactgaaagcaccattggtgtcagaggcacaattggttatgtcgcaccagaatacggcacgtcagggaaggtttctaccaatggtgatgtata
cagctttgggattatgcttctagagatattcgttgggaaggcgccaactgatgctgcgttcagagacggccttactctgcaggaattcgtcgccggggca
ttcccggagaagatagagcagattcttgatccggctctgctaccgattgaaggctcatcggacggaataaattccggttcagaagaaagcgtggtgcga
gttacagtgcgtgactgcctgctttccgccgtaaaggtcgggctcagttgctgcaggcaggcgccctgccaaagaatggtcatgaaggatgcggcggta
gagttgcgccggataagacatgcatgtgttcgtgcctgcggtcagtga
>g65470.t1
atggtccagaagtgggtctcgtacgcgctgcagcagtgcggccacgagttccaccttgatctgcgcctccactcgtacgacctttgccagcgcggcggtg
cggcgcacggtgaccaagacagagaatacctaaggggggaccggtaccagtcaaccatggggaggtccctcaggtttccaagatcgctcttctcctgc
gccgcgctgcgatcgctgcgcgtcgccgattgctacctcaacccgccacaagttgtctcttggccatcgcttgagacgctgcacctgacgacggtccacg
attcagaggaaaccatccaacggatgatcgctagctgtccacgccttgaagacctgatgctcgactcgtgctccaatgtgaggagaatctccgtcctcga

caagcgccttcgtcggttttcactccggtgctgccacgccgttgtgagcgtcagccttgacgcgtcggagctgcgggaactcgactaccgtggtgcggtg
cccgcggaatctctcttcatgtttcatggctcgctgaagaagatccgctcctgcaccatcgacttctgcggacctagcctttcgactgaggatgagctcgtc
aggttccgaaagttcctgggaaacttggtggcccccaggcacctgcacctgcagtcatggcggcttggttgcagcattgagagcgaattcgtcacaggc
ttcccggcattctccagcctgcagaagctcgagctcacgggacacgttgacagacacaccactgtctctgcagttcccaggatcctgcagcagaccccc
aaccttgaggtcctgacgctgttcctgaagccagtttacaaggaaagctcggaagcccaccttgagtacaatctagccaccatcccggatacgacagcg
aggctgtgcctacggcagcggctgagagagatcaacctggtgcactaccagggtagcgacgagcagaggatgctggccaatctcctgcttggcaatgc
cctggttctgcaggctctgtgcgtcgttttccctaaggcgtccctggggctgcagaccagaatgatgaatgagattaaacaaatggtggtgagcaagtcc
acagaaattttattcctgtaa
>g66223.t1
atggaattttcttgccacgccacggcgggcgccgaccgaccaataggcaggatgaatccaacgaaccgcagccggcgcgtgcgcctccactctcctct
gccgcgtgcaacttccctcgccttttacccgcgcgacaacggcatcgccgcagcagcaaaccgcaacagcgaagcaggggcggcccctccccacacc
caccccaccacaagccactacaccttgcggatggaggcgctgaagaagcaggccaacaagctccgggagcacgtagccaagcagcagcaggccgtt
cgcaagcagttcagtgcgcggtatactcaggatccttctcttgttgatgaagcagagcttgagtgccaccagaatctccaaaggctctacagcagtacca
gagcagctaaaaatttccaacgaaatatcatacgaggaattgaaggttttgttgctgttagcaccaaacagatggagatagtcaaaagactagctgagg
actgttgcagatatggaaacgacaatcagaattttggttttgctcttgcgggggcttctgtggagtttggcaaatcccataaccaaatagagaaagagcg
ggagaatcttcttaaagttcttggagagcaggttgatgctgagagagcttatcatcaaaatgctgctgatattctaaataaactccatgatgagatgctcc
atgctaagcatcataatgagtcagcaaaccactatgatgagcagtcatcaggacctgaatctgaaccaactacatcacatgtacgttcacagagtagca
catctgaagatccagtattgagcaaacttggtgaatctgcagatccagtattgagcaaactaggtgaatctgcagggaatggtcaggaggtgcattttgt
tggggaggtgattcatccattcgatgctcaggctgatggtgagctcagtattgccgtgggtgactatgttgtggtccgtcagagtagcacatctgaagatc
cagtattgagcaaacttggtgaatctgcagatccagtattgagcaaactaggtgaatctgcagggagtggtcaggaggtgcattttgttggggaggtga
ttcatccatttgatgctcaggctgatggtgagctcagtatcgccgtgggtgactatgttgtggtccgtcaggtagcatcaaatggttggtccgaaggtgaa
tgcaaggggaaggctggctggttcccatctgcctatgtagagcagagggacaaagccccggcaagtgctgaatttatggagccgagcctcacatctttc
tgctgccggtctgtcacgggctcggatgaggttgccacggttgttttctgcctcgacaagcacatggagacaggaaagatcaaaagctcacaagtgaaa
aacgcgagcggcggtgaggcacttcttgccttcaaaaaggcagttacaaattcagatggggtctttctaaattggcacgagcaagatgcagacccctgc
aattggaagggtgttggatgtgatagtcatagcaaaagggtggttaatctgattcttgcataccacaagctagttggtcccataccgccagaaattggaa
ggttaaatgagttgcaaaatttgtatctgcatggaaattatttaagcggatacatcccttcagaatttggtaatctcgtggaacttgaggcattggacttgt
ccagtaatacgttgagcggatctattccacattctcttgataagctgtcaaaactcaaattattcaatgtgtccatgaacttcctgacaggagcaataccat
cgtctggttcacttgccaatttcaatgaggcatcctttgttggaaaccttggtttatgtggaaagcagattaactcggtgtgcaaagatgcacttcagtcac
caagtggttctcagtcaccttctgcagattggatcagatattgtgaaaaccagatgcttgcattttatcaacaacaacaacaaagcctctaa
>g68571.t1
atgagccgggagggccggcgcctcgaccatggcggcggcggcggggacgacgtcgccggcggcgtcggggtcctcgtgctgtcgctggacctgctgg
gccaggtgctcgaccgcctgcgcgacccgcgggaccgcaaggcgtgccgcctcgtcagccgcgccttcgagcgcgccgaggcggcgcaccgccgcg
cgctgcgcgcgctccgccgggagccgctcccgcgcctgctccgcgcgttcccggcgctcgagcggctcgacctctccgcctgcgcctgcctcgccgcag
ccgtcgccggcgccgcaggggccggggacctccgcatccgcagggtgtgcctcgcgcgggccagcggggttgggtggcgcgggctggaggcgctcgt
cgccgcctgccccaggctggaggccgtcgacctctcgcactgcgtcggggccggggaccgcgaggccgccgcgctggcggcggcggccgggctcag
ggagctcaagctggagaagtgcctcggcgtcacggacatggggctcgccaaggtggtggttgggtgccccaggctggagaagctcagcgtcaagtgg
tgccgcgagatctctgacatcggcatcgatctgctcgccaaaaagtgtccagagctccgcagcctcgacatctcctacctacaggtgggcaatgagtcc
cttagatcaatatcctccctagagaagctagaggagttggcaatggttggttgctcatgtatagacgatgaaggcctggaactgctaagcaaggggagc
aactcactgcagagtgttgatgtatcaagatgtgatcacgtgacctcccagggattagcttcactcgtagatggtcgcaatttttccagaagctatatgct
gcagattgtttgcatgtgagttga
>g69256.t1
atggctgtggtgggagcatcagcgacgtgcttcttcctcttctcctcgtggttgtgcctctgctccaacgctcgtttgtctcctccaccggaaagcaacgcc
acggctgccgatcagctcgcgcttctctccttcaagtccttgctgaactctttcaacggctcgctggactcatggaacacgtccagccacttttgccgttgg
ccaggagtcgcctgcggccggcggcacccggagagggtggttgcgttgcgcatgccagagttcaacctgtcaggacgcatctcgccgtccttgggcaa
cctgtccttccttcgggagctcgaactccgcgacaacctactcagtggtgagataccaccgaagctcggtcggctcggcaggctagagctcctgaacct
gagtacaaacgctctcgttggtggaattcccccagccatagcagagtgcgccaacctcgtgatactagttctcagcaagaaccagcttcacggggagat
acccgctagccttggcgccatggagaacctatacatcctggacctccaagagaaccacttgtctggggagatcccccggtctctggcgaatctgccgtc
gattgagtacttgtttctatacagcaacatgctctccggtgagataccaccagagttgggcaacctgaccggcctccggcatctcgacctcatggacaac
atgctgtccggagctactccttcgtcgctgggcaagatgtcctacctatcctggctcagcttaggctataacagcctcagtggagagatcccggcatctat
ctggaacatctccaatctgtggggaatcaacatccaatggaacaacctcaccggtgtcattccggcaaacgcgttcagcaagctcgctgagcttcgaac
agtgtccatggacaacaatctgttctatggccctttaccagcctcgatagggaacgcctctcggctacatatgcttcagcttggccccaactccttcaacg
gtgtcattcctccacagattgggaggcttagaaacatgacaaagctgcttctgtccaacactttgcttggagctcaagatcctggagattgggagttcatg
acggcactggcaaactgctcccagctactaattttggacctgacagccaggccatcagcaggttggcagtgccgcagccagcgagcaatggagcaga
ggtacggcaacgagaaacagagtagtgcaacaaaggtctga
>g69638.t1
atgatgagcactagagtatatatagtagcagccggtctctcaaggaaaggaaggcatcaggaggagggaataaacaacatggcctcctccttgttggt
actcggccggaaagtggtcatcgtcctcctcctcgtggtagtggtgggcagggatgataatagcaatgccgctgaagccctgaaccaggacgggctcc
atctgctggaagcgaagcgcggcctcttcacagctcctggaagtgaggggaggttgcttgacgactggaaccccggcgacgcgacgccgtgcaattgg
acgggcatcacctgcgacgaagaagaaggagaaggagaaggaggtcatgtgacggagatctcgctgccggagtccaacctggcgggctccttcccc

gccgccttctgtcgcctacctcgcctccgctccatcaacctgtccttcaactacatcggccccgagctttctctttctctttctctatctgtcgatggagaaga
acagggaggaggatgcaggtccctggagcgtcttgacctgtcgatgaacgcgctggtgggcccgctccccgacgcgctcgccgacctccccgagctcc
ggtacctggatttgtcaagcaacaacttctccggcccaatcccggcctggtttgatggaggatccatgaggaggttggagtctctgtcgctggtgtacaa
cctccttgacggccacatcccggcgtctctctcctccatctccacgctccgggagctcaacctctcctacaaccccttctctccgggggaaacaccatcc
cgccggagctcggccggctctcctccctgcgcgtgctctggctcgccggatgcaacctcgtgggtcccatcccggcatcgctcggccgactcgccaacct
caccgtcctcgacctctccatgaactctctgtccggcggaatccctccggagatcgcgacggggctcaccagcgcgacgcagatcgagctctacaacaa
ctccctctccgggcccattccccatggattcggagggttgaggcagttgcgaaaccttgatttgtcgatgaaccggctgtcagggtccatcccggaggac
ctcttccaagccaagctactggagacggtccacctctacgccaaccagctcacgggtccgttgccggaatctctagccacaggtgccacctcgctggtg
gagctccgcctcttcgccaacaagctcaacggcacgctccccgccgatctggggaagaacgcgcccttggtgtgcgtcgacgtctccgacaacgccatc
tccggcgagatcccaaagggaatctgcgaccgcggcgagctggaggagctgctcatgctcgataaccatctctccgggtccatcccggcatccctgggt
cggtgccggagcctgcgtcgcgtgaggctctccaacaacaggctcaccggcgacgtgccttcagcagtgtggggcctgccgcacatgtcgctgctgga
gctcaacgacaaccatctctcaggaatcatctcccccgccatcgccggcgcggccaacctgtcgaagctcgtgctctccaacaaccgcttcaccggcac
gatcccatccgagatcggagcggcgaccaagctgtacgagatctccgccgacggcaacttgctctccggcccgctgccgtcgtctctcgccggcgagg
gcctcgcagacctcggcaggctcgtgctacggaacaactccctctccggcgcgctgctccaatccgtggccggttcttggaagaagctcaccgagctga
acctggcggacaacgcgttcacggggtccatccctcccgagctcggcgacctgccagtgctcaactacctcgacctctccgggaacaggctcaccggct
ccgtgccgatggagctggagaatctcaagctgaaccagttcaatgtctccaataaccagctcactgggcctctcccgccgcagtacgcctctgctgcgta
ccggaacagcttcctgggcaacccggagctgtgcacgcaagtcccagccctgttatgcgctgactctactggtagttcctcctcctcctcgcagcgcc
acccaacaatgagctgggccttgctgctgatgcggtgtatattcgtattcgcggccgtcgttctggtggccgggatcgcatggttctaccaccgggccttc
tcctcctcctccgataactctaagaagatgatgatgatgatgatgagtgagcggtccaaatggatggtgacgtcgttccacaagctgtccttcagcgagt
acgagatcctggactgcctggacgaggacaaggtcatcggcaccggcgcatccgggaaggtgtacaaggcggtgctcagcaacggcgaggtggtcg
cagtcaagaagctctggggtactaatgatgatcatgatgatgcttgtctagtcaacaaggacaagaacaagaacaatgacaaggacaacaaggacga
gagcttgttgttgttattcgatgcggaggtgcggacgctgggcaagatccgtcacaagaacatcgtcaagctgtggtgctgctgcgtcagcgctcattgc
aggctgctcgtctacgagtacatgcccaacggcagcctgggtgacctgctacacacaacaagcaataagctggactggcccaggaggtacaagatcg
ccctggacgccgccgagggcctgtcctaccttcaccatgactgcgtcccggccatcgttcacagggacgtcaagtccaacaacatcctcctcgacgcac
aattcggtgcacggctcgccgatttcggagtcgccaaggtcgtcgatgatgcccatcttccaaagtccatgtccgtcatcgcaggatcatgcggatacat
cgcgccagagtatgcgtatacacttcgcgtgaccgagaagagcgacacctacagcttcggtgtcgtcctcctggagctggtcaccgggaagccgcccgt
ggatccagagttcggggacaaggacctggtcaaatgggtgtgcagcaccatggagcagaaaggcgtggaacacgtgctggacaccaggctcctcctc
gactacaagcaacacatgctcagggtgctccacatcggactcctctgcgcaagctcactgcccatcaaccgaccgcccatgagaagggtcgtcaagat
gctgcaagaggttgctccgactccgaatcttactaattcccaaccccaatcgctctcgccgtactactactactacgaccctgacaccaccacatcctccg
atcaagggagcagcgtctag
>g71474.t1
atgtccatctcgacattcatggctacacatatctgcgatgaggccgcgctgctcgccttcaaggccgcggcgatcagcggcggcgggtacgacgacccg
ctcgcctcttggaatggcagcacgggcggatactgcagctgggagggagtaacgtgcggaggcagggaccaacgcgtggtggcgctcagccttcaat
ctcgtggactcactggcttcctctcatcggccgtcggcaacttgtcatcactgaggactctaaacctgagcttcaacaggtttaatggagacatccctgct
aacctcggccgccttcgtggcctccacgccctcgacctaagccacaacgctttctccggcgagttccccataaacttaacctcttgcatcaacctaaaggc
catacacctccgcttcaaccagctgcatgggtgtgtcccctctgagatcggggataaactcacgaggcttagagttctcagcctcgaaagtaacaacttc
actggagtcatcccggaatcgctaggcaacttaacaacactgagagctctgtcccttgcgttcaatcaactacacggcaccattcctcctagcctcggcg
acatcaccggcattcagtacctagcacttgccttcaacaacctctccgttgacaacaagaaacaaggaggtgagaagtga
>g71634.t1
atgaggagcctactccggccctcactgggcatccttgtgctcctcgctctgacgacggcggcgccgccggcagtcttggcgctgtcgcccgacgggcaa
gctctcctctctttcaagcaggcagtgaccgacgaccccacctccgccctctccgcatggtccgccaccgacgccgacccgtgccgctggccaggcatc
acctgcgccaacaccaccaccaccacctcctcctcctctccaccacgcgtcgtcagcgtggccatggccggcaagaacctgtcgggcaccctcccggcg
cagctcttctccctgccctccctccgccgtctcaacctccacggcaacctcctctccggcccgctccctccctccctctccaacgccacgtccctccgctccc
tcttcctctacgacaaccgcctcacgggcccggtccccgtacccgcgctctgcgccctgccgcacctacaaaacctcgacctctcacgcaacgcgctcac
cgggttccagtcccccctccgatctcgtcaaatgcacgcagctgcagcggctgctgttgggaagcaacgcaatctcgggtacggtcccgcgcggggtgtg
gccggagatggcgagtctccaggtgctggacctgtccagcaacaacctcacgggccccgtcccgccggacctcggccgcctgcccgcgctggccggg
acgctgaatttgtcgcggaaccacctcgccggcgccgtgccgccggagctgggccggctgccggtgacggtggcgctggatttgaggttcaatgacctg
tccggcgagatcccgcaggtggggtcgctcgcggggcagggccccaccgcgttcctcaacaaccccgggctctgcgggttcccgctccaggtcccatg
ccgcgccgcgccgccgtctgagtcctcctcgctcccgccgccgggatcgtcgtcgtcgtcgtacgctatccgcggcggcgaggagggaggaggag
ggccccgtcggccgccgatcaagagcggactcatcgtgctcatctccgtcgccgacgcggccggggtggccctcatcggcgtcatcgccgtctacgtat
actggaagctccgcgaccggcgacgccgccaacaccaagcggctgcggcgggacccgccaaaggcgacatggatggcaacgacgacgacgacgag
tcaagacgcggcggcctcttcgcgcgctgctgcgcgcgcgccgacgacgcgtcgtccgagctctccaacgatgatgacggcaagcccacaaacaaca
acaacaacaacaccaacaccggcggcggcggcaaggagggcgagctggtggcgatcgacaaggcgttcaggatggagctggacgagctgctgcgc
tcctcggcgtacgtgctggggaaaggagggaagggcatcgtgtacaaggtggttgtcgggaacgggaccacgcccgtggccgtgcgccggctcggcg
gcggcgccgccgcgcacgaccggtgcagggagttcgcggcggaggcggccgccgtcgggagggtcaggcaccccaacgtcgtgaggctcagggcct
actactggtccgcagaggagaagctcgtcgtcaccgacttcgtcaacaacggaagcctcgccgcagcgctgcgaggtcggtctggtcagccgagcctg
tcgtggtcgctgcggctgcggatcgccaagggcgcggcccgcgggctggcccacctccacgagtgcagcccgcggcggttcgtccacggcgaggtga
agccctccaacatcctgctcgacgccgactacaccgcgctggtcgccgacttcggcctggcccgcctcctcgccgtcgccggctgcaccgacgtgtactc
cgtcgccgcgggcggcggcggcgtcatgggcggcgcgctcccgtacgcccggccgcccggcgccgcgttcgaccggcccagcccgtaccgcgcgccc

gaggcccgcgcgcccggggcccggcccagccagaagtccgacgtgtactcgttcggcgtcgtgctgctggagatgctcacggggcgggcgccggagc
tacaggtgggcccctccccgtccgcgtcgtcgtcgttcccgctggggcccaccggcgagctccagcagcaagagcaggcgccggagatcgtgaggtgg
gtgcggcaggggttcgaggacgcgcggccgctggcggacctggccgacgccgccgtgcttcgggacgccggcgccaggaaggaggtggtggccgcg
ttccacgtggcgctcggctgcgtggaggcggacccggatcgacggccgaggatgaaggccgtgtccgagagcctcgacaagatcggggcgtag
>g71781.t1
atgccgtttcccaccgcgtcccccgcgaggcggcggcgcggcggcgggggcgcgggcctccggtgcgcgcccgtgctcctccttgtcctggcgctcgcc
gccggtgccgcggcgcagacatcgccgtcggatgcggaggcgatgcgggcggtggcgaaggcgctggacgcggacaagctcggctgggacgtctcc
ggcgacccctgctcgcccaagcggtgggacggggtgtcctgcgactcgtcgggccgcgtcaccgccatccaggtcgggaggcgcgggctcacgggga
ctctggcaccggaggtgcgcaacctcaccgcgctcgtccgcctcgaggtgatgctgaacaacatcaccggcccgatgccctccctcgcggggctctccc
agctccaggtactcctggcccacaactgcagcttcacctccatcccgccggacttcttccgcgggctcacaggcctcaacgtcgtagacgtcgaccacaa
cccgttcgccgcgtggacgctcccggccgacctcgttaactgcacctccctccagaacttctccgccaatggtgctggcgtcaccggcaccatcccggac
ttccttggcacgctgccgtcgctcgagctgctggcgctggcttacaactatctgtccggccccgtgccggaatctctgtctggcgcgccgcttgagcagct
ctggctcaacaaccaaagaggggacagcaagttaaacgggtccattagctttgtcaccaacatgacggctctgcagcaactgtggctgcatgacaaca
ccttctccgggcccttaccggacttcaaggggttcgataatctttgggatttgcagctccgtgacaaccaattcacggggccggtgccggacacccttgtc
aatttgaaggctttgaagacggtgaccctgaagaataatttccttcagggaccgaggcccgcgtttccgaagggtgtcgttgttgacatggacgatgag
aagttctgcttgcaagaccctggaaagccatgcgatccccgcataagtctgctgctcgaggtggctgctgggttcatgtacccaaagaggctagcagat
ggttgggtgggtaatgatccatgtaagaatagtttcactggtgttacttgcgatgctacaaccggcaacattacagtgctaaattttgcccatatgggattc
actggcatgatctcacctgccatcggaaagattacctcactgaaggtgctgaaactttctgataacaacatcactggaacggtgcctaaggaggttgcc
gcattgcctgcactgaatgaagttgatctgtcaaataacaatctttatgggccagtgcctacttttgctacaaaggtggtgccgataactgccggcaaccc
aaacattggcaaggatgctcctgctccaacagcaggatcaggtggcaccagcagtccatctggtaaaggtggtgatgagagcaaagaaaacaatggg
ggatcaggttcaccgtcgccttctgctggagtcattgcaggatcagtgcttggtttggttgctggattaggcctcattgctgccttaggtttatattgctaca
agagaaagcaaaggccttccggaagggttcagagtccacatgctacggtgatccatccacggcactcagggtcggatgacatggttaaaatcacagtt
gcagggagcaatgccaatggtggtgctcgtgcaagtgaggcatatagccaagggagtagtgatccacgtgacattcatgttgttgagagtggaaatat
ggttatttcaatccaagttctccgtaatgtgaccaacaacttcagtcaggagaacatccttggtcgaggtggttttggcgttgtttacaagggtgagcttca
tgatggtacaaagattgctgtaaaacggatggaggctggtgtgatgggtaacaaggggctaaatgaattcaaatcagagattgctgtcctgaccaagg
ggacactgagcgaacacctgtttgagtggtcagaacataatctgcagccattggaatggaaaaagcgactgagcattgcgcttgatgttgctaggggtg
ttgagtacctgcacagccttgcccagcagaccttcatccacagagacttgaagccatctaacatacttcttggtgatgacatgaaggcaaaggtggcag
actttggattggtcaggcttgcaccagctgatgggaagtgtgtctcagtggagacaaaactcgctggcacttttgggtaccttgccccagagtatgcagtt
actggacgagttaccacaaaagctgatgtcttcagctttggtgtcatcctgatggagctgattactggacgcagggcacttgatgagactcagcctgagg
acagcatgcacttagttacatggtttaggagaatgcagctcaacaaggacactttcaggaaagcaattgacccagttattgagcttgatgaggagacgt
ttgctagtgttagcacagttgcagagcttgctggccactgctgcgcaagagagccaaaccagaggcccgacatgggccatgctgtcaatgtgctctcaa
cactatcagatgtgtggaaaccatcnnnnnnttcattgctaaattttgcccatatgggattcgctggcatcatctcacctgccatcggaaagattacttc
gctgaaggtgctgaaactttctgataacaacatcactggaacggtgcctaaggaggttgccgcattgcctgcactgaatgaagttgatctgtcaaataac
aatctttatgggccagtgcctacttttgccacaaaggtggtgccgattactgccggcaacccaaacattggcaaggatgctcctgctccaacagcaggat
caggtggcaccagcagcaacagtccatctggtaaaggtggtgatgagagcaaagaaaacaatgggggatcagggtcggatgacatggttaaaatca
cagttgcagggagcaatgccaatggtggtgctcgtgcaagtgaggcatatagccaagggagtagtgatccacgtgacattcatgttgttgagagtggaa
atatggttatttcaatccaagttctccgtaatgtgaccaacaacttcagtcaggagaacatccttggtcgaggtggttttggcgttgtttacaagggtgagc
ttcatgatggtacaaagattgctgtaaaacggatggaggctggtgtgatgggtaacaaggggctaaatgaattcaaatcagagattgctgtcctgacca
aggggacactgagcgaacacctgtttgagtggtcagaacgtaatttgcagccattggattggaaaaagcgactgagcattgcccttgatgttgctaggg
gtgttgagtacctgcacagccttgcccaacagaccttcatccacagagacttgaagccatctaacatacttcttggtgatgacatgaaggcaaaggtggc
ggactttggattggtcaggctggcaccagctgatgggaagtgtgtctcggtggagacaaaactcgctggcacttttgggtaccttgccccagagtatgca
gttactggacgagttaccacaaaagctgatgtcttcagctttggtgtcatcctgatggagctgattactggacgcagggcacttgatgagactcagcctg
aggacagcatgcacttagttacatggtttaggagaatgcagctcaacaaggacactttcaggaaagcaattgacccagttattgagcttgatgaggaga
cgtttgctagtgttagcacagttgcagagcttgctggccactgctgcgcaagagagccaaaccagaggcccgacatgggccatgctgtcaatgtgctct
cgacactatcagatgtgtggaaaccatccgatccagattcggacgacagctatggtatcgacctggacatgacgctacctcaggcactgaagaaatgg
caagccttcgaggacagcagccattttgatggcgcaacctcttcattcgttgcaagcttggataacactcagacaagcatcccgacaaggcctccagga
tttgccgagtcattcacctccgctgatggaagatag
>g71931.t1
atgccggtcctcagcagcctcgacctgtcgtcgaacaagctctccggcgacatcccgccggcgctggccaagccgaccatcaccgccctcaacctctcc
tccaaccagctcaccggcaaggtccccgccgggctcgccaccgccgcctacgaccgcagcttcctcgacaaccccggcctctgcaccgccgccgccgc
tccgggactcctcgccggcgtgcgctcgtgttccgcgggatcatctcgaggaggaggaggaggagtctctcgcgggctccgaacgggcctcctcgccg
cgggcgccgcgctcctcgtcgtggccgcggcgttcgccttcttcgtcgtgcgcgacgtcaggaggcggcgtcgcctcgcgcgggagaaggagtggaag
ctcacgccgttccaggccctggacttcggcgagaaggccgtgctccgcgggctcacggaggacaacgtcgtgggccgcggcgggtcgggccgcgtgt
accgtgtcccctacacgaaccggcgcgacggcagcgccggcgtagtggccgtgaagcggatccgcgtcggcggcggcggcgacgagaagctggagc
gcgagttcgaggcggaggcggacgtgctcggccgcgtcaggcacaacaacatcgtcaggcttctgtgcctcgtcacgggccccgaggccaggctcctc
gtgtacgagtag
>g72157.t1
atgagccgccgctccaggagctccgccgcaacggcggcggcccttctcctcgcggcagtgctgctgcattgcgcggcggacgcggccacggacgcgg
agcgccgcgtcctcctggacttcaagtccgcggtgacgtccgacccgcgcggcgcgctgtcctcgtggacgccgacgggcgacccctgcgtggacttcg

ccggcgtcacctgcgacccctccacccgcgccgtctcccgcctgcgcgtccacggcgcgggcctctccggcacgctcgccccctccctcgcgcgcctccc
ttccctcgaggccatctccctcttcggcaaccgcctcaccgccaccatccccgccacattcgcctccctcgcgcccacgctccgcaagctcaacctcagcc
gcaacgcgctctccggcgagatcccgcgcttcctcggcgccttcccctggctccgactcctcgacctctcctacaacgcgctcaccggcgagatccccgc
cgcgctcttcaacccttgcccgccgcgcctccgctacgtctccctcgcgcacaaccgcctctcaggccccgtcccaaccggcattgccacctgcgcccgc
ctcgccggcttcgacttctcatacaaccgcctcaccggcggcctcccgccccgcctctgcgcgccgccggagctgagctacgtctccgtgcgcgccaac
gcgctctccggcgagatcaacggcctcgattcctcctgccgcagcatcgacctcttcgacgtcggcagcaacaacttctccgggcccgcgcccttcgcgc
tcctcgccatggtcaacatcacctacttcaacgtctcctccaacgccttcgacggccccatccccaccaccgcggcatgcggcgccaggttctcctacctc
gacgcgtccgggaaccgcctcacggggcccgtgcccgacaccctgctgacgaattgccgcagcctcagggttttggatttgggggcgaatgcgcttgcc
ggagacataccgcctgccgttgggacgctgcggtcgctctccgtgctccggctcgcgggcaatgctggaatttctggtgcaatccctcccgagcttgccg
ggattgagatgcttgtcacgctcgacctcgccggccttgctctcacaggacagattccacactccctcagccactgccagttcttgcttgagctgaatctct
ctggcaaccagttgcaaggaaccatcccagacacgctcaacaacttgacgtacctaaagctgcttgatctacacagcaaccgcctcgacggggacatt
cccctaacactcgggcagctcaccaagcttgatctcctcgacctctcacagaatcggctgacgggaccgattccttcagagctggggaacctctctaacc
tggcacatttcaacgtctccttcaatgacctctctgggatgatcccttctgcacaggtcttgcagcagtttggtgcctcggccttcataggcaatccacatct
gtgcggccctccgttgaacaatctctgtggtagtggtggtggtgggcaccgtaaacggttgggcgtatctgtcataatcgtcattgttgctgcggccctca
tccttatcgggatatgcattgtttgcgcgatgaacattaaggcttatatgcgcaagagtaatggtgtggaggacagcaaggaggaggaggaggttctcg
tgtctgagagcacaccgattgcatctccaggctcaaatgcgatcatcggaaagttggtgcttttcagcaagagcttgccttcaaggtatgaagattggga
agcaggaaccaaggctctcgttgacaaagactgcatcattggtggagggtcagttggcacagtgtacaaagccacctttgaaaatggcctatccattgc
tgtcaagaaactggagacacttgggaggctggaaaaccaggatgagttcgagcatgagatgggccagcttggcgtagcaaaggaacggttgagcaa
gaacattttgatgcaggagcagctcatctaa
>g72718.t1
atgtcgtcactgaccttgctgctgctgctattatgctccatatcactgtcgacgctactcatcgcggccgcagagacgggcgacgaggccgcgctgctgg
ccttcaaggccgcggcggtccacagagacggcgtgctcgcttcgtggaacgtcagcagcagcgccggcgggttctgcagatgggagggggtgacttg
ccagcaggggcgagtggtggcgctgagcctgccctcacgcggcctcgccggtgtgctctcgccggccgtcggcaacctgtcgtccctcaggactctcaa
cctcagctacaacgcgctccatggagccatcccggcgagcatcggccgcctgcaccgcctcgaggtcctcgacctgagcaacaacgccttctccggcg
agctccctgctgccaacctcacctcctgcaccagcctgcgggcgcctcgagggaccatccctcccagcctcggcggcatcccgggcctccgacgactca
ccattgccttcaaccgcctctcgggcgagctgcctgcctctatctacaacttatcctccttggaatggctgcaggtccagggaaacaaactccacggaga
aattcctgtagatatcggcagcaagttccccagtatacaaattcttagctttgctatgaaccagttcactgggtccatcccagcttcgctctccaacctcac
tactctgcaatggctcgacctctcgtacaatacgctcagtgggcatgtgcctagtggtcttggaaggctgcgagccctgcaaagtctcaacttgcgcatc
aacaggttacaagcagatgacacgctggggtgggaattcatagcttcactgtcaaactccagccagctcaaggagctacttctcagtcacaacgccgcc
ctcactgggcagtttccaaggataacggggccattctccaatacgaatagcatcagcgcgctgcatgaacttgttacccaacagaccaacaacagcaa
ctacccatcgaatctgccagtacatgtcgatgacgaggctgcgctgctctctttcaaagccgtggcgattagcagtgggtatgacgacccacttgcttcat
ggaatggcagcataggcggatattgcaactgggagggggtgaggtgccgtggcaggcaccagcgagtggtggcactcacccttccatctcatgggctc
accggcgtgctctcaccggccatcggcaacttgtcatcactcaggactctcaacctaagcttcaacggtttcagcggggacattcctgctagcctcagcc
gccttcgccgtctccatatcctcgacctaagccacaacaccttctccagcgaggtccccgtaaacttaacatcttgcatcaacctaaagaccatgcgccta
cgcttcaaccagctccgtggatgcgtcccgtctgagatcggtaacaaactgatgggtcttaagattctcaccctaggtgacaacaagctcaccggagcc
attccgacgtcgttgggcaacttatcatcacttgttacattggagcttgcatacaatcagctagaaggcaccatcccttccagtctcggcaacatcctggg
catccggtacttggccctcgcctacaacaacctatccgaagccagcgacaaggccgccactgtcagcagcagcggatacagcgatgctcttgcttcgtg
gaacggcactaccggcggatactgcagctgggaaggggtgacatgcggaggcaggcattga
>g73317.t1
atgcggctcatcttattgttcctctcgatctgggccgcgtctgccgccgccgcgggtgctggttcagacatggaggcgctgctggagttcggcagaggca
tccggcaagacccgtcccgccgtcaggccacccccctggaaccccaccagtgcgctggactccgacggctgccctgccgattggcatggtgtgcagtgc
aacggtggccagattctttccattgcattcgatggcattgggcttgttgggaaggctagcttatctgcgcttgcgaggatgccgatgctccagaacttgtct
ctgtcagggaacaagctggaaggggtcttgccgcgtgagctggggtctatggcttcgttgcagttgctggatctctccaacaacatgttctctggatcaat
tccctctgagctgaccaaattggcaggattaggatatctgaatctgtcttctaatggttttggtggcgcgttgctggtgggtttccggaacttgaggaaatt
gaagtacctggaccttcgcggcaatggcttcactggcaaagtgggcgacataattgcgcagttgcagagcccagtccatgttgatttgagctgcaacca
gttctcagggtccttggcatctatgtctgacaactcatctgcagttagcacgctgcagtatttgaatgtgagccacaatatgcttgctggaactctatttgat
agtgatccaatgcctttgtttgacagtcttgaggtcttcgatgcaagttttaatgcgctcagcggcaatatcccaccatttaactttctaatctccctcaagg
gacagagtgctcagagaagtgcagaacctgcaacagcagaaacacacgatgtatcattagaatcatcaccgactgctgaatatggagctgcatcactg
cctggaaaggacagacagcatgacgtcccagaggagctctcatctgctcctgctgagataattggtaggagttgccatgggacatcatacaaggctac
acttgataatgggtacgtgctgacagtgaaatggcttaaagagggctttgttaagagtaaaaaagaattttcccgtgaaataaagaagcttggtgctgtg
aaacatccaaatctagtctccttgcgaggctactactggggccccaaagagcatgaaaggatcatcatatcagactatgcagatgccacatctctgtcta
cttacttgtctgaatttgaagagcggaatctcccacctctatcagcaagccagcggctggccattgccattgacattgcccggtgtctagattacctgcac
aatgagcgggtgatcccacatggaaacattaagtcgtcgaatgtactgattcagaattccacttcatctgcgctggtcaccgactatagccttcacaggtt
gatgacgccgattggtatggccgagcaggttctaaatgctggtgccctcgggtactccccaccggagttttcgagcactagcaagccatgcccatctctg
aagagtgatgtatatgcctttggtgtcattctgcttgagttgctaactgggaagattgctggtgagatcgtctgtgtgaatgatggtgtggttgacctgaca
gattgggtgaggatgctggcactcgaggagcgtgtttcagagtgctacgataggcacattgtggaaggtgaaaactccgaaggcaccaccaaggcgct
ggatgacatgctgcgaattgcaatccggtgtatccgatctgcatcagagaggccggagatacggacggtgtttgaggacctcttgtccctttcgtcgtga
>g73432.t1

atgagcaacaacgcggtggtggtgccgttcctcctcctcctcgtttcctgccgcttcgccgccggcgaggtcggggaggaaacgaaggcggtggcggtg
gacccgtcgttccggttcccgaaccagcgcctgcgggacgcgtacgtcgcgctgcagacgtggaagcagcaggccatcttctccgaccccaaccgcct
caccgccaactgggtgggccccgccgtctgtaactacacgggcgtcttctgcgcgcccgtccctcgaggctctcccggcgccggggagctcgccgtcgc
gggggtggacctcaaccacggcgacatcgcggggtacctcccctcggagctgggcctgctcgccgacctcgccctcctccacctcaactccaaccgctt
ctgcgggctggtccccaccacgctccgccgcctccgcctcctcgccgagctcgacctcagcaacaaccgcctcgtcggcgggttcccctccgtcgtgctc
cgcctcccgtccctcaagttcctcgacctccgcttcaacgacttcgagggcccggttccccgccagatcttcgaccgcccgctcgacgccatcttcctcaa
ccacaaccgcctccgctccccgctccccgagaacatcggcaactcgccggcctccgtcatcgtgctcgccgagaaccacttcggcggctgcctccccgc
cagccttgggaacatgtccgacacgctcaacgagatcctcctcatcaacaacggcctcacctcctgcctcccgccggaagtcgggatgctccgggagct
caccgtcttcgacgtcagcttcaacgcgctcgtggggcctctgccggcggaggtggccgggatgcggaaggtggagcagctcgacgtcgcgcacaacc
gcctctccggcgccgtcccgcaggccgtctgcgcgctgccgcggctgcagaacctcaccatcgcctacaacttcttcaccggcgagccgcccgcctgcg
cgcgcgtcgtccggtccgacggcgacaggaaaaactgcctgcccaaccgccccgcgcagcgcacgccgcagcagtgcgccgcgttctacgcgcagcc
gccggtggactgctccgcgttccagtgcaagccgttccacccgccgcctccgcccgcgccggtgtaccccggccccttgccgccggtctaccccatgcca
tacgcctcgccgccgccgccgccgcgctaccgatga
>g73899.t1
atgctcaacctcgtagcggccgccggagggcgcgtactggagcgaagcagcagcctcaattggcgttgggtccgtggagattcttctccgacgccgtac
ttcgtctacgacggcggcgctccaggagagttaagcgcgagctcgctgcggatggaggcggaggaggcgagggagcaagcggcggctggtgcgcgg
gtgatcagccacctgacggtgctggacgccgtcgcggcgtatgacgtggaggttctgaaccagaagagctctgcttccgcggcaaagttgcattgttgc
agcatgcgtgcgcgcggctacgccgacgctggcgtcgacgagctcgcgtgggccgttcgcgcccgtccacgcgtgagaaactgccacaaacacaaga
tggcgagcacggggcggtcgatgctggtgtcgctgctgctcttcgcggtgacgctgtcgctgctcgagatgtacaaggccaagtttgcctcgtcggagct
catgacaatcgtcggcggcctcgtctgctccctcctcttcctgctcctcctcacctttattggaaactaccaagaatctagtggtgttagaactgggtgggg
tgcagttgttgtagcagaacttgttgctcttattgtagctggcactgtccaccgtgtctgtatcacaacatggtttgcgacagggaggaagagaataagta
agcaagaagaagaggaagaagcgttcgcggccagcttccaaggggaacgtgcttcagcttcgaaggaggacgtgcttccgatagccgtcgaagagg
aggatggaggcgccggcgcggcggcgtgtcgtgacgccgccgttgctgttgctggtgttggtggcggcgagccttcctgcggtcctctgctttccgctctc
caggctctgatgagcaaatggaagaactacccgtcgagctggtcgggcggcgacccctgcggcgcgcagtgggacggcgtcatgtgcaacggcggca
gggtgacgtcgctgcggctgtcaactgtaaaccttcagggcacgctcagcgacagcgtcggccagctcagccagctcgtctacctgtaa
>g74314.t1
atgagagcacttgcaaatttaatcaatatggtaaaccttgatcttgaaggttgcctaaatattcatggtggccttgttcacttaaaagacctgacaaagct
ggagtcgctaaatatgagatactgcaataacattgcggattcagacatcaagtacatatcaggccttgtgctattacaaagcttggtgctttctgatactg
aagttggtaccaatggactccgccatctgtctggtcttcgcaatttgcagagccttaatatctcatttaccttgatcacggatgatagtatgaagaagattg
ccacattgacttggctgaagtcaattaatctagataatcgccagattactgatataggcttggcagcacttacaggtcttagcgggttgactcatcttgac
ctttttggagctcgcataaccgactacggaacaaactgcttgagatgtcttcgcaatttgcagagccttaatatctcatttaccttgatcacggatgatagt
atgaagaagattgccacattgacttggctgaagtcaattaatctagataatcgccagattactgatataggcttggcagcacttacaggtcttagcgggtt
gactcatcttgacctttttggagctcgcataaccgactacggaacaaactgcttgagatttttaagaaccttcaatctcttgaaatctgtggtgggtcaat
caccgatgctggagtgaaaaatatcaaggatctgaaagctttgacattcctgaatctttcgcaaaatattaacctgacagacaaaactttggagctgattt
ctggtctcaaacacctaaaggacctgcagaacctgcggtcgttgtctcttgattccagtagggtgacagctgctgagatgaagaagctccaggcaacga
cgcttcctaatctgataagcgtgcgacccgagtag
>g74516.t1
atgtcgggcacctgctacctctccaccgtgctccccatcttgttcctgctggtactcaccggcgtgggcagcgtccagcccgccgctgccggcgaccgcg
acatgctgctggccatcaagaaggagtggggcaatccccggcaactcgcgtcttgggaccccgccgctgccgaccattgcagatggagaggcgtcacg
tgcgacgaaactactgatggcagcagcgccgggggagtcgtcgtgaagctgtctctctcgttgatgaacctcaacggctcagtccccgcgtctgtgtgc
gcgcttaagaacctcattcacctcgacctctccatcaacaagctcaccggtgccttcccggccgctgcgctctacacctgcacgcggctccgcttcctcga
tctctcacgcaattacttctccggtgcacttccaaatgacatcgacgggctgtcgcctgccatggagcacctcaatctctcaatcaacaacttcggcgacg
aggtgccgccgactgtgagcaggttgctggagctcaaatctctacggctgcacgccaacaatttcaccggtgcgtacccggcagccgagataagcgag
ctcgctgggctcgaggtgctcactttgagccgcaactcgtttgcaccaaggcccataccgagggagtttgcaaagctatccaacctgagcactcttagga
tgagggatatgaacctcaccggggagattcctgaggcgttcgccaacctcacggagctcagaacgatcgacctgtcttctaacctgctcacggggtcca
tcccgtcatgggtatggcaacacaagaagattgagaaggtttttctagacgacaacactctctctggtgagataacacgcaacatcacggcggccaact
tggctcacttacacctgtcctcgaatgagctcataggagagataccggcaagcatcggactactaccctgcctcgctcacataaatatttcatacaacca
gctatctggcgagctcccccaggagctcgggaaacacgggccactcgaatccttgacggtgcaccacaacaacctctctggccctctgccagagacga
tctgcgccaagggaaagctccgctatattctcgctggttataataacctctccggcgcgcttccggcaaacttaggagactgtgttcagctgaatttcctg
gtgctctccaacaaccgtttcgccggtgacttcccgatgaagatatggtcgtcccaaaatctgaagggtgtgataatcccaaacaacagcttcaccggca
ccctgccagacgagataccсttgagcatactctatatttatatcgagaacaacatgttctccggctccatcccaaaggcagctacggggttgaaggttttc
agcgcgacgaacaaccggttgaccggtgagttgccagtcgacatgaccaaccttgctaatctttcactcctggcattgtccggcaacagcatcacagaa
aatttcgccgtccgatgtacttcgttctcagactcttcccaaagacaacatcttctacccttaaccacgcacctctcgtcagtcatccgcagcccggacttc
caccgcagggcaatgttgcagctctctgctttgttcttgttactactattcgccggcggaggcagtgtccagtccgccgctgccggcgaccgtgacacgct
gctcgcgatcaagaaagactggggcagcccacagcagctagcgtcctgggaccctgccgccattgccaaccactgcagatggatcggcgtcacgtgc
aaaacccctggcggcagcctgggaggtgtcgttgtggagctgtccctccccatgatgaacctcaacggatcggtcccgccgaccgtgtgcgcgcttaag
agcctcacccacctcgacctctcctacaacaaactcaccggcgcctttccgaccgccgccctctacgcctgcatgcaacttcgcttcctcgatctctccagt
aaccgcttctccggccatctcccgatcgacatcgatgatctctcgcctgcgacgatggagcacctcgatctctcctggaaccgcttcgacggcgaggtgc
cagcggcggtaggaaggctgcgggcgctcaagtacctggcgttggaccgcaacagtttcaccggcgcatacccggtgcctgagataagtgagctcact

gaacttgaggaactctttctgagcgacattgcatccgcaggagggcccgtgccaagagagtttgtcaaccttaccaaccttagaaaactctggatgtcca
atatgagcctcaccggggaaatcccggatgcgttcgccaatctcacggagctcacgatgctcgtcatggattctaacaagcttacagtgttggtttag
>g74518.t1
atgcgccgctcgaagactaggccgtgtcaaacaacaatctctctggcccgttaccggagacactgctctaggggaaagctccaacgaatcttcgccgac
aacaataacttctctggcacgctccctgtcaacttaggagattgcttcctactgacagacctcgtgctccagaacaacagtttctccagcaatttcccgac
gaagatatggttgttgccgcagctgaccgtggtgttcctcaacaacaataacttcaccggaactctgccggacgagttatccttcaacgtttatagaattg
agatcggggacaacatgttctatggctccgtccctgcatcggctacagggcttcgcgtgttcaacgcaaagaacaaccggttcgatggagaattgccca
tcgacatgagcaaacttgccaatcttacggatctggtactgcccggcaacaggataactggtaccataccaaaatcgatcgaactactacaaaatcttgt
tcggctggacctcagtggcaaccggatatctggcgttgtgccctaa
>g74519.t1
atgtcggccaccttccacctcgccccgcccttgcttatcatcttattcctgctggtactcaccgctggaggcagcgctcacgaccgcgacacgctgctggc
catcaagaaggagtggggcaatccccggcagctcccgtcttgggaccccgccgctgccgaccactgcacatggagaggcgtcacgtgcgacgaaact
actgatggcagcagcgccggggcagtcgtcgtgaagctgtctctctcgttgatgaacctcaacggctcagtccccgcgtctgtgtgcgcgcttaagaacc
tcattcacctcgacctctccatcaacaagctcaccggtgccttcccggccgctgtgctctatgcatgcgcgcggctccgcttcctcgatctctcacgcaatc
acttctccggccccatctcggctggcatcgacgtcctctcgtctacgatggagcacctcaatctctctagaaacaacttcggcggcgaggtgctgccgac
ggtaggaaggctgcggaagctcaaatctctgtggttgcaccgcaacaatttcaccgaagcgtacccgacagaggagataagccagatcgctgggctcg
aggagctcactctgagcgacaactcgttcgcgtcgaggcccgtgccgagggagtttgcaaagctatccaacctgagcactcttacgatgagggatatga
gcctcaccggagatatccccgaggcgttcgccaacctcacggagctcagaacgatcgacctgtcttttaacatgctcacggggtccatcccgtcatgggt
attgcaacaccgaaagattgaagaggtttatctcagcaacaacgccttctccggcgagctgacacgcaacatcacggcgataaacttggttgacctgga
cctgtcctcgaatgagctcataggagagataccagaaagcatcggcaacctcaagaacttggcttcgatgtttcttgaccacaatcatttcaccggcaag
ataccgacaagcatcggactgctaccatgcctcactggcatctatctttcctccaaccagctatctggcgaggtcccccgagagctcgggaaacacggg
ccactagaaaacttgatggtgcagaataacaacctctctggtcctctgcgggagacgctctgcgccaagggaaagcttctctatatctttgctggtaaca
ataacttctccggtgcgctcccgtcgaacttaggggactgtgttctattggattacatcgacatctccaacaaccgtttttcaacgacttcccgataaaga
tatggtttctccgaaacatgcaagttgtgttgatccaaaacaacggcttcaccggcaccctaccagaggagatatccttcagcatctcctccattgacatc
ggggacaacatgttctccggctccatcccaaaggcagctacggggttgcagtttttcaacgcgacgaacaaccgactaactggtgagttgccagtcgac
atgagcaaccttgccaacctaacacagctgatactggccgggaacaggataactggtaccatgccgaaatcgatcgaactattacaacatcttgttcgg
ctcgacctgagtggcaaccagatatccggcatggtgctgtag
>g74689.t1
atgcctcctggcgtgagcaggaggcggccaccgccttcacttctgctgctgtgcgcggcggtggtgctcgcggcgtgcgtgggcggcgcgctggccgcg
gacgcgcagggcgcggcgctgctggcgtggaagcttacgctgcgcggcggcggcgcggcggagtcgctgggcgactggcaggagtcggacgcgtcg
ccgtgccggtggacgggcgtgtcgtgcaactccgcgggtggcgtgacggagctgagcctgcagtacgtggacctccacggcggcgtccccgacgacct
ctccgccgtgtccgccacgctgtcgcgcctcgtcctcaccggcaccaacctctccggccccatccctccacacctcggcgaccagctcccggcgctgacg
cacctcgacctcagcaacaacgcgctcacgggcccgatcccaaccagcctgtgccggcccgggagcaagctggagagcctccacctcaactcgaacc
acctggagggcgccatcccgtcgtccatcggcaacctgacgtccctccgcgagctcctcatctacgacaaccagctggacggcaccatcccgtcgtcca
tcgggcagatgagcagcctcgaggtgctccgcggcggcggcaacaagaacctgcagggcgccctcccgccggagatcggcgactgctccaacctcac
gatgctgggcctcgcggagaccagcatctccggcccgctccccgcgacgctgggccggctcaagaacctcgacaccatcgccatctacacggccctcc
tctccggcccgatcccgcccgagctcgggcaatgcaccagcctcgaaaacatctacctctacgagaacgcgctgtcggggtccatcccgccgcagctcg
gcaagctgagcaacctcaagagccttctgctgtggcagaacaacctcgtcggcgtcatcccgccggagctcggggcatgcgccggaatcacggtgctg
gatttatccatgaacgggctgacgggccacatcccggcgtcgctgggcaacctgacggcgctggaggagctgcagctgagcgggaacaaggtgtccg
gccccatccccgccgagctggcccggtgcggcaacctcaccgacctggagctcgacaacaaccagatctccggcgccatccccgcggagatcgggaa
gctggcgtcgctgcggatgctgtacctgtgggccaaccagctgacggggtccatcccggcggagatgggcgggtgcgccagcctcgagtcgctggacc
tgtcgcagaacgcgctgacggggcccgtcccgcgcgcgctcttccgcctcccgcgcctctccaagctcctcctcatcgacaacgcgctctcgggcgagg
tcccgcccgagatcggaaactgcaccagcctcgtccggttccgcgccagcggcaaccacctcgccggcaatatcccgcctcaagtgggcaggctcacc
agcctcagcttcctcgacctcgggtccaaccggctgtcggggtccatcccgcccgagctcgccgcgtgccggaacctcaccttcgtcgacctccacggg
aacgccatcgccggcgcgatcccgccggggctgttccgcgacatggcatccctgcagtacctcgacctctccgacaacgccatcgccggcgccatccct
tcagatatcgggaggcttgggtcgctcaccaagctcgtcctcgccgggaacaggctgtctgggaccatcccgccggagatcgggtcctgctccaggctc
cagctgctcgacctcggcgggaacgcgctgtccggcgcgatacccgcgaccatcggcaagatcccgggcctggagatcgcgcttaacctcagctgcaa
cggcttatccggtcccatccccaaggagttctccgggctggtccggctcggcgtgctcgacgtgtcgcacaaccgcctggccggtgacctccagccgctg
tccgcgctccaaaacctcgtggccctcaacatctccttcaacgacttcgcgggccgggcgcccgacacggccttcttcgcgaagctccccgcgagcgac
gtggaggagggcaacccggcgctgtgcgtgccgtcgcgctgcccgggcgacgcggccgccggtgaccgcgcggcaaggcgcgccgcccggatcgcc
accgccgtcctgctctccgcgctcgtggcgctcctcgccgcggccgcgttcctcctcctcagccgcctccgctcgtggcgcaagcagggaggcggcggc
tccgtgttcggcggcgaggacgacaaggacgcggagatgcttccaccctgggacgtgacgctgtaccagaagctggagatcagcgtgggcgacgtgg
cgcggagcctcacacccgccaacgtcatcgggcagggatggtcgggctccgtgtaccgcgccagcgtgccctcctcctcctctggcaacggcggcggc
gtcaccatcgccgtgaagaagttccggtcgcgcgacgaggccgcggcggaggcgttcgcgtgcgaggtgggcgtgctcccccgcgtgcgccaccgca
acgtcgtcaggctcctgggctgggccgccaaccgccgcacgcggctcctcttctacgactacctccccaacggcaccctcggcggcctgctccacggca
acggcggcgccgccgcggtggtggtgccggagtgggagctgcggctctcgatcgccgtgggcgtcgccgagggcctcgcgtacctccaccacgactgc
gtgccggcggtcatccaccgtgacgtcaaggcggacaacatactcctcggggagcggtacgaggcctgcctcgctgatttcggactcgccagggtcgc
cgacgacggcgccaactcgtccccgccgccgttcgccggatcctacggatacatcgctcccgagtacgggtgcatgaccaagatcacgaccaagagcg
acgtgtacagcttcggggtggtgctgctggagtccatcacgggccggcgccccgtggagccggcgttcggggagggccggagcgtcgtgcagtgggt

gcgggaccacctgcaccagaagcgggacccggccgaggtggtggacccgcgcatgcagggacgcccggacacgcaggtgcaggagatgctgcagg
cgctcggcatcgcgctgctctgcgccagcgcaaggcccgacgacaggcccaccatgaaggacgtcgccgcgctgctccgcggactccgcagcgacga
cggcgccgactccaggaaggtcagcggcgggcccaggccggactcctcggacaagtgggccgctcccgtctcgccgaccaagcccacaaccctcctc
ggttacccggcccaagcacggtcgctgtccccgtccagctcctcggcctactactcaacatag
>g75468.t1
atgacggttgtgtcagccttacctcccaatgtgcatgatgatacaatgcctcaatatatgtttacatactactacaagaacttggtcacggtgatgcagaa
gggattagaactaacactagtgaacatcctgtcagtcttcacgacccttgacctctcaaacaacaactttgaaagtaccatctcgaatgagattggtgac
ctgaagtcactcgtggggcttaacctgtcaaggaactcgttcagtggtgagatacctccccagatagcgaacatactgcagctggagtcactggatctct
cctacaaccagctttctggggagatccctcctgcaatggcacacttgtctttccttgaggtcctcaacctctcctacaatcacctattggggccaataccgc
agtccaatcagtttttgacattccaagacacatcgtatctgggaaatgatgggttgtgtgggaagccactgtcacatacttgtgacacaaaccacacacc
gccagctgctgagatgccaggctcttctaaagaggtgaattgggatgttttgtccattgaggtgggggtcatttggggcatggccattgtggttggaacta
cactactgtggagcaacgggaggacctgggtgtactggtacatggatatgtttctgctgtggattctgcagccatggatccgtcatcgtcgccactga
>g75734.t1
atggccgctgacgagcttgcactcctctccttcaagtccatgttgcactccgagaatggatctctcgacttgtggaacacgtccatccacttctgccgttgg
ccaggaatcgcctgtggccgccggcacccggacagggtggttgccttacgcatgccatcgtttaacctatcaggacgcatctcgccgtccttgggcaatt
tgtccttcctccggaagctcgacctccacgacaaccttatcacgggtgagataccaccggagctcggccgtctaggcaggttgcagtttctgaagctgag
catgaacactctcgttggtggaattcctccagccattgcagggtgcaccaacctcatgatgctagatcttagtcataaccagcttcacggggagataccc
gctaaccttggcaccatggagaatctgtccttgctggagctccaagataaccacctgtctggggagatccccttgtcactagcgaatctgtcatcgatcg
agtacttgtttctatacagtaacatgatctccggcgagataccaccagagttgggcaacctcacctgcctgagccacatcgacataatggacaacgtgct
gtccggagggataccgtcgtcattgggcaaggtgtccggactcatctggcttagcctgggctataacagcctcattggccagatcccagcatctatctgg
aacatctcctatctgtggggcatcaacatccaatggaacaacctcaccggcgccattccggcgaacgcgttcagcaagctccctaatctcgtaacggtgt
acatgggaaacaatctgttccacggccctttaccgccctcactagcaaacgcctctaagctacaacggcttcaccttggccccaactccttcagcggcgt
cattccttaa
>g76250.t1
atggattcattggagtccctggatctctcatggaaccagctttctggcgtgattcctcagagcatggcgtcattgcacttgctaaaccatctcaacatgtcg
tacaataatatttcagggaaggtacctccaggttctcagcttcaaacccttggtgatcaggatccatacatatttgctggcaaccactacctatgcagtcct
cttgttcctgggagttgttctgagcacaaggaaaatccagctgatcatgaaaaaacagatggccatgatatattgttgtatgtcttttcaggacttggcttc
ggatctgggtttgcagctgtgtggtggctacttatgttcagcaaggcagtaagcaaggtgtacatccagttcattgactccatcagtgacaatgtccgtgg
ctggataatcccactgaaggttaagtacttaagttga
>g76254.t1
atgctccagtcccgggtgaatggaggcggcggcgctgcagctgttagcccggccaccaacgacggcgctgtggccaccaagccttctctcagcaccgc
tgggaaggcaagctcgtgcatcccacgggaaagagatgcattgcttgccttcaaggctggcgtcacggcaccaggtgatgagctgcgctcctggcaaa
gcctggactgctgcaaatggagcggtgtggcctgcagcaacaagaccctccatgtcatcaagttggatgtcagccaattcgctctgaatgggaaaatca
actcatccttggctgctctgaccaggcttgtatatcttaacctgagccaaaatgactttggtgggatggctattccagaatttgtaggatccttgaagaaat
tgaagtaccttgatctttcgcgtgccaattttggtggaaagtttcctgagttatctggatttgggttggttcgatctcagtgcatccttagactggctacaag
tcaccagtaa
>g77523.t1
atgttcccgcgcgccgccgagctggtggcgctccttctcctccaacttgtcgcggcccttgccgccgacgacctggcgtcggacgctgcggctctacaag
ccttcctggccccgttcgggtcgtcggtcgtgcactggaacgcgtcccagcacccgtgctcatggacgggcgtggtctgcacgggcggccgcgtcacgg
gtctccacctcccgggcgacggcctccgcggcgccctcccggccggcgccctcggcgggctcaccaagctcgaggtcctctccctccgcttcaacgcgc
tgtccggcccaatcccgcccgacctggccgcctgcgtcgagctcaaggtcatcaacctccagtccaacctcctctccggcgagctccccgccgcggtgct
cgcgctgccgtcgctcacgcagctcaacctcgccgacaaccggttctcggggcgggtcccgccctccatcgcgcggaacgggaagctgcagcagctct
acctcaacggcaacctcctcacgggcccgctccccgacgtcaacatgccgtccctcaccgcgttcaacgtgtcgttcaacaacctgacgggccagattc
ccgggagcttcggtcgcatgccggccacgtcgttcctcggcatgtcgctgtgcgggaagccgcttccccgtgcagagtcgcgccgggatcggagctac
cgccggcgcagcccccgctctctcgcccggagcgggaggagggactgccgcatcgccgccggaccgccgcggaagccggcaccgcctcgccggcg
gcgcgatcgccggcattgtgatcggtagcgcgctcgggctcgccctcgtcgcggccgtgctcctcctcgcgtgccgccgccggccgcgcggcgccacgt
accgcagccaggacgcggtcgcggcggagctggcgctgcacagcaaggaggcgatgagccccaacgggtacacgccacgtgtctccaacgcgcggc
cgatgcatcctccttcgtccgcgccgccttcggttccgcctgcggtcacggtggggaagaagaagctcttcttcttcggacggatcccgcggccgtacga
cctggaggacctgctgcgcgcgtcggccgaggtgctggggaagggcacgtacggcaccacgtacaaggccgctatcgagtcggggccggtcatggcc
gtcaagcggctcaaggagacgtcgctgccggagcgcgagttccgggacaaggtancacgtacggcaccacccgccatcggcgggatcgaccacccc
aacgtcgtgccgctgcaggcttactacttcagcaaggacgagaagctcatggtgtacgagtacgtcgccatgggcagcctctcctccatgctgcacggc
aaccgtggctcgggccggacgccgctgagctgggagtcgcgccggcggatcgcgctggcgtcggcgcgcgggctggagtacatccacgcgacgggct
ccacggcggcgcacggcaacatcaagtcctccaacatcctgctcagccggtccgtggacgcgcgcgtcgccgaccacggcctggcccaccttgtgggc
ccgccgtcgacgcgcgtggcggggtaccgcgcgcccgaggtggtggcggatccgcgccgcgcggcgtcgcagaagggcgacgtgtacagcctgggc
gtgctgctgctggagctgctgacggggaaggcgcccgccagcgcggcgctgcgggacgaggaaggcgtggacctgccgcggtgggcccggtccgtg
gtgcgggaggagtggacctccgaggtgttcgaggcggagctgctgcgacacccgggcgccgaggaggagatggtggagatgctgcggctcgccatg
gactgcaccgccgcggcgcccgaccagcggcccgccatgcctgaggtcgtggcgcggatcgaggagatcgggcggaccgggtccgcgtccacggcg
cggtcggggaggagcgcgtccatcgacgaggccgacgaccggccgctcaggcccgccgggtccatcctacagagctga
>g77794.t1

atggcacccttgtcatcagaggtacaacgaagcaagagaactctgcacctcctcgtcctcctctgcttcgtcgttccatgcctcacccaaccggcggctcc
ttcccctgctccgacgacgctgcccctctctccgttcaacgaccgtctggacgcggcgtacatcgctctgcaggcgtggaagcacgccatcacggagga
ccccaaaaacctgaccgccgactggtgcgggcccttcgtgtgcaactacacgggcgtgtactgcgcggcggcgcccgacgacccacacgtgctcacgg
tggccggcgtggacctgaaccacgggcgcatcgccggcgagctcccggaccacctgggcctcctggccgacgtggcgttaatccacctgaactcgaac
cggttctgcggcacgttgccggagtcaatggcgcacatgcggctcctgttcgagctggacgtgagcaacaacctcctctccggcgcgttcccgtcgttcc
tcacctcgctgccgtcgctcaagtacctcgacctccgcttcaacgcctttgacggcgagctccccgccgccgtgttcgggcgcgagctcagcctcgacgc
catcttcgccaacaacaaccgcttcaacgtctcgctgaccacggcgagcctgaccaactcgacggcgtcggtgatcgtgctggccaacacggagctcg
ccggctgcctgccgccgaccatcggcgacatggccgacacgctggtggagctcgtactcctcaacaccagcatcagctcctgcatcccgccggagatc
ggcaagctgaagaagctcaaggtgctggacctcagccgcaacgagctcgccggagagctgccggagagcgtcggcgacatggagagcctcgaggtg
ctcaacgtcgggcacaacatgctctccggcgtggtccccgagaccgtctgcgagctgcccaggctcaggaacctcaccgtcgcggacaactacttctgc
ggcgagcccgtgtcgtgcctccaccacgtgcccctccgcgacgaccgcatgaactgcatccccgagaggccgcaccagcgatcccacgaggagtgcat
cgccttcgagcaccgcccgcccgtgcactgcgccgccgacggatgcattttgcttccgccgtga
>g77943.t1
atggctgtggtgggagcatcagcgacgtgcttcttcctcttgtcctcgtggttgtgcctctgctccaacgctcgtttgtctcctccaccggaaagcaacgcc
acggctgccgatgagctcgcgcttctctccttcaagtccttgctgaactctttcaacggctcgctggactcatggaacacgtccagtcacttctgcagctgg
ccgggaatcgcctgcggccaccggcacccggacagggtggttgcgttgcgcgtgccagagttcaacctgtcaggacgcatctcgccgtccttgggcaa
cctgtccttcctccgcgagctcgacctccgtgacaacctgctcattggtgagataccaccggagctaggccggctcggcaggctagagctcctgaacctg
agtacgaacgctctcgttagtggaattcccccagccatagcagagtgcgccaacctcgtgatactagttctcagcaagaaccagcttcacggggagata
cccgctagccttggcgccatggagaacctatacatcctggacctccaagagaaccacttgtccggggagattcccctgtctctggcgaatctgccgtcga
tcgagtacttgtttctatacagcaacatgctctccggtgagataccaccagagttgggcaacctgaccggcctccggcatctcgacctcatggacaacat
gctgtccggagctatcccttcgtcgctggggaagctgtccaacctcatctggctcagcttaggctataacagcctcattggccagatcccagcatctatct
ggaacatctcctatctgtggggcatcgacatccaatggaacaacctcaccggcgccattccggcgaacgcattcagcaagctttccaagctccgggcag
tgtccatggacaacaatctgttccacggccctttaccgccctccctagggaacgcctctcggctacaactgcttcagctcgcccctaactccttcagtggc
atcattcctccacagattgggaggcttagaaacatgacaaagctgcttctgtccaacactgcttggagctcaagatcctggagattgggagttcatgacg
gcactggcaaactgctcccagctactaattttggacctgactgccagtaa
>g79005.t1
atgcgaccgggtaggccgcgagtcggagccgccgccggcagcgtcgtcagcacttcagcggcagagcttgaccggccggcgtggcagcggaaaggt
gagttggttgcagctccttgcacgttcgcgttccgcattactgccgtgtcccgtccgtggtgccctgcgctgttcccgtgctgccctgtcgggcggggcggt
gtgctcgcactgtgcggagcttcgcgcgggagggagccaaaagctcgcgcccttttgcagatctgcttcggggaggtgcgcggcggtgtgctcctgggt
ccctccggatggcccgggggttcccgtgatttgagggctgaaacgatggggtaccgggctttgcagctgggtgtggtgctggcctttctgctggcctatg
gatcccagggtttgaaccatgaaggctggctcctcctggcgctgaagagccagatggttgacactctccaccaccttgataattggaacgcaagggatc
caacaccatgcgcctggagggggcgtcaactgctcgttgccgcccaacccagccgtggtgtctctcgacctcagcaacatgaacctgtcgggcatcgtcg
cacccagcattggaggcctggctgaactgacctaccttgatctgtcctttaatgagttctatggtaccatccctgcagaaattgggaattgctcgaaactg
gaggtgcttaacttgttcaacaacaatttcgttggttcaatcccacctgagcttgggaagctgtctaagctggtcactttcaatctgtgcaacaacaggctc
catgggtcaatacctgatgaaattgggaatatggcatcacttgagaacttggtgggctatagtaacaatctctcaggttcaatacctcattctattggcaa
actgaagaacctgagtactgttcggttgggtcagaacctcatatcaggcaacattcccgcttcgtctcggtga
>g79235.t1
atggtcctcaaagccgccgtctccgtcacctcgctgccgtcgcttccactccgccgccgcttcctccactctacgccttcatcgcgttccgcggctcgcagg
ctagctagggtctcggcggcgatggcgacggcggtgcagcaggctgtggtggtgggcggcgggcgcgtgggccgcgcgctgctggccatgggctcgc
cgggggctgacgtgctcgtcggccgcggcgagaaggtgcccgaggatgcgcccgggccgatcctcgtgtgcacccgcaacgacgacctcgacgccgt
gctcgagaacacacccaagtcgcggtggcgcgatttggtgttcttccagaatggcatgctggatccatggttcgagagcaaagggttagtggatgctaa
tcaggtcctggcctactttgctgtctcaaaacttggagagccaccagtggatggaattactgacaccaacccggagggcctgactgctgcatttggcacc
tgggctccagcagtagctgcccgcctgcaaaatggtggacttacctgcaaggtgcttgaaaaggaagcctttcagaagcagatgctggagaagttgat
atggatttcagctttcatgcttgttggtgcacggcatccaggtgctactgttggtgtggttgaaaaggactaccgatgtgaggttgcaagcctaatggctg
aattagcatctgctgcgactgcagagagggggcttacgttcgatgaaggcaagtctcttacacacctccattcttctgctactctgcacatatacatccag
tgcagtcgaccaagaagcagagtcacttcttcggtggaaatccaccttgtggccggccaaagatggcgccaccagcaacgggtcatcatcccctctgcc
cccgtggttgccccacaagccgttgtgttcatggtccggcatcaggtgcaacgctgtcgggcgtgtaaggagatcagcctcccgggcgctggcctcggt
ggcacccttggcgcgttggacttcactgtgttcccggcgcttaccaagcttaacctccgcaataacagcatcaccggcgccatcccggcgaacgtcacca
gcctgacgtaccttgacgtatcgcataacagcttgtccggggagataccggataccctgccgaaggcaatgccaatgagaggtgaccagatcgatgac
gaaggcgctatcggggatgtagcgtag
>g79312.t1
atggcggacccacctgtaccccctcctcctcttcctcctcctgctcctcctaatcccactcgccgccgccgccgcctcgtccgccgacctcgcggcgctcctc
gcgctcaaggccgcggtgacccacgacccaaccggcgcgctcgccgcctggtcctcttccgcctccgccgccagccactgcctctggcgcggggtcacc
tgccacccgtcctccaccgccgtggccgccatcgacctccgcggcgcctccctcaccggcgcgctccccgcctcccttactctcccgccgcgcctccgcc
ggctcgacctcgccggcaacaacttctccggcgccgtccccgccgccctcctcgcgtccccgaccctccgctcactcgacctcagcttcaaccgcctctc
gggcgccctcgaaatcccgcccgccaactcctcctcgtcgtcgtcggcgccgcccccctgcgccgcgctcgcccacctccgcctcgcgggtaacctcctc
gtcggcgggatcccctcggggatcgcgcaatgccgctccctccgcgtcctcgacctctcccggaacgtcctggagggtgacgtgccgccggcgctcggc
cgcctcgccgagctccgcgtcctcgacgtctcccgcaacagcctcaccgacagggtccccgccgagctcgccggctgcagggacctcgccgtgctcgtg
ctcaccaatctcacctcgtcgccggaggagcagccggagttcaacgcatttgttggggagctgccgcaggaggtgctgggcatcccggcgctgcaggtt

gtctgggcaccaggggccaaccttgatggccggttgcctgatgacaggaatggcacctgcggcctttgcgcgttgaatcttggacagaactacattgcc
ggtggggtgccccagtggctcgggcaatgccaagacttgacatttcttgatctcagttccaacaaattggagggcttaatgccggcagaactatcatttg
gatgtttcacttatatcaatatcagtggaaattctctgtcaggccctcttctttcatcgtcggatagcaaatgttcaagccacttgggcggtgatgatattgt
aatgcagtactatgatgaattggttgtgaatgcgctgattggcaaccctttcggttctgtattgggtattcctaacattgctctccatgacttcagcaacaat
ggatttggcggggcattgccatctattaccttgaggtgtccagctgaccctggcattggagttgcagtttttccaggaatcaagcagtaa
>g79561.t1
atgctccacaaggcggccgtgatcgacgccggcatgaaccagctcaccggcccgattccatcatcattctcctgcctcaacagcgtcgagcagctcaac
ctagccggtaaccgcctctacggagacgttcccgactcactctgcaagttcgcccgcccagccggccggctcgccaacctcactctgtccggcaactact
tcacgtccgtcgggccggcgtgcgcggcgctgatcaaggannnnnnnnnnnnnnnnnnnnnnnngctgatcaaggacggcgtgctcgacgtg
aagaacaactgcatcccggggctcgccaaccagaggcggccggtggagtgtgcggcgttccagagccagccgaagacgtgcccggcggcgagcact
caggtgacgtgccccgccgcagctgccacgaacgtggcggcgccggcggagaggaaggctgctagggactactccagctacgtgacgtacgctactc
tgcatgagtga
>g80149.t1
atgcttccctccacgcttcatgcttgcgtcctcttcacatatctccttactcttgcaatatcaagtcattctgcagaagctgtttacactgatgacaccagga
ctcggctacttgctgaagctgatgatgatcggatggctctcctgtgcgtcaagtcccagctatcagactccattgaagctttggcatcatnaggataagct
gtttacactgatgacaccaggactcggctacttgctgaagctgatgatgatcggatggctctcctgtgcgtcaagtcccagctatcagactccattgaagc
tttggcatcatggagcagcaacacctccaccgacatttgcagttggcatggtgttacttgcaataacacaagacaaccgtctcgagtcacagagctgaa
gctggagggctcacagctcggagagtatggtatgaacgaggagatatcagcctag
>g80153.t1
atgaataacgaaactctcagtgctccacctgttgagcatcttgatcttggttcaatggagctcagggcttccactagtgcatgttgggcggcggcaaggtt
cctgctgctggcagctattttgatgtacaacgccccatgctttgccttatccgttgccaaaccatcacctcctgctgccattgctgcccatagctcagaccgt
gctgtactcctcttgttcaagtcatgcataaccaaggatccctttggtgcgctcgcctcttggggcaatgaatcgctccatttctgcagatggccaggtgtg
gcgtgcgggaagcgcgggcagcgccgtggccatgtggtcggcctcagcctcggctcacttgggctggttggtaccatctctccttccatctccaacctca
cgcacctcagaaatctgcacttgccacataacaaattcaatggacgcataccacatgagcttggccttctcagtgatcttaagcatctgaacctgagtga
caactctcttgaaggagaaattccgagtgaactttctcgctgttcacaactccaaaccatcagtctgtggtataataaccttcaaggcagaatgccatcca
actttagccattgttccaacctaagaacaattgagtttttttgctaattatcttgaaggagacatacccagtgagtttggttccatcgagaatcttgagcttct
caaccttttcgataataacctcaatggaagcctcccccctga
>g80772.t1
atggtcatggagcaccattgctccgtcgtcctactagtcgcctccctcctccttgcaaggccgtcgtcgccctatgcgtggaacgccggtacggacactga
ccaccgcgccctcacgcagttccggtccctcatcacggaagacacatacggcgccttggcgtcgtgggacagcgtgggcagcggcggcaacatgtctg
ccgccacgccggcgtcgtattgccggtggcacggcgtgacgtgcggggcgcgcgggcgccgccgaggccgcgtcacggcgctggtactccgtgggca
ggacctgaccggctccatgatcgccccgtcaccgctcgccgacctcgcgttcctccggcggctcgacctctcccagaacagcctcagcggcggcgtgcc
cacgccgttgccggtctccctcgagtaccttaaccttagccgcaacgcgctgcgggggccggtgcccccggaactggggtcgctgcgccgcctccgggt
actcagcctcgaagtcaacaacctcactggcacagtccccgccgtccttggcaacctcacttccctcgccatcctcagcctcaccaacaactacctcagc
ggcgtcatccccagcaccctcggcaacctccgagccctcaccggcctctacctcaactataacatgctacgaggatccataccgtccacggtgttcaacc
tttcctctctccagcatcttgtcgtgcagaccaaccacctcaccgggactctacccccgaacgccggcagcaggctgcccagtctcaggctgctcagcgt
cgacgccaaccagctccacggcgccatcccggcgtccctctgcaacgcctccaagctggaggtgctccagatgctgaagaattccttctccggagtcat
accagactgcctcggtgctaacctcaagaacctctgggcattcgtactggacttcaaccagctggaagcaaatgttgatgctgattggaggttcatggat
agtctgaccaactgcagcaatataaaattcattggtctggccggaaacaagctacgaggtgtactacctgactcgatcgccaacctttcaacgaccatg
gagtccttgagcttatggaacaacacggtaagtgggaaaataccacagggaattggtaaccttgtcaatttaagaagaatttggatgggtacgaataac
cttagtggcaccatccctgactcacttggcaatcttagaatgttgagcgtcctgtacctctatgacaacaagctatcagggcaaatcccaccagcaatgg
gcaatctcaccgtgctcaacgacctgtccctgtttgataacatgcttacgggtcccataccttccagtcttggtaattgctctttggcggcgctggacctgc
accacaatcgccttactggtccaataccaaaagaacttgtcctcgtatcaacgctcacccaggcagattttcatgacaacatgttgagtggattgttgccg
tcggaagttggtcacctgataaatctccagggaatatctttcaagggaaaattccagactcaatag
>g80958.t1
atggctgctctttccattcttgttctggtgctgcttttcggggccgccgccggcgacgatgtggcggcgctgctcgagttcaagaagggcatcgcggaccg
gaccgaggacccggtgctgcggtcctggtccccgccggcgacaaccgaggccggcaacggcgccgcgggctgcccccaggcgtggcggggcgtggt
gtgcgacgacgacgccgtggtcggcctggctcttgacggcctcggcctcgctggggagctcaagctctccactctcagggacatgcgcgcgctccagaa
cctctccctcgccgggaacgccttctccggccgcctgccgcccgccatcggctacctcagctcgctgcgccacctcgacctgtctgggaaccgcttctac
ggccccatccccggtcgcctcgccgacctctccggtctcgtccacctcaacctctcccacaacaacttctcctccggcttcccaaccgacgggatccagc
agctccagaacctccgccgcatcgacatccgcaacaactcattctggggcaatgctagcgatctgctcaaagcgctccgcaatgctgagcacatcgatc
tgagccacaacctcttcaacgggcgggtggatttggagttggagagcttgagcagcatcgggaacacggtgaagtatctcaacatcagccacaacaag
ctcgagggtggcttcttccgaaatgagacggtgagggcgttcaagaacttggcggtccttgatttgagcaacaatgggattggtgggacagtgcccaag
ctagattcatggttctcgctggagattttcaaggttgccggcaatgggctatttgggatgatgccggaggcactcctccagaattcgatgcggctcattga
ggttgatctcagcaggaacggcttctcagatggtctgcctgctggaaatgatgattacactggagttggtcagagtcaaacatctcatggacataaggct
ggtgttagagttgcacttattgttggctgtatcggagctgtgttgctagtcatcttcatagcattggcattctatgtgaaagagttgctggctgaagctgctg
ttgaatatggctactctgatccaaaggaagtgactgaatccataagttctggtgtggttgagacttctgctgcagtccaggctcaggagtcatctccacgg
tctgcgttaccaacatcaccacattttaccgatcctaggttccatgaagaacctgttgcttttgaagtatactctccagatcgggtcactgccgcctgtgaa
ttctacaactttgaagagtcaactcctagaagatactcccgactgtcagtgagtcaacgtcttagaattgccattgacctggctcgctgccttcagttctta

caccatgagaagggcctgcctcatggcaacctaaagccaactaatatattcttgactggtcctgacctctctccaaagcttattgattatggtctacataa
gttcatgactccaagtggcattgctgagcaaatactgaatttaggagcattaggatatcgagccccagaactagcaaacacaaccaagcctgcaccatc
cttcaaggctgatgtgtatgcgtatggggtgattgtaatggagatgttgacgaggaagagtgctggagacatcatctcagggcagtctggcgcagttgat
ctaactgactgggttcagatgtgcaacagagaaggacggggaactgactgcttcgatcgagacatcacaggcttggaagaatctccaaggataatgga
cgagctgcttgctatctcacttaggtgtatccttcctgtaaatgagagacctaacatgaagacggtcgtccacaccgccgccggcgggttcgtgccgccg
tcggcgaccaccgctatccgcgctgcaaggcgcctcgctggtatccgctccagccgcaacgtgcagccctgcaactga
>g81110.t1
atgagggacatgggagagaagaggagacatcatggccatggccatgtcgttgggtcgtttggagctggtagacatgtagagcacgaggagaacaagc
acaaggaccagaagctagacatgagcggcatgaacatggaaaccatcccacacctcaccatgacactcggccacatcaccaccttggatctctccaac
aataacctacagagcattcctgaatcaataatcgcgaggctcctgaacgtagtcgtgctggacgtgcactccaaccagctgacgtcgctgcccaactcc
attggttgcctctccaagctcaaggtcctcaacgtctccggcaacctgctggagtccctgcccgccaccatcgaggaatgccgtttgctggaggagctga
acgccaacttcaaccagctgaccaagctgccggacacgctgggcttcgagctccacggcctccgccggttgtcagtcaactccaacaagctcacctccc
ttcccttctccacctcccacatgatagcactgcgtgcgctcgacgcacgcctcaactgcctccgtgcgctgcctgatgggcttgaaaacctgggcaacctg
gagacgctcaacgtgagccagaacttccactacctgcgtgagcttccctacggcatcggcctcctcgtctcgctccgggagctggacatcagctacaact
ccatctccgcgctaccggactccatgggctgcctcaccaagctggtcaagttcagcgccacgggcaacccgctggtgtgcccgcccatggaagtcgtgg
agcagagcctcgacgccatgcgcacctacctcagcgcgcggatcaacagcgccgaggatggcaagaagaagaagaggtcatggatgcccaagaag
ctggtcaagtacagcaccttcacggcggggatgatgacgcctggccgcgccaaggtgcatggcaacaacgacggcctcctcatgtcggattaccggtc
gattgatggccacatcgcctcgccggggttcctcagcatgttgtcgccccgaaggctcttctcaccacggaggaattcgcccaagcattag
>g81170.t1
atgcctgcgttcaacgcgtacggagatggcgggtgcctcgtctcggcgcctccggagttcgccggcctgttctgccgcggcgtgcagcagcggaagcgc
gcgctggtggccgcgtccgcggtcgccgctgcggcggcggcggagtgcgtgagggtgagcaagaagcagaggcagctgccgcagccgtcgctcgac
gcgctccccgacgagtgtctcttcgagatcctgcgccgcgtccccggcggccgcgagcgcggcgcctccgcctgcgtctcccgccgctggctcgcgcttc
tcggcagcatccgggcctccgagctcgaccaggccgctgccgcggcggcggcggtctctgagctgcccgacctcaacgaggagttcgtcatggaaga
ggaaaaggacgagtccccagcagatccctgcgtcgagagggctctcgagggtaaggacgccaccgacatccgcctggcggccatggccgtcgttgcc
gggtcccgtggcgggctccagaagcttgccgtccgcggcagccacccgacccgtggcgtcacggaccagggactcacggcagttgcacgcggcaacc
cgaacctctgctcgcttgccatctgggatgtgccgcttgtgaccgacgccggactcgctgagattgctgctgggtgccccttgctggagcgtctggatatc
tcttgctgcccacttgtcacagacaagggtctcgctgctatcgcgcagggttgccccaacttggtatcgctgaccatcgaggcatgctccggtattgaaaa
tgaaggcctcagggccattggacgctgctgtggcaaattgcaggttgtgaacatcaagaactgcgtgaacgttggtgatcagggaatctccagcctggt
gtgctctgctacagcctcgcttgccaagatccgactccagggattgaacattactgatgcttcactagctgttattgggtactatgggaaggctgtcactg
accttacacttgcccgtcttgctactgttggagagagaggggtttctgggtgatggctaatgctgctggtctgcagaatctcaaatgcatgagtgttaccgctt
gccctggagtcactgatcttgcacttgcttctattgccaagttctgcccaagcttgaagcagctttacctcaggaggtgtggacatgtgtctgatgctggtc
tcaaggctttcacagaatcagcaaaatcctttgagaatttgcagcttgaggaatgcaaccgggttactcttgttggcattctcactttcctcctcagctgca
cccagaagttcagggctctttctttggtgaaatgcatggggatcaaggatatctgctctgcacctgcacagcttcctctatgcagatcacttcgattcctga
caatcaaggactgcccaggtttcaccgatgcaagcttggccgtggttgggatgatttgccctcagttggagcaggttgacctgagtggacttggtgaggt
tactgacaatgggctccttccattgatccagtcttctgaggctggtctgatcaaggttgacttgagtggttgcaagaatgttacagatgtagctgtttcttcc
ctggtgaaggcacatggaaaatctcttaagaaggttaatcttgaaggttgcagcaagataactgatgcaagcctctttgccatctccgagagctgcactg
aacttgctgagcttgatctttcaaactgcatggttagtgactatggtgttgctgtcctggcatctgcaacgcacctcaagcttcgtgttctctcattgtctggc
tgctggaaggtcacccaaaagagcgtgccattcttgagcaacctgggccagtctttggagggtctcaatcttcaattctgcaacatgattggcaaccaca
acattgcatcattggagaagcagctctggtggtgtgacattcttgcctag
>g81384.t1
atgcggagctcgacgacagcagtgatgtttttggtgctcgccaccgtcgtcagctggccgctcctggcatcggcccagccggcgccgagcatgtcgccg
ccttccccgccgccggctgccacgaacaactcgcgcttggagaaggcctacgtggcgctgcaggcgctgaagcgcgccatcactgacgaccccaaga
agctgacgcacaactggtgcgggcccgacgtctgcaactacttcggcgtgttctgcgcgccggcgcccgacgacccttgccagcgcacggtggccggc
gtcgacctcaaccacggcgacctcgccggcacgctcccggaggagctgggcctcctctccgacctggccgtcttccacctcaactccaaccgcttctgn
nnnnnnnnnnnnnnnnnnnnnnnnnnnnnnnnnnnnnntatcagtgggagagatttcaagatcagtga
>g81385.t1
atgtttttggtgctcgccaccgtcgtcagctggccgctcctggcatcggcccagccggcgccgagcatgccgccgccttccccgccggcggctgccacga
acaactcgcggctggagaaggcctacgtggcgctgcaggcgctgaagcgcgccatcactgacgaccccaagaagctgacgcacaactggtgcgggc
ccgacgtctgcaactacttcggtgtgttctgcgcgccagcgcccgacgacccgtgccagcgcacggtggccggcgtggacctcaatcacggcgacctcg
ccggcacgctcccggaggagctgggcctcctctccgacctggccgtcttccacctcaactccaaccgcttctgcggcactctccccgagtccctccgctcc
ctccacctcttgcacgagatcgatgtcagcaacaatcagctctccggaggatttcccacccagcttctctgcctccccaacctccagtacgtcgacatcag
gttcaacaacttctgcggtgaggtgccgtcagccatctttgacaaaaagatcgacgctctgttcatcaacaacaaccacttcgagttctcgctgccgaag
agcttcagcaactcaacggcgtcggtgatcgtgctggcgaacctgccgcatctgggggctgcctgccgagcagcatcggcgacatggccgggacgct
caacgagctgattctcctcaacagtggcatctcctcttgcatcccgccggagatcggcaagctggacaagctcacagttctcgacctcagcttcaacagc
atcgccggcacgctgccggagaccattggcaacatgcgtgcgctggagcagctcaacgtcgcgcacaacatgcttgctggcgagatacccgagagcat
ctgcgcactgcctcacctgaagaacttcacctactcggacaacttcttctgcggcgagccacaccggtgcttcgaggtgccacgcatcgacgacaggca
gaattgcatcgccggacgcccagaccagcggtccggcgaggagtgcatcgcgttcctgcaccggccaccggtgcactgcgatgcgcacgggtgcatc
gcgccaccaacggcacatgcgcctccgccgccggtatactga
>g81795.t1

atgacatcgctatacctcctggatgcccattttaatgaactctgtggtctgccatctgcctttgggaagctcagcagtcttgaaattcttgaccttagcagc
aacttcagtgacctgaaagagctccctgcttcctttggtgacctgctgaatctgcgtgagctttacctgagcaacaaccagatccatgctcttcccgacag
ttttggccgtctggataaactggagaggcttaacctggagcagaatcccctgggcatgccaccatctgacattgtgaacaagggcgtggatgcagtgaa
agagtacatgtcaaagaggtggttggacatcngaggcgtttctgggagccttgtagggtaccttagcggaaatgagaagtcagaaaaagatgcatatc
tgaaccagcagttctga
>g84690.t1
atgagcacggccttaccgctcctgctcctgctgtccgcctcctcgtccatgtcgacggtccagggcgtcgaggctaacgacgcggcggcgcttcttgcgtt
caaggcagcagccgtcggtgacggtggctacaggcttctttcgtggaatgccagcagtaccggagagttttgcagctgggagggggtgacgtgccagg
gcaagcccaggcgagtggtggcgttgaacctctcatcccacgggctcaacggcgtcctgtcgcccgcgataggcaacatgtcatcgctcaaggtactca
acctgggatccaacatgttgagcggcgccatacctgcgagccttggccgtctgcaacatctcgagatcctcaacctcagcagcaactcgttatccggtga
catccctaccaacttgagctcctgcactagtctgactacaattgaactccagtcgaaccagctctacgggcgccttcccccggagcttggcaacaagctg
acgcgccttgaggtgctcaaactgtggaagaacaaccttaccggcaccatcccaacgtcattggccaacttgtcttcgcttagttttgtgtccctcgcattc
aaccagtttgagggcaccatcccctttggcctcggcggcattgcgggccttcgacacctcgatctcgcctacaaccatatttctggtgagcccccgcgctc
catttacaacttatcttcatttgaagtattacagcttgagggaaacatgctacatggagaaattcctaatgacattgggagtaggttccctagacttttgtc
gaaccagctctacgggcgccttcccccggagcttggcaacaagctgacgcgccttgaggtgctcaaactgtggaagaacaaccttaccggcaccatcc
caacgtcattggccaacttgtcttcgcttagttttgtgtccctcgcattcaaccagtttgagggcaccatcccctctggcctcggcggcattgcgggccttcg
acacctcgatctcgcctacaaccatatttctgaatggaggaggcagggacagattgaagcacgcattcgatgccaacaaggcatcagggaggacatcg
gggagggggaagtcaccgtggagagcagccaccaagacgccggcgacgtgggcgatagggactatccatcgtcaaagctccttttcttttga
>g84936.t1
atgtacctcgggctcggcaccaaccagctctccggccgcatcccgccgtcgctggccaacatcacgatgctcaggtggctcggactcgatcagaactttc
tggaaggcacggtccctgacgacttgtcgcgcctcaccaagctcatgtccttgaacgtctaccagagccacctcgccggcgagatcccgccgttcctctt
caacatgtcgtcgctgcaaggcttgacactcgccaacaacgcattcgagggcgagctccccgccgacgccggctacctctacctcggtgggaaccggc
tcaccgggccgatcccggcgtcgctcgctgaggcgtccagtctgacggtcctcagcctggccaacaacagcttcaccgggcaggtgcccacggcgatc
ggcaagctctgcttgcaggcgttgcagctgtcaaacaacgagctcacgacctccgacgccggcggctgggagttcttggagggcttgaccaactgcag
atcgctgtcggaaatctctctcgacggtaacaaatttactggagcaatgccgagctccattgcccacctctcgccgaacctccagacgctgaaactcggc
ggcaatggtggcatatccggagttataccgccgggcatcgggaacatcgtcgggctacagacgctggatttcgagtcgaatctcctatcgggcgtgata
ccggaaggcattgggaagctcaagaacctcaaagaactacggttgcagcacaacaagctaacaggccctattccatcctccattggcgacctcagcca
gctcctagtcatcgacctgagcaacaatttgctgaacggctcgatcccgcccagcctcggtgacctccagcagctcacactgcttaacctctccggtaac
gggcttaccggccacgtccccggagaactgttcaagctgtcgtcgctgtcctcggcgatggacctgtcagataaccggctggacgggacgcttccggga
gacgtcggccagcttgcgaaactcgcacttttggccttgtccggaaaccgcttctccggcgaggtgccgtccgagctgggcttgtgcgcgagcttggagt
ttcttgatctggacagcaatttcttcaccggaagcatcccgtcatctttcaaaaaataccggcagaactggagaacgtga
>g85270.t1
atgagtcagaatgaggctcgtcagcatgctatgaggattgaaagtgcatgctaccttgctgcagatgagctattcaggaaggagcctcatggtgatggt
acttctgctcgtcagctatatgcagctgaatctagcaggatggtgtgggaagttctgagtgaaaagtcagaaacaactgccaaagatgggactgctgac
accttgcttaatatatcatctaccaaggtttctactattggggcacttggagctagggcatttctcaagccactgactgaacaaaggcagtcatatacgag
gctttgcttcaggaacaggagctttggtctggatgctgccaatgttgctagaccagttcttgagtctatcaaagagcacatcacagagttggatatatcag
gttttgttgcttctaggcctgaggatgaagcccttggtgtgatgagcatattctctaatgctttgaagggttctaggcttagatatctgaacctctctgacat
tgctttgagtgagaagggtgtgatggcattcaaagagcttttgagctcacagaaagacctcgaagaactacacttgatgaacaatggtctgtctggtaaa
gctgcacgaattctatcaaagtttccttcaactgagaaactcaaggttcttcagatggtcaataattgtattggtgatgatggcgccgcatcaattgctga
gatggttaagcgctctcctaatctagtgaatttcaggtgctcagcagctaaaataggttctgatggtgcagtagccttggctaaggcgttagaaacatgc
attcatctggacaaacttgatctgagcgacaacttgtatggttatgaagcagcagcaactcttagtgaaagcgttacaaagcatcctgtcctcactgtgat
ccagatcaaggccacttcaaaatttgctcgtaaacttcaaaatctgaaatctgctgagaataaaattcttgactcagccaaacggaccttggatggtattg
tcaagaatgcattggaaggtagcaacaaagatctaagggcagctgatttgaacacaaacatgtcactgagggtcggtgctcattgcttctcacagtcta
acaccgctaagccaggttcgatgcatctgaactgtcaggcttctcatgaaggcattattggggtggaggatggtaagaacctgcccactggacaagaat
ga
>g85372.t1
atggtaaatggcttcatatcatcgtggatagcgttcgtcgcgctggtgcttcagctcgcagtgtatggctgctcggcggtcaatcttgaagggtcggcgct
gctcaagttccagtcaaaggtggaggaagacccgcatggcgccatggctggctggaagccccgtgacagggatccttgcagctggaacggcgtcagc
tgcgttgatggcagggttgtgatgctgtga
>g85480.t1
atggccgcgagcttcttggcagcggcggttgcggtgatgctggtacttgagatgacagcgcccgccattgccggcccggaggaggaagcggccctgct
gctcgccttcaagcgcgcgtcggtgtcaaccgacccgcgcggcgagcttgtgggctggtcggcgaactcctcctccgccagcgccggcgcgccgtgctc
gtgggccggcctctcgtgcgcgccacccccccgaggggcgcgtggtcggtgtgaacctcaccggcatgtccctcgccggcgagctccgcctcgacccgct
cctcgccctcccggccctccggcaactcctccttcgcggcaacgccttccacggcaacctctcccacgcctcctcgtcgccgtcctcgtgcgccgcgctcg
aggagttggacctgtcctccaacgcgttcaacggcacgctccccgcctcgttcttatccccctgctccgcgctccaatccctcaacctgtcgcgcaacgcc
ctcgtctccggcggtgaaaacggcgggtcctccttccccttcgccgcgctgccgtcgctgcggacgctcgacctctcccggaaccacctctccggcgagc
tgccggcgagattcgtggccgccgcgctgcccaatctgacccacctcagcatcgccgggaacaacttctccggcgacgtgtcggactacgacttcggcg
cgtgcgccaacctgacatttctcgactggtcctccaacggcctgagcggcacgaaattgccgcgtggcctcgccagctgccgccgcctcgagacgctgg
acctgtcggggaacaggatcctcgccgggccggtccccgcgttcctgacgggcttcccgtcgctgaagcggctcgcattggccggaaccgggctgtccg

gcccgataccggaggagctgggccagctgtgcgggcggatcgtggagctggacctgtcggggaacagcctcgtaggcgccctgcccgcgaccttcgc
caactgcacctccctccagctgctggacctcggcggcaaccagctttccggcgacttcgtctccaccgtcgtcagcaccatcccctcgctccgcgagctc
cgcatcccgttcaacaacatctccggcgagaacccgctgcccgtgctggcagccgggtgcccgttgttggaggtcatcgacctctcctccaacgagctcc
aaggggagatcatgccggacctctgctcgcggctgccgtcacttaagaagctttttctgcccaacaactacctcaacggcaccgtgccagcctctctcgg
cgactgcgccaacctggagtccatcgacctcagcttcaacctgctggtcggccacatcccgaccgagatcctggcactcccgaggctggtcgacctggt
catgtgggccaacggcctgaccggggagatcccagacacgctctgttccaacagcaccacgctggagacgctggtcatcagctacaacagcttcacag
gagtcatcccgccttcgattacgaaatgcgtcaacctcatctgggtctcgctctcgggcaaccgtctcacggggaccctgccccgggggctcggcggcct
ccagaacctcgccatcctccagctcaacaagaaccagctctccggccccgtgccggccgagctgggcacctgcaacaacctcatctggctcgacctcaa
cagcaacggcttcaccggcgcgatcccgcccgtgctggccgaccaggccgggctcgtcccgggcgggatcgtgtcggggaagcagttcgccttcctcc
ggaacgaggcgggcaacatatgcccgggagccggcgtgctcttcgagttcttcggcgtccggcccgagcggctcgccgcgttcccggccgtgcacctct
gcccgtccaccaggatttacacgggcaccacggtgtacgagttcgacaaaaacgggagcatgatcttcctcgacctctcctacaacggcctcaccggcg
ccatcccgcccagcctcgggaacatgatgtacctgcaggtgctcaacgtgggccacaaccagctcaacggcaccataccgtaccagttctccgggctca
aggccatcggagcgcttgacctgtccaacaaccatctcaccggcggcatcccccgggcctcggcggcttgaatttcctcgccgatttcgacgtctccaa
caacaacctgtccggtcccatcccttcgtccgggcagctcaccacgttcccgcagtcccggaagaaccagaaggctgaagagaagagaacagggtac
atcgagagcctccccacatccggcacatcaagctggaaactgtcaggtgtccgtgagccgctgagcatcaatgtggcaacatttgagaagccactcag
gaagctcacctttgcgcatcttctcgaggctaccaacggcttcagcgctgagaccctcataggctcaggggggtttggtgaggtgtacaaggccaagct
caaggacgggactgtcgttgccatcaagaagcttatccatttcacaggacagggtgaccgggagttcaccgcagagatggagactattggcaagatca
agcaccggaacctcgtgccattgctcggatactgcaagattggtgatgagaggctcctggtctacgagtacatgaagcacggcagtcttgatatcgtgct
gcacgacaaggccaaggctagtgtcaagcttgactgggcagcgaggaagaagatcgcagttgggtcagcgaggggcctcgccttcctccaccatagtt
gcatcccccacatcatccacagggacatgaagtcaagcaatgtgcttctcgacaacaacctcgaggcccgcgtttcggattttgggatggccaggctca
tgaatgcacttgatacgcacctgagtgtcagtacacttgctggcacgcctgggtatgtgccaccggagtactaccaaagcttcaggtgcaccacaaagg
gggacgtctacagctacggcgttgtgctcttggagcttctctcagggaagaagcccattgatccaaatcaattcggggacaataaccttgttggctgggt
caagcagatggtcgaggaaaacagaagcagtgacatctttgacccaactctgtcggaaacaaagtcaggggaggctgagctctatcagtatctcaaga
tagcttgcgagtgcttggatgaccggcctaaccggaggccaaccatgatacaggtcatggcgatgtttaaagagctacagcttgactccgacagcgact
tcctcgatggtttctccatcaactcgtcgactatagatgaatcagcagagaaatcgacgtaa
>g86297.t1
atgcaacgagcagctgcaaaactcctgctgctcatcacagcagcagtcgtcggaagcctctcggtcttcacccatgcactgcaactgcaacggccccat
gaaacagctgctggtaatggtaccaccagctgccggccacacgagagggatgccttgctggccttcaagcatggcatcactaaagatccgtacggcct
cctgacttcgtggcgaagcggcaaagattgctgccagtggagcggcatcacctgcagccgccatggccacgtcgtcaagcttgacctcaacaggacctt
gggtgatccgcctttggttggccagataagcctttcgttgctttctcttgagcatttggagtaccttgacctcagtgggaacttcctacagggacgtaacca
aagtgtgccaaaattctttagttttatgaaaaacttgcggcacctcgatctctcatatattcctttctctgctacagtgccacatttgttcggcagccttacca
agctggaatacctcaacctctccaattcatcatttttggtagtatgcctccacagcttggcaacctttcgaacttgcgatatcttgacctcattaggatgca
aactttgtattctgagtga
>g87061.t1
atgcctcctcctcccctcctgcttctgctcctcctcttcctcctggccccgccgcccgccgcgccgcagcccgtgcctgggggagcgggtgcgccgccggg
cccggcggaggacgacgtgcgctgcctccggggcatcaagtccgacctgaccgacccggacggccgcctcggcgcctggtccttcggcaacacctccg
cgggcgccgtctgcgacttcaacggcatcacctgctggaacccgcaggagtcccgggtcctctcgctctcgctctccgggttcaacctccagggccccgt
cccctcctcgctccagtactgccgcgccgccaccacgctcgacctctccgacaacgcgctccagggccagatcccgcccgcgctctgcgactggatgcc
cttcctcgtcaccctcgacctctccggcaaccgcctctccggcccgctccccgccgagctcgccaactgcggcttcctcaactcgctcaagctctccggga
acgagctctccggccagatccccgcctccctctcccgcctcgcccgcctcaagtcgctcgacctctccgggaacaagctcgagggccagatcccggccc
agctcggctccgccttctccaaggactctttctccgggaacaagggtctctgtggccgccccgtctcctcccgctgcggcgggctggggagcaccggcct
cggcatcgtcatcgccgccggggtcttcggcgccgccgcgtcgctgcttctcgcctacttcttctggcgctgcacggggaagagcaagggacgccgcca
ccacaagcgcggaggcagcgagtctggcggcggcgccgccgtggaggacggcagctggtgggccgagaggctgcgcgccgcgcacaaccggctgg
cccccgtgtcactgttccagaagccgatcgtcaaggtcaagctcgcggacctgatggccgccacgcaggacttcagcaccagccacatcgttgtcgccg
ggagctcacgggcaggaaccgcgtaccgagcggtgctgcgggacgggtccgccctcacggttaagcgcctgcactcgtgcccgctctccgagaaggc
gttcagggcagaaatggggcggattgggcagctgcggcacccaaatatcgtgcccctgctgggcttctgtcccgtgcctgggggagcgggtgcgccgc
cgggcccggcggaggacgacgtgcgctgcctccggggcatcaagtccgacctgaccgacccggacgggcgcctcggcgcctggtccttcggcaacac
ctccgcgggcgccgtctgcgacttcaacggcatcacctgctggaacccgcaggagtcccgggtcctctcgctctcgctctccgggttcaacctccagggc
cccgtcccctcctcgctccagtactgccgcgccgccaccacgctcgacctctccgacaacgcgctccagggccagatcccgcccgcgctctgcgactgg
atgcccttcctcgtcaccctcgacctctccggcaaccgcctctccggcccgctccccgccgagctcgccaactgcggcttcctcaactcgctcaagctctc
cgggaacgagctctccggccagatccccgcctccctctcccgcctcgcccgcctcaagtcgctcgacctctccgggaacaagctcgagggccagatccc
ggcccagctcggctccgccttctccaaggactctttctccgggaacaagggtctctgtggccgccccgtctcctcccgctgcggcgggctggggagcacc
ggcctcggcatcgtcatcgccgccggggtcttcggcgccgccgcatcgctgcttctcgcctacttcttctggcgctgcacggggaagagcaagggacgc
cgccaccacaagcgcggaggcagcgagtctggcggcggcgccgccgtggaggacggcagctggtgggccgagaggctgcgcgccgcgcacaaccg
gctggcccccgtgtcactgttccagaagccgatcgtcaaggtcaagctcgcggacctgatggccgccacgcaggacttcagcaccagccacatcgttgt
cgccgggagctcacgggcaggaaccgcgtaccgagcggtgctgcgggacgggtccgccctcacggttaagcgcctgcactcgtgcccgctctccgag
aaggcgttcagggcagaaatggggcggattgggcagctgcggcacccaaatatcgtgcccctgctgggcttctgtgttgtcgaagatgagaggctcttg
gtgtacaagcatatggangagaaggcgttcagggcagaaatggggcggattgggcagctgcggcacccaaatatcgtgcccctgctgggcttctgtgt
tgtcgaagatgagaggctcctggtatacaagcatatggagagcggggctctgtcaacggtgatcaagaagccagaggaagctccactggactggcca

acacgtcttagaattgcagttggggcggcacggggacttgcgtggttgcatcatgggttccaggttccacagattcaccagaacttgagctctagtgcgg
tgcttctagatgaggattatgaggctcggatcactgatgttgggctcacgaggttggtcaggatggcgcctggtgaaggcggtgatacgagtcctttcct
gaatggggactttggggagtttggatatgttgctccagagtacgctagcaatccagttggtaccgtgaagggtgatgtgtatgcattcggggtgatattgt
ttgaacttgtgagcgggcaggaggctagtgctgtggtaagtgatgcagcgggtgaagggttcaaggggacattggtggattgggtgaatcagctcaag
gcttctgggcggattggtgatgctgttgataagtcattgcgaggaaatggacacgagtcagagatcgaggagttcttgaagatagcttttgtatgtaccc
atgctcgcccgcgggacaggcactctatgtgccgagtttataactctctaaaaagcatcggagaccggcgtgatgtctcagagcagtttgacgagtttcc
actggcctataacaaggatgattcagacaacatgaagctgtga
>g87130.t1
atggcctcctactcctgcgccttcgttcccgtcctcctactcctactcctgtcctcggcgccgttcttcgccttctcctccgagccccgcaaccccgaagtcg
aggcgctgatcgccgtcagggacgagctggtggacccgcacggcgtgctgaacaattgggaccagtactccgtcgacccttgcagctgggccatgatc
acctgctccgccaagaacctcgtcattggcctgggagcgccgagccaggggttgtccgggactctgtccgggaggatcgccaacctcacccatctcgag
caagtgttgcttcagaacaacaacatcacgggccggctgccgccacagctcggcgcgctgccgcggctccagacgctggacctctccaacaaccgctt
ctccggccgcgtgcccgacacgctgggccgcctctccgggctccggtacctgcggctaaacaacaacaggctgtccggtgcgttccccgcgtcgctggc
caagatcccgcagctcgccttcctggacctgtcctacaacaacctcagtgggcctgttccggtgttccccgcgcgaaccttcaacatcgtgggcaacccg
atgatctgcgggagcaatggcagcgcgggggagtgcgccgccgcactcgcgccggtcagcgtgccgttcccgctggaatccactccgggcagcaaca
ggacgacaggcgcgtcgtcgacgaccaagccaaaggctggctccgcgcgtctgcccatcggcgtggggacaagccttggcgcctcctccctcgtgctc
ttcgccgtgtcctgcttcctctggcgccgaaagcgccggcactcctcctcgggcccttccgttctcggcatcctcgagcgaggaggcgggtgcgatctgg
aagaggacggcgttggcggggtggtggcgcggctgggcaaccttcgccagttcgggctgcgcgagctgcacgcggccacggacgggttcaacccgaa
gaacatcctcgggaaaggcgggttcggcaacgtgtaccgcggccggctcgccgacggcaccacggtggccgtgaagcggctcaaggacccggccag
cgcgtcgggcgaggcccagttccggaccgaggtggagatgatcagcctcgccgtgcaccgccacctgctccgcctcgtcgggttctgcgccgcctccgg
ggagcgcatcctcgtctacccctacatgcccaacggcagcgtcgcggcgcgcctgcgaggtaagccggcgctggactggtcgacgcggaagcgcatc
gcggtgggcgccgcgcgcgggctgctgtacctgcacgagcagtgtgaccccaagatcatccaccgcgacgtgaaggcggccaacgtgctgctggacg
agcaccacgaggccgtcgtgggcgacttcgggctggcaaagctgctggaccacggcgactcgcacgtcaccacggcggtgcgcggcaccgtgggac
acatcgcgcccgagtacctctccacggggcagtcgtcggagaagacggacgtattcgggttcgggatcctgctgctggagctcgtcacggggcagcgc
gcgctcgaggtcggcaagggctctgccggggtcgtgcagggacagtccaagggcgtcatgctggactgggtgagaaaggtgcaccaagagaagatg
ctcgacctgctggtggaccaggacctgggcccttactacgaccggatcgaggtggcggagatggtgcaggtggcgctcctctgcacccagttccagccg
tcgcaccggcccaagatgtccgaggtggtcaggatgctggagggcgacggcctcgccgagaaatgggaggccaccaaccgccccggatcggcagca
atggcacacgacgccctcggctgctacgactaccggaacgactccaatggctccgtgttcttcaacgatttctaccacgacaacgacagcagcctgagc
agtgacgaggcgcggtccatcgacatggtggaggagatggagctgtcaggaccaaggtag
>g87384.t1
atgggaggcggctgcggcgtcgcggcccggctccgtcttcccctcctcgtcccgctgctgctgctgctgctactcctggacctcggctgcggcaatggcg
gccggtgcctggcggcgacgctgctaccaccgcaagaaggtgagccgcagcttatgcggcagaatcttactggagttctgcctgatgaagttaccaatc
ttacttatctgagacatcttgacttgtcccgcaacttcattcaaggtccgattccagcatcatgggctaacctacctgttcttaatatgtctcttcagggaaa
tcgcatatctggaacactacctagggagcttgggcgcatgcccatgttgaaatctatacagctggaaggcaatctgattgagggtcctattccaccagag
ctgggtaatatcatcagcttggagcgatttcggattgatgggaacaacatatcagggaaaatacctagtttcataaagaactggcagcgggtcaacaga
attcggattgatgggaacaacatatcagggaaaatacctagtttcataaagaactggcagcgggtcaacagaatggatttaagcttcaacaagttgact
ggtcaaattccagcaacttttggaggaatgctggcactggcactacaatatttgtga
>g87734.t1
atgcttgttgaaaggatgaccaagaacctgtgcactgagtccatttttctaagaagtatggtcttttgggcaaggaagaggctcacgagaatgctaaaa
ggattgaagaggagtgcttcatgtctgcagatgagcatttcaagcaggagcctgatggtgatgggagttctgctgtgcagctgtatgcaaaggaaacga
gcaagatgatgttggaagttctgaaacgaggcccaagggcaactgtagtaccagaagcacctgaggttgatgcaccttgtcagcctgctgatactgtgt
ttgatatctctggtggcaaccgagctttcattgaggcagatgaagcaaaggaactactgagtctgctttccaaaccaggaagctcttataaaagaatctg
ctttagcaacaggagctttggtattggtgctgccaatgttgctgcaccaattcttgagtcaatcaaaaatcagctcacagaggtggacatctctgattttgt
tgctggaaggccagaggatgaagcccttgatgtgatgcgcatattctccaaagcattagaggggtctgtactgagatatttgaatatctcagacaatgcc
ctgggtgagaagggtgtcagggcattctcagatctcttgaaatcacaggaaagcctggaagaactctatgtgatgaatgatggtatatcagaggaagct
gcaaaagctctatctgagcttattccttccaccgagaatcttaaggtgctctgcaacaaggataggttctga
>g87972.t1
atgcgtacgcctgccgtcatgtcccgcagctggcagcttggcccggatcaaggtttagtggccgcgcgcgccggcttccccggctctccgatcactccgg
cattccccttccgggaggatgcaggtaccgttccaatcctcatcgttctgcctcggaagggctctccagtagaacaacggctaagtaattctaccctctct
gccggcgacgtctttgttattcggagcaaggtcgcgtacgccaagaaggctcctggcaacgactgtccgcccaaggtcatgcccgtggagttcatggac
atggcggacactgttcaggacgtcagcaaaggccgtggcaattccccaccagcgtgcaacacgaacgtcttcgttcaaccagccgccgacgtgtcggc
acaccggctgcgtgacgtgaacgaaggtaaggaggcctcgagtaacagagactcgtctgacgacgccgacctcgccgtcgtcgtcagccaagactcct
ccggttacagcagcggcggcaacagcaagaacaattctgtgactgtgagcgccggcgagtctcccatcattggccacatgtcgccgaagaagctgaag
cgctcgtgctccaacatcgagacggcgcggtccgtcccggtcccggaagagttcgacgacctgccggcgaagtcacgatcctacggcgacctgaaggc
ggcactgccttccggaaggacgatgtttttcaccaccaccccagcccggcggcgtgttccgccgaccgcgtgatgctcaggaggcggtcgtcgtccgc
gcaggtgctcccgtcccggagccggaagctgtggtggcgcctgttcctctggagccaccgcaaccgcaacctgcaccgccggggctccgccgccatgc
ctgcggctcttccgtcgactgacatgcacatcgccgcccaggagaatgaagggtacacgtccgacacgctggacgcggtcaccgctgctagcagggag
cccaagaacaaggaggtcgtcgtggaggaggaggaggacccggttcttcttccgagccagtgggtcgcgttctcctcggtggaccgggtcagcgcgtg
ggtgaacagcctccagaaaaacagctcctcgttccacgtcgtcgacgaggacgacgatgttgtggcggagcacgtcgacggcggcgcggtgcgcgcc

gtctggtgctccgagatcgaggagtgctcgacggtagcagccgagaagcatcacgcgcgggcgacgaagcggcgcgtccaggcgagcagcgtcgtg
cagaccgtcaacgtggtctcctccgtcgcgcacgtctccggcatggggctcaaggccgtcccgatgatcgccgcgttctcgaccctccgggcagtcaatc
tgtccgccaacttcatagctcacatccacccaggatcgttgccgaagggtctgcactcgctcgacctgtccaggaacaagatcgccagcatcgaggggc
tacgggagctgacgaggctgcgcgtcctcaacctcagctacaaccggatcactcgcatcggacatgtacgtacaagcgttactatctctgatctgatgga
gacgtctggggacttggagttatccgcatcgcaggaatcttgttcaatgcgcatagcgtcattggcatttagatctctgtcagatcgtcccctcgtgctaac
tcctaggatggtccaagcttcatcagacttgaggtccaaactgaaacctgctgttttaggcagcgcgccgcacctgcgtagctccaagtga
>g88156.t1
atggtaaacctacagtcaagctcaaggtatctnctcggcgccgtcgcggcgtgcatcctcctcgcgctcctcgcaacgccggcggcggggatccttgac
ccggtggacttcctggcgctgcaggcggtgcgtcggtcgctggacgacatgccgggctcctccttcttcgacgggtgggacttcaccgcggacccgtgc
gggttcccgggcgtcttctgcgacggcacccgcgtctcggcgctggcgctgggcgacccgcgggccgggtccccgggcctcacgggccggctcgaccc
ggccctgggccgcctctccgcgctcacggagctctccctcgtccccgggcgcgtgcagggggacctccccgccacgctcgcgtcctgcacgggcctccg
cttcctcgccgtcagcaagaacctcatctccggcggcatccccgacgggctcggcgccctgtccagcctccgcacgctcgacctcagcttcaaccagatc
tccggcgcgatccctccttccctcgcctccctcccgtcgctcaccaacctcatcctctgccacaaccagctctccggcggcatcccgaccttcccggactc
gtccccgctcctccgcctcgacctcaagcacaacgccctctccggcgccgtccccgccacactaccatcccagctgcagtacctctcgttacaatccaac
cacctgacgggccgggtggacgcggtcctcccgcgcctcacgcggctcaacttcctcgacctcagcatgaaccagctcgacggcccgatcccttcctcc
gtcttcgcgctgccgctctccgtgctgcagctccagcgcaacttcttcgcggggcccgtgcagccgtcgagcgacgtggcgatccccgtggtggacctga
gctacaaccggttctggggccaggtgtcgccgctgctggcgggggtggggcagctctacctcaacaacaacaggttcacgggcgacgtgcccgcgcg
gctggtgcaggagctcgtcggatccggggggctgcagctgctctacctgcagcacaacttcctcaccgggatcgagatctcgccctcctcgtcgctgccg
tccggggtctcgctctgcctcatgtacaactgcatggtgccgcccgtgtacgcgccatgccccatcaaggccggctcgcagaacacaaggcccgccgac
cagtgcccggagtggagggggtga
>g88157.t1
atgccgggctcctccttcttcgacgggtgggacttcaccgcggacccgtgcgggttcccgggcgtcttctgcgacggcacccgcgtctcggcgctggcgc
tgggcgacccgcgggccgggtccccgggcctcacgggccggctcgacccggccctgggccgcctctccgcgctcacggagctctccctcgtccccggg
cgcgtgcagggggacctccccgccacgctcgcgtcctgcacgggcctccgcttcctcgccgtcagcaagaacctcatctccggcggcatccccgacggg
ctcggcgccctgtccagcctccgcacgctcgacctcagcttcaaccagatctccggcgcgatccctccttccctcgcctccctcccgtcgctcaccaacct
catcctctgccacaaccagctctccggcggcatcccgaccttcccggactcgtccccgctcctccgcctcgacctcaagcacaacgccctctccggcgcc
gtccccgccacactaccatcccagctgcagtacctctcgttacaatccaaccacctgacgggccgggtggacgcggtcctcccgcgcctcacgcggctc
aacttcctcgacctcagcatgaaccagctcgacggcccgatcccttcctccgtcttcgcgctgccgctctccgtgctgcagctccagcgcaacttcttcgc
ggggcccgtgcagccgtcgagcgacgtggcgatccccgtggtggacctgagctacaaccggttctggggccaggtgtcgccgctgctggcgggggtgg
ggcagctctacctcaacaacaacaggttcacgggcgacgtgcccgcgcggctggtgcaggagctcgtcggatccggggggctgcagctgctctacctg
cagcacaacttcctcaccgggatcgagatctcgccctcctcgtcgctgccgtccggggtctcgctctgcctcatgtacaactgcatggtgccgcccgtgta
cgcgccatgccccatcaaggccggctcgcagaacacaaggcccgccgaccagtgcccggagtggagggggtga
>g88299.t1
atgtggtcgccgccgcccccgccgtggcggccgggccgcctcctccgctcccgcgcgctccccctgctgcttctggccgtcgtcgcggcgctggcggcg
ccggcgggggccaccctggcggacgacgcgcgggcgctgctggccttccgcgacgccgtggggcggcgcctgacgtggaacgcctccgaccccgcc
ggcgcctgctcgtggaccggggtcgcctgcgagaacgaccgcgtggcggtgctgcggctccccggctcgacgctcttcgggcaggtccccgcggggac
gctcgggaacctgacggcgctccacacgctcagcctccgcttcaacgtgctctccggcgcgctccccgacgacctcgcctcggcatccgcgctccggaa
cgtcttcctcaccgggaaccggctgtccggcggcttcccgcaggcgtttctcgcgctcccgggcctcctccgcctcgcgctcgaccggaacgacctgtcg
gggcccatcccggcggcggtcggcaacctcacgcgcctcaaggtcctgctcctcgacaataatcgctttgacggcgagattccggagatgacgctgccg
ctgcagcagttcaatgtgtcgttcaacccgctgaacggatccgtaccggccggtctacggtctcagccgcgctcggctttcctggggacgggcttgtgcg
gcgggcccttggggccttgccctggtgaggtgtcgccttcccctgcacccgctgggcaaccatcgtcgccgactgctccgaccggcggtggtggcacca
acaacaatggaaatgggggaaggagtggtagtaaggggaagaagctctctggtggcgccattgccggaatcgccgtaggctctgccttgggagctgc
gcttctcctattcctcctcatctgcctctgtcgcaggtccgagcgcaccaagacgcggtctctggagatgcctcctcctcctgcaacaacaactgctgctgc
tactggtggccggaaacctccagagatggccagtggtgcggccgtggcgccgttgaccacaataggccaccctaatgctcctattgctcagtcgacgtc
ggggaagaagctggttttctttgggtcagcggcctccgtggctccttttgacctggaggacctgctacgcgcttcggctgaggtgcttgggaaaggagcg
tttgggacgacatacaaggcggtgctcgagtccggggcaactgtggcggtgaagcggctcaaggacgtcacactttccgagcctgagttccgtgaacg
tattgctgagattggcgagcttcagcatgagttcatcgtgcccctacgcgcatactactacagcaaggacgagaagctacttgtttacgacttcatgccca
tgggaagcctttctgcagtcctacacggctctgccttgggagctgcgcttctcctattcctcctcatctgcctctgtcgcaggtccgagcgcaccaagacgc
ggtctctggagatgcctcctcctcctgcaacaacaactgctgctgctactggtggccggaaacctccagagatggccagtggtgcggccgtggcgccgt
tgaccacaataggccaccctaatgctcctattgctcagtcgacgtcggggaagaagctggttttctttgggtcagcggcctccgtggctccttttgacctg
gaggacctgctacgcgcttcggctgaggtgcttgggaaaggagcgtttgggacgacatacaaggcggtgctcgagtccggggcaactgtggcggtga
agcggctcaaggacgtcacactttccgagcctgagttccgtgaacgtattgctgagattggcgagcttcagcatgagttcatcgtgcccctacgcgcata
ctactacagcaaggacgagaagctacttgtttacgacttcatgcccatgggaagcctttctgcagtcctacacggtaacagaggttctggccgtacaccg
cttgactgggaaacaagatcaagcatcgccctagcagcagcccgtggtgtcgaatacatccactccacaagttcaacagcttctcacggcaacatcaag
tcatccaatgtgctcctgggcaaatcatatcaagcacgtgtatcagacaatggcctcaccactcttgttggcccatcatctgcaccatctcgcaccactgg
gtatcgtgcccctgaggttattgattcacggagggtaacccagaaggcagacgtgtacagctttggtgtcctcttgcttgagctactcacaggcaaggca
cccagtcaggctgctctcaacgatgaaggtgttgacctgcccagatgggtgcagtcggtcgttcggtcagagtggacttcggaggtgtttgatatggagc
tcctgagacatcagaacgttgaggaacagatggttcagcttcttcagcttgctatagactgtgttgcacaagtacctgaggcacgaccatcaatggcaca

tgttgttacgcggatagaggacatcaagaagtcgagtggaagcactgaagcaaaagatatcaaagaaggcgacgatcattcttcgagggctgaatca
gtcgacgcgccaaccaatctctttgcttcctaa
>g88578.t1
atgtttgttccgttccatgcaactgaaagccgaaataattccattgatcaccaacgatcgattaaggagcaggagcagaacaacctgagcgaaccttac
aagatccggaagctcccgatggatcggatcctcaagtctgccagggagtccggctccctcaacctctccaatcgctccctcagggttgatttatcaaaca
atcacctcgccgagcttccaaatagccttgccaaatgcctggccttgtcggagatcaaggcatcaaacaacaacataaccagaataccagatatacttg
ctggttgttccaagctcagcaagttggatctggaggaattggacttgagcggccttggtgtgaccagtgtaccaccggtggcatgggagacaggtgatg
tggtgaaacttgatctttctaaaaactcaatagaagatctgcccaatgagctgtcattgtgttcatcacttcaatctttggttttgtctaataacaagataaa
aaggtggcctcacacggtggtctcccttcccaatctgtcttccttaaagctggacaataatcctttggcagaggtatcatccactgatcttgtgtccctgac
caaacttgaagtgcttgatttaagtggcaatgcatctgcgcttcctgatccgtctgctgtttctgcattaccaaacctgcaggagctgtacctgagacgaat
gaaactccatgaatttcccaatgctttgctgagcttaaaacagctgaggattcttgacctgagtcagaacagccttacaactgttccagagcataagacg
cgctctgttggaacgaggaacgaaagcaatcctcaagtacctgaaagagaaattgcccactga
>g88580.t1
atggcggctgcggcggcggcgagcatgggcgtcgtgcgcgaggtgctcggcgacgacgtcgtcgaggaggtcgaccatcccatcatcgactacatcg
ctaacgtcctcgcagacgacgacttcgacttcggcttcccggacggccatggcatcttcgacgccctcggggaactactcatcgacgccggctgcgtcn
cttcccaatctgtatcatccactgatcttgtgtccctgaccaaacttgaagtgcttgatttaagtggcaatgcatctgcgcttcctgatccgtctgctgtttct
gcattaccaaacctgcaggagctgtacctgagacgaatgaaactccatgaatttcccaatgctttgctgagcttaaaacagctgaggattcttgacctga
gtcagaacagccttacaactgttccagagcataagacgcgctctgttggaacgaggaacgaaagcaatcctcaagtacctgaaagagaaattgcccac
tga
>g88751.t1
atggcagcactaggttgtgccctcctgatgcttctctccttaacctctcgtgccacctcttgcaccgagcaggagaagatcttcctcttccagtttctctaca
ggctttcacaagatggtggccttactgcatcatggcagaaagatagaaactgctacaagtgggaaggcatcacctgcaacggtaatggaggtgtcact
ggggtttctttggcatctaaaagacttgaagggcatatctcaccttctctttccaacctcaccaacatgatgcacctaaatctgtcatacaactcattgtctg
gtggccttccaccagaacttttgttctctggcagtatcagtgtccttgataatgttagcttcaaccgccttggtgggagtcttcaagatctcagcagcttaac
agtgtctgaaaagccactgcaggagaagatcttcctcttccagtttctctacaggctttcacaagatggtggccttactgcatcatggcagaaagataga
aactgctacaagtgggaaggcatcacctgcaacggtaatggaggtgtcactggggtttctttggcatctaaaagacttgaagggcatatctcaccttctc
tttccaacctcaccaacatgatgcacctaaatctgtcatacaactcattgtctggtggccttccaccagaacttttgttctctggcagtatcagtgtccttga
taatgttagcttcaaccgccttggtgggagtcttcaagatctcagcagcttaacagtgtctgaaaagccactgcagatctctcctgccatcgactcctactc
acgccctccacgccgccacgactgccgtcggcgctacgagctcctcccctccgtcactgatgccgacaagcacacgtccgacctctcagtggctaggga
tgcggacgaccacttccgcctccatggggatgcctccgacttggatttagatggcgagggcggctggagcaatggcttggggaaaaggattctgtcggc
gccacgtgctcctccccacctccctccttgcctccttctccgccgaccaacaactctgccacctcatcaactggggggcgcaccgcgaccccgctatgcg
ctcgacctgcctggcgctgccgggagtggcaggtggaggcgctggcagtggtgggtggtggtccgccccacgttctacccgcaacggcactccaaccg
gcaccgctgcatcctcgatttgtgccgccaccacgaggaggagtcggcgatagagaagaaggggacccaaacctggctgcggacgagcggcttgttg
gcggggacctag
>g88753.t1
atgcaaccacgtcaggtttcatacaaggaacacaaatccaaattgcccataccttcaattagcctagctcttctgctactggtatccttggcctctccaacc
agttcctgcacagagcaggagaggacctctctcctccagttcctcgctgggctctcccatgatgctggtcttgctgtatcatggaaggaaggaaaagatt
gctgcaagtgggaagggatcacctgcaaccggaatggaacggtcatcaaagtctctctaccttctaagggcctggaagggcacatctcacagtccctg
gagaatctcaccggcttgcagcacctcaacttgtcatacaactcactgtccggtggcctgccgcaagaattggtgtcttccagaagcatcattgttcttga
tgtcagctttaaccagctcaatggagacctgcatgacatgtcgtcttcaactcctggtcaacctctacaggtacttaatatctccagcaacctgcttacagg
acagtttacatccgtaacatggaaggggatggagaatctgatcacactcaacgccagcaataacagctttgttggacaaattccaagtcatttctgtaac
atatcaccatcattggctgtgcttgaactctgctacaacaaattcagcgggagtatcccaccagggctccgcaattgctccatgctaagagtgctcaagg
ctgctcacaacaacctcagtgggattctccctgatgaactcttcaatgctacctcattagagtatctctcttttgaaagcaatggtttacaaggaatacttg
atgggacacagatagtcaaacttagtaacctggtcatccttgatcttggaggaaacaagttcagtggcaagattccagactccataggtcagctcaaga
gactggaggagctccatttcgactacaatgccatgaacggggagctgccatcaactgtgagcaattgtaccaacctcataaccatggacttgaaggatc
tgctgaagaacctccggtctccacgaaacaaagaaataacaagggcaaagagattagccaaaagtagaagggaagataatggagatgttgaaacaa
cctcgttctactccagttcagagaatgcattggtaatggtgccacaaggcaagggagaagaaaacaaactcacattcactgacatcatgaaggcaaca
aataactttgccaaggagaacataattggatgtggaggttatggactagtctacgaggctgagctacctgatggctccaagctggcaatcaagaagctc
aatggtgaaatgtgcctcatggaaagggagttcacagctgaggttgaagcactctcaatggcccagcacaaaaatcttttgccactctggggttactgta
tacagggaaactcaaggttcctcatatactctttcatggagaatgaacaggagaaggcatctctacttcagttccttgccgggctctcccaggatggtgg
actagccacctcatggaaggaggctacagattgctgcaaatgggaaggatggtggactagccacctcatggaaggaggctacagattgctgcaaatg
ggaaggtatcacctgcagcagaaatgggaccgtcaccaatgtctctcattagcttctaggagccttgaggggcacatctcgcagtcacttgggaacctc
accggcctgcagtacctcaacctctccgacaacttgctgtcaggtggcctgccgctcgaattagtgtcgtccagaagcatcaatatcctcgacgtcagttt
taatcagctcagtggacacctgcatgagctgccatcttcaacccctggccagcctctacagctgtcaaatgggttaggtaacctgaagtctctctccttcct
gtcacttactggcaacaactttacaaatataacaaatgcacttcggatactgaggaacaccaagaacctcaccacccttcttattgggctcaacttcatga
atgaaaaaatgccagacgatgacaccatcgatggttttgagggtcttcaggttttggatattggagggtgcctggccctctccgccacaatggcaaactt
ccctgctaatccgcaccgcttccttcaccacatcgagattgaagatggaggcgcggatcgcattccgcgggccttcgtcaacctctcaggtgagccaatg
agaaggaatgaggagttcgtcatcgccgaggatgttgatgaagtgctggaggaaggtgactacgcagttgttctgcatcaggtctatcaattcctggttg
acgagttgtgcatcccggtcaggattagagtccgctttgtcagacatgaccgtggtttgaataggagggacaaggactacactcgctatgggcggatcc

tcatcttaggcttcccactggattataggactattgagcatgttgatcatgttgtgagctcctttgggaagatgttgcgatggcataacccacctaccaaac
ttggctatgttctagtaaatgcttatacaatgatccgcagtcagttccaagaagcctggttttcagacaaggcactaggcagggggctgcatcccctgac
caacatgaagcaaagcaggtacaagcaatggttgatcagcaccttaaccaggggcagggagaccaagtttgggaccagattgtgcaagggcagcag
caacagggcaatctgcagcatggagattggcctgcatggccagagcagcaagctttctatgcagcaaggtcaaggccatgagcatcactatctaccat
acaacctcaacaatggagagtctttcctcttcatccaggatggccaagcacagattcagcctcactgggtgcaaccatttgtctctgatgcactcaagagt
atgctaagagtggccttcccaagtgaggagcagcatcaagcatggtggaatcaggccatgaccagctcaaccatccagagtcagttaagctggtcagc
aaatggtgccctgagctgctccttcaatgtcactttcaacaatgttgctcctggcctcaacaacttcagcatagccagtaccagtcagtcaggaaattaca
caacagctagaggctcctcagttgtcatcaaagaactcaaccagcacccatag
>g88755.t1
atgccgccactccacctttctgataagacacatagcaatagattacccagacctaccgtaggccttgctcttctgctgctaattgctttggccaccaccacc
aattcatgtacagaacaggagaaggcatctctacttcagttccttgccgggctctcccaggatggtggactagccacctcatggcaagaggctacagatt
gctgcaaatgggaaggtatcacctgcagcagaaatgggaccgtcaccaatgtctcattagcttctaggagccttgaggggcacatctcgcagtcacttg
ggaacctcaccggcctgcagtacctcaacctgtccaacaacttactgtccggtggcctgccgcttgaattggtttcgtccagaagcatcaatatcctcgac
gtcagttttaaccagcttggtggagacctgcatgagttgccgtcttcaagccctggacagcctctacagtggacctatccccctga
>g88858.t1
atgcttgtagatccatctgtctatcccttcagagtaataccgataatacatgacacgctgaccgtgctcgacctctccggcaacgcattcaccggcgcgat
cccggcggctttgtcggggtgcacggggctcaggacgctgaacctgtcgtacaacgggttctcccggccgatcccggcttcggtcaccggcatcggagg
gagcctcgaggtgttggacgtctcgtcgaatcacctcacggggcagatccctgacacaatcgggagcgcgtgctcgtcgctgcgggttctcaaggtgtc
gagcaacaacatctcggggccggtcccggagtcgctgtcatcatgccgcgcgctccaggtgctggacgccgccaacaacaagctgtcaggccccatcc
cggccgccgtgctggggaacctgacgtcgctggagagcctccttctcagcaacaacttcatcaacgggacgctgccgggcaccatttcctcatgtgcca
gtctgaggattgctgacctcagcagcaataagatctctggagcgctgccggaggaaatctgttcctcgtccggcgatgcggcgctggaggagctccggt
tgccggacaacatgctcacgggtaccatcccgcccgggctggccaactgctcgaaattgagggtgatcgacttgagcatcaactacctgcgcggcgca
atcccgcgggagctgggccgactccgcggcctccagcagctggtgatgtggttcaacgggctggaaggccggatcccgtccgagcttggtcagtgccg
gcgtctccgaacgctgatcctgaacaacaacttcatcggcggcgacatccccgcggagctcttcaactgcaccaacctcgagtgggtgtcgttcacgag
caaccggctgaccggcacgatccgtccggatttcgggcgtctgacccgtctcgcggtgctgcagctggccaacaacagcctggagggcgccatcccga
ccgagctcggcaactgcagcagcctcatgtggcttgacctcaacagcaaccggctcaccggcccgatcccncatcccgaccgagctcggcaactgcag
cagcctcatgtggcttga
>g88889.t1
atggggcagcaaccagtcactgctgagtctgtgccagtggcatgcgtgacgtgcggcgcaaggggccggcgtcgtggccgggtcgtggcattggacct
cagcgatcaagatcttcatggtaccatttcaccttctataggcaacctcacctacctgagaaggctccagctccccatgaatcgccttggcggcaccatac
cttcagagcttggccgtctccttcaactcaggcatgtgaacctgagctacaatgctctcaaaggtggcatcccggcatcactgtcagactgcaagcaact
agagaacatcagcttggcaatcaacaacctctcaggagcaatacctgccgccatgggtgcttctatggaatggtattga
>g90340.t1
atggcctcctcctgcgccttcgctcccgtcgacctcctcctcctcctcctcctcctgtcctcggggccgttcttcgctttctcctccgagccccgcaaccc
cgaagtcgaggcgctgatcgccatcagggacgagctggtcgacccgcacggcgtgctgaacaattgggaccaatactccgtcgacccttgcagctggg
ccatgatcacctgctccgccaagaacctcgtcattggcctgggagcaccgagccaggggttgtccgggactctgtccgggaggatcgccaacctcaccc
atctcgagcaagtgttgcttcagaacaacaacatcacgggccggctgccgccgcagctcggcgcgctaccgcggctcgagacgctggacctctccaac
aaccgcttctccggccgcgtgcccgacacgctgggccgcctctccgccctccgttacctgcggctaaacaacaacagcctgtccggtgcgttccccgcgt
cgctggccaagctcccgcagctcgccttcctggacctgtcctacaacaacctcactgggcctgttccggtcttccccgcgcgaaccttcaacatcgtgggc
aacccgatgatctgcgggagcaatggcagcgcgggggagtgcgccgccgcactcgcgccggtcagcgtgccgttcccgctggaatctactccgggca
gcaacaggacgacaggcgcgtcgtcggcgaccaagtcaaaggctggctccgcgcgtctgccgatcggcgtggggacaagccttggcgcctcctccctc
gtgctcttcgccgtgtcctgcttcctctggcggcgaaagcgccggcactcctcgggcccttccgttctcggcatcctcgagcgaggaggcgatctggaag
agggcgttggtggcgtggtggcgcggctgggcaaccttcgccagttcgggctgcgcgagcttcacgcggccacggacgggttcaacccgaagaacatc
ctcgggaaaggcgggttcgggaacgtgtaccgcggccggctcgccgacggcaccacggtggccgtgaagcggctcaaggacccggccagcgcgtcg
ggcgaggcccagttccgaaccgaggtggagatgatcagcctcgccgtgcaccgccacctgctccgcctcgtcgggttctgcgccgcctccggggagcg
catcctcgtctacccctacatgcccaacggcagcgtcgcggcgcgcctgcgagggaagccggcgctggactggtcgacgcggaagcgcatcgcggtg
ggcgccgcgcgcgggctgctgtacctgcacgagcagtgcgacccgaagatcatccaccgggacgtgaaggcagccaacgtgctgctggacgagcac
cacgaggccgtcgtgggcgacttcgggctcgccaagctgctggaccacggcgactcgcacgtcaccacggcggtgcgcggcaccgtgggacacatcg
cgcccgagtacctctccacggggcagtcgtcggagaagacggacgtcttcgggttcgggatcctgctgctggagctcgtcacggggcagcgcgcgctc
gaggtcggcaaggcctccggcggggtcgtgcagggacagaagggcgtcatgctggactgggtgagaaaggtgcaccaagagaagatgctcgacctg
ctggtggaccaggacctgggcccttactacgaccgcatcgaggtggcggagatggtgcaggtggcgctcctctgcacccagttccagccgtcgcaccg
gcccaagatgtccgaggtggtcaggatgctggagggcgacggcctcgccgagaaatgggaggccaccaaccgccccggatcggcagcaccggcaca
cgacgcgctcggctgctacgaccaccggaacgactccaacggctccgtgttcttcaacgatttctaccacgacaacgacagcagcctgagcagcgacg
aggcgcggtccatcgacatggtggaggagatggagctgtcgggaccaaggtag
>g90442.t1
atggcgcaacaacctgcttcaggagcgcgtcctgcgctcctcctcctcgtcctgtccctcctcctctcctcctcctccctcctgatcgtcgccgggcagcag
gggtacgaccagcagcagccaccgtcgccatcaccggaggaggtgccgaagcagcagacggcggcgaccaacccgcggttgcagaaagcctacgt
ggcgctgcaggccctgaagcgcgccatcacggatgacccacacaacctgacctccaactggtgcggccccgacgtgtgcgcctacacgggcgtcttct
gcgccgccgcccccgacgacccgcacaccctcaccgtcgccggaatcgacctcaaccacggcgacctcgccgccaccttcccggaggagctcggcctc

ctcaccgacctcgccctcctccacctcaactccaaccgcttctccggcacgctgccggagtccctcccgaagctccacctcctacacgagctcgatgtca
gcaacaaccgcctctccggcgggttcccgcagcacatcctctgcttacccaacatcaagtacgtcgacatccggttcaacaacctctgcggcaccgtccc
cgccgccgtcttcgacaagcagctcgacgcgatattcctaaacgacaaccacttcgactttgagctcccgcacaacttcggaaactcccccgcgtcggtc
atcgtgttggccaacctcaaactccgcgggtgtattccacccgggatcgggaagatggccggcacgctgaacgagctcgtcgtgctcaactccgggttg
cgctcgtgtttgcctcaggaactcgggtggctccgggagctcaccgtgctcgacctcagctcaaaccagctgcagggtcagctgccggagtcgttggcc
gggttgcacaagctggagcagctcgacgtcgcgcgtaatgagctgtcgggtcacgtgccggaaggcatctgcgcgctgccgagcctgaagaacttcac
ctactcgtacaactacttctgcagcgagccgcagcggtgcttggatatccgcagcgtcgacgaccggcagaactgtatcgccggacggcctgaccagc
ggcccgtcgaccagtgcatggcgttcctgcaccgcccgcccgtccactgcgacgaccacggatgcttcgcgacgcccaacaagccgcagcccaagcca
cagcagccgcagccgcactactga
>g90558.t1
atgttgccccgcgcctacgcgggcgcttgcctccatttgctcctcgtgttgcttacttgctggggcttccaggcggcgcagtccaaaccggtggatgaggc
aaggctgttgcttcagatcaagagcatgtggggcgacccgccggtgctgacagcatggaacgcctcggcatcggccgcacactgcaggtggccctatg
tgcggtgcaacaaagccgggcgcgtctcaggcctctacctcgctgctgccaacgtctccggcccattcctagaagctatcgccagcctctcccgcctcaa
gcacctcgacctccggaacaacagaatcgctggcgtgttcccgacctcgctgtacagctgccgttctctggagtacctcgacctgtccatgaaccacttc
gtcggggagctcccggcagacatcggccttggtctttcggcaaacctgacccagctgtatctcggcaacaaccgattcaacggcaccatccaggcatct
ctctccaggctccgtaacctccggtttctcaaactgcaagacaaccttttcgtcggcaccatcccggcagagctcggcgatctgacaagcctccaggagt
tgtacctggggtacaatccgtttgatgctggtgagcttccgcgatctttcagaaagctagctagcctcagtgtcctcgctgtgtgggatagcaaccttgtcg
gagatttcccaagctttttggtggggatgcgggagttgaaggcactggccctcttcaacaactcattgaccggaagcatacctccagggatttggagact
caacaagctgcaagatttgtatttgtccaggaacaacctcaccggacacctagtggacgatggcttcgctgctatgagcttgaaatacatagacctcgga
cacaatcagctctcaggaatgctacctgactttgttgggcttttggagaacctcgccatattgtcactccccaacaacaacttcactggcaaaataccggc
gagcattgccaggttgccatcactgatggtcttgtggctggacaacaacaggtttaatggcacgcttcctccagagctcgggaagcactctttggacctg
gccaatgtttcagtggacaacaatgagctcacaggtgcaattccagagggattatgcaatggaggtaatctcgattccttctctacctcgagcaaccgat
tgaacggctccataccagtaggactcgccaactgcgccagtcttcaaacactgaggctcgacagtaaccagttgtcaggacaagtgccctag
>g90559.t1
atgttgccccgcgcctacgcgggcgcttgcctccatttgctcctcgtgttgcttacttgctggggcttccaggcggcgcagtccaaaccggtggatgaggc
aaggctgttgcttcagatcaagagcatgtggggcgacccgccggtgctgacagcatggaacgcctcggcatcggccgcacactgcaggtggccctatg
tgcggtgcaacaaagccgggcgcgtctcaggcctctacctcgctgctgccaacgtctccggcccattcctagaagctatcgccagcctctcccgcctcaa
gcacctcgacctccggaacaacagaatcgctggcgtgttcccgacctcgctgtacagctgccgttctctggagtacctcgacctgtccatgaaccacttc
gtcggggagctcccggcagacatcggccttggtctttcggcaaacctgacccagctgtatctcggcaacaaccgattcaacggcaccatccaggcatct
ctctccaggctccgtaacctccggtttctcaaactgcaagacaaccttttcgtcggcaccatcccggcagagctcggcgatctgacaagcctccaggagt
tgtacctggggtacaatccgtttgatgctggtgagcttccgcgatctttcagaaagctagctagcctcagtgtcctcgctgtgtgggatagcaaccttgtcg
gagatttcccaagctttttggtggggatgcgggagttgaaggcactggccctcttcaacaactcattgaccggaagcatacctccagggatttggagact
caacaagctgcaagatttgtatttgtccaggaacaacctcaccggacacctagtggacgatggcttcgctgctatgagcttgaaatacatagacctcgga
cacaatcagctctcaggaatgctacctgactttgttgggcttttggagaacctcgccatattgtcactccccaacaacaacttcactggcaaaataccggc
gagcattgccaggttgccatcactgatggtcttgtggctggacaacaacaggtttaatggcacgcttcctccagagctcgggaagcactctttggacctg
gccaatgtttcagtggacaacaatgagctcacaggtgcaattccagagggattatgcaatggagtttccaagacgctgaggctatatatacgtgcatccc
ggcactgccccatttacgcgaaaacacattcctattctttctgtcgagttgccttccgagtcttccccgtcctggcgatccacgcgcacggagcttcggga
gagcaggcctccggaacctctcccgtaaggcgacagctgctcgggtgtgtgggcgatcaggtttttggtgagtgcttgatggcgcgactactcaacctca
tcgacttcttcctgatccacgactccactccctccaggcgccgtgtccccgatctacaaatccacttcctccaggcgctgctcaaggaactgcacggcaac
aacattctacctgcatcaacttag
>g90562.t1
atgaaccgccttgtcggggagctcccggctgacatcggccttggtctttcggctagcttgaccacactgtacctcgacgacaaccgcttcaatggcacca
tccagacttctctctccaggctacgtaacctccgctttctcgcgctggacaacaaccttttcgtcggcagtattccggcggagcttggcgagctgacgcac
ctccaaactatgtacctaggggacaatccattcgatgctgccacacttccgccatccttcatgagtcttgtcaacggcatcgccactgctgcagaagttga
tcctctctag
>g90829.t1
atggctgtcgcagtactgctgcagctagcagtagtggtccagcttctctgcgccgtcgtcgcgtcggcacttaaccaggacggcatcttgctcctctccttc
aagctgtcgctggccggggacccgctgggttccctgtccgattgggggtacacggacgccaacccgtgctcgtggaacggcatcgtgtgcgactccga
gtcgcgcgtggtgagcgtggtcctcccgaacgcgcagctggtgggccccgtggctcgggagctgggcttcgtggagcgcctccggcacctggacctctc
cggcaacgcgctcaacggcagcgtccccgccgacctgttccgcgctccggagctccgcgtgctgtcccttgccggcaacggcctcaccggcccgctccc
tgagccacaagcgggccagctccgcagcctccgcgcgctcaacctcgctggaaacgcgctctccggcgccgtgccacgaaacctcgtgacgctgccga
gcctcacggccgtgtcgctcgcgggcaatttcttctccggcgaggtccccggcggggaggtctcgccggcgctgcagatcctcgacgtcagcgccaacc
tgctcaacggcaccctgccagcggacttgagcggcgccgggttgcggtacgtgaacctgtcgtcgaaccgtatcgccgggggggatccccgcggagatg
gcggcgcggctgccggcaaacgtcaccatcgacctcagctacaacaacctcaccggcgcggtccccaccgtggcgcccttctccacgcagcgggcgg
acgcgttcgaggggaacgcggagctgtgcggggcgccgctcgagaggctctgctcgttcgcgtcggcctctgccgtggagcccccgcggaacggcac
gaagtcgccgccggccatcgccgcgattcccaaggaccccaccactgaagccgcgttgccgggagacggcgacaccggcgcggcgtcgtcttcggga
aggaggaagcgtcttgtgaccatcgcggcggtcgccgttggtgacgtggcgggcgttgcggtgctcttcttggtggcgctgtacgtgtacaggttgagga
agcggagaggacatcgtcaggaggcggcgacgatgggggtcgtgtataagaagcccgaaccggaggacgctgtgggccggagcctctcctgctgtct
gcgcaacaacaacaacaacaaggccgacgacagcaaccgtgacgtcacggactacacgtccttcaccgccaaggaaaagaacaccatcaacaacca

agccgggcaggggaagaagaagggggtggtggtgacggtggacggagaaggggaggagctggagctggagacgctactcaaggcgtcggcgtac
atcctgggctccgccggcggcagcatcgtgtacaaggccgtgctgtcggacggcgccgtgctggccatacgcaggatcggcagcgacgacgccgccg
tgcggaggttcggtgacctcgaagcgcacatgcgcgccgtcgccaagctccggcacgggaacgtcctccggctgcgcgggttctactgggggccagac
gagatgctaatcatccacgacttggcagtcaacggcagcctcgccaacctctccgtcaagaggaagcctgggtcctcgccggtgaacctcgggtggag
tgcgcggctccgcatcgcacgcggcgtggcccggggcctcgcgttccttcacgacaacaagtgggtgcacggcaacgtgaagcccagcaacgtgctcc
tcgacgccgacatggagcccctcctcgccgacctcggcatccaccgcctcgtgcgcaccaccgctgactccggcacccaccaacacatgtcgttggtca
caactacagcattcaagttcgggaccaagcgttccgcgaagagcctccccgaccaagcaccggcgagcccgcttccctccgacgacgacacggcggc
acactaccgggcgccggagaagacgaagccgaccgccaagtgggacgtgtacgcgttcggcgtgctgctgctggaactgctggccggccgcgcgctc
accagcctcgagctgtgccagtgcgccgccgcgccggaagagaataaggcgcagacgatgatgcggatggcggactcggcgctgcgggcggagatg
ggaggcagggaggacgcggtggccagctgcctcaggctcggcgccgcgtgctgcaatttggcgccgaccaagaggccgtccatgagggacgcgctg
caggccatcgacaggataccggctcttgcctctgctgctgctgcttcctcttcctcctcctccagggaggcagcaggagctcggtga
>g90877.t1
atgtctccgttccacggatacagaggtgatggagctattggctcggctctcttcggcgccggcggcgtccgaacaaggaagcgcgtgttcgctgtggcg
gacgacgaggtcgtgacggccaagaggcagaagcagagggatgagccgtccctggacgcgctccccgacgcgtgcctcttcgaggtcctgcgccgcg
tgcagggcgcccgcgcgcggtgcgcctcggcgtgcgtctcccgccgctggctcgcgctcctcgccggcatccgcgcctccgaggccgtgctggccgcg
gcccccgccgtgccggacctcaaccaggagtaccttgccgacgacgacgaggacgagctcatgtgcgacgacggcgacagcgagaggaccttcgag
ggcaaggccgccacggacgcgcgcctcacggcggcggccgtcggcgacgcgctccgcggccgcctcgtcagcgtgtccctccgcgggagccacccg
acgcgcggcgtcaccgacgctggcctctccgcgctcgcccggggctgcccggcgctcacttcgctcgccctgtgggatgttccacaggtgacagatgcc
ggactcgttgagatcgccgccgagtgccgctccctggagcgtcttgacatcactggctgcccgctgatcacagacaagggcctcgtcgcggtcgctcag
gggtgcccagagttgaagtcgttgaccattgaggggtgctccggcgttgccaacgagggcctcaaggcaattggccggtgctgcgcaaagctgcaggc
ggtgaacatcaagaactgtgcacatgttgatgatcagggtgtgtcgggcctcatctgctcggcgaccgcttcattggccaaggtccggctccagggtttg
agcattactgatgcctctcttgccgtgattgggtactatgggaaggcaatcacagacctcaccctcgctcgcctccctatcgttggcgagagaggattttg
ggtgatggccaatgccctgggcctgcagaagctcagatgtacgactgtcgtctcctgcccggggctcacagatctcggcctcgcatctgtcgccaagttc
tgcccaagtttgaagcttcttcacctcaaaaagtgcagcaaggtctcagacggttgtcttaaggaagttgcggaatcagcaaaggttttggagaatctgc
agatcgaagaatgcaacaaggttactctcatgggcattcttggtttcctactcaactgcagccccaagtttaaggctctctctttggtcaagtgcatcggg
atcaaggacatctgctctgcacctgcacagcttccggtatgcaagtcgcttcgctccctgaccatcaaggattgccctggattcacagatgccagcttggc
catggtgggcatgatctgcccgcaattggagaatgtcaatttgagtggtctcggtgcagttaccgacaatggcttccttccattgatcaagagttctgaaa
gtggtngggcatga
>g91324.t1
atgatcccgctgctttccctctcctcttccgccgccgcaatggcggccgccgcccgcctcctcttcctccccttcctcgtcctcctcgccacagcggccgaa
ccggccacatctgtgaacgacgacgtgctcgcgctggtcgtcctcaagtccggcctctccgacccgacgggccgcctcgcttcctggtccgaggacgcc
gaccgcgcctgcgcctggccgggggtctcctgcgacccgcgcagggggcgcgtcgacgcgctcgacctctccgaggcgtcgctctctggccgcctccc
gcggtccgcgctgctccgcctcgacgcgctccgctccctcgcgctccccggcaacaacctctccggcccactccccgacgcgctcccccc gaacgtgcg
cgccctcgacctctccggcaacgcgctctccggggccatccccgcctcgctcgcgtcctgcggctcgctcgtgtcgctcaacctctcgggcaaccgcctc
accggcgccatcccggacggcatctggtcgctgccgtcgctacgttcggtcgacctctccggcaacctgctctcggggaatgtgcccggtgggttcccgc
ggagcagcgcgctgcgtgcgctggacctgagccgcaaccttctcgaaggggacataccggcggacgtcggcgaggccgggctgctgaagtcgcttga
tctcgggcacaactcgttcaccggcgggctgccggagtcgctcagtgggctgtccgcgctgagttttctcggcgttggtggcaatgctctatcaggagag
ctcccggcgtggattggggagatggcggcgctggagaggctggatttgtccggtaaccgcttcgtcggcgtcatcccggacggcatttcaggctgtaag
aacctagtcgaggtcgacctcagccggaacgcgttcaccggggagctcccgtggtggttgcttgggctgccgctgcagcgcgcttcggtcgccggcaat
gcactgtcagggtgggtcaaggtttccgatgatgctgccggaaccttgcaaactctggacctgtcaggcaatgagttcacaggtgctatcctgcctgaga
ttgggacctttgcaaaaatgcagtatctgaacttgtcctcaaattccatatcggggcagcttccagccagcattgaacagatggttttgctagaggtgttg
gatgttagtggaaaccagttggatgggaatctaccgccggagattggatccgtggtggcgctccagaagctggtgatggggaggaattcgcttactgga
ggcatcccggcgcagattggcacttgcagctcgctgattactttctcaaagaacaactcctgctatggagtcatgccaaagccaatcgttttcaatcctaa
ctcctcatctggtcccttgtcagaggatgccccaagttcctcaagcaaccagcaccacaagaaaatgctattgagcatctccacactcatcgccattgtg
ggtggagcagtcattgttattggcgtggtcaccatcacattgctcaacctccgtgcccgtgcaaaatcttctcgctctgcacttcccactgcattgtcggat
gattaccatagccaatcaacccaatccccagagaacggcgccaactctggaaagcttgtcatgttcggcagaggcagctcagatttcagtgctgatggg
catgcattgctgaatagggattgtgagcttgggcgaggaggatttggcactgtttacagggcagtcctcagagatggccagccagtggctatcaagaag
ctgacagtttcaagcttggtcaagtcagaggatgaattcaggcagcaggttaagcttctaggcaaggtgcggcaccataacattgttagcctcagaggc
ttctactggacttcctcgctgcagctcctcatatatgaatttgtgcctggcgggaacttgcatcagcacctgcacgagtgctcagaagagagctcgctctc
atgggtggagaggtttgacataatcatgggtgtagcccgggcacttgtccacttgcaccgccatggtgtaatccacttcaatctgaagtccagcaatgtct
tgctagacacaaatggcgagccaagggttggtgactatggtctagtgaacctgctgccgatgctggaccgctatgtgctgagcagcaagatccagagc
gcgttgggttacatggcaccagagttcacttgcagaacggtcaagatcacagagaagtgcgatgtctcaaagaacaactcctgctatggagtcatgcca
aagccaattgttttcaatcctaactcctcatctggtcccttgtcagaggatgccccaagttcctcaagcaaccagcaccacaagaaaatgctattgagcat
ctccacactcatcgccattgtgggtggagcagtcattgttattggcgtggtcaccatcacattgctcaacctccgtgcccgtgcaaaatcttctcgctctgc
acttcccactgcattgtcggatgattaccatagccaatcaacccaatccccagagaacggcgccaactctggaaagcttgtcatgttcggcagaggcag
ctcagatttcagtgctgatgggcatgcattgctgaatagggattgtgagcttgggcgaggaggatttggcactgtttacagggcagtcctcagagatggc
cagccagtggctatcaagaagctgacagtttcaagcttggtcaagtcagaggatgaattcaggcagcaggttaagcttctaggcaaggtgcggcacca
taacattgttagcctcagaggcttctactggacttcctcgctgcagctcctcatatatgaatttgtgcctggcgggaacttgcatcagcacctgcacgagtg
ctcagaagagagctcgctctcatgggtggagaggtttgacataatcatgggtgtagcccgggcacttgtccacttgcaccgccatggtgtaatccacttc

aatctgaagtccagcaatgtcttgctagacacaaatggtgagccaagggttggtgactatggtctagtgaacctgctgccgatgctggaccgctatgtgc
tgagcagcaagatccagagcgcattgggttacatggcaccagagttcacttgcagaacggtcaagatcacagagaagtgcgatgtgtatggttttgga
gtgctgatccttgagattttgacaggaaggaggccggtgcagtacttagaagacgatgtcattgtgctttcagatttggtcagagcagcacttgaggaag
gccggctggaggactgcctggatccacggctgtcaggcgagttctcaatggaggaggcgtcggtgatcatcaagctggggctggtttgcacgtcacag
gtgccttctcaacggccggacatggctgaggtggtcgctatgcttgagatggtgaggatcccccagggtagtagctcagaggatgaactggtttga
>g91563.t1
atggaagaggaaaaggacgagtcgcctgcagatccttgtgttgagagggtcctcgagggcaaggaggccaccgacgtccgcctggcggccatggctg
tcgttgccgggtcccatggcgggctccagaagcttgccgtccgtgggagccacccaaaccggggcgtcacggaccagggactcacggctgttgcacgc
ggcaacccgaacctatgctcgcttgccctctggggtgtgccgcttgtcaccgatgccggactcgctgagattgctgctgggtgccccttgctggagcgtct
ggatatctcttgctgcccacttatcacagacaagggtctcgctgctgtcgcgcagggttgcctgaacttggttgtgaacatcaagaattgtgtgaacgttg
gtgatcagggaatctccagcctggtgtgctctgctacagcctcgcttgccaagatccggctccagggattgaacattactgatgcttcactagctgttattg
ggtactatgggaaggctgtcactgaccttacacttgcccgtcttgctactgttggagagaggggtttctgggtgatggctaatgctgctgttctgcccaag
cttgaagcagctttacctcaggaggtgtggacacgtgtctga
>g91843.t1
atgtcgtcagaaatagcggagcactcaagtcaggaaaagctgatcgccgtgctggccaacgagcaagagacgctccggcgtataccatgggggaaca
caggcaaggtcatggagctgctgaagtcggtgagcgctgagctgagcgacccaggcatcgcgaacggcgcttcggacgccaagaagtgggtgcagc
aggtgacggagatacgccgggacgttgaggatatgctggacgagtcccgttccagctccagcagccgcaccgtcctctccacaacgctactggagagc
atgttcggcgtcttacggtctcgacgccggatggcgaagcaagtcaagaacatcaactcccgcatcgacgccatgaagctgaggctgtcgctgctggtc
aacctggacgacagggagtccccggcgaactccacgcggtaccggatagacgaccggcagctggacactctctcgttcgaggaggtcgaggccttgg
gcgtcgactcgtatcggaaggagataagggtgcggctgttggataaagctccggagctcaaggtgatatccgtcgttggcatagccggcatcggcaag
accaccctcgtgaggagcgtctacaacgagcccacggtgaggggccggttcagctgccatgcatggatcacctgcggcgcggcatcctcggcggctga
tctcctcaaacggatcgtgctgcaagtcttcctggagagaccaccggagctgccggcgagcgccgaccccatggagctggcggccatggtgggccgct
acctgagggacaagccgtacctagttgtcctggacgatatctggagctccgacgtttgggaccatctaagcgtcgcgctggccgacaacggcctcggga
gccggatcatcgtgtcttcacgggtgcttggcatagggagccaatgctggtgggtgtcggctaagtccagagagaccttcacacacagactcctggagc
cggaagactctctgcgcctcttccttcgcaaagccttcccgtccaccgaaaccatgatgctgtgcccaccagctgatccgaagctaaaaatgatcgcctt
gaagatcgtccgagagtgccatggcttgcccctgctgctcgtcaccttgggcgggctaatgtccaccaaggagccgtcggtccgggtgtggaatgatgtt
cttgaccagatgcacaagaccaaggacctccagctcacccttcctgtggtcctgtggctcgcttacaacgacttgcctagccgcctcaaggcctgcttcct
ctacttcgtccttttcccgaggacgtactgcgccaagcgcaccgcgctgatccgcctgtggatcgccgaaggtttcatcaacaaggaagacggcaagac
gctggaggaaaccgcggaggaatatctcatggagctgatccaaaggaaccttgtccatgtgacggaatactatgactacggcaaagtgaagagctgc
ggtgtccatgacatgttacgcgagatcatcattcttaagtcggaggaggagaactttgggactccgccgatcaccggagatgttaccaagctcctcggc
gccaacgtgcgccgcctgtccacggtcgacgccaaagaggacctcttccacgacatcatcaaggccaccaacgtccgcacgctcttcatgcttggcgcc
aactccgtcagcgcgtcttctcttcttgccttcatctctgaattcaagttactgagggtcttggacctggaaggtgcgccggtagatcgcttgcctgaagag
ctgcctgatggtctctacctccggtacctgagcctcaggaacactagggtcagtgacctgccaaaatcagtgaagaagttaactcacctgcaaactctgg
acctcaaaggaacagacgtctcaaatctgccttcagggatcaccaagctggagaacatccgccatcttcttgcttatcggtactacaccggacgccaccc
gccctactactacgcactcggcgtcacgctgccgcagggcataggactgctgacgaaactgcagaaattgacctacgtggaagccacgcggcacaatg
gaacgctggtagagctcggcaagctgacgcagctcaagaggttggggatcgtgaagctgaaaacaggtgacggtccgtgcctgtgctcgtccatagcg
aagatggctgagcttctgtccctgtcagccgcatcgatctccatcgatgagccccttgatctcagcgtgatcgatcctgctcctcagaggcttgagcgcct
ctacctgaggggccacttgctgaacctaccaggctggatcttctccctcaggagtcttgcgaggatccgattccgatggtccaggctgagtcaggagtcg
atcaaggagctccagtctctccctgtgattgaacttgcactaattcaggcatacgatgggacgacgttgcactttactgaaggtttcaacaggcttcagat
acttgaaattgaccatttgaccaatcttgaacacatgagccttggggcagcgatgcgcaacatccagaagatgtccatcaggtcttgcgagaagctaag
tacaatacctgacggagttgaaggtctgaaatccctcaaagaaatccatttatttgccatgccggaggttctggtgtcaagcataaaggagggaggacc
aaatcaagaaaaggtccagcatgtacccttcattcgcgtctacaatgagcaccgggacatttcttccataaatctttga
>g92767.t1
atgtcgggcacctgctacctctccaccgtgctccccatcttggtcttgctgccaatcgccggcaagggaagcgcccagccacgcgaccgcgacacgctg
ctggccatcaagaaggagtggggcaatccccggcagctcgcgtcttgggatgatccctcctccgccgaccactgcagatggagaggcctcacgtgcga
cgaaactacggatggcggcagcgtcggaggagccgtcgtgaagctgtctctcccggacctggaactcgaagggtcggtccccgtgtccgtgtgcgcgc
tcaagaacctcacccacctcgacctctccggcaacaacctcgccggcgcctttccggccgctgcgctctacgcctgcgtgcaactccgcttcctcgacctc
tcctacaacaaattttccggccccatctcggccggcatcgacgtcctctcgcctatgatggagcacctcaatctctccactaatcatttcaacggtgaggt
gccgccagcggtgggaaggctgtgggcgctcncggccgctgcgctctacgcctgcgtgcaactccgcttcctcgacctctcctacaacttcttctccggc
cccctctcgcccggcatcgacgtcctctcgcctactatggaacacctaaatctctccactaatcatttcaacggtgaggtgccgccagcggtgggaaggc
tgtgggcgctcaagtccctacgactagactacaacagtttcaccggtgtgtatccagcaaccgagataagcgagctcgctaggctccaggagctcgctc
tgagcaacaactcgttcacgccgaggcccgtgccgagggagtttgcaaagctatccaacctgagcactctctcgatgttgaatatgaacctcaccgggg
agatccccgaggcgttcgccaacctcacggagctcacggtactcgacctgtctacaaacatgctcacgggatccatcccggcgtgggtattacagcaca
ggaagcttgagaatgtttttctatctttcaatgctctctccggtgagctgacacacaacaccacggcgacgaacttggttttgctagtcttgacctcgaatg
agctcacaggagagataccggaacgcttgggcaacctcaagaagttgacttcgatgtttcttgacgagaatcatttcactggcaacataccgacaagca
tcggactgctaccacggctcactgacatcgatctttcctccaaccggctatctggcgagctcccccgggagctcgggaaacacgggccactccaaaact
tgctggtgcaatacaacaacctctctggccctctgcccgagacgctctgcgccaagggaaagctcagatatatcatggtcgataacaataacttctctga
tgtgctcccgacgaaattaggggactgcattctgctgcaatgtctgcagatcttcaacaaccatttctccggtgacatcccgatgaacatatggtcgtccc
aaaacttggtgggtgtgtggatccaaaataacagcttcaccggcaccctgccggacgagatatccttcagcatctctcagattgatatcggggacaaca

tgttctccggatccattccaaagtcagctacagggttgcagtttttcagagccacgaacaaccggctgactggtgagttgccagtcgacatgagcaacct
tgccaacctaacacatctgatacttgccggcaatagaataactggtaccataccaaaatcgatccaactattacaaaatcttgttcggctcgacctgagt
ggcaatcagatatccggcgtggtgccttag
>g92768.t1
atgtcgggcacctgctacctctccaccgtgctccccatcttggtcttgctgccaatcgccggcaagggaagcgcccagccacgcgaccgcgacacgctg
ctggccatcaagaaggagtggggcaatccccggcagctcgcgtcttgggatgatccctcctccgccgaccactgcagatggagaggcctcacgtgcga
cgaaactacggatggcggcagcgtcggaggagccgtcgtgaagctgtctctcccggacctggaactcgaagggtcggtccccgtgtccgtgtgcgcgc
tcaagaacctcacccacctcgacctctccggcaacaacctcgccggcgcctttccggccgctgcgctctacgcctgcgtgcaactccgcttcctcgacctc
tcctacaacaaattttccggccccatctcggccggcatcgacgtcctctcgcctatgatggagcacctcaatctctccactaatcatttcaacggtgaggt
gccgccagcggtgggaaggctgtgggcgctcaagtccctacgactagactacaacagtttcaccggtgtgtatccagcaaccgagataagcgagctcg
ctaggctccaggagctcgctctgagcaacaactcgttcacgccgaggcccgtgccgagggagtttgcaanccattgcgaaccggacaacactgatctc
ccttag
>g92769.t1
atgtcaggcacctactgcctctccccgtacgtgctccccatcttggtcttgctgctaatcgccggcaatggaagcgcccagccacgcgaccgcgacacgc
tgcaggccatcaagaagtactggggcaatccccagcagctcgcgtcttgggaccctgccactgccgaccactgcaaatggagaggcgtcacgtgcgac
gaaactacggatggcggcagcgtcggaggagtcgtcgtgaagctgtctctccggaggctaaacctcaacggctcagtcccggcgtccgtgtgcgcgct
caagaacctcacccacctcgaactctctatcaacaagctcaccggtgccttcccggccgctgcgctctacgcctgcgcgcggctccgcttccttgatctct
cacgcaatcacttctccggtgcacttccggatgacatagacgggctctcgcctgccatggagcacctcaatctctcttttaacaacttcggcggtgaggtg
ccgccgactgtgggaaggttgcgggagctcaaatctctacggctgcacgccaacaatttcaccggtgcgtacccggcagccgatataagcgagctcgc
tgggctcgaggagctctctctaggtcacaactcgttcgcgttgaggcccgtgccaagagagtttgcaaagctgtccaacctcagctctctcaggatgtgg
gatatgaacctcaccggggagatccctgaggcgttcgccaacctcactgagctcagaacgatcgacctgtcttttaacatgctcacggggtccatcccgt
catgggtatggcaacacaagaagcttgagaaggtttttctagatgacaacgctctctccggcgagctgacgaccaacatcacggcattgaacttggttg
cccttgacctgtcctcgaatgagctcataggagagataccagcaagcatcggactactaccctgccttgctgacatcaatatttcatacaaccagctatct
ggcgagctcccccgggagctcgggaaacacgggccactcgaatccttgatagtctacgacaacaacctctatggccctctgccagagacgctctgcgc
caagggaaagctccgctatatcgccgctgctaacaataacttctccgacgcgctcccagtaaacttaggggagtgcgctccgctgaatttcctgctcctct
cgaacaaccgtttctccggtgacttcccggcgaagatatggtcgatccaaaatatgaagggtgtgataatccaaaacaacaacttcaccggcaccctac
cagacgagatacccttgagcatcttcgagatttatatcgggaacaacatgttctccggctccatcccaaagtcagctacaggattgtatgtttttagcgca
gggaacaaccggttgaccggtgagtttccggtcgacatgaccaaccttgctaatctccagtacctcgaccttagtgacaaccgcataactggtaccatac
caaaatctatcgaagtattgcagaatctcgttacgcttaacttgaggggcaatcagatatccggcgtggtgccttag
>g92818.t1
atggccccatcctcccgccaccgcctcctcctcctcatcctcgccgtcgccgccgtctccaccacgccgaacatgacggacgcggaggcgcagaacatg
acggacgcggaggcgctgatgcagctaaagaagtccttcaccaactcctccctctcctcctggctcatcaccagcaccgacggcaacaagtccccc
tgctccccgggctcccacgagtggcacggcgtggtatgtgggcacggcggggtcacgggcctccggctgagcgggctgggcctgggcgggaccgtcg
acgtggacgcgctggcggccttcccgcgtctccggtccgtctccttcgcccgcaacaacttctccggcccgctccccggcttccacacccgcctcgccgc
gctcaagtccatgtacctctccgacaacgccttctcgggccccatccccgacgacttcttcgccggcctggcccacctcaagaagctctggctcgacggc
aaccgcctgtcgggccccatcccggcgtccatcgcgcaggccgcctccctcctcgagctccacctcgaccgcaacaacctcaccggcccgctccccgac
gcgccgccacccgcgctgcggtccttcaacgtgtcgcagaacgacctggaaggcgtcgtgccggagccgttccggaagttcgaggtggcacggttcag
cgacaacgagtacttgtgttacgtgcccacccacgtgaagccgtgcaagcgggaggagaccgtgccgagctcgttcaagcacgtggtgctggccgtcg
ccgtcgtgctcgtctccgccgtcgcgctcgccgtcgtcgcgtgcgcctgcaccgccagcggccaccaatcgaccaaccgcgtagtagtgaccgcccgcg
gcttcaacggcaaggtgcgtgacaacagcgacgacatggaggggttggaggagaagccgccggtgtacatgtcgaagcagacgtcggcgccgcaga
agcggagcacgtcgtggctggcgcggcgagccgggtcgtcgatgggcggtctagggcaccgtcgcgcggcgtcggcggcgaaggtggacgacctgg
gcgccacgggcgcgggaggaggtgacctggtgatggtgaacgagtgcaagggcgtgttcgggctggcggacctgatgaaggcggcggcggaggtga
tcgggagcggcgggctcgggtcggcgtacaaggcggtgatggcgagcggcgtggccgtggtggtcaagcgcgcgcgcgacatgaaccgggcgccca
gcaaggacgccttcgacgcggagatgcggcggctgggcgccgtccgccacgccaacctgctcccgccgctggcgtaccattaccgcaaggacgagaa
gctgctggtgtacgagtacatccccaagggcagcctccagtacgtgctccacggcgaccggggcatggactacgccgcgctcgccgactggcccaccc
gcctccgggtcgccgccgggattgcacgaggcgcggccttcctccacgtggcgctcgcggggacgaccacggcgacgacccacgcgccgcacggcaa
cctcaagtcggcgaacgtgctcctggcccccgacttcgagccgctgctggtggacttcggctacgcggggctcgtcggcgggcaggacccgcacgccg
cgccgccgccgtcaatgttcgcgtaccgggcgcccgagtgcgcggcggcgggcggccacgtggccccgggcgccaaggccgacgtgtactgcctcgg
cgtcgtgctcctcgagctgctcacggggaagttcccggcgcagtacctgcacaacgccaagggcgggacggacctggtcatgtgggcgacgtcggcca
tggccgaagggtacgagagggacctgctcgacccggccgtcgtcgcggggtggaagttcgccgtgccggacatggtgcggctcatgcgggtcgcagt
ggactgcgtcgaggcggacgcggagaagcggccgggcatgaaggaggccgcggcgagggtggtggaggcggtggtggcggcgatggcaacggtc
aaggagaggcgggaggagcaggccgccggcggcggcggagagggagacaggacgacgagctcgcacgccgcgtacgtgtatatcaacatccaac
agtgctccacagctcttgctgcactcagcacatttaaagccatctccccaaaattttaa
>g93681.t1
atgactcgcgccgcgcgcgggctcctcctccgggcgctgctgctcctctcgctggcggcggcgcccgggccggtggcggcgcagacgcctccctctcgc
ctcgcgtcgcgcgcggatatcgccgcgctctacgcgctgcggggctcgctgggcctgcgggcgcgggactggccgcgcaaggcggacccgtgcgcgg
cctggacgggcgtcggttgcagcggcggccgcgtcgtctccctcacgctcgcggggctccgccgcacgcgcctgggccgcctcgccccgcgcttcgccg
tcgacgggctgcgcaacctctcgcgcctcgaggacttcaacgcctccgggttcgccctgccggggcccatcccggcgtggctcggcgcgggcctcgcgc
ccaccttccgctccctcgacatctcctcctgcgacgtctccggggagatccccgcctcggccctcgcggggctcgacaacctcgccacgctcaacctcgc

ggggaaccggctgtcggggccgctcccggccgccgcgctcgccaacctcacgcggctcgccgtcctcaacctgtccggcaatgcgttctccggcgcgct
gcccgacgcggtctggtcgctcccggggctcgccgttctcgacgtgtcccgggccaacctcaccggcgcgttgccctcggcggggcttgcgctccccgc
caatgcgcaggtggtggatctgtccgggaacctcttgtacggcgccgtgccggactcgttcggccggctgtttagccgggtcgcgctggccaatatctcc
gggaactacttcgacggcaagctcggcgcgtctgatggtggtgggaatgtgtcgtctgagttgaattgcttccaagacgttccggggcagcggagccag
gccgattgccagcagttctatgccacgcgtgggttgccgtatgatggactggttgccccaccggcgcctgcaccttctgcgcccgccagaaagaagaag
cacaagaatttgaaatacatactgatcggggccattgttggaggcgtcctgctgatagccttggttgcggccctagtgttttgctttgtgtgctctgggagg
aggagcaggagaaccggcactgatcagatggaaagtgggacaccaagtgcacaggcgggggtatcaggtacaggcgcggctgcagttgctggtggc
tcacagccttctgcattgtccgcaaacatggcgaaggtgatttctaccatggtgtgctccaagatggaactgccgtggtggtga
>g94038.t1
atgaggccttcaccattaccaccactgacattactcctcgtgcattttattctcgtcttgttcgtcagtggcgtcgcgagcggcggcagtactggtaatggc
gatgacggcctgcgccgcgacgcggtggcgctgttgtccctcaaggccgcgctcaacagccacccgcaggcgctgccgtcgtggtcggcgggcaacg
ccgacgctgtgtgcgcgtggaccggcgtgcggtgcgccggcgggcgcgtagtcgccgtcgacatcgccaacatgaacgtgtccacgggcgcgcccgtc
tccgtcacgctgtcgagcggcctcgacgcgctcgagcacctctctctcgccggaaacaacatcgctggctcggtttcgttgtcctcgttcccggcgctcca
gcacatcaacgtctccggaaacgcgctcaccggcgggctggacggctgggccttgccgtcgtcgatgccgctcctcgaggtcctcgacgcctacgaca
acaacttcacctccccgcttccgctcggcgtcgcgtcgctcccgcggctccgccacctcgacctcggcgggaattacttcggcggcgagatcccgcgttc
gtacgggcggatgccggcgatcgagtacctctccttaaacggcaacagcctgaccggcgccatcccggccgagctcgggaacctcaccacgctcaag
gagctgtacgtcggctactacaacgcgttcgacggcggcgtcccgccggagctcgggcggctgcggaacctcacggtcctcgacgtctccaactgcgg
gctcaccggtggaatcccacccgagctcggggggctcgcgtcgctcgacacgctgttcctccacaccaaccagctcaccagcggcatcccacccgagc
tcggtaatctgacttggttggccgcattagacctctccaacaacgcgctcaccggcgagatcccccagtcgctggcgtcactcaccaacctccggctgct
caacctcttcctcaaccggctccacggcccggtgcccgacttcatcgccgcgctgcccaagctggagaccgtgcaactcttcatgaacaacctcaccgg
gcgcgtcccggccaacctcggcgccaactccgccgcgttgcagctggtggacctgtcctcaaaccggctcaccggcgggatcccatccacgctgtgcgc
gtcgggggcgctgcgcacggtgatcctgatgaacaactttttgttcgggcccgtgcccgagttgctggggtcgtgcgcgagcctcgcgcgcgtccggctc
ggccacaacttcctcaacggcagcctggcccagctcaacctctccaacaaccagctgaccggcccgctgccgcccacgctgggcaacctcacatccctc
cagacgctgctcgccagcaacaaccgcctctccggagccgtgccgtccgagatattcggcgcgctgaccgggctggtgaagctggacctgtccaacaa
cgcgctgtccgggccggtacccgagtcagtggggcggtgcggcgagctcacgtacctggacctcagccggaacaggctgtcgggcccgatccctgag
gccatcgccggaatacgggtgctcaactacctcaacctgtcgcggaacgcactcgacggcgccgtcccgggcgcggtagggtccatgcgcagcctcac
ggcggccgacttctcgtacaacgacctcgccggtcgggtgcccgacacgggacagctcgcctacctcaacgccacggcgttcacggggaaccgcagg
ctgtgcgggcccacagttcaccgcccgtgtggcagcagcgaggcgggcagtgggtccacgacgacgaggcgtggtggcgcgggtggggaggtgaag
ctggtgctggcgctgggattattggcctgctccgcggtgctgtgcgggcccacggttcaccgcccgtgtggcagcagcgaggcgggcagtgggtccacg
acgacgaggcgtggtggcgcgggtgaggaggtgaagctggtgctggcgctgggattattggcctgctccgcggtgttcgcggcggcggtggtgctgcg
cgcgcggtcgtggcgactgggcggcagcgactggaagttcacggcgttccataagattgactttggcatcgcggaggtgatcgagtgcatgaaggacg
gcaacgtggtgggccgcggcggcgccggggtcgtgtacgcgggccggacccgctccggcggcgccatcgccgtgaagcgcctcctccaggtccaggg
ctcctcgtcagaccgcgggttcaaggcggagatccgcacgctcggcagcatccgccaccgcaacatcgcggcgaccaacgtgctggtgtacgagttca
tgggtgggggcagcctgggcgagctgctgcacggcaagggcggcgcgttcctgggctgggaccggcggtaccgcatcgccgtggaggccgcgcgcg
ggctctgctacctccaccacgactgctccccgatcatcgtgcaccgtgacatcaagtccaacaacatcctcctcggcgacgaactcgaggcgcatgtcg
cggacttcggcctcgccaagttcctccgttccggtaccggcggcgcaacctccgagtgcatgtctgccgtcgcgggatcttacggatacatcgcgcctga
gtatgcgtacacattgagggtggacgagaagagcgacgtgtacagctacggtgtggtcctgctcgagctcatcacgggccggcgccccgtgggcgact
tcggggaaggcgtggacatcgtgcagtgggccaagcggaccaccggcggccggcgcgggaacgtgccgggcatcatcgatcgccggcttgacgcga
cgcccgccgaggaggtggcccacctcttcttcgtatccatgctctgcgtccaggagaacagcgtggagcggcccaccatgagggaggtggtgcagatg
ctcgccgaatacccgcaccacgcgtcgtccagccagacatcgccgtcgacgtcgtcatcaggcgcggcgaaggcagcggcggagaaggacagcagc
agccccgccaccgggaaggagccggccaactgcggctacaagctgttcccgggcctgctcgcctaa
>g94615.t1
atgagcaacaactcggtggcggcgctggtgccgttcctcctcctcctcctcgtctgctgccgcctcgccgccggcgaggtcggggaggagacgaag
gcggtggcggtggacccgtcgttccggttcccgacccagcgcctgcgggacgcgtacgtcgcgctgcagacatggaagcagcaggccatcttctccga
ccccaaccgcctcaccgccaactgggtgggccccgccgtctgcaactacacgggcgtcttctgcgcgcccgtccctcgaggcttccccggcgccgggga
gatcgccgtcgcgggggtggacctcaaccacggcgacatcgcggggtacctcccctcggagctgggcctgctcgccgacctcgccctcctccacctcaa
ctccaaccgcttctgcggcctggtccccaccacgctccgccgcctccgcctcctcgccgagctcgacctcagcaacaaccgcctcgtcggcgggttcccc
tccgtcgtgctccgcctcccgtccctcaagttcctcgacctccgcttcaacgacttcgagggcccggttccccgcgagatcttcgaccgcccgctcgacgc
catcttcctcaaccacaaccgcctccgctccccgctcccggagaacatcggcaactcgccggcctccgtcatcgtcctcgccgagaaccacttcggggg
atgcctccccgccagcctcgggaacatgtccgacacgctcaacgagatcctcctcatcaacaacggactcacctcctgcctcccgcccgaggtcgggat
gctccgggagctcaccgtcttcgacgtcagcttcaacgcgctcgtggggcccctgccggcggaggtggccgggatgcggaaggtggagcagctcgac
gtcgcgcacaaccgcctctccggcgccgtcccgcaggccgtctgcgcgctgccgcggctgcagaacctcagcatcgcctacaacttcttcaccggcgag
ccgcccgcctgcgcgcgcgtcgtccggaccgacggcgaccggaggaactgcctgcccaaccgccccgcgcagcgcacgccgcagcagtgcgccgcg
ttctacgcgcagccgcccgtcgactgctccgcgttccagtgcaagccgttccacccgccaccgccgcccgcgccagtgtacccgggtcccttgccgccg
gtgtaccccatgccatacgcctcgccgccgccgccgccgcgctaccgatga
>g94616.t1
atgagcaacaactcggtggcggcgctggtgccgttcctcctcctcctcctcgtctgctgccgcctcgccgccggcgaggtcggggaggagacgaag
gcggtggcggtggacccgtcgttccggttcccgacccagcgcctgcgggacgcgtacgtcgcgctgcagacatggaagcagcaggccatcttctccga
ccccaaccgcctcaccgccaactgggtgggccccgccgtctgcaactacacgggcgtcttctgcgcgcccgtccctcgaggcttccccggcgccgggga

gatcgccgtcgcgggggtggacctcaaccacggcgacatcgcggggtacctcccctcggagctgggcctgctcgccgacctcgccctcctccacctcaa
ctccaaccgcttctgcggcctggtccccaccacgctccgccgcctccgcctcctcgccgagctcgacctcagcaacaaccgcctcgtcggcgggttcccc
tccgtcgtgctccgcctcccgtccctcaagttcctcgacctccgcttcaacgacttcgagggcccggttccccgccagatcttcgaccgcccgctcgacgc
catcttcctcaaccacaaccgcctccgctccccgctcccggagaacatcggcaactcgccggcctccgtcatcgtcctcgccgagaaccacttcggggg
atgcctccccgccagcctcgggaacatgtccgacacgctcaacgagatcctcctcatcaacaacggactcacctcctgcctcccgcccgaggtcgggat
gctccgggagctcaccgtcttcgacgtcagcttcaacgcgctcgtggggcccctgccggcggaggtggccgggatgcggaaggtggagcagctcgac
gtcgcgcacaaccgcctctccggcgccgtcccgcaggccgtctgcgcgctgccgcggctgcagaacctcagcatcgcctacaacttcttcaccggcgag
ccgcccgcctgcgcgcgcgtcgtccggaccgacggcgaccggaggaactgcctgcccaaccgccccgcgcagcgcacgccgcagcagtgcgccgcg
ttctacgcgcagccgcccgtcgactgctccgcgttccagtgcaagccgttccacccgccaccgccgcccgcgccagtgtacccgggtcccttgccgccg
gtgtaccccatgccatacgcctcgccgccgccgccgccgcgctaccgatga
>g95267.t1
atggcttcctcactcatctcgctcgtcctcttgctcttccacgccgccgcggcaaccgcgcaggtcgtcgccggcggggacgcgccggtgccggagcttc
cgcggtcggagcttgcggccgtgttccgcgtgatggcggacctcctcggcgacccgacgtgggcgcaggcccacccgcgtccctgcacggacacgccg
tggccgggtctgcagtgcgaggtggaccccgacgacgcgcgccgcctccgcgccacgcgcctccacttcggccccgacgtcgccaccccgccctgccg
ttccggcgccgcgctcgccgccgacgcgctgcgcggcctgccccacctcaagacgctctccctcttctcctgcttcatcaacaccggcgcggcggcggcc
gtcgagctgccgccggcgctgttcgcgaacgcgtcgtcgtcgctggagcaggtcgtgctcaagtccaacccggggctgaccggcgcggtccccgccac
gctgggcggcgtgcgcggcctgcgcgtgctcagcctgtcgcagaacgcgttccgcggcggcatccccagggagctcggtggcctccccgcgctgcagc
agctggacctcagctacaacaacatcaccggcgagatacccgaggagatcggagggatggcgagcctgaccatcctcgacatgagctggaacggcat
caccggcgcggtgcccagcgccatcgggaagctccacaacctccagaaggcggacctgagctacaaccgtctcaccggccgcgtcccgccggagctc
ggctcgctgcagaacctcgtgttcctggacctgggccacaacgctctcacgggcccgcttccctccacgctggccgggctgggcaggctgcagtacctg
ctcctgcaggacaacccgctgggcacggcagtgccggcggtggtgggctcgatgcggcggctgcaggtgctgggcctgtcgggctgcggcctcacgg
gccccgtcccgttcttcgcgggcctgcgcagcctcaccgcgctgtcgctggaccggaaccgcctcgacgggcccatcccgccgggactcgccgcgctgc
ctcatctgggacagctcaacctcagccagaaccgcctcgcgggggagatcgccctgcccggggacttcgtcgcgcggctcggacggcgcctcgacgtg
cgcgggaaccgacagctctgcgtcggaaagaggctgcaggggacgaccggaggatacctcgtcggcgcgcggccgtgcgagggggggcgacgcggc
ggcggcggcggcacaggccgaggggtcgggcgccgccggcggctggcgacggggtttcgacgaggacggtgttgggatatgggcgcgtcacgttca
cgtccctgtcgtgttgttgtttctggtgtaccgtttgtga
>g96664.t1
atggcccaccggccaccaccgctcgcgcgcctcctcctccttctcgccgtggccgccagctacctcctccacctcctcgcgctcgctgacgccgcgcggg
tgctggcctcctcccaggcgcccgaggccgccgacgcgctgctcaagctcaagtccggcatcacggaccccaacggcgcgctcgccagctgggccgc
gggcaccagcccgtgcggcgacggcggcgcgtccacctgggccggcgtgatgtgcgacaaggacgtgggcgtgcacggcctgcagctggagaagat
gggcctgtcgggcacgctggacctggccgccctcaagcccgtccgcggccttcggtcgctgagcttcatggacaacgcgttcgcggggccgatgccgg
aagtcaagcacatcggcgggctcagggcggtcttcctgtccgggaacaggttctccgggcccatcccgggcgacgcgttcgccggaatggggtccctc
aagaaggtggtcctctcccagaacgggttctccgggcccgtgccggcctcgctcgccgacgcgcccaagctgctcgagctccgcctcgacgggaacaa
gtttcaggggaggatccctgatctgcggcagacggagctcaaggacgtcgatttcgcgaataatgagctcgagggggagatcccggccggcctcaaat
ccatcagcgccgacaagttcgccgggaacaagaagctctgcggcgcgccgctcgacgccaaatgcgaggcaccggcatcgccatctccgaagcaggc
gcagccgggagccgcagcagggaccacaccgacgcagccagcagcaccagcggacgacgccacgtcacgatcggccgccgacgacgccgtggcg
gcgcaggaacatccccagggccccaccgagcgctcggtttccacgggcgtcctcgccgcgttcctcggcacgctggcggtcgtcgccatcgccttcctg
gcgctgcagcggcggcgcgagtacgacaccaagaacttcgggccggcggcgtccacgccccccacccccaccaacaaggtcgagccgcacccgccc
aatgacagcgccgccaccagcccagcggcggcccataacgtcgcgccacgtggcgccggcgaggacggcagcagcggcggcggacgcgtggcagg
aggaggaggaggaggaggatgcggcagctcgacgcgcaaggcggtggagcaaggaaggctgacgtacgtgcgcgacgaccgcggccggttcttcg
agctgcaggacctgctcaaggcgacggcggaggtgctgggcgcggcgaacctgggcgtgtgctaccgcgccacgctcccggccggccactccgtcgt
cgtcaagcggttcaaggagatgaaccgggtcgggagggaggacttcgaggagcacatgcggcggctcggccggctcagccaccccaacctcctcccg
ctcgtcgcctactactaccgcaaggaggagaagctgctcatccacgactacgtgcccaaccgaagcctcgccaacctcctccacggcgaagggcgcgg
gctgaagagggcggtggtgcactgggcggcgcggctgaagatcgtcaagggcgtggcgcgggcgctgagctacctgtacgacgagctgtgcatgctc
acggtgccgcacggccacctcaagtcctccaacatcctgctcgacgccggcttcgacccgctgctcaccgactacgcgctggtccccgtgatgaaccag
tcgcacgccgcgcagctcatggtggcgttcaaggcgcccgagcggaaacagtttgggcggtcctccaagaagagcgacgtctggtgcctcggcctgct
catcctcgaggtgctcacgggccggcccgcggcccacgagccacccaagcccaccgctgccacggccgagggtggcgacgtgctcagcgcgctggcc
gcgacgccggagggggagtgggtggataacgtggtggacgaggacctgaggggcgaggggggcgagaacagggaggagatggtgaagctgatca
ggatcggaatggcgtgctgcgaggtcaatgtggaccgccggtgggagctcaaggacgccgtcgaccggatcgaggagctcaaggccaaggacagcg
ccaacgaggactactccttctactcgtcggtgaacgacgacgacgtcgccaatgattga
>g97002.t1
atggcgccgcagtcatcaccacgcggccatgtcctgctctgctgcctctgtctcgccttgtcctgcctcccgcgccacgccgggggcagagcgtcgtcg
tcgacgagggccagctcctcctgcagatcaagcgcgcgtgggggacccggcccagctcggatcatggaccgcggggaccacatcatcacgctgcac
caactggacctacgtatcctgcgacgccgcgggccgggtgacctcgctcgcgctccccaacgtcacgctcccggccggcgccgccgtccccgccgccat
cggcagcttggcgtccctcgcaacgctcgacctcagcaacaccagcgtcggcggcgtgttccccacctccctctacaagctcaccggtctcacccacctt
gacctctcgtcgaacagcctcggcggcgagctacccaccgatatcgaccggctcggagccaacctcacatacctcgccctggaccacaacaacttcac
cggcgaaatcccccccatcggtggggaagctcaggagcctgacctacctcgccctcaacgagaacttgctcaccggcacggtcccgccggagctgggcg
cgctgatcaacctcgaggcgctcaagctcgaggtgaacccgttcggtcccggggcgctgccggattcgttcaagaacttgaagaagctgaggacggtc
tggctgtcaaactgcaacctcacgggcgacttcccaggctatttcgcggagatgccggacatggagttcctggacctgtcggggaacgggctcaccggg

agcataccgcccgggatatggagcctcccgaagctgcagatcatgtacctgtacagcaacagcctctccggcgacgtcgtggtcggtaacaacggcac
gatgggcgcgacgagtctgatcgagctcgacatttccgagaaccagctgacaggaaccatcccggagagcttcgggagcttgatgaacgtcagggtcc
tcaacctcttctcgaacaacttctccggcgagataccggccagcctggcggggctgcggtcggtggtgatcatgaagctcttctacaacaagctcacgg
gaccgctcccggccgagctggggaagcactcgcccgtgctgcgggacatccagatcgacggcaacgacatctccggtcccataccggccgggatctgc
gacaaccgcgcgctgtggatcctctccgcttcgggaaaccggttaaacggctcaatcccggcgagcctcgccacctgcccggccctcatcagcctccag
ctccaggacaaccggctctccggcgaggtcccgccagagctgtggacggagacgaagctcatcaccctgctcctgcaggacaatggaggacttaccg
gtaccttaccggacaagctgttctggaacctgacgcggctgttcctcgacaacaaccggttcaccggcccggttccggcgtcggccacccggctccaaa
agttccacgccgcgaacaactcgttctcgggcgacatacccaaggcgttccccgcggggatggggatgctgcaggagctggacctgtcgttgaaccgg
ctctccggggccatcccgccgagcatcgcgtcgcttaacgacctcacgcagatgaacttcagcgggaaccggctcaccggcgagataccgccaacgat
cgggtccatgccggtgctcaccctgctcgacctctcgtcgaaccagctctccggcgccatcccgccggcgctgggatcgttgagggtcaaccagctcaa
cttatcctcgaaccagctcaccggcgagatccccgccgcgttcgccaccgccgcctacgagcagagcttcgcgggcaacccgaatctgtgcacgtccac
gcggcagctcctcactggcgtgcgcgcgtgcggggccacgtcatcgcccgaccgcgtgtcccctggcctccgcgcgggcctgctcgccgccggcgccg
cgctgctggtgctcatcgcggggctcgcgttcctcgccgcgcgcgacatcaagcgccggaagaggaacctggccgccgccgcggaggaggagtggaa
gctgaccccgttccagccgctggagttcagcgaggcggcgctgctacgggggctttccgacgacaacctcgtcgggaaaggcgggtcgggccgcgtgt
accgcgtcgcgtacgcgaaccgggccagaggtgggaccgtggcggtgaagcgcgtgtggacgggggcgaagctggacacgaccctggagcgggag
ttcgaggcggaggtgggcatcctcggccacatccgccacaccaacatcgtgaagctgctgtgctgcctgtcacgcgcggagaccaagctgctcgtgtac
gagtacatggactacggcagcctcgacaagtggctgcacgggcacaagtgggtggcgcgggacggcggtgtggcgacgacgacgagggtcccgtct
gtcaggcgcgcgccgctggattggccggcgagggtcagggtggctgttggtgctgcaagggggctctgctacatgcaccacgagtgctcgccgccgat
cgtgcaccgggatgtcaagtcgagtaacatcttgcttgattccgagctcaatgccaaggtcgccgacttcgggctcgccaggatgttggctgaggccgg
caagcccgacaccgtgtccgccgtcgccggaacgttcggatacatggctcccgagtgcgggtacacgaggaaggtgaacgagaaggtggacgtgtac
agcttcggcgtcgttctcctggagctgaccacgggnnnnnnnnnnnnnnnnnnnnnnnnnnnnnnnnnnnnnnnnnnnnnnnnnnn
nnnnnnnnnnnnnnnnnnnnnnnnnnnnnnnnnnnnnnnnnnnnnnnnnnnnnnnnnnnnnnncgccgggtacggcgacgagatcgaggccgt
gttcaagctcggcatcatctgcacgggcgcgcagccggccacgcggccgaccatgaaggacgtgctgcagatactgctgcggtgcgagcaggcgtac
cagaaaagcttggacgagaaggtcgcggagtttgacgcggcgccgctcatccaggtgcgaggaggcagccgacggaagcagttctcggatgctaaa
gtggtagatgatggtgaggccggctttgactccagcgtctga
>g98719.t1
atgcacgtccgggcgcgatgccagcagcaggaggccgcggcgctcctccggctgaagcagtccttctcttttactattggcccttcgttctgcagcaaca
gcgaggcatcagacgacacgaccattttattgccgtcgtggagggccggcaacgactgctgccgctgggaaggagatgggggatcaacattaactgtg
ccaattgcgggaattgagtattcgggatcacccactcagtatcaggtggcagtcgatgtcgccatgaaacagcagtacatgagggtctctaaaatcttta
gcgacctggtggtgattgacatgtcgaacaacagattcagcggctctattcccaggacggtaggcaaccttacgtccttgctcgtgctaaacatgtctcat
aacgcattgaccggcccaatccctggcgagcttggccgtcttgttcagattgagtcactcgacctctcctggaaccgtctcacgggggagatcccccggc
agcttgccgcattgacggcactcgagtggctcaacctctcatataatgacctcagtgggagtataccatcgggaagccagttctcgacgttcaccagcag
ctcgttccaaggcggcaacctagggctgtacggatgcccgcttcctgtgagatgcaatctgacgcagccatcaccaggcagcaaggtcctgccgccgcc
gtcaccgcaacagctggttcccgatggagcatcggctgaccgcaggttcgagcttatcgtgctctggctcttagtcggttctggctatggtgtaggcttcg
cactggcagttgtcttgcaggtggtcctcacccgcaggcgcaagaaaataatagcacggcaaagtgtttga
>g98814.t1
atggcgccagcggccggatcctcctcctccattgccatcctcctcctcctcctggggctgggcgtcctcgtctcccttgccgccgccgccatggaccccgc
cgagcgcgagaccctgttccgcgtgatggaatccgtctcctccgaccgcgactggcgctcggccgcgggcgccgaccccctgcgcctcccccctggccgg
gcctcgagtgcaagccatcctcctcctcctcgcagtctcagggcgggcggcccacgacgacgacgatgcacgtggcgcggctcgacttcggcgtgccg
cccaacccgacctgcaagccgacggcctccttcccgacggccgccttcgccctcccggagctccgtggcctgttcctcgtcgactgcttcaagaacccgg
acgcggccacgcccttcgcggtcccgcccgccgccaacctctcctcctcccgcctgcagcagctcagcatccgctccaacccgtccctctccggcacgct
gccgccgcagctcgccagcctccggtcgctgcaggtgctcaccgtctcgcagaacgcgatggtccggggcgagctccctccaggcgtcgccgacctcg
cgggcctcgtccacctcgacctcagctacaactcgctcacgggccccatcccgacccgcctcggcgagctccgcggcctcgtcggcctcgacctcagct
acaactccttctccggctccatcccgacccggctcggcgacctcacacaactccaaaaactcgacctcagctccaacaacctcaccggcgccgtcccgt
cctccctcacgggtctcaaaagcctcaccttcctcgcgctcagcaacaacgggctccgcggccggctcccggcgggcctctcggaccttcgagacctgc
agtacctgatcatggagaacaacccgatgggggcgccattgccgccggagctgggccgcatcgcgcggctgcaggagctgcggctggcaaactcggg
ctactccgggtccatcccggacacgttcggcaacctcgcgagcctcaccacgctgtcgctggagaacaacaacctcacgggccgcatccccgcggggc
tcacccgcctcaagcggatgtaccacctcaacctcagcaagaacggcctcgacggcgtcgtgcccttcgacggcgccttcctcaagcagctcggccgg
aacctcgatttgagcggcaaccccgggctctgcgtcgtcgcggacagggccgccgtcgtgcccgacgtcggcgtcggggtctgctccggcggtcacgct
gccgacgcctcctgcgcgagctcggccggtggcggcggtgggagagcggtccgccggggacagcaggtgacgacgacgacgagagggcggttcttc
ttctggcgacctgcggcgatgctgctgctcacgcacgccgctgtagcgtcgctgtgcagctgcattctcctctga
>g98881.t1
atgcgctgggtcagggtagtcgtgcgtgtgcaagtgattggtcagagacccacgcacgggccgcatcaccttgctctccctcctcaactcgtcgctctcg
ggcccctcccctgcgtgctacaccgcctcgatgccctcgcctccctctcactccccggtaacaacctcgcgggcctcgtgctccccaacctcttcgtcgca
ctcccgtgcctgcgctccctcgacctctcctccaaccgcctcgccgcccccgtccccgacgagctcttcacgcaaagccgctccatcagcaccctccttat
cggccctagggttagggtttggtatgggctatgctggcaggtggagattctgtga
>g99015.t1
atgccggcgccggccgtgggcttggccgtcctggtgctcctggccgccctgccgggacacctttccgacgacctcaactccgacgcgcaggcgctccag
ggcctgcggtccgcggtggggcgctctgcgctgacgtcatggaacagcacgacgccgacgtgccagtggcagggtgtcacctgcgagaacggccgcg

tggtggagctccgcctccccgccgccgggctcatgggcgccctcccctccggcgtgctcggcaacctctctgcgctgcgcacgctctccctccggtacaa
cgcgctgacgggccccgtccccgacgacctctcccgcctggtggagctccgcgcgatctacctccagcacaacggcttctccggcgagctccccgcgcc
gctgttcgcgctgaagaacctcgtgcgggtggacctggccgggaacaagttcaccggcgagatctcgccggacttcaacaagctggtccggctgggga
cgctgcacctcgagaacaacgacctcaccggcgagatccccaagctggacctccccgcattggagcagttcaacgtgtcttacaacaagctcaacggc
tccatccccaccaagctccggaagatgcccaagacagcgttcctgggtaccggactctgtggtggcccgctcggtctctgccccggcgagaccgcgccc
acgccggccggatcgcccgagtcgcagccgggtgccggcggcgccgctgacgtcggcggcggcaagaagaagaagctctccggcggcgccatcgcc
ggcattgccatcggatgcgtgttcggcgtgctgctgctcctggctctgctcttcttcctctgccggaagaagtcccccgcgcccaggtcgacggcggcggt
ggagaaggggcgtgacgtcggcatggccccgatggaagccgagcccaagggacagaacggctcagccggccacggtggctcagccgccgcggctg
cagcggcagccgtgcgagccgccgcaaccgcagcggcggcggcgaagaccgacggcgcgtcgacggggtcgaagaagctcatcttcttcgggccca
tggcggcggccccgccgttcgatctggaggacctgctgcgcgcgtcggcggaggtgctgggcaagggagcgttcgggacggcgtacaaggcggtgat
ggagagcggcgcggcggtggcggtgaagcggctcaaggacgtggacctgcccgagccggagttcagggagcgcatcgcggccatcggcgccgtgca
gcacgagctggtggtgcccctccgcgcgtactacttcagcaaggacgagaagctcctcgtctacgactacatgtccatgggcagcctctccgccctgctc
cacggtaaccgggcctccggccgtacgccgctggactgggaaacgcggtcggcgatcgcgctggcggcggcgcgcggcgtggagcacatccactcga
cgggccccacggcgtcgcacggcaacatcaagtcctccaacgtcctgctcaccaagaactacgaggcccgcgtctccgaccacggcctgcccaccctc
gtcggcccttccttctcgccgacccgggtctcgggttaccgcgccccggaggtcaccgacatccgccgcgtctcgcagaaggccgacgtgtacagcttc
ggcgtgctgctgctggagctgctcacgggaaaggcgcccacgcacgccgtcgtcaacgaggaagggctggacctgccgcgctgggtgcagtccgtgg
tcagggaggagtggacggccgaggtgttcgaccaggagctcctcaggtaccagaacgtcgaggaggagatggtgcagctgctccagctggccatcga
ctgctccgccactcaccccgacaggaggccaaccatggccgaggtcgccgcgcggatcgacgatatccgacgctccagcatcggagaccggcacgag
ggcgatgagccgtcgctataa
>g99337.t1
atggcggcacttgccgcagctctatccgccgtcctcttcgttctcgtggcggttgctgaagccgacaccgacgctgctgatgtagcggccttggggatct
ctactcctcttggaacagcccggctcagctcgcaggttggtcggcaggcggcggcgacccctgcggcgccgcgtggatgggtgtctcctgcacgggctc
cgccgtcacctccatagatgtatctttcaacaacttgaccgggaatcttccgatttctatgggctatctgtcaaaactttctagcctctatatgcaaaataat
caactatcaggcacaattgatatgctcagcaatctaagccttgcgacattaaatatcgcgaacaataatttcagcggcatgataccacaacagttcagct
caatcccaaatttgatgtacttgcatgaggtgtgcctgccccctgttgtacagagaaacttgaagtcatcaaatatccttcttgatgaagagaacagccca
catctgtctgactgtgggcttgctgctctatcaccaaatccagagagagagatttcgtctgagattattgggtctttcgggtacagtgccccagagtttgcg
atgtcagggacatatacagtaaagagtgacgtgtacagttttggagtagtgatgttagagctattgacaggccgtaagccgctagacagctcaaggga
gaggtcagaacagtctcttgtcagatgggctaccccgcagcttcacgatatcgatttacttgccaagatggtcgatccggcattggatggcatgtatcctg
caaagagcctatcccgtttcgctgacataatcgcaatctgtgtccagccggaagcggagttccgtccaccgatgtccgaggtcgtccagcaactggtgc
gcatgatgcagagggccagcattgtgcggcggcactcggatgatctgggattctcctacagagcccccgatcgcgaggtgggcgacatcgtctga
>g99395.t1
atggcgccgccgccgtacgcgctcctgctgctcctcctcctgctgctgcgcccgaccgcgcgcgtgctcgccaacatggaaggtgacgcgttgcata
gcttaaggaccaacttaaatgatcctaataatgttctacaaagttgggatccgactctggtcaatccgtgcacttggtttcatgttacctgcaataatgaca
acagtgttatcagagttgatcttggaaatgctgcactatcaggaactttggtcccgcaacttggccagcttaagaacttgcagtacctgcgtctcaacaac
aacagcctttcgggttcaattccaaaatcattaactgctatcactgccctccaagttctggatctgtcaaataacaatttatctggagaagttccatcaact
ggttctttttccttgttcaccccctatcagctttgcgaacaacccgaacttatgtggccctgggaccacaaaaccttgccctggtgctccccccttttctccgc
ctccaccgtacaatccgccaactcctgtgcaatcaccaggaagcagttcctctagtactggggcaattgctggaggagtagctgctggtgcggccttgct
atttgctgttcctgcaattggctttgcatggtggcggcgcaggaaaccccaagagcatttctttgatgtacctgctgaggaagatccagaggtccatcttg
gccagcttaaaagattttcactgcgtgagctacaagttgcaactgatagcttcagcaataagaacattcttggaagaggtggatttggcaaagtctacaa
aggaaggctggccgatggttcattagtagctgttaagagactgaaagaggagagaactcctggaggggaactgcagtttcagacagaagttgagatg
attagtatggctgtacacagaaatctgctgcgtcttcgtggattctgtatgacaccgacagaaaggttgcttgtgtatccatacatggctaacggaagtgt
tgcatcacgtttaagagagcggccaccatctgaaccccctctagattggcaaacaagaagaaggattgcattgggttcagctaggggcctgtcatattta
catgatcattgtgatccaaagattatccatcgagatgtcaaagctgcaaatattttacttgatgaagactttgaagctgtagtgggagattttggtttggcc
aaactaatggattataaggatacccatgtgacaactgctgtccgtggaacaattggtcacattgcacctgagtatctttcaacaggaaaatcctctgaga
aaactgatgtatttggttatggaattatgcttttggagcttattacaggacaacgagcctttgatcttgctcgccttgccaatgatgacgatgtcatgctgct
tgactgggtcaaggggctactcaaggagaagagattggagatgctggttgatccagatttacagaataactacattgacattgaggtggaatcgctgat
ccaggtcgcactcctttgcacacagggctccccaactgaccggcctaagatggcggaagtggttcggatgctggaaggtgatggtctcgcagagagat
gggaggagtggcagaagggccaagatgatgacattgcactcacgaccttcttctga
>g99460.t1
atggtgaggctcaccgccgacctgatatggaagagcccgcacttcttcaacgctatcaaggagcgtgagcttgacctccgaggtaacaagatcgctgtc
atcgagaacctcggcgccactgaggaccaatttgacactattgatttgtcagacaatgagattgttaagcttgaaaacgttccttacttgaatcgtcttgg
cactctgctagttaacaataacagaatcactcgtataaatcccaatcttggagagtttttgccaaagttgcatactctggtgcttacaaacaatcgcctga
caaatcttgcggagattgacccccctggcttctctcccaaagctacaatatctcagcttactggataatactgtaactaagcagcctgactatcggctttatg
tgatccataagttgaagcatttgaggatgctggatttcaagaaagtgaaacaacaggagagggttgcagcagcaaaaaagttccactcgaaagaggc
cgaggaacaggcaaagaaggtacctaccaaaacatttaatccaggggagttggcagatgctcaagacaacacaaaggaggcgcatgctcctaaagtt
gtcggcccgacacctgaacaaatcacagcaatcaaggctgctattgtgagttctcaaacactggaggaagttgcaaggctcgagaaggcattgagcac
tggtcaaatccctgcagaatttgctatgccaaagcctgatactaatatggctgaagcatcagaggaagctgacaagatggacacagatggccagaacc
aagagagtggagctggtgagcagaaacaaaatgatggaagcacgccaattgaagagttcgtttgctgtgtgcaggaggctgccatggcaggaaaga
aggcgagcccctcgaaggccaagggcaagacggtcgagaagaaggagagcagcccatcgaaggccaagggcaagacgccccaaaagatctga

>g99673.t1
atggatcaagagatagaggagaagctatggaacagagataacacactacacaaccaatcacttgttgttgaagtgcctgactccaggaacatgtcaaa
cacaaaggatattggtgaagaacctgaacgtggtaaaaaagttattgatgaggacccaaatgaactttctccaagggcttatagcattaagagaatcga
ggagtggatcaaccaaatcaatttcgatgatcttactgtgaatgagcaaggagaaagctcattttctgctttgggtgaatctcgtgagccagcagctggt
gttcctgctgtgcggcctgatgccaaaagtccacttggcatggagatagcctacacatatatttcaaagttaactcctgtttcatcttcggcgcagttggcg
aaccttggtttggttgcgatcccacgactgagtgcattctctggcttacgcctcttgaacttatcagggaactcaattggtctggcctcctgttcttcattga
aggagttatatctggctggtaacaagattagtgaggtagatggtcttcaccgccttctgaagctaaaagttctggacttgcgccacaacaagatttctaca
tccaaaggcctgggccagctagctgcaaattacaattctctagaagccattaaccttgacggcaaccctgcgcagaagaatgttggtgatgagcacctg
aagaaatacctcttaggcctgctgccaaaccttgtcgtctacaacaagcaccccataagagcagctggctcaaaagatgtctcagatcgtcattcccgca
aaatatcctcttcccatcgttctgaccgtggtgttagatcggatcgcaaatcttccaggctggtgggtgtctcatcttctcacaagacacagagctcacgg
catgcccgtactgggtacgcttcagtttcaggctctttgctgaagcattcaagggtgagaaacactcccatgccattgttggggtcaagaccagcaagtg
ctgtagatctgacaaagctagcccaaatggaaggaaaggcacagtga
>g100058.t1
atggccaaccggccagttctcctgatgaccgtactactgtctttccagctgctcatcacgacgtcactttgtcagacgtctcaggtgtccgaccgtgacctc
gcggcgctgatcaaggtcagggagcacttcggcaacccgaacctgttcccggggtggcggctcggtacgacctgcatctcgtggtggcccgcggcgtg
caacgagcagggccgcgtaaccaagctcttcctcgacaaccttctcggcatcaaggaacttctaagtgtcaacaccacgctcccgccggccatcagcga
actcgagatgctagagacgctgagcattatgagcattcccgacctgcacggccccatcccagaatcctacggcaacctcccgcacctctccctcttcaac
atcataggcaccaatgtctccggctcgataccggcgtccctctccctcaccaatctcacctccgtcagcttatccaagaacaagctcaccggcccgatcc
cgaggtcgctgcggaagctgccgtacctttcctacttcgacgcctcgtacaacgacctcatcgggaccattccgccgggtctggtgcgcagcacgcctga
cagcccggccgccctgcagctggatggcaaccggctgtcagggaccatacccagatcgtacagattcgagaggaactcgatggagttcagggtggcc
aacaaccggctcaccggagacgcgtcgttcctgttcgggcggcacaagacggtgtcagggaccatcgatttgtccgggaacaatttccggttcaacctg
acaggggtggagatgccgatgcatcttgggttcctgaacctgagccacaaccggatctacggcggcgtgcccgtgtcgctgagggagtccagggtctc
ggtgctggatctcagctataacgagctctgcggggagatacccaccggcgggcacatggtnnnnnnnnnnnnnnnnnnnnnnnnnnnnnnnn
nnnnnnnnnnnnnnnnnnnnnnnnnnnnnnnnnnnnnnnnnnntgcatcacgcatatttga
>g100059.t1
atggccaaccggccagttctcctgatgaccgtactactgtctttccagctgctcatcacgacgtcactttgtcagacgtctcaggtgtccgaccgtgacctc
gcggcgctgatcaaggtcagggagcacttcggcaacccgaacctgttcccggggtggcggctcggtacgacctgcatctcgtggtggcccgcggcgtg
caacgagcagggccgcgtaaccaagctcttcctcgacaaccttctcggcatcaaggaacttctaagtgtcaacaccacgctcccgccggccatcagcga
actcgagatgctagagacgctgagcattatgagcattcccgacctgcacggccccatcccagaatcctacggcaacctcccgcacctctccctcttcaac
atcataggcaccaatgtctccggctcgataccggcgtccctctccctcaccaatctcacctccgtcagcttatccaagaacaagctcaccggcccgatcc
cgaggtcgctgcggaagctgccgtacctttcctacttcgacgcctcgtacaacgacctcatcgggaccattccgccgggtctggtgcgcagcacgcctga
cagcccggccgccctgcagctggatggcaaccggctgtcagggaccatacccagatcgtacagattcgagaggaactcgatggagttcagggtggcc
aacaaccggctcaccggagacgcgtcgttcctgttcgggcggcacaagacggtgtcagggaccatcgatttgtccgggaacaatttccggttcaacctg
acaggggtggagatgccgatgcatcttgggttcctgaacctgagccacaaccggatctacggcggcgtgcccgtgtcgctgagggagtccagggtctc
ggtgctggatctcagctataacgagctctgcggggagatacccaccggcgggcacatggtccagttcaaagcggcggcgtacgaacacaacaaatgc
ctctgtggtacgccgctgccaccgtgcgccaacggccgctga
>g100859.t1
atgggcctaaatgggcttctcactgagattgatctgggccaatatgctggcctacgtagaatttgnnnnnnnnnnnnnnnnnnnnnnnnnnnnnnn
nnnnnnnnnnnnnnnnnnnnnnnnnnnnnnnnnnnnnnnnnnnnnnnnnnnnnnnnnnnnnnnnnnnnnnnnnnnnnnnnnnnnn
nnnnnnnnnnnnnnnnnnnnnnnnnnnnnnnnnnnnnnnnnnnnntgtgcctggacctgacgagctaccagctgcacgatctgaacgaggtggagattc
cacccacgctggaagagctcgacctcaccgccaaccgcctctcctccgtcgacccccgcataggccgcctcaccggactccgcaagctctccttccgcc
agaacctcctcgacgacgccgcagtcgcgccgctgtcgtcatgggaaacaattgctggtctgcaggagttggtccttcgagataacaaacttacaagaa
ttcctgatgccagcatcttcaagggtcttcttgtgttcgatgtatctttcaatgagatatcttccttgagtgggctatccaaggtttccagcacactgaaaga
actttatgtttcaaaaaatgaagttggaaagatggaagaacttgaacatttccatgcactagaaattcttgaacttggcagcaacagattaagggagttg
gtccttcgagataacaaacttacaagaattcctgatgccagcatcttcaagggtcttcttgtgttcgatgtatctttcaatgagatatcttccttgagtgggc
tatccaaggtttccagcacactgaaagaactttatgtttcaaaaaatgaagttggaaagatggaagaacttgaacatttccatgcgctagaaattcttga
acttggcagcaacagattaagggactgtattgcattagaagaactgtacctcagccataatggaatccaaaagatggaaggcctctctaccttgcagaa
cctccgtgttttagatgtttcatctaacaagataacagctatagaaaatattgaaagcctaaccaggttggaggacctgtggttgaatgacaaccaaata
ccatcacttgatggaatagaggctgcagtagctggttcccggcagaatctgacaacaatttatcttgagcgcaatccttgtgcaaaaactccaaactact
cagcaacattgaagagaatgtttccaaatcttgagcaaattgattcagatatcatagcatga
>g101040.t1
atggccgccaacctcctcctcctgtatctcctggccgccgtggcgtccatcgccggcgccgccgaggtgaacgcagtcctcgccttcaagcgcgcccttg
ttacaatcccgcccgaggccgcgggcttcttcgcgacgtggcacgacggggcgacatccccctgcaacttcaccggcctcgcgtgcgactcgcgcggcc
gcgtcaccgccgtctccgtgcgcggccagggcgtcgccacggattccgtgcccttcgacaaaatatgtgcctcgctcccggccctcaccgcgttggccct
gccggagaacgcgctcgccggcgacatcgccggcgtggccttgtgcaccaatctccatgagctcaccctgtcgttcaacggattctccggacccgtgcc
gaatctctctccgctgaagaatctgcgaattctgaacatttcatctaccagcctcgccggcccgttcccgtggaaatctctgctctccatgcgggacctcac
cgtcctcgccgtcggcgacaacccgttcctcgacccgacttccacgttccccgacgaggtgaccaagctcaccaacctcaccgcgctgtacatgtcggc
ggccaacatcggcggcgcgatcccgccgtcgatcggcgacctggtcaacctcgtcgacctcgagctcgccgacaacaacctcgtcggcgcgatcccac
cggagatcacgaagctgaccaacctccgaggccttcagctgtacaacaactcactcacgggccagctccccgccggcttcggcaacctcaagaagctc

cagtacttggacgcgtccatgaacaacctcaccggcagcctgttcgagctgcggtccctgacgcagctcgtgtccctgtacctcttcttcaacggcttcac
gggccacgtgccccaagaatggggggaattcaaggagctcgtggccatcttcctgtacagcaataacctcaccggcgagctgccgcagagcctcggg
agctgggcgcagttcagctccattgacgtctccacgaacgctctgtcggggcccatcccgccggacatgtgcaagcagggcactttgctcaagctccta
atgcgcgacaacaacttctccggcgagattccggcgacgtacgcgagctgcaagacgctgcagaggttccgcgtcagcaagaacaacctctccggcg
acgtgccagaggaactatgggcgctgcctaacctgactctgttagacctcgcgggtaaccagttcaccggcggtatcggcgacggcgtcgcgagagcc
gcggcgctcaccgaactctacctggccgggaacgggttcaccggcgtcataccgccatcgattggcgccgtgtcgaatctcgaggtcatcgacgtgtca
tcgaacgagctctccggggccatcccgacgagcatcgcctcctgctccaaacttagaacggccaaatttgccgggaacaagctgtccggtgtcatccca
taa
>g101041.t1
atgccacccgccgcccggcacctcctcctcctcctctacctcctggccgccgtggcgtccaccgccggcgccgccgaggtggacgccctcctcgccttca
agcgcgccctcaccaccatcccgccggatgccgcgggcttcttcgccacgtgggacggcgccgcggcctcccccctgcaagttcaccggcgtcgcctgcg
acccgcgcggccgcgtcaccgccgtctccgtgcgcgtccagggcgtcgcttcgaaatccgtgcccttcgacgacatctgctcctcgctgccggccctcac
cacgctcgccctgccggagaacgcgctcgccggagacatcgccggcgtggtcgcctgcaccaatctccaggagctcaccctatcgttcaacggattcac
cggacccgtgccggacttttctccgttgaagaacctgcgaattctgaacatctcatcgaccagcctcgccggcgcgttcccatggaaatctctgctctcca
tgccgcacctcaccgtcctctcgctcggcgacaacccgttcctcaagccgacgcccgcgttccccgacgaggtcaccaagctcaccaacctcaccgcgc
tgtacatgtctgccgcgaacatcggcggcgcgatcccggcggcgatcggcgacctcaccaacctcgtcgacctcgagctctccgacaacaacctccac
ggcgcgatcccaccggagatcacgaagctgaccaacctccagtacctcgagctgtacaacaactccctcaccggcgcgctccccgctgggttcggcca
gctgacgaagctgcagtacctggacgcgtccatgaacaagctcacgggaagcctcgccgaggtgcgtaccctgacgcgtctcgtctccctgcagctctt
ctacaacgggttcacggggcacgtcccgccggagctaggcgagttcagggacctggtgaacctgtcgctgtacaacaacaacctcaccggcgagctgc
cacagagcctcggccgctgggcccagttcaacttcatcgacgtgtccaccaacagtctcaccgggcccatcccgccggacatgtgcaagcagggcacc
atgctgaagctgctcatcctcgagaacaaattctccggcgagatcccggcgagctacgcgagctgcaccacgctgcagcggttcagggtcagcaagaa
caacctctccggcgcggtgcccgaggggatctgggcgctgccggcggtgaacgtgatcgacctcgaggggaaccggttcagtggcgggatcggcgac
ggggtcgggaaagccaaggcgctgacgatcctgcgtttggcggggaaccatttcactggtgccataccggcagcgatcggcggcgcgtcgaacctcg
aggtcgtcgacgtgtccaacaatggtctcaccggagaggtaccggagagcgtcggcaagctgtcccgtctgggcagcctcgacatttcgcagaattcca
tggtcgggcccatcccggctagcatcggcgcctgctccgcgctcagcaccgtcaatttcgcgcggaacaagattgatggggtgatcccggacgagttgg
gcgatctgccgcggctcaattcgctggatttgtcggggaacaggctctccggcggcgtgccggccagcctcgccggcctcaagctcagcttcctcaacct
gtcggataataagctcgtgggtcccgtgccggagccgctcgccatcgcggcgtacggcgagagcttcctgggcaaccccgggctctgcgccaccaatg
gacacttcctccgccggtgctcgccgacgggcaccgacggcgcgaacaaggcgcgcacggtcgtcacctgcgtgctggccgccatggccgtgctcctc
gcgttgctcggcgcggtcatgtacctcaacaagaagaagaaggctgctaataagcttggcggtgttggcggcaaggtgttgtggaacaagaaggggtc
gtgggatcttaagtcgttccgcgtgctggcgttcgacgagcgcgacatcgttgccggcgtgcgcgacgagaacctcatcggcagcggcgggtccggga
acgtgtaccgcgtcaagctcggcgacggcgccgtcgtcgccgtcaagcacgtcacgcgccacgccgcgccggcggcgacctcgaccaccgcggcgag
cgccaccatgctgccacggtcgaaatcgaccaccaataagacgtcggcgcggtggtgccgggagttcgacgcggaggtggggacgctgagcaggatc
cgtcacgtgaacgtggtgaagctgctgtgcagcatcaccagcgacgacggcgcggcgagcctgctggtgtacgagcacctccccaacggcagcctcta
tgaccgcctccactcggacgggctcggactgggcctgagctgggccgaccgccacgacatcgccgtcggcgcggcccgcgggctcgagtacctccacc
acgggtgcgaccggcccatcctccaccgcgacgtcaagtccagcaacatcctcctcgacgaggcgttcaagccccgcatcgcagactttgggctcgcc
aagatcatcaatagctcctcctcctccggtgccggtaacggtaacgactcctcctccgccggcgtcgtcgcgggaacgctagggtacatggcgccagag
tacgcgtacacgtggaaggtgacggagaagagcgacgtgtacagcttcggcgtggtactgctggagctggtgacggggcggcggccgatccaggcgg
aggacggcggcggcggcaataaggacctcgtcgactgggtggcgcggcgcctcgacaccagagacaaggtcgtctccctcatcgacgagcggatcac
ggaggactgggcgaaggaggaggcggtcagggtgctgcgggtcgccgtgctctgcaccagccgcgcgccgtcgatgaggccttccatgcgcaccgtc
gtgcagatgctcgaggacgccgccgtcggcagggagtacactgctgctgcactcgcgcaggcagggaagctactccaggtcaaggtcgtcacctaa
>g101042.t1
atggtggtcactctttcttcttggttgaataatcatccgccggcgttgctgtggtgtttcgtcgtcatcctcctgttggcaggagccgtcgcaagtgatgggg
cgttggtggtggacacgcaggcggcgtacctgtccaagatgaaggagcagttcgccgggccgggcatgtcgcagtgggacttctcatcatcgccaacc
tactactgcacgttccccggcgtcgcgtgcgaccacggcggcaacgtgacgggcatcgacgtgagctcatggcgcctcgtcggccgcctccctccatcc
atctgcgacgccatgccggctctccgcgagctcaacctcgcatacaacgacatccgcggcggcttccctctgggcgtcctcaactgctcctctctagagg
tcctgaacgtgagcttctccggcgtgtccggcaccgtgccgccggacctatccaagatgcaatccctccgcgtcctcgacatgtccaacaacctcttctcc
ggcgcgttcccggtggcctccatcggcaacctgacgacgctggaggtggtgaacatgaacgagaaccccgggttcgacgggtggtacccgtcctcctc
gacgttcgcgccgcttcgccgcctccgcgtcctcatcctctccaccacgtcgatgcgcggcggcgttccaccatggctcggcaacatgacctcgctcacc
gacctcgagctcagcggcaacgccctcaccggcgtgatccccgcctccctcggcaagctcacgaacctgcagctcctcgagctctactacaacaacctt
gacggcaccatcccggaggagctcggcaacctcacccgcctcaccgacatcgacttatcagagaaccggctcgtcggcggtatccccttctccctctgc
gcgctgccggacctccgggttctgcagctctacacgaaccggctcaccggtccgatccctcccgtgctcggcaactccacgcagctctggatcctctcgg
tctaccgcaaccagctcaccggcgagctcccggcggacctcggccggtacgcgccgctgaacgtgatcgaggtctccgagaaccagctcaccggcccg
ctcccgcccttcgcgtgcgccggcggcaacctccagtacatcctcgtcctcagcaacctcctcacgggggagatcccttctaggtacgcggattgcacga
cgctgctgaggttcagggtgagcaacaaccatcttgaaggcgaggtccccgcggggatcttcggtcttccccacgcctccatcgtcgacctctcctacaa
ccacctcaccggcccggtccccaagaccgtctccggcgccggtaacctgacgtcactcttcgcgtcgaataaccagatgacggggcccatcccggcgg
agatcgccgccgcgtcggcgctggtgaagatcgacctcagcaacaatgggttgagcgggcccatcccgagtgaactgggacggtacctcacgcggct
gaaccagctgtcgctgcagggaaacaggctgaacggcaccatcccggagtcgctggccgggttgcgcagtctcaacgtgctcaacctgtccgataacg
cgctctatggggccatcccggactcgctctgcaagttgttgcctaattcgttggatttctcgaacaacaacctgtcgggcccggtgccgctgccgctgatc
aaggaagggttgctggagagcgtggcggggaacccgggcctgtgcgtcgcgttccggctcaacctgaccgacccggccctgccgctgtgtcccagatc

gggaagcctccggcgcgggctcgccgggaacgcatgggtggtggcggcgtgcgcgctggtgtgcgtcgccgcggcgctcgtgctggtcaggaggtgg
ttgatgaaggcgaggtgcgacggagaccaggacggcggcgcggcggcgggggctcgccggcgtcgagctcgtcctacgacgtcaccagcttccac
aagctcactttcgaccagcacgagatcgtcgaggcgctcatcgacaagaacatcgtcgggcacggagggtccggcaccgtgtacaagatcgagctca
gcaccggcgagctcgtcgccgtcaagaagctctgggtctccacttccgccgcgcgaggacgacgactccaccctaataataaccatcatcatcatcaca
ccaacaagaacaacagcacgacgacgaagacgacgtggctgggcgaccgggagctgcggacggaggtggagacgctggggagcatacggcacaa
gaacatcgtcaagctctactgctgctacgccggcgcggacagcaacctgctcgtgtacgagtacatgcccaacggcaacctatgggaggcgctgcacg
ggtgctacctgctgctggactggcccaccaggcacagggtggccatgggcgtcgctcaggggctcgcgtacctccaccatgacctcatgttccccatcgt
gcaccgcgacatcaagtcgtccaacatcctcctcgacgccgatttcgagcccaaggtcgcagatttcggcatcgcaaaggtgctccatcaggccaccac
cgccgcaaagggaggaggagacgcgtccacaaccaccatcgcgggaacatacggatacctcgcaccagagtacgcctactcgtccaaggcgacgac
caagtgcgacgtgtacagcttcggcgtggtgctcatggagctggccacggggaggaagcccatcgagccagagttcggcgacaccagggacatcgtg
cactgggtctccggcaaggtggccgccggagccgaggccgaagcgctcgacaagcgactcgcgtggagcccgtacaaggaggagatggtgcaggcg
ctgcgcgtcgccgtccggtgcacctgcagcatcccgggactccgacgcaccatggccgacgtcgtacagatgctcgccgaggcagggccacggacca
ccatcaccaataaggacaacaataaggacaactctgctgcacaacagctgctgctcatgcccaaaccagacaccaccactccttag
>g101634.t1
atggcggcgccgcggtacgcgctcctgccgttcctcctgctgctgctgcgcccgaccgcgcgcgtgctcgccaacatggaaggtgacgcgttgcatagc
ttaaggaccaacttaaatgatcctaataatgttctacaaagttgggatccgactctggtcaatccgtgcacttggtttcatgttacctgcaataatgacaac
agtgttatcagagttgatcttggaaatgctgcactatcaggaactttggtcccgcaacttggccagcttaagaacttgcagtacctgcgtctcaacaacaa
cagcctttcgggttcaattccaaaatcattaactgctatcactgccctccaagttctggatctgtcaaataacaatttatctggagaagttccatcaactgg
ttctttttccttgttcacccctatcagctttgcaaacaacccaaacttatgtggccctgggaccacaaaaccttgccctggtgctccccctttctccgcctc
caccgtacaatccgccaactcctgtgcaatcaccaggaaaatcctctgagaaaactgatgtatttggttatggaattatgcttttggagcttattacagga
caacgagcctttgatcttgctcgccttgccaatgatgacgatgtcatgctgcttgactgggtcaaggggctacttaaggagaagagactggagatgctgg
ttgatccagatctacagaataactacattgacattgaggtggaatcgctgatccaggtcgcgctcctttgcacacagggctcccccactgaccggcctaa
gatggcggaagtggttcggatgctggaaggtgatggtctcgcagagagatgggaggagtggcagaagggccgagatgatgacattgcactcacgacc
ttcttctga
>g102613.t1
atggcttggataggaagggcgaggggcttgtcgggtggggctcaggcgacgccgcctgctgttcctggaccggcgtcacctgcgatcttgggagggtg
ggtggtcgggttggatctctacaacaagagcctacttggcggcatctcctcctccgtcgcctacctcgatggccttgtgacgctaaacctctccggcaact
cccttcgcggctcgccgccggcggagctcggcagcctggtgaggctgcgggtgctcgacctcagcgggaacgggctctacggctcgttcgcggcaagc
gacggcggcttcccggcgatcgaggtggtcaacatctcctccaacaatttcaccggcccccacccttcgttccccggcgcggcgaacctaacggttcttg
atatctccggcaacagctttaccggtggcatcaacgcctctgcgctctgtgttgcgccggtcgaaattctgcggttctcggagaatgcgttctccggtgaa
gttcccggtggctttggcctgtgcaaggcgctggttgagctctatctcgacggcaatggcttaactgggaacctacctagggacctgtacacgatgccga
agttgacgaggctcagcttacaggacaatcagctctccggcaacctcggcaatgggcttggtaacctctctcagcttgtgcagcttgacttatcctacaac
aggttcaccggtcccatccctgatgtgtttggaggtatgaggaggctggagtccataaacttggccacaaataggttctttggtgaattgcctccttccttg
tcaagttgcccaacgctgagggtaatcagtctacggaataacacactgtttggtgcgattgatatcaacttcgacatgctgccaaggctgagcacttttga
tgctggaagcaacaatttgactggtgatatacctcctggctttgcatcgtgcactgagttgaggacgctgaaccttgcaaggaacaagcttggtggcaag
ataccagagaccttcaaggatttgaggtccctgtcgtacctctcactaacagggaatggcttcactgatctgtcatcagcattacaaatcttgcagcacct
gcccaacctggcgagcttggtgctcaccaggaatttccgtggtggtgagacaatgccagtggatggcatcaatgggttcaagagcatccagtggggag
cttcctga
>g103167.t1
atggccgccgccgcccgcctcgtcctcgccctcgccctcgtgggcctcctcctcgccgcggccgcggctcccactggcgcgctgaccgacgacgtgctc
gcgctggtggtcttcaagacgggcgtctcggacccgtcgggccgcctcgccgcgtggacggaggacgacgaccgcccctgcgcgtggcctggcgtcgg
ctgcgacccgcgcacgggccgcgtcacctcgctctccctccccgactcgtcgctctccggtcgcctcccccgcgcgctgctccgcctagacgcgctcgcc
tccctctccctcccccgcaacaacctctcgggccccgtgctccccaacctcctcgccgcgctcccgcgcctccgctccctcgacctctcctccaaccgcctc
gccgcccccgtccccgacgagctcttcgcgcaatgccgctccgtccgcgcaatctccctcgcccacaaccagttctcgggctacatcccgtccgccgtcg
catcctgctcctccctcgtgtcactcaacctctcctccaaccgcctcgccgggcccattcccgacgggctctggtcgctgccctcgctccggtcgctcgatc
tctccggcaacgagctctccggaagcgtgcctggcgggttccccaggggcagctcgctgcgggagttggacttgagccgtaacctcctcgcctgggaga
taccggcggacatcggccaggcggcgctgcttaagtccctcgacttgggccacaatttgttcaccggtttcctgccggactcgctccggaggcttaccgg
tctgcggttcctcaacgccggcggcaatgcgctcacgggggacctgcccgcgtggatcggggaaatgtgggcgctcgagcggctcgacttgtcgggca
accgcttcacgggcgccatcccctacaccgtcgcgaactgcaagaacctggtggatgttgacatcagccgcaacgcgctcaccggcgagctcccgtgg
tgggtgttcggcctgccgctgcagcgcgtctcggtcgccgggaacaagctggacgggtgggtcaaggtgcccgacgacgcggcaatagcgctacggg
tgctggacctgtcgagcaacgggttctccggcgagatcccgccgcgcatcactgccttcgccggtttgcagtctctgaatctgtcctcgaattccttctcgg
ggcgtctaccggccggcattggcgggatgaggctgctccaggtgctcgacgtcagttccaaccggctcgacggagccgtgccgccagagattgggggc
gcggtggcgctccgggacctgcggatggggaggaattcgctcaccggacgcatcccggcccagattgggaattgcagctccctcattgccctggatttg
tcgcacaatggcctcacaggttcaattcctcacaccatgggcaacctgaccagcctccaagtggtgaatctttctcagaacaagctgaatggtacattac
cggtggagctatctaatctgcccagccttcacatctttgacgtctcccacaacatgctgtcaggggacctgccaaatagccgcttcttcaaccacatccct
gagcccttcattgcagaaaatgctggtctctgtagctcgcggaggaacgattcatgcagtgcagttatgccgaagcccattgtgctcaaccccaactcct
cgtccaacccctcttcgctggacactcccacggcccctagcaacatgcatcacaagaaaatcatactgagcatatccactctcattgccattgccggtgct
gctgctattgcagttggagtgatcacaatatctgtgctcaaccgccgtgtacgtgcccgggcatcagctccccgctcagctcctgctgttgcactatctgat
gattattttagccaatccccagagaatgatgctacctctgggaagcttgttatgtttggtaaaggcagccctgagttcagcgccggtgggcatgccttgtt

gaataaggattgcgagctcggacgtggaggctttggtgccgtctacaagacagtgctgagagatggacagccggtagccatcaagaagctcactgtct
caagcttggtcaagtctaaggatgattttgagcgacaagtcaagttacttagcaaggtgaggcaccacaatgttgtctcactcagaggattctactggac
ttcatcactgcagcttcttatctatgattacctgcctgggggaatttgcataagcacctgcatgagtgcactgaagataactcactctcttggatggaga
ggtttgacatcatcctcggtgttgcaagggggctgacatacctgcaccagcatggtatcgtccactacaatctcaagtccagcaatgtgctgttggacag
caatggtgagcccagggttggtgactatggtcttgccaagctgctgccaatgctagaccgctatgtcctaagcagtaagattcagagtgcacttgggtac
atggcgccagaatttgcatgcaagacagtgaagattactgataagtgcgatgtatatggctttggggtccttgtgcttgaggtcttgacaggtagaagac
ctgttgagtacttggaagatgatgttgtggtgctgtgtgatttggtcaggagcgctctggaggaaggcagactggaggattgcatggatccacgattatg
tggtgagttccccatggacgaggccctgtcgatcatcaagttgggcctcgtgtgcacctcacaggtgccgtccaaccggcctgacatgggcgaggcggt
gaacatacttgagctggtgagaagtccacaagatagtgctgatgatgagctggtttga
>g103963.t1
atggcgcggcagcggacggcgaccatcggcccccgcctggtgctatcaatggtggtctccctcctcgccgccgcgcttcccacggccgtggcgcagaa
cctggcgtccgaccgcgcggcgctgctggccctccgcgacgcgctggacccgggccgccgcctcccgtgggaccccgcggcgccaacaccatgtaac
aactggcgcggcgtcacctgcgacgcctccggcaaccgcgtcgtggagctccgtcttccggccaagtccctgaggggcacgataccatcgggcacggt
gggcaacctcaccgcgttacagaagctgtccctccggttcaacgccgtctccggcgcgatccccgccgacgtcggcaacctcgccgagctgagaggtct
cttcctccgtgggaacctcctcgacggcgagatccccgaggggctgttctcgctcgcgcggctcagaaggctcgacctcgccgggaaccggctcgccgg
cggcgtgtcgttggggttcaaccggctcacgcggctcgacacgctgttgctcgaggggaaccagctgcaaggagagatcccggaggggttcaacgtga
gcggcctcgcgcagaacggtttcaacgtgtcgttcaacgcggggctcgacggcgccgtgccggcctcgctcgccgggatgccggcgaccgcgttcctc
ggcacggggctctgcggcggcccgctcgccgcgtgcgccgcgccagacggcgagaagaagaagaagaggctgtcccggtgggcgatcgtcgggatc
atcgtcggcgcggtgctcctgatcctcctcgtcatggcgatcctcgccttaatccgctggcggcgccgccgcgcgccgccgacgtcgtcgtccgccgcgg
cggccgacgttcactcgggcaccgcgccgatcacggtcaccgttgccaggacccaccgggacgcgtccgtctcgcactcgcactcgttctccggcgcct
cgcctccggcggcgaagatcgccggcgagggcggcgagaagcagcagcagcagctggtgttcctgggcggcgcgccggaccggccctacgacctgg
acacgctgctgcgcgcgtcggccgaggtgctcagcaagggcgcgcacgggaccacgtaccgcgccacgctcgacggcggcgcgcccgtgctcgccgt
caagcgcctccgcgacgtgcgcgtccccgagcaagagttccgggagagggcggcggaggtcggggccatgcggcacgacaacctgcccaggctgag
ggcctacttctacggcagggaggagaagctgctcgtctacgacttcgtcggcaacggcgccagcctctcagccctcctccacggcaacggcgcgccgct
ggacttcacgtcccgggcgtgcatcgcgctggcggcggcgcgcggcgtggcgtacatccaccgcgtggggtccacccacggcaacatcaagtcgtcca
acatcgtggtcaccgccgggcgcgacggcgccttcgtcaccggccacggcgtcgccaagctcgtcggcgccgcctcctcatcgcggcgcggcaccggg
taccgcgcgccggagctctcctccgccggcgcctccgtgcctcttcggaagagcgccgacgtgtacagcttcggcgtggtggtgctggagctgctgagc
gggcgggccccggggccggacggcggcggcggcgtggacctgccgcgatgggtgcggtcggtggtgcaggaggagtggacggccgaggtgttcga
cgccgccatcgccggcgaggcccgcgtcgaggacgagatgatgcggatgctgcggctcggggtggagtgcaccgagcagcggcccgaccggcggcc
ggccatggacgacgtggaggcgcgggtcgagcgcgtcgtcgaggacgccgcccggaaggccgagttcagcagcaccgacggcagccggagcgtgt
cggcgtga
>g104181.t1
atggccgtcaaacattcggccattctgctcctcctcctccacctggcactctccttcgacctcgcgtcgacggcgctcaggccgcggcggcggcaggtcct
gctgcaggagagggccactctgctggccctcaagcgcgggctcacgctgccttctgcatccgccctggccgactggaacgagtccaacggccacgtct
gcagcttcacgggcgtcacctgcgaccggcggcgagagcacgtcgtcgggctcgccctcgccaacatgagcatctcgggcgccatcccggcggtcgtc
ggcgacctcgtgcgcctccggagcctcgacgtgtccaacaacagcatctccggggccatgccctcgtccctcggcaacctgacgcgcctcgagagcctc
gtcgtgcgcagcaaccgcatctccggcaccatccctctcgtcctcggcaacctcctgcgcctccagatcctcgacgtcgccaacaacctgatctccggcg
ccacgccgtctgtccttggcgacctctccaatcttcagaacctcgacgtgtccaacaacaatatctccggaaacatccctccgtcgatcggcaacctcac
gcgcctcgagaacctctacatgttcgacaaccgcatctccggagccatccctcggtccatcggcaacctctcgagcctgcagaacctcgacgtgtccgg
caacctcctctcggggcagattccagccgagctctccaacctccgcagcctcggcacgcttgatctcggcaacaatcagcttcgcggcggcataccgcc
gtcccttgccaagctgacggccatgtactacttcagccttcagcataactacttgtcaggcctcatccccgacgccatcttcctcaactgcaccattttggg
ggtcgtcgacctcggcgacaacgacctgtccggcgagatcccccgcgccgcctcgggcaccctcgccgacacttttgccgtcctcaacctctactccaac
aagctaaccggcacgctcccacggtggctggctaactgcacgattctactgatgctggacgtggagaacaactcgctcgccgacgagctcccgaccag
catcatctccggcaaacagtacctcaagtacctgcatttgtccaacaaccggttctggagccacgacggcaacaccaacatggagccgttcttcgccac
gctgtccaactgctcgcggatccaggaggtggaggccggcgccgtggggatgggcgggcggctgccgagccgcctcggcgcgatgctcccgcctaac
atgtggcacctcaacctggagatgaactccatcgtgggccccatcccggcggacatcggcgacgtgatcaacatgacgctgatgaacttgtcgagcaa
catgctgaacgggaccgtcccggcgtccctctgccggcttcccggactgcagcagctcagcctgtccaacaacaccctgacgggcgagatcccgccctg
catcggcaacgccacgcgcctcggcgagctggacctgtcgggcaacgcgctgtcgggaagcattcccagcagcgtcgggagcctcacccagctcacct
acctctccctgcagaggaacaggctgtcgggggagataccggcgagcctcggccggtgcgccagcctcatgcgcctcgacctctccagtaaccagctg
agtggggaggtaccggagcaggtggccggcattgtgaagatgacactgaacctgtcgcggaaccagctcgggggacagcttcccagaagcctcagca
agatgcagcaggtgcagacgatcgatctgtcatggaacaacttctccggcgcgatcttcgccggcatcgcggactgcgtcgagctgacggtgctcgacc
tatcgcacaactctctcgccggcgccctgccttcttccattagcggcctgcagagcctggagagtcttgacgtctccaacaacgcgctgactggcgagat
ccccgcgagcctgaccaaatgcaccggcttgaagcacctcaacctgtcgtacaacgacttcagtggcgtcgtgccggacaccggcgcgctggcgcggt
tcagcgtcctctcctacctcggcaaccggcggctctgcggtccggtggtccagcgcgcctgcaacgaaaggcgccaccatcgctcatggttccagtccc
ggaagttcctggtcatcctgtgcctctgcgccgcggccgtggcgttcgcgctgaccatcctctgcgcggtcagcttccgcaagatccgggaacggctggg
ggcggaggtgcgcgaggacaacatgttcttcatgatgggccgcctccgcggcggcggcggcggcggcgcgtcgccggtggcgatgaagtacaagtac
ccgcgcatcacgtaccgggagctggtggaggcgacggacgagttcagcgcggaccggctggtggggacgggcagctacgggcgggtgtaccgcggc
acgctgcgcgacggcaccatggtggccgtgaaggtgctgcagctgcagacggggaactccaccaagagcttcaaccgcgagtgccaggtgctcaagc
gcatccggcaccgcaacctcatgcgcatcgtcaccgcctgcagcctgccggacttcaaggcgctcgtgctgcccttcatggccaacggcagcctcgagc

gctgcctctacgccggcgccggcggggcggagctcagcctgctgcagtgggtcaacatctgcagcgacatcgccgaggggatgtcgtacctgcaccac
cactcgcccgtcaaggtcatccactgcgacctcaagcccagcaacgtcctcatcaacgacgacatgaccgcgcttgtgtcagatttcggcatctctcggc
tggtcatgagcgtcggcggcgtggtcaacaccgccgccgatgtcggcgcctccacggccaacatgctctgcggatccatcgggtacatccctccagagt
acggctacggctcaaacccgacgacgaagggagacgtgtacagcttcggcgtgctggtgctggagatggtgacgaggaggaagccgaccgacgaca
tgttcgaggccgggatgagcctgcacaggtgggtcaaggcccaccaccacggccgcgccgacgccgtggtcgacccggcgctggcgcggatggtgca
ggaccagacgccggagatcaggaggatgtcggacgtggccatcagcgagctgctggagctcggcatcctctgcacccaggaccacgcggcggcgcg
gcccaccatgttggacgccgccgacgacctggaccgcctcaagcggtacatgggcggcgagaccacggccaccttcgcgtcttcgctcggcttctcctc
cgcgacatttgaagacgatattgactggcgtaacaattaa
>g104414.t1
atggcatccttcctcctcctcctcctcctgctggttgcgtccgccgcctctccggcgctgtcccacccgccgccgtcgtgcaacgcggccgacctccacgc
gctgctggccgtgaagcagtcgctgggcaacccgaagacgctgtcgacgtggtccccctcctcccccgactgctgcaagtgggaccacgtccggtgcg
acgacgcgtccgggcgcgtgaacaacgtcttcatcgacggcgcggacgacgtgcgtggccagatcccgtcggccgtggcgggtctgacctcgctcatg
tcgctctcgctcttccgcctgccgggcctctccggcaccatcccgccctgcctcgccacgctccccaacctccagttcctcaccctctcccacaccaacgtg
tccggccccatcccggaccacctggcccggatccgcaccctcgactccgtcgacctctccaacaaccgcctcacgggctgcatcccggcagccttcgcc
gacctgcccaacctccgctccctcgacctccgccacaaccagctcacggggcccatcccggcgggcctcgtgcagggccagttccggtccctcatcctct
cctacaaccagctgtccggcccgatcccgcgcgacgacgcgcaggacgagatcaacaccgtcgacctgtcgcacaaccggctcaccggcgacgcctc
gcacatgttcgtccccggcaggcccatcgggaaggtggacctgtcgtggaacgacctcgacttcgacctcagcaaggccgtcttcccgcccgagctcac
gtacctggacctctcccacaaccgcatccacggcaccgtgccgccgtcgctggggaggctgtccacgctgcagaagctcgacctcagctacaaccgcct
gtgcgggccgctgcccaggttccacggcgtcatcaagcacggctgccaggcgtacgagcacaaccagtgcctgcgcggggcgcccatcgagacgtgc
caccggtactcgtag
>g104514.t1
atgagcacggccttaccgctcctgctcctgctgtccgcctcctcgtccatgtcgacggtccagggcgtcgaggctaacgacgcggcggcgcttcttgcgtt
caaggcagcagccgttggcgacggcggccacaggcttctttcgtggaatgccagcattaccggtgggttctgcagctgggagggggtgacgtgccagg
gcaagcccaggcgagtggtggcgttgagcctctcctcccacgggctcaacggcgtcctctcgccggcgataggcaacatgtcatcgctcaaggtactca
acctgggatccaacatgttgagcggcgccatacctgcgagccttggccgtctgcaacatctcgagaccctcaacctcagcagcaactcgttatccggtg
acatccctaccaacttgagctcctgcactagtctgaccacaattgaactccagtcgaaccagctctacgggcgccttcccccggagcttggcaacaagct
gacgcgccttgagtcgaaccagctctacgggcgccttcccccggagcttggcaacaagctgacgcgccttgaggtgctcaaactgtggaagaacaacc
ttaccggcaccatcccaacgtcattggccaacttgtcttcgcttagttttgtgtccctcgcattcaaccagtttgagggcaccatcccctttggcctcggcgg
cattgcgggccttcgacacctcgatctcgcctacaaccatatttctgaatggagaaggcagggacagattgaagcatgcattcgatgccaacaaggcat
cagggagaacatcggggagggggaagtcaccgtagagagcagccaccaagacgccgacgatgtgggcgatagggactatccatcaccaaagctca
caacaagacagcaacaccacattatccaacgagagacggaaatgagagcaccccacctctctcccaacccgccgccgacggccaatccggctccggc
gcccacagcaaggatccccagccccgggcccagtctctcaccactcgccacacctttccacccctcctccctgggtcgctccaagcacgaacgctggatt
agcagcccttcctcggtttcttcagatggtgagctatcggataggcgtgctccctcggaggtctcgtaccgggatgtggtagtccggagcctatctggtga
gctgtcggaacggcgtgctcctagaacaggctccagtggcggagatgcggcacagatctacagcacatcgaccaaggagaacaagctcagcccagat
ctagcctcgccgctgcagcggacccgcgcccgtgtgcgctcggagatccaaagggtctccgatgcggcactggtggacgacgggtggcgcgtggtga
gcccttactgcctggaggtgtggatagggtcagagctggatacaggaagttcaccaccaaggttgccgcggcgggtagcactgaggaacccagagttg
cagggtctggagatggatcggcttcaggaccagcccagcaacgccggcgcaacagaagacaacgaggtcggcgtggagccggacgccgagattgct
actgaggtggccgcaggattccccgccaccatccccaccaccgtcccctccgtcgggcgcaaccagtga
>g105691.t1
atgaggcggcggccaccacctttcgtcgcccgtgccgccgccgtgttcctgcttcttctgctgtcccccgccccgacctcccagctcggcctcggcgccgc
cattggcgcctggatcaacggcgcgccgccgccttctactgcctctgatcccatcccgtcctcctcctccgccggccagcagcagggtgagcagccgag
cccggagtacgcctcgctgcaggcattgaagtccgccatcaccgatgacccccgcggcgcgctgtcaacctggcacggtcccaacgtctgcgcctaca
agggcgtctactgctccgcctcccccgacgccgacgccgacgccgacgccacggtggtcgtcgccggcgtagacctcaaccggttcaacctccgcggc
accctccccgacgccgcggtggccggcctggcccacctcacgttcctccacctcaacagcaaccgcctctcaggcgccgtgcccaacgccctccgcgac
ctggcctacctcaccgagctcgacctcagcaacaacctcttctccggcgcgttccccacctcccctctcgtcgcgccctccctcgtctacctcgacctccgc
ttcaacgccttctccggcgccctcccgcccgacctcttcaccaaacccaacctggacgccgtcttcctcaacaacaaccagttcgacggcgccattcccg
acacgctctggtcctccccggccaccgtcatcaccctcgccaacaaccgcttcaccggccagctcccctccacctacggctacaccggtggtggcagag
tccgggaggcgctgttcctcaacaacaacctgaccgggtgcgtgccggaggcgctgggcttcatgcccagcatccaggtgctcgatttaagctacaacg
cgctcaccggccacctccccggcacaatctcctgcctgtccgggatcgaggtgctcaacgtcgcgcacaaccagctcacgggggagctcccggacctg
ctctgcgacctgcgccggatcaccaacctctccgtcgcgttcaacttcttctccgggatcagccagcgctgcgaccgcctcgccggccgcagcatgttcg
actttgtgggcaactgcgtgcccggaagggacatgcagcggccgcagccggagtgtgacgggttcggggggtgatggcgggctgagctgcctccgtatc
cccggggcgaggccggttgggtgcgccgaggcggcggtgtcggttggcgttggcgttggagtcagtgttgggggactgccgttcgggttgccaggcgc
gtcggccggcggcgtggtcaccgtctccgtgccttga
>g106584.t1
atgcaggggaaagggaaagaggaacagaggcatagcgaaagacagagcgtgtgggaagcagaggacgacgcagacgccgcgagcgcatgtggtg
gccgcgagctgccggagatttttcttcttcttccgcgttgggccgggttcgtcttccatgattcgggaaacgtggccatgtctcccttcctcatcggattcct
cagcctactcctcttcgcaggtgaggtggtggtggtgatcacagcgcagaacggcagcggcagcgacaaggatgttctcatcgacctcaagcggttcct
tcaggagaccaacaaggtgaaccgcggcgcctacgacgcgtggcctgagtccgacgcgtccccgtgcacatggcacggcgtgcgttgcgacgccacc
ggccgcgtgacgtcgctggacctctccggcgcgagcatctccggcccggccttcggcaacttctcccgcctcccggcgctcacccggctcgacctctccg

gcaacacgctccacaccgccggcgacgtcaaccaatgccgcggcctcacgcacctcaacctctcccgcaacctcatcgcggggccgctcgacgtgtcc
cgcctgacccggctgcgcatgctcgacgtgtcgggcaaccggctcgagggcgacgtctcggtaaatttcccggagatatgcgccgacctcgccgtgctc
aacgtgtccaccaacaggctaaccgggaacatcaccggactgtttgacggctgccacaagctcgagtacgttgacctcagctcgaataacttcaccggc
gagctgtggcccggcgtcgcgaggttcagggagttcagcggcgccgaaaacaagctcaccgggagcgtcccgccggacatgttccccgacggttgca
agctggagtctctggacctttcgtcaaaccggttcgtcggaaatttcccgggttccatcgccaagtgcgggaacttgacgtacctgtcgctgtgggggaa
tagcttcactggcatgataccggccgggatcggggagctcgcggcgattgagacgctgattctcgggaagaatcagttcaacagagaaataccgccgg
accttaccaactgctcgaggcttcagttcttggacgtcagcaccaattcgttcggaggggacgtgcaggaaatcttcggcaagtttgtgagcttgaggta
cctcgtgctgcaccacaacggctacaccggcgggatcgtcgcctcgggcgtgctccggctaccgctgctggccaggctggacctcagcttcaacaagtt
ctcgggagagctccctccagaggtggccgacatgaagtctctcaagtacctgatgctcgcctggaacaatttctccggtgccataccggcagcgtacgg
ccggctcagcgagctccaggcgctggacctgtcgtacaacaagctttccggtgagataccggcgagcgtgggcaacctcacgtcgctcctgtggctgat
gctcgccggcaaccagctgtccggcgagataccgccggagatcggcacctgcagcagcctgctgtggctgaacctggcggacaaccagctgagcggg
agcatcccgcccgagctggcggcgatcgggcgggacccggggccgacgttcgccaagaaccgcaacgacacgagcgtgctggccgggtccggcgag
tgccaggccatgaagcggtggatcccggccagctacccgccattcagcttcgtgtactcggtgatgacccgcgagaactgccgcggcatctgggaccg
catcctcaagggctacggcattgtccccatctgcaccaacacgtcctcgcccgtgcgctccgacaccatctccgggtacgtccagctgtccgggaaccgg
ctgtcgggggagataccggcgcagatcgggtccatgcggaacctcagcctgctccacctcgacggcaaccggctgactgggcggttgccggcagatat
cggcaacctcccgctcgtcgtgctcaacgtgtcgaggaacagcatttccggcgcgattccgtcggagatcggccgcgtccggtgcctcgagatgctgga
cctgtcgttcaacaacttctccggggagctgccggcgagtttgagccagctcacggagctgaaccggttcaacgcgtcgtacaacccgctcctctccggc
cccgtccccactacgggacagttcggcaccttcgacgagcagtccttcctcggcgacccgctcatctcgctgcggcaaggcaccggcaagcaaacgcc
actggaacccgcggaggttcaccgagctgtaaggcgtgacctgtctccgaaaaccatcgcggtgtggttcctcttctccctcctcaccgccttcatcgccg
gcgccctcgtgttcttcctctccaacctgcgcgcgcggttccccgtggaccaggaccccgaccccgagtcgtcgttgtccttctcgtgcgacgcgaaccac
cacaagtccggcaaatgcagcacgttacagatgatgacgacggcgtcgtcggggtcgtcttcgtggtccgcgacggaggaagggtgctgctccgcctc
gtccacggaaggcgtgcgcgtgttccggctggggaagacgacgacgtccttcacctaccgcgacatcgtgtccgccacgcgcaacttcgccgacgacc
tcgtcgtcgggcgcggcggctacggcgtggtgtaccgcggagtgctccccgacggccgcgccgtcgcggtcaagcggctggcgcggcaccaccagcg
cggtgaagacggcgacggcgagcgcgagttccgcgccgagatggaggtgctcgccgacggcggcgggtccactacctcgacggcggcagcctggag
ccgctga
>g106733.t1
atggcgtgggccgcggcggtggtgctggtggttgcggcggcggtgctttcaccggcctccttggcgtcggcgtccaactcggagggcgatgcgctgtac
gcgctgcggcaggcgctcgcagaccccggggcgtgctccagagctgggaccccacgcttgtcaacccctgcacctggttccatgtcacctgcaacgg
cgacggccgcgtcacccgcgtgtga
>g107729.t1
atggtgtcctccatggccgccgcgacaaccacccttctcctccttgccatcgccacccactgcacggccgccgccgactcctcctcatcttcttcttcgccg
gaagcagccgccctcctgaacctctcggccgcactgaccgacccctcgggctacctctccacgcactggacacccgacaccccgttctgctcatggccg
cgcctctcctgcgacgccaacgacgagcacgtcatcgcgctggacctctccggcctgaacctctccggtcccatcccgggcgcggcgctggcctccctc
ccgcacctccaatccctcaacctctccaacaacatcctcaactccaccttcccctccgagctcaccgcctccctcccccgcctccgcgtgctcgacctctac
aacaacaacctcaccggcccgctcccggcggccctccccaacctcacggacctctcccacctccacctcggcggcaacttcttctccggcgccatcccgc
ggtcttacggccggtggtcccgcctccgctacctcgccctctccgggaacgagctcaccggcccggtgcccccgagctcggcaacctcaccacgctgc
gcgagctctacctcggctacttcaacagcttcacgggcgggatcccccggagctcggccggctgcggcagctcgtccgcctcgacatggccagctgcg
gcgtctccggggaagttccccggaggtggccaacctcacctcgctcgacacgctgttccttcaaattaacgctttatcagggcggctccccgcggagat
tggcggcatgggttccctgaaatcgctcgacctgtccaacaacctgttcgtcggggagattcccgcgaccttcgcgtcgctcaacaatctcactctgctca
acctgttccgcaaccgcctcgcgggcgagattccggacttcgtcggcgagctgcccagcctcgaggtgctgcagctgtgggagaacaacttcaccggc
gggattccggcgagcctcggcgtggcggccaccgggctgaggatcgtcgacgtcagcaccaacaggctcacgggcgttctaccgtcggagctgtgcgc
ggggaagcagctgcagacgttcatcgcgctgggcaactcgctgttcggcggcatccccgacgggctcgcggggtgcccatcgctcacgaggatccgcc
tcggcgagaactacctcaacgggaccatcccggccaagctgttcacgctgccgaacctcacgcagattgagctgcacgacaacctgctctccggcgag
ctccggctcgacgacgccaagaaggtgtcgccgtcgatcggcgagctcagcctctacaacaaccggttaactggccacgtgccggacggcataggcg
ggctcgtcgggctgcagaagctggtgctcgccgggaaccggttgtccggcgagctcccgccggagatcggcaagctgcagcagctgtccaaggccga
cctgtccgggaacctgatctccggcgaggtgccgccggcgatcagcgggtgccggctgctcaccttcctcgatttatcgggcaacaagctggaaggga
ggatcccgccggagctcgccgagctccgcatcctcaactacctcaacgtgtcgcacaacgcgctggagggggagatcccgccggccatcgcggggat
gcagagcctcaccgccgtcgacttttccttcaaccgcctcaccggagaaatccccggcacgggccagttcgcctacttcaacgccacctccttcgcgggg
aacccgggcctctgcggcgcgttcctcggcccgtgcgcggacgaccgccacgtggtcgccgccacgtcggggttcgcctccctctcctcctcctccaagc
tcctcctcgtgctgggcctgctcgccctctccatcgcgttcgcggccggggccgcgctcaaggcccggtccatgagccggcggtcggcgtcggcccgcg
gcgcgtggcggctgacggcgttccagcggctcgacttcacggtggacgacgtgctggactgcctcaaggacgagaacgtgatcggcaagggcgggtc
ggggatcgtgtacaagggcgccatccccaacggcgcgggcgcgggggcggtggccgtgaagcggctgccggccatcgggcgggccggcgcggccc
acgacgaccacggcttctcggcggagatccagacgctgggccgcatccgccaccgccacatcgtgcgcctgctgggcttcgccgccaacaaggagac
caacctgctggtgtacgagtacatgcccaacggcagcctcggggaggtgctccacgggaagaagggcggccacctgacgtgggccacgcggtacaa
ggtcgccgtcgaggccgccaagggcctctgctacctccaccacgactgctcgccgcccatcctgcaccgcgacgtcaagtccaacaacatcctcctcga
cgccgacttcgaggcccacgtggccgacttcgggctcgccaagttcctgcgcggcagcaatgccggcggctccgagtgcatgtccgccatcgcaggat
catacggctacattgctcctggtacgttacagtttagtaccatttcgaattttctatgctgctttcagtga
>g107811.t1

atggcgacggagccgcgaatcttgctcctcctcgccgtcgtcgtggtgctcgccgctgatggcggggtcgtcgacgcggcggacccgccgctctcgccc
aagggactcaactacgaagtggcggcgctgatggcggtgaagagccgggtgcgggacgacaaggggctgatggccaagtgggacatcaactccgtc
gacccctgcacctggtccatggtcacctgctcccccgacggtttcgtcgtctcgctacagatggcgaacaacgagctgtcggggacgctgtcgccaagc
attgggaacctcagtcacctgcagacaatgtcactacagaataatagaatatcaggtcaaattcctccagagattgggaagctcacaaatttgaatgctc
ttgatctttctggtaaccagtttgttggtgatatcccaagttcactggggcaactgaccagactaaattatctgcgtcttgataggaataatttgtctggtca
gatcccagtagatgttgcaaagcttccaggtctcacattcctggacaacaactag
>g108307.t1
atggcgagcacggggcggtcgatgctggtgtcgctgctgctcttcgcggtgacgctgtccctgctcgagatgtacaaggccaagtttgcatcgtcggagc
tcatgacaattgtcggcggcctcgtctcctccctcctcttcctgctcctccttacctttattggaaactatcaagaatctaatggtgttagaactgggtgggg
tgcagttgttgtagcagaacttgttgctcttattgtagctggcactgtccaccgtgtctgtatcacaacatgtatcaagtttgcgacagagaggaagagaa
taagtaagaagaagaagaagaagaagaagaggaggaagcgttcgcggccagcttccaaggagaacgtgcttcagcttcgaaggaggacgtgcttcc
gatcgccgtcgcagaggaggatggaggcgccggcgcggcggcgtgtcgtgacgccgccgttgctgttgctggtgttggtggcggcgaaccttcctgcc
ggcctctgcgtcagcaatcctcaagatgtttccgccctccaggctctgatgagcaaatggaagaactacccgtcgagctggtcgggcggcgacccctgc
ggcgcgcagtgggacggcgtcatgtgcaacggcggcagggtgacgtcgcttatcaagtttgcgacagagaggaagagaataagtaagaagaagaag
aagaagaagaagaggaggaagcgttcgcggccagcttccaaggagaacgtgcttcagcttcgaaggaggacgtgcttccgatcgccgtcgcagagg
aggatggaggcgccggcgcggcggcgtgtcgtgacgccgccgttgctgttgctggtgttggtggcggcgaaccttcctgccggcctctgcgtcagcaat
cctcaagatgtttccgccctccaggctctgatgagcaaatggaagaactacccgtcgagctggtcgggcggcgacccctgcggcgcgcagtgggacgg
cgtcatgtgcaacggcggcagggtgacgtcgctgcggctgtcaactgtaaaccttcagggcacgctcagcgacagcgtcggccagctcagccagctcg
tgtacctggacctttcctacaacattggcctcagcggtccggtgcctgcttcgatcggaaatcttgcgcagctgacgacactgatcttggctggctgcagc
ttcaatggacagattccacgagaactagggaacctgcttcagctctcattcctggcactgaattcgaacaagtttacgggcacaataccacctacgcttg
gtttactgtcaaacctcttctggttggatctggctgataatcagctcactggaactatccccatctcatcaggaccgttacctgggcttgacctacttaccca
cacaaagcacttccatttcaacaagaaccagttgtctggaggacttacgggcctcttcaactctaatatgactctcatacacatgtaa
>g110406.t1
atgggcaccttcccattacggctcctcctgatcaccaccatttttcttccctcgtctttagcactgacggccgatgaggtggccaccattactcgccgtcagt
tcctcgccttcgagcggaagagcaatgatggtgttcacgtcgacgtcggcgttgatatcaagatcagcaacccgcttctgctcacagcacacaaggccc
ttcaggcgctcaaggatgccttgtactccgacccgaacaacttcaccgaaaattgggtcggccccgacgtgtgtgcctacaatggcgtgttctgcgtccc
gtcgatgtacaaccagtcggtaagcgccgtcgcgacgctggacatgaatggcgccgatgtcgcggggtacctgcccaaggagatcggccttatgaagg
accttgcagtgctccatctcaactccaaccggttctgtgggatcatccccgaggagatcagaaatatgacggagttgtatgaattcgacgtcagcaacaa
ccgcttcgtcggcccatttcctacggtggtacttggcgttcccaagcttagctacctcgacatcagattcaatgactttgacggcccgatcccgccagaac
tcttccgcaagccctatgacgccatcttccttaacaacaacaggttcagctccggcatcccagagactatcggcaaatcaaaggccacggtgattgtcct
ggcaaacaataacctcggtggatgcattcctcggagcattggtgaggctgctgccacacttgaccagttcatcttcatcaacaacagcctcagcgggtgc
cttccggttgagacggggctcctgactggcacgacagtgttcgacgtcagtggcaacgctctcaccggctcaatcccactaacgctggctagattatcca
aggttgagcagctagacttgtcacgcaacatgttcactggggaagtgccccggggtgtatgcaaggcccatgcagagaaccaaagttga
>g110515.t1
atggccagatcgacagtgcactttatttgtgccttcggcctcctccctatgcgtgctgacggcctcaggcctccaccccgcatgcgcccgcctaccgacgc
ccgccgccgcctccctgcactcgtcgacaaccgcctccctcccctctccgctttctttgcccagatccgccctcctggcccacgcatccgatcgcctcgag
ctcatggagcatcagcctcaaacattgctgaccaccttgcactcatgtcgttcaagtcacacataaggagtgatccatcgcgagccctgtcgacatgggg
caacctttcccttccaatctgccagtggcacggcgtggggtgtggcttgagtggacgtcgtcgtggtcgtgtggtggcgttggacctcgctgaactcaacc
tacttggcaccatcacccctgcgctgggcaacctcacctacttgaggcaattccatggcatcctgccgccggagctaggaagcatctcttccaacattgg
aagacttgtgaacctgaagaagctcgttctagaacgaaacaacatgacaggagaaatacccacagagataggtagccttgccaacctcaacgttctag
ctttgggtgccaatcagttctcaggaactattcccacttcacttagaaacctctcagcaatgactattcttactattccactaaatgaactagaaggaaacg
gtgctgcgggcacggccgccgccgcgacgaacgggttcgaggtggccatcaggatccgggagctcagcaactcgtgctggctgctggtcctcgtcgct
ctggcacggagcggcgccgccgaagactctggcgtccgcgacatgtgccggcgggcgagggtgaacggcgtgatacagaagccgatcacgctcccg
gcgctgagagctgaactccacagggtcctccagaacaacttaccaagatggatgggttgccatttgcatgcatag
>g110731.t1
atgtccgtctcaacattcatggctacacatgtcggcgatgaggccgcgcttctcgccttcaaggccgcggcgatcagcggcgggtaccgcgacgacccg
cttgcctcttggaatggcagcacgggccgatattgcagctgggagggagtaaggtgtggaggcagggaccaacaagtggtagcgctcagccttccatc
tcatggactcaccggcgtcctctcatcggccgtcggcaacttgtcatcactgagaactctaaacctgagcttcaacaggtttagtggagacatccctgcta
gcctcggccgccttcatggccttcacgctctcgacctaagccacaacgctttctcaggccagtttcctataaacctaacctcttgcatcaacctaaaggcc
atacacctccgcttcaaccagctgcaagggtgcgtaccctctgagatcggcgataaactcacgggccttagagttctcagcctcgaaaataacaacttca
ctggagtcatcccggaatcgctaggcaacttaacagcactgagagctttgtcccttgcgttcaatcacctacacggcaacattccttctagccttggtgac
atcaccggcattcagtacctagcactttccttcaacagcctctccggtgagctcccattctccttgtacaacctatcctcgttgcaagaacttcagctacag
aaaaacatgttacatggacgcattcctgctgatgttatccccatggcggccgcactctccctcaccacggtggctaccctcctcagccagccggtgacgg
agaagctcacgaagaccaatcatcccatctggaaggcgttggtgcttgcgaccctgcgcggtgcacaagttgctgggtaccttgatggtaccattgaag
aaccagcgaagaccaaggccggcgaaaacaaaatagacccttcggtcccgaaccctgagtag
>g110875.t1
atggcagcacttcccaagcagggcactgctaacactggcgttgtgctcttgacggttctcgtggccatggccatggtcttctcgtcttgtcatgcaagaga
ctactgccacgcgatctatccgtgcaactccgagatgtgcttctactactgccagaagaacaactacaagaacttccagacgctctgcatccccggaaa
gtactaccccagctgctgctgccgtgtgcctgtcattggtgccgcttcttcttcatctccggcgccgaattcatccaacgtcaacggcagcaacaccgacc

tggccgcgctgctggctttcagaggccagctctctgatcccctcgacatccttgcccgcagctggacaaccaatgtgtctttctgccgttgggttggcattt
cgtgtggccgccgtcggcagcgcgtcaccgtgctatcgttgccggacatgccacttcaaggagaactcagccctcacatcggtaacctttctttcctcaac
aagctcaatctcacaaacacaggcctcaccggcaccatcccttccgatctcggtaggttacaccggcttacctacctgagtctctttgggaatggcctctc
tggctccattcctgattcgatcccagactcgtttcaagatctcacaagcttagtgtcattagacctctcctacaataatctctctggtaccattccgaagttct
ggtatcgtgagcttgctctggccactgatcatttcagtgaaaataacctcttggggactggaagctttggtaaagtttttaaaggccaactgagcaacggt
ttagtggtggcaataaaagttctagatccgcagctagaacaggccagtaggagctttgatgcagagtgtcgtgtgcttcatatggcccggcacaggaac
ctgataaggatactaaacacctgctccaacttggagttcagagcactggtgctgcagtacatgcccaatggcagcttagagatgctgctgcactctgaa
ggtagacgccagttgggtttcctaaagaggctggacatcatgctagatgtggctatgggcatggaatatctgcaccatgatcatcacgatgttgtcttac
actgcgacttgaagcccagcaatgtactctttgatacggacatgacggggcatgtggctgacttcggcatcgcaaagttgctgttaggcaatgacaactc
gttgatcacagctagcatgtctggtactcttgggtacatggcaccagagtatggatctcttggaaaggcttcgcggaaaagcgacatgtttagctatgga
atcatgctccttgaagttttcactggaaagcgacccaccgatcctatgttcatcgctgatctgagcatcaggcagtgggttcatcgatcgtttccgtcggag
ctcatccatgttgtggacgagcagctggtgcaagatgcttcttcctcttcctgcaacatgaatgaatttcttccgccgatatttgagctgggtttgatctgct
caagagactcgcctgaccagaggatgtcaatgcgatgtggtggtgagactgacgaagttgaaagaagaatatgctaa
>g111066.t1
atggcggcgctcctcctgctcctcctcgtcctcgcactcccccaccaccaccagtagtagtaacggcgttgaacgcggagggcctggcgctgctggcgt
tcaagcaagcggcgacctcggacccgtaccgcgctctggcccgctggtccgagtccgacccggacccgtgcgcctggccgggcgtcacctgcgccaac
gccaccacctcctcctcctccaccgccgcgcggggggcggtggtggtgggcctggctgtggcggggaagaacatctccgggtacatcccctcggaact
cgggtccctcctcttcctccgccggctcaacctccactcgaacctcctcacgggggccatcccgtcctccctctccaacgcctcgtctctccgctcagtcta
cctctactccaaccgcctcaccggcccgctccccgccgccgcgctctgcggcggcgcccctcgcctgcagaacctcgacgtctcgggcaacgccctctc
cggccccatcccgctcgacctccgcgggtgccggagcttacaacgcctggtgctggcgagaaacaggttctctggggaggtgccggcggggatctggc
cggagctcggcggcagcctgcgtgagcttgatttatcatcgaacgacctcaacgggaccatcccgccggacctgggagagctcgggcggctaggcgg
cacgctgaacctgtcgcgcaaccggttcgagggcccagtcccgccggagctgggacggctgcctgcgacggtgaccctggacctgcggttcaacaacc
tgtccggcgcgatcccgcagaccgggtcgctggccagccagggtcccacggccttcctcaacaaccccgggctgtgcgggtacccgctccaggtgccct
gccgcgccgtcccccgcccaccctctcccccctcgccgcccgccacgaccacactaccgtccacctcctcggcgaggaggcagcgcgggacgatcaag
accagcctgatcgcgctcatctccgccgccgacgccgcgggcgtcgcgctcgtggggatcgtcctcgtctacatttactggaagctcaaagaccgcaga
cgagccgggaaggcggcggacggcgacggcagcgacagcagcggcaataagcagagcaacggcctctgctgctgcggcgcgatgtgggcccgcaa
cggcagcgggtccgacgagtctgacacgtcgtccacggaaaacaacgacgactccggcaagtacaccagcggcggcggaggaggaggaggaggag
gggaggaaggcgagctggtggcgatcgacaaggggttccgggtggagctggacgagctgctgcgctcctcggcgtacgtgctggggaaaggcggga
aggggatcgtctacaaggtggtggtggccaacggcaccacgccggtggccgtgcgccggttaggtggcggcggcggcgggcaggaccggtgcaagg
agtttgcgacggaggcgcggacagtgggaaggatcagacatcccaacgtcgtgcgactgagggcgtactactggtccgccgacgagaagctcgtcgt
caccgacttcgtcgccaacggcaacctcgccaacgcgctcagaggtcgtcctgggcaggccccccctgtcgtggtcggcgcgtctcaagatcgcccgcg
gcgcggcgcgcgggctagcgcacctccacgagtgcagcccgcggcggttcgtccacggcgaggtgaagccctccaacatcctcctcgagcacgacct
caccccgcgcgtcgccgacttcggtctcgcccgtctcctcgccgtcgccggctgcgcccccgacgcgccctcctccgcggccggcggcggcctcctcgg
cggcgccatcccctacgccaagcccccgcggccgcgcccgggtgggccgggcccgggcgggtacagcgcgccggaggcccgtgggccgggagcca
agccggcccagaagtgggacgtgttctccttcggcgtcgtgctgctggagctcctcacgggccgcgggcccggggaccacgcgtcgccgtccacgtcg
gcgtcgttctctgcgccggtgtcggggtccacggcggcgacagggacggaccgatccgggagcggggagcacgggccggtccccgaggtggtgcggt
gggtgcggcgcgggtttgaggaggacgcgcggcccgtcgcggagatggtggacccggcgctgctacgggccgggcccggcggcgcgccgccgccgc
cgccactgcccaagaaggaggtggtggccgcgttccacgtcgcgctggcgtgcacggaggtggaccccgagatgcggcccaggatgaaggccgtcgc
cgacagcctagacaagatcggctcatga
>g111755.t1
atggccgctgacgagcttgcactcctctccttcaagtccatgctgcactccgagaatggctctctcgacttgtggaacacgtccatccacttctgccgttgg
ccaggaatcgcctgtggccgccggcacccggagagggtggttgccttacgcatgccatcgtttaacctgtcaggacgcatctcgccgtccttgggcaatt
tgtccttcctccggaagctcgacctccgcgacaaccttatcacgggtgagataccaccggagctcggccgtctaggcaggttgcagtttctgaagctgag
catgaacactctcgttggtgaaattcctccagccattgcagggtgcaccgacctcatgatgctagatcttagtcataaccagcttcacggggagatacct
gctaaccttggcgccatggagaacctgtacttgctggagctccaagataaccacttgtctggggagatccccttgtcactagcgaatctgtcatcgatcg
agtacttatttctatacagtaacatgctctccggcgagataccaccagagttgggcaacctcaccggcctgagccacatcgacataatggacaacgtgct
gtccggagggataccgtcgtcgttgggcaagctgtccggactcatctggctcagcctgggctataacagcctcattggccagatcccagcatctatctgg
aacatctcctatctgtggggcatcaacatccaatggaacaacctcaccggcgccattccggcgaacgcgttcagcaagctccctaatctcgtaacggtgt
acatgggaaacaatatgctccacggccctttaccgccctcactagcaaacgcctctaagctacaacggcttcaccttggccccaactccttcagtggcgc
cattccttaa
>g111901.t1
atgagcggcggcaccgagaagcgttactgtctcgtggctacttgtgtcctcgcggcgatgacggtgttggctggaggagcacgcgcggaggtgacgga
gctggagctggaggagctccgcgacgagcggcgcgggctgctgctcctccgcgacgcgctgcgctcggcgctggacctgcactccaactggactggcc
cgccctgccacggcgtccacagccggtgggtgggcgtcgcctgcgacgccgacggccgcgtcgtgtccctcgccctctcccgcgcgcaactcacaggc
accctcccgcgcgacgcgctccgcggcgccacgcggctgtcggagctccgcctccgcgggaacgcgctgcgcggcgcgctcccggggttggacgggc
tgggccggctccgggttctggacctgtccagcaaccggttctccggccccatcccgcgtagctacgcttccgccgccgacgcggggggatgcgggcg
ctggagcgggtcgagctgcaggacaacctgctcaccggcggcgtcccggcgttcgcccagcgccggcttgtcgtgttcaacgtgtcgtacaacttcctcc
gcggcgaggtgccccgcgcgatgcggcggttcccggccaccgcgttcgggcataacctcgggctctgcggcgaggccgtgcgcgccgcgtgcccgcc
gccctcgtcttcgggatccgccggaagcgacggtactggtccggtcgtgacgaagccgggtggtcacggcaggatcgcgacgtggagcgtcgtcgcga

tcgcgctcatcgccgcgctggtgccgttcgcggccgtgctcatcttcctgcaccacacgaggaggagccggagccgccgggaggtccgcctcggtggtg
gccgcgccggagccgcaggcaacgtgacaggaggagcaggggaggacatcaaggacaaggcggcggaggagagcaaatcagggagcggcagcc
ggagcacggacttcttccgttgggacagggcgagcttcgacgtcgacgacctgttccggtcgacggcggagacgctcggcaagggccggctagggat
cacgtaccgcgtgacgctcgaccacgccaaaagcggcggcggcgtcgtggtcgtgaagcggctgcgcaacatggcgcgcgtgccgcgccgggagttc
ggccacaccatgcggctgctcggcaatctcccgcggcacgacaacgtcgtcggcctcgtcgcgtgctactactccggggaggagaagctcgccgtgta
cgagcacgtcccccccgggtgcagcctcttccagctgctgcacgagaacagaggcgaggggaggacgccgctgccgtggccgacgcggctggcgatc
gcgaagggcgtggcgcgcgggctggcccacctgcaccactcgctgccctacttccaccgcccgccgcacggcaacctcaagtcctccaacgtcatcat
ggccttctcctccaccgcaaatcatcaccaggcggcgaaagtggtgccgaagctgacggactacggcttccacccgctgctcccgcaccacgcgcacc
gcctcgccgcggccaagtgcccggagttcgcgcgcgggggcggtgggggcgggcggcgcctctcgtcgcgcgccgacgtgtactgcctcggcgtcgt
gctgctggagctagtcacggggaaggtgcccgtggaagaggacggcgacctggcggagtgggcccgcctcgcgctagagcacgagtggtccacgga
catcctcgacgtcgagatcgtcgccgaccggccacggcacggggacatgctgcgcctcacggaggtcgcgctgctctgcaccgccgtcgacccggagc
gccggcccaaggtgcacgacgtcgtcaggatgatcgacgcgattggcgttgcccgagacggggaacagccacgtaggctttattga
>g112035.t1
atggcggccacgccctctcccccgctgctcctcctcctcctcctccctctcctgcttcttctacccgccgcagcatccccgtccccttcctcccggaacg
ccgagctggacgccctaatggagctcaaggcggcgctggacccgccggggcgcgcgctcgcctcgtgggcccgcggcggcgacccgtgcggccgcg
gggactacttcgtcggcgtctcctgcgacgctcaggggagggtcgccaccatctctctgcaagggagggggctcacggggaccgtcccgcccgcggtc
gccatgctcccggggctcactgggctctacctgcattacaacgagctgggcggggagctgccgcgcgagctcgggcagctccccgacctcgccgagct
ctacctcggcgttaacaacctgtccggcaccatccccgtcgagctcggccgcctcggcgccctccaaggttgtgattttgagctcatgtgctcctgggatt
ggtgtgtactgcagctcggctacaaccagctctcagggagcattccgacacagctgggtcagttaagaaagctcactgttcttgctcttcagtccaacca
gctcactggtgcaataccggcatccctcggggacttaccggcattgacacggctggacttgagctccaatcacctctttggatcgatcccttcaaaacttg
ccgagattcctcagcttgtagcacttgacctccggaacaacacactttcaggaagtgttccctctggggctgttcttgccggaacaatcatcattgtagct
ggagcagcagcatgtggcctctctgtcttctcatggcaccgtcgacagaagcaaaaggtcggtagttctgttgagcatttagaaggccggctgagcctg
gaccaatcaaaggaaatgtatcaaaaaagcgcctcctcgctgatcaatgtcgagtattccagtggctgggatacttcgtcagaggggtcacaacatgga
actaggctttctccagagggttccccaagtgtccggttcaaccttgaagaagtcgagtgcgcaacacagtacttttcagatgtcaatttgctcgggaaga
gcagcttcgcagcgacatacaaagggatcatgcgggatggctcagttgttacggtcaagagcatcaacaagagcagctgcaagtcggaagaggccga
cttccttaaaggcctccgtatgcttacatcgctgcggcatgagaaccttgtaggcctaaggggcttctgccgctctcgagcaagaggggagtgcttccttg
tttatgaatttatggaaaatgggagcctttcccggtatctcgatgtcaagaacagcgtcgctgcaggtgcgacggtcctcgactgggccacgagggtctc
gatcatcaaaggcattgccaaaggcatcgactaccttcacagcagcaagccgaaccagccaccccttgtccaccagagcatctcggcagacaaggtcc
tcatcgaccacctcttcgtcccccacctctccggcgccggcctgcacaagctcctcgccgacgacgtcgtcttctccacactcaaggacagcgcggccat
ggggtacctcgccccggagtacaccaccaccggccggttcacggacaagagcgacgtgtacgcgttcggcgtcgtcgtgttccatgtgctcgccggga
gcaggaccgtctcgcacctccgcctgggcgccgagtccgggaggctggacgacctcgtggacccacgcctcggcggcaggttctcgcggcccgaggc
cgcgaagctcgccgggatcgcgctgctgtgcaccagcgaggcgcccagccagcgcccagccatggcggccgtgctgcagcagctcggcaccagcca
ggtcaatctggaacgtgtgnnnnnnnnnnnnnnnnnnnnnnnnnnnnnnnnnnnnnnnnnnnnnnnnnnnnnnnnnnnnnnnnnnnnnnnnn
nnnnnnnnnnnnnnnnnnnnnncgtggacccacgcctcggcggcaggttctcgcggcccgaggccgcgaagctcgccgggatcgcgctgctgt
gcaccagcgaggcgcccagccagcgcccagccatggcggccgtgctgcagcagctcggcaccagccagtga
>g112263.t1
atgaaccgcttcgtcggggagctcccagctgacatcggccttggtctttcggcgagcctgaccatgctgtgcctcgaagacaaccgcttcaatggcacca
tcccggcgtccctctccaagctccataacctcaagtttctcgccctggacagcaaccttttcgtcggcaccatcccagcagaactcggcgagctaacaag
cctccaggagctataccttgggaataattctttcactgctggcgagcttccgtcatcgttcaggaagctggccaacctgactaccctcgcagtgtggagct
gcagccttgtcggagattttccaacctttctagtggagatgccggagctggagtttctggacctcttcaacaactcgttgactggaagcatacctccgggg
atttggaaactaaacaagctgcaacaattggatttgtccaagaacaacctcaccggccacttgctagtggaggatggctttgcagctatgagcttgaaa
atgatatacctcagcaacaataagctttcgggaatgctccccgactttttgcacgtttggagaacctcaccgatttgtatctctacgacaacaacttctct
ggcgaaataccggcgagcattgccaggttgccatcactaagctatttgcagctgggtaacaacaggttcaatggcacgcttccaccagagctcggaaa
gcactctttgtacttgaccggtgtttcagtggataacaatgaactcactggttcgattccggaggggttatgtactggaggtaatctttggtacttcactgc
ctcgagcaaccgactgaacggcttcataccagcaggactcgtcaactgcgctggacttataaagctaaatctgggcagtaaccagttgtcaggacaagt
gccctag
>g113384.t1
atgatcatcgtggtcaccgtgttgctcggcattcaaccgccgtgccgcgccgagttcacggtggtggtgccggacacgtcggtcctcgtggacgcgccgc
agaccgggttctcggaccgggcccgcacggacccgtccgagcagcgcgcggtgcaggaggtgatggcggcgacggggaacgggtgggcgtgggac
atcccggacgtgtgccgcggccggtggcacggcatcgagtgcgtacccgaccgcgacgacgtctaccacgtggtctccctggccttcggcgcgctctcc
gacgacacggcgttcccggcctgctcccgcggcgcggcgctgtcgcccgccgtgctcgcgctcccgcacctccgctccctcttcttctaccgctgcttcac
ggccaacccgcagcccgtcccgcgcttcctgggccgcctgggcccggccttccgctccctcgtgctccgccagaacggcctcgtcggcccggtccccgc
cgagctcggcaacctctcggccctgcgcgtgctcgacctccacgggaaccacctctcctccgccatcccggcaacgatccagtccctcacccgcctcca
gatgctcgacctcagctacaaccgcctcgccgggcccgtgccccgcttcagcaggttcttccaacgcctcagcatcctcgacctcagccacaacgcgctg
cagggccgcgtgccgtccagcctcggccagtgccggtccctgctcaagatcgacctcagccagaaccgcctctccggcacgatccccgacgcgatcgg
cgacctgccgggcctcatgctgctggacctcagccgcaacgcgctgtctggcccgatcccgccggcgatcggccgcctctcctcgctgcggtcgctgatc
ctcagcgacaaccggatgcagctctccacgatccacggtgatttcttctccgagctgaaatcgctgaccacgctcgtgctctccgggacgggcctggccg
gatcgatcccggaatccatcggggagctgagcgagctgcgtgtgctgcgtctggacaacaaccagttcaccggcgtgattccggcgagcctccggcgg
ctggagcgggcgagcgaggtccgcgtcgacggcaaccggctcgtggggccgataccgttcggcaaggagatgatgtggaggctgggcaagaagctg

cgcgtcggcggcaacgaggcgctctgctacgacgccaagcaggaaggccttgagggtgtcgtcgcgcttgccggcgtcgcggactgcggcagcgtga
gaagccgcacgacgcagcacttggtctggagcaacagcactgtcgtaggtggccggggcggcgcagttggcagaatgaccactacgtcgccggctgc
tgcttccggcggccgcaactttggtgctgcttgtgtgttcgtggctctgcaccttgcatggtttctagcgttgtacctgtag
>g114104.t1
atggcctccttcttgttcctcccgctggtactagggaaagtgctaatcgtcgtcctcctcgtggtagtggtaggcagtgatgataatagcaatgccgctga
agctctgaaccaggacgggctccacctgctggaagcgaagcgcggcctcacactcgcagctgctgctccttccggccaattattactgggcgactggaa
ccccggcgatgccacgccctgcaattggacgggcatcacctgcgacgaagaagaaggagaaggagaaggaggtcatgtgacggagatctcgctgcc
ggagtccaacctggcgggctccttccccgccgccttctgtcgcctacctcgcctccgctccatcaacctcgccaccaactacatcggccccgagctttctc
tatctctatctctccatggaggaggaggaggaggatgcaggtcgctggagcggctggacctttcgatgaacgcgctggtgggcccgctccccgacgcg
ctcgccgacctcccggagctccggtacttggatttgtctagcaacaacttctccggcccgatcccggcatccttgggaggattcatgaggaggttggagt
cgctgtcgctggtgtacaacctcctggacggccacatcccggcctctctctcctccatctccacgctccgggagctcaacctctcctacaaccccttctctc
ccggcgcaaccatcccgccggagctcggccggctctcctccctgcgcgtgctctggctcgccggatgcaacctcgtggggcccatcccggcatccctcg
gccgactcgccaacctcaccgacctggacctctccatgaactcacttaccggcggcatccctccggagatcgcgaccgggctcgccagcgcgacgcag
atcgagctctacaacaactccctctccggccctattccccatggattcggcaggctgaggcagttgcggaacctcgatctgtcgatgaaccggctctcag
ggtccatcccggaggacctcttccaagccaagctactggagacgctgcacctctacggcaaccagctcacgggtccgttgccggattctctcgctacgg
ctgccacctcgctggtggagctgcgcctcttcgccaacaagctcaacggcacgctccccgccgatctggggaagaacgcgcccttggtgtgcgtcgacg
tctccgacaacgccatctccggcgaaatcccaaaggggatctgcgaccgcggcgagctggaggagctgctcctgctccacaactttttatccgggtcca
tcccggcatccctgggtcggtgccggagcctgcggcgcgtgcggctcgccaacaacaggctcaccggcgacgtgcccgcaccagtgtggggcctgccg
cacatgtcgctcctggagctcaacgacaaccggctgtcaggagtcatctcccccgccatcgccggcgccgccaacctgtcgaagctcgtgctctccaac
aaccgcttcaccggcagcattccatccgagatcggagcggcaaccaagctgtacgagatctccgccgacggcaacatgctctccggtccgctgccgtc
gtctctcgccgccgagggtctcgacgacctcggccgcctcgtgctacggaacaactccctctccggccagctgctccgatccgtggccggttcttggaag
aagctcacggagctgaatctggcggacaacgcgttcacgggctccatccctccggagctcggcgacctgcccgtgctcaactacctggacctctccgga
aacaggctcaccgggtgggtgccgacggagctggagaacctcaagctcaaccagttcaacgtgtccaacaaccagctcaccgggcctctcccgccgc
agtacgcgtctgctgcgtaccggaacagcttcctgggcaacccggagctgtgcgcacaagtcccagccttgctgtgcagcggtgacactgattcctctg
gttcctcctcctctcagcgccacgcaacaatgagggccttgatgcgctgcatattcattttcgcggccgtcgttctggtggccgggatcgcgtggttctacc
gggccttctcctcttactcctccagtaagaagaagaagaagaagaagatgatgatgatgatgatggaccggtcgaaatggatggtgacgtcgttccaca
agctgtccttcagcgagtacgacatcctcgactgcctcgacgaggacaaggtcatcggcaccggcgcatccgggaaggtgtacaaggcggtgctcagc
aacggcgaggtggtcgcagtcaagaagctctggggcaacaaggacaatgatgacgacagcctccagttattcgatgcggaggtgcggacgctgggca
agatccggcacaagaacatcgtcaagctctggtgctgctgcgtcagcgcgcacaacaacatgaggctgctcgtctacgagtacatgcccaacggcagc
ctcggcgacctgctgcgcgcaaggagcaacaatactaagctggactggcccaggaggtacaagatcgccctggacgccgccgagggcctgtcgtacc
ttcaccacgactgcgtcccggccatcgttcacagggacgtcaagtccaacaacatcctcctcgacgcacactttggtgcgcggctcgccgatttcggagt
cgccaaggtcgtcgatgcccatccaaatgcagcaggagcaccaaagtccatgtccgtcatcgcaggatcatgcggatacatcgcgccagagtacgcgt
atacacttcgcgtgaacgagaagagcgacacctacagcttcggcgtggtcctcctggagctggtcaccgggaagccgcccgtggatccagagtttttcg
gggacaaggacctgctcaaatgggtgtgcagcaccatggaccagaaaggcgtggaacacgtgctggacacgaggctcctcgactacaagcaacacg
tggtcagggtgctccacatcggactcctctgcgcaagctccctgcccatcaaccgaccgcccatgaggagggtcgtcaagatgctgcaagaggttgctc
cgactccgaatctttttaataattcccaaccccaatcgctctcgccctcgccctcgccctcgccctcgccctgctactactactaccacgaccctgacacca
ccacatcatccgatcaagggagcagcgtctactag
>g114672.t1
atgaggtaccgggctttgcaactcggcttggtgctggcctttctgctggcctatgaatcccagggtgtgaaccatgaaggctggctcctcctggcgctgaa
gagccagatggttgacactctccaccaccttgataattggaacgcaagggatccaacaccatgcgcctggaggggcgtcaactgctcgttgccacccaa
cccagccgtggtctctctcgatctcagcaacatgaacctatcgggcatcgtcgcgcccagcattggaggcctggctaaactgacttaccttcatttgtcatt
caatgggttctatggtaccatccctgcagaaattgggaattgctcgaaactggaggtgcttaacttgttcaacaacaatttcgttggttcaatcccacctg
agctcgggaagctgtctaggctggttactttcaatctgtgcaacaacaggctccatgggccaatacctgatgaaattgggaacatggcatcacttgaga
acttggtgggaatctcatatcaggcaaagttcccgttgagatag
>g115012.t1
atggccagccggccagttctcctgatgaccgtactactgtctttccagctgttcatcacgacgtcgctttctcagacgtctcaggtatcggaccgtgacctc
gcggcgctgatcaagatcagggagcatttcggcaacccgaacttgttcccggggtggcgccccggcacgacctgcatctcatggtggcccgcggcgtg
caacgagcagggccgcgtcaccaagctcttcctcgacaacctactcggcatcaaggaactactcaccgtcaactccacgctcccgccggccatcagcg
agctcgagatgctggagacgctgagcatcatgagcatccccggcctgcacggccccatcccggaatcctacggcaacctccggcacctctccctcttca
acatcatagccaccaatgtctccggctcgataccggcgtccctctcccggaccaacctcacctccgtcagcttatccaagaacaagctcaccggcccgat
ccccaggtcgctgcagaagctgccgtacctttcctacttcgacgcctcgtacaacgacctcgtcgggaccattccgccgggtctggtgcgcagcacgcct
gatagcccggccgccctgcagctagacggcaaccggctgtcagggaccatacccagatcgtacagattcgagaggaattcgatggagttcagggtgg
ccaacaaccggctcacgggcgacgcgtcgttcctgttcgggcggcacaagacggtgtccgggaccatcgatttgtccgggaacaagttccggttcaacc
tgacaggagtggagatgcccatgcatcttgggttcctgaacctgagccacaaccggatctatggcggcgtgcccgtgtcgctgagggagacgagggtc
tcggtgctggacctcagctataacgagctctgcggggagatacccaccggcgggcacatggtgcagttcaaggcggcggcgtacgaacacaacaaat
gcctctgtggaacgccgctgccaccgtgcaccaacggccgctga
>g115285.t1

atggcgtttcgctttgaagcagcagcaatactccttgctggtttactagcacttgcaacactggcaagctgcaatattgaaggtgatattctgtatgcgca
aaggatggagtggtatgacccaaataatgtgttggagagttgggatcctaccctcgtgaatccctgtacctggtttcatgtcacctgcaataatgataact
ccgtcatccgtgtggatttgggcaatgcaggcctcgggggctctctaattccagatctgggaagactgaagaaccttcagtacctgtaa
>g116428.t1
atggcgcggctcaccgaggagaaggcggcgctcgaggccggctcgaaaggaagcggcgccaccgtcctcaacctctctcatcgcgccctctccgatgt
gtcctgtctgagcagcttcaagaacctggagcgcctcgacctcggctacaactgcctcgtcacccttgagggtttgtcctcctgtgtcaatttgaagtggct
ttcagttgttgaaaacaagcttgtgagcttaaaaggtgttgagggcatgtcaaagctgcaagtgttgaacgccggcaagaacaaacttacaaaaatgga
tgaggtcacatctttaacaagcctcggagcattaattctgaatgataacaatatttcgtacatctgcaagcttgatcgactgcaacagctgaatacacttg
ttctctctaagaatcctgttgttactattggagatgctttggtcaaggctaagtctatgacaaagaaaataccttcagacttggccaaaaatgtcaaaatat
tgaaccttgatttagggaataacctgattgagaggagttcagatttgaaggtcctttccgaattgcgctacttgaggaaccttaatttacagggaaatcct
atttctgagaaagatggtcttgcgaaaaaggtcaagaaatttctgccaaacttgcgcatattgaatgcaaagcccattgaagccagctccaagaaagat
aagagccctatcgaatcaaatattccaaacaaagatgacaatatttatgatcatgatgctgttgagattagtacaaaaaagaaggataaaaagaagca
gtcaaagaaacaattaaaaggccctgaagagcatgatgtgaccacttgtaccccaagcaaatctgaaatggtacatggtaagggaacaaaaagggaa
aaagtagccatagaacatgctgataataacaaattaaagaggaagaatgataagtcttctgctaatgatactggtagaaatactggtagaaaggacaa
gaaagaagccaagaggacgaaattcattgatgaagaagatgctgatgaaattgacaacacagagatgtcatttgcagatttagtgttttccaaagtgga
cagtccagaacctaagctgaaggacaaaatccaagaagcagatggaaaacatgatggtttggtaattgatcatacaaaaaagaaaaagaaatcaaa
aggtgctgtcactatcaccgacttctcaactcttaagatgctatcctctgtaccggaggtgggggcaggcggactcgtactatcaggatgggatgattag
>g116787.t1
atgatccagaaccttaccttccttccctcaaaaagccttcccctgcaaaacccgcgggcctcctcatccctccccatctccgccaccacctcccagccgcc
gcgtccagcggcgatggagtcctccctcctcctctcctccactgcccgccccatcaagcccttccgcccgctcccgctcccgctccccttccccttcgcccg
ccgccgccacgcctccctcggcgcgaggcctccatctacgccgccgccacgcctcgtccgccgccggccgtctgtcgcgccgcgcgccgccgcgcacg
atgcgttcgacgacggttttcgaagcgggaacaggccgcccggggtcggccgcgggggcgcgggccggagggcctaccgcgaggcgcaaggggag
gcncgacgacggttttcgaagcgggaacaggccgcccggggtcggccgcgggggcgcgggccggagggcctaccgcgaggcgcaaggggaggcg
gccgtgccacccgcagcagctgccgcgagagccgtcgcgccctacgtcgtgcccgctggcgcggtgctcgtgctgagctttgtgctgaaaaagagtcg
aggaaaaatcttaacaagttctacaaagaactccggactttgaaaactgttaacatggcaggtcgtcagtttggggatgagggtctgtttttctagcag
aaagcttagcctataacaagagtgcagaagaggtggacttctctggcaatgggatcacagctgtggggatagaagcatttgatggtgttctccagataa
acacaactttaaaagcccttaatttatctgggaattccattggagatgaaggagctaagggagccaaagcaatttcaaatatgctgaagaagaataaga
caatacgtattctacagctcagcaacaatacaattgaatacagtgaaattcacttgcatggtaatgggattggaaacgagggggtacggaagatcaca
gttgtggatattggcaacaacaatattgggtcagaaggattacgaccggtcgccgagttcatcaaaaggacaaagtcgttactgtggttcagtctctaca
tgaatgacatcagcgatgagttggatttgagctataacccaattggtccggacggggtaaaggctttgtgcgatgtcctcaagttccatgggaagatcca
aacccttaagcttggctggtgccagataggtgtgccaggtgcagagttcattgctgattgcctgaagtacaacacaacattgtctacattggatttgcggg
caaatggtcttggagatgatggggctatatgcttggctcgaggtttcaagataatcaatgaatcgttgacgtcacttgacttgggtttcaacgagattaga
gatgatggagcgtttgcattggcacaagcacttaaggctaatgaagatcttgcggtcacttcgttaaatctttcaaataacttcttcactaaatttggacag
aaaagcatagatatatctcgcttggtgcttgcaatggtgtccaaagtgttctcatggcttcctcccctcctgctccttacggcggcaatcgtcgcagatggc
ttgacgcccaccacggcggcggcgttcaatgagaactacgcggtgcagtggggtgcagatggttatcacctagtcgatcacgggacggaggtcaatct
cgccatggatcaaagctctggcgctggttttcggtccaagtccacgtacggctctggtttcttccacatgaggatgaagttgcccaccggctacacggcc
ggcgtcgtgacaaccttctatcttatttcgcaaccggaaaacggtgagcacgacgaggtggacttcgagttcctgggcgacaaggacggcaagccaat
cacgctccagaccaacgtcttcgtcaatggccagggcaaccgggagcagaggctgcacctctggttcgaccctgccgccgacttccacgactaccaga
tcctctggaacccctatcagctcgtgatgttcgtggacgacacgccgatccgtgtgctgaggaacctgacgggcgtggtgcccgggtacgagttcccgg
cgaagcagacgatgctggtccgcgcgagcatctgggacggctccgactgggccacgaacggcgggcaggcaaaggtggactggtccaaggcgccct
tcacagcggggtaccagggcttcgacgtcgacgcctgcgccactgacggtgacagcggcagcggcgcgacgccctgcgactcgccggccctgtggtg
gaacggcgacgagtaccggaacgtcacggacgcacagagggaggcgtacgagggcgccaagaagtacatgtattacaactactgcatggatacggt
caggttcaacaatcatatgccgccggaatgcagctatgatttgtaa
>g117298.t1
atggctgctctttccatacttgttctggtgctgcttttcggggccgccgccggcgacgatgtggcggcgctgctcgagttcaagaagggcatcgcggacc
ggaccgaggacccggtgctgcggtcctggtccccgccgtcgaccaccgaggccggcaacggcgccgcgggctgcccccaggcgtggcggggcgtgg
tgtgtgacgacggcgccgtggtcggcctggcgcttgacggcctcggcctcgccggggagctcaagctctccactctcagggacatgcgcgcgctccag
aacctctccctcgctggtaacgccttctccggccgcctgccgcccgccatcggctacctcagctcgctgcgccacctcgacttgtctgggaaccgcttcta
cggccccatccccggtcgcctcgccgacctctcgggactcgtccacctcaacctctcccacaacaacttctcctccgggttcccaaccgacgggatccag
cagctccagaacctccgccgcatcgacatccgcaacaactcattctggggcaatgctagcgatctgctcaaagcgctccgcaatgctgagcacatcgat
ctgagccacaacctcttcaacgggcgggtggatttggagttggagagcttgagcagcattgggaacacggtgaagtatctcaacatcagccacaacaa
gctggagggtggcttcttccgaaatgagacggtgagggcgttcaagaacttggcggtccttgatttgagcaacaatgggattggtgggacagtgcccaa
gctagattcatggttctcgctggagattttcaaggttgccggcaatgggctatttgggatgatgccggaggcactcctccagaattcgatgcggctcattg
aggttgatctcagcaggaacggcttctcagatggtctgcctgctggaaatgatgattacactggagttggtcagagtcaaacatctcatggacataaggc
tggtgttagagttgcacttattgttggctgtatcggagctgtgttgctagtcatcttcatagcattggcattctatgtgaaagagttgctggctgaagctgct
gttgaatatggctactctgatccaaaggaagtgactgaatccacaagttctggtgtggttgagacttctgctgcagtccaggctcaggagtcatctccaa
ggtctgcgttaccaacatcaccacattttaccgatcctaggttccatgaagaacctgttgcttttgaagtatactctccagatcggttagtgggcgaattga
ttttcctggacagcactttggttttcacagctgaagacctgtcacgtgcaccagcagaggttcttggaaggagcagtcatggcaccacatacaaagctgt
gctacaaagtgggcatatgctgacagtgaaatggctgcgtgttggtctcgtgaagcataaaaaggagtttactaaggagatcaagaggatcggcacta

tcaggcatccaaatataattccatggagagccttctattggggtcctaaggagcaagaaagattaattatttcagactatgtcaatggcgatagcttggc
actctatctttatgagtcaactcctagaagatactcccgactgtcagtgagtcaacgtcttagaattgcccttgacctggctcgctgccttcagttcttacac
catgagaagggcctgcctcatggcaacctaaagccaactaatatattcttgactggtcctgacctctctccaaagcttattgattatggtctacataagttc
atgactccaagcggcattgctgagcaaatactgaatttaggagcattagggtatcgagccccagaactagcaaacacaaccaagcctgcaccgtccttc
aaggctgatgtgtatgcatttggggtaattgtaatggagatgttgacgaggaagagtgctggagacatcatctcaggacagtctggcgcggttgatcta
actgactgggttcagatgtgcaacagagaaggacggggaactgactgcttcgatcgagacatcacgggcttggaagaatctccaaggataatggacg
agctgcttgctatctcacttaggtgtatccttcctgtaaatgagagacctaacatgaagacggtctgtgacgagctgtgttccataacagtgtga
>g117408.t1
atggcacggaacgatcgtcacgtacaccatcagcttcacatcatcacctgcaaccctttcctcatgcaccagatgcagcctccactccacgtaacgctcc
tggcacgcgcctgcttgtcggcgctgttgcttctgcagcagctgtcctgcatgacagcacagccggcggcggcgcccggcgggaacagcagcgccttg
gcggccgacgagctcgcgctcctgtccttcaaggccgcgctgtcggacccggccgggtcgctcgcttcatggaacgcatccaaccgcatatgcagcaca
tggagcggggtgacgtgcggctcaggccgggtggtcgcgctgcggctggactcgctcggcctcgtcgggcccgtctcaccgttcgtcgccaacctctcg
ttcctgacgacgctggacctcggcaacaaccgccttgtgggccacataccgccgcagctcggccgccttagccggctgcagctgctcaacatcagcgcc
aactctctcgatggagacatcccctcggctctggggatgtgcacgggtctcgcgactctggttctcagctcgaaccgcctccacggcgggatcccagatg
aggtgggcgccttaaaaaacctcgtctacctgaaccttgaatccaaccgtttgtcggggcagatacctgcatctgtcggcaacctgagttccatccaaga
gttgcgcctcgcgaacaacaccttgtcaggagccatccctccgtctctgggtcagctgccaaacctctcctcgttcatcctctactacaacaacctgtcgg
gaccaatacctgaaaccctgtggaatatctcctccctgacaaggctaaacgtgttcgaaatgatctcaccggggagataccttcagacgtgttcgataga
gtccctcgtctccgtagcctcttcttga
>g117410.t1
atgcagatgcaactaccgcaagtagcagccttcgtgttggtggtgctactccgtcccggaggcagcagcgccttggcatcgtcgacgacatggtctgga
ggcaacagcgccttggcagctgacgagctcacgctcctgtccgtcaagtccgccctgtcggacccggccgggttgcttgcctcatggaacgcatccaac
cgcctatgcagctggcgtggggtggggtgtggccgtcggcaccccgccgggtggtcgcgctgctgctggactccttcggtctcggaggacaaatctcg
ccgtttctcgggaacctctcgttcctcaagtctcttgatctcggcaacaacagccttgtgggccggataccatggcagctcgggcgcctggggcaccttca
cgttctgaaccttagcgtgaactctcttcagggagacatccccactgctctgggcaggtgtatccaactcaggacgctcagcctcgaggctaaccacctc
cacggacttgtacctggggttcaacaagttatcaggagccatcccttcgtctctgggtga
>g117645.t1
atgcgcggcggcgtgcctccctggctcggcaacatgacctcgctcaccgacctcgagctcagcggcaacgccctcaccggtggcatcccaccttcgctc
ggcaacctccacaacctgcagctcctcgagctctactacaacaagctcgacggccccatcccggaggcgctcggcaacctcacccgcctcaccgacat
cgacttatccgagaacgccctcaccggcggcatccctttctccctctgcgcgctcccggacctccgcgtgctgcagctctacacgaaccggttcacgggt
cccatccctcccgtgctcggcaactccacgcagctccggatcctctccgtctaccgcaaccagctcaccggcgagctgccacccgacctcggccggtac
gcgccgcttaacgtgatcgaggtctccgagaaccagctcaccggccccttgccgcccttcgcgtgcgccggcggtaacctccagtacatcctcgtcctca
gcaacctcctcacgggggagatcccttctcgttacgcgtcgtgcacgacgctgctgaggttcagggtgagcaacaaccatcttgaaggcgaggtccccg
cggggatcttcggcctccctcacgcctccatcgtcgacctctcctacaaccacctcaccggcccggtttccagcaccgtctccggcgccggtaacctgac
gtccctcttcgcgtccaacaaccagatgacggggcccatcccggcggagatcgccggcgcggcggcgctggtcaagatcgacctcagcaacaacggg
ttcagcgggcccatcccgagtgagctgggacggtacctgacgcggctgaaccagctgtcgctgcaggggaactcactttccggcaccatcccggagtc
gctcgccgggctgagcagtctcaacgtgctcaacttgtcggataacgcgctctccggggccatcccggactcgctctgcacgttgttgcctaattccctgg
atttctccaataataacttgtccggcccggtgccgctgccgctgatcaaggaagggttgctggagagcgtggcggggaacccgggtctctgcgtcgcgtt
ccggctcaacctgaccgacccggcgctgccgctgtgtcccagatccggcggcgggctccggcgagggctcgccgggaacgcgtgggtggtcgcggcg
tgcgcgctggtgtgcgtcgccggggcgctcgtgctggtcaggaggtggttgatgaaagcgaggtgcgacggagaccatgacggcgcggcggcgggg
gggtcgccggcgaccagctcctcctacgacgtgaccagcttccacaagctcacattcgaccagcacgagatcgtcgaggcgctcatcgacaagaacat
cgtggggcacggcgggtccgggaccgtgtacaagatcgagctcagcagcggggagctcgtcgccgtcaagaagctctgggtctccacctccgccgcc
gcgcgaggacgacgaggagtccctactaatactaaccatcatcatcataccaacaagacgaacagcaacaacacgacgacgtggctgggcgaccgg
gagctgcggacggaggtggagacgctggggagcatacgccacaagaacatcgtcaagctctactgctgctacgccggcgcggatagcaacctgctcg
tgtacgagtacatgcccaacggcaacctctgggaggcgttgcacgggtgctacctgctgctggactggcccaccaggcacagggtggccatgggcgtc
gcgcaggggctcgcgtacctccaccatgacctcatgttccccatcatcgagctcagcagcggggagctcgtcgccgtcaagaagctctgggtctccacc
tccgccgccgcgcgaggacgacgaggagtccctactaatactaaccatcatcatcataccaacaagacgaacagcaacaacacgacgacgtggctgg
gcgaccgggagctgcggacggaggtggagacgctggggagcatacgccacaagaacatcgtcaagctctactgctgctacgccggcgcggatagca
acctgctcgtgtacgagtacatgcccaacggcaacctctgggaggcgttgcacgggtgctacctgctgctggactggcccaccaggcacagggtggcc
atgggcgtcgcgcaggggctcgcgtacctccaccatgacctcatgttccccatcgtacaccgcgacatcaagttgtccaacatcctcctcgacgccgatt
tcgagcccaaggtcgcggatttcggcatcgccaaggtgctccatcaggccaccactgccgcaaagggaggagctggagacgcctccacaaccacaat
cgcgggaacatacggatacctcgcgccagagtacgcctactcgtccaaggcgacgaccaagtgcgacgtgtacagcttcggcgtggtgctcatggagc
tggccacggggaggaagcccatcgagccagagttcggcgacaccagggacatcgtgcactgggtctccggcaaggtggccgccggagccgaggccg
aagcgctcgacaagcgactcgcgtggagcccgtacaaggaggagatggtgcaggcgctgcgcgtcgccgtccgctgcacctgcagcatcccgggact
ccgccccaccatggccgacgtcgtacagatgctcgccgaggcagggccacggaccaccaaggacaataaggacaactccgcacaacacctgctgac
gatgcccaaaccagacaccaccaccaccaccactccttaa
>g117646.t1
atggtggccactcatctttgttgtgtgaataataatcggtcggcgttgctggtgtggtgccttgtcgtcgtcgtgttcgcagaagccgtcgtaagtgatggg
gcggcgttggacacgcaggcggcgtacctgtccaagatgaaggagcagttcgccggtcctggcatgtctcagtgggacttctcatcgttgcccaactac
tactgcacgttccccggcgtcgcgtgcgaccacggcggcaacgtgacgggcatcgacgtgagctcatggcgcctcaccggccgcctccctcccggcgtc

tgcgccgccctgccgtccctccgcgagctaaacctcgcctacaacgacatccgcggcggcttccctctgggcgtcctcaactgctcctctctagaggtcct
gaacgtgagcttctccggcgtgtccggccccgtgccgccggagttgtcgaagatgccggcgctccgcgtcctcgacatgtccaacaacctcttctccggc
gcgttcccggcgacgtcgatcggcaacctgacgacgctggaggtggtgaacatgaacgagaaccccgggttcgacgggtggtacccgtcctcctcgac
gttcgcgccgcttcgccgcctccgcgtgctcatcctctccaccacgtccatgcgcggcggcgtgcctccctggctcggcaacatgacctcgctcaccgac
ctcgagctcagcggcaacgccctcaccggtggcatcccaccttcgctcggcaacctccacaacctgcagctcctcgagctctactacaacaagctcgac
ggccccatcccggaggcgnnnnnnnnnnnnnnnnnnnnnnnnnnnnnnnnnnnnnnnnnnnnnnnnnnnnnnnnnnnnnnnnn
nnnnnnnnnnnnnnnnnnnnnnnnnnnnnnnnnnnnnnnnnnaacctga
>g118191.t1
atggcgtctgggtccgcccgtcgcgtcctcctgctagccttgctgccgctcctcctcctcgcgccccggtcgtcgtccgcgccgacgaccaccttcccggg
cgacgccgccgcattagcgtcgctcaagtcgtcctccatcccgccgcaatcctgcctggcgacgtgggacttcgcccggcgcgacccctgcgccgcgttt
ccctgcggcctccgctgcgtcgcgccgcccaactccacccacctccgcgtcgccggcgtcgcgctcgacccggccgggtactcgggcgcgttcccgacc
cggctcctcgcctccctcccctacctcacgaccctctccctcgccggcaaccgcttccacggcccctcccggatggcgtcccgctcccaccaaccctccg
cgtgctcgacctctccagcaacgccttcacgggccccatcccgggctccctcttcacccgtgcgtcggaactgcaagagctctacctctcccgcaacgcg
ttctccggcgtcgtcccgccgcagctcgcgtcgctggcgtcgctcacgcggctcgagctccagcacaacgcgctcgcggggagcccgagccttccgccc
gggatgcgcgcgctcgcacacctcgacctcacggctaacaagctctccggcccgccgccgctcgacgcgctgcctccctcgctcgtgtcgttcgtggcg
cgcgataacgccttctcgggcgccgtacccgccgcgccgctcgccgctctcggctcgatccgcgttctggacctcaccggcaacgcgctgtcgggcgcg
gtgcccggcgccgcgctcgcgcacccgtcgctgcagcagctgcggctcggctccaaccagctcgacgcggtcgcggctcccaacggcggcggctgcg
cgtcgagccagctcgcggagctggacctcagcaacaaccggatcgcgggacggctcccggcgtgcctcggctcgatgccgcggctcgtggcggtggc
gctggaccggaaccggttcacggggcccgtccccgacgcgtacgccgcgcgcgtcgcggcgcaggaggccaccgacgagtgggtcccgttcgccagg
ctcatgctgcaggggaacttcctctgcggggcgctgccgggccggtggacgcagctcagggaggacgacggcgccaagctcagcgtcgcggacaact
gcctgccgaggtgcccgcgcaagttcttcttctgccagggcgcgccgcagaagaaccaggccgcgtgcccgaccaagtgcgatgccgccgcgatccgc
catgtccgcgagcacgaggatttgctgctggaaatgccctag
>g118914.t1
atggcccgaaataagctcgtggcgacgacgacgacggtgctgctgctactgacgacagtgctgctgatggccggcgctgagccgccggcgagcgagc
gttcggcgctgcaggcgttcatctccgggacgccgcacgagcgcgcgctgggttggaacgcgtcctcgtcggcgtgctcctggacgggcgtgacgtgc
gacaaagccaccaataactccaccgtcgtcgagctccggctccccggcatcggcctcatcggccggctcccctcgggcacgctcggccggctgaagag
tctccaggtcatctcgctgcggtccaaccggctctccggctccatccccgacgacctcttctccctcccgcggctccggtcgctcttcctccaggggaacct
cctcaccggcgcgctccccgccgacatcgccgggctttcgtcatcgctcgagcacctcgcactgtcccacaacaacctctccggcccggtccccttcgcg
ctcaacaatttgatgaagctcaagtcgctgatgctcgacggcaacagcttctccggcagcctcccgagcatcaccatccaccgcctcgacgtcttcaacg
tctccgataacgacctcaacggctccatcccggcgtctctcgcgcggttcccgccggaatccttcgccggcaacctccacctctgcggcgcgccgctcgt
cgaccgcccctgccacccattcttcccgccctcttctcctccctttcctcctcctcctggatcacctgatgatgagtctaacaaaaggaagaagctttccgc
cgcggcggtggtggccattgcggtggccgcggccggcgcggcgctgctcgcgctggtgttcctcgtcctctgcttggtccaccaccaccggcgaggacg
caagggtgaacaggatggcagcaagggggcgaccacaaccaccagtaggggaggcgtgacgccatcgtcgggggagatgggcacgggcggcgag
ttcacctcctcgtccaaggagatcagcgccgcggcggccacggcggcggggcttattaccaagctcgtcttcgtcggcggcaacaagaagcagcagag
cttcgacctggaggacctgctgcgcgcgtcggcggaggtgctggggaagggcagcctcggcacgtcctacaaggccacattggaggacgggaccacc
gtcgtcgtcaagcgcctccgcgacgtggcggcggcgcggagggagttcgccgcctgcgtcgaggcggccgccgccgtcgagcaccgcaacctggcgc
cgatgagagggtactactactccaaggacgagaagcagctcgtaggtggccgtggcaccggccggacggcgatggactggggagcccggatgcgcg
ccgcgctgtgcgccgctcgcggcgtggcgcatttgcacaccgcccacaacctcgcgcacggcaacatcaagtcctccaacctcctcctccggccagacc
ccgacgcggcggcgctctccgactacggcctgaaccagctcttcacatcatctgcaactccggcggcgcggcccattactagcgccgccggcattattat
tgggtaccgcgcgccggagctcgtggacgcgcggcggccgacgttcaagtcggacgtgtactcggtcggggtgctgttcctggagatgctgacgggca
agtccccgtcgtccggcgaggtggacctgccgcggtgggtgcagtcggtggtggccatggcgtgcgtcgccaccgcgcccgacgcgcgacccgacgc
cgccgacgtcgtcaggatggtcgaggagatcgctagccgcaccaacacgtcctccaggggcgccactccctga
>g119165.t1
atggcatcatcggcaccggccgcatggtcacagatcatgatcatcgtggtgaccgtgttggtcggcgttcaacggccgtgccgcgccgagttcacggtg
gtggtgccggacacatcctcatccgcggcgctcgtcgacgcgccgcggacggggttctcggaccgggcccgcaccgacccgtccgagcagcgcgcgg
tgcaggaggtgatggcggcgacggggaacgggtgggcgtggggcatcccggacgtgtgccgcggccggtggcacggcatcgagtgcgtgccggacc
gcgacgacgtctaccacgtggtctccctcgccttcggcgagctctccgacgacacggcgttcccggcctgctcccgcggcgcggcgctgtcgcccgccg
tgctcgcgctcccgcacctccgctccctcttcttctaccgctgcctcacggccagcccgcagcccgtcccgcgcttcctgggccgcctgggcccggccttc
cgctccctcgtgctccgccagaacggccacgtcggcccggtccccgccgagctcggcaacctctcggccctgcgcgtgctcgacctccacgggaaccgc
ctgtcctccgccatcccggcaaggatccagtccctcacccgcctccagacgctcgacctcagctacaaccgcctcgccgggcccgtgccccgcttcagc
aggctcttccaacggctcagtatcctcgacctcagcaacaacgcgctgcagggccccgtgccgtccagcctcggccagtgccggtcccttctcaagatc
gacctcagccagaaccgcctcaccggcacgatccccgacgcgatcggcgacctgcacggactcatgctgctggacctcagccgcaacgcgctgtccgg
cccgatcccgcccgcgatcggccgcctggcgtccctgcagtcgctgatcctcagcgacaaccggatgcagttcacgactcttcccggcggcttcttctcc
gggctgaaatcgctgaccacgctcgtgctctccgggacgggcctggccgggtcgatcccggaatccatcggggacctgagcgagctgcgtgtgctgcg
tctggacaacaaccagttcaccggcgtgataccggcgagcctacggcggctggagcgggcgagcgaggtccgcgtcgacggcaaccgcctcgtgggc
ccgataccgttcggcaaggagatgatgtggaggctgggcaagaagctgcgagtcggcggcaacgaggcgctctgctacgacgccaagcaggaaggc
cttgagggcgtcgtcgcgcttgccggcgtcgcggactgcggcagcgtgaggagccgcacgacgcagcacttggtctggagcaacagcactgtcgtagg
tagacgcggcggcgcggttgccagaatgaacactacgtcggctgctgcttccggcggccgcaactttggtggtgcttgtgtgctcgtggctctgcacctt
gcatggtttcttgcgttttgcttgtag

>g119681.t1
atggcgccaccggttactagagcagtactaggattattcatggcggtggtgctcttgtggatacaactggcggcggcggacaccgacggggacgccct
gcttgcgttcaaggccggcgtgaccgactcgaccggcgcgctggactcgtggaacgcgacgacgcccctgtgccaatgggccggcgtgacgtgcacc
gcgggccgcgtcacgtcgctggacgtctcggagcggcggctcaccgggacgctctcccccgccatcggcaacctcacgcacctcgaggttctcaacctc
acccgaaacggcgtctcggggcgcgtcccggcgagcctgggccggctgcggcgcctctcgtacctcagcctctgcgacaacgccttctccggcgcgat
ccccgacgcgctccgcaactgcacgggcctctccatcgcgttcctcaacaacaacgaattcaccggcggtgtgccggattggctcggcggcgcgtggcc
gaacctcaccgtcctccggctcggcacgaaccagctctccggccgcatcccggcgtcgatcgccaacctcacgcagctctaccgcctcgagctcgacca
gaaccttctggaaggcacggtccccgccgacctcgagcggctcaccagtctcctcttcttctccgtctaccagaaccggctctccggcgaggtcccgccg
ttcctcttcaacatgtcgtcgctggtaggcctggccctcgccgacaacgcgttcgaggggacgctcccctccgacgccggcgcgggctggcccaacctc
cagtacctcatcatcggcgggaaccgtctgacgggcccgatcccggcgtcgctggcgcaggcgtcgaatctgacggtcctcagcttggccaacaacag
cttcaccgggcgggtgcctcccgcgatcgggaggctctgcttgcaggtgctgcagctgtccaacaacgagctggaggcaaccgatgacgccggcgggt
gggagttcttggaggggttgaccaactgcggcgcgctgttcgagatctccctcgacggcaacaggttttccggagcaatgccgggctccatcgcgcgcc
tctcgccgcagctcggggtgctcgtcctcggcggcaatggcatatccggagctataccgccggggatcggcaacatcgtcgggttacagatgttggact
tgagctcgaatctcctctccggcgtgatcccggacggcattgggaagctcgagaacctgcagggacttcggttgcagcagaacaacctggccgggcct
gttccatcctccatcggcaacctcggtcagctgctacgactcgacctcagcgacaacctgctgaacggctccatccctccgagcctcggtaagctccagc
agctcaatctgctcaacctctccggcaacgagctcaccggccacgtccccggagaactgttcaccctgtcgacgctgtcgtcggcgatggacctgtcga
ataaccggctcgacggcgtgatcccgggagacgtcggccggctggtgaaactcgcgctcctggcagtgtccggaaaccggttctccggcgaggtgccc
tccgagctgggcaactgcgccagcttggagttccttgacctcgacagcaacctcttcaccggaagcatcccgtcgtctcttcgaaggctcaaggggctca
ggaggctgaacctcacgagcaacaggctgtcaggaaccatcccgccggacctcgcccagatctccgggctgcaggagctctacctgtcgcggaacga
cctgtccggcggcataccggcagagctggggaacatgagctccctgacggagctcgacctctcctacaaccacctcgacgggcctgttccggcgcgcg
gcggcgtgttcgcgaacgccaccgggtttcgggtggccggcaacggcgcgctctgcggcggcgccacaagtctccgcctgccgccgtgcccgccacca
ccatcatctactggttccacccgacgccgccaccacgtgttcctcatcaagatcgcgctgcccgtcatcgccggcgcggcgctctgcgtcgccgcgctac
tggctctgctctaccgccgtaataagaggaggaggaggaaatcgagaatcgcagacaccgcggcgcgaagcgtgctcaactgcaacagctacccgag
ggtctcgtacgccgacctggcacgagccaccgacaacttcgcgcgcgacaacctcgtcggcgcggggaagtacgggtccgtgtaccggggcacgctg
tcgctcaagacgaaggggagctccgtgcacgaggacgtggtggtggcagtgaaggtcttcgatctcgcacaactcggcgcttgcaacacttttgtttca
gagatcgaggcgctgcggagcgtcaagcaccgcaacctgatcaacatcgtcacctgctgctccagcatcgacgcggacgggaacgacttcagggccc
tcgtgttcgacttcatgcccaattacagcctacaccggtggctgcacccgagctttgtggacgccaagaaagggcgcgtcctcaccatggtccagaggtt
cgacatcgccgtcgacgttgccgacgctctgaactatctccacaacagctgcacgccgccgatcatccactgcgatctcaagcccagcaatgttctcctt
gccgacgacatgacggcctgtatcggcgactttggacttgccaagctcattcttgatccggggtgccacgctgtccaaagcagtgaaagcaccatcggg
ataagaggcacagttggttatgtcgcaccagagtacggcacgtcagggaacgtttctaccaatggtgacgtatacagctttggggtcgtgcttcttgaga
tcttcgtaggaaaggcgccaacggatgacgcgttcggagacggcctgacactgccggaatttgtcggcgcggcattcccggaccagatcgagcagatt
cttgatccagctctgctgccgattccagactcgtccgacgcatcgatttcagtttcagaagagagccaggcgtgtgttacggtgcgtgactgcttggtttct
gccataagtgtcgggctcgggtgctgcaggcagacgccgtaccaaagaatgtccatgagggacgcggcagtagagctgcgcctcatcagagatgcttg
tcttcgtgcttgcggacagtga
>g119752.t1
atgccttccgcctcctgccgcagctccgtcttcttgcccatcctcttcctggctctcagcgtcggcgctgccggcgccgacggggatgccgccgcaatgca
ggccctgcttcgtgagctgtcgccgccgggctggggcccgggcccggaccactgcgcgtggcgcggcgtcacctgcgcccctcccccggaccgcgccg
tcaccgccatcgacctgcctcgccgggggctcccggccctccccgccgccttccgcgccctcacgcgcgtcgaggagctccagatttccggcaacaacc
tgacgggcccgctcccgtcgtggctcgcgtccctccccaacctccgcgtcctctccgcctacgagaacgcgctgtccggcccgatcccgcccgggctgg
gccgggccgcgccgctccaggccctgaacctccactccaacgccctctccgggcccatcccgcccaccctcttcgcccgcgggacgctccaggtgctcg
tcctcacgctcaaccgcctaaccggccccgtccccgacgccatcggccggtgccgggacctctccaacgtccgcatcggcgacaacgccctctccggcg
ccatcccctcgtccatcggggacgcctccagcctcacctacttcgaggccaacgggaacctcctcaccggcgccatccccgcggatttcgcgcgctgcg
ccaacctcaccctcctcaacctcgccttcaaccgcctcgcaggggaggtgccggccgcgctcggcgggctgcggaacctgcaggagctgctggtggcc
gggaacgcgctcggcggcgcgttcccgacgcccctgctgcggtgccgcaacctcagcaagctggacctcagctacaacgccttccacggcgcgctgcc
cgacgacctgtgctacaactccttccacggcgtgccgcggatgcagttcctcgtgctcgaccacaaccgcttctccggccgcgttccacctggaatcgag
gggtgcacgcggctcctcgcgctgcagctcggcagcaacaacctcagcggggaggtaccgcccgagatcggcagggtcaagagcctgcagatcgcg
ctcaacctcagcttcaaccacttggtcggcccgctcccgcgcgagctcggccggctcgacaagctcgtcacgctcgacctgtccagcaaccagatatcg
ggacagatccctggagacatgagagggatgctcagcttgatcgaggtcaatctgtccaacaaccggctcaccggcgccataccggacttcgtgccgttc
cagaagagcgcgtcgtccagcttctccggcaaccccgggctgtgcggcgacccgctacacatcgcctgccgcgggtcggtttacgactcatcatccggt
tatgaattggaccacaacaggatatcgtaccgggtggcgctcgccgtcgcgggctcctgcgtcctcatcttctcgctggtgtcgctgctggtggcgctctt
catgtggcgcgagaggcaggagaaggaggctgccgaggccgagaaagcaggggaggtcgcggcgtccgccgtgttcgtcgacagcctgcagcagg
ccgtcgacttccagagctgcgtcaaggccacgttcaaggacgccaacatcctcagcaacggcacgttcagcgtcacctacgaggccgtcatgccgtcg
ggcacggtggtctgcgtcaagaagctcaagtcggtggaccgcgccgtcgcgcaccaccggtccaagatgatccgggagctggagcgcctcgcccaca
taacccaccccaacctcgtgcgccccatcgggtacgtcatctacgaggacgtcgcgctgctgctgcaccaccacgtgcccaacggcactctgcttcagct
actgcacggtgatgacgctgctgacgagggccggaaacctgactggccgaggctgctgtcgatcgccatcgacgtcgcgcaagggctcgcgttcctgc
accaggtcgccacgattcacctcgacatctcttcggggaacgtcttcctcgactcgcattacaatgcgctgcttggcgaagtcgagatctccaagctgctg
gacccgtccaagggcacggcaagcatcagtgcggtcgcggggacgtttggttacatacctccagagtacgcctacacgatgcaggtgacagtgtcagg
caacgtgtacagctttggggtgcttctgttggagatactgacatccaagctgccagcagacgaggggtttggggaaggtgtggaccttgtcaggtgggt

gcacaccgccccggcgagaggggagacgccggagcagatcatggacccgaggctcagcaccgtgtcgttcgcttggcgcaggcagatgctcgcggtg
ctccaggtcgcgatgctgtgcaccgagcgttcgccggccaagcggcccaagatgaagaaggtggtggagatgctgcaggaggccaggaagagctga
>g119894.t1
atgccaatgctgcgccgccagcacacgcgagcatacctctgcctcctactcgtattggtgccctgctgcgggtttcagcactacatggcagacgaggag
gcacagctgttgctccagatcaagagcgcgtggggcgacccgccggtgctggcagaatggaatgcttcggcatcggccgcacactgcatgtggcctta
catatggtgcaacagagccgggcgcgtctctggcctctacctcgctgatgccaacctctccggcccattcccagaagctatcgctgttagggaacgggta
ggctatgatagcatataa
>g120278.t1
atgaaccatgtgaaggagaagggcaagcaaggcttagtccaaaggaccggagcagtgttgaagggcagtgctcgcgccgtggccatgggcagcgcc
tgctcgaggaagaggggccagctgggcaacgaggatgttctctacagcgcgaggttctccaagagcggaagcttcaagtggctgctgcacacactgcc
ccgcagcaactccgatgtgcagcggaaggcacagggaccggggccggggcggtgcccgtcgcttgtggagctgtgcgtggccaaagttcgtgaggac
atgagcaggtattctgatttctcgctgctgcccagggatcttagtcagcagatattcaatgaactggtggagtcgaactgcctcacggaggcgtcgcttg
gagcttttcgtgactgcgctctgcaggtgagtcatgttgcaggactggcttcattggtattgttgaatctgagtcgctgcggcatatacgatcaaggctgtg
agaaaatggcaggctaccctgttgtttaccacactaactggcgctgccttgcagatttgatcaacttggagtgcttgaacttggactcatgcaagattggt
gatgaggctctaatacacctgaaaggcctcttgcagctaaaaagcctggagctttcagacactgaagttgggagcaatggacttcgacatctctctggtc
tgcggaatttgcagagcatcaatctctcttttacgctcatcactgacactagtttgaagaaaatttctggcttgagctcactgaagtctctcaatcttgacaa
tcgccagatcacagataacggtttagcagctcttgcaagtaaggttgtttggcttgatacaggtctcactgggttgacacaccttgatctatttggagctcg
aataactgatgctggcacaaactgcttcagatatttcaaaaatctacagtctctggaggtctgcagtgggttcatcacagacggcggagtgaagaatatc
aaggatctgaaagctctgacgctgcttaacctatctcagaacggcaacctcacggacaaaaccctggagctgatctccggcctgaccgcgttggtgtca
ctgaacgtgtccaactcccgagtgtccaactcgggcctccaccacctgaaactgctgcagaacctgcgttccctggccctggagtcgtgcagggtgacg
gcgaccgagatcaagaagcttcagctggccgatcttcccaacctggtaagcgtgcggccagagtga
>g120485.t1
atgccgtttcccaccgcgtcccccgcggggcggcggcgcggcggcgggggcgcgggcctccggtgcgcgcccgtgctcctcctgatcctggcgctcgc
cgccggtgccgcggcgcagacgtcgccgtcggatgcggaggcgatgcgggcggtggcgaaggcgctgggcgcggacaagctcggctgggacgtctc
cggcgacccctgctcgcccaagcggtgggacggggtgtcgtgcgactcgtcgggccgcgtcaccgccatccaggtcgggaggcgcgggctcacgggg
actctggcgccggaggtgcgcaacctcaccgcgctcgtccgccttgaggtgatgctgaacaacatcaccggcccgatgccctccctcgcggggctctcc
cagctccaggtactcctggcccacaactgtagcttcacctccatcccgccggacttctttcgcgggctcaccggcctcaacgtcgtagacgtcgaccaca
acccgttcgccgcgtggacgctccccgccgacctcggtaactgctcctccctccagaacttctccgccaatggtgctggcgtcaccggcaccatcccgga
cttccttggcacgctgccgtcgctccagctgctggcgctggcttacaactatctgtccggccccgtgccggaatctctgtctggcgcgccgattgagcagc
tctggctcaacaaccagaaagggaccagcacgttaaacgggtccattagctttgtcaccaacatgacggctctgcagcaactgtggctgcatgacaaca
ccttctccgggcccttaccggacttcaaggggttcgataatctttgggatttgcagctccgtgacaaccaattcacggggccggtgccggacacccttgtc
aatttgaaggctttgaagacggtgaccctgaagaataatttgcttcagggaccgagacccgcttttccgaagggtgtcgttgttgacatggacgatgaga
ggttctgcttacaagaccctggaaagccatgcgatccccgcataagtctgttgcttgaggtggctgctggattcatgtacccaaagaggctagcagatgg
ttgggtgggtaatgatccatgtaagaatagtttcactggtgttacttgcgacgctacaaccggcaacattacagtgctaaattttgcccatatgggattcg
ctggcatcatctcacctgccatcggaaagattacttcgctgaaggtgctgaaactttctgataacaacatcactggaacagtgcctaaggaggttgccgc
attgcctgcactgaatgaagttgatctgtcaaataacaatctttatgggccagtgcctacttttgccacaaaggtggtgccgattactgccggcaacccaa
acattggcaaggatgctcctgctccaacagcaggatcaggtggcaccagcagcaacagtccatctggtaaaggtggtgatgagagcaaagaaaacaa
tggggggatcaggttcaccatcgccttctgctggagtcattgcaggatcagtgcttggttcggttgctggattgggcctcattgctgctttaggtttatattgc
tacaagagaaagcaaaggccttccggaagggttcagagtccacatgctatggttatccatccacggcactcagggtcggatgacatggttaaaatcac
agttgcagggagcaatgccaatggtggtgctcgtgcaagtgaggcatatagccaagggagtagtgatccacgtgacattcatgttgttgagagtggaa
atatggttatttcaatccaagttctccgtaatgtgaccaacaacttcagtcaggagaacatccttggtcgaggtggttttggcgttgtttacaagggtgagc
ttcatgatggtacaaagattgctgtgaaacggatggaggctggtgtgatgggtaacaaggggctaaatgagttcaaatcagagattgctgtcctgacca
agaacttctccgccaatggtgctggcgtcaccggcaccatcccggacttccttggcacgctgccgtcgctccagctgctggcgctggcttacaactatctg
tccggccccgtgccggaatctctgtctggcgcgccgattgagcagctctggctcaacaaccagaaagggaccagcacgttaaacgggtccattagcttt
gtcaccaacatgacggctctgcagcaactgtggctgcatgacaacaccttctccgggcccttaccggacttcaaggggttcgataatctttgggatttgc
agctccgtgacaaccaattcacggggccggtgccggacacccttgtcaatttgaaggctttgaagacggtgaccctgaagaataatttgcttcagggac
cgagacccgcttttccgaagggtgtcgttgttgacatggacgatgagaggttctgcttacaagaccctggaaagccatgcgatccccgcataagtctgtt
gcttgaggtggctgctggattcatgtacccaaagaggctagcagatggttgggtgggtaatgatccatgtaagaatagtttcactggtgttacttgcgac
gctacaaccggcaacattacagtgctaaattttgcccatatgggattcgctggcatcatctcacctgccatcggaaagattacttcgctgaaggtgctgaa
actttctgataacaacatcactggaacagtgcctaaggaggttgccgcattgcctgcactgaatgaagttgatctgtcaaataacaatctttatgggcca
gtgcctacttttgccacaaaggtggtgccgattactgccggcaacccaaacattggcaaggatgctcctgctccaacagcaggatcaggtggcaccagc
agcaacagtccatctggtaaaggtggtgatgagagcaaagaaaacaatggggggatcaggttcaccatcgccttctgctggagtcattgcaggatcagt
gcttggttcggttgctggattgggcctcattgctgctttaggtttatattgctacaagagaaagcaaaggccttccggaagggttcagagtccacatgcta
tggttatccatccacggcactcagggtcggatgacatggttaaaatcacagttgcagggagcaatgccaatggtggtgctcgtgcaagtgaggcatata
gccaagggagtagtgatccacgtgacattcatgttgttgagagtggaaatatggttatttcaatccaagttctccgtaatgtgaccaacaacttcagtcag
gagaacatccttggtcgaggtggttttggcgttgtttacaagggtgagcttcatgatggtacaaagattgctgtaaaacggatggaggctggtgtgatgg
gtaacaaggggctaaatgagttcaaatcagagattgctgtcctgaccaaggggacactgagcgaacacctgtttgagtggtcagaacataatctgcag
ccattggaatggaaaaagcgactgagcattgcgcttgatgttgctaggggtgttgagtacctgcacagccttgcccagcagaccttcatccacagagac
ttgaagccatctaacatacttcttggtgatgacatgaaggcaaaggtggcagactttggattggtcaggcttgcaccagctgatgggaagtgtgtctcag

tggagacaaaactcgctggcacttttgggtaccttgccccagagtatgcagttactggacgagttaccacaaaagctgatgtcttcagctttggtgtcatc
ctgatggagctgattactggacgcagggcacttgatgagactcagcctgaggacagcatgcacttagttacatggtttaggagaatgcagctcaacaag
gacactttcaggaaagcaattgacccagttattgagcttgatgaggagacgtttgctagtgagaacatccttggtcgaggtggttttggcgttgtttacaa
gggtgagcttcatgatggtacaaagattgctgtgaaacggatggaggctggtgtgatgggtaacaaggggctaaatgagttcaaatcagagattgctgt
cctgaccaaggggacactgagcgaacacctgtttgagtggtcagaacataatctgcagccattggaatggaaaaagcgactgagcattgcgcttgatg
ttgctaggggtgttgagtacctgcacagccttgcccagcagaccttcatccacagagacttgaagccatctaacatacttcttggtgatgacatgaaggc
aaaggtggcagactttggattggtcaggcttgcaccagctgatgggaagtgtgtctcagtggagacaaaactcgctggcacttttgggtaccttgcccca
gagtatgcagttactggacgagttaccacaaaagctgatgtcttcagctttggtgtcatcctgatggagctgattactggacgcagggcacttgatgaga
ctcagcctgaggacagcatgcacttagttacatggtttaggagaatgcagctcaacaaggacactttcaggaaagcaattgacccagttattgagcttg
atgaggagacgtttgctagtgttagcacagttgcagagcttgctggccactgctgcgcaagagagccaaaccagaggcccgacatgggccatgctgtc
aatgtgctctcaacactatcagatgtgtggaaaccatctgatccagactcggacgacagctatggtatcgacctggacatgacgctacctcaggcactg
aagaaatggcaagccttcgaggacagcagcaattttgatggcgcaacctcttcatttgttgcaagcttggataacactcagacaagcatcccgacaagg
cctccaggatttgccgagtcattcacctccgctgatggaagatag
>g120597.t1
atggacctcgacgacgacgacgatgacgtgagcttcgccgacgacgatatctactactccgacaagccggcacgacgcctctatgacgaaaggttcaa
cgtgctgaacagcaactcctccaggcgcaacagctcccccggcgcggctttctgtaggctgctgtccctgaagatcctggacctttccaacaacaagctc
accggggagctgccggactgctggtgggacatgcaagctctgcagttcatggacctatccaacaactccttctcaggtgagatccctgcggctccgccg
ggccacaactgtactctcgagtcgctccatctgtccggcaacggcttcaccggcgtcttcccgccggtcgtcaatgggtgccgtgatctggcaactctgg
atattagtaacaatcagtttttcggtggtattcctccatggattggcgatggcggtgttcgatcgctgaaaatccttagccttgggtccaacaacttcacag
tgtag
>g120599.t1
atgccgacgccatcgcccgcccccgtgcgcctcctcctcttcctcctaatccagctcttcttcgccgtcctcgccaatgccgcgacgccgccgtcggaggc
ggacgcgctgctcgcgtggaagtccagcctcggcgacccgccggagctgtccacatggaccaacgccacctccctctgctccaaatggcgcggggtcg
cctgcgacacgtccggccgcgtctcctccgtcagtctccggaacctgggcctcgccggcaccctggacaagctcgacgccgcggcgttcccggcgctgg
ccgagctcgacctcaacgggaacaacctgtcgggccccatcccggcgagcctctcgcgtctgcgctccctctccaagctcgacctcggcagcaacagtt
tcgacggcagcatcccgccgcagctcggcgacctcacgggtctcgtcgagctccgcctctacaacaacaacctcgccggcgccatcccgcaccagctc
agccggctcccccgcatcgtgcacttcgacctgggctccaactacctgaccaacccggacagcagcttctccgcgatgcccaccgtccagtttctctcgc
tttacctcaactacctcaacggcagctttccggtcttcgtcctcggctgccgcaacgtcacctacctcgacctgtcgcagaacaccctgtccgggccgttg
ccggatacgctgccggcgaggctgcccagcctgaggcagctcaacctgtccgctaacgcgttctccggccgcatcccggagtctttcgcgaggctcacg
gggctccaggacctgcgcatcggcggcaacagcttcaccggcggcgtgccggagttcctcggctcgatgccggagctgcggatcctcgagctcggcgg
gtgcccgctcggtgggcgcctcccggcggcccttggccggctgcagaagctgcaccggctcgatgtgagccgggccgggctggtgtccactctgccgc
cggagctgagcaatctcaccagcctcgatttcatggacctctccgtgaacagcctctccgggagcctgccggtgacgtttgcccggatgcggaagatgc
gggagttcggcatttcgtcgaacaagctcaccggcgagatccccggggtcctcttcgcgagctggccggagctcatatcgttccaagtgcagaacaact
cgcttaccggcgagattccgcgggaggtcggcaagctcacgaagctcaatatcctgtacctcttctccaactacctcaccggccccattccgccggaggt
cggtgagctagtcaaacttcaggagatggatttgtcggtgaactcgctcacggggcccatcccgagctcgttgggcaacctcaaggagctcaccaggct
cgcgctctttttcaataatctcaccggcgccataccaccggagatcgggaacatgaagtcgttacaaatcttggacgtcaacaccaacaatctggaagg
cgggctgcccgttacttga
>g120600.t1
atgcccaccgtccagtttctctcgctttacctcaactacctcaacggcagctttccggtcttcgtcctcggctgccgcaacgtcacctacctcgacctgtcgc
agaacaccctgtccgggccgttgccggatacgctgccggcgaggctgcccagcctgaggcagctcaacctgtccgctaacgcgttctccggccgcatcc
cggagtctttcgcgaggctcacggggctccaggacctgcgcatcggcggcaacagcttcaccggcggcgtgccggagttcctcggctcgatgccggag
ctgcggatcctcgagctcggcgggtgcccgctcggtgggcgcctcccggcggcccttggccggctgcagaagctgcaccggctcgatgtgagccgggc
cgggctggtgtccactctgccgccggagctgagcaatctcaccagcctcgatttcatggacctctccgtgaacagcctctccgggagcctgccggtgacg
tttgcccggatgcggaagatgcgggagttcggcatttcgtcgaacaagctcaccggcgagatccccggggtcctcttcgcgagctggccggagctcata
tcgttccaagtgcagaacaactcgcttaccggcgagattccgcgggaggtcggcaagctcacgaagctcaatatcctgtacctcttctccaactacctca
ccggccccattccgccggaggtcggtgagctagtcaaacttcaggagatggatttgtcggtgaactcgctcacggggcccatcccgagctcgttgggca
acctcaaggagctcaccaggctcgcgctctttttcaataatctcaccggcgccataccaccggagatcgggaacatgaagtcgttacaaatcttggacgt
caacaccaacaatctggaaggcgggctgcccgttacnaaaagagacttcacatgcaccccctaatccattgtccccgaaagtcaattactgtggccactg
tacactcctaccccctccgcctgggctaaatttgcttgggaacactgggatctataatgagtataatccaaaatggaccgatccggcgcaccatgtttcca
ttgccaatatattccaagtcaaaggaaagaaccaagctggttgtttggttcgttgctacgatgcccttactaccgtcgatgccacagtaagatga
>g120975.t1
atggccggcggcggctgggccctcttcctcttcttggccgtgtccgtctgctgcgcggcggcgcaggagcccaacaccgacgccttcttcctctccaagtt
cttctccaagctgggccgcccggcgccgccgccgtcttcgaacaccagcgaggggccgtgctcgtggtcgggggtcttctgcgatggcgagggccggg
tggtgtccttctccgccgccgggatgggcctggcgggccccatccccgaggacaccgtcggcaagctggcccacctggagcgcctcgacctcagcggc
aaccgcctcaccgcgctgcccgacgacctgtgggagctcggcgccgccctcacggagctcaacctctccggcaacgccatcgccggcgcgctcaccaa
caacgtcggcaacttcgcgcggctccgctccctcgacgtctctggcaacgccttcgccggcgtgctacccccgcgctcggctcgctcgccggactgcgg
gtgctcaacgccagccacaacaaattccagggccaaattcccggcgccgtcgtctccgggtgtgagagcctcgtggcgatggatctctcttacaatgcg
ctggacggggacttgccggacctgtcgctgctccggtcgctgtcctacctcaacgtctccggcaaccggctcggcggctccgttgcaggggcgttccagg
agcagctcatggtcatcgacctcagcaacaacggcttctcgggcttgaatttcagcactgggtatgcaggctcatcactgacgtatctcgacttgtcagg

caatgagtttcaggggggaattcaccgttgttgctggtcggttcaggaacctgaagcatgtgaatcttgcaaacaaccggaggttgtccaatggcagtttg
ctcgtgtccatgggtgggatctccgggttggagtatgtcaatttgtcgagcactgggttgcgcgggcaaatccctgcagagttctcatctcggttggtggg
tttgaaggttcttgatttgtctcggaacaacatcagtggggttatcccagatttgagctcgcttcagctacaggtgctagacttgtcggtgaacaatctcac
cggtgagatacctgtgacgttggtgaagaagttgccgtccatggagcggttcaacttctcgtacaacaacctcactgtttgtgcctctgagctctccccgg
aggcgtttgcagctgcatttgctcggtccaggaatgactgcccaattgctgtcaacccggacaagatcaagaaacataggggaaagcgcaaggaaatg
aagctggcgttggccattgtgctctcgctgttctttgcagctcttgggttgctttgcttggccgttgcatgccggaggcggagaaagaggtgtgatacgca
gcccgtggttaagcaggtgtcattcaaggaagagtcagcaagtgtatcagggccatttgctttccagacagattcaactacatgggttgctgatgtgaag
gttgcgacttcggtgccggttgtcatctttgagaagccattgttgagcttcacgtttgccgatctcttagcagctacatcaaacttcgaccggggtacattg
ctggcagaagggaggtttgggccagtgtacagaggatttctccctggtggattccaagttgctgtgaaggtgctagtccatgggtcagtgatggaagac
caagatgctgcaagggagctcgagcggttgggacgaatcaaacatccaaacttggttcctttggctggttactgtctggccggggaccagagaatcgcc
atctatgagtacatggagaatggcaatttgcacaacttactgcatgacttgccactaggagttcagaccactgaagattggagtaccgacacatgggag
gataacaatggtggtgttgctactgaaaacatcacgccggagggtactgcaacatggatattccggcacaaaattgcattaggcgctgcaagggcact
ggcattcctccatcacggatgtattccgcagattgtccaccgggatgtgaagccaagcagcatctacttcgactcaactatggaacccaggctgtctgatt
ttggattgtccatggtcgctggtacaaacacagacaacgatctcttgcatcactccccaggttatgcacctccagagttctccaactcggagaatgtcatg
gcgaccgccaagtctgatgtctacagctttggagtcgttttgtttgaattggtcactggaaagaagcctcttggtgatgagtaccctgatcggaaggagg
aaagcctggttaactgggccagggcaatggtgaaggcaaaccttgggtcaagcatcatcgactcaaagattcgtgacaccgggctggagcggcagat
ggaggaggcgctgcggatcgcgtacctctgcaccgctgagctgccatccaagaggccagccatgcagcagatcgttggcttactgaaggacattgagc
ctaaaatatga
>g121130.t1
atggcgtcgttctcgaagaagactcgttacgtacaattgccagcagcccttgtcccgctgctagtggcagcagcattcgttgccgctccggcggcggcgg
cggccgcggtccctgactcgtcgacggcggtcgccacgcacctcgaggcgttgctggcgttcaagaagggcgtcacggcggacccactcggcgcgctg
tccaactggacggtggggtcaggaggcgttgccaccgccgccggtggccttctcccgcggcactgcagctggaacggcgtcgtgtgcgacgacgccgg
ccacgtcacatccatccagctcccggagacccagctccgcggcaggctgacgccgttcctcggcaacatctccacgctccaactcctcgacctgtcttcg
aacaacttcgtcggcgccatcccgccgcagctcggccgcctcgccgcgctcgagaagctcggcctctacgacaacagcttcaccggcggcatcccgcc
ggagctcggcgacctcgggagcctccaaatccttgacctagcccagaacactctccgcggcggaattccgagaggcctctgcaactgctcggcggtctc
ggcgctggccttggagtacaacaacctcaccggctcaatccccgattgcatcggagacttgtcaaacctcgagatgttcctgacctactacaaccagctg
gatggagagctgcctccatcattcgcgaagctcacgaagatggagacgctggacctcagcgacaaccagttctccggcgccatcccgccggctatcgg
caacttctccaacctcaacatcctccaactgaaggagaacaggttctccggtgccatcccgccggagctcggccggtgcatgaacctgagcgtgctcaa
catatacagcaaccggttcaccggcgcgatcccgagcgagctcgggtacctcacgaacctgctgtcgctgcgcctgtacgacaacgcgctgacgtctga
catcccgcgctcgctcgggcggctgacgtcgctgctgaacttgggcctctccatgaaccagctcacgggatccatccccaccgagctcggcgcgctccg
gtcgctccgcacgctgacgctccacaacaacagcctcaccggaactgtgccggcgtcgctgacccgcctcgtaaacctgacgtacctctccttcagctac
aactccctgtccggcccggtcccggaagaactcggctcgctgcggaacctccagaagctggtcatccaaggcaacgcgctctccggcccgatcccggc
gagcatcgccaactgcacctcgctggggaacgcgagcatggggaccaaccatttctccgggccgctgcctcccggcctcggccggctaccgaacctca
cgttcttctcggtaccgtacaacaacctgtccggcgagataccggaggacctcttcgactccgcgagcctccggacgctgacgctggccgggaacggct
tcaccggctcgctgagccgccgcgtcggccggctcagcgatctcaagctgctgcagctgcaggggaacgcgctgtcgggaccgatcccctcggagatc
ggcaacctgacgaagctcgtcggtctgaacctccggcggaaccgcttcgtggggcgcgtcccggattccatctccaacatgtcgtcgtcgctgcaagag
ctcgacctgggcgagaaccgtctcgacgggcccctccccggcgcggtgttcagcctgcggcggctcaccatcctcgacctcgcgtcgaacaggttcgcc
ggccccgtccccgacgccgtctccaaccttcgcgcgctgtccctcctcgacctctccaacaacgcgctcaacggcacgctccccgccgcgctgtccggtg
accagctcctctcgctggacctctcccgcaaccggttcactggcgccgtccccggcgcggtgctcgcgaaccttagcagcctccagacgttcctcaacct
gtcgcacaacgcgttcgacggcgagatcccgccggagatcgggaagctcgcgatggtccaggccgtcgacctgtccagcaaccggctgtccggcggc
gtccccgcggcgctcgccgggtgcttgaacctcgcccttctcgacctgtcggggaacgaactagccggcgcgctgccgccggggctgttcccgcagctc
gacgtgctcaccagcctcaacgtctccggcaacgcgctcgacggggagatcccttccaacatcgcggatctggagcatgtacaaacactcgacctgtcg
aggaacgcgttcacgggcggcataccgcccgggctggagaatctgaccagcctgaaatctctcaacctgtcctggaaccatttggagggggccgtgcc
ggacggcggcgtgttcaggaacatgagcgcgtcgagcctgcagggcaacgacgggctctgcgggtggaagctcctggcggcttgccagcgtcacagc
ggcaacaagcgggcgttctcgaggacgggcctcgtgatcctagtcgtgctgctggtgctgtccttactgcttcttctgctcctcgcgacgattacatgcgtc
gcgtacaagaaaaagcgaggaggaggcaacgacggcgccgccaggttctcggagacggtggtcgtgccggagctgaggaagttcacgtacagcga
gctggaccacgccacgcgttcgttcgacgaaggcaacgtcatcgggcgcagcgccctgagcacggtgtacaagggcgtgctcgtcgaatcaggcaag
gcggtggccgtgaagcggctcaacctggagcggttcccgtccagcggctccgataagtgcttcctcacggagctcgcggcgctgagccgcctgcgcca
caagaacctggcgcgcgtggtcgggtacgcgtgggagcccgggaagctcaaggccctcgtcctggagttcatggtcaacggcgacctcgacgctgcgt
tgcacgggcgcgacgcggcggcgcggtggacggtgcgggaccggctgcgcgtgtgcgtctcggtggcgcacgggctcgtgtacctgcacaccgggta
cgacttccccgtcgtgcactgcgacgtcaagccctccaacgtgctgctcgacgccgagtgggacgcgcgcgtcagcgacttcggcacggccaggatgc
tcggcgttcacctgaccgacgcagccgccgccgcgcagtccgcgacatcgtcggcgttcaagggaaccgtcggctacatggcaccagagttcgcgtac
atgaggaccgtctcgccaaaggccgacgtgttcagtttcggcgtgctggtgatggagctcttcacgaagcgccggcctaccggcaccatcgaggagga
cggcgtgccgctgacggtccagcagtacgtcgacaacgcactctcgaggggcctcgacggggtcgccgacgtcctcgaccccgacttgaaggtcgcca
ccgaggccgacctgtccacggcggcggacgtcctgagcctggccgtgtcctgcgccgcgttcgagccggcggagcggcctgacatggacgccgtgctg
tcgtccttgctcaaaatgagcaagatctctggagaagactag
>g121131.t1
atggcgtcgttctcgaagaagactcgttacgtacaattgccagcagcccttgtcccgctgctagtggcagcagcattcgttgccgctccggcggcggcgg
cggccgcggtccctgactcgtcgacggcggtcgccacgcacctcgaggcgttgctggcgttcaagaagggcgtcacggcggacccactcggcgcgctg

tccaactggacggtggggtcaggaggcgttgccaccgccgccggtggccttctcccgcggcactgcagctggaacggcgtcgtgtgcgacgacgccgg
ccacgtcacatccatccagctcccggagacccagctccgcggcaggctgacgccgttcctcggcaacatctccacgctccaactcctcgacctgtcttcg
aacaacttcgtcggcgccatcccgccgcagctcggccgcctcgccgcgctcgagaagctcggcctctacgacaacagcttcaccggcggcatcccgcc
ggagctcggcgacctcgggagcctccaaatccttgacctagcccagaacactctccgcggcggaattccgagaggcctctgcaactgctcggcggtctc
ggcgctggccttggagtacaacaacctcaccggctcaatccccgattgcatcggagacttgtcaaacctcgagatgttcctgacctactacaaccagctg
gatggagagctgcctccatcattcgcgaagctcacgaagatggagacgctggacctcagcgacaaccagttctccggcgccatcccgccggctatcgg
caacttctccaacctcaacatcctccaactgaaggagaacaggttctccggtgccatcccgccggagctcggccggtgcatgaacctgagcgtgctcaa
catatacagcaaccggttcaccggcgcgatcccgagcgagctcgggtacctcacgaacctgctgtcgctgcgcctgtacgacaacgcgctgacgtctga
catcccgcgctcgctcgggcggctgacgtcgctgctgaacttgggcctctccatgaaccagctcacgggatccatccccaccgagctcggcgcgctccg
gtcgctccgcacgctgacgctccacaacaacagcctcaccggaactgtgccggcgtcgctgacccgcctcgtaaacctgacgtacctctccttcagctac
aactccctgtccggcccggtcccggaagaactcggctcgctgcggaacctccagaagctggtcatccaaggcaacgcgctctccggcccgatcccggc
gagcatcgccaactgcacctcgctggggaacgcgagcatggggaccaaccatttctccgggccgctgcctcccggcctcggccggctaccgaacctca
cgttcttctcggtaccgtacaacaacctgtccggcgagataccggaggacctcttcgactccgcgagcctccggacgctgacgctggccgggaacggct
tcaccggctcgctgagccgccgcgtcggcnacaactga
>g121132.t1
atggaattcaacaacctgaccggcacgatcccgggctgcattgggaacctgtccaatctgaagatcttcgtggcctacttcaacaacctcgagggcgag
ctggcaccgtccttcgccgcgctcacgcggctgcagacgttggacctcagcgacaacaactttttttccggctccatcccgccggcgatcggcaacttctc
gaacctcaaaatcctccagctgttcgagaaccagttctccggcgcgattccgcgggagattggcgggtgcaggaacctgattatgctcaacgcatacag
caaccggttcaccggcgcgatcccaagcgaggtcggagagctcacgaacctgcactacctgcgattgtacgacaacgccctgtcttcggggatccctcg
ctcgctcggacggctaacggagctggctgtgcttgagatatcacagaaccagctcaccgggtcgattccgccggagctcggaatgctacggagcctca
ggcggttgagtttatcctataacaagctctccggcgacatacctgaggaactgttcgattgcgacgttctgctcgtgctgagcctggcggagaacagctt
cagtggcggactgagccgccgcgtcggccggctcagcgagctcagatggttggacctgcaggggaacgctctgtcggggatgataccggaggagatg
ggcaacctaacgaagctcacagtgctgctgctccggggaaaccgcttctttgggccgattgcgtaa
>g121133.t1
atggcgttcttcatcaacacttggcacgctcaaattcttcccgtgctagtagtaccagcgctgttcttggcgacgccgtcggccgccgtaccagacgcgtc
gacggtgtccgtggtgcagctcgaggcattgctggcgttcaagaagggcgtcactgcggacccgcgcggcgcgctgtccaactggacggtggggtcag
gaggcgttgccaccgccgccggtggccttctcccccggcactgcagctggaccggcatcgcgtgcgacggcgcaggccacgtcacctggaccggcatc
gcgtgcgacggcgcaggccacgtcacgtccatccgacttgtggagactcggcttcgtggcacgctaacgccgttcctcggcaacatctccacgctccaa
atcctcgacctctcatcgaacaactttgtcggcgccatcccgccacagctcggccagctaggtgatcagctggagcagctcatcctctacgacaacatct
tcagcggcgccatcccgccggagctcggggacttgaggagcctacaaatactggacctggccaacaacacgctccacggcggcatcccccgccgcctc
tgcaactgctcggcgctgtgggcgctcggcatggaattcaacaacctgaccggcacgatcccgggctgcattgggaacctgtccaatctgaagatcttc
gtggcctacttcaacaacctcgagggcgagctggcaccgtccttcgccgcgctcacgcggctgcagacgttggacctcagcgacaacaactttttttccg
gctccatcccgccggcgatcggcaacttctcgaacctcaaaatcctccagctgttcgagaaccagttctccggcgcgattccgcgggagattggcgggt
gcaggaacctgattatgctcaacgcatacagcaaccggttcaccggcgcgatcccaagcgaggtcggagagctcacgaacctgcactacctgcgattg
tacgacaacgccctgtcttcggggatccctcgctcgctcggacggctaacggagctggctgtgcttgagatatcacagaaccagctcaccgggtcgattc
cgccggagctcggaatgctacggagcctcaggcggttgagtttatcctataacaagctctccggcgacatacctgaggaactgttcgattgcgacgttct
gctcgtgctgagcctggcggagaacagcttcagtggcggactgagccgccgcgtcggccggctcagcgagctcagatggttggacctgcaggggaac
gctctgtcggggatgataccggaggagatgggcaacctaacgaagctcacagtgctgctgctccggggaaaccgcttctttgggccgattgcgtaa
>g121811.t1
atgcgggccggaggcggacggcgcctcgggccgcccggggttgaggtcctcgccttcctgctgctgatcgccgcggcggcattggtgccgcgtgcgcg
cgcggccaccgacccctccgacgttgctgcgataaatgaactttatgctgctcttggatcaccaactctgcctggatggacggcgagtggtggagatccc
tgtggtgagaagtggcaaggtgtcacatgtatcggcacaagtataaactcaatagtttttaatgctgcaaatttggggggacagctaggcggcctggag
aagttcacgacaatagctgaaatggccccacccccgctcccctgctctcaagttccattcaatgtgccgtga
>g121916.t1
atgtctcgtgccgccttcctgctcctcgtcacgctcgctgccaccgtcgcgccgccgtcggatgcgtcgtttgcggacgccggcgaagcggctgtcctcca
ctccttcctcgcctccctacctccggccgcccgccgcgtcctccgcccgttgtggaaggccagcgccgctgcaaccagcagccgcaacgggtcccgtgg
ccactgcgcgtttctcggcatcgaatgcgccgctgccggcgatgtcacagcggtcaatctctccggggcgggtctctccggcgaactcgcggcgtccgc
accgcggctctgcgcgctccgagcgctcaccacgcttgacctcaccggtaacaggttcacgggcgccgtccccgccgcgctcgccgagtgctccggcgt
cacgaccctcctccttggtggcaaccttctcaccggcgcggtcccgccggagctcttctcctcgcggaagctccgtaaggtcgacctcagtgtcaacaatt
tcaccggcgagattcccgtcccccgttcgccagttctcgagtacctcgacctcagcgtcaacgccctctccggtgctatttcgccggagctcgtcgcgctg
ccacagctcagccatctggacctgaacagcaacaacctgtccgggccagtgccggagttcatggcgcaatgcaggcttacctacctcagcctcttctcc
aaccagatcgccggtaacctcccccacagtctcagcaactgcggcaatctcaccgccttgtacctatccttcaatgagatcagtggcaaggtgccggatt
tctttgcctccatgccgaatttgcaaagtctgtatcttgatnctacctatccttcaatgggatcagtggcaagcattggtgagcttgtcaaattggaagaat
tggtggtgtcaaataacgggttcgctggacctgtaccagaggcaattgggaagtgtcagtccttgacgatgctatacttgaatggcaaccagttccctgg
cccaattccagcgttcattggaaatttaagtagattgcagaagttctctatagcggacaatggcatttctggaagaattccaccagagattggtaaatgc
gtggagttagttgagctccaacttcagaacaacagcctctatggggctgtaccatcggagatcagtaaccttacccagttgcagagactctatctattca
ataacttgcttcatggacatgttccttcagaattgtggcaattaccgaacatggtagaattgtacctcgacaataatagcttcagtggtgagttgcctgcac
agatcactcaaatgaggaagctaagggagatctcattgttcagcaattacttcacaggcatgttgccacaagccctggggttgaacacaacacctggac
ttcttcgcgttgaactcactggcaaccacttccatgggaagattccactgggtctctgcactggaggccagctcagccttcttgatcttggagataatcact

tcagtggtgggtttccagctgaaattgctaaatntcgcgttgaactcactggcaaccacttccatgggaagattccactgggtctctgcactggaggcca
actcagccttcttgatcttggagataatcacttcagtggtgggtttccagctgaaattgctaaatttgtgaacgtttcattcaatgagctcgctggtcagctg
cctgctagctgggctaagcttgcagctcggtcacctgaaggaaatgcaaggataattgtggcactagtagtatcggttcttgctatcgttattgctgggct
gtgcgctattcactacctagtgaagaaatcacagcgcttatcagcaagcaacagtcgtgccagtaacctggacacaacagaggaattgccagaagact
tgacctatgaagatatcctccgggcaacagacaattggagtgagaagtatgtcattgggcgaggccggcatggtacagtctaccgtacagaatgcaaa
cttggaaagcagtgggcagtcaagacagttgatctatcgcagtgcaagtttcccactgaaatgaagattctgaacacgatcaagcaccggaatattatc
aggatggacggctactgcatccgtgggagtgtcggcctaatgctttatgaatacatgcccagtgggaccctctttgatctgttgcatgcaaggaaggctc
aggttgatcttgactggatagcccggcatcagattgctcttggtatcgcgcaaagcctttcctatctgcaccatgattgtgtacccatgattgttcataggg
atgtcaagtcaagcaacatactgatgggcactgatctaacaccaaaactcacagatttggcatggggaaaattgtcaatgacaaggacactgatgca
acagtttctgtcatcgttggtacccttggctacattgctccagagcatggatactccacacgattatctgagaagagcgatgtctacagctatggtgtagt
gctacacgaaatcccttttcagaaagatgccggtggatccttcatttggtgacggtgtagacatagtggcatggttgagatcaaacctgaaacaggaaga
ccgctacagcatcataagtcacatggatgaggagatcatacactggcctgaagatgagcagacaagggcattggacttgctggatttggcggtttcttgt
acacaggttgtttgccagtcaagaccttccatgagggaagttgtaaacatcttaacgaagatacaaaagtga
>g121982.t1
atgttgagcagccagttctttctgaaaccatttgatagcagaaattatgaattgaaccgccacagcccacagaactgtggaatcccgggccacacaggc
aaaggatcaagcgtcaacaccgagcaaatcgacgccaggcagcagtacgccgccgccaccggacatgaggatgcagctgccgggtacgacgacaa
cgatgaggtcaacatgcttgctgtcgccggagattatggagccgaaggtgagaatgcagtcgtctttgaccgcgaccagctcctggcgggtgcgcccgc
gctgcagagccttgtcctctacggcaacgcgctgcgcgggcccctccccgctgacgcgctcgccggcctcgcctacctccagatcctcgacctctcctcc
aacgcgctcaacggcgccctccccgactcgatcctcaaatgccgccgcctgcggacgctcgcgctcgcgaggaacaacctcacgggcccgctccccgc
ggggctgggcatccaactgccggggctggagcggctcgatctgtcatacaatcgcttctccggaggcctccccgaggacctcgggaacctcaccaggc
tccagggcaccgtcgacctctcgcacaaccagttctccggctcgattccggcnccgaggacctcgaacggggcgctggagaaccggggccccacggc
gttcgtcggcaacccggggctctgcggcccgccactcaagaacccgtgctcgccggacgccgcgccctcgtccgacccgttggtgcccaaggacgggg
actcctcctccggggctggcagcggcaaagggaagaagagcaaggggttggggaagatcgccattgtggccatcgtgctgagcgatgtggtggggat
cctcatcatcgcgctggtcttcttctactgctactggagggcggtttcgtccaaggacaagaccggggttgccggttccaaaggatccaaatgtggtggc
agggattgcagctgcttcagccgggacgagtccgagagtccctccgagcagtacgaccttgtggcgttggaccaggaactgaggtttgatctcaatgag
ctgctcaaggcatcggcattcgtgctggggaagagcggcatcgggatcgtgtataaggttgttctcgaggacgggctcaccatggcggtcaggcgactc
ggggaagggggattgcagaggtttaaggaattccagactgaggtcgaggcaatcggcaaggtccgccatccaaacattgttaccttgagggcctacta
ctggtcttttgatgagaaattgctcatctatgattacattcctaatggaagcctctctgcagcaattcttggtagtaaacctgggacaatgacattcacacc
attgccatgggaagcccgtctaaacatcatgaaaggagttgccaagggaatgtcattcctgcatgaattcagccccaaaaagtatgttcatggggactt
gagaccaaacaatgtgctccttggaacaaacatggaggcatatatttcagattttggccttgggcgacttgcaaacattgctggagggtcgccttatgtg
caatcagatcgagttggtttagaaaagggtcagagccagccatcagatgcctcagtgagccctctggtgagcagagggtcatgctatcaagcaccaga
agcgctcaaaacgctaaaaccatcacagaaatgggatgtctactcttatggagtggtcttgcttgaaatgattactggcaggtcacctgctgttctcctgg
aaaccatgcagatggatcttgttcaatgggtccagttctgcatcgaggagaagaagccgtccgctgatgtgctggaccctttccttgcccaatactcgga
atgggaagacgagatgatcacggtactcaaaatggctctagcctgtgttcaggcaaaccccgagcggagaccgttgatgagacatgtcgcagagacc
ctggagcgcctcaatggttcaagctag
>g122119.t1
atggccaagcaggctctcgccgcgctggtaataataacaacgctcctcctcgcctgcttcccccgcgaggcgcggtgcgaggaatcctctgacgtgcgc
gcgtccctcgtgggcttcctcgtcgagctcgccggcggcgacgcgcagacggcgcgggacctccggtgggacgcgacggtccagccgtgcgacccca
cggcgccctggaccggcgtgacctgcggcggcaacgccggccgcgtccgggccatcgtgctcgagaaccaggggcccggcaagctgaccggcaccc
tgaacacgacgctgctgtgcgccgcgtccacgctgcgcgtgctgagcctgcagggcaacgcggtcggcggcggcctcgtggagggcgtcgcggcgtg
cagggcgctcacccacctctacctcgcggggaaccggctctccggcgccctcccgcccgcgctcgcgcggctcagggacctccacgtggtcgacgtcg
ccaggaacggcttctccggcgagatccccgcggggctcgccacgatcgggctcaagacgttcctggccaacgataaccggttcagcggcgccgtcccg
gacttcgacctcagagtgttcgacaagttcagcgtggccaacaacagcctcacgggacccatcccgaggaacgtgggcaagttccgcgacgccaggtt
cttcctgcccaacgccgcgggcatctgcgggcaacctgtcttggccgcttgtcaacgatcggcaccgtcaccacagccggactccgacttcgacgccgc
gccgccgcctagcaccgggtccaacagtaccggggagaggcggagcaagaagacgaaggtcctcatgtacctcgggtacgccctcctcggcgccgtc
atactggccttggtcacgtacctctgcgtcaagaagaggaggagcaagctgcgtaggaagttgttcaagcggggcgggggcaggcgcgtgtccgaca
gcagcagggtcaccacgacaaccgccacgacgtcggtgatcagcgcgagcaagtcggtgtactcgatcccgacgtcggtcgtgcagagccccgcggc
ggcggcggcgacgggaccagcagcagccgcgccgtcaccgtctctcgtggtgctgcggcggtccggcacggcggcgtcgatcacgtcggcggcggcg
gctgccgccgcgaaggagctgcggttcgaggacctgctcaagtcccccgcggagctgctgggccgcggccggttcgggagctcgtacaaggtggtggt
ccccggcggcgccgagctggcggtgaagcgcgtcaaggacggggtggtgggggaggccgagttcaggaggaggatggagcgggtggggcgcgcca
ggcacgccgccgtgctgccgccgctcgccttctactgcgccatgcaggagaagctcgtcgtctacgagttccagaccaacggcagcctcgcaaggctcc
tgcacggtaaacaatgctctccttgccccgcaacatttcgcgccctggtctctgctcctgcaccgtaa
>g122471.t1
atggtcgccgcgttcgccgactccgggcgagggcgcacgctcgtctcgctctccggcttccgtgacctcgcgcaggagtacatacccaccatcgcggtg
gtgtgcggcaacttgaggaccctcgacttgagctacgccccgatcacccccaatcagattgtcatgttcatcggccagtgcttcaatcttgagacgctatg
ggtgctcgactctgtgcgtgatgaaggactcgagactgtggcaatgtgctgcaagaagctccagtctcttcgcgtgctcccactggacgcgcacgagga
tgctgaggagctagtctcagagctagtctcagaggttggcctcgccgccatctcccggggctgccgtgacctccgttccatactgtatttttgccagacaa
tgaccaatgctgctgtcattgacatgtcgcaaaactgcccggagctcaaagtgtttcggttgtgtataatgggacggcacaagcccgaccatgccaccg
gggagcctatggatgaagggttcggtgccattgttcggaattgcagcaagcttaccaggctatccacatccggtcaccttaccgacagggcatttgagta

cattggcaagtatggcaagtccttgcggacactctctgtagcatttgctggaaacagtgatttggcgcttcaacacatcctccagggctgttcaaagctag
agaagcttgaaataagagattgtccattcggtgatgccggactcctctctggaatgcaccatttttacaatatgcggttcgtctggatgtcaggctgcaac
ctgaccctgcaagggtgcaaggaggtggctcggcaactgccacgaatggtggtggagttgataaatggccagccagagaatgaaaggactgaaggtg
tggacatcttatacatgtatcgctcacttgatgggccaagagaagatgtaccaccatttgtgaagatcctataa
>g123485.t1
atggcgcaaactgacgctgtcgagcttgcaacactcctaaaactcaagacagactggggcagcccttctgcgctcagctcatggagttcggagaactct
acctactgcaaatggtcagggattatttgcaacagcaatggccacgtcaccaccctctccttccataatttccacatagcccatccaattccagcctccatt
tgcagcctcaacgacctgtcacacttagacctgtcatacaacaacctcaccggcgacttccccacggcgctttacaactgctcggctctgcagtttctgga
cctgtcgaacaatgagttctccggcgcactgccggctgacatcgataagttgtcgccggggacgatggagcacctgaacctgtcgagcaatggcttcac
cggcaaggtgccgtcgggaattgccgagttcccgaagctcaagtcccttgttctcgacactaatagcttcaacggaagctatccggctaccgccatcagc
aaccttgttagcctcgagactctgacgctggcgagcaacccgttcatgccggggccagttcctgaagagtttagcaagctgacgaagctgaagacgctc
tggttgtcggggatgaacttgactggtgtcatcccggactccctctcggcgctcaccgagctgacgttattggacctgtcacaaaataaacttcatggtga
aatccccgggtgggtttggaaccaccagaagctgcagtgcctgtacctcttcgccaacaacttcatcggtgggatcggacccagcatcaacgccaccaa
catgatcgagctcgacctttctacgaacagtctcacggaaccgataccggatgctatcggtaacatgacgaacttgaagctgctgttcctgtacttcaac
aagattgccgggcccattccggcaagcgtggggcagcttccgaacctgagggacctccggctattcaacaacaagctgaaggggccgctcccgcctga
gctcgggaagcactcgccactgggcaacctagaagtgagccacaacatgctctcgggcaccgtcccggacaccctctgctccaacaagaagctctacg
acctcgtcctcttcaacaacagtttctccggcgagttcccggcaagcctgggcgagtgcgacacgctggacaacatcatgctgtacaacaaccacttcac
cggcgtgtttccagagcgggtgtggtgggcgttcccgaagctgaccaacgtgatgatccagaacaacagcttcacgggcactctgcccaccgcggtatc
ctccaacatctcccggatcgagatggggaacaaccggttctccggacccgtgccgtcctccgcgccgggtttgaacgctttcaaggcggagaacaaca
ggttctccgccgcgttgccaaccaacatgtccgggttggctaacctgaccgacctggacctctccgggaaccagatttccgggtcgatcccgtcgtccat
cctgtcgttgcgaaggatgacgtccttgaatctgagcggcaaccaactgtccggcgacatcccggcggcgatcgggtccctgcccgtgctcaacatcct
cgatttgtccgataaccagctcaccggaagcatccctagggagttcaacaacctccgcctcagcttcctcaacctgtcgtcaaacgaactcgccgggga
ggtcccgtcgtcgctgcagaaccaggcttatgaaggagcgttccttcgaaacccaggcctctgcgccacggtagtggacctgaacctgaacattcgggc
atgcgacggctacacaagaggccgcaacgaccagatgtcaaccggcctcaccatcctgttctcggtgctcgccggcgcgaccttcatcggcgtcgtggg
gtgcgtcgtcatcctggggaagaagaagaggcggcagcacgacacgaccgcgtggaagatgaccccgttccggaagctcatcgacttcacggagcgc
gacgtggtgacggggctccgcgaggaggacgtgatcggcagcggcgggtcggggaaagtgtaccgcgtcaagctcgctccctccggcacggcggtg
gccgtgaagcggctcaacaagggcggcagcggcaagtccgacgagaagctggagagggagttcgactcggaggttcaaatcctcggcgacatccgc
cacgccaacatcgtgagcctgctgtgctgcgtgtcgagcgatgagggcgacaggttgctggtttacgagtacatggagaacggcagcctcgaccggtg
gctgcaccgccgggacgccggcctcgcgccgctcgactggcccacgcgcctcggcgtcgccatcgacgcggcaaggggctcagttacatgcaccac
gagtgtgcgaggcccgtgatgcaccgggacgtcaagtccagcaacatcctgctcgacccggggttcagagccaaggtcgccgattttggctcgcgcg
tatcctcgtcaagtccggcgagcccgagtccgtgtcggccgtcggtggcacgttcggctacatggctccagagtgcgggcggggtgtgaaggtgaacg
agaaggtggacgtgtacagcttcggcgtggtgctgctggaactcgccaccgggcgtgtggccaacgacggcggcgcggagtggtgcctcgtggagtg
ggcctggcggcggtacaaggccggcgacccgctgcaggacgtcgtggacgcgaccgtacgggacagggacgccgcgttcgtgcaggacgccgtggc
cgtcttcgtgctcggcgtgatctgcaccggggacgacgcggcgtcgcggccgtcgatgaagcaggtgctgcagcagctggtccggtacgaccgcaccg
ccagcgtggcaggagcgtgccaggtggacggctgcgaggatggcgacgtcaaacgcgcggagctcacggcggggaagaaggcagctgcggcgaag
agctcggtggacgatggccgggcgtattgggacggtgacgaggagagtggcagcttcgtggctcatcctgcttag
>g123729.t1
atgagctgcttctctttcctgcttggcgggcagcagaagagatcaaaggttattcggagcaaggtcgcgcacgccaagaaggctcctggcaacgactgt
ccgcccaaggttatgcccgtcgagttcatcatggacacggcggacactgtcgacgacgtcgtcagcagaggccgtggcaattccccagcgtacgacac
gaacgtcttcgttcacacagcctccgacgagtcggcacgccaggtgcgtgacgtgaacgaagataaggaggcctcgagtaacagagactcgtccgac
gccgacctcgccgtcgtcgtcagccaagactcctccggttacagcagcggcgacaacagcaagaacaatgctgtcactgttagcgccgccgaacctga
cgccggcgagtctcccatcatcgcccacacgtccccgacacccgcttcgccgaagctgaagcgctcgtgctccaacatcgagacggtcccggaagagt
tcgatgacctgccggcgaagtcacgttcctacggcgacctgcacatcgcagcccagcagaatgaaggttacacgtccgacacgctggacgcggtcacc
gctgctagtagggagcccaagaacaaggaggtcgtcctggaggaggaggagggcccggttcttccgagccagtgggtcgcgttctcgtcggtggacc
gggtcagcgcctgggtgaacagccttcagaaaaacggctcatcgttccacgtcgtcaacgaggaggaggatgttgttgcagaggacaacggcggcgt
ggttcgcgccacctggtgctccgagatcgaggagtgcttgacggcagcagccgagaaacatcacgcgcgggcgacgaagcggcgcgtccaggcgag
cagcgtcgtgcagacgctcaacgtggtctcctccgtcgcgcacgtctccggcatggggctcaaggccgtcccgatgatcgccgcgttctccagcctccgg
gcagtcaatctgtccggcaactttatagctcacatccacccaggatcgttgcccaagggcctgcactcgctcgacctgtcgaggaacaagatcgccacc
atcgaggggctacgggagctgacgaggctgcgcgtcctcaacctcagctacaaccggatcactcgtatcggacatggcctgtcgagctgcacggcgat
cagggagctctacctcgcgggcaacaagatcagcggcgtggacgggctgcaccggctgctgaagctggcggtcctggacgtgggcttcaaccggatc
accacggccaagtcgctgggccagctcgtggccaactacgcctccctccgggccctcaacctggtgggcaaccccgtgcaggccgacgacacgctccg
cagggccgtggcgggcctcctcccgcgcctccagtacctcaacaagcagcccgtgaaggtgaagtcgcagcgcgcgcgggaggcggtaatggactgc
gtcgtcgcgcaggccgcgctcgggaacggcggcggcccgcgcctgtcgagctgcacggcgatcagggagctctacctcgcgggcaacaagatcagcg
acgtggacgggctgcaccggctgctgaagctggcggtcctggacgtgggcttcaaccggatcaccacggccaagtcgctgggccagctcgtggccaac
tacgcctccctccgggccctcaacctggtgggcaaccccgtgcaggccgacgacacgctccgcagggccgtggcgggcctcctcccgcgcctccagta
cctcaacaagcagcccgtgaagccgcagcgcgcgagggaggcggcccaggactgcgtcgcgcaggccgcgctcgccaacggctggggtggcgcgc
gcagcagtagcaggccgatgatggcgcggcgggcgagccacaagagccccgggtcgtcggggaaggggaagagccgcggcagcaggagcaggtc
catgacaaggagttga
>g123742.t1

atgtcgccatcctccgttgccgcgctcctgtccctgctcctgcttgtactactacccgtggctgcgacggtcgcggcggcggcggccatggacccggccg
agcgggagacgctgttccgcgtgatggaggtcgtctcctccgaccgcgactggcgcaccgagagccctgacccctgcgacgcgccgtggcccgggctg
gagtgcaagccatcggcggggaccccggcgcgcatgcacgtcacgcggctggacttcggcgtggagcccaacccgacgtgcagggacgcggcgacg
ttcccgcccgacgcgttcgccctgcctcgcctccagtccctcttcttcgtcgactgcttcaccaacccggccgccaccaccacgctcgtcctgccaccgcc
caccaacctctccgcggcgtccaccctgcagcagctcagcatccgggccaacccggcactatccggcacgcttcccccgagctggccagcctccggtc
cctccaggtgctcaccgtctcccagaacccgctggtccgcggcgggatcccgccgggcatcggcgcgctcacctcgctcgtgcgcctcgacctcagcta
caactcgcttaccggtccggtcccgagcgaggtcggcgagctgaagaacctggtcgggctggacctcagctacaactccctgtcgggccccatcccga
gccggatcagcgagctgcggcagctgcagaagctcgacctcagcgcgaacaacctcaccggcgccatccccgacaccgtcgccaacctcacttccctc
acgttcttcgcgctcagcaacaacggcctgaccggccgtttccctccgggcatctccgggctccggaacctgcaatacctgatcatggacaacaacccg
atgggcgtcccgctgccgtcggagctcggcggcctcgcgcgcctccaggagctgcggctggcgggctccgggtactcgggcccgatcccggcggcgtt
cgggcggctcgccagcctgacgacgctgtcgctgcaggacaacaacctgacggggcccatcccggcggcgctcagccggctgggcaggatgtaccac
ctcaacctcagcaacaacgcgctcggcggcgccgtgccgttcgacggcgcgttcctgagccggctcggccggaacctcgacctcagcggcaactcggg
gctgtgcatggaggaccggagcgtcctgcggggcgtcggcgtcggggtcggctcatgccgcggtggtgatggcagcggcgccggcggtgacacgtcg
gcggcggcggcgcgtcggggtacgggacgaggcgtgatgacggcgtggggttccggttccagagacggtttcccgttcgggctgctcggcccggcgtg
cgtgctcgtgttgcccgtcgtttttgccctgaacgagcacgcgccgtattga
>g123743.t1
atgtcgccatcctccgttgccgcgctcctgtccctgctcctgcttgtactactacccgtggctgcgacggtcgcggcggcggcggccatggacccggccg
agcgggagacgctgttccgcgtgatggaggtcgtctcctccgaccgcgactggcgcaccgagagccctgacccctgcgacgcgccgtggcccgggctg
gagtgcaagccatcggcggggaccccggcgcgcatgcacgtcacgcggctggacttcggcgtggagcccaacccgacgtgcagggacgcggcgacg
ttcccgcccgacgcgttcgccctgcctcgcctccagtccctcttcttcgtcgactgcttcaccaacccggccgccaccaccacgctcgtcctgccaccgcc
caccaacctctccgcggcgtccaccctgcagcagctcagcatccgggccaacccggcactatccggcacgcttcccccgagctggccagcctccggtc
cctccaggtgctcaccgtctcccagaacccgctggtccgcggcgggatcccgccgggcatcggcgcgctcacctcgctcgtgcgcctcgacctcagcta
caactcgcttaccggtccggtcccgagcgaggtcggcgagctgaagaacctggtcgggctggacctcagctacaactccctgtcgggccccatcccga
gccggatcagcgagctgcggcagctgcagaagctcgacctcagcgcgaacaacctcaccggcgccatccccgacaccgtcgccaacctcacttccctc
acgttcttcgcgctcagcaacaacggcctgaccggccgtttccctccgggcatctccgggctccggaacctgcaatacctgatcatggacaacaacccg
atgggcgtcccgctgccgtcggagctcggcggcctcgcgcgcctccaggagctgcggctggcgggctccgggtactcgggcccgatcccggcggcgtt
cgggcggctcgccagcctgacgacgctgtcgctgcaggacaacaacctgacggggcccatcccggcggcgctcagccggctgggcaggatgtaccac
ctcaacctcagcaacaacgcgctcggcggcgccgtgccgttcgacggcgcgttcctgagccggctcggccggaacctcgacctcagcggcaactcggg
gctgtgcatggaggaccggagcgtcctgcggggcgtcggcgtcggggtcggctcatgccgcggtggtgatggcagcggcgccggcggtgacacgtcg
gcggcggcggcgcgtcggggtacgggacgaggcgtgatgacggcgtggggttccggttccagagacggtttcccgttcgggctgctcggcccggcgtg
cgtgctcgtgttgcccgtcgtttttgccctgaacgagcacgcgccgtattga
>g123882.t1
atggatactgaaggcaatgagagtgggcaggctcaaaatactgacactggagctgccttgtctggttggaaggaccttcccatggagttgctgctgcgg
atcatgtcaatagttgcagatgatagaatggtcatcgtggcctctggtgtgtgcacaggatggcgtgaagctctaggttggggagtcactaaactttcgct
cctatggttaagttgtgttggatttgaggaaaaatatttgcttggcagcttctatattgggactccagatagtattagaatggatagtgcaggtccattgag
tggggaattgaacaactcgttcaatgaccttatggatactgaaggcaatgagagtgggcaggctcaaaatactgacactggagctgccttgtctggttg
gaaggaccttcccatggagttgctgctgcggatcatgtcaatagttgcagatgatagaatggtcatcgtggcctctggtgtgtgcacaggatggcgtgaa
gctctaggttggggagtcactaaactttcgctcctatgtattactgacaagggggtgaccagcttggcatcaggatgccctgaacttagggctctggactt
gtgtggttgtgttctgataacagatgagagcgtggttgccctcgcaaacgggtgcccgcacctgcgttccctggggctgtactactgccagaacatcact
gaccgcgccatgtactccctggccgcgaacagccgcatgcggagcaacaatggccatggctgggacgcggtgaagagcgacaacaaggacgggctg
gcgagcctgaacatcagccagtgcacggcgctgactccgccggcggtgcaggccgtgtgcgactcgttcccggcgctgcacacgtgcccggagcgcc
actcgctcatcatcagcgggtgcctcagcctcacctccgtccactgcgcctgcgcgctccacccgcaccgcgccggcggcagggccatcctctccaacc
agcacgcctattga
>g124196.t1
atgtcgacggcgaggcttctcgggatccttcttatgttcatgctcctcgtctcgagttctgaatccttttcagatgacgtgtttgcgcttctcgccttcaagag
agccattttcgaggacccgctttccaggctatcggactggaacgccagggacaaggacccctgcaaatggtccggcgtggggtgctcgccattcaaca
gccgcgtggttactatgattcttggtaccacagatcggatcactgagatctttgcagaaactgtcaaaatagccaagtgcaaagctagatcctctggatc
cgggaccagtgttccatggaccagagctgtcagctggaaggaaaacactgtaatctcgatcgacgatgatctcctaggaaatgtgccaaagataagcc
ggcatgagctttctgaagcctgcgaagatttcagcaacataattggatcttctcatgagacagtggcagaaatggctaggttgagtcacgaaaatgttgc
aaagatggcaggatactgcaaggagtccgatccattttcgaggatgctagtctttcagtacccagcaaatggaacactctatgagcatcttcacgatggg
gaaggatgtcagctatcttgggcgagacgaatgaaactagcgatgggtattgctcgagctgtcagatacttgcacaatgagcttcaacctccatttgctg
tcgctgcgctaacgtccagttcagtttacctaactgaagatttttccccaaagataactgatttcgagaggtggagatatctcgtgaccaaacctggactt
ggcttcggtggtgaagccaatggtggatttgtcagcagcgttacggattcgcagcacaagcgtttcatggacgtccaggctaacacattcgcctttggcg
tgattctgctggagctaatcagtggcagagccacagtttccaaagatacagcggatttggtggactgggcgaggaagcatttggaccagccggaggag
ttcagtaagctggtcgacccgaagctgcagagcgtgaaccaggagagcctgggcatcgtctgcaacgtggtgaacctgtgcatcgacccggagccgtc
gcggcggccgtccatgagcatgatcgcggccatcctggaggaaggcatcgaggcttcggcggccaccttgctcagggattcgtcattggcctgggctga
ggccgagctggccatctcctag
>g124627.t1

atgctctccggtggcctaccgatggatctgctgtcctccaacaacattgttgtcctcgacgtcagcttcaaccagctcaatggagatttgcaggacctgca
atcttcaactctccgacctctacaagtactgaacatctccagcaacttgtttacaggacggtttccatccaccgtatgggaagtgacgaggagcctggttg
tgctcaatgccagcaataacagctttactggccagataccaactaccttatgtgtcaccgcgccatccattgccgtgctccatctcagctacaaccaattc
aatggaagcatccctccaggactagtaatcagttag
>g124666.t1
atggcggtcagaagacacgcggctatagttgttcttgtgctcgccggggtgctgcacggcgttctgggcggcacgagccacgccgggttctccttctcca
ggaccggcaatgacgacctgaaaaacgtcacggcgccggctccttccgacgctctagaagaagcagcaggttgcggttgcggtccgacaccgccgcc
ttgggactttctgaacgagaagctggcggcgctgttcccggtgatccaggcgttcaagcgcagcatcacatgcgacccgctgggcgtgacgtcgacgtg
ggagggcaccaagctctgcgacagccactccggcgggaccgcgtacaggggcttctactgcgactaccctcccaccgcgaacacgacgctgaccatc
gcgtccatcgacttcaacggcttcggcctgtgcgccccgtccctcgccggcttcgtcgacgccttccccgacctggccctcttccacgccaactccaacaa
cttctccggcgccgtgcccgacctcaccaacctcccccttcttctacgagctcgacctctccaacaacaacttctccggcgcgtttcccgacacggtggtcc
cgctcggcggcctcctcttcctcgacctccgcttcaaccggttcgcgggcgccgtccccccgcccgtgttcggcatcaccctggaggcgctcttcctcaac
aacaacgggttcacgggccggatcccggacacgttgggaagcaccacggcccagtacctcgttgtggccaacaacaggttcacgggcccgatcccgc
ggtccatcttcaacacgtcggcccacctcgaggaggtgctcttcctcaacaacgacctctccgggtgcctcccctacgagatcggcctcgtggaagggct
cacgctcttcgacgcgggggggaaccagatcacggggcccatcccgctctcgtttgggtgcctggctgacgccgaggagatcaacctcgccgggaacc
agctgtacggccacgtacccgacgtgctctgcctgctggccaagaacgccaagctcaccaacctctcgctctccgacaactacttccactccgtgggac
accactgcatggagctcgtgcggagcagggtgctggacgtcaggcgcaactgcatcctgggattccccgatcagaggcccctgctcgagtgcacctgg
ttctacgcagacccgccgcagcactgcccattcgtgccgcacatcccatgcgatctgcccggctaccacaaaccgcatgccaacaatcccgcccttcttc
cggccgcgcatcgccaaggcggtggcaactga
>g125223.t1
atggcgacggggccgcgaatctcgctcctcctcgccgtcgtcgtggtgctcgccgctggtggcggggtcgtcgaagcggcggacccgccgctctcgccc
aagggactcaactacgaagtggcggcgctgatggcggtgaagagccgggtgcgggacgacaaggggctgatggccaagtgggacatcaactccgtc
gacccctgcacctggtccatggtcacctgctcccccgacggattcgtcgtctcgctacagatggcgaacaacgagctgtcgggaacgctgtcgccaagc
attgggaacctcagccacctgcagacaatcattataaaatag
>g125273.t1
atgattactgccctcaacacgctgtttactagcatgaattcacccgggcagctacagggttggaaggtgaatggtggtgatccatgtggagaatcgtggc
agggcatcacttgctctggctcctcagttatcgcaattaaattaccaagcttggggctctccggaacactgtcctacaacatgaacaacatggaatcact
agttgagcttgacatgagccaaaacaatctcggtaatggccagcagattccttacaatcttccaaataagaagcttgaaaggctcaatctggcaggaaa
ccagttcactggaactgtgccatactcgatatccactatgtcaaaacttaagtaccttgatctttccttcaattctctcactggtaatctaccgcaaagtttct
cttcattgccgagcctgaaaactctgtacttgcagaacaatcaacttacaggatccatcaatgttcttgctaatctccccttgatgatttgtga
>g125387.t1
atggcattctctggtctcgctctcgccctcctgctgctctcgctcgtctctcgtgccacaccgtgcacggagcaggacaagagttccctcctccagttcctt
gctcttctgtcccgggacggtggccttgctgtgttatggcagaacagcacaacagactgctgctcgtgggagggcatctcctgtaacggtgaaggggct
gccgtcgaggttgtgctagccaaaagagggctcgaagggcacgtctcaccagcccttggtgatcttaccggacttctgcgcctcaacttgtcccacaact
ctctgtctggtggactgccgctagatcacctgatgtcatccagcagaatagttgttatcgacgtcagcttcaatcgcctccagggagaactattcgagctt
caatcatcagtcacccatcgatggtcattgcaattgcaggtgctaaatatctccagtaacatgttcactggagaatttccatcaggaacatggaagtcaat
gaagaatctggtcgctctcaacgcgagctacaacagctttagcggtcaggtaccagcttctttctgccttgcctcggcgtccttcgccatgcttgaccttag
ttacaataagttcacaggcaacatccccccctaagctcgccagctcgtgcaccgagcaggagaagggctctctcctccagttcctcgccgggctctcgca
ggacaacggccttgctgcgtcctggcgaagtggctcaaactgctgcaagtgggaaggcatcacctgcggtggtaatggtaatggagcagtgaccgaga
tctccctggcgttaaggggtctggaaggacgcctctcgccgttccttggcaatctcaccagcctgcagcacctcaacctgtcgtacaactcattctcaggt
gaccttccgtcagagctgctgtcttccagcagcatcaccgtgcttgatgtcagtttcaaccacctcagcagggtcctgcaacatcagcaaaagcacaact
catcagtgccactacagaaacagaagcttgggttcatggctttcttcagccttgtcactcttgtacctctgttgctcctttccttggcctctccagccagctc
gtgcaccgagcaggagaagggctctctcctccagttcctcgccgggctctcgcaggacaacggccttgctgcgtcctggcgaagtggctcaaactgctg
caagtgggaaggcatcacctgcggtggtaatggtaatggagcagtgaccgagatctccctggcgttaaggggtctggaaggacgcctctcgccgttcct
tggcaatctcaccagcctgcagcacctcaacctgtcgtacaactcattctcaggtgaccttccgtcagagctgctgtcttccagcagcatcaccgtgcttg
atgtcagtttcaaccacctcagcagggtcctgcaacatcagcaaaagcacaactcatcagtgccactacaggtactgaacatctcgagcaaccattttac
tggagagttttcatcgatcttgtgggagaaaaagagtgatcttgtagcgctcaatgcgagcaacaacagtttccatggatggatgccgtcatccttctgc
gtcagttcattgtccttcgccgtgcttgacctctgccacaaccaattcagaggaaacatccctgccggactaggcaaatgctctgcgcttaaagtactcag
ggcagggcacaacaacctcaacgggacgcttccagatgaactcttcaacgcttcctcgttagagcatctctcctttgaggaaaatagtttgcacggcata
cttgacggtgcacgaataatcaacctccggaacttgtcagtacttaatcttgaagggaacaacttcgtcggcaggattccagaatctgtagggcacctca
agagactagagcagctacgtttgggagacaacagcatgtctggagagctaccatcaactctgagcaattgtacaaaactcatagcagttgacctcaag
cacaactatttcagtggagaacttaccagggttaacttctccaccctgaccaatctgaaaacattggatcttctgtacaaccatttcaccggagtaattcct
gaaagcatatactcttgcagcaatctgacagcactgcggctatctagcaacaaattacatgggcagctctcaccgagaataggagatcggaaaattga
aatcactcagtggactcatgttgagctttaa
>g126935.t1
atggcggcgctggcggcggcattgaccaccggtcttcttcttgtcctcgctaccgcgccggcgaaagcagacacggactccgccgacgctgcggcgctg
gtgaatctctacacctcctggaatagcccatcgcagctgactggttggtcggctggtggcggtgacccctgcggcgccgggtggcagggcgtcacctgc
accggcgccggcgtaactgagatgaatctagcaagcaacaatttctcaagcaaccttccatactctatagtaaacctggtgtcaattgtataccttaatct
tagccacaactcactgactcaagaaatgggtgatctgtttggaagcctcgattcactttcagagttagatgtgtctttcaacgaattgactggggatcttcc

caaatctattggttctttatcaaatctttcgagtctttatatgcaaaacaatcagttaacaggttctgtcaatgttctcagtggcctaagcctaactacacta
aacatcgcaaacaacaatttcagtggttggataccgcaagaattcagctccattccagatctaacggaaatcccggagcagagtcctgaaaatacctct
atggcaactgtgaaacttccgccccctgagaaaatgacaccggagaggatttacggtaaaaatggttctatgaagaagacaaaggttccagtaactgc
aatgccttatactgttgcttctctccaagttgctactaatagcttctgccaagattcccttctaggagaaggttcacttggccgtgtttacaaggctgattttc
ccaatggaaaggttcttgctgttaagaagatagacagctctgccctttcgttacaggaagaagacaacttccttgaggctgtatctagcatgtcgcgact
aaggcatccaaacattgtgccattaacaggctactgtgttgagcatgggcaaaggcttcttgtttatgagtacatcggaaatgggacgctgcatgatatg
ttacacttctccgatgaaatgagccggaaacttacttggaacatccgtgtgcggatagcattaggcactgctcgggcgctggaggttcactggtcagcaa
tttggtttgttattttcaggtctcgacagaagtgttttggagtagttatgctagagctattgacaggtcggaagccattagacagttctagagaaaggtcag
aacagtctcttgttaggtgggctacccctcagcttcacgatattgatgcacttgctaagatggttgatccagctctaaatggcatgtacccagctaaatca
ctctcccgtttcgctgatataattgcactctgtgttcagcccgaaccagagttccgaccaccaatgtcagaggttgtccagcaacttgtgcgcctgatgca
gcgggccagcatagtaaggcggcaatcaggggaagagctagggttttcatatagagccccagaacgtgaaggagacttgagagacatttcattctaa
>g127031.t1
atggggactccagggttcatggacttcagggtggtgcccctgccaacaatgttcaactacagctgcacaaacacgagcaggggaccatccatttcactc
acatgtaacggttgccggatccctccaagagaccactatgtgtcctggcagtttgttgatctaccggggcaaccggctgtggcggttgggttccaattca
acttgactgcaaaacaacatggcgttaatgaacatgtgagttttgtgagcggtgcgatgaattctgatggttatgttgatgatggcaagatgaagacgtt
caggggacaagattccagtgttctcaagatccagttgttccctcagatatacaataaacttggcaacttgaggctcttgcagccactggttcaggacttcg
ccgaaggttctgcattttcagatctaataccccatctctccttgtgcaagaaccaaggccgcgggatggccatgccttcgtttagccttgctcttgtgccgc
tgctctccttatccagcgcatgcacagagcaggagaagaccttcctgctccatttcctcaccgggctctcacaggatggcggccttgctgcctcatggca
gaatggcacggattgctgcacgtgggaaggcatcacctgcaatgaaaacagggacgtcactgaggtctatctggcatcaagaggcctggaagggcat
gtctcgccgtcccttggtagcctgaagggcatgctgcgcctcaatctgtctatcaactctctcagtggcagccttccattggaggtgatatcctccaccaac
atagtttctctcgatatcagcttcaatcacctcagtggggagctgcaagagctgccatcgtcaattcctagactacctttgcaggtatttaacatctcgagc
aatttatttacaggattgtttccaaaaaccacatggggaaagagtaggaatctggttgctctcaatgcgagcaacaaccgttttataggatggatgccttc
atctttctgcatcagcgcgccgtccttcacagtgcttgaccttagcaacaaccaattcagtggccatgttccctcaggactaggtaactgctctgcacttaa
agtgctcaaggctgaatacaacaacataaatgggactctcccagatgaactctttgatgctacctcattagaatacctctcactttctaataatcaactgg
aaggaacacttgatgatgcacatattaataagctcagaaacctgattttcttgaatttggcgatgactgggctcagtggcaagatcccagagtctatagg
ccagctcaagagactcgaggagcttcatctggaacacaataatatgtcaggagagcttccaaggtctctgagaaagtgcacaagcctcacaaccatca
acctcaggaggaacaaattccagggagagcttgccaacgtcaatttctcagccctatcaaatctgagaactttagatctttactcgaacaatttcactggt
acaattccagagagcatctattcatgcagcaatctgactgtcctgcggttatctgtaaacaagttccacggtcagttgtcaaagagaataggaaatttga
agtcccttgcctacatatcacttacccacaacaactttacaaatatcacaaatgctcttcacatcctcaagagctccaggaacatcaaggccctgctgatt
ggggtgaacttcaggcacgaggtcatgccgcaagatgaaaacatgtatggttttgagaaccttcaagtcgccatggtcgaagagctggcctcgctcatc
aaggataacctgcacagcaagcatctcgtcctatccaccgaggagtccctcatcgccatcctccatcaacagtgccgcctggacgacgacgacgatgc
ggaggataccatagagctccagcccaccggtgcctaccaccgcctcctcttgcatcggctcgccgacatatatgggtactactacgtcacctcggttcat
agtcagaccgtcctcgacctccttattacagacttgagatga
>g127342.t1
atgcggcgggctgccggcgttggcgccggtcgtcccatcctcctcctcctcctcgtcgtcgcagtgtgctgctccgctctcgtcccccgcgcgtccccc
gacctcgccgcggacagggcggcgctcctcgccctgcgcgacgcggtgggcccgcacctccggtggaacgcctcatcagcatcaccatgcgacggct
ggcgcggcgtgtcgtgcgacgccggcgggacgcgcgtgacgggtctgcagctcccggggtccagcctcgcgggccagctcccgccgcgagcgctggg
aaacctgacggcgctgcgcacgctgtcgctccgcctcaacgcgctgtccggcgggatcccggacgacatcgccgggtgcgcggagctcagggcgctgt
tcctccagggcaaccgcttcgacggcgagatacccgaggggttcttccagctcaacttgctgcagcggctcgacctctcggggaacctagtcgccggcg
gggtgtcgccggggttcaataagctgcgccggctcgccacgctttacttggacaacaacagactcaacggcaccttgccggctgatctcgacctcccga
agcttcagctgttcaacgtgtccgggaatggccagctcaccggtcccgtgcccgcctcgctcgccggtatgcccgcgagcgcgtttgcggggacagggc
tgtgcggcgcaccgttgttaaacccgtgcgccaacccgccctcgccgacgccgtctccgtcgccacagccagagcctccggctccggccgctcctgaac
atggaaaggcttcgaagctctctgccggcgccatcgctggcattgccgcgggctccgccgcggcgttgttggcgttgatcgccgcgatcgtcttcttcctc
tgtttccgacgaggtcgtcgtcagaggaggagtgagacgactgataagtctcccgacacgtcggcggacgtcgacgtttcaggctctcctccggtaccc
gtcaccgtggcgagcatttccacgaagcggtcgcggtcgcggtcctcgcggcagaccgctgcgtccggcaacaagaagctggtgttcgtgggggcgc
accggacactccgtacgacctcgagtcgctgctgcacgcgtcggccgagatcctcggcaagggctggctcggcacgacctaccgcgccacgctcgagg
gcggcgccgccgtgttagcggtgaaacggctccgcgaggcgccgatccccgagcacgagttccgcgaccgcgtcgccgcgatcgccgcgctgcggca
cgacaacctcgccacgctgcgcgcatacttctacagccgcgaggagaagctgctcgtctacgacttcgtcgacaacgcccggagcctctgctccctcctc
cacggcggcggcggcaacaacaacggttcgagcccggcgcgcctcgacttcgccgcgcgcgcgcgcgtcgcgctcgccgccgcgcgggggggtcgcg
tttatccacgcctcgggttcgggctcctgctcctggcacggcaacatcaagtcgtccaacatcctcgtccccgacgcggcgcgtgaccgcgccgtcgtga
cggaccacggcctgctccagctcgtggccgcgcacgtgccgctgaagcgcgtcacggggtaccgcgcgccggaggtcgccgacccgaaacgggcgtc
gcccgaggccgacgtgtacgcgttcggcgtgctgctgctggagctgctcacgggccggccgccggcgcggtcggtgccgggcagcgacgagggggtc
gacctgccggagtgggtgcgcgccgtcgtgcaggaggagtggacggccgaggtgttcgacgccgccatcgccgtcgaggagcgcgtggaggaggag
atggtgcggatgctgcgcctcgccgtggactgcaccgagcggcggcctgaccgccggccgggcatggccgagctggccgcgacgatagagcagatcg
tcgacagcgcggtacacaagactgccgacaacgtggacgatgacgatttccatagcatttcaccgtga
>g127367.t1
atgacggcgcacagcaccctcctcccattcctcttcctcgcgctcctcctctcccctcaactcctcgcctctgcggcgttcgtgagccgcggcctgagcgc
ggacgacgaggctcacatccgccgccggcagctcctccagtacggcaatggcggcggtagcagtaacgaggtggacccgtcgtacacgttcccgaac
ccgcggctccgcgacgcgtacatcgcgctgcaggcgtggaagcgcgccatcctctcggacccctacaacgtgacgggctcctggtggggtcccgacgt

gtgctcctaccacggcgtcttctgcgcgccctccccctccgacccctacctcaccgtggtggcctccatcgacctcaaccacgccgacctggcgggccac
ctccccgacgagctgggcctgctgggcccggacctggccgtcctgcacctcaactccaaccgcttctgcggcctggtcccgcgctccctcgcccacctcc
acttgctccacgagctcgacctcagcaacaaccgcctcgtgggcgggttccctcacgccgtgctggagatgccggcgctgcggtacctcgacctccgct
acaacgagttcgagggtcccgtccccagcgagctcttcgaccgccaactcgacgccatcttcctcaactccaaccggttcaagttcaggatccccgaca
acgtggggaactcgccggtatccgtgcttgtgctcgccaacaatgactttggcgggtgcctcccggcctccgtcgccaacatgtccgacacgctcaacg
agatcctcctcctcaacacggggctcaagtcctgcgtgcccagagagttcgggatgctcaccggactcaccgtgctggacctcagcttcaacagcctcat
gggccagattcctgaggagctggctaatctgcgcagcatcgagcagcttaacctcgcgcataacaggctcgtcggcgacgtgccggagggcatctgcc
acctgcctaaccttcagaacttcacctacagctacaactatatctccggcgagccgccggtgtgcttgcacgtgaagaacgtcgacgataagcggaact
gtatcccggaacgcccggagcagaggtcgcccgagcagtgcgagttcttcaagagccatcaccccgtcaactgcgacgctttcggatgcaagaagttc
gtgctgccgtcgccatcgccgccaccaccatctcctccgccaccttcgccatctcctccaccgccatcgcctccgccaccatctcctccaccaccttcgcca
tctcctccaccgccatcgcctccgccaccatctcctccaccaccttcgccgtctcccccgccaccttcacctcccccaccgtctcctccacctccgtccccat
cacctcctcctccttctccaccaccaccttccccacctccgccatctccatccccaccaccgccgtctccggtctactataactcgccgccgccaccttact
acgaggtgtcaccagaggaccggtacctctcgccgccgcctccggcctaccaggagtcaccgccgccaccatcctacgaggtctcacctgaggacagg
tacctctcgccgccgcctccagcttaccaggagtcgccgccgccgccatcctacgaggtctcgccggaggaccggtacctttcgccgccgccgcctcca
aggacgctctacaagcttcctgtctggaactacgcgtcgccaccaccgccggccactcttcaggaaccataa
>g127457.t1
atgggtgtgcggagctgcagcagtgaccgatgcaatggtggatcgagaaggaagccgctcctcctcctcctgggcgtcgttctcctgctgctgcagcag
gcggcgagcgggcaggagcttgacgacggcgtggtacccggtcaggcttcggacggcgtggtgatcgcgcaggcagacctgcagggtttgcaggcca
tccgtcagtcgctggtggacgcgcgcgggttcctgcgcggctggaacggtacgggcctggacgcctgctcgggctcctgggccggagtcaagtgcgtg
ctcggcaaagtcgtggccctccagctcccgttcaagggcctcggcggcgagctctccgacaaggtgggccagctctccgcgctgcgcaagctcagcctc
cacgacaacgccatcggcgggcaggtgcccgccgcgctcggcttcctccgcgacctccgcggcgtctacctccacaacaaccgcttcgccggcgccgt
gccgccggagctcggccggtgcgcgctcctgcggacgatcgacctccgaggtaacttcctctccgggagcgtccctccggctctcgccaacgccacccg
gctctacaggctcaacctcgcgtacaacaacctctctggcgtcgtgccgatcagcctcacttctctccccttcctcgtgtccctcgacctcgaccacaaca
atctcagtggcgagatcccgcccaccattggcaatctcagaagtctccgcgatctttctctcagtaacaatttgatcggtgggagcatccctcaagggatc
ggcaacctctcaaagctccacagactggatctttctgacaacctgttaggtggtagcctccctctgtcactcttcaacctcacctcattagttgagctcaat
ctcgatggcaatgacttcgaagggcccataccggagtccatcgacgggctcaagaacctgaccaagctgtccgtgaggagaaacgtcctcgacgggg
agattccagcgacggtcgggaacctctcggcgctgtcactgctcgacgtgtcagagaacaacctcaccggagagattccagagtccttgagcggcctc
gccaatctcacctccttcaatgtgtcttacaacaatctgtctggccccgtgcctgttgctctgtccaacaagttcaattccacctcattcattggaaaccttc
agctctgtgggttcaatggctcggccatctgcacttctgcttcctctcccttgacagcgccgtctcccccgctgcctttatcggagcggccgacacggaag
ctcaacaaaagggagctcatctttgcaatcggaggcatctgtttgctcttcttgctcctcttctgctgcgtcctcatgttctggaggaaagataagaaggag
agctcgtctcccaagaagaagggcgccaaggacgcggcctccaaggcggccggcgggaagcccggtgcgggcgccggctcaggcaaagacaaaga
cgccggcggcaagcttgtccatttcgacgggccgcttacgttcacggctgacgatctgctgtgcgcgaccgcggagatcctggggaagagcacgtacg
gtacggtgtacaaggcgaccatggaggacggcagctacgtcgcggtgaagcggctacgggagaagatcgccaagaaccagaaggagttcgagacg
gaggtgaacgcgctcggcaaggtccgtcaccacaacctgctcccccctccgagcctactacgtggggcccaagggcgagaagctcctggtcttcgacta
catgcccaaaggcaacctcgcctctttcctccatggtaataagctcatttcctcctcagtgcaatcaatgctcagagttcatatacaattactttag
>g127492.t1
atgggcgccgctcgcatggcggcgcctgcagctactctgctcctcctcctccttcacctggcgcgcgcagcagccgccgggcctggcgacgaggcc
gcggcgctgctcgcgttcaagcgcgcgtctgtggcccacgacccgcgcggcgcgctcgccgactgggccccggccgactcccgctcagcagcgtcgcc
ctgcgcctgggccggcgtctcgtgcgcggacggcagcgtcgaggcgctcaacctcagcggtatgttcctctccggccggctccacctcggcgccctgct
cgcgctcccggagctccggagcctcgacctccgcgggaacgcctttcatggcgacctcgcccaccggacctcgccgccgtgcgctctcgaggacgtgg
acctctcgtcaaacgccttgaacgggacgctacccagggcgttcttggtgtcctgcagcgggctgcggttcctcaacatgtccaggaacaccctcactgg
cggcggattccccttcccggcatctttacgacgcctggacatgtccaggaacaggctgtcagatgccggcttgctgaactactccctgaccggctgccac
ggggtgcagtacctcaacctctcggccaaccaattcaccgggggactcccggggttcgcgccctgcagcggggtcgctgttcttgacttgtcatggaacc
tcttgtcgggcgccctgcctgcgaggcttgtggccgctgcgccggtcaatctgacacacttgagcgttgccgggaacaatttctctggggacatcttgag
gcatcagtttggtggttgtgcaaatctcactctgctggattggtcatacaacagattgaacggcatggggttaccacctagtcttgccaattgccgccggtt
ggagacgcttgacttgtcctggaacaaacttctgtctggtccggtaccggtgttcttgggtggcttccaagcgttgaggcgacttgcattggccgggaaca
acttcacagggatcattccggagaagctgagcgtgctagaccttggcagcaatcagctgtctggggactttgtggccactgtcatcagcaaaatttcctc
cctccgtgtgctgcggctgccattcaacaatattacagggacaattccactgcctgcgcttgcagccgggtgccgttcgcttgaagttattaatcttgggtc
caacttatttgatggagaaatcatgccagacttgtgctcgtcattgccgtcactccgcaaactgcttctccccaacaataacattaatggaacactgccac
cgtcacttggcaattgcacaagtctcgaggtaattgacctcagcttcaacctgttggttggtcagattccaacagaagttttatttcttcctaagcttgttga
tctggtcatgtgggcaaaccgtctctctggtgaaataccagacaagctatgctccaatggcacaacactggagacgctggtaataagctacaacaactt
cactggaggcatccctccatccattaccagatgtgtgaatctcatatggttgtcacttgctggcaaccaactcaccgggagcgtgcctgcaggctttggca
acctccagaagcttgccatcctacagctgcacaagaattccctctctggtcaggtaccagccgagtttggcagctgcaccaacctcatctggcttgatctc
aacagcaataacttcactggctcaataccaccgcagttagcagcacaggcgggactcatcactggaggctttggcaacctccagaagcttgccatccta
cagctgcacaagaattccctctctggtcaggtaccagccgagtttggcagctgcaccaacctcatctggcttgatctcaacagcaataacttcactggctc
aataccaccgcagttagcagcacaggcgggactcatcactggaggtattgtttctggaaagcagtttgcattccttaggaatgaagctggcaatatctgc
cctggtgctggagtattgttcgagtttctcaacatccgtccagagcggctagcccagttctcacctgtgaattcgtgcaccccaacaaggatatacactgg
tacaacggtctacacctttagcaataatggaagcatgatctttctggatctctcatacaacagcctgactggtacaattcctgagagcttggggaatatga
catatcttgaggtccttaatttggggcataatgatcttacgggagcattcacagggctcaagacgattggtgtacttgatctgtcacacaatcacctcact

ggcctcatccctgcagaacttgggtgtctgcatttccttgcagactttgatgtctccaacaacaatctgactggtgaaatcccgacatcagggcagctcat
tacatttccagcatcccgctatgaaaacaactctggcctctgtggcattcccttgattccttgtgtgcacacttccagttctggaggtctgcctcaatcttccc
gtaatgtgcgaaagaagtttgatgaagaaagtgtgctcctcacagtggcactttcagtgatcatagtgctctcgcttatagtcactctgtacaaactcagg
agctcccctacaagtaaagctgaagagattcaaactgggtactgcaacagactccacgcatccaacaatttaagttggaagttttctggttctcgtgagc
cactaagcatcaatttggccatttttgagaatcccataaggaagctaacttatgcccacctgcatgaggctactaatggcttcagctcggaggcgcttatt
ggctcaggagggtttggggaggtttacaaggctaagctgaaaaatggcggtgttgtagccgccaagaagctgatgcattttacaggccagggtgaccg
ggagttcacagcagaaatggaaaccattggcaaaatcaagcatcgtaaccttgtaccgttgctaggctactgcaaaattggtgatgagcgtctccttgtt
tacgagtatatgaagcatggcagcttggatgtgctgcttcatgacaaggccaagaccgatgtgcaactcgattgggcatcaaggaaaaagattgcaatt
ggttcagcaaggggcctagcctttctccaccatagctgcatcccacacattatacaccgggacatgaagtcgagcaatgtgctgcttgatgataacttcg
acgcctatgtatcggatttcggaatggcacggctcatgaatgcactcgattcacatctgactgtgagtatgctctcaggaacacctggttatgtcccacct
gagtattgccagtcttgcatgtgcacaaccaagggtgacgtctacagttatggtgttgtgctcttggagcttctctcaggaagaaaaccgattgatccaac
cgcttttggtgacagtaaccttgttgactgggtaaagcagatggttaaggaggacagaggcattgagatattcgatcctatattgaccaccatgtcttgtg
aattggagctgtatcagtatctgaagattgcttgtcagtgtttgaatgatcagccaatccgtaggcctaccatgatccaggtcatggcaatgttcaaagag
ctacagattgattcagacaataacttcgttgatggcttctcaataagttcaccaaatatagaggaatcatatgagaaatcagtgtaa
>g127833.t1
atgaggatgaagccaaggctgagtcgctgcagcagcttcgtccatggcgtccctttgcttctgctgttcttgctgccacttctgtgttgtcattcttggaagg
cggctgcacaggctccgcagactgatccagttgaagcggcggcggtgaacgcgatcctgagcaagctggggctgagcgcgccgccgtcgtggaacat
cagcggcaacccgtgcagcggcgcggcgacggacgacacgtcactggacgacaacccggcgttcaatccggccatcaagtgcgactgctccgaccag
aacaacaccgtctgccacgtcatcagattgaagataaatatgctggacgcggttggtcccattccagaagagctgcggaacctcactcatctcaagaag
ttggacttcagaagaaattacttcactggaccgttaccagcattcataggagaattgactgctctagagactgtgggcatcaatgcattatcggggcctct
cccaaaggagcttgggaatcttacaaatctcaaatcactggcactgggctcaaatcattttaacggctcactacctgatgagctgggcaaattgacgaa
acttcaacagatgtacattgatagtaatgacttcagtggtccactaccatcaacattgtcccaactcaagaacctctcagtcttgtggacatcagacaata
atttcagtgggaagattcctgattatcttgggagcttgactaacttgactcaagtaatattgaggaactgcaagatatccgacacaattgcatcagtggac
ttcgcacggtttgggattttaaatttattggatctgagtttcaacaatatcacaggcgaaatacctcaatcaattttgaatctaccatcactgaatttcttga
atttggtggcaaacgactttgtgatggatagctccaatgacagtgttctgccatggggattgaactgtcttcagcggaatactccctgttttcttggttctcc
tcaatcctcctcctttgcggtggacagtggtggaagtagaactatatcaggttcagatagttccatgtatcaggctgataatgccactcttggtgctgcctc
atattatgtttctggatcacgtacatggggtgtcagcagcgttggaagattcatggatccaccaaatggaagttacataatctacagttcacgccagtttg
agaacacactagattcagcactgttccaaacagcaaggatgtccccatcatctttgagatattatggaattggactcgagaatggaaactacatggtcac
acttcaatttgcagaggttgactttccagacacagagtcatggagaagtagaggaaggaggatttttgacatctatgttcagggtgagcgcaaggagca
gaactttgacataagaaagacagcaggagggaaatctttcactgttgttaagaagcagtataatgtttcggtgatcaaaaacttcctcgaagttcatcttt
tctgggctggcaagggtacttgctgcattcctactcaagggtactatgggcctgcgatattggctttgagtgtaactccaagtactcacctgaattaa
>g128258.t1
atggccatggcagcaccacaccagcccaagcgccgccgctcctctacctctacctccctggctcagtctccgctggactcgctggccgacgagctcctgt
tcctggtcctggaccgcgtggccaccgccgacccgcgcgcgctcaagtccttcgccctcgcctcgcgcgcctgccacgccgccgaatcgcgccaccgcc
gcgtcctccgcccgcgccgcgccgacctcctccccgccttgctcgcgcgctaccccgccgccgtcctcgacctcaccctctgcccgcgcgtcgccgacgc
cgccttcgccgccctcgccgcggcgtcgccgtgcctccgcgccgtcgacctctcccgctcctcggggttccgcgccgccggcctcgccgcgctcgccacg
gcatgccccgacctcgccgacctcgacctctccaatggggtcgacctcggcgacgccgcggccgccgaggtggcgcggatgcacaggctccgaaggc
tctcgctgtcgcggtgtaagccggtcactgacatggggctcggttgcgtcgccgttgggtgccctgatctccgagacctgtctctcaagtggtgcattggg
gtcacggatttggggatcaagctcctcgctcacaagtgcaggaagctcaggacgctggatctctcctacgccatgatcacaaatgactcctttccagcca
tcatgaagcttcccaatcttgaggtgttgatactggtgggatgttctgggatcgatgatgatgctcttagtagtattgagacagattgcaacaaatcattga
aggtgcttgatatatctcattgtccaaatgcgactgatgtgggggtttcatctgtagtgaagtcgatacccaatctatcagaattgaatctttcatactgctg
tcctgttactcctactatggtaaacagcttccacagtattcctaatctgcagaccttgaagctggaaggctgcaaattcatggcaggtggactaagctcca
tcggaagattaaagaatctgctgaagctggacgttacttgttgccgcaatattactgatacttcactagcttccgtcgctagttcatgcacttccctcatctc
tctgagaatggagtcctgcacccatgtttctagtgaaggactcaaactgattggaaagcatcttcatcaactggaagagctggaccttactgacaatgat
ttggatgatgaagggctgacagctatcgctggatgtagcaaactctcaatcctaaaaattggcatatgcttgaagataagtgatgaaggtctcactcaga
ttggaaagtcttgcccagaactccgagacattgatttgtacaggtgcggagacattagtgatgatggcgttactcatattgcccaaggctgtccaaagct
agagtctaccaatctgtcctactgtacaggaataacagaccgttcactgatgtccctctccaaatgtgcgaatctgaacacattggagattcgtggctgcc
ccagggtttcatcctctgggatatcagaaattgcaatgggatgcaggctactttccaagcttgatataaagaaatgcttcgagatcaatgatgttgggatg
ctttacatttcccagttctctcatagcctccgtcagaacatgaccattgtacatttagcgggtatttcgcctaatggcttgatagctgctcttttggtctgtggt
ggtttgacaaaagtgaagctcaatgctgcattgaaatccatgatgcctcctcatatgctcagaaatgttgaggcacgaggctccagcggagtcttgcctg
ttaacttcttgaggggtaaacatgcagttattgagcagtaa
>g128404.t1
atggagccgccggcgccgcgcctcctggccgcggtcctccttgccgcgtgcctctcggcctccgtcgtcgtgcaggccgtcaccagcgccgaggcctcc
tacatcgcgcaccgccagctcctcgccctcaaccaatccaagtccaccatcgacggcatctcgccggacttcgagttcgacgaccgcgccggcgccgcc
tcgttcccgaacccgcgcctccgacgcgcctacatcgcgctgcaagcctggcgccgcgcattctactcggacccgagagggtacacctccaactgggtc
ggcagcgacgtctgcgcctacaccggcatcgtctgcgtcgccgccctcgacgacgccaccctcaccgtcgtcgccggcgtcgacctcaacggcgccga
catcgccgggtacctcccgccggagctcggcctgctcaccgacctcgcattcttccacatcaacaccaaccggttctgcggcatcatccccaaatccatg
tcacggttatccctcctccacgagttcgacgtgagcaacaaccgcttcgtggggcccttcccgatggtcaccctggaaatggcatcgctcaagtatctgg
atatccggttcaacgatttcgaaggggagcttccgcccgggttgttcgagaaggacctcgacgccatcttcgtcaacacgaaccggttcgtcgggccgat

accggagaacatgggtaattccacggcatcggtcgtcgtcttcgctaacaacgagctggttgggtgtattcccaagagtattggcaggatggctgatac
gttggacgagataatcttcctcaacaacaagctcgatgggtgcctgccgttggagatggggttgctccacaacaccagcgtcgtcgacatcagcgggaa
cgcgctggtgggcgcgctgccggagcagctcggggaattgaagaagctggagcagctggacgtgtcgcggaacgtgttcaccggcgtcgtgcacgag
tccatctgcgagctgcccatgctcgtcaacttcagcttcgcggccaactacttcaactccgaggccgtcaagtgcatgccctccgatgccgcgccggcca
gctttgacgataaaggaaactgcctcggcgcgctgcggcccctgcagaagacgccgctgcagtgcgcgccggtcctcgcgcgtcctgtcgactgcagc
aagcacgcgtgcgcggggtaccccaagagcggcccaccggagaagaagccgcccgtcatgccggggaagccgcccatggtgaaggtgccgccggg
accagtcgtcgtcacgcctcccaccgcggacactcccgacccgtctcccaagccgactccagagtttccctcgcccggcccggcacccaaggaggctg
atcctccgggggaacttcctagtggtctggaggcgccggagcttgcgcctgtgccggttactaagccgccaccggttgcagggtacgaggctcctccgc
ctcccaactccgcgtggctcccggtgtcgcctgagcgacccaaggccccgccgccgcaggcgcagccaccaattgagcctcctccggttccggtggtaa
gctcacccccaccacaggtgaaatctccaactctgccaccggtggtgtcttctcctccacctaaaccaccactgatctcatcaccgcctcctgtgcaacct
cctcctgtaccaaagccggttgcctcgccaccgcctaccgtgcagcctcctcctgcaccaaagccagtgttctcaccaccacctcctgtgcagcctcctcc
tgcacctaagcctatggcctcaccgcctcctcctgtgattccacctcctccaccagcacctgtggcctcgccaccgcctccagtcaagtctccaccaccac
cggcgcttgtgctctcaccaccacctccagtcaactccagtgagctctccaccacctccggtgaaatccccacctccacctgccccggtagcctcaccac
cacctcccgtgaaatcgccgccgccaccagctcccgtgagctctccacctccacccgtgatatctccacccccgcctgctccagtgaaatctccctcacct
ccagagaagttgttacctccacccactcccaagatctctccaccgccagaagagaagcctactcctccaccaactccggtgaaatcttccccaccagcc
gctccagtgttgcctcctccatctaa
>g128572.t1
atgagccgccgctccaggagctccgccgcaacggcggcggcccttctcctcgcggcagtgctgttgcattgcgcggcggacgcggccacggacgcgg
agcgccgcatcctcctcgacttcaagtccgcggtgacgtccgacccgcgcggcgcgctgtcgtcctggacggcgacgggcgaccccctgcgtggacttcg
ccggcgtcacctgcgacccctccacgcgcgccgtctcccgcctgcgcgtccacggcgcgggcctcgccggcacgctcgccccctccctggcccgcctct
cctccctcgagtccatctccctcttcggcaaccgcctcaccgccaccatcccgctcacattcgccgccctcgcgcccacgctccgcaagctcaacctcagc
cgcaacgcgctctccggcgagatcccgcgcttcctcggcgccttccctttgctccgcctcctcgacctctcctacaacgcgctcaccggcgagatcccctc
cgcgctcttcaacccctgcccgccgcgcctccgctacctctccctcgcgcacaaccgcctctccgggcccgtcccagccggcattgccacctgcgcccgc
ctcgccggcttcgacttctcatacaacgagctcaccgggggcctcccgccccgcctctgcgcgccgccggagctgagctacgtctccgtgcgcgccaac
gcgctctccggcgagatcagcggcgtcgaatcctcctgccgcagcatcgacctcttcgacgtcggcagcaacaacttctccgggcccgcgcccttcgcg
ctcctcgccatggtcaacatcacctacttcaacgtctcctccaacgccttcgacggccccatccccaccgtcgcggcatgcggcgccaggttctcctacct
cgacgcgtccgggaaccgcctcgcggggcccgtgcccgacaccctgctgacgaactgccgcagcctcagggttttggatttgggggcgaatgcgcttg
ccggagacataccgcctgccgttgggacgctgcggtcgctctccgtgctccggctcgcgggcaattctgccatttcaggttcaatccctcccgagcttggc
gggatcgagatgcttgtcacgctcgacctcgccggccttgctctcacaggacagattccacagtccctcagccactgccagttcttgcttgagctgaatct
ctctggcaaccagttacaaggaaccatcccagacacgctcaacaacttgacttacctaaagctgcttgatctacacagcaaccggcttgatggaggcat
tccccctaacactcgggcagctcaccaagcttgatctcctcgacctctcagagaatcggctgacgggacagattccttcagagctggggaacctctctaac
ctggcacatttcaacgtctccttcaacgacctctctgggatgatcccttctgcacaggtcttgcagcagtttggtgcctcggccttcataggcaacccgcat
ctgtgtggccctccgttgaacaatctctgtggtggtggtggtggtggtggtggtggtgcgcaccgtaaacggttgggtgtatctgtcataatcgtcattgttgct
gcagccctcatacttatcgggatatgcattgtttgcgcgatgaacattaaggcttatacgcgcaagaataatggtgtggaggacagcaaggaggagga
ggaggttctggtgtctgagagcacgccgattgcatctccaggctcaaatgcgatcatcggaaagttggtgcttttcagcaagagcttgccttcaaggtat
gaagactgggaagcaggaaccaaggctctagttgacaaagactgcatcattggtggagggtccgttggcacagtgtacaaagccacttttgaaaatgg
cctatccattgctgtcaagaaactggagacacttgggaggctcgaaaaccaggatgagttcgagcatgagatgggccagcttgcaacacaaacctcat
ttccaaaccaaatatctcaaatggagggagagtttgaagtgcctgagttcaaccctcgggagcgcgtcaagcagcagatttcagtcccgtttctctggga
ggtgaaacctggggcgccgaaatgggactgggtaatttccaagccaacgccatcagtgagtgcgtgcccatcatccccaaccccggcaaagcttgttgt
cagtgtgccgttccagtgggaagaaaagcctgggaagcctctacaagaagcatctccctttcatgcgccagctgattatgctggcttttcagcatcttctg
gctcactgaacccccttcgtagtggagggcgaagaggaatacgcgctcggttttgatatggaagcatttggttttccagacagtaaagacacttctggtgc
tgcagcatggatcggtggctccaaccgtcgtgacgactggtactcattttctgattctgaagacctgagcaactcgagcagtgatacttcagctcaagag
ttccagtttccacgggcaccctcagagaaaagctgggaggtggccaatgacaatgatctgggggtcacgattcagtttcccaaggcaccttcggagaga
agctgggaggtagccaacgatgatgatcagctgaccaattcttggagccctcctaggaacacggtgacgacactagaggagcttatgatgctgagccg
gagactgaggtgcggacaaggcttgccagttgatatcaggaagaagagcctctcctcagtggtaatgctctacacttcaaatttttcatttgtgcatcagc
aaacctga
>g128870.t1
atggctcgctccactgttctcctccacttccttctagtagtgttcttcacctccagttgtaccacactccaatcagcgatggcgcaaactgaggctgtcgag
cttgcaacactcctaaaactcaagacagactggggcagcccttctgcgctcagctcatggagttcggagaactctacctactgcaaatggtcagggatt
atttgcgacagcactggccacgtcaccaccctctccttccaaaatttccacatagccaaaccaattccagcctccatttgcagcctcaacgacctgtcaca
catagacctgtcatacaacaacctcaccggcgagttccccacagcggtttacagctgctcggctctccagtttctagacctgtcgaacaatgagttctccg
gcgcgctgccggctgacatcgataagttgtcgccgaggacgatggagcacctgaacctgtcgagcaatggattcaccggcagggtgccgtcgggaata
gccgagttcccgaagctcaagtcccttgttctggacactaatagcttcaacggaaactatccggctaccgccatcagcaaccttgttagcctcgagacgc
tgacgctggcgagcaacccgttcatgccggggccgtttcccgaagagttcagcaagctgacgaaactgaagacgctctggttgtcggggatgaacttg
actggtgtcatcccggactctctctcggcgctcaccgagctgacggtattggacctatcacaaaataatcttcatggtgaaacccccgggtgggtttgga
accaccagaagctgcagttgctgtacctctacgccaacaacttcatcggtgggatcggacccagcatcaacgccaccaacatggtcgagctcgaccttt
ctacgaatagtctgacggaaccgataccagaggctatcggtaacatgaagaacttgaagctgctgttcctgtacttcaacaagattagtgggcccattcc
ggcaagcgtggggctgcttccgaacctgagggacctccggctatttaacaacatgctgacggggccgctcccgcctgagctcgggaagcactcgccac
tcggcaacctagaagtgagccacaacatgctctcgggccacgtcccggacaccctctgctccaacaagaatctctacgacctcgtcctcttcaacaaca

gtttctccggcgagttgccggcaagcctgggcgagtgcgacacgctggacaacatcatgctatacaacaaccacttcatcggcgacttcccaaaacagg
tatggtgggcgttcccgaagctgaccaacgtgatgatccagaacaacagcttcacgggcactctgcccagcgcgatatcctccaacatctcccggatcg
agatggggaacaaccggttctccggtcccgtgccgtcgtccgcgccgggtctgaacgctttcaaggcggagaacaacatgttctccggcgcgttgccgg
ggaacatgtccgggttggctaacctgaccgacctggacctctccgggaaccggatttccgggtcgatcccgtcgtcgatcctgtcgttgcgaaggatgac
gtccttgaatctgagcggcaaccaggtgtccggcgagatcccggcggcgatcgggtccctgcccgtgctcaacatcctcgatttatccagtaaccagctc
gccggcgacatacctcgggagttcaacaacctccgcctcagcttcctcaacctgtcgtctaaccaactcgacggcgaggtcccgtcgtcgctgcagaac
gaggcttacgacagggcgttcctcggaaaccgtggcctctgcgccacgatggacgtgaatctgaacattccggcatgcggctacacaagagggcgcaa
ccagatgtcaaccggcctcaccatcctgttctcggtgctcgccggcgcgaccttcatcggcatcgtggggtgcctcatcatcctggggaagaagaagaa
gcggcagcacgacacgaccgcgtggaagatgacctcgttccggaagctggtggacttcacggagcgcgacgtggtgacgggcctccgcgaggagga
cgtgatcggcagcggcgggtcgggcaaggtgtaccgcgtccagctcgctcccggcggcacggtggtggccgtgaagcggctgtggagagaagcagcaa
gtccgaggagaagctcgagagggagttcgattcggaggttaagatcctcggcgacatccgccacgccaacatcgtgagcctgctgtgctgcgtgtcga
gcgatgagaatgacaggctgctcgtgtacgagtacatggagaacggcagcctcgaccggtggctgcaccgccgggacgccggcctcgcgccgctcga
ctggcccacgcgcctcggcgtcgccatcgacgcggcaagggggctcagttacatgcaccatgagtgtgcaaggcccgtcatgcatcgggacgtcaagt
ccagcaacatcctgctcgacccggggttccgagccaaggtcgcggattttggactcgcgcgcatcctcgtcaagtccggcgagcccgagtccgtgtcgg
ccgtcggtggcacgttcggctacatggccccagagtgtggccgtggggtgaaggtgaacgagaaggtggacgtttacagcttcggggtggtgctgctg
gaactcgccacggggcgtgtggccaacgacggcggcgcggagtggtgcctggtggagtgggcctggcgccggtacaaggccggcggcccgctgcag
gacgtcgtggacgcgaccgtacgggacagggacgccgcgttcgtgcaggacgccgtggcagtcttcgtgctcggcgtgatctgcaccggggacgacg
cggcgtcgcggccgtcgatgaagcaggtgctgcagcagctggtccggtacgaccgcaccgccagcgtggacggctgcgagaatggtgacgtcgaacg
cgcgcagctcacagcggggaagaaaggggataaggctgctgcggcgaagagctcggtggacgctagcccagcagcgtattgggacggtgacgagg
agagtggcagcttcgtggctcatcctgcttag
>g128882.t1
atgatcccaccgtcccttgctcaggcctccaagctccgggtgttgcggctggagtacaacagcctcgtgggtaagatcccggccaacttgagctatctgc
aggagctggaggtcttggatgttggcgcaaaccagatctcgggtgcgatcccaccgtcgctcggttcactctctaagctcacatacctggggttatacttg
aacagtctatctggaggtgtcccgccatcttttggtgttctgccggttgatattggcttcactctcccgaacctccagattctcctcctcagtcaatgccaatt
gagcgggcgaattccccactcgatagtaaatgcatctcagctcaagtatgtacagcttgatgacaatgagcttgaaggaacagtgccattggatggaaa
tgatttcaaagcattggtctacgaattcatgcctaatgaggacctcaacaagtggctgcaccacagtctcataacacaagatgagacacccacaaggag
gctgaccatgtctcagagggtgaacatcgcgcttgatgtcgctgaggcccttgattacctgcaccatcatggtcaggtgcccattgtccactgtgatttga
aaccaagcaatgttcttctggatgatgacttcgtcgcacatgtgggggactttgggctggcacggttccttcgtaagatggtgaacagttcaacagaaga
aagtactacttcagtgcgaatcaagggctccattggatatatccgtccagaatatgggatggatagcaatgtttccatccaaggcgatgtgtacagttttg
gaattatcttgtttgagatgttcacaggaaagaggccgtccgacagcttcttccaggaaggccaaactcttcaaagctatgttttagcttgttaccctgag
aggataatggaaatagttgatccaattctactgcctttggataatgaatttctcagtgaaggggaagcttcaaagcatcagtga
>g129060.t1
atggctttcttcagccttgtcactcttgtacctctgttgctcctttccttggcctctccagccagctcgtgcaccgagcaggagaagggctctctcctccagtt
cctcgccgggctctcgcaggacaacggccttgctgcgtcctggcgaagtggctcaaactgctgcaagtgggaaggcatcacctgcggtggtaacggta
atggagcagtgaccgagatctccctggcgttaaggggtctggaaggacgcctctcgccgtcccttggcaatctcaccagcctgcagcacctcaacctgt
cgtacaactcattctcaggtgaccttccatcggagctgctggcctccagcagcatcatcgtgcttgatgtcagtttcaaccacctcagcagggtcctgcaa
catcagcaaaagctcaactcatcagtgccactacaggtactgaacatctcgagcaatcattttactggagagttttcatcgatcttgtgggagaaaaaga
gtaatcttgtagcgctcaatgcgagcaacaacagcttccaaggatggatgccgtcatccttctgcgtcagttccttgtcctttgccatgcttgacctctgcc
acaaccaattcagaggaagcatcccggccggtcttggtgaatgctctgcacttatagtgctcagggcagggcacaacaacctcaacgggacgcttcca
gatgaactcttcgacgcttcctcgttggagcatctctcctttgaggaaaatagtttgcacggcatacttgacggagcacgaataatcaacctccagaactt
gtcagtacttaatcttgaagggaacaactttgtcggcaggattccagattctgtagggcacctcaagagactagaggagctccgtttgggggacaacag
catgtctggagagctgccatcaactctgagcaattgtacaaaactcatagcaattgacctcaagcacaactatttcagcggagaacttaccagggttaac
ttctctaccctgaccaatctgaaaacattggatctcctgtacaaccatttcattggacttcgcgggttttcatggcccgcgttcacgcaccgttccctctccc
gaccaccaatcctcctcctcggctga
>g129061.t1
atggctttcttcagccttgtcactcttgtacctctgttgctcctttccttggcctctccagccagctcgtgcaccgagcaggagaagggctctctcctccagtt
cctcgccgggctctcgcaggacaacggccttgctgcgtcctggcgaagtggctcaaactgctgcaagtgggaaggcatcacctgcggtggtaacggta
atggagcagtgaccgagatctccctggcgttaaggggtctggaaggacgcctctcgccgtcccttggcaatctcaccagcctgcagcacctcaacctgt
cgtacaactcattctcaggtgaccttccatcggagctgctggcctccagcagcatcatcgtgcttgatgtcagtttcaaccacctcagcagggtcctgcaa
catcagcaaaagctcaactcatcagtgccactacaggtactgaacatctcgagcaatcattttactggagagttttcatcgatcttgtgggagaaaaaga
gtaatcttgtagcgctcaatgcgagcaacaacagcttccaaggatggatgccgtcatccttctgcgtcagttccttgtcctttgccatgcttgacctctgcc
acaaccaattcagaggaagcatcccggccggtcttggtgaatgctctgcacttatagtgctcagggcagggcacaacaacctcaacgggacgcttcca
gatgaactcttcgacgcttcctcgttggagcatctctcctttgaggaaaatagtttgcacggcatacttgacggagcacgannnnnnnnnnnnnnnnt
aa
>g129062.t1
atggcattctctggtctcgctctcgccctcctgctgctctccctcatctctcgtgccacaccatgcacggagcaggacaagagttccctcctccagttccttg
ctggtctgtcccgggacggtggccttgctgtgttatggcagaacggcacaacagactgctgctcgtgggagggcatctcctgcaacggcgaagggggctg
tcgccgaggtcgtgctagccaaaaaagggctcgaagggcatgtcttgccagcccttggtgatcttaccggacttctgcgcctcaacttgtcccacaactc
tctgtctggtggactgccgctagatcacctgatgtcatccagcagaatagtcgttattgacgtcagcttcaatcgcctccagggagaactgcttgagcttc

catcatcaattacccatcgatggtcattgcaggtgctgaatatctccagtaacatgtttacaggaaggtttccatcaagaacatggaagtcaatgaagaa
tttggtcgctctcaacgcgagcaacaacagctttaggggtcaggtactggcttctttctgccttgcctcggcatccttcgccatgcttgaccttagttacaat
aagttcacaggcaacattcccccacagctagcagcattacaagaagcaggagcaggctcaggattcaggagcagcaggcagcaggtgtgggtagcc
atggcattctctggtctcgctctcgccctcctgctgctctccctcatctctcgtgccacaccatgcacggagcaggacaagagttccctcctccagttccttg
ctggtctgtcccgggacggtggccttgctgtgttatggcagaacggcacaacagactgctgctcgtgggagggcatctcctgcaacggcgaaggggctg
tcgccgaggtcgtgctagccaaaaaagggctcgaagggcatgtcttgccagcccttggtgatcttaccggacttctgcgcctcaacttgtcccacaactc
tctgtctggtggactgccgctagatcacctgatgtcatccagcagaatagtcgttattgacgtcagcttcaatcgcctccagggagaactgcttgagcttc
catcatcaattacccatcgatggtcattgcaggtgctgaatatctccagtaacatgtttacaggaaggtttccatcaagaacatggaagtcaatgaagaa
tttggtcgctctcaacgcgagcaacaacagctttaggggtcagggtctgttccaacagaagggcaactaa
>g129668.t1
atgcctcctcctcctctgctcattctcgtccttgtcctcctcgccgccgcttcggcgacctcggcgtcgccggagcgggacgtgtacgcgctgggcaagct
gaaggcggccttggccccgacgacccccactcacgcgctcgtggactgggaccccgccgctactccgccggcgcactgctccttctccggcgtgacctg
cgacgacgccggcacgggccgcgtggtggcgatcaacatcacggggattccgctccacggcgggtccctccctcccgaggtggccctcctggacgcgc
tggccaacctgaccgtggcggcgtgctcgctccctggccacatcccgccgaccatcgcgtccatgccggagctccgcttcctcaacctctccaacaacaa
cctcaccggccccttccctttacccgtccacggaaccgattacttcccgtcgctggaggtcgtcgacgtctacaacaacaacctctccggcctcctccctc
ccttggccagcaataaactcctccggtacctccatttaggcgggaactacttctcggggaccatcccggagagcttctcggagctcgcgtcgctcgagta
cctgggcctcaacggcaacgctctctccggccgcgtcccttcgtcgctgtcgcgcctgacgcggctgcgcgagatgtacctgggctacttcaaccagtac
gacggcggcgtccctcccgagttcagcaacctctcgtcgctggtccggctcgacatgagcagctgcaacctcacgggtcccatcccgccggagctcggg
aagctggccaacctcgacacgctcttcctcctcatgaaccgcctttcgggggagataccatctcaactcggcgacctcaggaggctcaagtcgctggac
ctctccatcaacgacctcggcggcgagatcccgcccagcttcgccaacctgaccaacctcgagctgctcaacctcttccggaaccacctacgcggcacc
atcccggatttcgtcgccgacgggttccccaacctcgaggtgctccaaatctgggagaacaacttcaccggcagtcttcctcagggtcttgggagaaaa
aacggccggctgaagaagctggacgtgacggggaaccacctcacgggcccgatcccgccggacctctgcgccggccgcaagctcgagctgctcgtgc
tcatggagaacggcctcttcgggcccattcctgagtcgctcggcgaatgcaagacgctgacacgggtccgcctcggcaagaacttcctcagtggtccga
tccccgccgggctgttcgacctcccccaggccgacatgctcgagctcaccgacaacctcctcaccggcgagctccccgacgtcatcggcggcgacaag
atcggcatgttattgttagcaaacaacgccattacaggccgcatcccgccggccatcggcaacctccccgcgctgcagacgctgtcgctcgagtccaac
aacttgtccggtccattgcctgcagagatcgggaaactccgaaacctcagccggctcaacgtgagcgggaacgcgatcgccgggaccatcccaccgg
agctcttcacgggctgcggctccctcgccgccgtcgacctcagccgcaacgccatctccggcgctataccggacggcatcacgtcgctcaagatcctgt
gcacgctcaacgtgtcccggaacatgctgtcgggccagctccccgccgggatctccaacatgaccagcctcaccacgctcgacgtctcctacaacatgc
tgtcgggacccgtgccgatgcagggccagttcctcgtcttcaaccagagctcattcgtcggcaacccaggcctatgcggcggcgggcccattattgacg
actgctcgtcgctagcatcatcatcatcgcacggaggagctgctgctagtcgtctctacaagaataagaagaagatgctgctgtggctggggatcatcgt
gttcgctgctgcgataataatcggcttcctcgtgagagggagaaagtttctagtcgacaaaacccggcggcaaggccaagggtggaagatgacggcgt
tccagaagctggacttctcggcggaggacgtggtggagtggctcaaggaggacaacatcatcggcaagggcggcgcgggcatcgtgtaccacggcgt
cacgcgcgccggcgtcgagctcgcaatcaagcgcctcgtcggccgtgggtgcggccacgaccacgacaggggggttcaccgccgaggtcaccacgctc
ggccgcatccgccaccgcaacatcgtccggctcctcggcttcgtctccaaccgcgagaccaacctcctgctctacgagttcatgcccaacggatcgctcg
gggagatgctgcacggcggcaagggcggccacctcggatgggacgcaagggcgcgcgtcgccgtcgaggccgcaaggggggctctgctacctccacc
atgactgcgcgccaaggatcattcaccgcgacgtcaagtccaacaacatcctcctcgactccgcgttcgaggcccacgtcgctgatttcggactcgcaa
agttcctcggcgtcggtgcggccgccggagccacgtcagagtgcatgtccgccatcgccgggtcatacggctacatcgcgccagagtacgcttacaccc
tccgcgtggacgagaagagcgacgtgtacagcttcggcgtggtgctgctggagctgatcaccggccgccgccccgtcggcagcttcggcgacggcgtg
gacatcgtgcactggtacgcttacaccctccgcgtggacgagaagagcgacgtgtacagcttcggcgtggtgctgctggagctgatcaccggccgccg
ccccgtcggcagcttcggcgacggcgtggacatcgtgcactgggtccggaaggtgactgccgagctgccggacacgtcggacacagcggcggtgctc
gcggtggcggaccgccgcctgacgccggagccggtggcgctgatcgtggacctgtacaaggtggcgatggcgtgcgtggaggaggccagcaccgcc
cggcccaccatgcgcgaggtcgtccacatgctctccaactccgctgcggcccagcccaacgacaccttctga
>g130772.t1
atgccgcccgccgccgctccgcttccccaccaccacctcgccgtcctcctcctcctccttctggcctccgcgccgcgccccgcggcgcccgcgtccaccg
accgcgcggcgctcctcgccttccgcgccgccctctccccgccctcccgcgccgcgctcgcctcctggagcggcccgctctcgccgacctggctcggcgt
ctcgctccacccggacgcccccgcccccgccgccgcgccgcccaccgtctccgcgctcgaactcccgggcctcaacctcactggcccgctgcccgcgcc
ggcgctcgcgctcctccgccgcctccgcacgctcgacctctccggcaacgcgctctcgggcgagctcccctgctccctcccgcgctcgctccaggcgctc
gacctctcccgcaacgcgctgtcgggcgccgtccccacctgcctgccctcctccctccccgcgctccgctccctcaacctctccgccaacctcctccgcct
cccgctctccccgcggctctccttcccctcccgcctcgccgcgctcgacctctcccgcaacgccatccccggcgcggtgcccccgcggatcgtggccgac
cccgacgcctccgcgctcctcctcctcgacctctcccacaaccgcttctcgggcgagatccccgccggtgtcaccgcgattcggagcctgcaggggctgt
tcctcgcgggcaaccagctgtccggggagatccctcccggaattggtaacctgacctacttgcaggtgctggatttgtcgcataatcggttgtccggtgc
agtgccagccggcctcgcgggctgcttccagcttctctacctgcggctcgggggtaatcggctctcaggggcgctgcgaccggagcttgacgcgcttgat
agtctcaaggttctggatgtgtccaataacaagatatcaggggagattccactgcccctggctggttgccgatctcttgaggtggtgaacttgtcaggaa
atgagatctcaggggagctcagtagtgctgtggccaaatggcagagcttgaggtttctgtcactggccgggaaccagctctccggccaactacctgatt
ggatgttctcatttccagtgctccagtggcttgatttgtctggtaataagttcgtgggtttcatcccggatggtgggttcaatgttagtgcagtgctcaatgg
tggaggtggtcaggggattccatcagaagaagtgcttccgcctaaattgtttgtttcagcatctgtggacacaatgtcatggcaattgaatctggggtacg
atcttcaggcaactactgggatagatctgtcaagaaatgagttccgtggggcgataccagaagggttggctgcaatgaaaggattggagtatttaaacc
tctcctgtaattacttggccggccagatccctgagggccttggagaaatggggaagttgcggacacttgatttttcgcataatgtgctgtcaggggaggt
gcctcctgtgattgcttccatgacagagcttgaggtgctcaacctctcttacaatagcctgtctgggcctttgccaacaactgatggtttacagaaattccc

aggagcattggctggaaacccagggatatacagtggggaagggtgccccgaggacgcaactctgcttcttgacgttaccatggggagcatggcgcctt
cgccgccaccgcagatgagcctggccgacctcaaggccttgtttgtgctcggccaaggcgccagaggcgtggtcttccacgtcgtgcctgccgacacaa
gaggcaaccccatggccatggcgctcaaggccatgtcccgcgccgccgcgcgtgacaagggccggggaccaggactcccggatggccaccggagga
tatggttcgagcgcgacgtgctgcgcgcgctccgtccctccgcggcgtgttcccgcgcggaagaagccgaggcgctggacgtggagaaggtcgtgcac
gaggtgttcggctctggcggcgctgctgatgaggagacgcgggcggtgaaggatggcaccgaggatgaaatagatgacgatttctccatatttttcgaa
cttgttgtgtttacctgcttctga
>g133025.t1
atggccgcggtgctcggcgcgttcgtgcctgacaccgcggtgcggtggcgcggcgtggtcacaggggaggtggcgcggtggatgggcgtcgcggcgg
aggccaagaaactggtgtcgcggctggatcgtgtgggcgccgccgtggaggacgccgaggcgcgagcggcgcgcggggacgaggccgccgcgcgg
tggctggccaatgtgcgcgccgtggcgtacgaggctgacggtgccgtggactggtgcagggtcactgcgcgccagctcaaggttcttcctcggctacta
tcttcatgctgcgacgcggacgagcccattgccgccaacatcaagagcctgaaccggaagctgcaggtcatcctgaaggagaagcacaggctccagct
ccgctcatccttgggcgatcatcaccacactccggtgcggacagtaccgaggcaccgggaatctcagcagggatccccgtctcccactaacagcgacat
catcggcacaaggatcgaggatgacgcgcatcagcttgagcaactgctgatggagaaggatggacaaatcagcggctgcaccatcgttgctatcaccg
ggcccgacggcattggcaagaccactttggcggcgaaggtgtaccgcagcgagaggatacggcgcagcttcgaggtgagggcatgggtgcgcatccc
caaggggcacacccgggccggcctgctctcgcaggtcatcgactcgttcggcagctacacgaaaggcggcgagagcgttgccgaccaaggcaggatg
ctgagccagttagtggagaagaagaggtgcttgctcgtgctcgacgacgtctggtacggtggggtgtggaaggaaatcttcaggagcccgatggagcg
cgccggccgcggcagcaaggtgctggtcacggcgcggcacggcagcattgctcgcgagatgggcgccggccacgtccaccgcgtgaagaagctcgg
cgtggaggatgactggcttctgctccgtgcggcggcccgtgtcgtcgacgaggcaacagccggcgagctgaacgacgtcggggagagtatcgttgag
aagtgcggcggcgtgcctctggcgatcaaggcggtggccggcgtcttgaggacgcggggcgcgaccaccggtgagtggacggaggtgctcaagagc
ccagcgtggtcggtgaaggggctccctgaggacgctatgaagtcactctacctctgctacgacgacctgccgtgccacctcaagcagtgcttcctgtact
gctcgatgttgccttcggacatcggtgtcgacaggcacgacctcgtgcagcagtggatatctgaaggctttgtgcagatcagagcagacgccagcatcg
aggaagtggccgaggagtactacgacgagctcgtgggaaggcaccttctccagaaggcagaggaagatgagagcggtggcgccacgaggtgctcga
tgcacgacacggtgcgtgctctggcacggatactctcacagggcgaggacttggtcggtgatgcacatcgacaggcagttgacggcgatgtccgtgaa
acagcagttgatctcgttccggagaacctggggaatcttgtatacctgaagtacctgaacctgtctgacacaaggattcaggccattccggagtccattg
gcaacctgtggaacctgaaattcctgctattacgaggatgcaagtcactgcataaactgccaaaagggatagagcatctgagagggcttagagatcttg
atcttgccggcacggtgattgatgatgtagcattcacagtaggccatctaagaagcctcacatcgctccagtgcttcgctgtgacgagcaaagaggcgc
aggccgcgcaggataggagcggttggcctctggatgagctcaagcacttgtctcagctgagaatacttcacattacgaaccttgagaaggccactcgtc
gatcagaagcagctgaaatggcacttgcgaccaaaaaaggactcagagagctcgagatgtcgtgcagcagcactgcaaggacgattcaggcaacgg
aggggatcaaatatatcgaggatatctttgaagaggcgaatccaccacaatgtctggagtcactgaagattgccaattactttggaacaaagtttccaa
gatggctgtcactgacctacctcccaaatctccgccatctctacatcatcgggtgtgacttctgccagtcctttcctccattaggccggctgccagagctga
ggtccctgtatatagcagactcttcagctctgaaggatattggggcagaattcatgggcactgaccaccagcatcaggtggtgctatttccaaagctgga
aaacctgcaattgcaagggttgcataagctcaagacctggatagacattgaggtaggggcatttccatctctgcaggcacttcagctcgagagctgccc
tgaactgcagcatcttcctgctggcctcagacaagtgacgtccctgacagagctgcgcatagtggacatggccagccttgaagctgtcgaagacattgc
ttcgctgagggaattgagtgtttggaatgctcccaatttgaagagtatatcaaacatgccttcccttgaagacctcaacatgtgtcactgcccagagttgc
aggtggtgaacatcgacgggctccaaacagtgcacatcttcgatcatgaattgaggacaatgccaagatggatcgagacaaaccatattaagagccag
attgcaactactccaaatgaaagtgcaaatgcaactccgacgtcagaaattccatcaggaaaggatgtgccaacgacatctgttggtgtcactactagt
agtgtaatcaatgaaacatctcaaactgtctatgttactgaaaccagtgaagatctggatagcagtttactttgcagcaaagagcaggcatccaataatg
aagcggaagaagtcgctagaacaatttgtgctactagtgctgtcagtaactctgttgatcaggaggaagatgccgaggtcgctgaaacaacttgtgcta
ctagtgctgtcagtagctctgttgatgaggagcaagacatcaaggtcactgaaacaattcgtgttactagtgctgtcagtagctctgttgatcaggagga
agacatcaaggccactgaaacagtttgtgcggttactaatgtcagcaattctgttgatcaggaggaagacattgagatcactgaaacaatttgtgctact
agtgctgtcagtaactctgttgatcaggaggaagatgtccaggtcgctgaaacaatttgtactactagtgctgtcagtaactctgttgatcaggaagaag
atgtccggatcactgtaacattttgtgctactagtgccgtcagtaactctgttgatcaggaggaagatgtcaaggtcgctgaaacaatttgtgctattagc
gctgccagtaactctgttgaccagaaggaagacgacaaaatcagtttgccaattcatgaagaatccaaagaaatcggtggcatacaaagtaattgtgat
tcaggggcttgcaaattgcctgccagtttggtttgcagcaagcaacagacacagaacacgctggaaactgtttctgtggagccaagtgatgacacagac
ggatccatgaagaaaatacttggatggcaagcagaattaccgagaagctcaccaacgaatgtgaggttggctcagtgaaggattcacttgctgaaac
agccatgaaaatcgcccgtcccacatccaagtcaacgcgcactatgtcacatgccattatcattgaagcagccccgaaaccagaagtcacaacggcaa
gcagattcgcagcgaccgcggcggcggtgaaggtgaacgatggccgcatcaacgacgatgacgacgacacactgggctccacggacgccgaggccg
aagaccctcaccaggcggcgccgaaggtgtacaccgccatgtgggccgataccgacacggacaccttgcgcgcccggttcttcgaatcgatgcggcgg
tatcgcaggatggcttcccgccggcgccggcgccgcgagaggaagcacggctccggggggagaagggttgatggggatgtgaggccctcggcgaca
ccggaggcggcggcgggggaggaagaggaggcgccggtctgcccgccggagccgccgaagcgggagattgtcagagtcagcaaggccgccatcaa
ggagaaggcgagcgcgttctcgtcggtgggctcctcctcggtgaagaacgaagatggcgaggcctgcggcgcgctgagcgcagcggtgttcaagggg
ttccacggctcagcaccagcggcaagagcggaggacagcgtcaccggcgacggcgagaaggccgtggaagatgatgatggctgcggtgtccggtttg
acattggggatacggagacgaggagcagccgcgagccctggaagaagcggaagagccccatcggcgacggccacgaggagcacaggagcagtgc
caaggccagacgcgtggtggtgctcggcgacgacccgcggccacggccgaggaggatgccgggccgcccgaggtccacgcgcggcggcagccagg
gcgagggcggccgcgccctgtacaaccactatgcaagcggaggcgggtggtggcacagcgacatggaaggcgtggacggcgaggaggtcgggtgg
acagacgacatgtgggaggggatggggtccgtctcgctcggcggcttggagtggcactag
>g133957.t1
atgacccaggaccaccaccagcgctccgtcaccgccaccaccggcgtggacgcgctgtcgctggacctgctcgcccacgccctctccaccctaactgac
ccacacgaccgcaagtcctgccgcctcgtctcccgcgccttcgccaccgccgaatccctctcccgccgcgccgcgcgcccgctcacccacgccgccctc

ccacgcgtgctccgcctcttcccttccctcacctccctcgacctctccgcctgcgccggcctcgactccacctccctctccctcctcccatccccgctccgcc
tcctccgccgcgtcagcctcgcgcgtgccagcggtgtgggcccgcggggtctgtacgcgctggtgacggcctgcccgaggctggaagccctcgacctgt
cacactgcgtcgcggcgggggaccgcgaggtggccgccctcgcgggcgccgaggggatcagggacctcgtgctcgataagtgtctcgaggtgacgga
tgttgggttggccaaggtcgccgttgggtgccctgggctcgaacggctcagcgtcaggtggtgccgcgagatctccgatattggggtcgacttgctcgcc
aagaagtgccggaacctgcgcagcgtcgacgtctcgtacctcaaggtcagcaacgaatccctcaggtctctttccactcttgagaagctagaacactta
gcaatggttagctgctcgttcatagatgacgatggcctgcaactgctgggtattggaggcagctcattacagagtattgatgtatcgagctttgcaacact
tgtcaaggtgctcagagctactaaccttaaagcttggcctctgctcaattgcagtgccatcactgatgatgggctggcagctttagctggtggctgtaaga
agatgaggatgctgaacctacgttattgcgaccaaatcactgatgctggtttgaagcatctaagctcactagaagaactgacgaacctggagatgagat
gcctggtgcgtgtcacaggcatcggaattgcttccattgccactggctgccctaacctgagagagctggacttgaagcgctgttactctgtagatgatgct
agcgtctgggctgttgcaagatactctcagaacctcagacagcttgacatatcctactgccaaatcacaggcttgggcctgtgccacctgttgagctccct
gaggtgccttcaggacttgaagatggtccacctctcctgggtctccattgaagggtttgagatggcgttgcgagccgcgtgtgggaggctgaagaaggt
gaagatgttgagtgggatgcggtccgtgctctccccggagctgctccatatgctgcaagcctgcggctgccgtgttcggtggatcgacaagccacttgtc
tacaagtga
>g134355.t1
atgcaaccgagctggcaagcagtcatcttctcgggggtggtactagtactcgtgttatctctttgcgacgcgtgctcgtcggaggagagggaggccctgc
tgtccttcctcgccgacctctcgccgcgtcccggcgacgacatcgccgcgtcgtggcgcgggtcgccggactgctgcacgtgggacggcgtgagctgcg
gcggcgacggcgcggtcacgcgggtgtggctgccgcggcgcgggctcggcggcacgatctcgccggcgatcgccagcctcgccgcgctcacgcacct
caacctctcccgcaacagcctcgacggcccgttcccggcggcgctgctgtcgctgtccaatgcctccgtcgtcgacgtcagcaacaaccgcctctccggc
gccctgccggacctgccggcggcgggcgacagcgtgctcgcgctgcgggagctggacgtgtcgagcaacctcctggcggggcagttcccgatcgcgtt
atgggagcacacgcccagcctcgtgtcgctcaacgccagcaacaacagcttccaaggttcgatcccttccttgtgcgcgacgtgtccgtcgcttgccgtg
cttgacctctcgatgaacatgttcggtggaagcgtccctcccgggttcggcaactgctcgcggctgcgcgttttcaacgccggccggaacaacatctccg
gcgaactccccgacgacctgttcgacgtgaagacgctggagcagctactgattccgtggaaccagatacaagggcggctggatcccggacgcatcgcc
aagctcaacaatctcgtgaagctggacctgagcaccaatgcgctgacgggggaactgccagggtccatcagccagttgccgaagctgaaggagctcc
ggctcgggaggaacaacctgaccggaacgctcccgccgtcgctgagcaactggacggcgctccggtgccttgacctcctgtcgaacagcttcgtcggg
gacctcggcgccgttgacttctcgggcctcgccaacctcaacgtcttcgacatggcctccaacaacttcacgggctccatgccgcggagcatctacacgt
gcaggtcgatcaaggcgctgcgcgtggccaacaaccagctgggcgggcaggtggcgccggagatcgggggcctgcaccagctgcagttcctctcgct
caccatcaactccttcaccaacatcaccggcgcgttctggaacctcaagggctgccgcaacctcaccgcgctgctcatgtcattcaacttctacggcgag
gcgttgccggacgccgaatgggtcggtgaccacgtcagcagcgtccggatgctggtgctggaagggtgccagctcacggggaagatcccgtcgtggct
gtcgaagctgcaggacctcaacgtgctgaatctcggcgggaaccggctgaccgggccgatcccgagctggctcggcgccatgaagaagctctactaca
tcgacctgtcgggcaaccagctctccggcgagataccgatgtcgctgatggatctgccgctgctggcgtcagaggaggccatggcggagttcaacccg
ggccacatgccgctcaccttcaccctgacgcccaacaacgggcaggccacaaggcgcggccgcgcgtactaccagatgtccggcgtggccacgacgc
tcaatttaagcgataaccagttcanatcagaggaggcgatggcggagttcaacccgggccacatgccgctcaccttcaccctgacccccaacaacggg
caggccacaaggcggggccgcgcgtactaccagatgtccggcgtggccaccacgctcaatttaagcgacaaccagttcaccggcgcgattcccagcg
cggtggggcggctcaagacgctgcaggtgttggacgtcagctacaacaacatctccgggagcatcccgccggagatcggcggcctggttaagctgcag
atcattgacctgcgacggaaccgtctgacggggacgatcccgcaggagctgaagcagctcaacttcctcgcggtgttcaacgtcgcgtacaacgacct
ggagggcccgatcccgacgggcgggcagttcgacgcgttgccggcgtggagcttcaaggagaaccccgggctctgcggcgacgtgatctccgtcccct
gttctaataagcggcgtgcacatggaggaggaggaggaggcgtggagggggaggattcctcgtcgtccaataagctagtaatcggcaacaaaatcgt
cgtcgctattgtcctcggagtttgctctggtgtggtcgcactgatcgtcttgctcgggtgcgtggtgatcaccgtcagaaggctgattttatcgaagaacgg
tgtatcatcagtccgcgacggcgggaaattcttcgcggaagataattcactgttcggtaactccatgtctgatgagttatacgacgactccaagggcaat
aagggcaccatcgtcttcatgtcggagcatgatgccaacaacaacaagagcgtcagttggatgacggcacgcgtctggccgtga
>g134964.t1
atggggagggcgggattcgccccctcctcctcgtcctcgtctccgccgccgccatggggacagcgacagccgaatccggcgatgcggccgccatcgc
cgacctagttaaatctctgtctttggatggcggcggcggcgatggctgcgacgtcgccggcatcaagtgcgacggctcggggcgggtcaccggcatca
accttgtcggcaagggtttgtcggggacgctgcccgcgtcgctgtcttccctcaccgcgctgcagtcgctgcagctccaggacaacgcgctcaccggcg
ccgtcccgtcgctcgccgggatgggctcgctcagcgacctcgccctcgacaacaaccggttctcctccctgcccgacgacttcctccaggggttgacctc
cctgacgtccctgagcatggatcagatcccctccttgaaggcctggcaaatcccggacgccattgccaactgcggtatgctccagaacttcacagcctcc
aatgcctctatcgttggttccttcccggcggttcttgcgaacctgacatcgctcaggctactgcggttgtcatacaacaaccttaccggccctctgccggcc
gagctgtccggatttgatgccctggagaccctgcaactaaacaaccagaattcggagaagctttcaggaccgattgatgtcctcacgaccgtgaagagc
ctgaaggtgatgcggcttcagtctaatgcattcacgggccctatcccagatttcagcgacttgcagctgaaggagttcaatctgagggacaatcacctca
ctggagtagtgccagcctcgttgacgaggatcgcgacacttacgagtgtctacctgtccaataacaatttgcaggggtcaaagccaactttcccagacc
atggtttggatacccacttaagcttgcacagtcatggccgggaaatgagccgtgtgctcaggatcccaattggttgggtgtctcttgtgctcactggccag
gtgcctgattttaagtcttcagtaaaggtgatggccacaggaaacccgtttggggaatcaggtgatgataatggtggacggagcagttccaaaggtggt
tcaagttcctcatcttcaaattctggaggtgcacctaattcgaagtcaaatgcaggcatgatcattggaattcttttagctgtcattcttcttgttgtttgtgtt
ggccttttctgcatcaccgaagcaagaagaatgttgacaagttcagtcccgtttcaaccaagagcccttctggtgaatctgagatgatgaagattcagg
tggttgagacaaatgacaatagcaacggaagttgttctgcagttccaactgatttttacagtcatgtgagttctgacagcacaaacattgctaatatctttg
agtcccatggaatgcaattgccaataaatgtgctgctaaatgcgacaaacaactttgatgaggacttcatcttaggtagagggggttttggggtggtcta
taaggggactctcaatggcaagctggttgctgtgaagaggtgtgacagtggcacgatggggactaaggggctgcaagaattcatggctgagattgatg
ttcttaggaaagtgagacaccgacatctggttgcactacttgggtactgtaccaatggcaatgagaggcttttggtctatgagtacatgtcaggtggaac
attgcgtgagcacctatgtgatcttcaacagagcggttacactcctcttacatggactcagaggatgacaattgctttggatgttgcccgggggatagaat

atttgcatggcttggcgcaggagactttcatccatagggatctgaagccttccaatattttgctggaccatgatttgagagccaaggtttcagattttggttt
ggtcaagcttgctaaagatacagataagtccatgatgacaagggtagctggaacatttgggtacctcgccccagagtatgctagtacgttcacctctccc
ttctatgataagggtttttcttgtga
>g135040.t1
atgaccaggatcagctgcttcccggccctgctgctcgccggcaggaggaagaagcaccacaaggtcgcggacgccaccaaggtcggattcaacgatg
ggtcgctcatcaaggttaagccggtggagttcatcgccgtcgtcgacgagccgggcaaggtcgtccctgcggccgcgctagacggaggtgacgagaa
ggtggaggacaacgaagagttggagttccacgccgccaacaagtccgacggcgccgacgtggcgccggaacaggccgcaacggacgcgtcgccga
ggctgatgcggtcgtgctccaacatcgagacgaaacgccccagcccggtgatgccggcccgatcccgctcatacagcgacctgatggacctgcccgcc
ggtctcgccacggagatcaagacaagcccgtttggcatgccggaggcgagcccggcgtcggtgaagacgtcgcgcacggcggaccgcgtgatgctga
agaagcggtcgtcgagccaggtgctcccgtcccggagcaggaagctctggtggcgcctcttcctctggagccaccgcaacctgcaccgcccaaggccg
gcggcgcgcccatcgacgcccggccagcacggcgggtacacctccgacacgcacgaggaggaccgcaagaataagaaggtcgtgacggtggagga
ggacgacggctcctcgccgtcgccgcagcagccgatcccgacgaaccaggagtgggtcgccttctgcgccgacaactcgctcctcgaccgcgtcagcg
cgtgggttagcagcatcgagaccgaccccttccacgtcgctccttccgaggacgacgacgacgacgttgaagccagtgatggtggcgactgcgcggcg
tcgcgcccgcgcgcgctcgaggtcggggagccgtcgtcggcgaagggggccacggcggcaggtccaggcgcgccgaggaggtggtccaggccaa
cagcgtcgtgcagtcactcaacgccttctccacggtggcgcacatttcagggatggggctcaaggtcgtgcccatgatcgcgcccttctccagcctccgg
tctgtcaacctctccagcaatttcatcgttcacatctccccaggatcgctgcccaagggcctgcactcgctggatctgtccaggaacaagataacatccat
cgaggggctccgggagctgaccaaactgcgcgtcctcaacctcagctacaaccgcatctcacgcattggtcacggcctgtccaactgcacggcgatcc
gggagctgtacctggccggcaacaagatcagcgacgtggagggcctccaccgtctgctgaagctggcggtgctggacctgagcttcaacaagatcacg
acggccaaggcgctgggccagctggtggccaactaccactccctcctggcgctcaacctcatcggcaacccggtccaggccaacgtcggcgacgacgc
actgcgccgggccgtgacggggctcctccccagcctcgcctacctcaacaagcaacccgtgaagccgcagcgtagcgcaagggaggtggccacggac
agcgtcgcacgcgccgcgctcaccggcggcggcgggcgcagcagccagcgcaggcgcgggtcacggcggctcggctccgggtcgggagacgggag
cttcaggagccggtccaggggcaagaaccatggctcgagctccatgaagaaatga
>g135072.t1
atggcgccagcggccggatcctcctcctcctccattgccatcctcctaatcctgaagctgggcgtccttgcctcccttgccgccgccgccatggaccccat
cgagcgcgagaccctgttccgcgtgatggagtccgtctcctccgaccgcgactggcgctcagccgcgggcgccgacccctgcgcctcccccctggccgg
gcctcgagtgcaagccatcctcctcctcctcctctcagtctcaggcggggcggcccacgacgacgaccacgatgcacgtggcgcggctcgacttcggcg
tgccgcccaacccgacctgcaagcccacggcctccttcccgatggccgccttctccctcccggagctccgcggcctgttcctcgtcgactgcttcaagaa
cccggacgccgcgaccccccttcacgctcccacccgccgccaacctctcctcctcccgcctgcagcagctcagcgtccgcgccaacccgtccctctccggc
acgctgccgccgcagctcgccagcctccggtcgctgcaggtgctcaccgtctcgcagaacgcgatggtccgtggcgaggtcccgccgggcgtcgccga
cctcgccggcctcgtccacctcgacctcagctacaactcgctcaccggccccatcccgacccggctcggcgagctccgcggcctcgtcggcctcgacctc
agctacaactccttctccggtcccatcccgacccggctcggcgacctcaaacaactccagaaactcgacctcagctccaacaacctcaccggcgccgtc
ccggcctccctcacgggcctcaaaagcctcaccttcttagcactcagcaacaacgggctccgtggccggctccccgcgggcctctcggacctccgcgac
ctgcagtacctgatcatggagaacaacccgatgggcgccgtgccgctgccggccgagctgggccgcatcgcgcggctgcaggagctgcggctggcga
actcgggctactcgggctccatcccggacacgttcggcaacctcgcgagcctcaccacgctgtcgctggagaacaacaacctcacgggccgcataccc
gcggggctcacacggctcaagcggatgtaccacctcaacctcagcaagaacggcctcgacggcgtcgtgcccttcgacggcgctttcctcaagcagct
cggacggaacctcgatttgagcggcaaccccgggctctgcgtcgtcgcggacagagccgccgtcgtgcccgacgtcggcgtcggagtctgctccggcg
gcggcggcggcgccgatgcgtcgtgcgcgagcaaggcaggcagcggcgggagatcggtccgctgggtgacgaggacgagagggcggttcttcttct
ggcgacctgcgatgctgctgctcacgcacgccgctgtagcgtcgctgtgcagctgcattctcctctga
>g135797.t1
atgtctctgctgctgctgctccgattactctgcttgtttctccttacagctgaggttgtggtgatagacgtgcgaggcggcgtcgggaaccgtactgacgaa
gtggaagtgctcatcaacctgaagcggttcttgcaggagaacaatacagtgaaacgcggcgcctatgacgcgtggccggagtgggactcgtctccatg
cggatggcgtggcgtggggtgcgacgcggacgggcgcgtcacatcactggacctctcgggctcgagcatctccggcccggcgttcggcaacttctcgc
ggcttacggagctcgcctggctcgacctctccgagaacacactccgcggcgtcggcgacatcaaccaatgccgcgggctcgtccacctcaacctctccc
gtaacctcatcagcgggcccttggacatgtctggcctcatcaggctgcggacgctagacgtgtccggtaaccggcttcagggcggcatcgcagcaaatt
tcccggcgatatgtcccgacctcgtcgtttttaacgtgtccaccaacatgctcaccggcaatatcactggcttattcgatgactgcggcaagctcgagtat
gtcgatctcagctccaataacttcagcggcgagctgtggccgggaatcgcgaggttccgtctgctaagcgtagccaagaacagcctcactggaagcatt
ccgccggccacgtttcgagaaggctgtaaactcgaatctttaggagtctctacaaaccagctcaccggagagttcccggattcaatctcgaactgtgcga
acctggggtacttatcgctgtgggggaatggcttcaccggcgtcataccggaaggaacgggagagctccctgttctccatactctgattcttgggaacaa
catgttcgaccgacatataccgtcgaatctggggaactgcacgaagcttcagttcttggacatcagcggcaacatgtttggtggggacctgcaagacgt
ctttggccggcttgcaagcttgaagcaccttattctgcaccacaacaacttcaccggcggaatcgtcgcctcggggttgctccagctgccactgctagcc
aggctcgacctcagcttcaacgacttctctggggtgctccctgcagaggtggccgacatgaaaaatctcgggtacctaatcctcgcctgcaacaaattta
gtggcgggataccatcggcgtacggccgactcgccgagcttcaggcgctggacctatcgtataacaagctcacgggcgcaataccggtgagcatagga
aacctctcgtcgctcttgtggctgatgctcgccgggaaccagctgtccggcgagataccgccagaaatcggcaactgcaccagcttgctctggctggac
ctagccgacaaccagttgactggcaagattccggcagagatggcggcgatcgggagggaccccggcccaacattcgccaagaatcgcaacgacccc
agcatgctagcgggttccggcgcgtgccaggccatgaaacggtggttcccagcaagctatcacccattcagcttcgtgtactctgtcatgacctgggaga
actgccgcagcatatgggacagaattctcaagggccacggcctcttcccgatctgcaccaactcgtcgtcgccggcaaggtcctacgtgatctccggtta
catcctgctgtctgggaatcggttgtatggagagataccaccgcagatcggtgaaatgcggaacctcagcttgctccacctcgacggcaaccggctcac
cgggcggctcccaccggagataggccagctcccgctcgtcgtgctgaacgtctcgaggaacaacatttcaggcccgattccgtcagagatcggccgca
tgcggtgccttgaaatgttggacctatcttacaactacttgtccggggagctgcccgaaagcctgagccagctcacggagctgaacaggtttaacgtgtc

ctacaacccgcttctctccggcagcgtccccgccactggccaattcggcactttcgacgagctgtctttcctcggcgacccactcatttcactgcggcgag
atatcggcaagcaaccaccaccggcagctgcgggagtgcccgcggttgcacggcgcgacgtgtctccaaggaagatcatgatgcgtttcttgttagcct
tcatcctcgtggccctcttgttgccaccagtggggttcatcgccttcttcgcgtacaaggtcccacatcttcatgaccaacctgcgtga
>g136185.t1
atggcgaagcttggcccaggtctgggtctggggtgcgaggcggcagaggggtcgcttgtgcccacccggaagcgggagtacaagccatgcggcaagc
acactgaggggaagcgcccgctctatgccatcggcttcaacttcatggatgctcgctactacgacgtctttgccactgtcggtggcaaccgcgtcacaac
ctaccgctgcctggagaatggtagttttgctcttctacaagcatatgttgatgaggatagctttgttggccatggtgattctataaatgagataagaaccca
ggcactgaagccttcactcatcatttctgcaagcaaggatgaatctgttaggctttggaatgttcatacagaccaagctcaggtgctcctccaactaaag
aagtccttcttcgatacatccaccaccagacttcagtcatggcaaaatggaaccgactgctgtctctgggagggtgttgggtgtgacacttcatctggcaa
tgtcacagtgctcgacctcaacaattgtggcttgttcagttatggcttggaccctgcaatcttcaacctcacatctcttcgacgcgttgaccttagtatgaat
gatttcagtcaaggaaagtcagagtttaacgtccctgctactggtttcgagatgttcgcattacttacccacctgaacctttccaactgtggtttacggggc
cagctttctggccctatccaagagttcaaccaggcactttcacagctggagtcagtggacttgagcaacaatgaattctcaggtgtgacaaataagcaca
agtcttctgatcatgttgatgtggtcctgttcctcttcgttgggctgggctttggcatcggatttgcatctgctatagtggtgaaatggggccagttcggcag
gtggattagtgcaactgcaagagcttcacgaacctga
>g136249.t1
atggccatggcagcagcagcgcaccaccagcccaagcgccgccgcttctctacatcccccgcccccctacgccgccacgccgctcgactcgctggccga
cgaactcctcttcctcgtcctggaccgcgtggccgccgccgacccgcgcgcgctcaagtccttctccctcgcctcccgcgcctgccacgccgccgagtcg
cgccaccgccgcgtcctccgcccgctccgcgccgacctcctcccggcggcgctcgcgcgctaccctgccgccgccaccctcgacctctccctctgcccgc
gcgtcaccgacgccaccctcgccgccgtctccgccgcgtcgtcgtcgtcgtcgtgcctccgcgccgtcgacctctcccgctcctcgggattccgcgccgc
cggcctcgccgcgctcgccaaggcctgcccggggctcgccgacctcgacctgtccaatggcgtcgatcttggcgacgccgcggccgccgaggtggcgc
ggatgcggcggctccggcggctctcgctgtcgcggtgcaagccggtcaccgacatggggctcggctgcgtcgccgtcgggtgccccgacctccgagac
ctctcgctcaagtggtgcattggggtcacggatttggggctcaaactcctcgcccacaagtgcaagaacctcaggacgctggatctctcctacggcatga
tcacaaaagactcatttccagccatcatgaagctacccaatcttgaggtgttgacactggtggggtgttctgggattgatgatgacgccctcagtagtatg
gagaaagattgcaacaaatcattgaaggtgcttgatatatctcattgtccgaatgcgactgatgtggggctttcatctgtagtgaagtcgatatccaatct
atcagaattgaatctttcttactgctgtcctgttactccttctatggcaagcagcttccacaggattgctaatctgcagaccctgaagctggaaggctgcaa
attgatggctgatgcactgagatccattggaaagtcttgtgtctctattagggagttaagtctgagcaagtgttctggagtgacagatgcagaactttcttt
tgctgtgtcaagattaaagaatttgctgaagctgaacattacttgttgtcgcaatattaccgatgcttcactagcttccatcactacttcatgcacttccctc
atctccctgagaatggagtcctgcgcccatgtttctagtgaaggactcaaactaattggaaagcactgtcatcaattggaagagctggaccttactgaca
atgatttggatgatgaagggctgaaagctatcgctggatgtagcaaactctcaagcctaaaaattggcatatgcttgaagataagtgatgaaggtctca
ctcagattggaaagtcttgccctgaactccgagacattgatttgtacaggtga
>g136256.t1
atgcgccgcatcatcctcgtgtcgccgtgcaggacgcccaccgtgctctgctccctttctgctctactagccgttgcaatcctcaccctcttcttcttgacta
acctctcatcaatgctccctgcttctctccctgcagcagcaatcgcctccctccctccctccctccattgctctcgagtctcaactcctagcgcacttcaccc
aaaactttctcctctgcttcctccgcttcgatccaagatgccgctgcttcagcggcgacctccgtcgctcgtcctccgtttctacgccgccacgctcctcctc
ttcctcctgctcgcgcctccgccggccaccgcgcagggccaccaccgccacgcggcgggcgacggcgtgatcatcagccaggcagactaccaaggcc
tacaagccataaagcacgacctctccgacccgtacggcttcctccaatcctggaacgcctccggcctcaccgcctgcagcggcgcatgggcggggatc
aagtgcgtcaagggcagcgtcgtcgccatcaccctcccctggaaaggcctggccggccggctctcggcccgcgggctgggccagctcacgggcctccg
caggctcagcctccacgacaacgccatcgccggcgagatcccctcatctctcgggttcctccccgacctccgcggcgtctacctcttcaacaaccgcttct
ccggccagatcccggcctccatcggcggctgcctcgccctgcagcagctcgacgccagcgggaaccgcctctcggggagcgtcccgcccgccgtcgcg
aattccaccaagctcatccgcctcaacctcagccgtaacgcgttggcgggggagatcccgaggtccgtcgtcgcctctgcgtccctcctcttccttgacct
ctccaaaaacaacctctccggccccatccccgacgcattcgccgccggctcctcctcatcctcatcctcatccaaagcatccatcacgggagactaccag
ctcgtcttcctcagcctggcgcacaacgccctcgacggacccatcccggaatcgctcgccaagcttcccaagctggaggaactagacctggctaacaac
aggctcaacggcaccgtcccgccgcagctcggcgcgctcgcggggctcaaggcgctcgacctctccgggaacgacctctccggcgagatcccggcga
gcttcgacaacctcaccgccaacctgcagtccttcaacgtctcctacaacaacctctccgggcccgtgcccttctccctcgcgcagaagttcggccgga
ctccttcgccggcaacgtccagctctgcgggtactccgccgcttccatcccgtgcccgtcgccggccccgtcgtctcccgccgaggagagcaataaccat
ggcggtagcaacaggaagaagaagctgatacttatcattgtgggcgtcgtcgcgggcctcctcctcttgctgctcctctgctgcctcctcctctgcttcctc
acaaaaaggaggtcctcaaccagcactagaacaaggagcgggaagcagcagcaggcggcggccaaggacgccggtggcggcgccgccgcaggag
gaggtggagggcgcggggagaagccaggagtaggggaaggggaggagtccggcggcggcgacgtgggcgggaagctggtccacttcgacgggcc
gctcgcgttcacggcggacgacctgctgtgcgccacggcggagattatggggaagagcacctacggcaccgtgtacaaggccaccctggaagacggc
agcctcgtcgcggtgaagcggctcagggagaagatcaccaagggccacagggagttcgaagccgaggcggccgtgctcggcaagatcaggcatccc
aacctcctcgcgctcagagcgtactacctcgggcctaaaggggagaagctgctcgtcttcgattacatgcccaccggaagcctctccgcgttcctgcacg
ctcgggctccaaacacgccggtggactgggcgacacggatgaccatcgccaagggcacgtcccgtggtctggcgtacctccacgacgacatgagcatc
atccacggcaacctcaccgccagcaacgtcctcctcgacgagcacaacaaccccaagatctccgacttcgggctgtcccgcctcatgacggccgccgc
caactccaacgtgctggcggccgcgggcgcgctgggctaccgcgcgccggagctgtccaagctgaagaaggccaacgccaagacggacgtgtacag
cctcggcgtcatcatcctggagctcctcaccggcaagtcccccgccgacaacaccaacggcatggacctcccccagtgggtggcctccattgtcaagga
ggagtggaccagcgaggtgttcgacctcgagctcatgcgcgacgccgcatcgggcaacgtcggcgacgagctcatggacacgctcaagctcgcgctg
cactgcgtggacccgtcgccgtcggtgaggcccgaggctcgggaggtgctgcggcagctggagcagatcaggccggggtcggattccggcgccggac
cgagcgaggaaggagctcacctgcccgtttctgcaggaggagacgactag
>g136521.t1

atggaaacgccgcgccgcgcgtgcgcgcgctcttacctcctccacctccacctccttctcgcgttgctgtccttgtgcggcctcctacgccttgcagcggc
gcagtccccaccgccggcggacgaggcgcagctgctgctccagatcaagcgcgcctggggcaacccctccgtgctggcggcatggaacgcctccacc
tcgtccgcgggcgcccactgctcgtggcccttcgtgcgatgcgacgcgtcggggcgcgtcacgaacctctccgtcgagaacaccaacgtcacgggccc
gttccccgacgccatcggcaacctcaccgcgctcacgcacctcgacgtctccaacaacaacatcaaagacgcgttcccgacctccatctaccgctgcgg
ctcgctgcagtacctcaacctctcccagaactacttcttcggggcgctcccggccggcatcggccggggtctcacggcgagcctcaccacgttggacatc
aacggtaacagcttcaacggcaacatcccggcggagctaggggagcttacgagcctgcagacgctgtacctgtcgagcaatccgttcgacgccggcg
agctgccggcgtcattcgggaagctgaccaacctgagcaccctgggtgcgtggcagtgcaacctcatcggaggattcccgagcttcgtcgtggagatgc
tggagctgaagtttctgtacctctacggaaactcgttgaccggaagcatacctccggagctttggagcctcaagaagctgcagcggttggttctgtacac
aaacaagctcaccggaaacttcgtggtggacaaatttgcagcggagagcttggaatggctggacgtctccaacaacacgctttctggaaccattccgga
catcttcgagaacttgaagaacctcacgagactgtacctcttcgccaaccagttctccggcgagataccggcgagcatcggccagttgccgtcgctcca
gatcttgaggctggaaaataacaggttcaacggcacgcttccaccggagctcgggaagcactcgccgagcttggatattgttgagctcgacgacaacg
agttcaccggtgggatcccggaggggctgtgcgacggaggcaagtttcggtccctcaccgccgggggcaaccaactgaacggttccataccggacgg
cctcgccaattgcgccgccctgctgtggctggggctggaaggtaacaagctgtccggggattttccagcggccctgatgacggcgacgcagcttggaac
cttgctgctacagaacaatcggctcaccggcagcctcccctccacgctgtacaccgccctcaccacgttacgcatcgagaacaatcaaatcggtggcgg
cgtcccggcggcagctgcagcgctcaaggtgttcaccgccggcaacaaccggttttccggcgagataccggcgggtctcggcgacggcatgccgatgc
tgaacaagctggacctgtccggtaaccagctctccggcgcgatcccggggagcctcggcaagctgagtcagctgacgcagatggacatgagcgggaa
ccagctcaccggagagataccggcggagctgggcgccatgccggtcctcagcagcctcgacctgtcgtcgaacaggctctccggcgacatcccgccgg
cgctggccaagccgagcatcaccgccctcaacctctcctccaaccagctcaccgggaaggtccccgccggtctcgccacctccgcctacgaccggagct
tcctcgacaaccccggcctctgcaccgccgccgccgccgatcctggactcctcgccggcgtgcggtcctgctccgcggggtcatcccgagaaggagga
ggaggaggagtctctcgcgggctccgcacgggcctcctatccgcgggcgccgcgctcctcgtcgtggccgcggcgttcgccttcttcgtcgtgcgcgac
gtcaggaggcggcgtcgcctcgcgagggagaaggagtggaagctcacgccgttccaggtcctcgacttcaacgagaaggccgtggtccgcgggctca
cggaggacaacgtggtgggccgcggcgggtcgggccgcgtgtaccgcgtgccctacacgaaccggcgcgacggcagcgccggcgtggtggccgtga
agcgcatccgcgtcggcggcggcggcgacgagaagctggagcgcgagttcgaggcggaggcggacgtgctgggccgcgtcaggcacaacaacatc
gtgcggcttctgtgcctcgtcacggggcccgatgccaggctgctcgtgtacgagtacctccacaacggcagcctggacgcgtggctccacgacggcgcg
cgggggcgcgcgccgatggactggccgacgagggtccgtgtggccgtgggcgcggcgcaggggctgtgctacatgcatcacgagtgcacgccgccc
atcgtgcaccgcgacgtcaagaccagcaacatcctgctcgactccgagttccgggccaaggtcgccgacttcggcctcgccaggatgctgctgcgcgcc
ggggcgcccgaaaccatgtccgccgtcgcaggatccttcggatacatggctcctgagtgcgcgtactcgaagaaggtgaccgagaaggtggacgtgta
cagcttcggcgtggtgcttctggagctgaccacgggcaagacggccaacgacggcggggagcacgggtgcctggcggactgggcgcgggaccacta
ccagtcaggagggagcatcgccgacgccacggacaagtgcgtcaggtacgcggggtacgccgacgagatcgaggtcgtgttcaggctcggcgtgctg
tgcaccggcagcgcgccgtcgtcgcggcccgccatgaaggacgtgctgcaggtcctggtcaagtgcggcgagcggacgcacgagaagagcaaaatg
gagcacagcaacggcgctaccccggagtacgaggcggcttcgttcctgctgccgcagcgagggagtcaccggaagcagctctccaaaggtacgggg
agcgacttcgacagcattgtctga
>g136656.t1
atggccgccgccggagaggacgacgcggtgtgggagcgcgccatcgccgcggcctccaaaaccgccgtcccctccgccgcagcgcccaagaccctc
accctcgacggcggcgtcaagtccaccacgggccgcctcccatcaccggctgtcctcgagcgcgtcgccgcctccctcgaggagctttccgtcgcgggc
gcgcgcctctcctcgctcgcgggcctcccgcgactcccggcgctgcgccgcctctcgctccctgacaaccgcctctccggcgccgccgcgctctccgcgg
tcgcggaagcctgcggtgccacgctgcgacacctcgacctgggaaacaatcggttcgctgatgttgaggagctcgtcccgctcgccaaggtcggggtc
gaggcgctcgacttgtaccagtgccccgtcaccaaggtcaaggggtatagagagaaggtcttcgcgctgattccaagcttgaagttcctggatggcgtc
gacgccgagggtaacgagcgattggagacggatgacgaagaggacgatgaggaggaggaagacgacgaaggagatgaggagggtgaaggtggg
gaagacgaggaaggagaagaggatgatggggacgacggggaaggggatgaagaagatggagaaggtgatgaggaggaagaagaaggcgatga
ggaggatgccgccgtcgggtgcccccgcattcgtgctaagctcatcgtggaccccagcataaaggccacggtggaggccgccgtcttcgacggcgcgg
tgtggagcgagaagttttcgactgtgacacaaacccgtagggtgctcgtcaaggggtggatgtaa
>g137104.t1
atggggcttcttgtgagtttattggttctgctcctgtttgcttcccctgcacttggccagctgccgtcgcaggacattctcgcgctcctcgcattcaagaagg
ggatcacccatgatcctgctggcttcatcaccgactcctggaacgatgagtccatcgacttcaatggctgcccggcttcctggaacggcatcgtgtgcaa
tggcgggagcgtggcgggagttgtgctcgatggccatggaatctcgggtgttgctgacctctctgtgtttgctaacctcaccatgcttgtgaagctctccat
ggctaagaacaacctctctgggagccttcccaccaatgtgggctgtttcaagagcttgaagttcatggacatctctaacaatcgtttctctggtccaattcc
tgaagatattggcaagcttcgaagcctgcaaaacctgtctcttgctgggaataacttttctgggccactgcctgactccattgacgggcttatgtcgcttca
gtcgctggatgcgagcagcaactctttgtctggtccattgcctgcaggtttgaaaggccttcgcagcttggttgcacttaacctgtcacgtaatgccttcac
gaagggtattcctgctgggcttggacttcttgcgaacctccagtctgtggatctgagctggaaccaaatggatggtggtgttgactggaaattcttgattg
agtcaactgttgctcatgttgacttcagcgggaacttgctgaccagtactacccctaaagagctcaagtttcttgctgacatttcagagacggttctatatc
tgaacctgagcaacaataagctgactgggtcattgatcgatggggttgagctctcgacttttgggaggctgaaggttcttgatctgagtaataatcagtta
tctggtgacctgccaggattcaattatgtttatgatcttgaggtcttgcgacttgcaaacaattcattcactggatttgtacctagtgggcttctgaagggtg
attctctcgtactaagcgagctggacctgagcgctaataacttgacaggtggcaaatcactcagtacagctgctaaaatagcaattatagcagccagca
ttgttgttcttgtcattcttatccttattgctatagtatgccactataagaagatctcacggcaattccctagctcggaaaaggtttcagacaagaacctcca
cagggctcctaaagatatggccagtacaaaagtaaaggacagcaaaggcggtttggtctcagcagatgagcttgttactcctggtaaaggctctacatc
tgaggcacaaagccaagaggagaagtcagcaggtggtggcttttcgccatcaaagggcagccgctactcttggtcaccggattctggagaggcatatg
gccaggaaggcttatcaaggttggatgtcagatcacctgacaggctagcaggagagttacatttccttgatgaaacaatcactttgacaccggaggagc
tttcaagggcaccagctgaagttctgggaaggagcagccatgggacctcatacagggcgacactggagaacggtgtgttcctgactgtgaaatggttg

agggaaggagttgcaaggcccaagaaagagttcgcgaaggaggccaagaaattcgcaaacatccggcatccaaatgtggttggcttgcgtgggtact
actggggtccaacgccacatgagaaactgatattgtcagattatgttgcaccaggaagtcttgcgagctttctatacgatcgaccaggaaggagaggtc
ctcctctgacctgggcacagcggctgaagattgcagtcgatgttgcgcgtggtctgaactaccttcactttgaccgagcaatgccccatgggaacctcaa
ggccaccaacatcctgctggacggccttgacctgaacgcccgtgttgctgactactgcctgcaccgcctgatgacccaagccggcgttgtggagcagat
tctggaccttggcgtactgggctaccgagccccggagctggctgcctccaagaaaccatccccttcgttcaagtctgacgtgtatgcctttggtgtcgtaa
tgctggagctcctgacgggcagatgtgctggggatgttgtctcagggtctgagggtggcgtcgacctctctgactgggtccggctgcgggtggcagaag
gccggggatcagactgcttcgacccagccatggcctcagattctgagaacccgcaggcggtgaaaggcatgaaggaggccctgggcattgcactgag
gtgcatccggccagtctctgagaggcctgggatcaagtctgtgtatgaggatctttcgtcaatctag
>g137230.t1
atgtacctctacgccaacaacttcatcggtgggatcggacccagcatcaacgccaccaacatgctcgagctcgacctttctacgaatagtctcacggaac
cgataccagaggctatcggtaacatgaagaacttgaagctgctgttcctgtacttcaacaagattagtgggcccattccggcaagcatgtcgctgcttcc
aaacctccaggacatccgactcttcaacaacatgctatccggaccactcccgcctgagctcgggaagcactcgccactcggcaacctagaagtgagca
acaacttcctcaccggcgagctcccggacaccctctgctccaacaagaatctctacgacctcgtcctcttcaacaacagtttctacggcgagttgccaatt
acccttggcgactgcgacacgctggacaacatcatgctatacaacaaccacttcattggtatgttccccggatcggtgtggtcagcgttcccgaagctga
ccaacgtcatgttacaaaacaatgacttcgctggtgttctaccaactgagatatcctctaacatctcacgaattgagatcgggaacaacagattctctggc
gcaattccaacatcggcaacaggactacgtgtgttcaaggcggagaacaactggttctcccatgggctaccggcggacatgagcaagctcgccaccct
cacgngttctcccatgggttaccggcggacatga
>g137540.t1
atggtggacgaggcgtgggagcgggcggtggaggcggcgctgcacgccggcggcgaggggagctcgtcgccggcgcggagcctgacgctcgacgg
cgccgtgaagtgcatgcacggccggctgccggcggcggagatactggagcggcaccagagcctggagcacctatcgatcgccggcgtcggggtcacg
tcgctcgaggggttcccgcggctgcggaacctcacgcggctcacgctctccgacaatcgcatcgccggcggcctcgaccacctcgtcgtcgcggggctc
tcctcgctgcgggacctcgacctcagcaataaccgtatccaggacgtcgacgacctcgcgccgctcgcggacctccgcctcgtctcgctcgacctctacg
agtgcccggtcacccgggtcaaggactacagatccagggtgttcggcttgatccggacgctcaagtatctcgacaagatggacgccgacgagaacga
gcgccccgagtccgacgannnnnnnnnnnnnnnnnnnnnnnnnnnnnnnnnnnnnnnnnnnnnnnnnnnnnnnnnnnnnnnnnnn
nnnnnnnnnnnnnnnnnnnnnnnnnnnnnnnnnnnnnnnnnnnnnnnnnnnnnnnnnnnnnnnnnnnnnnnnnnnatgaggag
gatgaggaggaggacgacgactatgagtagtgatgaggaggaagatggagttgaagaggacgatgaagatgatgaggatgatgaagaggaggttg
aggatgatgaggacgtggagccacagagcagcgggagggttgccatgatgacaggggaggctgggggagaggagattgatggtcatgatcacgga
gaagaggatgatgaggatgaggatggtgaaattggggaagaggatgacgagagattggaggatgatagggtttatgatgagggccacaacgacgat
gatgatgcagatgatgaggatgaagatacagaatatcttgttcagccgattgccaggccttcacctggcatggccattggaagtgacgagcctcaggcc
atggccatcaggagtgactttgatgctgctgatcctgatgatgccgatgaggacagggatgaggttgatgatgatgacgaagtggccacagatcagcct
tcatcctcacatggtgccaagcggaagagaggtgatgatccatcaggcagtggtgatgacagtgaggatgatggtgttgaagacttgaggcccttcaa
gcaccactga
>g137706.t1
atgtggggcaatagcaagaggaagggcctctctggcataatcaacttaagatttctgctcacaaccagttttctcatccatgctgttctccttcctatgtct
gtagctcagtctacagatgagcgagcactgcttgcattcaaggcctccatctccagcaacccaagcggggttcttggagcatggacgcccaccaatggt
attgtgaatgcaaccaacaacgtctgccggtggagtggcgtgtcatgcctctccagacaacaccctggccgagtcactgctctggagctcatgtcgtcca
acctgacaggtgtgatcagccccagcatagccaacctatctttccttcacacactcaacctcagcggcaacaacctccatggcagcatcccctcggagct
tggcatgtttcaccacctccaggtcatcagccttggtgggaattccctcactggtaagattcctgcatcgctcaccaattgtgctcgtctcactcatgtgga
gttacaacggaatggattgcacggtgagattccatcgaacttcagctactgtcgggaactccgggtgttcaatgtcagtgtgaacagcctgtctggtggc
attccaccatcgttcggggcattgttgaagcttgagttcttgggccttcatcggagcaacctcactggcggtattcccccatcattaggtaacatctcatca
ctcttggcgtttgatgccagtgagaattataaccttggcggtnnnnnnnnnnnnnnnnnnnnnnnnnnnnnnnnnnnnnnnnnnnnnnnnnnnn
nnnnnnnnnnnnnnnnnnnnnnnnnnnnnnnnnnnnnnnnnnnnnnnnnnnnnnnnnnnnnagccttggtgggaattccctcactggtaagattcctgca
tcgctcaccaattgtgctcgtctcactcatgtggagttacaacggaatggattgcacggtgagattccatcgaacttcagctactgtcgggaactccgggt
gttcaatgtcagtgtgaacagcctgtctggtggcattccaccatcgttcggggcattgttgaagcttgagttcttgggccttcatcggagcaacctcactgg
cggtattcccccatcattaggtaacatctcatcactcttggcgtttgatgccagtgagaattataaccttggcggtgacataccagatgctctagggagac
tcaccaagctcaactatcttaggctagcttctgccgggctcaacggcaccatcccaccttcgttatttaacatatcatccctgaggacacttgacttagga
aacaatgatctctctggtgttctgcctcccgacgtcggtattactctgcctagagttcaattcctttcactgtacaactgccagatcaaaggacgcatccca
tcatcgataggaaatgcaacagaactccgtctgattcagcttcattacaatgatttgcaagggactgttccacctgcaattggtaagctgaagaatcttga
ggtgctgaacctgcagttcaaccagcttgaggaaatatggaacaaggactggggtctgatggctgcactaggaaattgcagtaagctgttagcactga
gcctttccagtaacaagtttcatggtgtgctcccccttctcttgtcaacctcaccattggaatacaacagatacttatgtatggaaataggataagtggca
cagtttccccagcaattggcaagtttagcaatcttcgtgctcttgcgcttgctgataatgctctgagtggcaccatccctgacaccattggccgtcttcaca
gcatgattgggctagatgtatctggcaacgatatatctggtgagattcctcctatgcttgttgcaaacctcactcagcttgctctcctggatctgtcacaaa
atgaactccaaggaagcatacctgagagctttggggtcatgagaaatatcgcagttctggacctgtcttacaatcaatttactcgcatgataccgaaaca
gagtaacgagacagctcaggtggaaaacttgatggaacaacacttgaggttatcctatgatgaactgcagagggctactgatgggttctctgaggctaa
tctcataggtgttggaagctttggctcggtatacagaggaacactagaccatgaaaatcaggaagttgctataaaggtgttgaaccttctgcagcatggt
gcggagcggagcttcctagctgaatgtgactttgggctgtcacggtttgttcagggagcaaacaactcctcgttacagcagtcaagcagcactggtgga
atcaaaggcaccataggttacattccaccagagtatggaatgagcagtgacatatcagtggaaggtgacatctacagctacggggttctcctgctcgag
atgttcactgcgaagaggccaacagaccccttgttcgagagaggtcaaagcatcctcagctatgtcacgacagcaggaaccaccgagagcaaggatg
gcaacaagagatgccatcagaacgctaaccgctgcaacgtcccatgggagctcggcttcctttctcgcctcttgtacctggacctttgccacaactccttg

caaggcatgatcccaccatcccttgctcaggcctccaagctccgggtgttgcagctggagtacaacagattcgtgggtaagatcccagccagcttgagc
tatctgcaggagctggaagtcttggacgtcagcgaaaaaccagatctctggtgtgatcccaccgtcgctcggttcactcactaa
>g137846.t1
atggccaccacttcttcgccgctcaacagcggcctcctcctcctcctccctctcctcaccatcacctccgccgcgtcatccgcgccactccccctcctcgcc
ctcctctccctcaaatcctgcctccacgacccatccaactccctccaccaatggacctacgcatccgcggcctccgccggcgccacccactcgccgtcgc
cgccgtggtgcgcgtggccaggcgtctcctgcaccccaacaccaccggcgccatcgtcggaatcgacctctcccgccgcaacctctccggcaccgcca
tctcccccaccgccgcccgcctcctcgcgcccacgctcacctcgctcaacctcagcggcaacgcgttcaccggcgagctcccgccggccgtcttcctcct
ccgccggctggaggccctcgacgtcagccgcaacgccttcaacggcaccttccccgacgggattattgcgaagctggacgcgctcagagtgctcgacg
ccttcagcaactgcttcgtgggcccgctgccacgtggcctcggcgcgcttcggagactgggacacttgaaccttggtgggagctacttcaacggcagca
ttcccgccgagctcggcagcctccggcaactcaagttcctgcacctcgccggcaacgccctctccggaaacatccccaaggagctcggccagctcacgt
cgctggagcggatggagatcgggtacaacacttacaacagtagcattccaacggagttcggcaacctgaggaagctgcagtacctggacatcgcggt
ggccggcgtgacgggcccactcccagcgtctcttggcgccctcacgaacctccaatccctcttcctcttcaagaacaaactcaccggcaccatcccgccg
cactggtcaccccaccaccacctccgctcatcactacaagtcctcgacctctccgacaacgccctcgcgggccccatcccctccggtctcggcgacctca
ccaacctcaccacgctgaacctcatgagcaacgccctctccggcacgatcccgccctgtatcggcacgcttccgaatctcgaggtgctccagctgtgga
acaactcgttgacgggaaggttaccgccttccctcggcgcggggagcagaaggctcgtccgtgtcgacgtgtccagcaacgcgctctccggaccggta
ccggcgagtctatgcgccggtaaccggctcgcgcggctcatcctctttaacaaccggttcgactccgccatcccgccgagcctcgccgccaactgctcct
cgctgtggcgcgtccggctcgagtcgaaccggttttccggcgcgataccggccgggttcggtgggataaagaacctcacgtacctcgacctcattgcca
acgcgctcactgggggcggtataccggaagacctcgtgacgtcacccagcatcgagtacctgaacgtctccgggaacctggtcggtggcgtgttgccc
aacgccacgtggcaggcgccgaggatgcaggtgttctccgcggacaagtgcgcgctggtcggcgaagtcccgaagttcggcgccaacgggtgctcga
acctgtacaggctggagctggcggggaatgcgttgaccggagagaaatcccacgcgacattgggaggtgcaagaggctggtgagcttgcggatgcagcg
gaacgagatcagcggcgagatccccgcggggctggcggcgctgccgtcgatcacggaggtggacctgtcgtggaacgcgctgacgggtgctgttccc
cgcgggttcgccaactgcaccacgctcgagaccttggacgtctccttcaaccgcctctcgccgtcgccggcggcgtggtcttcgggcgaagaaggaagc
aacaacgtgatgtgggtggccgcggcggtggcgttcgcgggggtggcggtgctcgcggccaccgcgcgatggatgcaactgggacagggccccacta
ctacagtacggaacggcggcccaaaggttgggccgtggcggatgacggcgttccagaagctgggcttcacggcggacgacgtggcccggtgcgtgga
gctgctggcggggagtgatgccattgttggggccgggtcgtcggggacggtgtaccgcgccaagatgcccagcggcgaggtcatcgcggtgaagaag
ctgtggcagtcaccaacacaaaaggccacgagatgcaaacaaggcgacactcggatggtcgcggaggtggaggtgctggggcagctgcgtcaccgc
aacatcgtgcggctcctcggatggtgcaccaacggcgagaccaccatgctgctctacgagtacatgcccaacggcagcctccacgacctcctgcacgc
cgccaataatacttgcaacaaggctagtgacaatagtaaggaggaggcggtagtactagtagagctggactgggacgcgcggcgcaggatcgcgctt
ggcgtggcgcagggcgtgagctacctgcaccacgattgcctccccgccgtggcccaccgcgacctcaagcccagcaacatcctgctcgacgccgacat
ggaagcccgcgtcgcggactttggcgtcgccaaggcgctccacagcgccggagcgccgcccataatgtccgtcgtcgccggctcgtacggctacatcg
tgccagagtacacgtacacgctgcaggtggacgagaagagcgacgtgtacagctacggcgtggtgctgctcgagatcctgactggccggcgcccggc
ggaggcggagtacggcgaggggagcagcatcgtggactgggtgcggcggaaggtcgccgccggcgccgaggccgggctgcgcgaaatcatggcgt
gggtggcggacggcggcgaggcaacaagagacgagatggcgctggcgctgcgggtggcgctgctctgcacgagcaagtgcccgcaggagcggccg
accatgagggacgtcgtctccatactgcaggaggccaggcgtgatgaccggaacaaacccgcggcggtggtggtggccaacaaggaaacgcacaag
gtgctgaattgttag
>g138522.t1
atgtcggcgctcctcctgctcctcctcgtcctcgcactcccccaccaccactagtagtagtaacggcgctgaacgcggagggcctggcgctgctggcgt
tcaagcaagcggcgacctcggacccgtaccgcgctctcgcccgctggtccgagtccgacccggacccgtgcgcctggccgggcgtcacctgcgccaac
gccacctcctcctcctcctcctcctcctccaccgccgcgcgcggggcggtggtggtggtgggcctagccgtcgccgggaagaacatctccgggtacatcc
cttccgagctggggtccctcctcttcctccgccggctcaacctccactcgaaccgcctcacgggcgccatcccgtcctccctctccaacgcctcgtctctcc
gctccgtctacctctactccaaccgcctcaccggcgcgctccccgccgccgcgctctgcggcggcgcgccgaggctgcagaacctcgacgtctcgggca
acgccctctccggccccatcccgctcgacctccgcggctgccggagcctacaacgcctcatcctcgcgagaaaccggttcgccggcgaggtgcccgcg
gggatctggccggagctcggcggcagcctgcgggagctggacctgtcgtcgaacgagctcaacgggaccgtcccgccggacctcggcgagctcgggc
ggctaggcgggacgctgaacctgtcgcgcaaccggttcgacggccccgtcccgccggagctggggcggctgccggcgaccgtcacgctggacctgcg
gttcaacaacctgtccggcgcgatcccgcagaccgggtcgctggcgagccaggggcccaccgcgttcctcaacaaccccgggctctgcgggtacccgc
tgcaggtgccctgccgcgccgtcccaccgcccaccctctcgcccgcgccgccatcggcgaccaccacgccgatggcgaccacgtcgtcgaggaggca
gcgcgggacgatcaagaccagcctgatcgcgctcatctccgccgccgacgccgcgggcgtcgcgctcgtggggatcgtcctcgtctacatctactggaa
gctcaaagaccgcagagccgggaatggccgtgatggcagcggcaataagcagagcaacggcctctgctgctgcggcgcgctgtgggcccgcaacgg
cagcgggtccgacgagtccgacacgtccacggaaaacaacgacgagtccggcaagtacaccagcggcggagtcggagtcggaggcggaggggagg
aaggcgagctggtggcgatcgacaagggggttccgcgtggagctggacgagctgctgcgctcctcggcgtacgtgctggggaaaggcgggaaggga
tcgtctacaaggtggtggtggccaacgggaccacgccggtggccgtgcgccggttaggcggtggcggcggcggcggcgaccggtgcaaggagttcgc
ggcggaggcgcgggccgtgggaagggtgcggcatcccaacgtcgtgcgactaagggcgtactactggtccgccgacgagaagctcgtcgtcaccgac
ttcgtcgccaacggcaacctcgccaacgcgctcagaggtcgtcctgggcagtcccccctgtcgtggtcggcgcgtctgaagatcgcccgcggcgcggcg
cgcgggctggcgcacctccatgagtgcagcccgcggcgattcgtccacggcgaggtgaagccttccaacatcctcctggagcacgacctcaccccgcg
cgtcgcagacttcggcctcgcccgcctcctcgccgtcgccggctgcgcccccgacgcgccctcctccaccgctggcggcggcctcctcggcggcgccat
cccctacgccaagacccccgcgcccgcgccgggtgggccgggcccgggcgggtacagcgcgccagaggtccgtgggccaggagccaagccggccc
agaagtgggacgtgttctccttcggcgtcgtgctgctggagctcctcacgggccgcgggcccggggacaacgcgtcgccgtccacgtcggcgtcgttct
ccgcgccggtgtcggggtccacggcggcgacggggacggaccggtccggaagcggggagcacgggccggtccctgaggtggtgcggtgggtgcgg
cgcgggttcgaggaggacgcgcggcccgtcgcggagatggtggacccggcgctgctgcgggtcgggcccggcggcgcgccgccgccgccgctgccc

aagaaggaggtggtggccgcgttccacgtcgcgctggcgtgcacggaggtggaccccgagatgcggccaaggatgaaggccgtcgccgacagccta
gacaagatcggctcatga
>g139462.t1
atgtctcgactgctcctccgatcactctgcttgcttctcgtcacagaggtggtcgtgattggcgtgcggggcgaccgagacgacgaagtggaagtgctctt
ggagctcaagcggttccttcaggagaccaacaaggtgaaccgcggcgcctacgacgcgtggcctgagtccgacgcgaccccgtgcacaaggcacgg
cgtgggttgcgactccaccggccgcgtgacgtcgctggacctctccggcgcgagcatctccggcccggccttcggcaacttctctcgcctctcagcactc
acctcgctcgacctctccggcaacacgctccacaccgccggcgacgtcaaccaatgccgcggcctcacgcacctcaacctctcccgcaacctcatcgcg
ggtccgctcgacgtgtcccgcctgacccggctgcgcacgctcgacgtgtcgggcaaccggctcgagggcggcgtctcgatgaatttcccggcgatatgc
gccgacctcgccgtgctcaacgtgtccnnnnnnnnnnnnnnnnnnnnnnnnnnnnnnnnnnnnnnnnnnnnnnnnnnnnnnnnnnnnnnnnnnn
nnnnnnnnnnnnnnnnnnnnnnnnnnnnnnnnnnnnnnnnnnnnnnnnnnnnnnnnnnnnnnnnnnnnnngttgcgactccaccggccgc
gtgacgtcgctggacctctccggcgcgagcatctccggcccggccttcggcaacttctctcgcctctcagcactcacctcgctcgacctctccggcaacac
gctccacaccgccggcgacgtcaaccaatgccgcggcctcacgcacctcaacctctcccgcaacctcatcgcgggtccgctcgacgtgtcccgcctgac
ccggctgcgcacgctcgacgtgtcgggcaaccggctcgagggcggcgtctcgatgaatttcccggcgatatgcgccgacctcgccgtgctcaacgtgtc
caccaaccggctcacgggcaacatcaccggtctgttcgatggctgccacaagctggagtacgtcgacctcagctcgaataacttcaccggcgagctgtg
gcccggcgtcgcgaggttcagggagttcagcggcgccgagaacaagctcaccgggagcgtcccacctgccacgttccccgacggctgcaagctggag
tctctggacctgtcctcgaaccagttcgtcggaaatttcccgggttccgtcgccaagtgcggtaacttgacgtacctgtcgctgtgggggaacagcttcac
tggcatgataccggccgggatcggggagcttgcggcgatcgagacgctgattctcgggaagaatcagttcaacagagaaataccgccggagcttacc
aactgctcgaggcttcagttcttggacatcagcagcaattcgttcggaggtgacgtgcaggaaatcttcggcaggtttgtcaacttgaggtacctcgtgct
gcaccacaacggctacaccggcgggatcgtcgcctcgggcgtcctccggctaccgctgctggccaggctcgacctcagcttcaacaagttctcgggag
agctgcctccagaggtggccgacatgaagtctctcaagtacctgatgctcgcctggaacaatttctccggcgccataccggtggcgtacggccggctca
gcgagctccaggcgctggacctgtcgtacaacaagctttctggtgcgataccggcgagcgtgggcaacctcacgtcgctcctgtggctgatgctcgccg
gcaaccagctgtccggcgcgataccgccggagatcggcagctgcagcagcctgctgtggctgaacctggcggacaaccagctgagcgggagcatccc
gcccgagctgggggcgatcgggagggacccggggccgacgttcgccaagaaccgcaacgacacgagcgtgctggccgggtccggcgagtgccaag
ccatgaagcggtggatcccggcgagctacccgcccttcagcttcgtgtactcggtgatgacccgcgagaactgccgcggcatctgggaccgcatcctca
agggcaagggctacggcattgtccccatctgcaccaacgcgaactcgccagtaaggtcctacgcgacctccgggaccgtcaagctgtccgggaatcag
ctgtctggagagataccgctgcagattggtgacatgcggaacctcggcttgctccacctcgacggcaaccggctcacgggccggctcccccggagatc
agtaacctcccgctcgtcgtgctgaacgtgtcgaggaacggcatttccggcgcggttccgtcggagatcggccgcgtccggtgcctcgagatgttggac
ctatcgttcaacaacttctacggggagctgccggcgagtttgagccagctgaccgagctgaaccggttcaacgcgtcgtacaacccgctcctctccgga
cccatccccaccaccggccagttcggcaccttcgacgagcagtccttcctcggcgacccgctcatctcgctgccaccaggcacaggcaagcaaacgcc
accggcagctgcggaagtgcccgcggttgcacggcgcgacgtgtctccaaggaagatcatgatgcgtttcttgttagccttcatcctcgtggccctcttgt
tgccacctgtggggttcatcgccttcttcgcgtacaaggtccctcatcttcatgaccaacctgcgtga
>g139466.t1
atgataccggccgggatcggggagcttgcggcgatcgagacgctgattctcgggaagaatcagttcaacagagaaataccgccggagcttaccaact
gctcgaggcttcagttcttggacatcagcagcaattcgttcggaggtgacgtgcaggaaatcttcggcaggtttgtcaacttgaggtacctcgtgctgcac
cacaacggctacaccggcgggatcgtcgcctcgggcgtcctccggctaccgctgctggccaggctcgacctcagcttcaacaacatctccggcccggcc
ttcggcaacttctctcgcctctcagcactcacctcgctcgacctctccggcaacacgctccacaccgccggcgacgtcaaccaatgccgcggcctcacgc
acctcaacctctcccgcaacctcatcgcgggtccgctcgacgtgtcccgcctgacccggctgcgcacgctcgacgtgtcgggcaaccggctcgagggcg
gcgtctcgatgaatttcccggcgatatgcgccgacctcgccgtgctcaacgtgtccaccaaccggctcacgggcaacatcaccggtctgttcgatggctg
ccacaagctggagtacgtcgacctcagctcgaataacttcaccggcgagctgtggcccggcgtcgcgaggttcagggagttcagcggcgccgagaac
aagctcaccgggagcgtcccacctgccacgttccccgacggctgcaagctggagtctctggacctgtcctcgaaccagttcgtcggaaatttcccgggtt
ccgtcgccaagtgcggtaacttgacgtacctgtcgctgtgggggaacagcttcactggcatgataccggccgggatcggggagcttgcggcgatcgag
acgctgattctcgggaagaatcagttcaacagagaaataccgccggagcttaccaactgctcgaggcttcagttcttggacatcagcagcaattcgttcg
gaggtgacgtgcaggaaatcttcggcaggtttgtcaacttgaggtacctcgtgctgcaccacaacggctacaccggcgggatcgtcgcctcgggcgtcc
tccggctaccgctgctggccaggctcgacctcagcttcaacaagttctcgggagagctccctccagaagtggccgacatgaagtctctcaagtacctgat
gctcgcctggaacaatttctccggtgccataccggcagcgtacggccggctcagcgagctccaggcgctggacctgtcgtacaacaagctttccggtga
gataccggcgagcgtgggcaacctcacgtcgctcctgtggctgatgctcgccggcaaccagctgtccggcgagataccgccggagatcggcagctgca
gcagtctgctgtggctgaatttggccgacaaccagctgagcgggagcatcccgcccgagctggcggcgatcgggagggacccggggccgacgttcgc
caagaaccgcaacgacacgagcgtgctggccgggtccggcgagtgccaggccatgaagcggtggatcccggcgagctacccgcccttcagcttcgtg
tactcggtgatgacccgcgagaactgccggggcatctgggaccgcatcctcaagggctacggcattgtccccatctgcaccaacgcgtcctcgcccgtg
cgctccgacaccatctccgggtacgtccagctgtccgggaaccggctctcgggggagataccggcgcagatcgggtccatgcggaacctcagcctgct
ccacctcgaccgcaaccggctgacagggcggttgccggcagaaatcggcaacctcccgctcgtcgtgctgaacgtgtcgaggaacggcatttccggcg
cggttccgtcggagatcggccgcgtccggtgcctcgagatgttggacctatcgttcaacaacttctccggggagctgccggcgagtttgagccagctgac
cgagctgaaccggttcaacgcgtcgtacaacccgctcctgtccggccccgtccccaccaccgcccagttcggcaccttcgacgagcagtccttcctcggc
gacccactcgtctcgctgcggccaggcaccggcaagcaaacgccaccggaacccgcggaggttccccgagctgctgcaaggcgagtcctgtctccca
aaaccatcgcggtgtggttcctcttctccctcctcaccgccttcatcgccggcgccctcgtgttcttcctctccaacctgcgcgcgcggttccccgtggacc
aggaccccgaccccgagtcgtcgtcgtcgtccttctcgtgcgacgcgaaccaccacaagtccggcaaatgcagcacgttacagatgatgacgacggcg
gcggcgtcggggtcgtcgtcgtcgttgtccgcgacggaggaagggtgctgctccgcgtcctccacggaaggcgtgcgcgtgttccggctggggaagac
gacgacggccttcacctaccgcgacatcgtgtccgccacgcgcaacttcgccgacgacctcgtggtcgggcgcggcggctacggcgtggtgtaccgcg
gcgtgctccccgacggccgcgccgtcgcggtcaagcggctggcgcggcaccaccagcgcggcgaagacgacgacggcgagcgcgagttccgcgccg

agatggaggtgctcgcggagggcggcgggtccacgtggccgcacccgaacctcgtcacgctctacgggtggtgcctctccggctcgtccaagatcctc
gtctacgagtacctcgacggcggcagcctggagccgctcatccctgacgtggccgcgttcgggtggggcccgcgcctggcggccgcgaccgacgtggc
gcgcgcgctcgtgttcctgcaccacgagtgcgcgcccgccgtggtggtgcaccgcgacgtcaaggccagcaacgtgctgctggaacgccggcaggctg
gtggcgtgcgggccaaggtgacggacttcgggatggcgagggtggtccggcccggggagacgcacgtgagcacgatggtggccgggaccgtgggtt
acgtggcgcccgagtacggacagacgtggcgcgccaccaccaaaggcgacgtgtacagctacggcgtgctgctcatggagctcgccacgggccggc
gcgccgtggagtgcggtggcgaggaggcggagtgcctggtggagtgggcccggaggatggggaaagaagggcaagtgccgaggaaggaggaggc
gccgagcgcggtggcgtgggagctgctcgtgctcgggatgcggtgcacggtcgacgcgccgcaggagcggcccgacatgacggaggtgctcgccgc
gctgctcgacgtcgccgagaggtgtagataccaggacggcgtgcgactcgaattcacgtaa
>g139513.t1
atggcagcgccgcgccacctcctcgccgtggcggtgcttttcaccgcgttcgcctccgccgccgcgttcactgacccgcccgacgcgatagggcttttgg
gattatatcacacattggagtctccgtggcagctttctgggtggacattccagggtggtgacccctgcggcgagggtggtgaacctgggcaatggagag
gtgtttttgcaagggaccgtctgttgtcacaataaacattagtgggtttggagttggtggatggcttggcccagatttgctcaagttccatgagacttcctc
tagtaataaagacattacatccgttcccaagattaaaagttggtccaagacatctaaaaccattccaactgcaaagcagtttccagctgctgacattttgg
cggctacaaggaacttcaatgaagaatgccttatcggtgaaggtttcactggccgagtttacagaggtgattttcctgatggcaagattctggccatcaa
gaggattgacatggtggatttgtcattgtcagaacaagatgagttaatggacatgctttggaatatctccagattgaagcaccccaacatctgtgcgcttg
tgggatattgtgtggagtttggccattgtgctcttctatacgagtatgcagaaaatggttctcttgatgatattttgttttcagatgccatgaggtccagggc
tttgtcatggaaagctcggataaagattgctcttggagttgcctatgctctagagtaa
>g140718.t1
atggtggacgaggcgtgggagcgggcggtggaggcggcgctgcacgccggcggcgaggggagctcgtcgccggcgcggagcctgacgctcgacgg
cgccgtcaagtgcatgcacggccggctgccggcagcggagatactggagcggcaccagagcctggagcacctatcgatcgccggcgtcggggtcacg
tcgctcgaggggttcccgcggctgcggaacctcacgcggctcacgctctccgacaatcgcatcgccggcggcctcgaccacctcgtcgtcgcggggctc
tcctcgctgcgggacctcgacctcagcaataaccgtatccaggacgtcgacgacctcgcgccgctcgcggacctccgcctcgtctcgctcgacctctacg
agtgcccggtcacccgggtcaaggactacagatccagggtgttcggcttgatccggacgctcaagtatctcgacaagatggacgccgacgagaacga
gcgccccgagtccgacgacgacgacgacgatggggaaggcgatggcgacggcgacggcgaggaagacgatgatgacgacgacgacgaggatgag
gaccctgggagcggcgaggtggcgaacggcggcgtctctcattcgcggggcggcgtgccggcgcacccggtggaggtgaatggggtgattgatgttg
acgaggatgagagcgacgccgacgaggtcctccccaacggtggagtcgagcaccaccatggggtgaacggctttcgggtagcggcggttggaggag
ccccagatgaggaggatgaggacgtagatgaggaggatgaggaggaggannnnnnnnnnnnnnnnnnnnnnnnnnnnnnnnnnn
nnnnnnnnnnnnnnnnnnnngcgaggggagctcgtcgccggcgcggagcctgacgctcgacggcgccgtgaagtgcatgcacggccggctg
ccggcggcggagatactggagcggcaccagagcctggagcacctatcgatcgccggcgtcggggtcacgtcgctcgaggggttcccgcggctgcgga
acctcacgcggctcacgctctccgacaaccgcatcgccggcggcctcgaccacctcgtcgccgcggggctctcctcgctgcgggacctcgacctcagca
ataaccgcatccaggacgtcgacgacctcgcgccgctcgccgacctccgcctcgtctcgctcgacctctacgagtgcccggtcacccgggtcaaggact
acagatccagggtgttcggcttgatccggacgctcaagtatctcgacaagatggacgccgacgagaacgagcgccccgagtccgacgatgacgacga
cgacggggacggggacggcgacggcgaggaagacgatgatgacgacgacgacgaggatgaggaccctgggagcggcgaggtggcgaacggcgg
cgtctctcattcgcgcggtggcgtgccggcgcacccggtggaggtgaatggggtgatcgatgttgatgaggatgagagcgacgccgacgaggttctcc
ccaacggtggagtcgagcaccaccatggggtgaacggctttcgggtagcggcggttggagaagccccagatgaggaggatgaggacgtagatgagg
aggatgaggaggacgacgacgactatgaggtggaggatgatctcggggaggagatcgatgaggatgttaatggggaagatgctgtgattgaagtgca
tgatgtacagagtagtgatgaggaagatggagttgaagaggacgatgaagatgatgaggatgatgaagaggaggttgaggatgatgaagacgtgga
gccaaagagcagcgggagggttgccatgatgaccggggaggccggggcagaggagattgatggtcatgaccacggagaagaggatgatgatgatg
aggacggtgaaattggggaagaggatgatgagagattggaggatgatagggtttatgatgagagccacgacgacgatgatgatgcagatgatgagg
atgaagacacagaatatcttgttcagccaattgcccgaccttcacctggcatggccattggaagtgacgagcctcaggccatggccatcgggagtgact
tcgatgctgctgatcctgatgatgccgatgaggacagggaggaggttgatgatgatgacgaagtggccacagatcagccttcatcttcacatggtgcca
agcggaagagaggtgatgatgcatcaggcagtggtgatgacagtgaggatgatggtgttgaagatttgaggcccttcaagcaccactga
>g141423.t1
atggtcgccgcgttcgccgactccggccgagggcgcacgctcgtctcgctctccggtttccgtgccctcgctcaggagtacatacccaccatcgcggtgg
tgtgcggcaacttgaggaccctcgacttgagctacgccccgattacccccaatcagattgtcatgttcatcggccagtgcttcaatcttgagacgctatgg
gtgctcgactctgtgcgcgatgaagggctcgagactgtggcaatgtgctgcaagaagctccagtcccttcgcgtgctcccactggacgcgcacgaggat
gctgaggagctagtttcagaggttggccttgccgccatctcccggggctgctgtgacctccgctccatactgtatttttgccagacaatgaccaatgctgc
tgtcattgacatgtcgcaaaactgcccggagctcaaggtgtttcggttgtgtataatgggacggcacaagcccgaccatgccactggggagcctatgga
tgaagggttcggtgccattgttcggaattgcagcaagcttaccaggctatccacatccgggcaccttaccgacagggcatttgagtacattggcaagtat
ggcaagtccttgcggacactctctatagcgtttgctggaaacagtgatttggcgcttcaacacatcctccagggctgttcaaagctagagaagcttgaaa
taagagattgtccattcggtgatgccggcctcctctctggaatgcaccatttctacaatatgcgattcgtctggatgtcaggctgcaacctgaccctacaa
gggtgcaaggaggtggctcggcaattgccgcgaatggtggtggagttgataaatggccaaccagagaatgaaaggattgaaggtgtggacatcttata
catgtatcgctcacttgatggcccaagagaagatgtaccaccatttgtgaagatcctgtaa
>g141542.t1
atggagcgccatggctacaactacaaccaccaccgttgctacgtcgtcctcgtcgcctcccttgtctttgcattgccgtcttgggcttgggccgccacggg
aaacgcagtagcaggcagtgaccaccgcgccctcacgcagtttcggtccctcatcacagacgacccacggggagccttggcgtcatgggccggcggc
aacgcgtccgggccgtgcgggtggcacggcgtgacgtgcggggcccgcgggcgccgccgcgaccgcgtcacggcgctggacctccgcgatctgggc
ctggccggctccgtcgggtcttccctctccggcctcacctacctccggcggctcgacctcgccgagaaccgcctcagcggcggcgtgccctcgccgctgc
cgccctccctcgagcgcctcaacctcagccacaacgcgctgcacggcaccgtgccgcctgagctggggtcgctgcgccgcctccaggtgctcaatctcg

tccacaacaacctcaccggaacaatccccgcctcacttggcaacctcacctccctcacggtcctcagcctcaccaacaacaacctcgccggcgccatcc
ccggcgccctcggcaacctccaagctcttaccggcctctacctcaacgacaacatgctccaaggatccataccttctgaggtattcaacatctcctctctc
cagcaccttgtcgtgcagatcaacaacatcaccggaactctgcccctggacatcggcggcaggcttcccaacctcaggcgtctcgtcgttgactccaacc
agctccacggccgcatcccggcgtcactctgcaacgcctctaagctggagttagtccaaatgatgaagaattccttctccgggatcataccaggctgcct
cggagctaacctcccgaacctatggtcactgacactggacgtcaaccagctcgaagcaaatgatgatgcggattgggggttcatggatagcctgacca
actgcagccaactgaaggccattggtctgtccggaaacaagctgggaggtgtgctgcccagctcaatcgcaaacctttccacgagcatagagtacttca
gcataagtggcagcatgagtatggactag
>g141595.t1
atgggaagcagagcagcagccatactactggtgctgtctttgatcgtcctgtggcggtccgagaccggtgccgcgcagactgcgcagcgatgcggcgc
cggcgacctcggcgcgctgcgcggcttctccgccggcctcgacgcggcggtggacggctggccggtcggctccgacgactgctgcgcgtggcccggcg
tggtgtgcagaaacaacacagaagaggtaatcgggctggtgttgccgaaccatacgctacagggccaggtatcggcatcgctggccggcctcgccgc
gctgcgcgtgctgaacctctcgagcaacgcgctgcggggcccgctccccgcagacttgctccgccgccgcctcgaagtgctcgacgtcagcggcaacg
cgctctccggcaacgtcgaggagctcccgtccacgttgcgcgtgttcaacgtgtccaacaacgcgttcgccggcggccacccggtgctccgcggcggcg
gcggcgcggcgaacctgactcactacgacgcctcgggcaacgtcttcgacggcgccgtccaggctgctcggttgtgtcaaggttccccatctctgcgcgt
tctgcggctgtcgatgaaccggctgtccggcgcgttcccgttcgggttcgggcaatgccggtcgctcgtggagctggcgctggacgggaacgccatcgg
cggcgcgctccccgacgaccttttactgtcaagtctctacaaatcctcagcatccacaccaacgccatctccggcgggctgtcgccgctgttgttacgca
acctgagcagcctcgtccgcctcgacctctccttcaacgccttcaccggggaacttcccgacgtgttcgacgctctgtcgtcccaactccaagaattctcc
gcgcccagcaaccggttctccggcgagctcccggcgacgctctcgcggtgccggtggttgcgcgtgctcaacctccgcaacaacacgctcggcggcga
catcgtgaaccacctcgacttcaggaagctcaacgacctggcgtacctcgacctcggcgtgaacaagttgacggggcccatcccggcgagcatcccgg
agtactgtaaagggatgacggcgctgaacctgggaaggaacaggctcaccggcgagataccggcctccttcgccggcttctcgtcgctgtcgttcctgt
ccctcacggggaacggcttcaccaacgtcacgtcggcgttaacgacgttgcagtccctcccgaacctgaccagcctcgtgctcacaaagaacttcctcg
gcggccacgagtcgatgccatcatcatcaggcataaaaggttttccgagcatgcaagtgctggtgatcgccaactgcgagctgcgcggcacgatcccgt
cctggatcgcggggctccggaagctgcgagtgctggacatctcctggaaccgcctcacgggtcaaatcccaccatggatcggggagttcgaccgcctct
tctacctcgatctgtcgaataattccctccagggagagatccccggaaccctgctggcgcgcttaccgggtctcatcgattccttccagcagggagagat
ccccggagaggagttccccttcttcatccggcgcaacacgtcggtgaagggccggcagtacaaccaggtgagcagcttcccgccgtcgctcgtgctcag
ccacaaccacctcaccggcggcgtgcccaaggagctaggggagttgagcaaagtgcacatcgttgacctgagctggaacaggctgtccgggcccgtc
ccgacggagctgtccgggatgaggagcatcgagacgctggacctgtcgcataacgagctgtcgggaaccatcccggcgtcgctgaccagactcagctt
cctctcccggttcgacgtctcccacaacaacctctcagggcaggttcctgtcgggggggcagttctccacgttctctcgcgaggacttcgaggggaacccg
tttctctgtggaatccatgtcgcgccctgcgctctgcggaatcctaatgccaataatgaggtgacgcggaggagcggcagggcaaaaatggtggcggc
gatcagcgtgggcacggcgctgctcctgggcgtggtcgcggcggtgacgtggcgggcgtggtccaagcggcggcaggaggacaacaacaacgccag
ggtggcggccgattacgacgatgatcttgatttgatgaacaagaataagtcgacgatggtgctgctgttcccccaaactgacgacgacgaccagcagc
agcagcagcagacaactctgacggtggaggaggtggtgaaggcgacgggtaatttcgatgaatcccgcatcgtggggtgcggcgggttcgggatggt
gtaccgcgcgacgctccccgacggccgcgacgtggccgtgaagcgcctctccggcgagttcggccagatggagcgggagttccgggccgaggtggag
acgctctcgcgtgtgcgccacgccaacctggtcccgctgcagggctactgccgcaacaacaacaaggcggcggaccggctcctcatctacccgtacat
ggagaacggcagcctcgaccaatggctccacgagagacaccgatcctttccgtggccggcgaggttggggatcgcgcgtggcgccgccagagggctg
gcccacctgcacgccacgtcggagccgcgcgtgctgcaccgcgacgtcaagtccagcaacatcctcctggacgcgcggatggaggccaggctggccg
acttcgggctcgccaggctcgtggcgtgccccaccgacacccacgtcaccaccgacctcgtcggaacgctcgggtacatcccgccagagtacggccaa
tcctccgtcgccacgtaccgcggcgacgtgtacagcctcggcgtcgtcctcctcgagctcgtcacgggacgccgacccgtcgacatggcgcgccccgcc
ggcgacggacgcgacgtcacgtcgtgggcgctcaggttatggcgcgacgggaggggacacgaggtcatcgacgccaccagcatcgtccaagcccac
cacaacgaggaggcgctcaggatgctccacgtcgcgtgcgcgtgcgtcaacgagaacccaaagtcgaggcccacggcgcaacaggtcgtgcagtgc
ctcgacgccatcgccgcctcgtcgtcacagaacggcccgaaaagtagtgcctctatgtactag
>g142630.t1
atggaagccagagcgtcagtgctggtcctcgtagcagcagtgacactgctgtcatcattgtccgccgccggaggagccgtcgatgagcgggcggcgct
gctcgcgctgaaggcgggattcagtgacccgttgggcgcgctcgccgagtggaagaaggaggaggatgcctcgccgcattgccggtggagcggcgtc
gcgtgcgacgccggcgggcacgtggacagcctggacctctcagggaagaacctgagcggcaaggtctcttccgacgtgttccggctatcgtcgctccg
ggtgctcaacctgtcgtacaacgccttcgactcggtgcttcccaagtcgctggcgccgctgtccagcctgcaggtgctggacgtgagccagaactccttc
gaaggcgcgttcccggcgggcctcggctcctgcgccgacctggccaccgtcaacgcctccggcaacaacttcgtgggcccactccccgtggacctcgcc
aatgccacgtccctggaggtgctcgacctccgtggcagcttcttcgaaggcggcatcccggcggcgtaccggagcctgacgaagctcaagtttctgggg
ctctccggcaacaacatcaccggcaagatcccgccggagctcggtgagctggagtcgctggagagtctcatcatcgggtacaacgtgctggacggcgg
catcccgccggagttggggaacctggcgagcctccagtacctggacctcgccgtcggtaacctcgacgggcccatcccggcggagctcgggaagcttc
cgtcgcttacatcgctgtacctgtacaagaacaatctggaagggaagatcccggcggaggtggggaacatctcgacgctggtgttcctggacctgtcag
acaacctgctttcgggcccgatccccgaggaggtgtcgcagctgagtcacctccggctgctgaacctgatgtgcaaccacctggacggcacggtgccgc
cgtccatcggcgacaagatgacgagcctggaggtgctggagctgtggaacaactcgttgacgggcccgctcccggcgagcctgggccggaacgcgcc
gctgcagtgggtggacgtgtcgtcgaatctgttcacgggtcccgtcccaccagggatctgcgacggcaaggcgctggtgaagctgatcatgttcaacaa
cgggttcacgggcggcgtccccgccgggctagcgtcgtcgtgcgcgtcgctggtgcgcgtaaggatgcagggtaaccggctcaacggcacggtgcccg
tcgggttcgggaagctgccgctgctgcagcggctggagctcgccgggaacgacctgtcgggtgagatcccggttgacctcgccacatccagttcgttgt
cgttcatcgacgtgtcgcgcaaccgtctgcagtactcgctgccttccaacatcttcacgatgccgacgctgcagagcttcctggcgtcggacaacctcatc
tccgggtcaatccccgacgacttccaggactgtccggccctggcggcgctggacctgtcgaataaccggctcgtgggcacggtcccgtccagcctggcg
tcgtgtcagaggctggtcaagctcaatctccggcgcaaccggctagaaggggagatccccagcgcgctggccaagatgcccgccatggccatcctgga

cctctccgggaatgcactgacgggcaccataccggagagtttcgggagctcgccggcgctggagacgctcaacctggcgtacaacaacctcagtggcc
ccgtcccgggaaatggcgtcctgcgcaccatcaaccccgacgagctcgccggcaacccggggctctgcggcggcgtgctcccgccctgtaactcccgt
ggttacgacaacatggtgggcaacacgcggccacgcgtcactgcggcggcgcggctgaagcgcgtggcggtggggtggctcgtggggatgctggtgg
tggtcgccctggcagcggcgatgctcgccggacggcacgtgtaccggcgctggtacatgtacactgacgatgaggagaacgtggagtcaagtggtgg
cgcgtggccgtggcggctgacggcgttccagcgcgtcgggttcacgagcgcggacgtggtggcgtgcgtcaaggaggccaacgtggtgggcatgggc
gccaccggcgtggtatacaaggcccagctcccacgcgccaccatcgccgtcaagaagctctggcggccttcttctcaggcgcaggagacaccagagac
ggagactaggacggcggaggtgctcaaggaggtgaaccttttaggccggctccggcaccggaacatcgtgcggatgctggggtacgtgcacaacgac
agggacgccatgatgctctacgagttcatgcccaacggcagcctctgggacgcgctgcacgcccacccaacggaggagcagagccgcgctcatcagc
ttatggacgactgggtgtcgcgctacgacgtggccgccggcgtcgcgcagggcctggcctacctccaccacgactgccacccgccggtgatccaccgc
gacatcaagtccaacaacatcctgctggacgccaacatggagccgcgcatcgccgatttcggactggcacgcgcactggcccgcacaaacgagtcagt
ctccgtcgtcgcaggatcatacggatacatcgcaccagagtacgggtacactctgaaggtggaccagaagagcgacatctacagctacggcgtggtgc
tcatggagctcatcacggggcgccgacccgtggacccggagttcggggaaggccaggacatcgtgggatgggtcagggacaagatcaggaccaaca
cggtggaggagttcctcgacccaaacgtcggcggccggtgcgcgcacgtcagggaggagatgctgctcgtcctgcgaatcgcagtcctctgcaccgca
agggcgcccagggacaggccctccatgcgcgacgtcatcaccatgctcggcgaagccaagccaagaagaaagagcgccaccaccaccaacaacaa
cacaaagcctgccaccaccgtcgtggacaaggacaaaccagtcttcaccaccacgccggactcagactacgcctacctaagctag
>g143405.t1
atgtcccgacaccgcctcgccgtcaccgctgccgccgtggttgtcctcctcctgtcactctcgccgccgtgcgccgccgaccccgacgacgagaggtgcc
tgtcgagcctgcaccagtcgctgtcggacgcctccggcgggctccgtaactggaccaaggcggccttctcggcgccgtgcgagggcttcatctcccacc
tccagggcgtgacctgcaacaacgggcgcgtgtacaagctggccctcccggggctctccctgcgcggcgccatcccgccgcagctctccaactgcacc
aacctccagtccctcgacctctcctccaacgcgctctccggcgccatcccgccggagctctctgcgctgctcaacatcgcggtgctcaacctctccgccaa
cagcctcacgggcgccatcccgcgggagctcgccgcctgcgcctacctcaacgtcatagatctccacgacaaccagctctccggcaccatccccgacg
agctcggactactggtccgcctctccaccttcgacgtctcctacaaccgcctctccggtcccatccccgtgctcctcgccaaccgcacaggcgccggaac
ggccgcagtgggtaccgcgaggttcaatgccagctcgttcgtgggaaacaaggacctgtacggttacccgctgcctccgatgaagacgcgagggctgt
ccgtgctcgccatcgtcggaattggccttggaagnaggacctgtacggttacccgctgcctccgatga
>g143981.t1
atggaccctacgccgcagtcgcacccgatcctcgcctacgtgctgtcccgcctgccgtcgctgtcggcgcggacgacgccgcggtcctcctcgctctctt
ccccgcggtcgcgtgacctcgagcagccgtccccgcgcgcgccgttgggcgccgcggaggccgagctggtcgcgctcatgccggggctccgccaccc
gtccgtgctctcctccatgacgcgcgccgtggccgacgtcgccaccgcgcgcgacgcgatccggctcctcgggccccgacccgaccacgaggtcgtcg
acgcctcccgcgctgtcctcgtcgccgctgaatccgacgcctcagaggggaaggagggggacgagaaggtggcggcgagccgggaggtggtgcggc
tggaggaggagcacgaagcgtacggcgccatgctgcgggacgcggaggggaaactggagcgggtgtaccggatggcgatgcacgggagggacatc
caagaggcgagcaaggaggaggaggaggagcagggggctggggcggtggacgaggaggtggtgcgggtgctcagngccgtcctcgtcgccgctg
aatccaacgcctcagagggtaaggagggggacgagaaggtggcggcgagccgggaggtggtgcggctggaggaggagcacgaagcgtacggcgc
catgctgcgggacgcggaggggaaactggagcgggtgtaccggatggcgatgcacgggagggacatccaagaggcgagcaaggaggaggaggag
gagcagggggctggggcggtggacgaggaggtggtgcgggtgctcagggaggcggaggaggggaagccagtggagcgggtgtgcctcgccgacc
gccagctgcgcctcctgccggagcccgtgggccggatccgcggcctcctcgcgctcgacgtgtcccgcaaccaactcaaggtggtccccgatgctattg
gcggtctggaacacctggaaaaactccgcctgaattccaacgccctggtttctctcccggactccatcggcctcctctccaacctaaagatcctcgacgtc
tccggcaaccgcctaaaggttctccctgatagcatctcgaaatgcagatcactggtggagctggacgcgagctacaacgcgctggtgtacctcccgacg
ggcatcggccacgagctggtgaacctgcagacgctgcgcgtgcacctcaacaagctgcgctccctgccgtcgtccgtctgcgagatgcgttcgctgcgg
ctcctggacgcgcacttcaaccagctccacggcctgccgcccgccatcggccagctgtcggcgctggagacgctggacctgagcagtaacttcagcga
catgcgcgacctgccgccctccatcggcgacctggcgggactgcgcgacctcgacctcagcaacaaccagatccgcgcgctgccggactgcttcggcc
gcctcgccaggctggagcgcctgcgcctcgaccagaacccgatcgccgtgccgcccgcggaggtggtggcccggggcgtcgccgcggtgaaggagta
catggcgaagcggtgggccgaagccgtcgccgaggaggagcgtctgcgcgctggcgtggcggtggagagccccaaggcggccgccacgcccaggg
agtggctgacgcggagcgtgtcgtcgctgagcacctgggtgtcggacgtcaaggagaaggtcgttgggcaggacaaggtggcggaggaggacgagt
ttctccagcaggagttctga
>g144184.t1
atggcatctccgcggctggtggctctactggtgctcttcctcctcgtcgtcgcgtcagtggccgccgacgacgcccagctgctggagcagttcaaggcgg
ccgtgccgccgccaggccagccggacctccgccgctggaccgcagccgacggcgcctgcaagttcccaggcgcgcggtgccaggccggccgcctcac
gtcgctgtcgctcgcgggcgtcccgctcaatgccgacttccgcgccgtggccgcctcgctgctgcagctggcgagcctcgaggagctcagcctccgcgg
cgccaacgtcagcggcgcgcttgccgccccgccgcgctgcgccgccgccaagctgcgggcgctcgacctctccgggaatgccggcctgcgcggctccg
tcgccgacgtggacgcgctcgcggccgcctgcgccgggctgagggcgctcaacctctccggcgccgcggtcggcgcatccaaacccactagcaccaa
caccaacggcgtggggtcgggattcgccgcgctggactcgctcgacctctccggcaacaagatctccggcgacgccgacctgcggtggatggtggccg
cgggcgcgggcgcggtgcggcgcctggacctctccgacaacaagatctccggcgcgctgccggacctcgcaaactgctccggcctcgagcacctcga
cctctccggcaacctcatcgacggcgaggtcgccgcgggggcgctctccgactgcaagggcctcaagacgctcaacctctccggcaaccacctcgtcg
gcgccttcccgcccgacgtcgccgccctcccctcgctcctcgcgctcaacctctccaacaacaacttctccggggacctccccgccgacgccttcaccaa
cctgccgcacctcgccgtgctctccctctccttcaaccacttcaacggcaccgtcccggacacgctcgccgcgctccccgaactcgaggtgctcgaccta
agctccaacaccttctccggctcgatcccctcctccctctgccgggacccaaacgccgcgctccgcatgctctacctccagaacaactacctctccggcg
ccatccccgactccgtctccaactgcaccaacctcgagtcgctcgacctcagcctcaacctcatcaacggctcgctgcccgcgtcgctcggcgggctcgg
caacctgcgggacctcatcctgtggcagaacgagctggagggggagataccgccctccctcgccagcatgcacatgctcgagcatctcatcctcgacta
caacggcctcaccggcgccatcccgctggaactggccaagtgcaaggagctcaactggatatccctcgccagcaaccgcctctccggccccatcccgt

cctggttcggccagctcagcaacctcgccatcttgaagctcagcaacaactccttctccggtccgataccgcctgagcttggcgactgccagagcttggt
ctggctcgacctcaacagcaaccagctcaacgggtccattccgccggagctggccaagcagtccgggaagatgaatgtcggtctcgtcattgggcggc
cgtatgtgtatctccgcaacgacgagctcagcagtgagtgccgcggcacggggagtttgctggagtttaccagcatccggtccgaagacctcagccgg
atgcccagcaagaagctgtgcaacttcaccagaatgtacatgggcaacaccgagtacaccttcaacaagaatggatccatgatcttcctcgacttgtcct
tcaatcagctggactcggagatcccaaaggagcttgggaccatgtactacctcatgatcatgaatctcgggcacaacctcctgtctggcctcatcccgtc
ggagctagccggtgccaagaagctcgcggtgctcgacctgtctcacaaccagctggaagggcctatccccaactctttctcctcattgtccttgtcggag
atcaacctgtccaataatcagctgaatgggtcganccctgcccatctcccgccctccttcctcactcctcccccactctcttctcctacctcgtcgccacctc
ctcccctcaccgccgacccgcccttctttaatgattttaagctgctggagcagttcaaggcggcggtgccgccaccggtccagccggacctccgccgctg
gaccgcggccgacggcgcctgcaagttccccggcgcgcggtgccagagcggcggccgactcacgtcgctgtcgctcgcgggcgtcccgctcaatgcc
gacttccgcgccgtcgccgcctcgctgctgcagctggcgagcctcgaggagctcagcctccgcggcgccaacgtcagcggcgcgctggccgccccgcc
gcggtgcgccgccgccaagctgacagcgctcgacctctccgggaatgccggcctgcgcggctccgtcgccgacgtggacgcgctcgcggccgcctgcg
cgggattgagggcgcttaacctctccggcgccgcggtcggcgcatccaaacccactagcaacggcgtcgggtcgggattcgccgcgctcgacacgctc
gacctctccggcaacaagatctccggcgacggcgacctgcggtggatgctctccgcgggcgcgggcgcggtgcggcggctcgacctctccgacaaca
agatctccggcgcgctgccggacctcgccaactgctccggcctcgagcacctcgacctctccggcaacctcatcgacggcgaggtccccgcgggggcg
ctctccgactgcaagggcctcaagtcgctcaacctctccggcaaccacctcgtcggcgccttcccgcccgacgtcgccgccctcccctcgctcctcgcgct
caatctctccaacaacaacttctccggggacctccccgccgacgccttcaccaacctgccgcacctcgccgtgctctccctctccttcaaccacttcaacg
gcaccgtcccggacacgctcgccgcgctccccgaactcgaggtgctcgatctcagctccaacaccttctccggctcgatcccgtcctccctctgccggga
acccaacgccgcgctccgcatgctctacctgcagaacaactacctctccggcgccatccccgacgccgtctccagctgcaccaacctcgagtcgctgga
cctcagcctcaacctcatcaacggctcgctcccagcgtcgctcggcgggctcggcaatctgcgggacctcatcctgtggcagaacgagctggaggggg
agataccgccttccctcgccagcatgcacaagctccagcatctcatcctcgactacaacggcctcaccggcgccatcccgccggaactggccaagtgca
aggagctcaactggatatcactcgccagcaaccgcctctccggccccatcccgtcctggttcggccagctcagcaacctcgccatcttgaagctcagca
acaactcattctccggcccgataccgcccgagctcggcgactgccagagcttggtctggctcgacctcaacagcaaccagctcaacggctccattccgc
cggagctggccaagcagtccgggaagatgaatgtcggtctcgtcattgggaggccgtatgtgtaccttcgcaacgacgagctcagcagcgagtgccgc
ggcacggggagcttgctggagttcactagcatccggtccgaagacctcagccggatgccgagcaagaagctgtgcaacttcaccaggatgtacatggg
caacaccgagtacaccttcaacaagaatggatccatgatcttcctcgacttgtccttcaatcagctggactcggagatccccaaggagcttgggaccatg
tactacctcatgattatgaatctcgggcacaaccttctgtccggcctcatcccgtcggagctagccggtgccaagaagctcgcggtgctcgacctgtctca
caaccagcttgaagggcctatccccaactctttctcctcattgtccttatcggagatcaacctgtccaataatcagctgaatgggtcgattccggagcttgg
ttcccttgccacgttccccaaaagccagtatgaaaacaacactgatctgtgtggcttcccgctgccgaaatgtgatcccagtgccaggcaaagttcatcc
gacggcggatcgcaccggaggaagggaacgctcgcaggtactgttgctatggggatcctcttctccctcttctgtatatttggggtggtcatcatagcgat
agagagcaagaagcggaagcagaagaatgatgaggcaagcaccgcccgcgacatctacattgatagtcggtcacactctggcaccgccatctccaat
tggaggctttcgggtaccaatgccctcagcatcaaccttgctgcattcgagaagaacctgcagaagctcaccttggccgatctggttgaggccaccaatg
gcttccacaatgatagcttaattgggtctggtggttttggtgatgtttataaggcccagctgaaggatgggaggattgttgcaatcaagaagctgatacat
gtaagtggccagggtgaccgggagtttactgcagaaatggaaacaattggcaggatcaaacaccgcaatcttgttccgctcctcggctactgcaaggtc
aatgaagagaggctgctggtatatgagtacatgaagtatggcagcttggaggatgtgttacgtgaccgcaaaaggattgggctgaagctgaattggac
agcaaggcgaaagatcgctgtaggggcagcaaggggattggcattcctccaccacaactgcattccacatatcatccatcgagacatgaagtcgagca
atgtgcttattgatgagcaactggaagcaagggtatctgattttggtatggcgaggacggtgaatgtggtagacacacacttgagtgtgtctactcttgct
ggcactccaggttatgtaccgccggagtactaccagagcttccgatgcaccaccaaaggtgacgtgtatagctatggtgttgtattgctggagctgctca
ccgggaagccgccgacggactcgacagactttggcgaggacagtaatcttgtagaatgggtcaagcagcatacaaaggttaatattagcgatgtgtttg
atcctgagctgctgaaggaagatccaacactggagcttgagctgctagagcacctgaaaatcgcctgtgcgtgcttggataatcggccatcgaagcgac
cgacgatgttgaaggttatggcaatgttcaaggaaatccaggctggctccacggtggattccaagacttcctcagcttgcacggtctctaacgatgatgg
aggttttggtgtagacatgactctgaaggaagacaaggacgagaaggattag
>g145699.t1
atgcgcctcctcacgccgctcctcctcctcctcctcctcgccctcgccgcgggcgccggcgcggcgggcggcgacgcggacgcgctgctcgcgctc
aaggcggcgctcaccgaccccaccggcgcgctcgcctcatggaccgcctcctcctccgaccactgcgcctgggccggggtcacctgcgctccccgcgg
cgacaccgtcaccggcatcgacctctccgggctcaacctcaccggcacgctgccccccctcgctgtcgcgcctccgcgacctgcagcgcctctccgtcgcc
gccaacgccctctacggcccaatcccgccgtcgctgggccgcctcgcgcagctggcccacctcaacctctccaacaacgccttcaacggctccttcccg
gcgccgctggcgcgcctgcgcgcgctccgcgtcctcgacctctacaacaacaacctcaccagcccgctcccgctcgaggtcgcgcggatgccggcgct
ccgccacctccacctcggcggcaacttcttctccggggagatcccgcgggagtacggccggtggacgcggatgcagtacctcgccgtctccgggaacg
agctctccggcaggataccccccgagctcggcagcctctcaagcctcaaggagctctacattgggtactacaacagctacaccggcgggcttcccccgg
agctgggcaacctgacggagctcgtgcggttcgacgccgccaactgcgggctgtccggggagattccaccggagctcggccggctacggaagctgga
cacgctcttcctgcaggtgaacgggctcacggggaccataccgtcggagctggggaacctcgagagcctcagctcgttggacctgtccaacaacgcgc
tcaccggggagatacctgcgagcttcgccgagctcaagaacctcacgctgctcaacctgttccggaacaagctccgcggcaacatccccgacttcgtcg
gcgaccttcccggcctcgaggtgctgcagttgtgggagaacaacttcaccggcggcgtgccgcgccggctcggccggaacggacgcctccagctgctc
gacctctcgtccaacaggctcaccggcacgctgccgccggagctctgcgccggaggcaacctccagacgctcatcgcgcttggcaatgtcctgttcggt
gccatccctgactcactcggccaatgcaagtccttgagccgcgtccggctcggggagaactacctcaacggctcaattccgaaggggctctttgaattgc
ccaagctgactcaagtgaacgggctcacggggaccataccgtcggagctggggaacctcgagagcctcagctcgttggacctgtccaacaacgcgctc
accggggagatacctgcgagcttcgccgagctcaagaacctcacgctgctcaacctgttccggaacaagctccgcggcaacatccccgacttcgtcgg
cgaccttcccggcctcgaggtgctgcagttgtgggagaacaacttcaccggcggcgtgccgcgccggctcggccggaacggacgcctccagctgctcg
acctctcgtccaacaggctcaccggcacgctgccgccggagctctgcgccggaggcaacctccagacgctcatcgcgctcggcaatgtcctgttcggtg

ccatccctgactcactcggccaatgcaagtccttgagccgcgtccggctcggggagaactacctcaacggctcaattccgaaggggctctttgaattgcc
caagctgactcaagtggagctgcaagacaacctgctcactggcaatttcccggccatcgtcggcgttccagcacctaatcttggtgagatcagcctgtcc
aacaaccagctcactggcgcattgccggcgtctcttgggaacttctcgggtgttcagaagctgttgcttgatcagaacaagttctctggtgtcataccacc
ggagattggccggctgcagcagctctccaaggctgaccttagcagcaacaagtttgagggaggtgtaccaccggagattgggaaatgccggcttctca
cttacttggacttgagccagaacaacctctctgggaagataccaccggccatctctggtatgcggatactgaactacctcaacctgtcacggaaccatct
tgatggagagataccgccatccattgcgacgatgcagagcttgacggcagtcgatttctcatataacaacctgtccgggcttgtgccggggactggcca
attcagctacttcaacgccacttcttttgttggcaacccaggcctgtgcggaccataccttggtccatgcctcccgggtgttgctgcgattctgaaggcccg
ctcgttgaagaaagctagtgaagcgcgtgtatggaaactcactgcgttccagcgccttgacttctccagtgatgatgtgctggacagcttgaaggagga
gaacattatcggcaaaggtggtgctggaattgtgtacaagggggcaatgccaaatggtgagctcgtagctgtgaagaggctcccggcgatggtccgtg
gctcatcacatgatcatggcttctcggcggaaatacagacacttgggagaattcgtcaccgtcatattgtgcgcctgctgggcttctgctccaacaatgag
accaacctgctagtttatgaatacatgcccaatggcagccttggggagatgctccatggcaagaagggagggcacctgcactgggacaccaggtacaa
gattgctaccgaggctgctaagggtctctgctacttgcaccatgactgttcaccgctgatacttcatcgtgatgtcaagtcgaacaatatacttctcgactc
caactttgaagctcatgtagccgactttgggctagcaaaattcttgcaggactctggtgcatctgaatgcatgtctgccatcgctggctcatatggctacat
tgcgccagaatatgcgtacacactgaaggtcgatgagaaaagtgatgtctacagctttggtgttgtgctgcttgagctggtcactggaaggaagcctgta
ggtgaatttggtgatggtgttgacattgtccagtggactaagatgatgacagactcgaacaaagagcaagtgacgaagatcctcgacccaaggctctca
actgttcctcttcacgaggtgatgcatgtcttctatgttgcattactttgcattgaggagcagagcgtgcagcgcccgaccatgagggaggttgtgcaaat
gctgagtgagcttccaccgtcccccaagcaaggagaagaggtttcaaatgctggtgatggttctgcatctagtcctctacatccagcaccagcaggcac
gaatgaagcaccaaccgatgtagcgaaagatcagcaacagcagcaaacaagctcgcagccatctcctcctgatctcatcagcatttga
>g146414.t1
atgtcaatgtcgagcaccagctacctcttcccccctgctccttgccagtgccttgttcctgctgctgctagccggtgagtgccgcgcgcaggcagccgccgc
caactgggaccgcgacacgctcgtcgccgtcgagaaggattggggcagcccgccgcaggtcaagtcatgggaccccgccgctgaccactgcagctgg
cacggcgtcacgtgcggcggcgacgggggagctgtcacagagctctccctcccgagcctgagcgtgagcggctcggtctcggcgtccgtgtgcttgctc
aagagcctcgcccgcctggacctctcctacaacaacctcaccggtgcgtctcctaccgccacgctctacgcctgtgcaaacctgacctacctcgacctct
ccaacaacttcttttccgggcctctccctatcgacatggacgacctctcaccggcgatggagcacctcaacctctccaccaacaggttcgccggcgaggt
gccgcttgctgtagcccggctgccggcgctcaagtccctgatactcgacaccaacaacttcaccggctcctacccagcggcggagataagccggctcca
aggactcgagcggctcacgctgttcgcgccagcgcccgtgccgccggagttcgcccggctcaccaacctcacctacctctggatgtccaacatgagcct
cacaggagagatcccggaggcgttcgctagcctcacagagctctcgctgctcgccctgtacacgaacatgcttacgggctccatcccggcatgggtgct
gcagcacgggaagcttgcgcaggtttacctgtacagtaacgcactctccggcgagctgctgcacaacgtctcgatgcccaacttgcattgcaaccagttc
accggcacgattccggagaccatcgggctactaccgcggctaagcgacatccggctcttctccaaccggttctctggcgtgctcccggcggagttcggt
aagcactctctgctcagcaattttgaggtgtccaacaacaacctctccggcccactgccagagtcgctctgcgccaacgggaagctgtacagcatcgtc
gtcttcaacaataacttctccaggcaacctcccggcggacatgggagactgtgtcttgctggaatgcctgatgctctacaacaacttcgccagcccgcca
caagctccaggctgctcccgccagctggtgccacgtctgcggcgccaagagcgggttccaccggtgtcacgccatggagcggctggtcgagtccgtctg
cgtcctgtgcccgcacgtggtccacggctgcatctcccgtcggccttactaccgccgcgaatcgcacgcccgatactcgatatgttgtcatctcagtatttt
tttttctacgtggatga
>g146515.t1
atgctcgatctcggcaggaaccaactctccggggactttgtggttaccgtcaccagcagcatatcctcccttcgtgttctgcgcatgccattcagcaacat
caccgggccaaatccattaccagcattagcggctggttgcccattgcttgaggagatctcccttggctccaatgagctttatggggagatcatgcccaac
ttgtgcttgtcattaccactgttacggaagctcatcctcccaaacaattatctcagcggtgcagtgccaccgtcgcttggccactgtgccaatctagagac
aatccacctcagcttcaacctattggcaatcacctcactgataaagtgccctggcttcggaaacctcaagaagctcgccattttgcagcttaacaataatt
cgctctctggcttggttccaaccaaattaggcagctgcagcaacctcatctggcttgacctcaacagcaactacttcacaggcacaataccagaggaatt
aacagctcgggcagggctcatcgatgggggaatagtatctgggaagcagtttgcgttcctccagaatatggcagggaacatctgtcctggcgtgggtgt
gctgttcgagttccatgacatcagcccagaatggttactcaagttacctgccgtgaaatcatgcccatccacaaggatatatactggcacaacagtgtac
acatttcacaacaacgggagcatgaaacctcatacaatggtctga
>g146906.t1
atggagaagttcatcttcgaggagctggcgggcgacgcgctgcgcgaggtgctgcgcgcggtgaagggcaccttcctgtgccggtcgaacgcggagc
ggctccggcgcagcgtggagccgctgctcccgctggtgcagggcccgcacggccaccgcgagctgcgcgagctggcggcgcgggtgcgcgacgcgc
tggagctggcccgcagcgccgccgccgcgccgcgctggaacgtgtaccgcgccgcgcagctcgcgcggaggatggaggccgccgaccgcggcatcg
cgcggtggcttgcgcgccacgcgcccgcgcacgtcatcggcagcgtgcgccgcctccgcgacgacgccgacgcgcggatcggacggctcgagcgccg
cgtcgaggagatcgccgcggcatccgcctctgctagtcccccgccgccggccatgtccgtcgccgtcgcgccgccgccgcataaggccatggccgcttc
cgctgctgtgcctatggaggtcgtggggccgcataagggtgtggccgtggaggcaatgatgccgcagaagggcgtggtggccgtggcgcagcccaag
gaaggggtgatggccatggaggtggaggggcacgaggagggagctgggatggtgggcggcggcgtgaaggtggccaaggagtggatcaaggaga
aggtgatgagcgggggcggcgggtgggaggtggtcgggatctccggcatcggcggcagcggcaagaccacgctcgccatggagatcttcagagatc
ccaaggtccaagcctacttcagcgacaggatattctttcagacgatatcgcaatctgctaacttggaggccatcaagatgaatctctgggagcagatcag
cggcaacatggtggttgggacatacaaccagatcccggaatggcagctcaagttaggaccaagggaccgaggtccagttcttgttatccttgatgatgt
gtggtctctctcacagctagagcagctcgtcttcaagttcccaggatgcaagactcttgttgtgtcgaggttcaagtttccgacattggtaaagagtacat
acgagatgccgttgcttgatgaggaggaggctttatctgtcttctgccgtgccgccttcgatcaggagtgtatcccacaaactgctgacaagaaactggtt
aagcaggttgctgcagagtgcagaggtcttcctctagctctgaaggtcattggagcatcgttgcgggaacagcctcctaagatatggctgagtgccaag
aatcggttgtcacgaggagaggctatttctgactcccatgagaccaagcttttggatagaatggcggccagtgttgagtgccttgcagagaaggtcaga
gaatgtttccttgatatggggtgcttcccagaggacaagaagatccctcttgatgtgctgatcaacatttggatggagattcatgatctggatgagccaga

tgcttttgccatcttacttgagctatcaaacaagaaccttcttactctagtaaatgatgcacagaacaaagctggagatttgcacagtagctaccatgactt
ttcagtaacacaacatgatgttttgcgggatctagcgctacacatgagtggaagggattctctgaacaagcggaaacggctcgcaatgccaagaaggg
aagaatcacttccaagggattggctgaggaacaaggacacaccgtttgaagctcagatagtttccattcatacaggtgaaatgaaagaatctgactgga
ctcagatgagcttccctaaggcagaggtgcttgtcctcaatttcgcctctgatgtgtactgcctcccagcatttatctcaaccatgcagaacctgaaagcat
tagtgctgatcaactacggtacaagcagtgcagcccttgataatttatctgccttcacaacactcaatggcctcaggagtctctggctcgagaagatcac
actcccaccactgccaaagaccacgatccccctgaagaacctgcgcaagatttcccttgtcctctgcgaattgaacaacagtctcagaggatcaactatg
gatttgtcagtgacttttccgcgcctgtccaacctcaccattgaccactgcatagatttgaaggagctgccatccactatctgtgagatcagctcccttgag
agcatctccatctccaactgccatgatctcactgagcttcccctgaagaggttgaagtatcttgacatatctcagtgtattaacctgacagacctcccaga
ggagcttggtcacttgacgaacctggagaagattgacatgcgggaatgctcaaggctgaggagcctcccgaggtcatcgtcctccttgaagtcacttgg
tcacgtcgtgtgtgacgaggagacggcattgctgtggagggaggcggagcagtgtattaacctgacagacctcccagaggagcttggtcacttgacga
acctggagaagattgacatgcgggaatgctcaaggctgaggagcctcccgaggtcatcgtcctccttgaagtcacttggtcacgtcgtgtgtgacgagg
agacggcattgctgtggagggaggcggagcaggtcatcccggacctcagagtgcaggtggcagaagagtgttatgacctagactggcttgtagattga
>g147052.t1
atggcgcggccgcggccgccgggcccccatctccgcctcgccgtcgccattgccttcattgccgccgtcctctggtttcctccgcccgccgcgacgcagc
aggccgtcgtcgtccccgccgccgccgcagccccgcgctccgaggccgacgtgctgatcgcgttccgggacacgctgcgcgggccggacggcgcccc
gccgggcccgctgcgcacgtggggcacgccgggcccgtgccggggcaacgcgtcggcgtggtacggcgtgtcgtgccacggcaacggcaccgtgca
gggcctgcagctggagcggctgggcctggcgggcgcggcccccgacatcggggccctggccgtcctcccgggcctgcgcgcgctcagcctctccgaca
acgcgctcacgggcgcgttccccaacgtctccgcgctcgccgtgctcaagatgctctacctctcccggaaccgcatgtcgggcgtcatccccgagggca
cattcgggcccatgcgcgggctccggaagctctacctctccgggaacgagttttccgggccggtccccgggtccatcacctcgcctaggttgctcgagct
ctcgctcgccaataaccggttcgagggacccttgccggatttctcccagcccgagctgcggtttgttgatgtgtccaataacaatctgtgtggacccattc
ccaccgggctcagccggttcaacgccagcatgttcacaggtaacagtttgctgtgcgggaagccgttggacgtggagtgcgacgagtcgggagcgcca
cgctccggcatgtcgaagctcatgatcatcgccatcgtgatcatcatcctgggcgtgctcctgtgcgcgggtggggtaatcacgggcgtcctcggcagcc
ggcgcgggtcatcgcggcgccgccgcgcgccggccgagtcgatcggcggcggcgaccagacgccgtccaacccgaagctgcagaccgcgccggcg
gtgaacatcgagaacgcgcccgccgcggggcagccgcagccgcgcgccaccgcgacgggtgggcggcggccgcggcgcgacgagctggtgttcatc
aacgaggggcggcggttcgagatcgaggacctgctgcgcgcgtcggcggaggtgctcggcagcggcaacttcggctcctcgtacaaggccacgctct
gcgagggccccgccgtcgtcgtcaagcgcttcaaggacatgaacggcgtcgggagggaggatttctccgagcacatgcgccgcttgggtcgtctcgca
caccccaacctcctcccgctcgtcgcctacctctacaagaaggaggagaagctgctcgtcaccgatcacatggtcaatggcagcctcgcgcagctcctc
catggtaacaggagctcgatcttggattgggggaagcgtttgcggatcatcaagggcgcggcgcgggggctggcccacctgtacgacgagctgccgat
gctgacggtgccgcacgggcacctcaagtcctccaacgtgctcctggacgccgccttcgacccggtcctgagcgactacgcgctggtgcccgtggtgac
gccgtccatcgcggcgcaggtgatggtggcgtacaagtcgcccgagtgcgtggcgccacacgggaagccgtccaagaagagcgacgtgtggagcctg
ggcgtgctcatcctggaggtgcttacgggcaagttccccgccagctacctcggcggcaataataagcagggacggcagcagcagaacgccgacctcg
ccgggtgggtccactccgtcgtctccgaggagcgcaccgacgaggtgttcgataaggatatcacgggagcgagaggggcagaggccgacatggtcaa
gctgctccaggtcgggctcggctgctgcgacgacgacgtcgaacggaggtgggagctcaaaaccgtcatcgcgcgcatcgacgagatccccgtgccc
gccgagacggtcgttaccgtcgaggcagcgagctccggcgagcagcaccggccttaa
>g147214.t1
atggccggcgcggccgggcggctcctcctagccgtcctcctcgcggcggccgtggcgggccgcgccgacggcgcgaccgaggccgagacgctgctgg
ccttccggaccgcgctgcgtgggccgaggaacggcccgccgtcgccgctggaccactggctgaacacgcccgggccctgccccggcggcggcaactc
gccgtggtacggcgtcaagtgcaacccggacgccgaccaggtgctggtcctgcagctggagcacctgggcctgcagggcccagcgccggacctcgcc
gtgctcgcgccgctgcagggcctgcggtcgctcagcctggcccacaacaacctcaccggcgcgttccccgacgtgtccaagctccccgcgctcaagatg
ctctacctctccgggaacaggctcgccggcccgatccccgacgacgcgttcgcgtccctgcaggggctgcacaagctttacctgttcggcaacgccttca
ccgggccggtgcccggctccatcacgtcgccggggctgctggagctgcagctctccaagaacgggttcgaggggcccctgccggacttcgcccagaag
aacctgacgctcgtcgacgtctccgacaacaatctctcagggcccattcctcctgggctccaacggttcgacgccgccagtttcaaaggcaacaaggat
ctctgcgggccgccgctggatgtccccctgcccaaacccttcttccgtgacttccccgtcgtcgggatcgtcaaaggcctccggcagcatgaagacactga
tgatcatcgccatcgtggtggtcgcgatcggcggcttcctatttgccgccggcgtcttcgcggctgtggtcgcccggcgcaaccagcgacgctacgctgg
cggcactgaaacactcggcggcggcggaggtggtggcctggacgccgccaaggttagggtcctctccgctccggccgtcaagatcgtccaacaggtcg
gcgcggaacataacgccggcggcttggcgacgcccgggtccaaacgcggcgggcggcgcgacgacggcgggcgcctggtgttcatccaggagcatt
cgcgggcgcggttcgagctggaggaccttctccgggcctccgcagaggtgctgggcagcggcaacttcggcgcgtcgtacaaggcgaccctggtgga
cgggcccgccatggtggtgaagcggttcaaggacatgaacggcgtgggccgggaggacttctcggagcacatgcggcgcctgggccggctcgcccac
cccaacctcctccccgtcgtcgcctacgtctacaacaaggaggagaagctcttcgtcacggattacatggtcaacggcagcctggcccacctcctccac
ggcgggaggaggtcgagtttgccggcgctggactggccgaagcggctaaagatcatcaagggcgtggcgcgcgggctggcgtacctgtacgacgag
ctgccgatgctgacggtcccacacgggcacctcaagtcctccaacgtgctgctcgacgccgccttcgaggcggtgctgtcggactacgcgctggtgccc
gtggtgcgtccgcaggccgcggcccaggtgatggtggcgttcaagtcgcccgagtgcggcggcggccggcccagcaacaagagcgacgtgtggagc
ctgggcatcctcatcctggaggtgctcaccggcaggttcccggccacccacctccggcacggccgcgccgggacgacgacggacctcgccgggtgggt
ccactccgtcgtcagggaggagtggacgggcgaggtgttcgacagggacatgcgcgggaccaggagcgccgagggcgagatgctcaagctgcttaa
ggtcggcctcgggtgctgcgaccccgacgtggaccgccgctgggggatcagggaggccgtcgcgaggatcgaggagctcagggagcgcgacgccct
cgccggagacgacagcggcacccccttcctcctacgtcagcgacggcgagatcgtctcgtcgtcgcggcccgccgccaccggcgacatacactcccacc
actccgcgtag
>g147276.t1

atggctgcacatgtcggcgatgaggccgcgctgctcgccttcaaggccgcggcgatcagcggcgggtatgacgacacccttgcctcgtggaatggcag
tgcaggcggatattgcagctgggagggggtgcggtgccgaggtaggcaccagcgagtggtggcgctcagccttccatctcgcaagctcacaggcgtcc
tctcaccggccgtcggcaacctgtcgtcactcaggagtctcgatctgagctttaacgggttcaccgggaacatccctgctagcttgggacgccttcgtcgc
ctccacatcctcgatctaaaatataacaacttttcaggcgaatttccggtcaacttgacattttgcagcaacctaaagatcatcgacctcctctccaaccag
ctccatgggcgtgtccccactgagatcggcgacaaactggtgggccttttagttctcagactaagaaataactacctcaccaactccataccggcatcgt
tgggcaacttatcatcagtgagaactttgtccctctcgttcaaccagctagatggcaacatccctcctagcatcggtgacatcccgggcatactgtctcta
gacctggcctacaacaacctctccgctaccaagctcaacagggaatctctcagcaacccttcaaattctcagattggaagacactcagatctcaggaag
tatcccctcagccatcggcaatctcgttggccttcagatgatcagtgtggcaggtaa
>g147279.t1
atggcgacgcactcagtgaacctgttgctgctgtttctgctatcttccttcatctccatcttgacacctatggccgcccgagccaacgacgaggccgcgct
gctcgccttcaaggccgcagcaataaggggcgggtatggtgacccgcttgcctcatggaatggtagcgcaaatggatattgcagctgggaaggggtga
gatgtcggggctggaaccaacaagtggtggcactcaatcttccatctcgcgggctcaccggcgtcctctcaccggctatcggcaacctgacgtcactac
gaattctcaacctgaccaagaacgagttcagcggcgacatccctcctagcctcggtcgccttcaccacctccgcgtactcgacctgagccgaaacacctt
ctctggtgagatccccttaaacctgacctcttgcatgaacctcaagatcatggccctcgaacacaaccaactctgtgggcgcgtgccctcagagatcggt
gacaacctgatcgcccttgggactctccttctagagtataacaaccttactggagctatcccatcgtccttgggcaacttgtcatcacttgttgatcttgaa
ctaacgcacaatcagctagagggtaccatccctcctagcctcggcaacatccgaggcatccatttcttgagcctcgccgacaacaacctctccgacattg
gcagtagatttcctagcttgttggatcttagccttgctcagaaccagttcactggatccatccctaattcagaccacttccttttgaagttggtagcctaa
>g148165.t1
atgcaaagcagaacacaactgagagtgacgctatggctatgtcttgccttgtgcgtcctgcggctgcatgccttccccttccccatcccgttcgtcgatcc
ctacacgagccaacaagacgttgacgcgatcaacgacctgtacgcggcgcttggctcgccggacctcgacggctggacagactcaggaggagacccc
tgcaacgaaggatggctgggggtgcagtgtgatggcccaaacataaccgcaatagagcttcgaggtgccggcctgggagggaagctgagcgagaca
cttggatacttcacagccatcacaagactagatctgagcagcaaccaaattggcggggggataccgcagagcttgccacctgcagtcacgaaactaga
cctttcttcaaatgatttaagtggcgaactcccggattcaatggcgaagctgagctcactgtggacattgcatgtgcagaacaaccagctaaatgggaca
cttgatgcactaggggatcttcccctgagagacttgaatgtagagaacaatcttttctcgggatcaataccagaaaaactgctcgctatcccaaaatttct
aagaaatggcaaccacttcaccgtagtacctccgatccccggatcctctccaactccttctacacctccaaaccatgtgccggcaccacctgcagaaga
agagcctccagtcctttctggttcacatcctcctgtaaatgtaattccagacactccacaggttgaccgtccgagacacaacaacaaggtctcaccagcc
aaggctgccggattcagcatcctcgccgccggttcactcggcatcgctgtcgttgcgattgtgttcatggtatcgaaacggcgcagggaggggtctccca
atgcagatgcagaagagaagaaagagttgcctcaaagagattacgccaaggcggcagggtcatctgttcacccgtcgtctttcaagaagagcagcaa
cgatagcatcgtgtcagataagaatgttgcgtgcagttcagaagcgcaaccgtcgcaactcgctttcacgttcttcacggtcgcgtccttgcagcagcac
accaacagtttcggcgaccagaacctgatcagggagacctgtttcggcaagatttatctagcacaattccaagaaagcaaggtgacttggtggcttatta
ctgcacctcatcccaaaaagaaacacaccagttctgatcgacgtgtacacggtcaattcttcattgtgctatgctatattctcaacctcgaccatcatctct
tttgcgccgtccagtttgcagtgctgaagcttgacggcagcgcagcgaaaatgccggtcgccgagttcctgaagatcgttcagggcatcgccgagctcc
ggcaccccaacgtggaagaactcgtcggatgctgcgtcgaccacggccagaggctcctcgtctacaggcacttgagcgacagcactctcagcgacctg
atccattccgaagacgcgtatggctctgctacagaaaagaccctgccgtgggatgctcgggtagccgtggccctccaggccgcaaaagcactggagta
ccttcaccagaagcaggtggttcaccgccatttccggcctgaacatgtactggtggacgacgaattgcaagtatcggtgtccggcggcggtcttgccccg
ttcgtggctcttgagctcacggattactgcgacggcacgccggaagcggcggcgtgggcagccaagggcgacgtgtacagcttcggcgtcgtgatgct
gcagctcctcacggggcgcaagccgtacgacagctcgcgggcgcggggcgagaggcacctggtgccgtgttcgaggccgcggctgcacgacctcgcc
gcgctgaggaccatggccgacccgcgcctcggccatccgccgccggtcaggtccctgtcccgcttcgccgacatcatcggccggtgcatacagcatga
ggcggagttccggcctggcatgtcgcaggtggcgcaggacctgcgccgcgcgctggacgacgacgcgcgcgccgggagcagcggcgttgaatactcc
gctgacactccacttgtgttcacctatacgatcgattgtcatcggcccatcgaactcgtttgcttgtga
>g148594.t1
atgcgcgcctcatcactcgtcgagcaccgcgtcaccgtggccgtcgccgtgctcctcgccgcagcggcggcggcggcagcacggacgaagaacg
agtgccactccggcgacaaggcggcgctgctgtccatcaaatccgccttcggcaacgcctcctacttcaattcgtggacgcccgacaccccgtgctgcg
agtgggccaacgtcgcttgcgactcctcctcctcctcctcctccacggcgcgccgcgtcgtcgccgtctccttcctcgacgacgccggcctcgccggg
cccttacccggcgccgccatcgcccgcctcaccgcgctgcagcagctggtgctgagcaacgtgccgggcgtgaacggcaccatcccgcacaacctcac
ccgcctctccgccacactcagcttcctcaccatcacctccacgggcatctccggtccggtcccgccgttcctctccgagatcaccgcgctgggctacctca
gcctctcctccaacaagctcacgggccacatcccggcgtcgctcggggacatgcccaacctctacttcctcgacctcggctccaaccgcctcacgggca
ccatcccgccgctgctcctcagcagggcccccggcgacgcgtacctatacctctcccacaacaggctcgccggcggcgttcccgcggagttcgccgccg
tgaacttctggacgctcgacctctcgcacaacgggctctccggcgacgcctcgttcctgttcggggcgaacaagccgctggagaacctggacctgtcga
gcaacggcttcagcttcaacctctccgccgtgcagctgccggagaacctcaacagcttggagctgagccacaatgacatctacggggatataccggag
caggtggtcgacctcatgctggagtacttgaacgtcagctataatcgcctcagcggcacggtgcccgccggcggcaacatggcatggtttgaccagtcc
tgcttccagcacaacaaggggctctgtggaactccacttcctccctgcaaggagtga
>g148595.t1
atgctcgcctcgtcatcaatcaggcacggtgccaccgtggcagccgtcgtgctcctcgcggcagcagttgcggcagcacgggcgaagaatgagtgcca
ctccggcgacaaggcggccctgcttgccatcatgtccaccttgggcagcaacgcctcctccttccagtcctggactcccaacaccccgtgctgcgactgg
accaacgtcttctgcaccaacgtctcctccacatcccccctacacggcgcgccgcgtcatcggcttgtccttgcaggacgacgacgtcctcgccggcccgt
ttcccggagccgccattgctcgcctcaccgcgctggagcagctcctgatgatcaacgtgcccggagtgaagggcaccatcccgcacgacctcacccgcc
tgaacgccaccctttccttcgtggacatcagctccacgggcatctccggccagatgccgtcgtttctatccgagatcaccgcgctcggccacctcaggctc
tccaccaacaagctcacgggtcccatcccggcgtcgctcggggacatgcccaacctctacttcctcgatctcagcaacaaccgcctcacgggcacgctc

ccgaggctgctcttcagcaagagcaagacctctgatgagtactccgaaaagtacctctacctctcccacaacaagctcaccggccgcgtcccccccgag
ttcgccaccgtcaacttctggggcctcgacctctcgcacaacggattcaccggtgacgcatcgctcctgttcggcgtgaacaagacgctggactacctca
acctgtcgcacaacgtcttcggctttaacttctccgccgtgcagctgccggtgaacctcgacgggttggacctcagccacaaccgcatctacggcgacat
cccggaccaggtggtcaacctgacggtgacctatttaaacgtcagctgcaatcgcctcaccggcgtagtgcccaccggtggcaccatgttctggtttgat
gcatcctgcttccagcacaacaaggggctctgtggaactccacttccaccctgcaagcactga
>g148599.t1
atggcggcggcggcaccacggacgaagaacgaatgcgaccccgacgacaaggcggcgctgctggccatcaaggcgggattcggcaacgcctcctac
ttccaatcctggacttctgactacccctgctgcctctggggcaacgtcttctgcgacttctacacctcccccctacacggcgcgccgcgtcgtggccgtctcc
ttcctccgcgacactagcctcgtcggacctttgcccggcgccgccatagctcgcctcaccgctctgcaggagctcgacctgatccacgtgccgggcgtga
acggcactatccccccatgaactcgcccgcctctccgccaccctcagttccatcgacatctcctacacgggcatctccgggcccgtgccgtccttcttctcg
cagctaaagcagctcacctacctccgcctctccttcaactcgctcacgggccgcatcccggcgtcgctcggcgagatccccaacctctccttcctcgacct
cggccgcaaccgcctcacgggaaccataccacctggactgctcagtaagaccaacgacacgtcagacctgtgcctctcacacaacaacctcaccggcg
gcgtccccgccgagttcgccgccgtcaggttctcgagcctggacctgtcgcacaacgccctcaccggcgacgcctcgttcctgttcggggcgaacaagt
cgctggagaacctcgacctgtcgcgcaacacctttcagcttcagcctctccgccgtgacgctgccatcgaagctcggctcattggacctgaaccacaacg
gcatctacggcgagctaccgccgcaggtggccaacctccagtttttgaatgtcagctacaactggctcagcggcaaggttcccaccggcggcaacatgg
acaggtttgatcagtactgcttccagcagaaccgcggtctgtgtgggagtccactacctccatgctattag
>g148658.t1
atgaagatgacgacgccggcggcctcccgcgcgctcgttgccttactcatcgtcgccgtcgccgtcgccgacgacggttcgacgctactggagatcaag
aagtcgttccgcaatgtcggcaacgtactgtacgattgggccggcgacgactactgctcctggcgcggcgtcgtctgcgacaacgtcaccttcgctgtgg
ccgcactcaacttctctggactcaaccttcagggcgagatctctccagccgtcggcagcctgaagagcctcgtctcgattgacttgaagtcgaacgggct
gtctgggcagatccctgatgagattggcgactgttcatccctcagaatactggacttgtcattcaacaacttggatggggacataccgttttcgatgtcaa
agctgaagcaccttgagaacttgatattgaagaacaacaagttgattggtgcaatcccttcaactctgtcgcagctcccaaatttaaagattctgggact
gcgtggcaatcgattagaaggaagcctttctcctgatatgtgccagttgactggcctttggtactttgatgtgaaaaacaatagcctgactggggtgatac
ctgatacaattggaaactgtacaagttttcaggtcttggatctgtcttataatcattttactggaccaataccattcaacattggtttctacaagtggctac
attgtccttgcaatcgaacaactttacgggtccaattccttctgttattggcctaatgcaggcacttgcagtgctagaactcaatgataatcaacttacggg
gccaattccaccggagcttgggaagcttacaggcttgtttgacttgaaccttgcaaataaccaccttgaaggaccaattcctgacaacctaagttcatgtg
tgaatcttaatagctttaatgcttacgggaacaagttaaatgggaccattcctcaatccctacgtaaacttgaaagcatgacatatttaaatctctcatcaa
attatctgagtggtccaattccaattgagctgtcaaggatcaacaatctggataccttggatttatcctgtaacatgattaccggtccaattccatcagcca
ttggcagcttggagcatcttttgagacttaacctgagcaagaatggtctagtgggattcattcccgtggaatttgggaacttgaggagtgtcatggagatt
gatttgtcaagtaatcaccttggtggtttgatccccccaagaacttggaatgttgcaaaatctaatgttactaaaactggaaaataacaatataactggaga
tgtttcttcactgatgaactgcttcagccttaataccttgaatgtatcatacaataatttggctggtgttgttcctactgataacaacttctcacggttttcacc
tgacagccaccaggaaaaacctccgatttcaaaggctgcgatacttggcattgctgtgggtggacttgttatcctcctgatgatcttagtagctgtttgctg
gccacacagtccacctgttttcaaagacgaatccataagcaaaccagtgagtgtgcaccccaagctggtgatccttaatatgaacatggctcttcatgtg
tatgaggatataatgaggatgacagaaaatttgagtgagaaatacatcattgggtatggggcatcaagtacggtgtacaaatgtgttctgaagaattgc
aagccagtggcaataaaaaagctgtattctcactaccctcagagcttgaaggaatttgagaccgagctagaaactgttggtagcataaaacaccggaa
tcttgttagccttcaaggatattctctatcaccaattgggaacctttctcttctatgattacatggaaaacggcagcctgtgggatgttttacatgaaggtccg
tcgaagaagaaaaaacttgactgggagactcgccttcggattgctcttggtgcagctcaaggccttgcttaccttcaccatgattgcagtccacgaataa
ttcatagggatgtaaaatcgaagaatatcctccttgataaagattatgaagctcatcttacagacttcggcattgccaagagcttgtgtgtgtttcaaaaact
cacacctccacctatgtcatgggtactattggctatattgatcctgagtatgcacgcacgtctcgactcaatgagaagtctgacgtttacagctatggtatc
gttctgctggagctgctgactgggaaaaagccagtagataatgagtgtaatctccatcacatgatcctatcaaagacggcgagcaatacagtcatggag
actgtcgaccctgacattggagacacatgcaaggatctcggcgaggtgaagaaggtattccagctggcacttctgtgcaccaagcggcaaccgtcgga
tcggccgacgatgcacgaggtggtgcgcgtcctggactgcctagtaaatcctgacccgccggcaaagccctcgcatccaacggccttgccacagccat
cggtggtcccgagctacatcaatgagtacgtcagcttgcgaggcacgagcgtgctctcctgcgccaactcgtcgagcacctcggacgctgagctgttcct
gaagttcggcgaggcaatttctcaggacacggagtag
>g149785.t1
atgctgccgtcgcgggcgctgttcctgctgctcatgctagccctggcgccgttccatgcggcggcgcaggcgccggggtccgtgccgccggtggcgcgg
acgccggaggtgcaggccgagatcgacgcgcttctcgcgttccgtcgcggattgcgcgaccccctacggtgccatgtccggttgggacgcgtcctcgccg
tcggcgccctgctcctggcgcggcgtcgcgtgcgcgcccggcggcgccggccacgtcgtcgagctgcagctcccgcggctccgcctctcgggccccatc
tcgcctgcgctgggttccctcccctacctcgagcgtctcagcctccggtccaactccctctctggcgccatcccggcgtcgctctcccgcgtcaccgcgctc
cgcgccgtcttcctccagtccaactcgctctccggccccatccccagtccttcctctccaacctcaccaacctcgaaaccttcgacgtgtccggcaacctc
ctgtccggccctgtccccgcttcatttccctcgaccttgacgtacatcgacctctcctccaatgccttctccggcatcatcccggccaacatcagcgcctcc
gcggcgaacctccagttcctgaacttgtccttcaatcggctccgggggaccgtgccggcgtcgctgggcgccctgcagaacctgcattacctctggctgg
atgggaacctcctcgaggggaccatcccttcggcgctcgccaactgctcggcgctgctccacctcagcctgcaggggaactcgctccggggaatcttgc
ccaccgcggtggcggcgatacccacgctgcagattctctccgtgtcgcggaaccggctctccggcaccatcccggcggcggcgttcggcgcccagggg
aactcgtcgctgcgcatcgcgcagctcggcggcaacgagttctcagaggtggacgtgccgggaaagctcggtaaggatcttcaggtggtggacctcgg
gggcaacaagctggccagcccgttcccgacctggctcgcgggggccgggggcctgacgctgctcgacctctccggcaatgcgttcaccggcgacttgc
cgcctgacgtcgggcagctcactgcgctgctggagctgcgccttggcggcaatgctctgaccggcgctgtgcccaaggagattggcagatgcggcgcg
ctgcaggtgcttgaccttgaggacaacaacttcaccggcgaggtgcctgccgcattcggtggtctgacaaggctcagagaggtatatcttggtgggaac
tctttctccggcccaattccagcgagcttggggaacctgtcttggctggagaccctatccactccggggaacagcatctctggcggcctgtctggtgagct

cttcaagctggggaatctgactttcctggacctgtcggataacaacatcaccggagagatccctcctgccgtcggcaacctggccgctctccagactcta
aatttgagcagcaacgccttatctggccgcattccggcaaccattggcaacctgcagaacctgcgtgttcttgacctctctgatcagaggaacctctctgg
caatctccctgcagagctctttggcttgccacaactgcagtatgtgtcacttgctgacaactcgttctctggtgatgttcctgaaggattcagcagcctttgg
agcctgcagcatctaaatctctccagcaattctttcaccgggtcaatcccggcgacgtatgggtacctgccatcgctccaagtgctgtcggcctctcacaa
ccgcattactggggagattcctgccgagcttgcaaattgttccaacatcaccgtgcttgagcttagtggaaaccaactgaccggtaccatcccaagtgat
ctctcacgccttggtgagttggaggagcttgatctcagttacaatcagctttctggcaagataccacaggaaatttccaatgcctcgtcgcttgccattctc
aggcttgatgacaaccgtattggtggcgagataccagcctctctcgcaaacctctcgaagctgcagacgcttgacctgtcatcaaacaatcttacaggca
gcatccctacttcattggctcagattccagaacttcagtcactcaatgtatcacacaatgagcttactggagagataccggcaatgcttggctctcggtttg
gctccccttcagcatatgcctccaactctgacctgtgtggtccgccattggatagtgaatgtgatgagtaccggcgacggcgtaggaggcagaggatgc
agcgtctggctctattgattggtctggttgctgctgccgtggtgctccttgcgctgttctgctgttgctgcgtgttcagcttgatgaggtggaggcgccggtt
cattgagagccgtgatggtgtgaagaagaggagacgcagccctggacggggaagcgggtcgagtggcacaagcacggagaatggtgtcagccagc
caaggctgatcatgttcaattccaggatcacatatgctgatacagttgaggcgacaaggcagtttgatgaggagaatgtgctcagccgaggtcgccatg
gcctcgtgttcaaggcttgttactctgacggcaccgtgttggctatcttgcggcttccttccacctcttctgatggtgccgtggtgattgatgaaggctcatt
caggaaagaagccgaatctttgggcaaggtgaagcacaggaacctcactgtcctccgtggctactacgcagggccgccaccagatgttcgtctgctgg
tgtatgactacatgcccaacggcaatcttgccacactgctccaggaagcgtcccaccaagacggccacatcctcaactggccaatgaggcacctcatcg
ccctcggtgtctcccgcggccttgcattcctgcaccagtctggtgttgtgcacggcgacgtcaagccgcagaacatcctctttgatgccgacttcgagccg
cacctgtctgactttggtcttgaaacaatggtggtgaccgcaggagctgcagcagcagcggcagccgcatcaacatcagcggcaacaccagtgggctc
actcggctacgttgcccctgacgccgctgttgctgggcaggctacaagggaaggagacgtatacagcttcggcatcgtgttgctggagctactaaccgg
ccggcgcccgggcatgtttgccggggaggaggaagacatcgtgaagtgggtgaggcggcagctgcagcgtggcgcggtggccgagcttctggagcc
gggcctgctggagcttgatccggagtcgtccgagtgggaggagttcctgctgggaatcaaggtcggcctgctctgcacggcgccggacccactggacc
gcccggccatgggcgacgtggtgttcatgcttgagggctgccgcgtcgggccggacctcccgtcctccgctgaccccacctcccagccgtccccggcgt
ag
>g150120.t1
atggcaatgagcacggtgaccttactgctcctgctgttcgccttcacatccatgtccacgatcacgagcgctgatgctgacgatgcggcggcgcttctcgc
gttcaaggcagcagccattggcgacggcggctacaggcttccttcgtggaacgccagcgttaccggagggatctgcagttgggagggagtgacgtgcc
agggcaagccccggcaagtggtgacgctgagcctctcctcccgcgggctcaacggcgttttgtcgccggtgatcggcaacctgtcgtcgctcaccattct
tgacctgggctccaacatgttgagcggctgtcatacccgcaagtcttggccgtctacaatgtctcgagaccctcaacctgagcagcaactcgttctccggt
ga
>g150122.t1
atggcaatgcgctcactgagcttgctgctgttgctgatgctatctgcctccatgtatgtctcaacattcatggctacacatgtcgatgacgaggctgcgctg
ctctctttcaaagccgccgcgattagcggtgggtatgatgatccacttgcttcatggaatgacagcataggcggatgttgcaactgggagggggtgaggt
gccgcggcaggcaccagcgagtggtggcactcacccttccatctcacgggctcaccggtgtgctctcacctgctattggcaacttgtcatcactcaggac
tctcaacataagcttcaacggtttcagcggggagatccctgctagcctcagccgccttcgccgtctccatatcctcgacctaagccacaacaccttctccg
gcaagctccccgtaaacttaacatcttgcatcaacctaaagaccatgtgcctgcgcttcaaccagctccgtggatgcgtcccctctgagatcggtaacaa
actgatgggtcttaagattctcaccctaggtgacaacaagctcaccggagacattccgacgtcgttgggcaacttatcatcacttattaaattggagctcg
catacaatcagctagaaggcaccatcccttccaatctcggcaatatcctgggcatccggtacttgtcccttgcctacaacaacttatccggaaatactctg
accttagcacaaaggctcgatattgccatcgacatcatggatgctatggactatcttcataatcactgtcagccaccaattgttcattgtgatatcaagcca
agcaacattcttattgctgaagacatgagagctcgagttggagacttcggtatatcaaggatcctccccaaaagtgcatatagcaccctgtcaaattcga
atagcacaattggaataagaggctccattggttatgttgctccagatttttgtggacatcgtaatctgaacccaaggctaactacaggtggtctggctaca
tcctgcattggtgaaagatatctaaaacatgcaaaaggcttggtcgtctttgtgctgaagtccacgcacaatgatcaactcagaacagtatacaacgagc
acggggatggagctagcaccatcgacttaccggacaccaaccaccacccgtccatcgccggctggcttaagccagtaacatcaaccaaagcaacagg
aatgaccaccatgcgcccggtgaactgcatgctgttgctggtatccttctctatgactgcgacgttgctcgtggcggacgaggcggcgtcccttctcgcct
tcaaggccgccgccatcagcggtgccaatggcgacatgcttgcttcgtggaatggaagcagcgccagcaggttctgcagctgggagggcgtgatgtgc
ggccgcaagcaccggcgagtggtggccctgaacctctcctctcgtgggctcaacggagtcgtctcgccggccattggcaacctgacgttcctcaggtta
gtcaacctgagctttaacgggctcaatggggacatccctgcaagccttggccgtcttcatcgtctccaggtcctcgatctgagcaacaacgccttctctgg
caagctacccgccaacctgagctcctgcaccagattgaagtacatgtatctccgcttcaaccagcttcatgggcgcttgcctccagagcttggtgacaag
ctggcacatctcagggtgctgaacctacagaacaacagcctcaccggcaccatcccgtcttcgctggccaacttgtcatcactggaggccttgatccttc
cattcaaccatcttgagggaaccatccctcctgccctcggcagcatcccagggctgctgaatctcgaccttgcttccaaccacctatctggtgagctcccg
cactctctctacaatttgtcttcattggaatggttgcagctccagatgaatatgttccggggaggcatccctgccaatatcggcagcaggttccccaaaat
gcagatccttgtgttatctttcaaccagttcaccaggtcaatccccgcttcaatctccaacctcactacactccaatatctcggactctctggaaataggct
cagtggacgacagggaagggtgggaattcatcacttcactatcaaattgcagcaagctccagcaattatttctcagctacaacagcgctttcactgggca
gatgccaagttccatagggaatctctcaagtaa
>g150124.t1
atggcaacacacacacactcagtgagcttggtggtgttgttcgtgctatcttccgcacttgcggccgcaagagccaacgacgaggctgcactgcatgctt
tcaagctcgcagcgatcagcggcaggaatggtgaccctcttgcctcatggaacagcagcctaagcggctactgcagctgggagggggtgaggtgcag
gagcaggggcaggcaccagcgagtggtggccctcagccttccatcttacgagctcaccggcgtcctctcaccagccattggcaacctgacgtcgctacg
aactctcaacctgagcaagaacggcttcagcggggacatccctgccagcctcggccgtcttcaccacctccacatcctcgacctgagccgaaactccttc
tccggcgaggtccccacaaacctgacttcttgcatcaatctaacaattttgaacctcaacttcaaccagctccatgggtgcgtgtcctctgagatcggcga
caaactggcaggccttagatttctcggcctggcaaaaaataaccttacaggaacaatcccggcgtcgcttggcaacttatcatcacttattatcctgcaac

ttggattcaatcaattagagggcatcatccctcccatattcggtgtcatgagaggcatccaatttctagacctcgccttcaacaacctctctggatacttcc
atatccggagtgattcctaa
>g150125.t1
atgcagtcagtaatcctgttgttgctcctgccgttatcgttcctgataagttgctcggagcttgtggccacaaaagccggagatgaggccgcgctgctagc
cttcaaggccgcagcctacagcgatgcgcttgcttcgtggaacagaagcacagccggtggattttgcagctgggagggggtgaggtgtgggcgcaggc
accagcgagtggtggccctcagacttccatcttacgggctcactggcgtcctctcaccagccatcggcaacctgacgtcactacgaactctcaacctgag
caagaacggcttcagcggggacatccctgccagcctcggccgtctttaccaccttccttcatgtcctcgacctgagccaaaacactttttccggtga
>g150817.t1
atggccgccgcgtgcttccgcttcctcctccctctcctgttcctgttcctggctccgctcctctccgccgtcgcagccgcgggccgcaacgaggaagatgtc
cgcgcgctcatggcgctcaagcgtgcacttgaccccgtcggccgtgtcctgggctcctgggacccatccggcgacccatgcagcgactccttcgacggc
gtcgaatgcgaccccaccggccgcgtcacggccgtctcgctgcagggccgcgggctctccggcacccttccgtcctcagttgccgggctccggcggctc
caggggctgtacctccactacaacggcatcaagggcaccattccgcgggagattgggaaactgtccgagctcacggacctgtacctcgacgtgaacca
cctcaccggaccagtgcccgtggagattgcctccatggccagcctgcaagtgttgcagctgggttacaaccagttgacaggcagcataccaccccagct
aggcagcctgaataaactcaaagttcttgcaatgcagtcaaatcagctgactggagctattccggcaactcttggtgacctaacacagctgacacggct
cgacttgagcttcaacgaactgtttggttcaatcccatcgagaattggggagcttccattgcttgaagtctttgatattcgcagcaacacgctctccggga
gtgttccagatggattgaggcggctaaatggtggtttccagtatgcaaataacaaggagctttgtggagttggctttactttgctagatctttgcccatctt
cagaggatggcctgaaacccagcaagcccgagccttttggtccagatggcggaggtgttggatcatctggtgaagtcggtgatagctttaggttcaatct
agaggaggttgaatgtgcaacacagtatttctctgaggttaacttgctaggcaagagcagtttcgctgcaacatacaggggaatcctccgggatgggtc
agttgttgctgttaagagcctgaataaaacaagctgcaagcaagaggagtcggatttcttgcgtggtttgaagatgctcaccttgcttcgacatgagaac
cttgttagcttgaggggcttctgctgctccaggggaagaggggaatgcttccttgtctatgacttcatggtgaatgggtgcttgtcacagtatttggatgtt
actgacggtgccaacgtgaatgttcttgattggcctactagagtttcaatcatcagaggcattgcaaaaggaattgaatacttgcacgacaagagaagc
aataagccttcagtagttcaccagaacatatcagccgagaagatccttcttgaccaccacttcgccccacggttgtcggtcccaggactgcacaagctcc
ttgctgatgacgttgttttctcaaccttgaagaccagcgctgcaatggggtacctcgctcctgaatatgccaccataggtcgattcactgataagagcgac
gtctttgcatttggagttgtggttctccaagtcatcacaggcaagagggatgtctcacaactgaaggttggcacagccatcagcaaccttgaaggccttat
ggatggaaacctcaatggtgccttctctaggacagaagcagcaaagttcgccgctgttgctgcatattgcaccagcgaggtgccgagccagcggccga
caatggaagctgtggttcagcagctcagccactga
>g151473.t1
atggcaggctaccctgttgtttaccacactaactggcgctgccttgcagatttgatcaacttggagtgcttgaacttggactcatgcaagattggtgatga
ggctctaattcacctgaaaggcctcttgcagctaaaaagcctggagctttcagacactgaagttgggagcaatggacttcgacacctctctggtctgcgc
aatttgcagagcatcaatctctcttttacgctcatcactgacgctagtttgaagaaaatttctggcttgagctcactgaagtctctcaatcttgacaatcgcc
agatcacagataatggtttagcagctcttgcaagtctcactgggttaacacaccttgatctatttggagctcgaataactgatgctggcacaaactgcttc
agatatttcaaaaatctacagtctctggaggtctgcagtgggttcatcaccgacagcggagtgaagaatatcaaggatctgaaagctctgacgctgctc
aatctgtctcagaacggcaacctcacggacaaaaccctggagctgatctccggcctgaccgcattggtgtcacttaacgtgtccaactcccgagtgtcca
actcgggcctccaccacctgaaaccgctgcagaacctgcgttccctggccctggagtcgtgcagggtgacggcgaccgagatcaagaagcttcagctg
gtcgatcttcccaacctggtaagcgtgcggccagagtga
>g151854.t1
atggcaatgcttcgtcgcaagtacgcacgagcttacctccacctgctagtactcgtgttgctgccctgctgcggatttcagcgctgctgcgaggcaacgc
agtccaaaccggtggatgaggcgcggctgctgctccagatcaagagcgcgtggggcgacccgccggtgctgctggcatggaacgctacggcatcggc
cgcactctgcgggtggccctacgtgcgctgcaacagagccgggcgcgtctccggcctctacctcgctgatgtcaacgtctccggcccgttcccagatgcc
atcgccaccctctcccgcctcaggcacctcgacctctggaacaacagcatcgctggcgtgttcccgacctcgctgtaccgctgccgttctctgaagtacct
cgacctgtcatggaaccacttcatcggggagctcccggctgacatcggccttggtctatcagagagcctgaccacgctgtacctcaccagaaacttcttc
gtcggcaccatcccgcattcgctctccaggctccataaccttcggtttctcgggctgcacagcaacttcttcgtcggcaccatcccagcagagctcggcga
gctgacaagcctccaggagctgtaccttggggacaattcattcgctgctggcgagcttcctgcatccttcagcaagctgaccaacctgactatcctcgac
gtgtggagctgcaaccttgtcggagattttccgacctttctagtgcagatgccggagctggagattctggacctcgccaacaactcgttgaccggaagca
tacctccggggatttggagactcaacaagctgcaagttttgattttgtccgagaacaacttctctggtgaaataccggcgagcattgccaggttgccatca
ctgacggatttggacctgggtaacaacaggttcaatggcacgcttccaccagagctcgggaagcactctttggacctggctactgtttcagtggataaca
atgacctcactggagcgattccagaggggttatgtactggaggtcatctttggtccttcacggcctcgagcaaccgattgaacggctccataccagcagg
actcgccaactgccccagtctttcagaactgaggctggacaataaccagttgtcaggacaagtgccctag
>g152923.t1
atgacctccggcagcagtcgctgcgtgcaactcctaggagtgcacctgctccttcatgtcctcctaggagtgaacctctcttgctactccctcgccacctac
agaaacagcaaccgaaccatccgacgaccaccctcgtgccggccagaccaggcctcggcgctgctccagctcaaacattccttctccaccaacgggtc
gcaatgcactctcgcatcatggcggtccggcaccgactgctgccgctgggaaggcatcagctgcggggacgccgacggccgcgtgaccaccctcgac
ctggccgattgcggcctcgagagcggcggcggcctccacccggcgctcttcgaccttacgtccctgcgccgcctggacctcgccggcaacgacttcaac
gagtcgcagctcccctccgccgggatcgagcggctcgccgagctcgtctacctcaacctctccgctacggggttcgtaggcgagataccgcgcgggata
cggcggctcaggagactggtatccctggacctctccacctggatttaccttctcgaactggacgactatttcgtgccgctcggcgacgggcggtggcctct
cgtggagccggatattgggtcgttagtcgccaaccttagcaacctcaaagtgcttcgtcttgacagtgttgacttgtccagcaacggggcagcgtggtgc
gatggcttcgctaactccactcctcagctgcaagttctcaggttaccaaacaccggcctcgacgcgccgatttgtgggtcactgtccaccatccgctcgct
tgttgacattaacctctcatataacagactgcacggccaagttcccnctcctcagctgcaagttctcaggttaccaaacaccggcctcgacgcgcggatt
tgtgggtcactgtccaccatccgctcgcttgttgagattaacctctcatataacagactgcacggccaagttcccgagtcttttgccgatctgccaaacctc

agtgttcttagactttcttacaatcgccttgaaggatggttcccgacgaggatcttctggaacaataacttgacagttgttgatattagttacaattttgaaa
tttctggtattgcgtcatctgacttccaccaagagctcccctcctcaattggtgagctcacatccttgagattgttagaggtttctggggctgttgttctggca
tttggaatccgtctcaggagaatatgtcagggcatcatttttatgcgtcagaagaacatcttcatcctcgtataa
>g153108.t1
atgcggtgcgtccctggccgcgcggcggcagtgaatctcctcctcctcgccttgtggttctccgctctcctcccccgcgcgtcgcccgacctcgccgcgga
cagggcggcgctcctcgtgctgcgcgccgcggtgggcccgcacctccggtggaacgcgtcatccccatcaccatgcgacggctggcgcggcgtgtcgt
gcgaccccggcgggacgcgcgtaacgggtctgcagctcccggggtccagcctcgcgggccagctcccgccgcgaacgctgggcaacctgacggcgct
gcgcacgctgtcgctccgcctcaacgcgttgtccggcgggatcccggacgacatcggcgggtgcgcggagctcaggaagctgttcctccagggaaacc
ggttcgacggcgagatacccgaggggatcttccagctcaacttgctgcagcggctcgacctgtcggggaaccgagtcgccggcggggtgtcgccggag
ttcaataagctgcgccggctcgccacgctgtacttgcagggaaacagactcaacggcagcttaccggctgatctcgacctccctaagctccagcagttta
acgtgtccgggaatggccagctaacaggtcccgtgcccgcgtcgctggctgggaggcccgcgagcgcgttcgcggggacagggctctgtggcgcacc
gttgttaaacccgtgccccaacccaccctcgccgtcgccaacgccagcgccaccctcgccgccggctcctaatgctcctgaacgtagcaagaattccaa
gctctccgccggcgcgatcgctggcattgctgctggctccgcagcggcgttgttggcgttgatcgccgcgatcgtcttcttcctctgtttccgacgaggtcg
acgtcagaggagtagtgagccgactaataagtcccccgacacgtcggcagacgtcgacgtctccgggtctcctccggtacccgtcaccgtggcgagcc
tttccacgaagcggtcgcggtcacggtcgtcgcggcagaccacggcgtccggcaacaacaacaagcagctggtgttcgtgggcggcgcaccggacac
cccgtacgacctggagtcgctcctgcacgcgtcggccgagatcctgggcaagggctggctcggcacgacctaccgcgccacactcgagggcggcgcc
gccgtgttagcggtgaaacggctccgcgagtcgcccatcccggagcgcgagttccgcgaccgcgtcgccgcgatcgccgcgctgcggcacgacaacct
cgccacgctgcgcgcatacttctacagccgcgaagagaagctcctcgtctacgacttcgtcgacaacgccaggagcctctgcgccctcctccacggcgg
cggcagcagcagcaacagctacccttcgagcccggcgcgactcgacttcgccgcgcgcgcgcgcgtcgcgctcgctgccgcgcgggggatcgcgttta
tccacgcctcgggtccccgttcctcctgccacggcaacatcaagtcgtccaacatcctcgtccccgacgccgcgcgtgaccgctccgtcgtgacggacca
cggcctgctccagctcgtcggcgcgcacgtgccgctcaagcgcgtcacggggtaccgcgcgccggaggtcgccgacccgaaacgggcgtcgccggag
gccgacgtgtacgcgttcggcgtgctgctgctggagctgctcacgggccggccgccggcaaggtcggtgccgggcagcgacgagggggtcgacttgc
cggagtgggtgcgcgccgtcgtgcaggaggagtggacggccgaggtgttcgacgccaccatcgccgtcgaggagcgcgtcgaggaggagatggtcc
gcctcctgcggctcgccgtcgactgcaccgagcggcggcctgaccgccggccgggcatgcccgaggtggcggcgacgattgagcaggttgtcgagag
cgtggtacagaagactgccgacaacgtggacgatgacgatttccatagcatttcaccgtga
>g153166.t1
atggggcttcgcctcattgcggaactccagctgtttttcatcttcctccaggcgcaggtggggtgcagcacagctttgatcaacagtgaaggattggcgtt
actggaactgagtgcaagggtggaggctgatccccatggagccttccaggattgggatcccatggacagtaacccatgcagctggtctggtgtgcgatg
ctctgatggtaaagtagagattctgttcgcccgatacttgaatgtactgattgcagtcaaagaatttgagcatctggactggcctgcgaggatgaggatta
tcatgggaatcgcatactgtctccaatacacgcaccatgagctcgatccacctgttgcaataaatgatgtgtgctccgagacagtatacatgactgatga
ctatgctgccaagatggcagatgctagtatttggaaggaagtcgccaacaaagcaaagactgcaaaggaggacagtagcagccgttctgaagctcctc
ctgatcttgtcagtaacgtctactgctttggcgcacttctgatcgagatcatatctggaaagcttcccgaacaagacgatcctgaatccttgtgcaattggg
cttctgaatatctgaaagacaagagctacgccaagctcctcgacgactcactgaaagatcacaacagcactgagctcgaggccgtctgcgaggtgatg
cttgagtgcatcgatcccgatccgacgcaacggccggcgatgagagacgtcgtgggcaaactgagaccagctctcaacatcactcccgaggcagcgg
cgccgcggctgtcgccgctctggtgggccgagctcgagttactatccgtgaaggcaagctag
>g154149.t1
atggcggttccgcggggcgctgtggcggcggcggtcgttctgctgctggcgatgttggcttcgtctgcggcggcactgaacacggacgggctggcgctg
cttgcgctcaagttcgcggtgtccgatgaccccggcggggtcctcgggtcgtggcaggacgccgacgcggacccctgcgcgtggtcgggcgtcacctg
caccgacggcggcgggggccgggtcgccgccgtggagctcgggaacgcctcgctggcggggtacctcccctcggagctgtccctgctctcggagctcc
agacgctctccctcccctacaaccgcctctccggtcagatcccggtggccgtcgccgcgctgcagaagctggccgcgctcgacctcgcgcacaaccagc
tgtccggcccgatccccgagggcatcgggcggctgctctccctctcccgcctcgacctctcctccaaccagctcaacgggacgctgccgccggcgatcg
cggggctgccccgcctctccggcgtgctcaacctcagcgacaaccacttcaccggcgggatcccgccggagttcggcgggattccggtggcggtgagc
ctggatttgcggggaaacgacctcgagggcgagatcccgcaggtggggtccctcgtcaaccagggccccaccgcgttcgacggcaaccccaggctat
gcgggttcccgctcaaggtcgagtgcgcgggggagagggatgacgagccgaggatgccggagtcgggggccgggccgagcgaccccggcgccgcg
gcggaggtgcggcggccgcccccgaggcggcatgcgtcgtcgccgacggtgccgattctcgcggccattgtggtggtcgtcatcgtggcgggggtagt
gttgcagtggcagtgccgcaggcggtgcgcggcggcgacaggggacgaggaggagaaggggtcgtcggcgtcggcgaaggagaagagcgcggcg
gtgacgctcgcggggagcgaggagcggcggagcggtggtggtggtggagaggagggggaggtgttcgtggccgtggacgacgggttcgggatgga
gctggaggagctgctccgcgcgtcggcctacgtcgtcggcaagagccgcggcggcatcgtgtaccgggttgtgcctgttcgcggcgcggcagttgctgt
gcgacggctcagcgagcccgacgacggcggtgatagcggcggcgggtccgagtccgggtggcgccgccgccgcgcggcgttcgaggccgaggccgc
cgccatcggccgcgcgcgccaccccaacgtggcccggctccgcgcctactactacgcccccgacgagaagctgctcatctacgactacctccccaacg
gctccctccactccgctctccacggtggtccgacggcgtcgccgacgccgttgccgtggtcgacgaggctgtccatcgtgcagggcgcggcgcggggg
ctggcctacctgcacgagtgcagcccgcggcggtacgtgcacggctgcatcaagtcgtccaagatcctgctcgacgaggagctccgcgcgcacgtgtc
cgggttcggcctggcccgcctcgtcaccggcgccgcgggacactccaacaacaagaagcacgccggcgtcgcgctccgctccggcgccggcggggtg
tcgtacgtggcgccggagctgcgcgcgcccggcggcgcgacggcggcgacgcagaagggggacgtgtttgcgttcggggtcgtgctgctggaggccg
tgacggggcgggagcccgcggagggggagggcggcctcgacctggaggcgtgggtgcgccgcgcgttcaaggaggagcggccgctgtcggaggtg
gtggaccccacgctgctgggcgaggtgcacgccaagaagcaggtgctcgccgtcttccacgtcgcgctcgggtgcaccgaggccgacccggagatgc
ggccgcgcatgcgcgccgtcgccgagagcctcgaccggatcggtggctga
>g154151.t1

atggcagccaacgctctgcctctgctgctcctcgtcgccttgctcatcgccccgtcggcgcacgcggcgtcgtgctcggccgccgaccgcgacgcgctgc
tctccatccgcgcggcgctctccgaggacgcgcacctgggcgtcttctcctcctggacggcgaccaccgactgctgcacgaactggtacggcgtggcct
gcgaccccaactccggccgcgtggcgtacctctccctccgcggcgaggccgaggacgccgtgatggcccccgccggccaccccacctcaggcgtcatg
tcaggctacgtctccgacgcggtgtgccacctcgaccacctctccacgctcgtgctcgccgactggaaacgcatcacgggtcccatcccctcctgcgtcg
ccacctccctccctttcctccgcgtcctcgagctcccgggtaacgccctcaccggcgacatcccggcgtccatcggaaccctctcgcgtctcacggtcctc
aacctcgccgataaccagctcaagggacccatcccgacctccatcaccgcgctcgggtcctcgctcaagcacctcgacctcgccaacaaccggctcac
gggccgtgtcccgccggacttcgggaacctcgccatgctcagccgggccctgctggctcggaaccggctctcggggcccatcccgccgtcggtcgcgtc
catgccccggctcgccgacctcgacctctccgagaaccagctcaccggctcgattccggccgccctcggctcgagccgggtgctggcgtcgctctacctt
ggcgcgaaccggatctcgggaagtgttccggcgagcctgctcgggaacagcgggctgggcatcctcaacctcagccgcaacgccgtcgacggcgcca
tccccgacgcgttcacgccaaggtcgtacttcacgctgctcgacctgtcgcggaaccggctcacgggcgccgtgccacggtcgctcgccaccgcggcgt
acgtgggccacctcgacctcagccacaaccgcctctgcgggaccatccccgccggcgcgccgttcgaccacctcgacgccgcctccttcgcaagcaac
agctgcctctgcggcggaccgctcggcaagtgcacgtga
>g154435.t1
atgcctccacgctcgcgcgccactaccagagcgaggaggccgcttcttcttgctctgctcgcgtgcgccttcctgttcgcgccgtgccactgcgtcaacga
gcagggccaggcgctgctgcgatggaagaactcgatgaacgcgacggccggcggcgacgcgctcgcctcgtggaaggcgtcggacgcgtccccgtg
ccggtggctcggcgtgtcgtgcgacgcccgcggcgacgtcgtgtcactgagcatcaagtcggttgacctcggcggcgcgctgccgtcgttggacctccg
gccgctgcggtcgtcgctgaagacgctggtgctctccggcgccaacctcaccggcgcgatccccaaacagctcggcgacctcgccgagctgaccacgc
tcgacctcagcaagaaccagctctccggcgccatcccgcccgagctctgccgcctgggcaagctccagtcgctggcgctcaacaccaactcgttgcgcg
gcgccatccccgacgccatcggcaacctcaccagcctcacgcacctcacactctacgacaacgagctgagcggcgtcatcccggggagcatcggcaat
ttgaaaaaattagaggtgctgcgcgccggcgggaaccaggcgctgaagggcccgctgccgccggagatcggcgggtgcggcgagctcaccatgctcg
ggctcgccgagaccggcatgtcgggtagtctccccgacacgatcgggcagctcaagaaggtccagaccatcgccatctacacggccatgctcaccggc
tccatcccggagagcatcggcaactgcaccgagctcaccagcctctacctgtaccagaacgccctctccggcccgatcccgccgcagctcggccggctg
cgcaagctgcagaccgtgctcctctggcagaaccagctcgtgggcacgatcccgcccgagatcagcaactgcaaggacctcgtgctcatcgacctgtc
actcaacatgctgacgggccccatcccgcgcagcttcggcacgctgccgaacctgcagcagctgcagctgagcacgaacaagctgaccggcacgata
ccgagggagctctccaactgcacgtcgctcaccgacgtggaggtggacaacaacgagctcaccggggagatcgacctcgacttcccgcggctgggga
acctcacgctcttctacgcgtggcagaaccggctcaccgggcccgtgccaccgggcctcgcacggtgcgaggggttgcagtcgctggacctctcgtaca
acaacctcaccggcccgaacctcaccaagcttctcctcctcagtaacgagctctccgggttcatcccgccggagatcggcaactgcaccaacctgtacc
ggctccggctcaacgataaccggttgtccggcaccataccggccgagatcggcaacttgaagaacctcaatttccttgacctgggcaacaaccgcctcg
tcgggccgttgcccacggcgttatccggctgcgacaacctcgagttcatggacctgcattccaacgcactctccggctcgttgcccgacgagctcccccg
gagtctacagttcgtggacatctccgacaacaagctgaccgggctcctcggccccagcatcgggtcgctgccggagctgacgaagctgaacctgggca
agaaccggatctccggcggcataccgccggagctcgggtcctgcgagaagctccagctgctggacctgggcggcaacgcgctgtccggcggcatccc
gccggagctcgggaagctgccgtcgctggagatcgcgctcaacctcagctgcaaccggctctccggcgagctcccccgcagctcggcgagctcggga
agctcggcgcgctcgacctctcctacaaccagctctccggcagcctcgcgccgctcgccaggctggaggccctcgtcacgctcaacgtttcgttcaattc
gttctcgggcgagctcccggacacgccattcttccagaagctcccgctcagcgacatcgccggcaaccacaacctggtcgtcgtcggcgaccacggcg
acgcggccgcccgccgcgcgtcgtccgtctccgcgctgaagctggcgatgacggtcctcgccgtggccagcgcgctcctcctcgtcgccgccaccttcg
cactcgcacgcgcctcgcgccgccgcgacgacgggagcgccgccggtgtcggcggcgagggggcgtgggaggtgacgctgtaccagaagctggact
tgtcggtggacgaggtggtccggtcgctgacgtcagcgaacgtgatcgggacgggcagctcgggcgtggtgtaccgtgtgtccctgcccaacggggac
cacctcgccgtgaagaagatgtggtcgtcggaggaggccggcgcgttccgcaacgagatcgcggcgctgggctcgatccgccaccgcaacatcgtga
ggctcctgggctgggccgccaaccgcagcaccaagctgctcttctacgcgtacctccccaacggcagcctcagcgggttcctccaccgcggcggcgtca
agggcgccgccgactgggccccgcgctacgacgtcgcgctcggggtggcccacgccgtcgcgtacctccaccacgactgcctccccgccatcctgcac
ggggacatcaaggccatgaacgtcctgctcgggcccaggaacgagccgtacttggctgatttcgggctcgcccgcgtgctctccggcgccgtcgcggc
gggttccgccaagatcgactcgtccaagccaaggatcgctgggtcgtacggttacatcgcaccagagtacgcgtccatgcaacggatcacggagaaga
gcgacgtgtacagcttcggcgtggtgctgctagagatcctgacggggaggcacccgctggacccgacgctcccgggcggcacgcacctggtgcagtg
ggtgcgggaccacgtgcaggccaagcgcgccacggcggagatgctggacccgcggctgcgcgggaagcccgagccgcaggtgcaggagatgctgc
agctcttctccgtggcgctgctctgcatcgcgcaccgcgccgacgacaggcccgcgatgaaggacgtcgtcgcgctgctcaaggaggtcaggcggccg
gcggagtgcggcggcgacgaggggaaggagcagacggcgtgcaatggtgccgccacggccatgccgccggctgcgcagctggcgccggaccggag
cccgctgcccaagggcggctcgtcgagctgctcgttcgccatgtccgactactccagctga
>g154489.t1
atggccaagcaggctctcgccgcgctggtaacaataacgctcctcctcgcctgcttcccccgcgaggcgcggtgcgaggaatcctccgacgtgcgcgcg
tccctcgtggccttcctcgtcgagctcgccggcggcgacccgcagttggcgcgggacctccggtgggacgcgacggtccagccgtgcgaccccacggc
gccctggaccggcgtgacctgcagcggcaacaacgacggccgggtcaggaccatcgtgctcgagaaccaggggcccggcaagctgaccggcaccct
gaacgcgacccttctgtgcgcggcgtcgacgctgcgcgtgctgagcctgcagggcaacgcggtccgcggcggcctcgaggacggcgtcgcggcgtgc
agggcgctcacccacctctacctcgcgggcaaccggctctccggcgccctcccgcccgcgctcgcgcggctcagggacctccacgtggtcgacgtcgcc
gggaacggcttctccggcgcgatccccgtggggctcgccgggatcgggctcaagacgttcctggccaacgataacacgttcagcggcgccgtcccgga
cttcgacctcagagggttcgaaaagttcagcgtggcgaacaacagcctcaccgggcccatcccgaagaacgtgggcaagttcggcgacgccaggttct
tcctgccaaacgccgcgggtatctgcgggcaacctgtcttgcccgcttgtcaacaatcgtcgttcccgtcgccgtcgccgggcactgactccgacttcggc
gccgcgccgccgccgagcaccgggtccaatagtaccggagacaagggggcggagcaagaggacgaaggtcctcatgtacgtcgggtacgccctcctc
ggcgcggtcatactggccttggtcacgtacctctgcgtcaagaagcggaggagcaagctgcagaggaagttcaagcagggcgggggccggcgcgtg
acagacagcagcagggtgacgaccgccacgacgtcggtgatcagcgcgagcaagtcggtgtactcgatcccgacgtcggtcgtgcagagccccgcgg

cggcggcgacgggactagcagcagcatccgcgccgtcaccgtctctcgtggtgctgcggcggtccggcacggcggcgtcgatcacgtcggcggcggc
ggcggcggccgcgaaggagctgcggttcgaggacctgctcaagtcgcccgcggagctgctgggccgcggccggttcgggagctcgtacaaggtggtg
gtccccggcggcgccgagctggcggtgaagcgcgtcaaggacggcgcggtgggcgagggcgagttcaggaggaggatggagcgggtcgggcgcgc
caggcacgccgccgtgctgccgccgctcgcgttctactgcgccatgcaggagaagctcgtcgtctacgagttccagaccaacggcagcctcgaaaggc
tcctgcacggtaaacaatcctctccttgctctgcaacattttcgcacccctggtctctgctctgcgtcttcatcttacatgcatgcaaaagctga
>g154733.t1
atgctaccaatggtggtctccctcctcgccgccgcgcttcccagtgccgtggcgcagaacctggcgtccgaccgcgtggcactgctggccctccgcgac
gcgctggacccgggccgccgcctcccgtgggaccccgcggcgccgacgccatgtaacaactggcgcggcgtcacgtgcgacgcctccggcagccgcg
tcgtggagctccgtctgccggccaagtccctgaggggcacgataccatcgggcacggtgggcaacctcaccgcgctaaagaagctgtccctccggttc
aacgccgtctccggcgcgatccccgccgacgtcggcaacctcgccgagctgcggggcctcttcctccgcgggaacagcctcgacggcgagatccccga
ggggctcttcgcgctcgcgaggctcaggcggctcgacctcgccgggaaccggctcgccggcggcgtgccgttggggttcaataatctcacgcggctcg
acacgctgctgctcgaggggaaccagctgcaaggcacactccctgaagcgcttaacatcagcggcctcgcgccggggaggttcaacgtgtcgttcaac
gcgggtctcgacggccccgtgccggcctcgctgtccggcatgccggcgaccgcgttcctcggcacggggctctgcggcggcccgctcgccgcgtgcgc
cgcgccagacggcgaccacggcgagaagaagaagaggctgtcccggtgggcgatcgtcgggatcatcgtcggcgcggtggtcgtgatcctcctcgtc
atggcgatcctcgccgtgctccgctggcgccgccgccgcgcgccgccgccgccgcccgccgcggcggccgacgtgcactccggcaccgcgccgatca
cggtcaccgtggccaggacgcaccgggacgcgtccgtgtcgcactcgaactcgttctccggcgcctcgccggcgccgacgatcaccgacggaaacgg
cgacaagcagcagctggtgttcctgggcagcgcgccggaccggccctacgacctggacacgctgctgcgcgcgtcggccgaggtgctcagcaagggc
gcgcacgggaccacgtaccgcgccacgctcgacggcggcgcgccggtcctcgccgtcaagcgcctccgcgacgtgcgcgtccctgagcgcgagttcc
gggagagggcggcggaggtcggggccatgcggcacgacaacctgcccaggctgagggcctacttctacggcagggaggagaagctcctcgtctacg
acttcgtcggcaacggcgccagcctctccgcccttctccacggcaacaacggcgcgccgctggacttcacgtcccgcgcgcgcatagcgctggcggcg
gcgcgcggcgtggcgtacatccaccgcgtgggctccacccacggcaacatcaagtccaccaacatcgtggtcaccgccacgcgcgacggcgtcttcgt
caccggccacggcgtctccaagctcgtcggcgccgcctcctcatcgcggcgcggcaccgggtaccgcgcgccggaggtgtccaacgccgccgtgccgc
cgcggaagagcgccgacgtgtacagcttcggcgtggtggtgctggagctgctgagcggacgggcccccggccggacggcggcggcgtggacctgc
cgcggtgggtgcggtcggtggtgcaggaggaatggacggccgaggtgttcgacgccgccatcgccggcgaggcccgcgtcgaggacgagatgatgc
ggatgctgcggctcggggtggagtgcacggagcagcgggccgaccggcggccggccatggacgacgtggaggcgcgggtcgggcgcgtcgtcgag
gacgccgcccggaaggccgagttcagcagcacggacggcagccggagcgtgtcggcgtga
>g155978.t1
atgtctccgttccacggatacagaggtgatggagctgtcggctcggctctcttcggcgtcctaacaaggaagcgcgtgttcgctgtggcgggcgacgag
gccgtgacggccaagaggcagaagcagacggatgagccgtcccttgacgcgctccccgacgcgtgcctcttcgaggtcctgcgccgcgtgcagggcg
cccgcgcgcggtgcgcctcggcgtgcgtctcccgccgctggctcgcgctcctcgccggcatccgcgcctccgaggccgtgctggccgcggcccctgccg
tgccggacctcaaccgggagtacctcggcgacgacgacgaggacgagctcatgtgcgacgacgccgacagcgagaggaccttcgagggcaaggac
gctacggacgcgcgcctcacggcggcggccgtcggcgacgcgctccgcggccgcctcgtcagcgtgtccgtccgcgggagccacccgacgcgcggcg
tcaccgacgccggactctccgcgctcgcccgtggctgcccggcgctcacttcgctcgccttgtgggatgttccacaggtgacagatgccgggctcgctga
gatcgccgctgagtgccactcgctggagcgccttgacatcactggctgcccgctgatcacagacaaaggcctcgtcgcagtcgctcaggggtgcccgg
acttgaagtcgttgaccattgaggggtgctccggtgttgccaacgagggcctcaaggcaattggccggtgctgcgcaaagctgcaggtggtgaacatca
agaattgtgcacatgttgatgatcagggtgtgtcgggcctcatctgctcggcgaccgcttcattggccaaagtccggctccagggtttgagcattactgat
gcctctcttgcagtgattgggtactatgggaaagccatcactgacctcaccctcgctcgcctccctgtcgttggcgagagaggattttgggtgatggccaa
tgccctgggcctgcagaagctcagatgtatgactgtcgtctcctgccaggggctcacagatctcggcctcgcatctgtcgccaagttctgcccaagtttga
agcttcttcacctcaaaaagtgcagcaaagtctcagatggttgtctcaaggaagttgcgaaatcagcaaagattctggagaatctgcagatcgaagaat
gcaacaaggttactctcatgggcattcttggtttcctactcaactgcagccccaagtttaaggctatctctttggccaagtgcatcgggatcaaagacatct
gctctgcacctgcacagcttccagtatgcaagtcgcttcgttccctgaccatcaaggattgccctggattcacagatgccagcttggccgtggtgggcatg
atctgcccgcaattggagaatgtcaatttgagtggtcttggtgcagttaccgacaatggctttcttccattgatcaagagctctgaaagtggcttggtcaa
ggttgatttgaatggttgtgagaatctcacagatgcagctgtttctgccttggtgaaggcacatggaagttctcttgcacaccttagcctcgagggctgca
gtaagataactgatgcaagtctgtttgccatttctgagagctgcaaccagcttgccgagcttgatctttcaaattgcatggtcagtgactatggtgttgcgg
tcttatcttcagcaaaacagctcaagcttcgtgtcctctcattgtctggctgtatgatggtcacccaaaagagtgttcctttcttaggcagcatgtcgtcatc
tttggaggggctcaatcttcaattcaacttcattggcaaccgcaacattgcatcactggagaagcaactctggcggtgcgacatccttgcttag
>g157528.t1
atggcgccctcctccgccgacaaatcggttctcgagttcgtcgacctcaagaacaattccctctccggcgccattcccgcacagctcgccgcgctccctg
agctcagacaccttgacctgagcatgaaccaactgtccggtccaataccagacttcccaactcaatgcaagctcgaatccctccagctctacaacaatg
agatagccggtgagcttccccgcagcctcagcaactgcggcaatcttacagttctgttcctatccttcaacaagatcaccggtgcagtgccagatttcttc
gcatccctgccgttgctcaaaaagctttatctcgacgacaatgccttcaccggggagttgccggtgagcatcggtgagcttctcaatttggagaagctgg
tggcttcgacaaaccggttcaccggaccggtccccgggtcaattgggaagtattag
>g158246.t1
atgttatggaaggaagacagtgattgctgcaagtgggaagggatcacctgcaaccataatgggacggtcatcaaagtttcactgccttctagtggcctg
gagggacacatctcagagtccctggggaagctcaccggcctgcagcaccttaacttgtcctacaactcgctgtccagtggcctgccgcaggaattggtg
tcttccaacagcataattgttctcgatgtcagctttaaccagctcaatggaaacctgcatgagatgtcgtcttcaactcctggacagcctctacagtggga
gtatccccccagggctccgcagttgctccgagatcagagtgttcaaggctggccacaacaacctcagtgggactctccctga
>g158248.t1

atgccgccactccacctttcagataagacacatagcaacagattacctagacctaccgtaggccttgctcttctgctgctaattgctttggccaccaccac
caattgctgtacagaacaggagaaggcatctctacttcagttccttgccgggctctcccaggatggtggactagccacctcatggaaggaggctacaga
ttgctgcaaatgggaaggtatcacctgcagcagaaatgggactgtcactaatgtctcattagcatctaggagccttgaggggcacatctcgcaatcaatt
gggaacctcaccggcctgcagtacctcaacctgtccaacaacttactgtccggtggcctgccgcttgaattggtttcgtccagaagcattaatgtcctcga
cgtcagttttaaccagcttggtggcgacctgcatgagctgccgtcttcaacacctggtaatctgaagtctctctccttcctgtcacttactggcaacaacttt
acaaatataacaaatgcacttctgatgctgaggaacaccaagagcctcaccacccttcttattgggctcaacttcatgaacgaaaaaatgccagacgat
gacaccatcgatggttttgacggtgttcaggtattggatattggagagaaggctgcctatatggacccaagtgtctttgatctgcctgtttatgaagacca
atcacttcaataccgcgtaaccagtgctttccccaaagtgctgaagctagccattaacaaattcacaggtggcattccccctgagattgctcagttgaaa
gcccttactgaactcaatgtcagcttcaacaacttaacaggaccgattccccatcgatcagcaatctcacaaacctgcagaagctagacctatccaaca
acaatctcacaggtgtaatcccagctgggttggagaacctgtacttcctttccgcattcaatatttcaaacaatgacctagaagggcctattccaactgga
ggccagtttagtacatttcagaattctagttttgatggaaatccgaagttgtgtggctcttctcttactcatcaatgtggttcagctaaaacacctcagatct
tcacaaaacaaagcaacaagaagatcatttttgcaattgcattcggtgtgttctttggagggcttgccattcttctgttggtcgggcgccttcttgtgtcaat
gaggttaaagagtattacagtgaaaaatagaagggacactgatgaatatgccgagacaacttcattcaactccagttcagaacacacattagtgatgat
gccacgagtcaagggagacaaaaacaagttaacattcccggacattgtaaaagctacaaacaactttgacaaggaacacgtcattggctgcggtggtt
atgggctagtctacaaagctgagctacctgatggctgcaatcttgccatcaagaagctcaatggtgaaatgtgtctgatggaaagagagttcactgcag
aggttgaagcactgtccatggcacagcatgaccatcttgtgccttttgtggggatactgtcttcatggaaactcaaggctcctcatatattcattcatggag
aatggcagcctggatgattggcttcacaatagggacgatgatggtagtccatttcttgactggccaacgagactcaatattgcaaaaggagcaagtcgt
ggcctttcttacatccataatgtttgcaagcctcaaattgtccaccgtgatataaaatgcagcaacatcttgcttgacaaagaattcaaagcttatgttgca
gattttgggctcgcaagattgatccttcccaacaaaactcatgttacaaccgacttggttggcactcttggctacattccacctgagtacgggcaaggatc
ggtggcttctttgagaggcgatatatacagttttggagttgttcttcttgaactgctcacaggaaggcggcctgttacagccctgtcctcatcaaaagaact
cgtaccatgggtcatggagatgaggtcccagggaaagcagattgaggttctggagccaatactccaaggcacaaggcacgaagagcaaatgctgaa
gatacttgaagttgcttgcaagtgtgtgaaccataacccctttcatgaggccagccatcatggaagtagtctcgtgcctggaaaacttagatgctggcacg
taa
>g158639.t1
atggtcgtccatggcagctcgtcgcagcaatgccacgaggaggacgaggcagcgctactggcgttgaacgccggtctgggcagcccataccacttcga
gtcatggagcaccgacacgtggtgctgcgactggtacgacgtggactgcgacaacgtcaccggccgcgtcgtgggcctctccgtgttccaggacagca
acctcaccgccggcgccatccccgacgccgtcgccaacctgacccacctccgcagcctcactctgcaccacctcccggggctcaccggcgccatcccgg
agtccctttctctcctcgccaacctctccgtcctcaccatctcctacaccggcatgtcgggacccgtgccgtcctttttgtcgcagctcacctccctcactttg
ctcgacctctcttacaactcgttcaccggcgccatcccggcgtccctcgccgatctccctaacctcaccggaattaacctcagccgtaaccgcctcaccgg
cacaatcccacccctcctcttcagcaaattatcttcttcttcttctgataatgattcttctgataatgattctgatgaacagcagcagcagcagggaagaag
tgttaatctgtggctgtcgcacaacgagctctccggcagcatcccggcagagttcgcgtcggtgaactttgcgtacgtcgacctgtcaaggaacgccttc
accggcgacccgtcggcggcgctgttcggcgcggggagaaaaacgcagcacctggacctgtcaaggaacaagttcgcgttcagactcaccggcgctg
agctgccggagcagctcagtttcttggacctgagccacaacggcatccggggccgcatcccggagcagctggcaaatctcaccaatctgcagtcgttca
acgtcagctacaacaagctgtgtggccaggtgcccagcggtggggtcatgtccaggttcgacgcttacagcttccagcataacaagtgcctctgcggag
cgccgctccaggcatgccccaaaatgagctga
>g158640.t1
atgaaagctgcgtggggcgatcccaagcagctggcctcctgggcacccgactcctcctcttcctcctgctgcgacggcgactggcaccacgtccagtgc
gacgactccacctccaccaccactttgatgcggcgagtcaccagcctctccatcgacgacgatggtggtggtctcgtcgccggcgccatccccgacgcc
atcgcgggactgactcacctggagaaccttgacctgcgccgcctcccgggtctctccggcccgatccctccggccatcgccaggctgtcgaagctcgcct
tcctcaccatctccaggacggggatctccggccccgtgccgtcgttcctcggcgagctcacctccctcaccgagctcgacctctcctacaactccctctcc
ggctccatcccgccgtcgtcgctgccctgctgcaacctctggaccatcgacctcagacacaaccgtctcaccggatcactcccaaagctcctcttctccaa
tcatcctagtacaagcatcaacaaccgaggagaagaagaagaagaagaagatgatgatgagtacaattatctgatcgacctgaggctatcgcacaacagg
ctctccggacccatcccaccggactggtcgacggtgaggttcgggacggtctcgctgtcgcggaacgcgttgacgggggacccgtcggcggcgctgttc
ggcagggacaagccgctgcagatgctcgacatctcccgcaacgcattcagcttcaacctctccgccgtcgagttgcccgaggacctcgggttcatggac
ctcagccacaacgacgtctacggcggagtgccggagcaggcggcgaatctgcagtacctcaacgtcagctataaccgcctctgcggcgaactgcccac
cggcatggacaccttgttgttcgacaaatacagcttcatcaacaacacatgcctctgcggcgcccctctcggcccctgttaa
>g158884.t1
atgaccaggatcagctgcttcccggccctgctgctcgccggcaggaggaagaagcaccgcaaggtcgcggacgccaccaaggtcggattcaacgatg
ggtcgctcatcaaggttaaaccggtggagttcatcgcggtcgtcgacgagccgggcaaggtcgtccctgcggccgcgctagacggaggtgacgagaa
ggtggcggtgaaggcggaggacaacgaagagttggagttccacgccgccaacaagtccgacgacgccgatgtggcgccggaacaggtcgcaacgg
acgcgtcgccgaggctgatgcggtcgtgctccaacatcgagacgaagcgccccggccccgtgatgccggcccgatcccgctcatacagcgacctgatg
gacctgcccgccggtctcgccacggagatcaaggcaagcccgtttggcatgccggaggcgagcccggcgtcggtgaagacgtcgcgcacggcggacc
gcgtgatgctgaagaagcggtcgtcgagccaggtgctcccgtcccggagcaggaagctctggtggcgcctcttcctctggagccaccgcaacctgcac
cgcccaaggccggcggcgcgcccatcgacgcccggccaacacggcgggtacacctccgacacacacgaggaggaccgcaagaacaagaaggtcgt
gacggtggtggaggagtacgacggctcctcgccggtcccgacgaaccaggtccaggagtgggtcgccttctgcgccgacaattcgctcctcgaccgcg
tcagcgcgtgggtcagcagcatcgagaccgacccctttcacgtcgctccttccgaggacgacgacgacgacgttgaagccggtgatggtggtggccag
tgcgctgtcggggagccgtcgtcggccaagggggcccacggcggcaggtccaggcgcgccgaggaggtggtccaggccaacagcgtcgtgcagtca
ctcaacgccttctccacggtggcgcacatttcagggatggggctcaaggtcgtgcccatgatcgcgcccttctccagcctccggtctgtcaacctctccag
caatttcatcgttcacatctccccaggatcgctgcccaagggcctgcactcgctggatctgtccaggaacaagataacatccatcgaggggctccggga

gctgaccaaactgcgcgtgctcaacctcagctacaaccgcatctcacgcattggtcacggcctgtccaactgcacggggatccgggagctgtacctggc
cggcaacaagatcagcgacgtggagggcctccaccgtctgctgaagctggcggtgctggacctgagcttcaacaagatcacgacggccaaggcgctg
ggccagctggtggccaactaccactccctcctggcgctcaacctcatcggcaacccggtccaggccaacgtcggcgacgatgcgctgcgccgggccgt
gacggggctcctccccagcctcgcctacctcaacaagcagcccgtgaagccgcagcgtagcgcaagggaggtggccacggacagcgtcgcgcgcgc
cgcgctcaccggcggcggcgggcgcagcagccagcgcaggcgcgggtcacggcggctcggctccgggttgggagacgggagcttcaggagccggtc
aaggggcaagaaccatggctcgagctcgatgaagaaatga
>g159505.t1
atggcgcggccgttcatagtggcgatgatggccatggctctgctttcgtgcgtggcggcggcggacgacggggccgtgctgctggaggtgaagcgcgc
gctcgtcggggacccatcaggcccccctgtcgacctggtcctccggctcctcaccggacttctgctcgtgggccggcgtggcgtgcgacgacgcggggct
ccgcgtggtgggcctcaacctctccggcgcggggctgtctggcccggtcccgcgcgcgctggcccgcctcgacgcgctccaagtcctcgacctgtcgga
gaacgcgctggcgggccccgtcccggcgtcgctcggcgcgctgcggaatctcgagcggctcctcctcttctcgaaccacctcacgggcgagataccgg
cggcgctgggcgggctgtcggctctccaggtgctccggctcggcgacaacccggcgctgtcgggcccgatccccgcgggactcggcgggctcagcaac
ctcaccgtgctgggcctggcatcctgcaacctcaccggcccggtcccgaccacgctgggccggctcgcggccctcaccgcgctcaacctgcagcagaa
cgcgctgtcggggcccgtcccgccggagctcggcaacttggggaatctcgaggtgctcgcgctcgccggcaaccagctgaccgggaagattcctcccg
aggtcgggaggctcgccgcgctgcagacgctgaacctggggaacaattcgctcgacggcgctgtcccgccggagcttggagctctgggcgggctcga
gtacctcaacctcatgaacaatcgtctcaccggccgcatcccgcgcgagctcgccgcgctccaacgcgcgcgcaccattgacctctccgggaacctcct
ctccggcgagctccccgcggagctgggccggctgcccgagctcaacttcctcgtcctctccgacaaccgcctgtcaggcaccgtccccggcgacctctg
cggcggcgatgatgaagaggcagagtccattattaccagcagcagcctggaacacctgatgctctccaccaacaacctctccggctcgatcccggcgg
ggctgtcgcggtgccgcgcgctgacgcagctggacctggcgaacaacagcctgtcaggccccatccccgccgcgctgggggagctcggcaacctcac
cgacctgcttctcaacaacaacaccctctccggcgacctccccccgagctcttcaacctcaccgaggtccagacgctcgcattgtaccacaacaagctc
accggcgggcttcccgactccatcggccggctcggcgacctcgaggtgctgtacctctacgagaaccagttcacaggcgagattccggagtccatcggc
cggtgcacgagcctgcagatgatcgatttgttcgggaacaagttcagcggcagcatcccgtcgtccatcggcaacctgacccagctcgtcttcctcgacc
tccgtcagaacgagctgtccggcgcaatcccgccatccctcggcgcgtgcgcgcagctggaggtcctcgacctcgccgacaacgcgctctccggcgcc
atcccggacaccttgggcaacctccgctcacttgaacagttcatgctctacaacaactctctcacgggcccggtccccgacgccatgttcgcgtgccgca
acatcacgcgtgtcaacgtcgcgcacaaccggctcaccggcaccctcctgcccctcacgggcacggcgcggctgctctccttcgacgccaccaacaact
ccttctccggcgcgctcccggcccagctcggccggtcccgctccctccagcgcgtgcgcgtcggaagcaaccgcctcgcgggccccatcccgccctccc
tcggcgccttgccggagctcacgctgctcgacgcctccgacaacgcgctcacgggagggatccccgggacactcgcagagtgcgcccggctcagcgc
cgtggtgctcggccgcaaccggctgtcgggcccggtgcccgactggctcggcgcgctaccgcagctcggggagctcacgctctcgggcaacgcgttca
ccgggccggtccccgcccggatcagcaactgctccaagctgctcaagctctccctcgacagtaaccaaatcaatgggacagtgccgcccgagctgggc
gggctggcgtcgctcaacgtgctcaacctcgctggcaaccagctctccggcccgatcccgacgacgctgtcgaagctgaccaacctctacgagctcaac
ctgtcgcggaactacctctccgggacgatcccccggacatcggcaagttgcaggagctgcagagcttgctggacctgagcggcaacaacctgagcgg
ccacatccccgcgtccttgggctcactctccaagctggaggacctgaacctctcccacaacgcgctggtcggggaggtcccgtcacagctcgccgggat
gagcagcctcgtgcagctggacctgtccagcaaccagctccaaggaaaactgggggaacagttcgcgcggtggccgcaggacgcgttcctcggcaac
gccgacctctgcggcagccccttgcgaggctgtaacgccggcgggggaaacaaccggtcggcgctgcactcggcgtcgatcgcgctgatctctgcggc
cgtcacgctcgccgtcgtccttgtaatcatcattcttgcattactggccgcgcggcacagggggacaagacgccgttccggggaggtgaactgcgcggc
gtcgacgggaacgagctcctcgggcggcacgcaccggccgttggtggtgaagggctcggcgcgtcgcgagttccggtgggacgcgatcatggaggcg
acggcgaacctgagcgaccagttcgccgtgggctccggcgggtccgggaccgtgtaccgcgccgagctgcccacgggcgagaccgtggcggtgaag
cggatcgtcgtcgatgacagcgacgcgctgctggcccacgaccggggaggcttcgcgcgggaggtcaggatcctgggccgggtgcgccaccgccacc
tcgtcaagctgctcggattcgtcgcgtgccacgaccgtggcggcggcatgctcgtctacgagttcatggagaacggcagcctctacgactggctccacg
gcgacggcgaccgcaagaagcgggtgctcagctgggacgcgcggatcagggtcgccgcggggctcgcgcagggcgtcgagtacctccaccacgact
gcgtgccgagggtcgtgcaccgggacatcaagtcaagcaacgtgctcctcgacggcgacatggaggcgcacctcggggacttcgggctcgccaaggc
cgtcgcagagaaccggcaggctgccaaggactgcaccgaatcggcttcatgctttgcaggatcatacgggtacattgctccagagtgtgcttactccctg
aaggcgaccgagaagagcgacgtatacagcatgggcattgtgctcatggagctcgtcacggggctcatgcccacggacaagaccttcagcggcgaca
tggacatggtcaggtgggtgcagtccgggatcgacgcgccgcgcacggtgcgggaccaggtgttcgacccggccctgaagccgctcgcgccgcgcga
ggagtcgtccatgatggagatgctcgaggtggcgctgcggtgcaccaggaccgcgccgggggagaggcccacggcgaggcaggtctccgatctgctg
cgccacgtctcgctcgactactatcgagcaggggagcacaagcgttag
>g159792.t1
atgaagctaatacatttgcttcttgacaacaacaacttctctggcagcattccctcgactttaggccttcttagcccactagaagttctgcggtttgataaca
atcatcagctcactggtccggttcctagcaatatcaacaacctcaccaagcttgcagagcttcacatggaaaacaacaagctgacaggtccactaccag
atttaacaggaatgactgcactcagctttgtggacatgagcaacaacagcttcaatgcatctgctgttccagattggttcaccactttgccatctttgacct
cattgtaa
>g159880.t1
atgctgccgctctgcgtggcggccctgctcctcaccaccttcctgtgtcccattcccacctcgtcggagcccgacgccgacaagtccgcgctgctcgactt
cctcgccgccgtgggccacagcaactcccgcatcaactggcccaccacccctctcgcctgcggcggcgtcacccgggcgggctggacgggcgtgacct
gcagcgccgacggcgcccacgtcgtcgcgctccacctcccgggcctgggcctgtcgggccgcatcccgacgcgcacgctgggccgcctcgccgcgctg
caggtgctcagcctccgcgccaacaacctctccggccctctcccgcccgacctgctcgccctgcccaacctcacgggcctccacctccagcgcaacgcc
ctcgccggcccgctcccggcccagctcggcaccaccaccaccagtctcctccagacgctcgacctctccttcaacgccttctccggcgccctcctccccg
ccgcattctccaacctcacccacatcgtcgcgctcaacctctccaacaactccctctccggccgcgtccctgacctccgcctcccccagctccggttcctca
atctctccaacaaccacttcgatgggcccatccccgaatccctcctcaaattccccgacgccgccttcgcagggaacaacctcacccgcccccactctc

tcctcctctctcgcccaccaccaccactcccacgacaacctcctccaagaggagtagacacacgcgcaccccgcggctcggcgaggcggccatcctcg
ccatcgcggtgggcggctgcgcgttcctcttcgccgtggccgcggtgctcctcatcaccttctgcgccacaagagacaccaccgccaccagcaacagcg
gcgacgacggcatcgtcgtgtccaaggcggcggccgggaaggggaaggagtcgcccgagtccaaggcggtcatcatcggcagcggcagggccggg
gacggcaaccggatggtcttcttcgagggcgcctcgctcgcgttcgacctcgaggacctgctccgcgcctccgccgaggtgctcgggaagggcgccttc
gggacggcctaccgcgcggtgctcgaggacgccaccaccgtcgtcgtcaagcggctcaaggacgtcagcgccgggcgacgcgacttcgagcagcag
atggagctcgtgggacggattcgacacgacaatgtcgtcgagctgcgcgcatactactactccaaggacgagaagctgctcgtctacgattactacacc
agcggaagcatatccaacatgcttcacgggaagaggggagaggacatgatgcctttggattggcaaaccaggctaaagatagcacttggagcagcac
aagggatcgcacacattcacaatgagaacaatgggaaatttgttcatggcaacatcaaggcctcaaatgttttcataaacaagcaccaatacggctgca
tttcggatctcggcttggcggcactgatgaacccaatcaccggcaggtcccgttctctagggtactgtgcgccagaggtcactgacacgaggaaagcat
cacaatgcgcagatgtatacagcttcggcgtcttcatattggagctcctcaccggcaagtcgcctgttcaaataaccagcggaggcaatgaggtcgtac
acctagtgagatgggtgcagtctgttgtccgggaggagtggactgccgaggtatttgactccgagctgctgagataccccaacattgaggaggagatg
gtggagatgctccagatcgccatggcctgcgtctcaaggaccccggagcggcggcccaagatcccagacgtggtgaggatgatcgaggaggtgcgga
ggagcgacaccgggacgcggccttcgactgaggcctctactccggccgtagagggtcagagcagagcagagagctcatctgcggcccccctactga
>g160180.t1
atgcggttgctgttgtggatgcggtggtgggtggcggtcgccggcctactcgccgtgatcctgccgccggccaccgcgacgctctccccggccggaatc
aactacgaagttgtggccttgatggccatcaagatggagctggaggacccccacaacgtgctcgacaactgggacatcaactcggtcgacccctgcag
ctggaggatggtcacctgctccgccgacggctacgtctccgccttaggcctacccagccagagcttgaccggaaagttatcacccggcattggcaatctc
acgagactgcaatccgtgctgttacagaacaatgcgatatctggacctattcctggtaccataggaagactggggatgctaaggactcttgacatgtca
gacaatcaactcactgggagcatcccaaattcaattgggaatctcaagaatctcgactacctgaaattgaacaacaatagcttgtctggagttctacctg
attcacttgccaccattgatggtcttgctctcgtcatcgctggaaatccaatgatttgtggtcttcagtctggagacaactgctcttctgtgtcattggaccc
actctcttatccaccagatgatctgaagacaatatggtactga
>g160501.t1
atgcgcctcttcacgctgctcctcttcctcctcgccctcgccgcgggcgccgacgcggcgagcggcgacgcggacgcgctgctcgcgctcaaggcggcg
ctcatcgaccccaccggcgcgctcgcctcatggaccacctcctccgaccactgctcctgggccggggtcacctgcgctccccgcggcgacaccgtcacc
ggcctcgacctctccgggctcaacctcaccggcacgctgccccccctcgctgtcccgcctccgcggcctgcagcgcctctccgtcgccgccaacgccctct
acggcccaatcccgccgtcgctgggccgcctggcgcagctggcccacctcaacctctcaaacaacgccttcaacggctccttcccggcgccgctggcgc
gcctccgcgcgctccgcgtcctcgacctctacaacaacaacctcaccagcccgctcccgctggaggtcgcgcggatgccggcgctccgccacctgcacc
tcggcggcaacttcttctccggggagatcccgcgggagtacggccggtggacgcggatgcagtacctcgccgtctccgggaacgagctctccggcagg
ataccccccgagctcggcagcctggccagcctcagggagctctacattgggtactacaacagctacaccggcgggcttcccccggagctgggcaacct
gacggagctcgtgcggttcgacgccgccaactgcgggctgtccggggagattccaccggagctcgggcggctgcggaagctggacacgctcttcctgc
aggtgaacgggctcacggggaccataccgtcggagctggggaacctcgagagcctcagctcgctggacctgtccaacaatgctctcaccggggagat
accggcgagcttcgccgagctcaagaacctcacgctgctcaacctgttccggaacaagctccgcggcaacatacccgacttcgtcggcgacctccccg
gcctcgaggtgctgcagctctgggagaacaacttcaccggcggcgtgccgcgccggctcggccgcaacggacgcctccagctgctcgacctctcgtcc
aacaggctcaccggcacgctgccgccggagctctgcgcgggggggcaacctccagacgctcatcgcgctcggcaatgtcctgttcggtgccatccctgac
tcactcggccaatgcaagtccttgagccgcgtccggctcggggagaactacctcaacggctcaattccgaaggggctctttgaattgcccaagctgactc
aagtgaacgggctcacggggaccataccgtcggagctggggaacctcgagagcctcagctcgttggacctgtccaacaacgcgctcaccggggagat
acctgcgagcttcgccgagctcaagaacctcacgctgctcaacctgttccggaacaagctccgcggcaacatccccgacttcgtcggcgaccttcccgg
cctcgaggtgctgcagttgtgggagaacaacttcaccggcggcgtgccgcgccggctcggccggaacggacgcctccagctgctcgacctctcgtcca
acaggctcaccggcacgctgccgccggagctctgcgccggaggcaacctccagacgctcatcgcgctcggcaatgtcctgttcggtgccatccctgact
cactcggccaatgcaagtccttgagccgcgtccggctcggggagaactacctcaacggctcaattccgaaggggctctttgaattgcccaagctgactc
aagtggagctgcaagacaacctgctcactggcaatttcccggccatcgtcggcgttccagcacctaatcttggtgagatcagcctgtccaacaaccagct
cactggcgcattgccggcgtctcttgggaacttctcgggtgttcagaagctgttgcttgatcagaacaagttctctggtgtcataccaccggagattggcc
ggctgcagcagctctccaaggctgaccttagcagcaacaagtttgagggaggtgtaccaccggagattgggaaatgccggcttctcacttacttggact
tgagccagaacaacctctctgggaagataccaccggccatctctggtatgcggatactgaactacctcaacctgtcacggaaccatcttgatggagaga
taccgccatccattgcgacgatgcagagcttgacggcagtcgatttctcatataacaacctgtccgggcttgtgccggggactggccaattcagctacttc
aacgccacttcttttgttggcaacccaggcctgtgcggaccataccttggtccatgcctcccgggtgttgctggtaccaaccaccctgcccatggccatgg
tgggctgtccaatactgtcaagctgctcattgtgcttggcttgctggtttgctccattgtatttgctgctgcagcgattctgaaggcccgctcgttgaagaaa
gctagtgaagcgcgtgtatggaaactcactgcgttccagcgccttgacttctccagtgatgatgtgctggacagcttgaaggaggagaacattatcggc
aaaggtggtgctggaattgtgtacaagggggcaatgccaaatggtgagctcgtagctgtgaagaggctcccggcgatggttcgtggctcatcacatgat
catggcttctcggcagaaatacagacacttgggagaattcggcaccgtcatattgtgcgcctgctgagcttctgctccaacaatgagaccaacctgctag
tttatgaatacatgcccaatggcagccttggggagatgctccatggcaagaagggagggcacctgcactgggacactaggactctgatgcatctgaatg
catgtctgccatcgctggctcatatggctacattgcgccaggtcgatgagaaaagcgatgtctacagctttggtgttgtgctgcttgagctggtcactgga
aggaagcctgtaggtgaatttggtgatggtgttgacattgtccagtggactaagatgatgacagactcgaacaaagagcaagtgacgaagatcctcga
cccaaggctctcaactgttcctcttcacgaggtgatgcatgtcttctatgttgcattactttgcattgaggagcagagtgtgcagcgcccgaccatgaggg
aggttgtgcaaatgctgagtgagcttccaccatcccccaagcaaggcgaagaggtttcaaatgctggtgatggttctgcatctagtcctctacatccagc
accagcaggcacgaatgaagcaccaaccgatgtagtgaaagatcagcaacagcagcaaacaagctcgcagccatctcctcctgatctcatcagcattt
ga
>g162205.t1

atgtttccattgccaatatattccaagtcaaaggaaagaaccaaggtggttgtttggctcgtacccaccggtggccctttctccatccattccgcttccact
ggcgcccgcgagtcaacccgccacattgaccgccgcaagccactgagtggtgagaaccgtagccatccatcgcttaagaccaccaccacggggattcc
tccctcgccacaacgccgccaccctgcacgccggccaatgccgacgccatcgcccgcccccgtgcgcctcctcctcttccagctcttcttcgccgtcctcg
ccaatgccgcgacgccgccgtcggaggcggacgcgctgctcgcgtggaagtccagcctcggcgacccgccggagctgtccacatggaccaacgccac
ctccctctgctctaaatggcgcggggtcgcctgcgactcctccggccgcgtctcctccctccttctccggaacctgggcctcgccggtggcctggacaagc
tcgactcctcggcgttcccggcgctggccgagctcgacctcaacgggaacaacctgtcgggccccatcccggcgagcctctcgcgtctgcgttacctctc
caagctcgacctcggcagcaacagtttcgacggcagcatcccgccgcagctcggcgacctcacgggcctcgtcgagctccgcctctacaacaacaacc
tcgtcggcgccatcccgcaccagctcagccggctcccacgcatcgtgcacttcgacctgggctctaactacctgaccaacccggattacagtaggttctc
agcgatgcccaccgtgcagttcctctcgctctacctcaactacctcaacggcagcttcccggtcttcgtgctcggctgccgcaacgtcacctacctcgatct
gtcgcagaacaccttctccgggccggtgaccgacgcgctgccggcgaggctgcccggcctgcggcacctcaacctgtcctccaacgctttctccggccg
cattccggagtcgctcgcgaggctcacggggctccaggacctgcgcatcggggccaaccgcttcaccggcggcgtgccgaagttcctcggctcgatgc
cggagctgaggatcctcgagctcggcgggtgccctctcggcgggccgctcccgccggtccttggccggctgcagaagctgcagcggctctatgtgagct
gggccgggctggtgtcaactctgccgccggagatgggcaatctcaccagcctctatttcatggacctctccatgaacagcctctccgggagcctgccgg
cgacgttcgcccggatgcggaagatgcgggagttcggcatttcgtcgaacaggctcaccggccagatcccgggcgtcctcttcgcgagctggccggag
ctcatatcgttccaagtgcagaacaactcgcttaccggcgagattcccccagaggtcggcaaggcgacgaagctcaacttcctgtacctcttctccaaca
acctcacgggccccattccgccggaggtcggtgagctggtcaaccttctggagatggatttgtcggtgaactcgctcacgggccccatcccgagctcgtt
gggcaacctcaaggagctcaccaggctcgcgctctttttcaacaatctcaccggcgctataccgccggagatcgggaacatgacgtcgttacaaatcct
ggacgtcaataccaacaatctggaaggcgagctgcccgttacaatcacgaggctgaggaacctccagaacctcgccttgttcgacaacaaattcaacg
gcaccgtgccttcagacctcggcaaggggcttgctttggttgacgtcagcttcgccaacaacagcttctccggcgagctgccgcaggacctctgcgacg
gcctcatgctgcagaacttcacggcaaaccataacagtttcagcggcaagctgccaccgtgcctgaagaactgcacggagctgatccgggtccgattg
gaggggaacgatttttaccggtgacatatcggaggcgttcggagtccaccaccagttgaattacctggatatctccgggaacaagctaacgggccagct
gtcgagtgattggggtcaatgcactaatgtcaccaagttgcgcatggatgacaatcgcatatcgggcggcattccggcagcgtttgcgagcatggcgag
cttgcgagacctcagtctggctgagaacaacttcactggcgccattccacctgagctgggtggtctcagcttcttgttcaaactcaatctgagcggtaact
ccttttcagggccgattcctgcaaatctgggcaacaactccaaacttgagaaagttgatttgtcagggaacatgcttaatgggacgataccagtacacat
tggtaagcttagtcaactaacctatcttgatttgagcaagaacaagttgacggggcagataccaagtgagctaggcaatctggcccagctgcagagcct
gcttgatctaagcagcaattcgctgtctggtgatatccccctcaaatctcgtgaagctgacgagtctgcagaaactgaatgtgtcacacaacgacctcacc
ggctctatcccagcaggtttctctcgcatgtcaagcctggagacggttgatttctcttacaatcagctcaccggtgaggtaccatcaggcaatgctttcaat
aacactttggcttatgtcgggaatgtcggtctctgtggaaatgtgcaagcatgccgtaggaggccccgtgagcagaaggtgctggaagctaacacgaat
gatgcttatgagtccatgatctgggaaaaggagagcaagttcacgttcctcgatattgtgaacgccacagacagtttcaatgaatccttctgcattggaa
aaggagggttcggtagcgtgtacagggctgagctcccaagtggccaggtcgtggccgtgaagcgtttccatgttgcagagacaggagacatatctgaa
gcgagcaagaagagctttgaaaacgagatcaaggcactgacagaggtccgtcatcggaatattgtgaagctccatggcttctgcacgagcggagatta
catgtatctggtgtacgaatacttggagagaggcagcttggggaagacattgtatggggaggaaggcaagaagaagctggactggggcatgagggtg
aaagtgatccaaggggttgcccatgccatggcctacctgcatcatgattgcaacccatccattgttcaccgtgacatcaccgtgaacaacatcctacttg
agtcagaatttgagccacggttatctgattttggcacagcgaagctgcttggatcggcttctacaaactggacttctgtagcaggatcatatggctacatg
gctccagaactggcatatacaatgagggttactgagaaatgcgacgtttacagctttggagtcgttgctctagaggtgatgatggggaaacatcctggtg
acctcttaacctccttaccggctatgtcatcatcaaaagaagatgatctgttactcaaggacatactagaccagcggttggaccctccaacggcatgccg
taggaggccccgtgagcagaaggtgctggaagctaacacgaatgatgcttatgagtccatgatctgggaaaaggagagcaagttcacgttcctcgata
ttgtgaacgccacagacagtttcaatgaatccttctgcattggaaaaggagggttcggtagcgtgtacagggctgagctcccaagtggccaggtcgtgg
ccgtgaagcgtttccatgttgcagagacaggagacatatctgaagcgagcaagaagagctttgagaacgagatcaaggcactgacagaggtccgcca
tcggaatattgtcaagctccatggcttctgcacgagcggagattacatgtatctggtgtacgaatacttggagagaggcagcttggggaagacgttgtat
ggggaggaaggcaagaagaagctggactggggcatgagggtgaaagtgatccaaggggttgcccatgccatggcctacctgcatcatgattgcaacc
catccattgttcaccgtgacatcaccgtgaacaacatcctacttgagtcagaatttgagccacggttatctgattttggcacagcaaagctgcttggatcg
gcttctacaaactggacttctgtagcaggatcatatggctacatggctccagaactggcatatacaatgagggttactgagaaatgcgacgtttacagct
ttggagtcgttgctctagaggtgattatgggaaaacatcctggtgacctcttaacctccctaccggctatctcatcatcaaaagaagatgatctgttactca
aggacatactagaccagcggttggcccctccatcgggtgagcttgcagaagaaattgtgttcgtcgtgaaaattgcgcttgcttgcacccgggcaaatcc
tgagtcacggccttccatgcgatcagtggctcaagagatctcagcacatacgcaggcctatctttctgaaccgttccgactgattacagtaagcaagcta
acagactaccagaagtga
>g162206.t1
atggcgaggagcatggacctcgacgacgacgacgatgacgtaagcttcgccgacgacgatggttactactccgacaagccggcacgacgcctctacg
acgaaagattcaacgtgctgaacagcaactcctccaggcgcaacagctcctccggcgcggctttctgcaagctgctgtccctgcagatcctggacctatc
caacaacaagctcaccggggagctgccggactgctggtgggacatgcaagctctgcagttcatggacctatccaacaactccttctcaggtgagatccc
tgcggctccgccgggccacaactgtactctcgagtcgctccatctttccggcaacggcttcaccggcatcttcccgtcggtcgtcaatggttgccgtgatct
ggcaactctggatattagtaacaatcagtttttcggtggtattcctccatggattggctatggcggtgttcgatcgctgaaaatccttagccttgggtccaa
caacttcataggcgcggcgccggagatcgcccacccgctcctcgccgccggcgacgatgaggtgctcaacatcgcggcggcgctgctcagcttcttcct
gatccagacatggaagaaggcgctcgagaaggttggcttcgtctccaggcccttcaccaagcaggagaacaccgttatctag
>g162207.t1
atgtcgacgccaccagcgcgcctcctccacttcctcctccctctgtcccttgccatcatcctcaccgatgccgcgacgccgccgtcggaagcggacgcgc
tgctcgcgtggatgtccagcctcggcgacccgccggaactgtccacatggaccaacgccacctccctctgctctaaatggcgcggggtcacctgcgact
cctccggccgcgtctcctccctcagtttccggaacctgggcctcgccggcgggctggacaagctcgactcctcggcgttcccggcgctggccgagctcga

cctcaacgggaacaacctgtcgggctccatcccggcgagcctctcgcgcctgcgctccctctccaagctcgacctcggcagcaacagtttcgacggcag
catcccgccgcagctcggcgacctcacgggtctcgtcgagctccgcctctacaacaacaacctcgtcggcgccatcccgcaccagctcagcaggctccc
gcgcatcgtgcacttcgacctgggctccaactacctgaccaacccggacagcaggttctcagcgatgcccaccgtgcagttcctctcgctctacctcaac
tacctcaacggcagcttcccggtcttcgtgctcggctgccgcaacgtaacctacctcgatctgtcgcagaacaccttctccgggccggtgaccgacgcgc
tgccggcgaggctacccggcctgcggcacctcaacctgtcctccaacgctttctccggccgcatccccgcgtcgctcgcgagcctcacgatgctccagg
acctgcgcatcggggccaacagcttcaccggcggcgtgccggagttcctcggctcgatgccggagctgaggatcctcgagctcggcgggtgcccgctc
ggcgggcggctcccgccggtccttggccggctgcagaagctgcagcggctcgatgtgagccgggccgggctggtgtccgctctaccgccggagctggg
caatctcaccagcctcgttttcatggaactcgacatgaacagcctctccgggagcctcccggcgacgttcgcccggatgcggaagatgcgggagttcgg
catttcgtcgaacaggctcaccggccagatcccgggcgtcctcttcgcgagctggccggagctcatatcgttccaagtgcagaacaactcgcctaccgg
cgagattcccccagaggtcggcaaggcgacgaagctcaacttcctgtacctcttctccaacaatctcactggccccatcccgtcggaggtcggcgagat
ggtcgaccttctcgagctggatttatcggtgaactggctcacggggcccatccccagctcgttgggcaagctcaagcagctcacaaggctggcgctatat
ttcaacgggctcaccgacgctataccaccggatatttggaaacatgacggcgttgcaaatcttggacgtcaacaacaacaatctggaagcggcaccgt
gccttcagacctcggcaaggggcttgctttggttgacgtcagcttcaccaacaacagcttctctggcgagctgccgcagaacctctgcgacggcttcatg
ctgcaaaacttgacgataaatcacaacaatttcagtggcaagcttccgccatgcctgaagaattgcacacggctcattcgggtccggctggaggggaac
cacttcaccggcgacatttcagaggtgttcggtgtccaccccaagttgaactacctagatgcatcagggaaccaattcacagggaggctgtcatgggac
tggtcacaatcaagtctcatttttttagcatgaaggcaaactgcatatctgaactccagcaggcttag
>g162494.t1
atgtcgccatcctccgttgccgcgctcctgtccctgctcctgcttgtactactacccgtggctgcgacggtcgcggcggcggcggccatggacccggccg
agcgggagacgctgttccgcgtgatggaggtcgtctcctccgaccgcgactggcgcaccgagagccctgaccccctgcgacgcgccgtggcccgggctg
gagtgcaagccatcggcggggaccccggcgcgcatgcacgtcacgcggctggacttcggcgtggagcccaacccgacgtgcagggacgcggcgacg
ttcccgcccgacgcgttcgccctgcctcgcctccagtccctcttcttcgtcgactgcttcaccaacccggccgccaccaccacgctcgtcctgccaccgcc
caccaacctctccgcggcgtccaccctgcagcagctcagcatccgggccaacccggcactatccggcacgcttcccccgagctggccagcctccggtc
cctccaggtgctcaccgtctcccagaacccgctggtccgcggcgggatcccgccgggcatcggcgcgctcacctcgctcgtgcgcctcgacctcagcta
caactcgcttaccggtccggtcccgagcgaggtcggcgagctgaagaacctggtcgggctggacctcagctacaactccctgtcgggccccatcccga
gccggatcagcgagctgcggcagctgcagaagctcgacctcagcgcgaacaacctcaccggcgccatccccgacaccgtcgccaacctcacttccctc
acgttcttcgcgctcagcaacaacggcctgaccggccgtttccctccgggcatctccgggctccggaacctgcaatacctgatcatggacaacaacccg
atgggcgtcccgctgccgtcggagctcggcggcctcgcgcgcctccaggagctgcggctggcgggctccgggtactcgggcccgatcccggcggcgtt
cgggcggctcgccagcctgacgacgctgtcgctgcaggacaacaacctgacggggcccatcccggcggcgctcagccggctgggcaggatgtaccac
ctcaacctcagcaacaacgcgctcggcggcgccgtgccgttcgacggcgcgttcctgagccggctcggccggaacctcgacctcagcggcaactcggg
gctgtgcatggaggaccggagcgtcctgcggggcgtcggcgtcggggtcggctcatgccgcggtggtgatggcagcggcgccggcggtgacacgtcg
gcggcggcggcgcgtcggggtacgggacgaggcgtgatgacggcgtggggttccggttccagagacggtttcccgttcgggctgctcggcccggcgtg
cgtgctcgtgttgcccgtcgtttttgccctgaacgagcacgcgccgtattga
>g162495.t1
atgtcgccatcctccgttgccgcgctcctgtccctgctcctgcttgtactactacccgtggctgcgacggtcgcggcggcggcggccatggacccggccg
agcgggagacgctgttccgcgtgatggaggtcgtctcctccgaccgcgactggcgcaccgagagccctgaccccctgcgacgcgccgtggcccgggctg
gagtgcaagccatcggcggggaccccggcgcgcatgcacgtcacgcggctggacttcggcgtggagcccaacccgacgtgcagggacgcggcgacg
ttcccgcccgacgcgttcgccctgcctcgcctccagtccctcttcttcgtcgactgcttcaccaacccggccgccaccaccacgctcgtcctgccaccgcc
caccaacctctccgcggcgtccaccctgcagcagctcagcatccgggccaacccggcactatccggcacgcttcccccgagctggccagcctccggtc
cctccaggtgctcaccgtctcccagaacccgctggtccgcggcgggatcccgccgggcatcggcgcgctcacctcgctcgtgcgcctcgacctcagcta
caactcgcttaccggtccggtcccgagcgaggtcggcgagctgaagaacctggtcgggctggacctcagctacaactccctgtcgggccccatcccga
gccggatcagcgagctgcggcagctgcagaagctcgacctcagcgcgaacaacctcaccggcgccatccccgacaccgtcgccaacctcacttccctc
acgttcttcgcgctcagcaacaacggcctgaccggccgtttccctccgggcatctccgggctccggaacctgcaatacctgatcatggacaacaacccg
atgggcgtcccgctgccgtcggangaatctttgacactgcgactttctgtcttatacgtatag
>g162563.t1
atggctgcgaaggcggcgggagccctggcgttggcgctcatcctcgcgctcgcgggggccaactccgagggcgacgcgctctcggcgctgcgccgca
gcctgcgggaccccggcggcgtgctgcagagctgggaccccacgctcgtcaacccttgcacctggttccatgtcacttgcgaccgcgacaaccgcgtca
cccgcctagatcttggtaacctgaacttatccggtcatctggtgcctgagctagggaaattggagcatctgcagtatctggagctttacaaaaatgatatt
caaggaacaatcccatctgagcttggtaacttgaagaatctgataagcttggacctgtacaagaacaacatttcagggactatccctcctacccttggaa
atttgaagtcccttgtattcttctttgagaacaacccacgcttggaaggtccagagctgcaaggcctggcttggtcggtaaacgaaataccgaccacccc
tgaggccccgacccaacctggacgttgctgctcctcgccagcgccggcgcgcttcaaccacaccgcattcaccggcgttcgtctccacgctctgcgcgcc
gcggtgggcagcgtcctccgtctcgtcgcggaggcgcaacttgttggccggcccggcagcaacgccgtggcgtccacggctgttgcgggcggccttccc
gccatggccaacggcggcggaggagatatcggggagttcttcgattacatggggcggttgcagaaatacgaactctccggcattccacgcggcgtagc
caccgcctccgagtgtgaagtatccttctccgcacttgttgtgatcccccacccacttcccggttgttcatattgccggaccaaggggaaaggttcagctg
ctgcattcctgttcaatgagtatcatgacggagcaacactacaattcctgcctgttattgatgcaatgacttag
>g163184.t1
atggcctctccaaccctactgaacctgctttcgctgctctttctactgctgctcggaggagtcgtcgcctcggcctccggcggcggcgagcgcgaggcgct
gctgcagttcaaagcggcagtttcctccgaccccggcggcctgctgcgggactggacccaggcgtccgcggaccactgccgctggcccggggtgtcgt
gcggcgcgggcggcgaggtggtcgcgctcaacatctcctcctcgcccggccgcgtgctcgcaggagcgctgtccccggccgtagcagcgctgcggagc
ctccgcgtgctcgtgctgccgtctcacgcgctctccggcccgctccctcccgcgatctggtcgctgcgccgcctccgcgtgctcgacctctccggcaaccg

tctacaggggggagatccccgcggcgctcgcctgcgccgcgctccagacgctggacctcgcctacaaccagctcaacggctccgtgcccgccgcgctcg
gcgcacttccggggctgcgccgcctttcgcttgcctccaaccgcctcggcggcgccatacctgacgaacttggtggcgccgggtgccgcagcctgcggtt
ccttgacctctccgggaacctgctcgtcggcggcatcccccgcagtctggggaactgcagcgagctggtggcgctcctgctgtcgtccaacctgctggac
gatatcatcccgccggagattggccggctcaggaacttgcgggctttagacgtctcgaggaacagtttgagcgggcctgtgccagcggagcttggtggc
tgtgttcagctctctgtgcttgtgctgtccagtccatatacaccgcttggtgattcaaattcgtctggttatgaggatgcggacgacttcaactacttccaag
gagggattccggatgctgtcgcttcattgccgaagctgagggtgctgtgggcgccgagggctacattggaggggaaattgccagagaagttggcgatg
cagggggcctatgcttttctggctgatgggaatcatcttgagggtcagttgcagcctggcttctttgataagtgcaacagctcaaagggcttcattgtgga
agtgagcaacaacttgataaatggtgcaattcctgcagagattggctcactgtgcagttctcttgttgtacttggtattgctgagaagttggcgatgcagg
gggcctatgcttttctggctgatgggaatcatcttgagggtcagttgcagcctggcttctttgataagtgcaacagctcaaagggcttcattgtggaagtta
gcaacaatttgataaatggtgcaattcctgcagagattggctcactgtgcagttctcttgttgtacttggtattgctggtaatcaactttcaggtgttattcca
tcaagtatcgggcagttaagttatcttgtgagcttggatttgagtaggaaccaccttggtggtgcaattcctacgtctatgaagaacttgccacacttgga
gcgcctctctgtggctcataactttctgaatggcaccattcccacagacattaatcagttgcgcgctcttaaggttttggacctgtcatcaaaccttatcaca
ggggtgatccctgatgcccttgctgacctaaaaaatctcactgcactcttccttgataataataagcttactgggaaaatcccgacaggatttgctaactc
agcatcacttaccatgtttaatgtgtcgtacaacaatttgtctggtccagtgccaacaaatggcaacacgctgctcgtcagggtcgtggtttga
>g164146.t1
atgccgccgcccgccgcatcgaggctcgcgcttctcgtacgacgcctcgccttcttcgcgtccctgctgttgttgctggtgtccccgtgccactgcgtgaac
gagcaaggccaggcgctgctgcgatggaaggcgtcgctgcggccggcagacggcggcgcgctggcgtcgtggcgcgcgtcggaccccaacccgtgc
gagtggtccggcgtgacgtgcgacgcgcgcggcggcgtggccgagctgagcatcacgtcggtggacctgggcggcccgctcccgtcgaacctgcagc
cgctggcgccgtcgctcaagacgctggtgctgtcgggcaccaacctcaccggcacgatcccgccggagctcggcgacctcgcctcgctgaccgagctt
gacgtcagcaagaaccagctcaccggcgcgatcccgccggagctctgccggctcgcgaagctcgagtcgctggcgctcaactccaactccctgcgcgg
cgccatccccgacggcatcggcaacctcaccggcctgactcacctcacactctacgacaacgagctgagcgggcccatcccgcccggcatcggcagcc
tcaagaatttacaggtgctccgcgccggcgggaaccccgggttgaagggcccgctgccggcggagatcggcgggtgtagcagcctcaccatgctcgg
gctcgccgagaccggcgtctccggcagccttcccgacacgatcgggcagctcaagaaggtcgagaccatcgccatctacaccaccctgctctccggcc
gcatcccggagtccatcggcaactgcaccgcgctcaccagcctgtacctctaccagaactcgctctccgggccgatcccggcgtcgctcggcaatctcc
ggaagctacagactctgctcctgtggcagaaccagctcgtcggcgcgatccctccggagcttggacagtgcaaggagctcacgctcatcgacctgtcgc
tgaattctctcaccgggccgattccggcgagtctcggggaattgcccaacctccagcagctgcagctgagcacgaaccagctaacaggcgccatccctc
cggagctgtccaactgcacgtcgctgacggacatcgaggtcgacaacaaccagctgtcgggggaggtccgcatcgacttctcgcggctgcgcaacctg
acactcttctacgcgtggaagaaccggctcaccggccgcgtgccggccggcctcgccgagtgccccggcatgcaggcggtggacctgtcctacaacaa
cctcaccggctccattcccaaggagctctttggcctccaaaatctcacaaagctgctcctgctcaacaacgagctgtccgggctcgtcccaccggagatc
ggcgactgcaccagcctctaccgcctccggctgaacggcaaccgcctctccggcacggtgccggccgagatcggcaacctcaagagcctcaacttcct
cgacatgagcgacaaccacctcgtgggccccatacccgcggccatctccgggtgcgccagcctccagttcctcgacctccactccaacgcgctgtcggg
ttcactccccgacacgctcccccgcagcctccagctcatcgacgtctccgacaaccagctcgccggcccgctgagccccggcatcgggtcgatgccgga
gctgaccaagctgtacctgggacggaaccggctcaccggcggcatcccgccggagctcggcgcctgcgcgaagctccagctgctggacctcggcggc
aacggcttctccggcgccatcccgcccgagctcggggagctcccgtcgctggagatcgcgctcaacctcagctgcaaccggctctccggcgacatcccg
ccgcggttcgccgcgctcgacaagctcgccagcctcgacctgtcgcgcaaccagctgtcggggacactcgccccgctcgccgcgctgcagaacctcgtc
acgctcaacgtctcctacaacggcttctccggcgacctccccgacacgcccttcttccagaagctcccccctcagcgacctcgccggcaaccggcacctcg
tcgtcggcgacgggtcctcccggcacggcggcgcgctctcctcgctcaagatcgccatgtcagtgctcgccgtgttcagcgcggtcctcctggcggcggc
gacatacatgctggcccgcacccaccgcaataataccggcgccggcgggggaaagatggtccacggcgagggcggcccgtgggaggtgacgctgta
ccagaagcttgacatcgccatggacgacgtgctgcgggagctgacgtcgtcgaacgtgatcgggacggggagctcgggggtggtgtaccgcgtggag
acgcccaacgggtacaccttcgcagtgaagaagatgtggtcggcgtccgacgagtcggcctccgccgcgttccgcagcgagatcgcagcgctggggtc
catccgccaccgcaacatcgtgaggctgctgggctgggccgccaacggcgggacacggctgctgttctacacctacctccccaacggcagcctcagcg
gcctgctccacggcggcggtgggtcggggaagggcagcgccaacgacgagtgggccgcgcggtaccacgtggcgctcggcgtggcccacgccgtgg
cctacctccaccacgactgcgtgccggccatcctgcacggggacatcaaggccatgaacgtgttgctgggccccgcgtacgagccgtacctcgccgact
tcgggctcgcgcgcgtcttgtcatcggcgagccccaagcagcagctcaacgacaccatcaggcagccacgcatcgccggctcatacggctacatcgcg
ccagagtacgcgtcgatgcagcggatcagcgagaagagcgacgtgtacagcttcggcgtggtggtgctggagatcctgacgggcaggcacccgctgg
accccacgctcccgggcggcgcgcacctggtgcagtgggtgcgggaccacctgcaggccaaacgcgacgccgccgacctcctggacccgcgcctcac
cgcgcccggcgccgccgccgccgacgtgcaggagatgcgccaggttctgtccgtggccgcgctctgcgtcgcgcaccgcgccgacgaccgccccgcc
atgactgacgtcgtggcgctgctcaaggagatccgccgacccgccgccgccgcaggcgacgacgccacgaagacgcattcgcagccggcacccacg
cagccggtgtcgccggtgcggagcccgcactcgattggccactcgtccagctgctccttcgcctga
>g164904.t1
atgatgcgctggtcggaggcggcggcgcccagtctctttccagtcctcctcgcgttgcagtgcggggtggtcttcctgcagtgctccgccgcggcacccg
tggccgccgatgtgtcggcgctcatggcattcaaacgcgcgatcatcgatgatcctcggtctgtgctctcggactggactgacgcggatgggaacgcct
gcgactggcacggcgtgatctgctcctccccgcagggctccgtcatctctctgaaactgtcaaactcatctctcaagggatttattgcgccggcacttgga
cggctgagtttcttgcaagagctatatttggatcataacctgctttttggcacaatcccgaaacaaataggctcattgaggaacctgcgagtattggattt
gagttctaatcgactcgctggccctatcccttctgagataagtggcttgagcagcgtcagcataataaatttccattccaatgggttgaccgggaatatac
ctccggagctgggcaaactgcaaaatcttgttgaactgaggttggacaggaatagactcaagggatcaattccaggaagcactgctgccggtttctctc
ctgctgcaaataatggggaaggtgcccagttatcttggctcagacgaatgaaaatagccatcggtattgcccaaggtctaagaaataacataactttgtc
ttttgttgatcttccttccggatttgatgcatttatctatcatgtatacatcctgtccacctggctgctggctacttcagtagcaaaacctcttaccctggctgc
caagtacctccagcagccagaggagattgggaagctagtggaccccgaactgacgaatgtgaggactgaggacctcgctgtcctttgcagcgtggtga

gccggtgcatcgaccctgacccttccaagagaccgtcgatgcagatcatcacaggcgtgctggagaacgggatcgacctgtccgcggccgccatcctc
aaggagtcttctctagcatgggctgagctggccttggcgttgtag
>g165397.t1
atgtcgcccttcctactactcttcctgctcgttgccatcccgctcgccacctcggacacctcatccagcatcctcctttccttcctcgccgccctgccgtctga
atcacagcagctcctcctcccttcgtggcgagccaatgctggcaactcgacaccgggtccacattgcgcgtttctcggcgtcacgtgctccaccgttggc
gccgtggccgcgctcaacctctccggcgtgggcctgtccggcgagctatcggagtccgcgtcgctcctctgcacgctgccggaactctccgcgctcgacc
tcagctccaacaacttcaacggcgccgtgcccgccacgctcgccgcgtgctccggcgtcaccacgctcgtcctcgccgataacaggctctccggcggca
tcccgccagagctgttctcctcgctccagctccgcgacgtggacctcagcttcaacgcgctcgccggcgagatcctaatggcgccctcctccgccgacaa
atcggttctcgagttcgtcgacctcaagaacaattccctctccggcgccattcccgcacagctcgccgcgctccctgagctcagacaccttgacctgagc
atgaaccaactgtccggtccaataccagacttcccaactcaatgcaagctcgaatccctccagctctacaacaatgagatagccggtgagcttccccgc
agcctcagcaactgcggcaatcttacagttctgttcctatccttcaacaagatcaccggtgcagtgccagatttcttcgcatccctgccgttgctcaaaaa
gctttatctcgacgacaatgccttcaccggggagttgccggtgagcatcggtgagcttctcaatttggagaagctggtggcttcgacaaaccggttcacc
ggaccggtccccgggtcaattgggaagtgtcagtcgttgacgacactaaggctgaacgccaaccagttcacaggttcaattccccgccgtatgggtaat
ctcagcagattagaatggttaagtctcaaggacaattccatcactggctcaattccactggaactaggaaagtgcaaggaattggtcatgcttgaactcc
acaacaacaacctttccggaatcataccacccgagattaccgagctcagaaaattgcaggtgatgtccttgttcaagaacaggctccagggacctgtgc
ctgcggcattgtggcaaatgccggacatggaggagttggcgctctacaacaacagcctaagtggagaggttcctgcacaggttactcttatgaggaatc
taacagagttcaacttagcatttaacaacttcactggtgagctcccacaagctctggggttgaacacaacacaaggactcgttcggattgacctcaccgg
taacaacttccatggcgcaatcccgcatggtctctgcactggagggcgccttaatgttcttgatcttagattaaaccaatttagtgggagcattccgggtg
agattctcaagtgtcagtctctctcgagggtccgtcttggaaacaatcagttcagtgggatcttgccgtcagatttggggataaacactggctggtcgttc
gtggaattgagtggcaatcggtttcaggggagaataccaggtgtgtttggtttatggcgcaatctcacgatgcttgatttatctgaaaatagcttctctggt
ccaataccacgggaacttggagctctaagcaaccttgggactcttcgcttgtcatcaaacaagctgtctggcccgataccgaaggaattgggaaattgc
aagagccttgtccgtttggatctcgaaaataatttcctcaatggaaggatacctgcagagatcatagcccttggtagcctgcagaatctaatactcagtg
ggaataagcttactggacaaattccagattcttttactgcaaaacagggccttcttgaagtgcagcttgggggcaattcactggaaggtgccattccaag
cagcttagggaatcttcaagtcagcctgcgactgctaaacattagtaacaacagattgaacaagcaaataccaagtagcctcggcaaccttcagaatgt
agaaacacttgacttgtccgagaactccttatctggtccaattccatctcagctcagcaacatggatgcacttacttttgtgaacgtgtcattcaatgatctc
tctggtcaacttcctaatggctgggataagcgagcacaacaggcacctgaagggtttctggggaaccctcaattgtgcatacactccagcaatggccctt
gcttcaggaatttgtcaaggaaaaacagactgaggaatacacatattattgtggcactaatcgtatcaactctcgctgtcatggcttctgggctttgtgct
attcactacatcgtgaagagatcacagagcctagcagcaaatcgtgtttcaattcgtggtttggacacgacagaggaattaccggaagacttgacctat
gatgacatccttcgtgccacagataattggagtgagaaatatgtgattggaagaggtcggcatggcacggtctaccggacagagtttgctcctggtaga
cttagggcagtcaagacgattgatctttcactatccaaattccccattgagatgaagattcttaacatggtaaagcaccgaaacatcatcaagatggagg
ggtactgcatccgtggtaacgttggcctcattttgtccgagtacatgcccgaggggacactctttgagcttttgcatgaaaggaaaccgcaagtgcctctt
gactggaaggtccggtatcagatttccctcggtacagcacagggcttatcctatctacaccatgattgtgtgccaatgattgttcatagggatttcaaatc
aagcaacatattaatggataccgagttggtgcctaagattgcagactttggaatggggaagatagtaggagatgaagatgtagatgcaacagtttccgt
catagttggcactctcggctacatagctccagcatggatacaacacaagatcaacggagaagagtga
>g165518.t1
atggccgccggaaccgggagaagaagaagaagctgcagcagcagccacgctctgctcatcctgctcccgctgctccttgcactggttccgccgtcgac
gccgctgcagtcctcgcagacctggtccctcctcaagctccagcagctgctcaactaccccgccgtgctcggcacctggcacaaccacaccgacttctgc
tacggcggcgactacaagaccgcctccgccttcgtcgagtgctacggcgacagcgtcacgcagctccacatcatcggcccctccacctccaccccgcca
ccaccactccccaacacattcagcatcgacgccttcttcaccacgctctcccgcctcccggacctccgcgtcctcaccctcaccggcctcggcctgtgggg
cccgctcccggggaagctgtcccgcctctcctccctcgagatcgtcaacgtcagcggcaactacctctacggcgcgctccccgacggcctctcccgcctg
agcaacctccaaaccctaatcgccgacgacaacctcctctccggctccctccctccatggctcttcaccctcccttccctcgccgtcctcagcctccgcaa
caactccctccacggcgcgctcccttccaccctccgcgacgcgggggcactccgcgcgctcaccctcgccggcaacaacctcaccggcgacgtgccgg
atttaagcgcgctgaggagtctgcaggccgtggacctcgccaacaactcgttgggccccgcgttcccgcggttgggccggaaggtggcccgcgtcgac
ctcagcgggaacaggttcggcgacggcctgcccggcgacgaggtgggctcgttctacctcctcgaacgcctcgacgtgtcgcggaaccgcctcgtcgg
cccgttcccgccgcagctgctcgcgctgccctccgtcgagttcgtcagcgtcgcggggaaccggctcacgggactgctcgcggcgaacatgacgtgtgg
ggacaacctccggttcgttgacctctcgaacaacctcctcacggggaccctccccacctgcctcaccaccagagccgcaagctcgggaggcaagaatg
gtgatgattccaaggtggtgctcgccgcggcggcgaactgccttacgggcggcggcggcggcggcgacgggacgcagcatccggcagcgttctgcca
gaaccaggcgctggcggtgggcatcgtgccgcccgacgaggggagagggaaggggcgcgccgggaaaggggccgcgataatggcggggatcatc
gcggcggcgagcgccggcgcggtggtcgtcgccgtcggggtgttcttcggggtgaggaaggtggccgtcacgcgggccaaggcgcggccgcccaga
aggctcgtcgagcacgcgtccagtgcgtacccgtcgcagttcttcgccgacgcccgttacatttctcagacggtgaagctgggagctctgggcattccag
cgtacagatcgttctcgctggtggagctggaagctgcaaccaacaatttcgaggtgtcgtgtctcatgggacaggacgcgcacggccagatgtaccgcg
gaacgctgagcaacgggacgccggtgacgatccggtcgctgcgggtgaagcggagccagacgtcgcagagcttcaaccggcacatcgagatgatctc
caagctgcgtcaccggcacctggtcagcgcgctgggccactgcttcgagtacaacctcgacgactccaccgtcacgcagctctacctcgtcttcgagtac
gtgcagaacggcaacctcaggagcaggatctcgcaaggaacagaggggaggaagctctcctgggtacagaggatatcgaccgccatcggtgtggcc
aagggcatccagttcctgcacggagggatcatgcccggactgttcgccaacaacctcaagatcaccaacatcctcctggaccagaatcttgtcgccaag
atcggcagctacaacatccccatcctagccgaaacagcaaaatcagagggtggaagcaagcatgcacctgatagggtacccaacggtgataagatgg
acatatacgatttcggtgtgatactgcttgaggttgtatcaggcagacccatcacatccatatacgaggtggagatcatgaaagagcagctgcaaaagg
cgcagacgtcagaaggcccgacgcggcgtcggagcttcgtggaccagtcggtgagcaaggcgtgctccgacgagtcactgcggacggtgatggagat

ctgcctgcggtgcctggccaaggaggcggcgcagcggccgtcggtggaggacgtgctctggaacctgcagttcgcggcgcaggtgcaggacgactgg
cgcggggactcgcggagcagcgaagagtcgccgctctcgccgtcgcagatcccaagggagtcccactccagccggtcggcagcagatgacgcctag
>g165564.t1
atggagccgccggcgccgcgcctcctggccgcggtcctcctggccgcgtgcctctcggcctccgtcgtcgtgcaggccgtcaccagcgccgaggcctcc
tacatcgcgcaccgccagctcctcgccctcaaccaatcgaagtccaccaccatcgacggcatctcgccggacttcgagttcgacgaccgcgccggcgcc
gcctccttccccaaccctcgcctccgccgcgcctacatcgcgctccaggcctggcgccgcgcgttctactccgacccgagagggtacacctccaactgg
gtcggcagcgacgtctgcgcctacacgggcgtcgtgtgcgtcgccgccctcgacgacgccaccatcaccgtcgtcgccggcgtcgacctcaacggcgc
cgacatcgccgggtacctcccgcccgagctcggcctactcaccgacctcgccttcttccacatcaacaccaaccggttctgcgggatcatccccaaatcc
atgtcacggttgtccctcctccacgagttcgacgtcagcaacaaccgcttcgtgggacccttccccatggtcactttagaaatggcgtcgctcaagtacct
ggatatccggttcaacgacttcgaagggggagttgccgccggggctgttcgagaaggacctcgacgccatcttcgtcaacacgaaccggttcgtcgggcc
gataccggagaacatgggtaactcgaccgcgtccgtcgtcgtcttcgccaacaacgagctggtcgggtgtattcccaagagtattggcaggatggcag
atacattggacgagatcgtcttccttaacaacaagcttgatgggtgtctcccgttggagatggggcttctgcacaacaccagcgtcgtcgacgtcagcgg
gaacgccctcgtcggcgccctcccggagcagctcggggaactgaagaagctggagcagctcgacgtgtcgcggaacgtgttcatgggcgtcgtgcac
gagtccatctgcgagctgcccatgctcgtcaacttcagcttcgcggcaaactacttcaactccgaggccgtcaagtgcatgccgtctgatgccgcacttgt
gaacttcgacgacaagggcaactgcctcggcgcgctgcggcccgcgcagaagacgccgctgcagtgcgcgccggtgctcgcacggcccgtcgactgt
agcaagcacgcgtgcgcggggtacccaaagggcggtccaccggagaagaagccgcccgtcatgccggggaagccacccatggtgaaggtgccgcc
ggggcctgtcattgtcacgcctcccaaagcggacactcccgacccgtcgcccaagccgactccggagttcccctcgcctggcccggcgcccaaagagg
ctgatcctccgggggaacttcctagtgccgatggcctcaccgccgcctcctgtgatcacacctcctccaccggcacctatggcctcaccacctcctccaat
caagtctccaccaccaccggcacctatggtctcaccaccacctccagtcaagtctccaccaccaccggcaccagtggcctcaccaccacctcccgtgaa
atctccaccaccgccagctccagtggcctcaccaccacctccagtcaaatctccaccaccaccagttccagtgagctcgctgccacctccggtgaaatcc
ccacctccacctgccccggtagcctcaccaccacctcctgtgaaatcaccgctgccaccggctcccgtgagctccccacctccaccggtgaaatctccgc
cccgcccgctccagtgatatctccctcacctccagagaagtccttacctccacctactccaaagatctctccaccaccagaagagaagcctactcctcc
accaactccagtgaaatcttccccaccagccgctctggtgtcacctccaccttctga
>g165677.t1
atggaggccgccggccgcctcacgctcgccgcgtcgctcctcctcctcctcctcctaaccaccacctcaccaccaagaccagccttcgccctctccgacg
cggaggccaccgacatcgcgcggcggcagcgtcatctcctctccctccgctcccccctccggcgacctcccggacgacttcgagttcgacatccgcgtcg
atgtgaccttcgcgaacgcgcgcctccgccgcgcctacatcgcgctccaatccttccgccgcgccgtccactccgaccccaaaaacttcaccgccaactg
gcgcggcgccgacgtgtgtgcctacttcggcgtcacctgcgcggcggcgctcgacgacccgtcgctcaccgtcgtcgccggcgtcgacctcaacggcg
gcgacatcgcggcgtatctcccaccggaactcggcctcctcgtcgacgccgcgctcttccacgccaactctaaccgcctctgcggcgtcgtcccggaga
gcttcgcaaaactcgcgttgatgcacgagctcgacctcagcaacaaccgcctcgtgggaccgttccccgacgtcgtgttgaagatgcccgtgctcaagt
acctcgacttgcggtacaacgagtttgaaggccacgtcccccggggctcttcgagaaagacctcgacgcgattttcctcaacagtaactggttcgtcgg
gttcctgccggagaatttcgggaattcgactgcctcggtggtcgtgctcgcggacaacgcgttcgtggggtgtataccgaggagcgtgggccggatgga
cgccacgctggaccagctcatgctcaagggcaaccgcctcgacgggtgcatcccgccggaactcgctcatctccgaaacacaacggtggtggacgtga
gcgagaacctcctcgtggggacgctgcccgaccggctgcacgagatggccgggctccagcagctggacgtctccagaaaccggctcgcgggcgaggt
ggacgacaaggtctgcacgcttccggcgctcgccaacttcagcttcgccgacaacttcttcacccacgaggcggcggcatgcatgccgtcgtcgaacac
gcgcgtcaccctggacgacacggggaactgcatccgcggggagcggccggagcagaagtcgccggcggagtgcgcgccagttttatcgcacccggtg
gactgccggaccaacgtgtgttcgggtggcgggggtgggcctgtgccgccggtgccgaagaaggaggaacctgaggcggacccgccagggattggtc
cgaagccgcaggtggaggctccggagccgtcgtcagcggacccaccgggggagggccctcatgattacacggtcgccaccgccactggtgaaggctc
caccaccactggcaccggtcagttccccaccgccaccggtgaagtctccttcacctctgacaccggtcagctcacctccaccaccagtaaagtctcctcc
acctccatcaccggtcagctcacctcctccgcctgtaaaatcacctccaccaccagttccagtgaactcgcctcctccaccggtgaaatcacctccaccac
cggcgccagtgagctcacctctacctccgataaaatcccctccaacaacagcattagtgagctcacctcccctccgataaaatcccctccaccgccggc
accagtgagctcacctcccctccgaaaaaatcccctccaccgccgacaccagtgagctcacctccaccgccagcaccaatgagctcacctcctccacc
ggtaaaatcgccccacctccaacaccggtcagctcaccaccaccgcctgtgagatcacctccaccgccaacaccagtcagctcaccaccactgcctgt
aagatcacctcctccagcgagctcaccgccgccaccagtaaaatctccacctccaccggctccggtaagctcaccatctcctccagtaaaatctccgtca
ccaccagctccgatgagctctccacctccacctgtaaattcaccgccaccgccagctctggtgagctctccacctccacctgtaaaatcaccgccaccgc
cagctccggtgagctctccacccccaccagtaaaatttccaccaccccagctccggtgagctcaccgcctccaccaccagtcccatcacctctgccacc
ggcaccgtcgttgcctccaccagcagcagaggcggtcatcctgccaccgatcatatcacaaaattacgcgtcaccaccgccaccagtgttccaaggata
ctaagcgggcaggacacaccagcgaggtcaatcaatggaagaacgacgagggacagtgaccatcaggcgatcagagcacacgctctgcagggttga
>g166073.t1
atgtcaatgagcacggtgagcttactgctgctgctgtccatctccttgtccatatcatcgatcgctggagcagaggctgacaatgatgccgcggcgcttct
cgagttcaaggcagcagccgtcagcgatggcgggtatgcacttacttcgtggaacgtcagcagtgccatcgggttctgcagctgggagggggtcacgt
gtgaaggcaagctccaacgagtagtggcactgagcctccccttccacaagcttaatggcgtcctgtcgccagcggttggcaacctgtcattactcactgt
tcttgacctgagctccaacagcaacaacgccttctccggtgagatccctgccaacctgagctcctgcaccaacctgaccaccatgggactccagtccaa
ccagctccgagggtgcctcccccccgagctcggcgacaagctgacgcacctcaggatgctcaaactgtggaagaacaacctcaccggtgccatcccgg
tgtcattgggcaatttgtcatcgctcacttatgtgtccctcgcattcaaccggtttgagggcaccatacctcctagcctcggcgacattacaggtctccggc
accttgacctcgcctacaaccatctctccgttctgtaccgtctcgagaccctcaatctgagcaacaacgccttctccggtgagatccctgccaacctgagc
tcctgcaccaacctgaccaccatgggactccagtccaaccagctccgagggtgcctccccccgagctcggcgacaagctgacgcacctcaggatgct
caaactgtggaagaacaacctcaccggtgccatcccggtgtcattgggcaatttgtcatcgctcacttatgtgtccctcgcattcaaccggtttgagggca

ccatacctcctagcctcggcgacattacaggtctccggcaccttgacctcgcctacaaccatctctccgctgccaagttccatagggaatctctcaacgaa
tctacaaaaactcctcttgaatga
>g166492.t1
atggtgaggctcaccgccgacctgatatggaagagcccgcacttcttcaacgccatcaaggagcgtgagctcgacctccgaggcaacaagatcgctgt
catcgagaacctcggcgccaccgaggaccaatttgacactattgatttgtcagacaatgagattgttaagcttgaaaattttccttacttgaatcgtcttgg
cactctactagttaacaataacagaatcacccgtataaatcccaatcttggagagtttttgccaaagttgcatactctggtgctcacgaacaatcgcctga
caaatcttgcggagattgacccccctggcttctctcccaaagctacaatatctcagcttactggataatactgtaactaagcaacctgactatcggctttatg
tgatccataagttgaagcatttgaggttgctggatttcaagaaagtgaaacaacaggagagggttgcagcagcaaaaaagttccactcaaaagaggcc
gaggaacaggcaaagaaggtacctaccaaaacatttaatccaggagagctgacagatgctcaagacaacacaaaggaggcacatgctcctaaagtt
gttggcccaacacctgaacaaatcacagcaatcaaggctgctattgtgagttctcaaactctggaggaagttgcaaggcttgagaaggcattgagcact
ggtcaaatccctgcagaatttgctatgccaaagcctgacactaatatggctgaagcatcagaggaagctgacaagatggacacagatggtcagaacca
agagagcggggctggtgagcataaacaagatgatggaagcacgccaattgaagaggattga
>g166851.t1
atgccacgccacgcgccccaccacctcctgctcctcctcccgctgctcctcctctgcgcctcagtccccgcccatgtctcctccgcttcgtcgcctctctgcg
ccggccgccacgacgcggccaccgtcgcggccgcgttccggcacgtgcgcaatttccacccacagaggtccctgaacccgtgcgagccgatccgcga
gctgcgcctgccgtcgcggaacctgacgggcgccgtgtcgtggtcggcgctggccaacctgtcctctctggcggcgctggacctctccgggaacgcgct
gcagggcgccatcccgggcgggttctggcgcgcgccgtcgctccggtccgtcgacgtctcccgcaaccatctcggcggcgcgctccgcgtggctacgg
cgtcggccaacccgcgcctcgagtcgctcaacgtgtcgggcaaccgcttcagctccgtgtccggggtggacgcgctcgccgggctccaatcgctcgacg
tgtccaggaacaggatccatgccgtgccgcgcgggatgcggcgcctcacgcgggtgacacggctcgacctgtccgggaacgcgatgaaagggaggtt
ccccgccgacctgccgccgctcggcggggtgcgttccctgaacgtgtcgtacaacgagttctccggcgtggtggatcccgaagcggtgaagaagttcgg
gagatccgcgttcgtgcgcgccgggaactcgtcggctctggtgttcccccggccaacaacacgacgacgacggctccgcggcggccgtcgtcgcctct
tcatggaaaaagcaaaaaacacgatggtgccgggaggacgacgacggagaagaagccggcgaagaagaaggagaagagaagggtgcatctgag
ggtggtggcggtggcggtgatgtgcggcgcggcgtccctggccatgctgctgtgcctggtcgggtgcgtggcatgcggcaggtgccggaggaggagg
aacggaataaggaaggatgacgaggagaagaagacgccgcagtggagcgacgaccaggacgagatcgtggtgctcgcggcggcgacgaagggcg
cgtcggcggcgccggtggtgctgttcgagcggccgctgatggagctcacgctggcggacctggccgcggcaacctccgggttcggccgcgagtcgcag
ctcgccgagcgcggcggccgcagcggcgccgcgtaccgcgccgtgctccccggggacctgcacgtcgtcgtgcgcgtcgtggagggcgccgtggcgg
gcctcggggaggaggacgacgacccgtcggccgctgccaatgcgttccgggagctcgcgaggctccggcaccccaacattcttccgctcattggctact
gcattgcaggcaaggagaagctgctgctgtacgagtacatggagaaaggcgacctccaccggtggctgcacgagctgcccgcgggtcggcccgacat
ggacgacgccggcagcggcgacatctgggagacggcggaggacaagcggtccatctcggactggcccacgcggcaccgcatcgcgctcggggtcgc
ccgcgggctggccttccttcaccagggctgggccgggtcgggccggccgtggtgcacggccacctggtccccaccaacgtcctcctcggcgacgagc
tggagccccggatctccgacttcgggtttgggcacccgggcggcggcggcgacgagtgcacggcggagggcgacgtgtacgggttcggcgtggtggt
gctggagctgatgacggggcaggccaggtgggacgaggcgtccgtgagctgggcgcgcgggatcatccgcgacggcaaggggctggacatcgtgga
cccgaggatccgcgacggcggcggcgcggaggcggcggagcgggagatggtggagtgcctgcgggtggggtacctctgcacggcgcactcgccgga
caagcggccgacgatgcagcaggtcgtgggggtgctcaaggacatccgcccggcgcctgccacgcccaccaccaccaccctgcgtggtgaagcaatg
tga
>g167192.t1
atggacacctggttctggaacgtgccaaccctgcgttacctggacttctccgaaaactcgctgtccggcccgttcccggaatccctcggcaacatgacgc
gtctccgggagctctacctctacggcaacaacatggtcggcatgatcccggggactctgcagcgcctctgcagactacaggtggtggacgtgtccatga
accatatcaacggcgacatgtcagagttcatggaccggctcccgcgctgcgtgctgagccgcgttctaagggtgctgcatctcgcggctaccaacatgtc
cggcgaacttcctcggtggatcggcgacatgtcggagctcacgtccctcgacctgtcctacaacaatcttccaggggggtcttcctccaacaatcggcagg
ctctccaagctcacgacgctcctcctggtccgcaacaatctgaccggcaggttgacggagaagcatttttcgaatatggtgagcttggaattgattgattt
gtcgcagaactcaatgtcgatggaggtcaggccgagctggaggcctccgtgcaaattgctctacgcttacttggctgatgtccagatgggtcctctgttcc
ctgcttggatcaagcaccagccggacatcacgcaccttgacatttcacatgcagggagcggtggagttcgggacggacttcgcattgttcagaaaagct
atcttcacatcatcacgaaagggcaagatcgtctctacaccagcggagttatctacatggtgagccttgatttatctgacaatattctcagcggagagatc
cctgaagagatgtcatatcaaagtggactggtgaacctgaatttgtccaggaaccagttgaccgggacaatccctcagaacattagtgatttacaaaag
ctggagtcccttgacttgtcgatgaatgagctttcaggtgaaattccatccagtttgtccgacttgacttcattgggctacctgaacttgtcgtacaacaac
ctgtcaggaaggataccttcaggcaagcaactccaggtacttgccaatccagcatacatctacattggcaatgctggtctgtgcgggccacctctatcga
agaattgttcatctggagatgatagtaacaatagccatgcacctcgccatggagacaaagatttattcgaaatgttcttttaccttggtctggccgtaggt
acggtttcagaatcacaaggagaagataactag
>g167193.t1
atgcaacgtctctctgaggagcttatccgggacatgacaatgactcttttctttgctctcgccatctcgggcgtggacttgccgctcccggactgctgccgg
tgggccggcgtcgtctgtgacaacaacggcacgggccgggtcgttgagctccgtatccgcaacacgttcgctgacgccgacgccgccaaccctgggct
catcagcggcgagataagcacggcgctcaccaatctcacacggctcgagcacctggacctcagctgcaacatcttcggacccttgtcgatcccacggtt
cctgggctcgctctccggcctccggtacctcaacctctccggcacggacctcgtcggcgacatcccaccgcagctgggaaacctctcccggctggtctcc
ctcgacctgagttcctacggcctctactccggcgacctctcgtggctcatcggcttgtcctctctcgagcacctcgacatgagccacgtctacctcaaagc
gtccgtcaactggcagcgggcagtcaacacgctccttctctgcgcgtcatctccctctcggactgcgcgctga
>g167194.t1
atgcacgcggcagcaaaaatcctgtgtttttggcgctgtccgcgttcgggggcgccggtggctgcgtcccggccgaacgcgatgcgcttctcgccgtga
agcgtggcttctcaagcgacccggaaggcgtcctcgggtcatggagtctcaagctgaacccggactgctgccggtgggccggcgtcgtctgcgagagc

aacggcacgggccacgtcacagagctccgtctcaacaaccgcaacgtgttgggcagcaacggacttggcggcgagataagcccggcgctcaccaatc
tcacgaggcttcagtacctggacctcagcaggaacagccttgccgtgccaatcccacggttcctcggctctctatcaagcctccggcacctcaacctctc
cgaaatgggcctcgtcggcgaggtcccgccgcagctgggaaacctctcccgtctcgtctccctcgacgtcacctcctacggcctctactccggcgacctc
tcgtggatcaccggcttgtcctctctggagcacctcgacatgagcaacgtcaacctcaacgcgtccgttcactggcagcgcgacgtcaacatgctccctt
ccctgcgcgtgctcgccctcatcgactgcgcgctgaccgcaaccgtggcttcttcgtcgcgcaacaacctgacacggctgcacagtctcgacctgtccga
gaacggcatcaacacctcaaccgtggacgcctggttctggaacgtgccttcccttcgttacctgggcatctccggcaactggctgcccaacatgctgccc
ggcccattccccgaatccatcggcaacatgacgcgtctcaaggagctcgacctcttcggcaactacatggtcggcatgatcccggcgactctgcagcgt
ctctgcagcctgcaggtggtggtcgtatccgcgaaccagatcaacggcgacatgtcggagttcatgacccggctcccgcgttgcgcgttgaggcacctg
caagtgctgcacgtggcggcggccagcatgtccgggcacctgcccgaatggatcggcgacatgtctcagcttacggaccttgacctgtcctccaacaat
ctccaagggcgtattcctcgggggatcggccgcctctcaaagctcacaaggctcttcctggtccagaacaatctgaacggcagcctgacagaggagca
cttcgcagatatggtgagcttggagtggatcgacttgtcgcagaactccatgtcaatggatgtcagcccgagctggaggcctccgtgcaaattgttctac
gctttctttgctgatgtccagatgggtcctctgttccccgcttggatcaagcaccagccggacatcaagcaccttgacatttcgcatgcagggatagtgga
cacactgccgcaatggttttggaagtcgttttcagatgcagtatacctcaacatctctgtcaaccagatcagcggcagcctgccatcttctctgagattcat
gacctctgcatcagagatctatctcggagagaataatctcaccggctccatgccgttgttaccggagaagcttcttatctttgatctctctaggaactcctt
atctggtcccatcccatcagaattcggtggtcctgaattaaacatgttctcggggaccttaccagaatggatcggaaggaaacttccttccttgacacatc
ttcggctacgatcaaacatgttcacgggcatcgtccctactcagctcacgcagcttggcgacctgcaactcttggacctagctggcaacaagatatcagg
ttccataccccgttctctagggaacatgacaggaatgatgaaagagcacacaccagttgggttgaacccattggcagactacgacccatcggggaacg
atcgactggcaaatggactcccgttcctccacgtcacattagggaacggccttcccatcgtcacaaaagggcaagatcttctctataccaacggagttat
gtacatggtgagtcttgatctatctgacaatgttcttagcggagagatccctgaagagatgtcatttctaactggcatggtgaaccttaatttgtccagaaa
ccagttgaccgggacaatccctgagaacattagtgatttacaaaagctagagtcccttgacttgtcgatgaatgagctttcaggtgcaattccatccagtt
tgtccgacttgactgcattgggctacctgaacttgtcatacaacaacctgtcaggacagataccttcaggcaagcaactccagggtgttgtcgtcgacat
ccacatgatccagaggttgcatcgtcttaaaagattctaccacccaggtgatatttcttttgtgctcaagaactaa
>g167198.t1
atggcgatcctcttcttgttggcgctcggcggcgccggtggctgtgtcccagccgagcgcgaggcgctgcttgccgtgaaacgcggcttctccagcgatc
cggacggcgtcctcgggtcatggacgaccaaacactcaaactgctgccagtgggccggcgtagtctgtgacaacaacggctcggggcgccgtgttgca
gagctccgtctcagcaacgccgacgccgtgggcagctccgggctcagcgggaagataagcccggcattcaccaatctcacgcggcttgaatacctgga
cctcagctggaacagcctcggcggcaacgacgtgcggttcctgggctcgctctcccgcctccggtacctcaacctctctggcacggacctcgtcggcga
gatcccaccgcagctgggaaacctctcccggctcgtttccctcgacctgagcttctgcgtaaagctctactcccgcgacctctcctggctcaccggcttgt
cctctctccagcacctcgacatgagcggcgtcaacctcagcgcgtccgtttacttgcagcgcgacgtcaacatgctcccttcccttcgcgtcctggccctct
ccaactgcgcgctgagcacaaccgtgacttcttcatcgagcaccaacctgacacggctgcagaggctcgacctgtcctggaacagcataaactcctcaa
cagtgaatgcctggttctggaacgttcccacgctcaggtacctggacctctccggcaactcgctatctggtccgttccccgaatccctcggcaatatgacg
cgtctccaggaactctacctctacagcaacaacatggtcggcatgatcccggcgactctgcaccgcctctgtagcctgcagatggtggacctgtccgag
aaccagatcaatggcagcatgtcagagttcatggaccggctcccgcgctgcgcgctgaggcgcatgcaggtgctgcagctcgcgaagaccaacatgtg
cggggaacttcctcaaaggatcggcaacatgtcagagctgacgtccctcgacttgtccttcaacaaacttgcaggggaccttcctccaacaatcggtag
actttccaagctcacgaggctgtccctgatggataacaaccttaatggcagcttgacagaggagcacttttccaatatgatcccagaagagatctcatat
ctaaatggcatggtgaacctgaatttgtccaggaacaagttgaccgggacaatccctcggaacataggctacctgaacctatcgtacaacaacctgtca
ggaaggatacccacaggccaccaactccaggtacttgccaatccttcatacatctacattggtaatgttggtctctgcgggccgccggtgtccaagaaat
gttcatctggagatgatgataataatagcatccagactcctctccgtggagatagagatttaaccaagataacagtggccgtagggtttgtggtgggaa
actggctggtgatttgctccttgttgtttttgaaggcgtggcgatctgcttatttctggaccatcgacaaggcatatgacgtcctgtacgtgtttgtggcaat
aagattggcgaaacgcggcaagaagatcactagtaccaactcagcagattaa
>g167206.t1
atgcacgcggcatcggcgatcctgttcctgttggcgctgtccgcgttcggcggtgccggcggctgcgtccctgcagagcgcgatgcgcttctcgccgtga
agcatggcttctccagcgacccggacggcgtcctcgcgtcgtgggccgccagcaagttcccggactgctgccggtgggccggcgtcgtctgcgacacc
aacggcacgggccacgtcacagagctccgtctccgcaacgcgttcgccgacgccggcagccccgggctcagcggcgagataaacacggcactcacta
atctcacgcggctcgagtacctggacctcagccggaacagcctcggcggcatagcaaccttgccgatcccacggttcctcggctctctgtccagcctccg
gtacctcaacctctccggcacgggcttcgttggcgacatcccgccgcagctcggaaacctctcccgcctcgtctccctcgacatgagctcctacggcctct
actccggtgacctctcgtggctcgccggcctgtcctcctccctcgagcacctcgacatgaccagcgtcaaccttaacgcatccgttcactggcagcgcga
cgtcaacacgctcccttccctacgcgttctcgccctctccgactgcgggctaaggctaacgacgtcgtcgcaggtgccttcttcttcggcacacaccaacc
tgacacgtctgcagcgtctggacctgtcatccaacggcatcaacacctcgaccatggacgcctggttctggaacgtgccgacgctccggtacctcgacct
ctccggcaacgctctgtccggcccgttccccgacgccctcggcaacatgacgcgtctccaggcgctctacctcagcggcaacaacatggtcgggatgat
cccggcgactctgcagcgcctctgcagcctgcaggtagtggacgtttccgtgaaccagatcaacggcgacatgtcagagttcatggaccggctcccgcg
atgcgcgttcaggcacctgcaagtactgcagctctcggcgaccaacatgtccggggaacttcctcaatggatgggcgacatgtctgaactcacatacctt
gacctgtcgtctaacaaacttgcagggggcattcctccaacaatcggcaggctctcgaatctcacgagcctgttcctgctcaagaacaacctgaatggc
agcttgacagaggagcatttcgcagatatggtgagcttggagtggatcgacttgtcgcagaactccaacatgtcaatggaggtcacgccatcctggatg
cctccgtgcaaactggtctacgcttatttcccggacgtcaagatgggtcctcggttccccgcgtggatcaggcaccagccggacatcaagtaccttgaca
tttctcatgcaggcatagtagatacactcccgcactggttctgggagtcattcacagatgcggtttacctgaacatctcggtgaaccagatcagtggcag
gctcccaccttctctgagattcatgaactctgcactagctatctacctcgggtcgaacaacctcactggctccatgccgttgttaccggagcagctgcttgt
gttggacctctccagaaactctttatctggcacaattccatcggagtttggtggtcctgagttagtggagctagatgtctcctccaatataatcagcggagc
agtgccagcatccctatgccactttccaaacttgctgcatttggatctgtcaaacaacaatctgagcggccacctccctcggtgcccaaacatgtcatctg

atggcctcgggctcaccactctcatcttgtacaaaaacaatctttcaggcgggtttcctgtgttgttgaaacactgtcacgctatgacattccttgatctggc
acataacatgttctcggggacgttaccggactggatcggaaggaagatgccatccttgacacatcttcggctgcgctcaaacatgttcactggaatcatc
cctgctcagctggcgcaactaggtgacctgcaactcttggacctagccggcaacaagatatcaggttcgataccccattctctagggaacatgacagga
atgacaaaggagcacacgccacttgctctgaacccattgacaggctacggtgcatcagggaatgatagaattgtggacagtcttcctatcgtcaccaaa
gggcaggatcgtggctatactagcggagttatctacatggtgagccttgatctgtccaacaacattatcagtggagagatacctgaagaggtttcatctct
tactggcctggtgaacttgaatttgtcatggaaccagttgaccggaacaattcctcaagacattggtcatatacaaaagctggagtctctcgacttgtcca
tgaatctgctttcgggtgcgattccatcgagtctgtcagacttgacttcattaggccacctgaacttgtcctacaacaacctgtcagggacaataccttcag
gcaaccagctccaagcacttgccaatccagcatacatctacattggcaatgatggtctttgtggcccgcctctgttgaagaactgctcatctggagatgat
aatagcagtagccatgctccacgccatggagataaagatttatccaagatgtccttctaccttggtctagccgtaggggctatcgacaagacatatgacg
ttttgttggggaagcccggggataagagaactactagtaacccagcagattag
>g167405.t1
atgacgtcgagcaaaccgctctccacaagctcgttgcttcgtctgctttccctgctcctcttcctcttctccgccaacgtcgcctcctcgtccgccgccacag
actgcgaccccgacgaccgcgccgcgcttctccgcgtcaagtcccagctcggcgaccccgtgcggctgtcctcgtggcagccgtccaccaactgctgcg
cgtgggagcccgccgtcttctgcaacgccgagggccgcgtcacgggcctagccctctacgccctcggcgagctcaaggcgcccgtgccgccggcccta
ggcgagctcaccgcgctcgagatcctccagatcgagtccgtgcccggcctgtcggccccaatcccaccctccttctccaacctcacgcggctccgcgac
ctcgacatcaacggcacctccatctccggcccggtccccgccttcctctcctgcgccaacctcagcacgctcgtcatcgccaacagcaagctcgcgggcc
ccatccccgcgtccctggccgccctccctaacctccggtacctcgatctcagcggcaacatgctcagcggcgccatcccgcccgggctgctgcacggga
gcttcaggttcctcatcctctccaacaaccggctcacgggggagataccgcccgtcgactacagcgacgtcgacaccgtcgacctctcccgcaacgggc
tcaccggcgacccgacgccgttcctgttcggcatcacgaggcccgccgtcaagatcgacctgtcgtggaacgagctggagttcgacatgaccggggtgt
cgttcccgcaccacctccggttcctggacctcagccacaaccggatcacgggcagcgtggccaagtcgctgatggacgtcaacctcgagcattttgatgt
cagctacaacgagctctgcggcgagataccggcggggaggttcatggcggtgcacgggccggactgctacgcgcagaacaagtgcttgtgtggcgcg
ccactgacaccttgcgtctcaggcctgtag
>g167558.t1
atggcaacggccatcctcctcctccccttgccatcatcgtcttcgccgctctcgcggccgcatccacagcgccgctggacccgcggcagctgctggcttt
gcgcgcgctgggcctcggcgcgcggcgcgacccctgcggcgtcgcgccgccaggctcgatgaacttgtcctgcgacgcgagcctccccttccgccgcg
tcacctccctctccctcaccaactgctcggcgaccacatccgtctccaccgccgccctcgacgcgctggcgccctccctccgcaccctctccttctccgact
gccccgccgcgccgccgcgggccctcccgcccgaccagctcgctgccaacctcctctccttcacctgcacggcctcgctccgccgcctctccgcggtgtg
gctctcccgcctcgcgaacctcacggagctcaccgtcgccgacaccccgctcgcggccaccagctccccgacggagctcgccgtcgtggtgtcccacat
ggaccgcctcacccggctcaccgtctccaacgccaacctctccggcctcctcccgcaccactggcactgccccaacctcacccgcctcgacctctcggcc
aaccgcatcgcggggcccatccccgacacgctcacgctcctcgccgccatcacccacctcaacctcagctccaacgcgctcacgggcgccatcccgac
ctccatcggcgacctcatttcgctcaccacgctcgacctctccagaaacaccatctccggcggggttcccgacacggtctccaccctacccgagctggag
gtgctcgatctggggtccaaccgcctcaatggcagcatcccgctgttcctaacggagatgaaagggctcagggagctcaacctcgagaacaacgacttt
gacggcgtggtgccgttcggccccaagtttctgtcgcggctgcgggtgttcagggccgcgggcaacgccaagctctgctacaaccggtccgtgctgtcg
gcggagatggccgtcggggtggctccctgcgacaagtacgggttccccgtgctgccgccgccggccacggcgcagtcggagcggaacgccgactacg
acgacgggtacagggaccccgccgacgccgggggagacgccaggggcgggcccagcgtcgccgtgctcggggtcgccatcggcctctcctgcctcgc
cttcctcgtcatcctcttcgtctgcatttgcaaggtctgtcgctga
>g167798.t1
atggaggcgcctccgtcttcttccccgtccctcttgctcctgctcctcctgctcgtctccccttcatcttcgtctgccgtcctctcccccaagggcgtcaacta
cgaagtgcaagctctgatggcgatcaaggacctcctcaaagatccccatggcgtgttcaaggcctgggacataaactctgtggatccctgcagctgggc
tatggtcacttgctcaccggatagttctgtcactggactggaagccccaagcaagaatctctccggcatgctctccccaaacataggaaacctgaccaat
attgagactgttctcctgcagaacaacgacataaccggcccaataccaacagagattggcaagcttgcaaagctcaagaccctcgatctctccagcaac
cacctgtatggcgaaatccccacctctgtgggccaccttgaaagcctccagtacttgcggctcaacaacaacaccctgtctggtccattcccctcggcatc
agctaatttgtctcaacttgttttcctagacttgtcatacaataaccttagtggtccaataccaggatctttggcaagaacattcaacatagttgggaatcct
ctcatatgtggaacgactatggagaaagattgttacggcactgcaccaatgccagtgtcctacaatctgactatctcacagggtgctgcaccaccggca
aaatccaaaagccacaagtttgcagttgcatttggttctgccattggttgcatcagcttccttttccttgccgctggctttctcttctggtggaggaaccgtc
gaaaccgccagatccttttccatgtcgacgaccaacacatggaaaacgttagccttggaaacgtgaagcggttccagttcagggagcttcagtccgcaa
cagacaatttcagcagcaagaacatactaggtaaaggcggctttggaaacgtttacagagggaagctccctgatggaactcttgtggccgtcaagcga
ctgaaggacggcaatgctgctggcggggaggcacagttccagactgaagttgagatgatcagcttggcactgcacaggaacctcctcaggctctatgg
attttgcatgactgctacagagaggctactggtctatccatacatgtcaaatggaagcgtcgcatcgcgtctgaaagggaagccacctttggactggatg
accaggaagaggatagcccttggggcagggagagggttattgtacctgcacgagcagtgtgaccccaagatcatccacagggacgtcaaggcagcca
acatactcctggacgaatactgtgaggcgatcgttggcgacttcggtctcgccaagcttctcgatcaccgggattcacatgtcaccactgcagtgagggg
cacagttggccacattgcgcctgagtacctcagcactggccagtcttcggagaagactgatgttttcggcttcggcatcctgctgctggagctgatcactg
gacagaccgcgctggagttcggtaaatcagcaaaccagaagggagccatgctggactgggtgaaaaaaatgcaccaggagaagaagctcgacgtgc
tggtggacacgggcctgaagggcggctacgacaggatcgagctggaggagatggtgcaggttgcgctgctgtgcacgcagtacctccccgtccaccg
gccgaagatgtcggaggtggtgcggatgctggagggcgatgggctcgcggagcggtgggaggcgtcgcagcgcgcagactcgcacaagttcaaggt
gccggacttcaccttcagccggtgctattccgacctcaccgacgactcgtcgctgctggtgcaggccgtcgagctctccgggccgagatga
>g168016.t1
atgccgtctcgccgccgccaccgctgccgctaccctgtgctcctcctccttctcctgctacacctcctcaccaccgcctccgcggccggcgtctccccc
ggcgccgcatcgcactcgcagcccacgctgccgacgccggcccaagccgtgacgccatccgcggcgccggccaagaggggtacgccgaccgccgcc

gcaccgcacccgcggcccacgcaacctgcgccggcccaaccggtgcccccatccgcgactccgatggcaaggtcgccgacagccgcggcgccgcact
cacggcccactcttccctcgccggtcgcaaggccgccggccgccgaggcgccgcactcgcggcctacttttccctcgccagtccaagcagtggccccgg
cctcgaggccccccatggtcgccgccgccactcccctcgccgccttcctcgccaaggccgacccctcctcccacctccgcccgccgccctccgcctcccc
ctgctcccacccgggcgtcacctgctcctcctcaggccaaatcacccgcctcgtcctcgaatccctgggcctcaacggcaccttcgcccccgccacactct
ccctcctctcctccctccgcgtgctcagcctcaaatccaacgctctgcacggcccaatcccggacctctccggcctcgtcaacctcaaggcgctctacctc
gccaacaaccgcttctccggccccttcccctcctccctcgccgccctccagcgcctccgctccatcgacctctcgggcaaccgcctctccggcgcgctccc
acccggcatcgaggccgagctcccccacctcaccttcctccgcctcgacgccaaccacttcaacggcacgctcccgccatggaaccagaccgagctca
agacgctcaacgtctccttcaacgacttctccgggcccgtccccgtcacgcccgtgctcacccagatgggcgccgacgcgttcgaagggaaccccaagc
tctgcggccaggtcgtgcgacgcgagtgccacctcctcttcttccacggagacggcagcaacggctccgccgccgcggcgcccccggtccacagcgac
agcggcccgcagcgccaaagtttggatccgtccgatgcgcgcgcgaagaaatcgaggaagaggaggacgacggtggccgtcgcggccgccgctggg
tcggtcctcgcgctcctcctcctctgcgccatgattgcgaggagcaggaagcggaggcaccccatgcggtcgtcggcggcggcgtaccacccgagccc
caacaagaagagcgcgtcggacgcgggcagagacaacatcggcgccgacgtggggtacgtggagtgcgtgcgcgacgaggagacggcggcgatca
tggtgcccgaggagaaggcgcggaggctggagcggagcggctgtctcaccttctgcgcgggcgaggcggccagctacagcctcgagcagctcatgcg
cgcgtccgcggaggtgctcggccgcgggagcgtcgggaccacgtacaaggcggtgctggacggcagacttgtggtcattgtgaagcggctcgatgcg
gccaagattggacccgcggcgctggaggcggagacgttcgagcagaacatggatgccgttgggcggctgcggcatcccaaccttgtgccgctccggg
cgttcttccaggccaaggaggagcggctgctggtgtacgactaccagcccaatggcagcctctactctctaatccatggctggcacccggcagcgtcgg
cagaagaagaagaagaagaagaagaagaagaagaagaagaagaagaagaagaagaagagtgccagttgcgtgcgttcaccggcggggaagaag
aagcagttcaattcgattggattggattggattggatggggttccacctgttggcgatcgcggtggcgccgggattcctgcaggtgttccacctctcggcg
ccgtggctgcggccgctcaacctctgggctccctcggcgcgccacctgcccgaggcctgcgccgtcttctacgccgcgcccgcctcgcacgccgcctgg
ctgcgcgccgcctacgcgcgcggcaccgtctggagccaccgacgccgccgccgcgaagccgacgacgtcgacgagtacatccgccacgcgctcctca
gcgtctcctactga
>g168105.t1
atgacggcggcagcagcacggacgaagaacgaatgccaccccgacgacaaggcggcgctgctggccatcaaggcgggattcggcaacgcctcctac
ttccagtcctggacttccgactacccctgctgcatctggatcaacgtcttctgcgacccctacacctcccccctacaccgcgcgccgcgtcgtggccgtctcc
ttcctccgcgacaccagcctcgtcggacccttgcccggcgccgccatcgctcgcctcaccgctctgcaggagctcgacctgatccacgtgccgggcgtg
aatggcactatcccccatgaactcacccgcctctccgctaccctcaattccatcgacatctcctacacgggcatctccgggcccgtgccgtccttcttctcg
cagctgaagaagctcacctacctccgcctctccttcaactcgctcacgggccacatcccggcgtcgctcggcgagattcccaacctctccttcctcgacct
cggccgcaaccgcctcacgggaaccataccacctggactgctcagtaagaccaacgacacgtcatacctgtgcctctcacacaacaacctcaccggcg
gcatccccgccgagttcgccgccgtcaggttctggaccctcgacctgtcgcacaacgccctcaccggcgacgcctcgttcctgttcggggcgaacaagt
cgctggagaacctcgacctgtcgcgcaacacgttcagcttcagcctctccgccgtgacgctgccgtcaaagctcggctcattggacctgaaccacaacg
gcatctacgtcgagctaccgccgcaggtggccaacctccagaatttgaatgtcagctacaactggctcagcggcacggttcccaccggcggcaacatg
ggcaggtttgatcagtactgcttccagcacaactatggtctgtgtgggagtccactacctccatgctattag
>g168263.t1
atggcgccaccggtaactactagagcagtagtaggattaatattcatggcggcgctgctcttgtgcatgcaaccggcggcggcggacaccgacggtga
cgccctgctagcgttcaaggccggcgtgacggacccgaccgacgcgctggcctcgtggaacgccacgactcccttctgccggtgggccggcgtgacgt
gcacctcgggccgcgtgacgtcgctggacgtctcggagcggcggctcaccgggacgctctcccccgccatcggcaacctgactcagctcgaggttctca
acctcacccgaaacggcgtctcgggccgcgtcccggcgagcctgggccggctgcatcgcctctcgtacctcagcctctgcgacaacgccttctccggtg
cgctccccgacgcgctccgtaactgcacgagcctctccgtcgcgttcctcaacaacaacgaattcaccggcgtcgtcccggactggatcggcggcgcgt
ggccgaacctcgccgtcctccgcctcggcacgaaccagctctccggccgcatcccggcgtcgctcgccaacctcacgcagctctaccgcctggagctcg
accagaacctcctggaaggcacggtccccgccgacctcgcgcggctcgccgacctcggcttcttcgccgtctaccagaaccgcctctccggcgcgatcc
cgccgttcctcttcaccaacatgtcgtcgctggtaggcctgtccctcgccgacaacgcgttcgagggggagctccccgccgacgccggcgccggctggc
ccaacctccagtacctcatcctcggcgggaacaggctgacgggcccgatcccggcgtcgctggcgcaggcgtcgaatctgacggtgctcagcttggcc
aacaacagcttcaccgggcgtgtgcctcccgcgatcgggaggctctgcttggaggtgctgcagctctccaacaacgacctcgaggccgatgacgccgg
cggcgggtgggagttcttggaggggctgaccaactgcagcgcgacgttcgagatctccctcgacggcaacaggttttctggcgcaatgccgggctccat
cgcgcgcctctcgccgcagctcggggtgctcgtcctcggcggaaacggcatttccggagctatcccgcccgggatcggcaacatcgtcgggttacagac
gttggacttggggtcgaatctcctctccggcgtgatcccggacggcatcgggaagctcgagaacctgcagggattacggttgcagcagaacaacctgg
ccggacctattccatcctccatcggcaagctcggtcaactgctacggctagacctcagcgacaactttctgaacggctccattcctccgagcctcggcaa
gctgcagcagctcaatctgctcaacctctccggcaacgagctcaccggccacgtccccggagaactgttcagcctgtcgacgctgtcgtcggcgatgga
cctgtcgaataaccggctggacggcgtgatcccggcaggcgtcggccggctcgtgaaactcgcgctgttggcactctccgggaaccggttctccggcga
ggtgccgtcggagctgggcaactgcgcgagcttggagttccttgacctggacagcaacctcttcaccggaaccatcccgtcgtctctccaaaggctcaa
ggggctcaggaagctgaacctcacgagcaacaggctgtcaggaaccatcccggcggagctcgcccagatgtcgggcctgcaggagctgtacctgtca
cggaacgacctgtccggcgcaattccggcagagctggggaacatgagctccctcacggagctcgacctctcctacaaccacctcgacggcctgtccc
ggcggcgcgcggcgtgttcgcgaacgccaccgggtttcgggtcgccggcaacggcgcgctctgcggcggcgcgaaaagtctccgcctgccgccgtgc
ccgccaccatcatcatctactgagaattccacacgacgccgtcaccacgtgttcctcatcaagatcgtgctgcccgtcatcgccggcgcggcgctgtgcg
tcgccgccgcgctactcgctctgctctaccgccgccgtaataagaggaggaggagatcgagaatcgcagacaccgcggcgcgaagcgtgctcaactg
caacagctacccgagggtgtcgtacgccgacctggcacgagccaccgacaacttcgcgcacgacaacctcgtcggcgcggggaagtacgggtccgtg
taccgtggcacgctgtcgctcaagacgaaggggagctccgtgcacgaggacgtggtggtggcggtgaaggtgttcgacctcgcacagctcggcgcgtg
caacacttttgtttcggagatcgaggcgctgcgcagcgtcaagcaccgaaacctgatcaacatcgtcacatgctgctccagcatcgacgcggacgga
acgacttcagggccctcgtcttcgacttcatgcccaactacagcctagaccggtggctgcacccgagctttgtggacgtcaagaaagggcgcgtcctca

ccatggtccagaggttcgacatcgccgtggacgttgcagacgctctcaactatctccataacagctgcacgccgccgatcatccactgcgatctcaagcc
cagcaatgttctccttgccgacgacatgacggcatgtatcggcgactttggacttgccaagctcattctggatccgggctgccacgctgtccaaagcagt
gaaagcaccatcgggataagaggcacggttggttatgtcgcaccagagtacggcacgtcggggaacgtttctaccaatggtgacgtatacagcttcgg
gatcgtgattcttgagatcttcctaggaaaggcgccaacggatcctgatgcgttcggagacggcctgacactgccggaatttgtcggcgcggcattcccg
gacaagatcgagcagattcttgacccggctctgctaccgattccagactcatcggacgcatcgatttcagtttcagaagagagccaggcgtgtgttacgg
tgcgggactgcttggtttctgccataagtgtcggcctcgcttgctgcaggcagacgccgtaccaaagaatgtccatgagggatgcggcagtagagctgc
gactcatcagagatgcttgtcttcgtgcttgcggccagtga
>g168494.t1
atggtaatgttgctccgcgcctatgcgtgcacttacctccatcttctgctcatgttgctgccctgctgcggcctccatgcggcgcagccccaaccaccgga
cgagccgcggcttctactacagatcaagagcgtgtggggtgacccaccggttctggcatcgtggaacgtctcggccacggccgcacactgcaggtggc
cctatgtgaggtgcaacagagccgggcacgtctctggcctcttcctcgccaacactaaagtatccggcccattcccaggcggtgcaatcgccaatctctc
ttctctccggcacatcgacatcttcaacaacagtatcgccgacgtgttcccgacattgttgtaccactgccgttccctgcagtacctcaacctgtcttataac
ctcttcaacggggagctcccggctgacatcggccacggtctcgctgcgagcctcaccacgctggacctcagtggcaacgagttcagcggcaccatcccg
gcgtcactctccaggctccagaatctcaagtttctcgtgctagacaacaactttttgtgggcagtattccggcagagctcggcgagctgacgagcctcca
gactatgtatctagggggcaattcattcaatgctggcgagcttccaccatccttcgggaagcttgccaacctgagtaccctcgatgcacggaattgcaac
cttgtgggaagcatacctccggggatttggacactcaagaaactacaagaattaattctatacaaaaacaagctcacaggcaacgtagtgatggatgg
ctttgcagctatgtccttgaaacggatagaccttgcagataataatctctcgggaatcatccctggcatttttgggcatttagagaacctcaatggattaga
gctctacaaaaacaacttctctggcgaaataccgacgagcatcggctggttgccatcactgcaagcattgagtctaggcactaacaggttgaatggcac
gctaccatcagagctcgggaagcacgcttcagccctaaaatatgttgcggtcgatgacaacgagctcaccggcgtgattcccgagggtttgtgtgacag
aagacggctgcagtacttcatagcctcgagcaaccaactgaacggttccatcccagcaggccttgccaactgcgccactctgaaagcgctggtgctaga
tagtaaccagctgtcaggacaggttccccaggccatgacgtggactgtgctcacgatttttactgctagtaacaacctattttccggtgagataccagcag
gtcctgtgagcagcatgccgctactgcagaagttgaacctctccggtaatcaactatccggctgcatcccctag
>g168683.t1
atgggacccgtgccgaatggattccacagcgccaacatcgcaacactgaatttcaccaacaattacttctccggcgagatcaacttcgtcacgcatctgc
caagcgtcgccagtatccgcctcaacggtaaccaatttaccggaccattgccggacttcagcgacctgatagtactgcaggaactcgatgttgcacaca
acaggctcacaggcgtcgtgccagccctgctgccgaagatattgacctccgggtcaatatctctcgccggtaacctcttgcagggaccagtgccacagtt
cgagagtgaagtgcgagatgatgtcgccgaggcggccaaggctggatgcttctgccggctggattccggcccttgtgatccggttgtggactccttgttg
ttggtagctggggcactgcattcccctctgatcctcgccacgagttggaggcgcaatgacccttgtgcaggctggctcggcgtgcactgcagcgtcgaca
agggtcatcgcaaggtgaccggcatcaacctgtcccgcctcgccctcaacggcaccatcgacccggccctggcgtcaatcccgtcgcttaaatccatcg
tcctgtcaggcaacaacatcacggggacaatcccgcctgccatcgcgaagctaccgtctctgctcatcctcgatgtctccgacaatgcattggcgggtaa
aatacccaggttccaccgcggcgtggaggtttgggcagaagggaaccgtgggctctacagcgtcgcaccaccgatcatcaaagcatccacttcttttac
gttggttgtggctatcctagtatttgcattctag
>g169685.t1
atgaggaccctgttgcaaaatcccaatgatctgaaacggaacgctgctccatttttaatgctcggcgacacgcagtcgcaaaatcccgagttccggagtt
cccatcctcgtcatcccccgatgtcccgacaccgcctcgccgtcaccgctgccgccgtggttgtcctcctcctgtcactctcgccgctgtgcgccgccaac
cccgacgacgagaggtgcctatcgagcctgcaccagtcgctgtcggacccctccggcgggctccgcaactggagcaaggcggccttctcggcgccgtg
cgagggcttcgtctcccacctccagggcgtgacctgcaacaacgggcgagtgtacaagctggccctctctgggctctccctcggcggcgccatcccgcc
gcagctctccaactgcaccaacctccagtccctcgacctctcctccaacgcgctctccggcgccatcccgctggagctctccgcgctggtcaacctcgcc
gtgctcaacctctccgccaacagcctcacgggcgccatcccgcgggagctcgccgcctgcgcctacctcaacgtcatagatctccaggacaaccagctc
tccggcatcatccccgacgagctcggactactggtccgcctctccaccttcgacgtctcctacaaccgcctctccggtcccatccccgtgctcctcgccaa
ccgcacaggcgccggaacggccgcagtgggtaccgcgaggttcaatgccagctcgttcgtgggaaacacggacctgtacggttacccgctgccgccg
atgaagacgcgagggttgtccgtgctcgccatcgtcggaattggccttggaagcgggctgctcagccttgtcctgagcttttccgctgtttgcctttggctc
cgagctactcaccgaacggctgccacgcccagcgaggaaggcaagatctccaagctcatgccagattactga
>g171330.t1
atggcgcgcctcaccgaggagaaggcggcgctcgaggccggatcgaaaggaagcggcgccaccgtcctcaacctctctcatcgcgccctctccgatgt
gtcctgtctgagcagcttcaagaacctggagcgactcgacctcggctacaactgcctcgtcacccttgagggcttgtcctcctgtgtcaatttgaagtggc
tttcagttgttgaaaacaagcttgtgagcttaaaaggtgttgagggcatgtcaaagctgcaagtgttgaacgccggcaagaacaaacttacaaaaatgg
atgaggtcacatctataacaagcctcggagcattaattctgaatgataacaatatttcgtccatttgcaagcttgatcgactgcaacagctgaatacactt
gttctctctaagaatcctgttgttactattggagatgctttggtcaaggctaagtctatgacaaagaaaataccttcagacttggccaaaaatgtcaaaat
attgaaccttgatttagggaataacctgattgagaggagttcagatttgaaggtcctttccgaattgcactacttgaggaaccttaatttacaaggaaatc
ctatttctgagaaagatggtcttgccaaaaaggtaaagaaatttctgccaaacttgcgcatattgaatgcaaagcccattgaagccagctccaagaaag
ataagagccctatcgaagcaaatgttccaaacaaagatgacaatatttatgatcatgatgctgttgagattggtacaaaaaagaaggataaaaagcag
tcaaagaaacaattaaaaggccctgaagagcttgatgtgaccacttctaccccaagcaaatctgaagtggtacatggtaagggaataaaaagggaaa
aagtagccatagaacatgctgataataacaaattaaagaggaaggatgataagtcttctgctaatgatactggtagaaaggacaagaaagaagccaa
gaggacaaaattcattgatgaagaagatgctgatgaaattgacaacacagagatgtcatttgcagatttagtgttttccaaagtggacagtccagaacc
taagctgaaggacaaaatccaagaagcagatggaaaacatgatggtttggtaattgatcacacaaaaaagaaaaagaaatcaaaaggtgctgtcacc
atcaccgacttctcaactcttaagatgttatcctctgtaccggaggtgggggcaggcggactcggagtatcaggatgggatgattag
>g171945.t1

atggcacccttgtcatcagaggtacaaagaagcaagagaactctgcatctcctcgtcctcctctgcctcgtccttccatgcctcacccagccggctccttc
ccctgctccgacgacgctgcccctctctccgttcaacgaccggctcgacgcggcgtacatcgcgctgcaggcgtggaagcacgccatcacggaggatc
ccaagaacctgacatccgactggtgcgggcccttcgtgtgcaactacacgggcgtgtactgcgcggcggcgcccgacgacccgcacgtgctcacggtg
gccggcgtggacctgaaccacgggcgcatcgccggcgagctcccggaccacctgggcctcctggccgacgtggcgttaatccatctgaactcgaaccg
gttctgcggcacgctgccggagtcgatggcgcacatgcggctcctgtacgagctggacgtcagcaacaaccttctctggcgcgttcccgtcgttcctc
acctcgctgccgtcgctcaagtacctcgacctccgcttcaacgccttcgacggcgagctccccgccgccgtgttcgggcgcgagctcagcctcgacgcc
atcttcgccaacaacaaccgcttcaacgtctcgctgacctcggcgagcctgaccaactcgacggcgtcggtgatcgtgctggccaacacggacttcgcg
ggctgcctgccgccgaccatcggcgacatggccgacacgctggtggagctcgtcctcctcaacaccagcatcagctcctgcatcccgccggagatcgg
aaagctgaagaagctcaaggtgctcgacctcagccgcaacgagctcgccggggagctgccggagagcgtcggcgacatggagagcctcgaggtgct
caacgtcgggcacaacatgctctccggcgtggtccccgagaccgtctgcgagctgcccaggttcaggaacctcaccgtcgcgggcaactacttctgtgg
cgagcccgtgtcgtgcctccacgtgcccctccgcgacgaccgcatgaactgcatccccgagaggccgcaccagcgaccccacgaagagtgcatcgcct
tcgagcaccgtccgcccgtgcactgcgccgccgacggatgcattctgcttccgccgtga
>g172103.t1
atgccaccagcagacacggcaacgttgccccgcacctacgcgtgcacttacctccatcttctggtcgtgttgctgccctgctgcggcctccatgcggcgct
gccccaactagccgaggaggcgcggcttctgctccagatcaagagtgtgtggggcgacccgccggttctggcgtcgtggaacgtctcggccacggccg
cacactgcaggtggccctacgtgcggtgcaacaaagccgggcacgtctcaggcctcttcctcgccaacaccaaaatctccggcccattcccaggcagta
caatcgccagcctctctggcctcaggcacatcgatgtcttcaacaacagtatcgccgacgtgttcccgacattgctgtaccgctgccgttcgctgcagtac
ctcaacctgtcttataacctcttcaatggggagctcccggctaacatcggccacggtctcgctgcaagcctcaccacgctggacctcagtggcaacgagt
tcagtggcaccatcccggcatcactctctaggctccagaatctcaagcttctcgcgctagacaacaacctttttgtaggttgtattccggcgaagctcggc
gagctgacgagcctccaggctatgtatctaggggacaattcattcgatgctggcgagcttccgccatccttcgtgaagcttaccaacctgagcaccctcg
atgcgcagaattgcaaccttgtgggaagcatacctccggggatttggacactcgagaaactacaagaattaattctatacaaaaacaagctcacaggc
aacgtactgatggatggctttgcagctatgagcttgaaacggatagaccttgcggataataatctctcgggaaccatccctggcattttcgggcatttgga
gaacctctgtgtattagagctctacaaaaacaacttctctggagaaataccgacgagcatcggctggttgccatcactgcagctattgattgtaggcact
aacaggttgaacggcacgctaccatcagagctcgggaagcacacttcggccctaaaatatgttgcggtctatgacaacgagctcaccggcgtgattccg
gagggtttgtgtgacagaagccgactgcagtacttcatagcctcaggcaaccaactgaacggttccatcccagcaggccttgccaactgcgccactctg
aaagtgctggtgctagatagtaaccagctgtcaggacagattccccaggccataatatggactgtgctcacgatttttactgcgagtaacaacctgttttc
cggtgagataccagcgagtcttgtgagcagcatgccgctagtgcagaagttgaacctctccggtaatcaactatccggctggatcgcttag
>g172104.t1
atggcaacaccgcgccgccagtacgcgcgagcttacctccacctgcttgtactcgtgttgctgccctgctgcggatttcagcgctgctgcgcggcaacgc
ggtcccatccgatcgacgaggcgcggctgctgctccagatcaagagcgcgtggggcgacccgccggtgctgctggcatggaacgccacggcatcggc
cgcacactgcgggtggcccttcgtccggtgcaacagagccgggcgcgtctccggcctctacctcgctcatgccagagtctccggcccgttcccagatgc
catcgccagcttctcccgcctcagacacctcgacctcctgtacaacaacatcgctggcgtgttcccgacctcgctgtaccgctgccgttctctggagtacct
cgacctgtcatggaaccacttcgccggggagctcccggctgacatcggccttggtctttcagcgagcctgaccacgctgtacctccacataaacaggttc
aacggcaccatcccgcattcgctctccaggctccataaccttcggtttctcaggctgtccaacaacttcttcgtcggcaccatcccagcagagctcggcga
gctgacaagcctccagaagctgtaccttgggaataattcattcgctgctggcgagcttccgccatcgttcaggaagctgaccaacctgactgcccttgga
gtgtggagctgcaaccttgtcggagattttccgacctttctagtgcagatgccggagctggagtttctgtacctcttcaacaactcgttgaccggaagcat
acctccggggatttggagactcaacaagctgcaatatttgtcgttgtccgagaacaacttctctggtgaaataccggcaagcatcgcccggttgtcaaca
ctgattgatttggagctggacaacaacaggtttaatggaacacttccaccagagctcgggaagcactctttggacctggctagtgtttcagtggataaca
atgacctcaccggtgcaattccagagtggttatgcaatggaggtaatctctattccttctctgcctcgagcaaccgattgaacggctccataccagcagg
actcgccaactgccccagtctttcagaactgaggctggacaataaccagttgtcaggacaagtgccctag
>g172105.t1
atggcaacaccgcgccgccagtacgcgcgagcttacctccacctgcttgtactcgtgttgctgccctgctgcggatttcagcgctgctgcgcggcaacgc
ggtcccatccgatcgacgaggcgcggctgctgctccagatcaagagcgcgtggggcgacccgccggtgctgctggcatggaacgccacggcatcggc
cgcacactgcgggtggcccttcgtccggtgcaacagagccgggcgcgtctccggcctctacctcgctcatgccagagtctccggcccgttcccagatgc
catcgccagcttctcccgcctcagacacctcgacctcctgtacaacaacatcgctggcgtgttcccgacctcgctgtaccgctgccgttctctggagtacct
cgacctgtcatggaaccacttcgccggggagctcccggctgacatcggccttggtctttcagcgagcctgaccacgctgtacctccacataaacagagct
cggcgagctgacaagcctccagaagctgtaccttgggaataa
>g172117.t1
atgagcatctcgggcgccatcccggcggtcgtcggcgacctcgtgcgcctccggagcctcgacgtctccaacaacagcatctccggggccatgccgtcg
tccctcggtaacctgacgcgcctcgagatcctcgtcgtgcgcagcaaccgcatctccggcaccatccctctcgtcctcgggaacctcctgcgcctcgaga
ccctcgatgtcgccaacaacctgatctccggcgccatgccgtctgtccttggcgacctgtcgaatcttcagagcctcgacgtgtccaacaacaatatctcc
ggcaacatccctccgtccatcggcaacctcacgcgcctcgagaacctctacatgttcgccaaccacatctccggagccatccctccggccatcggcaac
ctctcgagcctgcagaacctcgacgtgtccggcaacctcctcacgggacagattccagccgagctctccaacctccgcaacctcggcacgcttgatctcg
gaaacaatcagcttcgcggcggcataccgccgtcccttgccaagctgacggccatgtactacctcagccttcagtataactacttgtcaggccccatccc
caacgccatctttctcaactgcaccattttgggcgtcgccgacctcggggacaacgacctgtccggcgagatcccccgcgccgcctcgggcaccctcgc
cgacacgtttgcggtcctcaacctctactccaacaagctcaccgggacactcccccggtggctggccntccggcgagatcccccgcgccgcctcgggca
ccctcgccgacacaacaactcgctcgccgacgagctcccgaccagcatcatctccggcaaacagtacctcatgtacctgcatttgtccaacaaccggttc
tggagccacgacggcaacaccaacatggaccccttcttcgccgcgctgtccaactgctcgcggatccaggaggtggaggcgggcgccgtggggatgg
gcgggcggctgccgagccggctcggcgccatgctcccgcccaacatgtggcacctcaacctggagatgaactccatcgaggggcccatcccggcgga

catcggcgacgtgatcaacatgacgctgatgaacctgtcgagcaatatgctgaacgggaccgtcccggcgtccctgtgccggcttcccggactgcagca
gctcagcctgtccaacaactccctgacgggggacatcccgccctgcatcggcaacgccacgcgcctcggcgagctggacctgtcggggaacgcgctgt
cgggaagcatacccagcagcgtcgggagcctcacccagctcacctacctctccctgcagaggaacaggctgtcgggggagataccggcgagcctcgg
ccgctgtgccagcctgatgcgcctcgacctctccagcaaccagctgagtggggaggtgccggaacaggtggccggcattgtgaagatgacactgaacc
tgtcgcggaaccagctcgggggacagctgcccaggagcctcagcaacatgcagcaggtgcagacgatcgatctgtcatggaacaacttctccggcgtg
atcttcgccggcatcgcggactgcgtcgagctgacggtgctcgacctgtcgcacaactcgctctccggcgtccttccttcttccattagcgtcctgcagag
cctggagagtcttgacgtatcgaacaacgaactgacaggcgacatcccagtgagcctgaccaaatgcaccggcttgaaacatctcaacctgtcgtaca
acgacttcagtggcgtcgtgccggagaccggcgcgctggcgcggttcggcgtcctctcctacctcggcaaccggcggctctgcggtccggtggtcgagc
gcgcctgcaatggaaggcgccggcaccgctcgtggttccagtcccggaagttcctggtcatcctgtgcctctgcgccgcggccgtggcgttcgcgctgac
gatcctctgcgcggtcagcgtccgcaagatccgggaaggaatcgcgacggcgcgcgaggacaacaacatgttcttcatgatgggccgtctccgcggcg
gcggaggcggcgcgtcgccggtggcgatgaagtacaagtacccgcgcatcacgtaccgggagctggtggaggcgacggacgagttcagcgcggacc
ggctggtggggacgggcagctacgggcgtgtgtaccggggcacgctgcgcgacggcaccatggtggccgtgaaggtgctgcagctgcagacgggga
actccaccaagagcttcaaccgcgagtgccaggtgctcaagcgcatccgccaccgcaacctcatgcgcatcgtcaccgcctgcagcctgccggacttca
aggcgctggtgctgcccttcatggccaacggcagcctcgagcgctgcctctacgccggcgccggcggggcggagctcagcctgctgcagtgggtcaac
atctgcagcgacatcgccgagggggatgtcgtacctgcaccaccactcgcccgtcaaggtcatccactgcgacctcaagcccagcaacgtcctcatcaac
gacgacatgaccgcgcttgtgtcagatttcggcatctctcggctggtcatgagcgtcggcggcgtggtcaacaccgccgccgatgtcggcgcctccacg
gccaacatgctctgcggatccatcgggtacatccctccagagtacggctacggctcaaacccgacgacgaagggagacgtgtacagcttcggcgtgct
ggtgctggagatggtgacgaggaggaagccgaccgacgacatgttcgaggccgggatgagcctgcacaggtgggtcaaggcccaccaccacggccg
cgccgacgccgtggtcgacccggcgctggcgcggatggtgcaggaccagacgccggagatcaggaggatgtcggacgtggccatcagcgagctgct
ggagctcggcatcctctgcacccaggaccacgcggcggcgcggcccaccatgttggacgccgccgacgacctggaccgcctcaagcggtacatgggc
ggcgagaccacggccaccttcgcgtcttcgctcggcttctcctccgcgacatttgaagacgatattgactggcgtaacaattaa
>g172243.t1
atgatgaactatgctgtaaagcagataagtgcaccaagttccaccgatgtggttgtcaagggttggattgcagaggtgcggatcctagctcaccgtgtcg
aggatgtcatcgacaaatattcatatcattcacttaaactggaggaagaaaatgctgtgaagaagatcttttccaaaccttattatgttgcagttttcagtg
acattgctgaggagattagcaagatagagaagaaaatagaaaacgttgcaaaaagaaaagaacgttggttgcagccatcgcagcttggtgccaatcc
gcttgctgatattgaaagaaagaagtctcaagactgcctattggatgttgttccagatgatattgtggggattgaagaaaataggatactactgactcag
tggttgtactccaaggagcaaagtggtatagtgataacagtatctgttttcagtgaaattgctgaggagattagcaagatagagaagaaaatagaaaac
gttgcaaaaagaaaagaacgttggttgcagccatcgcagcttggtgccaatccgcttgctgatattgaaagaaagaagtctcaagactgcctattggat
gttgttccagatgatattgtggggattgaagaaaataggatactactgactcagtggttgtactccaaggagcaaagtggtatagtgataacagtatctg
gagatcttagaaactgcttctgctactgtagtttgttccctgaggactaccaattctctcgtgacagcttagtgaggttgtgggtcgcagaaggtttttcagt
gcaaacacaacaaagcacatcagaggaggtggctgacaattatctcagggaactgatccagaggaatatgattgaagttgttgaccaagatgaattgg
gcagggtaagtacctgtaagatgcatgacctcgtccgtgagctggcactttctgttgctaaagaagagaagtttgggtttgcaactgattttgggaccatg
gtgaagatggacaagagtattcgtcgattgtcatcatgtggatggaaagacaaaacttcaccgaaggataaaatcgaactcccacgtctgcggacactt
gttgcagttggaataaatgcatcccctcatttgctatcttcagttttgtctgaatccgtgtaccttactgctatagagctgcaagattctgaattaactgaag
tgccaacatctatcggaaatctgtttaatctccggtacattggcctgcgccgcactaatataaaatcactcccagagtccattgggaagctctccaatctc
cacaccctggacataaagcaaactaaaatagagaagctaccacgaggcattgctaagatcaagaagctgcgtcaccttctagctgaccgatatgcgga
tgagaaacagtcagagttccggtacttcattggcatgcaagcaccagaagacctagccaacttgaatgaactacagactctcgagactgtgcaagcta
gtattgagttagcagcgcagctaaggaaacttgtgcaattacgaacaagtgataagaatgaagcactttgcctggaagctctcaaaccagagtcagaa
aacctccaccggttgataattcgagggtgcttcccacatctgaagacgcttgagttgaagaacatgaatgatgtcaataatctggagatcagggatggt
gcccttcctcagattgaaggtttatatgttgtgacacttccaaatctcaacaagattccccaaggcctcgaatccctacgttctttgaagaagctgtggctg
ctttatttgcaccaggacttcacatctcagtggaatagcttagtgaggttgtgggtcgcagaaggtttttcagtgcaaacacaacaaagcacatcagagg
aggtggctgacaattatctcagggaactgatccagaggaatatgattgaagttgttgaccaagatgaattgggcagggtaagtacctgtaagatgcatg
acctcgtccgtgagctggcactttctgttgctaaagaagagaagtttgggtttgcaactgattttgggaccatggtgaagatggacaagagtattcgtcg
attgtcatcatgtggatggaaagacaaaacttcaccgaaggataaaatcgaactcccacgtctgcggacacttgttgcagttggaataaatgcatcccct
catttgctatcttcagttttgtctgaatccgtgtaccttactgctatagagctgcaagattctgaattaactgaagtgccaacatctatcggaaatctgttta
atctccggtacattggcctgcgccgcactaatataaaatcactcccagagtccattgggaagctctccaatctccacaccctggacataaagcaaactaa
aatagagaagctaccacgaggcattgctaagatcaagaagctgcgtcaccttctagctgaccgatatgcggatgagaaacagtcagagttccggtactt
cattggcatgcaagcaccagaagacctagccaacttgaatgaactacagactctcgagactgtgcaagctagtattgagttagcagcgcagctaagga
aacttgtgcaattacgaacaagtgataagaatgaagcactttgcctggaagctctcaaaccagagtcagaaaacctccaccggttgataattcgagggt
gcttcccacatctgaagacgcttgagttgaagaacatgaatgatgtcaataatctggagatcagggatggtgcccttcctcagattgaaggtttatatgtt
gtgacacttccaaatctcaacaagattccccaaggcctcgaatccctacgttctttgaagaagctgtggctgctttatttgcaccaggacttcacatctcag
tggaataggtatggaatgcagcagaaaatgcagtatgttccagagctccacatttag
>g172356.t1
atgctgcagaacttcacggcaaaccataacagtttcagcggcaagctgccgccgtgcctgaagaactgcacggagctgatccgggtacggctggagg
ggaacgactttaccggtgacatatcggaggcgttcggagtccaccaccagttgaattacctggatgtctccgggaacaagctaaccggccagctgtcga
gtgattggggtcaatgcactaatgtcacgaagttgcgcatggatggcaatcgcatatcgggcggcattccggcagcgtttgggagcatggcgagcttgc
gagacctcagtctggccgagaacaacttcactggcgccattccacctgagctgggtggtctcagcttcttgttcagcctcagtctgagcggtaatttctttt
cagggccgattcctgcaaatctgggcaacaactccaaacttgagaaagttgacttgtcagcgaacatgcttaatgggacgataccagtatacattggta

agcttagtcaactaacctatcttgatttgagcaagaacaagttcacggggcagataccaactgacgagtctgcagaaactgaatgtgtcacacaaccag
ctcaccggttcaatcccagcaggtttctctcgcatgtcaagcctggagacggttga
>g172753.t1
atgcggcgctgctctcttaccataactccttgtcgcacaaggctcttgctcctcctcccaatagcagtagcagctgctgccttctccccaaccgtccatgcc
ctgcgccaccacttacctaccggcaacgccacctgcttgccgcgcgagagagatgctctgctggaattcaagcgaggaatcaccagcgacccgctggg
cgtcctcgcttcatggcaacaaggaggccagcatgatgtcgacaacgactgctgtcggtggggaggcgtccggtgcaacagcatcacgggccacgtcc
tcgagctaaggcttccgagtaaaacttatggcaccatgttggctggccagataagtccttccttgctctccttgtaccatctggagtacctagatcttagct
ggaacaacctcaccggccacatgccggagttcctgggccatctgagtaacttgagatacctcaacctgtccggcgtgccgttctctgggaaggtgccac
ctcagctaggtaacctctcgaagctgcacttccttgacatctcatccccttatcttgatacctcaggaatgaaaagactatttgatgatggtatggtcggct
tgggtctgaatatgcctgtgttcacgaagggccaagaacgttcctaccaaattgaaggagctgacatgcaccagctttggattatgacctactactaa

NB-ARC domain containing genes
>g427.t1
atggcaggtgcgctggtaagtgcttctacaggagtgatagaatccgtcctctgcaagctctcttcaatgttggagaaaggataccgcgggagcaaaagg
gtcgagaaggacattctgttcctacgaaatgagctaggcagcatgaacgccgtgatgcagaaacacgcgatgtccaaggagccagatttgcaagtgaa
ggcctggatgaaggaggtgcgggagctagcttatgacatcgaggatgtcgtcgatgccttcgtggatcaggttgaagagaaaccagatcagcctatag
gcatcaaagggtttatcatcaacagcgtccagaagctgagggaactggtctcaagaagcacgattgctgaagagattgaagaactcaagcaccaagtt
cttgaggtaagtgagcgcagaaaaaggtataaggttgatgagtatacatcaggggaaagtgctgagaccatagaccctcgtctgacagcgctatatgc
tgagattgatggactcgtcggtattgatgccccgatgaacaaaattatcaggttgctcactgaggataatgcagacggtgaattcaggcaacaggttaa
attggtgtccattgtcggatttggtggtattggaaaaaccactcttgccaaccaggtgtaccagaaaatcaagggacaatttgactgcgcatgttttgtatt
tatgtctcaaaaaccaaatatgaagaagattcttgtggatctactttctggacttggtccggcttgtaacatgtcggatgacgagattcaagtcatcaaca
gaacaagagactttctccatgacaagaggtga
>g639.t1
atggagctcgcggcagtggcgatgaagccactcatcgggaagctcggcaatctgctagcgtccgagctgactctcgagaagagactgcggaaagacg
tcgcctccctggagcgggagctgaaggtgatgtacgtgaagctcagggaggtggccgaggtgcctccagatcggcttgacgagggaaccaagctctg
ggcaagcgacgtgcgcgagctctcctacgacatggccgacgcaatcgacgccttcaccttgcacgtgggggacccgaccgacaccgggctccgaggg
ttcctcaagaggatggctcggcttttcagcaagggcaaggcgctgcacgagatcgccggcgccgtcagagacgccaaggacctcgccaaggagctgg
gtgagatccaccaaaggtacgctgggctcaagttgcaggatgtcagcgtcagcgctagcgcttctgacattgaccctcgtctgatggcaacattcaccg
aggccgcggaccttgtcggcgtcgacgacgcgagggacgagctgatcaaggtcttgtctaatcgctcggaagtggatgtgaagattgtctccattgttg
ggttaggtgggctgggcaagacgacactcgccaacgcggtatatgacaagatcaaagtgcagttcgtttgcagcgctttcgtttcggtgtctcgaaaccc
ggacatcactaggattttcaagaagatccttcatcaacttgacaaggaagagtttaaggacatcaatgaggcagtgagggatgttgatcaactcatccg
tgagctcaaacaataccttcagagtaagaggcggatgccaaaagggttggagaatctgacttccctagaagagctaactgggttgacaataatcagca
gtgaggattctgcagttgttgtaaaacaactcggcaacctcaccagactcaggatactcacattgtggtggcatgtggaggatggttgtgaagctttggt
acagacgcttggaaatttgcggaagctacagagtcttgatgtgtccatcgtaggcgggtgtggcaaattcatgcttggttgggcgccaccgccgcgcctt
cacaggttcttgtgcaggggctggcccatagcattgccgacgctgccggcatggatcaaaaacagttcgttgtccctaacatttctggaggtctcagtca
gacaggtggcccccaatgatctacaggtcctcgggacgctcccactgctacgtggcgtcagcctgtattcgaacgcccccgtcgaccaccacccgccgg
cacggatgacagtgaacactggcgcgttcccatgtgcgaggtggtgcgagttcgtgaacttcatgacggtgccgtccatgatcccaccgggcgccatgg
cacacgtgcagcaccttgagttcaatgttcgggtatggcatttcgacgacggagtaggtgggtttggatgggacgacctgcgcatggggcacctcccttc
gctggaggaggttactgtccacctgttgtacaggaggaaggactacgccgcccccggcggtagagcggatgcacgcggcgctgcggcaggccgcgga
agatcatcccaaccgcctcgcccttaaaatcgttgagagtgtcatctcgtcgccacggcttgtcttcctcgtcgtcgagtcggtctcacccaccgtcgtcaa
caacgtgatggtgctgcacgtcatcgccgcctcctctacccagtccagaaggaaacatgctggcgctgaactgtatgtcattgagcggtaccataatttt
aagatggtagatggcaaatctgacgtcgcgcaggctcatgagatacaatgcatagtgaaggaactcgaattgcttaagatcgtcttaactgagaagctt
gtggctgggggtattattgccaaattgtcttcaacttggaggaattttgccactactcttaaatacaagaggacgaacatatctgtctcagatttgatcgcg
tctcttgatgttgaggagaaagttcatgagaagaatggacgttctaaagttaacgagggaaacactattgccaatatggtgcatcaaccacaacattctc
atggcagtggtggtaagggcaagggcaagaacaataaacctaagcagaccactaccttcaagaagaagaataaggagaagagagcatgctttgtttg
cggttctaatgagcattgggcaaagaagtgtccagaatacaaaggaaggaagcctccacagcagaagtctatgaacatggtgaccagtgctggaaaa
aatagtggatcgcccgagattcctccgtcctaa
>g3897.t1
atgtggctgacggaggatgcgtttgacaacagtcggctaaaaatcatccccatcatcggaccaaagggcatgggcaagagcgttcttgcatggcaggtt
taccggcaccgcagcgtggtgggtaggttcgactgcagagcctggtgcaacgtcatcagtcgcaccagctcatggcctgacgtcgcagcacagctact
ggacagccttgagacgcgtgataacatcggcctgccgctgtggaacgatgacaaacccgtaatggagctcaaggcgcacctcagaggccgaaggttc
ctgctggttcttgacggtctggactatcaaccgacgcttacggcgatggaggagaccctgcgatggtgcccctacaccgccggcagcgtcgtggtggtg
accacatgtagtgagtccttggtcctcacctgtgacgcctacagcttcatgcacctcaagacccagccttccatagtggacgccttcatgaacagagctct
ggccatggttcccgatatgccttacagcatggcagtggttgttaggaagatcctgtcacggttctccacggaccccttctacatgaagatgcttatccatct
gctctatgtgaatccaagcaggaccacgcaagagcttcagggcctgtcaagcaggctagccgatgacgaaccactgaccaatgctatgcagatgttca
tgttcagctacgatgaattgcccagtcactacaagagctgcttgctgtatctgacaatcttccctcagggctgcaccataaggagaaagagcctgatccg
aagatgggttgtcgaaggtctgaggttaaagcttatcacggtgctggacctggaaggctgtcagggcctggaaaggcgccatttcaagatcatctgcaa
caaattatatcagctcaagtacttgtgccttaggagaactaatgttacacggttgcccaaggacataaacaagctccgttacttggagacgctggacgtc
cgggaaacccaggtgcaggatttcggcacagaacctctcgtgcttcaaatgctcaaacacttacttgctggtatgaaaaaagacatcacaatgtcaaac

acatccttttctgctgtttatatcccacaatgcgttgggatcatgagaaatctacaggtgctgtgccatgttgaagtctcccataacactgagttggatgag
attagccacttacagcagctcaggaagctaggtgtggttctgaatggcaaggacgttgatcttaagcatttacttgggacaatcaatgagttgagcagct
gtctccgctctctatcgatcagaatcaaggaactgcctccgccttactgggttgaagggctgcacatgccggatcccttgttcaaacgcccaccaaagttc
cttgagagcctcagcatttgcggcctaagaaatgaactgccttactggattcaagaactgcatcagcttgtcaagataaccctgagcgaaaccttcctga
cggagaatgctttaaggaccctgggccaactgaagggcttgcgctgtcttaggctccggcacaattcttttgtggcaactaagcttgcttttggggtgagt
gaattcagaaatcttaagtttctggtcgttgaaccaacccacatcaccaacataagcttcagtcgcaaagcagttccaaagcttgagaagattgtctggt
cattttctcacatacagtccatttctggaatcagaattaaggctaggaaacaggatgtgcttaagcgctcctttggtgccactttatga
>g4505.t1
atggcggaggccatagccatatccgtatctgcaaaacttgccatggcgctgtcccacagcgctgctgtgagcctgtcaaacctcttcggcgtcggctctg
acatcacggcaactgcacaggaacttgaagtcatgcgcgccttcctcaggtccgccgattcccagcaaggtatggacgccctcgcaacctcatggatta
agcaggtccgcgacgccgcctttgagctcgaggacgtcgcggacgaatgctcctacctctccggcaacggccgtagctgggtcaaagtcaggtcctgg
ctcgccttatcccgcaggctgcgcagggcacgcgagaggcttcaccagctttccgcagcgaaggagctctacggcatccgcctcgctgatggtggccct
gcagtccgaatcgcaaccgtcaacagccggacgcttgccgatagtgcgcatttcattagaaaggaggaggtcgtcggattcgatgatcacgagaagaa
gctactagagtgggttgcggaggatgccgagccacagcgaacactgatcgctgtctgcggcatgggcggcatcggcaagaccacccttgtgacgcgcg
tgtacaaggaggtagcaacgagccacttcgaatacgccgcgtgggtggccgtttcgcatggcttcacgacggacgacctccttggaaagatcctcaag
gagcttcgccgcaactctggtgctacaagtttaaacgccgagagcgacaccgacgcagactaccggtcgctcatggcggcggtgcagggtcatctttgc
aagagaaagtacctagtcgtgcttgacgatgtctgggacgcccacctttggggcaagctcctccacctagcgttcccggacgatgcagccggaagccg
agtggtcatcaccacgcgcagcaatcaagtggcaaaagcggcgacgctcgaccggaccatgatgctggagcctctgcagtggcatgaagcatggact
ctgttctgcaacgtcaccttcagggacgtgcccaaccgaacttgccccagtcacctggaggggattgctaccaatatgctgaagagatgccacggcttg
ccgatggcgattgtctccatcagcaaactcctagcgctgaggcagagaacagagttcgcctggaggaacgcctgtaacaacctcgtgtgggacaagag
cagcgacgatcttggaatcggggaggccgcaagtattctgaacctgagcatcgaagacctgccacaccacctcaagaaatgcttcttgagctgcagcgt
ataccccgaggacttgtggctcaagcggaagatattgatcaggagatgggttgcactgggcctcgtacaagataagccaggccactgtagcgcagagg
acgtcgcagatgaatacctagaccagctgattcaacgcaatctgatgcatccagttgccaagaacgagtttggtcgtgccaaacgttgtctcattcatga
tcttatcagggagctcattatccacaggtccagagaagaggaacggtactggcgatttgcaatgtgcaagcttctcactccaaacattaccgaggccaa
gaattag
>g4722.t1
atggagttcacgagggatattgtaatccccgcggctctgtccgtgatctttgagcggttattgtcgtcgctggtcgaccacttgccgcgtccgtcatcggcc
ggtgcccggtccgagcaccggcggcggctggagctgctgctgcaccagatcagcagcgtggtcgaggaggccgaggggcggcacatcaccaacccg
cacctcctcgtccagctcaagtcgctcaccgacgccatgtaccgcggccgcttcgctctcgaggccgcggaactcgacgacgtcataaaaaacaatgaa
atcagcgacaacgacgacgcgaaggatgatgactccgtggtgaccaccacgggcaagcgcaaattcgcgctctgctcgccgttcaacgccgccaagc
gtgtctgctttatcctgggcggcaaggactatgcgagggagaaggagaacactagggtgaccaccgtccttgaggatctggagactctgaccagtgac
tacacgccaatgttcgtcgagctgatgaaagggtacccccaccggcggtgtaataatgtgccccggccggtgaccacagcgctgttcatggaccgctgc
gtgttcggccgccacgtggagacggagcgcgttgtcgccttcctgttgcagcgcgcgcccatgtccaccggcctgagcgctctcgcggtggtcggcgcc
aagggagtcggcaagacgacactggtgaagcacgtgtgcgccgacgagcgcgtgcgcagccactttgcgctcatcgagtggttccagtggtgggagg
tgcagcagacgggctggcagcccggccagaccgagtggtccgagtacctcaccgccagatcgagtagggagggccgttccctactcgtgtttgagtgc
ccgtttccagtcgacgagtcggcctgggccacgctgttggcgtcgagcaagctcgccgaggggagcaagctcctcttcacgtgccagagcgcccgcctc
gcccggagtatcgggacggcggagccggtggtgctccgtcccttaccggaggaggagttttggtactacttcaaggccttcgcgttcggcggcgcggac
ccgggtgaccacccgagtttagtggcggtggcaagggacatcgcccgccaactacagtgcacgtttgtcgacgcgcggttgctacgggccaattttgac
gcccggttgtggcgcagggtccgggacgccatcgtcaggtgccaacgccaccctagattctcactctacgacgatgtcatgttggacgtccttaccatca
ggggttggaggctgatgcccgggtacggggagtcccggcccagcttcagtgagctcaccctgcaggacgtgatccgcgcagcgacctcttcttctggtt
ctaggcttgacggcactcatacggaggaaaccttgctcatccactattgcagggacacactctacaaggatttctggtataccatctgcattgaaaagga
cgcgcgaccgccgccacaaattaactacgacgatagtatagaagcacaataa
>g4724.t1
atggaggagtttacgagggaagtcgtggtccccgctgcactgtcgaccatctttgagcggatattctccttcctgttggacaagttcccgatgccgtcgtg
gggtggcggcgcccgggacacggaccgggcgcggctggagcagctgctgcggcgcatcggcagcgcggtggaggaggccgaggggcggcacatca
ccaacctgcacctcctcgccgagctcaagtcgctcatcgacgccatgtaccgtggccgcttcgccctggaggccgcggacctcgacgacgtcataaaaa
acagcgaaatcagcgacaacgacgacgacgacgcggggggtgatgatgactccgtggtgagcaccatgggcaaccgcaagttggcgctctgctcgcc
gttgaacgctggaaaaaagcgtgtccgcctgatcctgggcggtggcagcgacggcacgatgacgaagaagaggttgctcgtcctcaacgaggagctg
gaggcccttattaggggcgattacatgggggacttcatcctactggttcaaggctacccccaccggcggtgtaataacgtaccccggcccctgaccacg
acgctgttcatggaccggtgcgtgttcgggcgccacgtggagacggagcgcatcgtcgccttcctgctgcagagcgcgcccaccacctgcagccagaa
cgcactcgcggtggtcggcgccaagaaggtaggcaaggccacgttgctgaggcacgtgtgcaacgacgagcgcgtgcgcgaacacttcgcgcacatc
cagtggttccattgtgaggacgagtttgacgcgggcaagcagctcggacagaccgaacggtccttgtgcctcgccggcatgcgtagcatcctcgacga
ggagccgcggttcagttcgggccggtcccttttggtgttcaaggacgcgtggcccgtcgaggagtcggcctggaccacgctattggccgcgtcgagcaa
gctcgccgaggggagcaaggtcctcttcacgtgtcgcgacgtcaacttcgcccggaccctcagaacaacggaggagccggtggtgctccgccccttgc
cggaggaggcgttctggtactacttcaaggccttcgcgctcggcggcgcggacccgcgcgaccacccgcggatcacggcggtgggaagggacatctc
ccgccacctgacgaagagcacctttctcgacgcgcggttgcttggcacggtgctccgagccaacttggacgccccgttctggcgcagggtcctggccgc
catcctcaggtgccaacgccgccctaggtactcgctctacgccgacgtccttgccatcaggggctggaggctgatgcccggcgctgggtgtgcccgcc
taggttcagtcagctcactctgcaggacgtgttccgcgcagccacctcttcttctgcttctgggcttggcggcagtgatacggaggaattcttgaccatca
actattgcagggacacactgtacaaggatatctggtataccatcacctacgagctgcttcacgatgataataaccgcattaattaa

>g6003.t1
atggaggtcctgacgagccttatcccgaagctcgctgagcttctaatcggtgagtacaatctgcaaaaggaggtgaagggggtatcatattcctccaa
gctgagcttgagcacatcaaggccgcccttgagaagatctccaagacacctgtagaccagctcgatgagcaggacaagatctgggccagggatgtca
gagagctgtcctatgatatagaggataagatcgacacattcatggtgcgctgcaaggtcagcaagacggccaaagggcatggattcaagaaggccctt
gataggagccttgatttgttgatgcagcccaaaattcgccgtaagattgctactgatatcagagacatcaagggtcgtgtcaaagaggtgtcagaaagg
cgtgataggtacaagttcaacaacaatgatgttcctaaacctgtcagaattgaccctcgtttattcactcggtaccaaaaggctgtagatcttattggcatt
gatgcggcaacagatgatgtaatcagtattctgggggaagacaatgaagtgtccatgacacaagataagatagtttctatagttggatttggaggcctg
gggaaaactactcttgcttctgtggtgtatgaaaagcttagagccaaatttgactgctcagcctttgtctccgtttctcagactcctgacatggataaattgt
tgaaggatatgttctatcagcttacaaagagaagcaattcaagaataaatgttcttgaggaacttcgagaatttcttgagaacgagagtcttagatccctt
gaacagttgtttggatatgtgaacttgaatgagctcgtaaacgacaagtcaactaatgttatcgaagagctgggccttctagctgagctcagggtgcng
aattggattgggagtcttagatcccttgaacagttgtttggatatgtgaacttgaatgacctcgtaaacgacaagtcaactaatgttatcgaagagctggg
ccttctagctgagctcagggtgctgcgtatttgcttgctcactggtaaatggaatgacaagctggtggatagccaaggggctctgccgaagctcagaag
acttgcattcgatttccatgtgcgggaggcgagggaaatcgatggtaccgatggtggtttcaacttgggtctgggaaacctgccgtcgctcgagtatgtc
ggagtttcgttccgatctgcaggtgcaagccagcaggaggtcgaggaagcgaaggctgcgctgaagcaggcgactgaaatccaccccaatcgtcccg
aatttcaatag
>g9479.t1
atgccgccatcggtcgggtggtccgcctcggccttcgtcgccgccgtgctggcccggctcatccggaagggcctcgagctggtggccgagctcgacgac
gccggcacgggccacctgcgccgcctggagggcctcctcccgtccgtgtggcgcgtgctcgacgccgcggactccggcgccgtcgacatcgtcgtcgc
cggcgggcggccgctgcaggacctgctcgacgccgcctacgccgcggacgacgccctcgacgacctcgaccgcgcgctccacggtgccgcgggaga
aggagacgacggcgtcgaggagctgcagcgaagcccgagcgccgcgtggaagccgcgcagcccgctgaggttcctgctctgcttcagcccccacgg
agttcagtcgctagcactagcagcagcgctcatggcaagagctccgaagctgccaagaacagcaaagtcaacctcaatctcgacgcgttgcgggacgc
gctggagacgatggcacaagcggcgtacaggtgcaccaccgcgtacgagcacgtggagcccgagaggaactacgccaccgtcgtttccgggagcag
agcagaggagggagcagagaaggaagcagagaaggacgacatcttcggaagggaagccgaggtggaagcgatcctggaaaacgtgaggtccagc
gacgacccgcagtaccgtctcggtatcggcgtgctgcccgtcgccggggaccccggggtggggaagacggcgctcgtgcggttcatgttccaccacga
ggtcgtccgggcagagtttcccgtgcggatgtgggtgcacgtctccggcgagctcgggctaaggaagcggcgtatcgtggaacagatcatcaacggag
tcaccggctttgatgagcatcaagaagtcgatgatggcagggagcttcttgggatggaattggacgggaacaggttcttgctggtacttgatggtgtcac
ggacattgtcgagagtcagtggcaatatttgatggagttactgaagccagctgataggaggagcttgatcatcctgacaactcagtctcaaactgtggc
gacagcaatcggcaccatgccgacactgacgctgggcactctgggattcaaggagtacctcaagatgttcaagcatttcgcattcggcagcacggatg
aaaccgaagaatgcactctgctggatgacgattgttgggaagacgtggaggaggaggacgagcaggaggaattgtcacccatggaccagattgcttc
agagctggcaaagaagatgagccgcctacctttgccagcacaggcaatcgggcgggcgctgcgtttccggaaggacgatgaaggccactggagaag
cgtgctggaagacaagatctgggagggccaggatgtcggtgcccagatttccccggcattgtggctgtgctatcgtcatctggaccctcgcctcaagca
gtgcttcgcatactgtgcagtgtttcccggtgattatgtcttcaggaaggaagaggtggttcagacgtggatggctcaggggctgttatattctgatcagc
ccgcgacgaggttggaggacgtcggtggtgagtttttgatgaactgcagcacctcttgaaatcccttgaaacgctatcaatcaagaattgtgacaacgt
catctctgacattccagacgggttcaaggacctcactgcactgagggatatctccctatttgactgcccaaagatgctcgtcgataagtttcacacatcag
tgcgaacattggagatcagtgagtgcttcgttataaggggtgcatgggtgcaggaccaccctttcctcttctccgtttggaaactgaaaataacagggtg
ctcccatgtaagcagcagcgatcaggataacaggattgagcctttggattggttgaacagcatgatcaatgtgtacagccttcatctcgagaacacaaa
gatcctcagattgaacatgtttgatcagcttccatcacttgagaaccttgatgtcaatggatgtgattctttcttttctgatttgtatgatttcgcttggttgga
gaggctgcagaccatgtcgatcaggaactgcaaggaaatgagcgggcttccggagaatttctgcacgcttcctgagttggaggagctgtgtatagaga
actgtccagctattgaagtgttgccggagaatggccttccgacaactttgaagcgactttcgatctcaaaatgtggtccgctgctggctgacagatgcctt
gacgatgaagtcgatgggccaaagatttcaatggtagatgcggtttacattgatggccgttgcattcgaacgacacaaactgacatgtag
>g11002.t1
atggaggcgacggcggtgagcatcggcaaggccgtgctggccggggcggtggactatgccaagtctaccgtggctggagagattgcactgcagctcg
gcgtcgagcgtgacgtggggttcatcgccgacgagctggagatgatgcagtccttcctgatgacggccgacgaggagcgggaccagaacaacaaggt
gctcaacacatgggtgaagcaggtccgtaatgtggcgtacaatgtggaggacaacctcgtggatttcgaggttcacgcggagcgggagaagcctcctt
tgcttgggtgcatccctcgcaatccgtgcgaccgccgccgcatcgcaatggaggtgaaggagctcaaggccaaggtagaggacgttagcagcaggaa
tctccggtaccgtctcattaaggatgacaccatcgggggctccaagcctgtcgaccatggcgtccaacaaggtggcatggacgatgaggtgtggcgtct
caccatggagcaggagaaaccagatgtggatcttggccagctgatactcagcaaggtggttgatcttcaagtgatcgcggtatggggcaggagtggtg
attttgggaagacgtctgtaatctccaaagtctttgacgacacacgtgtaaaggcggagttcaaatgccgtgcctgggttggactgatgcgccctttcgat
ccaaaggaatttcttcggagtctactgtggcagtttcatgtgaattcttcttctcaagagcgagagattagtatggctcaggaaggaaaaactgttgggac
taatgtcttgctgaagatggacaagcttgggcaaagtgatttggtccatgagtacgatacacaggtcagtggcaacaagtacctgattgtgatcgatggc
ctttcaaagatagatgagtggcattccatcaaatcctacttccctgataagaagaatggcagtagggtcgttgtagctacgcagcaagttgaaattgcaa
gcttgtgccccgagcaaccatgccaaacgtcaaaactggagcaattttcatcgaatcaggtcctttacctgttccacaggaaggtaataagtaatctcca
tcagcacgttaataatctatatattgactgggtggcatacaatatgtaa
>g11123.t1
atgaatctggtgacgggagctgtgggcagcctcctccctaagctgctccaactcctcgggaatgaatacaatctacagaggggggttaaagaagaaagt
gaagtatcacgaacaggaactgcgcagcatcgatgccttgcttaagaaggtctcccacgtgccatcagaccagcttgatgaacagattatggtctgggc
tcgcgatatcagagaggcatcttatgacatggaggatatcctcgacaccattcttgttcatattgagggccgcaagcccaatgacctcagcaggcttcaa
cgtgcagtgaacaagatgaaaaacctgttctgcaaaatcaaggtgcgccatgacgtcggtaaagccatccaagatattatgaagcatctccaggaagtt
gccgaccggcgtgccaggtacaaaatcgaagaccatgtggtaaacagcacggccataaatacatcaacttcgaattctgatcctcgccttaaagctatg

tacaaagaagttacaagtttggttggcattgataaaccatgcaatgatctcttatccaagctgtctgacggccagggggattatatgtctcggcagaaga
agaagatagtatctattttcgggactggagggcttggtaagaccactattgccagagttgtgtatgacaagcttatagcatattttgaatgtcaggcttttg
tagctgttggtcggaatcctaacctagtgaaactcctcatggacattctctatgaacttgacaaaagcaagtattcagatattcataccaagaaagtagat
aaaaagcagcttatcgaccgccttcgagaattccttcagaaccagaggtatatcattgtcatcgatgacatatgggagactgaatcatggacagaagca
atggacctggcgttgcttgagaataattttggaagtagaataatcataaccactcggaacgctcaagttgccaaacaaattggtgatgaggtttacaata
ttcagccactttcacatggaaacgccataaagttattctatgcaaagaaagttgaagtgcgtatccgatgtgagggtgcgtctgctacagaggttggtga
agtggaggctgagctgaggaaagccaccatgggccatcccaaccgccccatgcttgaggtgtacagatggaatgaagacaaaataatcccaactgtc
caggatcaaaagctgtgccgccccctccgtccatgtgcacgatcaccgcaccacctccgccacccacgcgtgaagccaccacgccacagatccaactt
gccgtcgtgcgaggccatcctcgacatgcgcggcacacctaacaccagcatcccgacacacaacgcggccaccataccgcctccaatcccatgccgtg
atctccacggccgccccgccgcctaccggcaccgcttcctgctacgtgcagcccaacgacagatccagccctccaggcacagccacccgcctgcgagc
gaggagtcgacggccgcgcaccgaggccaccagcctgcgtggaagccatctcgtctcaaccaccacatgtag
>g11490.t1
atgggaaggctcttcctggtgctggagaaggagtacaacaagcacaagacccttgcacaagagacccggtcgatccagcaggatctgcgcatgatcg
ccgccgccatggacgatcagctccgcgccttgggccaccggagcgaccgcaccgccgtggcgaggctgtacagcgaagagatgctcgatctgcaaca
cgacatcgaggactgcgttgatcgcttcatgcatcgcctcaggtgcaagcagctcactggcggcggcgcagcgtcctccttggtgcgtcgggtcgctcac
gagctgaagaaagttcagagccgttcgagcttcgccgatgagatccagaagctcaagagccgcctcagggaagcacatcagcgggtaatcgacatgg
ttcctaccggcttcggcggcggtgcccaacccaacgagggatcgttatcagcactgcctcgtgtcgcgcgtaacccggtggggatcgagaagcctgtgt
catcagcactgcctcgtgtcgcgtgtaatccggtggggatcgagaaacctgtggaggagctgctctcgctgctagatgaggtggacggcgagcctgag
cagccgagggtcatctccatcgtgggcttcggtggattggggaagaccaccctcgcgagggcggtctacgacagccctcaagccagggaaaaattcga
ctgccgtgcttgggttgcggctgccaggggctcgccggagaccggcgatgggaacagagggatccttcgtgatataattaagcaaatgcgtccgaaag
atgccatgaatgttgacagccactatctagaggcctggatcaaagaaaacctcaaggatgtcaggcacttctttcacctcctggccagggtggtcgtcgt
cgacgcagcggcttctccggccgtcgtggacgtcatgacaaccatggctgctatgatgttcttggtcgccggatccccaaccgctgccgcggccgtcaa
ggccatggtggccgccgccagcgtcatggcagcggttacggtcaggatctag
>g13161.t1
atggacggcgcggcacaggctcttgtgagcaatgttgggcagctggttggggaggagtttcggcatctccgcggcgtgggtggcaaggtcgcttgcctg
aggaacgagctggccaccatcaacgccctcctgcgcatgcaatctgaagtcgacgagggttccgtggaccacttcgtccgggagtggatgaagcagct
acgggaggttgcctacgatgccgaggactgcgtccacctctatttttccgcgtcaggcgccggcagggtgaccacttcttcgtcaattgcaagcgcctcc
ttacgactcttatggctcgccatcgcctcgccggtgacatcagggagctccgtgcgcttgcttcctccatcaacgaacatcatgctcgttacggcttcagc
ctcgagccactgcgacgcacggctgcttcaggccctgtgcagcagcccttggcgcaatcggcgcgcccactccgctccgcggacaacgccgatgatga
cccaaatcatcatcatcaattcattggtaacaaggctcaggctatcttcctagcgaccaaggttaaggcacaaaatggtgagagtgataagcagctcaa
ggtgctctccatcgtgggcttcggggggctcggcaagacaacgctagcagtggagatctgtcggcagctggagagagagagtttccgcgccaggcgcaa
gtgtccgtgtcacagacattcagcagcaaggacctccacgcattgctcaagcgcctgcttcagcagatcgcacagcctagcaacgagcaagcggattc
cctggccggtaacatagacaccatgcctgtcgacgacctggaacgcgagctccaaaagagagttgagaacaagagcattttcccagaggattatatga
ttcgcaaggaccggcttttgaaaagatggatcgctgaaggattggttgctgagaatcgggggttgaccaagatggaggttgcggaaggctacttcaata
tcaaggccatgcctccaaaagtgggtgatctcgagcatttgcagacgcttgatgtacgtcaaacagacctcaaggatctgccaaagacaatcaaaaag
ctagagaagctggagcacctcttgttctctggcaagggtgagtcgtggtctggttggatgctgccccaagggattaataaaatgaaggcactacgccag
gtgaataaagcagcagtgatatatgaccccaaggtcgcccaggagataggtgaattggatcaattgcaagagctagttatctatgtcgacacgagaaa
ggaaatgggtccagaggttgtcaaaaatcttgcctcctcccttagcaagatgtactccctgcgatggcttgacattggcaatttcggctgtgacaaatggc
cttttgatccgataatgaaatttctacatgatgtagagtcaccaccacggctgctccgataccttaagatctgtggctgcatcgacagattgcctgactgg
gtggagtcactcagcgatcttgtcgagtttgacatagggtggacgtaccttaatggtgtccaactattcgacgtcatgtgtaagttacccaacctgaagag
ggtgtacctggggccatactttattcggaaaggagaaaatatggttgcacactgtagccagtcctttccggagcttaaggagctgactctaggctattctc
ctgaagttcctccagtttatagatttgagcaagggtccatgccaaagctcgagactctagtggtgtacttcggcgaccagcccaaggaaattgttggcat
cgagcatttgacgaaccttaaagaggtacagttcgatggttggagagaccaaatgaagcatgcactggaggaggtagaggagctgaataagaaacg
ggacgtgtccgaacacataactgttcgagtgaggtatgaggacgacatgtag
>g13264.t1
atggagttcgcaactggggcactgctctccaagctcgccaatctgctgcaagaggagtaccacctgcagaagaatgttaagaagaacatagagttcctc
atgaaagagctcgagactacccaggccgccctccgcattgtcggcgaggtgccagcggaacatcttaacgagctggtcaagatctgggcccgagatgc
tcgggagctgtcctacgacatggaagatgtcgtcgacagcttcctcgttcgcgtccagggcccagaccctctaagcaaaaggagctcgaaaaggttcat
tcagaagatgaagagtatgctcgccgtaggcaagcgccatgagattggtgaacagatcaaggacatcaaggaacgcgccgtagagctggcagcacg
ccgtgacaggtgcaaggttgatactgttaataatcataccatatctatggatcctcgcatgaaagctctatacaccacgacagctgaacttgtaggcattg
atgaaggaaagaaagaaataatcttcaaactgacaaagggagatgacatgtcagcacaattacaaaagataatctctattattggatttggaggacta
ggcaagacgacactagccaaagcagtgtatgacgagattaaattgcagttcgattgcacagcttttgttacagacctctaccacctatccattgcggtca
aggagatacaacagaaggatctcaatatcctcggcaggctaccgactctccgccagttagttctggagatgggccatacggatctcggaatccaaggg
ggattcatcattgattctgattcgttcccatgcctggtaaaatgccatttggagggatttacaggtcctctagtgtttcgtcaaggctctatgccgaggctca
aatatcttgcattcgacttccatgtgtgggcggtgagggaaattgcagacagtggtggcagctttgacttgggtctgcgtaacctgtcgttgctccagcta
gtcaatacttggttccgaggttccagcaaaaaggaggatttccaggaagcaaaggatgcgctggaccagaccactgaaatgcatcccagtcatcctga
cttccggtctcggattattgactggtgggtctccgttgggcagcgccatctctcgctctccctcaaggacgagaccctcctgcagctctccatcaccatcct
cccctccggaatttctaccggatcccctcacagcgtgcagctcatcgtgaacaactga
>g14611.t1

atgggatcgcttctcgccaagcttggtgccttgcttgagaaggacttcaagctagccaaagatgcgaagaaggaaatcatttctctgagagacgagatg
agcagcattaatgcttttctcatgaagctgtctgcaatggaggaaccagtcgatgtgcagctcagggagttgcgcagcaaggtgcgtgagctggcctat
gacatggaggactgtgtggacatcttcatgcacaaccttggctctaataagaaagggcttctgcatgggctgaagatgctcagggcacgctacgagacc
gccacactgatcaaaactctgaaggctcgggcggtgggaataggcaattggtatgagctgatgactaggctgcctgggtggcctcgcactgtgtgggtt
gaccctcgcatacaggcgctctacacagatgctgccattcttcaaggcatagatgacccaaaggagaaactcgtcaagctgctcaaggaggacggtgc
atgccgactgaaggttgtatctattgtcgggtttggtgggattggcaagacgactcttgccaaacaagtttacactaccatccagggccaatacgactgc
acagcttttgtgtcaatgtcccgaaatccagatttggccaaaatcttgtcagatataatcaaacagttcgttgggtggttttattcgagcctggatgatgag
aaacagctcattgaagaattccgggattgtctccaagacaaaaggggttgtgctggctggtcgggtcggccagatgagaagcctccgtacactatggat
cacgtgttgctggcggcgctcatgttcaacttcctgaacagggacgatatcggacacgactactacgcgccttgcagggactattcagagtggaggtaa
>g14703.t1
atggacgtggccgtcgagtgggcagcgtgggtggtgggcaaggcgctgggccccgccactgacgtcgtgttggaggcctgggcagctagcgtcgggc
tcggctctaacgtcgaggccctcaagaaagagctgctgtgcgtgaaggccttgctcgagcatacccgtggcaaagaaataagcaactccgcactagag
gaaatgatgcagaatctgcggaacaaggcgtacgatgcggaggacgtgctagatgagctggactacttccgcatccaggatgatctctacggcctcgc
cgaggccgccgatgtccaccccaaggcgtgggtggtgggcaaggcgctgggccccgccactgacgtcgtgttggaggcctgggcagctagcgtcggg
ctcggctctaacgtcgaggccctcaagaaagagctgctgtgcgtgaaggccttgctcgagcatacccgtggcaaagaaataagcaactccgcactaga
ggaaatgatgcagaatctgcggaacaaggcgtacgatgcggaggacgtgctagatgagctggactacttccgcatccaggatgatctctacggcctcg
ccgaggccgccgatgtccaccccaagggttgtggccacaacctcgtcctcaatgctcgccacagtgccaaggcggtggccaaacggctgctttactcgt
gcgcatgttcttcgcctgttgttggtgatgctgatccacctggcatgttggtaccttgcgatgaagcgccaaagtcggtgtttgatagggtggatgcctcta
tgaaaatgatggagattgtagagcaattgcaatcaatgcgcaaagtggtttcagagatacttacaccactagggccaggcctgataactacacgagaa
attggccataatcgctccagcaccatatctgaaagtacagaaccaaggctgtatgggagggacctcatcatgaagagtatcatacatgatatcaccaag
ggtcaatactgtgacaaggacgacctgactgtccttcccatttttggtcaaggtggcatagggaagacaactcttgcacagcacatatatcacaaccaac
aagtgcaaagccattttgatgtgaagggtcagctccgcaaattagaggtcatgtcgttggccccgagattcttcattggtacagatatcgtacagagtcc
gaaggatgaagatcaagtacggcttctttgccattcctccaaattgcaggagcttaggaccgatgatgtcacaggattccttaccaagcccatctgcact
gtcctatcttcctcccttaccagattactacttgacggggacaatgaggacgacgaggacgaggtgctctcttgcttcacagcggagcaagagaatgcc
cttcagcacctcacctccctccaggttctcatattttctcgtttctcccagaagcagtgcctcccagcagggctgcataaacttcccaatctacagatgttac
agatccgcaattgtgaactcatcagcagactacccagcgacggcctccccaattcactgcagatgttacacgttgaagattgctgcaacgaggagctaa
tacaagaatgcagaaagttcgtcaaggacaatcccagaatcaagctaataatctaa
>g14824.t1
atggatgaagtcaaatccaagttatctgaaaaggttaattatttgagggcgctgccagagaatcttgaggacgtgaagatggatttagaaatgatgagc
tgtgtaataaaagatttgggatctggagacctcagcaaagaagcagtacaaggatggattgggttgctgcggaaggtgtccttccgtgttgtggatgta
atggacaaatactcctaccacgcttttcagctgcagaaagaaagcttattcacaaggttctttaaaggaggatattatgtcaaggttttcagcgatgttgc
tgatgaggtagtcaacataaagaatgagatacagaaagtgaaagacctgcgaaaagactggcttcctatagatcaagttgttccacaaaatcctatgg
ctttagatcgaccggggtctcaaagcctcttcccggtactcgttcaagatgaagatctggttgggattaaagataaccagagtaagttgatcggatggct
cgactccaatgagccagatagcacagtgattacagtatcaggaatgggtggattaggaaagtccaccctagtttccaatgtgtacggccgggtgaaga
atagctttggagttaatgcatggatatctgtttcgcaaacctacgcagagcatgctctgctgagggaactactgtggaagatcagttgcactgacactga
gaagccacggtctataagtatggacaaaatggacacctttatgctgaaacaggaaataaagtcgaggcttgagggcagcggcnagagcatgctctgc
tga
>g15405.t1
atgtcgctggctgccttacggtgggtggcatcccctgtggtgaacaagctcctcgctaacgcttcaacctaccttgatgtggacctgacgcaagaacttca
agatctggagaccaccgtcctgccacagttcgacctgatcatcgaagcagctgaaaagagcccccacaggggcaaactgaaggcatggcttaagcga
cacaaagaagccttctatgacacggatgacctactggacgagcacgagtacaacctcctgaagcgccaggcgaagagtgggaaggatttctcgccgg
aagaagatgactcctttaagtcgactattttgaagcctcttcgtgctgtgaagagtaaagcatccaacttgctcccagaaaacagaaggataattcgaaa
gctgaacgaattgaaggtcatcctagcagaagttaaggaatttcgtgcaagtcttggcataccagctgtacagactaccgaaggcaccactataccgac
agaaacggttcctccgtccacatcattgccgcctcctgaagtgtttggtcgtgacatagatcgcgatcttataatagatcttcttaccaaggaaagtgctg
ctgacggctgctcaactaggtactcgggtgtggcaattgttggacatgcaggagccggaaaatcgacattagcgcagcatatctacaatgacgatagg
ataaaagaacattttgatgtcaggatgtgggtctgcatctcgcgcgagattgatgtccatcgtcacacacaagggattctagagtctgcgctaaactgga
agtgcccacatttcgataatcttgatacgctccagaaaacattaaagggcatactggaaaagtcaaagaaattcctcctggtcttggatgatgtctggttt
aaagaatccagtaaagagaaggaatgggacctactactacgtcctcttgtttctcagcaggccggaagcaaagttttagtaacctctgttagagatgtac
tcccagcagctctgtgctgcaatgaagtaattcgcctggaggacatggatgatgcagatttcttggcactcttcaagcatcatgccttctcgggagcaga
aataagagatcaatggttgcagctccagctagaagagatagcagagaagatagctaaaaggcttggacgttctccattggcagcaaaagttattggttc
ccagttgagcaggagaaagctggtttcgggaaggtttgttatgcacgacctccttcatgatttggcagagttactgtctacagaggactgcttcagattag
aagatgacaagaacatgaagaagttgcgtgtactgtatttgtcacttgacagtagcagcaaattgccagaatccattggtgacctgaagcaccttcggg
cttcgaagcttaacgatttccaggtgcccgatgcttatgtttatcacccatga
>g15554.t1
atggtgggcattgatgagcctagagatgcactcgtaagctggctgatggaagatgatccctgccttcgtgtgctggccatcgttggttttggtggcctcgg
caagaccacacttgcaaggatggtgtgtgagagcccaacagtgaaaggttcagttcccaatcttgagaggctgacgttgtcattcctgcgagaacctaa
ggatgggatctccggtctcaatattctccagaagctgaaggaggtagaggtctttggtaacataacattgtctgtgtggtacacaccacagttagtcacc
atatcctcctcggctccggggcgctgcgcggaccctcgtgcggcctgggtgtcgcactcgccagcgtccatgacggtgcgcctgctggtgtcactcggtc

gacccagcgccattgcactcgtggcccctgttgccaccacgctgctggcggccttcgtggggacacccgggcaccttcgtcgcagtcgcaaccccaagg
gcgcgcggggcacaggttga
>g16658.t1
atggaccttgtcgtcggcgcttccaacagcgccgtgaaatccctggtgaataagcttgggagcctgcttgcacaggagtacgccttgatcagcggcgtcc
gcgatgacatccagtacatcaacgacgagctggccagcatgcaggccttcctcaacaggatcaagcgagccgacgaccaggacgagcagcgtcaag
actggatgaagcaggtccgggaggtctcgtacgacattgaagattgcgtggacaacgtcagccaccgtctcgacggtgagccgcgcgggaccagtgc
gtggacgcacatttccaagaagtggtacgtgcttaagacgctgtacgcgcgccaccgcatcgccaccgagatcagcaacctcaagatccgggcgcaac
acgtcagcgagcggcgctccaggtacggggtagtgaatctgaaaaatgtcaaaggccgcacggaagcgaacgctaccagagaccatctggcacctcc
tccacagctcatcggcatcagggcgccggtgggcatcgaggacgccatgggagagctcgagccatggttcaccaaggcgaagacgctgcctactgat
cataatctgagatttcttgccatcgttgggtttggtggcctcggcaagaccacgctcgccatggcactgtaccagaagttcggtcttggtgacgagttcag
ttgcagagcctccgtcctcgcatcgnactga
>g19568.t1
atggactttgcgagggaagtcgtcgtccccgcggcgctatccgagatcgttggccggatattctcctgtctgattgacaacttcccgcggccgtcgtcggc
cggcgcccgtgacgcgcaccggcggcggctggagcgtctgctgggcaacatcggcagcgtggtcgaggaggccgaggggcggcacatcaccaacct
gcacctccttaatcagctgaagtcgctcaccgacgccatgtaccgtggccgcttcgcgctcgaggccaccgacctcgacgacatcaagaacggcagcat
actggacgacgatgacgcggacgacgccgcagtgaccactgctgcgggaaagcgctcgtcggcgctctcctcgccgttcgtcagcgccgccaagcgtg
cccgcgtgatcctgggctgcggcgacggcgcgacggagaagactagcaggctggccgtcgtcgtcgaggagctggacgccgtgacgcgcgactacat
gcacgacttcatcctgctcgtgcaaggctacccccggcggtgtaataatgtgccccggccgttgaccacgacgctgttcatggaccgttgcgtgttcggg
cgccacgtggagaaagaacgcatcgtggacttcctcctgcatcacgcgcctaccactaccggtaccggcctgagcgcgctcgcggtggtcggcgccaa
gaaggcaggcaagacgacgctggtgaagcacgcatgcgacgacgagcgcgtgcaaggccacttcgcgcgcgtcgagtggttcgagacgccggacgt
ggtgcgggccggtgggtgggccggccagaccatgtgggagagcggcggaccggagtacctcgcgggcgtgcgccgcatcctcagcgacgagcggcg
ggtgggcttgtcgggccggtccctgctcgtgttcgaagacgcgtggcccgtcgatgagtcggcatgggccgcactgatggcggcgtcgagcgcgctcgc
cgacgggagcaagctcatcttcacgtgtcgtgacgccgatctcgcccggattatcggaacgggtgcggagccggtggtgctccgccgcctgggggagg
aggagtactggtactacttcaaggcgttcgcgttcggtggcgcggacccgcgtgaccacccccggatcgcggccgtggcgagggagatctctcgcaac
atggaccgcattttcatcgacgcaagggtgctcggcacgctgctgcgggcaaacttcgacgcccggttctggtggaggatgctggacgccatggttagg
tgccagcgccgcccgctccatgtcggcgtcctgctcgacctactgccagtcagaggcaggatgcagtcgtacggatactgccggagcccgcccagatg
cactgtgctcagggccaacttcgacgcccggttctggcgcagggtccttgtcaccatcgtcacgtgccagcgccgcctccgccactgcaacaactacgtc
ttcagcttcctgctcaacctctttaccatcaacggttggaacatgtcatgggggacctctcggaccccgcccaagcccaatggtctcaccctcgaggacat
gctccgcgcagctacctcttcttctgcttctgggcttggcaacggtgatacggaggaagccttcaccttcaccttccgaaacagaagcccagccgccgcc
gctgcttcacgatga
>g19583.t1
atgtacgaggttgacgacgttctcgatgtgtgcatgattgagggcggcaagatcctcgcagacgaccacccccccacccccaaggtccggtgctccttg
gtgttctcctgcttcaagcacaaaggaccccgcaagttccaccatgaaatcgggttcaagatcagggacattgatctaaggctgagggaggttgaagag
gaaatgcccaggcttcctgctggatcactacaatcggattccaccagggattggttcagtgataacatatgtaagcactgccattccgcgattaagcctc
aggcagctggggcgcaagtccggaaggctgtgggtggccttgtgccaaggttgcttagagagggcaagaagaaggtggccgtgtttgccattgttggc
gcggtggggattggcaagacgacacttgctagggagatttacgacgatgacaggatgactgaaaatttccccatatgtgtgtgggtgaagatgtccaag
gatttatcagagatccgatgggagatggcgtggcgagaggcagaatactga
>g22070.t1
atggcggacctggtgctcggggtcgccaagtccctggtggacgggaccctgagcaaggcccagtcggcgattgaggaggagaccaggctgcgcgcg
agcacgcagcgcgacctggttttcatcgccggtgagttccacatgatgcagtccttcctcggcatcaccaacgaggagcacgtcaagaacggcgtggtg
agcacctgggtcacgcaggtccgcgacctcgcctacgacgtcgaggactgcatcgagttcgtcctccacctcgacaagaagcctgactggtggcgccg
cttcatctcctgctccttctgcaactgtgcagccaaggcgctcccccctggatgaggccgtcgccgacatcgagcagctcaaggccagggttgaggatgtc
agccgccggaacgcacgatacaaccaaatcattggcgacccgggcgtaataacgcagcagacgacgccacaggcaccagccagcggcgctggagc
gtcgtcgtccttcgacctgctcgctgaagaaggcaagcagcaagatgttctcaccaagctgatcatcaaggaaggcagtacagaccttcaagtgatctc
ggtgtgggcaactggtgctggcgaaggcgcaccatccaccaccatcgtcaagaaggcctacgacgatccaagaatccggtcaaccttcagatgccgg
gcttatgtgaagctggcgcatcccttcaatctccataagttcatcaggaccttgttggatcagttctatgccaactctagtgaacaggaacatggagcaat
ccgtcatgctgatgtcctcaagcgaatgaaggcggtcattgtggcggatgaagatctggtcaaggacttcatggatcatgtggagaaacaaaatttcctc
gttgtactgcaggatctgtcctccatggctgaatgggatgtcattcgaatgtacttccctgacagaagcaagggaagtcgtattgtggtgtccactcaaga
gtttgaggtcgccagcttctgcgtaggacctccatatgtgcagtggttttcagctgaccactctctatgtatcttcttcaagaaggtacaaaagataaatcc
aacattttctatggacatcgttgataatgacgtcgtgctaaatcaacttattccaaagtgtgggggcctcccaagagtaatagttgctacagctgatttttt
gttccaaatactatttgagtggccccaaaggtccgggataatgaacaaagaatttatgcagaacgtggagaacttactgaggccttgcatcttctacctg
tcaatcttctcaaagtctcaggccattcggcggcggcgtttggtgatgcggtgggttgctgaaggttactccaaggacagtagcaactctaatgccgaga
acaatggagaaaagatcttctccaagctcattgacttgagtatgatccaaccaccagagcggacagtcatcacaagaaacagaatggtcaagtgccaa
gtaaatgctttcttccacgaatatatcatctcacgaccaaaacaagagaacattaattttgcactggaggtccttacattggatggcagttgccggcacat
cgccgatcgcacaggacgacaccttgttatagagaaaagctgggagagaaataggactgtgtttgagagcattgacttctcgcggctacggtcactaac
ggtgtttgggaaatgggagtctttcttcatctccgacaacatgaaggttcttcgagtgcttgatctggagaatgcatctgatgtgacggataaagattttga
gaagatagtgaagtggatgactcgcctcaagttcctttctttgagaggttgttgtaagatttcccatctgccaaaatcactcgatgatctgaggcagatcc
aaattcttgatattagacacacgtccattgtcaccttgcaacagtccattatcaagctgaagaaactgcggtacattcgtgccggcactaatgccatacct
tgtaccttagtatccatgttgcctgaggtttgcagaagtcgccaaatagtaggtgtcactgtgccaccagggattgggaaactaaccttgcttcacacgct

cggagttgtgaacgtcattggtgataggggaagaccatcctgaaagagctcagaaatctcactcaattgcgcaagctcggagtatccagtgtcaaca
agaataacagagaagagttcatttctgccatctcttctctatttcagttggaatccttgtcaatgtggctcagcaatgataatgaaggttgtttggaagaca
ccaagttcttgcctccaaataacctgcagagccttaaaatttatgggctcgagggccagttgccaggatggactgcgaagcttggctatctcagaaagat
gcatttggagataacaaaattaaaggaagaatacctgaaacccctacgaaatttgaaagaacttggtattctacgtttatctttcaaggagcctcttgatg
gcaatcttgttttcagtgtcatattgatgggccaagaagtgcgctgttatgaagcaatcaagctcctcgagatctcttgcagctccaacctagctgtaactt
ttggagcacgagtaatgccaaacctggagctgctccagcttaactgctccagcctagatgtaacttttggttcagaagcgatgccaaagctggagctgct
ccagcttaagggttcccatgatgagaccgtactgcaggagttgaggagaaagctcgccgaccttccgaagagacctattctaaagcttttgtga
>g22336.t1
atggtggacatggctcttactggcttacggtgggcggcatcgccaattgttaaaaagctcatcgctgatgcttcaacctaccttggcgtcgacatggcgc
atgagctccaagaattggaggtgaccgtcctgccacagtttgacctagtgattgaagctgccgagaagagtccccacagggacaaactgaaggcatgg
ctccagagactcaaagaagccttctacgacactgaggacctgctggacgagcatgagtacaacatcctcaagcgcaaggtgaagagcgggaaggatc
tcttgctgaaagaggatgtcacctccattaaaacagcaattctgaagccttttcgtgctgcaacgagcagggcatccaacttgctcctagagaacagaag
actgattcgcaagatgaatgaactgaaggccacccttacggaagccaagagctttcgtgaacttcttggcttaccagctggagctggcaatactgcagg
atgccccgccgtaccagcaactgctgctcctgtggccacaacaacatcacttcctacttcaaaggtatttggtcgtgacaaggatcttgatcgtatcgtag
atattcttctcggcaaggcagcaacttcagatgagagtgcagctagctgctcgagtttgggcattgttggagtcggaggcatggggaagtccacgttggc
acaatatgtctacaatgacaaaagggtagaagaatattttgatgtccggatcttatttatcaaagagctcctttacagtagaccatga
>g22855.t1
atggcggaagcaatcgtctctgccgttgtcggcgaagccgtcagcagagtggtttcccttgtcacagggaatttcagtccccagcagagaaccgaagcc
aagctgcaaaggatatgccggttgctgatcagaatccacagcgtcgtggaggaggccaaagggcggcagatcgaaaaccagggcgttcttcagtggc
tctcccagctcatcgacggcgagtatgagggccgctacttgattgataccattgggtgcagtgaacacgagcttgatggtgacgagaaaaaaggttgtt
atgaggtatctcttccccaagcatccaccttgtccctgttcaatccggcgaagcgcatgcgtgttgcagcaagcgctgtgaagagcgtgctatcttgccgt
gttcgcggacctcgcgttgctgatggtgagattgaccgggtgctagagaacttgcaagggctgtctggtgaccttgtcgagttcatcatgctcgtccaag
ggtaccagccagcaatccgccggcctctggctacaaacatcttccttgaagggcagatgttcggtcggcatgttgagaaagagaggatcgtcaatttcct
cctgcatgacagtggtggtaactcgacagggcaattggatgtgctaccggttgttggcgacataggagtcggaaagacaaccttggtgcagcacgcttg
tgatgacccaagggtgcgcaaccacttccccgtaatcatgttattcagtttctcgtgtacctacgcaatcgcaacaagtgaagcaaccgttgttctacggt
caaaacatgtcatcggagaagctggaatagatatgaaagacccattgcagttgatcaagagggatttcagcaacaagagggtcctgatggtatttgag
gatgtcgacatgcgcgagaagcagatgctggaagagctgttaccaagcctgagatgtgccacgcaggggagcaagatcattttcacaaccaacaaca
ggcgtgttgcaagtatgggaacggttgagccgatcatactgaaagtcttaccctgcccagagtactggttcttcttcaaggcacacgcgttcgcaggcag
agacgttgaagagaaccacatgctcattgcagcaggcaaagccatagcgacgaagctaaatgggtcgttctttggtgcgaagatcgtcggaggattgc
tcaaggatcacccatatcctgcgttttgagtcaagagcaccaacaagagcaggaagagctctgctccattgcagaatggagagaaaaagattgacgga
ggagctaacactaacagcaggcaggttgcgccggagactcacttgggaactgtcgattatgatctcaccccagcaagatcaataacgtcccaaagcag
caagaccataccacctggttctcccaagctgctaaccctcggtgcaatcctgaaggctgagcctctgaagccgcccagtgcgcagaagaagcttgccga
cctcctgagggagtcacaagacgatgatagcgaggttgggggccccgacgacttgtcgaggggcgactcttttcgtgcggggcaggaggcggggcgg
tgctgcatgccgcagttggcgcgggcagttagctgcaatgggagagggaagaggaacaaatag
>g23824.t1
atgataggaaccgggggcatgctcgctgctgccatcctcaaggtggcggccgggaaggcggccgctgcagctggagatcggatcatgctgcagtgga
ggtttgagaaagacctggaatacatgagagacaccatggaatccatcgaggtggtgctggtggatgcggagaagcggtccatcgaggacaagtcggt
gcagctgtggctagagcgcctcacccgagcctcctacgacatctctgacttgtttgatgagtttgaggccaatgctgtcaagaaatcggcttttcgaaag
gcacgcgcgtcaactcagctaactacagactcgttctatttcaaagttccgacccccctgtctcacacttacttctgaggttggcatggcttgtaagatgaa
gaaagtcagggagaaactggagaatatctcaaatgaacgtcgcagatacagtttcatgacaagtaataacagctccaatgttcagcaagttatcaatg
aacgtgcaacatcttcaaatgtggtagaagctgatatcctaggaagggatgaagagaaacagaaagtgatagctcttttgatggaagcaagtatgtcat
cagagttcattgtccttcccatatatggcattggaggcattgggaagacaacattggcacagctgctttacaatgatacccatttcaaaaatcatggaaa
agcatggggccaattatcgtctacaagggagcaagatgctggtccatctgctcataggaacattctgatcgttttggatgacctatgggaggaagatga
cttcaagttggatgacctgaagaaatccctgaaaattatcgggaatggacacagagtgcatgtcattgtaaccacacgtgatgctggcattgctgagaa
agttcaaactattgaagcacacaagataggggccctgtctcttgatgtgtgctggactataattaagaagatagttggttttgacgcgaaacatgctaac
aacgaatggatggatgatgtagggaaggagattgcaaaaaagtgtggtggtgtggcattagcagctcgagctcttgggttcatgttgagatccagggat
gatgtcgatggatgggtttcagtaaaagacagtgctatctggaatgtttctaccccaggatatacaccttcggcgtacgataatgtccttgcatctttgaa
attgagctacatcagtataccaccacacttaaggctatgctttgcttattgtttagtctttccaaagggccacaagatggctaaagatgaactggtttacca
atgggcagctctaggtttaattacaccatctgatggagtctccatttggcagcatgtttga
>g24680.t1
atggcagctgtgttggaagctttggcgctctacgtgaaaaagatgatcacagacatggctgaggaagaggtgcacatgctgctgggcgtccccgatga
gatggacaagctggggcggaacctagataacattcaggcattcctagctgatgctgagaggcggcgcatcacggataggctcgtccaggggtgggtg
aacatgctcaagggcgtcatgtatgatgccactgacatcttagagctgggcaagcttaaggcagaggagaggcaggagtcaatgggtggcacgttggt
ggagaagatgcctggttgcttccagccgttattcttttgtttgcgcaatccagtcttcactcacaagattggtagcaaaatcaaggagttgaaccagcggc
tggatgagatccacaaggaggccgctaaattcaacttcactgccaacctaagttcctacacgaaacagaggactgaagctgaatactccagtagggag
atgacgtcagagttcattccatcggccatcgttggtgagaatatagagatggataccaagttgcttgtccaggagctagtcactgacgaaaaccatggc
aagaaggtggtttccatcgtaggcatgggtggcatgggaaagaccacccttgcccaaaatatcttcaaaagacacaaccataaaggagcacttcaaaa
tgaggatatggctgagcatcactcaacaattcaatga
>g24885.t1

atggcatccgatgacgacggactcctcacagacctgcgccaactccttgtcgatttcgcgttcggagccaagaagctcgccgcgccgctactccacccgt
tcgggccctccttagagccagttgccctcgacgatggcgatctcgagaagctgaaatccaagctcaggggcatccgcgccacgcttcaggccgcggaa
gatcgcgtggtggccgacgattttgtcaggctatggcttcgggagctcctggacctggagtacgcggcagaggacgtgctcgaggagctcgagttcgag
gccctccgcgcggcccgcctcgagcagttcaaggcccagctcctgcggtccagcgccggcaagcggaagcgtgagttcgcctcgatgttctcctttcct
ctcttcacctcaaggggaagatcggcaagataatggagcgctataacgagattgcaagggatagggacgcgctccggatgaggagcagcgacgggg
agaggcgggtcgaagccagccccatgacgccgaccagctgtttgacgaaatgccagcttcatggaaggcagcgagacaagaggcaagtaacagagc
tgctattttctgaagaagctgactacactggcgtgtattctgtggtgcctgtagtaggacctgccggtgttgggaagactagccttgttcagcacatctaca
acgacgaagccctcagctctaagtttgacataaagatgtgggtctgggatctggtttga
>g25366.t1
atggccgagttaatgatccgcccgctagtatccatggtgaatgagaaggcatccaacgaactgctggacatgtacagagtaatggaaggcatggagga
gcagcgcaagatcctagatcgccagcttccggcaatcctgggcatcatccagcatgtagaggagaaggtaaatccccaggttggagttggcgtctggct
cagaaacctcaagtcggtgtcctatgaggctaacgacgtctttgacgaattcaagtacgaggcacttcggcgagaggcccagaagaaggggcaccac
aaaatcaagggtttcttaagtttcccagctcgtaaccccattgtattccgttacaggatgggcaagaagctacgcaagattgtgcagaccatcgaagtcc
tagtcaccgagatgaataaatttgggttcagacacattcaacaagcaccaccatcaaagcggtggagagagacagatcccataatggttgactccgac
aaggatattatcagtagatccagagatgaagagaagaagaaaatcatgagaatactacttgatgaagcttgcggtaaggacctcacagtccttcctatt
gttgggatgggagggctgggcaagaccacctttgtgcaactcatttacaatgaccctgcgattgagaagcattttgagcttcggagatggtgttgcgtgt
caaatgattttgatgctgctaatattgccaacaatatctgtcagaccaatgtaaatgagtatagcggtcgtgaaaaagcattgtgggatctccagagact
gataagtggaaagaggtacctcattgtgttagatgatgtatgggacccgaatgatgataactggggaaaactgaagacctgccttaatcatggtgacaa
gggcagtgtagtactgacaacaactcgaaatgcaaaaatagctcaaagaatggccatgggtgtagccgaagcatataatattgaaaggctgagtgatg
agcacttgaaggaaattcttgagagtagagcattcagtttgcaaaaaccaaatcattgtgagctagatgaaattgttgataagattgtcgacaagaaga
caaccatcccaaaaccacaggagctgaaattttcaaggagctag
>g25840.t1
atggcggaccttgcagtagggatttccaagacggtagtggaggctctggtgaacaaggtggagaacgccatcaaggaggaggcggagcagtggcgg
attgtgcagagtgagactgtgttcatgagggatgaactggagatgatgaagtccttcctcaggaccgcggactggagacgaatcaagtgcgatgtggt
gaggacctgggtcaagcaggtacgcgatctgtcatatgatgcggaagatgtcattgagtccatcctcctcgtggacacaaagaggccgtcgtgttgcac
catatccttgtggcgccgcttgttggcatcatgctgttgctactcaggaacacaatctccacttgaccaagctgtcgccgagataaagctcctcaaatcac
gggtcgaggaggtgagtagtaggaatatgcgatacagccaactcaacgactccgcctccatgctcaccatgcctcaagagttggcgtctgtctctgctgt
tgctacagtagcagtcgtggacgtgctcaaggaggcatggtgcaacgcaaggatgcagggatttgtagacctcaccacgttggtcacgaggcacacta
ctgatgctattagagtggtctctttgtgggggacaggaggcgatcttgggttgttatccatcatcaaggaaacctatgaagggtcagatgaagttcaaca
aaagttcagaggtcgtgcctggatcagtctggggcatcgtcccttcaatcatctcgagttcatccgaagcttgatgcatcagttctacgaaaacacaccgt
catggaggcgttcgagagatgccgatgtcatgacaaggatgttgacggccagtcaaagcaccaatcctgtgaagcattttgcgcatctaattgaaaatt
ataggtaccttgttgtcctagaaggcttgtccaacttggctgagtgggaggccatccgggtgttcttacgagactgcaagaacggaagctgtatcgtggt
gtcaacaccgcaactcgaggtggcaacaaggctgtgcatggaaccctcccaggtattgccgctcagggtggtaccagatggtcaaataatttgtgcctt
catctgtaaggtacgtcctaagcattaa
>g26040.t1
atggattttgtgtcggggccgctcggaagtgtcgtcatcaagctcggcaagctgctcaaggaggagtacaaaatacagacggacctaaggaacaagat
catgcgtctctctcgagatcttgagagcatgcacgcggtcctccgcaaggtggctgatgtaccgccggagcaggtcgacgagccggtaaagctctgggc
gcgcgacgtccgggagctctcctacaatgtggaggacatcctcgacaccttcttcctggtgaacttcgacggccgtgaggcctgtgaccccaacaaattc
aagcgtgccgccaagaagatcggtaaactgttcagcaagagcaaggctcgccatcagattgctcgcatgatcgagagcatcaacaagcaagccgacc
tggtggctgagcgccatcgcaggtacagagctgaggatatcgtgtcaaagcctgccgcaacatctactaatgatgaccctcgttttgccgctatgcacaa
agatgtgacacaactcgtgggtattgacaagccaagggacgagctcgtatccagactatcgctcaaaggggggcaaggagtcaaaactgaagatgaag
atggtgtctgttctcggagctggaggattgggcaagaccacacttgcaaaagcagtgtatgagaagcataaaggagattttcagtgtgcggcttttgttt
cggttggccagaaacctgacttgcagaaagtctttcgagatattcttattgccctcggcaagaaggcatacaggaatttaaacattgaaatagcagataa
aaatcagctcgtttatcagctccaaaactacctcggaagcaagaggtatgcataa
>g26587.t1
atgcacgcggccctctgcaaagttgccgaggtgccgtcggatcagctcgatgagaaggtcaagctctgggtgcgcgacatccgggagctgtcctacaa
cattgaaaacatcctcgacaccttcctggtgcgcgtccagggtcctgaggcctgcgactcaaacaggtacaagcgcgccgcgaagaagattggcggcc
gggtgcggtcgcacagcttgcacctctcaatcgacaggcctgccagaagcaaggcttgccataagatcacgaccatgatccaaagcatcaacgagcaa
gccgatgcggttgctgaacggcatcgtaggtatggagcagaggatattgtggccagtcgtcttgccgcaacttcaaccgttgatcctcgtcttgctgctat
gtacgaggaagtgacacagctcgttggcattgaaaagccaagcgctgagatcatatccttgctatcagcccaacaaagggatggcgtgtcgaatatga
agacgaggatggtgtctattgtcggagttggaggattgggcaagaccactcttgcaaaagtggtgtatgacaagcttaaagtggatttcccacatacgg
cttttgttctggttggccagaatcctgacttgaagaaagtctttagagaccttcttatagatcttgacaaaaagaaatacatggatcccaaatggacaatg
ttggatgaaaagcaactcattgatgaacttcgagaatacctcagtgacaagaggtggccatggacactggccaacccttccacttcaccagcacttgtg
acaccattcgtcccgagcgagagcaaagatggtggtccaacactgacacaggcacaggagaaacatcagtaa
>g27041.t1
atggagaccgcggcggccgccgtgggcagcgtcctccgcaagctgcaggcctacctgagcgaccacaaagaagaagatcactgcggcggaggcgtg
aagcagctcggccgcatccacgccgcgctcctcggcaacgccggcgccctgccgagacagccccacgacgagccggcggcggcgtggacgggcgag
gcgagggagctggcgtacgacgtggaggacgccgtcgacggcctcctcgtcgcgtcgtcgccgccgggctccctcagccgcacgatcacggggcttct
acagagagccaccgatctttctagagcccgtcctcggaccccagctggcgttcctgcggccgccgccgtcgtcgaccaccctcgcaagaagcaccagct

gcctccaccgccggacggcgaggcgggcgtcgtcggcgttgatgatgccagggacgggctcatcaggaggctgcgcctgcgcgacgaagacgacga
gcaggtgaaagtggtctccgtcgtcgggcccgtggcgatgggcaagacgaccctcgctgcagcggcgtatggcgtgctcaagtcgaggttcgattgcg
cggcgttcgtctccgtcgggcttgatcccgacatcacccaggttttggagagagcgctacggcagcttggcgtcagcaaggaagccaacgctggtgaac
cgagggacgagtcctcgctcgtcagtcggctgagagagttgctacaaaacaagaggtacgcgtactggcaccacctcagagtctcagaaaattag
>g27433.t1
atgcaggtgcgcgaggcggcacaggaggtaggacgcacgctgggctgcgaagacgacgacttgctcgacagtggagtccctcaaaagatcggatca
cggtggccgtcgcggaagcggaagcggctctggcccaacgtggatctcgatctgcgccagcgctgggagttggatgcctgtatcgtccagatcaacga
gcgcctcggcgagatcaacaagagcaggagaaggttcaggttgcaggccggggacgggaggaggacagcgactcagccgatgcagcgcccaaggt
ttcttgaagcggtttcgcatcgcaacaagaggtccattggtcgcaccaaggagaaggcggcgatcatccgtgcgctgatttctaacagtaacatggatct
tcctgtgatttccatctggggaacggcaggaattgggaagacaacgctggcacgtatggtctacagtgagattcagaggatggtgcagatgggattctt
cttcttcgtcagcgccctggcgcacggctgcttgctccatgccgccagccgcgtggactacaccatcaagtacgggacccatgaggactatcaccgcgc
acgatctgtcgccacactgcagttcaacggccacctcgcgctgctcgtgattgctggtgcaactgtgtgtacgccttggtcctacccagggtaa
>g28459.t1
atgccttctctcctgagaaagttggatgctttcctgatgagtagtatttatgaactaggcgatgagatgcaaaatgatttcaggttccttagagctgagctt
gatgccatggatactttcctcaggaagatgtcggagatagaggagcactgcaagcaagcacagcgccaggcagtggttgtgcgagatctgtcctatga
cattgaagatcgcatggacaggctcatgctccttgagtctagtgacagaggtgaaataatagccaaggagattagaagcataatgaaacttatcgatga
ggttaaagaaaggcattcaaagtacactaagcaaattgacaatgaaatggagaccatctctatacagattcctgcggaccctcgcccacgcttcctctat
gcagatgtgtcggagctggtgggtattgaggtcctcatggatgacctcatctttatgttgacttgcggggaagatgaaggacggctgttgcaaagaatgg
tctctattgttggatttggagggtccggaaagacaactctggccaaacaactgtaccaagagcttggacctcagtttgactgctgtgcttttgtatcagttt
ccagaaagcccaacatcagctggacattgagaagtatgctttgtcagatccaaaatgacctgtgtgcctacgctgaagcatgggatgagaatcaactca
ttgatgagatcatggacttcctccaagataaaaggtatgctactccagtcaggcgtacaaaagaattttag
>g28755.t1
atggccgggtcgtcgctgcttctcggcttggtagtcagggtggccggcaaggccgccggcgccctcgttacggatgtcgctcgcatgtggcgcgtcgaat
cccaacgcgccatgctggagcgccagcttctggccgtgcagaagcgattggtcgacgcggaggactcgagtgaggccgccgtcaagaggtggaccaa
ggacctcaaggccgtcgcctacgaggccgagcacgtcctcgacgtcttccagtacgaggcgctgcgccgccaggcccggaggaccgccggctgctcc
ggccacgtccgtgataagtctcctctgttccggtacaaagggagcagaaagctcaagaaggtgctgcaaaagatcaatgtccttgtcgaggagatgaa
taagtttggtctcgtggaacgcaaggagccgccgcaagccgtgtttcggcagacgcactcggcgctcgacgactcggcggtgatcatcgggagggaga
aagacaaggaggcggtgatcaagatgttgctagaccaacgagataagcacaagtacaacgagcagagaccaaacgtgcaggtgttgcccatcactg
ggatggggggtctggggaagacgacgcttgcaaaggtggtgtacaacgaccacagggttcagaagcatttcgagttgaagatgtggtgcagcgtgcc
gcaagcaaaattcgacacatgttctgttttgaaatccatccttgaactggttaggaaagggcgatgtgacttgcctgacaccatcgagctgctgaaagag
cgtctacatgaagacattggtcggaagagaaagcattcagcaatggggtgcaagcgaaaccagagttggtcacagccagcaaaagtatcgtcaacaa
gtgcaagggtttgcctcttgctctcaagacaatag
>g30106.t1
atggctgtggagctcgcgtcggcggcgatgaccccgctcatcgagaagctcggcaatctgttggtgtcggagctgacgcttgagaagcgcgtgcggca
ggacgtcgcctccctccataaggagatgaaggcgatgcacgtggcgctccagcgggtcgaggtgcccccggaacagctggaccaggggaccgcgga
atgggcttctaacgtgcgcgagcactccaacgacatggaggacgctgtcgatgccttcaccctgcacttgggggagaatgacgaccatgccgcagcag
ggctgaggagccggtttcgcgggttcctagacaagacggttcgtcttttcagcaagggcaagtcgctgcaccaggtcgccgccgctgtcagggaggcc
aagagactcaccacggagctcgatgagatccaccagaggtacgctgggctaaagcccaaggacgccagcgttggtgcttcttccattgacgatcttgac
cctcgcctgaaggccatgtcgaccgaggcagcggacctcgtcggcgtagacggcgccagggacgaggtgatcaagatcctgtccaatgaatccaagg
tggatgtgcagactgtgtccattgttgggtttggcgggctcggcaagacaacgctcgccagagcggtgtttgacaagatcaagctgcagtttaattgctc
ggctttcgtttcggtgtctaagaaccccgacatcaccaagattttcaagaagatcctttatcaacttgatggggaaaagttcatcgacatcaacgaggcg
gtcagggattatgagcaactcgttcatgatctcaaagagttccttcatgataagaggtatttagggctgagacgtacttgcatccgtgaactaccggacg
aagtaggaaagcttcaattcctgcagacattggacgtaaggaggacgttacggggtacaagaattgccggcgaccatcactgggctgcagaaattgatg
tgccttcgtcttgactgggacacaaggctgccgaatgggttagggaatctgacctcactggaagagctgacgggtctgaggattggcggggaagattct
gcagccatcgtgacggagctcggccacctcaccgggctgcgggtgctcacattgaggtgggagaggctgaatatcaacgaagctctggtgcagtccct
tggaaacttgcgcaagttgcagagcctcgacatgtacgtcactactggcgggcgcggcgacctcatgcgtgattgggcgccgcctccgcgcctgcgcag
gttcttgtgtagagggcccacctgcgcgctgccgaagctgccggcgtggatgaagaagaacacgttgtcgtcatcgctcgcattcttggacatcttggtc
ggccaagtgggccccgacgacctgcaagccctcggggcgctcccgtcattgcgcggcgtgcgtctgcgctcgatgggccgcgtcgacgactaccgcca
cccgccgacgttgacggaggtcaacgccgacgcattctggtgcgcgagggcgtgcgcgttcctgaatttcgtgacggtgccgtccatgttccgtcgggg
cgccatgccgcgtgtccagcggctcgagttcaccgtccgggcgtgggattttgctgctagagaagggtttgggctggatgacttgtgcatggggcacctc
acttcgctggaggaggtccacatcgagctgtggtccacgaaggacgacgatgccgcggtggtggcggcggcgctgcggcaagcggcggaagatcac
cccaatcgcctcgccattcacatcaaacagagatcagctactgctaacccaatactgtcaatcatggcagatttgcacttctag
>g30258.t1
atggcgtccgacgacgacggactcctcacagacctgcgccaactccttgtcgatttcgcgttcggagccaagaagctcgccgcgccgctactccacccg
ttcgggccctcctcagagccagttgccctcgacgatggcgatctcgagaagctgaaatccaagctcaggcgcatccgcgccacgctccaggccgcgga
agaccgcgtggtggccgacgattttgtcaggctatggcttcgggagctccgggacctggagtacgcggcagaggacgtgctcgaggagctcgagttcg
aggccctccgcgcggcccgcctcgagcagttcaaggcccagctcctccggtccagcgccggcaagcgcaagcgtgagttcgcctcgatgttctccttttc
ctctcttcacctcaaggggaagatcggcaagatcatggagcggtacaacgagatcgcaagggataggacgctctccggatgaggagcagcgacgg
ggagaggcgggtcgaagccagccccatgacgccgaccagttgtttgacgaaatgccggcttcatggaaggcagcgagacaagaggcaagtaacaga

gctgctattttctgaagaagctgactacactggcgtgtattctgtggtgcctgtagtaggacctgccggtgttggaaagactagccttgttcagcacatcta
caacgacgaagccctcagctctaagtttgacataaagatgtgggtctgggtatgccaagagcttgatgtcttgaagttaacacggaaactcgctgaaga
ggctactgaatccccatgctgctttaccgagatgaaccagatgcatcgtgtcatcactgaccggctcaggggaagcggttcttgcttgttctcgatgatg
tatggaatgagagccagactgattgggtgagcttgcagatgctgtcttgcgaggccgtgatcccagtatcattgatgacagtcttatctccatag
>g31320.t1
atgggctgcgggagggccgcggcgcggcatgcggacggtgggctcctgcgtcgctgcccttgtagcagcacggatttgggcggggagcggggctctg
tctcccacagatctggccggggcgcaggacctttccgtacggtggatctgggccggggcgtggggaccaaggcggcggcgggggccagcccacgc
agtggggcgtcgcgatatctcttgctcgctggttgaagaaaaaagcaaagaactctcgcttactgatctcgtcatttaacaagaagccgagaacaagtct
tccagaaacgatggcgatggtggtagaaactttcatacccaacttcatcagcttactcacccagattgccgcaagagaagagtccgcagtccgcagatt
tgttgacgtccgtgaagagttcgagaggaccttggtcgaactcaaaaactttaacgctgacattgtctggaggagcaccgatggcactgtcacggcgtc
ccttagagacatcaaggatgtcctgtacaatgccgctgacatggacatctttcagctcaagtacgacaagagacgcgggggctgctatgccttctcccat
cgcatgttgcaaaccccgcgtgacatgaagtctctccacacagtcttgctactcaataaaaaactgaagcatattattgggcagagtgaacgataccgat
ttaccagctacatcaacccgtatgaggaccgaagggtgggccatcatgccagccgtatgaagcattcatccattgtccagttgtcagatgttgtgggggt
tcagatcgaagaaactatgtgggggcttttggacatcatgttacgagaaattgagaaccccggtgccaacatcaaaatcattgccatcgttggggccgc
cgggataggcaagagcacccttgcgaagaagatattcgactctaagatttgccacaaccattttgacttgccaatatggctgaacgtcactaggagcttt
gacaatgttgatctgctcaagagtattctcagcaacacagatgtatctgaattaccacatggcattggcaagatgaaaagtttgcagtacatcagcctcc
accactgtccaaatctggcacatcttcctaggagcattgtgaatctagagagattaagcagtgatgcatacgagcagcacaaccaagaggtatttgatg
agctccgtccttcgccctccttggaagatcttactattacaggatactttgggaggcatctaccagtgtggctgtcgccatcaaccctcgagaatttgagat
ggctgaaacttgagaacttgcgttcatgcacacagctcccatgtagttttggccagcttcccaatctggagtcgctctgcattaagcatgctctgaccatc
aggcgcattggagaagaattcctcgtcccttcctcggccttagtaagtgactctgacgaggaagaagtggaatccctcatcagtcgtcattttgattccgt
gactgcctctattattgtgttccccaagctgaggaagctagtcttctacgggatgcttgaatggaaagagtgggagtgggacgcgcagttggaagctatg
catgccttagaaaatgtacagatcagcaggtgcagattgggccgtctccctcctggccttgcatgccaggcaacagccttgagagagatcacaatagaa
aaggtcaaggatctcatctccctagagaatttcagttcagttgtggagctttatctgcattcagtctccaatttagagaagattgctaatcttcctaagatg
aaaaagcttaagatatccaaatgcccgaagctcaaggaactggaggcggtacatgcactttcgagcatcgaactgaaagatcaagaaatgagcacac
tcccagcatatctgcagaggccaatagactga
>g31804.t1
atggttaaggacaaggcatcaagctacctgttggacaagtacagggtgatggaaggcatggaggaacagcgtaagatcctggagcgcaagctgccgg
cgatcctggacatcatccaagatgcggaggagaaaggagcttttcggcctggagtaggagtctggctcaaagacctcaagacagtctcctatgaggcg
aatgacgtctttgacgaattcaactacgaggcgctacggcgagaggcccagaagaagggacaccacaggatcaaggctttgacaagtttcccagctcg
taaccccattgtatcccgttacaggatgggcaagaagctacgtaagtttgtacaggctttcgaagtcctggtcaccgagatgaataaatttgagttcaga
cacattcaacgggcaccaccgtcaaagcagtggagagagacagatcccataatggttgactccgacaaggatattatcagcagatccagagatgaag
agaagaagaaaatcatgagaatgctcctcgatgaagcttgcagcaaggacctcacagtccttcctattgttgggatgggagggttgggaaagaccacc
tttgtgcagctcatttacaatgactctgcaattcagaagcattttgagcttcggagatggtgttgcgtgtcacatgattttgatgctgctaatattgccaaca
acatctgtcaaaccaatgtaaatggcaatagaggccgtgaaaaagcatttcaggatctccagagtctgattactggaaagaggtacctcattgtgttag
atgatgtatgggacccggatgacgataagtggggaaaactgaagacctgccttaatcacggtgacaagggcagtgtagtactgacaacaactcgaaat
gaaaaaatagcccgcaaaatggtcatgggtgtagctgaagcctataatattgaaaagctgagtgatgagcatttgaaggaaattattgagaatagagc
attcagttcgcaaaagccaaatcactgtgagctagataaaattgttgataagattgtcgacaggaagacaaccatcccaaaaccacaggagctaaaat
tttcaaggagctag
>g31807.t1
atggctgagttaatgatcggcccgctagtctccacggtgaaggagaaggcatccagttacctgctgcaccagtacagggtgatggaaggcatggacga
gcagcgcaacatcctggagcgcaagctactggcaatcctggatatcatccaagatgcagaggagaaagaagcttcccgacctggagtaagggcctgg
cccaaagaactcaagacagtctcctatgaggccaacgacatctttgacgagttcaagtacgagacgctacggcgagaggccgagaagaaggggcac
cacaggaccaaggttttgacaagcttcccagctcacaatcccattgtattccgttacaggatgggcaagaagctacgtaagattgtgcagaccatcgaa
gtcttggtcaccgagatgaatacatttgatttcagacagcaggcaccaccatcaaagcagtggaggaagacagattccataatggttgacttcgataag
ggcattgttagcagatccagagacgaagaaaagaagaaaatcataaaaatactgcttggtgaagctagcaacatggatctcactgtccttcctatttctg
ggatgggcggcctgggaaagactacttttgttcaactcatttacaatgaccctgcaatcaagaagcactttgagcttcgtaggtggtgttgtgtttcagat
gattttgatgccgctaatattgcaagtaccatctgccaggccagtgtaaatgacaacagaggtcgtgaaaaagcattgcaggatcttcagagtttgataa
gtggaaagaggtacctcattgtgttagatgatgtgtggaacccagataatgatatgtcgggaaaactacggagctgccttaagcaaggtggcaagggc
agtgcagtactgacaacaactcgtaatgcagaaatagctcggagaatgtccatgggtgcagctcaagcccataatattgaaaagttgagcgacgagac
ttcatacctgtgcaggaagatgaccatcctgaagctgtgggagaggaaattttcaaggagctaa
>g32066.t1
atgcatatcctgctgggaaactgcatgtttggccgccagatggaagcggaacttgtaatcaacttcctgttgcagacacaacctcatcaagacctggag
gtcctgccaattgttggtcctggtagtaaaatcataatcacaagccggtctgacaaggtcaaaaggctaggaacaacagaaacagtaacactgaaata
tctgtccaatgaagcatactggtatttcttcaagacacttacatttgggagcacagatccagaagctcaccctaggcttgcatgcctggccatggagata
gcaagggtgttgaatgggaccttccttagtgcaaccgtcaccgcctgtttgctgagagacaactttgaaatccttttctggagaaaggttctggccttcttg
agacggctcatccagaagcatgtcttcagagtcggtagtaaaatcataatcacaagccggtctgacaaggtcaaaaggctaggaacaacagaaacag
taacactgaaatatctgtccaatgaagcatactggtatttcttcaagacacttacatttgggagcacagatccagaagctcaccctaggcttgcatgcctg
gccatggagatagcaagggtgttgaatgggaccttccttagtgcaaccgtcaccgcctgtttgctgagagacaactttgaaatccttttctggagaaagg
ttctggccttcttgagacggctcatccagaagcatgtcttcagagtcgaagaggaggttccaaatgtaacaatccaagacctgatgtacggaagcctca

agtgttatggcaaattcgagttgctagcatggaaatctcggttacctccttactatagctatgtctgtactggtgagattcaagagcaaaagcgggcgac
gccgctcctcaccgcgctgctccccaccacgccccattgctccccaccgtgccccgccgctccccaccgtgctccaccatgccctacggcgctgctttcca
tcacgccccaccgctccccatcggtggccccaaatcgggatgacagcggcagcggcggcctcaaatcgaggaggcgggagtggaggagcgggatgg
cgagcgggaatgggggagtgggagcggaggcaaggtga
>g33341.t1
atggccgagcttgttgccaacatgctggtcaggccagtagtgtccttggtgaaggagaaggcctccaactatctcctagacaagtacaagatcatggag
ggcatggaggagcagcacaagatcctgaagcgcaagcttccggccatccttgacgtcattgctgacgccgagaagcagtcctcccaccgagaaggag
ctaaagcatggctcgaggagctcaaatcggtggcgtacgggacgaatgatatcttcgacgagttcaactacgaggcgctccgccgtcaagccaagaag
aagggaaaattggattctcgacatcaaaagatgagcgacaagctccgccgggtcgtggaaaccatcgaggtccttgtgtctaagatgaatgcgtttggg
ttcaagtactgggaaaaagcaccggcatccaagcagtggaggcagacgggttccatcattgttgaccctgataatattgtcaccagatcaagaatcaaa
gagaagcaggagattgttcagatcctcaccgacaagagcaacgacgaagctctcacggtcgttcccatatttggaatgggtgggttaggcaaaaccac
cctggctcagctcatctacaacgacgctgaggtcatgaaccatttcgacctacgcatctgggtttgcgtgtcagacgagttcgatgtttgtgaccttgttaa
caagatctgcaactctactgagaacattcttgacattgcactgcagaagctcaaggaaaaaatcgaacaaaagaggtactatacaaattga
>g33868.t1
atgaaggtactcgaggatttggttgctccagcagtcgtctccgatctcgtgagcaggttaatatccttcctcacttcccaatatgtgaagaagaggacaag
tcagaagggcacggcacgacggcttcagctgcttaccttcaagatccatgctgtcgtggacgaagcccagcagagggaccttgccagcaaccgaagg
ctgcttttgtgggtcaagaagctcatagagggcatgtaccttgcatactacatgctagacatcagcacacatcatgtcgatgatcaaggatgtgcgctgg
tgaccagatcctggtccttcggcgtaaatcatcccagaaagagaccatgccttatcggggatgacaactgtggaattgatcaactgagtgctgtactcag
aagattggaagttgttgctgctgacatgaaggagtttattgtactactggcctgttgccctagaatatcactgccgccgccgttctcaagttccagctccga
cgataaggtcatgtttggccggcttgttgagagggagcagataatcagcttcttgctgcagcctaggagtaacttgggcgtgctacctatagttggtgatc
cggaagctgggaagggcactatcgtcaagcatgtctgcagcgacgagagggtgcgcggtcattttgcggtcattgtttactcctatgggtcttatcttgtt
caggagaattgtggtcaagatatgttgaggacactaaggattggtggtcatgtacttcacgagtcatcggcctcaggacatgagaggcatctcattgtga
taaaaaatacatttgaagtagtaatcaacttggaagtatggacgatactctgtgaatctctaagactcagagctcccggaagcaaggttatagtcatcag
cgagaaagacaacatcgcggaccttgggactgccatggcaatgcgtgtgaatccattgccatgggaggagtactggtacttctttatgtctctcgcgttc
ggtggcaacaatgatcaagaagagcacagcgcagagctggctgtgctaggtaggcagattgcagcagtactgcacgggtcattctttggagcaaaggt
gctcggtggcatactcaggatgaacctcaataagaaattctgggagggtgtgctcgacgttgttaacaacttcaatgaaactatgcaaggagatggtgtt
gaccacttctcgaagatcagggttgccaatatggttttgaagacgctccctctgcctctgaagcttaagagtgctcttcagcagactcgaagcgatgattt
ggagttgacaggaatgaccgtccaagaactattgcactctgcccctgttcctcatagaaaggagatgaggattattctttgggaatctgcctgcccaccg
aattatcgatacatagttgtctgcgagaaggtgaaagcagagcacgcgtgtgtggagacagagaggaagaggcggagaaaccagcaatag
>g34427.t1
atggagctcgcagcagcagcgatgaccccgctcatagagaagctcggccacctgctggcgtccgagctgaatctcgagaagagcgtgcggaaagacg
tcgccacccttgagcgggagctgaaggtgatatacgtgaagctcagggaggtgtccgaggtgcgcccggaccggatcgacgaggggaccaagctctg
ggctgccgacgtccgcgagctctcctatgacatggcggacgccatcgacgccttcaccctgcgtgtgggggaggttggccccaccaacaccgggctcc
gtgggttcctcgacagaacggctcggctgttcaccaagggcaagcagctgcaccaagtcgccgacgccattagagatgccaaggatcgtgccaagga
gctgggtgagatccaccaaaggtacgccgggctcaagctgcaagacaccggccacaacgcttctgacattgatcctcgcctgacggcaatgtacaccg
aggttgtggatcttgttggcgttgacggcgcaagagatgagctgatcaaagtcctgtctgatcccttggaagcggatgtcaagactgtctctattgttggg
tttggtggccttggcaagaccacactcgcaaaagtggtgtatgacaggatcaaagtggagttcgattgtgcagcttttgtttcggtgtctaggaatcccaa
cattaccaggatttttcagttgatcctttttgaatttgacaagaatcgctacgccaacaccaacgtagcgttatgggatatcaaacaacttattgatgagct
cagaggcttccttcaggataagagtttgtcgttgctcacgttcctggacgtcttgatccgccaggtgcgtcctgaggatgtacatgtcctcggtgcgctgcc
gtcgctgcgcatcgtccgcctgtggtcggagggccgccactcggtggcagggatgaagaaggtgaacgctggcgcattcccgtgcgcgaggatgtgcg
cgttcatgcactttgcgacggcgccatccatgttcgctccagacatgaagcgtttcttacatgtgctttctctgaatctcaactctgtggttccaagttga
>g34434.t1
atgctcgccgccgctgtcctcaagttggtggcgacaaaggtggccgaagccgccggagatcggttcatgctgcagtggaggtttggcgaggacatgga
gattatgaaggagacgatggaatccatcgcggcggtgctcaaggatgcggagaggcggtccatcaaggaagaggcagtgcggctgtggctcaagcg
cctcactggagtctcgtaccgtatcactgatatttttgatgagttcgaagtcagtagcatcaggaaatcagctttacgaaagttcaaagttctgaacccct
atctcacacttgcttctgaggttcgcatggctggtaagatgaaggtggtcagggagaaactggatagtatctcaaatgaacgtgacaaatacagtcttgc
aacaagcagcagctccaatgatcagcaagttatcaatgaccgtgcaacatcgccagaagtgatacaagcagaaatcatagggagggatgaagagaa
acagaaaatagtggctcttttgagtacctcatcgaacttcattatccttccaatatgtggcattgggggcattggcaagacaacattggcacaactgctttt
caatgatacctatttcaaggactacaaaaaagcatgggtatatgtctcccagacattcgacttgaacacaattaaagagagtgtaagatcacaacttcg
gctggagcaggggcagctagccaagtcacaggagcagaatgccggttcacttgctcctaagagcattctgattgttttggatgatctgtgggagcaaga
tgacatgaagatggatgacctgaagaagtccctaaaaatggtcgggaagggacacaaagtgcatataattgtaaccacacgtgatgctggcattgctc
agaaaattcaaacagttgaagcacacaatataaaggccctagctcctgaggtgtgttggactataatcaagaagatagctgactttgaaaatagatctg
acaaagagatgttgaaggatatagggaatgagatttcaggaaagtgtggaggtgtggccctagcagctcgtgctgttgggttcatgttgagatccaggg
acatcgatggatgggtttcagtaaaagacagtgctctctggaatgtttctacgccaggatatacaacttcggcgtacgacaatgtgcttgcatctttgaaa
ttgagctacagcagtatgctgccacacttaaggttatgcttcgcctattgtgcagtcttcccaaagggccacaagatggttaaagatgaactgatttacca
gtgggctgctctaggttttattatgcgatctgacggaatctccattcaacagcatggcgaggcctacattaagcaactactggacatgtccttctttcagag
gtcagagtataggtttgttcggaaatatgatacattgttcactatgcatgatctggtgcatgacctcgcaagatcgatcatgggggatgatcttcttcacgt
cagcaaggagttcatcaagtgtggacgaagcagctgccgctatgccttccttgcagactgcggcaagccactgaattcatatgtcacttatcctgacaat
attagagcccttcattttctgggaagtggtggaactggacattcttctgtaggattttcgaccgctagccttcgaaagctgcatacgttggacctcactgac

tgcttcgagctgaaggagctaccaaaaaacatggaccgtatggacagcctgaagtttctgattggggtcgactacgagatcatcccaatcaacaaaaat
ttgatccctttgccaaatttcatagtacatgatgtggaaggggaacagagcagcaacctcattctgctgaaggatgtaaatcctgacgagttgaaaataa
gtcgtcttgaaaacgtgaagtcaaaagaggaagcacggagaattgaactgagaggaaaacaaactctgacagatttggcacttgattggactagagg
aacaaatggatacgtagaggacatggaactgctaagagagctagtgccaccaaagtacaactga
>g35763.t1
atggctatggtgagctcttccacgggcccaataaactcagtcctcgacaagctccccgccagaccagagttcagtggcttgaggaaggcacttgggggc
atgaaggaacagatgctgggattctgtgtcccaggggtggtgacgagcaggctggcccggcgatggctgctacaactccgtgagattgtatatgatgtt
gatgaatggctcgacgaacgtctcatccattctgggtgccggttccagtggcagccatggtcgtcctctaatgacttggctcaaatccagtacttcgaggt
ccaaatcgctgatgcgcagaatcgtggcaaaacgtttggagttcaccttgagctcagcagaatggtcaacgtggtcccagagaatgaaaagccgcagt
tgaaagatggattttcttgcagacagatgcctcaccttgtgggtctcagttgctcagaaaacgagattgtccagtatcttatggatgatgagcaggaactc
aaggttatcgctcttcttgggccaggaggccttggcaagaccactcttgccagggaggtgtttaagaagcaacaaagcaaattcgactgcgcagcattt
gtgtatgttggctggaacccatcaaggaatgctgttcttactgacatcgcccgtcaggtgatgctgaacactctactcccccaagatgagaatattgttttc
agattgtatgagttcctcgaaacaaagaggtacacccttcttcattctctcggatag
>g37093.t1
atgaagcttggcggtctgctcgccgacgagtgtgctcgtctcaaaggtgtccaaggtgaggttatctcccttcagtccgagctgaaaagcatgtgtgccc
ttcttcaaaagtgtgcaacaagggagaagctggacgtccaagtgaaggcctggacaaaggaggtgagggagctcgcctacgacatcgaggactgcgt
ggacatgtttgtccacgaagttggtaccgaggagcacccgggaggcatcaagggattcttcagcagcatccttcatcgtctgaagacgttgggcgcaag
gcacaacattgcggagaaaatccaagaacttaaggcccgtgccatcgaggtcagggagcagcgtgaaaggtacatgttcgatgatggttctaccacca
gctccaatgataaagcgatagacatagatcctcgtctctctgctattttcgcggagcaggctgaccttgtgggcgttgatggcccaagggacgagctagt
gaggcgtctggtggagggaaaggctaggcacatgaagcgtcgcatggtgttgtctatcttcggctttggtggcttgggcaagaccacacttgcccacga
ggtccgtcgcaagattgggaaccaattcgattgccaagcttatgcatcagtttcacaaacagttgacttgaagaaaattctcttggccatatgctctggcg
tgaccaagaacgacaacgatgaatctgtcaaagttggagaaacatgggacgaggaaagacttattgaaaagacacgagagcttctttaccaaaagag
accctgctag
>g37425.t1
atggcggacttcgcgcttgggctgaccaagaccgcgttggaggggaccctgagcagggtcaagtcggccattgaggaggaggcgaagctgaaggtg
acggtgcagcacgacctggtgttcatcactggggagtttcagatgatgcagtccttcctcaatgtggccaccaaggagcgtgtcaacaatgaagtggtg
aagacctgggtgaggcagctccgcgacctggccttcgatgtcgaagactgcgtcgagctcgttgtccacctggacaacagctcgacctggtcatggatg
tggcgcttggtcccgtcttgcatggcaccgccgcgtcacctggatgcggcggtggctgagataaagctgctcaaggccagggtcgaggaagtgagcca
aaggaatgcacgctacaacctcatcagcgactcagcctccaaacatgtctcatcaccagccccgcctggagcagcattcgacatccagagagagatat
gggagtctgacgggaagagatgcaccatggataacctccaacatttaatcaccaccgaaggcgatggccgtcaagttatctcactgtgggaaagcact
ggaggtgatattggggccgcttccattttgaggaagctgtacagtgaccagacagtctgccacaagttcagaagatgcgcctgggttaagctggtgcat
cccttcaacccaggtgagttcctcgggagcatgctgactcagttttgctcgagctctcaagatccaacaaaccctggtgatgacttcccaaggagagtaa
gggcagcgttcgcggtggaggacgatttcatgaaagcacgggaggagctcgagcagcttcttagtgaccagacgtaccttgtcatactggaagacgttt
caaccatcgtggagtgggaattcatcagctgcttcctgcccaacaacaagaaccacaaccgaattgtcgtgtcaaccaagcaactgaagattgcaactt
caagcacaggagagccgaaccaagtttttgcgcatcgtaatggatttggagccagggaggcttctcgcaactgccttgaggaattacgcgagtgcgcc
aagaaaattgtagacatttggcctatggacaaagtcgagcgcatacaagaaatatcaagaaacaagggtggtgtgacctccgtgtggggtattgctgg
tgttggaaaatcaagttttgtcagacaagaatactattggacattcattgatgctcatgaacccccctagtttgtcgtcgtattatccagaatatagccgtgc
aaaatatgacaagtacagctgggtcgatgcgcccaatccgttcaatttggtggaattcgcccgtcgcttatttgttgattttcattcagatgactatcaaac
aaagcaaacggcagcaattagtatggtggaaggtctggacccaatccaagggtgccgtaagcttctggatggaggaaattgcttaattgttattgatga
tctgcgctccacagaggactgggacacgataaaagctgccttgccccaacttggcagcatcggctccactgtggtcgtgattacatctcaagcaactgtg
gccaggcattgcgtcaatgatcaaagtcaaatggttaacatcaaaggtctagatgcctacactgcctttgatctcttcaaaaaggtgtgtgtttag
>g37784.t1
atggccgagctcgcgttcggcgccgtgagctcactgctgggcgtcatccgaagcgaggcgcggctgcttggcggcgtcaagagcgacgttcagttcatc
tccgaggagatggagagcatgaaaagcttcctggcgcatctttccaggactacggcaccaccatctcgtcgcgagcacaacgagcaggttcgcgcctg
gatgagccaggtgcgtcacctcgcccttgactgcaacaactgcatcgacctctacctgtcccgcatcaaccccgacatctacctcgccaggggtggcctc
cggcactaccttctgtggcctaactggttcctgcacaagaaaatagcgcagcacagcgcggctgtccagctgagcgagctcaaggagcgagcgcgcg
acattggcaagcggcggttgaggtacggcgtggagataccgtccaaggcggctgctgaagcaggctgtgaagaagacaacgatgaagaagaagaag
gcgtggatggcttgccggcaacagtagaggcacgccacttgctcagcgcccagcatgatcctgtggactacttcaataacaagatagccaactgggta
aaggaactcaagggaagcaataattccgaaaaggcggcgggagcagtaataccatccatcgccgttgttgcgccaggtaattgtgaagatgcctgcgc
tcttgcgcaaggagctctggcttcagccaagaagcttaatttccgcactgtcatgatcgacatcccgagactgcactaccttggtgaacccctaggatca
gaggaaatcctctgctacatttggcgagagctcgagctcgagcatgccaaatcctcgtccagttcttctcagcagcaaggcacaacaagcagtcagaga
gaggccaaggagcaacatatttattcttggcgaagcacgagtaataaattggatattttccatgaaaagaggaaggtgattcattattacatcaggaaa
aatatccaggcagaggttgacgaaaagcttgcatggatcaagagcaagatcaggaaactgacggatgacgatctggaccaacgcaaaattatcaatc
tatctctacctgcactactcctgctgctgaacgagtaccaggcagggaagaagaaaatcctgctacctcctactgcgtcgtgcgaaataatacttaggga
aacagccattaagtttaaagcgtatatggaagcaaaagaactcgaagaagcaagggcaaagcggccggtgacccagatctatctcgaacctgccgaa
tatgaagacatcctgcgggaggttttcctacaggcccaggagcaatacacagctcctgctagcaccagtgcattgggtgaggaccaaatcgaagtaaa
gatacgtgaggctaaaagagaaatcctactggaggtggaaaagatgctggaagggaaaactggcaagaaacaagcaacaggagtactagtttgcca
ggatgaaatcacacaagccagtactgcagaaaccaaggatgaaattgcagaaatcgaggagaagatcaagaaaatccagcagaagattgaggcga
caacgaagatcaaatatatcatgaacaacattcatgattacttgaatgatcatgatctacacatcttaatcatcctctacatcggtcaggactatattctga

gatgggacgagaccagatatgctttaagcctactgcattgcgttgctggtgcagtaatcgtgacctcctcaacagacacccaacgggccaaagaatatt
gcagtccaccgctggagcctatggagtgttctactgttggcctctaccataatattgtgctcgagcttaaaagaaagcagctagagcagatggatgaag
agggtgacagacacattgattcccatgtttttcgaagcattttggacgagtgtgcaccatatgaattcaccatgaagatttttgttcatgctttgtacgcca
accccaagaggaacaatgtacaactgagcgagttgcacagaactctgcaggtttctccaaaatctttgaacagtactgctatgaagatgatcaagttctc
atacaacaatttgcctgaagaatacaagtactgcttggtgtacctagcaatcttcccacctggacatcaaatcaggcggtcaacgttgatacaacggtgg
gttgcagaagggctcataacaagggaagattggcctacctcagtgcgccaggccgaaagatgttttgacgcactcacagatcacttgcttgtttgccctg
ctgatattggtgttacaggcgaggtcaagagctgcatggttggtaatgtaatccatcaatttattgccaagattgccaagaaacaaggcatcatggagac
acgttgcttgtctcaccacttctccatatttaactatctcagactccatagctctgagacaatctgtggtttcttgcagaagctctctaaatcatctcaattat
cggtgctcaaagtgctagatcttgaaggttgccaatgctttgccgggaagaacaagcaatacctcaaggacatctgctgtaagatattactgctcaagta
tctgagcctaaggagaacaaatattacccacctgcccagtgatattaacaacctccatgagctggaggttttggatatccgtcagactgacgtgcctcca
tctgcaacaaggagtttccggctcctaaagctaaagcgcttgctggctggtctcactggcgtttcaagtgttgacattcctgaaaaggttggaaatatgga
agagatggaggtactgtccagcgtcaagccacgatctagtgaagacttgaaagacatcggagccctatggcacttgaggaagctaggtgtggttattg
aggacaagagtgctcacctcaagagcttgctttacgcaatcagttttcctcggtgcctccagtccctgacaatcactcttaccccgtccaaagataaggac
actccttctagtggagagttaccaaatcagatggcccctaagatatataccccacttaagcatcttaagagtctaagcattaggggaaccacaataaac
aagcaatttctaccactgttgttcacaaatcatggccaacttaccaaggtaactctaaataacaccaggctggggcagaaagatcaggatatccttgca
gacctgcccacattgcactatctcaggcttcgatacgaggcatatgatgagagtacgctcaccttcaaaaagaagaaatttcggaatctgaagtacctcc
tcgttgagggctccaacattactgacatcagctttgatggtggagctcttgagcttgagaagatcgttttgaccttcacaagcttaaagcttactggagtcc
agaaccttaccaaactgtgggagcttgagctgaacaataatacttccaccaatatcaccaccaccaccaccaccagcaacttcttgctttcaatattcaa
agattccatacctaacatagccaagttgactcttcgtggtaccctcctgaagcaagacgatctacgagattttgccaaggaatcaaacatatgctgccta
atgctcttggacaagtcttatgtcgaaagacagcttaacttcaacaaagatgaattcccaacgctcaaccttcttattctcaactgctctgacatcaccaat
gtcagctttgacagtggatctgccccctaagataagtaagatcgtttggtttttcaaggaaatggagtccctctatggcatcaataatcttcagagactgag
ggagctcgagttcaatggtgacctgatacctcctgaggtgaaagaagacattagaaaacataagaacaaaccttatcttttacataatacactagaaaa
tagagaccaagcaacaggaaacacaaatgactag
>g38021.t1
atgtcgataaangcattccagaatttcaatggaagccgcatcattatcactacacggcagagccatgtggcggctcttgctcccccaacacgtcgccttg
atctccagccactgagcaagactcatgcttttcatctcttctgcagaagagccttttacagcaagaaggattataagtgtcccgaggagcttgtgatagtt
gctacttctatagttgataggtgccagggcttgcctctggcaattgtatcaataggaagcctcttgtcttcaaggccagagacaccatacacttggaacca
aacgtacaaacaacttcgaagtgagctatcaaaaaatgataacattcgagcaattctgaacctgagctaccatcatctatctggagaactcagaaattg
cttcttgtactgcagcctattccctgaggattacccaatctcacgtgagaaccttgtgaggctgtgggtcgcagaaggctttgtgatgagcaaaggcaag
agcacagcagaggaggtggctgagggaaatctcatggaactattacagcgcaatatgcttgtaattaaggagaatgatgagcaaggtagagtcagta
cgtgtatgatgcatgacattgtacgagatctggctcttgaaattgcaaaagaggaaagattcggcgcagctagtgactgtggtacaatggttcagatgg
ataaggatgtccgtcgcctatcatcatatggatggaaagacaaaactgaaataataataaagcttccacgccttcgaactctagtttcacttgaaacagt
atcgtcatcccctgacatgttatcgtccatttttctgaatctacctatctgaccgttctggaactacaagattctgaacttactgaagtgccagcgtctattg
ggactctgttcaacttacgctacctcgggttacgacgtaccaaggtcaaggctctccctgactctgttgagaagctatacaaccttaacactctggatatc
aagcaaacaaaaatacagaggctacctcaagggattgcgaaggttaagaagctacaacaccttatagccgacagacatgatgatgagaaacagtca
gcgttccggtactttgttggaatgccagcacccaaagatctgtccaacatgttagaactgcagactcttgagaccgtggaagccagcaaggacttggct
gagcagttgaagaaactgatgaagctaaggactctgtggattgacaatttaagtgctactgattgtgcaaatatttttcgaccttgtcaactatgccactc
ctttccagcttgcttctatctgcttgggacgagaatgcggcactctgcttcgatactctgaagccatgttccacagaactccataagttgattattagggg
cgatgggccaaaggaacattggagtgcccgatattttgtggctacgggacgcatcttaagtatcttgctctaagctggtgtcaccttggtgaagatccact
ggagatgcttgcaccgcacttgcctaacctcacatacttgaaacttaacaacatgcacagtgcaaagactttagttctttctgcaggatcttttccgtacct
gaagacgcttgtcctgaagcacatgcctagtgtcaaccatctggtgataaacagtggtgcccttccatcccttgaaggtatgtacattgtgtctctgaccg
agctggataagatcccccaaggtatcgaatcacttcgccacctcaagaagctctggctgctcgacctccacaaagatttcatagctcaatggcatgaga
accggatgcaccagagggtgcagcatgttccagagattcgtgtttag
>g38035.t1
atggcggacctggtgctcggggtcgccaagtccctggtggacgggaccctgagcaaggcccagtcggcgatcgaggaggaaaccaggctgcgcgcg
agcacgcagcgcgacctggtgttcatcgccggtgagttccacatgatgcagtccttcctcggcatcaccaacgaggagcacgtcaagaacggcgtggt
gagcacctgggtcacgcaggtccgcgacctcgcctacgacgtcgaggactgcatcgagttcgtcatccacctcgacaagaagcctgactggtggcgcc
gcttcatctcctgctccttctgcaactgcgcagccaaggcactccccctggatgtggccgtcgccgacatcgagcagctcaaggccagggttgaggatgt
cagccgccggaacgcacgatacaaccaaatcattggcgacccgggcgtaataacgcagcagatgccacagccaccagccggcggcgctggagcgtc
gtcgtccttcgacttgctcgctgaagaaagcaagcagcaagatgttctcacccagctgatcatcaaggaaggcagtacagaccgtcaagtgatctcggt
gtgggcaactggtgctggcaaaggcgcaccgtccaccaccatcgtcaagaaggcctacgacgatccaagaatccggtcaaccttcagatgccgggctt
atgtgaagctggcgcatcccttcaatctccataagttcatcagggccttgttggatcagttctatgccaactctagtgaacaggaacatggagcaatccgt
catgctgatgtcctcaagcgaatgaaggcggccatggtggcggatgaagatctggtcaaggacttcatggatcaggtggataatcaaaagttcctcgtt
gtactgcagggtctgtcctccatggctgaatgggatgtcattcgaatgtacttccctgacagaagcatgggaagtcgtattgtcgtgtccactcaagagtt
tgaggtcgccagcttctgcgtaggacctccatatgtgcagtggttttcggctgaccactctctatgtattttcttcaagaaggatcctcaaaatgacacagc
tagtagcccagcatccccggaccaggtggacctcaaaggacgcacatctgaaatgcttgagcttcctatttatctatctaaggctcttatggaagacttgc
atgtgatgtccgtgtggggaatagctggtgctgggaaatctgctcttgttaaagcattctaccaaaaacaactacaggcacacccatcctcatttcaaaa
gcacaacttactgaggccttgcatcttctacctgtcaatcttctcaaagtctcaggccattcgacggcggcgtttggtgatgcggtgggttgccgaaggtt
actccaaggacagtagcaactctaatgccgagaaaaatggagaagatctcttctccaagctcattgagttgagtatgatccaaccaccagagcggaca

gtcattacaagaaacagaatggtcaaatgccaagtaaatgctttcttccacgaatatatcatctcacgaccaaaacaggagaacattaattttgcactgg
aggtccttacactggatggaagttgccggcacatcgccgagcgtacaggacgacaccttgttatagagaaaagctgggagagagataggaatgtgttt
gagagcatcgacttctcacggctacggtcactaacggtgtttgggaaattggagtctttcttcatctccgacaatatgaaggtacttcgagtgcttgatctg
gagaatgcatctgatgtgacggataaacattttgagaagatagtgaagtcaatgactcgcctcaagttcctttctttgagaggttgttgtaagatttcccat
ctgccaaaatcactcgatgatctgaggcagctccaaattcttgatgttagacacacgtccattgtcaccttgcaactgtccattatcaagctgaagaaact
gcggtacatccgtgccggcactaatgccataccttgtaccttagtatccatgttgcccgaggtttgcagaagtcgccaaatagtaggtgtcacggcgccg
ccagggattgggaaactaaccttgcttcacacgctcggagttgtgaacgtcattggtgatagggggaagagcatcctgaaagagctcagaaatctcact
caactgcgcaagctcggagtatcaagtgtcaacaagaataacagagaagagttcatttctgccatctcttctctatttcagttggaatccttgtcaatgtg
gctcagcaatgataatgaaggttgtttggaagacaccaagttcttgcctccaaataacctgcagagtcttaaaatttatgggctcgagggccagttgcca
ggatggactgcgaagcttggctatctcagaaagatgcatttggagataacaaaattaaaggaagaatacctgaaacccctacggaatttgaaagaact
tagtattctacgtttgtctttcaaggagcctcttggtggcaatcttgttttcaatgtcatattggagggccaagaagtgcgctgttatgaagcagtcaagctc
ctcgatatctcttgcagctccaacctagctgtaacttttggagcacgagtaatgccaaacctggagctgctccagcttaactgctccagcctaaatgtaac
ttttggttcagaagcgatgccaaagctggagctgctccagcttaagggnnnnnnnnnnnnnnnnnnnnnnnnnnnnnnnnnnnn
nacctggatgcattcctattttcgtcactgcccagacttactga
>g38742.t1
atggaggctgcggtggtgagcgcatcctgtggcgtcatggcctccctgcttgggaagcttgctgatttgctcacagacaagtacaagttgatcaaagaa
gctaaggggcagatcatgttcctcaaagccgaactggagaccatgtatgttttccttcaaaagatgtcagacacagaggagcccgacgtgcaagccaa
gtgctgggcgaatgatgtgcgtgagctgtcctatgacattgaggacagcatcaacgagttcacgctccgcttagagtgtgagtccagcagcaagccaca
ggggttcagggggttcatcaacaggagcataaccttgctgactaccatgaacattcggcatgggatcgccaaagagttagaaggatttaagagtcgtgt
catggaggtgagcgagcgatacaagaggtacaagattgatgacactactgtctccatggcaaacaacacaagcatcgatcgtcggttattggcactcta
tgcagagtcttcaggtcttgtcggtatcgatggcccaagggaacagctgattcagttgatggatgaacaaggtacgcctgcaaatcagctaaaggttctt
tctattgtgggatttggaggtcttggaaaaactacacttgcgaatgagatttatcgcaagctagaagggcagttccagtgccgagccttggtttctgtgtc
ccagaaaccaaacatgaggaagattctgagatccatgctattacaagctggctttgtagcccctgagcacaccgatgtggaaatatgggacgagcttga
attgattgctgccctccggaaattcctctttgagaagaggtattttgttgtaatcgatgatatatgggatgcttctgcatgggatattatccgatgtgctcttc
cagaaaacaggaaagtacctgcttcttcctgccagctccaaagattccgtgcacccggtttcttgagggttcccaagtggattggccaactccataacct
ctatgacctaaagcttattgttaaggaagtgttggaggatgacattgaaatactatcacagctgccctcacttacctacctcgtcttgatcttcaaggaag
caccagaacacaagattgtcatccgtgaaaacgggttccttgttctgaagcatttgaaagtagtttgtaacaagatatcatttctgaccttcgaggcaggg
gcgatgaccaagcttgaagggctcaagctatgttttaatgcgcacggatgggacaggcacggcgctgctcttgccggcatggaatacctatcaggcctc
aaggtcatctttgtagacattggaggccgtcatgccaaggaatccaacataagagcggcacggtccgcactgaggaatgctattgacatgcatccggg
gcgtcctgcagctaagatcaagtgccatgacgatgaaaccttcggtttcgatgaagtggatgaggagtgcgatgactcgtgctgttag
>g38858.t1
atggagggtgcagcccatagtcttgtgagcaacggagggcagctgctggccgagcagtaccggcagctccgcggcgtccgcagcgaggtggccgagc
tgcgggacgacctggccaccatgaacgccctcctccggatgcagtccgaggccgaggacggcgccgcggaccacttcgtccgcgagtggatgaagca
gctgcgcgagctcgcctacgactcggaggacaccatcgacctctaccggctgcgcgtcaagtgccgcccgggagacaacggcgcgaccaggtttcgg
catctgctgcggacactcttctcccgccgccgcctcgccgccgagatcaacgccctccgcgcgcgcgccgttgccatcggcgatcgccacgcgcggtac
ggtgtcgaccgcgaggcgctccgccgctgccgctccgccgtatctgcggcaccgccgctggcgccccgccgtgcaaacaataatgccgatgcccatca
gctggttggcatcgacggccaggtccacgcactggccgggcggctcaaggcaagggccgacggagagcggcttctcaaggtgctctccgtcgtcggat
tcgggggccttggcaagaccacgctggcccttgagctgtgccggcgggtagaggcggatttcgagcgccaggctatggtgtcggtgtctcaggcgttcg
agcccggcagggacctcaggccactgctcaagcgcctgcttcgccagctcgtcaagcccacaacggacaatgaccaaggcatcaaggaagagggaa
ctctgggagacatcgaaggcttagatgacgataagctggccaaaaagctcgaggagcttctcaacgacaagaggtag
>g39069.t1
atgaatctgtgggagcagatcagcggcaacatggtggttgggacatacaaccagatcccagaatggcagctcaagttaggaccaagggaccgaggtc
cagttcttgttatccttgatgatgtgtggtctctctcacagctagagcagctcgtcttcaagttcccaggatgcaagactcttgttgtgtcgaggttcaagttt
ccgacattggtaaagaatacatacgagatgccgttgcttgatgaggaggaggctttatctgtcttctgccgtgctgccttcgatcaggagtgtatcccgca
aactgctgacaagaaactggttaagcaggttgctgcagagtgcagaggtcttcctctagctctgaaggtcattggagcatctttgcgggaacagcctcct
aagatatggctgagtgccaagaatcggttgtcacggggagagactatttctgactcccatgagaccaagcttctggacagaatggcggccagtgttgag
tgcctttcagagaaggtcagagaatgtttccttgatatggggtgcttcccagaggacaagaagatccctcttgatgtgctgatcaacatttggatggagat
tcatgatcttgatgagccagatgcttttgccatcttacttgagctatcaaacaagaaccttcttactctagtaaatgatgcacagaacaaagctggagattt
gcacagtagctaccatgacttttcagtaacacaacatgatgttttgcgggatctagcgctacacatgtgtggaagggattctctgaacaagcggaagcg
gctcgcgatgccaagaagggaagaatcacttccaagggattggctaaggaataaggacacaccgtttgaagctcagatagtttccattcatacaggtg
aaatgaaagaatctgactggactcagatgagcttccctaaggcagaggttctcgtcctcaattttgcctccgatgtgtactgcctcccagcatttatctcaa
ccatgcagaacctgaaagcattagtgctgatcaactacggttcaagcagtgcagcccttgataatttatctgccttcactacactcaatggcctcaggagt
ctctggctcgagaagatcacactcccaccactgccaaagaccacgatcccccctgaagaacctgcgcaagatttcccttgtcctctgcgaactgaacaac
agtctcagaggatcaaccatggatttgtcggtgacttttccacgcctgtccaacctcacgattgaccactgcatagatttgaaggagctgccatccactat
ctgtgagatcagctcccttgagagcatctccatctccaactgccatgatctcactgagcttccgtatgagctgggcaaactgcattgcctaaacattctac
gagtgtatgcctgcccggcattgtggcgactcccagcatctgtgtgcagcctgaagagattgaagtaccttgacatatctcagtgtattaacctaacaga
cctcccagaggagcttggtcacttgacaaacctggagaagattgacatgcgggaatgctcaaggctgaggagcctcccgaggtcatcgtcctccttgaa
gtcacttggtcacgtcgtgtgtgacgaggagacggcattgctgtggagggaggccgagcaggtcatcccggacctcagagtgcaggtggcagaagag
tgttatgacctagactggcttgtagattga

>g39278.t1
atggcagacctgacgctgaccgccatcgagccgctgctaggcctcctgacaacggcggtcaaggatgaggcccggctcctgcgcggcgtccagggcg
acatccagttcatcaaggacgagatggacagcatgaacggcttcctgatgcacctgaccaagacgcagacagagcacaacgaccagcaccgcgcctg
gatgaagcaggtccgcgacatttcttacgtcgcgcaggactgcatcgagctctacctgcgcgacctcaccccgccggaggacggcttcttggccttcctg
gcccatctccctacactcatatgcacctgctgcaagcggcgcgacctcgccaaggagctccgcgagctcaaggaccgggtgcgcgaggtcggcgagc
ggcggctgcgctacggcgtctccgtcccagaaacggccaagtacaagcccgagcaggacggcaaagacagcaaggtggccgaggagaagagggac
aagttcctgcatgccctggaagaagatgccaacaaggcttctcctccgtccttcgacaaagcaatcagccttctcccgggtgatctcaattccgaagcaa
aaagggacgaaataaatgaaatcctacagatgtgtgtggactttgattgcaagaagatgtttctctgtgctctctttgcttacccctacgtgagcaaagaa
gaactaaagactagtattctggacaagaagaacaagttgacgatggagggcagtagtgcagtggatgatgctgtgaggaaggaggtgatgcttttttc
ctacagcaagctgtccacacattacaagggctgtcttcagtacctgactgctttcagcaatgaaaaaagcatcagtaggacaagcctggtcaggcgatg
ggccgccgaaggcctcgtggcaagggacgacgggaatgaaacaatggaagaggcaggtgaacgctgcttcgatgagctggtgtttcgtggctttgtcc
gtcctgtaacacctgctgttcctggcctgaaaatcaagagctgccatgtggagggattcgtctgggactttatcttcggcatggccaaaagtgaaaactta
gtgggcgcactgccgacccaccttcgtcaccagcattccatccgagagattgctcaacgggaactactgccacagcaccagcaggaaagacagagac
cgtctccattgtggaacatctgcccaagaataaagcagcggcgtcagtatgacgatgacaacatggctgtggaggacaagcggaatcccatggatgac
ctggcgcatctcctcaaaaacctacctgaagagtaccggctcaacgtgttggatctaggaggatgtactgccttgaaggaccgccatctccagggcatc
atctccaaggtgccctggcttaagtacctgagcgtcaggaacactcaagtttcaaggctgccaaaggaaatcaacaagctccggcgcctggagacgct
cgacattcggcagaccaagatcaaggacgccggcacgaagcatatcgtcctcctcaggttgaagcatctccttgccgggaacatcaacaagtgccacg
agaacgagtcactcagcaccgtgcagatgcctcgcaaaattgggatgaccacggaggtactgcgccatgtccagatcttgaagggaaaagctgaactt
gctatgcttaccaatctgaagcagctaaggaagctgggtgtggttattgaaggcagtacagataacatgaagtgtttgcctagagcaattgaaaaatta
agcgaatgcctgcgctctctgtcggtctggatcaatatgccgacgacggatgacactgttgccggcaacgacagcggcttcattcttggcgatgacaac
aatgagaagggggcactaatgactcattatctcccaagaatcttcagagtctaaacatcaagtcttattttaagaaagacggccccggcggcaagcta
ccaccgtggatcaaggggttaaatcgactttccaaggtcactctacgcgacacccggctgtcggagagcagtatgaaaatattcggccagcttgaaagc
ttgcgctgcctccggctccgtcgtggttcttacaatggtactagtatcatccttcacaggggagagttccagaagctccgcttccttatgattgatcaggtc
acggccaccaccagaatcgaattccatgaacacgcagccccggagctcgagaagatcacttggaccgtagacgagaggatggaggccaaggaggat
acgctttccgggataaagtaccttgataacttgaaagagctcgagctaaacggcgactgggtcgacctccaaggccatataaacaaagtgcttgccgat
catccgaagcgtcgccccttctcacgtgctag
>g42155.t1
atggcgaaggaggaggtgcatatgctgcttggggtccccaatgagatggaaaagatgaacatcaagctcggggatctaaagaagttcctcgcggatg
ctgacaggaggaacatcaccgaagaacatgtccacggttgggtgaaagagcttagaggtgccatgtatgatgccactgacatccttgacttgtgccaac
tccaagccatgaaacaaggcccaagccgggacacgggatgtctcaacccgttgctcttctgtttgagaaaccctaaccatgcccatgacatcgcacatc
gtctcaagaagctgaatgaaaagctagatggcatcagcgagcgcagcaaaaccttcaacttcatcaacctcacttcatataaggaccaaggaggaaa
gatagaatcctcccgtcttccaagccgcgagacaacaggggagcccgagttggaagtggttggtgagaagatcatggaggacacaaggaatttagtg
gagttgctcacatgtaaagaaaagacaataaatgaagacaaaaaagtcatggttttcgcaattgtgggagttggtgggattggcaaaaccaccctcgc
ccaaaatatcttcaatgatgaaatcattcaacaaaagtttgacaagaaaatatgggtgagtgtgaaccaagattatacagagattgatctattaatgaga
atcatagaagctgcaggagggaaccaaagggctggaaatacaaaggtcgtgttgcagcaaactcttaaggaaacattgaaaggttgcaagactttgct
ggtgatggatgatgtttggaaccaccaagcatgggagaatgtcctgaaaactccaatgacaaaagcctctgctcaaggtagttgtgtccttgtcaccaca
aggaattaccgcattgcccgaggcatgatggcagaggaaccataccaccctattaacaaactagaaccagaagatgcctggtctttgctcaggaagca
ggagggctgtgccatcagatccctgaaaccaatgcacgcgtcggcgatggaggaagaggagcccatggtctccgtgctgcagtgccgctcaagcagc
acccatggaggaaaggttgccgcggctgcggcggcgcgctctttggccttcttccgtcgagccgatccagatcgtttcgtcattgatcgaggtgtccactc
cgtgttccttgaccgtgcggcgaccgtcgaccgggagtccctgacggctgccatgacaagcaagtggatgttgtgcacgcggatggacgtggaggagg
tgaagtag
>g42169.t1
atgacggaggcaatcgtctctgccgttgtcggcgaagccgtcagcagagtggtttcccttgtcacagggaatttcagtccccagcagagaaccgaagcc
aagctgcaaaggatatgccggttgctgatcagaatccacagcgtcgtggaggaggccaaagggcggcagatcgaaaaccagggcgttcttcagtggc
tctcccagctcatcgacggcgagtatgagggccgctacttgattgataccattgggtgcacaagcgctgttggtgctgatggtgagattgttgaccgggt
gctagagaacttgcaagggctgtctggtgaccttgtcgagttcatcatgctcctccaagggtaccggccgatccgccagcctctggctacaaacatcttc
atggaagggcagatgtttggtcgacacgtcgagaaggagaggatcatcaacttcctattgcatgacggtggtggtaagtccatggggcaattggctgtt
ctaccgattgttggtgatatcggtgttggaaaaacaaccctggtgcagcatgcctgcgatgaccccagggtgcgcaatcacttctttctaatcatgttattc
aatttcacatgtacctatgcaattgcaacgagtgaagcaactgttgttctatggtcaaaacatgtcatcggagatcctggagtaaacattaaagaaccatt
gcagttgatcaagagagattttggaaacaagaggttcctgatggtatttgaggatgtcgatatgcgcaagaagcagatgttagaagaattgttgccaag
cctgaggtgtggcaagaagggaagcaagattgtttttacaactaacaacagacgtgtcgcgagcattggaacagttgaactgatcatactaaaggtgtt
gccacaaccacagtactggttcttcttcaaggcacacgcgttcaggggacgagatgttgaggagaacccgaggcttgtagcagccggaaaagcgattg
tgaggaagttaaatgggtcattctttggtgcgaagatcgtccgagggttactcaaggaccacccagatcccaagttttggtgtaaggtcttgaggagcaa
cattgggggtctgtcgttgctgggtgatggtcttgcttacattgcagatttggtagaaaatttgctgccgaaaaatgttgacatgtgccatgtaaccatttct
aaggatcagtactctcagactgaactcgccaggttccaggatctgtgtgactcgtag
>g43234.t1
atggagcacgcgacgcagaacctcgcaagcaatgttgggcagctgctggctgcggagtatcggcagctccgtggcgtgggccgcgagatcgctgagc
tgcgcgacgatctggccaccatgaacgccctcctccgcatgcaatccgaggccgaggacggtgccgttttccacttcgtccaggagtggatgaagcagt
tgcgggagctcgcctacgattcagaggactgcatcgacctgtacctgctccgtatcaagtgccgcccgggcgaaggcgtgcgcgccaggttgggacgc

ctgctcgagaccttcttcccgcgccgcagcctcgccggccaaatcagcgagctccgcgcccgcgccgtcgccatcagcgagcgccacgcaagccgtgc
gaacgagcctgggcacaacctggtcatcagaatcgtggagcaggtcgacgccctggccaaccaactgaaggcgtctgtacaaggggaccagtatctg
aaggtgttctccgtcgtgggcttcgggggcgtcggcaagaccacgctggccatggaggtgtgccagcggctggagccggagttcccgtgccaggcgtt
cgtttcagtgtctcaggcgttcgatccaagcagtgatctgaaggcgctgctcaagcgtgttcttgaacagatcgtcaagcccaaaacagagaacgagaa
aggcatcaaggaagaggagtcgcttcgtgacatcgacagcttggatgctgatctcctggccaaaaaactcgaggacaaagtcaaggacatgagagag
gatgagctagtggcacgcatgaagcataaatttccggtgctcagggacctgcggtttggaggtgctctgcccaaagttatccggtttgaggaagggtgc
atggaaaatcttgagacgcttgaactccgtattcgcagccgcgagttggagactagcattattggcatcgagcgcttgacgaaccttaaaatgatcacgc
tcaagggtgacaaagatagccctgcactgtaccatggactgtctcaagggtga
>g43977.t1
atgcatgctgctcgccgtgcttggattgacaagcgtgttgtggaggcactggaggaggtgaacaaacttatcgacgaaggtaggggtttcaagaaattt
gggttcaagcccctgccagatattgttgagcacctgcctcagattgagacatttgggcttgagagcatgctgactcagctccatgatctgcttgagaaga
gtgactcgagcgtaattggtgtgtggggtgaaggaggtatcggtaagacaacactccttcacatcttcaacaatgatcttggaaggaaggcccatgact
atcaggtaccatcatggttccttcangaaggaggtatcggtaagacaacactccttcacatcttcaacaatgatcttggaaggaaggcccatgactatca
gaaaatggacacatcatgttattcagaactgctagatcgaactgaagattttgcagcttaa
>g44340.t1
atgaacgagctcaagagcaagcgcgacgacgtgaagcgcatggttgactccgccgagcggcagggcatggaggccacgagccaggtcatgtggtgg
ctggagtgcgtcgccgcgctggaggtcgccgccgcgcggatcgtggacgagtaccaggcgcgcctgcagctcccgccggaccgggcccccgggatga
aggccacctaccacctcagcaagaaggccgacgagacgctcgccgaggccaccagcctcaaggagaagggcgacttccacaaggtcgccgacgagc
tcgtgcaggtccgcttcgaggagatgcccagcgcgcccgtggtcggcagggacgcgctgctccaggagctccacgcctgcgtccgcgacggcgacgtc
ggcatcgtcggcatctacggcatggccggggtcggcaagaccgcgttgctcaacaagttcaacaacgagttcctcgtcaacacccccgacatcaatgtc
gccatctacatcgaggtcggcaaggagtacaacctcgacgacatccagaggatcgtcggcgaccggctcggcgtgtcctgggagaacaggacgccca
aggagcgcgccggggtcctctacagggtgctcagcaagatgaatttcgtgctgcttctggacgatgtctgggagcccctcaatttcaggatgctcggcat
ccccgtgccgaagcagaattccatgagcaagatcgtcttgaccacgaggattgaagacgtgtgcgaccgcatggacgtccgccgaaagctcaagatg
gagtgcctgccatgggcgccctcctgggagctcttccgtgacaaggttggggagcacatcatgagtgcgaccccggagattcggcagcaggcgcaggc
gctggccatgaagtgcggcgggctgcccctcgcgctcatcactgtcggccgggcgatggccagcaagcgcactgcaaaagagtggaagcacgccatc
actgtgctcaagatcgccccatggcagctccttggcatggagatggatgtccttgtgcctctcaaggacagttacgacaacttgcccagcgacaagctaa
ggctctgcctcctgtattgctctctgttcccagaggaattctccatttctaaggattggatcattgcctactgcatcggtgaaggtttcatagatgaattgta
cactgagatggatgagatatacaacaaggggcatgaccttcttggtgatctcaagattgcctctttgttggaaaaaggtgatgatgaggatcatatcaga
atgcatcccatgcaggcgcaggcgctggccatgaagtgcggcgggctgcccctcgcgctcatcactgtcggtcgggcgatggccagcaagcgcacgg
caaaagagtggaatcacgccatcacagtgctcaagatcgccccatggcagcttcttggcatggagatggatgtccttgtgcctctcaaggacagttacg
acaacttgcccagcgacaagctaaggctctgcctcctgtattgctctctgttcccagaggaattctccatttccaaggactggatcattggctactgcatcg
gtgaaggtttcatagatgaattgtacactgagatggatgagatatacaacaaggggcatgacctgcttggtgatctcaagattgcctctttgttggagaa
aggtgatgatgaggatcatatcagaatgcatcccatggtccgtgccatggccctatggatagcgtcagagtttggccagaaagagaccaaatggctcgt
ccgtgccggagtcatgctgaaggaggcaccaggtgcagagaaatggagtgatgctgagcggatttccttcatgcggaacaacattcttgaactgtacg
agaagcctaattgccctttgctcaagaccctcatgctacaaggattcagttcattggttgagttgcagtacctggatttgtacaacacaaacatcagggca
ctgccaagggagctcgggtcgctggtgaccctgcggttcttgcttctctcacatatgccactggaaatgatcccaagtggcgttatatgcagtctcacaag
gctgcaagtcttgtacatggacctcagctatggagattggaaggttggtgagagtggaaatggtgttgattttcaggagcttgagagcctgcgtaggctc
aaggctctcgacatcactttacagtcgcttgatgccctggagcggttgtcaaggtcatttcgccttgctggttccacaaggtacatactgataaagacttgt
tccagcctaacaaagattgaacttcctggaagcaacctctggaagaacatgactggactgaagagggtgtggatcgctagctgcagcaacttagagga
ggtaatcattgatggtagccaagaaactgttcggggcaatgcgctccctcatgccatcttgcaggtgcaggctcgggctggacttgtcgatgacgagca
gcccatccttccaaacctgcatgatgtcattcttcagggccttcataaggtaaagatcatatacaaaggcggatgcgtccagaacctttcatcgctgttca
tctggtattgccatggactcgaggagctgattactctcagtggtgaagaacaagaaacgactgccagtggaggcgaacaagctgctggattcaagatc
atcacaccattccctaacctcaaggacctgtatctccatggcttggcgaaattcaggaggatgagcagcagtacatgtacgctacgattcccttcactgg
agagcctgaagattgtcgagtgcccgaatctgaaacagctgaaactttctgctggagggctgaacgtggtacaatgcacgagagaatggtgggatggg
ctggagtgggacgacgaggaagtcaaagcgtcttatgaaccgctcttccgtccaatgcactga
>g45563.t1
atggagaccgcgacggccgccgtgggcagcgtcctccgcaagctgcaagcctacctgagcgaccacaaagaagaagatcactgcagcggcgacgtg
aagcagctcagccaggagttcgggcacatccacgccgcgctcctcggcagcgccggcgcccaggcgccgccgccggggcagccccacgacgagccg
gcggcggcgtggacgggtgatgcgagggagctggcgtacgacgtcgaggacgccgtcgacgacctcctcgtggcgtcgtcgctgccgggctccctca
gccgcacgatcacggggcttctccagagagccaccgatctttctagagcccgtcctcagacgccagctggcgttcctgcggccgccaccgtcgtcgact
accctcgcaagaagcagcagctgcctccaccgccggacggcgaggcgggcgtcatcggcgttgatgacgctgacgccagggacgggctcatcagga
ggctgcggctgcacgacgaggccgccgccggcgatgaggacgaccaggtgaaggtagtctccgtcgtcgggcccgtggggatgggcaagacgaccc
tcgccggagcggcgtacggcgtgctcaaggcgaggttccattgcgcggcgttcgtctccgtcgggctcgaccccgacatcaccgaggttcttgagagcg
cgctccagcagcttggcgtcagcaaggaagccaacgctggtgaaccgagggacgaggccctgctcgtcagtcagctgagagagttcctacaaaacaa
gagggtatccgtgcgtgtccgtggtggtggaaggaaagcagaggatgttctgtcttcaaaacgaaggaaagcagaggactctgcttctctcggcagca
acagcatgagcgtgcctcaactgaggtccctcactgttttcagccctgccactcttgagaaaagcattgatctgaagatgtgtagtaagtttcttcgtgtgt
tggacctagaaggatgcgatctttcagagtctgaacttcttccggagcaccttggaagtttggttcacttgaggtacctaggcctacgagagacacgcat
cgcctctgtccctgaagaaatcggcaacctcaagtttctgcagacactggacctggacgagaacctcaaggtgttcgacctgcccgcgagcatcactcg
tctcggagagctcacgtgcctgcgtgtctccactctgacgcggctgccgagcgggatcgggagcctgacggcgctggaggagctctcggatgtctcgac

ccggtacacgccggacctggtgaacgagctgggcagcctgacgaagctgaggctccagacgctggaggtctacgccgacagcggcggcgacgagtc
cgagaagaagaagcagctgtgcgaagaagaagaagcagaagaagcctggtgggccccgccgcgcagcctcgtcgagttccgaggcggcgcgttga
acgcgtactggagcccggtggcgcggctgctgctgccgccgctgcggcgcttcgacgccgccaccgccgcggccgcgccgaagaacctggccgtcctc
gtcgcgaaggtcgccgagctgcggcagcgcgacgtcgacgcgctcgggacgctgccggcgctgcgcgtcctgcggctggagccgtacgccacggcgg
aggcgatccacgtgtccggcggcggcggcgcgtgcttcccgcgcctgacggagtgccggttccgggacgccgacgtggcgcccgtgttcggtcgcggg
gccatgccgcggctaaggaggttggagatctgcttcaatgtgcggaagacagtcgacctcggcaacggcgggttcgagttcgggctggagaatctgct
ctgcctcgaggaagtctcagtctacgtcggatgccaggagtccaccgagccggaggccgaggccgcggaggcggcgctgcggcgtgccgccgatggc
caccctaaccgcgccgccttcgacgtgatcacgttcggcgaagagctcatgtgtttcgacgattga
>g45745.t1
atggaatttgccacggccctgcttcccaagctcgccaagctgctgcaggaggagtacaacctgcagacgagcgcgaaaaagggaatcgagttcctcca
cagtgagctcgtgaccatgcaagccgccctccgcaagatcggcgaggtgccaagagagcagctcgacgacctgcgaaggatctgggcccgcgatgtc
cgggagctgtcctacgatatggaggacatcgttgacagtttcatggtggacgtcgagggtcctgatcccccagcaaaagaggcgctcgaaagatcttc
aagaagatgataagaaagttttcttcattcattgtattggtggtttccttggacggcctgcacaggtacaagcttgatgctagtattgctcctgccaataga
accgctgttgaccctcgcatcacagcaatgtacaccaagatgactgagcttgtgggcattggagaggcaaaggagcaagtaatcgcgaggttgataaa
gggagatgctgatgaccacaaggaaaggatagtagtctccgttgctggttttggaggactaggcaagacaacacttgccagagcagtgtatgatgaga
ttaaaaaagattttgcttgcacggcttttgtttcggtgtctcgcaatcctgacgcgaagaaacttctcaaggacatcctgtatgagcttcacaaaggaaga
ggacatcctggtgaaaatttggatgaaataaagcacctcattgatcttgtccgtgattttctctgtgacaagaggtacctcattgttgttgatgatatatgg
gataaagatccatgggataaggacccaccggagattactggacaccgttccgaccacgggtactgcaacatgctcgtcccgctcaccggccgcctctg
a
>g48729.t1
atggccttttttcgaagctggtattgagtgttctgttgcagagctactgtggatgctccttgagctgctctctggcgtcagcggtggcgactggcaccacctc
cagtcgcagctcaggaccatacatgacaggatcgacgtctgctacgaatccctcgacaactccgatgatcaggcttgggcctgggtcacccaagctcga
gagctcgcttacgacatggatgattgtatctgctttcgcaggtactacaacaacggcagtgccaacccctcgttgtccggttttctcgagtggattcgtgg
cctcaaagatatctcccacaaattacgtgacctagaggctcgttttaatgcactgaaacaggaggtgcggtctcactctatcttcgatggccaagcgggg
gcagtgcctgagcccatcgacgaccctgagccggtccacctcgtcggtcaccacgcggcaatgggcgagttggtcaggatggtcatggatagcggtga
caaggctgaacgcaagatcatttccattgccgggatggccggctctgggaagactacactcgccgcaacagtgtacctgcaggtcagagaacagaacc
gcttccagtgccgagattttgtctccgtcggacagaagccagacctccgtgggactctcaagaaaatgctctcgcagctttggggacgggctcggtggg
agcgaggagattga
>g48730.t1
atggcctttttcgaagctggtattgagtgttctgttgcagacctactgtggatgctccttgagctgctctctggcgtcagcggtggcgactggcaccacctc
cagtcgcagctcaggaccatacatgacaggatcgacgtctgctacgaatccctcgacaactccgatgatcaggcttgggcctgggtcacccaagctcga
gagctcgcttacgacatggatgattgtatctgctttcgcaggtactacaacaacggcggtgccaacccctcattgtccggttttctcgagtggattcgtgg
cctcaaagatatctcccacaaattacgtgacctagaggctcgttttaatgcactgaaacaggtgcggcctcgctccatcttcgatggcgaagcgcgcgca
gtgcctgagcccatcgacgatcctgagccggtccacctcgttgataccgaaagcctcgtcggtcaccacgcggcgatgggcgagttggccaggatggtc
atggatagcggtgacaaggctgaacgcaagatcatttccattgccgggatggccggctctgggaagactacgctcgccgcaacagtgtacttgcaggt
cagagaacagaaccgcttccagtgccgagtttttgtctccgtcggacagaagccagacctccgtgggactctcaagaaaatgctctcgcagcttgggga
cgggctcggtgggagcgaggagattgaccaactcgtcgtgggcattggagacctactgaaggagaagaggtag
>g49968.t1
atgtatgaaggctgctacttggtcgatacttttgggtatcggatgctgcagcaaaagatgaacaacaatcaggttggtagtcaccttgccttgaccaaatt
aagtccagccaagtgcttctgcttctccactacaaggatcaacaggggtcttcaaggtgatgggaggaaggagattcagaagatgctagaaagcctgc
ataacatcatcaatgaaatggcagattttgtcgccttcttgaattcctgtcctcccgtcacctatcagccttatagcaagtacctgattatggagaagtctat
gtttggccaccaagcggagatggagaaggtcatcagcttcttattgcagccagagtctccagaaaggttgacaatggagacattcatttctgcggtcctc
agcgatcttatcagcagatcagtatccctcgtcatcgataattactatcggtggcacaagggcgaggatgaaaacctgcaacggcttctacagatgctg
ctgcgggttcaagtcattgtcgaggaggccgaggagcgacatattacaaaccgtgcaatgctgcagcaacttcagatgctgagagagacgatgtacaa
aggcttgtacttgcttgatacttctaggagaatgaacatcacatttcaaggtgacaagttgaaggaggttcagaaaatgctaggaaacctgcatagcatc
atcaacgatatggcagagttcattgtgttcttgaaatcctacccacccatcagccgtgagccttatagcaagtacttatttctggagaagtgtatgtttggc
cgtcaggcagagatggagaacatcatttgcttcttgttgcagccagagcctcccggcgtagaaagattacaagtgcttccgatagtaggtccgccaaga
gttggcaagagcactcttgttgagcatgtcttctgcgatcagagggtgcgcaaccacttctcttcaataattctatgcagtggagatcctactgctcctgaa
ggcagtgaagttgtcaaaaagcaaatgtctagttcacgtggaagatcacttattgtcatggagcttgctgtagattttagtctaaacaagcagcattaca
gaaagttgcactttttaagaagccacatgccgcctggaagtaagattatcattacaagtagatcagagagtatcctgaaacttggaacatcggaacctat
tagactgacatttttgcctcaagaagcatactggtacttcttcaaggtgatggcctttggaagcacaaatcctgaagtgcacccggagctagcatcaata
gctatggaacttgcagcagagctagatggatccttcttggtcgcgaacctcatctgtggcctgctgagaactaatatacaaactcggttctggcgcaaga
tccttgaactgcataaacaccacgttgagagaaatcgccatctctttggtgaacatccccatactcttcttcataaaaaaaatcaggtcgtatatgtttggt
gtctctctgatatttcgatgaggctcaaagttcattactgtgagaaactttatcatccaaatgaggttccaaatataacgttgcatgatgttatgattggaa
gtgcaaaaggtcatgggaaatttgatgttgtggcctggaagtcatgcatacccccataccacaaccacttgatgacctgtgaaatagaggcaccacgag
acatgatgggcaagaagaagcggcctcacttcatggtgtga
>g49969.t1
atgccagaagatgcagagcacatgcttcaacggctgcagcgcgtgctgctccgtattgacgccatggtcgaggaggccgaggggaggcacatcacca
gcaaggcgatgctgcgccagcttcagctgctcaggcaaggcatgtaccatggccactacatgctcgacaccttcagatgcagagaccatggaggcgac

gaagaggtaaacggtggccgtgctgttgccttattacggcctcgattcagtacagccaagcggcgcctcttctcccccgtcaacgacgatacggataatc
agcagaacaccatgctagacgccgatagcttgaggaagctggagaaggtggtcgacggcctcgagatgctgatggggaacatgccagagttcgccgt
gcttttgggggggataccctcctcgcatctgccggcagccgtacggcacctacttgatcctcgacaaggtcatgttcggccggcacatggagaaggaaac
catcctcaattttcttctacggccagaggctacaggcgatgaaaatcctggcgtccttcccatcgtgggcgaagccagggttgggaagagcacgcttgta
gagcatgtatgccttgacgagagagtgcgcaactacttcagatttattgtcttcttcagtagtgataatcttggttgtggaaatatggctgctctgagagaa
attggtgtgatcaagcatcaagatcttacgaccgggttgcaaggaaggtcacttgccgttattgagcttacaggtgacatagaggaggagacatggaga
aggctgtcctcctctgcaacaagcagcatgggtcatggaagcaagatcataatcacaagcagatcagagaagatagttgctctcggaacaacgcacgc
tctaagactgaaggttctgccgagagaagctttctggtacttcttcaaggcgcttgcgtttggaagcgcggaccctgatgatcaaccgaagctcttgtccc
taggcatggagatctcgacgcttctgaacagcgttttcttaagagcaaatattgtgtcaagcctgctgagaaccaaccagaatgttcagttctggcacag
ggtgcttcagtgcttgagagattacacaagcaaacatgtctgcacgtttggtaagcatccttcagatcttgtgtccaaaagccagcctgtgtacccatgga
gtatggtcaggagccagaatgtcgttgagatctccaaattctatcagaagccttctccatatcatggtgtccccaagctaagagtacaagaatttttaaca
gaatgtgtaatagatcatgggaagttcgacatgttaatgtggagatctaacatacctccctactatgcttacgccgcgagttgtgtatcagagccgtcgag
atgctcaatggcaaacaagaagcgccctcgacacgcgagggagagatga
>g49970.t1
atggagccgcttctttcggcaatcgtgagcgatcttctcggcagagccctgtccacggtgattcagaggtacagccgagctaaggcagaagagacaga
gcagaagctccaacggctgcagcgcctgctactccggatcgaggccacggtcgaggaggcagaggggcggtacatcacgaaccagacgatgctccg
tcagctcgagatgctcaggcaaggcgtgtatggaggccactacatgctcgacaccttcaggagagaacctgcagcagcgacatggatgtgcagaagacc
gagcgcgtcggaaagctggagaagatgctcgacggtctcgagacgctgatgggcgacatgcaggagttcgtcctgttcttggagggataccctcgcat
ctgccggcagccttacatcacgtacctgattcttggcaaggtcatgttcggccggctgatggagatggagactgtcaccagcttcctgctccggcctgaat
tcaatcctggtgtgcttcccattgttggcgcagcgaggtccggcaaaaaaacgcttgttgagcatgtctgccttgatgagagagtgcgtacctacttctcg
tcgatcgttttcttcacacgacacgatcttggagccggaaacatggcagctctgaaaggcactggtgtgattaagcatcaagatcgtactgcttcctctca
tggaagatcacttgctgtcattgagctggtcaaggacatggacgaggagacgtgggaaaggctgtactcctcggcatcaagcttcatggagcacggta
gcaagatcataatcacgagccggtcagagaagatagtggctttcggaacagtacaagctctgaggctgaagtatctgactgaagaagcttactggtact
tcttcaaggcactcgcattcggaagcgcaaacccggaggatcaaccgaaacttgcgtccataggaatggagatcgccgtgctgctgaaaggttccttct
taggagcaaacatagtgggaagcctaatgagggccaacctgagcgttgagttctggagcagggtgctcaagtgcttgaaagattttacaagcaaatac
ctgctcatgtacggagaggatcctacggatctcttacggagggaccaacctgtctgcatatggaggatggccaggactcagcatgccattacaatctgc
aaagtttaccggatgcctgcccctcagaactgtgttcccgagctaccgacggcacaagatatattaacaggacgtgttacagatcagggtgatttctgca
tgaagcgtgagaaggacaaggtgaaaataaaagctgtatgcaagaagctcgagacggcggctatgcaaatttttgtggaccatggatga
>g49971.t1
atgctgctccggattgacgccaccgtcgaggaggctgaggggcggttcatcaccaaccaggcgatgctccttcagctcgagacgctcaggcaaggcat
gtacgaagggcactatatggtcgacaccttcatgtacagaggccatggagaagacgatgaggtgagtgctggccgtgccgtcgccttgcctagattcag
tccagccaagcttttcttcccgttccccgtcaccagcaacacgaagaacatgacaattgacgccgagaccgtgaagaagttggagaagatgctcgaca
gactcgacacgctgatgggagacatggaggagttcgtcctgttcttggagggatatcctcgcatcttccggcaaccatacagtacctacttgattcttgat
aaggtaatgttcggtcggcagatggagaaggaaacgatcatcaatttccttctccggtcagaagctgccggcgatggaagcccaggcgtgcttccggtc
gtcggtctagcgagggtcgggaagagcacgctcgtcgagcacgtctgccttgatgagagggtgcgcgggtacttctcgtccatcgtcttcttcactggcg
gtgaccttattggtgccggaaacacggctgctctgaggaacagcggtgtagtaaagcatcaggatcttacggctgcctcaccctcgcatgggagatcac
tcgtggtcattgagctcgacggggacatggaagaggagacatggagaaggctgtactcgtcggcagcaagcatcatggagcacggaagcaagatcat
agtcgcaagccggtcagagaaggtaacatgtttcggaacagctcaggctctgaggctcaagttcctgcctcaagaagcttactggtacttcttcaagac
gctcgcgttcgggagcacgaaccctgacgatcatcctaagctcgcatccctaggcatggagatcgcagcgacgatgaaaggttcgttcttgggggcaa
acatagtgggcggcatgatgagagacaacctcaacacacagttctggcgcaggttgtttcagcgcctccgcgagttcacaaacaaacacttgatcgcgt
acggtgagcacccgacggatcgtattcagaaaggccaccctgttgacatatggaggatggccaggacttggcaccccgttacaatctgcaaaatctttc
aggaccgctcttaccagcagcatgctgtccccaagctaactctgcaagacattgttacggggtgcgccacagatcaggcggtgtttggcacagtagcat
ggaggtcaagcatacctccctattacacttacctggcgacttgtgcatcccagacgatcaccaaatgttcaatggtcagcaagaagcgccctcggcagg
tgagggtctga
>g50061.t1
atggcaagcgccttgtctgggatgatgacctctgtcatcagcaagctcacaaccctgcttggggaagaatacgcgaagctgaaaggcgtgcaaaggga
ggtgaattttatgagggatgagctgagcagcatgaacgccctccttcagaggctggccgaggtggacagtgttgttgatgtgcagacgaaggagtgga
ggcgacaagtgaaggagatgacttatgatatagaggactgtatcgataattttacacattgcattggacacaatggcacggctgattctgtgggccttgt
ccataggttggttcaaaaccttaaggcattgagagcacgctgtcgaattgccaaccaaattcaggagctcaaggcacgtgttgaagatgctagcaagcg
ccgcatgagatataagcttgatgagcgtgcctttgaatctattgatgcaactgtgactgatcctcgtttgccttcgctctatgctgaaccggaagggcttgt
tggcattgaccagcagaaagatgacatcaccaggctgttaatggagggggaaggaacatcaatgcagcaactgaaggtgatatctatcgtgggtcctg
gggtcttggtaaaactacacttgcgaatgaggtgtaccgtgaattagaaggcaaattccagtgtcgagctttagtttctctgtcacagcaacccgatata
aagaagatattgagaaatatactctcccaaatcagtcagaaggaatgtggtaacatagaagtgtgggatgaggaaaagttgatcaattcaatccgaga
atttctgaagaacaagaggtaa
>g50914.t1
atgtcagaggagatatccaagctgagaagcaagtatttgtctgcaggttcttcatcaaacccctccgtggcatctccggccgtgaacaactccccagcgt
catttgactcggatacccctctggctgatctggtgggtatggaagggcccctgagtgaccttctggaccttatccgcgaagctgatgaaggccaaccggg
aaagctcaaggtgatctccattgttgggtttggcggtttgggcaagaccgtcctcgccaggaacgcatacaaccgtgtagccgtcggcgagctgtatga
accacgcatatgggttcgcgcatcggagaaggacgcaaggtgtgttctcaaggagatactcaaacaagttgaatccggcatgcaagatcatgattatg

acgatacctccgatcccaacaagctcatcacaagtctcagaaattgcctcaagaccaagaggttcttcatcgtaatcgatgatatgcgcacagagttctg
gaacatcatcaaaaatgccttcccagatgatacgggaataggcagcattctgatggtgaccacggctattcattccgttgcaaatgcggtcagctccatc
aacggccatgtgttcctaatgagaactcttgaggaggagcactcaagacgattgttctgtaaagaggcatctttggatttctgtgaacctgcagcggaac
cagtgttgaagaaatgcgatggtcttccccttgctctcattaccatagcccagctgttgcaaagtaaaagtcagctaacatccaaaggatgtgcagatct
gtgccggaacatgggtgaacatgtggagaaggaagagacgttggcgagaatgaacttgaagaaggtgaaagtccggtgtgagtccactgcggacgg
cagcaacgacttcgcacatctttcaaggccaattcaggagttcatccagagaggcacagacataaatgatgcccgttcactctctgtggatttcgagggg
ttctcacagaagttcctggattttcatctggaggacgattcctgctgtcttaactcgctcaagctacaaggcaagctatacagcctacctccatttgtcacg
cagctgcgtagtattactgaactgtgcatttcatcccctggtgacctgaacttggatcttcttgactccttgatcaatttgagctccttgcattacctcaaact
catcggaagtcatctggacaggaaggagctctgccacatctcgagtccctctggttgctctgtaaagttctaa
>g51147.t1
atgtggggcatcgacagcgaccgcgagaagctggaggacttcctggtgtacgtgagccgcatgctggctcacgccgaggtgaaggccgagacggacc
ctgccgtcaagaggtggatgaaaaatctcaagactgtcatgtaccaggccgacgacgtcctcgacaatttcgagtacgaggcgctgcgccgcgaggcc
cagggtgcccggtccatgactagcaaggtactgagcaacttcactcgccacaacacgcttttgttccgccttaaaatgagcagggagctcaaaaatgtg
cttgacaagatcgagggattagtatcagacatgaatcggttcgccctggtggagcatgaggtggtgccgcaagttgtgtgccggcagacacgctcagg
gctggacatgtcagaggagatcctcggaagagaagatgaaatggagaaagtggtgaagttgttgcttgaccagcaagatcagcagagtgtgcaggtg
cttcccatcattgggatgggaggtttgggcaagaccacactagcaaagatggtttacaacgacaacagagttcagaagcactttgagttgaagacatgg
tactgcgtgtcagagaacttcgaagctactactgtggtgagatctgttatcgaactggccacaaaagacagatgcaagctgcctgacaacattgagctgt
tacaaggacgactgcagcaaagggctgaaagtgttgcctgcagggatggagggtctcacttcccttga
>g51148.t1
atgcgcaccggcgagcttgactttgccgccatggtctattttgtgggcgagcacggcccaagcctccatttggtgggcgagctcggcccaagcctctgtt
ctgagggcacattggatcccaccgccatggccaggggcgcaggaagcttggctccggtcaccatggccagacgcgccgggaggcgagccctccgtcg
ctattcatcgttcatccatgccatggagcagacgcatcccggacgcagtgcgaacgactcagcgaggagggccgcccgcgcgctcgtcagctgcgcct
ggggccttggcgcccctcgccgttgttcggcgcacgtcggctggtggtggccgtgccggcggacccaccgcgacttccgcgccctagctgccctccgtct
cctcctcaggtcgcgctctcttcatctcccgcctccctatactacctgcaagcagctattgaacaattctacaatcatggcagcgtcgctgctcctgcccgt
ggtgagctcggtggccagcaaggccgccgacgagctcgtccaaaccgtcacccgcatgtggggtgttgacgccgaccgcggaaagctggaacgtcgg
ctgctggccgtgcagtgccttctggctgacgcggaggtgaagagtgagaccaaccctgccttccggagatggatgaaggatctcaaggctgtcgcctac
caggctgatgacgttctcgaccacttccagtatgaggcgctgcgccgccaggcgcagagtcgtaagtccatgaccagcaaggtactgagccacttcaca
aacaacagtcctcttttctttcgcttgaaagttagcagggggctaaagaaagtgctagacaagattaatgagcttgttgaggagatgaacaagtttggcc
tggtggagcgtgcggaggtgccacatgttccctatcggcagacacactcattactggacgaatctgaagagatctatggcagagacaaagacaaggaa
ctggtggtaaagctcttgctggatcagcaaagtcagcagaatgcgcaggtgcttcccatcattgggatgggggtctgggcaagaccacacttgccaat
atggctagcatggctgagtcgctgctgctcgccgtggtgaccagggtaacaggcaaggccgccgatgtgcttgtccagagcgtcactcgcatgtggggc
gtcgacgccgaccgccacaagctacaacgccggctgctgaccatgcagtgcatgttggccgatgcagaggtgaaggaggagacaaaccctgccgtca
ggaggtggatgaaggaacttaaggccgtcgcttacctggctgatgacatcctcgacaatatacagtatgaggggctgcgcctggaggcccaggatggc
gcagcatgcaagatacagaggtacttcaccctccatagcccgtttctgttccggctcactgtgaataggaatctgaacagaatcctcaagaaatttgatg
agctgatcctggagacgcgcatgttaggacttgttgagcgcatggaggtgcctcaacctctgcatcagcaggtgtactcaacacttgacgagtctgcag
agatctttgttcgagttcacgaaaaggagatggtggtgaagctgttgctggaccaggaagataacaagaaagtgcaggtctcaggcagaccagaggc
aatcaaaacatcagcccgaccagcatcacctacggtcaactcttcatcaaatagaacctttgtcaatgctgctccagccatccccaaagagaagaacca
acgtcacacagtagacaagagatcaagtctcaaggttcccatgaatgggcagcagaaggcagttgctgcagctcctagaagcagcggtacaacgaag
aaacactcggagaggtgttacgcaagccaaacctgcagcacaagtgcagtcaagggagtgacagaacaagatttcctcttcaaggcaccgaagaatc
tgattacaagtggacccatgttccggcgccatgttccatcggcggacagagcacggactaaaattcctgggttcggagcagatgtcaaaaaggaaaatt
gcaacgcaagaaggcagtcatgtccaccggctgcaagaaggggcataaaggagcagcagttagagggcaggcagaagcagaatgtactaacgccc
agagggagggaggagtctgcttggagcaagggaacaagaataacgcatgggaagaaagagcagagaccggaacctggaaccggtatccaggcaa
agaagtga
>g51286.t1
atgctgagaggtggtgccatggccaggaacctggtctccgaggtcagggcggttgcatacgaggtggaggacatcattgacaaagctgaaatcttgag
gaggcaaagcaaccccaggatatcaattagaggggccatttccaaatatgcttgctttcccatctatctaactcatctccaccgtcttggagcaaggata
gattcagcaaatgcatggatgaaaaccatatttgaggaccatgagaatctaaggactgctgcttctgcaattgcagaaccaagcggtgacatcacccct
ggtgacatcagcccacaacggcaattaattcacccagactttgatgagaacgttgatgtgattggatttgatgatcaagttgataagatcaagaatgact
tgcttgatggagaaaatattaatctaactattgtttctattgtcggtcttggtggggcaggcaaatcaacaatggctatgaaggtgtattgtttggatgcag
taaaggaacactttgacgttcataattggatccttgtgtcacatagatttgtacttcgtgatctgttgaaagagatggcaaagagatttataccttttggtat
cttggaagctactgtagcatcccaacaacctgatgatcttgaggagcatgggctgaagaagttgcttcataactttctacagagaaaaagatacttgatt
gtattggatgatgtctggagccgtgatgactgggataagcttacatccatcttccctgatgcgaagaatggtagcagagttgtcttgaccactcgaaatg
aggttgttgcaaagcatcccttcaatgctaggatccataaacttaagctcctaaatgaagaagcaagcaaagagttgcttcttagaacggctgttccatg
gtataatatagatggaagcagtagtagtcatgnnnnttag
>g51287.t1
atgctgagaggtggtgccatggccaggaacctggtctccgaggtcagggcggttgcatacgaggtggaggacatcatagacaaagccgaaatcatga
gaaggcaaagcaaccccaggatatcagttagaggggccatttccaaatatgcttgctttcccatctatctaactcatctccaccgtcttggagcaaggat
agattcagcaaatgcatggatgaaaaccatatttgaggaccatgagaatctaaggactgctgcttctgcaattgcagaaccaagcggtgacatcacccc
tggtgacatcagcccacaacggcaattaattcacccagactttgatgagaacgttgatgtgattggatttgatgatcaagttgataagatcaagaatgac

ttgcttgatggagaaaatattaatctaactattgtttctattgtcggtcttggtggggcaggcaaatcaacaatggctatgaaggtgtattgtttggatgca
gtaaaggaacactttgacgttcataattggatccttgtgtcacatagatttgtacttcgtgatctgttgaaagagatggcaaagagatttatacctttttggt
atcttggaagctactgtagcatcccaacaacctgatgatcttgaggagcatgggctgaagaagttgcttcataactttctacagagaaaaagatacttga
ttgtattggatgatgtctggagccgtgatgactgggataaacttacatccatcttccctgatgcgaagaatgagggtttcatcccgctggtcagaggcga
ggaagaagctgtcaagtgcatcgatgagctagtgcggcgatgtataatccaagtggagcaaagggcgacctctgggagggtcgtgacaatcaaggtg
caagacatcctacgccattgtgggatcgagcgagctcgaaaagaagggtttataaaggactgttgcaccatggatgacatggagacagcctactcaga
ggagacggtggagttctaccgtgtggttctgcacggtttcctggctcaggaagttggtatctacaacagaaaacttcgcactatattagccttccatctctc
tggagtccaaataggaaaatcattccaaggtttccgtcacctgcgcgtgctttacttgcagtgcttggatgaggaagtgtttctaccaagggagattgacc
agatgagatatttgaggtacttgggctttgggggcaccagcacatatcatctgcctacctccattgggaacctcctgagccttgagaccatggatgctata
ggagggagaatataccacatccctggatctatatggaagatcccagcactgaagaggttgcacatcgtctgtgctagaggctggtcagtgccacgggtc
agtttacagtccaagatgcaagtgatggtattcagttcggctgctgacctccacagccaattagccccaaacaacataattttggaagctaaacggataa
tggacgcaacaatacagcaattatcaaagaagaagaaccccaatctgtcatgtatttttggaatgacatgtaacacggacgaggcaggaagccatatg
aacattgtgggaaggcgcaaggaaggactccagttgaccaatgacctcccggattttgaggacatgaatcatatcacggtgctcaaattgcggtgcgct
aatctgctcaacactgaccagaagatgctagagatcgggaggatgacgttcctcaaggtgctggagataggagaacagtcatacacaggatcagtgct
gacgtgtccttctggcagcttcagttctcttgtacaattggtgctctacgatcttgcactggagcattggaagatggaggttcaatccatgatccgtctcag
ggaggtgacgctttgcaaatgccccaatctgctttaccttcctgatggtttttgcggctcccgcatctttgcaagctacacctcatcgcaatgcctcgtgct
tgctacgagcgtgatacagttagggagctgaagactatgcgttgcctggtctttgaatcagttgacgagagagatttcaagcacctagatcttccgtga
>g52506.t1
atggatgaagtcaaatccaagttatctgaaaaggttaataatttgagggcgctgccagagaatcttgaggacgtgaagatggatttagaaatgatgagc
tgtgtaataaaagatttgggatctggagacctcagcaaagaagcagtacaagtatggattgggttgctgcggaaggtgtccttccgtgttgtggatgtaa
tggacaaatactcctaccacgcttttcagctgcaggaagaaagcttattcacaaggttctttaaaggaggatattatgtcaaggttttcagcgatgttgct
gatgagaacacgaaaatggctgaggtggagttcgttgcaatctcaaagatagctacagccttgggagataaggtcatggatgaagtcaaatccaagtt
atctgaaaaggttaataatttgagggcgctgccagagaatcttgaggacgtgaagatggatttagaaatgatgagctgtgtaataaaagatttgggatc
tggagacctcagcaaagaagcagtacaaggatggattgggttgctgcggaaggtgtccttccgtgttgtggatgtaatggacaaatactcctaccacgc
ttttcagctgcaggaagaaagcttattcacaaggttctttaaaggaggatattatgtcaaggttttcagcgatgttgctgatgaggtagtcaacataaaga
atgagatacagaaagtgaaagacctgagaaaagactggcttcctatggatcaagttgttccacaaaatcctatggctttagatcgaccgggctctcaaa
gcttcttcccggtacttgttcaagatgaagatctggttgggattaaagataaccagagtaagttgatcggatggctcgactccaatgagccagatagcac
agtgattacagtatcaggtatgggtggattaggaaagtccaccctagtttccaatgtgtacggccgggtgaagaatagctttggagttaatgcatggata
gttgtttcgcaaacctacgcagaacatgctctgctgagggaactactgtggaagatcagttgcactgacactgagaacccacggtctataagcatggac
agaatggacacctttatgctgaaagaggaaataaagtcgaggctccagggcagcggcaaatgtttggttgtcttcgatgatgtctgggacagacacgc
gtatgaaagtgtgcaagatgttttcaagaatctccaatcatgtcgtgtcgtcatcacgacacggaaagatgatgttgcatcgcttgcatccttgggatata
aaattcagctccagcccttggacagagctgacgctctccgtctattctgtgcaagggcctttaggaacacggtcgaccggaagtgccccactgatcttga
aaatgtggcttccaaaatggtggatagatgccaaggtctgccactagcacttgtctccatgggcagcctcatgtcatcgaagcaacgaacagagtacgt
ctggaatcaagtgtacaatcagtttcgaagtgagctcttgaaagcagacgatgtccaggcaattctgaagctaagctacaacgacttaccagggaacct
tagaaattgcttcctatactgcagtttgtttcctgaagattacaccatagcgcgggagagccttgtcaggcagtgggttgctgaaggctttgcaatggcca
ctgagaacaacacaccggaggatgttgctgagttaaatctcatggaactcatcaccagaaacatgctgcaggtagtggactatgatgagcttggtaggg
tgagcacctgtaagatgcatgacattgttcgtgacctggctctttcgactgcaagaaaggagaagtttggttcagcaaatcatcgtggtgcaatgttactt
atggaaaaagatgttcgtcgcttgtcatcgtacggatggagagatagtgattcttcgacagaagatttgccatgcctccgcacctttatgtcttttgaggcc
gtgacgtccactacccaaatgttatcctcgatttttactggttccccttaccttacagttctcgagctgcaagattctgcaatcactgaagtgccagcatcta
ttcagaacttgttcaatctacgttacattggcttgcggcgcacaagggtcaagtcactgccagattgtattgagaaattgtcaaacctacagaccctggac
atcaagcaaacaaaaatagaaaagctacctcgaggtatagctaagctcaagaagctgcggcatctattagctgacagagtcatcgacgagaagcaaa
gaagtttccaatacttcgtcggggtacagccacccacggatctttcaaacttgttggaattgcagacactcgagacggtggaagctagtgatgaccttgc
agaccaactggagaaaatgaacaggctgcagagcgtgtggattggcaacgtaaatgctacccattctgcaaaactttttttgtagcctgtcgaaggtgcc
acttctttccagcttgcttctaaatgcatgtgatgtggatcagcccctttccttggaagctctcaacccacagtctagacagctccacagactcattgtcag
aggacgctgggctgctgggatgctgcagtgcccaatgtttcagtttgatggccatgggaagaacctcaagtatcttgctctaagctggtcttgtcttgtgg
aagacccactgctactgcttgcaccccaggtgccgaacctcacttatctcagccttaacaaggcgagcagtgtagagaccttggttgtttctgatgggtg
cttcccaaagctgaagacacttgtattgaagaacttggagaatgtcaacaagattacaattggaaaaggtgcccttaaaagtatccaaggattgtatgtt
gtggcgctgcccaatctggacaaggttcctcaagacatagaataccttatctcactgaagaagctgtggcttctctatcttcacggtgacttcaaagttca
gtggggcgagaatgggatgcacaagaagatggcacatgttctggagcttcgagtctag
>g53955.t1
atggaggatgacacgcgtcaacttgagcaactgctgatggagaaggatggacaaaccagcggcggcaccatcgttgctatcaccgggcccgacggca
ttggcaagaccactctggcggcgaaggtgtactgcagcgagaggatacgacgcagcttcgagacgagggcatgggtgcgcatccccaaggggcaca
cccgggccggcctgctctcgcaggtcatcgactccttcggtggctacacgaaaggcagtgggagcgtcgccgacctcggcaggacgctgagccggtta
gtggagaagaagaggtgcttgctcgtgctcgacgacgtctggtacggcggggtgtggaaggaaatcttcaggagcccgatggagcacgccggccgcg
gcagcaaggtgctggtcacggcgcggcacggcagcattgctcgcgagatgggcgccggccacgtccaccgcgtcaagaagctcggcgcggacgacg
gctggcttctgctccgcgcggcggcccgtgtcgtcgacgaggcagcggccggcgagctgaacgacgtcgggaagagcatcgtcgagaagtgcggcgg
cgtgcctctggcgatcaaggcggtggccggcgtcttgaggacacgggcagcgagcgccggtgagtgggcagaggtgctcaagagcccggcgtggtcg
gtgaaggggctccctgaggacgccatgaagtcgctctacctctgctacgacgacctgccgtgccacctcaagcagtgcttcctgtactgctcgatgttcc
cctcagacctcggtgtcgacaggcacgacctcgtgcagcagtggatatcagaaggctttgtgcagatcagagcagacgccagcatcgaggaagtggc

cgaggagtactacgacgagctcgtgggaaggcaccttctccacaaggcagaggaagatgagagcggtggcgccacgaggtgctccatgcacgacac
ggtgcgtgctctggcacggatactctcacagggcgaggacttgaccggtgatgcacatcgacaggcagttgacggcgatgtccggtccgtgccacggc
gtatctcctttccaggaagaaacttgactgccataccagagaagattctgaagttcgagggattaagaacgctgcttcttcagaggaacccgctgacaat
ggaaggaagcatatttactaggctgcagcatctgagaatcttggatctcagtgaaacagcagttgatctcgttccggagaacctggggaatcttgtatac
ctgaagtacctgaacctgtctgacacaaggattcaggccattccggagtccattggcaacctgtggaacctgaaattcctgctattacgaggatgcaagt
cactgcataaactgccaaaagggatagagcatctgagagggcttagagaccttgatcttgccggcacggtgattgatgatgcagcattcacagtagga
catctaagaagcctcacatcgctccagtgcttcgctgtgacaagcaaacaggcgcgagccgcacaggataggagtggttggtctctcgatgagctcaa
gcacttgtctcagctgagaatacttcacattacgaaccttgagaaggccactggtcgatcagaagcagctgaaatggcgcttgcaaccaaaaaaagact
cagagagctcgagatgtcgtgcagcagcactgcaaggacgatccagacagtggaggggatcagacattttgaggatatctttgaagaggtgaatccac
cacaatgtctggagtcactgaagattgccaattactttgggacaaagtttccaagatggttgtcactgacctacctcccaaatctccgccatctcgacatc
atcgggtgtgacttctgccagtcctttcctccattaggccgcctgccagagctgaggtccctgtatatagcagactcttcagctctgaaggatattggggc
agaattcatgggcactgaccaccagcatcaggtggtgccatttccgaagctggaaaacctgcacttgcaagggttgtataagcttaagacctggacaga
cattgaggtaggggcatttccatctctgcaggcacttcaactcgagagctgccctgaactgcaacatcttcctgctggcctcagacaagtgacatccctg
acggagctgcgcatagtggatatggccagccttgaggctgtggaagacatctcttcactgagggaattgagcgtttggaatgctcccaagttgaagtcta
tatcaaatatgtcctcccttgaagacctcaacatgtgccactgcccagagttgcaagtagtgaacatcgacgagctccaaacagtgcacatcttcgatca
tgaattgagggcaatgccaagatggatcgagacagttgcttctaggcttcaatcactgaatttcacgagcacggttgtgctgctaaaaaggtgtttggttg
atggtccggactggccaatgattaaggatatcaagcaagttaatggatactccactggttccagttatatacactataccaagagtccttacatgtttgaa
agcaatgtgaatattgatatcgaagagaaagatgaagatcctgatgatgttgatcaatgttcagtttcatcttcagacactggttatcaagaaatacagg
gtttctttgactcaaaagtagtaaatacaggaactacaaggtctaaagacaatgtgaaaagtaacacggaggattccataccaagtctcactcatcgtc
gcttgcataagctggcagaagtccctgaggaggacgaggttgaggacagtgtacattcagctttgcgttttccttctaatccaattagagttacaccagat
atgaaaaaagttcgtcctgttttcactgatgataatgtcaagaacaatgatcttagttcagggtcaaaagctacccaaaaccatattaagagccagattg
caactactccaaatgaaagtgcaaatgcaactccgacgtcagaaattccatcaggaaaggatgtgccaacaatatctgttggtgtcactactagtagtgt
aatccatgtaacatctcaaactgtctatgttactgaaaccagtcaagatctggatagcagtttactttgcagcgaagagcaggcatccaataatgaagca
gaagaggtcgctagaacaatctgtgctactagagttgtcagtaactcttttgatcaggaggaagatgcggaggtcgctgaaacaacttgtgctactagtg
ctgtcagtagctctgttgataaggaggaagacgtcgaggtcaccgaaacaattcgtgttactagtgctgtcagtagctctgttgatcaggaggaagacgt
caaggccactgaaacagtttgtgctactactgccgtcagcaattctattgatcaggagaaagacgtcgaggtcgctgaaacaatttatgctattaatgct
gtcagtaactctgttgatcaggaagaagacgtccgggttgctgaaacaatttgtgctactagtactgccagtagctctgttgatcaggaagacgatgtcc
aggtcgctgaaacaatttgtgctactagtgctatcactgtcagtaactctgttgatcaggatgaagacggcaaggtcgctgaaataatttgtgctactagt
gctatcactgtcagtaactctgttgatcagagggaagacgtcaacatcacttcactaattcaagaagaatccaaagaaatcagtgccagtgaaagtaat
cgtgatgatttgggggcttgcaaattgcctgccagtttagtttgcagcaagcaacagacacagaacacgttggaaaatgtttctgtggaggcaagcgat
gataccgatggatccatgaagaaaatacttgggatggcaagcagaatcaccgagaagctcaccaacgaatgtgaggttggctcagtgaaggattcgc
ctgctgaaacagccacgaaaatcgcccgtcccgcatccaagtcaacgcgcaccatgtcgcatgtcattatcactgaagcagccccgaatccagaagtc
acaacggcaagcagattcgcagcgactgctgcggcggtgaaggtgaacgattgccgcatcaacgacgatgacgacgacgcgctgcgctccacggac
gccgaggccgaagactcttaccaggcggcgccaaaggtgtacaccgccatgtgggccgataccgacacggacacactgcgcgcccggttcctcgaat
cgatgcggcagtatcgcaggatggcttcccgccggcgccggcgccgcgagaggaagcacggctccggagggagaaggtggagcgtcggcccggcg
actgttgccgccgtcctcctagttttctctgtggcgcagctgctgttcatcttcgggatgtaccggaggctcacgagtcaaaactag
>g54533.t1
atggaaggaatcatggagagtgctgccacgggggcgatgaactccatcctcgccaagctcgcggagctgctgagggaggactatcagatgcagaagg
gcatgaggcgcgatatcactttcctcaaggacgaactgagcagcatgaacgcgctcctggagaggctggctgatgccgaggctcttgatccgcagacc
aaggagtggaggaaccaggtgagggagatgacctatgacatcgaagattgcatcgacaactacatgctccagctgcaacatgaacaacagaggcctg
ggagtagtggggtcatggggttctttcttgggtatgtacagaaagtgaaggagctcgtcgcgcatcacgaaatcgctgatcagattcaggagctcaggg
ctcgcattgtggaggccggccacagaaggaagaggtacaagattgatgctgctctgaattcttgtgatcctaaaatgatccttgtagatcgtcggttgcc
ggcactgtatgcagagttggatggccttgttggtattagtggtccgagagatgaggtcatcaaattcttagtggatgatgagcaggggatgaaggtggtg
tcaattgtagggtgtggaggattaggaaaaactactcttgctaatcaggtttacaggaaaattgccaagcaatttgattgccacgcttttgtgtcattgtcc
cagaaccctgacatggggaggatctttcggtccatactagtgcaagtcacggaggatgagtgtgataacgccaaagcttgtgatgaggaacagctcat
caatgaattgagggatttcttcaaggacaagaggtatataattacgataagttcgtatacagatggatga
>g54581.t1
atggaggctgaaagcgccgttggctcccttttgatgaagctcggcggtctgctcgcggacgagtgtgctcgtctcaaaggtgtccaaggcgaggttctct
cccttcagtccgagctgaaaagcatgtatgcccttcttcaaaagtgcgcaacaagggagaagctggacgtccaagtgaaggcctggacaaaggaggt
gagggagctcgtctacgacttcgaggacggcgtgaacatgtttgtccacgaaattggtaccgaggagcacccgggaggcatcaagggattcttcagca
gcatccttcatcgactgaagacgttgggcacaaggcacaacatcgcggagaaaatccaagaacttaagtcccgtgtcatcgaggtcggggaacagcgt
gaaaggtacatgttcgatgatggttctacctccagctccaacgatcaagcgatagacatagatcctcgtctctctgctattttcgcggagcaggctgacct
tgtaggcattgatgccccaagggacgagctagtgaggcgtctggtggagggaaaggctaggcacatgaagcgttgcatggtgttgtctatcttcggcat
tggtggcttgggcaagaccacacttgcccacgaggtccgtcgcaagattgggaaccaattcgattgtcaagcttatgcatcagtttcacaaacagttgac
ttgaagaaaattctcttggtcatactctctggcgtgaccaagaacgacaacaatgaatctgtcaaagttggagaaacatgggatgaggaaagacttatt
gaaaagacacgagagctcctttaccaaaagagtgataagtgggactcttggtacccctccaagtataaaacaaaacaaatgagctttgttagaacaag
accctgctag
>g55725.t1

atggatacggcggtggttccggccaccggcgtcgtcaacgacggcgcgcttctggtgtgggcttccaatgttggcctgggtcgcaaggttgagcagctc
attgccgctcgcagggacctgagttcggtgctcgcagaggcgcaggggaaggagatccagaaccagacgctgctgctgcacctcagagaagcctgga
atagggcgtgcagcgccaaggacctcctttgcgagctggactactaccgcatccacggagagatggagcagcctgacgataagctcttgcatggtatc
gtggaccaaaattttcttctctccgcacctggtagtgacattaagttttctaacaatgatatagctgaagacgcttccacagaaaaagttcctgttaggaat
gaaggttcagctacaccaacagctgagatcatcccatacattaccaacacaatggtagttgctaacactggcacagtatcccattatgagattcttggca
aggatatctcatgggagataagtgaacagatcgaggaatgctgcaggatcactgggcatgtgcgcaaggctcttgagctcgaaaatcttgattaccata
tcatgcagatacacaacagcacaaagtcagactatcgggagtcatcaccttatcatactgagcccaaagttcatgggagggaccaagagagggacttc
atcataagcaagttaacaaatgaggaatctactacgaaaaaattctctgtcattgccatcactggtcatggaggtgttggaaagactactctggctaggc
tggtcttcaacaactccgtagtgtcaaaacattttggcatactattatgggtttacgtctctgttcactttgatcaagttaagattatgcgtgaactgttagat
acattgtgtggcgatagtcatgaaaacaatacgaaatcagatgagctccaagaaaagcttgagtacatgttgtga
>g55756.t1
atggcattggccatcatccggccgctgctggggctgctacgcaatgaagccaagctgctgcggggcgtccgcggcgacgtgcagttcatcaaggacga
gatggagagcgtgaaagggttcctcctgaacctggcggcaaggaccagggaagggggccgcgccgatcaccaggtccgggcatggatagagcaggt
catggagctcgcctacgactccaagaactgcgttgaggactacacccagagcggccgcatgagcccgcacggcgggcggggcctcatgcgtcgcatc
aagtgggccgtgcggctgccgcggcggatacttgtccgccgccgcatcgccgcccgcatccgacagctcaaaatccgggcccgggacgtcggccagc
ggcagaagcggtatgctgtcgccgttccaccgccactcaagatggacaccacaacaagaacaacaaccggagctgttaccatagacttctggaatgat
ccgcaggctggtagtggtggcccaagagggcagaatcatcatcagcaaccatcgtcccacccgcccaaagattctcctcgttgggccgtgctctccgcc
gacatcctgacggaggccaaacaggagctgcttcagtggctggcagtgaaaccagaagaccaccacacaaggccgtggccaagcgtcctcgctgtcg
taacgccggacggggtaaatggagcttctcttgcagaccaagtgtacaagcactgtgaaaatcccttcaataatggagacctcaagttcgactatgttgt
ccgcatcaaaatcaagagtccctccttcctgctggtcatactgcttgacatgcttcgccaactccaacctcactacttcatgtctccggggaacggcaccg
agacttgggatctggcacaagccaccgagaagctcaaggagttattgaggggaaaagcttgctgcttgttctttctgatttggatcatccagatatatg
ggactacatcaaacaatcactggagaaactggagtgctccgccggtagtgctgtgctgttctccaccaacaacaagaagctggctgacagatgctctcc
ggacgaatgtaagatctacacgcttgttggtttctactatcagaaggcagtctccctggttcctagcaaattcgaccccaaagacaatcagcctctgcgg
gggaccatccgggaagtcttgacgaaatgcaaaccggatgtctattgcatgaacctcttgcttcgtgcccttctcagcaacccaaataggacggtgcaa
gagctcaagagcctgtcctggagccttgccccagctcagtgtcccaccaaaattgacaagcacaaaagaatgctcatgttctgctaccacgggttgtcc
aatgaatacaagaactgcttgtggtactccactgcttttactagaggaaccttcaaggttcggagggccagcttattgaggagatgggtcgctgaaggct
tgataaagagcggaggccagtactccacggaggaagctgagaggtgcttcgatgcgcttgttacccagaagcttcttcttccctctgaacttgatagtac
aggcaggataaagagttgtggcctggatcctttgtttgcatcaatggttactgacgtcgaagcagatgaggcattcctggatgccaaccaaatgccggct
gacttggaactccacttctccatccgcaacggaatccaactccgaaggatgttgcagataaatagcagcacgacagatagcaccgtttctctgaagaat
aagaagacagaacagcaaatgaagatgatggcgttcttgaaccaccttccttcttcttcaagcttccggcttctaagagtcttggagcttgaaggtttcaa
aggctttaataagcgccacctcaacaacatatgcaagatccaccagcttaagtacttgtcccttcgggacactgatgtcgtgcaacttcccaaggaaata
gataagcttgagcagttggagacatttgatatccgaggtaccaaggtgcaagagctcaacgctgtgctgcctatgctaaagcatcttctggctggcagc
atcgattataaacagggccatggagacaatgtgaagtccaacgagctgtttaccactgtgcgcatgcctaaggatgtttcaaagatggacaagttggag
attttgtcccatgtcaaagtctatagcagtggtaaagatttggccaacattggtgacaaaatgaagaagatgaggaagctaggcgtagtcctctgcggta
agaaagctaacatgaaggatttgttccatcagattgatgaactcaacgaatgtcttgtctccttgtcaatccgtgtggaaccatcaatcaaatgggacac
ggttgatggtgctacagtgacaccgccgaggcttcttgaaagcctaaccatctgcgatgtcaggggctggttgccagcgaggataaatgagctccatcg
gcttgtaaagattactctgcgtggcacatatctgaaagagggtgcccttgccatccttgggagcctgggaagcctacttcgcctgaggctccgattccatt
catttgccgaggccgcactagccttcagaaatggacagtttggtaaactcatagagcttgtgatcgaagatgacgatcttagaagcctcagatttgaaca
tggaactgcccccaagctcagtacgatggtttggtcttttactcgcatggattcaattacagggatcaagaaccttgtgagtttgcagcgccttgagctta
acaaaggaaaatgtgactcggctgggctagaagaactgcagcaagacatttctacacattgtaaaaaggttcgtttcagcctcaatccaccagaaaata
atcaaggatcagaagccgatgccgctgttaccgcccgttga
>g56678.t1
atggagccggtgtcaagtgcagtggtgagcgagctggtgagcaggtccatctcgttcctcttctcccggtgcgagaaccggcgggccgcggaggctca
caataacctgcagcggctgcgccacctgctgctccggtgcgccacggtcgtcgaggaggccgagcggcgccacgtcaccaaccgggccatgctgcag
cagctccaggccctccgggacgaaacgttccggggccactacgtcctcgacgccgtccgcctcaaggatcataacaacaacgtcgagaaggagaagg
aggagcacgaggagaaggactcggcggcgggccgccgaaccttcgcgctgtccatgttcaacgcggcgaagcgtgtgcgttattacccgggtggtggt
ggcgactcatcgtcatcatcatcgtcggccatcagcagggcgagctcatcatcgagcagggaggagctaccgcgcgtggtgcacagcctggaatccat
gatcggcgacatgagggagttcgtcgtgttcctgacgagctacccgccgctgctgcaccggcggcagccttacagcgcccacatgtcagtggacaagt
gcatgtttggccgccacatggagtgggagacggtcatggagttcctgctcaaaccggacccccaccaccgcctgggcgtgcttccgatcgtcggcccgg
ctcacatcgggaagagcacgctcgtcgacaacgtctgcgacgacgagagggtgcgcggacacttctccttgatcttgttctacaacggcagtaataatg
gaatcaaagatgagactacggtagccggtctcagagccaagtgtgccgtcaagcaccggaacaacgacgaggcttcatcaggggagaggttgctgat
cgtcgtggagctcttagacgaggacgacgtggacgacgagacgtggaagaagctctccgattccgctgaaacaagcttgccacacgggagcaggatg
atcattacgggcagatgcgacaacatcgcgaggctcggcgggacaaggcagcaggcgcttcggctcagatgcctgcccgtggaagcctactggtactt
cttcaagatgatggtgttcgggagtgacgacccagagcagcacccgaggatggcgtcgctagcgttagagatggcgcacgtgatgcaagggtcctact
tgttcgcgtacgttggtgccatactgctgagggacaacttccacgctagaagctggttccggattctgtcgaggttgagacagtacttgcagaggaacgt
ctccttgattggggagtacccggatgacatcaaggcgaccaaggatcaacctcgatacacgtggagcctactccaggacaaacctgacacgtacttcat
gctatataagctttaccaaagaagccccgctgaagagaaggcctcggagataacaatgctagatctgctcgtcggaactcctcaaccgcgagggaaac
acgagatcttgttctggaaatctcgaattcctccctactatagttacatgagtacatgtgacatttgcgacatgtga
>g57088.t1

atggcggaagctgtcgtgggagtgctgatcagtaagctcggtgaggccttgttgagcgaagcagctgcctatggtgcatccttgttttgcagggaagctt
ctgccctcaagggtctctttagtgagatccgcagggctacgggctggctggagattatgaaggcctacctgcaagattcggagaaattcagggatacta
gcaacactactgacgcctttgtcaagaagatccgaggtctagcgttcaggattgaagatgttgcggatgagttcaagtacaagctagaggacggaaag
cacggtggatttgctgccaagatgaacaagaggatccgacatgtcaaggtttggcaccgtctaacttatgagctccgtgatatcaatgctgatcttgaag
acgctgccaaacaggccaacctttgtgccatgccaattatagagaggaccggtgcaggcaattattatcatgctggatcaattaatccaacttcctgtttt
gcaagggatgaggatctggtgggtatcgaggataatgcagagaagttgaaagggtggttgctagatatggaagaaaagaagagcagaatcgtagca
atttgggggatgggtggagcaggtaaaacaactttggttggtcatgtctacaagtctgtgaaagaggattttgattttgctgcttggctgaaccatgtgga
tatcgaagaaggtgcactggagagccttgttgacctcatgtttattcaatgtccagaactgaagcaccttcctcacggcattgagtatcttgcagccctta
aggaactacgcttggaagacactgcaacgcagctcatagagaagcttaggaaaaaacatgaagcagatgaagagtttatgaagatcagccatattag
aaaagtgtttgttatgctaactgagaaaaacatatgggaaagagttcgctga
>g58781.t1
atgtcgctgccgccatcggtcgggtggtcggcctcggcctttgtcgccgccgtgctggcccggctcatccggaagggcctcgagctggtggtcgagctcg
atgatgccggcacgggccacctgcgccgcctggagggcctcctcccgtccgtgtggcgcgtgctcgacgccgcggactccggcgtcgtcgccggcggg
cggccgttgcaggacctgctcgacgccgcctacgccgcggacgacgccctcgacgatctcgaccgcgcgctccacggcgccgcgggagaaggagac
gacggcgttgacgagcggcagagaagcccgagcgccgcgcggaagccgcgcagcccgctgagcttcctgctctgtttcagcccccccacggagttcagt
cgctagcactagcagcagcgcccatggcaagagctccagagctgccaagaacagcaataatgtcaacctcaatctcgacgcgttgcgagacgcgctg
gagacgatggcacaagcggcgtacaggtgcacgaccgcatacgagcacgtggagcccgagaggaactacgccactatcgtttccgggagcagaaca
gaggagggagcaggggaggaagcagagcaggacgacatcttcggaagggaagcagaggtggaagcgatcctggagaaggtgaaggcaagcgac
gacccgcactaccttctcggcatcggcgtgctgcccgtcgccggggacgccggggtggggaagacggccctcgtgcgcttcgtgttccaccacgaggtc
gtccgagccgagttccccgtgcggatgtgggtgcacgtgtccggcgagctcgggctaaggaaacgacgtattgtggaccagatcatcaacggagtcac
cggctttgatgagcatctacaagaagtcgatgatggcagggagcttcttgggatggaattagacgggaacaggttcttgctggtacttgatggtgtcacg
gacatcgttgagactcagtggcaatatttgatcgagttactgaagccagctaataggaggagcttgatcatcctgacaactcagtctcaaactgtggcga
cagcaatcggcacgatgccgacactgacgctgggcactctgggattcaaggattacctcaagatgttcaagcatttcacattcggcagcacggatgaaa
ccgaagaatgcactctgctgggtgacgattgttgggaagacgtggaggaggaggaagagcaggagccggaggaattgtcacccatggaccagattgc
ttcagagctggcaaagaagatgaaccgcctacctttgccagcacaggcaatcgggcgggcgctgcgtttccggaaggacgatgaaggccactggaga
agcgtgctggaagacaagatctgggagggccaggatgtcggtgccgggattcccaccgcattgtggctgagctatcgccatctcgaccctcgcctcaag
cagtgcttcgcatactgtgcagtgtttcccggtgattatgtcttcaggaaggaagaggtggttcagatgtggatggctcaggggctgatatattctgatca
gcacgcgacgaggttggaggacgtcggtggtgagtttttgatgaactgcagcacctcttgaaatcccttgaaacgctatcaatcaagaattgtgacaac
gtcatctctgacattccagacggcttcaaggatctcactgcactgagggacatctccctatatgactgcccgaagatgctcgtcgataagtttcacacatc
agtgcgaacattggagatcagtgagtgcttcgttgtacggggcgcatgggtgcaggaccacccctttcctcttctcagtttggaaactgaaaataacaggg
tgctcgcatgtaagctgcagcgatcaggataacaggattgagcctttggattggttgaacagcctgatcaatgtgtacagccttcatctcgagaacacaa
agattctcagattgagcatgtttgatcagcttccatcacttgagatccttgatgtcaatgggtgtgatgctttcttttctgatttgtatgatttcgcttggttgg
agaggctgcagaccatgttgatcaggaactgcaaggaaatgggcgggcttccggagaacttgtgcacgcttcctgagttggaggagctgtgtatagag
aactgtccagctattgaagtgttgcccgagaatggccttccgacaactttgaagcgactttcaatctctaaatgtggttcgctgctgactcacagtgtattg
cccggaacgccgctggagcgcagcccgtcgccaccgctacccgccccgctcattgagaccacgccgaaggtgagccctcctgcctcccttcacgcgat
gcgcctcctccgcgacccccctgtgcttttacctctccgttgtgtcccccgacgccgtgcccctggctcgccgtggccgtgcgctccatggccactatccagg
cgtggccagccagcccggccgtgatggccttaggtcttag
>g60089.t1
atggatgctcttgtgtctgcggtggcgagcgacctcatcggccggttcatctccttcctcatcgggaagtaccaggagagcagcgcagctgacatctccg
acaccatagtcaggctgcagcgagcgctgctgcgagctcgcgtcgtggttgaggaagccgaggggcggcagatcaccaaccgagcgatgctgcagca
gctcaaccagctgagacgagagatgtgcggagccgcatacgcacttgatgccttcaggtggcgagataggaggtcacaagccacggtgaggctatctc
gagcgcaagcaaggcctccttcgagcagcgattgctctgactttttatccgccatggtgggaaccctggaagccacgctaggcgacatgagagagttcg
tggtgctcctcaatggctgcccccgcttcactaggcagccatacagtggctacctgttcgtggagagctgtatgtttgggcggcagatggagaaggaag
aaatcatcagcttcttatcgcggccatctcaagatctggatgtcctcccaatcatcggcccgttcggtgtcgggaagcggaccttggtggagcacgtctgt
ctcgatgagagggtgcgtgagcgcttcggcaagattcatcgtctcagaagtactgatcttgatctatacagttacgggcatcattggagctcattcgactt
caccacaatgttgctgatggtgatcgatatggtcaacaatgatccggatgccgaggagtcatggaggagattccactcagcggttcaccgccgcgcgca
cagagggagcaaaatcatagtcgtcagcaggacggaggcgacgcactcgagtctgggcactgtctcgccgctcaggctgcgcccactgcgccgggag
gacctttggtactttttcagggcgctggccttcggggccgcggaccctgacgagcacccggacctcgcgcatctcgctatgaccatgtgcacttgcatca
acggcggcttggcgctattcaccgtggccaacatcgtcgccgggtcgctgcgtgccgatcccagcgtgcgctcctggcgccgtttgttcaaggtgatcttt
gagacggcagtgctgcagttcggcacagccgctgacgataagcccgaaaactactactacctctgcagtccgctgggagacgctggtgcttcttgcttgt
tctacaacaggcgcaagttgaccaatgtggcgcggagcgagctgcccaaagtgacgatgctggagatgctcaccagcggctccctcccctccacaggg
aagacgcagtttgatgtgctcatgtgggaatctcgcattcctccgtatgcaagctacgtcgccacgtgtgatacagacacagctcatcatcagcaggtgg
tagcacttaagaagccacttcttaacaagaggaggagagaccaccaagaagcagatagccctagtaactag
>g60801.t1
atggactctgcgatcctcacggtaatgaacaagatcggcgaggtcctcagcctggccgcggccgagtaccggcgcgtggcggacgtggacgcccagg
tgagggaccttcgttccaagctgcagttccttatggcctggatccagatggcagaagaccggagccgcgaccagctggtgcgcgtctgggtcagccag
atacgcgaggccgtctgcgccgccgaggacatcgtcgaggagtactgtcatgagatgttcatgtcgcggcggccaggcctcgagacgacggaagcaa
gacgacgatggcagcctctctccacctggtttccttttcctgtcaaggaggtgtccgtacggtacagcttgtctcaacggatatccggcgtcctgcttcagc
tcgagggcatccggagcaacagtctcttagacatgcccaacagagtggacggcggcgtccccattttcgacaggccatctatctattcagcaaccatgtt

tgaggagatatatgtggatgagtacacaaatgccataaggacaacgcttcttgatgacaatcgcaagcaccgagctattgtcacgattacaggggaga
gcgggattggcaagagtacggtggcaaaggagttcctccaggatagggtggtattgcggcattttgacatttgtgcttgggtgtgcttgccaccagaatc
taggctggacaggttctgggatcaaatttaccagcaggcgcaagaacagatacattgtgacagcactaaacgagaagatgtgaagcagttgttggtga
gaaggaagtatcttgtggttctggatggaatggacaacatggttaaactgaaatccctacttcgggagctgccacctgatggcgccaatggtagtggga
tactgggcttctctattgcaaagaccacttga
>g62063.t1
atgtcacttggtcgccggcagaaagtggttttcacgacgagaaaccagaccatctgcgccgagatgaggtgtgctggcaacaccatccagatgcagca
gcccttgggtgaagatgatgcatggaacctcttcagatatcatgttacagatgaaatcatcgattatcatactgagatcaaggagcttgccaagaagatg
attgcaatttgtcaaggtttcccagacgccttacaaatcattggtagaaccatgtcaatcaggagcgatgtgaagctagcctatgatttgctcaagcaga
cattacacaccgaaacacaaggtttggatgacaaaggttgcccctgctgtttgcgcatttttgaagatgaaatctag
>g62974.t1
atggctgacaccatcctcacttccttgatccatgaaatagcctctcttcttgccaccggagtagctggacagtgctggagcctcgtggccacgggtcctga
tgtccgttggctacgcgacgagctccacagcatgcagctcttcttgcaagaaatggagcaatccaacggtgacagcagcatggccagacaagcatgga
tcgaccagatgagggatatcatgctcgattcggaggacgtcgctgatgtcttcgatgccagtcaggtgagtggttgtggcattctaggcaacctcggtgc
acggcgtgaagttgggactcggatcaggcgcatccggacccagctgagcgatatatcacgccgtcggttagattatgcaattccaaccccaaagcctgc
aaattccattgataataaatgggtccacaacctcttagcatcatctccgctgattcatgacaaagataccgtaggtctggatagaggcctcgacgagttg
cttcagcacgtcatggctgggggctcagagttaagcgtcatctccttagttggcatgggtggtgttgggaagaccacccttgcaaagaaggtgtacaatc
acccagatgtgaagaagcacttcaactgtagttcatgggtctatgtttcgaacacaatggagctacgagatgtcctacgccagatggccaagggtctga
ttaggatactctctgccgacttgagctctctcaatgagagccagctccaagaactgctcttgagtggtcttgacggtttgcgatttttgcttgtgctcgatga
tgtctgggaccagggggtctgggatgtgatcaagctggtattgcccatgaataacaaagggagtcgggtcctagtgacatcgcgtaacaccatcgtggc
agagtcggttgttgaagtgacgaaccatgtccatcaacttcagccactgacatttgaagattcctataatttgttttgcaaaagggcatttgtgaaaggtg
ggatatgtcctgatgatctgaaggagaccgcgacaaacattgtgaagaagtgtgctggtctgccccttgctattgttgccgctgggagcatgatgtccag
gaaagaaacgacagacatagaatggaggcatgtccttgcaagcattcaaaaggacctaagcaacggtgaaacaggagtccatcaaacactccttaag
ttgatccgtttatggattgctgaagggtttgtgcaagagaaagatggtgagacacttgaaacaactgcagagaagtacctcatggagctcattaacaga
agcatgctttgcaaccttacgaagctgcgccttagcttctcgcagctgaaagatgaccctcttccagttctaggtctattgccaaatttgcagttcctccag
ctgaacaatgcataccagggaaaactgtaa
>g64631.t1
atggccgaccttgtcctcgggttggccaaaaccgtggtgaatgggacggtgattatggccaagtcggccattgaggaggaggagaagctgaagaaaa
gcgtgcagcgtgacctgatgcttatttctgacgaatttgagatgatgcattcctttcttagtgtggccaatgacaatgtccctgatgatatggcaacgacct
ccgtgaggcaggtccgtgaaatggccttggatgttgaggactgtatcgagtccatcattcacttggaggacaagtcaacctggtggcggagcatgcttcc
atcctgcataccagcgcatatgccagcagcggccctgcatagcgccattgcagatatagagctcctcaagaccaggcttgaggccatggtccagagga
atgctcgttacaggcatataggtgactccacaacgaagcctgtggagaagatgcctcaacaggccatgtccaacagcgcatcggcctcaacaattgat
gatgaagctcgggatctgatcgagttgatcaacaatgaagatgacaaacttcaagtcatctcagtgtttggtaacgtcgggacgatgtccgtcatcaag
gaggcctataccaatcaagaaatctgccaaaaattcagatgccgtgcttgggtaaggttcatgcatccgttcaatccgcaggagttcatgaggagcttg
gtggctcaattctgtggccacgaaagcactgtaaatcccagcgaggaaatgatggcagcagcagaaggcgtacacgttagggacttcatgaagctaat
gagccatcggaattacctggtaatccttgaagacgtgtcaaccatggatgactgggaggccgcaagggtttacctgccggacaagaaaaatggcagct
gcatcattgtgaacacgcaacagatggaaatggcaaccttgtgcgtgggacatccttaccgagtggcggagctggaaaagtactcagctgatcattcag
tctttgccttttacaaggaggtatgttcgtctgtttggacatctgtattctaa
>g65225.t1
atggccgagatactggccactatggtggtcggtccactggtgtccatggtgaaggagaaggcgtccagctacctcctggagcagtacaaggtgatgga
gggcatggaggagcagcacgaggtcctcaagcgcaagctgccggccatcctggacgtcatcgccgacgcggagatgaaggcggcagaacaccgag
aaggcgcaaaagcttggcttgaggcagtccggaaggtggcgtaccaggcgaatgatgtcttcgacgagttcaagtacgaggcactccgccggaaagc
caagaaggaggggcactaccgcaagctcggcatgcatgtaataaagctcttccctactcacaaccgtattgtgttccgttacaggatgggaaacaagct
ccggtttattctacaagcaatcgaggnnnnnnnnnnncaagctcggcatgcatgtaataaagctcttccctactcacaaccgtattgtgttccgttacag
gatgggaaacaagctccggtttattctacaagcaatcgaggtcctcatcgcagagatgaatgcctttcggttcaaattccggccacaacaacaactttcc
atgaagtggaggcagactgattctaacatcattgaccccatggagattgccagtagatcacgagctgaagacaagcagaaggtggtcgatacattgctt
gcccaaggaagcaatactgatctcacacttcttgccatctttggaatgggagggttgggcaagactaccttagctcagctcgtttacaatacgggagcac
ttccagatgcaactctgggtctgtgtctctga
>g65359.t1
atggagctcgctgcggcagcgatcagcagcctcctccccaagctgggctctcttctcaccggcgagtataagctgcacaagggggtgagggggagat
caggttcctgcaagccgaaatggagatgatgcaggctgccctcaagaaggtgtccaagctgccgcctaatcagattgatgatgatgtcaagatctgggc
gagagacttgaaggagctgtgctacgacatcgaggacagtgtcgacgatttcttggtgcgnnnnnnnnnnnnnnnnnnnnnnnnnnnnn
nnnnnnnnnnnnnnnnnnnnnnnnnnnnnnnnnnnnnnnnnnnnnnnnnnnnnnnnnnnnnnnnnnnaggcgaagtgg
ttcctgcaccggacgtggtggacgaggttttgtgagatgatcggatgtggagctctggatcacggtgtacccagtccacaagccacaatgtaccagcaa
ggcgcgctcatggagctcgctgcggcagcgatcagcagcctcctccccaagctgggctctcttctcaccggcgagtataagctgcacaagggggtgag
ggggagatcaggttcctgcaagccgaaatggagatgatgcaggctgccctcaagaaggtgtccaagctgccgcctaatcagattgatgatgatgtca
agatctgggcgagagacttgaaggagctgtgctacgacatcgaggacagtgtcgacgatttcttggtgcgtatagatgcacctatttatgccaaccaa
aaggcttcaggaggttcttcgatagaaccttgggcttgttaacaaaggccaacaaccgccgtcatatcgccactgacatcaaagaaatcaagaggcgc
atcgaggaggttgctactaggcgagagaggtacaagtttcaagatactgcactgcagcctgacgagaagcccatcgatcctcgcgtgctggctagcttc

gtagatgcagcaaagctcgtcggcgttgaaggccctacagagaagattgttagcctgctaacccatgggaagggcggacaaaagcagaatctaaagg
tcgtgtcaattgttggagttggaggcctaggcaaaacgaccattgccaattctgtgtatcaaaagcttgggggagaatttcattgtcaagcttttgtctcgg
tgtcgcttaaacccaacgtaaaacagattttcagcagcatactacgccaaattacccaagacacgtgttccaatgctggagagaaggaacccgaggaa
cttataagatgcatcaggaaattcctcatggataagaggtatgttacatgcctagctagaatgactaaagtttaa
>g66196.t1
atggagctcgcggcagtggcgatgaagccactcatcgggaagctctgcaatctgctagcgtccgagctgactctcgcgaagagcctgcggaaagacgt
cgcctccctggagcgggagctgaaggtgatgtacgtgaagctcagggaggtggccgaggtgcctctagatcggctcgacgagggaaccaagctctgg
gcaagcgacgtccgcgagctctcctacgacatggcggacgcaatcgacgccttcatcttgcacgtggaggaccccaccgccaccgggctccgcgggtt
tctcaagaggatggctcgtcttttcagcaagggaaaggcgctgcacgagatcgtcggcgccgtcagagacgccaaggacctcgccaaggagctgggt
gagatccaccaaaggtacgctgggctcaagttgcaggatgtcagcgtcagcactagcgcttctgacattgaccctcgtctgatggcaacattcaccgag
gccacggaccttgtcggcgtcgacgacgcgagggacgagttgatcaaggtcctgtctaatcactcggaagtgaatgtgaagattgtctccattgttgggt
taggtgggctgggcaagacgacactcgccaacgcggtatatgacaagatcaaagtgcagttcgtttgcagcgctttcgtttcagtgtctcgaaacccgg
acatcgctaggattttcaagaagatccttcatcaacttgacaaggaagagtttaaggacatcaatgaggcagtgagggatgttgatcaactcatccgtg
agctcaaacaataccttcagagtaagaggtacgcaatctaa
>g67655.t1
atggaaactattgctaatatggtaccaggtgctgccgccataatatgtcatgctagtggattgctgtttccacacctggcatatcctttcaatgctgagaag
aatgttggcaacctgaaaggatggtgtagaagattggaagcaaggaggaatgatattgagcttcgggttgagaatgctgagaggaagcaaagaattt
gcaagaatgaagtaaaagaatggctggaaactgccaagcatgcaataaatgaagtagataaaatagaagatgcgtataaaaagagagctatgtgttt
tcatggatggtctcctaatgtcattgggcgctaccagatcagcaaaaaagccaacaagaagctgacagaactgaaggacgtgttcgaaagaggtgaat
ttgatgatgttgcttatgataaacctccagcttctgttgtaccctggcctgttggcacctcagtcattggaatgcaaccttatctaattaaggtcatgtcctat
ctgagggacgataatatcccactcatagggatatgtggaatgggtggagtcgggaagacaacgctgctaaagctcatcaacaatcaatttctcagttca
gttgaagacgactactcgatatggaacattgatctggtgaactgctggattgggctgggtctaataccgatcggaagagctatctgtgaaagccacaat
gacggatatggcgtcattggggtactgaaaagtgtgtgcttattagaggagggggatgtgaaacatactgaggtcaggctccatgacaccatacgtgac
atggcactctggattgcatcagagaagggttggcttgtggaggcgggtgttaggatgaaaaagagcaagtactcaggatttgaaaacgaattcagtgg
aagctttaaagatggcaaggaaagtaaagagttcagcctaacagagctggagtgcttccgcaatggcctggcccttggagaaataggggcaaattcta
atgacttggaagcagcatga
>g67666.t1
atgcccgcggcggctccgtggccggtctcgcaggacctccccggcctcgccggccgagcccatgccctctccctcagcgctggcgccgacctgggga
cctcgctgacgcgctcctccggatcgagcccgtcgcttgcgagctcgagctgcggccagggggccacgaggcgcccgcgctgctcgcctggctcgtcg
agctgaagggcgcagtggccgatgcggaggacctgctcgacgaggtccacatccgccggcggggccaggccggcccggtcgcgctctcgatctgcgt
cggtgccgccttccctaaccccaggcgcggaagctgcgggggctggtcgggaggctcgaccgcgtccgcgagggctcggatcagctgcggggcggc
ctcggccgcgtggcggtggagtgcggggtgcgatcgcccaaccgcgtgacgggttccttcctcgtggagcgcagggtaattgggcgccagaaggagtg
cgacgaggtagtcaacaggctcattaatgatctaggcgtggagaaatgttcgtcgtctgctccggttgttgcactagtaggccatggagggatggggaa
gaccacggttgcccagtgcgtctacaacgacgcgaggatcgaagcacactttgatctgagagcctgggtctgcgtctgggaccggtcagacgaggccg
agctcacaagggagatactgcaatccatcggcagtgcagatgaaacattgcgcgacgatggtatggccagtttggagaggttgcaggagaggcttga
agaatcggttgcgtccaagaggttcctcctggttcttgatgatgtttgggtcgatgaaggcaagactgtgcaggagaacagggctgcatggagcagggt
gttggctcctcttagatttgctgggagtgggagcaaggtcttggtgaccactagggtgaagttagtagctgaggttctgaacgccacaattcaagagcac
atacaggttggcaaagctgcgaacattgaaatgccggaaagcattagcgagttggttcatctccgctaccttgctttgtgctacataaggttggtgaggg
acatgattggtgtctaa
>g67667.t1
atggaggtcggctggctggtgtgcccgatcatcaagatcgtcatcgacaaggccaggagctgcgcctccgagaggatcaagtggctgggcagcggca
gcgtcggcgatgcgctgagacaggttgagcagatgctggtccagctcagcaccgtggcgggcgttgtcgagaagcggacctcaacgggcaccagtgc
ggacctccgcgcgtggctgcagcagctcaaggacgccgtgtacgacgccgacgacgtcctcgacgacttcgacggctcggcgccgcagcccgggtcct
acgtcgccacggtcggcaagcggatcctcggcaccgacgaacccctcaacaggctcaaagaagtcctcaagacgctggagacagtctacgcaagctc
cgacaagctcatgcaggcggcgcagctggaggcgtccgggtcgggggagctcagcggccagtacagcctggacagtcgcgccacgggctctgtgtgc
cgtcaggagcaggtggtgttcggtcgggataaggagctgcgagacatggtggcctggctcgtcggcgcatccgatggagatgcacggcccttgttgtcc
gtccccatcgccgtgatcatgggccatggcgggatggggaagacaacgctggcgcagcaactgttccaggaccagacagtggcctccaccttcgacct
caaaatctgggtacgccccaaagccacggacaacacgcttgagctcgtgacacagatcctccagtccgcggaggtcgaggtgcctgatcgcaccaac
aacttagattgcctgcagaagagactccagaaagccgtctcatcgcgcaggttcctgctggggatcgacgacgtctggaacatggaggacacgaagg
agaaggagcatctctaccgcgagatgtggaccaaggtgctggcacctctcagcgaagggaaaacaacggggagcaagattgtgatcaccagccggc
aaaggagcgttgccaagttgttggatgcaaggaaggaggtctggctggacggtttgcgggacgatgacatctggtcgctgttaaagaggagcgcttttg
cagaagaggacatcgacaagcaccctcaactgcagcatatcgggaggcagattgctgaaaagctcagagggtcgccattggcggcgatggccgtcgg
acagatgctgaaagatagccgcagcaccaccgagtggagaaaggtgcttgacatgaacatctttgacaatgtctcctcaacgctagaactgtgttacca
gaatctgccgaagcacctacagccctgcttcgcaatctgcagcatattccccaagaactggaagttcaagcgagagaggctggtgaagatttggatggc
tcttgatttcatcgtaacagctggtggggtgccattggaggaagagggaggcaagtactttgatcagcttgtggacaggtcattcttccataagcagaag
gtggggcgtcggaggtactattacatccacgacctcatgcatgatttggccgagaaggtttcttggtctgattgcataagagttgaaggcatcaagaaag
agattccaaagacagttaggcacttgtctgtgttcagtgatgccatggaaatgctccagagcccatgtgatctcaaaagaaagtgggaggtcctgcctcc
tctcgggcagcttccatgccttaagaatctttccctgccaccttcaacagcaacagctctttga
>g68297.t1

atggatctggtggtgggaggttcgggatccaccgtgaagtctctggtgagcaagctgggcagccttttggcccaggagtacgctctgatcaggggcgtg
cgcggcgacatccagtacatcaacgacgagctctgcagcatgcaggccttcctcaccaacctcggcccccgcgacaaggaccaggacgcccagatcc
aggactgggcgaagcagatccgggacatcgcgtacgacatcgaggactgcgtcgatgacttcgcccatcggtttcccgacgaccctggcggttctgcc
atcaagatgtggatatacgagatccgtacgtggtatcctcgccgcggcatcgccggaaagattggcgaactcaagaaacgggccgagcatgtgggca
cgcggcgcaccagatatggcgtccgcgacccgcggccccgacaggacaccggtacgtcggccggcgaagctgcatacctcgccgcggagaatcagg
tgtcaagctgtcagcttgtgggcgtcaaggagcccgtgggcatgactgacgctatcaggaaactcgagctgtgggtgaaagaacccaagaaggagag
gcgcgtgctgtccctcgtcggattcggcggtgttggtaagaccaccattgccatggcgctgtaccggagatttgggggtcagttcgagcaccgggcggt
agtcacggtgtctcagaaatttgatttggggacggtgctccagagcatattaagccaagtcatgccgcaggtggccctaaaagaggaaaaaggcaggc
ggccgagcaagattgaaactcttgagaatcgactcaagaaagatctgctgcagcatctgaaaggaaagagcaggaagctgctgatccgtcggttgatc
gcagaaggttttgtaagtgagaagcatgggcagagtatcgaggaacttgcagagacatatttcaatcagttgattagaaggaaaatggtacgagctac
aaaacatagcagtaatggtaaagtgaagacctgccaagttcatgacatggttcttgagtatattatatccaagtcaagtgaagagaattttatcaccgta
gttggtgggcactggctgatgccaacgccaaggaacaaggtccggaggctatccatgcacaacagtgatgccaagcatgtgaaagatacaattggca
aaattaacttgtctcatgtgcgatcagtgaccattttcgggagcttggaccagctgtcttccctttctttcaactttggaatagtgcaagtgcttgatcttcaa
ggctgcaagggcttcaagaagcatcatgtgaaagcaatatccaaaatgctacttgtgaagttccttaacctccgggggacagacattaaagagcttcctt
ccaaaatcggaaggctcaagtatttggaaactctcgacatacgggagacaaatgtcagagagctgcctgattctattgtcgagctcgagaagatttgta
acatacttgggggaaataaacacacacgggaaacactcaagctgccaaaagaaatcgggaagaaaccgatgaaatccctgcgtatactatcagggat
tgagattgtcggagaatccagtgccatgctggacctccatgaatacaccgcgctgaagaagcttgccatttacaagctgaatatccaggagaaggatcc
aggcttcaagaatttcgtctcctccattgaataccttggcggctgctcactccagactctttcaatcgaagatgaggtctcagacacgatcaactcgctag
attatctgagctcccctcccaagtacctcactgggcttgagctgcacggcatgctgaccaaagttccccagtggatccatcagcttggcgacctcagcaa
gttaactctttccgtgacagtccttcggacggacactctcgagctcctgagcaagctatcgtcgctgttttctctcaccttctccttcagcgaagcgaagcc
agctccacatctcgccgacatccttcacaggaacaaatcagactcggaaggagagattgtcgttccggatgaaggatttgagggcctgaagctgctgcg
ctttgccacgccaatcctgcccctgctgatcttctcggagaaggcgatgccgcaggtacagagggtggagatgaggttcaaggtgctggaaggtgtctt
cggcatggacaaccttgcggggctccaagagttgcacctgcgagtgagcgacaaggccggtaaattcaccaagtcggcgctggctcacctggcgaag
caggcgaggaagtacgcccaggcgcccagagtggtagttgatgaatactacgacgtgctcaactga
>g69465.t1
atggctgagggagttcttgcctcgaacatcgtcaaggaggtgctggccaagattggttcctcgatttggtcggagctcgcgcttctgaggagcttcaggg
ccgacatgagagccatggagcgcgacctcgccaccgtccaagacgtcctctttgacgccgaggcgcgcggcggcggcggggacagcgccgtccgtga
ctggctccgtaggctcagggtcgtctcccacgacatcgacgacctcctcgacgagtgccggacggacctttgtgccgcccgccgccaccggagcgagg
acacgatctgcggcatgtccagcaacccctccgtcttccgttcattcgccctggcacacaggctgaggtccctgaggcgcaagctggacgccgtcgccg
ctgggcgtgacaggctgcggttgaatcccaacgccttcccaccggcccacccgcctgttcctcccagaccggagaccatctctaaggtcgatgaatcca
agacggtcgggcggaccactgacaaggagaagctcatgaggctggtcctcgatgccgcctgcgaagaggacgtttctgtgatcccaatcgtcggtttcg
gcggcctcggcaagacgactctggcgcagctggtgttcaacgaccggcgtgccaatgacgaggtgtttgaccctcggatctgggtctccatgtccgttg
acttcagcatcaggaccctggtccaacctatcgtgagcgcgaccaaggagaactgcgacattgccaatctggaagccgtggccagcttcctgtcaagg
acgttcacggggaagaagtatctcctggttcttgatgatgtatggagtgagaaccaagaggagtgggagaagctcagaatgctcctcaaggacggcaa
gcgtggcagcaagatcattgtgacaacgcggagtaaaaaggttgccaagattgtgcgaacagtgccgcctttcatactcaagggcatctcagacgatg
attgcttcgagctgttcaggtgcaaggcttttgaagaaggggaggaggatctgcatcccaagttggtcaggataggcaaggagattgtacaaaaatgtg
gcggcgtgccactggcagcgaaggctcttgggagcatgttgaggttcaataagagtgaacaatcatggatggctgtgaaggacagtgaggtgtggcag
atggagaaggaagagacaatcttgccgtctctcaaacttagctacgaccaagttgcacctggtctgaagcaatgcttcgcttattgttctgtatttccaag
gaatcgtgagattgacagggaaaagcttattcagcaatggatttctcttggcttcattcaacctgcaaagtatggctaccaaacagtttctgaccgagcg
aatgactgtttcgagcacttattgtggatgtctttccttcaagaagtagaggagcatgatctatcaaagaagaaactagaggaagacggcaatgtgaag
tacaagattcatcacttggtgcatgaccttgcacaatctgttgcaggggatgaagttcagacaatcaattagtag
>g69826.t1
atggctaaggctaatcagggagaccaatccatcatccacgcgctgcgagatgcgctgctgcagttcgccgtcaagtccaagaagctcgcgttgccaatt
ttggagccgttcgggcgccggacccccgaaccggccaccgtggacgccgacgagctgagggcgctcaagtccaagctgcagcggattcgcgccgcgc
tccgcgacgcagagcgtctctccgttagcgaccactccgtgcggctatggctgtccgagctcggcgacctcgagcagcgcgccgaggacgtggtcgag
gagctcgagtacgagtcccgccgcgcagcccagctggaggagctcaaggccgacctcctctgcgccgccgcgacggggaagcggcggcgggaggtg
gcgctgttgttcaccgccgcgccggcgaggcggctccgccgcaaggtcgacgacatctgggcgaggtacgaggagatctcgtcggacaggaggaagc
tccgtctgcggcccggcgacggcgcgcacagccccgcggctgccgcgctccaaccgagcagcgcccttctccgttgccagctccacgggcgggaccac
gacgtggagaaggtcgccgcgctggtggtgagcgggcagcagcacgatgggggaagagctacgccgttgtgcctgtcgtggggatggccggcgtc
ggcaagacggcgctggtgcagcacgtcttccacattggggccgtcaaatcacgctttgaaatgatgctctggatttgggtctcgcaggaggtcgacgtc
atcggcgcgacacgcaaaatcgtggaggcgatcaccaggtctcgccccgagtgcggagagctcagcacgcttcacgaactcatagttgaaaacatgg
cagggaagaggtgcttgattgtcctggatgatgtgtgggatgacaatcccagccactgggactgcctgatggcgccgctgagccgctgcacgcttggga
gcacagtcgtcgtgacgacgaggagctggaaggtggccaggatggtgagccccaaggtgtatcatctgaaatgcttgtcagatgacagctgctggctc
atgtgtcggcaacgggcactgccgaacagcaacgtggacattgacccagaacttgtcagtataggtgagaaggttgcaaggaagtgcggaggcttgc
cattggcggctgaggctgttggtagtgccctgagcacctccattagccggaaacactgggatcaagtcctggaaagtgacttatgggctgataatgagg
tgaaaaatctggtactgccggcactgaaggtgagctatgatcacctgtcgatgccactaaagcgttgctttgccatttgttcattatttcccaagggcttta
ttttcgacaaagatgccctggtccagctatggatggcgctgggttttgtggatgctgtagggggcactcccgaacttattggcagttggtacttcaatgac
ttggtgtcaagatgcttctttcaaccttcccggtcccatcatgatggcgaagggaagttcatcatgcatgacttgtatcaggagcttgctcagtttgtttcag
gcagtgaatgtaggatggtacagaacaataattcgatgaaaacggacgagagccctcggcatttgtcctttgtctctgaggagttccattctggtaaaga

actgcacttcaactgctctcatgattatcgtgagctacggacattcctgtttcttgcaagaacagaacagaaccatgatgggatcccatgcaaagcaatg
attccatctggacttgtcacagattttgaatacataagagctctagatttgagcaacactaacatcatggagctgccaaagtccattggaaatctgataca
tctaaggtttgttgggccagaatttttggtgatatcagtaatatggcttcgagcagcagcagtaaggtctgtaacacgtttcctgcactggagtccctaac
gttcaggaacatggaagcttgggatgaatggttcggcgtcaggagtgaacacttccccaatcttaaatatctcggcattgctaagtgtaggaagctgaa
gctattgcccaagttcacttcagaaccggagctgaggatccggcattgtgacctactgcaaatgcacttgtgccagatctcattattttttccagtgttcag
caaataa
>g70404.t1
atggaggcgacggccgtgagcttggctcggtccgtgctggacggcgtcgtgacaagcgtccggtccgccgtcgccgacgagatcgcgctcctcctcgg
cgtaccgcaggaggtggagttcatccgcagcgagctggagatgatgcggtctttcctcatggtggcctccgcgtatcccgaggctcccgagacgttcag
aacatgggtgaagcaggtgcgcgacctcgggtacgacgtggaggactgcctcctggacttcgcgcactacgcggccagaacgtcctcgtcgtcgcgag
ctggctccttcctcccgggctccgtcgccgagcgccaccgcatcgcggagcggatccggggcctcaaggccaccgtggaggacctgaaccagcggaa
ccagcgttaccgcatcgctatcgacctccccgcggcccgcgcggagcagaaggcggctgctatgcttcccgaagatgacatgctcgcgttccaggagtc
cgacatcatcggccgggaaagcgagaaggctaagctgaccaagctcatctcaagggcagagcagcccgagccggagcccgagcccgagccagagg
agccaaaggaggaacaacaagtgctgccactgccacgtacgtcatggtggtcaatgtcaacattattaacttcttattggaggatcaattacatggcacc
tagaacagggacgaccagtcctcggccgcgtccagtcccagcgcgcccttcttcttcttcccccgccccgtcggcagcagcgacggagcgctgcg
cgtggtgtcggtgtacgggatgggcggcatggggaaaacgtcgctggtacggatggtgcacaacgacccggagctcctggacgagttcgacatgggc
acgtggatcaccgtaccgcacccgctcgacaacccggaagtgttcaggcggcggctaaggaaggagctcggcttaaaccacgaccagaacatcgagg
agcacctgcgggacaaacggttccgggtcgtcgtggatgacctgcttagccaggacgagtgggagaacgtcaggaaagttttgtgggtggagaacaa
caagggaagccgtatcatcgtcacgacgcggcgggaggacgtcgcacggcattgcgcgagggaggggaatgtgccggagggacgcgagcttatcta
cgagctcaaggcactgggggaccacgagtccatggacctcctctgccgtaaggtgcgtcttcctttatttacaaccttctcttctgtcagtgtgtga
>g71079.t1
atgttgaatctggatagggtggccgaaaccgcagcgggtggcagtatatcgaatattgtgaagaaactcgcttccttatcgctgggtcaactctccaagc
ttagggaggtttctagcaacatcgaatctctcgaggatgagctgcagatcatccatgctgttcttctggacctatccaatcaagagcatcctgataatcga
ttaaaactttggatggacaaggttagggagcttgcctacgacatcgaggatgcaatcgacatgtttgtgctggcatcagaaaatgagcatgtgcgcaga
gttgaggagtcacgggatccaaactttatagaaagaatcatgactaccattcgaaggtgcactgattcaatcaaaagcttgccatctgactaccagattg
ccaccaagatccaaggacttaaggaccgtgttgtagcagcagaagaccagcacaagaggttgaagacagtggacaatagtgatttctcaggctatgat
gccattgctatccgggatgtggctcgatattcgaatccatctagccttgtgggtatagatgctccaagtgaagacatcatccgcatgctcactgaggga
gcagctcctcagtgcatcaacacatggtcgtagcaattgttggatttggaggcctaggcaagacaacacttgccaaacatgtatttgacaagattgctgc
acattttaattgcacagcatttgtgtctgtgtcacgaaaaccagatataaagaagcttttaggagatatacttttggatttcgggtcaggtgacaggctga
aggacctgcggcaacttgatgaattacagcttgcaaaaaaactccggaaatacatcattggaaagaagtatgaacctgctgcttccctagtactgtatta
catttga
>g73371.t1
atgtgcccatggccagtatggtggccgaacgccttgtatgatgcccaccgctgggtctccgacgtctgcgacatgtacaacatcggccatggctgctgct
tctgccttagtacatctcatagatttttggatgctactgtaatcactgatgctacgttcaggtcgtacagaatcttccatgatccctgcaggtataagaagc
aactttgtggatccagctccgacattgggcttgcccgctatggcgtggaggctgatagggagggtggcttccatcttgtcggtatccgtcgcccagcgaa
gaagctgattcggtggatcacggccgctgacaggagcttgagagtgatcactatccttggccccgccggcattggcaagaccactcttgccacggagat
ctaccggcagcttcagagccaaactgaaggtcgcaaccattttgagtgtcacgttagggttcagatgtccagggtgccagacatcaagaatgtgctcga
gcagatcctagtccaggtttcacaatttcccacagaggtgaattatgaggattatgacaaaaaggagctgaagcttgagaagttcaaggtcacagcag
gaagatttgcaagtgttcccaagtggattagcaagctcgattctctatctttcttgcaaatcaccctctgcggattagggacagaggatctgaagatactt
aaagacatgtccacattgcagtgccttgtactgggcttggaattcattcctagggaagagatagtgattgaatccgggggcttcagtgagcttatcagatt
ctgcgtggattgcccagtgccatggctcagattcaaagatggagctatgcggaggcttgcataccttgaactgcaagtctgctcaggtccagcaagacg
agaaggtgcagttccttcaggtatttgcaatctccaaaacctctcggagatagtcatgcattacaaccaaaagtggttcactgatggctcccacgtcaag
aagactcttgaagccgtgaaaaacgaggttgccaaacattacaaccgtatcgacctcgtcatcaagggcaccaaaattgatgatgctcagacgttcgat
gaaacagaatggaagattgaaattcaaaatgacgctgacgtgaaaccgatgaccatgttcaagcaggtaattggaaatcagtatgggattgaagctga
agtggaggagatcaatgcacttagggatgatttcaagctcaagcctgtggaggtcattagaagatttgtgaggctattcgaggatcccaagtatgagtc
catcatcacctacaccttgcaaggtgacctcaagaccaagaccatcaagggcttnctattcgaggaccccaagtatgagtccatcatcacctacaccttg
caaggtgacctcaagaccaagaccatcaaggatgtgcttggaaagatcactgctttcgagagctaccagttgggtttggatgatccacaaccattcaac
cttgctcttgaagccgaaaagctagagctgagcaagagcaagaagaaggtgatcgaggatgattga
>g73377.t1
atggaccttgtcgtcggcgcttccaacagcgccgtgaaatccctggtgaataagcttgggagcctgcttgcacaggagtacgccttgatcagcggcgtcc
gcgatgacatccagtacatcaacgacgagctggccagcatgcaggccttcctcaacaggatcaagcgagccgacgaccaggacgagcagcgtcaag
actggatgaagcaggtccgggaggtctcgtacgacattgaagattgcgtggacaacgtcagccaccgtctcgacggtgagccgcgcgggaccagtgc
gtggacgcacatttccaagaagtggtacgtgcttaagacgctgtacgcgcgccaccgcatcgccaccgagatcagcaacctcaagatccgggcgcaac
acgtcagcgagcggcgctccaggtacggggtagtgaatctgaaaaatgtcaaaggccgcacggaagcgaacgctaccagagaccatctggcacctcc
tccacagctcatcggcatcagggcgccggtgggcatcgaggacgccatgggagagctcgagccatggttcaccaaggcgaagacgctgcctactgat
cataatctgagatttcttgccatcgttgggtttggtggcctcggcaagaccacgctcgccatggcactgtaccagaagttcggtcttggtgacgagttcag
ttgcagagcctccgtcctcgcatcgcagaagttccacctccagactgttctgaggaaacttttggagcagttccatgagcagcgggcggatgcctccaag
aatgacctccaaggaattgaagactggaaagacgagcagctcatacgcaaactccgggtacagctatctgggaggaggtacactattgtggttgatga
catatggtctgtatcagcatgggagaagatcagggattctttgcctaacggtggtagcatagttgtgactacaaggtttaactcagtagctgaagcctgc

cggcggcggcaaggggaggttcacaagctcaaaccactcactgatgaaagctcctaccgcttgtttcatgaaataatcgtcaatgctgattattctgacc
aacgtcctttgctaactgctcaaggctgccaaatcagtaggaagcgtctgatcagaagatggatagctgaagggttcctctttgaaaaacatgggaaga
ctgtcaaggaagtcgctgaagattgtttcagtgaactgattggcagaaacctgattcgagcagtcaacaacagcagcaacggcaaggtgaagaactgc
cagattcatgacatggttctggagtacattgtatccaagtcaagtgacgagaatttcatcactgtggttggtggtcactggcagacgccgtttccaagcta
caaggttcgacgtctgtcggtccaaaagaatgatgggaaagannnnnnnnnnnnnnnnnnnnnnnnnnnnnnnnnnnnnnnnnnnnnnn
nnnnnnnnnnnnnnnnnnnnnnnnnnnnnnnnnnnnnnnnnnnnnnnnngctggcaacaagaacaagcggcatgcgctgatattaactga
agggatcacaaagatgactgccctgcagacactgtccggggtagagatctgtactggtttgggaaaggaacctttaagaggcttgcagaacctcacca
accttaaaaaatttaccatctataaggttggaaaactcactaacgaagagttgctgctgtctgccatagagcatctgagtagttgctcgctcaagtatctt
ggaattgacgatgacttcactggcttccttgacaggttactaaatgcttctcaagcaccacctgagcaccttcataccctcggtctgtgtggcatgttgtcc
caattgccaaagtggattaatcaccatctgtacaacctcgagaagctgactctgtcattaacctcactcacaagggacgccatgctgaacctcgcaatgc
ttcctgagctgttctctctcaccttctttcttgatactacgaaagcgaatcaaggagtcctagagatgctaagtaataatacgcttaaatcaggagggaag
atttttgtgctagctgatggattcagaaagcttaaactgttgcgcttcatgacaccggtgctgccacttctaagcttcatggatggggcaatgccagagctt
gagaggcttgagctgaagatcaaatttattgaaggcatttacggcctggaaaaccttgaaaacctccaacaggtgctcttgactgtcagcagcaaggct
ccggatgatgctagagaaaaggcttctcagatcaagagattaacaaggctgatgaccaatgcgcccattgtggtgtacaatgaatcttcagaagagta
a
>g73378.t1
atggccttttcgaagctggtatggagtgttctgttgcagagctactgtggatgctccttgagctgctctctggcgtcagcagtggcgactggcaccacctc
cagtcgcagctcaggaccatacaagacaggatcgacgtatgctacgaatccctcggcaactccgatgatcaggtttgggcctgggtcacccaagctcg
agagctcgcttacgacatggatgattgtatctgcattcgcagctactacaacaacggcagtgccaacccctcgttgtccggttttctcgagtggattcgtg
gcctcaaagatatctcccacaaattacgtgacctagaggctcgttttaatgcactgaaacaggaggtgcggtctcactctatcttcgatggccaagcggg
ggcagtgcctgagcccatcgacgaccctgagccggtccacctcgtcggtcaccacgcggcaatgggcgagttggtcaggatggtcatggatagcggtg
acaaggctgaacgcaagatcatttccattgccgggatggccggctctgggaagactacgctcgccgcaacagtgtacctgcaggtcagagaacagaa
ccgcttccagtgccgagtttttgtctccgtcggacagaagccagacctccgtgggactctcaagaaaatgctctcgcagcttgggggacgggctcggtg
ggagcgaggagattga
>g73379.t1
atggatggtgtgaagatgatactcaacatgagctacaacgatctgccactacatctcaagtcatgtctactgtatctgagcatatttcctgagaattacat
ggttactgttaagcggctgatgaggctatggactactgaacgaataataattgacactggctgttatgtgactgagaatgaattagcaaaaacatacctc
ggcgagctcaacagaagatatatggtacagcttctgtatcccaacaataatcttcttgcccccccaaggtactgcaggctccaccccgtgatacatgattt
cactgtttgcaaatccaaggaagagaactttgtcactttagtggatgctcagcaccaacatgccccaatccaccacaaaactatccaccggctatcaatc
cagagcagcgacaagcaggatgatgcactggcgccaaataatgtcactatgaacttgtccagtgcaaggtcagtcaccgtcttcggtcaggcaagcgc
cactcctagcctgacttgtctgccatcatttgtgcgggtgctggaccttgaaggctgcaacggtctagtatgccttgatggcctactcaatctgcggctgct
gaggtacctgagccttagaggcaccgacgtcagtgagctcccggcaacaatcgggatgcggctaaggtacctggagatactcgacgtgggatccaca
aaagtgaaagccttgcctagaagcgttggggcgctgcggttactgagatacctcagccttagaggcgctgatgtcaaagagctcccggattcaaagga
actgattcaaaggaaacaattacgtggataccagagaccatccggcgttgccggagtctaa
>g75289.t1
atggttttcgtgacgggggccctgggcagcgtcatccccaagctggccctgctgctgaaggaggagtacaagctacagacgggcctgaggaagaaga
tcgagtctctctcacgagatctggagagcatgcacgcggccctccgcaaggtagccgatgtgccgccggagcagctcgacaatgaagtcaagctctgg
gcacgcgacgtccgtgagctctcctacgacatggaggacatcctcgacaccttccttgtgcgtgtccatggctgcgaggcccgacgagaccccgacagg
ttcaggcgtgccgccaagaagatcggtaagctgttcagcaagagcaaggctcgccatcagattgctggcatgatcaaaaccatcaatcaacaagcaga
ggtcgtggcagagcgccatcgcaggtacagggccgaggatattatgtctaagcctgcggcaccacggactgttgaccctcgccttggggctatgtacaa
agaaatgacacagctcgttggcattgaaaagccaagtgatgagctcatatccatgctatcgcctctagggagtgatgagatgaagatagtgtccgttgt
cggacctggaggattgggcaagaccactcttgcaaaagcagtgtatgacaagcttaaagggcattttcagtgtgcggcttttgttccagttggccgggat
ccgaatttgaagaaagtctttatagacatccttatagagcttaacaattaa
>g75290.t1
atgagtaatatggatctcatgaaaggggcggtgggcagcctcataggcaagctcgggaagcttctcgaggaggagtataagctgcagaatgataata
gcgtgtcgaagaaaattggatctctcataaaagagctcgactgtgctcacgccaccctccgcatggtggcagaggggccgccggaccagctcgacgag
caggtcaggatctgggctcgcgagatcagggaggcatcatacgacatggaagacgtcctagacgctttcatggtcagtgcccagtatgaccatgagta
caaggaccacgaaggattgtttgaacgcctcaagaagatgatgattggtgtctatgacatggtggtgagtggccctttcaaacgtcgtaagattgatgtc
acaatcgatgacatcaacaagagactggaagagttgacaaagcggcatcgaaggtacacaatcaatagtatcgtgtctaaacctttaccaaagtcgac
tgttgacccccgtctggcggctatgtacacggaagtgacacaacttgttggcattgaccagtcgagcactgagcttatacgcattctatggcctctaggg
gacaacaacgaaaccggaaagaagatgaagacggtgtctgttgtcggagttggaggactgggcaagaccactcttgttaaagtagtgtatgacaagct
taaagtggattttcaatgtcgagctttcgtacctgttggccggaatcctgacttgaagaaagtcttaagagacattctcatagcgctgaacaagaagaag
tacatgaattcagatatcacaatattggacgaaaggcagctcattaatgaggtccgagaatacctcgaaagcaagagtcccttggatctccctaacctg
acctacttggacctgcacctttgccctatagaaagccaacacctacaaatcctaggtgaatttccagagctccgtgatctcaagcttaatacaaaagcaa
ctgctagaaatgtcacaaaggtgacggtagctagtgctgccactgattgctccttcaagaagttgagatactgcatgctgaattattcaatggtccagttt
gtgcttaacaaagactcgactgtttcatttactatgtggaatggagcggctgacgtgacctttggttctaacacaagggacgacagagttgcaccagctg
ttatgcccaaccttgtagagatctttttttacctccgagtgcagcatatgattgataggaactgtagttgtgacaatctcggcctggagtacctcccttccct
tcagaaagtcaaagtgcttttcatgaccgagaacaccgatcctgatggcaggaagaaagtgaaggatgcacttaagaacgcaatggaagtccatccca
acaaacccaccgtggatttcagctacattgcaactcgtttccatcactag

>g75291.t1
atggatcttgtggcagggccgctaggcaacgtcatcatcaaactcggcaagcttctcaaggaggagtacgacctgcagacaggagtgaggaagaaga
tcatgcgcctctcacgtgatctggagagcatgcacgcggccctccgcaaggtcgccgatgtgccgccggagcagctcgacgatgaagtcaagctttgg
gcgtgcgatatccgggagctctcctacgacatagaggacatcctcgacaccttcttcctggtgcgtgtcgaggaccgcgaccccaacaatttgaagcgt
gccgccaagaagatcggtaagctgttcagcaagagcaaggctcaccatcagattggtggcatgatcaaaaccatcgatcaacaagcagaggtggtgg
ccgagaggcatcgtaggtacagagccgaggatattgtgtccaagccggccccaccgcggactattgatcctcgccttaaggctatgtacacagatgtga
cacagcttgttggcattgaaaagccaagcgatgagctcatatccatgctgtggcctcttgggggtgatggggtgaagatggtttccattgttggagctgg
agggttagggaaaacaactcttgcaaaaacagtatatgacaaggttaaggtgaattttgactgtacagcttttgttccagttgggcgggatcctgacctc
aagaaagtcttcagagatattctcataggacttgacaaggacaaatacatggatcctagattcacaatactggatgaaagacaactcatagatgagatc
cgagaatacctcgagaataagaggtacttcgtcgtcattgacgacatatggaagactgatacttggaaaataattgaatatgctttggttcagaataaca
atggaagtagactgattacaacgactcgtcactatgacgttgctgatcaagctggtgcggtttacaagcttgaaccacttccagttgatcaatcagaaaa
gttattttacaaaaggatattcggtgctgatggaaaacgtttcaattatcagctgaaggaggatgagaactttgttagtatcgtctcaggagataatgaag
acatatcgttgccaaaaagagtacgcagattggcccaccaaaacagaaaggtggtgctagatcatgaggatgctcaagcagatctgcggcacgatag
gcgtatgctagtgaccgatgggttaattgagaagttgacctcgctagaggaactgcggggtatcacgctggggcaatttgtgaaggacctgggcaacct
gcatgaacttagggtgctcgagatttatttggggtcgtctttcgaggagttagacgggagtatgagtatagaatcagaatcagaattggtggtgtctctag
gcaatctgcacaagatccagcatgatgagaactttgttagtatcgtctcaggagataatgaagacatatcgttgccaaaaagagtacgcagattggccc
accaaaacagaaaggtggtgctagatcatgaggatgctcaagcagatctgcggcacgataggcgtatgctagtgaccgatgggttaattgagaagttg
acctcgctagaggaactgcggggtatcacgctggggcaatttgtgaaggacctgggcaacctgcatgaacttagggtgctcgagatttatttggggtcgt
ctttcgaggagttagacgggagtatgagtatagaatcagaatcagaattggtggtgtctctaggcaatctgcacaagatccagcatcgtatggatgagc
agagtctgagaaacctcggcaagttgccggagcttcgtgacctcaagctaagtgtgtggtccacgataacaataattattcgtgcctctgaggatggttg
ttgtttccagaagctggtatccttgacgattcctggagctgcaatcctgtttctgctcaatgaggactcgactatttcatttacgatctggtatttcacggac
gacgtggcatttgattctgaaaagagggacgagtgcatagtacgtgcaccgactgtcatgccgaacctccaactgctcgacttccaggtcggagtccag
accttcattcgtaacatgggtagttctgaccgtctcggcctggagtatctgccttctcttcggaaagtcaaagtagaactctggtgtagcaatgccttcgtt
gatgaggtggagaaagaggaggctgcactgaggcacacaattgaagcccatcccaatcggcccacgctagaaatgcatagagagggtcgaattaaa
acaaggagtcgaccagccactcatgatttagagatagaagaagacgaggaggacgtgggatcatcgatggataatgaggacgtggctaccaggtca
agaggaggtggtcactga
>g76723.t1
atggccgggtcgtcgctgcttctcggcttggttggcagggtggccggcaaggccgccggcgccctcgtcacggatgtcgctcggatgtggcgcgtcgaa
tcccagcgcgccatgctggagcgccacctcctggccgtgcagaagcgattggtcgacgccgaggactcgagtgaggccgccgtgaagaggtggacca
aggacctcaaggccgtcgcgtacgaggccgagcacgtcctcgacatcttccagtacgaggcgctgcgccgccaggcccggaggaccgccggctgctc
cagccacgtccgtgacaagtctcctctgttccggtacaaagcgagcaagaagctcaagaaggtgctgcacaagatcaatgtccttgtcgaggagatga
atacgtttggtctcgtggaacgcaaggagccgccgcaagcagtgtttcggcagacgcactcggcgctcgacgactcggcggggatcgtcgggaggga
gggcgacaaggaggcggtgatcaagatgttgctagaccaacgagataagcacaagtacaacgagcagagaccaaacgtgcaggtgttgcccatcac
tgggatgggaggtctggggaagacgacgtttgcaaaggtggtgtataacgaccgcagggttcagaagcatttcgagttgaagatgtggtgcagcgtgc
cgcaagcaaaattcgacacatgttctgttctgaaatccatccttgaactggttaggaaagggcgatgtgacttgcctgacaccatcgagctgttgaaaga
gcgtctacatgaagacattggccagaagaggtatctgttgattcttgacgatgtatggaatgaagaggagaagaagtgggaggacgacctcaagacac
tgttgtgttcttctgttggtggaccaggaagcagaatagttgtcaccagccggagccaacaagtcgcctctataatgggcacccttccaccatatgagcta
tcatgtcttagtgaggatgattcgtggaaactattctcgcagaaagcattcagcaatggggtgcaagagaaagcagagtttgtcacagccggtaaaagt
atcgtcaacaagtgcaagggattgcctcttgctctcaagacaataggtggcttgatgagctcaaaacagcagaaagcattcagcaatggggtgcaaga
gaaagcagagtttgtcacagccggtaaaagtatcgtcaacaagtgcaagggattgcctcttgctctcaagacaataggtggcttgatgagctcaaaaca
gcagcaatgttttgccttctgtgcaatcttccccaaggaccatgagatggacaaggacatgttaatccagctatggatagcaaatagttttgttcaagaag
agccaacaacagatctggaacagaaaggttgggttgcaagcaaatatgatgatgccttcagaagaccagcctag
>g76793.t1
atggccggagtaatggttagcgtcgccacaggcgtgatgacctccgtcgtcgagaagctgacgatccttctcggcggcgagttcaatcttgctagcgac
gtcaggcgcgggatccgcttcatgaaagatgagctctgcagcatgaacgctgcgctgcagaggctggcggacgtggacgacgaccagatcgacgtgc
agacaatccagtggaggagcaaggtgcgtgagctttcctatgacattgaagactgcgtcgaccgtttcatgcttcttcacggctgcgacaaggccaaga
aaaactttgtgaagagcaccctgcgtaagatctttccattgtgggggggaccgccaaatcgccaaagaaatccaggacctcaaggcccgagttatcgag
gagaaggaaaggcgtgatcggtataacgtcgatcaatacatcaacgacatgcatcagcgggtgtgcctcgacccgcgagcaccaacgctgtatgagg
aggcgagagatcttgtaggcatccatggtcctcgtgaggagatcatagggtggctcaaggatgaagagaggcagctgaaggtggtgtccattttcgga
attggaggacaagggaagactactcttgccatggaggcctaccgcaaaactcaagaacccttcgattgccgtgcttcagtaactgtctctcgagctcttg
atatcaacaagcttctcagggatatactctttcaaatcaacaggggcgagtatgaccgangcctaccgcaaaactcaagaacccttcgattgccgtgctt
cagtaactgtctctcgagctcttga
>g77246.t1
atggtggacatggctcttgctggcttacggtgggcggcatcaccaattgttaaaaagctcatcgctgatgcttcaacctacctggatgtggacatgacgc
atgagctccaagaactggaggtgaccgtattgccacagttagacctggtgattgaagctgctgagaagagtccccataaggacaagctgagggcatgg
ctccagcggctcaaagaggccttctacgacactgaggacctattggacgagcatgagtacaaccagctcaagcgcaaggcgaagagcgggaagagt
cccttgctggaagaggatgccacttctgttaaatcaactattctgaaacctttttcgtgcggtgaagagcaaggcatccaacttgcttccggagaacagaa
ggctgattcgcaagatgaatgaactgaaggccatccttgctcaagccaagggttttcgtgaacttcttggcctatcaatgggcagttctgcggggtgccc
cgctgtaccaacaaccgctgctcctgtggcgagaacaacatcactccctacttcaaaagtgttcggtcgcgacaaagatcgtgactgtatagtagatatc

cttctcgacaaggcaacagctgttgaggaaagttcaaccgattgctctaatttggccattgttggagcaggaggcatggggaagtctaccttggcgcagt
atgtctacaatgacaagaggatagaacaacattttgatgtcaggatgtgggtctgcatctcgcgtaaacttgatgtccatcatcacacgcgggagattat
cgagtccgcagctaaaggcgaatgcccacgtgtcgataatcttgacactctacagtgtactgcagaatttgaagaagttacgcactttgtgtttatgctttt
acaatag
>g78869.t1
atgggggaagagcaaaaagctccgagttttcttgatgtgcccaaggatatccccattgccaccaagtccctcaccatcaggaccagcgccgccggatgc
gccagcggctccgaccggtccaaccccatctcgccggcactctccctcacgccgcacctctactcgccgtcgccgccgtcgtccgcgttcgtgtcggcgc
ttcagtccccgtacatctcccctcgagtcctcgacccgccgctcccgccggcggcgcagccgcgccaagacgccaaggccgccggcgtcagcgcaacc
accaccgcggcgccgtcgccgacgtcatgctccaacggtggagactcggaggatttggacgcgcccagcgcgtcccgcacgccgccgtcggagcgct
acgactccagcggcatcgacgcgcccaagatttccgatggcggcggcgggggcggcggcgtgggtccccttccgccgcgcgtgtcgttttcgttccccg
tgccgcgggtgtccttcacaaggggctccgtggcgtcgccgtcctccaacaccaagctccggagctgcgacgtgtacattggctaccatggcaacgtcg
gcctcggcaggttctgcaagtggctcaagtcagagctcgagctgcagggcattgcatctttcgtcgccgaccgtgcaaagtactcagacacacagagcc
atgagattgcagaccggattatttgctcggtcgcattcggtgttgtggtggtcacgatgtcaagtttcctcaatccatttagccttgaggagattagattttt
tgctcagaagaagaacctagtgcctatattgtttgacactgagccctcagatattgctgggctttttgatggtaaactggaggataaggaggggaagga
agcatttgaagggctaatgcggtgccatgaattcaagctcgaggccaatgagagcaattccagaaactgcgtgtcaaggacggtcacattgctgcggtc
gaagctgggccggaagaatattgctgagaaggagaatgaagcatctgagggcctgccttttccacgcaaccggcattttgtggggagggagaaggag
cttaccgaaattgaaggactgttcttcgggtccactgtggatatccaagaagtggactgcccaaggggttctatcgcaaatgacagatctagtggtgtat
ctgatggttttgctgacgaggagagtgattctgcgaggacatctaatgccagatacatcagtttggagatgcgcaagtgcaaggagccgacattggagg
cttggattgatccggtgatcgagttgtcttcgggtaaagggagaggcctccagaagcaaagatcaaagcaccggaggtcaaggtttcggtgcaacagc
aaaagctatggcagtgccaacgttatctgtatcaatggctcttcaggtatcggcaagactgaactggcattggagtttgcttacaggtactctcagcggta
caaaatggtactgtggattggaggcgaagctcggtatttgaggcagaacatcctcaatttatctatgaatctgggattggacatcagtgctgaggctgag
aaggagaggggtagaattaggagctttgaagagcaggagttcgatgcgttccagcgggtgaagcgggagcttttccgtgatgtgccctatttgcttgta
attgataaccttgagagtgagagggattggtgggaaggcaaggatctccatgacttcattccaaggaacactggagcaactcatgttattgtgaccaca
cgtttgccacgtgtgatgaatcttgaaccaatgcaattaccacaactctcgtacattgacgcgatggccttgatacaggggaagaggaaggactatcctc
ctgaggaagcagaagttcttaggaagtttgatgagcggctaggccgactgagctttggcctatgggttgttggatctctgctgtctgagctcatgattgcg
ccttctactctgtttgaggctgttgagaggatatcattgagcgacaatttgttcccaattggtgccaatgatgatggcttctgccgcaacaattctttcttgat
aaaggtcttagtcttctgttttgcattgatggaccgggcaaaaggaggaagccttacctcaagaatggtcatcgctggctcttggttagctcctgctcctgt
ctcatctacactattggctgccacagcaagcaagctaccaatgaaaggcagtggtatgcacctgtttggtgaatccctcaagactgcatttctatgtggca
cacattgctttctagctcctaatggacggaaggcagaggtagagtcggcactcttacttgttaatcttgggctggcaaggaaggctaatcgacatccagg
ttgctggatccaattccatccgatcacacagctctttgggaagatcaggggaggtttagcactgacaactgcggcggtgaatggagtaatgagggctgg
caacccatcagtatactccgaccacctttgggctagtgcattccttgtgtttggtttcaagtctgagccacctgctgttcaacttaaggcggttgacatggt
actcttcataaagaagacagcactgcccctggcaatcgactctttcatgacattctcaaggtgtggctcagctttagagctgcttaaggtgtgcaccaacg
ttcttgaggaagtggaaaaatcttatgcgtctcgtatgcaagactggaaccgtggatcattatgctggaggaagaagctgcaaccaaaccaccgtgtcg
atgaatttgtctggcaggaggtgactcttctcaaagcgacattgctggagacaagggcaaaactgctgctccgaggtggactgtttgacactggtgagg
agttgtgcaggacctgcattagcatccggacagtcatgcttgggcatggccatgcccaaacactagctgctcaggaaacgttggcaaagctggtcagat
acaggagtaaggtatga
>g79116.t1
atggcaggcattgcggtgagcatttccatgggtgtgatgaagcccgtcctggccaagctaggtgccctcatgggtgacgagtacaagcagctcaaggg
gctgcggaaagaggtctccttccttgagcgtgagctcagtgcaatgaacgctctccttgagaagatggatgctgctgatgaactcgacccgcagtccaa
gaattggaggaaagatataattgagatgtcctatgacattgaagactacattgatcactttgtagaccatattggtgaatctgaagacaaggtgggaatt
ctccagaaagcttctcactatctgaaaacattcaaggatcgtcgtcgcttagcaaatcagttccaggagatcaagacactagtgattgaagcaagcgaa
cgcagaaagagatacttgcttgatgagtgtatctcacataccaatccaatagtcgtcgatccacgtttatcagcactttatacggagacagcaagcctag
tgggagttagtggtcaaaaggaagagcttgtcaaatggataatggatgaagagcaacaaataaaggtgatgtcaatagtagggcttggaggtttaggc
aagaccacacttgcgagtgaggtgcatcgtgaagttggagcaaagttcaattacaaggcatttgtatccatttctcaaaaaccagacatgatgaggcta
ctgaacagcatattatcacaacttgagcaacagggcaagaggatgtcttcacatgtttgtgagatggacctaatcaacaacataagagaacatctgcaa
gataagagagttccgagatggataggggacctgcataacctctgtgagctgagcctttgggtagagcatataggggaggaagatgtcggtgtgattgg
gaagctaccctccctcgtccaactgtacctacggatcccaggcatgacatctggaagtatcacagtaggtggctctaccgggttcaggtttctcaagaatt
ttgacttcgattgtgatgggatgtcctgcctgaaatttgaggcaatggcgatgcccagcctcgtcaagctccgactgattcttgatggaaagcgatggga
caagggtacaccaattgggctccaacacctctccagcctcaaggaaatccatctacggatgacagcgggctcgaaattggcctgggatgctttccagaa
agctgctgatgctcacccgggtggcccggcatttacctttgttcacgtaatttatcttaggtga
>g79172.t1
atggcggcagtgctgggcgcgttcgcgtccaagctggcgttcatcatgatgggcatggccaaggagaaggtggagatgctcctcggcgtgccgggcga
gatcaccaagctccagacaactatgggcgacctgagccgcatcctggctgacgcncgctctctatatacaaattcggagtcctctctaccacttttgctct
tgccaccccatcccctgaccatggcggcagtgctgggcgcgttcgcgtccaagctggcgttcatcatgatgggcatggccaaggagaaggtggagatg
ctcctcggcgtgccgggcgagatcaccaagctccagacaactatgggcgacctgagccgcatcctggctgacgccgacaggacgcgcattcagggctc
ggctacagaggggtgggtgagggagctcaaggatgccatgtacgatgccgatgacatccttgacctctgccagatcatggagtgtggagaagatccaa
caacaactacagcagccactccgaagaccatttcaaggcgctggaacatccagaagttcttttgctttcgacaccctgttgttgcacacgaaatagggaa
gaagatccaggcgctcaacaagcgcctgctggacatctcggaaaggagctctcgctttggattcctgatccaacagattaactcttcctccggccactcc
accaacacggctgccacttcgtctttgctccactgcaatagaatgaccgggccgagcatcatcaaatctgctattgtcggtgaaaagattgagcaagata

caaagaaaattgttgatctactacttgtcaaggtggacactcgtgtggaagagaatacggttgttgcagctgctatcacaggcgctggcgggataggca
agaccacccttgccaggatggtgtacaataacacctccgtggctgaacatttcgacaagatgatctggttgagtgttactaaggaagtcagtgagatcg
gtatgatgcagagagttatagctgcttttggggccagctacgatggccttgcaggagacattgctttgatggagaatgttttaaagatgctggttaggca
gaagaagtttttgctggtgatggatgatgtgtggaccgagaaggtgtggaacgatctacttagagtaccacttaatggcggtgcttctggaagtcgagtt
ttggtgaccacaagagacgaaaaggttgcttgccgaatgaatgcgcaccatctccaccgaattgacaagctggaagcagaagatggttggattctactc
aagaagcaggtatactctctgtctcatgagacattttctgtcaaatcagaaacaattatggaaatcagataa
>g79354.t1
atggctatggagctcgcatcggcggcgatgaccccgctcatcgagaagctcggtgagctgctggtgtcggagcttacgattgagaagcgtgtgcggca
ggacgtcgcctccctggagcgggagctgaaggcgatgcacgtggcgctccaggggattgaggtgcccccggaacagctcgatgaggggactaagctc
tgggctgccgacctacgggagctctcctacgacatggaggacgccatcgacgccttcatcctgcaattgggagaggatgatgatgaccctgcagcagc
ggggctgaggagtcgtttccgctggtttctggacaagacggttcggctgttcagcaagggcaaggcgctgcaccagattgccgccgctgttagtgaggc
caagagactcgctaaggagctcggtgagatccatcagaggtacgctggactcaagcggaagcacgtcggtgatgcttcttctgctgacattgaccctcg
cctaatggcaatgtacactgacaccacggatattgtcggtgtcgatggcacgagggacgagctgatccaagtcctgtctgatcgctcggaaatggatgt
caagaccgtctccattgttgggcttggtggactgggcaagacgacgctcgcaaaagcggtatatgacaagatcaaagtggaatttgattgcggggcttt
cgtttcggtgtctcggacccccaacatcaccaagattttgcagttgatcctgtttgaacttgacaagcaaggctatacaaacatcaacgagatcttcaggg
atgatacaaaacaactcattgataagctcagagactttcttcagaataagaggttgccaaaaggatttgggaatctgacttccttggaagagctgactgg
gttgaaaattgacaaccgtgataatgttgcagctgtcatgagcgagctcgggcacctcaccgggctcagggttctcgagttgtcgtggggcacacatgat
gttagtaaagctttgatacagtcgcttgggaatttgtgcaagatgcagagtcttgatgtgtacgcctatgggatcgataaggaactcatctgtggctgggc
gccaccaccgcgcctccgtaggttcttgagcactggtctggccagcacgttgccagcgtggacgaagaacacctcgtcgtcgctcacgttcttggacgtc
ttcttactcaaggtgaggcccgaagatattcgagccctcggagcgctgccgtatctgctcaccatccgcctggagtcgcaatcccaaaactgcttcgaca
gcccgcaggatagaatggaggtgaacgcgttcccgtccatgaggtcgtgcaagttcgtgcgcttcaggacgatgccgtccatgttcccccgggcgcca
tgccgcaactgcagcagcttgagttcaccgtctggccatcggatttcgccgacggaggaggagtgtatgagttggaggaccttcgcatgggacacctcc
ctttgctggaggaggtcggagttgagctgttgtacaggatcatgaaggaggaggacgccaaggtggtggaggcggcgctgcggcaagcggcagaag
accacccaaaccgcttcgccctgcgcgtcgacaagtgcgacagggacgtcctcattctttcagctagggacggagcttga
>g79372.t1
atggatctgctcaagaagctctcctccgcgctcggcttcacggagaagatcggcggcgcggtcgtcgatatgctcttctccacgggcgcccgcctgtgga
acgtggaggaggaggccgagaaactgcggcgaatggagaagcgcattcgcgcggtgctcaccgacgccgagcagcgccgcttcgtcgacgacgact
tcgtcaagctctggctgcaggagctccgggcggccgcgttcgacgtcgacgccctgctagaccgtctcggcaccatggctgctgtctccagggtggccg
ccgcggagccatcgcggaagcggaagcggctctggcccaacgtggatctcggtctgcgccagcgctgggagctggacgcctggatcgtccagatcaa
cgagcgtctcgacgagatcggcaagagcaggaaaaggttcaggttgcaggccggggacgggaggaggacggcgactcagccgatgcaacgcccaa
ggtttcttgaagctgctgcgcatcgcaacgagaggcccattggtcggaccaaggagaaggcggcgatcatccgtgcgctgatttctaacagtaacatgg
atcttcctgtgatttccatctggggaacggcaggaattgggaagacagcgctggcacggatggtctacagcaattcagaggtagagaactttttcactga
caggatttgggtttggttaccggatgtgtgtgatgtgaaggcagccactaagatgatcattgaagctgtgactggaaagaagtgcaagcatctaagcttg
gacatattgcaggaacagctgcgtgatcatctgcgcaaaaagcagttcatgctggtgattgataacgtttgggccaagggctttcaattttgggaatttct
gaagccttcattgacggttggagaggaaggaagcaaggttcttatcactactcaagatgaaagagtgtccaacatgatgtccaatattctgaacttgcat
ttagagggtctggaagtaaatgaatgctgggaaatccttaaagtttatgcttgctctggctggaacagcaattatcagcatgatttgcagtcgattgggca
gaggattgccacaaactgccgaggttccccattagcagcaaagtcacttgggatgcttttgtctgatgctaacggacagaaagagcagtgggaaatcat
actgagtgacatgcagttccttgaagatgatagaaatacagacattatcttggctagtttgcagataagctatcaacacctgtcgtatcaactgaaacag
tgctttgccttttgttctatgtatccttttggttttgaatttgagaaggaggagctgctcagactttggatggccgatggtcttgttaagagcaatggaagga
gaagggttgagatggaagcaaacagattctttgatgaactcctatggaggtcatttttgaaacttcccagacctttccaaatcagaagttcagagtgcc
aaatctgatgcttgatgttgcacggcgtgtttctagatatgaatctctgactctggaccctgagagttcacaggttactgagcatcctgagtgggttcgtta
tgcaactatcttgtgccaaaatgatgaacctcttgcactcgagaagatttatcggtatctcagccttcggaacactttgataaaggcacttccagaaacag
tctgcaacctattcaatttgcagacacttgacctcaatgactgttactggctcatggatttgcctgaaggcttgaaccgcttagttaacttgcgacacctctg
tttacatctcgactgggatagagttactcctttcagatcaatgccaagcggcattgacaagttgcaatcacttcagactctttccaggtttgttgtaacctct
agagatggaggcaagtgcaacattaatgagttgaagaacttgaagatccggggagagctttgtattcttaatttggaagctgccagtagtgatggtgcc
acggaggccaatatgcttgggaaggaatatttgcagaaactgatgctgaaatggagtgaagtctgcaaggacgagcagcagcaacacatggaggata
gtgagaggaaaattgaggcactctgtccacataccaaccttaagcatctacgcatcgagaactaccctggaaggaaacttccttcttgggtggataaga
tcaaatttctggtttccctggagataatttcttgccctaggctcactgaattttctgtggagactctgccatttctcaggaatttgaagatttgtcagtgtgct
gatcttgaggttcttccaaaaggtttatgtaacctagaactcctccattgcctggaaatagatggcgccccgcatctgagaataagcgcagaggacacat
tgccaaggaacatcggccaattggctgtaagtggatgcagcgccctggagaactggtgcattaatgaaggcgctgagagagtgcagcaaattcctgaa
aaaaggattgaatttggcaatagaagtggaagagacatatga
>g79903.t1
atggctagccaagaaaagcgcgtgttcgtcgccaacctcaaggcctccctggaatccttcctcttcgccgccaggcgccttgccaagtccgccctcgcgc
ccctcggcccgtcctccgagccgtgcgccctcgtcgacgacgaccagctccgccgcctcgaagccaagctccagcgcgtccgcgcggcgctccgggac
gccgagcgcctcgctgcggacgccgccggagagtccgccgagctctggctccgcgagctccgcgacgttgaatgcggcgtcgaggacatcctcgaga
aggtgcagtttgaggcccttcgctcctcccttgtggaggacctcggcgacgacgacgacgacggccgcggcggcgatccggccaagaatcgggcccg
ccaacgcgaggtcggtgccttgctctcttactcgccgctgagctctctcggcctcaagatcaagaagatttgggagaagtacgaggaaatagcctcaga
ccgcgaggcgcttcgcctgacaccggaggacggtgtggttcgaccgtgttcctccgctcgcccaccaacctcgtccctaagctccggtgagctctacggc
cgggaggatgatctccagcgcctccttgagcttctcttgtccggggaatgcggtggccgcgtgttcacggtcgtgtccgtcgtcgggatggccggcgtgg

gaaagacggcccttgtccagcacgcatgcagcgaccgaagggtcagggatttcttcgactgctatgtatggatttactcgggccaaggtgttgatgtggt
gagcgtgaccaagacgatggtgcaagcgtgcggaagggatacccgtgacatcactgaactgaggttgctgcaaggtctgctcgtagatcttctgaagg
ggaagaggttcttgattgtgctcgatgaggtttggagcattgaaaggtctgtgtgggagctgctcgagcttcgttccaggaggacgatggctagccaag
aaaagcgcgtgttcgtcgccaacctcaaggcctccctggaatccttcctcttcgccgccaggcgccttgccaagtccgccctcgcgcccctcggcccgtc
ctccgagccgtgcgccctcgtcgacgacgaccagctccgccgcctcgaagccaagctccagcgcgtccgcgccgcgctccgggacgccgagcgcctc
gccgcggacgccgccggagagtccgccgagctctggctccgcgagctccgcgacgttgagtgcggcgtcgaggacatcctcgagaaggtgcagtttg
aggcccttcgctcctcccttgtggaggacctcggcgacgacgacgacgacggccgcggcggcgatccggccaagaatcgggcccgccaacgcgaggt
cggtgctttgctctcttactcgccgctgagctctctcggcctcaagatcaagaagatttgggagaagtacgaagaaatagcctcagaccgcgaggcgctt
ggcctgacaccggaggacggtgtggttcgaccgtgttcctccgctcgcccaccaacctcgtccctaagctccggtgagctctacggccgggaggatgat
ctccagcgcctccttgagcttctcttgtccggggaatgcggtggccacgtgttcacggtcgtgtccgtcgtcgggatggccggcgtgggaaagacggccc
ttgtccagcacgcatgcggcgaccgaagggtcagggatttcttcgattgctatgtatggatttactcgggccaaagtgttgatgtggtgagcgtgaccaa
gacgatggtgcaagcgtgcggaggggatacccgtgacatcactgaactgagcttgctgcaaggtctgctcgtagatcttttgaaggggaggaggttctt
gattgtgctcgatgaggtatggagcattgaaaggtctgtgtgggagctgctcgagataattctgtag

>g82891.t1

atggaggtcgggtggctggtatgcccgatcatcaagattgtcatcgacaaggccaggagctgcgcctccgagaggatcaagtggctgggcagcggca
gcgtcggcgaggcgctgaaccaggttgagcagatgctggtccagctcagcaccgtggcggacgccgtcgagaagcggacctcaacgggcaccagcg
cggacctcctccgcgcgtggctgcagcagctcaaggacgccgtgtacgacgccgacgacgtcctcgacgacttcgacggttcggcgccgcagcccggg
tcctacgtcgccacggtcggcaggcggatcctcggcaccgacaagcctctcaacaggctcaaggaagtcctcaagacgctggaggctgtccgcgcaag
ctccgacaagctcatgcaggttgtgcagctggtggcgtccgggccgggggagctaagcggccaccaaaccccgcacggacgggtcacgggctctgtg
tgccaccaagagctgctcggtcgcgacaggcagctgcgagaaatggtggcctggctcgttgacccgtccgatggggatgcgcagaccatgtccgtccc
cattgccgcgataatgggacacggcggaatggggaagactaagctggctcagaccctgttcaatgaccagacggtggtctccaccttcgacctcagga
tctgggtccagccttccgccattgatggcgagttcgagcttgccaagcaaatcctgcaatctgcagacgtcgacgtgcccagcggcatggggagcttcg
actggttacagcagaaactcatcgagaccatctcgtcgcataaattcctgcttgtgatagacaatgtctggaacattgaaaacatggcggagcttgagta
ccgagagatgtggtccaaggtgctggaaccaataatgaaccaagggaagaaaacggagagcaagattgtgctcaccacccggcgaaggatcgttgc
gagattgctttgtgcaagcaaggaggtctggatgaaaagcttgcaggccgatgacatctggtccctgttaaagaggtgtgcctttggcgaggaagacat
tgagaagcagcaccccgtgctgtggaacatagggaaagaaattgctcaaaagttaaagggctcacccatggcagcaaaggccatcggacaaatgctt
agaggcaaccacagcgtcacccactggagcaagatcacctacagccatgcttcgcaatctgtagcatatttccaaagaacacgagattcaagcgagat
aaactggtaa

>g82892.t1

atgcccgcggcggctccgtggccggtctcgcaggacctccccggcctcgccggccgagcccatgccctctccctcagcgctggcgccgacctgggga
cctcgccgacgcgctcctccggatcgagcccgtcgcccgcgagctcgagctgcggccagggggcacgaggcgcccgcgctgctcgcctggctcgtcg
agctgaagggcgccgtggccgatgcggaggacatcctcgacgagctccacatccgccagtgccgccagcccggccccgcctcgctctcggcctgcgtc
ggtgccgccttccctaatctccaggcgcggaaggtgcgggggctggtcggcaggctcgaccgcgtctgcgagggttcggcgcatctgcgtggtggcctc
ggccgcgtggcggtggagtgcggggtgcggtcccccaaccgcgtgacgggttccttcctcgtggatcgcagggtagttgggcgccagaaggagtgcga
cgaggtagtcaacaggctgattggtggtcttagcgaggagaaatgttcgtcgtctgctccggttgtggcgttgattggccatggaggcatggggaagac
cacggttgctcagtgcgtgtacaacgatgtgaggatcgaaggacactttgatctgagagcctgggtctgcgtctgggaccggtcagacgaggccgagct
cacaagggagatactgcaatccatcagtggtgcagatgaaacattgtgcgacgatggtctggccagtttggagaggttgcaggagaggcttgaagaat
cggttgcgtccaagaggttcctcctggttcttgatgatgtttgggtcgatgaaggcaagactgagcaggagaacagggctgtgtggagcagggtgttgg
ctcctcttagatttgctgggagtgggagcaaggtcttggtgaccactagggtgaagttagtagctgaggttttgaacgcaacaagtgtggttcctcttgat
ggtttaaggagctgtgattgttggttgctgctcaaggaggctgcattcggtgcggaaaccgtggattttccaccttacttgcaggagattgggagagccat
ttctgcaaagcttcaagagcacatacaggttgggaaggctgcgaaccttggtagttcacagaagctcagcattgtcctccggtcgttttccctgtaa

>g87177.t1

atggagggcgcagcccagaccctcgtcaaaaatgctgggaagctgctggctaccgagtatcggcagctgagcggcgtcggcggcgaggtcgttgagc
tacgcgacgatctagacaccatggacgccctcctccgcatgcagtccgaagcggacgagggcgccgtggaccacttcgtccgagtgtggatgaagcag
ttgcgcgaggtggcctacgatgccgaggactgcatcgacctgtacctgctccgcatcaagggccgcgtcagcgacggcgtgttcgcgaagttgaagca
ccggctgaggaccctcttgcctcgccgccggctcgccagcaaaatcagctccctccgcgccagagccgtcgcaatcagtgagcggcatgcgcggtacg
gcgtcaaccgcgacgcgctgctgcgccgctcccctgctgcttcatctgccgctccgatgctgatggcctctactggagatgggcaagaagcgctaagcct
tagcaatagtgccaacgacagcagccgtcaccaggtcattggcatcaatgaccaggtggagtcgctggccgagcggctcaaggccgtgtctgcagag
ggagaacgccatctcaaggtttgctccatcgttgggtttgggggcgtcggcaagactacgctggcaacggagctgtgccgggtgctgaaggctgacttc
ccgtaccaggccttcgtctcggtgtctcaggcattcgacccaagcaaggacctcaagccgctgctcaggcgcgtgcttgagcagatcgtcaagactaaa
gcgggtgtcatggaagaggggtccctgggaaacattgataatttggattcaaatcagctggccaaacaactcgaggagaggctcaaggacaagaggt
aa

>g87182.t1

atggagggcgcggccgagacccttttgagcaatgcagggcagctgttggccgaggagtaccggcagctcagtggggtcggcaaccaggtcgctgagc
tgcgcgacgacttggccaccatgaacgccctcctccgcatgcactccgaggccgaggacaggaccgtggaccacttcgtccgggagtggatgaagca
ggtgcgtgagctcgcctacgatgcggaggactgcatcgacatgtaccggttccgcatcaagtgccgcccgggcgatggcctgcgcgccaggttcaaac
acctgttcatcacgctcttcccgcgccgcggcctcgccggcaaaatccgctctctccacgcccgcgccatcgccatcagcgagcggcacgcacggtatg
gcgttaacctggatgcgctgcgccgctcctctgctgctttatctgtcgctccgatgctgacgtcctccgcaccagcgcaagagcacagccttgccaacggt
gacgctggccaccgccaggtcatcggcatcaaggatcaggtcgacgagctggccaagcggctcaaggccgcttctgtagagggaaacggccatctca

aagtgttctccatcgtcggatttgggggcgtcggcaagactacgctggccacggaggtttgccagctgctggaggcggacttcccgtaccaggcgttcgt
ctcggtgtctcaggcattcgacccaggtagggacctcaaggagctacccaagcgcgttcttcaacagatcgtcaagcccaaagcaggcaagaagaatg
gcatcatggaagaggagaccctcggagagatcggaggcttggatgcaaatcagctgatgagcaagaaactccacggcaagagatggatcgccgaag
gattgatctcagagaagcggggtttgacaccaatggaggttgcagaatccttcttggatgagctcttggttaggagcatgattgaagctgacagtttgga
gcacttcacgggcgagcagttttaccgggtgcacgacatgcttctcgaggtcatggtgtccaagtcccttgaggccaactttgttagcctgcaaggagga
caatatgatgggatgtcgtatgacaagattcgtcgtctttcaattcatggcaacataaacggtccagactctccacccaagggaaaggtgacgcgtcacc
gaggtgccgaggaggttaatgtgcagcatcttcgatcactgagcacgtttcagctccaagggcacaagctcctcgatgaactaggcaagttcaccttgct
gagggtccttgatctggaaggttgcgggggtgtgacaaacatgcatgttaggtgggatattatgtggaccttgccccaggggctcagcaagatgaaagc
attgcatagtgtggccgtagcacgtcttggaaacaacctcaaagttgctgaagagttgggtgaattagaacaactgcagaggctatgcttgttcatcggt
ccagagaccatcggtgaggatgttctcgaaaagcttgcgttatccctgagtaagagaagccatagttttccggtgctcaggaacctagtgtttggcggca
ttctgcctaaagttctccgaatcgagaaagggtcaatggaaaacattgagaccattgaattgcgcgttccttacaatgatgagttggagtggagcatcga
gcacttgaccaacctcagaaaggctacgctcaagggcaacaaggatagcccttcactagatcttgcacttgaacagatgaaggctgagaatgataagc
gccccaagcccaatcaatttcaggttgcagtgaaatatgtagattga
>g87555.t1
atgagccccgcgctccagatgctcgccgatgcagagcagctcaaccctcaaatgaaattatggagggatcaggtacgcgagctggcctatgatattca
ggattgcgtggatgcctttatggttcgtgttgataatggggacggtgggcctttgggattcttggggttatataacaagttcaagaaattgatacaacggc
atgagatcgctaccgagactgaagaactcaagactcgtataatagagataagtcagaggcacaagagatacaattttgtcgagccgacatccaacgtt
gctaggacttttgttgaccctcggctgccagcgctctacgaggagatagataagctcgtgggcatcaaggatcctaagaagcgggtcattgagttgttaa
acataaacaataatgggtcatctacaaaacttaaagttgtctcacttactggttgtggaggtattggtaaaactactcttgcaaagcaagtctatgatact
gttaaaaatcaattctcgtgtgcagcatttgtttcaatttctcgtagtcctgatgtgagaaagattctaagagacattgctataggagttggttgcactgatt
acccacccgatggtcatgagcagcagctcattgataaacttaggaaacacatcgagcacaaaagggcatgtgggacctcactagagtttgagccaaga
tcaatgccaaagcttgagcacgtcaaccttggtttcctgatgcatagcatgaagtgcctgaatggttcttctaattttggcatccagcacctctccgctctc
agcgaggttgtgttcagaattagagataacttcatgatgcataaaggggaatatgttaagacggatgctgatgatggcacagttaaatgtgttgccgga
gccgttaatgctgccatcgaggtagtattgaaagggctcacattgtcctctccattgcagctagcacaatttattttttgtgtttag
>g87764.t1
atgttgatccagaggtacatgcaattcatgccagaagatgcagagcacatgcttcaacggctgcagcgcgtgctgctccgtattgacgccatggtcgag
gaggccgaggggaagcacatcaccagcaaggcgatgctgcgccagcttcagctgctcaggcaaggcatgtaccgtggccactacatgctcgacacctt
cagatgcagagaccatggaggcgacgacgaggtaaacggtggccttgttgttgccttgcggcctcgattcagtgcagccaagcggcgcctcttctcccc
cgtcaacgacgatacggataatcagcagaacaccatgctagacgccgatagcttgaggaagctggagaaggtgctcgacggcctcgagacgctgatg
gggaacatgcaggagttcaccgtgcttttgggggggataccgtcgcatctaccggcaaccgtacggcacgtacttgatcctcgacaaggtcatgttcggtc
ggcacatggagaaggaaaccatcctcaattttcttctagggccagaggctgcaggcgatgaaaatcctggcgtccttcccatcgtgggcgcagccagg
gttgggaagagcacgcttgtagagcatgtatgccttgacgaaagggtgcgcaactacttcagatttattgtcttcttcagtagtgataatcttggtactgg
gaatacggctgctctgagaaaaactggtgtgatcaagcatcaagatcttacgaccgggttgcaaggaaggtcacttgccgttattgagcttacaggtga
catagaggaggagacatggagaaggctgtactcctctgcaacaagcagcatggggcatggaagcaagatcataatcacaagcagatcagagaaaat
agttgctctcggaacaacgcacgctctaacactgaaggttctgccgagagaagctttctggtacttcttcaaggcgcttgcttttggaagcgcggaccctg
atgatcaaccgaagctcttgtccctaggcatggagatctcaacgcttctgaatagtgttttcttaagagcaaatattgtgtcaagcctgctgagaaccaac
cagaatgtccagttctggcacagggtgcttcagtgcttgagagactacacaagcaaacatgtctgcacgtttggtaagcatccttcagatcttgtgtccaa
aagccagcctgtgtacccatggagtatggtcaggagccagaatgtcgttgagatctccagcttctatcagaagccttctccatatcatgatgtccccaag
ctaacagtacaagaatttttaacaggatgtgtaatagatcatgggaagttcgacatgttaatttggagatctagcatacctccctactatgcttacgcggc
gagttgtgtatcagagccgtcgagatgcccaatgccaaacaagaagcgccctcgacacgcgagggagagatga
>g87919.t1
atggatctggtagcgagagccgtgggcagcctccttccgaagctgcttcaggtgctggagcgtgaatacaagctgcagacaggcctgaagaagaacat
ggagtctctctcagaagaactgaagcatatccatgccttcctccacaaggtatcagaggtgccatgggaccagctcgacgagccagtgaaggagtggg
caagcgaaatcagggaggcatcctacgacatggaagacatccttgacacttacctcgtgcgcatcgagggctgtgacgattgtgagcccgccggccgg
agcaggctgaagcacgccatgaacaagatggccaacgtgttcagtttcagtaaggggaaggcacgccacagcgttggtacttccatccaaagtatgat
gaagcgactccaggaggtggccgaccgacgtgccaggtacaaaattaacgaccttttggtgaactcttcagcaacgacgtccacctctgatcctcgcct
taaagccatgtacaatgaagttgcaaacttggtcggcatcaacgagtcaagcaaggacctcatatccaagctatcagccggccagggggggtgatatga
cccgccggaagaagacggtctcaattttttgggattgggggccttggtaagaccactcttgccaaagcagtgttcgacaagcttagaccccactttgaatg
tggggcttttgtacctgttggtcggaatcccatcctgacaaaagtactcgaggatattctctatgaacttgacaagaccaagtatgaggatattcatacca
agaagagggacaaaagacagctcattgacaacctccgagaattcctcgtgaacaagaggtag
>g87926.t1
atgggcagcctcctccccaagctgctcaaactgctcggggatgagtacaagatgcagacggggatgaagaagcaagtgaagtttatttcagaggagct
tggggacatctatgattttctccacaaggtttccgacgtgccatggtaccagctcgatgctcagactaaggcttgggcgcgcgaggcgagggaggtatc
ctaccagatggaggatgtccttgataccttccttgtgcatgttcaggagcgggagcctgccagcccgagcaggctcagacacgccttgcaaaagatggt
tagcctgttcagaaagggcaaggcaaatcgtgacatcggttacgccatcaaagacatcaaaaaccgactccagcatttggccaaccggcgtgccaggt
acaggattgatgatattattgcaaacccaactgcaactatgtcgactgttgatcctcgccttcaagctatgtacaacgaagttgcaaagttggttggcatc
aacaagccaagggatgaccttatatgcaagttgtctgcccaaggtgatgatatggcccttccgaggaagatagtctctatctttgggaccggagggctc
ggaaagaccactcttgctaaagcggtatatgaaaaacttagaccggattttcagtttggggcttttgtaccagttggtaggaacccaaacattgcaaaaa

ttctcatggacattctttatgatcttgacaagaacaaatatgcggatattcataccaagaacagagacgaaagacagctcaccgacagcctccgagaat
ttctcacggacaagaggtacaaattacccactcaaactttacactaa
>g87931.t1
atggttagcgcttacactggggcagtgaactccgttataacgaagctcgcgaccctgatgggagagcggtggtacattgagctgacaggcacggatga
gcaggagctgatcttgcgagatgagctccaggtcatgagcgctctacttaagcagctgtcagacattctttacctgaacaaagaaatggtggagtgcag
gaatcacatgagggaattgacatacgacattgaggactctattgatgatctcctctctgttaacaaagagggcagtagttctactcagaagagggttatg
ccccatgactttattgaccaaattcaaaagctcaatgcacaactcgagatggcagacctacaaaacacagtgagcagggctgaaaagcgcacttttgg
accaaaacatatgcctagggaacaatggctatccgaactctactatgatgagagactgaaactacaagagagggatcagaacagtgtagaatcgatg
aggaagttgcccagcatcaattatgatgatctttcttatgaaatgaagacttgtcttttgtatttgagcagtttccctgccgatgaagtaatcatgaaggac
agacttatacgcaggtgggcagctgaagggtttcttccaaaacgaagcatggaagcttggtgggagacaggggagaactactttttcgaacttatcgcc
agaaaactgattgaaccagtatatggtgaggtggaggaagatattctgccgacaggttgtaaaatcagtgattatgttcatgatttcatcacatcgctgtc
cacttacgagaattttgtcaagcccgggacgcatgaacggagcatcatcagcaaggacggctttcggtccctcagggtgttctggtacgactctctgttt
accggtaggatggggctgcagtttaaagcaggagccatgccgcagcttcgaaggcttcggctagagttcaacccaaggaaaactatgtccatatccgg
cggtgtcgatttcggcattcagtttctcacttgcctcgcacgatttgatgccatcatcgactattatggtagcacagatgcatcgctcgaggctgtggaggc
tgccatgaggggacaagtatctcagaatcccaacaatcccatgatggaaataaaaagaagacgagaggaacttctggagggtgtagagaacaaaga
tcgcaagtccccaatgttcccatactga
>g89169.t1
atgcaggtggtggagctatcttacgacatcgaggatcatgttgatacttttatggtgcgggttgatgcccacataccaaacaagctgcgtggcgtcaagg
gtcttatcgacagaagtggcaatttgatgaagaagacaagcattcgacgcaagattggccttgctgtcaaggacatcaaaagccacatcacagaggtg
agtcagcggcgtgacaggtacaaacttgacaacattacctcaaagcctgtcggcacaactattcatagccttcgtctatcagctttatacaacgacatga
caaagctcgttggcatagaagagaagagtgatcacttggtcaagaggctaatgaaggggcatcaggcttccaggggccaactagagattgtctctatt
gttggttttggaggattaggcaagaccactctggcaaaaatagtttacgacaagattaaagatcaattcgactgtggagcatttgtctctatatctcttaat
ccaaatatggagaagattttcaagagcatgatccatcaaattgacatgtctgagtatagtaaaatcaatgatgcaacattggatgaagtggccctcattg
aaaaacttagagaaatccttgaaaagaggtatgtacaatcttag
>g89388.t1
atggctgacgcgggagtgacgggggtggcggcgaagctgggagagctggcggcggcggaggcgacggcactgttgcgcgtggacgcggagatccg
ggcgctgcgccggaagctggcctacctgcaggcgctggtccgcggggccgaccgccagcgccgcggccgcgccaccgagctgctcctgctctggctgc
gcgacaccagggaagtcgccttcgaggtcgaggacgctgtcgacgaattccacctccgcatcgagctcgccgacctccgtgccaaacgacgccgccgc
gggtggtggggctggcacctcgccgccgtcgacctcgtccagggccttgccgcgcagattgttgttcgtcatgggctgtcaaatcagatactgaagatca
atgaaaggatcaatgaacttaatgagaacaaggaaacatatcagattgaaagttctccttgtgaaatttggagttcatcatcagttgagactgatccaga
gtggtacgaggaaggatatgtcgcaggctccagactgtgtgagtttgcgctcctcaaaggccaaatcatcaacaaagaggaaaatgtttttcaccgagc
tgtcgtctcaattctgggggagtctggcattggaaagacaacgcttgcaaagaagttgtaccaacaccccgatgtcatgaaacacttcgaggtgcatgc
atggatatgtcttccacctcgtatcaggtttgaagactatatagaaattatgtacgcgcagatcagttcacaggttccagaagcacctaaaaatgttgtac
tactttcacctgactacggagagaccatcaataaggaaaacaagcttcgacagcaccttcggggcaggagccgaatcttactcacatcacggctcaatg
tgaaagaaatcagtcatatggacccacatattgctcccgtagagctgacttgtcttgacatggtacatgaaaatgttattgttcaccctgatcgtttggttc
gtctctggattgctgagggttttgtggtgccaaagaaggcagaaaccctggaggatgttgggtttcactatctgaaagatctggtctcaagagggttagt
ccaggtcatggagaaagatggtggaggaagcatcaagctggtagccattcataatctgcttcatgcctttgtggagtgtgaagcacaggactcttgtttc
cttgaaatccaccaccatacaaatgtgaagaccaacgcagcagctcaagttttggtggattccaacctcagacactatgggggctctagatttctaagag
tcatcgatctgaatggtctaaagatgcaaaggctgccaaatgaaattggaagcataatccatttgagagctagggtttcatctcctctctctcctcttct
cccacccacacgccgctgccgccggtcacctcgccgccggccaccgccgccatggccggcaggcctcctcctcctctttccctcctctccccgcctcctat
gccgccgccaccgaccccgccgccgccgctccggtgccgctatcgcgagatctgccgccgtcgccgcgggatccgccgctatcgccgcagatcggggc
agtgccggtgtggatctggtcacggccgtgggtgccgtcgtcggccacgcgggcgcggatttcgccgtcacgggccatgtgggggtcacgggcgccac
gggcgaggctgtgccgccggctcacgccgctgctgcatgcgcccgggcagacgccgacactgtcaccgtcgccgccatcactgctagccaagatccga
ccgtcgtcgtcatag
>g90503.t1
atgaagcagctgcgcgagctcgcctacgatgcggaggactgcatcgacatgtaccggttccgcatcaagtgccgcccgggcgatggcctgcgcgccag
gttcaaacacctgttcatcacgctcttcccgcgccgcggcctcgccggcaaaatccgctctctccacgcccgtgccatcgccatcagtgagcgccacgcg
cggtatggcgtcaacctggaggcgctgcgccgctcctccgctttatctgtcgctccgatgctgacggcctccacaccagcgcaagagcacagccttgcc
aacggtggtgctggccactgccaggtcatcggcatcaaggaccaggtcgacgagctggccaagcggctcaaggcgtctgcagagggagaaggccat
ctcaaagtgttctccatcgttggatttgggggcgtcggcaagactacgcttgccacggaggtttgccagctgctggaggcggacttcccgtaccaggcgt
tcgtctcggtgtctcaggcattcgacccaggcagggacctcaaggagctgcccaagcgcgtgcttcagcagatcgtcaagcccaaaacaggcaagaa
gaatggcgtcacggaagacgagaccctcggagagatgggaggcgtggatgcagatcagctgagcaacaaactccacggcaagaggtaa
>g90506.t1
atggagggcgcagcccagaccctcgtcaaaaatgctgggaagctgctggctaccgagtatcggcagctcagcggcgtgggcggcgaggtcgttgagc
tacgcgacgatctagacaccatgaacgccctcctccgcatgcaatccgaggcggacgagggcgccgtggaccacttcgtgcgggtgtggatgaagca
gttgcgcgaggtggcctacgatgccgaggactgcatcgacctgtacctgctccgcatcaagggccgcgtgagcgacggcgtgttcgctaagttgaagc
accggctgaggactctcttgccccgccgccggcttgccagcaaaatcagctccctccgcgcccgagccgtcgcaatcagtgagcgccacgcccggtac
ggggtcaaccgcgaggcactgctgcgccgctcccctgctgcttcatctgccgctccgatgctgacggcctctactggagcggggcaagcgctaagcctt
agcaataatgccaacgccaccagccgccaccaggtcattggcattaaggaccaggtggagtcgctggccgagcagctcaaggccgcgtctgcagacg

gagaacgccatctcaaggtgttctccatcattgggtttggaggtgtcggcaagactacgctggccacggaggtgtgccggctgctgaaggctgacttcc
cgtaccaggcgttcgtctcggtgtctcaggcattcgacccaagcaaggacctcaagccgctgctcaagcgcgtgcttgagcagatcgtcaagaccaaag
cagcgaacgacaagggtgtcatggaagaggggtccctgggaaacattgataatttggattcacatcagctggccaaagaactcgaggagaggctcaa
ggacaagaggtaa
>g90676.t1
atggacatcgtcgtgggagccgcgctgtcgggcatcatagacgcgctgccgggcaagctcggcgggctgctggagcaggagtacgagctgctctccg
gcgcccgcggcgacgtggagttcctccagatggagctgtcctccatgcgcgccgccatccggcactgcgagtccctcgacaaccccgacgcgcagacc
gggacctggatcggccgcgtccgcgagctcgcctacgacatcgaggactgggttgacctcttcggcatccgcgtcgacggccgtgcccccacctcctcc
gggttcttcgggtggctccgccgcggggcgaagaagctcgcgacgatgcccgaccgccacgtcatcgccaacgagctcagggacctcaaggagcgcg
tcgtcgagctgagcgagcagcgcaaccgatacaggtgcgacctggccgtatccgccccacccagcaccgttgacccgcgcctggtcgcgctcttcgcg
gacccaggcagcctcgtcggcctcgccgagccggtggaggaggtgaccaggatggtcgccacgaacaccgccggcaggacggggatcaagattgtc
tccatcgtcgggatggctggcgccgggaagacgacgctcgctaacacggtgtacaagcagctcaaagcgcagaagggcttcgattgctatgctttcgtt
tccgtgggtccgaaaccagaaaatatctgcaaggttctcacggacatgctctggaaacttgggggcgggcagcaccggggaatcgtcgacattaacca
actcatcagacagctcagagaagtgctggagaagaagaggtaa
>g92943.t1
atgaagatagccgactggataaaggaacttaagggaagcaataattccgaaaaggccgcgggagcagtaacaccatccatcgccgttgttgcacctg
gtaatcgtgaagatgccctcactcttatacatggagctctggctgcagccgagaagcttaatttccgcactgtcatgatcgacatcccgagactgcactac
cttgatgaaccgctagggtcagaggaaatcctctgctacatatggcgagagctcgagctcgagcatgccaaatcctcgtctacttcttctcagcagcaag
gcacaacaagcagtcagagagaggccaaggagcaacatattcattggcgaagcacaagtaataaattggatatgttccatgaaaagaggaaggtgat
tcgttacatcaggaaaaatatccaagaagaggttgacgaaaagcttgcatggatcaagagcgagatcaggaaactgagggatgacaatctggaccaa
cgcaaaattaccaatctgtctctacctgcactactcctgctgctgaacgagtaccaggcagggaagaagaaaatcctgctacctcctactgcgtcgtgcg
aaataatacttagggacatagccattaagtttaaagcgtatatggaagcaaaagaactcgaagaagcaagggcaaagcgggtgacccagatctatctc
gaaccttccgaatatgaagacatcctgcgggaggttttcctacaggcccaggagcaatacacagctcctgctagcaccagtgcattgggtgaagacca
aatcgaagtaaagatacgtgaggctaaaagagaaatcctactggaggtggaaaagatgctggaagggaaaactggcaaaaaacaagcaacaggag
taccagtttgccaggatgaaatcgcacaagccaatactgcagaaaccaaggatgaaattgcagaagtcgaggagaagatcaagaaaatccagcaga
agattgaggcgacaatgaagatcaaatatatcatgaacaacattcatgattacttgaatgatcatgatctacacatcttgatcatcctctatatcggtcag
gactatattctgagatgggaggagaccagatatgctttaagcctactgcattgcgttgctggtgcagtaatcgtgacctcttcaacagacacccagcggg
ccaaagaatattgcagtccaccgttggagcctatggagtgttctactgttggactctaccataatattgtgctcgagcttaaaagaaagcagctagaaca
gatggatgaagagggtgacagccacattgattcccatgtttttcgaagcattttggacgagtgtgagccatatgaattcaccatgaagatctttgctcatg
cattgtacgccaaccccaagaggaacaatgtacaactgcacgagttgcacagaacccttcaggtttctccaaaatctttcagaagtactgctgagaaga
tgatcaagttctcatacaacaatttgcctgaagaatacaagtattgcttggtgtacctagcgatcttcccacctggacatcaaattaggcggtcaacattg
atacaacggtgggttgcagaagggctcataacaagggaagattggcctacctcagtgcgccaggccgaaagatgttttgacgcgctcacagatcactt
gcttgtttgccctgctgatattggtgttacaggcaaggtcaagagctgcgtggttggtaatatgatccatcaatttattgtcaagatagccaagaaacaag
gcatcatggagacacattgcttgtctcaccacttctccatatttaactatctcagactccgtagatctgagacaatctgtggtttcttgcagaagctctctaa
atcatctcaattatcggtgctcaaagtgctagatcttgaaggttgccaatgctttgccgggaagaacaagcaatacctcaaggacatctgctgtaagata
ttactgctcaagtatctgagcctaaggagaacaaatattacccacctgcccagtgatattaacaacctccatgagctggaggtattggatatccgtcaga
ctgacatgcctccatctgcaacaaggagtgtccggctcctaaagctaaagcgcttgctggctggtctcactggcgtttcaagtgttgacattcctgaaaag
gttggaaatatggaagagatggagaaagatctggatatccttgcagacctgcccacattgcactatctcaagcttcgatgcgtggcatatgttgagagta
agctcaccttcaagaagaagaaatttcggaatctgaagtacctcctcgttgagggctccaacattactgacatcagctttgatggtggagctcttgagctt
gagaagatcgttttgcccttcacaagcttaaagcttactggagtccagaaccttaccaaactgtgggagcttgagctgaacaataatacttccaccaata
tcaccaccaccaccaacaacaacaacttcttgctttcagtattcaaagatttcatacctaacatagccaagttgactctttgtggtaccctcctgaagcaa
gacgatctacgagattttgccaaggaatcaaacatacgctgcctaatgctcttggacaagtcttatgttgaaagacagcttaacttcaacaaagatgaat
tcccaaagctcaaccttcttattctcaactgctctggcatcaccaatgtcagctttgacagtggatctgcctctaagataaataagatcgtttggtttttcaa
ggaaatggagtccctctatggcatcaataatcttcagagactgagggagctcgagttcaatggtgacctgatacctcctgaggactgcaacaactgcat
cgacctctacctgtaccgagggcatcctgacatccgccgtctccggggccgcctccggcgctacctctcctggcttccgtggtttttgcgtaatatggtcac
gcagcacaatgcggccgtccagctgcgcgagcccaaggagaaggcccatgacgtcggcgagcggcagcagaggaacggtgtcgaggtgccagcga
aggcagcggctatgttgttgccgttcctacttcctgatgctgcgcctccaacgcctgctgccataggagaggacgatgaagacagtgaccatcatcaact
gcaggtggtcgccactgatcattctagtcgcccaacatcccttttcgagacacacactcttgatgacttcttgaaggtgaaaatagttgattgggtgaagg
aaattgagcaaggcaaccctgcaaatgctgggccggtgatagatttagaaggttgtcattgctttggtgggacaaaccagcacttcctcaaggacatct
gcaacaagattatactactcaagtaccttacacagctgcccagtgaaatcaacaaccttcatgcactggaggcactggatatccggtatacccaggtgc
ctacatttgcaacagaacatgtcctgctactgaagcttaagcgcctgcttgctggtcacattaatccaagcccaagcagtactgacatcagcaaggttgt
ccatgttcctgttaagatcaaacaaatatcgaacatggaggtgatagatttagaaggttgtcattgctttggtgggacaaaccagcacttcctcaaggac
atctgcaacaagatcatactactcaagtaccttacacagctgcccagtgaaatcaacaacctccatgcactggagttactggatatccggtatacacagg
tgcctacatttgcaacagaacatgtcctgctactgaagcttaagcgcctgcttgctggtcacattaatccaagcccaagcagtactgacattagcaaggtt
gtccatgttcctgttaagatcaaacaaatatcgaacatggagggctccaacatcactgtcatcagctctgaaaatggaacagctcctgagctcgagaaa
attgttttgtccttcactggctacttgaagtttgtttctggactcgaccatgttccaaaactaaaggagctagaattgactaacaacagcaggttgctttcat
tatttgacactgcaaaccaattagccaagttgacacttcgtggcacattgctcaagcaagatgatctggaaatccttgccaagaaatcaaacatacgca
acttagccctcctggacaggtcttatagcaaaagccaacttatcttcaatgaagatgagttcccaaattccaatcttcttattgtagattgctctttgatcac
caagatcagtttcaacagaaaatctgctcataaccttgagaagatcgtctggtcattccgtgtgatggaatctctaaccggcattgaccacctttcaaaat

tgaagaagctcgaattgcaagggcgcacaccgatccgcgccgtcaccggcagaatnccctccatctgcgcctgcggtgctcatcacgcggtgcagtac
tgcgccgttgtcaggcccgcctcctccccaatccgttcgagccatcgaccaatttggatcctgtccaatgctgaagcatcatttctggacgggatgccctc
cgtcagggcgcacaccgatccgcgccgtcaccggcagaatcccagccaagggcaagggcgcaatgcgttcgttgccntcctctatctgcgcctgcggt
gctcatcacgcggtgcagtactgcgccgttgtcaggcccgcctcctccccaatccgttcgagccatcgaccaatttggatcctgtccaatgctga
>g93782.t1
atgcgtactgaggttattgaggctgacacaattgatgcagctgctgaacgaattcttgacgtgctgaaggaagacagtagaagcatcagtggcaaaga
caatgtcatctacttcgatggctgggacgggctgggggcaactaccgtccttcgaaatgtggcccaacgcctcgctgtggccgtagagccccatgctgg
actgcgttttgatcggataatccacatcgactgctccaagtgggagagcaagagagctgtgcaaagagcgatagcagagcaactgaagctaccagatg
aggtgatggagatgttggaccaacaagatgaggaggacgactaccacgggctagagcaagggtcccgtgcagaggtgccacaagttgtcagagaga
tctaccaaagtatccagaactctaggttcctggtgatctttttcaatggaagcaatgaagaaattgatctgactaacttgggcttccatctatactcgagca
acaaggtgctgtggacatttcaagggtctttgcggctcatgtccaagaagaagatcgatggggccatgaagacaacaactggtacaggtacgacaggt
gtttttctatcagctgcatcagtccatgaggatgggttctggtcagactttgtgcgccaagaggctgctgagttagttacatga
>g94580.t1
atgctgattggcaagatcggtgcagccttggcgaacaaagcagcaacctacggtgcttccctgcttggcaaggaagcctctgctctcaaggatctctttg
ctgagatccgtaaagctgagacggagttggaaagcatgaaggcctacctgcatgatacagagaagttcaaggattcatgtgagtctactggcatctttg
tcaagaacatacgagatctagctttccagattgaagatgttgtcgatgagttcacgtacaagcttgaagacaagcatggaggcttcttagccaagatga
aacagcggatcaagcacgtcaagagttggcaccgtcttgctcttgaactccgtagtatcaatgatcagcttaaagaggctacggaaagaaggaaccgc
tatgccatcacaggatatgttggaggcagtgatcctaatgatgtatcaaaccatcaaatcacaacttttgcgagagaagaggatcttgttggtgttgagg
ataaagcagaacaactgaagcactggctaataagtgatttggaagaaagaaagcataaaattgtcacggtttgggggatgggtggagcaggtaagac
ttctctggttgagcttgtctataagcttgtaaaggatgattttgatgttgctacatgggtaactgtgtcaaagtgctaccaagtagaggacttgctgaagaa
gattgccacagggttgggcatcacaaatgatattagcaacgtggaaaagaggagtttatttgaggccatctcttggaggaccttccatatgagttga
>g96342.t1
atggccgaggcggcctctgcagcggccggcggcgccgctaacctcgccatccaggaggtctcgttcctgtgtggcgtccacgacgaggtggagtccttg
agagatgacctcaggtcgatgcaaaccttcttgagcgtggaagcccagcgtggaggcggagggaacgcggtcacggcggattctgtccgccgggtca
ggcatgcggtgtacgaagcggagaacatcatcgatgacgcggactatagagccaagaggaacagcaggcgcaagggcatgcttggtgccatatcga
ggtacgcccgtaaacccatggatctagtggcccgtcacaagcttggtaaggatgttctccagtggaggaggaagatccaggacatcaagtctagcaga
gagtttctggatgccatcaacgctgaccggctcgtgactgcaagcacaccacagcctaggactcatcgtgcatgcctctcgcgcctcgctgcagatcccg
acaacgaggcggcccttgttggactcaaaggagatgttgagcgcatagaggaatggctgaaagatgaagacaacacgcaagtcaccgtcatctccat
agtagcgatgggtggggcagggaagaccacactggcgcgcaaagtgtacaactcagctgctgcgaaagagcattttgacgcttttgctttcgtttccatt
tcacaacagtttgaggtgctttctgtgttgaaagaaatcgcaatgcaagctatggggattaacaagagggtcagggaatttgataagattggccaggga
catgagctggagaacatgggggaacaagagctggcagggacgttgcgcagttttcttgaaatgaaaagccctgccggtgcgttcccacggctgcggca
cttgagactctatggggtccaagttcacaagctgagatttcagaccggtaccatgccgaagctcgtccaattgacatttggtttcggccacatgacgacg
gttccagatggactgttcgacctgccgtccctagaaaaattgaaccttcacataatggaaaatgtgttgcccagggagattcacgaactggtcgagggc
aaaggttggctcttcgtacgtgatgtgaaacctgagaagctgtatcagtga
>g97573.t1
atggagggcatggaggagcagcacaagatcctgaagcgcaagcttccggccatccttgacgtcattgctgacgccgagaagcagtcctcccaccgag
aaggagctaaagcatggctcgaggagctcaaatcggtggcgtacgagacgaatgatatcttcgacgagttcaactacgaggcgctccgccgtcaagcc
aagaagaatgggcacaccaccaagctcggcgtgctgggtgcagtaaagctctttcccacacataaccgtgttgtgttccgttacaggatgagcgacaag
ctccgccgggtcgtggaaaccatcgaggtccttgtgtctgagatgaatgcgtttgggttcaagagattgttcagatcctctaccgacaagagcaacgacg
aagctctcacagtcgttcccatatttggaatgggtgggttaggcaaaaccaccctggctcagctcatctacaacgacgctgaggtcatgaaccatttcga
cctacgcatctgggtttgcgtgtcagacgagttcgatgtttgtgaccttgttaacaagatctgcaactctactgagaacattcttgacancatctacaacg
acgctgaggtcatgaaccatttcgacctacgcatctgggtttgcgtgtcagacgagttcgatgtttgtgaccttgctaacaagatctgcaactctactgag
aacattcttgacagtgcactgcagacgctcaaggaaaaaatcgaacaaaagaggtactatacaaattga
>g99056.t1
atgccttccctcctgagaaagttggatgctttcctaatgagtagtatttatgaactaggcgatgagatgcaaaatgatttcaggttccttagagctgagctt
gatgtcatggatactttcctcaggaggatgtcggagatagaggagcactgcaagcaagcacagcgccaggcagtgattgtgcgagatctgtcgtatgg
cattgaagatcgcatggacaggctcatgctccttgagtctagtgacagaggtgaaataatagccaaggagattagaggcataatgaaacttatcgatga
ggtgaaagataggcattcaaagtacactgagcaaattgacaatgaaatggaggccgcatctatgcagattcctgcggaccctcgcccgcgcttcctcta
tgcagatgtgtcggagctagtgggtattgaggtcctcatggatgacctcatctttatgctgacttgcggggaagatgaaggactgctgttgcaaagaatg
gtctctattgttggatttggagggtccggaaagacaactctggccaaacaactgtaccaagagcttcgacctcaatttgactgctgtgcttttgtatcagtt
tccagaaagcccaacatcaactggacattgagaagtatgctttgtcagatccgaaatgacctgtgtgtctacgctgaagcatgggatgagaatcaactc
attgatgagatcgtggacttcctccaagataaaagatacgcatcaggtggcagcgtgtttcttcttcctcccacccacagctcctcttcttcctcttgccgtc
gtgccgccgaccgccggcgagtcggcctcgaatccttctacaatccctcgggaggcatcggccccagcaacccagaccccataagccgccggcttgaa
ccgccgaccggcggcacggcggcgccctcaccgccacgctccgtcaccgctccacctcactctcctcaccgccagcactaa
>g99286.t1
atggagctcgcggcagcagcgatgaccccgcttatagagaagctcggccacctgctggcgtccgagctgaatctcgagaagagcgtgcggaaagatg
tcgccaccctcgagcgggagctgaaggtgatgtacgtgaagctcagggaggtgtccgaggtgcgcccggaccggatcgacgaggggaccaagctctg
ggctgccgacgtccgcgagctctcctatgacatggcggacgccatcgacgtgtgggggagagatgccaaggatcgcgccaaggagctaggtgagatc
caccaaaggcacgctgggctcaagctgcaagacaccggccacaacgcttctgacattgatcctcgcctgacggcaatgtacaccgaggttgtggatctt

gttggcgtcgatagtgcaagagacgagctgatcaaggtcctatctgatcccttggaagtggatgtgaagactgtctctattgttgggtttggtggcctggg
caagaccacactcgcaaaagtggtgtatgacaggatcaaagtggagttcgattgtgcagcttttgtttcggtgtctaggaaccccgacatcacccggatt
tttcagttgatcctttttgaatttgacaagaatcgctacgccaacaccaacatagcgttgtgggatatcaaacaacttattgatgagctcagaggcttcctt
caggataagaggtacgcatcaccaattaagtactactag
>g99892.t1
atggcgctcctcaccgaggcgagcctcatcgacgacatgcggaacttcctgggcgacttcgcactcagggccaagcggctggcggcgccgctgctgca
gccgttcggcccctcatccgagccgagcaccatcgacgacggcgagctcgacaagctgaaatccaagctcaagcgcatcaacgctactctacgggac
gctgagaacctcgtggtgtccgacgaatccgccaagctctggcttcgggagctccgggacctggagcacgccgctgaggacgtgctcgaggagctcga
gttcgaggcgctccgggcgacgctcctcgagcccttcaaggctcagctgctccgctccagcgccgtcaaaaacaagcggaaggtcaggtcgatgtact
cctctgctcccgatcgcctcaagcgcaagatcgccagcaacatggagcggtacaatgagattgcgagagacagggacgcgctccgcctcaggagcag
cgacggggagaggcggcaggagggcagcgccatgatgccgacgagctgtttgatgaaatgccaccggcttcatggaagggagctcgacaagaggcg
ggtcgttgacctgctatcttctgacaaagctaactgtagtgatgtatattctgtcgttcctgtggttggaccggctggtgttgggaagactagtctagttcaa
gatatctacaacgatcaggatttcagctctaagtttcatacaaaaatgtggctctgggtgtgccaagagttggatgtcttgaagcttacacggaaacttgc
tgaagaggctactgagtctccttgtggctttgccgatatgaaccagatgcaccgtgtcatcactgatcggctccaggggaagcggtttttgcttgttctag
atgatgtatgggacgggagctgctggatgagcttgcagatacaccaattggggttcctctctgatactaattgctggtgcaaaggcttgccattagctgc
aaatgcagctggtaatgttttgtccagtgcaattgataggaaccattgggaggctgttgagcaaagctatttgtgggacagtgatgtagtcggacagatc
cttcctgctcttctggacaagctggttcgcctatggttagcacaaggctttgttgaagcagacaaagaacgccagcctgaagatattgcttcaaagtactt
tgatgacctagtggagaagtttttctttcagagatcaccatatcatcaagagaggtactcgtaa
>g100095.t1
atgtttaaccctgcaaagcgtctccgtaagtgcagcggccgttcccagattgctggagagctgcaggggattcttgaaggcctagaaactatcattcaag
atgcagccgagtttgtccagctctctggcagataccctcgtcttgctcgccagccctacagcatgtatttgctcttggacaacttcatgttcagtcgtcagat
ggaaatggagcacatcatgaacttcctgcaccatggtgcacaggatcctagtgccgagcatctgggcgtcctaccgattgttggccccgtaaatgttggg
aaaagcaccctagttgaacatgtgtgcatggatgagggggtgcgcggccacttctcgcagattatgtttatcagtggaagtgatcttagcggtgaaggc
atggtgactcttgcaaatgagaccggtgtgatcaagtatgagaaccaatcagggtcagctgaagccaggatactgatcatagttgaagctgatagggа
catcaacgaggattcgtggcagaggttgtactcagcatcaaaagctcgcctggcacgtgggagtaaaattattgtgacgactcgatctgacaagataat
cggattgggaaccacgctgcctcttaggctttaa
>g100160.t1
atggcagatcttgtagtaagcgcggcgacaggagcgttgacgcccctgctcaagaagctcgctgctgcgctggttgaagactacaagcagttcaaggg
tgtgcgtggtgagatcaattcactcactgaagagcttccagccatgcatgcatttcttctcaagatgtcggaggaggaggatccagatgtgcaggataaa
gtttggatgaaggaggtgcgggagctatcctatgacatggaggacagctttgatgaattcaggcttcgtgttgatgacagatctactaatccagaaggcc
tcattgacaaattcaacaacttcgtgagcaaaaccaagactcgccgtcggattgccaaggcaattgatgatctaaagatccaaatcaaagaggttggtg
aaagaaatgcaagatacaagacacgggagatcatatccaagacaagcaacgcctatgtagatcggagagctcttgctgtatttgagaatgtttcaaag
cttgtgggtttggagaggcccacgaatgaagtgatcgaagtgatgatgacaaagaagaatggatttccatcgcagatgccaaaggaggtgcgggagc
tatcctacgatatggaggacagctttgatgaattcaggcttcgtgttgatgacagatctactaatccagaaggcctcattgacaaattcaacaatttcgtg
agcaaaaccaagactcgccgtcggattgccaaggcaattgatgatctaaagatccaaatcaaagaggttggtgaaagaaatgcaagatacaagacac
gggagatcatatccaagacaagcaacgccgatgtagatcggagagctcttgctgtatttgagaatgtttcaaagcttgtgggtttggagaggcccacga
atgaagtgatcgaagtgatgatgacaaagaagaatggatttccatcgcagatgccaaaggttctttccattgttggatcaggggggcttaggcaagacga
ctcttgcaaaccaagtatatcagaagctcaaaggaaactttgagtccaaggctttcgtctcagtgtcacggaatcctgacctgtccaatgttctacgtaac
attctgtatcaagtaaacaaggaatctcatcctaacatacaagactgggatatacatctacttatcagcaaaatcaatgattctctgatggacaaaaggg
gctccccgtggcacagcgccagtagcaggaggtgggagaggctgctagatccatgccgagggaggggccaccgccaagggagagatccacaatgca
ttggtggatgggccatcaagggaggagcccactga
>g100574.t1
atgttcagacttccagggatgctcgaggggttgctgcagcgccacggcaccactctgcccaggggcgccgaggatgagatcccactcatcaagcgtga
tcttgaggaaatcataaccattctcaagggactaggaggggccggccgttctaagatggccgtcagttgctggatgaaggaggtgcgcgagctgtcgta
cgacatggaggacttcatcgacgagtacgagcatgtcagatctcgttcctccacgcctcgacgtaaccgcaagctgaggcagcgcttgtggacggcaa
acaagatcagggagttcagtgtgcgggcgcaggaggcgcttcgacggcatgccatatatcaactgggttccaatactgccagcacttccggagcaaga
cgggatgatgcatcctctagctcccggcttccggcgatgcttttgcacgacgacgtacttgttggtattgacgcctcgatggacaagctcgaggaatggct
catgacgatgcatgattatgaagagcatcagaagcttagggtggtgtccgttgttggacccggaggagttggtaagacagccctagccaacgagctgta
ccgcaagattggccactgcttcgactgccgggcttttgtgcggacttcccagaagcctgatatgaggaaggtcacatctgcagtataa
>g102295.t1
atggaggccccgaagacagagcactttgctgcagttaagaggattctgtgctacctcaccggcactcattctcacgggctctgctacaagcaaggaaga
atgaagaacgccaagcttgtcggttactctgacagtaacatggctggcgatattgacacatgcaagagcaccagtggcagcatcttcttcctcggtggct
gcccaatcagttggggatacatggcaccagaattctatgataaagttatcacagcgaagtcggacatatatagtcttggtgtaatattcacagagatact
gacaggacagaagggatattccacgcaaaacaatgtaacggtctcagctgttattgtgaagattgatgcagttctagcagatgaagctgccaaagcag
tggtagaaaaacatcatggagaagctgtggaactgcaaaacaagatacggaacataagggagagactgacgttcatggctaagatgtccaaggtctg
catgaacagtgatgcttggtgtgaggaagtgcggaatctggcctaccgtgttgaggatatgatagacaaatacttttattactcatttaaaatgaaggat
gaatcgtttcttaaaacgatcatcactgaatcgcacaatgcccatgtttttagtgaaatagcagatgagttggtcaaggtagacatggagattgaaaata
ttaaacaaattaatgatatgaagtcgccgtcatccctggatgttcttgatgatattattgagaaggaaagacagaggtcccaagtcagcttccaagaattt
cttaaacatgaagatcttgtgggcattgaggacaatagaagaatgctcaccgaatggctgtactttgacgagctaagcactgttataacggtgttagcgg

tctcagctgttattgtgaagattgatgcagttctagcagatgaagctgccaaagcagtggtagaaaaacatcatggagaagctgtggaactgcaaaaca
agatacggaacataagggagagactgacgttcatggctaagatgtccaaggtctgcatgaacagtgatgcttggtgtgaggaagtgcggaatctggcc
taccgtgttgaggatatgatagacaaatacttttattactcatttaaaatgaaggatgaatcgtttcttaaaacgatcatcactgaatcgcacaatgcccat
gtttttagtgaaatagcagatgagttggtcaaggtagacatggagattgaaaatattaaacaaattaatgatatgaagtcgccgtcatccctggatgttct
tgatgatattattgagaaggaaagacagaggtcccaagtcagcttccaagaatttcttaaacatgaagatcttgtgggcattgaggacaatagaagaat
gctcaccgaatggctgtactttgacgagctaagcactgttataacggtgttaggtatgggcggattgggaaaaaccgtcctggttgctgatgtttataagc
atgaaatgatcaattttccttctcatacgcttgtcttggagtatatggatgatgtggacaacttggttcttcgatatggcgcgcttccaatgattgaagccct
gtacatcatgtcactaacgaagctggataaggtccctcatgggttggaatcccttgcccacttgaagaagctttggctactgaatctgcacacgaatttca
gtgctcagtggcacaagaatggaatgcacaataagatgcagcatgttacggagattcgcatctag
>g105632.t1
atggatcttgtgaccgggacaattagcaatctcattcccaagcttggggagctgcttaaagaggaatacaatctgcagaagggcgtgaggaagcgagt
cgagtatctgcaaatagagcttgagagcatgagcaccgtcctccgcaaggtggcccagctgatgccggaccagcttgatgagcaggtcagagcatggg
cccatgaagtcagaatggtgtcgtacgacatggaagacatcctcgacaccttcctagttcacgtggagggccaggagcctgctgcatcggataggttca
aacgtctcctgaagaagatggccaaactgttcaacaagacaaaggcacaccataagattgcaggtgccatcaaagatatcaagaagcaactccagga
ggtggctaaccggcgtggtaggtacatgcttggtgatattatggctaaatctactgtaacaattgttgatcctcgtctatcggctctgtataatgatgtgac
aaagcttgttggcatcgacgaggcaagtgatgagctcatatccatgctcttgtgccaaggggatgaagggctatacagggacaaggtgaagaaagtct
ctattcttggacctggtggattgggcaagaccactctggtcaaaacagtgtatgagaaacttaaaatgggtttttgctgtggagcctttgttcctgtcagcc
ggaatccagactttaagaaagttttcaaggatattctcattgatcttgacaaacaacattacacaacatgttttaatatgatgatactagacgaaagacag
ctcattgatgagctacgaggattcctcaagaacaaaaggtacttcattgttattgatgacatatgggagatacaatcttgggaaacaatcaaattggctct
gattgataacaatcgtggaagcagaatagtaacaactacttctgggaggcagaaggctttgtcctccctcgacatctccgacgtctgctactag
>g105638.t1
atggcggagacagcgatcacgacggtgcttgcgaaggtggctgagctggtggcgtgggaagctgcggtgctgctggaggtgggcgacgacgtgcgtc
tcctccgcgacaagctggagtggctgcacaccttcatccgcgacgcggaccggcggcggcggctccgcgacgacgagttcgtggcggtgtgggtgcgg
cagacccgcgacgtggccttcgaggccgaggacgcgctcgacgacttcctccaccgcgccggccgccggcggcgctcgtccgggcccgggtgcggcg
gcctcctctggtggcgcgggtgcgccggccaggtcgccctgcaccacgacctgtacggccgcatccgacggatctacaagcggctcgacgagatctca
accaaccgcgcctcgttcaacatcgagcacacgcccgccgcgcccgcgtgggtcgcgtcatcgtcctccgccaccacactcgccgcatgggacgatctg
gaggagtacacggtgggtttggagaattacagcgacatcctgaaagggcagctgctggacgattccgccgtcccctcgcgcgcggtggtgtccatctcc
ggcgagagcagcatcgggaagacgacgctggcgcggaaggtgtacgggagccccgaggtgcgcaaccactttgcgatccgcacgtggacggtgctc
ccgcacaagagccgtcccgccgacgtgctcggcgacatccacgggcaggccacctcccagctgcggcggtgttccctgtcgtcgtctcagcatcagca
gcagcagtgcgcggcgggggggaggaatcggaggtgacgtgagcaaccagctgtacaacaagctgacgggtcggcggtacctggttgtgatcgacgg
cagcgtgggcgtggcggagtggaacagcctgcgcgcgtcgctccccgacgagggcaacggcagccgcgtggtcctgatcacggactcggcgggcctg
gaggtgttgggcccgacccccccacgagcccgtggtgctggcccctctgagcccggaggacacgtacgggctgttccggcgtcgcgtgttcggcccgcg
cggcggcgactgcccacgccagcaccggtcgcgctactaccaggacgtgttccgcatcacgcgcggcctcccgctctccgtggtcgtcctcgccggcgt
gctccggtccaagtcgctccccgccgagtgggaccaggtgatggcccagctgggccccgcacgcgccgagccgccgcaccggagctgccagcgcatc
atggcgctggcgttcgaccacctcccgcaccacctcaagtcgtgcttcctgtacctggctgcgatgcgtgagggcgcgcacgtggacgcggcgcggctg
gtgcggctgtgggtggcggaagggttcgtgcgccccaggcgcgggaccaccatggaggaggtgggccaggggtacctcaaggagctcatctcccggt
gcatggtgcagctggtccaccgcgacgagttcggcgccgtgcgcaccgtggtggtgcacgaccgcctccacgccttcgcgcaggaggaggcccagga
ggcgtgcttcgtggagagccacgacagcaccgacgtgctggcccccgccaccgtgcgccgcctcgccgtgctcaccgacacgtacgtccagctcggcg
acgcgctgcccaagctgcgctccatcgtctgcgacttcgtggagggccgtcgtcgcgtcgtccagtgcagcgacgcccatgacctgggattcctccacg
cgtccaggttcctccgcgtgatcgacatccaggggctggagctcaacaagctccccaacgagatcggctccatgatccacatcaggtacctgggcctcc
agtgcagcggcaccatggagaagctcccgaccaccatcgccaacctcgtcaacctgcagtcgctcatcctcagacgcggcaacggcggcggcctcctg
gaggtcacctccgcgttctggaggatccccacgctgcgccacgtcgtcgcgcccttcgcgctgccctgcgccgccgccatgggcgacctgcacggcctg
cagacgctccacggcgtgcagccacgtggctggcccaacagcacatgtaacagcaatccattaggcaataataaggccgccaacctccggtcactgga
gctgagcgggctgacggcggcccacgccggcccgctagaggccgcgctggagagcctggacctgctcgtgcacctggtgctcaacggcgagtcgctg
ccgtcgagcgtgttcaccatcccgagcccgcgccggctgcagagcctcaagctgcagggcccagtggacgagccggactcggcggcggaggacgtgc
gctacatccggcccaacctggccaagctgtccatgtggaacaccatggtgggccacaagttcgtcgccatgctggcggacctgcccaacctggccgag
ctcatgctcatgtgcggcgcctacgacggcccacgcctcgcgttcggcgggttcccgagcctgcaggagctcacgctcggattgcccgacctggaggag
tggacggtcagcgacgccgccatgccggggctcgcgtggctcgggctatacagctgcgccaagatgcggatgctcccggaggcgctcgccgggatga
aggacctcgaggagctggtgctctacagaatgcccgacatggtcaccaggatcaaggagggaggaggacaggatcaccacaaggtcaagcacgtgc
ccgtcatccagaccatctggtag
>g105810.t1
atggccgaccgcgacgggaagcttgatggcacggtggctgggtgggcgtcggtcagcgactattgccgacacgtcatccccaagctggccctgctgct
gaaggaagagtacgacctgcagacgggcgtgaggaagaagatcacgcgtctgtcaagggatcttgagagcatgcacgcggtcctccgcaaagtggc
cgatgtaccgccagagcagctcgacgatgaagtcaagctctgggcgcgcgacgtccgggagctctcctacgacatagaggacatcctcgacaccttcc
tggtgcgagttgatgcccacggggctcgcgacccaaataggttcaagcgtgcagccaagaagataagcaagatgttcagcaagagcaaggctcgcca
tcagattggtggcatgatcaaaatcatcaacgagcaagccgatgtggtggccgagcggcatcgtcgatacagagccgaggatattgtggccaagcctg
ccacaacatcgactgttgaccctcgcctggaggttatgtgcaaacaagtgacacagctcgttggcattgaaaagccaagcggcgacctcatatccatgt
tatctgtttcttgtccccaaggaaacgacgggtcaaaaatgaagatggtttctgttgtcggaactggtggattgggcaagacaactcttgcaaaaacagt
atatgacaagcttaaagtggattttcagtgtatggcttttgttccagttggccgggaacctgatttgaagaaagtctttatagacatccttatagagcttaa

caaggacaaatacatggatcccaaatatagattattggatccacatcatctcattaaggagctcagagaattcctccaagacaagagcgtcatccccaa
gctggccctgctgctgaaggaagagtacgacctgcagacgggcgtgaggaagaagatcacgcgtctgtcaagggatcttgagagcatgcacgcggtc
ctccgcaaagtggccgatgtaccgccagagcagctcgacgatgaagtcaagctctgggcgcgcgacgtccgggagctctcctacgacatagaggaca
tcctcgacaccttcctggtgcgagttgatgcccacggggctcgcgacccaaataggttcaagcgtgcagccaagaagataagcaagatgttcagcaag
agcaaggctcgccatcagattggtggcatgatcaaaatcatcaacgagcaagccgatgtggtggccgagcggcatcgtcgatacagagccgaggata
ttgtggccaagcctgccacaacatcgactgttgaccctcgcctggaggttatgtgcaaacaagtgacacagctcgttggcattgaaaagccaagcggcg
acctcatatccatgttatctgtttcttgtccccaaggaaacgacgggtcaaaaatgaagatggtttctgttgtcggaactggtggattgggcaagacaact
cttgcaaaaacagtatatgacaagcttaaagtggattttcagtgtatggcttttgttccagttggccgggaacctgatttgaagaaagtctttatagacatc
cttatagagcttaacaaggacaaatacatggatcccaaatatagattattggatccacatcatctcattaaggagctcagagaattcctccaagacaaga
gcgtcatccccaagctggccctgctgctgaaggaagagtacgacctgcagacgggcgtgaggaagaagatcacgcgtctgtcaagggatcttgagag
catgcacgcggtcctccgcaaagtggccgatgtaccgccagagcagctcgacgatgaagtcaagctctgggcgcgcgacgtccgggagctctcctacg
acatagaggacatcctcgacaccttcctggtgcgagttgatgcccacggggctcgcgacccaaataggttcaagcgtgcagccaagaagataagcaag
atgttcagcaagagcaaggctcgccatcagattggtggcatgatcaaaatcatcaacgagcaagccgatgtggtggccgagcggcatcgtcgatacag
agccgaggatattgtggccaagcctgccacaacatcgactgttgaccctcgcctggaggttatgtgcaaacaagtgacacagctcgttggcattgaaaa
gccaagcggcgacctcatatccatgttatctgtttcttgtccccaaggaaacgacgggtcaaaaatgaagatggtttctgttgtcggaactggtggattgg
gcaagacaactcttgcaaaaacagtatatgacaagcttaaagtggattttcagtgtatggcttttgttccagttggccgggaacctgatttgaagaaagtc
tttatagacatccttatagagcttaacaaggacaaatacatggatcccaaatatagattattggatccacatcatctcattaaggagctcagagaattcct
ccaagacaagaggtaccttggcctgaatggaacaaaaacttgtaagctcccggaaggagtaggagctctgaagttgctgcagacactggactttgatc
tcgtgtttaacaaagagaaggaagatgcgcctgacaaattgccatctagcttgggtctgctaacagatttgatctgcctacgtgctggactcgcgatggt
gccgaatggggtaatcaagaagctgacgtccctagaggaactacatatgtcaactacttatgacgataagcacaacaaagcaggttactacacctcga
ggcagtttgtggaggacctgggcaaccagtgtgaactcagggtgctcatggcagattttggggtgggggaaggggaagaagagtgcacagaattcga
tttggtgaagtctctaagcaatctaaaaaagatccagcatctaaaactggactgttggatcgaaatggcagatcagtccttgtggaacaccgtggtgctt
ccacaatctctccgatatttggttgcagaagacattcgctttcctgcgctgccatcatggattaattcctcagctcttcccaacctcactaggttgaacctga
aggtaggtaatatggatgagcagggtctgaaaatcctgggtggactgccagagctccgtcacctcagacttgcagtcatgagcaagcgcctgaaactct
tcgactatgtggacggctccttccaggcgctgccacagttcatcaccgaaggcgctggtgaatctgcacgtcaggtcccgaacactgagttcctgcactg
gttcacgcagaaccagatcgtgctcagtgcccttgtctcgtccatgaacgaagacatgctgggacagatgacccagtacactactgctcgcgccatctg
gatggcgctgcacgccatgttctcctcgcagaaccgtgcccagatcatgtag
>g106835.t1
atggctgaccccatcctcacttccttgatccatgaaatagcatctctgcttgccaccagagtagctggacagtgctggagcctcgtggccacgggttctga
tgtccgttggctacaagacgagctccacagcatgcagctcttcctgcacgaaatggagcaatccagcggtgacagcagcatggccagacaagcatgga
tcgaccagatgagagatatcatgctcgattcggaggacgtcgccgatgtcttcgatgccagtcaggtgagtggttgtgacattataggtaacctcggtgc
acggcgtgaagttgggactcggatcaggcgcatctggacccagctgagccatatatcacgccgtcggttagattatgcaattccaaccccaaagcctgc
aaattccattgataataaatgggtccacaacctcttagcatcatctccgctgattcatgacaaagataccgtaggtctggatagaggcctggacgagttg
cttcagcacgtcatggctgggggctcagagttaagcgtcatctccttagttggcatgggtggtgttgggaagaccacccttgcaaagaaggtgtacaatc
acccagatgtgaagaagcacttcaactgtagttcatggtctatgtttcgaacacaatggacctacgagatgtcctacgccagatag
>g107535.t1
atgcccaactctgatgcccggccggcctgtcgattggtcgctaaatacaggccaccaccaccaccaccatatcaaaataaccccaaagcaaagcaatc
aacaagcacacccaccatcgccatggaatttgcgactgccctgcttcccaagctcgccaagctgctgcaggaggagtacaacctgcagacaagcgcaa
ggaaaggcatcgatttcctctacagggagctcgagaccatgcacgcggtcctccgcaaaatcagcgaggtgccaagagagcagctcgacgacctgca
aaggatctgggcccgggatgtcagggagctatcctacgacatggaggacattgtcgacaccttcatggtggacgtcgagggtcctgatccccccagca
aaaggggcgccagaaagatattgaagaagatgatcagaaaggtcgcaaaggccatggctcgccgtgaggtcgcccaggagatcaacgacatcaggg
agcgtgttagggagctggccgagcgacgtgagaggtacaagcttgatgatattgctcctgccaacaaaacatttgttgaccctcgcataagagcactct
acaccaaggtgactgagcttgtgggcattggtgaggcaaaggaacaagtaatcgcaaggttgacaaagcaagatgctgacgagcaggaaagaattgt
agtagtctccattgctggttttggaggaataggcaagacaacacttgccaaagcagtatatgacgagattaaaggacagtttgattgcacggcttttgttt
cggtgtctcgcaatccagacgcaaagaagcttctcaaggacatcctctatcaacttcacagaggaggtgcacatcctggtgcagatttggatgaaataa
ggcacctcattgatcttgttcgtgattttctccagaacaagaggtacctcattgttattgatgatatatgggataaagaaccatgggataagacactggac
ttatcagaaactagagtattagaactgccatcgagtgttattactggtctaggacaattgatgtgcctacgttgctatcgcgtgggcacaaggctgccaga
tgggctgaagaagttgacctcccttgaagtgctagagcaggtggatctgacgcctaaatgcattgcggaagagctgtgccatctaactcaactgagggt
acttgaggtctgggtcacattatctacaaaggtggcggtggctgctgctgctgccgatgatgatgatgatgatgatgatgaggaggaggaggaggagg
aggaggaggaatggacgacgtgtagtgaagctctattggaatccctaggaaaactgaccaaatttgaatgtcttcacattagtgttcgtcctgatggatg
caatctcaatggctcaatggcggagccactatgcaacctacgccggctttgcatccgtaatgctagagtggtgcccacttggattaggcctgcatggctt
ccaagcctatgctacctggagatattggtgaagcacgagcgaagggacgacatccaagtcctgggcaccctgccgtgtcttggtcatctaaaattttgtg
tgctaaaaggagcagtggaagaggagccggcgtga
>g108394.t1
atgcggagccccaccgtatcgccgtacggcgccgccttcctcggcgacgacgtcgaccccgaggccatcaccgacgacgacgactgccgccgggagg
acagcctcgttgccgactccagctccgtggtacccactctcctcctctcaacatttcaccacaacgtcttcacatcaaagctatccactccacccatggctc
gaattgacccaatatgggctggattcctttcaaggggcaagcagcagggggccatctccatagtgtccatggcgatggtgctcgaatccttcgtgaagc
gatgcacggcgtcactggaggacttcgccggccaggaagcctgcgccgccctcggcatcggggacgacgtcagggccctccttgccactctctggcgc
atcgaggccatcgtctcccacgaggagcgcaggaggctgctgagcaccaaagtcgacgcctgggtggcgcagctcaaggattccatgtacgaggttg

acgacgttctcgatgtgtgcatgattgagggcggcaagatcctcgcagacgaccaccccctacgcccaaggcccggtgctccttggtgttctcctgctt
caagcacacaggaccccgcaagttccgccatgaaatcgggttcaaggtcagggacattgatctaaggctgagggaggttgaggaggaaatgcccagg
cttcctgctggatcagtacactcggattcgaccaggaattggttcagtgataacatatgtaagcactgccattccgggattaagcctcaggcagctgggg
cgcaagtccggaaggctgtgggtggccttgtgccaaggttgcttagagagggcaagaggaaggtggccgtgtttgccattgttggcgcgatggggattg
gcaagacgacacttgctagggagatttacgacgatgacaggatgaccgaaaatttccccatatgtgtgtgggtgaagatgtccaaggatttatcagagg
tagctttttgaagaagataatcaggggtgccggtgccaacgtcggggacacagagaacaaggaggaactccttggcctcctcagtcattgcgggtttg
ctgaggtcacgaggcaagagcaaggctga
>g109274.t1
atgaaaattgatgaaaagcttgacaagaccaccatgagtagtgatactctagctcggccacagaaaggtggccagctgaaacaaaaggacatggacc
aactggagctagacgtaatatttcagctgctgcacaaattaccagagtgggacgacaaaagcatcgataaaatagccataaagtttaaggagcatatg
gaagcagacaaaaagggcaacaggcttaaagagcaaatggggagaggaggaggagaaacaacaaccaagcatatgaaggaaaaagaagacggt
gatgaaacaaccaaccatgtggaggaggcacaagaagaagccaaccatatggaggagggcaaggaggaggaggaggaggaggaggaagtcacc
aaacaacatagggcaccgattcgtctcaatgtaagccagtatgcacacatcctgcagcaagtgttcctgacctcccgcagcgaaaacctgcaagacca
gcagcaagatgtatcgtcaaccaagcatgctataaggaccacaacaactacgttgggtgaggatcaaataaaaaaaatgatcgaccacgcaaaacaa
gagatcttgtgggagctgcaggaaggcatatatgaacagagtcaagcgaggggtaaacctgatgtcctacgtccaaacacagtagctatttttgaaga
aaccgagaagaattctttcgaaaataaagaaattgagaagaagatgcacaaaatgaaggagttaaaagagcagctggagatcaaatggttcgtggac
aagattaaacatcacttgaagaatgactgtcccctgattatcctcagaattgataagatgatgggtgtatccacatggaatgatatcagatatgctttggg
cctgttacaatgcggtgctgatttactgattgtcaccaccacaaaggacacccacctggctaaagagtattgctatccatcaccgggggaacctatagac
tattctcttgctggcctctactatgatacagtgctcaagcttactaaccagaatgaagacaactacgacaccaagatttttcacgacatcttgaaggagtg
tgagccacatgaattctgcatgaagatcttcactcatgctttatatgctaaccccaaaaggagcaatgaagagttacacaagctgcacagaaacctgca
ggatttgccaaaatcattcaacgtcagtgctaagaagatgttcatgttctcttacaatgatctccctaaggagtacaaatcatgcttgttgtacctagctat
ctttcctctgggacacaagatccggcggtcaaccttgattgcacggtgggttactgaagggctgacattaaaggaagattggcccagttctgtcagtcag
gccaaccgatgtttcgatgcactggtctaccggtgcctagtttatcctgctcatattggtgtcacagggaaggtccagagctgcgtggtagataaggaag
gtccagagctgcgtggtagataa
>g110319.t1
atggaggcgacggcactcagtgtgggcaagtccgtgctagatggagcactaggatacgccaaatctgccctcgcggaggaggtggccttgcagcttgg
catccagcgtgaccatgctttcatcagggatgagctccagatgatgcaggctttcttgatggctgcacacgatgaccaagacaagcacaaggtcctcat
ggcctgggtgaagcaagttcgagacgtggcctatgatgcggaggactgcctccaagatttctccatctacctcaaggagccatcttggtggcgtctcccc
cacacgctgcgagagcgacgccgcatcgccaagcagctgaaggagctaagagctagagtagaggatgttagccagagaaacctacgctaccaactc
actaagagctccagatttaatactgcaactgttgctgagatgtctagcattacaactgcaacggtatttggtgttggtgaagcacgacgtgctgcgagac
aagaggaaccaaaagaggatcttgtccgcctgatcaacaaggagggagaggatcttagagtgatcgccttgtggggaacaaatggtgatcttggcctg
acaaccatcatcaatgcggcctatgagaattcagatatcaaaaagaaattttcgttccgagcatggataagaatcttgcacccgttcaatccaagtgact
tcatccagagtttggtgaagcaatttcgattatctgaggggggttgacattctattggagaaggacaagacagggcatgatttggctatggagttcacagg
atacgtaaaggagaagcgttatctcattgtgcttaatgacctatccacctttgaagagtggaacgtgatcaaaacatgttttcccagcagtaagaaaggg
agctga
>g110881.t1
atgctgccatccaagctgctggagctgctcaagaaggagtaccagctgcagaagggcgtcagggaagatgtcaagttcctctcacgggagctggaga
gcatgtacaccgccctccgcaaggtggccatggtgccgtgggagcagctcgatgatcaggtcaaggtctgggcacgtgaggtcagggaggcgtcctat
gaaatggaggatgtgcttgacaccttcatcatccgtgctggcagcggccctgagcccgcgcatatggtccaacgactcatgaagaagatgggcaagct
gttcgacttgagcaaggtcatggctcgacgcgatatcgcagctgctatcgagggcatcaagctgcaacttcaagagatgtctgacaggcgtgacaggta
caaggttgatggtattgtggtccatccttctgccgcagcaaatgtcgatcctcgcctctcgcatctctacataaaagaatcacagcttgttggcatcgatga
gcccagggaggagctcataaagatgctgtccatacagccggactcccacatatccgatggcaaggagatgaaaattgtctccgttgtcgggctcggag
gattgggcaagaccacacttgccaaggcagtgtatgacaaagtcaatctgcaatttgattgcagggcttttattccagtgggccagaatcctgacccaag
gaaagtactgagggacattctcattgatatcgaaaataacaacaagacaaatattatctttgacattgagaagacgaggtctgctcttgttgatctgatgc
aattagatgaaaggcagctcatcaacgaactccgtgaacatttgaaaggaaaaagagaaaactttgttgttgtattgaatggaccagagatgtaccatg
gtgtcggcaacaagtgctctacacggcagtttgtgaaggagttaggcaagctgacaaagctaagggtgctcatgaccgggatgactctacaggacgaa
gagcagggaagagagtttttggagtctctgaggaaactgcacaagatacagtctataaatatttcattaccgaatggattcttaccgatggatgtgaata
agatggaacctaagtttgtcctctcttcaaatctccgtgtcctggatttatttaatctgcagttttcaaagctgccggcatggattaatgcacaaaatcttcc
caacctctgccatttgacggtggtggtgtttgacgaggacgagcaggatctcgcagttctcgggaagtttataaggctccgtaagctcgcacttgcagtt
ataagagtccatagattaaaacttgcagcttgtggatctgtcggcttccagaatttgacatcatttgaagtcactgtaccgttaaaatttgcacatggagct
atgccaagtcttgaatacatcaatttcagcatcccggtggcggaattgaaggatgttggcatcaatcttgagtttggcttagagaacctttcatctcttcga
tctgtacacgtcaacatcgattgtgctgatgcttgcgaaatggaggtgaatgatgcagaggctgtgctcaagaatgcaatggatatccaccccaaccatc
cagcatgtgaagttaaaaagaccggagaaaataaaatgggccccgcggatgcactctcaaggatggaaaatcggcaatgtcataagattgtgaaggc
ttccacactcagtgccgacatcaatgttcaacaagagctccataacgcattgtataatgaggcagcggatgtggcacgttccatgccgaaggagcaccc
ctgcgttgcaaagttagcactgctccccaagaacctcgccggcgacccagtcggaagtctagaccgtgcatgtgagctgttggcgatggatcaactgat
tgaaaaaatggagacccaggaacgactactcgaggaatacaacgttgaaattccgcgaatggctacaattctggaggaactgaaaactgcgctcagc
ctgcaggtggaggatgatggagtgcgcaagatctgtgatctgttgccgagggtctcaccagccccagcggccaaccgtaaagactga
>g110882.t1

atgagcaatatggatctcatgaaaggggcggtgggcagcctcataggcaagctcgggaagcttctcgaggaggagtataagctgcagaatgatggcg
tgtcaaagaaaatcgggtctctcagaaaagagctcgagtgtgctcacgccaccctccgcatggtggcagaggtgccgccggatcagctcgacgagcag
gtcatgatctgggctcgcgagatcagggaggcatcgtacgacatggaggacgtcctagacgctttcctggtcagtgcccagtatgaccatgagtacaag
gaccacgaaggattgtttgaacgcctcaagaagatgatgattggtgtctatgacatggtggtgagtggcccctttcaaacgtcgtaagattgatgtcacca
tcgatgacatcaacaagagactcgaagagttgacaaagcggcatcaaaggtacacagtcaacagtatcgtgtttaaacctttaccaaagtcgactgttg
acccccgtctagcggctatgtacacagaagtgacacaacttgttggcattgaccagtcgagcactgagcttatacgcattctatggcctctaggggacaa
caacgaaaccaaaaagaagatgaagatggtgtctgttgttggagttggaggactaggcaagaccactcttgttaaagtagtgtatgacaagcttaaagt
ggattttaaatgtcgagctttcgtacctgttggtcggaatcctgacttgaagaaagtcttaagagacattctcatagggctgaacaagaagaagtacatg
aattcagatatcacaatattggacgaaaggcagctcattaatgaggtccgagaatacctcgaaagcaagagaccacgattctacttcgtgccatatgca
aacaattga
>g110883.t1
atggatcttgtggcagggccgctaggcagcgtcatcatcaaactcggcaagcttctgaaggaggagtacgacctgcagacaggagtgaggaagaaga
tcatgcgcctctcacgtgatcttgagagcatgcacgcggcgctccgcaaggttgccgatgtgccgccggagcagctcgacgatgaagtcaagttttggg
cgtgcgatatccgggagctttcctacgacatagaggacatcctcgacaccttcttcctggtgcgcatcgagggccgcaaccccaacaatttgaagcgtgc
caccaagaagatcggtaagctgttcagcaagagcaaggctcaccatcagattggtggcatgatcaaaaccattaatcaacaagcagaggtggtggcg
gagcgccatcacaggtacagggccgaggatattgtgtccaagccggccccaccgcggactattgaccctcgccttaaggctatgtacacagatgtgac
acagcttgttggcattgaaaagccaagcgatgagctcatatccatgctatggcctcttgggggcgatggggtgaagatggtttccattgtcggagctgga
gggttggggaagaccactcttgcaaaaacagtatatgacaagattaaggtgaattttgactgtacagcttttgttccagttgggcgggatcctgacctca
agaaagtcttcagagatattcttatagggcttgacaaggacaaatacatggatcctagattcacaatactggatgaaagataa
>g110925.t1
atggacgttattttgggggcactgccgaacctcctacccaagcttggagagctgctcgtcggagagtataaactgcaaaaggggatgaagggggagat
caggttcatccaagccgagctccagagtatgaagggtgccctcgagaaaatctcaagtacaccaagagaccaacttgataaccaggacaagatctgg
gccatggatgtgagagagctgtcctacgacatagaagacagtgttgacgcattcatggtgcacggaaaaggaagtgaactgactgggccacgtggctt
taaagttttcattgatagatgctatgatttattgacacagtttcaagttcgtcgtagcattgcgacagaaatcagagatatcaagaggcgcgtcatagagg
tgagtgagcggcgtgacaggtacaagatcgacactgcgactgcaaagcctgtcataattgatcctcgtgtgttcgttcagtataagaatgttacagagct
tgttggcattgacgaggcaagagatgaggttatcaagattctgatggaaggcaatgaagtgtacaggaaggaagacaagataatttcaattgttggctt
tggaggcctaggaaagacaacccttgctaatatggtgtatggaaagcttacggcacagtttgattgcacagcttttgtttcagtgtcacagactcctggca
tggataaaatattcaaagacatgctctatcaactttctgatagatgtattgcaggcgtcaatgttatcaacgaacttaaagaattcctcaagcagaagag
gtatgtactctacctagaatactag
>g112712.t1
atgaagggagtcatggtgagcgttgctacaggggcgatgaattctgtactcgagaagctcaccacgctattaggtaaggaattcagcttgttgcagagt
ggggtgaagcacgacaagctgagctgcatgaacgcccttttggaaaacatggccgacatggaggtgatcgacccacagatgaaggattggagaaatc
aagtgagggagatggcatacgacatagaggattgcattgacgtctacgtgctccagcagagacacagtggaatcttgggattcgttcgtgactatgtcc
agaaagtcatggaacttgtaggtcaccatggagttgctcggcagataagagagctcaaggatcgcattgttgaggcaagtcatagaagaaagaggtat
aagctcgaccctgaagttgaaactagaaccacatcaattgatcctcgattacctgcactctatgttgagtcatcggatcttgttggtattgatgttccacgg
gagaatcttattaactttctagatgatggagaactttcactgaaggtgatttcaattgtgggatttggaggattaggggagacaaccctagcaaaggaag
cttataagaaactctctgtcaaatttgattgccatgctctggtgtcagtgtctcagaaacctgatgtgaggaagattctatggagcatactatgtcaattga
agaaccagaattatgccaacaccaacccaggggatgaggagtatctcatagatgtgctatgggatctcctcaaggacaagagcacacttgagttatatt
ag
>g112988.t1
atggaaggcaatgaagtgtacaggaaggaagacaagataatttcaattgttggctttggaggcctaggaaagacaacccttgctaatatggtgtatgg
aaagcttacggcacagtttgattgcacagcttttgtttcagtgtcacagactcctgacatggataaaatattcaaagacatgctctatcaactttctgatag
anttggcattgacgaggcaagagatgaggttatcaagattctgatggaaggcaatgaagtgtacaggaaggaagacaagataatttcaattgttggct
ttggaggcctaggaaagacaacccttgctaatatggtgtatggaaagcttacggcacagtttgattgcacagcttttgtttcagtgtcacagactcctggc
atggataaaatattcaaagacatgctctatcaactttctgatagatgtattgcaggcgtcaatgttatcaacgaacttaaagaattcctcaagcagaaga
ggtatgtactctacctagaatactag
>g113074.t1
atgggcagcctcgtccccaagctagctgaactcctgaacaaggagtacaagctgcacaagggagtgagaaaggcagtcagttctctctcccgggagct
ggacagcgcgcacgccgccctccgcaaggtggccgaggtgccgccggagcagttgaatgagcaagttaggaattgggcccgcgaagtcagggaggc
gtcgtatgacatggaggatgtcctcgacaccttcctcgtgcgcgtcgaggagggccctgagcacacctaccctgacggggtgaaacgcctcttggagaa
gataggcagctggagcagcatttttaggaaggtaaaaatccgccatgagattgctaaggcgatcggcgacatcaataagcaactagaggaggtggcc
gagcgccgtggcagatacagggttgatgatattattgctgcccctgtcataacatcgattattgatcctcgcctttcgactctatatgaagatgccacaaa
acttgttggcatcgacaaggcaagggacgagctcatatcgatgctgtcatcctcggcgcagcatggtgcctccaacaagaagatgatgatagtttctatt
gttggacttgctgggttgggcaagaccactcttgcgaaaacaatatacgacaatattcaagtggattttgattgttgggcttttattcccgtaggccaaaat
gctcacccggagaaagtttttagggacatgctcatcgatcttgacaagacacgctgcacgatgaacccaaattttttaatatttgatataaggcagctcat
tgacgaactccggttagtactggcaaacaagagcgaggccaagggcaggttccagcagatacacgaggcctaccaggtgctgtcgaacgacaagag
gagagcgctctacgacgccgggatgtacgacccgctcaacgacgaccaggaggaggtcaacggcttccacgacttcctccaggagatggtctcgctca
tggccaccgtcggaagagagtgggagaagtatagggatgcacgtgtcgtggatggccctgtgcctcccccgcttcctgtgctgcaatgcaagtgtggtg
aaccagcacaagttcagcagtccagacactatttgatggtgggtcgagcatactacatgtgtaaagagaagcctctttctgatgtgtccagttgtcatttt

tccaatggattgacggccctgacaagtatgacccacgcatcatgttgttcccgtatgacaacaaagcgtcatcgccgtacgctgagtttgagcgttgggt
acccctgccgccggatcctgaaccaatggaagaagatgagatgttagcggatggatggcgtttgtgtgactttcaagaatatgtttatggtccgaagcca
ctttgggccgaggccgggttggtgtggtaccttctgaacttggatatgggtacttttacgggcacaagaaggtgtgattgggaatcttttcatggtgacat
catgattataaacaacactgagagaatgctatcggaggagaaagcaaaatatctgcagaagaaaagagatgaagttcgtcgggaatatggtgttcca
agtccacccagttggattgttacaaccatcaagcacgagatgaacactgactccgagggagctttgatgtattggaggaggaacaaggacaagtttcct
gatgtagcgcagtggaaaacattaacatcatatatgaaagaggagggcattagttgttatggcatgtggaaacatagtacagacttgatgcggtgggcc
agaagtacattggatgatcttcatgatcccgtggtgcaggagcgatggaggatggaccaccagttgaagagggaggaggaggtaagacaagagaat
gcaacacgggaggccgcacgacaagagaggagcgatggaggatggaccaccagttga
>g113448.t1
atggcggaggccatagccatatccgtatctgcaaaacttgccatggcgctatcccacagcgctgctgtgagcctgtcaaacctcttcggcgtcggctctg
acatcaccgcaactgcacaggaacttgaagtcatgcgcgccttcctcaggtccgccgattcccagcaaggtatggacgccctcgcctcctcatggatca
agcatgttcgcgacgccgcctttgaactcgaggacgtcgcggacgagtgctcctacctctccggaaacggccgtagctgggtcaaagtcaggtcctggc
tcgcgctatcccgcaggctgcgcagggcacgccagaggcttcgccagctttccgcagcgaaggagctctacggcatccaccccgctgattgtggtcctg
cactccgaatcgcaatcgtcaacagccggatgcttgccgacactgcgcatttcattagaaaggaggaggtcgtcgggttcgatgatcacgagaagaag
ctactagagtggattgccgaggatgccgagccacagcgaaaactgatagctgtctgcggcatgggcggcatcggcaagaccaccccttgtgacgcgcgt
gtacaaggaggtagcaacgagccacttcgagtacgccgcgtgggtggccgtttcgcaaggcttcacgacggacgacctcctcggaaagatcctcaggg
agcttcgccgccactctggtggtagaagcttaaacgccgagatcgacaccaatgcggactaccggtcgctcatggcggcggtgcagggtcatctttgca
agaggaagtacctagtcgtgcttgacgatgtctgggacgcccacctttggggcaacctcctccacctagcgttcccggacgatgcagccggaagccga
gtggtcatcaccacgcgcagcaatcaagtggcaaaagctgcgacgctcgaccggaccatgatgctggagcctctacagtggcgtgaagcatggactct
gttctgcaacgtcaccttcagggatgtgcccaaccgaacttgccccagttacctggaggggattgctaccaatatgctgaagagatgccaaggcttgcc
gatggcgattgtctccatcagcaaactcctagcgctgagggagagaacagagttcgcctggaggaacgcctgtaacaacctcgtgtgggataagagca
gcgacgatcttggaataggggaggccgcaagtattctgaacctgagcatcgatgacctgccacaccacctcaagaaatgcttcttgagctgtagcgtat
accccgaggacttgtggatcaaaaggaagatattgatcaggagatgggttgcactggacttcgtagaagataagccaggccactgtaccgcagagga
cgtcgcagatgaatacctagaccagttgattcaacgcaatctgatgcatccagttgccaagaacgagtttggtcgtgccaaacgttgtctcattcatgatc
ttatcagggagctgattatccacaggtccagagaagaggaacggctaaatggcaagggcaaaaagagcgagaagtacaacaacaaaaataagccc
aacaagaagccatacaacaaggggcaagctcatattggtcaagagtacatctctgatgatgaggatgatgttcccctgtgcttcatggccaagaaaag
caagggagcaacctcgaattag
>g114459.t1
atggatgctgtgacgggggcgatggccaacctcgcccccgagctactggagctgctccaggaagaatacaggctgcagacgggcttgagggagcaga
tcgagtccgtgtcaaaggagctggagagcatccacgcggccctcggcaaggtggccgacgtgccgtcggatcagcttgatgagcagtccaagatctgg
gcgcgcgacgtcagggaggcctccttcgacatggaggacgtcctcgactccttcctcttgcgcgccgggggacgagcccgtgatcctccggctgatacg
ggcaggctccgccgagccttaaagaagatggcccatctgttcagtaaggccaaggcacgccgtgacattgccggcaccattggaaacatcaagaagc
aactcaaggaggcgtccgagcggcgtattaggtacagaatcgacgacactgtggcaaagcctgctgcttcgtcctcgactgttgatcctcgcctctctgc
tctgtacacacaagcgtcacggctggttggcgtggatgagccaagagacgcgctgatcaagatgctgtccctaggggacactgatgttgcagacaaga
aactaaaggtagtttctgttgtcggagctggaggatttggcaaaaccactctagtgaaggcagtgtatgacaagcttaaaccgcaattcgattgcgggg
ctttcgttccggtaggccggaacccagacttgaagaaggtcttgcgggatgttctgattggtcttgacaaggaaaggtttacaaaattcgatctgttgata
ttagatgagaggcagctcattgatgaactccgggagttcctacttagcaagtggtacgcttcactatcccagcactag
>g115756.t1
atggctgtggtcctggatgcttatgcatgctatgtcgctaacctgcttatggagatggcgagagaagaggtgggagtgcttttgggtgtctccagtgagat
tgatagcatgggcatcaagcttggagacctcaaaaacttgcttgccgatgccgacagaaaaagcatcaccgactatagggtgcaggggtgggtgagg
gagctcaaggatgctatgttcgacgccactgacatcctggacctgtgccagctcagggccatggagtgtggcccaaccaccatgagcacagccagctg
ctgcaaatcagtgctcttctgcctgcagaatccccccttcgcccattatatcggcggtcgcattagggtactcaaccgaaagctagatgacatcagggag
aggagtgctcagttcagattcgtcaaccttgtctcctatgaggaccataacagtaaggtgacctcacactcagctgctaaccgtgagacgacaggagag
ccttttcggccgaatgtggtggtcggtgagaagataaaagaagacacaaagggacttgtggaaatgctaacagaaaaatgtggtagcagcagggaca
tcatggttgtggctgttgtgggtgtcggcggcattggcaagaccacccttgcccgggagatcttcaaccacgagggtatcaaagacagttttgacaaga
ggatatggctgagtgtcaatcaaaattttgacaaggccgaactgctcagaactgccatcactcttgctggggggagaccaccatggcgagaaagcaatg
gctgtgctccagccagcccttgctactgccttggcagggaagaagctcttgctggtcatggatgatgtatggtgccacaaggcatgggaggatgtgctcc
aaacacccttagtcaatggtatgcttcaagaaggtagccgagtcatcatcaccacaagggatgaaagagtcgctcgagcaatgaaagcagagcaacc
ctaccatcatgtcgacaaactggggctcgaggatgcctggtcattacttaagaagcagcttctgcaggttgatcacgccccagcgatcacgcatgtggg
ggctgatttccagcgaccctacagccatccatctcacataggtgtagcgtttccgagactgcataggctggagtttattggaatggtggaatgggaggag
tgggagtgggcggagcgagccgatgtgcaagtcatgcctgttttagatctgctcgtgctgaagaggtgcaacttatggtcccttcctactggcctagcctc
tcatgcgaggatgctgaagaagttattcgtatatgaggtccagcaccttagttctctggagaacttggtttccattgttgagcttgaggtgtttgacaaccc
caacctggagaagatcagaaacctccccaaattgcaaaagcttaccattgttaagtgccaaaagatgaaaatactgtatggcatgcctgcaatccaga
agctcgagctggaggattactgcatggataccctcccaacatacttgcagaatatgagatgcaggtggagtctggcaaagcttgcgggtcttaccgatt
atcctgtagaacaagaaggtcggacaagaggagccgacagcgcctggatccagggcagcgctcgatag
>g116145.t1
atggtcctggggccaaagtatgagctgatgtcaggagcgaggaccgacatcctcttcctccggtctgagctcgagagcatgcatgccttcctcgagaag
ctcaccacggtgcagggcccagactcacaggtcaggtgctggatgaaggaggtgagggagctggcctacgacatggaggacagccttgacgagttca
tgcaccgtgtcgaagccgatggcactgccgccacttctctccatggctcaagcagcctcatggggtttgtggggcgcatcaagcagctcttgagaaccg

gttggacacatctccgccttgccaatgagatcaaaggcctaaaagcccgcgccgtcgaggtcagtgagcggcgatcaaggtacaagcttgtggaggac
atatggatgccaggaacccacatgtctgctgatcctaggatcactgtcctgtacgcagatgcgccagatctggttggcattgaacgttccgcaggcgaca
tagaaaagtggttagtggatgaaataagcacgctaaagatgctctcaattgttgggttcagcggtctgggtaagactactcttgccatggaggtgtaccg
cagagttgggggcagattcaactgtcgagctttcgtaaccgtgtcgcaaaagcttgacatgaagaagctcttcaagaacttgctgttgcaggtttctcata
atgaggccgaccatttggacaactgggaggaggttcaacttattcgcaaggtcagggagtgcttactaaataaaaggtaa
>g116146.t1
atggtcctggggccaaagtatgagctgatgtcaggagcgaggaccgacatcctcttcctccggtctgagctcgagagcatgcatgccttcctcgagaag
ctcaccacggtgcagggcccagactcacaggtcaggtgctggatgaaggaggtgagggagctggcctacgacatggaggacagccttgacgagttca
tgcaccgtgtcgaagccgatggcactgccgccacttctctccatggctcaagcagcctcatggggtttgtggggcgcatcaagcagctcttgagaaccg
gttggacacatctccgccttgccaatgagatcaaaggcctaaaagcccgcgccgtcgaggtcagtgagcggcgatcaaggtacaagcttgtggaggac
atatggatgccaggaacccacatgtctgctgatcctaggatcactgtcctgtacgcagatgcgccagatctggttggcattgaacgttccgcaggcgaca
tagaaaagtggttagtggatgaaataagcacgctaaagatgctctcaattgttgggttcagcggtctgggtaagactactcttgccatggaggtgtaccg
cagagttgggggcagattcaactgtcgagctttcgtaaccgtgtcgcaaaagcttgacatgaagaagctcttcaagaacttgctgttgcaggtttctcata
atgaggccgaccatttggacaactgggaggaggttcaacttattcgcaaggtcagggagtgcttactaaataaaaggtaa
>g116574.t1
atggtggtgggtgcttcggccgtggccgtcgccggagtggcgatgagcactgtttgccgaaagctcaccgccctgctgcaagaaaaaaagtgcgaact
gaagggactcaggcgacacatcatcttccttcgggaagagctccagcagatgcaagctttcctgttgattttggagtcggagggcaatcctcatagaga
atattattcgagagaacttcgggagctgtcctacgatattgaggacagcgttgacacgcttatgctcgagcagcaaccatggggcttaaggatgcacgg
acctaggagtttcaccgacaggatcatgggagtttcaaagggggtcatgttccgcgtcctgatggccaagcagcttaaagaattcaagtccaaattgaa
ggaactaagcgagtggcgggataggtacaagatcgacagggctgcaccaggaaagagcagcgtgcttgtcgcagatcctctttccccgatgctcgatg
tggattcgtcgagccttgtcggtatttacggtcagcgggattatctcgccaagctgctgttcatgcgggacaagaacgacgagtatcctgattcagtaac
gtggtatagggtgctccccattgttggacctccaggcctgggaaagactagccttggtaaagaggttgtctgcaaaatccaggcacactttgactgcctg
gcttgggtatcaatgtcaccaaatcgcagtttcaggaacatcatggaggaaatacttcgccaagttgaggagccatgctgcggcagagtccgtgtcaaa
tcagatattatcggaagcatcagagaatcccttcagcataagaggtacccgtttgctgaacttctcatcccgtatgtccagtggcgagtctag
>g116763.t1
atgaagcaggtccgggaggtctcgtacgacattgaagattgcgtggacaacgtcagccactgcctcgacggtgagccgcgcgggagcggcgcgtcga
tgtacatttgncaggtccgggaggtctcgtacgacattgaagattgcgtggacaacgtcagccactgcctcgacggtgagccgcgcgggagcggcgcg
tcgatgtacatttgcaagaagtggtacgtgcttaagacgctgtacgcgcgccaccgcatcgccaccgagatcagcaacctcaagatccgggcgcaaca
cgtcagcgagcggcgttccaggtatggggtggtgaacctgaaaaaggtcaaaggccgcacggatcaagcgaacgctcctagagaccgtctggcacct
cctccacagctcatcggcatcagggcgccggtgggcatcgaggacgccatgggagagctcgagccatggttcaccaaggcgaagacgctgcctcctg
atcataatctgagatttcttgccatcgttgggtttggtggcctcggcaagactacgctcgccatggcactgtaccagaagttcggtcttggtgacgagttca
gttgcagagcgtccgtcctcgcatcgcagaagttccacctccagactgttctgaggaaacttttggagcagttccatgagcagcgggcggatgcctccaa
gaatgacctccaaggaattgaagactggaaagacgagcagctcatacgcaaacttcgcttacagctatctgggaggagcccacagtatttgcactgcg
accgtatggtcttcagcccccatacggcagtattatga
>g117128.t1
atgagtgccgccctggagatggtgtctgagtcagaggaagagaatccccgagtaaaggaatggatgagtcagttgagggagctgtcctatgacgtcga
ggattgcattgaaatcttcacgcactgcctaggcaatgttgacacctgcgatgggttcattcacaagattataaacaaggtcaccactctgaaagcacat
tataacattggtaatcagattaatgagcttaaggaacgtgctctggagattaatgagcttaaggaacgtgctctggaggtcagtgataggcgtaagagg
tataaacttgatccatccgcctcctccccaaggtctcttgttattgatcccaggttacctgcgctctttgaggagacagatagacttgtgggtattgataccc
aaatggacaaacttgtcaagttgcttacagatggcattgattcgtattcacagcgtaaggttgtgtcaattgctggacttggaggattaggcaagacaac
acttgctaatcagatattacacaagtttcttaagagcagcagtccacttacagaagatcaaaatcagaacattgcaaagatgcaggaaaaccattatctc
aaagcatttgaatatctacaactcgtgcatatgaatagagaatatcttcagaatagaagggtttcagaagtggagacagcccgggtgaccattaaaaat
gcagctgctctccttcccaaccgtccaagacatgaaattcatatattcggagacgaggaaatggtaaaggacaaggagcggaggtga
>g117502.t1
atggatatcgtcgtgggtgccgcgctgtcgggcatcatggacgcgctgccgggcaagctcggcgggctgttggagcaggaatacgagctgctctccgg
cgcccgcggcgacgtggagttcctccagatggagctctcctccatgcgcgccgccatccggcactgcgagtccctcgacaaccctgacgcgcagacgg
ggacctggatcggccgcgtccgcgagctcgcctacgacatcgaggactgggttgacctcttcggcatccgcgtcgacggccgtgcccccacctcctccg
ggttcttcgggtggctccgccgcggcgcgaagaagcttgcaacgatgcccgaccgccacgtcatcgccaacgagctcagggacctcaaggagcgcgtc
gtcgagctgagcgagcagcgcaaccgatacaggtgcgacctggccgtatccgcctcgcccagcaccgttgacccgcgcctggtcgcgctcttcgcgga
cccaggcagcctcgtcggcctcgccgagccggtggaggaggtgaccaggatggtcaccacgaataccggcgggaggacggggatcaagattgtcttc
atcatcgggatggctggcgcagggaagacgacgctcgctaacgcggtgtacaagcagctcgaagcgcagaagagcttcgaatgctatgctttcgtttc
cgtgggtcggaagccagaaaatatctgcaaggttctcacggacatgctctggaaacttgggggcgggaagcaccggggaatcgacgacattaatcaa
ctcatcaggcagctcagagaagtgctggagaagaagaggtaa
>g117954.t1
atggaggcgacggcggtgagcgtcggcaaggccgtgctggctggcgcggtggactacgccaagtctaccgtggctggagagattgcactgcagctcg
gcgtcgagcgtgacgtggggttcatcgccgatgagctggagatgatgcagtccttcctgatgacggccgacgaggagcgtgaccagaacaacaaggt
gctcaacacatgggtgaagcaagtccgtaatgtggcgtacaatgtggaagacaacctcttggatttcgaggtccaggcggagcgagagaagcctccttt
gctgggctgcatcccccgcaacccgtgcgaccgccgccgcgtcgcaatggaggtgaaggagctcaaggccaaggtggaggacgttagcagcaggaa
cctccggtaccgtctcatcaaggatgacaccaccaggggcaccaagcctgtcgaccatggcgtccaacaaggcggcatggaggatgaggtgtggcgt

gtcaccatggagcaggagaaaccagcagacgtggatcttggccagctgatactcagcaaggtggttgatcttcaagtggtcgcggtatggggcaggag
tggtgattttgggaagacgtctgtaatctccaaagtcttcgatgacacacgtgtaaaggcggagttcaaatgccgtgcctgggttggactgatgcgccctt
tcgatcccaaggaatttcttcggagcctactgtggcagtttcatgtgaattcttctcaagagccagagattagtatggctcgggaagggaaaactgttgg
gactaatgtcttgctgaagatggacaagcttgggcaaagtagtagtgatttggtccatgagtacgacacacaggtcagtggcaacaagtacctgattgt
gatcgatggcctttcaaagatagacgagtggcattccatcaaatcctacttccctgataacaagaatggcagtagggtcgttgtagctacgcagcaagtt
gaaattgcaagcttgtgcccggagcaaccatgccaagcatcaaaactggagcaattttcatcgaatcaggtcctttacttgttccacaggaaggtaataa
gtaacctccatcagcacgttaatactctatatattgactgggtggcatacaatatgtaa
>g118417.t1
atggccgaccagacgctaaccgccatcgagccgctgctaagcctcctgacaacggtggtgaaggaggaggcccggctcctgcgcggcatccacggcg
acatccagttcatcaaggacgagatggacagcatgatcggcttcctgatgcacctgaccaagacgcagacagagcacaacgaccagcaccgtgcctg
gatgaagcaggtccgagacatttcatacgtcgcgcacgactgcatcgagctctacctgcgcgacctcaccccgcccgaggacggcttctgggcatgcgt
ggcccacctccctacactcatacgcacctgctgcaagcgccgcgacctcgccaagcagctccgcgagctcaaggaccgggtgcgcgaggtcggcgag
aggcggctgcgctacggcatctccgtccccaaaaaggccatgtacaagcccgagcaggacggcaaagatagcaaggccgaggagaagagggagaa
gttcctgcatgccctggaagaagatgccaaggcttctcctccgtccttcgacaaagcaatcagccttcttccgggtgatctcaaatctgaagcagaaaag
gaaataaatgaaatcctacaaatgtgtgtggactttgattgcaagaagatgtttctttgtgctctctttgcttacccccacgtgaccaaagaagaactaaa
gactagtattctggacaagaagaagaagttgacgatggagggcagtgcagtggatgctgtgagaaaggaggtgatgctttttcctacagcaagctgc
ccacacattacaagggctgtcttcagtacctgactgctttcagcaatgaaaaaagcatcagtaggacaagcctggtcaggcgatgggccgccgaaggc
ctcgtggcaagggacgacgggaatgaaacaagggaagaggcaggtgaacgctgcttcgatgagctagtgtttcgaggctttgtccatcctgtaatacct
gctgttcctggcctgaaaatcaagagctgccgtgtggagggattcgtctgggactttgtcttcgccatggccgaaagtgaaaacttagtgggcgcactgc
cgactcaccttcgtcaccagcgttcaatccgagagattgctcaacgggaactactgccacagcaccaggaaagacagagaccgtgtccattgtggaac
atctgtccaagaaagcagcatctgcaggatgacgatgacaacatggctgcggaggataagcgccatcccatggatgacatggcgcatctcctcaaaac
cctacctgaagagtaccggctcaacgtgttggatctaggaggatgtactaccttgaaggaccgccatctcgagggcataatctccaaggtgtcctggctt
aagtacctgagcatccggaacactcaagtttcaaggctgccaaaggaaatcaacaagctccggcgcctggagaccctagacattcggcagaccaaga
tcaaggacgccggcacgaagcacatcgtcctcctcatgttgaagcatctccttgccgggaacatcaacaagtgccacgagaacgagtcactcagcacc
gtgcagatgcctctcaaaattgggacgaccacggaggtactgcgccatgtccagatattgaagggaaaagctgaacttgatatgcttaccaacctgaag
cagctaaggaagctgggtgtggttattgaaggcagtaaatataacatgacatatttgcccagagcaattgaaaaattaagcgaatgcttgcgctctctgt
cggtctggatcaatatgccgacagacaacactactgacagcaacgacagcggcttcattcttggcgatgacaacaatgagaagggggcactaacga
ctcattatctcccaagaatcttcagagtctaaacatcaagtcttattttagggaagacggccccgggggcaagctaccatcatggatcaacgggttaaat
cggctttccaagatcactctacgcgacacacggctgtcggagagcagtatgacaatattcggccagcttgaaagcttgcgctgcctccggctccgtcgtg
gttcatacaatggtactagtatcatcctgcacaagggagagttccagaagctccgcttccttctgattgatcaggtcactgtcaccaccagaatcgaattc
catgaacaagcagctccagagctagagaagatcacctggaccgtagacacgaggatggtggccaaggaggatacactctctgggataaagtaccttg
aaaacttgaaagagctcgagctaaacggccactggatcgacctccaaggccatataaacaaagtgcttgccgatcatccgaagcgtcgccccccttctca
cgtgcttgttcccaatcttcgagcttactatcacggaaatgtga
>g119488.t1
atgcttacagagatcttgaccattgtggatgtcattgagaagcgagagatcaagaatggcaaccagaggaagctgctaggcatacttaaaaatgccatt
tacagcgccgttgatgtgctggacagctttcaatacatggtcctgcactccaaggtcatcagacaatctacagtgactcttgttaccacatcctttgttgag
atgggtaaacgtatgctggggactaccaagttccggagaaagcttactgatattttggagaaactgggtgaggtcaaaacgtctgctgataccctgttga
gagtagttacttttgacaaggctactgcaaagttattgcttgtaacacagttgcgtgtgacttcacctttgaaggaaaagaatcatatttatggtcgaaga
gctgaacttgataagttgagagatatgttgctggaggtaagtgacaatagtgaacctggacctagtgagtcaagtgttcctgttatatctattgttggtgtt
ggtgggattggaaaaactagcctggcacaagtggctttcaatgatgaacagatacgcatgaactttacccacagaatttgggtttctgtgtctgatagtta
tgatgaggttagaatgctcttcatgaaaagatggagggaaagaagtttcttcttgtgctggatgatgtttggtatgacgaagacaggacaacatgggag
aatgagcaactatggagcagagtattgtgttctttaa
>g119725.t1
atggccgacctcgccattgggttttccaagacggtggtggagggactcgcaaacaaggtccaggaggccatcaaggaggaggcagagcaatggcag
atagtggagcgtgatctggtgtttatcacaggcgagttcgagatgatgcagtccttccttgacgttgccgatgaggagcgcatcaggaacaatgtggtga
ggacatgggtcagacaggtccgtgatctgtcctatgacgttgaagattgcatcgagttcgtcgtccacctggaagcaaagcagtcgtggtggctccgtct
ctggaacatggttccatcctgcaactgctgcaagacgacagaacctcagcaacggcctatcgaccaggctgttgctgagataaaacagctcaaggccc
gggtcgaggatgtcagccagagaaacatgcgctacagcctcatcagcgactccggctccaagccttcggcacagtcccgttcaccggtcaatgtagca
gcagtcgacatggttgtggaggcaagggaagccgcgagtaggaatcgtggcctagtggatctgactaagttgatcaacgacgacgccactgagcttct
cgtcatctcgttgtggggatcaggaggcgatcttggcatcacgtccataatcaaggatgcctatgaagaaaaagaagtttgtgataccttcaaattccgt
gcttggatgaagctggttcatcctttcaatccccaaaagttcatccggaccttggcagctcagttttatacaaactctggtcaaggatacgcctctccagg
ggaccttggtgaggagtttgtgcagcgaaacaatgcgaaaaaataccttgtcgttcttgaagatctgtgcagcatggctgagtggcatgctgtcaagact
tacctgccagacctgcggaaggggagcaggatcgtcgtctcaacacggcaacttgaaatcgcaaggctgtgcacggggagaccgtaccaagtatcgg
agctcaggaagttctcacatgatcactctgtctgcgtcttctacaaggagcagatcagccctgaagacccaaaagtggagacatag
>g119835.t1
atggctagccaagaaaagcgcgtcttcgttgccaacctcaaggcctccctggagtccttcttcttcgccgccaagcgcctcgccaagtccgccctcgcgc
ccctcggcccgtcctccgagccgtgcgccctcgtcgacgacgaccagctccgccgcctcgaagccaagctccagcgcgtccgcgccgcgctccgggac
gccgagcgcctcgccgcggacgccgccggagagtccgccgagctctggctccgcgagctccgcgacgttgagtgcggcgtcgaggacatcctcgaga
aggtgcagtttgaggcccttcgctcctcccttgtggaggacctcggcgacgacgacgacgacggccgcggcggcgatccggccaagaatcgggcccg

ccaacgcgaggtcggtgctttgctctcttactcgccgctgagctctctcggcctcaagatcaagaagatttgggagaagtacgaagaaatagcctcaga
ccgcgaggcgcttggcctgacaccggaggacggtgtggttcgaccgtgttcctccgctcgcccaccaacctcgtccctaagctccggtgagctctacgg
ccgggaggatgatctccagcgcctccttgagcttctcttgtccggggaatgcggtggccacgtgttcacggtcgtgtccgtcgtcgggatggccggcgtg
ggaaagacggcccttgtccagcacgcatgcggcgaccgaagggtcagggatttcttcgattgctatgtatggatttactcgggccaaagtgttgatgtgg
tgagcgtgaccaagacgatggtgcaagcgtgcggaggggatacccgtgacatcactgaactgagcttgctgcaaggtctgctcgtagatcttttgaagg
ggaggaggttcttgattgtgctcgatgaggtatggagcattgaaaggtctgtgtgggagctgctcgaggtacctctgcgggttgcgctgcaaggaagca
gagtgataattactagccgcaatgctgaggtcggcatgatcatggggtgtgatcaaaggcaccagttgagattgaattgtttgtctgatgccaactgttg
gttgatctgcaagagttatgcatttgatgtcaataagatgcaggtgtgtcctaaattggcttcacaaagcgaggagattgttacgatcatcttccaccaca
tttga
>g119872.t1
atggcggaatcgttgcttctccccctggtgcgtggcgtggtcggcaaggccggcgatgcgctcgtgaagcgcatcactcatatgtggggcatcgacggc
gatcgtgagaagctggaggattggctggtgtacgtgagccgcatgctggctcacgccgaggtgaaggccgagacggaccctgccgtcaagaggtgga
tgaaaaatctcaagactgccgtgtaccaggccgacgacgtcctcgacaatttcgagtacgaggcgctgcgccgtgaggcccagggtgcccggtccatg
acctccaaggtactgagcaacttcactcgccacaacccgcttttgttccgccttaaaatgagcagggagctaaaaaatgttttggacaagatcgagggat
tagtatcagacatgaatcggttcgccctagtggagcgtgcggaggtgccgcaagttgtatgtcgacagacacgctcagggctggacttgtcagaggag
atccttggaagagaagatgaaatggagaaagtggtgaagttgttgcttgaccagcaagatcagcagagtgttcaggtgcttcccatcattgggatggga
ggtttgggcaagaccacgatagcaaagatggtttacaacgacaacagaattcagaagcactttgagttgaagacatggtactgtgtgtcagagaacttc
gaagctactactgtggtgagatctgttattgaactggccacaaaagacagatgcaagctgcctgacaacattgagctgttacaagggcgactgcagca
agcgagccctccctcgctgttcatccatgccatgctgggtggggattggccaactggagcagacggcgcctgcaagcagctattgaacaattctacaat
catggcagcgtcgctgctcatgcccgtggtgagttcggtagccagcaaggccgccgacgagctcatccaaaccgtcacccgcatgtggggtgttgacgc
cgaccgcggaaagctggaacgtcggctgctggccattcagtgccttcttgccgacgcggaggtgaagagtgagaccaaccctgccgtccggagatgga
tgaaggatctcaaggctgtcgcctaccaggctgatgacgttctcgaccacttccagtatgaggcgctgcgccgccaggcgcagagccgtaagtccatga
ccagcaaggtactgagccacttcacaaacaacagtcctcttttcttccgcttgaaagttagcaggggactaaagaaagtgctagacaagattaatgagc
ttgttgaggagatgaacaagtttggcctggtggagcgtgcggaggtgccacatgttccctatcggcagacacactcattactggacgaatctgaagaga
tctatggcagagacaaagacaaggaactggtggtaaagctgttgctggatcagcaaagtcagcagaatgtgcaggtgcttcccatcattgggatgggg
ggtttgggcaagaccacgcttgccaagatggctgatgacgttctcgaccacttccagtatgaggcgctgcgccgccaggcgcagagccgtaagtccatg
accagcaaggtactgagccacttcacaaacaacagtcctcttttcttccgcttgaaagttagcaggggactaaagaaagtgctagacaagattaatgag
cttgttgaggagatgaacaagtttggcctggtggagcgtgcggaggtgccacatgttccctatcggcagacacactcattactggacgaatctgaagag
atctatggcagagacaaagacaaggaactggtggtaaagctgttgctggatcagcaaagtcagcagaatgtgcaggtgcttcccatcattgggatggg
gggtttgggcaagaccacgcttgccaagatggttaaccgtgttctgatctacaacggcgagaaggatgtggatgtgaacctcgtggaaccgatgtactg
gctcaaatttgacaatatctga
>g119945.t1
atggatctgctcaagaagctctcctccgcgctcggcttcacggagaagatcggcggcgctgtcgtcgacatgctcttctccacgggcgcccgcctatgga
acgtggaggaggaggccgagaagctgcggcgcacggagaagcgtatccgcgcggtgctcacagacgccgagcagcgccgcttcgtcgacgacgact
tcgtcaagctttggctccaggagctccgggcggccgcgttcgacgtcggcgccctgctggaccgtctcggcaccatggccgccgtctccagggtggccg
ccgcggagccgtcgcggaagcggaagcggctctggcccaacgtggatctcggtctgcgccagcgctgggagctggacgcctggatcgtccagatcaa
cgagcgcctcgacgagatcgacaagagcaggaaaaggttcaggttgcaggccggggacgggaggaggacggcgactcagccgatgcagcgcccaa
ggtttcttgaagcggctgcgcatcgcaacgagaggcccattggtcgcaccatggagaaggcggcgatcatccgtgcgctgatttctaacagtaacatgg
atcttcctgtgatttccatctggggaacggcaggaattgggaagacagcgctggcacggatggtctacagcgattcagaggtagagaactttttcactga
caggatttgggtttggttaccggatgtgtgtgatgtgaaggcagccactaagatgatcattgaagctgtgactggaaagaagtgcaagcatctaagcttg
gacatattgcagcaacagctgcgcgatcatctgcgcaagaagcggttcatgctggtgattgataacgtttgggccaagggctttcagttttgggaatttct
gaagccttcattgacggttggagaggaaggaagcaaggttctgatcactactcaagatgaaagagtgtccaacatgatgtccaatattctgaacatgca
tttagagggtctggaagatgagctgctcagactttggatggccgatggtcttgttaagagcaatggaaggagaagagttgagatggaagcagacagat
tctttgatgaactcctgtggagatatgaatctctgactctggaccttgagagttcacaggttgctgagcatcctgagtgggttcgttatgcaactatcttgtg
ccaaaatgatgaacctcttgcactcgacaagatttatcggtacctcagccttcggaatactttgataaaggcacttccagaaacagtctgcaacctattca
atttgcagacacttgacctcaatgactgctactggctcatggatttgcctgaaggcttgaaccgcttagttaacttgcgacacctctgtttacatcttgactg
ggatagagttactcctttcagatcaatgccaagcggcattgacaagttgcaatcacttcagactctttccaggtttgttgtaaccgctagagacggaggca
agtgcaacactaatgagttgaagaacttgaagatccggggagagctttgtattcttaatttggaagctgccagtaatgatggtgccacggaggccaata
tgcttgggaaggaatatttgcagaaactgatgctgaaatggagtgaagtctgcaaggacgagcagcagcaagacatagaggatagtgagaggaaaat
tgaggcactctgtccacataccaaccttaagcatctacgcatcgagaactaccctggaaggaaacttccttcttgggtggataagatcaaatctctggttt
ccctcgagataatttcttgccctaggctcactgaattttctgtggagatgctgccatttctcaggaatttgaagatttgtcagtgtgctgttcttgaggttctt
ccaaaaggtttatgtaacctagaacgcctccattgcctggaaatagatggcgctccacatctgagaataagcggagaggacatattgcgaaggaacat
gggccaattggctgtaagtggatgcagcgccctggagaactggtgcattaatgaaggcgctgagagagtgcaacaaattcctgaaaaaaggattgaat
ttggttactga
>g119970.t1
atgcagtgcttcctcagcaacctgagcaacagtgacgagggccacgacgagcagacggaggggtggatgaagcaggtccgcgacgtcgcctacgac
atagaagactgcgttgatgacttcgcctacagcctcgaacccgaccccggcggcaacgactggctgaccgtggtccgcaggaccctgtaccagatccg
aacatggaggcttcggcacaaaatcgccggaaagatccgcgagctcaagaaaggcgcgcagcacgttggtgatcggcgcagcaggtatggcgtccct
gatccaaaacctggcaagaagaagagtcacttgggtggtgcaacggggtatctcgccgctgagcatcaggagacaacccgtcagctcatcggcatcat

gcagcccgtgggactgaaggagatgccagatctggagaagtggatctcctttgatgagagcaggaagcagcgaggggtgctgtccattgtcggatgtg
gtggtgcaggaaaaacaacaattgcaatggcgctctaccggaaactcgggccgcagttccagtgccgggcgatggtcaccgtgtctcagaaggcggat
gctgaagtcgtcctcagggacatactggctcaagtcaagccgaaggccagcaacggggaacagcaaaccaaacatagcagccgtgccgtctccgag
aataagattcctgttgtcggaagcatattgagccgaattagtctaccgtctagaaaaccagaggataatgctggaatcgccgggcaggacaagctaca
ccaaataaaaacggagctgtataagcaccttgaaacaaacaggaccctgtaccagatccgaacatggaggcttcggcacaaaatcgccggaaagatc
cgcgagctcaaggaacgcgcgcagcatgttggtgatcggcgcagcaggtatggcgtccctgatccaaaacctggcaagaagaagagtcacttgggtg
gtgcaacggggtatctcgctgctgagcatcaggagacaacccgtcagctcatcggcatcatgcagcctgtgggactgaaggagatgccggatctggag
aagtggatctcctttgatgagagcaggaagcagcgaggggtgctgtccattgtcggatttggtggtgcaggaaagacaacaattgcaatggcgctctac
cggaaactcgggccgcagttccagtgccgggcgatggtcaccgtgtctcagaacgcggatgctgaagtcgtcctcggggacatactggctcaagtcaa
gccgaaggccagcaacggggaacagcaaaccaaacatagcagccatgccgtctccgagaataagattcctgttatcggaaggataatgctggaatcg
tggggcaggacaagctacaccaaatcaaaacggagctgtataagcaccttgaaacaaacagatgttgaagagttgcctgcaactataggacagatgg
aaagaataaataacatatttggtggagataagagaacacggaaaacattaaaacttcctgaagagctcaaggaaacaatgaagaacctgcgcatatt
gtctgggattgaaatagtggatgggtcgacggctgcatcagacctcggttacttcactgggctgaagaagttggaccctggactcaatgtcatcttatcc
aacagacttaagagctcttga
>g120191.t1
atggaagcgacggcagtgagcttggcgcggtcggttctggacggcgtcctgagcagcgccggctcggccgttgccgacgaggtcgcgagcctcctggg
agtgccgaaggaggtggacttcatccgcaacgagctggagatgatgcagtctttcctcaaggtggcctcctcgtcgggccaccccgagcgcaacgaca
tcgtgcgcacgtgggtgaagcaggtgcgcgagctcgcctacgacgtcgaggactgcctgctcgacttcgcgctctacgccgccagaacgtcctcgtcgt
cgtgctcgtgcagcaacccgtggctcccgagcgcggtcgcggcgcgccaccgcatcgcgtcccggatccgggacctcaaggccagcgtggaggcgct
gaaccagcgcaacctgcgctaccacatccacatccccgcgggctcgggggaggaggcggcgcacgcgtcatcgtcgtcgtcgtccgtgctcccggacc
cggaccaccaccacgccgagctcgatttcgaggcgtcgggcatcatcggccgggacagcgacaaggaggagctgacccggctgctgctgctcatctcc
gccgggagttgcggcgcgctgggtgtggtggccgtgtgggggatgggcggcatggggaaatcgtcgctggtgcggatggtgcacaacgacccggtgc
tcctggacgagttcgactgcggcgcgtgggtcaccgtgcggcacccgctcgaccacccggacctgttcaggcggcggctcaggaaggagctgggcctg
ccgccggcggccggtggcggcgaggacgtggacgtgcaggattacctgcgggagaagcggtacctggtcatcgtcgacgacctgctcggccacgacg
agtgggagaacgtgtggcaggtcttcccgcgggacaaccgcagggggagccggatcatcgtcacgacgcggcgggaggacgtggcccgccagtgcg
ccgcgcatgcggcggaggggcaccgccatgtctacgagctcaagccactggggaaggagcagtccatggacctcctctgtcggaaggtctacaagac
aactgactaccctttgccagaagacatggccaagcaagccagctatatcttgaagagatgccggggtcttcctcttgccatatctacaattggaggtctg
ctcgccaacaggcctaaaacaagcattgaatggaggaacctgcacgagcatcttggagccgagctggagtctgacctccgcaacatcaccaacgtgat
tgtctcaagttatgacggtttgccgtatcacctgaaatcgatcttcttgtatctcagtgttttccctgagaaccatgagataaggcgcacccgcttgttgag
gaggtggatggcagaaggctacatagccaagaaccgtgacatgcctgtggaggatgtagcagagcgcttctttagtgagctcataaacagaagcatga
ttcaaccttcaaagaccagccctggagtaagagccgatcgttgtcgggttcatggcatggtgttgcagatcatcctattcatgttcactgaagagaacca
actattcctcatagagaagcactccaatgaggttccacaaatgttggacctggaggatacagaaaatctgaatgatgacgacctcaagcacatagggg
agctgtaccacctaagatacttgtgcttgagagggacaaaaatttcgagacttccatcttctttgcagaaacttcgatacctggagacattggacatccaa
gacaccaaagtgacacggctccctgacggcatcgccaaacttgagaagctgcgttatcttcttgccggtgtcaatttggcgagggatttactgctcaagg
tggaggagttaggcatggacacccaaaaggccaacgtttttggaaacatcacttccagtctatgctacaagtgcaatgagtactgtatggtttctgggct
ggaccaactcagtgtaagggctcctgaaggaatagagaagttaaggaacttgcatatgttggggatggtcaacgttggtcagggcagtggtgtggcag
ggaggctcaagaagctgacaaatttgcgaagttcactcaatgatctagcgaaggtgaagctactgggaacacaactcgatcaggaagacattgtgcac
cttcaaaacttagctaacctggtttcgttaggtctgtgggtgaactcctacatagggatgtcgttgtgttttagcactggtacattcccaaagctcaagttcc
ttgatatcgacgggttacagaagattgaaacggcttacattatccctattgccttctag
>g121122.t1
atggatttcgctgtgggtgcatccgagagcgcgatgagctcgttgctcggcaagctcggcagtctcctcgcccaggagtacatgctcattagcagcgtcc
ggagcgagatccagtacatgaacgacgagctcgccagcatgcatgccttcctccgcaagctaggccgaacatctgtggccggagctgcccacgatgag
cagaccaaggactggatcgagcaggtccgtgatgtcgcctacgacatcgaggattgcgtcgatgacttcgcgcaccgcctcggccgccaaccccgtgg
cgagggcctcctcatcagcctccgccgcgcgtggtacactatgaccacgctttggtcacgccgggacattgccgcccagatcatcgacctcaagaaccg
ggcgcaggatgtcggagaacgccgctcaaggtacggcgtgcaggaccctaaacacgacgttgatgacaagctgagcgggacgacgccgcgcccgtt
aacgatggaacaccaagccccggcaccgcagctcgtcggcacgacaaagccggtaggtcaggaggtggcaatcgccagacatgggccatggctcac
ggagccacaggccgagggtgacctaaggatcctggccatcgtcgggtttggcgggcttggcaagacgacaatggcgctggagctactccgaaggttc
ggagagaagttcgaataccgggcgtttgtgcaggtgtcgcagaagctcaacttgacgtccctgctgagtactattctcaagcaggtcacgccacaaaat
gaacccaagcgcagagctaacggtggcgtcaatgggctggagagccgtgccaatggttaccaaggatggaacgtcaagcagctcaagcaaaagctt
gaagcacaactccaacaaaagaggtga
>g122400.t1
atggacgtggcgaaggaggaggtgcatatgcttcttggggttcctgatgagatggaaaagatgaatgccaagctcggagatctcaagaagttccttgcg
gatgctaacaggaggaacatcactgaagagcatgtgcatggctgggtgaaagagctcagaggtgtcatgtatgatgccattgacattcttgacttgtgtc
agctaaaagccatgaaacggggccctagccgggacatggggtgtctcaaccccttgctcttctgcatgagaaaccccaaccatgcccacgacatcggg
catcgtctcaagaagctgaatgaaaagttagatggcatcagcgagcgtagaaagaccttcaacttcatcaacctcacttcatatgaggaccgaggagg
aaagatagaatcatcccgtttaccttgtcgcgagacaacaggggaggccgagctggaagtggttggtgagaagatcagggaggacacaaggaattta
gtggagttgctcacatctaaagagaagacaataaatgaacacaacaaagtcatggtttttgcaattgtgggaattggtggtatgggcaaaaccaccctc
gcccaaaatatcttcaatgatgaaatcatccaacaaatattcaacaagaaaatatgggtgagtgtgaaccaagattatacagagactggtctattaatg
agaatcatagaagctgcaggagggaaccaagaggctgggaatacaaaggtcgtactgcagcaaactcttaaggaaacattgaaagggtgcaagact

ttgctggtgatggatgatgtttggaacctctgggcatgggagaatgtgctcaaaactccaatgacaaaagcctcagctcaaggtagttgtgtccttgtca
ccccgagggactaccgcattgcccgaggcatgatggcagaggaaccataccaccatattaacaaactagaaccagaagatggctggtccttgctcaag
aagcaggctgcccaggatgtcgtggccttgagaagtggtgcacgtggctga
>g122404.t1
atggtgaaggaggaggtgcatatgctgcttggggtccccaatgagatggaaaagatgaacgtcaagctcggggatctcaagaagttcctcatggatgc
tgataggaggaacatcaccgaagagcatgtgcatggctgggtgaaagagcttagaggtgccatgtatgatgccactgacatccttgacttgtgccaact
caaagccatgaaacgaggcccaagccgggacacgggatgtttcaacccccttgctcttctgcttgagaaatcccaaccatacccatgacatcggacatcg
tcttaagaagctgaatgaaaagctagatggtatcaacgagcgcagcaagaccttcaacttcatcaatctcacttcatatagggaccaaaggggaaaga
tagaatcctcccgtcttccttgccgcgagacaacaggggagcccgagttagaagtgattggtgagaagatcagggaggatacaaagaatttagtggag
ttgctcacatgtaaagagaatacaataaatgaacacaacaaagtcatggttttcgcaattgtgggagttggtgggattggcaaaaccaccctcgcccaa
aatatcttcaatgatgaactcatccaaagaaagtttgacaagaaaatatgggtgagtgtgaaccaagattatacagatattgatctattaatgagaatca
tagaagctgcaggagggaaccaaagggctgggaatacaaaggtcgtgttgcagcaaactcttaaggaaacattggaaggttgcaagactttgctggtg
atggatgatgtttggaaccaccaggcatgggagaatgtcatcagaactccaatgacaaaagcctccgctcaaggtagttgtgtccttgtcaccacaagg
gattaccgcattgcccgaggcatgatggcagaggaaccataccaccctattaacaaactagaaccagaagatgcctggtctttgctcaggaagcagag
ccttttcctcaccgtgactccttaccttcttctcatcgccgccacccaggccaagcaactgggcgccctcaacaccctcgtatga
>g122911.t1
atggcagaggcgatcattgggcctctggtgggtaagctccaggagcttgccctcagcgaggccagggcgctggtggccgtgaacgatgacatccaga
gcctccgggacaagctcatgtggatgcaggccttcctgcgcgacgctgatccacggcgccgcgtcgccccagacgaggctagccgggtgtggctgcag
cagacgcgcgacgccgccttcgacgcggaggacgccgtcgaccagtacttcctcctggtcgacctgtcgagggacaatttgcttcagcctcttcttgtga
gtcgtgagaagaagcacaaagaactcgactctactctgcgtgatggcattgctcgtccaaaggttatctctgtcatggggcaaagtggagtgggcaagt
caactcttgtaagagatgtgtacgaaagtctggcaatcaagaatcattttgatgagcaagcattggcaacttttccaccttattccaatgcctctgatatcc
ttaaactaatacttagggatctaacagaagatgatttcacattgtctgagaaagaagtcaacacaaaacttaatcaggagttaaaagagaaaaaatatt
tggtggtgatagatggtgaggttagcaccacagaatggaaacacattcttgctgctcttccatatgaagaaggcagtagaatagtgagaatgtcaaaag
agagactagcagagccaccaggaaactttgatcatgttgtcatccaacttgaccatctagataaggatggtaccatagaactgttccagaagagagttt
gcagggaagaaagtagcccaaagtacaatgaagctgtgcaagatggtgttcgaaacattcaccagcaagatatttttgacaccacagaggggctgcc
actagcaattgttcttctttccggccttcttcgaacaaaggagtttccagtggagtggaaagctgtatttgatcatcttagatctaagcaatcaaagcggct
tgacagcatactatccctctgctttgatgatctaccttatgacttgaaatcttgcttcctttattttgctgcactgccaacaaacatgctaattgaggcacaa
gatttaatatgcatgtggatggctgagggcttcctaagagcaaaggaagggatgacaatggaaaaggttggctatcgttatttgaaggagttgatcgca
aggcatttaatcaaccttgagcccatggatgagaatagtcccggggtggagcttgtgtctatccaaagcaaagtccattctttttgcagactgaagcac
aagaagtaaattttgtggaaatccatagcagtgatgacattccagctttgtcatcagctcgtcgcctctccctacaaaaccgcacggataaatatgctgct
attagcaaccatatgccaaaacttcgatccatattgtctaattttgagaaggaagaaaaaagcaaggaagatgaaagcagcaacgaagaggaacccg
agatgcaatctactacaacttggctacagtgcacacaatatcaaaccaagcacaagaagaacgacttcaaatcctacatacgacaacttctacaagaat
caaagttccttcgtgtcatcaacctacaaggccttgaagttggggacaagttaccaaatgaaattggtgatgtagtgcaccttcagtaccttgcagtaac
atcatgttctttgagagtgataccatcatcggttggaaggctcactgggcttcagacacttgatgttcgagacactgcagttgagaggctacctgagtcat
tttggaaaattatgtcactcagacatgtgtttggtcatgaccttgtcttgcctaaacagattggtgacctaaagaacctacaaacacttgatactataaaac
ctgataattatggatgggatagaattacacttgccaaactgaccaatcttcgttctctgttcatttgggaactctccaaaggtcatgtgaatgcactctcta
ctgctctaagaaaactcaaacaccttgtgacacttaccatacatggtaattgtattccctccagtgtgttcacaagcccctcacttcgacgtcttgagatta
tggagttggatggaatacttgatatgacgtctgaaccagaggacatcaaatcatgcctgcccaatctacttctcttgtcactagaggaaacaaaggtttc
acaagaatttatcaataagttagctgagctgcctttcctagctggtctcactttggatgttggatcatacaagcatgaacaactcgtattctatgccaatgg
atttcatagcttaaaaaggttaacaattgacttggtagaactgaagaaacttgaaatacatgaatcagcacttcctaagcttgtcgatttggatattcttga
atatgataatctggaaattgagattataggtaagttcaacattgttgacaagctccttggtgaggatgaaaaccttcataagaagatcaaacgagctcca
cataagaagggaaggcactga
>g123564.t1
atgctctcctttggaccaccagaaatgacggaggcagtacttcttgccctcacaaagattggtaatgttttggcagatgaaactgcccaggcattggtca
acagactgtccgaaaagattaataatctgagaaacctgaaagacaagatagaaaaaattaggaagcaactgacaataatgaataaagttgtacggca
gattggcacagtatacctcagtgatgaagttgtaaggagttgggtcggggaggtgcgaaaagtggcctaccgtgttgaggatgtaatggacaagtactc
atattattctcttcaaatggcagaagaatggttcctgaagaagtactttatcaaaggatcacattatgtcttggtgttcagtcaaattgccaatgaggtggt
caaggtagaaagcgagattcagcaagttatagaacttaaggagcagtggttgcatccatcccagcttgttcctgaccagcttactgagatggaaagaca
acggtcccaagacagcttcccgcaacttgtgaaagatgacgatcttgtggggattgaaggttacaggagaatgctcacagaatggttatattctgatga
gcttgacagtacagtgataacagtatgtggtatgggtggattgggaaaaagtaccctcgttacaaatgtttatgaacgtgaaaagatcaactttccagtc
catgcatggatggttgtgtcccagacctacacattagatgctctattgaggaaacttcttaggaagatgcgtgatgcattccagaatttcaatggaagccg
catcattatcactacacggcagagtcatgtggcggctcttgctcccccaacacatcgccttgatctccagccactgagcaagactcatgcttttcatctctt
ctgcagaagagccttttacagcaagaaggattataagtgccccgaggagcttgtgatagttgctacttctatagttgataggtgccagggcctgcctctg
gcaattgtatcaatagggagcctcttgtcttcaaggccagagacaccatacacttggaaccaaacgtacaaacaacttcgaagtgagctatcaaaaaat
gataacattcgagcaattctgaacctgagctaccatcatctatctggagaactcagaaattgcttcttgtactgcagcctattccctgaggattacccaatc
tcacgtgagagccttgtgaggctgtgggtcgcagaaggctttgtgatgagcaaaggcaagagcacagcagaggaggtggctgagggaaatctcatgg
aattattacagcgcaatatgcttgtaattaaggagaatgatgagcaaggaagagtgaatacatgtatgatgcatgacattgtacgagatctggctcttga
aattgcagaagaggaaagattcggcacagctagtgactatggtacaatggttcagatggatgaggatgtccgtcgcctatcatcatatggatggaaag
acaaaactgaaataatgataaagcttccacgcctccgaactctagtttcacttggaacagtatcgtcatcccctgacatgttatcgtccatttttctgaatc

tacctatctgaccgttctggaactacaagattctgaacttactgaagtgccagcgtctattgggactctgttcaacttacgctacctcggattacggcgtac
caaggtcaaggctctccctgactctgttgagaagctatacaaccttagcactctggatatcaagcaaacaaaaatagagaggctacctcgagggattgc
gaaggttaagaagctacaacaccttatagccgacagatatgatgatgagaaacagtcagcattccggtactttgttggaatgccagcacccaaagatct
gtccaacatgttagaactacagactcttgagaccgtggaagccagcaaggacttggctgagcagttgaagaaactgatgaagttaaggagatcttttcc
gtacctgaagacgcttgtcctgaagcacatgcctagtgtcatccagctggtgataaacagtggtgccctttcatgccttgaaggtatgtacattgtgtctct
gaccaagctggataagatccctcaaggtatcgaatcacttcgctacctgaagaagctctggctgctcaacctccacaaagatttcatagctcaatggcat
gaaaagcggatgcaccagagggtgcagcatgttccagagattcgtgtttag
>g123925.t1
atggaggcgacgggcgtgagcttggctcggtccgtgcttgacggcgtggtgacaagcgtccgttccgccgtcgccgacgagatcgcgctcctcctcggc
gtaccgcaggaggtggagttcatccgcagcgagctggagatgatgcggtctttcctcatggtggcctccgcgtatcccgaggctcccgagacgttcaga
acatgggtgaagcaggtgcgggacctcgggtacgacgtggaggactgcctcctggactttgcgcactacgcggccagaacaacgtcctcgtcgtcgtc
gcgtgctgctggctccttctccttcctcccgggcgccgtcgccgagcgccaccgcatcgcggaccggatccggggcctcaaggccaccgtggaggacct
gaaccagcggaaccagcgttaccgcatcgctatcgacctccccgcggcacgcgcggaacagaaggcggctgctatgcttcccgaagacgacatgctc
gcgttccaggaggccgacatcatcggccggcacagcgagaaggctaagctgaccaggctcatctcaagggcagagcagccggagccggagcccgag
cacgagccagaccagccagaggaggaggaacaagcagtgctgccactgccacgtaggtcatggtggtcaatgtcaacattattatggacttcttattgg
aggatcaattactacatggcacctagaacagggacgaccagtcctcggccgcgcccactcccagcacgcccttcttcttcttcttcttcttcccttcgc
cccgtcggcagcggcgacggagcgctgcgcgtggtgtcggtgtacgggatgggcggcatggggaaatcgtcgctggtgcggatggtgcacaacgacc
cggagttcatcgacgagttcgacatcggcccatggatcaccgtgccgcacccgctcgacaacccagaagtgttcaggcggcggctcaggaaggagctc
ggcttaaagcacgaccagaacatggaggagtacctgcgggacaaacgattccgggtcgtcgtggatgacctacttagccaggacgagtgggagaacg
tcaggaaagttttgtgggtgggcaacaacaagggaagccgtatcatcgtgaccacgcggcgggaggacgtcgcacggcattgcgcgagggaggga
atgtgccggagggacgcgagcttatctacgagctcaaggcactgggggacgacgagtccatggacctcctctgccgtaaggtctacaaaacagctact
tacactttgccggaggacatggcggagcaagccagacacatcttaagcagatgccatgggcttccccttgccatatctacaatagggggtctcctcgcc
aacaggcctaaaacaagtatagaatggagaaatttgcacgagcaccttggagcagtgctggagtccgacctccgcaacatccctgaggtgattgtctc
aagttatgatggcttgccatatcacctgaaatcaattttcctgtatcttagtatcttccctgagaaccatgagatcagacgtacccgcctgctcagacggtg
gatggcagaaggatacatagcaaagaaccgtgacatgcctgtagagaatgtaggagaacgtttctacaatgagctccttaacagaagtatgattcaac
catccaaggtcatccctggggatgttatagctgatcactgccgagttcatagcatggtgcgccacataatcatatacaagtccactgaggagaaccaact
attcgtcattgagaagaactcctatgaggttccacaaaaaatgcggttgttacgagtgctggacctggaggacactgtcaatctgaagaatgatgacctc
aagcacataggagagctacaccatttaagtatcttccctgagaaccatgagatcagacgtacccgcctgctcagacggtggatggcagaaggatacat
agcaaagaaccgtgacatgcctgtagagaatgtaggagaacgtttctacaatgagctccttaacagaagtatgattcaaccatccaaggtcatccctgg
ggatgttatagctgatcactgccgagttcatagcatggtgcgccacataatcatatacaagtccactgaggagaaccaactattcgtcattgagaagaac
tcctatgaggttccacaaaaaatgcggttgttacgagtgctggacctggaggacactgtcaatctgaagaatgatgacctcaagcacataggagagcta
caccatttaagtctaagagctccggaaggaattgaaaaattaaagaacttgcacatgttgggggtggttaatgttggtaatggcaatggtgtggcaggg
aggctcaggaagctgacaaccttgacaaacttgcaaaggctgcctgaatggatcggttcacttaacaatttaatgaaaatggagctgctaaagacaag
gttggaggaaggagacattacgcgccttcaggacctgcgcaacctggcttttctaggcctgtgggagaactcctacataggggtgttattgcgtttccgc
actggcactttgccaaagctcaagttccttgacattgacgggctgaatacgattacaagagtggaaatcatggagggcgctatgcctgagcttgagcaa
ctctgggtgaacaactgcccatcaatccatgacgacggttctggattgtccggggtaccatacctgctggacctgaatgagcttcttctgaagaaatgtg
gtcgagtaaaggtgaagggccgtagagaggtagctgtattcgtcctggattatagggcagagtacatagttacatgcttgagttcaaggcttcaagtgg
accaaatgaacgcctccaacgacacggcctgcggaacaaccttcgttgcatcctatgttcccagctgcctgagcacattgatcatcttctgctcggatgc
gtcgtga
>g123926.t1
atggaagcgactgcagtgagcttggcgcggtcggttctggacggcgtcctgagcagcgccggctcggccatcgccgacgaggtcgcgagcctcctggg
cgtccccaaggaggtggagttcatccgcaacgagctggagatgatgcagtcgttcctcaaggtggcctcctcgtcgtcgggccaccccgagcgcaacg
acatcgtgcgcacgtgggtgaagcaggtgcgcgagctcgcctacgacgtcgaggactgcctcctcgacttcgcgctctacgccgccagaacgtcctcct
cgtcgtcgtcgtgcagcagctcgtggctcccgagcgcggtcgcggcgcgccaccgcatcgcgtcccggatccgtgacctcaaggccagcgtggaggcg
ctgaaccagcgcaacctgcgctaccacatcggcgtctcgggctccggggaggaggcgcacgcgtcgtcgtcgtcgtcgtcgtccgtgctcccggacccgga
tcaccaccacgacgccgcggagctcgatttcgaggcgtcgggcatcatcgggcgggacagcgacaaggaggagctgacccggctgctgctgctgatct
ccggcggcggatccgggaccgggagttgcggcgcgctggtgggcgtggtggccgtgtgggggatgggcggcatggggaaatcctcgctggtgcggat
ggtgcacaacgacccggtgctcctggacgagcggcggctcaggaaggagctgggcatgccgccggcggccggcggcgaggacgtcgacgtgcagg
attacctaagggagaagcggtacctggtcatcgtcgacgacctgctcggacacgacgagtgggagaacgtgtggcaggtcttcccgcgggacaaccg
cagggggagccggatcatcgtgaccacgcggcgggaggacgtggccaggcagtgcgccgggcatgcggcggaggggcaccgacatgtctacgagc
tcaagccacttgggaaggagcagtccatggacctcctctgtcggaaggtctacaaaacaaccgactacccctttgccagaagacatggcgaagcaagcc
agctatatcttgaagagatgcaggggtcttcctcttgccatatctacaattggaggtctgctcgccaacagacctaaaacaagcattgaatggaggaacc
tgcacgagcatcttggagccgagctggagtctgacctccgcaacatcaccaacgtgattgtctcaagttatgacggcttgccgtatcacctgaaatcgat
cttcttgtatctcagtgttttccctgagaaccatgagataaggcgcacccgtttgttgagaaggtggatggcagaaggctacatagccaagaaccgtgac
atgcctgtggaggacgtagcagagcgcttctttagtgagctcataaacagaagcatgattcaaccttccaagaccagccctggagtaagagccgatcgt
tgtcgggttcatggcatggtgttgcagatcatcctattcatgttcactgaagagaaccaactattcctcatagagaagcactccaatgaggttccacaaaa
agacatggccaagcaagccagctatatcttgaagagatgcaggggtcttcctcttgccatatctacaattggaggtctgctcgccaacagacctaaaac
aagcattgaatggaggaacctgcacgagcatcttggagccgagctggagtctgacctccgcaacatcaccaacgtgattgtctcaagttatgacggctt
gccgtatcacctgaaatcgatcttcttgtatctcagtgttttccctgagaaccatgagataaggcgcacccgtttgttgagaaggtggatggcagaaggct

acatagccaagaaccgtgacatgcctgtggaggacgtagcagagcgcttctttagtgagctcataaacagaagcatgattcaaccttccaagaccagc
cctggagtaagagccgatcgttgtcgggttcatggcatggtgttgcagatcatcctattcatgttcactgaagagaaccaactattcctcatagagaagc
actccaatgaggttccacaaagtaagatacggcacctcgtagtatccagatggaagaggaggggcgagaagctgttaggcataaatatgtcatatgtc
cgatcattgactattattggagagtgcccctcatctatcatctctccaaaactgcgactgttgcgagtgttggacctggaggatacagaaaatctgaatga
tgacgacctcaagcacataggagagctgtaccacctaagatacttgtgcttgagagggacaaaaatttcaagacttccatcttctttgcagaaacttcga
tacctggagacattggacatccaagacaccaaagtgacacggctccctgacggcatcgccaaacttgagaagctccgttatcttcttgccggtgtcaact
tggcgagggatttactgctcaaggtggaggagttaggcatggacacccaaaagaccaacgtttttggaaacatcacttccagtctatgctacaactgca
acgagtactgtatggtttctggggtggaccaactcagtgtaagggctcctgaaggaatcgagaagttaaggaacttgcacatgttggggatggtcaacg
ttggtcagggcagtggtgtggcagggaggctcaagaagctgacaaatttgcgaaggttaggggccgcagggtttaacgaagaagaagggcaggagc
tctggaagtctataggcaagctcaatcgattgcaacggcttgaaatgcgctcggaatcaattaagtttcttgcagcaatggatgaatcgacagtgccgag
gcatctagtttcgctgaggctgtgtggtaatcttggcagtctgcctaaatgggtcagttcactcaatgatctagcgaaggtgaagctactgggaacacaa
ctcgatcaggaagacattgtgcaccttcaaaacttacctaacctggtttcgctaggcctgtgggtgaactcctacataggggtgtcgttgtgttttagcact
ggtacattcccaaagctcaagttccttgatatcgacggattacagcagattgaaacggtaacaattgagaaaggtgcgatacctgagcttgaacagctc
tgggttaacaagtgcacatcgctgcgtgacgacaatgatggactgtctggagtgcaatacctgccgaacctgaatgagctacttctaaagaaatgtggt
gagaaaaaaaggctgatagagacattgcagcgacaagtcaacaaacatgttagacgcccaaagttccttatcggcaagtcgattccacaaactaatcc
gaatgtgtctacgagcacaacaactgagcagtga
>g124019.t1
atggcggagggagttcttgcctcgaatatcgtcaaggaggtgctggctaagattggttcctcgatttggtcggagctcgcgcttctgaggagcttcaggg
ctgacatgagagccgtggagcgcgacctcgccaccgtccgagacgtccttttcgacgccgaggcgcgcggcggcggcggcggggacagcgccgtccg
tgactggctccgtaggctcagggacgtcgcccacgacatcgacgacctcctcgacgagtgccggacggacctttgtgccacccgccgccgcgggagcg
aggacacgatctgtggcatctccaccaacctctccgtcttccgttcattggctctggcacacaggctgaggtccctgaggcgcaagctggacgccgtcgc
cgccggacgtgacagactgcggttgaatcccaacgccttcgcacctgcccacccgcctgctcctccgaggccggagaccatctccatggtggacgagtc
taagacaatcgggcgggccgccgacaaggagaagctcatgaggctggtcctcgattccgccagcgaagaggatgtgtccgtgatccccatcgtcggat
tcggcggcctcggcaagacgacgctcgcgcagctggtgttcaacgaccggcggaccaacgatgaggtgttcgacccccgaatttgggtttccatgtccg
ttgactccagcatccgaacgctggtcaagccgatcgagagcgcgaccaaggagaggtgtgatcttggcaatctggattccgtggccagcttcctatcga
gaacattcacagggatgaagtacctgctggttcttgacgacttgtgtagcgagaaccaggaagagtgggagaagatgaagcttgtcctcaagggcggc
aagcgcggcagcaagatcattgtgacaacgcggagtaaaaaggttgccaagattgtgcgcactgtgtcgcctttcatgctcaagggcctctctgacgat
gattgctgggagctgttcaggtgcaaagcttttgaacaagggcaggaggatctgcatttagtcagaataggcaaggaaatcgtcaggaaatgcggcgg
catgccactggcagcgaaggctcttgggagcatgttgaggttcaataagagtgagcaatcatggatggctatcaaggacagtgaggtagaggagcatg
atttatcaaagaaggaagtagaggaagatggcaatgtcaaatacaagattcatgacttggtgcatgaccttgcacaatctgttgcaggggaggaagttc
agacaatcaactgcttttgcgagtactaa
>g126598.t1
atgcaagcttttctcaaggacctggatgacaaaatgcttagaggtggaggcagtgccatggccaggaacctggtgtcagaggtcagggaggttgcata
tgagttggaggacgtcattgacacagctaatatcttgcagagggacccaaagacatcaatcagtggggccatttctaaatatgcatgcttccccatctat
ctcactcatctccacaagctgggaacaaggatagattcagcaactgcaaggaataagaccatctttgaagactttgagaggcacaatattgttgccagt
gcaattagagaagtacaacaggtttacatcacagaggatgacattgtccaacattggcgatctgttcaccccgaatttggtgagaaagttgatgttatta
gatttgatgagcagattgaacatatcaagagtgatttgctggacaagcataataggcaccttactgttgtatccattgtcggtcctggcggggcaggcaa
atcgataatggctaagacggtatatggttttgctgcagtaaagggccactttcaagtcacttcttggataactgtgtcccagagattcatacctcgtgatct
attgaaggaggtggttaagcatacaattggggtccagcaagccgaatcgcttgagaagaagtctgaagaagaggtgaagaagttacttcacgacttcc
tacagaggaaattgtacttgatagtgttggatgatgtatggagcaccgctgtctgggatataatcagagatgctttccctgatgaaaagaacggaagtag
agtcatcttcaccacacggaatgaggttgttgcacaacacccacatacaaggaagaaactgtacaaaccgaagctcttggatagaaaagaaagcactc
agttgcttctcagcatgtctctccaagaatatatgcttgatggcaacagtaacaggtctgcagctgcagaacaaagcttggataaattaaaggagctagg
tgaagatcttgccgtgaaatgccgtggcctacctctagcccttgttgttctaggagggtacctgagaaggaatctcgatgttgccgagtggaggagatta
acaagcagtatggattggcatactatgatcagtggtgagaaggtcataggtgctgtactggatctcagttactatgacatgccgagcaacctgagatcat
gcttcatgtacgccacagccttcactgaagacttccttattgacattcaagttctaataggcctatggattgccgagggtttcatccctctggtcagaggtc
atactcagaatgaagtagccgtcaattacatcaccaagctggtacaacgaagcatgatccagcaaggtacatttgttcagaagctgaaggagaagggc
tgtgtggtctccatctcttctgatgaggaagcattccagcatatcaatgttccgtttttctttctcagtcttagttactctgctgctctttactatgccatgaagt
ga
>g126604.t1
atggcccaacaggctgttttcacagtgctggaacgggccagcagcattgcagttgaagaggcaaaattcttgctgggggcctctgacaaggtagagttg
gcgaagatgcaactcatgcaaatgcaagggtttctcaaggacgcagacgacaagatgctgagtggtggcgccacggccagggtcttggtctcccaggt
cagaggggttgcatatgaggtagaggatatcatggatagtgccaacatcttgatgaagaaaaatgaccccaagacgtccaccaaaggtgccatttgta
aatatgcttgcttcccagtccacctcactcatctccacaacctgggagcaaggatagatacagcaaatgcgaagatgaaaaaaatcttcgatgatttcca
gaagctcaacattgccgctgctgcaatttcagaaccaccgcaggattatattacccaggatgacgccatccaacaatggcgatcagttcaccctgacttt
gacgaggaagttaatgtgttaggatttgatgaccagatcgaacagatcaaggatgacttgctggacagagggaacaaaaacctaactgttgtttcgata
gtcggtcctggtgggtccggtaaatcaacaatggccaagaaagtgtatgctttggctgcactacacttcgaagttcatgcttggataactgtgtcgcaga
gacctgtacctcgtgacctcttgacagagatggttaacagagttgtacctagtgatgtcctgaaaaagatgattaagcgtatcaccatgaaggaagctga
agagctcaaggagctgacacagcgtcagatgattaagttgcttcaagacttcctgtgggagaaaaggtacctgtttgtgttggacgatgtatggcatcga
gatgtctgggattcaatcagattcacttttcctgacgataagaacagcagcagaatagttctgacaactcggagtcaggctgttgcgcagcaccctaata

caaggaggaaaacctacgaaccgaagcacttggataaagaagaaagtgtccagttgcttctgagcatagccttgccagaatacaacctggatgatagc
ggtaacaataatgctgcagctatggggcaaaatttggctgagttaaagaagcttggtgaagatcttgcagtgaaatgtggcggtcttcctcttgccattgc
tgttgtggggggctaccttctaaggaatcttaatgttgctgaatggaagaggttgactagtagtgtggattggcatgatatgatcagtacggagaaggtc
attgacgccacactgaatctcagttactatgacatgccgagccatctcaagtcatgcttcatgtacaccacaaccttccccgtagactcccttatcgatgtg
cgagttttagcaaggctatgggttgcagaaggtttcataccgcatgtcagaggccacacccgcgaggaagtagccatcaaatacattgatgaactggtg
caacggtgcatgatccaagtggaagaaagggcgtacacggggaggaccacggccgttaaggtgcatgacatcctccggaactgggggataaggcgg
gctcgaatagaagggtttatgaaagattgctataccatggacgacatcaaggcagcctacacagaggagttggtggaagcttatcgtatggctctccat
ggcttcatgctggagacagagttcattgcctccatgagacagctccgctctctagtagcctttagtttccacacgtcacaggtggggaaatcgctccagg
atttgcatcatctgcgtgtgctttacttacacagctccggatgggaagtatttttgccaagggaaattggtcggatgagatacttgaggtatcttggctttg
ggggaaactgcacatatcatctcccttcctctgtcggggacctcctaagcctcgagattgttgatgccacaggaggacggatacacaacatccctggatc
actctggaagatcccaacactgaagcaagtgcacatctttcgtgctcgaggttggtccatgccacggatcagctcgcagttcaaggtgcatgcgattgta
ctcagctcaatttctgacctgcacacccgttcaacagagaccaacgatattctgaaagcttggcgtataatggaagcgacaaaacaacaattgtcagga
gacaaaaatcccaacctctcatgttgcttcgggatgacatatgacatggtgacagggaaccagatggacattgtgggaaagtgcgtggctgatctccgg
ttccccaatgatttcctggactttgagatgatggataatgtcaccgtgctgaagatacgatgcgccaagctactcaacaccgatcagaagatgaaagag
ctcagcaggatgagattcttgatggtgcttgagattggggagcagtcatacacagagtcggtcctcgattgtcctggtggcagcttcaagtgtcttgtcaa
attggtcctctatgaccttgcattgaaggattggaagctggggagtggatccatgatacatctcagagagctgatagtttgcaaatgccccaatctgagtt
accttcctgaggagttgttgcagcttctgagtcttaggaagctagacctcatcgagatgtctacaagttgctaccagcaaggcacagttgcttcggagctc
atgaagaagggctgccatgttttcaagtcagctgaagcaaaaaatttccagcatctcgatcttccgatccccgaatttacttttaggaggtttcaggagtg
a
>g127709.t1
atggccgagatactggccaccatggtggtcggtccactggtgtccatggtgcaggagaaggcgtccagctacctcctggagcagtacaaggtgatgga
gggcatggaggagcagcacgaggtactcaagcgcaagctgccagccatcctggacgtcattgccgacgcagagttgcaggcggcagaacaccggga
tggagcgaaagcttggcttgaggcagtccggaaggtggcctaccaggctaatgatgtcttcgacgagttcaagtatgaggcactccgccggaaagcca
agaaggaggggcactaccacaagctcggcatgcatgtaataaagctcttccctactcacaaccgtattgtgttccgttacaggatgggaaacaagctcc
ggttgatcctacaagcaatcgaggtcctcatcgcagagatgaatgcctttcggttcaaattccggccacagcaacaactttccatgaagtggaggcaga
ctgattctaacatcgttgaccccatggagattgccaagagatcacgtgctgaagacaagcaaaaggtggttgatacattgcttggccaaggaagcaata
ctgatctcacagtgcttcccatcattggaatgggagggttgggcaaaacgaccttagctcagctcgtttacaacgactccaaaattcgggagcactttca
gttgctactctggatctgtgtctctgataattttgatttgcatttgttggccaaaagtatagttgaagcagctatcaaagagaagaagaatggttctgaaac
agatagaaaaccatcgctagagagccttcgagaagtgttgagtggaaagaggtaccttctcatattggatcacgtctggaaccgtgatgttagtcaatg
gaaaaagctcaagtccagtcttcagcatggaggcatcggtagttctgttctgacaacaactcgtgatgaagcaatcgctcagttcatgggtacaactgca
tcctacaaactccacctcctggatgaaagcttcataaaataa
>g127710.t1
atggctgagatactggtcactatggtggttgggccactggtgtccatggtgaaggagaaggcctccagctacctcctggagcagtacaatgtgatggag
ggcatggaagagcagcatgaggtcctcaagcgcaagctgccagccatcctggatgtcattgccgacgcggagatgcaggcggcagaacaccgggaa
ggagcgaaagcttggcttgaggcagtccgcaaggtggcgtaccaagcgaatgatgtcttcgacgagttcaagtacgaggcactgcgccgcaaagcaa
agaaggagggacactaccgcaagctcggcatgcatgtaataaagctcttccctactcacaaccgtattgtgttccgttacaggatgggaaacaagctcc
ggttgattctacaagcaatcgaggtcctcgtcactgagatgaatgcttttaggttcaaatttcgaccgcaacagcaaccttccatgaagtggaggcaaac
tgattccaacatcgttgaccctatggagattgccagtagatcacgagctgaagacaagcaaaaggtggtcgatacattgcttggacaaggaagcaata
ctgatctcacagtgcttcccatctttggaatgggagggttgggcaagacgaccttagctcagctcgtttacaatgactccaaaattcgggagcacttccag
ttgctactctgggtctgtgtctctgataattttgatgtgcatttgttggccaaaagtatagttgaagctgctgccaaagggaaggctactgtaacaaatgaa
aaatcagcactggacagacttcgacaagaagtgagtgggaagaagtaccttctcgtattggatgacgtctggaaccacgatgttagtaagtgggaaaa
gcttaagtccagtcttcagtatggagggatcggtagttctgttttgacaacaactcgtgatgaaggaattgctcagttcatgggtacaactaaatcataca
aacttcaccatctgaatgaaagcttcataaaagaaattatcaaggcaaaagcatttagttcagaagaagaaactgctaccgagctgattgaaatgtacg
tggtggagcttattttatctaactgcagcaaaattgagaagcttcctccactatggcagttaccagctctacgaattcttgacttggtccgaatggaaaag
ctacattgcttgtgtgctgatgatcctcccttcacatttaagaaactgaaggcacttggcttgtgtgagatgtcaaatctggaggcatggtgggacataaa
tgaggaacagacgcctccgtcatgctcggtgatgcattacagccactgcgtcaaggcgttcgtggcctactacatcgacgccagcgtagtaggtcgacg
agctcttctggcttccccaaggagttga
>g127711.t1
atggtcatgacgggtgctacggctgcagcgacagccggagcggcggtgagctcccttctcacaaagctcgacgccctgctgcttgaagaaaattacaa
atccaaggaagcaattcacatcacgttcctccgtgacgagctccagcagacgcaacgcttcctcgagcttttggagacagagcgtgatcccgatgggaa
atctttctcgagaaggcttcgggagctgtcgtacgacatcgaggacactgtggacgcgatcatggtcaagaagcagcagcagcaaccatcatcaggctt
gttgaaatcgtcacacaggtcgtggaggagtttcgtcgccaggatcttggaatttccaaacagggtcgtgttccgcttggcaatgctcgaccagcttcag
gagcacaagtcaagactgaaggaactgaccagagagctcaaggacaggtacaggtacagggccaagatccgcagatcagcagcaactccaaggat
caacgtggaggttgcgtcttcctgccttgtcggtgtcgacggtcagcgggatcgtctcgtgaagctgctgctcgaggttgatgagaagaacaatgatgcc
aggagggtcctctccattcttggatatccaggggtgggaaagactacccttgccagacaagttttatgcaagatccaaccatacttcgactgcgtggcgt
gggtgtcaatgtcgccgtatcgcagtgtcacaaacattctcgaggatattcttcgccaagttgatgatggggtgcaagtggaccggtatatcaccaaagc
catcagagaatcccttctacacaaaaggtacggtctgctatccaatgtcctttcttcgtttcctgatgattgcattgtcgcacaatag
>g127928.t1

atggcggcagtgctgggagcgttcgtatccaagctagcgttcatcatgatgggcatggccaaggacgaggtggagatgctcctggatgtgccgggcga
gatcaccaagctggaaacgacgcctggcgacctgagcagcatcctggctgacgccgagaggagacgcatctgcagcacggccgtagagaggtgggt
gagggagctcaaggatgccatgtacgacgtcgatgacatccttgacctctgcctgatcatggagggtggtggagaagatcccacagcctcagcagccg
ctccgaagaccaaaccaaggagctggaagaagatacccaccaagttattttgctttcgaaaccctgtcgctgcgcacgaggttgggaagaagatccag
gcgctcaacaagcgactgtcgggcatcgcggagaggagctctcgctttggattcatcatccaacagattaactcctcggccaacacaacaactgccact
tctttgtccaacagcaataatagcagaatgaccgggccgagtatcatccggtctgatgttgtgggtgagaagattgagcaagatacaaaaaaaattgta
gatctactgatcaagaaggtgggcgttcgtgtgggatcaaaccaagagaacgtggttgttgctgctgctatcacaggcgtcggcgggataggcaaagc
aacccttgctaggatggttttcaatgatagcatggtggaggaaaacttcgacaggaggatctggttgagtgttaacaaggaagtcaaccaaaccagtgt
gttacagaatgttattgctgctctcgggtgcagctacgatgcctccatgggagatacggccctgctcgagcatttgttgaagctggcagtgcggcaaaag
aagttcttgttggtgatggacgatgtgtggagcgagagtgagaatgtgtggagtgatgttcttagagcagcgcttaatgatggtgccccctggcagtcgtg
tcttggtcaccactagaaatgatggagtcgcgcgcaagatgaaggcacaacatctccaccgggttcaaaagctagaagaacaagatgcttggattttgc
tgaaaaatcagtatgacatcaatccactactgaaaattctagagctgacaaagaattga
>g128170.t1
atggagacacttctctcagcaatcgcgagcgatctcgttagcagagccctgtctactgtgatacagaggtacagacggtccaaggcggaagagacaga
gcataagctccagcagcttcagcgcttgctgctccggatcgacgccaccgtcgaggaggctgaggggcggtacatcaccaaccaggcgatgctccttc
agctcgagacgctcaggcaaggcatgtacaaagggcactacatgctcgacaccttcaagtacagaggccatggagaagacgatgaggtgagtgctgg
ccatgccgtcgccttgcctagattcagtccagccaagcttttcttcccgttccccgtcaccagcaacacgaagaacatgacaattgacgccgagaccgtg
aagaagttggagaagatgcttgacagactcgacacgctgatgggagacatggaggaattcgtcatgttcttggagggatatcctcgcatcttccggcaa
ccatacagtacctacttgattcttgacaaggtaatgttcggtcggcagatggagaaggaaacgataatcaacttcctgctccggccagaagctgccggc
gatggaaacccaggcgttcttccggtcgtcggtctagcgagggtcgggaagagcacgctcgtcgagcacgtctgccttgatgagagggtgcgcgggta
cttctcgtccatcgtcttcttcactggcggcgacattattggtgccggaaacacggctgctctgaggaacagcggtgcagtaaagcatcaggatcttacg
gctgcctcgccctcgcatgggagatcactcgtggtcattgagctcgacggggaaatggaagaggagacatggagaaggctgtactcgtcagcagcaa
gcatcatggagcacggaagcaagatcatagtcgcaagccggtcagagaaggtaacatctttcggaacggctcaggctctgaggctcaagttcctgcca
caagaagcttactggtacttcttcaagacgctcacattcgggagcgcaaaccctgacgatcatcctaagctcgcatccctaggcatggagatcgcagcg
atgatgaaaggttcgttcttgggggcaaacatagtgggcggcatgatgagagacaacctcaacacccagttctggcgcaggttgtttcagcgcctccgc
gagttcacaaacaaacacttgatcgcgtacggcgagcatccgacggatcgtattcagaaaggccaccctgttcacatatggaggatggccaggacttg
gcaccccgttacaatctgcaaaatctatcaggaccgctcttaccagcagcatgctgtccccaagctaactctgcaagacattgttacgggatgtgccaca
gatcaggcggtgtttggtacattagcatggaggtcaagcatacctccctattacacttacctggcgacttgtgcatcccagacggccacaagatgtccaa
tggtcagcaaaaagcgccctcgacaggctagggtctga
>g128171.t1
atgctcgacaccttcaggcaaggcatcgacagcgtcagaggtagccgcactgttgccttgtcaagattcagattattaatcaggagaacctgcagcagc
agcagcatggacctgcagaagaccgagctcgatgccgagcgcgtcgggaagctggagaagatgctcgacggtctggagacgctgatgggcgacatg
caggagttcgtcctgttcttggagggataccctcgcatctgctggcaaccttacatcacgtacctgattcttggcaaggtcatgtttggccggcagatgga
gatggagaccgtcaccagcttcctgctccggcctgaattcaatcctggtgtgcttcccattgttggcgcagcgaggtccggcaagagcacgatcgttgaa
catgtctgtcttgatgagagagtgcacgatcttggagctggaaacgtggcagctctgaaaagcactggtgtgattaagcatcaagatcgtactgcttcct
ctcatggaagatcacttgctgtcattgagctggacaaggacatggacgaggagacgtgggaaaggctgtactcctcggcatcaggcttcatggagcac
ggtagcaagatcataatcacgagccggtcagagaagatagcggctttcggaacagtacaggctctgaggctgaagtatctgactgaagaagcttactg
gtactttttcaaggcactcgcattcggaagcgcaaacccagaggatctaccgaagcttgcatccataggaatggagatcgccgtgctgctgaaagattc
gttcttgggagcangctttcggaacagtacaggctctgaggctgaagtatctgactga
>g128687.t1
atggcagaggctgttgtgggactgctgatctgcaagctcggggaggccttattgaacaaaggagcagcctatggcgcgtccctgctttgcacggaaact
tccgcactcaagggtctctttggtgagatccacagagccatgggctggatggagatcatgaaggcctacctgcatgactcggagaagttaagggatacc
aaagagaccactgatacatttgtgaagaagatccgcggtctagcatttcggatagaggatgttgtcgacgagtacgagtacaagctccaagatggcaa
acatggaaggtttgcagccaagatgaagaagagattgcagcacggcaaggcttggcgccgtctagctcgggagcttcgtgatatcaatgccgaccttg
aagacactgcaagacagaagaacctttgtgccgtgccagaaatggcgaggtacggtggaggcaatggccatgatgctggatcacttaatccaaccttg
tgtttcgcgagaaatgaggatctggtgggtattgagaataatgcagagaagttgaaggggtggatactagatagtgaagtgaagagcaaaatcgtcac
agtttgggggatgggtggagcaggtaaaaccactcttgttgatcatgtctacaagattgtgaaagaggatttcgatgttgctacttgggtaactgtgtcaa
agagctaccaacttgaagacctgctgaagaagattgccagagggttgggtatctcagtccttgatgttagtgacagtgaaatgagaagcttaggggatg
ccatcagtaaccatcttcaagaggattatgaaataaagaggaagagattaattaggcattggattactgctggattcgtcaaggaagaagagaacaat
acattggaacaagtggcggagggattcttgaatgagcttgaactatacatacagggtgctccagagctcagccaagttgaaatagaagaaggtgcact
gaaaagccttgttttgttggagctgtcaggctgtccagagttgaaatgcctgcctcgtggcactgagtaccttacagcccttgaggaattatgtttgctaga
tacctcagaggagctcatagagaagcttaggcaagaacgtgatgcagatgcatgcaatgaagagcttatgaagattaggcaaattagaagggttgttg
ttaagtga
>g128717.t1
atggagctgcagctggcggccgtgctcggctcgctcgccctcggcggcgcgcttctggcgctactcttcggcaagtggtggcagccgctggccgacgcg
gacaagcgcgtgaaggagctggccgacgaggtggacgcgctgctccgcctgcgcgctgacctgctcgggctcgacgcacaaccgccgtcggatgcgg
tacgcgcgtgggcgcggcgcgtgggtgaggcgcaggacgaggcggcgtccgtccgcgcgcggcacggcgccggcgggcagcagctctacgtggtgc
gcctggtgcagtacctcctcctctcctccggtcccgacaacgccgacctcgtccaccgccagctcaagtccgcccgcgccctgcacgagcagggcgccg
cgctcctcgacgccgcgccgcaggcgccgccgccactgctccgccagcccgaggacctcgacgagagcgacctccccgcggagacaggccccattcg

ggcccacctcatcgggatgctccgtttcctcggcgactgcgatgcggccctcggcgtgtggggcgcggggggcgtgggaaagtccacggcgctgaggc
tggtgcgcgaggtgtgcggccgggtcgcgcggttcgaccacgtcctcgtcgtggccgcgtcgagggactgcacggtggcgaagctgcagaaggaggt
ggtggccgtgctcgggctgcgggatgcgcccacggagcaggcgcaggcggcgggaatcctgggcttcctgagggacaagagcttcctcctgctgctgg
acggcgtgtgggaccgcctcgacctcgagcgggtcggcatcccgcagccgctcggcgtggtggccggaaaggtcaggaaggtggtagtggcgtccag
gagcgaggccgtgtgcgctgacatggggtgccgtaagaagatcaagatggagtgctggaacgaggatgatgcgtggagcctgttcgaagccagcgtt
ggcgacgacgccatcggtcgccaccctcagatttcctcatcacttgctagacaggttgctgcagaatgcagaggattgcctttgtccctcgttaccgtcgg
ccacgccatgtcaaagaagagcacaccggaggactgggccgaggcactcgacactctgaaatcgtcgctgcagcagcaaacgggcatgccgggccc
agacaagagcacacacgctctattgaagttctgcttcgacaacctggagagcgacacggtgcgggagtgcttcctgaccaccgcgctgtggcccgagg
accacaacatctccaaggacgagctggtgcagtgctggaccgggctgggtcttctcccggagcacgccgacgtcgacgaggcccaccggccgggca
ctcggtgctcgccgccctggagtccgcgcgcttgctggagtgcggcgacaaccaccggtacaacctgttcccgtccgacacgcacgtccgtgtgcacga
cgccgtccgcgacgcggcgctccgtttcgcccccggcaagtggctggtccgcgcgggcgccgggctccgcgatcccccgcgcgacgaggcgctgtggc
gcgtcgcgcgtcgcgtctccctgatgcacaacgccatcgacgacgcgccgccggccagggcactcgcggaggagcagccggcggaatcgctgatgct
gcagcgcaaccgcgcgctgccgaggcggatgctgcaggcggtgcagcacttcagtaagctgacgtacctggacctggaggacacgggcatcgcgga
gtcgttccccgtggagatctgctgcctggtcagcctggagtacctcaacctgtccaagaacaagatcctgtcgctgcccgtggagctgggtaacctcacc
cagctcaagtacttttacctccgcgacaactactacatccagatcaccatcccgccgggtctgatcgcgcggctcgggaagctccgcgtcttcgaggtgt
tcaccgccagcatcgtctccgtcgcggacgactacgtcgcgccggtcatcgacgacctcgaggctaccgccggcgcgaccgtggcgtcgctctcgctcg
ggttatggctggacagcacccgcgacgtggagaggctggcccggctcgcgccgcgcgtgcgcgcccggtgcctgcacctgcggaggctacagggcgc
gcgggcgctgccgctgctgtcggcgcggcacgccccggagctcggcagcgtgcaggaggggctccgcgagctggtggtgtactcggccgacgtggag
gagctggtggccgacgcgcgcgcgccgaggctggaggtgatcaagttcgggttcctcaccaggctgcgcgtcatggcgtgggcgcccgccgcgtcggc
cagcctccgggaggtcgcgctcggcgcgtgccacagcctcacgcacctcacctgggtgcagcacctcccgtgcctcgagacgctcaacctcagcggat
gcaacaggctcaccaggctcatgggcgggcccgaggagggaggcagcgacgcggaggaggtggtcgcgttcccacggctcaggctgctggcgctgc
tgggcctgccgaagctggagggcatacgcgccgccgatggggagtgcgccttcccggagctgcggcgcgtgcagatcaggggggtgcccgcggctgaa
acggtttcagatgcagccggtgcgcgggcaggagggacaggtgcggatcgagtgcgacaagcactggtgggaggcgctgcagtgggccaacgacga
cgtcaagtcctgcttcgtcaccgtcttctga
>g129389.t1
atgaacattaatgatgagaaagaagttcacaacttgaagactgttggaaaggagattgttcggaaatgtggttgccttcctcttgcaatcagaattattgc
tagtgttctagcaacaaaggagacaactgaaattgaatggctgaagattctaagcaatgatgcttggtccatgaataagctccctaatgaacttagagat
tggacaatgtaccgtgatgaacttgtcagattttgggatgttaagaacatcgagaagatctttgagcagccaatccctccaaggaacctagaaaagcta
attattgaaggattctttggtcagagatatcccacgtggcttgctacaggccatttgtcttcaatgcaactcttacacctcataaactgcaagtcctgtttgc
accttccagcaattgggcaactccccaacttgaaatatctgaaaatcaatgcagcaacagcaattaccaagattggacctgaatttgtaggatgcgggg
tgggtaatactgaatctgcagaggtagtagctttccccaagcttgaaatactgatcatcgaggacatgcccaattggaaggactggacctttgttgttaa
aggagaaacaacaacaataggtatggcggtgggagatgatggagctaaaatgaagcaaataagagaaaccccgcctccaaggatgcaacttctgcc
tcgtttgaaggaattggtacttgtccgctgccccaagctaagagctctccctgggcagcttggacaggaggccaccagcttgaaggagattctattaaga
gatatgaatggccttaaggtggtggagaaccttccattccttgcagaggagcttgtaatagtaggatgtgaagacctagagaaggtctcgaatctttgcc
aagtgagacggctacatgtacagctctgtccaaacttgaggaggagcgccatcaacgccagtgcgaagacctggatgtctacaattggtcatccttga
>g129629.t1
atggaggacgcagcccagaccctcgtaaaaaatgctgggcagctggttgctgcggagtaccggcagctcagcggtgtgggcggcgagatcactgagc
tacgagacgatctagacacgatgaatgccctcctccgcatgcagtcggaggctgaggagggcgccgtggaccacttcgtccgggtatggatgaagcag
atgcgcgaggtggcctacgatgccgaggactgcatcgacctttatcttctccgaatcaagtgccgcatgagcgacggcgtgttcgccaagttcaagcac
ctgttggagactttcttctcgcgccgccgcctcgtcggcgaaatcggcgctctccgagcccgtgccatcgcaatcagcgagcgccatgcgcggtacggc
gtcaaccgcgaggcgctgcgccgttcccctgccttttccgccactccgatgctgactgcatctggaccggctcatgcgctcggctcttccaacggcactga
gcatcatcagctagtcggcatcaagtgccaggcggacgccctggtcgatcggcttaaaaagaaggtagatggggaacagcatctcaaggtgttctcca
tcgtgggatttggtggtgtcggtaagacgacgctggccaaggaggtgtgccggctgctggagatggagttcccacaccaggcaatggtttccgtgtctc
aggcgttcgatcccggtagggaactcacggaactgcacaagcgtatatatctgcaggtcatcagggtcaaaacagagaatgagaggggcatcaagga
agaggctaaccaggggcatttcgagcacctggatgtccagaagctgaatgagatcaatacatggagttcggataagctggcacagaagctccaggaa
tatctgaacgataaaagcatctttccggaggattatgagatcaggaaggatcgactcttgtgtagatggattgccgaaggattggttctggagaagagg
ggcttgactctgatggaggttgcagaatcttactttgatgagctcctgtacgatgtcttgtgtgagttgcccagcctcaagaccatcggcattcagcagaa
gtgctatgttgaccatgagcttgtggcacgcaataaacacaggtttccggagctcaccaacctgtatgttgcatgcaactacgaaactcccaatgttattc
ggtttgaggaaggatccatggcaaaactcgagacacttctgtttaacttcactaaccaggagaagagtattgttggcatcgagcacctgaagaacctca
aagaggtacacctatggggagacaaggataaccatacactatgtcatgcactggaggtgctgaaggatgagaacaagaggcgcagtattgagtccaa
aaatcagtttcagattgtggtgcgataccagtga
>g129946.t1
atggcggctacccttgccagcactgcggtgggcgcggtgctaggagctgtgaccaagaacgtggtggctcaggcattcaagcttctggagaagagatg
cgagctgtggaaggacttccccaacgacgttgacttcatcaaaagagagctgctcatgatcgccggagccgaggaggatcagctgtctggaaagggtg
atccgagagccgtgagtagcatatccatggaagagatgcgagatcttgcgcacgacatcgaggactgcctcgatcggatcctgcgctatgctgaaggc
aagatggacccattatcattgtcgtcgtcatcatcacttattcagcgattcatagcagtcagcggtcctccatatgcgacggagatcaagaagctcaagg
agcggctgaaggccgcacatcagcggaaacatgactacaatatcaacggcagccaaccggccgccgccacctcttcggcgttcccgcgattcatagca
gtcagcggtcctccgtatgcgacggagatcaagaagctcaaggagcggctgaaggccgcacatcagcggaaacataactacaatatcaacggcagcc
aaccggccgccgccacctcttcggcgttcccggtgatcacagaggttaaacctgtgggcatcgaaaaacccaagagagagatcatcgagttgctcgag

gacatcgaaggccgaccggaacacctgaaggtgatctccatcgtggggttcgccggctcagggaagccaacgctcgcaaaggcagtgtaccaggacc
ccgatgtcgtccgccgattcccttgccgagcctntcaagcctgggttctagcatcagagctcagtggtgatatcaagggtcttctgacggcgctccttgag
aagctccgtccaggcgaacctgtaaggggcgatcagcaacagctccgaaacgatatcgcagagctactgaatactacgaggtacctcattgtacttgat
ggcatcggggagcagcaatgtaagctgcaacaagttctccttactaactacagcagcctacctggccatcctctcaagacgtgcttactgtacatctgtgt
gttccccaatggccgtaccatcagcagcagcagtctcataaggcgatggttagctgaaggatatgtccagcgtcaacatccacgcagtgctctgctgatt
gcagatgaaaactttaaggaactgattgaccggaatatccttcgacagatagattcaagctacagtgcaaaagtgaaaacttgcagagcccagggcat
tatgcacgaattcatgctgcagatggccatgtcagctaaattcataacatctcttgatgatccagagagaagcagttaccgccatctcttttggagaatc
attcaagtagcaaggcactggacatgaagcattgtcacaaaagtaaagcaagtgcatccagcagcactgagaaattccgtgctaggtctcttacaatct
gtgggagtgcaggaaaagctgttgcagatttgaccaagtgtgagctgctccgagtcttggatctggaagaatgcaacgatttggaggacgaccatatga
gtggcttacacaagctatggcatctgaagtatctgagtcttggggccacaattagcagacttccaaggaagtttgagaaattacattgcttagagacgct
cgacctgcggaagaccaagatagaaacattgcccttggaagtagtcaagctgccccacctcacgcacctgttaggaaagtttaagcttttgtttggaag
gaaggacttgaagatgaatgaagcacacaaagctgcacagtgcccggaacgatacgatgacatcttcttcatgggcagtgtcctggccctggagacag
cattccgtgaagacctgatatga
>g130415.t1
atgaagctcagcatggtcctggggccaaagtatgagctgatttcaggagcgaggaccgacatcctcttcctccggtctgagctcgagagcatgcacgcc
ttcctcgagaagctcaccactgtgcaaggcccagactcacaggtcaggtgctggatgaaggaggtgagggagctagcctacgacatcgaggacagcc
ttgatgagttcatgcgccgagttgaagctgatggcactgccgccacttctctccatggctcaagcagcctcatggggttcgtggggtgcatcaagcggct
cttgaggactggttggacacatcttcgccttgccaatgagatcaaaggcctaaaagcccgcgccatcgaggtcagtgagcggcggtcaaggtacaagc
ttgtggaggacatatggatgccaggaacccacgtgtctgctgatcctaggatcactgtcctgtacgcagatgtgccagatctggttggcatcgaacattc
cgcaggcgacatagaaaagtggttagtggatgaaataagcacactaaaggtgctctcaattgttgggttcggcggtctgggtaagacaactcttgccat
ggaggtgtaccgcagagttgggggcagattcaactgtcgagcttttgtaaccgtgtcgcaaaagcttgacatgaagaagctcttgaaggacttgctgtc
gcaggtttctcacaatgaggctgaccatatggacaactgggaggaggctcaacttattcgcaaggtcagggagtgcttactaaataaaaggtaa
>g130416.t1
atgcaaagaggtctaagtgttgaggatgtcgcagagggctacttcaatgagctcatcaatcggagcatggtccaaccaattgatataaactgggatggt
agggctaatgcctgtcgagttcatgatatgatgnagcggcggtcaaggtacaagcttgtggaggacatatggatgccaggaacccacgtgtctgctgat
cctaggatcactgtcctgtacgcagatgtgccagatctggttggcatcgaacattccgcaggcgacatagaaaagtggttagtggatgaaataagcaca
ctaaaggtgctctcaattgttgggttcggcggtctgggtaagacaactcttgccatggaggtgtaccgcagagttgggggcagattcaactgtcgagctt
ttgtaaccgtgtcgcaaaagcttgacatgaagaagctcttgaaggacttgctgtcgcaggtttctcacaatgaggctgaccatatggacaactgggagg
aggctcaacttattcgcaaggtcagggagtgcttactaaataaaaggtaa
>g131904.t1
atggaagctctaacttgcctcctccaaaaacttgctgagctggtcgccggtgaatactgtctacaaacggaggtgaagggggtatcatattccttaaag
ctgagctcgagcacatgaaggctgccttggagaaaatctcaaagacgccaatagaccagcttgataagcaggagaagatctgggctagagacgtgag
agatctgtcctatgagatagaagacaagatcgacacattcttggtacgctgcaaggggaacaaggcgccggtccaagaacatggcttaaagaaggtc
attggtaagagccttgatttgttgatgcagccaaggattcgccgcaagattgctaccgacatcagacacatcaagagccgtgtgaaagaggtgtccgag
aggcgtggcaggtacaagatcgacaatgatgttgttaagtccgcaacatatgatcctcgtttgttcactcggtacatgaaggagacagaacttattggtg
ttgatcaggcaagggatgaagtaatcaagattctaatgcaatgcaatgaagtatccaagactgaagacaagatactgtccattgtcggatttggagggc
ttggaaaaacaacccttgctaatatggtgttcgaaaaacttagagtacagttcgattgctcagcttctatttcggtgtctcagactcctgacatggagaaa
ctattcaaggacatgttctataaacttgccaagagatgtaatgaaagcattgatcttattgatgaactcagagaattcctccacaagaagagattactgg
agaacatataccactctgtgagtgataacgctctgctcaccatggtgggatctaagatgtga
>g131905.t1
atggagttggcaactggagcactgggcactctcctccccaagctcgcccagctgctgcacggtgagtaccagctgcagaagaatgtgaggaagaacat
cgagttcctcaccaaggaactcgagaccacacaggccgcactccgcgttatcggtgaggtgccaacggaacaggtcaatgagctggtcaggatctggg
cccgggacgctagggagctgtcctatgacatggaggatgtcgtcgacaccttcctggtgcgtgtcgatgggccggatcctccaagcaaaaggagctcc
aaaaggttcattcagaagatgagaagtatgcttgccataggcaagcgccatgaaatcggggaacagatcgaagacatcaaggaacgcgtcgtggagc
tggcagcgcgccgggacagatgcaaggttgatactgttaataattatcatatcacatctgtggatcctcgcataaaagctttatatgtcaagacgactga
acttgtcggcattgaaaaggcaaggaaagaaattatcttggagctgacaaagggagatgacaagtcatcacaagtacaaaaaatagtttctattgttgg
atttgggggactaggcaagacgacacttgccaaagcagtatatgacgagattgaatcacgatttgattgcacagcttttgtcacggtatcaagaaatcca
gacataaagaagtttctcaaagacatgctctatgaacttgacaagggaaagttgtttgggtttgtagcgcctgtggtgcttcagcaaggtgcgatgccgc
tgctcagaacgcttcaatttggtgtccaagtgcgggaggcgaggaaaatcgctggcagcagcggtagcttcgattttagcttggggaatctgtcatcgct
ccagaatgtctatgttttttttcaatatgaatacgccagcgaggatgaggtggaggaagcgatagctgcggtgagaaaggaggctgaaatccatcccaa
tagccccacccttgaaatgtataataaaggtctgcgaatcaggtga
>g134614.t1
atggagcttgcaatgggggccctgtgctgccttctacccaagctatatgacctcctgaaagaagagtacaacctccagaaggggggtgaagaagaacat
caagttcttgtcgaaggagcttgagagcacgcacgctgccctctgcaaggtggcccaagtgccgatagaccaactcgatgagcaggtaaagctctggg
cacgtgatgtgagggagctatcctatgatattgaggataccatcgacacctttatgatgcgtgttgaaggccgccgcgagtctgcccatcccaacatgct
caagaagtttggcaagatggccatcaacttgttcaagaaggaccttcagatcgcgcataaagtggatgaaatcaaggctcaagtccaggacgtggctg
accggcgtgacaggtacaagattgacagtattgctgctgccaatgttgtggcaacctccatcgatcctcgcttgtcagctctgtacagaaatgacagaga
gctcgtcggtgtccaggaagccagggacgagctgatccaaatgttgacagcagaggggattaatgtgtccaaaaagcagaaactgaagacgatatcc
atcgttgggtttggaggattaggcaagacaactcttgccaaagcagtatatgacagacttcgaacaaactttgactgcagggcttttattgcagctggca

gaaatcctgacttgaagaaagtcttcagggacattctcattgatattgataagcaaaagtacatggatgtcaacgtgacagtactggataaaaggcagc
tcataaacgaactccgtgaacttcttcataacaagaggtacctcatcgttattgatgacatatgggatatatcagcctgggcaatgattatgaatgctttgt
gggtagctgaaggttttgtccatgagaaacacggaactgctggattgtttgagacaggggaggtatacttaaatgagctcgtaaaccgaagcatgatcc
aggcagtggaggtccaaggcgaaggtggtgaacacaataagcggcgatcagtttcagaaagctgcaatattcacaggctagccatgcagaaaacaa
gtgtggagcacaaccgttgggtagcggcaggccgcatgtcacaaatgaggtccatcgttgccagtttctgtgatatcagtatgaagcttccactcttgag
attccaagtgctacgggtcctagctctggagtactgtgaatttaccacagaaagacatcttaagcatcttgaaagtttggcacacttgaggtacctcggat
tgaaccacacacctactgttgagctcccagaagagatagggcgtctcaagtttcttcagacactggacttgcagaaaaccggcatagaagaacttccct
cgagcatcggccagttaacacaactgatatgcctgcgtggtgattacacgacgagagcaccgccagatgggatcatctcgaagctgacatccctggag
gagctgtggatatttccagcttcagccgacaggttcatgagctttgtgagggagctgggtaacctgtgggagctgaggactctccggactcgcattgttgt
ggaggaccaagggcacgagcgagctttgctggtggactgcctgcgcagcctacgcaagctacggtctgttaagattctgagtgagggaaacagcactg
tcaagtgggatgaggaagcaggaacaggctatttccacaagttgagatcctgctgcatcaggaacgcatcaaggcgggaggctgtgctgatctatcag
ccgcccaactaa
>g134615.t1
atggagcttgcaatgggggccctgtgctgccttctacccaagctatatgacctcctgaaagaagagtacaacctccagaagggggtgaagaagaacat
caagttcttgtcgaaggagcttgagagcacgcacgctgccctctgcaaggtggcccaagtgccgatagaccaactcgatgagcaggtaaagctctggg
cacgtgatgtgagggagctatcctatgatattgaggataccatcgacacctttatgatgcgtgttgaaggccgccgcgagtctgcccatcccaacatgct
caagaagtttggcaagatggccatcaacttgttcaagaaggaccttcagatcgcgcataaagtggatgaaatcaaggctcaagtccaggacgtggctg
accggcgtgacaggtacaagattgacagtattgctgctgccaatgttgtggcaacctccatcgatcctcgcttgtcagctctgtacagaaatgacagaga
gctcgtcggtgtccaggaagccagggacgagctgatccaaatgttgacagcagaggggattaatgtgtccaaaaagcagaaactgaagacgatatcc
atcgttgggtttggaggattaggcaagacaactcttgccaaagcagtatatgacagacttcgaacaaactttgactgcagggcttttattgcagctggca
gaaatcctgacttgaagaaagtcttcagggacattctcattgatattgataagcaaaagtacatggatgtcaacgtgacagtactggataaaaggcagc
tcataaacgaactccgtgaacttcttcataacaagaggtacctcatcgttattgatgacatatgggatatatcagcctgggcaatgattatgaatgctttgt
gggtagctgaaggttttgtccatgagaaacacggaactgctggattgtttgagacaggggaggtggtgaacacaataagcggcgatcagtttcagaaa
gctgcaatattcacaggctag
>g134616.t1
atggattttgtgtcgggaccactcggaagtgtcgtcatcaagcttggcaagctactcaaggaggagtacaaaatacaaacgggcgtgaagaagaagat
cacgcgtctctcacaggatcttgagagcatgcacgcggcccttcgcaaggtggccaatttacctccggaggaactcgatgagacggtcaaactctgggc
gcgtgacatccgggagctctcctacaatgtggaggacattcttgacaccttcttcctggtgaacttcgacagctgtgggtcccacaaccccaacaaattc
aagcgtgccgccaagaagatctgtaaactattcagcaagagcaaggctcgccatcagattgctcgcatgatcgaaagcatcaacgagcaagccgacct
agtggctgagcgccatcgcaggtacagagctgaggatatcgtgtccaagcctgccgcaacatctactaatgatgaccttcgttttgccgctatgcacaaa
gatgtgacacaactcgtgggtattgacaagccaagggaggagctagtatccagactatcgctccaaggggacaatgagtcaaagctgaagatgaaga
tggtgtctgttctcggagctggaggattgggcaaaaccactcttgcaaaagcagtgtatgagaagcataaaggggattttcagtgtgcggcttttgtttcg
gttggccagaaacctgacttgcagaaagtctttcgagatattcttattgcccttggcaagaaggcatacagaaatttaaacatcgaaatagcagataaa
aatcagctcatttatcagctccaaaactacctcggaagcaagaggtacttcatccttatcgacgatgtatgggagactgcgtcttgggaaataatcagat
tagctttcataaagagtgggagtggaagcagattgatcacaactactcgtaaactagaagttgccgaggaagttggtgagatttatgagcttgcaccact
tcctccagagaaatcaaaaatgttattttatatgagcatattcagtgctaaaggcagatatattgataatcagctggacaagtgggtagctgaaggttttg
ttgaaaagggcagaagaactggatttttgaggttggagagggatacttcaatgagctcatagatagaagtttgatccaggcagtgcggtcagaagag
aatggcattgtatatggctgtcgtgtccatgatatggtgcttgatcttctacggatcatttcacaggaagaaaactttgttaccntcaacgagcaagccga
cctagtggctgagcgccatcgcaggtacagagctgaggatatcgtgtccaagcctgccgcaacatctactaatgatgaccttcgttttgccgctatgcac
aaagatgtgacacaactcgtgggtattgacaagccaagggaggagctagtatccagactatcgctccaaggggacaatgagtcaaagctgaagatga
agatggtgtctgttctcggagctggaggattgggcaaaaccactcttgcaaaagcagtgtatgagaagcataaaggggattttcagtgtgcggcttttgt
ttcggttggccagaaacctgacttgcagaaagtctttcgagatattcttattgcccttggcaagaaggcatacagaaatttaaacatcgaaatagcagat
aaaaatcagctcatttatcagctccaaaactacctcggaagcaagaggtatgcagaagctacctaa
>g135015.t1
atggttagtgcttcaatgggagtgataaaatctgtgctcaccaaatttgccaccttgttggagaagaagtacaaactgatcaaagatgtgaagaaggat
atcatttccttgagagatgagttgagcagcatgaatgctctgcttacgaagctctctgacatggaggcacttgatgtgcagcagaaggaatggcgtgac
aaggtgcgtgaattggcttatgacactgaggactgcattgatatctttatgcataaaattggtgatggtggtgccaaagcaggaattggaaaccgactaa
aggaagcaaaagcacgctacaatatagctggtcttattgaagaactaaagtctagggcggtagaaataagtgatcattgcagtagatacaagctagat
gagggcatctctgggtctagtgggcatcatgctgtggctattgacccacgaatccaagctctttacgtcgaacctgccaaccttatgggtgtagatgaac
cgagaagtaagatcatcagatggttgatggaggaggaaatgaaacaactgaaagtgatatctattgttggatttggaggtatgggcaagaccaccctt
gccaaccaagtctataataagtgcaaagaacactttgactgcaccgctttcatatcagtgtcacaaaatccagatttgatcaaggttctcataaatttatt
gtcagaattgggatatcatgtgtcccacttcgattag
>g135281.t1
atgcgctggcggaggagcatattagtggtgtcgagatcctcaacgcgcgccttcctggccgctggccgcgccgccgatagcccggacgcccacccagc
tgagagcgttggccttgcccctgccacagccaatgcgcggacggacgcccttggcgccgtcctacggccggaggctgtcggggtaaggaacagggtc
agcggtgaggaagaggtgcacggaggcgctgtgacggcggccatggctgctacgaggcggggctcgagccaggctatgccgcggcttcgggcggag
acgaggcggtgccggcgcgccttggcggcgaggaagcagaacagaagaggagaggaacaacacctgaagatggctgctgcagtgcgtcaggcgcg
ccgtggcggtcaggatctgcaatgcaaaatctccatgccgacagtgctccatgaggaggacaacctgcaaagaactctcctccgggcacaggtcatag
ccgatgaggccatggggtaccagtctgacaacaaggacgatgccaagaaccagatggaagcagaacttgtaatcaacttcctgttgcagacacaacct

catcaagacttggaggtcctgccgattgttggtcctggtaaagtcggcaagagcacccttgtcgcccatgtttgcaaggatgaaaggatccaaggacac
ttctcagaagtcttcttcttgcataatcatgattttagagatgatgagctggagcttgttactttcatagaagcatgtgcaatgaaacatcaaaatctgccat
cgcagtcaaacacagacaggagggtacaccgttag
>g135513.t1
atggcggagctcgccgttggcttgaccaagtcggtggtggagggggcagtgaccaaggcccaatcggcaatcgaggaggaagcaaagcttcggaag
agtgcgcagcgtgatctggtgttcatcacgggcgagttccagatgatgcagtccttcctctccgtggccaacgacgaacgcgtcgggaacctggtggtg
atgacctgggtgcgtcagatccgcgagctggccttcgacgtggaagactgcatcgagttcgtcgtccacctggacaagaagtccgacaagtggcgccg
ctttctccattccatcatcccgccgtgcattcccacatcggcgctgccgctggacgaggccgtcgacgagatcgagcagctcaaggcacgggtcgagga
cgtcagcgcaaggaatgctcgctacaacctcatcagcgacactggatccaagcccgtcgtgcatcatcagcaggcagcgccaaacgctgccgtcgcaa
ccgcagcgctcaacttgctcctcgacgagagggacgccagcaaaaggcagcagggggacctcacccaattcatcacccatgaagacgatgaccttca
agtgatctcagtttggggctcaagcggcgatcttggcaccacgtccctcatcaggaaggcctacagtgaccaggagatctgcacgaccttcgccagacg
tgcctgggtgaagctcatgcatcctttcaatccctacgagtttatccactctttgatggctcagttctacgaagaaacttgcaaagatcagggacgtaccg
tcggtctacatgtcctgaaaaagatgaagggcaccgaagcagagctcttcgaggagttcgaacagcaagtcgaaaagaataggtatcttgttgtcttgg
aggatctattgaacatggtagagtgggatgctatcaggacgttccttcctaacatgaagaatgggagctggataattgtgtccactcaacaatctgaagt
agcaagattgtgcgtcggaaatcctacccaagtactggaactgaggcagttctcgccagagcactctgtttatgccttcttcaaggagatccgcgagctg
gccttcgacgtggaagactgcatcgagttcgtcgtccacctggacaagaagtccgacaagtggcgccgctttctccattccatcatcccgccgtgcattc
ccacatcggcgctgccgctggacgaggccgtcgacgagatcgagcagctcaaggcaagggtcgaggatgtcagcgcaaggaatgctcgctacaacct
catcagcgacactggatccaagcccgtcgtgcatcatcagcaggcagcgccaaacgctgccgtcgccaccacagcgctcaacttgctcctcgacgaga
gggacgccgccaaaaggcagcagggggatctcacccaattcatcacccatgaagacgatgaccttcaagtgatctcagtttggggctcaagcggcgat
cttggcaccacctccctcatcaggaaggcctacagtgaccaggagatctgcacgaccttcaccagacgtgcctgggtgaagctcatgcatcctttcaatc
cctacgagtttatccactctttgatgtctcaattctacgaagaatcttgcaaagatcagggacataccgtcggtctacatgtcctgaaaaagatgaagggc
accgaagcagagctcttcgaggagttcgaacagcaagtcgaaaagaataggtatcttgttgtcttggaagatctatcaaacatggtggagtgggatgcc
atcaggacgttcctccccaacatgaagaatgggagctggataattgtgtccactcaacaatctgaagtagcaagattgtgcgtcggaaatcctacccaa
gtattggaactgaggcagttctcgccagaccactctgtttatgccttcttcaaggagtcccctgaggccaaggaaactgcatcccggcgtatgattggaa
gtaaaaaccccattcaagagtgccgtgctcttttgaaacaacaacgttgtctcgttgttattgatgatctgcagaccaaggaagaatggaacttgatacg
agataatctcatatcaccatctgccagtgttatcattgtcattacaactgaagcaagcatcgccacacattgcgcagataacgaagagcttgtattcaatg
ttaaaggtctacaggctgacgcagcatcggatctcttcaacaatgagtatgaggaggcctttaaagactctgcagattga
>g135632.t1
atggcggagatgtcgctggctgccttacggtgggtggcatcccctgtggtgaacaagctcctcgctaacgcttcaacctaccttgatgtggacctgacgc
aagaacttcaagatctggagaccaccgtcctgccacagttcgacctgatcatcgaagcagctgaaaagagcccccacaggggcaaactgaaggcatg
gcttaagcgacacaaagaagccttctatgacacggatgacctactggacgagcacgagtacaacctcctgaagcgccaggcgaagagtgggaaggat
ttctcgccggaagaagatgactcctttaagtcgactattttgaagcctcttcgtgctgtgaagagtaaagcatccaacttgctcccagaaaacagaagga
taattcgaaagctgaacgaattgaaggtcatcctagcagaagttaaggaatttcgtgcaagtcttggcataccagctgtacagactaccgaaggcacca
ctataccgacagaaacggttcctccgtccacatcattgccgcctcctgaagtgtttggtcgtgacatagatcgcgatcttataatagatcttcttaccaagg
aaagtgctgctgacggctgctcaactaggtactcgggtgtggcaattgttggacatgcaggagccggaaaatcgacattagcgcagcatatctacaatg
acgataggataaaagaacattttgatgtcaggatgtgggtctgcatctcgcgcgagattgatgtccatcgtcacacacaagggattctagagtctgcgct
aaactggaagtgcccacatttcgataatcttgatacgctccagaaaacattaaagggcatactggaaaagtcaaagaaattcctcctggtcttggatgat
gtctggtttaaagaatccagtaaagagaaggaatgggacctactactacgtcctcttgtttctcagcaggccggaagcaaagttttagtaacctctgttag
agatgtacttccagcagctctgttctgcaatgaagtaattcgcctggaggacatggatgatgcagatttcttggcactcttcaagcatcatgccttctcggg
agcagaaataagagatcaatggttgcagctccagctagaagagatagcagagaagatagctaaaaggcttggacgttctccattggcagcaaaagtt
attggttcccagttgagcaggagaaagctggtttcgggaaggtttgttatgcacgacctccttcatgatttggcagagttactgtctacagaggactgctt
cagattagaagatgacaagaacatgaagaagttgcgtgtactgtatttgtcacttgacagtagcagcaaattgccagaatccattggtgacctgaagca
ccttcgggcttcgaagcttaacgatttccaggtgcccgatgcttatgtttatcacccatga
>g135773.t1
atggatctcgtgacgggggcgctgggcagtgtcatccccaagctggccctgctgctgaaggaagagtacgacctgcagacgggcgtgaggaagaaga
ttacgcgtctttcacgtgatcttgagagcatgcacgcattcgcattgctccgcaaggtgccgccggaacagctcgacgatgaagtcaagctctggtcgcg
cgacgtccgggagctctcctacgacgtggaggacatgcttgacaccttcctggtgcgcgtcgaggcccgcgaccccaacaggttaaagcgcgccgcca
agaagataactaagatgttcagcaagagcaaggctcgccatcagattggtggcatgatcaatatcatcaacgagcaagccgacgtggtggccgagcg
gcatcgcagatacagggccgaggatactatgaccaagcctgccacaacatccactgttgaccctcgcctggaggcaatgtacaaagaagtgacgcag
ctcgttggcattgaaaagccaagcggtgacctcatatccatgttatctctttcttctccccaagggaatgacgggtcaaaaatgaagatgaagatggtgt
ctgttgtcggaactggaggattgggcaaaacaactcttgcaaaagcagtatatgacaagctcaaggtggattttcagtgcacggcttttgttccggttgg
ccgggatactgatttgaagaaatccgtcctgctcgccgggacgacggcggcgctggcagtagcaccggtagccacggcgcgggcggcgtcgctcccgt
tctgcgcgcagtcgctgccgccgcgggcccgggcgagggactacgtgacccgggcggagaaggtgcgccttctggtgaacaacgcggcgggggttcc
caggctcggcgtggccaggtacgagtggtggtcggaggcgcttcacggcgtgtccgacaccgggcccggggtccacttcgccggcgcgttcccgcagg
tccgctgtgggagctcgtcggaaaggtactacgtcgtgctttccttcctgtgcctttggctttacctaccttggtggcgccacccactgcattgtgttggcaa
gtgtgtggtggcgccattgctgctggtcaagcgcctcctccctcactcgggctccatgctctcggcctgggggtcgctctag
>g135891.t1
atgggcagcctcctccccaagctgctcaatttgctcggggatgagtacaagatgcagacggggatgaagaagcaagtgaagtttatttcagaggagctt
ggggacatctatgattttctccacaaggtttccgacgtgccatggtaccagctcgatgctcagactaaggcttgggcgcgcgaggcgagggaggtatcc

taccagatggaggatgtccttgataccttccttgtgcatgttcaggagcgggagcctgccagcccgagcaggctcagacacgccttgcaaaagatggtt
agcctgttcagaaagggcaaggcaaatcgtgacatcggttacgccatcaaagacatcaaaaaccgactccagcatttggccaaccggcgtgccaggta
caggattgatgatattattgcaaacccaactgcaactatgtcgactgttgatcctcgccttcaagctatgtacaacgaagttgcaaagttggttggcatca
acaagccaagggatgaccttatatgcaagttgtctgcccaaggtgatgatatggcccttccgaggaagatagtctctatctttgggaccggagggctcg
gaaagaccactcttgctaaagcggtatatgaaaaacttagaccggattttcagtttggggcttttgtaccagttggtaggaacccaaacattgcaaaaat
tctcatggacattctttatgatcttgacaagaacaaatatgcggatattcataccaagaacagagacgaaagacagctcaccgacagcctccgagaatt
tctcacggacaagaggtacaaattacccactcaaactttacactaa
>g137260.t1
atggatctcgtgacgggggcgctgggcagtgtcatccccaagcttgccctgctgctcaaggaagagtatgacctgcagacgggcgtgaggaagaagat
cacgcgtctttcacgggatcttgagagcatgcacgcagtactccgcaaggtggcgcaggtgccgccggaacagctcgacgatgaagtcaagctctggg
cgcacgacgtccgggagctctcctacgacatagaggacatcctcgacaccttcctggtgcgtgtcgaggaccatgaggcctgcgaccccaacaggttc
aagcatgccgccaagaagataagcaagatgttcagcaagagcaaggctcgccatcagattggtggcatgatcaaaatcatcaacgagcaagccgacg
tggtggccaagcggcattgcagatacagagccgaggatattgtggccaagcctgctataacgtcgactgttgaccctcgcctggaggctatatacaaag
aagtgacacagctcgttggcattgaaaagccaagcgctgaccttatatccatgctatctctttcttctcctctaccgaatgacgggtccaaaatgaagata
aagatgaagatggtctctgtcgtcggaactggaggattgggcaagacaactcttgccaaagcagtatatgacaagattaaggcggattttcagggtacg
gcttttgtttcggttggccgggaacctgatttgaagaaagtctttatagacatccttatagagcttaacaaggacaaatacatggatcccaaatatagatt
attggatccagaacagctcattaaggagctcagagaatttctccaagacaagagctacttcacacaacttcatgttatgtga
>g141066.t1
atggtaccacctctggagcaagcggcggcggccgccttgctgaacaacgttatgggcaagctcttcgaagctctgggactagtggagatgtccaggat
gctgcgggacctcaagccgcagagcgagtccctactgcaggacctccgcatgctcgcggcggccgttgacgacgagctagccgcctccaggggcgcc
cgccgctccgccgtagcccgggcctacagcgtggagatgcgcgctctgacgcacgacgtggaggactgcgtcgagcggtttgtacaccgcgtcgccgg
ccttgacggcgcatcgtcgcggctccgccgcgccgtgcgtgcggtgaccacgctccgcacctgctaccggttcgcgacgcagatcaagaggctcaaga
agcgcgtggaggaggcgagggcccgggtcctcagtcctcccgacggcggccagccctccggcggcagaggcgttgcgagacggcgcacagccgacc
acgccgcgcgccaccccgtcggcatcgagaagcccatggaggagctcctcgcgctgttggagttggacaaggacgaaggggagggccaccaatcgc
agccgagggttatcgccatcgttggatttggcgacgtggggaaaactaccctcgcgagggcagcgtacgacgcacccgcagtcgccgatgcgttccctt
gccgcgcttgggtccccgttggctcaccggaggaaggggaggcggcggggattctggagaagatacaccagcaacttcttccggggcagcaatattcc
caatttttgctcaccgagtacctcaaggataaaaggtatttgattgttattgatgacgtcgatgacatcgaggaggagcagtgggacactatcacatcag
ccttcgaaggaaatgctgagttggtgcagggttcagaaacacttctgaagaaatgtgatggccttccacttgcgcttgtcagcatggccagacaattaag
cagtgaagatgaacctactgggagattctgctcagaattatgcaacaaactgggttcttatctggaacgagaagatgttaccccgccagaggagtcatt
acatgaggagaccttcatccaagggcagcaagctgaggaggcacctggaccttcttctgtgcagcatatgcctctttctacctgcaacaattccagattc
agcagtgaaattgatgatcccgcacgtcaggggcattgttcacaggaagctgcaattgagactagcgtctttgaccatggccaactgctgatcaaggag
cctttcgtacatattcctttgccgatgaaacaggaagataggccactgcaatcaagatcgactgacatactatgctctacttctgatgatggaggtcaaag
ggatcatctgaggtgcagtgaggccccagagaagcctgttgctccggacgaatcaacttctaggacgtctacgggcgggtgcagtgtggtggggtggc
tcgctgcaggactgaggaacagggaggcgtcgggccggctcagaacaggcaggcggcgcatggagcagggaggcttcaaggcggctcggataagg
caggcgacgagtggagcagcgaggcatcgggtggtacaagcaggagacacaaggagcgggcctgggtgcagaggcagggtctgggcaaagaggt
gggtcggcacacccgctcgcgctgagccgtcggcgcgcgccgcaatggcgcatccacccgcccactaa
>g142764.t1
atggacggcgcggcacaggctcttgtgagcaatgttgggcagctggttggggaggagtttcggcatctccgcggcgtgggtggcaaggtcgcttgcctg
aggaacgagctggccaccatcaacgccctcctccgcatgcaatctgaagtcgacgagggttccgtggaccacttcgtccgggagtggatgaagcagct
acgggaggttgcctacgatgccgaggactgcgtccacctctatttattccgcgtcaggcgccggcagggtgacaacttcttcgtcaattgcaagcgcctc
cttacgactcttatggctcgccatcgcctcgccggtgacatcagggacctccgtgcgcttgcttcctccatcaacgaacatcatgctcgttacggcttcagc
ctcgagccactgcgccgcacggctgcttcaggccctgtgcagcagcccttggcgcaatcggcgcgcccactccgctccgctgacaacgccgatgatgac
cccaattatcatcatcatcatcatcatcaattcgttggcaacaaggctcaggctatcttcctagccaccaaggttaaggcactaaatggtgagagtgata
agcagttcaaggtgctctccatcgtgggcttcgggggggctcggcaagacaacgctagcagtggagatctgtcggcagctggagacagagtttccgcgc
caggcgcaagtgtccgtgtcgcagacattcagcagcaaggacctccacgcagtgctcaagcgcctgcttcagcagatcgcacagcctagcaacgagc
aagcggattccctggccggtaacatagacaccatgcctgtcgacgacctggaacgcgagctccaaaagagtgttgagaacaagaggtatctcatcttg
attgatgatgtatggagcatagctgcttgggatgcgatccgatccaagttaccagacagcaactgcggttgtagaatcattgtgaccacccggatggatg
atgtggcaaaagcatgcagtgctgctaatgatggttacatccatcgtatgaagaagctggacgagaaagactcagagcaattgttcgtcagcaaagca
tttggctctggaaactcttgcccccaggatttggaagctgcaatgagaagtatcttaaaaaaatgcggtgggctaccgttggccattgttagcattgccag
cctattggcaacctataaacctccagaaggcaaagatatgtgggaaaaagttcagaaatccattggttcacagatggagactaaccctaccctcgagg
ggatgagactaatactcacactgagctataaccacctacctcatcacctcaaggcttgtgtaatgtatcttagcgttttcccagaggattatatgattcgca
aggaccggcttttgaaaagatggatcgctgaaggattggttgctgagaatcgggggttgaccaagatggaggttgcggaaggctacttcaatatcaag
gccatacctccaaaagtgggtgatctcgagcatttgcagacgcttgatgtacgtcaaacagacctcaaggatctgccaaagacaattaaaaagctaga
gaagctggagcacctgctgttctctggcaaaggattgcctgactgggtggagtcactcaccgatcttgtcgagtttgacatagggtggacgtaccttaat
ggtgtccaactattcgacgtcatgtttcctccagtttatagatttgagcaagggtccatgccaaagctcgagactctagcggtgtacttcggcaaccagcc
aaaggaaattgttggcatcgagcatttgacgaaccttaaagaggtacagttcaatggttggagagaccaaatgaagcatgcactggaggaggtagag
gagctgaataagaagcgggacgtgtccgaacagataactgttcgagtgaggtatgaggacgacatgtaa
>g142885.t1

atgaccggggcaattagcaatctcattcccaagctcggggagctgcttaaagaggaatacaatctgcagaagggcgtgaggaagcgagtcgagtatct
gcaaatagagcttgagagcatgagcaccgtccttcgcaaggtggcccagctgatgccggaccagcttgatgagcaggtcagagcatgggcccatgaa
gtcaggatggtgtcgtacgacatggaagacatcctcgacaccttcctagttcacgtggagggccaggaacctgctaccttggataggttcaaacgcctc
ctgaagaagatggccaaactgttcaacaagacaaaggcacgccataagatcgcagatgccatcaaagatatcaagaagcaactccaagaggtggct
gaccggcgtggcaggtacatgcttggcgatattatggttaaatctactgtaacaatagttgatcctcgtctatcggctctgtataatgatgtgacaaagctt
gttggcatcgacgaggcaagtgatgagctcatatccatgctcttgtgccaaggggatgaagggccatatatggacaaggtgaagaaagtctctattctt
ggacctggtggattgggcaagaccactctggtcaaaacagtgtatgagaaacttcaaatgggtttttgctgcggagcctttgttcctgtcagccggaatc
cagactttaagaaagttttcaaggatattctcattgatcttgacaaacaacattacacaacatgttttaatatgatgatactagacgaaagacagctcatt
gatgagctacgaggattcctcaagaacaaaaggtacttcattgttattgatgacatatgggagatacaaacttgggaaacaatcaaattggctctgattg
agaacaatcgtggaagcagaatagtaacaactactcctggcaggcagaaggctttgtcctccctcgacatctccgacgtctgctactag
>g142889.t1
atggcccagctgggccccgcgcgcgccgagccgccgcaccggagctgccagcgcatcatggcgctggcgttcgaccacctcccgcaccacctcaagtc
gtgcttcctgtaccttgccgcgatgcgtgagggcgcgcacgtggacgcggcgcggctggttcgtctgtgggtggccgaagggttcgtgcgcccgagacg
cgggaccaccatggaggaggtgggccaggggtacctcaaggagctcatctcccggtgcatggtgcagctggtccaccgcgacgagttcggcgccgtg
cggaccgtggtggccgaggacgcgctcgacgacttcctccaccgcgccggccgccggcggcggcgctcccccgggtccgggtgcggcggcctgctct
ggtggcgcgggtgcgccggccaggtcgccctgcaccacgacctgtacggccgcatccgacggatctacaagcggctcgacgagatctcaaccaaccg
cgcctcgttcaacatcgagcacacgcccgccgcgcccgcctgggtcgcctcctcctcctccgccaccacactcgccgcatgggacgacctggaggagta
cacggtgggtttggagaattacagcgacattctgaaagggcagctcctggacgattcagacgtcccctcgcgcgcggtggtgtccatctccggcgagag
cagcatcgggaagacgacgctggcgcgcaaggtgtacgggagccccgaggtgcgcaaccacttcgcgatccgcacgtggacggttctcccgcacaag
agccgccccgccgacgtgctcagcgacatccacgggcaggccacctcccagctacggcggtggcccgtctcctcgtcgtctcagcatcaccagcagca
gcagtgcgcggcgggaggcagtggcggtgacgtgagcaaccagctgtacaagaagctgacgggtcggcggtacctggttgtgatcgacggcagcgtg
ggcgtggcggagtggaacagccttcgcgcgtctctccccgacgagggcaacggcagccgcgcggtcctgatcacggactcggctggcctggaggtgct
gggcccgaccccccacgagcccgtggtgctggcccatctgaccccggaggacacgtacgggctgttccggcggcgcgtgttcggcccgcgcggcggc
gactgcccgcgccagcaccggtcgcgctactaccaggacgtgttccgcatcacgcgcgggctcccgctctccgtggtggtcctcgccggcgtgctccgg
tccaagtctctccccgccgagtgggaccaggtgatggcccagctgggccccgcgcgcgccgagccgccgcaccggagctgccagcgcatcatggcgc
tggcgttcgaccacctcccgcaccacctcaagtcgtgcttcctgtaccttgccgcgatgcgtgagggcgcgcacgtggacgcggcgcggctggttcgtct
gtgggtggccgaagggttcgtgcgcccgagacgcgggaccaccatggaggaggtgggccaggggtacctcaaggagctcatctcccggtgcatggtg
cagctggtccaccgcgacgagttcggcgccgtgcggaccgtggtggtgcacgaccgcctccacgccttcgcgcaggaggaggcgcaggaggcatgct
tcgtcgagagccacgacagcaccgacgtgctggcccccgccaccgtgcgccgcctcgccgtgctcaccgacaggtacgtccagctcggagacgcgctg
cccaagctgcggtccgtcgtctgcgacttcgtggaggggagccgtcgtcgcatcgtccagtgcagcgacgccgacctggggttcctccacgcgtccaag
tacctccgcgtgatcgacatccaagggctggagctcaacaagctccctgccgagatcggctccatgatccacatcaggtacctgggcctccagtgcagc
ggcaccatggagaagctcccgaccaccatcgccaacctcgtcaacctgcagtcgctcatcctcagacgcggcaacggcggcggcctcctggaggtcac
ctccgcgttctggaggatccccacgctgcgccacgtcgtcgcgcccttcgcgctgccatgcgccgccgccatgggcgacctgcacagcctgcagacgct
ccacggcgtgcagccacgtggctggccaatcaacaaaatcgcaaacccgttaggaaacaaggccgccaacctccggtcgctggagctgagcgggctg
acgacggcccacacgggcccgctcgaggccgcgctggagagcctggacctgctggtccacctggtgctcaacggcgagtcgctgccgtcgagcgtgtt
caccatcccgagcctgcgccggctgcagagcctgaggctgcagggcgcagtggacgagccggacggcggcgcggcggcggaggacgtgcgatacat
ccggcccaacctggccaagctgtccatgtggaacaccatggtgggccacaagttcgtggccatgctggcggacctgcccaacctcgcggagctcatgct
catgtgcggcgcctacgacggccaacgcctcgagttcggcgggttcccgagcctgcaggagctcacgctcggattgccggacctggaggagtggacg
gtcagcgacgccgccatgccggggctggcgtggctcgggctctacagctgcgccaagatgcggatgctcccggaggcgctcgccgggatgaaggaac
tcgaggagctcgtgctctacaggatgcctgacatggtcaccaggatcaaggaaggtggaggacaggatcaccacaaggtcaagcacgtgcccgtcat
ccagaccatctggtag
>g145096.t1
atggcgggagtcatggtgagtgcatccaagggggtgatggactctgttctttcaaagctcaatgatctggtttgcgacacttgcactaacctcataagcgt
gtccaaggatattctgtttctcagagacgagctcacctacatgaacactctgctcaagaaattggaggataatgaacaactcgacccacaagcaaagaa
ttggaggaaccaggtgctagagctgacctatgatattgaagattccattgatgatttcaagagcaatgcgacaagtgtcaatgccaaacctagccttgtc
agtaaggtttccaactttgtcattaatttgagggctcgtttggatactgctatgcagattaaggatctcaagactcgtctgcacgacataagtgatcgtcgt
aacaggtataaaatagattattgcacctccggtactacaatagagatcttagatgcccgtctaccggcactctacaatgaggcagacagccttgtcggta
ttgcaaatccaaaggaagagcttctcaatcggttactggacgaggatcagaagttgaaggtggtgccaatagtaggtacaggaggtttaggcaaaacc
acgcttgccaatgtggtgtatggtgaagttaaagaacatttcagctgcaaagcatttgtacctgtttctcgcactccagatatgttgaggatactcactagc
atgctgtcacagctacatcagctagatgaaatgcctcgtgcttatgatgtgcaatatctcgtcaatggccttagaacatttctacataacaagaggtaa
>g145097.t1
atggacgccgttattgctaagctcagtgaactggttgggggaacttgcgctgacctcttaggcgtatcccaggatattctgtcactcaagaacgagctca
gaggcatgaacgttctgctcacgacgctggatgacaaggagcaactcgatccacaactgaggaattggaggaaccaattggtagatactgcctacgat
attgaagattgcattgatgatttcaagaacaacacgacaagtgttgatgccaaggctagcttcatcagcaacgtttacaactttttgatgaatttgagggc
ccattatgatactgccaaacagattaaggtgctcaagactcgtatgcaggaaataagtgagcgccgtaagagattgaagatagatgatagcacctcca
gtactaccataatagagaccttagatcctcgtatgccagctctgtacaacgaggcagacagccttgttggtattgacaacccaaaggaagagcttatcg
aatgggtactggatgaggatcaaaagttgaaggtggtgtcgatagtaggtagaggaggtttaggcaagaccacacttgccaataaggtgtattgtgag
gtcggggagcaaggaggtttaggcgtgactacacatgctaagaggtttacacacaaagcatttgtgtcagtttctcgcactccagattggaaaaggata
ctcaccgaaatgctgagggggacctatcctctggtgcttccgatgtgcaatcgctcatcgatagccttagacaacatttatatgacaagaggcttgaga

gacttgagattagttgtgtggccttccccagagttcccaagtggatcggcagtctccgtgacctccgcaaattgacacttgtggtggatgagatttctaact
ccagctggaaggacgttgccatcatcgggatgctgccctcccttgtgtttctcaacttgcagatcacaggagtccctgctgaaaggattgtgataacggg
gtttcctgttctcaagagatttatatttgactgtgatgggatatcacgcctttcgtttgaggcaggggccatgcccaatctgtgggtgctggagttacaattc
catccgggtcaatgggaccaagctgcaccagcagggctggagcacctccgtagccttgaggaaatcactgcaactaaggtacaatatttttcttcccta
atgcgagaaagctgcaccagaggagaggaagccgagaaagcgaacgtgctgatcagggtcgtgttccaggaagctgttggcgctcacccgactagcc
caacatttaccttccagcccaaaaggtggctggggcgtctctctacatga
>g145098.t1
atggcgacagttgtggtgacagcatacaagggcgtgattgagtccgttcttgctaagctcaaagagctcatgacaggggagaactgcagcaaccttgta
cgaggcgtatccagcagcgacatcctgtttcttagggatgagctacccgccatgaacgctgtcctcaagaagctggaggatgctgatgatcttgatgtgc
aggccaaagattggaggaaccaagtgagggagatggcctacgacattgaagactgcatcgatgatttcagaaccaacgctgcaagcgtcgacaccaa
ggctagctttcttgacaaggcttctcattttctcaaggcttgcagggcacatcttgatgctgcctggcaaatcaaagagctcaagagtcgtctgcaggaga
taaatgaacggcgtaaaaggtacaaagttgaagatcacatctccagtactaactcggtgatagttgatcctcggatatcggcattctataaggaggcag
ccagccttgtgggcattgatagcccaaagagggagcttatcaagatggtgatggatgaagagaagcatttaaaggtcatgtctattgtcggctttggag
gtttagggaagaccacccttgctagccaggtgtatcgtgaagttggagggcagttcaactgcaacaaggcctttgtgtctgtttctcagaaaccagatgt
gcacaagggggctttgccggatggtattggaaaggtgaaatcgctactcactttacatggctttaatttaccggtgagctcgttagaaaatatttatggcc
ttggtgagctcaccaatctatcagatttgtctctccattgtggcaaagaatcccctgaatctaatacacttggttggatgactgctctgagttgttcccttga
gaagctcggcagcctcaaagggttttcagtgagatctagtactcttagctgcaatgcagatgctctgagcagctggtcctcacctccttttctgaatctgg
agcagtttgatctattggactggaccttctccagagctcctaggtggatcagccatctctgtagcctccgtgagctggcacttggagccaaccatatactc
caggaagatgttgacatgattgggacaagactgcactttcttgtctatctcagcctgcgcatcgtcaccggtaaccaccagaagaagttaaggatgatca
cgatatcagggtccacgggattctcagctctccggtttttctgtctcgactccaatgagatgttgtgcctggcatttggggctggtgctatgcccaagctcc
gacggctgttactagcactggatccacggaaatgggacaaggccacacctgccggactcaaccacctcccctgccttgaagaaatccgagtactgaca
gtatcatcaccagcttgttgtgggggatctgaatccgtgaaagacaagtctgagctgatcaaggaggtgttccaggacgccgttaacgcactcccaact
ccaccagcgtttactttgctcccaaggttccggtccctgtcagaccacgtaaactgttgtaaaatcaacatggaaacatttgcgtacaaatag
>g146083.t1
atggctatggtgagctcttccacgggcccaataaactcagtcctcgacaagctccccgcccgaccagagttcagtggcttgaggaaggcacttgggggc
atgaaggaacagatgctgggtttctgtgtcccaggggtgatgacgagcacgctggcccggcgatggctgctacaactccgtgagattgcatatgatgtt
gatgaatggttcgacgaacgtctcatccattctgggggccggttccagtcgcagccacggtcatcctctgatgacttggctcaaatccaggacttcgagg
tccaaatcgccgatgcgcagaatcatggcaagacgtttggagttcaccttgagctcagcaccatggtcaacgtggtcccagagaatgaaataaagcctt
ggaagccacagttgtcttgcagacagatgcctcatcaccttgtgggtttcagttgctcagaaaatgagattgtccagcatcttatggatgatgagcagga
actcaaggttatcgctgttcttgggccaggaggccttggcaagactactcttgccagggaggtgtataagaagcagcaaagcaaattcgactgcacagc
atttgtgtatgttggctcgaacccatcaagggatgctgttcttactgacattgcccgtcaggtgatgctgaacactctactcccccatgatgagaatattgt
tttcagattgtatgagttcctcgatacaaagaggtacacccttcttcattctctcggatag
>g146636.t1
atggaggtgacaccattgagcgtgggcaagtcggtgcttgatggagcactcggctatgccaaatctaccatagcggaggaggtggccttgcagctcgg
cattgcgagcgaccatgctttcatcagggacgagctcgacatgatgcggtctttcctgatggtagcgcatgaggagcgaaatgagcaaaataaggtgct
cacaacctgggtgaagcaagtccgcgatgtcgcttacgacacagaggactgcctccaggattgctccatttatctccagaggccatcatggtggcgcctt
cctcgcacgctaggggaacggcgccgcattgcaaagaagatgaaggagctgagagccaaagtcgaggacgtgagccagaggaatttgcgctacggg
ctcatcaagggctcttgctccaagcttgctactgcccctgtgcattccgacatggtcagtgctacaatgtctagcatggaggaatcatggcggcaacagg
acaaagcaaaggagggccttattcaactgacggacaacaaggatgaggaccttagagtcatagctctgtatggggcaagtggtcttctaggggagacg
tccgtcgtaaaaaaagcctacaatgatctgaaggggaggaacaggttcaaatgccatgcctgggtcaggatcgcgcaccctttcaacccaacagagttt
ctccaaaatattatcaggcaattctatgtcgattcacttgatgaaatggcaaacgcacaaaacaaatctgatccttccatccaagatcttctgaagacgg
gaggatga
>g146717.t1
atgcaggtcgtgacaggggcgatgagcaccctcctgcccaagctggccaccctgcttacggaggagtacaagctgcagaggaacaccaagggcgaa
atcatgttcctcaaggccgaattggagagcatggaggctgctctgctcaagatctccgaggctcctgtagataatccacccgatcatcaagtcatgatct
gggcaagggaggtcaaggagctgtcctacgatatcgaggacagcatcgacacattcatggtgcgcatcgagacccatgcgccagaaagaacaaaca
cgcataactttcggaatttctttgagaaaaccatcgatctcttgacaagagccaagatccgtcacaagataggcacggaaatcataggcatcaaaaacc
gtatcatggatgtcggtgagcgtcgtgacaggtataaggttcatagtgttgctacaaagacgattgcacagacagtggacagccttcggttagcggctct
gtacaaaaagacgaccgagctagttggtgctaaacgaaacaccaaagaattagttgagttgctgatggtaccagatgagaatcaacggcgcagaatt
gtctcagtagttggcattgggggattaggcaagaccaccattgcaaaaatagcatatgactggctcaaagggcgcttccaatgcggagcattcgtttcct
tgtccctcactcctaacatggttaatgtcttcagtaacatgttccatcaactaggcctcaagcctgaagcaacatgggatgaagcacagctaatcgacga
gctaagaaaatttctttcgggcaagaggtatgagagatactcagtaatttaa
>g147017.t1
atgaaagagtgggcaagcgacatcagggaagcatcttacgacttggaagacatccttgacaccttcctagtgcgtgtcgagggttgcgagcttgctggc
gtgagcaggctagagcgcgccttaagaaacatgaccgacgtgttcagtttcagtaaggggaaggcacgccacgatgttggctcttctatccaagatatc
atgaagcaactccaggacatagccgaccggcgtgccaggtacagaatcgacgacgttgtggtgaactctgctgtaacaatgtcaactagtgatcctcgt
ctcaaagctatgtacaacgaagttgggagcttggtcagcattgaagaaccaagcaaggacctcatatacaagctgtcagctagtcagggggggtggccg
gaagaagaagacagtctcaattgttgggactggaggacttggtaagaccactcttgcccaagcagtgtttgacaagcttagagccgatttcgaatgttg

ggcttttgtacctgttggtcgaaatcctagccagacaaaagtactcggggacattctctatgaacttgacaataacaagtatgcagatattcatacaaag
aacagagataaaaggcagctcatcaacaacatccgagaatttctgctaaacaagaggtga
>g147796.t1
atggctgtggagctcgcgtcggcgactatgaccccgctcatcgagaagctcggcaatctgctggcgtcggagctgacgcttgagaagcgcgtgcggca
ggacgtcgcctccctccataaggagatggaggagatgcacctggcgctccagcgggtcgaggtgccaccggaacagctggaccaggggaccgcgga
atgggcttctaacgtccgcgagctctcctacgacatggaggacgctgtcgacgccttcatcctgcacttgggggagaatgacgaccatgccgcagcaac
agtagggctgaggagccggtttcgtgggttcctagacaagacggttcgtcttttcagcaagggcaagtcgctgcacctggtcgccgccgctgtcaggga
ggccaagagactcaccacggagctcgatgagatccaccagaggtacgccggcgttggtgcttcttccattattgacgatcttgaccctcgcctgaaggc
catgtcaaccgaggccgcggacctcgtcggcgtagacggcgccagggacgaggtgatcaagatcctgtcgtccgatgaatccgaggtggatgtgcag
actgtgtccattgttgggtttggcgggctcggcaagacaacgctggccagagcggtgtttgacaagatcaagctgcagtttaattgctcggctttcgtttc
ggtgtctaagaaccccgacatcaccaggattttcaagaagatcctttatcaacttgatggggaaaagttcagcgacatcaacgaggcggtcagggatta
tgatcaactcgttcatgatctcaaagagttcctacataataagaggtatttagggctgagacgtactcacatccgtgaactaccggacgaagtcggaaa
gcttcaattcctgcaaacattggacgtaagaggaagtcacggggtacaagaattgccggcgaccatcacggggctgcggaaattgatgtgccttcgtct
tgactgggacacaaggctgccgaacgggttggggaataataatctgacctcactggaagagctgacgggtctgagaatcggcgggcatgacgattctg
cagcaatggtgacgacggagctcggccacctcaccgggctgcgggtgctcacattgagatgggagaggccggcgaatagtatcgacgaagctctggt
gcagtcccttggaaatttgcgcaagttgcagagcctcgacatgtacgtcaatggcgggcgcggcggcctcatcatgcgtgactgggcgccgcctccgcg
gctccgccggttcttgtgcaaagggcccaccagcgcgctgccgacgctgccggcatggattatgaagaacacgacgtcgctcgcgttcctggacatgtg
ggtcggccaggtgggccccgacgacctgcgggccctcggggcgctcccgtcgctgcgcggcgtgcgcctgcgctcgaggggccgcgtcgacgacgac
gaccgccaccggccgtcggcgttgaccgctggtgcgttcccgtgcgcgagggcgtgcgcgttcctgcatttcacgacggcgccgtccatgttcctgcagg
gcgccatgccgcacgtccagcggctcgagttcaccgttcgagcgtgtgattttggtgctagtgctagaggagggtttgggctggatgacctgcgcatggg
gcacctcacttcgcttgaggaggtccacgtagagctgtggtccaggaaggaggacgacgccgctgtggtggcggcggcgctgcggcaagcggcggaa
gatcaccccaatcgcctcgccattcgcatcaaccagagatcagggggcagaatgtacctgtcgttcaccacgcacaccaacttgtga
>g148733.t1
atgatgcagtccttcctctccgtggccaacaacgaacgcgtcgggaacctggtggtgatgacctgggtgcgtcagatccgcgagctggccttcgacgtg
gaagactgcatcgagttcgtcgtccacctggacaagaagtccgacaagtggcgccgctttctccattccatcatcccgccgtgcattcccacatcggcgc
tgccgcttgacgaggcggtcgacgagatcgagcagctcaaggcacgggtcgaggacgtcagcgcaaggaatgctcgctacaacctcatcagcgacac
tggatccaagcccgtcgtgcatcatcagcaggcagcgccaaacgctgccgtcgcaaccgcagcgctcaacttgctcctcgacgagagggacgccagc
aaaaggcagcaggggggacctcacccaattcatcacccatgaagacgatgaccttcaagtgatctcagtttggggctcaagcggcgatcttggcaccac
gtccctcatcaggaaggcctacagtgaccaggagatctgcacgaccttcgccagacgtgcctgggtgaagctcatgcatcctttcaatccctacgagttt
atccactctttgatggctcagttctacgaagaaacttgcaaagatcagggacgtaccgtcggtctacatgtcctgaaaaagatgaagggcaccgaagca
gagctcttcgaggagttcgaacagcaagtcgaaaagaataggtatcttgttgtcttggaggatctattgaacatggtagagtgggatgctatcaggacgt
tccttcctaacatgaagaatgggagctggataattgtgtccactcaacaatctgaagtagcaagattgtgcgtcggaaatcctacccaagtactggaact
gaggcagttctcgccagagcactctgtttatgccttcttcaaggaggtcagccagggactaaaaactcgtcaattactttcaattaagaagatgccttaa
>g150979.t1
atggcagatcttgtagtaagcgcggcgacaggagcgttgacgcccttgctcaagaagctcgctgctgcgctggttgaagactacaagcggttcaagagt
gtgcgtggtgagatcaattcactcactgaagagcttgcagccatgcatgcatttcttctcaagatgtcggaggaggaagatccagatgtgcaggataaa
gcttggatgaaggaggtgcgggagctatcctacgatatggaggacagctttgatgaattcaggcttcgtgttgatgacagatctactaatccagaaggc
ctcattgacaaattcaacaatttcgtgagcaaaaccaagactcgccgtcggattgccaaggcaattgatgatctaaagatccaaatcaaagaggttggt
gaaagaaatgcaagatacaagacacgggagatcatatccaagacaagcaacgcctatgtagatcggagagctcttgctgtatttgagaatgtttcaaa
gcttgtgggtttggagaggcccacgaatgaagtgatcgaagtgatgatgacaaagaagaatggatttccatcgcagatgccaaaggttctttccattgtt
ggatcaggggggttaggcaagacgactcttgcaaaccatgtatatcagaagctcaaaggaaactttgagtccaaggctttcatnnnnnnnnnnnnn
nnnnnnnnnnnnnnnnnnnnnnnnnnnnnnnnnnnnnnnnnnnnnnnnnnnnnnnnnnnnnnnnnnnnnnnnnnnnnnnnnnnnnnnn
nnnntaaagcttggatgaaggaggtgcgggagctatcctacgatatggaggacagctttgatgaattcaggcttcgtgttgatgacagatctactaatc
cagaaggcctcattgacaaattcaacaatttcgtgagcaaaaccaagactcgccgtcggattgccaaggcaattgatgatctaaagatccaaatcaaa
gaggttggtgaaagaaatgcaagatacaagacacgggagatcatatccaagacaagcaacgccgatgtagatcggagagctcttgctgtatttgaga
atgtttcaaagcttgtgggtttggagaggcccacgaatgaagtgatcgaagtgatgatgacaaagaagaatggatttccatcgcagatgccaaaggttc
tttccattgttggatcaggggggttaggcaagacgactcttgcaaaccatgtatatcagaagctcaaaggaaactttgagtccaaggctttcatctcagtg
tcacggaatcctgacctgtccaatgttctacgtaatattctataccaagtaaacaaggaatctcatcctaacatacaagactgggatatacatctacttat
cagcaaaatcaatgattctctgatggacaaaagggactataacatttttcctgggttttcgaatgaatctcatgtgccgaatgggaggaagtacttggctg
cagcagcgatggggaactaccgattcattaacaacaagtgcaaggcagttgttgtaacgtctcgctcctacgaggccgggcccacttacacctggcatc
tctag
>g152403.t1
atggatgctcttgtgtctgcggtggcgagcgacctcatcggccggttcatctccttcctcatcgggaagtaccaggagagcagcgcagctgacatctccg
acaccatcgtcaggctgcagcgagcgctgctgcgagctcgcgtcgtggttgaggaagccgaggggcggcagatcaccaatcgagcgatgctgcagca
gctcaacctgctgagacgagagatgtgccaagccgcctacgcactcgacgcgtttaggtggagagataggagatggagaaggaagaaatcatcagct
tcttatggcggccttcccaagatctggatgtcctcccgatcatcggtccgctcggtgtcgggaagcgngtccgacatggttgggagcctggaagccctgc
taggcgacatgagagagttcgtcgtgctcctcaatggctatccccgcttcactaagcagccatacactgcctacctgtgcatggagagctgtatgtttggg
cggcagatggagaaggaagaaatcatcagcttcttatggcggccttcccaagatctggatgtcctcccgatcatcggtccgctcggtgtcgggaagcgg
accttggtggagcacatctgtctcgatgagagggtgcgtgagcgcttcggcaagattcatcgtctcagaagtaccgatcttgatctatacagctacgggc

atcattggagctcattcgacttcacgacaaggtcgctgatggtgatcgatatggtcgacggtgacgtggatgccgaggagtcatggaggagattctactc
agccattcgcctccgcgcgcatatcgggagcaaggtcattgtcatcagcaggatggaggcgacgcactcgaatctgggcactgtcacgccgctcaggc
tgcgcccactgcgccgggaggacctttggtactttttcagggcgctggccttcggggccgcggaccctgacgagcacccggacctcgcgcatctcgctat
gacgatgtgcacttgcatcaacggcggcttggcgctattcaccgtggccaacatcgtcgccgcgtcgctgcgtgccgatcccagcgtgcgctcctggcgc
cgtttgttcaaggtgatcttcgagacggcagtgttgcagttcggtacagccgctgacgataaggccgagcactactactacctctgcagtccgctgggag
acgctggtgcttcttgcttgttctacaacaggcgcaagttgaccaacgtggcgcggagtgagctgcccaaggtgacgatgctagagatgctcaccagcg
gctccctcccctccgcagggaagacgcagtttgatgtgcttatgtgggaatctcgcattcctccgtatgcaagctacgtcgccacgtgtgacacagacaa
agctcatcatcagcaggtggtagcacttaagaagccacttcttaacaagaggaggagagaccaccaagaagatgaccctagtaactag
>g152405.t1
atgctgctgcagctcagtcagctgaggcgagagctgtgccaagctgcctacgcgctcgatgccttgaggtggcgagataggaggtcacaagccacggt
gaggcgacctcgagcgcaagcaaggcctcctccgagcagcgattgctccgaatttttatccgccatggtgggaagcctggaagccgcgctaggcgaca
tgagagagttcgtggtgctcctcaatggctgtccccgcttcactagacagccatacagtggctacctgttcatggagagctgtatgtttgggcggcagatg
gagaaggaagaaattataagcttcttatcgcggccttctcaagatctggacgtcctccccatcatcggtccgctcggtgtcgggaagcggaccttggtgg
agcacgtctgtctcgatgagagggtgcgtgagcactttgtcaaaattcatcgtctcagaagtaccgatcttgatgatctccacacccctgatcatcatcat
catcactggagcttgttcgacatgaccacaaggtcactgacggtaatcgatatcgtcgatgacaacacggacgccgaggtggcctggaggagattctac
tcagccattcgcctccgcgcggcgcacatcgggagcaaggtcatcgtcatcagcagggcggaggcgcacgcgagcccgggcaccgtgccgccgctca
ggctacacccgccgcgtcgtgaggagctctggtacttcttcagggcgctcgcttccgggcccacggaccccaaagagcacccgggcctcgcgcgcatc
gccatggcgctgtgcgagggcgtctgcgacttcgcgctgttcgccgccgccaacgtcgtcgccgcgtcgctgcgtgccgacctgagcgcgcgctcgtgg
cgccgcgtgctcaggggtgtacgacgacgttgtcaggaatgggttggatga
>g153152.t1
atggctaacaaggtgtatggtttggctgaagtaagtggacgcttcaatgctcgtgcttggataattgtatcccagagatttgtagagcgtgatctgttgag
ggagatggttaagcgcaccatgggggctcgactagctgaagagctcgacaagatgaaccagagtgaggtgaagaagatgcttcgtgatttcctacgg
ggcaggaggtacttgataatattggatgatgtatggaaaacagatgtctgggatatgatcagcgacgcctttccagctgataacaagaatggcagcag
ggtcattctgaccactcggaatgaggctgttgcaaatcatcccaatgcaaggaagaaaatctacacaccaaagctcttgaatgaaaaagaaagtgccc
agttgcttcttagagcagccattccagaatacattttggatgccttccctgagtactcttatattgacgtgcgtcttctatcaaatctgtgggtcgcagaggg
tttcatcccacttgtcaaaggacacacacgtgaggaaattgccttcgggtatgtcaacgagctggtgcagcgatgcatggtccaagtggagaaacggac
gccctgtgggaggatcttgctgataaaggtccatgatgtcctacgtgactggggaattggtcgagctcgaagagaagggcttatcaaagattgccatga
tgcagaagacatagaggcagactactcagaggagaaggtgaaagcttaccgcaaggatgacgtctatctgcccaaggagattggtcggatgagatat
ttgagacgctggtcagtgccatggatcagttcacagtccaaggtccatgtgacgatattctatacggcatatacatattaccgttctgaacaggttgatat
agctaaacgggtcatggaagctacaaggcaacaattatctgagaaaaagaaactcaaccggtcatactgctttgggatgacgtatgttgccaatgggg
gtgatcaaatagaaattgtgggaaatagccggtcagagaatcagttccgcaacgtccttgcaagctttggagagatgaaaaatatccgcgcgctgaag
atgtgctgcaccagtcttctcagcgatgaacagaagctacaggagctggtgtgcatgagatggcttcaagttctcgagataggggagcagtcatacaca
gggcaggtcataactttcccacatattagtttcccttatcttgaacagttgacactccacgatcttgcagtggaggaatggagaattgaggatggggcaat
gatcaatctttgggagctaacgctttgcaagtgctccaatctgaagtgccttcctgaggggctgtcattgctcccacgtctaaagagggtgaagctcatcg
tgatgcctacaggttgcccggagagtacagttgctagggggttggagaaaaaagattgtcaggtctttgtctcatccgacgagaaagatttccagcatct
ggttattccgtga
>g154233.t1
atgattgatgtggcgaaccaagagatctcttcgttcctccgtgttaagtccaactggagtgatcttgataaggccagggacagcttgcgggccatcgaga
tgatggttaaagcagaggtcacccgggagctagacaagctcaacatctgtgatcctcaggtgcaggtgtggttgaatcgtgtggaagagctacaactgg
acacgattgatgaggattacagccaatttatgaggtattcttgcctctgccagtgcaccatgcatgctgctcgccgtgcttggattgacaagcgtgttgtg
gaggcgctggaggaggtgaacaaacttatcgacgaaggtaggggtttcaagaaatttgggttcaagcccctgccagatattgttgagcacctgcctcag
attgagacatttgggcttgagagcatgctgactcagctccatgatctgcttgagaagagtgactcgagcgtaattggtgtgtggggtgaaggaggtatcg
gtaagacaacactccttcacatcttcaacaatgatcttggaaggaaggcccatgactatcagatgggtgcacagaggagcctgatcaaaatgcaagttt
tagatgctgctgctgcatggaacctgttcttgagcaagctgagtaacgaagcttctgcagcagttaaatcacccaattccaacaatgttgttagggagcg
tgccaaggtaatatgcaaaagttgtggaggtctaccacttgcactgaatatcgtgggaactgcagtggcgggcttggaggaaccagatgactggatttc
agctgcagatgcaattactgtcaacatggacagcattgatggtgtggatgaaatgtttgctcagctgaaatacagctatgacaggctcacacccactcag
cagaagtgctttctgtactgcgctcttttcccggaatacggatctattaaccagtggttcaatgtcatcaaaggtcaaaatgcaccatgtaaccaggcgcc
tggggatttggttggttaa
>g154522.t1
atggctctcgtgacgggggttctgggaagcgtcatccccaagctcggcgagctgctcaaagaggaatacgacctacagacgggcgtgaagaagaaga
tcgtctgtctctcacgtgatctggagagcatgcatgcggccctccgcaaggttgccaaggtgccgtcggaccagctcgacgagcaggtcaagctctggg
cgcgcgacgtccgggagctgtcctacgatgtcgaaaacatcctcgacaccttcctggtgcgcgtccagggtcctgaggcccgcgacccaaacaggtac
aagcgcgccgcaaagaagattggtaagctgtttaccaagagcaaggctcgccataagatcgcaagcatgatccaaagcatgaacgagcaagccgatg
tggtggctgaacgacatcgcaggtacagagctgaggatattgtggccagtcgtcctgccgcaactttaaccgttgatcctcgtcttgctgctatgtacgag
gaagtgacacagctcgttggtattgaaaagccaagcgctgagatcatatccatgctatcggcccaacaaagggatggcgtgtcgaatatgaagatgaa
gatggtgtctattgtcggagttggaggattgggcaagaccactcttgcaaaagtggtgtatgacaagcttaaagtggattttcaacatacggcttttgttct
ggttggccaaaatcctgacttgaagaaagtctttagagaccttcttatagagcttgacaaaaagaaatacatggatcccaaatggacaatgttgggtga
aaggcaactcattgatgaacttcgagaatacctctgtgacaagaggctaagcatttga
>g154527.t1

atggagaagatcgccagttcggcagtggatggcgcgcttagcatagccaaatccgccgtggtggcagagctcgccctgcagctcggtgtgaagggcga
tttggagttcataaaggatgaattcgagctgatgcgaacttttctggtgacttccaacgaagcgcacggaaaggatttggtggcaagggcttgggcgcg
gcagatccgcgacgtcgcctacgacttggaggactatatcgaggatttcgcaattcatatcggtaaatcatcatggtggtggtgctccccttccaaggtcc
tgaaccggcgtcgcatcggcgccgagatgaagaagctgaggaccagggtggacaacgccagcaaaaggagaacactgtatggcatggtccctttgcc
accaccaactgaatctgtgctacctccggggcaatacgtctctacactgtctggtcaggaggctgccgatgcacagcatggagaagatctgaaaggacc
atctggagttgttcttggcaagctgctgggcaagcgagttacctcgctcatggtagtctcgatatgtgcaagaaccccgacgacgctgattggggagacc
tacgacgagctggccatgaccggggagttccattgccgggcatgggtgacggtatacaatcctttcaatctgaccgagttcatccgcagcttgctcaggc
aatttcaagcgaatgcttctggggaagaacaacaggttgtggaaggacccacaacagtcgatgtgcaagaaaacttagagacgatgggtacccaaga
acttatcaagtcccttggacgaaacctcgaccgcaagaggtacctgcttgtgctcgacaacatttccacgcgggaggaatgggattccataaaactgta
ccttcgaccggacctcaacaacgcgagtcggatcatagccatctccgagagggacgacgtggctaaatactgcgctggcggcaagagaagtaactctt
tctgcctctccagaaacggggcagttgtttctgctttctgcaacacggtaaataaatga
>g155916.t1
atggaggaagagcaaaaagctcggagctttcttgatgtgcccaaggatatccccattgccaccaagtccctcaccatcaggaccagcaccgccggatg
cgccagcggctccgaccggtccaaccccatctcaccggcactctccctcacgccgcacctctactcgccgtcgccgccgtcgtccgcgttcgtgtcggcg
cttcagtccccgtacatctcccctcgagtcctcgagcctccgctcccgccggcggcgcagccgcgccaagacgccaaggccgccggcgtcagcgcaac
caacaccgcggcgccgtcgccgacgtcatgctccaacggtggagactcggaggatttggacgcgcccagcgcgtcccgcacgccgccgtcggaacgc
tacgactccagcggcatcgacgcgcccaagatttccgacggcggggtgggtccccttccgccgcgcgtgtcgttttcgttccccgtgccgcgggtgtcctt
cacaaggggctccgtggcgtcgccgtcttccaacgccaagctccggagctgcgacgtgtacattggctaccatggcaacggcggcctcggcaggttctg
caagtggctcaagtcagagctcgagctgcagggcattgcgtctttcgtcgccgaccgtgcaaagtactcagacacacagagccatgagattgcagacc
ggattatttgctcggtcgcattcggtgttgtggtggtcacgatgtcaagtttcctcaatccatatagccttgaggagattagattttttgctcagaagaaga
atctggtgcctatattgtttgacactgagccatcagatattgccgggctttttgatggtaaactggaggataaagaggggaaggaagcatttgaagggct
aatgcggtgccatgaattcaagctcgaggctaacgagagcaattccagaaactgcgtatcaaggacggtcacattgctgcggtccaagctgggccgga
agaatattgctgagaaggagaatgaagcatctgaggtctgccttttccacgcaaccggcattttgtggggagggagaaggagctgaccgaaatcgaa
ggactattcttcgggtccactgtggatatccaagaagtggactgcccaaggggttctatcgcaaatgacagatctagtggtgtatctgatggttttgctga
cgaggagagcgattctgcgaggacatctaatgccagatacatcagtttggagatgcgcaagtgcaaggagccgacattggaggcttggattgatccag
tgattgagttgtcgtcgggtaaaggaagaggcctccagaagcaaagatcaaagcacaggaggtcaaggtttcggtgcaacagcaaaagctatggcag
tgccaacgtaatctgcatcaatggctcttcaggtatcggcaagactgaactggcattggagtttgcttacaggtactctcagcggtacaaaatggtactgt
ggattggaggcgaagctcgatatttgagacagaacatccttaatttatcgatgaatctgggatcggacatcagtgctgaggccgagaaggagaggggt
agaattaggagctttgaagagcaggaattcgatgcattccagcgggtgaagcgggagcttttccgtgatgtgccctatttgcttgtaattgataatcttga
gagtgagagggattggtgggaaggcaaggatctccatgacttcattccaaggaacactggagcaactcatgttattgtgaccacacgtttgccacgtgt
gatgaatcttgagccaatgcaattaccacaactctcctacattgacgcgatggccttgatacaggggaagaggaagaaggactatcctcctgaggaag
cagaagttcttaggaagtttgatgagcggctaggccggctgagctttggcctatgggttgttggatctctgctgtctgagctcatgattgcgccttctactc
tgtttgaggctgttgagaggatatcattgagtgacaatgtgttcccaattggtgccaatgatgatggcttctgtcgtaacaattctttcttgataaaggtctt
agtcttctgttttgcattgatggaccgggcaaaaggaggaagccttacctcaagaatggtcatcgctggctcttggttagctcctgctcctgtctcatctac
acttttggctgccacagcaagcaagctaccaatgaaaggcagtggtatgcacctgtttggtgaatctctcaagactgcatttctatgtggcacacattgct
ttctcgctcctaatggacggaaggcagaggtggagtcggcactgttacttgttaatcttgggctggcaaggaaggctaatcgacatccaggttgctggat
ccaattccatccgatcacacagctctttgggaagatcaggggaggtttagcaccgacaactgcagcggtgaatggagtaatgagggccggcaacccat
cagtatactcagaccacctttgggctagtgcattccttgtgtttggtttcaagtctgagccacctgctgttcaacttaaggcggttgacatggtattcttcat
aaagaagacagcactgcccttggcaatcgactctttcatgacattctcgaggtgtggctcagctttagagctgcttaaggtgtgtaccaacgtccttgagg
aagtggaaaaatcttatgcatctcgtatgcaagactggaaccgtggatcattatgctggaggaagaagctgcaaccaaaccaccgtgtcgatgaatttg
tctggcaggaggtgacgcttctcaaagcgacgttgctggagacaagggcaaaactgctgctccgaggtggactgtttgacactggtgaggagttgtgca
ggacctgcattagcatccggacagtcatgcttgggcatggccatgcccaaacactagctgctcaggaaacgttggcaaagctggtcagatacaggagt
aaggtatga
>g157583.t1
atggatcttgtggcagggccgctaggcagcgtcatcatcaaactcggcaagcttctgaaggaggagtacgacctgcagacaggagtgaggaagaaga
tcatgcgcctctcacgtgatcttgagagcatgcacgcggcgctccgcaaggttgccgatgtgccgccggagcagctcgacgatgaagtcaagttttggg
cgtgcgatatccgggagctttcctacgacatagaggacatcctcgacaccttcttcctggtgcgcatcgagggccgcaaccccaacaatttgaagcgtgc
caccaagaagatcggtaagctgttcagcaagagcaaggctcaccatcagattggtggcatgatcaaaaccattaatcaacaagcagaggtggtggcg
gagcgccatcacaggtacagggccgaggatattgtgtccaagccggccccaccgcggactattgaccctcgccttaaggctatgtacacagatgtgac
acagcttgttggcattgaaaagccaagcgatgagctcatatccatgctatggcctcttgggggcgatggggtgaagatggtttccattgtcggagctgga
gggttggggaagaccactcttgcaaaaacagtatatgacaagattaaggtgaattttgactgtacagcttttgttccagttgggcgggatcctgacctca
agaaagtcttcagagatattcttatagggcttgacaaggacaaatacatggatcctagattcacaatactggatgaaagataa
>g158460.t1
atggcggaaatcgggggcatgctcgccgccgctgtcctcaagttggcggcaacaaaggtggccgaagccgctggagatcggttcatgctgcagtgga
ggtttggcgaggacatggagaatatgagggagacgatggaatccatcgcggcagtgctccaggacgcggagaggcggtccatcaaggaagaggcag
tgcggctgtggctcaagcgcctcactggagcctcgtaccgtatcaccgatatttttgatgaattcgaagttagtaggaccaggaaatcggctttacgaaa
gttcagagttctcacacttgcttctgaggttcgcatggctggtaagatgaaggtggtcagggagaaactggatagtatctcaaatgaacgtgacaaatac
agtcttgcaacaagcagcggctccaataatcagcaagttatcaatgaccgtgcaacatcgccagaggtgatagaagcagaaatcatagggagggatg
aagaaaaacagaaaatagtggctcttttgagtacctcatcggacttcattatcgttccaatatgtggcattgggggcattggcaagacaacattggcaca

actgcttttcaatgatacctatttcaaggactacaaaaaagcatgggtatatgtctcccagacatttgacttgaacaccattaaagagagtgtaagatcac
aacttcggctggagcaggggcagctagccaactcacatgagcagaatgccggtccacctgctcctaagagcattttgattgttttggatgatctgtggga
acaagatgacttgaagatggatgacctgaagaagtccctaagaatggtcgggaagggacacaaagtgcatataattgtaatgacacgtgatgctggca
ttgctcacaaaattcaaacagttgaagcacacaagataaaggccctagctcctgaggtgtgttggactataatcaagcagatagctgactttgaaaaca
gatctgacaaggacatgttgaaggatatcgggaatgagatttctgcgaagtgtggaggtgtggccctagcagctcgtgctgttgggttcatgttgagatc
cagggacgtcgatggatgggtttcagtaaaagacagtgctctctggaatgtttctacgccaggatatacaacttcggtgtacgacaatgtgcttgcatctt
tgaaattgagctacagcagcaacaccatgacatccctcctgaggtccagacgtgttagaactatccgcgacttcatgcatgaccttgcaacatcgatcat
gggggatgatcttctttatgccagcaaggaattcatcaagtgtgggcaaagcagctgccgctatgccttccttgcaggctgcaagtacaactga
>g158462.t1
atgcttgccgccaccgccctcgagttggcgctggaaaaggtggccgaagccgccagcgatcgggtcatgctgcagtggaggtttggggaggacatgg
agaccatgagggagacgatggaatccatcacggcggtgctcaaggatgcggagaggcggtccatcgaggaagagacggtgcggttgtggctcaagc
gcctcactggagcctcgtatcgtatcacagacatttttgatgagtttgaagtcaataccaacaggaaatcggctttgcgaaagctcaaaattctgaaccc
ctgtcttaaacttgcttctgaggttcctatggctggtaagatgaaggagatcaaggacatactggataatattgcaagggaacgtcaaaattatagtttca
caacaagtagcagctctaatgatcagcaagttatcaatgaccgtgcaacatcatcagagacatttgacttgaacaaaattaaaaagagtgtaagatcac
aacttcagctggagcaggggcagctagccaacgcacatgagcaggatgttagtccacatgctcctaagagcattatgattgttttggatgatctgtggga
gcaagatgacatgaagatggatgacctgaagaagttcctaaaaatggtcgtgaagggacacaaagtgcatataattgtaaccacacgtgatgctggca
ttgctcagaaaattcaaacaattgaagcacataagatagaggccctgtcttctgaggtgtgctggactataattaagcagatagttgactttgaaaacag
atctgacaaagagatgctgaaggatatagggaatgaggtagcaggaaagtgtggaggtgtggccctagcagctcgtgctattgggtccatattgagatt
cagggatgttgatggatgggttgcagtaaaagacaatactttgtggaatgtttttacgccgggatctacaacttcggcgtatgacaatgtgcgggcatctt
tgaaactgagctacagcagcatgccaccgcacttaaggttatgcttcgcctattgtgcagtcttcccaaagggccacaagatggctaaagatgacctgat
ttaccaatgggcttccctaggttttactatgtcatctgacagaatctccattcagcagcatggcgaggcctacattaagcaactattggacatgtctttcttt
cagcggtcagaatcgcagtttgatacaaaagatgttacattcttcactatgcatgacctggtgcatgaccttgcaaaatcggtgatgggggatgagcttct
tgatgccagcaaggagttcgtcaagtgtggcgcaagcaactgccgctatgccttccttgcagactgcagcaagccactgagttcatatgtcacttatcct
gacaagattagagcccttcgttttctaggaagtggcaaaactggacattgttctattggattttcaaccgctaagtacctcagagtcttggaattaggga
aagctccataaagaagttgccgtcttctattggtcaactgaaactattgagatatctcaatgctccagggatcaaagatcgagtgattcccagttgtataa
ccaagctctccaaattgatttacctcagcctccgtgaatctgatgagcttttagcattgccagagtcgtttggagaaatggaagctctgatgtatcttgattt
gtctggttgtaagctataccagaagccttgtgtagcctaa
>g159286.t1
atggacgctgttctgtcatcggcggcgagcgagctcattagccggctggtctcctacctcatcaggaaataccaaaatcgcagagccaccgataatact
gccatcaggctgcagcaaacgctgctacgagctcgggtcgttgttgaggaagccgaggggcggcagatctccaaccgagcgatgctggagcagctca
accagctgaaacgagagctgtaccgagcagcctatgcgatcgatgccttcatgtggcgggatagaagatcacacgccaagttgagccgatatcgatcg
cacacaaggccgcctcttagcagcgattgctctgaccttctaccgaccatggtgggcagtctggaagctgctctgagcgacatgagggagtttgttgtgc
tcctcagtggctgcccccgcgtcaccaggcagccatacagcacctacctgttcatggagagctgcatgtttgggcgacagatggagaaggaagaaatc
atcagctttttatcgcggccttctcaagatctggatgtcctcccgatcatcggcccgcttggtgtcgggaagcggaccttgatggagcacgtctgtctcgat
gagagggtgcgtgagcgcttcaccaagattcatcgtcttgctagtgacgagcttgatgatcttcagaaccctgaccatcattggagcttgtttgacaactt
ctccacaaagtcactgatggtgatcgatatggtcggcagcaacacagatgccgaggagtcatggagaaggttccactcagctgttttccagcaggcaca
cagagggagcaagatcttaatcatcagtaggatggaagggcactcgagcctgggcactatcccggtgctcaggctgcacatgccgcaacgggaggag
ctttggtacttcttcagggtgctcgccttcggggccgcggaccctaacgagcgcccggatcttctacgcattgctatggcactattcaaaggcactagcag
tgacttcgctgttctattcgccgccgccaacaccatcgctgcagcgctgcgtgcggacctgagctcgcgctcctggtgccggttgctcaaggtattttctga
ggcgacgatgttgcaggccgggcactattacatctccctgccggtgaagggcgcaccgggttcttcttgcttgttctacaacgggcgcaagttgactagc
atggctctgaacgatctgcccaaggtgacgatgtcggggctgctcactagcggctccccctccccacaggggagacacggtttgacgtgcttgtgtgg
cagtctcgtattcctccgtatgcaagctacatcgcaacatgcgacatagacgaaactcatccgaagacggtagcactcaagaaacgacttctaaacaag
aggaggagagaccaccaagaagatggtactagtaactag
>g160091.t1
atggaggcacttggatggatcgtgtcggccgggagcaacgtccgggaagagactcagctcaaggacgaggtggaccgcctccggatcaccttgccca
aggctcgcttcctcatcggtcgtggcgagtggtgcaggttcaagaacaaggagctggcagaacttgtcactcagctcaaagacagcacctacgatggtg
aggacctgctccgcgagctggatgaccaggagctgcggcaaaagatcgaggacgctggccgtaccagggcacgtcaggtcttgtcttcttctctgaatc
ttgcaagaaactttatccacggtaacaaaacaagggtgaaggaggcccagaataagctggacaaggcagtggtagcgatagaaggcgtgcttaattc
catgggttttgaatcagagcagttcatgccagagacaagttcggttgtcagtgctcctcaagtgttcggccgtgataaagaacgagatgcgttaatggag
atgttgggcgtgccgattgggcgcgtggacaaagttgatcaggtgatcgagcaattgggagtgcccctcaccagggatggtggaggcaagcagtcatc
tgccgggtccaagggaccaccagacgaagcaggcagtaccacatctagtgccaaacgactaaaaattggttgtggcagggtcggatttcctcagacca
gctgcaccggcgacgtttctgtcttgacggtttatggtattggcggggtggggaagacaaccctggcgcagctcatctacaatgatccgagggtggaac
acaattttccagagaagatatgggtctgcgtttcggacctctttgacaagagaaggataacaaaggagatccttgaatcaatctctagggaaaagtaca
accactcatgcagtttaagtactcttcaagaaaagttgaagaaggagctggagcggtgcccaaagttccttctagtgttggatgacatttggccaaatgc
taatgacgcatgggaggagttttatgcaccattgaggactggacctgaaggcagcatgatcttagtgaccacaaggagagtgaattgtgggaactacc
gcatcgagataatgagatattaccagcccttcaactga
>g160231.t1
atgaagctcgcggcagcggcgatgacccctctcatagagaagcttggcaatgtgctggcgtccgagctgacgctggagaagagtgtccagaaagaca
tagcctccctggagcgggagctgaaggtgatgaccggccctgagctcagggaggtagctgaggtgccccaggaccggctcgacgaggggaccaagc

tctgggcaagcgatgtgcgcgagctctcctacgacatggcggacgccatcgacgccttcaccttgcacgttcctcgacgaggggaccaagctgctgggt
tgaggagccggctccgcgggtttctcgacaagacgacatggctgttcacaaagggaaaggagctgcaccagatcgctggagccgttagaggcgccaa
ggacctcgccaaggaattgtgcgagatccaccagaggtacgctgggctcaagctccaagacgccagcactggcgcttctgacattgaccctcgcctga
cggcaatgtatagcgaggtcgcggacctcgtcggcatcgacggcgcgagggacgagctaatcaaggtcctgactgatcgctcgatagtggatgtgaag
actgtctccattgttgggtttggtggactggggaagacgacgctcgccaaagcagtgtttgacaagataatagtacagttcgattgcggggctttcgtttc
agtgtctcgaaaccctgacatcaccaagattttgcagttgatcctgtttgaactggacaagcaacgctatgcaaacatcaacggggtcgtcagggataat
atgaaacaactcattgatgagcttagaggctttcttctggataagaggctaccaattaaagggttggggaatctgacttccttggaagagctgggtgcgg
tgacaatcgacggcgatgacgctgcagctgtggtgagcgagctcgggcacctcaccgggctcagggttcttaaattattatggaatacagaggttattg
gtgaagctttcgtacagtcgcttggaaatttgcgcaagttgcaaagtcttaatatgagcctcagtggtgagcgctgcgacctcattcgtagctgggcgcc
gccgccatgcctccgcaggttcatatgcaacggcggctggatcagcgtgctgccggaatggatcaataataactgttgtccgtcgctcacgttcctggac
atctgtgtcagcgaggtaggcgccgaggacttgaagacccttgggacgctgtcgtcgctgcgtgtcgtccgcctgcgcaatttgggacgcatcgatgaa
ggcgaccgtttgctagaggtgaacgccaacgcgttcccgtgcgcgaggaactgcaggttcattaacgttgtggcaacgccatcgatgttcccccgggc
gccatgccgctggtgcagtgcctgcggttctgcgtccgggcgtgggacttcgccggcggaggagggtttgggttggatgacctgcgcatggggcacctc
ccttcgttggagaaggtcgaagtcgatctgtggtacagggaggaggaggacgacaccaaggtggtggaggcggcgctgcggcaagcggtggaagac
caccctaaccgcctcgccctagacatccacaagtggaaatgctggaggccgagccaggcgtag
>g160233.t1
atgtacaccaaagcctcagacctcattggcatcgaggacatgaaggatgagctaattnaaaaggatgccggcggcgcttcttcatctgctgacattgac
cctcgtctgacggcgatgtacaccaaagcctcagacctcattggcatcgaggacatgaaggatgagctaattcagatcctgtctgatccctcggaagtg
gatgtaaagactgtgaccattgttgggtttggtgggctgggcaagacgacgctcgccaacgtagtgtatgacaagatcaaagtgcaatttgattgcaag
gctttcatttcagtgtctcagaaccccgacattactaggatatttaaaaagatccttcatcaacttgacaaggaagagttcaaggacatcaatgaggcgg
taagggatgttgatcaactcacccatgatctcaaagggtaccttcagagtaagagtgggaatacaaggctgccaaaagggtttgggaatctgacttccc
tggaagagctatgtgagttgcccatcgacgatgatgattctactgctactgtcgtgaaagaactcggccatctcaccgggctgagggttctcaaattgttg
tggatgggggcggataccgttggtgaagccttggtacaatcgcttggaaatatgcgcaagctgcaaagcctttacatgtcggtctatcaaggaagcagc
gacgtcatgtgtgactgggcgccgccaccgcccctccgcaggttcttgtgcactggtctgaccagcgtgctgccgacgctgccagcgtggatgaagaac
aactcgttgtcgctcacgttcgtggacatctcgctaagccaggtgagacccgaagatctgcaggcacttggagcgctgtcgtctctgcgcgccatccggc
tgtggtcgcagagctgctttcacaacccgcagatgaggatgaaggtgaacgccggcgggttcccgtccacgagggcgtgcgcgttcctggacttcgcg
acggtgccgtccattttcgccccgggcgccatgctgcacgtgcagcagcttgagttctgcatttgggtttggaaatttaccagcggaggaagcgggtttg
ggttagaggatctgcgcatgggacacctcccttcgctggtggatgtcgaagtcgacctgtggtacatgcatgagaacgacgccgcggtggtggagcgg
gttgaggcggcgctgcggcaagcggcggaagaccaccccaatcgcattgccctgcgcgtcaacaagtgcatggtcagatcgagacggagttgggcat
gggaatag
>g161558.t1
atggcgatggtgctcgaatccttcgtgaagcgatgcacggcgtcactggaggacttcgccggccaggaagcctgcgccgccctcggcatcggggacga
cgtcagggccctccttgccactctctggcgcatcgaggccatcgtctcccacgaggagcgcaggaggctgctgagcaccaaagtcgacgcctgggtgg
cgcagctcaaggattccatgtacgaggttgacgacgttctcgatgtgtgcatgattgagggcggcaagatcctcgcagacgaccaccccctacgccca
aggcccggtgctccttggtgttctcctgcttcaagcacacaggaccccgcaagttccgccatgaaatcgggttcaaggtcagggacattgatctaaggct
gagggaggttgaggaggaaatgcccaggcttcctgctggatcagtacactcggattcgaccaggaattggttcagtgataacatatgtaagcactgcca
ttccgggattaagcctcaggcagctggggcgcaagtccggaaggctgtgggtggccttgtgccaaggttgcttagagagggcaagaggaaggtggcc
gtgtttgccattgttggcgcgatggggattggcaagacgacacttgctagggagatttacgacgatgacaggatgaccgaaaatttccccatatgtgtgt
gggtgaagatgtccaaggatttatcagaggtagctttttttgaagaagataatcaggggtgccggtgccaacgtcggggacacagagaacaaggagga
actccttggcctcctcagttctgctctttcaaagaggtttctgattgtcttggatgacttggacagcccgggcatatgggacaatctgctcaaagatccgat
gggagatggcgtggcgagaggcagaatactgatcacgacacggaacgaggaagtggcaacaagcatgaaggcaactgtccaccgagttgaaaaaa
tggacactgagagtggctgggatttgttgtgcaaacaagtccttcccgagtgcaacacagaagagcttgtggcactgaaaaatgtcgggattaagattg
tggacagatgtgaaggtcatcctctggcaatcaaggtcattgcgggtttgatgaggtcacgaggcaagagcaaggctgagtgggagacgatcctgaga
agtgattcttggtccatgcaattggtccttccagaagtaccacgagctctatataatgtccgattcagcgctttgaccttgtgcgtcattggattgctgaag
gcgttgtaa
>g163942.t1
atggcggataccatcatcatcgctgccattggcgatgcggtcagtagagtaatttctcttctccttgggcacttcaatcaacaacagagcagtgaggcca
agctagccacggtttgccacatgctaatcaggatccacagcgttgtcgaagaagccaaagagcggcagatccacaaccacggcaccttgcagtggctc
tcggagcttattgatgccgagtaccagggtcgttacctgctagacaccatagagtgtgacaagaacaacgaacaacatcaacatggtaaagtttcctat
gtttctaccacgtccttgttcaatccatcaaagcgtgtgcgggttggggagagcactatgaggagattattgccctggcaccatgattttgatgtttatgag
attgatagggtgcatgagaggttgcgggacatgtctagtgatctcagggagtttatcatgttactgcaaagctgccagcaaattagccggcctctagtta
caagcatattccgagatggtcagatgttcggtcgacatgttgagaaggagagaataatcaacttcttgctccacaccagaggtggttcaacaggcaaag
tggaagtattgcctattattggtgacgacgggactgggaagacaaccttggttcagcatgcttgcgatgatgctagggtgcgcagacactttgcggtgat
catgctctgttgtacctacacgatcacggcagacagaggaactcctgttctacgatcgaaacttagtattggtgatggtggaataaatctaaatgaccca
atgcagttattcaagggtcagctcggggacaagaagtttttgatggtatttgaggacgttgacatgaaaaagatgcagatgctagaagggctcttgcca
agcatgggatggagcaaagaggggagcaaagcagagttgtag
>g164407.t1
atgcaagccttcctcacagacctggatgaaaagatgctgaggggtggtgccatggccaggaacctggtttgtgaggttcgggaggtcgcgcaagaggt
ggaagacatcattgacactgcaaacatactaaggaggcaaagggaccagaagaagtccatcagggtggccatttccaaatacgcttgcttccccgtct

atctcgctcatctccacaagatagtaacaaggatagattcagcaacagcaagaatgaaaatcatctttgatgactttcagagacataacattgtcactac
tgcaattggagaagatgaaaggtgtttcaccaaggaggatgaaatcatccaacactggcgatcatcagcccaccctgacctcggaaagcaagtggatg
tgattggatttgaccaccagattgaacagattaagggtgacttggtggacacattgaacaagcagctatctgtattttccatactcggtccaggagatctg
ctcaaggagatggttaagcgtattatgccagtccggcaagctaaagagcttaacaagaagacccagagtgaggtttatgctctatatacaactaggga
gatggccgaacaagctgttttcacggtgctgcaacggggcagtagcattgcagttgatgaagccaaatacttgttgggcgtctttaacaaactggagtta
gccaaacaacaactcgtgagcatgcaagccttcctcacagacctggatgaaaagatgctgaggggtggtgccatggccaggaacctggtttgtgaggt
tcgggaggtcgcgcaagaggtggaagacatcattgacactgcaaacatactaaggaggcaaagggaccagaagaagtccatcagggtggccatttcc
aaatacgcttgcttccccgtctatctcgctcatctccacaagatagtaacaaggatagattcagcaacagcaagaatgaaaatcatctttgatgactttca
gagacataacattgtcactactgcaattggagaagatgaaaggtgtttcaccaaggaggatgaaatcatccaacactggcgatcatcagcccaccctg
acctcggaaagcaagtggatgtgattggatttgaccaccagattgaacagattaagggtgacttggtggacacattgaacaagcagctatctgtattttc
catactcggtccaggagatctgctcaaggagatggttaagcgtattatgccagtccggcaagctaaagagcttaacaagaagacccagagtgaggtga
agaaaaggcttcatgatttcttacagaggaggaggtacttgataatattggacgacgtatgggaaggagatgcctgggatgtgataagtgacgctttcc
cggacatggaggattggaaaattgaggacggatgcatgatcagtctcatggagctaattctttgcaagtgctccaatctgcgttaccttcctgaggggct
gtcaatgctcccacgtctcaagagggtgaagctcattgccatgtcagcgagttgttatcaggaaagcagagttgctagggagcttgataacaagggctg
tgtggtctctgtctccaccaacgagaaagatttcaagcatctggatatgccaatccaagctttttttggggccagtgagtgccgaggaccgtacctccgta
gtccaaccagcatgccgagtccacacgccctcctccgtggaggccggtgttgacgcgcaagcctcacatcgttccccagatggccacctctgtcggtcc
acggcgcacaccacatccccccctccatggaagctgccgccgccgccacgccaaggggctcagcacgtctgtcgaaggagccgccaccagcacaagg
gggccagccgccgcgccccgtgcgtcaggccttcgccaggagtactatcagattcgtccatcaatcaaccaaatctga
>g165266.t1
atggcagacacctcggcggccgtgagcgtggccctctcgaagctcgccgccctgcaggacgttaatcagtacaagatctcgcggcgcgtgcggcggca
catcgcggccatcgcggactcgctccgggagacgcacgccgccgtggcgctggcctcggaggcggaggcccagcacgagctgcacacggctcagtcg
tccaaccatggcagttcatcatcaggactggtcggagaaacgatgctcatgttgcagcgggtcatcgaccagctggaggacctcgaccccccggtcggc
ggtccgagccgtcccgatcagcgttccggtggacgacgacgacgcgcgccgccgccgtcctcccccaataacggaggcggcgccgtacggtgtcgtcg
tgggcgtgggcccgcagagagaccaggtggtcgagatgctgcgtttgggagaggaggaggaggagctcagggtggtcaccattgccggaccggtcg
gcatggggaagacgacgctggctcacgaggtgtaccgcacggtgggaccatccttcgactgccgggcttgggtgtccgcatcacggagttacggtgag
atgaacatcttgatcgacattctttgccaagttgaggaggaggatcatccgcatggcggcggcggcgtgaccgatctgcatctcatggagaagatcgat
ggaatcctgcaagataaaaggtacctcattgttctggataatgtgtcgacgtcaacagcccggaacaccgtcgtgtctgctttgccatgcaatgctctgg
gcagcagagtggtcgtgacgacgagcgacagttttggccaactgaatttgagaaactggccagtttggtggaacaaccacgcctacggcatgcaatct
ctcaggtcgtacgacgatgactgtgataagctgttgttccatacaagagtcttcggctccacgggcagctgtcccgcagaactggcagatgtttccaaca
agatcgtgtcggaatgctccggtgtgccgctagcccttactctcgtctcaggtttactggcgaacaagccgtgcacgcgggtgatatgggagcaagtgtg
tgatttcattgctggttggaatcgtggtgatgcagacaaagggatgaggaacattgtactgctcagttaccatggccttccccactatctgaagacttga
>g165278.t1
atggctatgattcttgacgccttcgtccccattctcggccgtatggttgcagatgcggtgagggcgaagtttgacatgctcgtcggtgttcctgccgatatg
caaaagctggagaccatattggaggacctcggcaacgtccttgttgacgccgagcggaagcgcatcactgacacggtcgttgatggctgggtccggga
gctgaaagatgtcatgtacgacgctgacgatgtcctcgaccagtggatgatggaggcccgtagcagccatgcggcgaagcggtcctcctctgctgcag
gcggctgctgcgtgcccctcttcacgtgctttcgtgaccccgtttttgctcacgacatgtccaggcaaatcaaggagctgaatcggaggctggagagcgt
caccaagcggagctccatgtttcatttcgtcagggcttcatccgcctccccttggcactggcagcgaccacgaccagcggcggcgcggaagacgagctc
ggtgttggtccacgccgatctcgtcggagacaagatagaagaagacgcagtcacgctggtggacgcgttgaccagcgacgaccaccgtgaaaacgtg
cttgtactggccatcacaggcgccggtgggatcgggaagaccacccttgccaagagggtcttcgacgaccagcgtgtgcgagacgagttcgatctgcg
ggtatgggtgtgcgtgtcgcaggaggtgaacgaggctgacctcctgtggtcggtcatcggcggcgtaggaggcacgcaccagctgcacctgcacgacg
acgccgcagacaagtcgtcgctcgagcccgtgctccagcgcgctgtctcaggaaagaaggccctgctagtgatggacgacgtgtggagcgacgaggc
ttggaacgtgatgctccgggacgcgttcaaggccggcgtccgtggcggcagccgagtcctcgtcacgacgaggaacgagatggtcgcccggcagatg
aaggccacgtacatccaccgcgtggagaagctgcagcctcaagacggctggaaattgctcaagaaccagattagatactttcttcatcccatattacttg
tcattttagtcttagatgcggtgagggcgaagtttgacatgctcgtcggtgttcctgccgatatgcaaaagctggagaccatattggaggacctcggcaa
cgtccttgttgacgccgagcggaagcgcatcactgacacggtcgttgatggctgggtccgggagctgaaagatgtcatgtacgacgctgacgatgtcct
cgaccagtggatgatggaggcccgtagcagccatgcggcgaagcggtcctcctctgctgcaggcggctgctgcgtgcccctcttcacgagctttcgtga
ccccgttttcgctcacgacatgtccaggcaaatcaaggagctgaatcggaggctggagagcgtctccaagcggagctccatgtttcatttcgtcagggct
tcatccgcctccccttggcactggcagcgaccacgaccagcggcggcgcggaagacgagctcggtgttggtccacgccgatctcgtcggagacaagat
agaagaagacgcagtcacgctggtggacgcgttgaccagcgacgaccaccgtgaaaacgtgcttgtactggccatcacaggcgccggtgggatcggg
aagaccacccttgccaagagggtcttcgacgaccagcgtgtgcgagacgagttcgatctgcgggtatgggtgtgcgtgtcgcaggaggtgaacgaggc
tgacctcctgtggtcggtcatcggcggcgtaggaggcacgcaccagctgcacctgcacgacgacgccgcagacaagtcgtcgctcgagcccgtgctcc
agcgcgctgtctcaggaaagaaggccctgctagtgatggacgacgtgtggagcgacgaggcttggaacgtgatgctccgggacgcgttcaaggccgg
cgtccgtggcggcagccgagtcctcgtcacgacgaggaacgagatggtcgcccggcagatgaaggccacgtacatccaccgcgtggagaagctgca
gcctcaagacggttggaaattgctcaagaaccagagtgtccaggtgttcaacagattattccctccattctga
>g165787.t1
atgctgatggagatggcgatagaagaggtgcgtatgctgcttggggtccctgacgagataaacaagatggacgccaagctcggggatctcaagaagtt
ccttgcggatgctgacaggaggaatatcaatgatgagagtgtgcaggggtgggtgaaagagcttaggggcgccatgtatgatgccactgacatcctcg
acttgtgccacctcaaagccatgaagcagggtccaagtcgagacatagggtgtttcaaccccttgctcttctgcatgagaaatcccctccatgcccatga
catgggtagccgtctcaggaagttgaatgagaagctagacgacatcagcaagcgtagcaccgccttcaacttcatcagcctcacttcatatgaggaccg

aaaggtaaagatagaatccttctttcttgctaaccgtgagacaacagcggagctcgacaagttgggtattgttggtgagaatattgaggaggacacaag
atatctggtggaattgctcacgatgaaggataaaataattgatgagcacaagaacgttgttgtttttgccattgttggagttggcgggatcggaaaaacc
actctcgcagaaagtatcttcaacaacgaaaccatcaaacaagattttgaaaaaaaaaatatggttgagtatcaaccaaaacttcaatga
>g166671.t1
atggaggcgacagcactgagcgttggcaagtccgtgctagatggagcacttggatacgcaaaatcggccctcgcggaggaggtggccttgcagcttgg
catccagggcgaccatgctttcatcagggacgagctccagatgatgcaggctttcttgatggctgcacacgatgaccgagacaagcaccaggttctcat
ggcctgggtgaagcaagttcgagacgtggcctacgatgcggaggactgcctccaagatttctccatctaccttaaggacccatcttggtggcgtctcccc
tacacactgcgagagagacgccgcattgccaagcaactgaaggggttaagagtcagagttgaggatgttagccagagaaacctacgctaccaactctc
taagagccccagctttaaggctggaactgttgctgagatgtctagcattacagctgcaacggtatttggtgttggtgaagcacggcgtgctgcgagacaa
gaggaaccaaaagaggatcttgttcgcctcatcaacaaggagggcgaggatcctagagtgatcgccttgtggggaacaaacggtgatcttggcctggc
aaccataatcaatgcggcctacgagaattcagatatcaaaaagaaattttcactccgagcatggataaggatcttgcatccgttcaatccaaatgacttt
atccagagtttggtgaagcaatttcgatcatctgagggggttgacattctgttggagaaggacaagacagggcatgatttggctatggagttcacaggat
acgtaaaggaaaagcgttatctcattgtgcttaatgacctatccacctttgaagagtggaacgtgatcaaaacatgttttcccagcaaatga
>g166683.t1
atggcggaagcgctgcttctctccgtcatgcgcggcgtggtgggcaaggccggcgacgtgctcgtgcagcgcgtgacccgcatgtggggcgttgatgg
cgaccgcgagaagatggaggaccggctggtgtacgtgagccgcatgcttgttgacgccgaggtgaaggccgagacggaccccgccgtcaagaggtg
gatgaaaaatctcaaggccgtcgcctaccaggccgacgacatcctcgacaacttccagtatgaggcgctgcaccgcgaggcccagggtccccggtcca
tgactagcaaggtactaagcaacttcacccaccacagcccgcttttattccgcctcaaaataagctgggagctcaagaatgtgctggacaagttggagg
gattagtatcagacatgaatcagtttggcctactagagcgtgcggaggtgccgcaagttgtgaaccggcaaaaacactcagcactggacaagcatgag
gagatatttggcagaaacaatgacaaggagatgatagtgaagctgttgctcgaccatcaagatcagcaaagtgtgcaggtgctgcccatcattgggat
gggaggtttgggcaagaccacactagcaaaactggtttacaatgacagcagggtcaagaagcactttgaactaaagatgtggtgttgtgtgtcagaaa
attttgaagcaactgctattgtgagatctgtcattgagttggcaacaaaaggaacatgtgacctacgtgacaacattgagttgttgaagggaagactgca
ggaagtcattggccgcaaaaggttcctactcattcttgatgatgtgtggaacaacgagcaacacaagtgggaggaggacctgaagccgctgttgtgttc
ttgcatcggtggatcaggaagcacgatagtcgtgacaagcattggaatgcttcttgctcccgaatgcccgctgcggtcctccgccgcgttccaaggcccc
ccggctcagtccaaggccagcttcccgcttgctcgattcatcgccatccaaaaccaggcgagcagcatcggccttaaccttacggcggcgatgcccactt
cacttcgtgcgtcgtggcggatcaagccggtggtcggcgcggccaggcagcatccgtcttggaggatcggcagtgatggggagaagcgtcgcaccccc
agtagcagacctactggccgtgcacgcggagtgtttgcagaaatgccgcactgggccggcggctcacatcgagaccccgtagtggtgtggtttaatttg
ttcttgcaatttatccgcttttcaaagcaaccaagaaattaa
>g166684.t1
atggctgctgagtggctgcttctccccgtggtgcaaggcgtggtcggcaaggcagctgatgagctcgtccagagcatcactcgcatgtggggcgttgac
gacgaccgtgagaggctggagcgcctgctgttgtacgtgcagtcactgctgcctgacgcagaggcgaagagtgagaccaaccttgccatcaggacgtg
gatgaaggcactcaaggccgtcgcctacaaggctgatgacgtcttggatgatttccagtatgaggcgctgcggcgtgaggcacaaggaaaccagtcca
tagcaagcaagttacttagcaatttcacttcaaaagatcgacttgtgctccgttataaggcgagtagggaccttaaggatgttctcgacaagattgatga
gctagcgactgagctcagtaaatttggcctggcggagcgtgtggaaatacagcatgttccgtatcggcagacacactcattgctggacaagtctgagga
ggtctatggcagagatgatgacaaggaagcgttggtgaagctgttgctcgatcagcaaaatcggcggaaggtgcaggttcttccgataattggaatgg
gtggtttgggcaaaacaacgctagccaagatggtgtacaacgacagcagggttcagaaacactttcagttgaagatgtggcactttttccccaaggact
atgagatagagaaggacattctcatccaactatggatggcaaacgaaatggagattggcttggtgatccctgaaaacatgtactatagagagcagagc
atgaatccgatccgacatcttcccctgcatgttgaccaagtgggatcgccggcgaagctccgcgccgccaccgcgtcgacaacctcttga
>g167095.t1
atggatagcagattaacgagcacccatcacgccgagctgcttgagccttgtggatcagcgtgggcagcagggtgcgatggctccttctatactaagatt
gtgatccatgtgaacatgacgatgacgtgcagcaaaagccatgttttaagtttagtctccaagataagtggagtgaaagccatgagtttcatggatgaca
agaacatgttgacagtgctaggcaatagcgtttattgcaaggacattgagacctgcctgcggaagaaggtcggtgtcgcgacgatcatggagttggac
aacaggggcgtgcagttgcagccggcaatggtcggtgccgccggggagcagcaaatgccgaccaacaggaggccgcgtactcgcagcagtggcatc
cgggctattactgctataatctctagattcatgtgcctacgagctgaggatacggacgtcccggaggagctggctgagacaggccccaggtcactctgg
cgtctggtcagcgcggatacaccattgtggcaaactggtgaaaacatattcacgacgccggacccgctgctcctgcagctgcagcaccagccggcggc
ggttccagtgcgacgcaggcggcgcaccttcaacatgtccaaggagcacaggaaaggtacgcacgctcgagccatggcggccgtcctgaatgctttgg
cggcctacgtaaccaagttgataacggacatggcggaagaaaaggtccatcttctgctgggtgtctcggacgagatcgacaagcttgggcagaaccta
gataacctcaaggcgttcctagccgatgccgaaaggcagcgtatcacggatcagcttgtgcagaggtgggtaaggatgttgaaggacgtcatgtatgat
gccactgacatactggagctgatccagctcaagggtgaaaagtggcgggagtcaatgggtggaagcgtggagaagatgcctcgtcgttgctaccagcc
atttcttttgtgcctgcgcaatccattgttcgctcacaagattggaagcagaatcaataagctgaaccagcggctgaaccacatccatggagaggctgct
aaattcaacttcacagcagcgagtctcagctcctacctggaaaaaaggaggatgctcattgaggctgaatactccagccaaaagatgacatcagagttc
attccatccaccatcgtcggtgagaaaatcgagagggatacaaaattgcttgtccaagagctaatcatggatgacaactttgacatgaaggtggtatcc
attgtaggcatgggcggcatgggaaagaccacccttgcccaaaagatcttcaaagatagtgccatagacgagcacttcaaactgaagatatggttgag
cattactcagaactttgacgcagttgagctgttgagaacggcaatcaagcatgctgggggagaccctggtaaggagcaagacaatactctcctcacgc
agaccgtcactaatatattgtccacaagcaagtttttgctggtattggatgacatatggagtgttagagcatgggaatgtgtgcttagtgtcccagttaca
aatgccagccataaacaacctggaagcaaggtcctggttactacaagatttgaagatctagctccacggttttatgatgccttctaccaacaccacgtta
gccccctcgacgatgaggacgcgtggtccatgctgaagaggcagctgccacaacggcctaatcaggtcagtctgcccatgccgtga
>g168148.t1

atgggaaggctcttcctggtgctggagaaagagtacaacaagcacaagagccttgcgcaagagacccagtcgatccagcaggatctgcgcatgatcg
ccgccgccatggacgaccagctccgcgccttgggccaccggagcgaccgcaccgccgtggcgaggctgtacagtgaggagatgctcgatctgcaaca
cgacatcgaggactgcgttgaccgcttcatgcatcgcctcaggtgcaagcagctcactggcggcggcggcggcgcagcgtcctccttggtgcgtcgggt
agctcacgagctgaagaaagttcagagccgttcgagcatcgccgatgagatccagaagctcaagagccgcctcagggaagcacatcagcgggtaatc
gacatcgttcctaccggctccggcggcggtgcccaacccaacgagggatcgtcatcagcactgcctcgtgtcacttgtaacccggtggggatcgagaag
cctctggaagagctgctgtcgctgctagatgaggtggacggcgagcctgagcagccgagggtcatctccatcgtgggcttcggtggattggggaagac
caccctcgcgagggcggtctacgacagccctcaagccagggaaaaattcgactgccgtgcttgggttgcggctgccagggactcgccggagaccggc
gatgggaacagagggatccttcgtgatataattaagcaaattcgtccgaaagatgccatgaatgttgacagccactatctagaggcctggatcaaacaa
aacctcaaggatatcaggtacttaattgtcattgatgacatccggatggatgaatggaccaccataaaggtagcctttgagaacaattgtagaagcagc
agagtactgcttactacaactattcagtcagtggctagcatgtgcagccatggcaatggttatgtgtaccaaatgaacagcctcggcgaagaagactcta
ggaaaatagcctttccgtggctctgctcacctgagttgaagcaaggttcaacagcactattgcgaaaatgtgatggccatccactcgctcttgtctgtgttt
ccgactatctgaagagctcaagtgaaccaacaggagagctttgtggaacgttatgccgcaacttgggcttccatctgaatgagatgcatggccatgaca
acttcgcagatctcagggaagtccttatcgataactacgacagtttctccggccatgctctgacttgcctgttgtatcttggcatcttcccaaacaatcatcc
attcaagaggaaagttataataaggcggtggttagccgaagggtatgcacgaactgattctcttcgtgatgaacaggacatcgctgatgagaacttcaa
caagcttatcgactggaacatcattcagccagttgatacaagaaacaattcaaaagcgaagacctgcaaagctcatgggatcgtgcacgagtttgtgca
gcacaagtccatgatgcagggatttctcgcaaaattatctcacgattacccaagacccagtgccagtgctaataatgcgcgccacatttctgtccattctg
gcaaaccaacagaatgcctgccaactatcgagcaaggagctttgccaaacctcgtatcactccagctgctctgccggggtctggttggtgtttcaagtat
taacatcagaaacttcaaacatctcaaagaaatcacaattgacgacaaagtggctggacaaacaagacaagactgggaactggcagcaaagaacca
cccaaataggcctcgtgttttgctactcaaaacgtgcgatcccacggaaagcaaggaacaagagtactttgctatggtggagaagcgaaaaaggcgtg
aaaaagcctatccagcttcagatgatggactggattctagtctgaagaaggtgagactttcagagcctcagttcttcacgtttccatgtgatcatccgtca
agtggtggcaacgatgagattaacgccaccatccgatag
>g172421.t1
atggatctggtgagagaggccatgggcagccttctccccaacctgcacaagctgctcgggtacgagtacaagctgcagacggggatgaagcagcaac
tgaagtttatcacacgggagcttgagatcatcgacgctttcctccacaaggtctccgacgtgccatggtaccagctcgatgcgcagaccaaggcctggg
cgcgagaggcaagggaggtatcctatgagatggaggatgtcctcgataccttccttgtgcattttcaggaccgggagcccgccaacccgagcaggctc
cgacgcgccatgaagaacatggctagcctattcagcaagggtaaggcacgtcgtcacatcacttctgcaatccaagacatcaagaatcgactccagaa
tctggccgaccagcgtgccaggtacagggtcgatgatattatggtgaaccatcctgcaactacgttgactgtggatccgcgccttcaagctatgtacaaa
gaagttgcaaacctggttggcatcgacaagccaagggattacataatatccaagctgtctctttatggcgatgatatggcctgctcgagaaaaatagtct
ctatttctgggaccggagggatcggtaagacgactcttgccaaagcggtgtatgacaaccttagagcgtacttcccacgtggggcttttgtacccgttggt
cggaatcctaacatggcgaaactactcatggatattctttatgatcttgacaacatcaactatgcggatgttcattccttcaagaacagagatgaaaggc
agctcattgacagcctccgagaaattctcaaggacaagaggtatgcatttactaggaaaataccattgtag
>g172564.t1
atggaggttgtgatgggagcacttcctaatctcctccccaagcttggcaagctgctcgtcggcgagtataatctgcaaaaggatgtgaagaatggaatc
acattcctgaaatctgagcttgagagcatgcaggttgccctagagaagatctccaagacaccagcagaccagcttgacgaccaagacaagatctgggg
caggcaggtgagagagatgtcctatgacatagaggacagtattgacacatttatggtccagggcaaaggcagtgagctgtcggacagtcagcgtggtt
tcaagaagttcattgataggagcctcgatttcttaacacggactaggattcgccgtaagattagtaaggatgtcagagacatgaagatccgtgttgaaga
ggtcgccaagcggcatgataggtacaagatgggaggtgttgatgcaaagccaagcatggttactattgatcctcgtttatggggtcagtacaagaaagc
agaagagcttgttggcatcaacgagacaagagatgagataatcaagaacctaaccaaaggggatggaatggcaaaacatcgaggcaagatagtttc
catagttggctttgggggggctagggaaaacaacccttgctaatgcaatatatcaagagatcagtgcaacatttgaatgccgagcttttgtttctgtgtctc
aaactcctaacatgaggaatcttttcaaggatatgctcaaggaccttgctggcatcaatactgaaatgacgggtgagggaagactcatcaacgagctca
gacaatttcttcaagataagaggtatgaaattaaggatcacataacatactag

Protein Kinase domain containing genes

>g256.t1
atgggctttgtgcgccttcctcaatctgatttttggggttctgatctagactacatgccgtcgacatctctggatgcttgcgaagctctgtgcttggcaagat
gctcctgcctggcctttgagtacaaatcagataacaatggttgcttcttgaaatctgttctcttaaatggaaagactgttcctgggtaccctggcactgcac
acatcaaggttcctgagagctttttatctgaaacatcttctgattctgaatatactgaattccataactgtgatgagtcaagcaggcaagtagaagttcaa
atatttgattctaatgtaagaaatggtggaaaggctacgatgtggtcctactactatgggtttctggcggcgttctttttcatagagctctgcttcattgcttt
tggttggtggttcatcacaagaaaacattcagagcaatcagagatgtggacagcggaggaaggttacagggttgtaacaaatcatttccgcaggttttc
caatatggaacttaggaaggccactaaaaatttcaaggatgagcttggtcatgggagatatggttctgtatacaagggtatcctacctgacaaacgcat
aattgctgtgaagaagctcaatgatgtaagccaaggagaagatgagttcgagacagaggttagtgtgatagggcggatctaccatatgaatctggtga
gagtatggggtgtttgttctgaacagaagcaccggttgcttgtctacgaatatgtggaaaatggctcactggccatgttcttgtttggtaacaatggattgc
tgcaatgggaccagaggtacaagattgcagttggtgtggccaaagggttggcgtaccttcaccatgagtgcttggactggatcatccattgtgatgttaa
gcctgagaacatactactggacgaggattttgaacccaagatcagcgactttggggtcgcaaagctgctacagagagatcaaactgatccaaatgtgtc
gaaggttcgtgggacaagaggctacgctgctccagaatgggcctttaacattccgataaacgaaaaggtggatgtctatagctatggggttgtgcttctt
gaattagtcatgggatcaagggtatcagagttgagatcaaatggctacggtgattcagaggctgtactgagacagttaatgtggacaattgtggagaag
ctaaaatacggtggtcaatcatggagtgctgagtttgtagatcctaagttaaatgttgacaacgcctag
>g333.t1

atgctgctgctgcttcagcatttgtacctgggcgctatgctcctcctcacgatgatcacacatggcagcactgcagcaccaacaggagacagagaccgg
ttcatctacaacggcttcgcgggtgcgaacctcactcttgacggcgtggcagcggtgacgccagccggcctcctggtgctcaccaacggcagcctccag
tcgaagggccacgcgttccatccgtccacgctgccgttccgggaccccaacgcccggaacgccaccgcggcgcggtccttctcgaccaccttcgtgttc
gccatttacgggcaatacgcggacctcagcagcaacgggctcgccttcgtcgtcgccgcggacagggccgtgctgtccaacgcgatgccgggccgcaa
ccacgtcggcgtggatgtcaacagcctcaagtcgatcaacgccaccgatgccggatactacgaagacgacggcacggagcagttccagaacctgagc
ctgatcagccggaagcccatgcaagtctgggtggactacgacggcgcggccacgcaggtcaccgtgaccatggctccgctaggcttgccccggcccaa
gacgccgctgctgcaggcctccatcgacctctccgacgtcgtgcgggccaaggcgcacgtcgggttctcgtcggcgacgtccagcctcgtctcgcgcca
cttcgtgctcggctggagcttcgcgctggacgggccggccccggcgctgaacatcgcagggctgccagccttgccgcctgcagggccgaagccgcgtt
ccaaggtgttggagatcgtgctacccgtagcgtcagcggcgctggtgcttgccgtgggcatcgcgatctacaccttggtgcggcggcggatcaagtacg
ctgagctgcgcgaggactgggagctcgcgtttggcccgcaccggttcgcgtacaaggatttgttccacgccaccagtgggttcagtgacaagaagctgc
tcggtgcaggagggtttggaagcgtgtacaagggcgttcttcgcaagacgggcatggaggtcgcggtgaagaaggtgtcacatgagtcgaagcaggg
cctcaaggagttcatctccgaggtcgtgagtatggggcgactgaggcaccgcaacctcgtgcagttactcggctactgccgccgcaaaggtaaacttct
cttgatctacgactacatgccaaatgggagccttgacagacacctttatgatcaaagcaagagtgcattggattggcctcagagatttcacatcataaga
ggagtggcatctggcctgttgtatctccatgatgactgggagcaagtagtcatccatcgggatgttaaggcaagcaacgtgctcatcgacggggaaatg
aatggaaggctaggagactttggcctcgcacggctttacgatcatggatcccatgcgcacactacacatgttatgggcactatgggttacctagccccag
agttaggccacaccggcaaggcaactccagccacggatgtcttttctttcggggcgttccttctggaggtcacctgtgggcgaaggcctgttgagaaaga
tgggctcaacaatcgtgtcatgctcgtggattggaaaacaaactatgccatccacgcttgtacccttgctcttcctgctcctcctgatccatgtggcctggt
ccgccggagaaggccagtttgtctaccaagggttcacctcatcagcgaaccttactctggatggcctgngctcttcctgctcctcctgatccatgtggcct
ggtccgccggagaaggccagtttatctaccaagggttcacctcatcagcgaaccttactctggacgacctcgctgtcgtgacgcccggcggcctcctgg
cgctcacgaacgccacgatccagaagaaagcccacgccttccactccactcctctccactttctcaacaagtccttctcgacgtgcttcgtgttcgccatc
gtgtccagctacgacgggctcagtgatcacggcctggccttcgtgttttccccgaccaccaacttctctgcggcgaaagcgggccagtacctgggcctcc
tcaacgccacaaacggcacggcgagcgaccacgtcttggcggtcgagctcgacaccatcatgaaccccgagttccgcgacatcaaccccaaccatgtg
ggcgtcgatgtcaacagcctcatctcggagcaagcccagcccgccggctactacgacgacgcggcaggcggcgctttgagagaactgaagctaaaca
gtcgcaagccgatgcaagtgtgggtggattatgacggcaacgccaggcagctcaacgtgacactggccccggtgaaagtgccaaagcccaagaggcc
tcttctctccacgcccgttaatctgtccagcatcatggcggataagatatatatcggcttctcgtcggcgacgggcgtcgtggccacgcaccactacgtgc
tcggatggagcttcagcttggacggaccagctcccccctatagacttctccaggcttcctgccctaccacgggtgggtccaaagcctcggtccaagctctt
ggacgtcctgctgccgctcaccacagcgttgttcgtcgctgcggtgctatcagcagtcttcctgatcttatggaggagacaccggtacgctgaggtgaag
gaagattgggaggacgagtttggtccgcagcgtttcgtatataaagatttatttcacgcaactgatggcttcaaggataggaacttgctcggcgtcggag
ggttcggcaaagtatacaaagggctactttcagcgaccaatttggagattgcagtgaagaaagtctcacatgattcgcggcaaggagtgagggagttc
gtcgccgaggtggtcctcgcagatggctgcacggtggcgaaagggatgagcacagcgatcagtaggatggacttgttgaatgacgccttttcctgggtc
gatgtgaagggatttgttttgggtatcgatgaatga
>g403.t1
atgaaagatggaaagctgtatgcagtgaaggttttgaataagcgttacatgatgaaaatacatgttgtacaatcagtaactgccatgacagatgttcttc
gggaagatgacgatgatatgcttacgagatctcctggtactcttatttttgctgcgccagagtgctatgaaggttcaccctaccatggcagggcatctgat
atatgggctgttggtgttactttatattgtatgatttctgggcactacccattccttggagacactctgcaggagattcaagataagattgtcaatgatccg
gtgcaaatacccggggatatgaacccacaactcgctgatttgctccaaagacttctctgcaaagatcctggagatcgtatcaccctgcaggctgcatctg
tgcatccttgggttgctggcgacgaggggccagtccctgaatacatctgtagatgtggctttggccgcaggaagacggaagaatttggggaagaagta
caatag
>g473.t1
atgcccaatggcagcttacagatgctgctgcactccgaaggtagacgacagttgggtttcctaaagaggctggacatcatgctagatgtggctatggcc
atggaatatctgcaccatgagcatcacgatgtggtcttacactgcgacttgaagcccagcaatgtactctttgatacagacatgacggggcatgtggctg
actttggcatcgcaaagttgctgttaggcaatgacaactcgttgatcacagctagcatgcctggaactcttgggtacatggcaccagagtatgggtctctc
ggcaaggcttcgcggaagagcgacatgttcagctacggaatcatgctccttgaagttttcactagaaagcgacccaccgatcctatgttcgtcgcggatc
agagcatcaggcagtgggttcgtcagtcgtttccatcggagctcatccatgttctggacgagcagctcgtgcaagatgcttcttcctcttcatgcgacatg
atgaatgaatttcttctgccgatatttgagttgggattgatctgctcaagagactcacctgaccagaggatgtcaatgagcgatgtggtggtgagactgac
gaagatgaaagaagaatatgctaaatcgacatcagtagcaaggcagaccgttcaatag
>g475.t1
atgcccaatggcagcttagagatgctcctgcactctgaaggtagaagctgcttgggactcctcaagaggttggacatcatgctagacgtgtcaatggcc
atggaatacctgcaccatgagcattatgaagtgatcttgcactgtgacctgaagcccagtaacatactctttgacgaggacatgacggcacatgtctctg
actttgggatcgcaaagttgctattgggtgataacaactccgtcaccactgcaagcatgcctggaacacttgggtacatggcaccagagtgtggatatct
tggaaaggtatcacgcaagagcgacgtattcagctatggaattgtgctccttgaagtatttactggaaggcggcccacagatcctatgtttgacgggga
aacgaccattcggcaatgggtttatcaggcatttccaactgcgcttacctctgttctagatgagcagctgcagcaagatgctatttgttcaatctttggcct
gaatgacttccttaagccggtatttgagctgggtttgctctgctcgagtgacgcgcctgaccgaaggatgtcaatgagagacgtggtggtggcgctgaa
gaagatcaaaaagaattatgcaaaatcgacgatgcagagtgttgcctgggtcaatttgcaaatgctcccggccgtcgtcctcgcttccggcgacgacct
cctgtcgccgccggcgtccgcgaacccctcctccatgcgttccgaccaatcctccgtcgattccgctggtccaacctcttcctccggcctcccagcgtgcct
actgcctatccgcgctggctgcgcgtga
>g619.t1
atgtctggctttgagctgtcaaagatgacaagaaaggacaagatgacttttaatggtagccaaggcacaatgggatacactgcaccagagtgttctgta
gatggtgagcccatcacggccaaagccgatgtctatagttatggcatgatgctgctcgagatcatcactggccgatcaaacaaggaagttaaagattca

gtatttatagacagctggtactgtcccaagtgggtgtatgagaaagtatatgaagagcggcgtctgcaggacatcttagatgaacgcattgtcccatttg
aggcctacattgacccccaaagcatgtccatggtggagcggatggtgaagacggccatttggtgtgttcaaagccgtgcagtgatgcgaccttcgatgg
acaatgtcatcaagatgctccaaggctccattgatatcaccaaaccaccaaagccatacattttgttaccgtaa
>g931.t1
atgcggcagaggcaagcggttgcacgcggtgaggcggggcggccctggcgactatgcggggcgaggaaggacgaccctgcggtggaagctgcagt
ggcggcgcggggccatatgcagaaactgactggtgttgctgggagcctgcatatggggctcccgagattctacatggaaaactactggcagaaggtag
acatatgggctgttgctgtactcctgcatgtgctactgatgagcactcttccatttcaatggaacgctgctgaagctatctttgatgccataaagaccatcg
agcttgatttttcacagtggtcagtgggcaacggtagcacttcttgcacgtga
>g1100.t1
atgatcacgtttcgccacgacgttgacctgcccctggttgatgccaacgggacgacgatcaagctgctggtatacgagttcgttgagaacgaatcgttgg
acaagcacctgttcgtcggcgcagagcacaggccgccgttgccgtggcgcgagcggtacaggatcgcgctgggcacggcccggggtctggcctacct
ccaccacgagtgcctcgagtgggtcatccactgtgacgtcaagccggagaacatcctggtgacacgggagttcgacgccaagatcgcagatttcgggc
tggccaagctgtccggcaggagagacggcggcgccggcggcgtgcagctctcgcacatgagggggacgacggggtacatggcgccggagtgggcg
ctgaacctgcccatcaacgccaaggtcgacgtgtacagctacggcgtcgtgctgctggagacggtgatcgggaggagggtcgcggaccagaagacgc
cggaggggcagccgctggagatgccgcggatcgttcaggcgctgaggcgggttgtggacagcggggacgtcgcggccttggtagatggcaggttgca
ggggcagttcaatcctcgacaggcattggaaatggtgaggatttccttggcgtgcctggaggagagaagcagcagtaggccgacgatggacgacgtc
gccaaggctcttaccgcgtacgatgatgaggacgagcaccctgcgtacaagtcgtag
>g1297.t1
atgtcctataagagctccatctgtgctattctcttcctgctcccgctcatcttctccaccgacgatgggttcgtgttcaatgggttcaacggtagttccggct
gggtcatggatggtggttcggatgtcctgccgaacggcatccttgcccttggcaacgctttctattcttctccgttccagatgcgtaaccacagtgacggtt
ccgtattctccttctctgccactttcgcatttgttatacctcctgccaccgggagtggcagagacggaatcgcctttgtcctggccccaaacatcagcttaa
gcaccgtacgaacttatggcggaggtatgtttggtcttcttagccccggtgacaacggcaatccgtcaaatcacctcctctgcattgagctagatacactt
tacaatgaaaagttcgctgatatagatgacaaccatgtcggcatcgacgccaacagcttgaactcctcacgatcttctccagcaggctattacaccgata
acccattctctgatttgcacccgctgcggctgtccagtggcagggagatgcaagtttggatagactacgaccatgggctcatgcagctcaacgtcagtct
gtctctgatcccgaatcctaaacccaagcacccattgcttgctcacaacatcaatctttcacaagtgttcttggatcacatgtatgtcggcttctcatcatca
gcgtggggtattattcatggttatttcatcctcggctgcagcttcaaggtaaacacaaaatctgcaaaacttgactactccaagctgccgaaagtaaacg
catcccaatcccttgtccacgctgctgtgcctccaaatactattagtcgattcatggcccattattcagttcttattggttttgggatatttgtattactacttgt
ggccttcatcaccctatcagtgagaagatgccgctaccgagagatccgtgaggattgggagatcgagttcggcccacatcgtttctcgtacaaggatcta
ttccatgcaacagacggctttaaggataggctgttgcttggagcgggaggattcgggaaggtatataaaggagtgcttccatcatcaaagctggaggtt
gcagtgaaggtcatgtcccatgattcgaagcaagggatgaaagagttcatcgcagaggttgtaagtatgggccgccttcggcatcgtaaccttgttcaat
tacttgggtactgtcgtcgcaaaggtgaactgctgctagtctacgagtacatgtcaaatggcagccttgataagcacttgtatgatctggagaagccttca
ctgaattgggctcacaggtttgagatcatcaagggtgttgcatcaggcctcctttatctgcacgaggaatgggagcaggttgtaatacaccgtgatgtca
aggcaagcaacgtgcttctggatgaagagatgaatggagagctaggtgactttggcctagctaggttgcacgaccatggagtcgaggcgcacacgac
atgtgtggcgggaactagaggctacatctcccccgagttagccaggcttggaaaggccactaaagcaacggatgtgttcgctttcggtgcgttcatccta
gaagtcgcttgtggcaggcgaccaatcacagtgaattcactcggcgaaccgatagtcttggtggattttgttctcaaatgctggcaggatgatttgatagt
ttgtgttattgatcctagattgggtggagaatatgtggaggaggaagcagagctggtgctgaagcttggacttctctgttcgcacccagctccggtaggc
aggccgaacatgaggcttatcatgcagtatctatgtggagatgtgccatttccagaaatgcccaatgtttatctggatatccggagtataaacgagctac
aagttgaaggcggcttcagtgaagaagatgtacagtgccgatcggtgcagaccagtataactggtctctcaggaggaaggtga
>g1310.t1
atgggcacccacggctacgccgccccggagtacatactcaccggtcacttgacggcgaagagcgacgtgtatagcttcggcgtggtgctgctggagctt
ctcagcgggcggcggagcgtcgacaagcggcggcgcggacgggagcagcacctggtggagtgggcccggccgtacctgcgtcggccggagcggct
gcaccgcgtgatggacccgagcctggacgggcagtactcaaccaaggccgcgcacaaggcagccatggtcgcctaccactgcctgcacagcgtgccc
aagtcgcgccccaccatgcgcgacgtcgttgacgcgcttgagtcgctgcttcacatgtgcagcgacgttcccgcgggcccgttcgtttacacggtcccttc
ggcgccggcagatgacggaaaggcaacagaggaggaggtggccaagaagagccatgtcgcgtcgtcggccgtggacgccgagggtaatctgcgga
cgaggaaccaacggtacgcgagctcggtgacggggcacaagagctcctctccgacgcagagcagggacagaggagcctaa
>g1400.t1
atggattactacgagcggctcggcaagatcggggagggcgcttcggcgatcgtctacctagcccgcgacctccgcactggcgagaccgtcgccgtgaa
gcgcctccgccccggcctcggcggcgacggcgacgtcgacgaggagaccttccaacgcgaggtgcgttgcctcgaggcgtgtcgcggccacccgtcc
ctcgtcgcgctccgcgccgcgcactgggacccacgcgccggcgggggagcgttcctcgtgatggagcacatcgggccgagcctggcgcgcatcatga
aagaggaccgcgcgggccggcctttccccgaggctgaggcccgccgcgtgatgcggcgactgctggagggcgtcagtgcgatgcacgggctcgggtt
cctccaccgggacatcaagcccgacaacgtcctcgtccgcggcgactttgacgtcgtcaaaatctgcgacttcgggctgtctcgcgccacgtcgtcacca
gccgccgccgcgccgcggtacacgtcgggcgtcgtgacgctgtggtaccgcgcgccggagctcatgctgggctcgacggagtacgacgcgggtgtgg
acacgtgggcgctcggctgcgtcatggcggagctcctcaccggcggcaagccgctgttcccggggcggtcggagatggaccagctgaaccgggtgttc
gacacggtcgggatgcaggacatgaggtcgtggccggggttcgcgaggctcccgcgcgccgagtccgcgctctgtcggcgcagcaggccgcccggcc
ggctccgggagatgttcccggagctgtcggctgccgggttcgacgtcctcagcgggctgttgagctgtcggccggaccggaggctcaccgtggtggag
gcgctgcggcgcccgtggttcgacgaggtcgtcgtggtagcgccgccgcctgtggcccagcacgatcagccgcgtgttacttgctgctgcgccggttac
agcaccgacgtttccagtgtcgccgaggcaatcttggcatag
>g1685.t1

atgcgctggtggaagcgttcctcctcccccctcccgctccgcctccgcctccacgccctcgtcccccgcacgctcctccacctcccgcctcatcggcgggag
caaccgtcgcgatggcggcggcgggagtgccgaatcgcagccgaaacttgttaggacgccgcggatgcgctacgtcgtcgacgactccgaggtcggg
ccctccggactcgggctggatgccgaccccgccaggagggaatcggcgggcgggttcgccctcgcgaccgcgagctcgacgccgatctcgaggtcgc
cgagcaacatggaggtggcgccgttgaggtcgtcatcgacgcccttgcttcttccgctgccgctgcctggcgaggggggagttgtcctgccgcgtatctgg
gaggccgcttccctcgcctgcgtccaggatggttgatggggactggaccgtgtcggcggcggaggcgccaggggggtttagagactgggagtgaaagg
attccgccgttgctcgcctcgaggcatactggagctctttgtgccatgaaagaggtcaatataattccagatgatgctaaatcagctgagtcgctgaaac
aattggaacaggagataaagtttcttagtcaatttaagcatgaaaacatagtgcagtactacggcagtgaaacaattgaagatagattctacatatacct
ggaatatgtccatcctggttctattcataaggatatcaaaggggccaatttgttagttgacatcaatggtgttgtcaaattggctgattttggaatggctaa
gcatttgagtactgcagctcctaatctttcattgaaaggaacaccatactggatggctcctgagttgagtactgcagctcctaatctttcattgaaaggaa
caccatactggatggctcctgagatgttaatccttatcatataa
>g1859.t1
atgagcgcggagctgaagagggactacgagatcggcgaggagctgggccgcggccggttcggcgtggtccggcgctgcacgtgccgcgccaccggc
gagtccttcgccgtgaagtcggtggaccggtctcagcttggcgacgacctggaccgcgagctggccgaggtggagccgaagctggcgcagctggcga
gcaagggcaacccgggcgtggtgcaggtgcacgcggtgtacgaggacgagacgtggacccacaccgtcatggacctctgctccggcgccgacctggt
ggactggctccgcctccgccgcggcgcgccggttcccgagcccgtggcggccgagatcgtcgcgcagctggcccaggccctcgccatctgccaccgcc
gcggcgtggcccaccgcgacgtcaagcccgacaacgtcctcgtcgacgacgccgccgaggccgaggagaacgaggaggccccgcgcgtgcggctcg
cggacttcgggtcggcggcgtggatcggggccggcgggttggggacggtggaggggctggtggggacgccgcactacgtggcgcccgaggtggtgg
cgggcgaggagtacggcgccaaggccgacgtgtggagcgccggggtgctcatgtacgtgctgctctccggcggcgcgatgcccttcgtcggcgagtcc
gcggcggaggtgctcgcggccgtgctgcgcgggagcgtcaggttcccgccaaggctcttcggcggcgtgtcgccggcggcaaaggacctcatgaggc
gcatgatgtgccgtgacgtgtggaggaggttctccgcagagcaagtcctcgctcacccctggatcgtgagtggcggaggagcccgggcggcgggtacc
tga
>g2316.t1
atggagtccagcggggaggagctcttgaagaagatccgggagctggaggtggggcaggcgcagctcaagcaggagatgtccaagctcatgcccagc
gcaggggagcggcggcgctcgcagtctgtctcgccgcggcgaggcgtgcctgtgccgccgccgccgcctgcaaaggcgaagccgcctactaggcggc
tgtccggcgggttcgagggcggcccgcgcgcgtggtcccgtgggtcggcatcgttctcgcactcatcgccgctgcagcgggagggccgcgccacgggc
gccggagacgccgggaccagctccgcgctgccggagcggcagtactccagggtgctgcaatcgctagggcagtccgtccacattcttgacctcgacgg
caggatcatatactggaatcgttctgcagagaatctatttggttatcctgcatcagaagcccttggtcaggatgctctcatgcttctagttgattcgcatga
ccacaatgtggtaaatgatatcttccgacgcatttctatgggcgagagttggactgggaagttttcagttaagaacagggcaggagataggttttcagct
gttgccaccaacacccctttctacgatgaggatggcagtttggtggcccttggtcaggatgctctcatgcttctagttgattcgcatgaccacaatgtggta
aatgatatcttccgacgcatttctatgggcgagagttggactgggaagttttcagttaagaacagggcaggagataggttttcagctgttgccaccaaca
cccctttctacgatgaggatggcagtttggtgggctcttgcgggacagtatatcatgctttgtggtatggctcggatgtggctgttaaagttttctccaagc
aagaatattcagaagatgtgatacaaacttttcgacaagaggcgaggggtatgaattatcttcaccattccagcccgcctatcatccatcgagatttaaa
atcatctaacctgttggttgataagaattggaccgtgaaggtggcagactttggtctttctcgtctcaaacgtgaaacttttctaacaaaaactggaaaag
gaacagcggtgagtaccgggagcagcagtgccgatgaggtcgcgctcctgttcttcaagtccgcgctatgggacccggccgggtcgccacatccaacc
gcctctgcagcttag
>g2359.t1
atgcacagctatgatcctccatatgctcataatgatgttaagcctggtaatgttcttatcactcgtcatagaggacaagcacctattgcaactttaatggat
tttggaagtgcaaggcctgcaagaaaggaaatccgttctcgttctgaggctttacagttacaggaatgggctgctgagcattgctctgcaccttatcgcg
cacctgaattgtgggactgtcccagccttgctgatattgacgagagaacagacatttggtctttaggatgcaccctttatgcaatcatgtacaatgtttcac
catttgagtatgctcttggtgaatctggaggaagcctgcaacttgccattgtcaatgggcagttgaagtggcctgcaggacccaatcctccttaccctgaa
gaacttcggcagtttgttatctggatgcttcagcctcaacctgcaatgcgccctcacattgatgatattcttcttcatgttgacaagcttattacaaaataca
agtcttaa
>g2451.t1
atgcaaccggacccgagcgggaacgccaatgcgaaggggaagctgccgcagccggtgacggcgccggcgccgtcgtccggccggccggcgtccgtg
ttgccgtacaaaacggcgaacgtgcgcgacttctaccgcatcggtaagaagctggggcaggggcagttcggcaccacgtaccagtgcgtggacaagg
ccgatggcgcagagtacgcgtgcaagtccatccccaagcgcaagctgctgtgccgggaggactacgaggacgtgtaccgcgagatccagatcatgca
ccacctttcggagcaccccaacgtcgtgcgcatccgcggcgcttatgaggatgcgctcttcgtgcacatcgtcatggaaatctgcgccggtggtgagctc
tttgatcgcatcgtcgcaaagggacactacagcgagcgtgcggcggcgaagctcatcaagaccatcgtcggggttgtggaggggtgccactcgcttgg
tgtcatgcaccgggacctcaagccggagaacttcctctttgccagcacagcagaggacgcacctctcaaggctactgactttgggctctccatgttctac
aagcccggtgataaattctctgatgtcgttgggagcccctattatgtcgcaccagaggtgcttcagaaatgctatggtccagaagctgatgtctggagtg
ctggggttatcctgtacattttgctttgtggagttcccccattttgggctgaaactgaagcaggaatcttcaggcagattctacggggaaaacttgattttg
aatctgaaccctggcctagcatctctgatagtgccaaagatctggtccgtagtatgcttactcgtgatcctagaaagagatatagtgctcatgaagttctc
tgtcacccatggatcattgatgatgccgttgcacctgataagcctattgattctgctgttttgtcaaggctgaaacatttctcagcaatgaacaagctcaag
aagatggcattgagggttattgctgaaagcttgtcagaggaagagattggaggcctcaaggagctgttcaaaatgattgatactgacaatagtgggact
ataacatttgatgagctgaaaaatggcttgaaaagggttggctcagagttaacagagaatgagatccaagctttgatggaagcagctgatattgacaac
agtggaaccatcgactacggtgaatttatcgcagctacattgcacatgaataaactagagagggaggaaaacttggtgtcagcattctcgtttttcgaca
aagatggaagtggctttataaccatagatgagctgtcacaagcatgccgtgaatttggtcttgatgatgttcaccttgaggatatgattaaagatatcgat
caaaacaatgatgggcaaattgactacagcgagttcacggcaatgatgaggaagggcaacgctggttgtgcaacaggtaggaggaccatgaggacc
aatttgaatcttggtgaactcttgaatcccagcaacagctag

>g2493.t1
atgtcgtcttgggacgacgacgacgagtctgcgtcggcgtcggcgtcggcggcgaccaccgacgtggagctgctcaagcgggcgtggcgcaacgaga
aggcggcgccggaggtcctccgcttcgacaaggacctcgtcgaccgcgaggagacgctggatgacttcgccgacagcggcgtcgacgacctggtggt
ctcgctctaccagatggacctcgaccgcacgctcttcctcctccgctcctacctccgactccgcctccaaaagatcgagaaatacacaatgcacatctca
agatctcaagacctcaaggaccggctgtcggagcaggagcagaagtttgccaagagttgcggggagatcatggaaaagcatctgaagcagtccgtgc
tgtccaagctgccgtacgggtacggggacgagtttgtgacgaaacagtcactgtcgagcacagaggatgatatgactcctttcctcagcagccttgttgt
cccatttcgtgctggatctggctttgtaagatccaagcagcttgctggcccctctcggcaaagatcctcgcttgggtggttcttggtgacttcggggaggat
gcagcaagatcagaagaagaaggcagcatcggagatggactttttcacagagtatggcgagggaagccggtataaaatagaagaggtcataggaaa
aggcagttacggtgtggtatgctctgctctggacactcatactggtgaaaaggttgctataaagaagattaatgacatcttcgagcatgtctccgatgca
actcggatacttcgtgagatcaagttgcttagactgctgcggcatccggatattgtggaaatcaagcatattctacttcctccatcaaggagagaattcag
ggatatatatgttgtttttgaactcatggagtctgatttgcaccaagttataaaggctaacgatgacttgaccccagagcattatcaatttttcttatatcag
ttgcttcgtggattgaaatacatacatacagaactattgactgggaagcctcttttccctgggaaaaatgtggtacatcaacttgatataattacagatctc
ctgggaacaccttctccagaagcaatatctaggattcgaaatgagaaggctaggcgctatttgagcagtatgaggcggaaaaagcctataccgttcac
gcacaaattcccaaatgcagatccacgtgcactaagtttattagagcgaatgctagccttcgagccgaaagaccgaccgagcgcggaagaggctcttg
ctgatccttatttcaagaatatagccaatgtggatagagaaccttctgcacagcccgtcacgaagctggaatttgaatttgagaggcggagggtcacaa
aggaggacataagagaactcatatatagggagattctggagtaccatccaaaaatgttgagagaatttcttgagggaaccgagtcaagtggtttcatgt
atccaagtgcagtagaccaatttaaaaagcagtttgcataccttgaagaacattatgccaagggatcgacaggaactccgcctgagaggcaacataatt
cattgccacgaccttctgttatctattcagacaaccgaccacaaagcacggtgggtatcactgaggatctttcaaaatgtttaatcagagaaaatgcaca
aaggacgcagcaatattctgctgcagttagtaccagtaaatttccacaccatgttcctcaaggtgctgttgcaaggcctggcaaagtagttggttcagtgc
tgcagtacaactccaatccagcgtctgctgctgagcaatatgagcaacgaagggtagctagacacccagctgttggtccaaacaatataccttctggaa
gctcgtacccgagaagaaatcagacgtgcaagagtgagactggtgaggctgataggattgatgtaaaccaggctgggcagcaaaaaccatatgcagc
aaacaaactacctgctactgtcgatggtcgtggtggccactggtag
>g2509.t1
atgaagatcgcggaggacgtggcggcggggctgctgcatctccaccagtcgagcatcgtccacggcaacctgaagccgttcaatgtgctcctgggccc
ggactttgagtcatgcctcaccgactatggactactcatgatgatgatgatgcttcccgaccatgtcaatgatgcatcttcaacctcgctctttttctcgtgc
ctatcatggacatggtcattcctcatatggctttga
>g2546.t1
atggacatcgacctggaccgtgcgcgcgcgctgcgcgtcctcgggcgcggcgccatgggcaccgtcttcctcgtcgccgacgacccttcctcggcgtac
ctgccatcccggtacgccctcaaggtgctcgacaagcgcagcgccaagcgcgacgccgcccgtcgcgcgcggtgggaggtctccgtgctgtcgcgcct
ggcccaccacccgcacctcccctccctcctcgggtgcgccgagaccgacgacctcctggcgtgggccatcccctactgccccggcggcgacctcaacg
agctgcgccacgcgctccccgaccgcgtcttctccccggcctccgtccgcttctacgtcgccgagatcgtctccgcgctcgccgaggtccacgccgccgg
catcgtctaccgcgacctcaagcccgagaacgtcctcctccgcgccgacggccacgtcaccctcaccgacttcgacctctcccgcctcctcccgccgcgc
caccactcctcctccgcatccacctcgccctccccgccgccgccacacccattccgcggcggcggcgagtacaagtacaaccgccgcacacgcgccac
cgcgaggagcgactccgtggtgggccaggcctccaagccgccggagccctggtcgtcgtcgtcctactcctcgccgcggcagcacctgcagaacctgg
tgcgcttcctcatgcggagcgacgggccgggccggaaggccaagtcggcccgcgtgtcgcccaagcccgcgagcttcggcggctcatcctcgtcatca
tcatcgccgtgggggaaatcgtactcgttcgtgggcacggaggagtacgtggcgccggagatggtgcgcggcgagggccacgggttcgcggtggact
ggtgggccgtgggcgtgctgatccacgagatggcattcggccactcgccgttcaagggccgggaccgcaaggacacgttccgcaacgtgctgcaccg
ggagctcgagttcccggtggacacgcgccgccggatgccggagctcaccgacctcgtcgcgcgactgctggagaaggaccccgccaagaggctcggg
ttcgcgggcggcgccgacgaggtccgggcgcacccgttcttcgccggcgtcgcgtgggacatgctcaccgagctctcgcgcccgccgtacatcccgcc
gccggccgacgacgccgacgccagcgccggcgaggggttcgacgtcagggaccacttcaagaacgttcaccaggcgccgccgccgccggaggttga
tcggacgtcgaggagctcggcggacttctcggcagagttctaa
>g2757.t1
atggatgttgacgccggcactcgtgccaagcgtgccgccacaggaggggaagaacgcaccgtggttgcctcccgtgtcgcggccatctgcgacatgat
cgagcatcatcgcgaggccgggaaaccactaacctccagccaagtcgctacaatctcctccatgatcgacgacgtcgccggtaccaaggctccgccac
cgaggaccggaagaaggagcagtaaaaaaaagcggtgcatgatcagcgttgggtcgtacgaggacgagggcgtggtcggcgagggccggttcggc
gtcgtgttccgcgcgcgccaccgcggcacgggccaggacgccgccgtgaagtcctaccgctccgactacgacgacagcattaaagacgacgggaccc
gcatgctcctgcgggaggcgtgcttcatggcggcgtgcggggcccacccctcggtcgtcggcttccacgccgtggcgtgcaacccttggagctccaata
atggcggctactccctcgttatggagtacgtgggtcccagcaacctcgggcaagtcctcgacgaccgagaaggccggccgttgccggaggccgaggc
gcggcggatgatgcggcaactcctggacggggctgcggcgatgcaccagcacggcatcgtgcaccgggacatcaagcccgggaacatcctcgtcgtc
gctgacaacaacgtcaagatcggcgacctggggctggccaagtctatgcacgagaaggcggcgcccttcttcatggccgggacgaggggctacatgg
cgccggagatgctgctgtggaccgaggaccacgacgcggccgtggacgcgtggtcgctcggctgcgtcatggcggagctcgtcaccggcaagatgctt
ccgttcgagggcaaggacgagatggaccagctatacaagattttcgacgtggtcggcgtgcccgggaagagggcgtggcgggccatgaagccgcac
gaggacctcgacgacgacgtgcagcagtggagggcgaggcagcggaggctgggccaccgcaaccgcctgcgcgagatggtcccggaggaggtgtt
gtccagggatgggtttgaggtcttgaaagggctcctgaccactgatcccaagaagcgcctgacagcagcggacgctctccggtgcccgtggttcgccaa
caacagcaacgacgacgtcgtcgatactcctgtcgcagacactgccgctcccgattccaaggtcaacaagtcgtggtcactggtttggcagatcgccat
gtcattcgttgggcgtgcgctagaattagttagccccaagggtctactcaagtga
>g3077.t1
atggaggcagtacataagattgccgagcctaaagactctttagtcgtcactactcggaaggttcagagtttggagccgccggtaccgataaaggcttca
tggaaagggaagaactccgagcagcatgaagagaaggattttctagctgatggcgaggagagcttcaggagcctggattcctcagatgagggtggcc

ggagctccttctctggggctagccaccctccagagccaatagacatggatattatgaagacagtatatgttgcaatcgatgaggagaagtccgagccac
ctgtgtgtttagtgcgaggcctgtccatgaaggggcctttcatagatgactcattggtccgtgttactggtgcaaaagcaaatgtggtggtagctgcaagc
ggtgctgaaggtttggctgaagagaggaaggtgtctagttctgcagtcgcatcgctggccacaacacgctcatctcaggctacatctttacctcaggact
cggaggagaaggaatgtgtttgggatgcttcactccctcccagtggaaatgttagccctcacagtagcattgacagcatgggtgtggtcaccgccatga
gcactatgaacagctgcacaagtacatataaaaatgaagccatagttagtgaagcaatgcttactgtagagagaaactctggaagtgtaaagggcatt
cgagtggatatgctagaaagtgcaaaaactagcatgagtcgagcaagtgacagcagtggtgttagcgatgacagcagctggagccatataactggag
gcgccaataagcctcacaaggggaatgatcctagatggaaggcaattcatgctgtacggactcgtgatggtgtgcttgggatgagccatttcagattact
caagcgcctgggctgtggtgatattggtaccgtatacctctcagaattaagtgggactcggtgctactttgcaatgaaagtaatggacaaggcatcgttg
gcaagtaggaagaagctgaacagggctcaaactgaaagggagattctacaacttctggaccacccattccttccaactctgtacacacactttgagacc
gacaggttctcttgtttgatggaggcagtacataagattgccgagcctaaagactctttagtcgtcactactcggaaggttcagagtttggagccgccggt
accgataaaggcttcatggaaagggaagaactccgagcagcatgaagagaaggattttctagctgatggcgaggagagcttcaggagcctggattcct
cagatgagggtggccggagctccttctctggggctagccaccctccagagccaatagacatggatattatgaagacagtatatgttgcaatcgatgagg
agaagtccgagccacctgtgtgtttagtgcgaggcctgtccatgaaggggcctttcatagatgactcattggtccgtgttactggtgcaaaagcaaatgt
ggtggtagctgcaagcggtgctgaaggtttggctgaagagaggaaggtgtctagttctgcagtcgcatcgctggccacaacacgctcatctcaggctac
atctttacctcaggactcggaggagaaggaatgtgtttgggatgcttcactccctcccagtggaaatgttagccctcacagtagcattgacagcatgggt
gtggtcaccgccatgagcactatgaacagctgcacaagtacatataaaaatgaagccatagttagtgaagcaatgcttactgtagagagaaactctgg
aagtgtaaagggcattcgagtggatatgctagaaagtgcaaaaactagcatgagtcgagcaagtgacagcagtggtgttagcgatgacagcagctgg
agccatataactggaggcgccaataagcctcacaaggggaatgatcctagatggaaggcaattcatgctgtacggactcgtgatggtgtgcttgggatg
agccatttcagattactcaagcgcctgggctgtggtgatattggtaccgtatacctctcagaattaagtgggactcggtgctactttgcaatgaaagtaat
ggacaaggcatcgttggcaagtaggaagaagctgaacagggctcaaactgaaagggagattctacaacttctggaccacccattccttccaactctgt
acacacactttgagaccgacaggttttatgctgcagaagtacttttagctctggagtatctacacatgttgggagttgtctacagggatctgaaaccagaa
aatgttctagtgcgtgatgatggccacataatgctttcagactttgacctctccctgagatgtgccgtctctcctacactcatcagaacatcagcttttgatt
ctgatcccagaagggcgggtggtttctgtgtgcaacctgcatgcatggaacctacatcagcatgtattcagcctgcatgcttcttgcccaaaattttcggtc
agaagaacaagaaaaagactagaaaaacaagatctgagctgggacagagtaccaccaccctacctgagcttgttgctgagccaacatcggctcgatc
aatgtcgtttgtcgggactcatgagtacttggcccctgaaatcatcaaaggcgaaggccatggtagtgctgttgattggtggacctttggcatcttcctgc
atgagttgctatacggcaaaacaccatttaaggggtcaggcaaccgtgccacgttgtttaacgtggtcggccagcagctaagatttccagagtcaccat
ccacaagctactcgagcagagacctaatcaggggctcctggtcaaggaaccacaacaccgcctcggcgtgaagcggggcgcgacagagataaagc
agcacccgttcttcgagggcgtgaactgggctctcattcggtgcagcactcctcctgaggtccccaggcccgtcgaagccgagctgccagtgaagtatg
gggtggcggaggcgatcgggagcaacagcaagaggatagctggtgcggacgtgaagtcgggcgggaagtacctggactttgagttcttctag
>g3239.t1
atggccgcgaccccgccgtcgtcgcgggacccgtcgccgcagccacggcggccgtcctcctccgccgccgcggccggggccgccacgaagcgcggg
gggctcctgctcgggcgctacgagctcggccgcctcctcggccacggcaccttcgccaaggtctaccacgcgcgccacgccgacacgggcgagtccgt
cgccatcaaggtgctcgacaaggagaaggccctccgccacggcctcgtcccgcacatcaagcgcgagatcgccatcctccgccgcgtgcgccacccca
acatcgtccgcctcttcgaggtcatggccacccggtccaagatctacttcgtcatggagctggtccgcggcggggagctcttcgcgcgcgtcgcgaaag
gaaggctcaaggaggacaccgcgcggaggtacttccaacagctcgtctccgccgtcgggttctgccacgcgcggggcgtgttccaccgcgacctcaag
ccggagaacctcctcgtcgacgagcgcggcgacctcaaggtctccgacttcggcctctccgccgtcgccgaccagtcgcacaccgacggactcctccac
accttctgcggcacgccctcctacgtcgcgccggaggtgctcgcgcgccgcggctacgacggcgccaaggctgacatatggtcctgcggcatcatcctc
ttcgtgctcatggcgggatacctcccgttccacgaccagaacctcatggccatgtaccgcaagatttacagaggggagttccggtgcccgaggtggttct
caaaggatcttacaggcctattgaatcgtcttcttgacaccaacccggagacgaggatcaccattgctgagatcaaggagagcaggtggtttcagaagg
gataccagccggttaggttctatgtcgaggatgaccaggtgcatagcttggcagatggtgagaatgaggtgccagaggggagcctagcgagccccc
gtctccagcgccgccaccgcagcaagaggacgaaggcgaggattgtgggtgggaatcagactcgtcagtagcatcctgtccagccacattgtccacgg
aggagaggcgacggcctgctgggtctctcacgcggccagtcagtctcaacgcgttcgacatcatatcgttctccaagggattcaatttgtcaggtttgttt
gaggagaaaggcaatgaagtgaggtttgtctcagcacatcccatgcagacgattatcacaaaattggaggagattgcaaaggtgaagaggttctcggt
tcggcgaaaggactggcgggtcagcatagaaggtaccagggaatgccagaaaggattattgacgattggagctgagatatttgagctcacgccgagc
cttgtggtggtcgaggtgaagaagaaggcaggggacaaagaagagtatgaggagttctgcgagaaggagttgaaacctgggatgcaacatctcgtgc
accatacggcctcggttccggacataccttctgattctgagtag
>g3240.t1
atggccgcgaccccgccgtcgtcgcgggacccatcgccgcagccgcggcggccgtcctcctccgccgcagccgcggccggggccgccacaaagcgcg
gggggctcctgctgggccgctacgagctcggccgcctcctcggccacggcaccttcgccaaggtctaccacgcccgccacgccgacacgggcgagtcc
gtcgccatcaaggtgctcgacaaggagaaggccctccgccacggcctcgtcccgcacatcaagcgcgagatcgccatcctccgccgcgtgcgccaccc
caacatcgtccgcctcttcgaggtcatggccacccggtccaagatctacttcgtcatggagctggtccgcggcggggagctcttcgcgcgcgtcgcgaa
aggaaggctcaaggaggacaccgcgcggaggtacttccaacagctcgtctccgccgtcgggttctgccacgcgcggggcgtgttccaccgcgacctca
agccggagaacctcctcgtcnnnnnnnnnnnnnnnnnnnnnnnnnnnnnnnnnnnnnnnnnnnnnnctgatcatttgagccgagccgatcggggg
gcgcggttgggtaggtcgctggcggtttttgtttttgcggctttcacgggggccggcgcggttgggtag
>g3293.t1
atggaaacagagaagaatggtaacattctgctgcaaaggtatgaggttggtagattacttgggcagggcacttttgctaaggtttaccatgctaggaac
atagtgacatcacagagtgttgctatcaaggtgattgataaagacaaaattttcaaggttggtctaatggagcagatcaagcgggagatatctgtgatg
aaattagtgaggcaccccaacattgtacagctttatgaggtcatggctaccaaatcaaagatatattttgttcttgaatatgtcaagggtggtgaattgttt
aacaaaatagctaaaggtaagttaagagaagatgcggctaggaagtacttccagcagttgataagtgctgttgatttctgccatagtagaggtgtatac

catcgtgatctgaagccagaaaacctacttgttgatgagaatgggaatctgaaaatttcagattttggattgagtgctcttgctgagtcaagacgccaag
atggccttctacacacaacttgtggtactccagcatatgtggcgcctgaggttatcagccgaaaagggtatgatggtgccaaggttgacacatggtcttg
tggggtgatcctgttcgttctcatggctgggtatctgccttttcaagattccaatttggtggagatgtatagaaaaattgggaaagcggagttcaagtgcc
cgccttggtttccatctgatgatctgtggaacatggaaacagagaagaatggtaacattctgctgcaaaggtatgaggttggtagattacttgggcaggg
cacttttgctaaggtttaccatgctaggaacatagtgacatcacagagtgttgctatcaaggtgattgataaagacaaaattttcaaggttggtctaatgg
agcagatcaagcgggagatatctgtgatgaaactagtgaggcaccccaacattgtacagctttatgaggtcatggctaccaaatcaaagatatattttgt
tcttgaatatgtcaagggtggtgaattgtttaacaaaatagctaaaggtaaattaagagaagatgcggctaggaagtacttccagcagttgataagtgc
cgttgatttctgccatagtagaggtgtataccatcgtgatctgaagccagaaaacctacttgttgatgagaatgggaatctgaaaatttcagattttggatt
gagtgctcttgctgagtcaagacgccaagatggccttctacacacaacttgtggtactccagcatatgtggcgcctgaggttatcagccgaaaagggtat
gatggtgccaaggttgacacatggtcttgtggggtgatcctgttcgttctcatggctgggtatctgccttttcaagattccaatttggtggagatgtataga
aaaattgggaaagcggagttcaagtgcccgccttggtttccatctgatcatcatgttcttaagttgcacctgccatcaattgtagttgattaa
>g3454.t1
atggttgactcccggtgggtggtgttgtggctacttttcctcttcttctcctacggcctgcttggagccgccgccgcctccgccggcgatggcaggtttctct
actccggctttgcaggctcgaacctcgacctcgacggcacggccacggtgaccccgaggggcaaactccagctgaccaacggcaccgtgcagctgaa
aggccacgcgttccacccgacttcgctgcgcctccgccgcaacaacaatgccacggtccggtccttctcttcgtccttcgtgttcggcatcctccccgccg
cgcccggcgggttgggcgggcacggcatcgcgctcatcgtcgccccgaggaaggacttcccctcgggtatagctagccagtacatggggttcctcaac
aagaccagcaacggcaacgcctgcaaccacatcttcgccgtggagctggacaccatcctcaaccccgacttccacgacctcgacaacaaccacgtcgg
cgtcgacatcaacaacctcacctccgtcgaggccgccaccgccgggtactacgacgacgtcaccggcgagttcaggagcctcaccctcatcagcggcg
aggccatgcgggcgtgggtggactacgacggcgcggccaagcgggtcgacgtcacgctggcgccggccggcgtggcgaaaaagccgaggaggccg
ctggtgtcggccacggtcgacctttcaacggtcatcaccgaccaagcctacatcgggttctcgtcgtccacgggcacgctgagcacgcgccactacgtg
ctcggctggagcttcggcgtcgacgagccggctccggaactcgacgtggccaagctacccaagctacctcccatcgaaagacccaaggaggatgtacc
aaagattctgttgacaatagctctgcctgtagtctccggggcgctcaccttcgcgatgctcgccacctgcgtcctcctgctcctgcggcggcggtaccgct
acgtggagcttcgagaagactgggagatcgagttcggggcgcaccggctctcgcacaaggatttgtttcacgccacccacgggttcaaccccaagaac
ctcctcggggcaggagggttcgggagagtgtacaagggagtcctccccaattccggcacggaggtggccgtgaagcgggtgtgccacgagtcacggc
aggggatgaaggagttcgtggcggaggtcgcgagcatcggccgcctccggcaccgcaacctcgtgcagctgctcgggtactgccgcctcaaggacga
gctcctcctcgtctacgactacatgcccaacggcagcctcgacaagttcctctacaataataataacgctcatcatggcgacaacaacaacaagcagca
gctgccagcgctgagctgggcgcagcggttccagatcatcaagggcgtcgcgtcgggtctgctgtacctgcacgaggagtgggagcaggtggtggtcc
accgcgacgtcaagcccagcaacgtgctcctcgacgccggcatgaacgcccggctcggcgacttcggcctcgccaggctgcgcggccacggcgccgg
cgccgagctgcagaccaccgtcgtcgcggggacgttcggctacatcgcgccggagctcgcgctcaccggcaaggcgtcgccgctcaccgacgtcttcg
ccttcggggccttcctgctcgaggtggtcacgggcaggagacctatcgacgacggcgggaacggcggcggcggaacggtcatgctcatcgactgggt
gttcgagcactggcgcagggagacgctcgtcgaggtggtggaccccgggattattactcggggagatcaatacgacgttaatgagctcagcctggcgtt
gaaggttgggttgttgtgctcgcagccgatggccgatatgaggccgagcatgagacaggtcatgcagtacctccaaggggacgcttcacctcccgacct
gagcacggcggctcagcacatgggcctaagcatgttggcgttgctgcagaaccaagggttcgactcctatgtcatgtcctccagttcatcgtcctcctcct
cgatgaagagactagacacttctctctcgacgggtcgctga
>g3716.t1
atggactatccgtgggagatgtacacgctcaaggagctgctgcaggcgaccaacaacttcaacgataacaacaagctgggcgagggcgggttcggca
ccgtctactggggtcgcacgtccaagggcatcgaggtgccggtgaagcggcttaaggcgatgacggccaaggcggaaatggagttcgccatcgaggt
ggagatcctcggccgggtccgccacaagaacctgctcagcctccgcggcttctacgccggcggcgaggagcgcctcatcgtctacgactacatgccca
accacagcctcctcacccacctccatcctcaccgtggcgcccccggatcccagcaacatgaacccctcgactggccacgccgcgtcgccatcgccgtcg
gcgccgccgagggcctcgcgtacttgcaccacgaggcgaacccgcacatcatccaccgcgacatcaaggcgagcaacgtgctcctggacgcggactt
ggtccccaaggtggccgacttcgggttcgccaagctcatcccggacggcgtctcccacctgacgacgcgcgtgaagggcacactgggctaccttgcac
ccgagtacgccatgtggggaaaggtctccgagagctgcgacgtctacagcttcggcgtgctgctgctggagctcgtcagcgcgcgccgcccgctcgag
aagctccccggcggcgtcaagcgcgagatcgtgcagtgggcggcgccgctcgtcgagcgccgcaagtgggagcgcctcgcggacccgcgcctcgccg
gccggttcgacgcgcagcagctccgcgccgtcgtcgagacggccatgctctgcacgcagagcaccgccgagtgcaggccggccatgactgaggtcgt
cgagatgctcaagtacggcggcgtcgagaggcggaccaaggagatcgtccccgtgctggcggcgagcagcgaggagacgaccacagaactggaca
gcgtcaccggcagcagcgaaccgctcgacaggcggagctggaagcttaccaatctgagatga
>g3770.t1
atggtagctcatattggagacttcggcctagcaagcttcaagtttggttccatggcatcatcttttaccaattcgaattccacttcttcggttgcaatcaaag
gaaccattggatatgttgctccagaatgtgcgggaggtggtcaagtctcgactgcctcagacgtctacagcttcggagttgttctcctcgaagtgttcatc
cggaggaggccaacggatgacatgtttaaggaggaaatgagcatcacaaagtttacagagatcaacttccctgagaatgtgttgcagattgttgaccc
gcagctcttgcaagagctggacctcagcatggagattccaacgaccatcaaggaaactggagctgagattctgcagtccgtgctggaggttggactctg
ctgcaccaggacttcccccaaagagcgcatcaacatgcaggttgccgctgctaagctgcatgggatcagggacgcctatcttagaggaaacttaagga
ccagttga
>g4005.t1
atgaaggagttgtcgtacgaggaggtggaggcggcgacgggcggattcgcggagaagaacctcgtcgggaagggcagccatggcagcgtgtacagg
gcgaagctgcgtggaggaggcggaagaggcaggacggtcgtcgtggccgtgaagagggcgtcgcacgggcagggcgagacgaagctcgccaacg
agatcgccgtgctctccgccgcgccgcgccacccgggcgtcatcggcatcgtcggggtcgtcgcgtctccgcctcctcatgcgccgccggccaaggaca
aggaggaggaggacgtggggcactcgaagaagacgacgggggggggggggggggcccccgacccccccggggnnnnnnnnnngggggg
ggggggggggggggggcgctccgtcagccgctgctggtcatggagttcatgccgcacgggtccctccacgacctgctgcaccggtccccgcgcccgc

cgccgtggccgcgccgcctggagatcgcgctcgacgtggcccgcgccgtgctggcgctgcacgccgccaagccacgtggcatcatccaccgcgacgtc
aagtccgccaacatcctcctcggccgcgacggccgcgcgcgcctcgccgacttcagcctcgccgtgatcatggtcgccgaccgaggagaccgccgcgc
gtgtgacgacgtggacgcggtgggcccggcgccggcggggacgatcgggtacctggacccgtgctacacggagccggggcggctgggcccggagag
cgacgtgtttagcttcggcgtggtgctgctggagctcgtgagcaggcgcaaggtgatggacgtggacgcctgcccgtcgtccatcgtctcgtgggccgg
gccgctgctcgccgcgggccgggcccgggacgtgctcgacgcgcgggtgccggccccgcccgccgcctcgcgggccgagcgcgccgtcgtccgggtg
ctcgacgtcgcggcgcggtgcgtgtcggagcgcgtggagcggcggccgggcatggcggaggtggtggcggagctggacggcgccatcgagggcgcc
gggtggcgccgccgccgcgggccacgtggcgtgatggagagcgtgcgcaggcgcgtcgtggcgtgggggcagcacgtgaggagccggagggtcag
ggcggccaaggtcgagtgcacagagcactccggcagcggcggggcaccggaacgcctgccgtcgtcagtggcgaggatccgtgtgctgcacaagca
ttgcattcaaacagcacaaatgatcgaacatcccaattgctcgatgccaaggaaagggcaacataatgagttaattgcgagagagtccaaggaatcct
ccagtcctcggggatccttggaatttctcgttgaccaattcagctga
>g4092.t1
atgtcagactcggagctgtcccggagcacggtggtgttcgggctccgcatgtgggtgctcgtcggcatcggcgtcggcgccgccttcgtgctgctcctcg
ttctcatctccgtcctctgcctcctcgccttccgccggaggcggcggaagcggccgaacggctccgcccagcagctccccaccacggcccctcccaaga
acgccgcgacggtcaaggcgcccaaggacatccaagaagtgccctcccacgccgccgccgccgccgcgaagatgccgctcgcgcaggtgctccaact
ctccacgccctcgggacccatccagatcgccaccggcaaggagcaccacatcacgtacccggagcagcagcggcaccacagccaccggagcgacgc
ggggtcatcacacggcagcggcgagggccgaggcggcggcgcggggtccgcgccaccgggagtaccggaggtgtctcatctcggttggggccactg
gtacacgctcaaggagctggaagcagcgaccgagatgttctccgacgagaaggtgataggagagggcggctacgggatcgtgtaccatggcgtgctc
gagaacggcacgcaggtcgctgtcaagaacttgctcaacaacagggggcaagcagagaaagaattcaaggtcgaggtcgaagcaattggacgtgtg
cggcacaagaatcttgtgcgccttctaggatattgtgctgagggtaatcagaggatgcttgtttacgagatgcttgtttacgagtatgttgataatggaact
ttggaacaatggctgcatggagatgttggacccataagtcctcttacatgggatcataggatgaagattatactaggaacagcaaaagggttaatgtatt
tgcatgaaggacttgaaccaaaagtggttcaccgtgatgtgaagtcaagcaatatccttttggacaagcactggaatgctaaactctctgatttcggactt
gcgaagcttctaggttcagaacggagttatgtcactacgcgagttatggggactttcgggtatgttgctccggagtatgcagggactggaatgttgaacg
aaacaagtgatgtatatagttttggcattcttattatggaaataatttctggtagggtaccagtggactacaacagaccaccgggagaggttaatttagtt
gattggcttaagacaatggtgagcagccgaaattctgatggggttgtggatccaaagatgtcaaagaagcctacttctagagcactgaagaaggcattg
ctagtggcattacgatgtgtagaccctgatgctagtaagaggccaaggatgggacatgtcattcacatgcttgaagtcgaggatttcccatacagagac
gaacgccgagggagtagagctcctgggcaatcaagattgccagacaagctggtagttggaacaagccaccatgatactgtcaatgacgaggataagg
aaacaaatgcagagccgtttagatggagaaacccagaggactag
>g4406.t1
atggggatacagaggtacaagctcttcttgtgcgggtgcggcgtcaatgcggccgccgccgcacgcgacctggacggggacgacgacgacttggact
gctgctccggcgccgacgcgttcggtgagaaggggtgcggcggcgaggccggggcgccccggcgcctgtcgctggcgcaggtggaggcgatgaccg
gcaggttcacctccgccgtggtcggcgagggcgggttcagcaccgtgtacctcgcccgcctctccggcgcgcttgccgccgtcaaggtccaccgcagca
gcgagcgtcttcaccgcgtgttccgccaggagctcgacacgctcctccgcgtccgacacccccacatcgtccgcctcctcgccttctgcgaccagcaaga
ggaaggcgtgctggtgcttgagttcgcggcgaacgggaacctgcacgagcgcctccacggcgggggcaagtcgtcggggacgctgccgtgggcgcg
gcgcgtgtgcgtggcgctgcacgtggcgcgggcgctggcgcacctgcacgacgcgtgcgagccgcaggtggtgcacggcgacgtgaaggcgtcgaa
cgtgctcctggacgcgtcgatgcgggccaagctctgcgacttcgggtcggcgcgggccgggttctccgccgccgtgcggccgcggccggcgggccgga
ccaccatgctgggctcgcccgggtacgtggacccgcactacatccggtcgggcgtggtcaccaagaagagcgacgtgtacagcttcggcgtgctgctg
ctggagctgctcacggggatggaggccttctgcgcgggggaggggcggctcctcaccgccgtcctcgcgcccaggctcaagggcggaaacccgggcg
acgcgaggggggttggtggacgacaggctcggcacggcgtacgacgcggcggaggccgacgcggtggcggcgctcgcggcggcgtgcgtcggcgag
aacccgagcctgcgcccgtccatggccgacgtggtgcgcaccttggagcagagcgcgcacgggtcaatctccgccgtcgaggggagatcggacggac
gcgggaagctgtga
>g4994.t1
atggtggagcccatggcgcgacactacttctggcggctggtctccgccgtgcggtactgtcacgcgcacggcgtgttccacagcgacatcaagcccgag
aatctcctcctggacgccgccggcgacctcaaggtcgccgacttcggactcggcgcctactacgcctcctttgaccaccagcagcaggaccaccacctg
cgccacatcctgtgcgacacgccgacttacatggcgccggagattctaagcaagagagggtacgacccgaccaaggtggacatgtggtcgtacgacat
ggtgctgttcgtgcttcccgcctgtgttgctcagtag
>g5151.t1
atggaggaggagaaggtgttcgtggcgttgccggcggagttcaaggccgggcaatcgacgctgtcgtgggcgctcagccatttcggcggcggcggatc
cacgatcgtgatcgcacatgtgcatgtcccgtcacagatgattccagtcagtaggtgttcttccttctccttctccttcacatctcaatctcatctctccaaag
ctcttaattatctgtatctagcattccttccttcttttgctgaaagaatcagctcgacaaattcaaccgttgccgattttgataattttcagtgggaggtgaaa
tgcgagaaactcatgtttgagaaggaagatgtcgtttcgggtctgatggaccttattgcctcgcatggagtcacgaacctggtgatggcagctgctgcag
ataggcactactcaagggataatggagtggagatttcacgagctgtttcacctgggtcttctgttcactcgacgtcagttggctcttggacgcagaaaga
aaggcaggttgctttcggagcggaaagggagaagccgggaggaggcgagacccctgtgatcccaattaagccactccccgaaacggcaaactccctg
accgccctccgcccctcctccaaccgcccgtcgccgccgcccgccgcgatgccgcccacgccgccggagccggagacggacccagagttcgtag
agctcgaccccaccggccgctacggacggtacacggaggttcttggcaagggcgccttcaagacggtgtatcctttgatgtctgatttggatttattctgt
cggcacagatacaaggcctttgatcaacttgaagggctagaagttgcttggaaccagatcaaagttggtgatttacttcggaacaatgatgatttggaga
ggctgagatcagaagtgcggttgctcaagaccctcaagcataagaacataatcaagttctacaattcgtggcttgacaagaaaaacaacaacatcaatt
tcatcaccgaggtcttcacatcaggaacattgcgccagtttgtgctcctgtgctctctgattctaattactgccattaaacatcgtagatatgcacaagctgt
actaaacacctgaagcatgacagattcacatccaagtgatcactacgtacatgaatccatggatcctcatgatgcaattggtcgaattatcacagtcgag
agtcaacggaaggatttgaatacaatatttttgaagctgagaattgctgattcaacaggtcatgcccaaaacatccacttcccatttgatatcgaagcag

acacttcaatcagtgttgcaactgagatggtcgtacagctggatctcacagaccaagatgtaacagccatagcagaaatgatagatgctgaaatacgtg
ctcacattcctgattgggcagtagaggaatcagttgacaatcaaggggatgaagctgctcattctgaggcccatagttcagacagtgatgagggaatct
ccgaattgcgtgatgagcctgatgctgcacacaacggcttcgtacaggaacagcttccttctgggcggaagtactggtcagactcacccaggagagac
aatgatacacctctaccagtgctgaacccacaaacaggaagcatgacagatccacatccaagtgatcactacgtacatgaatccatggatcctcgtgat
gcaattggtcgaattatcacagtcgagagtcaacggaaggatttgaatacaatatttttgaagctgcgaattgctgattcaacaggtcatgcccaaaaca
tccacttcccatttgatatcgaagcagacacttcaatcagtgttgcaactgagatggtcgtacagctggatctcacagaccaagatgtaacagccatagc
agaaatgatagatgctgaaatacgtgctcacattcctgattgggcagtagaggaatcagttgacaatcaaggggatgaagctgctcattctgaggccca
tagttcagacagtgatgagggaatctccgaattgcgtgatgagcctgatgctgcacacaacggcttcgtacaggaacagcttccttctgggcggaagta
ctggtcagactcacccaggagagacaatgatacacctctaccagtgctgaacccacaaacaggtgatagcgtttcaaatggaatagagaaaatgaatg
atgccaataactttatctacagtgcaaaacataaggacaatcaatctcctgctgtttctgttcaccctgtggaagatatctgtgagggaatatcctcatctg
tgggattgtcaaagctttcggtggtcgacaggatctctggaggggcttctgttagtactagccctcactcttctgatgttgagtggcaatgcatttcagcag
atgttactgagaagttggaaaacctgttagctcagcaacaagaggagcttaatgcgcttcaaaagaagcacaacgtagacatacaagacatcttgaaa
agtgtacctgcggaggatcgtgaagaaaccttgactaggtgccgcttgaagatggatcagcaaaacaaagcaagccaactttag
>g5164.t1
atgatgtaccgagtcaggaagcctccgtatgcgactggtgaaggcttcagccttcagctattcattgtttcgggcactgaagaacaagcagacatgtcgg
ctgccgttgcaattatatcctcagtcgctggaaccgccacggtagtagttgcgatcgctctgatcagaagatgctgcgtggtgaagaggaagatgaaga
agaaactgaagaagaaagagatgcagaggaggagcctggaggcgcaagctgccaccagcactgccgtggacgacgtggtgatcgaggtggggccg
gtggagaagttcctgaacgagatcctgagcgagaggccgatgcgcttcagctccgagcagcttgcggcctgcacacggaacttctcgtcggagctggg
gtccggcggcttcggcgtcgtgtacaagggcgagctgcccaacggcctggccgtggccgtgaaggtcctgaaggcgtccgtgatgaagaagcgggtc
gaggagcagttcatggcggaggtggggaccatcggccggacctaccacgtcaacctcgtccgcctctacggattctgcttcgacagcgacaccaaggc
gctagtctacgagttcctggagaacggctccctcgacaactacctctatggcggcaatgatgatgatgaggaaggcggcacagaggagaagaagctg
gagtggagcacgctgcacgccatcgctgtggggacggcgaaggggatccggtaccttcacgaggagtgccagcagaagatcgtgcactacgacatca
agccgggcaacatccttctgaacgccgatttcaccccgaaggtcgccgatttcgggttggcgaggctggcggagcgggagaacacgcacatgacgctg
accggcggcaggggcacgcccgggtacgcggcgccggagctgtggatggcgatgccggcgacggagaagtgcgacgtgtacagcttcgggatggtg
ctgttcgagatcctggggcggcggcgcaactacgacgcgggcaaggccgaggagagcagggagtggttcccgcggtgggtgtgggagaggtacgag
cgggaggagctggagtgcgtgctggacgacgccgggatcggggaggaggacagggagaaggccgagatgatgtgcaaggtggcgctgtggtgcgt
gcagttccagccggcggcgcggcccaccatgagcagcgtcctgcgcatgctcaaggggacatggccatcgtgccgcccgtgaaccccttccactacg
tcatggatagcggcggctcgacgagctcgacgacgtggagcggcaccaacatgagcagcggtggctacttgaccaccgttggaagagacagtgagat
ttcagtaagccctgccgccaagtcgacgagcgcgatgattgaagacactgataagtcgactgatgctgtgtag
>g5165.t1
atgtgctccgagatggccatcaagatgatgatgcaaagagacagaggaggaggcatacgcgcggcgtcgatcagagacgagcccagcagcagcgg
cgtcaacgaggaggcgggcgacgtggagatggggagcatgagctacttcttcgaggacatccagaacgagcgcccggtccggttcagctcgcagcag
ctccgcgccttcacccgcaactacgcgcacaaggtcgggtccggcgggttcggcgtggtgtacaggggccggttcccgaacggcggcgcggcggtgg
ccgtgaaggtgctgcacagcacgctggggaagcgcgcggaggagcagttcatggcggaggtggggaccatcgggcggacctaccacgtgaacctgg
tccgcctctacggcttctgcttcgacgcgtcggtgaaggcgctggtgtacgagtacatggacaacgggtcgctggacgggtacctgttccacccggcgc
ccgagaagaagggtgtcgggttcgggaagctccacgacgtcgccgtggggacggccaaggcgctgcggtacctgcacgaggagtgcgcgcagcgga
tcatccactacgacatcaaacccgagaacgtgctcctcgatgcgaacctggaacccaaggtgtcggacttcgggctcgcccggctctgcgacagggag
gacacgcacctgaccatcaccggcgcgcggggcacgcccgggtacgccgcgccggagctgtggatgccgctgcccgtcacgcacaagtgcgacgtgt
acagctacgggatgctgctgttcgagatgctcgggcggcggcgcaacctggagctgggacgattgcacagccggtccgagagccaggagtggtaccc
gaggtgggtgtggcaccggttcgaggcgggcgacacggacgccgtgctggcgcgcgccgtggcggcgggggagaggaaggagataatcagggaga
aggccgaacgggcgtgcatggtggcgatgtggtgcgtgcagtaccggcccgaggatcggccgtccatgggcagcgtcgtcaggatgctggaaggga
ggaccagataccccgccgcggaacccgttcgcgcacctgtcagcctacgactctggtgccgccaataacctgctgcagtcgtcgtccgggtccggga
gggctcgtatgcttcttccagccatgtttttagcgctaggtag
>g5194.t1
atgtcgccgccgcctccgccgctgctgcgcgacgagctgtcgcgcaggacggcggtgctgggcctgcgcctgtgggtgctcgtgggcctcgccgtcggg
ctcgccttcctcctcctcctcgcgctcatctccctccacctcgccgcggcgcggcggaggcggccgaggaagggcgtcccgcgcgcggcggcggcggt
ggcgccgccgctctcccccgcggccaccaccaccatcccgccggtgtccaaggagatccaggaggtggccgtccacgtgggctccctccgccactacc
tcgagatgggccacgcgttcctcaccaaggacgccaccaacggcgcgcagcagcagcagcagcacgacggcgacggcggcgagtccgtggggacc
gcgcacgggtcgcagcgcgtgtacatcgaggccggcaagggccgccgcatggtcgcggcgtgcggcgccgacctggaggccggcgccgacgtggcg
gtggccgtgggccccgaggtgtcgcacctcgggtggggccactggtacacgctccgtgagctggaggaggccaccgccgcgttcgcgcccgagcacg
tcgtcggcgagggcggctacggcatcgtgtaccgcggcgtcttcgctgacggctaccaggtcgccgtcaagaacctcatgaacaacaggggccaggcc
gagcgggagttcaaggtggaggtggaggcgatcgggcgcgtccgccacaagaacctcgtccggctgctgggatactgcgccgagggagcgcaccgg
atacttgtgtatgagtacgtcgacaatggcaacctggagcagtggctgcacggtgacgtcgggcctgtcagcccgctcacctgggacatccggatgaac
gtcgtgctcggaacagccaaagggatcacctacttgcacgaggggctggagccgaaggtggtacacagggacatcaagtccagcaacatactgctgg
acaggaggtggaaccccaaggtgtctgatttcggcctcgccaagctcctcggatccgatagcaactttgtcaccacaagagtgatgggtacatttggtta
tgtggcaccagagtacgcgagcaccggcatgctcaatgagaggagcgacgtgtacagcttcgggatccttataatggagatcatctccggtcgaagcc
cggtcgactacgccagacctcctggagaggttaaccttgtggaatggctcaagaacatggtgtccaacagggactacgaagcaattctggaccctaaa
ctgccggaaaagccttcgtcaaaggcactgaagaaggccctgttggtcgcactgaggacggcggtgctgggcctgcgcctgtgggtgctcgtgggcct
cgccgtcgggctcgccttcctcctcctcctcgcgctcatctccctccacctcgccgcggcgcggcggaggcggccgaggaagggcgtcccgcgcgcggc

ggcggcggtggcgccgccgctctcccccgcggccaccaccaccatcccgccggtgtccaaggagatccaggaggtggccgtccacgtgggctccctcc
gccactacctcgagatgggccacgcgttcctcaccaaggacgccaccaacggcgcgcagcagcagcagcagcacgacggcgacggcggcgagtccg
tggggaccgcgcacgggtcgcagcgcgtgtacatcgaggccggcaagggccgccgcatggtcgcggcgtgcggcgccgacctggaggccggcgccg
acgtggcggtggccgtgggccccgaggtgtcgcacctcgggtggggccactggtacacgctccgtgagctggaggaggccaccgccgcgttcgcgcc
cgagcacgtcgtcggcgagggcggctacggcatcgtgtaccgcggcgtcttcgctgacggctaccaggtcgccgtcaagaacctcatgaacaacaggg
gccaggccgagcgggagttcaaggtggaggtggaggcgatcgggcgcgtccgccacaagaacctcgtccggctgctgggatactgcgccgagggag
cgcaccggatacttgtgtatgagtacgtcgacaatggcaacctggagcagtggctgcacggtgacgtcgggcctgtcagcccgctcacctgggacatcc
ggatgaacgtcgtgctcggaacagccaaagggatcacctacttgcacgaggggctggagccgaaggtggtacacagggacatcaagtccagcaacat
actgctggacaggaggtggaaccccaaggtgtctgatttcggcctcgccaagctcctcggatccgatagcaactttgtcaccacaagagtgatgggtac
atttggttatgtggcaccagagtacgcgagcaccggcatgctcaatgagaggagcgacgtgtacagcttcgggatccttataatggagatcatctccgg
tcgaagcccggtcgactacgccagacctcctggagaggttaaccttgtggaatggctcaagaacatggtgtccaacagggactacgaagcaattctgg
accctaaactgccggaaaagccttcgtcaaaggcactgaagaaggccctgttggtcgcactgaggtgcgtggatcccgattcacagaaacggccaaag
atgggacatgatcgacgaactcttcgaccgggcaatggcagccccctcaccctggagaaggcacgaacgccaggaaagccggtggcagggtcatgtg
acagcagctgctacgagggcaacacgaccactgccagcacgccctcaagactggtccaggacatgtag
>g5473.t1
atgggtcgccgccacctcttggccgccgtcgccgccaccatcgtcgccgtcgcgttcctcgcgcctcacgccgccggctacccgtggcccatgtgcggca
cgacaaacaacttcgcgcccaacagtacgttccagaccaatctgaaccgcatcgccgccacgctacccaacgccaccgcctcgtcccgcgacctctac
gccaccgcgagagccggcaccgtcccggagcaggtctgggccatggcgctctgccgcagcgacatcaacgccacggagtgccgcacctgcatcaccc
aggcgttcagtaacgcgcagacctcgtgcgcctcttaccgggacgcgtccatctactacaacccctgcatgctccactactccgacgtgcacaccctccc
cgacgacgacactggccccaccaccgacttctaccccatcaccaacaacgggaacgtcacctccgacccggaacggttcaacgaaatccttacggccc
tcgtcaacgccaccgccgactacgccgcgttcaactccaccaaaaggtttgcgaccggggaggccgacttcaaccaggagttccccaaggtgtacacg
gtggcgcagtgcccgcccgaccagccgccggagcggtgccggaagtgcctcgccgggattatcagggacaccctgccggtcttcctgggccatatcgg
aggcagggtgctctgggtcaactgcacctaccgattcgagacctcgcccttctacaatggaccgccgattgtgcggctggcgtcactgagctccggagc
gcctgcgcctgcgcccgccgttgaggagccggcaacggccgggggagggggaagaaagtaccatgttccgggcgtggttcttgccgtggtgctggctg
ttctagcagccttgaacctgctggtcgcctgcctctgtttctggaggaggcggggaccaagtgccgaaccgaagaagccataccccaagtattctacag
aagcagaggacgccgaaatggtggaatccctgatgatcgatgtctccactctacgagcagcgaccggggatttcgacgacagcaacaagctcggcga
aggcgggtttggcgcggtgtacaagggcgtcctccccggcggcgaggagatagcagtgaagcggctgtccgacagctcgaagcagggagacgatga
gctgaagaacgagcttgcgttggtggcgaagctgaagcacaagaacctggtcaggcttgtcggcgtctgccttgatgagcaggagaggctgctcgtct
acgagtacgtccccaaccggagcctcgatctcatccttttcggcagtgaggatgagaatgaggaaagacgcgagcagctggattgggagcagaggta
caggataatcaacggcatcgctcgaggattgcagtacctccatgaggactcgcagctcagagtaatccaccgggatctcaaggccagcaacattttact
cgatgccaacatgaaccccaagatctcggactttggcctcgcgaggatcttcacccgagaccagacgcaggccgtcaccaacaccatagcgggaacct
gtggatacatggcgccagagtacgtgatgcgtgggaactactcggtgaagtcggatgccttcagcttcggcgtcattgtgctcgagatcgtgacaggga
ggaagaacaacgatacctacacctccaaacagtcgggagatctcttgaacactgtatgtgcaaactga
>g5689.t1
atgcacaacatggagggagacggcggcggagcagggtgcctggggctgggccaaggcggggaggcgcagatcatcaagggcacgcacacccatgg
cggccgctacgtgcagtacaacatctacggcaatctcttcgaggtctccgccaagtacgtcccgcccatccggcccgtcggacgcggcgcctgcggcat
catatgtgctgctgtcaacgcgcagactcgtgaggaggttgccatcaagaagattggaaacgccttcgacaatcagatcgacgccaaacggactttgcg
agagattaagctgcttcggcacatgaatcacgaaaatgttatttcaataaaggacatcatacgcccaccaaggagggagaatttcaatgatgtttatatt
gtctacgagttgatggatactgatcttcatcaccttctgcgatcaaaccagccactaaccgatgatcactgtcagtatttcgtctatcagctgcttcgagga
ttgaagtatgtgcactcggcaaatgtcttgcacagagacctcaggcctagcaatctgctgctcaatgccaaatgtgaccttaagattggggactttggcct
ggcaaggaccacaactgaaaatgacttcatgatggagtatgtggttactcgatggtacagggcgcctgaacttctgctgaactgctgcgagtatactca
agctattgatatgtggtcggtaggctgcatccttggtgagattgtgacacgggagcctttatttcctggaaaggattatgttgatcagctgaggctaataa
ctgaggttatttcaataaaggacatcatacgcccaccaaggagggagaatttcaatgacgtttatattgtctacgagttgatggacactgatcttcatcac
cttctgcgatcaaaccagccactaaccgatgatcactgccagtatttcgtctatcagctgcttcgaggattgaagtatgtgcactcggcaaatgtcttgca
cagagacctcaggcctagcaatctgctgctcaatgccaaatgtgaccttaagattggggactttggcctggcaaggaccacaactgaaaatgacttcat
gatggagtatgtggttactcgatggtacagggcgcctgaacttctgctgaactgctgcgagtatactcaagctattgatatgtggtcggtaggctgcatcc
ttggtgagattgtgacacgggagcctttatttcctggaaaggattatgttgatcagctgaggctaataactgagatcacaggaagcactcctttgaaaga
tactaactttttctctttttttggcattgtagttgatgaggctctgtgccacccttacttggcatcccttcatgagataaatgatgaacctgtttgtccagcacc
tttcagcttcgatttcgagcagccatcgctcaccgaagaagatatcaaggagatcatatggaaagagtctcttaagttcaacccagatccaattcactaa
>g5746.t1
atgcctacatttcttgcacttcttaccatcaccattgctactatcagctccaactatgcttctccggtggattcacatgaaggcgaccgcactgcccttctct
ccttcaagtccggcgtttggggcaacctatctgactggagctcccttggatcaatgtgcaattggactggtgttgaatgtgattcaagagggcgcgtgatt
catctctttctctctcactctaacctcaccggcgtcatctccccggcaatcggcaacctttctgtgctcgagaggcttgagctcgacaacaaccaactctcg
ggaatcctcccaccagagctcggaatgctgtcatcgttgttagagctgagcctttataacaacttcctggaagggacaattcctgaaacgcttggccttct
gagaaacctgacctacctctccctcgagtctaacaatatcaaaggcgaccacactgcccttctctccttcaagtccggcgtttggggcaacctatctgact
ggagctcccttggatcaatgtgcaattggactggtgttgaatgtgattcaagagggcgcgtgatgcatctctttctctctcactctaacctcaccggcgtca
tctccccggcaatcggcaacctttctgtgctcgagaggcttgagctcgacaacaaccaactctcgggaatcctcccaccagagctcggaatgctgtcatc
gttgttagagctgagcctttacaacaacttcctggaagggccgattcctgaaacgcttggccttctgagaaacctgacctacctctccctcgagtctaaca
atatcaatggtagcatccctgaggctgtcgtatgtaactgctcctccttgacttacattggcctaagcagcaactccctcaccggtgaaattcctttcaccg

cacactgccggctcccaggcctgaacgaactcgttctctacgagaacaagcttgttggagccatcccgccgtcgatattgaacttcacaagcttagagtg
ggttcttgacgtttctttcaatagccttaccggcgtgcttcctcagtctttgcaggcagcgacggtgctctggtttgccaacttctcctacaacaacttctccg
gatccgtcagtgccgcggtcggaatggagagcgatcatccgaggatctcatatcgtgagcttgttgatgcgaccgacggcttttcggaagtgaatttgat
tggcaaaggaggatatggacatgtctacaagggcgtactccgtgaaggaatagtagtcgcagtcaaagtcctgcaccaggaccatgccagtgaggtg
atttctggcagcttcgtgagggaatgccgggtgctcaggagcatccggcaccggaacctcatccgtgtcgtgacggcgtgcagcacgccggacttcaag
gccgtcgtgctgccgttcatgccgaacggcaacctcgacagcctcgtccacgggcctcccggaggaggtgataagccggcagcacggcggcgcctag
accttgacttgctgctcagcatcgccagcgacgttgcagagggcgtggcgtacttacaccaccacgcccccgtcaaggtcgtgcactgcgacctgaagc
cgagcaacgtcctcctcgatggcgacatgaccgccatcgtctccgatttcggcatctcgaaactggtcaaggatgcaagagatccagagatgagctacg
cgtccatgacgtccgcatccagcagctccattactcggctgttacaaggctctgttggttacattgcacccgagtatgggttgggagggcggccatccac
gcaaggtgatgtgtacagcttcggggtgatgctcctggagatgatctccgggaagaggccgaccgacgtgatctccgaggaggggcacagcctgcac
gagtgggtcaggatctgctgcctgcaacagcacgaagaggacgccgtcgtcgagcgctcgctgctgcctggtgatccggttcccaggcacggcgaggt
tatggaggtcatcgtggagcttctcgagctcggcgttgcatgcactcagctcgtgcctaacatgaggcccagcatggacgacgtggcgcacgagatcgg
ttgcctaagggacggtacatggaggaagtacaggggcatacctgaatactga
>g6122.t1
atggttctcacagactacgatggcacggtggtttggcgtgttgatggtgacttcagtgttcggtttgctcagctcttggatactggaaatcttgttatgaaa
gacacaggtgaatggaagcaaaggtttaatatcgctgtaggtgttgcaaagggtttgtcctatcttcaccatgagtgtttggagtgggtggtccactgtga
tgttaagccagataatatactgcttgaccaagattttgaaccaaagattattgactttggtttggccaagttactaaaaagacatggatccagtcaggtta
catctcaagtaaggggaactgttggttacattgctccagagtggatctccggcctgcctatcactgcaaaggttgatgtctatagttacggtgttgtgctgc
ttgaactagtatcaggaattcgcgcctcagacatgtctaagggcgtggatgatgacttgcacttggaagtcaggaagcttgttagtattcttgccgacaa
agtgaacagggaggatcagtcatggatagctgaatttgcagattacagattggatggtcaattgaactgtttgcaagcaagaacgctgatcaagttagc
tgtttcatgtttggaggaagacaaaaacaaaaggcccaccatggaaactattcttcagaccctgctttcaagtgctgaagctgatggcgcaaatgaaac
aatttattga
>g6208.t1
atgcgacgagatgacgcaggcggcggcggcggcactgggttccacgagctcttcgactccgtgcgccggtccatctccttccgccccgccgccgccgc
ccccgcggagccccccgccatccccttcggtggcgggggcgggggcgggatcggcgtacggattagttcctgccttagaaagtcgcgggggatgggcc
tgctcgggctcatctccaagagcccctccccgccgcgccgcctgctgccgccgacgcccgacgagtcgggcggcggcggcggagggggggcggtgcgg
cggagatgggaaaggagaagagaacccgccgattcggtggcggaaaggggagctgatcgggtgcggcgcgttcgggcaggtctacctcgggatgaa
cctggactctggcgagctcctcgccgtcaagcaggttttgatagggactagcaacgccacacgggagaaagcccaagcacatattaaagagcttgaag
aagaagtgaagctgctaaagaacttgtcgcacccgaacattgttgaagacacactaaatattttgctggaatttgtgcctggagggtccattcagtcactt
ttagggaagcttggttcattccctgagcctgtcattaggaaatacactaaacaaattctgcaaggattggagtatctacataacaatgcaataatacata
gagatataaagggtgcaaacattctagttgataataaagggtgcatcaaacttgctgattttggagcatcgaagcaagtggctaagttgcacccatttgt
tacaggagaattggacaacctgcagtcagtaaatcgcgctgcacacaaggaaacttccgttgataagattcctgcacatgatatgccaaatgtcttgggt
ttaaatcattcgtccaactggtcgactcttaactccaacaaatcaacaaatgccaagcccttatgggagggtggttgtgacgatgatgacatgtgtgtgtt
cgctgacaaggatgactatcgagcagttggatctagctataatcctatgtctgaaccatttgataactgggaaaacaagtttgacataagtgcagagca
aaattctcgtcaatcgacagagtttagtggattagccaatcatactgaaagcagcataactgagaatgattttactttcccttgtgagggaagttgtgaag
atgacgatgtactgacagaatcaaagattaaggcatttcttgaggagaaggcccttgacctgaaaaagctacaaacacctttatatgaggagttctaca
acacagtgaatgcagggaattgtcagggagctgagcaaacatccaagggaaaatttancctgaaaaagctacaaacacctttatatgaggagttctac
aacacagtgaatgcagggaattgtcagggagctgagcaaacatccaagggaaaatttacaaatagcccaaaattgccccctcgaggaaagtcatctc
caagcaagatgaaaggaggcttgtcaccaacatgtgacaatttgaataatacaagtcctgtaagatgcagcaagcaattcccaagcagtggtgtagag
aatagcagaattttgagagaaatagcatccccacaactgaacgagtttgcggataaaagccatcttgatgtcccagatagcccaagcatcagcttcgct
gagaggcagaagaagtggaaagaagagttggaccaggagcttgagagggaaagagtgatgagattggctggtggcggtaaaacaccatctccaaat
agaggccctaatgtgaaaagagcgccgtgctga
>g6344.t1
atggcggggctggaggaactgaagaagaagctgcaacccttgctgttcgatgactcggacagcatcagcacccgggtgcccgtcccggaggatgcct
gcgattcctatgtggtttctgataatggaactgttaatttactgagtagatcattgggtgagtataacatcaatgagcatggatttcacaaacgagttgctg
ggccagatgagtcaaattctgatgagaaggcttaccgatgtgcctctcacgatatgcacatttttggtgccattggtagtggtgccagtagtgttgtggag
aaagctatatttattccagtacatcgaattcttgccttgaagaagataaacatatttgagaaggagaagaggcaacaaattctgaatgagatgagaaca
ttatgcaaagcctgttgttatcctggtttagttgaattccagggtgcattttacatgcctgactcaggacaaataagcattgctcttgaatacatggatggtg
gttcattagctgatgttataaaggtcaagaagtctataccagaaccagttcttgcacatatgctacaaaaagtattgcttggtctgcggtacttgcatgaa
gtaagacatctagtgcacagagatataaagccagcaaatctgctgataaatctcaagggtgaggcgaagattacagattttggtattagtgctggtttgg
acaatacgttggctatgatactggatgacccatcaccaacaccaccagaagatgctttttcatcagaattttgctcattcatcaatggttgcttgcaaaaa
gatgctgatgcaaggcccacatgtgagcagcttatgtcgcacccattcctcaagaggtatggaagaaatgatgttgacttagaagcatacgtcagaagt
gttgttaatccaagagaaaggctaaagcaaatagcagagctgatgttgatggtgtcccaaacatctagagctgaaactgcctga
>g6528.t1
atggcgatgatggtggatcctcccaatgggatcggggtccaggggaagcattactacactatgtggcagacactatttgagattgacaccaagtatgtg
ccaatcaagcccattggaagaggagcttatggaattgtttgctcatctataaaccgtgagaccaacgagaaagtcgcaataaaaaagataaacaatgt
ctttgacaaccgtgtagatgcactaaggacgctgcgggagctgaaactccttcgacacttgtgccatgagaatgttattgcactcaaggacataatgatg
ccagtgcatagaaggaacttcaaggatgtctacttggtttatgaactcatggacactgatctgcatcaaataatcaagtcatctcagccactttcaaacga
ccactgccagtactttcttttccagctacttcgaggcttgaagtaccttcattcagcaggtatactccatagagacctaaagccagggaacctcctggtga

atgcaaattgtgacctgaagatctgtgatttcggacttgcccgcacaaacaacactaaaggtcagtttatgacagaatatgtggttactcgctggtacag
ggcccctgagctcctactttgctgtgacaactatggcacctccatagacgtctggtctgtgggctgcatctttgcggagttacttggccgcaagccgatatt
tccaggaactgagtgcctcaaccagctgaagctcatagtcaatgttcttggcaccatgagtgaggctgatctggagttcattgacaacccaaaagcccg
caagtatataaagacccttccatatacccccggcattcccctcatcagcatgtacccgcaagcacatcctcttgccattgatctgttgcagaagatgctag
tctttgatccctccaaaaggattagcgtaacggaggctctcgagcacccttacatgtctccgctgtatgatccaagcgcaaaccctcctgctcaagtgccc
atcgacctcgatatagatgaaaatcttggtgtagagatgatccgagaaatgatgtggcaggagatggtccactaccaccccgaggccgtaacaggaat
gagcatgtga
>g6529.t1
atgacagaatatgtggttactcgctggtacagggcccctgagctcctgctctgctgtgataactatggcacctccatagacgtctggtctgttggctgcat
ctttgctgagctacttggccgcaagccgatatttccaggaactgagtgcctcaaccagctgaagctcatagtcaatgttcttggcaccatgagtgaggct
gatctggagttcattgacaacccaaaagcccgcaagtatataaagacccttccatatacccccggcattcccctcatcagcatgtacccgcaagcacatc
ctcttgccattgatctgttgcagaagatgctagtctttgatccctccaaaaggattagcgtaacggaggctctcgagcacccttacatgtctccgctgtatg
atccaagcgcaaaccctcctgctcaagtgcccatcgacctcgatatagatgaaaatcttggtgtagagatgatccgagaaatgatgtggcaggagatgg
tccactaccaccccgaggccgtaacaggaatgagcatgtga
>g7346.t1
atggacgtctacagcttcggggtcatccttttggagctcatcaccggaaagccagcggagcagccaatggccgacgactccgtcgacatcgtcaggtgg
gtgcggcggcgggccaacgtgacggacggagcgttgcagattcttgaccccagcatctccagcacggcgcagcaggggatgcaggcggccctcgagc
tcgccctgcgttgcacgtccgtgatgcccgaccagaggcctgccatggacgaggtcgtccggtcgctgcagccactgtgtttcagcgttcatccacagac
gccgctgtcaaccgaaactgctattaatccctga
>g7459.t1
atgcgcaggacactcatcaacgccgtcctcttcccggcgctcgccgtggtcgccgcgctggtagtcctctacttcgtccgccgctgccgccggcgccgcc
gccgccgccgcgggggcgtgctgccctcccacggcggcggcggcgcgcgggcggaccggtaccaggcgggcgggtcgggcgggtacgcccccggc
ggggaggaggcgctggtgcggttccccggcggcgaggcgctcacggtggccgcgatcctcgaggcgcccggcgaggtggtcgccaagtcggcgcac
agcacgctgtaccgcgccggcctgagcgcgggcgaggccgtcgcgctgctccggttcgtgcgcccggcctgcgccgcgggcgccgaggaggccgtgg
ccgcagcgcgggtgctcggcgccgtgcgccacgccaacctcgtcccgatccgcgcgctctacgtcggcccgcgcggggagaagctgctcgtgcacccg
ttctacgccgccggctcgctccgacgcttcctgcaagaaggaatcaatgattcacagagatgggagataatttgcaagctatccattggaattgtcaagg
gattggaccacctccacgctgcatcgctgaaaccaatcattcacggcaacctcaaaaccaataacatcatgcttgacgctgatttccagcccaggatatc
tgactttggcctctatctcctgctgaatcctgctgctgctcaagagatgctagagacagctgcagtgcaggggtacaaggccccagagctgatcaagatg
agggacgtcactagggaaagcgacatctacagcctgggggtgattatgctcgagatgcttgctcaaaaggaggtgccgaatgatcggcagctgaatgc
tcgtgacatccatttgcctgcctctttcaaagatctggttctcgagaggaagatatcagatgctttcagttccgaccttatcaagcaaagcaagaattctgg
gaaggaggagaatctaaacgcatacttcgaattggcaaccgcttgctgtaacccatcaccatcattgagaccggatactaagaaaatactcaaaaggc
ttgaagagatagcaagataa
>g7514.t1
atggccagcgtgagctcccgactctctgccctctgcgccaagatccaaggcctcgcagagccaggaacagacgaggcgcttcggcgcgtgagcatggc
gtgcatctgcgtcgcgatgcacgatcttggcaaagcggcgatgccatcatcatcaaggtcaacaacaaggaccacgaccaggaagaggaaatccctg
accccaatcacactctcgcagttcgttgacgacgagtgtatgctcggggagggtgacagcggcttcgtttccaggtttcatgaccgcacgaccggcaag
accttggccgtgaaggccctcttcccgctgccgtacgaagaagaaggatcggaggacgacatggccacctttaaccttcgcgtgctgcgtgaggcgtgc
ttcatggcggcctgtcgcggccacccttccctcgtccgcctccacgccgtctgcatagaaccagacacggaccggtactgtctcgccatggagcacgtcg
gcccgaccctggacgatgtcaacgggcagcggcgccgcaatggcgtgccgttccgggagcacgaggtgcgtcgcataattcggcaggtcctgagcgg
ggccaaggcgatgcacgaccgcggcatcgtgcaccgcaacatagtctcggagaacgtcctcgtggccgcgtgcggcgacgtcgtcaagatcgggggga
tttgggcaggcgacttgcgtatcggagacagtcgacccttgcgaaaacacgttgtgggacaacagtgcgcccgaggtgctgttcaagggacaaggcgc
gtgtcagagtgtgctcctcgactcgtggtccatcggctgcttgatggcggagcttctcactggctatccgctctttatgtatgcaaaactcaacgtgatatt
ccgtgtgctcggctccccggacaagggcgatttggaggatatcaagcctcaagaccttgaccgtgctgaagcggtggtgcgtgaatggagagcccggc
agcaagtatggaagaagctgcggttcggcaacaggctgcgtcgtgaggttccccgtaaagttctatccgacgacggattcgaggtcctccggggactc
gttgccttaaatcccaagaagaggctcacggcggatgccgcgctccaactcccgtggtttgccaacgatgacaacgctccggccactaccagtgtcttg
aggccacggccatttagatggttgttgattgccggtgtgccagcactgctatttttggttggtttttgtttgctttggcggcaccagatacaattgatatag
>g7515.t1
atggccagcatcagcgcgctactctccgccctctgcgagaagatccgaggtctagctgcgccaggaacagacatggagcttcggcgcgtcagtatggc
gtgcatcttgatcgcgatgcacaatctgcgcagcacaagaaggaccatgaaaacgaggaaatccatgaccccctattagcgctttgcagttcgtcgacga
cgagggccgccgccgtcggctcgggggcggcctccgtgcgtgcgttacaaaagtgcggcaccgcacgacgggcaagacattcgccatgaagaccctc
tacccgctgccgcaccaaggactggacgacgcggccatctataatcttcgcgtgctgcgggaggcgtccttcatggcggcctgccgcggccacccttctt
tcgtctgcctccatggcgtctgcatgaaacccaacaccgaccagcagtactgcctcctcatggagcaggccggcccgagcctgtacgaggccacgctgt
accggcgcctccatggcacggcgtttcgggagcaccaggtgcgccgcatgatgcggcaggtcctgagctgggcgaaggcgatgcacgacctcggcat
cgtgcaccgcaacatagtcgcggagaacgtgctcgtgggttttagtagcttcaccgtcaagatcgggggattcgggcaggcgacgtgcacgtcggaga
cggacgtcccgtgcaacgtcgcggtgcgtgacgaggcgccggagttgttgcttcggggacgaggcgcatctgagagcgagctcctcgactcgtggtcc
atcggctgcttgatggggaagctcctcacgaacaggacgctgtttgagattgaggcgaatgaccgcgacaacacggcgagagcacagctctgcagga
tattcaacgtgctcggcgtcccgggcaagagagccatgcaggagatgaaggctcgtaattttgagcttgctaaacaggtgcgggaatggcgagcccga
cggtcaagtgtagggaagacgaacaactacaggctgcgccagctggtccccccctgaggttctatccgatgacggattcaaagtcctgcgaggactcctt

atgtgcaatcccaagaagaggctcacggcgggtgccgcgctccagctgccatggtttgccgacaacgacaagtccccagccgctgggatctcacagca
agtcagtggcccggtatcgtga
>g7517.t1
atggccagcgtcagcgcgctactccccgccctctgcaaaaagatccgaggtgttgcagagccaggaacagatatggagcttcggcgcgtcagtatggc
gtgcatcttcgtcgcgatgcacaatctgggccacgccaggaagaccaccaataaaggagaaaatccattacccctgttagcacctcgcagttcttgga
taacgagtatcctctcgggacgggcatcagcagcaaggttagcgaagtgcagcaccgtacgagcggcaagaccttcgccatgaagaggctggacgag
caggacgacgacgttggggaggaggaagacgatgtttgttttaacctccgcgtgctgcgtcaggcgtgcttcatggcggcctgtcgcggccgcccttcc
ctcgtccgcctccacgctgtctgcatagacccagcagacacggaccggtactgcctcctgatggagaacctcggcctgagcctgcacaaggttctatcgt
gcatgcgccactacaagccgttcctggagcatgaggtccgccacatgatgcggcagatcctgagcggggccaaggcgatgcacgagctcggcatcgtc
caccgtggcataaactcgaagaacgtccttgtggcagcgatcggctacatcgtcaaaatcgggggatttgggcaggcgacgtgcacgtcggagacgg
acgtcccgttccttaacgaggtgtgggaggaggcgcccgagttgttgctccaaggacgaggcgcaaacgagagcgagctcctcgactcgtggtcgatt
ggctgcttgatggcggagctcctcacgggcagcgcgctgtttgtggtgaagaaccgcgagaacacgaaggaagcgcagttccacaggttactggacgt
cctcggcgtcccgggcaagaaaaccatgcaggagatgaagcctcgaaaccttgagcttgctaatgaggtgcgtgaatggagagcccgacggccaaga
gtagggaagaagaagccgcacaacaacaactacaggctgcgcaagctggtccccccctgaggttctatccgacgacggatttgaggtgctccaaggtct
tgtcacgttcaatcccaagaagaggctcacggcggctgacgcgctccaactgccatggttttctgacaacaacggtgataagactgataactctccggc
cgctgaggcctcgcagcaagtcagccgcccggtatcgaggccgtgcccgtttacatgctggttgctgctcattgggctgctacttctatgtttggtggtttt
tgtttgcatcggcagcagataa
>g7518.t1
atggcgagcgtcagctcgcaactcgctgccctctgcaacaagatccaaggccttgctgaaccaggaacagacatgtcgcttcggcacgtcagtatggcg
tgcatctgcatcgcgacgcacaatctgggcaacgcggcggtgccatcaaggatcacgaggacgaggaaatccctgccccttgtcagcaccttgcagttc
cgtgtcgacaacgaggagtatctgatcatggagggcgaccgcggttttgtttataaggtgcaccaccgcatgaccggcaaggccttcgcctctcttccga
gtactagcctgtcaaggccacccttcctcgtccgcttctatgccatcggcaaattccccgtgcccgaccagtacttcctcctcatggagcacgtcggcccg
agcctgcgcgaggtcttgacgaaccagcgccgcggcaagccgttccgggagcacgaggtgtgccgcatgatgcggcaggtcatgaacggggccaag
gagatgcacgaccgtggtatcgtccaccgcggcattcacccggagaacgtccttgtggttgctggtggtggtgacgacatcaagatcgggggattcgga
caggcgacgtgcacgtcggagacggaggttccgccgcgcagcaacaccatgtgttactgcacgccgcctgatgtgttgcttaagggcagggcgcgct
tgggagtgagctcgtggactcgtggctcatcggctgcttgatggcggagcttctcacggtcaaggtgctgtttgaggcgacggaccacgaggacgagtc
ggaccagctccacaagatattcggtgtgctcggtgtcccggacaaggaagctatgaaggatatgaagcctcaggaccttgacctcgctagagaggtga
gcaaatggagagctcgacgacggggagcagggaagctgcggagcgacagcacgctgcgcaagctggtccccagtaaggttctatccaatgacggatt
cgaggtcctccaagggctccttacgtgcaatcccagaaataggctaactgctgttgccgcgctgcaacttccatggtttgctaatgatgacagctctccgg
gtaccgctgccatccctaagcaaatcgtcaccgtcgggtattga
>g7519.t1
atggatcagtacgagatcatggagcagattggccgtggcgcattcggtgctgccatccttgtgaaccacaagattgagaagaaaaagtatgtgttaaag
aaaatccggcttgcaaggcagacagagcgttgccggaagtctgcccatcaagagatggctctgattgcccgcctgcagcacccatacattgtcgaattc
aaggaggcttgggttgagaagggatgctatgtctgcatcgtcacgggttattgtgaaggtggagatatggatgaactgatgaagaaatcaaatggtact
tactttcccgaagaggttgttggaacaccaaattacatgtgcccggagctgcttgcagatattccctatggattcaaatcggacatctggtctcttggatgc
tgtatgtatgagatggcagctcatcgccctgccttcaaggcttttgatatggctgggttaataagcaagataaatcgatcttccatgggtcccttgcctgtc
tgctactcggtgtctatttctgaacaaaagtttgttcccaaggataatatgcaatccagcaaacaggaacagagcaccatcacatgccgcagtgatgag
gataagttcactgtccaagaattcctttcatcagcaccagcagttgctccttccgtttcatcagcaccagaagttgctccttctgttttgtcagcaccagaaa
tagctccttctgttctaaccccacaaggcacatcactggaggaacttacttcgattcagtcatcgaagaaagaagtaattcctcattcaaatccttcagttc
atgatgttgaacagaatataagacacaatacattgtgtgttagtgacaagaagcctacatcagcaagtgttgaaagggaagctcatagtacggatatca
tcaagcatctgaatgttgtaagagaagatgtcaatgtacggagcagttcatcaaatgctctgtcttcaggacttcccttggtcgttgccagatcaaatattt
cagaggctaatactgtcagtaagatatttgtttcttcagacgtggcaaaattgtctactattacatccgaggccagcaatagaattgagacactccctgct
aaggaaacacttgatgtcacatcatttaggcagcgagccgaggcccttgaagggctcctggagctttctgcagagcttcttgaaaaccataggctggag
gaacttgccattgttttaaagccatttgggaaagataaggtttccccacgagaaactgctatctggctggctagaagtttcaaagggatgatgaatgacg
aatcaggtcgcatgtcactttga
>g7634.t1
atgaccgagcaactgaagcgcgactacgagatcggcgaggagatcgggcgcggccgcttcggcgtggtccaccgctgcgtctcccgcgccacggggg
aggccttcgccgtcaagtccgtcgaccggagcgcgctcgccgacgacctggaccgcgcgctggcggagctggagcccaagctggcccgcctcgccgc
cgcggggaacccgggcgccgtgcaggtgcgcggcgtgtacgaggacgaggcctggacccacaccgtcatggacctctgctcgggccccgacctgctc
gactggatccgcctccgccgcggcgcgcccgtcccggagcccgtcgcggccgcgctcgtgtcgcagatcgccgaggcgctcgcatgctgccacagccg
cggcgtcgcgcaccgagacgtcaagccggacaacatcctcctcgacctcaatgaggacgacgcctcgccgccgcgggtgcggctggcggacttcggg
tccgcggcgctcgtcggcggcgggcggcgcgccgagggactggtgggcacgccgcactacgtggcgcccgaggtggtggcgggcggggagtacggg
gagaaggcggacgtgtggagcgccggggtggtgctgtacctgctgctctccggcggcgcgctcccgttcggcggcgacacgcccgccgaggtgctcgc
ggcggtgctgcgcgggatcccgaggttcccgcccaggctgttcgccggggtctcgccggcggccaaggacctcatgaggcggatgctttgccgcgacg
cttctagaaggttctccgccgagcaagtcctctgtacgtactgctctgcatcacgttgttattag
>g7985.t1
atggggcgcgggcgctccaggaagaggaagaagaaggggtcccaggcttggccggacctcgacgaccatggcggcggtggtgcgggtgaggagcc
ggcggcgctcttcccggccggcatggcggtgccgatccacgaccaccgcaagaagggctttgccttatcattggacgatgccaccaccgtcaaggaca
tcaaggaggacgggctggtggaggaggacggcgacgtgccgcgccggcggggcttgttggtgcgcgaggcgttccacgacgagggaaggtgggccg

gcgtcgtcagcgacgtgcggcggccggaggcggggaatgacggcggagacgcgccgccgcggcgagtgtgcgtcgccaccaccgggggcctgctga
atttcgaggagaccacgggcgagcagctggtcccacctgcagagacggtccccatcggcggctctgccgctccggccgtcgtcggcaagttcgacctc
gccggtgccaattatgccaccacctcaagatcccagtacgggcgaggaagaaaaatcggcgcgctactgcaagaagaggaggatgtggtcgccgag
gtctcattctcaaggccggtggccaggagatcccgtcagccgggatcccaccgctgctgggaccttcgtacccaatctggagctccatggtctctgtgcc
tcctcgaggacaatccccatgctgcttcacggcgggattccatggccgccgccataggggcgagaacctcctcagttactgcctctaaaaagctcaatat
ctgctgccctgacacctttgttctcctgctctggctctctgctgtttgtatgctagctgctgccgaggtgcctccaggtggctgccagcaggaaaggatcca
gattgcccatcaagtggctgaggctctcacatacttgcacaattgggcattcccacccattctccatggggacgtcaagtccgacaacatcctgatggat
cataactacaccgtaaagatttcagactttggttcttccacattagctccaagtaacgaggcacagtttatgtcccgggttcagggtacttttgggtacctt
gatcccgagtatatgcagacaggtcgcttgacagccaagagtgatgtgtacagttttggagttgttattttggagcttctgacacgcaaaaagcctttgaa
tcttcaacgtcctgaagacgaggatacaattgttggaaaatttatgtttgggacagatgaacagaggatcagttatatggatgatcagatcaagagtgac
agcaatatgatgataataaatgatatttcagatctggcacagcacaacctggaggaggaggatcaatttctggaccaaatgaggaggaacaattctgc
agcttctcaagagggtggacttgtctcccccacgcctattgaggagtatgacatagttgtaagcacagcaggaatggatgctgctaacaagaaacacaa
gaaaacgcctgccacatatgctaccctccgcagaactataaaggccaagaaaggaagagatgggttcgagccaccctcagctgaagggccaaccatg
aggagcaagaaggccagatcggcaacagcctccacccctgcaaatgagatgggtaaacaactctcatccatcctctcatttgcttctgaatatcttggac
ctcttgacctatctaaaattgccaaaatctggtggcatcgaccctga
>g8447.t1
atggagctagttggcaaggttggccagcatcagaatgttgtcccattgcgtgcttactattactccaaggatgaaaagctactggtgtatgactatgtcca
gttgggtagcctttctgctgctttgcacgggaacaaagctgctggaagaactccaatggactgggagactagagtaaaaatagctctaggtgctgctcgt
gggatggcttatcttcatgctgagggtggtgggaaattcatccatgggaacataaagtcaagtaacattcttatttcacaggaaatcagtgcttgtgttac
cgagtttggcattgcccagcttatggccccgcctcatattcacccgcgactcatcggttaccgagcacctgaggtcctcgagaccaagaaaccaacaca
gaaatctgacgtctacagctttggtgtactgctccttgagatgctaacaggaaaagcccctcttagatctcccggtcgcgaggatttcatcgaacaccttc
ccaggtgggtgcaatctgtggttcgagaagagtggacatcagaggtttttgatgttgacttgttaaagcacccgaacattgaggatgagatggtacagat
gcttcaggttgcaatggcatgtgttgctgttcttcctgaccagcggcctcggatggaggaggtggtcggcagaattgaagagatccggaactcgtactcg
gataccaggacgtcacctgaggacaagatctag
>g8795.t1
atggacaagtacgaggaggtgcgggacatcgggtcgggcaacttcggggtggcgcggctgatgcgcaaccgcgagacacgcgggctcgtcgccgtc
aagctcatcgaacggggccaaaggattgatgagaatgtgtaccgggagatcgtcaaccaccggtcgctgcggcaccccaacatcatccagttcataga
ggtgatccttactccaacgcatcttgcaatagtgatggagtatgcagctggtggtgaactctttgatcggatcgtcgatcgtggacggtttagtgaagatg
aggcgagatatttcttccagcagttgatctgtggtgtgagctactgccaccacatggtgatccttactccaacgcatcttgcaatagtgatggagtatgca
gctggtggtgaactctttgatcggatcgtcgatcgtggacggtttagtgaagatgaggcgagatatttcttccagcagttgatctgtggtgtgagctactg
ccaccacatgcaaatatgccatagagatttgaagctggagaatgttctcttggatggaagtccagcaccacggctcaagatatgtgattttgggtactcc
aagtcatcagtactacattcaaggccaaaatcagcagtggggacgccagcatatattgcaccagaggtgctttctcggcgggagtatgatggtaagctt
gcagacgtatggtcttgtggagtgactctttatgtcatgctcgtgggagcctacccatttgaagatcaggacgacccaaaaaatatcaagaaaactattc
agcgaatagcagcaattcaatataagatcccggaccatattcacatatctactgattgcagacagctcatttcacatatctttgtcagcaatccattgagg
agaattaccatgagagaaataaagagccatccgtggttcctcaagaacttgccaagggaacttacagaagcagcacagtcaatctactacaggggag
gaaacagtgttcctactttctcagaccaaagcactgaagaaatcatgaacattgtcgaagaggcaagaaccatgcccaaatcgtcgacatcgggatat
ggttatggttacgagtactcggatgaggaggaaaaggcagaggagaacgaacccgaagaggaggaagaagaagatgagtgtgataagagggtcag
ggaggttcgcgagagcggagagctggatatggcctcgctgcgcatataa
>g8924.t1
atgcctaatgatgaatctgctttgcaggtctttgaaccatttggccaagtggagcttgtacagctgcctacagatccactgactgggatgtgcaaaggtta
cggtttcattcagtttgccaggcttgaagatgcaaaagctgcacagagcttgaatggacagcttgatattgctggcagagttataaaggtttcagctgtta
ctgatcatgtgggtgctcaagttagtggaacaactgctggagatttagatgacgacgaaggtggaggcttggctttaaatgcaagctccagggccgctc
ttatgatgaaattggaccggagtggtactgcaaccagcttaactggtggtattggttccactggtctgaacgcctccatggctgtaccagctgcatcagtt
gtcggggctgctcctgcagcttccccttttccacaaccaacaattcctggaccagtttctggggcatctttgcttcctgtcatcactcagcctgtgggcatgt
ctacacctaccgaattcctattgcttaagaacatgtttgacccagcagtggagacagatcctgattttgacttggatattaaagatgatgtccaagatgaa
tgttctaagtttggtttagtgaagcacatttttgttgacaagaatacggctggctttgtgtacctgcactttgatagtgtgatggcagcatcgaaggcacaa
caggcacttcacggaagatgagaagggtgtgccggttatggactggagcaggtgttcgccattgatatcgtgctcggctccgccaagggactcacctac
ctgcacgaagattgccatcgcaggatcattcactgtgaaatcaaggccgcaaacaaccttttggatgaaaattttgaggccaaggttgcagacttggga
gttgggagttggtggaggggttggggcgtcgccgtcctcgacggcggcggactggcgtga
>g9044.t1
atgctgcccgggtgctgcctccctccgtgcgtccgccgcgacaagcgcgacccggagacgggccgagccggcggctcccgcgtctcggacgaacccg
ccctgtcgtcgtcatcatcgttggggaagagcggcggcgccgggtcggccacgaagcggttcgcgtggtcggagatcgagtcggtgacggcggggttc
tcgtcccgcgtgatcgggcaaggcgggttcagcacggtgtacctggcgtcgctctcgtcgtcccgcctgggggccgtcaaggtgcagcgcagcaccgg
cagcgagcgcctccaccgcgcgttccgcgcggagctcggcgttctcctctcgctgcgccacccgcacgtcgtccgcctcctcggctactgcgacgaccg
cgacgagggcgtcctcgtcttcgagtacgcgcccaacggcgacctccacgaccgcctcttctcatcccacgccgcggggaccgagaagaaggcccgcc
tggcggcggcgctcacgtggccgcgccgcatggcgatcgcgttccaggtggccatggcgctcgagcacctccacgagggccgcgacccggcggtgat
ccacggcgacgtcaaggcctccaacgtgctcctcgacgccaacctcgacgccaagctctgcgacttcgggttcgcgcgcgccggcttctccgccgcggt
ccagcccacggcccggtcgtcggcccggcccgtcatgatgggctcgccgggatacgtcgacccgcacttcctcgcatccggcgtggccaccaagaaga
gcgacgtctacagcttcggcgtgctgctgctcgagctcctgaccggacgcaaggccttctgctccgacacgggccgccggctcaccgccacggtgtgcc

ccgcgctcgaggaaggcgagctcgccagcatggtggacccgaggctgcgaggcgattacgacgccgacgaggccgcgaccgtggcggtgctcgcgt
cacggtgcgtcagcgacagccccgggctccgcccgtccatggcggaggtggtgcgcgagttccaggagaagaccaccgcgctgttctccgccaccgg
caccaaaccggccggcaagacggtttag
>g9054.t1
atgggaacctatggttatgcagctccagactatgttgagacaggccatctaacaacaaagagtgatgtttggagctttggagtggttctatacgagatcc
tcactggacggcgctcaatggaaagaaaccgtccaaaaaatgaacagaagcttctggaatgggtgaggcagtatcctgttgaaagtaaacgattcatc
aagattatagacacaaggctcgatggtcagtactctaagcagggcgctagagaaattgcaaagctggccaatagctgccttgcaaagcaaaggaagg
accgccccacaatgaaagaggtggtcgagagcctgaagcgagtgatgcaacgcaaggagttggatggggatgtcggttcttcaggtgagagttcgcc
acctgataaggtgtttgggaaaactactgcagaaaatgttgctgtggcatcagcaaggcggcgtatgcttcacctggctgccctaggtgagaatgcaaa
cagcattgcaaaaaggagattcatgctcatgagggctgcggctgctccaactcctacataa
>g9090.t1
atgaggctgctcgtccccgacggcgcgcggcgccgggggctgccgttcgaggagacgatgacggcggcgtcacccactccggctccggctccggctcc
ggcggggagcgggaggacggtggtggtggggatcaggcgggacgccgccagccgagagctcctcacctgggcgctcgtcaaggtcgccaatgccgg
cgaccgcgtcgtcgcgctccacgtcgccgccgccgccgacggattgttgatgggagtggatgagcggagaagagctgcggacgcgctctcgtcggtgc
tcgccgtctacgacggcttctgcaacctcaagcaggtctgtggaggatcgtctgttcggaaaactctgatcaaggaagcagcttcctgcggcgccgccc
atctgatcctcggcgtcgccaagaattcccggtcattcggatcctcttctacttcagttgccaagtactgtgcaaagagagttccgtcagggtgctcggtcc
ttgctgtgaacaacggcaaggttgtttaccacagagatgcaggaaatgcgacgcaccatgaacttgaccaaaccactatcacaatgccagagactcca
aggaggagctgccggaagctcttgtcgtccatgataggagagaagctttgggatgaatacggaaagggctgcagatcgccgtctcgtgctatcaccatt
ccagcaaggtctcccacgccgccaaaagaagtttcattagcattggtcccaacgaatggttacagacgagaatcgcctgaagcagcaacaggctggcc
cctcctaaggaagaaattcttgccggaccggaaggcttccctgcctgacaagtcgaagatgtctgttgtgcagtgggcaatgcggcttcctagccgctac
tcagcagtttcaccagtccattctgagtacagaaccagaacattgaattccactgcaaataccagtcatattctccgtgaccgggttgctggccctttacg
aaacaattcaggatggtcttctgtagtgattgaagaactagagaaagaaacaccagaagagcttaccttactcaaagagaaattcacatcactttattct
tccttcagttacagtgagcttgcaaagatcacatctgatttctcacctgagtgtatagtcggacagggtggcactagccgagtttacagagggtgtttggt
agatggcagggaactggcagttaagatcctgaagtactctgatgaagtactgaaggagtttgtctcagaaattgagattgtcagctctctcaaccacaa
gaacgtcatatccctcattggtttctgcttcaagaacgatgatcttctgttagtgtatgagtacttgcaaaaaggcagcctcgaagaaatgctgcacggta
aaaaaggaggccagagcatatttggttgggccgagaggttcaatgtggctgtgggagttgctcatgctctagattatcttcacggtaaggacaatagtc
gtccagtgattcatagggatgtcaagtcatcaaacatcctcatctcagagtgcttcgagccaaagttatcagactttggccttgcagtctgggctgctgat
gtgacttcccagatgacatgcaatgacgttgcaggaactttcggatacctagctcctgaatacttcatgcatggcaaggtgaacaacaaaattgatgtat
atgcttttggcgtagtcctacttgaactcatttcaggcagaaaaccactttgcactggttgtccaaaagggcaagagagccttgtaatgtgggcaaattcc
atcatacaaggaggaaagctgacacaacttgtagatcccaacttacctactgaaggccatactgctgaggttgagaagatgacccttgctgcttccttgt
gcatcagaccagcacctcaaaaccgtcctcaaattgatgctgttctgaagttacttgaaggtgacaacgcagtcctcaagtgggctagatcagaagccg
gactggcatgcgacagcgatggtgatgaatgtgtgatgacaccgccagcaccagggagtaacacaaacatccaatcttacatcaatctcgcatttgatg
ttgaagatgatgctgcctccgtcagcagcaacgatttcatcacagcaaacacatcccttgaagagtatctccggggaagatggagccggtcatctagctt
cgattga
>g9279.t1
atgggcaagcttggtgctggccactaccaccgccatgatcacgttggcagtgacaattgtgaagggtgtgctgaggtttatacctgctcctctccactctg
cggttgctgtggtggggggctgaagaatggggacgacctggagatggtcaggagctcgacctccaccttgtatgagagataccagatcatagatgacc
agacggagatacaggatgattgtgcccatgatgggtttcagttgaagcagcccgaggatgttatactcgagtgcggtttgctgaggggtcctgcacttga
agatgaccgtttggagcagagtgttgtggagaaggaacttcaaatgctcagctcgtttgatactgatgctgatgtcgatcctgtttctattattgctggaag
atattatgtcactgagtatctaggctcagctgctttcagcaaggtcgtacaagcacatgatcttcagacagggatggatgtttgccttaaaataattaaaa
atgataaagatttctttgatcagagcttggatgagataaaactcctgaagttcgtgaacaaatatgatccattagatgagcaccatgtgttgcgcctctat
gactacttttattatcaggaacaccttttcattgtcactgaactactgcgagcaaatctgtacgagtttcagaaatataatgaggagtctggtggtgaggtt
tattttacattgcccaggatacaggtattatttcctaatgaaccagtgtccataatgcttgctcaaatgattgggataattggcccaattgacatggaaatg
ctagaactagggcaggaggcgcacaagtatttcactgacgattatgacctttttaccaagaatgaggaaaaggccaacagctga
>g9531.t1
atgccgtcgtccgacgattctccggcggccgccacccaatccagctacgcgaaggtcctgcagggccagtacgagctgggccgcgtcctcgggcgcgg
cggctcctccaaggtctaccgcgcgcgcgacctccgcacaggcgccctcgtcgccgtcaaggccgtgcggaagccaccgccgccgcgcttcccgtcgt
cgagctcgccggaggacgcgacggcggcgcgccgatccgtggagcgtgagctcctcgcgctccgccgcgtgcgcggccacccgcacgtcgtccgact
cctcgacgtcctcgcctcccgctccgccgtctacctcgtcctcgacctcgcccgcggcggcaccgtcgcctcagccgtcgagcgcggcggcgccgccga
cgagcccggggcgcggcgcctcttcggccagctcgccgccgcggtcgcccacgcgcacgatcgcggcgtgttccaccgcgacgtgaagccggacaac
ctccgcctcgacgagcacggggacctcaggctcgccgacttcgggctctgcgccttcgccggcggcaacgcgggcgagctcgacggcatcaccctcac
ggcgacggcgtgtggaaccccggcctacgtggcgccggagatcctgctcaagaagcgttacgacccgcgcaaggctgacgtgtggtcgtgcggcgtc
gtgctgttcgtcatcgcggcgggttacctgcccttcaacgacggcaaccacatgggcatgtaccggaagatctgcgccgggaggttccgctgccccaag
tggttctcgccggagctccggtgccttctcgaaaggatgatggatccggtacccgatacgcgcattgggatcggtgaagtcttggatcacccttggctac
aacacgatggagtgtcatttggtaagattattccagtccgtgcttctcaacctactccggaagtggtgaaatgggagatcgaactggagcaaacaagag
agctcaatgcatttgacatcctcacattcgcatcgggatgcgatcttaccggattgattgggcctttaccggaccggattctatttgcgatacatggtgtgg
gtgtaagatcagtgttggacaagattgcagagctagggagagaagatggtttcatggtgaggagaaagggtgaagcagaatatagaggcgctctgct
ggagaccatggatggcaaatttgttgcacaggttagagttcatcgactgcatgaacaaatgttaatggttgaggcagaaagagctagtattgatgaagc
atctggtttttgggaaggacttcaatcaagtctaaagttctcagccaactga

>g9577.t1
atgcaatacaaatcttgcgtgatgagcttatgcaagaagggcgtgaaggacgcgactcccgggccggccctgagctggaaccagcgggtgaagatcg
cctacggcgcggcgaggggtctcgagtacctgcacgagaaggtgcagccggccatcgtgcaccgggacgtcaggtccagcaacgtcctcctcttcgac
gactacgactccaagctcgccgacttcaacctcaccagccagccgcccgacggcgccgcgaggctgcactccacccgtgtcctcggcacattcggctac
catgctcccgagtacgcgatgacgggagtattaacacagaagagtgatgtgtatagctttggagtgatccttctcgagctactgaccggccggaagccc
gtcgaccacaccttgcccaagggacagcagagtcttgtaacttggatggcagccgtggcggcgttgtgcgtgcaatacgagtccgattttcggcccaac
atgaccatcgtggtgaaggcgctacaacctcttctcaaagccgcagcagggccccccagtacatga
>g9581.t1
atgaaaagtgacctgtttatttcaccgtcgaagtcaactggcagcgacgaggtgaagctgcaagagggcggcaacgccttcgactgcgatgaaccaga
gatggaagggaacttagagaaagggactggtcccaagtggttcagctacgacgagctcgccatcgccaccgataacttctcccaagagcacaagctcg
gccaaggcgagttggtggccacatcaaagcagcggaggaaggagttcgccgccgaggtgcggatcatcagccggctccggcaccctaacctagtgca
gctcatcggctggtgccataacgtcaccgacgatgtgctcctcctcgtctacgagctcatgcccaatggcagcatcaacttgcacttgcacctctacagcg
acgatgacgcgatgcccatgccatggccgcgcaggcacgaggttgtgctgggcctgggctcggcgctgctgtacctgcaccagcaggattgtgagcag
tccgtggtgcacaggaacatcaagcccaccaatgtgatgctggacgcggcgttcaacgccaagctcggcgacttcgggcttgccaccctcctcgatcat
gcccggagcccgcccaccatgtatctcgacgggaccatgaggtacacatacatggaccccaagtacatggtcaccggaatagccaatgcagaggcag
acgtctacagctttaatttggtgccatcctcctcgatcgagatcgcctgcggccgacggccacagggggacacggggctgggcctagacgtgcgtgatc
ggcctcgtgcaatgggtgtgggagcactacggcagaggagccattcttga
>g9801.t1
atgcggcatcgggaggaagcatgtggaaatgcttcgacgcgtgcaccaggggccttactcgccttcccgctcagcattcccgggacggccttctacaag
tgcatgcaaggtcgcaacaatgtcatgcgtacgctgaagcggcagcttgacgagcgcaggaacgcggcggcagcaatgcgggagaccgtggatttct
tccacctcgtgatcgacgagctggacaggcctgactccgtgctgaacgagaacatcgcgctagacctgttgttcttgctgctgtttgccagccacgagac
cacttccatagggctgacagccatactcaagtttctgtcgaacaaccccaaagccctgcaggaactgacgggcggtttcggcgccgtcgtcaaggcgcg
ccaccgcgtcacgggccagttcgtcgccatcaagcgcctcgccaaacccggcgaggggcaatcggaggtgatgcgggagaagaccttcctcgaagcc
tgcagaggcaaccccttcgtcgtcggcttccacggcgtggtccgcgcgcccgactccatggagctccgtctcgtcatggaccacgtgggccccagcctc
cgcgacttcctccacctgcgcggcatcacgtggcagctgctgcagggcgtcaagaggatgcacgccatccacgtcgtgcaccgcgacatcaagcccga
gaacatcctcgtcagcgccgaccagaacctcctcaagatctgcgacttgggccttgccctgcacatgtccgacgcgccgccgtacgaggaggccggca
cgaggtggtacatggcgcccgagatgctgctggggatgcacgactatgacgcgctcgtcgacgactggtcgctgggatgcgtcatggccgagctcgtc
aacggtagagtactcttctccttctcgggcgacaacagtagagacggccagatatgctccatcatcgacgtgctcggcgtgccgttcgcgaccggcgttc
tgcctcgactacggtaccggcagaggcgaagcctgctgcgtcacaagttccctgccacgatgctgtcccagcaaggtttcgaggtcctcagcggcctcc
tcacgtggaaccccgacaagcggctcacggccagcgccgccctcaagcacccatggttcgacgatctcaacgcccctactgcttcgccggcaaggaac
aagatgacggacccgctgatgatttctcccagcttgcacaagagccgcaagatacattgcgcctga
>g9876.t1
atgcttgtgaggtcatcatctgtaggcagagatgagcccagcaggccttctggtccctgtgcacaaagctgcattgatccgctgtgcatccagccatcct
ggagcaattcttcttgtttcacgccacggctggttccttcgacaacatcaaggacacggaagcctagagctgaccagcagaaaccatcacttccgcagc
ttgttgttgagcccactgatgcgaggtcaaattcatttgttgggacccatgaataccttgccccagagatcattagaggagatggccatggcagctcggtt
gattggtggactctcggcatcttcctttatgagttgttgtatggcaagacaccattcaggggacctggcaacgaagaaacactttcaaatgtgatctcgca
gggcttgaagttccctgatagcccagctgttagcttacatgcacgaaacttgatcagagggttgcttgtgaaggagccagagcatcggcttggctcgtca
agaggagctgctgagatcaagtggcatcccttcttcgaaggcttgaactgggccttgatccggtggaccgcaccaccagagacgccgaagacgtttgat
gcagcaactcttgcaacagctcgcaagaaaaaggaaggtaaatgtctagagttccggctgaatggtgatgatatcgagtttgagctcttttag
>g9975.t1
atgaatcagagtcaagttcgtgttatgggagcaaatgctgcacctgaagaccctgggaagacagttgtccttattgatcttgtgccaatgggtgaaaaat
tcgacaattcgacagcattgctagtatttgaaaggttttggcataagcagatcatcataaaccgtgcgcattttggaaactatgatgttttatatgttcaat
acccaggtcctgggtctgtagtggtcgggagcaggataggaagcaggcctatttcagcatcaccgtccttcagctcaggcatggtgacatacaaaggat
ctgccaaaacgtttagcttggctgagatggagagagctacacagggatttgacgagtccagaattattggtgagggtggttttggacgtgtatacaaag
gtattcttgaggatggggagcgggttgctgtcaagattctgaaaagggatgaccaacaaggatcagacaagggcactgctcggctcgattgggatgct
aggcttaaagttgcacttggtgcagcacgtgcacttgcttatctacatgaagattcaagtccacgtgtcatacatcgtgacttcaaatcaagtaacatctta
ttggaatacgacttcaccccctaaggtgtcagactttggcctagcaagaacagctttgggtgagggaaatgagcatatttcgactcgtgttatgggaacttt
tgggtatgttgctcctgaatatgcaatgactgggcatcttctagttaagagtgatgtctacagctatggtgttgttcttcttgaacttcttacaggaaggaag
cctgtagatatgtcaagacctccaggacaagagaacttagttgcatgggctagttctcttctgacaagccgagatgggttggaagcactcatagatcctt
cacttgggagtagcgtcccatttgacagcattgcaaaagtagcagccattgcttccatgtgcgttcagcctgaggtggatcagcggccatttatgggga
ggttgttcaagctctgaagttggtgtgcaacgaaggcagtgagtttaatgaatccagaagctttagccaagatttgcatatccaggatgccgagattata
ggcacaatgagtccggatatggacgtcgactctgcagagctgtttacttcatctgcacgttatgatgctatggatgactctggttcttttcgacgatataca
agttcaggacctctaagaacaggcagaacaggacacggtaaggacaggggcttgtcaacaggtagctccagtgagcacgttggtcttcagagataca
ggactgattcagaatag
>g9985.t1
atgggcaactgctgcccgggctccggggatgcggagcccgccgccggcgacgcctcgtcccgcctccacgccgcctccctcaaggccggcggcagcg
nnnnnnnnnnnnnnnnnnnnnnnnnnnnnnnnnnnnnnnnnnnnnnnnnnnnnnnnnnnnnnnnnnnnnnnnnnnnnnnnnnnnnnnn
nnnnnnnnnnnnnnnnnnnnnnnnnnnnnnnnnnnnntcccccatccgccgccgccgccccgcgcgccatgggcaa
ctgctgcccgggctccggggatgcggagcccgccgccggcgacgcctcgtcccgcctccacgccgcctccctcaaggccggcggcagcggcagcggc

agcgggccgacgtccgcgcccacgccgaccaagccgccggcgccgatcgggcccgtcctgggccggcccatggaggacgtccgcagcatctacacc
gtgggcaaggagctgggccgcggccagttcggcgtgaccagcctgtgcacgcacaaggccacgggcgagcggttcgcgtgcaagaccatcgccaag
cggaagctgtccaccaaggaggacgtggaggacgtgcggcgcgaggtgcagatcatgtaccacctggcgggccagcccaacatcgtcgagctcaag
ggcgcctacgaggacaagcagtccgtgcacctcgtcatggagctctgcgcgggaggggagctcttcgaccggatcatcgccaaaggcaagtacacgg
agcgcgccgccgccgcgctgttgcgcaccatcgtcgagatcgtgcacacgtgccactcgctcggcgtcatccaccgcgacctcaagccagagaacttcc
tcctcctcagcaaggacgagcacgcgccgctcaaggccaccgatttcgggctctccgtattcttcaagcagggggaggtgttcaaggacatcgtgggaa
gcgcctactacatcgcgccggaggtgctcaagaggagctacggaccagaggcggatatctggagcgtcggtgtcatcctctacatcctactctgcggag
tgcccccgttctgggcaggcaagttcatccctgttcaatcggagcacggcatcttcaacgccatcctccggggacaggtcgacttctccagcgacccatg
gccgcgcatttcctccggcgccaaggatctcgtcaggaagatgctcacctctgaccccaagaagaggatctcggcctacgacgtcctcaatcatccttgg
atcaaggaagacggcgaggcgcctgacacgccgctggacaacgctgtcatgaacagactcaagcagttcagggcaatgaaccagttcaagaaagcc
gcgctcagggtcattgccgggtgcctgtcggaggaagagatcaaggggctcaaggagatgttcaagggcatggacgctgacaacagcggcaccatca
ccgtggacgagctgcggcgagggctggccaagcagggcaccaagctctcggaggccgaggtggagcagctcatggaggccgccgacgcggacggg
aacggcacgatcgactacgaggagttcatcaccgcgacgatgcacatgaaccggatggacagggacgagcacctctacgccgcgttccagtacttcg
acaaggacggcagcggctgcatctccaaggaggagctggagcaggcgctcagggagaagggcctgctcgacggccgggacatcaaggacatcatct
ccgaggtcgacgcagacaacgacgggaggatcgactacagcgagttcgtggcgatgatgaggaaagggaaccccgaccccaaccccaagaagcgg
cgcgacgtcgtgctgtag
>g10089.t1
atgaagccgcgggagctcctggagcgcctggcccgccccttctcctcgtccgcctcgcggcggcgggaggagaagcaggcggaggaggaggcggcg
aacctggaggcgatcgcggcgcgggagaaccgcgcgttccggtacgaggcgctcgaggcggccacgcgcgggttcgcggagaagaaccggctcgg
gcggggcgggttcggccccgtgtaccgcggccgcctcgaggacggccgcgaggtggccgtcaagctgctcggcgccgggtcgcggcagggcgccaa
ggagttccggaacgaggccacgctgctgtcccgcctgcagcaccgcaacgtcgtcaacctcttcggctactgcacgcgcggcgccgacgacaagctgc
tcgtctacgagtacgtctccaacgagagcctcgacaagatcctcttctcttcctcacccggcacccgcggcagccacagcagcggagacctgtcgcggc
gcgcggagctgacctggccgcgtcggcacgagattgtagtgggcgtggcgcgcggcctgctgtacctccacgaggacgcgcacacgcccatcatccac
cgcgacatcaaggccagcaacatcctcctggacgaccgctgggtgcccaagatcgctgacttcggcatggccaggctcttcccggaggccggcgacgg
ccgctcccgcgtccacacccgcgtcgcgggaaccaacggctacatggcgcccgagtacctnnnnnnnnnnnnnnnnnnnnnnnnnnnnnnnnnnnnnnnnnnnnn
nnnnnnnnnnnnnnnnnnnnnnnnnnnnnnnnnnnnnnnnnnnnnnnnnnnnnnnnnnnnnnnnnnnnnnnnnnnnnnnn
nnnnnnnnnnnnnnnnnnnnnnnnnnnnnnnnnnnnnnnnnnnnnnnnnncccgcggctgcggccggacatgaagcgcgtgg
tgatcatcctgtccaagaagcagagcacgctcgaggagcccacgcggcccggggtgcccgggtcgcggtaccggcggaggtcccacggcctgcgtgg
ctcatcgcactactccgtcggctcgtcgtccggcaccagctcgccgtcgacgtcggcgacgtcgcacgcgtcggcctcggcatcgaacgcgatgacgac
gtccagcacgcacaccatgaggagccagggcttgccgtcgcaccgagaagagcacgagtag
>g10375.t1
atggacgtggttgctccggccaccgcgatcacggggaaaacgaaagacttgactgcaatcgacagtaccaaaattacaacggcctctgtgctctgcga
gcggaactacatacatcactctggatgcggcagcaccccccattttctgtgccgttgccggcgacgtgattacctacagcatcccggatttcaacgacacc
tcccgacgtgatgactactacatagcaaccagcacaagcttcggtcccatggccttgtccgatttcaacttcgagggattcgtgttactctcagatgtaat
ctacttccggcgtggaggcgtccttcaacagcagcttcacccttgtcgctggcgccccgatccggccaacgctaccgccgcaagtctcgccttcgtcgag
gtcaattgggtgaggagatggaagaaggagcaggacaagctcgccaagatcatgcaggacctccccggtgtgcccaccaagattgagtttacagaca
taaaaagggctaccagaaacttccacgacaccatgaagctagggaaaggaggatttggggccgtctaccgatgcacgcttcctgcttctgcttccagga
ccggggaaccaatggaggtggcagtcaacaagttcctcgccgaggttagcatcatcaaccgctggtcctaccacaagggagtacccgtgcttgtctacg
agtacatgacaaatggtagcttggaccaacatctatttcgaaggagagaggaggatgctacaatttcgcactgggacacacgctacaacattgtgcgc
gatatagctaccggcctgcactacgtccgtcatgagcacgagcccatggtgcttcatcgcgacatcaaggccagcaacatcatgctcgactctacattcc
gtgcccgtctcggcgactttggcattgcctgcaccgtcgctgctaataagagctatgtcaatggcatcgccggtacctttggctacataggacctgactat
gcaaccactggaacggccacgcgcaagactgacacttatcctcgaggttgttactggcaagaagaatggcggatgtgcaacctcatgacattgttttga
>g10552.t1
atgaagaacaagaagcgcgaaagcctccgccgccggatagcaacaatggcgtcgtcgcaggggcggttgccggagtccgaggtgcgcgagtacatg
cgccagctcctgcgcgcgacgaagcgggcccacgccaggggtctcgtccacggcgacatcaccccggacaacatcgtcgtggtgggcccgccggaca
acggcaagacgaaggtcgagctccgcggcttctcgcgcgtgtcgccggtgaaaggcccggatgggcgatgcatcctgcgcacgacgatgcagtaccg
ctcgccggagctgctcaactacatcccctgctttctcctgtgcgagggccccgccgctgacgtgtacgcgctcggctgcgtcatgtacgagctcatcacg
ggagacccccctgttcaaggcggacacggcgggggagttgttcgaggaggcggaggagctgcacgacgcgctgatcgagctggaggaaggctacgac
ggcctcgacctgggggacctatcgctgcacggcaagcagctacagaattattccctgaagctgcagtaa
>g10594.t1
atgaatgttgtggttggacggctcttggatctaagagcgactcttgaagaagcaaaagaaaaggccttgaaaaaatgggaccggcgtgatacagacaa
tattgccgccaggtcagcgctgcatacgatcaggaatcttggaatattcagtagatttgcacccaactctaggaagtatagtgtgttggaggacctcccc
gccggcggtcaccgacggttctcgtacgcggagctgaaggcggcgactaaggacttctccgaagtggtgggcagaggctcgtacgggacggtgtacc
gcggcgagctgcccggccggcgcgcggtggcggtcaagcagctgcacggcgtgggaggcggcgatgccgagttctgggccgagtacatgcccaatg
gatcactcgacaagtacctcttcgtcgcgtctggttcctccaatggtgaagcagaggctacactgcaagagcaagctccactgcttgacctgcacacccg
ttacggcatcgccctgggcgtggcccgcgtcctcgcgtacctccacgaggggtgcgtacactgggtgctgcactgcgatattaggccagagaacatcct
actagatgaccacttccaacccaaggtgtgcgactttgggttgtccaagatgaaggacaggcaggacaaggtcaccgtgtcagacatccatcaggagc
tcgactttggatcatcccatacgacgagcaaacctgatacgatgcccatttctgggatccggggcgcgctcgggtacatggcgcccgagtggttcatcca
gcgggagcccatcacggcgaaagctgacgtctacagattcggcatggtgctgctcgagatcgtctccggcaggcgcaactacgggttcctgcaggaat

cggtgggatccgaggactggaacttccccgagtgggcttacgagaaggtctacgtcgagcgccggatcgacgacatcctcgacccgcgcatcgctgca
tcatacgacgatcccatcgtcgagcgcatggtcaagactgcaatatggtgcctgcagtaccgcgccgagatgcggccgtccatgggtatggtggccaa
gatgctcgagggctccgtcgagatcacagagccagtcaacccaacaatcttctgtgtccagaacgactag
>g10595.t1
atgaatgttgtggttggacggctcttggatctaagagcgactcttgaagaagcaaaagaaaaggccttgaaaaaatgggaccggcgtgatacagacaa
tattgccgccaggtcagcgctgcatacgatcaggaatcttggaatattcagtagatttgcacccaactctaggaagtatagtgtgttggaggacctcccc
gccggcggtcaccgacggttctcgtacgcggagctgaaggcggcgactaaggacttctccgaagtggtgggcagaggctcgtacgggacggtgtacc
gcggcgagctgcccggccggcgcgcggtggcggtcaagcagctgcacggcgtgggaggcggcgatgccgagttctgggccgagtacatgcccaatg
gatcactcgacaagtacctcttcgtcgcgtctggttcctccaatggtgaagcagaggctacactgcaagagcaagctccactgcttgacctgcacacccg
ttacggcatcgccctgggcgtggcccgcgtcctcgcgtacctccacgaggggtgcgtacactgggtgctgcactgcgatattaggccagagaacatcct
actagatgaccacttccaacccaaggtgtgcgactttgggttgtccaagatgaaggacaggcaggacaaggtcaccgtgtcagacatccatcaggagc
tcgactttggatcatcccatacgacgagcaaacctgatacgatgcccatttctgggatccggggcgcgctcgggtacatggcgcccgagtggttcatcca
gcgggagcccatcacggcgaaagctgacgtctacagattcggcatggtgctgctcgagatcgtctccggcaggcgcaactacgggttcctgcaggaat
cggtgggatccgaggactggaacttccccgagtgggcttacgagaaggtctacgtcgagcgccggatcgacgacatcctcgacccgcgcatcgctgca
tcatacgacgatcccatcgtcgagcgcatggtcaagactgcaatatggtgcctgcagtaccgcgccgagatgcggccgtccatgggtatggtggccaa
gatgctcgagggctccgtcgagatcacagagccagtcaacccaacaatcttctgtgtccagaacgactag
>g10820.t1
atgacggccgcggcgccctcgccgccaccgcagctgagccttgatgggctgaaggcggtgtccgtgctggggcgcggcgccaagggcgtcgtgttcca
cgtcgtgcccgccgcgggcgaggacggcggcggcagggcgatggcgctgaaggcggtgtcgcgggacgcggcgcggcagaagaaggcgtcggcg
ggggacggcgacgggcaccgccggatctggttcgagcgggacgtgctcctcgcgctgcgccacccgctgctgccctccctccgcggcgtcctcgccac
cgacgccgtcgtcggcttcgccatcgaccgatgcggcggcggcgacctcaacgcgctccgacgccgacagactgagaagatgttctccgactccgtca
tacggttctacgcggcggagctggtgctggcgctggagcacctgcacagcatcggcatcgtgtaccgggacctcaagccggagaacgtgctcatccag
gacagcggccacatcatgctcgtcgacttcgacctctccacgcggctcccggcgccgccgcaggacccgccggaggccgcgccgatctgtcctcccaa
accggcggcgcctccggcctccaccgcaccaccgttctacgcggcggagctggtgctggcgctggagcacctgcacagcatcggcatcgtgtaccggg
acctcaagccggagaacgtgctcatccaggacagcggccacatcatgctcgtcgacttcgacctctccacgcggctcccggcgccgccgcaggacccg
ccggaggccgcgccgatctgtcctcccaaaccggcggcgcctccggcctccaccgcaccaccgtcccctagccgccgccgtcgcgggaagccgagga
agcccgccgcggcgctctgtttctcgttccgcactggcgggggcgcaaaaccggccgcgcagtcggactcgctctcgccgccgtcgacctcgcggacg
gcctcctcgtcttcatcgtcctcgtcctcgacggcgaccacggtctcctcctccgctaagtccaactcgttcgtgggcaccgaggactacgtggcgccgga
gatcatcgccggccgcggccacgacttcgccgtcgactggtggggcctcggcgtcgtgctctacgagatgctctacggccgcacgccgttccgggggca
gaaccgcaaggagaccttctaccgcgtcctcgccaagcagccggagctcgtcggcgagcagacgccgctgcgggacctcatcgcgcgactcctcgag
aaggaccctgccaagcgcgtcggagcgcgggcggtcaaggcgcacccctcttcttccgcggcgtcgactgggaccgcatcctccacgtggcgcgcccac
ccttcatccccgcgccgccgccgccgctggacgaggagggggacgaggcgctcgacgtcgagaaggtcgtccgtgaggtgttcgccaccaaggcgga
gagcgagaaggcttcgccggaggggacgacggcgtggggagggggagagatccgtccaaagaggacaccgatttctccgtgtttttctga
>g10941.t1
atgcatatggcgcgctcatcagcccatgctaataagctctctgtagctcacaacaaatttgcactgctgctgctgctgctctgcgcggcgtcaaccaccat
ctccgttgccgtggccgccggcgactggtttacctacagcatcccagcgttcactggcaccagcaaagacttgatggtgttaaccaacatgacctacgta
cacagcagcctggagagcttgacaccccaggagctcagcggtgagggcattgtgcaactctcccagtttgtcgagttctggcgtcctactgatgacctcg
aggcgtccttcaacacccgtttcacgctcatcacccgggaagcccccgtctcgttcatcgtcctcgaggagcgaagactgccgggcggccccgtttcacc
cggtggccccaatcccaccagggcgaacggaagcctcgcctttgttatggtcgatgcggtgaggtcgtacggcccgcaaaaactcacagccagtctca
acgtcaccgtcactccccacggcagcgccacggccgtcaccgtgtgggtcgagtaccaagcctacgagcacctcttgtccgtgtacgtctcccaaattgg
cgagctgaggccgccgaacgccctcgtcaacacatcactcggagcgggtgggcactggagcaccgcgaacgcgtccgtcggcttcttcgcgggcagg
atcagtgtcctcaacgccggagttcaggactggaacctgactgtggtgttcccgctgtcaagcaactctgacggttcacgaggcttgagttttgctgccat
attatccctggtccttggattggcagctgccattaccatcatcacagctactgcagtatacctgtatctcaactccaaatacaggaggtggaagaatgagc
tggacaagctcgccaaggtaatgcaggacctccccggaatgccgacccagattgagttcactgagatacagaaggccaccaggaacttccatgacac
gatgaagcttgggaaaggaggatttgggtccgtctaccggtgcacgcttcctgcttctgcttcgaggacggggcaagcaatggaggtggcagtcaaga
agttcacgcaggaagttgaggaccggcgctatgatgacttcctcgccgaggttagcatcatcaaccgtctgcgtcacaagaacatcgtcccactagtag
gctggtcttacgacaagggagtacccgtgcttgtctacgagtacatgacaaatggtagtttggaccagcatctatttcggagaggtggcaacaatgggc
atgatggtcacatggacgctgcaatttggcaatggcacacccgttacaccattgtgcgggatgtagctaccgggctgcattacgttcatcatgagcacga
gccaatggttcttcaccgcgacatcaaggcgagtaatatcatgatcgactctagcttccgtgcccgtctcggtgactttggcattgcctgcacagttgctgc
taataagagctatgtcaccggcatcgccggcacatttggctacatagcaccagactatgcgatgagtcacaaagctacacgccagaccgacatttatgc
gtttggggtactcatcctcgaggttgtcacagggaagaagaacggggatgtcctacctgattatgatcatatcacagaatgggtttggcatcttcaccgt
gagggaatgctgctccaggctgtggacagcaatctcgccttttgggacaagaatcgggacgatatcatttcgatcgatgaggctaaacgtctgcttctcc
tgggattagcatgtactaatcctaacccatctagtcgcccaagcatggaggaggttgtgcacgtgatcaccaactgtactggatgtggctgtgtctga
>g11787.t1
atgatgcggcagctcctggacggggccgcggcgatgcacaggcacggcatcgtgcaccgggacatcaagcccgggaacatcctcgtcgttgctgatgc
tgacaacggcgtcaagatcggcgacctggggctggccaagtctatgcacgagaaggcggcgcccttcttcatggccgggacgtggggctacctggcgc
cggagatgctgctgtggaccgaggaccacgacgcggccgtcgcgtggtcgctcggctgcgtcatggcggagctcgtcaccggcaaggtgctgccgttc
gagggcaaggacgagatggaccagctctacaggatcttcgacgtggtcggcgtgcccgggaagagggcgtggcggggcatgaagccgcacgagga
cctcgacgacgacgtgcagcaatggcgggcgaggcagcggaggctgggccaccgcaaccgcctgcgcgagatggtcccggaggaggtgtngaggg

cgtggcggggcatgaagccgcacgaggacctcgacgacgacgtgcagcaatggcgggcgaggcagcggaggctgggccaccgcaaccgcctgcgc
gagatggtcccggaggaggtgttgtccagggatgggtttgaggtcttgaaagggctcctgaccactgatcccaagaagcgcctgacagcggcggacgc
tctccggtgcccgtggttcgccaacaacagcaacgacgtcgtcgtcgatactcctgctgtcgcagacactgccgcttacgattccgcggtcaacaagtcg
tggtcactgtcactggtttggcagatcgccatgtcattcgctgggcgtgcgctagaattagttagccccaagggtctactcaagtga
>g12084.t1
atgataattgatgagcaacaaaagaaagaatttggaaaggagatgctcattctatctcaaatcaatcataggaacattgtcaagctcctcggctgctgcc
tcgaagttgaagtccccattctggtgtacgaatacatcccaaatggaacattgttccatctatttcatgggaactttaattgtcacatctctttggccacacg
ccttcgaatcgcgcatgagtcagctgaggctctagcctatctccattcgtgtgcacacccaccaatcatccatggtgatgtcaagtccactaacatcctcc
tggatagtgagtacacagccaaagtctcggactttggtgcatctatcctagcaccaaccgatgagtctcaattcgtcacatttgtccagggaacttgtggg
tacctcgaccccgagtacatgcaaacatgtcaattgacggacaagagtgatgtgtatagttttggagttgttctattggagttgctcacacgaaaaaaac
ccttcaaccttgaagggcctgaagacgacaagagcctagcagtgaggtttatttctgtatcaaaaaagggcagacttgaggacatactagacaatcaa
atcgtgagcaacgagaacatgggggttctcgaagagattgcagaattggcaaatcagtgcttagagatgtctggtgtgaataggccatcgatgaagga
aatttctggaaagctcgataggttgaggaaggccatgcagcatccatgggcacaacagaaccctgaggaaatggagtccttacttggggaatcatccg
tggctaactcagagatggctaacacaggacacttcagcattgagaagaaagctttcaagagcctagagtcagggcgttga
>g12107.t1
atgaggaggactcttctccaagctggtccggtcaccggcgacgcaggaaacacgctggcaacgccggccaccgaccttctcccccgtcgtcgtcgcc
gccggcagacgcttccccgccgccgcccgactcctcctcttcgtcttcgaccccacctccagcgtcatcctcctccgaaccaccaccaccctctcctgaca
cccccccgccggatgcctctcctccgccgccggagctcgccccgcctcctccgtcacccgtcgacgcggcgcctcccccgccggacgcctctcctcctcc
gccgccggaggaatttccgcccccaccaccggcgccggtgccggagtcctctccgccgcctcctcccccctcccgacgtcgcgccgcccccacccgacaa
cgccgcgccgccgctcgccccaccgccgccgtccgctcccccgccgacaaagtcagacgccccgccgccttcttccatcccctcgacgccgccgcctcc
tcgacctcccgccgcgcaaccgaccgcgtcaccgcccgcggtcgccgcgccaccgccgccaccagtgggcaacaacaaccacgacgctcctgccccg
cgggcccacccaggttcaacgtccaccaccaccgcgctctcgcctcctccgccggcaacagacagcggcggcggcggcggcgggctgagctccggca
cgacggccgcagtcgcggtggtggtggtgctggcgttcctgggtctggccggcgcgttcgtgtgcatgtcgcggcggcggagcaagcgtcggcagctg
ggcgaccggtactacgccggcttcgccccgccgccggcgtacacgccgcagcacctctccggcgaggcgccgttcctgcacgcgccttccggtcccgg
ctcggccaacttcagcggcgggcccggcggcatgtcatcaggcgccgccagccaaatgtacgggcaccagcagcagatgccgccgctgcagccgccg
ccgtggatgcaggcggcgtcgttggcgaactacagcgcgtcgatcgggagccagatgccgccgcggagcgtggcgccgtcgtcgggcgagatgagcg
tgggcaacaccaaggcgttctcgttcgacgagctgtacgagatcacgggcgggttcgcgcgggacagggtgctgggcgagggcggcttcgggtgcgt
gttcaagggcacgctcctcggggacggcggcaaggtggtggccgtcaagcagctcaagggtggcggcggacagggcgagcgcgagttccaggcgga
ggtggagatcatcagccgcgtgcaccacaggcacctcgtctcgctcgtcggatactgcatcgcggaggatcacaggctcctcgtatacgacttcgtatcc
aacgacacgctgcaccaccacctccatggtcacccgaggatcatccacagggacatcaagtcatccaacatcctgctcgatgatcaattcgaggctcag
gttgcagactttgggcttgcccggctggcagagaacgatgtcacgcacgtctccacacgtgtcatgggaacattcgggtacttggctccagagtatgcgt
cgacagggaagctcaccgagaaatcagacgtcttctccttcggcgtcgtgctgctggagctcatcaccggccggaaacctgtcgactcgtcgcgtcccct
tggcgacgagagccttgtggagtgggcaaggcctctattgaaccgcgcgatcgagaaccaggagtttgaagggctagtcgacccgcgcctcgatggg
aactttgatgacgtcgagatgttccatgtcattgaagccgcggcagcttgcatccgccactctgctgccaggaggcccaagatgggacaggttgtccgg
gtccttgacagcttgacagacatcgatctcagcaacggagtgcagcctgggaagagccagatgttcaatgtggccaacacggccgacatcaggcagtt
ccagaggatggccttcggtagtcaggatttcagctccgaatatagccagtccagatcaagcataagcaaagatttctag
>g12154.t1
atgcggtcgagcttgatcgccctgtacatcaccatctgttccgtgctgttcatcgtgtccaagatgctcatctcgttcctgctgtacaagaaatgggcgcgc
aagaccaagaagaggatcatcgagaacagcctggaaggaggcaagatggtgatgttccgggcggcggcgacgacgaacaagtcgttgttcttgagc
atgatcatggggctgagcagcaaggacatgatcggctccggcgggtacggcaccgtgtacaggcttcgggtggatgagaagacggccttcgcggtga
agaagctgaaccggggcggcgccgagatggagcgtgggttcgagcgcgagcttgaaaccatgggcgacatcaagcatcgcaacatcgtccctctctg
cggctactacacggcgcctcacttgaacctcctcatctacgagctgatgcccaacggcagcctcgatgccatgctccatcatcatctccaccatctccctg
atgatggtctccatctccgtggcagcatgatcagcaacaaaaacaacaagaagctaatatcgttgttcttgagcatgatcatggggctgagcagcaagg
acatgatcggctccggcgggtacggcaccgtgtacaggcttcgggtggatgagaagacggccttcgcggtgaagaagctgaaccggggcggcgccga
gatggagcgtgggttcgagcgcgagcttgaaaccatgggcgacatcaagcatcgcaacatcgtccctctctgcggctactacacggcgcctcacttgaa
cctcctcatctacgagctgatgcccaacggcagcctcgatgccatgctccatcatcatctccaccatctccctgatgatggtctccatctccgtggcagcat
gatcagcaacaaaaacaacaagaagctaatagtaatggattggcctgcgaggtacaagatcgcgttgggcgttgctcgaggcctctcctacctccatca
cgactgcatccctcacgtgatccaccgtgacatcaagtccagcaacatcctgctcgatcacaacatggagcccaggctctgcgactttggcctcgccacg
ctcctcaaccccaaccacagccacgtcaccaccatcgtcgcaggcaccttcggctacctcgcgccagagtacttcgacaccggaaaggccaccaccaa
gggagacgtctacagctacggcgtcgtcctgctcgagcttctcaccggcaagaggcccaccgacgagtccttccttgaaaatggcaccaggcttgtcac
atgggtcaaggagaccatggaagacaagggggaggagcacgccgtcgaccaatctttgacgtcattccccgccgacgaggtcaagttcgtcttcgccg
tcgcagacaagtgcctcgactctgatccatgcaacagaccaaccatggcacaggtcgtcaacatgctccagcaacgcaaccactcataa
>g12174.t1
atggcggtggcggcgccggggcagctcaatctcgacgagtcgccgtcatggggctcccgcagcgtcgactgctttgagaagctcgagcagatcgggg
agggcacatatgggcaagtgttcatggcgaaggagatgcagacgcaggagattgtggcgctcaagaagatccgcatggacaacgagcgcgagggct
tccctatcacggccatccgcgagattaaaatcctcaagaaactacaccacccgaacgttatccagctcaaggagatcgtcacctcgccagtcgagggta
acaagtacaaggggagcatttacatggtcttcgagtatatggatcatgacttgacagggttggccgacaggcctgggatgcggttcaccataccacaga
ttaagccagagcagctgaccaaaatatttgaactttgtggcacacctgatgagctgaactggccgggtgtcacaaaaatgccgtgttttgaccggcatgc
tcttgatttactagagaagatgttaacattggatccttcacagagaatatcggcaaaagatgcacttgatgcggagtacttttgggctgatccccttccag

cagatcctaaaagactaagaaaaagcgtcagcagcagcgacaagctgaagaagcggccaagcgacagaaaacacagcatcctcaaccacattcgc
gtttgccaccaatccagcaacctggccaaccgcatgcacaaaacaggccaggtcagcctccaatggcagctggaagccatcattatgcaaagccacgt
ggcccgggagggccaaacaggtacccgcagggtgggagccaaggtggaggataccaaaatcgcggaggacaaggtggtggctatggcagtggccc
ttatcctcctcctcagcaaggccgaggccctcatccataccctggtggagcgatgggtggaacaggtggtggtggccccaggggtgggactggctatgg
ggttggaggaacaaactatccgcatgctggtccttatggttcctctggtccgcccagaggaccgaactataaccaacaagctggctctcgcaaccagca
gcagtatggaaactggcaatagtacaccatcatag
>g12191.t1
atgccgcgcctcatcctcttcctgctcgccgccgcctccctcgccgcgcacaccggcgccgaggacgacaagcgctacaacgcgagcatgtgcgaggc
gtcgttcccctgcggctcccacgtgaacatccactacccgttcttcctcgccaacgcgaccacggccatcgacgcatactcctactgcgggtaccccggc
atggccgtcgcctgcgacgacggccgcgccacgctacggctcaaggaagacaactacacggtcctgaacatcgactacgacaaccacaccgtgacgg
tcgccgacgcggacgtgctcgacggcggcggcggcggcggcgactgcccgcgggtcaagcacaacgtgaccatcccggaggagacgtggctcaacc
tctccaccgccgccaacgacaacctcgtcttcttcttcgactgcgccttcgccgccggaacccccaagccgccgccggcggccttgcccccgatcaactg
cagcggcttccaggaaggaagcgggatgacgtctttcgtggccgtgcagcccgacgtgccgccgaaggacagcttgccgggggcgtgcagggcggtg
atcaccgtgccggtcctgaaatactggctcgatgagtacttgccgtggctgaactatgacgggtacggcaaggtgctgaagcaggggttccagctgacg
tgggaccccagcgcggggccctgcttcgtgtgcgaggactcccgtgggcagtgcagctacaaccagaacggcgagttcctcggctgcttgtgctccgac
ggccgcgtgcgcaacccgggctgcgagccaatgcactgcctgcgcttagcaccactcctcttcatcgtcctcctcgcatcactgccaccttccaggccac
aacaaccctacacttacttccggtacagcaactgccggccaacaccataccgatgcggctcagttcagttcgacatcggctacccgttctcggtgaacgg
cgtggaccggcccgactactgctcttaccccgggtaccgcctctcctgcacgaacggcaagctggtgatcaccatgaaaagcgacgagttccaggtcac
gagcgtcgactacgacaaccacctcctcaccgtcatcgaccagagcctagctgatgagcaggcatgcttgcagccctaccggaacacaaccatcgatg
aggccatgttcacgtacaccgaccgggacctgttcctgacggtgtacatcaactgcagcgccagatcatcgccgtttcctttcgcatacgatctcttctcgt
gcttgtctgggggccgatcctactatagcctggacaatggcacggtggccccggatttgctggggtcatgcagctcaacactggttgttccctacaactc
aaccatggccgcctcactggctgccgggagttctagtcttggggacgcgataaaagccggtttcacggtgtggtggaaggagggaacctctatagctgc
cggtgttctgttcgtgctcctggtggtggtgtcgttcctgtacatccgcaagaggagagagtacgagatgacctcgtcctcgagcctcctcaagtacaccg
cctccggcggcacgccgcgttcgaacaagtggagcatcagcgacacggagtccggcagcgtccacgacctgcagacgcaccacttcacgtacgagga
gctggaggacgcgaccgacggcttcagcgacgagcgcgagatcggcgacggcggcttcggcaccgtgtacaaagggcaactccgcgacggtcgcgt
cgttgccgtgaagcggctgtacagcaacagcctccggcgcgtggagcagttcgttaacgaggcggcgatcctgtcgcggctgcggcacccgaacctgg
tggcgttgtacgggtgcacgtcgagccgaagccgcgcgctgctgctggtgtacgagctggtgcccaacggcacggtcgcggaccacctgcatgggtcc
cgcgccgcggagcgcgcgctggcgtggccgctccgcctgcgcgtcgccgtcgaggccgccgccgcgctggcctacctccacgccgtggacccgcccgt
cgtgcaccgcgacgtcaagaccagcaacatcctcctcgacggcagcttccacgtcaaggtcgccgacttcgggctgtcgcggctgttcccggccgacgc
cacgcacgtgtccaccgcgccgcagggcacgcccgggtacgtcgacccggagtaccaccagtgctaccagctcacggaccgcagcgacgtctatagtt
tcggcgtcgtgctggtggagctcgtctcgtcgaagccggcggtggacgtgacccgggaccgcgacgagatcaacctggccgccatggccgtggccaa
gatccagcgcggggaggtggaccagctggtggaccgcgagctcgggttcgacgacgcggaccaggcgacgagaaggacgatgacgatggtcgccg
agctcgcgttccggtgcctgcagcagaacggggagatgcggccggcgatcagggaggtgctcgacgcgctcaggagcatacaggaggaagggttcg
ggaagaaggtcaacacgccaccgtccccggacacggtgcatgctccgtgggacagcatcagtacaacgcccagcgtaagccagtga
>g12379.t1
atggtttcccgccatgccccatcttttcccgccctctcccattgttttcctgccctgccctactatggcgtccaccccagctcgcctcagtccattatggacat
gttgggaccaagcctctgggatgtatggaataataactcccactcaatgtctgttgaaatggttgcttgcattgctatagaggcaatttccatactagaga
agatgcattcaaaagggtacattcatggtgatgtaaaacctgaaaactttttgcttggacctcttggcactcctgaagagaagaaacttttccttgttgacc
ttggtttagctactagatggaaagaacctggtacaggagagcatgttgaatatgatcagaggccagatgtcttcaggggaactgttagatatgcaagtg
ttcatgctcatttgggaagaatcggaagcagaagagatgatcttgaatcccttgcctatacacttgtatttcttcttcgtggtcgccttccttggcaaggata
ccaggaatggataatggaacaatgggagaaaaactattacattactgcactagcaggtgcaaacaatggcagttctttagtgatcatgtcaagaggaat
gaataagtaa
>g12532.t1
atgtggctgaataaccgggctgatgcagttgaagcccttggatggccagatcgtctgaagatttgtcttggttctgctcatgggcttgcatttctacatcat
ggcttcgtgccccatatcatccaccgtgacatgaaatcaagcaacattctactggatgagaacttcgagcctagggtctctgactttggtcttgccaggat
aatcagtgcatgtgaaactcatgtgagtacggacattgctggaacttttggctacataccaccagaatatggccaggccatgaaatcctctacgaaaggt
gatgtctacagcttcggtgtcgtcatgctagagctgctcactggacgtccacctacagggcaagaggaggtggaagggggaggaaaccttgttggctg
ggtgcgttggatgattgcacatggcaagaaaaatgaactgtttgatccatgtttgccagtttcaagtccgtggtgggagcagatgttgtgtgttctttccat
tgctcagaactgcacggttgacgagccatggaagaggccgaccatgatggaggtggtgaagaacctcaagatggccgaggctatggaacgtggacct
ctggtggtgaaagtctctaccctctag
>g12533.t1
atgttgaaagaaatgttccttgatggaaattccttttctggacaactgagccctgctattggtcagcttcaagagctcaggaagctttcgatacctgtgaat
gcaatctctggtgtccttcctctggaattgggcgctctgcagaacctggaatttctggacattcacgcaaatgcattaagcgggctgataccagcaacttt
cagtaacctgtctaggctcttgcaccttgacgcgagccaaaataatctcagtggatccatattcccaggaattacttcgatggcaaacctcttgacaattg
atctctcatcaaatagtttgacggggccactgcctaagcagatcgatgatattctgaaagccacagaaaatttcagtaaggtgcacatcataggggatg
gtggctttggcactgtctacagggcagcgctccctgaaggtcggagagtcgcaatcaagaggcttcatggtggacatcagttccaaggtgatcgtgaat
tcttggctgagatggagacaattggaaaggtgaaacatccaaaccttgttcccctacttggctattgtgtttgtggtgatgaacggttcttgatttatgagt
atatggagaatggaagtcttgagatgtggctgaataaccgggctgatgcagttgaagcccttggatggccagatcgtctgaagatttgtcttggttctgc
ccatgggcttgcatttctacatcatggcttcgtgccccatatcatccaccgtgacatgaaatcaagcaacattctactggatgagaacttcgagcctaggg

tctctgactttggtcttgccaggataatcagtgcatgtgaaactcatgtgagtacggacattgctggaacttttggctacataccaccagaatatggccag
gccatgaaatcctctacgaaaggtgatgtctacagcttcggtgtcgtcatgctagagctgctcactggacgtccacctacagggcaagaggaggtggaa
gggggaggaaaccttgttggctgggtgcgttggatgattgcacatggcaagaaaaatgaactgtttgatccatgtttgccagtttcaagtccgtggcggg
agcagatgttgtgtgttctgtccattgctcagaactgcacggttgacgagccatggaagaggccgaccatgatggaggtggtgaagggcctcaagatg
gcccaggctatggaacgtggacctctggtggtgaaagtctctaccctctag
>g12548.t1
atggcgcgcgaggagtggagcatgtcggactttgagatcggcaagtacatcggcgagggaaagttcggcaaggtctacctcgcacgggagaagaag
gtcggccctcgaaacccttgcatccagtccctttcatttcccccaatttttacttgtcctccgcggcggctaatctgcgatcgatccagagcgggtacgtg
gttgcgctcaaggtgatgttcaaggagaagctggaaaagtaccggttccacgcgcacctgcggcgggagatcgagatccagagcggcctcgaccacc
ccaacgtgctccgcctcttcgcctggttccacgacgaagagcgtatcgtcctcgtcctcgagtacgccgcccgtggcgagttgtacaaggtccttcgctcc
gctagccgcttcagcgagagcacggccgccacctatgtcgcgagccttgctggtgcattggcatactgtcacaagaagcaggtcatccatagagacatt
aagccagagaatttgctactcgacattgaggggcgacttaaaattgcagattttggatgggcagttcggacaaatgctaaacggcacacactctgtggt
acaatagattatctggcaccagagatgattgagaaaaaagcgcatgatcatgctgttgacaactggaccctaggaatcctgtgctatgagttcttatatg
gtaagccaccttttgaagccgaggaacaagatgataccctgagaaggattgttaacgtggatctgacattcccttcaactcattatgtatctccacaggct
aaggatctcatttccaagcttctctactaa
>g12550.t1
atggagctcgtcgccgtcaaatacatcgagcgcggcgagaaggtcattttaacgccaacccatcttgctattgtgatggaatatgcatctggtggtgagc
tttttgagagaatatgtaaaaatgtgcgattcagtgaagatgaggcacgctatttcttccagcagcttatctcaggagtaagctactgccattcaatgcaa
gtatgtcaccgtgatctgaagctggagaatacactgctggatgggagtgatgctcctcgcttgaagatatgtgacttcggctattccaagtcttcagttctt
cattcacagccaaagtcaactgttggaacaccggcttatattgcacctgaagttcttttgaaaaaagaatatgatggcaagatagctgatgtatggtcttg
tggtgtaaccctctatgttatggtagttggcgcatatccttttgaagaccctgaagagcctaagaacttccgcaagacaattcagcgaatcttgaatgttc
agtactcaattccagataatgtgaacatatctccagagtgcaggcatctaatttcgaggatatttgttggtgatcctgctatgcggatttcaatccctgaaa
tccgaaaccatagttggttcttgaagaaccttcctgctgatctgatggatgatgatagcatgagcaaccagtacgaggagcctgatcagccgatgcaga
ccatggatcagatcatgcaaattttgacagaggccaccataccacctgcttgttctcgcagcataaacgccctagctgatggactggacatggacatgg
acgatgacatggatgacctcgagtccgactctgatcttgatgttgatagcagtggtgagatcgtatcgtgcatgcttaaaggggaggagatgaagcgag
agcgctggaacgcgtacagtgcagagcctgacagcctgatcgaggagaggaaggaggttctggacatggtcaggaacgagtttgaacaagaaaaat
aa
>g12991.t1
atgtctccctccccgtcgcgctccctcctccacctccaccacctcctcttgggcttgggcttgggccggcgccggcgccggcgcggcgacaacagcaaca
ctagcatgggctgcgcgcagggaaagccctcgcggggctcgccggcccgcagcgacggcccacgcgggctggaccgcctgatgcgcgacaacgcgt
accggcccgcatcgagccgcaccaccacctcccgcctctccgacccgctccccgtccccgcctggcagcagccgccgccgccagccgccaataaggag
cccgcggccggcagcacgggaacggataagaagacgacgccggacgacgatgacgtggcggcggcgcaggcgccgacgacggccacgccgccgc
tgccgccgcggcgggaggacgagctggtggacgggtggcccacgtggctgctggacaacgtcccccgcgaggcgctccacggcatcgtgcccaaga
gcgccgacgcctacgacaagatctacaaggcacgcgtgggccaggggacgtacagcaacgtgtacaaggcgcgtgagcgcgggacgggtcggatcg
tggcgctgaagaaggtgcggttcgacacgtcggagtcggagagcgtcaagttcatggcgcgggagatgcggatcctgcagcgcctggaccacccaaa
cgtgatcaggctcgagggcatcgccacctcaaggatgcaccgcagcatctacctcgtcttcgacttcatgtactccgacctcgcaaggctcatcccgcgc
gccgccgaacaacgactcaccgagccgcagatcaagtgctacatgcagcagctgctgtcgggcctgcagcactgccacgagcgcggcatcctgcacc
gggacatcaagggctccaatctgctcatcgaccgccacggcgtcctcaagatcggcgacttcggcctcgccaactactacggccgcaggccgctcacc
agccgcgtcgtcacgctctggtaccgcgcgcccgagctgctgctcaactccaccgactacggcgtcggcatcgacctctggagcgcaggctgcctcctc
gctgagatgttcttcggcaagcccctcatgcccggcagaacagaggtgagcatatctcaatctgtactgcacgactctgctacgtgctactgctag
>g13044.t1
atgcttctgcacaggcacgggatcctctgctgcgggtgcgggggcagcactgccatcgcggctggaggagaaggaggaggaggaggaggaggagcc
tggaataataaggacgatgagtcttcgtcatcgtccgtctcctactcagccaggcagctgtcttgggcgcaggtggaggcgatgacttgcggcttcactt
ccgccgtggtcggcgagggcgggttcagcacggtgtacctcgcccgtctcgccggcgggtccctcgccgccgtgaaggtccacaaccgcagcagcagc
agcgagcgtctccagcgcgcgttcaagcaagagctggatgcgctcctccgcgtgcggcaccaccctcacatcgtccgcctcctgggcttctaccacggc
gtcgaccaaggcggcgtcctcgtcctcgagttcgcccccaacggcaacctccacgaccgcctccacgccaacaaggacatcatgccgtgggcccggcg
cgtgtccgtgggcctgcaggtggcccaagcgctcgagtacctccacggcctgcaggtggtccacggcgacgtcaaggcctccaacgtcctgctcgacg
cgtccatggccgcaaggctctgcgactttggctccgcgcgggccgccaccgggttggccctcctccgcccaagggccgtcatgggctcgccagggtacg
tggacccgcactacatccgctccggcgtgctcaccaacaagagcgacgtctacagcttcggcgtcctgctgctcgagctcgtcaccggcgtacagccctt
ctccgacggcggcgggctactcacggccgccgtaagttcatctattagtaaaagcgacgtcgccaataataagctcgttgaccaccggctgggatgccg
gtacgacgccgacgaggcggcggtcgtgctggcgttggcgttggcctgcgtcggggacaaccccagcctccggccgtccatggccgacgtggtgcgaa
ctctggagcaaaacgcgcaggcctccatctccatctcatcgaccgtcagaagaagatcggacggagccagaaagctactactataa
>g13082.t1
atgcgccacctccccaagcaccccaacatcgtcaccctcagggacacctacgaggacaacaatgccgtccacctcgtcatggagctctgcgagggcgg
ggagctctttgatcggatcgttgcccgtggacactacacggagcgcgctgccgccttggtcactaagaccattgtcgaggtcgtgcaggtgagattggct
tcattgttcgtgcaggttcattga
>g13117.t1
atgggcaactgctgcgtgacgcctaacggcgccgatggcggcggcaagaagccgaagcagcccaagcagaagaagggcaagaagccgaacccgtt
ctcgatcgactacaaccggtcggcgccgccgggctcgaggctggtggtgctgcgggagcccacggggcgggacatcgccgcgcggtacgagctgggc

ggggagctcggccgcggcgagttcggggtcacctacctctgcaccgaccgcgccacgggggaggccctcgcctgcaagtccatctccaagaagaagc
tccgcacccccgtcgacatcgaggacgtgcgacgggaggtcgagatcatgcgccacctccccaagcaccccaacatcgtcaccctcagggacacctac
gaggacaacaatgccgtccacctcgtcatggagctctgcgagggcggggagctctttgatcggatcgttgcccgtggacactacacggagcgcgctgct
gccttggtcactaagaccattgtcgaggtcgtgcaggtgagattggcttcattgtngatgatttcatgtaagttttga
>g13118.t1
atgggcaactgctgcgtgacgcctaacggcgccgatggcggcggcaagaagccgaagcagcccaagcagaagaagggcaagaagccgaacccgtt
ctcgatcgactacaaccggtcggcgccgccgggctcgaggctggtggtgctgcgggagcccacggggcgggacatcgccgcgcggtacgagctgggc
ggggagctcggccgcggcgagttcggggtcacctacctctgcaccgaccgcgccacgggggaggccctcgcctgcaagtccatctccaagaagaagc
tccgcacccccgtcgacgtcgaggacgtgcgacgggaggtcgagatcatgcgccacctccccaagcaccccaacatcgtcacgctcagggacacctac
gaggacgacaatgccgtccacctcgtcatggagctctgcgagggcggggagctctttgatcggatcgttgcccgtggacactacacggagcgcgctgct
gccttggtcactaagaccattgtcgaagtcgtgcagatgtgccataagcatggagtgatgcacagagatctcaaaccagagaacttcttgtttgcaaaca
agaaggaaacagcggctctcaaggcaattgattttggtctgtccgtatttttcactccaggtgaaaggttcactgagattgttggaagtccttattatatgg
ctccagaggtactgaagagaaattatggcccggaagttgatgtttggagtgcaggagtgattctttacattcttctttgtggggttcctccattttgggcag
aaactgaacagggtgttgctcaagctattattcgttcggtcattgattttaaaagagacccatggccaagggtctccgataacgccaaagatcttgtcag
gggaatgcttaatccagatccgaaacggagattgacagctcagcaagtgcttgatcatccttggctgcagaacataaagaaggctccaaatgtcaattt
gggtgaaactgttaaggccaggcttcaacaattttctgtgatgaacaagttcaagaagcatgcgctcagggtcatagctgatcatctttcagtagaagag
gctgctgacataaaagatatgtttgagaaggtagatcttaacaaagatcagatgattagttttgatgagctgaagcttggtctgcataagtttgggcacc
aaatgcctgatgcagatgtccaaatacttatggaagctgctgatgccgatggaaatggaaccttggactatggagagtttgttactctatctgttcaccta
agaaagattggcaatgacgaacatctgcataaggcgtttgcttactttgatcggaatgagactggatatatcgaaatcgatgaactccctgatgacctgg
gacaaaatcctgaagatgttatcaatgccatcatacgtgacgtggacactgataaggatggcaagataagctacgatgaatttgcggcaatgatgaag
gctggaacagactggaggaaagcatcaagacagtattcaagagaacggttcaccaaccttagcctaaagctgcaaaaggacggatcgttgcagatga
cgagtacccggtag
>g13341.t1
atggcctcctccccttgtcacactgacaccaccaccaccgcgcccgcgttgtcgtcaccgccgcccaccaagcctccgaaccgcgccgccaccatgctc
gaccacccttcgtaccccgacgccgctgtctccagcgtcagcagctcctcgagcagcagcagcagcggtggaagcagcctggccgaccggtcgtcgac
gttctcgctggactcgtcgtcggccgccgcgacgccctcgtcctccccgccgcggccgcaccgggccagcgacgtggcctgggcccccatccgcgggc
gggactccgctctgggcccgcgcgacttcaccctcctccgccgcgtgggcgccggcgacattggcacggtgtacctctgccgcctcgataccaaagcca
ccgaggcggagggcagcgtgtccacgtcatcgtgcgagtacgcgatgaaggtggtggaccgccgcgcgctggcgcggaagggcaagctgtgccgcg
cggcggcggagaagcgcgtgctccggcgcctggaccacccgttcctccccaccatgttcgccgacttcgacgcgggccccgactactcgtgcgtggtga
tggagttctgcccgggtggggacctccactccctccgccaccgcgccgcgctgccgggccgcaggttcccgctggcctccgcgcggttctacgccgcgg
aggtgctcctggcgctggagtacctccacatgatgggcatcgtgtaccgcgacctcaagcccgagaacgtgctcatccgcgccgacggacacatcatg
ctcaccgacttcgacctctccctcgaggccacctcgtcgccctccctctccatggacctggacgaggacacaacagctccgacgtgcctgcccatcccgg
agctgcagctgctgcgtctgaagcggtggaggcggcgagcagtgccaccacagccgcggccgcggttcgtggcggagccggtggacgcgcggtcga
gctcgttcgtgggcacgcacgagtacgtggcgccggaggtggcgcgcggcggaggccacggcgcggccgtcgactggtgggcatacggcgtgttcct
ctacgagctcatctacggccgcacgccattcgtaggggacaccaacgaggccacgctgcggaacatcgtgcgacgaccgctccagttccccaccaacg
gcaacggggacggaagcagcgacgccgcggcaagggacctcatcgccaggctcctggacaaggacccgcgcaccaggctcgggtccaggcgcggc
gcagcagacgtgaaggcgcacgcattcttcaagggcctcaacttcgcgctgctacgctccgccaccccgcccgtcgtgccgccgcctgtcgcgacgcac
cggtgcgacgacaataaggcggccgacgtgcaccagctgttggaccacttctga
>g13621.t1
atggccatggatgtggccatggtgctcgccatcatcttcttctgcatcctcatgctctcctccgccgccatcgccttcctcctcatccgctactgccttcgcc
ggaggaccagtagtagtgctgccgaccccgaggtagctgtaccagtagcagtagcaccaccggagccggaggaggagcccgtgctgtccttatttcca
tcgtcggagaaggagaaggagaccttgttgttggagccgcgcaggctgacgtggcgggaggtggaggcgctgaccggcggcttcgacgaggctgccg
tggttgggcgcggcggctccagcatcgtctacctcgccaggctccggcgcggcgcgcccgtcgccgtcaaggtgcaccgctggtgcggcggcgagcgg
cggctgcgcgcgttccggcaagagctcgacctgctccgccgcatccaccacccgcacatcgtcgccctcctcgcctactctgacgaccacgaggaagg
gggtgcactggtcctggaatacctcgccggcggcacgctcgccgaccgtctccacggcggcgccattattgccccgccgctgccctgggcgcaacgcat
gcgcatcgtccacgacgtggcctgcgccctggagcacctccacgacgcctccaccggcgtcgtgcacggcgacgtgtcgtcgtccaacgtgctcctcga
cggcggcggcgctcggctctgcgacctgggctcctcctgcgaaggcttctccgccgccgtggcgccgtccagggcggccgtgggctcgccggggtacg
ccgacccccttcttcctccgcaccggcatcgtctccaagaagtccgacgtgtacagctttggagtgctgctgctcgaggccatcacgggcatgccggccgc
cggatccggattggacggcacatcagagtacctgacggctcgggtgctgccacgtgtcaggaggcagggtgtggatggcctcgtggacagcatgctag
gagaggactacgacgtcgaggaggccggcgatgtggccaggatcgccgtggagtgcgtggcggcgcagcccgggctccggccgaccatggaccagg
tgcgagtggccattgccgagaaggcagcgaggtccattgccaaggctgattacggtcaccatatacagttgagcaagctgctggagataacatga
>g13776.t1
atggagaaaggagggggtggactgaaaatgttcaacatagtttcagcgtggaacaagaggagaagaagcagatcgctggatcagctaaatccatgg
gtgtacaagccagctgagctgtggcaggtgaaggagcagagcccgccgccgaaaaagcgcaactacacgatggtgttcacgctcaaggagatggag
gaggcgaccaacatgttcagtgaccgtaacctcatcggcaagggcggctttggccgggtttacaggggcgtgctgaaggatggccagatcgtggccat
caagaagatggacctgcctacatccaagcacgccgacggcgagcgcgagttccgggtggagatcgacatcctgagcagactagaccaccccaacctg
gtcacgctgatcggctactgcgccgacgggaagcaccggttcgtggtgtacgagttcatgcccaggggcaaccttcaagacatcctcaatggcattggg
gaggtgaagatattggactggccggtgcgcgtgcggatcgcgctcggcgcggcgagggggctggcttaccttcactccagcacggccgtcggcgtccc
cgtcgtccacagggacttcaagtccagcaacatcctcctgaccgaacactttgaggcaaagatctcagattttgggctcgccaagctgatgccgcagga

cctggacctgcacaccgccaccagagtgctgggcacgttcggctacttcgaccctgaatacgcactgacagggaagctgacgctgcagagcgacgtgt
acgcgttcggcgtggtgctgcttgagctgctcaccgggcggcgcgccatcgacctgagccaggggccccaggagcagaacctcatcgtcaggatccac
caggtggtgggtgaccggaagaagctgcgcaaggtggtggaccgggatatggcgaaaggcacctacactttggagtcggtgtccatgttcgcccgcct
cgccgcgcgctgcgtctgcttcgagagcgccggccggccgtccatgcaggactgcgtcaaggagctccagttcatcatgtacgccaacatgaagatatg
a
>g13864.t1
atggggttcgccaagcccatagagggcgggcgctcaccttctgtgtctgccgccaaggctgcgcacttcgaggacgaagtgctgctcggggccgcgctt
ggcggcggggttgcgaccggtgaggggatggaccttctccagaactcgcgaggtgagggggcggcgcgcgatggggcgtcatcgagctcttccggc
ctcgcggcgaacgagacgacggggtggcgaccgccatgtacgagaagcaggagaagatcggcgagggcacgtacggggtggtgtacaagggtaag
aaccgtcaaaccaacgagacgatcgcgctcaagaagatccgcctcgagcaggaggacgagggcgtcccctccaccgccatccgcgagatctccctcc
tcancaagaaccgccaaaccaacgagacgatcgcgctcaagaagatccgcctcgagcaggaggacgagggcgtcccctccaccgccatccgcgaga
tctccctcctcaaggagatgcagcaccggaacatcgtcaggctgcacgacgtggtgcacaacgagaagagcatctgccttgtctttgagtacctcgacc
tcgacctcaagaagcacatggactcctccccggacttcaagaaccaccgcatagtcaaatccttcctctaccagattctgcggggcattgcctactgcca
ctcgcaccgcgtgctccaccgggatttgaagccgcagaacctgctgatagatcgccgtaacaacctggtgaagctcgctgactttggattggcaagggc
attcggcattcctgttcgaacatttactcatgaggtggtgacactgtggtacagagcacctgaaattcttcttggtgcaaggcattattccacccctgttga
tgtatggtcagttggttgcatttttgctgaaatggtaaaccagaagccactatttcctggggattctgagattgatgagctcttcaagatcttcagaattatg
ggcaccccgactgaagagtcttggcctggtgtcgcttcattacctgattacaagtcaactttccccaagtggacgccagtggatattgcatctttggtccc
aacacttgaacctgctggaattgatcttctctcgaaaatgctatgtttagatccaagcaagaggatcaatgcccgggctgccctcgagcacgagtatttc
aaagatcttgaagttgcatag
>g14009.t1
atgggaattgaggaattgaggattgttgaagaaggatggggatgggagtatgggacttggaacatacggacactcgacccgatttgccccggccgtct
cacgggaccttcagatctggctgtacccaccttccgcgacaggcccaggaaagcgacccgcgaagccgcgtgcgccgtgcaatcgcctcctcgcggaa
accggaacaggcagaagaaagatgagatcttttcctcccgcccgcaagccattcctctcccagtcgtcccacccatgggaacgtcggcggacggcgcc
attaaaccctcagattggggatttcctgccggaggaagcatttcaagggagctgcctttgggaggaactcttccagctctgaaggtttcagttttgagtgc
caagtttggtggtggaagccctcaggctggagatttcctccctgaagacgcccgaggagccggctttggaaggaatttcctgccaaccctgaggttggt
gatttcctccctctcaggacgcaagggcaggcttgcttcggaggggctcttccagttcccagggtttgctgatggatatgttgaggaggatcgctactccg
atttgaagaaatttgacaagccatggcctgaggtgaacgcatttaagcccactgctgctggtattttgaggcgggggttggacccgaactccatctctgt
ccttgagcggaagactgcagaccttagggatcattacatcattggtcggaggcttggtcaggggcagtttggtacaacatacctgtgcacagagataag
tactgggtatgaatatgcatgcaagaccatcccaaagcgtaagttcatcaccacggaggatgttgaggatgtgcgccgtgagatccggataatgcacca
tttgtcaggccacaagaacgttgttgcaatcaaagatgtatatgaggacagtcaggcaatacacattgtgatggaggtctgtgctggtggggagcttttt
gacagaattcaggagaaggggcattacagcgagcaaaaggccgcagagcttatacgaattattgttagcattgtggctatgtgccactcacttggggtg
atgcatcgtgatctaaagccagaaaatttcctccttttggataaagatgatgatttgtcaatcaaagcaattgattttggtctatctgtgttcttcaaaccag
gtatgatatcaaatgtgatgcctggaaatgcattctttgttgtttgttttaaaacatga
>g14038.t1
atggcggtggagcagcagcagcagggcggggaggaggcgttcgaggaggtggacccgacggggcggttcgggcggtacgccgacgtcctgggcct
gggctccgtgaagaaggtgtaccgcggcttcgaccaggaggaggggatcgaggtggcgtggaaccgcgtgcgcctccgcgcgctggcggaccgcga
cccgggcatggtggaccggctccacgcggaggtgcggctgctgcgctcgctccaccaccgccatatcatcgggttccacaaggtgtggctggaccgcg
acgccggcgtgctcaacttcatcaccgaggtctgcacgtcggggagcctccgcgagtaccgccagcgccaccgccacgtctccgtcaaggcgctcaag
aagtgggcgcgacagatcctcgaggggctcaaccacctccacacccacgacccctgcatcatccaccgcgacctcaactgcagcaacgtcttcatcaat
ggcaacaatggacagatcatggcggtggagcagcagcagcagggcggggaggaggcgttcgaggaggtggacccgacggggcggttcgggcggta
cgccgacgtcctgggcctgggctccgtgaagaaggtgtaccgcggcttcgaccaggaggaggggatcgaggtggcgtggaaccgcgtgcgcctccgc
gcgctggcggaccgcgacccgggcatggtggaccggctccacgcggaggtgcggctgctgcgctcgctccaccaccgccatatcatcgggttccacaa
ggtgtggctggaccgcgacgccggcgtgctcaacttcatcaccgaggtctgcacgtcggggagcctccgcgagtaccgccagcgccaccgccacgtct
ccgtcaaggcgctcaagaagtgggcgcgacagatcctcgaggggctcaaccacctccacaaccacgacccctgcatcatccaccgcgacctcaactgc
agcaacgtcttcatcaatggcaacaatggacagcaaacggccgggctaacgccgccgcggcacgtgtcgcaggtcaagatcggcgacctcgggctgg
cggcgatcgtggacaagacgcacgtggcgcacaccatcctcggcacgccggagttcatggcgccggagctgtacacggagacgtacaccgagtcggt
ggacatctactcgtacggcatgtgcgtgctggagatggtcacgcgcgaggtgccctacgccgagtgcggcagcgtcgtgcagatcttccacaacgtcac
ccgcggggtgccgcccgccgcgctcaagcggctcaaggaccccgagctgcgcggcttcatcgagcgatgcatcggccagccgcggaaccggccatcg
gcggccgagctcctgcaggaccccttcttcagcggcattggtgaggacgacgacgacgcgatcaccgacgcggccagcgtcgtcgccgctgccggctc
gcccgtgccgcgccccaggagctacgtcgacgacctcgccgggctccgcctggattga
>g14049.t1
atggcttcagcaggtgtggcctcatctgggtacaagaacagcagcagcactagtatgggtgttgagaagttacctgatcagatgaatgaattaaagata
cgggatgataaggaagttgaagcaaccatcatcaatggcaaggggacagaaactgggcacataattgtgacaactactggtggcaagaatggtcaac
caaaacagacagtgagctacatggctgagcgcattgtaggtcaaggttcttttggaatcgtcttccaggctaaatgcttggagacgggtgagactgttgc
cataaagaaggttcttcaagataagcgttacaagaaccgagagctgcagaccatgcgtcttcttgaccaccctaatgttgttgctctgaagcattgtttct
tttcaacaactgaaaaggacgaactatatctgaatttggttcttgagtatgtgcctgaaacggttcatcgagttgttaagcattacaacaagatgaaccaa
cggatgccacttatttatgtgaagctctatatgtaccagatctgtagggcactggcctacattcacggtagcattggtgtttgccacagggatatcaagcc
acaaaatcttctggtccttgggactcccacaagggaagaaattaagtgcatgaacccgaattacaccgagttcaaattcccacaaattaaagcacaccc
gtggcacaaggtattccacaaaaggatgccacctgaagctgttgatcttgtgtctaggctgctccagtactcccccaatctacgatgcactgctgtggag

gcacttattcacccgttctttgatgagcttcgagaccccaacactcgccttccaaatggtcgatttctgccacccctcttcaacttcaagcctcacgagctg
aaaggagtcccagcagacattgttgcgaaattgatcccagaacacgcgagaaagcagtgcccttacgttggagtatga
>g14183.t1
atggaggccatcagcagccggaagcgcttcgccgtgggcaacctggaggaggagtacgagccgatatgcgagctcggcaagggcggcttcggctcc
gtcgtgaaggctcgccaccgcggcactggccatctcgtggccatcaagcgcctcgccaatcccggcgagggccaatcggaggtgatgcgggagaaga
ggttcctggaggcgtgcagcggcaacccgttcgtggtggggttccgcggcgtggtccgcgcccccgactccatggagctccgcctcgtgatggaccacg
tgggccccagcctccgcgacttcctccgcctgcgccgcgccgacgaggacacgccgctcccggaacccacggtccgcgccatcatgtggcagctgctg
cagggcgccaagcggatgcacgcggtccacgtcgtgcaccgcgacatcaagcccgacaacctcctcgtgagcgcccaccagaacctgatcaagatct
gcgacctcggccttgccctgcacttgtgcgacgcgccgccgtacacccaggccggcacgacgtggtacatggcgcccgaggtggtgctggggaaaccc
gactacgacgcgctcgtggacgcctggtcgctcgggtgcgtcatggccgagctcatcaacggtgaagtcctcttcttcccgggcgacaacgacaacgac
aacggcagcgaagcagcaggccagcaaatccgcgccatcgtcgacgtgctcggcgtggccgtggttctcgaccacgccgttcgccaccgggggttcg
aggtactcagcggcctgctcacgtggaaccccgacaagcggctcacggccagcgccgcgctcaagcaccccctggttcgacgatctcaacgcccctgct
gcgccggcaagaatcaacgaggacctggcgtcggctttgcccaacaacatgaaggacccgctgacgatgatttctccaaacttgcacaagaggcgca
agatccaatgtgcctga
>g14233.t1
atggaggatatggttcttgcccttgatgcgcccggatctcccaactcgcactggtacacagtacatgttaagcacgagcaacctgaacaactagagcct
atttgtgttgtcccaccagagatgccgacatctgaaagctacatatcatttgcatctaaagcaccaaattgtactgaaactggtgaagtagaggcagatg
aagatgagcctgaacttcctaatgtgtttgacaaagaggaggagtatgttggcgttaacgacgaatacatacagataagtcaaggtttagagctgaatg
cgggcatgaaggttgtccatggcgtatacatgcttctgtgctacaggatggtagaacagtacagaaagcgtaaggctacatcagaggacgacactcag
aaccaaatgtccaagaagcaaaagtcagccaagaagaagaaaacaccaaagaagaagaagaagatgccaaagaagaagaagacacccaagaaa
aagaaggctatggctgcatcaagtgcaccacctccgagagtcatcaagaagcagactacctcaactctttattgtgtccattatcacgtgtccagttacat
ttccagtcatggcaccacccatccgccactgcctcctcgccgtcctcctccctctcctcctcgttgccgccgctgcggataccccctgttttcttctccttcccg
cccttctccctccgcaacctcaccctcctcggcggggcctcgctccgcgccgcctccgtctccctcccgccgccctcctcccacgcgctcttcccccctgccg
cctccgtctccctcccgccgccctcctcccacgcgctcttcccccctgccgctcccgttccctcccaacgcctccttcaccacctccttcctcttcgctgcgccg
gcctccgcgcgccccgcctcccgcctctccttcgtcctcctcctcgacggggccgagggcccctcgacgaaccggtccctcccgctccaggtcatcctcg
acgcgtccgcgaaccgcgtctccgcgttctccgccggcgcggaactcgccgcgaactccacgggggccgtggacctgcggaacggcaacggggtcgg
ctcctgggtcctctacgatgcgcgccgggcgcgcgtcgaggtgttcctcagccacgccagcctgcggccgcagacgcccgcgctcgccgccgacgcca
cgggcctcgccgcccgtttcgccgagttcatgttcgtcggcctcgaggtatcgtcagacaacgcgaccagccgcgacggcggcttcgtcgtcgagagct
ggaccttcctcaccgccgggctgcctgccgtcgatctcgcgtcccacgccgcgcacagcgtatccgacagcgtccgcagcgcgcccgcgtctgtccgtct
cgccgctcatggggatgcgcgccgcaggaggttggcgttggggctcgccatacccttgcccattctatttctcggcgcactcatggtgtttgctgtaatgtt
cctgaagaagtggaggtggggtactgctgcaggacttaacgaggtagttgggtcaagggcgccaggcaagccgaggcagttcatgtaccaggatctct
tctcggccatgatgggatttgatccgtctatgatgattggcagcggtggctttgatacagtgtacaaagcagtgtgcccacgctcgggggtcacgtacgc
ggtcaaaaggtccaggcggtcccgggagagctacaatgagttcaacgctgagctcaccataattgctgacctgaagcaccctaatctggttcagcttca
aggttggtgcacggagagggacgaactgctgcttgtctatgagttcatgtcgaatggcagcttggacgaggctctgcacccttgttcaggcgcacagca
cgatgttactcttggttggtctcagcggtacaatgtggctgctggcattgcatctgtggtggcatacctacatgagaaacatgacaagcaggtgatccata
gggatataaagagcagtaacatactccttgactcatctttcagtccaagattgggagactttggacttgctaggttaaaggatcctaacacgagccctca
atcaaccctggcagcagggactattggttaccttgcacctgagtatctccagatggggaaaaccacagaaaagagtgatgtctacagctttggggttgt
gctgctggagatctgcacaggcaagcgaccaatagagagagaggcacctgacagtatgaacatgttgaatgttgtggattgggcttggaatttgcactc
aaaaggaaagctcttggatgcggcggatgcacacttgaatggggagtatgatgccaagcaaatgatgcgtctgcttcttctaggtttgagttgtgtcaat
ccgttttcagaggagagacctttcatgaggacagttctggggatactggagggcaagaatgagctactttctgttccaagaaagaagccgttgcttgtgt
ttgtttccaatgcaccgattgatcttgaggggatagtttcagaatgcagtcagagtacaatttcaagtaatatgtatcaactgaaaatcgacatacctata
cctaggtaa
>g14412.t1
atgcccgtcatcggcgccgctcgcgtccggggcaggaggagttgcggcactgagtggaaggcgccgaggcggctggatcttgcgcggctacggcaag
gacacgccatcaaatcctccaagacgcaggaggcgttgaaattgtggagaatcgaggagagcgactcagacttcacgctctacgacttcaatgagatg
gcggccgcaacaggcaacttctccgtctcgaatcgtctcggcagaggtggctttggccccgtctacaaggcaagtggaccagcagttgacctgagaca
cggccagcttcatgacggggtggagatcgcggtgaagcggctttcagcacaatcattgcagggactggtagaattcaagaacgagatccagctgatcg
ccaagctgcagcacaccaacctggtccgcctggtcggatgttgcgtccacgaggaggagaaaatgctggtctacgagtacatgcccaacggaagcctc
gacttcttcctctttgacccagtgcgaggacggttgctggactggaagaagcggatacacataatcgagggaattgtgcaggggctcctctacctacata
agcactcccgggtgcgcatcattcaccgggacctcaaagccagcaacattctgctggacaaagacctcaatcccaagatttcagacttcggcatggcaa
ggatcttttgtgcgaacattacgaaagccaacaccaacagggtcgtcggcacctag
>g14785.t1
atgaagattctgaacacgatcaagcaccggaatattatcaggatggacggctactgcatccgtgggagtgttggcctaatgctttatgaatacatgccca
gtgggacactctttgatctgttgcatgcaaggaaggctcaggttgatcttgactggatagcccggcatcagattgctcttggtattgcgcaaggcctttcct
atctgcaccatgattgtgtacccatgattgttcatagggatgtcaagtcaagcaacatactgatggacactgatctaacaccaaaactcacagattttggc
atggggaaaattgtcaatgacaaggatactgatgcaacagtgtctgtcatcgttggtacccttggctacattgctccagcatggatattccacacgattat
ctga
>g14911.t1

atggggtgcttcgccttcaagagcggcaagagcaggaggtccaagaggcagaaggcagttggtgcgccggggtccgattccggcgggcagaagagc
aaggcgtcgtcggcgtcgacgccgtcgaggagcatacaggagctgtcggaggagaggggcgcgcagcggctgcgggtgttcgacctggaggagctc
agcagcgccaccaacggcttcagccgctcgctcaagctcggcgaggggggattcggctccgtctaccgcgccttcttccgctccgccgccggcggccgc
gtcgtcctcgccagcaaggcgtcgtcggcgtcgacgccgtcgaggagcatacaggagctgtcggaggagaggggcgcgcagcggctgcgggtgttcg
acctggaggagctcagcagcgccaccaacggcttcagccgctcgctcaagctcggcgaggggggattcggctccgtctaccgcgccttcttccgctccg
ccgccggcggccgcgtcgtcctcgccgtcaagcgcctcaaccagcgcagccgccagggacacaaacaatggctggctgaagtccagttcctgggcgtt
cttgagcacccgaatctggtaaagcttatcggctactgcgcagtggattcagaagcaagcaacgacaggctgctggtgtatgagttcatgcccaacaag
accttggacgaccacctgttcaaccgagcccatcctcctctcccgtggagaacgaggctgcagatcatgattggagccgcgcgggggctcgattatctg
catgaaggggtgccagaagttcaggtgatctacagggatttcaaagctgccaacatccttctggactctgaattcaagccaaaactttcagattttgggc
tggcaagggagggaccaaccgaaggcaaaacacacgtgtccacggcggtggttgggacgcacgggtacgcggcgccggactacatcgagacgggg
catttgacagtgaagagcgacgtgtggagcttcggcgtggtgctgtacgagatcctgacggggcggcggtcgctggatcggaaccgcccgccggagg
agcagaagctgctgggctgggtccgccgccacccgccggacggccagagcttcaggacgatcatggacccaaggctgggcggacggtacccgccgg
ccgccgcgcgcgaggtggccaggctcgccgaccgctgcctcgtcaagaaccccaaggagcggccggccatgagagacgtcgtcgacgacctcgaga
gaggacacaaacaatggctggctgaagtccagttcctgggcgttcttgagcacccgaatctggtaaagcttatcggctactgcgcagtggattcagaag
caagcaacgacaggctgctggtgtatgagttcatgcccaacaagaccttggacgaccacctgttcaaccgagcccatcctcctctcccgtggagaacga
ggctgcagatcatgattggagccgcgcgggggctcgattatctgcatgaaggggtgccagaagttcaggtgatctacagggatttcaaagctgccaac
atccttctggactctgaattcaagccaaaactttcagattttgggctggcaagggagggaccaaccgaaggcaaaacacacgtgtccacggcggtggtt
gggacgcacggttacgcggcgccggactacatcgagacggggcatttgactgtgaagagcgacgtgtggagcttcggcgtggtgctgtacgagatcct
gacagggcggcggtcgctggatcggaaccgcccgccggaggagcagaagctgctcggctgggtgcgccgccacccgccggacggccagagcttcag
gacgatcatggacccgcggctgggcgggcggtacccgccggccgccgcgcgcgaggtggccaggctcgccgatcgctgcctcgtcaagaaccccaag
gagcggccggccatgagagacgtcgtggacgacctcgagaaagtgctgcagatggagcctccgccgccggctgacaaaaagaaagaaggcgatgg
caacaagcctgctttcccgtcgtcggccaagaggtga
>g14993.t1
atgcgggagaagaccttcctcgaagcctgcagaggcaaccccttcgtcgtcggcttccacggcgtggtccgcgcgcccgactccatggagctccgtctc
gtcatggaccacgtgggccccagcctccgcgacttcctccacctgcgcggcatcacgtggcagctgctgcagggcgtcaagaggatgcacgccatcca
cgtcgtgcaccgcgacatcaagcccgagaacatcctcgtcagcgccgaccagaacctcctcaagatctgcgacttgggccttgccctgcacatgtccga
cgcgccgccgtacgaggaggccggcacgaggtggtacatggcgcccgagatgctgctggggatgcacgactatgacgcgctcgtcgacgactggtcg
ctgggatgcgtcatggccgagctcgtcaacggtagagtactcttctccttctcgggcgacaacagtagagacggccagatatgctccatcatcgacgtgc
tcggcgtgccgttcgcgaccggcgttctgcctcgactacggtaccggcagaggcgaagcctgctgcgtcacaagttccctgccacgatgctgtcccagc
aaggtttcgaggtcctcagcggcctcctcacgtggaaccccgacaagcggctcacggccagcgccgccctcaagcacccatggttcgacgatctcaac
gcccctactgcttcgccggcaaggaacaagatgacggacccgctgatgatttctcccagcttgcacaagagccgcaagatacattgcgcctga
>g14994.t1
atgatgcaggccacattggaatacatgatgccggtgccatcaaggcggatgcacgacgctgaagtgtgcagcacgatgggcggtttcggcgccgtcgt
caaggcgcgccaccgcgtcacgggccagttcgtcgccatcaagcgcctcgccaaacccggcgaggggcaatcggaggtgatgcgggagaagacctt
cctcgaagcctgcagaggcaaccccttcgtcgtcggcttccacggcgtggtccgcgcgcccgactccatggagctccgtctcgtcatggaccacgtggg
ccccagcctccgcgacttcctccacctgcgcggcatcacgtggcagctgctgcagggcgtcaagaggatgcacgccatccacgtcgtgcaccgcgaca
tcaagcccgagaacatcctcgtcagcgccgaccagaacctcctcaagatctgcgacttgggccttgccctgcacatgtccgacgcgccgccgtacgagg
aggccggcacgaggtggtacatggcgcccgagatgctgctggggatgcacgactatgacgcgctcgtcgacgactggtcgctgggatgcgtcatggcc
gagctcgtcaacggtagagtactcttctccttctcgggcgacaacagtagagacggccagatatgctccatcatcgacgtgctcggcgtgccgttcgcga
ccggcgttctgcctcgactacggtaccggcagaggcgaagcctgctgcgtcacaagttccctgccacgatgctgtcccagcaaggtttcgaggtcctca
gcggcctcctcacgtggaaccccgacaagcggctcacggccagcgccgccctcaagcacccatggttcgacgatctcaacgcccctactgcttcgccg
gcaaggaacaagatgacggacccgctgatgatttctcccagcttgcacaagagccgcaagatacattgcgcctga
>g15077.t1
atggtggcagcagcagcggagatcaggcactggacgcggctgcagacgctgggccgcggcacgtcgggcgccgtggtgtccctcgcggcggacgcc
gcctcggggcagctcttcgcggtcaagtcggcgcgcgcgccttccgccgcctcggcggcgctgcggcgggagcaagcggtgatgtcgggcctctcctc
gccccacgtcctccgctgcatcggcggcgccggcgaggaggacgagtaccacctcttcctcgagtacgcccccggcgggtcgctggccgacgcggccg
cgcggaatggcgggcgtctcgacgagcgcgccgtcgttgcgtacgcggccgacgtggcgcgcgggctggcgtacctccacgcgcgctcggtcgtgcac
ggcgacgtcaagccacggaacgtggtggtcggcgcggacggccgcgccaagctcgccgatttcgggtgcgcccggattgcggcggcgggggagccc
gcgcggccgatcggcggcacgccggcgttcatggcgaccgaggtggcgcgaggggaggagcagggcccggccgcggacgtgtgggcggtcgggtg
cacggtcgtcgagatggccaccggacgcgcgccgtggagcggcgtcgtggacgacgacgacgtgttcgcggccgtgcacaggatcgggtacaccgac
gccgtgccggaggtgccggcgtggatgtccgccgaggggaagagcttcctcgcctgctgcttcgcgaggaacgccggggaccgacccacggcggcgc
agctgttggagcacccgtttctggcatccgcaagcaatgaagcgaagtcgacatgggagttgaagtgggtgtccccgaagagcacgctggacgccgca
ttctgggagtcggaagagaccgacgacgatgatagcagcccggcagccgagaggatcaagtcgttggcgtgctcatccttcccggactggtgggactg
cgaatcgagcggctggatcgacgtgctcggcggcggcggcgcggagaccgaggcggtggtccatgtggcgcgcagcgcgccaagtaaggtgtcaag
cgccgtggcggtgccagctggagagatgggtgtcggcggcggcgtcgtctcttgctgtgatgatgatgatgaacaacaactggaaacagaggaggcg
ctgctgcagttcggtgcagctgctgcagatggtccatcgcttccagagatgtga
>g15078.t1
atggagatatcggtcagcggccggtggacacgcgtccgcacgctgggccgcggcgcgtccggcgcggtggtgtggctcgccgccgacgaggcctcgg
gcggcgcgcttttcgccgtcaagtcggcccccgcggacgcgccggcgggggagcagctccggcgcgaggcgagcgtcctgtccgcgctccgctccccg

cacgtggtcccctgcctcggcttccgcgccgacgacggggagtgccagctcttcctcgagtacgcgcccggcggcacgctcgccgatgcggcggcgaa
gagcgtcgggggccgcctcccggagcgcgacatcagagcctacgcttgcgacgtggcgcgtgggctggcctacctccacgggctggagctcgtgcac
ggcgacgtcaaggcgcgcaacgtggtcctcagcgccgacggccgcgccatgctcgcggacttcggctgcgcacggccaacagagtcaacccggccga
tcggaggaacgccggcgttcatggcgccggaggtggcgcgcggggaggagcagggcccggccgccgacgtgtgggcgctggcctgcacggtcgtcg
agatggccacgggccgcgcgccgtggagcgacacggacgaggtcctcgccgcggtccaccggatcgggttcaccgacgccgtgccggaggtgccgg
cctggctgtccgcggaggcgaagcacttcctcgcctgctgcttcgcgaggaacccacgcgacaggcccacggcggcgcagctcctggagcacccgttc
ttggcgtcggccggctgcggcgccgtcaaggtggaggaggtgtcgtccgcgaaatgggtgtctcccaagagcacgttggacgccgcgctctgggagtc
cgacaccgacgacgacgaggaggaggatgttgacatttcagagaaccccgctcagagaatcaaggccttggctggcccctgctcggccctgccggatt
gggactccgacgaagcaggctggatccaagtgctcaacgagcaatgtgaagcctgcaatttggtgttcgtaccaaccaaagaggcggctgaggagatt
gccgtcggcggggacgagattagctgctctgtctatcgaccagcagcagcagcaaaaactgcgtcctcgcagtga
>g15079.t1
atggactccaccaccagcatcatcgcgaagcagctcaggcgagtccgcactctgggccgcggcgcgtcgggcgccgtggtgtggctggcctccgacga
ggcctccggcgagctgctggccgtcaagtcggcgcccgccggcgtggccgcgcagctgcggcgcgagggccgcgtgctcgagggtctccggtcgccg
cacatcgtgccctgcctcggcgcccgcgccgcacccggcggcgagtaccagctcttcctcgagttcgcgccgggcgggtccctcgccgacgaggctac
caggtttggcggccacctcccggagcaagagattcgtgcgtatgccggtgacgtggcgtgcgggctggcctacctccacgcgcgctcggtcgtgcacg
gcgacgtcaaggccaggaacgtggtgatcggccccgacggccgcgccaggctcacggacttcggctgcgcgagaccagccgattcaacgcggccgat
cggcgggacgccggcgttcatggcgccggaggtggcgcgcggggaggagcagggcccggccgccgacgtgtgggcgctgggctgcacggtcgtcga
gatggccacgggccgcgcgccgtggagcgacgtggacgacgtcctcgccgcaatccaccggatcgggttcacgggcgccacgccggatgtgccggcg
tggctgtcggaggaagccaaggacttcttggacgggtgcttttccagacgtccctgcgacagggccacggcggcgcagctcgtggcgcacccgttcctc
gcctgcgccgttcgcgactgcaagccagggctggggaaagacgaatgctcgtctcccatgaccacactgcatgacgcgttctgggattccgacactgac
gacgaggaggccgacgaggcgtcgacgagcccggccgagaggatcgcggcattggccacctcggccttcccggactgggactccgacgaaggctgg
atcgacctggagctggagcactccgcctccaaatctgccgacgcgatgacggggccgatggacaccgcggactgcttcgtctgggcggaaccattatc
cgacgacgaagacttggagcaattccccgccgcggaactcgtcactggccatcgcccaaacagcacagcagactgcttcgatgtgaacagagcatca
aacggagcgtcggagcaattcgccggcgcggcagatgtgatcagcgacctcccgcgcgttgccggagcaggcacgaggccaaagcagccaccacgg
catcatctcatcagggccgtcgcgatcgaagaatga
>g15080.t1
atggacgccgccgccgcagggaagaagcagctccggcgggtccgcactttgggcagcggcgcgtcgggcgccgtggtgtggctcgcctccgacgacg
cctcgggcgagctgctggcggtcaagtcggccggctcgcccgttgcggcggagcagctggcgcgcgaggcgcgcgtcctctccggtctcagttcgccg
cacgtcgtgccctgcctcggcgtccgcgccggggacggcggcgagtaccagctcttcctccagttcgcgcccggcggttccctcgccgacgtggccgcc
agaagcggtggtcgcctttccgagcgggaaacccgggcgtacgccgccgacgtggcgcgcgggctggcctacctgcacggactctctctggtgcacgg
cgacgtcaaggcgaggaacgtggagtgcgaaaggcattcaagtgtgaatgtagacagtgggggggtggcgaagcagctccggcggatccgcacgct
gggccgcggcgcgtccggcgccgtggtgtggctggcctccgacgaggcctccggcgagctgctggccgtcaagtcggcgccggccggcgtggccgcg
cagctccggcgcgagggccgcgtcatggaggggctctcctccccgcacatcgtgccctgcctcggcgcccgcgccgccgccggcggcgagtaccagct
cttcctcgagttcgcccccggcggctccctggccgacgaggccgccaggtgcggcggccgcctcccggagcaagagatccaggcgtacgcgggggac
atcgctcacgcgctggcatacctccacgaattatcgctggtgcacggcgatgtgaaggcgaggaacgtggtcatcggcgccgacggccgcgccaggct
cacggatttcggatgcgccaggccagcggagtcaacgcggccaatcggaggcacgccggcgttcatggcgccggaggtagcgcgaggggaggaaca
gggtccggccgccgacgtgtgggcgctcgggtgcacggtcgtcgagatggccacgggacgcgccccgtggagcgacatggacgacgtccttgctgca
atccaccggatcggctacaccgacgccgtgcccgaggtgccggcgtggatgtccgagggggccaaggacttcctgacctgctgcttcgcgaggaacgc
cagcgaccggtgcacggcggcgcagctcttggagcacccgttcgtcgccttcgccgcacgcgacttcgacgacgcgcggccggcgaagcctgagcac
ctgtcgcccaagagcaccctgcacgacgcgttctgggactttgacaccgacgacgagacagacgacgacatgtcaacgagcccggccgagagaatca
gcgccctggccacctcagcgttgccggactgggactccgacgaaggctggatcgacctccaggacgagcaccaccaaaccgttcacgcgacgccgga
gacatccgaggcggcgccggctgcagagtacttcctctggttccaaccaacggaggcagagtctgagcaatccgtcgccttcacagatgccacagtgg
atctcccgcagattctagtagtaacacaaactgatgtcagcgttaggccggatgctttcagtgaatgagacgatattcgtgttaaaaaaagatatgttgt
gagcttgttaatctcaagattggttggctttga
>g15211.t1
atgcatcggctgtcctgctcctgcaagtttgcggacccagacagcgtcgcatcctccgcggcgacgacggcgacggcgacggcgcgccgcgtgcggcc
ggacacgatgagctgtggcagcgagggggccgagacggcagcagcggtggcagcgaggagttcaggcggccgcggcccagcaaggtcgcggctg
atgatgtggtagcgccagaacggtgctccgattccataacgccggcacggtgttcggatgccaccacgccggcgtcctggatcgacaggaagctgcttg
ttgatcccaagacgctgtacgttggggacaaaattggggagggagctcatgggaaggtctacaaggggaagtacggtgatcaaattgtagcaataaa
ggttcttaaccgtggcagtacacctgaagaaaaggcaacacttgaagctcgtttcattcgtgaagtaaatatgatgtgcaaagtgaaacatgagaatctt
gtgaagttcattggagcttgcaaggaaccattgatggtgattgttagtgaactactaccagggatgtcactgaaaaattatttgaacagtattcggcctaa
tcaactagatacacacactgcaatcagctatgcactagacatagctcatgcattggaatgtttacatgccaatggcataatacatagagacctgaaacct
gataatttgctgctcacagcaaaccgtaagaagctgaaacttactgactttggacttgcccgcgaagaaactgtgaccgaaatgatgacagctgaaact
ggaacttaccggtggatggccccagagctatacagcactgtcacacttcggcgcggcgagaaaaaacattatacaaataaagtggatgtatacagcttt
ggcattgttttgtgggagttactgaccaataaaatgcctttcgaaggcatgtcgaatctacaggctgcatatgctgctgcatttcagttcattggagcttgc
aaggaaccattgatggtgattgttagtgaactactaccagggatgtcactgaaaaattatttgaacagtattcggcctaatcaactagatacacacactg
caatcagctatgcactagacatagctcatgcattggaatgtttacatgccaatggcataatacatagagacctgaaacctgataatttgctgctcacagc
aaaccgtaagaagctgaaacttactgactttggacttgcccgcgaagaaactgtgaccgaaatgatgacagctgaaactggaacttaccggtggatgg
ccccagagctatacagcactgtcacacttcggcgcggcgagaaaaaacattatacaaataaagtggatgtatacagctttggcattgttttgtgggagtt

actgaccaataaaatgcctttcgaaggcatgtcgaatctacaggctgcatatgctgctgcatttcagcaaaagcgcccagctttccctgaggataccccc
caggagctggtgttcatagtacaatcatgctgggttgaggaccctagcatgcgacccagtttcagtcagatcatccgaatgcttgagactttcctcctgtc
gatacctccaccagcaacatcagaatcaaatgaagatgaggaatcagaagaaacaaagtcgtcattgagcagtaggagttcctctggttcctcacttgt
ttcgcgtgcaacaagcaagctctcggtcgtccgccatctcttcgcctcaaagaaggcaagcaacgggaaggcatga
>g15320.t1
atggagagcgagatggcggatgaacgggtgatggcggccgcacgaggcgggggcgggccgctggccgcggtctcggaggaaggggaaggggagc
ctgaggtcgctccggcggcggtgggctcgagcaagaccatggagcgcgtggcggccgcgaagaagttcatcgaggaccactaccgcgcgcagatga
agaacatccaggagcgcaaggagaggcgcttgattttagaacaacagctagcttcttcacaagttcccagggaagagcagataaacttaataaaggat
ttggagagaaaggagactgaatacatgagacttaaaaggcacagaatttgtgtggatgactttgagctgctcaccatcattggaagaggtgcttttggg
gaggtccgactatgtcgggagaagtcttctggcaacatttatgcaatgaaaaagctgaaaaagtctgaaatggttgtcaaagggcaggtggagcatgtt
agagctgagcgaaacttgctggctgaagttggtagccactgcattgtgaaactgtattattctttccaagatgctgaatacctctaccttatcatggagtac
cttcctggaggtgacatgatgaccctccttatgagagcggatacgttgactgaagatgtggctcggttctacattgctgagactgttcttgccattgaatcc
attcataaacataactatattcacagggatataaagccggacaacctacttctagataagaatggtcacatgaagctgtcagattttgggctatgcaagc
caatcgactgttcaaaactgtctaccttgaatgaagatgaacccatgagtgatgaaaaccttagggagtccatggacattgatcgttcgttctcagatac
cgcaaatggtaggcggtggagaagtccaaatgaacagcttcagcactggcagaagaacaggagaaaactggcattttctactgttggaacacctgact
atattgctccagaggttctactaaagaagggatatggagtagagtgtgactggtggtctcttggtgcaatcatgtatgaaatgcttgttgggtacccgcca
ttttattctgatgatccaataactacatgccgaaaggctcatccttggtttaaaggagttgaatggagtaagctttatgaaatggaggcgacatttaaacc
tcaagtaaatgatgagctggatacacaaaatttcatgaagtttgaggaatcaggcccagctccagcaagaacaagttctgggccctcaaggaagatga
tgctcaactctaaagatcttagctttgtggggtatacatacaaaaattttgaagctgtgaaaggcttgcatcagtctgcagtcaggcccagctccagcaa
gaacaagttctgggccctcaaggaagatacaacagacatggactcatcatcagaaccgaacggaagggacacgcacatgcgcactgtttcatccgat
gaccctatggtgcaatga
>g15632.t1
atgaagctccgtcctctctcattctccgcgcgcaccgcgccgtcctcgccgttgaatccggggagcgacaagactcagaggaggaggacggtggtggt
ggcgctccgccgcgacgccgcggggagggagctcctcacctgggcgctcgtcaaggccgccgccgccggggaccgcgtcgtcgcgctccacgtcgctt
cttccgcatcagccgaggatgatgccgcggcggaaaagggaagggtcgccgtcgagacgctggggtccttgctgggcgcgtacagggggttctgcga
gcgcaaccagatcgagctgcagctcacggtccgcgaggcgccctccatcaagcgggccctcgtcgccgaggccaccgactatggcgcggcgcacctc
gtgctcggcgtcaccaagacctcctcttcctccttctccaggccttcgtccaggtcgtcggccaccgcggtggcgcggtactgcgcgaggagggtgccgg
cgagctgcgccgtcaccgccgtcagcaacggtgtcgtcgtctaccaacgagacgccaccgccgccgtacagcagcaacagcatcatcaccaccagca
gccgcagctcagccattacagctcaatggtagagacgccgcggaggctgtaccgcaagctgttcgatgtgacgatgaccggcggagacaacaaatcc
caggacgacctggcgatcgaccagtaccacggccgggccacgtgccagaacatgtcggtgtcgatgagcgcgctggtctcccccagactgcggtcctt
gactccgttgaggtctcagaagaagccggaggtggccgccggctggccgctcctcaggaaggacttcaagtcagccttgttgccggaggactcgtcgtc
gtcggagatctcggtggttcagtgggcgatgaagctgccgacacggtgctcccaagtgtcgccggtggattcggaacaaatttccagtgacaaggaag
tcgatttgggtaagctgtccccagagatcgaggaggaggaagggaatcaagttccagaagagctcgtcgcgctcagagaaaagtacacgattttcagt
tacagcgagcttgccaagatgactcacaacttctctccagatcgactggtcgggaaaggcggcgcgggccgggtgtaccgtgcgcgtaccgacgacgg
cacggagctggccgtgaagctcctgaaaccctcggacacggccgcccgcgagttcgcgtcggaggtcgacgtcctcagcttcgtcgcccaccgccacg
ccatgtccctcgcggggctctgcgtcgacgacaggcgcctcgcccttgtctacgactacgtgcgccgcggcagcctggaggaggtgctgcacggcggc
ggcgcgttcgggtggccgcagcgggtcagggtcgccgtcggcgtcgccagcgcgctggagtacctccacgccggcgacggagaacgaaggccggtg
atccatcgggatgttaagtcgtccaacatcctcgtcggagatgacttcgaacccaagctatgtgactttggcctcgcgatctgggcggaggacgcggcgt
cgcaggtcaccggcgacgacgtcgccggaacattcgggtacctggctcccgagtacttcatgcacggcaaggtcagcgacaagatggacgtgtacgc
gttcggagtcgtcctcctcgagctcgtctccgggcggaagccgctcagcgcagccggcgggcaaaagggacaggagagcctcgtcatgtgggccacct
ccgtcgtgcaacaaggaaagctcatggacctcgtcgacccgaccctgccccatgccggcggcgaggtcgagcggatggcgctcgccgccgcactctgc
atccggcgagcgccgcaacgaaggccgaccatgagcaacgtcctgaagctgctcaccggcgacggcgaggcggtcaagtgggccaagtcgcagatc
ggcgtggcggttgacgacgacgagtgcggcgtggcaaattcgccggacaagagcgacatccagtcgtacatcaacctggcgctgctcgacgatgacg
acgccgggtccgtcagcagcgtcgacttcctcggcgccaacatgtcgctggaggagtacatgaagggaagatggagccgctcgtccagctttgacgac
tga
>g15713.t1
atggggatacagaggtacaagctcttcttgtgcgggtgcggcgtcaatgcgtccgccgcacgcgacctggacggcgacgacgacttggactgctgctcc
ggcgccgacgcgttcggggagaaggcgtgcggcggcgaggccggggcgccccggcgcctatcgctggcgcaggtggaggcgatgacgggtcggttc
acgtccgccgtggtcggcgagggcgggttcagcaccgtgtacctcgcccgcctctccggcgcgctcgccgccgtcaaggtccaccgcagcagcgagcg
gctccaccgcgtgttccgccaggagctcgacacgctcctccgcgtccgacaccctcacatcgtccgcctcctcgccttctgcgaccagcaagaggaagg
cgtgctggtgctggagttcgcggcgaacgggaacctgcacgagcgtctccacggcggggtcaagtcgtcgggcacgctgccgtgggcgcggcgcgtgt
gcgtggcgctgcacgtggcgcgggcgctggcgcacctgcacgacgcgtgcgagccgcaggtggtgcacggcgacgtgaaggcgtccaacgtgctcct
ggacgcgtcgatgcgcgccaagctctgcgacttcgggtcggcgcgggccgggttctccgccgccgtgcggccgcggcccgcgggccggaccaccatg
ctgggctcgcccgggtacgtggacccgcactacatccggtcgggcgtggtcaccaagaagagcgacgtgtacagcttcggccgacgcgatacagaga
ggcagcaagcagcagagcagcctcacaagcaagtcgtctccttccccctttctcctcctctcctgtgccgtgtgtgcaagaaaccatttcaaggcagggg
aagaaggaagggagaagcgaaagcgagaaactttcttaagatggggatacagaggtacaagctcttcttgtgcgggtgcggcgtcaatgcgtccgcc
gcacgcgacctggacggcgacgacgacttggactgctgctccggcgccgacgcgttcggggagaaggcgtgcggcggcgaggccggggcgccccgg
cgcctatcgctggcgcaggtggaggcgatgacgggtcggttcacgtccgccgtggtcggcgagggcgggttcagcaccgtgtacctcgcccgcctctcc
ggcgcgctcgccgccgtcaaggtccaccgcagcagcgagcggctccaccgcgtgttccgccaggagctcgacacgctcctccgcgtccgacaccctca

catcgtccgcctcctcgccttctgcgaccagcaagaggaaggcgtgctggtgctggagttcgcggcgaacgggaacctgcacgagcgtctccacggcg
gggtcaagtcgtcgggcacgctgccgtgggcgcggcgcgtgtgcgtggcgctgcacgtggcgcgggcgctggcgcacctgcacgacgcgtgcgagcc
gcaggtggtgcacggcgacgtgaaggcgtccaacgtgctcctggacgcgtcgatgcgcgccaagctctgcgacttcgggtcggcgcgggccgggttct
ccgccgccgtgcggccgcggcccgcgggccggaccaccatgctgggctcgcccgggtacgtggacccgcactacatccggtcgggcgtggtcaccaa
gaagagcgacgtgtacagcttcggcgtgctgctgctcgagctgctcacggggatggaggccttctgcgcgggggagggacgcctcctcacagccgtcc
tcgcgcccaggctcaagggcggcgacgcgagggggatggtggacgacaggctcggcacggcgtacgacgcggcggaggccgacgcggtggcggcg
ctcgcggcggcgtgcgtcggcgagaacccgagcctgcgcccgtccatggccgacgtggtgcgcaccctggagcagagtgcgcacgggtccatctccg
ccgtcgaggggagatcaaacggccaagggaagctgtga
>g16078.t1
atggaggagaggtacgaggcgctcaaggagctgggcgccggcaacttcggggtggcgaggctggtcagggacaaacggaccaaggagctcgtcgc
cgtcaagtacatcgagaggggaaaaaagatcgatgagaatgtgcagagagagattatcaatcaccggtcacttcgacatcctaatatcgtacggttca
aggaggtaattcagaaatcattaactctatcttttgtttga
>g16311.t1
atgaaccatccaaacattgtgaagctcaaggaagttataagagagagtgatacattgttctttgtttttgaatacatggagtgcaatctctatcagctgat
gaagaacaggggaaagcctttctcggagactgaagtccgaaattggtgctttcaagtattccaagctctgaatcacatgcatcaacgtggatattttcac
cgtgatcttaaacctgaaaatctgctggttacgaaggaactcatcaagatagcagattttgggcttgctcgtgagatttcttctgaaccaccatatacgga
atatgtgtccactcgttggtaccgtgcccctgaagttctgctacaagctactgtctataatgctgcagttgttgcggctaaaggagctgttgatcagaaga
atggtaggagattttccacaggacctgtcaccaatgggaggcccacagttaattacttgtatgcaagcactaatgctccagcaagagctattggtgtgca
aaggaaactggaactggatcatcaggtcccagagagtaaccacaaattgacaaaggcaaacacaatgaatcagccttggtctagacaaccaccagct
aggagcaatggaaattatctcatcaaggaccagagtcctcgtgcacctgatcttgctgagaagctgtctcagctgtcaatgggccctaatagggtgtcgg
gtttggcttccgagaggtttacggatcttaaggctagaaccaatgggaacaccatcaagcaccccttgcctgtgggatccagggcatggcatggcccaa
ccgacccctttcgccgcccatacgagatgccaggtgacaggaatttccttccgaggaagcttgttagctga
>g16602.t1
atgatgtcaccatctcctctccagcgcctcctcctcctcctcgccgcagccgcgtctgccgtgtcgccggcaggaggcggttgcagcaccggctgcg
acgtcgctgttgcctactactccaccaagccgaaccagaacctcacttacatcgggtcactctttggcatcgaagactatcgtgcgctggcggcctacaa
cgcaggtcatcccaaccaggatttcgtccctgccggcgagcctgtcctcgtcaatttcacctgccgatgcctagcgcttccaggcgcgccgtcatccacct
acctcgccggctccttcccctacagggtctccccaggggacacctactccagcatcgccgccgtctacaataacctcaccactgccgcctggctggagg
agaccaactgctcctgcggggatccggcagtttcgccggacctccgtatgttcctaacctacccgctcgctggcatctcaggcctatttgggctatgcata
atcctcctcgcattcttcctgtgctacaagagatattatggcatgatgctgtggcaaagagggtcaagaaatccatcaaggattgaatcattcctgcaaa
agcaaggaacttcacatcccaaaaggtacagttactcagatgtgagaagaatgaccaaatctttcacccacaagcttggtcaaggtggatatggagctg
tttacagaggaaacctgcctgatggcggagagatagctgtcaaattgctgaaggacacagagggagacggggaggaattcatgaatgaggtggctag
catcaacagaacatctcatgtcaatgtggtcactctcttaggattttgcctgcaaagttcaagcagagctcttgtgtacgagtacatgcccaatggttcact
cgagcaatacacttttggcaactattctgctgaaggacaccagactctaacctgggataggttatttgatattgttgttggaattgcacgagggctggagt
atctccacactgggtgcaacactcaaatcgtgcattttgatatcaagcctcaaaacattctgttggatcaggacttctgtccaaagatcgctgattttggaa
tggcgaaactgtgccaacaaaggagagcaaaatctccattgctggtgcaagagctctgccgccggttgagctccgccacagccccgcctcccccacg
tcggtgcgcctagagctccagctcctccgtcagccgagatctcgaggccgccgcagccccgccgcacccagcgtcggcacgcctagagctgccgctcc
gctgtcgcgcgccgcccgagatcctccgccgcgcacccgaggtccttctcctgcgccgtcgcacgcgccactcgagcttctcctccgctgccacgcgtca
cccgaggtcctcctccgccgcgcgcccaaggtcctcctccgccgcgcgcccgaggtcctcctcctccgcccgtgctcctcctcctgcgctgctgcgcatgc
acccgagattgcggcggcgggaggcagcctcgatgcagcggcttgggcgacacgaagcagcagcggccgggcgagcacaaagcaacggcggcgg
ctgctgggcacgaaggagaagcggctgggcaagcgcgaagcagccgcggtggcgtcagacgagcacgagacggcggcgcaagacctagtttag
>g16742.t1
atggatcatgtcgtcgggggcaagttcaagctcggaaagaagatcgggagcggatcgtttggggagctcttccttgctgtgaatgtgcagactggagag
gaggtcgccgtcaaactggaaaatgtgaagacaaagcacccgcagcttcattacgagtcgaagctatacatgcttctccaaggaggaactggcatccc
gcacttgaagtggttcggtgtcgagggggagtataatgtgatggttattgatcttctggggccaagtcttgaggacttgttcaactactgcagtagaaagt
tctcgctgaagactgtgcttatgctcgctgatcagatgattaacagagttgagtacatgcatcaaaagggttttcttcaccgtgatataaagcctgataatt
tccttatggggcttggtaggaaagccaatcaggtatacataatagactatggtcttgcaaagaagtatagagaccttcaaacccacaagcacattcccta
cagagagaacaagaacctcactggaactgcacgatatgcaagtgtcaatactcaccttggcgttgacaagccagattattcttacctgaagagactatt
ccgtgatctctttatccgggaaggataccagtttgattatgtttttgactggaccatcttgaaatatccccaaatcggctccaatccacggatgaggagtg
agagaactagtggtgctgctggaccttctatggacaaaatagaaaaaaacccaggtgaagcatcaggtagaaggaatcctaatgcttctcttaatcaga
gtgacaattatgcgcagcgaccacgagaaggcgtatccatgtcactgaaggaaattcacagaccgggctggggaacggactgtggagaggcctcgta
catcctctcgcacaggcagtgcttcgcggagagctattgcgtcaagcagcaggctggcctcatctgcagaaccaaaccgggctggggaacggactgtg
gagaggcctcgtacatcctctcgcaccggcagcgcttcacggagagctattgcgtcaagcagcaggccggcctcatcggcagaaccaaggccaagtcc
tag
>g16888.t1
atgcggaaagggatccgaggagatctcctcgtcctcctcttctgcagcttggtggcgctgcttccggtggtgtgccatggcgcggacatgccgctgggat
cgaccctctctccaggtaactctgcggcggcttggtcatcccccaacagcaccttctcgctgtccttcacggcgtcccccagctcgccgtccctcttcgtgg
cctccctgacctacgccggcggcgtccccgtgtggtccgccgggtccggcgctgccgttgactcgggtggctccctccgcctgacctcgaccggcgacct
ccagctggtcaacggatccggcgccgtggtctggtcgtccaacaccggcggtcgcggcgtgtcgtcggcggccgtccaggagagcggcaacctcgttc
tgaagaacgcgacgacgacgctgtggcagtcgttcgaccacccgaccgacacggtggtgatgtcccagaacttcacctccgggatgaacctcacctcg

ggctcctacgtcttcaccgtggaccgcggcacgggcaacctgaccctccggtgggcccaggccggcaccacggtgacctacttcaacaaggggtacaa
ctccaccttcaccgggaaccggacgctgagcgcgccgacgctgacgatgcagaccaacggcatcgtctccctcaccgacgggtcgctgtcgtcccccgt
ggtggtcgcgtacagcagcaactacggagagagcggcgacatgatgcggttcgtgcggctcgactccgacggcaacttccgcgcgtacagcgccacg
cgcggcagcaacagcgcgacggagcagtggtcggcggtggcggaccagtgccaggtgttcgggttctgcggcaacatgggcgtgtgcagctacaacg
gcacgtccccggtgtgcgggtgcccgtccctcaacttcgtgttcaacaacccgtccaaccctcgtgaagggtgcaagcggacggtggacctccagagct
gcccgggcaactccaccatgctcgccctggacaacacgcagttcctcacctacccgcccgagatcaccaccgagcactcatgcgtcgcctccaccgcgc
tctccgacggctcagggctctgcttcctcaaggtgtccagcttcgtgagcgcgtaccagtccgccgcgctgcccagcacgtccttcgtcaaggtctgcttc
ccgggcgtgtccaacccgcccgccacgggctccgggcccgccgggtcaccgcgcggcaccgtccgcggctgggtggtggccatcgtcgtgctgggcg
cggtggccgggctggtcctctgcgagtgggccctgtggtggttcttctgccgtcacaaccccaaatacgggccggcgtcggcgcagtacgcgctgctcg
agtacgcgtcgggcgcgcccgtgcagttctccttccgggagctgcagcgatccaccaagagcttcaaggagaagctgggcgccggcgggttcggcgcc
gtgtaccgcggcgtgctggcgaaccgcaccgtggtggccgtgaagcagctggaagggatcgagcagggggagaagcagttcaggatggaggtggcc
accatcagcagcacacaccacctcaacctcgtccgcctcatcggcttctgctccgagggacggcaccgcctgctcgtctacgagttcatgaagaacggc
tccctcgacgcgttcctcttcggggaggcgccgccgggcgggaagctgccatggccggcgaggttcgccgtcgccgtcggaaccgcgcgagggatcac
gtacctccacgaggagtgccgcgactgcatcgtgcactgcgacatcaagccagagaacatcctgctcgacgaccagtacaacgccaaggtctccgatt
tcgggctcgcaaagctcgtcaaccccaaggaccaccgacaccgcacgctcaccagcgtgagaggcacgagggggtacctcgcgccggagtggctcg
ccaacctgcccatcacggcgaaatccgacgtctacagctacgggatggtgctgctggagattgtcaccggccaccgcaacttcgacgtctcggaggag
acagggaggaagaagttctccgtatgggcgtacgaggagtacgagaaagggaacgtcgctgacgtcatcgacaagaagctcccccccggggaggac
atcgacatggcgcaggtgcatcgcgcgctgcaggtgagcttctggtgcatccaggagcagccggcgcagcgaccgtccatgggaaagtcgtgcaga
tgctcgaggggatcatggagctcgagaggccgccgccgccaaagtcctccgacagcttcctgagtagcgtcaccacggcgagtggcagcgccagcgg
cgtcaccacgagcatggtctccaccttcgcgtcgtcagccccggtggcgccaacgccgtcccccaatctgggtctggagcgggaggagattgctgcgtt
ggaccgctcggcgtcggtgaaccgtgacagagtgtcccgggcgcagcgatccacgcagccatacatgacaatgtaa
>g16982.t1
atggcgcggctcgacgggctcatgagcaacctcacatggaaggccgcgcatgggaacaaccccgtatgttcgcggccggggcggtcgaccacacgc
cattggtgaagatatacggcatggcccagtgcaccggggacctcggcggcgacgactgctacagctgccttgacagagctgtcgcgtatatcccgacct
actggggtaggaagcagggcggccaggccatcttgtggagttgcttcgtccggttcgagtcggccattttctataatgtccacgctgtcgagattgccatg
tcgtcgtcgccagcgctggcgccggttccggcgccgggagacggatcgcccaactacagcaaccactcaagcaaaggaagcaccggaattagtcaga
cggtcagaacagctcttcttgtctcagttcctgttgctgtaacgctgctggtgttcatgtttgttgctgtcttgacctacaagaagaatagaaaaccataca
ggcgtgtctgggttgcaagcaacagacttggagatgaagaaactggaagtctagaacccttccagtatgatttgagtactctgcgggccgccactgaca
acttcttagaacaaaataagctcgggcaaggcgggtttgggccagtttacaaaggtatgctacaaaatggtaaacacattgcagtgaagaggttgtcag
caacatcggagcaagggcaagcggagatgaagaatgagattgtcctagttgcaaaactccaacacaagaacctggtgcgcctacttggcttctgcatg
gaacaagatgagaatctcctggtctacgagctcctcagtaacaagagcctcaacaaatttctttatggtccgacaagaaaggagctaagctgggcaca
aagatacaaaattatcaaagggatcgggcgagggcttttgtaccttcacgaggattcaaggttgaagatcatccatcgcgatctcaagccgggcaacat
cttgctagatgtagacatgaaccccaagatctcggattttggcttggcaaagctgttcaacgttgattcgagcgtgaaatacacaatacaccaagctgga
acttacggatacatggcaccagagtacgtcttgaatggcatcttctcagccaagtcagatgttttcagctatggcgttcttgtcttggaagtcgtcacctgt
aggcgaccttctgaagagctcctaaaatttgtatggagacactggaggcaggaaaacgtgccaccgttgttggaaagttgcccaaccgacgagcatgg
gcagagggagatgctaaggtgcatccacatcgggctcctatgcgtccaggatgaccctgaactccggccccgcatggcggacgtcgatctcatgctca
agagccacttcatgacgttggttgcacccactgagccggtcttcgcagccaccggccagaggccaagagtggctgctaatgagccatccatcaacaaa
gtttccatctcagacgtggagacccgttaa
>g16983.t1
atgcccaggcttcttccgctcgtgctcctctgctcgttgtctcttctggccgcaataacgacctacgctgaagacagtatcctcttcgagtgcgagagcgac
acaaactacacgcgtggcagcgcgttccaggccaatctgaaagccctcctctcttctctcccctccgccgccgcggcgtcccagggcttcgccgagaac
gtcaccggtgccgcgcccgaccaggctttcggcctcgcgcagtgccgtcgtgacgtcagtgcgtccgtctgcaacaagtgtgtaaccaaattggcacag
aagctctacacaggatgcctaggcaagaggagtgccatcatcgtctccgatacctgccagcttcgatactccaacgtgagcttctttggtgaagccgaca
cgaggttattggagtattttgctaccccatggaacgcgacggagccggagctgttcatgagacggctcgacgagctcatgagcaacctcacatggaag
gctgcgcatgggaaccccgtatgttcgcggtgggtgcgttcgaccacacgcccttggtgaagatatacggcatggtccagtgcaccggggacctcggc
agagacgactgctacagctgcctcaacacagcagtatcgcatatccttacctactgggataaggaggcggacggccatgccatcttatggagttgcttc
attcggttcgaccgggtccttttctataacgtccatgctgccgagatagccatgtcgtcgccagcggcgctggcgccggttccggcgccgggagacgggt
catccaactacagaaaccactcaagccaaggaagcactgatgttgctaccggtgcaggaattagtcacacggttagaagagcgcttcttgtctcggttc
ctgttgctgtaacgatgctggtgttgatgtttgttgctgtcttgacctgcatgaagaacacaaaaacatacaagcgtgtccgggttgcaagcaacagactt
ggagatgaagaaactggaagtctagaacccttccagtatgatttgagtactctgcgggccgccaccgacaacttctcagaacaaaataagcttgggca
aggcgggtttgggccagtttacaaaggtatcctacaaaataggaaaaatattgcagtgaagaggttgtcagcaacatcggagcaagggcaagcggag
atgaagaatgagattgtcctagttgcaaaactccaacacaagaacctggtgcgcctgctcggcttctgcatggaacaagatgagaatctcctggtctac
gagttcctcagcaataagagcctcaacaaaattctttatggtccgacaagacaggagctaagctgggcacaaagatacaaaattatcaaagggatcgg
gcgagggcttctgtaccttcacgaggattcaaggttgaagatcgtccatcgtgatctcaagccaggcaacatcttgctagacgtagacatgaaccccaa
gatcgcggattttggcttggcaaagctgtttcacattgactcaagcgcgaaaaacacaatacaccaggctggaacttatgggtatatggcgccagagta
catcctgaatggcatcttctctaccaagtcagatgttttcagctttggagtccttgtcctagaagtcatcaccagccgacggccttcagaagatctcctaaa
atttgtctggaagcactggaggcaggggaatgtgacaccgctactggaaagctgccaagctaacgagcatgagcagcaggagatgttaaggtgcata
cacatcgggctcttatgcatccaggacgacccagaactccgacccccctatgggggctgtcggcctcatgctcaacagcccctccatggctctcgatgcac

ccaccaagccaatctttgcaaccactagcgagaagccaaaagtggacaccgcagagccgtcaatcaacaaagcttccatctctgacttggagccacgt
taa
>g16986.t1
atgaccaacctcacggcgacggcggcgtacgtgtcccggctcaagttcgcggccgccgcggtggaagtcgcgcccttggtgaccatctacgggatggc
ccagtgcacgcgggacctcgacgacgacggctgcgaccgctgcctccgcaccgccgtcggggccatcccgaaatgctgcgaggggaagcaaggcgg
gcagatcaactatcggacctgctccgtccggttcgacgtggagccgttctacaacgccaaggctgccgaggcggcaatgttaccggcaccgtccccgg
gaagcacaggaagcgacaggcgcacagtgaggacggctcttctcgtgtcaatcgctgtagccgtcacgctgttggtggtggtgctggttgctctgtatat
ccgcaagaggaacaggaagctacacaggcacgcgcagattgcaagcgccaggcatggagatgatgattacgaggaggaagaaatgaggagttcag
agtctctcctgtacgatctaagcactctgagagctgccacagacaacttctcagaagaaaataagcttggggaaggtgtatttgggccagtatacaaag
aacatgagaggctcctcgtctatgagttccttagcaacaacagcctcgacaagatcatatttgatcctgcgcggcaacatgagctgcgatggggacaaa
ggcacaagatcatcgagggaatcggtcgggggctcctctacctacaccaggactcgaggttgacaatcatccaccgtgacctcaaggcaagcaacatc
ttgctggacaaggacatgaaccccaagatctccgacttcggactggcgaagctattcgacgtggacttgagcgtggggaacacgagccacattgccgg
gacatatggctacatgtcaccagagtacgccctacatggcatcttctcggccaaatccgacgttttcagctatggcgttcttgtcctcgagatcatcatag
gacgacgtaacacacatgtgcaacaatctgaggatctgcttagctttgtctggaggtgctggagacgtgggagcatcagcgagctgcttgatggctgcc
cgacggacgggaggcagccgcaggaggttctgaggtgcgtccacgtggggctgctctgcgtccaggaggacccgcagctccggccagggatggcatc
cgtcgtcatcatgctcaacagccgctccatcacgctgccagcacccactgcgccggccttcgttgttccccgccatgttgtggcagccgtcgacgacg
tgcccgagaggagcattgatagcgatgcgccaagggaggaagctcgttggccatcgatcaactatgcttcttctgtctctgacttggagccacggtga
>g16987.t1
atgtatcttcacgaggattcaagattgacgatcatccactgtgatcttaagccgggcaacatcttgctagatgtggacatgaaccccaagatctcagattt
tggcttgaccaagctttttcgcattgacttgaacgttgcaaacacaagctgtattgctggaactcgctatggtatccttgtcctcgaagttgtcaccggtcg
gcgaccttctcaagatctcctaaaatttgtctggaggcactggaaccaggggaatgtaccaccgctactggacagctgcccgatggatgagcctgaaca
gcagcagcagatgttgaggtgcatccacatcgggctcctctgtgtccaggatgacccggaactccgacctcgcatggcggatgttgttcttatgcttaac
agccactccatgacgttggttgtgcccaccgagcctgtcttcacagccactagcgagagacccagagtgctcgctccagagtcgtccatcaatgaagca
tctgtctccaacatggagccacgttag
>g17182.t1
atgacccagataccttctgcatgcaaagacatcaaggaagtaaggacggtggaccagttttcagccaatgattttattgtacatgatgggcttctactcg
ccattcagaatgagcctgagcctgtggagtcagttgatagagaagcaattgagtcagctcaggaggacaagttaaggaaaaaagacgagaataatctt
tctggctcatttcgtgttacggatgacacagatggaattcaaactatttccctcagtgaacagtctcctgctgacactgcaccattagcaggcttacctgag
ttttcttatcttggttggggccattggttcactctcagagatctggagcttgctaccaaccgtttttcaaaggacagtgtaattggcgagggtggatatggg
gttgtgtatcgaggcagattatcaaatggcaccccagttgctgtcaagaaaatccttaataatttaggacaggctgaaagagaattcagagtggaagtt
gaggcaattggccatgtgcgtcacaagaatctggtccgccttttgggttattgtgttgagggtactcaaaggatgcttgtgtatgagtatgttaataatgg
gaatcttgaaagttggctccatggagaactgtcccaccttgcttacttacatgaggcaatcgaaccaaaagtggtgcaccgagacatcaagtccagtaa
tatcttgattgatgatgagtttaatgccaaaatatcagactttggtttggccaagatgcttggtgctggtaaaagtcatattgctactcgagttatgggtacc
tttggatatgttgcacctgagtatgctaacagtggacttttgaatgaaaagagtgacgtgtatagctttggagttgtcttattagaagctattacaggtcga
gatccaattgattatgagcggcctccgaatgaggtaatgtttgtctgccttgcttacttacatgaggcaatcgaaccaaaagtggtgcaccgagacatca
agtccagtaatatcttgattgatgatgagtttaatgccaaaatatcagactttggtttggccaagatgcttggtgctggtaaaagtcatattgctactcgag
ttatgggtacctttggatatgttgcacctgagtatgctaacagtggacttttgaatgaaaagagtgacgtgtatagctttggagttgtcttattagaagcta
ttacaggtcgagatccaattgattatgagcggcctccgaatgagatatctaatgctgataattctgtggagaatctgatatag
>g17596.t1
atgtcgcaggtgtcgtcgctgtcgccgtcgctgtcgggcgacgaggaggtcgaagaagaggaggaggacgagggcgtcgacggataccgcaagggc
gggtaccatgccgtccgccccggagacctcttcgccgccggccggtacgtcgcccagcgcaagctcggctggggcaacttctccaccgtctggctcgcc
ttcgacgtccagttacagaaatatgtggcccttaagatccagaagagtgctccagagtttgcccaggctgctctccatgagattgagttcttgacggagat
cacaaagagggacccgtcaaattgcaagtgcatcattcagctgatcgatcacttcaaacatacaggaccgaatgggcagcatatctgcctcgtctttga
gttcctcggtgacagcttgcttaagctggtacagtacaaccgctataaaggcattggaatggatcgagtgaagcgaatatgcaagtcgattttggtagga
cttgattatttgcacagcgagcttggtatcatccattcagacttgaagcttgagaatgttctccttgtttcaacaatcgatccatcgaaggaccccatacgc
tctgggcttaaacctaaccttgagaggcctgaagggaatcctaatggagaagctggtcttaacccaattgagaagaaactcaagatgagagcaagacg
ggtacttgcaaagcttgcggagagaagaaaatcggttgcagagtcttcacgtgcagaaaggagcttagatgggattgatctgacttgcaagattgttga
cttcgggaatgcttgttgggctgacaagcaatacacagattttattcagacaagacagtacagggcgccagagattattttaggtgcaggagatgctaa
gaactctgatattaagccccacactggaagcatcaccaacagagatgccaaaagctctaatataaagccagaaagtggaagcatcactaaccaaaatt
ccaaaacaagtgatattaagcccaacactggaagcatcaccagcagagatgaaaagaacagtgatgtgaacaactctgaaagtgttgtcaacagaga
tgtgaagcgcagcatccgaagtgttgtcaatagttatatcaaaaactttgatatgaaaagcaacactggaagcataggcaatgatacaaagaactcatt
agataataaacccagtgctagaagtgttacaaatgttgatgatgccaagggtacgagtgtaaagccaaacagtagaagtgttgaggacaatgatgcca
tcagtagtaaagtgaagccccacactggaattgttgccaacagcgattccaaggatgttgatatacaaagtaatattgaaaatgttgccagcagtgagg
acaacaatatcgatacgaaacctaacattggtcgtgttgctgcaagtatacagaggttggagagcagcttgagtaaagtgcaaaccgggagatatcgtt
aa
>g17952.t1
atgagtgcagggaaagcgaagccgcagctgccgccacactccaagaacagctcctctacagaggacgccctgaagctgtggcgggtcgaggagagc
ggcaccgagttcactctctacgacttctccgacctggccgccgccacgggggacttctccgacgagaaccggctcggcagaggcggcttcggtcccgtg
tacaagggcaggctggctgatggcgcagagatcgcggtgaagcggctggcggcgcattccgggcagggtctagaggagttcaagaacgagatccag

ctgatcgcgaagctgcagcacaccaacttagttcgcctcgngctgcagcacaccaacttagttcgcctcgtaggctgctgcgtccaggaggaggagaa
gatgctggtatacgagtacatgcccaaccgcagcctcgacggcttcatcttcgaccagcagcgcgggacgctgctggactgggagaagcggctgcata
tcatcgagggcatcgcgcaggggctgctgtacctgcacaagcactcccgggtgcgcatcatccaccgggatttgaaggcaagcaacattttactcgata
aagacctcaaccccaagatatccgacttcggtatggccaggatcttcggcattaacatgaccgaggctaacaccaacagggtcgtcggcacatag
>g18065.t1
atggctctcgtccgccagcgccgccagctcccgcacctcaccctcccgctcgaccacttccccctgcgcctgccaccacagccgcagccacagcccgcc
gccgccgcgccgtccacctcgatctccgactacgagcggctctccgtcctcggccacggcaacggcggcaccgtctacacggcgcgccaccgccggtc
ctccgcgaacccgccggtcgtcgcgctcaagatcttcgccgcggcgggggacccttccgcggcgcgggaggccgagatcctcgcgctcgcctccgacg
cgccccacgtcgtgcgcctgcacgccgtggtgccctccgccggtgaggcgcccgcggcgctggcgctggagctgatgccgggcggctccctcgcgggc
ctgctccgccgactgggccggcccatgggcgagcggcccatcgccgccgtggcccggcaggcgctcctgggcctggacgccctccacgcgctccgcgt
cgtccaccgcgacctcaagccgtccaacctgctcctcggcgccgcgggggaggtcaagatcgccgacttcggcgccgggaaggtgctgcggcgccgg
ctcgacccgtgcgcgtcctacgtcggcacggcggcgtacatgtcccccgagcggttcgacccggaggcctacgccggcgactacgacccctacgccgc
cgacgtgtggagcctcggcgtggccatcctcgagctgtacctgggccacttcccgctgctgcccgagggccagagacccgactgggccgcgctcatgtg
cgccatatgcttcggcgaggcgccggagccgcccgccgccgcgtcggaggagttcagggacttcgtcgcgcggtgccttgagaagaaggccggacga
agggcgtcggtggccgagctgctcgaccaccccttcgtcgcagagagggacgcggccgacgcgcggcaggcgctcgccgcgcttgttgcccaggcgg
acgacctgtag
>g18088.t1
atgagcggcggcaggtgcaagagcaaagacttcggcgcggccgcttactgggacgcccgctactcctccgtctcccccgctggcggtggtggcgggtt
cttcgactggtaccagacgtaccccgctctgcggccgctcctccgcgcccgcgtcccggcctcctcccgcgtcctcatgctcggctgcggcaactccctgt
tgtcggaggacatggcaaacgatggttatgaggacattgtaaatattgacatttcatctgtggtcattgagcagatgaaagagaaacatacagatattcc
acaactaaaatatatgcagatggatgttagggacatgagtgtctttgataatgaatcctttgattgtgtcctcgataaaggaaccttggatgctatgatgt
gtgctgatgatgcccccgatggtgcttccaaaatgctagaagaagtggcaaggattcttagaccatgtgggatctacctgttgccgagccagctcgcgcc
gcaccgccctcgtcgcctccccgcgaaggcggggctccccttctcccgcgccaccgtcacccccgcgtggaccgacctactcacccacccggcccggc
gcagctcaccctcccgcttcccccgccggacgcgccgccgcccatcgcaaggacgatggcgagcggcagcggggacgacgactcgggcgggaagct
ggtggtggaccgctaccgaaagcgcgaggttctcggagagggcacatacggagtcgtcttcaaggccacggacaccaagactgggaacacagttgcg
atcaagagagaattcgattagggaagtacaaagaaggcgtaaacttcaccgcattgagggaaattaaactgctcaaggagctgaaagatcctaacattat
agagctgattgattgtttcccatacaagggaaatttgcaccttgtatttgagttcatggagacggacttagaagctgtcatacgagacaggaacattgtgt
tatctccagctgacacgaaatcatatgttcacatgatgctgaaaggtctagctatctgccataagaaatgggtgctacacagggatatgaaaccaaata
atttactcatcggtgctgatgggcagctcaagcttgctgactttggtctggcacgcattttcgggagcccaggaaggaacttcactcaccagggttctagt
gacattgatcaactagggaagatcttcgcagcttttggaactccaaagtcttcccaatggccagagatggtataccttccagattatgttgagtatcaattt
gttgctgcacctccgcttcgttcactgtttcctatggccagtgatgatgctctggatcttctgtcaaaaatgtttacatatgacccaaaagcaagaatatca
gctcagcaggctttagatcacaggtactttacatcaacaccacctccaacaaaaccatcactgcttccaaggccaccgccgaagggagattctgggaat
aacaagataccagatcttaatctccaggacggtcctgtggtactgtccccgccaagaaagttacgcagggtgactgctcatgaaggcatggagggcaa
tatgcagcggccagataaagtggaagagcatccttctgggccgaggcacattggtgacatgtctagtcaaagttctagaataccaatgtcaatagatatt
ggagctgtgtttggcacacgaccagctcctagaccaaccttgaacagtgctgacaagtctcatttaaaacggaaacttgagatggatccggaatttggat
atgcagagtaa
>g18272.t1
atgactggccagtataccctgaaaagtgatgtttacagctttggagtagtcatgctggagctcctgacaggccgtaagccgtttgacagttctaagcctc
ggtcagagcagtcacttgttaggtgggcaactccccagttgcatgacattgatgcattggaccggatggttgatccggcactcaagggtctataccctgc
taaatccctatctcgtttcgccgatgttgttgcactgtgtgtccaggtatga
>g18516.t1
atggtcaccatggtggccgtcgtcatcggaggagaccatattgacgtcggcgcaacccgggcaacagcgaggccgcctctccagaacctccagttcag
gatgacggcacctccccggctcagggcccgccatgcgtcgttgactgaagatgaagctacagtttcaaggattgtcaatgacggacctctcttggggtct
ggcacgctgcgtctccatttccgacgttggactaggcaggcctttgcctcaggagcttccctgccagttctcgtcgatgtcgagatgcgcggcatcccggc
gcacgcctgggagatgtctacggccgagaacttgctcaacccttacagctggccgcaactgctccacccggccaccaggaatcgggaggattactcgg
tttttcgggtattagcctggtgcttcaagccggattccttcccaagttctcatgatcttcacgtggtggaaccgtccgtcggcgttatcaaagtcccaccag
gcaagccgactctgtgctacccaatctcgttctcggatatttcgatgaaatttcaccgaaattgttttcctagcccggggtcgacaacgacgaacctttctt
ctcgggtggtgatgacaactccgaccatcacagacgccaatggcagcgcccgtaaggccgcagttgtcgaggcgaacggacctgaaatcgactccgg
gaagacaaggaccctcgagcctattccctcttacgtgtcgcgggaggcgatcgtcatcgagctgaatgggcttagggttcaggaagacctgctcagtgc
ttcaacacagaatgttgtcgttatagagcctatcccctccggcgggctgctggtggtagccaccgtcgaggcgggtggctcggacgccggcgatgtgac
ggaagctatcacagcagacgggcccacaaccggttgcgtccccaaggaggcacccatcaattctgaagacgagacgtgcacacccccacctcggtca
ctacgtatggcacagccagcggaattcgtgcaaattaaccaagcacctctcatattgcatgcctcggttaccgatgcgcgtgatgttgtttcaaatgggcc
aatgggggggagaaagtaccatgttccgggtgtggttcttgccgtggtgccggctgttctagcagcctttaacctgcactacggaaaaaccttggattat
cacctcccgggcggcgaggagatagcggtgaagcggctgtccgacagctcgaagcagggagacgatgagctgaagaacgagctggcgctggtcgcg
aagctgaagcacaagaacctggtccggctcatcggcttctgccttgagcaagaggaaagactgctggtctacgagtacgtccctaaccgaagcctgga
tctcatcctcttcggtgctgctgagaatgaggagaggcgcgagcagctcgattggaagcagaggtacaggatcatcaacggcatcgctcgaggcctgc
aatatctccatgaggactcgcagctcagagtaatccaccgcgatctcaaggccagcaacattttactagacgcaaatatgaatcccaagatcacagact
ttggccttgcaaggattttcagcagggaccagacgcaggccgtcacaagccgcgtcgttggaacgtatgggtacatggcgccggagtacgcgatgcgc
gggaactactcggtgaagtcggatgtcttcagcttcggcgtcatcgtgctgaagatcatcacgggcaggaagaacaacgatagctgcaactccaaaac

gtcgggtgacctcttgagcactgtatgggagcactgggaggccaggacgataatggagctggtggacccgtctatgaagggcagcttcccagagggcg
acatactgagaggctttcacattggactgctctgcgtccaagaagatccggcggcccggccgatgatgtcgtcgatggtgatgatgcttagcaatgaca
cggtcacccgccatgccccgtccaagccgaggttcttcgccagaaacaacggcgctaacacgaccgggtcaacggcagcgtcggtgcagggttag
>g18665.t1
atgacatcacagcagaagctgaaaaggcaagctggacatgtgtgtgcgcagcacatccacgaggggaatggaactctagcagcagtttgttgtctaac
tgctcttcgtcctgccattgccaacaagatgaaaatagcagtacagggtcatggaactgcacatgcgcctccgacaggagccttcggaaggaaggaca
tgctgtgttgcacgacaggagctgcttcacttcatgtaactgcacatcaggttggtgagagtggcacgttccccggtgtaatcatccggttttccttttcgg
agttggaacaagcaactgggaaattctcagacgagcaactgattggcgtgggtggatccagcaaggtgtaccgaggccagcttgctgatggcaaagtt
gtggccgtgaagaagcttaggcctctaggaggtgccgacgaagactatgagttcctgtccgagatcgagctgctgtcgaggctgaaccactgccatgtg
gtgccactgctggggtactgctcggagagtcaagggcggcagctggagcggctgctggtgtttgagtgcatgtccaatggcaacctgagggactgcctt
gacctgaagcaagggaagaaggcgatggagtggcagactcgtgtgagcgtggcgctgggcgccgcgagggggctggagtacctgcacgaggcggc
cgcgccgcgcatcctccaccgtgacatcaagtccaccaacatcctcctggacgacaagttcagagccaagatcactgatctcggcatggccaagtgcct
gatgaacgacggcgtgacaagctgctcgagctccccggcgcggatgctgggcacgttcgggtacttcgcgccggagtacgccatcgtggggaaggcg
tcgctcaagtcggacgtgttcagcttcggcgtggtcatcctggagctcatcaccggccggcagcccatccacaagtcgccgtccaccaggaccgacgag
agcctcgtcatctgggctacgtcgcggctgcaggacagcaggctggtggtgtcagagctgccggacccgacgctgcagggcaagttcccggcggagg
agatgcagatcatggcgcacctggcgagggagtgcctgcagtgggacccagagtccaggcccaccatgacagaggtcgttcagatcctctccaccatc
gccccactccaccacggcacaaagcgacggaacctacccatgacattcaacctcaccaccacgccgcctcacgatcacgaggacatggagaggcagc
aggaatgctcgtcgtccgtccaatggaaagggcgcgcgtcctggcctgctgacagagggcacacttctaacaagggcgccggagccgtgatgtccggc
gagctgctcaacgggatgctgctcatgagcccgcacgacgacgaccatatggtggacctcacggagccccggctggagacgttcacgcagccagcaa
tcttcagatga
>g19074.t1
atggcagaatgtgaagctctgagaagtttaaggcaccgcaatctcgtccctatcctaactgcctgctcgagcattgattctggaggaaatgatttcaaag
ccttggtttatgaattcatgcctaacggtagccttgactctttgttatacccaaaggaagatggcacacatacttcatgttatctgaccctagctcaaaggtt
ggccattgcttgtgacattgcaaatgctttggaatatctgcaccatggtagccagaggtccattgttcactctgatctgaagcctagcaacatcctactcg
gcattgatatgactgcccacatcagcgattttggcctggcaagattcttggatattgcaagcacaactagtacagttgcagtgaagggaaccatcggtta
cattgctccagagtatgcaactggtgggccgctgatgactcctggtgatgtgtactccttcggaatagtcctacttgagatgtttattgggaggcgaccta
ctgatgacatgttcaaagatgggttaagcatcgtgagctttgtagaggcatggtcgaccgaccatatactggagatagtcgatgccgggctgaaggaag
aaatcaatgattttggagatccaaatgaaagcatggtaacggttgtggactgcgtgcaatctgtggtgaggatcggactctcctgcactcgccgactccc
taacgagaggatgaacatgattgaagtagcccagaagctgcaggccctcgggatgaatatgggagatgaagctctgcaagtaatgccttaa
>g19110.t1
atgaagcgatcagccgccgccgccatcaagcacgtcatcgcggcggacggcgacccggagaaggagctgatgcaggaggcgcgcttcctcgactcct
gcgccggcatccctttcgtcgtcggctaccacagcgtcatccgcgacccggccaccaagaacctctccctcgtgatggagtacgtgggccccggcatcg
cccacttcctccgctcgcacgattctgcgccaccgcatctcgacgagtccacgctacgtgccgtgatgtggcagctgctgactgccacggcggggatgc
acgcgcgcggcgtcattcaccgcgacatcaagccggagaacatcctcgtcacggaggaccatggctccgtcaagatctgcgatttcgggctcgccatgt
ccgcctccgacccgccaccgcatgagcccgccgggaccctctggtacaaggcccccgagatgctccttgaccttccagtctacgacgcgcgcgtcgatg
cctggtccctcggctgcgtcatggccgccatgatcaaaggcagcgctctcttccagggatgctacgaggacgggcagctctgcgccatcttcgacgtgct
cggcgtgcccgacgacgacgcatggcccgggttctcctccacggcgttcgccactgtcgtgcttccggaactcgacgtgcagaagcagagctggctccg
tgagctgtttcccgagaccatgctgtccgaccaaggattcgagctcctcaacggcctcctcacctgcaatccggagaaccgcctcacgtccgccgccgc
gctcaagctcccttggttcgccggcgtcgacgccacggaactgccaatcaagaagaagcaggtgccgtcgtcgatgttgcccaagaacacgaagaag
ccgccactgtcgaagcagaggacgaggaagcccccatcctgcagtgcgcctga
>g19310.t1
atggcgggctgcagccggaaacgcttcgccgtcggcagcacagaggagtacgaggagacgtgttgcctaggcgagggcggctacggcaccgtcatc
aaggcgcgccaccgggtcacgggccagttcgtggccatcaagcgcctctgccatcccggccaaggccaaccggcggtgatgcgggagaagagctttc
tggaggcgtgcagcggcaacccattcatcgtcgggttccacggcctcgtccgcgcgcccgattcaatggagctccgcctcgtgatggagcacatcggac
ccagcctccgcgacgtcctcctccaaaccgccgacgacgagccgccgttcccggaggccacggtgcgcggcatcatgtggcagctcttgacgggcgcc
aagaagatgcacgggctccacatcgtgcaccgcgacatcaagcccgagaacatcctcctcagcgccgacgatcagagcgtcgtcaagatctgcgactt
cgggctcgcgctgcgcatgtcaaacgacgcgccgccatattatcaggtgggcacgggctggtacatggcgccggagatgctgctgggaaagaccgact
acgacgccctagtcgacacgtggtcactgggatgcgtcatggcggagctcatcgatgggtgcgtcctcttccatgccttgaatgatgaagggcagttgtg
tgccatcatcgacgtgctcggcgtgccggacgccagagcgtggccgtggttgtcttccacgccgttcgccaaggaactgctgcctcaactacagtacgc
gcggagacgaaacggactgcgcaagcgcttccctgagacgttgctgtccgagcaagggttcgaggttctcagcggcctgctcacgtggaaccccgaca
agcggctcacggccagcgccgcgctcaagcaccccctggttcgccgccgatctcaacgcccctaggccggcaaagaacaaggaagatttgacatccac
gttgcccaagatgatggatccgctaattgttcgtccgaatctgcacaagaggcagaagctgcaatgtgtctga
>g19501.t1
atgatccattcgcagctgtggtaccgctcacctgccagtatcgccaagcccgccgccgccgccgccgccgccgtcgtcgtagcggcactcgcgtcc
tccttccttgccctccctcctccgcgcgccgccccggtcgccgcgggagccaggtccatcatgtcgaaggctagggtatactccgacgtcaacgtcctgc
gccccaaggagtactgggactacgaggcgctcaccgtccaatggggtgagcaggatgactatgaagttgtcaggaaagttggaagaggtaaatatag
tgaggtctttgaaggcatcaatgttaacaataatgagaaatgcatcattaagatccttaagcctgtcaagaaaaagaaggctcttgattactgccattctc
aaggcattatgcaccgagatgtcaagccccataatgtaatgatagaccatgagctgcgaaaacttcgcctgatagactggggtttggctgagttctacca
tccaggcaaggagtataatgtccgtgttgcatcaaggtatttcaagggacctgagcttcttgttgatttacaagactatgattattctttagacatgtggag

tcttggttgcatgtttgctggaatgatatttcgcaaggaaccattcttctatggccatgacaatcatgaccagcttgtgaagattgcaaaagtgcttggaac
agatgcactgaatgcttatctgaacaagtatcacattgagcttgacccccaacttgaagctcttgttggaaggcatagtagaaaaccttggtcaaagttc
attaatgctgataaccaacatctagtatcacctgaggccatagattttctggataagcttctacgctacgatcaccaagacaggcttactgcacgtgagg
ctatggcacatccctacttccttcaagtgagagctgcggagaacagcaggcctcgagcacaatag
>g19529.t1
atgggccaatgctgcgccaagggcggcgacccggccgccgccgccggcgacgacaaggaccgccccgcgtcgtcgtcgtcggagcccaagccggag
gcctccagccgcggcggcgcgaacgccgccccgccgcgctcatcgtcgtcttcctccaaccacccggcggtgggcccggtgctgggccggcccatgga
ggacgtgcgcgcgacgcactcggtggggaaggagctgggccggggccagttcggcgtgacccacctgtgcacacagaagcccacgggggagaagct
ggcctgcaagaccatcgccaagcggaagctctccacccgcgaggacgtggacgacgtgcgccgcgaggtccagatcatgcaccacctctccggccag
cccaacgtcgtcgtcctcaagggcgcctacgaggacaagcacaacgtacacctcgtcatggagctctgcgccggcggcgagctcttcgaccgggtcat
cgccaagggacagtacacggagcgcgccgcggcggcgctgctccgcaccatcgtgcagatcgtgcatacctgccacgccatgggagtcatgcacagg
gatatcaagccggagaatttcttgctgcttagcaaggacgaggacgcgccgctcaaggccacggatttcgggctatccgtcttcttcaaggaaggggag
gtgttcagggacatcgtaggaagtgcctactacatcgcgccggaggtgcttaagaggaagtacgggccagaggcggatatttggagtgttggagtcat
gctatacatcttccttgctggagtgccgcccttctgggcggagaatgagaatggcatcttcaccgccatcctgcgtggccagattgatctcgccagcgac
ccgtggccaaagatttcgtcgggggcaaaggatcttgtcaagaagatgctcaccatcaaccccaaggagcggcttacggcgttccaggtcctcaatcac
ccgtggatcaaagaagatggagatgcacctgatacgccacttgacaacgttgttctcaacagactcaaacagttcagggccatgaaccagttcaagaa
agcggcactaaggatcatagctgggtgcctatcggaagaggagatcaccgggctgaaggagatgtttaagaacatcgacaaggataacagtgggacc
attacgcttgaagagctcaagaacgggttggcaaagcacggaacaaagctgtcggacggtgaaattcagcaactcatggatgcagctgatgctgatgg
caacggattgatcgactacgatgaatttgtcaccgcgacggtgcacatgaacaaactggacagagacgaccacctctacactgcattccagtatttcga
caaggataacagcgggtacattacaaaagaagagcttgagcaagctttgaaggagcaaggattatatgacgccgaggagattaaggacgttatctca
gatgctgatgctaacaatgacgggaggatagattattcagagtttgtggcgatgatgaggaaaggtactgctggcgctgagacggcgaaccccaagaa
aaggcgcgacctagtactgtag
>g19699.t1
atggatcatgtcgtcgggggcaagttcaagctcggaaagaagatcgggagcggatcgtttggggagctcttccttgctgtgaatgtgcagactggagag
gaggtcgccgtcaaactggaaaatgtgaagacaaagcacccgcagcttcattatgagtcgaagctatacatgcttctccaaggaggaactggcatccc
gcacttgaagtggttcagtgtcgagggggagtataatgtgatggttattgatcttctggggccaagtcttgaggatttgttcaactactgcagtagaaagt
tctcgctgaagactgtgcttatgctcgctgatcagatgattaacagagttgagtacatgcatcaaaagggttttcttcaccgtgatataaagcctgataatt
tccttatgggtcttggtaggaaagccaatcaggtatacataatagactatggtcttgcaaagaagtatagagaccttcaaacccacaagcacatcccta
cagagagaacaagaacctcactggaactgcacgatatgcaagtgtcaatactcaccttggtgttgagcaaagcaggagagatgatttagaatctctgg
gatatgtccttatgtactttcttaggggcagcctaccatggcaggggctgaaagcaggtaccaagaagcagaaatatgacaaaatcagtgagaaaaag
atgcttactcctgtggaggtactctgcaaatcttacccatcagagtttgtctcctactttcactactgtcgttcattacggtttgaagacaagccagattattc
ttacctgaagagactattccgtgatctctttatccgagaaggataccagtttgattatgtttttgactggaccatcttgaaatatccccaaatcggctccaat
ccacggatgaggagtgaaagaactagtggtgctgctggaccttctatggacaaaatagaaaaaaacccaggtgaagcatcgggtagaaggaatccta
atgctactcttaatcagagtgacaattatgcacagcgaccacgagaaggcgtatccatgtcactgaaggaaattcacagaccgggctggggaacggac
tgtggagaggcctcgtacatcctctcgcaccggcagcgcttcacggagagctattgcgtcaagcagcaggccggcctcatcggcagaaccaaggccaa
gtcctag
>g19732.t1
atgggcaacgtctgcgccgggtcgcggttccccaagctcggctccatctccggctccatctggccgtcgcgcgcctccacgccgtcccagcccaccacc
acctcccgctccgtccccgtcgtccaggtccaggccagcgagtccacctccaccgatttgaagccacctccggtcccggcacccgccgcacaaaccaca
cctcctgcccccatcgtcatctccgaaccaccacgacactcatcatcacaatcagagccctcccggccacaaccaaaaagagacgacgcgtcccaaca
accggcaccttcctcctcgtcatcgtcgtcgtcgcagccgccgccgcagccacaggggaaaaagaagcccgcgcacatcaagcgcatctccagcgcgg
gtctgcaggtggaatccgtgctacgtcgcaagaccgagaacctcaaagacaagtacagtttgggacgaaaactcgggcagggccagttcggcacgac
gtacctgtgcgtggacaaggccacgggccgggagcacgcgtgcaagtccatcgccaagcggaagctggtcaccgacgaggacgtggngggacgaa
aactcgggcagggccagttcggcacgacgtacctgtgcgtggacaaggccacgggccgggagcacgcgtgcaagtccatcgccaagcggaagctgg
tcaccgacgaggacgtggaggacgtgcggcgcgagatccagatcatgcaccaccttgccggccaccccaacatcatctccatcgtcggggcctacgag
gacgccgtcgccgtgcacgtggtgatggagctgtgtgcgggcggggagctgtttgatcggattgtgaggaggggacattactcggagcggcaggcggc
ggcactggcgcgggtcatcgttgcggtcgtcgagtcgtgccactcattgggggtcatgcaccgggacctcaagccggagaatttcctgtttgttgggaat
gaggaggacgcgccgctcaagaccatcgatttcggactctccatgttcttccggccaggtccgtgtgcttatctccgtgttgagttgttgtaa
>g20055.t1
atgggtctctgccatggcaagccaacgcaaatcccggagcctgaggcggaagaagatccccatgtagcctccggcgccggcgacgccgccggtgccc
ggaacggcgcctccgcgccgtcgccagcgcctgcgacgaagccgggcacgccgaagcagcccaagttcccgttctacctaccgagcccgctcccgcc
gtcgagctacaagggctcgccggcgaactcgagtgtggcatcgacgccggcgcgcggagggttcaagcggccgttcccaccgccatcgccggcgaag
cacatccgcgcgctcctggcgcggcggcacggctcggtgaagcctaatgaggcgtccatccctgagggcggcgagccggagctcggcctggacaaga
gcttcggcttctcaaagcacttcttcgccaagtacgagctcggcgaggaggtcggccgcggccacttcggctacacctgctctgccagggccaagaagg
gcgagcacaaagggcaggacgtcgccgtcaaggtcatccccaaggccaagatgacaactgctattgcaattgaagatgtcagaagagaagttagaat
attgagttctctgtcaggccacaacaacctagtgcagttctatgatgcttttgaggatgaagataacgtgtatatagtcatggagttatgtaaaggaggcg
aactccttgatagaatattggcgagaggtggaaagtattctgaagaggatgcaaaggttgttatggtacaaattttgagtgttgtatcattttgccatcttc
aaggtgttgtccatcgtgatctgaaaccagagaatttccttttctcttcaaaggatgaaacttctgccttaaaggtcatagattttggcttgtctgacttcgt
gaagccagacgaaagactcaatgacattgttggcagtgcttactacgttgctccagaggtgctccaccgatcttatggaatctttcgagctgtcctgaag

gcagagccaagttttgatgaggctccatggcccactctcactgcagaagctaaagactttgtaaaaaggcttcttaataaggattaccgcaagagcatg
actgccgcacaagctctcacaagtgaaagttccgttagatatgataatttacaagcttatgagagcttacatcagttcgtcctcactgcgaaaatctgcttt
gaggacgaaagactcaatgacattgttggcagtgcttactacgttgctccagaggtgctccaccgatcttatggaatctttcgagctgtcctgaaggcag
agccaagttttgatgaggctccatggcccactctcactgcagaagctaaagactttgtaaaaaggcttcttaataaggattaccgcaagagaatgactgc
cgcacaagctctcagccatccatggatccggaatgctcagcaagtgaaagttccgttagatatgataatttacaagcttatgagagcttacatcagttcgt
cctcactgcgaaaatctgctttgagggccctggccaagacattgacaacaaatcaactcttctatgtaagggagcaatttgaattgttgaatccaaacaa
gaatggttatatctcgttgcaaaatttgaaatcggcattggtgaaaaattccacagatgcaatgaaggactctagggttgttgactttgttaacacgatgg
aagctttggatacgtgggaacagcacgctaggcgtgcatatgagctgtttgataaggaaggcaaccggcctattgtgatagaagaacttgcatcgaag
cgagcagaactactgaaggccgaacacagctcgtcgtcctctggcctgccgccggcagctgaaggtgacgatgacgacaataattcctccgcgccgcc
gctcgtcgtgaccgatccggcatcggcggcatcggccgaagatgacgtcgacggttctcccacgatctccatcgcggtgacctccttcttcttccgctcgc
cgtccttgtcgtcgtcggtctcccgcacgagcctaccccctttgaggcggaacgtgatgcttggccctgatgccgctttccatctgttggttccgcaggctc
ggtcgaggcccttgatggttccgtatggccagatatcgtggtcatgtgacaggactcatcaggcggcgccgacctga
>g20106.t1
atgtacagtctcacatttgtcaacttctctggtaatgacattggcatggagagcttagcaaattgtgtggcagcaggaatttgccctggaaatggtattga
tcatatggtggttcattcatctcgtcgagtactaagagcagcgatcatctgtgtgatacttgctgtcatcattgcagtaattgttttggtagtttatctgagat
ggaagctattgagaagcaagccagaggctattatcccgatcagcaagtccaaggctagtgttgagccaacatcaagtgatgaactcctagggaggaa
gtcacgggaacctttgagcatcaacgtcgctacatttgagcatgcattgttgagggtaaccgcagatgatattctgaaagccacagaaaatttcagtaag
gtgcacatcataggggatggtggcttcggcactgtctacagggcagcgctccctgaaggtcggagagtcgcaatcaagaggcttcatggtggacatca
gttccaaggtgatcgtgaattcttggctgaaatggagacaattggaaaggtgaaacatccaaaccttgttcccctacttggctattgtgtttgtggtgatg
aacggttcttgatttatgagtacatggagaatggaagtcttgagatgtggctgaataaccgggctgatgcagttgaagcccttggatggccagatcgtct
gaagatttgtcttggttctgctcatgggcttgcatttctacatcatggcttcgtgccccatatcatccaccgtgacatgaaatcaagcaacattctactggat
gagaacttcgagcctagggtctctgactttggtcttgccaggataatcagtgcatgtgaaactcatgtgagtacggacattgctggaacttttggctacat
accaccagaatatggccaggccatgaaatcctctacgaaaggtgatgtctacagcttcggtgtcgtcatgctagagctgctcactggacgtccacctaca
gggcaagaggaggtggaagggggaggaaaccttgttggctgggtgcgttggatgattgcacatggcaagaaaaatgaactgtttgatccatgtttgcc
agtttcaagtccgtggcgggagcagatgttgtgtgttctgtccattgctcagaactgcacggttgacgagccatggaagaggccgaccatgatggaggt
ggtgaagggcctcaagatggcccaggctatggaacgtggacctctggtggtgaaagtctctaccctctag
>g20409.t1
atggaaaaggcagttcccagtatctccgccgcctcagtgctctcctttgtttcaggccgggtgcctcgcggcttgccgcggccacccgaacgtggtcgag
accgcgacgtggccgtggacgctgccacgggcgctgccttcctggtcatggagttcgtgggccccagcctgcagcaggcccggggagggtggttattct
cggagcgcgaggcccgcgcgtgcatgcggcagctcctcggcggcgcagtgcggatccacgccgccggcctggtccaccgcgacgtgaagcccgaca
acgtgctcgtgggccccagcggcgagctcaagatctgcgacttcgggatggccacgccggccaagggaggcagggcgaagccgtacgccgactga
>g20410.t1
atggccgctgttgctgtgatgggcaccaccacccgcaagcgctcatcccccgacgccgccagctcatccaccgccggcgatcagaagaagaagcgtgc
gcgcgtcacgttcgggaacatctacgactacgagaagcttgaggtcctcggcaagggcacctacggcgtggtcctcaaggtgcgcgactggagcacgg
gcgagacagtggccgtcaagtgcttccgcggcgacaaggacaaggacaacaaccccgacctccgcgccgtgttccgcgaggccgggtgcctcgcggc
ttgccgcggccacccgaacgtggtcgagatccgcgacgtggccgtggacgctgccacgggcgctgccttcctgatcatggagttcgtgggccccagcct
gcagcaggcccggggagggcggttattctcggagcgcgaggcccgcgcgtgcatgcggcagctcctcggcggcgcggtgcggatccacgccgccgg
cctggtccaccgcgacgtgaagcccgacaacgtgctcgtgggccccagcggcgagctcaagatctgcgacttcgagatggccacgccggccaaggga
ggcagggcgaagccgtacgccgactggtgggccagcacgccgggtaccggcaaggaaaaaaatttcggtataaaaaaaactattggtgatcaccgat
aa
>g20666.t1
atggacggcggggcgcagccgccggacacggagatggcggacgccggcggcggtgcgggaggagggcagcagccaccgccacagcagccggcgg
ggggcggaggcatgatggacaacatccaggcgacgctgagccacggcggccggttcatccagtacaacatcttcggcaacgtgttcgaggtcaccgc
caagtacaagcccccatcctccccatcggcaagggcgcctacggcatcgtctgctcggcgcttaactcggagaccggggagcaggtggcgatcaaga
agatcgccaacgccttcgacaacaagatcgacgccaagcgcacgctccgcgagatcaagctgctgcgccacatggaccacgagaatattgtggcgat
aagggatatcataccgcctccccagagggaggcattcaatgatgtgtatattgcgtatgaattgatggatactgatctacaccaaattattcgctcgaatc
aagctttgtctgaggaacactgccagctcatcgggacaccaaatgaggctgatctggattttgtaaatgaaaacgcgagaagatatatccgccagcttc
cccgtcatccaagacaatctttccctgaaaagtttccacatgttcaacctttagcaattgacctggtggaaaaaatgcttacatttgatcctaggcaaaga
ataactgaagaacaaatgaaggatctaatctaccaagaggctcttgcattcaacccggagtaccagtag
>g20789.t1
atgaagatagcccatggaactgctaaggggctcgaatacctccacgagaaggcaaatccacctgtcatttaccgagatctcaagtcccccaacatactc
atcgacgaagactacaaccctaagctctcagacttcgggctagcaaagcttgggcctgttgagggaaagacgcacatttcaactcgagtgatgggaac
atatgggtattgcgctccagaatacataagaacaggccagctaactgtcaagactgatgtgtacagttttggggtcttcctacttgagctgatcacggga
agaagagctgtggatagctccagaccaccaaatgaacagatcctggttaactgggccaagcccatgctcagagacaggaagaggtgccatgagcttg
tagaccccttctcaggggtgactacccagagaaggatctgggtcaagctctgggtgtagcagcaatgtgcctacaagaggaagcctcagtccggcca
tacatgagtgatgctgttgtggcactgggattcctcgcagaagtgcctgctggctatgagaagaaccccagtaccgtacctcaaaagaaacaagttgaa
gatccctctttaagtagtagtgctaagcaagataagagcacatatgatcgtcaaaaggctgttgctgaggccattgaatggggctccttgaggcagaaa
cagaaagctaaaagtcaaggcattgtatcagaaccaaagggcaacagattgtga
>g20971.t1

atgagcggcggcgggtgcaagagcaaagacttcggcgcggccgcttactgggacgcccgctactcctccggttcctccgctggcggtggcggcggcgg
cggcgggttcttcgactggtaccagacgtaccccgctctgcggccactcctccgcgcccgcgtcccggcctcctcccgcgtcctcatgctcggctgcggc
aactccctgttgtcggaggacatggcaaatgatggttatgaggacattgtaaatattgacatttcatctgtggtcattgagcagatgaaagagaaacata
cagaaattccacagctaaaatatatgcagatggatgttagggacatgagtgtctttgatgatgaatcctttgattgtgtcctcgataaagcgcagccgag
ccagctcgcgccgcaccgccctcgtcgcctccccgcgaaggcggggctccccttctcccgcgccgccgtcaccccccctcgcgggccgacctactcaccc
acccggcccggcgcagctcaccctcccgcttcccccgccggacgcgccttcgccgatcgcgaggacgatggcgagcggcagcggggacgacgactcg
ggcgggaagctggtggtggaccgctaccgaaagcgcgaggttctcggagagggcacatacggagttgtcttcaaggccacggacaccaagactggg
aacacagttgcgatcaagagaattcggttagggaagtacaaagaaggcgtaaacttcaccgcattgagggaaattaaactgctcaaggagctgaaag
atcctaacattatagagctgattgattgtttcccgtacaagggaaatttgcaccttgtatttgagttcatggagacggacttagaagctgtcatacgagac
aggaacattgtgttatctccagctgacacaaaatcatatgttcacatgatgctgaaaggtctagctatctgccataagaaatgggtgctacacagggata
tgaaaccaaataatttactcatcggtgctgatgggcagctcaagcttgctgactttggtctggcacgcattttcgggagcccaggaaggaacttcactca
ccagggttctagtgacattgatcaactagggaagatcttcgcagcttttggaactccaaagtcttctcaatggccagagatggtataccttccagattatg
ttgagtatcaattcgttgctgcacctccacttcgttcgctgtttcctatggccagtgatgatgctctggatcttctgtcaaaaatgtttacatatgacccaaaa
gcaagaatatcagctcagcaggctttagatcacaggtactttacatcaacaccagctccaacaaaaccatcactgcttccaaggccaccaccgaaggg
agattcagggaataacaagataccagatcttaatctgcaggacggtcctgtggtactgtccccaccaagaaagttacgcagggtgactgctcatgaagg
catggagggcaatatgcagtggccagataaagtggaagagcatccttctgggccgaggcacattgatggcatgtctagtcagagttctagaataccaat
gtcgatagatgttggagctgtgtttggcacacgaccagctcctagaccaaccttgaacagtgctgacaagtctcatttaaaacgaaaacttgagatggat
ccggaatttggatatgcagagtaa
>g21024.t1
atgggcaactgcgggacgagagaggagaatgccgtcgttcctgcgcacgcgcaagataggaagcacacccgcacctcatcagatacaagtgatcctt
caacacctaggaaaatcgaagatgccaagaacatttccatttacaacaatgtgatttccttcacattgtttgagctcgagacaatcacgaagagcttccg
tgctgattatgttcttggtgaaggaggatttgggactgtttacaagggttacatagacgagaatgtcagggttggtctgaagtcattgcctgttgcagtca
aggtgctcaacaaagacggacatcaagggcacagagaatggcttctcgagacaatcacgaagagcttccgtgctgattatgttcttggtgaaggagga
tttgggactgtttacaagggttacatagacgagaatgtcagggttggtctgaagtcattgcctgttgcagtcaaggtgctcaacaaagacggacatcaag
ggcacagagaatggcttgactatactgctaaactgtctgattttggcttagcaaaggctggccctgaaggtgatgagacccatgtatcgactcgggtgat
gggaacttatggttatgcagcccccgaatatgtgatgacagttgcaggactaatggttttactccagaaactgatgtctttatga
>g21034.t1
atggagatcgtggagaagtacgagaagctggagaaggtcggggagggcacctacggcaaggtgtacaaggcgcaggacaaggcgacggggcagct
ggtggcgctcaagaagacccgcctcgagatggacgaggagggcatcccgcccaccgcgctccgcgagatctccctcctcaacctcctctcccactccct
ctacgtcgtccgcctcctcgccgtcgagcaggccgccaagaacggcaagcccatcctctacctcgtcttcgagttcctcgacaccgacctcaagaagttc
atggacgtctacaaaagggggcccaacgcgaggccgctacccaccgacctcgtcaaggtggtaccactttcgaattactactcgttgttcatccactatt
cctccctaattcagttcaactaccagatgcagaatgtactaccgtgcaatgcttcatannnnnnnnnnnnnnnnnnnnnnnnnnnnnnnnnnn
nnnnnnnnnnnnnnnnnnnnnnnnnnnnnnnnnnnnnnnnnnnnnnnnnnnnnnnnnnnnnnnnnnnnnnnnnnnnncccatcctctacc
tcgtcttcgagttcctcgacaccgacctcaagaagttcatggacgtctacaaaagggggcctaacgcgaggccgctacccaccgacctcgtcaaggtgt
tatgcaaaggtgttgcacactgccacggccatggtgttcttcacagggatttaaagccacaaaacctgttggttgacaaggagaaggggatactgaaaa
ttgcagatctggggcttggtagagcttttaccgtgccaatgaaaagctacacccatgagattgtcacactctggtatagagcacccgaggtgttgcttgg
agcaagacattactcaactggtgttgatatgtggtccgttggatgcatctttgctgaaatggtcagaaggcagcctctattccctggtgactcagaattgc
aacagttgcttcacattttcagattgatgggaactccgaatgaggagcagtggcctggagtgactggattgagggactggcatgagtttccgcagtgga
agatccagagtttggcccgcgttgttccaacgctggaacctgaaggcattgacctgctatcaaaaatgcttcagcttgatccggcaaacaggatatcagc
taaagctgcaatggagcatccttacttcaacagccttgacaagtcgcagttctag
>g21561.t1
atgagatatacattttgcaactatgtaggattaagaggacaaccattggacttttcagagcgtctcaatattgctattgacatagttcataccatcgcctac
ttacatggatacacagaccaccccatcatccaccgcgacatcaagtcgtccaacattctcctaacagatcaccttcgagcaaaggtcgccgattttgggt
tcgcacgcctagccccggagaaccctgaagtgacgcacgtctcgacggtggtgaaggggacagcgggatacgtagaccccgagtacctgcgcacgaa
ccagctcaccgaccgcagcgacgtctactccttcggcgtcctcctcgtcgagctcgtcactggccgacgcccaattgagcgcggccgcggccgccacca
acgcctcaccacccaatgggtaaagcaatcacaccctaccagtgtttttgggacatggaaacgtagtgtgggtacggtgttcgacgaaattaccttgtct
gcgcaggcgctgcggaagtggagggagggcgacacggtggtggcgatggacccccggatgcggcggggcagcgcggtggtggcggcggtggaaa
aggtgatggcgctggcggccgagtgcacggccccggatcgcgcggcgcggccggccatgcggcggtgcgccgaggtactgtgggccgtgaggcgcg
acttccagcaggagcagcagcgcgcggcggcggcggccgctgcgtccgcgagcgcaggagcgaggcggctcgatggctccacgtacgcgctgccat
ccgtgtcgaggttgaggtag
>g21702.t1
atgcggctcaggcagctgcttgctggatggcagatcactctatcagatgtttttggtccatatgagcttctcaatttcacgcttggttcctatgaagacgta
ctctcaaggttttctatcaaggtcgatggtgtgaagtgcttcacatttgaagaaatggctttggcaacaagtgattttactgctcaaattggtcaaggaggt
tatggaaaagtttataaaggaaatctagctgatggaacagcagtggcaatcaagcgtgcacatgaagattctctgcaagctaaaactgaaagacctct
gagctttggtcggagattgcatattgcgcttggtgctgcaaaaggaattctctatctacatactgaggcaaatccacctatatttcaccgagatgtcaagg
ccagcaacattctactggactcaaagtttgtagccaaggtggctgactttggtnacattctactggactcaaagtttgtagcaaaggtggctgactttggt
ctttcgagactcgctccggtgcctgacatcgaagggactttgccggctcatatctccactgttgtcaagggcaccccaggctatcttgacccagaatattt
cctgactcataagctgacagaaaggagcgatgtatatagccttggtgtcgtgtttcttgaactattgactggaatgaaaccgatccagcatggcagaaat
cttgttagagaggtaaacacggcttaccaatcaggtgacgtctccaagatcatcgacagccgggtgtcctcttgccctccagaatgcaccacaaggttcc

tctccctggcaataaaatgctgccaggacgagaccgaggcgaggccttacatggtcgacattgtccgagagcttgagaccatccttggcatgatgcctg
agggagagagcctccaatccgaaacaacagagacgggctcaggagcgctgacaaagtccatgtcctcctcgaccaccaccggagccctcttcgtatca
tcccacacctccggcagcgacaacgcgacaaatagtggcatcccctccgggacggtggcgcctcggtag
>g21762.t1
atggcgtcggcgtcccgcaagcgctcggcgcccgaggaagcccgtcccgcggcgacggcttccaagaagcgatcacgctacaacttcagcaagatcg
acgagtaccagatgctggaggagctcggggagggcacgttcggcgtggtcgtccgggcgcggcaccgccgcacgggcgagacggtggccgtcaagt
gggtccgcggcggcgcctccgagtccgggtccgacctcgacgcggtcgtccgcgaagccggctgcctcgccgcatgccgcggtcacccgtcggtggtc
cagatcagggacatcgcctccgacggcaacacgggcgacctgttcctggtcatggaactcgtcgacggccccagcctccgagactggctcacgcggcc
cgtcccggaggccacggcccgcacgttcatgcgtcagctcgtgagcgcggcggcgacgatgcacgccgccccgatggccacgatccaccgcgacatc
aagccggagaacgtgctcgtcacccccggctgcgaggtaaagctctgcgacttcgggtgcgcgacgccggcgaggcccctgggcaagccgtacccgg
agcggcgcgtgggcacgatgccgtactgcgcgccggagcagctgatgggtatccggtgctacgccacggccgtggacgtgtgggcgctggggtgcgt
catggcggagctcctcgccggcgcgcccctgttcgcggccgacacggaggacgacgtgctcgtgcagatgctggacctgcgggacgggatcgccacc
gtggggctcaaggccttcgatggcgtggtggagctgtcgacggccgggcgcgagctccttgccggtctgctcagcttcgaaccgggcgcgaggctcac
ggcggcacaagcgctcaagcaccgctggttcacggaggaggtggcagagcccccggccgccgcgaaggcagagtttcctggatttgtgccactcttct
cggcagcatag
>g21787.t1
atggctggtcgccctcatggtgacctcgccgttctcctgcttctgtcttttgcattggtcctcgccctcggtttcttgctttcgccgccgcctgaacacctgat
gctggtcgtggccgtggccgtgcttgcggtcgaggtcgccggcgccgtagagttcacctacaacggctttggtggcgcgaggctgtcccttgatggcata
gcgaccgtgacgccgtcgggacttctcctcctcaccaacgacaccaacatgtccaagggccacgcgttccacccggacccagtcacgttccgccgcac
gccgaattcgacgggggatagtatgccgtccttctcgacgacgttcgtgttcgccatcgtgtcggagttcctggacctgagcacgagcgggttcgtgttcc
tgatctccccctccacggacctgtccacggccatgccgcagcagtacctgggcctgttcaaccgcagcgacaacggcgacccgcgcaaccgcgtcttcg
ccgtggaattcgacaccgtccgcaacccggagttcgccgacatcgacaacaaccacgtcggcgttgacgtcaacagcctcaactccaccgccgccgcg
accgccgggtactactacgacggcggggaggagggggggtcgttccagaacctgagcctgatcagccgggagccgatgcaggtgtgggtggactac
gacggcaaggcctcggtggtcaacgtcacgctggcgcctgcggggaatccaaggccgaggaggccgctgatttccacccaaatcgacctctccaccgt
catctccaacgaggcctacctcggcttctcgtcggcgtccagcatcgttctcgtgaagcactacgtgcttggatggagcttcagcctcggcggcacgccg
gcgccgccgctggattacagaaatctccccaagctgccgcgcatcgggcccaagccccagtccaaagcgttaaccgtcgctctgccgatcgccaccac
gttgaccgtcctcgccgccgtcgccgccgggttcgtgctccaccgtcgaagaatcaagtacgccgagctccgcgaggactgggagacggagttcgggc
cccaccggttcgcgttcaaggaccttcaccacgccacggccgggttcagagacacgcgcctcctcggcgccggtgggttcggccgcgtgtacagaggc
gtgctcccttcgtcggggaccgaggtcgcggtgaagcgcgtgtcccacgagtcgcggcaggggatgaaggagttcgtggccgaggtggtcagcatcg
gccgcctccgccaccgcaacctcgtgcagctgctcgggtactgccgccgcaagggcgagctgctgctggtgtacgactacatgcccaacggcagcctc
gacaagcacttgcactcgtcgcacggcggctacgagtacgacgtccagctggactggccgcggcggctccacgtcatccggggcgtcgcggcggggc
tgctgtacatgcacgaggactgggaccaggttgtggtccaccgcgacatcaaggccagcaacgtgctcctcgacgccgagatgaacggccgggtcgg
ggatttcgggctcgccaggctgtacgaccacggcgccgacccgcagaccacgcacgtcgtgggcaccatgggttacctcgccccggagctcgtgcgaa
cggggaaggccaccacgcgctccgacgtgttcgccttcggcgcgttcctactcgaggtcgcctgtggacgcaggcccattgaggaagaggatgacgac
gacaggttcgtgctcgtggagtgggtgctcggccactggcacgagggcgccatcgtcggcgcggtggacgcgaggctcgggtcggcgttcgacgcgg
ccgaggcggacctggtgctgcggctcggcctcgcgtgcctgcacccggcgccggcggcgaggccgaccatgcgccaggtcatgcactacctcgacgg
cagcgcgacgttgccagacctgccggagacgtacgccacattcaacacgccggcggccatggagatctacaaaccaaagttcgactcgtggtccacct
ggcggtccacgacgtccacggccacgagcggcgccgccatgtcagacatcgctctctccggtggaagatga
>g21992.t1
atggccagcgtcagcgcgctactccccgccctctgcgaaaagatccgaggtcttgcagagccaggaacagatatggagcttcggcgcgtcagcatggc
gtgcatcttcgtcgcgctgcacaatctgggcaacgccaggaggaccaccaaaacgaggaaatccatgagccctgtcagcacctcgcagttcttgcatct
ggtctggacagacatcagcgagatggttaccaaagtgcagcaccgtacaagcggcaagaccttcgccatgaagaccctcgacccgctgctgcacgga
gtattggacgacgacccggccatggtcatctataacctccgcgtgctgcgtcaggcgtgcttcatggcggcctgccgcggccacccttctatcgtccgcct
ccacgccgtctgcaaagaaccagacaccgaggagtaccgcctcctgatggaacacctcggcccaagcctgtccgacgttctggcgtgccagcgccgcc
acaagccattcccggagcacgaggtccgccacatgatgcggcagatcctgagcggggccaaggcgatgcacgaactcggcatcgtccaccgcggcat
agtttccgagaacgtcctcgtcgccgcgggcggcgacgtcttcaagatcgggggctttggccaggcgacgtgcgtctccgagacggacgtcccattcga
aatcctgggtagggacgactgggcacccgaattgctgctccaacaacgaggcgcatacgagagtgagctcctccactcgtggtcgattggtcgcttgat
ggcggagctcctcacgggctgggcgctgtttcagccgatttggcatgacgagtccaagggagctcagctctacagtatattcgacgtgctcggcattccg
gacaagagagccatgcagcgtatgaagcctcatgaccttgagcttgctaaaaaggtgcgggaatggagagcccgacggccaagagtagggaagaag
cagcagaacaataactacaggctgcgcaagctggtccccccctgaggtcctatccgacgacggattcagagtcctgcgaggactccttacgtgcaatccc
aggaagcggctcacggcggctgacgcgctcgaactgccatggttttccaacaacaacgacgataaccctccggccgctgctgaggtctcgcagcaaat
actgattgagtcctatacatatttgcaatggaccaacgcacaagacaaacgttaa
>g22370.t1
atgggcaacacctgcgtcggccccagcgcggcgggccgcagcggatttctcgcctccgtcaacctgtggcggcccgcgcagcccgccgccgccctccc
gccgccctcctcccccgcctccgacaaggcgcccgagcccgtcacaatccccgcctccgagcactcctcccaccactcctcccgatcctccgaccagcc
gcagacgccctccgcggagccgcagacgcaggacaataatcctccggcgaagaggcccgtgcccaaggtgaagcgcgtccagagcgcgggcctcct
cgcggactccgtcctcaagcgcgacgtcaacaccgcccggctcaaggatctctacaccatcggcaagaagctagggcaggggcagttcggcaccacc
tacctctgcgtcgagaaggccaccggccgggagttcgcctgcaagtccatcgccaagcggaagctgctcacggaggaggacgtcgaggacgtgcgcc
gcgagatccagatcatgcaccaccttgcgggtcacgccaacgtcgtctccatcgtcggcgcctacgaggacgcggtcgccgtgcagctcgtcatggagc

tctgcgccggcggggaactattcgacaggattatccaaaggggacattactccgagaaggccgccgcgcagctggcgcgggtgatcgtcggcgtcatc
gaagcctgccactcgctcggtgtgatgcacagggatctcaagccggagaacttcctgttcgtcaaccagaaggaggactcgccgctcaaggccattgat
ttcggtctctccatctttttcaaaccagaacaggattccccccccgcctatctggtgaatatatactga
>g22373.t1
atgggtaacacatgcgtgggccccagcatcaccaagaacggcttcttccagtccgtgtccacgctgatgtggaagacgccgcaggacggcgacgcggc
gctgcccgccgccgccgccaatggctccgaaggcggcagcccgggccggtcgcagcccaccaagcccgaagtgcagagcaaggccccggagcccat
gaagatcgccgcagcccaacaaccggatgagtcgccgccgccgcccagcaataagcagcagcaggacacgaagccaaacgccggcgccggcgagg
cgcccagcaacaagccgcggcccaaggttcctcaggtgaagcgcgtgtcgagcgccgggcttctggtgggctccgtgctgaagcgcaagacggagaa
cctcaaggagaagtacagcctgggccgccggctcgggcagggccagttcgggacgacgtacctgtgcgtggagcgcggcacggggaaggagctggc
gtgcaagtccatcctgaagcggaagctggtgaccgacgacgacgtggaggacgtgcgccgtgagatccagataatgtaccacctggcgggccacccg
aacgttatctccatccgcggcgcgtacgaggacgccgtcgccgtgcacctcgtcatggagctctgcgccggcggggagctcttcgaccggatcgtgcag
aggggacactacaccgagaggaaagccgccgagctcgcaagggtcatcgtcggcgtcgtcgaggtgtgccactccatgggcgtcatgcatcgggatct
caagcctgagaacttcctcttcgttgacaacaaggaggaggccgcgctcaagaccattgactttggtctctccatcttcttccgcccaggtcaagtattca
cggatgttgtcggtagcccatactacgtcgcgccagaggtcttgaaaaagaaatatggtcccgaggcagatgtctggagtgctggtgtgatcatctacat
tttgctgtgcggtgtgccaccattttgggcagagaacgagcagggtatatttgaagaggttttacatggaaaacttgactttcagtcagagccgtggccta
gcatctccgaaggtgccaaagatcttgtgaggagaatgcttgttagggaccccaaaaagagattgacggctcatgaagttctacggcatccatgggttc
aggttggcggtttggctcctgacaagccgctggattctgctgttctgtctcgcatgaaacaattctcggctatgaataagctgaaaaagatggctcttagg
gtcattgcagagaacttatccgaggacgagatcgccggcctgaaagaaatgttcaagatgatagacacggacaacagtgggcacatcacctacgagg
aactgaaggtcgggttgaaaaaagttggtgccaacctccaggaatccgagatccaggcactcatggaagctgcggacgtggataacagcggcacgat
agactacggcgagttcatcgcggccactctgcacctgaacaaggtggagagggaggaccacctgttcgctgcgttccagtacttcgacaaggacggca
gcgggtacatcacgcccgacgagctgcagctggcgtgcgaggagttcggcctgggcggagacgtccagctcgaggagatgatccgagaagtggacc
aagacaacgacgggcgcatcgattacaacgagttcgtggcgatgatgcagaagcccacgctggggctcccgaagaagtccgggggtctgcagaaca
gcttcagcatcggcttcagggaggcactgaggatttcgtaa
>g22468.t1
atggggaactgcttggttagtgggagctcccacatcaagaaggtctcctccaatgtaaagccagattctcccaagatccagagcccgtcggtgagggac
cggagcgaggagcagaagctgccgtcgaaccccgtcgaggtggaggccctgcgtcgcggcgcgtcaacggcgaagcgcaacccgctggtggccttct
catatgcggagctgcggaaggttgccaacgatttccgtcaggacgcgctgatcggcggcggcggcttcggccgcgtgtacaagggcgccgtgggtgag
acgacgctgctgcccgtcgccatcaaggtccacgacggcgacaacagcttccagggccaccgcgagtggctggccgagcggcccgtcatctaccgcg
acttcaagacctccaacatcctgctggacgcggccttcaacgccaagctctccgacttcgggctcgccaaggccggcccggaaggggacagctcccac
gtgtccacacgcgtcatgggcacctacgggtacgcggcgcccgagtacgtcatgacgggccacctcaccgccatgagcgacgtgtacagctacggcgt
cgtgctgctggagctgctcacgggccggaggtcgctggaccggtccaggccgcacagggagcaaaccctcgccgactgggccatgccggccttgacg
cacaagaagcgggccatggccatcgtggaccccaggctcaccgccggagacgaggacctaccagctgccaggtccgtgcacaaggtcgccatgctcg
cgtaccactgcctcagccgcaaccccaaggcaaggccgctcatgcgtgacgtcgtcgcgtcgctcgagccgctccagcaaccgccggttgcgtga
>g22819.t1
atgacggccgcggcgtcctcgccgccaccgccgccgccgcagctgagcctggatggattgaaggcggtgtccgtgctgggtcgcggcgccaagggcg
tcgtgttccacgtcgtgcccgccgcgggcgacgacggcggcagggccatggcgctgaaggcggtgtcgcgggacgcggcgcggcagaagaaggcgt
cggcgggggacggcgacgggcaccgccggatctggttcgagcgggacgtgctcctcgcgctgcgccacccgctgctcccctccctccgcggcgtcctc
gccaccgacgccgtcgtcggcttcgccatcgaccgctgcggcggcggcgacctcaacgcgctccgacgccgacagaccgagaagatgttctccgactc
cgtcatacggttctacgcggcggagctggtgctggcgctggagcacctgcacagcatcggcatcgtgtaccgggacctcaagccggagaacgtgctca
tccaggacagcggccacatcatgctcgtcgacttcgacctctccacgcggctcccggcgccgccgcaggacccgcccgacgccgcgccggtctgtcctc
ccaaaccgactcctccggcctcacaaccgtccccgagccgccgccgtcgcgggaagtcgaggaagcccaccgcggcgctctgcttctcgttccgcacc
ggcggaggcgcaaaaccggccgcgcagtcggactcgctctcgccgccgtcgacctcgcggacggcctcctcctcgtcatcgtcatcgtcctccacggcg
accacggtctcctccgcgaaatccaactcgttcgtgggcaccgaggactacgtggcgccggagatcatcgcgggccgcggccacgacttcgccgtcga
ctggtggggcctcggcgtggtgctctacgagatgctctacggccgcacgccgttccgggggcagaaccgcaaggagaccttctaccgcgtcctcgcca
agcagccggagctcgactacgtggcgccggagatcatcgcgggccgcggccacgacttcgccgtcgactggtggggcctcggcgtggtgctctacgag
atgctctacggccgcacgccgttccgggggcagaaccgcaaggagaccttctaccgcgtcctcgccaagcagccggagctcgtcggcgagcagacgc
cgctgcgggacctcatcgcgcgactcctcgagaaggaccccgccaagcgcgtcggcgcgcgggcggtcaaggcgcaccccttcttccgcggcgtcga
ctgggaccgcatcctccacgtggcgcgcccgcccttcatccccgcggcgctggacgaggagggagacgaggcgctcgacgtcgagaaggtcgtccgt
gaggtgtttgcgaccaaggcggagggcgagaaggcttcgccggagggggacgaaggccacagcggcggcgacggcgtggggagggggagagatc
cgtccaaagaggacaccgatttctccgtgtttttctga
>g23424.t1
atgcccagcaaggcgctgacgttcagggagctgggcgccgcgaccaacgggttcagcgagagcaacctgctcggagaaggcggcttcgggcgcgtgt
acaagggggtcctcccggacaccaaagaggtgatcgccgtgaagcagctggacaggaacgggttccagggcaaccgcgagttcctcgtcgaggtgct
gatgctcagcctcctgcaccaccccaacctcgtcaagctgctcggctactgcaccgacgccgcccagcgaatcctggtctacgagtacatgcccaaggg
ctccctggaggatcaccttctagacctgccaccaaactggaagccgttgccgtggcacacgcggatgcagatcgccgtggacgcggcgaagggcatcg
agtacctgcatgaggtggccaacccgccggtgatctaccgagacctcaaggcgtccaacatcctcctggacgcggacttcaacgccaagctgtccgact
tcgggctcgccaagctggggcccatcggcgaccagagccacgtcagcaccagggtcatgggcacgtacggctactgcgcccccgagtacgccatgac
cggcaagctcaccaagatgtccgacatctacagcttcggcgtcgtgctgcttgagctcatcaccggccgccgtgccatcgacagtgcgatgcagtccga
ggaacaggtccttgttcagtgggcggcgcctatgctgagagacaagaaacggttcatgcggctggccgatccattgctggggaggaagttccctccca

agggtctgtaccaagcgctcgcggtcgcgtcgatgtgcttgcaggaggacgccagcagccggccggtgatcagcgacgtcgtcgcggcgctctcgttc
cttgcggacccgaaatactaccctccagaaggcgcggcgaagaaggccacggaagagagcagtaaatcgaatgagaacatagaaaaatttagtggt
agcagtcctcccatgacagacattgtttctgagatgagatcagacgatgagatcaagcagagatga
>g23487.t1
atggctagctccagtaacagtagtattatctacaccttggagaaagctgcagtgatctgttccttgcttgccgttgttgttgctgcagatgttggaggagga
aggcactgccctccttcctcttgcggccatcttcgaaacatatcctaccctttccgtcttcaaggcgatccacgacagtgcgttgctactcctcgtccatggt
acgacctctcttgcagcagcagtggcaaggccaccattcagatcaacacaagaaagtactacgtaagtagcatcgactatactgctgaggacttcttgg
tcatcgacgccaccgtgcaggatgatgatgcaaacagcagctgccctcttcctcgtgccgatgatcacccttaccctccttacttaaaatggcttgcgtctt
tgttaaggttaatcttggggtcactggtggtattcatcttccttgcccacaagtactggaaaacaaagatcacaatcgatgcagtagagaagttcctccgg
atgcaacagatggttggtccaatgaggtatgcctacacaagcatcattgccatcacaggccattttcgggacaagctgggccaaggaggctacggttct
gtatataaaggcgtactactcccaggcaatgtccatnccattacaggccattttcgggacaagcttggccaaggaggctatggctctgtatataaaggtg
tactactcccagggaatgttcacgttgctgtcaaaatgttgtctggtaactccaactgcaatggagatgaattcattagcgaggtctccacaattggtagg
atccaccatgtcaacgtggtgcgtctggttgggttctgctcagaggaaatgaggagggcactagtctatgagtatatgcctcggggctctcttgataagt
acatcttctcatcagagaagagtttctcctgggacaagctgaacgagatcgctttgggcattgctagagggatcaactacctacatcagggttgcgatat
gcagattctgcactttgacatcaagccgcacaatatccttcttgatgacaactttgtccccaaagttgctgattttggtcttgccaaattatacccaaggga
caagagctttgttccggtgagcgcagctcgaggaacaataggttacatagctcctgaaatgatctcccggagctttggtgtcatatcaagtaaatctgac
gtttacagcttcgggatgctactgctagaaatggctggaggaagaaggaatgcagacccaaatgcagcaaactcaagtcaagcatactatccatctcg
agtgtacatgcaactaactcggaaagaaacatgtgggatctctgatgacatgcataagttggagaagaaactttgtattgtcggattatggtgtattcag
atgaggccgtctgatagaccaacaatgagcgaggtgatagagatgctcgaatgtggtagcgatgatctgcaggtacccccaagccattcttctgtgat
gatgagcaacaccctggagtagagtcctatcatatgtcgtctgatctgactacaatctctgagatgcaagaggacgatgacgacgatagcatatgtctgt
ttcaaagctacaagtaa
>g23563.t1
atggaggagcagaactcatggcttaggaggaccaagttctcccacactgtctactcgagggtggatcctcgccgggtacctattgctccacttagcaag
gatattgagcggaaagtgcagaaatttgcaactatgggaaagtccatgtcaatgcctgttgatcgggacaatgaagatacagggactgcacttaagca
ctctcctagcttgcctctagtccgatcctcgcttcagcttgatagtgataaggctaacaagccgaagaaagcgagcttggaaattcctttaagctccctga
tgaacttggggaactccaagggttcgagggcaaagagcctcatcaagagcccaagttcgacattgctcctcagctacttgaacaaggcagagattcctt
taagcccatcaaggaaccaggagaattcaaagggtccgagggcaaggagcctggtcaagagcccaagttcgatgttgctcctgagctacttgaacaa
gtcaccttcaatccaaggaattgtacaccgtgacattaagcctgagaatattctctttgatgagaacttctgcgtgaagattgctgattttggtattgcctgc
gaggagactttgtgtgatgtgctagtggaggatgagggcacttacaggtggatggctcctgagatgatcaagcaaaaggcatacaacaggaaggtgg
acgtcaatagctttgggttgctcatatgggaaatggtgtccggaagaattccttatgagaacctgacccctttccaggtggcttatgctgttgccaatagg
aatttgaagccaacaattcctcccgaatgcccctcagctctcaggcccttgattgagcagtgttgctcgttgcagcctgacaagaggcctgacttctggca
gattgtgaaagtactagaacagttccagtctgttctcgcgcagggcggctgcctcgatacactgaagagcggcacctgccaggatcacaagaagcggc
ttctgcattggattcagaaactgaagccatcgcatggcacctga
>g23565.t1
atggggtgcgtctgcggccgcccctctgtcgtcgacgacggcccgtgccagacgacgcccccgccggcgtccaagctctctgcggcggcggctggagt
ggcggcggtgaggagggaggaggaggtgctgaagcagcagcagcaattacagcatgccagggtggggagccgtagggaggaggtgctggagagg
cggcgggcgatgttggcgatggcggcggcgtgtcaggcgaggagcccggtgccgaggacggtggagggagagcaggtggcggcggggtggccgcc
gtggctcgtcgccgtcgcgccggaggccgtgcgcggctgggtgccgcgccgcgccgagtcgttcgagaagctcgacaagattggccagggaacatac
agtaatgtctaccgagctcgtgatcttgaaaaggagaagattgttgctctgaagaaggtgcgctttgataatttggaacctgagagtgtgaagtttatgg
ctagggaaatcctcattctgcgacgtcttgatcatccaaatgtcatcaagctagagggcctagtaacatcaagaatgtcctgcagcttatatcttgtttttg
agtatatggagcatgatctagctggactagcttctttccctggagcaaaattcactgagtctcaggtgaagtgttacatgcaacagctccttcgtggtcta
gagcattgtcatagtcgtcatattttgcaccgtgacatcaagggttccaatctcttgattgacaacaggggcatattaaagatagcagattttggattagc
aagtttttcgatcctgaacaacggcaccctttgactagtcgtgttgtcacactttggtacaggccaccagagcttttgcttggtgccactaattatggtgtt
gctgtagatctctggagtgctggctgcattcttgctgaactatatgctggcaagcctatcatgcctggtagaacagagttgacccagcagatcgtggaac
tgcatcatctgcgcttcaaagtgaggcaaggtgctgctggaggaaagcatcataagcatgaccctgaaaggcgaagtagagaatcaagagctgtccct
gctcctgacgcaaatgcagaactagtgtcgtctttgcagaagaggcaagcccaagctaataccaagagcaggagtgagatgtttaatcccagcaagga
agactctgcttctggttttcgaattgaaccactcaggcaaactcctgttattgaatcttctgaagactcacgacatgcctacccgaccagaaccttccattc
tgggcctttggttaatcagaataacccatcaaaggctggacgtggcaaaagtggtgaacttcaggttcctggtgctgccaatgtctcagtggttgtatcg
acaagagcaggtcttcgacccgataacagcaataggacaatgataactcaagctgaagcttttgctcatggacggaggctgtctgagtctatcaacgag
catttcagtaatagtgggaagtatgaccaagtattccagaaggatgaaaggaacgtccgggtggatggagcaattggctatggatccaagggcaacaa
gatccaccattccggaccccttacatgcccttcaggcaatgtcgatgagatgctgaaggagaacgatcgccaaatccaagaagtcttcaggcgaaccc
gggtggagaagtccagagtgaggaatgggcatcaccaccccagcgattttggtgccatcccggtctacccttctagtcgttccagctatcagtctgtgca
gcagtag
>g23674.t1
atggcgggtttggaggacctcaagcggaggctgcagcccttgtttgtcgactctgaagccaatggcgagcccgccgccgggactctcgccggctgcga
ggccctgcgctacttgcacgaagtgaggcatgtcgtgcacagagacataaaaccagaaaatttacttttaaatctcaagggtgatgcaaaaattacaga
ctttggtgtgactgctggtctgtttgactctatgagcatgtgtgctaccttcgttggcactgtcacatatatgtctcctgagagaattatgaacagctgctac
tcatattctgctgatatctggagtcttgggctaacagtgctggaatgtgctactggaagattcccatatgatgtaaatgggtgcctcagcgatctcatgctg
cagatactggatgacccatcaccaataccaccaaaaggtgtttattcaccagagttctgctcgttcatcagtgcttgcttgcagaaagatgctgatgcaa

ggcctacatgtgcgcagcttctgtcacatcccttcatcaagagatacaagaaaactggtgtggacttggccgcatatgttaaaagtatttatgatccaact
gaaatactaaggcaaatagcagatatacttgttgtgcattactacctgacttttgatggttccaataatgtatggcggtacatgaaaacattctatagaga
agaatctgttttcagagaaacaaaggaggcggcgatgcaggcagctgggtctctctcgttttctgcgttcggcacgcggcctccgggctccggccttccc
cgtttcaccaagcggagagcgcgcgtccaatcaccaatggcgcactcgtcaaccaggatcacgccgccctga
>g23788.t1
atgcctcctggcgtgagcaggaggcggccgccttcgctgctgctgctgctgctgtgcgcggcgtgcgtgggcggcgcgctggccacggacgcgca
gggcgcggcgctgctggcgtggaagcttacgctgcgcggcggcggcgcggcggagtcgctgggcgagtggcaggattccgacgcgtcgccgtgccgg
tggacgggcgtgtcgtgcaactcggcgggtcgcgtcacggcgctgagcctccagtacgtggacctccacggcggcgtccccgacgacctctccgccgt
gtccgccacgctgtcgcgtctcgtcctcaccggcaccaacctctccggccccatccctccacacctcggcgaccagctccctgcgctgacccaccttgac
ctcagcaacaacgcgctcacgggcccgatcccggccagcctgtgccggcccgggagcaagctggagagcctccacctcaactcgaaccacctcgagg
gctccatcccgtcctccatcggcaacctcacgtccctccgcgagctcctcatctacgacaaccagctcgacggcaccatcccgtcgtccatcgggcagat
gaccagcctcgaggtgctccgcggcggcggcaacaagaaccttcagggcgcgctgccgccggagatcggcgactgctccaacctcacgatgctgggc
ctcgccgagaccagcatctccggcccgctccccgcgacgctgggccggctcaagaacctcgacaccatcgccatctacacggccctcctctccggcccg
atcccgcccgagctcgggcaatgcacgaaccttgaaaacatctacctctacgagaacgcgctgtccgggtccatcccgccgcagctcggcaagctgag
caacctcaagagcctgttgctatggcagaacaacctcgtcggcgtcatcccgccggagctcggggcctgcactggaatcacggtgctggatttatccat
gaacgggttaactggccacatcccggcgtcgctgggcaacctgacggcgctggaggagctgcagctgagcgggaacaaggtgtccgggccgatcccc
gccgagctggcccggtgcggcaacctcaccgacctggagctcgacaacaaccagatctccggcgccatccccgcggagatcgggaagctggcgtcgc
tgcggatgctgtacctgtgggccaaccagctgacggggtccatcccggcggagatgggcgggtgcgccagcctcgagtcgctggacctgtcgcagaac
gcgctgacgggcaccgtcccgcgcgcgctgttccgcctcccgcgcctctccaagctgctcctcatcgacaacgcgctctccggcgaggtgcccccgag
atcggcaactgcacgagcctcgtccggttccgcgccagcggcaaccacctcgccggcaatatccctgctgaagtcggcaggctaaccagccttagcttc
ctggacctcgggtccaaccggctgtcggggtccatcccgcccgacctcgccgcgtgccggaacctcaccttcgtcgacctccacggaacgccatcgcc
ggcgcgatcccctcggggctgttccgcgacatgcccgccctgcagtacctcgacctctccgacaacgccatcgtcggcgggataccttcagatatcggg
aggctcgcctcgctcaccaagctcgtcctcgccgggaacaggctgtccgggcccatcccgccggagatcgggtcatgctccaggctgcagctcctcgac
ctcggcgggaacgcgctgtccggcgcgatcccgcccagcatcggcaagatcccggggctcgagatcgcgctcaacctcagctgcaacgcgttatccgg
tcccatccccaaggagttcgccgggctggtccggctcggcgtgctcgacgtgtcgcacaaccggctggccggcgacctccagccgctgtccactctcca
aaacatcgtggccctcaacgtctccttcaacgacttcgccggccgcgcgcccgacacggcattcttcgcgaagctccccgcgagcgacgtggaggagg
gcaacccggcgctgtgcgtgccgtcgcgctgccctggcgacgctgaccgcgaccgtacagcgcggcgcgccgcccggatcgccaccgccgtgctgctc
tccgcgctcgtggcgctcctcgccgcggccgcgttcctcctcctcagccgccgccgcttgtggcgcagcaagcagggaggcggcgggtccgtgttcgac
gacgacaaggacgcggagatgctgccaccatgggacgtgacgctgtaccagaagctggagatcagcgtgggcgacgtggcgcggagcctcacaccc
gccaacgtcatcgggcagggatggtcgggatccgtgtaccgcgccagcgtgccttcctcctcctcctcgggcaacggcggcggcgtcaccatcgccgtg
aagaagttccggtcgcgggacgaggccgcggcggaggcgttcgcgtgcgaggtcggcgtgctcccccgcgtgcgccaccgcaacgttgtcaggctgct
gggctgggccgcaaaccgccgcacgcggctcctcttctacgactacctccccaacggcaccctcggcggcctgctccacggcaacggcaacggcgccg
ccgccgcggtggtggtgccggagtgggagctaaggctctcgatcgccgtcggagtcgccgatggactcgcgtacctccaccacgactgcgtgccggcg
gtcatccatcgtgacgtcaaggcggataacatccttctcggggagcggtacgaggcgtgcctcgctgatttcggtctcgccagggtcgccgacgacggc
gccaactcgtccccgccgccgttcgccggatcctacggatacatcgctcccgagtacgggtgcatgaccaagatcacgaccaagagcgacgtgtacag
cttcggggtggtgctgctggagtccatcacgggccgccgccccgtggagccggcgttcggggagggccggagcgtcgtgcagtgggtgcgggaccac
ctgcaccagaagcgggacccggccgaggtggtggacccgcgcctgcagggacgcccggacacgcaggtgcaggagatgctgcaggcgctcggcatc
gcgctgctctgcgccagcgccaggcccgacgacaggcccaccatgaaggacgtcgccgcgctgctccgagggctccgcagcgacgacggcgccgact
ccaggaaggtcagcggcgggcccaggccggactcctcggccaagtgggccgcacccgtctcgccgaccaagcccacaaccctcctcggttacccggc
ggcccaagcgcggtcgctatccccgtccagctcctcggcctactactcaacgtaa
>g23878.t1
atgatgggtagaagaagaagcaaatggcgaggcgcgctgctggttgtggtgctctgcctctgcgtggtctgcggcgctgccgccgtggacgagcaggg
cgcttcgctcctcgcgtggaaggcgaccctgcacggcggcgacgacgccctgccggactggaagtccggcgacgcgacgccgtgccggtggacgggc
gtgacgtgcgacgccgacggcggcgtcacggagctgaacttgcagttcatcgacctgttcggcggcgcgcccgacaacctcaccgccctgggccccac
actaacacgcctcgtcctcaccggcgtgaacctgacgggccccatcccgccgtcgctgggctcgctgccggcgctggcccacctcgacctctccaacaa
cgcgctgacggggcccatcccggcggcgctgtgccggccggggagcaagctggagacgctacacctcaactccaaccgcctcgagggccccatccct
gacgccatcggcaaccttgcgtcgctacgggagctcatcttctacgacaaccagctcgccggccgtatcccggcggccatcgggcggtgcgcgagcctc
gaggtgttccgcggcggcggcaacaagaacctccagggcgcgctccccgcggagatgggcaactgctcgaagctcaccatgatcgggctcgccgaga
ccagcattaccggtcccttaccggcgagcctcggccggttgaagaacctcaccacgctggccatctacaccgcgctggtttccggcccgatcccaccgg
agctcggccagtgcgagagcctcgagaacatctacctctacgagaacgcgctgtccgggtccatcccgccgcagctggggaagctcacgagtctcacc
aacctcctgttatggcagaaccagctcgtggggaccatcccgccggaactcgggtcctgcaccgggctcaccgtcgtggacctctccctcaacagcctg
accggccatattccaacctcctttggcaacctatcgtctttacagcagctgcagctgagcatgaacaagctctctggtacggtaccggcggagctcgccc
ggtgcggcaacctcaccgacctggagctggacaacaacgcgctcaccggcagcatcccgtcggccctcggcgggctcccgtccctccgcatgctctac
ctctgggcgaaccagctgaccggcacggtaccagcggatctcggccagtgcgtgagcctcgaggcactggacctctccaacaacgcgctcacgggccc
catcccacactcgctcttccgcctcccgcgcctatctaagctgctcctcatcaacaacgccctctccggggagctcccccgcgaggtcggcaactgcacc
tcgctggtccggtttcgggtcagcggcaaccgcatcgccggtacggtgccggccgagatcggccggctcgggaaccttagcttcctcgacctcggctcc
aaccggctgtcggggtctgtaccggccgagatcgccgggtgccggaacctcaccttcgtcgacctccacgacaacgccatcagcggcgacctccctcc
gggcatgttcaacgacctgttatctctccagtacctcgacctctcccacaacgccatcaccggcgccctcccaccgagcgtcgggatgctcacctcgctc
accaagctcatcctcaacggtaaccggttttccggcccggtgccgcccgaaatcgggtcgtgcacccggctccagctactcgacctcggcggcaacgcg

ttcgaaggcgagataccggggagcatcgggaagatcccgggactggagatcgcgctgaacctcagctgcaacgcgttcaccggcgcagtcccgccgg
agttcgcggggctggcaaggctcggcgtgctcgacgtctcccgcaaccgcctctcgggcgacctccacccgctcgccgcgctgcagaacctcgtggcg
ctcaacgtctccttcaacgcgttcgtggggaggctcccggagacgccattcttcgcgaagctccccaccagcgacgtcgagggcaacccggcgctgtgc
ctgtcccgctgcaaggatggcgacggcgaggatgaccataaccgcgaccgcgccgaacgggtcgccgtcgccgtgctcctctccgcgctcgtggtcctc
gtcgccgccgcggcgctggtcctggtccggcagcagcggcggcggcgcgcgggagaggaggcggagaaggccggcggtgaggagatggcgccgcc
gtggaacgtgacgctgtaccagaagctggagatcggtgtggccgacgtggcgcggagcctcacgccggcgaacgtcgtcgggcagggctggtcgggt
tccgtgtaccgcgcgagcctcgcctccccgcccggcttgaccgtcgccgtgaagaagttccggtcgtgcgacgaggcctccgtggaggcgttcgcgtgc
gaggtcagcgtgctgccgcgcgtgcgccaccgcaacatcgtccggctgctcgggtgggcgaccaaccgccgcacaaggctcctcttctacgactacctc
cccaacggcacgctcggcgcgctgctccacggcggcggcgcgccggtggtggtggtcgagtgggaggtgcggctcgccatcgccgtcggcgtcg
ccgaggggctggcttacttacaccatgactgcgtgccgggcatcattcaccgtgacgtcaaggccgagaacatcctcctcggggagcggtacgaggcg
tgcctcgccgatttcgggctcgccaggttcgccgacgagggcgccaactcgtcgccgccgccgttcgccgggtcatacggctacatcgctccggagtat
ggatgtatgaccaagatcaccaccaagagcgacgtgtacagcttcggtgtggtgctgctcgaaatgatcaccggaaaacggccactcgattcggcgttc
ggcgaggggcagagcgtggtgcagtgggtgcgcgaccacctgtgccggaagcgcgagcccatggaggtcatcgacgcgaggctgcaaggcaggtcg
gacgcgcaggtgcaggagatgctccaggcgctcggcatcgcgctgctgtgcgcgagcccaaggcccgaggaccggcccgtcatgaaggacgtggcg
gcgctgctgcgtggaatccagcacgacgacgccgccgaagcacgcaaaggaggagggagtggagccggagccgaagcggaggtgggggtgagga
agtggacggacccgaagcaacccatttcgcccaccaagttgatggccctcgctcgaccagctgctgctgtccaaacacagaccgaaacccgggcgagt
tcaggttcacagagcctcctcaacgaccgggaataa
>g24120.t1
atggggaggagcgggagcatcaagcgccccgcggcagctgcttcggcggcgccgccgtcgttcacggtgaagcccggcgactaccggctcatggagg
aggtggggtacggcgcgcacgccgtcgtgtaccgcgcggtcttcctccccaggaacgaggtcgtggcggtcaagtgcctggatctcgatcagctcaata
acaacataatcgcataccctgatggttttgaggagcccgtcattggctccattctaaaggaaacacttaaggctctggaataccttcacaaccaaggtca
aatccatcgtgatgtcaaggctggtaatatccttatcgatagtgctggtgcggtgaagcttggagactttggtgtttctgcctgcatgtttgatagaggtga
taggcaaagatctaggaatacatttgtgggaacaccttgctggatggctccagaagtgcttcagcctggcactggatataactttaaagctgatatatgg
tcatttggaatcactgcacttgaactagcacatggccatgctcctttctcgaaataccccctatgaaggttcttctgatgactctccagaatgcaccgcct
ggtcttgactatgaccgcgacagaagattctcaaagtgcaacagtaatcaactgaaggaactctgcaatagctcagtatcatcgtcaattctaattcccc
atcttgagaaccttgtgcagcagacaatgtttcagcaggaactgatcatgaacctattaggtggcttgcaacagaatgagaaagtagatggagcacag
cctggagtatcggagaatgatagaatcgcttctacagctaactctgagaaggaacggtcattacttgttacaatttcagaattacagtctaggatgataa
cactgactgacgaactaattgcagctaagttgaaacatgttcagttgcagcaagagctaaatgcattgtactgccgggaagaaattgaagacattaggg
acgaggacaatgaagaaacataa
>g24154.t1
atgcttctccaaggggaactggaattcctcatttgaagtgggttggagtggagggggagtacaatgccatggtcattgatcttcttggtccaagtttgga
ggatttgttcaactactgcaacagaaagttctctcttaaaacagtacttatgcttgctgatcagatggtttatgtcattgactatggccttgcaaagaagta
ccgggatctccaaactcataagcacataccatatagggtagaatacatgcacacaaggggtttcttcatcgtgatatcaagccagacaacttccttatg
ggtttaggccgtaaagcaagccaggtttatgtcattgactatggccttgcaaagaagtaccgggatctccaaactcataagcacataccatatagggat
acacttgtgaagcttacatgttttctttgtttccatagggagaacaaaaatctcacaggaacagctcgttatgccagtgtaaatacccatcttggagtaga
acaaagcaggagagatgatttagagtctcttggctatgtgctgatgtatttcataagaggaagccttccttggcaaggcctgaaagctggcacaaaaaa
gcaaaagtatgataaaattagtgaaaagaaaatgctaactccagttgaggttctttgcaaatcttacccatcagaattcacttcatactttcattattgccg
atctttgcgattcgaagataaaccagactattcatatttgaaaagactcttccgtgatttgttcatccgtgaagggtaccagcttgattatgtatttgattgg
acagtatcacagcaagctgcggatagtagcaggttgcgggtgggttggctggaccatctgcagaacgagctgaacggactgcaggctgttgactccga
tagaagaaggcattcatcatctcggaatggaagcacgtcaaggaaggcccttctgtcaagcagcagacaaggttctggagatcccagtgacccaaatc
gcagtcacctactccctaccagcagtagcagcagccgcccatcaactaatcagaggctccaccagtcaactgggcttgagggcaggaccgcatcatttc
caaaatctggaagaaatgtctatgatgaccccactatgaggacctttgaacgccttactattagcgctgaaagaaggaaatga
>g24329.t1
atgagcaggaccggcaaggccaccacggccaccgacgtctacgcgttcggcgcgttcctcttggaggtggcgtgcgggcggaggccgttggagttcag
cgacaacgccgactcgccgcggctcgttgacctcgtcctcgagcactggaaggcggggcggatcgcagacgcgagggacccgaggatgatcgtcggc
aagtgcgaggaggacgatctcgagttggtgctcaagctgggtctgctctgctcgcaccctgacccgcggcagcggccgaccatgaggcaggtcacgca
gatgatggaaggcgccgcgcccgtgccggagaccgcgccggaggatctagactacaatggcaggttgttctatagcgagtccttcgacgagttcgtca
ccatgttcccaaccacgtcacagaccaccaccaccgtcaccacgcagccgtcttcttcctactcctccgatgaccaacgacacctcatctccggttga
>g24330.t1
atgccaggaccaagccttgcgaagcagctggtggccatcgtcctcctcgtcctcgccggaggaggtatcatcatcggagcatcatcatccgccgccgcc
gccgagttcgtcttctccggcttcgcgcgcgagaacgtgaccaccagcggcgccgccacggtcacctccacgggcctcctccaactcaccaacgacacc
aacgaggtgttcgggcacgggttctacccggtccccgtccgcttcaagaactccaccaccggcgcgccgctctccttctccaccacgttcgtggccgccc
tcgtgccgcgctacccggaagcccacggccacggcctggccttggcgctcgcgccgtcggcccagctcccgggcgccctacccggcaagcgcctcggc
ctcttcaacgcctcggacgacggcgtaaccgccgggggagtagcagcagcagtagcagtaacaaggtcgtcgccgtcgagcttgacacggcggtgg
acgcggagttcggcgacgtcgacgacaaccacgtcggcgtggacgtccacggcccgcggtccgtcaacgccacccccgccggccacgtgggcctcag
cctcatcagcggcgcgccgctgcaggtgtgggtcgagtacgacggcccaagcgcgcggatcgaggtgaccgtgggccccgccggcgcgccgaggcc
cgggagcccactcgtgtcgtgcgtcgtcgacctcgcgtccgccgtcgccgacgagaccttcgtcggattctcggcggccaacggcgccgccgcgagctc
ccactacgttctcggctggagcttccgtctcggcggcggcggcctcgcgccggagctcgacctcgcgaagctccccacgctcccatcaagaccaaccaa
gaagaagacgccggtgctactggtggtggcgctgctcctcctttccgtcgtggtcctcctattggtatcagcagcaatcgcagcagtcctcgtccggcgg

cggcggttcgccgaggaggaggaggaggactgggagatggagcggtacggcacgcaccggatcaggtacagggacctccacgtggcgacggacgg
gttccgccacgtcatcggcgccggcggcttcggcaccgtgtaccgcggcgtgctcccggcgacaggagaggaggtggcggtgaagaaggtggcccac
gactcgcggcagggcctgcgcgagttcgtggcggaggtggcgagcatgagccggctgcgccaccgcagcctggtccacctcctcggctactgccgccg
ccgcggcgagctgctgctcgtctacgactacatggtcaacggcagcctcgacaagcgcctcttcgacgaccccgttgaccacgaccaccagccgctga
gctgggcccagcgcgggaaggtcgtccgtgacgtggcggcggggctgctgtacctgcacgaagggtgggagcaggtggtcgtgcaccgcgacgtcaa
ggcggccaacgtgctgctcgacgccgccatgaacgccaggctcagcgacttcggcctcgcgaggctctacgaccacgggaccagttcgcagaccacg
cacgtggtggggaccatcggatacatcgcgccggagatgagcaggaccggcaaggccaccacggccaccgacgtctacgcgttcggcgcgttcctctt
ggaggtggcgtgcgggcggaggccgttggagttcagcgacaacgccgactcgccgcggctcgttgacctcgtcctcgagcactggaaggcggggcgg
atcgcagacgcgagggacccgaggatgatcgtcggcaagtgcgaggaggacgatctcgagttggtgctcaagctgggtctgctctgctcgcaccctga
cccgcggcagcggccgaccatgaggcaggtcacgcagatgatggaaggcgccgcgcccgtgccggagaccgcgccggaggatctagactacaatgg
caggttgttctatagcgagtccttcgacgagttcgtcaccatgttcccaaccacgtcacagaccaccaccaccgtcaccacgcagccgtcttcttcctact
cctccgatgaccaacgacacctcatctccggttga
>g25032.t1
atggccgccaccggcaacgcctcttcgctccccgggagcctcctcctcctcctcccctcctcaccatcacctccgccgcctcatcagcaccactccccct
cctggccctcctctccctcaaatcctccctccacgacccatccaactccctccaccaatggacctacgcatccgccgtctccgcaggcgccacccactcac
cagcttctccatggtgcgcgtggccaggcgtcacctgcaccccaaacaccaccaccggcgccatcgccggaatcgacctctcccgccgcaacctctccg
gcaccgccatctcccccaccgccgcccgcctcctcgcgcccacgctcacctcgctcaacctcagcggcaacgccttcgccggcgctctcccccccggccat
cttcctcctccgcaggctccagtccctcgacgtcagccgcaacgccttcaacggcacgttttcagatggtgttggtcagctgggcgagctgagactgctc
gacgccttcagcaactgctttgtggggccactgccacgtggcggtctcgtcgcgttgcgtaggctggaacacttgaacctcggcggcagctacttcaacg
gcagcattcccgccgagctcggcagtctccggcaactcaggttcctccacctcgccggcaacgcgctctccgggaccatcccgaaggagctcgggcag
ctcacgttgttggagcggatggagatcgggtacaacgcttacaacagtaccattcccaaagagctcggcaacctgaggaagctgcagtacctggacat
cgcggtggccagcgtgtcgggcccactgccagcgtccctcggcacactcacgcacctccaatccctcttcctcttcaagaacaaactcaccggcacgat
cccgccacactggtcgtcccaccacaacctccactcatcatcactgcaagtcctcgacctctccgacaacgccctcacgggccccatcccctcgggcctc
ggcgatgacctcaccgacctcacaacactcaacctcatgagcaacgccctctccggcacgatcccgccgtccatcggcgtgctccgaaatctcgaggtg
ctccagctatggaacaactcgttgaccggaaggttaccgccgtccctcggcgcggtgagcagacggctcgtccgcgtcgacgtgtcgagcaacgcgct
ctccggaccggtaccggcgggtctctgcgccggtaaccggctcgcgagactcatcctctttaacaaccggttcgactcgtccatcccgcccggcctcgcc
gccaactgctcctcgctgtggcgcgtccggctcgagtccaaccggtttaccggcgcgataccggccgggttcggaaacatcgccaacctcacctacctc
gacctcagtgggaacttgctcacgggcggaggtataccggaagacctcgtgacgtcagccagcatcgagtacctgaacgtctccgggaaccccgcact
cggcggcgcgttgcccgacgccacgtggcaggcgccgaggatgcaggtgttctccgcagagaagtgcgggctggtcggccaagtccccaagttcggc
ccccacgggtgcgcgaacctgtacaggctggagctggccgggaacgagctggtcggagagatcccggaggacattgggaggtgcaagaggctggtg
agcctgaggatgcagcggaacgagatcagcggcgagatccccgcgggtctcgccgcgctgccgtccatcacggaggtggacctgtcgtggaacgcgc
tgactggtggtgtcccccccgggttcgccaactgcaccaccctcgagaccttcgacgtctccttcaaccgcctctcgccggcggcgtctccacgcgccgg
cgaggaagaaggaaggcacacggcggcggcggcggcgatggtgtgggcggcggtggcgttcgcgggtgtggcggtgctcgcgggcaccgcgcgat
ggatgcaactgggacagggctccaataccgtacagaacggcggcccacgggccgatagcaacgccggcgtggtcaaggttactgggccgtggcgga
tgacggcgttccagaagctgggtttcacggcggacgacgtggcccggtgcgtggagctggtggtggggagtggtggcatcatcggagcggggtcg
tcggggacggtgtaccgcgccaagatgcctaacggcgaggtcatcgcggtgaagaggctgtggcactccacagcacaaaaggaggcggcatcggag
cagcagcaggcgacgagatgcaacaaaaggaggatgcttgtggcggaggtggaggtgttgggccagctccggcaccgcaacatcgtgcggctcctcg
ggtggtgcaccaacggcggcgagaccaccatgctgctctacgagtacatgcccaacggcagcctccacgacctcctgcacggcgccaatactactact
gccaaaaaggctactagtagtaataataaggcggtagtactagagctggactgggacgcgcggcacaggatcgcgctcggtgtcgcgcagggcgtga
gctacctgcaccacgattgcatcccggccgtggcgcacagggacctcaagcccagcaacatcctgctcgacgccgacatggagccgcgcgtcgcgga
cttcggcgtcgccaaggcgctccatggcaacggaggagcgcccataatatccgtcgtcgctggctcatacggatacatcgcaccagagtacacgtaca
cgctgcaagtggacgagaagagcgacgtgtacagctacggcgtggtgctgctggagatcctgacggggcggcgaccggcggaggcggagtacggcg
aggggagcaccatcgtggactgggtgcggcggaaggtcaccgccggcggcgaggccgggctgcgcgaaatcacggcgtgggcggcagacgagacc
tgctgtgacgacgaggagcgagacgagatggcgctggcgatgcgggtggcgctgctgtgcacgagcaagtgcccgaaggagcggccgtccatgaga
gacgtcgtctccatgctgcaggagaccaggcgtgaggaccggaagaacaaggccaacaaggaaacgcacaaggtgatgaactgttag
>g25167.t1
atggcgacgctccagcaccagcagaagccggcggcgccgatgacgacgacgagcggcggggcccgcgcgatggacctgtacgagaagcttgagaa
ggtcggggagggcacctacgggaaggtgtacaaggcgcgggagaaggcgacgggccggatcgtcgcgctgaagaagacgcgccttcccgaggacg
acgagggcgtgccgcccaccgcgatgcgcgaggtctccctgctgcggatgctgtcgcaggacccgcacgtcgtgcgcctcctcgacctcaagcagggc
cagaacaaggagggccagaccatcctgtacctcgtcttcgagtacatggacaccgacctcaagaagttcatccgcggctaccgcgccaaccacgagaa
gatccccgcgcacatcgtcaagatcctgatgtaccaactgtgcaagggcgtgtccttcatccacggccacggggtgctgcaccgcgatctcaagccgca
caacctgcttatggaccgcaagaccacggcgctcaagatcgctgatctcggcctcagccgctccttcaccgtacccctcaagaagtacacccatgagat
cctgaccctgtggtacagggcgcctgaggacgacgagggcgtgccgcccaccgcgatgcgcgaggtctccctgctgcggatgctgtcgcaggacccgc
acgtcgtgcgcctcctcgacctcaagcagggccagaacaaggagggccagaccatcctgtacctcgtcttcgagtacatggacaccgacctcaagaag
ttcatccgcggctaccgcgccaaccacgagaagatccccgcgcacatcgtcaagatcctgatgtaccaactgtgcaagggcgtgtccttcatccacggc
cacggggtgctgcaccgcgatctcaagccgcacaacctgcttatggaccgcaagaccacggcgctcaagatcgctgatctcggcctcagccgctccttc
accgtacccctcaagaagtacacccatgagatcctgaccctgtggtacagggcgcctgaggttcttcttggcgccacgcactactccacgccggttgaca
tatggtccgtcggctgcatctttgctgaattgatcaccaacacggctctcttccctggtgattccgaggttcagcagctcatgcacattttcaagttgctggg
taccccaaatgaggaggtgtggccaggagtaagcaagctgccgaattggcacgtgtacccccagtggaagccctccaagctggagactcttgttcctg

ggctcgatgccgatggctacgatttgcttgagaaaatgctgcaatacgagccgatgaagcggatctctgccaagaaggccttggtgcacccatacttca
acggcgtgaacaaggagcagtactga
>g25367.t1
atgtcttgtgcttacttgatccaccaaaagaggagcctcgctgctgtgaaacagaggtacttcaggcagcacgggggcctcttgttgttcgaggagatga
ggtcaaaacaagggctctccttcacattgttcaccaaggaagagctccaagaggcgaccgacaaattcgatgagcggaatgtgcttggcaggggagg
gaacggcaccgtctacaagggagcactgagagacacaagattggttgcagtaaagaagtgcaagataatcaacgcgagacaggagaaagagttcgg
gaaggagatgctcatcctgtcccagatcaaccacaagaacgtggtgaggatatatggctgctgcctcgaggtggaagtccccatgctcgtctacgagtt
catcccaaacggcacgttgtaccagcttatccatggtcggccccatggctcacgcatttccttcacgacgcgcctgaagatcgcgcatgagaccgctgaa
gccctcgcgtacttgcactcttgggcttcgccaccaatcatccatggagatgtcaagtcacccaacatactagtcgatgaggattataccgcaaaagttg
cagactttggtgcttccacattggccccgacagatgaagctcaatttgtcacatttgtccaagggacttacggttacctcgatccagagtacatgcagacc
agcaaattgacgagtaagagtgatgtgtacagtttcggtgttgttctgttggagttgcttacatgcaggaaggccatgaaccttcaggcacttgacgagg
agaagaacctctcatcacatttccttcaagccgtgagcgagaacaggcttgatgagatactggatgaacagataagaggtgaacacagtgtcgagctg
atcgagcaggtggcggagctggccaaggagtgtctcgagatggctagcgacaagaggccttcaatgcgggaagttgcagaagagcttgatagaatca
ggaaactgttgcggcatccttggggccaacaaacttcagacgacgagcgaaaggccttgctatctggatcaccaagcacgtgccctgaagtagaacta
agtaatgggtttgttagtctcactgattcagcttacttgggaattcagtccccacgtaccgacataaagcccgactcgccgtgcaaggagggcttcccact
agcccatgaagcttacaagacacacctggacgttcatgaggagaaggccacttatgctgacctaattttagatgaagaaactgatgatgatgatgagta
catccctgactctgatgaggatgaccaggatgactttgctattgatgatgaagttggtgtgacatttgaggatggaattacttttaagaaggcaattagac
actatgctattttgaatgagattgaaatgcacctcactatagtgaatctacaagatgagactacactaggtgcatcagctcttcagaagaggcttgagga
gaagcatttggtcaaattgtcctactacaaggattga
>g25390.t1
atgatgggagctctgcaggtgcagagcaatggtgaggacgggctcttcttccccgaccccgaccctgggtatgtcgaggtcgatcccacaggcagatat
ggcaggtacaatgaggttctcggcaagggctcctccaagacagtgtaccgtgcgttcgacgagtatcgtggcatggaggtcgcgtggaacaaggtcca
gctccacgacttcctccgcagcccgggtgacctggagcgtctgtacggcgagatccacctcctcaagtccctccgccaccgtcacgtgatgcgcctccac
gcctcgtgggtggacgcgccccgccgcgccgtcaacttcgtgacggagctcttcacctccggcacgctgcgtcagtaccgtcgtcgccacccgcgcgcg
cgcgccgccgccgtccggcggtggtgccggcaggtcctcgacggcctcgcctacctccacgcgcgcggcgtcatccaccgcgacctcaagtgcgacaa
cctcttcgtcaacggcagccagggccaggtcaagatcggcgacctcggcctcgccgccgtcctccgccgcggaggaggaggagggcagaggtgcgtc
gtgggcacgccggagttcatggcgcccgaggtgtacgcggaggcgtacgacgagcgggccgacgtgtactcgttcgggatgtgcgtgctggagatggt
cacgctcgagtacccatacagcgagtgcgatcaccccgtgcagatctacaagaaagtcacatccggagtgaagccggcggctctgcagaaggtgagg
gaccccatggtgcggcggctcatcgacaagtgtctggcgccggcggcgcgaaggccgtcggcgatcgagctgctcaacgacgacccgttccttcagac
ggaggacgacgggttcatcagctacggcgacgacgacggcgactacggcgccatgtacaattacctccaccagcctgcgtgtctgctggactaccacc
ccgccggcggtagcaacggctcctcctcggcgacgaacggcggcggcggtgatgatgaggaggatcagccggcgccggaggacaggtactgggact
gcgtcgaggatgaggatgatgacgagaggaggtttcaagggatcgacttgctgttcaacgagcacgaggacgacgagcacgtcgccggagtggatat
cgcgatcaaggggaagaggatggacgacggcaccatcttcctccggctccgcatcaccgacaagaacgacccagggctggtccggaacatctacttc
ccgttcgacactgagtctgacacggcgctgagcgtggcgacggagatggtcggggagctggacatcacggaccacgaggtcacgcacatcgccgaca
tgatcgacggcgaggtcgccgcgctgctgccgcactggacgcgggggggccgccggcacgatcaacgacgacgacgcgtgctgccgcaactgccgcc
agtccaccagcgcctcctccggcggctcgctcgccgactacgtgtcgcgccgccgcgggtgccggtgcgccgagctgcacggccggttcgaggagatc
acggtccaagccgacgaggagcaggtgcagttccagagctccgggggcagctccgacgactga
>g25417.t1
atggattctgcacgaagttggttccagaagttccagccgagggacaaatccaagagtccagcagtgcccgccagccatggaaaggacccgggaaagc
cccaatggatgatgccccatctagtgcgactaaacagagggttgccgctgcaaagcagtacattgagaaccactacaagactcaaatgaagtccctg
caagataggaaagagagttggttccagaagttccagccgagggacaaatccaagagtccagcagtgcctgccagccatggaaaggacccgggaaag
cccccaatggatgatgccccatctagtgcgactaaacagagggttgccgctgcaaagcagtacattgagaaccactacaagactcaaatgaagtccct
gcaagataggaaagagagggatatcaagccagataatctattgctggatcgcagtggccacctgaagctttctgactttggtttgtgtaaacctttggac
agcagtaattttccaaatttgaatgacccagattatacatctggaaaaggtgccaaacctttgactgataacaccagtcggttgggcaatcctcctgcatc
taggcgtacacagcaggaacaattgtcacattggcaaaagaaccgccggatgttggcatattcaacagttggcacacctgattatattgctccagaagt
gctactgaagaaaggatatggaatggagtgtgactggtggtcccttggtgctatcatgtatgaaatgcttgttggttatcctccattttattcagaggatcc
aatgtcaacctgcagaaagatgcttccttccaaagatgcaaactttgttggatatacatacaagaactttgaaattgtgaatgatgatgaagttgcagga
attggttag
>g25425.t1
atggctgccgctgcccccgccgcctcctccccggcgccgcagcccatcattgccacgcccaccgacacctctgctgcccctccgccttccctctcgccgct
ggctgcttctccacgctcccccggttgcccttgcccacacccttgccttcgcctccgccgccggcactctcgcccccctcccacacccttgccttcgcctcc
gccgccggcgctctcgccccctccctcacccttaccttcgccttcgcctccgccgccggcgctctcgccccctccagcaccctcacctgcatcagcccttc
caagccaagaagcaggacgacgccttctgctgcgtccccggcgcctgctcctaccaatgaatatgatgctccccttctccggccgtcgtgctgccatca
tcaccgagtcgccacaagcacttgcccacgcctgccacggcagctgacccggcccacccaaagaatcccagtcccagcaagtcctcctcgagtggaag
gcttagtactggtgttatcataggcatcggcggtgcggtggtcgctcttcttgttcttagcctgatagcagctgctgttttgtacaccaacaagcgtaggcg
caagagggaagcaaatggttaccgtgctggattcatgtcacctacctctcctttctcctctcagccgccatcaggtggctcatcagttgacccttctgtcca
aaccagatatagtgccgggacccctaggctgcaaccgtgcgtatctgatattagcatgggcaattccaggttcttcgcgtatgaagagctctatcagatt
actgatggtttctcggttcagaaacttctgggcgaaggaggttttgggtcggtatacaagggctgccttcctgatggacatggaaggcctgttttggaatg
gactgccaggattaagattgctgctggtgctgctcgtggaatagcatacctacatgaagactgccatccaaggatcatccatcgagacatcaagtcttca

aacattttgttggataacaactttgaggcaaaggctaggccattgctttctcgagctgttgacacaggtcatttggaagggttgattgacccccaacttga
gaagaacttcaatgagctagagatgttccgtatgattgaggctgctgcagcatgcatccgacattcagcgtcaaggaggcccagaatgagccaggtgg
tgcgagttcttgatagcttagcggatattgatctaacaaatggtgttcagcctggacaaagtgaacttttcaatgtcgcccacacagaagagatacggat
gttccagcggatggtggctggcacacaagatgatagctccgacttgagccaatacagttggaacagccacagtagaggaggtactgacgctgctccaa
gctccaggatcttgtga
>g25501.t1
atgggcaagcttggtgctggccactaccaccgccatgatcacgtcggcagtgacaattgtgaagggtgcgctgaggtttatacctgctcctctccactct
gcggctgctgtggtggggggctgaagaatgaggacgacctggagatggtcaggagctctagctccaccgtgtatgggagataccagatcatagatgat
cagacggagatactggatgattatgtccatgatgggtttcagttgaagcagagaggggatgttatacttgagtgcgatatgccgaggggtcctgctcttg
aagatgaccatttggagcagagtgttgtggagaaggaacttcaaatgctcagctcatttgatactgacgctgatgtagatcctgtttctaatcatggggta
catgatgtcgtgggcattgatttgctcaatggcggcatggacaaaaatatgaaaagcagctgtgataaagaatacctaaaacaaggccataggatcca
gttattcccggaaagcagctaccctgatgattcctatgactttggagatattgggtcactaaatgcagatgtgcaacattctactgttcccaaggctgagg
acgattcacaaacaaatattgatcttgctctctctaattttcaacgagaatatgagtcacgttcttatagggctcctgaggtcatcttgggcctgccatatg
atcagaggattgatatttggtctcttggctgtattctagctgaactgttcactggtgagtcacgttcttatagggctcctgaggtcatcttgggcctgccata
tgatcagaggattgatatttggtctcttggctgtattctagctgaactgttcactggtgaggtattatttccaaatgaaccagtgtccacgatgcttgctcaa
atgactgggataattggtccaattgacatggaaatgctagaacttgggcaggagacgcacaagtatttcactgacgattatgacctttttaccaagaatg
aggaaaaggccaacggctga
>g25754.t1
atgtctgcgtccaagagaactaatagttccaccaccaccgggaagctctcaacaatcagcaccagtaccttcatgccatcaacggtcagtggtgttagc
acggatgacgattttgtagagggccagattctcgagtctccaaacctcagaatctacacctttgtggaactcaagaatgccaccaagaacttcaggcctg
agacagtgctcggggagggcggcttcggcaaggtttacaagggatggatcgacgagaagaccatgaacccgtccaggagtagcacaggcatggtgg
ttgccgtcaagaagctcaaccccgagagcgtgcagggaatagaacaatggcagaatttcaatgcgaagctatcagatttcggattggccaagcatggt
ccggatggtgaagagtctcatgtgacaacaagagtcatgggcacttatggctatgcagctcccgagtatgtttctaccggccacctatatgtgaagagtg
atgtgtatggcttcggcgttgtgctgctggaaatgctctctggcctaagggcactggaccggagccgccccagtgagaaggtgaacttggtgaactggg
caaagccattgctggtcgataagaggaagctgagccaggtaatggacagccggcttgagggacaataccattccaaaggagccctccaggcagctca
actcacactcaagtgcctgaacggtgaccccaagagccgaccatccatgaacgaagtagtagagaatttggagaagattgagtcgatgaagagcaag
acgagggagcccaggcacagctcctcgctcactcgcggtcaggcacattcaccgagaagggacagctctaggaagagctccaggggcagatag
>g25896.t1
atgggtgtggtctcttctttcacggagtgggcaaacaagctgctgacccgcgtccaccgcgacctaatccacgccatcggcacgcacgctgcgttgatga
cgacgacggtgcccgacaaccaccaccgcaccatggccgccgccaccaccaccagcgacgaccctgccaccgtcgagaacttcctccgcgaggtcgc
gcacgacaacccggtccggttcaccccgcagcagctcgccgccatcacccgcggctactcctccccgctcggctccggcgggttcggcgacgtgtacaa
gggcgcgctccccaacggcgtcgcggtggccgtcaaggtcctccgctccggcgaccacggcgggccggggcggcggtcggcggcggagcagtcgttc
atggcggaggtggggaacataggacggacccggcacgtgaacctcgtccgcctcctgggcttctgctaccaccaccaccacgcccccaccaccaccct
ccgcgcgctcgtgtacgagttcgtcgagctcggcgcgctagacaccttcctcctccaccacgactacgccggcgtcgaagccttacgcggcatcgccgt
cggcgtcgcgcgggggatccggtacctccacgaggagtgtggcgagcagaagatcgtgcactacgacatcaagcctggaaacgtgctcctggacggg
gacttgacgcccaaggtcgccgacttcgggctggcaaggctggtgggcccggacgccacgcacgtcacggtgtccgggccccgcggcacgcccgggt
tcgcggcgcccgagatgtggtcgcggtcggaggtcacggagaagtgcgacgtgtacagcttcgggatgctgttgttcgagatcgtgggaaggaggag
gaattttgatgaggcagttcccgagagccagcggtggtttcctgagatggcgtggaacaagtaccaggaagggaagcttgtggacctcgtcctctcgtc
gtcgtcgaggaggaggaggaggagcagtaatcagagtgttgttgttgctgcagtggatgaggaggaggaggaggtggtgcggcggggagagattgt
ggagaggatgtgtgagacggcgttctggtgcgtgcagcagcggccggaggcgcggccgccgatgagcgtggtggtgaagatgctcgagggcgagat
ggatatcccgccgccggtcaacccgttcccgcatctcacggcgttgcctgcggcggccatggcgtggacgacgacggtggtgagcggcggggacacg
gcaacgaccacgacgaagggcttgtctgagcgcagcgatgatctcattcggctttga
>g25917.t1
atggagatcgtggagaagtacgagaagctggagaaggtcggggagggcacctacggcaaggtgtacaaggcgcaggacaaggcgacgggccagct
ggtggcgctcaagaagactcgcctcgagatggacgaggagggcatcccgcccaccgccctccgcgagatctccctcctcaacctcctctcccactccct
ctacgtcgtccgcctcctagccgtcgagcaggccgccaagaacggcaagcccatcctctacctcgtcttcgagttcctggacaccgacctcaagaagttc
atggacgtctacaaaagagggcccaacgcaaggccgctccccacccaactcgtcaagttatgcaaaggagtggcacactgccacagccatggtgttct
tcacagggatttaaagccacaaaacctgttggttgacaaggagaaggggatactgaaaattgcagatcttgggcttggtagagcttttaccgtgccaat
gaaaagctacacccacgagattgtcacgctctggtacagagcacccgaggttttgcttggagcaacacattactccactggtgtcgatatgtggtctgtt
ggatgcatctttgctgaaatggtcagaaggcagcctctattccctggtgactcagaattgcaacagttgctgggaactcccaatgaggagcagtggcctg
gagttagtggattgagggattggcatgagtttccgcagtggaagacccagagtttgacacgtgtagttccaacgctggaacctgacggcattgacctgct
atcaaaaatgcttcagcttgatccggcaaacaggatatcagctaaagcggcaatggagcatccctacttcaacagccttgacaagtcgcagttctag
>g25932.t1
atgcatgcagctgccatggcgccgccgttgaccctcctcctcttcctgtgcttcgtgtcgtccgagctcgccgccggcgtccatggccagcatgtcgacac
tctcgggttcatcagcatcgactgcggcatcgatgagaacgcgtcgtacggcgaccagtcctcgcgcggcctgcgctacgtctccgacaccgggttcat
cgacgcggggctcaacggcgacgtcaacccgccgtacaacatcaaaggcctggccgaccgctacctcaccgtccgctacttccccggcgccgccggct
cgcgcagctgctacacgctccggccggtcaccaagggaggcaggtacctcgtccgggccaccttctactacggcaactacgacgattttgacaagctcc
ccgtcttcgacctccacctcggcgtcaaccgctgggtcaccgtcaacgtcacctccgctggccgggcttacatcttcgaggcggtggtggtgtcaccggc
tgacttcttccaggtgtgcctcgttaaccgagggctgggcgcaccgttcatttctgggcttgacctgaggccgctcggggataaaatgtacccggaggcc

accgtcaaccagtcgctggcgatgctcaaccttccccggccgtcggcgacatacagcttcaaccggtaccatttctggcggccggccagcacgtacaat
atattcaggtacccatttgatccgttcgacaggctctggcagtcctatggtgatgttgaggaatggacaaacataacaacatcaacagccgtcgatgtct
ccaacatcagcagtttcgatacatcatcaaagatactgtggagcgctgccaccccagtgaacagaacccagatcgacttcacatggagtccggattcct
ttgtaaacaatgacaacacaacatacctcctgttgctgtactttgctgagttacagagactgtcaagcaatgcactgagggagtttgatatccttgttgac
aatgctacatggaacggaagccagcgctacaccccaaagtacctttccgcagagcttgtaaaaaagatggtgcaaggatcaagtcaacacacagtctc
actagttgccacgacagatgcaacacttccaccaatcctgaatgcgttcgagatatattcagtgctgcctatgactgagcttgcgacaaatgatgcagat
gccagcgccatgatgacaattcgcacaaagtatgcactgaagaaaaattggatgggtgatccttgtgcgccaaaagcatttgcttgggatggcctgaac
tgcagttatacttcttctggtcctgcatcgataacaactgtgggcttatcatctagtgggttgagtggtgacattgatgcttcttttggtgatttaagatacct
ccaatccctggacctgtcaaataacagcctatctggtttggtacctgatttccttgcacaaatgccatcactaatgttcctgactgataacaatgcaaacct
gtgtgatgatggtgtgtctaattgtgagacagcgatcaagaaaatcaacaggatacttgtcacctacaaggaactaaagctcatgacagcgaacttcaa
agaagaaatagggcgaggaggatttggttctgtcttcctaggccacctggagaatggaagtcctgttgctgttaagatgtgttcaaaaacaacgtcaca
aggggataaagagttctcaacagaggctcagcacctgactagaattcatcatagaaaccttgtttccatggttggatattgcaaggaggagaaacatct
aggcctcatatatgaatacatgcatggaggaaacctagatgaccgactgagaggtttggagtatctacataaggcatgccaaccaccactgatccatag
ggacgtgaagacaacaaatattctgctatctgctgatcttgaggcaaagatatctgactttgggctcatgaaggtgtttgctgatgaattcaggacccac
atcacaacccaaccagcaggtaccctggggtatctggatcctgaatactacaatacatcccgaatcagtgagaagagtgatgtgtacagctttggagtt
gtactactagagcttatcacaggccaaccaccagcagtcactatcagtgacaccgagagcatccacattccacgatgggtgcgtgaaaagctctcagtg
ggtgaaattgagagcattgctgatcggaggatgggaggagagtatgatgtcaactctgtctggaaagtcgcagaattagcattgcagtgcatagagcg
gccatctcatgaacggccaacaatgactgaagttgtattggaggtcaaggagagcttggcaccggagttgttgcattcaatggggtactacagttcagct
ccgagcagcacagtcgacctcagtgcaacaagtattgacatgcggagtgatgcacaagcaagtgatgcaagacaggatactatgtttgagttggaaaa
gcttggcgatggatcagtaactcgcatggaacctgcaccacgatga
>g25972.t1
atggcgagcaccaacgccgccgaggaggaggcgccggcgacggtgggcggatacgagctgcgggagcgccttggagggcgccctccgtccacgtcg
gtctggcgcggggtgtcgcgttccaccggtgcccccgtggcggtgaagcaggtacggctcgccggcctccccgcccgcctccgcgacagcctcgactg
cgaggtccgcttcctcgccgccgtcagccaccccaacatcatccgcctcatcgatgtcatccagacccagagcttcatgtacctcgtcctggagctgtgc
gagggaggggacctggcggcctacatccaccgcaacgggagggtagaggagcgcgtcgccaggaatttcatgaaacaaataggagatggcttacaa
gtgctacgaaggcaccatgtcgttcacagggacttgaaacctcagaatatcctcctctcatctccaggcagcgatgcgatcatcaagatatcggattttg
gcctagcaaggtctgccactggtcgtgtacattcttgtcaccctatatcctga
>g26272.t1
atgcgcccgccgtgcttctccatcctcgcggtcctcctcctcctcgccgtccatctcccgcccgccgccgcggccaagcaccgccccgcgtccacctccac
ctccccagccttctacccctcgtacctcggcatctcatgggcgagcaacctcacgctcctaggctccgcctcgctcctccccggagccgccgccgtcgctc
tcaccaccccgtcccgcgacggcatcggcgcgggacgggctctcttctcggagcccgtccgcctcctcctcccctcctccgcggcacctgcttccttctcc
acccgcttcaccttccgcatcacgcccgcgcccacctacggcgacggcctcgccttcctcctcacctcctcccgcaccttcctcggcgcctccaacgggtt
cctgggcctcttcccttcctcctccgcctccgacgatggcgaggccgaccccgcggcgtctccaccgtcgctgtcgagttcgacacccaccgcgacatc
gcgctgcgcgaccccgacggcaaccacgtcgcgctcgacgcggggtccatcttctccgtcgcgtccgcgagtcccggcgtcgacctcagggccggcgt
gcccatcaccgcctgggtggagtaccgcgcgccgcgccgccgcctcgccgtgtggctctcgtactcttcgtcccgccgccccgagaagccagccctctct
gttgacgtcgacctctccggcctcttgcgcacgcacatgtatgccggcttctcggcgtccaacggcaacggcgctgcgcttcacgtcgtcgagcgctgga
ccttccgcaccttcggcttcgcagccaaatcatcccaggctccgccaccgtccgagcccccaatgccgcccagcaagccgctgccgcttacaggaagct
accacctccaccgccaccacttcctattgttcaaggtgcttggtggagtcctctgtggcgtgatcttgcttgttcttgtcgtcggtggctctgttgtctgcctc
tacaagccaattcgccgcacaaaacaagaacctaggctggtgcctaatgaggacacacctcttgggacaatgcctattgaagtggtgcgggaagcaac
aaaggacttcaacagtggcaatgtgatcggcattggtggttctggtgccactgtgtatgagggagtgctgtctggatcaagggttgctgtgaaacggttc
cagcaggcttattattcatgcagaaaaacatttgtgagtgagcttaccgccatgcttagctgccctcatcaccccaatcttgtaaggctgagggaatggta
ttacggtaaggatgagctcgtgcttgtttatgatttcatgcccaatgggactcttgacagtgcactgcatgcatcgggtggagcaacacttccctgggagg
cacggttcaggtcagtacttggcatcgcctcggctcttcaacatttgcatgatgggtgtgagcaccggattctgcatcgtgatgtcatgtcatcaaatgtga
tgctcgatggagagttcaatgcccgacttggtgattttgggcttgctcgcctaatgagacatggtgggaggccattgacaacacagccagcaggcacag
ttgggtaccttgctccagagtacgtgtattcaggtgttgcaacggaacacactgatgtttacagctttggtgtgctagctctagaggtggccactggccgg
cgtcccattgaaagggaagtctctgttgttgactgggtatgggttctttggggtcgacggaggcttgttgatgcagcagaccagcggcttcagggacggt
ttgttgcagaggagatgcggcgggttctgcttgtgggtctgtcttgtgtgcatccagatttccggaagcggcctggtatgcgaagggtagtcaggatgctt
gatggatctgcacctttgatagtgccgccagataagaagcctccagttatgctgcagatgcgagtaaatcaagcttcatcaatgaattccatggagactg
tcaatacggcattttacagctgtcgctga
>g26575.t1
atggcttcagcaggtgtggccccatctgggtacaagaacagcagcagcaccagtatgggtgttgagaagttacctgatcagatgaatgaattaaagat
acgggatgataaggaagttgaagcaaccatcatcaatggcaaggggacagaaactgggcacataattgtgacaactactagtggcaagaatggtcaa
ccaaaacagacagtgagctacatggctgagcgcattgtaggtcaaggttcttttggaattgtcttccaggctaaatgcttggagacgggtgagactgttg
ccataaagaaggttcttcaagataagcgttacaagaaccgagagctgcagaccatgcgtcttcttgaccaccctaatgttgtagctctgaagcattgtttt
ttttcaacaactgaaaaggatgaactatatctgaatttggttcttgagtatgtgcctgaaacggttcatcgagttgttaagcattacaacaagatgaacca
acggatgccacttatctatgtgaagctctatatgtaccagatctgtagggcactggcctacattcacggtagcattggtgtttgccacagggatattaagc
cacaaaatcttctggtgaacccacacactcaccaacttaaactatgtgactttgggagtgcaaaagttttagtcaagggagaaccaaacatatcatacat
ttgctcccgatactatagggcacctgagctcatttttggtgccactgagtatactacagcgattgacatttggtctgcaggatgtgttcttgctgaacttatg
ttaggacagcccttgtttcccggtgaaagtggtgtggaccagcttgttgaaatcatcaaggttcttgggactcccacgagagaagaaattaagtgcatga

acccgaattacacggagttcaaattcccacaaattaaagcacatccgtggcacaaggtatttcacaaaaggatgccacctgaagctgttgatcttgtgtc
taggctgctccagtactcccccaatctacgatgcactgctgtggaggcactcattcacccattctttgatgagcttcgagacccaaacactcgccttccaa
atggtcgatttctgccacccctcttcaacttcaagcctcacgagctgaaaggagtcccagcagacattgttgcgaaattgatcccagaacacgcgagaa
agcagtgcccttatgttggagtatga
>g26709.t1
atgatggcaatcaatggctttataattgcggctgttgctgttggattcgtcatatttctgctagctgctgtgatgttcactagatgcttttcaaagaggaagg
agaagaaagatggagtgaattacaatggcgacagaatggttgatggtgtgaggattgagaagcacaaagaggatatcagtagtggtgtccagatggc
tcaaaagaataagttggttttcttggatggatgcagttacaactttgacttggaggatttactgagagcttctgcggaagttcttggcaaagggagctatg
gaactgcctacaaggcaatacttgaggatggcactaccgtggtcgtcaagaggctaaaggacgttgtggcaggaaaaagggaatttgagcagcagat
ggagctcattgggagggttggcaaacatgcaaacctcgtacgttttcgtgcctactactactcgaaggacgagaagatagttgtctatgagtatagttct
actggcagcttctcagctttgttgcacggtattaaaggcattgctgagaagaccccattagattggaatgccaggatgaagattatacttggaacagcac
gtggtattgcacatattcattcagaaggtggctccaagctcgcccatggcaacattaagtcaaccaatatcctgctggatcaagaccacaatccttatgtc
tcagactatggactgagcgctttgatgagccttccgatccacccttcccgaattgttattggttaccgtgctccagagacctttgaatctaggaaattcacc
cataagtctgatgtttacagctttggtgttctcctcatggagatgttgactgggaaagccccctccagtctcagggacaggatgattgtgttgatctgcca
aggtgggtgcattccgtggtccgagaggagtggactgccgaagtgtttgatgtccagctgatgaaatatcccaacatagaagatgagctggtccagatg
ctccatatcgccatggcgtgcacgtcatggtctccagaccgacgtccgacaatggtcgaggtgatcaggacgatggaggagcttcggccgtctgcttca
gagaactacacttcttga
>g26719.t1
atggctagaccagctatggtagtggttggagggcaggacggctcgacggcgatggcgccgccggccacgacggtggtggcaggcggttgggctagtg
atggaggcaggggcgttgagtttggaccagttaacgtggaggtggtagtgggcggcggactgacggctcctgtggccggcaagacaagggcagaggt
cggcggcgagggcgtcaaggttggacgggttaacgtggaggtggtggtgaacaacggatcctatgattttggaatatcaaaattcttctgtgatcagaa
acttgaaactcgtgccacgagtgctataggatcacttggttacatggcaccagaatatttaagcggcaatatagtctcaaaaatggcggacatatacagt
ttgggtgttataattatgcagattataacagggcgcagggtcgatgtaacatcttgtgaagacattgtggaacctttgatggagtgcaacaataatatgtg
ggaccaggaatcacactataataaaaacgaaggaacattcgaggagattgtcgaggaaataatattaatgggagacgctgatgatgatcagaatggc
tttccagatgaggaagaaatgccgccaagcgtaatgaatgcaaagctattagtaggagcaacagagaaggaggagctcggagagatgggcgcgcgt
agtggcccggccgcagcccgggaggagacaacggaggagatggttgcgagaattggtggaatggtgcgggcggcgctggacgcagtgagggcggc
ggcagggttgtttccaggtctgtggaaggcgatcgcccgtaaattggagaagctccgggcatgcctacttaacccgtcctgctacccttgtcaccttgaa
aaatacgacactgcgctctcccgcgaggtgctgcaatcggttgcggacatgctcgccgcagcgaccgacatcgcggagcggtgtcgagcacctctgac
tgcaggcaagctgcagttgcggagctccatcgacgcggtcactgtgaagctggacgtcagcctccgcgactgcgagctgctcctcaagatcggtgagct
gtatgatgactcctcatcaccgcctcccctggacgacattactgcatgtaccagtgtccgggagctgcagcgactgttggcgttgctgcagatgagccac
acggcggccaagaagctcgctttggatgggcttctcgaggcgctgcacaaggatgagaagagtgtgatcttcatgctccatgccgccaacgtatccacc
atggtgcagctgcttaccgcgtcgtggcccatggtcgtccgggagaaggccgccactgtggtatgccacatggcggggagcagcagcttcaaggacct
gctcgtgtcggagggcgccctcgtgccgctcattcggcttgccgagtccggcggcctcgtgggccgccagaaagccatggtcgcgctacatcatctgtcc
tccaagtggtacagcaccgcgcgggccatcgtgtcgcacggcggggtccgcctgttcctcgagatgtgccgccagacgagcgttgattccgtgtcccag
tctgccgcggccggcacgctcaagaacatcaccgtggcgccggatttccggcaggcgctggccgaccatggaaccgtccgcgtcatggtggacctgct
gcgccgcggtgatgctgcgccggaatccaaggagcacgccgtgcagttcctgcagaacctcacggcacggtacaacgatgacgggctgcggcggtcc
gtcctgtcggagggcggcctgctcgccctgcttctctacctccgtgacaatgacgaggcggtggcggtgaacgcgatccgcaacctcgtcgatgtcatct
ccaccactagcggctcggcagatgatgacacgacgacgatgaagcgcgtggcgggcgagcaggggtgcgtgccgctactcgtgcggatgattcagga
ggacggcaactccgacagcgcgcgcgaggtggccgcgcaggcgttggccagcctggcgacctaccctcccaatgtaagggcgatgaccaaggacga
caagtgcgtggccggactggtgcagctgctggaccagagccctgatgacaacaccgcaaaggagtacgccatccagtgcctcctctcgctcgcctcca
ccaagcgctgcaggaagctcatgatctcgcacggcgctaacgtgcaccttaggaagctctctgacacggacgttgaaggcgccaccgagctgctacaa
aggctggaacgcggcatcctccgaagcctctttagtggtagcaagcagtaa
>g26750.t1
atggaccgcagagaatcctccaccatttcgccgcccttcaccggccaccgcaggcgccccaggccggacgccgacgcggaggaggcgtcgtccacga
agcgccacaagtctacccgtgatcatcctcaccacggggacatgggtgctctggcggaggccgtgggggcggtcgcgcttgtcgctacgccctcatcgg
ctgctggggggccggtgacggacgaggtcgaggacggtgaggatttagaccagggcgtcgcctccgcggaagcggggagggagctgggtgctgatc
ccggtgacgcgggggcagcatccggcgctcgcgtacctcctccgcacaaggaaggtggtttccgcatcgaggagaatgctcattatgacaattgggat
gatgcacaaggatattacaaataccgcattggggaagtgctatgtgggcgttatgagattttggcaggacatggtaaaggagttttctccaatgttgtcc
gggcaaaggatcttaaagctggcaaggatgacccttacgaagtagctatcaaaattatatgcagcaatcctataacggtatactcaaagcagctattca
tggcgttgaagcatcttaaaaagtgtggtgtcctccactgtgatatcaaaccagataatgtgctggtaaatggggttgcaaatgttctaaagctgtgcgat
tttggtagtgcttcgtctgctggaatgaatgcaatcacaccatatcttgtgagccgcttctaccgagcacctgagattagtcttgggttgccgtatgatcac
ccattggatatgtggtcagttggctgctgcttgtatgaactatacacaggagaagtcctatttcctgggaagacgaacaatgatatgcttcggcttcacat
ggaattgaagggccccttccctaagaagatgcttcgaaaggtaaatggggctgcaaatgttctaaagctgtgcgattttggtagtgcttcgtctgctgga
atgaatgcaatcacaccatatcttgtgagccgcttctaccgagcacctgagattattcttgggttgccgtatgatcatgcattggatatgtggtcagttggc
tgctgtttgtatgaactatacacaggagaagtcttatttcctgggaagacgaacaatgatatgcttcggcttcacatggaattgaagggtcccttccctaa
gaagatgcttcgaaagcaaaacaagccattgaattcgcagaatgtagctgatgcgctacagaagtttaatcttaagaagactgcagtacaaaaggcac
tggacgcattggctgacagtggacagatttccttcaaggagtatggcaagcagaaaatttacatcgctcggcaagatcaattcgacattccaaatgcgg
aagaacttgaggagctgaagaaagcaaatagcaggttacaggaagaactcgctgatcaaaagaaagcaattagtgaggttgaatctgaggtgcgag
gtctgcagtcaaacttgactctggaagagatcaagtcggaggagactaagttacaaagtgaagtccaggaaatggaagaaaaactaaataaactgcg

gaatggagtcatcttggtgaaacctgaggacaagaaggtcattgaggattcctttgctgagaaagttaaccaatggagaaagcggaagcggatgttca
aggagctttgggataatattactgagaacagcccaaaagatcagaatgaatttaaggaagaacttggtctcgagtatgatgaagatgttgatgtgaaca
tgcagtcttacactgacatgctggcaagtctcaataaaaagcgaaaaatttcccgctga
>g27040.t1
atggcgatcggcggcggcggcggcggcggcgggagctggagcatccacggccggcccgacgtcacctcccgctacgaggtcctcggccgtgcgggct
ccggcgcctacgccgacgtctaccgcggccgccgccgctctgacggcgccaccgttgccctcaaggaggtccacgacgccgtcagcgcccgccgcga
ggccgacgccctcctctcagtctcctccccgcacgtcgtcgcgctcgtcgaccacttccccggcggcgactgcgacgacgacgtcctcgtcctcgagtgg
ctcccgctcgacctcgccgccgtcgtgcgccacgccaggcgccgtgcccgtgacgatgggaccggcatacccgtcgcgcagctcaagcggtggatgct
gcaggtgctcgagggggtcgccgcgtgccaccgcgccggcatcgtgcaccgcgacctcaagccggcgaacctgctcatctccgaggacggggtgctc
aaagtcgctgactttggtcaggccaggatacttcagcagacaacacctacttacctggatatgccacaaggtgaggaaagttctggtgtagagccgtgg
gtttcacaacaacaaccagagttacaaggaacagaaaaggaaccatcatctcatggatctgacgttcatctcaagcggtggatgctgcaggtgctcga
gggggtcgccgcgtgccaccgcgccggcatcgtgcaccgcgacctcaagccggcgaacctgctcatctccgaggacggggtgctcaaagtcgctgact
ttggtcaggccaggatacttcagcagacaacacctacttacctggatatgccacaaggtgaggaaagttctggtgtagagccgtgggtttcacaacaac
aaccagagttacaaggaacagaaaaggaaccatcatctcatggatctgacgttcatgtaagtcaggagccagagactctcactgcagctgactaccta
catgagctggaccaactccgagccaaatccagcgatgttgacaaaatgagtctgcaagatggaaatgcatcatgtcttgccacgtgcagcacaggaga
catcgaagatgatccattccgagcctcttacacatatgatttggaaggaatgggggaagagtctggtgccttcacatcctgtgttggtacaaggtggttta
gggctcctgagcttctatatggatcaacaaactatggactggagattgacctctggtcgctaggatgtattttggctgagttgcttaatttggagcctatat
ttcccggcatatctgatattgatcagataagtagaataattggggtcctgggcaacatcacagaagaaacctttccaggttgttcaaatttaccagactac
aacaaaattttctttaacaaggttgagaagccaacaggtctggaagtatgtttgcctaatagatctgcttctgaggttagtatcattaagcaactgctttgc
tatgacccagcaaagagggtaagtgcagctgtcctgttgaatgaccggtacttcacggacgaacccttgcctgcacctatagaaggattacaagtccca
gcttcaaaggatgatgatgatgataacagctctatggaagaatgggcaaataacaggaatggtggttcagattcagactttgacgagtttggtaccatg
gatgtatccagaactgacaagggtttcagcatacgcttttcctga
>g27130.t1
atgctgtatggttgttacagttttggaaacaatccacttctttgtggtcccggtacttcaaagccctgtcctggggctcccccgttttccccacctcctccgta
taatcctccatcgcctccagctcaatcgacaggtgcctctagcactggaccaattgctggaggtgttgctgctggtgcagcattggtttttgctgttcctgc
aattgcatttgcaatgtggcgccgtcgcaaacctgaagagcatttcttcgatgtgcctgctgaggaggatccggaagttcatcttggtcagcttaaaaagt
tttcactgcgggaacttcaagtcgcaactgataatttcagcaacaagaacattttaggaagaggtggttttggaaaagtgtacaagggaaggcttgctg
atggctcgttggtagcagtgaaaaggctaaaagaggagcgtacacctggtggtgagctgcagtttcagacagaagttgagatgattagcatggcagtc
cacaggaatcttctccggcttcgtggtttctgcatgacgcctacagaacgattgctagtctatccatacatggctaatggcagtgtagcatcacgtttacg
agagcggcaaccatctgaaccacctcttgactgggatacaagaagacggattgcattgggatctgcaagaggactctcttacttgcacgaccactgtga
tcctaagatcatccatcgtgatgtcaaagcagccaatattcttttggatgaggactttgaggcagttgtgggtgattttggtcttgcaaaacttatggatta
caaagatacccatgtaacaacagctgttcgtggaacaattggacacattgctcctgagtatctttccactgggaagtcctctgaaaagactgacgtttttg
gctatgggatcatgcttctggagcttattactggacagagggcatttgatcttgctcgtcttgcaaatgatgatgatgttatgctgcttgactgggtgaaag
gactgttgaaagagaaaaaggttgagatgttggtggacccagatctgcagaacaactaccaagagacagaggtggagaacctgatccaggtggcgct
gctctgtacacagggctccccattggaccgtcccaagatgtcggaggtggtaaggatgctcgaaggtgatggattggcggagagatgggatgaatggc
agaaggtggaggtggtgaggcaggaggctgagtcggcccctctccgtaatgactggatcgttgattccacttataaccttcgtgcagtggaactgtccg
gtccaaggtga
>g27229.t1
atgtcgtcgtcgccggcgccgacctcgcctccgggcccgcccccgtccaacgccacggcgcctccccccctcgccatccgcggcagcgacgccccttcc
tccccgtcgccaccggccgagaccccgcctccgcccccgcgacgccctccgcgccgccaggcacgccctcgcccccgtcgccgaccgcgactacctc
cccaccgaccccgggctccacgccctcgacgtcctcgccgccgtcatcggactcctcctcctcctccgggctcaagagcggcgtcgtcgcggggatcgc
ggtgggcggggtcgtcgcgctgctgctcgcatgcctcctctgcgtctgcctcttcaagaagaagaggaagaagcgccatcacccgcacccgcccccgcc
gccgccgccgcacatgcactactacggccacccaccgccgccgcccccgccgcacaaaggggatcagtatggcgcgacgtaccagaactggcagcat
aatgcccctcctccaccaccacctaaccacgtggtcaatatgcagcatccttcccctccacctcctcctcctcctcctgttaatgcgcacagcagtggttcc
gcttcaaataactcaggcagtgacggccgtcctccactttcaccttcccctgcctttggcttctcaagaagcaccacattcacttatgagcagttggcggc
ggcgaccgacggattttctgatgctaacctgctcggacaaggtgggttcggctatgttcacaaaggagtgctgccgaatggaaaagaagttgctgttaa
gcaattgagagatggaagtggtcagggagagcgtgagttccaggcagaggttgagattatcagcagagtgcatcacagacatcttgtgtctttggtcgg
ctattgcagttctggggacaagaggctgctcgtctatgaatttgttcccaacaatacattggaattccacctacatggctatcctaggatcattcatcgtga
cattaaggctgcaaatattcttcttgatttgagatttgaagctaaggtggcagattttggacttgcgaaattgacttccgacaataacacacatgtatcaac
cagagtaatgggcacatttgggtaccttgctcctgagtatgcttcttctggcaagctcaccgagaaatcagatgtcttttctttcggagtaatgcttctgga
gctaataactggtcgtcgtcctgccaggcctttaatgacaaaagcatttgaggacggcaatcacgatgatttagtggatcctcggttgggaaatgagtac
aataataatgagatggaaagaatgatagcctgtgctgctgcatgtttattcaactattttcctgctacttcaatgcaattaaaggttgttcgggccctggag
gggatgtatcgttggatgatctgaacgaaggcgtccggccaggccacagccggttcatggggtcatacagcagctcagattacgacagcggacagta
caacgaggatctgaagaagttcaagaagatggcgctcggcagcagcggcctgcagagcagccagcagacgcccaccagcgagtacggccagaacc
catccgtatcgagcagtgatggccaccagacgcaggaaacagaggcggggagcgtaaacaaagccggagacagccaatcttcgtaa
>g27252.t1
atgggcaactgcgggacgagagaggagaatgccgtcgttcctgcgcacgcgcaaggaggatttgggactgtttacaagggttacatagacgagaatg
tcagggttggtctgaagtcattgcctgttgcagtcaaggtgctcaacaaagacggacatcaagggcacagagaatggcttgactatactgctaaactgt
ctgattttggcttggcaaaggctggcccagaaggtgatgagacccatgtatcgactcgggtgatgggaacttatggttatgctgcccccgaatacgtgat

gacaggtcacttgactgctagaagtgatgtctacagcttcggcgtcgtccttctcgagctcttgactgggagaaagtctattgacaagtcacggcccagc
agggagcagagtttggttgactgggcccttccgaagctctgtgacaagaggaggcttctccaaattatggaccctaaactggagggacaatatccagtc
agagccgcttacaaggcttgcagccttgcatattactgcttgaatcacaacccgaaggcaaggccgcttatgagtgatgtcgttgagacactcgagcca
ctgcagggcagcggtggacgtgatggaggtggtcaatcttctggccttactgactacagagttcgccgcaagctaacagggaacattgtccactacagg
gccatcccaaaccccaagtgctcccctgctgttccgacatgccgagtgaggtga
>g27619.t1
atgctgatgggcaagtacgagatggggcggacgctgggggaaggccacttcggcaaggtgaagctggcgcggcactccgagaccggccaccccttc
gccatcaagatcctcgaccgacaccgcatcctcgccatgaagatcgacgagcagataaagcgggagatcgcaacgctcaagctgctcaagcacccca
acgtcgtccgcctcttcgaggtttcagcaagcaagactaaaatatacatggtcctcgagtatgtaaatggtggggaactgtttgacaaaatcgcattgaa
aggtaaactgtcagagaaggcaggcagaaaattattccaacaactaatggatgctgtaagctattgccacgagaagggagtttatcatagggacctca
aggtaagcactgacctgtatttgaaacttgacgtcttctga
>g27641.t1
atggcgattgctactgagtcgtgcggcggcagtgaggacaaggctgcatcacatgcaaatgaggagaagcgttgggtgctgccagattttgaggttgg
aaagccacttggcaggggaaagtttggccatgtttacttggcaagagaaaaaaggagcaatcagattgtggctttgaaagttcttttcaagagccaact
gaaacagtctcaagtcgagcatcagcttcggcgtgaagttgaaatccagagtcacctaaggcaccccaatattctacgcctgtatggttacttctatgac
caggtacataatagataa
>g27650.t1
atgttcgctaggcggtgcaccagctacgggatggctgcggcggcagataggaaaaggaatgaagaaggagataggatccgtgttgtaatgaggaag
gggtgggacgggagccgtacagggaggaacgcccccgtgccactttcaggggtgctcggcagcggcaacttcggctcctcgtacaaggccacgctct
gcaagggccccgccgtcgtcatcaagcggttcaaggacatgaacggcgtcgcgagggaggacttctccgaccacatgcgccgcctcaggcgactcgc
ccaccctaacctcctcccgctcgtcgcatacctctacaagaaggaggagaagccgctcgtcaccgaccacatggtcaatggcagcctcgcgcaagctcc
tccatgcgcgatcttggattgggggaagcggttacggatcatcaagggcgcgacgcgggggctggcccacctgtacgacaagctgccgatgctgacgg
tgccgcacgggcacctcaagtcctccaacgtgctcctcgacgcctccctcgaccccgtcctgaccgactacgcgctggtacccgtggtgatgccgtccat
tgcgacgcaggtgatggtggcgtacaagtcgcccactaccggaaaccaatga
>g27702.t1
atgccttcccgccttctcctccagcagctgccgccgcccgcgccgcctcctcctcccctccacgaccaccaccgccacggcgccctcctcgccgccaccg
ttgcctccgccgcggcagccttcttcctcctccttctcgtggtggtcctcctgctcctcctccggcgccgatactggcgcctccggccgacgctccccttctc
ccctcctcctgccccggcgcgcccgctccgtcgctactcccgtcgcgccctccgccgcgccaccgccgggttccatccctcccgcctcctcggccgcggc
gccgcctcccccgtctacctcgccaccttcccctcctcgtcctcttcaacctccctcgcggccgtgaagacgcgcgcgtcaccgcacgagctccacctcct
ggcgtcgctgccggactccccgcgcgtcgtctccctcctcggctactccggctccggcgacggaaaagaagacgaccgcgccacgctgtcactggtgtt
cgagtacatgccgcaggggtccctccaggcggcgctgttcggcggcgacgagtctcggttcctggactggccgcggcgggtgggcgtggtccgcgacg
tggcccgcgcgctggcgttcctccacggcgagtgcgcgccgccggtggtccacggcgacctcaagcccagcaacgtcctcctggacgccgagttccgc
gccaagcttgcagacttcgggctcgcgagattcaagactcctgaagtcattgcttccgtcgctggtggtggggaccaagacttcatgagccaggagctc
ggcgaggcaggggaccacctctccaccacgacagcgacagcatctgtggctggcggcggcggcgctaataagggggacgagtcttctgggccggcg
agcgcgtgggggaaggagtggtggtggaagcaggacggcagcggcgagctcgattccaaagattacgtggccgagtggatcggcagccaaatcaac
tgtcccgggaggaacccagattgggcagacgacaacgatcagaacaagcactcgccctcggccgccacggacgagcacgccgcgtcgacctcgccg
gaggaggacgagaagaagcagaccaccgccgatgccaaatcaggcaatgctattgattccaagaaggagaccgcgacccagatgcgggagtggtg
gaaggaggagttcttcgaggagatgagcaagaaagccggcaccggcgcgggcagcttcgacaagcgccaccgcagcggcggcaccaagccgtggc
tccgttccatcagcatgaacaatgccgagccggggagcggaggcctcgacctcagcagcttccgccggaagcggagccggcggcagcgcggccggtc
cgccggcgccggcagcgacgccgccaacaacaacgacctcctcagccgggagctgagcagcacgacgagcatgcgcggcacggtgtgctacgtcgc
gcccgagtgcggcggcgaccacctcgacctgctggagaaggctgacatctacagcttcggcgtgctggtgctggtcatcctgtcgggccggcggccgct
gcacgtgctggcctcgccgatgaagctggagcgggccaacctcgtgggctgggcccgccagctcgccagagcggggaacgtgctcgacctcgtcgac
gacagattggacggaggctacgatagggagcaggccgccatgtgcgtgcatctcgcgctgctctgcctgcagcgccagccggagctcaggcccgaca
gcgcggacattgtcaagatgctcgacggggagatggaggtgccgcacgcgccggtcgtagagaactcgcagtcgccgagggtccgcccgttcccgag
gtcatcgcgacgagcggcgccgcagcaaaatgacacggccgccgccctcgagtga
>g27841.t1
atgccgtcgtggtggaagcgctccaagtccgccttccaccgccccgtcgccgcctcggcctccacgccgacctcccccgcgcgggcctccacctcgcgc
gcccaggccgccgccggccgcctcggcgacgccggggaccttctcgccgctccgccacggctcacgcggcagcggaagctgcgccacgtcgacgaca
tcgacgtcgtgcttggcgacctcggaatcggcgacgacgcgctgccgtcctcgccgccgccgccgcagaggagccgctcctccgcgtccgaggccctc
gggcccccgatctccgccccgatctcgaggtccgctagctccagggagagcggggccccgccgccgaggtcggcgtcgtcgcccgtgcttcatccgct
gccgctgccgtcgcagaggttcccggaatttgaggcgccggagccgccgtccagggtcgccgatgggtggggcgagagggcgacgccggtaccgtcc
tccaatgcgccgagttggaatgattttgctcttgtgtgtgatagagacaatggtaggacaagggatatcaaaggagcaaacctgttagttgatgttcaag
gtgtagtgaaattggctgactttggaatggccaagcatctaagtactgcagcccctaatctttcattgaaaggaacaccatattggatggccccagaggt
cggttcacggcagtacggacggtggagccacgcggcggctgcggaggcgccgatggcgactgcctcgcggtggacggagacgggcagccgggcac
cgggcgagcagcagtgcgagcaggcgatggctgtcgtgagcgaggtcggttcacggcagtacggacggtggagccacgcggcggctgcggaggcgc
cgatggcgactgcctcgcggtggacggagacgggcagccgggcaccgggcgagcagcagtgcgagcaggcgatggctgtcgtgagcgaggtgctcc
agcgatga
>g28083.t1

atgttctcgtggggccgtaagcagtcctcctccgccgcctcgccgtcctcgtcctcctcctcgagccgccgccgcggcggggcggacggcgggatggatt
cgagcatccgcggcggcggcgggagcgggagcgggagccgcggacggagcccgcggctggatcgccggaacgcggcgaagcacatcgagtacga
ggtcgggggcgcgggggcgtccgcgccggcgttcgcgtcgtggtcgtcgtcctcgtcggcggagcagcggtccccgggtctccgcccgtcgcgctcgct
cgacctggcgcccggcccgtccggcgccgacttccgcatcagcgggagcaccgagggggaaatcgacgagctctgccgcagcctggggctctccggg
cccgaggacttcgccatcccgatcgacgcctgggaggcgcgcaagccgcggtgcaactccgacctcctaccccgctcccgccccgcggcgtcccctgc
cgccggccaactctcgtcagtcgcccactcagtctccgccccggaggcccagctccaccagcttctctccgtccctgctccaattcctgaggagtccctcc
actcctcgtccacttccaccgccaccgactcggtggaggagcctaccgttgcgccgcccgtggaatcccccaaggccgaccccgtcgttgctgttgtggc
tcccgtagcggccttgcctctcccgtcaccaaggaggggaggtggcgaggtggggatacgtggcacccggcctcccttactctccccgcctcctccaatc
accgcactcgcaccgccaccgggaaggaggtcgtctgttgctcatgacatgactgggtctgcttgggatattctggagtcgtttgctcctagggaagaga
aaggtgaactgggacaggctcatgaacgaattgatccttctcacacgtcggactcagaggaggaggatgttgaggatgagtcggcagcagtgggagg
ggaactgaaagaaatgagaataggagagaccttcgaagggtttaccggaacttcttcgttatcgacaaccaatgatgacgatgcatcaagtacgaata
cggaggcaatgttcatcatctcaccaaatggcaagtttaagcggaagattaagtcttggatgcgtggtgctcttctcggaagcggctcttttggaatggtg
tatgaggggatcagcgatgagggtgcattttttgctgtgaaggaagtatctttgcttgaccaaggcagcaatgcacaaccatcaattctcgcacttgaac
aagaaattgcacttcttagtcagtttgaacatgagaacatagtccagtattatggaactgacaagatgtcaaaaattaatatgctaagatcatgcaaagg
aagtgtttactggatggcacctgaggttattaatcctaaaaagacctatgggccgtcagccgatatatggagccttggctgcactgtgttggaaatgcta
acccgtgaaataccatttcccaacgtggagtggattttcgtttccactcctgctgcatga
>g28352.t1
atgggaagcagagcagcagcgatactattggttgtgtctttgatcgtcctgtggcggtccgagatcggcaccgcgcggactgcgcagcgctgcggcgcc
ggcgacctggaagctctgcgcggcttctccgccgggctcgacgcggcggtggacggctggccggtcgcctccgacgactgctgcgcgtggcccggcgt
tgtgtgcagtaacgcggaggaggtgatcggtctggtgctgccgaaccggacgctacagggccaggtgtcggcgtcgctggccggcctcgccgcgctcc
gcgtgctcaacctctcgagcaacgcgctgcggggcccgctccccgcagacttgctcctccgcctccgccgcctcgaggtgctcgacgtcagcggcaacg
cgctctccggcaccgtcgatgagctcctcctcccgtcgtcgatgcgcgtgttcaacgtgtccaacaacgcgttcgccggcggccacccggtgctccg
cagcggcggcgcggcgaacctgacccactacgacgcgtcgggcaacggcttcgacggcgccgtccaggctgcccggttgtgtcaaggcgagtcccca
tctctgcgcgttctaaggctgtcgatgaaccggctctccggcgatttcccgttcgggttcgggcaatgccggtcgctcgtggagctcgcgctcgacggga
acaacatcggcggcgcgctccccgacgaccttttcagtgtgacgtcgctgcaaatcctcaccgtccacaccaactccgtctccggcgggctctccacgcg
attacgcaacctcagcagcctcgtccgcctcgacctctccttcaacgccttcacggggaaactaccaaacgtattcgacacgctccccgccctcgaggaa
ttctcggcgccaagcaaccggttctccggcgagctgccggcgactctctcgcggtgccggcggctgcgcgtgctcaacctccgcaacaacacgctcgcc
ggcgacatcgtcaacaatctggacttcaggaagctaagcaacctggcgtacctcgacctcggcgtcaacaacttgacgggtcccatcccggcgagcat
cccggagtactgtaaagggatgacggcgctcaatctcggaaggaacaggctcaccggcgagataccggcctccttcgccggcttctcgtcgctgtcgtt
cctgtccctcaccgggaacagcttcaccaacgtcacgtcggcgttactgacgttgcagtctctccccaacctgaccagcctcgtgttgaccaagaatttcc
tcggcggccaggagatgatgccatcgtcaggcataatcacaggtggtttccgaagcatggaggtgctggtgatcgccaactgcgagctccgcggcacg
atcccgtcctggatcgcggggctccggaagctgcgagtgctggacatctcatggaaccgcctcacgggccaaatcccaccgtggatcggggaatttga
ccggctcttctacctcgacgtgtccaacaactccctccacggagagatccccggaaccctgctggcgcgcttaccgggtctcatgaattccgacgactcc
ggggtgcaggagcaggagcaggcggtgcaggacttccccttcttcatgcggcgcaacacgtcggtgaagggccggcagtacaaccaggtgagcagct
tcccgccgtcgctcgtgctgggccacaaccacctcaccggcgccgtacccaaggagctaggggagttgaccaaggtgcacatcgttgacctgagctgg
aacaggctgtccgggcccataccgacggagctgtccgggatgagcagcgtcgagacgctggatctgtcccataacaatctctccggcacagtcccggc
gtcgctgaccagactcagcttcctctcccggttcgacgtctcccacaacaacctctccggccaggtccctgttggtgggcaattctccaccttctcgcgcg
acgactttgaggggaacccgtttctctgcggaatccatgtcgcgccatgcaccgggcatagcaaggagccaccggaggctgttgatacgaagcggagg
aacgcgggggcgtggtggcggcggcgatcagcgtgggcacggcgctgctccttgccgtggtcgcggcggtgacgtggcgggcgtggtccaggcggc
ggcacgaggacaacaacaacgccagggtggcggctgattacgacgatgaagatctcgaattgatgaacaagcagaacaagtcgtcgacgatggtgg
tgctgttcccccaaattgacgacgacgacgaccagcagcagcaagaaagtttgacggtggaggatgtggtgaaggcgacgggtaattttgacgagtcg
aggatcgtagggtgcggcgggttcgggatggtgtaccgcgcgacgctccccgacggccgcgacgtcgccgtgaagcgcctctccggcgagttctacca
gatggagcgggagttccgcgccgaggtggagacgctctcgcgtgtgcgccacgccaacctggtcccgctgcagggctactgccgccgcgcgggtggc
aacaacaacaacaacaaggcggcggaccggctcctcatctaccctttcatggagaacggcagcctcgaccagtggctccaccaccaccacgagtccc
aatcccttccgtggccggcgaggttggggatcgcgcgcggcgcgtcaaggggactggcccacctgcacgccacgtcggagccgcgggtcctgcaccg
cgacgtcaagtcgagcaacatcctcctggacgcgcggatggagccccgactcgcggatttcgggctcgccaggctcgtggcgtgccccaccgacacac
acgtcaccaccgacctcgtcggaacgctcgggtacatcccgccggagtacggacactcgtcggtggccacgtaccgcggcgacgtgtacagcctcggc
gtcgtcctcctcgagcttgtcaccggacgcaggcccgtcgacatggcgcgacccgccggcgacggacgcgacgtcacgtcgtgggcgctcaggatgtg
gcgccagggcagaggccacgaggtcgtcgacgccaccagcatcgtccaagcagaccataagagggaggccgtcaggatgctcgacgtcgcgtgcgc
atgcgtcaacgagaacccaaagtcgaggcccacggcgcagcaggtcgtacagtggctcgacgccatcgccgcctcatcgtcagagcacgacaggccg
aatattactggctctatgtag
>g28366.t1
atgcaaaatctccggttgttctcgtacaaggacatcaggatagcaacgaaaaacttccatcagtgcaacaaactaggccgaggtggctttggaacggtg
tacaagggagttttgagagacggtacttcatttgctgcaaaggtcctgtcttctgaatctgatcaagggatcaaggaattcttggctgagatcgagagcat
ttctgaagtcaagcacgcgaacctcgtcaggttgctgggctgctgcatccagaggagaaacaggatcctgatctacgaatacctcgagaacaacagcc
ttgaccatgcgttgcaggcttcaggtttggagaaaggagtaacgactctgagttggagcgctaggtcggacatctgtatgggcaccgccaaggggctga
gctacctccacgaggagcacgagccgaacattgtgcaccgggacatcaaggctagcaacgttcttcttgacgggaacaacaagcccaagatcggtga
ctttgggctggcgaagctgtttccggacaacgttacccacatcagcacaggcgtggtcggaacaaccgggtacttggcaccagagtattttgtgcacgg
gcagctgaccaagaaggccgacgtctacagcttcggggtgctcatctttgagatcgtcagcgggaggaggatctcgcagaccatccggtcagatacgtt

tctagtgcgcgaggcgtgggtgctgtacaaacagggcagcctgctggacatggtggacgcgagcatggaggactacccgaaggaagaggtgctccgg
ttcatccgggtggggctggcctgcacggaaaaaaaattggtgcacaccgtccgcaaaccttcagtttgcagacccgatctcgattgccgccacgtggag
aatatgtga
>g28550.t1
atggagagcgagatggcggacgcgccgcagcagcaggcggcgacgggcgtcaccgcggagccgcttgcggcggtggcggaggaaggggacggcg
cggaggggaacgagacggtggggtccaccctcaccatggagcgcgtcgccgccgcgaagaagttcatcgagaaccactaccgctcgcagatgaaga
ccctccaggagcgaaaggagagacgctttaaactggagagacagctagcttcttctcaagttcccaaggagcagcaaatcaatttaataaaagacctg
gagcggaaggaaactgagtacattcgacttaaaaggcacaaaatatgcgtcgatgactttgagctgctcacaatcattgggaggggtgcttttggagag
gttcgactatgtcgagagaagacgtccggcaacatctatgcaatgaaaaaactcaagaaatctgatatggttgtcaggggccaagtagagcatgttag
agccgaaagaaacttgctggctgaagttgctagtcactgcattgtgaagctttattattcttttcaagactcagaatacctttaccttattatggagtacctc
ccgggcggtgatatcatgacccttctcatgagagaggataccttaactgaacatgtggctagattctacattgctgagacaatccttgctattgaatccat
tcataaacacaattacatccacagagatattaagcctgataacctgcttctagacaagaatggtcacatgaagctatcagattttggtctgtgcaagcca
atcgattgttcaaagctctcaaccttgaatgaggaggaacctatgggtgaagacaacatgacagaatcaatggatgttgatagttctttgtctgatactgc
aaatggtagaacatggagaagtcaacacgaacagcttcagcactggcagatgaacaggagaaaattggcattctcaactgttgggacgccagactat
attgctccagaggttctgctaaagaagggatatggaatggaatgtgattggtggtccctgggtgcaatcatgtatgaaatgcttgttggatatccaccatt
ttatgctgatgatccaataactacttgccgaaagattgtgcactggaagaaccatttgaaatttcctgaggatgcaagttggaaaatcctcctaccagaa
caggttcaggaccctctagaaagtcttggcttctcgatttcttcaggttcaggtgcatcagtcacctaa
>g28591.t1
atgatgacgagcgcgagcgtgagagccttcacctggaaagagcttcaccaagccaccaacggcttcgacaagctgctgggcaaagggagctttggcg
aggtgtaccagggcacgatccggtcccaccagcttgtggcggtgaagaagctcgtcgactccaacgactacaccgagaaggagttcaccaacgaggt
gcagtccatcgggcagatccaccaccgcaacctggtgcgcatgctggggtactgcaaggaaggcaggcaccggatgctcgtcttcgagtttatgccgg
gcggctcgctccgcaccgccctcttcaaggacgacgacgacgacgaaaacaagcagcagcgccgcccgccgtggccgtggcgcgccgacgcgg
cgctcgccatcgcccgtgggctcgggtacctccacgacgggtgcatcgccccgatcatccactgcgacatcaagcccgacaacatcctgctcgacggcc
gcggcgtcccgaggatcaccgacttcggcatctccaagctgctggggagccaccaggtgcacgccaccgtcaccaacgtcaggggggaccagaggcta
catcgcgccggagtggcttcgtggagacgcgcgcgtggacacccgggctgacgtgtacagcttcggtgtcctgctgttggagataatttgctgccggag
gtgccaggagccggtgacgggcacggacgatgacaatgacgacgagacggtcacgctgttcgggtgggcggcgcagctggtgagcgcccggaggac
ggagctgatgctgctacaccacgacgccgtcaacgccacggaagatctggagagagtggagcggttcgcgcgcgtggcgttgtggtgcatcgatccta
acccggcgatgcgcccgaccatgcaccaggtggtgcggatgctggaggccagtgatgagcaggttcagtctcaggccctgcctgaccctcccggctgc
tacctggaatcgtcgcccttaatccctcatcttgcaggccaccgatcagtttga
>g28595.t1
atgggaagtaagagatcgaacctggactggccaacacgctttgatatatgcttgggcattggtaggggtctggcttatctccatgaagagtctagcatcc
gtgttgtgcacagagacataaaggccagtaatgtcttacttgacgcaaatctcaaccctaagatctcagattttgggctggccaaactttatgatgacaa
gaagacacatgtcagcacaaaagttgctggcacatttggttatcttgcacctgaatacgccatgagaggtcatatgacggagaaaattgatgtgtttgcc
tttggtgtggttgtattggaaactttagctggaaggccaaactttgacaacacgcttgacgaggacaaactttacattttagaatgggtctggcaactgta
cgaggaaaagcatcctctagacgtggtagaccccaagcttacagagttcaacgccgacgaggtgctccgagccatccatgtcgccctcctctgcaccca
ggggtcgccccaccagcgtccgtccatgtcacgggccgtctcgatgctcacgggggacatggaggtgggcgaggtggtgaacaagcccagctacatc
accgagtggcagatcaagggcggcaacaccggcacctacatgagcagcaatgtcagcgggcagccaagcatggcagcaagggcggccgcgtccga
caattcgtcgtcgttcctcagctcttga
>g29027.t1
atgactgccgcgaggacgcaaccgccgcagcagtcgccggcgcggcccgtggccaaggcgcccccgccgccgcgccagcacgcgcgtccaccacct
ccttctccttctccttctccttctcctccgccgccgccttctcctcggcctccgccgccgcgccaccagcacctcccgccgccttttcatcacgcgccgccgc
agccgcggcaccagggcgggccttcgtcgtcaacgtcagtgctgggcgtagacgtcccgcacgtcggctggctctcgcagcgcacgcccgtcctcggc
ctccgcggcggggtcctcgccgccgtcgcggccgtgatcgcgctgctctgcatctgcgcctgcctctgccgccggtgccggcgccgaacgccacgcctc
gccccgtgcccgcaccgccgtcctctcaaacaccgcgtgcacgcgcaccaccaggccacgacaacgccgaccggcggcaaggatggcgacgacgtc
gaggcggcgcggtggcgcccgccgacgttcgacccgccgatcgaggccatcaaggcggagcagaaggcgccgctgatgctggcgcgcggccccgcg
gggaccagcggcgagacggaagccagcgagagcgccgccggcggcggcgaagcggggccgcccgaggcggcgcggcgcgggtggggccggagg
ttctcgcgaagggagatagaggaggccacgtgcgggctcgccccgggaaacgtgatcggggagggcgggtgcggcgtcgtctacagaggcgtgctcc
gggacaacaccgccgtcgccatcaaaaacctccacaacaacaggatgcttgtgtaccagtacatggacaacagcaatctggacaagtggctgcaccat
gatgacagtgaaatcagcccactgacctgggacatcaggatgcgcatacttcttggaactgctaaagggttggcctaccttcacgaagggttggaaccc
aagatagtccatcgcgatatcaaatccagcaacattcttctcgaccggcactggaactccaaggtgtcggatttcgggctcgccaagcttttgtgctcgg
agaggtcctttgttatcactcgtgtcatgggaacgttcgggtatgtagcgcctgaatacgcgaaaaccgggaagttgaacgagaggagtgacgtttata
gcttcggagtgctcgtcatggagatcgtcacgggaaggactccggtggattacaccaggccggccgatgaggtcaacttggtagactggctgaagcgc
atggtcgccgagaggaaggtggaggaggtgctggaccccaggctgccggagaacccgccttccaaggcgctgaagcgggtggtcctcgccgctctcc
ggagcttaaacttgcccgggatttatctcctcatgcgtcggactgctacgacgacagatgataaaaggttgttatgcgagggaaaatag
>g29317.t1
atgcctcccgacggcgaggccgccgccgacctggcggcggacgagctgcagagcctcagctttggctcctcggagcgctcccgctccgcgtccaccgt
ctccaccgccaccgcctcctgctccacctcctactccggccccatcgtcccgccgccgccgccgcgcgccaccaccaacctcccctccgccgccgccgtc
ccgcggttcggaaccgtcgcgctctccgacatccggttcctgaagcggctcggcgcgggggacatcggcagcgtgtacctcgccgaggtcaagccgca
gcaggggaagaacaacgggagcggcagcgcgtccggcgcgctggtggcggcgaaggtgatggacaggaaggagctcgaaggaaggaacaagga

ggggcgggcccgcacggagcgcgagatcctggaggccgtcgaccaccctttcctcccgcgcctctacggcgtcgccgacggggaccgctggtcgtgcc
tcctcaccgagttctgccccggcggcgacctccacgtcctgcgccagcgccagcccaaccggcgattctccgaggccgccgtcaggttctacgcggcgg
aggtggtggccgcgctggagtacatccacatgatcgacatcgtgtaccgggacctcaagccggagaacgtcctcgtccgcgccgacggccacatcatg
ctcaccgacttcgacctctccctcaagtgcgaccccatcgcgccgactccggcgcacgtcatctccgaccccgtcgccctctccggccactcctcatcatc
cacatcctgcaccatttcatcgtgcatcgtccccgccgtgtcctgcttccagctcttccccggccgcggccgacggcaccgccgccaccgttggcgccgta
acaaaaagacttcttccaataatgttggtggttgtggaagcagtagctttccatctggaggtagcggcaacatggacctggagttcgttgccgagccggt
ggagctccgatccatgtcgttcgtgggcacgcacgagtaccttgcgccggagatcgtctccggcgagggccacggcagctcggtggactggtggacgc
tgggcatcttcatctacgagctcctgtacggcgccacgccgttcaaagggtacgacaacgagatgacgctggccaacatcgtggcgcgcgcgctcgag
atccccaaggatccctcggtctcgtcggcggcgagggacctcgtcaccgcgctgctcgccaaggacccggcgcgccggctcggcgccaccgtcggcgc
cgcggcaatcaagcgccacccgttcttcaacggcgtcaactgggcgctgctccggtgcgccgcgccgccgtacgtgccgccgccgttcaccgtggcga
aaaatgttaagaatgccgccggtgtcgccggcggcggcaacaacgatgacgacgaggtgtctgatgaggacagttgtcccggcacgcccgtggagta
ctactaa
>g29366.t1
atgggggaggaggagtgcgcggagggggaagcgaggccgaggccgagggcgaatcggagggtggtgggggactacgagctgcagaggctggtgg
ggaagggctcgttcgcaaaggtgttccgcgcggcgcaccggcgtaccggcgcgcgcgtggcggtgaaggcgatcgatcgtgagctcgttgacaagcg
cgtccacgagggtatcctccaggagagggagatcctcaacagcatctcccaccccaacatcctccgccttctcgacaccatcgatacggggaaggtgct
gtacttggtgttggaatactgcgatggaggcgacctcggttcctttctgaataagcatgggcggctgccggaggcgactgccaaggacttgatgcggca
gctcgcggaagggctgaaggtgctgagggggaggaacatcgtgcaccgggacctcaagccacagtcagacttgtggagtgtcggggtcattcttttcc
agttagtcacaggacagctgccatttccaggaagtaactattaccagctgcgccagaacatcctgacttctgaaaaactacattttccttcagcagttgaa
cctgatttatgccaagattgcattgatctgtgcaagagactgctgctccttgatccagagaagagaataagctttgaggaattcttcaatcacaagtttttg
gcaacaaggtaa
>g29397.t1
atggcctctccaaccctactgaacctgctttcgctgctctttttactgctgctcggaggagtcgtcgcctcggcctccggcggcggcgagcgcgaggcgct
gctgcagttcaaggcggcggtcgcctccgaccccggcgggctgctgcgggactggacctcggcgtccgcggaccactgccgctggcccgggttgtcgt
gcagcgcgggcggcgaggtggtcgcgctcaacatctcctcctcgcccggccgcgcgctcgccggcgcgctgtccccggccgtcgcagcgctgcggagc
ctccgcgtgctcgcgctgccgtctcacgcgctctccggcccgctcccgctcgcgatctggtcgctgcgccgcctccgcgtgctcgacctctccggcaaccg
tctccaggggggagatccccgcggcgctcgcctgcgccgcgctccagacgctggacctcgcctacaaccagctcaacggctccgtgcccgccgcgctcg
gcgcacttccgggtctgcgccgcctttcgcttgcctccaaccgcctcggcggcgccatacctgacgaacttggtggcgccgggtgccgcagcctgcggtt
ccttgacctctccgggaacctgctcgtcggcggcatcccccgcagtctggggaactgcagcgagctggtggcgctcctgctgtcgtccaacctgctggac
gatatcatcccgccggagattggccggctcaggaacttgcgggctttagacgtctcgaggaacagtttgagcgggcctgtgccagcggagcttggtggc
tgtgttcagctctctgtgcttgtgctgtccagtccatatacaccgcttggtgattcaaattcgtctggttatgaggatgcggacgacttcaactacttccaag
gagggattccggatgccgtcgcttcattgccgaagctgagggtgctgtgggcgccgagggctacattggaggggaaattgccagagaagttggcgatg
caggggggcctatgcttttctggctgatgggaatcatcttgagggtcagttgcagcctggcttctttgataagtgcaacagctcaaagggcttcattgtgga
agtgagcaacaacttgataaatggtgcaattcctgcagagattggctcactgtgcagttctcttgttgtacttggtattgctgaaacccatgcaacaacag
gtgtagctggaacttttggatatgttgctccagaatatgcaatgacttgccgtgtttcagacaaagctgatgtatatagttacggtgttgtcctgatggagt
taatctcagacaagaaagccttggatccatcattttctccttatggcaatgggttcaacatagttgcttgggcatgcatgctgctccgtcaaggccgtgctc
gtgagttcttcattgatggttgttcaacggttgaagcaactgcagcctccaattcgtga
>g29473.t1
atggcattattggcctacacgtccttactcatcaccttggccctcgcgccgctcaccgccggcgagcccctgggccagtcatgcgacaccagctacaact
acacgacgaacagcccctaccaatccaacctccagcgcgtcgccgccacgctccccaggaatgcgtccgcctccagtacgctcttcgacaccgcgaca
ctgggcgccatccccgacaccgtctacgcgctcgcgctgtgccgcggcgacgtgaacgcctccgcctgcgagtcctgcgtcacgggcgccttcgtggac
gcgcagaacctctgcgccttcaaccaggacgccaccgtgttctacgacttgtgcctcctccgctactccaacaagaacttcctcaccttccccaccagcg
acggccgcggggattacatgatcctcagtagcaagcagaacgtgaccgcgccgttcggcgtgttcgacgcggccgtggccgtgctcctcaacgccacc
gccgactacgcggcggcggcgaactcgtcgaggaggttcggcaccgctgtgcagggattccagactttcgacagcaagaacccgaagctctatggact
ggcgcagtgcacgccggacatggcgccggccgactgccggagctgcctctcgggaatgcttcaactgacgctcagcgatttcagtgggaagacgggg
ggcaggatcctcacgctgcggtgcaattacaggtatgagcagtacgccttcttcagcggcagaccgctgcttcagctgccagagccagccgtcggggc
gccgccggaaccggcgccaccggtgaacgtgaagccacctgcctccagaggagatccagagaagcgtagtgagcttgactggggaaagagatttaa
aatagttaatgggattgctcgaggcttgcaatatcttcatcaagattctcaagtaaagataatccaccgggacctcaaagcaagtaatgttttgttagatt
ctgactacaatcctaagatctctgattttggcttagcaaggctatttggaagtgatcaaatacaagatgtcacaaattgcgtcgtcggaacctttggttaca
tgtcgcctgagtatgctatgcgtgggcattactcgatcaaatcagacgtgttcagcttcggagttctgatcctagagattgtcaccggaagaagaaacgg
tggctcttatagcactgaggacagtgctgacctccttagtttggtgtgggagcactggacgacaggaatagtgatggagatcatggatccatccttgagc
cgcctcgcttcaagggaccagatggtgaagtgcatccaaattggcctcctgtgtgttcaagacgatcccatggataggccagtgatgtcgatggtgaac
gtcatgcttggcagcaacacggtgactctcaatactccaaaaaagccagtattttgcgtccgaaaaaatggtacaaacttgaacatgtactcggatgtat
accaaggttctcaattcacaaacagggtgaggtctcctgatgtgccaagccacaaaatgactgggaaagggggagtgcgtccaaatggtggaagcc
gacaaaggagggtgtgttgaagatcaacgttgatgaagccttcaatcctgcatctggtgtggcaggtcttggcgtcatcatccgagaccatgttggcaa
cctctgctcacagtgtggcgggtaatcttcaactgccagtatgcggaagaggctaaggctacggcatgtcttgatggcgtgcgcctggcggggagatgg
cctgatcgatgtatcgtcttggagtccgactgcgcctcgtcgcctcgatag
>g29620.t1

atgacgacggccgtctggaggcatgatgttggcttcctagtcgtcgtgctcctgcttcgctcgccggcggcaaaggcagggagcggcgggaactcgtcg
ccgtcgtgcccgctggacctgcgctacgtgtcgacgctcccgtgggaccgcgcgccgtgcctgccgccggtaaccaaccgcacggcctgctgcacgac
gctgctctccgtgctgggcgtgggcctggcgtcgcgcctgcggtccacgggccgcttccgcctcccgtccgccccggcctccgccgcctgcctgcgcgcc
ttctccgccgcgctcgccgcgccgcccgtctctctcccgggctcctcccgcgtggtgtcgggctgcttcccggacccggcccagctcgccatctccccggc
ctactgcgccggcgtcaccgacgccgcgcagtacgaggcggtcgtcggaaactcctccgcggcgcagctgaacgcgtcgtgcggcgccgacctcgcgt
ccatgtcggtgtgctcgcggtgcctcgccgccggcatcgacgcctcgtcgcggctcacggtggccgcgaacgacgggtccaagtcgctcaactgcttct
acctcaccgtgctctacgccgccggcatctccagctcctccggccccgactctctcgccaccgccaactgcgtcctcggcctcgctctctccacccctttgt
tgtcgtctcccacttcttcttcccctccttcttcttccagctccaccaaccacaccaacatcgcggtcgccaccaccatcccgatcgcctccgtgctgctcgt
tttcctcatcatcgctctgcttctcctctctaggaagaagcggcacgacaataacaacagaaagggcatcatccacctgtcgcggcgggaccggcatcgt
cccaacacggggtcggtgctgttcgacgtgggcgagctggcgcaggcgacgggcggcttctccgatcgccacgtgatcgggcgcggcgggttcggcgt
ggtgtaccgcggcgtgctcccgggggacggcaccgtcgtggccgtgaagaagatgctggacccggacgtggacgacgagttgaccaacgaggtgga
gatcatcagccagctccggcaccgcaacctggtcccctccgcggctgctgcatcacttccgacgatgatcaccaaggcggcgggaagcagatgttcct
cgtctacgactacatgcccaacggctccctggaccagtacatcttcagtactgaagatgccaactccaagcggctcttgttgtcgtgggcgcagcggag
aagcgtgatcctggacgtggcgcgggggctggagtacctgcaccacggcgtgaagcccggcatcttccaccgcgacatcaaggccagcaacgtcctcc
tcgacgccgacatgcgggccagcctcgcggacttcgggctcgcccggcggagccgcgagggccagtcccacctcacgacgcgcgtggcgggcacgc
acgggtacctcgcgcccgagtacgcgctctacggccagctcacggagcgcagcgacgtgtacagcttcggcgtgctggtgctggaggtcatgaccgcc
cgccgcgcgctcgacctggccgaccccgcgggcgtcgtgctcgtcaccgactgggcctgggagcgcgtcagggcgggccggcccggggacgtgctcg
cggaggccctgcgcggggataataataataataataatcatgaggcggccatggagaggttcgtgcgcgttgggatcctgtgcgcgcacgtcacg
gtggcgtgccggcccaccatgcccgaggcgctgaggatgctggagggcgacgtggacgtgcccgagctgcccgaccgcccgcagccgttcgggttcg
gggccagaggatcgcgttcgacgccgccaacttcagtgtttcctcggtgatgagcggaccgttcgtggactttggcgatatgctcaggtga
>g29704.t1
atgaaaacgaagcgggatgactggtggatcacacctttccaagatctggattatgatgatttggccattacccaggagctacaggaggagaacctcgtt
ggccgtggtggctcaggaaacgtgtaccgtgtcacatacaccaaccggtacaatggcagcactgggacggtggccataaagcagatacagaatgcca
ggaggctgaatgagaagctggagcgagagttcaaatccgaggcatatatccttgggaacatccggcacaacaacatcgttaagctgcagtgttgcatc
tccactgcagagtcaatgctcctcgtgtacgattacatggacaatggcagcctggacaattggctacatggccatgttctctttgctggccactctatgtcc
gtgacatgtgcgccgctggactggccgacaaggctcagagtgtctgttggtgccgcgcaagggttgtactacatgcaccatgagtgctcgccgcctatc
attcaccgtgatgtcaagacaagtaatatcctgttggattcggagttccgagctaaggttgctgactttgggttggctaggatgttagtgcaaacagggg
agtccgagacgatgtccgcagttgctggatcgtttggttatatggctccagagtatgcttacacaaagaaggtgaacgcaaaggtggatgtctacagctt
tgggatcgtactcctggagctcaccaccggcaagagggcctatgacggcggcgagcatggttgcctggcacaatgggcatggcaccactatcgatcag
ggggaagcatccctgatncgagcatggttgcctggcacaatgggcatggcaccactatcgatcagggggaagcatccctgatgccacagacaagtgc
atcagatatgcagggtaccctggagagattgacactgtgtttaggctag
>g29807.t1
atgtacctcgtcctggagctgtgcgagggaggggacctggcggcctacatccaccgcaacgggagggtagaggagcgcgtcgccaggaattacatga
aacaaataggagatgggttacaagtgttacggaggcaccatgtcattcacagggacttgaaacctcagaatatcctcctcctctctaggcagcaatgc
catcctcaagatatctgattttggtctagcaagcttgcattctgatgctgttgatatatgcaataagctactgtgctctaatccagtgaaaaggctgtctttc
caagaattcttcgaccacagcttcttcagagtataa
>g29907.t1
atggggtgcgcggcgtctcggcccggcgtggcggcgtcgccggggtacgacgcgtcgacgtcgtcgaggtacacggcctccggcagcctgtcggcgtc
ggcgtcgatctggacccgtcccgtgcgcctgggctccgacgaggacgaggagtatcaagaccgcaagccggcagcgccgcaaccccggcgcggcg
tcgcgtggacggcgagcaggcggcggcggggtggccgtcgtggctgagcgcggtggcggcggaggcggtgcaggggtgggtcccgctgcgggcgg
aagggttcgagaagctggagaaggttgggcagggtacgtacagcagcgtgttccgcgcgcgtgagctgcgcacgggccgcgtcgtggcgctcaagaa
ggtgcggttcgacagcgtggagcccgagagcgtgcggttcatggcgcgcgaggtggtcctgctccgccgcctgcagcgccaccccaacgtcgtcgggc
tcctcggcatcgtcacctcccgctcctcgcgcgccgtctacctcgtcttcgagtacatggagcacgacctcgcgggactcacctcctcacccgacgtcaca
ttcaccgagccccagatcaagtgctacatgcggcagttgatggaagggctagcgcactgccacgcgcgcggggtgatgcaccgggacatcaagtgcg
cgaacctgctggtgagcaacgccggcgagctgaaggtggccgacttcggcctggccacccaattctccacttcttcttcatctccgtcaagtgctacgct
gacgagccgcgtggtgacgctgtggtaccggccgccggagctgctgctggggtcgacggcgtacggccccgccgtggacctgtggagcgccgggtgc
gtgttcgcggagctccacgcgcggcgcccggtgctgcagggccgcacggaggtggagcagatccaccgcatcttcaagctctgcgggtccccgcccga
cgagttctggcggcgaggtggtggtggtgcccacgccgccgtgttccgcccgcaccagccgtacccgagccggctgcgtgacacgttcgccgcggcca
tgccggaccccgcgttccggttgctccaaaccttgctcgcgctcgaccccgacgcgaggggcaccgccgccgccgcgctcgactccgactattttagga
cggcgccgttggcgtgcgatccctcgaccctgcccaagtacgcgcccaacaaggagatggacgccaagattcgagaagattctacaaggaggagcaa
cggcaggacccacgccggcgacgcggcgaagcgggggcacaacagcatggtgctccaggacgccagccagcgccacgtgcacgccgaggaatccc
tcccgccggcggcgaggagggacagcggcggcgaggcaaggctgatcgtggacctgcagcccgtggcggcgatcagcaagcggcacgacgtcgag
caccaccaccaccacgccaggaattcatcctacagcttcaacgggccggcgcagctcgccgcgtccacgggcttcgcgtgggccaagaagccccgac
ccgacgcggcgaagaggagcgcctccaggtgcccgaggcccaacagcggcagcaacggggaaggagccacgaccacggcgccgtacgaggcgga
gaagcaggagatgatcaggcagtgggcgcaggtcgcggaggccttcagctcgtctgacgctcacaacggcaggttcaggcagacggtggacgtcaa
gcagctcaagaccgtgaagcacaaggggcagatggacagggtggacttctcagggccgctgctgtcgcagccgcggcggatcgacgagctcctgga
gaaccacgagcaccagatccggcgagcagaaagcaaaagggagcagcattga
>g29981.t1

atgtaccaagaactcaggaagaagcttgcggagcatgaagccaactatgttgaagctccacaagccaaagaagacatcacgaagatggattctggta
tattagacaaagctcaaggtcatggagaaccaagcaaggcctctgatattgaattggatgatgacctcatgacagaggatgaggaggacctgcatgaa
gattcacactgaatcaacttctctgccaaatccctcgatcctctggcacgagtgaagcttattgcatcgtggattaagtactacggaactgagcctggtat
acattatcttcgccgtccttccttctattatgaggacgacgtcaacgatgccaagaagagcgaagtatcaccggcaaacacgactagcacagcacagag
tgcaagctttcgcgaacctagagcaaagtcaggcgttttgctgtggtgcaagcggaccaagaagctcccttctttgcgagccgaggtcaaggcatcgctt
tcaccaatcttcacactgccccgcatttggttgtctgaactctgccgctcgactcccagacggtacagccacagtgaggttgccattgcgacgcaggggtt
cactgacgaggcgaagtgtgggagaggtggattcggcccggtgtaccgaggctatctcggtgatcaggaccgccacgtagccatcaaggtgctgtctc
aaggatcttccattcagggagccaaggagttcgaggccgaggtgaatatcatgacgagcctgaggcaccggaacatcgtccaactccttggctggtgc
gacgctcaggaccacagagacctcttgcttgtctacgagtttgtgccaaatggtagcctcgacaagcatctctatgatccacagaggatactcacctggt
ccaacaggtacaaggttgcgcttggcgtgggctctgccatactctacctccacacggagtgcgggcagtgcgtcgtgcacggcgacatcaagccggca
aacatcatgctggacacctcctgcaacgccaagctcggggacttcgggcaagcaaggttcatcgaccacgacgccgacccgcgaaccacgcatgtcgt
ggctgggacgctcgggtacatcgacccggagttcgtgaacaatcggaggcccagcgccgagtcggacgtctacagcttcggcgtcgtccttctcgagat
ggcatgcggcagacggccaacgacctcgacgcagcagtcaaacaggacgccggcgctggtgaactgggtccgcgacatgtaccgccggaactcgatt
cttggagcggcggaccggcggctggacggcgagttcgacgagcggcagatggtgcacgcatcaggagcagagccagcgaccgaccatcgcgcaag
ccatggatgtgctgcgacgggaagatgccgaattgcctgtcctcctaggtccggatatgcaatctattgctga
>g30006.t1
atgatcatgggaggagcactggagatggagagcaggaagaaggcgaagatcatcctcgccggcgcggcgttggccgtgctggcgaccatcgagctg
accctcttcctctgcttccgcctgtcccgtcccttctacctctccacggcatgcgtcctacccgtcgcgctggcgtcgaccgccgccgcgctgctctactgct
cctctcaccacggccgggcccaccgcatgtcccggcggcgccccgcgctgcacgaacacgacggcggcggcgaggaggaggttgggcacgtggagt
acagcttcttccggaaggtcgccggcctcccgcggcggttctcgctggcgtcgctggcggcggcgacggacgagttcgggtgcgtggtggggcgcggg
tcgtcgggcaccgtgttccgcggcgtgcttgacgacggcacggcggtggccgtgaagcgattggatgggccgtcggcccgcctggcggagcgggagtt
ccgggccgaggcgggcgccatcggcggcgcgcagcacgccggcctcgtccgtctcctgggcttctgcctcgtcccgcacggcgggccaaggttcctcg
tctacgagtacatggagcacggctccctggacaagtggatcttcccttcttcttcctcgtcgttgtcggcccaccagcagcagcaggacaggtggctgac
gtggcggcagcggcgacaggtggcggtggacgtggcgagggcgctggcgtacctgcacggggacctgcgtggcgggaacaaggtggtgcacctgga
cgtgaagccggagaacatcctcctcgacgacgccttccgcggcgtgctctcggatttcgggctgtccacgctgatgggaaaggaccagagccgcgtcgt
caccacggtgcgcgggaccacggggtacctcgcacccgagtggctgctcggcgtcggcgtcaccgacaagtccgacgtctacagcttcgggatggtcc
tgctggagatgctcggcggcaggcggaacatacaagtaatcgacgaggaagcagatattgaagtcgtccaagggtcgaggagaaggcagaggtggt
cgtactttccgaagatcgtggcggagaaggtgagggaggggagggtgatggaggtggtggacccgcgggttcggggagacgcggcggcggacgag
gcgggggtgaggcgggtggcgcacgtggcgctgtggtgcgcgcaggagaaggcgagcgcgcggccggacatggcgcgcgtcgtggagatgctgga
ggcgaagggcggagcgacgacggaggtggaaccgccgccgccgtcggagatgatcttggtcgacatgctcgcactcgaccccgaccggccaccgttc
gggttgcccgtgccgccgtcgtcgggcgtgtcgtcggcgatgagccgcaccgactccttcgcgctctcctacctctcggggcggtaa
>g30007.t1
atgttcagcaacggagagcagaattatttcagcccgtgcagctatgggttcgtcgtcgatggggactggttcagctttgacccaagatatgccagattat
cttattttaaagaagattacggagacggtgtacctttggttcttgactgggtggttgacaacgaaacatgcataaaagccaagaacctaccatcctacgc
atgccaggcggcaaacagcaattgcatcgacgcgctcgatgactccggatacctctgcagctgctctcaaggatatgacggaaatccgtacctaaaag
gaggctgccgagacatcaatgagtgtgataatccatcgctgtatacctgcaacggtttggccatcagtgtagttttcatcattgcgtgtatcttcgttgtga
tgatcaagtgtgagaaaaggaagctggcaaaagagaaggagcgattcttcaaacagaatggcggtcagatactgtatcagcaaatcctctccagaaa
agtcgacacggtgataatattcacagtggaagatctgaagacggcgacaaacaattttgataagagcagagagctgggcataggggtcatggtgca
gtctacaagggtattctgagagacaacaaggtggttgctgtgaagcgctcaaagattagcaatgtgacgcaagcagaagaattcgtgcaagagataat
tatactttcacagatcaaccacaggaacattgtcaggcttctaggctgttgcttggaagtggaagtacccatactagtatatgagttcatcccaaacggca
cgctctttcacttcatccacggtaaacaacaacctgtttccctggaagctcgtctccggattgcccaagaatctgctgaagcacttgcatatatgcatctgt
ccatcagtcatcccattgtccatggagatgtgaagtctctcaacattctcttggatgaaaactacatggcaaaagtaaccgactttggggcgtcaagattg
cttcccaaggatgaagctcagctcatgacaatagtgcaaggaactctaggttacctagaccctgagtacttgcaagagcgtaagctaacggagaaaag
cgacgtctatagctttggagttgtgcttctagagctaatcacaaggaagacggcgatctactcagaaggcactgatgagcaaggaaaaagtctcgcgtc
atccatcatacaagcaatgaaggagaacaaagttgaaggcatgttggacaccagcataatgtgtgtggggatggaggagctgctccaagaagttgtgg
tgctcagcagccagtgcttgagtatcaaacgggaagagaggccatccatgagccaagtggccgacaaacttaaagctatccgaagtacctggagcaa
gctactgttactgaagcataatgaaactgaacttctaattcaggattcaggcgaggatgccgcttgcggcagcctgtcgcccagcatgtactggacggc
gggaatgatgggaatggatatagaaacaccagttgcgaacaacagaagtacttacagcatgggatag
>g30008.t1
atgatccgtggagtgctccttggcgccaccaagtacgagcactacgggagcgcgtgcggctcattctgtaaccgggatagcaccgaaagtagcacgga
atgcactggcatagggtgctgccagatttccatccctcctaaccttcgggctatgaacttctccttcttaacaatagatggcatcatgaactactccgctgt
cagaagctacaacccatgcagctacgcgttcgtcgctgaggcgggctggttcaatttcagcacttcatatgtcacaaccagagaattcgtatcccagtac
ggaaccgacggccatggggtacctatgctggtcgattgggctattgttggtaacgggaattgcgtggaagccaagaagacgaacgcatcatcttatgcc
tgccgtgcgatgaacagcgaatgcatcgacgcgccccatggcctagggtatcgctgcgtctgctctcaaggttatgatggaaatccctacctggacgat
gggtgccaagatatcgatgagtgccagcctccatacgagtatacctgccacggtgaatgcataaacaccaatggaagctacaactgttcatgcccacat
ggaagtcagagtgaggacccaaagaaggaaccttgcactaaaatccctatccccagtcaactcagtcagaaaagaaagctagcaaaagaaaaggag
agcttcttcaagcggaatggcggccagatattataccagcaaatcctctctcaacaagtggacacagtgacaatattcaccatagaagatctgaagaca
gccgcaaacaacttcgaccagagcagagaactaggaacagggggtcatggcactgtctacaagggcattttaagggacgacagagtagtagctgtga
agcgatcaaaggtcatgaatgtggtcgagaccgaagaattcgtgcaggagattatcatactctcgcagatcaaccacaggaacgttgtacggcttctcg

gctgctgcttagaagtggaagtacccatattggtatgcgagttcattccgaatggcactctgttccatttgatccatgacactgatggcagaccacctatgt
cgttggaagcccgtctcaaaatcgcccaggaatctgcagaagcacttgcgtatctgcatctttccaccaacagccctatagtacatgcagatgtgaaatc
tctgaacattctcctagatgaaaactacatggccaaagtgaccgactttggtgcttcaaggatgctcccaaatgatgcggttcagttcatgacaatggtgc
aggggactttaggttatttagaccctgagtacttgcaagagcgcaagctaacgcagaagagcgacgtgtatagctttggagttgtgcttctggagctgat
cacaaggaagacggcaatctactctaaaggccttgagcgaggaaaatgcctggcatcctccttcatacaagcaatgaaggagaacagggttgaaggc
atgttggacaccagtataatttgtgtgggaatggaggagctgctccgagaagtcgctgaactgggcagccagtgcttgagcatcaaaggagaagagag
ccatccatga
>g30079.t1
atgagaagggtgcagaggcagctggagatgcttgcccgggtgaggcatcagaatgtgatggccctcaaggcgtatgttcgcgagcctgactgcctctc
gctcgtgtacgacttcgttcctggaggcagcctagaggatatgatgaagagggtgaggtcgcagcaggtcagcctcggctgggatgcaaggagcagg
attgctgttgggattgccaagggtttgaggcacttgcattttgagtccaatccaaggatactacactacgacctcaaaccatcaaatgtgttgctagatga
aggttttgaacccattttgacggattgcggggtctcaaggctgattgcagcaggttcaggtgatccagaactatgcagcggcctctacgctgctccagag
tgctaccagaacagcaggtacacagataagagtgatgtctatagtcttggcatgatcctgggagtgctgcttactggtagagacccaacagatacatttt
tctccggcgaaactggccggggtggcctggctagatggctgcggcatatgcagcaatctgcagatccaaaagaagttctggacagtagcattctatggg
aggaaggagaagaagaggaaatgcttatggctatcagggttgccattatctgcctctcagattcaccagccgatcggccatccagcgatgaacttgtcg
ctatgctaatgcagcttcacagcttgtga
>g30465.t1
atggtgggggttggcggcgggggcggggcgctgcggcgcgtcggcaagtacgaggtggggcggaccatcggagaaggcaacttcgccaaggtcaa
gttcgcgcagaataccgagactggagagagcgtcgccatgaaggtgctcgaccgatcttccatcctcaagcacaagatggccgaacaggtgaacattt
tccccctctccttccttcccgatccttgcctaggggcacggttgagaaatagtgcgctcaagcatttaatcctccgtattgatctcaccccacgaaatgttg
ctgctgatgaggggtcagtagtcttgaggttagggttctctggtttccatgtgaagttctga
>g30565.t1
atggagcaaggaggtgggatggtgtgcaagaacccttccaaaaccctgccgccgctcatgtccattcttctcctcgcgctcgccttcctcttcctgtgcgc
gccgccggcctccgcccagccgctccactcggagcccatggccacgcaggccccaccgccctcgcccccgccgccgcaatccaagattccccgcgcgc
aggccggcgccgccgcccgcctgcaccgcctcgcgctcggcgtcctcttcggctccctcaccggcttcctcctcgccctcgccttcctctacgctatccgtg
tcgcaatcctccacgccaagaacgcgcccgcaatcatcagggggcccgtctccttcaccccacaaatttcccccaagaacctcctggccgcgctaccct
ccgcgcagccgctcgcccatggcccccatggcaagtactacaagctgtctcttgacaatgacctcaccgtcgccgtcaagcggctcgacgcagtaaacc
gccctgaggcctcgccgtcagtgtccccaaacacatccaagtctgacatgagaagggtgcagaggcagctggagacgcttgcccgggtgaggcatca
gaatgtgatggccctgaaggcgtatgttcgggagcctgactgcctctcgctcgtgtacgacttcgttcctgggggcagcctagaggatgcgatgaagag
ggtgaggtcgcagcaggtcagtctcagttgggatgcaaggagcaggattgctgttgggattgccaagggtttgaggcacttgcattttgaatccaatcca
aggatactgcactccaacctcaaaccatcaaacgtgttgctagatgaaggtttcgaacccatattgacggattgtggggtctcaaggctgattgcagcag
gttcaggtgacccagaactatgcagcggcctctacgctgctccagagtgctaccagagcagcaggtacacagataagagtgatgtctatagtcttggca
tgatcctgggagtgctgcttactggtagagacccaacagatgcatttttctctggtgaaactggccggggtggcctagctagatggctgcggcatatgca
gcaatctgcagatccaaaagaagttctggacagtagcattctatgggaggaaggagaagaagaggagatgcttatggctatcagggttgccattatct
gcctctcggattcaccacatgatcggccatccagtgatgaacttgtcgctatgctaatgcagcttcacagcttgtga
>g30801.t1
atggcggcgccgcatcgaccccgcccgcggccgcggccgcagctcgcgcgcatcaacgccatgaggcactcctactccgccgaggaggacgaggcc
ccggcggccgacctcggcgccgacttcgcgtcgcagaccagcttccgcatccgcggcgggcgcggcgcggaggtcgccgacctcttccgcaagcttgg
cctctccggccccgaggacttcaccatcccccggccgtctacgccgccgcgatggcgcacatccccaactcctcctcccgccgccgcggccagtcgca
ggagctgggcgcgggtgccgaggggtccgcgccgcaggagcttccaggaacttcgggtaatgacgtcacggtcggaatcggaacaaggctagaggc
ggccgatgtggggaaggcagacgtgttggccaccgaaccggtccaactccaacccgaggttattcaagttgctgcacaatctaacaggagagggggg
tctggaactgaatcgcgcagcagagaagtcgaattggacaccgcggaagcttcgagaagaggcgtggccgccgaggtgaagcgggggacagcttac
gaagaaaaggggaagagcgttgcggccaaggtggatcggttgcgagtggagaggacagaagcggtggtcgtggagacggcaagggagactaccgg
cgccgtagttgaggttgcggcggagtccacttctcgcagcgttgaacattactatttctcgccgtccccgcacaggcggattaagagaaccatcacgtca
tggatcaagggccagcatttggggagcggatcgtttgggtcagtgtacgaagctatcagcgatgatgggtttttctttgctgccaaggaggtatcgttggt
tgatcagggactcaatgcaaaacaacgaattctgcagcttgagcatgagatctctctcttgagtcgtttggaacatgaaaatatagtccagtactttgga
acagataaggctatctataaggattttgataactgtccagcatga
>g30850.t1
atggagcagtacgaggtgttggagcaaattgggaagggttcgtttggctccgcgctgctggtgaggcacaaggttgagaggaaaaggtatgtcttgaa
gaagatccgccttgcgcgtcagactgacagatgccgccgatcggcacaccaagagatggagctgattgcaaaagtaaggaacccttacattgtggagt
acaaagagtcctgggtggagaagggttgttatgtgtgtatcgtgattggttattgcgagggaggggacatgtcagaggctatcaagaaggctaacgata
accatttctcagaagagaagctgtgtgtgtggcttgtgcagctcctaatggcacttgattatttgcatgtcaatcacatccttcatcgggatgtcaaggttg
tgggaactccaacttacatgtgccctgaacttcttgctgatattccatacggttccaaatctgacatctggtcccttggctgttgtatctatgagatgactgc
tctcaagccggcatttaaagcatttcgtccaccctccaggtcttcagtggaccgaatggttcaagcagcacgaagaatgtcactccccctgtcaatggtc
aaaacttctgaaaagaagcagggccatgacattcttaaaagactgaaatctcctgatgtttcagttaacgctccacgaatcgacagaattgctgaattcc
cactaccatcttttgaagacccattgcattccatcacgaagatcatgtcgccatctatcattgaccaatccataaccaaggacaagtgcactttccaagtg
cttcgtagtgacattgaacactacgttgacactcctgatatcgactttcgtgtcgatagttcaccaaggagctcatcagattgggagcaaaagaggttca
acacgagatcttaccaacagcgagctgaggctcttgaaggcttgcttgagttcagtgcacagttgctgcagcaagagaggtttgaggagctgggaattt
tgctgaagccttttggacctggcaaggcgtctccaagagagacagccatatggttgtcaaagagttttaaaggaacagggttataa

>g31264.t1
atgccgccgccgccgcgccggctcagtcatggccgcagcttcctgctcctcctcctccttgtcctcctgctcgcgcggtgcacccccgcgtcggc
ggccacgctctcgaccttcgccctggccaaggccgccgacacgaccatagtgtgcgggctcctcccttccaatttatccccactcctcgtggacctgaact
gcacggcggccggcggtgaccacgcgcggcaggagacgtacccttcgtcccaccccttctccgcgctcgccggcggggaagacttcctctgcgcggtg
ggtcccacctccagcgccggcgaggtggacatgcgttggtgggacctgtcgcggcacggcaacgacagccgcccgaagcgcgtgtacgcgggcccgc
cgctccgcgcgctggactcgggcgagtaccgcgtctgcggcgtgctggcctccggcgagctccactgctggcgttggcgcgacctcgccctttccagga
gcctccagttctccgccgtcgccgtcggggatggcttcgtctgcggcatcgtcgccgagggccagcagtctgccggctccatccgctgcttcggcgacga
cccggccgacgaggccgtggcgggcgcgccgcgcgacgaggggcagagctacgacgtggtggaggcgtgcgggagccgggcctgcgcgctgaccc
ggggcggcggggtccgttgctggggccgcggcgcgccgacggtgggcccggacggcgacgccgggttcgcggcgctcgcattgggggagaccggcg
cgtgcgggctccgcaccaacggcaccatccgctgcttcggcgaggatgtctcgtcgccgccggaggagctggcccggctgcagtacgtgggcgtgcag
gcgcagggggattgcttctgcggcgtgctgatggccaactactcgctggtgtgctggggcgggtccgagttccacgccaccaaccggctcgtgttcggc
cgcgtgctgcccggcccgtgcgtcgacatgtcgtcgtgccagtgcggcgtgctgcccgggtcggccagcctgtgcgcggacgggctctgcgtctgcgtc
gactgcgcgttcgagctcaacgtcgccgtgcccaacgcgactattctgcccggtgggaacgggaacaacagcagcagcagaagaaaccggaggacc
atgtggatcgtcatcggcgcggcgggcggcgccctggtgctgctgctggccgcattgcagggcgcgctggtcctctggttccgccgccgcaaacgcaac
aactccaccaacgacggcagcgagcagcggcagcggctcatgctgcagcgccctggctcgttcagcatcagcgccgtggaacgcttcaccatcgagg
cgctggccgccgccaccgacaacttctccgatgcgcggcggatcgggacgggcagcttcgggtccgtgtaccgcggcacgctgccgtcgggtcgcgac
gtggccatcaagcgcgccgaggagccggcctccgcgcgcccgtccacctcacgccctcttcgccgccgcgaccgcgaggccgccttcgcgtccgagct
caccgcgctggcccgcgccaaccacaagaacatcgtgtgcctcctcgggtgctgcgccgtggatgacgacgacgacgagtccgatgccccaggcgag
cgcgtgctggtgtacgagttcatggccaacggcaccctccacgaccagctccacgggagcaaccctttggcccctcccgtggcgtcgtggcgcggacg
actggccatcgcgctggacgccgcgaggggatcgagtacatgcacgtgtacgccgtcccgcccgtcatccaccgcgacgtcaagtccgccaacatcc
tcctcgacgacgcatggaccgccaaggtcgccgacttcggcctctcctccgtcctcgacgccaccaccgccgcacactgccccgacaacaacaacaat
gccggcgacacgccaccgccacctgtctacacgggcgggacggtggggtacatggacccggagtactaccggctgcagcacctgacggacaagtccg
acgtgtacagcttcggcgtggtgctcctggagctgctctcaggcctgcgcgtggtgcagcggtacgcggagagcgcgacgccgcgcaacgtggtggag
ttcgcggtcccgctcatcctcgccgacgacgtggcacgcgtgctggacccgcgcctacccgcgcccaacgaagaggaagctgaggcgctcgcctacgt
ggggtacctcgccgccgactgcgtctctcccgtgggatgcgaccgcccgtccatgacggaggtcgtcgacgggctcgagcgcgctctcgccgtgtgctc
gccgcccgcgcagctgtcgcgcgtcgggaccgccgccaggcgcgcgctctccaggtcagggacagaccacttcgacctcaccgacaccgagtga
>g31278.t1
atggatgacaatgagctactgctcgtctacgagttcatggcaaaggggagcttggagaaccatctcttccgaagaggtgcagtttgcgaatcactgcctt
ggagcctcaggctgaaaattctcatcggtgcagctcgtggtctcgccttccttcattcctcagaaagacagatcatctacagggactttaaggcttccaac
atcttattggattcgcatttcaatgcgaagctgtcagatttcggattggccaagcatggtccggatggtgaagagtctcatgtgacaacaagagtcatgg
gcacttatggctatgcagctcccgagtatgtttctaccggccacctatatgtgaagagtgatgtgtatggcttcggcgttgtgctgctggaaatgctctctg
gcctgagggcactggaccggagccgccccagtgagaaggtgaacttggtgaactgggcaaagccattgctggctgataaaggaagctgagccatgt
aatggacagccggcttgagggacaataccattccaaaggagccctccaggcagctcaactcacactcaagtgcctgaacggtgaccccaagagccga
ccatccatgaacgaagtagtagagaatttggagaagattgagtcgatgaagagcaagacgagggagcccaggcacagctcctcgctcactcgcggtc
aggcacattcaccgagaagggacagctctaggaagagctccaggggcagatag
>g31683.t1
atgagagcagaatgtaagctccttggtgaaggcaaatatggaacaacattcaaggccacgctggagaatggtgttgtgtttattgtaaagaggtcaaag
gttgagaatgtggcaaagactgtgttcgaggaacgtatagaagcaataggtgctattgagcatgagcttatagtgccattatgtgggtattatttcagca
aggacgagcagttgttgatatataattactttggcaacggcagcctctcttcaaatcttcatggaagcagaatcaacaaagtggagccagctggttggga
gacacggtcagccatcgcactctcttcagctcgtgctgtggcttacatccactcaacaaatccaaaagcatcccatgggaatatcaaatcctccaacatt
cttcttactgggagctacgaagcccacatctcagagcatggcatcaacactctggtcagaacaccagcatcatgtattacatataatacagctccagagg
ttaaggacatgcaaaatgtttctcagaaggctgatgtgtacagctttggtgtcttactgcttgagctgctcacaggcgtatcgccatgtcccacatacagtt
ccgagggacatgatttggttgcctgggatatggcagcccctcaggagcaacgggaaacgcgagtgttggatcaaaagcttcttaaggacaacggcatg
gtagaagagatgttgcagtttcttcaacttgcaactcagtgttgtgagaagagacccaacttgagaccagtgatgtcggaagttgttcagaagattgagg
agatacaaagtttaattgttgggaacaggcagcccatgtggagcagtgaagaggaatag
>g31895.t1
atgacaagtgctactccaaggcagagtagcacttcacgcttgttcttccccttgtccttctccttggctctcctgtgctgcattgccgtgtgcaatgcagccg
gcgacgaggccgcggcgctgctagccatcaagtcgtcgctcgtcgaccccttgggcaagctccagggctggaatgccgcgtcagcctcgtcgcattgtt
cctggaatgccgtgaagtgcgccaagggcgtcgtcaccggcctcaacctcgccggcatgaacctgagcggcactatcccggaggatatcaagggcctc
tccgggctcacttcgatcatcctgcagagcaacgcgtttgccgacgagctgccgccggtgctcgtctccatcccgaccctccaagagctcgacgtcagcg
acaacaacttcggcggccacttccctgccggcctcggcgcgtgcgcctcactgacatacctcaacgcgtcgggcaacaacttcgccggcccgcttccgg
cggacctcggcaatgcgacggcgctcgagaccatcgacttcaggggcggcttcttctccggcacgatcccgaaatcctacggcaagctcaagaagctc
aagttcctgggcctctcaggcaacaacctcggcggcgctctcccggcagagctgttcgagatgtcggcattggagcagctcatcattggctacaacgag
ttctccggcgcgattcctgccgccatcggcaacctcgccaatctccagtacctcgacctggcgatcggcaagctggaaggtcctctccccgcggagctcg
gccggctaccgtacctcaacaccgtctacctctacaagaacaacatcgacggaccgatccccaaggagctcggcaacctctcctccctcgtcatgctcg
acctctccgacaacgcgctctccggcaccatcccatcggagctgtcgcagctgacgaacctgcagctgctcaacctcatgtgcaaccggctgaagggcg
gcgtcccgtcgggcatcggcgatctccccaagctggaggtgctggagctctggaacaactccctcaccggcccactgcctccctccctgggccgcgcgc
agccgctgcagtgggtggacgtgtcgacgaacgcgctgtcgggccccgtgcccgcaggcctctgcgacagcggcaacctgacgaagctgatcctcttc
aacaacgcgttcacgggcccaatcccggcgggcctgaccacgtgcgcgacgctcgtccgcgtgcgggcccacaacaaccgtctcaacggcacggtgc

ccgcggggctcgggcggctgccgcggctgcagcggctggagctcgcgggcaacgagctgtcgggggagatccccgatgacctggcgctgtccctgtc
gctctccttcatcgacctctcccacaaccggctgcggtccgcgctgccgtccaacgtgctctccatcccggagctgcagacgttcgcggcggcggacaac
gagctcaccggcggcgtccccgacgagatcggcgggtgcccggcgctctccgcgctcgacctctccagcaaccgcctctccggcgcggtcccggccag
cctcgcctcctgccaacgcctcgtctcgctcagcctccggaacaaccggttcacggggcacatccccgccgcgatcgccatgatgtcgacgctctccgtg
ctcgacctctccaacaacctcctcaccggcgacatccccagcaacttcggaagctcgccggcgctcgagatgctcaacctcgccaacaacaacctctcc
ggtcccgtgccgtcgacggggctgttgaggaccatcaaccccgacgagctcgccgggaacccgggattgtgcggcggcatcctcccccgtgcgggtc
aacctcccttcgcgcctcgtcgtctgatgaggcggctgacacgcggcggtcacacgtgaagcacgtcgcggccgggtgggcgatcggcatctccgccg
cgctcgtggcgtgcgtcgccgcgttcctcggaaagcagctgtaccagcgctggtacgtcaacggcgggtgctgcgacgacgccgggctcgaggagga
cgggaccggcgcgtggccgtggcgcctgactgcgttccaaaggctgagtttcaccagcgccgaagtgctcgcgtgcatcaaggaggacaacgtcgtgg
ggatgggcggcacgggggtggtctaccgtgccgagatgccacgtcaccacgccgtcgtcgcggtcaagaaactctggcgcgcggcggggtgccctga
ccaggaggccgccgccaccataacagacatggaggccggcggcgagttcgcggcggaggtgaagctgctggggcggctgcggcaccgcaacgtggt
gcggatgctgggctacgtcagcaacaacatggacacgatggtgttgtacgagtacatggtcaacggcagcctgtgggaggcgctccacgggaagggg
aaaggcaagatgctgctggactgggtgtcgcgctacaacgtcgcggcgggcgtcgcggcagggctcgcatacctgcaccacgactgccgcccgccgg
tcatccaccgcgacgtcaagtccagcaacgtgctcctcgacaccaacatggacgccaagatcgcggatttcggactcgcacgcgtcatggcccgggct
cacgagaccgtctccatggtcgctggttcctacggttacatcgcgccagagtacgggtacacgctgaaggtggaccagaagagcgacatctacagcttc
ggggtggtgctgatggagctgctcacggggcggcggccgatcgagccggagtacggcgagacgcaggacatcgtgggctggatcagggagcggctg
cgcagcaacagcggggtggaggagctgctcgacgcgagcgtcggcggccgcgtggaccacgtcagggaggagatgctgctggtgctgcgcatcgcg
gtgctctgcacggcaaagtcgcccaaggaccggcccaccatgcgggacgtggtcaccatgctcggcgaggccaagccgcgccggaagagcagcagc
gccaccgtggccgccaccgtcgtggacaaggacaagccggtgttcacgacgtcgccagactccgattacctgtag
>g32049.t1
atgtccccgccgccgcgcgccgccgtgctgctgtcgctgcttctgctccaacttggctcggcccttgccgccgacgacctggcctcggacgccgcggcgc
ttcaggccttcctggccccgttcgggtcgtcggtcgtgcactggaacgcgacccagcacccgtgctcatggacgggcgtggtctgcacgggcggccgcg
tgacgggtctccacctcccgggcgacggcctccgcggcaccctccccgccggcgccctcggcgggctcacgaagctcgcggtcctctccctccgcttca
acgcgctgtccggcccaatccccaccgacctggccgcctgcgtcgagctgaaggtcatcaacttccagtccaacctcctctccggcgagctccccgccg
ccgtgctcgcactgccggtgctcacgcagctcaacctcgccgacaaccggttctccgggcgggtcccaccctccatcgcgcggaacgggaagctgcag
cagctctacctcaacggcaacctcctcacgggccctctgccaaacgtgaacatgccgtccctcacgtcgttcaacgtgtcgttcaacaacctgacaggcc
agattccagggagcttcggccgcatgccggccacgtcgttcctcggcatgtcgctgtgcgggaagccgctcccgccgtgcagcgcgccgccgggatcg
gagctaccgccggcgcagtctcccgcgctctcgcccggagcgggaggaggggctgccgcatcgccgccggaccgccgcggacgtaggcaccgcctc
gccggcggcgcgatcgccggcattgtgatcggcagcacgctcgggctcgcattcctcgtcgcggccgtgctcctcctcgcgtgccgccgccggccgcgc
gccacgtaccgcagccaggacgcggtcgcggcggagctggcgctgcacagcaaggaggccatgagccccaacgggtacacgccacgcgtctccaac
gcgcggccgatgcatccaccttcgtccgcgccgccttcggtcccgcctgcggtcacggtggggaagaagaagctctacttcttcggccggatcccgcgg
ccgtacgacctggaggacctgctgcgcgcgtcggcggaggtgctggggaagggcacgtacggcaccacgtacaaggccgcgatcgagtcgggcccc
gtcatggccgtcaagcggcttaaggagacgtcgctgccggagcgcgagttccgggacaaggttgccgccatcggcgggatcgaccaccccaacgtcgt
gccgctgcaggcttactacttcagcaaggacgagaagctcatggtttacgagtacgtcgcgatgggcagcctctcctccatgctccacggcaaccgtgg
ctccgggcggacgccgctgagctgggagtcccgccggcggatcgcgctggcatcggcgcgcgggctggagtacatccacgcgacggggtccacggc
ggcgcacggcaacatcaagtcgtccaacatcctgctgagccggtccgtggacgcgcgcgtcgccgaccacgggctggcccacctggtgggcccgccgt
cgacgcgcgtggcagggtaccgcgcgcccgaggtggtggcggacccgcggcgggcggcgacgcagaagggcgacgtgtacagcctgggggtgctg
ctgctggagctgctgacggggaaggcgcccgccagcgcggcgctgcgggacgaggagggcgtggacctgccgcggtgggcccggtccgtggtgcgg
gaggagtggacctccgaggtgttcgaggcggagctgctccgccaccagggcgccgaggaggagatggtggagatgctgcggctcgccatggactgca
ccgtcgcggcgcccgaccagcggcccgccatgcccgaggtcgtcgcgcggatcgaggagatcgggcggaccgggtcggcgtccacggcgcggtccg
ggaggagcgcgtccatcgacgaggccgatgaccggccgctcaggcccgccgggtccatccgacagagcttatga
>g32373.t1
atggagaagtacgagctgctcaaggacatcggctccggcaacttcggggtggcgcggctgatgcggaacaaggagaccaaggagctcgtcgctatga
agtacatcccgcgggggctcaagattgacgagaatgtggcgagggagatcatcaaccaccgctcgctgcggcaccccaacataatccggttcaaaga
ggttgtgctcacgcccacgcacctggccatcgtcatggagtatgccgctggcggcgagctgttcgaccggatctgcggtgccgggaggttcagcgagga
tgaggcgaggtatttcttccagcaactgatttgcggtgtcagctactgccacttcatgcaaatctgtcaccgggacttgaagctcgaaaacacgctgctg
gatggcagcccggcaccgcgccttaagatttgtgacttcggctactccaagcaaatctgtcaccgggacttgaagctcgaaaacacgctgctggatggc
agcccggcaccgcgccttaagatttgtgacttcggctactccaagatggcagatgtttggtcctgtggagtgaccctttatgtgatgctggttggtgcata
ccttttgaggatcccgacgatcccaagaatttcagaaagacaattgggagaatagtatctattcaataccaaataccagaatatgtccacatatcccaa
gattgcaggcagctcctctcgaggatcttcgttgcgaatcctgcaaagagaataacaattagggagattagaaatcacccctggttccttaagaacttgc
ctagagagcttacagaagctgcacaagcgaagtactacaagaaggacaacagcacacccacctactctgatcagactgttgaagagatcatgaagat
tgtagaggaggctcgcacaccacccaaattatctactcctgtggctggtttcggttgggctgaggaagaagagcaagaagatggcaagaaacctgatg
atgaagaacatgatgaagaggatgaggaatatgatggtgaggacgagtatgacaagcaggtgaaggcagtacatgccagcggtgattttcaacatct
gataaaaggaagcaactag
>g32998.t1
atggagagtagaagtaatggttattatgaggagaaaaaggcacttctgattttcagagatatctgcaatgctgtttttgccatgcatgggcagtcgccgc
ctattgcacatagggacctgaaagctgaaaatgtccttctgggatttgatggtgcatggaaaatatgtgattttggaagtacgtccacaaatcataaatg
ctttgaaaagccagaagagatgggcattgaagaagatatcattagaaagcatacaaccccagcctacagggcccctgagatgtgggatctatacaga
agagaagttatttgtgaaaaagttgacatttgggtctggttctgtgttaatgaactactgcctcttgagttactgaaagatttacctgatggggcttcacca

gccatttccatggtttcacgagatgaagattttgactcaagcagtctccatgctgggacttctgctgctagtaaggcatctgatcttgaactggagttgtcc
agtctgaaggagcagttgaagaaaaccacattagagaaggacgagatgacagccaagtatgaaaagctatctgcaatctgccgatcacaacgtcagg
agattcaagagttgaagcgcacttttgccgagacaacgcctctgtcaaacaaagttagctcaaggacacatgattctgtgcctcagcaaaaggaaaag
atagaaggaactgtgtgggagcttgaacaaggaatgcttgcaagtaactcttcttcagtatccaattctgaggtcaagacatggcaggcattccctgagc
cgaaagctcaggctcgaccaaagaacatcgccatcatcagcgtcaagcgcttctgcacacgtcagcaggcctgctgcaaaggggagtgggagttcctc
gcagagatttag
>g33080.t1
atgggtcttggtgtcatgatgggtagaagaagaagcaaatggcgaggcgcggtgcttctggcggtgctctgcctctgcgtggtctgcgccgccgccgcc
gtggacgagcagggcgcggcactgctcgcgtggaaggcgacgctgcgcggcggcgacgacgcgctgccggactggaagtccggcgacgcgtcgccg
tgccggtggacaggcgtgacgtgcgacgccgccggcggcgtcacggagctgaacctccagttcatcgacctgttcggcggcgcgcccgacaacctcac
cgcgctgggccccacactaacaagcctcgtcctcacgggcttgaacctgacgggccccatcccgccgtcgctcggcgcgctgccggcgctggcccacct
cgacctctccaacaacgcgctgaccgggcccatcccgccggggctgtgccggcagggcagcaagctggagacgctgcatctcaactccaaccgcctg
gagggccccatccctgacaccatcggcaacctagcgtcgctgcgggagctcatcttctacgacaaccagctcgccggccgtataccggcggccatcgg
gcggtgcgcgagcctcgaggtgttccgcggcggcggcaacaagaacctccagggcgcgcttcccgcggagatcggcaactgctcgaagctcaccatg
atcgggctcgccgagaccagcattaccggtcccttaccggcgagcctcggccggttgaagaacctcaccacgctggccatctacaccgcgctgctttcc
ggcccgatcccggcggagctcgggcagtgcgagagcctcgagaacatctacctctacgagaacgcgctgtccgggtccatcccgccgcagctcggca
agctcacgaggctcaccaacctcctgttgtggcagaaccagctcgtggggaccatcccgccggaggtcgggtcctgccccgagctcaccgtcgtggac
ctctccctcaacagcttgaccggccatattccaacctcctttggcaacctctcgtctttacagcagctgcagctgagcatgaacaagctctccggcacggt
gccggcggagctcgcccggtgcagcaacctcaccgacctggagctcgacaacaacgcgctcaccggcagcataccggccgcgctgggcgggctggta
tcactccgcatgctctacctctgggccaaccagctgaccggcacggtaccggtggatctcggccggtgcgcgagcctcgaggcgctggacctctccaac
aacgcgctcacaggccccatcccacgctcgctcttccgcctcccgcacctctccaagctactcctcatcaacaacgccctctctggggagctccccgccg
aggtcggcaacggcacctcgctggtccggttccgagtcagcggcaaccgcatcgccggcacggtaccggccgagatcggccggcttgggaacctcag
cttcctcgatctggggtcgaaccggctgtcggggcccgtaccggccgagattgccgggtgccggaacctcaccttcgtcgacctccacgacaacgccat
aagcggcgagctccctccggggctgttcaacgacctgctgtcgctgcagtacctcgacctctcccacaacgcgatcaccggcgccctcccgccaggcgt
cgggatgctcacctcgctcaccaagctcatcctcaacggtaaccggctttccggcccggtcccccccgagatcgggtcgtgcacccggctccagcttctc
gacctcggcggcaacgcgtttgaaggcgagataccgggaagcatcgggaagatcccgggtctggagatcgcgctgaacctcagctgcaacgcgttca
ccggcgccgtcccgccggagttcgcggcgctggcgcggctcggcgtgctcgacgtctcccgcaaccggctctcgggtgacctccacccgctcggcgcg
ctgcagaacctcgtgtccctcaacgtctcgttcaacgcgttcgcggggcggttgccggagacgcccttcttcgcgaagctccccaccagcgacgtcgag
ggcaacccggcgctgtgcctgtcccgctgcgccggcggcggcggcggcgaggatgaccacaaccgcgaccgcgccgcacgggtcgccgtcgccgtg
cttctgtccgcgctcgtggtcctcgtcgcggcggcggcgctgatcctggtccggcagcggcggcgcgcgggggaggaggcagagaaggccggcgccg
gcggggagatggcgccgccgtggaacgtgacgctgtaccagaagctggagatcggggtggccgacgtggcgcggagcctgacgccggcgaacgtca
tcggccagggctggtcgggttccgtgtaccgcgcgagtctagtctcttccggcggcgtcaccgtcgccgtgaagaagttccggtcgtgcgacgaggcct
ccgtggaggcgttcgcgtgcgaggtcagcgtgctgccgcgcgtgcgccaccgcaacatcgtgcggctgctgggctgggcggccaaccgccgcacgcg
gctcctcttctacgactacctccccaacggcacgctcggcgcgctgctccacggcggcggcggtggtccggtggtcgactgggaggtgcggctcgccat
cgccgtcggcgtcgccgaggggctggcttacctccaccacgactgcgtgccgggcatcattcaccgcgacgtcaaggcggagaacatcctcctcgggg
agcggtacgaggcctgcctcgcggatttcgggctggcaaggttcgccgacgagggcgccaactcgtcgccgccgccgttcgccgggtcatacggctac
atcgctccggagtatggatgtatgaccaagatcaccaccaagagcgacgtgtacagcttcggggtggtgctgctggaaatgatcaccggaaaacgacc
actagactccaacacgttcggcgaggggcagagcgtggtgcagtgggtgcgcgaccacctgtgccggaagcgcgagcccatggaggtcatcgacgcg
aggctgcaaggccggtcggacgcgcaggtgcaggagatgctccaggcgctcggcatcgcgctgctcctcgaggcgctggacctctccaacaacgcgc
tcacaggccccatcccacgctcgctcttccgcctcccgcacctctccaagctactcctcatcaacaacgccctctctggggagctccccgccgaggtcgg
caacggcacctcgctggtccggttccgagtcagcggcaaccgcatcgccggcacggtaccggccgagatcggccggcttgggaacctcagcttcctcg
atctggggtcgaaccggctgtcggggcccgtaccggccgagattgccgggtgccggaacctcaccttcgtcgacctccacgacaacgccataagcggc
gagctccctccggggctgttcaacgacctgctgtcgctgcagtacctcgacctctcccacaacgcgatcaccggcgccctcccgccaggcgtcgggatg
ctcacctcgctcaccaagctcatcctcaacggtaaccggctttccggcccggtcccccccgagatcgggtcgtgcacccggctccagcttctcgacctcg
gcggcaacgcgtttgaaggcgagataccgggaagcatcgggaagatcccgggtctggagatcgcgctgaacctcagctgcaacgcgttcaccggcgc
cgtcccgccggagttcgcggcgctggcgcggctcggcgtgctcgacgtctcccgcaaccggctctcgggtgacctccacccgctcggcgcgctgcaga
acctcgtgtccctcaacgtctcgttcaacgcgttcgcggggcggttgccggagacgcccttcttcgcgaagctccccaccagcgacgtcgagggcaacc
cggcgctgtgcctgtcccgctgcgccggcggcggcggcggcgaggatgaccacaaccgcgaccgcgccgcacgggtcgccgtcgccgtgcttctgtcc
gcgctcgtggtcctcgtcgcggcggcggcgctgatcctggtccggcagcggcggcgcgcgggggaggaggcagagaaggccggcgccggcgggga
gatggcgccgccgtggaacgtgacgctgtaccagaagctggagatcggggtggccgacgtggcgcggagcctgacgccggcgaacgtcatcggcca
gggctggtcgggttccgtgtaccgcgcgagtctagtctcttccggcggcgtcaccgtcgccgtgaagaagttccggtcgtgcgacgaggcctccgtgga
ggcgttcgcgtgcgaggtcagcgtgctgccgcgcgtgcgccaccgcaacatcgtgcggctgctgggctgggcggccaaccgccgcacgcggctcctct
tctacgactacctccccaacggcacgctcggcgcgctgctccacggcggcggcggtggtccggtggtcgactgggaggtgcggctcgccatcgccgtc
ggcgtcgccgaggggctggcttacctccaccacgactgcgtgccgggcatcattcaccgcgacgtcaaggcggagaacatcctcctcggggagcggta
cgaggcctgcctcgcggatttcgggctggcaaggttcgccgacgagggcgccaactcgtcgccgccgccgttcgccgggtcatacggctacatcgctcc
ggagtatggatgtatgaccaagatcaccaccaagagcgacgtgtacagcttcggggtggtgctgctggaaatgatcaccggaaaacgaccactagact
ccaacacgttcggcgaggggcagagcgtggtgcagtgggtgcgcgaccacctgtgccggaagcgcgagcccatggaggtcatcgacgcgaggctgc
aaggccggtcggacgcgcagcgcgagcccatggaggtcatcgacgcgaggctgcaaggccggtcggacgcgcaggtgcaggagatgctccaggcg

ctcggcatcgcgctgctgtgcgcgagcccaaggcccgaggaccggcccgccatgaaggacgtcgcggcgctgctgcgtggcatccagcacgacgacg
ccgccgaagcacgcaaaggagggggggactactgcagcagccggagctgaaccggaggttatgctctatgtttag
>g33480.t1
atggccggcgaggaggcgccgctgggcgctctcaacctgtccgagtacgcccctgccggcgcccgcacagttgactgctacaggcgcatccgcaagat
cggcgagggcacctacggagaggtgtttgaggctgtggatatcattactggggaaagagcagcactcaagaaaataaaattggatgatggtaaagaa
ggggtactgcacatggaacaggaggatcagatgatcacatttataggggagatatctacatggtttttgaatacatggatcacgatttga
>g33742.t1
atgctgccctcgatgtcgtgctgcctgcgcctcttcatcctcatcgcgttcgccactctgtcaactgaagcaaacctaaactacaattatactggtgatgcg
ttaccgacctatctcgactgcccctccctagcacctgatcagccatcaagaaacgacaccgccgacaacggcaactttcggaacaacgtcgtcgtgctg
ttggagaaccttccgatcgacgtggcttccggtggtggcttcgcctccctcgaaagggtctgcctcatggccttcgcgcaggagatcaacgccaagtgta
gcggagaccggcgcgccgccgcccttagaggtaggtgcttcctgcttattaacgacaccaaggcatcctccacggaaggctacctgcggttattgaaca
acgtacacaacgtctccgacaaggtggctttccagagaacgtaccatgagctcatgggaagcctctctgaccgtgccgccaacggcacgccccagcct
acgtcgacggcgcggttgttcgcgacgggacaggctgtctacgacagcaacgccacgaacggcaccatatacgggatgatgcagtgcatgccggacg
cggaggaggagtgctctcggtgcttgaagacctcgtccgcgtatttctttgggcaacagggaatgactgtgttcgggcacaactgctacctgcgcatgga
aatctacccgttctatgaccttgccctcaatgcacagcaacgtccggcgaacttggtgccgcctacccaagaaagacaggcacttgctctgggtttttttg
ttgctggggtcattgtggtggttgttctgtacaagaggaaggcctggcagaaggtaacactaccagatgattgtagcacggaagaggatatcagaatcg
ttgacctggagcagcttaatctacagttgctgagagcagcaacagacaacttctctgagaaaaacaaacttggggagggaggctttggagaagtcttta
agggtacgttaagagccggagaacagatagcagtgaagaggctctcaaagcattcctcgcagggattccacgagctgaaaaatgagctggtgcttgct
gccaagctcaaacacaagaacctcgcacctctcgttggagtttgcctgcaagaagaaaagctacttgtctacgagtacatgcccaacagtagcttggac
acttttctttttgacccagtcaaacggcaggagctggattgggccaaaaggttcatgatcatctgcgggatcgcacggggccttcgttatcttcatgagga
gtcacgtctcaaggtcatccacagggatctgaaaccaagcaatgtgttacttgatgcagatatgaaccccaaaatatctgattttggcctcgctagggctt
tcggcggagatcagtcccgagatgtaactagacggcctgctggaaccctcggatacatgtcaccggagtatgcctattgtggtcatgtctcgaccaagtc
cgacatgtttagctttggtgtgatcatactagagatggtcactgggcagaagagcaacagcacctacgagtgcctggattccacctctctactgagctat
gcatggaagaagtggagttcagggtcgtggacggatgtggtggacacgtcactgaaggggcagtacccccagagcgaggtcctcaactgcttggtcgt
ggggctgctgtgcgtccaggaaaacccagctgacaggccggatgcgtccacggtggtgctccttctaggaaggtcaaattccatgcctgatgaaatcaa
ccagctggagccctctaggccggccttcttctttggctccagcagaagaggcttcttggatgtggctactaatgggagctccagcgatgaccagatcaga
gatggaaaaccgccttcaaagaatgtgatgaccatttcggattttcaggggaggtag
>g33911.t1
atgccagcgacgcctcgtgacgcggcggccacagaaaatcagcgcggccaagcgaagccagaccagcactctgccttggcttcttcttcgctgcttcgt
cttccccagcccagcccgttacgctgccgctgccgctctcctgctccgttctgcagtcctctccaccgacccaatcgccggcgctttcccccgtcgagccc
gagctccgtattcggcctccaagtctcctcacccgtaccagatccgcccgctcgcgcggcggctgcctccctgctcgcgccgctccagatccgcgggcgg
tgctcctccgccgccgtcgagattcgccaccgtgcgccggtactgacgctgctggcagcccgagatcagctgcacgacgcgccccgtggcgtcaccag
aaggtttccagttgatcccaatgagtacaagttatatgaggaaattggagaaggtgttagtgccacagtataccgagctctctgcgtccctcttgatgttg
tggttgccatcaaagtgcttgaccttgagaaatgcagcaatgatcttgatgggataagacgggaagtgcaaactatgagtttacttgatcacccgaacct
tcttgctgcacactgttcatttacaaatggacacgagctttgggttgtcatgccttatatggctgctggatctgctctccacatcatgaagacttcttttccgg
aaggatttgatgagccggtcattgcaacacttctacgggaagttctgaaagctctcgtctacctacattctcaagggcatattcatagagacgtaaaggc
tggaaacatcttaatagatacaaatggagctgttaagctaggagattttggagtgtcagcctgcatgtttgatactggaaatagacaaagagcaagaaa
tacatttgttgggaccccttgctggatggctcctgaagtcatgcaacaactgcatggttacgattacaaagctgatgtttggtcatttgggataacggcgtt
ggagctagcacatggtcatgctccattctcaaagtaccctcccatgaaggtgttgcttatgaccttgcaaaatgcaccaccaggtttagaccatgagagg
gacaagcggttttcaaaggctggaaacatcttaatagatacaaatggagctgttaagctaggagattttggagtgtcagcctgcatgtttgatactggaa
atagacaaagagcaagaaatacatttgttgggaccccttgctggatggctcctgaagtcatgcaacaactgcatggttacgattacaaagctgatgtttg
gtcatttgggataacggcgttggagctagcacatggtcatgctccattctcaaagtaccctcccatgaaggtgttgcttatgaccttgcaaaatgcaccac
caggtttagactatgagagggacaagcggttttcaaagtctttcaaggatttggttgccacatgcttagtcaaggatccgcgcaagcgcccttcttcaga
aaagctgttgaagcattctttttttaagcaagcacggacagctgaatttcttgcacgaagtattgttgatggcctccctccgctgggtgaacgctttaggga
attgaaggccaaagaggctggcttacttctcaataataagcttggtgcagagagcaaagagcagctatcacagaaagagtacatacggggcatcagtg
gttggaatttcaatctagaggacttgaaaaatgcagctgcccttctagataactcaaatggcacttgtcattatgatgtcagtgataacaaaattaaggat
gactcacaagacacttgtgttcatccagaagataattaccagaaaatggtgaaccatgttgcttctggaatgcctgaagaggaggatgagatacaaga
agttgaagatttgaatgatgctctgtcctcatcttttccaagccgacccctcgaggcactaaaggatgggtttcgtcagaaaaatccaagcatcaggaac
cgcagtggtcctttattgttccgtccagtaaaggattcacgtgcccatttacctggtagcctcatccgcatgcagcagcagggcaaatctacctattggag
cagtgcgcccaaatcccaagtcctcatcaattcttccagcattgcaatttttgatgcagcaaaatactatgcaaaagatgcttctgaagcaagtgtagcca
gttcgtctcagtcttctggagtgtttgttaaggaaaaggaattgcagtcatacgttatccatttgcagcaaagtgtcactgaactaaatgaggaaatacaa
cgactaaagctccgaaacaatcagctcgagcagcagatcaatgcattgtcaaaaacagatgaaaggtcccaaactgaggataaccagcaatga
>g34089.t1
atgggaaacgcatgcggcggctcccttagatccaggtatctgccgagcttcaagacctccgcctcgcagcgccacgactccgaccacagcgccgccgc
cgactcgccgaagaagccctcccgccccgccacgcccccgccgccgcgacggacgcccacgcggcacccgcgccgggcctgccgccgccgccgcc
cggcatgaggcgcggcggcgcggcgcccccggacctcggttccgtgctgggccaccccaccccgaacctccgtgacctctacgccgtgggccgcaag
ctcgggcagggccagttcgggaccacctacctctgcaccgacctcgccacgggcgcccagtacgcctgcaagtccatctccaagcgcaagctcatcac
ccgggaggacgtcgacgacgtgcgccgcgagatccagatcatgcaccacctctcgggacaccgcaacgtcgtcgccatcaagggcgcctacgaggac
cagctctacgtccacatcgtcatggagctctgcgccggcggggagctcttcgaccgcatcatacagcgcggacactacagcgagcgaaaggccgcag

agctcacgcggatcatcgtcggggtcgtcgaggcgtgccactcgctcggggtcatgcaccgggacctcaagccagagaacttcctgctcgccaacaag
gacgatgacctctcgctcaaggctatcgatttcggcctctccgtcttcttcaagccgggtgagctgctgctcattactgattcatattccgacttgattactg
tttcggtcaacgattgtgctgtcaatgatatgcgactaannnnnnnnnnnnnnnnnnnnnnnnnnnnnnnnnnnnnnnnnnnnnnnnnnnnnnnnn
nnnnnnnnnnnnnnnnnnnnnnnnnnnnnngtgggccgcaagctcgggcagggccagttcgggaccacctacctctgcaccgacctcgccacg
ggcgcccagtacgcctgcaagtccatctccaagcgcaagctcatcacccgggaggacgtcgacgacgtgcgccgcgagatccagatcatgcaccacct
ctccggacaccgcaacgtcgtcgcaatcaagggcgcctacgaggaccagctctacgtccacatcgtcatggagctctgcgccggcggggagctcttcg
accgcatcatacagcgcggacactacagcgagcgaaaggccgcagagctcacgcggatcatcgtcggggtcgtcgaggcgtgccactcgctcggggt
catgcaccgggacctcaagccagagaacttcctgctcgccaacaaggacgatgacctctcgctcaaggctatcgatttcggcctctccgtcttcttcaag
ccggaaacgcagcaaggaatatttgatgctgtattgaaaggtgacattgatttcgattctgacccgtggcctgtgatatctgatagtgccaaagacctga
taaaaagaatgctcaatcctcgccctaatgaacgcttaacagcacatgaagttctatgccatccatggattcgtgatcatggagtagcacctgatcgtcc
acttgatcctgctgtcctatctcgcatcaagcaattctctgcaatgaacaagttgaagaagatggctttgcgagtaatagctgagagtctctcagaggag
gaaattgcagggttgaaagaaatgttccagaccatggacactgacaacagtggcgcgattacatatgatgagctaaaagaaggattgagaagatatg
gctccacactaaaggatactgaaattcgtgatcttatggacgcggctgatattgacaacagtgggacgattgactacatagaattcattgctgcaacatt
gcatctcaataaactggagcgtgaggaacatctggtggcagcatttacatattttgacaaagatggtagtggttacatcactgtggatgagctgcaacaa
gcttgcaaagagcataacatgccagatgcttttcttgatgatgtcattaaagaagctgatcaggacaatgacggacgcattgactatggagagtttgttg
ccatgatgaccaagggcaatatgggagttggtcgaagaacaatgagaaacagtttgaatatcagcatgagggacgcacctggtgcattttag
>g34176.t1
atgggcaactgcttcaccaagacgtacgagatacccatcaccacggagccgtcggatcggcggcgcgcgccgtcgtcgtacggctaccagccgccga
ggagccccgatcagcgcaagtcccgcgacgtgccggtgatgacctcgcgccggccgtccttccgtccgccgccgcccttccggccgtcgagcacgacg
ggcaccgggtcgtcgtcggcgtcgtcgatgccccgtcgcgcgccctcctccggcgaggtgggcccggtcctgcagcggccgatggtgaacgtgcgcac
gctgttccatctggagcggaagctggggagcgggcagttcgggaccacgtacctgtgcacggagcgcgagacggggctccggtacgcgtgcaagtcc
gtgtccaaacgcaagctggtgcgccgcgccgacgtggaggacatgcgccgggagatcaccatcctgcagcacctcagcgggcaggccaacgtggcc
gagttcaagggcgccttcgaggacgccgactacgtgcacctcgtcatggagctctgctccggcggggagctcttcgaccgcatcaccgccaagggcac
ctactccgagcgccaggccgccgccgtgtgccgcgacatcgtcaccgtcgtgcacgtctgtcacttcatggggggtcatgcaccgcgacctcaagccaga
gaatttcctgcatgccagccccgcggaggacgcgccgctcaaggccatcgatttcggactctccgtcttcatcgaagaagaacatccatggctcaaaga
aggtggtgcatcagacagacctatcgacagtgcagtcctatcaagaatgaagcaattcaaggcaatgaacaagttaaagcaattagcactcaaggtaa
tagcggagaacctatcaccagaagaaatcaagggattgaagcaaatgttcaataacatggatactgacaagagtggcacaatcacagttgaagaact
gaaggaaggactgacgaaactagggtcgaagattagtgaagcagaggttcagaaacttatggaagcagttgatgtagacaagagtggcagcattgat
tatacagagttccttactgacatgatgaacaaacataaagtggaaaaggaggaggatttgctccgtgcatttcagcacttcgacaaagatagcagcgg
gtacataacaagagatgaactggaacaagccatggaagagtatggaatgggtgatgaggcaagcattaaacaagtactggatgaagttgataaagat
aaggatgggaaaattgactatgaagagtttgtggaaatgatgaggaaaggaagctatacctga
>g34202.t1
atgggctttgttcgccttcctcaatctgatttttggggttctgatctagactacatgccatcgacatctgtggatgcttgtgaagctctgtgcttggcaagat
gctcctgcctagcctttgagtacaaatcagataacaatggttgcttcttgaaatctgttctcttaaatggaaagactgttcctgggtaccctggcactgcat
acatcaaggttcctgagagctttttatctgaaatatcttctgattctaaatatactgaattacataactgcgatgagtcaagaaggcaagttgaagttccaa
tatttgattctaatgtaagaaatggtggaaaggctacgatgtggtcctactactatgggtttcttgcggcattctttttcatagagctctgcttcattgctttg
gttggtggttcatcgcaagaaaacattcagagcaatcagagacatgggcagcggaggaaggttacagggttgtaacaaatcatttccgcaggttttcct
atatggaacttaggaaggccactaaaaatttcaaagatgagcttggtcatgggagatatggttctgtatacaagggtatcctacctgacaaacgcataat
tgctgtaaagaagctcaatgatgtaagccaaggagaagatgagtttgagacagaggttagtgtaatagggcggatctaccatatgaatctggtgagag
tatggggtgtttgttctgaacggaagcaccagttgctggtctacgaatatgtggaaaatggctcactggcaatgttcttgtttggtaacaatgggttgctgc
aatgggaccagaggtacaagattgcagttggtgtggccaaagggttggcgtaccttcaccatgagtgcttggactggatcatccattgtgatgttaagcc
tgagaacatactactggacgaggattttgaacccaagatcagcgactttggggttgcaaagctgctacagagagatcaaactgatccaaacgtgtcaa
aggttcgtgggacaagaggctacgctgctccagaatgggcctttaacattccaataaatgaaaaggtggatgtttatagctatggggttgtgcttcttga
attagtcacgggttcaagggtatcagagttgagatcaaatggctacggtttcgttgatgctagaggtggctactatgtgcatggagaaagagaggagtc
aaagaccaaccatgaatga
>g34382.t1
atggggagcgacaccgagacggagaagaagaaggcccccgtggcgctggcacctatcgcgaagcccctcgccgggaagaagctctgcaagcgaac
cctcaagctcgtgcgacgagcctcggaggcaaagtacctgaagcggggagtcaaggaggtggtcaagagcatccgccgtgggaacaaagggccggc
aaacgccctcctctctcccaaaggcgtaaactatgaagttcaagcgctcatgatgatcaagaactatcttaaggaccctcatggtgtgctaaagaactgg
gaccaagactctgtcgatccttgcagctggaccatggtcacttgttcaccagagaaccttgtcactggcctagaagctccaagtcagaatctttctggcct
actctccccaagcatagggaatttgacaaaccttcagacaattcttctgcagaacaacaacataaatgggttaatcccagaagatattggcaagttaac
aaagctcaagacacttgacctgtccagcaaccatttctccggtgaaatcccaagctcggtggggcaccttgaaagccttcagtatctggatctgtcctac
aataatttgagtggtccaatcccagggtctctgacgaagacattcaacatagtagggaatcccctgatctgcgaagcaagcacgggacaagattgttac
agaactttaccaatgccgatgtcctacagcctgaatgacacacagggtatgcagtcattaactaaggtgacacacctgtttgatgttactattttcgccac
tgcagaacaacacatagaaaacgtcaacctcggaaatatgaagaggtaccagttcagagagctccaagctgcaactgaaaacttcagcagcaagaac
atattagggaaaggtggctttggaatcgtttaccgcgggcagctagcagatgggactgttgtagctgtcaagaggctcaaagatggtaatgccgcaggt
ggggaagcacaggttcaaactgaagttgagatgatcagcttggcggtgcacaggaacctcctcaggctctacggattctgcatgactaccagcgagag
gctactggtctatccgtatatgtcaaatggaagtgtggcatttcgcctgaaagggaagccaccactggactggacaaccagaaagaggatagcacttg
gggcagcacgaggtttactatacttgcacgagcagtgtgaccccaaaatcatccatagggatgtaaaagcagcgaacatacttcttgatgactgctgtg

aagcaatcgtcggagattttgggctggcaaagctcctggaccaccatgactcacatgtcaccacagcagtgaggggcactgttggccacattgccccgg
agtacctctccactggccagtcgtccgagaaaanagagtatctctctactggccagtcgtccgagaaaactgatgtctttggtttcggaatcctgcttctg
gagttgatcactggccagactgcactggagtttggaaagtcatcaaaccagaagggagcgatgctagactgggtgaagaagatgcaccaagagaag
aatctggatatacttgtcgacaagggcctgaagagcaactacgaccgcattgagctagaggagatggtgcaggtagcgctgctatgcacccaatacctt
cctggccataggcccaagatgtcagaggtggtccggatgctcgaaggggatgggctcgcagaacggtgggaggcatcacaaaactccgagtcacaca
agttcaaggtgccagaattcaccttcggccgctgctactctgacctgacggacgactcgtcattgctggtgcaggcagttgagctctctgggcctagatg
a
>g34517.t1
atgtccaaggcacgggtctacaccgacgtcaacgtcctgcgccccaaggagtactgggactacgaggcgctcaccgtccaatggggtgagcaggatg
actatgaagttgtcaggaaagttggaagaggaaaatacagtgaagtttttgagggcatcaatgttaataataatgagaaatgcattattaagatcctca
aacctgttaagaaaaagaagatcaaaagagagatcaaaatacttcagaatctctgtggaggccctaatattgtgaagctgcttgatattgttagagatc
agcattcaaaaacacccagcttgatctttgaatatgtaaacaacacagacttcaaagtattgtacccaacattgacagattatgatattcgctattacatc
tatgagttactcaaggccttagattactgccactcacaaggtattatgcaccgagatgtgaagcctcacaatgtcatgatagatcatgagcttcggaaac
tccggctgatagactggggccttgctgaattctatcatcccgggaaggaatataatgtccgtgttgcatccaggtacttcaagggacctgagcttcttgtt
gacttacaagattatgactactctttggacatgtggagtcttggctgcatgtttgctggaatgatattccgcaaggaaccatttttctatggccatgacaac
cacgatcaacttgttaagattgcaaaggtacttggaacagatggactgaatgcttatttgaacaagtacagaattgagcttgaccccccagctagaagcc
cttgttggaaggcatagcaggaagccctggtctaagtttattaatgcagacaaccaacatctagtatctcctgaggccatagatttccttgacaagcttct
tcgttatgaccaccaagacaggcttactgctcgtgaagctatgacaaccttatcctcctgcacgcccttcgtccccgccgctgccctgcacttctccccgcg
cgagcgagctcgcttcccttcgtccgttggcgcctccacttctcccccgcgcgagctcgtcgccctgtgctcctccaccgccgccaccctccacttctccct
cgcgcgagcatcgccgccctgcgctcctcccctgccgtcatcgcctcctcccccactgccgtcgcttcctccccctcgtgagcccgctacccttcccttctc
ccccgccaccatcggctctcccccctcgcgagcctgccgtcctcccttctcccctgtcgtcgtcgactcctccccctcacaagcctgcgagctcggtgcgca
gctcagtcgagctcgcgccgtccgacctgccgagccagcacttcagtccaagctcgatgacggcgccacttcttcctccccgtggagctcggcacactcc
ccctcccctggcgcctccacttcttcctcctccccgtgcgagctcgccgggtttgggtatgggcgctgccggatttgggatggaaatgtctggcgcgaggt
ag
>g34547.t1
atggcggcactggccgccgctctagccgccgtcctcttcgttctcgtggcagttgctgaagccgacaccgacgccgctgatgtagcggccttggggaac
ctctactcctcttggaacagcccggctcagctcgcaggttggtcggctggcggcggcgacccctgcggcgccgcgtggatgggtgtctcctgtacgggct
ccgccgtcacctccatctatatgcaaaataatcaactatcaggcacaattgatatgctcagcaatctaagccttgcgacactgtacttgcatgaggtgtgc
ctgccccctgttgtacagagaaacttaaagtcatcaaatatccttcttgatgaagagaacagcccacatctgtctgactgtgggcttgctgctctatcacc
aaatccagagagagagagatttcgtctgagattattgggtctttcgggtacagtgccccagagtttgcgatgtcagggacatatacagtaaagagtgacgt
gtacagttttggagtagtgatgttagagctattgacaggccgtaagccactagatagctcaagggagaggtcagaacagtctcttgtcagatgggctac
cccgcagcttcacgatatcgatttacttgccaagatggtcgacccggcattggatggcatgtatcctgcaaagagtctatcccgtttcgctgacataatcg
caatctgtgtccagccggagccggagttccgtccaccgatgtccgaggtcgtccagcaactggtgcgcatgatgcagagggccagcattgtgcggcgg
cactcggatgatctgggattctcctacagagtccccgatcgcgaggtaggcgacatcgtctga
>g34784.t1
atgagagaaccaacagaggttcgttgggccggcggccggccgcggaccggcgctcgcggagtggtcgccacgcgcgccccacattccgagcccaag
ccgcgaactgaccgccgggagaccgcggcccggatatcccgcgcgggtggcagggggagcaagtgcgcgctcgccgcacgcgtggccgtccgttcg
gggacgatggaggatcgcacgggcgaggcgggggagggggaccggcgcgcgggccgccacggcggtggcaatgattgggctcgcacccgggagctt
tgaccggcagactcacctcacctgtgcccaaaaattccggctgagttcctcccgctcagggctcaccattttcttcaaagcttctaaattcacggggacct
tcgctggtggtgactctggctccaaaaggccaaaatttgatcaggagggagcaggagatattgttatcgaaccacacctaaccgaggacaaagctgtg
aggatagaccaagaattgtcatcatcgtcgaatagagatgctgaggcaagcacatctactagcatgaagcctgttcaaactgaagaagcaagggcag
atcttcttccaaaagaactgaatgatatgacaattagtgatgataaagttgacggccataatgacaaggaaagtgaaggggttactgttgatggaaatg
gaactgagacaggacagatcatcgtgacaacaataggaggccataatgggaagccaaagcagaaggtctcatacatggcagaacgggttgtcggta
ctggttcgtttggtgtagtttttcaggccaagtgcttggaaactggtgagactgttgcaattaaaaaggtcaatcctcatacacatcaactcaaaatttgtg
attttgggagtgctaagaaactggtcccgggtgagcctaacatatcatacatttgctcgcggtattatagggctcctgaacttatatttggagctacggaa
tataccactgcaattgatatctggtctgttggttgtgtaattgctgagcttatgattggtcagattttgggtactccgacaagagaagaaatcaggtgcatg
aacccaaattactcagaattcaagttccctcagattaaagctcatccctggcacaagctttttggtaaacgcatgccgccagaagctgttgaccttgtgtc
aaggctacttcagtactcaccaaatttgcgctgcacagctgttgatgcttgtgctcatccgttctttgatgagctccgggaccccaaggcctgcttgccaaa
tggaaggccactaccaccattgttcaacttcacagcagctgaattagaagggctccctattgaacttgtccatcggattgttcctgaacacatgaggaaa
tga
>g35317.t1
atgttcaagttcctcaaggaggtggtcgcgggatccggatcgggcctcaaggacttcccctacaccatcggcgagccctacgcctccgcctggggctca
tggacgcaccaccgaggcaactcaaaggatgatggatcgcctgtgtcaattttttctctgtcagggagcaatccacaggacaggcatttggttgctggtc
gcaatggtgtgaagagattacgaacagtgcggcatccaaatatcttatcctttctccacagcaccgaagctgaaattcctgatggacctgccatgaaaca
cacaatttatattgtaacagagccagttgtgccactttcagagaaggtcaaggaactgaacctgggcggtacacagagagatgagtactttgcatgggg
acttcatcagattacgaaagctgtgagctttctcaataacgattgcaagcttgttcatggaaatgtatgcttggccagtgtagtagttactcaaacacttga
ttggaagcttcatgctttcgatgttctttcagaatttgatgccaacaatgaagcctctggtagccccatgctgtacaagccaatggagctgacaaagtcag
actgggctacaatcagaaaatcaccttcgtgggccattgattcttggggactggggtgtttaatctatgaactcttttctggtgcaaagctggctaggaca
gaggaccttcgaaatactgcttcaatcccaaagtctttacttccagattaccagagactcttgagttccgcacctactcgtagactaaatccttcaaaactt

attgataacagcgggtgtttaatctatgaactcttttctggggcaaagctggctaggacagaggaccttcgaaataccgcttcaatcccaaagtctttact
tccagattaccagagactcttgagttccgcacctactcgtagactaaatccttcaaaacttattgataacagcgagtttttccagaacaagttagtagaga
ccattcagttcatggaagttctgaatttaaaggatagcgttgagaaagatagctttttccggaaactcccaaatgtagccgagcagcttcctcgtgagatt
gtcctgaaaaagttgctccctgtattggcttctgcgcttgagtttggttcagccgctgctccggctttgactgttttgttgaagatgggatcctggcttccatc
tgaccaatttagtgccaagatattgccaacaattgtcaagctgtttgcttccaatgaccgggctatccgcgttagtcttttgcagcacatagatcagtttgg
ggagtcattgacagctcaaactgttgatgaacaagttttcccacatgttgccactggcttttctgatacttcttctattctccgtgaactgacattgaagtca
atgcttgtattggcaccaaaattatcgcagcgcacaatctcaggatctcttttgaagtatctctctaagctacaggtggatgaagaacctggaatcagga
caaacactacaattcttcttgggaatattgcaaattacatgaatgatggggtttgtctcaagaacactactatcatttttgtttgtattcgttgctga
>g35557.t1
atgtcggacggcggcggcggtgacgagtacaagcgcgaggagtcggtggccctactggtcatcatctccctcgctgcggtggccctcctctccctcatc
gccgccttcgcctactactgctacatcacccgcaaggtctcccgccgcctgcactcgctcaatctccccaagcgctcctcgtcccctcccccttctctgcgg
ccgccgccgccgccgccccaggggaaggagagcccatccagcaactccgccagcggcggcggggcgggggccaccgcggtggtggtgggtgg
ggagaggggcgtgcaggtgttcagctaccggcagctccatgcagccacgggtgggttcgggcggggccacatggttgggcaggggagcttcggggcc
gtgtaccgcggggtgctcgccgacgggaggaaggtcgcggtcaagctcatggaccgccccgggaagcagggcgagcaggagttcgagatggaggtg
gagctgctgactaggctccgatcgccttacttgttgagtctgattggtcattgttcagaaggtggacaccggctactggtgtatgagttcatggccaatgg
cggcctccaggagcatctctatccaagagatctctccctgaacatctgtgtcatgctttctcaacatgtgatgcttatgcaggtggagctgctgactaggct
ccgatcgccttacttgttgagtctgattggtcattgttcagaaggtggacaccggctactggtgtatgagttcatggccaatggcggcctccaggagcatc
tctatccaagagatacctgtggtgggatctcaaaattgaactgggatacaagactgagaatagcacttgaagcagcaaaaggtctggaatatcttcatg
agcgcgttaatccacctgttatacacagagacttcaagagcagcaatattcttttggacaaagatttccatgctagggtttcagactttggtcttgccaag
cttggatctgatagagctggtggtcacgtctcaactcgagttttaggaacgcaaggctatgttgcaccagagtga
>g35590.t1
atgtcaggctcggacacccgctaccctctcttccccccttctccccgccttggttctgcttctggtcgccggcgggtgccgcgcgcagcccgccgccgccgc
cggcgaccgcgacacgctcctcgccgtgaagaaggattggggcagcccggcgcagctcaattcatggaattcctcttcctccgaccactgcagctggca
cggcgtcacctgcgccgccgggtcagtcacggggctgtcgctcccgagcctgaacctgagcggctcggtgccggcgtcggtgtgctcgctcaagagcct
cgcccggctcgacctctcctacaacaacctcaccggcgcgttccccgccgccgcgctctacgcctgcgcgaacctcaggtacctcgacctctccaacaa
cctcttctcgggacccctccccgccgacatcgacgggctctcgccggcgatggagcacctcaacctctccacgaaccggttcgcgggctcggtgccgcc
ggctgtggcgcgcctgccggcgctcaagtccctggtgctcgacaccaaccacttcgccggcgcgtacccggcaaaggaaatcagccggctcgccgggc
tcgagcggctcacgctggccgacaacgacttcgcgccggggcccgtgccgagggagttcgccagtcttaccaagctcacctacctgtggatgtcaaata
tgaacctcaccggagaaattccggacgcgctcggcagtctcacggagctcacgatgctcgccatgtcggggaacatgctcaccggcgccgtcccggcg
tgggtgctgcagcacgagaagctggaggtggtgtacctctacgacaaccggctctccggtccgcttccgcgcaacgtgtcggcggcgaacgtggtgga
gctggacctgtccatgaaccagctcaccggaaccataccggacagcttcggcaacctcaagagcctgttcctgctgttcctctacaagaaccagctcac
cggcacgattccggcgagcatcggcctccttccgcggctcggcgacatccggatcttcttgaaccagctctccggcgagctgccccccggagctgggcaa
ccactcgccgctcggcaatttggaggtgtccaacaacaacctctccggcccgctgcgggagtcgctctgcgccaaggggaacctgtacgacatcgtcgt
cttcaacaacagcttctccggcgcgctccctgcgaacctgcgcgactgcgtccggctcaacaacctgatgctctacaacaaccggttcacgggcgagttc
ccggcggagatctggtcgttccccaagctcaccaccgtgatgatccagaacaacagcttcaccggcacgctgccgcccgtgatatccttcaacatgtcg
cggatcgagatgggggacaacttgttcaccggctcgataccgacgtcggccaccaagctgcacgtgttcaaggccgggaacaaccggttcaccggcg
aactgccggccgacatgagcaagcttgccagcctgacggatttcctggtgcccgggaacaggatatccggctccataccgggttcgatcaagctgctgc
agaagctgaattcgctcgacctgagcggcaacctgctttccggcccgatcccgccgggcacgatcgggcagctcccggtgctcaacatcctggacctat
ccagcaacgagctcaccggcgacataccgtcggatatccacaacgcgttcatctcgctgaacctgtcgtcgaaccggctcacgggcgaggtgccggcc
gcgctgcagatcgcggcgtacgaccgcagcttcctcggcaacgaccgcggcctctgcgcgcggaccgggtcggggacgggactcccggcgtgctccg
gcgggccaggcgggcacgacgcgctgtccaggggcctcatcgtcctcttctcgatcctcgccggcgtggtcctggtcggcagcgtgggcatcgcgtggc
ttctgctccggcggcgcaaggacatccgcgaggtgaccgactggaagatgacggcgttcacgcagctggacttcaccgagtcggacgtgctgagcaac
ctccgcgaggagagcgtgatcggcagcggcgggtccgggaaggtgtaccggatccacctcgccggcggcaacggaggaagcaggatggtggcggtg
aagaggatctggaactcgaggaagctggacgcgaagctggacaaggagttcgagtccgaggtgaaggtgctgggcagcatccggcacaacaacatc
gtcaagcttctctgctgcgtctccagccaggaagctaaacttcttgtgtacgagtacatggagaaccgcagcctcgaccggtggctgcaccaccgcgac
agggagggcgcccctgtaccgctggactggcccaccaggctcgccatcgccatcgacgccgccaaggggctcagctacatgcaccacgactgcgcgc
agcccatcgtgcaccgcgacgtcaagtccagcaacatcctgctcgacctcgacttccaggccaagatcgccgatttcggtctcgcgaggatcctcgtca
actccggcgagccggagtccgtgtcagcgatcggtggaacgttcgggtacatggcgccagagtacgggtacaggcccaaggtgaacgagaaggtgg
acgtgtacagcttcggcgtcgtgctgctggagctcaccaccggcaaggtcgccaacgacagcggcgccgacctgtgcctcgcggagtgggcgtggaa
gcggtaccagaagggcgcgccgttcgaggacgtggtcgacgaggccatccgtggggagccggcgttgatgcaggacatcttgtcagtgttcactctcg
gtgtgatctgcacgggggagagccccctgacacggccgtccatgaaggaggtcctgttccacctcatccgatgcgacaggatgtccgccgaggcgcag
gcgtgccaggtcgactacgacgccggcggcggcgcgccgctactggaggtcaagaagaaaggcagcgggcggcggagcatgtcgagctccggcag
gtgggatgaggacgacgacgacagcggcaacttcgtcgtgcacgtggtctag
>g35767.t1
atgcccaaggttgcagactttgggtcatccaagcacatgttaaagtccacgtacgtcagatccgtgaaaactgataggaactacttggatcccgtgtaca
tgaagacagaccgtttcaccaccaagagtgatgtctacagctttggtgtggtgctcctggagctcatcacaagaaagacacccaagtacgacggtaaca
acagcctctcaattgacttcgtacagacctgcaaggtggagggcaacggaaggaagatgtacgacacagacatcttgatgaatgatggcaatgctcaa
tcatctcaagtttacattgagtgcctggacaagattggggaactcgcggtgcgatgcctcaaggaagtcgacgtggatgagagaccgaccatggagag
gtggtggaggagcttaaggaagtcaagttaa

>g35770.t1
atgcccaatggcagcttacagatgctgctgcactccgaaggtagacggcagttgggtttcctaaagaggctggacatcatgctagatgtggctatggcc
atggaatatctgcaccatgagcatcacgatgtggtcttacactgcgacttgaagcccagcaatgtactctttgatacggacatgacggggcatgtggctg
acttcggcatcgcaaagttgctgttaggcaatgacaactcattgatcacagctagcatgcctggaactcttgggtacatggcaccagagtatgggtctctt
ggcaaggcttcgcggaagagcgacatgttcagctacggaatcatgctccttgaagttttcactggaaagcgacccaccgatcctatgttcatcgcggatc
tgagcatcaggcaatgggttcgttga
>g35811.t1
atgtacagtagcattctcggcgtcgtcgtgccaacagggatgagcagcaggaagatgaagagcctcctgaatctgaccacggcgcaattcggattcga
gaaggaggagggcgtgctctgcgagagcgaccgcagcatcgtcatgaaagtgcggcaccgcgtggccggaaagaccttcgtcatcaaggaacttcag
ctgctcactacaacagaagaagatgaagctgaagaggagaagattgcgctacgtgtgcttcgcgaggcttgcttcatggcgtccacccttccctgcctg
ctggaagtcgtcaggagccagcgccgcgacgagcccttcccggagcaggacgtgcggtgcatcgtgaggcaggtgctcggaggcgccatggcattgc
acgaacgcggcgtcgtccacggcgccatcaacacggagaccgtcctcgtcgccgattacgccaacctcgtcgtcaagatcggcggcgccggccaggc
caccatgggcaacgccgccataaaagagggcgactcgtggtccatcgggtgcttgatggcggagcttctcacgggcaagccgctgtttgatgaggaac
aagggagggaggaagaaagcctccacgagattttccacgtgctcggctacccggcggcggacaaggagaccatccatcaggtcatcatcaagctcag
cagcaacaaccacgaccaggagacgagcaccaagaaccggctgcgcgagctggtccccctgagattctctccgacgacggattcgaggtgctccaa
ggactcctcacctgcaatccaaacaagaggctcaccccgccgccgcattacaacgtccatggttccatcaccttcacgccgctccagaatccacaaca
actgcactcgtttctctttctcaggtcggctgcccactagcactagtaaagagcactgtaaacaacgctaggcctaaagctcattgtaactaa
>g35923.t1
atggccgcatcgctggctgccgatccgaccttctgcgagcgcttcctgcgcgagggcaggaccctggcgcggctgtcgcaccccaataccgtgaccatc
cacgacatcggcaatgtcggcagcctgtactacatggccatggagtacctgcccaacggcacgctcaaggagcgcatcgccgcgggcctgagcatcg
accaggggctgctgtggatccgccagatcgcctcggccctgggcatcgaccaggggctggtgtggatccgccagatcgcctcggccctgggttacgcc
cacgcccagggcatggtgcaccgcgacgtgaagccggccaacatcctgttccgcgccgacggcaccgcggtgctcagcgacttcggcatcgccaagg
cgctggacgatcgcacccagttcacccaggccggcttcgccgtcggcacgcccagttacatgagccccgagcaggcccgcggcctggacatcgatggc
cgtgccgacctctacgccctcggtgtggtgctctacgaaattctcaccggcaagctgccttataccggcagcgattcgctgtccacggcgctcgcgcacc
tgaccgagcctttgccggcgctgcccatgcgatag
>g36482.t1
atggttgtgatctgtggcggcattgacgactgctgctgctgggctttgcggttccccttgctgtgcgcgcgcgggaggccgagatgcggcgtcttgctct
cctggtgcttcttcttgcttcggttcctggatccagaggtactactctggctcctgctccagctgttagtgattctcctgcaaatcaaggacaaaattctact
cctcctgaacctgtcattgctcttgctccagtgatccttccaacagcaccgcctgctccaccagtaagtcctgccccggagaagggagctgttagccctgc
tgttcccacccagccgcaaaatgctccgattccagtaactccccctaaagagtacagtgcaacccctccaattgctgatgcacccccctgatcccactggc
gatgttcccccgccagtagctccgccacaaggtgcagtcgaaaatcccactccaatacttcctgggacacctgcattactgccttctgttcaggctcctgc
tccatctgctgccctcaagcctaatccaccagtcgtgcctcctccgtctgtcaacaatcaaccaagcatgccgattaatcccactccagtacttcctggga
cacctccattgctaccttctgttcaggctcctgctccatctgcagccttcaagcctaatccaccagtcgtaccgcctccatccgagaacaatcaaccaagc
aggccggttgggtcagttccacatgttaaccctccgaccgcatcgccgtttactgcagaagcaccacaacagaaggcagctgctccaagtagcaagcat
atggatgggcaccatatttctcctattattccaccaatccctcctgaatcagagccagaagcaccatcagctcatgctatctgggcactacctccaccacc
acctaatctaggacaggcaaaaacattcaaatttgctgagattgacaaggccacagatggctttgatgattcaaaggtacttggagaaggtggctttgg
atgtgtctaccagggcacacttgaaaatggaacgagggttgcagtaaaggttctgaagagatatgatggccagggagcggaccgtgagattgctccac
ttgattggaatgcccgaatgaagatagccctgggggctgcacgagcacttgcatatctacatgaagattcaagcccttgtgtgattcatcgtgatttcaaa
tcgagcaacattcttcttgagcaagatttcacaccaaaagtgtcggacttcggactagccaggactgcaagaggggaggggagccagcacatctccac
tcgtgttatgggaacattcggctatgttgcaccagaatacgccatgacagggcatcttcttgtaaagagtgatgtatacagttatggagttgtattgcttga
gctcctcactggtaggaagcctgtagacatgtctcagcctgcagggcaagaaaaccttgttgcatgggctcgccccccttctgacaaatgtgctgagccta
cggcaagctgttgatccgcttcttgggcctagtgtaccacttgacagtgtagcaaaagcagccgccattgcacaaatgtgtgtgcaacctgaggtcgcac
atcgtcccagtatgggcgaagttgttcaggccttgaaacttgtttgcagtgagggtgatgacggtcttggatcgggaagattcagccaggagttgccggt
acaaactacaacagtttatgatgccactggcatggaagcggagagggttctgttatctgagatatttggctcgacacctgtcttcactccagctgctgaga
caagttctttccacaagcaatccagctcaggccctctgatgacaggcaaaaacaggaagttctggcagaggttgcgaagcttgtcaaggggggagcatg
agtgagcatggtttctcaccagattatgagacacattcgcagtatagtggttga
>g36543.t1
atgtcacacctttcagatgctggactggcaagcttcatccccgatgctgaattccaggcagcagagcagagtgctggatacactgccccagaggtggac
atgactggtcagtacaccctcaagagtgatgtatatagttttggagttgtcatgcttgagctattgacaggacgtaggcctttcgacaggtacgcgctcag
aactggtttcagcttctga
>g36901.t1
atggcgggcaccccgtgcttgagccgtctcctcctcctggtgggcaccatcctgtcgctcctgatgccgacgagccatgcgcgcgcgtgcccgccctgcg
gctcgacgcccgtgccgtacccgctcagcacggcggccggctgcggcgacccggcgtacaaggtccggtgcgcggccaatagtaactcctcgacgctc
ttcttcgacgcggtcaacggcacctcgtacccgatcacctccatctccccctccgcgcagcgcctggtggtgtccccggcgccgttcgagtccccgtccgc
ctgcgtctcgcgtggcgcgcccgcggcgcgaggggtgcagctggacccgtccctgcccttcaacgtgagctcctccaacaccatcatgctgctcaactg
cacggccgcgctgctgcagtcgccgctcaactgctcctccgcctcgctctgccacgcgtacgccaacgccacggcgccggcgtgcgcgcctctgccgct
gtgctgcacgttcgtggccgggggatcgtccacgtcgtacagcatcagggtgtcgccgcggttctgcagcgcgtaccggagcttcgtggggctggaccc
gacgcagccgccggctacgtggggaagccgcctcgggctcgagctgcagtgggccacgcccagggagccgctctgccgcacgcaggccgactgcga
ggaaggagccaacgccacctgcgccgatgatccttcctcccccggcacctccacctccaggcgatgcttctgcgtgcccgggctcacgtggagcccccct

cgccggcgcgtgccagcagaatccttcggattgccagaggaccggagactgtggaggatccaaccacgcgcctctcatcgcaggcctcgtgtgtggcc
ttggcggcgctctgctggtcgccgcagcggcgcttttcgcctaccggcggcagcggcgcatccggctagcccgggagaggctggccaaggagcgtgag
gagatcctgaacgccaacaactcgagcggccgcaccgccaagaacttctccggccgcgagctgaagcgcgccacgggcaacttctcccacgacaacc
tcctgggctcgggcggcttcggcgaggtgtacaagggcgtgctcggcgacggcaccgtggtggccgtgaagtgcgcgaagctgggcaacaccaagtc
cacggagcaggtcctgaacgaggtccgcgtgctgtcgcaggtgaaccaccgcagcctagtgcgcctgctgggctgctgcgtggacctggaccagccgc
tcatggtgtacgagttcatccccaacggcacgctcgccgaccacctgtacgggtccatgatgagccgcccgccgctgccgtggcgccgccgcctcgccg
tcgcgctccacacggcggagggcatcgcgtacctgcacttctccgccgcgccgcccatctaccaccgcgacatcaagtccagcaacatcctcctggacg
atcggctcgacggcaaggtctcggacttcgggctgtcgcggctggcggagcccgggatcagccacgtgtccacgtgcgcgcaggggacgctgggata
cctcgacccggagtactaccgcaattaccagctcacggacaagagcgacgtgtacagcttcggcgttgtgctgctcgagctgctcacctccaagcgcgc
catcgacttcacccgcggggccgacgacgtcaacctcgccgtgcacgtgcagcgggcggcggaccaggagaggctcatggatgtcgtcgacccggcc
atcaaggacggcgccacgcagctggagctcgacaccatgaaggcgctcggattcctcgccctcggctgccttgaagagcgccgccagaacaggccat
ccatgaaggaggttgccgaggagatcgaatacatcatcaacattgaggctggcggcaatgccattcatagtcactga
>g36982.t1
atggagcggtacgaggtgatccgggacattgggtctggaaactttggtgtggccaagctcgtccgcgacgtccggaccaaggacctctacgccgtcaa
gttcatcgagagggggctgaagcaagtgtgccacagggatctgaagctagaaaacacccttttagatggaagtgttgctcctcgacttaagatttgtgat
ttcggttactccaagtcttctgtgctgcactctcaaccaaaatcaactgttggcacacctgcgtacattgctccggaggttctctctagaaaagaatatgat
ggaaaggttgctgatgtttggtcatgcggagtaacactatatgtgatgcttgttggtgcatatccttttgaggaccctgatgatccaaggaactttcgcaa
gacaatcagcgttagtacaccaattctccgtcttcatgatattcatgaaaaataa
>g37113.t1
atggctgctctccttgctgcttttgtcatcctctctttgcttgctctacccttgcatgccgcacatcacagtatcctgccgctcaaatcgtttctcgccgtcgaa
gagtacaaaaccaagattctgcagtcacgggacggcacattctcctgtggcttttaccaaatctacaccgacgcctttaccttctcgatatggtactccaa
ttcggtgaacaagacaaccgtctggagtgcaaaccgtggcagccctgtgcgctcctgggggtcggctgtcaccttgcgcaaggatggaagcatcgtact
cacagactacgacggcacagttgtatggcaaactgaaggtaatttcccaaatgttcaatatgctgagctactggattctggaaatcttgtcctgaagaatt
ctagtggtgatattgtttggaaaagttttgattcaccgacagatacatttcttcccacacagctcatcactgatggaataaagagggagcagaggtcatc
acaagtgtgggccgctgaggaaggttacagggtaatgaccaatcatttccgaatgtacagttacagagagctggttaaggcaactgagaagttcaagc
acgagattggatggggaggtacaggggttgcctacaaggggatattggatgatggtcgagctgttgtcataaaaaagctgggaaatctggtcagcaga
tctgggactaaccaaaatgtatcgcgggcgcgaggaaccattggctacattgctccagagtggatatctggttcaccaataactgcaaaagtcgatgtat
atagctacggagttgttcttcttgaactggtatctggaatgagggtttttgatttggtgaaagatgaggatgagaagttgcatgtgatgctgaagaattat
gttaagatgctttctgataaattgcatgggcaggacccattttggctggaagaattcatagatttcagactgggtggtgaactcaacaactcgcaggcag
aagtgatgatcaaggtggctatctcctgcttggaggaagaaatgaagaaaaggccaacaatggaatcagtagtcgaaatccttcttgatgttgctgaaa
tcaatgaaacaacaacgtgttga
>g37335.t1
atgctcctccgcacccgtgccaccgctcagctcttccgcctcctctccccgaccatcgacggcgcgcgaggctgcacggtagggcgtgtcgtcgtgcctg
acggaccctccgagatagtcgagtccaccgaagaggaatttgacagtgcttctgttgattgctccggcttgcggcaaattaaagtgaaacctgaggagg
aaaacggcaagtgtcgtgattaccctgtcgaagatgacctggaccaacttcttaaagccatcgacagcaggaccttccgccgggcacttagtcctgcca
atactggtggagatgcgctaggaaagagttctctgaagaagccatctagatcaggcttgtctcaaaattcaggaatcgggatatcctcgaagactgtta
atatgaagcaagccctgaggaggctgtgcatttcccaggcatcagaaatggctgcaatgaagaggctatcaatgtcacctggatcttcaagttcatctga
agctggaaccatccacaggctttatgcctctttgatggtccaaagtaatgatgaaaattatcttcatgatgatgaaaagatgaatcttattgaaatatcact
caccccagggaagattgataagtgttcatctagaggaacaagcgagttcagtgaagactgtgattttgagacagctgacggaagtgcagtcacttccat
ccgttctgtatcttccacatcagctgaggtaaggaagatcaggattcaagatgtcatcagtggcgaccctgtagatatcgagaactcctttgtggaaaat
agtggtgatttgcatgttcttcgtcaaagacaacctggaagaagctttccagagccagcagcaaggttttatgtcgcagaagttctccttgctctggaata
tcttcacatgctgggggtcatataccgcgatctaaagccagagaacattctagttcgtgaagatggacatataatgctgtcggactttgacctctcactga
ggtgctctgttaaccctgtgctccttcggtcatcctctatagctgcaaatcaccaaccaaagaaacttaccggaccatgtgcagagaactactgcattaat
tcgtcatgcctccagccctcttgtgctcaaacatcttgcttcacaccacgcctaccatctgttccaaagcctcggaagcctaaatcttcccagaaaaggct
ccctcaacttgttgtagagcctattgaagcacgttctaattcttttgttggcacacatgaataccttgcaccagaaatcattaaaggcgatggccatggaa
gtgccgtggattggtggacctttggtgtcttcctttatgaacttttgtatggcaagacccctttcagaggacctgggaatgatgaaaccttagcaaatgtgg
tgtcccagaatctccggttcccagacaatccatctgtgagcagcaacgccaaagatctcatcagaagtttacttgtaaaggagccagagaaccgactgg
gatctttaagaggggcagctgagatcaagcagcaccctttcttcgatggcttgaattgggccctgataagatctgctgctccaccggagactcagccttg
caatgttgtgactttggcaaaggcacagaagaaagaaggcaaatgtcttgagttcagaagtaactccgaggacctggaatttgaggttttctag
>g37867.t1
atgcctcccagcggcggcactgctccgctactcctcctcctcttgccactgctgctgctgctctgccacgccaatgccggctcgctggagtccgacgtcgc
ggcgctctcggatttccgcctcgccgcggaccgcacgtccgcgctgacgagctggaacctgtccgcgaacccgtcccccctgcgcatcctggcgcggggt
cacctgcgccggcggccgcgtgacgcgcctggtcctcgaggacctcggcctcgccggctccgactccctctcgacgctcgcgaggctggacgggctcc
gcgtgctcagcctcaagcgcaacgccctctccggctccatccccgacctctccccgctcgcggggctcaagctcctcttcctcgcccacaacgcgctgtc
cggcccggtcccaccctcgctcgggaagctctaccgcctgtaccgcctcgacctctccttcaacaacctctccggcgaggtcccgccggagctcaaccg
gctcgaccggctgctcacactcaggctcgactccaaccgcctcaccggcggtatcgacgccttaaatttaccgaggctgcaagagttcaatgtgtcccac
aattccatgtcggggaagatcccgtccccgctggccacattccccgccgcggcgttcgacgggaaccccggcctgtgcggcgcgccgctcccgccgtgc
aacgcgtcggaggcggggcggtgtcctccatcgacggtggtctcctcgccgtacgcgaagccggcgggcgcggagacgccgccggaaagtgggaag
gggaagatgagccgcgccgccgtggtggccatcgtggcgggggatttcgcggtggtggtcctcgtcgccgccctcctcttctgctacttctacccgcgcc

tctccaaccggcgccgcctccaacaaggggagaaaatcgtctactcgtccagcccgtacggcgccgccggggtcgtggccgcggcgggggggtggtgc
tgccggcggcggcggcggcacgttcgagcaccgggggaagatggtgtttctggaggacgttacgaacagcagcggcgggtcaagacggttcgagctg
gaggagctgttgcgcgcgtcggcggagatgctggggaaaggcgggtccggcacggcgtacaaggccgtgctcgacgacggcaccgtcgtcgccgtg
aaacggctgcgcgagacggctcccggcgttgtgggaggtgcgtcgtcgaggaaagagttcgagcaccacatggcggtccttggtcgactgcgccaccc
caacgtcgtgccgctcaaggcgtactactacgcccgcgacgagaagctgcttgtgtacgagtacatgcccaatggcagcctcttctccatcctccacggc
aagcccggggcgcacgccgctggagtgggcggcgcggctgcgcatcgcgtcgggcgcggcacggggtctggcgtacatccaccacgcgggccggcg
gcggacgacggggtcgccgcacaagctggcccacggcaacgtgaagagcaccaacatcctcctcgacaagtccggcgcggcccggctggccgactg
cggcctggcccnnnnnnnnnnnnnnnnnnnnnnnnnnnnnnnnnnnnnnnnnnnnnnnnnnnnnnnnnnnnnnnnnnnnnnnnnnnnnn
nnnnnnnnnnnnnnnnnnnnnnnnnnnnnnnnnnnnnnnnnnnnnnnnnnnnnnnnnnnnnnnnnnnnnnnnnnnnnnnngcctggcccac
ctggtcccgccgtcgccgcgctccgcggggtaccgcgcgccggaggcgccgccgccgcaccggccctgggcgtcgcagaagggcgacgtgtacgcgc
tcggcgtcgtgctgctcgagctcctcacggggcggttccccggcagcgagctgcccaacggcggcgtggtggcggagctgccgcggtgggtgcagtcc
gtggtccgggaggagtggacctccgaggtgttcgacctggagctcatgaaggacaggggcatcgaggaggagatggtcgccatgctgcagctcgcgc
tcggatgcgcggccgcggcgcccgagcagaggcccaagatcggctacgtcgtcaagatgatcgacgaggtccgcgcctgcggggaggcgtcgtcgcg
ggaggagtcgtccatggacgagtcctccggcgtatctgactcgcccgccgtgtcggaaggcggcgcgattagcggctaa
>g37881.t1
atgcgcttgctgctgtctctgctactcgtctccttcctccgagcggcggcggcggcagcggctaggccggcaaatttgtccagctgcttgcccaagaaatg
cgggaatctggacatttcctacccgttctggctggaggagcccagcggggcgcgctgtggatcgccgtcgttccagctcaagtgcaacagcagcggcg
cgttcctgagccgctccgtgctccagtcctaccgggtggtcagcatcttcgtccataacaactccttccatgtggtcgacgacaacctcccgctcgccacc
ggctgcccggcgccattcttcaacatctccctcggaatagggttggcgccgttcctcatcagcacaaccaactaccagctgcttttcctctcgaaatgtaa
ggagccatcaccgtcggtcccgcctgggtttcgtcgcatgccttgcaacaacctctccttcgtcaacgatggtgtggccaccggaaacgaccatgttgcc
aggatgaagaacgggttcctcctcaagtggacaggattgtcaactgattgccccggatgcgtggcaagcggcggggagtgcatgtacggcgacaacg
gcgtcgggttcgcctgcaagtgctccgatggcatgcatcatgtgaaatgcgtcgcatctggagttggaggtggtgccttattggtagtatgcgtcctcttt
atatctggcaaaagcacaagaggaggaaacatggcagagcttcaagtgacctcatgcgtagtggatcttcgttccattcttacagcaaggaccttgaat
tgggtggttctccccatatcttcacttatgaggaacttgaagcagctactgatggatttagtggcttaagggaacttggtgacggtgggttcggtactgttt
acaaaggaaaactccgggatggaagagtagttgcagtaaaacgcctttacaagaacaactacaaacgggttgaacaattcataaacgaggtagacat
cttgtcccgcctactccacaagaacctcgtgaccctatatggctgcacgtctcgtattagtcgtgatcttctcctggtgtacgagtacatcccaaatggaac
agttgcagaccatcttcatggaccccgtgcggcagaacgtggccttacatggcctttacggatgaacattgccatagaaacagcagacgcgctagcata
cctccatgcagtggaaatcatacaccgtgatgtcaagaccaacaacatattgctggacgacagcttccatgtcaaagttgccgactttgggttgtcacgtt
tgtttcctcttgaagtcactcatgtctcaactgttccgcagggcacaccaggctatgttgacccggtataccaccagtgctataagctaactgataagagt
gatgtgtacagcttcggtgttgtgctggtagaactcatatcctctaaacccgcggttgacatgagcaggagccacagtgagatcaacttggcgaacatg
gcactcaacaggattcagaaccatgaagttgagcagttggtcgaccctgagattggctatgagacagatagcgagacaaagagaatgatagatcttgt
cgccgagctggcttttcagtgcttgcagatggaaagggatttgagaccatcgatcaaggaggtagtggagggcacaccaggctatgttgacccggtata
ccaccagtgctataagctaactgataagagtgatgtgtacagcttcggtgttgtgctggtagaactcatatcctctaaacccgcggttgacatgagcagg
agccacagtgagatcaacttggcgaacatggcactcaacaggattcagaaccatgaagttgagcagttggtcgaccctgagattggctatgagacaga
tagcgagacaaagagaatgatagatcttgtcgccgagctggcttttcagtgcttgcagatggaaagggatttgagaccatcgatcaaggaggtagtgg
agggcacaccaggctatgttgacccggtataccaccagtgctataagctaactgataagagtgatgtgtacagcttcggtgttgtgctggtagaactcat
atcctctaaacccgcggttgacatgagcaggagccacagtgagatcaacttggcgaacatggcactcaacaggattcagaaccatgaagttgagcagt
tggtcgaccctgagattggctatgagacagatagcgagacaaagagaatgatagatcttgtcgccgagctggcttttcagtgcttgcagatggaaaggg
atttgagaccatcgatcaaggaggtagtggagagaccagagaacactgagaggcagcaggagagttcattcgattcgatggctggaaggctgcacaa
gccgaaagcgaagagcttttggatcctggtgcgccgccttctctgccggaagaatcgtaaggcaaatcctgcagcagcagaggaggagaagagcggc
ctcctcagcagaagctctttggaggagctcctgatgacggatgccgcccaggaggatgccgccaagaaacacgggcaggcgccagtggaggcgaca
ggcggctggcctgggcttcaccttcaccgtccggtaacggctcggtcacaggtggcatgcggaggccgtgacggcgcggccatgcaccggagattcat
gttcggcggcttccggcggcgacttctcatgaggaggccgtggcggccggtgctcgtcgccatcccggagtga
>g37921.t1
atgacggacgcggaggcgctgatgcagctgaagaagtccttcaccaactcctccctctcctcctggctcatcaccagcaccgacggcaacaagtcc
ccctgctcgcccggctcccacgagtggcacggcgtggtgtgcggcggcgggctcggcggcgtgacgggcctgcggctgagcgggctgggcctgggcg
ggaccatcgacgtggacgcgctggcggccttcccgcggctccggtccgtctccttcgcccgccgcgcggcgtcggcggcgaaggtggacgacctgggc
ggcgcgggaggaggggacctggtgatcgtgaacgactgcaagggcgtgttcgggctggcggacctgatgaaggcggcggcggaggtgatcgggagc
ggcgggctcggctcggcgtacaaggcggtgatggcgagcggagtggccgtggtggtcaagcgcgcgcgcgacatgaaccgcgccgcgagcaagga
cgccttcgacgcggagatgcggcggctgggggccgtgcgccacgccaacctgctcccgccgctggcttaccattacaggaaggacgagaagctgctg
gtgtacgagtacatccccaagggcagcctccagtacgtgctccacggcgaccggggcatggactacgccgcgctcgccgactggcccacccggctccg
cgtcgcagccgggatcgcacgcggcgccgcgttcctccacgtggcgctcgcggggaccaccaccacgacccacgcgccgcacggcaacctcaagtcg
gcgaacgtgctcctggcccccgacttcgagccgctgctcgtcgactttgggtacgcggggctcgtcggcggccaggacccgcacgccgcgccgccgcc
gtcaatgttcgcgtaccgggcgccagagtgcgcggcggcgggcggcaacgtggcgccgggcgccaaggccgacgtgtactgcctcggcgtcgtgctg
ctcgagctgctgacggggaagttccccgcgcagtacctgcacaacgccaagggcgggacggacctggtcatgtgggcgacgtcggccatggcggaag
ggtacgagcgggacctgctcgacccggccgtcgtcgcggggtggaagttcgccgtgccggacatggtgcggctcatgcgggtggccgtggactgcgtc
gagaaggacgcggagaagcggcccgggatgaaggaggccgcggcgagggtggtggaggcggtggtggcggcgatggccacggtcaaggagaggc
aggaggagcagccggccgccgccgccgccggcggagagggagacaggacgacgagctcgcacgccgcgtacgtaagggaaggatcgatgcaacg
ggtcaccagcgtcgtcgagcggtcgtcgaggcgaggcagtaacgattactcgtacggcatctcgtga

>g38214.t1
atgcaaacttggaaagcagtgggcagtcaagacanaagttgtgccagtaacctggacacaacagaggaattgccagaagacttgacctatgaagata
tcctccgggcaacagacaattggagtgagaagtatgtcattgggcgaggccggcatggtacagtctaccgtacagaatgcaaacttggaaagcagtgg
gcagtcaagacagttgatctatcgcagtgcaagtttcccactgaaatgaagattctgaacacgatcaagcaccggaatattatcaggatggacggctac
tgcatccgtgggagtgtcggcctaatgctttatgaatacatgcccagtgggaccctctttgatctgttgcatgcaaggaaggctcaggttgatcttgactg
gatagcccggcatcagattgctcttggtatcgcgcaaagcctttcctatctgcaccatgattgtgtacccatgattgttcatagggatgtcaagtcaagca
acatactgatgggcactgatctaacaccaaaactcacagattttggcatggggaaaattgtcaatgacaaggacactgatgcaacagtttctgtcatcgt
tggtacccttggctacattgctccagcatggatactccacacgattatctga
>g38464.t1
atggaggctattagcagccggaagcgcttcaccgtcggcaacctagacgactacgagccgatatgcaagctgggcaagggcggattcggcgccgtcg
tcaaggctcgccaccgcgccatgggccagctcgtggccatcaagcgcctcgccaaacccggagagggacaatcagaggtgatgcgggagaagagttt
tctggaagcgtgcaacggcaacccgttcgtcgtcggcttccacagcgtggtccgtgccctcgactccatggagctccgcctcgtcatggaccacgttggc
cctagcctccgagacttcctccaccaccagcgccgctccagcgacacgccgctcccggagacaaccgtgcgcggcatcatgtggcagctgctgcaggg
ggtcaagaggatgcacgtgatccacgtcgtgcaccgtgacatcaagccggagaacatcctcgtcagtgccgacaagaacctcctcaagatctgcgattt
cggccttgccctgcacatgtccgacgcgccgccgtacaaggaggccggcacgaggtggtacatggcgcccgagatgctgctggggatgcacgactat
gacgcgctcgtcgacgactggtcgctcggatgcgtcatggccgagctcatcagcgggagcggcgtgctcttccccggcgacaacaacgaaggcaaga
cgtggctgtggttctcgaccacgccgttcgccaccggcgttctgcctcgactacggtaccggcagagacgaagcctgctgcgtcacaagttccctgccac
catgctgtcccagcaaggtttcgaggtcctcagcggccttctcacgtggaatcccgacaagcggctcacggccagcgccgctctcaagcacccctggttc
cacgatctcaacaacgcccctactactgcttccccggccgcaaggcacaagatgacggacccgctgatgatttctcagaacttgcacaagaggcgcaa
gattattctgttagaattatatgggcttggcccattaatatttataataattactagaaaatctcaaaggctcatttatgagtggtga
>g38480.t1
atggtggagcccatggcgcgacactacttctggcggctggtctccgccatgcggtactgccacgcgcacggcatgttccacagcgacatcaagcccga
gaatctcctcctggacgccgccggcgacctcaagagggtatcgtactttgtctccttcatctataaaatcagccacgcccgtcacccctggcctcgcacgt
ggttccccaatccaagagccgacgacaattccaacgaggactctgccatcaagcacgaggcggccatcctccgtcgtctccatcaacggtgcgtggttc
ggctccacgagatccgagcgacacgaaagaaggtccacttcatcctcgacctcaccgccgacggcgagctcttctccctcgtcgatgcgtccaggcgca
tggtggagcccatggcgcgacactacttctggcggctggtctccgccatgcggtactgccacgcgcacggcatgttccacagcgacatcaagcccgag
aatctcctcctggacgccgccggcgacctcaaggtcgccgacttcggactcggcgcctactactcctcctttgaccaccagcagcaggaccaccacctg
cgccacatcctgtgcgacacgccgacttacatggcgccggagattctaagcaagagagggtacgacccgaccaaggtggacatgtggtcgtacgacat
ggtgctgttcgtgcttcccgcctgtgttgctcagtag
>g38590.t1
atgcggcagggtgctgcacgcttgggcgatgcatgctgcattcaggtcgcgcccccgtctactagtgtggcttctgcttgcgcgtattgtctacttacagta
ttgtctgtcatcaatggtaaaggttacctctgcaactgcaccgaaggctaccatggtaatccttaccttcctgatggatgccaagatattgatgaatgtgc
aaatttgccatctccatgccctgggcactgcaaaaacaaacctggaagtttctggtgcccaaatggttcatcgcactctcgcattgtagtgctagttgttg
gtccaagcattggggctgtaattgttgtcataaccattacgtgtctatacctgatccgtgaaaggaagaagttggcaaatatcaagcagaagtatttcca
acagcatggtgggatgctgctgctgcaggagataagcttaaagcaaggcactacattttcaatcttcacagaagctgaactcatcgaggcaacaaaca
agtttgaagacaagaacatccttggccgtggtgcccatggtattgtctacaggggcatgctcaagaacggtagtctcatcgcagtcaaaaggaacgatg
gctgccgcgacatcccattctcgactcggttacacattgcccttgagtctgctcagaccttagcttacctgcattcatgggcatcgcctccgatccttcacg
gtgatgtgaagtcttccaatatacttctcgatgagaactatgttgcaaaggtatcagactttggagcatcaattgtggcaccgactgatgagtcccaattt
gtcacactggtgcaagggacctgtgggtacctagaccccgagtacatgcagacatgccagctgactgataagagtgatgtgtacagctttggcgttgttc
tcttggagctgctcacccgcaagaaggcattgaaactcgatggccctgaggatgagagaagcctttctcagcgcttcctcaacgccatgaaggaggac
agactcatggacgtcatagacgatggcatcaagagtgatagggacactgggttgcttgaagaggttgcagagcttgcgaggcagtgcctggacatggt
cggtgagatgcgaccgtcaatgcgagatgtcgccgagaaactggacaggctgagcaaggtcatgcagcagcagcacacgttggtgccagtacaacat
gaccctgaagagatggagagcttacttggggagtcatcgatggcctgcgtggagttgatcggtactggaaatttcagcatgaagaagaggattgtgca
aaggctactagaatctgggcgttag
>g38641.t1
atggctgcgtgctgtgcttctctgcttcttctcaccttgattcatctgattttgcgcatttcagcccgcgatttcctctccacaggcggctccctctccgtcgag
catagttccgatgtgctacattcacaagatggaacttttagctgtggcttctacaacatctccccgaatgcctccaccttctccatatggttctccaaatcat
acgagagaaccgtcgtctggagtgcgaatcctctccaccctgtctacacctggggatctagagtcaagttacacactgatgggagcatggtattgaagg
attatgatggccagattgtgtggaccagcaatgtgagtgtttctgatggggaacatgttcaaactcagctcctgaacacagggaacctagtaatgaagc
gaaaaggtggtacaatcctgtggcaaagctttgattctcctactgataccttgctacccactcagagcattactgctactaaaaagctgatatctaccaac
aggttactagatcctggcaactacagcttccattttgatgatcaatacttgctttctctattttatgatcggaaggatatctcctttatctactggccaaatcc
aagtaggaatgtctgggacaagcttagaatgccattcaacagcaccactactggggctcttgatagccggggccattttcttggaagtgacaatgcaac
cttcacagcggctgactggggtcctgggatcatgaggaggctaacattggattatgatggcaatctcagattatatagcctgaataaggcagacggaag
atggtcagtcacatggatggcgtttcctctgctctgcaacccatcgatccacgtgatcggagcaaaggctgcgaacaaaagatcaacatcagttgtgat
gcacggaaggtacacctacaaagagttggtgacagccactgaaaactttaaggatgagcttggaaagggggcatcaggcattgtatacaaaggagtc
ctgaaagacaatagggcagtagctgtgaaaaagttgaaagacataaaccaagaatacattgagaatggttcgttggataaaaccttatttggtacaga
gggttcagaaatcttacttggatggaagcaaagatttaatatcgctctaggagtggctagaggattggcatatcttcatcacgagtgcttggagtggatc
atccactgtgatgtgaagcctgaaaacatactgctggataagaacttggtgcccaagattgccgactttggcgtttcgaagctcctgaagagaggtggat
acaatataaatgtttcgcagatccaaggaactagaggctatttagcacctgaatggatttccagtctcccaataacagcgaaggttgatgtctacagctt

cggagtggttctactggaactaatgaaaggagcccgtgtttcagacatggaagggaatgaggatgaggcggtggaaatggtcctgggaaggacggtt
aggatgcttaaagagaagttacagtttgatagcagtgaagagtattggatcaatgacttcattgacaccagattaaatggccaattcaacaagttgcaa
gccaaaacaatgatgaaacttgctgtttcgtgcttggaagaagacaggggcagaagacctaccatggaagacgtggcgcagatgctggtttcaattga
tgatgctagtggcccatctcggcagctgtaa
>g38691.t1
atgggctgcgtgcacggccgccccgcgacctccagccccgtcgcggtctccgcctcgcgccgccgggaccacccgtccactgccgcggccgtttcccag
ccgcagcaggagggggtcgcttcctctgccgcgccggcggaggcggcgccggaacaggcggcgggtgagaaggccgagaagccggcgcaggtcaa
gcgggagaggcggtcgcggtcgtcgaggtcggccgccgccgcggcgcacgccgaggtccgactcgggggcagcttcgccaacaaggcgcgcgggg
agcaggtcgccgccggctggcccgcctggctctctgccgtcgccggcgaggccatcgacggctggaccccgcgccgggccgactcgttcgagaagatc
gataaggtgcgccttctcctcctcctcctcatcttcccctgcttcgtgatccatgccgcgcgtgcgccgaggcggaaaattgaatcttttttttttcttctctct
accccatctctctctcagatcggccaaggcacgtacagcaatgtgtacaaggcgcgggattcgctgagcggcaagatcgtggcgctcaagaaggtgcg
cttcgacaacctcgagccggagagcgtgcgcttcatggcccgcgagatcctcatactccgccgcctcgaccaccccaatgtcgtcaagctcgacgggct
ggtcacctcgcggatgtcctgcagcctctacctcgtcttcgactacatggtccacgacctagccggccttgccgccagccccgacatcaagttcacgctg
ccccaggtcgagatcccattttgctga
>g38725.t1
atggatcccgtggaggcggaggagcagcagacggagcccccggacgaggacgacgaagaggaaggctacgccgagaaagatcccaccgggcgat
tcatccggtatgacgaggtcgtgggatatggggccgtcaagatcgtctacaaagccttcgacaagctggagggtgttgaggttgcatggagccaatcac
ggataaatgattctgtcatgggttgctcaaaaaagatggagcaactgaacactgagatccaactgttggggaaactaaggcacaagaacattgtgaag
ttgtttgcctcatgggttgacgaggacaaggggattgttaacattgtcacagagtacttcacatcaggcaacttgagacagagcagcagaacttttacaa
taaaaaaggccgtacccagtgctgagagctcccgcactgtgcggggtctggggaagggtttcagcggcaggccttacccccgcctgtgcaatgcgggg
agaccgcggctcgaacccgggacctgttggttacaggcgggtgaatttctgctgaaaggacatatggatgttgcgaaccctgttgttctaagtctaagat
ttcctgatccatacggtggatttaagactgttgagttcccattggacgtggctaaggatacaggcctctcggttgccgtggaaatggctgaacagtttgaa
ctgccacatgggagcattgagatcatatctgagctgattggtgcgtttatgctcgtcctgattcagtattggaggaggtcttgtgctgccatgccataa
>g39068.t1
atggataggacggcggcgacgacggggccgctggggatggagatgccgataatgcacgacggggaccggtacgagcacgtcaaggacatcgggtc
cggcaacttcggcgtcgcgcgcctcatgcgcaaccgcgcctccggcgagctcgtcgccgtcaagtacatcgaccgcggcgagaagatcgacgagaac
gtgcagcgggagatcatcaaccacaggtcgctgcggcatcccaacatcatccgattcaaggaggttatcctgacgccgactcacctcgccatcgtcatg
gagtacgcctctggaggagagctcttcgagcgcatctgcagtgccggaagattcaacgaggacgaggcccggttcttcttccagcagctgatttctggg
gtcagctactgccactccatgcaagtatgtcatcgtgacctgaagctcgagaatacattgctggatggaagtaccgctccacggcttaagatctgcgact
ttggttattcaaagtcatccgttcttcattctcaaccaaaatcaacagttggtactccagcttatattgccccctgaagttttgctcaagaaagaatatgatgg
caagattgctgatgtgtggtcatgtggtgtaaccctctacgtcatgttggttggcgggtacccattcgaggacccggaagatcctaggaatttcaaaaag
acaattcagaaaatattgggtgttcagtatgtgattccagattacaggatcaccatgcctgaaataaaaagtcatccttggttcgttaagaatctcccagc
agacctcatggatgatgccacaatgagcagccagtatgaggagcctgaccagcctatgcagaacatgaacgagatcatgcagatactagcagaggca
actataccagccgctggtacccgtggaatgaaccagtttttggttgacagccttgaccttgatgatgatatggaggatctagactcagaccttgacatcga
cattgagagcagtggagagatagtgtatgccatgtga
>g39328.t1
atggcgacgtacgggggccggtacatgctgtacaacgtgtacggcaacctcttcgaggtctccgccaagtacaccccgcccatccgccccatcggccgc
ggcgcctacggcattgtctgcgcggctgtcaactcggagacaggggaggaggttgcgatcaagaagattggcaatgccttcgacaaccatattgacgc
caagcggacgctcagagagataaagctgcttcgccacatggaccatgagaacatcattggtttaaaggacataatacggccacctactaaagagaatt
ttaatgatgtttaccttgtttcggagttgatggatactgatcttcatcagatcattcgctcaaatcaatcgctgactgatgatcattgccaggtctgtggcca
tgttgcctcctccagcttatgctag
>g39398.t1
atgcgcatcgtcaccgcctgcagcctgccggacttcaaggcgctcgtgctgcccttcatggccaacggcagcctcgaccggtgcctctacgccggcgcc
ggcggggcggacctcagcctgctgcagcgggtcaacatctgcagcgacatcgccgaggggatgtcctacctgcaccaccactcgcccgtcaaggtcat
ccactgcgacctcaagcccagcaacgtcctcatcaacgacgacatgaccgcgctcgtctcagatttcggcatctcacggctggttatgagcgtcggcgg
cgtggtcaacaccgcagacgtcggcgcctccacggccaacatgctgtgcggatccatcggctcctga
>g39429.t1
atggagtgccttgccgagatgccccacgcgccgctcgaccgccgcccgcgcaagcggcagcggatgggctgggacgtcggccccgccgagttcaatc
agatccagatgggattttgcggtcaagaagttgcacatgccatcagtgctgtggcactggggctctcttcgggtgttgtttcttctcaggggaaccaagag
ctccctcgtctcggttcccctccattgagagaagatgacaaggatggtcattatgtttttgctgtgggagataacctcacacctcgctataagattaatgca
aaaatgggtgaaggtacttttggtcaagtattggagtgttgggatagggaaagaaaagaaatggtggctatcaagatcatcagaggaataaaaaagta
cagggatgctgcaatgattgaaattggcatgcttgagcagcttggtaaatatgaacaaagcagatccagttgtgttcaaattcggaactggtttgactat
cgtaaccatatctgtattaacctggtcatgcaaaatgttgaccaatctgctggggaattgatcaatctattacaagggctgctgaagtatgatccagctag
ccgtctaactgctgaagaagcgctgaggcatccattcttgacaggagtgagcgagcagagaagatga
>g39711.t1
atggcagttctgtttatcactgcgttgtggctttcagtccggaggaagaaaaagacagtgaaaagatacgacaacaacgcatcgctagcagagatcccc
atcgtgtcgaaggaaatcaatgttgacagagtcgatgcacaaagtctgcatgacagcgaagtgccattcatgccggtgcaagataaatatgcacagat
gaagggtgcagggcacttggcagagagtagatctgtggatgtcgacacgttcagccaatgcagctcagtttacaacatagagaaggctgggagttcat
actctgaggattacagcagctctgggccggctaggaaaggcagctcaccatacgcatattcagcttcgccgcttgttggtttgcctgagttatcacatttg

ggatggggccactggtttactttgagggacctggagtacgcaacaaatcgatttgcaaagagtaatgtccttggagagggtggctacggagttgtctac
aagggtcgcctggtaaatgggactgagattgcggtgaagaagatccttaataatgtaggtcaggcagaaaaggaatttcgagttgaagttgaagccat
cggtcatgttcggcataaaaatctggtgcggcttctgggctactgtgtggaagggatacacaggatgctcgtttatgagtatgtcaacaatggcaaccta
gaacaatggcttcatggtgctatgagtcaacatggtgtacttagttgggaaaaccgtatgaagattctccttggcactgcaaaagcgcttgcatatcttca
tgaggccatagaccccaaagtcgtacatcgagatatcaagtctagtaatatcttaatcgatgatgaattcaatagcaaggtttctgattttgggttggcca
agcttctgaactctgataaaagccatatcaacacaagagtgatgggaacatatgggtacgttgcacctgaatacgcaaacagtgggatgttaaacgaa
aagagtgacatttatagttttggagtggtgttgttggaatctgtgacagctagggatccagttgactacagtaaacctgccgaagaggtgaatcttgttga
atggcttaaaatgatggttaccaataagagggcagaggaggtagtggatccaaaccttgagatcaagccgtctaagcgtgcccttaagcgggcgatctt
ggttggactgaagtgtgtcgatcctgatgctgacaagaggccaaagatgagtcatgttgtccaaatgcttgaggcggttcaaaatgcctatcgacagga
tcagaggaagctcagccaggtgggaagcatggatatcgagtcacagcagtcactagaggaggtttcaaacagtgctgatgcctgcagctccaatcctg
aacaatcatag
>g39845.t1
atggttaacgggtggcttactgggctgagctccaatggcgtcctcgccttctccctctccgggcaccatcaaggccaccgtgccgccatccactacaacc
ccatcgccggcatctacaactccgccaccgtccacttccacgccggactcttcggctccggtgacgcagcccaatgccaccccagccgacccctcctcct
cgccgccggccgctcccccctccacttcctcccgcagccgtgccacccccgcaacccgcttccccgccgccctcatctccgccacctccggtcaccgtgcc
gccaccgccagtaattgttgcttctccaccaccggccccaccggccccggccgccgtagtcccgccaccctcaccgccggttgcagtgccgcctccagct
accccctgctgcccctccaaaggcttcgccttcagtctcccccgtggtcgcgtcgccgcccccatccaatttgccggcacccgacccaccggcagaaccaa
ccccgccgacagtagtgcagtcacctccgccaccacagcgtcaccggccacgaacgcctccatctcagccagaagctccggccctggcgccaccacct
tcttgccagccccgggcacgacggttgatccagccagtccagcaacgaccagcagccctggttccaacaagtcatcaaatcccaccggacagggcatg
gccccctcaaataacaatggtggaatgtcatctggtgcgaaggcaggcattggcattgtcgtcgctcttcttgtgcttagcttggttggagctgcattttgg
tacaagaagaaacggaggagagtgcatggttatcatgctgggtttgtgatgccttcgcctgcctcctctcctacacaagtgttagggtattcagctaaaa
ctaactacaccattggcagtcctgaatcaaaagaatcaatgccagagttcagcatgggcaactgtcggtttttcacctatgaggaattgtacaagatcac
aaatggcttctcagctcagaatctgcttggggaaggtgggtttggttcagtgtacaagggttgcttggctgatggaagggaggtcgctgttaagaaactg
aaagatggcagcggacaggggaacgccatccacgtataatccaccgagatataaaatcctctaacattttgctggatgataactttgaagctcaggtc
gctgattttggtctagcaaggttggctttggatgcctgcacacatgtaactacacgggtgatgggaacttttgggtacctagcccctgagtacgcatcaag
tggcaaactgaccgagagatctgatgtattctctttcggtgttgttctcttggagcttattacaggcagaaagcctgttgatgcatcaaagccgctgggtg
atgaaagccttgttgaatgggctagaccattgcttgggcaagcacttgagactggcaatgtgggggaactggtggaccagagacttgacaagaactat
aatgaagttgaattattccgtatgattgaagcggctgcagcttgtattcggcattcagcatctcggagacctagaatgagccaggtggtgcgggctcttg
acagtttagctgatgtcgatctgaccaatggtgttcagccaggtcagagcgagttgttcaatgttgcaagtactgcagagatcaggctgtttcagcggat
ggcatttggaagccaggatttcacaactgatttcagccattctagctggaacagccaaagccaaagccagagccagagtagaggttttgacgcctctgg
ttcgaggcacatgtaa
>g39990.t1
atgagcgcggagctgaagagggactacgagatcggcgaggagctgggccgcggccggttcggcgtggtccgtcgctgcacgtgccgcgccaccggc
gagtccttcgccgtgaagtctgtggaccggtctcagctgggcgacgacctggaccgcgagctggccgaggtggagccgaagctggcgcagctggcga
gcaagggcaacccgggcgtggtgcaggtgcacgcggtgtacgaggacgagacgtggacccacaccgtcatggatctctgctccggcgccgacctggt
ggactggctccgcctccgccgcggcgcgccggttcccgagcccgtggcggccgagatcgtcgcgcagctggcccaggccctcgccatctgccaccgcc
gcggcgtggcccaccgcgacgtcaagcccgacaacgtcctcgtcgacgacgccgccgaggccgaggagaacgaggaggccccgcgcgtccggctcg
cggacttcgggtcggcggcgtggatcggggccggcgggttggggacggtggaggggctggtggggacgccgcactacgtggcgcccgaggtggtgg
ccggcgatgagtacggcgctaaggccgacgtgtggagcgccggggtgctcatgtacgtgctgctctccggcggcgcgatgcccttcgtcggcgagtcc
gcggcggaggtgctcgcggccgtgctgcgcgggagcgtgaggttcccgcccaggctgttcggcggcgtgtccccggcggccaaggacctcatgaggc
gcatgatgtgccgtgacgtgtggaggaggttctccgcagagcaagtcctcgctcacccgtggatcgtgagtggcggaggagcccgggcggcgggcact
tga
>g40220.t1
atgcggctgccgctcgcggtgctggtcctctgcgccgccacgcttctcccgccacccgcggcgatcgcggcaggtatgcaccaatttttggttgcttacag
ccctgtcatccagatttcagcacgaggtaaacatctgctaccatggaaaaacaggatacaaattgccattgatgttgcaaatgcgttggagtaccttcatt
tctattgtgaccctcctctgtaccatggagacgttaagcctagcaatgtcctcttggacaagaactaccgagcaaagcttgccggttgtggtccttcacgc
tgctcaggtggtgctggtgacactagtggtagttccactcctgtgaatgccaaggtccaagcagctcccggctacgtggaccccgagtacgcggtgacc
caggagctgaccccgaagagcgacgtgtacagctacggcgtgctcctgctggagctggtcacgggtaaggccgtgatccaggacgacggcaacaaga
gcctggtggagtggtcccgcgagctgatcggcacggactaccgtctccacgagctggtggacccggcggtggcggacgcgttcgacctggacgagctg
caggtggtcgccgacgtgatccactggtgcacccaccgcgacggcggcgcgcggccgtcgatgaagcaggtgctccgcatcctgtacgagcggctgg
acccgctgtcggggcggttcgcgcgcgccgtggagggcgaggagggctactactactcccaccacggcggcgctgggcgggccagtaaggggaatgt
tggtggcggcggggaggtggtccagttcagcggcgaggcggcgtggctgccgtcgtcgagcagcacgacgcggtcgcactgcagccgcagcgtgctt
ctggagagcggccactcgccggagccggaggagtccccggcgctttgtgatgccgggcgggatccaccctgcgagacgacggggcagggcccggtg
atcgcctgcacgagagtcaggagacggatgaatcgtggcagtgccagtgccacgcgtggagtgactgctgctgtggagtggagtgaactgaagttcag
cgtccctatgcgtcggtggttgtatcgtatcgccgcattgatttgcttggggaacatggcctcgcatgacatgttttactggagcgtgcagtcaccaaaaat
gcccttttcccatctcgactgccgcttgcttgccgagaggctgtttcaacttgcagctgaaacttgcgggagcaagaaaggcagcctacgcgcgtgtcgg
tag
>g40321.t1

atgaacgatgtactttcccgtctccaaatcatcgacacagatcagaactgcaaaagcaaagcctctgaattcgccggacacggggaattgcacagcaat
gaggaaacaagccgcaacggctcgactccacgcttcccccacgacaggccgccggttttcttcttcgggagagtgccgcggccgtacgacctcgacga
cctgctgcacgcctccgctgaggttctcggcacgagcaggtacagcaccacgtacaaggtcacgttggagtccggcacggttttagcagtgaagcgtct
caggtgcccctcgtcgccagagcgcgagtttcgggacaaggctgcggagattggtggcattgatcaccccaacgtcgtcccccctacaagcgtactgctt
cagcaaggacgagaagctcatggtgtacgagtacgtggccatgggtagtctcttctccatgctttgcgagaaccgtggcttcgagcagtcgtcgctgagt
tgggagtcgaggagacgcatcgcgctggccacggcgcgcggcctcgagcacattcacgcgacgggctccagggtcattcacggcaacatcaagtcct
ccaacatcctcctcagccagacggtggacgcacgcgtggcggaacacggcatcgcgcacctcttcgacccggaggcagttgagccttcgagcggcgtc
ggctaccacgcgcccgaggtcgagggggacccacgcaagctctcgcacaagtccgacgtgtacagcttcggcgtgctgctcctggagctgctcacagg
cgagatgccgacgcctaacgaggacctccctctgtgggtgcggtggtccatggtggaggaggagtggacgtccaagttgttcgatgatgagctgctcgg
agaacccggcgccgaggaggggatgatggagatgctgcagctggcatttgactgcaccgcggacttgccggacaagcggccagcaatgccggagat
cctggggcgcttggcagagcaaacactttag
>g40799.t1
atggcaaatggtagtcttgataagtatctttacggcgaggagaacaagccgttacttggttactgtcggagaaaaggtgaactgcttttggtctatgacta
catggcaaatggtagtcttgataagtatctttacggcgaggagaacaagccggtattaggctgggagcagaggtttcagattatcaaagatgtggcatc
cggcttgttctacctccatgagaaatgggagaaggttgtcatccaccgggacatcaaggcaagcaatgtgctcctcgacaccgggatgaatgcgcattt
gggcgattttggccttgcaaggttgcatgaccatggaactgatctgcaaacaactcatgtggttggcaccatgggctacatcgccccggagctggcgag
gtcaggcaaggcatcccctctcactgatgtcttcgcctttggtacgttccttcttgaggtcacctgcgggcggcgaccggtgtgcaacaccgtgcagcacg
gccggaagcttttggttgatcgggtgctcgagtactggaacaaaggagcactcatggagacagtggatccaaagttgatgggcatctacaaccgtgag
gaggcacacctggtgctcacgcttgggctgatgtgctctgacccggtcccaagagacaggcctacgatgcggcaggtcatgcagtacctggacggcga
cgcgccgcttccggagctgacgccgtcgaacctgagcttgctgagcatgatgcagagtgaagggttggtgttgaaccagtcctcccagcagtatccgtg
gtcagggattagcatggcaacgatgacgccagacataacagaaggaagatga
>g41167.t1
atgccagagttgcgtagtggagtccgacaatctcggttgaaggcaaagaaggttgaggatctcgtggctcaggacccagctgagaacttggtcgcggc
ggcccctacggtggcaggaaggcggggtagaggaaggggtgggaggggtggagggagaggaacagcgcggggaagggcagcaggaagaggga
gagctgttccagttattgacttggaccctgaacagccttgtgaggtccttccgggagctgctcttggtgctggtgctgcaggtggagctgctcttggtggtg
gtgctgcagttggtgataaggttgtgaagatgaatggtggtagtgctgagaagaatgctggaggtgatgatgaagggactacaactccagtcccagag
aaggttcaagttggccattctccgcaatacaaggttgatcggaagttgggtaagggtggctttggacaagtttatgttggcagaaggatttctggtggaa
cagagcgccctggacctgatgcttatgaggtaatggatattctaggacctagcctttgggatgtttggaactcttttgggcagacgatgacacctaacatg
gttgcttgcatcgcagtagaagcaatctctatacttgagaagctccatgcaaaggggtttgttcatggagatgttaaaccagagaactttctacttggcca
acctgggtctcctgatgagaaaaaactttttttgattgatcttggtttaggaactggtttttcatcccaagtttatgaactctcacccatattccttcataagg
attggattatggagcagtgggagaataactattacatcagtgcaatagcaggtgcaacaaatggaagttccctggtggtcatgtcaaaaggtagtagaa
ctagatttcctttatccaagtga
>g41340.t1
atgttgagagggtgcggcctgttcgggtacgcccgccgcggcggcgacctccggaagcgcggcgagatgggcggcgcgagctcccgcgtcgcgccgg
cgggattcgacgacgatagcggcgcggcgaggcagctgacgtgggcggaggtggaggccgcgacgggcgggttctcgtcgcgggtgatcggccacg
gcgggttcagcaccgtgtatctcgcctcctccttcccttccggcggccgcgtcggcgccgtgaaggtccactgcagcagcgagcggctccaccgcgcgtt
ccggcaggagctcgacgtgctcctctccctccgccacccgcacatcgtccgcctcctcgccttctgcgacgtcgacgacgagggcgccctcgtcttcgag
tacgcccccaacggcgacctccaccacaagctccactccaacaacgacgacgttcttccgtggtcacggcgcgtggcggtggcgttccagctggcgca
ggcgctagagcacctccacgaggaccgtgcggtggtccacggcgacgtcaagtcatccaacgtgctcctcgacgccgccatggacgccaagctctgcg
acttcggctccgcgcgccgcgtcgacgccacggcgagcaagcagcgcggcggcggtgccggccgccacgccgtggtggggtcaccgggatacgtgg
acccgcaggtgctccggtccggcgtggcaaccagggagagcgacgtgtacagcttcggcgtgctgctgctcgagctcctcacggggaacgaggccgt
gtgccgcgagacgggccacaggctcactgccactctggggccacaactcagagaagccaaggtgtccgacgtggtggactcgaggcttggggacaag
tacgacgccgcggaggcggacgccgtggcggagctcgccgcgacgtgcgtcagtgacagccccgggctccggccgtccatggccgacgtcgtgcgcg
tgctccgggagaagaccgccgtcgttggatccaaatcagaccgcagatga
>g41603.t1
atgcctcgaatccagcaccattgctcgtccctgctcctcgtcaccatcgtcttcgttcacctatcggcagtagtcttggtgcacggggaagttgccaatggc
gtccacctggacctccccgccctcctcgccttcaaatcctacaacccaaacgccaccgcgctggcgacctggtccggcccgaacccgtgctcgggctcct
ggttcgggatcaaatgcaacaagggccgggtagcgggcgtcttcctggacggcgcgtcgctgtccggcgccgtggcgccgctgctccggctgacccag
ctgaggccgtgtagcgggcgtcttcctggacggcgcgtcgctgtccggcgccgtggcgccgctgctccggctgaccccgccgtggcgctcgccgtccgg
aacaactccctgtccggcgcgctcccgccgctggccgacgccacgagcccgcggctgcggcacctgctcgtggcgcgcaaccagctcaccggaggcct
caacgtctcccggggcgctctgctcacgctgcgggcggagcgtaacgggttcagcggcgggctcggggcgctgcgcgttccgacggtcaggagcttca
acgtgtccgggaacaagctcgccggagagatctccggcgagctggcccgattcccgagctccgcgttcggcgggaacctcgctctctgtggccggcctc
ttccgagatgcgttcgtgcttacagtgcactaggagatacagcaagtggcaactcaaatgtcactgttgcagctcaatctcctaatagtgcagaaccgaa
cgggagcttcagtaaacttagcatgactgcgctgatggccaccggcatcggcaacgcggtgctaatcacagtctcgctaaccatctcggttgccatgttc
gtgtacatgaagcgaaagctccggtccgccaaggccccttcactcttcttcgacgaggaagtgtccgacggcaacaagaggcggacgacaacgacga
cgaacagggacggcgacggcaccaagtgcccggcggcgaagagcagcggcgggctggtgtgcttcgagggcggcgaggcgctgcggctggagagc
ctgctgaaggcgtcggcggaggtgctgggcaagggcgcgtcgggcagcacgtacaaggcggtgctcgaggacggcgccgtcgcggtggccgtgaag
cgcctcagcgcgctgcggttcccggccggcagcggcaggggcaaggcgttcgaccggcacatgcgggtcgtgggccgggtccggcaccggaacgtg
ctcgggctccgggcctactgcaactccaacggcgagcgcctgctcgtctacgacttcctacccggcggcagcctccaggccgcgctgcaagcccacggt

ggggacgtgaggagcttggactgggcggcgaggaagagcgtgctgctcggcgcggcggaggggctcaactacatccacacgttcccggcgcggccg
gcgctggtgcacgggaacgtgaagccgtctaacatcctgctggacgagcgaggcgccgcgtgcgtgtcggagtgcggggtcatgcgctacgccacca
acgtgcagcactccatcctcccgcaggcggcgttcctggacctgtcggcgtcggcgtcgagcagcggcggcgggtggcacgggtacgcggcgccgga
gttggcatccacgacgagggcgatgcaggagtcggacgtgtacggattcgggatggtgctgctggaggtggtcaccggttgtcgcaaggccggtgacg
gctgcgaggaggaggaggaggacgacgagacgatggggtgggtcaagatcgggatgctgtgcactgctgaggcgccggaggagaggcccaggatg
gcgcaggtgctgtccatgatgagcgagctcatgtga
>g41788.t1
atgagctgcttcggcctgttcaagcggaagccgaggagcggcgaggggtcggtgcagcgcggcgggtccgcgccgccgtcgggcccggcaacgacg
accgggtcggggtcggcggtgagcacgagccggtcggacgactcgggtgcggtgcggcccctcagcaagtcgggcgggtcgtcgcagtcgcagcac
agcatctcgtcgctgtacgaggagcggggccacggccagctgcgcgcgttccagtacgaggagctccaggccgccacggccgacttcggccgcgcgc
agaagctcggcgagggagggttcggcagcgtctacaagggcttcatccgcgccgccgaccccagggggggaccgcgtagccgtcgccgtcaagaggct
taaccagcgaagcctgcagtcgggcgggtcgtcgcagtcgcagcacagcatctcgtcgctgtacgaggagcggggccacggccagctgcgcgcgttc
cagtacgaggagctccaggccgccacggccgacttcggccgcgcgcagaagctcggcgagggagggttcggcagcgtctacaagggcttcatccgcg
ccgccgaccccagggggggaccgcgtcgccgtcgccgtcaagaggcttaaccagcgaagcctgcaggtaatctacagggacttcaaaacgtcgaacat
tctactggataaggacttcagagcaagattgtcagactttgggctagccagggaggggccaactggaacagacactcacgtctcgactgcggtcgtcg
ggacgcacgggtacgcggcgccggaatacgtggacacgggtcacctgacgtccaagagcgacgtgtggagcttcggcgtggtgctgtacgagatcct
gacggggcggcgctcgctggaccggaacaagccggcggcggagcagaagctcctggagtgggtggcccagttcccgccggacagccgcagcttccg
ggtgatcatggaccccaagctccgcggcgagtactcggtgaaggccgcccgggagatcgccaagctggccaacagatgcctgctcaagaacgccaag
gagcggcccaccatgaccgaggtggtcgaggtgctcaggcaagccgtgcaggcgcacgccgagccggctcgtggggacataaacaggaacggaag
cgggaaggggaagaaggccaacgcggtcacgacgccgtccaggagcgacgtgtggagcttcggcgtggtgctgtacgagatcctgacggggcggcg
ctcgctggaccggaacaagccggcggcggagcagaagctcctggagtgggtggcccagttcccgccggacagccgcagcttccgggtgatcatggac
cccaagctccgcggcgagtactcggtgaaggccgcccgggagatcgccaagctggccaacagatgcctgctcaagaacgccaaggagcggcccacc
atgaccgaggtggtcgaggtgctcaggcaagccgtgcaggcgcacgccgagccggctcgtggggacataaacaggaacggaagcggcaaggggaa
gaaggccaacgcggtcacgacgccgtccaagaggtga
>g41896.t1
atggcgctgaacatgaagacgctgacgcaggcgctggccaaggcgtcggcagtgatcgagaaaacggtgacgacgacggtgcaggaggtgacgggt
ccgcgaccgctgcaggacttcgagctgctagaccaggtcggctcgggcggaccggggctcgcgtggaggatctacacggcgcgaccgcgggagggc
gcgccgtcgacgccctacccggtcgtctccgtctgggtgctcgacaagcgcgcgctcgcagatgcccgggcgcggtcggggctgtcgaagtcggccga
ggacgccttcctagacctagtccgcgccgacgcggcgcggctcgtgcggctgcgccacccgggcgtactccacgtcgtgcaggcgctcgacgagacca
aggccgccatggccatggtgaccgagccgatcttcgcgtccgtggccaacacgcttgggtccttcgacaacgtcggcaaggtgcccaaggagctcaag
ggcatggaaatggggcttctggagattaagcatggattgctacaagttgctgagaccttggattttcttcataacaatgcccaccttgcccaccgagctat
atcacccgagaccgtctttataacttcaagtggatcttggaagcttggtggtttcggttttgctgtatctgctgaccaagccacacgtggtttgacatcaac
acagcaattccattattcggactatgatgttgaggatacagctttacctcttcaaccatccttgaattacactgcaccagaattggtccgaagtggagattc
caaagttggctctgcttgtgacatgttcagctttggatgtctggcttaccacctgatagctcgtagaccacttctggactgccataacaatgttaaaatggt
atgtgttgaatctgggtga
>g41922.t1
atggcggtggcggcccccgggcagctcaacctcgacgaggcccccctcctggggctcccgcggcgtcgactgctttgagaagcttgagcagatcggcga
gggcacctacgggcaagtgtacatggcgaaggagactggcacgaacgagatcgtcgcgctcaagaagatccgcatggacaacgagcgcgaaggctt
ccctatcaccgccatacgtgagatcaaaatcctcaagaaattgcaccataagaacgtcatcaagctcaaggaaatcgtcacctccccaggcccagaga
gggacgagcaagggaagccaatcgacggcaacaagtacaaagggagcatttatatggtcttcgagtatatggatcatgacctcactgggcttgcggat
aggccagggatgcggttcactgtgccacagattaagtgttatatgaggcagttgcttacaggcttgcattattgtcatgtcaatcaagtgctccatcgtga
tattaaaggatctaacctcttaatagataatgagggcaacttgaagctagcagattttggccttgcaagatcattctcaagtgatcacaatggaaacctta
ctaaccgtgtgatcactttgtggtatagacctccagagttgcttcttggaagcacaaaatatggtccagctgttgacatgtggtctgtgggatgtatttttgc
agagcttctcaatgggaagccaatattgcctggaaagaatgagccagagcagctgactaaaattttcgagctttgtggcaccccagatgacttaatatg
gcctggtgtcacaaaaatgccatggtacaacaacttcaaacctccccggccattaaagagacgtgttaaagagttctttaaacattttgatcggcatgcg
ctggacctgttagagaagatgttgactttagatccatctcagaggatatcagcaaaagatgcccttgatgcagagtacttctggactgatcccttaccttg
tgatccaaaaaggtacaatctgatggcttgccaaagtacgaagcatcacatgaattccagacaaagaaaaaacgtcagcaacaaaggcaagcagag
gaagctgcaaagcgtcaaaaaatccagcacgctcctccacattctcggctgcctccaatccagcagccagggcaggcacacccaatcaggcccggcc
agggtatgcacaatgtaccacccgtgggggctggcccaagccatcactatgcaaagccccgagggccaggaggacctaataggtacccacagggtgg
gaatcaaggtggagggtacaatccgaaccgtggaggacagggtggtggctacggtagtggcccatatccgcagcaaggacgaggacctcctccttac
cctggtggtggtccacgtggtagcggtggcagtggatatggaagtggcgggccaaatttcccacaaggtggcccgtatggtgcatctggtccgggtcga
gggccaaactatagtcaaggtggttctcgcaatcagcaacagtatgaagcatgattttctccaaaatccaaatttatgggaaaatggcttcgacaaatgc
ctcggcatcaaaaggttgaaggtcttccaccccttcgccgacaccaacaaatttcactggaatcccaagctcatctacaacgctaacctgtga
>g42207.t1
atgcccgcaccaccgcacgccgcatcgctgctccctcttatctccctctcctattcattcaccctctcgtcgcttgcgacgtgcaccaccagccgcgccacg
gccatgcgaccggcggacgcggacgcgctctccccgccggcgccgtcccccgtcgccgccgacgacctcgcgccgtggccggcgacccgcggggac
gacggcggcggcggtggtggtcgggcggcgagccctttgttcaccatcctgccggtctcggcgttcgggattggggtcgtgctgctcgtcgccgtggcg
gtcgtgctggtggtgacgcggcggggcaagccgacccggaccgcggacgccggcggcgagagctgcagcggcgacggcaagcccgccgcgcctcc
gtccagctgcggcagccacaccaccaccaccaccaccaggtgctacgccgccgccggtaagcaaagcacgccgccgaaagtcgctgcgcgctcttcgtg

cattgcgagtcagtcgcgtcgatgaagaagcttactgtattggtttgttgctctgctgctgcgggcggtgttggtgcaggcgtcgggtgcatctacggcgc
ggggaggctgggcctgggcttcgctgcggcgcagcatcagcaccagcaccagcaggcggcgcggaggagccggggcgcgcaggtgttcacgtaccg
ggagctggagcgcgcgacggacgggttcagcgaggccaacgtggtgggccggggcgcgtacggcgtcgtcttccgcggccggctcaacgacggcac
gcccgccgccatcaagcgcctcaggctggaccaccggcgccagggcgagcgcgagttccgcatcgaggtggacctcctgagccggatggactcgccg
ttccttgtgggcctgctgggctactgcgccgaccagagccaccggctgctagtgttcgagttcatgcccaacggcagcctcaagggccacctgcacccgt
ctccgccgccgcccgccaccggcgacggcagccatccgctgccgcggccgccgctggactggcagacgcggctgggcatcgcgctggactgcgcacg
cgcgctcgagttcctgcacgagcacaccagccccgcggtgatccaccgcgacttcaagtgcagcaacatcctgctcgaccacaactaccgcgcccgcg
tctccgacttcggcatggccaaggtcggctccaacaaagccgacgggcaggtcgtcacccgcgtgctcggcaccacaggatacctcgcgccagagtac
gcgtcaacagggaagcttacgaccaagtccgacgtgtacagctacggcgtcgtgctcctcgagctgctgaccggccgggtgccggttgacacgaagcg
gccgcccggacagcatgtccttgtttcctgggcccttccacgtcttacaaaccggcagaagcttgtggagatggtggaccctgcgttgaaaggacagttc
gccctcaaggatctcgttcaggtcgccgccattgccgcgatgtgcatacagaccaaggcggagtaccggcccctgatgacggacgtggtgcagtcact
gatccccatcgccaagactaccccggccatatcctgctcctccacgccgctcagacctctgcaccacgtcatctacatgagccctcacttagccaacaag
tcctag
>g42215.t1
atggcggtcggcggcgacgacgacatggagagggacttcgccgccaggctgcgcctcgcgcattcgcccgcgcccgcggcctcgtcgaccccgcctg
cttcctcctcccccacagtcggcgggatcgccttccgcgcgccgcaggagcagttcactgtcgatgacttcgagctcggcaagatctacggcgtcggctc
ctactccaaggtggtaagggccaagaagaaggacactggcaacgtctacgcgctcaagatcatggacaagaagttcatcaccaaggagaacaagatc
tcctacgtcaagatggagcgcattgtgctcgatcagctcgaccacccgggcgtcattagactcttcttcaccttccaggacacctactccctctacatggc
gctcgagtcgtgtgaaggcggggagttgttcgaccagattgtccggaaaggccggcttcttgaggacgaggcacagttctatgctgctgaaattgttgat
atactagagtatttgcacagcgttggcctaattcatcgggacgtcaagccagaaaacctgctgcttacttctgatggccacatcaagattgctgattttgg
tagtgtcaagcctacaaaggatactccaatcaaagtcctcccgaattcaactagtaagacaaatcattaa
>g42216.t1
atggcggtcggcggcgacgacgacatggagagggacttcgccgccaggctgcgcctcgcgcattcgcccgcgcccgcggcctcgtcgaccccgcctg
cttcctcctcccccacagtcggcgggatcgccttccgcgcgccgcaggagcagttcactgtcgatgacttcgagctcggcaagatctacggcgtcggctc
ctactccaaggtggtaagggccaagaagaaggacactggcaacgtctacgcgctcaagatcatggacaagaagttcatcaccaaggagaacaagatc
tcctacgtcaagatggagcgcattgtgctcgatcagctcgaccacccgggcgtcattagactcttcttcaccttccaggacacctactccctctacatggc
gctcgagtcgtgtgaaggcggggagttgttcgaccagattgtccggaaaggccggcttcttgaggacgaggcacagttctatgctgctgaaattgttgat
atactagagtatttgcacagcgttggcctaattcatcgggacgtcaagccagaaaacctgctgcttacttctgatggccacatcaagattgctgattttgg
tagtgtcaagcctacaaaggatactccaatcaaagtcctcccgaattcaactaatgaaagggcttgtacatttgtgggaacagctgcatatgtaccacct
gaggtcctgaactctgcccctgcaacttttgggatgttgatccaagcaaacggccgggtgcaggacctgatggttatgcttccttaa
>g42331.t1
atggagtgcctcgccgagatgcccaagcggcggatgggctggggcgtcggtcccgccaagttcaatcagattcagataggattttgtggtcaagaagtt
gcacatgccgtcagtgctgtggcgctggggctctcttcgggtgttgtttcttctcaggggaaccaagagctccctcgtatcggttcccctccgttgagaga
agatgacaaggatggtcattatgtttttgctgtgggagataacctcacacctcgctataagattaatgcaaaaatgggtgaaggtacttttggtcaagtat
tggagtgttgggatagggaaagaaaagaaatggtgcctatcaagatcatcagaggaataaaaaagtacagggatgctgcaatgattgaaattggcat
gcttgagcagcttggtaaatatgaacaaaagcacatccagttgtgttcaaattcggaaagggggttaattaaggaaagtagagcccattag
>g42633.t1
atgcaagagaagagctttctcggggcgtgcagtggcaacccattcatcatcggtttccacggcctcgttcgcgcgcccgattacatggagcttcgcctcg
tgatggagcacgttggccccagcctccgtgatgtcctcctccagagcgccgatgacgacccgccattcttggaggtgacggtgcgcggcatcatgtggc
agcttctgacgggcgccaagaagatgcacgagctccacgtcgtgcaccacaagatcaagccggagaacatcctcgtcgacatggaccagagtgtcat
ggtaaagatctgcgacttcgggctagcccagcacatgttcgacgcgctgtcgtacaatcaggtgggcacgaggttgtacatggcgcctgaggtgctgct
gggaaagaccgactatgacgccctcgtcgacacgtggtcactaggctgcatcatagcagagctcatcgacgggtacgtcctcttccacgccttgaacga
caaaggccagttgtgcgccatcatcaatgtgctcggcgtgccgaacgctagagtgtggccgtggttgtcttccatgccgttcgccaacggactgctactt
gacctacggtacggatggaggcgaagcctgctgcgtaaggggttcctcgagacggtgctgttcgagcaaggtttcgaggtcctgaatagcctgctcgcg
tggaaccttgacaagcggctcatgtcatcctgcgcacccgagaaattagaaaatgctcaacctgcgcacccgggaaaacaatga
>g43195.t1
atgcctcgaatccaccattgctcctccctgctcctcgtcaccatcttcgttcacctatcgctatcggcagtagtcttggtgcacggggaagttgccaatggc
gtccatctggacctccccgccctcctcgccttcaagtcgtccaacccaaacgccaccgcgctggcgacctggtccggcccgaacccgtgctcgggttcct
ggttcgggatcaaatgcaacaagggccgggtggcaggcgtcttcctggacggcgcgtcgctgtccggcgccgtggcgccgctgctccggctgagccag
ctgagggcgctcgccgtccgaaacaactccctgtccggcgcgctcccgccgttggccgacgccacgagcccgcggctgcggcacctgctcgtcgcgcg
caaccagctgagcggaggcctcaacgtctcgctgggcgctctgctcacgctgcgggcggagcgtaacgggttcagcggcgggctcggggcgctgcgc
gttccgacggtcaggagcttcaacgtgtccgggaacaagctcgccggagagatctccggcgagctggcccgcttcccgagctcggcgttcggcgggaa
cctcgctctctgtggccggcctcttccgagatgcgttcgtgcttacagtgctctaggagatagtagtgccgatacaaatgttactattgcagctcaatctcc
tagtgcaggtgcagcaccgaacgcgagtgcgagtgcctctttgagcaacgggaagtttagtaaggtcagcttgactgcgctgatggccaccggcatcg
gcaacgcggtgctaatcaccgtctcgttatccatctccgtggccatgttcctgtacatgaagcgaaagctccggtccgccaaggccccttcactcttcttcg
acgaggaagacgacggcaacaagaggcggacgacgacaacaacgaacagggacgacggcaacaagtgcccggcggcgaagagcagcggcgggc
tggtgtgcttcgagggcggcgaggcgctgcggctggagagcctgctgaaggcatcggcggaggtgctgggcaagggcgcgtcgggcagcacgtaca
aggcggtgctcgaggacggcgccgtcgtggtggccgtcaagcgcctcagcgcgctgcggttcccggccggcaggggcaaggcgttcgaccgccatat
gcgggtcgtcggccgggtccggcaccggaacgtgctcggcctccgggcctactgcaactccaacggcgagcgcctgctcgtctacgacttcctccccgg

cggcagcctccaggccgcgctgcaagcacacggtgggggcgtgaggagcttggactgggcggcgaggaaaagcgtgctgttcggcgcggcgcaggg
gctcaactacatccacacgttcccggcgcggccggcgctggtgcacgggaacgtgaagccgtccaacatcctgctggacgagcgcggcgccgcgtgc
gtgtcggagtgcggggtcatgcgctacgccaccaacgtgcagcactccatcctcccgcaggcggcggcggcggccgggctgttcctggacctgtcggc
gtccgggtcgaccggcgggtggcatgggtacgcggcgccggagctggcagccacgacgagggcgacgcaggagtcggacgtgtacggattcgggat
ggtgctgctggaggtggtcaccggttgtcgcaaggccggcgaggagggcgaggaggaggaggacgacgagacgatggggtgggtcaagatcggga
tgctgtgcactgctgaggctccggaggagaggcccaggatggcgcagcgcctgctcgtctacgacttcctccccggcggcagcctccaggccgcgctg
caagcacacggtgggggcgtgaggagcttggactgggcggcgaggaaaagcgtgctgttcggcgcggcgcaggggctcaactacatccacacgttcc
cggcgcggccggcgctggtgcacgggaacgtgaagccgtccaacatcctgctggacgagcgcggcgccgcgtgcgtgtcggagtgcggggtcatgcg
ctacgccaccaacgtgcagcactccatcctcccgcaggcggcggcggcggccgggctgttcctggacctgtcggcgtccgggtcgaccggcgggtggc
atgggtacgcggcgccggagctggcagccacgacgagggcgacgcaggagtcggacgtgtacggattcgggatggtgctgctggaggtggtcaccg
gttgtcgcaaggccggcgaggagggcgaggaggaggaggacgacgagacgatggggtgggtcaagatcgggatgctgtgcactgctgaggctccg
gaggagaggcccaggatggcgcaggtgctgtccatgatgagcgagctcatgtga
>g43227.t1
atgctgcgccgcctcctccttgagcttcccctcctctcactccacctcctcctcctcttctccgccgcctcctatgccgcctccgtcaactcagaggctgc
gttcctgacgtcgtggctgaacacgacggcgtcgcggccgccggactggtctccgtcggccgcgtcgccctgcaagtggtcccacatcacctgcgacaa
caacaacaaggtgacctccgtgagcttccagtccgtccacctcgcggtctccctccctccgggcctctgcgccgcgctcccttcgctgacgtcgttcgtgg
cctccgacgcgaacctgaccggcgccgtcccggccgacctccacctatgccgccacctcgccgtgctcgacctgagcaacaacgcgctaactggcccc
atccccgcgtcgctcgggaacgcgtcgtcggcgctggagacgctggcgctcaactcgaaccagctctccggccccatcccgccgtccctcgccccctcg
ctcaagaacctgctcctcttcgacaaccacctctccggcgagctcccgtcctcgctgggcaacttgcagctcctggaatccctgcgcttgggcgggaacc
gcgacctctccggccccatccctgagtcgctctccaagctctcgaacctcgccgtgctgggcctcgccgacaccaagatctccggcgcgctccctgcgtc
cctcggccagctccagaacctcgagacgctctccgtgtacaccacctcgctctccggctccatcccgccggagctcggcaactgctccagcctcgcatac
atctacctgtacgagaaccagctctccggcccgctcccttcctccctcggcaacctcccccggctgcagaagctcctcctgtggcagaacgcgctgtccg
gacccataccggactcgttcgggaatctcacctcgctcgtctcgctcgacctctccatcaactccatctccggcgcgattccgggttcccttggtcggctgt
cggatttgcaggacctgatgctcagcgacaacaacatcacgggcaccatccccagctccctcgccaacgccacctccctggtgcagctgcaggtggac
accaacgagatctcggggctcatcccgccggagatagggacgaggctctcctccctgcaggtgctcttcgcgtggcagaaccagctggaaggcgcgat
cccgccgacgctgtcctcgctcaccaacctccaagcactcgacctctcgcacaaccacctcaccggcgccatcccgccgggcctcttcctcctccgcaac
ctcaccaagctgctcctcctctccaacgacctgtccggcgcgttgccgccggagatgggcagcgcggggaacctcgtccggctccggctcggcgggaa
ccggctcgcggggacgattccgggttccgtctccgggatgaggagcgtcagcttcctcgacctgggaagcaaccgcctcgcggggacgattcccccgg
agatcgggaaatgcgcgcagctccagatgctggacctcagcaacaacgcgctcaccggcacgattccggagtctctcgcggatatccatggtctccag
gagatcgacgcctcgcgtaaccggctcacgggcgccatccccgacaagcacatggagacgttgagccggctggttctgagcgggaattccttatccgg
cccgatccctgcgtccctcgggcagtgccgcagcctggagctcctggacctgagcgacaactcgctgagcggcccgatcccggacgcgctgtgcgggc
tggacgggctggacatcgcgctcaacctgagccggaacgggctcacgggcccgatcccgcgcaacatctccgcgctttccaagctgtcgattctggacg
tgtcgtacaacggcctggacggcagcctggcgccgctggccgggctggacaacctggtcgcgctcaacgtctcccacaacaacttctccgggtacctcc
ccgacaccaagctgttccgccagatgtccgcgtcgggactcgcggggaacgccggcctctgcaccaagggcggcgacgtatgcttcgtcaccgtcgac
ggccgcccggtgacgagctcatccgagtcagaggaagcggcgcgcgtgcggaggctcaagctggccatcgcgctgctggtcaccgccacggtcgcca
tggtgctcggcatggtcggtatcctccgcgcgcgacgcatcggcgggaaggctggaagaagcagcgactccgaaaccggcaacggcagtaataacag
agaccagctggcgtggccgtggcagttcacgccgttccagaagctgagtttcagcgtggaccaggtggtgcggagcctcacggacgccaacgtcatcg
ggaaaggctgttccggcgtggtgtaccgcgtcagcatcgacaccggcgaggtcatcgcagtcaagaagctctggcccaacacgcaccctgccgccgcc
aacaaggacgacgacgcggcggccaacaccatccgcgactccttctcggcggaggtgcgcacgctggggtccatccgccacaagaacatcgtgcggt
tcctcgggtgctgctggaacaagaccacgcggctgctcatgtacgactacatgcccaacgggagcctcggcgccgtgctgcacgagcgaggaaggtgc
ggaggtggcgcgcagcagctggagtgggatgtgaggtaccggatcgtgctgggcgcggcgcaggggctcgcgtacctgcaccatgactgcgtgccgc
ccatcgtgcaccgggacatcaaggcgaacaacatcctcatcggactcgatttcgaggcctacatcgcggatttcggcctcgccaagctcgtcgaggacg
gcgatttcgggaggtcctccaacaccgtcgccggatcatacggttacatcgcaccggagtacgggtacatgatgaagatcacccagaagagcgacgtg
tacagctacggcgtggtggtgctggaggtgctgacggggaagcagcccatcgacccgaccatccccgacggcctccacgtcgtcgactgggtgcgccg
ctgcaagggcggcgccgacgtgctcgacccggctcttaaatgccggaacagctccgaggtcatgggcgtcgcgctgctctgcgtcagccccacgcccg
acgaccggcccaccatgaaggacgtcgcggcaatgctcaaggagatcaggctcgagcgggaggactttgcgaataatgtcgatctgctactcaaggg
cggcggcggcggcgcgtcgccgcctcgtcgtcttcccaacaacaacactacggcggtaccaaaggggacggccacgtcgtcgtccacgtccagcacg
ccgccgtcgtaccggcaaggtccaagcaacagcaacagctgcagtagcagcagcttcgtctctgctgccaccatctactcatcggcggcgtccaaggc
caagacaccgtttggctga
>g43753.t1
atggcgggtagcggcagcgaagtcaagtacccgctgaatgccgagtcgtaccgcctactttgcaagatcgggagtggcgcgagcgcggtggtctacaa
ggccgtgtgcctgccgctgggctcggccgtggtggccatcaaggccatcgacctggagcgttcccgcgcaaacctcgagaacgtgtggcgggaagcca
aggccatgaccctgctttcccacagcaacgtgctccgcgcgcactgctccttcaccgtcggcagtcacctttgggtggtcatgccgttcatgggtgccggc
tcgctgcactcgatcctaacacacggcttcgccgacggcctcccggagccgtgcatcgccctcgtactcaaggacacgctgcgggcgctgtgctacctc
cacgagcagggccgcatccaccgtgacatcaaagcgggcaacatcctggtggacgcccacggctctgtcaagctcgccgactttggcgtctctgcgtcc
atctacgagagcgagacctcctccatgcatgcgccatcaccggtacccatcagctcatcatcttacttcagcgacttggccgggacgccgtactggatgg
ctcccgaggtcattcactcgcacgtcggctatggcatgaaggctgacatctggtcgttcgggatcacggccctcgagcttgcccacggccggcctccgct
gtctcacctgccgccatccaagtcgatgattatgagaatcaccagccgcgtccgcctcgaggacgccgagatgatctccaggaggaagaaattctccaa
agcattcagggacatggtttcttcctgtctctgccgggagccggcacggaggccatcggcggagaagcttctccggcatccgttcttcaagggctgtcgt

tccaataatgactaccttgtccgcaacgtgctcgtcgccgtcccgggcatagaggagaggtgtaataaggatactaacctctgcggctgtgccaggggt
gcagcacgttgcgtgtcgtcatgctgtcacaccaacgtcgcaaagaaccggcggattagcggctggaacttcaacgaggataacttacagctcgatcca
accgacgacgggactgacgagcagaggtatcgttttcctttcgtgaaaaatgaccccgtcgagagagctagcaataattcgactggtggagagtgtga
aggcagccagacccggcaacgggcgagaataataggagtaatggaagttgtgataccgcaactgatgactatcctggagagcctcaagatgcagcta
gggatggtcatgcacgtactagaatgcggcggcttccacgtcaacgacagtgggtgtggaagagcacctgcaagagaggagagggaagaaatgctg
cttttctacgtgcggcagcttgagcagagggtggaggagctgacaacgcaagttgacgaggagatggccaggaatggccggctagaggcgcagctgc
aggcgagggttaatgacaaaaacataaatccatcgcagatatctgggagcagttag
>g44029.t1
atgaagatcgcggaggacgtggcggcggggctgctgcatctccaccagtcgagcatcgtccacggcaacctgaagccgttcaatgtgctcctgggccc
ggactttgagtcatgcctcaccgactatggactactcatgatgatgatgatgcttcccgaccatgtcaatgatgcatcttcaacctcgctcttgtaccgggc
cccggaggctcatcgtactacagctttcacggcggcgtccgacgtgtacagcttcggagtgctcctcctggagctcctcacggggaggacgccgttcca
ggacctcctcatgttcatgaatgaggacgaccacataaccagctgggtgcgcgccgtgcgtgaggaggagagggacaccgactcctcctccggtggta
ccggtgctgctgccgacgagaagctcgccgccttgatcggtatcgcggcggcgtgcgtggccgcggacccggaaaggcggcccaccacggcggaggt
gctccggatggtgagagaggctagggccgaagcaatatcctcctccaacagcagcgatcgctcacctgctcgctggtccgacgccatcctcgccgcgc
cgtcgcagccatttcccacagactactga
>g44036.t1
atggagaggtacgaggtgatcaaggacataggatcgggcaacttcggggttgccaagctggtccgggacgtgaggaccaaggagctcttcgccgtca
agttcatcgagagggggatgaagattgacgagaatgtccaaagagagatcatgaaccacaggtcattgagacatccaaacatagttaaattcaagga
gtcctctgtattgcactctcagcctaaatcaactgttggcactccagcttacattgctccagaggtccttgctagaaaggaatatgatggaaaggttgctg
atgtttggtcctgtggagtaactctttacgtgatgcttgttggtgcttatccctttgaagatcctgatgaaccgaggaattttcgcaagacacttactcggat
tctcagcgtacaatatgcagtccctgattttgtccgagtttcaatggagtgcagacatttgctgtcccggatcttcgtggcaaaccctgagcaacgaataa
ccatcccggagatcaagaacgacccatggttcctgaagaacctccccatcgagatgaccgacgagtaccagacgaacctgcagatcatcgacatgaa
cgttccctcgcaaagcctagaggagatcatggccgtcatacaggaggcccggaagcctggcgatgggctgaagctcacagggcagcttccgggacttg
ggagcatggacctggatgacattgatgtggacgacatcgacgttgatgacagcggtgatttcgtgtgcgccttgtga
>g44056.t1
atgggaggttgccacgcgaagccgctcacccacgacccggacaactcgccgccgcacgcggcgccggcgacgccgctcccggagggccgcacctcc
gcgtccgccacgcccgcgaagaagcagcactgggcgtcgtcgcccttcttcccgttctccacgccgagcccgagtccggcgcaccacctcttcggctcg
tccgcggcgtccccgcgcaagtcccctgcaccgcccggcgcgggctccgcgcccaccacaccggccagacggctgctgcggctgcccttcccgccgcc
gtcgcccgccaagcacatcagggcggcgctcgcgcggcggcacgggccgtcgcggccgtccatccccgaggaaggtggcggcgaggggggacggca
gcggcagggggctcgacaaggggttcgggttcagtaaggggttcgcgaccaagtacgagatgggcgatgaggtcgggaggggacacttcggctaca
cctgtgccgccaaggtgaagaagggggcgcgcaagggcgagtctgttgccgtcaaggtcatccccaaagccaagaatttcctttttacttcgaaagatg
agaattcccaacttaaggctatcgactttggcttgtcggattttgtaaaaccagatgaacgcctaaatgatattgttggaagtgcttactatgttgctccag
aagttctgcatagtcgtccattttgggcacgcactgaatctggcatattccgttctgttctcaaagctgaccccagctataatgaggcaccatggccttctc
tgactccggaagcaatggattttgttaagcgtttgctgtgcaaggatccacgtagaaggatgactgcagcacaggctttaagtcatccatggatcagaa
attataatgacattaagctgccgttggacgtccttatattccgacttatcaaagcttatatccgttcctcatctttacgaaaagctgctttgagggctctatc
gaagactttaactgttgatgaacttttctatctgaaagcacagttttccttgttggaaccagacagaaatggatgcattactcttgataatatcagaacggc
attaacaagggaggccactgatgcaatgaaagaatcacgagttcaagagattcttgtttcgttgagcgctcttcagtacagacgaatggacttccaaga
gttctgtgcagcagcagttagtgtgcaccagctcgaagcattggatagatgggagcaacatgcccgatctgcttatgaatattttgagaaggatggcaat
cgtgctattgtaattgatgaactggcttctgaattgggtctcagcccttccgtgccattgcatgttgttctgcaagattggatcagacatactgacgggaaa
ctgagctttcttggttttgtcaagctattgcatggcatgtccagcaggtccctgtcaaaggtgaggtag
>g44590.t1
atggctctcgtccgccagcgccgccagctcccacacctcaccctcccgctcgaccacttccccttcgcctgccgccgcagcctcagcccgccgccgccg
cgccgtccacctcgatctccgactacgagcggctctccgtcctcggccacggcaacggcggcaccgtctacaaggcgcggcaccgccggtcctccgcg
aacccgccgctcgtcgcgctcaagatcttcgccgcggcgggggacccctcggccgcgcgagaggccgagatcctcgcgctcgcctccgacgcgcccca
cgtcgtgcgcctgcacgccgtggtgccctccgccggtgaggcgcccgcggcgctggcgctggagctgatgccgggcggctccctcgcggggctgctcc
gccggctgggccggcccatgggcgagcggcccatcgccgccgtggcccggcaggcgctcctgggcctcgacgccctccacgcgctccgcgtcgtccac
cgcgacctcaagccgtccaacctgctcctcggcgccgcgggggaggtcaagatcgccgacttcggcgcggggaaggtgctgcgacgccggctcgacc
cgtgcgcgtcctacgtcggcacggcggcgtacatgtcccccgagcggttcgacccggaggcgtactccggcgactacgaccccctacgccgccgacgtg
tggagcctcggcgttgccatcctcgagctgtacctgggccacttcccgctgctgcccgagggccagcgacccgactgggccgcgctcatgtgcgccata
tgcttcggcgaggcgccggagccgcccgccgcggcgtcggaggagttcagggacttcgtcgcgcggtgccttgagaagaaggccggacgaagggcgt
cggtggccgagctgctcgaccaccccttcgtcgcagagagggacgcggtcgacgcgcggcaagcgctcgccgcgcttgttgcccagacggacgacct
gtag
>g44653.t1
atgccctcgcgccagcttcccttccctgctccgccgccgctgcctttcccgcccccacacgcgcttgcccaagaccatcaccaccaccgccgccgcaactt
catcgcggccggcgcatcagcggcggcggtcgcggtggctctggcggcgctgctgtggtggtggctgcggagaaggaagcgggcggcgggggagga
cggggcggggcctctgcagcgcttgtcgtacaggcggctgcggcgcgccacgggagggttcgcggcggcgaacaagctcgggcagggcgggttcgg
ccccgtcttccgcggcgcgctcccccggtgaagggggggcagaggagggggcgcggcggcccgtggccgtcaaggtcatggacgccgcggggtcgctc
cagggagagcgcgagttccacaacgagatcgctgtcgcctcccacctcctctcctcctcccctcctcctcctcttccatcctgctgccgttcgcctactcc
atgtccgagcaggggcaggggcaggcgcggccgcggcggacgatgctggtgtacgacctcatgcccaacgggtcgctgcaggacgcgctgctgggg

cgccgccgcgcggagctcgtggcggagtggccgcgccggctcgcggtggcgcgcgacgtcgccgccgcgcttcactacctccattccgtcgttacgcc
ggcggtcgtgcacggggacgtcaagcccagcaacgtcctgctcgacgccgacctgcgtgcccgcctcgccgacttcggtctcgcccagttcaaatccga
tcctgacgccgacggcaagccggagagcggcgtgatcgctgaaggccaagtgatggctcatcaaggggacgacgatgccattgcgcatcatcctcctg
atggcggatgcgatgacgacgtctcggtagtggctgagagcacggtcactactacggtgaatggggagggaaacgttgcgcccaacaagtcgccaga
ggatgatgagggattcacatctgcatcaccggcggcagaggctgcgtccacatctgggtttgacaggaccagtgtcgacagtggtatcaactgcaatgg
tggctcacgtactggtactggcactgactggtggtggaaacaggacaacggtactacggccagcatcggcgtgaaggactatgtaatggagtggattg
gatcagagatcaagaaggagcgtcccaagaatgactggattgcagatgcagcagcaaccaataatcctatagtaccagagaggaagaagaagcaga
aggcaagggcgcgagaatggtggcgtgaggattatgtcgatgagctggacaagaagcagaaacgacgggcacttgctaagaaatcaaagagtgagc
aaacggggttgcaatggtgggagagggatcttgatgacgacttggatgagaagggtccgtccaagtggaatctggtgaagagctggagtcgtaggag
gagcagcattagcaccagcaatggcaatagcggcattaactggtgggtgaatggtgcgagaagcagcagccgtgattgggcaagtggtgatttcgtgc
ccaagagcggtggtacagtgagcagcacgccgagcatgcgtggaaccgtatgttatgtggctccagagtacggtggaggtggccctgtgtccgagaaa
tgtgacatctacagctatggcgtcctgttacttgtcattatctccggccgccggccattgcaggtgacggctttacccatgtcagagtttgagaaggcaag
ccttatatcatgggcaaagcaccttgcccgtgccagccgcttgcttgatcttgttgatcccgccatgcgtgatgttaaccgtgatgaggcactgctctgcat
tactgtcgcattgctctgcatccagaggtctccagcgcgtcgcccgtcgagtgaagagctgattgcgatgctctcttgcgagggagaactgccacaactc
ccactggagttctcgccgtcgccacctggtgggttccctttcaagtccaggaagaaaggtcgttga
>g44837.t1
atggtgctcgtgaggatgttgaagagcaaggacacggcggcgctggctaacgcgcaggatggtgggtcctccggttccgacaggggtgagggcggcg
acgtgcaggatggcagtttgatccagtggctgaccactgattggctcctcatggatgctggcattgcaaacataaaatggtgtggcattgatggtgatga
aaatattcttgttattgatctccttggaccaagcctagaggatctttttgtttattgtggccgtagattctcactaaagactgtcctgatgttggcagatcaga
tgattacaaggatagaatttatgcattctaaaggatatttgcacagagacatcaagccggataattttcttatgggccttggtcgaaaagctatccaggtc
tacatcatagacttcgggcttgcaaaacgataccgtgatgcccacactaatcgccatataccttatagggaaaataaaaatttgaccggaacagcccgc
tatgctagtcgtaatactcatcttgggattggttttcagtttgattatgtatatgactggacattgttgaagtgtaagcagtcccagaaggctaaggcccaa
caaggatgctctcaagatgcaggtgtaagtagccgtcctgttgcaacaaatatggacaagcatcaagttagtgtttcacgcccagtcgaagcttctggtc
tgcttgaaggagagcaacgcccggcaatcagaatgcagttcagatctagtgctgagaacagtcgttcaaataatatgcaccctgagagatcgagagtg
gttgcaagccctgataatgcattgctgcaacccacattgcttggtcgttctgatactccaaggaacaatgttacaatctcaaaaacacaggggctcaatg
atccccacattcgtggaagtaacagtccagggccctctaaccacatttag
>g45070.t1
atgccactgccaccagaagatggcgaccggacctacgcgtcctcgtatcgtgacaatgccacagcgtacagtccaagaccaaccgctgcttccgtcga
gaattctgttgtcttgaagctaccagacgccggccgtgagctgtggcacgcgatggagcctctccgccgcttcctccacctcctcctaccacttctcttcct
cctcgccgccgtctcctatgaccacgcggccgtcctcgccgccgacgagttcagctacaacggctttgggggagcggacctgacccttgacggcatgtc
cgccgtggcgccgaacggcctgctggtgctgagcaacggcacctcccagatggccgggcacgcgttccacccgacccccatccgcctccgctccaccc
cgggcggcgccgtgcggtccttctcctgcgcgttcgtgttcgccatcgtgtccaacttcacggtgctgagcgacaacggcatggcgttcgtggtggcgcc
cggcacgcggttgtcgaccttcaacgcggggcagtacctggggatcctcaacgtgaccgacaacggcgcggcgtccaaccgcgtgctgttcgtggagc
tggacaccatgctcaacccggagttccaggacatgaacagcaaccacctcggcgtgaacgtgaacagcatgcggtccctgcacaaccacaccgcggg
gttctacgacgacgacgacgccacggggacgttcaacaacctcagcctcatcagccggcagccgatgcgggtgtgggtggactacgacggcgccacc
acgcggctggacgtgaccatggcgcccctggacgtggcgagcaagcccaggaagccgctcatctcggccccggtgaacctgtccggcgtcgtggacg
acaccgccttcgtgggcttctcggccgccacgggggtgatctacacgcggcactacgtcctgggctggagcttcgcgctggacggggcgccggccccg
ccgctggacgcctcgggcctgccgaggcttccgcggttcgggcccaagccccggtccaaggtgctcgagatcgtgctgcccatcgccaccgcggccttc
gtcctcgctctggtcgtcgtcgtcttcctgttcgtgcggcggcggatccggtacgcggaggtgcgcgaggactgggaggcggagttcggcccgcaccgc
ttctcgtacaaggagctgtaccacgccaccaaggggttcaagaacaagcagctgctgggcacgggcgggttcgggcgggtgtacaagggcgtgctca
agaacaaatccaacctggagatcgccgtcaagcgcgtgtcccacgactccaagcaggggatgaaggagttcatcgcggaggactgggaggcggagtt
cggcccgcaccgcttctcgtacaaggagctgtaccacgccaccaaggggttcaagaacaagcagctgctgggcacgggcgggttcgggcgggtgtac
aagggcgtgctcaagagcaaatccaacctcgagatcgccgtcaagcgcgtgtcccacgactccaagcaggggatgaaggagttcatcgcggaggtgg
tcagcatcggccacctccgccaccgcaacctcgtgcagctgctgggctactgccgccgcaagggggagctgctattagtctacgactacatgtccaacg
gcagcctggacaagtacctctacgacaaggccaagccggtgctcgactgggagcagcggttccagatcatcaagggcgtcgccgcggggctcctctac
ctgcacgaggactgggagcaggtggtggtccaccgcgacgtcaaggccagcaacgtgctgctggacggcgagatgaacggcaggctgggcgacttc
ggcctcgcaaggctgtacgaccacggcgtggacccgcagaccacgcacgtggtgggcaccatggggtacctggcacccgagctggtgcgcacgggg
aaggccacgcccgtgaccgacgtgttcgcgttcggggtgttcgtgctggaggtggcctgcgggcggcggccgctcgggtgcgtggcgcccgacgacca
gaacgtgctgctggactgggtgcaggactacgaccgcaagggcgcggcgctcgacgccgtggacgcgcgcctgtgcggaaagtacgacgccgacga
ggcgcggctggtgatcaggctggggctgatgtgcgcgcacccgctgcccgacgcgcggcccgggatgcggcaggtggtgcagtacctggagagcgac
gcgccgatgccggaggtgccgcccacctacgtcagctacaccatgcttgcgctcatgcagaacgacggattcgactccttcgccatgtcgtacccctcca
ccgtcacgtccggcgtcagccccgtctccggtgccttctccgacgtctccggcctctccggcggaagatga
>g45270.t1
atgatgatttgctgctggtgcggcgcaaaggaggatacatatggggcaccgcccagctaccacccagcagctcctgcgtcacggaacaatgcaggccc
aggcaagccaagaggacccaatgcgccgagacctggcggcccaccgaaggttctccccatcgacgtgcctgccatttccatgggcgagctgaacaac
atcaccgggaacttcgggcagggcgccctggtaggtgaaggctcctacggccagatctaccgggccacactcacctccggggagctcgtcgccatcaa
gaagctcgatcccagcgtgtcaaaagacaccgtcgctgacttctccgctcagctgtcgatggtgtcgaggctgaagaacgagtacttccttcagttgatg
ggctactacctcgacgactgccaccgggtcctcgtctaccagttcgcctcgcacggctctctgcacgacactctgcacggcaagaagggtgtgaaggac
gcgacgcccgggccggccctgagctggaaccagcgggtgaagatcgcctacggcgcggcgaggggtctcgagtacctgcacgagaaggtgcagccg

gccatcgtgcaccgggacgtcaggtccagcaacgtcctcttcgacgactacgactccaagctcgccgacttcaacctcaccagccagccgcccgacgg
cgccgcgaggctgcactccacccgtgtcctcggcacatttggctaccatgctccagatgatgactacaaaatcaatcaaaaagacttaacagtgatagg
tgtgaaggacgcgacgcccgggccggccctgagctggaaccagcgggtgaagatcgcctacggcgcggcgaggggtctcgagtacctgcacgagaa
ggtgcagccggccatcgtgcaccgggacgtcaggtccagcaacgtcctcttcgacgactacgactccaagctcgccgacttcaacctcaccagccagc
cgcccgacggcgccgcgaggctgcactccacccgtgtcctcggcacatttggctaccatgctccagagtacgcgatgacgggagtattgacacagaag
agtgatgtgtatagctttggagtgatccttctcgagctactgaccgggcggaagcccgtggaccacaccttgcccaagggacagcagagtcttgtaactt
gggcgacgccaaggttgagcgaggacaaggttaagcaatgcgtcgatccgaagctgggggatgactatcctccaaaagcagtcgcaaagatggcag
cggtggcggcattgtgcgtgcagtacgagtccgatttccggcccaacatgaccatcgtggtgaaggcgctacaacctcttctcaaagccgcagcagggc
cccccagtacatga
>g45564.t1
atggcgatcggcggcggcggcggcgggagctggagcatccacggccgccccgacgtcacatcccgctacgaggttctcggccgtgcgggctccggcg
cctacgccgacgtctaccgcggccgccgccgctccgacggcgccaccgtcgcacttaaggaggtccacgacgccgtcagcgcccgccgcgaggccga
cgccctcctcaccgtatcctccccgcatgtcgtcgcgctcctccaccacttccccggcggcgactgcgacgacgacgtcctcgtcctcgagtggctcccgc
tcgacctcgccgccgtcgtgcgcgacgccaggcgccgtgcccgtgacgatgggaccggcataaccgtcgcgcagctcaagcggtggatgatgcaggt
gctcgaggggggttgccgcgtgccaccgcgctggcgtcgtgcaccgcgacctcaagccagcgaatctgctcatctccgtggatggggtgctcaaagtcgc
tgactttggtcaggccaggatatttcagcagacagcgcctacttacctggatatgccacaaggtgaggaaagttctggtgtagagccatgggtttcacaa
caacaatcagagttacaagggacagaagaggaaccatcatctcatggatctgatgttcatgtaagtcaggagccagagactctcactgcagctgacta
cctacatgagctggaccaactccgggccaaatccagcgatgttgacaaaatgagtctgcaagatggaaatgcatcatgtcttgccacatgcagcacag
gagacatcgaagatgatccattccgggcctcttacacatatgatttggaaggaatgggggaagagtctggtgccttcacatcctgtgttggtacaaggtg
gtttagggctcctgagcttctatatggatcaacaaactatggactggagattgacctctggtcgctaggatgtattttggctgagttgcttaatttggagcc
tatatttcctggcatatctgatattgatcagataagtagaataattggggtcctgggcaatataacagaagaaacctttccaggttgttcaaatttaccag
actacaacaaaattttctttaacaaggttgagaatccgacaggtctggaagcatgtttgcctaatagatctgcttctgaggttaatatcattaaacaactg
ctttgctatgacccagcaaagagggtgagtgcagctgacctgttgaatgaccagtacttcacggacgaacccttgcctgcacctatagaaggattacaa
gtcccagcttcaaaggatgatgatgatgataacagctctatggaagaatgggcaaataacaggaatggtggttcagattcagactttgacgagtttggt
accatggatgtatccagaactgacaagggtttcagcatacgcttttcctga
>g45664.t1
atggcgtcaggcaagtctctcctcgtcctcgtctcgctgctcctcctcttgtttgtttccactgttcaaggctacgacgccggccgccatgctgctattgcgc
gcttggccgtgtcgcgccgttcccgggtgggaacccggcacttgcagcacggccggaccacatcgccgcaccggtacgtgcttgcggagaagaacgg
cagctcatcatcaccacccgcgacatcatccaacaccacctcgccccctgcggccgagcagcagggcaagcgccaccgcagccacaagcaccgcgtg
cgcaactggatcatcggcttcgtggtgggctccatcgcgggggtcatctccgggctggtgatgtccgtgctgttccggctggttctcaactgcgccaggg
gccggtaccggcgccgctccggcacggtgatcttcacgccgaagctgatcaagcgggccgaccagctggccttcctggagaaggaggatgggctggc
gtcgctgacggtgatcgggagcggcgggtgcggcgaggtgtacaaggcgcagcttcccccgagcgtgagggcgaggagcccaagttcatcgccatc
aagaagatcaagctgcagcccagcaacgagagcgccccgccgcagaacctgagcgacgaggagagccggcagctggacaagtggtcccgccagat
ccagtcggagatccgcaccgtgggccacatccgccaccgcaacctgctcccgctggcggcccacgtgccgcgacccgactgccactacctcgtctacg
agtacatgaagaacggcagcctgcaccacgcgctcagggcccatcatcgtgcaccgggaccatcagaagatgccggagacactagcactagcaataa
tagcagcagcggaggaagcagcgtcggcacgatggtggcgacggcgctgccgtggccggcgcggctgcgtgtggcggtgggcgtggcggcggggct
cgagtacctgcacctgtcgcagcgcccgccgatcgtgcaccgggacctgaagccggccaacatcctcctcgacgacgacatggaggcccgcatcgcg
gacttcgggctggccaaggccatgcccgacgagcacacgcacttcacggcgtccaaggtcgccggcacgctgggctacatcgcgcccgagtaccacc
agacgtacaagttcacggtcaagtgcgacgtgtacagctttggcgtcgtgctggccgtgctcgccacggggaaggagcccacgcacgacttcttcacgc
aggtggacgacatcggcctcgtcaagtggctcaggcgactcatgcaggacggggacctcgcacaggccatcgacccggccatcgcaggacaaggctt
cgaagagcagatcctcctcgtcctgcgcatcgccgtcttctgcaccgccgacgacccaaaggagcggcccacggccaaggacgtacggtgcatgctcg
cacagatcaagagctag
>g45863.t1
atggaggccatcagtaggcggaagcgcttggccgtcggcgtcctggaggagtacgagccgacgcggtgcctcggcaagggcggcttcggctccgtgg
tggaggcgcgccaccgcgtcacgggccagaccgtggccatgaagcgtctgatcggcaatctcggccaaggacgacaatcatcggcggtgatgcggga
gaagagcttcctggaggcgtgcagcagcaaccccttcgtcgtcgggttccacggcgtggagaagacgacgccgcctctcccggcggaggaggccacg
gtgcgcgccatcatgtggcagcttctgacgggcgccaagaagatgcacgcgatccacatcgtgcaccgcaacatcaagccggagaacattctcgtcga
cgtcgaccagagagtggtcaagatctgcgacttcaggatcgcgctgcgcatgtcccacgcccttcgcagcgaggactag
>g45972.t1
atgggctacgggtacgtggatcccttcctgccgccgcagcaggccgccgcggcgtcggcgtaccagccgcagaagcaggcggcggtgtcggtgttcgt
cgcgccgcaggggggacggatacggccagccggcgccgagaccgccgggctgtccgttcggctcctccgcttcggccccgccgacgtcgtaccactcct
tgccgccggcgacgtcggctccgccggtgagcagcccgccgccggcgtcgcctccgcccgagccgttgccgccgccggcattgtcgcctccacctcccg
acgcgccccctccggcgctcccgccgtcgctgccgccgacccctcccccgtcgccgcctccggtcgaggtgagcgccccgcctccgccgctggcgacg
gaccagccgcgcgttcagcctcgcgtgtacccgtcgccgccgccgccgtcgcttcccccgccgccgccaccgtccgtctcgccgccggcgccggctcca
tctctcccaccgtctccgtcccctgctccagccgcacgtgctccagctcccgcgcggcatgctttccactcgcctcccccgccgagaattgctcctccgccg
cctccacgacatcacgtcaagccacattactcgcccgggaagtcgcattcgaactctacccacgcagctagtagcagcgggaagaacatcgagatatc
gagggaagcggccaccaccatcgtagcaatcgctggtcttgcaatgctcagcttcatcggtgccaccatttggtttgtcaagaagaagcgcaggcgggt
agagccaccagcgtcactgcccacacagcaaccagctccgccgccaccgcccaactacattccatcttctgctggatcctccttagcatcagggtatcct
ttcatgagatccagtaccgggagtcacgggtttccatactcaccagctgattctggcattgggtactccagaatgctgttcacgccagagaatttggcag

caatcacaaatgactttgcagacgaaacacttttgggagaaggtggattcgggtgcgtgtacaaaggaattttgccggatggtcgccctgtggctgtca
agaagctcaagattggaaatgggcaaggcgagctcagtgaaacatctcttgattggcgaacaagggttaaagttgctgctggagcagctcgtggaatc
gcttacctgcatgaagattgtcatccacggattattcacagagatattaaatcatctaatattttattggataacaattttgaagctcaggtttctgattttgg
actcgcaaggttagcggctgattctaatacacatgtgactacacgtgtcatggggacatttgggtatttggctccagagtatgccttatcaggcaagttga
cagcaaaatctgatgtatattcttttggagttgttcttttggagcttattacaggaagaaaacctgttgatgcttctcagccgttgggagatgagagccttgt
tgaatggatccccgacgcccgcaacttcaccaatcaccaggtcaccaaccagttcctgccgcaggaagcttcattgcgccgcgcgcgccgatcaatctc
cgctgcccaaagcttcaacgtgctgcagacgggtccctgccgccgtattgagctctatggaaacacaaggctagttagtaggccatgtcttcaaagagt
agaggaggtggacgccgtggtcgtggccgtggccatggaagacatactattgcagaatatgacatag
>g46040.t1
atgaaggtgatggacaaggggtcccttgcggggcggaacaagctggcccgcgcgcagacggagcgcgagatccttggactgctggaccacccgttcc
tgcccacgctctactcccacttcgagaccgacaagttctgctgcctcctcatggagtactgcagcggcggcaacctccattcgctccgccagaagcagcc
caacaagcgattcacagaggacgccgccaggtaa
>g46117.t1
atggacgcggcggcgaagaagatcacgcggagcatctcgcggcagttctcgtcgggggcggcgcgggtatggcgccagctgtcgctggacccgcaca
cgccgcgccgcggcggggcggcggcggcggcggccctgcccacgcggttcgcgatcgcgaggcagtcgtcgctggacccgacgccgcggtcggagg
gcggcgggtcggcgcacgacctggcggtgccggataacctggacgccacgatgcggctgctcttcgccgggtgccagggggacgccgccggggtgca
ggagctgctccagtccggggtcgacgtcgacagcatcgacctcgacggccgcaccgcgctgcacatcgccgcctgcgaggggcagggggaggtcgtg
cggctgctgctcgactggaaggccaacatcaacgcccgcgacagatggggaagcacgagtaccatcnnnnnnnnnnnnnnnnngtcgacgtcgac
agcatcgacctcgacggccgcaccgcgctgcacatcgccgcctgcgaggggcagggggaggtcgtgcggctgctgctcgactggaaggccaacatca
acgcccgcgacagatggggaagcacgggtgatttaaccagctatatgaatgaaaaaggtcgcctgaagccccacaaagccatcagatttgctcttgac
attgcaagggggctgacttatctccatgaatgcaagcctgatccaatcatacatggcaatctatcaccgaaaaacatcgtgcgggacgatgagggaaa
acttaaggtggcagattttggatcacttaatctgattaaagtctcagacgataaagtacaaatggctcaaccaataacaaaatttgatggtgtatacattg
cccctgagatgtacagaaatgaaccatttgacagaagtgtagatgtatttgcatttggtctcattctttatgagatgattgaaggaactgctgcttttcacc
caaaaccatcagaggaagcgatcaagatgatttgtttggaaggtgtatacattgcccctgagatgtacagaaatgaaccatttgacagaagtgtagatg
tatttgcatttggtctcattctttatgaggctaatacaagaatgctgggatccgacgccttccgtcaggccagctttctctga
>g46330.t1
atggagaagtacgagccggtgcgggacatcgggtccggcaacttcggggtggcgcggctcatgcgcaaccgcgagacccgcgagctcgtcgccgtca
agtgcatcgagcgcggccaccggattgacgagaatgtgtacagagagatcatcaaccaccagtcgctgcgccacccaaacatcattcggttcaaggag
cttgcagacgtatggtcctgtggggtgactctttatgtgatgcttgtgggcgcgtacccatttgaagacccagatgaccccaagaacatacgcaagaccg
ttgagcaaataagcaaagtacagtacacgataccagattatgtccacatatcaacagagtgcagacaacttattgcccgtatctttgttgccaatcctgc
gaagaggatcacaatgaaggaaatcaagagccatccatggttcttgaagaacctgccaagggaacttacagagacagcgcaagctatgtactacagg
agggataacaggcaaataagcaaagtacagtacacgataccagattatgtccacatatcaacagagtgcagacaacttattgcccgtatctttgtcgcc
aatcctgcgaagaggatcacaatgaaggaaatcaagagccatccatggttcttgaagaacctgccaagggaactcacagagacagcgcaagctatgt
actacaggagggataacagggtgccttcttattcagaccaaacgtcggaagaaatcatgaagatcgtccaagaagctaggaccatgcctaaatcatcc
agatcagggtacgaatcgagctatgaggcaacagatgatgaagaaaataaggaagaggaacacagaccaggagagaataaaggcgaatacgata
gaagggtcaaggaggttggcagaactcaaggattcagtggcttatgcaattttggctacagaactgcttctgtttggtccttctccctgtaa
>g46523.t1
atgcccgtggtgaggaccgggagtatgcgccggtcttcggcccccgtgccgccgccggtgccggccttcactgcgtcggccggcgactaccgcctcctc
gaggaggtcggttacggcgcgaacgccgtcgtgtaccgcgcgatattcactccaactaaccgggtggtcgctgtgaagtgcctggatctcgatcgagtc
aacagtaacctcgatgatgttcgaagggaaactcaaaccatgagcttgatagatcaccctaatgtcatcaggtcttactgctcatttgttgtggatcataa
cctctgggtgataatgccattcatgtcagagggttcatgtctgcacttaatgaagattgcatatcctgatggttttgatgaatcaatcatcgcttcaattta
aaggaaacacttcaggctctagagtacctccataggcaagggcatattcacagggatgtcaaggcaggtaatattcttattgatagtccgggtgtagtca
agcttggagactttggggtctctgcttgcatgtttgatagaggtgataggcaaagggctaggaatacgtttgtgggaacaccatgctggatggccccaga
agttctccagcctggaaccggatataacttcaaagcggatatatggtcatttggaattactgcactggagctagctcatgggcatgctcctttttcaaagt
atcctcctatgaaggttcttcttatgacccttcaaaatgctccaccaggtcttgactatgaccgtgataaaagattctcgaagattcatgatgacgatccag
ctgaattaaaagaggatgatgacattgctaaaataaatgacattgataaggatgtatctcgtcgaagtcatttcggaatgctgccatcatcaagtggaaa
taattgcagtgaacaaacttctgctgcagtaaatccatgtcaaagcggtcctgaaataagtgagacattggcttccaaccttgttcttgctgatagtgaac
agaaagctgacgaacttagagacaaaggaccagaatttgatgcaattccctcaacatcaaatcaggatcctgatggaaaaacctatgggagtgatgtt
agacaaaaacaaaggacttactctggaccaattctgcaatctggttatgtaggctctttgggagataagcccaagcgtaatatggtggagataaaaggc
cgcttttcagtgacgtctgagaatgtggatcttgcaaaggttcaagaaattccagggggtagtgtctcacgcaagttgaaagagcttcaagaagagcta
aatgcgctgtactcccaagaagaaattgtcgacatcagagaggatgaaaatggagaaatctga
>g46612.t1
atgcagcacgatcaacgcaagaagagctccgcggaggcagagtttttcacggagtatggggacgcaagtcgatacaggattcaggaagtcattggta
aaggaagctatggggttgtatgctctgcaatagatgtccacaccggagagaaagtagccatcaagaagatacatgacatctttgagcacatatctgatg
ctgcacggattctccgtgagatcaagcttctgagactcctaagacatcctgacattgttgagatcaaacatattatgctgcctccatcaagaagggacttc
aaagatatttatgttgtttttgaacttatggagtctgaccttcatcaggattatgttgcaacaagatggtatagagctccagagctctgtggatccttcttttc
aaagtatacaccggccattgatatttggagcattggatgcatctttgctgaggtgctaacaggaaagccgttgtttcctggtaaaaatgttgtccatcagtt
agatttgatgactgatcttctgggaaccccatccttggataccatttctcgggtctgcaatgagaaagcaaggagatatctgagcagcatgagaaagaa
agaaccaatctcattttcacagaagtttccaaatgcaggtcctttggccttggatcttttgcaaaggctgttagcatttgatccaaaggaccgtccaactgc

agaagaggcattggctcatccatacttcaaagggctagccaaggttgagagagaaccatcttgtcaaccaatcacaaagatggagtttgagtttgaacg
tagaagagtgacaaaggaagacataagggagctgatattccgtgagatactggaataccatccacatgctgttgatcaattcaggaagcaatttgctca
tctcgaagagaataatggaaatggccctgtgattccgatggaaagaaaacatgcttctcttcctagatctacaattgttcactctactccaatccctgtca
aggaacaaccccgtattggctcgtcaagagacaaggcttcacctgatgagtcctacagaaatcctcgtgagacagacagattttccggcaatgccccga
gaacctcacaagctccccatagagtgccagcagcgagaccgggaagggttgttggtccagtaatgccatatgaaaatgggagcaccaaagacccta
tgacagacgaaagtttgcattgaattcaggatatcctccccaacaacaaatccaacaaacgtatggttattatcagacatccggcaaggtagcttgctct
gagcagtcgcaggctgaaagcagggcctgcaagtga
>g46670.t1
atgcctgattccgtggcgctgctgcagaaggacgcgcgcggtggtggtgcggcggcgatggtagtggagggcggcgtgcgggagatggactcgccgc
ggttccgcgcgatcctccgcgcgacgagcgggcggcggaagcgcgcgccggaggtgaagagcttctcccacgacctgagcgaccgcggcggcggcg
gcggcgtgggccccatgatgcgcaagatcatggcgcagcgctgcggaggagaggagttcgcgggcgccgtgcgcgccgagttcacgcggctcaagg
ccgaagtagactccgagctgggcgtcttcgccggcgacctcgtcggcgtcctggcccgcgccgcctccgacgacgacgaggaatcccgcgtggcgttg
gaggacctgctggtggtggcgcagcggtgcgcggagatgagtccggaggagttctggtcccggtgcgagggcgtcgtgcaaggtctggacgaccggc
ggcaggagctgggcggcggcggcgcggcgaagcaggcgcacacccgcgtgctgttcatcctcacccggtgcacgcggctcctccagttccgcaagga
cgccgctgccggcgctggtggtgtggaggaggagggagaagggagggaggagggtgggcggcgcgtgctgcggatgcaccagctcagcgacctcg
ggctgttcccgttgtccggcggcgagctcgggcgtaagagcacgtctagcctcaccgagctcaaggacaggctcatccgccgccgcatgctcgagcat
aagcacctcaccctcgacttgttcccccgcctatctcctcctcatcaaacaacaataacaaccatggatcgtctgaccattcccccaagatcgcgtcgtg
gaagaagctcccctcccccgccgagaagaacaagaacaagaattccagcacaacaacaaccgatgccaaggtcgtcgcggcggcggaggaggagt
cgaagaagacggtgaccgtcttgcacaacaaggcgtcggtggaggagatcgtggagcgcgtgggcgcggcgagcatccacccggaccaccaccaca
ccctgccgagcgaggagcagcaacatgagcaggcgcagcaggtgatggtggtggacggcaagccgcggatgatctgccgcgtgtgcgacttcgagat
ccccatggcctgcgccgagggccacttcgtcgtctgcacgctcgccgaccgatgcgacggcggcaagggcctcaccgccgaccagcgcctcctccgcg
tcgccgacgtgctcgaccgcgtgctcgcctgcttcgccgactcttcctcctcctcctccagctccaacgccgccgaccacgacgcgctcgcacagctc
ctcgccgtcccgtccgccgagctcttctcagagggcgcgctggtgacgccgacgggcggcgggggagcctgccgcagtcgccgctgccgacgccgg
cgcggctgaacaacgcggcggcggagacgaagcagcagcaccgtttcgggttcgcggacctggagagcttccagcaggtggagagcctggcatccat
cgcgcgaggggtggaagccgtgcgcgcctccgcctacgcctcactcgaagacctcagcgccttcctcgaggacctcaacgccgtgatcgacacgcgcc
gcgtcgacgcgctcgtcgtcgagaccttcggccgccgcatcgccaagctcctccaggagaagttcctgctgctgtgcgcccacatcgacgacactgacg
ccgccctcaccatgggacccatcgacgaggacgggcccacagtgtcgtctgagagcacgagcaccacgtcacgagccatgactgccaccagcaatgg
caatggcggcgtcatcaaggaccgcacctccatcgaggacttcgagatcatcaagcccatcagcaggggcgcgttcggccgcgtcttcctcgcaagga
agcgcgtcacgggggacctgttcgccatcaaggtgctcaagaaggcagacatgatccgcaagaacgccgtcgagagcatcctcgccgagcgcgacat
cctcatctccgcgcggaatcccttcgtcgtgcgcttcttctactccttcacgtgcagggagaacctctacctcgtcatggagtacatcaacggcggggacc
tctactccctcctccggagcctcgggtgcctcgacgaggacatggcacgaacgtacatcgccgaacttgttcttgcgctggagtacctgcactcgatgaa
cgtgatccaccgcgacctcaagcccgacaacctgttgatctcccgcgaaggccacataaagctgacggacttcgggctctcaaaggtcggtctcatcaa
cagcacggacgacttctccggcccggacgtgagcagcgttctcgtcggcgaccaccacccgacggacgcggagcaccgcgctcagaagcgggagca
gcggcagaagcagacggccgtgggcacgcctgactacttggcgcccgagatactgctcggcatgccccatggtcctactgcagattggtggtccgttgg
catcattctctttgagctccttgtggggattcctcccttcaatgctgaacatccacaggctgcatttatccctgctacagatgatgagtatgacacgagcta
ctttgcgtgccgccacgcctggggcatcgcagacgagcaagtcaatacggcttccaatgagtatgatgacagaagcgagaccagcagcatgagctgtg
gcagcagcccacatagttgtgactatgaggaagatggtgatgagtgtggtagcatggaagaattcggagcgccatcgctatcggtgaagtattctttca
gcaacttttccttcaagaacatttctcagttagcgtcgatgaactacgatctgataacaaagcacaacgaggatcctcttcactcttcaaaatcttga
>g46871.t1
atgaacttctacaaacctgactttgacatcggtttcaacacaaccaaaatatggaggttcaaccgatgcaactatgctgtgttgatggagacaacaacat
tcaacttcagtagcacgtacatcaacacaaccaagttcaacgacacaagtgctgggcgggtgcctgtggtgatcgactgggccataaggaatggtggg
acatcttgtgagattgcgaaacggaatgagacgggaacttatgcatgcatcagtagcaatagtgtgtgcatggactccatgaatgggccagggctatct
gatgtgacagagggctcttgctctggcattggctgttgccagacagcgataccgaagggtatgaacttctacaaacctgactttgacatcggtttcaaca
caaccaaaatatggaggttcaaccggtgcaactatgctgtgttgatggagacaacaacattcaacttcagcagcacgtacatcaacacgaccaaattca
acgacacaagtgctgggcgggtgcctgtggtgatcgactgggccataaggaatggtgggacatcttgtgagattgcgaaacgaaatgagatgggaact
tatgcgtgcatcagtagcaatagtgagtgtgtggactccatgaatgggccagggtatttctgcaactgctccaaaggttacgatggcaacccatatcttcc
aaatggatgcaaagaaactgagcttgtacaagccacaaataactatgacaagagcagaatacttgggaaaggtggtcaaggaacagtctacaaagg
acttgtcaaagacaatatgcatgttgcaattaagcgatgtgcgttggttgatgaaagacaaaagaaggaatttggtcaagaaatgctcatactctctcaa
atcaatcacaagaacattgtcaaactcttgggtgcttgtctagaggtcgaagttccaatgttagtgtatgaattcatcccaaatggtacactatacgagctt
atccatggcaagaaacaagctttgcggatctctttcagcaccctcctccgaatcgctcatgaagcagctgaaggacttaacttcctacattcttacgcatc
accaccaattcaccatggtgacgtgaaaagtgccaatatccttcttgatgaaaactacacggccaaagtgtcagactttggagcttcaatattggcacca
tccgatgaagatcaatatgtcacagtggttcaaggaacatgtggttacctcgaccctgaatacatgcaaacattccaattaacaaataagagtgatgtct
atagctttggtgttatccttttggaggtcctcaccggtcaagtaccgctaaaattgaatgggcccgaggagcagagaagtctgtcatcaaatttcttgtctg
ctatgaaggagaataatcttgatgtattattgccaagccatataaaagagcatgagagcactgaactgatcaggggacttgcagagctggccaagcaa
tgcctggacatgtgtggcacgaatagacctgccatgaaggagatcgccgatgagctcatcagattgagaaagctttcgcagcatccatgggtacaaac
ggacacacaaccagagtctcgaggccttcttgctgggacaccaactactggttttgacgacatagagatttgtgcctctgggtatcctatgctagacagt
gataacctgcccatgaaccccatgagttcgtattatgggaggtaa
>g47025.t1

atgggctgcgtcgcgtccaagaacgccgtctcggtgacgccggcggccgactcgtccggggcgctgagggaaagggtccagccgcgggagccgcag
cccgtggcgctgccggtgccggcggcggccgtgtcggtgtcttccctgcggagctcgtcgtccgcggcggcgaggcgggtggagaaggtcgtcaagga
cgatgcggaggagcagcacgggaaggtcgtcgccgtgtccgccgcgtcgcggagcttccggctgcggagcctgcggaagagcctggagggggagca
ggtggcggcggggtggccgccgtggctcagcgccgtcgccggggaggccatccaggggtggatcccgctcaaggccgactccttcgagaagctggag
aaggtactcctttacttagtatttgaatacatggagcacgatcttgctggccctttgctcctccccagacataaaattcagtcaagcacagctcaagtgctac
atgaaccagttactgtcagggctggagcattgtcattcacgtcacgtagttcatcgtgatatcaagggtgctaatctccttgtgaataatgaaggagttct
caagatagctgatttcggtcttgctaattactttgatcccagcaaaaaccaccctctgaccagccgtgttgttacactatggtaccgaccacctgaactgct
actaggttcaactcactatgatgcagctgttgacttgtggagtgttgggtgtgtatttgctgagatgtttcgcgggaagcctatattgcagggaagaactg
agtttttcaagacaaaaccttacgcatgcgaaccatcgagcttgcctaagtatgcacctaacaaggagatggatgcaaagttccgagaagatgtgctca
ggagaaaagcaagagcccatgggccagatgcatcgaagaaatcttcaaggctcagcagagcaccaagagaacctactgcaattactaagcaaatga
acaaaccagagggtgatcatgatcaaggtgagaaggttgaatattcaggccctttgctgtctcagacacacaaggttgatgaactgttacagaagcatg
agcgccacatccggcaagttgttcgaaaatcatggttcaggagaggatggaagcaagacaagtga
>g47095.t1
atggagcggtacgaggtgatccgggacattgggtctgggaactttggtgtggccaagctcgtccgcgacgtcgggaccaaggagctctacgccgtcaa
gttcatcgagaggggggctgaagcaagtgtgccacagggatctgaagctagaaaacacccttttagatggaagtgttgcacctcggcttaagatttgtga
tttcggttactccaagtcttctgtgctgcactctcaaccaaaatcaactgttggcacacctgcgtacattgctccagaggtcctctctagaaaagaatatga
tgggaaggttgctgatgtttggtcatgcggagtaacactatatgtgatgcttgttggtgcatatccttttgaggaccctgatgatccaaggaacttccgcaa
gacaatcaccagaatactcagtgtacagtactcaattccagactatgttagaatttcaatggaatgcaggcatctgctgtcccggatatttgttggtaatc
ctgagcagcgaattaccattccagagatcaagaaccacccatggttcctgaagaacctgcccatcgagatgactgacgagtaccaacagcagatgaag
ttggcagacatgaacaccccggagcaaagcctagaggaggtcatggcgatcatccaggaggcgcggaaaccaggggatgacctgaggcttgccggg
caggtaccctgccccctaggaagcatggaccttgatgacactgatttggacgatattgacgacattgacatagagaatagcggcgatttcgtttgtgcaa
tgtga
>g47734.t1
atgctcccgccctcgcgccgcgacttccgcgacatctacgtcgtcttcgagctcatggagtccgacctccaccaggtcatcaaggccaacgacgaactct
cggccgagcaccagcagttcttcctctaccagctactccgcggcatgaagtacatccacgccgccaatgtcttccacagggacctcaagcccaggaaca
tccttgccaatggcgattgcaaactcaagatttgtgacttcggccttgctcgggtgtcgttcaatgataccccttctgcgatattctggacggtaacctga
>g47939.t1
atggagccgtcccgtgacgccgccggcgcgcggcggtggggggatggggaggacgaaggaggcgccgcggtgccggcggaggacggcggcgggg
aggtgagcctgcgggagtggctggaccggccgggccgagcggtggaggcggccgagtgcgtccacgtgttccgccaggtggccgaggcggtggccg
tcgcgcacgcgcagggggtggccgtcggcagcgcacgcccgtcctgcttcgtcgtctcgccgcccttcgcgcgcgtcgccttcatcgagtccgcctcgg
gctccgacgcctcgggcggctcctgctcgggctccgacgcctccgatgacgccgacccggacgcctcgccgccgcggcggggcgacggcggcgccgc
ctccggggaggaccgcgccgcgaaggggttcccgctcaggagcgtgctggcgatggagctcacctggtacaccagccccgaggagtcggacgacag
cggcgctacctttgcctcggacgtgtacaggcttggtgtgctcctgttcgagttgttttgcacctttgagacgatggaggagaagatgcgggcaatggca
aacctgcggcaccgtgtcttgccgccacagctgctgctcaagtggcccaaggaagcatccttctgccagttgctgatgcacccagtgccggaaaccagg
cctaaaatgagtgaggtgctacaaagtgaattccttaatcaatcacggaataaccttgaagagcgtgaagcggctcttaggttacgtgaggagatagaa
gaacaggaactactgctggattttcttcagcagttgcagaaaagaaagcaggatatagcagacaatttgcaggacactgttgcctttctctcttctgatat
caatgaggtactgtaccagcaatctgctcttgggcattgtggaaccttcttatccgatttggataaagaggtgtgctctgaaactgttgaagatcagagtg
attgtggatccaggaagcgtttcagaccagagatgcaaggtgttgatgcggaggaacagaatcgtagtctggaagaaggttccaggacggtgccgtcc
tctgtgttgcagaaaagaaagcaggatatagcagacaatttgcaggacactgttgcctttctctcttctgatatcaatgaggtactgtaccagcaatctgc
tcttgggcattgtggaaacttctcatccgatttggataaagaggtgtgctctgaaactgttgaagatcagagtgattgtggatccaggaagcgtttcagac
cagagatgcaaggtgttgatgcggaggaacagaatcgtagtctggaagaaggttccaggacggtgccgtcctctgtggtttgggatgttacaaggagt
caagtttttgttgagatgagggagcatgagaggcgtgtgtggtcggtggacttctcaactgtggacccaacaaaattggttagtggtagcgatgatggtt
ctgtgaagctgtgggatatgaatcaggcaattctacttttacacctgctagtcccagttcagcttccatgcctgacgtacgcgtctaatcagtcccagttca
gcttctatgcctga
>g48184.t1
atggaccacaaccggcggcagcacacgacagcgctggccggcacctttggctacatggatcccaaatgtgccataaccagccgtgcaagcactgaat
ccgacgtgtacagcttcggcgttgtcctcctcgagattgcttgtggccggcggccaatctgtccagaggaagacgagagcaaggtccagcttgtttattg
ggtgtggaagctctacgagagtgtcaggctccttgacgccgtggacccgcggctgacctcccaatttgacgtggaagagatgaaacgcgtgatggttgt
tgggctctggtgcgtgcaccaggacagtggcttccggccgtcgatcaggcaagccatgaacgtgctccagttcgaggcccctacaccaatcctgcctca
tgaaatgcccctgccactgtactacagatcggatggcacgtcatcagcgaccagttccggcgagggaggagaatcattgatgtctgggccatcggcaa
ggaaccacagtcacaatgatcatcacatatgttcgtaa
>g48234.t1
atgagcgccggcgaggaggcgctgcggagccagtacgtgatcggcgacgagatcgggcgcggccgcttcggcaccgtccgccgctgcttctccgccg
cgacgggggagcccttcgcggtcaagtcgacggcgaaggcgccgcttcgcgagtcggacccgctggacctggcgctggcggagcaggaacccaagg
tccacctcctggcgtccgcgtcgccgcccgcgagcgcgcacctcgtcgcgctccacgccgcctttcgaggacgccgacgccgtccacctcgtcctcgacc
tctgccccggcggggacctcttctccctcgtctccgcccgcgcgccgctcccggagcccgaggccgcggggctcatggcccagctcgccgacgcgctcg
ccgggtgccaccgccgcggggtcgcgcaccgcgacgtcaagcccgacaacgtcttcttcgacgccgccggggtgctcaagctgggcgacttcggatcc
gcggagtggttcggggacggccgcgccatggcgggactcgtcgggacgccatactacgtggcgcccgaggtggtggcggggagggagtacaacgag
aaggttgacgtgtggagcgccggcgtcgtgctctacatgatgctctccgggaccgtgccgttctacggcgcatccgcgggggagatcttcgaggccgtg

ctccgaggcaacctcaggttcccgccgcgcgccttcgccaacgtctcgccggaggccaaggacctcatgcgacgcatgctctgcaaggacgtctcgcg
caggctctccgccgaacaagtcctaagtaagaagcacgccatcccctcccctcggatttcttcctactctatctatttccgccaccaacaccacataccac
atgcagcagatctggatacttctaaaactgtttgctga
>g48403.t1
atggtgctccaggtggttggtggtcggagccggccgcctcggcgtactagcgccgcccacttgccgtacggccatgccaattccaagggcgttgcggcg
ttcaattgggagtcatggagcaagggaggatccgggtggtacgatttcctgcgcggccaggtggacgacatcgccggcgccggaatcacccacgtgtg
gctgccgccaccgtcgcactccgtggacgcgcagggctaccttccgggtcggctctacgacctgaacgcgtccaggtacggggacgaggcgcaactcc
gtgccctcgtggcggcgttccaccgccggggagtgaagtgcctcgccgacatcgtcctcaaccaccgcacggcggagcgcaaggacgggcgcggcgt
ctactccgtgttcgagggcggcacgcccgacgaccgcctggactgggggccccacatgatctgccgcaacgacagctactccgacggcacggggaac
gccgacacgggcctggactacgagccggcgccggacctggatcacctcaacacccgcgtccggagcgagctcgccgactggctcaactggctcaagg
ccgacgtgggattcgacggctggcggctcgacatggccaacggctactccgcggccgtcgccggcatgtacgtcaacgccaccgcgcccgacctcgcc
gccgccgagatatggacggacctggcgtacgacaaggctgacgggaagccgagcgctgaccaggacacgcaccggcagaacctcgcggcgtgggt
ggacgccgtcggtggccccgccgccgcgttcgactacacgaccaagggcatcctccaggccgcgctcaacttcagcgaggtgtggaggatgcaggac
gctcaggggaggccgccggggctggtgggcctgcggccggcgcagaccgtcacgttcgtcgacaaccacgacaccgggtccaagacgcagcacagc
tggccattccccccggagaaggtgctgcagggctacgcctacatcctctcgcaccccggcatcccgtgcatcttctatgaccatttctttgatccgaccat
gaaagacgacatagctacgatgataaaaatccggacgcggaacaagatcggcccacgagctcgctgcgcatcctgctcgctcaaaacgatgcctac
gtcgcggagatcgacgggaaggtggtgaccaaggttggagctaggtacgatgtcacgaggactgttcctccggagtttgcgttgaccgccagcgggaa
cgatttcgcaatatgggagaacaacaggtccagcggcaatgtgcaacccaacacagagccggcgtcggcgtcgttcacaggaagaaggtcgtcgcgg
aggtgggtggtcccagtcgccgccacggttgcgccgttcgcggtcttgctcgcgtgcagtgcggcagtggtgctcctgttgcggcggcgccaaaagcac
aactccgacgagcagtaccagtactccgacgggggggaaagcagcagcggagttcgagaaaggggtggaagtgggaccgaggcggtacagctaccg
cgcgctggcggcggcgacgggtaacttcgcggaggaaaaccggctcggtcgcggcggcttcggccccgtctaccgcgggcgcctcgaaggccaccaa
ggccgcgacgtcgccgtcaaggtgctcgcgccggagtcgtcgtcctcctccgcgcagggccgcaagcagttcgaggcggaggtgcggatcatcagcc
agctccggcaccgcaacctcgtccagctcctcggctggtgcgacgacgacaagggccgcctcctgctcgtctacgagctcgtcccgggtggcagcctcg
acacgcacctccacggcagccgccgcgtcctgacgtggccggagcgccaccgcatcgtgctgggcctcggcgccgcgctggcgtacctgcacgagga
gtgggaccagtgcgtggtgcacggcgacatcaagccgggcaacgtcctcctggacgcgtccctgggcgtcgccaagctcggcgacttcgggctggcca
ggctcctcgaccgcgccgcgggcccgcggaccacccgggtggtgggcggcaccatgggctacatggacccggacctgctcagcacgcgccggcccag
cacggcctccgacgtctacagcttcggcgtcgtgctgctggaggtggcctgcggcaggcgcgccacggacgaggcgctggctgagtgggtgtgggacc
tccgcgaccgcggcgccgtcctggaggctgccgacgcgaggctgggcggcgagttcgacgcgtgggagatggagcgcgtgctgctcgtggggctctg
gtgctcgcaccccgtgccggcggaccgaccctcctccatcgtgcacgccgtcaacgtgctgcagtcccgggatgccgcgccgccggacctccccacca
aagtgcatcgtgccgccgccgccggattcagtggctacatgcactgcttgtcgtccgtcggcagcgttgcggagcaaaataacaaatga
>g48450.t1
atgtcgtcgtcgccgcccgccgcgccggcgccgacctcgccgccaggcccgcccccgtccaacgccacggcacctccccccttcaccacccgcagcagc
gccgccccccttcctccccgtcgccaccggccgagacgccgcctccgcccccagcgacgccctccgcgccgccgccgctaccgagcgggtccggcggcg
tccccgggaccccaggctccacgccgccgccgcctccaggcacgccctcgcccccgtcgccggccgcgactacctccccgccgaccccgggctccacg
cccggaactccctcgtcggactcttcctcctcctccgggctcaagagcggcgtcgtcgcggggatcgccgtgggcggagtcgtcgcgctgctgctcgcct
gcctcctctgcgtctgcctcttcaagaagaagaggaagaagcgccaccacccgcacccgcccccgccgccgctgccgcacatgcactactacggccac
ccgccgccgcctccgcctccgcccccgccgcacaaaggggatcagtatggcgggacgtaccagaattggcagcataatgcccctcctccaccaccacc
taaccacgtggtcaatatgcagcatccttcccctccacctcctcctcctgttaatgcgcacagcagtggttccgcttcaaataattcaggcagtgacggcc
gtcctccactttcgcctgcccctgcctttggcttctcaagaagcaccacattcacttacgagcagttggcagcggcgaccgatgggttttctgatgctaatc
tgctcggacaaggtggctttgggtatgttcacaaaggagtgctgccgaatggaaaagaagttgctgttaagcaattgagagatggaagtggtcaggga
gagcgtgagttccaggcagaggttgagatcatcagcagagtgcatcacagacatcttgtgtctttggtcggctactgcatttctggggacaacaggctgc
tcgtctatgaatttgttcccaacaacacattggaattccacctacatggctatcctaagataattcatcgtgacattaaggctgcaaatattcttcttgattt
gagatttgaagctaaggtggcagattttggacttgcaaaattgacttccgataataacacgcatgtttcaaccagagtaatgggaacatttgggtacctt
gctcctgagtatgcttcttctggcaagctcaccgagaaatcagatgtcttttctttcggagtaatgcttctggagctaataactggtcgtcgtcctgccagg
cctttaatgacaaaagcatttgaggacggcaatcacgatgatttagtggatccccggttaggaaatgagtacaataacaatgagatggaaagaatgat
agcctgtgctgctgcatgtgttgttcgggccctggagggcgatgtttctttggatgatctgaacgaaggcgtccggcctgggcacagccgcttcatgggg
tcatacagcagctcagactacgactccgggcagtataacgaggacctgaagaaattcaagaagatggcactcggcagcagcggcatgcagagtagcc
agcagacgcccaccagcgaatacggccagaacccatccgtgtcgagcagtgatggccaccagacgcaggaaacagaggcggagagcgtgaagaaa
gacggagacaaccaatcttcgtaa
>g48899.t1
atgggcctctgctcctcctccaccgcgtccgcgcaccgtgccggtgccgcgccggggaagaaggagaacaagggcgccggaggcggcagccggggg
atcgtggcgtgtgggaagcggacaaacttcggctacgacaaggacttcgaggcccggtactcgctcggcaagctgctgggccacggacagtttggcta
cacctacgccgccgtcgaccgcaactccggtgagcgcgtcgccgtcaagcgcatcgacaagaacaagggattaggagtcatagttgcctctcatttctg
a
>g48953.t1
atggcggggttggaggacctcaagcggaggctgcagcccttgttcgtcgactctgacgccaatggcgagcccgccgacggccctctcgccgactgtga
ggccctgcgctacttgcacgaagtgaggcatgtagtgcacagagacataaaaccagaaaatttacttttaaatctcaagggtgatgcaaaaattacaga
ctttggtgtgactgctggtctgtttgactcaatgagcatgtgtgctaccttcgttggcactgtcacatatatgtctcctgagagaattatgaacagttgctac
acatattctgctgatatctggagccttgggctaacagtgctggaatgtgctactggaagattcccatatgatgtgaatgggtgcctcagcgatctcatgct

gcagatactggatgacccatcaccaaaaccaccaaaagatgttaattcactagagttctgctcgttcatcagtgcctgcttgcagaaagatgctgatgca
aggcctacatgtgcgcaggtgatgtga
>g49337.t1
atgagtacaaccaaggtgaagagacgtgtggggaagtatgagcttggccggaccataggcgagggaacatttgcaaaagtcaggttcgcacgggac
accgagactggcgacccggtggccatcaagattctggacaaggagaaagttctcaagcacaagatggttgagcaggtgatgggaagtaaaacaaag
atctacattgtgttagagtatgtaaccggcggcgagctctttgatacaatcgttcttgaggatcaaggttatgatggtgcgatggctgatctgtggtcatgt
ggagttatcctgtttgttctactagcagggtatttgccttttgaggactctaatcttatgacgttgtataagaaaatatcaaatgcagtgtttacatttcctcc
ttggacatcgtttcctgccaagaggttgttaacaagaatccttgatccaaatccaataacgttgaggctttcaaaggtcaaagcagggaggaagggaaa
cctcaatgtagccaccgagatattgcaagttgcaccctctcttcacatggtagaagtccgaaaagcaaaaggtgacactctggaatttcacaagttctac
aagaacctttccaagaccttaaaggacgttgtctggaaatccagtgacatgcaaatgcagcccacttcttag
>g49580.t1
atggccgcgagcttcttggcagcggcggtggcggtggtgctggtacttcagttgacggcgcccgccattgccggcccggaggaggaagcggccctgct
gctcgccttcaagcgcgcgtcggtgtcaaccgacccgcgcgacgagctcgcgggctggtcggcgaactcctcctccgccggcggcggcgcgccgtgct
cgtgggccggcgtctcgtgcgcgccgcccccccgacgggcgcgtggtctccctcaacctcagccgcatgtccctcgccggcgagctccgccttgacccgc
tcctcgcccttccagccctccggcaactcctccttggcggcaacgccttccacggcaacctctcccacgcctcctcgtgcgccgcgctcgaggagttgga
cctgtcctccaacgccttcaacggcacgctccccgcctcgttcttatcccccctgctccgcgctccaatccctcaacctgtcgcgcaacgccctcgtctccgg
cggcggcggtgggtccttcgccttcgccgcgctgccgtcgctgcggacgctcgacctctcccggaaccacctctccggcgagttgccggcgggattcgt
ggccgccgcgccgcccaatctgacccacctcagcatcgccgggaacaacttctccggcgacgtgtcggactacgacttcggcgcgtgcgccaacctga
cggtcctggactggtcctccaacgccctgagcggcacccggctgccacgtggcctcgccagctgccgccgcctcgagacgctggacctgtcggggaac
aggatcctcgccgggcccgtccccgcgttcctgacgggcttcccgtcgctgaagcggctcgcattggccggaaccgggctgtccggcccgataccgga
ggagctggggcagctgtgcgggcggatcgtggagctggacctgtcggtgaacagcctcgtgggcgccctgcccgcgaccttcgccaactgcacctccc
tccagctgctggacctcggcggcaaccagctctccggcgacttcgtctccaccgtcgtcagcaccatcccgtcgctccgcgagctccgcctcccgttcaa
caacatctccggcgagaacccgctgcccgtgctggcagccgggtgcccgttgttggaggtcatcgacctctcctccaacgagctccacggggagatcat
gccggacctctgctcgcggctgccgtcacttaagaagctttttctgcccaacaactacttcaacggcaccgtgccaacctctctcggcggctgcgccaac
ctcgagtccatcgacctcagcttcaacctgctggtcggccacatcccgaccgagatcctggccctcccgaggctggtcgacctcgtcatgtgggccaacg
gcctgaccggggagatcccagacacgctctgttccaacagcaccacgctggagacgctggtcatgagctacaacagtttcacgggagtcatcccgactt
cgattacaaaatgcgtcaacctcatctgggtctcgctctcgggcaaccgcctcacggggaccctgcccagggggctcggcggcctccaggagctggcc
atcctgcagctcaacaagaaccagctctccggccccgtgccggccgagctcggcacctgcaacaacctcatctggctcgacctcaacagcaacggcttc
accggcgcgatcccgcccgagctggccgaccaggccgggctcgtcccgggcgggatcgtctcgggaaagcagttcgccttcctccggaacgaggcgg
gcaacatctgcccgggagccggcgtgctcttcgagttcttcggcgtccggcccgagcgcctcgccgcgttcccggccgtgcacctctgcccgtccaccag
gatctacaccggcaccacggtgtacgagttcgacaacaacgggagcatgatcttcctcgacctctcctacaacggcctcaccggcgccatcccgcccag
cctcgggaacatgatgtacctgcaggtgctcaacgtgggccacaaccagctcaacggcaccataccgtaccagttctccgggctcaaggccatcggag
cgcttgacctgtccaacaaccatctcaccggcggcatcccccccgggcctcggcggcttgaatttcctcgccgatttcgacgtctccaacaacaacctgtcc
ggtcccatcccttcgtccgggcagctcaccacgttcccgcagtcccggtatgctaacaactctgctctctgcggcatccctttacctccctgtggtcatgat
accggccgaggaggaggccagccacctgtctcgcgtgatggaaagaggaaggttattgaagccagcatccttgtcgggatagtgctctccgtgatcat
gcttctcatgctgctggtcactctctgtaagctcaggaagaaccagaaggctgaagagaagagaacagggtacatcgagagcctccccacatccggca
catcaagctggaaactgtcaggtgtccgtgagccgctgagcatcaatgtggcaacatttgagaagccactcaggaagctcacctttgcgcatcttctcga
ggctaccaacggcttcagcgctgagaccctcataggctcaggggggtttggtgaggtgtacaaggccaagctcaaggacgggactgtcgttgccatca
agaagcttatccatttcacaggacagggtgaccgggagttcaccgcagagatggagactattggcaagatcaagcaccggaacctcgtgccattgctc
ggatactgcaagattggtgatgagaggctcctggtctacgagtacatgaagcacggcagtcttgatatcgtgctgcacgacaaggccaaggctagtgtc
aagcttgactgggcagcgaggaagaagatcgcagttgggtcagcaaggggcctcgccttcctccaccatagttgcatcccccacatcatccacaggga
catgaagtcaagcaatgtgcttctcgacaacaacctcgaggcccgcctcaccacgttcccgcagtcccggtatgctaacaactctgctctctgcggcatc
cctttacctccatgtgctcatgataccggccgaggaggaggccagccacctgtctcgcgtgatggaaggaggaaggtcgttattgaagcgaccatcctt
gtcgggatagcgctctccgtgatcatgcttctcatgctgctggtcactctctgtaagctctggaagaaccagaagtctgaagagaagagaacagggtac
atcgagagcctccccacatccggcacatcaagctggaagctttcaggtgttcgtgagccactgagcatcaatgtggcaacatttgagaagccgctcagg
aagctcacctttgcccatcttctcgaggctaccaacggcttcagcgctgagaccctcgtaggctcaggagggtttggtgaggtgtacaaggccaagctca
aggatgggactgtcgttgccatcaagaagcttatccatttcacaggacagggtgaccgggagttcaccgcagagatggagactattggcaagatcaaa
caccgaaacctcgtgccgttgctcggatactgcaagattggtgatgagaggctcctggtgtacgagtacatgaagcacggcagtcttgatatcgtgctgc
acgacaaggccaaggctagtgtcaagcttgactgggcagcgaggaagaagatcgcagttgggtcagcaaggggcctcgccttcctccaccatagctg
catcccccacatcatccaccgggacatgaagtcaagcaatgtgcttctcgacaacaacctcgaggcccgcgtttcggatttcgggatggccaggctcat
gaatgcacttgatacgcacctgagtgtcagtacacttgcaggcacgcctgggtatgtgccaccggagtactaccaaagcttcaggtgcaccacaaagg
gggacgtctacagctacggcgttgtgctcttggagcttctctcagggaagaagccgattgatccaaatcaattcggggacaataaccttgttggctgggt
caagcagatggtcaaggaaaacagaagcagtgacatctttgacccaactctgtcggaaactaagtcaggggaagctgagctctatcagtatctcaaga
tagcttgcgagtgcttggatgaccggcctaaccggaggccaaccatgatacaggtcatggcgatgtttaaagagctgcagcttgactccgacagcgacg
tcctcgatggtttctccatcaactcgtcgactatagatgagtcagcagagaaatctacgtaa
>g49693.t1
atgacagcgctgcacccccttctcctcctcctcctcctcgctgccgccgcgcccgcctccgcgtccacgctcgccgtctccggcggcgccggcaacccgc
ccaccgtctgcggcgtcgcggaattcaacggcaccctcctctgcgcgccgctcctcgctggaggccgcaccaccaccaactcctcctcctcctactccgt
cgtgccgttcctctccttctccgaggtctccgccggccgggacttcatctgcggcctcaccgccctcggcaacgcgctcttctgctggccacccggcgcgc

cgtcgacccagctccgccgcgtgtacaacggccccaacgcgctcaccgacctcgccgtcggagccgaccacgtcgcggcgtacgaccgcgcggcgcg
ccggatcctatggtggcgcggcgtggggcggttcccgcagttcgcctccggcgcgttccgctccctcgtgtccggcgacgggttctcctgcgccgtgcag
accaacgactcctccatccgctgctggggcccgcgcggtagcgccgtgcaggccgccttctccccctcttccacaaccacctactcgtacctcgccgccg
gcgggacccgcgcctgcgccgtggaggacgcgaccggcgccgtccgctgctcgggcgggaccaccgtgccggtggcggtggtcccgggtgagctgtt
cccgtacgggctcgccgtgggcgactcccacgcgtgcgggctcctccgccccaaccacaccgtcgcgtgctggaccctcggcggcgccgggcccacca
ccaccaccccgacgctctactaccccgccctcgggacctcgttcgagctcctcgtcgccgacggcaactccacctgcggcgtcacgtccgccgacttctc
cgtgctctgctggtcgtcggcgagcatcgatgccgccgtcacgccgctgctccgcgcgggggtgctccccggcgcgtgcgtcgccgacgcgagtgaatg
cgactgcgggttcctcccgcagtcggcgcagctctgcacggggtccggcggcggcgtcgtctgcaaacgcctctgcgacgccgcctcgccgccgtcgg
ctccgtcctctcccccgccgccgccgcccgagacggcgagtagtagtaaggataacaacaggagggtgtcggggcggtgggtggcgttcgtggtggtg
ggcgcggtgggctgcttcgcgggcctctgctccatcgtctactgcctcctcttcggcttctgcagcaacaagcgggtccacaactccgtccagcccaacat
caccaccactgctaccccggccgccgctgacactaacaacaacaacaacaacaacgctgggcctggaggaggcccaagcccatacgggtccccgaa
cgggtcgcgggctcgcaacatcttccgtcggcagctatcccgcgtgatgacgcggcagcgcagcggtccctcctccttcaaggacgccgccacccactc
gtcgtcggtcccggaggagttcagcttcgcgcagctggacgccgccacgaaagggttcgcggcggaggcgaagatcggcgagggcagcttcggcgcc
gtgtaccgcggcaagctccccgacgggcgcgaggtggccatcaagcgcggcgtctcctcgtcgtcgacgacggcggggagaaggttccacttccagg
agaaggagagcgcgttccggtcggagctggccttcctctcccggctccaccacaagcacctcgtcgggctcgtcggatactgcgacgaggacgacgag
cggctcctcgtgtacgagtacatgaagaacggcgcgctcttcgaccacctccacccaaaggcaatgtcagagtcgtcgtcgtcgccggtggtggcgtcg
tggaagctgcgggtgaagattctgctggacgcgtcgcgcgggatcgagtacctccactcgtacgccgtgccgcccatcatccaccgcgacatcaagtcc
tccaacatcctgctcgacgccgcctggaccgcccgcgtctccgacttcggcctctccctcatgggccctccgccggagacggaccaccactcggcggtg
aaggccgccggcacggtggggtacatggacccggagtactacggcctgcaccacctcaccaccaagagcgacgtctacggcttgggcgtcgtcatgct
ggagacgctcacgggaaagcgcgccatcttcaaggacgaccaggctggtgggagtgggacccccgtcagcgtggtggaccacgccgtgccggccgtc
gtggcgggcgagctgggccgggtgctggacccgcgcgcgccggagcccgcacccaacgaggccgaggccgtcgagctcgtcgcgtataccgccgtg
cactgcgtcaggttggaagggaaggaccgacccgccatggccgacgtcgtcgccaacctcgagacggccttcgcgctctgcgaggggagcgccggcg
gtggcggcgggttcgggaacagctcctccagcgccagcatctccgtcacgtccatggaccgctcgggagcgctctag
>g49852.t1
atggattcgacgccgcggaagcgcaagggcgcgccgccggcgtgctccgccgcgcgctccctccaagatttggcgtcccgcaagcgcttctgccgcga
atcggaccccattccccgcgccccgacggcggcccccgcggggcaccacccgccgcggtgatgacggcgccggccgcgagcggcgcgtcggcgtc
ggcggtccccggccgcgggcacaagcggaaggtgggctgcatcgactccgccacgcggctcggccggcggaagcgcctcgacagcgagtacgagct
cggcgacgagatcgggcacggcaagttcgggcgcgtgcgcgcctgccgcgcccgggtccccggcggcgaggacgaggagctcgcgtgcaaggcgct
gcccaagggcggcggcgacacggcccaccgcgagatggaggtcatgcagcacctctcgggccacccgggcgtcgtcacgctcaaggccgtcttcgag
gacgccgacagcttctacctggtcatggagctctgctccggagggaggctcctcgacgaggtcgccagggacggcaggctctccgagcgccgcgccgc
caatgtcgtcagggagctcatggccgtgctcaagtactgccacgagatgggcgtcgtgcatagggacatcaaaccggagaatgttctgctcaccaagg
acgggaggctgaagcttgcggattttggattggccgtgcggatttctgatggtcagaaacttaatggtgtcgctgggagtcctgcatacgtggctcctga
gattctacttggtagctactcacagaaggtagacatatgggctgctggtgtgctcctgcatgtgctactcatgggcactcttccatttcaaggcaactctgc
tgaagctgtctttgatgccataaagacagttgagcttgattttcacagtgttcagtgggcatcagtatcgcttcttgctcgtgatctcatgagccaaatgctt
aatcgagattcttcttcacgatttagtgctgatgaagttctcctgtcgtacttgggtgcttctgtcaagcacaccatcgtcacccgcaacctctgtggggact
ggggacacggcgcgcaaggcgcaacaaggctcatgcttgacgactgccccgagtgcatgaaccgatga
>g50039.t1
atggggccacggccgctggcgctgctgctgcttctgtgctgcctgtgcttgctccccaccccggcgcactcccagaaccggacggcggcgccggcgtcc
gtggaaggattcaactgctcggcgaacagcacgtatccgtgccaggcgtacgcgctgtaccgcgctggcctgggcggcgtgccggccgacctggccgc
catgggcgacctcttcggcgtgagccgcttcaccatcgcgcacgccaacaacctctccaccacgtccacgcccgcgcaggggcagccgctgctggtgc
cgctgcagtgcgggtgcccctcgggctcgcccaacgccttcgcgccgacgcagtaccagatcgcctccggggacacgttctggatcgtgtccaccacca
agctgcagaacctcacgcagtaccaggccgtggagcgcgtcaacccagcgctcgtccccaccaagctcgaggtcggcgacatggtcacgttccccgtc
ttctgccagtgcccgccttcctccgccaacaacgccaccgcgctcgtcacatacgtcatgcagccgggcgacacctacgcctccatcgccgcagacttcg
ccgtcgacgcgagttcgctcgtctcgctcaacgggcccgagcaggggacgcagctcttctcggagatcctcgtgccgctgcgccgccaggtgccgcagt
ggctgcccccaatcgtgctccggaccaacgcctccggcgcgccggcgtcgccgccgccgtccgcggcccccggcacgaacgcgaccaccagcgatcg
gaacggggtggtcaccgggctggccgtcgggctgggcgtggtgggcgcgctgtggctgctgcagatgctgctactagtctgcctgtggaggcgcctca
gggcgaataagggccggaacggggaccaggtggcgggcggcgtggagggcggcaggttcggtaagagcgggtccggcggcggcggggagcggtt
cctggtgggcgacatatcagagtggctggacaagtacagggtgttcaaggtggaggagctggagcgcggcacgggcgggttcgacgacgcgcacctc
atccagggcagcgtctacaaggccgacatcgacggcgaggtgtacgccgtcaagaagatgaagtgggacgcctgcgaggagctcaagatcctgcag
aaggtgaaccacagcaacctggtgaagctggagggattctgcatcaacgcgtcgacgggcgactgctacctggtgtacgagtacgtggagaacgggt
cgcttgacgtgtggctgctggaccgcggccgcgcgcgccgcctcaactggcggtcgcggctccacatcgcgctggacctggcccacgggctgcagtac
atccacgagcacacgtggccccgcgtggtgcacaaggacatcaagagcagcaacgtgctgctggacgcccggctccgcgccaagatcgccaacttcg
ggctcgccaagacgggccacaacgccgtgaccacccacatcgtgggcacgcaggggtacatcgcgcccgagtacctcgccgacggcctcgtcaccac
caagatggacgtgttcgcctacggcgtcgtgctgctcgagctcgtgtcgggtcgcgaggccgtgctcgacgagtcaggcgagccgctctgggcggacg
cggaggagcgggtgttcaggggggagggaggacaggatggaggcgcgcgtcgccgcgtggatggaccccgtgctcgccgagcagacgtgcccgccg
gggagcgtggccagcgtcgtgggcgtcgcgcgggcgtgcctgcaccgagacccggccaagcgacccagcatggtcgacgtggcatacacgctgtcca
aggcagacgagcacttcgctgactactccggcgagagcctgcccgttgacagcagcggcgaggtgaaccacagcaacctggtgaagctggagggatt
ctgcatcaacgcgtcgacgggcgactgctacctggtgtacgagtacgtggagaacgggtcgcttgacgtgtggctgctggaccgcggccgcgcgcgcc
gcctcaactggcggtcgcggctccacatcgcgctggacctggcccacgggctgcagtacatccacgagcacacgtggccccgcgtggtgcacaaggac

atcaagagcagcaacgtgctgctggacgcccggctccgcgccaagatcgccaacttcgggctcgccaagacgggccacaacgccgtgaccacccaca
tcgtgggcacgcaggggtacatcgcgcccgagtacctcgccgacggcctcgtcaccaccaagatggacgtgttcgcctacggcgtcgtgctgctcgagc
tcgtgtcgggtcgcgaggccgtgctcgacgagtcaggcgagccgctctgggcggacgcggaggagcgggtgttcaggggggagggaggacaggatgg
aggcgcgcgtcgccgcgtggatggaccccgtgctcgccgagcagacgtgcccgccggggagcgtggccagcgtcgtgggcgtcgcgcgggcgtgcct
gcaccgagacccggccaagcgacccagcatggtcgacgtggcatacacgctgtccaaggcagacgagcacttcgctgactactccggcgagagcctg
cccgttgacagcagcggcgaggtcaccgcacggtga
>g50228.t1
atggcgccttcgccgccgccgcagatgagccttgccgacctcaaggccttgtcggtgctcggccaaggcgccagaggcgtggtcttccacgtcgtgccc
gccgacacagcatcatcctccgcccctgcaggcaaccccatggccatggcgctcaaggccatgtcccgcgctgccgcgcgcccacaaggtccgggcacc
aggactgccctgcggcgcagacatggacggccaccggaggatatggttcgagcgcgacgtgctgcgcgcgctccggcacccgctgctcccgtccctcc
gcggcgtggtggccaccgacgccgtggtcggcttcgccatcgaccggtgccccggcggcgacctcaagtccctcctccggcgccgcccgcggttcccg
gactcagtgatccggttctacgcggcggagctggtgctcgcgctggagcacctccacggcctcggcgtcgtgtaccgcgacctcaagcccgagaacgtc
ctcatccagggctccggccacatcatgctcgtcgacttcgacctctccaccacgttaccacctcctcctcccccacctccaccggacgccgccacctcctc
gcctacttcaccttcttcacgccacctaataatcaaccacagcaagaagaagaagaagaagaagaagaagaagaagaagatggcatcgatgatctcctcgt
gctgcttcttattaccctcctcccgccgcaccacggagtcatcgtaccggtcgtcgcgcggctcgtcggcgccacagcaccacaagtcgaagtcgttcgt
gggcacggaggactacgtggcgccggagatcgtggcgggcagcggccacgaccacgccgtcgactggtggggcctcggcgtcgtgctgtacgagatg
gtgtacggggccacgccgttccgggggcagagcacgagggagacgttccgccgcgtgctcgacgcggcgccggagctgcccggggagcgcacgccg
ctgagggacctcgtcgcgcgcctgctcgagaaagacccgcggaagcggctcggcgcgcccggcgtgaagcggcacgccttcttccgtggcgtggact
gggaccgcgtactcgacgtggcgcgcccgccgttcatcccggcgccggacgagtacaacggcggcgacagttcccgcggggaagaagccgaggcgc
tggacgtggagaagatcgtgcacgaggtgttcggctctggcggcgctgctgatgaggagacgccggcggtgaaggatggcaccgaggatgaaatag
atgacgatttctccatatttttctaa
>g50390.t1
atgttcaagaggtgcttctgctgcgtcgccggcgatttcgagtcggagccaggcgccgccgcagcacccgtgggtcgtcggagatcgactcagtcgagg
gcttccaagaacaggaacatggactatccgtgggagatgtacacgctcaaggagctgctgcaggcgaccaacaacttcaacgataacaacaagctcg
gggagggcgggttcggcaccgtgtactggggtcgcacgtccaagggcgtcgaggtgcgtgagttcgtcttgtcacatgtcgcatgttggccggccggtg
catccgagatgttgacgaaggtgcatacgtgtgataaccagatcgcggtgaagcggcttaaggcgatgacggccaaggcggagatggagttcgccat
cgaggtggagatcctcggccgggttcgccacaagaacctgctcagcctccgcggcttctacgccggcggcgaggagcgcctcatcgtctacgactacat
gcccaaccacagcctcctcacccacctccatcctcaccgtggcgcccccggatcccaacaacatgaacccctcgactggccacgccgcgtcgccatcgc
cgtcggcgccgccgagggcctcgcgtacttgcaccacgaggcgagcccgcacatcatccaccgcgacatcaaggcgagcaacgtgctcctggacgcg
gacttggtccccaaggtggccgacttcgggttcgccaagctcatcccggacggcgtctcccacctgacgacgcgcgtgaagggcacgctggggtacct
ggcgcccgagtacgccatgtggggaaaagtctccgagagctgcgacgtctacagcttcggcgtgctgctgctggagctcgtcagcgcgcgccgcccgc
tcgagaagctccccggcggcgtcaagcgcgagatcgtgcagtgggcggcgtccctcgtcgagcgccgcaagtgggagcgcctcgcggacccgcgcct
cgccggccggttcgacgcgcagcagctccgcgccgtcgtcgagacggccatgctctgcacgcagagcaccgccgagtgcaggccggccatgactgag
gtcgtcgagatgctcaagtacggcggcgtcgagaggcggaccaaggagatcgtccccgtgccggcggcgagcagcgaggagacgaccacggacctg
gacagcgtcaccggcagcagcgagccgctcaacaggcggagctggaagcttaccaatctgagatga
>g50524.t1
atgctcaaggttgcagctgagtgccacctgcatggtttggttcatcgggacatgaaaccagagaatttcctactcaaatcaacaaaagaggactcaccc
cttaaggctacagattttggtctttcagacttcataagaccagggaaacaattccgtgacattgttgggagtgcctactatgtagcaccagaagtactcaa
gccacatgattgggtcagagaaggaggtaaggcatctgaaataccattggatatatcagtattacacaatatgcgacaatttgtgaaatacagtcgcttc
aagcaatttgctttaagggcattggcatcaacactaaatgcagaagagctttctgatcttcgtgaccagtttgatgccatcgatgttgacaaaaacggca
caatcagtctggaggaactgaagcagattgacagtaacacagatgggttggtcgattttgaagagtttgttgcagcgacactacatgtgcatcagctgg
cggagcatgatactgagaagtggagatcgttgtctcaagctgcatttgataaatttgacgttgatggagatggttatattacatctgatgaattgagaatg
gtatcttaa
>g50595.t1
atggcaacccataccagcatcaggtgttctgctcggtttgcttctgcaactcttgtcatcctcatatgcagtacctgcagttgcttgcagttcaattacccca
cattcgacacagccagcaaagctgatttcagcttcagtccaggctcaggaatagcaaacgggtccctgcaaatcacaccaaccaccggagacatgagc
caccgatcgggaagagtcctctacgcgagagaaacactcaagctgtggaacagggagcggacagcgttgtcgtcgttcgagacagagttcatactca
acatcctcccccagaacaggacaggagaaggtatggctttcatcctcaccaacaacccgtcgctaccgaacaacagcagcgggcagtggctcggcata
gtcaacaaccagacggacggcgcggcggcgaaccggatcgtggccgtggagttcgacacgaggaagagcagcaacaagtacgaccccgacggcaa
ccacatcgggctcgacatcaacggcatcaaaactgtgtcgccgtaccctctcaacaacctttccataatcctttccagtgggtctgatgtacgggtcagga
tacagtacgatggtaccctgctgtacatagacgcatggcagaactggtggactttcagttatggctgggctgttgatctgtcgaggtatcttgtggaggac
atcactgtcggctttgcagcttcaaccggcgactttacccagttgaaccagatcaggtcttggaattttagcacaacgggcgacggtgacagtaaacag
aggagcacgggcaggctgaatttggttctcggcatcctcctaatccccttggtcatctctggatcgttcctggcgttctacgcgtggagaaggctgacgcg
gcagaggaggcttgcctatcacaacctcgagaagatgatcgacgcgcacggcccagtgcgattcaagctacgtgagctcaggaacgcaacatgcaac
ttcagttccgaccgcaagctcggcagaggcggcttcggcaccgtgtaccttggttacctgagcaggatggccggcatggaggtggccgtgaagcgggt
gtcgacgagcagtaactcatcaagaagaggggagcaggagttcgtcgcggaggtgaacacgatcagcaagctctcgcaccgcaacctcgtgaagctc
attggctggtgccacgagaggggcgagctgttgctggtctacgagtacttccccatgggcagcctggacaaactcctttacaatgatggcagagaaaag
gcggatctgacctgggaccggcggtacaggatcatctgcggcgtggcgtcggcgctggactatctgcaccacggcagcagcaagcgtgtcttgcaccg
cgacgtgaaggccagcaatgtgatgctggacgccgagtacaacgcgaagctgggcgattttggtctcgcccgcgtggtccagcgcgacggcgccacg

caccactcgacgcaggcggtggccggcacacgggcatacatggcgtacgaggccttcttcaccggccgggccagcctcgacacggacgtgtacgcgtt
cggggtgttcgtgctcgaggtgatcagcgggaggaggcccagcagctctgtctcgtaccgcaacgacgggaacggtgatgacttggacgtgtcaacaa
cgagttgcaggcgagggcacgggcaggccatgtacatcgtggactgggcgtggaggctgtacggggaagggagagcattgcacgccgcggacgccg
cgctgaacggggagttcgatccggcgcaggtggagtgcgcggtgaggctggccctggcgtgctgccacccgaacccgagggagaggccgtcgatga
aggcggtggtgcaggtgctggtcggtggagccaaggcgccggatcccccccttccagaagccggcgttcgtgtggccacctgagggcgagcgggaaga
catggagctgccgcatgtcgggctgctgttcacgggagggcactccagcttctgttccatgacctccaactccatcactggcagagcagtcaattcccaa
gttccggtcccgctgcttctcggcgtccaggcacatgacccggcggacacgctcccgcgcgcgaggcggcaacaacgacacatgtcccccaacaatgt
tcacccgcccggcgccggagcactcgtggccgccacaaggagccaccagatggagcactactga
>g50906.t1
atgcgcctccactgcctcgtcgccgtcctcctcctcctcgccgccggcgccgccgcggcgggcgacgcggacgcggacgcgctgctggcggtgaaggc
ggcgctggtggaccctgccggcgcgctcgcctcatggaagaagaacgccacccccctgcacatggctgggcatcacctgcaacgcccgcggcgccgtc
atcgggctggacctcgcgggccgcaacctctccggcccggtcccaggcgcggccctctcgcgcctggcccacctggcccgcctcgacctcgccgcgaa
caccctctacggccccatcccgccgccgctcgccgcgctccgcttcctcacccacctcaacctctccaacaacctcctcaactccaccttcccttcctccct
cttccccggcggcctccgcgtgctcgacctctacaacaacaacctcaccggtgcgctccccgcgggcctccccgcggccgccccccttgctgcggcacctc
cacctcggcggcaacttcttctccggctcgatcccgcgcgagttcgggcgctggtcccggctgcagtacctcgccgtctcggggaacgagctctcgggtc
ccgtcccgccggagctggggaacctcaccagcctcaacgagctctacatcggctacttcaacagctactccggcgagctccccgcctcgctcggcaacc
tcacgcgcctcgtccgcctcgacgccgccaactgcggcctctccggcgcgatcccgccggacctcgggaagctcgccaacctcgacacgctcttcctcc
aagtgaacaccctcgccggcgccatcccgccggagctgggccgcctcgccagcctcagctcgctcgacctctccaacaacgcgctctccggcgagatc
ccggcgaccttcgccgagttgaaaaacctcaccctcctcaacctcttccgcaacaagctcaccggcagcgtccccgacctcgtcggcgacctcccgcaa
ctcgaggtgctccagctctgggagaacaacttcacgggcggcgtcccccgccgcctcggccgcaacggccggctccggctcgtcgacctctcctccaac
cgcctcaccgggactctcccccccgacctctgcgccggcggcaagctcgagacgctcatcgccctcggcaacttcctcttcggctccatcccggcgtccc
tggggaaatgcgggtccctcacccgcgtccgcttaggggagaactacctcaacggctccatcccagaggggctcttcgagcttcccaacctcacgcagg
tcgagctccaggggaacctcctctccggcggcttcccgtcgcccaaatccgcggcggcggcggcgccgaacctcggcgccatcagcctctccaacaac
cagctcaccggcgcgctgccgcccaccatcggcaacttctccgggctccaaaagctcctcctcgaccacaacaagttcaccggcgcggtgccgccgga
gctcggccggctgcagcagctgtccaaggccgacctgagcggcaacgcgttctccggcgaggtcccgccggagatcgggaaatgcaggctgctcacc
tacctcgacctcagccagaacaacctctccggcaagattcccccggccgtctccgggatgcggatcctcaactacctcaacctgtcacggaaccgcctc
gccggggagataccgccgaccatcgccggcatgcagagcctcacggcggtggacttctcgtacaacaacctgtccgggctggtgccggcgacggggc
agttcagctacttcaatgccacatctttcctcggtaaccccgggctgtgcgggccctacctcgggccgtgccacgtcagcggcggcggcgctggaacgg
agcggtcggggcgctcatcgcacggtggcggcggcatgtccaacacgttcaagctgctcatcgtgctgggcttgctcctctgctcgatcgcgttcgcggc
catggcgatcctcaaggcgcgggcgctgaagaaggcaagcgaggcgcgcgcgtggaggatgaccgcgttccagcggctcgagttcacctgcgacga
cgtgctcgacagcctcaaggaggagaacgtgatcggcaagggcggcgccggggtcgtgtacaagggcgccatgcccgacggcgaccacgtcgccgt
caagcgcctctcggcgatgagcggccgcgccggcggcgggtcggcgcacgaccacggattctccgcggagatccagacgcttggcaggatccggcac
cgctacatcgtgcggttgctgggattctgctccaacaacgagaccaacctgcttgtgtatgagtacatgcccaatgggagcctaggggagctgctgcac
ggcaagaagggtgggcacttgcactgggacactcggtacaagatcgccgtcgaggccgccaaggggctcagctacctccaccatgactgctcgccgc
cgatcctacaccgcgatgttaaatccaacaacattctgcttgactctgagtttgaagctcatgttgccgatttcgggctcgccaagttcttgcaggactccg
gcacgtcgcagtgtatgtctgccattgccggctcctatgggtacattgctccagagtatgcttatacgctcaaagtcgacgaaaagagcgatgtctacag
ctttggggtcgtgctgcttgagctggtcacgggaaagaagccagtaggggagttcggggacggcgtggacattgtccaatgggtcaagacgatgacgg
actcgaacaaggagcaggtgatcaagatcatggaccctagactctcgacggtgcccgtgcacgaggtcatgcacgtcttctatgtcgcgttgctctgtgt
cgaggagcagagcgtgcagcggcctacgatgcgggaggtggtgcagatgctcagtgagctccccaagccaactgatgagaggcagggagacgagct
ccctggtgatggagatggttctgcatctgaggagcctccagtgtcgtctgaatctgttgaagcacccacaaacgaagccaaagagcagcagcaaacga
gctcgccgtcttcaccagctcctgatctcatcagcatctga
>g50929.t1
atgcacccgaagctgtcgcgggccgcgcgcaggctgctctgctgcgccggggccgccgccggggaagacttgtctgacgacgggagtggatcgttga
ggtgggtgttctctctgagggagcttcgatcagcgacaaacagcttcaattacgataacaagattggagaagggcctcttggaagtgtgtattggggac
aagtctgggatggttctcagattgctgttaagaggttgaagaatgcaaaatatggcaccgaagcggaatttgcttccgaagtcgagatattaggaagaa
taagacacaagaacctcctcagtttgcgtgggtaccttcaccaccatgcaacacctcaaacaattcatgggagcatcaaggctaccaatgttctactgga
ttcagactaccaggcacacgttggtgactttggtctgataaggcttattcctgatgaaatggaccatgaaaagatagccagtgaaaaccaacgaggcta
tattgctccagagtacattatgtttggcaaaccaacagcaggacgcaacttcagcctcgaggcgctgcctaccgacctgccgctctttggtgatattgacc
cataa
>g51012.t1
atggcggcggcggcggcagaagctgcaatggctcaaccggcgttccccgtcgcgccccacgagttcttccgccggcctcctcctcctcatcatcacg
agacgaggcccaagccggcggcggcgtcaaacgaggcggagcagacgatgcgtctgtcggacctggaagagatctgccacctcggcgcgggcgcca
gcggcgtggtcaccaaggtccaccaccgccctaccggcgcggtgttcgcgctcaagacgaccttcttccccgaccgcggcgtggacgacgacgagcag
acggaggccgaggcgctccgccggtcggccgggtccccgcacgtggtccggtgccacgccgtgctcgccgatcccgacgagaacgtgctcgcgtacgt
gctcgagctaatggacgccggcacgctcggcgccatcctcaagaaacgcggaaaccgcggcctcccggagcccgccctcgccgaggcggccgcgcg
gtgcctggacgggctggcccacctccacgcccgcggcgtcgcgcacctcgacgtgaaccccgacaacctcctcgtgaacgcaaaaggggacgtcaag
gtcggcgacctcagcgagtccaggaccttcctcgaccgcgcgggggcggcgcgccgcgtgcacgtcgccgtcggcaagacggcgtacatgagccccg
agcggttcgcgcccgtcggtgcccgcgaggggccgcgcggcgccgcggccgccgacgtgtggggcctcggcgtcactgtcctggagctgttcacggg
gcgccgcgccttcctccccgcggcggagcgcccgcagtacgcggacctgaggcgggccatctgcgacggggaggtgcccgccgtgccggagggctcg

tcgcccgagctgcgcgggttcgtggcagcgtgcctgcaaaaggaccccaggcggcgcgccaccgtgacgcagctgcaagcgcacccgttcgtcgcgc
gccgcgacgtcgaggagtcgcgacgcgcgatgcgggagatcgtcgccgagaccaagaaggagtag
>g51044.t1
atggatcagtacgaggtgctggagcagatcggtaaaggagcatttggctcggctctgctggtcaggcacaagctggagaagaagaagtatgtgctgaa
aaagatcaggcttgcacgtcagactgaccgcactcgccgatctgcccaccaggagatgcagctcattgccgcagtgaggaacccattcattgtagaata
caaggattcatgggtggataagggttgctacgtctgcattgttatagggtactgtgagggaggagacatggctgaagccatcagaagagttaatggtac
ccacttctctgaggagaaactttgccagtggctagtggagcttctcatggcccttgattatttgcatgcgaatcacattcttcaccgtgatgtgaagtgctc
caatatatttctcacaagaggtcaagatataagacttggtgactttgggcttgcaaaagtattgacccctgatgatctagcatcatcggtcgtaggaacac
caagccatatgtgcccggaacttcttgctgatataccttatggtaccaaatcagatatttggtcactaggatgctgcatgtatgaaatggctgcgctgaaa
cctgcatttaaagcttttttttcaggaacaccagtcaacagaatcgctcgacaagcacgcagagcgtcgctcccactgccagcattcgagacacctaagc
gcagcatcagcatcctagagcagtttacatcccctgacatctccgtgaattcacctcgaatcgacagaattgctgagtttccactcgcttcatcagatgaa
cccttattctcaatcgccaaggcatcgtcagctcacggctcatgctcagccaccccaccatccatcaaccgatcactcaccaaggacaggtgcacggtg
caaaccctccgtgccgccgacgatgacagccggagcgacgagtcctcgggccgcaacgccactgccgcagtctcgagccgcggctctacggactcca
cgcagcggcggttcgatacgacgtcgtaccagcagagggctgaagcgctggaaggcctgctcgagttcagcgcgcagctgctgcaacaggagaggta
cgaggaattggggatcctcctgaagccgtttggcccagagaaggtgtcgcccagggagacggccatctggctcactaagagcttcaaggagacaggg
aatccagtattaacatcaggaggtagcattagaacggaagtgtctgagacagcccggccgttcctcccgacgggccgtcccaagtttcagcgctgggtg
gaaagcagtccgtcatcagatggacctacggctcccctatctgggctggggtcgtacaaggaggcgctcctcgcctcgtcctctcctccagcggcggctc
cgaagccgccggtccccgcacctgccccgcgggtagtaatcaagtcggaggtggtgggtaacctgcggccccagcccgcggtcgacgtcgaaggatg
gcagacggtcacgaggtgtagccgcccacgccggggtcctggtcctcgccgtcgtgaggtgccggcggacctgcggggacgctgcttcaactgcttctc
caccacccaccgcgccgccagctgtagtagccgcactcactgctttcggtgcggcctgccagaccaccgctcgtacttctgtccctccaagcccgccgag
tccggccaccctgctcgtggtggtatgttgatttggtggcctgtgcagaaggggggagaagacggcgccaccgatggaagccgacggccctcaacaaca
ccagcaggaaacgcagaatgggccgccacgtcgtcggcctagacgcaggcgacgtcgccccacgcggcgggatgtcggccccgctccttcggtttctg
agcatgaggctacggcgtcggaattcagtggtgcaatggttccgcccagcccggccggcattcgagctaggccgctgtgtgttattgatcgtacagata
gcatcgctcgtgccgaggccgacctccgtagggcccttctgatctctgaggtgagcaaccgaccggcggtgtcgaagcagcaggtcattgaagagatc
gtggcagaattcgacgtcaatgaggcggagatgttcatctgtcctgctgcgcctgaagattttctgttgattctaccgaacatgagacggcggacagagt
ccataatcatggccagccccttcatgccccggggttcagcctcctcaccaagcggtggacgcggctag
>g51365.t1
atgctctcacgaatactgaaaagagaaggaaacatcgccttcttcgagagaggagacattatgaaggccacacgtagctttcacaaggccaacattgt
cggggaaggagcagacgggaccgtctacagggggattatcagcaccggtggcggcaccaccaccactgtcgcggtgaaacgatgcaagcggattga
taaaagcaggaaggaggagttcgtacaggagcttgtcatactctgccgcgtcaatcatccgaacatcgtcaagcttctcggctgctgccttcatttcgaa
gcgcccatgctcgtgtacgagttcatgcataacggaaccttgaacgatctgcttcacgggagctcaaggcggtgcgtgacgctggcgacccggctgcgg
attgcagcggaggtcgctgaggcactcgcccacttgcactcgccgccgcacaccacgcttcacggtgacgtgaagccggaaaacatcctcatgggcga
gggctgggtggccaaggtgtccgactttggttgttcaacaatcgatgacaacgttcaggtcgtgcccaagggaacgctggcttaccttgacccagagttt
ttgcaggacttccagatcacagacaagaccgacgtctacagcctaggggtggtattaatggagctattgaccagaaagaagccacctgcaaaacaaca
gatgaacctgaggataatatttcaggagtccatggcnccgcacaccacgcttcacggtgacgtgaagccggaaaacatcctcatgggcgagggctgg
gtgtccaaggtgtccgactttggttgttcaaccatcgatgacaacgttcaggtcgtgcccaagggaacgctggcttaccttgacccagagtttttgcagga
cttccagatcacagacaagactgacgtctacagcctaggggtggtattaatggagctattgaccagaaagaagccacctgcaaaacaacagatgaacc
tgaggataatatttcaggagtccatggcgaatgggacgctcagtgagctcttggacactgacataatcgtcgaggaaggcggcatgagggttattcatc
aagctgccgaactagccaaccgatgcactgcttttccagcgcagcagctcgcacgctacaatatcgagcagcatgccataccactgtaccagcagagg
aagttcctagaggatgcggcggccagtcacgacgagcctttgatgcattggctgcgggaggcatcacaccagaagcacgctgctgacgtgacaccggc
ttccactgctgcggaacctgccaccggccgtcgtaccagctgctgggcattgcggagggagcgggacatgctgctctcctcgatgagcttccagtatga
gatcatgagccaggaatttggattgagggggccaattag
>g51660.t1
atgggctgcctgccttgctttgggtcgggcggcgagggggcggccaagaagggtggcgcgcgcaaggacgcctcatcggatcgccgcgtgtcccggg
acggatcagataaatcaaaaccacagggtggatcagaatctaaaaaggatgcagtcatcctgagggatgggaacaatcaacatattgcagcacacac
tttcacttttcgtgagcttgctgctgccactaagaacttcagacaagattgcttattgggtgaaggaggctttggccgcgtttataaaggacgtttggagaa
tggacaggctgttgcagtgaagcaacttgatcgcaatgggcttcaaggaaatcgggagtttctggtcgaggttttaatgcttagtctattgcatcatgaca
atctggtcaaccttattggatactgtgctgatggggaccaacgtcttcttgtatatgagtttatgccattgggatcacttgaggatcatttgcacgatatccc
tcctgataaggaacctcttgactggaatacaaggatgaagatagctgctggtgctgccaagggcttagaatacttgcatgacaaggcaagtcctcctgtt
atttaccgggattttaagtcatcaaacattctacttggtgaagggtatcatccaaaactatcggatttcggccttgcaaaacttggccctgttggtgacaag
actcatgtttcaacacgtgttatgggaacatatggctactgtgccccagagtatgcaatgactgggcaacttacggttaaatctgatgtatatagttttggt
gttgtattccttgagctgattactggccgcaaagcaattgacaacaataaaccgcatggcgagcaaaacctagttgcctgggtatgttctttctctactta
a
>g52003.t1
atgacatgtgggacgccggcatacacggtgccagaggtgctgcagcggcgcgtgtacgacagcgcgaaggtggatgcatggtcgctaggagtggtcc
tcttcgtgctcctcgcaggggaaggggtgcgaccgcggaggcagaggggaaggggtagcaaggctgcggcggagaggaaggcagtgatcggagag
gagcagagccagccaaacacctcagctccagaaattttcggagcagccaactctaaggaatttgaggagctccagttcgtttttcagagcacctcaaa
ctcagcttcgaggaccagagaaacatggagttggagaaaattacccgcactgtcatcggttacataaagaaaacgattcgtttcttcttttctcccctacc
gacgctctcctccgccttcttcctcccgccgttcccagccctgccccacttcgccgctgctnnnnnnnnnnncccccttcttcctttcattgccccaccccg

ccttgctccgccgctgcccccccccccctcgtgccgccctgctctaccgttgcctcccctgctcgctccgccttcgcctcgattcgccccatgccgtcaccc
gcctcgattcacaccacctccgcctcaattcgccccatgccgtcacccgcctcaattcatgtcgcctccgcctcgattcgccccacgccgtcacccgcact
gcctcgagctggccatgtcattgcttgtcgtcgcccgtgccaccctag
>g52148.t1
atggacaagtacgagctgctcaaggacatcggcgccggcaacttcggcgtcgcaaggctcatgcggcacaaggagaccaaggagctcgtcgccatga
agtacatcccgcggggacagaagattgacgagaatgtggcgagggagatcatcaaccatcgctcgctgcggcaccccaacatcatccggttcaagga
ggtgctggtcacgccgacgcacctggcgatcgtgatggagtacgccgcaggaggcgagctgttcgaccggatctgcaacgccgggaggttcagcgaa
gacgaggccaggtacttcttccagcagctaatttgcggtgtgagctactgccacttcatgcaaatttgccaccgagacctgaagctggagaacacactg
ctggacggcagcccggcgcctcgactcaagatctgcgacttcggttactccaagtcgtcgctgctgcactcgaagcccaagtcgacggtgggcacgccg
gcgtacatcgccccagaggtgctctcacgccgggaatacgacggcaagaggataatgtctatccagtacaaaataccggagtatgtccatgtatctcag
gactgcaaggatatgcttgcaaaaattttcgtcgcaaaccctgcaaagagaattacaatcagggaaatcaggaaccacccctggttcctaaagaacttg
cccagagaactcacagaagccgcacaggcaatgtactacaagaaggataacagtgccccaacctattccgtccagtcagtcgaggagatcatgaaga
tcgttgagcaggcgcggacgccacctccttcctccacccctgtggctggctttggttgggtagaggaggatgaagagaagaatagcaagaaaccagag
gagaaacaggaggaagaagaggatgctgaagatgaatatgagaaacaggtgaatgaagtccgcgccagtggtgaatttcagatcagctaa
>g52168.t1
atggaaaagggctcctcagtgggaggttcttcaggtggtgagaatgacagtggaataaacggttttgctattggaggaattgtgatagctgtgcttcttg
ccgctctgattcttttgtccgtattgaagaggaagaaatcatctcctgtctcatcccattactatacggatgagtcaggtcataacccactagttgatgaatt
caaaggtcataaggaatcttcagtggtcgacatgaagtcactggagcaaccttcagcagtaggcagtaggacaccatccacagttcctcgcaagtcaat
cagtgacaatgagttcgagaacaaactaaactattcgaggcggagtgcagagccaatcagcctcatgacttatgcatcatcagacctggaggcagcca
ctggcagcttccatagtagcaggctactaggtcaagggacaatcggtcacgtttacaaggcaaaatatgccgatggacaggtgctggcagttaagaaa
tttgatccattgagcttctcaggaagcagcgacttcatggatcttgtggacagcgtttcaaagttgcgccatgcaaatatggctgagcttgttggttactgc
tcggagcctgggcactacatgctggtttatgactaccacatgaatgggtccctttatgacttccttcacttatcggacgactacagcagaccgttgacttgg
gacacccgagttcgaatcgctcttggcacagctcgtgctttggagtacctacatgaagtctgctcgcctccggtgacccacaagaacataaaggcatcta
atgtgttgcttgatgctgatctcaatcctcacctctccgactgcggatttgcgtacttctatgaggatacaagtgagagcctaggaccagggtacaatcct
ccagagtgcacaagaccatcaggctatgtgatgaaaagtgatgtctactgcttcggtgttgtcatgcttcagctattgaccggccggaagccctacgata
gctcgaagcctaggatggagcagtccttggtcaactttgtgactccacaacttcatgacattgacgccttggctgcactggcagatcccgctctacgagg
cttgtacccaccaaaagcattgtcccgttttgctgacgtcctctcacgctgtgttcagtccgacccagaattccgaccatccatgtctgaggtggtgcagtc
gctgctccaatgcatccagcgagccatcagcaacaagaggatgggtggccaccgcagtgtctcacaacggagtgacgattcggattggtga
>g52213.t1
atggtcgataagacgacggccgtggtcatcggggtcagcgctggcgtcgtcgccgccgtgctcgcggcggcgtgcgtgttcctggccatctggctgtac
cggcggcgggcccgcgtcgccacccggacgcggtcgatggagtcgagcacggtgacgctgcgggatggccccagcagcctggcgtccagcgtgtcca
tctcggtggtctccgagagcgtggccgactgggcacccccgccggagaagcgcgccgcgttctgggcgtggaggggcggcggccacaacggcaggg
aatcgccgttgtccgtctccgggatcccgaaataccattacaaggatctgcagaaggctaccagcaactttaccacaattctggggcaaggatcgttcg
gtcctgtgtacaaggctgtaatggctactggtgaagtggtagctgtaaaggtgctcgcgagcgattcaagccaaggggaaagagagttccagactgag
gctgttccacctgttattcacagagacctaaagtctgataatatacttttggaccattcaatgaaagccaaggttgcagactttggtctgtcgaaggagga
agtatatgatggaaggaagtcaggcctcaagggcacctatggatatatggatcctgactacatgtccacaaataagttcacaaagaagagtgacgtgt
atagttttggcataatactttttgaactaataacaggcataaatccacaacaaggtttaatggagtacattaatctgcaattggagcgggtgatggaagg
gttgattggggtgagatactag
>g52260.t1
atggctgccatcaaagaggagtccgactacgacagcagccggtcctcgctgacggcaccgggatcccgccggagctggatcagcgacattggcagct
cctccagctcgatctcggtgcgctccttgggcggcggctgggacacgtcgtgccgccacaagccccacaaggcgaaccaggccgagtgggaggccat
caggcgcgtccgcgccgccaccggccgcgtcgggctggagcacttccgcctggtgcggcgcctcggcagcggcgacctcggcaacgtgtacctctgcc
agctccgggagccctggtccacggggtgcctgtacgccatgaaggtggtggacaaggacgcgctggcgttccgcaagaagctgcggcgcgcggaggt
ggagcgtcagatcctgcgcacgctcgaccaccctttccttcccacattgtacgccgacttcgacgcgtcgcactacgcctgcctcgtcatggagttctgcc
ccggcggggacctgcacgtggcccggcagcggcagccgggcaggcggttcagcatcgcctcggcgaggttctatgcggcggagacggtgctggcgct
ggagtacctgcacatgatgggggtggtgtaccgtgacctcaagccggagaacgtcctggtgcgcggcgacggccacatcatgctctccgacttcgacct
ctccctcaagtgcgacgtcgtcccgcggctgctccgccacaacagcctccctcgccacaccgccgccgatccagcccccaagccgtcatcctgcgtcgc
gccgatccagcccgtgctgacgtgcttcttcaacggcgtgcacaagtgccaaaagcccgccggcggtaataacggcagcaaggcggagagcccgtcg
tcgtcgttggatcccgaggacgacgacgacgacagtagtagtaataacaacaacaacaacaacaacccggcggagctggtggtggagccggtgtcgg
cccggtcgaggtcgttcgtgggcacgcacgagtaccttgcgccggaggtgatctccgggcagggccacggcggcgcggtggactggtggaccctggg
cgtgttcatgtacgagatgatctacgggcggacgccgttcaagggcgagacgaacgcgcagacgctggccaacatcgtgaagcagccgctgagcttcc
cgcgcgcggcggtggcgagcgggcgggagtgggacgagctgctgcgcgcgcaggacctcatgacccggctgctggccaagaaccccaggaggagg
ctcggcgggtgcacggggtccgccgaggtgaagcgccacggcttcttcaagggcgtcaactgggcgctcgtcaggtccgtgcgcccgccgcacgtgcc
caagaaggtgaccccgccgccgctgccgcagaagaagaagagggtggtggtgatgagcaggaaggagcggcaggagccgttcaagccccggaacg
acgacaaccgtttcgactacttttaa
>g52329.t1
atgacccagataccttctgcatgcaaagacatcaaggaagtaagggcggtggaccaattttcagccaacgattttattgtacatgatgggcttctacttg
ccattcagaatgagcctgagcctgtggattcagttgatagagaagtaattgagtcagctcaggaggacaagttaaggaaaaaagacgagaataatctt
tcaggctcatttcgcattacggatgacacagatggaattcaaactatttccctcagtgaacagtctactgctgacattgcaccattagcaggcttacctga

gttttcttatcttggttggggccattggttcactctcagagatctggagcttgctaccaaccgtttttcaaaggacagtgtaattggcgagggtggatatgg
ggttgtgtatcgaggcagattatcaaatggcaccccagttgctgtcaagaaaatccttaataatttaggacaggctgaaagagaattcagagtggaagt
tgaggcaattggccatgtgcgtcacaagaatctggtccgccttttgggttattgtgttgagggtactcaaaggatgcttgtgtatgagtatgttaataatgg
gaatcttgaaagttggctccatggagaactgtcccaccttgcttacttacatgaggcaatcgaaccaaaagtggtgcaccgagacatcaagtccagtaa
tatcttgattgatgatgagtttaatgccaaaatatcagactttggtttggccaagatgcttggtgctggtaaaagtcatattgctactcgagttatgggtacc
tttggatatgttgcacctgagtatgctaacagtggacttttgaatgaaaagagtgacgtgtatagctttggagttgtcttattagaagctattacagactttg
gtttggccaagatgcttggtgctggtaaaagtcatattgctactcgagttatgggcacctttggatatgttgcacctgagtatgctaatagtggacttttga
atgaaaagagtgatgtgtatagctttggggttgtcttattagaagctattacaggtcgagatccaattgattatgagcggcctccgaatgaggtgaatctt
gtcgattggcttaaaatgatggttgcaaaccgacggtctgaagaagtagtggatccaaaccttgagagaaggccttcaacaaaggagctcaagcgtgc
ccttttgacagctctgaggtgcattgatttgaatgctgagaagagaccaaggatggatcaggtggttcggatgttggattctaatgaacccataccacaa
gaggtatga
>g52449.t1
atgatggtacgacgacgagcgacaaaggtccaaggccttgagggcggcgtcatagccttcaagtaccgtgaactgcagtccttcgccaacaacttctct
gacaagctcggcggcggctccttcgggtcggtcttcaggggccagctccccggccgcaccaccaccatcgccgtcaagaagctggaggggctctggca
aggcgagaagcagttccgcgcggaggtgagcaccctgggcatgatccaccacgtcaacctcatccgcctgctcggattttgctccgactccggcaaccg
gaataagctgctcgtgtacgagtacatgcccaacggctcgctggatcggcacctcttccgcgcaacgctcagctctcgtatcgccgtcggcatcgccaag
ggactcgcctacctgcacgacaagtgccgggattgcatcatccactgcgacgtcaagccggagaacatcctcctggatgcggaatttgtgcccaaggtt
gcggacttcgggctcgccaagctcctccgccgggacacgagccgcgtgctgaccaccatgcgcggcaccatcgggtacctcgcgcccgagtggattag
cggcgagcccatcactgccaaggccgacgtgtacagttacgggatgatgctgttcgagatcgtgtccggaaggaggaacgccgaccacgggaagact
gagtccgaacaagagccgtcattgggtagtgattacgacgccgcagcggatgagttcttccctgttctggtggcgaggaggctagcggagggtgtgca
aggtcgcctgctggtgcatccaggacgaggctga
>g52650.t1
atggaagttgtaactggagctctaggttatgaagcgcctgaatacttacaagctggacagttgacagcgaagagtaacgtatggagctacggcgtactt
ctctaccagctcatcactggccgccagcccattgaccggaaccgtccaaaagcggagcagaagcttctggtgtgggtgaaaccatatatctccagcgtc
aagcggttctccaaaatagtagacccgcggttggaggggcactacaacctcaagtacgtgataaagctgggcagtgtggcgaaccgatgcctagttcg
ggagccgaagtcccgcccaaagatgtgtgaggtttatgagatggtacagaagattgtcgacgtcgacagtgtggatgtcacggcacagcatcatcctca
gcgctaccatggcttggttcctgaatcaagtgcaaagcggtcaaataaaagatag
>g52662.t1
atgaccgaaggggccgagatcacccagtggctggacgctgcgcgcggcggcgatcgcgacgcgctcgatcgtgtgctcgccacgctctaccaggagc
tccaccagatggcacggcgccagctcgccggccagcacgggcatacgctcgatgcgaccgcactggtgcacgaggcctatctcaaattgatcggccgc
cacgaggcacagttcgacgatcgcgcccatttcttcgcctatgccgcctcggccatgcgcagcgtggtcgtcgactacgcgcgccagcgtctggcgcag
aagcgcggcggtgacctgcaccgcgtgaccgagctgccggagaacatcgagagcggcctcaagctcgacgaggacacgctggcgctggacatcgcg
ctgacgcggctggccgcggtcgacacgaaactggcgcaggtcgtggagctgcgttacttcgccggcctgtccgagctggagatcgccgcgctgctgga
cctcaccgcgatggacggcgcgcgctggcagcggctgtctccggccctcgacgccctgctcgaactggacgccgaagcacgcgtcgccgcgctcgcgg
tgcatcgacgcgaagacccggcgttcgccgatgaactcgaaacgctgctggcgctggaggccgaccgcgacgatttcctcgccgagcnctcacgggg
ggggcggctgtctccggccctcgacgccctgctcgaactggacgccgaagcacgcgccgccgcactcgcagcccatcgacgcgaggacccggcgttc
gccgatgaactcgaagcgctgctggcgctggaggccgaccgcgacgatttcctcgccgagccgatcgtgtccacgctcggcggcgtgctgccgggcca
ccatgtcggcccctacgcactggaacggctgctcggcgaaggcggcatgggccaggtctggctggcgcgccgcgcggacggtctgtacgagcgccgc
gtcgccctgaagctgctgcgccccgggctcgccgatccgggcctgcgcctgcgcttcacccgcgaacggcagatcctcgcgcggctcgagcatccgca
catcgcgcgcctgctcgacgcgggcatcagcgccgaccaccagccctatctcgcgctcgactacatcgacggcgaaccggtcaccgactggtgccgcc
ggcaccggccgggcgtgtccgaacgcgtgcggctgttcctgcaggtctgcgatgcggtcagccacgcgcacaccaatctgatcgtgcatcgcgatctca
agccgtcgaacattctggtcaccacgctcgacgacatccgcctgctcgatttcggcatcgccaaactgctcgacaccgccggccacgcgcccgagcag
acgcgcaccggcgtgcgcgccttcaccctgcactacgccgcgcccgagcagatccgcggcgagccggtgaccacgctcaccgatgtctattcgctggg
cgtggtgctgtacgagctgcttgcaggccgcaagccctaccagccgtcccatcccagcgacgcgcagtgggaacaggcgattctcgatgccgatccac
cgcgcccgtcgctggccctgcagcgcgcgcaggacgacgggctcgaccccgtgcagcggcggcgcatggcgcgcgcgattgccggcgacctcgaca
ccatcgtgctgcgcgcgctcgccaagctgccggcgcatcgctatccctcggtcgaagcgctggcgctggacctgcgccgctggagcgagggcaagccg
atcctcgcgcggccgcagcatctgggcnnnnnnnnnnnnnnnnnatggtcgcggtga
>g52802.t1
atgaaggttgctcttggggcagcaaaaggtctagctttccttcatgaagaagcggaaagaccagttatttaccgtgattttaagacatctaatattctgctt
gacgcggagtacaatgcaaagctctcagatttcgggcttgcaaaagatggtcctgtaggtgataaaactcatgtctctactcgagtaatgggtacctatg
gttatgctgctccggaatatgtcatgacaggtcatttgacatcaaagagtgatgtctatagcttcggtgtggttcttctcgagatgatgtcggggcgcagg
tcaatggacaagaatcggcctaacggtgagcacaaccttgttgaatgggcgcgtcctctcctcggagagagacagcgcttctacaagctaattgaccct
cgcctggagggtaatttctcagtcaaaggtgcccagaaggcagcacagctagctcgtgcctgccttagtagggaccccaaagcacggccgctgatgag
ccaggtggtcgaagctctcaaaccacttattaacctcaaggacatggctagctcctcctacttctaccagacaatgcaagccgagaggatggcacactc
gagcagcatgaacggcagaaacagccacggcatcaagatgcagggcccatttgctcgcaatgggcagcagcctatgagaagcctgtcagatggcccc
cgtgcttccccattccgttactcgccgaagccgaatgtgaagtaa
>g52872.t1
atgtccgactcggcgtcgatggcggccgccatcgaggcgcggttcagcaaccgcgacctcatcggccgcggctcatttggcgacgtctacaaagggttt
gacaaggagcttagtaaggaggttgcaataaagggctcccgtttttacctctacgggatagggttcagtgctgcaaatattctcctcactgagagcggag

atgtgaaggttgcagattttggtgtttcagcacagttgactaaaacaatgtctcggagaaagacattcgttggaactcccttttggatggcacctgaggtt
attcaaaattctgaagggtacaacgagaaggcagatatctggtctttgggtataactgctatagaaatggcaaaaggtgaaccccctttggcagatattc
atcccatgagagttcttttcatgatacctcgtgaaaatccacctcagaggcctagtgctaaggagcttcttaagcatcgtttcattaaaaatgccagaaaa
actgcaaagcttctggagagaataagggagcggccaaaatttgctgcaaaaaacagcttggatgccacacaaaatgggcgaacacatattgaagaag
atatcagtacaggtactatcaaggtaacagtcaagaaagctgttggccgggattttcctgacagatcggagggcacaggaactgttcgagttgggtcaa
ggccacctcaaatttcctccaccaaggatggtagatccgatatctctcatagtccaaatactccaactagtacttctgaccgagaaaatcagtggaggcc
atcatggaggggatctgatgaatcacttgcatcaagcttgtctcagagagaggtgcatagtgaacgtggggagctagaaagttctacagaagatgctac
tggggacaagtttgaacagcctaccgtccatgcattccttgattcgttgatggatctagaacatgaattacctggatcgtgtgaggtactgattggcagaa
tgctacaccggctaggaagcttgaaggattcctcgttgcaaggtcttcaggagatggcagcttcggctttctctaagaaatctgatcctcctttggaacca
gcgagcagcaaaaaacaagctaacacgccaacattggcagctccaactgtaagcccgctggcgaggttccttcttacaaggtggcaaaaccaagtgtc
gcaagaccttaattctgtttga
>g53242.t1
atgggctgctccttctcggggctcaacgctctctacgatgcgtcgacaggcggcggcgacgtctggatcaacgagcgccgcttccgcgtcctccgtcag
atcggcgagggcggcttcgtctatctcgtcagggagctgcagccccagtccgacgctgcgctcagcaggcgcgcctcccacgtcccagaggatggaac
atatgctataaagaaggtgctgatacagagcaaggagcagctggatctggtgaaagaggagatccgtgtgtcgtcactcttcagccaccccaatctgct
cccgcttctcgatcacgccgtaattgcagtcaagctttgtgaaggactgaagcacatgcacagctatgatcctccatatgctcataatgatgttaagcctg
gtaatgttcttatcactcgtcatagaggacaagcacctattgcaactttaatggattttggaagtgcaaggcctgcaagaaaagaaatccgttctcgttct
gaggctttacagttacaggaatgggctgctgagcattgctctgcaccttatcgcgcacctgaattgtgggactgtcccagccatgctgatattgacgaga
gaacagacatttggtctttaggatgcaccctttatgctatcatgtacaatgtttcaccatttgagtatgctcttggtgaatctggaggaagcctgcaacttg
ccattgtcaatgggcagctgaagtggcctgcaggacccaatcctccttaccctgaagaacttcggcagtttgttatctggatgcttcagcctcaacctgca
atacgccctcacatcgatgatattcttcttcatgttgacaagcttattacaaaatactag
>g53314.t1
atgagcgtgtccgccgggagaacgcgggtggggaggtacgagctgggccggacgctcggggaaggcaccttcgccaaggtcaagttcgccaggaat
gtcgagaccggcgagaatgtcgccatcaagatcctcgacaaggagaaggtgctcaggcacaagatgatcgggcagagattggagataaggttcatca
agcgttttgtttttctgttcacgattccctcccggcgcgcagagtggagtcgattccttgtgcctcttga
>g53565.t1
atgacgactgtgggtacggatactcgggtcttgctgatgggacgacactacctgagcaagctgttgggcaagaggagttttgccaaggtgtacgagatg
cgacatgccgagaccgacgagaaggtgaccatcaagttcatggagaaggataaccttgccaagtccggtgccaagcggcaacagatcatgagagag
atcgacatcatgcgtcgtgtgcgtcatcccaacatgatctacctttga
>g53568.t1
atgnggccgaagcggtaccactaccgcgagctcgctgtcgcaaccggtgacttcagcgacgagaacaagctcgggagaggaggtttcggcagcgtct
accaaggcaacatcgtccaaatggacggctccaccaagcaagtggctgtgaagaagttctcgtcagagacgtcgtcccagggacgcaaagagtttga
ggccgaggtgaagatcataagccgtctcaggcatcgcaacctggtgcagctgctgggctggtgcgatagcgccaaaggcctcttgcttgtctacgaact
ggtgccagaagggagccttgataaacatatccatcacaacaccaggttcttaacctggtctgagaggcacaagatcattatgggactagggtcggctct
gagttacctgcaccgagaatgggatcaatgcgtcgtgcacggtgacatcaagccgagcaacataatgctcgactcgtcctacaacaccaagttgggag
actttgggctcgctcgactggcggaccatntatccttctaa
>g53597.t1
atgggcaactcgtgccgcggctacaccacaagcttgtacgattgcgactaccagcacagcagcacctcctattcgtcgtcgtcgtcgaatttctccagca
ggcgcagcagcagcagcatcgtctgtggaggcggcggcatgcgcgggcaccagcaacagctttcgtccccgacggccgtgctcgggcacgtgacccc
accgctgcgggagctctactcgctggggcggaagctcgggcagggtcagttcggcaccacgtacctgtgcacggatctggccacgggcgcgtccctgg
cctgcaagtccatcgccaagcgcaagctgctcaccccggaggacgtggacgacgttcgccgcgagatccagatcatgcaccacctcgccggccacgg
cagcgtcgtcaccatcaagggcgcctacgaggacccgctctacgtgcacatcgtcatggagctctgcgagggcggcgagctcttcgaccgcatcgtca
accgcggctacttctccgagcgcaaggccgccgagatcgcgaggatccaatag
>g53660.t1
atgagcagcagggacgacggcgcggtggaagttcctgagctcactctgcgcgagctcaacaatgctaccctctccttctcggaggaacggcttatcggg
gaagggttctgctccaccgtgttcatggcgtcgctgcggggcggcaaggacgccgtcgcgaagaggatcgacctgcggccctacggcgccgccgtcgc
atctgactacaagcggcatgtatcagccgcgtcgcggctgaggcacgagaatgtggtccgtccggctgctcgggtactcgatcgtcaccgccgaccaa
atcgtgctgatctacgagttggctaccatgggaacccttcacgacgtcctacacgtctgagctgggcgcagcgcgtgcggatcgcgctgcacacggcca
aggggctggagtacctgcacgatggggtgcagccgccggtgacgcacgggagcgtccggtccagcaacgtgctgctgttcgagggcttcagggccca
gaccgatggcaacgacatgttcaagatcgcctcggcatacaacgagcacaccggttgcgagccggtgggttacattgcggcagagggttttatgatgg
gtctgactcggaagagcgatgtgttcggcttcggcgtggtgctgctggaactcttgacagggaggaaggcgttcgaccagacgttgccggtgccgatcc
tggtgaaatggataccgatactcctctga
>g53809.t1
atgtctgggctctctgcctcatcggatactgtcggcagctcgcgcggcgtcggcgaacggagccggggcgacgacgacgaccgcggcagccgctacg
aacagaagtcgctgcagtggccggctcacgggacgggctccacccaacccaacgatcgattcacgcagccgggcccacggaacaccgagtccacca
acgtctacaacgccaaaagaaaaggaaaaaactcgtacagctccataaacggcgcagcggctgcctacgccacgctccgcttccctccactccgtcgc
cacctccctcccctccccaccgcaacgggccggagacgcgacacggccccaccgccgccaatggacgccgccggcccaccgcccgagccctcccccg
ccgcggctgacgtcagcgtcagcggcgcaggcaggaacaacaacagctccctcctcggtctgccgcccgccgccgcggccgcggccgccgcctcgct
cgtcatcctcctcgtcctcgcggcggcgggcgccgccgcgttcttcacgcgccggcgggggggccaggccgccctcgctctcccgcgtcgagcacgcgcc

ttcctcggggtcctcccacccggcgtcctcgtccgcgcgcaaggagaaggtcgccgtcggcggaagctcctccgacgcggcaagctcgtccgccgccg
cgagctcgctggagtcgccggcaaggaggaaggtggcggaggtcagggtcggcgtggagatggggtgggggcggtggtacgacctcgcggagctgg
aggcggccactggcgggttccgcgcggagaacgtcgtcggcgagggaggctacggcacggtctaccgcggcgtgctcgccggcggcgaggtggtgg
cggtcaaggacttgtttgatcacaaaggccaggctgagaaggagttcaaggtggaggtggaagccatcggcaaagatgctcgtgtacgagttcgtcga
gaacggnaggtggtggcggaggtcagggtcggcgtggagatggggtgggggcggtggtacgacctcgcggagctggaggccgccacgggcgggtt
ccgcgcggagaacgtcgtcggggaaggagggtacggcacggtgtaccgcggcgtgctcgccggcggtgaggtggtggcggtcaaggacttgtttgat
cacaaagggcaggctgagaaggagttcaaggtggaggtggaagccatcggcaaagtgaggcacaagcaccttgtgggactcatcagctactgcgca
gaagggcctaaacggatgctcgtgtacgagttcgtcgagaacggcaacttggagcagtggctgcacggcgacgtcgggcctgtcagcccgttgacgtg
ggagatccggatgaagatcgccgtcggaacagccaaaggcatcgcgtacttgcacgaaggcttggaaccgaaagtggtgcaccgcgacatcaagtcc
agcaacattttgctggacaagaaatggaaccccaaggtgtcggactttggaatggccaaggtcctgggcgccggctccagctacgtgaccacccgtgt
catgggcacattcgggtacgttgctcccgagtacgcgtcgacgggcatgctgaacgagagcagcgacatctacagcttcggcgtgctgctgatggagct
catctccggtcggagccccgtggactacaaccgccccgccggcgaggtgaacctggtggagtggttcaggggaatggtgggcagccggcgcgtggag
gatctggtggacccgcgcatcgcggcggcgccgccgccgcgggtgctgaaccgggcgctgctggtgtgcctccgctgcatcgactccgacgcgcacaa
gcggcccaggatggggcagatcgtgcacatgctcgagggcgacgagttccccttccgctccgagcaccggtcgccgcgggcgtcgcaccggacgtcg
gcggcgagcccgcggccgtcgctcctctcccagaacgtcggcggcgccgaggacaccgacaagtccatgtggaggtga
>g53821.t1
atggcgtcggcagcggcggcgcggttcctgctcgcggcggtgatcctcctcgtcgtcctcaatccgacgacaccggcggacgacggcgcagcagcggc
ggcagcgctgctagagttcaagtccgcgctgcaagacccagacgggcgcctcatgagctggaccaccgccgcgggcagcgacccgtgctcatgggcc
ggcattgcctgctccgccaccggcgacgtcaccggcatcaccctcccgaacctcaacctccacggcaccctctccaccaccctctgccattccctcccttta
cctcgccgtcctcaacctctccacgaacgcgctctccggcgaggtcccctccgccctctcctcctgcgccgcgttccacacgctcgacctcagcaccaac
gccttccactcccccatcccgccttccctcggcaacctcccgcgcctccgccgcctcttcctcagcgagaacctcctgtccggccccatcccgccgtccat
cggcaacctctccgccctcgaggagctcgagatctacagcaacaacctcaccggcgcgatcccctccacggtgggcgccctccgccgcctccgcgtcct
ccgggccgggctcaacgcgctctccggcccagtcccggccgccctgggccggtgcgcgggcctccaagtcctcggcctcgcgcagaaccgcctctccg
gcccgctccccggcgccgcgctatcccacctccataacctcaccaccctcatcctctggcagaacgcgctgtccggcgaaatcccccgcgagctcggcc
gatgcacgagtctccagatgctcgccttgaacgacaacgcgttcacggggagcgtccccgccgagctcggaaacctccctgacctcgccaagctctac
atctaccgcaaccgcctcgacggcgtcgtcccgcccgagctggggaacctacgcgccgcgctcgagatcgacctctccgagaaccggttgcgggggac
cgtcccgcgggagctcggccggatccggacgctgcggctgctctacctcttcgagaaccggttggaggggaccatcccgccggagctcggccggctcc
gccgcgtccgccggatcgacctctccatcaacagcctcaccggcgagatcccggtggagctccaggaactccccagcctcgagtacctcgagctcttcg
acaaccagctccacggcgccatccctcctttactcggcgccaacagcaacctctccgtactcgacctttccgataaccagctcaccggcaccatcccgcc
ccacctctgcaagcaccagaagctcatcttcctcagcctcggctccaaccgcctcgccggcaacatcccgcccggagtcaaggcgtgccggacgctcac
gcagctccggctcggcggcaacttgctcaccgggagcctccccgtggagctcgccctgctgcagaacctctcctcgctcgaaatgaaccagaaccggtt
ctccggcccaatcccgcgggagatcggcgagttcaggaccgtcgagcggctgattttatccaacaactacttcctcggatacatcccggccaccatcgg
caacctcaccgagctcgtcgccttcaacatctcatctaaccaattaaccggaccgatccctcgggagctggcacggtgcacgaagctccagcggcttga
cctcagccggaactccctcaccggagcgatcccacaggagatcggttctctggtgaacctggagcagctgaagctatctgacaacggcctgaacggga
ccgttccgggcagtctcggaggcctgtcgcggctcaccgagctgcagatggggggggaaccgtctgtccggtcaaatcccggtcgagctcggcgagctc
gcctccctccagatcgctctcaatgtcagctacaacatgctctccggcgagatcccgtcgcagctcggcaacctgcgcatgctggagtacctgtacctca
acaacaatgagcttcaagggaaggtcccttcctcattcggggagctctcgagcctcatggagtgcaacctctcctacaaccacctcgtcggccgctccc
cagcacgcccttgttcgagcacctggaccaaagtaacttccttggcaacaatggcctctgtttcatgaaagggagtacatgtcagggttcatcaccatttt
caagcaacgaagaggaagcacagaagaagcgctacctcagggagaagatcatcagcgttgtctccatagtcatcgcgttggtttcgttggtgctgatcg
cggttgtttgctgggcattgagagccaagataccggagcttgtatcaagcgaagagctgcgtagaaccaggttctctggccctcaatacttcatcaagga
acgggtgaactatcaagagcttatgaaagcaaccgagagcttctcagagagtgctgtgattggaaggggtgcttgtggcacggtttataaagctgtcat
gccagacggccggaagatcgcggtcaagaagctgaaaactcaaggagaaggctcgaatatcgacagaagcttccgagctgagataaccacgcttgg
aaatgtcaggcaccgcaacatagtcaagctctatggtttctgctctcatcaggactcccatcttatcctgtatgagtacatggcaaatggtagccttggag
agttgcttcatggcagcaaggatgcctgcctactggactgggacacacgttaccggatcgcgctaggtgctgctgaaggcctgcggtaccttcacagtg
actgcaagccgcaggtcattcaccgtgatatcaaatcaaacaacatacttctcgatgagatgatggaggctcatgtgggggactttggtttggccaaact
tattgacatttccaactccaaaacaatgtctgctattgctggttcatatggctacattgcaccagagtacgctttcaccatgaaggtcactgaaaaatgcg
atgtgtacagttttggagttgttttgttggaactattgaccgggcagtctccaatccaacctcttgaaaaaggtggagatcttgtcaatttagtaagacgga
tgatgaatagtatgacaccaaactcagaagtgtttgacagcagactgaatctgaactcgaagagaactgtggaagaaatgtctctggtgttaaaaattg
ctctgttctgcaccaatgagtcgccatttgataggcctactatgagagaagtcatatccatgctgatcgatgctagagcatccgcctacgattcattttcat
caccagcatcagagaaggaccctatagaagatgatgtttctttgaaactttag
>g54013.t1
atgaggctagcgccacctccattaccggttcggtgctcctccgtcctggtcgtgctgctgctcgcatcgtcttcgtcgtcggccgatgggagggacccgcc
gccttcttccccgggtgcttcgcctgtcgccgagcgcattctcgccgccgccgctgccggtggtgccaatgggaccactccggcggttccggcgccgcct
cccgttgtgatcatcgtggagaggcaccaccatttccaccgtgagctcatcatcgcctccgtcctcgcctccatcgccatcgtcgcgatcatcctctccatg
ttctatgcctggatcctgtggcggcgatctcgccgcttgcccgacggcaaaggcgcccggagctcaggcgctgcaaggggaatgacgctggtgccgat
cctgagcaagctcaactcaatgaagacgagcaggaaggggctcgtggcaatgatcgagtacccgtccctggaagcggcgacaggcaagttcagtgag
agcaatgtgctcggtgtcggcgggtttggtcgcgtctacaaggcggtgttcgacaggggagtcaccgcggcggtgaaaaggctggaaggcggcgggc
cggagggcgagaaggaattcgagaatgagctggatttgctcggcaggattcgtcacccgaacatagtgtcccttctgggcttctgtgttcctgaggga
atcatttcattgtttatgagctgatggagaagggatccttggaaacacagctgcacgggccttcacatggagcagccctgagctggcacatccggatga

agattgcgctcgacatggcaaggggattggagtatcttcacgagcactgcagtccaccggtgatccatagggatctgaagtcatctaacatacttttaga
ttcagacttcaatgctaagatttcagattttggtcttgcagtgaccagtgggaatcttgacaagggaaacatggagctttctgggacattgggttatgtag
cccctgagtatttattggatgggaagctcactgagaagagtgacgtatacgcatttggcatagtacttcttgagctgctgatgggaaggaagcctgttga
gaagctgagtcaatctcagtgccaatcaattgtgacttgggccatgcctcagctcactgacagatcaaaacttcccaacataattgacccggtgatcagg
gacatgatggatccaaaacacttgtatcaggttgcagcagtggctgttctatgcgtgcaaccagaacccagttacagaccgctgataacagatgttctcc
actctcttgttcctctagtgcccgtggaacttggagggacactaagggttgcagagcctccgtctccaaaccagaacaattttcctcgttga
>g54155.t1
atgagctgtggcagcgaggggggccgggacggcagcaacggtggcagcgaggagtacaggcggccgcggcccagtaaggtcgcggctgatgatttg
gcggcgccagaacggtgctcggattccatagcgccggcgcggtgttcggatgccacagcgcctgcgtcctggatcgacaggaagctgctagttgatcc
caagacgctgtacgttggggacaagattggggagggagctcatgggaaggtctacaaggggaagtacggtgatcaaattgtagcaataaaggttctta
accgtggcagtacacttgaagaaaaggcaacacttgaagctcgtttcattcgtgaagtaaatatgatgtgcaaagtgaaacatgagaatcttgtgaagt
tcattggagcttgcaaggaaccattgatggtgattgttaataatttgctgctcacagcaaatcggaagaagctgaaacttactgactttggacttgcccgt
gaagaaactgtgaccgaaatgatgacagctgaaactggaacttaccggtggatggccccagaggctgcatatgctgctgcatttcagtatactgatgtg
agccatattctcttgtttatacaacagcaaaagcgcccagctttccctgaggatacccccccaggagctggtgttcatagtacaatcatgctgggttgagga
ccctagcatgcgacccggtttcagtcagatcatccgaatgcttgagactttcctcatgtcgatacctccaccaccaacttcagaatcaaacgaagatgag
gaatcagaagaaacaaagtcgtcattgagcagtaggagttcctctggttcttcgcttgtttcgcgtgcaacaagcaagctctcggtcgtccgccatctctt
tgcctcaaagaaggcaagcaacgggaaggcatga
>g54373.t1
atgggcaactgctgcggcgcgccggcgacgccgggcggccgcaagaaccggcggaagcagaaggcgaacccctacaacgtctcgtacaaccgtgg
ggcggcgccgccgccggcgcgtccggggctgatggtgctgcgggacccgacggggcgggacatgaacgcgcggtacgagctcggcggcgagctgg
ggcgcggcgagttcggcgtcacgtacctgtgcacggaggtggccacgggggcgcggttcgcgtgcaagtcgatatcgaagaggaagctgcggacgcc
ggtggacgtggaggacgtgcgccgggaggtggacatcatgcgccacatgccgtcgcaccccaacatcgtcagcctcagcgccgcctacgaggacgag
gacgccgtgcacctagtcatggagctctgcgagggagggggagctcttcgacaggatcgtcgcgcggggccactacaccgagcgcgccgccgccgccg
tcacgcgcaccatcgtggaggtggtccagatgtgccacaggcatggtgtcatgcacagggaccttaaaccagagaacttcttatatgcaaacaagaag
gagagttcccctctgaaagcaattgattttgggctgtctgtgttcttcaggcctggtgagcgctttacagaaattgtaggcagtccatactacatggctcca
gagagactgaacagggagtag
>g54451.t1
atgtacaaggctaaaagagctgcattatcaccaaaggtgaagcgccgtgttgggaaatatgagcttggacggaccatcggggaaggcgcctttgcaaa
ggtccggtttgcgaagaacactgaaactggagaacccgttgcaatcaaaatccttgacaaggagaaggtgcagaagcacagattggttgaccagatta
ggcgtgaaatttgcactatgaagttaataaagcatcccaatgttgtccgacagtatgaggtgatgggaagtaaagcaagaattttcattgttctcgaatat
gttactggcggagagctttttgaaatcatagctaataatgggaggctgaaggaggatgaagcacgtaaatactttcaacaactgatcaatgcagttgatt
attgccatagtaggggagtatatcacagagacttgaagttagaaaatttgctgctcgatgccggtggaaatctcaaagtatctgatttcggcctgagtgct
ttaaaagagcaagtgaaggctgatggtctgcttcacacaacttgtggaactcccaattatgttgctcctgaggtgatcgaggatggaggctatgatggtg
cgactgctgatgtctggtcttgcggagtaatccttttttattcttcttgccggttatttacctttcgaggatgacaacatcattgctctatataaaaagatttctg
aagctcagttttcctgcccctcttggatttctgcgggagctaagaacctgattaccagaattcttgatcccaatcctacaactgaccagcatgtcaaagag
gaaactgaagatcagccaacctcaatgaatgcatttgaactgatttcactgaaccaggcactgaatctggacaatttgttcgaggcaaagaaggagtat
cgaagagagacaagattcacatcacaatgtcctccaaaggaaatcatcacaaagattgaagaagctgcgaagcctctaggatttgacgttcagaagaa
aaaatacaagatgcggatggagaaccccaaagcaggtagaaaaggcaatctaaatgttgcaactgaggtttttcaaattgcgccgtctctacacgtagt
tgaggtcaagaaagcgaagggagatactcttgagttccaaaagttctacagaagtttatcgacgcaactgaaggatgtggtgtggaagtgcgacggcg
aggtcgacggcaacagcgcagcggcatga
>g54631.t1
atgcccaactacatctggcattccagtagagaaatacagcttcaggccctttcttcccctgggagtgattattctggtagctggtttagtgatcattggccag
gcactgatgatacacaagcttcgcagcatggtgccgcggggcgcgggcgcgagcgcgaggccatcgccagatgagacctcccggacggcggcgcgc
cgggaagaggcgacaccaccagcccgcgcaaacgacaccgtggccgtgggcgagaccgcggcgggtacacagcagcagctgcgccggcgccgtcc
tcatgtccacattgatgtacctgggcgacaggcgtcggcccacgcctcgttccttgcccctgacaaggacctgccatttgccttgcctgcttccgccgcgg
cccagccgccgccgccagtggcggagcttgaccgcgtcgggagcggcgctggtggcaccgtgtgggaagtactgcatgggcccacgggtcgcaccta
cgccctgaagatcctgcacggtcagcattacgaagacacgatccggcagcaaataaacagagagattgcgattcttctcatcacagacagtccatatgt
ggtgaaatgctatggcatgtatgaaaacgctggtaaacttcagatcttgcttgagttgatgtccggttctcttgatggcctccgcatatatgacgaggcca
tcctggcgcatgcaagtcgctcgccagacatctggagctttgggttaacgattcttcaactgcacctgggaaatttcccgtacggcgaagacgatgccgt
gcagaatgccgccaaggactccgtgccagacgcctcgcaggacacccccgcgccggaaggcgctgtgcaggagtgcgccgtgcagggcgccgctct
ggacagagccacgaaggactacgccgcgcactag
>g54926.t1
atgctggagggtgcacactggcggttgtccagtatccatgctactctacaagcatcggctgcaattgaggtacatgagggccccacaacagcaagtcaa
aattttgatgatgagagcatggtcattcggtcctattctttccatgatctggagctgttcacagaggggtttgctgaagagctaggaagaggagcatatgg
taccgtgttcaaaggtgttttgaccaacagtaacatggacattgcagtgaagagacttgagaggatggcagaaaatggtatacgtcgtttgcttgtgtac
gagtatgttccaaatggatcccttgctgacctcctcttcaagtcagatgcactgcctaattggaacatcagggtttcgattgcactggatgttgcaagggg
gctacagtaccttcatgaagagatagaaggacctataatacattgtgatattaaaccagaaaatatactcattgacagcaccgtaagggcaaagatagc
agactttggcctctccaagctgctgattggggacgagacaaagactttcattggcatccgaggcacgcgtggatatcttgcaccagaatggagcaagaa
catggcaatcactgtgaaggcagacgtttacagctatggcatcatgcttcttgagatgataagctgcaagaagagcatggagctgaagctagctggtga

ggaatgcaacatttccgagtgggcttatgagtatgttatatccaacgacttaaagaaagtggcagctggagactctgtcaacaaagtcgagctggagag
gatggtacggattggcatttggtgcacgcagaatcagccagtgatgcggcctattatgaagaatgtcgttcagatgatggaaggaagcactgaagttcg
acaacctccaccgcctgcgtcattttcacagtcactgatgcggtcttga
>g54933.t1
atggagccacgcatcgggaacaagtttcgtgtgggccgcaagctgggaagcggctccttcggggagatctacctcggtaccaacgtgcagaccaacg
aggaggtggcgattaagctcgtttacattatagattttggacttgcaaagaaatacagggatacatcgactcaccagcacattccgtatagggagaata
agaaccttactggaacagcaagatatgcaagtgtaaatacccatcttggaattgaacaaagccggagggatgacatggaatctcttggatatgtactga
tgtacttcttgagaggaagtctgccatggcagggtctaaaagctggtaataagaaacagaagtacgagaaaatcagcgaaaggaaaattgctacttct
actgaggccctatgccgtggatatcctaccgaatttgcttcttactttcactactgccgctcactacgttttgaagatgcaccagactatcaatatctgaag
agattgttcagagaccttttcattcgagaaggctttcaatttgattatgtttttgactggaccattctaaagtatcaacagtcgcagatgaccagtgctcctc
cccgtgcaattgttagtacaatcactactcaccatctatgcttttcttga
>g55246.t1
atgcgacgagatgacgcaggcggcggcggcggcactgggttccacgagctcttcgactcggtgcgcaggtccatctccttccgccccgcggccgccgc
ccccgcggagcccccgccatccccttcggcgacgggggcgggggcgggatcggcgtgcggattagctcctgccttcgaaagtcgcgggggatgggc
ctgctcgggctcatctccaagagcccctcgccgccgcgccgcctgctgccgccgtcgcccgacgagtcaggtggcggtggcggaggggggcggtgcg
gtggagaggggaaaggagaagagaacccgccaattcggtggcggaaaggggagctgatcgggtgcggcgcgttcgggcaggtctacctcggtatga
acctggactctggcgagctcctcgccgtcaagcaggttttgatagggactagcaacgccacacgggagaaagcccaagcacatattaaagagcttgaa
gaagaagtgaagctgctaaagaacttgtcgcacctgaacattgttaggtatcttggtactgttcgggaggaagacacgctaaatattttgctggaatttgt
gcctggagggtcgattcagtcacttttagggaagctcggttcattccctgagcctgtaattaggaaatacactaaacaaattctgcaaggattggagtatc
tacataacaatgcgataatacatagagatataaagggtgcaaacattctggttgataataaagggtgcatcaaacttgctgattttggagcatcgaagc
aagtggcgaagttggttgctcttctatttcatgttgggaccacaaagtcacacccacccatacctgaacatctctccccggaggccaaagattttctgctg
aaatgcctgcagaaggagccagagctgagatctactgcatcagatttgttacagcacccatttgttacaggagaattggacaacctgcagccagtaaat
cacgctgcacacaaggaaacttccgttgataagattcctgcacatgatatgccaaatgtcttgggtttaaatcattcgtccaactggtcgactcttaactcc
aacaaatcattaaatgccaagcccttatgggagggtggttgtgacgatgatgacatgtgtgtgttcgctgacaaggatgactatccagcagttggatcta
gctataatcctatgtctgaaccatttgataactgggaaaacaagtttgacataagcgcagagcaaaattctcatcaatcgacagagtttagtggattagc
caatcatactgaaagcagcataactgagaatgattttactttcccttgtgagggaagttgtgaagatgacgatgtactgacagaatcaaagattaaggc
atttcttgaggagaaggcccttgacctgaaaaagctacaaacacctttatatgaggagttctacaacacagtgaatgcagggaattgtcagggagctga
tcaaacatccaagggaaatttttcaaatagcccaaaattgccccctcgaggaaagtcatctccaagcaagatgaaaggaggcttgtcaccaacatgtga
caatatgaataatacaagtcctgtaatatgcagcaagcaattcccaagcagtggtgtggagagtagcagaattttgagagaaatagcatccccacaact
gaacgagtttgcggataaaagccatcttgatgtcccagatagcccaagcatcagctttgctgagaggcagaagaagtggaaagaagagttggaccag
gagcttgagagggaaagagattctggatggactgggctggacaagcccaacctcgtctcgcatcctgccgatgctgctattcttcctccccctcctgcac
gctgtagccagggcaccaccgaccttccgccgcccatcgcgcttctctcccttccctctccggtgctctccgcttccgccatggcccagcccgccttcctct
cctcgctccgctcccgcctccgttctccggcgccgccgtcgccgcactcgcgtcaccagcctagccgcggctaccacgtcgagctcggtgctcgcgaga
aagcgcttttggaggaggatgttgctttgaagagattcaaatcatacaagaacagtgtgaaacaagtctcgaaggttgggaatgttctcaccctcgtagt
tgctgttgcttttgcttgtggcagcagcgccatggcggcttctctgtttcaaatggcaggcacagcgaggtttcgttgcgcagatccagaccttccatttca
gatcggacggcatgtcccagccgacagtgcagcatgtgaaacccctcgaacagataaggagccccgagaccgagaggagacaaccgaagagcaga
gaagtccggtgatggcaaccaaggcgtgggcagcggccgcggccttaaaccctcatcctctccgcgctgtcccctcatcttctcccgtccagctcggcgt
ctccgcgcggcgcacggcgccgagggagcacagcctccgcagcagctggcagaagccggttaagttcgtgtgccgccgcgccaagaatgcgggcttc
gacgactacaagttcccagatcccatccccgagtttgccgagcaggagacgagcaagttcagggagcatatggcgtggcggttggagcagaagaaag
atgactacttcggggagcatgtcgaggagatcgttgacatatgcacccaggcatggacgctcctggttcatccgttcctggacatgaagggtgagatca
aagagaggggccttcctggtgcacctcaagctgcacgggcagcgatcgcatgggctgagaagaacattgacaaggattggaaggcctggactggaga
atactag
>g55352.t1
atgggtggatgcttctcgctggaggagcaccggctcccaagaaggccagctgaagctgctggaccagatggattggggaagtgcaaatctgattctaa
agctagaactgttcttgctcctccaaaagatgtggaggacttgcaagtagaaggatatgggaatgttaatattttcacttacaacgaattgagagcagcc
acaaaaaactttcgaccagatcaaattcttggagagggtgggtttggagttgtgtacaaaggtgtgattgatgaaaatgttagagcaggatttccttcaa
gacaggtggctgtgaaagaactaaatccagaggggtttcagggagacaaagaatggttggattacaatgctaaactgtcagactttggtcttgcaaga
acaggacctagtggagatcaaacacatgtgtcaactcgagttatgggaacctatggctatgcagctccagaatatgtcatgactggccatctgaccgct
agaagtgatgtctatggctttggtgttgtacttctagagatgatcattggaaggagggctgttgacaagagccgcccgactcgtgagcataacctagttg
agtgggcacgcccccttctcgtccataaccggaagctgttcaggattattgatccaagaatggaagggcagtactccaccaaagctgccattgaggtgg
caagccttgcataccgctgcttgagccagaaccccaaaggacgaccaaccatgagtcaagttgtcgagacctttgaggcagtgcagaacatgcctgaa
tgccaagatgttcttcttcagaattgcatcacaggtgctgtaacgctctatgaggttcccaaggaacctgcagaaagtgttgagccggagaaggccaag
ctggagccgacaataaaacctgacgtggtgcctcctgtgaacggaaagccagtccccacgagcaggagaacacgtccagggaatggccggagcaag
agcgagacagctttggagtccaagctgtacattcccccgtctccggactctgacggacctggcctagaggtgctagcatctccttccagaaatggaaata
tcaaggatcctcctgatgaggatttgtacaagatataa
>g55778.t1
atgctcagcgccaggtggtacgaggaggtgtgcaagctcggcgagggcacgttcggcgccgtggtcaaggcgcgcagccgctccacgggtcagcacg
tcgcggtgaagaaacctcgccaatctggcccggacaacgacgtcctctgcgacctcctgtgcgaggcctgcatcatggcggcctgccgaggccactcct
caatcgtcggctaccgcggcgtcgctcgcgatccacgtaccaaggaacaagaatactctatcgtcatggactgcgtcgggcggcggaatctccacgac

gctctttaccaccgcggccagccgttcccggaggcggacgtgcgccgcatcatgcgcgagctcctgtgtggcgccaaggcgattcacgggaacggcat
tgtccaccgggacatcaagccggccaacattctcgtcaccgccgaaggcttcgttaagatcggggattttgggtctgccaaatctcttgtggagaagcaa
ccacccttcggcatcgccggaacgatcgggtacatggcgcctgaggcattagtgaagaacgaggaccacggcacggaagctgacatgtggtccctcg
gctgcataatggcggagctcctcaccgacaagcagatgttcgatggggatgatgaggctgagcagctgttcaagattttcgacgtgctcggcgttccgg
gcgagaaggcgttgaaggcccaggtgctcatcgacgaggtgaagcagcaacgggcgtggcaaaggcgattcgggcaccgcaacctgctgcgcaaga
tggtaccggaagaagtgctctcccgggacgggttcgaggtcttggaaggcctccttgcgtcagacccgaggaagcggctgacagcggacgctgcactc
aagcttccctggttcacagagcacgacctcaacaaagtcgcagtcgtcagtcactcgactgttgttcctaacaccgagaatacggtcacgaaaccttggt
cacggatttggaacatggcaatgtcattcgccgggaaggcgctaggattgcttagggccaccagcgcatcgtag
>g55784.t1
atggagatgagggataacttccttcggcccccaacgccctccccagaggaagccggagccggaaagagccttaggatccccaccgctggggacacgg
cggccaccaacctgacatcgtcctcgggttcttcctcgtccctgacgcagtccccaacgagcttcctgcgcgagcttcaggtcgctgttaagcggcagag
gcctcccggttcagtgcagtccaacaatccaagagcggtccgagtcctggtgcccagagctgagcgggtaaagaaaactggtgccagcccttctgcac
agcagaaccttgaacttgaagggaaggttacgcagactcagaggggcttgttaggaccctccaggctgcaaaatgtgactcctggtcagcagaaagat
gccagcagtgttaaattggggtctgctactccagatcagttgatttcagcaacatcatcgatgttgggaactttaactgattcctgtgatcagagtgctggc
cagaaatgtcagcagaagagtgaggttatgttggtggataagagattgaagccatctttggaagtttcttcatctggattagcacctcaagatgcattctt
gggggaaagcttcaagaaagaacaattttactcagctggtccacagttaacttctcagagaaataattttcctgcaaagcaaggggaacatgatcacca
tgcgaatcatcaggagctcaaaattgctgatgcagcagttgatatggatatcaagtatgatgcagttaatttgtctgaaagagggatagaggacgtttgg
cctactgtccaaagtatacgggcttcacagggtgaccaatctgcttcctctgcgaagatgccagaatctgctgtggaatcatctggaggtgtgccaggtc
ttggccatcaaaaacagactgcaggtgcaacaggtgttggagattggaatcctgatgatcatcaagttcataatccggggtcttgtgccgatgaaaaag
ctgtttctggtacccgcggtcgttttccttctgaagggttatctgcaaatgatcagtctacatcaggcagggatggtggtggctcccgggcgaacaaagtt
cagaaggagcgacacaagaagaattttgatcctaatgtattcttcaaggtgaacgagaagctttatcagaaacttggtaaaataggatccgggggtagc
agtgaagttcacaaagttatatcagcagagtgctcaatatatgctttgaaaaagatcaagcttaaaggccgtgactaccctactgcccatggcttttgcc
aagaaattgagtacttaaataagttgaaaggaaagagcaatatcatacagctcattgattatgaggtcactgataaaagcttacttcaagaaggttccat
gtcacccagggacggaataatcaaggatgatcactacatttacatggtcctggagtatggagagattgacttagctcatatggttgcccagaagtggaa
ggaaaggaacaactcaaatatgaaaatagatgaaaattggctacgcttttattggcagcaaatgcttgaagctgtcaatacaatacatgaggagcgaa
tagtgcactctgatctgaagcctgccaactttatgctagtgaggggctcgcttaaacttattgactttggcattgccaaagctataatgaatgatacaaca
aacatccaacgtgattctcagtgggcatgctaa
>g55907.t1
atggccggcggcaataatatggcctcatcacgtcgtctcttcatcgtctacgtcatcatgtcactgatctgccgcacagcggttgctgaggacgacaccat
cctgcggccgcatgggccatcttgctcaaggacgaacaactacacccgcagcagccagtacaagaagaacctggacgagctcctcgccgcgctcccc
gtggaagccggcgccaacggctggttctacaagggcagcgccggtgcgggctctgaccaggtgtttggcctcatgatgtgcttcgccgacagcagcgg
atcgctgtgccaggactgcctgacacgcgcttccgctgggatcgcgtcgtcggtgtgcccgggaagccggaacgtgagcgcgttgtacgacgcgtgcgt
gctccggtacgcggacgcctccttcttctccgtggccgacaccgacgtggtgtggaacgtgtctgttaccggtggctcttccttgatctacagcagcgtcg
acatgctggcgtggtggggcctgatgaatcggctcgcccatagggcggccgactcgccgctgctcctggccgacgggagcacgccgttcaacagctcg
gcggaggatatgtacggcctgtcgcagtgcacgagggacctgacgccggggcagtgcgagtcgtgcctgaacaggtacgcggacttgctgccgctgc
cggacaggttcccaaaaacctccggtggcgcgatcaaggggtacagctgctatgtcaggtacaatattggggctttcgacatcactcttccgccgcgga
aatcacctccagcattagcattattggtgatcggcctgtttgtcggctccgtctcaatattggtcagtctgggcttctcgacatggcttcgtctccgacggca
gcgaaaacgggctgctaataaactgcttcaggaatcgaggcagcaagagctggaagagggaaacttattcaacgacgaaccggagatggaagacga
attcgagaaagggaccgggccaaagcggttccgctacggcgagctcgccattgccacggacaacttctccgacaagcacaagcttggggaaggcggg
ttcgggtccgtgtaccgtggcttcctgacggagatgtccctcgaggtggcgatcaagcgtgtgtccaagagctcgaagcaggggagaaaggagtacgc
ctcagaggtgcggatcatcagccggctgcgccatcgcaacctggtccagctcatcggatggtgccacggcggtggcgagctgctcctcgtctatgagct
catgcccaacggcaaccttgacaagcacctccacaacagcaccaggagcggcgacgacgcgttgccgatgccatggccacgcaggcacgagatcatt
ctagggatcgcctcggcaatcctgtacctgcaccaggagtgggagcagtgtgtggtgcacagggacatcaagcccagcaacgtgatgctggatgcaac
gttcaccgccaagctcggcgacttcggcttggcacgcctcatcgacgacggccggcaatcccacactacaggcgtcgccggcacgttcgggtacatgg
acccggagtgcatggccaccggcagggccagcgtggagtccgacgtgtacagctttggcgttcttctcctcgagatcgcgtgcggccggcgcccggcg
gttcgcgtaggtgaggatgacttcatccatctggtgcagtgggtgtgggacgtgtacagcggcaggagcgtgctcgatgcggcggacatgcggctcgg
cggcgagttagatggccgggaggtggcgtgcacgatgctcgtggggctatggtgtgcgcacccggaccccggactccggccgacaatcaggcaggcc
gtcaacgtgctccggttcgaggcgccgccaccgagcctgcccgcgaagatgccggttgcaacctacgggccgccggctagctgttctagcaacgctgc
atcattgacgacagaaggaacagcaggtggcggcggcaccggcacttggcgttccaccgcgacggagatgtcgtcgttgtccagtccggtgaaagaa
ctgtgtatacttcgactttag
>g55908.t1
atggccggcggcaataatatggcctcatcacgtcgtctcttcatcgtctacgtcatcatgtcactgatctgccgcacagcggttgctgaggacgacaccat
cctgcggccgcatgggccatcttgctcaaggacgaacaactacacccgcagcagccagtacaagaagaacctggacgagctcctcgccgcgctcccc
gtggaagccggcgccaacggctggttctacaagggcagcgccggtgcgggctctgaccaggtgtttggcctcatgatgtgcttcgccgacagcagcgg
atcgctgtgccaggactgcctgacacgcgcttccgctgggatcgcgtcgtcggtgtgcccgggaagccggaacgtgagcgcgttgtacgacgcgtgcgt
gctccggtacgcggacgcctccttcttctccgtggccgacaccgacgtggtgtggaacgtgtctgttaccggtggctcttccttgatctacagcagcgtcg
acatgctggcgtggtggggcctgatgaatcggctcgcccatagggcggccgactcgccgctgctcctggccgacgggagcacgccgttcaacagctcg
gcggaggatatgtacggcctgtcgcagtgcacgagggacctgacgccggggcagtgcgagtcgtgcctgaacaggtacgcggacttgctgccgctgc
cggacaggttcccaaaaacctccggtggcgcgatcaaggggtacagctgctatgtcaggtacaatattggggctttcgacatcactcttccgccgcgga

aatcacctccagcattagcattattggtgatcggcctgtttgtcggctccgtctcaatattggtcagtctgggcttctcgacatggcttcgtctccgacggca
gcgaaaacgggctgctaataaactgcttcaggaatcgaggcagcaagagctggaagagggaaacttattcaacgacgaaccggagatggaagacga
attcgagaaagggaccgggccaaagcggttccgctacggcgagctcgccattgccacggacaacttctccgacaagcacaagcttggggaaggcggg
ttcgggtccgtgtaccgtggcttcctgacggagatgtccctcgaggtggcgatcaagcgtgtgtccaagagctcgaagcaggggagaaaggagtacgc
ctcagaggtgcggatcatcagccggctgcgccatcgcaacctggtccagctcatcggatggtgccacggcggtggcgagctgctcctcgtctatgagct
catgcccaacggcaaccttgacaagcacctccacaacagcaccaggagcggcgacgacgcgttgccgatgccatggccacgcaggcacgagatcatt
ctagggatcgcctcggcaatcctgtacctgcaccaggagtgggagcagtgtgtggtgcacagggacatcaagcccagcaacgtgatgctggatgcaac
gttcaccgccaagctcggcgacttcggcttggcacgcctcatcgacgaaggccggcancaaaaattatactcacatatgtgtatgatttttgcatacaca
ccaatttgcatcataaaaatcttcaaggttgttaaaataaaaatcaattaa
>g55942.t1
atggggtcgtctggttgtagctccgagatagtcgagtccaccgaagaagaatttgacagtgcctcatttggttgctctggcttgcggcaaattaaagtga
aacctgaggagaaaaacggcaagtgtcgtgattaccctgtcgaagatgacctggaccagcttcttaaagcgatcgacagcaggaccttccgccgggca
cttagtcctgccaatactggtggagatgcgctaggaaggagttctctcaagaagccatctagatcaggcttgtctcacaattcaggaatcgggatgtcct
caaagactgttaatatgaagcaagccctgaggaggctgtgcatttcccaggcatcagaaatggctgcaatgaagaggctgtcaatgtcacctggatctt
caagttcatctgaagctggaaccatccacaggctttatgcctctttgatgatccaaagtaatgatgaaaattatcttcatgatgatgaaaagatgaatctt
attgaaatatcactcaccccagggaagattgataagtgttcatctagaggaacaagtgagttcagtgaagactgtgattttgagacagctgacggaagt
gcagtcacttccatccgttctgtatcttccacatcagctgaggttatggatattgaatacctgatcaatagaaagaagatgctgcgagcacaggctgaaa
gggaaatactagaaatgctagatcatccgtttcttcctactctttatgcccattttaccacagacaatctctcatgcttgatcatggagtactgtcctggtgg
tgatttgcatgttcttcgtcaaagacaacctggaagaagctttccagagccagcagcaaggttttatgttgcggaagttctccttgctctggaatatcttca
catgctgggggtcatataccgcgatctaaagccagagaacattctagtccgtgaagatggacatataatgctgtcagactttgacctctcactgaggtgc
tctgttaaccctgtgctccttcggtcatcctctatagctgcaaatcaccaaccaaagaaacttaccggaccatgtgcagagaactactgcattaattcgtc
atgcctccagccctcttgtactcaaacatcttgcttcacaccacgcctaccatctgttccaaagcctcggaagcctaaatcctcccagaaaaggctccctc
aacttgttgtagagcctattgaagcacgttctaattcttttgttggcacacatgaataccttgcaccagaaatcattaaaggggatggccatggaagtgcc
gtggattggtggacctttggtgtcttcctttacgaacttttgtatggcaagacccctttcagaggacctggcaatgatgaaaccttagcaaatgtggtgtcc
cagaatctccggttcccagacaatccatctgtgagcagcaacgccaaagacctcatcagaagtttacttgtaaaagagccagagaaccgactgggatc
tttaagaggggcagctgagatcaagcagcaccctttcttcgatggcttgaattgggccctgataagatctgctgctccaccggagactcagccttgcaat
gttgtgactttggcaaaggcgcagaagaaagaaggcaaatgtcttgagttcagaagtaactccgaggacctggaattcgaggttttctag
>g55973.t1
atggccaccgtgtacgtcgcgacggacctgaggctcgagcggcgcgtcgcgctgaaggtgatgcacggccacctctccgacgacaccgtcttccagaa
ccgcttcatccaggaagcgcggtcggccgcgcggctcgccgacccccacgtcgtcaacgtcttcgatcaggggcaggacggcgagatggcgtatctcg
tcatggagtacctccccggcatcacactgcgcgagctgctgcgcgaacagaagcgcctgacgctcccgcagacgatcacgatcatggatgcgatcctg
tccggtctctcggcggcgcaccgcgccgggatcgtgcaccgcgacgtgaagcccgagaacgtcctgctggccgaggacggccgcatcaagatcggcg
acttcggcctcgctcgcgcgacgagcgcgaacaccgccaccgggcagatgctgctcgggacgatcgcctacctggcgccggagttggtgacgcgggg
cacggcggacgcccgcagcgacatctacgcgctcggcatcatgctgtacgagatgctcgcgggcgaacagccctacaagggcgaacagccgatgcag
atcgcctaccagcacgcgacccactcggttccgcgcccgagcgtgaagaaccccgcggtgccggagcagctcgacgagctccgcctccgcgagatcg
agaagcagctcggcgtcttcccgcaggtggtgcgctccacccccaccggcacgaaggtcgtcgtggacgactcgctcgactccgacgaggtgacccgt
gtcatgccgggcaccttcacggcgccgagcgtcacgacggaagtcgacaacgccacgcgcctgcgcccggcccccgggtcgctcgtgcgcgtaccggc
cgtctccgggatga
>g56334.t1
atgaaagtgaaagacttacataatcaagatgatggtgcatctagcactggagcgattgctggcggtgctgctgctggtgctgcattgttgtttgcagttcc
tgctatcggatttgcattgtggcgccgtcgtaaacctgaagaacatttctttgatgttcccgccgaggaggacccagaagttcatcttggacaacttaaga
ggttttcattgagggagcttcaagttgctactgataattttagcaataagaacgttctaggaagaggtggttttggaaaggtgtacaaagggagattgac
ggatggttcattagtagcagttaagagactaaaagaggaacgcacacctggtggagagctccagttccagacagaagttgaactgattagcatggcag
tgcataagaatctgcttcgactccgtggattctgtatgactcctacagaacggttgctagtctatccatacatggccaatgggagtgttgcatcacgtctga
gagaacgccagccagatgatccgccacttgactgggaaacaagggctaggattgcattgggatctgccaggggactctcctacttgcatgaccactgtg
atcctaagatcattcaccgcgacgtaaaagctgccaacattcttctggatgaagactacgaagctgttgtaggcgactttggcctcgccagacttatgga
ttacaaggacacccatgtaacaactgctgttcggggaacgattggacatattgctccagagtacctctccacagggaagtcgtcggagaagaccgatgt
tttggctacggaatcatgcttctggagctcattactggacagagggcatttgatcttgctcgacttgcgaacgatgatgacgtcatgctgcttgactgggt
gaaggggctgctgaaggagaagaagctggagccgctggtggacccggacctgcagggcgggtacgtggaccacgaggtggagtcgctgatccaggt
ggcgctgctgtgcacgctgggctccccgacggaccggcccaagatgtcggaggtggtgcggatgctggagggcgacggcctggcggagcggtggga
ggagtggcagaaggtggaggtggtccggcaggaggccgacctcgccccgcaccacaaccagtggctcgtcgactccacctacaacctcaggcccgac
gagctgtccggcccgaggtga
>g56651.t1
atgacccccctatatttctctctatctttgattctgctcatcaaccacgctattcctgctatcgcagctcatgtcaacaagattggcatccctttagggtccca
aattgatgcgggtggtgtgcaaagttgggtatcaccttcaggccgctttgcttttggcttctatcccaatgctgaaggcttctccatcggagtttggattgtc
atcggtgcatccagaattgttgtctggacggccaaccgggatgatcccccggtctctggtggctcgattcagttaacctatggtggttcacttcaatggatt
ccagcgaacgcgggaagccagtggaagtccatatctgccactgctactcaggctacttctgctgcgatgttggacactggcaactttgttttgtatggcat
gaataagcagatcatatggtccacatttggttctccaacggacaccttattaccaggccagaatctacctcctggtagccagctctcctcaagtgtatctg
atactaaccatgccacaggcaagtatcgccttctcaaccagcaagatggcaaccttgtgatgtatcctgttggcgcaatcgaccctggcagctcatattg

gaatacaggcacttttggcagtttctacctcttcacactctcattggatcccaatgtatccatgctactctacaagcatcggctgcaattgaggtacatgag
ggccccacaacagcaagtctcagaatttgatgatgagagcatggtcattcggtcctattctttccatgatctggagttgttcacagaggggtttgctgaag
agctaggaagaggagcatatggtacggtgttcaaaggtgttttggcgaacagtaacatggacattgcagtgaagagacttgagaggatggcagaaaa
tgactttggcctctccaagctgctgattggggacgagacaaagactttcactggcatccgaggcacgcgtggatatcttgcaccagaatggagcaagaa
catggcaatcactgtgaaggcagacgtttacagctatggcatcatgcttcttgagacgataagctgcaaaaagagcatggagctgaagctagctggtga
ggaatgcaacatttcagagtgggcttatgagtatgtcatatccaatgacttaaagaaagtggcagctggagactctgtcaacgaagttgagctggagag
gatggagatggtgtggaagagctggggcggcagcctcgtttccgtcctcctcatggcatcgtcgtctctactctgctccggcgccgtcatcttttctgagg
ccccgcggcgagtctccgggtcgtcgtccgccttcttcgccttccgggttgcgcagagtggcggtatgtcttgcttgggttgcgccctcacctgcaaggta
ggcatctga
>g57087.t1
atgagttccaaggtcagaagaattgagaggcagcaaaggccatgtggcacccgacagcacagagcaaacaccgagagggggagcgggtccgaggc
gccaatccatccatccatccatccattcgatttgatagacggcggcggcggcggcggcggcgccatgaggctgctcgtccccgacggcgcgcggcgcc
gggggctgccgtgcgaggaggaggccacaacggcggcgtcccctgctccggcggggcgcgggaggacggtggtggtggggatcaggcgagacgcc
gccagccgggagctcctcacctgggcgctcgtcaaggtcgccaatgccggcgaccgcgtcgtcgcgctccacgtcgccgccgccgccgacggattgtt
aatgggagtggatgagcggagcagggccgccgacgcgctctcgtcggtgctcgccgtctacgacggcttctgcaacctcaagcagattaatttggagct
caaggtctgtggaggatcgtctattcggaaaactctgatcaaggaagcagcttcctacggcgccgcccatctgatccttggtgtcgccaagaattcccgg
tcattcggatcctcttctacttcagttgccaagtactgtgccaagagagttccgacaggatgctcggtccttgctgtgaacaacggaaaggttgtttacca
cagagatgcaggaaatacgacgcaccacgaactttaccaaaccactatcacaatgccagagactccaaggaggagctaccggaagctcttgtcatcc
atgataggagagaagctttgggatgaatacggaaagggctgcaggtcgccatctcgtgcaatcaccatgccaacaaggtctcccacgccgccaaaag
aggtttcattagcattggttccagcgaatggttacagacgagaatcgcctgaagcagcaacaggctggcccctcctaaggaagaaattcttgccagacc
ggaaggcttccctgcctgacaagtcgaagatgtccgttgtgcagtgggcaatgcggcttcctagccgctactcaacagtttcgccagtccattctgagta
cagaaccagaacattgaattccgctgcaaataccagtcatattctccgcgaccgggtcactggccctttacgaaacaattcaggaaggtcttctgtggtg
atcgaagaactggagaaagaaacaccagaagagcttaccttactcaaagagaaattcacatcactttattcttccttcagttacagtgagcttgcaaag
atcacttctgatttctcacctgagtgtatagtcggacagggtggcactagccaagtttacagagggtatttggcagatggcggggaactggcagttaaga
tccttaagtattctgatgaggtactgaaggagtttgtctcagaaattgagattgtcagctctctcaaccacaagaacgtcatatccctcattggtttctgctt
caaaaacgatgatcttctgttagtgtatgagtacttgcaaaaaggcagcctcgaagaaatgctgcacggtaaaaaaggaggccagagcatatttggttg
ggccgagaggttcaatgtggctgtgggagttgctcatgctctagattatcttcacggtaaggacaatagtcgtccagtgattcataggatgtcaagtcat
caaacatcctcatctcagagtgctttgagccaaagttatcagactttggccttgcagtctgggctgctgatgtgacttcccagatgacatgcaatgacgtt
gcaggaacttttggatacctagctcctgaatacttcatgcatggcaaggtgaacaacaaaattgatgtatatgcttttggtgtagtccttcttgaactcattt
caggcagaaaaccactttgcactggttgtccaaaagggcaagagagcctcgtgatgtgggcaaattccatcatacaaggaggaaagctcacacaactt
gtagatcctaacttacctactgaaggccatactgctgaggtcgagaagatgaccctagctgcttccttgtgcatcagacaggcacctcaaaaccgtcctc
aaattgatgctgttctgaagttacttgaaggtgacaacgcagtcctcaagtgggctagatcagaagccggactggcatgcgacagcgatggtgatgaat
gtgtgatgacaccgccagcaccagggagtaacacaaacatccaatcttacatcaatctcgcatttgatgttgaagatgatgctgcctccgtcagcagca
acgatttcatcacagcaaacacatcccttgaagagtatctccggggacgatggagccggtcatctagcttcgattga
>g57173.t1
atgctgatgggcaagtacgagatggggcggacgctgggggaaggccacttcggcaaggtgaagctggcgcggcactccgagaccggccaccccttc
gccatcaagatcctcgaccgacaccgcatcctcgccatgaagatcgacgagcagataaagcgggagatcgccacgctcaagctgctcaagcacccca
acgtcgtccgcctcttcgaggtttcagcaagcaagactaacatatacatagtcctcgagtatgtaaatggtggggaactgtttgacaaaatcgcattgaa
aggtaaactgtcagagaaggcaggcagaaaattgttccagcagctaatggatgctgtaagctattgccacgagaagggagtttatcatagggacctca
aggttcttctcaaccgaggttacgatggctccatgtctgacatatggtcctgtggtgttattttatacgttatgcttacagggaaccttccgtttgatgatga
aaatgtggttgccctctatcaaaaggtatga
>g57224.t1
atgttcaattccaggatcacatatgctgatacagttgaggcgacaaggcagtttgatgaggagaatgtgctcagccgaggtcgccatggcctcgtgttca
aggcttgttactctgacggcaccgtgttggctatcctgcggcttccttccacctcgtctgacggtgcagtggtgattgatgaaggctcattcaggaaagaa
gctgagtctctgggcaaggtgaagcacaggaacctcactgtcctccgtggctactacgccgggccgccaccagatgttcgtctgctggtctatgactaca
tgcctaacggcaatcttgccacactgctccaggaagcatcccaccaagacggccacatcctcaactggccaatgaggcacctcatcgccctcggtgtct
cccgcggccttgcatttctgcaccagtccggtgttgtgcacggcgacgtcaagccgcagaacatcctctttgatgccgacttcgagccgcacctgtctgac
tttggctacaagggaaggagacgtatacagcttcggcatcgtgttgctggagctgcttaccggccggcgccccggcatgtttgctggggaggaggaag
acatcgtgaagtgggtgaaacggcagctgcagcgtggcgcagtggctga
>g57258.t1
atgatgggtcgccgcctcttggccgtggccgccgccaccatcgtcgccgtcgcgctgctcgcgccgcacgccgccggccacccgtggccgatgtgcggc
acgacaaccaacttcgcgcccaacagccctttccagaccaatctgaaccgcatcgccgacacgctacccaacgccacggcctcgtcccgggacctcta
cgccaccgcgagagccggcaccgtcccggagcaagtctggggcatggcgctctgccgcagcgacatcaacgccacggagtgccgcacctgcatcacc
caggcgttcatcgacgtgcagacctcgtgctcctttctacaaagacgccacaatctactacgacccctgcatgctccactactccgacgtgcacatcctctc
cgacgacgacaccggcccgaccaccgacttctacaccatcaccaacaacgggaacatcacctccgacccggaacggttcaaccaaatccttacggccc
tcgtgaacgccaccgccgactacgccgcgttcaactccaccaaatggttcgccaccggggaggccgacttcaaccaggagttccccaaggtgtacacg
gtggcgcagtgcccgcccgaccagccgccggagcggtgccggaagtgcctcgccgggattatcagggacaccctgacggtgttcctgggccatatcgg
aggcagggttgggtcaactgcacctaccgattcgagacctcgtccttctacaatggaccgccgattgtgcggctggaagaaggtcttcattgctctcatta
ttgcatccatcacggttttctgtgttatgctggcacatgatgcgctcaagcagtggaggaccgaggaggacggctcggctttcacactgtacgacttctct

gaactggctgacgcgacggatggcttctccatgaagaaccggcttggcacaggtggcttcggccccggcaaccttggcgatggggttgagatcgcggt
gaagcggctggcggcacactccgtgcaggggctcgtggagttcaagaacgagatccagctggttgccaaattgcagcatgaaaatttggtccgcctgg
ttggctgctgcgtcgaggaggaggagaagatgctggtatatgagtacatgcccaacggaagcctcgatttcttcatctttggactcctctacttgcataag
cactcccgggtgcgcatcatccaccgggacctcaaggcaagcaacatcttgttggataaaggcctcaatcccaagatctcggacttcggcatggcaagg
atcttcaacaacaatatcacagaagctaacactaacagggtcgtcggcacatacggctacatggcccctgagtatgcatcggagggcatcttctctgtca
gatccgacgtgttcagctttggcgtgttgttgcttgagattgttagcggaaagaggaacactgactatcaccattttggaggtttcgtcaacctcatcggat
atgcatggcagttgtggagtgaagggagagcggttgagctgatcgacctggcgctgggcgactgcggtgacacagcgtgtatcatgcgctgcatcaat
gtggcgcttctatgcgtgcaggaaaatgccatggaccggccgacgatgacggaggtcgcggtgatgctgtgtagccacgggacagccctgaaggacc
caaggcaaccaccgcacttccatctgaggttcaacgacaacagtgatggagaggacggggcggagacagagaaacaaccgcgcttcattgtgtcatt
cagcagccacgacatgactatcactacctcgactaccacagggatggaagaaggtagatag
>g57288.t1
atgcatagcccacgagaaggtgtgactactagtcgcatcatcctcctgtgctgcctagttccaataatcaccccggcatgctgcagtggtgtctccggcta
cgatcggaggagaggcgacgtagttagcttctccttcccgtcgttcgcgcatgtggacaagggcgacagaccgttcgatctatcctgggatgccggcat
cgtggatggtgcccttcacctcaccgtcgacgatgccaaccagccactggttaggtaccctccggacccgcggaggcagctcggacgcatcatcctccg
tccaagctttctgctgtcggttgcatcgtcctcctcccgcaacccgtcacaggatgcatccttcaacacctccttcaccatgagtgtcagccgtnnnnnn
nnnnnnttcgatctatcctgggatgccggcatcgtggatggtgcccttcacctcaccgtcgacgatgccaaccagccactggttaggtaccctccggac
ccgcggaggcagctcggacgcatcatcctccgtccaagctttctgctgtcggttgcatcgtcctcctcccgcaacccgtcacaagatgcatccttcaacac
ctccttcaccatgactgatgcatccttcaacacctccttcaccatgagtgtcagccgtcgcctgtcgagcaaccaagacatggccgcaaccaagaccaag
agcaagagcgatggcgatggcggcggcagcggacttctattccagttactccctgacatcatggctgagttctccagagtaagatcctacagctataga
tacaacggctactacagatcctcagatagcttgtccagcgacaacacctatagttccctggctagcttgaccaccagtggaaacatctccgtcgacatgg
gtagtacacgcacaacttatgaaggttacaatggtctgtatgtcctcttcacgccagccacaccggcgaactacaccgtgtggattgattacgacggcac
gagccagataatatgggtgtacgtgggcgagggcagcaagcccaaacctgctcaagcaacactaggcgccctcaacatcagcggcatcttcagatgg
ccggactatggttcctacttcggtttaatggcctcgaatgacggcagcaacagcttgccctcttgccagccggtcatctatagctggaacctgacggtgg
acaagctctacaaacccggacgtttccacatggcaccatggttcctggccattgtgctgtgttcgggtcttgtagcaacaattgcggccatccttgtgttgt
ttcggaaaattatcgcacgcgccttggcgtccagataccgggctctcaggatgaagctgaggctgtccagggccctgcgtcggcttccggggataccca
gggagttcaagtatgctgacgtcaaaaaggccaccaagaacttccaccaaagtttgttgctcgggaaaggaggcttcggtgccgtgtacaaagggaca
atacaaattaccaccaccgatggtgatggagaagaagacgacggacaacgacaccggagacccgccgacgtcgccgtgaagaagttcacgcgcaag
gacaaccgcggctaccaagacttcctggctgaggtcgccgtcatcaacaggctgcgacataaaaatattgttccactcaccggttggtgctacgagtat
ggaaagctgctacttctatatcagtacatgcccaacggcagcctggatcaacatctcttccacaaggaccgtcaccagcactttcctacgctccagtggg
agaagcgctacagtgtcatcagagacgtggccgccggactgcattatgcccaccacgagtacgagcgtgtggtactccatcgtgacatcaaggcaagt
aacatcttgctcgacgacgccttccgaggccggctcggagactttggcctagcccgtgtggtgggcttcaacaagaactccatcaccgacatgggcatc
gcgggcacctggggcttcatcgcgccggagtacgccgtcagccacagagccacacgccagaccgacgtctacgcctttggggtgcnnnnnnnnnn
nnnnnnnnnnnnnnnnnnnnnnnnnnnnnnnnnnnnnnnnnnnnnnnnnnnnnnnnnnnnnnnnnnnnnnnnnnnnnnnnnnnnnnnnnnnnnn
nngcaaagttggtgctacgagtctggaaagctgctacttctatatcagtacatgcccaacggcagcctggatcaacatctcttccacaaggaccgtcac
cagcactttcctacgctccagtgggagaagcgctacagtgtcatcagagacgtggccgccggactgcattatgcccaccacgagtacgagcgtgtggta
ctccatcgtgacatcaaggcaagtaacatcttgctcgacgacgccttccgaggccggctcggtgactttggcctagcccgtgtggtgggcttcaacaaga
actccatcaccgacatgggcatcgcaggcacctggggcttcattgcaccagagtacgccgtcagccacagagccacacgccagaccgacgtctacgcc
tttggggtgcttgtcctggagatcgtcaccggcaggaggtcactgagcgcggcggacgctcccttcccgctgctcacggactgggtatggtggcttcacc
gggagggtaggctgctggaagccgtggacgatgtgctcatcgccgacgcgttcgatgccgacgaggttactcggatgcttctgcttggtttggcctgcag
taatccaaaggcgtcggaccgacctagtatggctgaggcggtgcaagtcgttgccaagtcgatgccgccaccgccagttccgatggcgaagccaacct
tcttgtggccgcctgaaggagagcttcagctactgatggaggatgccgacgatgacctcgtcgggagcatgggctatgatgattctgagtggagccaca
gggaggaggcacggacccacggccgtggttttgtgattagtatcgggtcgttggagatcaccgtgggcaggtctggtaaggcacgaaccctgtga
>g57290.t1
atgccgccgcccacgccggcggacaacctcgccgtgcaggaggagaccagcgtgttcgtggtgatcggcatcatcctgcttgtgatcatgctggtcacc
agcgccatggtgctcatgctgcggcaggacgagatgaacagcacccgggcgccggcggcgtgggactaccctgccggcgtcggcgctggaggaagg
tctcccgtggtgtcgccgacgaggcgtgccgccgagatggtggccgtggacgtggccggcgggtcaagccacggaggaggagggtcgcagagcggc
agccggcggatgggcgagttcgtgctcatgcgcgacgacatccccccgttcgggttgccggagctgatgaaggcgtccgccgaggtgctcgggaacgg
cacgctcgggtcggcgtacaaggccgccatgcgcaacggcgtgaccgtggccgtgaagcgcatgcgcgacatgaaccgcgtggggcgcgtcgagttc
gagcagcacattctcatgctcagcgagctgcgccaccccaatgtgctccccccctgtcggatatcactatcgcaaggaggagaagctcatcgtctccgag
tacatggcacgcggcagcctcctctacgtcctccacggggaccagagtccgaacaggttggtcttggactggccagctaggttgaaggtggccgtgggc
gtggcgcgcggcatggcgttcctccacgagaagctcggcatcgcggcggggaggttggtgagcatggacggcgccgacttcgacgcgccgccgccgc
ctccgccgcacgggaacctcaagtcggggaacatcctgcttgactccaacctggagccgcggctggtggactacggcttcttcccgctggtgaacgccg
cgcaggcgccgcaggccatgttcgcgttccggtcacccgagggcgccacgcgcggcgtcgtgtcggcgcgctccgacgtgtactgcttcggcgtcgtgc
tgctcgagctcgtcacggggaggttcccgtcgcagtacctcaccaccgcgcgcggcggcaccgacgtcgtgcactggtcggcggcggcggtgtccgag
ggcaacgagcgggaccttatcgaccctgccgccgccgccggaggaggtgacggcgctgtaagactgctacgggtgggtgttcagtgcgccagcgccg
agttggagtgccggccaagtatgttggaggcggcgaggatggtggaggagatcgccgccggtggtgcgtcgtga
>g57551.t1
atggcgatcgccactgagtcgtgcggcggcagcgaggacaaggctgcatcacatgcaaatgaggagaagcgttgggtgctgccagattttgaggttgg
aaagccacttggcaggggaaaatttggccatgtttacttggcaagagaaaaaaggattgtggctttgaaagttcttttcaagagccaactgaaacaatct

caagtcgagcatcagcttcggcgtgaagttgaaatccagagtcacctaaggcaccccaatattctacgcctgtatggttacttctatgaccagactcgtgt
ttatttgatcctggaatatgctgccaaaggagagctgtacaaggagctgacaagatgcaaacatttcaatgagagacgttcagctacttacatcgcatca
ctggcaagagcactcatttaccttcatgggaagcatgtcatccatagagacattaaaccagagaatcttctaattggagctcagggcgagctgaaaatt
gcagactttggctggtctgttcatacattcaatcgaagacgaacaatgtgcggaacgctagattacctgccacctgaaatggttgagaagactgagcat
gattaccatgttgacatctggagcttgggaatactctgttatgagttcctctatggagccccgccttttgaagcgaaagaacattccgagacatatcgaag
gatagtgaacgtggacctgaagttcccattgaaaccgtatgtttctgctgccgcgaaggacctaatttcacaggatagtgaacgtggacctgaagttccc
attgaaaccgtatgtttctgctgccgcgaaggacctaatttcacagatgcttgtgaaaaactctgcgaaccggcttcctctgtataa
>g57679.t1
atggtgcgaccggacaagaagagggtggtggcgcgcattgcagcaggcggatcagaggagcgctcaacggcgcggctggacgtgaggataacggc
ggtgcgcggagcatcagcagggccaagacaagaggaccagacgagtatcgtgtattcgtgtggctgggcggtggaattccaggatgcgttgaggga
gcagatggtgcgaccggacaagaagagggtggtggcgcgcattgcagcaggcggatcagaggagcgctcaacggcgcggctggacgtgaggataa
cggcggtgcgcggagcatcagcagggccaagacaagaggaccagacgagtatcgtgtattcgtgtggctgggcggtggaattccaggatgcaaacg
gattaaccatttggatctcaaaccagcaaacgtgctgatgggtggtgctcagatggagccaatgattacagattttggtatatcaaggcacaatgatgga
actcagtctacaatagtaactaagaacgccaaaggaacactgcggccggagaaactgccagtggagcgtccaaagtggaagctagcgttccggagca
caccaaagctccccatcattacggaccagaggatcgaggatgccaacggcaagccgctagagttgatcctcgtggacgcaggagacagcggcgatgc
atcatcggcggctgccgtcgtcgtcccaaaggcattggaaatcgagttggtcccggtgttaggtaacttcccacggggccactgctgggacgctgacga
ttttcagaagggcatcgtggcggatgtagaagggaagcccccgctcctggctggccagtgcagactcacaatgtgggacgggcgtgccaccgtgaacg
agctctcgttcaatcacgattttctcaagtgtggctgcctgctccgcatcggcgtgcgggttatcccgggctcctacaacggcgccagcaggatccttgag
gccatgactgaggccttcatggtccaaggccgcaacggtggtagtgacgacgacgaggtgcacaataataatagcaacaagcaccacaagtggcagc
tggtgttccagtgccagccgcggatcccgatccacgtcggcaggaacatcagggacgtcatcggcaacccaatggaggtcatccttgtggacaccaaa
actgggtcgccgtcgtcggtgctcccgaaggaggagctggacatcagagtggtggcgctcgatgccaagctccccatgcgcatggactggtcaggcga
cgaattccagagggccatcgtgaagcagggccaaggggaaggagaagggccgctcctctccggtgacgtcagcctcaccatgaagaagcacgacga
cggccgtgtcgccgtgaacgagctccggttcacggacagccacgtggcattgctcatcggggcgtgcgtcgtgccgggcggcggatataataacgacg
acaggatcctcgagggcatcaccatggccttcatggtccaagacagcggagagatgaacaggaagtactcccgtcctgaacttgaggatcaaataatg
ggagaaggcatggatgacgacacatgggaggagattaccgggcacgccaggaagtgcgccctccctggccacaaggtgtacgcctacagcgcggcg
cacgccaccatctacgtgacctccatcttcgacctcgtcaaggtcgaattcgacggcatggtcccgctgcagaagctccacgaggtccaaaagagactc
gtgcagcagttgatgcgagaggcgtacgagcatcgccacagcctccagaaggtggaactgacgtggaagctggcgttcaagagcccgctacggctcc
ccatccacaccggtagcaggatcacggatgccacgggcaacccgctggagatcatcctcgtggatgcgagaaccgggtcgccgttacagttcccgatg
gagctgctggagatcactgtggctccgcttttaggggacttcccccgtacgacggcaaagaagactggtccgccgaagaattcgaggcggccatcgt
gaggccgcgcacacgccatcttgatcttccgctcctcaaatccgacttggtcttcaaaaaggaggacgggcgtgtcaccgtggacgagctgcagttcac
ggacgactcctcctgggtccgctgccgcaagttccgcatcggcgcttatgtcgtcctgggcgggcgctacgatggcgaatttaggattgttgaggccatg
accgaggccttcgaggtccacgatctgaaccggaagcactaccctcccgcacgcagcgacccggtttggaggctggagatgatcgataaggacggag
cattccacagggagctgacgcgcaacaacatcgacactgtccaagagttccacagtatgctaatgctcaagcccaacgagctgcgcgcgattgtgggc
gacggcatgacggacggaatgtgggtgtggacaaccagccacgccatggagtgcgaccctggggacaaggtgtacgcgtacaacgggtcccacggc
accatctacgtggactccatcttccgtcgactcttgaagcagatcgtcattgacggtgtggaatgtcagctgccgtggcagccagacaaggcccagacg
atgctcgtgcggcagctacttctggaagcgtacgagcaccgccacaacctccacgaggtcgttgatgcggccatggaactctcctacgacaacactatc
gaacttgaatatctgctgtcccgtgcacaacgatacgacgccgacgataacgacgatggcggcaaccgcgctcagccactcatacaccacgacgagtc
ctcaaaccctaaactcattctctcttctcctctcttcccagccgccgcctctcctcccttctcttctttccctctacctaggagctcgacccctctccctagcgg
cgacctcgccctcaagctgtgtggtggcgacgagctctcgcantctttccctctacctaggagctcgacccctctccctagcggcgacctcgccctcaag
ctgtgtggtggcgacgagctctcgcacgcgggctgggcggcggcggcggcctcatgcatacgagctgcacggcgagcggcggtgacctcgtgcacgc
agcctgcgcagcgagcagtggcatcttcgtgtacgagggctgtgtga
>g57893.t1
atgaaggagttctcgtatgaggaggtggaggcggcgacgggcggcttcgcggagaagaacctagtgggcaagggcagccatggcagcgtgtacagg
gcgaagctgcgtggaggaggcggaagaggcaggacggtcgtcgtcgccgtgaagagggcgtcgcacgggcagggcgaggcgaagctcgccaacg
agatcgccgtgctctccgccgcgccgcgccacccgggcgtcgtcggcatcgtcggggccgtcgtcgcgtctccgccgccgacagccgcagaggagga
ggaggagaaggagaccgggcactcgaagaagacgacgggggtggtgggcccgctccgtcagccgctgctggtcatggagttcatgcccaacgggtc
cctccacgacctgctgcaccggtccccgcgcccgccgccgtggccgcgccgcctcgagatcgcgctcgacgtggcccgcgccgtgctggcgctgcacg
ccgccgagccacgtggcatcatccaccgcgacgtcaagtccgccaacatcctcctcggccgcgacggccgcgcgcgcctcgccgacttcagcctcgcc
gtcatcatggtcgctggccgaggagaccgccgcgcgcgtgacgacgacgacgatgacgtggacgcggtgggcccggcgccggcgggcacgatcggg
tacctggacccgtgctacacggagccggggcggctgggcccggagagcgacgtgttcagcttcggcgtggtgctgctggagctcgtgagcgggcgca
aggtgatggacgtggacgcctgcccgtcgtccatcgtctcgtgggccgggccgctgctcgccgcgggccgggccggggaggtgctcgacgcgcgggt
gccggccccgcccgccgcatcgcgggccgtcgtccgggtgctcgacgtcgcggcgcggtgcgtgtcggagcgcgtggagcggaggccgggcatggcg
gaggtggtggccgagctggacggcgccatcgacggcgccgggtggcaccgccgtcgcgggccgcgtggcgtgatggagagcgtgcgcaggcgcgtc
gtggcgtgggggcagcacgtgaggagcaggagggtcagggcggccaaggtcgagtgcgcagagcactccggcagcggcggcgcaccggaacgcct
gcccgtcgtctgcggcgtggatccgtgtgctgctgcactagcattgcattcaaacagcacaaatgatcgaacatcccaactgctcgatgccaaggaaag
gccaacgcaatga
>g58172.t1
atgcagcagcttggtggtccctcgagatatgcctacacggacatcattgcaatcacaggacatttcagagataggctcggccaaggaggatacggctct
gtatacaaaggcattctgttcccaggcaacatacatgttgccatcaagatgcttatcaactccaaatgcaacggagaagagttcatcagcgaggtctcca

caattggtaggatccatcatgtcaatgtggtgcgtctcataggattctgctcggaagaaacaaggagagcactagtatatgagtacatgcccaatggttc
tctagataggtacatcttctcttcagagaagagtttctcttgggacatgctcaacatgattgctttgggcattgcgaggggtatcaactacctgcatcaggg
gtgtgacatgcagattctacactttgatatcaagccacataatattcttcttgatcgcaactttgtcccaaaagttgctgattttggtcttgccaaactatac
ccgagggacaacagtttcgtgccattgagtgccatgcggggaacagtaggatacatagctccagagatggtgtcacggagctttggtgtcatttcaagc
aaatctgatgtgtacagttttggaatgctgctattggagatggctggagggagaagaaatgctgatccgaacgtaacaaactcaagtcagaattactac
ccatcttgggtgtatgatagactgattgagcaacaagtgggagagatatctgtcattaatatgcatgaattggaaaagaagttgtgcattatcgggctct
ggtgcatccagatgaaatctcatgatcggcctacaatgagtgaggtcatagaaatgctccagtctgacatcaatgtgctggagatgccttcgagaccgtt
cttttgtgacggccacgagcaggtttctgcgttggattcttattatttgtcctcccaactggatgatatagaggaagatgatgagtga
>g58173.t1
atgcttggtggtccattgagatacgcatacacagatatcattgcaatcacaagtcatttcagggagaaactcggccaaggaggatacggcagcgtatac
aagggtgtcatactcctaggcaatgcacatgttgccgtcaagatgctgagcaactccaaatgcaatggagaggagttcatcagcgaggtgtccacaatt
ggcagtatccaccatgtcaatgtggtgcgtctagtggggttttgctctgaagaaatgaggagggcgcttgtatatgagtatatgcctaatggttctctcga
caggtacatcttctcttcggagagaaatttctcatgggacaagctcaatgagattgcgttgggcattgcaagaggtatcaactacctgcatcagggatgt
gacatgcaaattctacactttgatatcaagccacacaacatacttcttgatcagaactttgttccaaaagttgctgatttcgggctcgccaaactgcttccg
agggacaatagtttcgttccactgagcgctgtgtggggaacgatcggatatatagctccagagatggtgtcacggagctttggcgccatttctagcaaat
ctgacgtgtatagctttgggatgctactgttggagatggctggagggagaaagaacgcagatccgaatgtagcaaactcaagtcagaattactacccgt
cgtgggtgtatgacacactaaagaagcaacaagtgggtgaggtttctgctattgttgatatgcatgtgctagagaagaagctgtgtattgttgggctctg
gtgcatccagatgaggtctcaggatcggccaacaatgagcgaggtcatagaaatgctcgagtccaacgtcaatggcctggaaatgccttcgaggccat
tcttttgtgacgaggcgctgcttgcggcgttgcagggcgtgttcacggcggacccgtcgtcgatgccgatcgactacgccgtccagtctgcgtcgcggac
gcgacggctgtga
>g58549.t1
atgcaccccgaggtggcgcacacggcgcagtcgcgactcaaagccgagacgggaacgagcagctacgctccccacctccacaattcgtgcctctcccc
catctccgccgccccaagcgcacggctcccgaggcgacgaatccggcgtcctcctcctccaccgccgctccggtccccgccgccgatcgccgatgttca
agttggtaattctccagaatacataacagagagaaaattgggtaaaggtggttttggtcaggtatatgttggtagaagggtatctggtggaactgcccgt
gttggtcctgatgcctatgaggtcgcattgaaattggaacatcggaacagtaaaggatgtaactatggcccccccatacgagtggcaagtttatcagatgt
ctcctcatatggccgcttgcattgctgtagaggctatatcaattcttgagaagcttcattctaaagggtttgtgcatggtgatgtgaagccggagaatttcc
tgcttggtcagccaggatcacctgacgataagaagctttaccttatagatcttggcttaggaactccttacacgcagcaatcttacaaggtcagtgaatca
tttccttacaagtggatcaataaaaagtggaaagaaggttttcatgtaacatcaatggccactgctggaaatcgctggggagtagtcatgtcaaggaact
caggctattctagtcaggttgtagagctggacttcctgtatccaagtgaaggcattcataggcgatgggagactggatacaggataacttccacagcag
ctaccaatgaccaagctgcttttatcttgagcataccaaaacgaaagccgatggacgagacccaagaaactcttcgtacgtctgcctttcccagcaacca
tgtcaagtggagaggcaaaagtgtcgagcctgccgctcgccgtcgttggctccgccccacgccgcctcgtcttgccccgccgctggccccatcgctcgcc
gccacgctcatccccgcgccgcctcgtcccgccccgccggcggccccgctggccacaccgctcgccgccgtgctcgtccccgcgcgccgcaagccacc
tccccgcccacgccaccgccaagctctgcccgtgccgtcgcgcgctgcctccgcgctcgcctcccgcgcgctgcctccgccctcgcttcccgcgcttcact
cccgcgcgcttcgccagctcctccagcggatgtaa
>g58551.t1
atggcgcactataaaagaaggacgaacgaacttttcagaaagaacggcggttcagtgcttcaagagctggaaaacctcaggattttcaccagagagg
agcttaagaaaatcacaaagaacaattctcatgtcctcggcaaaggaggttctggtcacgtgtacaaggggattcttgaggacggcacaatggtggca
gtgaagacggcgatcgaggtcgatgaagctcgaaaggaggatttcacaaacgaagtgatcatccagtcacagatgatccacaagaacatcatcaagc
ttttgggttgctgcttggaagtcgatgtgccaatgctggtctacgagtttgctcccaaagggaacctctacgacatcctccatggcaacaaggccaaccg
ctaccaacggcttcgtctccctctggacttgcgcctggacatcgcagccgaatctgcagagggattgaaatacatgcactcgtccacaaacaacaccatt
cgacacggtgatgtcaaggcagccaacatacttctcgacgaacatttcatgccaaagatctcagatttcgggacgtccatgctgctgtcgagaggcgag
gacttcaccgcgcttgtggtcgggagcatgggctacatcgacccggtgttcagccaaaccgggcttctaacgcagaagagcgacgtctacagcttcgga
gtcgtgctcctggagctgatctgcaggaagccaaccatatacaacgagcactgcagcctcgtcattgagttccgtagatctcacgagaagaacggtaat
ggaagggcgatgtttgacgaggacattgcagtgacggaagaagacgtgtttgtgctggaggaaatgggtaggctcgcgatagaatgcctggaagagg
aggttgaagaccggcctgacatgaaggaggtagcggaacggcttgtgatgcttaggagagccaggaaccatggacagtggaactacgggagccccg
ctcatcactgtcagaagaacaattttgcagcagccctcgggaccgatgttagcagaacgacaagcagcagcaccgtgccattgattccagcaatgaga
caagaattccctaacctttag
>g59145.t1
atgtatgcgaatgtttgggtccgtgcaggtcgttctggccagccgagcctgtcgtggtcgctgcggctgcggatcgccaagggcgcggcccgcgggctg
gcccacctccacgagtgcagcccgcggcggttcgtccacggcgaggtgaagccctccaacatcctgctcgacgccgactacaccgcgctgctcgccga
cttcggcctggcccgcctcctcgccgtcgccggctgcaccgacgtgtactccgtcgccgcgggcggcggcggcgtcatgggcggcgcgctcccgtacgc
ccgaccgcccggcgccgcgttcgaccggcccagcccgtaccgcgctccagaggcccgcgcgcccggggcccggcccagccagaagtccgacgtgtac
tccttcggcgtcgtgctgctggagatgcttacggggcgggccccggagctgcagggtccctccccgtccgcgtcatcgtcgtcgtcgacgttcccgctgg
ggcccaccggcgagctccagcagcagcaagagcaggcgccggagatcgtgaggtgggtgcgtcaggggttcgaggacgcgcggacgctggcggag
ctggccgacgccgccgtgctgcgggacgccggcgccaggaaggaggtggtggccgcgttccacgtggcgctcggctgcgtggaggcggacccggatc
gacggccgaggatgaaggccgtgtccgagagcctcgacaagatcggggcgtag
>g59182.t1
atggcgagagatctaagcgaaacgagaaaacgcggtaccttcaaggccagtgccgggactcggtgcacggccatcctccgcgcctccaccggtggcc
gcggcgtcgtccgcgtgcgggtccatggcgtcacggcggggaggggaagcttccggatgcggcgatcgggcggggaggtgggtggcgccgccggcg

actggccgttcgttggttggccggcggagacgacgacgagatcgaggcggcgccctattatcccgtgccggtggacacctgcacgcggcgcgagtgtc
ggcgtcgcagtcctgtggtaccccggtggtgccccaaaatcttcttctgcggaggcgaccaagaaacggggatttgctcttcttgatttcctttgtcctgct
ggaggtgctgagcctggtcatgagatgggcttcttggcgtgcttgttccggtgtcctcaggaggaagtggatgaggagaacggcgacgagaaggaag
gggagcagttcaggatgaatcatcaaatcgcctcagatcttcgacccgatcaagaacctctagattggaaaacacggatgaagatagctgctggtgca
gctgctggtttggaatacctgcatgataaggcaaaccctccagttatctaccgagacattaaaccgtcaaacattcttcttggtgaagggtatcatgcaaa
attgtctgactttggacttgctaagcttggccctctgggtgataagactcatgtgacaacccgtgtcatgggaacgtatggttattgtgctccagaatatgc
ctcaaccgggaagctaacaattaaatctgacatctatagttttggtgtcgtgttccttgagctcatcacagggcgaagggcacttgacagtgaccggcct
cctggggaacaggacttagttgcatggcttgaaaagaccatgggcgttatcctggggaagctacgacttcagaagaagaagacgcgacggcgcgccg
gggcctgcgcggagacagcgagcatcaagacgatgacggtgacgaggagcaccgcggtgaggatgtcgtcgttgactgacagaaggcagaacttcc
ccatgcgcgctttctcaacttctgcctggtga
>g59197.t1
atggtctacattacctccatgaggaatgccaaccacatggattttggaatatcaaaatttttctgtgatcagaaacttgaaactcgagccacgagtgctat
aggatcatttggttacatggcaccagaatatataagcggcaatatagtctcaaaaatggcggacatatacagtttggacgttataattatgcagattata
acagggcgcaaggtcgatataacatcttgtgaggacattgtggaacctctgatggagggcaacaatatgcgcgacaaggaaccacactacagtaaaa
aagaagcaacaattgaggtgattgttgaggaaagaatattaatgggagacactgaagatgatcggaatggttttctagatgaggaagaaatgccgcga
agccttattaatgcaaagctattaggcggagcaacagagaacgaggagcacggagagatgggtgacccggccacggcaggggaggagatggttgtg
agagtaagtggaatgattgtagcagcgctggatgcagtgagggcagcggcagggtttccagggcggtggaaggcgatcgtcactaccctggagaagc
tccgggcttgcctagttaacacatccagacaccttgggcagctcgaatcatatgacaacgccctctgccacgaggtgctgcagtcagtggcagatacgc
tcgtcgcaatgaccgacatcgcagggcagtgccgagcgcctatgaccgcgggcaagctgcattttcagagctccatcgatgcggtggcagtgaagctg
gacgtcaacctccacgactgcaagctgctcgtcaagatcggcgagctctatgataacgcttcatcatcgcatcccctggaggacactagtacttgtgtgg
agctgcaacgactgttggtgtgggtacggaggagccacacggaggcgaagaacctcgcactggacaggcttctcgaggcactgcacaaggacgaga
agagcgtcgtggcctccgtgctccaccatggcgacatctccgccatggtgcagctgcttgcgacgtcgtggcccatggtcgtccgagagaaggccgcca
cggtggtgagccacgtggtggggagctgtcgcctggacctcctcaggtcagagggcgccatcccgccgctgatcaggctggccgaatcgggcagcctc
gtgggccgccagaaggctgcggtcacgctgcaccacctgtccaccaggttgaacagcaccgtgtcggccatcgtgtcacacggcggcatccgcccgct
agtcgaaatgtgccgccatgagaggggtgattccgtgtcccagtttgctgcggccggtacgctcaagaacatctccgcggagccggatatccggcagtc
actggccgacaatggaaccatccgcgtcatggtggacctgctgggtcgcgatgatgttgtgctggaatccaaggagcacgccgtgcggtgcctggcga
acctcacggcacggtacaacgatgacaagctgcggcggtctgtcttgttggaaggcggcatgtgtggcctgcttctctacctcaacaacaatgaagact
ggaacaaggcggcggtgaacgcgatccgcaacctcgtcgatgtgatcaccaccagcagctcggcgggtgacacaacaacaatgaagcgcgtggcgg
gcgagcagggttgcgtgccgctactggtgcggatgatcctggagcacaactcagacagtgtgcgcgaggtggccgtgcagatgttggcctgccttgcg
acctaccctcccaatgtaagggagatgaacaaggacgacaagtgcgtggccggactggtgcagctgctggacccgagccgttacaacaccgccacca
acgagtacgccatccagtgcctcctctcgctcgcctccagcaccaagcgctgcaggaagctcatggtctcgcacggcgccaacggtcacctcaagatgc
tctccgacatgaacgtccaaggcgccaccgagctgctccaaaggctcaaacgcggcatgctgcaaagcctcttcagtagcagcaagcagtaa
>g59531.t1
atggtcgccgcggtgcatccggcctccgcgactccgccggcagcggcggaggagtcggacgtggagacggaggcgtcgtcgacgtgcgcgcccaact
cgagcctcagttcaggcagcagcaccagcagcctctcccgcctctccttcgactgctccgcgctctcctcctcctctcctcctccgccacctgcacctcctt
catcatcaccacggccccaccggtcctccgacccggcgtgggccgccatcctcgcggcctcaaaaacatccgccccgctcggcccaaaggacttcaag
ctgatccgccgcatcggcggcggcgacatcggcaccgtctacctctgccgcctcctcacccccaccaaccacctcttcgcgatgaaggtggtggaccgg
cgcctggccgccgcgaagcgcaagctccaccgcgccgcggcggaggcccgcgtcctgcaacgcctcgaccacccgttcctccccaccctctacgcctg
cttcgacgccgggccacggttcgcgtgcgccgtcaccgagttctgccccggcggggacctccacgcgctccgccaccgcgtgacggcgaggcccggac
gacgcgcgttctccgccgccgccgccaggttctacgccgccgaggtgctcctcgcgctcgagtacctccacatgctcgggatcgtgtaccgcgacctca
agccggagaacgtgctcgtcagggatgacgggcacgtcatgctcaccgacttcgacctcgcgctcgagtccacctcccacccgtcgtctcttctcctcct
caatgacgacgagccagacacggcgagcgccaactgcttctcgttcacgcggcgccgtcgccgcaaggccacttccgccccgggctcgagaacgccg
ccgccgtgcttcgtggtggaggcgcgttcgcggtcgttcgtgggcacgcacgagtacgtggcgccggaggtggccgccgggggacccacggcgccg
cggtggactggtgggcgctcggcgtgttcctctacgagctgctctacggccgcacccccttcgccggcgccgacaacgaggccacactccgcaacgtcg
tccgcgccccgctcgcgttcccgtcgtccacctgcggtggcccgctcgacgcggcggcgcgggacctcgtagcggcgttgctggtcaaggaccccgag
aagcggctgggggcgaggcgaggcgccgcggaggtgaaggcccacccgttcttcgacggggtgaatttcgcgctgatgagggaggcgacgccgccg
gtcgtgccgggcaatgcggcgaaggcgaaggtggacgcggcgaggaggtcgctgtcgtgcaaggccgtgacggcgaaggtggacggcgtgcggcg
gtcgcagtcgtgcaaggccagggacgccgggttcgacctcttcttctga
>g59734.t1
atggcggcgccgcatcggccccgcccgcggccgcggccgcagctcgcgcgcatcaacgccatgaggcactcctactccgccgaggaggacgacgag
gccccggcggccgacctcggcgccgacttcgcgtcgcagaccagcttccgcatccgcggcgggcgcggcgccgaggtcgccgacctcttccgcaagct
cggcctctccggccctgaagacttcaccatcccccggccgtctacgccgccgcgatggcgcacatccccaactcctcccgccgccgcggccagtcgct
ggagcttggcgcgggcgccgaggggtccgcaccgcaggagctaccaggaacttcaggtagtgacgtcacggtcggaacaaggctagaggcggccga
tgtggggaaggtagccgtgctggccaccgagccggcccaactccaactccaacccgaggtatctcacaggagagggggtctggaactgaatcgcgc
agcagagaagtcgaattggacaccgcggaagcttcgagaagaagcgtggccgccgaggtgaagcgggggacagcttacgaaggaaaggggaaga
gcgttgcggccaaggtggatcggttgcgagtggagaggacagaagcggtggtcgtggagacggcaagggagactaccggcgccgtagttcaggttgc
ggcggagtccacttctcgcagcgttgaacattactatttctcgccttccccgcacaggcggattaagagaaccatcacgtcatggatcaagggccagcat
ttggggagcggatcgtttgggtcagtgtacgaagccatcagcgatgatgggtttttctttgctgccaaggaggtatcgttggttgatcaggactcaatgc

aaaacaacgaattctgcagcttgagcatgagatctctctcttgagtcgtttggagcatgaaaatatagtccagtactttggaacagataaggctatctata
aggattttgatagctgtccagcatga
>g59736.t1
atggcgggatggcgggagatgcccagatccggggtgccatggatccgactcccctgtgacggattcgtggggatgcggcgtttggtgctcccgaccaa
gctggtggttgtgctcgacggcgttgtggctgagtctgagacagttgatgcggaggagttctggcaactggccgctcatgttcaacatgggggcactact
ccgagcgcgctgcagcagcgctgtgcagggagatcgtctgtcgtgcatacatgccattccatgggggtcatgcacagggacctcaagccagagaactt
cctcttcctcaacaaagcagaagactcgccgctcaaagcaacagactttggactatccgtattcttcaagccagctgcccgatcgagaaacatggagtc
ggtatacgaacttatcgacgcgcaggagacatcaggattcttcgatgttctcgccgagtaccggcggcgcccggttcctgaagggaaaaggttcttcgc
aatgaaagtgcggccgcgcgcaaggtctaataataagcaggcacgcacgcacaagtgctgcatcatggctcgcaaccggttcggcgtctggtcgcag
gtgctgcagcgctacgtgaggaagacgcggtacctcaggttcaccagggtcggctgcgaggtggcgcagctggacaggatctgcagcccgctgatca
agcagtacggcatgcacgcgtgggcggagtaccagccgtggcgagaggagatattggcgatggacgccgtggtgaagaagatctacttcgattatgc
gcccccgtggatccaacggcagattaggctacgagcgaggccgagttggaggcgagcgctggcaatgtgttga
>g59931.t1
atgagcgtgtccgtcgggagaacgcgggtggggaggtacgagctgggccggacgctcggggaaggcaccttcgccaaggtcaagttcgccaggaac
gtcgagaccggcgagaatgtcgccatcaagatcctcgacaaggagaaggtgctcaggcacaagatgatcggacagataaagcgtgagatctcgacc
atgaagctcatcaggcacccgaacgtcataaagatgcacgaggtgatggccagcaagacaaagatttacatcgtgatggaacttgtcactggtggtga
acttttcgacaaaattgcttcacgtgggaggctgaaagaggatgatgccaggaagtatttccagcaactggtcaatgctgttgattactgccacagcaga
ggcgtgtaccaccgtgatctgaagcctgaaaatcttctgcttgatgctagtggcactctcaaggtgtcagatttcggactgagtgcactttctcaacaagt
ccgagaggatggtcttctgcacacaacctgtggaactcccaactatgttgcccccgaggtccttcgnaatatttttccatatctttctgtcaacttgcagga
ggatggtcttctgcacacaacctgtggaactcccaactatgttgcccccgagccaaggctgatctttggtcatgtggagtga
>g60235.t1
atgggcctgttctcgtcggcggccaagagatgcaacgacggcaagaggctcctccgcggcgcgtgcgcgggctgctgttcgtctgccgtgtgtggtggt
ggccgcggcaaggaggaggcctcgacgtccgcgccggcgccgacgacgccctccgcgcctgatcataagaagaagaggtggaggaagaggaagtt
ctggaggaagaaaaagaaggcgaggaaggacggcagcaaaggcaattccaacggcgagatcaggaggtctggtgagagcgatctcgccgacctcg
tgaacaatatctcggcacagtcggacgtttgcaagaacgtgagtgcggccgaagagattctgcgcaatagcaaccagaacatgcccagcaaggcgct
gacgttcagggaactgggcgccgcgaccaacgggttcagcgagagcaacctgctcggagaaggcggtttcgggcgcgtgtacaagggggtcctcccg
gacaccaaagaggtgatcgccgtgaagcagctggacaggaacgggttccagggcaaccgcgagttcctcgtcgaggtgctgatgctcagcctcctgca
ccaccccaacctcgtcaagctgctcggctactgcaccgacgccgcccagcgaatcctggtctacgagtacatgcccaagggctccctggaggatcacct
tctagacctgccaccaaactggaagccgttgccgtggcacacgcggatgcagatcgccgtggacgcggcgaagggcatcgagtacctgcacgaggtg
gccaacccgccggtgatctatcgcgacctcaaggcgtccaacatcctcctggacgcggacttcaacgccaagctgtctgacttcgggctcgccaagctg
ggccccatcggcgaccagagccacgtcagcaccagggtcatgggcacgtacggctactgcgcccccgagtacgccatgaccggcaagctcaccaaga
tgtccgacatctacagcttcggcgtcgtgctgcttgagctcatcaccggccgccgtgccatcgacagtgcgatgcagtccgaggagcaggtcctcgttca
gtgggccgcgcctatgctgagagacaagaaacggttcatgcggctggccgatccattgctggggaggaagttccctcccaaggggctacaccaagcgc
tcgcggtcgcgtccatgtgcttgcaggaggacgccagcagccggccggtgatcagcgacgtggtcgcggcgctctcgtttctcgccgacccgaaatact
accctccagaatgcgcggcgaagaaggccacggaagagagcagtaaatcgaatgagaacatagaaaaatttagtgctagcagtcctcccatgacag
acattgtttctgagatgagatcagacgatgagatcaagcagagatga
>g60353.t1
atggcggcggacgcgtctgcgtcggggaggaagacgcgggtggggccgtacgagctggggaagacgatcggggagggcagcttcgccaaggtcaa
gctcgcccggaactcgcgcaccggcgacgtctgcgccatcaaggtgctcgaccgcaaccacgtcctccgccacaagatggtcgagcagataaagcgg
gagatttcgacaatgaagctaatcaaacatccgaatgtggtccagttgcatgaggttatggctagcaaatcaaagatatacatggttcttgaatttgtcg
atggaggggagctatttgataagatcgtcaattctgggaggctaggagatgatgaagcaaggagatactttcagcaacttataaatgcagttgactatt
gccatagccgtggagtgtaccatagagatttgaagccagagaatctgcttctggattcacatggatctcttaaagtctcagactttggtctgagtgcgttt
gcaccacaaacaaaatgtacaccacaagagatcatgtctaaaatagaggaagcttgtggaccacttggtttcaatgtgcggaagaaagattataagat
gaaattaaaaggtgacaagtcaggaagaaaaggacatttatctgtagcaacagaggtttttgaggttgctccaacacttcacatggttgagcttcgtaa
aactggtggagacacactggagtttcataataattga
>g60426.t1
atgccgagaggcgtcgcaacgcagctgccgttgtggctggtggtgctgctgctgccgcgcgcggcggacgcgagctgcgcacggagctgcgggggctt
aacggtgcgccacccgttcggcttctcccgcggctgcgacgtacagctgggctgcgaccacgcggcgggcgtcgcgtgggtcggggaccagcgcgag
ctgggcctggtcctgcgcaacgtcacggagcgcgcgctcatcgtcgacctgcgcccggactgctcccgcgggctcaacgcctccctcgcggcgctcttc
gctgacaggtacgcgcccagcacgcggaacgcgctcgtcgtgagctcgtgcgccgccgccgcccgcgccaccaccaactgcagcatcccgccgggga
agtacctcggcggcgagaacaggtcgcactgcagcaaagccgccgcggattccatccgctgcgtccctccgccgtactactccaacaacaacgccgtc
gtccaccgcgagttactattcttgaacagaaccgagatgctggagctcggtaaggagtgccagggcctggtgaccgccgtgagctactcgccgtcgccg
tcgccggcgattctgctgggcgcgctggacctcgagtggtgggtgcccggccgtgccgatgctcggtccacgccaactgcacctcgttcaccgcgccg
acgcagcagcaggcgttccggtgcgactgccttcaggggttcgagggcgacggcttcgccgacggcgccggttgccggagaggtgtgtgtttcggcgc
catggtgacgtgcgtgacgtgcgtggtgtaccacctgctgaagcggcgttcggcgtcgatgcgcagcaagcggagcacgaagcggctcctgtcggacg
cctcctgcgcggtgcccttgtactcgtaccgcgagatcgagcgcgccacggacgggttctcggaggcgcagcgcctgggcacgggcgcgtacggcacc
gtgtacgcggggcggctcagcgacagccgcctggtggccgtgaagcgcatcaagcagcagcgcgacggcgacggcgacgtgatgaacgaggtgaa
gctggtgtgctgcgtgagccaccggaacctggtccgcctcctgggctgctgcatcgagcagggccagcagatcctcgtctacgagttgatgcccaacgg
cacgctggccgagcacctgcagcgggagcgcggcccggccgccatgccctggaccatccgcctccgcatcgccgccgagacggccaaggccatcgcc

tacctccactcgggctcgggcgtgcgcccgcccatctaccaccgcgacatcaagtccagcaacatcctgctggaccacgagtacaactccaaggtcgcc
gacttcgggctgtcaaggacgggcaaggtctcctcctcctcttcctcctcctcttccctgtcactggactacgcgtcccacatctccacggcgccgcaggg
cacgcccgggtacgtggacccgcagtaccaccagaacttccacctctccgacaagagcgacgtgtacagcttcggcgtcgtgctggtggagatcatca
ccgcgatgaaggccgtcgacttcacacgggtgcccagcgagatcaacctggcgcagctggccgtggacaggatcgggaggggnnnnnnnnnnnn
nnnnnnnggctttggcagtgtctgtttcggcgccatggggacttgcgtgacgtgcgtggtgtaccacctgctgaagcggcgttcggcgtcgatgcgca
gcaagcggagcacgaagcgtctcctgtcggacgcgtcctgcgcggtgcccttctactcgtaccgcgagatcgagcgcgccacggacgggttctcggag
gcgcatcgcctgggcacgggcgcgtacggcaccgtgtacgcgggtcggctcagcgacagccgcctggtggccgtgaagcgcatcaagcagcagcag
cgcgacggcgacgtgatgaacgaggtgaagctggtgtgctgcgtgagccacagcaacctggtccgcctcctgggctgctgcatcgagcatggccagca
gatcctcgtctacgagttcatgcccaacggcacgctggccgagcacctgcagcgggagcgcgggccggcggcgatgccatggaccgtccgcctccgc
atcgccgccgagacggccaaggccatcgcctacctccactccggcgtgcacccgcccatctaccaccgcgacatcaagtccagcaacatcctgctgga
ccacgagtacaactccaaggtcgccgactttggtctgtcaaggacgggcaaggtctcctcctcctcgtcgtcttcttccctggacgcgtcccacatctcca
cggcgccgcagggcacgcccgggtacgtggacccgcagtaccaccagaacttccacctctccgacaagagcgacgtgtacagcttcggcgtcgtgctg
gtggagatcatcaccgcgatgaaggccgtcgacttcacacgggtgcccagcgagatcaacctggcgcagctggccgtggacaggatcgggaggggg
ctcgtcgacgacatcgtcgacccgtgccttgacctacacagggacgcgtggacgctcagctccatccacaaggtcgcggagctcgcgttcaggtgcctg
gcctaccacagcgagatgaggccgtccatggccgaggtcgccgacgagctggagcagatacagctcagcgggtgggcgccgtcggcggacgacgcc
gcgtttatgtccacgacctcgtcgctctgctcctcggcgccctccacgcgcgccacggagaggtcgtgggggaatggcaggagccggaataaggaggc
gggcgtggtggccgtgcaagatgctactaccgccaagtgcgcggcagtggagtcgccggtgtccgtgcaggacacgtggttcagcgacaagagctcg
ccttcctccaatagcttgttgggaaattaa
>g60494.t1
atggcgctgaaggtcgccaagctgatggaggagctccagaagcaaagggaactcaaggagacatacaaggccaggctcgagagcacgcaggggta
cctcaggttctgcctggaggtcgcgcaggagcacggcttcctgcacctcatgtctgacaacgcgcagccgccgtgctcgccgcgccgtcacgacgccga
agctgaacctgcgtcgacggaggaggacgacgtcgaaggagacgaaatagccgagacaccgccgagcgacccgtacctggccgccacgcgggacc
tcgcggtgcagcacggctggtccatcacgccggacgagatcgagctgcacgaggtgatcggccggggatcgacggcggacatacaccgggcgacgt
ggcgggggctggaggtggcggtgaagtgggtccggccggacttcctccgcaccaacccgagcggcgcggccttcgtcgcgcaggagctggacgtgct
gtcccggcagcggcacccgcacgtgctgcgcctgatgggcgcgtgccttcacccgcccgagcgctgcttcatcgtgaccgagctgctcggcggcgcca
cgctcggggagtggctgcacggcggaagggagaggcggccgcggcgggcgtcgtcgtcaccctggccgccgccgccgccgctggaggacagagtga
gcagggcgatggaggtcgcgctggccatgcggtacctccacgagcagacgccccgggtcctgcaccgcgacctcaagcccagcaacgtgctgctcga
cgcgggcatgcgcgcccgggtcgcggacttcggccacgccaggttcttgccggacgggaaggaggcgctcaccggagaaacgggaacctatgtgtac
atggctcctgaagtgattcgctgcgagccgtacactgagaagtgcgacgtctacagtttcggcgtcatactgaacgagctgataactgcagaacatccct
acgttgaaacaagctacggccctaatcgcattggaagtagcagctggaagattacggccgagactcccagaaagtga
>g60563.t1
atgaagagcctgcagtgtttcaaacagagtgggggcaatggtggtgggggcatgggaagcggctggagcggcggctgtcgctgggcgagtacaag
aaggcggtgtcgtggtccaagtacctggtggctccgccgggcgccaggatccggggcggcagcgaggagctgtggagcgccgacctgtccaagctgg
agatccggagcaagttcgccacgggccgccacagccgcgtctactccggccggtacgccggccgcgaggtggccatcaagatggtcagtcagccgga
ggaggacgccgcgctcgccgccgagctcgagcgccagttcgcctctgaggtcgcgctcctcctccgcctccgccaccccaacatcatctcgtttgttgca
gcatgcaaaaaaccaccagtttttgtatcatcactgagttcatggcaggaacctacaggtggatggctccagaaatgatcaaagagaaacatcatact
aggaaagttgatgtgtacagctttggaatcgtcatgtgggagttattaacatctttggttccatttagcgagatgactcccgagcaagctgctgttgcagtt
gcactgaagattccaaaccagctttctttctctcctattgaacagctgcggattcctccggtcaggctcaaatttatttcagtgatggccgttgagcggtgt
agggtctggtggccgcgccagcagccgcagctcgagcaggactctggtcccgtgtcgcacatcctttttggatggctcttcactgccgtcggctccctcg
acgccgtcgtctctgcgactattcctcaggatcagatcttgcggtcatttactacgctcgacgccctacagaagagaagaaaatacccttgtgaagagac
caaggtagcggcttag
>g60645.t1
atggagcggtatgagttggggagactgttggggaaaggcacatttggcaaggtgcactatgcaaggaaccttgagtccaatgggagtgtcgccataaa
gatggttgataaggacaaagcactgaaggttgggctttcggagcagataaaacgtgagatcacaacaatgcggttggtagcacataagaacattgtcg
agcttcatgaggtcatggcaacacggaacaagatctactttgttatggagtatgtgaaaggtggagaactctttgacaagattgagaaaagtggccgcc
tcacagaggaaatagcacataagtattttcagcagcttattagtgcagttgatcactgccacagtcgaggtgtgtttcaccgagacttgaagcctgagaa
cctgctgctggatgagaatgagaacctgaaggtctcagactttgggctgagtgcactttcagaatcaaggagacaagatggcttgctccatacgacctg
tggaaccccagcatatgtagctccagaggtgatcagcaagacaggctatgatggtgcgaaatcagatatatggtcttgtggtgctgtcctatttgttcttg
ttgctggttatctccctttccaaggaccaaacttgatggagatgtatcggaagatacaacatggtgatttcaggtgtcccagttggttttcacacaaactca
aaaagcttctgtacaagatcctggaccccaacccaaacacaagaatttcaatccagaagataaaagagtctacatggttccgaaaaggtcctaatgaa
acccgtgcagtaaaagagagaactcctagtgaaaatgccatcactaatactacttcaatacatggtactgcaaggcgcaagaagaatgctcctgaaga
tgtgaagcccctgaccgtgacaaacctgaatgcctttgaaattatctctctctcagcagggtttgatctgtctggcttatttattgaaaaggactgtagaaa
ggagacaagatttacatcagataagtctgcctcagctatcatctcaaagcttgaagatgtcgcaaagaccctgaatcttagggttaggaagaaggataa
tggtatagtcaagattcaagggcggaaggaaggaaggaatggtgtcatgcagtttgatgcagagatttttgagatcacaccttcccatcatcttgttgag
ctgaaacaaacaagtggtgatttaattgagtatcggaaactattggaagaaggcatccggccagcgctgaaagaaatagtctgggcctggcatgggga
tgatcagcaacagaagcaagagtag
>g60659.t1
atggctgacagataccaagtcctcttaagaactgtacagctcaagacctctgtgacagtacaacttgcactagttggctgccatgtccgtgttttagatttc
ttcatcatgcaagacctagaagagggaaccttgtttgacgatgacctggaggatgacttcaagaaagggactagcccaaagcggttcaactacggcga

gctcaccactactaacaacaacttctccaacgagcacaagctcggggaaggcagcttcggctcggtgtaccgtggcttcctgacggagatgtccctcac
gattgcgatcaagcgagtgtcaaagagctccaaaaaagggaggaaggagtacgcgtcggagtttcatatcatcagccagctctag
>g60683.t1
atggccttctccccgcgcaccccatggtcgcgctccaggaagcccgacgtctactccaccgtggtcgtccacggcgatgacgacgaagacccccgcgg
cggtgggaggcccgcctctggggtggaggacgatgaggaggaggaggacccctcctcgctgcccccgctcctccagcgactccccaaggacttcggc
ggcgcctccttcgatgatgatgacgacccctactcctccgaccccgacgacgcgtccctctccgccaccgtcgtggtcaagcgcggcgcgcccgcgtcc
acctctgcctactcccgatccccgttcctggatctgcggcgctccagcccccgcgcctccgaggatgacccgtactccaccttcgtagtacactccacctc
gcgctcgggtggcagcccgcgggagtccgcctccggcaccttcatccgccgctcgggtggctcgaccagtccccgcgagtccgtctccggcacattcat
ccgccggacgggcagaccgtcgagcccccgcgagtccgtatcaggcacattcatccgccgcacgggcagcccttcgagcccagttgaatcgtactccg
gcaccttcatacgccataccggcaccgactccagtccccgtgagtccggccccgaaggtggcgggggatttgggtcttcattctggactccagcagtgg
agcaattggaagagcgtcggcaaccatcgcctctgatgcaggaacagttaaggcgaaaaccgtcggtgagctcagtgccagagagcattactaggga
ggacccttccaccaagtacgagctgctccacgagcttgggaaaggttcgtatggcgcagtgtacaaagcaagggatcttaggacgcaggagcttgtgg
ctgttaagatcatctccttaacagaaggggaagaaggatatgaagatataaggggagaaattgagatgttgcagcaatgcagtcatccaaatgttgttc
gctactttggaagttatcaaggggaggaatacctatggataattatggagtactgtggtggtggaagtgttgccgacttgataggcgtcacagaggaac
ctcttgatgaatctcaaatagcttacatatgcagggaagcacttaagggccttgcttacttgcactcgatttttaaagtacatagagacattaagggtgga
aatattcttctaacggagcagggtgaagtgaagttgggtgattttggtgtagctgcccagctgacaagaaccatgtcaaagcgcaacacggaagaagg
atatgaagatataaggggagaaattgagatgttgcagcaatgcagtcatccaaatgttgttcgctactttggaagttatcaaggggaggaatacctatg
gataattatggagtactgtggtggtggaagtgttgccgacttgataggcgtcacagaggaacctcttgatgaatctcaaatagcttacatatgcagggaa
gcacttaagggccttgcttacttgcactcgatttttaaagtacatagagacattaagggtggaaatattcttctaacggagcagggtgaagtgaagttgg
gtgattttggtgtagctgcccagctgacaagaaccatgtcaaagcgcaacacgttcattggtactccgcattggatggcaccggaggtcattcaagaaa
gtcgttatgatggaaaggtatcctcccctcttctcgcggtgtatagctactga
>g60793.t1
atggttgactccccgtgggtggtgttgtggctacttttcctcttcttctcctacggccttggagccgccggcgatggcagcaggtttctctactccggcttct
ccggcgccaaccttgacctcgacggcacggccacggtgacgccgagaggcaaactcgagctgaccaacggcaccgtgcagctgaaaggccacgcgt
tccacccggcttcgctgcgcctccgccgcaacaatgccacggtccgttccttctcttcctccttcgtgttcggcatcctcccctccacgcccgacaggttgg
gcggtcacggcatagccctcgtcatcgcaccgaggaaggacttcccctcgggtatagctagccagtacatggggtttctcaacaagaccagcaacggc
aacgcctgcaaccacatcttcgccgtggagttggacaccatcctcaacccggacttccacgacctcgacaacaaccacgtcggcgtcgacatcaacaa
cctcacctccgtcgccgccgccaccgccgggtactacgacgacctcaccggcgagttcaggagcctcaccctcatcagcggcgaggccatgcgggcgt
gggtggactacgacggcggcgccgccaagcgggtcgacgtcacgctggcgccggccggcgtgacgaaaaagccgagtaggccgctggtgtcggccg
tggtcgacctgtccactgtcatcaccgaccaagcctacatcgggttctcgtcgtccacgggcacgctgagcacgcgccactacgtgctcggctggagctt
cgccgtcgacaagccggctccggaactcgacgtggccaagctacccaagctacctcccatcgaaagacccaaggatatacatgtacccaagattctgg
tgacgatagctctgcctgtattcgccggggtgctcgccttcgccacggtcgcgacctgcgtcctcctactcgtgcggcggcggaaccgccggtacgtgga
gctgcgagaagactgggagatcgagttcggggcgcaccgcctctcgcacaaggatctggtacacgccaccgacgggttcaaccccaagaacctcctc
ggcgcgggcgggttcgggagagtgtacaagggagtcctccccaattccggcacggaggtggccgtgaagagggtgtgccacgagtcacggcagggg
atgaaggagttcgtggcggaggtggcgagcatcggccggctccggcaccgcaacctcgtgcagctgctcgggtactgccgcctcaaggacgagctcct
cctcgtctacgactacatgcccaacggcagcctcgacaagtttctttatggccatggcgataacaacaacaacaacaagcagcagcaggttctgagctg
ggcgcagcggttccagatcatcaagggcgtcgcgtcgggtctgctgtacctgcacgaggagtgggagcaggtggtcgtccaccgcgacgtcaaggcc
agcaacgtgctcctcgacgccggcatgaacgcccggctcggcgatttcggcctggccaggctgcgcggccacggcgccgtcgccgagctgcagacca
ccgtcgtcgcggggacattcgggtacatcgcgccggagctcgcgatcaccggcaaggcgtcgccgctcaccgacgtgttcgcgttcggggcattcctgc
tcgaggtggtcacggggaggagaccggtcgaggaggacgggaacggcggcggaggcacggtcatgcttatcgactgggtattcgagcactggcgca
gggagacgctcctcgaggtggtggaccccgggattactactcacggagctggagatcagtacgacgttaatgagctgagcttggcgttgagggtcggg
ttgttgtgctcgcagccgatggccgatatgaggcccagcatgagacaggtcatgcagtacctcgaaggggacgcttcacctcccgagctgagcccagc
ggctcagtacgtgggcctcagcatgttggcgttgctgcagaaccaagggttcgactcctacgtcatgtcctccagttcatcgtcgtcctcctcttcggtgaa
gagactaagcacttctctctcgaggggtcgctga
>g61453.t1
atggcttccggccttttccggcgctggaaaaaaggctttcatccttttaaggagcaggagaacgagttcgagaaagggaccgggccgaaacggttcag
ctaccgcgagctcgccgtcgccacaaacaacttctcggaccagcttgtgctcgggcaaggcgcgtttggatcggtgtacaaaggcttcctgcaggacat
gagcctcgcggtggctattaagcgtgtgtccaagatctggtatcaggggaggagggagtacgccactgaggtgcgtgtcatcagccgcctccggcacc
gccacttgatgcagctagtcggttggtgccacggcggcgacgagctgctcctagtctatgagttcatgcccaacggcagtctcgacaagcacctccaca
gcgccaacatgtcgcccctgccatggccgtgcaggcacaaggttgtgctcgggatcgcctcggctatcttgtacctgcacgaggatgccgagcaaagcg
tggtgcacagggacatcaagcccagcaacgtgatgctggacgcctccatcaacgccaagctcggtgacttcgggctcgccaggcccatcgaccacgac
cgggactcacacagcacggccatcatgggcacggtcgggtacattgacccggagtgcatcgccaccggcagtagcagggtcaccgtcaagtctgacat
ctacagcttcggcgtcctcctcctcgagatcgcctgcggacggcggccgatcgtggccatgggggaagaagtggtctacctcgttaaatgggtgcggga
atcttatggcagagggatcattcttgacgccgccgatgcacggctgaacggagaattcgacgttgaagagatggagagagtgatggtcgtcgggctct
ggtgcacggaacctgacgagagcctcaggccatcgatcaggcgggtcgtcaatgtgttgcggtcggaggcgccgctgccgagcctgccagcacgttat
gctttcgattacacatcttcaatcgctacagggggaagcggcagcacgggaaccactcagtccactgaggccgtcaatatgctgcagtcggaagtgccg
tcacagatgccggtggcgactttcctgccaccacctgatgttttcgattgcagttcttcacttgcaacgggggccagcagcagcggcggcggcaggagc
acgggcaccctgtttattggcgatcataaccctataggaggactgcaaatggactccgaagaacatgcacgagatctcttggagagcatgctacttgat

gcaagtgtagagccgacagatatgccactatcattgctaaaatccatcacgaataatttttctaatgatcgacaaattggcgttggtggatttgcagtggt
ttacaagatgcatcccaaggactcgaatggatga
>g61455.t1
atgtgggtacactatgacatcaagcccagcaacatgatgctggacgcggccttcaatgccaagctcgacgacttaggcctcacgcggctggtcgaccac
gctaggggttcgcacaccgcgccgcttgcccagaccatggggtacttggacctagagtgcatggtcactggcatgaccaacgctgaatcggatgtctac
aacttcagggtcatgctcccggagatcgcttgcggccgccgaccgatggtggtcatcggggaagatgtgatccgtctcttgcaatggacgtag
>g61456.t1
atgtacatcctggcgcagtgcacgagggacctaacgacgggcagtgcatgcagcctctttacgcagcaagacctagaagagggaaccttgttcgacga
tgacctggaggacgacttcaagaaagggactggcctaaagcggttcgactacggcgagctcaccactactaccaacaacttctccaacgagcacaagc
tcggggaaggcggctttggctcggtgtaccgtggcttcctggcggagatgtccctcgcggttgcgatcaagcgggtgtcaaagagctccaaaaagggg
aggaaggagtacacctcggagctcatgcccaacggtagtctcgatttacatctccacaacagtggtagggacgacgaggacgctctgccaatgccgtg
gtcgcgcaggcacgagatcgtgctcaggatcgcctcgtccatcctgtacctgcaccaggagtgggaacagtgcgtggtgcaccgagacatcaagccca
acaatgtgatgctggacgcctccttcaacgccaagcttggcgactttgggctcacgcggcttgtcgaccattcccatggcttgcacatcatggtgctcgcc
agtacggtcgggtacatggacctggagtgcatggttaccggcagggccgacactgagttggacgtctatagctttggcatcatgctcctcacgatcacct
gcggccgccagtcgatggtgctcatcagggaagacgtgatccacctcgtgcaatgggtgtgggagtcccactactgtacaggaaccattctagatgccg
ccgaccagtactatcaccgacgcatcgcagaacgaagagtttga
>g61625.t1
atggcctcctccccacacaccagtgccaccaccgcgcccgcgtcgtcgtcaccgccgcccaacaagcctccgaaccgcgccgccaccatgctcgacca
tccttcgaattcgtaccccgacgccgcagtctccagcgtcagcagctcctcgagcagcagcagcagcagcagcgctggaggcagcctggccgaccggt
cgtcgacgttctcgctggactcgtcgtcggccgccgcgacgccgtcgtcctccccgccgcggccgcatcgggccagcgacgtggcctgggcccccatcc
gcggacgggccgccgctctgggcccgcgcgacttcaccctcctccggcgcgtgggcgccggcgacatcggcaccgtgtacctctgccgcctcgagagc
agcaataacaagcagcgtgacgacatgggcagcgtgacgacgtcatcgtgcgagtacgcgatgaaggtggtggaccgccgcgcgctggcgcggaag
ggcaagctgtgccgcgcggcggcggagaagcgcgtgctccggcgccttgaccacccgttcctccccaccatgttcgccgacttcgacgcgggtcccga
ctactcgtgcgtggtgatggagttctgcccgggcggcgacctccactccctccgccaccgcgccgcgctgccgggccgcaggttcccgctggcctccgc
gcggttctacgccgcggaggtgctcctggcgctggagtacctccacatgatgggcatcgtgtaccgcgacctcaagcccgagaacgtgctcatccgcgc
cgacggacacatcatgctcaccgacttcgacctctccctcgaggccacgtcgtcgccgtccctctccctcgacctcgaggacaacaacgcggccgagcc
accggcggcgccgacgtgcctgcccatcccggagctgcagctgctgcgtctgaagcggtggaggcggcgcgcagcgccgccacagccacggccgcg
gttcgtggcggagccggtggacgcgcggtcgagctcgttcgtgggcacgcacgagtacgtggcgcccgaggtggcgcgcggcggagggcacggcgc
cgccgtcgactggtgggcatacggcgtgttcctctacgagctcatctacggccgcacgcccttcgtaggggacaccaacgaggccacgctgcgcaacat
cgtgcgacgcccgctccagttccccacaaacggcaacggggacggaagcgacgacgccgcggcgagggacctcatcgccaggctcctgcacaagga
cccgcgcaccaggctgggatccaagcgcggcgcagcagacgtcaaggcgcacgccttcttcaagggcctcaacttcgcgctgctacgctccgccaccc
cgcccgtcgtgccgccgcctgccgcggcgcaccggtgcgacgacagggcggccgacgtgcagcagctgttcgaccacttctga
>g62116.t1
atggctgcggcgcggcacgggagccacaggggccgcgactctgtgagggaacgggagcttgacctggagcgttctaggaggagcaaggagtatcac
caccaccgccggcaccggagccgcgaccgcggctgggaccggcgtagcgacggaggccacagcaggtggcgggagctgtccgacaggtacagtcg
ccaccgctcaccgctgaggagcaggctgtcagggagagtggaggacagggagcctggcgagctctcgaacgggagtggctcagaggagtctcgtga
gcgttcactgaagacgagagcacggagggagaatggtgttgtgaaagtctgcaggtatggtgccgctctgtctccaagtaggaagaggaagcattcac
cagtccaggatgcgaatgttttgaagctgcaggcaacagattctatgaggagcagaatggaggtagagctccctcagccttcaccacctcctgtatctg
atgcgagtactgtggctacggccagtgagtgctcaccaatgaatttggatatgtcaattgtttcacatgaggcaaagcggttacctgatcatgagaagaa
taagatcacggaaggggatgagtacccaatgatgagaaatattttcacttcaagatgggctgacgctgaagaggaggaagaggtggttgtgcctcaaa
agaagaaaagtgtaccacctgctaatgcagttgaaaaaatatcttgggagagagctacaagcctagagcctggagaactgctggtgatgtcaggagg
aaattcttccatttcccccagctccatgttggtgcaagaaagtgaaaatgaagatcttgaagaagatgagggtaactgtatggatgttgcagtggagcac
gacattgattctcctgctggttgtttgctcgacactgatgttgagagcaatgcatgtaggtctggaacacctgagaatgtgcagccgccacgaagggctc
cggaattacttttaggagcaaaagagtattcaactgctattgacatgtggtcactgggttgcataatggcagaactcttgaccaaaaaaccattatttagc
gggaaaactgagatagctcaactaaatgagatgtttaggatgcttgggacacccaatgatgagatatggcctgattattccaaattacctggtgctagag
cccaatttgtaaagcaaccgcgaaataggttaagggagaagtttccagctgtatcattcactggagggttgacattatccgaatctggattcgacctgct
aaataggttgctggcatttgaccctgagaagcgcatatcagcagatgctgccttgaaccacgagtggttccgtgaagttcctctgcccaaaacaaagga
tttcatgccaacacttcctgctctgaatgaacaagacaggcgcttcaaaacatatgtgaagagccctgatcctctggaggagcaactgatgaaaaaaca
ggggagcataagagatcgagggctatttggttga
>g62158.t1
atggcggcgcggaggaggctgcttgccgaggcgccaccgggggaccaccacaagcgcggcgcgccgccggcggagtggactgaggggtacctcaa
cgggtggctgtcgcagcccacgggggtgttcggcctccgcctctgggtgctcatcggcatcgccgtcggcgccgccatcgtgctggtgctcctcctcatct
tcgtctgcctctcccgccgccgccgccgccgcgaggacctcgccgccaatctctacctctccgaccccaagctgctcaagcagcacatgcagcaggcca
cgcccaccaaggacatcccggaaatcgtccgccgacagcagcagcagacgccgccgccggccgcgcggcccgcggtgcagctcgccaaagcggag
cagacgccgccgacacagcagcagcagccacagagggcgcaggtgccggtggtgccgacactgcccacgcggaaaacgccgggcagcggcatgtc
ggccacgacgagcggcgggagcgagcggggcttgggcacgccgaggagcaccgggagcgccggcgcggggccggaggtgtcgcatctcgggtgg
gaccactggttcacgctacgggagctggaggaggccacggatgggctcgccgaggagaatgtgatcggggagggaggctacggtatcgtgtaccgg
gggacgttgcagaactccgccacgattgccgtcaagaacctgctcaataacaggggccaggctgagaaggagttcaaggtggaggtcgaaacaatcg
gccgtgtccggcacaagaatctagttaggttgctcggctactgcgtagagggtgcttataggatgcttgtgtacgagtacgtggacaatggtaacctcga

tcagtggcttcatggtgatgttggggaagtgagcccactaacctgggacatcaggatcaacattattctcgcaaccgccaaagggctggcatatcttcat
gagggcctggaaccaaaggtggtccaccgtgacatcaaatctagcaacattcttcttgaccagcaatggaatgctaaagtttcagattttgggctcgcaa
agctcttgtgctcagagacaagttatgttacaacccgtgttatgggaacctttggctatgtggcacctgaatacgccagcactggaatgttaactgagag
gagtgatgtttatagctttggtgtacttataatggagattatcactggtcgatctcctgtagattatacaagagcagctggagaggtgaacttggttgaatg
gctcaagaccatggttgctgaaaggagagcagaggaggataagaagcctggaagggaagttcagcctcagtcatcagacagatatagctctaagga
agatggaagcttcagcaaacgtgagaatcaccagtacagatga
>g62237.t1
atggggaacacctgctccggtccaacgccccctcggaccgccatggtttcttcgactccgtctccctcgccgtgctctggcgcccacccgccgccgcca
gggccgagccccttctccatcggactccaggccctccgcctcctcgacgacttcctccaggcctcccgagtccgtcaccttcaccgatgccgagcactc
cccccacgcctcccctccgcgccggaccctaacggcaagcccaggcctaaggtgaagcgcgtgcagagcgccggcctccttgtcggctccgtcctga
aacgcaactccgagcggctcaaggacctctacaccctgggcaagaagctgggacaggggcagtttggcaccacctaccagtgcgtcgagaaggccac
cgggaaggtattcgcctgcaagtccatcgccaagcggaagctcgtcagcgaggaagacgtcgaggacgtgcgccgcgagatacagatcatgcatcac
ctctccggccacccgaatgttatctccatcgttggggcttatgaggacgccgttgcggtgcaccttgtcatggagctgtgtgcgggaggggaactgttcg
atagaatcatacagcgggggcactactctgagaaagctgctgcacaattggcacgggtgatcattggagtggtcgaggcatgccattctctcggggtga
tgcacagagacctcaagccagagaatttcttgtttgtgaaccacaaggaggatgcaccgctaaaggcgatcgattttgggctctccatattcttcaaacc
aggtgaaacatttttttgatgtcgttggaagtccgtactatgttgcacctgaagttctgttgaaacactatggctgtgaggtggatgtgtggagtgctggtgt
aataatttatatcttgctgagcggagtccctccattttgggatgaatctgaacaaggaatatttgaacaagtcctgaaaggtgatcttgacttttcttctga
gccctggcccagcatctcaaggagtgcaaaggatttggtcaggaagatgttaatccgtgatcccaaaaagagattgactgcacatgaagctctatgtca
tccatgggtttgtgttgatggagttgctcctgacaaacctcttgattctgctgtcttaaccagattgaaacagttttctgcaatgaacaaattaaagaagat
ggcccttagggtcattgctgagaatttgtctgaagatgaaattgccgggttgcgagaaatgttcaaaatgctggacactgacaacagtggccaaattac
attggaagaactaaaaagtggcttgaagagagttggtgctaacttaaaggactctgaaattacaacgttaatggaggcggctgatattgataacagtgg
ttcaattgattatggagagttccttgctgctactttgcatctgaacaaagttgagcgagaagataatctctttgctgcgttctcatattttgataaagacggc
agcggttacattactcaagatgagctacagaaagcatgtgaggagtttggtataggagatgaacaccttgaagatattatcagagacattgatcaagat
aataacttcattctgccatttgagagagagagagagagagagagagcagccgccggcactggacgtggagccgctgcttgttcctccagctggaccag
cagcaatactgcgctgcggttccccatcgaagcagtggccgcccaacagcagcagagcctccgcctcctcttcaccttcggagcagcggcaacctgcc
agcagcagagccttcgcctccgcctcctcttcaccttcggagcagcggcaacctaccaggagcagagcctccgcctctgcctcctcttcaccttccgagc
agcggcaacctgccagcagcgcctcaacctccgcctcctcttcttatgctccatgtccagctcgtaa
>g62762.t1
atggagaagaaggacaaagacgctcaccaggtgatcatgggcgggcggtaccgcctgggccggctcctcgggcgcggcaacttcgccaaggtgtac
aaggcccataggacgtcgacgggcgaggccgtggcgatcaaggtgttcgacaaggaggccgcgcggcggtccggcacggcgggcctggtgacgcgc
gaggcgggggcgatgcggcgcgcgcggcacccgcacgtggtccggctccacgaggtgatggccacgcgctccagggtgtacttcgccatggagtacg
ccggcggcggggagctcctggagcggctcgcccgcgtcgcggcgcgcgggctcccggagcccgtcgcgcggcgctacttccggcagctcgtcgccgc
cgtcgggttctgccacggccgcggcgtgttccaccgcgacctcaagccggagaacctcctcctcgacgccagaggcgacctcaaggtctccgacttcgg
gctcagcgccgccgtggacctcgacggcggcgcacggctgcgcggcgacgcgctcctgcacaccgcgtgtggcacgccggcgtacgtcgcgcccgag
gtgctcctgaagtgtggctacgacggcgcgaaggcggacatctggtcgtgcggggtgatcctgttcgtgctcctagcgggctacctcccgttcaacgac
accaacctcgtgtgcctgtaccgcaagatcatggacggcagctacaggtgcccgcattggttctccgtcgaggcgcgcaagctcctggcgcggctgctc
gacccgaaccccaacaccaggatcaccatcgccgagatcaaggcccacccttggttccagaaaggcgcctcctcctcctcctgctgccctctcaccgac
gagccactagtactcctcacgaataccgagaccggcgccccgctgctcggcaaggaagcgtgcaaggcgcaccaccaccgccgtgacgacgacgac
gacgacgaggaagacgagacgcggcagaggaagcggtccaaggtggccgccgaatcgtcgtcgccgaggaccagtaacgccgtcacgccgaggag
catgaacgcgttcgacatcatcgcccggtcgagcgggctggacctgtcgacgatgttcgacgcggagcacgacaggtccgacgaggcgcggttctcca
tcaccggggagaccgcgagcgcgatcgtgtccaggatggaggagatcgcggaggcgcgcgggttcagcgtgacggtgaaggagacgggcagggtg
gtgatggaattggaaggccgccagtgtcaggacgcgcggggaggcgcgctcgccgtggaggcggagatcttcgaggtggcgccgtcggtgcacgtgg
tggagctcaggaacgcgggcggcgactccgtcgggttccgggagttctgcacgcgggacctgaagccgtcgctgggcgacgtcgtgtggtcgtggcag
ggcggtgactcgccgccgccctcgttcgttccggcggcccgtgccgaagagaggacgactactgatgattgttga
>g63403.t1
atggcggcggcggcgtcccggaagcgtccggcgcccgaggaagcccgtcccccgtcgaagcgggcgcgctacaagttcagcaagatcgacgactac
gagctgctcgagcagctcggggagggcgccttcggcgtggtgttccgtgcgcggcaccgccgcacgggcgaggcggtggccgtcaagcggatccgcg
gcggcggaggctgcctcgacgcggtcgtccgcgaggccggctgcctcgccgcgtgccgcggccacccgtccgtggtccagatcagggacgtggcctcc
gacgccgccacgggggacctctacctggtcacggagctcgtggacggcccctccctccgcgactggctcacgcggccgctccccgaggccgtggcgcg
cggcttcatgcgccagctcctcggcgcggcggcgcggatgcacgccgcgccgatggtccaccgcgacatcaagcccgagaacgtcctcgtcggcggct
tcggcggcgagctcaagatctgcgatttcgggtgcgcgacgccggccaagacgccgtacctgggggaggggtgcgtgggcacgctgcagtactccgc
gccggagcagctcgagggcggccagtgctacggcgcggccgtggacatgtgggccctggggtgcgtgatggcggaactcctcgtcggcgcgccgctg
ttcacggcggacacggaggccggcgtgctccagcagatcgaggagctgcgagacgggatcgaggccgtgggattggaagccgtcgacgacctgctg
gagctgtcgccggccgggcgtgaggtgctcgccggcctgctgagcttcgagcctggagagaggctcacggcgacagaggcgctcaggcaccgatggt
tgacggaggagaaggcagagtgccccgtcgcagcaaacgcagagttccctggcttcgttccactattctcagcggcatag
>g63909.t1
atgggtgcatacgtgcaggtgggccaggggacgtacagcaacgtgtacaaggcgcgtgagcgcgggacgggtcggatcgtggcgctgaagaaggtg
cggttcgacacgtcggagtcggagagcgtcaagttcatggcgcgggagatgcgcatcctgcagcgcctggaccacccaaacgtgatcaggctcgagg
gcatcgccacctcgcggatgcaccgcagcatctacctcgtcttcgacttcatgtactccgacctcgcaaggctcgtcccgcgcgccgccgaacaacgact

caccgagccgcagatcaagtgctacatgcagcagctgctgtcgggcctgcagcactgccacgagcgcggcatcctgcaccgggacatcaagggatcc
aacctgctcatcgaccgccacggcgtcctcaagatcggcgacttcggcctcgccaactactacggcggcagccgccgcaggccgctcaccagccgcgt
ggtcacgctctggtaccgcgcaccagagctgctgctcaactccaccgactacggcgttggcatcgacctctggagcgcaggctgcctcctcgctgagat
gttcttcggaaagccactcatgcctggcagaaccgaggtcgaacagctgtacaggatcttcaaccttctgggctcgccgccggatgactactggcggaa
gcagaaggtgtccgccgcgttccggcctacaaagatgtacaagtccaccatggccgaacggctccgcgacctgccgccttcggcacgtagactgatcg
ccacgctcctcgcgctcgacccggccgcccgcggcacagccgcccaggcccttcagagcgacttcttcaacaccccgccgctgccgtgcgacctctcat
cgcttccggtcgtgtacaaggaggaacccgaggatcgtactacctcccacgatggaaggaagccaaagctgagacaacgctcgcatagacggaggg
acacgagactcaaaaccgaggaagaacgggcagacgaaccaaagatcaacagcgggtctccagacaaaccggtgaaggaagacagactaaaaac
tgaagcagcaaaatcaggccaagaatcagctggagctacaatcgtcaagaaggcttcctccggcgttcaggaatcaccagacaaccccgtcgtcaac
gcgtcgaaacggttttcaggtagcccagctcaggtgctgccacaaccacatgagacttcacctgctgcgcctcaggatcagccgctgccaaggggaag
cagacaccaccactcaggcagcgacgaagatcacgagaacgaccggcctcttctggctctggacgatgatgaggtcgacggcgagccgtcgggcagc
ggcagcgtcattgtcctcaaccgcagcttggaaatggagagcaggacggcttccatgacggactacgacgcagcagtcgcggcgctacgtggatcag
gagaactcacctccaagccgtgcgtacttgtggatcacgtgtag
>g64164.t1
atggcggcggcgcctctcctggtggtggtggtggtgcttgtggcgacggtgggtgcggaggcgcgcatggtgccggtggagtacctgtaccctcccttc
aatctgagctacttccacttcatcgacaccaacggcgtgttccttctgtctccccactccaacttctccgccgccgtgtacaacgccgggtccatcgcctcc
tccgacacgcagtcgcgcttcttcttctccgtggtccacagcgccacccgcaccccggtgtggaccgccacctctccctccggcgggtccaccatcctgg
agtcggtgatcctgtccctctcgtcgcgaggcctctccctcttcgacccctcctcctcttcttcctcctccggggcgtggtcgacgccgccgctcccccgtcc
cgcgacggcgctccggctcctggacacgggcgagctgcggctcatcgacggcgagaacgcgacgctgtggtcctcgtacgagcggccgacggacacg
ctggtgccggggcaggagctcctccgcgttggtggtggcggggtgcccctgctcacgtgttcgacgtcggagcaggacctcactccgggcgcctaccgc
ctcgtcctcacccgcaacgacgcgctcctgcagtggggccccaccaacgtcacctactgggccctctcctccgaccccgccgccgtccaggactccacc
aacgacgtcgcctccatggtcgccaacgccactggactctacctcctcgccgccaacaaaagggacaccgtatacaggctgctcttctctctcctctg
caggcggtggtgtcctcatcgtcaaggttgacccctccggccgcctgcgcgcgatgacgctgaccgccggcggcagcagcagcgtctggtggagcgcg
ccggccaacgactgcgacctccctctgacgtgcgggtccctcggcatctgcaccaactccggcggtgccggcaacgggtccacctgcgggtgccccga
cgcgttcagcacctactccagcggcggatgcgcgccggccgacggatcctccgtccttccggtggacggtacctgcaaggaagaaaacaagtacagc
agcagctatgttgggctgggcgccgggatcggctacctccccaacaaattcgcggtgcccgacacctccggcgactcgctcccggcgtgccgcgacctc
tgctcggcaaactgctcctgcctgggcttcttctaccgtaattcttcctcgaaatcctgcttcctcctgcacaatcagatcggctccgtgttccgcagcacca
ccgacaatctgcagatcggcttcatcaagacgacgcttccccctaattcgccgcctcaccggagcaacaagaataataataagctgagcttcatcacca
tcgtgttcggcatcgtgctccccaccgtggcggccgtcttcgtcgccttcctgctctacgccatggccgcgcactggctcaacaagaagaagcctccttgt
cctcctcatcagctcaacaaaaagagcggcggcggcggcagctggttcaggctgccggcgatgctgtcgtcctcctcctcctcatcatcatccaggagg
agggggaatcaacaagtggatcaagaggacaaggaaaaggatgaggacgaggaggtgctcatcccgggcctgcccacccggttcacgtacagcgac
ctggacaccgccaccgacgggttccaatggcagatcgggtgcggcgggttcggctccgtgttccgaggcgagctccccgaccggagcaccgtcgccgt
caagaggatgaacaacacccaacaagggcagcggcgccgcgagtttctgacggagatcgccgtcatcggcaacgtgcaccacgtgaacctggtgaa
gctgcggggattctgcgccgagggcacggggcgccacctgctggtctacgagttcatgaaccggggatccctggaccacagcctcttccgagccaccct
agtcatggagtggccggcccgcctccgcgtctgcgtcggcgccgcccgtggcctggcatacctccacgccggctgcgaccgcaagatcctccactgcga
cgtcaagcccgagaacatcctcctcgacgaccatggcgccgtcaagatcgcagactttggcctcgccaagctcatgaccccggagcagtcggggctctt
caccaccatgcgaggcacaagagggtacctggcgcccgagtggctcatgaacgcgcccatcaccgacaaggctgacgtatacagcttcggaatggtg
ctcctcgagatcgtcagaggcagaaaaaactcctcctacaacaacaagaaagaagaagaagacgacgaggagtcgtcctcggaggagagaagctcc
gactccacctataataagaagaccaagaagatgatgagcaggagcagctacttccccgcgctggcgctggacctgcacgaacagggccgctaccacg
agctcgtcgaccccaggctcgagggccgggccaacgtcgacgaggtggcccgggtcgtcaaggtcgcgctctgctgcctccacgaggacgccgcgct
ccggcccaacatgacagccgtctccgccatgctcgacggcaccatggacgtcgccgacccgcgcgtcgacctcctccgctacctcaggatgtacggcc
gcggcctcgtcgacgtgcggcccctccccgtcggctggatggacgacgacactgatggaaagagaggctccaacaccaccgccggagccagcagca
gctggtcgccgccgtcatgcatctcggcgcagcagctctcaggacccagatga
>g64190.t1
atggacgagtcctaccaggaggagcaaccgttcgccgctgctgctaccacgacttcctacaagatcgacccgaactggtacatcgacacaggggctac
cgatcatatcactagtgatttggattgtctggcgcttcgtgatcagtaccatggtggtgaaaccgtccaagtcgacaatggagaagggcttttaagcatg
caaaatctccgggtgttctcgtacaaggacatcaggatagcaacgaaaaacttccatcagtgcaacaaactaggccgaggtggctttggaactgtgtac
aagggagtttggagagacggtacttcatttgctgcaaaggtcctgtcttctgaatctgatcaagggatcaaggaattcttggctgagatcgagagcatttc
tgaagtcaagcacgcgaacctcgtcaggttgctgggctgctgcatccagaggcgaaacaggatcctgatctacgagtatctcgagaacaacagccttg
accatgcgttgcagggtttggagaagggagtaacgactctgagttggagcgctaggtcggacatctgtatgggcaccgccaaggggctgagctacctc
cacgaggagcacgagccgaacattgtgcaccgggacatcaaggctagcaacgttcttctcgacgggaacaagcagcccaagatcggagactttgggc
tggcgaagctgtttccggacaacgttacccacatcagcacaggcgtggtcggaacaaccgggtacttggcaccagagtattttgtgcacgggcagctga
ccaagaaggccgacgtctacagcttcggggtgctcatcctcgagatcgtcagcgggaggaggatctcgcagaccatccggtcagacacgtttctggtgc
gcgaggcgtgggtgctgtacaagcagggcagcctgctggacatggtggacgcgagcatggaggactacccgaaggaagaggtgctccggtacatcc
gggtggggctggcctgcacgcaggtgacgccgagcagccgccccacgatgcgccaggtagtggccatgctgtcggggcccgtcgcgctgcacgagct
ggacatgcgcccgccgagcttcgcggagcactgcggccagagtccgagcccgaaacgcccgcgcgaagtcccgttggtacgctcgtcgcctaaatttc
ggtggccgccggcagcgggaagctctacctcgttcactttcaatgaggttgctccgcggtag
>g64299.t1

atgcggaaagggatccgaggagatctcctcgtcctcctcttctgcagcttggtggcgctgcttccggtggtgtgccatggcgcggacatgccgctgggat
cgaccctctctccaggtaactctgcggcggcttggtcatcccccaacagcaccttctcgctgtccttcacggcgtcccccagctcgccgtccctcttcgtgg
cctccctgacctacgccggcggcgtccccgtgtggtccgccgggtccggcgctgccgttgactcgggtggctccctccgcctgacctcgaccggcgacct
ccagctggtcaacggatccggcgccgtggtctggtcgtccaacaccggcggtcgcggcgtgtcgtcggcggccgtccaggagagcggcaacctcgttc
tgaagaacgcgacgacgacgctgtggcagtcgttcgaccacccgaccgacacggtggtgatgtcccagaacttcacctccgggatgaacctcacctcg
ggctcctacgtcttcaccgtggaccgcggcacgggcaacctgaccctccggtgggcccaggccggcaccacggtgacctacttcaacaaggggtacaa
ctccaccttcaccgggaaccggacgctgagcgcgccgacgctgacgatgcagaccaacggcatcgtctccctcaccgacgggtcgctgtcgtcccccgt
ggtggtcgcgtacagcagcaactacggagagagcggcgacatgatgcggttcgtgcggctcgactccgacggcaacttccgcgcgtacagcgccacg
cgcggcagcaacagcgcgacggagcagtggtcggcggtggcggaccagtgccaggtgttcgggttctgcggcaacatgggcgtgtgcagctacaacg
gcacgtccccggtgtgcgggtgcccgtccctcaacttcgtgttcaacaacccgtccaaccctcgtgaagggtgcaagcggacggtggacctccagagct
gcccgggcaactccaccatgctcgccctggacaacacgcagttcctcacctacccgcccgagatcaccaccgagcagttcttcgtggggatcaccgcat
gccgcctcaactgcctgtccgggagctcatgcgtcgcctccaccgcgctctccgacggctcagggctctgcttcctcaaggtgtccagcttcgtgagcgc
gtaccagtccgccgcgctgcccagcacgtccttcgtcaaggtctgcttcccgggcgtgtccaacccgcccgccacgggctccgggcccgccgggtcacc
gcgcggcaccgtccgcggctgggtggtggtcatcgtcgtgctgggcgcggtggccgggctggtcctctgcgagtgggccctgtggtggttcttctgccgt
cacagccccaaatacggtcccgcgtcggcgcagtacgcgctgctcgagtacgcgtcgggcgcgcccgtgcagttctccttccgcgagctgcaacgctcc
accaagagcttcaaggagaagctgggcgccggcgggttcggcgccgtgtaccgcggcgtgctggccaaccgcaccgtggtggccgtgaagcagctgg
aagggatcgagcagggggagaagcagttccggatggaggtggccaccatcagcagcacgcaccacctcaacctcgtccgcctcatcggcttctgctcc
gagggacgccaccgcctgctcgtctacgagttcatgaagaacggctccctcgacgcgttcctcttcggcgacgcgccgccggggggggaagctcccctg
gccggcgaggttcgccgtcgccgtcggaaccgcgcgagggatcacctacctccacgaggagtgccgagactgcatcgtgcactgcgacatcaagcca
gagaacatcctcctcgacgaccagtacaacgccaaggtctccgatttcgggctcgcaaagctcgtcaacccaaaggaccaccgacaccgcacgctcac
cagcgtgcgcggcacgagggggtacctcgcgccggaatggctcgcgaacctgccaatcacggccaaatccgacgtctacagctacgggatggtgctg
ctggagattgtcagcggccaccgcaacttcgacatctccgaggagaccgggaggaagaagttctccgtctgggcgtacgaggagtacgacaaggga
acgtcgctgacatcatcgacaagaagctccccccggggaagacatcgacatggcgcaggtgcaccgcgcgctgcaggtcagcttctggtgcatccag
gagcagccggcgcagcgcccgtccatggggaaggtcgtgcagatgctcgaggggatcatggagctcgagaggccgccgccgccaaagtcttcagac
agtttcctgagtagcgtcaccacggccagtggcagcgccagcggcgtcaccacgagcatggtctccaccttcgcgtcgtcggccccggtcgcgcccacg
ccgtccccgaatctggagcgggaggagattgctgcgttggaacgctcggcgtcggtgaaccgtgacagagtgtcacgggcgcagcgatccacgcagc
catacatgacaatgtaa
>g64360.t1
atgatggaggccctcagccggaagcgcttcgccgtgggcaacctggaggaggagtacgagccgatatgcgagctcggcaagggcggcttcggctccg
tcgtcaaggcgcgccaccgcgtcacgggccagctcgtggccatcaagcgcctcgccgaccccggcgaatgccaatcggaggtgatgcgggagaaga
ggttcctggaggcgtgcagcggcaacccgttcgtggtcgggttccgcggcgtggtccgcgcccccgactccatggagctccgcctcgtgatggaccacg
tgggccctagcctccgcgacttcctccgccttcgccgcgccgaggacccgccgctcccggaacccacggtccgcgccatcatgtggcagctgctgcagg
gcgccaagcggatgcacacggtccacgtcgtgcaccgcgacatcaagcccgacaacctcctcgtcagcgccgaccagaacctggtcaagatctgcga
cctcgggctggccctgcacatgtgcgacgcgccgccgtacgagcgggcgggcacgacgtggtacatggcgcccgaggtggtgctggggaagcccgac
tacgacgcgctcgtggacgcctggtcgctcgggtgcgtcatggccgagctcatcaacggggaagtcctcttcttcccgggcgacaacaacaacggcag
cgaagcatgccagcaaatccgcgccatcatcgacgtgctcggcgtgccggacggcaaggcgtggccgtggttctcaaccacgccgttcgcaacaggg
gtgctgcctcggctacggcaccggcagaggcgaagcctgctgcgccacaagttccctgccacggtgctgtcccagcaagggttcgaggtcctcagcgg
ccttctcacgtggaaccccgacaagcggctcacggcgagcgccgcactcaagcacccgtggttcgacgatctcaacgcccctactgctttgccggcaag
gaacaaggaagacctgccgtcggcgttgcccaacatgaagcacccgcggacgatgatttctccaaacttgttgcacaagaggcgcaagatccaatgcg
cctga
>g64361.t1
atgtggcagctgctgcagggcgccaagcggatgcacacggtccacgtcgtgcaccgcgacatcaagcccgacaacctcctcgtcagcgccgaccaga
acctggtcaagatctgcgacctcgggctggccctgcacatgtgcgacgcgccgccgtacgagcgggcgggcacgacgtggtacatggcgcccgaggt
ggtgctggggaagcccgactacgacgcgctcgtggacgcctggtcgctcgggtgcgtcatggccgagctcatcaacggggaagtcctcttcttcccggg
cgacaacaacaacggcagcgaagcatgccagcaaatccgcgccatcatcgacgtgctcggcgtgccggacggcaaggcgtggccgtggttctcaacc
acgccgttcgcaacaggggtgctgcctcggctacggcaccggcagaggcgaagcctgctgcgccacaagttccctgccacggtgctgtcccagcaagg
gttcgaggtcctcagcggccttctcacgtggaaccccgacaagcggctcacggcgagcgccgcactcaagcacccgtggttcgacgatctcaacgccc
ctactgctttgccggcaaggaacaaggaagacctgccgtcggcgttgcccaacatgaagcacccgcggacgatgatttctccaaacttgttgcacaaga
ggcgcaagatccaatgcgcctga
>g64377.t1
atggcgcgggagtggagcatgtcggactttgagatcggcaagtacatcggcgagggcaagttcggcaaggtctacctcgcacgggagaagaagagc
gggtacgtggttgcgctcaaggtgatgttcaaggagaagctggaaaagtaccggttccacgcgcacctgcggcgggagatcgagatccagagtggcct
cgaccaccctaacgtgctccgcctcttcgcctggttccacgacgaagagcgtatcgtcctcgtcctcgagtacgccgcccgtggcgagttgtacaaggtc
cttcgctccgctggccgcttcagcgagagcacggccgccacgtatgtcgcgagcctcgctggtgcattggcatactgtcacaagaagcaggtcatccat
agagacattaagccagagaatttgctactcgacattgaggggcgacttaaaattgcagattttggatgggcggttcgaacaaatgctaaacgacacac
actctgtggtacaatagattatctggcaccagagatgattgagaaaaaagcgcatgatcatgctgttgacaactggaccctaggaatcctgtgctatgag
ttcttatatggcaagccaccttttgaagccgaggaacaggatgataccctgagaaggattgttaacgtggatctgacattcccttcaactcattacgtatc
tccacaggctaaggatctcatttccaagcccttctgctgggaaccggaccggtttccgtga
>g64379.t1

atggcagcgccgacgccggatcgggcggcgctgacggtggggccgggcatggacatgccgatcatgcacaacagcgaccgctacgagctcgtgcgc
gacatcggctccggcaactttggcgtcgcgcgcctcatgcgcgaccgccgcaccatggagctcgtcgccgtcaagtacatcgagcgcggcgagaagca
agtatgtcaccgtgatctgaagctggagaatacactgctggatgggagtgatgctcctcgcttgaagatatgtgacttcggctattccaagatagctgat
gtatggtcttgtggtgtgaccctctatgttatggtagttggcgcatatccttttgaagaccctgaagagcctaagaacttccgcaagacaattcagcgaat
cttgaatgttcagtactcaattccagataatgtgaacatatctccagagtgcaggcatctaatttcgaggatatttgttggtgatcctgctatgcggatttca
atccctgaaatccgaaaccatagttggttcttgaagaaccttcctgctgatctgatggatgatgatagcatgagcaaccagtacgaggagcctgatcagc
cgatgcagaccatggatcagatcatgcaaattttgacagaggccaccataccacctgcttgttctcgcagcataaacgccctagctgatggactggacat
ggacatggacgatgacatggatgacctcgagtccgattctgatcttgacgttgacagcagtggtgagatcgtgtatgcaatgtga
>g64609.t1
atgttttccaagcttctactcgttccgctcctcctgatccatgtggcctcggccaccgccgccgatgaccagttcgtttaccacggcttcgctaaagcgaac
ctggcggtggatggcgttgccgtcgtgacgcccggtggcctccttgaggtaaccaacaccacggaccaggcgaaaggccacgccttccaccccatccc
tctccacttcctcctcgaccagtccacatcaaggaggaatgccaccgcgtctacagcttcgacgaacactactaccttgctgcgatccttctcgacgtgct
tcgtcttcgccatcgtgtccaagtacgaggggctgagctctcacgggctcgccttcgtggtcgcctcgaccaagaacctctccacggcgaactcggggc
agtacctgggcctcctcaacgcaaccaccagcgacacagcgagcgaccacatcctcgcggtcgagctcgacaccatcatgaaccccgagttccgcgac
atcaacagcaatcacgtcggggtcgacgtcaacagcctcgtgtctaagccagcccatccggccggctactacgacgatgatgccggcggcgtgtttcga
gagctgaagttaaacagccatgagccgatgcaggtgtgggtggactacgacggccgggctagacaactgaacgtgacgctagctccggtccgagtgc
caaagcccaagaagcctcttctctccacggccgttgatctttccgttgtctttgcggatccgatgtatgtcggcttctcggcggggactggagtcttgtcta
cgagtcacaacgttctcggatggagtttcagcttcgatgggcctgctcctgctcttgacatcaccaaactacccgtcctgccacggttgggtccaaagccc
cagtccaaggtcttggatgtcattctaccactcagcaccgcatcgatcatcaccacattgctagcaaccatcttcctcatcatttggaggaggcgcatcta
ctccgaagtgcgggaagactgggaggaagagttcggtccgcatcggttcgcatacaaggacctgtttcatgctaccggtggcttcaaggatcggaattt
actcggcgtcggagggtttgggagagtatacaaagggatacttcgggcgtcccatctggaaattgcagtcaaaaaggtctcacatgattcaaggcaag
gagtgagggagttcatcgccgaggtggtgagcatcggccgcctccgacaccggaaccttgtgcaattgctgggctactgcagacgcaagggtgaactt
ctattagtgtacgactatatggcaaacggcagtcttgataagtacttatacgatccaagcgagcccgctctttcgtggcaacagaggtattggatcatca
aaggtgttgcatcaagcatcctgtatctgcatgaagactgggaccaagtggtcatccatcgtgatataaaagcgagcaatgtgctcttagaccatgacat
gaacgggcgtctcggtgactttggccgagccagactgtatgatcacagcacggaccctcagacaacccatgttgttggaaccatgggttacctcgcacc
agagctcgtacgtgatgggaaggcaactccagccaccgatgtgttcgcattcggcgcattcctcttggaggttgtgtgtgggcggaggccaattgagcg
caacagccacggcaattccatggtgctggtcgactgggtgatggagtgccacggcaatggtacaatcctcaacgcggttgacccgcgacttgggtgga
agtacaacgccgaggaggtaaacctcgtgctaaatctgggtatgatgtgttctcatccgctgcccggtgcacgacccagcatgcggaaaattgttcagt
acttggagtcagatcacccggtccctgacctatcgccgagctattcgagctatgacatgttgagactaatgcagatccaaggatttgattcttatgctacg
tcgagcggtctgtctgtggccagcattggtgcatcatcttcgagtgttttctcagaggggaggtga
>g64611.t1
atgtctgccatgattgtatccttcatcgccctgctattcctcacgcacgtgccctcatccgtcggcgacggccagattttctaccaaggcttcgcctcagcg
aagcttagtctggacggcaacgccgtcgtaacacccgccgggctcctgatgctcaccaacactacggaaatgcagaaaggccacgcgttccaccccac
tcctctccacttcctcaacaagtccgctgcaacggcgacggcgcggtccttctcggcgagcttcgtcttcgccatcgtgcccacccgcgaagggctcagc
gaccacggcatcgccttcgtggtctccccgaccatgaacttctccaaggcgaacgccggccagtacctcggcctcttcaacgccacaaacggcacggc
gagcgaccgtatcctggcggtcgagctcgacaccatcatgaacccagagttccaagacatcaacagcaaccacgtcggcatcgacatcaatagcctca
tctcggagcctgcccagccggccggctactacgatgaagcagcaggcggtgccttgcgagaactgaagctaaacagccgcaggccgatgcaagtatg
ggtggactacgacggaaacgccaggcagctcaacgtaacgctttctccggtgaaaatgtcaaagcccaagagacctctactctccgctaccgtcgatct
atccggtttcatggcggacccgatgtacgtcggcttctcgtcggcgacgggcatcgagtccacgtaccattacgtgctcggatggagcttcaacttggac
ggaccagctccgcctctcgacttctccaagctccccgtcgtgccacgcgtgggtccaaagccccggtccaagctcttggacgtcctgctgccactagctg
cagcgttgctcgttgccgcagtgcttgctgccgtcttcttcttcctgtggagaaagcggcggtatgccgaggtacgcgaagattgggaggacgacttcgg
tccacaccggttctcatataaagatctgttccacgccactgatggcttcaaagatagaaacctacttggagttggagggttcgggagagtgtacaaagg
gctgcttccggcgtccaatttggagattgcagtgaagagagtgtcccatgactcaaggcaaggagttaaggaattcgtcgccgaggtggtgagcattgg
ccgcctccgacacagaaatctcgttcaactactaggctactgcagacgtaaaggtgaactcctcttggtgtacgattacatggcaaatggcagtcttgat
aaatacttgcatgatcaacacatgccgactctttcttggcacacgaggtatcaaattatcagcggagttgcagcaagcctagtgtatctacacgaggact
gggagcaagttgtcatccatcgagacgtgaaagcaagcaatgtgctactggacgccgagatgaacggcaggctcggtgacttcggtttggccagactg
tacgaccacggcaccgatcctcagacaactcgcgtcgttggcaccatggggtaccttgcgccggagctcgtgcgcaccgggaaggcgacgcccttaac
ggacgtgttcgcgtttggcatgttcctcttggaggttgtatgtgggcataggccaatttatcgcgatgaacacgaccgtcggatcatgttggtagattggg
tgattgagcatcatcacaaaggctcaatcctggatgtcgtggatccaaggatagtggggaagtacgaaacggaggaggtgactcttgtgctgaaactg
ggcttgatgtgcgcgcacccaatgccgaacgagcggccaaccatgcggagaatcatgcagtacttggactccacggaacaggttcctgatctgtcacc
aagctatatgagctatggtacgatgacgctgatgcagagcgaagggtttgattcgtacgtcatttcgtctcagccagcatctatgacaaacagtgtatcct
cctctgtggctggtctctcaggggggagatga
>g64612.t1
atgatgtttcatcatctctacctaattactctgctcctcctcgtcctgcctgctgatcgctgcatcacagcagtcgcagctgccgacggcgatggcagaccg
ttcatctacaatggcttcaagggtgcgaacctcaccctcgacggctcagccacggttacgccgaacggccttctgatgctgaccaacggcagcatccag
atgaagggccacgcgttccatccatctccgttgcctttccaacaagatggccagaacagcagcgcccccggtccttctcgaccaccttcgtgttcgccat
cttcgggccgtaccccgaccggagcacccatggcttcgccttcttcatcgctgcgaacagggcggtgctgtccaccgctatgacgagcaagtacttggg
cctcctcaatgacaacaacgacggcaaccagagcaatcacgtattcgccgttgagttcgacactctcttcaacaccgagctatttgaccccaacggcaat
cacgttggcgtcgacatcaacagcttgaagtcacacgttactgctaacgccggttactacgacgacggcaccgggcagttcaggaacctgagcctggtc

aaccgaaaggccatgcaagcatgggtggactacgatggcgtggccacgcagctcactgtgaccatggcccctctgggactagccaggcccaagaagc
cgctgctgcagactactgtgaacctgtccgaagtggtgcaaagcacggcgtatgttgggttctccgcagcgacgggcatcctcttctcacgccactacgt
ggccggctggagcttcgcgctggacggaccggccccggcgctgaacatcgcagagcttccaaacttgcctcccgctgggcccaagcccagatcgaag
gtgttggagatcgtgctgcccatagcgtcagcgacggtggtgttctcagtggggatcgtgatctacaccttggtgcggcggcggatcaagtacgcggag
atacatgaggactgggaggttgcatacgggccgcaccggttctcgtacaaggattcgttccatgccaccacgggtttcagtgacaagcagctgctcggc
gcaggaggatttggaagtgtgtacaagggcgttctccgcaaacccaacatacaggttgcggtgaagaaggtgtcccatgagtccaggcagggcatga
aggagtttgtcgctgaggttgcgagtatgggacgacttaggcaccgcaaccttgtgccgttactcggctattgccgccgcaaaggtgagcttctcttgatc
tacgattacatgccaaacggtagcctcgataaatacctatacgacccaagcaagagcccgttggattggtctctcagattccacatcattagaggagtgg
cgtccggtctactgtatctccatgaggattgggaacaagttgtcattcatcgcgatgttaaggcaagcaatgtgctccttgatgttgaaatgaacggacg
gttaggagattttggactcgcgaggttgtacgaccacggagacgatgctcatactacacatgttgtgggcactatggggtacctagcccctgaactgggc
cacactgggaaggcaactccggcaaccgacgtctttgcttttggggcttttcttctggaggtcacctgtggaagaaggcctattgagcacgatgaacaca
acaattgtgttgtgctcgttgatcggataatggagtattggcgcaagggttcgatcatcaacgccatcgacccgaggattccagatgggtataaccctga
agaggtctctcttgtactaaagcttgcgctattatgcttgcacccattacccaatgcgaggccaaccatgcgtcaggtcacgcggtaccttgacggcgac
atgatccttccggacatgtcaccagcttacctcgacttcaccttgatggataggatatatgggacagggttgaaccagaatgtggtgtcatgcatgtcatc
cacgagcgtaggttccatttctgagctttcgatcggcagatga
>g64736.t1
atggatttcagcccgcaacgaaatagcaatgaatacaatcgtagcagcttcggggatgacgggtatgagttcaccgcagactccatcaccccgaaaca
acagcaacctgcaaattcttcgcaggacaagaaatccaagcccaagaacaaaccggatgaagtcgacaaagtgagcccgcaaggcgcaacaaaca
atccaggccacaagaccacgacgccagcgcaaggctccgatcatgccggagccatgtcggcggcggcggccacgacagcaagcgccggcaaccata
ataatactgaagcctccagcaacaacaaccaggaaagtggcggcgcgcggagcaacagcatggagagcagcagcggcggcgcgccgtcggcgcac
gtgcgtcgccacaccggcggcgacagccggtgggacgcgatccagctggcgacgtcccgcgaggctgccctgaacctgtgccacttccggctgctgaa
gcggctcgggtacggcgacatcggcagcgtgtacctggtggagctccgcggcacgtccgccttcttcgccatgaaggtgatggacaaggcctccatcat
cagccgcaacaagatggcccgcgcgcagacggagcgcgagatcctcggcctcctcgaccaccctttcctccccactctcttcacccacttcgagaccga
caagttctactgcctcgtcatggagtactgcagcggagggaacctccattccctccgccagaagcagcctggcaagcatttcacggagcccgcagccag
gttctacgctgctgagatactgcttgcactggagtacctgcacatgctggggatcgtgtacagggacctgaagccggagaacgtgctggtccgtgaaga
cgggcacatcatgctgtcggacttcgacctgtcgctgcggtgcagcgtgtgcccgacgctggtcaagtcctcctccgtgcactccaacaacaacaacgg
cagcagcaacagcgtggtcatccgaggagacgccggcgacggcgacgtggtgagcggcaaccagggcagctgcatccagccctcctccttcttcccg
cgcatcctgccgcgccgcagccggaagccgtccaggagcgacgtgggcggcggcgggccgcccgcggtggagttcaacgccgagcccacggacgcg
cggtccatgtcgttcgtgggcacgcacgagtacctggccccggagatcatccgcggcgagggccacggcagcgccgtcgactggtggacgctcggcat
cttcatctacgagctgctgcacggctgcacgccgttcaagggcgccggaaaccgcgccacgctgtgcaacgtcatcgagcagccgctcaggttcccgg
ccgacgggatcgggcccgggccgcagcccagcaacgccgcgcgggacctcatccgcggcctcctcgtgaaggagccgcacaagaggatcgccttca
ccaggggcgccacggagatcaagcagcacccccttctttgagggtgtgaactgggcgctggtcaggagcatgaccccgccgtcggtgccggacccggt
ggatttccggcaatacatcgaggccagggagaagaaggcgccgccggagaatgctgcagcggctggtggtccgccaacggcgaaggccaactccga
gtccggtactgactttgagtatttctag
>g64816.t1
atggcctcgtcgccgcacagcacaacaattctctgtgtggctgtggcaagaagagcccaagagcaccacttcttgttcgatcgatcgccgcacaacaag
atgagaggactcgttccatctcgtgtcctggcgacggcggcggcgtcccgcaagcgcaagcgatcaagaagcttggcggcaatgaagaacaagaagc
gcgtcgtcccccgcagcgcgggcggccaaagcctccgccgccggatagcaacagtggcgtcgtcgcgggagcggttgccggagaccgaggtgcgcg
agtacatgcggcagctcctacgcgcgacgaagcgggcccacgcgaggggtctcgtccacggcgacatcaccccggacaacatcctcgtggcgggccc
gccggacgacggcaagacgacgaagctcgagctccgcggcttctcgcgcgtgtcgccggtgcggggcccggacgggcggtgcatcctgcgcacgacg
atgcggtaccgctcgccggagctgctcaactacatccccctgctttctcctgtgcgagggccccgccgctgacgtgtacgcgctcggctgcgtcatgtacg
agctcgtcaccggcgatcccctgttcaaggcggacacggcggaggagctgttggtggaggcggaggagctgcacgacgcgctgatcgagctggagga
aggctacgacggcctcaacctaggggaactgtcgctgcacggccagcaggtgctctccggcctgctgcagttcttcgactgcgagaggctcacggccg
cagacgcgctcaagcacccctggttcacggaatcgccgaggacgaagacgaccaggagaagccgcgccgaaggcagagtggaagacccggaagcc
agcttcggtagtccaacacgaagcggctcttga
>g65167.t1
atggccttgggatggatgagccgggtggatccgatcaccgccgtttcccagctcgccggcgtggacgctgtgagcctgatcaccatgatcatcgagcgg
gtggagaaggtccggaggaacaagcacgagtgccgtgcgctggccaaggacgcggagaagatcaagcaggtcctggcgcaggcgtcgctgaggcc
ggacccagcgatcgatgacatcatcaaggagctggaggcgacgctgagggaggcgtgcgagctcgtcgcgtcctgcgaggccaccagctacctccgc
cgcttcttccgcagcaacaaggtggccgagcgcttcgagcgcgtcaggcagagcatccagctctacatcctgctcctcccggtgctctaccacttcgaca
ccaccaagcgcctcaagaggatccagcacatggtccagcagctccagcccgtgtctgaaacagagtccgcagaggcgcagagtggctgccctgccac
cacgcccaacaccaccgccatgactagtctcgacacaccacaccgccctgggttgactcccgcacactgcgccatgcggcatgtaccgggcaagcgcc
acccttgcgcagatggaacaaatggcgaatatttgaactggggtacattgtttccgataattgagggaatagctcagggggtggtgtatcttcacttgca
ctcagggctaaatataatccacagagatctcaagccgagcaatatcctgctggactctgaaatgaatcctaagatttctgattttggaacagcaaggttt
ggtcatctggacaagaacacgagggcagatgtcattgcaggaacatatggttacatggcttcggagtattcaagcaaaggtatcttctcagccaagtca
gatgtgttcagtttcggcagcatgctgcttgagatactaagtggaaaaagaaatggcacgtgctacaaaagcagattcaggaaggggaagttattgagt
ttgcacgaatatgcatggtacctgatgtttatggaggatgcaccaaagaagctgaatcagctgatccatccatccttgtactgcgaacgagggcctcgag
cagagcacgtcacgaggtgcgtgcacatcgcgctattgtgtgttcaggaggatccagcggaccggccctccatgaaggacgtcgtccttatgctcaacg

gcggggccagtatggcagggcagctggtgccgcccaaaaaaccggcacaccggtacggcgacggcaagatgatgccgactttggcagagttgttgcg
tgatgacctcgagcaatgcaacaagacaatggtagctgcacgcgcacccaacaagacactgcacgcgcatccatatagataa
>g65304.t1
atggagatcgtggagaagtacgagaagctggagaaggtcggggagggcacctacggcaaggtgtacaaggcgcaggacaaggcgacggggcagct
ggtggcgctcaagaagacccgcctcgagatggacgaggagggcatcccgcccaccgcgctccgcgagatctccctcctcaacctcctctcccactccct
ctacgtcgtccgcctcctcgccgtcgagcaggccgccaagaacggcaagcccatcctctacctcgtcttcgagttcctcgacaccgacctcaagaagttc
atggacgtctacaaaagggggcctaacgcgaggccgctacccaccgacctcgtcaaggtggtaccactttcgaattactactcgttgttcatccactatt
ccgacctaattcagttcaaattaaactag
>g65421.t1
atgtggcccgcgtggatggcgccggaggtggcgcgaggcggcgcggcgacgccggagtccgacatgtggtccctgggctgcacagtgctagagctac
tcatggggaagcgcccctggtcggagcacggcggcgcgtccaaggtcagcgagctgctcttcctggtcggctttggcgggacgcgccccgctttcccga
cttccctgtccgatgcatgccggaacttcctcgacaagtgcctctgccgcaatgtcgggcagtggcggacctgtgagcagctgctgcaccacccgttcct
cctccttgtctcttcggacgacgatgacggcgccgatgagtcgtccacgtgtccttctcctcgtgcggtcctcgattgggccatggcagactcaaactctg
aggcggtggatgatgaactggagctcgaggacgaggaggtcatggtgcgtgccaaggggcgggtcgccgtattggtgcgtcctcaaacaggccacac
gcgagcggggagaagctgggctagggccagtagcaccacttgggcggccaacatctag
>g65684.t1
atgaggatgcttgggacacccaatgatgagatatggcctgattattccaaattacctggtgctagagcccaatttgtaaagcaaccgcgaaataggttaa
gggagaagtttccagctgtatcattcactggagggttgacattatccgaatctggatttgacctgctaaataggttgctgacatttgaccctgagaagcgc
atatcagcagatgctgccttgaaccacgagtggttccgtgaagttcctctgcccaaaacaaaggatttcatgccaacacttcctgctctgaatgaacaag
acaggcgcttcaaaaaatatgtgaagagccctggtcctctggaggagcaactgatgaaaaaacaggggaacataagagatcgagggctatttggttg
a
>g65964.t1
atgacgacggccgtgtggaggcatgatgttggttttctcgtcgtcgtgctcctgcttcgctcgccggcggcaaaggcagctggcggcgggacctcgtcgc
cgccgtgcccgctggacctccgctacgtgtcaacgctcccgtgggaccgcgcgccgtgcctgccgccggtgtcgaaccggacggcctgctgcacgacg
ctgctctccgtgctgggcgtgggcctggcgtcgcgcctgcggtccacgggccacttccgcctcccgtcggccccgggctccgccgcctgcctccgcgcct
tctccgccgcgctcaccgcgccgccggtctctctcccgggctcctcccgcgtggtggcgggctgcttcccggacccggcccagctcgccatctccccgac
ctactgcgccggcgtcaccgacgccgcgcagtacgaggcggtcgtcgggaacgcctccgcggcgcagctcaacgcgtcgtgcggcgccgacctcgcg
tccatgtcggtgtgctcgcggtgcctcgccgccggcatcgacgcctcgtcaaggctcaccgtggccgccaacgacgggtctaataagtcgctcaactgct
tctacctcaccgtgctgtacgccgcaggaatctccagctcctccggccctgactccctcgccaccgccaactgcgtcctcggactcgctctctccacctctc
ccacttcttcccctgcttcttcttccagctccaccaaccacaccaatatcgcggtcgccaccaccatcccgatcgcctccgtcctcctcgtttttctcatcatc
gctctgcttctatggaggaagaagcggcacgaaaacaacaacatcagaaagagcatcgtccacctgtcgcgggaccggcgtcccaacaccgggtcgg
tgctgttcgacgtgggcgagctggcgcaggcgacgggcggcttctccgatcgccacgtgatcgggcgcggcgggttcggcgtggtgtaccgcggcgtg
ctgccgggggacggcaccgtggtggccgtgaagaagatgctggacccggacgtcgacggcggcgacgacgagttcaccaacgaggtggagatcatc
agccagctccggcaccgcaacctggtcccgctccgcggctgctgcatcacttccgacgacgatgatcaccaagaagacggcggcaagcagatgttcct
cgtctacgactacatgcccaacggctccctggaccagtacatcttcagtactgaagatgccaactccaagcggctcttgttgtcgtgggcgcagcggag
aagcgtgatcctggacgtggcgcgtgggctggagtacctgcaccacggcgtgaaacccggcatcttccaccgcgacatcaaggccagcaacgtcctcc
tcgacgccgacatgcgggccagcctcgcggacttcgggctcgcccgccgcagccgcgagggccagtcccacctcaccacccgcgtggcgggcacgca
cgggtacctcgcgcccgagtacgcgctctacggccagctcacggagcgcagcgacgtgtacggcttcggcgtgctggtgctggaggtcatgaccggcc
gccgcgcgctcgacctcgccgaccccgccggcgtcgtgctcgtcaccgactgggcctgggcgcgcgtcagggcgggccgggccggggacgtgctcgc
ggaggccctgcgccgggataataataataataataatgaggtcatgaagaggttcgtgcgcgttgggatcctgtgcgcgcacgtcacggtggcgt
gccggcccaccatgcccgaggcgctgaggatgctggagggagacgtggacgtgcccgagctgcccgaccgcccgcagccgttcgggttcgggggcca
gaggatcgcgttcgacgacttcgacgccggcaacttcagtgtttcctcggtcatgagcggaccgttcgtggactttggcgatatgctcaggtga
>g66177.t1
atgcaccgtggttgcctgtcgtgtcgcggccatctgcgacatgatcgagcatcatcgcgacgccgggacacccctaagctcgagccaagtcgctacaat
ctcgtccatgatcaacgacgtcgccggtaccaaggcgccgccgccgaggaccggaagaagaaaaagcgatgcatgatcagcgtggggtcgtacgag
gacgagggcgtggtcggcgagggtcggttcggcgtcgtgttccgcgcgcgccaccgcggcacgggccaggacaccgccgtcaagtcctaccgctccg
actacgacgacggcattaaagacgacggagcgccaataatggcggctactccctcgtga
>g66199.t1
atggggtgcgtctgcggccgcccctctgtcgtcgacgacggtcggtgccagacgacgcctccgccggcgtccaagctctcggcggcggcggctggagt
ggcggcggtgaggagggaggaggaggtgcggaagcagcagcagcaattacagcatgccagggtggggagccggagggaggaggtgttggagagg
cggcgggcgatgatggcgatggcggcggcgtgtcaggcgaggagcccggtgccgaggacggtggagggcgagcaggtggcggcggggtggccgcc
gtggctcgtcgccgtcgcgccggaggccgtgcgcggctgggtgccgcgccgcgcggagtcattcgagaagctcgacaagattggccagggaacgtac
agtaatgtctatcgagctcgtgatctcgaaaaggagaagattgttgctctgaagaaggtgcgctttgataatttggaacctgagagtgtgaaatttatgg
ccagggaaatcctcattctgcggcgtcttgatcatcccaatgtcatcaagctagagggcctagtaacatcaagaatgtcctgcagcttatatcttgtttttg
agtatatggaacatgatctagctggactagcttctttcccaggagcaaaattcaccgagtctcagttgactccaagtgttgccaatacgcaaggtgctgct
ggaggaaagcatcataagcatgaccctgaaaggcgaagtagagaatcaagagctgtccctgctcctgacgcaaatgcagaactagtgtcgtctttgca
gaagaggcaaacccaagctaataccaagagcaggagtgagatgtttaatcccagcaaagaagactctgcttctggttttcgaattgaaccaatcaggc
aaactcctgttattgaatcttctgaagactcacgacatgcctacccgaccagaaccttccattctgggcctttggttaatcagaacaactcatcaaaggct
ggacgtggcaaaagtggcgaacttcaggttcctggtgctgccaatgtctcagtggttgtatcgacaagagcaggtcttcaacctgataacagcaatagg

acaatgataactcaagctgaagcttttgctcatggacggaggctgtctgagtctatcaacgagcatttcagtaatagtgggaagtatgaccaagtattcc
agcagaaggatgaaaggaacggccgggtggatggagcaattggctatgggtccaagggcaacaagatccaccattccggaccccttacatgcccttc
aggcaatgtcgatgagatgctgaaggagaacgatcgccaaatccaagaagtcttcaggcgaacccgggtggagaagtccagagtgaggaatgggca
tcaccaccccagcgattttggtgccatcccggtctacccttctagtcgttccagctatcagtctgtgcagcagtag
>g66202.t1
atgaacttggggaattccaagggttcgagggctaagagcctcgtcaagagcccaagttcgacattgctcctcagctacttgaacaaggcagagattcct
ttaagcccatcaaggaaccaggagaattcaaagggtccgagggcaaggagcctggtcaagagcccaaagaatcaccccatccctctggaaaagatca
tatccattgttcttgatgttgcacgcggactggagtacatacactctcaaggaattgtacaccgtgacattaagcctgagaatattctctttgatgataactt
ctgcgtgaagattgctgattttggtattgcctgcgaggagactgtgtgtgatgtgctagtggaggatgagggcacttacaggtggatggctcctgagatg
atcaagcagaaggcatacaacaggaaggtggacgtctatagctttgggttgctcatatgggaaatggtgtctggaagaattccttatgagaacctgacc
cctttccaggtggcttatgctgttgccaataggaatttgaagccaacaattcctcccgaatgcccctcagctctcaggcccttgattgagcagtgttgcgcg
ttgcagcctgacaagaggcctgacttctggcagattgtgaaagtcctggaacagttccagtctgttctgtcgcagggcggttgcctcgatacgctgaaga
gcggcacctgccaggatcacaagaagcggcttctgcattggattcagaaactgaagccatcgcatggcacctga
>g66239.t1
atgcacaggagaggtgggaaatattctgaagaagatgccaaggttgtcattcatcaaattctaagtgttgcttcattttgccatcttcagggtgttgttcac
cgggatctgaaaccagagaatttccttttcatgtccaaggatgagaattctgccttgaaagtcatagactttggtttgtctgactttgttaggccagatgaa
agacttaacgatattgttggaagtgcatactatgttgctccggaggtgctccaccgatcttacggcaccgaggcagatatgtggagcattggagtgattg
cgtatattttgctttgtggaagccgccctttctgggcacggaccgagtcaggaatatttcgggctgtcctaaaggcagagcctagttttgatgaatccccat
ggcctaacctttctgctgaagcaaaagactttgtaagaaggctgctcaataaggattaccggaaaaggatgactgctgcacaggccctctgccatccat
ggatctgtggcggccaagaagttaagattaacttggacatgattatttacaggcttatgagggcttacataagttcatcctctcttcggaagtctgctttaa
gggctatgcacaggagaggtggaaagtattctgaagaagatgccaaggttgtcattcatcaaattctaagtgttgcttcattttgccatcttcagggtgtt
gttcaccgggatctgaaaccagagaatttccttttcatgtccaaggatgagaattctgccttgaaagtcatagactttggtttgtctgactttgttaggcca
gatgaaagacttaacgatattgttggaagtgcatactatgttgctccggaggtgctccaccgatcttacggcaccgaggcagatatgtggagcattgga
gtaattgcgtatattttgctttgtggaagccgccctttctgggcacggaccgagtcaggaatatttcgggctgtcctaaaggcagagcctagttttgatga
atccccatggcctaacctttctgctgaagcaaaagactttgtaagaaggctgctcaataaggattaccggaaaaggatgactgctgcacaggccctttgt
catccatggatctgtggcggccaagaagttaagattaacttggacatgattatttacaggcttatgagggcttacataagttcgtcctctcttcggaagtct
gctttaagggcattgacgaagaattccacaagtgcaatgaatgattcaagggttcttgatttcgttaacacaatatgcaatgtccagtaccgaaagcttg
attttgaggagttctgtgcttcggctattagtgtttatcagatggaaggtctggagacctgggaacaacatgctcgagaagcatatgaattatttgacaag
gagggaaatcgaccaattctaatggaagaacttgcatcggtatag
>g66251.t1
atggatcccgccgccgcacacgaccccaacgcgccctgctccccgctctccaccacgaacaccgacctcctctcccctcacttccctccctccctcgccg
acggcgccgccgcgctggacctctccttcacctcgaccgcctccgcctccacctcctccttcacgacggccaccaccttcagcgcgcgcagctcgctgtc
cctcccgtccttctcctcctccacctcgctctccccgcggccacactcctcctcctcctccacgcactggacccacctcgccgccgcccgcgccgccaccc
ccgacggcgtcctccgcctgggccacctccacctggtccgcgagctaggccacggccacctggcccgcgtcttcctctgccgcctcaagagcgcgccgc
cgagctccccgctcttcgcgctcaaggtggtcgacgtccgcgacgacgacccgtcccgcgtgtcccacgtgctggccgagtcccgcgtgctctcctccct
cgaccacccgttcctgcccaccctgtacgcgcgcctcgacgcgggccgatacgcgtgcttcctcatggactactgctccggcggggacctccacgccgt
gctccggcgccgccccggcgggcggctcccggtggccgcggcaaggttctacgccgccgaggtgctcctcgcgctcgagtacctccacgcgctcgggt
tcgtgtaccgcgacctcaagcccgagaacgtgctcctccgtggcaacggccacgtcgtgctctcggacttcgacctcgcgctcccggcgtccgtcgagc
ccgccgtgcgacgccgccacgtgcggaatggtatgaagcagagtcgaaggcgcagcaggaagaagatgaacaccttcttcttgtcgtgcttctcatctg
gtgggaataataataacaaggacaccgacgaggaggaggaagacagcgtggtggacgccaaggagcggttcgagttcgtggcggagccgacgggc
gcgagctccaaggactgcgtgggcacgcacgagtacctcgcgccggagctcgtcagcgggagcggccacggcaacggcgtcgactggtgggcgttcg
gggtgttcctgtacgagctcgtgtacggccgcacgccgttcaagggggcaaccaaggaggccacgctcaagaacatcctgtccaggcaggtggcgtac
ccgcacctcgacaacaccgaagacgtggcgcagctcaaggacctcgtcgcgaggctgctggagcgggacccgcgccgccgcatgggcgcgtcgcgc
ggcgccgcagagatcaagaggcacccgttcttcgccggcgtggactgggcgctcatacggtgcgtggcgccgccggtcgtgccggacagggacgccg
ccactggcgggaataagccggcggggaagcagctggggagctggagcagcatgggacgcggcagcagcgccagcagcaagaggaagagcagcag
cttctccaggaagtccaattacgaggagcgccagggggttttccgcaagctcatgacctggagccacgagaaccggtccagcaagaccgccaccaag
accagcaaagtaaaactatga
>g66283.t1
atggagaagagcagcagcagcaggcaggaggacgccgcgccgtcgtgggcgcccagcaaggcctcggcgttccgccgttatgttgttgcagcggctt
cggcgcggccgcaggaggcgtctccgtctgctgctggcaatggggctgccgcccgcgtgagcagccttcatggcgtcaggaggaagccgtttgttgtca
gatcaacggcggacatcattcaaacatttgaacggtgcaaccctgagtttaagtactcagaggcactaaacccgaaacgttttctcactaatccctctac
tccagctcataataatgggcttgataatgcgaattgggacctcatattgtatgtcaacttggaattggtcaatgagacgtcgaaccggaggttcattgtca
aggaaatgcttggtcaaggaacatttggtcaagttgttaaatgctgggacacagaaagcaatggctatgttgctgtaaaggccttttcaaaacagatatt
agatgccatggttgtgatgagagatgcgggcatcattcattgcgatctaaagcctgaaaatatcctcttagctcccaatgccacaacagcagcagcagt
gaaagttattgattttgntttttttgagcattggatgttttatttgtag
>g66332.t1
atggagagcgagatggcggatgaaccggtgacggcggtcgcggaggagggggaaggggagactgtggcggctccggcggcggtgggctcgagcaa
gaccatggagcgcgtggcggccgcgaagaagttcatcgaggaccactaccgcgcgcagatgaagaacatccaggagcgcaaggagaggcgcttga
atttagaacaacagctagcttcttcacaagttcccagggaagagcagataaacttaataaaggatttggagagaaaggagactgaatacatgagactt

aaaaggcacagaatttgtgtggatgactttgagctgctcaccatcattggaagaggtgcttttggggaggtccggctatgtcgggagaagtcttctggca
acatttatgcaatgaaaaagctgaaaaagtctgaaatggttgtcagggggcaggtggagcatgttagagctgagcgaaacttgctggctgaagttggt
agccactgcattgtgaaactgtattattctttccaagatgctgaatacctctaccttatcatggagtaccttcctggaggtgacatgatgacgctccttatga
gagcggatacattgactgaagatgtggcttgcttctacattgctgagactgttcttgccattgaatccattcataaacataactatattcacagggatataa
agccggacaacctacttctagataagaatggtcacatgaagctgtcagattttgggctatgcaagccaatcgactgttcgaaactgtctaccttgaatga
agatgaacccatgagtgatgaaaaccttagggagtccatggacattgatcgttcgttctcagataccgcaaatgaggttctactgaagaagggatatgg
agtagagtgtgactggtggtctcttggtgcaatcatgtatgaaatgcttgttgggtacccaccattttattctgatgatccaataactacgtgccgaaaggc
tcatccttggtttaaaggagttgaatggagtaagctttatgaaatggaggcggcatttaaacctcaagtaaatgatgagctggatacacaaaatttcatg
aagtttgaggaatcaggcccagctccagcaagaacaagttctgggccctcaaggaagatgatgctcaactctaaagatcttagctttgtggggtataca
tacaaaaattttgaagctgtgaaaggcttgcatcagtctgcagatacagcagacatggactcatcaacagaaccgaacggaagggacacgcacatgc
gcactgtttcatctgatgaccctatggtgcagtga
>g66407.t1
atgttacttgaatggaagcagcgatttaaaattgctctgggattggccaaaggattggcataccttcaccatgaatgcttagagtgggtcatccactgtga
cgtgaagccggagaacatacttttggctgaaaacttggagccgaagatcactgactttggcctcgcaaaacttcagaacagaaatggatccaataaaa
atgtgtcacggatccatggaactaggggctatgtggctcctgagtgggtttctagcctcccaataacagcgaaggttgatgtctatagctttggagtggtg
cttctagaattgctaaagggagttcgcatctcacagtgggcagcaaatgaagatgaggaagttgagatggtccttacaaggactgttaggatgcttgaa
gagaacatcaacgagcagtcatggatcactggtgtcattgattccagattgaatggtcagttcaacagcttgcaggcancactggtgtcattgattccag
attga
>g66416.t1
atgggcaactgctgcggcgcgccggcgacgccgggcggccgcaagaaccggcggaagcagaaggcgaacccctacaacgtctcgtacaaccgtgg
ggcggcgccgccgccggcgcgtccggggctgatggtgctgcgggacccgacggggcgggacatgaacgcgcggtacgagctcggcggcgagctgg
ggcgcggcgagttcggcgtcacgtacctgtgcacggaggtggccacgggggcgcggttcgcgtgcaagtcgatatcgaagaggaagctgcggacgcc
ggtggacgtggaggacgtgcgccgggaggtggacatcatgcgccacatgccgtcgcaccccaacatcgggagctcttcgacaggatcgtcgcgcggg
gccactacaccgagcgcgccgccgccgccgtcacgcgcaccatcgtggaggtggtccaggtga
>g66771.t1
atgtataaaactaagagatctgcttcacttaaagtgaagcgccgtgttgggaagtatgagcttgggcgcacaattggggaaggaacctttgctaaggtt
cggattgcaaagaacacggagactggggaacatgttgctatcaagatccttgacaaggcaaaggttctgaagcacaaattggttgagcagttagagaa
cttgctactggatattgctggaacccttaaaatttctgattttgggttaagtgctatatctgagcaattgaaggctgatggattgctgcacactacatgtgg
aacggctaactatgttgctccagaggttatcaatgacaagggctatgatggtgccactgcagacctttggtcttgtggggtaatcctttttgtgttgcttgc
aggatatctgcctttcgaggatgacaatactgtctccctctataaaaagatctcaggagctcaatttacttgcccctcttggttttctgctggagcaaagaa
gctgatctgtagaattctggatcctaatccttcaactgtaagtgacccttga
>g67384.t1
atgttgttcttcacctccgtcgccggggcgcagctcggatctggaaacttgctgatcgacgacgccgagctcctttggtcatacaggcaacagatgtctg
gctttgagctgtcaaagatgacaagaaaggacaagatgacttttaatggtagccaaggcacaatgggatacactgcaccagagtgttctgtagatggtg
agcccatcacggccaaagccgatgtctatagttatggcatgatgctgctcgagatcatcactggccgatcaaacaaggaagttaaagattcagtatttat
ancggcgtga
>g67513.t1
atgaatggaatgagtataagagatgacaaggaagttgaaactgttgttgtcaatggcaatggaatggaagttggtcatactattgtgacgactgttggtg
gaagaaattcacaaccgagacagactattagttacacggcagagcgcattgtggggcaaggttcatttggagttgtattccaggccaggtgtctggaaa
ctggtgagagagtagctataaagaaagttcttcaagatgcaaggtacaagaaccgtgagctgcaaacaatgcaagttcttgatcacccaaatgtcgtgt
gcttgaagcattacttttgctccacgacagataaggacgagctatacctcaatttggtgcttgaatatgtaccagaaacagttcaccgtgttattaaacatt
ataataagatgaaccaacggatgccattgatatatgttaaactgtatatgtatcagatttgtagagctttggcttacatgcacaactgcgtaggagtatgc
cacagagatataaagccacaaaatattctggtgaacccacatactcatcaactgaagctgtgtgattttggcagcgcgaaagtcttggtgcaaggggaa
ccaaatatttcatatatctgttccagatactacagagctccagagctcatatttggtgctacagagtatacaacagctattgatgtgtggtctgctggttgt
gttcttgctgagcttcttctagggcagcccgttttccctggggacagtggcgttgatcagcttgttgaaatcatcaaggttctaggtacaccgacacgcgat
gaaattaaacgcatgaaccccaactacaccgagtttaaattcccacaaatcaaagctcacccatggcacaagactattagttacacggcagagcgcatt
gtggggcaaggttcatttggagttgtattccaggccaggtgtctggaaactggtgagagagtagctataaagaaagttcttcaagatgcaaggtacaag
aaccgtgagctgcaaacaatgcaagttcttgatcacccaaatgtcgtgtgcttgaagcattacttttgctccacgacagataaggacgagctatacctca
atttggtgcttgaatatgtaccagaaacagttcaccgtgttattaaacattataataagatgaaccaacggatgccattgatatatgttaaactgtatatgt
atcagatttgtagagctttggcttacatgcacaactgcgtaggagtatgccacagagatataaagccacaaaatattctggtgaacccacatactcatca
actgaagctgtgtgattttggcagcgcgaaagtcttggtgcaaggggaaccaaatatttcatatatctgttccagatactacagagctccagagctcatat
ttggtgctacagagtatacaacagctattgatgtgtggtctgctggttgtgttcttgctgagcttcttctagggcaggttctaggtacaccgacacgcgatg
aaattaaacgcatgaaccccaactacaccgagtttaaattcccacaaatcaaagctcacccatggcacaagattttccacaaaaggatgccatctgaag
ccgtagatcttgtttctcgtcttctgcagtattcaccaaacctccggtgcactgcattagaggcgttaatccacccattcttcaatgaactacgggatccga
acacccggttaccaaatggtcgttcccttccccagctcttcaattttaagcctaatgagctgagaggactaccaatggagtttctcgtgaaattagtccctc
agcatgctaagaagcagtgtgccttcttgggaatatga
>g67679.t1
atgcaactaacacttggtggtccactgagatatgcgtatacagacatcattgcaatgacaagtcattttagagagaagcttggccatggaggatacggct
ctgtgtataagggtgtcctcctcccgggcaatttacctgttgccatcaagatgctaagcaactccaaatgcaatggagaggagttcatcagtgaggtctc

cacaattggcagaatccaccacgtcaatgtggtgcgtcttgtagggttctgctcagaggaatcacagagggcgcttgtctacgagtacatgcccaatggt
tctctcgataagtacatcttctctctggagaagactttctcttgggataagctcaccgaggtcgctctgggcattgcaaggggaatcaactacctacatca
gggttgtgacatgcagattctgcactttgatatcaagccacacaatatacttcttgatcacaactttgttccaaaagttgcagattttggcctcgccaaact
gttcccgagggacaatagtttcgttccatngtccgtacgagctgacatcattgcaatgacaagtcattttagagagaagcttggccatggaggatacggc
tctgtgtataagggtgtcctcctcccgggcaatttacctgttgccatcaagatgctaagcaactccaaatgcaatggagaggagttcatcagtgaggtctc
cacaattggcagaatccaccacgtcaatgtggtgcgtcttgtagggttctgctcagaggaatcacagagggcgcttgtctacgagtacatgcccaatggt
tctctcgataagtacatcttctctctggagaagactttctcttgggataagctcaccgaggtcgctctgggcattgcaaggggaatcaactacctacatca
gggttgtgacatgcagattctgcactttgatatcaagccacacaatatacttcttgatcacaactttgttccaaaagttgcagattttggcctcgccaaact
gttcccgagggacaatagtttcgttccattaagtgctatgcggggaactattggatacatagctccagagatggtgtcacggagctttggcgtcatttcga
gcaaatccgatgtgtacagctttgggatgctattgttggagatggctggagggcggaggaatgctgatccgaactttgcaaactcaagtcagaattactt
tccgtcctgggtttatgacaaactaattgggcaagaagtaggcgagatatctattgttgatatgcatgagctggagaaaaagctttgcattgttgggctct
ggtgcatccagatgaagtctcatgatcgtccatccatgagtgacgtcatagaaatgctcgagtctgatatcaatgggctggaaatgccttcgaggccatt
cttttgcgacggtgttgagcaaatttctgcagtagattcttattatgtgtcgtccgaactcgatgatatacaggaggaggatgagtga
>g67700.t1
atggcggtggagcagcagcagcagggcggggaggaggcgttcgaggaggtggacccgacggggcggttcgggcggtacgccgacgtcctgggcct
gggctccgtgaagaaggtgtaccgcggcttcgaccaggaggaggggatcgaggtggcgtggaaccgcgtgcgcctccgcgcgctggcggaccgcga
cccgggcatggtggaccggctccacgcggaggtgcggctgctgcgctcgctccaccaccgccatatcatcgggttccacaaggtgtggctggaccgcg
acgccggcgtgctcaacttcatcaccgaggtctgcacgtcggggagcctccgcgagtaccgccagcgccaccgccacgtctccgtcaaggcgctcaag
aagtgggcgcgacagatcctcgaggggctcaaccacctccacaaccacgacccctgcatcatccaccgcgacctcaactgcagcaacgtcttcatcaa
tggcaacaatggacagatcatggcggtggagcagcagcagcagggcggggaggaggcgttcgaggaggtggacccgacggggcggttcgggcggt
acgccgacgtcctgggcctgggctccgtgaagaaggtgtaccgcggcttcgaccaggaggaggggatcgaggtggcgtggaaccgcgtgcgcctccg
cgcgctggcggaccgcgacccgggcatggtggaccggctccacgcggaggtgcggctgctgcgctcgctccaccaccgccatatcatcgggttccaca
aggtgtggctggaccgcgacgccggcgtgctcaacttcatcaccgaggtctgcacgtcggggagcctccgcgagtaccgccagcgccaccgccacgtc
tccgtcaaggcgctcaagaagtgggcgcgacagatcctcgaggggctcaaccacctccacaaccacgacccctgcatcatccaccgcgacctcaactg
cagcaacgtcttcatcaatggcaacaatggacagcaaacggccgggctaacgccgccgcggcacgtgtcgcaggtcaagatcggcgacctcgggctg
gcggcgatcgtggacaagacgcacgtggcgcacaccatcctcggcacgccggagttcatggcgccggagctgtacacggagacgtacaccgagtcgg
tggacatctactcgtacggcatgtgcgtgctggagatggtcacgcgcgaggtgccctacgccgagtgcggcagcgtcgtgcagatcttccacaacgtca
cccgcggggtgccgcccgccgcactcaagcgactcaaggataccgagctgcgcggcttcatcgagcgctgcatcggccagccgcggaaccggccatc
ggcggccgagctcctgcaggatcccttcttcagcggcattggtgaggacgacgacgacgcgatcaccgacgcggccagcgtcgtcgccgctgccggcc
cgcccgtgccgcgtcccaggagctacgtcgacgacctcgccgggctccgcctggattga
>g67711.t1
atggcggcggcggcggcggtggggagatggtgggcgctggtcgtcgcggtggcgttgctggtcgggccgggacgggtcgtcgccaacaccgagggtg
cctctagtactggagcaattgctggaggtgttgctgctggtgcagcattggtttttgctgttcctgcaattgcatttgcaatgtggcgccgtcgcaaacctg
aagagcatttcttcgatgtgcctgctgaggaggatccggaagttcatcttggtcagcttaaaaagttttcactgcgggaacttcaagttgcaactgataat
ttcagcaataagaacattttaggaagaggtggttttggaaaagtgtacaagggaaggcttgctgatggctcgttggtagcagtgaaaaggctaaaaga
ggagcgtacacctggtggtgagctgcagtttcagacagaagttgagatgattagcatggcagtccacaggaatcttctccggcttcgtggtttctgcatg
acacctacagaacgattgctagtctatccatacatggctaatgggagtgtagcatcacgtttacgagagcgccagccatctgaaccacctcttgattggg
atacaagaagacggattgcattgggatctgcaagaggactctcttacttgcacgaccactgtgatcctaagatcatccatcgtgatgtcaaagcagcca
atattcttttggatgaggactttgaggcagttgtgggtgattttggtcttgcaaagcttatggactacaaagatacccatgtaacaacagctgttcgtggaa
caattgggcacattgctcctgagtacctttccactgggaagtcctctgaaaagactgacgtttttggctatgggatcatgcttctggagcttattactgggc
agagggcatttgatcttgctcgtcttgcaaatgatgatgatgttatgctgcttgactgggtgaaaggactgttgaaagagaaaaaggttgagatgttggt
ggacccagatctgcagaacaactaccaagagacagaggtggagaacctgatccaggtggctctgctctgtacacagggctccccattggaccgtccca
agatgtcggaggtggtaaggatgctcgaaggtgatggattggcggagagatgggatgaatggcagaaggtggaggtggtgaggcaggaggctgagt
cggcccctctccgtaatgactggatcgttgattccacttataaccttcgcgcagtggaactgtccggtccaaggtga
>g67791.t1
atggcgccgcgccgcgggactttgctcccgctggcgctcgttgccgcgctggcggccttgtcgtgcgtggcggggcaaggccgtcccgtgaccgacgac
ggcgcccagacggcgccgacgccgtcaagcttcaccccgaaggacagcttcctgatcgactgcggcagcgccgacgccgtgaacacctctgacggca
agtcgtacaagacggacgcgcaggcgaaccacctcctggctgccaaggacgcgttccgcgtgtccgacccgaaagccgacgtgccttggcccatctac
gccaccgcgcgcgtgttcaaggaggaggccgtgtacagcttccctctcgccgtccccggctggcacttcatccgcatctacctcttcccgctcaagggcg
gcggcgacgtggacttgtcgtcggcgaccttcagcgtctccaccgacgtcaacgtgttgctccacggcttcacggcggaggcgaagcccgtgatgaag
gagtttctggtgaacgcgacggagaaccacctcgcgctacggttccagccgctcaagggttccgccgcgttcgtgaacgcgatcgaggtggtgaacgc
gccggacgaggtggtgacggactcggcgctgtcggtggggccgccgctgggcccggtgggcgggctggcggagcaggcgtaccaggttgtgtaccgg
gtgaacgttggcgggccgggtcttgggccgggcaacgacacgctgggccggacctgggaggccgacggggcgtacgtgcagggcccgaaaggcgc
ggtggaggacaagtcggtgccgacgagcacggtgaagttccccgacggcgtgtcgaggctggtggcccccgcatcggtgtacgcgagcgtggcgcac
atgggcaactcggagaccaccagccccagcttcaacctgacgtggaagatggacgtggacccctcgttcgggtacctggtccgcctcttcttctgtgaca
tcgtgagcgagtccaagaacgacctctacttcaacgtcttcctcaacgggcggaaggccgtctccgggctggacctctccaccgtcgccggcgacctcg
ccgcgccgtactacaaggatttcgtggtgaattcctccaccatcccggcggacaacaagctgagcgtgcaggtggcgccgctgggggaggacacggg
gaaggtggacgctttgctcaacggcatcgaggtgctcaagatgagcaactccgtgggaagccttgacggcgagttcggcgtggacgggaagaaggcg
gacgacgggacggggagcaggaaggccgtcgccgccgtcgggttcgcgatgatgttcggcgcgttcgccgggctgggcgccatggtggtgaagtggt

acaaacggccgcaggactgggagcggcgcaacagcttctcctcgtggctgctccccatccacacggggcagtcgttcaccacgagcaaaggagggtc
cggcgggtacgggtcccaccgcagcgggaacaccttctcctccaccatgggcctgggccgcttcttctcgttcgcggagatccaggcggccaccaaga
acttcgacgagaacgccatcatcggggtgggcgggttcgggaacgtgtacctgggcgagatcgacgacgggacgtgcaaggtggccgtgaagcggg
ggagcgccgagtcggagcaagggatcaacgagttcaacacggagatccagatgctgtccaagctgcggcaccgacacctcgtctccctcatcggctac
tgcgacgagaaccaggagatgatcctcgtgtacgagtacatgcacaacggcgtgttccgggaccacatctacggcggcaaggacgacgtgccgccgc
tgacgtggaagcagcgcctcgagatctgcatcggcgccgcgcgcgggttgcactacctccacacgggcaccgcgcaggggatcatccaccgcgacgt
caagaccaccaacatcctcctggacgagaacttcgtggccaaggtgtcagatttcgggctctccaaagacgggccagggatgaaccagctccacgtca
gcaccgccgtgaagggaagcttcgggtacctggacccggagtacttccggtgccagcagctgaccgacaagtccgacgtgtactcgttcggggtggtg
ctgctggagtcgctctgcgcgaggccgcccatcgacccgcagctcccgcgcgagcaggtcagcctcgcagagtgggggatgcagtggaagagaaag
ggactcatcgagaagatcatggaccccaagctcgcagggacggtcaaccaggagtcgctcaacaagttcgccgagacggcggagaagtgcctcgcc
gagttcgggagcgacaggatctccatgggcgacgtgctatggaacctcgagtacgcgctgcagatgcaggacgccaacccgccgcagcaggaaggc
gacaaccaagactcgcccgaccacggctcttcatccaccgagagcagcgtcggcggcgtcaccgccgtgccagacgcgtccaccacctccgccggcg
agctcttccagcagctcgccgacatgaaggggagcgaagaggacgggggcagagagaaaaacagtactcgcagcncgccaccccgccgcagcag
gaaggcgacaaccaagactcgcccgaccacggctcttcatccaccgagagcagcgtcggcggcgtcaccgccgtgccagacgcgtccaccacctccg
ccggcgagctcttccagcagctcgccgacatgaaggggaggtga
>g67801.t1
atgaccgccgccggttggggcggcggcggagtaggaggagagcgcgccgacgccaagtacaactcgtacaaggcgccggggttgcgcggcgccatc
ctggaggcggcgcacgtctcgtgcctcgaggaccgctacgcgctgggcccacagctcggcttgggccagttcggcgtcatccggtcctgttccgacctg
gtcaccggcgagccgctcgcctgcaagtccatcgccaaggaccgcctcgtgtcccccgacgacgtcagaggcgtcaagctcgagatcgaggtcatggc
gcgcctcgcgggccaccccaacgtcgttgacctcaaggcgctgtacgaggacgaggagttcgtgcacctcgtcatggagctctgtgccggcggggagc
tcttccaccgcctcgagcagcgcggatgcttctcggagcacgaggctgccgtgctcttccgctatctcatggaggtggtgacactttgccatagcaaagg
aattgttcaccgggacctcaagccggagaacatcctgttggtcagcaagtcgccgtcgtcaccgatcaagcttgcggactttggtctcgccacctacatc
caacctggtcgaagcttgagtggcatggtaggaagcccgttctacattgcgccggaggtgctttctggtgggtacaatgaggctgctgatgtgtggagc
gctggggttatactttacatcctcctgagtggcatcccgccattttgggggaaaaccaagtcgaagattttcgaatgtatcaagtcaacggagctgcgatt
ccctcctgatccctgggatacggtttctgattcagctaaggagctgatcatcgggatgctgcagcgcgatccaaaactgcggctcacggccaaggaagt
tctagagcattcatggatgcaagaacacgcggaccagtctcaggattcctgcagccactgccacgagattggcctccgaagggaggagcaccctggct
cgtgctccttctccacgccgctggcttcacggagccgtgatgtgagcttcaataccggcggtccgatcgcctgccagagcctgtccgaggaggcgtgctc
ccctacgttcgcctgcaggtcttccttctccgcattcgttgctgccgacagcgctgctccgccttgcacaacatctgggttctcgtttgagggcacaactga
agccaccggcatcgcgttcccctctatgccgagcttctcgttcttctgcgcacaagaatgcagcgagcccgagccgtcggtctccggtgatgccaggggt
gatgagaaagctcggatgtcagaggctgcgaggacggtgaagtccgcgagcaacacatcgagggccgtgggcggcatgaacagcaggaggaacca
cacgatcggggcaggcgagcggcatgacctggacgtggcggtggcggagtccgttatacggtgggcgtcgtgcaccaacttctccacgacgcactccc
tccgggcctcgctcgtgtgctag
>g67824.t1
atgccgccgccattgccgctcctctgctcctccgtcttcctgctgctcctcctgtgccgcccattggtggccactgggaggaccatacggctggatgcgtcg
tcttcgtcttctccggggccgtcgcctcagtcggtcaacggcagcgccgccgtggttcctgcggtggcggcacctcctcccacagtggtcgtagtggtgg
agcggcaccaccacctccgcagggagcttatcgctgccatcgtcgtctcctctgtcgccgccgtcgcggttttcctcaccgcaatgtatgcttgcatcctgt
ggatgcgatcacgccaaaccctcgactacaaggacacccagagttcagataccgccaggatcgcatttgtgccaatgttgaacaacttcaactcattca
aggcttgcaagaagggggctccggcgatgatggattacacctccttggaggtggctactgggaaattcagtgagagcaatgtgcttggagctggtgggt
ttgggtgtgtgtacaagggtaactttgatggaggagttgttgctgctgtgaagagattgagtggcggtggaaaggaatgtgagaaggaatttgagatttc
agatttcggcctagcagtgactagtgggaatcataacaaagggaatctgaagctttctggaacattgggttatgtagcccctgagtacttattagatggg
aagttgactgagaagagcgatgtatatgcgtttggagtggtacttctagaacttctgcttggaaggaaacctgtcgagaagacgactgactctcaatgcc
aatcaattgttacatgggccatgcctcagttaactgatagatccaaactccctaacataatcgatcccatgatcaaaaacaagatggatctgaagcacct
gtaccaaatcttcgagtacaacggaagcgccgtggtggccatggtgggcaagaactgcttcgcgatcgccagtgaccggaggctcggcgtgcagctgc
agacgatcgggactgacttccagagggtgttcaagatccacgacaagctctacatcggcctctcgggcctcgccacggacgcccagacgctgtaccag
aggctggtgttcaggcacaagttgtaccagctaagggaggagagggacatgaagcctgagaccttcgctagccttgtttccgccctcctctatgaaaag
agggaattagcaaaagattttgtggtgtctggcactgcttcagagtcgctttatggtgcttgtgagtccatgtacaagccagacatggcagaagttgttga
caaggagcgaagagagacgaagaggctcaacggcggcgacaatggtcgccggtggctcgacgccgccgccgcgcctgttgagctcgacctcctgact
ggcatggtcgggtcggtcgaccaccaggcaaaggcaaccgttggcgtggcgcagtcgttgccggtgctcgtcgtgccggacatggcgtcgccgtccgg
gctggagccgccgcagctgttcgcgtgccactactgccggcgccagttctacagctcgcaggcgctcggcggccaccagaacgcgcacaagcgtgag
cgcacgctcgccaggcacctctccgccgacgtgctggctccgctcgccgtccacggcggcgcgttcgcggcggcgccggcggcgctgggctggatgca
cgccgtgaacggccaggcggacggaggggaggagctgccaaagcttgacttgacgctgaagctgtga
>g68206.t1
atggatatggaggaggaggaggaggtagagtttgagcaacgacgtcaaggagaagaagagcagcaggtggtgtactgcgccgtggggaaggaggc
gggtcgggaatggaaggccaacctgctgtgggtgcttgcaaaccttccccggagcaagcgcctggtgttcctgcacctccaccgcccggcctcaaggat
caacatgatgggagcatgggtcccagtgagccagcttgcggagcaggaggtgactgcatacagacagttggaggaagggaagatgggaaaagtctt
ggacgatctcttagacatttgcaaaagtcaaaaggtcaatgcgagcaagacaatcatcgccagcgacgacactggtaggggtcttgtccagtttgttga
tgatcatggtgtcacccaactcgtcatgggcgctgcatctgatcgggcgtaccacagggaggcgagcgagggatgtcaatgtcatagtcatagaggag
gagaatcatcgacaggcagcagcacaagtccgagatcaagcagcgtttcagactactcaataagatggaagttgtcgccgcgcgtgcagagcgagac
gcaagaatcaaaggacgcgacgacaacatcatcgagggataatgccatggatggcaagcagctggaggcggagaatattttgaatgatcgagcaact

gacgtcgtggctggatcgacgtctggagttgcggggggacctgaagaggaaggaccaggaggcggtgaagattcttcggaggatgacgaagcgtcgt
tgtatgagaagctcaaggacgcgatgatggaggcggagcacctgaagcaggaggcgtacgaggagacccggcggcgtcagatggcggagcgcgag
ctcgcggaggcgaacaggcaggccgaggaggcggagatgttgtacgagagggaggcgaagcagcggaaggaggtggaggagatgcttggtcgtga
gcgcgcagcgatggagcgcgacaggcaggagctggaggagatgctggagctggtagagaaggtgaaggagaggagcgcggagctggaggcgcag
atcgccgggtcggagcgcgtgatgagggacatggaggccaggctctcggactcgtacgccgtcctcgacacgctcatcgtcactactactacttgttttt
cttcttctttttcttcctcttcctcttccttcccggcgacgagggaggatccaccacatccacacgacgacgacgacgaagaagaagaagaagatgatga
tgatgatggggaagcatttcgatggttccattcgtcggagattgaggaggccaccaaccatttcgacgagtcgttgaggatcgatggtggtggcgatgg
tgggcgaggcaaggtgtacagagcgtgcctgcgtggcgatgatgttgccgtgaaggtgttcattatgagtcccagcatcgcggcggacgagaaccggt
tcaggcgcgaggtggaagccgttaaaagggtgagtaataggcagccgtcgtcgggaggacttgtgaaggtggttggtgcgtgcccggacccggaggc
gcgcgccgtggtgtacgagctggtgcccggcggcgggagcctggaggatcgcctcgccggcatgccgtggcacgtgcgatgcgccgtcgcgcaccgc
acctgcgcgacattagcagccctcctccactcgcactcctcacacggcgatgtcaggccggccaacatcctccttcatcttgatgatgcttcgagttcgtg
caagctggccgggctcggcacgcgccgcctggttgttgaccaccagccgcacaggaatcggaggaggcagtacatcgacgacgtgtactcggtgggc
gtgctgctcctccgcctcgtcaccgggaaaccgccgttcctggccatgaaggcggcgcacgaggctgctgctgcatcatccaacaacaccaccaccacc
accaccaatactagcagctggcaccacgtcgtggacccacgagcttgctggcccatccaccgcgccagggaggtggcgctcatcggcctcaaatgctg
cggctaccaccaccactaccggcggagaccatcattattattagagctgctgcaggaggccatgaccgtgctggagggtgccatcagcgccgcgcccg
gaaggtcaccgtcggtcatgtcggatggcgaggccgccacgccgtcctacttcctctgcccgatacttaaggaggtgatgagggacccgcagatcgcc
ggagacggattctcctacgaggccgaggccatcaaggagtggctccacagcggacatgacacgtcgcccatgaccaacctcaagctcctcacccgcg
acctcacacccaactacgcgctccgctccgccatccaccagtggcgccaacgccacagatatttggatggctag
>g68355.t1
atggggttattgagctcggctaatagatgtggctgcaagaacctcctgagctgcaacctcctgcgttgtgcgtgctgctgcacctggatccgccatatttg
tggcagtaaggaggcaacacatgaggcgtcaacatcagagacaaagagaaaaaagaagaagaggaagtggctgcgtggtatttgtggtggaactgt
ccgagaagcagaggagccattgacatcggagtcaaaaaagaagaagaagaaccccacgacaccggagccagagaagtatctcactccgaactctca
gcctctgtcgtggcatacacggatgaagattgcatgtggtgcagccagaggcattgagtacttgcatgagatagccaacccaccagttatctaccgtgat
ctcaaggcctcaaacattctcctggatggaagctttaatgcaaagttgtcagattttggcctggcaaagcttggcccggttggcgacaaaagtcatgtca
gcacaagggtgatgggcacatacggctattgcgctcctgaatatgccatgtctggcaagctaacaaagatgtcagacatctatagctttggcatagtcct
cttggaggcagcaccattcttcagggataagagaaagtttgtgaaaatggctgatccaatgcttgatggaaagttcccccctgaaaggagatccagatgc
tagaattgagacaatggttgatgatagttga
>g68419.t1
atgttctcgtggggccgtaagcagtcctcctccgccgcctcgccgtcctcgtcctcctcctcgagccgccgccgcggcggggcggacggcgggatggatt
cgagcatccgcggcggcggcgggagcgggagcgggagccgcggacggagcccgcggctggatcgccggaacgcggcgaagcacatcgagtacga
ggtcgggggcgcgggggcgtccgcgccggcgttcgcgtcgtggtcgtcgtcctcgtcggcggagcagcggtccccgggtctccgcccgtcgcgctcgct
cgacctggcgcccggcccgcccggcgccgacttccgcatcagcgggagcaccgagggggaaatcgacgagctctgccgcagcctggggctctccggg
cccgaggacttcgccatcccgatcgacgcctgggagaagtgcaagccgcggtgcaactccgacctcctaccccgctcccgaccctcggcgtcctctgcc
gccgaccaactctcatcagttgcccactcggtctccgcaccggaggcccagcttcaccatcttctctccgtccctgctccaattcctgaggagtccctccac
tcctcgtccacttccaccgccaccgactcggtggaggagcctaccgttgcgccgcccgtggaatccccaaaggccgaccctgccgttccggttgtggca
cccgtcgcggccttgcctctcccgtcaccaaggaggggaggtggcgaggtggggatacgtggcacccggcctgccttactctccccgcctccgccaatc
accccactcgcgccgccacccggaaggaggtcgtttgttgctcatgacatgactgggtctgcttgggatattctggattcgtttgctcccagggaagaga
aagatgaactgcgacaggctcgtgaacgaattgatccttctcacacgtcggactcagaggaggaggaggatgttgaggatgagtcggcagcggtggg
aggggaactgaaagaaatgagaataggagagaccttcgaagggtttaccggaacttcttcattatcgacaaccaatgatgacgatgcatccagtacga
atacggaggcaatgttcatcatctcaccaaatggcaagttcaagcggaagattaagtcttggatgcgtggcgctcttcttggaagcggctcttttggaat
ggtgtatgaggggatcagcgatgagggtgcatttttttgctgtgaaggaagtatctttgctcgaccaaggcagcaatgcacaacaatcaattctcgcactt
gaacaagaaattgcacttcttagtcagtttgaacatgagaacatagtccagtattatggaactgacaaggaagaatcaaaattgtacatttttattgagct
tgttacacaaggttctctttcatcgctctatcaaaagtataaactacgagaatcgcaagtctctgcatacacaaggcagattcttaatggattggtttacct
ccatgagagaaatgtggtccacagcaaaaataatgggaaccaggagaaatga
>g68431.t1
atggagacgagggataacttccttcggcccccaacgccctccccggaggaagccggagccggaaagagccttaggatccccaccgctggggacacgg
cggccaccaacctgacatcgtcctcgggttcttcctcgtccctgacgcagtccccaacgagcttcctgcgcgagcttcaggtcgcttttaagcggcagag
gcctcccggttcggtgcagtccaacaatccaagagcggtccgggtcctggtgcccagagctgagcgggtaaagaaaactggtgccagcccttctgaac
cgcagaaccttgaacttgaagggaaggttacgcagactcagaggggcttgttaggaccctccaggctgcaaaatgtgactcctggtcagcagaaagat
gccagcagtgttaaattggggtctgctactccagatcagttgatgtcggcaacaccatcgatgttgggaactttaactgattcctgtgatcagagtgccgg
ccagaaatgtcagcagaagagtgaggttatgttggtggataagagattgaagccatctttggaagtttcttcatctcaattagcacctcaagatgcattct
tggggaaaagcttcaagaaagaacaattttactcagctggtccacagttaacttctcggagtgataatgtagggatcactgttgatagtagaatggaca
atatgttgtcttgtctggattctgtttccttgatggaagaaaataattttcctgcaaagcaaggggaacatgatcaccgtgcgaatcatccagagctcaaa
attgctgatgcagcagttgatatggatatcaagtatgatgcagttaatttgtctgaaagaaggatagaagaggtccctgatccagtgacccgttgctctgc
cattggttcatccgtcactgctgtatcaattcattcagggcctactgtccaaagtatacaggcttcccagggtgaccaatgtgcttcctctgcgaagatgcc
agaatctgctgtggaatcatcagaaggtgtgccaggtcttgaccatcaaaagcagcctgctggtgcaacaggtgttggagattggaatcctgatgatca
tcgagtgcataatctggggtcttgtaccgatgaaaaagctgtttctggtacccgcggtcgttttccttctgaagggttatctgcaaatgatcagtctacatc
aggcagggatggtggtggctcccgggcgaacaaagttgagaaggagcggcacaagaagaattatgatcctaatgtattcttcaaggtgaatgggaag
ctttatcagaaacttggtaaaataggatccgggggtagcagtgaagttcacaaagttatatcagcagagtgctcaatatatgctttgaaaaagatcaag

cttaaaggccgtgattaccctactgcctatggcttttaccaagaaattgagtacttaaataagttgaaaggaaagagcaatatcatacagctcattgatta
tgaggtaccattttacgtctcatctccaattttggtttatgttccagcataa
>g68493.t1
atgtgtgatgagagtggcacgttccccggtgtaatcatccggttttccttatcggagttggaacaagcaaccgggaaattctcagacgagcatctgattg
gcgtcggtggatccagcaaggtgtaccgaggccagcttgctgatggcaaagttgtcgccgtgaagaagcttaggcctctaggaggtgccgacgaagac
tatgagttcctgtccgagatcgagctgctgtcgaggctgaaccactgccatgtggtgccactgctggggtactgctcggagagccaagggcggcagctg
gagaggttgctggtgtttgagtgcatgtccaatggcaacctgagggactgcctggacctgaagcaagggaagaagcccatggactggcagactcgtgt
gagcgtggcgctcggcgccgccaggggggctggagtacctgcacgaggcagccgcgccgcgcatcctccaccgtgacatcaagtccaccaacatcctc
ctggacgacaagttcagagccaagatcactgatctcggcatggccaagtgccttatgaacgacggcgtgacaagctgctcgagctccccggcgcggat
gctgggcacgttcgggtacttcgcgccggagtacgccatcgtgggaaaggcgtcgctcaagtcggacgtgttcagcttcggcgtggtcatcctggagct
catcaccggccggcagcccatccacaagtcgccgtccacaagaaccgacgagagcctcgtcatctgggctacgtcgcggctgcaggacagcaggctg
gtggtgtcagagctgccggacccaacgctgcagggcaagttcccggcggaggagatgcagatcatggcgcacctggccagggagtgcctgcagtggg
accccgagtccaggcccaccatgactgaggtcgttcacatcctctccaccatcgcccctcctcttcacggcaccaagcgccggaacctccccatgacctt
caacctcaccaccacgccgcctcacgacgacgacatcgagaggcagcaggaatgctcgtcgtccgtccaatggaaagggcgtgcgtcctggcccggt
gacagggggcacaagggcgccggagccgtggtgtccggcgagctgctcaacgggatgctgctcatgagcccgcacggccggagcacctggcggcca
ccgccgccgcacgaggacgagccggtggacctcacggagccccggctagagacgttcacacaggcagcaatgttcagatga
>g68606.t1
atggatctcctcaaagggcatcctaatgtggtcacactggttgcccatgatgtttttgacatgggccgtacaaaagaggcactgcttgtgatggagttctg
cgagaagtccttggtgagtgcaatggagcgtagaggtagtggttattatgaggagaagaaggcacttctgattttcagagatgtctgcaatgctgttttc
gccatgcatgggcagtcaccaccaattgcacatagggacctgaaagctgaaaatgttcttctgggatttgatggtgcatggaagatatgtgattttggaa
gcacgtcaacaaatcataaatgctttgacaggccagaagagatgggcattgaggaagatatcattagaaggcatacaaccccagcatacagggcccc
tgagatgtgggatctttacagaaaagaagttattagtgaaaaagttgacatctggacaaattctgtgaaagatcccttcaacagttttgtagcagattttg
actccaacaatctccatgctgggacttctgttgctggtaaggcatctgatcttgaactggagctttctagtctgaaggagcagttgaagaaaaccacatta
gagaaggatgagatgacagccaagtatgaaaagctttctgcaatctgccgatcacagcgtcaggagattcaagagttgaagcgaactcttgctgagag
cacacctccgtcaaacaaaatcagctcaagggcacatggttctgtatctcagcgaaaagaaaagatggaaggaactgtgtgggagcttgaacaagga
atgcttgcaaccaacttttcagcacctagttctgaggccatggcatggcaggcattccctgaaccaaaagctcaggcccggccaaagaacatcaccatc
gtcaacgtcagcggcaaccgctgctgcacaggtcagcaggcctaa
>g68626.t1
atggcggcggacgcgtctgcgtcggggaggaagacgcgggtggggccgtacgagctggggaagacgatcggggagggcagcttcgccaaggtcaa
gctcgcccgaaactcgcgcaccggcgacgtctgcgccatcaaggtgctcgaccgcaaccacgttctccgccacaagatggtcgagcagataaagcgg
gagatttcgacaatgaagctaatcaaacatccgaatgtagtccagttgcatgaggttatggctagcaaatcaaagatatacatggttcttgaatttgttga
tggaggcgagctatttgataagatctttgtagatgctgctgtttacgtgttctga
>g68680.t1
atgcggccgcatctccccttcctcctcctcatcctcgccgcctgccttccgccgccggccgcgtgtcacaaccctcccctccgccgccgcaccaccacca
cccgcgccagtcgaagaacagcagcaccgtggtgaccgccgcgctcatctccgccgcgtcggtgctcgcgctgctcctgctctacctctgcgccgccat
cgccgtccgccgcttccgccgtccgcgcggcgcggtgggcctcgagccagccgcggcggcgggctcctcctcccgcgccgccgcgttcctccgccgcc
acgggctccaccaccaccggcccaactttacctacgagcagctccgcgccgccaccgccggcttcgacgccgcccgcaagctcggcgacggcgggttc
ggcgccgtcttcctcgcccacctcccccgggcggccgccccgccgccgtgaagcgcctccacctcccgccctccccgtcgccgtcgtacccctccgccg
ccgccaccatcaccaagtccttctgcaacgaggtgctgatcctctccgcgctgcgccacccgcacctcgtccgcctccacggcttctgcgccgacccgcg
cgcgctcctcctcgtctacgacttcgtccccaacggcaccctctcgcaccacctccaccgcctccgcagggccggggccgctgctgggccctcaccacca
cccctcccgtggcggacccggctcgcgatggcggcccagatcgcgtcggctctcgagtacctccacttcggggtgaagccgcccgtcgtgcaccgcga
cgtcaccacctccaacatcttcgtcgaggccgacatgcgggcccgcctcggcgacttcggcctctcgcgcctcctggccccgccggacgcctgcgccac
cggcgccggcgccgcccgcgagctcgtctgctgcaccgcgccgcagggcacgccggggtacctcgacccggactaccaccgctccttccagctcacgg
agaagagcgacgtgtacagcttcggcgtcgtggtgctggagctcgtcacggggctcaggcccgtcgacgtgggaagggagcgcagggacgtcacgct
cgccgattgggtggtggccaagatccaggtcggcgagctcagggaggtcgtcgacccgccggtgctcggcgaggggcccggcgtgatggcgagcgtc
gaggccgtggcggagctcgcgttccggtgcgtggcgccggacaaggacgaccggccagacgccagggaggtgctcgccgagctcaggaggatccaa
gggatgctccccgagcttcctcccgggcacaaggggtcttga
>g69295.t1
atggcgtcgctgctcggcaacggctggtacggctactcgtcggggtcggagggggaggacgaggacgaggggacggagggttaccggaagggcgg
gtactacgccgcccgccccggggaccgcttcgccggcgggaggtacgtcgcgcagcggaagcttggctggggcaacttctccaccgtctggctcgcct
acgacaccctccaaagccggtttgtggcgctcaagatccagaagagcgcgagggattatgcacatgcggctctccacgaaatcgagctgctgtcggcg
gtagccaaaggcgacccaatgaattccaaatgcgtggtgaagttgctggaccagttcaagcacgctggccccaacggtcagcacgtttgcctggtcacc
gaattcctgggggacagcttgctccggctcatacggtacaaccggaacaagggcattggcctgaacagggtgaaggaaatctgccgatcgcggtttgt
ggcgctcaagatccagaagagcgcgagggattatgcacatgcggctctccacgaaatcgagctgctgtcggcggtagccaaaggcgacccaatgaat
tccaaatgcgtggtgaagttgctggaccagttcaagcacgctggccccaacggtcagcacgtttgcctggtcaccgaattcctgggggacagcttgctcc
ggctcatacggtacaaccggaacaagggcattggcctgaacagggtgaaggaaatctgccgatcggtgctggtcggccttgattacctgcacagcgag
ctcggcatcatccacactgatttgaaacctgagaacgttcttctggtttcgacaattaacccggccaaggacccggtgcgctctgggttcacaccaatcct
tgatctgtctgtggggaaccagtatggcaggacagtgatcagttttagtgagaagatgcttaggatgagagcaagacgtgcagtggcaaagatttcgca
gaggcgggtttcgcttggcggggttctggctgaagtagagaaggagagaagcctggatgggatcagcttgaagtgcaagattgtcgatttcggtagtgc

ctgctgggctgatcagcagctttctggtgaaatacagacaaggcagtaccgagctccagaggttatcatcggttccgggtactcatattctgctgacctgt
ggtcatttgcctgcatggcctttgagcttgccactggtgacttgttgtttgctcccaagaattgccagcgatgcagcgaagatgagatgtaa
>g69364.t1
atgtcgccgccgccgccgccggcttccccgccgctgctgcgcgacgagctgtcgcgcaggacggcggtgctgggcctgcccctgtgggtgctcgtgggc
ctcgccgtcgggctcgccttcctcctcctcctcgcgctcatctccctccacctcgccgcggcgcggcgcaggcggccgaggaagggcgtcccgcgcgcg
gcggcggcggtggcgccgccgcactcccccacggccaccaccaccatcccgccggtgtccaaggagatccaggaggtggccgtccacgtgggctccc
tccgccactacctcgagatgggccacgcgttcctcaccaaggacgccaccacgcagcagcacgacggcgacggcggcgagtccgtggggaccgcgc
acgggtcgcagagcgtgtacatcgaggccggcaagggccgccgcatggtcgcggcgtgcggcgccgacggcgtggaggccggcgccgacgtggcg
gtggccgtgggccccgaggtgtcgcacctcgggtggggacactggtacacgcttcgtgagctggaggaggccaccgccgcgttcgcgccggagcacg
tcgtcggcgagggcggctacggcatcgtgtaccgcggcgtctttgccgacgggtaccaggtcgccgtcaagaacctcatgaacaacaggggtcaggcc
gagcgggagttcaaggtggaggtggaggcgatcgggcgcgtacgccacaagaacctcgtccggctgctgggatactgcgccgagggagcgcaccgg
attcttgtctacgagtacgtcgacaatggcaacctggagcagtggctgcacggtgacgttgggcctgtcagcccgctcacctgggacatccggatgaac
atcgtgctcgggacagccaaagggatcacctacttgcacgaggggctggagccgaaggtggtgcacagggacatcaagtccagcaacatactgctgg
acaggaggtggaaccccaaggtgtctgatttcggccttgccaagctcctcggatccgatagcaactttgtcacaaccagagtgatgggtacatttggtta
tgtggcaccagagtacgcgagcaccggcatgctgaatgagaggaacgatgtgtacagcttcgggatccttataatggagatcatctccggtcgaagcc
cggtcgactacgccagacctcctggagaggttaaccttgtagaatggctgaagaacatggtgtccaacagggactacgaagcaattctggacccaaaa
ctgccggaaaagccttcgtcaaaggcactgaagaaggccctgctggtcgcactgaggatcgacgaactcttcgaccgggcaatggcagccccctcacc
ctggagaaggcacgaacgccaggaaaatcggtggcagggtcatgtgacagcagctgctacgagggcaacacgaccaccgccagcacgccctcaaga
ctggtccaggacatctctgcacccggtctccagtaccggaaaccgcatctggggtgctgaggaatag
>g69424.t1
atgggcttcttggcgtgcttgttccggtgtcctcaggaggaagtggaggaggagaacggcgacgagaaggaaggggagcagttcaggatgaatcatc
aaatcgcctcagatcttcgacccgatcaagaacctttagattggaaaacacggatgaagatagctgctggtgcggctgctggtttggaatacctgcatg
ataaggcaaaccctccagttatctaccgagacattaaaccatcaaacattcttcttggtgaagggtatcatgcaaaattgtctgactttggacttgctaag
cttggccctctgggtgataagactcatgtgacaacccgtgtcatgggaacgtatggttattgtgctccagaatatgcctcaaccgggaagctaacaatta
aatctgacatctatagttttggtgtcgtgttccttgagctcatcacagggcgaagggcacttgacagtgaccggcctcctggggaacaggacttagttgc
atgggctcgaccattgtttaaagaccaaagaaaatttcccaagatggctgatcctttgttaaaaggtcacttcccaaggagggggtttataccaggccttg
gctatcgctgcaatgtgtttgcaggagaaagccaaaaatcggccttccatgagggaagttgctgctgccctctcctatttagcatcacaaacatatgaaa
gaaacaacactgctgctcgccataacctggttggtccttcatcaagagttctggatgatcagattaaccaagacacaacgatgcctagtcaacagggag
ctcaaatacctgaaggaagctggagtggttcacatcgtgctggaaggggcagagttgctccgaatggcattgatagagagcgtgcactcgcagatgct
aatgtatgggccgaggcctggaggcgccaggagaaggcaagcatagtgcggtga
>g69739.t1
atgaatgaagggaaggcgaagcagcagctgctgccacactccaagaacagctccactacagaggaggccctgaagctgtggcggatcgaggagagc
ggcaccgagttcacgctctacgacttctccgagctggctgccgccacgggggacttctccgacgagaaccggctcggcagaggcggctttggccccgtg
tacaagggcaggctggctgacggttccgagatcgcggtgaagcgtctggcggcgcattccgggcagggtctagaggagttcaagaacgagatccagc
tgatcgcgaagctgcagcacaccaacttagttcgcctcgtaggctgctgcgtccaggaggaggagaagatgctagtgtacgagtacatgcccaaccgc
agcctcgacggcttcatcttcgaccagcggcgtggcccgtcgctggactgggagaagcggctgcacatcgtcgagggcatcgcgcaggggctcctgta
cctgcacaagcactcccgggtgcgcatcatccaccgggacctcaaggccagcaacattttactcgataaagatctgaaccccaagatctccgactttgg
catggccaggatcttcggcaccaacatgaccgaggccaacaccaacagggtcgtcggcacatag
>g69902.t1
atggcttcggatccggagttggccgccggcgagcagccggagccgcccgacgaggacgagtacgaccccgacgtcgaggaggtcgaccccaccgga
cgctatttccggtacaaggaggttctgggatcaggggccttcaagagagtctacaaaggcttcgattcggtggacggcattgaggtggcatgggccaaa
gtggagataaccgacaggatcatgggatccccgaaagagttgcagagactcaagacggagatccagctgctgaggtcactccagcacaagcacatcc
tcaagctctacacctcgtggattgacaagaagaaggggacagtgaacatggtcactgagttgttcacctctgggaacttgagggagtga
>g69957.t1
atggctcgccgctcagtggtggtggtggtgctcgttgtcgtcgttgccggcgttgtgggcattggcgaggccaaggtgtacaacccgacggaacagatc
cttgtgaactgcgggtcggcgacggacggcctggacggggacgggcggaagtggatcgcggacacgaacgagaactcgtggctgcccgactccggc
aagtcgtccctgatggcggcggcagacgacatggagcagtcgctgccgtccaccatcccctacatgactgcgcgcgtgttcaccgtggaggcctcgtac
aacttcacggtgaacaagaacgagcggcactggctgcgcctccacttctacccggcctgctacaacggcctctccgccgacgacttccagttctccgtga
ccacctcgacgggcctgaccctcctccgcaacttcagcgcgtacctgacgggcaaggcgctgacgcaggcgttcctggtccgtgagtactctctgccgc
cgacgccgtccgggttcgtgaccgtgacgttcacgccgatgccgctgggcaacgacacgtacgccttcatcaacggcatcgagctgatctccatgccgg
agatcttcggcgacccggcgaccatggtcgggttcgcggaccagacggtggacttcgcggcgtcgacgctgcagaccatgtaccgtctgaacgtgggc
gggtcgcagccgctgatcccgccgtccaacgactcgggtctcacccggacgtggtacgacgacacgccgtacgtgttcgggcccacgcagggtgtggt
gtaccaggcccctcacctccacatcgactaccagagcgacaccgccgagtacgcggcgccgcccgacgtgtaccgcgggtcgcggtccatgggcacc
gacgcgcgcgcgaaccagaactacaacctgacgtggctcatggcggtggacggcaacttcacgtacgtggcgcggctccacttctgcgagctgctgct
gacccgcgccaaccagcgcgccttcgacgtgtacgtgaacaaccgcacggcgcagtctgacgcggacgtgatcgggatggccggcgagcgaggcgc
gcccgtgtacaaggacttcgcggtgcacgtggcggacgagcctggcgccgacgaggccatgtgggtggcgctgcacccgtcggtggcgcttcgcccgc
agttctacgacgccatcctcaacgggctggaggtgttcaagctcaacgacagcacgggtaacctggccgcgcccaacccggagccgtcccggatgctt
gccaaggcggagctgggtcgcggcgaccacggcacgcaccaccacgaggcggaggagcggcaccacgacatggcgtgggtgatgggcggcacggc
gggcggcgccgccgcgctcggcatcttcgccgccgcctgcgtggccgtgtaccacgaccggaagaacatgagccgggacgccgcggcgggcggctca

cacacctccgggctgtggctgccgctgtaccactcccacaccagcggcaagtcgtcgggtcacctggcggccaacctggcgggcatgtgccgccacttc
tcgttctccgaaatcaaggccgccacaaagaacttcagcgagtcgctggtgatcggggtgggcgggttcgggaaggtgtaccgcggcgtgctggaagg
gggaaccaaggtggccatcaagcggtccaacccttcctccgagcaaggcgtgcacgagttccagacggaggtggagatgctgtccaagctgcggcac
cggcacctggtctccctcatcggcttctgcgaggacgcgggggagatgatcctcgtctacgactacatggagcacggcacgctgcgcgagcacctgtac
agcggcgggaagccgccgctgacgtggcggacgcgcctcgacatctgcatcggcgccgcgcgggggctgcactacctccacacgggcgccaagtac
accatcatccaccgcgacgtcaagaccaccaacatcctcgtcgacgacaactgggtggccaaggtctccgacttcggactctccaagtcgggacccac
caccgtcaaccagacccacgtcagcaccatggtcaagggcagcttcggatacctcgacccggagtacttccgccgccagcagctcaccgacaagtccg
acgtctactccttcggcgtcgtgctcttcgaggtgctcttcgcgcgcccggcgctcgacccgaagcaggagagggagaagatgtgcctcgcggattacg
cgatcatctgccagcgggaaggcacgctgcacgaagccgtcgacccggtcatcagggaccagatcgcgccggaatgcctacggaagttcgccgacac
ggcagagaagtgcgtcaacgaggtgggcaccgaccggccatccatgggcgacgtgctgtggaacctcgagttcgcgctgcagatgcaggactcgttc
gagggcatcaccggaagcagccgggggcgctccgtcgagggcggcgagagcagcggcggccccacggcggggctagagacgacgacgggcgacg
acagcgtcggaagcgccggcagcgtcaccacgacggccagggcgcacgagacgcgcgtcgtcgtcgaggagacggacgacgagctggccaacagc
gccgcattctcgcagctcgtgcgacccaccggacgggatgtgatgtgggttcgagacaaggataagatgtcttggatggatttctatgatgaatggagt
gcagaaatcaaacgagctagttggcaggagttggctgttacattctgggacaagaaagctggttacaccatcatccaccgcgacgtcaagaccaccaa
catcctcgtcgatgacaactgggtggccaaggtctccgactttggcctctccaagtcaggacccaccaccgtcaaccagacccacgtcaacaccatggt
caagggcagctttgggtacctcgacccggagtacttccgccgccagcagctcaccgacaagtccgacgtctacgagacaagatcgtctgcctttgcacg
agtgaagtcagatcgacgtcgggagcacatggactgtctacgcaaatcgaacgcccaacacacgtcgcactcctcccacgtggactccaacgctagca
gcctcgtgatccgtggcccatnnnnnnnnnnnnnnnnnnnnnnnnnnnnnnnnnnnnnnnnnnnnnnngataagatgtcttggatggatttctatg
atgaatggagtgcagaaatcaaacgagctagttggcaggagttggctgttacattctgggacaagaaagctggtcctagcactgttgttgatgtgatttc
ctcctctgtcatggctgcttgctcagaattgattgctggtaatgttgttgtgaatgctgaggttgctaatgctgtagtggtagataaaactgatgttacttgt
gaacctcatcctacatataaagagtacaccatcatccaccgcgacgtcaagaccaccaacatcctcgtcgatgacaactgggtggccaaggtctccgac
tttggcctctccaagtcaggacccaccaccgtcaaccagacccacgtcaacaccatggtcaagggcagctttgggtacctcgacccggagtacttccgc
cgccagcagctcaccgacaagtccgacgtctacgagacaagatcgtctgcctttgcacgagtgaagtcagatcgacgtcgggagcacatggactgtct
acgcaaatcgaacgcccaacacacgtcgcactcctcccacgtggactccaacgctagcagcctcgtgatccgtggcccattaggcctagtaggcaagt
atctgttgcttcacgcgagtaaaaatccttcctccttcattcgtcgcatcggccgcaagggcaaatataaaattccccaaattgattggtcaatctcctcca
tgaatcgagcggcaacagtctga
>g70340.t1
atggcgctgtggaacggcgtgggtcaggtggccaacatcgtgcagctcaccggtgtggatgcgtatgggctcatctccatgatcgtggaggctgccgag
acagtcaagaggaaccgtgagacctgccagctgctgtcacgccgcgtgaggatgatcgggtgcctcctgcagcagctccagagttcccagctgatgca
gcacatggagacgaggatcccgatggaggagctggaggagacgctccagcacgcttacatgctcatcgcgtcctgtcgggacagcacctacctgcac
aactgctttaggggaggaaaacagtccgagcagcttcgcgaggtgcagaacgatatcgccttctacctccagctcttcccccttgtcagcttcgtcgaca
ccacccgtaacttggagcagtctattacaagagacagcccttcctgctccgagggaaagctgtctaatggtcttgacattgctatcaaaagacacaatgt
atactcagttcaagaggtgtctgttatgggcacaactggctatgcagatcctgaatatattgctactggaaagatctcggagaaggcagatgtgcatagc
tttggtgtcgtacttcttgaaatcattagtggaaacccattgtggtttttggaagatgatactgaaagagtgctacttcctgattatacgcaagaaacatttt
gcgaaccttga
>g70548.t1
atgcgaccggcggacgcggacgcgctctccccgccggcgccgtcccccgtcgccgccgacgacctcgcggacgcgcggctcgcgccgtggccggcga
cccgcggggacgacggtagcggcggcggcggtggtggccgggcggcgagccccctgttcaccatcctcccggtctcggcgttcggcatcggggtcgtg
ctgctggtcgccgtggcggtcgtgctggtggtgacgcggcggggcaagccgacccgcaccgcggacgccggcggcgttgagagctgcagcggcgac
ggcaagcccgccgcgccgccgtccagctgcggcagccacaacaccgccaccaccactaccaggtgctacgccgccgccggcgtcgggtgcatctacg
gcgcggggaggctgggcctgggcttcgcggcggcgcagcatcagcatcatcaggcggcgcggaggagccggggcgcgcaggtgttcacgtaccggg
agctggagcgcgcgacggacgggttcagcgaggccaacgtggtgggccggggcgcgtacggcgtggtcttccgcggccggctcaacgacggcacgc
ccgccgccatcaagcgcctcaggctggaccaccggcgccagggcgagcgcgagttccgcatcgaggtggacctcctgagccggatggactcgccgtt
cctggtgggcctgcttggctactgcgccgaccagagccaccggctgctggtgttcgagttcatgcccaacggcagcctcaagggccacctgcacccgtc
cccgccgccgccatccgccggcgacggcagccacccgctgccgcggccgccgctggactggcagacgcggctgggcatcgcgctggactgcgcgcgc
gcgctcgagttcctgcacgagcacaccagccccgccgtgatccaccgcgacttcaagtgcagcaacatcctgctcgaccacaactaccgcgcccgcgtc
tccgacttcggcatggccaaggtcggctccaacaaggccgacgggcaggtcgtcacccgcgtgctcggcaccaccgggtacctcgcgccagagtatgc
gtccacagggaagctgacgaccaagtccgacgtgtacagctacggcgtcgtgctcctcgagctgttgaccggccgggtgccggttgacacgaagcggc
cacccgggcagcatgttctggtttcgtgggcccttccacgtcttacaaaccggcagaagcttgtggagatggtggatcctgcgttgaaaggccagttcgc
cctcaaggatctcgttcaggtcgccgccattgccgcgatgtgcatacagaccaaggcggagtaccggccgctgatgacggacgtggtgcagtcactgat
ccccatcgccaagacaaccccggccatatcctgctcctccacgccgctcagacccctgcaccacgtcatctacatgagcccgcacttagccaacaagtc
ctag
>g70592.t1
atggccgacatggcattcggcggcgtggagaagatcatcaagatcgcgctcgccatcaaggaggccgtgaatacggttaagcacaacaagaaggagt
gcggcggcaacatcgaaaagtgcgtggctcgatgcactgctctcttgaagcggctggaggagcagacggatacgatgaaggaccaggtgatgcatgg
cccgctggaggatgtggcggagagcctcgaggaggcccttcaactcgtcacgaaatgccaacggaagcactacgtgagccattcgtggaaggcaggg
gacatggcaaaggagctgcgtcgggtgcaggacgatatactgcggaaactccaggggatagcccaaggagtacattacctacatgagcaacgcattg
tccatatggatctgaagccatcgaacatcctctttgattccgatatgaatcctctggtcggtgattttggagtagtagctacgatgttggaccatggtgatg

atgagatcattagtgacgactctttatatggcacaccgcgatacatagctccagaatttatcggagaaggtattatatcggtcaagtgtgacgtgtacgcc
tttggtctcattttcatggagaccatcagcagtatgaatagatccggagaagaatatctagatcactacgattcagttgcatggcttgacctttga
>g70695.t1
atggacggcggggcgcagccgccggacacggagatggcggacgccggcggcggtgcgggaggagggcagcagccaccgccgcagcagccggcgg
ggggcggaggcatgatggacaacatccaggcgacgctgagccacggcggccggttcatccagtacaacatcttcggcaacgtgtttgaggtcaccgcc
aagtacaaacccccatcctccccatcggcaagggcgcctacggcatcgtctgctcggcgcttaactcggagaccggggagcaggtggcgatcaaga
agatcgccaacgccttcgacaacaagatcgacgccaagcgcacgctccgcgagatcaagctgctgcgccacatggaccacgagaatctcatcgggac
accaaatgaggctgatctggattttgtaaatgaaaacgcgagaagatatatccgccagcttccccgtcatccaagacaatctttccctgaaaagtttcca
catgttcaacctttagcaattgacctggtggaaaaaatgcttacatttgatcctaggcaaagaataactgattggtggtcctatgttgaaagttga
>g70696.t1
atggacggcggggcgcagccgccggacacggagatggcggacgccggcggcggtgcgggaggagggcagcagccaccgccgcagcagccggcgg
ggggcggaggtatgatggacaacatccaggcgacgctgagccacggcggccggttcatccagtacaacatcttcggcaacgtgtttgaggtcaccgcc
aagtacaaacccccatcctccccatcggcaagggcgcctacggcatcgtctgctcggcgcttaactcggagaccggggagcaggtggcgatcaaga
agatcgccaacgccttcgacaacaagatcgacgccaagcgcacgctccgcgagatcaagctgctgcgccacatggaccacgagaatgagtccaaaac
aaaatttgtcggagcgtaa
>g70829.t1
atggatcaaggactgttatgtgatagaccaaagctaaacatggtgcctcctagagccggtgagtggccaaatatggtgggtggcggcggcctagtgaa
gtcagtggtgtcgttgtacaagacggccatgccagggaaaagagggcggcccagtgcagctgaggagtccaggagcggtcgagggagagttccttat
gctggagagcacgtgttggcctatatgtggaagcactacttttccgctaaggatgagtatgatcgggatggcaggatcttcgacaacaaggcggaacgt
gtgctcggggagctgttggatttctacagatgcgagacgggatacaagaacagagcaaagatcgtggctcaccatgcatgtcctaagctcgtgaggaa
catgttttacgaggcacatatccaggctgtgattgtgtaccacgtccagcaccttaaagttaaggtcaccaaaactcaagcgagaaacatgaagcttacc
agagatgaatacatgcaggtgcctacgtggtggtgtgctaccaatgcgtctacctgggaagccgttgtggacaagtggtgcgcggagggctgggagga
gacgcataatgcttgtcgggagcgtcgtttgctgatgccagttccagcacaccatcaagggaatctttcccttaagcaatatgcggcgagatggtcggtg
tcacatgatggtcgcccttgcacccagttcgtggcatttgctatagcatacaagggcaaggccacagcgcccgatgtccagtacaacacggaagccccg
cccacagcgtacagcaattcctccgtccacacccgcctcacagagtacacatcgatgggaagggatatccatgggcccgagtgggatcctatcacccat
gatcttgatgaggaagtcgtgatgagggtgggaggaggcaagaagcatgggcggtactggattggagaaggcatgcttgacacggcgactacgccca
ctctctccgcgatacgagcaaggagcacgagctcaatcccggctatacgccctcgaccgaacactacacagtcccagtcccagctcgctgtagagagg
aaggcaagggaggacatggaacaggatttcgggaggaggctcaagcaccaccaacagcaattgactcagtgggatggcatcagttggctagagttta
ggacactgcactatctcatccattcgcacgacgataacaaagacatcaggactctagatatccgtctgaaaattgctaccaaatctgctgaagcactggc
atacctgcattcacttaaccatccaatcatgcatggcgatgttaagtctgccaatattcttcttggtccagatctctcccctaaggtctccgattttgggtgct
cgatgattacatcaacagttgaaaatattcaagtagtaaagggcacacttggctacttagatccagagtacctgttgaagtttgaactgactgataagag
cgacgtttacagctttggtgtccttctccttgaactcttgacacgcaagaaggttatatctaaggagaaggagtgcctcgccttggttttccaggaggcttt
gaagaagggaaaccatcatgagcttctggactctgagatcatagtagatgaccaagacaacatggacgttgtgcatcaaatagcacatgtggcagctc
gatgcctggttatgcctgctgagcacagaccaacaatgagggaggtgggagaggaactcaagctactggagggtgtggtaaaacagaggccaggag
tattgtttaacagtgataggctattaatggtgccagaaaggtcagctaccaacatgtcagagttctattctggggaagaaaccacggactttgnaggcca
ggagtattgtttaacagtgataggctattaa
>g70992.t1
atgatgcgcgcgaccatggcgatgtgctgcagcttagccgatttgcgcctcgtcgccgttctacaattactggtggtggtggcaataagttcgcaagtgg
ccttggcgaccaacaacctgacctcgggagacacactgacgccgccgggctacatcaccagcccgtcgggcgtgttcgccttcgggttccgtgcgctgg
actcggacccgaccaagttcctcctcgccacgtggttccgttccggcaacagtaacctctccccggcgccgccgccgcagtcggtggtgtggttcgcgaa
gcaatctccctcgggcgccactccgttggccacgacggagtccgtgctgagcgtcacttccgacggcgcgctcgcgctcaccgacgccggcaaccggc
cgctctggaacgccacctcgacgaagcagcagcagggctccgtcgtcctcgcgctcgccgactccggcaacctccgtttactcggcgacgacggcgtc
accgtgctgtgggagagtttctggtacccgacggacacgctgctgcccggtcagtcgttgaccctcgacgaccggtcgcaggggaagctcttcgccaag
cgcgcggacgccgagttcaccgccaccggtcggttcagcatgggcatccaggcggacggcaacgtcgtgctgtacgtggacctcccctccggaaacaa
ctccaacaacgcgtactggcaggcgtacagcaacagccccgacggcagcaacacgacggtcaccttcgacgaccgtggccggctcagctacactctc
cacaacggcaccgtgcacagcctcatcaccacgagctcctccgctcccggcgcctacttccagttcgccagaatggacccagacggcgtcgtccgcatc
tacgcccgctccagcaggaacaacaacaacaacagcacgagttggtggcttgtttctggcgcgttccccgacgacgggtgcaacaagaggacgtcgg
ggttgcagggcatgtgcggcccggggtctttctgcgtcgagactaccgaccgtctccggtgcgagtgcccgaccgggtacaactacaccgacacgcag
cacaacgacagcggctgcgcgccggcgttcgaaccgcagagctgcggcgacggcggcggggagaaggatggcgggtccttcgagctcgtggagctg
cagaacgtcacctgggaggcctccatatactacaagaagctgtcgtcggtcacggaggacgagtgccgggccaactgcctcgccgactgcttctgcgc
ggccgcgctggtgtccaactccgagtgcgtggagctggcggcgctcaccaacggaaggcgggcgaacgacgtcacgaccaaggcgttgatcaaggtc
cggtcttcgccgcggacaataggaactaccaccagcagtccctacaaaatcctcacggtttgtttggccgctctcttggcgatcaccgtcggcgggctcc
tggcacagcgctactacctcagcaggaagaacaggggaggagagacccagcggctgctgagcgtcagagctttcacctggaaagagctccaccaag
ccaccaacgggttcgagaagctgctgggcaaagggagcttcggcgaggtgtacaaggggacgatccggtcctccccttatccgcgcctcgtcgcggtg
aagaagctcgtcgacgcgaacgcgtacaccgagacggagttcgccaacgaggtgcagtgcgtcgggcagatccaccaccggaacctggtgcgcatgc
tcgggtactgcgaggaaggcaggcaccggatgctggtcttcgagttcatgcccggcggctcgctccgcgccgccctcttcgacgacgacgtcgagcgg
aggccgccgtggccgtggcgcgccgaggcggcgcacgccatcgcccgcgggctcgagtacctccacgacgggtgcagcgtgccgatcattcactgcg
acatcaagcccgacaacatcctgctcgacggccgcggcgtcccgaggatcaccgacttcggcatctccaagctgctggggagccaccaggtgcacgcc
acggtcaccaacgtcagagggaccagagggtacatcgcgccggagtggctccgcggcgacgcccgcgtggacaccagggcggacgtgtacagcttc

ggcgtcgtgctgctggagatgatatgctgccggaggtgccaggagccggtggcgggcgcggatgatgacaatgacgagacggtcacgctgttcgggt
gggcggcgcagctggtgagcgcccggaggacggacctgatgctgcacgacatcgtcgacgccgcggaggatctggagagggtggagcgcttcgcgc
gcgtggcgttgtggtgcgtcgagcctaacccggcgtcgcgtccgacaatgcaccaggtggtgcacatgctggagaccagcgatcaggccctgaactag
>g71113.t1
atgcactcgtccgcttcgccaccgatactacatggtgacgttaaaaccgcgaacattctacttgatgacaagctcacagccaaagtttcagatttcggtac
atcaaaacttgcaccaacagacgaggccgagatagcaacattggttcaagggacttgtggatacctagatccagaataccttattacatgccagctaac
tgataagagtgatgtgtatagcttcggcgttgtcctactggagcttctaaccaggaagaaggctttgtactttgaggggtcagaagaagacaggtccctc
gtatcatgcttcacgacagcaatgaaggctggccgacatgaagagcttttggacggacatgtaagaaacgagataagagccgaggtgctagaagaaa
ttgcttacctcgtgatgcgatgcctcagccttagcagagaggaacgaccgacaatgaaggagactgccgagaagttggagatgctaaggagataccag
cagcatccgaggtttcaagctgatggtgatgcagaggagaagcagcacttgcttggtatggaacagcggaaccttcctttcagacaagatgtcattgat
cttgaagagggcagcacatatacttttagtatgtag
>g71115.t1
atgagcgccggcgaggagttgctgcggagccagtacgtgatcggcgacgagatcgggcgcggccgcttcggcaccgtccgccgctgcttctccgtcgc
gacgggggagcccttcgcggtgaagtcgacggcgaaggcgccgcttcgcgagtcggacccgctggacctggcgctagcggagcaggagcccaaggt
ccacctcctggcgtccgcgtccccgcccgcgagcgcgcacctcgtcgcgctccacgccgccttcgaggacgccgacgccgtccacctcgtcctcgacct
ctgccccggcggtgacctcttctccctcgtctccgcccgcgcgccgctcccggagcccgaggccgcggggctcatggcccagctcgccgacgcgctcgc
cgggtgccaccgccgcggggtcgcgcaccgcgacgtcaagcccgacaacgtcttcttcgacgccgccggggtgctcaagctgggggacttcggatccg
cggagtggttcggggacggccgcgccatggccgggctcgtagggacgccgtactacgtggcgcccgaggtggtggcggggagggagtacaacgaga
aggtggacgtgtggagcgccggcgtcgtgctctacatgatgctctccgggaccgtgccattctacggcgcgtctgcgggggagatcttcgaggccgtgc
tccgtggcaacctcaggttcccgccgcgcgccttcgccaacgtctcgcccgaggcaaaggacctcatgcgacgcatgctctgcaaggacgtctcacgca
ggctctccgccgaacaagtcctaaggcacccctggattgtgacccgcggaggcagtgcggcggtaaactaa
>g71140.t1
atgtgcggcacgcttgcattggtggaccgtcgcctcgccggagaagaaagccggcactacccaccggagtacccaaacccaaccgctggcggacaag
acaagcacggcgccgcggcaaccgacacacacaggcacatgcacatgcaccactctcatcagcccatgcttactattgccatatgctgcggtcgcctcc
gtctcgtcctcgtctcgtctcgtctcgtctcgcgcatggaacggaatggaacggaattgatgaagaagcacgaccggacagggacaggcctttagtctcgg
gcggcgtgacgggccttgtcccgggcgcagctgaaaaagcaaaagtgaagagcaatgcacttgcaagagaggcatcagcgccaaaggatgccaatg
gcaatgtcatatctgctcagactttcaccttccgcgagcttgccaccgcgacaaggaacttcaggccagagtgcttcctgggtgagggagggtttggacg
tgtttacaagggccgtctggagagcacaggccaggttgttgctataaagcagcttaacagggatgggcttcaaggaaacagagaatttcttgtagaagt
cctcatgctcagtttactgcatcatgataaccttgttagtttgattggttactatctaccaccagataaggaggccttggattggaacacgaggatgaaaa
ttgcagctggtgctgccaaaggacttgagtaccttcatgataaagctagtccaccagttatttatagagatttcaagtcatcaaacattctattggctgag
ggtttccatccaaagctatctgactttggtctagctaagctgggccctgttggcgacaaatctcatgtctctacacgtgtgatgggaacatatggttattgt
gcaccagaatatgctatgacaggacaactgactgttaagtccgatgtgtatagctttggagttgtcttgcttgagttgattactggtcgtcgggctatagac
agcaccagaccacatggagaacagaaccttgtctcatggcagtccagaaagaagacgacggacgctttcagctttactgatgcacggtggatgttcgc
cgcgcccccgcctgtacaagcgtgcatgggactctttctacacgagaacgtggctggggccagtgcagtggtactacagcttttagcttcactgcgcatg
ctgctggctgctccacacgtcgtcccgtccgcggacgctgccaacaagaacaagcaagcatctagccagaggtactctcgcagcctcaacgtggcgct
cgccgtacgttga
>g71475.t1
atgagcctgggcaacctcaacactctgcagttcattaatctcgagcataacagcctctccgaagaaattccacggtcactgtacaacctgtcgtcgttgat
cttctttgccgttggtggcaacaatttacgtgaccacattcccgtggatattggcgacaagtttcctggaattggaactttcagcgtggaaggtaaccagtt
cagtggagaggacatgagtgcccctgttgcagattttggcatatcaaaagtactttcagagaacgcaatcaatgccgagaacaactcaaatagcactat
tggaataagaggctccattggttatgttgctccagagtatggcgaaggttcttctgtgtcaactcttggtgatgtatatagcctcggaatattgctgctcga
gatgtttacaggaagaagcccaattgatgatatgtttagagaaaaacaaatcccgccgcgcctctcccgcgaacgcatcgcttttggcttgcctcctccgt
cctcccgtgcggcgccgccgtggtcgtccccgcctcggtcggccgtctccctcccgtcgagccgttcggtcttcccctaccgccccgcctcggtcgtctgc
ctctgcctccctcccgccgagctcatgaactccctgacggtggcactcagcatcgcggcaacaaggttactcatcagcctggccccacacatcggtcaca
ccatcgcagaaaggctctgcaagacacaggggcagccgagcaagctgattaaaagtatcagttacaccaggcgcgtcacggaacggcaagctgcatc
ggcgactctgctctcaaggttaccttacagaaacacctcgttcaatgtcgcgctcgtggaggaacacgtacgccatgccgaccataatgagcgtgataa
aggagatggagaaaggcacagcgagaacgagcaggcatgccgtgccgttcatggagggcctcgtcggtgcgctagtgcggttcaccgctactctgtac
agtacagcccagaagtgctgaaggcggccatggatcacaacctgacgtcggtgctcgctgatctgctcggaggagcagctggaaacgacgaggtgca
acggctagctgtcgtggggctggagaacctcagctacatctccattaagctgtcccagccaccacaccacctgaagagctatcttcaaagaagacaatg
a
>g71586.t1
atggagaaggacgccgccgccgtggcgctcggtggtggtgcgccggcgatggtagtggagggcggtcgggagatggactcgccgcggttccgcgcg
atcctccgcgcgacgagcgggcggcggaagcgcgcgccggaggtgaagagcttctcccacgacctgagcggccgcggcggcgccggcgcccccatg
atgatgcgcaagatcatggcggcgcagcgctgcggccctggcgtagaggagttcgcgggagccgtgcgcgccgagttcacgcgcctcaaagccgaag
tagactccgagctgggcgtcttcgccggcgacctcgtcggcgtcctggcccgggacgagcactcagacgacgaggaatcccgggtggcgatggagga
cctgctggtggtggcgcagcggtgcgcggagatgagccccgaggagttctggtcccggtgcgagggcgtcgtgcagggtctcgacgaccggcggcag
gagctgggccccggcggcggcggcgcggcgaagcaggcgcacacgcgcgtgctgttcatcctcacccggtgcacgcggctcctccagttccgcaagg
acgccgctggtgtggtggaggaggaggaggagggaggagggagggaggagggtggccgccgcgtgctgcggatgcaccagctcagcgacctcggg
ctgttcccgttgtccggcggcgacctcggccgcaagagcacctccagcctcaccgagctcaaggatcggctcatccggcgccgcatgctcgagcacaa

gcacctcaccctcgacttgttccccccgcccatctcctcctcatcgaacaacaacagcaacaaccatggatttggtgaccattcccccaagatcgcgtcgt
ggaagaagctcccttcccccgccgagaagaataagaacaagaatcccaccgatgccaaggttgtcgcggcggcggacgaggagtcgaagaagacgg
tcaccgtgttgcacaacaaggcttcggtggaggagatcgtggaacgcgtgggcgcggcgagcatccactctgacgggctgctgccgagcatccacccg
aacgagcagcagcagtacccggagcaggcgcagcaggtgatggtggtggacgggaagccgcggatgatctgccgcgtgtgcgacttcgagatcccc
atggcctgcgccgagggccacttcgtcgtatgcacgctcgccgaccgatgcgacggcggaaagggactcaccgccgaccagcgcctcctccgcgtcgc
cgacgtgctcgaccgcgtcctcgcatgcttcgccgactcctcatcctcctcgtcgtcatcctccagctccaacgccgccgaccacgacgcgctcgcgcag
ctcctcgccgtgccgtccgccgagctcttctcagagggcgcgctgctgacgccgacgggcggcagcggagggggaggaagcctgccgcagtcgccgc
tgccgacgccggcgcggatgaacaacaacgcggcgggggagacgaagcagcagcaccgtttcgggttcgcggacctggagagcttccagcaggtgg
agagcctggcgtccatcgcgcgtggggtggaagccgtgcgcgcctcctcctacgcctcgctcgaggacctcagcgcgttcctcgaggacctcaacgccg
tcatcgacacgcgccgcgtcgacgcgctcgtcgtcgagaccttcggccgccgcatcgccaagctcctccaggagaagttcctgcttctctgcgcccacat
cgacgacgacgccaccctcacgacgatgggccccatcgacgaggacgggcccatcacactgtcggagagcacgagcactagctcgcgcgccatgac
cagcaataacaacggcaactgcggagtcatcaaggaccggacctccatcgaggacttcgagatcatcaagcccatcagccgcggcgcgttcggccgc
gtcttcctcgcgaggaagcgcgtcacgggggacctcttcgccatcaaggtgctcaagaaggccgacatgatccgcaagaacgccgtcgagagcatcct
cgccgagcgcgacatcctcatctccgcgcggaaccccttcgtcgtgcgcttcttctactccttcacgtgcagggagaacctctacctcgtcatggagtaca
tcaacggaggggacctctactctctcctcaggaacctcgggtgcctcgacgaggacatggccaaaacgtacatcgccgaacttgttcttgcgttggagta
cctgcactccatgaacgtgatccaccgcgacctcaagcccgacaacctgttgatctcccgcgaaggccacataaagctgacggatttcgggctgtcaaa
ggtcggtctcatcaacagcacggacgacttctccgggccggacgtgagcagcgttctcgtcggcgaccaccacccgacggacgcggagcaccgcgctc
agaagagggagcagcggcagaagcagacggccgtgggcacgcctgactacctggcgcccgagatactgctcggcatgccccatggtcctactgcgg
attggtggtctgttggtatcattctctttgagcttcttgtggggattcctcccttcaatgctgaacatccgcaggctgcctttatccctgctacagacgatgag
tatgacacgagctactttgcgtgccgccatgcctggggcatcgcggacgagcaagtcaatacggcttccaatgagtatgatgacagaagcgagaccag
cagcatgagctgcggcagcagcccacatagttgtgactatgaggaagatggagacgagtgtggtagcatggaagaattcggagctccatcactgtcgg
tgaagtattcttttagcaacttctccttcaagaacatttctcagctggcgtcgatgaactacgatctgataacaaagcacaacgaggatcctcttcactctt
caaaaccttga
>g71757.t1
atggatagattcaagttgattaaggaagttggtgatgggactttcggcagtgtatggcgtgctataaataaacagaacagtgaagttgttgcggttaaga
aaatgaagaaaaagtattattctttcgaggaatgtatgagcctgcgtgaagtgaagtctttgcgccgcatgaatcaccctaacattgtcaagctcaagga
ggttatcagagaaaatgatatcttatacttcataatggaatacatggagtataatctctatcaacttatgaaagatagagtcaagcccttctcagagtcag
aagttcgcaactggtgctttcagattttccatgctctcgcttacatgcatcaaaggggctactttcatcgtgaccttaagcctgagaatttgctggttagca
aagatgtcataaagctggcagactttggtcttgcaagggaagtctcatcagcaccaccatacacagaatatgtctcaactcgctggtatcgagcaccgg
aagtgttgctccaatcatctgtttatgattctgcagttgctgatgagattctcaagatatgcaatgttatcggcagtccagatgagcaatcatggcctgaag
gattgtctcttgcagagaccatgaagtataaattcccacaggtcaaaggtaatcaactgtcagaggtgatgacctctgctagtagcgaggcaattgatct
catttcatcactatgcacgtgggatccttgcaagagaccaaacgccacagaagtcctccagcatgcattctttcagggttgtacatatgttccacctcctgt
caggccaaaagttggaggacctcctaaaacgcctccgggtgttggagcaaaaggaatttcagagcacagtgttgctcgaagattctctactggaacact
atctgcaatgaaaagcaatgcatctgtcaaatcaaacgctttatctaagactgggaatcctgtactcaggcattcacgcagtttgcctgaaactggccga
gcaatgcacaaggtttcagccatcaccgataagctttcccatatgtctgtgacctccagaacacgcagtactgtgaagcctcctgccctaacgttgaagg
ctgggcatggcaaatcggacttcctcgggaagtctgatgagatccctccagcaaagaggttgacacgaaaattggtcagctaa
>g71886.t1
atggctgccgctgcccccaccgcctcctccccggcgccgcagcccatcattgccacgcccaccgacacctctgctgctgcccctccgccttccctctcgcc
cgctgctcctccacgggggagctcccccggtcgccgccgcccacacctcctcctcctcctactcccaggcccttgccttctccttcgcctccgccgccggt
gctctcgccccctcccacaccctcgcccccctccagcaccctcacctgcatcatcagccccttccaagccaagaagcaggacaccttctgctgcgtcctcg
gctcctgctcctaccaatgaatatgatgctccccccttctccggccgtcgtgctgccatcatcacggagtcgccacaaaaacttgcccacgcctgccacgg
cagctgacccggcccacccaaagaatcccagtcccagcaagtcctcctcgagtggaaggcttagtactggtgtcatcataggcatcggcggtgcggtg
gtcgctcttcttcttcttagcctgatagcagctgctgttttgtacaccaacaagcgtaggcgcaagagggaaacaaatggttaccgtgctggattcatgtc
acctacatctcctttctcctctcagccgccatcaggtaacttgttctgcctctacctgagaaccaagaaactgctgggcgaaggaggttttgggtcggtat
acaagggctgccttcctgacggacatggaaggcctgttttggaatggactgccaggattaaaattgctgctggtgctgctcgtggaatagcatacctaca
tgaagactgccatccaaggatcatccatcgagacatcaagtcttcaaacattttgttggataacaactttgaggcaaaggctaggccattgctttcacga
gctgttgacacaggtcatttggaagggttgattgaccccccaacttgagaagaacttcaatgagctagagatgttccgtatgattgaggctgccgcagcat
gcatccgacattcagcgtcaaggaggcccagaatgagccaggtgatgcgagttcttgatagcttagcggatattgatctaacaaatggtgttcagcctg
gacaaagtaaacttttcaacgtcgccaacactgcagagatacggatgttccagcggatggtggctggcacacaagatgatagctttgacttgagccaat
acagttggagcagtcgcagtaggggaggtacagacgctgctccaagctccaggatcttgtga
>g71932.t1
atgcaccacgagtgcacgccgcccatcgtgcaccgcgacgtcaagaccagcaatatcctgctcgactccgagttccgggccaaggtcgcggatttcgg
ccttgccaggatgctgctgcgcgccggggcgcccgaaaccatgtccgccgtcgcaggatccttcggctacatggctcctgagtgcgcgtactcgaagaa
ggtgaccgagaaggtggacgtgtacagcttcggcgtggtgctgctggagctcaccacgggcaagacgcccaacgacggcggcgagcacgggtgcct
ggcggactgggcacggaaccactaccagtcaggcgggagcatcgccgacgccacggacaagtgcgtcaggtacgcggggtacgccgacgagatcg
aggtcgtgttcaggctcggcgtgctgtgcaccggcagcgcgccgtcgtcgcggcccaccatgaaggacgtgctgcaggtcctgatcaagtgcggcgag
cagacgcaccagaagagcaaaatggagcacagcaacggcgccaccccggagtacgaggcggctccgttcctgctgccgcagcgaggcagtcgccgg
aagcagctctccaacggcacggggatcgatttcgaagaggagcgacttcgacagcattgtctgacaagatga
>g72393.t1

atggaccgggcggcggtgacggtggggccggggatggacatgccgataatgcacgacggcgaccggtacgagctcgtccgtgacatcggctccggc
aacttcggcgtcgcgcgcctcatgcgcagccgcgccgacgggcagctcgtcgccgtcaagtacatcgagcgcggcgagaagattgacgagaacgtgc
agcgcgagatcatcaaccaccggtcgctgcgccaccccaacatcatccgcttcaaggaggtcatccttacgcccacgcacctcgccatcgtcatggagt
acgcctcaggaggggaactcttcgagcgcatctgcaacgccggcaggttcagcgaggacgaggtccagaccctttctgccttcttcctgtcacctctgca
gtgctcactgaatcaaacatcagtgctgccctctgttgacgttccttcgtcggttcttcattcacagccgaagtctactgtaggaactccagcatacattgc
tcctgaggtccttctgaagaaagaatacgatggaaaggttgctgacgtgtggtcatgtggggtaacactttatgtgatgctggtcggtgcatatcctttcg
aggatcctgatgagcccaagaatttcaggaagacaattcagagaatactgggtgtgcagtactcaattccagattatgtccacatatctcccgagtgccg
agatcttatttcaaggatatttgttgcggacccatctactagaatcaccatacctgagatcacaaaccatccatggttcacgaagaacctcccagctgact
taatggatgagaacacaacgagcaaacagtatgaagagcctgatcagccagtgcagagcatggatgagatcatgcagatactggccgaggcgacaa
taccggcagctggttctcgaatcaaccagttcttaaatgatggtcttgacctcgatgacgacatggacgatctagattcagatgccgatctcgacttggaa
agcagcggggagattagaatactgggtgtgcagtactcaattccagattatgtccacatatctcccgagtgccgagatcttatttcaaggatatttgttgc
ggacccatctactagaatcaccatacctgagatcacaaaccatccatggttcacgaagaacctcccagctgacttaatggatgagaacacaacgagca
aacagtatgaagagcctgatcagccagtacagagcatggatgagatcatgcagatactggcggaggcgacaataccggcagctggttctcaaatcaa
ccagttcttaaatgatggtcttgacctcgatgatgacatggatgatctagattcagatgccgatctcgacttggaaagcagcggggagattgtatatgcg
atgtga
>g72581.t1
atggaggagaggtacgaggcgctcaaggagctgggcgccggcaacttcggcgtcgcgaggctggtcagggacaagcggaccaaggacctcgtcgcc
gtcaagtacatcgagaggggaaaaaagatcgatgagaatgtgcagagagagattatcaatcaccgatcacttcggcatcctaatatcgtacggttcaa
ggaggtagcagacgtttggtcctgtggcgtcacattgtacgtgatgctggttggatcatatccatttgaagatcccgaggatccaaggaactttcgcaaa
acgatcagcagaattcttggcgtgcaatactccatcccggactatgtgagggtgtcttccgactgcagacgcctactatctcagatatttgttgccgatcct
tcaaagaggatcaccatcccggagataaagcagcacccttggttcctgaagaacctgccgcgggagatctcggagagggagaaggcgagctttaagg
acaaggaggccgtcgagccgacgcagcccgttgacgagatcatgcggatcgtgcaggaggccaagaccccaagcgacatgtccaacttggaccccg
cgctgctcgccgagatggccgagctggagagcgaggaggaggatgcggaggccggcgatgatacctactga
>g72642.t1
atggcaaagcaaaacttgtgctcaagtagccatactcatcagctgctgattctcctacaagcaatcatcctgatactatcagtgcacgcccctggtgcca
cacctctctccttcagctacaacgccttcgaccccaatgacttccatccggaggccgatgcagcggtcaccgccggcagaattgagctgctcggagatg
agtttaatgcacgtgcccgaggccgggcttggcacaagcaagcggtgcagctctgggacgccaccaccggcgaggcggcaagcttcaccgcaggctt
caacttcagcatccaatcggtggttaataagagcgctgccgagaccggccacggcatgacgttcttcctcgcgccatacgtgccggacctcccgcagga
atcctacgacggcggcctcggcctcatcgacgggaaccagcaggcggctactaccaacgcctccggtgacgcaaggtttgttgccgtggagtttgacat
ccacaagaatgcttgggatcccagcagcgaccacatcggcatcgacgtgaacaacatggattcacatggtgaatacacgatcttgccagacggcagcc
tcgtggatggcggcatgatgtcggcagtggtcatctataacaagggcacgaggaggctggatgttgctctgatggtcagaaatgttacttacacgttggc
agccaccgtggacttgcgaagcttgctcccggaacaggtggccgttggcttctctgcagcgacaggcgacaagtacgccggcaaccacaccgtattgtc
gttctccttcaattcgacgttgccaacgaaaaacagcaccgcccttcctgcgtcctcaaccggcaagaacaccgccatagagctcaccgccggtctggtc
gctggtgcggttcttgttctattgctcgttgccgccgtcgcggtgctactagttcaacgatcaagaagaaggaggcaggaacgggacgaagagaagctg
actacagacggcgatgactccctggacgctgtcgatgacaaggatttcgagtccggcaccggacccaggccgatcccttacgctcaactcaccgcggc
gacgagggactttgcggaggacgggaagctggggcagggcgggtcgggctcggtgtaccgggggcacctcaaggagcatgaccgtgacgtcgccat
caaagtgtttgccaggggagcgtccatggaggggaggaaggagtaccggtcagaggtcactgttatcagccggctacggcaccggaacctcgtgcag
cttattggatggtgccatggccggagacggctgctgctggtctacgagctcgttagcaatggcagtctcgatcgccacctctacgacatggaggcgacg
ctgacttggccgatgaggtaccagattatacttggactgtcctccgccgtgctgtatctacatcaggaatgggaccagtgcgttgtccacggcgatatca
agccgagtaacatcatgctcgacgagtccttcaatgccaagctcggtgacttcggcctcgcgcggctcatcaaccatggcatgagccttcagacaatga
ctgctgtcgccggtacaccgggctacctcgacccagagtgcgtcattaccggtaaggcctccactgagtccgacatctacagctttggtgtcgtgctacta
gaagtcgcctgcgggcggcggcccatggcaccgcctgcggcgggtgaggggaaggatggccaggtgttccggctgatcgagtgggcctgggacatgt
acgggcaagggacggcgctcaatgctgcagatgagcggctggaaggagtgttcgactcgtgggaaatggaacgggtggtggccgtggggctctggtg
cgcgcatccggaccccaagatgcgcccggggatccagcaggctgctgaggcactacggtccaggaagttcaggatgccgttgctaccgcccaagatg
ccagtcgccgtgtacctgcagccattcggatcttgcacgatgcagtttagtgaaaccaccacatctgtcgggtaccagattatacttggactgtcctccgc
cgtgctgtatctacatcaggaatgggaccagtgcgttgtccacggcgatatcaagccgagtaacatcatgctcgacgagtccttcaatgccaagctcggt
gacttcggcctcgcgcggctcatcaaccatggcatgagccttcagacaatgactgctgtcgccggtacaccgggctacctcgacccagagtgcgtcatt
accggtaaggcctccactgagtccgacatctacagctttggtgtcgtgctgctagaagtcgcctgcgggcggcggcccatggcaccgcctgcggcgggt
gaggggaaggatggccaggtgttccggctgatcgagtgggcctgggacatgtacgggcaagggacggcgctcaatgctgcagatgagcggctggaa
ggagtgttcgactcgtgggaaatggaacgggtggtggccgtggggctctggtgcgcgcatccggaccccaagatgcgcccggggatccagcaggctg
ctgaggcactacggtccaggaagttcaggatgccgttgctaccgcccaagatgccagtcgccgtgtacctgcagccattcggatcttgcacgatgcagtt
tagtgaaaccaccacatctgtcgggtcttctattaccatgccatacacgtcgacgacaagccgtgaaactgtaatggggtcttccaactactcaccgccg
gctgtcagtgaaaacttctcatctagaagtgtagactag
>g72681.t1
atgccgcaccgccgccgaacgcttaacccggtgcccgcgctccccgtccctgccttccacctcgccgccgacgactcgcggctgccgctcctcgccgcc
gacttctcgctgctgcagccgcccctcgacgcggccccggcgtccgccgagtggagcggcggcagcgggttcacggccagctcggacgcggccacca
ccaccgcctccgcgtccaccgccaccgcgccggggtcctccctcctcgacgacggcgggagggacacctgggtccgccgcgccagggaagggtacta
cctccagctgtccctcgccatccgcctcacctcccaggccttcctggccggcgcacccgccccgcccgagctcctcctccgctgcggcaccggcgatgcc
gaccaacgtgacgtcgccgctgatccggaggccgtctcctaccggctttgggtgaacgggtgcctgtcgtgggccgacaagatcgcgcacgggttctac

aacatcatgggcatcgacccgcacctgtgggccatgtgcaacgccgcggaggagggccgccggctcccgacgctgccggcgctgcgggcggtggacg
ccagcgagtcgtcgctggaggtcgtgctcgtcgacaagcacaccgacgcggtgctcgccgacctcgagcgccgcgccctcgagctccaccgcgcgctc
ggcctcacgctcgacctcgtccgccgcctcgccgtcctcgtctccgaccacatggggggcgcgttgagatcggaggacggtgacctgtacatgcggtgg
aaggcggtgagcaagcagctcaggaagcggcagaagtgcgtcgtcgtccccatcggcggcctgtccatcggcttctgccggcactgtgccatccttttc
aaggaactggcggatttcattggcttgccgtgccggatcgcgcaaggttgcaagtactgctctgcgcctcaccgttcctcttgtcttgttaaaatcgacag
cgagaggagatatgtaagggaatatgttgtggaccttgtagttgatccaggaagcatcaccagcccggactcatccatcaacggacaattgctgacctc
cgtcccttcacctttcaagactatgtgtacagcaggatcagcaaacagtgcagcaccagttgcttgtaatcaagctatggttgatgaccgatgcaacaac
acggtgctgtcaacctcccagtattcagctgctaggctctgcaccgtggaggtgaaaaactctgttcacgcggctagcagagagggcttgcacccgaaa
tgcagtcagatcatgcagaacggccactgtaacggcgtatccgcgctggaagtatcggcggcacaattaaaggttatggacattaataatgagaatgg
aaacaaggagaaccttatggctgtttcgcttcgaaaacgactgagcattgagccgtctctcgctccggattggcttgagatatcatgggaagagcttgag
ctcaaggaacgcgtaggcgccggttctttcggcacagtttatcgtgctgattggcatggttctgatgttgcagtgaaggtacttacagaccaggatgttgg
tgaagcacagctgagggaattccttagagaggcaaaaggcatcaactatctgcactgcctgaatcctccaattgttcactgggacctgaagaccccaaa
catgctggttgacaagaactggtctgtgaaggtaggtgactttggcttgtccagatttaaggcaaacaccttcatatcatccaaatcagttgctggaacac
cagaatggatggcaccagaatttctccggggcgaaccatccaacgagaagtgtgatgtgtatagcttcggggtgattttgtgggagcttctgacaatgc
agcaaccatggagtggcctcagccctgctcaggcaatcttccacccatttattctgttcatctgttgctga
>g72799.t1
atggctgtggcggcgccggggcagctcaacctcgacgagtcaccgttgtggggctcccgcagcgtcgactacttcgagaagctcgagcagactgggga
gggcacatacgggtacgcatctatcgaaaccccccaaagtctcccccaatacgccacagcatccgtcccgatcggccgcgtcgacgcgttgtttcttgtg
cattttgtggttgtttctaggcaagtgttcatgtcgaaggtgctcaagaagatccgcatggacaatgagcacaagggcttccctaacacggccatccgcg
agattaaaatcctcaagaagctgcaccacccgaacgttatccagcacaaggagatcgtcacctcgcccgttgagggtaacaagtacatgggctag
>g72860.t1
atgtcgggttgcgacagcgaagtgaagtacccgctgaacgccgagtcgtaccgcctacttttcaagatcgggagtggcgccagcgcggtggtgtacaa
ggccttgtgcctgccgctgggctcggccgtggtggccatcaaggccatcgacctggagcgttcccgcgcaaacctggacgacgtgtggcgggaggcca
aggccatgaccctgctttcgcacagcaacgtgctccgcgcacactgctccttcaccgtcggcagccacctgtgggtggtcatgccgttcatgggcgccgg
ctcgctgcactccatcctgacacacggattcgccgacggcctcccggagccgtgcatcgctctcgtactcaaggacacgctgcgggcgctgtgctacctc
cacgagcagggccgcatccaccgtgacatcaaggcgggcaacatccttgtggacgccgacggctctgtcaagctcgccgactttggcgtctccgcctcc
atctacgagagcgagacttcctccatgcatgcgccatcagcaccggttctcagctcatcatcctacttcagcgacttggcggggacgccgtactggatgg
ctcccgaggtcattcactcgcacgtcggctacggcatcaagnnnnnnnnnnnnnnnnnnnnnnnnnnnnnnnnnnnnnnnnnnnnnnnnnnnnnn
nnnnnnnnnnnnnnnnnnnnnnnnnnnnnnnnnnnnnnnnnnnnnnnnnnnnnnnnnnnnnnnnnnnnnnnnnnnnnnnnnnnnnnnnnnn
nnnnnnnnnnnnnncggcggagaagcttctccggcatccgttcttcaagggctgtcgttccaataatgactaccttgtccgcaacgtgctcgtcgcc
gtcccgggcattgaggagaggtgtaataaggatactaataacctctgcggctgtgccggcaggggtgcacgttgcgtgtcgtcatgttgtcacaccaac
gtcgcaaagaaccggaggattagcggctggaacttcaacgaggataactttgagctcgatccaaccgacgacgggaatgacgagcagaggtgtcgtt
ttcctttcgtcaaaaatgacctcgtcgagagagaaccagcaataattcaactggtggagagtgtgaaggcagccagacccggcgacgggagagaataata
ggagtaaaggaagttgtcataccgcaactgatgactatcttggagagcctcgagatgcagctagggatggtaatgcacgtactggaatgcggcggcttc
cacgtcaacgacagtggttgtggaagagcacctgcaagagaggagagggaagaaatgctgctttgctacgtgcggcagctagagcagagggtggag
gagctgacaacgcaagttgacaaggagatagccaggaatggccggctagaggcgcagctgcaggcgagggttaatgtcaaaaacataaatccatctc
agacatctgggagcatcagttag
>g73108.t1
atgctccactgccgcggcccgctcacagaaccagagacgcgccggataatgcggcagctgctcaccgccgtggaggcgatcgtgcacggagacctca
agcccgagaacgtgctgattgggaaagggccggacggccacgtcatctacgtcatctgcgacttcggactatcggtcttcgtatccgagccacgaggta
acataccggggacacttcactacgcagcgccagagcagctgcttaatgaagagtgcgggccaccggtggatgcctgggctctcgggtgtgtcatgggt
gagctcctcactgggaagccgctagtcgaggaatgctgtgaggtgctgcaatggacccgtacagtgctcttcttcggagtctctgatgaggtatcgctca
tgaacaatggtttcgaccattccacgcctagcaagctgcgaggctccgttcctgaagtggtgctatcgccggatgggttcgacgtcctgttcggcctgctc
tgctacaatcccaacgacaggctaacacccgcagcagcattgcaaatgccatggttagctcttcaggaagaggaggctgagtaa
>g73242.t1
atgactggaaactatgaaaactttcttggtaagggatcgtttggtaaagtttataaaggaacactcaaggaccaaactacagtggcagttaagacgtac
gctactgcagttgacaaagacaaagtgaacgaattcgtggaggaggtgaagattcaatctaaaatgattcacaagaatgttttaaggctcaagggctgc
tgcttggaggtgaaatttcctgttttggtctacgagtatgctgctgaaggaaacctccgagacattatcgatcgtagaaatagaaaaatgatccagttccc
tctagagcgacgtttggaaattgcaataggggcagctgaaggattagcatatatgcacgcgtacacagcaagcatcatacagcatggagatgtcaaac
cagacaatatactcctcgatggtgagttcgtgccaaaattggcagattttgggttatcaaagcagcttgctccaggcaagcagtatgtgacacatgtggt
ggggtccatcggttacatggatccggtgcttctaaagaaagggaggcaaactccagcgaatgatgtatatagctttggagttgttctcttggagctcatta
ccagaaaggccattgaacctgatcattctagtggagagtgcacactcgttgctaagtttcggctagtctctgaggaagaaagtagatggcgtgaaatgtt
agaccaagacatcacagcggaagacgatcttccagtgcttgaaggaattgtcagattagcaatcaagtgcttagcggatgaaaaccgtccagatatgg
ctgaggtagcaagacaacttgcggtacttaagaaggactggaaagatggcaagaaaaacagccagactggccatcacccggaggagacaactgctg
atgatgatgctaagagcacagtgcgttctccaagtgccagccaaacttga
>g73354.t1
atggtttcctcggagataacctctgtcacagctgctcatgctcaaggccccaagctcttcagagggaagataattctacctggggggcctccagatgttgt
cccatctgaaaatgttgagttcgacttttctgatgtctttggacctactgcagtccatactcccacagaagtgagtatgctcacaccggacagcccggcac
cttttgctgagtcaaacgatgaggtttatgatgatcctgttgtcattgtcaagcgatctcattctcttgttggtccatcatctcttgtcagccaatcattgccat

ttagcaagcttacactacgcgagacagagagctcagtggaactttcagagtgctcttccaaagaaaaggaagctaaccaagggccacttggtgacgag
gagctggataatgcaaaaaaggagaatgaaggtgttggccttgatgattttgaagtcttgaagcttgttggccaaggggcatttggaaaagtatatcag
gtgagaaaggaaggcacttcagaaatatatgcaatgaaagtcatgaggaaggacaggattttggagaagaatcatgctgagtacatgaaagctgaga
gagatatactgacaaaagttgaccatccctttgtagtgcagctgaggtactcatttcagacaaagtatagactttaccttgtcctggacttcataaatggg
ggccatctcttcttccagctttaccaacagggtttgtttagagacaaagttcagcagaagattgtgaaggagaagatcaagctgccggcatttttgtctag
tgaagtccactctctactgaaagggttactgcacaaagaagctggcaggcgcctgggaagtggaccaggaggcagtgacgaaataaagaaccacaa
gtggttcaaatcaataaactggaagaggcttgaggcccggcagatccagccaagcttccgcccgaacgttgctggcaagacctgcatcgcaaactttg
acgagtgctggacgagcatgccagtgcaggactctccggtggccagccccgttgctgctgacagcaacttcgtggggttcagttatttactgcacaaag
aagctggcaggcgcctgggaagtggaccaggaggcagtgacgaaataaagaaccacaagtggttcaaatcaataaactggaagaggcttgaggccc
tgcagatccagccaagcttccgcccgaacgttgctggcaagacctgcattgcaaactttgacgagtgctggacgagcatgccagtgcaggactctccgg
tggccagccccgttgctgctgacagcaacttcgtggggttcagttatgtgaggccgactccattccttcaaaagccgagtcctctaggctga
>g73415.t1
atggagcatgtcatcggagggaagtataagcttgggaggaaaattgggagtggatccttcggagagctatatcttggtgttaatatacagaacggaga
cgaagtaggaataaagttggagcctgtgaaaacaaaacatccacaactgcactatgaatctaaagtttatatgctactgcagggtggaaatggtatccc
acacctcaagtggtacggtgtcgagggggagtacaatgttatggtgattgatcttcttggcccaagcttggaagatatgtttaactgctgcaacaggaag
ctttcaatgaagacagtgcttatgcttgctgatcaactggtatgctcattccactccatatgcgacctcatgctgaccccctag
>g73571.t1
atgcttgccggccgctatgccgccgccgccgtcgccctggcgctgctgctgctgtcgccaccagcggcggtgtcattggtgccaatctgcgaccagcag
agctcctacacggctaacggcacgttccaggcgaacctgaacagcctcatcgcaacgctgaccaccaacgcctccgcctcgccgacaggattcgccac
ggccagcgtcggcacgctgaccgaccaggccaacgggctggcgctctgccgcggcgacacgaacacgtcaacctgcgccgcctgcctcgcgacggcg
ttccagaacgcgcagcagacctgccccctaaacaagggggtcaccgacgtggaggacgcctgcgtcctgcgcttctccgacacgcagttcctcgatttc
ctcaaccagagccagtgggtcgtctcggagctggtgtatgaggactggttcaccgccgccgtcaatgcgacctacgccgccctcatcgacagcgcggtg
gcggcgaccaattcgacgagaaagtacttcgccactgcggagatggcattcaaccccaagatctacggggtcgcgcagtgcacgccggacctgacgc
cggttcagtgccggagctgtctgggaatgttccgcgacgagaccatggcgcggcacatgagcgggcggcccccgtccaactacgctggggcggtgtgg
tgcttgctcaggtacagcgtcgtctcgccggtttacgagggccgggcgatgctgcagcttgccgcaccgccggaaccagcacccacgctttcccctgcta
ctccagagtccagaacagggacaaaagagattgctggtatcgcggcgggcatcgctggttccgttgtgttgattttgattctttcagtctttctcttccttcg
cttccagtccaggcgaagaaggatcaaggcgacagagccactgaagaagatagggaattcacaatgcccagtcttcgatgtgtcgacattgcaagag
gctaccgaaaacttctcagagaagaataagcttggagaaggcggttttggtacggtgtacaagggaatactctctgatggccaagagatagcagtcaa
gacacttttaggaagaaccggggatgcagtggatcagctaaacaatgagatggagtacaacatcattctaggaattgccaaggggatattgtatctcca
tgaggactcaaccatgaggatcatccaccgagatttgaaggctagtaatattctcctcgacgacgacatggaaccaaagatcgcggattttgggctggc
gaggctgctaacagaaggtcacacccgtagtcagacaaccaaaagggtcgggacattaggctacatgccaccagagtacgcgatgcaggggcgcgt
gtcgcccaagatcgacattttcagctttggagtgttgatccttgagattgtaacgaggcggagcaactgcagctacaacgatcatagcactgtgaatctc
ctgagcgatgtctggcgtcacacgacgagtgggtcgctatcgcaaatgctggaccaatcgctagatgaatatggtcgaaaccaggcgctgcgatgcatc
cacgtcggtttgctgtgtgtccaggtggaccctgatgacaggcctgacatatcaactgtcgtattcatgttaaccagggatggcatggagctgccgccgc
cggagaagcctgcattctgcttcacgggagagtctcctacacggatcgacaggtctagtttcatttcggaacaggacatctccttgaatggtcttacattt
acagagccataccctagatag
>g73726.t1
atgggcaactgctgctgcgggggggtccaagcaggatcccgccgcagagccggcgtcctccggctccagccgccccgtgggcagcacgacgacctcgg
tctcgccgtcctcggctccggcgcccatgaagccccccgccgcggtgggccctgtcctggggcggcccatggaggacgttcggagcacgtacagcatc
ggcaaggagcttgggcgcgggcagttcggcgtgacgtcgctgtgcacgcacaaggcgacggggcagaagctggcgtgcaagacgatcagcaagcgg
aagctgtcgaccaaggaggacatcgaggacgtgcggcgcgaggtgcagatcatgtaccacctctccggccagcccggcgtcgtggagctcaacggcg
cgtacgaggacaaggcctccgtccacctcgtcatggagctctgcgccggcggcgagctcttcgaccggatcatcgccaagggccactatacggagcgc
gcggcggcggcgctgctccgcaccatcgtcgagatcgtgcacacgtgccacgccatgggcgtcatccaccgcgaccttaagcccgagaacttcctcctc
ctcagcaaggacgagagcgcgccgctcaaggccaccgacttcggcctctccgtcttcttcaaagaaggggaggtgttcagggacatcgtcggcagcgc
atactacatcgcgccggaggtgctgaagcggagctacgggccggaggccgacatatggagcatcggcgtcatcgtctacatcctgctctccggcgtccc
cccgttctgggcaggcaagcgatctgttgtcttcagagagacgcacggcatcttcaattccatcctgagggggcaagtcgacttcaccagcgacccctg
gccgcgtatctcgtctggtgccaaggaccttgtcaggaagatgctcaactcggaccccaagcagcggatatctgcgtatgacgtcctcaaccatccgtg
gatcaaagaagacggcgacgcaccggacacaccgctggacaacgcggtcctcggccggctcaagcaattcagagctatgaaccagttcaagaaagc
agcgctaagggtcatcgccggatgcctgtcagaggaggagatcaaggggctgaaggagatgttcaggagcatggacgcggacaacagcggcaccat
aaccgtggacgagctgcggcgcgggctggccaacaagggcaccaagctcagcgaagccgaggtccagcagctgatggaagctgccgacgcggacg
ggaacgggacgatcgactacgacgagttcatcacggcgacgatgcacatgaacaggatggaccgggacgagcacctgtacacggcgttccagtactt
cgacaaggacagcagcgggtacatcaccatcgaggagctggagcaggctttgagggagaagggcctgctcgacgacggccgggacatcaaggacat
catatcggaagtcgacgccgacaacgacgggaggatcaactacacggagttcgtggcgatgatgaggaaaggggacccggaggtggccaaccccaa
gaagcgccgcgacgtcgtgctatag
>g73981.t1
atgagctgcttcgtgtgcttcggatcggcccaggatggggaccccaagaagcccggcgcggacgccaaggacgcgcgcaagggcgccccgccggat
cgcgtggtgacccgggttggatcagatcttccacctgacaaggaacctcttgactggaatacacggatgaagatagctgcaggtgcagctaagggctta
gaataccttcatgacaaggcaagccctcctgttatttacagggatttcaagtcatcaaacattcttcttgatcttccacctgacaaggaacctcttgactgg
aatacacggatgaagatagctgcaggtgcagctaagggcttagaataccttcatgacaaggcaagccctcctgttatttacagggatttcaagtcatca

aacattcttcttggtgaaggatttcacccgaagttatctgattttggccttgcaaaacttggcccggttggtgacaagacccatgtttcgactcgtgtgatg
ggtacttatggctactgtgccccagaatatgcaatgactgggcagctcacagttaaatctgatgtatacagctttggtgttgtgttccttgaactgattacg
gggcgtaaagcaattgacaacaccaagccccagggggagcaaaatcttgtggcatgggctcggccactattcaaggaccgtaggaagtttccacaaat
ggctgatccaatgcttcaaggtcgcttccctatgaggggtctataccaggccctagctgtggctgccatgtgtttgcaagagcaagctgccacacgccct
catattggtgatgttgtcactgccctctcatatcttgcttctcagacatatgacccaaatgctcctgttcaacgtagtcggagcaattcatctactcctaggg
cgaggaacccttctgggtggaacgatgatcagcgcagtgtgcgctcaccaaaccatccttctccagacttgcggcggagggaagctgcaaggacttct
aagtatggagcagaggttagaaggaccagctctgctggtgagtctggtaggagatcaggattggatgacatggacatgacaggttcacaagtgggtag
cccagcacaaactggaagaagaagagaagcttcaaggacggcggacaggcaacgtgctatcgcagaagccagaatgtgggggggagaattcaagag
agcgatcgaatggacttggtagcttcgatagcacaaatgagtga
>g74529.t1
atggcgtcatcgttgtccgaggaagaggaggccttcgagcacacgcttctggtggtgcgcgaggtgtcggtgtacaagatcccgccccgcaccaccag
cggcggctacaagtgcggcgagtggctgcagtcggacaagatctggtcgggccgcctccgcgttgtgtcctgcggcgaccgctgcgagatccggctcg
aggaccctgccacgggagagctcttcgccgcctgcttcgtgctccctggacagagggagagcgccgtcgagaccgtgctcgactcctcccgctacttcg
tgctccgcatcgaggatggccgggggaagcacgccttcgtcggcctcggcttcaacgagcgcaacgaggcgttcgacttcaacgtcgcgctgtcagac
cacgagaagtacgtcaagagggagcaggagaaggagaccgtcggtgaggagagcaacgaaagccagattgatatacacccggctgtcaatcaccg
gctcaaggaaggtgaaaccattaggataactgtgaaaaacaagccaacagctggagctggcatgctttcatctgctggtttatcaggaggggctgctgc
gaaacctaaagcaagtatgcttcttgcgccaccacctggtgcagctgggaagcttcggtctcctcttccacctccacctaatgatcctgcagttgcaagga
tgaattctggaaagaatactggaatcagagccccaaaggaacccaccaagcgaaacgatgatcctctttcagatctttctgctataaaggaaggtgaaa
ccattaggataactgtgaaaaacaagccaacagctggagctggcatgctttcatctgctggtttatcaggaggggctgctgcgaaacctaaagcaagta
tgcttcttgcgccaccacctggtgcagctgggaagcttcggtctcctcttccacctccacctaatgatcctgcagttgcaaggatgaattctggaaagaat
actggaatcagggccccaaaggaacccaccaagcgaaacgacgatccgttttcagatctttctgctataaagtgtgatcatagttggtgccaatcgattc
tgctgccgcttctctttcatgcaatggattcagcgaggagctggttcaataagctccaaacgagagagaaatccattggcaagaagaaagaattgcctc
caaacggcaaggaatcgagtgatgacgcgccttcaagcgctaccaagcagagggttgctgcggccaagcagtacattgagaagcactacaaggagc
aaatgaaacatctgcaggacaggaaagagagacggtgtagcctagaaaagcaattagcagacgccaatgtatcggaagaggaacaacacaacatcc
tgaaacagtttgagaagaaagaaacagaatacatgcgattgcagaggcataaaatgagcgtcgatgattttgacttgttgacaatgataggcaaaggg
gcatttggtgaggtcagagtctgcagagagaagaccacagggaatgtctatgcaatgaagaagcttaaaaagtcagaaatgcttcgtcgtggtcaggt
tgagcatgtgagagctgaaagaaatcttcttgctgaagtggatcatcactgtattgtcaaactatattgctctttccaagacagtgagtatctgtaccttat
catggagtaccttcctggaggtgacatgatgacactgctcatgaggaaagacacactaactgaagatgaggccaggttctatgttggtgagacagttct
tgcgattgaagcaatccacaagcacaattacatccacaggtatgtgagcaactaa
>g74966.t1
atggattcgacgccgcggaagcgcaagggcgcgccgccggcgtgctccgcggcccgctccctccaagatttggcctcccgcaagcgcttctgccgcga
atcggaccccccatcccccgcgccccgacggcggcccccgcaggacaaccccccgcggcggtgatgactgcgccggccgctagcggcgcgtcggcgtcg
gcggtccccggccgcgggcacaagcggaaggtgggctgcatcgactccgccacgcggctcggccgacggaagcgcctcgagaccgagtacgagctc
ggcgacgagattgggcacggcaagttcgggcgcgtgcgcgcctgccgcgcccgggtccccggcggcggcgaggaggaggagctcgcgtgcaaggc
gctgcccaagggcggcggcgacacggcccaccgcgaggtggaggtcatgcagcacctctcgggccacccgggcgtcgtcacgctcaaggccgtcttc
gaggacgccgacagcttctacctcgtcatggagctctgctccgggggaaggctcctcgacgaggtcgcgagggacggcaggctctccgagcgccacg
ccgccaatgtcgtcagggagctcatggccgtgctcaagtactgccacgagatgggcgtcgtgcatagggacattaaaccggagaatgttctgctcacca
aggacgggaggttgaagcttgcggattttggattggccgtgaggatttctgatggtcagaaactgaatggtgtcgccgggagccctgcctatgtggctcc
tgagattctacttggcagctactcacagaagcacaccatcgtcacccgcaagcgcaacctctgtggggacaggcatgcttga
>g75066.t1
atgtcaccatcgtcgctgagatactatggcattggccttgagaatggaaactacactgttgatcttcagtttgcagagttttcctttccagactcacagacc
tggcaaagcagaggaagaagagtttttgacatatatgtgcagggtgatctgaaagaaaagaatttcaacataaggcagacagcaggtggaaaagact
tgtcttacaaccagctcactggtagttttccatcttgggctaccgagaacaatttgcaattctcccaacagttccagaatgcactggattcagaactatttc
agactgcaaggatgtcaccatcgtcgctgagatactatggcattggccttgagaatggaaactacactgttgatcttcagtttgcagagttttcctttccag
actcacagacctggcaaagcagaggaagaagagtttttgacatatatgtgcagggtgatctgaaagaaaagaatttcaacataaggcagacagcagg
agacagtggcttgagcctcaactggtcaacacgatttgagatcattgtgggcatagccagaggcctagcatatcttcatgaggagtccagcgtgcgcat
agttcacagggacatcaaagctagcaatatcctacttgacactgatctaaccccccaaaatctcagattttgggcttgccaagctctacgatgagaagaag
actcatgtcagcaccaaaattgcgggcacattgggctacctggcgcctgagtatgcaatgaaaggccacctgacggaaaaagtagacgtttttgcattt
ggggtggtcgtattggagactgttgctggtcgctcaaacaccgacaactcactggaggaagacaaaatgtttctctttgagtgggcctgggaactctatg
aaaaggggacaagcactcggcattgtggacccaaggctcgaagaagaattcgagagcgaggaggcactcagagtcatcaaggttgctctcctctgtacc
cagggctcacctcaccagcgccctacaatgtccacggtcgtggccatgctcaccagagacttcgagatgactcaggtcgtgacgaaaccgaggtacat
cactgagtggcagctccgtggtgggaacagctatgttaccactgactactctagcgatacgacgcttgagttcagcgagcagagggatgcgattgcgcc
tctcacaccttcgccggcggtcacgggagttattgatgggggaaggtga
>g75132.t1
atgggtggctgctactccgcgtacgcctgctcccgcaagctccgcagccgcatctccctcgtcctccccgtcaccgaccgcgacgcctcgcccgcgaccc
cgcgcgtgaagaagatcaagtccacgaagaagaaggacgaggcggcggaggagctggtgcccaacaccacggcggcggacttcgcgcggcggtac
gtgctggggaaggagctgggccgcggcgagttcggcgtcacgcgacgctgccgcgacgccgccacgggggagtcgctcgcgtgcaagaccatccgc
cgccaccgccgccgcggcacgcaccaccagaccgccgcgcgccgcatggtcgccggcgccgtccaccaggacgccgcggccactaatgcggcggac
gtgcggcgcgaggtggccatcatgcggcgcatgtcgtcggcacgcggcggggacgccgtggtggtgcggctccgcgaggcgtgcgaggacgccgcc

gacggcgccgtccacctcgtcatggagctctgcgagggcggcgagctcttcgaccgcatcgtcgcgcggggtcactactccgagcgcgccgccgccac
cgtcttccgcaccatcgtcggcgtcgtcaagctgtgccactccaacggcgtgatccaccgtgacctgaaaccggagaactttctgttcgcgagcaagga
ggaggacgccccgctcaaagtgatcgacttcggcctgtccgtcttcttcaaccccggcgaccggttcacggaggtggtgggcagcgcctactacatggc
gcccgaggtgctgaagcggagctacggcccggaggtgaacgtgtggagtgcgggggtgatcctctacatcctgctctgcggcgtgccgccgttctggg
gcgacaacgacgagaagatcgcgcaggcgatcctgcgcggcgggatcgatttcaaccgggagccgtggccccgcgtgtcggcgagcgcaaaggacc
tcatcagaaggatgctggacccggacccggccacgcggcccacggcgaggcaggtgctggaccacccgtggctcaagaacgccgactcggcgccca
acgtgtcgctcggcgacgccgtgcgcgcaaggctgcagcagttctccgccatgaacaagttcaagaagaaggcgctcggggtggtggcgcggaacct
gcccgtggaggagctggacaagtacgtgcagatgttccacctcatggataaggaccacaacggcaacctgtcgctggaggagctcaaggaagggctc
cacatcaacggggagccggtgcccgagtcagagatcaagatgctcctcgaggccgccgacacggacggcaacgggacgctggactgcgacgagttc
gtcaccgtctcgctgcacctcaagaagatgaccaacgacgactacctggccgccgcgttcaagcacttcgacaaggacggcagcggattcatcgagct
ggaggagctcagggaggcgctcggaccaggggaccagcagctcatccaggacatcatacgcgacgtcgacaccgaccaggacggtcgcatcagcta
a
>g75314.t1
atggactacgtgatcgggggcaagttcaagctcgggaagaagattgggagcgggtcgtttggggagctctacctcggcgtgaacgtgcagaatggcg
aggaggtggccgtcaaactggaatctgtaaaatcacggcatcctcagcttcactatgaatcaaaactgtacatgcttttgcaaggggaactggaatcc
cacatctgaagtggtttggggtggagggtgaatacaatgtcatggtgattgatcttcttggtccaagtctagaggacttatttaactactgcagcagaaag
ttctctctcaaaacagtactgatgcttgctgatcagatggacgtaaagcaaaccaggtttgtgctacctgnnnnnnnnnnnnnnnnnnnnnnnn
nnnnnnnnggctcgggggccgcgaggatggactacgtgatcgggggcaagttcaagctcgggaagaagattgggagcgggtcgtttggggagctc
tacctcggcgtgaacgtgcagaatggcgaggaggtggccgtcaaactggaatctgtaaaatcacggcatcctcagcttcactatgaatcaaaactgtac
atgcttttgcaaggggaaaacaaagcaggagagatgacctggaatctcttggttatgttctgatgtatttcttaagaggaagtctcccctggcaaggtct
caaagctggcacaaagaagcaaaagtatgatagaatcagtgaaaagaaaatgctgaccccagttgaggtcctctgtaaatcttatccaccagagttta
catcatacttccattactgtcgatcactacgttttgaagataaaccagattattccttcttgaagagactcttccgtgacttatttatccgtgaggggtacca
acttgattatgtatatgactggaccatcatgaaatatcctcagtttagagataaaaacaaacttcagtctagtgggaagacaagtggcttggcaggccct
tctgcagaacggactgaaagaactgcaggctttggaaaacgttatagtcttggcaagcgtattgctgagctgctggaggatctagctgggctaattgag
gaagggaaccaattcaaaacatttgcatccaagcgtttaccagaatttgtcgaggagcggccacggacacaaacctttggcattgaaccggtcctgag
agatttaaggaaatcttttgacagcattgatgtgagcatcattggagtttggggtccagggggtgtggggaagaccacactcctcaacaccttcaacaac
gagctcaagacatggggcagggattaccaggtggctatcatggtcgaggtgtccaactcgggaactctaaacaaagcagccatccaacgcacaattac
agaccgccttggtttgccatggaatgacacagagacagaggaggctcgtgccagattcagggagtaa
>g75440.t1
atggcggtggcggcccccgggcagctcaacctcgacgaggcccccctcctggggctcccgcggcgtcgactgcttcgagaagcttgagcagatcggcga
gggcacctacgggcctgctttcccctacgcgacgcgagcgaggtatcaggttccggcggccatggcggtggcggcccccgggcagctcaacctcgacg
aggcccccctcctggggctcccgcggcgtcgactgcttcgagaagcttgagcagatcggcgagggcacctacgggcaagtgtacatggcgaaggagac
tggcacgaacgagatcgtcgcgctcaagaagatccgaatggacaacgagcgcgaaggcctcaacctcgacgaggcccccctcctggggctcccgcggc
gtcgactgcttcgagaagcttgagcagatcggcgagggcacctacgggcaagtgtacatggcgaaggagactggcacgaacgagatcgtcgcgctca
agaagatccgaatggacaacgagcgcgaaggcttccctatcaccgccatacgggagatcaaaatcctcaagaaattgcaccataagaacgtcatcaa
gctcaaggaaatcgtcacctccccaggtccagagagggacgagcaagggaagccaatcgacggcaacaagtacaaggggagcatttatatggtcttc
gagtatatggatcatgacctcactgggcttgcggataggccagggatgcggttcactgtgccacagattaagtgttatatgaggcagttgcttacaggct
tgcattattgtcatgtcaatcaagtgctccatcgtgatattaaaggatctaacctcttgatagataacgagggtaacttgaagttggcagattttggccttg
caagatcattctcgagtgaccacaatggaaaccttactaaccgtgtgatcactttgtggtatagacctccagagttgcttcttggaagcacaaaatatggt
ccagctgttgacatgtggtctgtgggctgtatttttgcagagcttctcaatgggaagccaatattgcctggaaagaatgagccagagcagctgactaaaa
tttttgagctttgtggcaccccggatgacttaatatggcctggtgtcacaaaaatgccatggtacaacaacttcaaaccaccccggccattaaagagacg
tgttaaagagttctttaaacattttgatcggcatgcgctggacctgttagagaagatgctgactttggatccatcacagaggatatcagcaaaagatgcc
cttgatgcagagtacttctggaccgatcccttaccttgtgatccgaaaagactaagaaaaaacgtcagcaacagaggcaagcagaggaggctgcaaa
gcgtcaaaaaatccagcaccctcctccacattctcgtctgcctccaatccagcagccagggcaggcacacccaatcaggcccggcccgggtatgcaca
atgcaccacccgtgggggctggcccaagccatcactatgcaaagcctcgagggccaggaggacctaataggtacccacagggtgggaatcaaggtgg
aggctacaatccgaaccgtggcggaggacagggtggtggctacgggagtggtccatatccacagcaaggacgaggacctccgccttaccctggtgga
ggtccacgtggtagtggtggcggtggttatggaagtggcgggccaaatttcccacaaggtggcccgtatggcacatctggtccgggccgaggaccaaa
ctatagtcaaggtggttctcgcaatcagcaacagtatggtaactggcaataatagggcaagcaacatcacggccttgaaatgctgtttcagttggactat
gtatttgcaatgtttgtaa
>g75579.t1
atggagccgcgcgttggcaacaagtaccgcctcgggcgcaagctcgggagcggctccttcggggagatctacctcggtacaaacgtgcagacaaacg
aggaggtcgccattaagctggaaagtgtgaaaacaaaacatccccagttgctgtatgagtcgaagctatataggatactgcagggaggaactggaatt
ccaaatgttaagtggttcggagttgagggtgactataatgttcttgttatggacctcttggggccaagccttgaagaccttttcagcttttgtagcagaaag
ctgtccttgaagactgttttgatgcttgctgatcaaatggtatacatgattgattttggactggctaagaaatacagggacacttcaacacaccagcacatt
ccatacagtcttccatggcagggtctaaaagctggaaccaagaaacagaagtacgagaaaattagcgaaaggaagaaaactacttcacctgaggctg
ctgcagcgggtcaaagctccgggatggttcctatgacaaataataatagaccatctgaggaggaaaggcaaaaggaaccgtctccaacagacaatgc
agaggagggaaggcagtgcacaccggagctcgcccgcccgcccgtggccgcgccgccagcagctcgcccgctgcggaaggagctcacccgctgccg
ccagcagcctcgctctgcgcacgccgccacctctgctcgcctgtcgctctgccgcctcgtcatccttgccgcgcatcggcccgctcgcacgccgctctgcc
gcctcgtcgtcctcgctgtgcgccagcccgctcgcatgccgctctgccggcgatcctggccgcgccttgtcccgcgcgcgccggccccgtgcgcctcgcc

gtcctcgttgcacctcggctgcaggtccgatggcggcggtggaggaggaagcggctccccgcacgcccgcctcaccgcgcacgcctgcctcctcgcga
gctggaggcgtcggtcgcgtctgcgtgagctggtgccgcagccccccatccacacaacgccgccccccgcgtgagctggcgctag
>g75627.t1
atggcgccgccgttcgccgtcgatgtcgtctgctcggagggtacgagggtgaccgaccggcggaagggagcccagcagatcatcgccgggcactcgt
cggcccagaatgtagctgggcttacgttcaaaggcaatatcccagattctattggtgagatgaagaaacttggggagattcatttggaccacaacatgat
gtctggggagcttccatcaactctgagcaactgcagagaactcataaccatggacctcaagaacaacagattcagcagagaactctccaaggcaacaa
gtaactttggcaaagagaatatcataggctgtggaagttatgggctagtctacaaggctgagcttcctgatggcttcaaggttgccatcaagaagctcac
tagtgaaatgtgcctgatagatagggaattcagtgcagaaattgatgcactctccatggcgcatcatgaaaatcttgtgcctctctggggttactgcatcc
agggaaactctagattcctcatatattcttacatggaaaatggcagtctggatgattggcttcacaatatgaatgatgatgcttccagctcatttcttgact
ggccaaggaggctaaggatcgcacaaggcgcaggccatggtcttatatatatccatgatgtctgcaagcctcacattattcaccgtgacatcaagtcca
gcaacattctattggacaaagaatttaaagcctatgttgctgattttgggctatccagattgatccttcccaacaaaactcatgtcacaactgagttggttg
gcacatttggttatatccccccagagtatgggcaaggttgggtggctactctgagaggtgatatgtacagttttggagtagtcctgcttgagctgctcaca
ggcaggcggcctattccaatctcatcaatatcaaaagaactcgtccagtgggtacaggagatgagatctgaaggaaagcagattgatgttttggatccg
acgctaaggggcacagggcacgaagagcaaatgttgaaggtgcttgatgtcgcttgtcagtgcgtgaaccataatccttccatgaggccaacaataca
agaagtagtcgcctgtctggacagcatagatgccagccagaagatgcaaaatttagtcaaacaagcaatgatgtga
>g75628.t1
atgcagcagcaactcaattcctcgtgcaagaagtacagaaatggatttttcgttcctttgtcaattcttgtgcttgtgcttatgctcttctccttcatccgtcct
gcaagctcctgcacagagcaggagaagagttccctcctccagttcttcgctgggctctcgcacgatggtgggcttgccttgtcatggcagaatggcaccg
attgttgcacttgggaaggggttgcctgcggcacaaacagggtcgttactgatattaccctgacttcaaagggccttgagggacacatctcagcatccct
gggcgagctcaccagtctgttacgcctcaacttgtcccacaacttgttatatggcagcctacctctggagctgctatcatccaacagcatcatcgtccttga
tgtcagcttcncctgggcgagctcaccagtctgttacgcctcaacttgtcccacaacttgttatatggcagcctacctctggagctgctatcatccaacagc
atcatcgtccttgatgtcagcttcaatcacctcaatggagacctgcgtgacctgccatcgtcaacccctggccggccccttcagtggcagcattcctcccg
gacttggtaattgctccatgctcaaagtgctcaaggctggccgcaacaaactcagcggaccactcccaggtcagctcaacagacttgaggagctccatt
tggaccacaacagcatgtccggggagctgccatcaacactgagcaattgcacgaatctcatggcaatcgacctcaagagcaacatgttcagtggagag
cttcagaagatccttaagagttcttggaatctcacttcccttcttattggaaccaatttcaagggtgagaccatgccagaagatgaaacaattgagggtttt
gagaatcttcagttagacttgtcaagcaaccatctcacgggtgaaatcccatcagcactgaacaacctgcacttcctctctgcattcaacatttctcataat
aacctggaagggcctattcctgatggaggccagcttagtacattctcaaattctagctttgaagggaatccaaatttgtgcggtactattctccatcgtagt
tgtggttcagcagaagcaccattgtcattcagaaaagaacgcagcagaaaagccactttggcgattacatttggtgtgtcagttggagttgttgttctttt
actgctggtgcttctccttgcctggttcaagggtagaagtttgataatcaaacgcaagaactgcaacaacagagatgaagaagaaacctcccacatgtc
cgattcagaacagtcactagtaatagtgccaagaggcaaagacggcgaaaacaagctcagatttgctgatattgtaaaggcaacaaataactttcacc
atgataacatcataggttgtggaggctgcggactagtctacaaggctactttgcctgatggctccaagctagccatcaagaagcttaatggtgaaatgtg
tctcatggaaagagaattcactgcagaagttgaagcactatccatggcgcaacatgacaatcttgtgccactgtggggttactgcatacatggagattca
aggctcctcatatattcctacatggagaatggtactctggatgattggctgcacaacagagacgatagtgccagctcctttctcaattggccaatgcggct
caaaatcgcacaaggagcaagctggggccttgcttacatccatgatgtctgcaagcctcacattgtccaccgagacatcaagtccagcaacattctactt
gacaaagaattcaaagcatatgtggcagattttgggctctcaagattgatccttgccaataaaactcatgtcacaactgaactggttggcaccctgggtt
acattccgccagagtacgggcaaggttgggttgccacactgagaggtgatatgtactgttttggaatcgtcctgcttgagctgctaacaggcagacggcc
tgttcaagtcatgccttcatcgaaagaacttgtgaagtgggttcaggagatgaaatcagaagggaagcaggtcgaggtccttgatccagcattgcgagg
aagtagtgtcctgcctggacagtatagaccccaaactgcaaatgcaaaattcagttaa
>g75678.t1
atgaagggagccggcggtgctggcgatcgatcgagcaccaccggtggtgcggcggcggcgattgatgcgtgcgagaagggcgtgctccagggccgg
tacgagctgggccgcgttctcggccacggcaacttcgggcgcgtccacgcggcccgcgacctccgcacgggccgcagcgtcgccgtgaaggtgctgtc
caaggacaaggtggtccgcgccgggatgatggagcagatcaagcgcgagatcgcggtgatgaagcgcgtgtcccaccccaacatcgtcgagctccac
gaggtgatggccacgcgcaccaagatctacctcgccctggagctggtccgcggcggggagctcttcgcccgcatcgtccgcgccggccgcgtcaggga
ggacgtggcccgccgctacttccgccagctcgtctccgccgtgggcttctgccacgcgcgcggcgtcttccaccgcgacctcaagcccgagaacctcctc
atcgacgacaagggcgacctcaaggtggtcgactttgggctcagcgcgctggcccaccacgcgcgcggcgacggactcctccacacgctctgcggcac
gcctgggtacgccgcgcccgaggtgttcaggaacaagggatacgatggcgccaaggccgacctgtggtcgtgcggggtgatactctacgtgctgcttg
ccgggtcgctgccgtttcccgatgataatattgcgtccatgtttaagaagatgagccgaggggattacaagtgcccgccgtggctgtccacggacgcgc
gccgccttatccccaggctgctcgaccccaaccccgacacgcgcatcaccatcgcgcagctcgtcgagacgccgtggttcaccaggtcgtccgtcgcga
gaccgcccatgcccatcgtggctgcggccgcggagcagccggcggcggacgatgcggcgaaggacaaggacgagccggagacgctgaacgcgttc
cacctgatctcgctgtcggcggggttcgacctctcgccgctgttcgaggggcactcctcctcctcctcctcggcggcggcgcgggagggaggcgggatg
cggttcgcgacgcgggagccggcgagcggcgtggtctcgcgcctcgaggaggtggcggcgcgcggcggcggccggatgcgcgtgaccaagagcgg
cgcgcgcggcgtgcggttcgagggcgccgaggggccaagggacgcctcgccgtggccgccgacatcttctgcgtcgcgccgtcggtgctcgtcgtcg
acgtcaagaaggacgccggcgacacgctcgagtaccgctccttctgcagcgacgagctccgcccggcgctcaaggacatcgtctggggcgccgagcg
gccgccggccgccgccgccgtcgtgtga
>g75938.t1
atgcccaaagagcaaactgcaggccttgagggccgcaacatcgatgtcgccaatgtcaaggtccacgtccgagaagtcatagctgagggtgggttctc
atgtgtctatgctgcaagagacatggtgaatccaacaaagcagtacgcgctaaagcacatgatcatccaggacgaggagactcttgagctggcccaaa
aggagatcacggtgatgcggtcactcaagggacacccgaatgtggtcactcttgttgcgcatgcaatacttgatatgggtcggacaagggaggcactg

ctggttatggagttctgtgagaagtcactagtttctatgttggaagggagaggtgctgcatactttgatgaggagaaggtggcgctgatctttcgtgatgt
ttgcaatgccgtctttgctatgcattgccaaacactgccagtggctcacaggtaa
>g76085.t1
atggccggcgccaagagcagcaagaagctggccggctcgccgctcctgggcaagtacgagctcggcaagctcctcggccgcggcaccttcgcgaagg
tgtaccacgcgcggtccctcgacggcggcgacgacccggtggccatcaaggtcctggacaagtcccagctgacctccaccggcatgatcacccgcgtg
ctccacgaggtctccgccatgcgccgcctccgccacccgaacgtgctccggctccacgaggtcctcgccacccgcaccaagatctacctcgtcatggag
ctggccccgggaggggacctcctgacccggctcgcctccctgccctcgcgccgcctccccgagcacgccgcccggcgcgtgttcctccagctcgccaac
gccctctcctactgccacgcgcgcggcgtgtctcaccgcgacgtgaagccgcagaacgtgctcatcgactcccagggccagctcaaggtctccgacttc
gggctcgccgcgctcctccccgactccctccgcgacgacgggcggctccacacggcgtgcgggaccccggccttcacggcgcccgaggtgctccgccg
caaggcctacgacggtgccaaggccgacgcgtggtcgtgcggggtcatcctcttcgcgctgctcgccgggagcctccccttcgacgacgccaacatccc
ggggatgtgccgccgcgcgcagcgccgcgagtacgcggtcccggcgtgggtgtcccggcccgcgcgccggctcatcgcgcggctgctcgacccgaac
ccggacacgcggctcgcggtgggggacctccccgaccacccctggttcaagcggtcgctcagcgtggactcgcagctcggcgggctcatcgcggggc
agcccgagcgggacctcgcgttcgccgcggcgccgcacgcgctcaacgcgttcgacatcatctccatgtcgcccgggctcgacctgtcggggatgttcg
gggagagcaggcgggtcagggagaagaggttcatgacgacggccacgctggaggagacggtggagcggctcggccgggcgggggacaggctcgg
gtacttcctcgtggggaagaagggcgtcgaacgcctgccgatcggcggcgtgcaggggatcgtggccatgtccatggaggtgtcggaggtgtcgcggg
agctcatgctcgtcgagctcaggctggagagcggcgacggcgatgaggcccaggcgttcggatgggacgacctcagggtcgagctcggggacgtcgt
catggcgtggcacgtctgtgaagacggttaa
>g76257.t1
atgggccagaacctgtctacaatgatcaagaacactgcccctgttggaactttctgctatatcgacccggaatatcaaagaaccggtgtactatcgatga
agtctgatgtttacgctctaggcattgtgatattgcagttgctgacagcaagatcacccatgggccttgctcacactgtcgagactgcacttgaagatggc
tgctttgttgacatcctggatgcaactgctgggcagtggcctcggaatgaaacgcacgaattggctgtcctggccctcaggtgttctgaaatgaggcgca
aagaccgtcctgacttgaatgagcaggaagtgatggatgatccatatgttgcatctgatggatacacttatgatcgtaaggcgattgagttgtggctaag
tataaatgacaagtccccaatgacgaacctgcatttactgaacaagagcctgataccaaatcactcgcttcgttctgcgatcatggactggaggtcgaaa
agcaaatga
>g76816.t1
atggatcgaatcgtcggcagcaagtacaagctcggccgcaagatcggtagcggctccttcggggagatctacctcgcgacgcatgtcgacacctacga
gatcgtcgccgtcaagattgaaagcagcaaaaccaatcatcctcaattgctatatgaggccaagctgtacaatgctctccagggaggaactggtattgc
aaatataaaatggtgtggcattgatggtgatgaaaatattcttgttattgatctccttggaccaagcctagaggatctttttgtttattgtggccgtagattct
cactaaagactgtcctgatgttggcagatcagatggaaagcagcaaaaccaatcatcctcaattgctatatgaggccaagctgtacaatgctctccagg
gaggaactggcattgcaaacataaaatggtgtggcattgatggtgatgaaaatattcttgttattgatctccttggaccaagcctagaggatctttttgttt
attgtggccgtagattctcactaaagactgtcctgatgttggcagatcagatgattacgaggatagaatttatgcattctaaaggatatttgcacagagac
atcaagccggataattttcttatgggccttggtcgaaaagctatccaggtctacatcatagacttcgggcttgcaaagcgataccgtgatgcccacacta
atcgccatataccttatagggaaaataaaaatttgaccggaacagctcgctatgctagtcgtaatactcatcttgggatcgagcaaagccgcagagatg
atctggaatccattggatatgttcttctttatttcctcaggggaagccttccttggcagggcttaaaagcagcaaccaagaaacagaagtatgacaaaat
aagtgacaagaagctttcaactccgatcgagcaagatgcaggtgtaagtagccgtcctgttgcaacaaatgtggacaagcatcaagttagtgtttcacg
cccaatcgaagcttctggtctgcttgaaggagagcaacgcccggcaatcagaatgcagttcagatctagtgctgagaacagtcgttcaaataatatgca
ccctgagagatcgctttaa
>g76956.t1
atgagggagctgcgcatcctggtgctggttctgctgatatccttgccgtgcttccccgcatccgatcgtcaaggtgaggatgatcgacgaattgcatttgg
ccagctcaaaagatttgcatggcgagaactacagattgcaactgacagtttcaatgaaagaaatgttcttggtcagggaggttttgggaaagtttacaa
gggagtacttccagatggtactaagattgccgtaaaacggctgactgattatgagagtcctggtggggaggctgccttcttgcgtgaagtggagctcatt
agtgttgcagttcaccgcaatctgctaaagctgattggcttttgcaccacacagacagagcgccttctggtctatccatttatgctgaatttgagtgttgcct
gccgcttacgagatttcaagcctggagaacccatcttagactggcctacaaggaaacgggtggctattggcacggcacgtggcctggagtatctgcatg
aacactgcaaccccaagatcatacaccgtgatgtgaaagctgcaaatgtattgcttgatgagtattttgagcctgttgttggtgattttggcctcgcaaag
ctagtagatgtgcagaagacatctgtgacaactcaagtccgtggaactatgggccacatagcgcctgagtacctatccacagggaagtcgtctgaaag
gactgatgtttttggctacgggatcatgcttctggagcttgtgaccggacagcgcgccattgacttttcgcgtttggaggaagaagatgatgtgctattgct
tgatcatgtaaagaagctacaaagagaagggcagcttggctctattgtggacggcaacctgaatcagaattatgaagaggaggaacttgaaatgatta
tacagattgcattgctgtgcacacaagcatcacctgaggaccggccatccatgtcagaagtggtccggatgctggaaggtgagggcctggcagagcgg
tgggaggagtggcagcaggtggaggtgacaaggaggcatgagtatgagcggatgcacaggagatttgaatgggttgaggactccatctataacccgg
aagcaatagagctgtctgcaggtagataa
>g77184.t1
atgatcatgggaggagcattggagatggagagcaggaagaaggcgaagatcatcctcgccggcgcggcgttggccgttctggcgaccatcgagctga
ccctcttcctctgcttccgcctctcccgtcccttctacctctccacggcatgcgtcgtatccgtcgcgctagcgtcggccgccgccgcgctgctctgctcctc
ccatcacggccgggcccaccgcatgtcccggcggcgctccgtgcttgacggcggcggcggcggccaggaggagggactggacgtggagtacagcttc
ttccggaaggttgccggcctcccgcggcggttctcgctggcgacgctggcggcggcgacggacgagttcgggtgcgtggtgggtcgcgggtcgtcggg
caccgtgttccgcggcgtgctcgacgactgcacggcggtggccgtgaagcggatggacggcgggccgtcggcccggttggcggagcgggagttccgg
gccgaggcgggcgccatcggcggcgcgcagcacgcgggcctggtccgcctcctgggcttctgcgtcgtcccccacggcggcccacggttcctcgtctac
gagttcatggagcacggctccctggacaagtggatcttcccctcttctccttctccttcttcttcccacggcggtagtaaccatcggtggctgacgtggcgg
cagcggcggcaggtggcggtggacgtggcgagggcgctggcgtacctgcacggtgacctgcgcggcgggaccaaggtggtgcacctggacgtgaag

ccggagaacatcctcctggacgacgccttccgtggcgtgctctcggatttcgggctgtcaactctgatgggcaaggaccagagccgggtcgtcaccacc
gtgcgcggcaccacggggtacctcgcacccgagtggctgctcggcgccggcgtcaccgacaagtccgacgtctacagcttcgggatggtcctaatgga
gatgctcggcggcaggcggaacctgcaaataatcgaggaggccggtgaagtcgtggtggaagggtcgtcgtcgtcgaggaggaggaggtggtc
gtatttccccaagatcgtggcggagaaggtgagggaagggagggtgatggaggtggtggacccgcgggtgcgggaagaggcggcggcggcggacg
aggcgggggtgaggcgggtggcgcacgtggcgctgtggtgcgcgcaggagaaggcgagcgcaaggccggacatggcgcgcgtcgtggagatgctg
gaggcgcggggcggagcggcgacggaggtggagccgccgccgccgtcggagatgatcttggtcgacgtgctcgcgctcgaccccgaccgggcgccg
cggccgttcgggttgccgcccgtgccgtcgtcgggatggaatacgggcgtgtcgtcgtcggcgatgagccgcagcgactccttcgcgctgtcctacctct
cgggacgatga
>g78254.t1
atgtcgagcgccgaagtggacgagagccctcccgctcccgcacgtggcaccaagcggacgcgagctccacctcacgcactcatcaccaaggacacag
cgaagttggcgttgctgaccttcatcctcatgtgctacctgaccgtcgaactcctctaccctccttcccgatccacccttactccgacgagcgttacgtacc
caaaattcgcgaaggccgccgtcgagaaagccaccgccatcagcaacgtggtgagcaccaacgagtacagctacacgggcagcaaggtcctcggca
acggcacgttcggctacgtcttcacagcgcgccaccgcgtctccaacgacgtggtcgccgtcaagtccctcaggaccgaccgcgaccacgacggcaac
aacaggtcctacaccgccggcgccgccgagatcctcggcgaggcccgcgccctcgccgccgcgggcggccacccgtcggtcgtcgcgctccgcgcca
tcgccgtccacccgacgacgggggagctgtacctcgtgatggaccacgcggggcgctcgctgggcctagagctctaccgcaaccgcagcggcgcgcc
gttcccggagcgcgacgtgcggcgcgcgatgcggcggctcctcgccgccgtcgcgcacatgcactcccgcggcgtcgtgcaccgggacgtcaagccg
gagaacatcctcgtcgtcggttccggtagtgccggcgccggcttgttattggacggcgtcaagatatgcgacctcggggtcgccatgttcgccgacgag
ccgggaccgcgcgtgcgcgtcggcacgagccagtacatggcgccggagatcctcgcggggaggacggactacggcgacaaggtggacatgtggtcg
ctcggctgcgtcatggccgagcttctcactgggcgaccggtgttctacgctccgaacgacgacgacgcggcgcaaatgcgagtgatcttcgacgtgctt
gggccgccgtgcccgggatacaactcttccgagcagcacgacgatgacaaggtagcgacgactctgcagaacgtgagcgaccaccgcaaccggctgc
gcgacttgttccctgagcagaacttgtcagatgaaggcttcgatgttttgaaggggcttctctcatgtgacattgaccagaggttgtcggcgaccgatgcg
ctccggctgccgtggttcgcctctgacgccggtgaagaagctccatctgcgctgcaccttccacttcgggatagggcgtcttggacgtggatttttcccat
cggaggggcatcgcaccggagagagaaaatttaacaacatattaa
>g78393.t1
atgccggccccggcggtgctgaagctcgtcgtcctcctcgttctgctcgcccggccctcctccaccgccggcgccgggaacgcgacggcggaggcgtgc
ccgctggacctgggctacgtgcnggcggtgctgaagctcgtcgtcctcctcgttctgctcgcccggccctcctccaccgccggcgccgggaacgcgacg
gcggaggcgtgcccgctggacctgggctacgtgcggacgttcccgtgggacccgtcgccgtgcggcggcggcacgccgcgcaacatgacggcctgct
gccagacgctgctctctgtgctggccatcggcatcgccggccgcctccgcgccacgggccgcttccgcctcccctccgcctccgcatccgccgcctgcct
cgaccgctacgcggacgagctcgccggcgcgccgctggacctcccgggctcctccctcgtccccgcctgcttcccgtcccccgaccagttcgccatcacc
ccgtcctactgcgccggcgtcactaccgtcgcggacttcagggccaccgccgggaacgactccgtcgcggccctggattccgcgtgcgggcccgacct
gaccacgtcgcaggtgtgcctgccctgcctcaacgccggcatcggcgccgcgtcgcgcctcaccgcggcggcgggcaacgattccaagtccgccccat
gcttctacctgaccgtcctctacgccgccggcgtcgccaacaaggccggccccacctacccgcccaccgccgcctgcgccctcggcctgggcctctcctc
ccctccgcccagttcgtccaaatccaaggaaacggccaccatctacgccaccacaatcccaatcgccttcgtcctcctcctcgcatcagtcctcctcgcat
tcttcgtctggaggaagaggaggcacgatgctaacagcaagaagaaaactaagatgatgaatcacaagatctccgaggaaggctcgatggagcggc
ggtcgcacccgcggcccaacacggggtcgatcctcttcgacatcgcggagctcgccaaggccaccggcggcttcgcggagcgcaacctcatcggccg
cggcgggttcggggccgtgtaccgcggcgtgctcgcggacggctccgtggtggccgtgaagaagatgctggacccggacgtggaaggcggcgacga
ggagttcaccaacgaggtggagatcatcagccacctccggcaccgcaacctggtcccgctccgcggctgctgcatctccgacgacgacgtcgaggaag
gcaagcagcggttcctcgtctacgactttatgcccaacggcgcgctcgaggacttcatcttccgcgacaaggaacccgcgtcgaagcggccgccgctga
cctggtcgcagcggcggaacatcatcctcgacgtggcgcgggggctcgagtacctgcactacggcgtgaagccggcgatctaccaccgcgacatcaa
ggccaccaacatcctcctcgatggcgacctgcgcgcgcgcgtggcggacttcgggctggcccgccggagccgggagggccagtcccacctcaccacg
cgcgtggcggggacgcacggctacctcgcccccgagtacgcgctgtacggccagctcacggagaagagcgacgtgtacagcttcggggtgttggtgct
ggaggtcatgagcgcgcgccacgtgctggacatgaccgccccggccgggcccgtgctcatcaccgactgggcgtggacgctcgtcaaggccgggcag
gcgagggaggtgnnnnnnnnnnnnnnnnnnnnnnnncgcgcgaatattgaattcaagagagataagataaatataacccgtgcacgcaaaa
gcgttggccgtagcgtagccgcccccgcttaccgctccttgtccccacccactctccgccccgacgtcagcggagccgccgccggccgagcacgccaac
tattcggtgaaatgccggccccggcggtgctgaagctcgtcgtcctcctcgttctgctcgcccggccctcctccaccgccggcgccgggaacgcgacgg
cggaggcgtgcccgctggacctgggctacgtgcggacgttcccgtgggacccgtcgccgtgcggcggcggcacgccgcgcaacatgacggcctgctg
ccagacgctgctctctgtgctggccatcggcatcgccggccgcctccgcgccacgggccgcttccgcctcccctccgcctccgcatccgccgcctgcctc
gaccgctacgcggacgagctcgccggcgcgccgctggacctcccgggctcctccctcgtccccgcctgcttcccgtcccccgaccagttcgccatcaccc
cgtcctactgcgccggcgtcactaccgtcgcggacttcagggccaccgccgggaacgactccgtcgcggccctggattccgcgtgcgggcccgacctg
accacgtcgcaggtgtgcctgccctgcctcaacgccggcatcggcgccgcgtcgcgcctcaccgcggcggcgggcaacgattccaagtccgccccatg
cttctacctgaccgtcctctacgctgccggtgtcgccaacaaggccggtcccacctacccgcccaccgccgcctgcgcgctgggcctggggctctcctcc
cctccgtccaattcgtccaaatccaaggaaacggccaccatctacgccaccacaatcccaatcgtcttcatcctcctcctcgcatcagtcctccttgcattc
ttcctctggaggaagaggaggcacgatgctaacagcaagaagaaaattaagatgatgaatcacaagatctccgaagagggctcgacggagcggcgg
tcgcacccgcggcccaacacggggtcgatcctcttcgacatcggcgagctcgccaaggccaccggcggcttcgcggagcgcaacctcatcggccgcg
gcgggttcggcgccgtgtaccgcggcgtgctcgccgacggctccgtggtggctgtgaagaagatgctggacccggacgtggagggcggcgacgagga
gttcaccaacgaggtggagatcatcagccacctccggcaccgcaacctggtgcccctccgcggctgctgcatctccgacgacgacgtcgaggaaggca
agcagcggttcctcgtctacgacttcatgcccaacggcgcgctcgaggacttcatcttccgcgacaaggaacccgcgtccaagcggccggcgttgacct
ggtcgcagcggcggaacatcatcctggacgtggcgcgggggctcgagtacctgcactacggcgtgaagccggcgatctaccaccgcgacatcaaggc
caccaacatcctcctcgatggcgacctgcgcgcgcgcgtggcggacttcgggctggcccgccggagccgggagggccagtcccacctcaccacgcgc

gtggcggggacgcacggctacctcgcccccgagtacgcgctgtacggccagctcacggagaagagcgacgtgtacagcttcggggtgttggtgctgg
aggtcatgagcgcgcgccacgtgctggacatgaccgccccggccgggcccgtgctcatcaccgactgggcgtggacgctcgtcaaggccgggcaggc
gagggaggtgttcgacgaggcgatgctgtcggcgggcacggcggcggagagcccgcgcggcggggccatggagaggttcgtgctcgtcgggatcct
gtgcgcgcacgtcatggtggcgctccggccgaccatcaacgaggcgctcaggatgctggagggggacatggatgtgccggagctgcccgaccggccg
ctgccgtacgggcacagcgtcatgttcagcgaagccgggagcaacttcagtgcttcgccggcgttcagtggcccgttcatggacaatggggacatgctc
aggtga
>g78417.t1
atgcacggcgccggcgtcgtccaccgcgacatcaagccggacaacatcctcgtcggagaagacggcgcgttcaagatctgcaacctcgggatggcgg
cgccggtgaaccccgcgggggtgccgtgcgaggaggaccgcgtgtgcacgctgtggtaccgcgcgccggagctggtgatgggggtccgagcctacgg
caccgccgtggacgtctggtcgctgggatgcgtcatggcctag
>g78418.t1
atggcggcggtgacgacccgcaagcgcgccgcgccggaaggagccttgttccccgaggcagccggcaagaagcgcgcgcggttccagttcgggagc
atctacaactacgagaagctggaggtgctcgggtcgggcagctacggcaccgtggtgaaggcgcgtgacctgcgcacgggcgagaccgtggccgtca
agtggatccgccgcggcggcgacggcgccaccgacctccgcgcggtgcatcgcgaggccagctgcctcgcagcgtgcggcggccacccgtccgtggt
ccagatcagggacgtggccgcggacgaggccacgggggacgtgttcatcgtgatggagttcgtgggctccagcctgcaccgcctcgtcgcgcgggctc
ggccctccggcttctcggaggcccaggcccgcggctgcatgcgccagctcctgcgcggcgcggagaggatgcacggtgccggcgtcgtccaccgcga
catcaagccggacaacatcctcgtcggagaagacggcgcgttcaagatctgcgacctcgggatggcggcgccggtgaaccccgtaggggtgccgtac
gaggaggaccgcgtgtgcacgctgtggtaccgcgcgccggagctggtgatgggggtccgagcctacggcaccgccgtggatgtctggtcgctgggatg
cgtcatggccgagctgctcgccggcgtgccgctgttcgagggcgccgaggatgaggacgacatgttcgccagggtgatggagcttcggattgagatgg
tggcggcggagtgccgcgcgttcgagggactgcccgggttgtcggaggccgggcgggagctcctgtgcggcttgctgagctttgagcccgaccagcgg
ctcaccgcggcggaggcgctcgatcaccagtggttcgaggaagaggacgagcccgtgtcctcttttctgctgtctccgaagcagccaaatggtttcatca
acttcttctag
>g78435.t1
atgtccgactcggcgtcgatggcggccgccatcgaggcgcggttcaacaaccgcgacctcatcggccgcgggtcatttggcgacgtctacaaagggttt
gacaaggagcttggtaaggaggttgcaataaaagttatcgatctggaagaagcgttcgccacaatagctgtgttgctgcctaggcgacaggctgcaaa
tattctcctcactgagagtggagatgtgaaggttgcagattttggtgtttcggcacagttgactaaaacaatgtctcggagaaagacattcgttggaactc
ccttttggatggcacctgaggttattcaaaactctgaagggtacaacgagaaggcagatatctggtctttgggtataactgctatagaaatggcaaaagg
tgaacccccttttggcagatattcatcccatgagagttcttttcatgatacctcgtgaaaatccacctcagaggcctagtgctaaggagcttcttaagcatcg
tttcattaaaaatgccagaaaaactgcaaagcttctggagagaataagggagcggccaaaatttgctgtaaaaaacagcttggatgccgcacaaaatg
ggcgaacacatattgaagaagatatcagtacaggtactatcaaggtggatagggcaaaagaaacatctcagactttgagatcggagggcacaggaac
tgttcgagttgggtcaaggccacctcaaatttcctccaccaaggatggtagatccgatattcctcatagtccaaatactccaactcgtacttctgaccgag
aaaatcagtggaggccatcatggagggaatctgatgaatcacttgcatcaagcttgtctcagagagaggtgcatagtgaacgtggggagctagaaagt
tctacagaagatgctactggcgacaagtttgaacagcctgcagtgcatgcattccttgattccttgatggatctggaacatgagttacctggatcgtgtga
ggtactgattggcagaatgctacaccggctaggaagcttgaaggattcctcgttgcaaggtcttcaggagacggcagcttcggctttctctaagaaatct
gatcctccttcggaaccagtgagcagcaaaaaacaagctaacacgccaccattggcagccccaactataagcccactggcgaggttccttcttacaag
gtggcaaaaccaagtgtcgcaagaccttaattctgtttga
>g78483.t1
atggtcggatccgccgccgccttctgcgcctcgtgggtcgcctcccgcatcgagcctcgagtcccacatggccggatctgccgcgtcatgagaggagtg
gtggaaggaggtgaaggcgacggccggccttcatttttgccagacaacagaagcccggaggtcagcgtcgacgtgccggaggtggcgacgcgcgag
ctgaaaaaggccacgcgtcccttctcagagaagacgctgactggcgagggggcgctacgccgaggtttacaggacgtcgttaccgagcggcacgaccg
tcgctgccaagagactcaaggcgccctcgtcccaacgctggaaggcttccgacgccgcgttcctcatgcgccaggtgtcagtggcgtcgaggctgagg
caccacaacctggtacgcctcctcgggtacaacatcaccgccgacctctgcgtgcttctctaccagaggttgagaagggaggcgccgcatgggcggct
ggccaaagcgggccgcagctggccgacgctgagctgggcgcagcgcgtgcgggtcgcgctggacgcagcgaacgggctggcttacctgcacggtgc
aggggtgacgcaccacgacgtccggtccaccaacgtgctgctgttccagggattcagggccaagatcggggactatgacctcttcaagcagctgccgg
agaaggacgtggagaacgattcatggtgcatgctttgggcgttacaggttcgctaccacgggccaagtgacccagaagggcgagacgtgttctgctttg
gggtcgtgcttctcgagctactgacggggaacaaggcagaagactccaagttgaaattgggcgcgtcgccaccgaccgcctccctcgggcgcgccgcc
gccggccgcctccatcgcgcacgccgccacctccctcggacgcaccaccgccacccgtctacctcgcacgcgccgccgcccgaaaacgcctctcccgc
gcgcgcccgcgccgccgtcgggcgccccccctcgcgtgcgccgctgccgcctctggccacctccctcgcgcacgccgccgccgccaggacccggctccc
cccgcgcgcgccgctactcctcccccctcctctcacgcggggatccgcatggcggcgtcctcctccagtcggatccggcgcgccttgctcctcctcctcctc
ctacgcggggatccgcgcagcggcgtcctcctccggtcggatccggcgtgccttgctcctcctcctggacctcaaggacggtcgcgctggagcaggacc
tcgtcggcggtggcccgcagcggtgctgctctccgcctctgtcggcgccctccccctcttctccaccaccggcgcgcaggggagcacctccttctccttcc
tcctcggcggtggcgcgcctcgctcgtctccgtggaggccgcgcgcaggggtggttgggaggcgtgtagggcccgtgtgatgagggtatag
>g78519.t1
atgggcaacacctgcgtcggccccagcgcggcgggccgcagcggatttctcgcctccgtcaacctgtggcggcccgcgcagcccgccgccgccctccc
gccgccctcctcccccgcctccgacaaggcgcccgagcccgtcacaatccccgcctccgagcactcctcccaccactcctcccgatcctccgaccagcc
gcagacgccctccgcggagccgcagacgcaggacaataatcctccggcgaagaagcccgtgcccaaggtgaagcgcgtccagagcgcgggcctcct
cgcggactccgtcctcaagcgtgacgtcaacacagcccggctcaaggatctctacaccatcgacaagaagctagggcaggggcagttcggcaccacct
acctctgcgtcgagaaggccaccggccgggagttcgcctgcaagtctatcgccaagcggaagctgctcacggaggaggacgtcgaggacgtgcgccg
cgagatccagatcatgcaccacctcgccggccacgcaaacgtcgtctccatcgtcggcgcctacgaggacgcggtcgccgtgcagctcgtcatggagc

tctgcgccggcggggagctattcgacaggatcattcaaaggggacattactccgagaaggccgctgcgcagctggcgcgggtgatcgtcggtgtcatc
gaagcctgccactcgctcggtgtgatgcacagggatctcaagccggagaacttcctgttcgtcaaccagaaggaggactcgccgctcaaggccattgat
ttcggtctctccatctttttcaagccagtggaatcatttactgctcatatgggtgttgcagaacaggttattgctgagagtttatccgaggaagaaattgca
gggttgaaagaaatgtttaaaatgcttgacagtgacaatagtggccatataacattggaggaattgaaaactggcctgcagagagttggagctactttg
atggactcagaaattgatgctttgatgcaagcagcagatatcgacaatagtggcacaattgattatggggagttcattgctgcaactttgcatataaaca
aagttcagaaggaggataagctctttgcagctttctcatattttgacaaagacggcagcggttacataactcaagatgagctccaaaaggcatgtgagg
agtttggtataggagatacccggcttgaggatattattggggacatcgatcaggataatgatggacggatcgactacaatgagtttgtcgcaatgatgca
gaaaggagataatccactagggagaaagggtcaaagtgcggattttggtcttggtgaagcactgaagcttcgataa
>g78522.t1
atggggaacacgtgcgtgggccccagcatcaccaagaacggcttcttccagtccgtctccacgctgatgtggaagacgccgcaggacggcgacgcgct
gcccgccgccgccaatggccccgaaggcggcagcccgggccggtcgcagcccgccaagcccgaagtgcagagcaaggccccggagcccatgaaga
tcgccgcagcccaacaaccggatgagttgccgccgcccaaaaccaagcagcagcaagacgcgaagcccaacgccggcggcgaggcgcccagcacc
aagccgcggcccaaggttcctccggtgaagcgcgtgtcaagcgccgggcttctggtgggctcggtgctgaagcgcaagacggagaacctcaaggaga
agtacagcctgggccgtcggctagggcagggccagttcgggacgacgtacctgtgcgtggagcgcggcacggggaaggagctggcgtgcaagtccat
cctgaagcggaagctggtgaccgacgacgacgtggaggacgtgcgccgggagatccagataatgtaccacctggcgggccacccgaacgtgatctcc
atccgcggcgcgtacgaggacgccgtcgccgtgcacctcgtcatggagctctgcgccggcggggagctgttcgaccggatcgtgcagaggggacact
acacggagaggaaggcggccgagctcgcaagggtcatcgtcggggtcgtcgaggtgtgccactccatgggcgtcatgcatcgggatctcaagcctga
gaacttcctcttcgttgacaacaaggaggaggccgcgctcaagaccattgactttggtctctccattttctttcgccctggtcaagtattcacggatgttgtc
ggtagcccatactacgtcgctccagaggtcctgaaaaagaaatatggtcccgaggcagatgtctggagtgctggtgtgatcatctacatcttgctgtgcg
gtgtgccaccattttgggcagagaacgagcagggtatatttgaagaggttttacatggaaaacttgatttccagtcagagccgtggcctagcatctccga
aggtgccaaagatcttgtgaggagaatgcttgttagggaccccaaaaagagattgacagctcatgaagttctacggcatccatgggttcaggttggtgg
tttggcgcctgataagccgctggactcggctgttctgtctcgcatgaaacaattctcggctatgaataagctgaaaaagatggctcttagggtcattgcag
agaacttatccgaggacgagatcgccggcctgaaagaaatgttcaagatgatagacacggacaacagtgggcacatcacctacgaagaactcaaggt
cgggttgaaaaaagttggtgccaacctccaggaatccgagatccaggcactcatggaagctgcggacgtggataacagcggcacgatagactacggc
gagttcatcgcggccactctgcacctgaacaaggtggagagggaggaccacctgttcgcggcgttccagtacttcgacaaggacggcagcgggtacat
cacgcccgacgagctgcagctggcgtgcgaggagttcggcctaggaggggacgtgcagctcgaggagatgatccgcgaagtggaccaagacaacga
cgggcgcatcgattacaacgagttcgtggcgatgatgcagaagccgacactggggctcccgaagaagtccggcggtctgcagaacagcttcagcgtc
ggattcagggaggcgctgaggatcgcgtaa
>g78546.t1
atgggggcggtgatcaccggggagtggcagcgcgggccggtcatcggccgcggcgcgtcggcgacggtgtcgatcgccgccgaccgtcgcacgggc
gcggtgttcgcggtcaagtcggtggaggcggcgcgcgcgggcgtcctgcggcgcgagcgcggcgtgctcgacgccgtgggcccgtcgccgcacgtgg
tgtcctgcctgggctccggcgcctcggcggacggcgtttgcttcgatctgttcctggagtacgcgcccggcgggtcgctcgccgacgaggccaggcggc
ggcgctgcgactgcgaggaggacgacgaggcgtgggtccgggcccggtccgcggacgtgctacgcgggctggcccacgtccacgccaccgggtggg
cccactgcgacgtgaagggccgcaacgtgctcctcggcgccgacggccgcgccatgctcgccgacttcgggtgcgcgcgctggaccggcgacgaagg
cagcagtaatgctggcagcggattggggatcaggggcacgccggcgttcatggcgcccgaggcggcgcgcggggaggcgcagggcttggcggcgga
cgtgtgggcgctcgggtgcacggtgatcgagatggccgcgggcggcgcggcgccgtggacggggcggttcgcggaccccgtggcggcgctgcacca
cgtggcgcactccggcgaggtccccgagccgcccgcgtggctctccgacgaggggaaggactttttggccaggtgcctcgtcagggaccccgccaagc
ggtggaccgcggcgcagctgctccaccaccccttcgtcgccgccgccgccggctctgattcctctgctgcggccgccaagggcgctccgatcgagcagc
gggtgtcgccgaagagcatcctcgatcaagcattctgggaggaatgtgagtcggaattgggatcggactcgtcggcaaccgacgaatccgagacgac
ggcggcggcggcgctcaccccccgccgaccgcgtcggcgcattggctggtggcggcgcgcctgactggacatggatcagcggcgaggaggactggatc
accgtcttcgccgccgacattgccgctcctgacgaactcgacgctccaacgcccccccaaattcgacgcggacaccgcagaatcattcgacgcggatgcc
ggaggaatcagcatggcgggagcgtccgaggagccgccgcacattgttgttggcggcgagcacatgggcgcggacgcctcctcgtcacgtggcggcc
gcggtcacggaggcggcgcgagctccagcgccggatcacgccgcggtgatcgcggcggtagtaacaatgagtgtggttgtaccagcggtaactccgc
aagcgcgagtgataataataaagtagtagtacaggcgacggacggaggccaccacgagcacgacaggagggaggagaacaggcgttggtcgcaag
cacactccaatccgtcgcaagctatatgcctcgcgtgccgtccaagcctcctcctcctcctcttccttcccaactcgtga
>g78548.t1
atggcggcggcggcctcccgcaagcgtccggcgcccgaggagcaccatcccccgacgacggccgccaagaagcgggctcgctacaacttcaacagg
atcgacgactacgagatgcttgaggagctcggggagggcaccttcggcgtggtggctcgggcgcggcaccgccgcacgggcgagacggttgccgtga
agtggatccgatgcggcgtctccggcggtcccgacctcgacgccgtcgtacgcgagagcggctgcctcgccgcgtgccgcggccacccgtcggtggtc
cagatcagggacgtggcctccgacgagtccacgggcgacctctacctggtgacggagttcgtgggcccctgcctccgcgactggctcacgaggccgct
ctccgaggatgtcgcccgcgacttcatggcccagctgctcagcgccgcggcgaggatgcacgccgccccgttgatccaccgcgacatcaagcccgaga
acatcctcgtcagcgccggatgcgacctcaagatctgcgatttcgggtgcgccaagccggcgaagccgcccgggaagccgtacccggagccgctcgta
ggcacgctgcagtactgcgctccggagatgctgatgggcgatcggtgctacggcacggccgtcgacatgtgggcgctggggtgcgtcatggccgagct
cctcgtcggcgctcccctgttttcggcggatacggaggaggacatgctcgaggagatcacggagctgcgagatcggatcgccgctgtgggattggaag
ctatcgacgacgacctgctggagctgtcaccggccgggcgcgaggtccttgctggtttgctgagattcgagcccagggagaggctcacggcgaaagag
gcgctggagcaccggtggttcaaggaggaggcagaatcccctgccgcggcgaaatcagagtttcctggctttgtgccactgttctctgcagcatag
>g78573.t1
atgatgggagctctgccggtgcagagcaatggtgacgacgggcccttgttccccgaccccgaccctgggtatgtcgaggtcgatcccacaggcagatat
ggcaggtacaatgaggttctcggcaagggctcctccaagaccgtgtaccgtgcgttcgacgagtatcgaggcatggaggttgcctggaataaggtccat

ctccacgacttcctgcgcagcccgggttacctggaacgtctgtacggcgagatccacctcctcaaatccctccgccaccgtcacgtgatgcgcctccacg
cctcgtgggtggacgcgccccgccgcgccgtcaacttcgtgacggagctcttcacctccggcacgctgcgtcagtaccgccgtcgccacccgcgcgcgc
gcgccgccgccgtccggcgatggtgccgtcaggtcctcgacgggctcgcctacctccacgcgcgcggcgtcatccaccgcgacctcaagtgcgacaac
ctcttcgtcaacggcagccagggccaggtcaagatcggcgacctcgggctcgccgccgttctccgccgccgtgaaggaggaggaggaggagggcag
aggtgcgtggtgggcacgccggagttcatggcgcccgaggtgtacgcggaggcgtacgacgagcgcgccgacgtgtactcgttcgggatgtgcgtgct
ggagatggtcacgctcgagtacccatacagcgagtgcgatcatcccgtgcagatctacaagaaagtcacatccggagtgaagccggcggctctgcaa
aaggtgagggaccccatggtgaggcgcctcatcgacaagtgcctggcgccggcggcgcgaaggccgtcggcgatcgagctgctcaacgacgacccgt
tccttcaaatggaagacgacgggttcatcagctacggcgacgacgatagggactacggctccatgtacaattacctccaccagcctgcgtgtctgctgg
actaccaccccgccggtgatgaggaggatgatcagccgccggaggacaggtactgggactgcgtggaggaggatggtgatgatgatgatagtaggtt
ccaagggatcgacttgctgttcaacgagcacgaggacgacgaccacgttgccggagtggatatcgcgataaagggggaagaggatggacgacggcac
catcttcctccggctccgcatcaccgacaagaccgacccagggctggtgcggaacatctacttcccgttcgacacggagtcggacacggcgctgagcgt
ggcgacggagatggtcggcgagctggatatcacggaccacgaggtcacgcacatcgccgacatgatcgacggcgaggtcgccgcgctgctgccgcac
tggacgccgggtatgatcaacgacgacgatgctccggacgacgacacgtgctgccggaactgccgccagtccaccagcgcgtcctctggcggctcgct
cgccgactacgtgtcgcgccgccgcgggtgccggtgcgccgagctgcacggccggttcgaggagatcacgttccaggccgacgaggagcaggtgcag
ttccagagctcggggggcagctccgacgactga
>g78960.t1
atgagctgcttcgtgtgcttcggatcggcccaggatggggaccccaagaagcccggcgcggacgccaaggacgcgcgcaagggcgccccgccggat
cgcgtggtgaccagggttggatcagatattccacctgaaaaggaacctcttgactggaacacacggatgaagatagctgctggtgcagctaagggctt
agaataccttcatgacaaggcaagccctcctgttatttacagggatttcaagtcatcaaacattcttcttggtgaaggatttcacccgaagttatctgatttt
ggccttgcaaaacttggcccggttggtgacaagacccatgtttcgacacgtgtgatgggtacttatggctactgtgccccagaatatgcaatgactgggc
agctcacagttaaatctgatgtatacagctttggtgttgtgttccttgaactgattacggggcgtaaagcaattgacaacaccaagccccagggggagca
aaatcttgtggcatgggctcggccactattcaaggaccgtaggaagtttccacaaatggctgatccaatgcttcaaggtcgcttccctatgaggggtctat
accaggccctagctgtggctgccatgtgtttgcaagagcaagctgccacacgccctcatattggtgatgttgtcactgccctctcatatcttgcttctcaga
catatgacccaaatgctcctgttcaacgtagtcggagcaattcatctactcctagggcgaggaacccttctgggtggaacgatgatcagcgcagtgtgcg
ctcaccaaaccatccttctccagacttgcggcggagggaagctgcaaggacttctaagtatggagcagaggttagaaggaccagctctgctggtgagtc
tggtaggagatcaggattggatgacatggacatgacagaatatgcaatgactgggcagctcacagttaaatctgatgtatacagctttggtgttgtgttc
cttgaactgattacggggcgtaaagcaattgacaacaccaagccccagggggagcaaaatcttgtggcatgggctcggccactattcaaggaccgtag
gaagtttccacaaatggctgatccaatgcttcaaggtcgtttccctatgaggggtctataccaggccctagctgtggccgccatgtgtttgcaagaacaa
gctgccacgcgccctcatattggagatgttgtcactgccctctcatatcttgcttctcagacatatgacccaaatgctcctgttcaacgtagtcggagcaat
tcatctactccgagggcgaggaaccctgctgggtggaacgatgatcagcgcaatgtgcgctcaccaaaccatccttctccagacttgcggcggaggga
ggctgcaaggacttttaagtatggagcagaggttagaaggaccagctctgctggtgagtctggtcggagatcaggattggatgacatggacgtgacag
caatccagagcgccggggcggcggcaggttatcgacatgagctggagcctgggctgattacagttggcgtcgatgtcactgttcacgatggtggatacg
ccaggatgtccactgtgatcagtcctaaggcgccatccatgaaacacctttccaccgtcagagcctgggttgatttttccaaagacgtggttgagtga
>g78984.t1
atggtcgcgcaatcaaagcgccgtgaaacaattatggccagatccaccagtttcctgattgctctgctcatgaccctcgtgctgtgctgttcagcatcggc
gtcggttccgtacacgctgggcaccggcgcgtccctggccgtggaagaccatgcgggtgccttcttgatgtcccccgacgccaccttctcctgcggattc
cggcaagccggcgagaacgccttcacattctccatctggtacacccacgccgcggagaagacggccgtctggacggccagccccggcaacctcgtca
acggccgggactcgaagatcacgttccgccgcgacggcaacctggccctggtcgacgcgaacggcaccacggtgtgggataccaagacgaaggga
aggacctgaccctctccctcctcgactccggtaacctcgtcatcaccgacaacccgtcatctcccggccgcgccgcgtggcagagctttgactcgccgac
ggacacgctggtcccgacgcagccgctgaccaaggacacgaggctcgtcgcgggggggattcatcctctactacgaccacaccaacaacctgctgcgg
ctgctctacgacgggccggtgatgtcaagcaactactggcccaacccggacaacaacgcgttcaagaacggtcggatcacgtacaacagctccaggct
cggcgttttggatgactccggcgtgttcatgtccagcgacaacctcacggtccaggcttccgacctggccaacccggggtcaagcggcggctgaccgt
cggccccgacggcaacgtgcggatccacagcctgaacaattcgagcggtaggtggacggtgacgtgggcggccacggcgcagccgtgctccgcgca
cgggctgtgcggcgagaacgccctctgcgagtacgtgccgaggctgcggtgctcgtgcccggccgggttcgagatggttgaccgccgggactgggag
aaaggatgcaggcccgtgttcaattccctcaactgcagcaaaggaggagcgcagcagttcaagttcgtgaggatgccgcacaccgacttctacgggtt
cgacctggggtacatccagtccgtcacgttcgagcagtgcgccaagacctgctccgacaactgcctgtgcgcggcctttctcgtacagggtcgacgggcg
cggcctctgcttcccgaagttcaggctgttcaacggcttcaggtcctcgtccttcttcggcaacatatacctcaaggtcccaatcgacttcaacatctccgc
gccggcggtcgtccagcggagcagcgtcgggctcgaggcctgcgattccaaagcgccgcggatcgtcatggctgggtcgccggacacgctgttcgcga
cgcccaggaacgacatgaagtggtcctacttcttcgtgttcgtgggaatacttggcctgctggtccttctcttcatcgtgacgggctggtggttcctctcca
gcaagcacagcatacccagctcgctggaggccgggtaccggatgatggtcgcggcggggcagttccggcggttcacgtatcgggagctcaaggacgc
cacggggaacttcaaggaggagctggggcgaggcggctccggcgtcgtgtaccgcggcgtgctcgatgaggggaagaaggtggtggcggtgaagaa
gctaacacaaaacgtggcgcagggcgaggaggagttttgggcggagatgacggtaatcgggcggatcaaccacatcaacctggtccggatttggggg
ttctgctccgagggaaagcgcagcaagctactggtgtacgagttcgtggagaacgagtcgttagacaagcacctgttcgtcggcacggagcacaggcc
gccgctgccgtggcgcgagcggtacaggatcgcgttgggcacggcccggggcctggcctaccttcaccacgaatgcctcgagtgggtcatccactgtg
acgtcaagccggagaacatcctggtcacacgggagttcgacgccaagatcgcagatttcgggctggccaagctgtccggcaggagggacggaggcg
ccggcggcgtgcagctctcgcacatgagggggacgtcggggtacatggccccggaatgggcgctgaacctgccaatcaacgccaaggtcgacgtgta
cagctacggcgtcgtgctgctggagacggtgatcgggaggagggtctcggaccagaagacgccggaggggcagccgctggagatgccgcggatcgt
tcaggcgctgaggcgggtggtggacagcggggacgtctcggccttggtggatggcaggttgcaggggcagctcaatcctcgacaggcattggaaatg

gtgaggatttccttggcgtgcttggaggagaggagcagcagtaggccgacgatggatgacgtcgccaaggctcttaccgcgtacgatgatgaggacga
gcaccctgcgtacaagtcgtag
>g78985.t1
atgttggtgcgtgcgctggagatgggtgtcacctcatatcatggccatgcacgcatcctcatccagctgcggcgaaggcgggagcgcacccatgagag
cgttggcaacgcatcatcgagcgttggtgcatccttgtcagtggaggagcatgataaaaccttcctcgtttcaccaaaccatatattttcttgtggtttctac
aaaggtggcaaaaacgccttctacttctccatctggttcaccaacacctttgataaaactgtcatctggacagcaaactctggcgttccagtcaatggcca
tggttccaagatatatttcaacgaagatggcaacttagtactctatgatgtaggtggctctactgtctgggagagtaagatgacccttggcaaggcagca
acagttcatctccttgaatctggtaaccttgttataaaaaactccagcaatgctgtcgtgtgggaaagctttgattcacccacggacaccttgcttcctttac
agccccttaccaaaaacaagaagctgatatcaagctactacagactgtattatgataacgacaatgtcctgcggcttatgtatgatggaccacaggtctc
aagcatctattggcctagtccagatagcaagatgtttgacaatggacggacaagctacaacagctccaggatcgcaatacttgacaaaaagggccgttt
cacttcgagtgatgggctcaacattgtgacatcagattttggccccaccagtgtatggagaaggctaacaattacccaagatggcaacctcaaaatttac
agcttgaatgtatcaaacgggagctggatcattacatggcaagccctcatacttgtctgtaaggtacatgggttatgtggaaacaatggaatttgtgagc
agtcaccaaatcctcaatgctattgtcctccgggttatgagatggttaatccacagaactggaccaaaggctgccaaccaatgttcaacaacatcaacca
agccaaggagcaagttaagtttgtcagattacatcatactgacttttatggctttgatatgggctataatgcatcaaatatttcattctatgattgcaagaa
atattgttcaagtgcaagttcatgccttggcttttcatataagttgaatggaaaaggtgaatgctacaccaaaagtctgctcttcaatggccataaaggtc
ccttctttcgtggaagcttctacatcaagaaaagaaacaacagcaacttctccacaatctcagcagcagcagcccaacagagtcatcttatctgcaaga
acatttcagaggttgtagttggacctgcatatggcgtgcagaaaagcagcatcaagtggtcgtactattatgcatttgtagtagtactgggtagtttagag
attttctttatgttaacagcatgcttatttcttcgtagcaagcaaaatattccaaaaacagcactggaaggatataggcagatcacaaaccagtttaggag
gttcacataccgagaattgaaggaagcaacaggaaatttcaaggaagagatcggacggggtggttcaggattcgtttatagaggggtatccagtagca
gccaagttgtcgctgtgaagaagctgacaaatgcaacacaaggcgatgaagaatttcagggggaaataactctgattgcaaagatcaaccatatcaat
ttgctcaggatttggggattttgctcagaaggcaaacataaactactagtgtacgagtatgtggagaacggatcactggacaaacacctcttcagcacta
ctaactgcaatcagttacttggatggagtcagaggttcaagattgcactgggaacagcaagagggttggcatacctccaccacgagtgccttgagtgga
ttgtccattgtgatgtgaagcctgaaaacatccttttgaccagagaatttgaagtcaagatagcagattttggtctggcaaagttgtccaacagagactgt
tcaggattccatttctctcgtatgagaggaacaacaggttacatagctcctgagtgggcatctagcttgccgatcacagcaaaggtcgatgtatacagct
acggtatcatgcttcttgagatcgtgatgggaacaagggtttcaaatcagacaacacaagatggacaaccgctggagttgagggacattgtgcaaata
ctcgagcaggttgtagctagtggagaaccatcacatatagtggaccctaagctacgaggtcagttcgatcatcatcaagcaatggaaatggtgggagta
tccttatcatgcatgggggaaaggagccagaggcctacaatggattcaattgtcacagtgctcatgtcatgtaatgaggacgatgaccagactatctatg
cctga
>g79793.t1
atggggtacatagacccggagctcctcttgaccggccgcgccggcccggagtcggacgtgtacagcttcggcgtcgtgctcctagaagtcgcctgcggc
cggcctcccgtcgtgttcgaccaggagctggaggagcagggcaagacgcgcctcgtggagtgggtatggagtctttacggacgaggcgccgtgctgg
aggccgccgacgagcggatgggcggcgatttcgacgccggcgagatggagcgcgtgatggtcgtcggactcgcgtgcgcccacccggactgcagcct
ccggccgtccatcaggcaggcgatgagcatgctccagtgtgaagcgacgttgccgatactgcctgccaagatgcccacgcccaagtatttgtga
>g80100.t1
atggcctggtgcgccctgcgcagccgcgtcccccgctcccgctctcgctcgcggcgaccgccgccgccgcctcggagccccacagcctgctcctgcgc
tccctcctctccaccgcggcctcgcgccacgggcatcaccgccgccgccggctcggccccaccgtctacgccgctgcggccgccgccgccgccgccga
gacgccgctgcccatgacgccgcgcatcagccgggccaggcggcaccctgggggcgccgcgtccctggcgcgcgtctacgccgacgccaacgcccag
cggcccaaggagtactgggactacgagtcgctcgacatccagtggggggagcaggatgggtacgaggtgctgcggaaggtcggaggggcaagtac
agcgaggtgtttgagggcttccggcccgggagtgacgataaatgcgtcattaagatcctcaagcctgtgaagaagaagaaggcactagattattgtcat
tcacgaggtatcatgcaccgagatgtcaaacctcacaatgtcatgattgatcatgaaaagcgccaacttcgtcttattgattggggtctcgccgagtttta
tcatccaaagatggagtacaatgccagagttgcttcaagatgttacaagggccctgagcttcttgtggatttgttagattatgattactcgttggatttgtg
gagccttggttgcatgtttgcagcaatgatattcagagtggaccctttctttagcggccaggataattatgaccaattagtgaaaataaccgaggttctgg
gcaccaaagatttctataactaccttgaaaaatacggccttgaacttgacccgcagctggaaagacttgttggaagacataacaggaaaccatggtcca
agtttgtaagctctaggaacagacacctagcaagccctgaggctatcgatctggtggataggctcctccgctacgatcaccaagaaaggcctacagcta
aggaagccatggctcatccatatttcaatccagtaagaagttcggaaagcagcaggacgaactctcagtag
>g80163.t1
atgaagtcggtgtaccggggcttcgacgaggcccggggcgtggaggtggcctggaaccaggcatccttaaccgacgtcctccgcacgccggacgccg
tccagcgcatgtacgcggaggtgcagctcctccgcagcatccgtcacgactccatcgtctccttcctggcgtcatgggtacacgtccccacccgcaccttc
aacttcatcacggagctcttctcctcgggcacgctccggtcctaccgcctcaagtacccgcgtgccagcctcctggccgtcaagtcgtgggcccggcaag
tcctccgcggcctggcccacctccacgcccgcggcgtcgtccaccgcgacctcaagtgcgacaacctcctcgtcaacggccacctcggccaggtcaag
atcgccgacctcggcctcgccgccgtgcttaacctcaccggcgacggcggacggcggcggagcgtggtggggacgccggagttcatggcgcccgaga
tgtacgacgaggagtacgatgagcgcgtcgacgtgtacgcgttcgggatgtgcgtgctggagatggtcaccctgcagttcccttacgccgagtgctcca
acccggcgcagatttacaagaaggtcaccagtggcaggctccccgacgcgtttttagggtggaagacgacgaagcaaggcggttcattgggaggtgc
ttggttgctgcctcccaacggccgtccgcggcggagctgctgatggacccgttcctccttgatgatgtggatgagaatgatgatgccacgtcgtcgcctc
gtcgtccccttccgccagctcctgatcttgtgcatcctacgttcacaaccagcagcaataatgccgtcatcactgacgacgacgacgacacatcgtgctc
atcctcgttattctcggatgatcatcatcaggaggaggaagaagaacgaccaccaccaccggttcaacaagaaaggagtgacgacataatgatgacc
atcaccggcaagctgaacgccgaggaggacaccatcttcctcaaagtacagatcacagacgacacatgcgggtacgttcggaacatctacttcccgttc
gacatggccagcgacacggcgacggaggtggccacggagatggtcaaggagctggacatcaccgaccgggaccccgcggagatcgccgccatgatc
gagcaggagatccacaggctcatgcccgccggcagagagcagcacgagtactcgtactacgcgggtcatgaacatgatgacgacgaaaaccaggag

cagcctcctcctcccccgttctattacctctcctcttctcccacctcgtcccagggagggtccctctacggtggtgtggggtcctcgtcctctggcggcttctc
tggcccgcatggcagtccctggtccaaaggtcaccaagaagatgaggcgctgcaacaagcaccacctggtagtggtaaacaagtagaaagcaggccc
agcagtagctcctcaaaggctgcccaggctaccaagtccaccaggttcggcccagatggaatcagcagaagcagaagcccacgtagagcagatacg
gcccaaatgcagcagctgtcatcgctagcgcaccagctgcagcggcagtgcggcatgtcaccgcagcagcagcagccgcacgccgggcggccgcgg
cagcgcaccggcggcgaggacgacgacgaagaccgccggaggaggaggatgagcaggaaccgttcgatggtcgacatgcggagccggctgctgca
ccggacgctggtcgaggagctcaaccgccgcctcttcttcaacaccgtcggcgccgtcgagaacatcggcttccgcgcgccggccggatacggcggct
cctcgtcgtcgtcctcgaccgcgcgtgggcgcggccgcgccgaccaacgtggcaggaggagcaacaaagaagatgataagaatcagcggtacttcat
gctttag
>g80513.t1
atggtggccatggaagaggctccatcacaagctgcccttgctctgtacttattcacacttgtcgccgctgccaccaccatcttgaatctcgtcgcccccgg
ctgtgctgacgtgcctttccatccttaccggccccacacctttggcaccttcttcctcctcctcctcctcctcctcctcctccggattcgacgactgatatgcct
gctgcaaccatcaacccagacccagcgaccgacggcgcgtctgttgcatggcttggcttgcggacaaacactggccaccaacgcaacgagcgcggcc
ggtgcatgcgacgcagcagcaaccaaagcatttgccccggcccagctctggatgctagtctatgagtacgtgaacaacggaaacctggagcaatggct
tcatggagctatgagtcattgtggctcgcttacatgggaagcccgcataaagattcttcttgggactgcaaaagcgctggcttacttgcatgaggcaattg
aacccaaagttgtgcaccgtgatatcaaatcaagcaatattttgattgacgatgagtttgagtccaaagtatcagattttggcttagccaagcttcttggtg
ctggaaagagtcatgttaccactcgggttatgggaacatttgggtacgtggccccagagtatgcaaacactggactcttgaatgaaaagagtgacattt
acagcttcggggttgttctcctagaggcaattacaggaagggatcctgttgactatggtcgcccagcaaatgaggtgaatctggttgactggctaaaact
gatggttgccagcaggcgctcagaggaagtagtggatcccaccatagagacacgaccttcaacgagagctctgaaacgtgcacttttaacagctctga
ggtgtgtagatccagattcagaaaagagaccaaagatgggtcaagttgtccggatgctggagtcagatgatccaatgcctcgtggggacagacgatct
aggcacaaccgtggagggagtacagaaatagattcacaaagagacaactctgatacagagaagagtgataatccagattctaaacctagcaggagc
gaagcatcatcgtctaaatga
>g80538.t1
atggcgcgccacccagagatcgttgatcacaccggcgacgagccacacgacaacgaccgtcgctccggctcgtccgccgggaaccacgcttggcttcc
gcagatggacctttgcagcggccatttcggcatcgccaacaccaccgatccgtccgttttcctgttgctgctggtctctctcccaggccggccaggagatc
agttggtgcaccatcctggaagcaaaagggatgcgaatcgcacggcaccctggacttcggttccactggaacaggggacgcgtaagatgttggaaaat
ggaagacacatccgtcatgctggactgtgcccttctggacctaggcggtaccgccactgtgagcttgcggcagcgacccacggattctccgagcaggg
gaagattggccgaggaggcttcggctcggtgtaccagggctacctgcccgacaaagactgtcatgtggctatcaaggtactgtctcaagaatcctccgt
gcagggagtgaaggagtttgaatctgaggtgaaggtcatgtcgaggttgaggcaccggaacatcgtccagctgctcggctggtgtgatggtcccaaag
accttttgcttgtttacgagtttatgtccaacggaagcctcgacaagcacctgtatgataatccttggaggatactatcctggtccgagaggtacaacatt
gcccttggtgtgggttctgccatgctgtacctccacacggagtgcgagcagtgtgtcgtccatggggacatcaagcctgcaaacatcatgctcaactcgt
cctttcaggccaaggttggagactttggcttagcgaggctactcgaacatggagccgactcacaaaccacacaggtcgtggcagggacaatcgggtac
atcgatccagagctcgtgaacaaccggaggcggagcgccaagtccgatgtctatagctttggcattttattgctcgagattgcctgcggcaggcgactgt
catcaaaggcagtgctatcgaacggcgcctcggtgcatctcctgaattgggtccgctacatgtaccagcagaactcgattcttgaagtggcggaccggc
ggttggacggcgagttccacgagcaccagatgaaccgcgtgctcgttacaggcctttggtgcgcgcatcgcgatcagagtcagcgtccatcgattgccc
aagccatggatgtcctgcgccgtgaagaggccgagctgcctatctttggtccggaagtggacaatcgttatgaagttccttcactggaagagcgagcttt
tgttgaattgtcaggagagggctcccgttgtgaggattcacctgctgaaacagagtaccacacttttgagggatccatttga
>g80632.t1
atgagatatacattttgcaactatgtaggattaagaggacaaccattggacttttcagagcgtctcaatattgctattgacatagttcatgccatcgcctac
ttacatggatacacagaccaccccatcatccaccgcgacatcaagtcctccaacattctcctaacagatcaccttcgagcaaaggtcgccgattttgggt
tcgcacgcctagccccggagaaccctgaagtgacgcacgtctcaacggtggtgaaggggacagcgggatacgtggacccagagtacttgcgcacgaa
ccagctcaccgaccgcagcgacgtctactccttcggtgtcctcctcgtcgagctcgtcaccggacgacgcccaatcgagcgtggccgcggccgccacca
acgcctcaccacccaatgggcgctgcggaagtggagggagggcgacacggtggtggctatggacacccggatgcggcggggcagcgcggtggtggc
ggcggtggaaaaggtgatggcgctggcggccgagtgcacggccccggatcgcgcggcgcggccggccatgcgacggtgcgccgaggtgctctgggc
cgtgaggcgcgacttccagcaggagcagcagcacgaggcggcggcggccgcggcggccgcgagggcaggagcgaggcggctcgatggctccacgt
acgcgctgccatccgtgacgaggttgaggtag
>g81740.t1
atggagaaaggagggggtggactgaagatgttcaacatagtttcagcgtggaacaagaggagaagaagcagatcactcgatcagctaaatccatgg
gtgtacaagccagctgagctgtggcaggtgaaggagcagagcccgcccccgaaaaagcgcaactacacgatggtgttcacgctcaaggagatggag
gaggcgaccaacatgttcagcgaccgtaacctcatcggcaagggcggctttggccgggtttacaggggtgtgctgaaggatggccagatcgtggccat
caagaagatggacctgcctacatccaagcacgccgacggcgagcgcgagttccgggtggagatcgacatcctgagcagactagaccaccccaacctg
gtcacgctgatcggctactgcgccgacgggaagcaccggttcgtggtgtacgagttcatgcccaggggcaaccttcaagacatcctcaatggcattggg
gaggtgaagatattggactggccagtgcgcgtgcggatcgcgctcggcgccgcaagggggctggcttacctgcactccagcacggccgtcggcgtccc
tgtcgtccacagagacttcaagtccagcaacatcctcttgacagaacactttgaggcaaagatctcagactttgggctcgccaagctgatgccgcagga
cctggacctgcacacgaccaccagggtgctgggcacgttcggctacttcgaccctgaatacgcactgacagggaagctgacgctgcagagcgacgtgt
acgcgttcggcgtggtgctccttgagctgctcaccgggcggcgcgccatcgacctgagccaggggccccaggagcagaacctcatcgtcaggatccac
caggtggtgggcgaccggaagaagctgcgcaaggtggtggaccgggacatggcgaaaggcacctacactttggagtcggtgtccatgttcgcccgcc
tcgccgcgcgctgcgtctgcttcgagagcgccggccggccgtccatgcaggactgcgtcaaggagctccagttcatcatgtacgccaacatgaagatat
ga
>g82246.t1

atggcagccgcaacctccacctccgtcctcctcctcttcctcctctcgccactgatcgccgcctcaatctcgccgccgactgaagaagcgcagacaccgc
aagaccttctcctcgacttcaggtccaccctgcacgacccgtccgccgcgctctcctcctggtcgcgcgccaccccttactgcaactgggcccacgtgac
ctgcaccagcgccacggccgccgccaacgccaccgtctcggtctccctctccctcgacggcctcggcctctccggcgagttctccgccgcctcgctctgc
cgcgtgcccggcctcgcctccctcaacctcgcatccaacgccttcaaccagaccgtcccgctggagctcgcgcgctgcgcctcgctcgcgtcgctcaacc
tcagcgccgccgggttctggggcccgctcccggaccagctcgccgcgctcgcctccctcgtctcgctcgacctcagccgcaacagcatcgagggccagg
tgccgcccgggctcgccgcgctgggggcctcgagacgctcgacctcggccgcaaccagctctccggcgtgctccacccggctctgttccgcaacctca
ccagcctccacttgctcgacctgtccaggaaccagttcctggagtccgagctgccgtctgagatcggcaagatgagcggcctccggtggctgctcctgc
aggggtcagggttcatcggaacgatccccgaatccttccttgatctggagcagctcgaggtcctcgacctgtccatgaacaacctcactggagtcgtgcc
gcccgggttgttcggccgcaagcttcagaagctgatgaccctggatttgtcccagaacgggttgtccgggccgttcccggatgacatcgccaagtgcttg
atgctgcagaggttcgaggtgcacgacaacgcgttcaccggaacgctccccgccgggctctggctgctgccagacctgcgcgtgatccgcgcccagaa
caaccggttcgccggccggttaccggagttacccggcgggcagtctcggctcgagcaggttcagctcgacaacaacagcttctccggcgggatacctc
ggagcatcggccaggttcgcaccttgtaccggttctccgcctccctgaacgagctcagcggcggcttgccggacaacctctgcgactccccggtgatga
gcatcgtaaacatctcgaggaactcgctctccggcacgatcccggagttcaggaactgcaagaggctggtgtctctgtacctgtccggcaacggcttca
ccgggccgataccggcgtcgctcggggacttgccggtgctgacgtacatcgacctgtccagcaacaacctcaccggtggcattcccgtggagctacag
aacctgaagcttgcgctgctgaacgtgtcgttcaaccagctctctggccgtgtgccggcgtctctgatctccggccttcctgccgtgtttctccaagggaac
ccggggctctgcggtcctggattgccaaacgattgcgatgcgcctttgaggaagcatcagggtctagtactggctgcaaccgtggtgctgttggtcaccg
gagtgatgctgctcgctgtaggagcatttgcagcgtggagaaggttgcacggcagcaggtcctccccatgtaagctggtgctttttcaacctatcacgatt
actggagaggaattgtttgctggatttcacgagaagaatgtcatcggcaggggagcatttggaaaggtttatctgattgagctgaaagacgggcaaaat
gtcgcggtgaagaggctggtcaattcaggtaagcaaactttcagagcagtgaagaatgagatgaaactgcttgcgaaaatccggcacaagaatatcgc
aaagatcctaggattctgctacatggaggacgagatcagcgtcatctatgactacttgcagaggggaagcttgcaggacatgatctacgcacccaaatt
cacaatgggttgggacaaacgggtaaggatcgcactgggagtagctcaagggttggcacaccttcaccatgatcatactcctcgcgtgctgcatagag
atctgaagtcgagcaatgtactgctcagtgatgaatttgagcccagacttaccggatttggaaaagatcgcgtcgttggagncgagacgctcgacctcg
gccgcaaccagctctccggcgtgctccacccggctctgttccgcaacctcaccagcctccacttgctcgacctgtccaggaaccagttcctggagtccga
gctgccgtctgagatcggcaagatgagcggcctccggtggctgctcctgcaggggtcagggttcatcggaacgatccccgaatccttccttgatctgga
gcagctcgaggtcctcgacctgtccatgaacaacctcactggagtcgtgccgcccgggttgttcggccgcaagcttcagaagctgatgaccctggatttg
tcccagaacgggttgtccgggccgttcccggatgacatcgccaagtgcttgatgctgcagaggttcgaggtgcacgacaacgcgttcaccggaacgctc
cccgccgggctctggctgctgccagacctgcgcgtgatccgcgcccagaacaaccggttcgccggccggttaccggagttacccggcgggcagtctcg
gctcgagcaggttcagctcgacaacaacagcttctccggcgggatacctcggagcatcggccaggttcgcaccttgtaccggttctccgcctccctgaac
gagctcagcggcggcttgccggacaacctctgcgactccccggtgatgagcatcgtaaacatctcgaggaactcgctctccggcacgatcccggagttc
aggaactgcaagaggctggtgtctctgtacctgtccggcaacggcttcaccgggccgataccggcgtcgctcggggacttgccggtgctgacgtacatc
gacctgtccagcaacaacctcaccggtggcattcccgtggagctacagaacctgaagcttgcgctgctgaacgtgtcgttcaaccagctctctggccgtg
tgccggcgtctctgatctccggccttcctgccgtgtttctccaagggaacccggggctctgcggtcctggattgccaaacgattgcgatgcgcctttgagg
aagcatcagggtctagtactggctgcaaccgtggtgctgttggtcaccggagtgatgctgctcgctgtaggagcatttgcagcgtggagaaggttgcac
ggcagcaggtcctccccatgtaagctggtgctttttcaacctatcacgattactggagaggaattgtttgctggatttcacgagaagaatgtcatcggcag
gggagcatttggaaaggtttatctgattgagctgaaagacgggcaaaatgtcgcggtgaagaggctggtcaattcaggtaagcaaactttcagagcag
tgaagaatgagatgaaactgcttgcgaaaatccggcacaagaatatcgcaaagatcctaggattctgctacatggaggacgagatcagcgtcatctat
gactacttgcagaggggaagcttgcaggacatgatctacgcacccaaattcacaatgggttgggacaaacgggtaaggatcgcactgggagtagctca
agggttggcacaccttcaccatgatcatactcctcgcgtgctgcatagagatctgaagtcgagcaatgtactgctcagtgatgaatttgagcccagactt
accggatttggaaaagatcgcgtcgttggagagaaggcgtaccagatttcattggcttcgaatattaattacaggtgctacgttgcgccagagcaaaac
tgcaccaagaagccaacgcacctcatggacgtttacagcttcggagtcatccttctggagctcatcaccggaaagccagcggagcagccagcggccga
cgactctgtcgacatcgtcaggtgggtgcggcggcgggccaacatgacgggtggagcgttgcagattcttgaccccggcatctccagcacggcgcagc
aggggatgcaggcggccctcgagctcgctctgcgttgcacgtccgtgatgcccgaccagaggccggccatggacgaggtcgtcctgtcgctgcagcca
ctgtgtttcagcgttcatccacagacgccgctgtcaaccgaaactgctattgagccctga
>g82484.t1
atgcacaatggcaccctcaaagagcatcttcacggacgcgacaagcacattagttggatcaagcgacttgagattgccgaggatgcagcgaaaggaa
tcgagtaccttcatactggatgcacgccctcgatcatccacagggacatcaagacaagcaacatccttctagacaagcagatgagagcaaaggtgtct
gactttgggttgtcgaaattcgtggctgaggactctcatgcatcaaccaacgtgcgtgggacactaggctacctagacccgcagtga
>g82705.t1
atggccatggggaggatctacccccttcctcctcgccgccgccgcgctgctgctcgcccagcccgcgtcgcccttctccttcacgtacaacttcacctcccc
ctcggacacgccgccgccggggatcgccttccagggggacgccttcttcaacaaggcgatccgcctcacccgcgacgagcgcatgggccccatcacca
gcagcgccggccgcgccttctacgcgcgcgccgtcccgctcgccgacccggtctcccgccgcccggcctccttcgccaccgccttcgccttctccatcgc
ggcgcccgacccggccgccgcctcgggcgacgggctcgccttcttcctctccccgttcccctccgcgctccccgaccgctccgcgggcggcctcctcggc
ctcttcaactcctcctcctccacccgcgcccccatcgtggccgtcgagttcgacacctacaggaacgactgggaccccagcgacgaccacgtcggcgtc
gatctcggcgggatcgtctccgccgccaccgccgactggcccaccagcatgaaggacggccgcagggcgcacgcgcgcgtcgagtaccacgccgac
gaacacaacctcaccgtcgcgctcgcctacggcgacgacctccccgccgtcctgctctggcacgccgtcgatctgagggactacctgccggactccgtc
gccgtcgggttctccgccgccacgggcgaggccgcggagctgcaccaggtgctctactgggagttcacctccaccgtcgatcccaaagaggaatcaat
cgtcctctgggtcgtgctcggactctgcgcgctgcttctcgtgctcgtcgccgcaggggtcgtctggttcgtgaaggaatggagaaaagcaggggaaca
gctggtcgacggcgacatcgacatcgacgacgtgatgggcggcgactacgacgagctcgccgacgaattcgtcgtcgagagcgggccgagacggttc
cggtacggcgaactggcggcggcgaccaggaatttcgccgaggatcgcaagctcgggcagggcgggttcggcgccgtgtaccggggctacctcaagg

agctgggccagcaggtggccatcaagcgggtgtccaggggctccacgcaggggcgaaaggagtacgcggcggaggtgcgcatcatcagccagctgc
gccaccgccacctcgtccgcctcgtcggatggtgccacgaccgccgcggcgacttcctcctcgtgtacgagcacatgcccaacggcagcgtcgaccacc
acctctacggcaagggaaagggcggcgcgcccctcgcctggcccacgcgctacgacgtcgtgctcggcctcgcgtcggcggtgctctacctccacgag
gaatgcgcccagtgcgtcgtccaccgcgacatcaagcccagcaatgtcatgctggacgccgccttcagcgccaagctcggtgactttggtcttgccaag
ctcgttgagcatggcagccagccatacaccactgtcctcgccggcacattgggctatttggcgccggagtgtgtgatgactgggaaggccagccggga
gtcagacgtgtacagcttcggagtcgtggcgctggagatcgcgtgcgggcgccggccggcggagccgaacgaggagccaagcaaagcgagactggt
gccttgggtgtgggagctgtatggcaagaactcccttcttgaagcagcagactggaggctgaagggggagtttgacggggagcagatggagcgtctga
tgattgtgggattgtggtgtgctcatcctgactatgcacataggccgagcatcaggcaggccctgaacgtgctgaaattcgaggcgccattgccagtgct
tccgccgaagatgccggtgccgacgtacttcccccgttaccggagttggttgatccgatttccgtccaaggcagcacgaccaccgatggccctggtgtg
agtgattttggatcgtcagggtctcacaaggccggaaaaggatcatcagtgacggataggctactggagccatga
>g82907.t1
atgtgggcgcgtctctggtgcaccatgccggcgaccgagaggccgccgttcacgcccccacccgcaccgcccgcgccgccagagtacggccctcctcc
tgaaccatcatccgtcgcggcactatacgcgctggccggtgatttccttgaccgcgccaagacggcgctactggccaccaacgggccgacggcgacgg
cttctgatgtcgtcacctccgccaccgtcactccgagcgcacgccgtgtcgtggccgaggtcacggcccctgcctccacggcgaccccagccgcgccgc
agaaaagcgactcgtggaccttgagttctagcgcggtgcactggatcatcgcagccggtgcggtagttgcggtggttctggttctgtgcgtggtaacggt
cttggtccgaaggaggagacggcgccggcggcgtcggccgcccaccatggaggcgcctcagctgccaccagtgatgatgtaccacaaagacggccc
gacacgtccagcagcacttcagcaagcgccatccgagcactacttcgcgcagcaccaccgccccacgcctccccacaccagcggcacgttctcggact
ccggctccgccgtcgacgtcgtcacggagctgccggccggcgggtcgcacacgtacgagcagctcgccgccgcgacggacgggttcgctccgggcaa
catcatcgggcaaggcgggttcgggtgcgtgtacaggggacggctggacggcgcggaggtggcgatcaagaagctcaagacggagagccggcagg
gcgaccgcgagttccgggcagaggtcgagatcatcagccgcgtccaccaccggaacctcgtcacgctggttggctactgtatctactccgacgagagg
ctgctggtctacgagtttgtccctaacaagacgcttgactcccacttgcatggttatcccaaaatcatacatcgtgatgttaaggcgtcaaacattcttcttg
atcataattttgaaccgaaggttgccgattttggattagcaaagtatcaaccaggtgaccatactcacgtttccacaagagtgatgggaacttttgggtac
atagctcctgagtttttgtctagtgggaaactgactgacaaagcggacgtatttgcatttggtgtcgtcctcttagaacttgtaactggaaggctaccggtt
cagtcatcccaatcgtacatggatgacacattagttggctgggcaaggcccttgcttttgcaggctgtagcggacggcaatcttgaaaaccttatggatc
cagaaatcggaagcgattacgatccggccatactgatgcgaatggtcgagtgtgccgcagcctccgtgcgccaatcagcgcatcagcgtccaaccatg
gttcagatccttaaatacttgcaaggagaaatgcgcgaagatgatccaaattgtgtcttcaaaattacttccgccgacgattattcatacagctccggcat
ggactctggtgaatccgtggtagtaccaaggccaagaagaacgcagcaaagtcagggaaagactagcaccgactacagcagcagcgagcaaggcc
ctggagacaaaccaaactggagctctgcaagcggttggtga
>g83359.t1
atgcccatggagcagtacgaggtggtggagcagatcggccggggcgcctacggctccgcctacctcgtccatcacaaggctgagcgaaagaggtacg
tgatgaagaagatccgcctgtccaagcagaacgacaagttccagcgcaccgcataccaggagatgtccctcatggcgagcctcagcaacccgtacatc
gtcgagtacaaggacggctgggtcgacgaggtcgtaggaaccccaaactacatgtgcccagaaatactgtcagacataccgtatgggtacaaatctga
tatatggtcacttgtgaagcccaccaaaagtaacctgagagacaagcagtcaagaaagccaagcagtggcagaaagcgaattgtcaaggccagtgga
tccaatgaagcgttagaaactgcagcagagcacagtggagaaacaagagacagctccaccaacttttctgatgtatcaactattggtacccaggatgcc
tgcatttctcacatgcctacagatactgatgcaagacacaaggagcagcagatcagtgacgctttatcaattcaacatactgaggataacttgacggca
atgactgaccaacagattgatgagaccattagacttaaatctataagaaccagtaaggtaggagatgaggccgtgattagtattgcaaatcacaagctt
actgaggcaccgataccaaatgaggaattaacaattgtagtagtacaagaacaaaagaaggacgtgaaaacactcccttaccaagaagcaaagcca
ggcttagatgacccatctgtgacagaggaatcatcaccgattagcacgctaaaattggcacacacagatagcacacctgctgagtgggatcacctcaa
catagttcagcagagagctgatgctctggagtcacttctcgagctttgtgcgaaacttctggagcaggagaggctcgaagagcttgcaggtgttcttcga
ccatttggtgaaggagccgtgtcatctagagagacggcaatatggctgacaaaaagtctgatgactccaccaagataa
>g83503.t1
atggccgccgacggaagagcgcgctccctgtcgccgcccaagcaccgccgctccaccgatgacgcccagctagatgcctcgtcggcaaagcacccga
agcatgccgctgctgctatagacgtcgatgcagattcatcttaccgtagcagagattatcggaacaatggaagagagtggatgacgagtcgtgttgtca
gtgatcgacatagcgacagtgatcttcttatatctaccacgagcggaccggtcaacaagggtgagagatcgacagctactggtgtaactgttactgaac
cacctgctgtaacacgcaaaacgggcaagggtggcgcttcgcacatccagagaaatgaccatcatgacaattgggatgatgcagaggggtactacag
ttaccggtgcggtgagttgctgaacggacgttacgcaatccaatcagggcatgggaagggtgtgttctccacagtcgtgcgggccaaagatcttcaagc
tgggaaagatgatcctgaacaggtttccatcaaaactatccgcaacaatgatacaatgaacagggcatcactgtgtgaagtttatttctattttcagcctg
acaatattctgttcgatgacaccaagaaactgctgaagctatgtgatttcggcagcgctatgcttgctggaatgaacgaggtgacaccttatcttgtcagc
cgctggtatcgggcgccctag
>g83671.t1
atgcgacgagtggtttgcttccggaagcttcgtggcgcggctggtgcgagcaccgggccgtgcggtctcggcgacccactgagcccaggcttgcagctt
gccgtcgcagcggcgacagcggctgcaggagacaaggctgtggccctgacggggatgcagatgggcgcagagacggtgaaggcggctcctgcgctc
gtggcttttctgcagaactcgcccacgaagccacatgtgagcaagtactacttcaagaagaggaccagtagccaaagcagaaatggcaaggacgata
cagaagggaactccaggatcgagcccaggtcgcccttgagcggacagagtctgaccatgcgcggcacctccacataccactctggagccttctacgag
atcgatcacgagatgctgcccccaaaatccccaatccatctgaagtccataagagtggtaaaggtgagcgagtgcacgagcctggacgtcacggtgtg
cttcccgtccctcctggcgctgcggagcttcttctccgcctaccagacgtcccgcagcgggccggagctcgacgagcgcttcgtcatgagcgtcagccac
gccgcccgcatcttgcgccgccgggtggctgaggaggagctcgagggcgaggtacacctggaaagcttctggctcgtcaagcccctcctctacgacttc
ggcgtgtcccagcagacgccatcaagtgctgcgtcgccgtcctcgccgcccgctccgcaggcagctaataagctggctccagcagccggctcctgtctg
ctcaccaccttgaagtgcgatggcaccgggtggggcgtgaggcgccgtgtcaggtacatcggccgccatcatgatgatcccaaggacttcgacggcta

cgaaacagaggccggcgtcagggaggaggagccgcgtccggcaacgcgggagatcaggaggagtccaagaaactgcaagaggaagcgggaggc
ggaacgaagcagcccaggcacgcacagagacgaacgaaataagaagaaaaaggttcagggccgcagcaagaagagccccgtggagaagaagcg
caccgttgaggccaaggatggtgaccctcggcgcggcaaagaccggtggtcagccgagcgctacgccgcagctgaaaagagcctgcttaacattatg
ctctcccgtggtgccagatacggcgagcccgtgatgcggccgcacatggccggccgggtgccagatggcagcgctcaccggttccgacgccgccataa
cgcagatggtgccatggagtactggctggagccggccgagcttgctgatatacggcgggaggccggtgtgtctgatccatactgggtgccaccgccgg
gatggaagccaggtgacgacgtgtcctccgttgccggcgacttattggtcaagaggcaggtggaagagctcgccgaggaggtcaatggcgtgaaaag
gcacatggagcagctattgggaaaggatgggaaatttgatgcagaaagagcttacatttcattgaaggagaaataccagactgttatcagggtcaatg
aaaaacttgagaagaaggtgttgtctttgaaggacaagtacaagcatgtggttcaaatgaatggtgagctgaagaatgaggtgttatccttgaaggaca
aatttgaacatataggtgacaagaatgacaagctggaggagcagatcacctctctctccagctcctacctttctttcaaggatcagatactgccgctgctg
aagatggaaggaactaatcaggtggaagtgtctccccgtgccggcattcgagaagctcttcctcgcacatcactgtatgctgctagcggcgatcagatg
acccaccctgcagatggaatcgtcgtccaaggtggtcctgaccgtgcggcgaggaagaacagcttccgcatctgcaagccacagagcacgttccagtg
gccaagtatggcgtccggcatcaccaccagcgggggtgccagcaacggctgccctaggcctgggctccctcgcggcaccagccgcctcagcagtgctg
ggccagggacgggtctcccgccgtcgtcgcgagctccgtttgaggtgatggccacaccgcccggccctgatgagcacatgacgcgtggcgccctcacc
acgccgccctctgcatcgtccaccacgctgcagctgtccctgcccagcccgaggtcacccctccagccacaacagctatttggtgctactgctactagtg
ctgctgttcggttcagcacctcacctgggaagcagcatctgtccggactcctcgtcctcgccgcctactccctgcgctgccagcctgctggagagaaaga
ggggcatcttcaacgcagacgcgggagggatcggtttggtgggcaccgagctggccctggctactccctcctactgctgagtttctgcatccaacgcga
catctcgcaagagctggcgcatcgcccccgcagcgccgaggagtacgaggaggagccgtgcagcagcctcggcaagggcagctacggcgccgtcgt
gaaggcacaccaccgcgtcaccggcaaggccgtcgccatcaagcacgtcatcgctgcggacggcgacccggagaaggagctgatgcaggaggcgc
gcttcctcgagtcctgcgccggcatccctttcgtcgtcggctaccatggcgtcgtccgcgacccggccaccaagaacctctccctcgtgatggagatgca
cgcgcgcggcgtcatccaccgcgatatcaagccggagaacatcctcgtcacggaggaccacagcgccgtcaagatctgcgattttgggctcgccatgt
cgacctccgacccgccgccgcacgagcctgccgggacgctctggtacaaggcccccgagatgctcctcgacatccagttctacgacgcgcgcgtcgat
gcctggtccctcggctgcgtcatggccgccattatcaagggctccgcgctcttccagggatgctacgaggacggacagctctgcgccatcttcgatgttct
tggcgtgcccgaccatgagacgtggcccgggttctcctccacggcgttcgctaccgtcgtgcttccggaattggacgtgcagaagcagagctggctgcg
tgagctgttccccgagacgatgctgtccgaccaaggattcgagctcctcaacggcctcctcacctgcaatccggagaaccggctcacgtccgccgccgc
gctcatgctcccttggttcgccggcgtcgacgccacagaactgcccatcaagaagaagcaactgccgttgacgttgcccaacaagacgaagaagctgc
cgctgtcgaagcagaggaggaagcccatcctgcagggcgcctga
>g83673.t1
atgatgtggcagctgctgacggccgcagccggaatccacgcccgcggcgtcatccaccgcgacatcaagccccagaacatcctcgtcgccgacgacct
cagcgccgtcaagctctgcgacttcgggctcgcgattcgggcctccaacctgccgccgacgctcgacatgcccggcagcctcgacgcgcgcgtggacg
cctggtcgctgggatgcgtcatggcatcggtcgtgaccgggaggtcggtgttcaacgcgctcaatgacccgcccgacgaggagctcttcgcgatcctcc
gcgtgctcggcgtgccggacgaggacgcgtggccggggttcctggacacgccgttcgccgtgagctggatgccgaagctggacctgagacagcctag
cgtgatgcgcgacatcgtgcccgagacggcgctgtccgaccaaggcttccaggtgctcagcggcctcctcgtctgcaaccccgaaaggcggctcacgg
cggccgacgcgctcaagctcccctggttcgcccaccaggacgcacgggagacgactttgctagtaccaaacaaagcacaagcgctgctagccctttga
>g83693.t1
atgaaaattgctcttggtgctgccaaaggcctagcttttcttcatgaagaagctgaaagaccagtgatatatcgtgatttcaaaacttccaacattctttta
gatgcggactataatgcaaaattgtctgatttcggacttgctaaagatggtcccgagggagataagacacatgtctcaactagagtcatgggaacatat
ggatacgccgctcctgagtatgtgatgacagagagtcttcttggtgagggaggtttcggctgtgtctttaaagggtggattgaggagaatggaactgctc
ctgttaagccaggcactggactaactgtcgctgtcaagacactcaaccatgatgggctgcaaggacataaagaatgggtggcagaagttgattttcttg
gaaaccttcaacatccacaccttgtgacattggactataacgcaaaattgtctgatttcggacttgctaaagatggtcctgagggagataagacacatgt
ctcaactagagtcatgggaacatatggatacgccgctcctgagtatgtgatgacaggtcacttgacatcaaagagtgatgtatacagctttggtgtagtg
ctactagagatgatgactggtagaaggtcgatggataagaacaggccaaatggggaacacaacctggttgaatgggcacggccctatctcggtgaaa
ggcgccgattctataagcttgttgatcctcgtttggacggaaacttctccataaaaggtgctcagaagactgctcagctggcccatgntggtgaaaggcg
ccaattctataagcttgttgatcctcgtttggatggaaacttctccataaaaggtgctcagaagaccgctcagctggcccatgcgtgcctcagccgtgatc
ccaaggctaggcctctgatgagccaagtggtggaagttctgaagcctctccagaacctcaaggacatggccagcagctcctacttcttccagtccatgc
aacatgagcgcggcgcgtccctcacgaatccacatggcagtcaaagcatgaaggtgcagagctcctttgcacggaacgcccagcagccaatgaggag
cctctcgtatggcccgcatgnatgtccctcgcgaatccacatggcagtcaaagcatga
>g84040.t1
atgaagatgacgacgccggcagcggcctcccgcgcgctcgttgccttactcatcgtcgccgtcgccgtcgccgacgacggttcgacgctgctggagatc
aagaagtcgttccgcaatgtcggcaacgtactgtacgattgggccagcgatgactactgctcctggcgcggcgtcctctgcgacaacgtcaccttcgctg
tggcagcactcaacttctctggactcaacctccagggcgagatctctccagccgtcggcagcctgaagagcctcgtctcgattgacttgaagtcgaacgg
gctgtctgggcagatccctgatgagattggcgactgttcatcgctcagaatactggacttgtctttcaacaatttggatggggacataccgttttcgatatc
aaaactgaagcacctcgagaacttgatattgaagaacaacaagttgattggtgcaatcccttcaactctgtcgcagctcccaaatttaaagattctggga
ctgcgtggcaatcgattagaaggaagcctttctcctgatatgtgccagttgactggcctttggtattttgatgtgaaaaacaatagcttgactggggtgat
acctgatacaattggcaactgtacaagttttcaggtcttggatttgtcctataatcattttactggaccaattccattcaacattggttttctacaagtggcta
cattgtccttgcaatcgaacaactttacaggtccaattccttctgttattggcctcatgcaggcacttgctgtgctagaactcaatgataatcaacttacgg
ggccaattccaccggagcttgggaagcttacaggcttgtttgacttaaaccttgcaaataaccaccttgaaggaccaattcctgacaacctaagttcatgt
gtgaatcttaatagctttaatgcttacggcaacaagttaaatgggaccattcctcgatctcttcgtaaacttgaaagcatgacatatttaaatctctcttcaa
attatctgagtggtccaattccaattgagctgtcaaggatcaataatctggacaccttggatttatcctgcaacatgatcaccggtccaattccatcagcca
ttggcagcttagagcatcttttgagacttaacctgagcaagaatggtctactgggattcattcctgcggaatttgggaacttgaggagtgtcatggagatt

gatttgtccagtaatcaccttggtggtttgattcctcaagaacttggaatgttgcaaaacctaatgttacttaaactggaaagtaacaatataactggaga
tgtttcttcactgatgaactgcttcagccttaataccttgaatgtatcatacaataatttggctggtgttgttcctactgataacaacttctcacggttttcacc
tgatagtttttaggtaatccgggactctgtggatattggcttgattcgtcgtgccgttcttccatcctccaagaaaaacctccgatttcaaaggctgcaata
cttggcattgctgtgggtggacttgttatcctcctgatgatcttagtagctgtttgtaggccacacagttcacctgtttttaaagacgaatccataagcaaac
cagtcagcaatgtgcctcccaagctggtgatccttcatatgaacatggctcttcatgtgtatgaggatataatgaggatgacagaaaatttgagtgagaa
atacatcattgggtatggggcatcaagtacggtgtacaaatgtgttttgaagaattgcaagccagtggcaataaaaaagctgtattctcactaccctcag
agcctgaaggaatttgagaccgagctagaaactgttggtagcataaaacaccggaatctagtcagccttcaaggatattctctatcacccattgggaac
cttctcttctatgattacatggaaaatggcagcctctgggacgttttacatgaaggtccatcgaagaagaaaaaacttgactgggagactcgccttcgga
ttgctcttggcgcagctcaaggccttgcttaccttcaccatgactgcagtccacggataattcatagggatgtaaaatcgaagaatatgctccttgataaa
gattatgaagctcatcttacagacttcggcattgccaagagcttatgtgtttcgaaaactcacacctccacctatgtcatgggtactattggctatatcgat
cctgagtatgcacgcacttctcgactcaatgagaagtctgatgtttacagctacggtatcgttctgctggagctgctgactgggaaaaagccagtagata
acgagtgcaatctccatcacatgatcctatcaaaaacggcgagcaatacagtcatggagactgtcgaccctgacattggagacacgtgcaaggatctc
ggcgaggtgaagaaggttttccagctggcactcctgtgcaccaagcggcaaccgtccgatcggccgacgatgcacgaggtggtgcgcgtcctggactg
cctagtaaaccctgacctgccggcaaagccttcgcatccgacggccttgccacagccatcggcggtcccgagctacatcaacgagtacgtgagcttgcg
aggtacgagcgtgctctcctgcgccaactcgtcgagcacctcggacgccgaactgttcctgaagttcggtgaggccatttctcagaacacggagtag
>g84330.t1
atgccttgctttgtcatcggagccgcacctcaccgcctgttccacggcaccaccactcctgcaccgcgcgagacgccgacgagaccgccactcccacca
ccagcaccaccggaggtagagtcgtcggacttcgacttcatcggcgtcctcggcgagggcgggttcgcccgggtgttcaaagtgcgccaccgccgcac
ttgcgaggtgttcgcgctcaaggaagccttcaacccgtccccggacgcggaggaggaggccgaggtgctccgccgcgccgccagttccccttcaccgc
acgtggtgcagtgccacgcggtcttcctcgggggcaacgacgacgacaacaacggcgcgctggtggtgagcgtgctggagctcatggacgcgggctc
cctgcgggccgtgctccgccgtctccagcggcgcgggttcccggagccggcgctggccgaggcggcccaccggtgcctgatgggcctggcccagctgc
actcccgcggcgtggcccacctcgacgtcaagcccgacaacttcctctgcaacgcgcgcggcgacgtcaagatcggcgacttcaacgcgtcccgcgtc
gtgtacggacgcgcgggggaagccctcgtgctgttcctgggccgcccgcccgtctggcccgacgccgtcgagaagcccaagaagtgcgactggaagg
aggtcatctgcgaccgggacccgccgtccgtgcccgagtacgtggacgcgtcgccggagctgcgacacttcgtcgccgcgtgcctgcataaggacccg
gcgcggcgcgccagggtgccgcacctgctcaggcaccccttcgtcacgggccgtgacgtcgaggcgtccagccgcgcgctgcgagacctcatcgtgga
gaacctgtag
>g84417.t1
atgcagcagcagcagcagtacgtcatgggcgtgccaccgtcgcaacagtcacagtcgccggtgatgtggggacgcggccgcctcagccactggtcatc
atcgaacatgccgatgtggaatagggtgctaggattgcactggtcatttgatagtgctcctttagatactatcacgatcttgccgcaggagcttgttcggg
ccacagataattttagcgacaacaacctgctgggggctgggagtttcggcaaagtattcaagggccgattagacaccggtttggtggttgcaataaagg
taatagacatgcagctagaaaaggcaattggaagcttcgatgccgagtgccgtgtgcttcgcatggctcgccaccggaacctgataaggataataaac
acctgctccaacctggattttcgagccctagtgcttgagtacatgtccaatggaagcttggagatgctcttgcactctgaaggtagaagccacatggggtt
ccttagaagaatggacatcatgctagatgtatcaatggcaatggaatatctgcatcatgaacatggcttggtggttctgcattgtgacttgaagcccggc
aatgtgctatttgatgacgaaatgacagcgcacgtagtagacttcggcatcgcaaagttgctattgggtgacgacaactccatgatcgtttcaaccatgc
ctggaacacttggatatatggcaccagagtatggatctcttggtaaagcatcacgcaagagcgatgtgttcagctatggcatcatgctttttgaagtcttc
gcagggaagagacccacagatccaatgttcgagggagaactgagcattacgcagtgggttcatcaagcatttccatctgagcttcactctgttgtggac
aaccggctgctgcaggatgtcttttcttattcaggcgacctgaacgagttactgtccccaatatttgaggtcggtttgctctgctcgactgggtcacctgac
caaaggatgtcaatgagtgatgtagtggcaacgcggacgcggatcagaaaagggctacacctaatatcgaaggacaacgtccacgcccgagcgcaa
ccatga
>g84613.t1
atgcggttcacttacaaggagctccaggaagcaacaaacaacttttcaaacaagctcggccaaggaggttttgggtctgtttaccttggtacactgccag
atggaagtcgcatcgctgtaaagaagctagagggcataggtcaagggaagaaagaattccgttctgaggttactatcattgggagtatccaccacatcc
atcttgtcaaactccgagggttttgtgcagagggcacatacagacttcttgcatatgaatacatggcaaaagggtccctagataggtggatatttcggaa
cagtggtgattcctccctgctggactgggacacaaggtttaatattgcacttggaacagcgaagggattggcatacctccatcaggattgtgagtccaag
atcattcactgtgacatcaaacctgagaatgttctacttgatgagaacttccttgcaaaggtgtctgactttggtcttgcaaagttgatgaccagggaaca
gagccatgttttcacgacactcaggggcacacggggctaccttgcacctgaatggatcaccaattacgctatctcagagaagagcgatgtgtatagcta
tgggatggttcttcttgaaatcatcagtgggcggaagagctttgatcctgttgaagtttcagagaaagttcacttcccctcatacgctttcaagaagctag
aagagggtgaacttcgcgatatctttgatgccaagctaaaatacaatgacaaggatgagcgacttgagattgcaatcaaggttgccctgtggtgcatcc
aggatgatttttatcagcgaccgtccatgtcgaaggttgtccaaatgctagaaggtgtatgtgacgtgccccagccaccaatatcctcacaaattggatc
ccggctctatgcaaatgccttcaaatcgagcagtgaagagggcacatcatctggaatgtcagactacaacagtgatgccttactttcagctgtgcgtcttt
ctggtcccagatga
>g84684.t1
atgggctccgccccgtcgtcgcgtgtccagagctcgtcgggggaggcgccgttcttcacggacttcggcgaggcgagccggtacgaggtcacggaggt
ggtcggcaagggcagctacggcgtggtcgccgccgccgtcgacacgcacaccggcgagcgcgtcgccatcaagaagatcaacgatgtcttcgagcac
atctccgacgccacccgcatcctccgcgagatcaagctgctccgcctcctgcgccacccggacatcgtccagatcaagcacatcatgctcccgccctcgc
gccgcgacttccgcgacatctacgtccagttcttcctctaccagctactccgcggcatgaagtacatccacgccgccaatgtcttccacagggacctcaa
gcccaggaacatccttgccaatggcgattgcaaactcaagatttgtgacttcggccttgctcgggtgtcgttcaatgatacccccttctgcgatattctggac
gtatactcctgcgattgatatttggagcataggatgtatatttgctgaaatgcttacagggaagcctctctttcctggcaagaatgtggtacaccaattaga
tctcatgactgatcttcttggcactccttcagcagaaacactttccaagattttggagtaccatcctcagatgttgcaggagtatctttgcggaggggatat

ggcaaactttgtgtacccaagggaaaaagtaaatggcaacgggaatgagcttgagaagccaaatgcagactacgtcataaaattgcatgagtttcgct
acgtgctcgcccctctccttgcagcttgtgctgatgagcaatga
>g84988.t1
atggcgccgcggcgcttgttcgtcctcgtcctcgccgtcgtctcgttgttggctggagggatagcactagtggctggccaggcatgctcgaccacgggca
acttcacttccggcagccagtaccaggtaaacctcctcaatctcatcggcgacctgccaaagagcaccattgacaacggcggcttcgccgaaagcacg
gctggcagcgcaccggacaaggtatttggccttgccatgtgctacgccgaccgtgacgcgacccagtgccgagactgctttcgggatatgtatagagat
gttgtgcagagcaagtgcctcttcagccgggaggccaaggcctgctacgacgcgtgcttcctccgctactcggacctgtcgttctccggttttgccgatct
cggcatcgatcactacgtttggggggtgaattcctatgtcagcgacatggagagcatgaacgccagccggtggagtctgatgaccgggctggtgccgg
aggccgcgagctctccgctgcggttcgccaatgacagcaaggagtacacggactcgcagggcagcacgcaggtgatgtacgggctggcgcagtgcac
gagggacctgagcgccagcgagtgcacaaggtgcctcaacaggttcgtggcggagctctcgagctcgcgtccgaacaacacctacggcacggtcaag
ggctacagctgctacgtggcatatcgggtcgggatcgacctcggcatcaccattcttcccgtgacagccccgccgccaacgccggttcatcctccggatc
cgtcgcctctcccagccccccgagcaattcttcagcagcactgattgccggcgtgactgttggttcagtggtgttcataatctgcaccggcatcttggttt
ggttcctactgcggcgctgcaggaagaagccgagacagattgaacacgatgcgacagacgagaaggctctgcaggacgagttcgagaaacaggcgg
ggccaaggcggttccgctacggcgagctcgaagttgcaactagcttcttctccgaggacaagaaactcggcgaaggtggctttgggtcggtgtaccga
gggtacttgcaagacacggaccttcatgttgcggtaaagagaatttcccagaactctgaccagggtcgaaaggagtacatttccgaggtcaagatcatt
agccagctgaggcatcgcaacctagttcagctcataggctggtgccatgacggcagcaagctcttgcttgtctaccagcttatgcccaacgggagcctg
gacgctcatatccacgaccagaacaacatcatgtcatggcagctaaggtatgagatagtgctcggaatcggttccgcacttctgtacctgcatcaagact
cggagcagtgcatcttgcaccgtgacatcaagcccagcaacattatgctggacgcgtcgttcgtcgccaagcttggagacttcggtctggcgaggctcat
cgaccgcgatcgtcagtcctacacaaccgcgcaggccggtacaatgtggtatatggatccagagtgtatgttctctggcacggcaagcacatcatccga
cgtttacagcttcggtgtcgtgctccttgagatcgcctgcggccggcggccgatcgttgttgtatcaaatactgaagaatacgccagcatccacctggtcg
attgggtctgggaacattacggccgcggtaggattcttgacgcagcggacgtgcgactgaacggcgaattcgatnccgcggtaggattcttgacgcgg
cggacgtgcgactga
>g84989.t1
atgaagatccccaaccctgttttgttgagtgcattccgctgggggcagggtagcagagctgtatctgtgaagcatgtcctactgtcctgatttgtaattcaa
attgacggaccgaggagattccgctacagcgagctggcggtggcaaccggcttcttctcagaggatgagaagcttggcgaaggaggtttcgggcatga
gatcgtgctcggtctcggttccgcgctactctacctgcatcaggactggaagcagtgtgttctacaccgtgacatcaagccgagcaacgttatgctcgac
gcaaccttcaacgccaaactcggcgacttcggcctcgcgaggctcgttgatcacgataggcaatcgcacacaacagcacttgccgggacaatgggata
catggacccagagtgcgttctcactggcgcagccagcgccgcgtctgacgtctacagcttcggcgtcgtcgtcctcgagatcgcgtgcggcaggcggcc
aatcgtggtggagcaagatactgaagaacacaacacggtgcaccttgtccagtgggtatggaagttctatagccgtggaaggatcatggatgcggctg
acccgcagctaaactgcgagttcgacagcgacgagatggaacgcgtgatggccaccgcgctatggtgcgcgcaccctgaccgcacaatgaggccatc
tattaggcaggcggtcaacgtgctgcgccttgaggcgcccttgcccactctgcccgcagagatgccagttgccatgttcatgccaccggtaacaggcttc
cattctggatccggccttgcaagcagtaccaccagcggcagcgccgggactacacgttcaagcattgtgacggaagcttcttcattgttgagatga
>g85046.t1
atggagtcgctgccgcggaagcgcaagggcgcgcggggcgcctgctcgctggccggatccttccacgacgcgcccgacgcccgcaagcgcacctgcc
gcgagcccaagccgcgccccgagaagaagaagccccacgccgccgatgacgaggcctccgcccggggcgccggcggcggcgtagtgatgacggcg
cccccgcgagcgggcgggcggcgcccgacagccccacccgcggtctgaagcggaagctgggctgcatcgagtccgccacgcggatgggccggaag
aagcggctcgagagcgagtacgagctcggcgccgagatcgggcagggcaagttcggctccgtccggatctgccgcgccaaggccggcggcgaggag
ttcgcctgcaaggcgctgcccaagaacggcgaggagacggtccaccgcgaggccccatccccgcgcatggagtcgctgccgcggaagcgcaagggc
gcgcggggcgcctgctcgctggccggatccttccacgacgcgcccgacgcccgcaagcgcacctgccgcgagcccaagccgcgccccgagaagaag
aagccccacgccgccgatgacgaggcctccgcccggggcgccggcggcggcgtagtgatgacggcgcccccgcgagcgggcgggcggcgcccga
cagccccacccgcggtctgaagcggaagctgggctgcatcgagtccgccacgcggatgggccggaagaagcggctcgagagcgagtacgagctcgg
cgccgagatcgggcagggcaagttcggctccgtccggatctgccgcgccaaggccggcggcgaggagttcgcctgcaaggccctgcccaagaacggc
gaggagacggtccaccgcgaggtggagatcatgcagcacctctcgggccaccccggcgtcgtcacgctcaaggccgtctttgaggacgccgacaagtt
ctacctcgtcatggagctctgcagcgggggtcgcctgctcgacgagattgcccgggagggcaagttctccgagcagcgcgccgccattgtgatcaagg
accttatggccgtcgtcaagtactgccacgagatgggcgtagtgcaccgggacattaaaccggagaatattttgctcaccaagacagggaggaccaag
cttgcagactttggactcgctgcacgggttactaatggtcagaaattgtctggtgttgctgggagcccagcatatgtggcacctgaagttctttcaggaag
ctattctgagaacgtagatatttggagtgctggtgtgctactccatgtactactgcttggttcacttccatttcaaggaggttctctggatgctgtcttcgag
gctataaagacagtcacccatgggtcctgttttacacagaatgtcccctga
>g85082.t1
atggccaatgggagtgttgcatcacgtctgagagaacgccagccagatgatccgccacttgactgggaaacaagggctaggattgcactgggatctgc
caggggactctcctacttgcatgaccattgtgatcccaagatcattcaccgtgacgtaaaagctgccaacattcttctggatgaagactacgaagctgttg
taggcgactttggcctcgccagacttatggattacaaggacacccatgtaacaacggctgttcgtgggacgattggacatattgctccagagtacctctc
caccgggaagtcgtcagagaagaccgatgtttttggctacggaatcatgcttctggagctcattactggacagagggcatttgatcttgctcggctcgcg
aacgatgatgacgtcatgttgcttgactgggtgaaggggctgctgaaggagaagaagctggagccgctggtggacccggacctgcagggcgggtacg
tggaccacgaggtggagtcgctgatccaggtggcgctgctgtgcacgctggggtccccgacggacaggcccaagatgtcggaggtggtgcggatgct
ggagggcgacggcctggcggagcgctgggaggagtggcagaaggtggaggtggtccggcaggaggccgacctcgccccgcaccacaaccagtggc
tcgtcgactccacctacaacctcaggcccgacgagctgtccggcccgaggtga
>g85545.t1

atgcaggaggagcgtggcaggatgggcgccgccgcagaagaccgacacgccacggcggcagtcgtcagagctcggcaccgcactactacttgtata
gctggctctgccatggccccggtccccgcctccacaacggcacgatccgcctctcccgcgacccaaaaatccgcacccnctgacccggccgccgccctc
cgcttcgactacgcgacgctcaccctgggcagcctgacgctcctgggcgacgcgcacctcaagaacggcacgatccgcctctcccgcgacctccccgtc
cccacctccggcgccggccgcgcgctctacgcctccgccgtccccctcgccgcgggcttctccacccagttcgccttcaccgtcgccacgctcaacaagg
gctccgtcggcggcgggctcgccttcgtcgtcgccaccgacgccgccgcccccggcgccgccggcgccttcatcggcgtcgaccccgccgccgacgcc
gtcgccgtcgagttcgacacgctcttggacccacagttcggggacgtcaacggcaaccacgtcgggttggatctcgggtccatggtctccgccgccgcc
gccgatctcggggatgtcggggtcgacctcaccagcggcagaaccgtcaacgcctggatcgagtacaaggaagggaggatggaggtgttcgtgtcgt
acgcggcgaagcggccggcgatgccggtgatttcgagtcccgtggagttgggaatgagctcggcgtttgttgggttctcggcttccacgcagggtagca
ccgaggcgcacgccatccagtggtggagcttctccacaaccgaaccgccgagccagccgccgcccgtggtggtggtgatgcctccgccgccgccgccg
ccggcgagcgtcatcccggtgctcccggcgccgctgcttcccccgccgacaaccaccacgacggtcacggcttccgcgccgacggggtccgtcgcggc
ggcgagcgctccggcggcggcggcgcgctccgccacggcaggaggaggaggagcctcagcggcgacgcaccaggcggcggtggccggcgcggcg
acggcgggcgccgtggtggccgcgtcgttcgcggggctcgcgctctgggcgctggcgcggcggcggcgacggaagagggctgtttcagcggcggcgg
cgaccaagcacggcaacaacgggagcagcagcagcctggcggcgctggcccggtccccgcgcgagttcagctacaaggagctgagcgccgccaccc
gcgggttcgacgcgtcccgcgtgatcggcaccggcgcgttcggcaccgtgtacaagggcatcgtccccgacaccggcgccatggtcgccgtcaagcgc
tgcaccaaccacaacgcgaacagcaacaacaaccaagaggaggcccggtccgagttcctctcggagctctccatcatcgcgggcctccgccaccgca
accttctccgcctccagggctggtgccacgccggcggcgagatcctcctcgtctacgactacatgcgcaacggcagcctcgaccgcgccctcttcgatca
ttcctccaacaccaaccctccgttgccgtggcgccaccgccgggagatcctcgccggcgtggcctccgcgctggcctacctccaccacgagtgcgaccg
ccgcgtgatccaccgcgacgtgaaatccagcaacgtcatgctcgacgaggcctaccgggcccgactcggcgacttcggcctggcccgccaagcggac
cacggcgcgtcccccgacgccaccgccgcggcgggcaccatgggctacctggccccagagtacctcctcacgggccgggccaccgaggccaccgac
gtattcagcttcggcgccctcgtcctcgaagtcacctgcggccgccggcccatcatcgacacctctaccaccaccaccaccaccgaggcacgacgatgc
aacaacctcgtcgagtgggtgtggggcctgcacggggaaggccgggtgctggaggcggtggacccgtggctgggcggcgagttcgacgagggcgag
gcgaggcgggccgtgctggtgggcctggcctgctcgagcccggaaccggcgctgcggcccgggatgcgggccgtcgtgcagatgctgagcggggag
gcggacccgcccttcgtccccgcgacgaggccgtccatgaccttgagcagcgccgccaaccagcagcagctgctgctcagcctgcaggacagcgtctc
cgactacaacgcgctgctcgggctcaacctctccgacgactcctactcttccgaggactccatgagcagcgcgtccctcaccagcgcgctaagacgcgg
cggccacgacatcgggttcagtagcaccgcaggtgaccaccccaggtga
>g85589.t1
atgcggctcaggcagctgcttgctggatggcagatcactctatcagatgtttttggtccatatgagcttctcaatttcacgcttggttcctatgaagacgta
ctctcaaggttttctatcaaggtcgatggtgtgaagtgcttcacatttgaagaaatggctttggcaacaagtgattttactgctcaaattggtcaaggaggt
tatggaaaagtttataaaggaaatctagctgatggaacagcagtggcaatcaaacgtgcacatgaagattctctgcaagctaaaactgaaagacctct
gagctttggtcggagattgcatattgcgcttggtgctgcaaaaggaattctctatctacatactgaggcaaatccacctatatttcaccgagatgtcaagg
ccagcaacattctactggactcaaagtttgtagccaaggtggctgactttggtctttcgagactcgctccggtgcctgacattgaagggactttgccggct
catatctccactgttgtcaagggcaccccagtcgatggtgtgaagtgcttcacatttgaagaaatggctttggcaacaagtgattttactgctcaaattggt
caaggaggttatggaaaagtttataaaggaaatctagctgatggaacagcagtggcaatcaaacgtgcacatgaagattctctgcaagctaaaactga
aagacctctgagctttggtcggagattgcatattgcgcttggtgctgcaaaaggaattctctatctacatactgaggcaaatccacctatatttcaccgag
atgtcaaggccagcaacattctactggactcaaagtttgtagccaaggtggctgactttggtctttcgagacttgctccggtgcctgacattgaagggact
ttgccggctcatatctccactgttgtcaagggcaccccaggctatcttgacccagaatatttcctgactcataagctgacagaaaggagcgacgtatata
gccttggtgtcgtgtttcttgaactatttactggaatgaaaccgatccagcatggcagaaaccttgttagagaggtaaacacggcttaccaatcaggcga
catttccaagatcatcgacagccggatgtcctcttgccctccagaatgcgccacaaggttcctctccctggcaataaaatgctgccacgacgagactgaa
gcgaggccttacatggtcgacattgtccgagagctcgagaccatccttggcatgatgcctgagggagagaacctccagtccgaaacaacggagacga
gttcaggagcgctcacgaagtccatgtcctcctccaccaccaccggggccctcttcgtgtcatcccatacctccggcagcgaccacgtgacgaacagtg
gcatcccctccgggacggtggcgcctcggtag
>g85592.t1
atgcaacatcttaaagttattgcgtttgtggttgcttgtctcctctttgcaagaattcctcatgccaaaggtgctgatctaaactctgacaagcaggctcttc
tcgcatttgccgcgtcactgcctcatggcaggaagctcaactggagctcgaccacccctgtctgcacttcttgggtgggggtgacgtgcacaccagataa
gagccgtgtacacacactacgcctacctgctgtaggactcttcggtccaatacttcggatactcttggtaagcttgatgcccttgagaaattctcagcaa
attccttcttgggcaatgcttttctttgtgggtttcctttggaaccatgccctggaactgcacctttctccttctccagcattgccaccatcgcccaataagacc
aagaagagcttctggaaaaagatcagaactggtgtgataattgcaattgctactgtaggtggggtactgttacttatcttgatcctcctgctcttgctatgt
attttcaagagagaaagaaacactccgaacccaccacagcttcgtccaaaggaaaggctgttgcaggtggcagagcagaaaagcctaaggaagactaca
gcagtggtgttcaggaagcagagaggaacaagttggctttctttgagagcagttcatacaattttgacttggaagatttactgagggcctctgctgaagtt
ctgggcaaaggaagttatggaaccacctacaaagctgttcttgaggatggcaccacagttgtggttaagagattgaaggaagtggttgtgagcaagaa
ggacttcgaacagcagatggagatggttggcaggattggtcagcaccaaaatgttgtgccattgcgtgcttactattactccaaagatgaaaaactgtta
gtgtttgactatgtcccgtctggtagccttgctgccgttttgcatgggaataaagctgccggacgagatccgttggactgggagacaagagtgaagatat
cacttgatgtagctcgtggtatttctcatcttcatgctgagggtggtggcaagttcatccatggcaacatcaaagcatcaaatgtccttctatcactgaacc
ttgacggctgtgtctctgagtttggcttggcacaactcatgaccaatgcacagagtccggcacggcttattggataccgagcacctgaagtccttgaaac
taaaaggccgactcagaagtctgacgtctacagctttggtgttttgcttctggaaatgctaactggaaaagccccactgagatctcccggacgcgaggat
tccattgagcaccttccaagatgggtgcagtccgttgttcgagaagaatggaccgcagaggtcttcgacacggacttgatgaggcatcccaacgtggag
gatgagatggttcagatgctccaagtcgcaatggcatgcgtggccgttgtccccgatcagcggccaaagatggaggaggggcctctcatggcatcgaa
gcgtatcctcaaggaattgaaggacttgcaaaaggatcctccaacatcatgcagcgcaggtcctgctggtgaggatatgttccactggcaggcaaccat
catgggtccacctgatagtccatatgctggaggtgttttcttagtgaacatccatttcccccggactaccccttcaagcctccaaaggtatcttttaagac

gaaggtcttccatccgaacatcaatagcaatggaagcatatgcctcgatattcttaaagagcagtggagccctgctttgacaatttctaaggttttgctttc
aatctgctccttgcttactgaccccaaccctgacgaccctcttgtccctgagattgctcacatgtacaagacagatcgatccaaatatgaagctacagccc
gcagctggacacagaagtatgccatggggtga
>g85619.t1
atgggcaacgtgtgcttctgcggcacgacgtccacgtcccctgacgagcctccatctgatcccaaaaaatccgtagcgccgccgcaacagcaaggcaa
gaggccggcgacgcctccaagcagcagccatgaaaagagccccactcccacccccaggccgaagcagaagcagcagcaacagcaacccgagcccc
gtacgcgcgccaagcccataaagcccaacccgtacgactgggcatcgccgtcgtcgtcgcgcgtgctggacggcgtggtcccgcaccacccgcgtctc
cgcgtgacggacaagtacacgctgggccgcgagctgggccgcggcgagttcggcgtgacgcgtctggcgacggaccgggccacgcgtgagcggctg
gcgtgcaagtccatccccaagcgccgcctccgcacggccgtggacgtgggcgacgtgcggcgcgaggtggccatcatggcgtcgctccccgaccacc
cgtccctggtgcgtctccgcgcggcgtacgaggacgccgacgccgtgcacctggtcatggagctctgcgacggcggggagctgttcgaccgcatcgtg
gcccgcgggcggtacacggagcgggccgcggcggcggcggcccggacggtggccgaggtggtccgggcctgccacgcgcacggggtgatgcaccg
ggacctcaagcccgagaactttctgtacgaggggcggggtgatgacgcgcggctcaaggccatcgactttgggctgtcggtgttcttccggcccgggga
gaggttcacggagatcgtgggcagcccgtattacatggcgcccgaggtgttgcgacggagttacgggcccgaggtggacgtgtggagcgccggggtg
atactgtatatcctgctgtgcggggtgccgccgttctgggccgagacggagcagggcgtggcgagggctatactgcgagggagcctggacctggaccg
cgagccctggccccggatctcccacggggccaaggacctcgtcaggcagatgctgcagatggaccccaagaagcggccaaccgcgcagcaagtcctc
gagcacccgtggcttcagaacgcgcggaaggcgccgaacgtgccgctgggcgacgtggtgcgcgcgcggctgcagcagttctcggccatgaacaagt
tgaagaagaaggccatgcgggtcatcgcggagcacctgtccgtggaggaggtggaggtgatccgggacatgttcgcgctcatggacacggacaatga
cggcaaggtcacgctgcaggagctcaaggcggggctcaacaaggtcggatccaagctcgccgacccggagatggagctgctcatggaggccgccga
cgtgaatggcaacgggtacctggactacggcgagttcgtggccatcaccatccacctgcagcgcctctccaacgacgaccacctccgcacggcgttcct
cttcttcgacaaggacagcagcggctacatcgagcgcgacgagctggccgacgcgctcgccgacgactccggccaaaccgacgaagccgcgctcaac
aacgtcctccgagaagtagacacggacaaggacggtcggataagtttcgacgagttcgttgccatgatgaaagccggcacggactggaggaaggcgt
cacgacagtactcaagggaacggttcaagactctgagcaacagcctcatcaaggacggctcgctcgccatggcgcactga
>g85790.t1
atgctcctcagacctccctcagtccctcctcgcctcgtcactgtcgtccgcatcccgctggaaggcgaactgcctctgccaacggtaacgggaaagggca
accacaaggcaattcgttcccgggctcgaacccattcgaaggcggcggcgcactcgtcgctccggctcgtccctttccacctagctccgccgcaaaccct
aaccctctcactccgccccacctccatggaccgcagagaatcctccaccatttcgccgccctccaccggccagcgcaggcgccggaggccggacgccg
acgcggaggaagcatcgtccccaaagcgccacaagcctacccgtgatcgtcctcaccactgggacatgggtggtgtggcggaggccgtggaggcggt
ggcgcttgtcgctacggcctcatcggcggctgggggccggggacggatgaggtcgaggacggtgaggaattagaccagggcgttgcctccgcggga
gcggggagggggccgggtgctgatcccggtgacgcgggggcagcatccggcgctcgcgtacctcgtccggttcaaatatcagaggatgtatttgtcga
tgatatttttggagaatcacctattgcagttcagaaatttcacaaggaaggtggtttccacatcaaggagaatgctcattatgacaattgggatgatgaac
aaggatattacaaataccgcattggggaagtgctatgtgggcgttatgagattttggcaggacatggtaaaggagttttctccaatgttgttcgggcaaa
ggatcttaaagctgggaaggatgacccttacgaagtagctatcaaaattatatgcagcaatcctataacgtacaaggctggaaagcaagaaatttctat
attagaaaaattggcaagtgcaaatcccgaagacagacggcactgtgtgaggttcttttctagcttcatgtataggaaacatctctgcttggttttcgaat
ctcttcacatgaatcttcatgaggttctaaagaaatttggtcgtgatattggccttaagttgactgctgtaagggtatactcaaagcagctattcatggcgtt
gaagcatcttaaaaagtgtggtgtcctccactgtgatatcaaaccagataatgtgctggtaaatggggttgcaaatgttctaaagctgtgcgattttggta
gtgcttcgtctgctggaatgaatgcaatcacaccatatcttgtgagccgcttctaccgagcacctgagattagtcttgggttgccgtatgatcacccattgg
atatgtggtcagttggctgctgcttgtatgaactatacacaggagaagtcctatttcctgggaagacgaacaatgatatgcttcggcttcacatggaattg
aagggccccttccctaagaagatgcttcgaaaggttcaacctattgttgatgaatga
>g86357.t1
atgggcagcgtcggcggcgggttgctcctcctgcttctgctcctgctccgcgcggcctccgccctccatgagttagaagttgaagttgcagctggcacatt
tcttaaacaaagccaagtgaaaattatggctgcagttgcaagtgctgataaggctaagggaatgctgaactgggatgtcaggatgaaaattgctcttgg
ggctgctagaggcctagcgtacctacatgaagattcaaaccctcatgttatacaccgtgacttcaaggccagcaatatattgttggaggaagatttcact
cctaaggttacagattttggtttggcaagggaagcatccaatggaactcaacccatatctactagggtgatgggtgctgataaggctaagggaatgctg
aactgggatgtcaggatgaaaattgctcttggggctgctagaggcctagcgtacctacatgaagattcaaaccctcatgttatacaccgtgacttcaagg
ccagcaatatattgttggaggaagatttcactcctaaggttacagattttggtttggcaagggaagcatccaatggaactcaacccatatctactagggt
gatgggtacttttgggtatgttgctccagaatatgccatgacgggacatcttcttgttaagagcgatgtctacagttatggggttgtcctgctggagctttta
tcaggaaggaagcctgtaagcatatctgattccaaggatcctgagaaccttgtgacttgggcacgtcctcttctatgcaataaggagggcctggagacg
ttaatcgatccttctctagaagggaagttcaattttgataatgtagccaaagtggcatccatcgcttccatgtgtgtgcatactgacccatcgcaaaggcc
attcatgggtgaagtggtccaggcattgaagcttctgtacaacgatgcagatgaggcttgcgatgattcttacagcccaagggattcatcagacccagat
ggtgactatcatgggggcctggtctttgagagtggcagctggggcattggaacctctgggtgtttagagtaccggaattccttaccatttgtaaacatgga
atacagttcgggccgaatagaaggacaacgagaccctcatgccacactgtcaatgggctcccaagtgcagttaccagtgctgcagaatagatcaggac
ccttgcggacgaagaaaaagctcccctccttctaccgttctagaggcagcattagcgagcatggacacctgccacgccattga
>g86528.t1
atggaggaggagaaggtgttcgtggcgttgccggcagagttcaaggcggggcagtcgacgctgtcgtgggcgcttagccatttcggcggcggcggatc
caccacgatcgtgatcgcgcatgtgcatgtccagtcgcagatgattccagtgaaatgcgagaaactcatgtttgagaaggaagatgtcgtttcgggtctg
atggaccttattgcctcgcatggagtcacgaacctggtgatggcagctgctgcagataagcactactcaagggacaatggagtggagatttcacgagct
gtttcacctgggtcttgtgttcactcgtcgtcagttggctcttggacgcagaaagaaagacaggttgctttcggagcagaaagggagaagccgccgcga
cgaggaggaggcgagacccctgtgatcccaattaagccactccccgaaacggcaaactccctgaccgccctccgcctctccaaccgctcgtcgccgcc
gccgcttgccgcgatgccgcccacgccgccggagccggagacggacccggagttcgtagagctcgaccccaccggacgctacggacggtacacgga

ggttcttggcaagggcgccttcaagacggtgtatccttttggtgtctgatttcgatttattctgtcggcacagatacaaggcctttgatcaacttgaagggct
agaagttgcttggaaccagatcaaagtcggtgatttacttcggaacaatgatgatttggagaggctgagatcagaagtgcggttgctcaagaccctcaa
gcataagaacataatcaagttctacaattcgtggcttgacaagaaaaacaacaacatcaatttcatcaccgagggtgaaaagcctggttcactggctaa
ggttgatgatcctcaggtgaaattctttatagaaaagtgcattgccaaagcccctcaaagactctcagcaaaagagttattattggatccctttctcctag
atgttgatgacgaaaggatattctatccactacatcaaagcggtaatggatcagatgctgctggcaattcaaacccaagcacgagttacagatacaaca
gagcggcatcatctgtcgaccgccatgaccacacaggaagcatgacagatccacatccaagtgatcactacgtacatgaatccatggatcctcgtgatg
caattggtcgaattatcacagtcgagagtcaacggaaggatttgaatacaatatttttgaagctgcgaattgctgattcaacaggtcatgcccaaaacat
ccacttcccatttgatatcgaagcagacacttcaatcagtgttgcaactgagatggtcgtacagctggatctcacagaccaagatgtaacagccatagca
gaaatgatagatgctgaaatacgtgctcacattcctgattgggcagtagaggaatcagttgacaatcaaggggatgaagctgctcattctgaggcccat
agaagcatgacagatccacatccaagtgatcactacgtacatgaatccatggatcctcgtgatgcaattggtcgaattatcacagtcgagagtcaacgg
aaggatttgaatacaatatttttgaagctgcgaattgctgattcaacaggtcatgcccaaaacatccacttcccatttgatatcgaagcagacacttcaat
cagtgttgcaactgagatggtcgtacagctggatctcacagaccaagatgtaacagccatagcagaaatgatagatgctgaaatacgtgctcacattcc
tgattgggcagtagaggaatcagttgacaatcaaggggatgaagctgctcattctgaggcccatagttcagacagtgatgagggaacctccgaattgc
gtgatgaacctgatgctgcacacaacggcttcgtacaggaacaacttccttctgggcggaagtactggtcagactcacccaggagagacaatgacaca
cctgtaccgatggtgaacccacaagcaggtgatatcgtttcaaatggaatagtgaaaacgaatgatgctgatgaccttatcaacagtacaaaacataag
gacaatgaatcccccgctgtttctgctcattctgtggaagatatctgtgagggaatatcctcatctgtgggattgtcaaagccttcggtggtcgacaggat
ctctggaggagcttctgttggtactagtcctcactcttctgatgttgagcggcaatgcatttcagcagatgttactgagaggttggaaaacctgttagctca
gcaacaagaggagcttaatgcgcttcaaaagaagcataacgtagacatacaagacatattgaaaagtgtacctgcagaggatcgtgaagaaaccttg
actaggtgccggttgaagatggatcagcaaaacaaagcaaaccaacattag
>g86792.t1
atgggctacgggtacgtggatcccttcctgccgccgcagcaggacgccgcggcgtcggcgtacgagccgcagaagcaggcagcggtgtcggtttttgt
cgcgccgcaggggggacggatacggccagccgcagccgcagccggcgccgaggccgccgggctgcccgttcggctcgtccgcctcggccccgccgac
gtcgtaccactccttgccgccggcgacgtccgctccgccggtgagcagcccgccgccggcgtcgcctccgcccgagccgttgccgccgccgccattgcc
tccgtcgccgccaccgccggcattgtcgcctccccctcccgacgcgccccctccggcgctcccgccatcgctgccgccgacccctcccccgtcaccgcct
ccgatcgaggtgagcgccccgcctccgccgctggcgacagaccagccgcgagttcagccccgcgtgtacccgtcgccgccgccgccgtcgcttccccc
gccgccgccaccgtccgtctcgccgccggcgccggctccatctctcccgctgtctccgtcccctgctccagccgcgcgtgctccagctcccgcgcggcat
gctgtccactcgcctcctccgctgagaattgcctctccgccacctccacgacatcacgtcaagccgcattacttgcccgggaagtcgcattccaactctac
ccatgcagctagtagcagcgggaagaacatcgagatatcgagggaagcggccaccaccatcgtagcaattgctggtcttgcaatgctcagcttcatcg
gcgccaccatttggtttgtcaagaagaagcgcaggcggatagagccaccagcgtcactgcccacacagcaaccagctccgccaccaccgcccaacta
cattccatcttctgctggatcctccttagcatcagacgggttctacttacgatcaccggggtatcctttcatgagatccagtaccgggagtcacgggtttcc
gtactcaccagctgattctggtattgggtactccagaatgctgttcacgccagagaatctggcagcaatcacaaatgactttgcagacgaaacacttttg
ggagaaggtggattcgggtgcgtgtacaaaggaattttgccagatggtcgccctgtggctgtcaagaagctcaagattggaaatgggcaaggcgagct
cagtgaaacagctcttgattggcgaacaagggttaaaattgctgctggagcagctcgtggaatagcttacctgcatgaagattgtcatccacggattatt
cacagagatattaaatcatctaatattttattggataacaacttcgaagctcaggtttctgattttggactcgcaaggtatttggctccagagtatgccttat
cgggcaagttgacagcaaaatctgatgtatattcttttggagttgttcttttggagcttattacaggaaggaaacctgttgatgcttctcagccgttgggag
atgagagccttgtcgaatggatcccagacgcccgcaacttcaccaatcaccaggccaccaaccagttcctgccgccggaagtttcattgcgccgcgcgc
gccgatcaatccccgctgcccaaagcttcaacgtgctgcagacgggtccctgccgccgtattgagctctatggaaacacaaggctagttacgaggccat
gtcttcaaagagtagaggaggtggtcgccgcggccgtggccgtggccgtggaagacatgctattgcagaaaatgacatag
>g86983.t1
atgcctaatggaagccttgacactcatatccataaccagaacaatgtcatgtcgtggtccctcaggcatgaggtcgtgctcggtctcggttccgcgctact
ctacctgcatcaggactgggagcagtgtgttctacaccgtgacatcaagccgagcaatgttctgctcgacgcaaccttcaacgctaaactcggcgacttc
ggcctcgcgaggctcgttgatcacggcaggcaatcgcacacaacagcacttgccggaacaatgggatacatggatccagagtgcatcctcacgggcg
cagccagcgccgcgtgtgacgtctacagctttggtgtcgtcgccctcgagatcgctgggtatggaagttcaatagccgtggaaggatcatggatgcggc
tcacccgcagctaa
>g86997.t1
atggcggctgtgacggccaccacccgcaagcgctcgtcccccgacgccgccagctcgtccaccgccggcgaccagaagaagaagcgtgcgcgcgtca
tcacgttcgggaacatctacgactacgagaagcttgaggttctgggcgagggcacctacggcgtggtcctcaaggcgcgcgaccggcgcacggggag
accgtggccgtcaaatccgcgacgtgccgcggacgccgcaacgggcgccgccttcctggtcatggagttcgtgggccccagcctgcagcaggcccag
ggagggcggttattctcggagcgcgaggcccgcgcgtgcatgcggcagctcctcggcggcgcggctcggatccacgccgccggcctggtccaccgcg
acgtgaagcccgacaacgtgctcgtgggccccagcggcgagctcaagatctgcgacttcgggatggccgcgccggccaagggaagcagggcgaagc
cgtacgcctactggtgggccggcacgccggggtaccgggcccctgagcagctcgcgggcaacccgagctacgggcccgccgtcgacatgtgggcgct
cgggtgcgtcatggcggagctgctcacgggggaggtcctgttcgacacggagacggaggaggacatgctcaacatggccatggacatgctcggcgag
ttcaccgagctcgggataagcgcgttcgacacgttggaggcgttccggggcctgccgcccgagatgtccatggccgggcgcgaggtccttgctggactg
ctcaacttcgacgccgccgagaggttgacggcggcggacgcgctcaagcatcgctggttcaccgaggatttgcctgatgatgacacgactcagacgcc
gccgccgccgcagagtctgagcgatgatcgtccgttcttccacttcttgtag
>g87188.t1
atggccgccattagctctcgcccctcccttctggttttgtcctgcctctactgctttgcgctgttgacaatccatgtccacccggctggctcgctatccttcaa
cctccgcttctcccaaccccaaagcccagatattgctcagttgataggctgcactggcgacgcgttcatctcgccggaaacgcttgagctgacacgaaac
aggcgcgatcaaagcagcacaaatagcaccggccgggcgaagtacaagcggcaagtgcggctatgggatgctgccaccggcgagatcgccagcttc

actaccacattctccttcaaaactactctggacccgagcacgcaacagcccggcgatgggatggctttcttcctcgcgcatttcgactcagatatccccgc
cgacagcgaagggggcggcttgggcctcctcccgccggtcaacggcaccggcaacagcagcatcatcgcggtcgagtttgacaccttttcgaatcgga
ggtacgccgatatcagcagcagccatgtcgccatcgacatcaactctctcaattccacggcttccacggacaccacctcacagacgaagaacctgactt
cgggttacacgatggtggcgactatcaggtacgaaaataccaccaaattactggatgtcgatctgaggatcaacgacacatcgtacaaggtaaccgcg
acggttgatctgagggagtacctgccagagcatgtcgccgtggggttctcagccgctagcgagcagcaccaggaccgcccggcggcgatcaagatgtt
ctcgtcggagtcgtcggcgcaggggaggaaagagtttgaggcagaggtgaggatcatcagccggctcaagcgtcgcaatctagtgcagctcttgggtt
ggtgcgacagtcgcaaggggctcctgcttgtctacgagctggtggctgagggcagcctcgacaagcacctcagcagctccaataagagattacataca
tggccagaaaggtaccagatcatccttggcttgggctgggcgctgcggtacctccaccatgagtgggagcagtgtgtcgtgcacggggacatcaagcc
gagcaatatcatgctggacgactcgctccgcgccaagctaggagacttcggcctggcccggctcggcgaacacggcgcgaggtggcacaccaccaag
gccgtgctgggcacagcggggtacatcgacccggagttcgtcaacacgcgccaccccagcatcgtgtccgacgtctacagcttcggcatcgccctgctc
gagatcgcctccggccggcctccggtgattctgcaggacagcaggccgcccttcatcctgctgaaatgggtatggagtctgtacggccggaacgccatc
ctcgacgcagctgatgagcgactgatgaggggcgacgagctcgacgagaagcgcatggagcgtgtgcttgtcgttgggctgtggtgcgcgcacccgg
accaaagcgaacggccgtcgatcgcggaggcaatgcaggtcctgcagtcggaggacgcgaggcttccggcgctcacaccgcagatgtacaaaacgg
tcacggatttcgccgtcgccggccgtgcgtacggtgctttgtccatcgaaagctacagtggtggatcctcgacaacgacgaccggcgactccaagctttc
ctccggctcgtccaccacagccttgctccgtgattcaaacgagagagcttga
>g87277.t1
atggctgagtcggaagcggggtcgtcgtcgaggcggcggacggaggaggaggaagaggcggtcgcgccggtgatggaggcggacccggcggcggt
tgggagggggaagggcgaggctgaagaggaggacgacgacgtggagagcagcgactacacgtcggaggacgagggcacggaggactaccgacg
cggcgggtatcacgccgtccgcgtcggcgacaccttcaagcagggcgcctacgtcgtgcagtccaagctcggatggggccacttctccaccgtctggct
cgcctgggacaccgcccgctccagatatgttgccctgaaggtgcagaagagtgctcagcactacaccgaagcagccatggatgagatcaagatattga
agcagattgctgatggtgacccagatgacactcggtgtgttgttaagcttcttgaccacttcaagcactcaggccctaacgggaaccatgtgtgcatggt
gtttgagtttcttggtgataacttgctcaccctgataaagtacacaaactatcgtggaatgccacttccgatggtcaaggagatatgtcgctacgtgctcat
tggccttgactacctccaccgcgagctttctattattcacactgaccttaaacctgagaacatattgctcgagtccaccattgacccctcaaaggatccatg
gaaatctggtgtgcccttggttctaccttctgcaaagacagatgaaccacccccaaaggcatcagcaccatcaacaaatggtggcctcacgaagaacc
agaagaagaagatccggaggaaagccaaacgtgcagctgttacaacttcagaaggcactggtgctgtggcatctgctgacacagatgggtcagacga
tagaggagatttgggtacagcaaatgagggcagtcctaggaaggatggagacaagaagagaggcacaggagatagacggggcggcaaagagaga
tatgttgccctgaaggtgcagaagagtgcgcagcactacacagaagcagccatggatgagatcaagatcttgaagcagattgctgatggtgacccaga
cgacactcggtgtgtggttaagcttcttgaccacttcaagcactcaggccctaacgggaaccatgtgtgcatggtgtttgagtttcttggtgataacttgct
caccctgataaagtacacaaactatcgtggaatgccacttccgatggtcaaggagatatgtcgctacgtgctcattggccttgactacctccaccgcgag
ctttctattattcacactgaccttaaacctgagaacatattgctcgagtccaccattgacccctcaaaggatccatggaaatctggtgtgcccttggttcta
ccttctgcaaagacagatgaaccacccccaaaggcatcagcaccatcaacaaatggtggcctcacgaagaaccagaagaagaagatccggaggaaa
gccaaacgtgcagctgttacaacttcagaaggcactggtgctgtggcatctgctgacacagatgggtcagacgatagaggagatttgggtacagcaaa
tgagggcagtcctaggaaggatggagacaagaagagaggcacaggagatagacggggcggcaaagaggggatgttctgtttgatccccatagtggt
gacaattttgaccgggatgagttttgttgcatag
>g87427.t1
atgggcacgtacgggtactgcgcgccggagtacctccgcacgggccggctcagcgccaagtccgacgtgtacagcttcggcgtgctgctcctcgagctc
atcacgggccgccggcccatcgacaccagcaggcccgacggcgagcagagcctcgtcgcgtgggccgccacgctgctctccgagcccaagaagatg
gtggacccgaggctcgtcgtcgccatgcaggcccccgcgcccgccgagctcaaccaggccgtcggggtcgccgtcatgtgcttgcaggggaaccacgc
gctgcgacccgtcatggccgacgtcgtcacctcgctcgcgttcctcgccgccaacactgatgattcatcttcttcttctttctga
>g87514.t1
atggcaatgtcgatcggtgagtggatacggggcccggccatcgggcgcggctcctcggccaccgtctcgctcgcggtggaccaccgcacgggcgccgt
cttcgcggtcaagtccgtcgcggacgccgcccgcgcggaggagctgagacgcgagcacagcatccttcaggacctcgactcgccccacgtggtgcggt
gcctgggtctggtggactgcgacctgttcctggagcacgcggcgggcgggtccctcgccgacgagatcaagcgtctcggtgccggcgtcgaggaaggc
ctgatccggtcccgggcccgcgacgtgctccttggtctggcccacgcccacgcggccggggtggtccactgcgacgtcaaggcccgcaacgtgctcctc
gccgccgacgggcgcgccgtcctcgccgatttcggatgcgcgcgccgaatcggcattagtggcggcaaggtaatgggcggcacgccgatgttcatggc
gccggaggcggcgcgtggggacgagcagggtcccgcggcggacatctgggcgctggggtgcacggtcgtggagatggccacgggcggcggcgccc
cgtggtggccgcggttcgccgaccctatggccgcgatgcaacaccggtggacggcggagcagctgctccggcacccgttcgtggccgccgctgctggc
gactccaacaattctgtcccaattgtcgctgggaaggaggagccgttcgtgtccccgaagagcgtcctcgaccaagcgctgtgggacgactccacggc
gtgcgtcactgccggcgcgagcacaatgaccgcaacggcgccgatcgacagagtccgtgaattggctgccggcgcgccggtggtgccggactggacg
tgggacgcgacctggatcaccgtccacagcgacgacgacgacgtgcgttcgacttcaccggaggctgagcatgtcaagatttctggtttttccttccccc
taccgattgagcgtggccatgtgaggcgcgtgactggcaattag
>g87686.t1
atggtacaccataaaagaaggtcgaacgaacttttcaaaaagaacggcggttcagtgcttcaacagctggaaaacctcaggattttcaccaaagagga
gctcaggagaatcacaaagaacaattctcatgtcctcggcaaaggaggctccggccacgtgtacaaggggattcttgaggacggcacaatggtggcg
gtgaagacggcaatcgagttcgacgaagctcgaaaggaggatttcactaacgaggtgatcatccagtcacagatgattcacaagaacatcatcaagct
cttgggttgctgcctagaagtcgatgtcccaatgctggtctacgaatttgctcccaaagggaacctctacgacatcctccatggcaacaaggccaaccgc
catcaacagcttcatctccctctggacttgcgcctggacatcgcagctgcatctgcagagggattgaaatacatgcactcgtccacgaataacaccatac
gacatggggatgtcaagtcagccaacatacttctggaccaaaacttcatgccaaagatctcagatttcgggacgtccatgctgctgtcgagaggcgagg
acttcaccgcgcttgtggtcgggagcatgggctacatcgacccggtgttcagccaaaccgggctcctgacgcagaagagcgacgtctacagcttcgga

gtcgtgctcctggagctgatctgcaggaagccaacaatatacaacgagcactgcagccttgtcattgagttccgaagagctcacgagaggaacggcag
tggaatggcgatgattgacgaggacattgcggtgacggaagaagacgtgtttgtgatggaggaaatgggtaggctcgcgatagagtgcctcgaagag
gaggttgaagaccggcctgaaatgaaggaggtagcggaacggcttgtgatgcttaggagagccaggaaccatggacagcggaactacgggagcccc
gctcattactgtcagaagaacaatgttggaggagccctcgggaccgatgtcagcagaactacaagcagcaccaccgtgccgttgattccagcaatgag
acaagaattccctaacctttag
>g87861.t1
atgcatgtgcatagcccataccacttgtacgtgtacgcggtgctggtgttcgaggcacttgccacgttcgtggggcaggtctccgtgctctccctcatcgc
gattttcggcgcggcaaccacagctctgacgacgacggcgaggaaggcggtgacgctgctgctgtcgtacctcatcttcacgaagccgctgacggagc
agcacgtcacggggctcctcctgataaccatggggatcgtgctcaagctcctgccggagaacaaggagaccggccagcggcggcaccaccattctga
cgatacgcaggggaagggcgacagagtggtagaagaggagaagacatcattgtacgagaagcaggagaagatcggggaaggcacgtacggggtg
gtgtacaaggcgctggacaaggtcaccaacgagacgatcgcgctcaagaagatccgtctggagcaggaggatgaaggcgtcccatccacagctatcc
gcgagatctctctcctcaaggagatgaatcatggcaacatcgtcagatacttcgaggcgttgcttactgtcattctcatagagttcttcatcgagatttga
>g87933.t1
atgtggcgcaacaagggcagcggcgcggcgtcgcagcggtggaccagcgacgagtccatctccatctccccgcgctgcgtcctcgacgacgactccac
caccacgagccagagcaccaccaccacgtcagcattccccgtcgtcacggcgacgtacaccgacccgccgccgtccacgtcaccgacgacgccgcca
cgtcaccagccgacgtcacgccgcgacgggcagtcgtggaaggcggtggcggaggcgtggcggtcgcggtcccgtcgccgcctctccgcgggcctcc
cgaccatgtcgtcggccctccgccgcctgagcatccgccggcccacggagcccaccaccaccacgacggactcggcccacgagttctgccgcgtggtg
cggcccacgctgcgcaccttcacgctctcggagctcaagaaggcaacgcgcaacttcagccgggacaacgtggtgggccggggcgggttcgcgcggg
tgtacaggggcagcctgccgggcggcgagctggtggccgtgaagcggctgacggggggccgggaccgggccgagggcttcctggccgagctgggcc
acgtcgtcaacgtcagccaccccaacatcgcgcggctcgtcggggtgggcgtcgacggcggggagcacctcgtgttcccgttctcccgcctcgggtgcc
tctccgggaagctccacgggaacggtggagcggcggcggcgatgacgtggcaggagaggtatagggtggctgtggggacggccagagggttggagt
atcttcatgagcggtgtgcgaggcggatcgtccatcgggatatcaagcctgcgaatatcctcctcatggacaactacgagccactgatctgcgacttcgg
tctggcgcggtggctgccggcgcagctgacgcacctgcaggtgacggtgttcgagggcaccttcggctacgtcccgccagagtacaccacgcacggcg
tgttcagcgagaagacggacgtgttcgcgctcggcgtcgtgctgctcgagctcctcaccggccggcgagctgtcgacgccgccaagctcagcctcgcga
ggcagttcttggacgacggcgaggaggacgagatccccaagatggccgacccagcgatgggcggccggtacgacggcaagcagctccgcgacgtcg
cgtgggcgggcaagctctgcatcaacacctcgccggaccacaggccgagaatgagccaggtcgtgcaaattctacaaggagtggggtcgtgccaccg
aagcgaggggcagcagcagctcagcaacggtgagctgcatcagcacggtatgaacggctatgatgccgtgcggtacacggacgatctgagccggcat
aagaagctggccttcgacttcgacgcggagtcgccccgttcatgtgcgggcacgccacgcgttaattag
>g88519.t1
atgtggttcacggagaaggggaagaaggcgggggcgtccaagcccaaggggaggaagcgggagttccccatccgagcggcggactacgagctgct
cgagcccatcggtgacggcgccaccgccgtcgtgcgccgggcgcggtgcctgccgcacgacggcgagatcgtagccatcaagatcatgaacatgtcg
cagcgcaccgagtccgacgtgatcaatgcgtcagaggaagtcaagaccatgatcatgactgaccatccaaatcttctcagtgcctactgctccttcaccg
agggtgaaaccttgtggatcgtgatgccctacatggccggtggttcgtgctaccacctgatgaaatcttcgtacccaaaagggttcaaggacgaaagctt
catagcgtttgtgttgcgcgaagcactgaaaggattagaatatcttcatgagaatggtcacatacatcgggatgtcaaggctggaaacatacttcttgat
caagagaaaggagtgaaattatcagattttggagttacagccaatgaatatgtatgttcgtattctaagtcagagaacaattacagggcagacatctggt
cctttggcataactgcagttgagcttgctattggtcatgctccattttctagtcaacctccagcaaaggtctttctcatgacattgacgcatgcccctccatc
ccttaacaacacaaaggagaagaaatttacagaagaggagtatataagaggtgtgagtgagtggaactttgatattgaagatttaagagcacaagctg
caaggtactctgatgaggatgaagcagaagctgttctacgctttgtatttgaaattgataccgtcaacgaaactagttcttcacaggagttacaagaaca
acgttgcactaagagtgatgagaaaatgatgtattattaa
>g88552.t1
atggtttggtggaggagaatcgtgttgttgttgctcctgttggtcgctgacgtcgcggccggtagtggtggtggcgatgcggaggccctgctgagtctcaa
atcctctttggacgggtcggatgtgctgccgtggcgggccgagacggtggcgggcctgtgctcgtggtgggccggcgtgcggcagtgcgacgggcagg
gccgcgtgaggaagctggtgctggagggcctgaacctgacgggttccctgacggcggcggtgcttgcgcttgcgccgctgtcggagctgcgcgtgctg
agcctgagatcgaacgccctgagcgggcccatccccgacgcgagagcattcccccccaacctgaagcttttgtacctcgccgacaaccgcctggaggg
ccgcatccccggcagcctcgccgccctccaccgcgccaccgtcatcgtcctctccggcaaccgcctctccggccagatcccagcgtccctggccgccatt
ccgcggctcacgtcgctgctcctggaccgcaacatgctcacggggcccatcccgccgctccggcagcgcacgctccgactgctcaacgtctccggcaac
cgcctctccggcgagatcccggcgtccctcgccaccaggttcaacgcctcctccttctccctcaacgcgggcctctgcgggcctccccctattctctgaggg
caacccccccgcgtgcaatcattttgttgcccaggcgccatcaggttcgaattcgaacgcggcggcggcggcggcggcgccgctgccctcactaggctc
gcgccatggcaacaaggcggccatcgtggccggcgccactgtcgcaggagcggtggtgctggccatcctggtggcggcggcggtggtggtcatcagg
agaaacaagaccagggttgtggtggacgacgcctcttctccaacttctcctcctcctcctactactactggagggcggaatggtaaacaagatttattac
acgaggacgaggaggcacaacctcctactagtactactactagtgctacaggaggaggaggaggaggaggaggcgtcgtttgggagcgcgagggca
tcggcaagctggtgttcacggagaatgagatgctctacaccctggaggacctgctgcgcgcgtcggcggagacgctgggccgcggcgaggtcggaag
cacctacaaggccatcatggagaccggattcatcgtcaccgtcaagcggatgcgagacccttccaactcatcaccatcgtcgtcgtccatggactttgcc
cgccgcgccgaggagctgggctggctgcgccaccccaacattgtgccgctcagggcatacttccaggccaaggaggagaggctccttgtctacgatta
ctacccaaacggaagcatcttcacactcgtccatggctgtcagggtcccgatttggcagagacagtcaaacttgacattcacagggctgatactggtact
ggatcgcgggcggcgagcaagggaaagcctcttcactggacgtcttgcatgaagatcacggaggacgtggcggcggggctgctgcatctccaccagt
cgagcatcgtccatggcaacctgaagccgtccaacgtgctcctgggaccggactttgagtcatgcctcaccgactatggactactcatgatgatgatgct
tcccgaccatggccatgctgcatcttcaacctcgctcttgtaccgggccccggaggctcatcgtactacagctttcacggcggcgtccgacgtgtacagct
tcggagtgctcctcctggagctcctcacggggaggacgccgttccaggacctcctcgtgttcatgaatgaggacgaccacataaccagctgggtgcgcg

ccgttcgtgaggaggagagggacaccgactcctcctctggtggtaataccggtgctgctgccgacgagaagctcgccgccttgatcggcatcgcggcg
gcgtgtgttgccgcggacccggaaaggcgacccaccatggcggaggtgctccggatggtgagagaggccagggccgaagcaatgtcctcctccaaca
gcagcgatcgctcacctgctcgctggtccgacgccatcctcgccgcgccgtcgcagccattttccatagactactga
>g88622.t1
atgaaggagttccttgcagagatagccattattggccgcatccgacatcgtaatcttgtgcaattacttggttactgccgaagaaaagatgaactccttttt
gtttacgcctacatgccaaatggtagcctcgataaatatctgtacgacagcgatctatgtacaggaactctgacctggttacagaggttccatgttctcaa
aggtgtggcaaccggattgctctatctccacgaggggtgggagaaggtagtcatccatagagatatcaaggcaagcaatgtgctcctcgacaaagaca
tgaacggccaactaggtcattttggcctggcaaggttgtacgaccatggcactgatacacaatccacacatgtggttggtaccatgggatacctagcccc
agagcataaacctatcaaggagaatgttcaaggcagcggacacaccgtgctggccgactgggtactccagcactggcgcaacgggtccctcatggcg
actgtggacaggaggctccagggcgattacaacgttgaggtagcaggattagtcctgaaactaggtcttatgtgctcacacccgtttccaaatgcaagg
ccggacatgggtcaggtcgtgagctacctcgacggcgtggtgccgcttcctgagctgacaccgacagacctgagcttcgacgtgccagcgatgatgca
gaaaaaagagtttggtatgtcggccgaatcgtatcctgatcttgtgacgagctttggcacaatctccagcatctctggaggaagataa
>g88888.t1
atggcagacaagggtaactctttcttgctcatgctgcttttccttgtgaccactgtgtcgatcgatcagccaacctccacggctgatgctgacactcacact
gaccgccttgcactgatgtccttcaagtcactcataaggagagacccatcatcggcactggcttcatggggtggcaaccagtcagtctctccgtgtcagt
ggcatggcatgacatgcggtgcgtgggggcggcggcgcggccgtgtggtggcgctggacctcagcaacctggatcttcttggcaccatgtcgccctcta
taggcaacgtcacttacctgagaaggctccagctccccatgaatcgccttggcggcaccataccttcagagcttggccgtctccttcaactcaggcatgt
gaacctgtgctacaatgctctcaaaggtggcatcccggcatcactgtcagactgcaagcaactagagaacatcagcttggcaatcaacaacctctcagg
agcaatacctgccgccatgggtgctctgtccggtctgcgcatactccaactgccctacaacaagcttgatggaccaatacctcacgctagcctgcaatat
ttgaagttggagggaaacttcctacaagggatgatcccggcatcaattgagcagctgaaaggcctcgaggtacttgatttttcacacaataacttctcca
gagacattcctcagtttcttggcaacatgaaagggctttctagattgaacctatcattcaactacttcgagggcgaagttcccaaagatggaatatttctc
aatggccaagacttcaaggctcttgtgtatgagtttctaccaaatggaaatttagaccaatggctacaccaacaacctgaagagaatgttgaagaaaag
gtgttaaatatcttcaaaaggctcagcattgccattgatgtggcttctgcacttgattatcttcaccaacacaggtcatcgccaattattcattgtgatctca
agccgaacaatattctccttgacagcgacttggtcggtcatgttggtgactttgggcttgcaaggatcctgcaccaagaccatagtgaaatgtcagaaaa
atcaagtggctgggctacaatgagaggaacaatcggctatgccgccccagacagcggcacgcacaagactcccacgcgacggcgtgcacggtcgttt
gatcgatgccggagcacgccgacgagccgctgctgccgcaacagcagcagagcggcccgcccccgccggaggcggaaggcgacgcgggcggccgc
cgcagctgccggcgcatcacgtggtccatcccttgcccttgccttcctcctcttcaagctcaactgcggcgtggccgtgtaccaaggccagcgcgtctccg
tcttcgtgagcgactgcctcctcggcctcgtcatctgcctcaagtactacgacaaggctgagcccggctcgcagctcagggaccggctcaaagtggctat
ctggttgcttatgacggcgcttgcccttttctttgcttacaacgtcgccaccgcaatgcaccccgccgtcgccgtggttgcctgcttgatggcgttgggcac
agtcgctggagggtcttactacctccctgatccatag
>g88933.t1
atgcccccgacggcgagtctgccgccgccgccgccgacctggcggcggacgagctgcagagcctcagtttcggctcgtccgagcgttcccgctccgc
gtccaccgtctccaccgccaccgcctcctgctccacctcctactccggccccatcacccctcccccgccgcgcgccgccaaccacctcttctcctcctccg
ccgccgctcccgtcccgcgcttcgggaccgtggcgctctccgacatccggtttttgaaaaggctcggcgccggggacatcggcagcgtctacctcgccg
aggtcaagcagcagcagcagcaggggagcaaagacggaagcagcagcggagtcctggtggcggcgaaggtgatggaccggaaagagctggcggg
gcggaacaaggaggggcgggcccgcacggagcgggagatcctggaggccgtggaccaccccttcctcccgcgcctctacggcgtcgccgaggggga
ccgctggtcgtgcctcctcaccgagttctgccccggcggcgacctccacgtcttgcggcagcgccagccgcaccgaagattctccgaggccgcagtcag
gttctacgcggcggaggtggttgccgcgctggagtacatccacatgatcgacatcgtgtaccgggacctcaaaccggagaacgtcctcgtccgcgccg
acggccacatcatgctcaccgacttcgacctctccctcaagtgcgaccccaccgtcgtccccacccccgcccacgtcatctccgaccccgtcgccctcac
cgccggccactactcatcatccaatacgtcatgcaccatctcctcctgcatcgtccccgctgtctcctgcttccagctcttcccgggggggccgtggccgcc
ggcgccgcaacaaaaagtcttcttccagtggcgctggtggtggaaacagtagctttccatctggaggcggtagtggcctggacctggagttcgttgccg
agccagttgagctccggtccatgtcattcgtgggcacgcacgagtacctcgccccggagatcgtctcgggcgagggccacggcagcacggtggactgg
tggacgctgggcgtcttcatgtacgagctcctgtacggcgccacgccgttcaagggccacgacaacgagacgacgctggccaacatcgtggcgcgcgc
gctcgagttccccaaggcggcgcccgttcccgtctcgtcggcggcgagggacctcgtcgccacgctgctcgccaaggacccggctcgccgacttggcg
ccaccgtcggcgcggcggccatcaagcgccacccattcttcagcggcgtcaactgggcgttgctccggtgcgccgcgccgccgtacgtgccgccgccgt
tcaccgtgaacagcgtcaaggctagtgctgctgataatgccaacgacgacgtgtcggatgatgacagttgtcccggcacgcccgtggagtactactaa
>g89559.t1
atggagggaggcggcggcggagcagggtgcctggggctgggccaaggcggggaggcgcagatcatcaagggcacgcacacccatggcggccgcta
cgtgcagtacaacatctacggcaacctcttcgaggtctccgccaagtacgtcccgcccatccggctcgtcggacgcggcgcctgcggcatcatctgtgct
gctgtcaacgcgcagactcgtgaggaggttgccatcaagaagattggaaacgccttcgacaatcagatcgacgccaaacggactttgcgagagattaa
gctgcttcggcacatgaatcacgaaaatgttatttcaataaaggacatcatacgcccaccaaggagggagaatttcaatgacgtttatattgtctacgag
ttgatggacactgatcttcatcaccttctgcgatcaaaccagccactaaccgatgatcactgccagtatttcgtctatcagcttctacgaggattgaagtat
gtgcactcggcaaatgtcttgcacagagacctcaggcctagcaatctgctgctcaatgccaaatgtgaccttaagattggggactttggtctggcaagga
ccacaactgaaaatgacttcatgatggagtatgtggttactcgatggtacagggcgcctgaacttctgctgaactgctgcgaatatactcaagctattgat
atgtggtcggtaggctgcatccttgcatcccttcatgagataaatgatgaacctgtttgtccagcacctttcagcttcgatttcgagcagccatcgctcacc
gaagaagatatcaaggagatcatatggaaagagtctctcaagttcaacccagatccaattcactaa
>g90117.t1
atggaggcgcagtggctcgccgagtacccgcaccagagcgccgacaaacggccgcgcaagcggccccggcttgcctgggatgttccgccgacgctct
tccagacacccaaggaactaattaatggtaattttgcacctccatttctgccaccgccacaatttttttacactggacctccgccgcggaatctgtcgcctc

cttggagaccagatgacaaggatggacactatgtgttcgcagttggagagaaccttacaccgagatataggatactcagcaagatgggtgaaggaac
attcggacaagttttggaatgctgggacttggaaaaccaagaaacagttgctatcaagattgtccgctccctccaaaaatatcgagaagctgctatgata
gaaattgacgtgcttcagagacttgggaagcatgacttcactggcaaccgttgtgtgcaaatccggaattggtttgactatcgtaatcatatatgtatagt
ttttgaaaggcttggtccaagcttgtatgattttctgcggaagaatagttaccgctcttttcctatcgaccttgttagggattttgccagacaaatattagag
tctgttgcatttatgcatgatttgcgacttattcacacggatctgaagccagagaacattctccttgtctcatcggagtctgttagggtgcctgattacaagg
tttctgtacggccacccaaggatggttctttctttaagaacgttccaaaatctagtgctatcaagctgattgattttggaagcacaacatttgagcaccaag
accacaattataatgtggaagctgatgagttccctgtgaacttccacttagattaa
>g90328.t1
atgaagggagccggcggtgctggcgatcgatcgagcaccaccggtggtgcggcggcggttgatgcgcgcgagaagggcgtgctccagggccggtac
gagctgggccgcgtgctaggccacggcaactttgggcgcgtccacgcggcccgcgacctccgcacgggccgcagcgtcgccgtgaaggtgttgtccaa
ggacaaggtggtccgggccgggatgatggagcagatcaagcgcgagatcgcggtgatgaagcgcgtgtcccaccccaacatcgtcgagctgcacga
ggtgatggccacgcgcaccaagatctacctcgccctggagctggtccgcggcggggagctcttcgcccgcatcgtccgcgccggccgcgtcagggagg
acgtggcccgccgctacttccgccagctcgtctccgccgtgggcttctgccacgcgcggggcgtgttccaccgcgacctcaagcccgagaacctcctcat
cgacgacaagggcgacctcaaggtggtcgacttcgggctcagcgcgctggcccaccacgcgcgcggcgacggcctcctccacacgctctgcggcacg
cccgggtacgccgcgcccgaggtgttcaggaacaagggatacgacggcgccaaggcggacctgtggtcgtgcggggtcatcctctacgtgctgcttgc
cgggtcgctgccgttccccgatgataatatcgcgtccatgtttaagaagatgagccgcggggattacaagtgcccgccctggctgtccacggacgcgcg
ccgcctcatccccaggctgctcgaccccaaccccgacacgcgcatcaccatcgcgcagctcgtcgagacgccgtggttcaccaggtcgtccgtcgcgag
accgcccatgcccatcgtggctgcggccgcggagcacccggcggacgatgctgcgacggcggagaaggacgagccggagacgctgaacgcgttcca
cctgatctcgctgtccgcggggttcgacctctcgccgctgttcgaggggcactcctcggcgacggcgcgggagggcggcgggatgcggttcgcgacgc
gggagccggcgagcggcgtggtctcgcgcctcgaggaggtggcggcgcgtggcggcgggcggatgcgcgtgaccaagagcggcgcgcgcggcgtg
cggttcgagggcgccgagggaggcaagggccgcctcgccgtggccgctgacatcttctgcgtcacgccgtcggtgctcgtcgtcgacgtcaagaagga
cgccggcgacacgctcgagtaccgctccttctgcagcgacgagctccgcccggcgctcaaggacatcgtctggggcgccgacccgcagccggccgcc
gccgccgtcgtgtga
>g90378.t1
atggagcagtacgagaagcaggagaagatcggggaaggcacgtacggggtggtgtacaaggcgctggacaaggtcaccaacgagacgatcgcgct
caagaagatccgtctggagcaggaggatgaaggcgtcccatccacagctatccgcgagatctctctcctcaaggagatgaatcatggcaacatcgtca
ggttacacgatgttatccacagtgagaagcgcatatatctcgttttcgaatacctggatctggacctgaagaagttcatggattcttgtccggagtttgca
aagaacccaacattgattaagggattttctggtcccactggacgtgcacaacatacaaaagatgctatccgcgagatctctctcctcaaggagatgaatc
atggcaacatcgtcaggttacacgatgttatccacagtgagaagcgcatatatctcgttttcgaatacctggatctggacctgaagaagttcatggattct
tgtccggagtttgcaaagaacccaacattgattaagaccaatgtcaatgttgagcagcaaactaatctgacaaaggcatag
>g90444.t1
atgtacaacaacgcctccaccgagttcctcttcaaagcctctctgtcttcacagattaattacaagaagtttgaagctgatgtggagtcaggagcatcgta
tagttcaaagctgattttgaatcaggattcaggagcaccagaaattttgaggctgactttgaatcaggtgcatcacaaaatggagcagtacgaggtgttg
gagcaaattgggaagggttcgttcggctccgcgctgctggtgaggcacaaggttgagaggaaaaggtatgtcttgaagaagatccgccttgcgcgtca
gactgacaggtgccgccgatcggcacaccaagagatggagctgattgcaaaagtaaggaacccttacattgtggagtacaaagagtcctgggtggag
aagggttgctatgtgtgtatcgtgatcggttactgcgagggaggggacatgtcagaggctatcaagaaggttaacggtaaccatttctcagaagagagg
ctgtgtgtgtggcttgtgcagctcctaatggcacttgattatttgcatgtcaatcatatccttcatcgggatgtcaagtgctcgaatatatttctcaccaagg
accaaaatataaggcttggtgactttgggcttgctaaaattctaacttccgatgatttagcttcttcagttgtgggaactccgacttacatgtgccctgaact
tcttgctgatattccatacggttccaaatctgacatctggtctcttggtaaagttcttcacaatattcagcttcatctcttgagagtattaattgctgaattccc
actaccatcttttgaagatccattgcattccatcacgaagatcatgtcgccatctatcattgaccaatccataaccaaggacaagtgcactttccaagtgct
tcgtagtgacactgaacactatgttgacactcctgatatcgaccttcttgtcgatagttcacccaggagctcatcagattggaagcaaaagaggttcaac
acgaggtcttaccaacagcgagctgaggctcttgaaggcttgcttgagttcagtgcacagttgctgcagcaagagaggtttgaggagctgggaatcttg
ctgaagccttttggacctggcaaggcgtctccaagagagacagccatatggcagctattttga
>g90518.t1
atggttgcgcaacgctgcggcggcagcttctcgctgttgctgctgctcggtggcgcggccctgctcgcggaggccgcgagggttgcgttctccgacgtga
caatgtcgccgccgccttcgccgtcgccggcagcggaaacgccgtttgttcccgatgtggcgtttcctcctgcggctataaggatccataccgagcaagc
taaccgttaccaaagacaaatcctccttgctgtcatcttggcacttgttgtggtcattgtgatgatggtttcagcaatttttgcatgggccttctggaggaaa
tctcagggagctctacactccaaggacatcaaaacatcaaacttcaatgcaaagatatcagattttggtctcgcaatatgtggtggaaaccttgacaaat
gcgccatgatgccttcaggaactgttggctatgttgcgcccgagtatcttcttgacggccaattaactgagaagagtgatgtgtatgcttttggggtggttc
ttcttgagttgttacttggaaggaagccggtggaaatgattggtgaaactcattgtcaatctattgtttcatgggacactaaaccaaaaacttttgtgtga
>g90827.t1
atggatgggaagcctgggtcctcgccggtgaacctcgggtggagtgcgcggctccgcatcgcacgcggcgtggcccggggcctcgcgttccttcacga
caacaagtgggtgcacggcaacgtgaagcccagcaacgtgctcctcgacgccgacatggagcccctcctcgccgacctcggcatccaccgcctcgtgc
gcaccaccgctgactccggcacccaccaacacatgtcgttggtcacaactacagcattcaagttcgggaccaagcgttccgcgaagagcctccccgac
caagcaccggcgagcccgcttccctccgacgacgacacggcggcacactaccgggcgccggagaagacgaagccgaccgccaagtgggacgtgtac
gcgttcggcgtgctgctgctggaactgctggccggccgcgcgctcaccagcctcgagctgtgccagtgcgccgccgcgccggaagagaataaggcgc
agacgatgatgcggatggcggactcggcgctgcgggcggagatgggaggcagggaggacgcggtggccagctgcctcaggctcggcgccgcgtgct
gcaatttggcgccgaccaagaggccgtccatgagggacgcgctgcaggccatcgacaggataccggctcttgcctctgctgctgctgcttcctcttcctc
ctcctccagggaggcagcaggagctcggtga

>g90828.t1
atggatgggaagcctgggtcctcgccggtgaacctcgggtggagtgcgcggctccgcatcgcacgcggcgtggcccggggcctcgcgttccttcacga
caacaagtgggtgcacggcaacgtgaagcccagcaacgtgctcctcgacgccgacatggagcccctcctcgccgacctcggcatccaccgcctcgtgc
gcaccaccgctgactccggcacccaccaacacatgtcgttggtcacaactacagcattcaagttcgggaccaagcgttccgcgaagagcctccccgac
caagcaccggcgagcccgcttccctccgacgacgacacggcggcacactaccgggcgccggagaagacgaagccgaccgccaagtgggacgtgtac
gcgttcggcgtgctgctgctggaactgctggccggccgcgcgctcaccagcctcgagctgtgccagtgcgccgccgcgccggaagagaataaggcgc
agacgatgatgcggatggcggactcggcgctgcgggcggagatgggaggcagggaggacgcggtggccagctgcctcaggctcggcgccgcgtgct
gcaatttggcgccgaccaagaggccgtccatgagggacgcgctgcaggccatcgacaggataccggctcttgcctctgctgctgctgcttcctcttcctc
ctcctccagggaggcagcaggagctcggtga
>g90918.t1
atgccggcgtggtggaagaagggcaggagcaagagcaagggcgcgggcgccgccgcggcggcggcgggctcgatttcgtttccggctggcgaagg
ggaggagaaggtgggcggcggcaataggaagaaggcgagcagcttcgacgaggcgctgctcgccaaggccggccgcgggaagcagcaagcggtg
gtagggcacccgctgccgcggccggcgtccttgccggcgccgctgccgtccccgtcggcctcggcctccgcgtccgcgtcggcgtccagcgggggcag
ctcgtcgctggggtcatcggcggcgtccgacgagccgccggatctcggggcgtacagtgaaggcggtcaaatgtgtgcaattaaagaggttaaggtca
tttctgatgattctaactcaaaagaatgtctcagacagcttaatcaggaaatcgttctgttaagtcagctgtcacatccaaacattgtgcggtactatggca
gtgatctgtctaatgagaccctctcggtctatctcgaagatattaaaggagcaaatatactcgttgatcccaatggtgacatcaagcttgctgactttggta
tggccaagcatatatcagcatacacttctatcaagtccttcaaaggaagcccgtactggatggctccggaggttattatgaatagtaatggctacagcctt
tccgtggacatttggagccttggctgcaccattctcgagatggctactgcaaagccaccttggagtcagtatgaaggggtagctgctatatttaaaattgg
gaacagcaaagacatacctgatatcccagaccatctttcttctgatgcaaaaaacttcttgaaactctgtttgcaacgtgatcctgcggcccgtcctacag
ctgctaagttgatggatcatccttttgtcaaggaccatgctacagttaggagttccaagtctagcatgacaagggatctgtttgcaacttctactgatggg
aaaaatagcacgaaggagatgatcttcaggatgcagacaaccactgaatctgagaggagtagagctatgaaagccgctgcttcaattaaaggggtgg
aatctgtgacactcgccggcgaggaaaagaacctgctgctgataatcggcgttggacttggcctgagccacatcaacactttgtccaaaaaggtgggcc
acgcagagctgatcgagttgcggacctcgggcttcctctccaccgtgcacgcgtaa
>g90959.t1
atggacgcgccggccgtgatcgggcagtggacgcgggtgcgcacgctgggccacggcgcgtcgggcgccgaggtctacctcgccgccgacgccgcct
cgggcctcctcttcgccgtcaagtccgtcccgccgcgcggggcggcggcgctggcgcgcgagcatgaggtgatgtcctgcctgccgcgctcgccgcac
gtggtcgcgtgcgccggcggccgcgccgcccgcgacggctcgtaccagctcttcctcgagttcgcgcccggcgggtcgctggccgacgccgcggcggc
ggcgatgagggacggcgggcggctcggggaggacgaggtcgggcggttcgccgccgacatggcgcgaggggcctcgcgcacgtgcacgccgccgggg
tcgtgcacggcgacgtcaagccgcggaacgtcgtcgtcggggccgacggaacggccaagctcgccgacttcgggtgcgcgcggagggtggagaagg
ggtcgccgatcctcggcggcacgccggcgttcatggcgccggaggtggcgcgcggggaggagcagggcccggcggctgatgtctgggcgctgggctg
cacggtcgtcgagatggccaccggccgcgcgccgtggagccacgtcgtggacgacgtggtcgccgtcgtgcggatgatcgggtacaccgacgccgtg
ccggacgtccccaagtggctccccgcggaggcgaaagatttcctgggcaagtgcctgtgccgagacgccagcgagcggtggacggcggcgcagctgc
tggagcaccccttcattgccgccgccgccggctgcgacgacgcgacggcggaagtcgactgcggcaagggaaaatgggtgtcgcccaagagcacgct
ctgtttcgacgcggcgttctgggagtccgacaccgacgacgaggacgacgagctctcacacggcgcggcggagaggatcaagtcgttagcatcctctg
tttctaccagtctgccggactgggtctccgacgacgacgagggttggatcgatgtcatctccaccccaaccgaatcgtcttcagactacgcggtgcccgt
cccggcagaagggaagaccgccattgacgacgctgttgtcagcaaagaagaagaagaagaattgagcaacgcggagatatttttgttccggttccgcg
gctaa
>g91052.t1
atgcttatcctgcctttgcactacttggccactctgctcctgctcatcacgtcggcacgccgctgcgctgcggacgacgctactggcgtccatggtggcca
gttcgtctacaacggcttcaaggatgcgaacctggacctcgacggcgcggccatggtgacgccacgcggcctcctggagctgaccaacagtaccctcc
agatgaagggccacgcgttccacccgtccccgctgccgttccgggacccgagctcccagaacaccaccgccgcgcggtccttctccaccaccttcgtgt
tcgccatctacgggcaatacgcggacctgagcagccatggcatggccttcttcgtcgccgcgagaagagaggtgctgtccaccacactgcccagccag
ttcctaggccttctcaacagcgccgacaatggcaaccggagcaaccacatcttcgccgtggagctagataccatagccaacgccgagttccatgacatg
agcagcaaccacatcggcatcgatgtcgacagcctggtgtcggccaaggcggccaacgctgggtactacgacgacggtacggggcagttccacaatc
tgagcttgattagccggcaggctatgcaagtctgggtggactacgacggcggggccaaacagctcacggtgaccatggcccctctaggtgtgaccagg
ccgaagaagccgctgctgcggaccgtcgtcgacctctccgacgttctacagggcctggcctatgtcgggttcacgtctgcgacaggcgtactcttgtcac
gccactttgtgctcggctggagcttcgccctggacgggccggccccggagctgaacattgcagcgctgcctgccttgccgccagcggggcctaagcccc
ggtcgaaggtgttggagatcgtgctgccgatagcgtcagcgacgctggtcttggccgtcggcgttgtgatttacaccttggtacagcggaggctcaaata
cgccgagctgcaagaagattgggaggttacctatgggccgcacaggttttcctacaaggacttgttccgtgcgaccaaggggttcagcgacaagcaatt
gctggggacaggcgggtttggaagagtgtacaaaggcgttctccgcaagtccagcatggaggtcgcggtgaagaaggtgtcacatgagtccaggcag
ggcatgaaggaatttgttgctgaggtcgtaagtattggacgactgcggcaccgcaacctcgtgcagctgctcggctactgccggcgtaaaggtgaactt
ctcttggtctacgattacatgccaaacggcagcttagacacatatttgcacgcgcgaagcaagggtacactagattggtctcaaagattgcagatcatca
gaggagtggcgtccggcctgctgtatctgcacgaggattgggagcaggtcgtcatccatcgcgatgttaaggcgagcaatgttctgctggacagcgag
atgaatgggcgattaggagattttggccttgcaagattgtatgatcatggaactgatgcacagaccacgcacgtggtcggcactatggggtacctagcg
ccagagctaggacataccggcaaggcagctccatcaactgatgtctttgctttcggcgcgttcctttttgcaaatcaattgtgggcgacggccagtcgcatt
agatgagcaaaacaatcgtgtattgctcatcgattgggtgattgaacaatggcacaacggactgatcatcaacgcggtagacaggagcatgcccagtg
gcttcaatcctgaagaggtctctctagtactaaagcttggtttattatgctcgcatccattgccggtcacgaggccaactatgcggcaagtcatgcagtac
cttgacggtgatatgcccctcccagacttggcacaggcctacctcggcctcgccatgctgaagagtatgcatagcacagagttcaagcataatgtgatat
catgtgtatcatcatcgacaagcgtgggtgccatttctaatctctcgggaggaaggtga

>g91053.t1
atgtctgtcttgattgtatccttcatcgtcctgctcttcctcacgcacgtgccctcatccgtcggcgacggccagattttgtaccaaggcttcgcctcagcaa
accttagtctggacggcaacgccgtcgtaacacccgccgggctcctgatgctcaccaacaccacggaaatgcagaaaggccacgcattccaccccact
cctctccacttccttaacaagtccgctgcaacggcgacggcgcggtccttctcgtcgagcttcgtcttcgccatcgtgcccacccgcgaaggactcagcg
accacggcatcgccttcgtggtctccccgaccatgaacttctccaaggcaaacgccggtcagtacctcggcctcttcaacgccacaaacggcacggcca
acgaccgcatcctggcggtcgagctcgacatcatcatgaacccagagttccacgacatcaacagcaaccacgtcggcatcgatatcaatagcctcatct
cggagcaggcccagccggccggctactacgatgatgcagcaggcggtgccttgcaagaactgaagctaaacagccgtaggccgatgcaagtatgggt
ggactacgatggcaaggccaagcagctcaacgtgacgctttctccggtgaaaatgtcaaagcccaagagacctctactctccgctactgtcgatctatc
cagtttcatggcggacccgatgtacgtcggcttctcgtcggcgacgggcatcgagtccacgtaccattacgtgctcggatggagcttcaacttggacgga
ccagctccccctctcgacttctccaagctccccgtcgtgccacgcgtgggtccaaagccccggtccaagctcttggacatcctgctgccgctagctgcag
cattgctcgttgccgcagcgcttgctgccgtcttcttcttcctgtggagaaagcggcggtatgccgaggtgcgcgaagattgggaggacgacttcgatcc
acacctgttctcatataaagatttgtttcacgccaccgatggtttcaaagatagaaatctacttggagttggagggttcgggagagtgtacaaagggcag
cttccggcgtccaatttggagattgcagtgaagaaagtgtcccatgattcaagacaaggggtcaaggagttcgtcgccgaggtagtgagcattggctgc
ctccgacaccgcaaccttgtgcagttactaggctactgccgacgtaaaggtgaactgctgttggtgtacgattacatggcaaatggcagccttgacaagt
atctacatgatcaacacatgccgactctttcttggcacacgaggtatcaaattatcagcggagttgcagcaagcctagtgtatctacacgaggactggga
gcaagttgtaatccatcgagacgtaaaagcaagcaatgtgctactggacggcgagatgaacggcagactcggtgacttcggcttggctagactatatg
accatggcactgatcctcagacaactcatgttgtgggtgccatggggtaccttgcgccagagctcgtgcgcacggggaaggcaacaccctttacagatg
tgtttgcgtttggtgtgttcctcttggaggttgtgtgtgggagaaggccaatttaccgcgatgactgcgacagccgggtcatgttggtagattgggtgattg
agcatcaccacaacggctcaatcctggacgtggtggatccaaggatagtcgggaagttcgaaacagaggaggtcatccttgtgctgaaactgggtttg
atgtgtgcacacccattgcccaacgagaggccaagcatgcggaccgtcatgcagtacttggattccagggaaccggttcctgatctgtcaccaagctat
atgagctatggtacaatgacgctgatgcaaaacgaagggtttgattcgtacgtcatttcatcccatccatctatgacaaacggcgtatcctctatgactgt
tctctcaggtggaaggtga
>g91054.t1
atgaacggcagactcggtgacttcggcttggctagactatatgaccatggcactgatcctcagacaactcatgttgtgggtaccatggggtaccttgcgc
cagagctcgtgcgcacggggaaggcaacaccctttacagatgtgtttgcgtttggtgtgttcctcttggaggttgtgtgtgggagaaggccaatttaccgc
gatgactgcgacagccgggtcatgttggtagattgggtgattgagcatcaccacaacggctcaatcctggacgtggtggatccaaggatagtcgggaa
cttcgaaacagaggaggtcatccttgtgctgaaactgggtttgatgtgtgcacacccattgcccaacgagaggccaagcatgcggaccgtcatgcagta
cttggattccagggaaccggttcctgatctgtcaccaagctatatgagctatggtacaatgacgctgatgcaaaacgaagggtttgattcgtacgtcattt
catcccatccatctatgacaaacggcgtatcctctatgactgttctctcaggtggaaggtga
>g91102.t1
atgcaccgccagaaagttgctgaggttgaaaagctcacgcagactgtcagagagctagaagaagctgttcttgctggtggtgcggctgcaaatgctgtg
agagactatcagcggaaagttcaggaaatgaatgaggaaatgaaaacccttgaccgtgagctagctcgtgcaaaggtttcggcaaatagggttgcagt
tgtggtggcaaatgagtggaaagatggcaacgataaagtaatgcctgttaaacaatggctggatgagcgaaggattctgcaaggagaaatgcagcag
ctccgggacaagctcgctatagctgaaagggctgcaagatcagaagctcaactaaaagataagttccagctacggcttaaagtacttgaggaaggatt
gagaatgtctgcaccccggactagtgtctgtgccacacggcgccagtctattggaggtgctgatagtttaaccaagagcaatggttttctgtctaaacgc
acatcattccagatgagatcctcagcatctaccacaacaactctagttaaccatgcaaaaggggcatctaagtcctttgatggaggctgtagatcacttg
aacgttacaagggacttgtaaacgggaacattatgaatatatcgactgattccagtgaagacaaggagtcaaacaactcagatgagaagcccagtgaa
tttccgtccacggagtcagaggacaaggttgccatcaaattcgagcacactagcagcaagggctgcaactacggccctccaggcgagtggcaggtcta
tgccatgtcagcagaaatggtggcctgtattgcagtggagtccatttccatcctcgaaagcatgcattcgaaagggtatgtacatggagatgtcaaacct
gagaactttcttctcggtcagccttcgactcctcaagaaaagaaactttatcttgtggatcttggattagggtga
>g91554.t1
atgggcaagtcgacgtcgtggaccgcgatcttcggcggccttggctgcttcagctacttccacgccgaccgctccggcgaccccagcaagaacaacaa
cacgcccatgccactaccgtcaccggaggacctgtctctttcactggccgggttgacgcccgacgtgatggcgttcacggtggaccagctccgggcggc
gacgcgggacttctcgatgagcaacttcgtcggcgagggcgggttcggcctgtatacaagggccgcgtcgatgagcggatcaagcccggcgtgcgg
aagccgcaggccgttgccgtcaagctgctcgacctcgagggctcgcagggccacaaggagtggctggactacacggcaaaactgtcagatttcggcct
agctaaggctggacctggggaagacgagacacatgtgtccactagagtcatgggcacgcagggctatgccgctcctgaatatgtcatgacaggtccag
ctactggcgggcctagccaggggaatgttgcaaggccatga
>g91599.t1
atggacaacgccatggacggtaacaacagcagaagcaatgggaggagcacgacgaagagcagcagtagtagtaataacaagaagaacctgcttct
gggcaagtacgagctgggccctctcctgggccgcggtaccttcgctaaggtgtacctggcccaccccgtcggcgccggcgacaacagcccgccggtgg
ccgtgaaggtgatcgacaaggcggaggtggcgcgtacgccgggcatggcgccgcgcgtgctcggtgaggtcgccgccatgcgccgcctccgccaccc
gcacgtgctccggctccacgaggtcctcgccacccgcgccaaggtctacctcgtcatggagctcggccccggcggcgacctcctctcccgcctcgcctcc
ttgccgccgtcacggaacggccgccggtgcctccccgagcacgcctcccgccgcgcgttcgcgcagctcgtcgacgccctggcccactgccacgcccg
cggggtggcccaccgcgacgtcaagccccagaacgtcctcctcgacgccgacggcaacctcaagctctccgacttcggcctcgccgcgctcaccatca
ccgacggcgacgaatccgactcctccgactcggcgtccggcggcggagacctgcttctaaggacggcgtgcgggacgccggcgtacgcggcgccgga
ggtgctgcggcggcgcgcgtacgacggcgcgaaggcggacgcgtggtcgctgggggtggtcctcttcgtgatgctcgcggggaggctgccgttcgacg
acgccaacgtggcggccatgtgcgcgcggcagcgctgccgcgaccacgcgccgctgccggactgggtgtccccgccggcgcgccgcctcgtgcgccg
cctgctcgacccggacccgtccacccgcctcgccgtcgccgacctcgccgcgaaacacccatgggtcatcaggcgctcgctcagcgtcgactcgcggct
cgccgggctcgtcgccggcgcgccggaacgcgccgccgagttccgtacgccggcggcggtcaacgcgttcgacatcatctccatgtcgccggggctcg

acctgtccggcctgtttaacaccaacaaggggaacggcgataagaagaggttcttcatgaccacggcctcgccggagacgacggtggagcggcttggt
cacgccggcggcaagctcgggtacgtcgtggtgccggggacgaagaaaggagtcgactgcctgacgccggtggggccaggacgcgcggcgatgatg
tcggtggagatgtcggaggtggcgccaccgctgatgctcgtcgagctctggatcgacggcgatgaagagcggcagaggttcgggtgggaggagctga
ggcaggagctcggggacgtagttagggcctggcatagctgtcaagacttctga
>g92145.t1
atgggttcgtcggcgagttcgtgctgcggttccgagaaggtggagcaagggtgtgtgaactcgtcgtcgatgagcagcagcacgtggaggatattcag
ctacaaggagctgcacgcggcgaccaacgggttcagcgaggagaacaagctcggcgagggcggcttcggcagcgtgtactggggaaagacctccga
cggcctccagatcgcggtgaagaagctgaaggcggcgagcaacaactccaaggcggagatggagttcgcggtggaggtggaggtgcttgcccgtgtg
cgccacaagaacctcctgggcctccgcggctactgcgcgggagccgagcagcgcatgatcgtgtacgactacatgccgaacctgagcctgctctcgca
cctccacggccagttcgccggcgaggtccgtctcgactggcggcggcgcatcgacgtggccgtgggctcggcggagggcctcgtgtacctgcaccacg
acgccgcgccgcacatcatccaccgcgacatcaaggccagcaacgtgctcctggactcggacttcgcgccgctcgtcgccgacttcgggttcgccaagc
tcgtgccggagggcgtgtctcacatgaccacccgtgtcaagggcacgctgggatacctcgcacccgagtacgccatgtgggggaaggtctcgggggcc
tgcgacgtctacagcttcggcatcctgctgctggagctcgtgtcgggccggaagcccatcgagcggctgccgtccggggccaagcgcaccatcaccga
gtgggccgagccgctcatcgcgcgcggccggctcggggacctcgtcgacccgcgcctccgtggcgcgttcgacgcagcgcagctcgcgcgcgtcgtcg
agtgcgccgcgctctgcgtgcagggcgagcccgaacggcggcctgacatgcggaccgtcgtgcgcatcctccgcggcgacagcgacgccatcgccgt
tgtgggcgaacagccggtgaggatgcagagcgtcaagtacgcggaccgactcatggagatggacaagagcagcgacgacgaggacgacgaagaca
ttgatgacgatgacgatgacgatgacgacgaggaggtggaggagtactctctgatgatggacgacaagagcagcatgaacttcggggcgttcggcgc
catgccggtgcagaccatgaacgacccctacgccaagaggttttcatcaggaaatgctaatcatcatgccatcaagatatga
>g92146.t1
atgggttcgtcggcgagttcgtgctgcggttccgagaaggtggagcaagggtgtgtgaactcgtcgtcgatgagcagcagcacgtggaggatattcag
ctacaaggagctgcacgcggcgaccaacgggttcagcgaggagaacaagctcggcgagggcggcttcggcagcgtgtactggggaaagacctccga
cggcctccagatcgcggtgaagaagctgaaggcggcgagcaacaactccaaggcggagatggagttcgcggtggaggtggaggtgcttgcccgtgtg
cgccacaagaacctcctgggcctccgcggctactgcgcgggagccgagcagcgcatgatcgtgtacgactacatgccgaacctgagcctgctctcgca
cctccacggccagttcgccggcgaggtccgtctcgactggcggcggcgcatcgacgtggccgtgggctcggcggagggcctcgtgtacctgcaccacg
acgccgcgccgcacatcatccaccgcgacatcaaggccagcaacgtgctcctggactcggacttcgcgccgctcgtcgccgacttcgggttcgccaagc
tcgtgccggagggcgtgtctcacatgaccacccgtgtcaagggcacgctgggatacctcgcacccgagtacgccatgtgggggaaggtctcgggggcc
tgcgacgtctacagcttcggcatcctgctgctggagctcgtgtcgggccggaagcccatcgagcggctgccgtccggggccaagcgcaccatcaccga
gtgggccgagccgctcatcgcgcgcggccggctcggggacctcgtcgacccgcgcctccgtggcgcgttcgacgcagcgcagctcgcgcgcgtcgtcg
agtgcgccgcgctctgcgtgcagggcgagcccgaacggcggcctgacatgcggaccgtcgtgcgcatcctccgcggcgacagcgacgccatcgccgt
tgtgggcgaacagccggtgaggatgcagagcgtcaagtacgcggaccgactcatggagatggacaagagcagcgacgacgaggacgacgaagaca
ttgatgacgatgacgatgacgatgacgacgaggaggtggaggagtactctctgatgatggacgacaagagcagcatgaacttcggggcgttcggcgc
catgccggtgcagaccatgaacgacccctacgccaagaggttttcatcaggaaatgctaatcatcatgccatcaagatatga
>g92161.t1
atgcagccatcacgctcccagcacgccagcatcaagccgcagccgcagcatctgccgagatcccacacgcgctcgcgcagccaggcgcagtcggctg
tcgcgccgctggatccgggctccgacgactccgagtaccaggatttccggttccacttcgtccccgaggtgttcgacctccacccgatgggcggcggcg
gcgtcggtgggggagagaggaagaacggcggcgagattcacaaaacgacgacgacggagaaggtcctgtccttcgagttcgacaaggtgaggctca
gcattgcgtcgtccagcgacgaggaggaggagttgggggaggcgccgccccggagctccttctccggcgccagccacccgccggagccggtggacg
agatgatggacaccgtcttcgtcgccgtcaacaccaccaccaagccggcaccgacggccaacaacaacaagccgtgggacgcgtccccgccgccgcc
ggagagcggcgcggcgagcccgcacggcagcagcagcacggacagctccagcggcgcggcggccacggtcaccagcgccgcgccgagcttcgcgc
tcaccagcgccaagaccagcgtcagcagcagcagcgccgccagcgacggcagcggctggaccaccagcaccgccggcgccagcaagccgcacaag
ggcggggacccgaggtggaaggccatcctggcggcgcgcgcaagggacggcccgctcgccatgggcagcttccgcctgctgcgccgcctcggctgcg
gcgacatcgggacggtgtacctgtccgagctcaccagcgccggcggcgggaaggagaggccgtgctggttcgccatgaaggtgatggacaaggcgtc
gctggagagcaggaggaagctcagccgcgcggagacggagcgcgagatcctgcagatgctggaccaccccttcctccccaccctctacgcgcacttc
gagacagacaggttcgcctgcctcgtcatggagttctgccccggcggcgacctacacgcgctccggcagcgacagccgggcaagcatttccccgaaca
cgccgcgaggttctacgccgcagaggtgcttcttgctctagaatacctgcacatgcttggagtggtgtacagagacctgaagccggagaacgtcctggt
cagggaagacggccacatcatgctctccgacttcgacctgtccctccgctgcgccgtctcgccaacgctggtgcgatcgtccccgcacgccgacccgca
gacctgcgcgcagccggcgtgcatccagcccacatgctttatgcccaggctcttccacccgcggagcaagaagaacaccaccaagaaacccaagagc
agcagcagcagcgacagcaagcatcagcaccaggcgtccctgggccttcccgagctcgtggtggagccgacgggcgcgcggtccatgtcgttcgtgg
gcacgcacgagtacctcgccccggagatcatcaagggggaaggccacggcagcgcggtggactggtggaccttcggcgtgttcctgcacgagctgat
gtatgggaagacgccgttcaagggccagacgaaccgcgccacgctgttcaacgtcgtgggccagcagctcaagttcccggagtgcccggtcacgagc
aacgccagcagggacctgatcaaggggttgctggccaaggagccgcagagccggcttggggtgaagaggggcgcagccgagatcaagcagcaccc
gttctttgaaggcgtgaactgggcgctcatacggtgcagcacgccgcccggcgtgccgagggccgtcgagacagttgcggtggcggcggtgcccgtgg
cgaagccgccgccgccgccggtggagagagtggcgccgccgatgaacaataacagcagcagcaagaggatggcaggtggtgcagatgtggagtctg
gagggaagttcctggacttcgagttcttttag
>g92367.t1
atgacagcgctgcaacccctcctccttctcccggcgctcctcctcctccagctcgcctccgcgtccacgctcgccatctccggcgacggcaacccgccca
ccgtctgcggcgtcgcggaatacaacggcacgctcctctgcgcgccgctcctcacgagcggcagcaccaattcctcctactccgtcgcgccgttcctctc
cttctccgaggtctctgccggccgcgacttcacatgcggcctcaccgccctcggcgccgcgctcttctgctggccgcccggcgcctcctccacccagctcc
gccgcgtctacaacggccccaacccgctcgccgacctcgccgtcggcgccgaccacgtcgcggcgtacgaccccgccgcgcgccggatccgatggtg

gcgtggcggcgtgggccgccggttcccgcagttcgcctcgggcgcgttccgctccctcgtgtccggcgacgggttctcctgcgccgtccagaccaacga
ctccgccgtccgatgctggggcccgcgcggtagcgccgtgcaggccgccttctcctcccccctcctccaccaccaccaacaagtcctcctactcctacctc
gccgccggcgggacccgcgcctgcgccgtcgagggcaccaccggcgccgtccactgctcgggcgggacaaccgtaccggtggtcccgggtgatctgtt
cccgtacgggctcgccgtgggcgactcccacgcgtgcgggctcctccggcccaaccacaccgccgcgtgctggaccctcggcgacaccaccccgacgc
tgtactacccggccctcggcaccccgttcgagctcctcgtcgccgacggcaactccacctgcggcgtcacgtccgccgacttctccgtcctctgctggtcg
tcgacgagcatcgaggccgccaccacgccgctgctgcgcgcgggggtcctccccggcgcgtgcgtcgccgacgcgagcgaatgcgactgcgggttcct
cccgcagtcggcccagctctgcgcggggtccggcggcggcgtcgtctgcaaacgcctctgcgacgccccctcgccgccgtcctctcccccgccgccgcc
gccgccgccggagacggcgagtagtggtaaggatgacaggaggagggtgtcggggcggtgggtggcgttcgtggtggtgggcgcggtgggctgcttc
gcgggcctctgctccatcgtctactgcctcctcttcggcttctgcagcaacaagcgcgtccacaactccgtccagcccaacatcaccaccattgctactac
cccggccgccgctgacactaacaacaacaacaacaacggcgggcctggaggaggcccaagcccatacgggtccccgaacgggtcgcgggcccgca
acatcttccgtcggcagctgtcccgcgtgatgacgcggcagcgcagcgggccctcctccttcaaggacgggcactcgtcgtcggtcccggaggagttca
gcttcgcgcagctggacgccgccaccaaggggttcgcggcggaggcgaagatcggcgagggcagcttcggcgcggtgtaccgcggcaagctccccg
acggccgcgaggtggccatcaagcgcggcgtctcctcgtcgacgacggcggcgagaaggttccagttccaggagaaggagagcgcgttccggtcgga
gctggcgttcctgtcgcgtctccaccacaagcacctcgtcgggctcgtcgggtactgcgacgaggacgacgagcggctcctcgtgtacgagtacatgaa
gaacggcgcgctcttcgaccacctccacccaaaggcaatgtcagagtcgtcgtcgtcgccggtggtggcgtcgtggaagctgcgcgtgaagatcctgct
ggacgcgtcgcgggggatcgagtacctccactcgtacgccgtcccgcccatcatccaccgcgacatcaagtcctccaacatcctgctggacgccgcgtg
gacggcccgcgtctccgacttcggcctctccctcatgggcccccgcctgaaacggagcacgagtcggcccaccactcggcggtgaaggccgccggca
cggtggggtacatggacccggagtactacggcctgcaccacctcaccaccaagagcgacgtctacggcttgggcgtcgtcatgctggagacgctcacg
gggaagcgcgccatcttcaaggacgaccgcgacggtgggagtgggacccccgttagcgtggtggaccacgccgtgccggccgtcgtggcgggcgag
ctgggccgggtgctggacccgcgcgcgcccgagcccgcgcccaacgaggccgaggcagtcgagctcgtcgcctacaccgccgtgcactgcgtcaggt
tggaagggaaggaccggcccgccatggccgacgtcgtcgccaacctcgagacggccttcgcgctctgcgaggggagtgctggtgctggtggtggtga
gcgcggcggcgggttcgggaatagctcctccagcgccagcatctctgtcacgtccatggaccgctccggagcgctctag
>g92705.t1
atggcgcgacgacgagcaacagataagggcggcgtgttattcaggggccagctccccgaccgcactaccatgatcgcggtgaagaagctggagggg
ctcggtcagggcgagaagcagttccgcgcgcggagctcagttggagtgcgcggcacgggatcgccgtcggcgtggccaaggggctcgcctacctaca
cgacgagtgccgagtccgcatcatacactgcgacgtcaagccggagaacgtcctccgagttcgcgcccaaggtggcggacttcgggctcgccaagctc
ctcggccgggacttgagccgggtgctcaccaccatgcgcggcaccgtcgggtacctcgcccccgagtggatcggcggcgaggctgtcaccgccaagg
ccgacgtgttcagctacggcatgatgctgttcgagattgtgtctgggaggaggaacgtcgtcgggcacgggaagaccgattcatcatcgtcggggacta
gcggcgaggccaacaccgcagctgcggaaaccaccacgttcttccccgtgctggcggcggccgaggaggtgaggcgtttattgaaaacttgtaaaata
ctgacaagaaaatag
>g92835.t1
atggttttgaataagccttacatgatgaaaatacgagttgtacgatcagaaactgcaatgacagatgttcttcgagaggatgatgatgatataatttgga
gatctcctggtacacctgttttcactgcaccagagtgctgtcaaggttcagcctaccatggtaggccatctgatacatgggcagttggtgttactttatatt
gtatgatttctgggcactatccattccttggagatacattgcaggaaacttatgataagattatgaacgattctgttcaaatacctgacaacacgaacccc
caccttgctgatctgctccaaaggcttctctgcaaagatccaggagatcgcattaccctgcaggccgtggctgaacacccttgggttgttggggacaagg
ggccagttcctgaatatatctgtagatgtggctttggccgcaggaagattgattttcaggaacaagaacaataa
>g93216.t1
atggagatggcgggtgcggcagaggcggcgacgccgggtcaggagatgcacaaggggcggagcaacaccatcctcctccccatcgtcggcctcctct
tcgcctacctcgcgtaccgcctcctccgcccgcgcttcccggccctccgccgcctcacggaccgcctcccgttccgcgcgccggcgtgcctccgccgccg
cgcgcccagcaacgtgctgctcccttacttcgtccccatcgccgaccgcctcggcgcgctgccgtacctgggccccatcgccgagcggctcggccaggg
gggcccccacaaccaccgcgccgaggcgctcgtcaagttccccggaggagaaacgctgtcggtggctgccatcctcgaggcgcccggggaggtggtg
gccaagtccgcgcacagcacgctgtaccgcgccgccgtgcgcgccggggaggccgccgtgctgctccggttcgtcaggcccgcgtgcgccgtcgggg
cggaggaggcgtccgccgtggcgaggcggatcggcgccgtcacccacccgaacctcgtgccgctccgcgcggtgtacgtcgggcccaaggggggaga
agctgctcgtgcacccgttctacgccgccggctcgctccaacgcttcctgcaaggcttggaaaaaccgatggttcatggcaacctcaagacaagcaaca
tactgcttgatgctaactatgagtccaagatatcggactacggactataccttctgttaaatcaggccgctacccaggagatgcttgaagcatctgcagc
gcagggatacaaggcacccgagctgttcaagatgaaggacgcctcgcgggagagtgacatctacagcttgggagtggttctacttgagctgcttgctca
aaaggagtccgcagatgataaccggccaaaggcaagggacatccatttgcccgtctcctttaaaaacctggttcttgagaggaagatgtccgacgcgtt
cagctctgatcttgccaagcactgcaagaagtcagggaaggagaagaatctgaatgcgttctttgagctggccactgcttgctgtagcccttcaccatca
ttgagacccaacacgaagagcatactcaagaggcttgaagagatagcgagatga
>g93590.t1
atgcggcccctcgcgctgctgcttctcgcccacctcgccctcctggcggcggcgccggcggcggaggccaagggcggcggtgccggtctggacgacga
cgtgctgggcctgatcgtgttcaaggccgacgtgacagacccggacggccgcctcacgacgtggagcgagtacgacgcgcgggcctgcaactgggcc
ggcgtcacctgcgacgcgcgcacgggccgcgtctccgcgctctccctcgccggcttcggcctctccggcaagctgggccgaggcctgctccgcctcgag
gcgctccagtcgctctccctcgcgcgcaacaacctctccggggacgtccccgcggacctcgcccgcctcccggccctccagacgctggacctcagcggc
aacgccttcgccggcgcaatcccgaacgggctcttctcccggtgccgcgcactccgcgacgtctccctcgccgggaacgccttctccggcgacatcccg
cgggacgtcgccgcctgcgccacgctcgcgtcgctcaacctctcctccaaccgcctcgccggcgcgctgcccagcgacatctggtcgcttaacgcgctg
cgcacgctcgacctctccggcaacgccgtcacgggcgacctgcccgttggcatcagcaagatgttcaacctccgggcgctcaacctgcgaggcaaccg
cctcaccggcggcctccccgacgacattggggactgcccgctgctcaggtcagtggatctcgggtccaactcgctgtccggtaatttgccggagtccctg
cgccggctctccacttgcacgtaccttgacttgagctcgaacgagttcactgggagtgttccgacatgggttggagaaatggcgagcctggagatgttca

acctccgggcgctcaacctgcgaggcaaccgcctcaccggcggcctccccgacgacattggggactgcccgctgctcaggtcagtggatctcgggtcc
aactcgctgtccggtaatttgccggagtccctgcgccggctctccacttgcacgtaccttgacttgagctcgaacgagttcactgggagtgttccgacatg
ggttggagaaatggcgagcctggaggtgcttgatttgtcggggaacaagttctcaggggagattccgggatccattggtgggttgatgtcgttgaagga
gttgaggttgtctgggaatgggttcactggtggcttgccggagtcaattggtgggtgcaagagcctgatgcacgttgatgtgagctggaattccctaacc
ggtgcactacctggttgggtctttgcgtctgctgtgcaatgggtgtcggtgtcggagaacacattgagcggagaagtatcagtgcctgcgaatgcatcttc
ggagctccggggtgtggacttgtcgaacaatgtcttctctggagttatcccatcagaaatctcgaagctgcagaatttgcagatgttgaacatgtcatgg
aattcattctctggaagtattccgcccagcattttggcgatgaaatcgctggaggctcttgatttgactgccaaccgtataaatggaagcattcccgctgtc
attggaggggactcgttgaaagagttgaggctgggga agaactccctcacaggcaaaatcccgccacagattggcaattgttcctcgcttgcatcattg
gatctttcacgtaacaatctaacaggaacaattccagaaacaatagccaatctcgccaatcttcagactgctgatctttctcgaaaccagctcactggtg
gtctgccaaagaatctatctaaccttccccacctcctgcacttcaatatctcacacaatcagctctctggggatctccctcctggcagctttttttgacacaat
ccccttatcatctgtggcggacaatcctggcctatgtggtgcaaagctcaactcttcttgtcctggtgtccttccaaagccaattgtgttgaatccagattct
tcaactgatccgctgtcacagactgtgcctgtgcctgctggtggcccaagacataagaaaaccatactgagtatctcagctcttgttgcaattggtgctgc
tgtcctaattgctgttggtgtcattacaattactgttcttaaccttcgagtgcgtaccccttggatctcattccgctgctgtcctggaactctcagatggatatc
ttagccagtcacccacaactgatgtaaacgccggcaagcttgtaatgtttggaggaggaaatccagaattcagtgccagtacccatgctcttctgaacaa
ggactgcgagcttgggcgtggtggtttcggcaccgtctacaagaccaatcttcgagatgggcaacctgttgccatcaagaaactcactgtatcaagcttg
gttaaatctcaagtagaatttgagagggaagtaaagatgctgggcaaactacatcatcgcaaccttgttgcactcaagggatattactggacaccgtca
ctgcagcttcttatttatgagtttgtctctgggggtaacttgcataaactgttgcatgagtcatcaaccacaaattgcctctcctggaaagaaaggtttgat
ataattcttggtatagcaagaagtctggctcatctacacaggcatgatattatccactacaaccttaaatcaagcaacattctgcttgatggatcaggcga
ggccaaggtgggtgattatggactggcaaagctacttccaatgctagaccggtatgttttaagcagcaaggtacagagtgcacttggttacatggctcca
gaatttgcatgcagaactgtgaagatcactgagaaatgtgatgtgtatggttttggtgttattatccttgagatcctaacggggaggacgcccgtggagt
acatggaggacgatgttattgttctttgcgatgtggtgaggggtgctttggacgaagggaaggtggatgagtgtgttgatgagaggctttgcgggaaatt
tcctccggaggaggccattccaatcatgaagcttgggttggtctgtacttctcaggttccttccaatagaccagacatgtatgaggtggtcaacatcttgg
agctgatcaggtgcccccaggatagtcctgaaactgaactaggttaa
>g93669.t1
atggaaaggtacaaattgataagggagataggtgatggaacctgtggaaacgttttcatggcatataatatagaaacaaacgaaattgtcgctgttaaa
aaaatgaagagaaaattttaccagtgggaagagtgcattagtctgcgagaagtgaaggctcttcagaaactaattcaccctaacattgtgaagctgaag
gaggtgacgatggaaaaccatgagctgttcttcatctttgaacacatggtcgctgttaaaaaaatgaagagaaaattttaccagtgggaagagtgcatt
agtctgcgagaagtgaaggctcttcagaaactaattcaccctaacattgtgaagctgaaggaggtgacgatggaaaaccatgagctgttcttcatctttg
aacacatggaatgtaatttatatgatgtcataagagaaaagcaagttgctttctccgaaggagatatacagaatttcatgctccagatacttcaaggtctt
gcgcacatgcataataatggatatttccaccgggatctgaaacctgaaaacttgttggtgtcaaatagtactataaaaattgctgactttgggttggcaag
agaagtatcttccaaccctccttacactgactatgtttccaccagatggtatcgagctccagaggtgctgctgcaatcttcagcttacacacccgctattgg
tctgtggccgctggtgtcatcagaccgtcccatcggtgacgttccggccatgccatcatggccacaggcttatatggttgacagccaatcttccttggcag
gatactctggttctccgttcggcctgtctttacagccgaacctgttggagaaccattcgttaggacccatccggcaggtcaacttcttctga
>g93825.t1
atgttcaggccgtcccgggtgtgggccgtcgaggaagggtacaagctcatcaccagcaacttccggcggtacacctacgcggagatcaagcgtgccac
ggccaacttcaccgacgtcatcgggagcggtggctccggcgtcgtttacaagggggtcttgggggacgacagggtggtggccgtcaaggtgctcagga
acgtgagccagaccgagcaggagttccagtccgagctcagcgtgatcgggagaatctaccacatgaaccttgtcaggacgtggggatgctgctccgag
ggcaagcacaggatcctcgtctacgagcacatcgaaaacggctcacttgccaagatgctgtttgacagggaggaggattattcatcggacaccagcgtt
cttggttgggatcagcggttttcgatcgcgttgggtgtggccaaagggttggcgtaccttcacagcgagtgtttggagtggatcatccattgcgacatgaa
gcccgagaacatattgttggaccaggacctggagcccaagatcaccgatttcgggctctcgaaactgctcaaccgagatggttcagacggagatttctc
acggataagagggaccagagggtatatggcaccagagtgggtgtctagtttgccgatcacggaaaaggttgatgtgtacagctacggagttgtgctcct
tgagctagtaaagggagtccggatttcagagtgggtggtcgacggggttatgtttgctgagatggatacgagggtacttgtcaagatgatccaagataa
gatgcatattgcccatgatcaagaaacatgtttcaaggatcttatcgactctcgactcaatggcgaattatttaatcgtgtgcaggcaaaagcgatgctta
agattgccgtttcatgcctggaggaagatagggcgaaaaggccaaatatgagatctgttgtggaggcacttctatcagttgaagacgaaacaaggttat
aa
>g94323.t1
atgaagtccagcaacattctattggatgagaacatggagtcgagagtctctgactttggacttgcaagaataatcagtgcatacgagactcatgttagca
ccaatgttgccggcacgcttggatacattcccccggagtacggtatggcaatgaaatgcactgcaaaaggggatgtctacagctttggagttgtcatgct
ggaggtgctcacaggacgaccacctacagggcaagagatggaagaaggtggtgggaaccttgtcggctgggtgcgatggatgatagcaaatggtcg
gcagagcgaancagggcaagagatggaagaaggtggtgggaaccttgtcggctgggtgcgatggatgatagcaaatggtcggcagagcgaattatt
tgatccttgtttgccagtttcagggctgtggcgagaacagatgtcccatgtgctcgccattgctcggaaatgcactgtcgatgagccatggaagaggccg
accatggtggaggtggtgaagggcctcaggatgattcagctgatgaagcatgaacctgatgacctccaggaacatgtagagcaggcatga
>g94978.t1
atggctacgcggttccgtgaccccctggattccggaaacaggctccgtcgtgctgcgcctgcgcgtgctgattttgggagcagctctccttaccgtgctggt
cgagcttactgggctgagctccaatggcgtcctcgccttctccctccccgggcaccatcaaggccaccgtgccgccgtccactacaaccccatcgccggc
atctacaactccgccaccgtccacttccacgccgggctcttcggctccggtgacacagcccaatgccaccccggccgactcctcctcgccgccggcagct
ccccctccacttcctcccgcagccgtgccacccccgcaacccgcgtcgccgccgccctcatctccgccgcctccggtcaccgtgcccccaccgccagtag
ttgttgcatctccaccaccggccccaccggccccagccgccgtagtgccgccaccctcaccgccggttgcagtgccgcctccaccgacacctgcagtcc
ccccgaaagcttcgccttcagtctcccccgtggtcgcatcgccgcccccatccaatttgccggcacccgaccccccggcagaaccaaccccggcgacag

tagtgcagtcacctccgccaccacagcgtcgccggccacgaacgcctccatctcagccagaagctccggccctggcgccaccaccttctgctctgattct
tcgtcagtgccagccccgggcacgacggttgatccagccagcccagcaacgaccagcagccctggttccaacaagtcatcgaatcccaccgggcagg
gcatcacccactcaaataacagtggtggaatgtcatctggtgcgaaggcaggcatcggcattgtcgtcgctcttcttgtgctcagcttggttggagctgca
ttttggtacaagaagaaacggagaagagtgcatgggtattcagctaaaactaactacactgtcggcggtcctgaatcaaaagaatcaatgccagagttc
agcatgggcaactgtcggtttttcacctatgaggaattgtacaagatcacaaatggcttctcagctcagaatctgcttggggaaggtgggtttggttcagt
gtacaagggttgcttggctgatggaagggaggtcgctgttaagaaactgaaagatggtggtggacagggggaacgccatccacgtataatccaccga
gatataaaatcctctaacattttgctggataataactttgaagctcaggtcgctgattttggtctagcaaggttggctttggatgcttgcacacatgtaacta
cacgggtgatgggaacttttgggtacctagcccctgagtacgcatcaagtggcaaactgactgaaagatctgatgtattctcttttggtgttgttctattgg
agcttattacaggcagaaagcctgttgatgcatcaaagccactgggtgatgaaagccttgttgaatgggtggtgcgggctctcgatagtttagctgatgt
cgatctgaccaatggtgttcagccaggtcagagcgagctgttcaatgttgcaaatactgcggagatcaggctgtttcagcggatggcatttggaagcca
ggatttcacaactgatttcagccattctagctggaacagccagagcttgagtcagagccagagccagagccagagtagaggttttgatgcctctggttcg
aggcaaatgtaa
>g95063.t1
atggagtggctgcgcgggaaatgcatcggcaagggcgccttcggcacggtccacctggccgttgaccggaccacggaccgcgccttcgcggtgaagt
ccgtcgacgccgggaccgcgccggcggcagcgctggcgtgcctcgagaccgagataagcatcctccggcgtctgagctcgccgtacgtcgtcgcctac
ctcggcgacggcgcgacggggaagacccgggacctgcacatggagctcctgcccggcgggaccgccgccgacgcggcggcgcggggccacggcct
cggcgagcgcgccgcccgcggcgtcctgcggcgcgtggccgccgcgctgcggtacctccacgacgacgccggcgtggtccacggcgacgtcaaggg
ccggaacgtgctccttgacggcgtcggcggcgcgaagctcgcggacttcggcgcggcgaggatgttggtctccgacgcagcgctgccgccacgtggcc
cgcgcgggacgcccgcgtggatggcgccggaggtggcgcgcggcggcgcggcgacgccggagtccgacgtgtggtccctgggctgcacggcgctgg
agctgctcacgggacagcgcccctggtcggagctcggcggcgcggccgaggtcggcgagctgctcttcctcgtcggcttcggcgggacgcgccccgcg
ttcccggcctccctgtccgacgcgtgtcgggacttcctcgacaagtgcctccgccgcgacgccgggcagcggtggacctgcgaccagctgctgcgccac
ccgttcctcctccctgtctcttcggacgacgacgacggcgccggcgagccgtctccgcgtccttctccccgcgcggtcctcgattgggccatgtcggactc
ggactccgaggcggtggacgacgaatcggagcccgaggatgacgagggggtcatggcgcgcgccaagggggcgggtcgccgaattggcggcgtcctc
aaaggggccgcgcgcgagcggggagaagctggactgggggaccagcagcgccacctgggcggccgacacgtggggcccactgccaatttcggacg
cgccaggaaccgtgccgccatccgcatcaaactccgacgcgggaaatggcaccgcgggctggcccgcctcgagtgccgcctccgccgaagctggccg
tgatcatcaagtcggcggtgggttactgcgcgtccacgaccgtcctgcggctgctaatttttag
>g95360.t1
atgccgtctcgccgccgccaccgctgccgctaccctctgctcctcctcctcctgctacacctcctcaccaccgcatccgcggccggcgtctcccctggcgc
cgcatcgcacccgcagcccacgctgccgacgccggcccaagccgtgacgccatccgcggcgccggccaagaggggaacgccggcccaagccgcga
ccccctccgcggcgccggccaagaggggaacgccgaccgctgccgcaccgcactcgcggtccaagctgccggccccagttcaagcggtggccccatc
cccgacgccggtcgcaaggccgccgaccgcccccgcgccgcactcgcggcccacttttccctcgccggtccaagccgtggccccggcctcgaggcccc
ccacggtcgccgccgccgctcccctcgccgccttcctcgccaaggccgacccctcctcccacctccgcccgccgccctccgcctcccccctgctcccaccc
gggggtcacctgctcctcctcaggccaaatcacccgcctcgtcctcgaatccctgggcctcaacggcaccttcgcccccgccacgctctccctcctcgcct
ccctccgcgtgctcagcctcaaatccaacgtgctgcacggcccaatcccggacctctccggcctcgtcaacctcaaggcgctctacctcgccagcaacc
gcttctcgggccccttcccatcctccctcgccgccctccaacgcctccgctccatcgacctctcggggaaccgcctcgccggcgcgctcccgcccggcat
cgaggccgcgctcccgcacctcaccttcctccgcctcgacgccaaccacttcaacggcaccctcccgccctggaaccagaccgacctcaagacgctcaa
cgtctccttcaacgacttctccgggcccgtccccgtcacgcccgtgctcacccagatgggcgccgacgcgttcgcggggaaccccaagctctgcggcca
ggtcgtgcgacgcgagtgccacctcctcttcttccacggagacggcaccaacggctcctccgccaccgcggcgcccccggtccacagcgcggccgcca
gcgacagcggcccgcagcgccaaagtttggatccgtccgatgcgcgcgcgaagaaatcgaggaagaggaggacggcggtggtcgccgtggcggccg
ccgcgggctcggtcctcgcgctcctcctcctctgcgccatgattgcgaggagcaggaagcgccgtcaccccatgcggtcggcggctgccgcgtaccacc
cgagccccaacaagaagagcgcgtcggacgcgggcagggacaacatcggcgccgacgtggggtacgtcgagtgcgtgcgcgacgaggagacggcc
gcgatcatggtgcccgaggaaaaggcgcggaggctggagcggagcggctgcctcaccttctgcgcgggcgagaccgcgagctacagcctcgagcag
ctcatgcgcgcctcggcggaggtgctcggccgcgggagcgtcgggaccacgtacaaggccgtgctcgacggtaggctcgtcgtgattgtcaagcggct
cgacgccgccaagattggacccgcggcgctggaggcggagaccttcgagcagaacatggatgccgttgggcggctgcggcatcccaaccttgtgccg
ctgagggcgttcttccaggccaaggaggagcggctgctggtgtacgactaccagcccaatggcagcctctactctctagtccatggtaagtactgtatttt
ttcttcgtttaggttgtga
>g95475.t1
atgtacttcgactcaactatggaacccaggctgtctgattttggattgtccatggtcgctggtacgaacacagacaacgatctcttgcatcactccccagg
ttatgcacctccagagttctccaactcggagaatgtcatggcgaccgccaagtctgatgtctacagctttggagtcgttttgtttgaattggtcactggaaa
gaagcctcttggtgatgagtaccctgatcggaaggaggaaagcctggttaactgggccagggcaatggtgaaggcaaaccttgggtcaagcatcatcg
actcaaagattcgtgacaccgggctggagcggcagatggaggaggcgctgcggatcgcgtacctctgcaccgctgagctgccatccaagaggccagc
catgcagcagatcgttggcttactgaaggacattgagcctaaaatatga
>g95723.t1
atgaactcctccggctggggccaagatgcaggatctctcccgatatcccacaagttgataaagatacacgtgcctattggtatgtggaatccgcccagg
aacatattgtccgaaactacctcccncatgccgacgcggttcacctacacggagatcgcgacgatgacggacaacttcggaacgaaaatcgggtccgg
cgggttcggctccgtgtacaaaggggagctcccgggcggggaaggcctcgtcgcggtgaagaagctggaggccatcggcgtgcaggccaagcggga
gttctgcaccgagatcacggtcatcggcaacatccatcacgtgaacctggtacgcctccgcggcttctgcgcggagggatcgcgccggctgctcgtgta
cgagtacatgaaccgcgggtccctggaccggtccctgttcggcgcaacacggggcggcccgtgctggagtgggggagcgcgtggaggtggcgctg
ggcgcggcgcgcgggctggcctacctccacacggggtgcgaccacaagatcgtccactgcgacgtgaagcccgagaacatcctgctggccgacggcg

ggcaggtgaagatcgcggatttcggtctcgccaagctgatgtcgcccgagcagtcggcgctcttcaccacgatgcgcgggacgcgcgggtacctcgcg
cccgagtggctcagcaacgccgccatctcggaccgcgccgacgtgtacagcttcgggatggtgctgctggagctggtgcgcgggaggaagaacaggg
gcggcgaggagggcaaccacgtcgtcaccgtcgtcggcgaacagtcctcggactggccgtccgccgggtggagcagcgtggcggcgtcgacgccga
gcgggaccatcgatggcggggacgagtactttcccatggttgcgatggacctccacgagcaggggaggtacctggacctggtggacccgaggctgga
aggccgggtggacgaggccgaggtggcgcgcgcggtccgcgtcgcgctgtgctgcctgcacgaggacccggccctccggcccagaatgtccgccgtg
gtgcggatgctggaaggcagcgtgccggcgccggagccgcgcgtcgaggcgctcggattccttcgcctatacgggaggggatacgccgcgccggcct
ccacgaccgccatggccggcacgtcgggctccacggccacgccgtcaacgcccggcgcgtcgtcccaatccccgctgaatgactcatcgcaagacgtg
tcattgccaaggtag
>g95724.t1
atgccgacgcggttcacctacacggagatcgcgacgatgacggacaacttcggaacgaaaatcgggtccggcgggttcggctccgtgtacaaagggg
agctcccgggcggggaaggcctcgtcgcggtgaagaagctggaggccatcggcgtgcaggccaagcgggagttctgcaccgagatcacggtcatcg
gcaacatccatcacgtgaacctggtacgcctccgcggcttctgcgcggagggatcgcgccggctgctcgtgtacgagtacatgaaccgcgggtccctgg
accggtccctgttcgggcgcaacacggggcggcccgtgctggagtggggggagcgcgtggaggtggcgctgggcgcggcgcgcgggctggcctacct
ccacacggggtgcgaccacaagatcgtccactgcgacgtgaagcccgagaacatcctgctggccgacggcgggcaggtgaagatcgcggatttcggt
ctcgccaagctgatgtcgcccgagcagtcggcgctcttcaccacgatgcgcgggacgcgcgggtacctcgcgcccgagtggctcagcaacgccgccat
ctcggaccgcgccgacgtgtacagcttcgggatggtgctgctggagctggtgcgcgggaggaagaacaggggcggcgaggagggcaaccacgtcgt
caccgtcgtcggcgaacagtcctcggactggccgtccgccgggtggagcagcgtggcggcgtcgacgccgagcgggaccatcgatggcggggacga
gtactttcccatggttgcgatggacctccacgagcaggggaggtacctggacctggtggacccgaggctggaaggccgggtggacgaggccgaggtg
gcgcgcgcggtccgcgtcgcgctgtgctgcctgcacgaggacccggccctccggcccagaatgtccgccgtggtgcggatgctggaaggcagcgtgcc
ggcgccggagccgcgcgtcgaggcgctcggattccttcgcctatacgggaggggatacgccgcgccggcctccacgaccgccatggccggcacgtcg
ggctccacggccacgccgtcaacgcccggcgcgtcgtcccaatccccgctgaatgactcatcgcaagacgtgtcattgccaaggtag
>g96025.t1
atgaccaaatttttgctcacaagcttggcaaaggaggctatgggaccgtttacagaggtaacctgcctgatggacgtgagatagcagtcaagatgcta
aaggacaccgagggcgacggcgaggatttcatgaatgaggtggctagcatcagcaaaacatctcatatcaatgttgttactctcataggattttgtcttc
aaggttcaaagagagctctcctgtacgagtatatgccaaatggctcactcgataagtacacttttggcagcaactctgttgaaggagacaacactttatc
atgggataagctgtttgatattgttgtcggaattgggcgagggctggagtacctccacattgggtgcaacacccgaatagtgcattttgatatcaaacctc
aaaacatcctgttagatcaagacttctgtccaaagatttcagattttggattagcaaaactgtgccgaaaaaaggagagcaaaatctcaattgccatgcc
aagaggcacggtaggctacatagcacctgaagtaatttatagaaactgtggagcagcgagcagcaagtctgatgtgtacagttacgggatggtggttct
tgagatggtcggagcaaggaagcaaatcgaagtaagtaccgatagcagcaccaagtattttccacagtggttgtatgacaacttggatcaattctgtgg
agccaccacctgcgagatcagcagtgagagcacggagctcgtgaggaagatgactgttgttggtttgtggtgcatacagttgagatctgatgatagacc
ttccatgagtaaggttcttgagatgttggaaagcagcacggtggacttgcagatgcctccaaagtcctttcatgctagttaa
>g96026.t1
atgcgagcgcactccgaactcggccttgcagcgagacctagcaccgtctacaccaggcacaagtcctctgtattgcactctcagcccaaatcaactgtta
gcacaccagcttacattgctccagaggtccttgctagaaaggagtatgatggaaaggttgctgatgtttggtcctgtggagtaactctttacgtgatgctt
gtcggggcttatccctttgaagatcctgatgaaccgaggaatttttgcaagacacttactcggattctcagtgtacaatatgcagtccctgattttgttcga
gtttcaatggagtgcagacatttgctatcccggatcttcgtggcaaaccctgagcaacgaataaccatcccggagatcaagaaccacccatggttcctga
agaacctccccatcaagatgaccgacgagtaccagacgaacctgcagatcatcaacatgaacgttccctcgcaatgcttggagatcatggccgtcatac
aggaggccatcatggccgtcatacatacaggaggcccggaagcctggcaaatgggctga
>g96097.t1
atgtttgctccgagagaagaagtaatggatttggcatcggtcaacgaggagctggccgagatcgacggacagatcggcgatattctccgcgcattgca
aaatgggttccagaagctggacaagatcaaggatgccagccgccgaagcaggcagcttgaagagctcacagatgagatgcgagattgcaaaaggct
tatcaaagactttgatcgagttaccaaagatgaggctgggcatactgatccagataccacaaaaatgctgcatgagagaaagcagtcaatgatcaagg
aattaaactcttttgttgctcttaagaagcaatatgcaagtgaaaacaaacgagttgatcttttcgatggtccaagtgttgaagatggttttggtgaagag
aatgtcctgttggcatcaaatatgaccaaccagcagttaatggatcaaggaaatcaactaatggatgagactgatcaagctattgcacgatctaaacag
actgagcaaatgagcagagtaattaatgagctggattccatccatttctccattaaaaaggcatctcaattagtgaaagaaattggtagacagatgggg
tgcttcgccttcaagagcggcaagagcaggaggtccaagagccagaaggcagttccctcggggtcggattccggcgggcagaagagcaaggcgtcgt
cggcgtcgacgccgtcgaggagcatacaggagctgtcggaggagaggggcgcgcagaggctgcgtgtgttcgacctggaggagctcagcagcgcca
ccaacggcttcagccgctcgctcaagctcggcgagggggggattcggctccgtctaccgcgccttcttccgctccgccgccggcggccgcgtcgtcctcgc
cgtcaagcgcctcaaccagcgcagccgccagggacacaaacaatggctggctgaagtccagttcctgggcgttcttgagcacccgaatctggtaaagc
ttatcggctactgcgcagtggattcagaagcaagcaacgacaggctgctggtgtatgagttcatgcccaacaagaccttggacgaccacctgttcaacc
gagcccatcctcctctcccgtggagaacgaggctgcagatcatgattggagccgcgcgggggctcgattatctccatgaaggggtggcagaagttcag
gtgatctacagggatttcaaagcttcgaacatccttctggactctgaattcaagccaaaactttcagactttgggctggcaagggagggaccaaccgaa
ggcaaaacacacgtgtccacggcggtggttgggacgcacgggtacgcggcgccggactacatcgagacggggcatttgacagtgaagagcgacgtg
tggagcttcggcgtggtgctgtacgagatcctgacggggcggcggtcgctggatcggaaccgcccgccggaggagcagaagctgctgggctgggtcc
gccgccacccgccggacggccagagcttcaggacgatcatggacccaaggctgggcggacggtacccgccggccgccgcgcgcgaggtggccaggc
tcgccgaccgctgcctcgtcaagaaccccaaggagcggccggccatgagagacgtcgtcgacgacctcgagagagtgctgcagatggagcctcctcct
ccgccgccgctggctgacaacaagaaaggaggcgatgccaacaacaagcctgctttgccgtcgtcggccaagaggtga
>g96309.t1

atggacgacgaggaagattatggcgccgcacccggaggggaacgtgccctcacagaagtagcatgggaaacggcctcggtggccactcaactcgca
gccatcctcgacacgatcgagcaccaccgcggaaccgagaaaccgatgagcccgagccgcgtcgcccacatctccgccatgatcgacgacctcgctgt
caccctcaccgccgctgctctaggcatccgcagacggaggaggatgcttagcgcccggtggtacgaggaggtgtgccgggtcggcgagggcgggttc
ggtaccgtggtcagggcgccgcaccgctcgacgggccagcacgtcgccgtgaagacgccgcgccgatctgggccggggggccacgaggtcatcatg
gacctcctgcgcgaggcctgcttccttgcggcctgccgggcccacccctcgctcctcgtgggcttccgcggcatggttcgccgtgttgatactagtagcag
gggcgaagaagagtactccatcgtgatggactacatcgacgggtccagcctcgcccacatccttcacgagcgtggccagccgttcccggaggaagaa
gtgcgccgcatcatgcggcaactcctgcgcggcgccagcgcgatgcacgagaacggcatcgtccaccgggacatcaacccttccaacattttcgtaga
cggtgacgatgagggcatcatcaagatcggagattacgggtgcgccaagtcgacggctgaggcgcgcccgccgttctgcgttgccggaacgaacggg
tacatggcgcccgaggtcctggtggaggacgcggcgggccatggcacggcggccgacatgtggtcccttggctgcgtgatggcggagctgctcaccga
cacgctcatgttcgacggggacgaggacgccgagcagctgtgcaggatcttcgacgtgctcggggttccggaggacgaggaggcattgcggccccgg
gtgctcgaggacctggtgcggcagcagcgagtgctgcagcggctggtcgggcaccgtcaggacctgctgcgcaggatggtgccggaggagatgctct
ccctggacgggttcgaggtgttgagaggactgcttgcgtgtgaccccaggaagcggctcacggcacacgaggcactccgacttccatggttcacagag
cacgcggtcaccacgaaatcgtggtcagctgtgcttttgcagatggccgggcgtgtgctaggatggcttagggccaccaccgtaccgtggggcgcatgt
ttatgccattaa
>g96652.t1
atggctgcgctgttctaccttctcgctcttccattgctctccttccagttttgctcttgcgctttatcatggcgcatcatgaccactggctcgcacataagagc
agaagaccatgaaaaaatctttctcctgtcaccagacaccaccttctcctgtgggttccatgaacttggaacaaatgctttcaccttttccatctggtacac
ctacaccaataacagaaccgtcgtttggacggcaaatccctactccccaaagggtggctactctccagtgaacaagtatggctcaagaatttcgctaaa
ccgtgatggtaacctgatcctcacggataccaatgggtcgacggtgtgggaaagcaagacatcgtcaggcaagggcacgacagttaccctccttaaca
gtggcaaccttgtgataaatgattccagcaacaacatcgtatggcagagcttccattcaccaactgacaccttgctcccggggcagaacctgacaaaag
acacaagaaccgtcgtttggacggcaaatccctactccccaaagggtggctactctccagtgaacaagtatggctcaagaatttcgctaaaccgtgatg
gtaacctgatcctcacggataccaatgggtcgacggtgtgggaaagcaagacatcgtcaggcaagggcacgacagttaccctccttaacagtggcaac
cttgtgataaatgattccagcaacaacatcgtatggcagagcttccattcaccaactgacaccttgctcccggggcagaacctgacaaaagacacaagg
ttagtctatggttaccataatctatattttgacaacgacaacgtattacggctgttgtacgacggtgtagagattacaagcatctactggccaagtccagat
tacaatcccgaaacgaatggccgcaacagatttaatagcaccagaatagcagttctagatgacatggggaattttgtgtcaagtgatgggtttaagata
caggcttcagatttaggtccaggaatcaagcgaagaatcacaatggattatgatggtaatttcagaatgtacagcctgaatgcgtcaacaggggaatgg
gtcatcacgggtcaggctgtaatacagatgtgctatgtgcacgggctatgtgggaagaatgggctctgtgactacacaggctggtattttttctcaagaa
gcatactatacccaaatcaatggaagatggatacaagatgattacaaaccagttcagacgctttacctaccgagaattgaaggaagcaactggaaagt
tcaaagaagagcttggcagaggaggcactggaattgtttatagaggactgcttgaagataagaaaatagtggcagtaaagaagcttacagatgttcgc
cagggagaagaagaatactgggcagaagtgactcttattggtaggattaatcatataaatttggtcaggatgtggggtttttgttcagaagggacaaga
aggttgttagtatatgagtatgtggagaacgagtctctggacaagtatctctttggcgagaggagtacagaaagcctgcttaattggagccaaagatac
aggattgcgctgggcacagcaaggggtcttgcttatcttcatcatgaatgccttgagtggattgttcattgtgatgtgaagccagagaacatactcctaac
tcgagatttcgatgccaaaatagcagactttggactagccaagctagcaaagagaggtagcgctagtttcaatttcacacatatgagaggcacaatggg
ctatatggcaccagaatgggcactgaatttgcccatcaatgcaaaggttgatgtttacagctatggagtcgtgcttctggaaattgtgactggaatcaga
gtttcaaatggcatgacgttagatgaaagatgtgtagattttctgcaggttgtccaggaggctaaacagattctggctactgggaatgtcagtgatatagt
tgataacaggctgcatggccatttcaaaccagagcaggcgattgcaatggtgtcagtagctctttcttgccttgaagaaagaagcaagaggccaacaat
ggatgaaattgtcaaggtactcatgtcatgtgacgatgaagacgacttccaccctgcctattcatattga
>g96687.t1
atgatgatgggagccacgaccaccgtgtctgactggccggactacgcggaagtcgaccccaccggccggtacggacggttcaaggatgttctcggcaa
gggcgcgtccaagaccgtttacagagcgttcgacgagtatcaggggatggaggttgcgtggaaccaggtgaagctgcacgacttcctgcagagccccg
aggacctggagcggctctactgcgagatccacctcctcaagacgctcaagcaccgcaacatcatgaagttctacacctcctgggtcgacgtctcccgcc
gcaacatcaacttcatcaccgagatgttcacctccggtaccctccgccagtacagacagagacacaggcgggtgaacatatgggcggtgaagcactgg
tgccggcaaatcctcagcggcctgctgtacctgcacagccatgatccacccatcattcaccgggacctcaagtgtgacaacatcttcgtgaacggcaac
cagggggaggttaagatcggcgacctcggcctggcagccattctccgcaagtcccacgctgtccactgtgtaggcacaccggagttcatggcgccaga
ggtgtatgaggaggaatacaacgagctggtggacatatactcatttggaatgtgcgtgctcgagatggtcacctttgagtacccgtacagcgagtgcac
gcacccagtgcagatctataaaaaagttacctctctagtggtttatttcgtagggtactaa
>g96774.t1
atggacaagtacgagctgctcaaggacatcggcgccggcaacttcggcgtcgcaaggctcatgcggcacaaggagaccaaggagctcgtcgccatga
agtacatcccgcgaggccagaagattgacgagaatgtggcgagggagatcatcaaccaccgctcgctgcggcaccccaatatcatccggttcaagga
ggtgctggtcacgccgacgcacctggcgatcgtgatggagtacgccgccggaggcgagctgttcgaccggatctgcaacgccgggaggttcagcgaa
gacgaggccaggtacttcttccagcagctaatttgcggtgtgagctactgccacttcatgcaaatttgccaccgagacctgaagctggagaacacgctg
ctggacggcagcccggcgcctcgactcaagatctgcgacttcggttactccaagtcgtcgctgctgcactcgaagcccaagtcgacggtgggcacgccg
gcgtacatcgccccggaggtgctctcacgccgggaatacgacggcaagaggataatgtctatccagtacaaaataccggagtatgtccatgtatctcag
gactgcaaggatatgcttgcaaaaattttcgtcgcaaaccctgcaaagagaattacaatcagggaaatcaggaaccacccctggttcctaaagaacttg
cccagagaactcacagaagccgcacaggcaatgtactacaagaaggataacagtgccccaacttattccgtccagtcagtcgaggagatcatgaaga
tcgtcgagcaggcacggacgccacctccttcctccacccctgtggctggctttggttgggtagaggaggatgaagagaacagcaagaaaccagagga
gaaacaggaggaagaagaggatgctgaagatgaatatgagaaacaagtgaatgaagtccgcgccagcggtgaatttcagatcagctga
>g96832.t1

atgaccgaatcgattcccactgcgccttacgacgcgtccgccgaggaccttttcgacggccgctaccgcctgggacgcgtcctcggacagggcggttac
gcgcgcgtctatgaagcggtcgacacgtcgctcggccgcaccgtcgccctcaaggtgattgagggcgagggagcagacccctcggatgccgcgcgcg
tgcgctccgagatccgcctgctcgcgtcgctgtcgcacccctcgctcgtcaccctctacgacgcgcgcctggcggcgactcccgcgtacctgtcgatgga
gctcatcgccggtcccaccctcaccgatctcatcaaccgcggaccgatgtcggccgaagacgtcgcccgcaccggcgtcgagatcgccgaggcgttcg
cggtgatccacgcgcgcgggatcgtgcaccgcgacgtcaagccgtcgaacatcctcatccgtcccggcgcccactccggagagaagccgcgannnn
nnnnnnnnnnnnnacgctcgccgacttcggcatcgcctcgctggtgggcgccacgcgcgtga
>g97105.t1
atgcttgccggccgctatgctgccgccgccgtcgccctggcgctgctgctgctgtcgccaccggcggcggtgtcaatggaccctatctgcgacaaaaact
tctacacggccaacggcacgttccaggcgaacctgaacagcctcgccgcagttctggccgccaacgcctccgcctcgccggcaggattcgccacggcc
agcgtcggcacactgcccgaccaggccaacgggctggcgctctgccgcggcgacacgaacgcgtccacctgcgccgcctgcctcgcggcggcgttcc
aaaacgcgcagcaggcctgcccccctggacaaggggggtcaccgccatcgaggacgcctgcgccaacgggctggcgctctgccgcggcgacacgaacg
cgtccacctgcgccgcctgcctcgcggcggcgttccaaaacgcgcagcaggcctgcccccctggacaaggggggtcaccgccatcgaggacgcctgcgtc
ctccgcttcgccggcacgcagttcctcgacttcctccgaccggaccagtggatcgtctcggagatagttcctgcgagcagcacgtccattgggagcgtca
acgcctcggatgcctggtacaccgccgccgtcaagacaatctacaccgccctcatcgacagcgcggttgcagcgaccaactcgacgaggaagtacttc
gccactgctgagatggcgttcaaccccaagatctatgggctcgcgcagtgcacgcccaacctgacgccggatcagtgccggggctgtctgggactgatc
cagaatgtgacgttggcgcagcagatgagcgggcggcccctgtcaaacgaaggtggggtggcgtggtgcttgctcaagtacagcgtcgtctcgccggt
ctacgagggtcgggccatgctgcagcttgccgcgccgccggaaccagcacccacgctttcccctgctactccagagtccgtaacagggaggaaacaga
tttctgccggaattgtggcaggcatcgccggctccgttgcgttcatttttactatttcagtctttgtcttccttcgcttcaggcgaatgatcaaggcaaggaa
gaacaaacacccaccaacgaagatagggaacgcgcaatacatggccttcgatttggcgacgttgcaagaggcgactgaaaacttctcagagaagaat
aagcttggtgaaggtggttttggtacggtatataaggggatactctctgatgggcaggagatagcagtcaagacactcttaggaagaaccgggcatgc
attggatcagctgcacaatgaggtgcaggtactagcacagcttcagcacaagaatcttgttaggttactgggcttttgctcgcatcaggatgatacactg
ctcgtctacgaatacatcaagaacgggagcctcgacaactttctttttgataagagtaagggaaatgcactccactgggagctacagtacaacatcatta
ttggaattgccaaggggatattgtatctccatgaggactccagcatgagaatcatccaccgagatctaaaggcaaataacattcttcttagtgacgacat
ggaaccaaagatcgcagactttggactggcgaagctgctagtagaaggccatacccgtacccagacagccagagcggtcgggacattaggatacatg
gcaccggagtacgcgatgcatgggcgcgtgtcgcccaagatcgacatcttcagcttcggggtgttagtccttgaaatcgtgacgaggagaagcaattgc
agctctgacgattatagtgctgtgaatctcctgagcgatggtaagggaaatgcactcaactgggagctacagtacaacatcattattggaattgccaagg
ggatattgtatctccatgaggactccagcatgagaatcatccaccgagatctaaaggcaaataacattcttcttagtgacgacatggaaccaaagattgc
agattttggactggcgaagctgttagtagaaggccatactcgtacccagacagccagagcggtcgggacattaggctacatggcaccggagtacgcga
tgcatgggcgcgtgtcgcccaagatcgacatcttcagcttcggggtgttagtccttgaaatcgtgacgaggagaagcaattgcagctctgacgattatag
tgctgtgaatctcctgagcgatatgttggaccaatcgctggatgtatacgctcgagaccaggcgatgcgatgcatccacgtcggtctgttgtgcgtccag
gtggaccctgacgataggcctgacatatcagccgtcctcttcatgttaaccagggatggcatggagcttcaaccgccagaggagccagcattcttcttcg
caaaaggatcagcttcacggtcagatgggcaaagcagctatgtatatgatcggtctagttttattttggaacaggacatctctgtgaatggtcttacagta
tcagagccgtaccctagatag
>g97187.t1
atgaggggggaagaagccagtcagtgagctcaaggtcgccgtgccggcgcaggagaccccctgtcgacaagttcctgacggcgagtggtacattcaagg
atggtgaactacggcttaatcaaagtggcttgcggctgatctccgaggaaaatgtagatgacagtcctgagcggattagtggaagttcgtatgactaca
agagtgacatatggagtttgggcttagtaatacttgaatgtgccatcggccggttccccctatatacctccagaaggagaagattggttaagcttttatgaa
ttactggaggcaatagttgatcagccaccaccttctgcacctgcagatcagttttctccagaattctgctcatttatctcctcatgcatacaaaaggatcca
gcagagaggatgtctgcttcagaactcttggatcaccccttttatgaagaagtttgaggataaggatttagacctgcggactctggttgagaacctgcagc
ctcccatgaatataccggagtag
>g97274.t1
atgaatgttaaggggcaaattggatatatggctccagaatatttgtacagaggtgaaatctccaccatgtcagacatatacagtttaggcatgataattct
tgagaccacgacaggacagaaacatttgccaactcctgcagacagggcatcaaggaaatttgttgaaaatatacaaaacaactggaagaaacctggc
cacattacagacaagtatccatcactagaaatgaatggctacaaccaagaatttggaggataa
>g97298.t1
atgtcaaacctcaagcttccaatcttcttcctcctggcctccatcctcgccgcatccaccgaagcacaagagttcacctaccaaggcttccacccctccgg
cgcggggaccaacccgaacatgaccctgaacggcgtgacggagctccgcgccgacggcatcctccggctgacgaacgagacctcccgcctgatcggc
cacgccttctacccctcccctctccgcctcgtcaccagctccggcggcaaggcggtctccttctccacctccttcgccttcgccatcgtgccggagtacccg
aagctaggcggccacggcctcgccttcgtggcggcgccggacccgcggctccccggcgcgcttcccagccagtacctgggcctgctcagcgccggcga
caacggcaacgcgacgaaccacgtgttcgccgtggagttcgacaccgtccaggacttcgagttcggcgacatcaacgacaaccacgtcggcgtggatc
tcaatagcctggcgtccaacgcgtccgcctccgccgcgcctgtgaacctcaagtccggcgagaccgtggtcgcgtgggtggattacgacggcgatgca
agggtgctgaacgtgtcgatttctacgacggcggagaagcccgcggcgccggtcgtctcgttccgtgtcgatttggggaaggtgttgttggaggagatg
tacgtcgggttctcctcgtcgactggcctcctcgcgagctcccactatttgatgggctggagcttccgtctcggaaccggcgggaatgccgaagcgccgc
cgcttgacctgaaatcgctgccgtcgcttccacggccgaagcaggaagggaacagcacgaccaagatcctggcggcgacgttctcggcgttcgtgggg
gtggtgctcctcgccggagcaacggcgtacgcggcgcaccggtacaagcaccgcgacgtggtggagccgtgggagctggactacggcccgcaccggt
tcaactactcggagctccggcgcgccacccgcgggttccgcgaccgcaaccacctcctcggcgtcggcgggttcggcaaggtgtaccgcgccgtgctg
ccgtcgtcgggagagacggtggccgtgaagcgcgtctcgcacgggtcgaggcagggcctgcgcgagttcgtggcggagatcgcatctattggaaggc
tccgccaccgcaacctcgtccagctccagggctggtgccgccgccgcgccgacctcctcctggtctacgacttcatgcccaacggcagcctcgacgccc
acctcttcctcgataagtccaccaaccactccgacgccgacgagaagcacaagcagcagcagcagctgctgacgtgggcagtgcggtggcaggtgct

ccgcgacgtggcgtcggcgctgctgtacctgcacgaggggtgggaccgggtggtgctccaccgggacgtgaaggcgtccaacgtgctgctggacgcc
gggatgcgggcccggctgggcgactttgggctggcgaagctccacgagcgtggggccaacccgagcacgacgcgggtggtggggacgctcgggtac
cttgcccccgagctcacgtggacggggaaggccacgcccgccgccgacgtgttcgcgttcggggccctcgcgctcgaggtggtcgcgggccgccggcc
catcgagccgagggccccgcccgaggagctcgtcctcgccgagtgggcatgggagcggttccttgacgagcctggggaggtgcctgagccgccgccg
ccgccgccgccaccgcccgcgtgctccggtgaggtcggatttgatgactttgtgcactcctacccgtcttcgtccttcgagccggtgggcggcgccggcg
cgggagccgccgggatgtcgttcgagacgggcgtctcgtcggtcgctacgttcccgtactcgccgctgtccatgcgctcatcccacgtcagcgtctga
>g97317.t1
atgccggcgccggccgtgggcttggccgtccttgtgctcctgaccgccctgccgggacacctttccgacgacctcaactccgacgcgcaggcgctccag
ggcctgcggtccgcggtggggcgctccgtgctgacgtcatggaacagcacgacgccgacgtgccagtggcagggggtcacctgcgagaacggccgcg
tggtggaggtccgcctccccggcgccgggctcatgggtaccctcccctccggcgtgctcggcaacctctcggcgctgcgcacgctctccctccggtacaa
cgcgctgacgggtcccgtccccgacgacctctcccgcctggtggagctccgcgcgatctacctccagcacaacggcttctccggcgagctccccgcgcc
gctgttcgcgctcaagaacctcgtgcgggtggacctggccgggaacaagttcaccggcgagatctcgccggacttcaacaagctggtccggctcggca
cgctgcacctcgagaacaacgacctcaccggtgagatccccaagctggacctccccgcattggagcagttcaacgtgtcctacaacaagctcaacggc
tccatccccaccaagctccggaagatgcccaaggactcgttcctgggtaccggactctgtgggggcccgctcggcctctgccccggcgagaccgcgccc
acgccggccggatcgccggagtcgcagcctggtgccggcggcgccgctgacgtcggcggcggcaagaagaagaagctctccggcggcgccatcgcc
ggcattgccatcggatgcgtgttcggcgtgctgctgctcctcgccctgctcttcttcctctgccggaagaagtcccccgcgcccaggtcgacggcggcggt
ggaaaaggggcgtgacgtcggcatggccccgatggaaaccgagcccaagggacagaacggctcagccggccacggtggctcagccgcggctgcag
cggcagccgtgccagcagccgccgcaactgctgccgcggcggcgaagaccggcggcgcgtcgacggggtcgaagaagctcatcttcttcgggcccat
ggcggcggccccgccgttcgatctcgaggacctgctgcgcgcgtcggcggaggtgctgggcaagggcgcgttcgggacggcgtacaaggcggtgatg
gagagcggcgcggcggtggcggtgaagcggctcaaggacgtggacctgcccgagccggagttccgggagcgcatcgcggccatcggcgccgtgcag
cacgagctggtggtgcccctccgagcatactacttcagcaaggacgagaagctcctcgtctacgactacatgtccatgggcagcctctccgccctgctcc
acgggaaccgggcctccggccgtacgccgctggactgggagacgcggtcggcgatcgcgctggcggcggcgcgcggcgtggagcacatccactcga
cgggcccgacggcgtcgcacggcaacatcaagtcctccaacgtcctgctcaccaaaaactacgaggcccgtgtctccgaccacggcctgcccaccctc
gtcggcccttccttctcgccgaccagggtctcgggttaccgcgccccggaggtcaccgacatccgccgcgtgtcgcagaaggccgacgtgtacagcttc
ggcgtgctgctgctggagctgctcacgggcaaggcgcccacgcacgccgtcgtcaacgaggaagggctggacctgccgcgctgggtgcagtccgtgg
tcagggaggagtggacggccgaggtgttcgaccaggagctcctcaggtaccagaacgtcgaggaggagatggtgcagctgctccagctcgccatcga
ctgctccgccacgcaccccgacaggaggccaaccatggccgaggtggccgcgcggatcgacgagatccgacgctccagcatcggagaccggcacga
gggcgatgagccgtcgctataa
>g97334.t1
atgaccatgctagtcatcctgtacttgatgatcttttccgccctgaacctcccagcaattggcgccattgactccctcaccttgggccaatcactcccatgg
aaccagacactggtctccaagggcggcaatttcgagctgggactcttcaagccgggcaactccaccaagtactatataggcatatggtacaagaaggt
ctccaaacaaacggtcgtgtgggtggcgaaccgggagcgcccgatcagtgaaccctcggcctcccggttcacgctaagtgatcgcggcgagctgcagc
tcctcgcgacgacgccgtcgaactcgaacacgctgctgtggtcgtccaacgcgtcctcgccgtcgccgcgcagcaccgtggccacgctccaggacgac
ggcaacctcgtggtgcagagcaacgcgacgacgacgacatcgaccgttccgacgtggcagagcttcgaccaccccacggacacgtggctccccggag
ccaggctggggtacgaccgggaacgcggcgtgcacagcttcctcacgtcgtggacggactccgggaacccggcgccgggagcgttctccatggagat
cgacccaaacggggagcccaagttcgacctgctcgccggcggcgcgcaccggtactggacgacgggactctgggacggcgagatcttcgtcaacgtg
ccggagatgcggtcgggctacttcgtcggcgtcccccacgcgcctaacgccagcgtcaacttcttcagctaccacgaccgggtcctcatgggcatcggc
cacttcaagctggacgtgaccgggcagatgcggcggcggcagtggaacgcgctggacgggaagtggatcctcttctgctccgagccccacgacacgt
gcgacgtgtacggctcgtgcggggccttcggcgtgtgcagcaacaccaccgaccccgcgtgcgagtgccccgcggcgttcgcgccgcggtcacggag
ggagtgggagttggggaacacggccgcggggtgcgagaggcgggtacggctggagtgcgccaccgacgggttcctcaagctgccgcacgccgtgca
gctcccgaacggctcggtggaggcttctggagcagcacagagcgacaaggcgtgcgagctcgcctgccgacgagactgctcctgcacggcttacctct
acgatggtacgaagtgctccgtgtggaacggagagctcgtcaacctcaggacgctaccaaacgatgatcaaggcgacgacggctcatcccagaccgt
gcttcacatacgtgtcgcggcctcggaggtgccgccggcagcaacgcattcctggaagaaatcgatggtgatgctgaccagcggagcagcggccgtgg
tcttcctcctggcgtgtctcgtgatggtggtcgcggtggtgctgctgctgcggaagcggcggggcaagggcaaggttacgtcgacgcaaggctcgctgc
tgctgttcgactgccgcgccgtgagggcggcgacccgggacttctcggagaagctcgggagcgggagctttgggtccgtgttcaagggtgcgctctcag
acgcgacgcccgtggcggtcaagacgctggacgggctccggcagggagagaagcagttccgcaccgaggtggtcacgctcggcatgatccaccacgt
caacctcgtccgcctacgcggcttctgctccgaggggaacaagagggcgctcgtgtacgactacatggccaacggctcgctggacgcgtacctgttcaa
gaacggctcgagcaccaaggtgttgagctggagccagaggttcggcatcgctgtcggcgtggccaggggcctggcgtacctccacgagaagtgccgg
gagtgcatcatacactgcgacatcaagccggagaacatcctcctcgacgaggagctgggcgccaagctcgcggacttcggcatggccaagctcgtcgg
ccacgatttcagccgtgtcctgaccaccatgcggggcacgctcgggtacctcgcgccggaatggcttgcaggcggtgcggtcaccgccaaggccgacg
tgtacagcttcggcttgctgctgctcgagctcgtctcgggccgccggaacaacagctcatcgtcccagggcgcctgctctggcgtgtactttccggtgcat
gctgcggtcaggttgcaagccggcgacgttgttagcctgctggacgagaggctggccagggataccaacttgaaggagcttgagagggtctgtaaagt
cgcgtgctggtgtatccaggacgacgaaggcgaccggccgacgatgggcctcatcgtgcagcagcttgaaggtgttgccgacgttgcgttgccgccga
tcccgtcgcagcttcacatgctggcaatggcgaatgtctgcgtcagtggaaacgaaaaggatgaattctgttga
>g97370.t1
atgctgtcagattttgacctttcacttcggtgttcagtaaacctaactgtgatcaagtctgccaatccagggctagatgcactgcagaggaataatggagc
ttactgtgtccaacctacttgcatcgagccatcctgcattcagccctcatgtgttgctccaaccacttgttttgggccacggttcttctccagatcaaaatca
aagaccaagtccaagtccaaaaaggagaagtcaaagcctgaggttcaaaaccaggaaaacctattccccgagctcattgctgagccaaccgatgctc
gttctatgtcctttgttggtacccatgagtacctggccccagaaattataaaaggggagggtcatggaagcgctgtggattggtggacctttggcatattc

ttgtatgagctgttgttcgggaagacccctttcaagggttcgggcaatcgggctacacttttcaacgttgttggtcagcctttgaggttccccgagtctccg
atagttagcttctctgcgagggacatgataagaggattgctggtcaaggacccacagcaccgacttggctacaagcgtggtgctactgagataaagca
gcatcctttctttgagggcgtgaactgggctctcataagatgtgcaagccctccagatgtaccaaagccggttgagctcgactgccgtccaaagcaagtg
tcattgacaaatgggaaagttgcgccagttgccaaccaaaagggtccagataactatctagagtttgaattcttctag
>g97633.t1
atgggcgcttgcttctcctccgcctccgctgccaccgccgccgccgggccgcccaaggcggagaagaggaagcgccgcggcgccgcctcgccggacg
gacaggggaaggcggtgcgggtggagttcggctacgacagggatttcgaggcgcggtacgaggtcggcaggctcctcggccatggacagttcgggta
caccttcgccgccaccgaccgcggctccggggaccgcgtcgccgtcaagcgcatcgacaaggccaagatggaccgccctgttgccgtggaggatgtg
aaaagagaagtgaagattcttaaagcacttcaaggacatgagaatattgttaacttctacaatgcatttgaggatgattcgtatgtgtatattgtgatgga
gtaa
>g97782.t1
atggcacaggctgcgtccagcattgcggaggtcaataggctggggctcgcgttgcagcaggagaagttgcactacgaggaggcggacaaggcggctc
aggactctaccgcatgttgcgcagacctcgggtcaaagctgacagcggtcgagggctccctgcgtcaggaggtcgaggagaagggcgcacttcgcgct
ctgatgggtcgagcggtgcaggagcttcaggtggacctccctctcgacgtcgttcctgctgaacatgtcagcaggtcaaagaaagaattgtgcgctgca
aacttgtccaccgtctggaagatcagcggagccgacccaaccgcgggcagttatgctactcaatacgccgccaaccctgtgcttctccgccccatcgca
gcgccggaataccgtaacctgtatttcggtgccggattctactgcgttaccccgtgcaacaccttcgtctttggggtctacgctgtttccactgtgaaccag
agtgatgcgtacaagctcaatgccaacgcaaactcggtgatgcttgtctggtcggcgaaccgtgaccaccaggtccagaagagtgcaaccctcaacttc
accggcgacggtgatctcattctccgggacgccgatggcagcctcgtctggtccagcggcacatcaggccggtctgtcgttgggatgaacatgacggag
tccggtaacgtggtgctcttcgaccaggacaacaagtcggtgtggcagtccttcgatcatccggtggattctctgctccccggccagcgtctactcgtcgg
gaaaagcctcactcccactgcaaacttgtccaccgtctggaagatcagcggagccgacccaaccgcgggcagttatgctactcaatacgccgccaacc
ctgtgcttctccgccccatcgcagcgccggaataccgtaacctgtatttcggtgccggattctactgcgttaccccgtgcaacaccttcgtctttggggtct
acgctgtttccactgtgaaccagagtgatgcgtacaagctcaatgccaacgcaaactcggtgatgcttgtctggtcggcgaaccgtgaccaccaggtcc
agaagaatgcaaccctcaacttcaccggcgacggtgatctcattctccgggacgccgatggcagcctcgtctggtccagcggcacatcaggccggtctg
tcgttgggatgaacatgacggagtccggtaacgtggtgctcttcgaccaggacaacaagtcggtgtggcagtccttcgatcatccggtggattctctgct
ccccggccagcgtctactcgtcgggaaaagcctcactcccaacatcgccaagggactatcttatcttcatgaggactgtaggcagagaattgctcatctt
gacatcaaactgcagaacatccttgtggatgacaacttcgatgctaaagttgctgacttcgggttatgcaagctgatagacaggggccagagtcgtgtg
gtgactagaatgagaggcacacctggatacttggcgcctgaatggttgacctcacaaatcactgagaagggtgatgatgaaatgcacatgcacaggga
agaagttattcagatgatgaagttcgcaatgtggtgtttgcagattgattacaacaagaggcctcaaatgtcggttgttgtcaaggtactagaaggaaca
atgaacgtggaaactaatatagagtataactttgtaactatggttgcaaccagccttggcaatgctgggaagctggcgtcatcaggtccacttctagcgt
cgcatttatcaggtcctaggtga
>g97806.t1
atggaggcgccgccggggccggatcccatggcgctggacgcgcctgcgcccgcggccgcatccgccgcccagccggcccctgtcagcaaggagaag
ttgcaggcgctcaagacgatgaaggacgagaagggcgagacggtcacgggccacatcatctccaccaccatcggcgggaaaaacggcgagccgaa
gcagactatcagctacatggcagagcgtgtcgtgggtacaggttcttttggaatcgtcttccaggctaagtgtttggagacgggagagactgtggccatc
aagaaggtgcttcaggacaagcgttacaagaaccgcgagctgcaaattatgcgggcgatggatcaccccaacgtcatctgcctgaaacactgcttcttt
tcgacaacaccaagggacgagctgttcttgaacctcgtgatggagtatgtcccggagacgctgtaccgcgtgcttaagcactacagcaacgccaacca
gagaatgcctcttatctacgtcaagctctacatgtatcagctttttagagggctagcttatatccataccgttcctggaatctgccacagggatgtcaagcc
acaaaacgttttgctggatgcatgtgcacacccccttctttgatgagctacgggcacctaatgctcgcttgccaaatggccggccattccctcctctattcaa
cttcaaacatgaactggcagatgcctctcccgaccttatcatgaggctcgtaccagagcatattagacggcagaatggccacaactttggccatgctgg
gagctag
>g97892.t1
atgcatcggtggatcatcgccagctgcggcgacatgcggccggcggcgccccggccagcccgccgtcgagatgagcctcctccgcagcagagggcgt
cgtcccctcccaagcttcggaaggtcgggtcggaggggacgctggctattccaaaggacgtggaggagttccggaccatgtcggcgtacgggcacctc
aagctgttcacacacgacgagctcaggcaggccaccagagacttccatccggaccagatcatcggcgaaggtggctttggcgtcgtctacctcggggt
catcgatgatcggaatggctaccctgtcgccgtcaaggagctcaatccggagggcctacaaggggacagggagtggctgacagaggtgagttaccttg
ggcagtacagccacccgaacctggtggagctcatcggctactgctgcgaggacgatcaccggctgctggtgtacgagtacatggccaagggaagcctc
gagaaccacctcttccgccgctcctgcaacctgtcgtggacgacaagggtcaagatcgtgcgtgacgtggcgagggggctcgcattcctccacggcgc
cgaccgccccatcatctaccgagacttcaagtcctccaacatcttgctcgacaaggatttcaacgcgaagctgtcggatttcgggctggccaaggaggg
gcccatgggcggaaagacgcacgtgtccaccagggtcatgggcacctacggctacgccgcgccggagtacatggccaccggccacctcacggtcatg
agcgacgtctacggcttcggcgtcgtgctgctggagatgctcgtgggccgacgagcgctggagcaaagccgggcgggggcccgcgacggcagcctgg
tcgactgggcgcggcccatcctcatccggcccaagaagctcgagcggatcgtggaccggcggatggccgagctcggcggggcccggagcttggagcg
cgtggcccgcgtcgcgtacgactgcctcagccagaaccccaaggtcaggcccgccatggccaaggtcgtgcagacgctcgacgccgtcctcgccgccg
acgacgaccatgacgatggagacgcagccggggtcgacgatgctccgccacgggtcaagatcgtgcgtgacgtggcgagggggctcgcattcctcca
cggcgccgaccgccccatcatctaccgagacttcaagtcctccaacatcttgctcgacaaggatttcaacgcgaagctgtcggatttcgggctggccaa
ggagggccccatgggcggcaagacgcacgtgtcgaccagggtgatgggcacctacggctacgccgcgccggagtacatggccaccggccacctcac
ggtcatgagcgacgtgtacggcttcggcgtcgtgctgctggagatgctggtgggccgccgggcgctggagcccagccgtgcgggggcccgcgacggc
agcctggtcgactgggcgcggcccatcctcatccgacccaagaagctcgagcggatcgtggaccggcggatgggcgagctcggggcccggagcatgg
agcgcgtggcccgcgtcgcatacgactgcctcagccagaaccccaaggtcaggcccgccatggccaaggtcgtgcagacgctcgacgccgtcctctcc
gctgccgacgacgacgttgaggcgtggcgccaaatcgattga

>g98089.t1
atggacaagaacgagaaggagaagaaggagggcgccacggtcacccatggtgtgtgtccagcggtgggcctgcgcgctgccgccttcgtcattgcgg
ccctcctctgctccacggcatcgctggcgctcaccccggacggtacggagctgcatttcctcttcaatcgatttgcaatgaactcctcccgcccgcccttac
tcatcgtctcggttgcaggcgaagctttgctggagctcaagctggccttcaacgccaccacccagcggctcaccacctggaggccgtccgaccccaatc
cctgcggctgggagggcatctcctgctccctacccgacctcagagtccaatccataaatctgccctacatgcagcttggcggcatcatttcgcccagcatt
ggcaagctcgacaagctccagagactggctctgcaccagaacagcttgcacggatcaatccctccagagattaagaactgcaccgagctcagagcaat
ttatctgagagctaactacctgcaaggaggcatcccttcagagattggcgagctcatccacctcactatcttggacttgtctagcaatctgttgaggggca
caattccagcatcgattggaagcctcactcacctgcgttttctgaatctgtccaccaatttcttctccggtgaaatcccgaatgttggtgtccttggaacctt
caaaagcagctcgttcgttggaaatctggaactttgcgggctgcccatccagaaagcttgccggggaacccttgggtttcctgcagtgctaccacattct
gatccactctcctcatctggtgtttctcccatcaacaacaacaaaacatcacatttttgaatggcattgtgattggttcgatgtcaaccttggctcttgcctt
gatcgcaatacttggcttcctatggatttgcttgttgtctaggaagaagagtattggtggaaattatgtaaagatggacaaacaaggtgttcctgacggtg
cgaagcttgtgacgtaccagtggaaccttccctattcatcgagtgagatcattagaaggttggagttgcttgatgaagatgatgtggttggctgcggggg
atttggcacggtgtacaaaatggtgatggatgatggcacagcatttgcggtcaagaggattgacatgcaccgtgaagcgcgtgataggacttttgagaa
ggagcttgaaatcctgggcagcattaggcacatcaaccttgtcaacctgcgaggctactgtaggctcccaacagccaaactactcatatatgattttgtg
gagcttggcagcttggattgctaccttcatggagatgcacaggaggaccagccattgaattggaatgcacgtatgaaaatagctctgggttcggctcga
ggtttagcatatttgcaccatgactgctcacctggtattgtccacagagacatcaaagccagcaatatccttctggatataagtttggagcctcgagtatc
agattttggcctagcaaggctgcttgtagacaatgctgcccatgttaccactgttgtggctggcacctttggatacttagcaccagagtacttgcaaaacg
ggcatgctactgataaatcagacgtgtatagctttggcgtgcttttgctggagcttgtgaccggaaagaggccaaccgattcatgcttcatcaagaaggg
actgaacattgtcggctggctgaacacactgacgggggagcaccggttggaggacatagtcgatgagagatgcggcgacgtggaagtggaagcggt
ggagtcgatactggacatcgctgcgatgtgcactgatgcagaccctagccagcggccgtccatgagcgccgtcctgaagatgctggaggaggagatcc
tgtctccctgcatgagcgagctttgctacgagcagcacctagagctatga
>g98183.t1
atgggcgggctgtgctccaaggatgccgtcctggacgcgccgccgcccgcgccggtcccggcccacgcgctggtccccgtggccacgctgcagaagg
cgcccagccactcgctgaagcagctcatcgcgctggcccccatggacgacgacgcctcctccgtcgtgcacgccgtcctctcccgcaccgactccaacg
ccaagcccaacaagccgggcggcaacaccaaggcgagcggcggcgcggcggcggcggagaagaaggcggtggagaagacggcgcctcccgtggt
ggtcatctcgtcactcagcaagtcgtacagcacggcgggggcgccgacgcaccaccgccgcgacaccgtggacggcaataatggcgtcggcggcgcg
gcggcggtgatctccagcatgccgcaggggttctccggcgagcacgtcatcgccgggtggccgtcgtggctcacctccgtcgccggggagatcgtgga
agggtggctgccacgccgcgccgacacgttcgagcggttggacaagatcggtcagggcacgtacagcaacgtgtacaaggcgcgtgacctggagacc
ggcaagatcgtggcgctgaagcgggtccggttcgtgaacatggacccggagagcgtgcggttcatggcgcgggagatccacatcctccggcgcctgg
accaccccaacgtcatcaagctcgaaggcatcgtcacctcccggctgtcccacagcctctacctcgtgttcgagtacatggagcacgacctggcgggtct
cgccgcgctgccgggacagaggttcacggagccgcaggtgaagtgcttcatgacgcagatcctggaagggttgcgccactgccacgcgcgcggcgtg
ctccaccgcgacatcaagggctccaacctcctcatcgacgacaacggggtgctccgcatcgcggatttcgggttggccacgtttttcgaccccaaccgg
aggcagcccatgaccagccgcgtcgtcacgctctggtaccgcccgccggagctcctgctcggcgccaccgagtacggcgtcgcggtggacctgtggag
caccgggtgtatcctcgccgagctgctcgcggggaagcccatcatgccaggacagacagagatcgagcagctgcacaagatcttcaagctctgcggct
ccccgtccgaggactactgggccaaggcgaagctgccggacgtgacgctgttcaagccgcagcggccgtaccgccgcaagatcgccgagacgttcaa
ggacttctcccccacggcgctggcgctcctcgacacgatgctcgccatcgagccgtcggcgcggggaacggcggcgtccgccctcgacagcgagttctt
caggaccaagccgctggcgtgtgacccggccagcctgcccaagtacccgccgagcaaggagtacgacgccaagctccgcggccagcaggaggcctc
caggcaaaactcgaacgcgatcggcggcaagggctcgcagtccatcaaacaggggagagacgacaaggtcgtcgcgtcaggtaccaaggagtcgtc
cgcggcggagcagcaccggcggcaggtgcgcgcgaaccagaagagcacgagccaccactacagctcgcaggaggacagcgtccctgggttccgcat
cgagccgccggccgtgggccgcgcgggccccgccacgatgcagaccgccggcggcgggttcgggtccacgtggtacggccggaacgagacgcaca
aggcggcggtgtcgcgcacgtccagctccgtccgggtctcgcaccaggcctcctcgcaccggtcgcggggcaccgacctgcacccgagctcgtcggcc
gcccggaacgccaactccaggtacaaccggctcgacgtcgccgagccggcaaacgccatggaccgcccgggttcgtcccacaacaaggacggcggc
atgagggacagggacgcctcatcagctgggttcggggcgaagaacaagaggattcactactcggggccgttgatgccgccgggtgggaacatggag
gacatgctccgggagcacgagaggcagatccaggaggcggtgcggaaggcgcgggtggagaaggagaggaccaacaggcaccaccattactga
>g98425.t1
atgatcgacggtgtcgcgaacgatgatgtcgtgctgccgggcgccctcctggcgggccggtatcgcgtcgtcgagccgatcggttcgggcggcatggc
gcgggttttcctcgcgcgcgacgaggccctcggacgccccgtcgctgtcaaggtgctgcggcccgagctgaccgacagtgccgcgtccgatcgtgcgg
cgatcgagacgcgcctgctcgcctcgctcaatcacccgggcctggtcaccctgttcgacgctcatctgacggccgaaccgcgcttcctcgtgatggagca
cgtcgtcggcacgacgctggcgcagcgaatcgcgacaggtgtgctcgacgagggcgagctcgcagcgctcggcggccagctcgcggacgcactgcac
gtcgtccacgacgcggggatcgtgcaccgcgacgtgaagccgtccaacatcctcctcgcccggtcgacggtcccgggggttcccctgcgcgccaagct
ggccgacttcggcatcgcccacctcctcgacagcgaacgtgtcacctcaccgagcctgctgatcggcaccgccgcttatatcgctcccgagctgctgcac
ggggccgacccggccccgccgtcggacatctacgccctcggcctcgtgctgctcgaggcggccacgggtgagcgcgcgttctccggatcggacggcaa
tcgcggccagctgctggcacgtttgaccgcccgaccgcgctcgaggtcatgtcacggctga
>g98454.t1
atgagggagctgcgcgtcttggtgctggttctggtgatatccttgccgtgcttccccgcatccgatcgtcaaggcgaggatgatcgacgaattgcatttgg
ccagctcaaaagatttgcatggcgagaactacagattgcaactgacagtttcaatgaaagaaatgttcttggtcagggaggttttgggaaagtttacaa
gggagtacttccagatggtactaagattgccgtaaaacggctgactgattatgagagtcctggtggggaggctgccttcttgcgtgaagtggagctcatt
agtgttgcagttcaccgcaatctgctaaagctgattggcttttgcaccacacagacagagcgccttctggtctatccatttatgctgaatttgagcgttgcc
tgccgcttacgagatttcaagcctggagaacccatcttagactggcctacaaggaaacgggtggctattggcacggcacgtggcctggagtatctgcat

gaacactgcaaccccaagatcatacaccgtgatgtgaaagctgcaaatgtattgcttgatgagtattttgagcctgttgttggtgattttggcctcgcaaa
gctagtagatgtgcagaagacatctgtgacaactcaagtccgtggaactatgggccacatagcgcctgagtacctatccacagggaagtcgtctgaaa
ggactgatgtttttggctacgggatcatgcttctggagcttgtgaccggacagcgcgccattgacttttcacgtttggaggaagaagatgatgtgctattg
cttgatcatgtgaggcttatttcctttgttttcaaggtaaagaagctacaaagagaagggcagcttggctctattgtggacggcaacctgaatcagaatta
tgatgaggaggagcttgaaatgattatacagattgcattgctgtgcacannnnnnnnnnnnnnnnnnnnnnnnnnnnnnnnnnnnnnnnnnn
nnnnnnnnnnnnnnnnnnnnnnnnnnnnnnnggaactatgggccacatagcgcctgagtacctatccacagggaagtcgtctgaaagga
ctgatgtttttggctacgggatcatgcttctggagcttgtgaccggacagcgcgccattgacttttcacgtttggaggaagaagatgatgtgctattgcttg
atcatgtgaggcttatttcctttgttttcaaggtaaagaagctacaaagagaagggcagcttggctctattgtggacggcaacctgaatcagaattatgat
gaggaggagcttgaaatgattatacagattgcattgctgtgcacaagcatcacctga
>g98523.t1
atgggtctctgctatggcaagtcagcggcggctccggagccggcgggggtggaggaagcctgcgtagccaacggcgccggtgacccggtcccggaca
gcgtcgcgtcgccggcgaagccccggacaccgaagcagcccaagttctcgttctacctgccgagcccgctcccgccttcgagctacaagggttcgccg
gcgaactcgagcgtggcttccacgcccgcgcgcggcgggttcaagcggccattcccgccgccgtctcccgccaagcacatccgcgccctgctggcgcg
gcggcacggctcggtgaagccgaacgaggcttccatccccgagggcggggagccggagctgggcctggacaagagcttcggctactcgaagcacttc
tcggctaagtacgagctcggccgcgaggtggggcgcggccacttcggctacacctgcgccgccaaggccaagaaaggcgagctcaagggcgaggac
gtcgccgtcaaggtcattcccaaggctaagatgacaactgctattgccatagaagatgtcagaagagaagtcaaaatattgagttctttgacaggtcac
agcaacctagtgcaattttatgatgcttttgaggatgaagaaaatgtatacgttgttatggagctatgcacaggaggtgaactgctggacaggatcttgg
ctagaggtggaaagtattctgaagaagatgccaaggttgtcattcatcaaattctaagtgttgcttcattttgccatcttcagggtgttgttcaccgggatc
tgaaaccagagaatttccttttcatgtccaaggatgagaattctgccttgaaagtcatagactttggtttgtctgactttgttaggccaggttaa
>g98813.t1
atggagttcgaagtcgacgacgccgccaggctggaccaacgcgcgcggtgcgccggcgccgggaacagctgccgtacgcggaagccatgcggcgtc
ggacggggagaacgtggtggcgccgggccggcagtctccgccacggccgcgcgccgccaccaccgactcgtacgcccgccgtcgcccgtcgtggca
gaggagttcgacctagatttcgacccgtcttccccgctgggcgtcaacgccgatcgtaacctttgtagtacacggcggagcaattcatcagacgaaaaa
gactggtttcccggtgtagcgaagtgccgcatctttagcgtgagacccggcgacaacgctcgcgccctcgtggagtcagccgtctgcagcgtcgttgca
gttttcgacggcgacctcgatgctctcctctccgccagacagcgagaacgttgctactacatggcacgaagcccgtgccgctcccaccactgtggcacca
accgaccagcaagaggccaggcgagggtgtcgtgccgtgcggtgcgaagcatcatcaggtacaccatggagggagctggcagagatgtcaatccctt
gagcggctaccgaatcggcaaaaccctggggattgggtcgtttggtaaagtcaagatcgcggaacatatattgactggccataaggtggcgatcaaaa
tcctcaatcgccggaagattagaagcatggagatggaagagaaagtcaagagagaaatcaagatactgagattatttatgcatcctcatatcatacgtc
tttatgaggtcatagatactcctgctgatatttatgttgttatggagtatgtgaagtgtggagagttgttcgattatattgttgagaaaggtagactgcaag
aagaagaagctcgccgctttttcagcagtttgatgaggaaacactaagtgatgttatcaagatgggttttgacaaaattcagctaatcgattctctgcag
aacaggttgcaaaatgaggcgacagttgcatattatttactattggacaacaggcttcgtacaaccagtggttatcttggagctgagtttcaagaatctat
ggactcttctttcgcacatgtaacttctgaaacgccaacttcagcaactgaacttaggctgcatgggcatatggaatctcctgggtttggcttgaggcaac
attttgcagctgaaaggaaatgggcccttggtcttcagatggagtcctag
>g98955.t1
atgcacacagaagcagcggcaatggatcccgccgccgcgcacgaccccacggcgccctgctccccgctctccaccaccaccacgaacaccgacctcc
tctcccctcacttccctccctccctcgccgacggcgccgccgcgcttgacctgtccttcacctcgaccgcctccgcctccacctcctccttcacgacggcca
ccaccttcagcgcgcgcagctcgctgtccctcccgtccttctcctcctccacctcgctctccccgcgcccacactcctcctcctcctcctcgcactggaccca
cctcgccgccgcccgcgccgccaccccgacggcgtcctccgcctgggccacctccacctggtccgcgagctgggccacggccacctggcccgcgtctt
cctctgccgcctcaagagcgccccgccgagctccccgctcttcgcgctcaaggtcgtcgacgtgcgcgacgacgacccgtcccgcgtgtcccacgtgct
gaccgagtcccgcgtgctctcctcgctcgaccacccgttcctgcccaccctgtacgcgcggctcgacgccggccgctacgcgtgcttcctcatggactact
gctccggcggagacctccacgccgtgctccggcgccgccccggcgggcgcctcccggtggccgcggccaggttctacgccgccgaggtgctcctcgcg
ctcgagtacctccacgcgctcgggttcgtgtaccgcgacctcaagcccgagaacgtgctcctgcgtggcaacggccacgtcgtgctctccgacttcgacc
tcgcgctcccggcgtccgtcgagcccgccgtgcgcaagcgccatgtgcggaatggtaagaaacagagtcgaaggagcagcaggaagatgaacacctt
cttcttgtcgtgcttcacatctgctggcaaaaacgacaaggacaacgaggaggagagcatggtggacgcgaaggagcggttcgaattcgtggcggag
ccaacgggcgcgagctccaaggactgcgtaggcacgcacgagtacctcgcgccggagctcgtcagcgggagcggacacggaaacggcgtggactgg
tgggcgttcggggtcttcctctacgagctcgtgtacggccgcacgccgttcaagggggccaccaaggaggccacgctcaagaacatcctgtccaggca
ggtcgcgtacccgcacctcgacaacgccgagtcgtccgacgacgtggcgcggctcaaggacctcgtcgggaggctgctggagcgggacccgcgccgc
cgcatgggcgcgtcgcgcggcgccgcagagatcaagaggcacccgttcttcgccggcgtggactgggcgctcatacggtgcgtggcgccgccggtcgt
gccggacaaggacgcggccgccgcgggaaagccggcggggaagcagctggggagctggagcagcatgggacgcggcagcagcgccagcagcaa
gaggaagagcagcagcttctccaggaagtccaattacgaggagcgccagggggttttccgcaagctcatgacctggaaccacgagacccggtccagc
aagaccaccaccaagatcagcaaagtgaaactatga
>g98982.t1
atgtccatgaatcacctcaccgggcagataccggaggacatgttcagggcgccgagcctcgagagcctccacgtatatcagaacaatctgactgggcg
gttgccggagaccctggggacggcgccaagtctgtcggacctcaggattttcagcaaccagcttgaagggccgctgccaccggagttggggaagaatt
gcccgctcgagttcctcgacacgtcggacaaccggctgtcgggtcccataccggcgaatctgtgtgcttccgggaagctgagccagatcatgctgttgg
ataacgagtttgaaggtgtcattccggcggaattggggcaatgccggacgctgttgagagtgcgtctgcagagcaacaggctgtccggccctgtgccgc
cggaactctggggactgccgggtgtcattctgcttgagcttcgagataatgcattatcagggactattcatcctgccattgctggtgccaagaacttgtgc
aagctgctcatacaggacaaccagttcactggtactctgcctacagagttgggggcattgtccagtctagcagagttggtggcttccaacaatggcttct
ccgggccgctgcccacttcgctcgccgagctctctgcactttacaagcttgatctcagcaacaactccctctccggtgggatcctgcctggattttctaagt

tgaagaatctgtcgcagatggacctttcatataatcacctgagtgggagcatccctcctgaggttggagatattggaaatgtaaacttacttgatttgtcg
aacaatgagctttctggtggagtgcctgtgcaattggaaaatcttaggctgtctcatttcaatctatcatataacaagctctcggggcctgtacctggttac
ttcagtgacttggcgtaccgagaatgcttcttgggtaaccctggcctgtgctatgggttctgtcagggcaatggtaattctggtaacaaccggtctacactc
gtcaggttaattgttgtcattctcattgccgcagcgatcgtcctactcatcagtgtcgtgtggcttggttacaagtgcagaacccacgggggtgttgctgct
gaatccggtgatggaaaatctacctgggtgctcacatccttccacaagatggatttcagtgagagagacatagtgaatagcctccatgaaaacaatgtg
attggtcagggtgcagcaggcaaggtctacaaggctgttgtcgggcctcacggcgaggccatggccgtcaagaagctctgggcgagcagtgacccaa
gtgggagaaatgacacctttcaggcggaagttgagacattaagtcaagtacggcacaagaacatagtgaagcttgcttgctgtatcacaaacagagtc
tgcaggttgctcgtctatgagtacatgcccaacggcagtctgggggactttctccacagtgccaaaagcagcatcttggcctggccgatgagaacccac
gggggtgttgctgctgaatccggtgatggaaaatctacctgggtgctcacatccttccacaagatggatttcagtgagagagacatagtgaatagcctcc
atgaaaacaatgtgattggtcagggtgcagcaggcaaggtctacaaggctgttgtcgggcctcacggcgaggccatggccgtcaagaagctctgggcg
agcagtgacccaagtgggagaaatgacacctttcaggcggaagttgagacattaagtcaagtacggcacaagaacatagtgaagcttgcttgctgtat
cacaaacagagtctgcaggttgctcgtctatgagtacatgcccaacggcagtctgggggactttctccacagtgccaaaagcagcatcttggcctggcc
gatgaggtataagattgcttgcgatgctgctgaaggactcagctacttgcaccatgactgtgtgccccctatcatccacagagatgtgaagtcaaacaac
attttgcttgatgcggagtttggtgcgaaggtttcagattttggtgttgcaaagacacttgggaatggacctgccactatgtctgtcattgctggctcttatg
gatacatcgctcctgaatatgcttacactctccgtgtaactgagaagagtgacatatacagctttggcgttgtgcttctggaacttgtcactggtaagaag
ccgatggcaccggagatcgatgagatggaccttgtgacatgggtgtctgctaatgttgagcagaatggaccagagtccgtgctcgatcagaccctcgcc
gagcagtttaaggatgagatgcgcaaggttctgaggattgggcagctctgcgtggcaaaacttccaaacaaccgcccaccgatgagggttgtggtgaa
gatgctgcaggaggtcaagggtgcgatccagccaaaggtgaatcaggaggcatcccctgccatctga
>g99007.t1
atggggtacctctcctgccgggcggactcgtccgtggcgacgtgccgctccatcacggccatctcgccgctccccatctcgcgccgctcgggctccggct
cctcccggtcgtccaggcggaggccgccggcgctacctcccccatcgagcggttcgcgtacgcggagctggaggcggcgacctcccacttcgcggag
tcggcgctgctgggccggggcagccacggggccgtgtacaaggcggtgctcccctcgggccgcgccgtcgccgtcaagcgcccctccccgcgccgcg
ccgaggtggacaacgagatccgcatcctctcctccctgcgcggcccgcgcctcgtcaacctcctcggcttctccgaccccgggcccgcctcccccgccg
gctcctcctcctccgctgcccgcctcctcgtcgtcgagtacatgcccaacggcacgctgtacgacctgctccactccagcccgcgcccccccgggtggcc
gcgccgcctccgcctcgcgctgcagacggcgcgggccctccgcgcgctccacgacgccgacccgcccgtcatccaccgcgacgtcaagtccgccaac
gtcctcctcgacgccaacctcgacgcgcgactcggcgacttcggcctcgcgctgcgcgtgcccggcaagcgactacccggggacgccgccccggccac
gcccgcgcccgcgggcacgctcggctacctcgacccggcctacgtcacgcccgagagcctcagcaccaagaccgacgtcttcagcttcggcatcctgct
gctcgagatcatgagcggacgcaaggccatcgacgtgcagcactcgccgccctccgtcgtcgagtgggcggttcccctcctgcggaaggggaagtgg
gcggttcccctcctgcggaaggggaaggtggccacgctcttcgacccgcgggtgccgccgccgcgcgacccggccacgcgcaaggacctggccgcgc
tggcggccagctgcgtgcgctcctgcagggagcgccgcccgtccatggccgacatcgtcgaccgcctcgtggtcctcagcaaggccgtgtctgccaag
gtatggaacggacttgcagatgggctcgcggtggtcggcaatccttgcgcggtcgtcgatgtccagaagaccatctccaagcgagctgccgccagcag
tagagctgaatcggagcgggagtcgacttcagtgattgcatttgacgatgacgatgatgataaggaagaagaggaagaagaggcggttgaacaggcc
cttgaggatcaggttccactgggtgattcacgatag
>g99051.t1
atgatcaaagatggcaacctacactgtgcatcaaccatagcgggcaaaggccactgcatcgtttactcagctgatgtggggagcagaaggggacgcac
gtacggggatgaggagaccgcgaggatcgcctacctgggacgaacccgacgaaggccaccacaagtccacgcgaaggcgttcaattgggagtcatg
gagcaagggaggatccgggtggtacgatttcctgcgcagccaggtggacgacatcgccggcgccggaatcacccacgtgtggctgccgccgccgtcg
cactccgtggacgcgcagggctaccttccgggtcggctctacgacctgaacgcgtccaggtacggggacgaggcgcagctccggtccctcgtggcggc
gttccacagccgcggggtgaagtgcgtcgccgacatcgtcctcaaccaccgcacggcggagcgcaaggacgggcgcggcgtctactccgtcttcgag
ggcggcacgcccgacgaccgcctggactgggggccccacatgatctgccgcaacgacagctactccgacggcacagggaacgccgacacgggccgg
gactacgagccggcgccggacctggaccacctcaacgcccgcgtccggagcgagctcgccgactggctcaactggctcaaggacgacgtgggattcg
acggctggcgcctcgacatggccaacggttactccgcggccgtcgccggcatgtacgtcaacgccaccgcgcccgacctcgccgccgccgagatatgg
acggacctggcgtacgacaaggccgacgggaagccgagcgccgaccaggacgcgcaccggcagaaccttgcggcgtgggtggacgccgtcggcgg
ccccgccgccgcgttcgactacaccaccaagggaatcctccaggccgcgctcaacttcagcgagctgtggaggatgcaggacgctcaggggaggccg
ccggggctggttggcctgcggccggcgcagaccgtcacgttcgtcgataaccacgacacggggtccaagacgcagcacagctggccattcccgccgg
agaaggtcctgcagggctacgcctacatcctctcgcatcccggcatcccgtgcatcttctatgaccatttctttgatccgaccatgaaagacgacatagct
acgatgataaaaatccggatacggaacaagatcgggccgacgagcacgctgcggatcctactcgctcaaaacgacgcctacgtcgcggagatcgacg
ggaaggtggtgaccaaggttggagctaggtacgatgtcacgaggactgttcctccggggtttgcgttgaccgccagcgggaacgatttcgcaatatggg
agaacaacaggtccagcggcaatgtgcaacccaacacagagccggcgtcgttcacaggaagaaggtcgtcgcggaggtgggtggtcccagtcgccg
ccacggttgcgccgttcgcggtcttgctcacgtgcagtgcggcattggtgctcctgttgcggcggcggcaaaagcacaactccgacgacgcgcagtact
ccgacgggggaataaagcagcggcggagttcgagaaaggggtgggagtggggccgaggcggtacagctaccgcgcgctggcggcggcgacggg
caacttcgccgatgcgaaccggctcgggcgcggcggcttcggccccgtctaccgcgggcgcctcgaagtcgaaggccaccaccagggccgcgacgtc
gccgtgaaggtgctcgcgccggagtcgtcgtcctccgccgcgcatggccgcaagcagttcgaggcggaggtgcggatcatcagccagctccggcacc
gcaacctcgtgcagctcctcggctggtgcgacgacgacaagggccgcctcctgctcgtctacgagctcgtcccgggggcagcctcgacacgcacctc
cacggcaccgggaaactcctgacgtggccggagcgccaccgcatcgtgctggggctcggcgccgcgctggcgtacctgcacgaggagtgggaccagt
gcgtggtgcacggcgacatcaagccgggcaacatcctgctggacgcgtccctgggcgtggccaagctcggggacttcgggctggccaggctcctcgac
cgcgccgcgggcccgcggaccacccgggtggtgggcggcaccatgggctacatggacccggacctgctcagcacgcgccggcccagcacggcctcc
gacgtctacagcttcggcgtcgtgctgctggaggtggcctgcggcaggcgcgccacggacgaggcgctggccgagtgggtgtgggacctccgcgacc
gcggcgccgtactggaggccgccgacgcgaggctgggcggcgagttcgacgcgtgggagatggagcgcgtgctgctcgtggggctctggtgctcgca

ccccgtgccggcggaccgaccctcctccgtcgtgcacgctgtcaacgtgctgcagtcccgggatgccgcgccgccggaccttcccaccaacgtgcatcg
tgccgccacgcccgggttcagtggctacatgcactgcttgtcctccgtcagcagccttgcggagcaacatggaaaatga
>g99073.t1
atggtgaaccacagcaacctggtgaagctggagggattctgcatcaacgcgtcgacgggcgactgctacctggtgtacgagtacgtggagaacgggtc
gcttgacgtgtggctgctggaccgcggccgcgcgcgccgcctcaactggcggtcgcggctccacatcgcgctggacctggcccacgggctgcagtaca
tccacgagcacacgtggccccgcgtggtgcacaaggacatcaagagcagcaacgtgctgctggacgcccggctccgcgccaagatcgccaacttcgg
gctcgccaagacgggccacaacgccgtgaccacccacatcgtgggcacgcaggggtacatcgcgcccgagtacctcgccgacggcctcgtcaccacc
aagatggacgtgttcgcctacggcgtcgtgctgctcgagctcgtgtcgggtcgcgaggccgtgaaccacagcaacctggtgaagctggagggattctgc
atcaacgcgtcgacgggcgactgctacctggtgtacgagtacgtggagaacgggtcgcttgacgtgtggctgctggaccgcggccgcgcgcgccgcct
caactggcggtcgcggctccacatcgcgctggacctggcccacgggctgcagtacatccacgagcacacgtggccccgcgtggtgcacaaggacatc
aagagcagcaacgtgctgctggacgcccggctccgcgccaagatcgccaacttcgggctcgccaagacgggccacaacgccgtgaccacccacatcg
tgggcacgcaggggtacatcgcgcccgagtacctcgccgacggcctcgtcaccaccaagatggacgtgttcgcctacggcgtcgtgctgctcgagctcg
tgtcgggtcgcgaggccgtgctcgacgagagcggcgagccgctgtgggcggacgcggaggagcgggtgtttcgcgggagggaggacaggatggag
gcgcgcgtggcggcgtggatggaccccgtgctcgccgagcagacgtgcccgcccgggagcgtggccagcgtggtgggcgtcgcgcgggcgtgcctgc
accgagacccggccaagcgccccagcatggtggatgtggcctacacgctgtccaaggcggacgagcacttcgccgactactccggcgagagcctgcc
cgttgacagcagcggcgaagtcaccgcacggtga
>g99233.t1
atgacgtcggcgtcccgcaagcgctcggcgcccgaggaatcccgtcccgtggcgacggcgacggctgctgccaagaagcgatcacgctacaacttca
gcaagatcgacgagtacgagatgctggaggagctcggggagggcacgttcggcgtggtggtccaggcgcggcaccgccgcacgggcgagacggtg
gccgtcaagtgggtccgcggcggcgcctccgagtcggagtccgacctcgacgctgtcgtccgcgaggccggctgtctcgccgcgtgccgcggtcaccc
gtcggtcgtccagatcagggacgtcgcctccgacgacgacacgggcgacctgttcctggtcatggaactcgtggacggcccctccctccgcgactggct
cacgcggcccgtcccggagaacacggcccgcgcgttcatgcgccagctcctgagcgcggcggcgacgatgcacgccgccccgatggcgacgatccac
cgcgacatcaagccggagaacgtgctcgtcacccccggctgcgagctaataaagctctgcgacttcgggtgcgcgacgccggcgaagccgctcggga
agccgtacccggagcggcgcgtgggcacgatgccgtactgcgcgccggagcagctgatgggcatccggttctacggcacggccgtggacgtgtgggc
gctgggctgcgtcatggcggagctcctcgccggcgagcccctcttcgcggcggacacggaggacgacgtgctcgtgcagatgctggacctgcaggacg
ggatcgccgccgttgggctgaaggccttcgatggcgtggtggagctgtcgacggccgggcgcgagctccttgccggtttgctcagcttcgaaccgggcg
cgaggctcacggcggcggaggcgctcaaacaccgctggttcacggaggaggtggcggagcccccctgccgccgcgaaggcagagtttcctggctttgt
gccactgttctcggcagcatag
>g99618.t1
atgacccttggccagctgaaccaccgcaacctggtgaagctcgtcggctggtgcgacggtggccagaacagcaggccactgctcgtgtacgagctgat
ggcgaacaagagtgtcgacaagcacctccacgacgagtcgacggagaagacgtcactgccatggccggaaaggtataagatcgtgatcggcgtcggt
cgcgccatagaatacctccacgctggctgccacaatctcatcatcctccaccgcgacatcaaacccagcaacgtgatgctgaacgccgccttcgagccc
aagcttggggactttgggctcgtgaagcgggtctacaccgggcagggctccctagaaaccgccatgttcgggacctacgattacatggaccctagttgc
atcgccaccagcacggtgagcaccgcgtctgacatgtacagcttcggtgtgctgttgctggagatcgccactggcaagataccgngtcgccaccagca
cggtgaacaccgcgtctga
>g99721.t1
atggcgggatcgggggagaaggcagcgggcagtggaggagtggagaagcgcaagtaccccattggatgggaggactacgagctgtacgaagaggt
cgggcagggcgccagcgccgtcgtgtaccgtgcgctctgcaagccgctcgatgagatcgtcgccgtcaaggtgctcgacttcgagcgcaccaacagcg
acctgaacaatatcatgcgtgaagctcagacaatgattcttatagaccatcctaatgttgttaaggcacattgttcatttgcaaaggaccaaacgctttgg
gtagttatgccgtacatggctggagggtcgtgtcttcacataatgaaatcagtctatcctactggtttcgaggaagccgtcattgccacggtgcttcgtca
agtcctgataggtttagagtaccttcatcaccacgggcatatacatcgtgatgtgaaggcaggaaacattcttgtcgattctcgtggtggaattaagctcg
cagactttggggtttctgcttgcctttttgattctggtgacaggcaaagggccagaaacacctttgtgggaactccttgctggatggcaccagaagttatg
gagcagctacatggatatgatttcaaagcagacatatggtcatttggaataactgcacttgaacttgcgcatggtaacgctcctttttcgaagtaccctcc
catgaaggtcttgcttatgacacttcagaatgctcctccaggcctcgaccttgagagagataagaaattctccagggatgagtacaaaaggggtattag
cagctggaacttcgatatggacgacctcaaatctcaagcatcactggttacagagtgtgatgacaatatctcttgcaaagattcagatgcattatcgttct
tcgacatggacagtttgcaggagcaagcaccagaaggacttcatttatcaagagatttcacaatgaagtatgatatagacaccgtgaaacttctcccga
agctgtttccaaggcacctaaatcagcagagtcacccaccgggcctacaaaagtgtcacagaacaaatactaaacgcaatgaagttgctcagttactcc
gacatggcttcaccgtccatcccttccatggcttcgccgtacattccttccgcaagtcgctcaatatctccatcatctgcattatcagttga
>g99822.t1
atgcgctggtggaagcgttcctcctcccctcccgctccgcctccgcctccacgccctcgtcccccgctcgctcctccacctcccgcctaatcggcggtag
caaccgccgcgatggcggcagcgggagtgccgaatcgcagccgaagcttgttaggacgccgcggatgcgctacgtcgttgacgactccgaggtcggg
ccctccggactcgggctggatgccgaccccgccaggagggaatcggcgggcgggtttgccctcgcgaccgcgagctcgacgccgatctcgaggtcgc
cgagcaacatggaggttgcgccgttgagatcgtcgtcgacgcccttgcttcttccgctgccgctgcctggcgagggggagttgtcctgccgcgtatctgg
gaggccgcttccctcgcctgcgtccaggatggttgatggggactggaccgtgctggcggcggaggcgccaggggttttagagactgggaatgaaagg
attccgccgttgctcgcctcgagagttatggcccaaactgtacaaaagactcatggccacaacggcttacagttaaatgggcctacatgtggtcagcgg
aggaaggcttttaaagagaagttccaagatacaaactccgctgaaactttgaacttcaggttgaacattcctgccaaaagtgctccaagcagtgggtttt
cgagccctgtgcagagtccccgaagattgagtaatgtagacttcccatctgctgcattctccgtccaagataccaatgtatggtcagcgcggtcagcctg
gtcatatgatatattgggatcttcacctccttgtgcctcacctgaaacatttgcggctggtcatgagcgttcccctcgctctagtccactaagaagtccagg
tctaatatcaagaaatccaagtggaccccatcaccaatgcatccaaagctgtacccggagaacaatgtttctcggtctgaaggccatggaagtgccag

ttttcatccgttacccctccctcctgcttctgtaagcccaaagcagacaaatggtagccaccaagtagttccaaaagttgagatgccgtcagtggctggtc
agtggcaaaaaggaaaactccttggcagtggcacattcggatgcgtatacgaggcgaccaacaggcatactggagctctctgtgccatgaaagaggtc
aatataattccagatgatgctaaatcagctgagtcgctgaaacaattggaccaggagataaagtttcttagtcaatttaagcatgaaaacatagtgcagt
actacggcagtgaaacaattgaagatagattctacatatacctggaatatgtccatcctggttctattcataaaatgcagcgcccttgcctgtgcatcacc
tgcattgctaccttcaacatagaattcacaccagctctgatgatcttctgcatgtcctccaagtacaaacctgcccagtag
>g99869.t1
atgtctgtggaggccgtgaagcagaatgttctgggggacaatggcaaaccagctgctgatcatacagttgacccagttcctgaaaaggccaacaagat
ggctaacaatgcaccagagtttgccgctactaaggaggtgttagaggagcaaaagcccacccattggcgtcaagagtcttctgcatctatgttggacaa
gggtccatctagtgtttgctcagactctggtgtgctggatgggctattgactccagaggaagattctggggagctgaaacagattaaaaacatagatggc
agtggtaaccaagaacagaagatatcgcaaaagagcagcacgagtgagagctttgccactgccaaagaggggacctgcacactcttcggcaaaggc
agcctggaaaacgtttctctgagcaagcagctaaaagttctccttgcattggaatacctgcacatgcttgggattatatatcgtgatttgaagccagagaa
tgtccttgttcgggaggatgggcatatcatgctgtcagattttgacctctcccttcgttgtgccgtaagcccgactgttgtcaagtctgccaaccctggacc
agatgcattgcagaggaacaaccaagcatactgtgttcagcctgcttgtatcgagccatcctgcatacaaccatcatgtgtggctccaactacttgctttg
gtccccggtttttctcctccaagtctaagaaggagaagaagtcaaagccgaagccgaagcctgagattgtcaaccaagttagtccactgccggagctca
tagcagaaccaactgatgctcgctccatgtcatttgttggcacccatgagtacttagcgcctgaaataatcaagggagaaggtcatggcagtgccgtgg
actggtggaccttcggtatattcctgtatgaactcctgttcggtaagacccctttcaaggggtcgggaaaccgggctacacttttcaatgttgttggtcagc
ccctgcgtttcccagagtccccaattgtgagtttctcagctagggacttgattagaggattgctggtcaaggacccacaacatcgtcttggctacaaacgt
gggactaccgagataaagcagcacccctttttcgagggtgtgaattgggcactgatacgatgtgcgagccccccagaggtacccaagcctgttgagctg
gattgccgaccgaagccagcagcagctaatgataaggttgccccagctgcaagtcagaagggcccagataattatctggagtttgagttcttctag
>g99903.t1
atggcgcttagccctggaattctcgccttagcaatcattggtgtgatcacgactggcgtagtgacagtcgttaccatagttgcgatctacatgtgctccaa
gatggccatcgagatgtacgtgcaaagagacagaggaggaggaggcatacgcgcggcgtggaacagagacgagcccagcggcggcggcgtgaac
gtcgaggcgggcgacgtggagatggggagcatgagctacttcttcgaggacatgcggaatgagcgcccggtccggttcagctcgcagcagctccgcg
ccttcacccgcaactacgcgcacaaggtcgggtccggcgggttcggcgtggtgtacaggggccggttccccaacggcggcgcggcggtggccgtgaa
ggtgctgcacagcacgctgggcaagcgcgcggaggagcagttcatggcggaggtggggaccatcgggcgtacctaccacgtcaacctggtccgcctc
tacggcttctgcttcgacgcgtcgtcggtgaaggcgctggtgtacgagtacatggacaacgggtcgctggacgggtacctgttcgacccgccgcccgag
aagcgaggggtcgggttcgggaagctccacgacgtcgcggtcgggacggccaaggcgctgcggtacctgcacgaggagtgcgcgcagcggatcatc
cactacgacatcaagcccgagaacgtgctcctcgacgcgaacctggagcccaaggtgtcggacttcgggctcgcccggctctgcgacagggaggaca
cccacctgaccatcaccggcgcgcggggcacgcccgggtacgccgcgccggagctgtggatgccgctgcccgtcacgcacaagtgcgacgtgtacag
ctacgggatgctgctgttcgagatgctcgggcggcggcgcaacctggagttgggacgattgcacagccggtccgagagccaggagtggtacccgagg
tgggtgtggcaccggttcgaggcgggcgacacggacgccgtgctggggcgcgccgtggcggcaggggagaggaaggagataatcagggagaaggc
ccaacgggcgtgcatggtggcgttgtggtgcgtgcagtaccggcccgaggatcggccgtccatgggcagcgtcgtcaggatgctggaagggggaggag
cagatacctccgccgcgcaacccgttcgcgcacctgtcagcctacgactctggtgccggtgccgccaataataacctgctgcagtcgtcgtccgggtccg
gggacaccaccaataacaccaccccggcctcgtatgcttcttccagccatgtttttag
>g99904.t1
atgccgacgacggagaagtgcgacgtgtacagcttcgggatggtgctgttcgagatcctggggcggcggcgcaactacgacgccggcaaggtcgagg
agagcagggagtggttcccgcggtgggtgtgggagaggtacgagcgggaggagctggagtgcgtgctggacgacgccgggatcggggaggaggac
agggagaaggcggagatgatgtgcaaggtggcgctgtggtgcgtgcagttccagcccgcggcgcgccccaccatgagcagcgtcctgcgcatgctca
agggggacatggccatcgtgccgcccgtgaacccattccactacgtcatggacagcggcggctcgacgagctcgacgatgtggagcggcaccaacgt
cagcagcggtggctacttgaccaccgttggaagagacagtgagatttcagtaagccctgccgccaagtccacgagcgcgatgatttcagtaaacactg
ataagtcgactcatggtgtgtag
>g99905.t1
atgaagaagaagctgaagaagaaagagatgcagaggaggagcatggaggcgcaggctgccaccagcactgccgtggacgacgtggtgatcgaggt
tgggccggtggagaagttcctgaacgagatcctgagcgagaggccgatgcgcttcaggtccgagcagctggcggcctgcacacggaacttctccacgg
agctggggtccggcggcttcggcgtcgtgtacaggggcgagctgcccaacggcctggccgtggccgtgaaggtcctgaaggcgtccgtgatgaagaa
gcgggtcgaggagcagttcatggcggaggtgggcaccatcggccggacctaccacgtcaacctcgtccgcctctacggattctgcttcgacagcgaca
ccaaggcgctggtctacgagttcctggagaacggctccctcgaccagtacctctatagcggcaatggtgacgaggaaggcggccgcaaggagaagaa
gctggagtggagcacgctgcacgccatcgctgtgggtacggcgaaggggatccggtacctgcacgaggagtgccagcagaagatcgtgcactacgac
atcaagccgggcaacatcctcctcaacgccgatttcaccccgaaagtcgccgatttcgggttggcgaggctggcggagcgggagaacacgcacatgac
gctgaccggcggcaggggcacgcccgggtacgcggcgccggagctgtggatggcgatgccgacgacggagaagtgcgacgtgtacagcttcgggat
ggtgctgttcgagatcctggggcggcggcgcaactacgacgccggcaaggtcgaggagagcagggagtggttcccgcggtgggtgtgggagaggta
cgagcgggaggagctggagtgcgtgctggacgacgccgggatcggggaggaggacagggagaaggcggagatgatgtgcaaggtggcgctgtggt
gcgtgcagttccagcccgcggcgcgccccaccatgagcagcgtcctgcgcatgctcaagggggacatggccatcgtgccgcccgtgaacccattccac
tacgtcatggacagcggcggctcgacgagctcgacgatgtggagcggcaccaacgtcagcagcggtggctacttgaccaccgttggaagagacagtg
agatttcagtaagccctgccgccaagtccacgagcgcgatgatttcagtaaacactgataagtcgactcatggtgtgtag
>g100480.t1
atggcggagttcctcttcttgtctgccttgagtctcctgatcctcgcacctcgtgctacctcggtctcctttagctttaacttctccagctccggtgacccatg
cgatgtcgagctccgttgtgagcgcgacacacgtataggcccgggatggctggagctaacaaagaacgagatcagaggtgaccccttcagcgtcggtc
gcgcatcttacgcgcgtccggtgccgctctggaacaacaccaccggcgagctggccagctttacctccaacttcacgttccagatcaagcccaaaaacg

agaccttctaccgcagctgtaacatgagcggcgacggaatggctttcttcctcgcgcactacccatcgaggattcctcccaacagctttggctcgaacct
cggcctcttcaacgacagcaacaacctcgaccgcagcggcgtggatcgggttgtcgcggtggaatacgataccttcttcaatgaccagtgggaccaaa
gctcaaatgatttgggcatcgacatcaattccatcaagtcccaggcctccacgccagtgaccaagggcctggtctccgaagatgccattatggctgccac
tatcagctactacaacctcacaggtatcctaacagccaatctccagatcgctggtgaagtgtatagtgtcagctcgccagttaacatgaaagagaacttg
cccagtgaagtggcggtcggcttttccgcggccactggtatgtgtatcgagctgcaccaggtgctctcctggtcgttcagctccactctgggaaccgcaa
gctccaaggttgtatctggatcggggcgacgccggctactactaaagctccacggcaacactgatggtgattcttctgacggagaacacgagcaagctg
aattcgagagaagggtgggtcctagaaggtaccactacaacgagctggccacggcgacgaataacttcgcggaggaggagaagctcggccgtgggg
gattcggcaacgtgtaccggggtcgactcgtcagcgagcaggactgtccggtgatggccgtaaagatgttctctgctgagtcgtcggtgcaggggaga
aaggcgttcgaggctgaggtgaagatcattagccgcctgcggcatcggaaccttgtgcagttgctagagtgggatcagtgcgtcgttcatggtgacatc
aagcccagcaacattctactcgactcgtcacacagcaccaagctcggggatttcggactggccagactcgtcgaccacggagcgggaccgcgtacgac
gcaggtcgtgatgggcacggcagggtacatagacccggtgttcattcgcacccgacggccgagcaccgagtccgatgtttacagcttcggcgtcgtgct
cctggagatcgtcacggggcggcggccagagatggagaacccggacaaggtcatcccgctgatcaggtggatctgggacctgtacgacagaaacgct
ctcatggaagcattggacgagaggttggttgccggaggcagctggctcggtggcgaagatgaaggcggctgcgggtggcaggtgcggcgtgtgctca
ttgtcgggctgtggtgcgcgcacccggacccgggtctgcgaccgtccatcgtggaggctatgaacgtcctgcagtcaaaagacatgacattgccggttc
tttcacggcaactatataacaggactacgtccggcttctcccatggatcgtctggctacgacatactggcggcggggaccaatatttccagcgatgaaat
gtcttggacgctgagtggtggtagatga
>g100658.t1
atggacgacccgatcctggcgctgatcctcaccgccggcgcctccctcgccgtcgccatcccctgcctcctcgccgcctacctctgccgccgccgccgca
gcaaggctcccgtcaagccggcggcgcagcctgagctccccgtctccgttccggcctcgatcgcgcatcaagactcctcgtcgtggtccttctacggctc
gtcggcggctgactcctccctcgcgaagctctctctggctgacctcgcggcggcgacaggcgggttctccccgtcgaacatcatcggcgacggcagctt
cgggttcgtgtaccgcgccgagctccccgacggcggcggcgcggtcgccgtgaagcggctcgagacgcacggcgcggcggggaaccgtgagttccg
cgcggagctggaggtgctgggcagcctgagccacccgaacctcgcccgcctcctcgggtactgcgccgccggcgccgaccgcatcctcgtgtacgagc
tgctccaccggggcagcctcgacgcctggcttcacggcgacggtgggcccgacgacgcgcctggctccaggtgcgtgctcacgtggccggcgcgtctc
cgtgtggcgcgcggcgtcgcggcggcgctcgcgttcctccaccatgatcgccggccgccggtgctgcatcgggatatcaaggcgagtaacgtgttgctt
ggggacgggttcgaggccaagctcgcggatttcggacttgcaagggtcgtcaagggaagccctgcggcgtcgcacgtcagcacgcaggctgccggga
ctgccgggtatctagcgcccgagatatgggctggtgtgggcgcgactgcgaaagcggatgtgtacagctttggcatcctgatgatcgagatagtaaccg
gtcaccgaccaagttggccggtgaagcccaacgtcgaggagaaggaggaggttgatttggtagactgggcgagggagaaaatacaagcaggcaaag
catcggaggtcctggattgccggatagggattgacgaagggcaagggaaggagacggaggagatgaagggtctcttggacatcgcgcggcagtgta
cggagggtgttcctaaggataggccgaccatggaggaggtggtggccatgctgaacaagctttga
>g100730.t1
atgcagcaggagcagcccaagaagagttctgcagaggccgagtttttcacagagtatggcgatgcaaaccggtacaagatccaagaagtcatcggta
aaggaagctatggtgttgtgtgctctgccattgatttgcacactcgacagagagtggcgatcaagaagatacacaatatctttgagcacgtctctgatgct
gcaaggatcctccgtgagattaaacttctgagactcctaaggcatcctgacattgttgagatcaagcatattatgttacctccttcgagaaaggacttcaa
ggatatttatgttgtttttgagctgatggagtctgatctccatcaagttataaaggccaacgatgacttgacaaaagagcattaccaattctttctctatcaa
ttgctccgtgccctcaaatacattcatactgtgaacttaactgcagctaatgtttatcaccgtgacctgaagcccaagaacattttagcgaattctaactgc
aaactgaaaatatgtgactttggactagcacgagttgcattcaatgatacaccaacaacagtcttctggacggattatgtggcaacaagatggtacaga
gctccagagctctgtggatccttcttctcaaaatatacaccggctattgacatttggagcattggatgcatctttgctgaggtattgactgggaagcctctg
tttcctgatggattaggatttgctgagtttgttgttccctccatcctcggcaggttcggaatgagaaagcaagaaggcattgcgtga
>g100737.t1
atggcccgcccccatctcctccccgcgctcctcctcctccttctgcttcccgttccgctccgctcccagcccgcctcctcgccgccgccgcaatgcgcgctc
aacttcactgcgctgcgccccttcctcgcgccgccgctcccgtccgacggcgcctcccgctgcgcgctcgccacacagtccgtctccttcctcctctccctc
cacctcgccgccacgggctccttcgttctcccctccaacgcctcctcctgcttcgccccgttccgcgccgtgctccccttcccgctccctgcatcctcctgtg
gcgggctgtcgagcgggatcgactccctcctcgccgcccccggatgcggcaacgtctccacgctcgccgaatttaacgcgctcgtcccgcccgccgcgc
gcgccgacatggacgccagctgcgaccgcgacctctcagccgtccccgactgcactgcctgcaccaccgcgctcagcaaggccgccgccgcctacctc
ctgcccggatccggtaacgccgggagcaacaacgtcaccggctgcgtgcagtaccccttttatctacgccggcgccaaggccagcccgcgcggccccac
cgacccggccacagccttctgcctctacctcctcagggtcaaccctcccaccgcagcccgctccggcgtcgcccccctgggtctacggcgtggcgttcggg
tgcctcggggccaggatagccatcgggatggcgaggggggctggcctacctgcaccgtggcgtgcagccagccatcatccatagggatatcaaagcga
gcaacatcttgctcgatgacgaatttgaggccatggtggctgattttggactagccaagtttgcacctgaggggatgacacatgtcagcactcgggttgc
tggaacactgggttatgtcgcccccgagtatgcactctatggccaattgactgagaagagtgatgtctatagctttggagttgtgcttcttgagcttctgag
tggtaagagagcattcatctctctcagtgagggccagagcttcgcgcttactgattgggcttggtccttggtgcggaggggaaagacgatagatgtgatc
caagaaggaatggttgaccctggtcctactgagataatggagaagtatgttcttgtcggcgcgttgtgcacacatccccaactttacgcaaggccaaca
atggaacaagctctgaagatacttgaggcggattcagctccaagtcctttaattattccagagaggccactcccagttgttgcaaaccttggtgagattg
agaggtcggcaagcggcagcggctcaggaccagctaccagtgattctggcacccgccatgctccacatctttacctcctcgccaactgttctcaacacg
gtggcaaggctgggtga
>g100808.t1
atgcaggttttggtggactataatggcgagacgaagcagatcaacgtgacactagctcccttcagaatgaccaggcctctccgaacgctactctcagcc
gtgtatgacctctcaacggtgctcactgacgtggtgtatcttggattctcggcggcaactggtcgtgtgaattcactttactgcgttcttggctggagcttca
gcataaactacgtggtgtatctgtattatgcaactggcggattcaaggaaaggcgcctactaggagaaggaggttttgtggaattttacaaaggagtgct
tcagacatctaagctggaggttgtggtgaagagggtgtcccatcaatcaaaacagggagtgaaggagttcattgcagagatagccagtatcggccgca

tccgacatcgtaatcttgtgcaattacttggttactgcagaagaaaagatgaactctttttggtttatgcctacatgccaaatggtagcctcgataaatatc
tgtatgacagcgatctgtgtaagggaactctaacctggtcacagaggttccattttgtcaaaggtgtggcatccggattactctatctccacgaggggtgg
gagaaggtagtcatccatcgagatatcaaggcaagcaatgtgctcctcgacaaagacatgaacggccaactaggtgattttggcctggcaaggttgta
cgactacggcactgatacacaatccacacatgtggttggtgtaccatgggatacctag
>g100887.t1
atgatgatgatgatgacgggcggcgcggagatgaagcggctccggacgctgggccgcggcgcgtcgggcgccgtggtgtcgctgtacgcggcgggc
gaggacgagctcgtcgcggtgaagtcggcgcggggatcctcgggcgccgccgcgcagctgcggcgcgagtgggaggtcatgtcctccctccgctcgc
cgcacgtgctcccctgcctcggtttccgcgccgccggcggagaggaggaggagtacgagtgccagctcctcctcgagttcgcgccgggcggctcgctg
gccgacgcggcggctcggagcggcgggcggatcgaggagcccgcggcgcgggcgttcgcggccgacgtggcgcgcgggctcgcctacctccacgac
gccgcgtccaccgtgcacggggacgtcaaagcgcggaacgtggtgctcggcggcgacgggcgggccatgctcgcggacttcgggtgcgcgcgggcc
gtgggatccgagaaagggcgccggcccatcatcggcggcacgccggcattcatggcgccggaggtggcacgcggggaggagcagggccccgaggc
ggatatctgggccctgggatgcaccgtcatcgagatggtcactgggcgcgcgccgtggagccacgtcgtggacgacgtggtcgccgcagtgcggatga
tcgggtacaccgacgccgtgcccgaggcgccggcgtggatgtcgccggaggggaaggactttttggggaagtgtctgtgccgggacgccggcgagcg
gtggacggcggcgcggctgctggagcacccgttcttggcgtccgctgattgtggagtcaaggcggaggatgtgaagggcaagtgggtgtcgcccaag
agcacgctggattccgcattgtgggagtccgacgccgacgacgaggacgacgacgagctgctgccggagagcccggccgacaggatcaaatcgttgg
cggggccatgctcggccttgccggagtgggaatccgacaacgatgatggttggatcgaagtgtgcagcagcagcagcagccactctgaaatttccgac
gcggcggcgtccgtcagccaagaggtgaaatttccggcgcactgtgaaattcctgacgcggtggtggtggccacgtcgtccgagcagacgccgcaccg
tgactttcccgatctgccggaggtgtcgatgcctatgcgcaccggcaacagcaggatattttag
>g100888.t1
atggacacggcgccgctcgtgatcgggcagtggacgcgggtgcgcacgctgggccgcggcgcgtcgggcgccgaggtcttcctcgccgccgacgccg
ccacgggccgcctcttcgccgtcaagtccgccccgccgcgcggggcggcggcgctggcgcgcgagcaggaggtgatgtcctgcctgccttgctcgacg
cacgtggtcgcctgcaccggcggccgcgccgcccgcgacggctcgtaccagctcttcctcgagttcgcgcccggcgggtcgcttgccgacgcggcggc
ggcggcgatgagggccggcgggcggctcggggagggcgaggtccagcggttcgccgccgacgtggcgaggggcctcgcggacatgcacgccgccg
gagtcgtccacggcgacgtcaagccacggaacgtcgtcgtcggcgccgacggccgcgccaagctcgccgacttcgggtgcgcgcggcagtgggtgga
aaaggggtcgccgatcctcggcggcacgccggcgttcatggcgccggaggtcgcgcgcggggaggagcagggccccgaagcggatatctgggcgct
gggctgcacggtcgtcgagatggccaccggcaaagcgccgtggagccacgtcgtggacgacgtggtggccgccgtgcggatgatcgggtacagcgac
gccgtgccggatgtccccaagtggctccccgcggaggcgaaggatttcctgggcaagtgcctgtgccgagacgccagcgagaggtggacggcggcgc
agctgctggagcaccccttcattgcctctgccggctgcggtgacacgacggcggaagtcgactgcagcaaggggaaaaaatgggtgtcgcccaagag
cacgctggacgcggcgttctgggagtccgacaccgaagacgaggacgacgagctctcacacgacgcggcggagaggatcaagtcgttagcctcctcc
tctgtttcgaccagtctgccggactgggtctccgatgacgacgagggttggatcgatgtgatctccaccccaaccgaatcgtcttcggactacgcggtgct
cgtcccggcggaagagaagacagccattgacgacgacggtgtcagcaccgatggagaaggaggagaattgagcaacgcggagcgccgcccgcggc
cgcgtagccgtgaccgcgactccggcggtgaaaagcacaggaacgccgcggcggccagtcccgtgccggcgctcaccagcttgcccgccacgccacg
cgccgaagcttccaacgcagagaaagatcgcgttgacacggaaagcgcggcgcccgagtcagcgcggacgtag
>g101153.t1
atgcagctgctgctcgtctacgagttcatgcccaacgggagcctggccgatcacctgtccgcaaggtcgacgcggccggcgtcgtgggcgatgaggctc
agggtggcgctcgacacggcccgagggttgaagtatctccatgaagaatctgaattcaagattatatttcgtgatctgaagccttcaaacattcttctcga
cgagaactggaatgctaaactgtccgatttcggcttggcgagattaggaccacaagaagggagccatgtctccacagcggtggtgggcactatcggat
acgcagcaccggaatatatccatacaggacgcctcagcagcaagaatgacatatggagctacggcgtggtgctttatgagcttcttacaggtcggcggc
ctatcgacaggaataggcccaggggcgagcagaatcttgtggattgggtgaagccctactcttccgatgtcaagaagcttgaggctgtaatggatccaa
ggcttcaagggaactatagcataaaggcagcagccaagctcgcatcagtggcaaataagtgccttgtgcgtcatgccaggtacaggccgaagatgagt
gaggtgctggagatggtgcagaagatcgttgacagcagtgaacttggaacgccagagcatcccctgaccgggaattcaaaggaattagttagtgatga
[truncated: 557,999 more chars]
